# Impact of the chemical modification of tRNAs anticodon loop on the variability and evolution of codon usage in proteobacteria

**DOI:** 10.3389/fmicb.2024.1412318

**Published:** 2024-08-05

**Authors:** Sebastián Delgado, Álvaro Armijo, Verónica Bravo, Omar Orellana, Juan Carlos Salazar, Assaf Katz

**Affiliations:** ^1^Facultad de Ciencias, Universidad de Chile, Santiago, Chile; ^2^Facultad de Ciencias Químicas y Farmacéuticas, Universidad de Chile, Santiago, Chile; ^3^Programa Centro de Investigacion Biomédica y Aplicada, Escuela de Medicina, Facultad de Ciencias Médicas, Universidad de Santiago de Chile, Santiago, Chile; ^4^Programa de Biología Celular y Molecular, Instituto de Ciencias Biomédicas, Facultad de Medicina, Universidad de Chile, Santiago, Chile; ^5^Programa de Microbiología y Micología, Instituto de Ciencias Biomédicas, Facultad de Medicina, Universidad de Chile, Santiago, Chile

**Keywords:** codon usage, tRNA, RNA modifications, genome evolution, proteobacteria, threonylcarbamoyl adenosine (t^6^A), glutamyl-queuosine (GluQ)

## Abstract

Despite the highly conserved nature of the genetic code, the frequency of usage of each codon can vary significantly. The evolution of codon usage is shaped by two main evolutionary forces: mutational bias and selection pressures. These pressures can be driven by environmental factors, but also by the need for efficient translation, which depends heavily on the concentration of transfer RNAs (tRNAs) within the cell. The data presented here supports the proposal that tRNA modifications play a key role in shaping the overall preference of codon usage in proteobacteria. Interestingly, some codons, such as CGA and AGG (encoding arginine), exhibit a surprisingly low level of variation in their frequency of usage, even across genomes with differing GC content. These findings suggest that the evolution of GC content in proteobacterial genomes might be primarily driven by changes in the usage of a specific subset of codons, whose usage is itself influenced by tRNA modifications.

## 1 Introduction

The genetic code is extremely well conserved in nature, with only minor variations between species that are phylogenetically very distant (Koonin and Novozhilov, 2017). For instance, although the last common ancestor of proteobacteria [also called pseudomonadota (Oren and Garrity, 2021)] lived about 2,400 to 3,200 million years ago (Battistuzzi et al., 2004), most proteobacteria share the same genetic code (Elzanowski and Ostell, n.d.), with the exception of some insect parasites with very small genomes such as *Candidatus* Hodgkinia cicadicola where UGA has been reassigned from Stop to Trp (McCutcheon et al., 2009). Notably, although the meaning of each codon has been strongly conserved, the frequency with which each codon is used (the “codon usage”) may vary within a wide range between diverse proteobacteria. For instance, while 11.3% of codons in the genome of the betaproteobacteria *Candidatus* Nasuia deltocephalinicola are AAT (coding for Asn), in the gammaproteobacteria *Pseudoxanthomonas suwonensis* only 0.2% of coding sequences correspond to this codon (Choi et al., 2013; Bennett et al., 2016). Nevertheless, while codons such as AAT may vary within a wide range of codon usages, here we report that other codons are restricted to a very narrow range of possible frequencies of usage.

The enormous variability of codon usages has interested the research community for decades and a series of factors have been proposed to affect its evolution. Given that bacterial genomes are mostly composed by coding sequences, the frequency of codons usage is intimately related to the percentage of guanines (G) and cytosines (C) that compose each genome (GC%) (López et al., 2020). This implies that organisms with a higher GC% tend to prefer codons with a higher number of C plus G and a lower number of adenine (A) and thymine (T), particularly in the third position of the codon (Iriarte et al., 2021). The GC% of genomes depends in part on the mutation rates between each nucleotide. Although mutations occur mostly at random, some trends are observed due to factors that either promote or restrict specific nucleotide changes. For instance, enzymes involved in DNA replication and/or repair are known to present biases, being more efficient in protecting from some mutations (Iriarte et al., 2021) [e.g., in *E. coli*, MutL protects preferentially from A:T to G:C mutations (Lee et al., 2012)]. Similarly, environmental conditions also favor some mutation trends [e.g., in *E. coli*, UV promotes G:C to A:T mutations more frequently than other mutations (Shibai et al., 2017)].

In addition to biased mutation rates, bacteria are confronted to diverse selective pressures that also model the evolution of codon usage and GC%. For instance, it has been proposed that codons decoded by the most abundant tRNAs are translated more rapidly. Such increased speed would affect the amount of produced protein, but also the rates in which erroneous amino acids are introduced in the nascent peptides. Correspondingly, the frequency of codon usages in highly expressed genes is correlated to the concentration of the corresponding tRNAs in proteobacteria like *E. coli* and to a great extent also to the number of genes coding for cognate tRNAs (Ikemura, 1985; Dong et al., 1996; Rojas et al., 2018; Iriarte et al., 2021). Furthermore, the correlation between codon usage and tRNA concentrations is higher in bacteria that replicate rapidly, suggesting a stronger selective force for efficient protein production in such bacteria (González-Serrano et al., 2022). Coincidentally, it has been observed that selective pressure for codon bias is relevant mainly in rapidly replicating bacteria (Sharp et al., 2010).

Additional factors may affect codon usage evolution. For example, alterations in codon usage (Hu et al., 2013; Buhr et al., 2016; Iriarte et al., 2021; Moreira-Ramos et al., 2022) and tRNA concentration (Fedyunin et al., 2012; Arias et al., 2020) may affect the co-translational folding of proteins. Similarly, environmental conditions also exert selective pressures on codon usage evolution. For example, in prokaryotic thermophiles there is a preference to use AGR over CGN codons to encode Arg (Iriarte et al., 2021). Thus, GC% and the frequency of codon usage would depend on a balance between bias in mutagenesis and selective pressures to which each specie would have been exposed.

All of the factors we have discussed here are general, indicating that most codons should vary within a similar range of possible usage frequencies. Nevertheless, as mentioned above, here we report that while the usage of some codons in proteobacteria vary within a broad range of frequencies, others are restricted to a much narrower range. Moreover, our analyses suggest that evolutionary variability is strongly affected by the chemical modification of the anticodon stem loop in tRNAs.

## 2 Results

### 2.1 The usage of some codons is restricted to a narrow range of frequencies

In order to study the evolution of codon usage we selected proteobacteria as a model group as this is one of the best represented groups in genome databases. We defined a limited collection of 1,484 genomes from diverse proteobacterial classes as described in the methods section. The frequencies of codon usage from each gene were calculated. The average of codon usage in either all genes from each genome or a subset of genes expected to be highly expressed was used for all the described analyzes. We note that we used the frequencies of codon usage for all our analysis, not codon usage bias (CUB) another commonly used parameter where the frequency of usage of each codon is normalized by the frequency of usage of all codons coding for the corresponding amino acid. CUB is useful in several cases, but may distort data interpretation. For example, if the usage of an amino acid encoded by two codons decreases, CUB may show that the preference for one of the codons increases, while the frequency of usage of both may decrease. Similar misinterpretations may occur if the usage of one codon remains constant, while the usage of the other varies.

**FIGURE 1:**
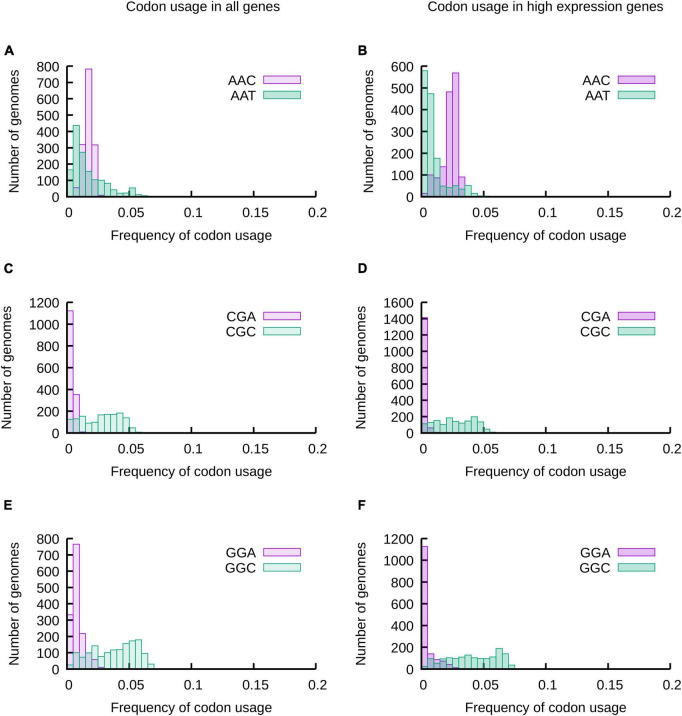
The usage of most codons may vary in a wide range of values, but some codons are restricted to a narrow range of codon usages. The frequencies of usage of all codons in 1484 species of proteobacteria were calculated considering all genes of their genomes **(A,C,E)** or a subset of genes expected to be highly expressed **(B,D,F)**. The distribution of codon usages for Asp codons AAT and AAC **(A,B)**, 2 of the 6 Arg codons (CGA and CGC) **(C,D)** and 2 of the four Gly codons (GGA and GGC) **(E,F)** are presented in the histograms. The data for all codons is presented in Supplementary Figure 1.

**FIGURE 2:**
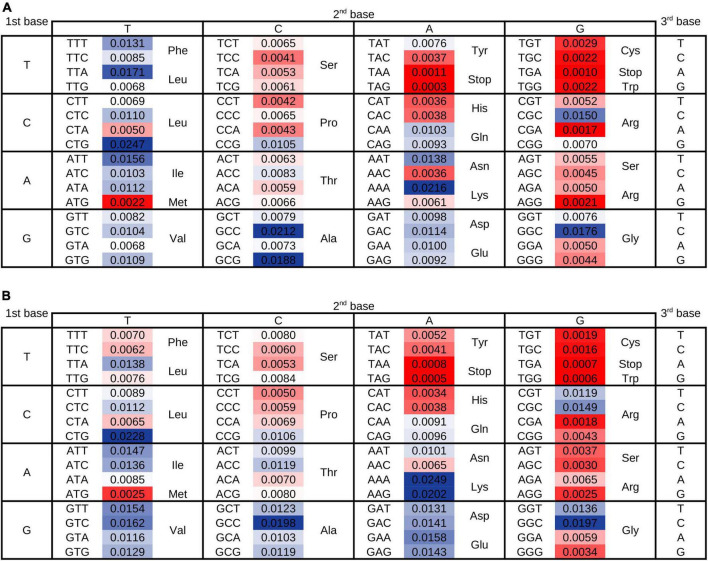
Variability of codon usage in proteobacteria. As an estimation of the variability in frequencies of codon usage in proteobacteria the standard deviation of codon usages in the complete population was calculated. Data is presented in the context of the genetic code and values are colored with respect to variability, where the most variable codons are highlighted with blue colors and the least variable codons in red colors. **(A)** Standard deviations of codon usages considering all genes of genomes. **(B)** Standard deviations of codon usages considering a set of genes expected to be highly expressed. Variability estimation based on the distance between values at percentiles 10 and 90 are represented in Supplementary Figure 4.

As a first approach we analyzed the distribution of codon usage frequencies in histograms and phylogenetic trees (Figure 1 and Supplementary Figures 1–3) as well as by comparing standard deviations (SD) (Figure 2) and the distance between frequencies at percentiles 90 and 10 (Delta 90-10) of histograms (Supplementary Figure 4). Analyzing these representations of the data, it is evident that while the frequency of usage of some codons may vary within a wide range of values [“Variable Codons,” for example AAT (Asn), CGC (Arg) and GGC (Gly)], other codons vary in a very narrow range [“Low Variability Codons,” for example, AAC (Asn), CGA (Arg) or GGA (Gly)] (Figure 1 and Supplementary Figure 4). Variability of codon usages showed only small variations when comparing codon usages in highly expressed genes vs. complete genomes (Supplementary Figure 5), with the exception of TGG and TTT where we observe a reduced standard deviation in codon usages of highly expressed genes (∼3.9 and ∼1.9 fold decrease, respectively) and codons AAG, CGT, GTT, GGT and GAA where we observe an increased standard deviation in codon usage of highly expressed genes (∼3.3, ∼2.3, ∼1.9, ∼1.8 and ∼1.6 fold increase, respectively).

### 2.2 Genome comparison reveals that the presence of tRNA modification enzymes correlates with broad variability of codon usages

We were intrigued by the fact that the usage of some codons vary widely, while others are restrained to a narrow range of frequency values. To get some insight regarding the genome features that allow wide variability of codon usages, we performed an unbiased screening of genes that could explain the wide variabilities observed for some codons. As the usage of the most variable codons correlate either positively or negatively to the GC% (see below), we randomly selected 25 bacteria with high and 25 bacteria with low GC% (above 67% and below 32% GC, respectively, listed in Supplementary Table 1, histogram of GC% in Supplementary Figure 6) which also present extreme codon usage values for variable codons. Genes present or absent in each group of genomes were obtained using Roary (Page et al., 2015), a program that groups genes based on the percentage of identity in blastP (Altschul et al., 1990) comparisons of translated genes (in this case with a 40% identity threshold, given the long evolutionary distance between the analyzed genomes) and Scoary, a program that calculates gene enrichment statistics. The analyses showed that genomes with high GC% differed from those with low GC% in a series of genes, including several related to translation (Supplementary Table 2). Interestingly, the strongest correlations were observed for two enzymes involved in the chemical modification of the tRNA anticodon loop: *tilS* involved in the synthesis of tRNA(Ile)-lysidine (k2C), and *tsaB* involved in the formation of threonylcarbamoyl-6-adenosine (t^6^A). *gluQ* involved in the hyper modification of queuosine (Q) with glutamate forming glutamyl-queuosine (gluQ) also presented a very low *P*-value (below 1e-10). More than 25 additional genes involved in tRNA modification presented a weaker, but statistically significant, association to GC% of the analyzed genomes (Supplementary Table 3).

Chemical modifications have been shown to have diverse effects in tRNA biology, ranging from folding stability and resistance to nucleases to alterations in codon-anticodon interactions (El Yacoubi et al., 2012; Björk and Hagervall, 2014; Tittle et al., 2022). Given that the efficiency of codon translation depends on codon-anticodon interactions and that the enrichment of efficiently translated codons is expected, particularly in highly expressed genes, we speculate that the presence of these modifications allows the alteration of codon usages and thus, genomic GC%. Supporting this idea, Novoa et al. (2012) have previously proposed that the contrasting codon preferences between eukaryotes and bacteria depend on inosine and 5-oxyacetyluridine modifications present in tRNAs from each phylogentic group.

### 2.3 The presence of genes involved in modification of tRNAs is correlated to changes in codon usages

The confidence in these results is limited by the fact that Roary and Scoary algorithms were designed and tested to work with related organisms having small evolutionary distances. Additionally, we are comparing only extreme GC% cases and, given the long evolutionary distance, some genes with similar functions were not grouped in Roary analyses. Thus, further generalization of any conclusion might be controversial. To further support the potential role of tRNA modifications on codon usage evolution, we selected the tRNA anticodon modification genes that showed the lowest *P*-values in the Scoary enrichment analysis. We then screened for the presence of these genes in our complete collection of proteobacterial genomes using RPSBlast using diverse sets of sequence patterns (motifs) published at the Conserved Domain Database (CDD) (Wang et al., 2023) (Sets of patterns listed in Supplementary Table 4). Additionally, we screened for the presence of other genes involved in the pathways for each modification. Four genes involved in t^6^A formation in bacteria were screened: *tsaB*, *tsaC/tsaC2*, *tsaD*, and *tsaE* genes. TsaC/TsaC2 catalyze the formation of threonylcarbamoyl-adenylate -an activated intermediate- before the transfer of the threonylcarbamoyl group to adenosine 37 in tRNAs decoding ANN codons. This last step is catalyzed by TsaD with the support of accessory proteins TsaB and TsaE. These last two proteins are not essential for t^6^A synthesis and are absent in some models (Thiaville et al., 2014, 2015; Su et al., 2022). Additionally, we analyzed the presence of two genes involved in the synthesis of GluQ: *gluQ*, coding for GluQRS that modifies Q with glutamate in tRNA*^Asp^* (Blaise et al., 2004; Salazar et al., 2004), and *tgt* a gene essential for the replacement of G by Q, a key step for the introduction of Q to tRNAs (Iwata-Reuyl, 2003) (Given that the name abbreviations used for GluQ modification and *gluQ* gene are similar, in the figures we use GluQRS to refer to the protein coded by *gluQ* gene). Finally, we screened for *tilS* required for ligation of lysine to cytidine in the synthesis of k2C at position 34 of tRNA*^Ile^*_*CAU*_ to restrict wobble pairing and force ATA instead of ATG translation (Nilsson and Alexander, 2019). We further selected the case of *tusE* and *mnmA* as examples of genes with lower correlation scores. *tusE* and *mnmA* are involved in the synthesis of 2 thiouridine (s^2^U) in the wobble base of three tRNAs: tRNA*^Glu^*_*UUC*_, tRNA*^Gln^*_*UUG*_, and tRNA*^Lys^*_*UUU*_ (Shigi, 2014).

**FIGURE 3:**
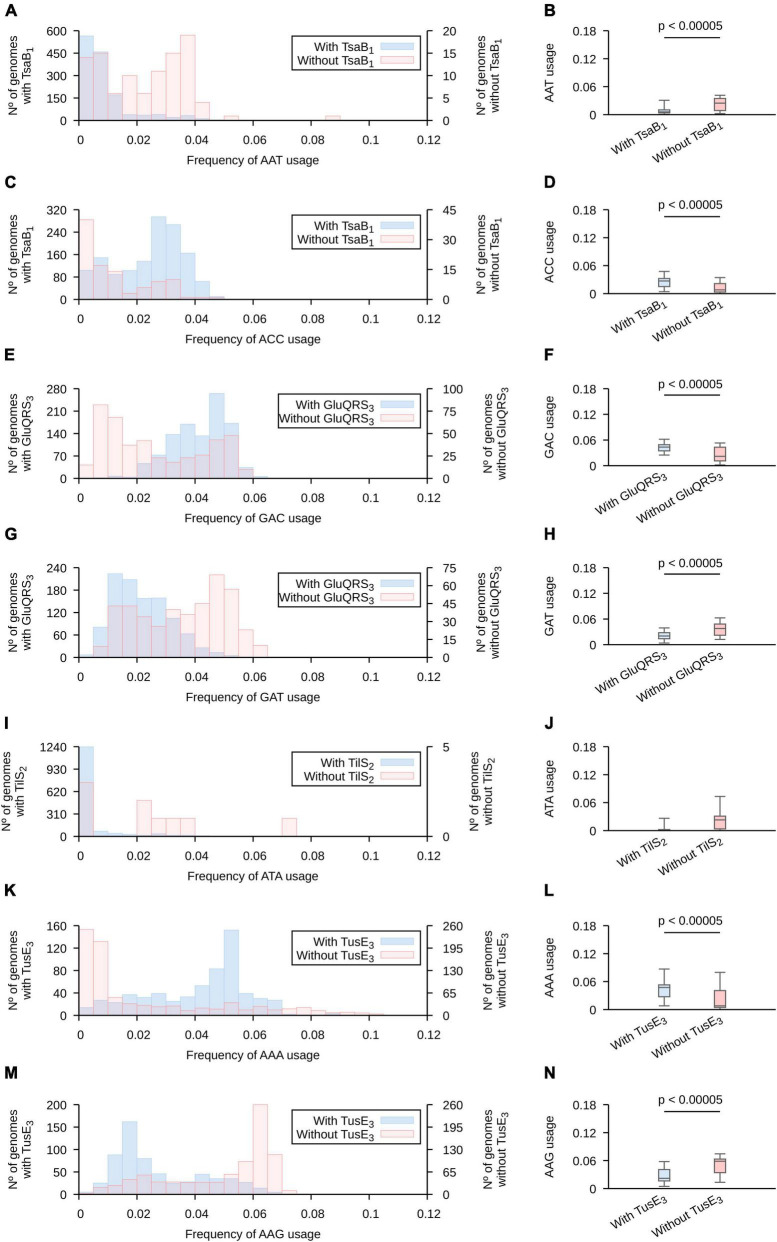
The presence of genes involved in tRNA modification is correlated with altered frequencies of codon usage. The collection of 1,484 proteobacterial genomes were separated in groups, based on the presence (light blue) or absence (orange) of the indicated pattern in any gene of the analyzed genomes. **(A,B,C,D)** Genes found using TsaB motif 1; **(E,F,G,H)** genes found using GluQRS motif 3; **(I,J)** genes found using tilS motif 2; **(K,L,M,N)** genes found using tusE motif 3. See Supplementary Table 4 for a detailed description of the used patterns and Supplementary Figures 7–14 for the complete analyses. Here, only the profiles that allowed the highest number of matches were included. Left: Codon usage in a subset of genes expected to be highly expressed is presented is in superposed histograms. Right: The data is represented in a boxplot. A horizontal bar was added when the two populations are different based on Wilcoxon-Mann–Whitney and *p* < 0.00005. Whiskers include 95% of data. **(A,B)** AAT; **(C,D)** AAC; **(E,F)** GAC; **(G,H)** GAT, **(I,J)** ATA; **(K,L)** AAA; **(M,N)** AAG.

Based on the screen results, we constructed histograms comparing the frequencies of usage of each codon in genomes that contain and those that lack a copy of each of the analyzed genes (Figure 3 and Supplementary Figures 7–10 for analyses based on highly expressed genes and Supplementary Figures 11–14 for analyses all genes in tested genomes). We focused our analyses in histograms produced for highly expressed genes and the patterns that allow the identification of the highest number of representatives of each tRNA modification gene (Supplementary Table 5). Based on these analyses, we observed for *tsa* genes (required for t^6^A) that, as a general rule, ANA and ANT codons tend to present lower frequencies of usages in genomes that lack either *tsaB* or *tsaE*, while ANC codons (with the exception of AGC) present the inverse trend. The presence of these genes produce only subtle effects on the usage of ANG codons. Given the presence of t^6^A in most tRNAs decoding ANN codons, and that *tsaB* and *tsaE* are non-essential for t^6^A formation (Thiaville et al., 2014), we hypothesize that *tsaB* and/or *tsaE* presence may enhance t^6^A formation and, thus, increase the fraction of tRNAs decoding the analyzed codons that are modified with t^6^A. This would alter the velocity of ANN codons translation and potentially alter the expression of highly expressed genes.

In contrast to the wide number of tRNAs modified with t^6^A, only tRNA*^Asp^* is known to carry the GluQ modification (Dubois et al., 2004; Salazar et al., 2004). The presence of *gluQ* was correlated to a higher usage of Asp codon GAC and a lower usage of Asp codon GAT. The presence of *tgt* was also correlated to high usage of GAC and low usage of GAT. In the case of s^2^U, we concentrated our analysis in Glu, Gln and Lys codons decoded by tRNAs known to carry this modification. Genomes with *tusE* tend to present higher frequencies of usage for codons Lys AAA and Glu GAA and lower usages of codons Lys AAG and marginally Glu GAG. Gln codons CAA and CAG were not significantly affected by the presence of *tusE* gene. Thus, the presence of tRNA modification genes do correlate with codon usages. Finally, we analyzed the frequency of ATA usage in genomes where *tilS*. Although the genomes lacking *tilS* tend to present a higher frequency of usage of ATA codons, this trend is not statistically significant based on the threshold used in this work (*p* < 0.00005). Possibly, the lack of statistical significance is because only 9 genomes do not present a copy of this gene. Furthermore, given that bioinformatic identification of genes is not free of errors, it is possible that some of these 9 genomes present a *tilS* gene that has diverged too far from available protein motifs to identify its sequence. In fact, we were able to identify potential *tilS* genes in 2 of these genomes using tblastn (Altschul et al., 1990).

We note that the described codon usages are variable among organisms that either present or lack a given tRNA modification gene. This is expected as, in addition to tRNA modifications, other factors must affect codon usage evolution as mentioned in the introduction. But, tRNA modifications may explain in some cases why codon usage distributions are multimodal. For instance, analyzing AAA usage in highly expressed genes of proteobacteria we observe a bimodal distribution with modes at 0–0.5% and 5–5.5%. Separating the populations based on the presence of *tusE* (involved in s^2^U formation), we observe two monomodal distributions (Supplementary Figure 14). Thus, *in toto*, these results show a strong correlation between the presence of tRNA modification genes and the preferred frequencies of codon usage suggesting a role of these modifications in making possible a wide variability of codon usages.

### 2.4 The size and GC content of genomes is correlated to the presence of genes involved in t^6^A and gluQ modification of tRNAs

**FIGURE 4:**
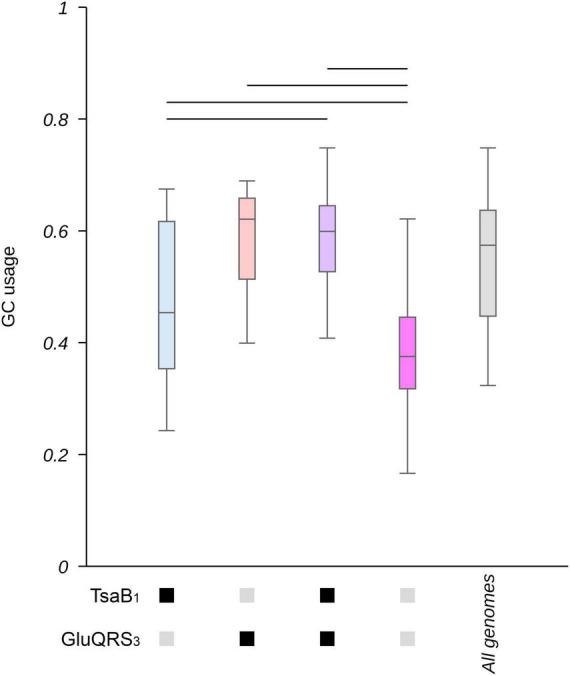
Genes involved in t^6^A and GluQ formation impact the GC content in proteobacterial genomes. The collection of 1,484 proteobacterial genomes were separated in groups based on the presence or absence of genes involved in GluQ and t^6^A modification (based on motif 1 for TsaB and motif 3 for GluQRS, see Supplementary Table 4 for a detailed description of the used patterns). The content of GC as a fraction of all nucleotides in each genome was calculated and data presented as boxplots. Blue: genomes that present TsaB, but lack gluQ. Orange: genomes that lack TsaB and present GluQRS. Purple: genomes that present TsaB and GluQRS. Pink: genomes that lack TsaB and GluQRS. Gray: all genomes. Additionally, the presence of genes coding for TsaB or GluQRS is indicated by black boxes below the *X*-axis. Their absence is indicated by gray boxes. A horizontal bar was added when *p* < 0.00005 based on Kruskal-Wallis test and Dunn’s post hoc test. *P*-values were adjusted using the Holm’s method. A box representing the GC content in the complete collection of genomes is added as reference and was not included in statistical analyses. Corresponding histograms can be found at Supplementary Figure 16.

Codon usage is correlated to GC% in the case of variable codons (see below). Thus, one would expect that if tRNA modifications are required to allow alterations in the frequency of codon usage, these would be also correlated to changes in GC%. As shown in Supplementary Figure 15, the most evident effects on GC% were observed for genes involved in GluQ and t^6^A modifications (particularly when analyzing the patterns that allowed a higher number of genes to be identified), which coincidentally are two of the genes that showed lower *P*-values in the enrichment analysis of genomes with extreme GC% (Supplementary Table 2). We further studied the combined impact of these tRNA modifications on GC% by selecting two representative patterns based on the number of genes these were able to identify: pattern 1 for *tsaB* and 3 for GluQRS. As shown in Figure 4 and Supplementary Figure 16, when both genes are absent, GC% tend to be lower, while the presence of either *tsaB* or specially *gluQ* is associated to higher GC% values.

Low values of GC% (Almpanis et al., 2018) and the loss of tRNA modifications enzymes (Hansen and Moran, 2012; Manzano-Marín and Latorre, 2016; Quaiyum et al., 2024) have been each independently associated to small genomes sizes. Thus, we analyzed the relation between genome size and the presence of tRNA modification enzymes. As shown in Supplementary Figure 17, the lack of genes coding for GluQRS, TsaB or TsaE is strongly correlated to small genome sizes, mostly below ∼2 Mbp. Further analyzes of the combined effects of GluQRS and *tsaB* shows that genomes with GC% below ∼40% tend to lack these tRNA modification genes (Supplementary Figure 18A). As the number of genomes presenting a gene for GluQRS, but lacking *tsaB* is small, we further confirmed *tsaB* absence in these genomes by tblastn analyses and a search in annotation databases such as BV-BRC (Olson et al., 2023). We found four potential false negatives. Eliminating these genomes from our database did not alter significantly our analyses (Supplementary Figure 18B) . Furthermore, given that intracellular bacterial symbionts have been proposed to potentially acquire functional proteins from their hosts (Tamames et al., 2007), it is possible that some of the smallest genomes in our database do present some tRNA modification activities that we have predicted to be absent based exclusively in sequence analysis. Thus, we repeated some of our analyses excluding from our database the 7 genomes that presents 400 or less CDS. As shown in Supplementary Figures 19–22, eliminating these genomes did not altered significantly our results.

### 2.5 Codon usages variability and the genetic table

Our results show that the evolution of codon usage in proteobacteria is strongly affected by the existence of enzymes capable of modifying the tRNA nucleotides. We were also intrigued by the factors that may explain the restricted variability of low variability codons. We hypothesized that the uneven distribution of variability in the genetic code table may be related to properties of encoded amino acids. For instance, ATG and TGG probably present a low variability of usage (Figure 2 and Supplementary Figures 1, 4) because these are the only codons that encode for their respective amino acids (Met and Trp). In consequence, any variation in codon usage would be accompanied by an alteration of the abundance of the Met or Trp in the proteomes. Amino acid abundance in proteins depends on diverse selective pressures that would indirectly affect the frequency of usage of their matching codon. For instance, the fact that Met may be easily oxidized (Vincent and Ezraty, 2023) probably limits the frequency with which the corresponding codon can be used in the studied genomes. Similarly, Cys usage is also restrained to a low usage by its reactivity (Knoke and Leichert, 2023; Luo et al., 2023; Tikhomirova et al., 2024; Wang et al., 2024). So, although there are two codons coding for this amino acid, their usage must vary within a small range in order to maintain a low total usage of Cys in the proteobacterial proteomes (Figure 2 and Supplementary Figures 1, 4). In contrast to codons coding for Met, Trp or Cys, the low variability of codons AAC (Asn), CGA (Arg), AGG (Arg) and GGA (Gly) is difficult to explain by amino acids properties considering that other codons for the same amino acids vary within a wide range (e.g., AAT for Asn, CGC for Arg or GGC for Gly). While this difference in variability of codon usage decreases in cases such as Asn codons when analyzing highly expressed genes, the difference remains when analyzing the usage of other codons such as Arg and Gly codons in highly expressed genes (Figures 1, 2 and Supplementary Figures 1, 4). Thus, although amino acids properties may partly explain the low variability of usage in some cases, it is not sufficient to explain the full diversity codon usage variability ranges.

When analyzing the variability of usage of each codon in the context of the genetic code table (Figure 2 and Supplementary Figure 4), we observed that the frequency of usage tend to present a lower variability for codons at the top and right of the genetic code table, that is, in codons beginning with T and especially for those with G in the second position of the codon. In contrast, codons beginning with G and partially codons presenting a T in the second position tend to present highly variable codon usage in proteobacteria. Nevertheless, these are not absolute trends as codons CGT (Arg) and CGC (Arg), GGT (Gly) and GGC (Gly) present high variability of codon usage.

It has been proposed that C at the third (wobble) position tends to be preferred in the case of synonymous codons that may use either C or T in such position (Higgs and Ran, 2008). Variability of codon usage does not seems to follow this tendency. In some cases, frequency of codon usage in all genes vary in a wider range of values for C ending codons (e.g., Arg CGC vs. CGT; Gly GGC vs. GGT; Ala GCC vs. GCT; Thr ACC vs. ACT or Leu CTC vs. CTT), while for others the greater variation is observed for codons with wobble T (e.g., Asn AAT vs. AAC; Tyr TAT vs. TAC; Phe TTT vs. TTC or Ile ATT vs. ATC). In a third group, differences are negligible (e.g., for Cys TGT vs. TGC, Ser AGT vs. AGC or His CAT vs. CAC). Analyzing codons of highly expressed genes, we observed that differences between Tyr, Phe and Ile codons are reduced. So, in this case there are mainly two groups, one where C at wobble position correlates to a larger variability of codon usage, and one where differences in variability are not observed. The only exception is Asn AAT that varies in a wider range than Asn AAC. Thus, although the properties of encoded amino acids and the sequence of codons seem to have a role in the evolution of codon usage variability, they do not explain by themselves the full range of variabilities observed in codon usage.

### 2.6 Low variability of codon usages is independent of GC% and the number of tRNAs

**FIGURE 5:**
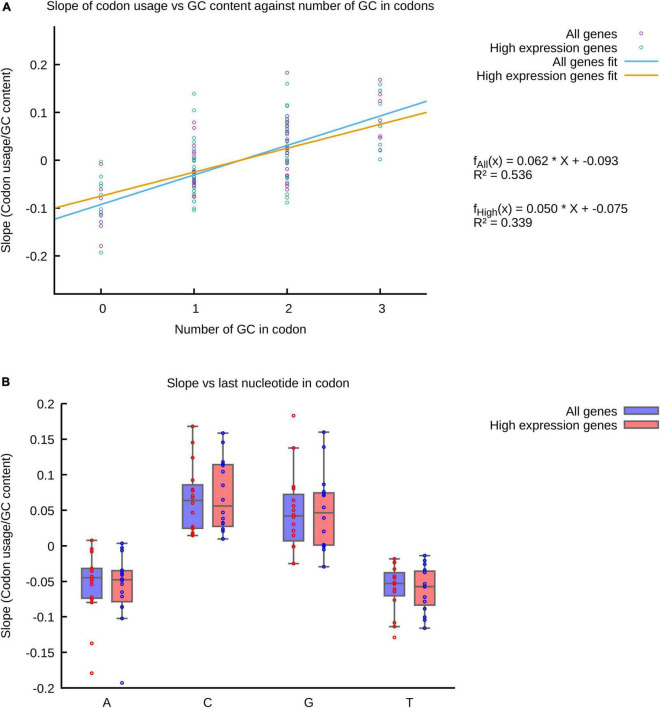
The usage of some codons is independent of genomic GC%. The slopes of linear regressions in plots of codon usages vs. GC% (Supplementary Figure 17) was used to estimate the impact of GC% on codon usage. **(A)** Slopes of codon usage vs. GC% are compared to the number of G or C in the analyzed codons in a scatter plot. Each point represents the slope of codon usage vs. GC% plots. **(B)** Slopes of codon usage vs. GC% are compared to the identity of the last nucleotide in codons (third or wobble base position) in box plots. Each point represents the slope of codon usage vs. GC% plots. Note that in both **(A,B)** some of the codons present a slope ≈ 0 in plots of codon usage vs. GC%.

As discussed above, the frequency of usage of codons is usually correlated to the GC% of genomes, as well as to the number of genes coding for tRNAs decoding each gene. Nevertheless, when we compared codon usage to the GC% of genomes we found that only variable codons usages correlate to GC%, while poor or no correlations were found for low variability codons. Thus, while the usage of many codons strongly correlate to GC% (Supplementary Figure 23) and the global trend is a positive slope in codons rich in G or C or codons with these nucleotides at the third position (Figure 5), for some codons the usage is not affected by the GC% of genomes, even when presenting 2 or 3 G/C. When comparing codon usage to the number of genes coding for tRNAs with complementary anticodons, we were also unable to find any obvious correlation to usage variability (Supplementary Figure 24). This is most evident in cases such as the Asn, encoded by AAC and AAT. Both codons are translated by tRNA*^Asn^*_GTT_, translating AAC by perfect complementarity and AAT by wobble interactions. The gene for tRNA*^Asn^*_ATT_ (perfectly complementary to AAT) is absent from most analyzed genomes. Thus, changes in tRNA*^Asn^*_GTT_ concentration should in theory affect the frequency of usage of both codons. But, while AAT is a variable codon, AAC is a low variability codon (Figure 1).

## 3 Discussion

In this work we investigated the variability of codon usages in proteobacteria and its relation to diverse factors. Based on these results, two observations are remarkable:

1.- Variability of codon usages is correlated to the presence of tRNA modification enzymes. tRNA concentration is usually considered one of the most relevant changes to allow significant alterations in codon usage, as increased codon usage is usually related to increased speed of translation of the corresponding codons (Ikemura, 1985; Dong et al., 1996; Iriarte et al., 2021). Interestingly, we did not find a correlation between codon usage and the number of genes coding for tRNAs, which are considered a good proxy of tRNA concentration (Dong et al., 1996). Nevertheless, such correlations are mainly observed in rapidly replicating organisms (Sharp et al., 2010; González-Serrano et al., 2022). Thus, given the wide scope of organisms analyzed in the current study included bacteria that replicate slowly, it is not surprising that we have not found such correlations. Instead, we have found a correlation between the presence of diverse tRNA modifying enzymes and codon usage or GC%. Further experimentation will be required to confirm if there is a cause-effect relationship between tRNA modifications and codon usage. Nevertheless, one has to consider that increasing the concentration of tRNAs may have a limited impact on translation efficiency. For instance, in proteobacterium like *E. coli* that present a GC% of ∼51%, the most abundant tRNAs are at a concentration of ∼30 μM (Dong et al., 1996), a concentration expected to almost saturate its ribosomes [K_M_ = ∼4.6 μM (Thompson et al., 1986)]. Thus, increased tRNA concentrations may have limited effects in the rates of translation of the corresponding codons in some cases and would not be enough for increased codon usage or adaptation to extreme GC%. Changes in K_M_ values are possible, but should affect tRNA selectivity in addition to translation speed. Alterations in the anticodon stem-loop modification patterns may also allow for modified codon-anticodon affinities (Saint-Léger et al., 2015; Nilsson and Alexander, 2019; de Crécy-Lagard and Jaroch, 2021), a property that should alter translation speeds (Saint-Léger et al., 2015; Smith et al., 2022) thus, possibly also codon usage (Saint-Léger et al., 2015). As mentioned previously, other researchers have previously shown that alterations in tRNA anticodon stem-loop modifications [inosine (I) in eukaryotes and 5-oxyacetyluridine (cmo^5^U) in bacteria] correlate with the differences between bacterial and eukaryal frequencies of codon usage (Novoa et al., 2012). Additionally, Diwan and Agashe (2018) found correlations between the predicted presence of four tRNA modifications and either the number of tRNA genes, codon usage bias (CUB) or GC%. Although they did not included gluQ and t^6^A nor codon usage variability in their study, their results also strongly support the relevance of tRNA modification in codon usage evolution.

Interestingly, correlations between GC% and the presence of some modification enzymes seem to be correlated to genome size. It is difficult to define at this point which of these factors is a cause and which a consequence. The reduction of genomes require the loss of genes and these may include tRNA modification genes (Hansen and Moran, 2012; Manzano-Marín and Latorre, 2016; Quaiyum et al., 2024). We hypothesize that the loss of *gluQ* and/or *tsaB* will alter translation efficiency, which in turn may exert a selection pressure to alter GC% and codon usage, thus reestablishing a balance with the new translation efficiency profile. Alternative hypothesis are also possible. For instance, a selection pressure to reduce GC% may allow the loss of some tRNA modification genes or codon usage and the loss of tRNA modification enzymes may be independently related to genome reduction, but have no cause-effect relation between themselves. Discriminating between these scenarios will require further research.

2.- The frequency of usage of a subset of the genetic code is restricted to a narrow range of codon usages. Codon usage is usually considered to be strongly correlated to the GC% of genomes. Our data mostly confirm this view, however, it also highlights the previously unreported observation that certain codons are restricted to a narrow range of possible usage frequencies, consequently having no correlation to GC%. As discussed previously, in some cases the low variability of codon usages might be related to the requirement of a low usage of the encoded amino acid. Nevertheless, in cases such as Asn AAC or Arg CGC, this explanation is unlikely because other codons for the same amino acids do vary within a wide range of possibilities. In these cases, we expect that low variability is a consequence of the decoding process. Such expectation is supported by the trends we have observed in the genetic table, for instance, that low variability codons tend to have a T at the first position and G in the second position of the codon. Perhaps, the ability of G and U to pair with two nucleotides (G with C and U and U with A and G) (Varani and McClain, 2000) increases the probability of errors during codon-anticodon pairing, thus, restraining the usage of such codons to a narrow (mostly low) range of usage frequencies. Given that tRNA modifications may affect codon-anticodon pairing and reduce error rates in translation (Agris et al., 2017), their presence could broaden the range of biologically possible codon usage frequencies. Unfortunately, such explanations do not consider all low variability codons and further research will be required to fully understand the low variability behavior of some codons.

Altogether, our results support the previous idea that the evolution of codon usage and the GC content of genomes depends in part on changes to the translation machinery. Nevertheless, our results also highlights that such evolution is mostly restricted to a fraction of the genetic code. The usage of other codons remains mostly unchanged independent of other factors such as the GC content of genomes or the existence of tRNA modification enzymes.

## 4 Materials and methods

### 4.1 Selection of proteobacterial genomes

The file “assembly_summary.txt” was downloaded RefSeq (O’Leary et al., 2016) ftp site^1^ on May 30th 2018. This file contains descriptive data for all genomes sequence at RefSeq. Using the TaxID number of each genome, taxonomy information was obtained from Entrez (Gibney and Baxevanis, 2011) using Bioperl (Stajich et al., 2002; Stajich, 2007) taxonomy module (Bio::DB::Taxonomy). Based on the taxonomic information and the data available at “assembly_summary.txt,” the sequence and annotations for a single genome from each proteobacterial specie available at the time at RefSeq were downloaded. Only genomes with sequencing levels equivalent to “Complete Genome” were used, giving preference to genomes annotated as “reference genome” over “representative genome” over other genomes. When needed, we randomly selected one of the genomes with equivalent priority. Based on these criteria, 1,484 genomes were selected for further analyses. RefSeq accession codes for these genomes are listed in Supplementary Table 6.

### 4.2 Analysis of codon usage, GC% and tRNA genes

Gene sequences were obtained from RefSeq annotations and the percentage of codons corresponding to each triplet in each gene was calculated. The average codon usage per genome was then calculated and used as the “codon usage” for all analyzes. Genes annotated as “pseudogenes” or presenting a number of nucleotides not dividable by 3 were excluded from the analysis. The sequence of genes coding for translation elongation factors EF-Tu and EF-G, translation initiation factors IF-1, IF-2 and IF-3, 50S ribosomal subunit protein L7/L12, 30S ribosomal subunit proteins S1 and S6, RNA polymerase subunits alpha and beta (RpoB and RpoA) and chaperons DnaK, GroEL and ClpB were selected to estimate the codon usage in highly expressed genes, based on the estimations published by Karlin and Mrázek (2000) and Karlin et al. (2001). 71 of the genomes (4.8% of the genomes collection) lacked one or more of the screened genes. Given most of these genes are essential, this is most probably due to sequencing errors that introduced stop codons in the sequence of this genes. Given the high number of analyzed genes in these 71 cases codon usage of highly expressed genes was calculated based on the genes that were found. Codon usages as well as GC% were calculated using inhouse Perl scripts (Supplementary files). The number of tRNA genes were estimated using tRNAscan-SE 2.0 (Chan et al., 2021) which allows differentiation between initiator and elongator tRNA^Met^. Initiator tRNA^Met^ was not considered in the analysis of this work. When present, plasmid sequences were included in all analyzes. Codon usages calculated in this work can be found in Supplementary Tables 7, 8. The content of each nucleotide in genomes may be found at Supplementary Table 9 and the number of genes coding for each tRNA in Supplementary Table 10.

### 4.3 Phylogenetic trees

Phylogenetic trees were constructed using CVTree (Zuo, 2021) using the five amino acids method and based on the protein sequences of the 1,484 selected proteobacteria and two additional genomes used as outgroups (*Clostridium botulinum* A and *Bacillus subtilis* str 168, RefSeq genome codes GCF_000009045.1 and GCF_000063585.1). Graphical representations of these phylogenetic trees with their corresponding codon usage graph bars were constructed using the iTol web platform (Letunic and Bork, 2021).

### 4.4 Screening of genes enriched in genomes with extreme GC% and their presence in proteobacterial genomes

The genomes of 25 bacteria with GC% above 67% and 25 bacteria with GC% below 32% were randomly selected (listed in Supplementary Table 1). Genomes were re-annotated using Prokka 1.14.5 (Seemann, 2014) with default parameters. Then, genomes were compared using Roary 3.12.0 (Page et al., 2015), setting a threshold of 40% for BlastP identity given the large evolutionary distance between analyzed species. “-s” parameter was used to prevent splitting of paralogs. Further analysis was performed with Scoary 1.6.16 (Brynildsrud et al., 2016) to screen for genes with statistically significant associations to the GC% of genomes. In addition to analyzing gene enrichment, Scoary considers for statistics calculations an estimation of the number of times a gene-trait association has appeared during evolution (Brynildsrud et al., 2016).

Genes involved in the formation of four selected tRNA anticodon loop modifications were screened in proteobacterial genomes using RPSBlast (BLAST^®^ Command Line Applications User Manual, 2008) based on the set of patterns listed in Supplementary Table 4 obtained from the Conserved Domain Database (CDD) at NCBI (Wang et al., 2023). Motifs searches may lead to a low number of false negatives which may alter the results interpretations in some cases. To prevent misinterpretations of our results due to false negatives, we additionally searched for *tilS* and *tsaB* using tblastn (Altschul et al., 1990) analyses against the genomes where these genes were not found. 11 genes selected from genomes with a range of GC% were used as a query. A small number of potential blast matches were found, always with a coverage below 50% of the gene length. Eliminating the corresponding genomes did not greatly affect the conclusions of our analyzes. For an example, please compare Supplementary Figures 7–25.

### 4.5 Scripts

Diverse scripts were used to analyze codon usage, construct databases, perform simple statistical analyses and draw plots. All scripts are written in Perl 5. Some of these use Perl modules (Statistics::Descriptive, Statistics::Basic, Statistics::LineFit, Statistics::Ttest and Statistics::Test::WilcoxonRankSum) or external programs [tRNAscan-SE (version 2.0) (Chan et al., 2021), Gnuplot (version 5.2) (Williams and Kelley, 2015) and rpsblast (version 2.8.1+) (BLAST^®^ Command Line Applications User Manual, 2008)]. Scripts and their description can be found in Supplementary files.

## Data availability statement

The datasets and Supplementary material generated for this study can be found in the “Repositorio de datos de investigación de la Universidad de Chile” at https://doi.org/10.34691/UCHILE/AYDRZL.

## Author contributions

SD: Data curation, Investigation, Methodology, Software, Validation, Visualization, Writing – review & editing. ÁA: Investigation, Software, Visualization, Writing – review & editing. VB: Conceptualization, Data curation, Methodology, Writing – review & editing. OO: Conceptualization, Resources, Supervision, Writing – review & editing. JS: Conceptualization, Formal analysis, Methodology, Writing – review & editing. AK: Conceptualization, Data curation, Formal analysis, Investigation, Methodology, Project administration, Resources, Software, Supervision, Validation, Visualization, Writing – original draft, Writing – review & editing.

## Footnotes

1
ftp://ftp.ncbi.nlm.nih.gov/genomes/refseq/bacteria/
