# Supplementary material for: Impact of the chemical modification of tRNAs anticodon loop on the variability and evolution of codon usage in proteobacteria
Source: Front Microbiol. 2024 Aug 5;15:1412318. doi: 10.3389/fmicb.2024.1412318 (PMC11332805; doi:10.3389/fmicb.2024.1412318)
Supplement: Supplementary file 1 [file Data_Sheet_1.zip › Supp_figures/Fig_S25.pdf]

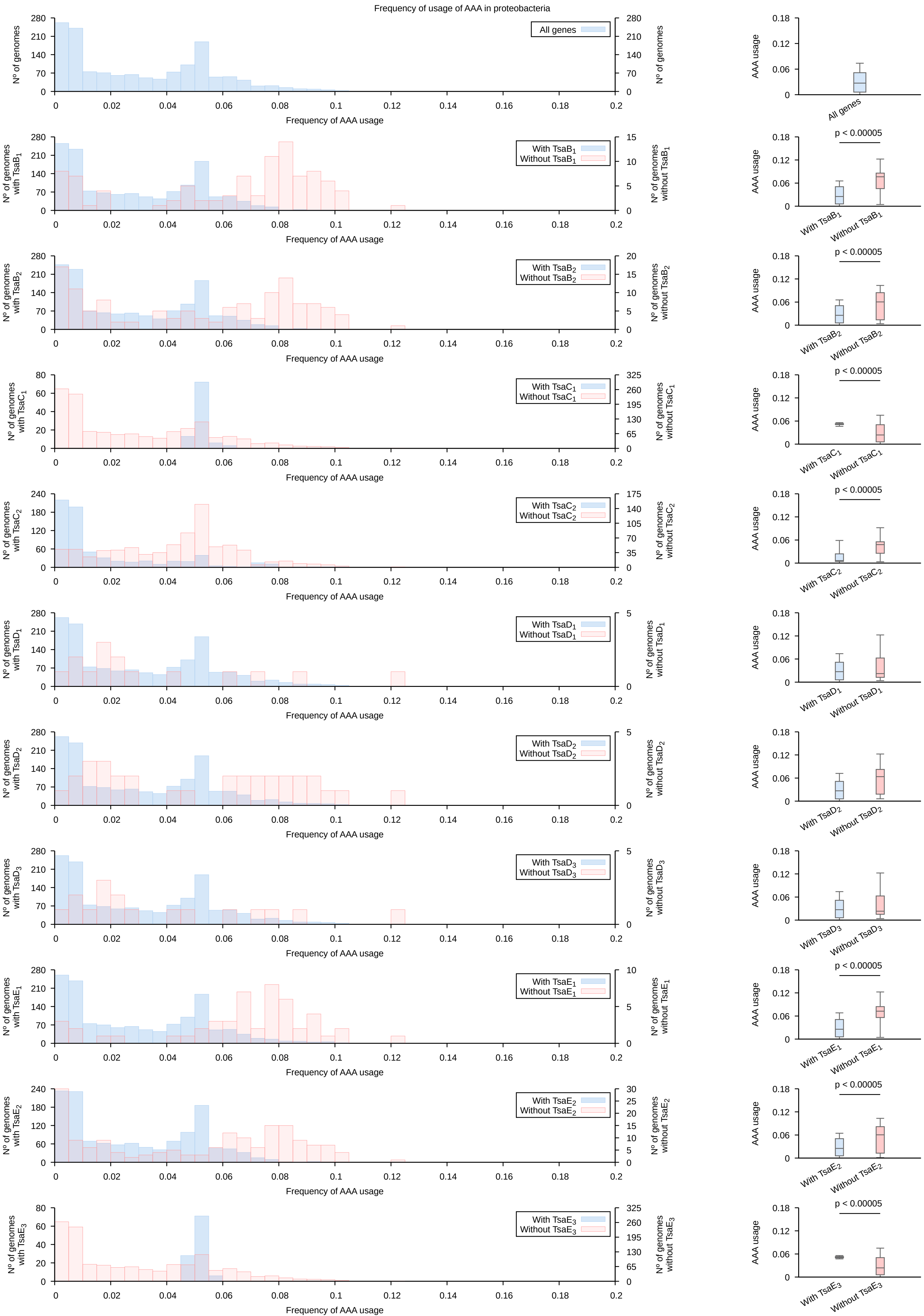
Frequency of usage of AAA in proteobacteria
All genes
Nº of genomes
Frequency of AAA usage
AAA usage
With TsaB$_1$
Without TsaB$_1$
Nº of genomes with TsaB$_1$
Nº of genomes without TsaB$_1$
p < 0.00005
With TsaB$_2$
Without TsaB$_2$
Nº of genomes with TsaB$_2$
Nº of genomes without TsaB$_2$
p < 0.00005
With TsaC$_1$
Without TsaC$_1$
Nº of genomes with TsaC$_1$
Nº of genomes without TsaC$_1$
p < 0.00005
With TsaC$_2$
Without TsaC$_2$
Nº of genomes with TsaC$_2$
Nº of genomes without TsaC$_2$
p < 0.00005
With TsaD$_1$
Without TsaD$_1$
Nº of genomes with TsaD$_1$
Nº of genomes without TsaD$_1$
With TsaD$_2$
Without TsaD$_2$
Nº of genomes with TsaD$_2$
Nº of genomes without TsaD$_2$
With TsaD$_3$
Without TsaD$_3$
Nº of genomes with TsaD$_3$
Nº of genomes without TsaD$_3$
With TsaE$_1$
Without TsaE$_1$
Nº of genomes with TsaE$_1$
Nº of genomes without TsaE$_1$
p < 0.00005
With TsaE$_2$
Without TsaE$_2$
Nº of genomes with TsaE$_2$
Nº of genomes without TsaE$_2$
p < 0.00005
With TsaE$_3$
Without TsaE$_3$
Nº of genomes with TsaE$_3$
Nº of genomes without TsaE$_3$
p < 0.00005

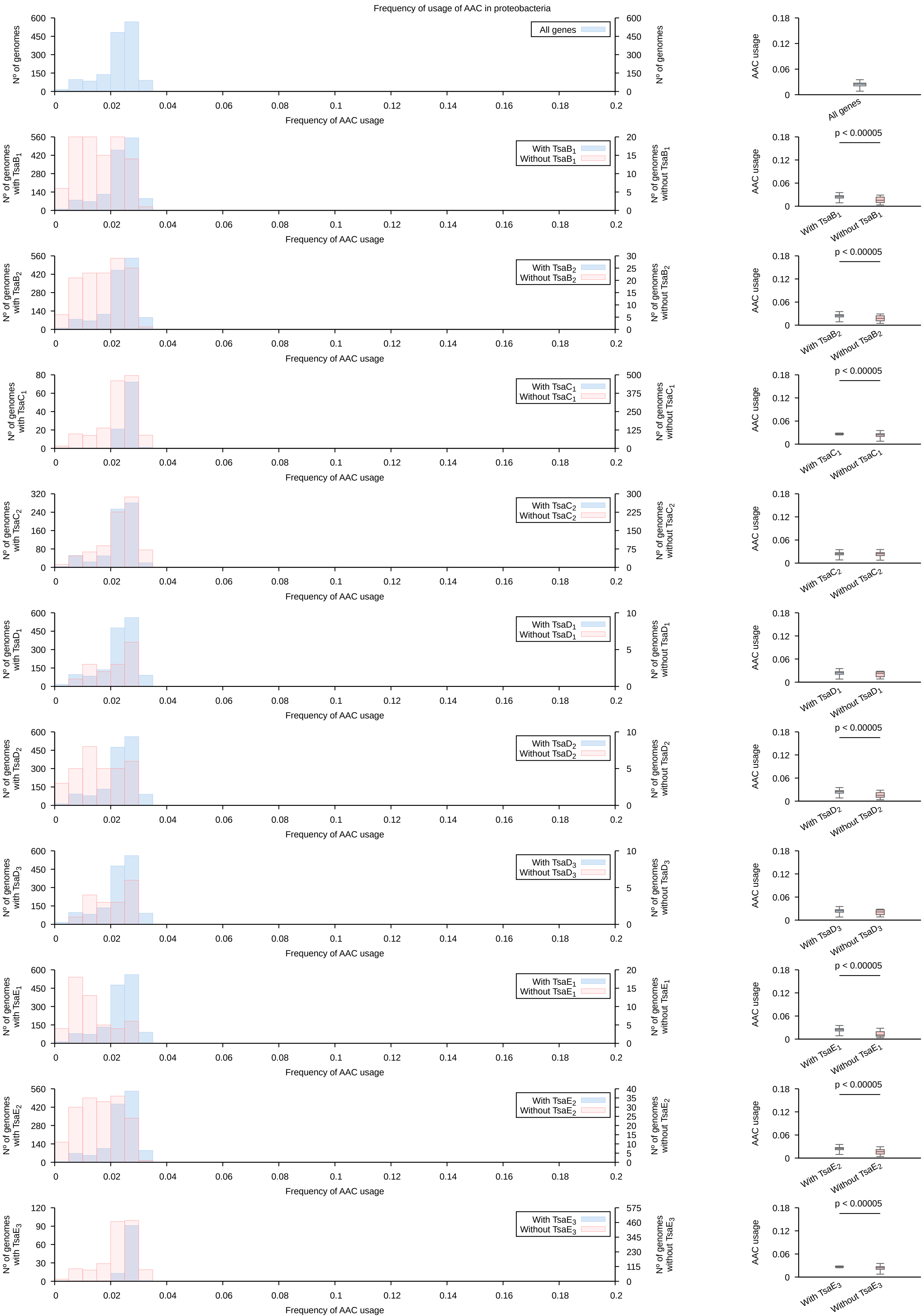

Frequency of usage of AAC in proteobacteria
Nº of genomes
All genes
Frequency of AAC usage
AAC usage
With TsaB1
Without TsaB1
Nº of genomes with TsaB1
Nº of genomes without TsaB1
p < 0.00005
With TsaB2
Without TsaB2
Nº of genomes with TsaB2
Nº of genomes without TsaB2
With TsaC1
Without TsaC1
Nº of genomes with TsaC1
Nº of genomes without TsaC1
With TsaC2
Without TsaC2
Nº of genomes with TsaC2
Nº of genomes without TsaC2
With TsaD1
Without TsaD1
Nº of genomes with TsaD1
Nº of genomes without TsaD1
With TsaD2
Without TsaD2
Nº of genomes with TsaD2
Nº of genomes without TsaD2
With TsaD3
Without TsaD3
Nº of genomes with TsaD3
Nº of genomes without TsaD3
With TsaE1
Without TsaE1
Nº of genomes with TsaE1
Nº of genomes without TsaE1
With TsaE2
Without TsaE2
Nº of genomes with TsaE2
Nº of genomes without TsaE2
With TsaE3
Without TsaE3
Nº of genomes with TsaE3
Nº of genomes without TsaE3

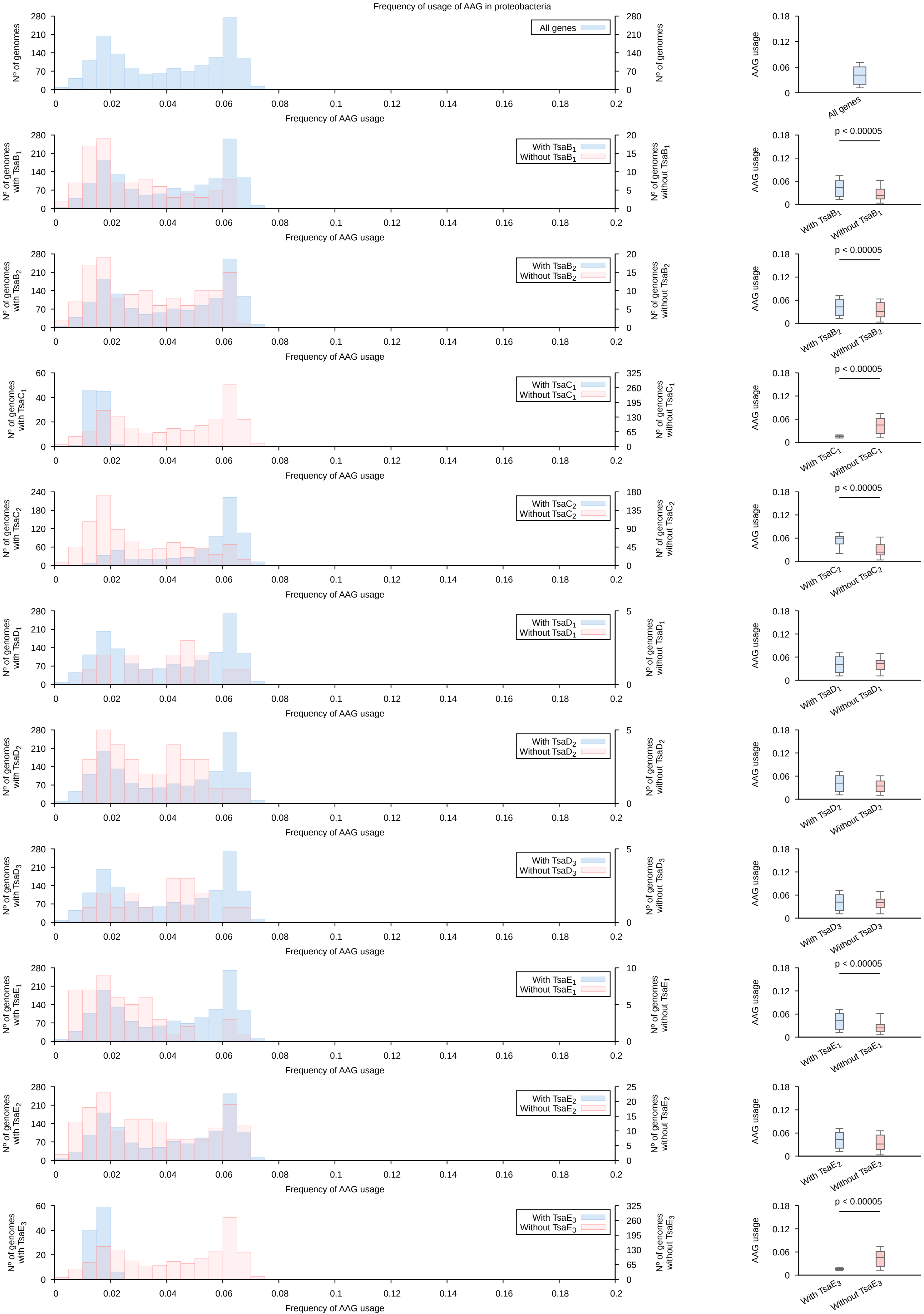
Frequency of usage of AAG in proteobacteria
Nº of genomes
All genes
Frequency of AAG usage
AAG usage
Nº of genomes with TsaB1
Nº of genomes without TsaB1
With TsaB1
Without TsaB1
p < 0.00005
Nº of genomes with TsaB2
Nº of genomes without TsaB2
With TsaB2
Without TsaB2
p < 0.00005
Nº of genomes with TsaC1
Nº of genomes without TsaC1
With TsaC1
Without TsaC1
p < 0.00005
Nº of genomes with TsaC2
Nº of genomes without TsaC2
With TsaC2
Without TsaC2
p < 0.00005
Nº of genomes with TsaD1
Nº of genomes without TsaD1
With TsaD1
Without TsaD1
Nº of genomes with TsaD2
Nº of genomes without TsaD2
With TsaD2
Without TsaD2
Nº of genomes with TsaD3
Nº of genomes without TsaD3
With TsaD3
Without TsaD3
Nº of genomes with TsaE1
Nº of genomes without TsaE1
With TsaE1
Without TsaE1
p < 0.00005
Nº of genomes with TsaE2
Nº of genomes without TsaE2
With TsaE2
Without TsaE2
Nº of genomes with TsaE3
Nº of genomes without TsaE3
With TsaE3
Without TsaE3
p < 0.00005

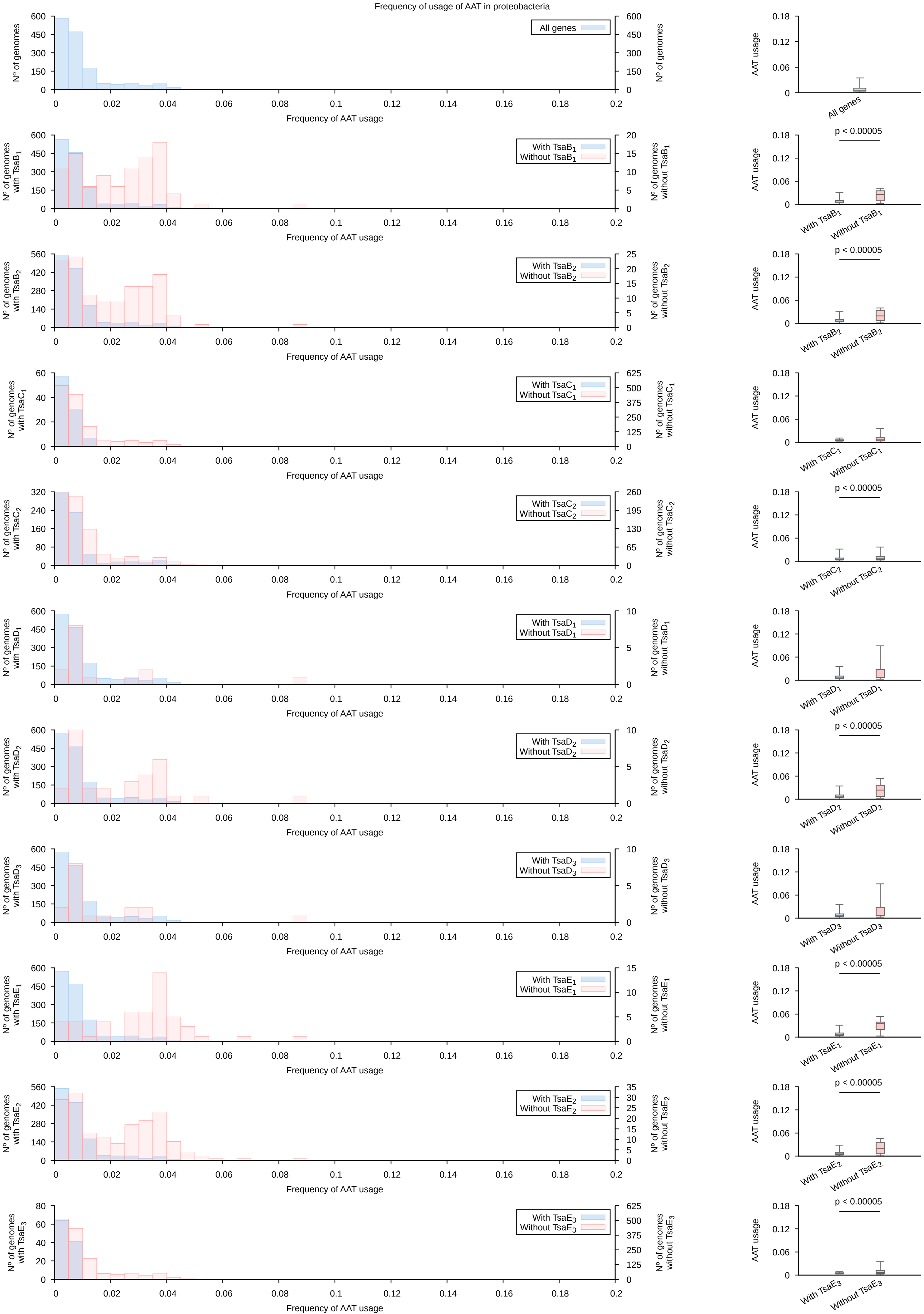
Frequency of usage of AAT in proteobacteria
All genes
Nº of genomes
Frequency of AAT usage
AAT usage
With TsaB1
Without TsaB1
Nº of genomes with TsaB1
Nº of genomes without TsaB1
p < 0.00005
With TsaB2
Without TsaB2
Nº of genomes with TsaB2
Nº of genomes without TsaB2
With TsaC1
Without TsaC1
Nº of genomes with TsaC1
Nº of genomes without TsaC1
With TsaC2
Without TsaC2
Nº of genomes with TsaC2
Nº of genomes without TsaC2
With TsaD1
Without TsaD1
Nº of genomes with TsaD1
Nº of genomes without TsaD1
With TsaD2
Without TsaD2
Nº of genomes with TsaD2
Nº of genomes without TsaD2
With TsaD3
Without TsaD3
Nº of genomes with TsaD3
Nº of genomes without TsaD3
With TsaE1
Without TsaE1
Nº of genomes with TsaE1
Nº of genomes without TsaE1
With TsaE2
Without TsaE2
Nº of genomes with TsaE2
Nº of genomes without TsaE2
With TsaE3
Without TsaE3
Nº of genomes with TsaE3
Nº of genomes without TsaE3
0
0.02
0.04
0.06
0.08
0.1
0.12
0.14
0.16
0.18
0.2
0.06
0.12
0.18

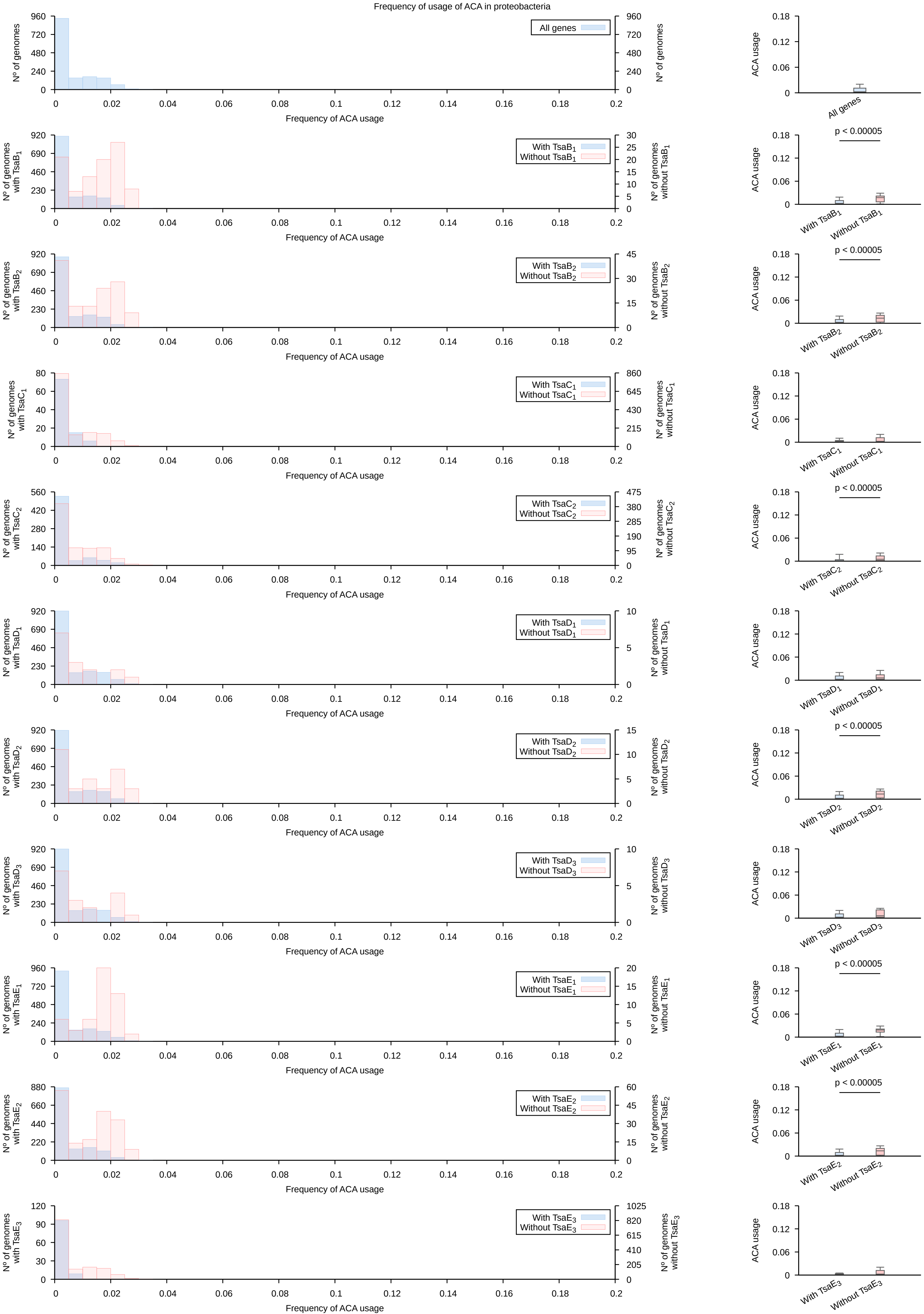
Frequency of usage of ACA in proteobacteria
Nº of genomes
All genes
Frequency of ACA usage
ACA usage
Nº of genomes with TsaB1
Nº of genomes without TsaB1
With TsaB1
Without TsaB1
p < 0.00005
Nº of genomes with TsaB2
Nº of genomes without TsaB2
With TsaB2
Without TsaB2
p < 0.00005
Nº of genomes with TsaC1
Nº of genomes without TsaC1
With TsaC1
Without TsaC1
Nº of genomes with TsaC2
Nº of genomes without TsaC2
With TsaC2
Without TsaC2
p < 0.00005
Nº of genomes with TsaD1
Nº of genomes without TsaD1
With TsaD1
Without TsaD1
Nº of genomes with TsaD2
Nº of genomes without TsaD2
With TsaD2
Without TsaD2
p < 0.00005
Nº of genomes with TsaD3
Nº of genomes without TsaD3
With TsaD3
Without TsaD3
Nº of genomes with TsaE1
Nº of genomes without TsaE1
With TsaE1
Without TsaE1
p < 0.00005
Nº of genomes with TsaE2
Nº of genomes without TsaE2
With TsaE2
Without TsaE2
p < 0.00005
Nº of genomes with TsaE3
Nº of genomes without TsaE3
With TsaE3
Without TsaE3

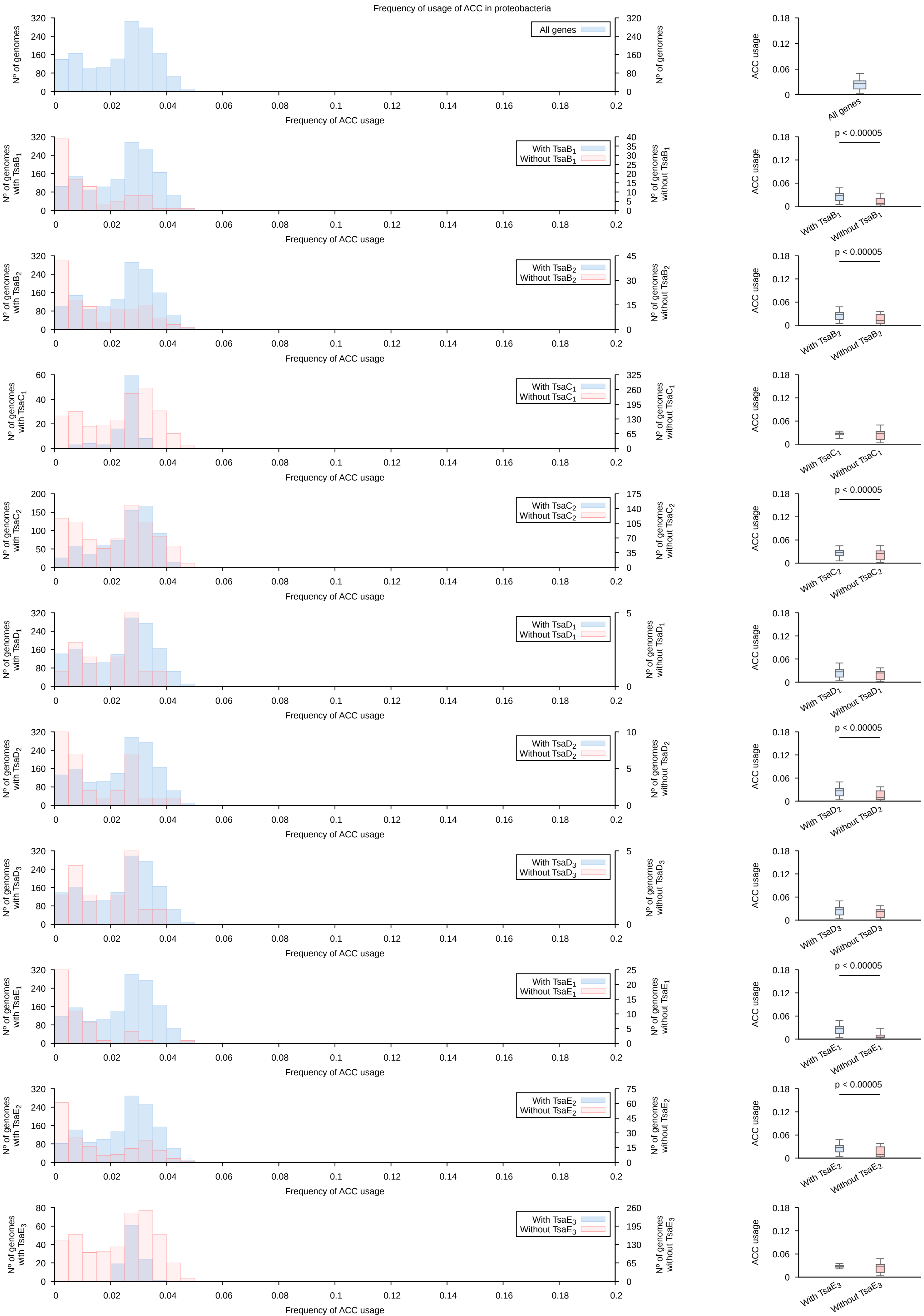
Frequency of usage of ACC in proteobacteria
All genes
Nº of genomes
Frequency of ACC usage
ACC usage
With TsaB1
Without TsaB1
Nº of genomes with TsaB1
Nº of genomes without TsaB1
p < 0.00005
With TsaB2
Without TsaB2
Nº of genomes with TsaB2
Nº of genomes without TsaB2
p < 0.00005
With TsaC1
Without TsaC1
Nº of genomes with TsaC1
Nº of genomes without TsaC1
With TsaC2
Without TsaC2
Nº of genomes with TsaC2
Nº of genomes without TsaC2
p < 0.00005
With TsaD1
Without TsaD1
Nº of genomes with TsaD1
Nº of genomes without TsaD1
With TsaD2
Without TsaD2
Nº of genomes with TsaD2
Nº of genomes without TsaD2
p < 0.00005
With TsaD3
Without TsaD3
Nº of genomes with TsaD3
Nº of genomes without TsaD3
With TsaE1
Without TsaE1
Nº of genomes with TsaE1
Nº of genomes without TsaE1
p < 0.00005
With TsaE2
Without TsaE2
Nº of genomes with TsaE2
Nº of genomes without TsaE2
p < 0.00005
With TsaE3
Without TsaE3
Nº of genomes with TsaE3
Nº of genomes without TsaE3

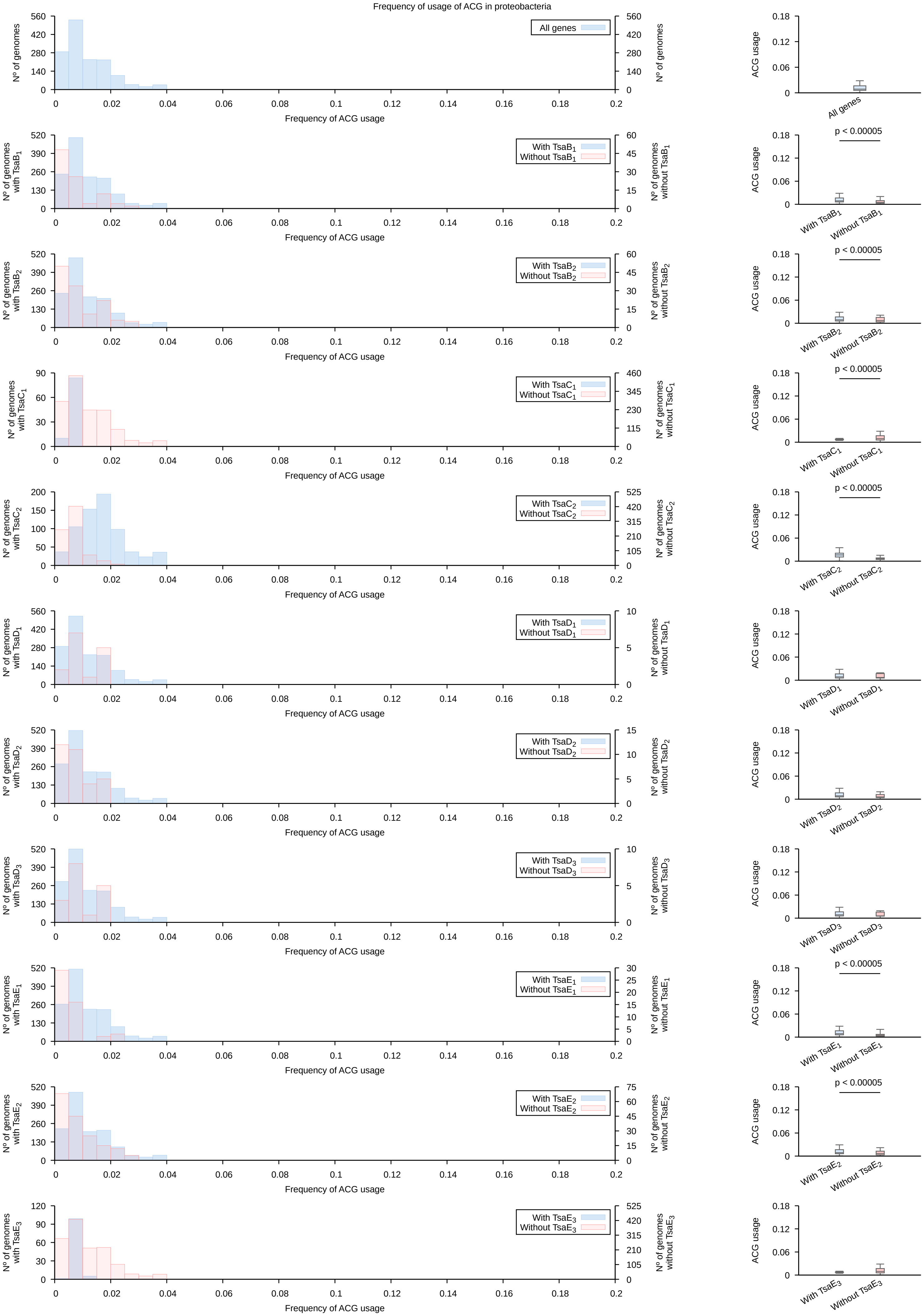

Frequency of usage of ACG in proteobacteria
Nº of genomes
Frequency of ACG usage
All genes
ACG usage
Nº of genomes with $TsaB_1$
Nº of genomes without $TsaB_1$
With $TsaB_1$
Without $TsaB_1$
p < 0.00005
Nº of genomes with $TsaB_2$
Nº of genomes without $TsaB_2$
With $TsaB_2$
Without $TsaB_2$
p < 0.00005
Nº of genomes with $TsaC_1$
Nº of genomes without $TsaC_1$
With $TsaC_1$
Without $TsaC_1$
p < 0.00005
Nº of genomes with $TsaC_2$
Nº of genomes without $TsaC_2$
With $TsaC_2$
Without $TsaC_2$
p < 0.00005
Nº of genomes with $TsaD_1$
Nº of genomes without $TsaD_1$
With $TsaD_1$
Without $TsaD_1$
Nº of genomes with $TsaD_2$
Nº of genomes without $TsaD_2$
With $TsaD_2$
Without $TsaD_2$
Nº of genomes with $TsaD_3$
Nº of genomes without $TsaD_3$
With $TsaD_3$
Without $TsaD_3$
Nº of genomes with $TsaE_1$
Nº of genomes without $TsaE_1$
With $TsaE_1$
Without $TsaE_1$
p < 0.00005
Nº of genomes with $TsaE_2$
Nº of genomes without $TsaE_2$
With $TsaE_2$
Without $TsaE_2$
p < 0.00005
Nº of genomes with $TsaE_3$
Nº of genomes without $TsaE_3$
With $TsaE_3$
Without $TsaE_3$

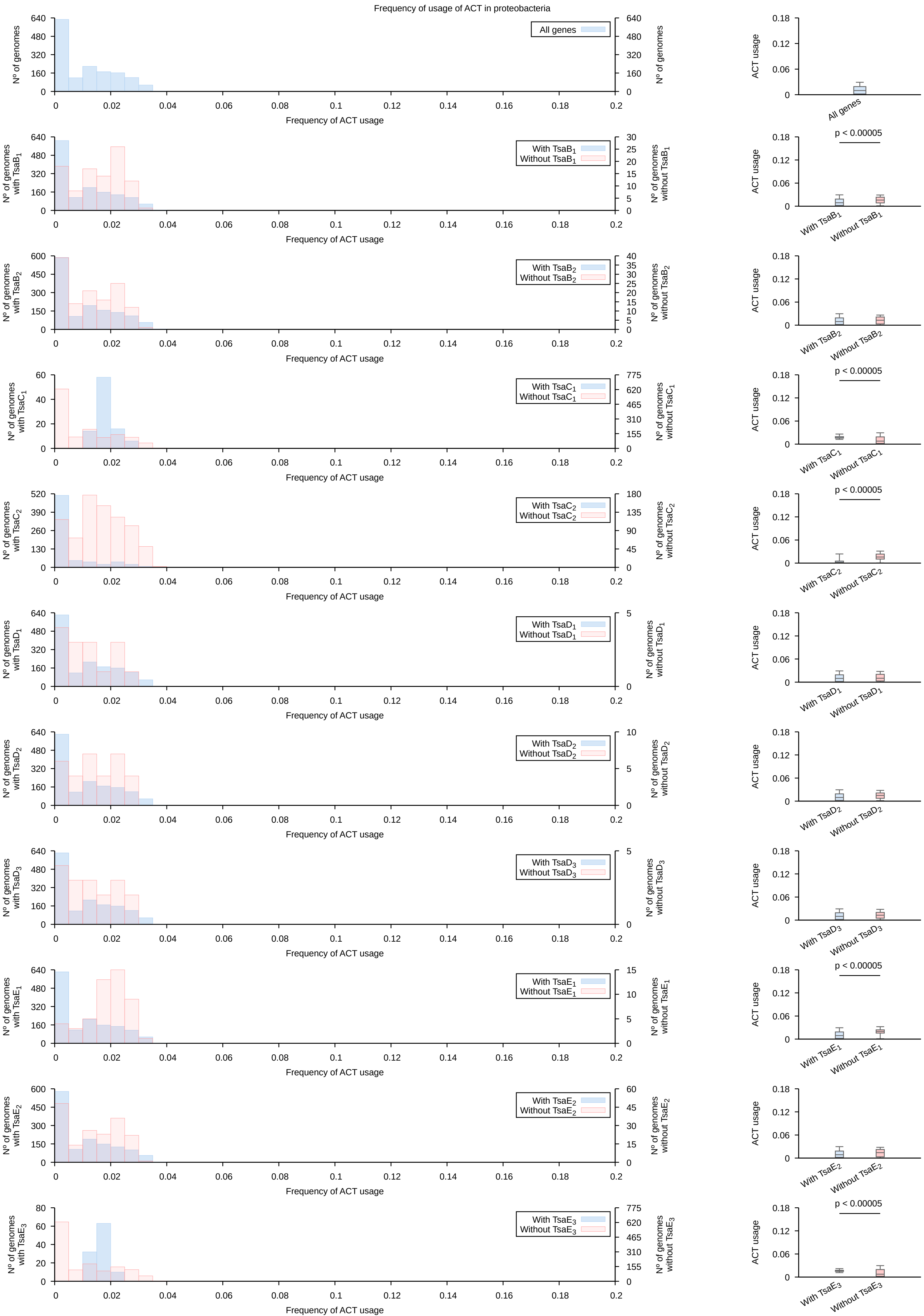

Frequency of usage of ACT in proteobacteria
All genes
Nº of genomes
Frequency of ACT usage
ACT usage
With TsaB1
Without TsaB1
p < 0.00005
With TsaB2
Without TsaB2
With TsaC1
Without TsaC1
With TsaC2
Without TsaC2
With TsaD1
Without TsaD1
With TsaD2
Without TsaD2
With TsaD3
Without TsaD3
With TsaE1
Without TsaE1
With TsaE2
Without TsaE2
With TsaE3
Without TsaE3

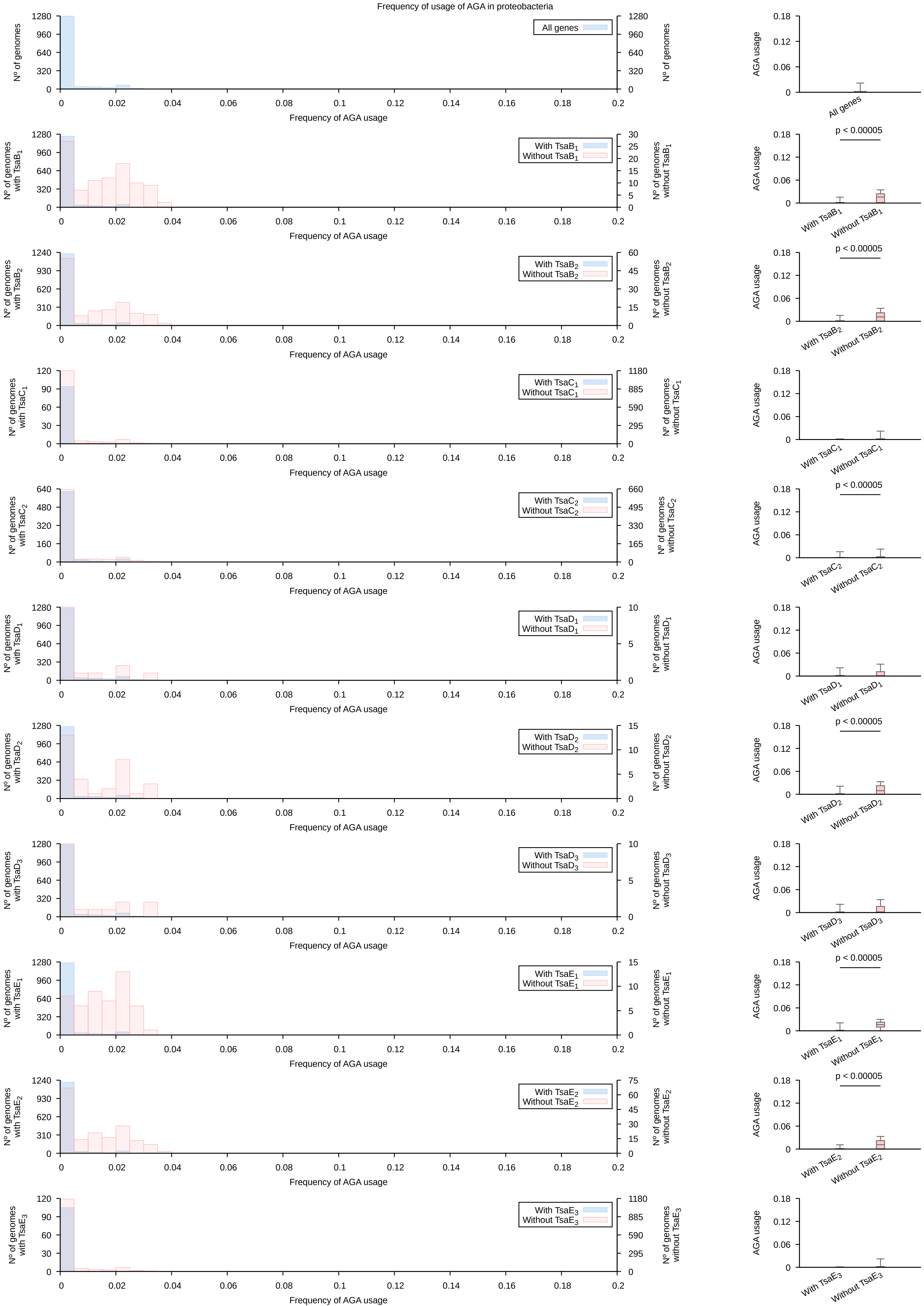

Frequency of usage of AGA in proteobacteria
Nº of genomes
All genes
Frequency of AGA usage
AGA usage
Nº of genomes with TsaB1
Nº of genomes without TsaB1
With TsaB1
Without TsaB1
p < 0.00005
Nº of genomes with TsaB2
Nº of genomes without TsaB2
With TsaB2
Without TsaB2
p < 0.00005
Nº of genomes with TsaC1
Nº of genomes without TsaC1
With TsaC1
Without TsaC1
Nº of genomes with TsaC2
Nº of genomes without TsaC2
With TsaC2
Without TsaC2
p < 0.00005
Nº of genomes with TsaD1
Nº of genomes without TsaD1
With TsaD1
Without TsaD1
Nº of genomes with TsaD2
Nº of genomes without TsaD2
With TsaD2
Without TsaD2
p < 0.00005
Nº of genomes with TsaD3
Nº of genomes without TsaD3
With TsaD3
Without TsaD3
Nº of genomes with TsaE1
Nº of genomes without TsaE1
With TsaE1
Without TsaE1
p < 0.00005
Nº of genomes with TsaE2
Nº of genomes without TsaE2
With TsaE2
Without TsaE2
p < 0.00005
Nº of genomes with TsaE3
Nº of genomes without TsaE3
With TsaE3
Without TsaE3

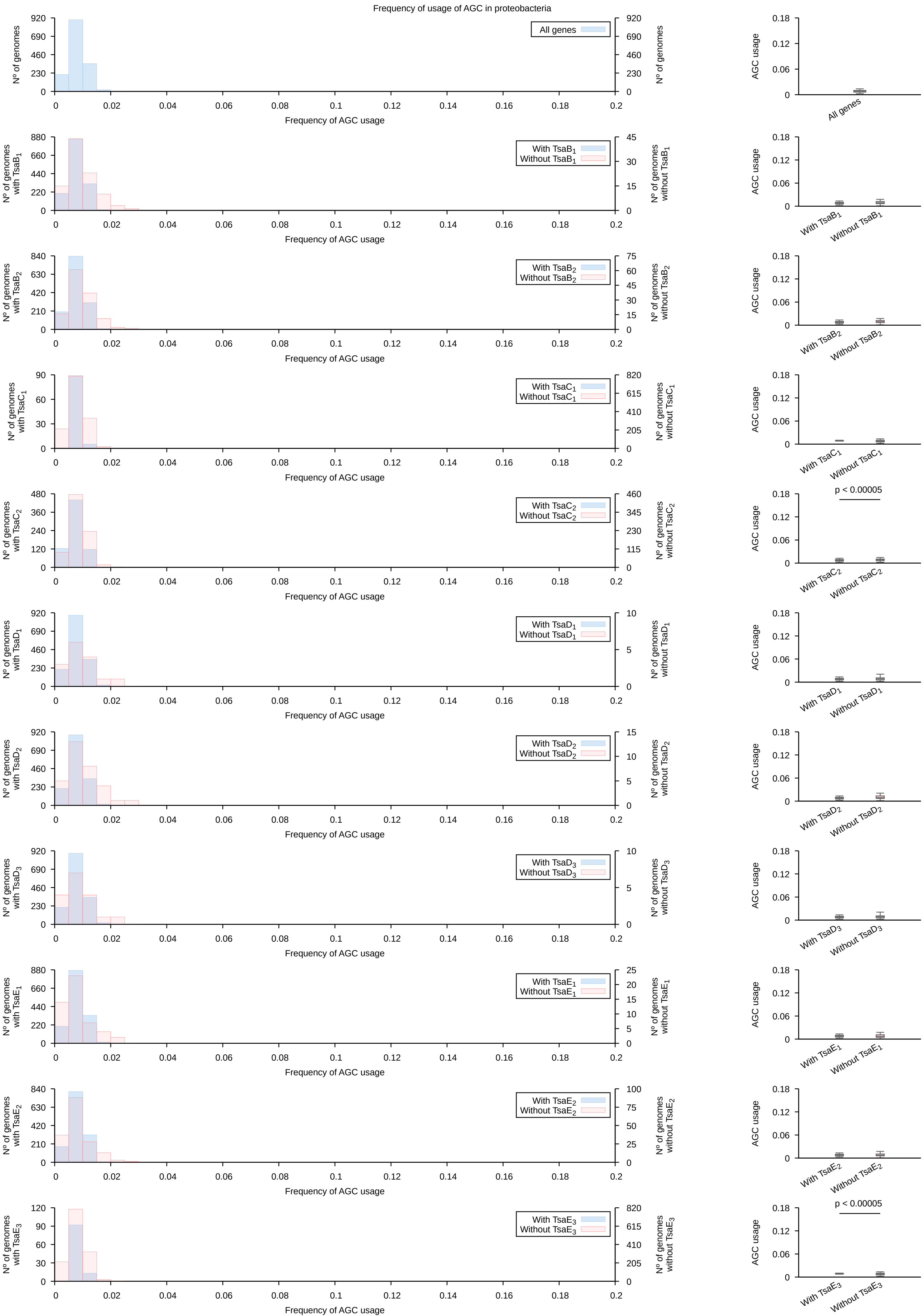

Frequency of usage of AGC in proteobacteria
All genes
Nº of genomes
Frequency of AGC usage
AGC usage
With TsaB$_1$
Without TsaB$_1$
Nº of genomes with TsaB$_1$
Nº of genomes without TsaB$_1$
With TsaB$_2$
Without TsaB$_2$
Nº of genomes with TsaB$_2$
Nº of genomes without TsaB$_2$
With TsaC$_1$
Without TsaC$_1$
Nº of genomes with TsaC$_1$
Nº of genomes without TsaC$_1$
With TsaC$_2$
Without TsaC$_2$
Nº of genomes with TsaC$_2$
Nº of genomes without TsaC$_2$
p < 0.00005
With TsaD$_1$
Without TsaD$_1$
Nº of genomes with TsaD$_1$
Nº of genomes without TsaD$_1$
With TsaD$_2$
Without TsaD$_2$
Nº of genomes with TsaD$_2$
Nº of genomes without TsaD$_2$
With TsaD$_3$
Without TsaD$_3$
Nº of genomes with TsaD$_3$
Nº of genomes without TsaD$_3$
With TsaE$_1$
Without TsaE$_1$
Nº of genomes with TsaE$_1$
Nº of genomes without TsaE$_1$
With TsaE$_2$
Without TsaE$_2$
Nº of genomes with TsaE$_2$
Nº of genomes without TsaE$_2$
With TsaE$_3$
Without TsaE$_3$
Nº of genomes with TsaE$_3$
Nº of genomes without TsaE$_3$
p < 0.00005
0 0.02 0.04 0.06 0.08 0.1 0.12 0.14 0.16 0.18 0.2
0 0.06 0.12 0.18

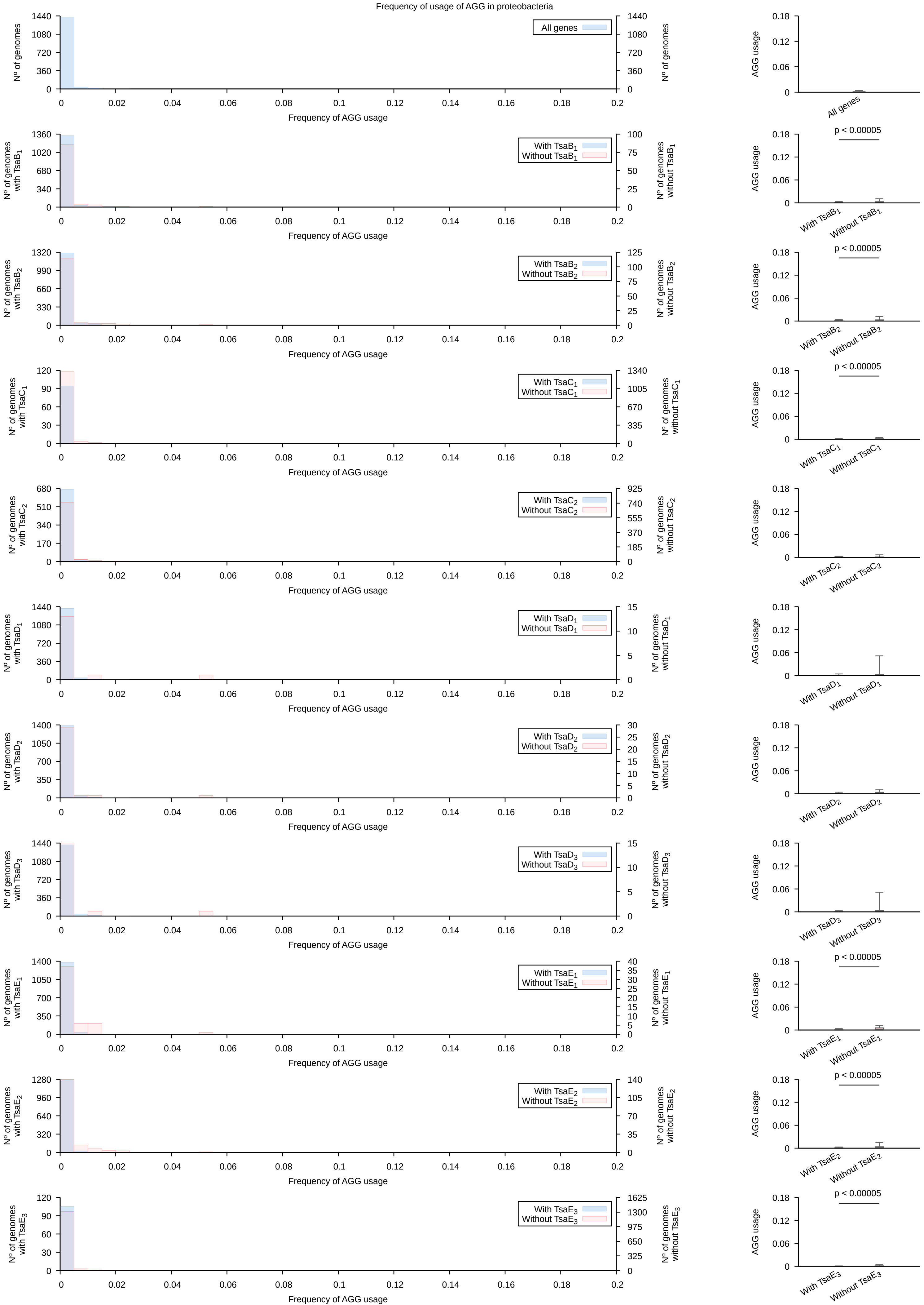
Frequency of usage of AGG in proteobacteria
Nº of genomes
All genes
Frequency of AGG usage
AGG usage
Nº of genomes with $TsaB_1$
Nº of genomes without $TsaB_1$
With $TsaB_1$
Without $TsaB_1$
p < 0.00005
Nº of genomes with $TsaB_2$
Nº of genomes without $TsaB_2$
With $TsaB_2$
Without $TsaB_2$
Nº of genomes with $TsaC_1$
Nº of genomes without $TsaC_1$
With $TsaC_1$
Without $TsaC_1$
Nº of genomes with $TsaC_2$
Nº of genomes without $TsaC_2$
With $TsaC_2$
Without $TsaC_2$
Nº of genomes with $TsaD_1$
Nº of genomes without $TsaD_1$
With $TsaD_1$
Without $TsaD_1$
Nº of genomes with $TsaD_2$
Nº of genomes without $TsaD_2$
With $TsaD_2$
Without $TsaD_2$
Nº of genomes with $TsaD_3$
Nº of genomes without $TsaD_3$
With $TsaD_3$
Without $TsaD_3$
Nº of genomes with $TsaE_1$
Nº of genomes without $TsaE_1$
With $TsaE_1$
Without $TsaE_1$
Nº of genomes with $TsaE_2$
Nº of genomes without $TsaE_2$
With $TsaE_2$
Without $TsaE_2$
Nº of genomes with $TsaE_3$
Nº of genomes without $TsaE_3$
With $TsaE_3$
Without $TsaE_3$

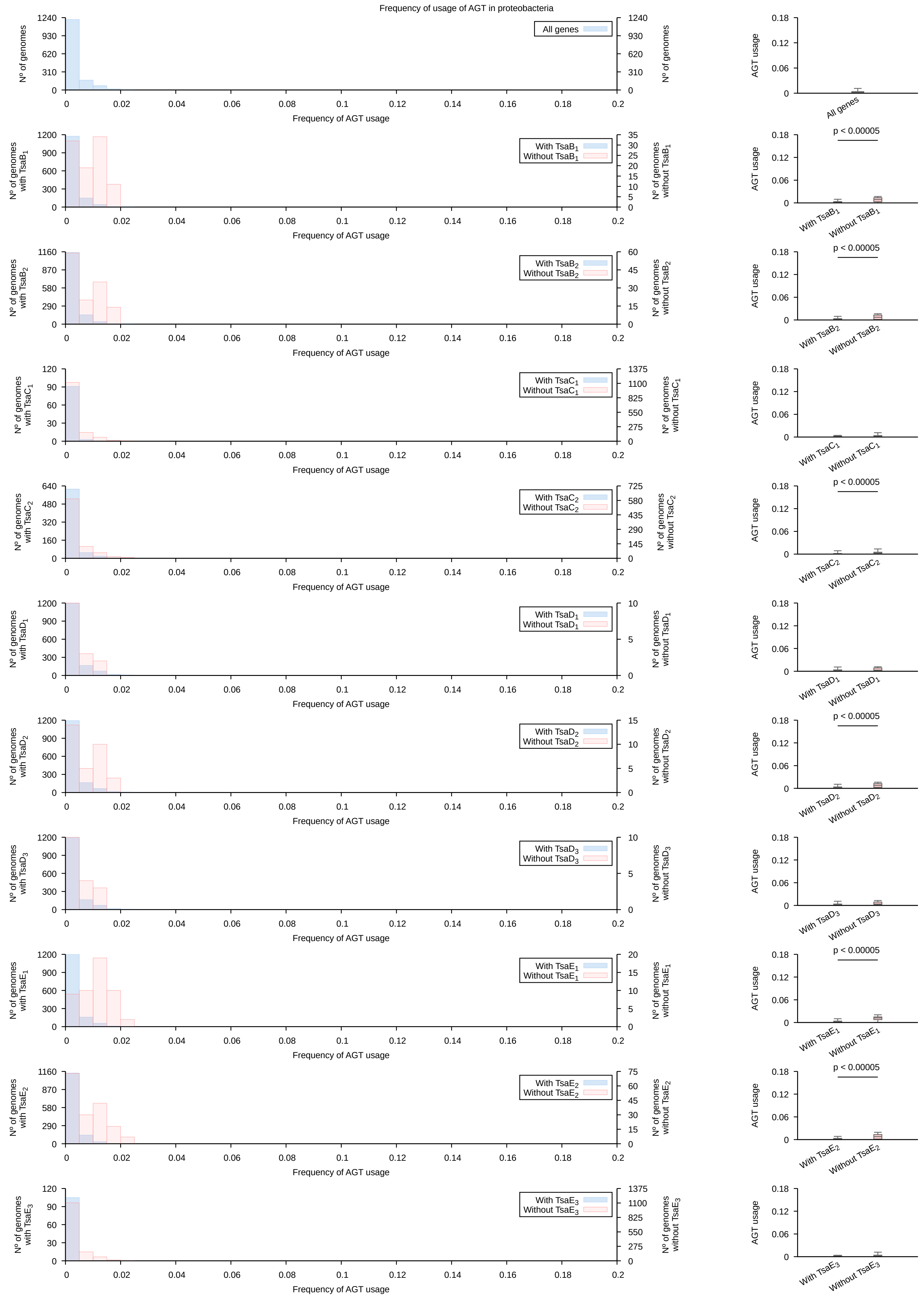
Frequency of usage of AGT in proteobacteria
All genes
Nº of genomes
Frequency of AGT usage
AGT usage
With TsaB$_1$
Without TsaB$_1$
Nº of genomes with TsaB$_1$
Nº of genomes without TsaB$_1$
p < 0.00005
With TsaB$_2$
Without TsaB$_2$
Nº of genomes with TsaB$_2$
Nº of genomes without TsaB$_2$
p < 0.00005
With TsaC$_1$
Without TsaC$_1$
Nº of genomes with TsaC$_1$
Nº of genomes without TsaC$_1$
With TsaC$_2$
Without TsaC$_2$
Nº of genomes with TsaC$_2$
Nº of genomes without TsaC$_2$
p < 0.00005
With TsaD$_1$
Without TsaD$_1$
Nº of genomes with TsaD$_1$
Nº of genomes without TsaD$_1$
With TsaD$_2$
Without TsaD$_2$
Nº of genomes with TsaD$_2$
Nº of genomes without TsaD$_2$
p < 0.00005
With TsaD$_3$
Without TsaD$_3$
Nº of genomes with TsaD$_3$
Nº of genomes without TsaD$_3$
With TsaE$_1$
Without TsaE$_1$
Nº of genomes with TsaE$_1$
Nº of genomes without TsaE$_1$
p < 0.00005
With TsaE$_2$
Without TsaE$_2$
Nº of genomes with TsaE$_2$
Nº of genomes without TsaE$_2$
p < 0.00005
With TsaE$_3$
Without TsaE$_3$
Nº of genomes with TsaE$_3$
Nº of genomes without TsaE$_3$
0 0.02 0.04 0.06 0.08 0.1 0.12 0.14 0.16 0.18 0.2
0 0.06 0.12 0.18

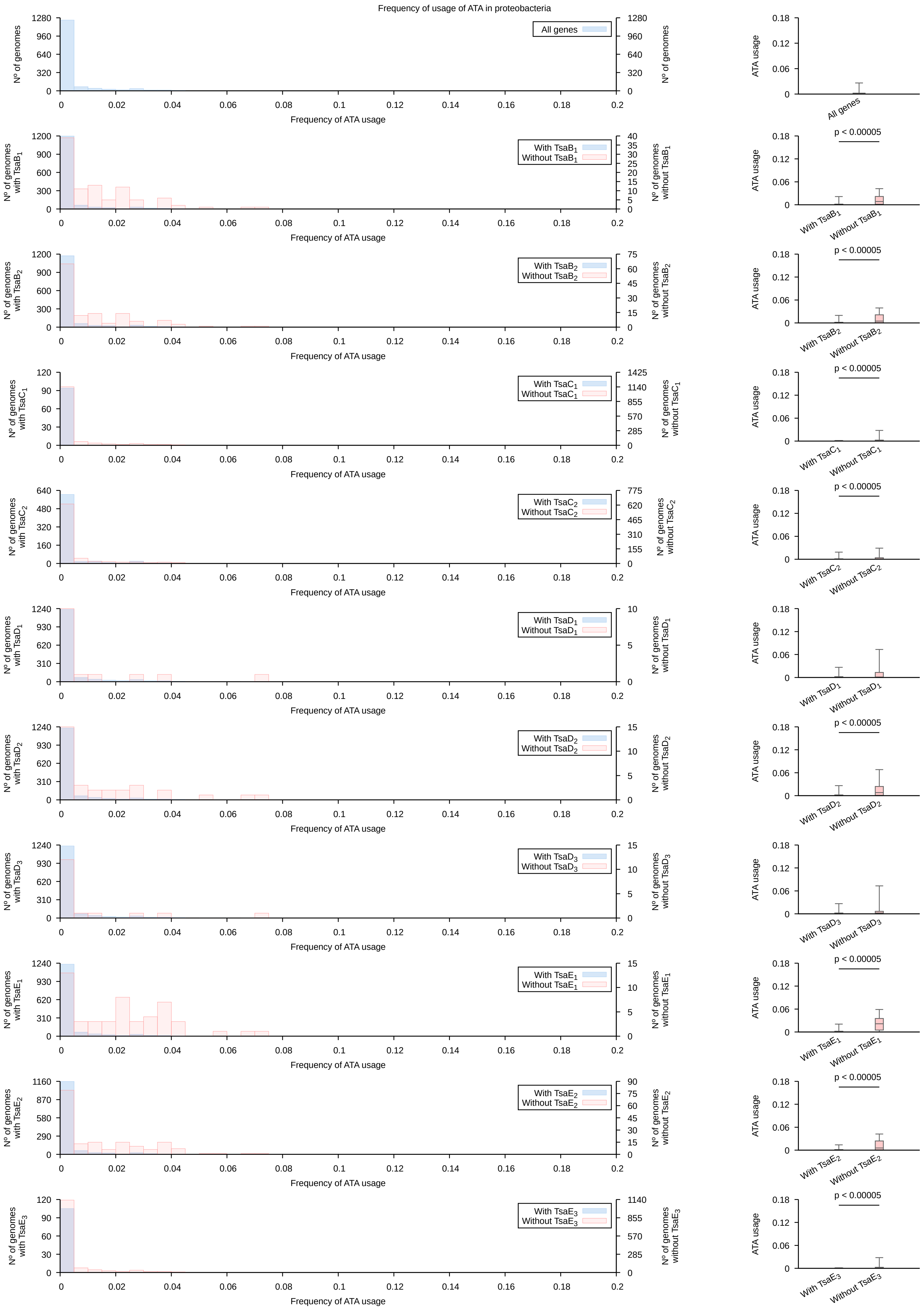

Frequency of usage of ATA in proteobacteria
All genes
Nº of genomes
Frequency of ATA usage
ATA usage
With $TsaB_1$
Without $TsaB_1$
Nº of genomes with $TsaB_1$
Nº of genomes without $TsaB_1$
p < 0.00005
With $TsaB_2$
Without $TsaB_2$
Nº of genomes with $TsaB_2$
Nº of genomes without $TsaB_2$
p < 0.00005
With $TsaC_1$
Without $TsaC_1$
Nº of genomes with $TsaC_1$
Nº of genomes without $TsaC_1$
p < 0.00005
With $TsaC_2$
Without $TsaC_2$
Nº of genomes with $TsaC_2$
Nº of genomes without $TsaC_2$
p < 0.00005
With $TsaD_1$
Without $TsaD_1$
Nº of genomes with $TsaD_1$
Nº of genomes without $TsaD_1$
With $TsaD_2$
Without $TsaD_2$
Nº of genomes with $TsaD_2$
Nº of genomes without $TsaD_2$
p < 0.00005
With $TsaD_3$
Without $TsaD_3$
Nº of genomes with $TsaD_3$
Nº of genomes without $TsaD_3$
With $TsaE_1$
Without $TsaE_1$
Nº of genomes with $TsaE_1$
Nº of genomes without $TsaE_1$
p < 0.00005
With $TsaE_2$
Without $TsaE_2$
Nº of genomes with $TsaE_2$
Nº of genomes without $TsaE_2$
p < 0.00005
With $TsaE_3$
Without $TsaE_3$
Nº of genomes with $TsaE_3$
Nº of genomes without $TsaE_3$
p < 0.00005

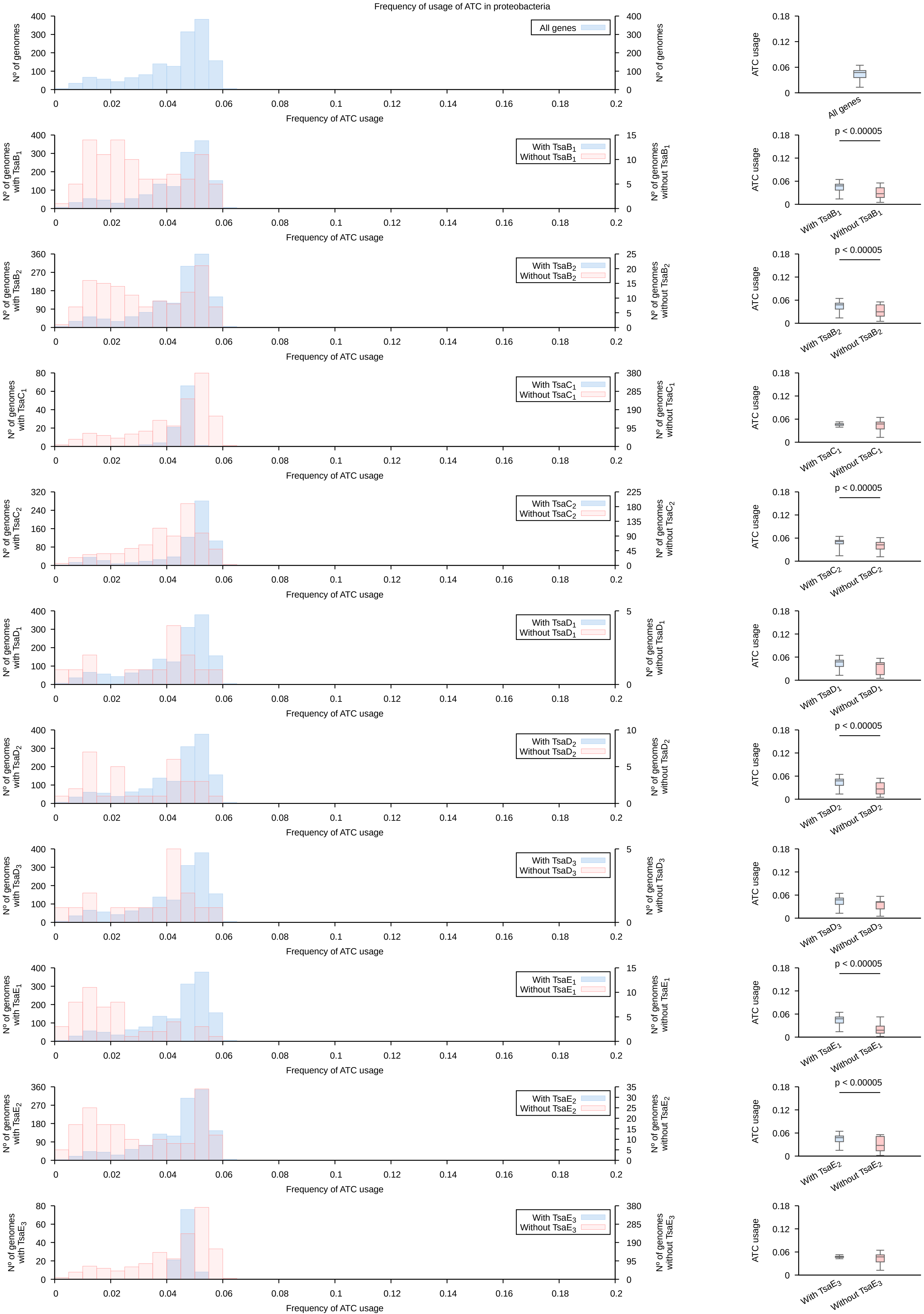

Frequency of usage of ATC in proteobacteria
All genes
Nº of genomes
Frequency of ATC usage
ATC usage
With TsaB1
Without TsaB1
Nº of genomes with TsaB1
Nº of genomes without TsaB1
p < 0.00005
With TsaB2
Without TsaB2
Nº of genomes with TsaB2
Nº of genomes without TsaB2
p < 0.00005
With TsaC1
Without TsaC1
Nº of genomes with TsaC1
Nº of genomes without TsaC1
With TsaC2
Without TsaC2
Nº of genomes with TsaC2
Nº of genomes without TsaC2
p < 0.00005
With TsaD1
Without TsaD1
Nº of genomes with TsaD1
Nº of genomes without TsaD1
With TsaD2
Without TsaD2
Nº of genomes with TsaD2
Nº of genomes without TsaD2
p < 0.00005
With TsaD3
Without TsaD3
Nº of genomes with TsaD3
Nº of genomes without TsaD3
With TsaE1
Without TsaE1
Nº of genomes with TsaE1
Nº of genomes without TsaE1
p < 0.00005
With TsaE2
Without TsaE2
Nº of genomes with TsaE2
Nº of genomes without TsaE2
p < 0.00005
With TsaE3
Without TsaE3
Nº of genomes with TsaE3
Nº of genomes without TsaE3

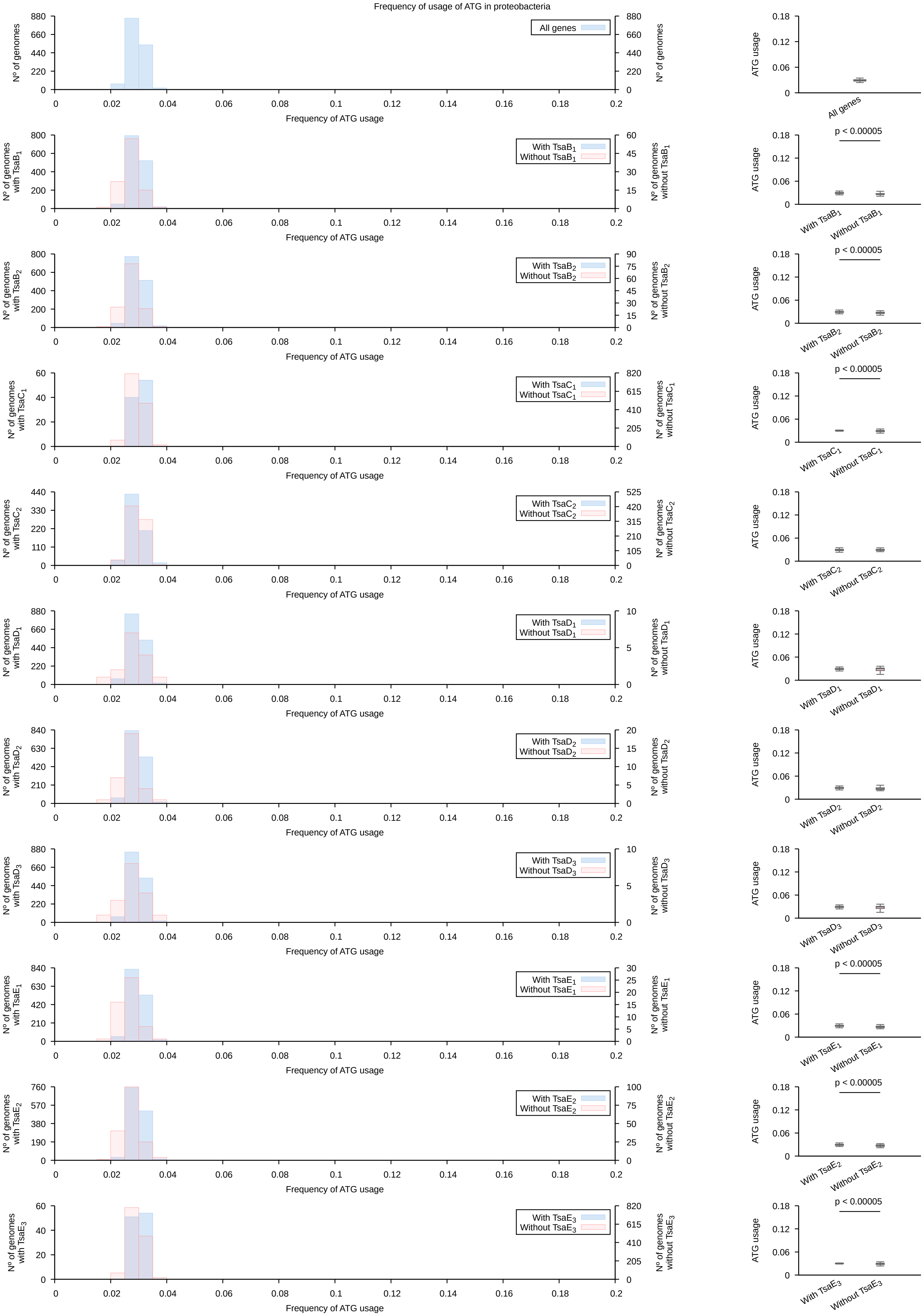
Frequency of usage of ATG in proteobacteria
All genes
Nº of genomes
Frequency of ATG usage
ATG usage
With TsaB1
Without TsaB1
Nº of genomes with TsaB1
Nº of genomes without TsaB1
p < 0.00005
With TsaB2
Without TsaB2
Nº of genomes with TsaB2
Nº of genomes without TsaB2
p < 0.00005
With TsaC1
Without TsaC1
Nº of genomes with TsaC1
Nº of genomes without TsaC1
p < 0.00005
With TsaC2
Without TsaC2
Nº of genomes with TsaC2
Nº of genomes without TsaC2
With TsaD1
Without TsaD1
Nº of genomes with TsaD1
Nº of genomes without TsaD1
With TsaD2
Without TsaD2
Nº of genomes with TsaD2
Nº of genomes without TsaD2
With TsaD3
Without TsaD3
Nº of genomes with TsaD3
Nº of genomes without TsaD3
With TsaE1
Without TsaE1
Nº of genomes with TsaE1
Nº of genomes without TsaE1
p < 0.00005
With TsaE2
Without TsaE2
Nº of genomes with TsaE2
Nº of genomes without TsaE2
p < 0.00005
With TsaE3
Without TsaE3
Nº of genomes with TsaE3
Nº of genomes without TsaE3
p < 0.00005

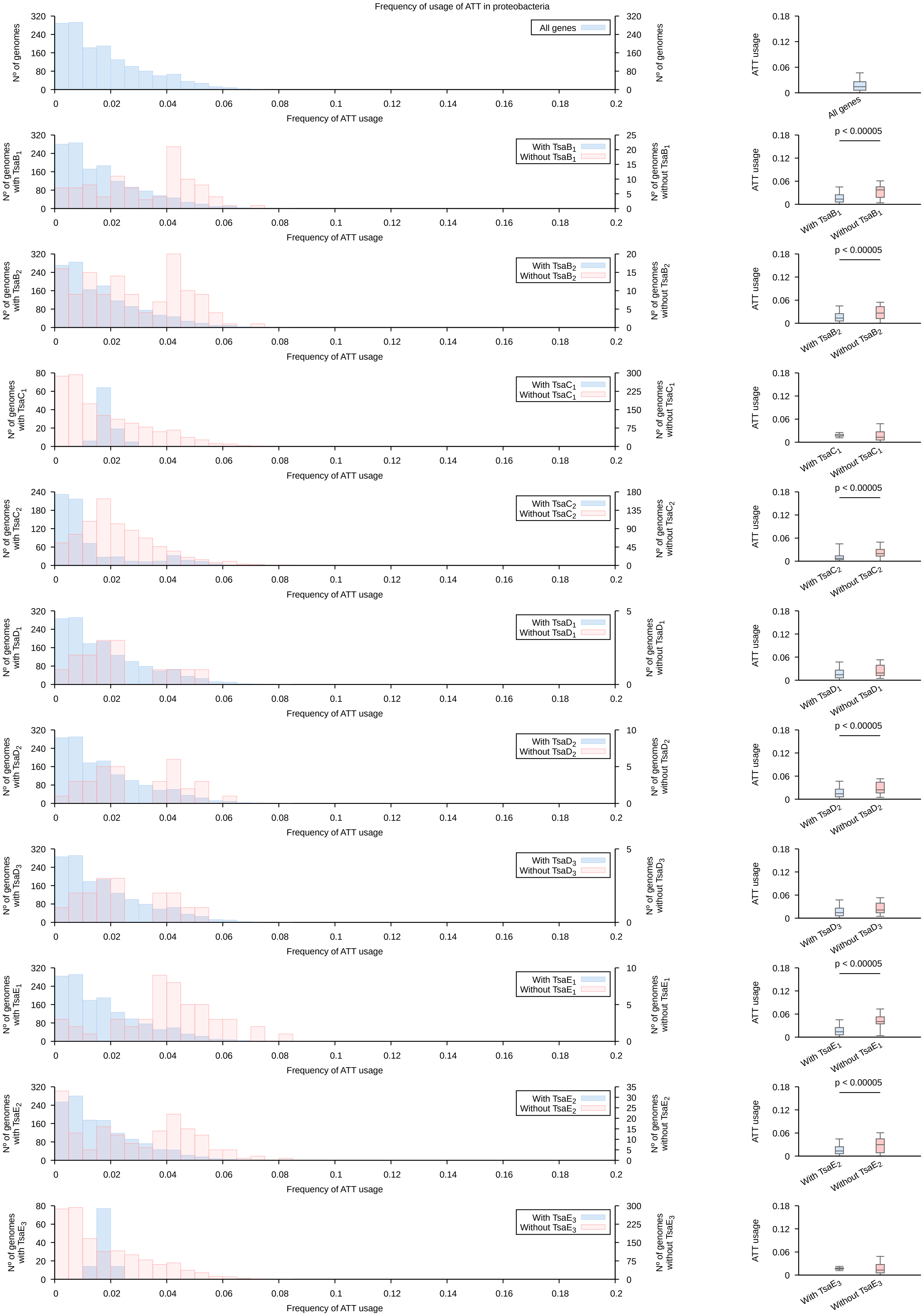

Frequency of usage of ATT in proteobacteria
Nº of genomes
All genes
Frequency of ATT usage
ATT usage
Nº of genomes with TsaB1
Nº of genomes without TsaB1
With TsaB1
Without TsaB1
p < 0.00005
Nº of genomes with TsaB2
Nº of genomes without TsaB2
With TsaB2
Without TsaB2
p < 0.00005
Nº of genomes with TsaC1
Nº of genomes without TsaC1
With TsaC1
Without TsaC1
Nº of genomes with TsaC2
Nº of genomes without TsaC2
With TsaC2
Without TsaC2
p < 0.00005
Nº of genomes with TsaD1
Nº of genomes without TsaD1
With TsaD1
Without TsaD1
Nº of genomes with TsaD2
Nº of genomes without TsaD2
With TsaD2
Without TsaD2
p < 0.00005
Nº of genomes with TsaD3
Nº of genomes without TsaD3
With TsaD3
Without TsaD3
Nº of genomes with TsaE1
Nº of genomes without TsaE1
With TsaE1
Without TsaE1
p < 0.00005
Nº of genomes with TsaE2
Nº of genomes without TsaE2
With TsaE2
Without TsaE2
p < 0.00005
Nº of genomes with TsaE3
Nº of genomes without TsaE3
With TsaE3
Without TsaE3

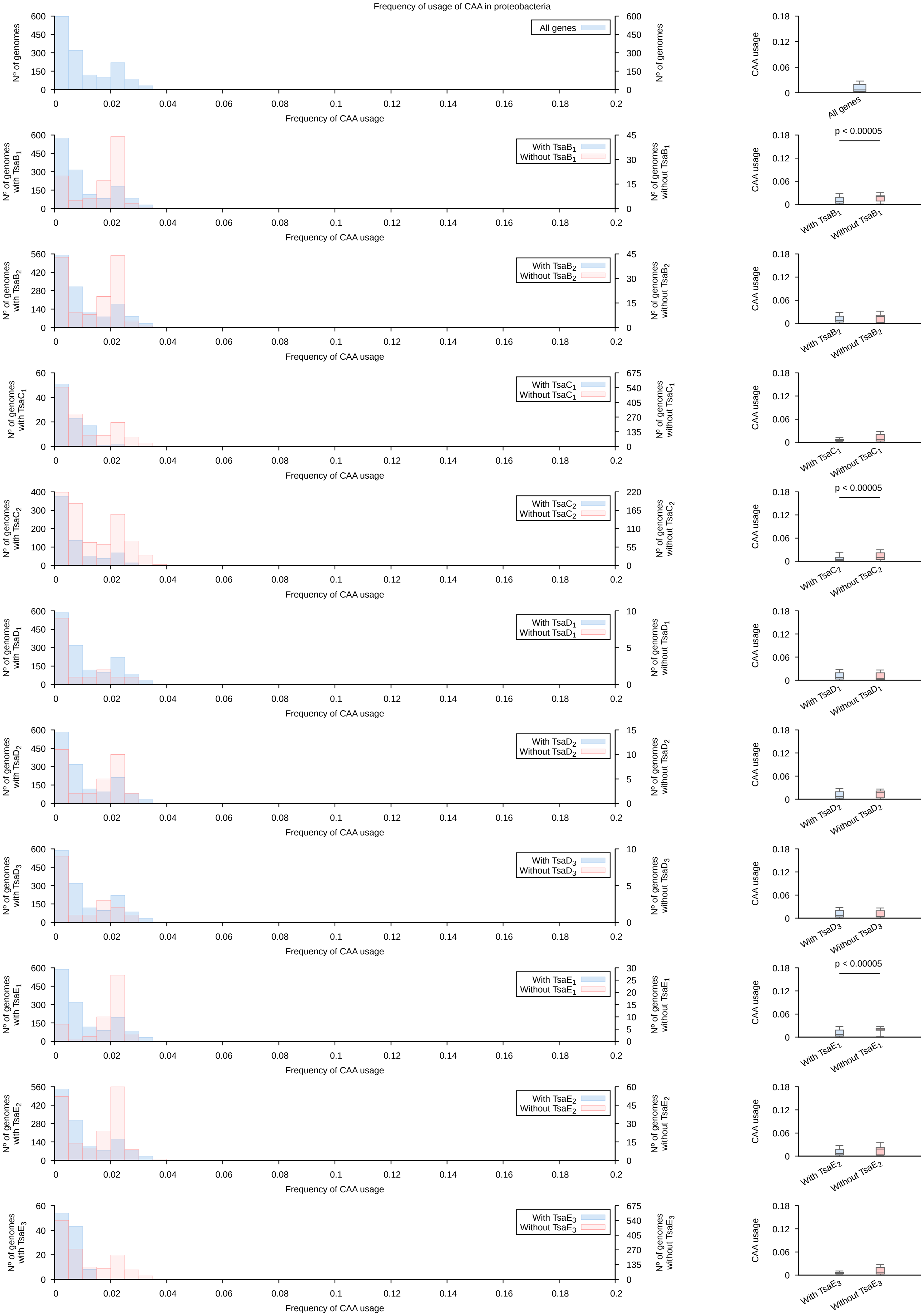

Frequency of usage of CAA in proteobacteria
Nº of genomes
All genes
Frequency of CAA usage
CAA usage
All genes
Nº of genomes with $TsaB_1$
Nº of genomes without $TsaB_1$
With $TsaB_1$
Without $TsaB_1$
p < 0.00005
Nº of genomes with $TsaB_2$
Nº of genomes without $TsaB_2$
With $TsaB_2$
Without $TsaB_2$
Nº of genomes with $TsaC_1$
Nº of genomes without $TsaC_1$
With $TsaC_1$
Without $TsaC_1$
Nº of genomes with $TsaC_2$
Nº of genomes without $TsaC_2$
With $TsaC_2$
Without $TsaC_2$
p < 0.00005
Nº of genomes with $TsaD_1$
Nº of genomes without $TsaD_1$
With $TsaD_1$
Without $TsaD_1$
Nº of genomes with $TsaD_2$
Nº of genomes without $TsaD_2$
With $TsaD_2$
Without $TsaD_2$
Nº of genomes with $TsaD_3$
Nº of genomes without $TsaD_3$
With $TsaD_3$
Without $TsaD_3$
Nº of genomes with $TsaE_1$
Nº of genomes without $TsaE_1$
With $TsaE_1$
Without $TsaE_1$
p < 0.00005
Nº of genomes with $TsaE_2$
Nº of genomes without $TsaE_2$
With $TsaE_2$
Without $TsaE_2$
Nº of genomes with $TsaE_3$
Nº of genomes without $TsaE_3$
With $TsaE_3$
Without $TsaE_3$

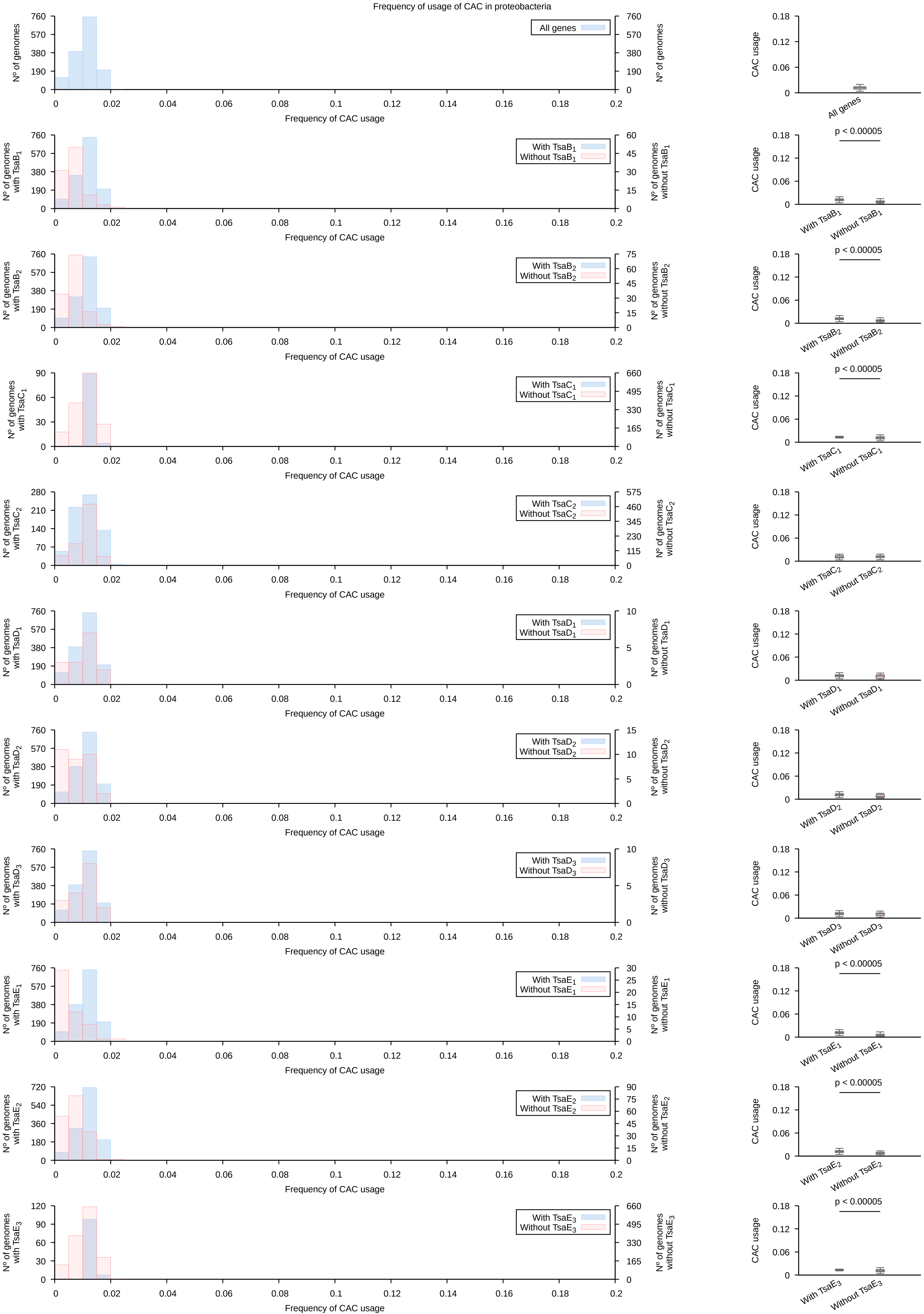

Frequency of usage of CAC in proteobacteria
All genes
Nº of genomes
Frequency of CAC usage
CAC usage
0
0.02
0.04
0.06
0.08
0.1
0.12
0.14
0.16
0.18
0.2
With TsaB1
Without TsaB1
Nº of genomes with TsaB1
Nº of genomes without TsaB1
p < 0.00005
With TsaB2
Without TsaB2
Nº of genomes with TsaB2
Nº of genomes without TsaB2
p < 0.00005
With TsaC1
Without TsaC1
Nº of genomes with TsaC1
Nº of genomes without TsaC1
p < 0.00005
With TsaC2
Without TsaC2
Nº of genomes with TsaC2
Nº of genomes without TsaC2
With TsaD1
Without TsaD1
Nº of genomes with TsaD1
Nº of genomes without TsaD1
With TsaD2
Without TsaD2
Nº of genomes with TsaD2
Nº of genomes without TsaD2
With TsaD3
Without TsaD3
Nº of genomes with TsaD3
Nº of genomes without TsaD3
With TsaE1
Without TsaE1
Nº of genomes with TsaE1
Nº of genomes without TsaE1
p < 0.00005
With TsaE2
Without TsaE2
Nº of genomes with TsaE2
Nº of genomes without TsaE2
p < 0.00005
With TsaE3
Without TsaE3
Nº of genomes with TsaE3
Nº of genomes without TsaE3
p < 0.00005

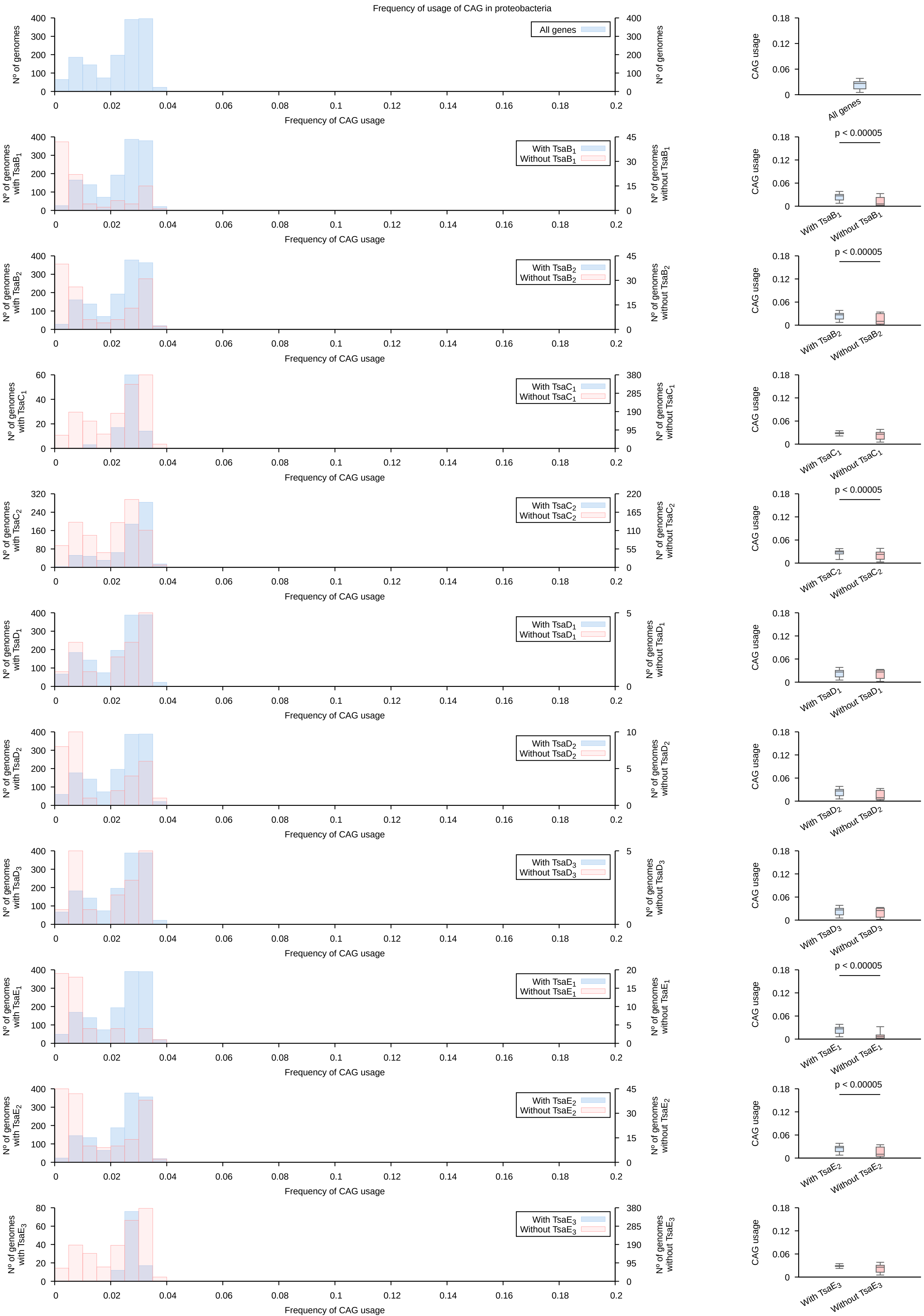

Frequency of usage of CAG in proteobacteria
All genes
Nº of genomes
Frequency of CAG usage
CAG usage
With TsaB1
Without TsaB1
Nº of genomes with TsaB1
Nº of genomes without TsaB1
p < 0.00005
With TsaB2
Without TsaB2
Nº of genomes with TsaB2
Nº of genomes without TsaB2
p < 0.00005
With TsaC1
Without TsaC1
Nº of genomes with TsaC1
Nº of genomes without TsaC1
With TsaC2
Without TsaC2
Nº of genomes with TsaC2
Nº of genomes without TsaC2
p < 0.00005
With TsaD1
Without TsaD1
Nº of genomes with TsaD1
Nº of genomes without TsaD1
With TsaD2
Without TsaD2
Nº of genomes with TsaD2
Nº of genomes without TsaD2
With TsaD3
Without TsaD3
Nº of genomes with TsaD3
Nº of genomes without TsaD3
With TsaE1
Without TsaE1
Nº of genomes with TsaE1
Nº of genomes without TsaE1
p < 0.00005
With TsaE2
Without TsaE2
Nº of genomes with TsaE2
Nº of genomes without TsaE2
p < 0.00005
With TsaE3
Without TsaE3
Nº of genomes with TsaE3
Nº of genomes without TsaE3

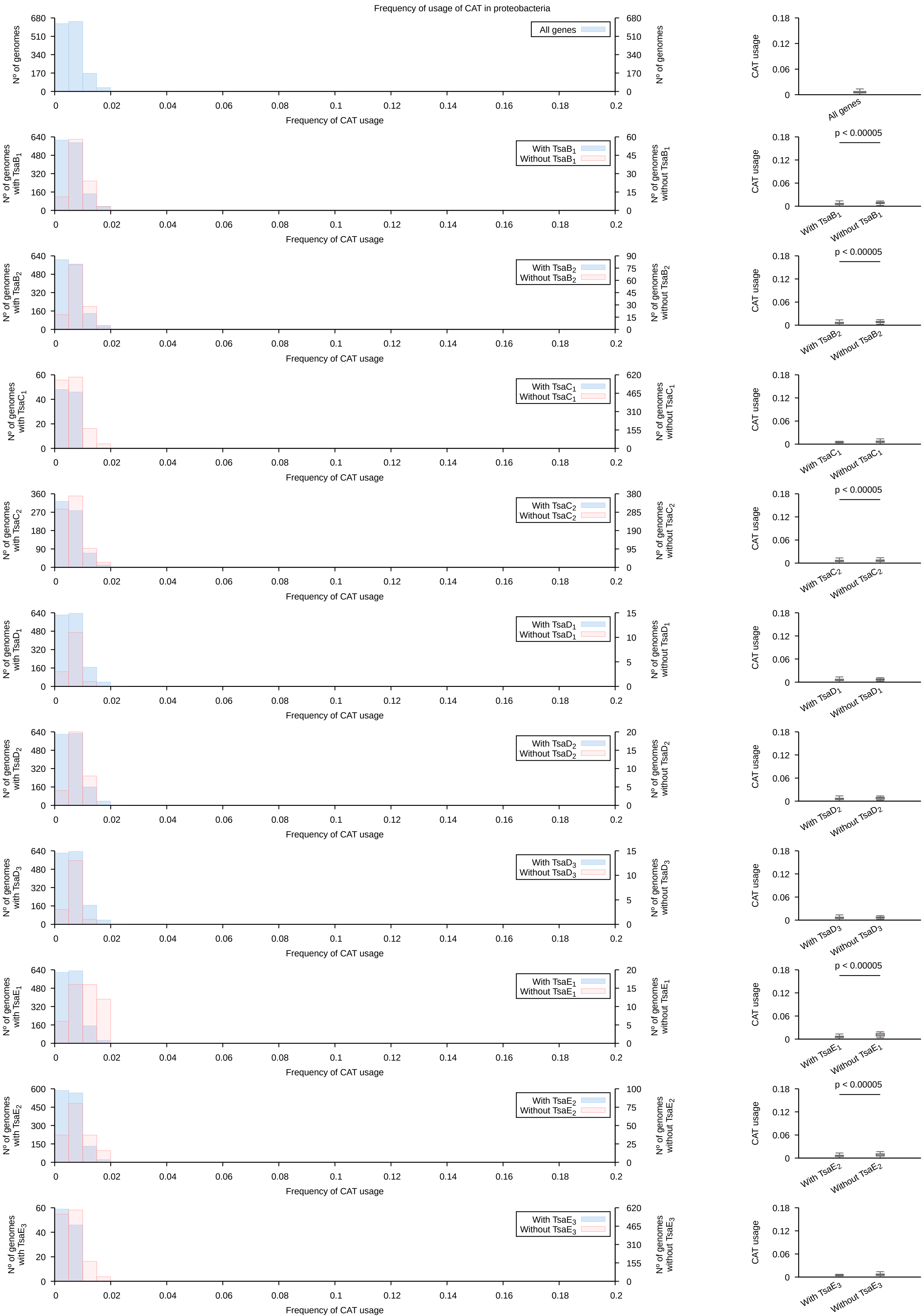

Frequency of usage of CAT in proteobacteria
All genes
Nº of genomes
Frequency of CAT usage
CAT usage
With TsaB1
Without TsaB1
Nº of genomes with TsaB1
Nº of genomes without TsaB1
p < 0.00005
With TsaB2
Without TsaB2
Nº of genomes with TsaB2
Nº of genomes without TsaB2
p < 0.00005
With TsaC1
Without TsaC1
Nº of genomes with TsaC1
Nº of genomes without TsaC1
With TsaC2
Without TsaC2
Nº of genomes with TsaC2
Nº of genomes without TsaC2
p < 0.00005
With TsaD1
Without TsaD1
Nº of genomes with TsaD1
Nº of genomes without TsaD1
With TsaD2
Without TsaD2
Nº of genomes with TsaD2
Nº of genomes without TsaD2
With TsaD3
Without TsaD3
Nº of genomes with TsaD3
Nº of genomes without TsaD3
With TsaE1
Without TsaE1
Nº of genomes with TsaE1
Nº of genomes without TsaE1
p < 0.00005
With TsaE2
Without TsaE2
Nº of genomes with TsaE2
Nº of genomes without TsaE2
p < 0.00005
With TsaE3
Without TsaE3
Nº of genomes with TsaE3
Nº of genomes without TsaE3

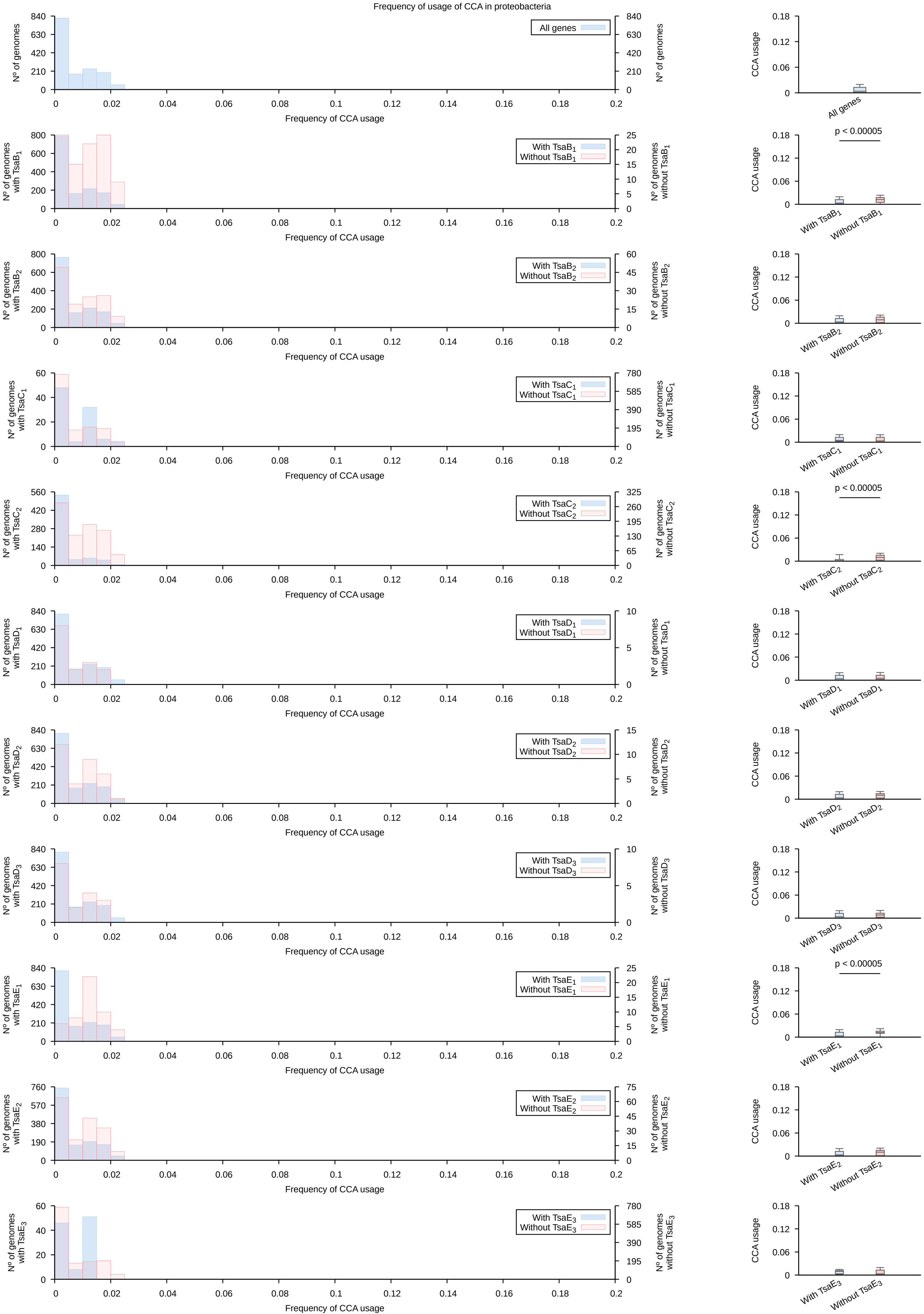

Frequency of usage of CCA in proteobacteria
All genes
Nº of genomes
Frequency of CCA usage
CCA usage
0
0.02
0.04
0.06
0.08
0.1
0.12
0.14
0.16
0.18
0.2
With $TsaB_1$
Without $TsaB_1$
Nº of genomes with $TsaB_1$
Nº of genomes without $TsaB_1$
p < 0.00005
With $TsaB_2$
Without $TsaB_2$
Nº of genomes with $TsaB_2$
Nº of genomes without $TsaB_2$
With $TsaC_1$
Without $TsaC_1$
Nº of genomes with $TsaC_1$
Nº of genomes without $TsaC_1$
With $TsaC_2$
Without $TsaC_2$
Nº of genomes with $TsaC_2$
Nº of genomes without $TsaC_2$
p < 0.00005
With $TsaD_1$
Without $TsaD_1$
Nº of genomes with $TsaD_1$
Nº of genomes without $TsaD_1$
With $TsaD_2$
Without $TsaD_2$
Nº of genomes with $TsaD_2$
Nº of genomes without $TsaD_2$
With $TsaD_3$
Without $TsaD_3$
Nº of genomes with $TsaD_3$
Nº of genomes without $TsaD_3$
With $TsaE_1$
Without $TsaE_1$
Nº of genomes with $TsaE_1$
Nº of genomes without $TsaE_1$
p < 0.00005
With $TsaE_2$
Without $TsaE_2$
Nº of genomes with $TsaE_2$
Nº of genomes without $TsaE_2$
With $TsaE_3$
Without $TsaE_3$
Nº of genomes with $TsaE_3$
Nº of genomes without $TsaE_3$

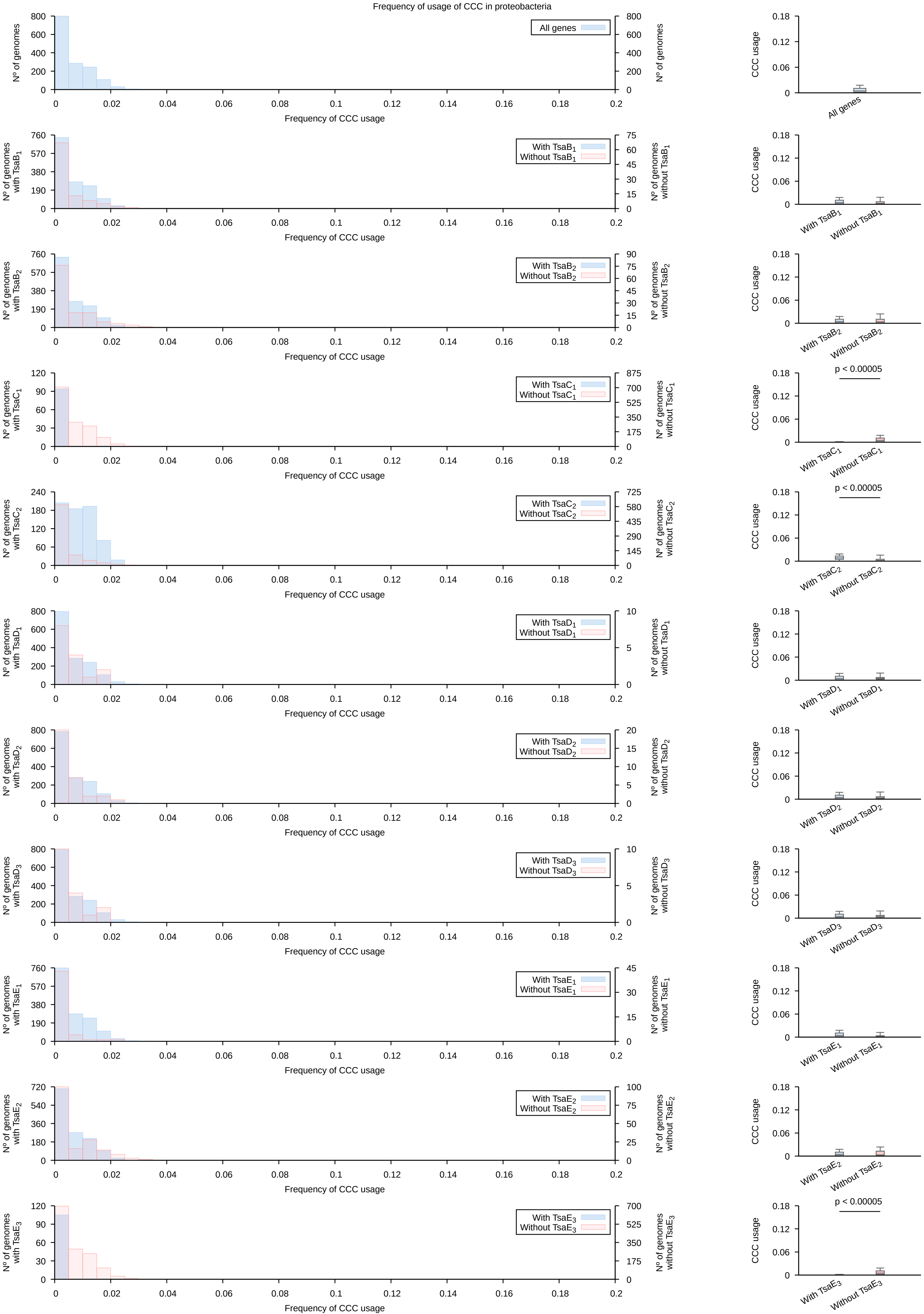
Frequency of usage of CCC in proteobacteria
Nº of genomes
All genes
Frequency of CCC usage
CCC usage
Nº of genomes with TsaB$_1$
Nº of genomes without TsaB$_1$
With TsaB$_1$
Without TsaB$_1$
Nº of genomes with TsaB$_2$
Nº of genomes without TsaB$_2$
With TsaB$_2$
Without TsaB$_2$
Nº of genomes with TsaC$_1$
Nº of genomes without TsaC$_1$
With TsaC$_1$
Without TsaC$_1$
p < 0.00005
Nº of genomes with TsaC$_2$
Nº of genomes without TsaC$_2$
With TsaC$_2$
Without TsaC$_2$
p < 0.00005
Nº of genomes with TsaD$_1$
Nº of genomes without TsaD$_1$
With TsaD$_1$
Without TsaD$_1$
Nº of genomes with TsaD$_2$
Nº of genomes without TsaD$_2$
With TsaD$_2$
Without TsaD$_2$
Nº of genomes with TsaD$_3$
Nº of genomes without TsaD$_3$
With TsaD$_3$
Without TsaD$_3$
Nº of genomes with TsaE$_1$
Nº of genomes without TsaE$_1$
With TsaE$_1$
Without TsaE$_1$
Nº of genomes with TsaE$_2$
Nº of genomes without TsaE$_2$
With TsaE$_2$
Without TsaE$_2$
Nº of genomes with TsaE$_3$
Nº of genomes without TsaE$_3$
With TsaE$_3$
Without TsaE$_3$
p < 0.00005

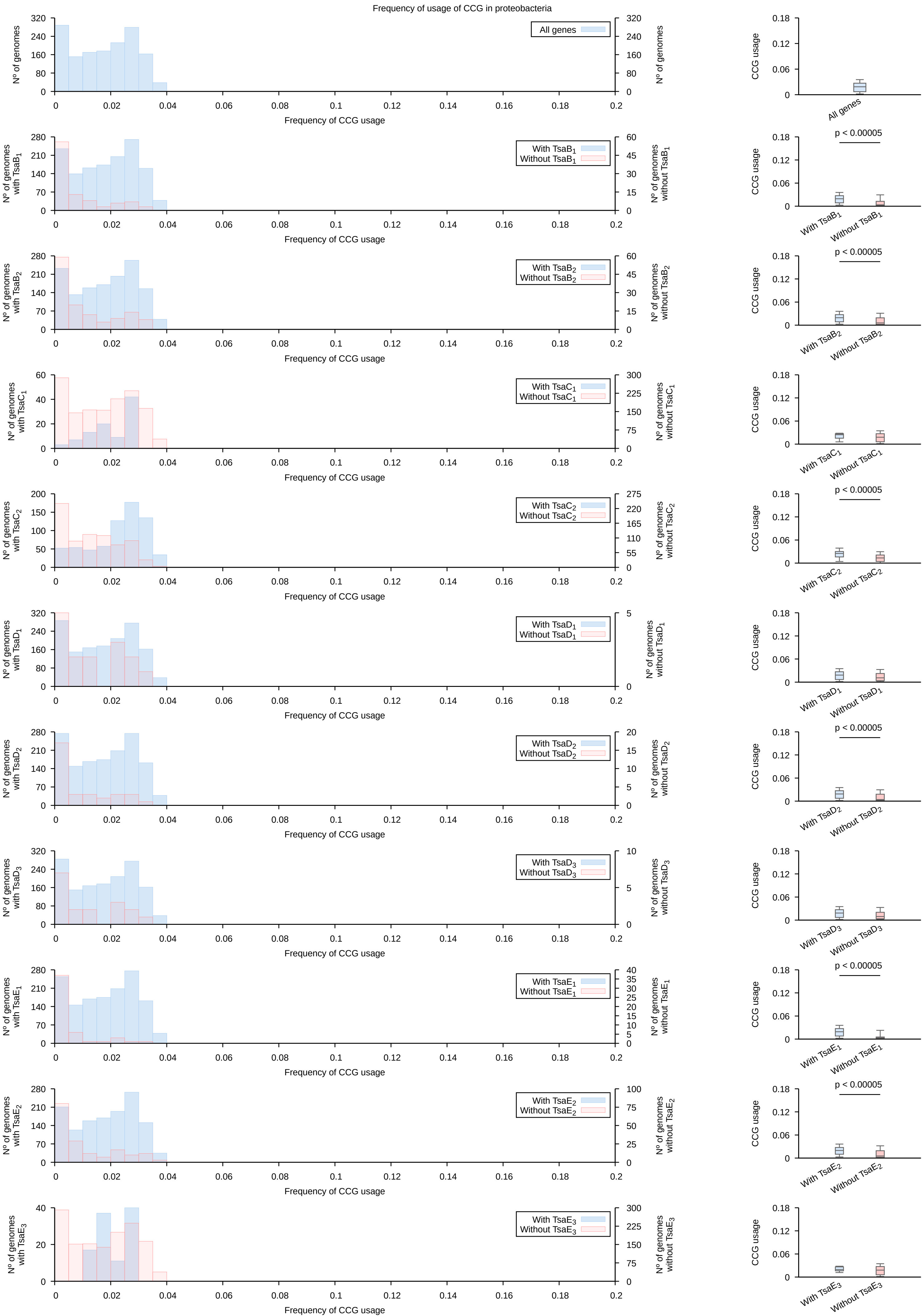
Frequency of usage of CCG in proteobacteria
All genes
Nº of genomes
CCG usage
Frequency of CCG usage
With TsaB1
Without TsaB1
Nº of genomes with TsaB1
Nº of genomes without TsaB1
p < 0.00005
With TsaB2
Without TsaB2
Nº of genomes with TsaB2
Nº of genomes without TsaB2
p < 0.00005
With TsaC1
Without TsaC1
Nº of genomes with TsaC1
Nº of genomes without TsaC1
With TsaC2
Without TsaC2
Nº of genomes with TsaC2
Nº of genomes without TsaC2
p < 0.00005
With TsaD1
Without TsaD1
Nº of genomes with TsaD1
Nº of genomes without TsaD1
With TsaD2
Without TsaD2
Nº of genomes with TsaD2
Nº of genomes without TsaD2
p < 0.00005
With TsaD3
Without TsaD3
Nº of genomes with TsaD3
Nº of genomes without TsaD3
With TsaE1
Without TsaE1
Nº of genomes with TsaE1
Nº of genomes without TsaE1
p < 0.00005
With TsaE2
Without TsaE2
Nº of genomes with TsaE2
Nº of genomes without TsaE2
p < 0.00005
With TsaE3
Without TsaE3
Nº of genomes with TsaE3
Nº of genomes without TsaE3

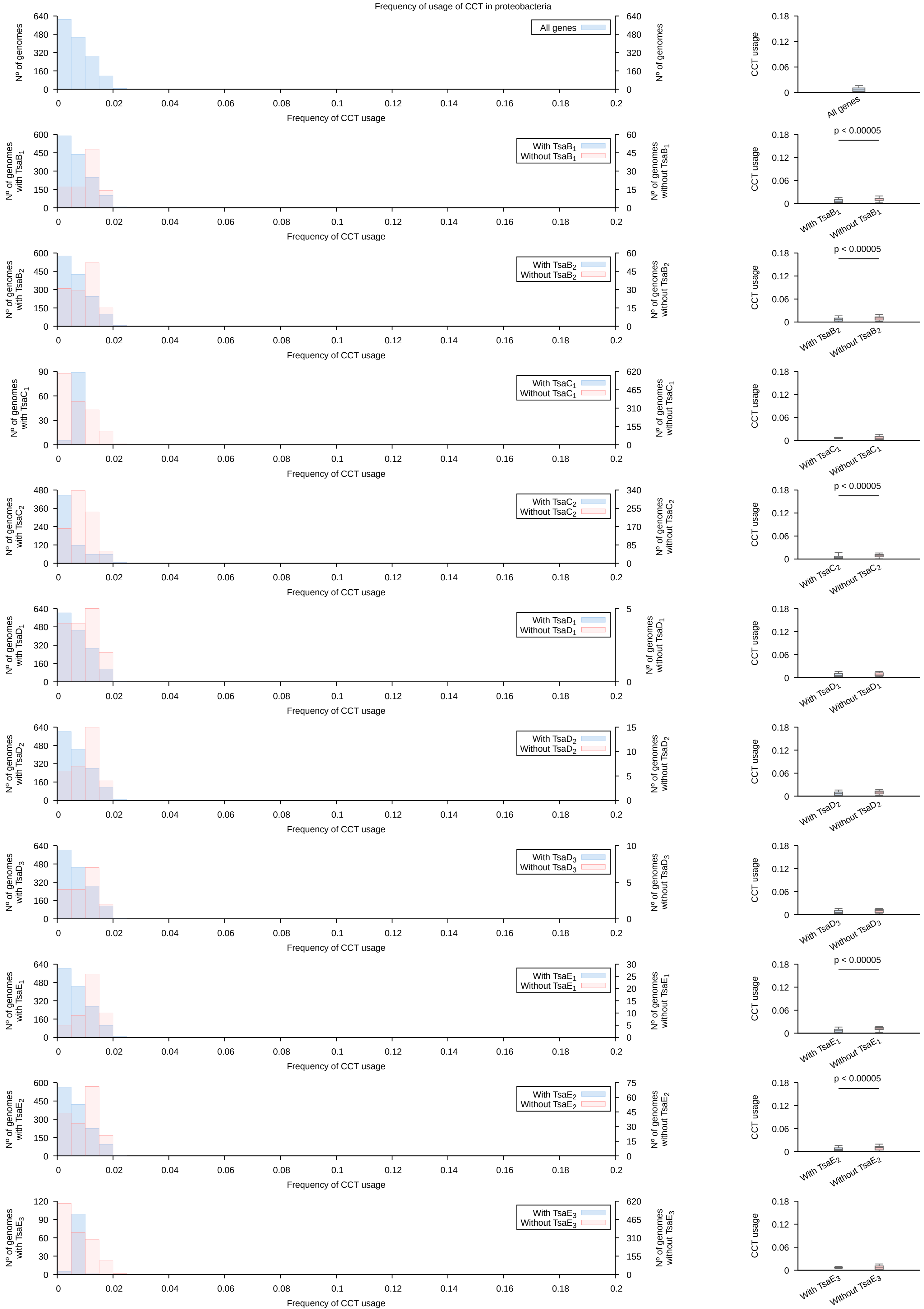
Frequency of usage of CCT in proteobacteria
Nº of genomes
All genes
Frequency of CCT usage
CCT usage
With TsaB1
Without TsaB1
Nº of genomes with TsaB1
Nº of genomes without TsaB1
p < 0.00005
With TsaB2
Without TsaB2
Nº of genomes with TsaB2
Nº of genomes without TsaB2
With TsaC1
Without TsaC1
Nº of genomes with TsaC1
Nº of genomes without TsaC1
With TsaC2
Without TsaC2
Nº of genomes with TsaC2
Nº of genomes without TsaC2
With TsaD1
Without TsaD1
Nº of genomes with TsaD1
Nº of genomes without TsaD1
With TsaD2
Without TsaD2
Nº of genomes with TsaD2
Nº of genomes without TsaD2
With TsaD3
Without TsaD3
Nº of genomes with TsaD3
Nº of genomes without TsaD3
With TsaE1
Without TsaE1
Nº of genomes with TsaE1
Nº of genomes without TsaE1
With TsaE2
Without TsaE2
Nº of genomes with TsaE2
Nº of genomes without TsaE2
With TsaE3
Without TsaE3
Nº of genomes with TsaE3
Nº of genomes without TsaE3

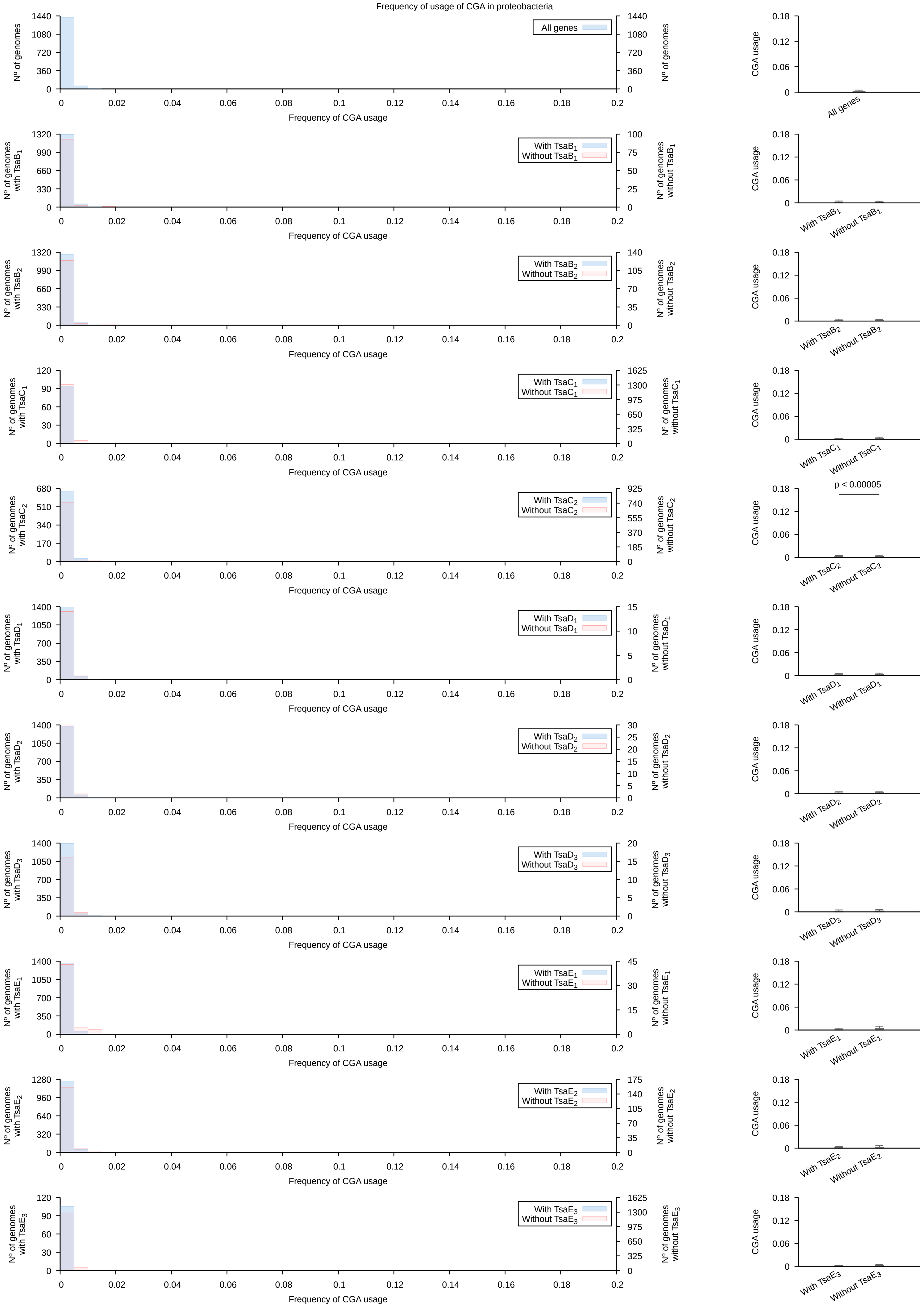

Frequency of usage of CGA in proteobacteria
Nº of genomes
Frequency of CGA usage
CGA usage
All genes
Nº of genomes with $TsaB_1$
Nº of genomes without $TsaB_1$
With $TsaB_1$
Without $TsaB_1$
Nº of genomes with $TsaB_2$
Nº of genomes without $TsaB_2$
With $TsaB_2$
Without $TsaB_2$
Nº of genomes with $TsaC_1$
Nº of genomes without $TsaC_1$
With $TsaC_1$
Without $TsaC_1$
Nº of genomes with $TsaC_2$
Nº of genomes without $TsaC_2$
With $TsaC_2$
Without $TsaC_2$
p < 0.00005
Nº of genomes with $TsaD_1$
Nº of genomes without $TsaD_1$
With $TsaD_1$
Without $TsaD_1$
Nº of genomes with $TsaD_2$
Nº of genomes without $TsaD_2$
With $TsaD_2$
Without $TsaD_2$
Nº of genomes with $TsaD_3$
Nº of genomes without $TsaD_3$
With $TsaD_3$
Without $TsaD_3$
Nº of genomes with $TsaE_1$
Nº of genomes without $TsaE_1$
With $TsaE_1$
Without $TsaE_1$
Nº of genomes with $TsaE_2$
Nº of genomes without $TsaE_2$
With $TsaE_2$
Without $TsaE_2$
Nº of genomes with $TsaE_3$
Nº of genomes without $TsaE_3$
With $TsaE_3$
Without $TsaE_3$

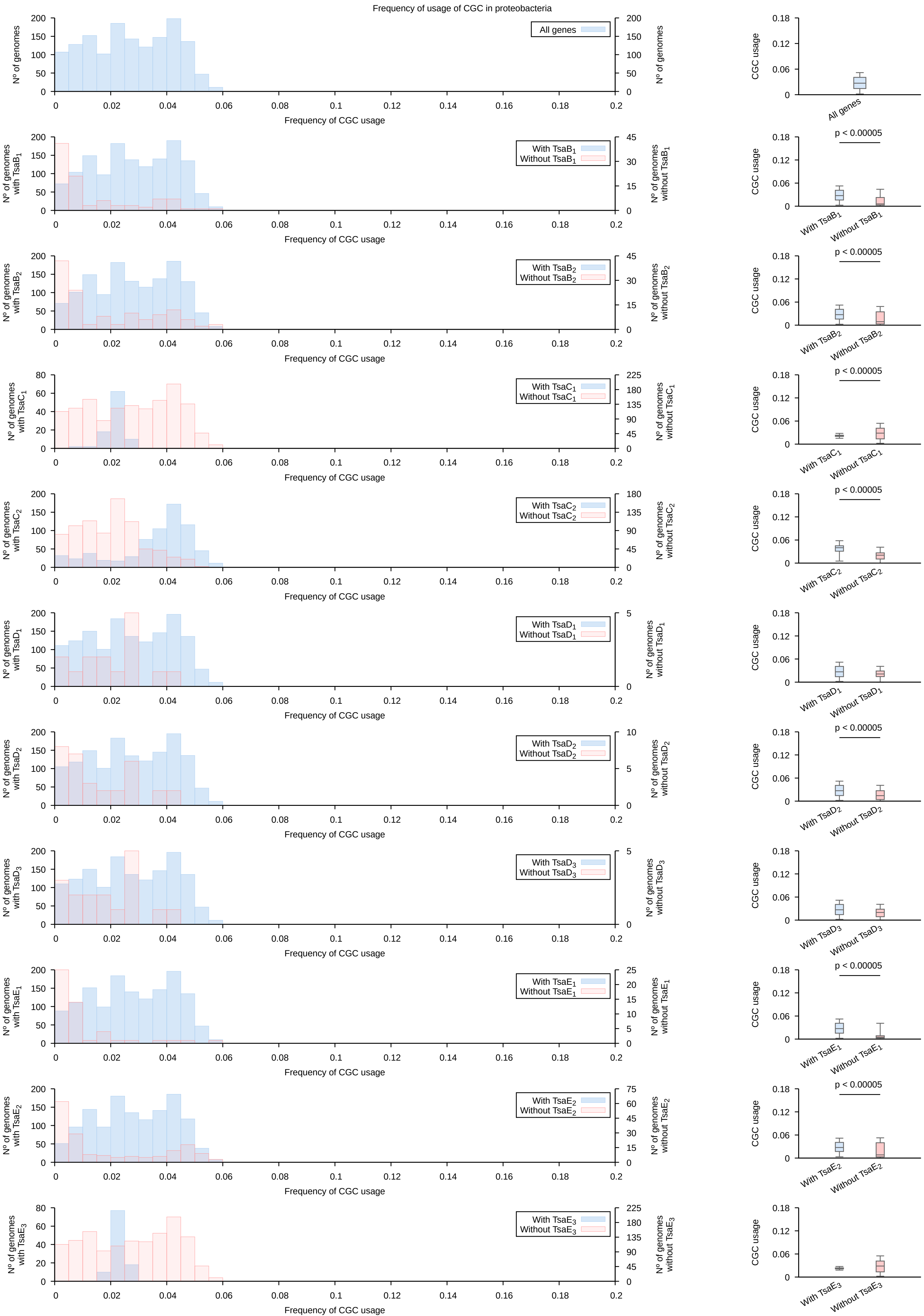
Frequency of usage of CGC in proteobacteria
All genes
With TsaB1
Without TsaB1
With TsaB2
Without TsaB2
With TsaC1
Without TsaC1
With TsaC2
Without TsaC2
With TsaD1
Without TsaD1
With TsaD2
Without TsaD2
With TsaD3
Without TsaD3
With TsaE1
Without TsaE1
With TsaE2
Without TsaE2
With TsaE3
Without TsaE3
Nº of genomes
Nº of genomes with TsaB1
Nº of genomes without TsaB1
Nº of genomes with TsaB2
Nº of genomes without TsaB2
Nº of genomes with TsaC1
Nº of genomes without TsaC1
Nº of genomes with TsaC2
Nº of genomes without TsaC2
Nº of genomes with TsaD1
Nº of genomes without TsaD1
Nº of genomes with TsaD2
Nº of genomes without TsaD2
Nº of genomes with TsaD3
Nº of genomes without TsaD3
Nº of genomes with TsaE1
Nº of genomes without TsaE1
Nº of genomes with TsaE2
Nº of genomes without TsaE2
Nº of genomes with TsaE3
Nº of genomes without TsaE3
Frequency of CGC usage
CGC usage
p < 0.00005

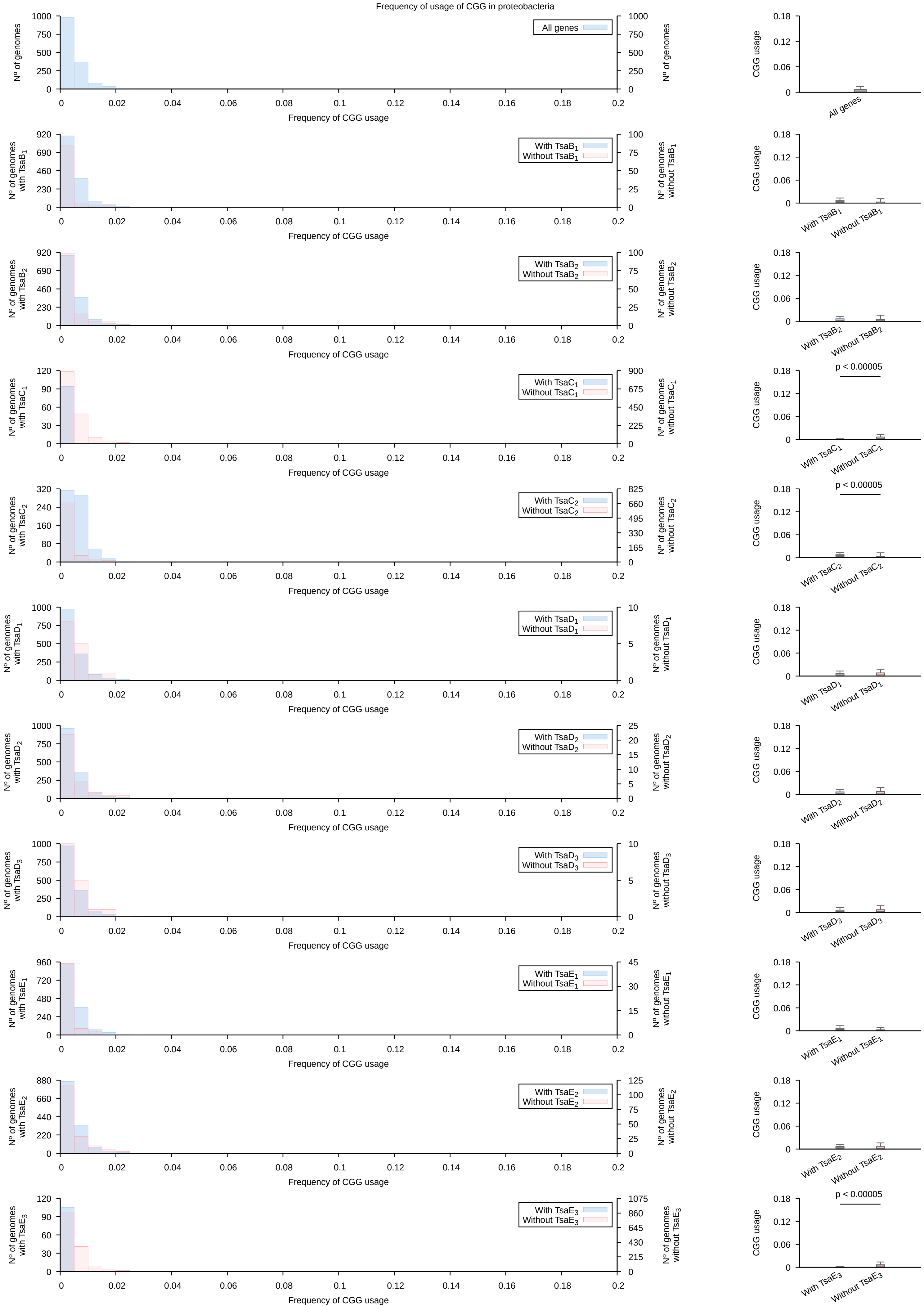

Frequency of usage of CGG in proteobacteria
Nº of genomes
All genes
Frequency of CGG usage
CGG usage
Nº of genomes with TsaB1
Nº of genomes without TsaB1
With TsaB1
Without TsaB1
Nº of genomes with TsaB2
Nº of genomes without TsaB2
With TsaB2
Without TsaB2
Nº of genomes with TsaC1
Nº of genomes without TsaC1
With TsaC1
Without TsaC1
p < 0.00005
Nº of genomes with TsaC2
Nº of genomes without TsaC2
With TsaC2
Without TsaC2
p < 0.00005
Nº of genomes with TsaD1
Nº of genomes without TsaD1
With TsaD1
Without TsaD1
Nº of genomes with TsaD2
Nº of genomes without TsaD2
With TsaD2
Without TsaD2
Nº of genomes with TsaD3
Nº of genomes without TsaD3
With TsaD3
Without TsaD3
Nº of genomes with TsaE1
Nº of genomes without TsaE1
With TsaE1
Without TsaE1
Nº of genomes with TsaE2
Nº of genomes without TsaE2
With TsaE2
Without TsaE2
Nº of genomes with TsaE3
Nº of genomes without TsaE3
With TsaE3
Without TsaE3
p < 0.00005

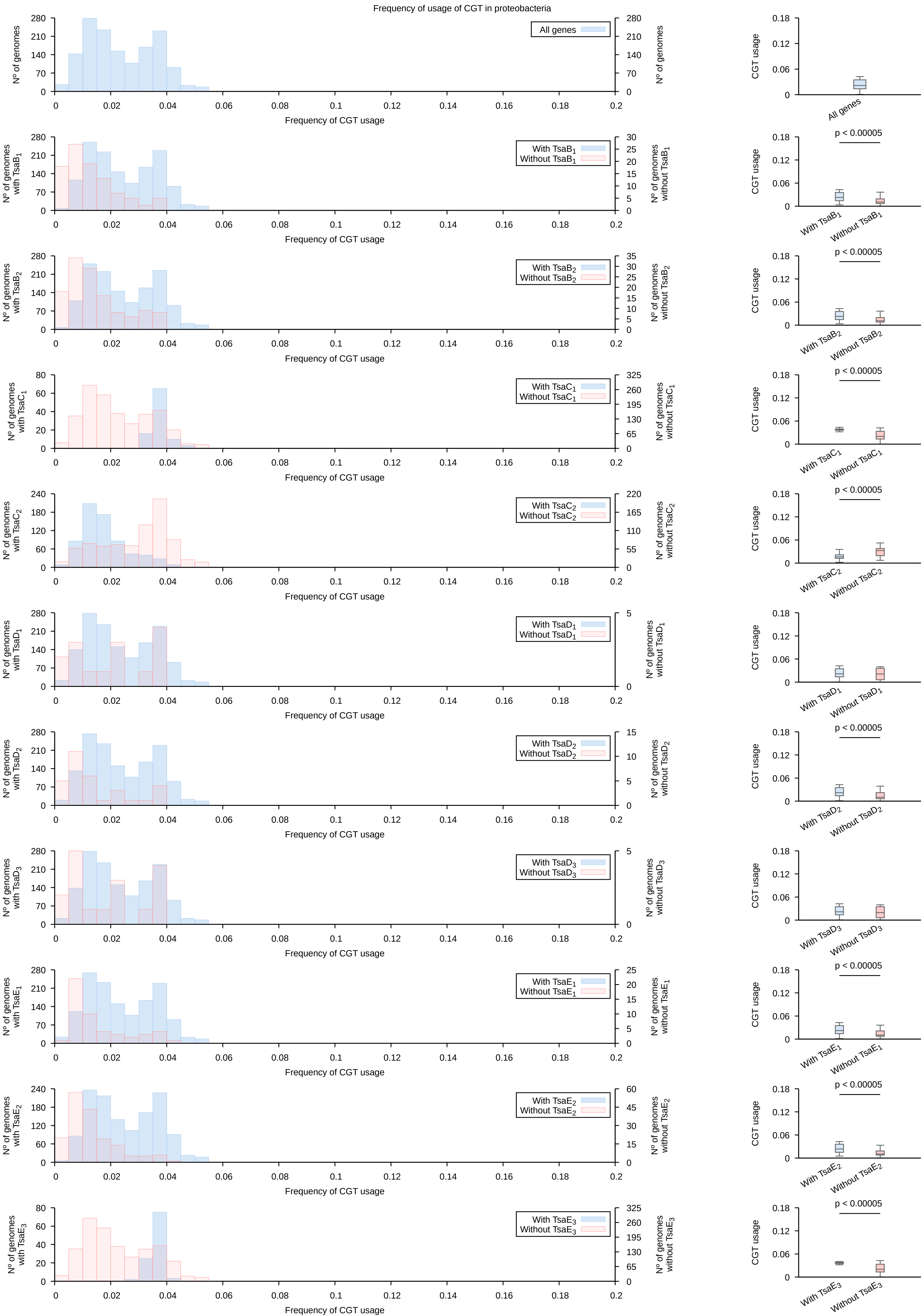

Frequency of usage of CGT in proteobacteria
All genes
Nº of genomes
Frequency of CGT usage
CGT usage
With TsaB$_1$
Without TsaB$_1$
p < 0.00005
With TsaB$_2$
Without TsaB$_2$
p < 0.00005
With TsaC$_1$
Without TsaC$_1$
p < 0.00005
With TsaC$_2$
Without TsaC$_2$
p < 0.00005
With TsaD$_1$
Without TsaD$_1$
With TsaD$_2$
Without TsaD$_2$
p < 0.00005
With TsaD$_3$
Without TsaD$_3$
With TsaE$_1$
Without TsaE$_1$
p < 0.00005
With TsaE$_2$
Without TsaE$_2$
p < 0.00005
With TsaE$_3$
Without TsaE$_3$
p < 0.00005

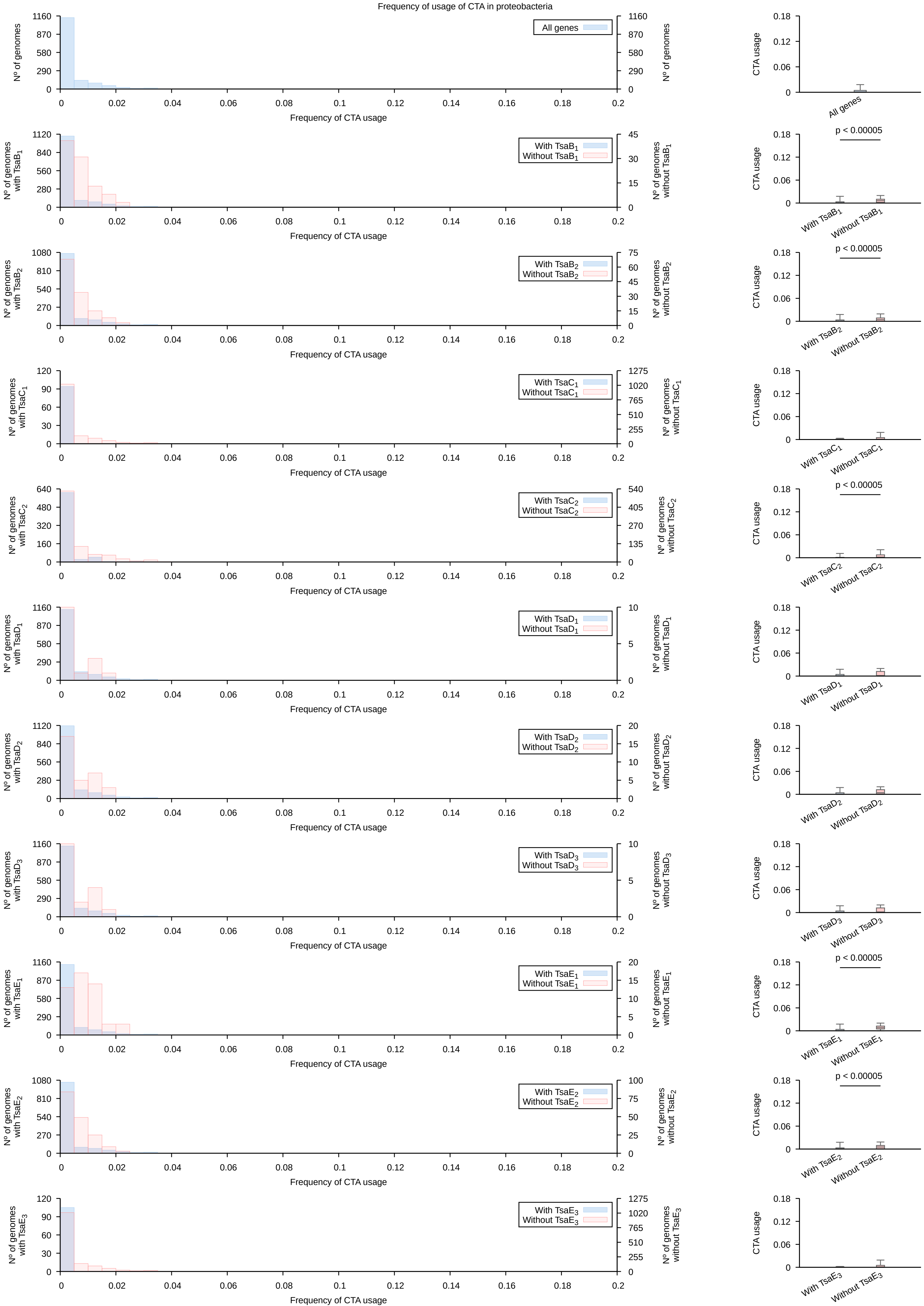

Frequency of usage of CTA in proteobacteria
All genes
Nº of genomes
Frequency of CTA usage
CTA usage
With TsaB$_1$
Without TsaB$_1$
Nº of genomes with TsaB$_1$
Nº of genomes without TsaB$_1$
$p < 0.00005$
With TsaB$_2$
Without TsaB$_2$
Nº of genomes with TsaB$_2$
Nº of genomes without TsaB$_2$
With TsaC$_1$
Without TsaC$_1$
Nº of genomes with TsaC$_1$
Nº of genomes without TsaC$_1$
With TsaC$_2$
Without TsaC$_2$
Nº of genomes with TsaC$_2$
Nº of genomes without TsaC$_2$
With TsaD$_1$
Without TsaD$_1$
Nº of genomes with TsaD$_1$
Nº of genomes without TsaD$_1$
With TsaD$_2$
Without TsaD$_2$
Nº of genomes with TsaD$_2$
Nº of genomes without TsaD$_2$
With TsaD$_3$
Without TsaD$_3$
Nº of genomes with TsaD$_3$
Nº of genomes without TsaD$_3$
With TsaE$_1$
Without TsaE$_1$
Nº of genomes with TsaE$_1$
Nº of genomes without TsaE$_1$
With TsaE$_2$
Without TsaE$_2$
Nº of genomes with TsaE$_2$
Nº of genomes without TsaE$_2$
With TsaE$_3$
Without TsaE$_3$
Nº of genomes with TsaE$_3$
Nº of genomes without TsaE$_3$

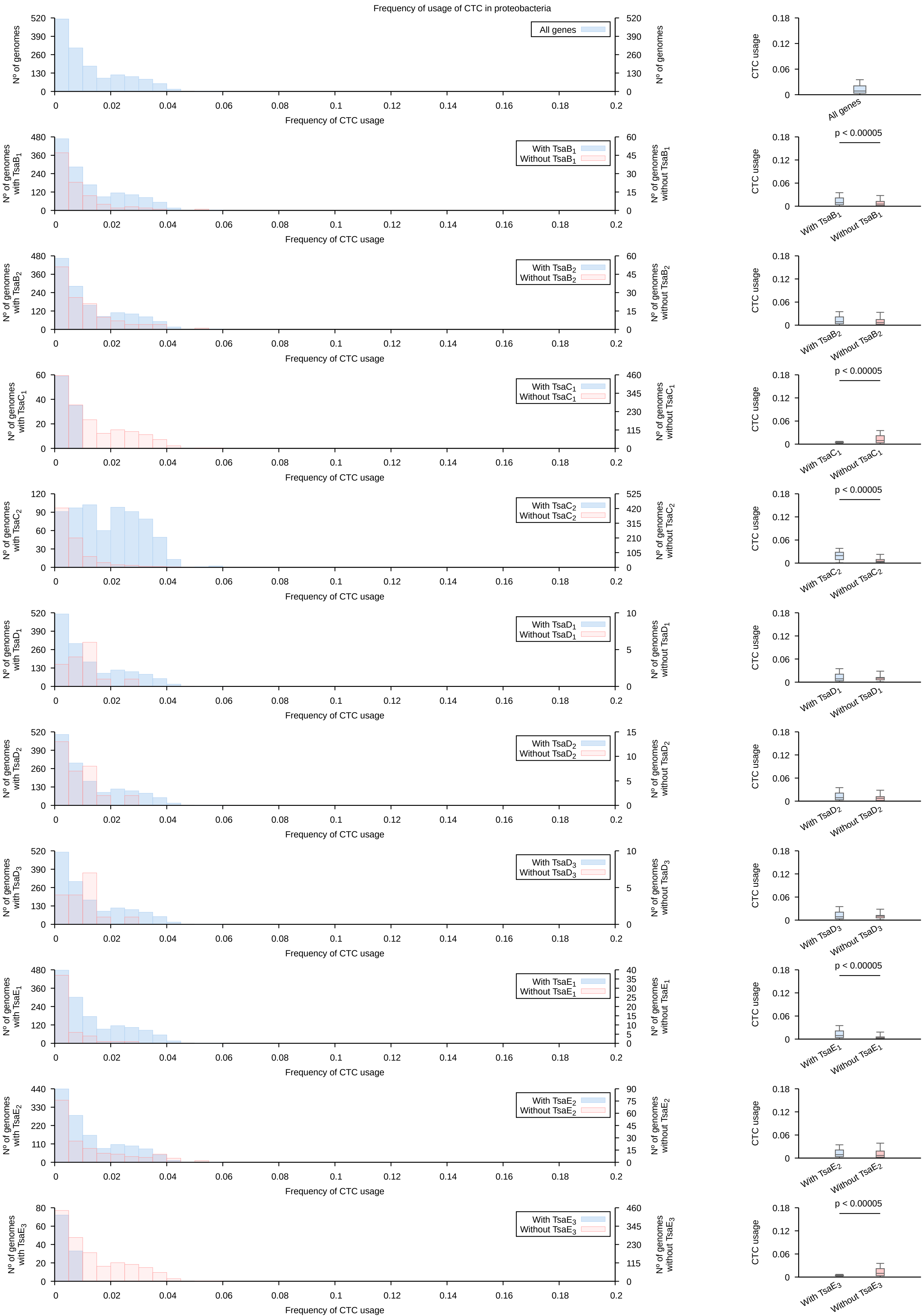

Frequency of usage of CTC in proteobacteria
Nº of genomes
All genes
Frequency of CTC usage
CTC usage
All genes
Nº of genomes with TsaB$_1$
Nº of genomes without TsaB$_1$
With TsaB$_1$
Without TsaB$_1$
p < 0.00005
Nº of genomes with TsaB$_2$
Nº of genomes without TsaB$_2$
With TsaB$_2$
Without TsaB$_2$
Nº of genomes with TsaC$_1$
Nº of genomes without TsaC$_1$
With TsaC$_1$
Without TsaC$_1$
p < 0.00005
Nº of genomes with TsaC$_2$
Nº of genomes without TsaC$_2$
With TsaC$_2$
Without TsaC$_2$
p < 0.00005
Nº of genomes with TsaD$_1$
Nº of genomes without TsaD$_1$
With TsaD$_1$
Without TsaD$_1$
Nº of genomes with TsaD$_2$
Nº of genomes without TsaD$_2$
With TsaD$_2$
Without TsaD$_2$
Nº of genomes with TsaD$_3$
Nº of genomes without TsaD$_3$
With TsaD$_3$
Without TsaD$_3$
Nº of genomes with TsaE$_1$
Nº of genomes without TsaE$_1$
With TsaE$_1$
Without TsaE$_1$
p < 0.00005
Nº of genomes with TsaE$_2$
Nº of genomes without TsaE$_2$
With TsaE$_2$
Without TsaE$_2$
Nº of genomes with TsaE$_3$
Nº of genomes without TsaE$_3$
With TsaE$_3$
Without TsaE$_3$
p < 0.00005

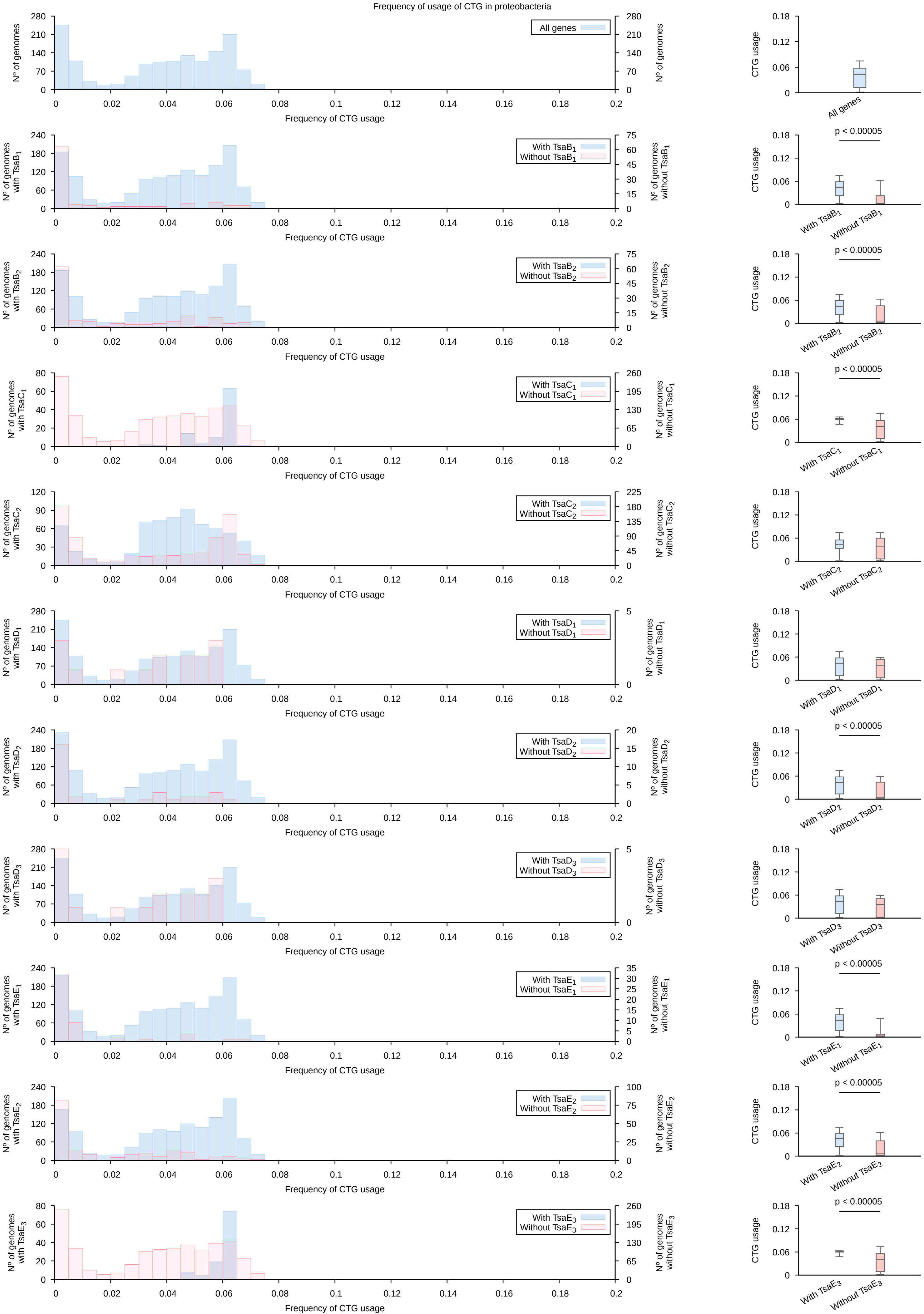
Frequency of usage of CTG in proteobacteria
Nº of genomes
Frequency of CTG usage
CTG usage
All genes
With TsaB1
Without TsaB1
With TsaB2
Without TsaB2
With TsaC1
Without TsaC1
With TsaC2
Without TsaC2
With TsaD1
Without TsaD1
With TsaD2
Without TsaD2
With TsaD3
Without TsaD3
With TsaE1
Without TsaE1
With TsaE2
Without TsaE2
With TsaE3
Without TsaE3
p < 0.00005

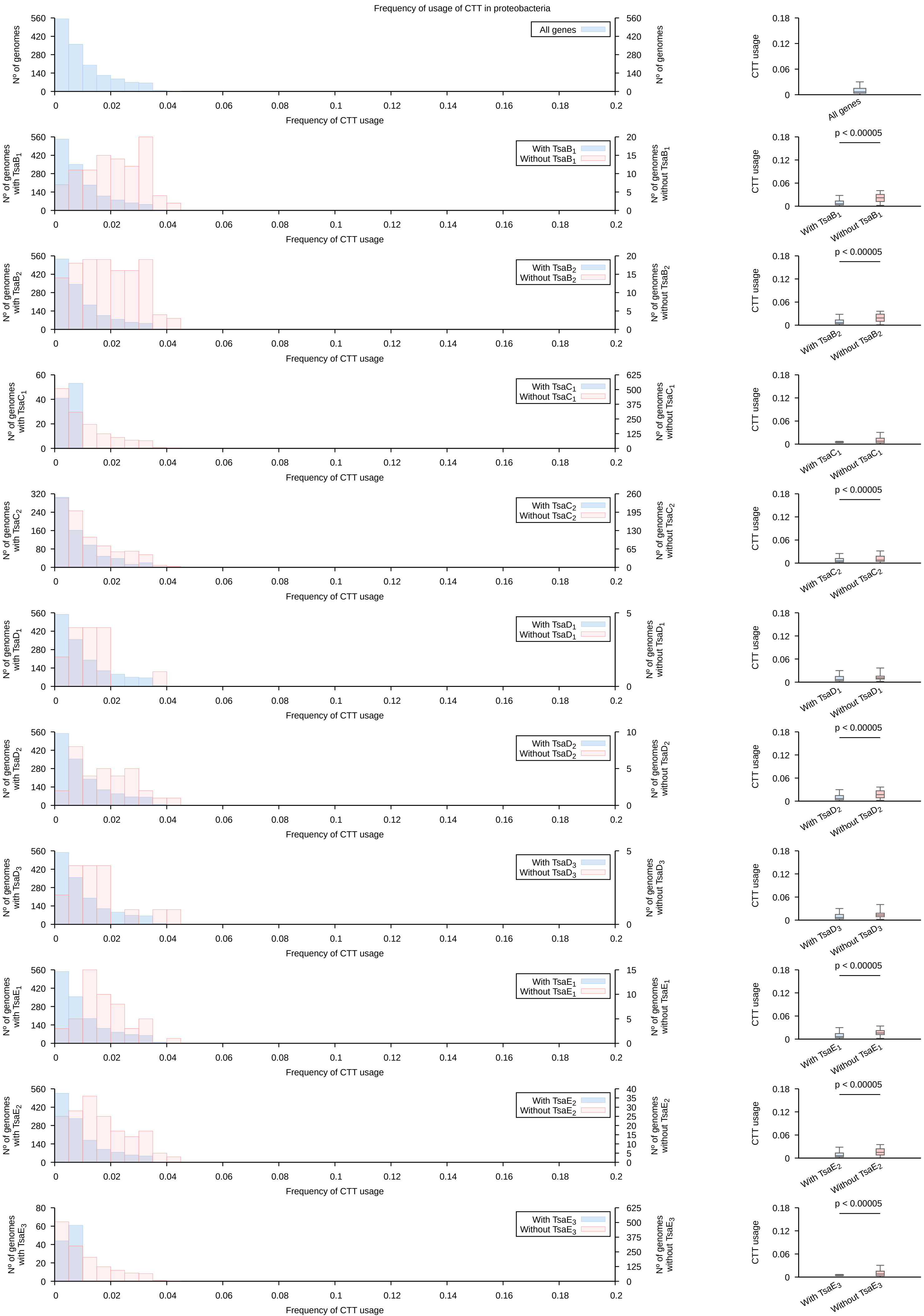

Frequency of usage of CTT in proteobacteria
Nº of genomes
Frequency of CTT usage
All genes
CTT usage
p < 0.00005
Nº of genomes with TsaB$_1$
Nº of genomes without TsaB$_1$
With TsaB$_1$
Without TsaB$_1$
Nº of genomes with TsaB$_2$
Nº of genomes without TsaB$_2$
With TsaB$_2$
Without TsaB$_2$
Nº of genomes with TsaC$_1$
Nº of genomes without TsaC$_1$
With TsaC$_1$
Without TsaC$_1$
Nº of genomes with TsaC$_2$
Nº of genomes without TsaC$_2$
With TsaC$_2$
Without TsaC$_2$
Nº of genomes with TsaD$_1$
Nº of genomes without TsaD$_1$
With TsaD$_1$
Without TsaD$_1$
Nº of genomes with TsaD$_2$
Nº of genomes without TsaD$_2$
With TsaD$_2$
Without TsaD$_2$
Nº of genomes with TsaD$_3$
Nº of genomes without TsaD$_3$
With TsaD$_3$
Without TsaD$_3$
Nº of genomes with TsaE$_1$
Nº of genomes without TsaE$_1$
With TsaE$_1$
Without TsaE$_1$
Nº of genomes with TsaE$_2$
Nº of genomes without TsaE$_2$
With TsaE$_2$
Without TsaE$_2$
Nº of genomes with TsaE$_3$
Nº of genomes without TsaE$_3$
With TsaE$_3$
Without TsaE$_3$

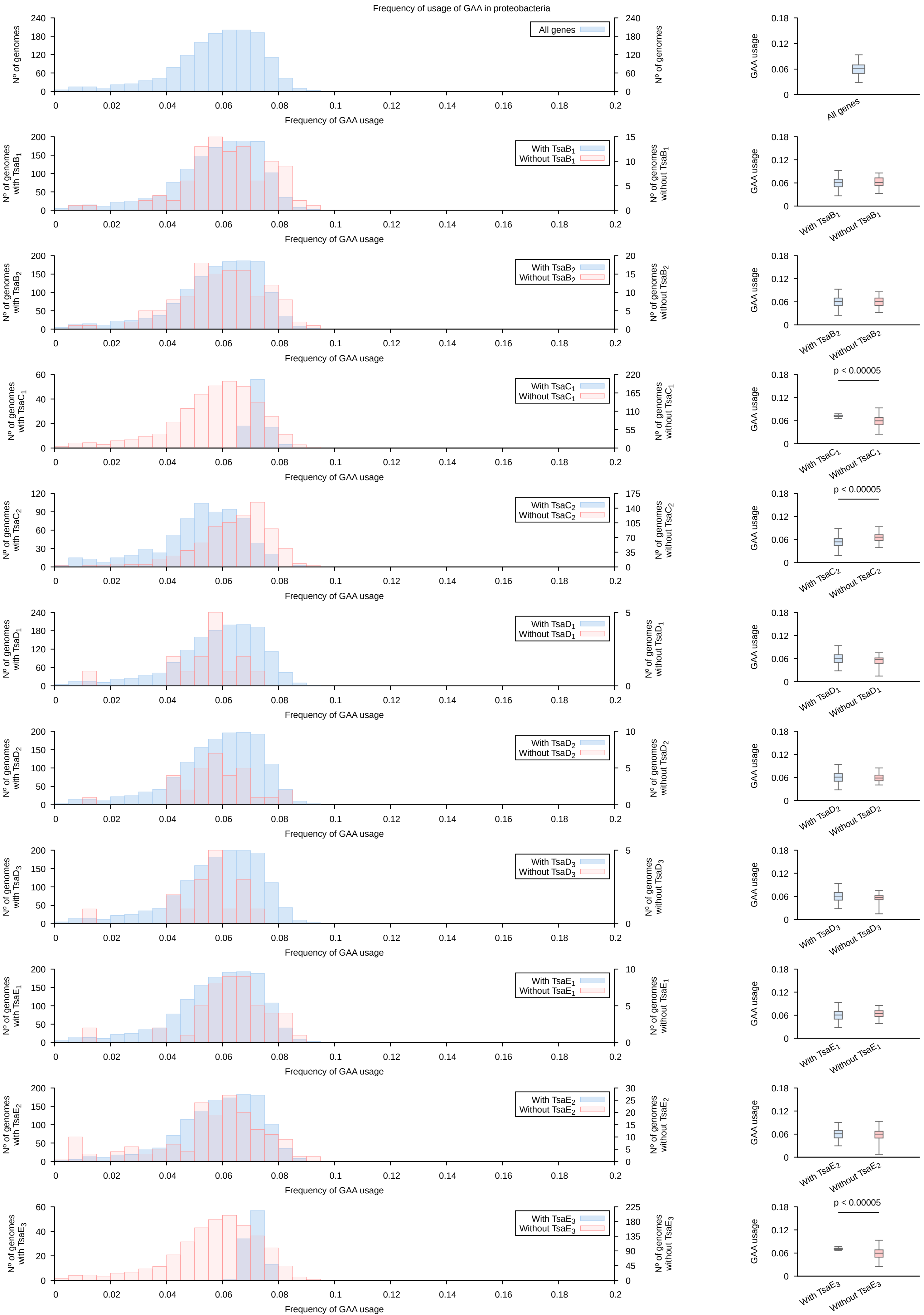

Frequency of usage of GAA in proteobacteria
Nº of genomes
All genes
GAA usage
Frequency of GAA usage
Nº of genomes with TsaB$_1$
Nº of genomes without TsaB$_1$
With TsaB$_1$
Without TsaB$_1$
Nº of genomes with TsaB$_2$
Nº of genomes without TsaB$_2$
With TsaB$_2$
Without TsaB$_2$
Nº of genomes with TsaC$_1$
Nº of genomes without TsaC$_1$
With TsaC$_1$
Without TsaC$_1$
p < 0.00005
Nº of genomes with TsaC$_2$
Nº of genomes without TsaC$_2$
With TsaC$_2$
Without TsaC$_2$
p < 0.00005
Nº of genomes with TsaD$_1$
Nº of genomes without TsaD$_1$
With TsaD$_1$
Without TsaD$_1$
Nº of genomes with TsaD$_2$
Nº of genomes without TsaD$_2$
With TsaD$_2$
Without TsaD$_2$
Nº of genomes with TsaD$_3$
Nº of genomes without TsaD$_3$
With TsaD$_3$
Without TsaD$_3$
Nº of genomes with TsaE$_1$
Nº of genomes without TsaE$_1$
With TsaE$_1$
Without TsaE$_1$
Nº of genomes with TsaE$_2$
Nº of genomes without TsaE$_2$
With TsaE$_2$
Without TsaE$_2$
Nº of genomes with TsaE$_3$
Nº of genomes without TsaE$_3$
With TsaE$_3$
Without TsaE$_3$
p < 0.00005

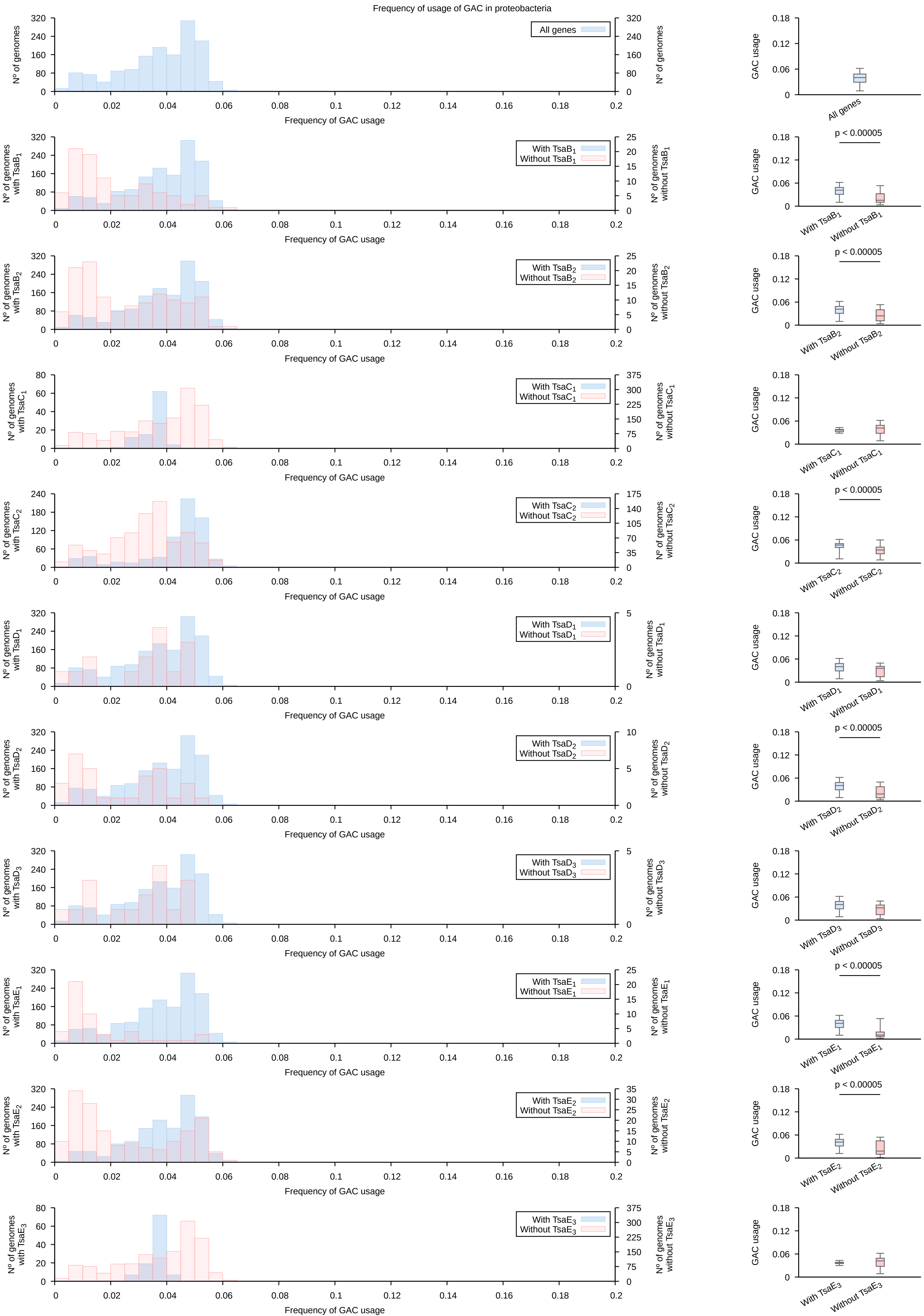

Frequency of usage of GAC in proteobacteria
Nº of genomes
All genes
Frequency of GAC usage
GAC usage
Nº of genomes with TsaB$_1$
Nº of genomes without TsaB$_1$
With TsaB$_1$
Without TsaB$_1$
p < 0.00005
Nº of genomes with TsaB$_2$
Nº of genomes without TsaB$_2$
With TsaB$_2$
Without TsaB$_2$
p < 0.00005
Nº of genomes with TsaC$_1$
Nº of genomes without TsaC$_1$
With TsaC$_1$
Without TsaC$_1$
Nº of genomes with TsaC$_2$
Nº of genomes without TsaC$_2$
With TsaC$_2$
Without TsaC$_2$
p < 0.00005
Nº of genomes with TsaD$_1$
Nº of genomes without TsaD$_1$
With TsaD$_1$
Without TsaD$_1$
Nº of genomes with TsaD$_2$
Nº of genomes without TsaD$_2$
With TsaD$_2$
Without TsaD$_2$
p < 0.00005
Nº of genomes with TsaD$_3$
Nº of genomes without TsaD$_3$
With TsaD$_3$
Without TsaD$_3$
Nº of genomes with TsaE$_1$
Nº of genomes without TsaE$_1$
With TsaE$_1$
Without TsaE$_1$
p < 0.00005
Nº of genomes with TsaE$_2$
Nº of genomes without TsaE$_2$
With TsaE$_2$
Without TsaE$_2$
p < 0.00005
Nº of genomes with TsaE$_3$
Nº of genomes without TsaE$_3$
With TsaE$_3$
Without TsaE$_3$

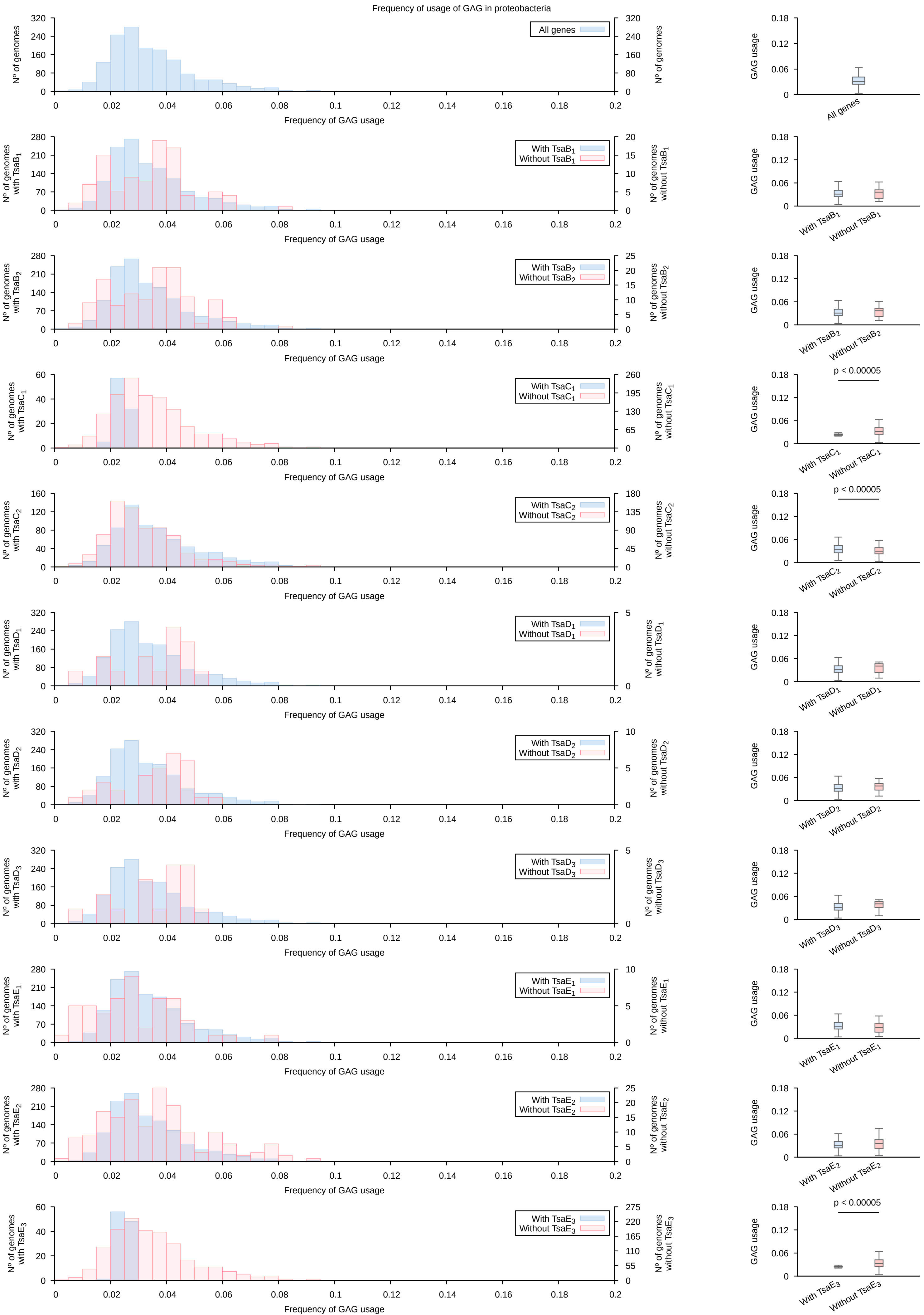

Frequency of usage of GAG in proteobacteria
All genes
Nº of genomes
Frequency of GAG usage
GAG usage
With TsaB$_1$
Without TsaB$_1$
Nº of genomes with TsaB$_1$
Nº of genomes without TsaB$_1$
With TsaB$_2$
Without TsaB$_2$
Nº of genomes with TsaB$_2$
Nº of genomes without TsaB$_2$
With TsaC$_1$
Without TsaC$_1$
Nº of genomes with TsaC$_1$
Nº of genomes without TsaC$_1$
p < 0.00005
With TsaC$_2$
Without TsaC$_2$
Nº of genomes with TsaC$_2$
Nº of genomes without TsaC$_2$
p < 0.00005
With TsaD$_1$
Without TsaD$_1$
Nº of genomes with TsaD$_1$
Nº of genomes without TsaD$_1$
With TsaD$_2$
Without TsaD$_2$
Nº of genomes with TsaD$_2$
Nº of genomes without TsaD$_2$
With TsaD$_3$
Without TsaD$_3$
Nº of genomes with TsaD$_3$
Nº of genomes without TsaD$_3$
With TsaE$_1$
Without TsaE$_1$
Nº of genomes with TsaE$_1$
Nº of genomes without TsaE$_1$
With TsaE$_2$
Without TsaE$_2$
Nº of genomes with TsaE$_2$
Nº of genomes without TsaE$_2$
With TsaE$_3$
Without TsaE$_3$
Nº of genomes with TsaE$_3$
Nº of genomes without TsaE$_3$
p < 0.00005

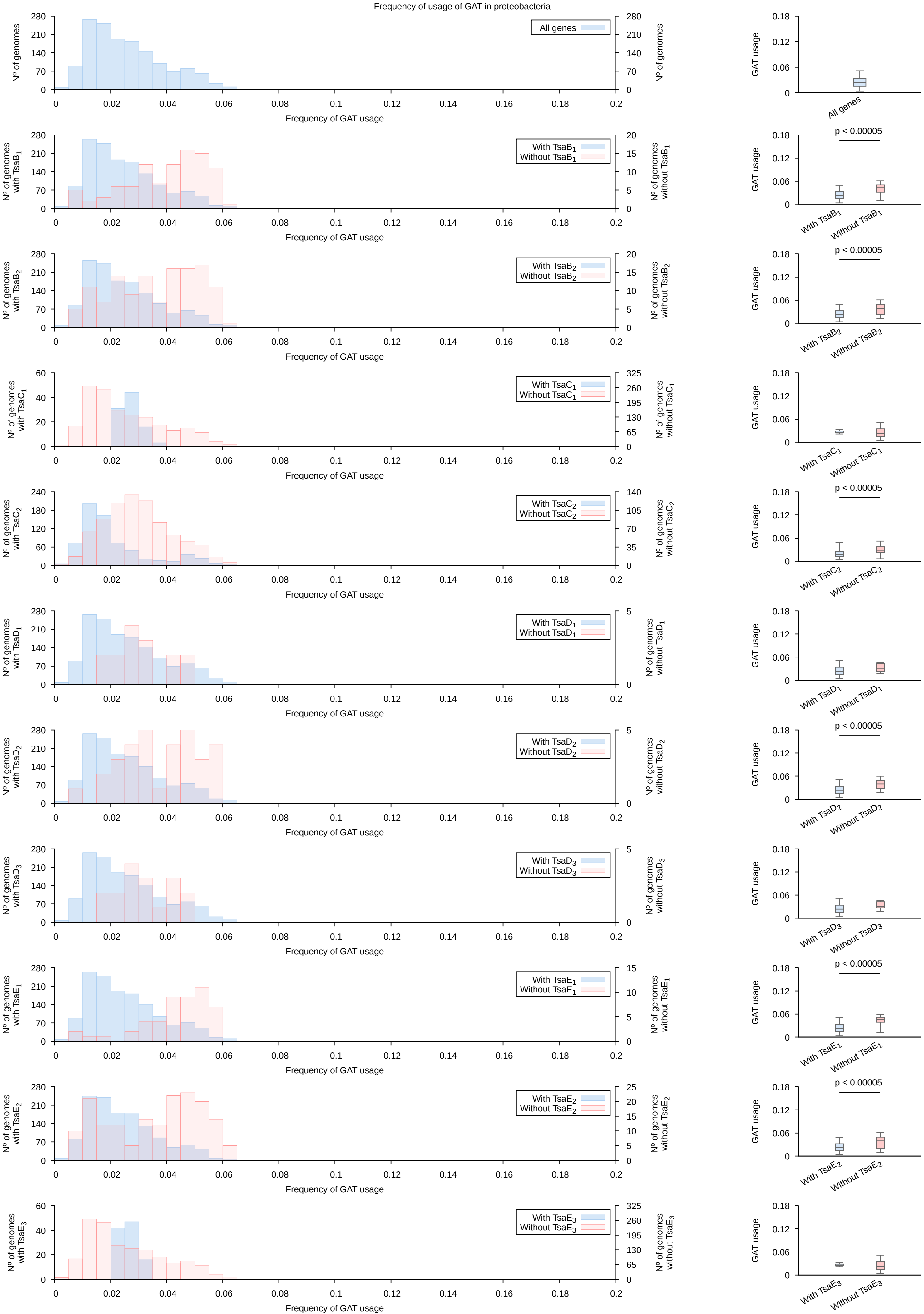
Frequency of usage of GAT in proteobacteria
All genes
With TsaB$_1$
Without TsaB$_1$
With TsaB$_2$
Without TsaB$_2$
With TsaC$_1$
Without TsaC$_1$
With TsaC$_2$
Without TsaC$_2$
With TsaD$_1$
Without TsaD$_1$
With TsaD$_2$
Without TsaD$_2$
With TsaD$_3$
Without TsaD$_3$
With TsaE$_1$
Without TsaE$_1$
With TsaE$_2$
Without TsaE$_2$
With TsaE$_3$
Without TsaE$_3$
Nº of genomes
Nº of genomes with TsaB$_1$
Nº of genomes without TsaB$_1$
Nº of genomes with TsaB$_2$
Nº of genomes without TsaB$_2$
Nº of genomes with TsaC$_1$
Nº of genomes without TsaC$_1$
Nº of genomes with TsaC$_2$
Nº of genomes without TsaC$_2$
Nº of genomes with TsaD$_1$
Nº of genomes without TsaD$_1$
Nº of genomes with TsaD$_2$
Nº of genomes without TsaD$_2$
Nº of genomes with TsaD$_3$
Nº of genomes without TsaD$_3$
Nº of genomes with TsaE$_1$
Nº of genomes without TsaE$_1$
Nº of genomes with TsaE$_2$
Nº of genomes without TsaE$_2$
Nº of genomes with TsaE$_3$
Nº of genomes without TsaE$_3$
Frequency of GAT usage
GAT usage
p < 0.00005
0
0.02
0.04
0.06
0.08
0.1
0.12
0.14
0.16
0.18
0.2

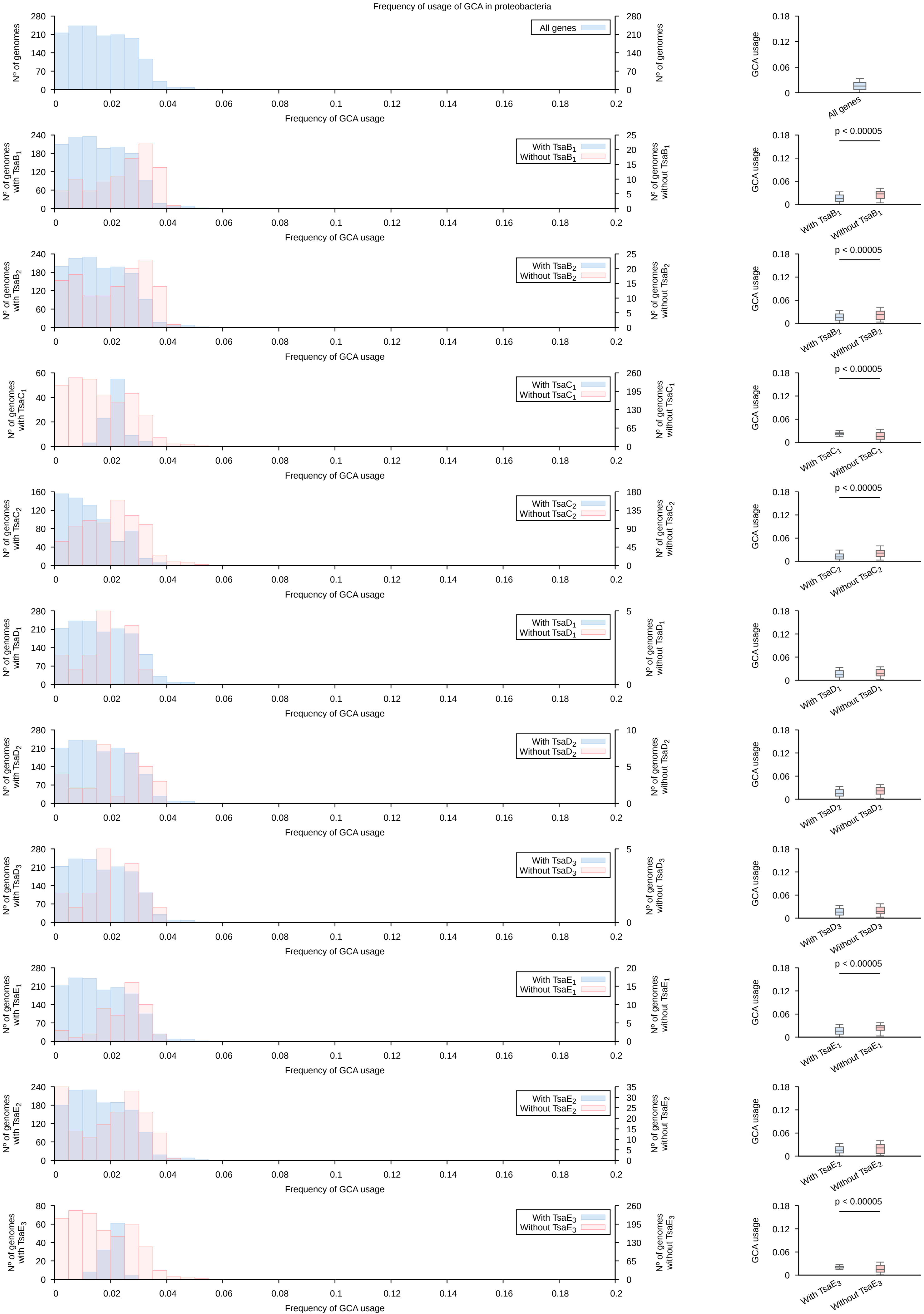
Frequency of usage of GCA in proteobacteria
Nº of genomes
All genes
Frequency of GCA usage
GCA usage
Nº of genomes with TsaB1
Nº of genomes without TsaB1
With TsaB1
Without TsaB1
p < 0.00005
Nº of genomes with TsaB2
Nº of genomes without TsaB2
With TsaB2
Without TsaB2
p < 0.00005
Nº of genomes with TsaC1
Nº of genomes without TsaC1
With TsaC1
Without TsaC1
p < 0.00005
Nº of genomes with TsaC2
Nº of genomes without TsaC2
With TsaC2
Without TsaC2
p < 0.00005
Nº of genomes with TsaD1
Nº of genomes without TsaD1
With TsaD1
Without TsaD1
Nº of genomes with TsaD2
Nº of genomes without TsaD2
With TsaD2
Without TsaD2
Nº of genomes with TsaD3
Nº of genomes without TsaD3
With TsaD3
Without TsaD3
Nº of genomes with TsaE1
Nº of genomes without TsaE1
With TsaE1
Without TsaE1
p < 0.00005
Nº of genomes with TsaE2
Nº of genomes without TsaE2
With TsaE2
Without TsaE2
Nº of genomes with TsaE3
Nº of genomes without TsaE3
With TsaE3
Without TsaE3
p < 0.00005

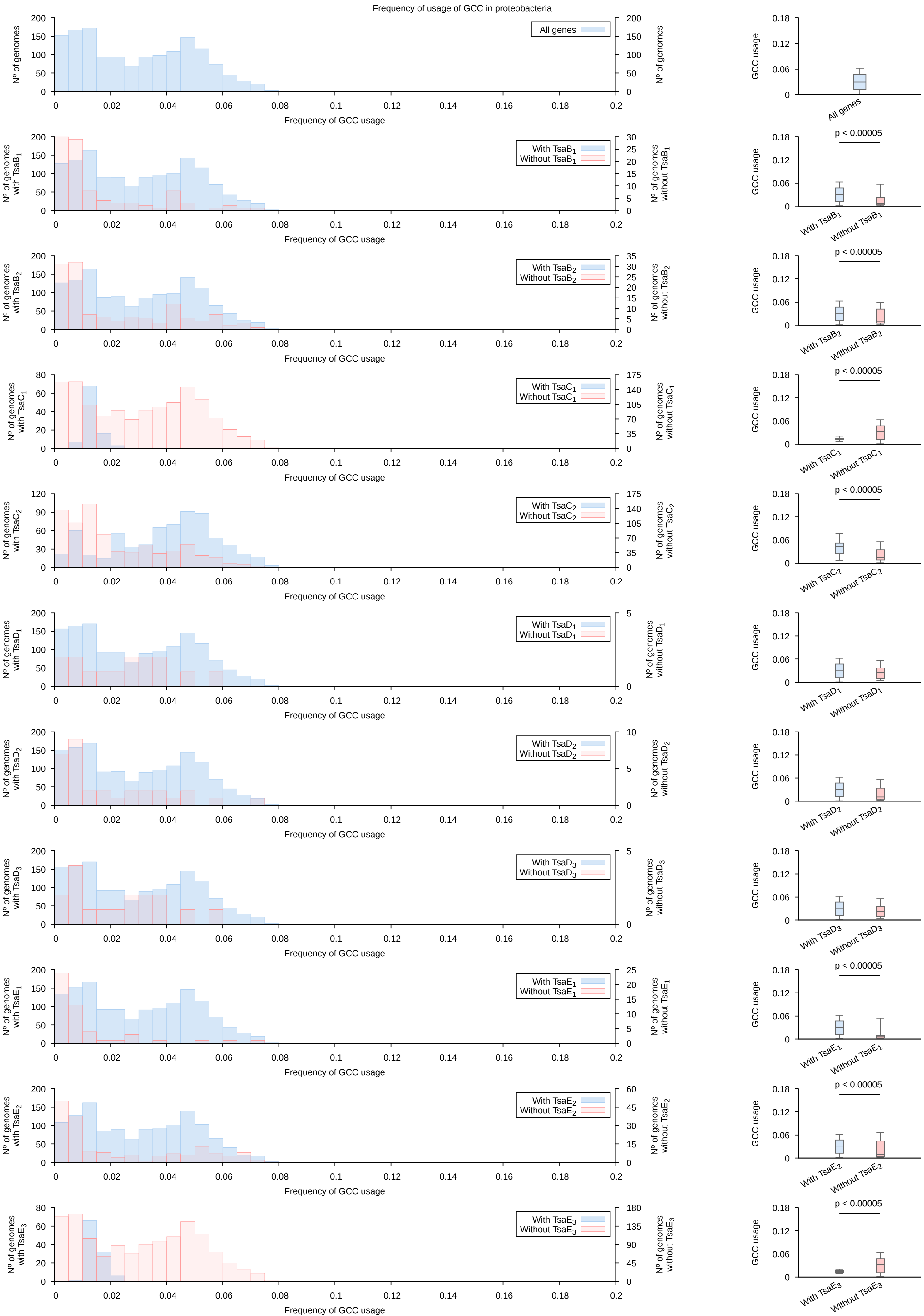
Frequency of usage of GCC in proteobacteria
Nº of genomes
All genes
Frequency of GCC usage
GCC usage
Nº of genomes with TsaB$_1$
Nº of genomes without TsaB$_1$
With TsaB$_1$
Without TsaB$_1$
p < 0.00005
Nº of genomes with TsaB$_2$
Nº of genomes without TsaB$_2$
With TsaB$_2$
Without TsaB$_2$
p < 0.00005
Nº of genomes with TsaC$_1$
Nº of genomes without TsaC$_1$
With TsaC$_1$
Without TsaC$_1$
p < 0.00005
Nº of genomes with TsaC$_2$
Nº of genomes without TsaC$_2$
With TsaC$_2$
Without TsaC$_2$
p < 0.00005
Nº of genomes with TsaD$_1$
Nº of genomes without TsaD$_1$
With TsaD$_1$
Without TsaD$_1$
Nº of genomes with TsaD$_2$
Nº of genomes without TsaD$_2$
With TsaD$_2$
Without TsaD$_2$
Nº of genomes with TsaD$_3$
Nº of genomes without TsaD$_3$
With TsaD$_3$
Without TsaD$_3$
Nº of genomes with TsaE$_1$
Nº of genomes without TsaE$_1$
With TsaE$_1$
Without TsaE$_1$
p < 0.00005
Nº of genomes with TsaE$_2$
Nº of genomes without TsaE$_2$
With TsaE$_2$
Without TsaE$_2$
p < 0.00005
Nº of genomes with TsaE$_3$
Nº of genomes without TsaE$_3$
With TsaE$_3$
Without TsaE$_3$
p < 0.00005
0 0.02 0.04 0.06 0.08 0.1 0.12 0.14 0.16 0.18 0.2
0 0.06 0.12 0.18

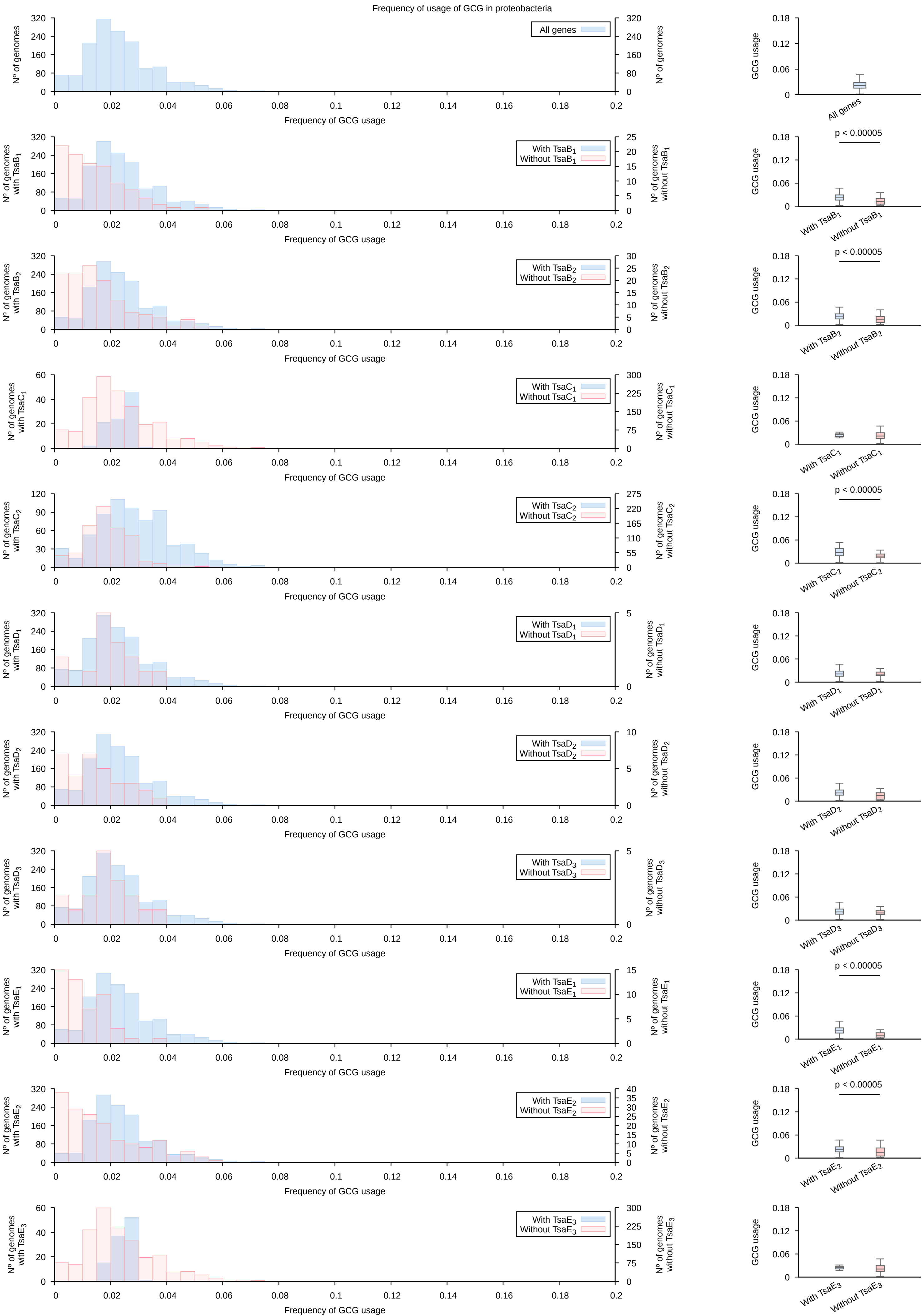

Frequency of usage of GCG in proteobacteria
All genes
Nº of genomes
Frequency of GCG usage
GCG usage
With TsaB$_1$
Without TsaB$_1$
Nº of genomes with TsaB$_1$
Nº of genomes without TsaB$_1$
p < 0.00005
With TsaB$_2$
Without TsaB$_2$
Nº of genomes with TsaB$_2$
Nº of genomes without TsaB$_2$
p < 0.00005
With TsaC$_1$
Without TsaC$_1$
Nº of genomes with TsaC$_1$
Nº of genomes without TsaC$_1$
With TsaC$_2$
Without TsaC$_2$
Nº of genomes with TsaC$_2$
Nº of genomes without TsaC$_2$
p < 0.00005
With TsaD$_1$
Without TsaD$_1$
Nº of genomes with TsaD$_1$
Nº of genomes without TsaD$_1$
With TsaD$_2$
Without TsaD$_2$
Nº of genomes with TsaD$_2$
Nº of genomes without TsaD$_2$
With TsaD$_3$
Without TsaD$_3$
Nº of genomes with TsaD$_3$
Nº of genomes without TsaD$_3$
With TsaE$_1$
Without TsaE$_1$
Nº of genomes with TsaE$_1$
Nº of genomes without TsaE$_1$
p < 0.00005
With TsaE$_2$
Without TsaE$_2$
Nº of genomes with TsaE$_2$
Nº of genomes without TsaE$_2$
p < 0.00005
With TsaE$_3$
Without TsaE$_3$
Nº of genomes with TsaE$_3$
Nº of genomes without TsaE$_3$

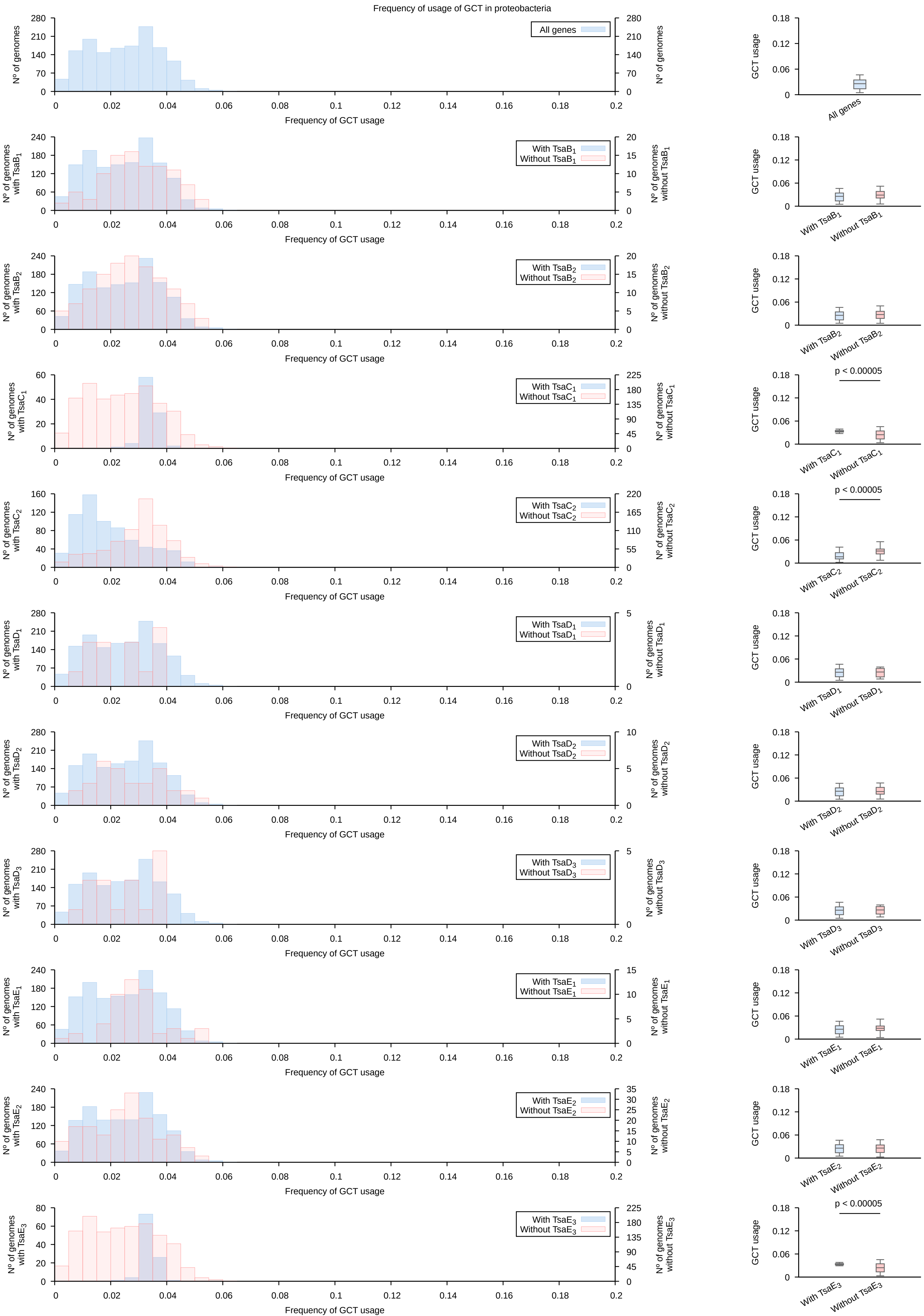
Frequency of usage of GCT in proteobacteria
Nº of genomes
All genes
Frequency of GCT usage
GCT usage
Nº of genomes with TsaB1
Nº of genomes without TsaB1
With TsaB1
Without TsaB1
Nº of genomes with TsaB2
Nº of genomes without TsaB2
With TsaB2
Without TsaB2
Nº of genomes with TsaC1
Nº of genomes without TsaC1
With TsaC1
Without TsaC1
p < 0.00005
Nº of genomes with TsaC2
Nº of genomes without TsaC2
With TsaC2
Without TsaC2
p < 0.00005
Nº of genomes with TsaD1
Nº of genomes without TsaD1
With TsaD1
Without TsaD1
Nº of genomes with TsaD2
Nº of genomes without TsaD2
With TsaD2
Without TsaD2
Nº of genomes with TsaD3
Nº of genomes without TsaD3
With TsaD3
Without TsaD3
Nº of genomes with TsaE1
Nº of genomes without TsaE1
With TsaE1
Without TsaE1
Nº of genomes with TsaE2
Nº of genomes without TsaE2
With TsaE2
Without TsaE2
Nº of genomes with TsaE3
Nº of genomes without TsaE3
With TsaE3
Without TsaE3
p < 0.00005

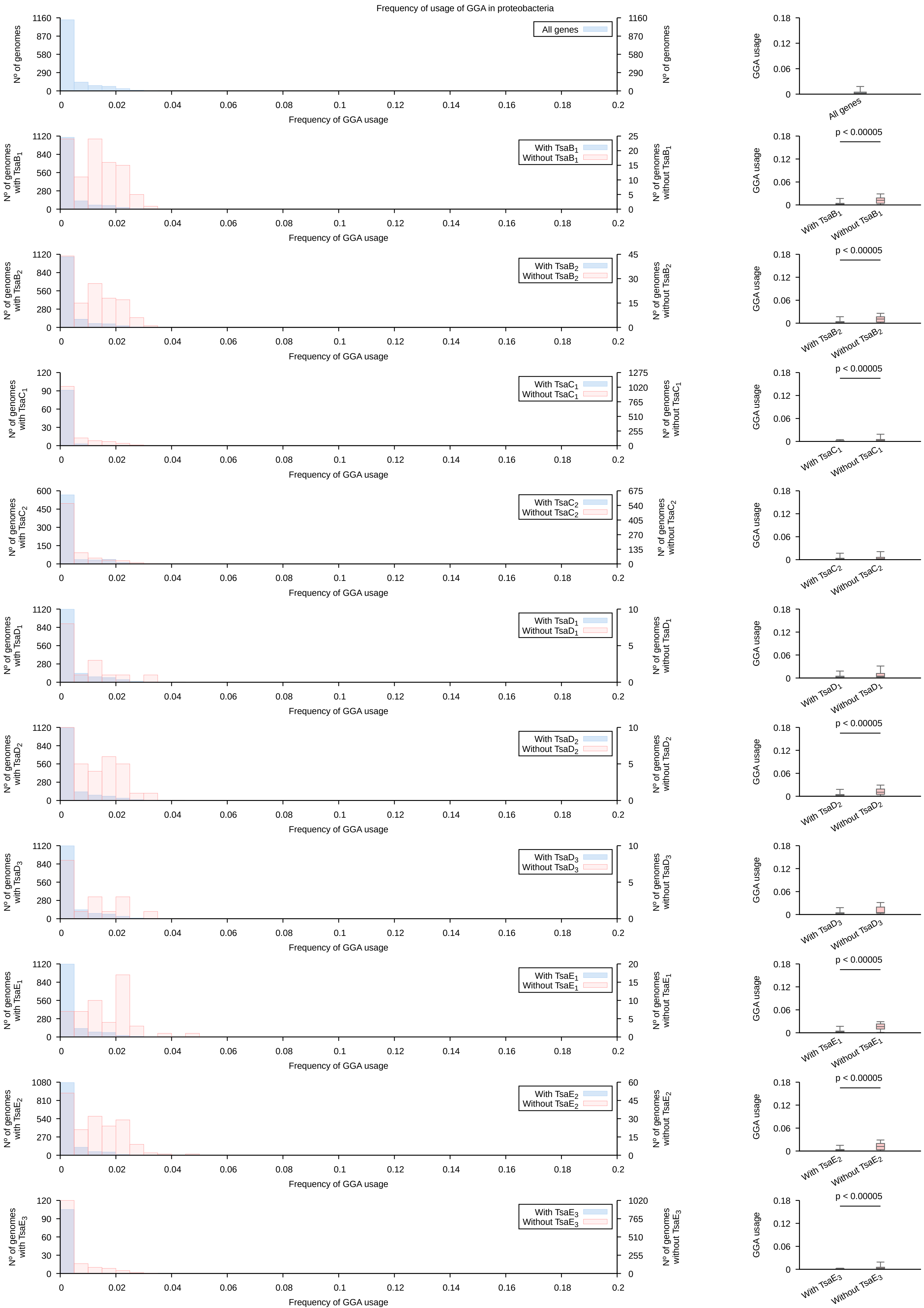

Frequency of usage of GGA in proteobacteria
All genes
Nº of genomes
Frequency of GGA usage
GGA usage
With TsaB$_1$
Without TsaB$_1$
Nº of genomes with TsaB$_1$
Nº of genomes without TsaB$_1$
p < 0.00005
With TsaB$_2$
Without TsaB$_2$
Nº of genomes with TsaB$_2$
Nº of genomes without TsaB$_2$
p < 0.00005
With TsaC$_1$
Without TsaC$_1$
Nº of genomes with TsaC$_1$
Nº of genomes without TsaC$_1$
p < 0.00005
With TsaC$_2$
Without TsaC$_2$
Nº of genomes with TsaC$_2$
Nº of genomes without TsaC$_2$
With TsaD$_1$
Without TsaD$_1$
Nº of genomes with TsaD$_1$
Nº of genomes without TsaD$_1$
With TsaD$_2$
Without TsaD$_2$
Nº of genomes with TsaD$_2$
Nº of genomes without TsaD$_2$
p < 0.00005
With TsaD$_3$
Without TsaD$_3$
Nº of genomes with TsaD$_3$
Nº of genomes without TsaD$_3$
With TsaE$_1$
Without TsaE$_1$
Nº of genomes with TsaE$_1$
Nº of genomes without TsaE$_1$
p < 0.00005
With TsaE$_2$
Without TsaE$_2$
Nº of genomes with TsaE$_2$
Nº of genomes without TsaE$_2$
p < 0.00005
With TsaE$_3$
Without TsaE$_3$
Nº of genomes with TsaE$_3$
Nº of genomes without TsaE$_3$
p < 0.00005

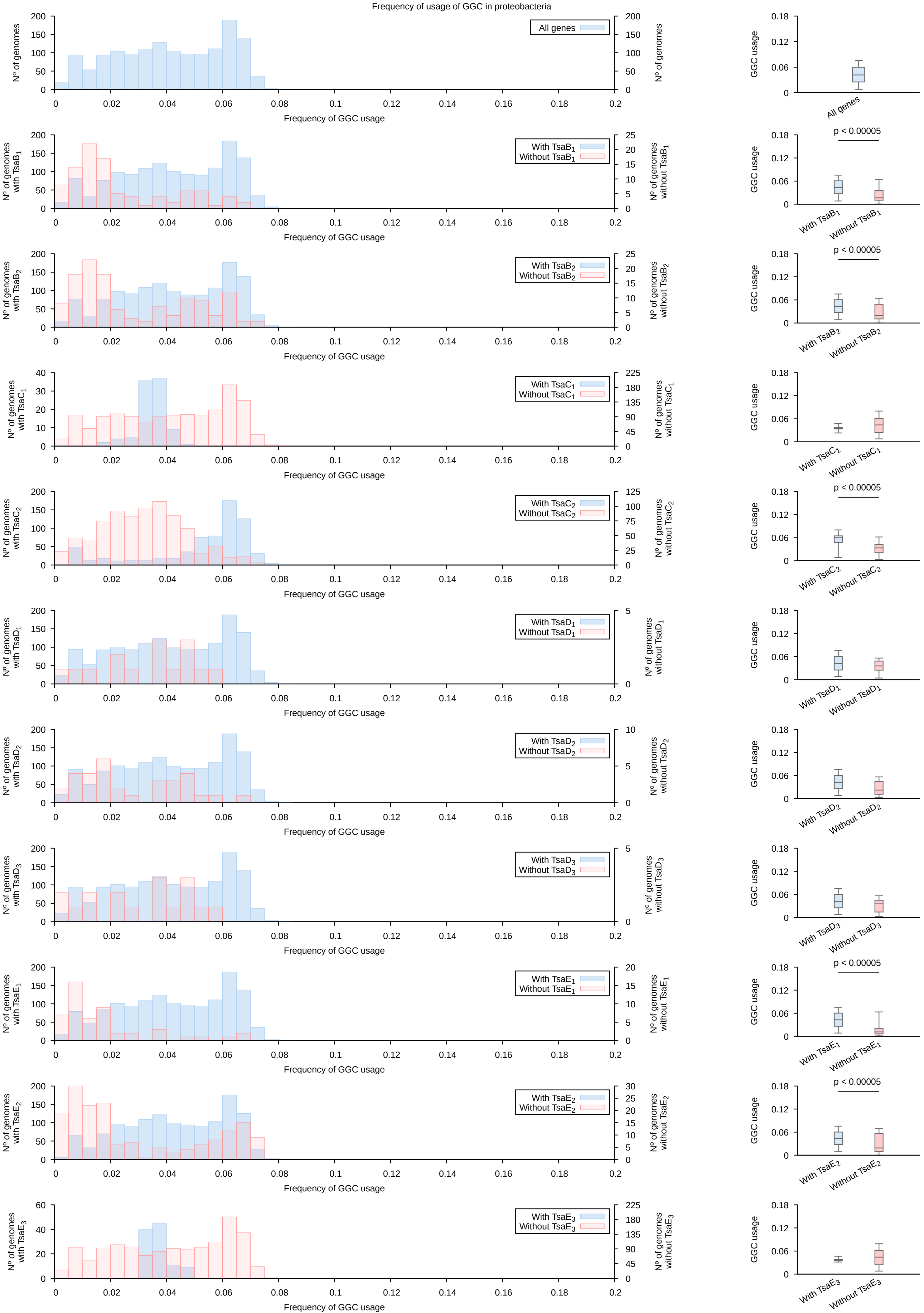

Frequency of usage of GGC in proteobacteria
Nº of genomes
Frequency of GGC usage
GGC usage
All genes
Nº of genomes with TsaB1
Nº of genomes without TsaB1
With TsaB1
Without TsaB1
p < 0.00005
Nº of genomes with TsaB2
Nº of genomes without TsaB2
With TsaB2
Without TsaB2
p < 0.00005
Nº of genomes with TsaC1
Nº of genomes without TsaC1
With TsaC1
Without TsaC1
Nº of genomes with TsaC2
Nº of genomes without TsaC2
With TsaC2
Without TsaC2
p < 0.00005
Nº of genomes with TsaD1
Nº of genomes without TsaD1
With TsaD1
Without TsaD1
Nº of genomes with TsaD2
Nº of genomes without TsaD2
With TsaD2
Without TsaD2
Nº of genomes with TsaD3
Nº of genomes without TsaD3
With TsaD3
Without TsaD3
Nº of genomes with TsaE1
Nº of genomes without TsaE1
With TsaE1
Without TsaE1
p < 0.00005
Nº of genomes with TsaE2
Nº of genomes without TsaE2
With TsaE2
Without TsaE2
p < 0.00005
Nº of genomes with TsaE3
Nº of genomes without TsaE3
With TsaE3
Without TsaE3
0 0.02 0.04 0.06 0.08 0.1 0.12 0.14 0.16 0.18 0.2
0 0.06 0.12 0.18

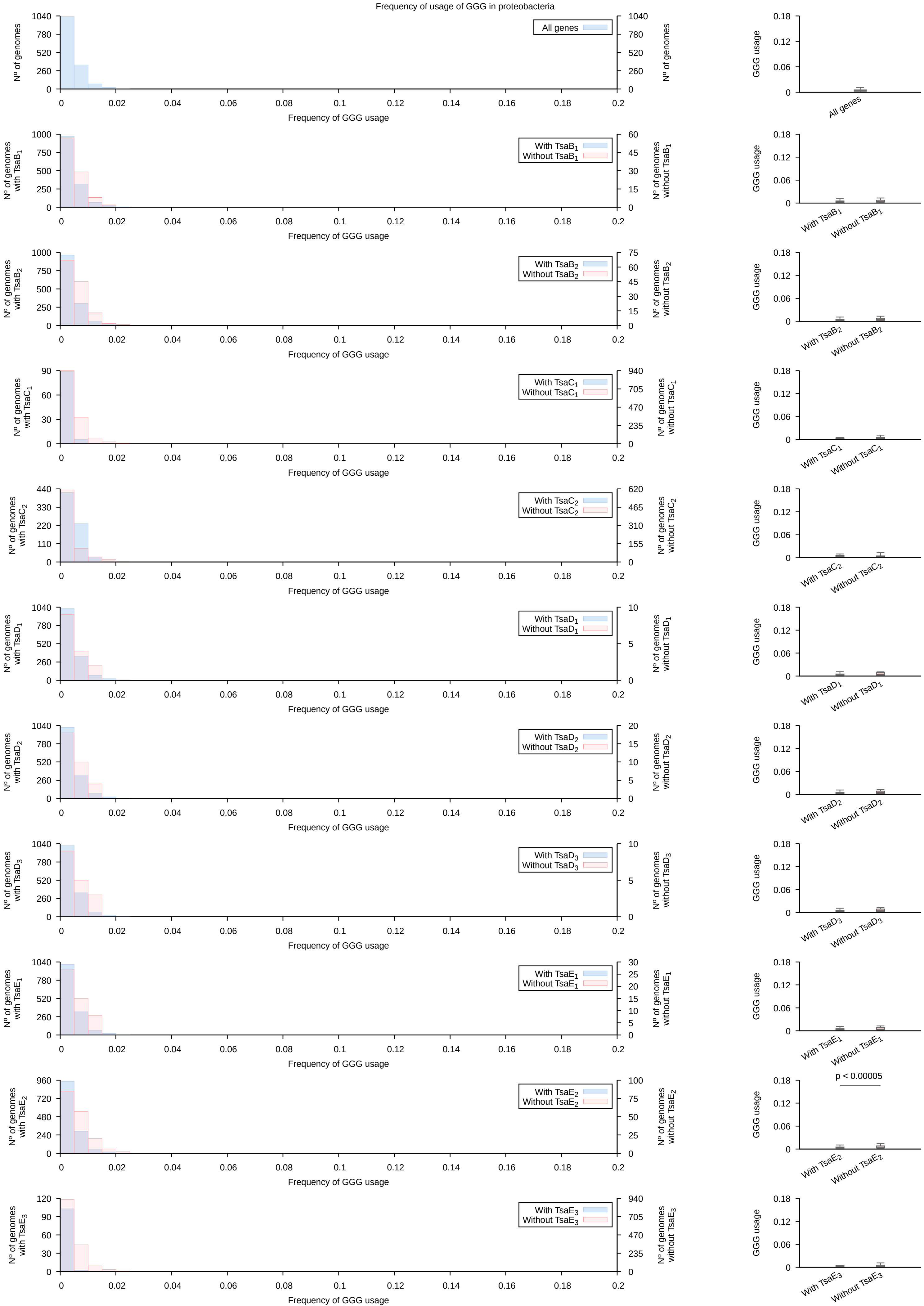
Frequency of usage of GGG in proteobacteria
All genes
Nº of genomes
Frequency of GGG usage
GGG usage
With TsaB1
Without TsaB1
Nº of genomes with TsaB1
Nº of genomes without TsaB1
With TsaB2
Without TsaB2
Nº of genomes with TsaB2
Nº of genomes without TsaB2
With TsaC1
Without TsaC1
Nº of genomes with TsaC1
Nº of genomes without TsaC1
With TsaC2
Without TsaC2
Nº of genomes with TsaC2
Nº of genomes without TsaC2
With TsaD1
Without TsaD1
Nº of genomes with TsaD1
Nº of genomes without TsaD1
With TsaD2
Without TsaD2
Nº of genomes with TsaD2
Nº of genomes without TsaD2
With TsaD3
Without TsaD3
Nº of genomes with TsaD3
Nº of genomes without TsaD3
With TsaE1
Without TsaE1
Nº of genomes with TsaE1
Nº of genomes without TsaE1
With TsaE2
Without TsaE2
Nº of genomes with TsaE2
Nº of genomes without TsaE2
p < 0.00005
With TsaE3
Without TsaE3
Nº of genomes with TsaE3
Nº of genomes without TsaE3

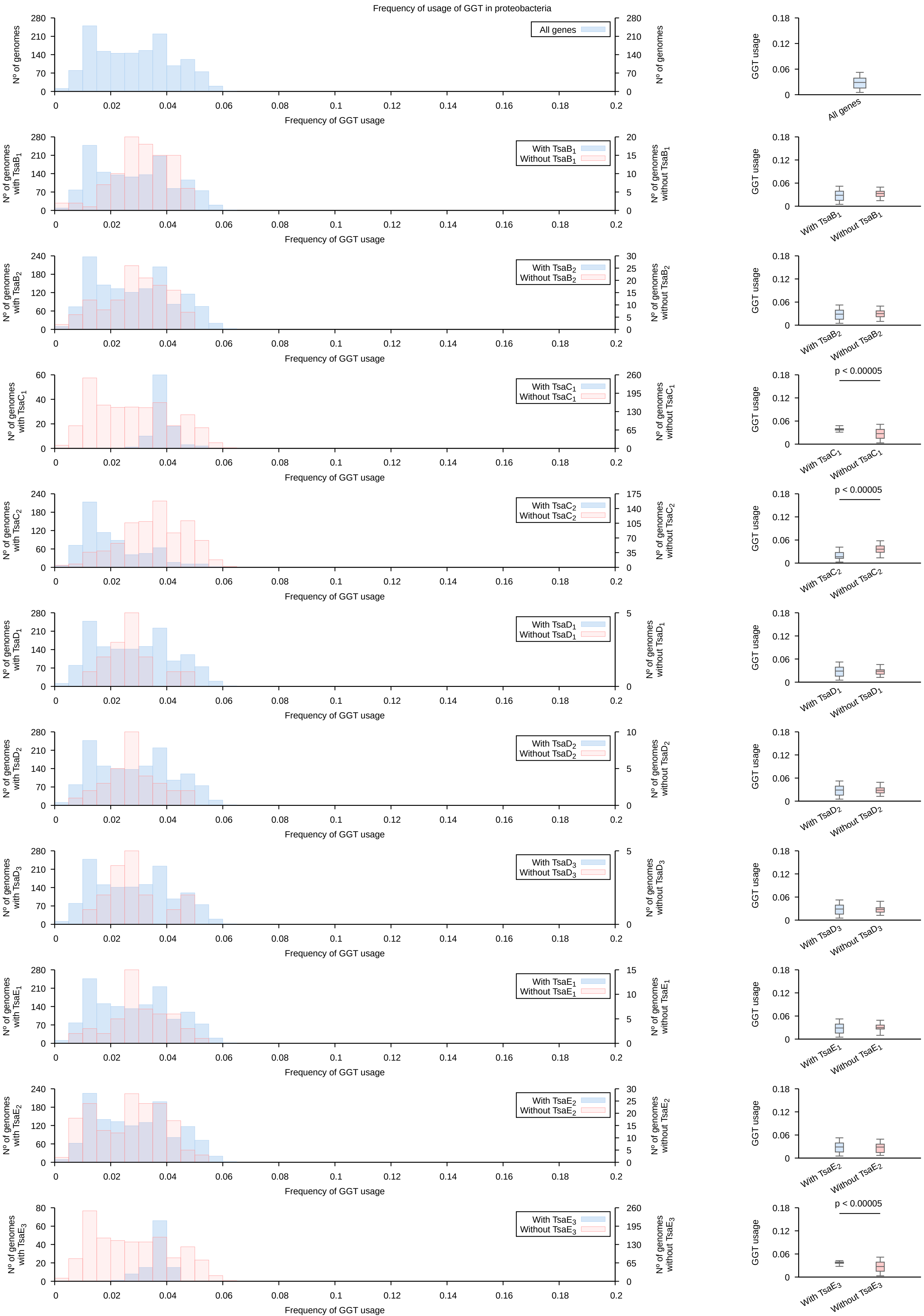
Frequency of usage of GGT in proteobacteria
Nº of genomes
All genes
Frequency of GGT usage
GGT usage
Nº of genomes with TsaB$_1$
Nº of genomes without TsaB$_1$
With TsaB$_1$
Without TsaB$_1$
Nº of genomes with TsaB$_2$
Nº of genomes without TsaB$_2$
With TsaB$_2$
Without TsaB$_2$
Nº of genomes with TsaC$_1$
Nº of genomes without TsaC$_1$
With TsaC$_1$
Without TsaC$_1$
p < 0.00005
Nº of genomes with TsaC$_2$
Nº of genomes without TsaC$_2$
With TsaC$_2$
Without TsaC$_2$
p < 0.00005
Nº of genomes with TsaD$_1$
Nº of genomes without TsaD$_1$
With TsaD$_1$
Without TsaD$_1$
Nº of genomes with TsaD$_2$
Nº of genomes without TsaD$_2$
With TsaD$_2$
Without TsaD$_2$
Nº of genomes with TsaD$_3$
Nº of genomes without TsaD$_3$
With TsaD$_3$
Without TsaD$_3$
Nº of genomes with TsaE$_1$
Nº of genomes without TsaE$_1$
With TsaE$_1$
Without TsaE$_1$
Nº of genomes with TsaE$_2$
Nº of genomes without TsaE$_2$
With TsaE$_2$
Without TsaE$_2$
Nº of genomes with TsaE$_3$
Nº of genomes without TsaE$_3$
With TsaE$_3$
Without TsaE$_3$
p < 0.00005

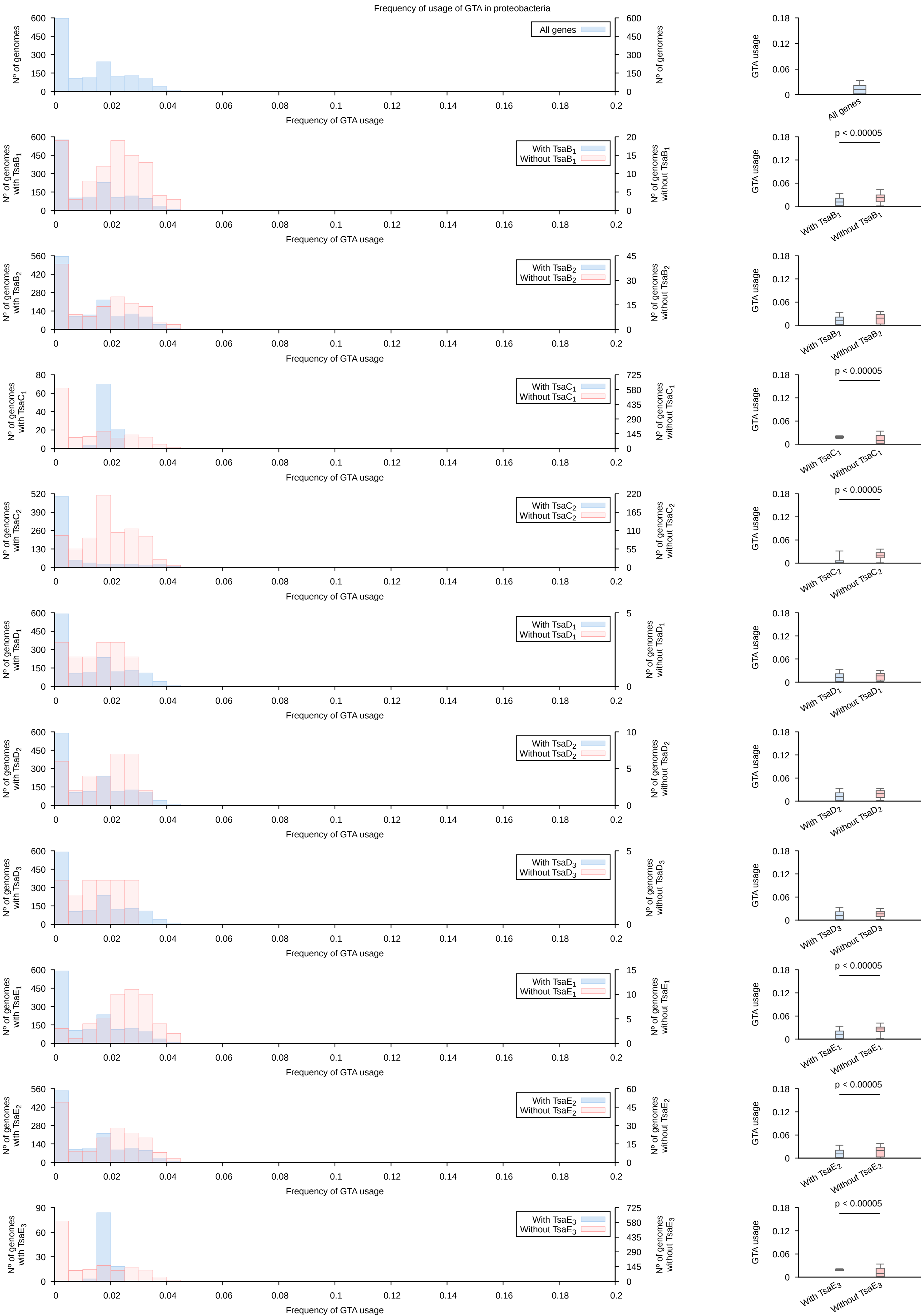
Frequency of usage of GTA in proteobacteria
All genes
Nº of genomes
Frequency of GTA usage
GTA usage
With $TsaB_1$
Without $TsaB_1$
Nº of genomes with $TsaB_1$
Nº of genomes without $TsaB_1$
With $TsaB_2$
Without $TsaB_2$
Nº of genomes with $TsaB_2$
Nº of genomes without $TsaB_2$
With $TsaC_1$
Without $TsaC_1$
Nº of genomes with $TsaC_1$
Nº of genomes without $TsaC_1$
With $TsaC_2$
Without $TsaC_2$
Nº of genomes with $TsaC_2$
Nº of genomes without $TsaC_2$
With $TsaD_1$
Without $TsaD_1$
Nº of genomes with $TsaD_1$
Nº of genomes without $TsaD_1$
With $TsaD_2$
Without $TsaD_2$
Nº of genomes with $TsaD_2$
Nº of genomes without $TsaD_2$
With $TsaD_3$
Without $TsaD_3$
Nº of genomes with $TsaD_3$
Nº of genomes without $TsaD_3$
With $TsaE_1$
Without $TsaE_1$
Nº of genomes with $TsaE_1$
Nº of genomes without $TsaE_1$
With $TsaE_2$
Without $TsaE_2$
Nº of genomes with $TsaE_2$
Nº of genomes without $TsaE_2$
With $TsaE_3$
Without $TsaE_3$
Nº of genomes with $TsaE_3$
Nº of genomes without $TsaE_3$
$p < 0.00005$

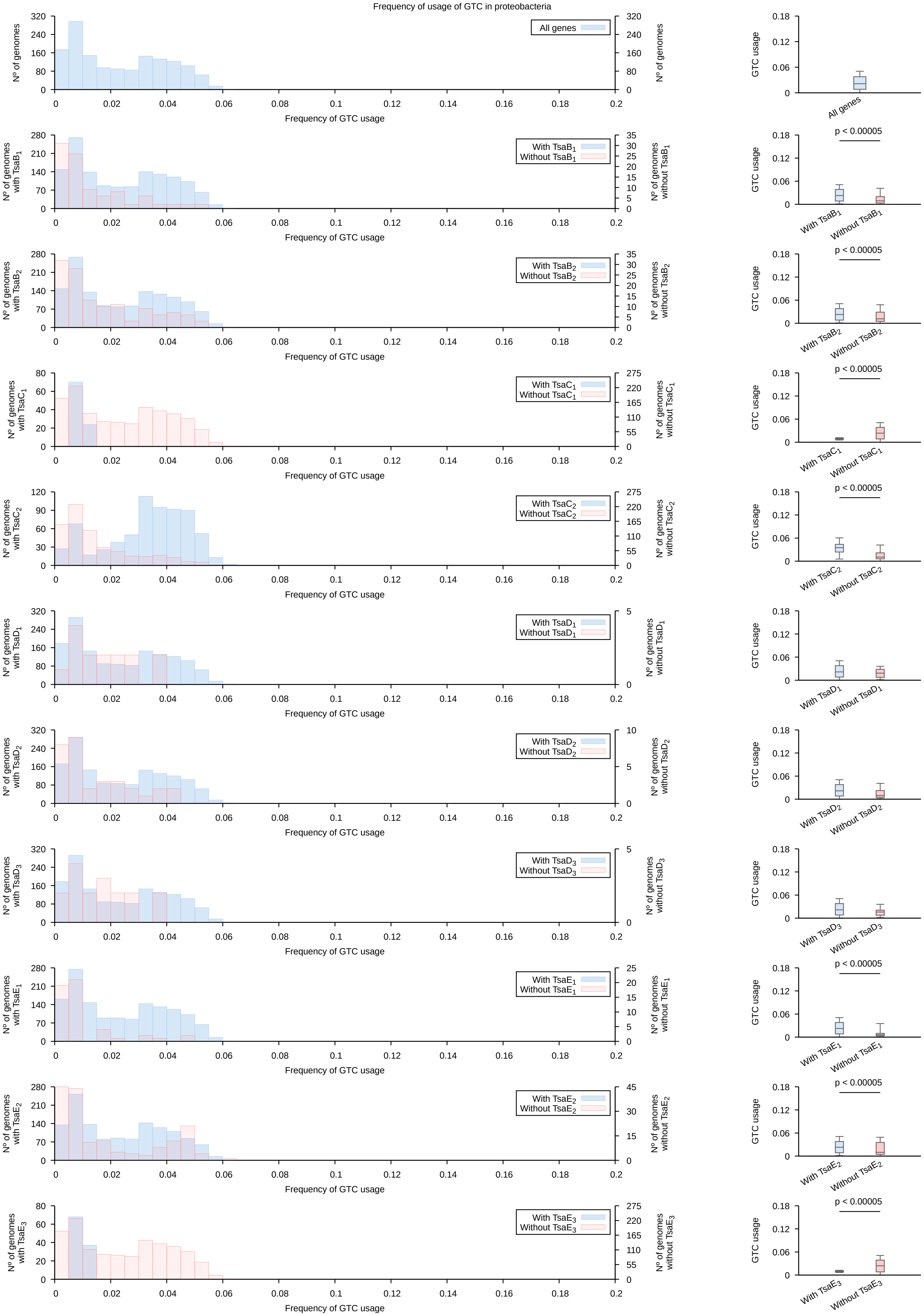

Frequency of usage of GTC in proteobacteria
Nº of genomes
All genes
Frequency of GTC usage
GTC usage
All genes
Nº of genomes with TsaB1
Nº of genomes without TsaB1
With TsaB1
Without TsaB1
p < 0.00005
Nº of genomes with TsaB2
Nº of genomes without TsaB2
With TsaB2
Without TsaB2
p < 0.00005
Nº of genomes with TsaC1
Nº of genomes without TsaC1
With TsaC1
Without TsaC1
p < 0.00005
Nº of genomes with TsaC2
Nº of genomes without TsaC2
With TsaC2
Without TsaC2
p < 0.00005
Nº of genomes with TsaD1
Nº of genomes without TsaD1
With TsaD1
Without TsaD1
Nº of genomes with TsaD2
Nº of genomes without TsaD2
With TsaD2
Without TsaD2
Nº of genomes with TsaD3
Nº of genomes without TsaD3
With TsaD3
Without TsaD3
Nº of genomes with TsaE1
Nº of genomes without TsaE1
With TsaE1
Without TsaE1
p < 0.00005
Nº of genomes with TsaE2
Nº of genomes without TsaE2
With TsaE2
Without TsaE2
p < 0.00005
Nº of genomes with TsaE3
Nº of genomes without TsaE3
With TsaE3
Without TsaE3
p < 0.00005

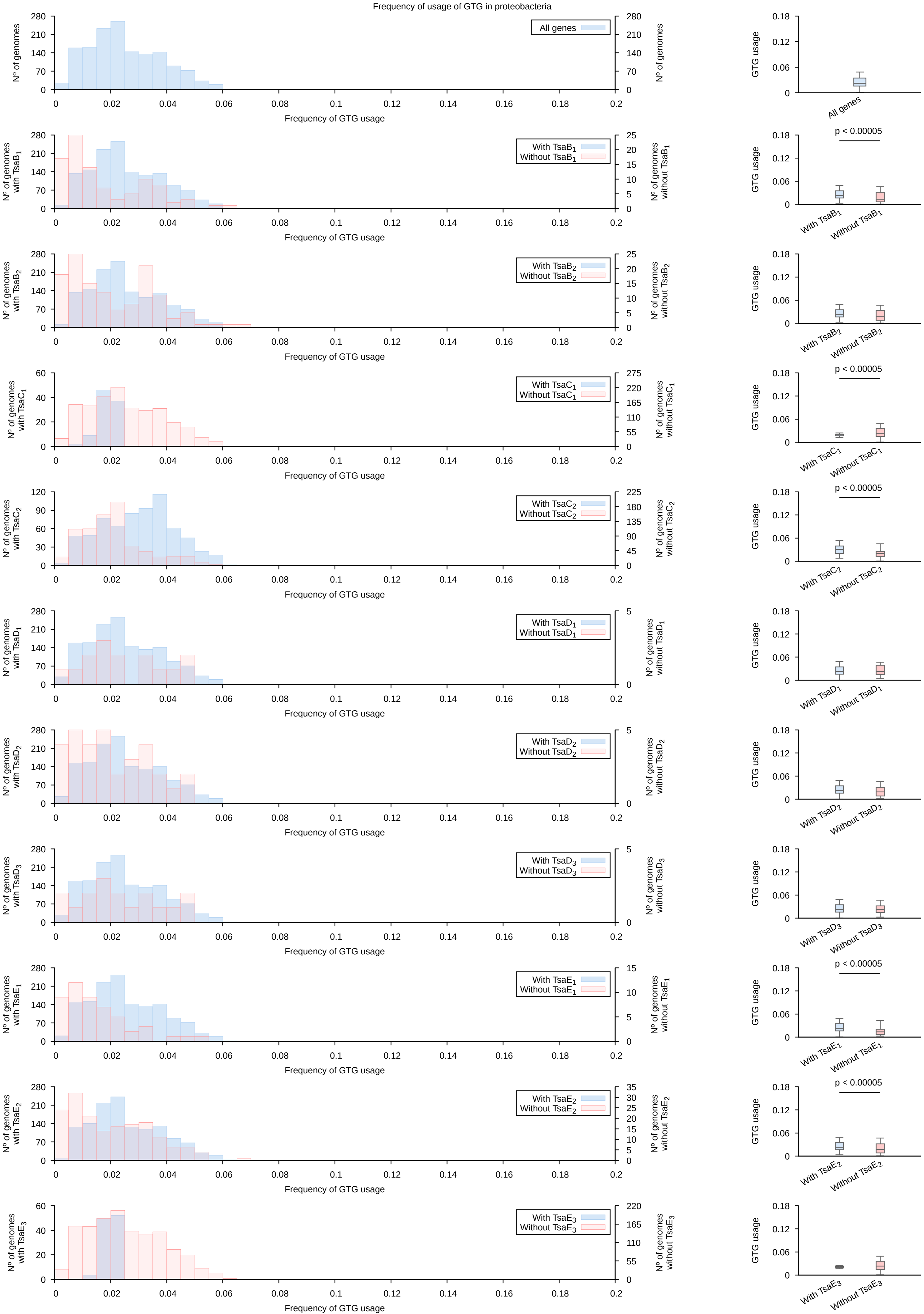
Frequency of usage of GTG in proteobacteria
Nº of genomes
All genes
Frequency of GTG usage
GTG usage
Nº of genomes with TsaB$_1$
Nº of genomes without TsaB$_1$
With TsaB$_1$
Without TsaB$_1$
p < 0.00005
Nº of genomes with TsaB$_2$
Nº of genomes without TsaB$_2$
With TsaB$_2$
Without TsaB$_2$
Nº of genomes with TsaC$_1$
Nº of genomes without TsaC$_1$
With TsaC$_1$
Without TsaC$_1$
p < 0.00005
Nº of genomes with TsaC$_2$
Nº of genomes without TsaC$_2$
With TsaC$_2$
Without TsaC$_2$
p < 0.00005
Nº of genomes with TsaD$_1$
Nº of genomes without TsaD$_1$
With TsaD$_1$
Without TsaD$_1$
Nº of genomes with TsaD$_2$
Nº of genomes without TsaD$_2$
With TsaD$_2$
Without TsaD$_2$
Nº of genomes with TsaD$_3$
Nº of genomes without TsaD$_3$
With TsaD$_3$
Without TsaD$_3$
Nº of genomes with TsaE$_1$
Nº of genomes without TsaE$_1$
With TsaE$_1$
Without TsaE$_1$
p < 0.00005
Nº of genomes with TsaE$_2$
Nº of genomes without TsaE$_2$
With TsaE$_2$
Without TsaE$_2$
p < 0.00005
Nº of genomes with TsaE$_3$
Nº of genomes without TsaE$_3$
With TsaE$_3$
Without TsaE$_3$

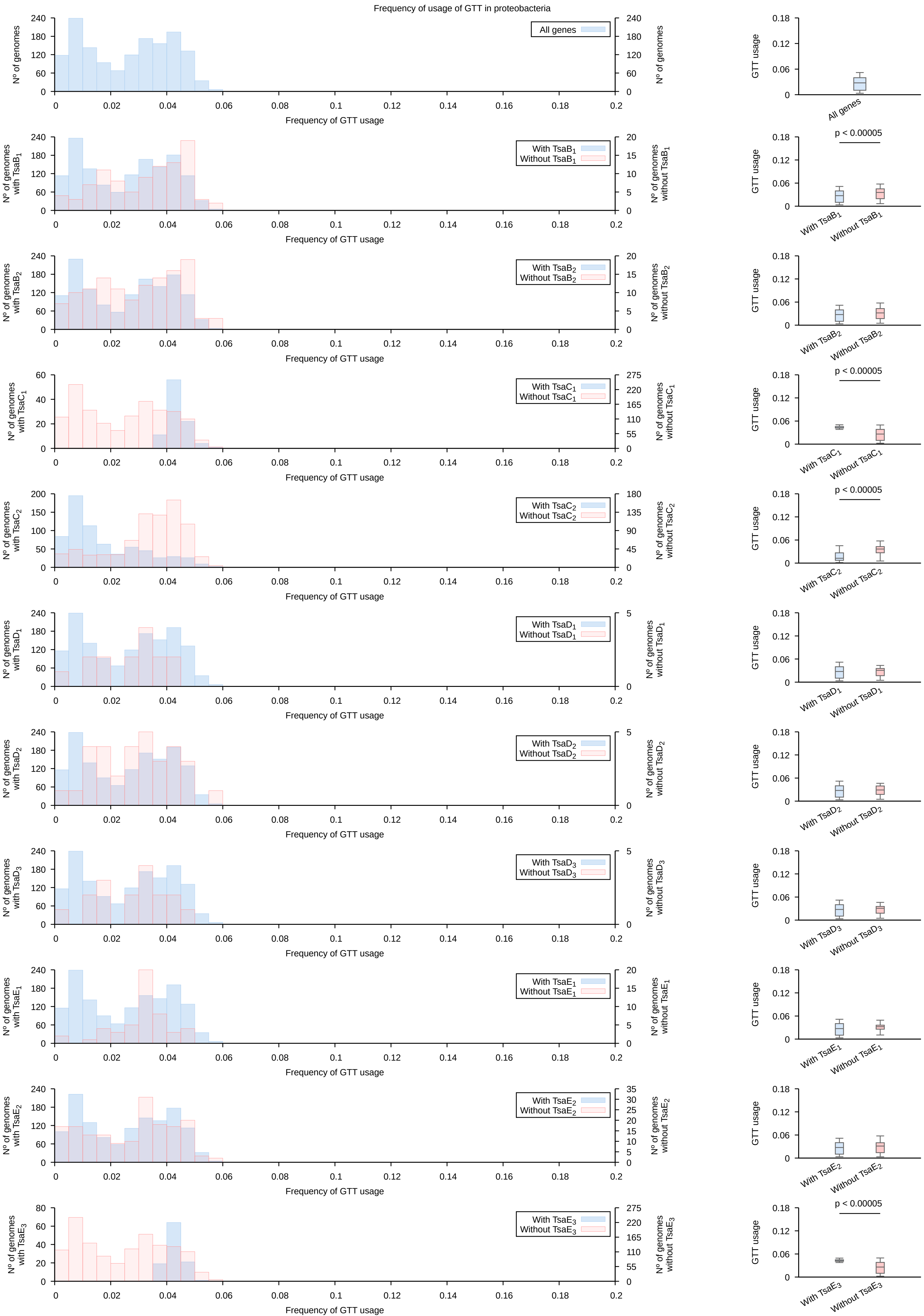
Frequency of usage of GTT in proteobacteria
Nº of genomes
All genes
Frequency of GTT usage
GTT usage
Nº of genomes with TsaB1
Nº of genomes without TsaB1
With TsaB1
Without TsaB1
p < 0.00005
Nº of genomes with TsaB2
Nº of genomes without TsaB2
With TsaB2
Without TsaB2
Nº of genomes with TsaC1
Nº of genomes without TsaC1
With TsaC1
Without TsaC1
p < 0.00005
Nº of genomes with TsaC2
Nº of genomes without TsaC2
With TsaC2
Without TsaC2
p < 0.00005
Nº of genomes with TsaD1
Nº of genomes without TsaD1
With TsaD1
Without TsaD1
Nº of genomes with TsaD2
Nº of genomes without TsaD2
With TsaD2
Without TsaD2
Nº of genomes with TsaD3
Nº of genomes without TsaD3
With TsaD3
Without TsaD3
Nº of genomes with TsaE1
Nº of genomes without TsaE1
With TsaE1
Without TsaE1
Nº of genomes with TsaE2
Nº of genomes without TsaE2
With TsaE2
Without TsaE2
Nº of genomes with TsaE3
Nº of genomes without TsaE3
With TsaE3
Without TsaE3
p < 0.00005

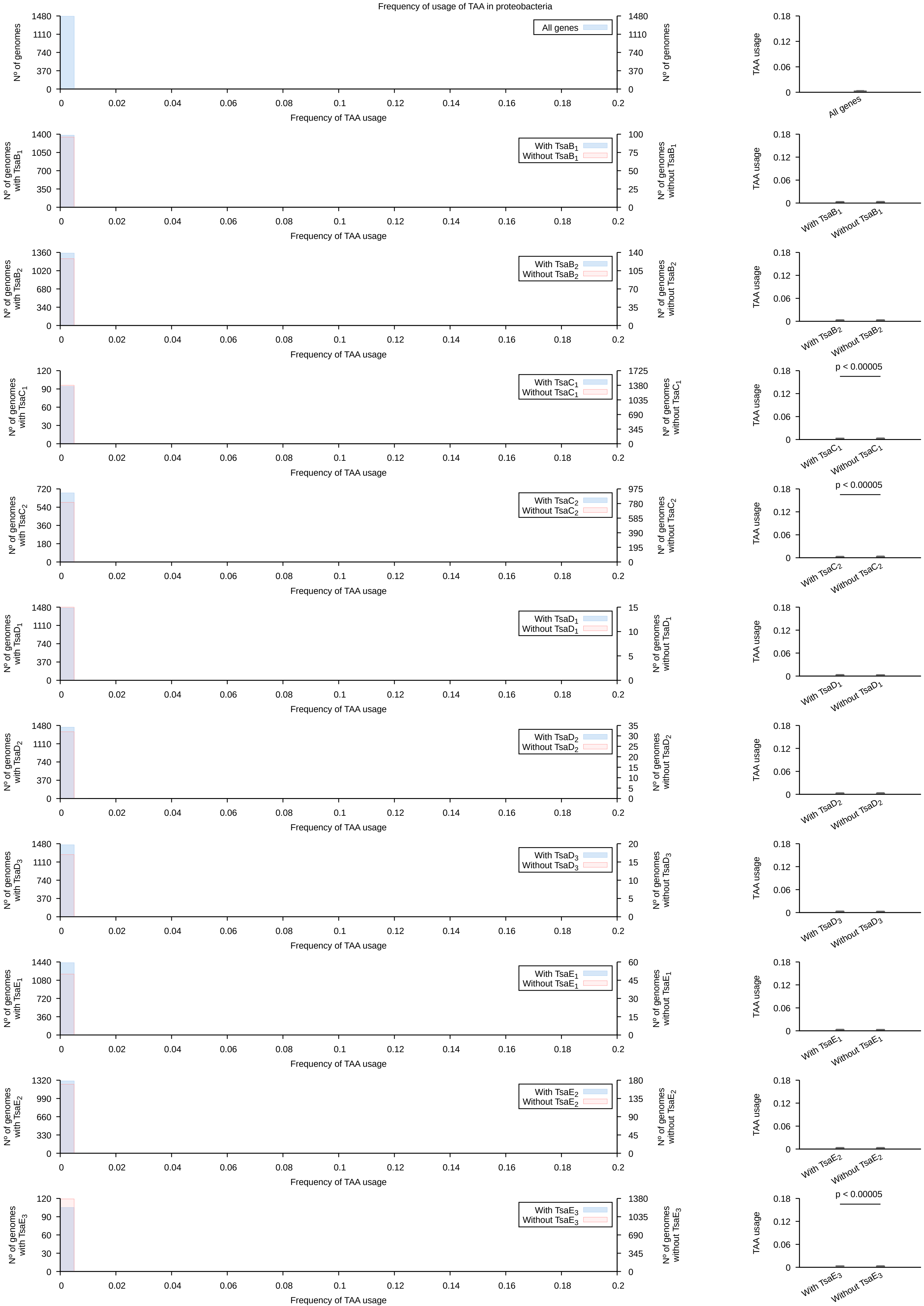
Frequency of usage of TAA in proteobacteria
Nº of genomes
All genes
Frequency of TAA usage
TAA usage
Nº of genomes with TsaB1
Nº of genomes without TsaB1
With TsaB1
Without TsaB1
Nº of genomes with TsaB2
Nº of genomes without TsaB2
With TsaB2
Without TsaB2
Nº of genomes with TsaC1
Nº of genomes without TsaC1
With TsaC1
Without TsaC1
p < 0.00005
Nº of genomes with TsaC2
Nº of genomes without TsaC2
With TsaC2
Without TsaC2
p < 0.00005
Nº of genomes with TsaD1
Nº of genomes without TsaD1
With TsaD1
Without TsaD1
Nº of genomes with TsaD2
Nº of genomes without TsaD2
With TsaD2
Without TsaD2
Nº of genomes with TsaD3
Nº of genomes without TsaD3
With TsaD3
Without TsaD3
Nº of genomes with TsaE1
Nº of genomes without TsaE1
With TsaE1
Without TsaE1
Nº of genomes with TsaE2
Nº of genomes without TsaE2
With TsaE2
Without TsaE2
Nº of genomes with TsaE3
Nº of genomes without TsaE3
With TsaE3
Without TsaE3
p < 0.00005

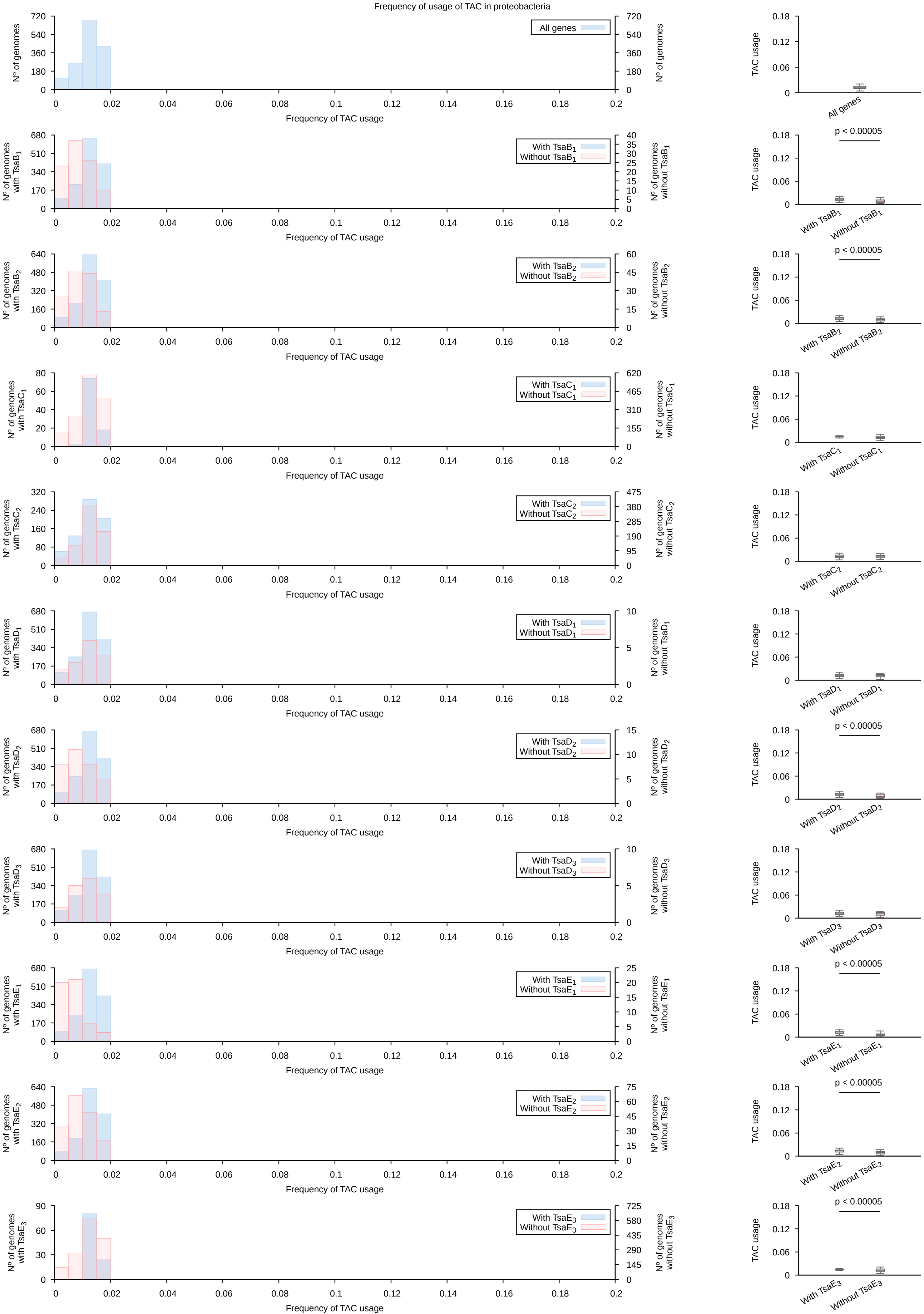

Frequency of usage of TAC in proteobacteria
Nº of genomes
All genes
Frequency of TAC usage
TAC usage
Nº of genomes with TsaB$_1$
Nº of genomes without TsaB$_1$
With TsaB$_1$
Without TsaB$_1$
p < 0.00005
Nº of genomes with TsaB$_2$
Nº of genomes without TsaB$_2$
With TsaB$_2$
Without TsaB$_2$
p < 0.00005
Nº of genomes with TsaC$_1$
Nº of genomes without TsaC$_1$
With TsaC$_1$
Without TsaC$_1$
Nº of genomes with TsaC$_2$
Nº of genomes without TsaC$_2$
With TsaC$_2$
Without TsaC$_2$
Nº of genomes with TsaD$_1$
Nº of genomes without TsaD$_1$
With TsaD$_1$
Without TsaD$_1$
Nº of genomes with TsaD$_2$
Nº of genomes without TsaD$_2$
With TsaD$_2$
Without TsaD$_2$
p < 0.00005
Nº of genomes with TsaD$_3$
Nº of genomes without TsaD$_3$
With TsaD$_3$
Without TsaD$_3$
Nº of genomes with TsaE$_1$
Nº of genomes without TsaE$_1$
With TsaE$_1$
Without TsaE$_1$
p < 0.00005
Nº of genomes with TsaE$_2$
Nº of genomes without TsaE$_2$
With TsaE$_2$
Without TsaE$_2$
p < 0.00005
Nº of genomes with TsaE$_3$
Nº of genomes without TsaE$_3$
With TsaE$_3$
Without TsaE$_3$
p < 0.00005

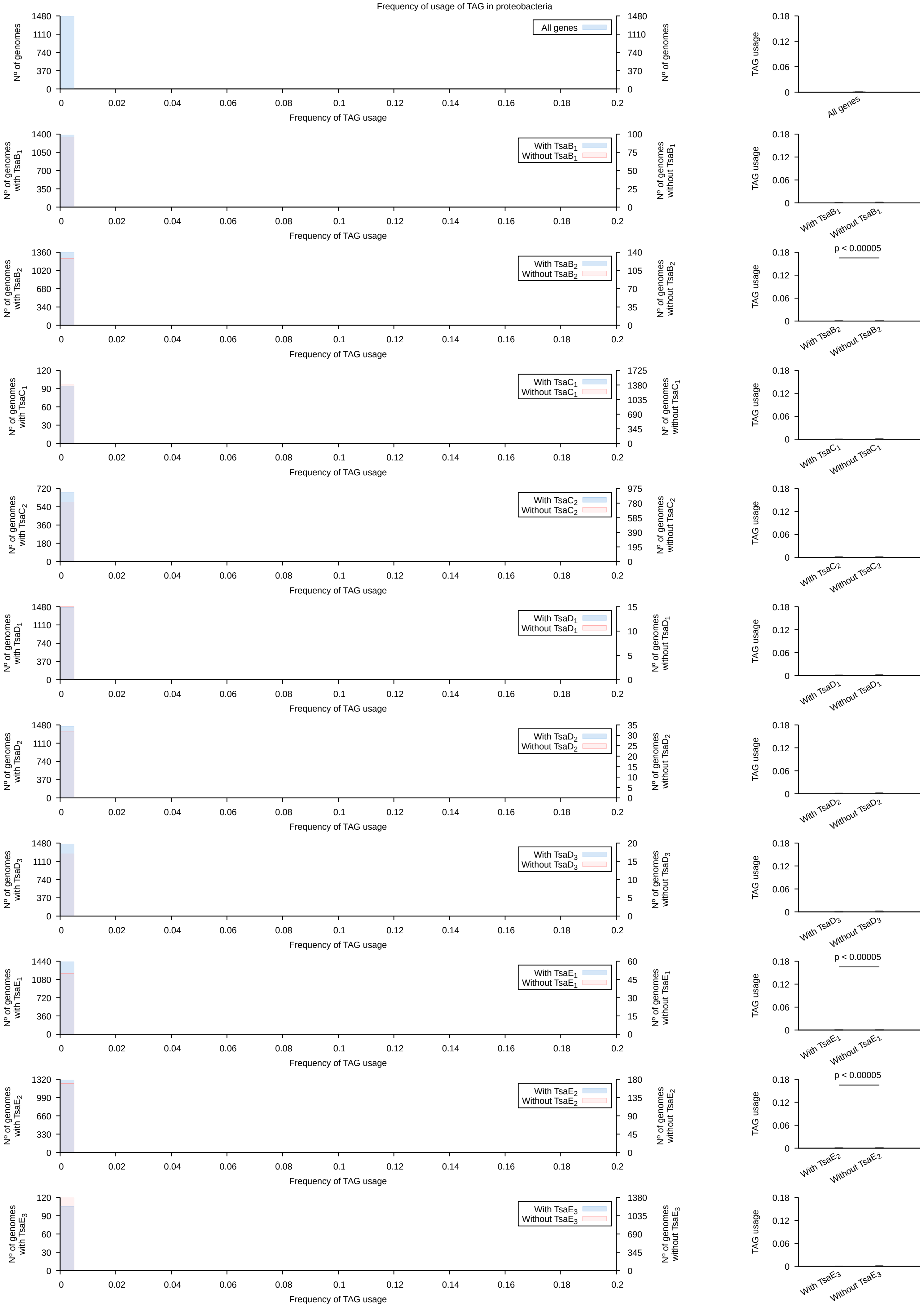
Frequency of usage of TAG in proteobacteria
Nº of genomes
All genes
Frequency of TAG usage
TAG usage
p < 0.00005
0
0.02
0.04
0.06
0.08
0.1
0.12
0.14
0.16
0.18
0.2
Nº of genomes with $TsaB_1$
Nº of genomes without $TsaB_1$
With $TsaB_1$
Without $TsaB_1$
Nº of genomes with $TsaB_2$
Nº of genomes without $TsaB_2$
With $TsaB_2$
Without $TsaB_2$
Nº of genomes with $TsaC_1$
Nº of genomes without $TsaC_1$
With $TsaC_1$
Without $TsaC_1$
Nº of genomes with $TsaC_2$
Nº of genomes without $TsaC_2$
With $TsaC_2$
Without $TsaC_2$
Nº of genomes with $TsaD_1$
Nº of genomes without $TsaD_1$
With $TsaD_1$
Without $TsaD_1$
Nº of genomes with $TsaD_2$
Nº of genomes without $TsaD_2$
With $TsaD_2$
Without $TsaD_2$
Nº of genomes with $TsaD_3$
Nº of genomes without $TsaD_3$
With $TsaD_3$
Without $TsaD_3$
Nº of genomes with $TsaE_1$
Nº of genomes without $TsaE_1$
With $TsaE_1$
Without $TsaE_1$
Nº of genomes with $TsaE_2$
Nº of genomes without $TsaE_2$
With $TsaE_2$
Without $TsaE_2$
Nº of genomes with $TsaE_3$
Nº of genomes without $TsaE_3$
With $TsaE_3$
Without $TsaE_3$

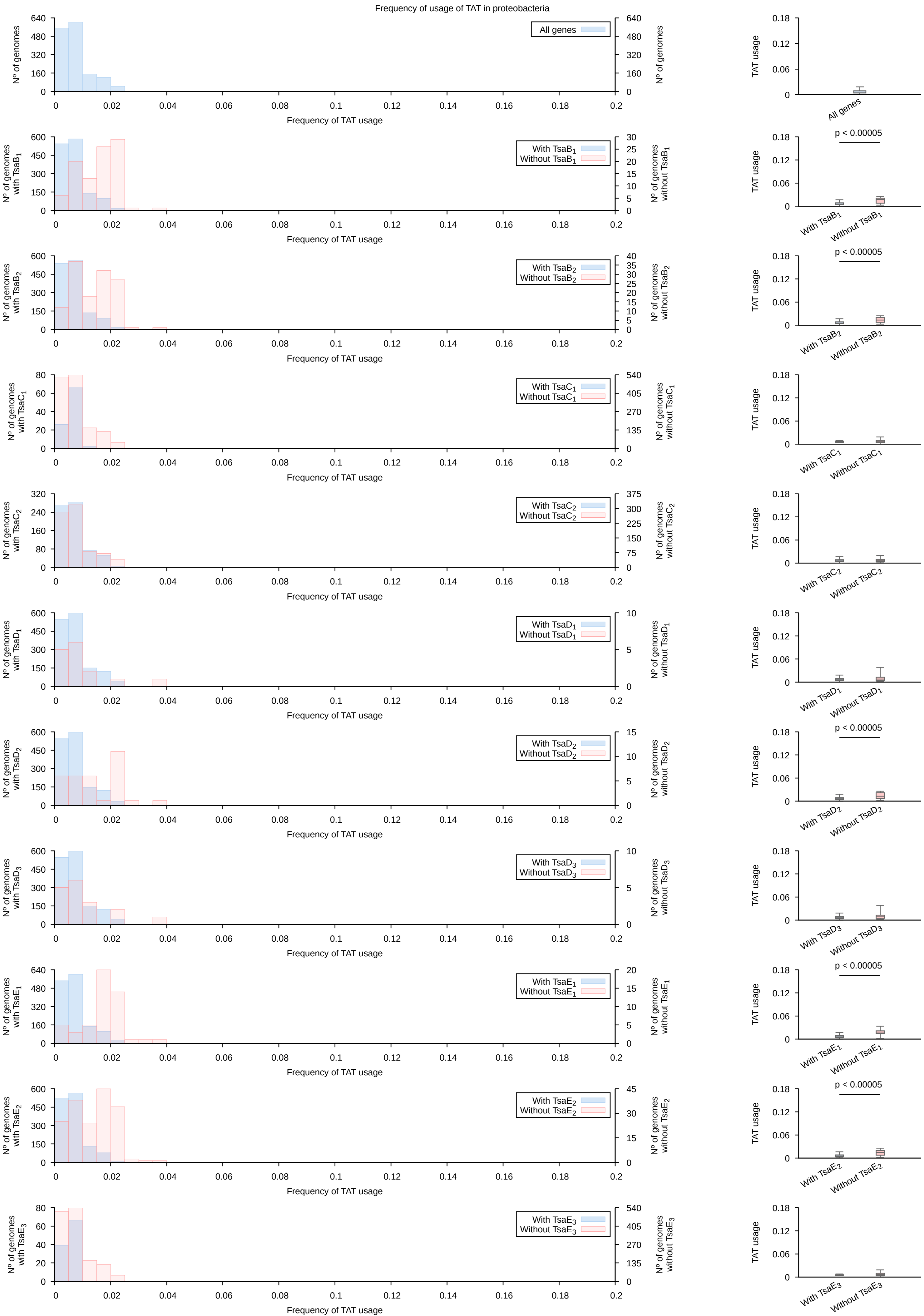

Frequency of usage of TAT in proteobacteria
Nº of genomes
Frequency of TAT usage
TAT usage
All genes
With TsaB$_1$
Without TsaB$_1$
Nº of genomes with TsaB$_1$
Nº of genomes without TsaB$_1$
p < 0.00005
With TsaB$_2$
Without TsaB$_2$
Nº of genomes with TsaB$_2$
Nº of genomes without TsaB$_2$
p < 0.00005
With TsaC$_1$
Without TsaC$_1$
Nº of genomes with TsaC$_1$
Nº of genomes without TsaC$_1$
With TsaC$_2$
Without TsaC$_2$
Nº of genomes with TsaC$_2$
Nº of genomes without TsaC$_2$
With TsaD$_1$
Without TsaD$_1$
Nº of genomes with TsaD$_1$
Nº of genomes without TsaD$_1$
With TsaD$_2$
Without TsaD$_2$
Nº of genomes with TsaD$_2$
Nº of genomes without TsaD$_2$
p < 0.00005
With TsaD$_3$
Without TsaD$_3$
Nº of genomes with TsaD$_3$
Nº of genomes without TsaD$_3$
With TsaE$_1$
Without TsaE$_1$
Nº of genomes with TsaE$_1$
Nº of genomes without TsaE$_1$
p < 0.00005
With TsaE$_2$
Without TsaE$_2$
Nº of genomes with TsaE$_2$
Nº of genomes without TsaE$_2$
p < 0.00005
With TsaE$_3$
Without TsaE$_3$
Nº of genomes with TsaE$_3$
Nº of genomes without TsaE$_3$

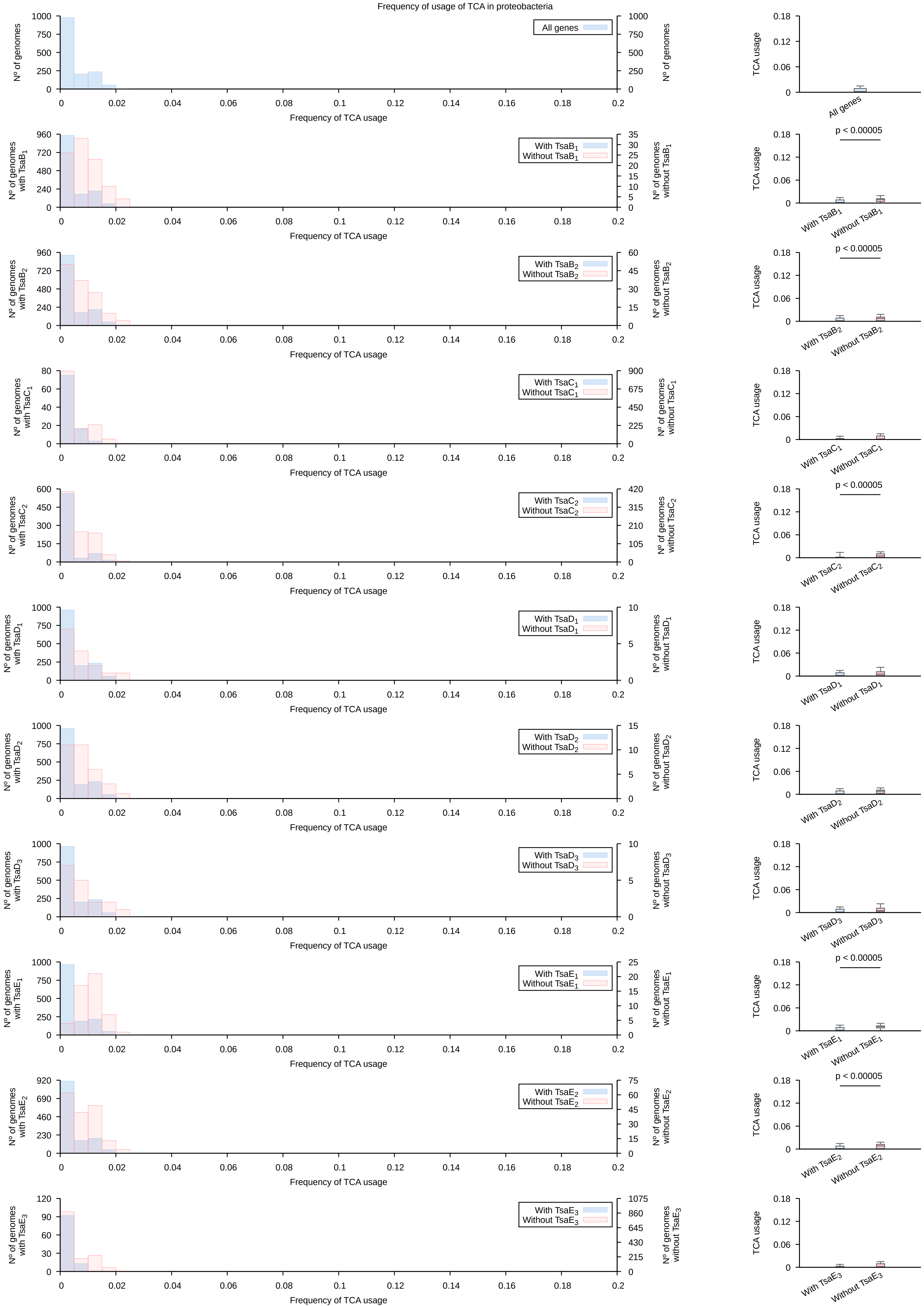
Frequency of usage of TCA in proteobacteria
Nº of genomes
All genes
Frequency of TCA usage
TCA usage
Nº of genomes with $TsaB_1$
Nº of genomes without $TsaB_1$
With $TsaB_1$
Without $TsaB_1$
$p < 0.00005$
Nº of genomes with $TsaB_2$
Nº of genomes without $TsaB_2$
With $TsaB_2$
Without $TsaB_2$
$p < 0.00005$
Nº of genomes with $TsaC_1$
Nº of genomes without $TsaC_1$
With $TsaC_1$
Without $TsaC_1$
Nº of genomes with $TsaC_2$
Nº of genomes without $TsaC_2$
With $TsaC_2$
Without $TsaC_2$
$p < 0.00005$
Nº of genomes with $TsaD_1$
Nº of genomes without $TsaD_1$
With $TsaD_1$
Without $TsaD_1$
Nº of genomes with $TsaD_2$
Nº of genomes without $TsaD_2$
With $TsaD_2$
Without $TsaD_2$
Nº of genomes with $TsaD_3$
Nº of genomes without $TsaD_3$
With $TsaD_3$
Without $TsaD_3$
Nº of genomes with $TsaE_1$
Nº of genomes without $TsaE_1$
With $TsaE_1$
Without $TsaE_1$
$p < 0.00005$
Nº of genomes with $TsaE_2$
Nº of genomes without $TsaE_2$
With $TsaE_2$
Without $TsaE_2$
$p < 0.00005$
Nº of genomes with $TsaE_3$
Nº of genomes without $TsaE_3$
With $TsaE_3$
Without $TsaE_3$

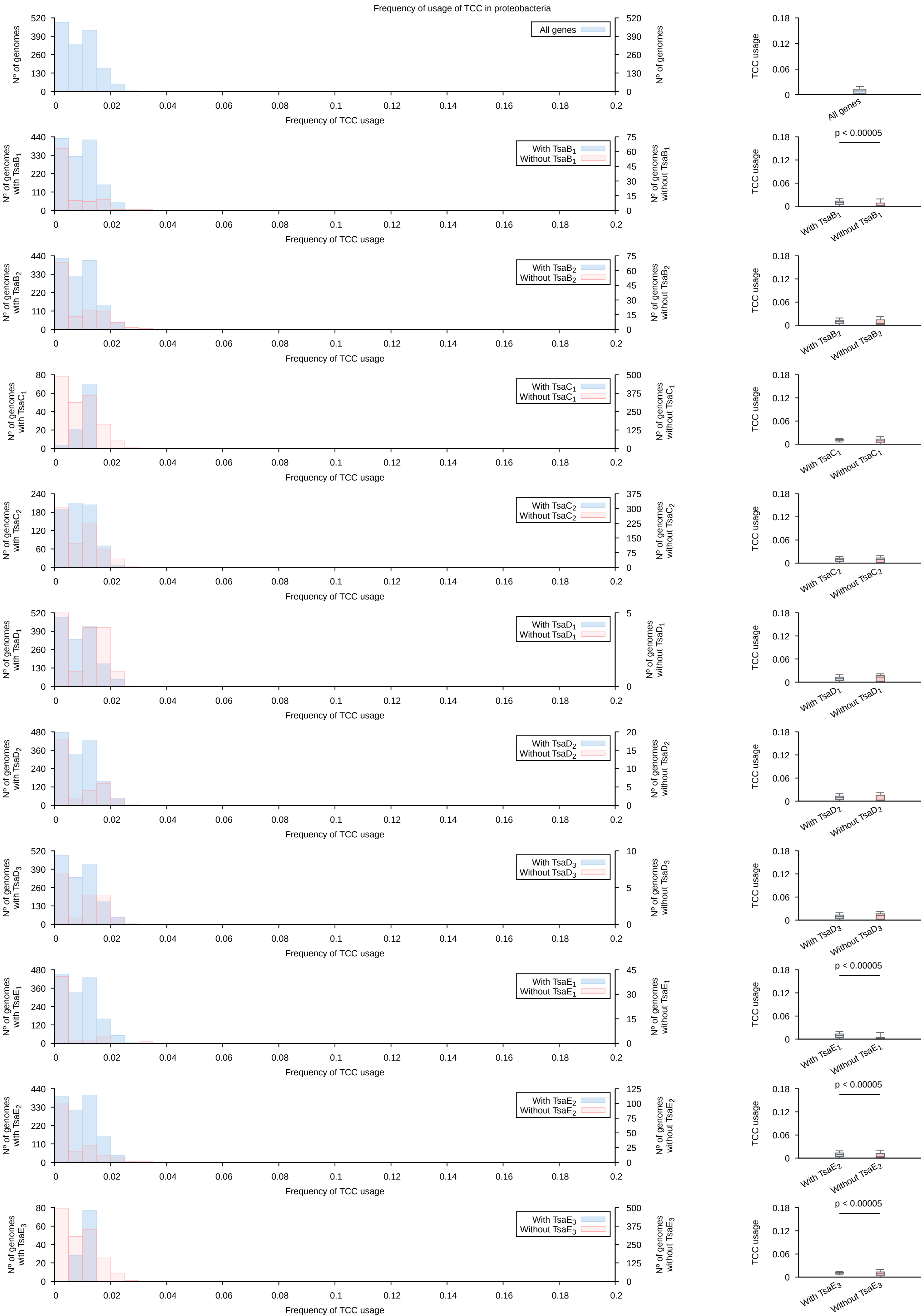
Frequency of usage of TCC in proteobacteria
All genes
Nº of genomes
Frequency of TCC usage
TCC usage
With TsaB$_1$
Without TsaB$_1$
Nº of genomes with TsaB$_1$
Nº of genomes without TsaB$_1$
p < 0.00005
With TsaB$_2$
Without TsaB$_2$
Nº of genomes with TsaB$_2$
Nº of genomes without TsaB$_2$
With TsaC$_1$
Without TsaC$_1$
Nº of genomes with TsaC$_1$
Nº of genomes without TsaC$_1$
With TsaC$_2$
Without TsaC$_2$
Nº of genomes with TsaC$_2$
Nº of genomes without TsaC$_2$
With TsaD$_1$
Without TsaD$_1$
Nº of genomes with TsaD$_1$
Nº of genomes without TsaD$_1$
With TsaD$_2$
Without TsaD$_2$
Nº of genomes with TsaD$_2$
Nº of genomes without TsaD$_2$
With TsaD$_3$
Without TsaD$_3$
Nº of genomes with TsaD$_3$
Nº of genomes without TsaD$_3$
With TsaE$_1$
Without TsaE$_1$
Nº of genomes with TsaE$_1$
Nº of genomes without TsaE$_1$
p < 0.00005
With TsaE$_2$
Without TsaE$_2$
Nº of genomes with TsaE$_2$
Nº of genomes without TsaE$_2$
p < 0.00005
With TsaE$_3$
Without TsaE$_3$
Nº of genomes with TsaE$_3$
Nº of genomes without TsaE$_3$
p < 0.00005

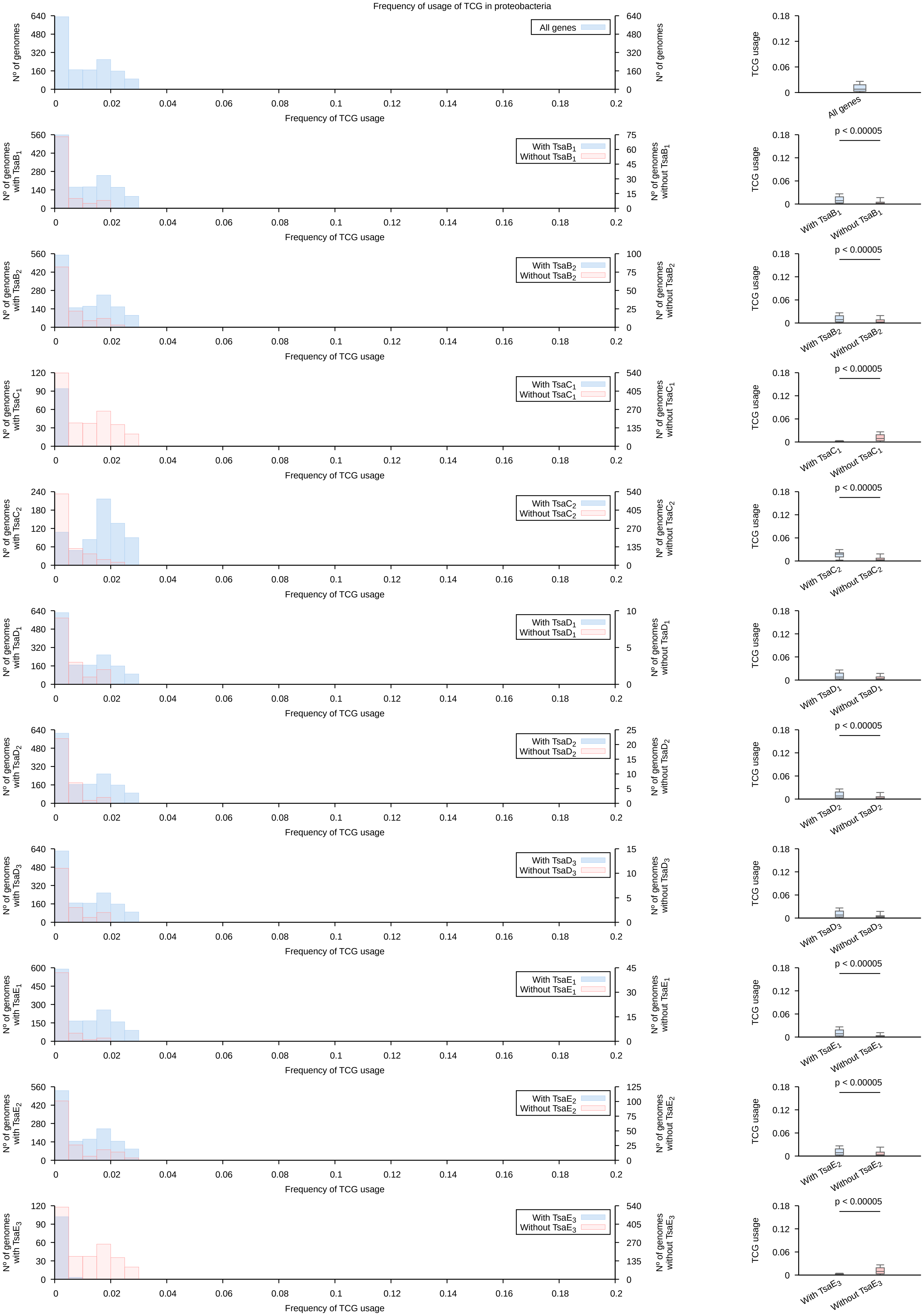
Frequency of usage of TCG in proteobacteria
Nº of genomes
All genes
Frequency of TCG usage
TCG usage
Nº of genomes with TsaB1
Nº of genomes without TsaB1
With TsaB1
Without TsaB1
p < 0.00005
Nº of genomes with TsaB2
Nº of genomes without TsaB2
With TsaB2
Without TsaB2
p < 0.00005
Nº of genomes with TsaC1
Nº of genomes without TsaC1
With TsaC1
Without TsaC1
p < 0.00005
Nº of genomes with TsaC2
Nº of genomes without TsaC2
With TsaC2
Without TsaC2
p < 0.00005
Nº of genomes with TsaD1
Nº of genomes without TsaD1
With TsaD1
Without TsaD1
Nº of genomes with TsaD2
Nº of genomes without TsaD2
With TsaD2
Without TsaD2
p < 0.00005
Nº of genomes with TsaD3
Nº of genomes without TsaD3
With TsaD3
Without TsaD3
Nº of genomes with TsaE1
Nº of genomes without TsaE1
With TsaE1
Without TsaE1
p < 0.00005
Nº of genomes with TsaE2
Nº of genomes without TsaE2
With TsaE2
Without TsaE2
p < 0.00005
Nº of genomes with TsaE3
Nº of genomes without TsaE3
With TsaE3
Without TsaE3
p < 0.00005

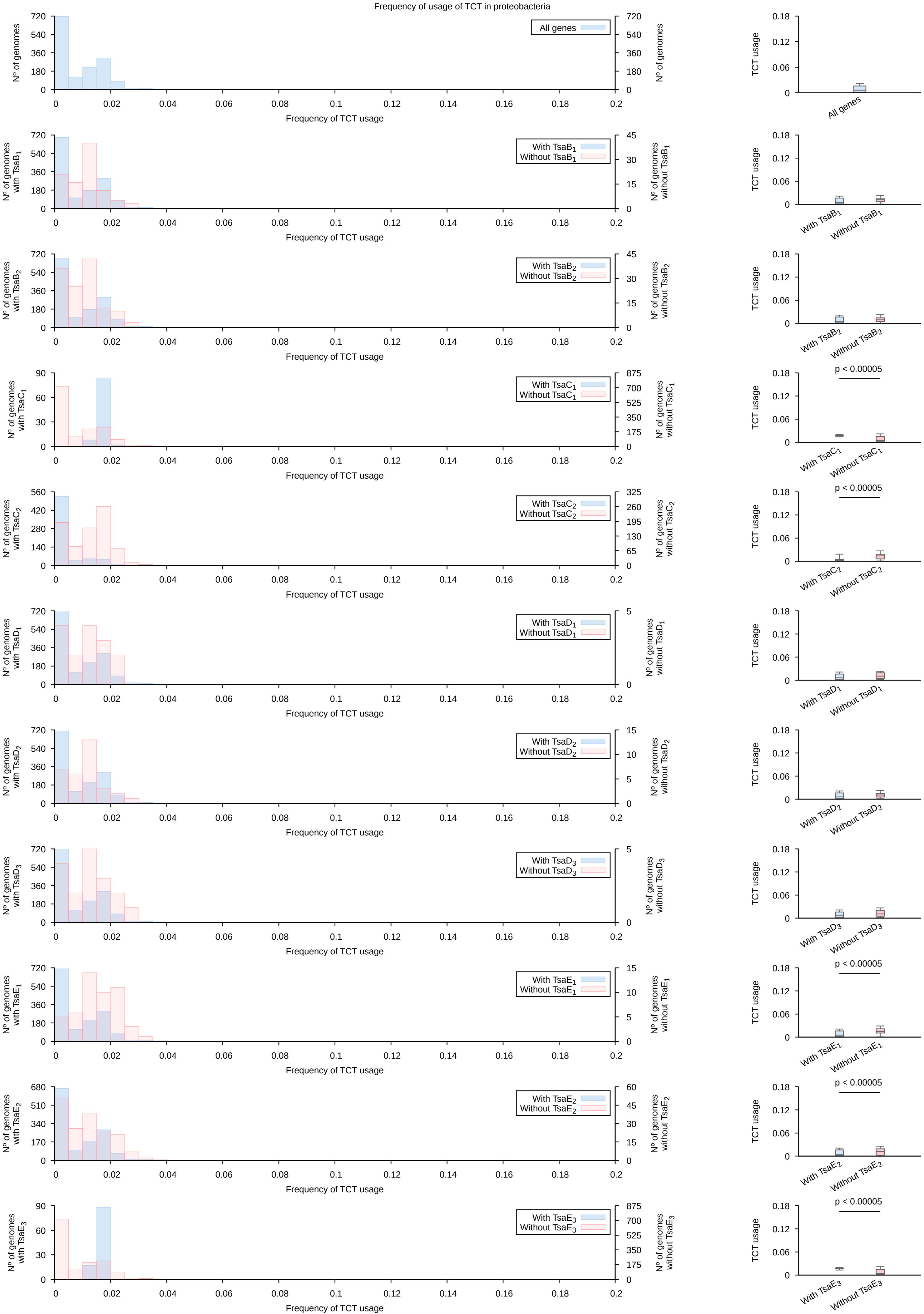

Frequency of usage of TCT in proteobacteria
All genes
Nº of genomes
Frequency of TCT usage
TCT usage
With TsaB$_1$
Without TsaB$_1$
Nº of genomes with TsaB$_1$
Nº of genomes without TsaB$_1$
With TsaB$_2$
Without TsaB$_2$
Nº of genomes with TsaB$_2$
Nº of genomes without TsaB$_2$
With TsaC$_1$
Without TsaC$_1$
Nº of genomes with TsaC$_1$
Nº of genomes without TsaC$_1$
p < 0.00005
With TsaC$_2$
Without TsaC$_2$
Nº of genomes with TsaC$_2$
Nº of genomes without TsaC$_2$
p < 0.00005
With TsaD$_1$
Without TsaD$_1$
Nº of genomes with TsaD$_1$
Nº of genomes without TsaD$_1$
With TsaD$_2$
Without TsaD$_2$
Nº of genomes with TsaD$_2$
Nº of genomes without TsaD$_2$
With TsaD$_3$
Without TsaD$_3$
Nº of genomes with TsaD$_3$
Nº of genomes without TsaD$_3$
With TsaE$_1$
Without TsaE$_1$
Nº of genomes with TsaE$_1$
Nº of genomes without TsaE$_1$
p < 0.00005
With TsaE$_2$
Without TsaE$_2$
Nº of genomes with TsaE$_2$
Nº of genomes without TsaE$_2$
p < 0.00005
With TsaE$_3$
Without TsaE$_3$
Nº of genomes with TsaE$_3$
Nº of genomes without TsaE$_3$
p < 0.00005

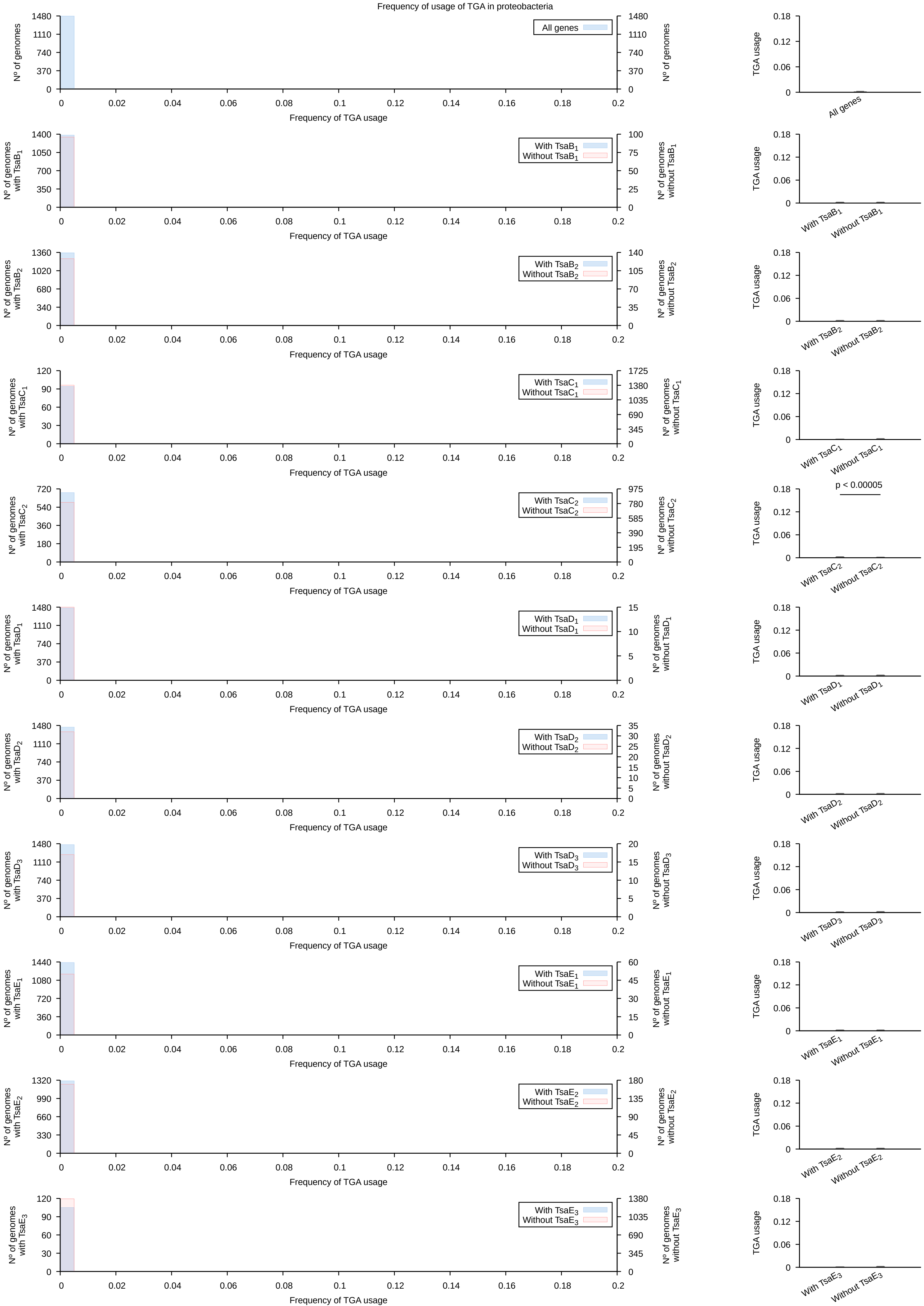
Frequency of usage of TGA in proteobacteria

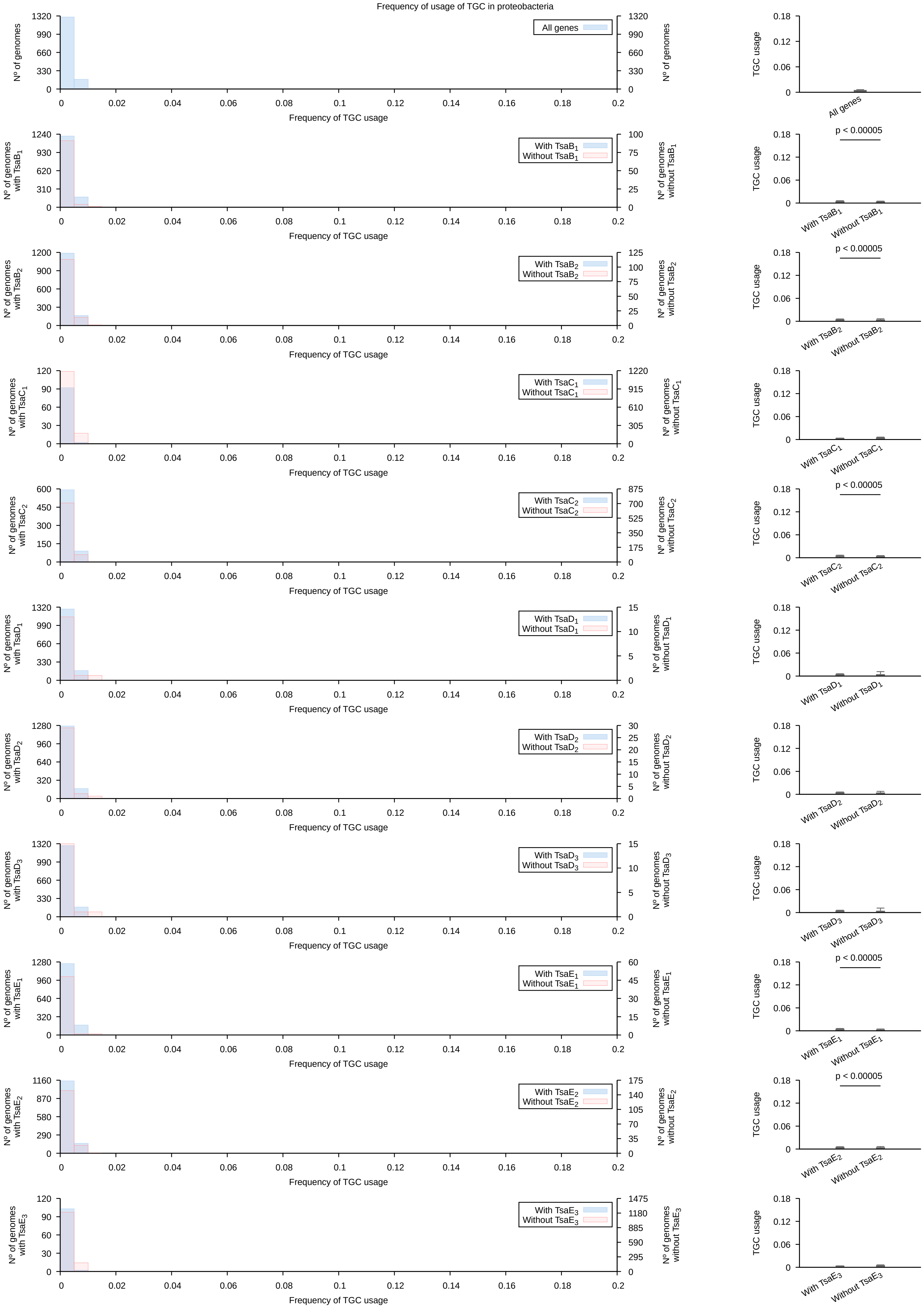
Frequency of usage of TGC in proteobacteria
All genes
With TsaB1
Without TsaB1
With TsaB2
Without TsaB2
With TsaC1
Without TsaC1
With TsaC2
Without TsaC2
With TsaD1
Without TsaD1
With TsaD2
Without TsaD2
With TsaD3
Without TsaD3
With TsaE1
Without TsaE1
With TsaE2
Without TsaE2
With TsaE3
Without TsaE3
Nº of genomes
Frequency of TGC usage
TGC usage
p < 0.00005

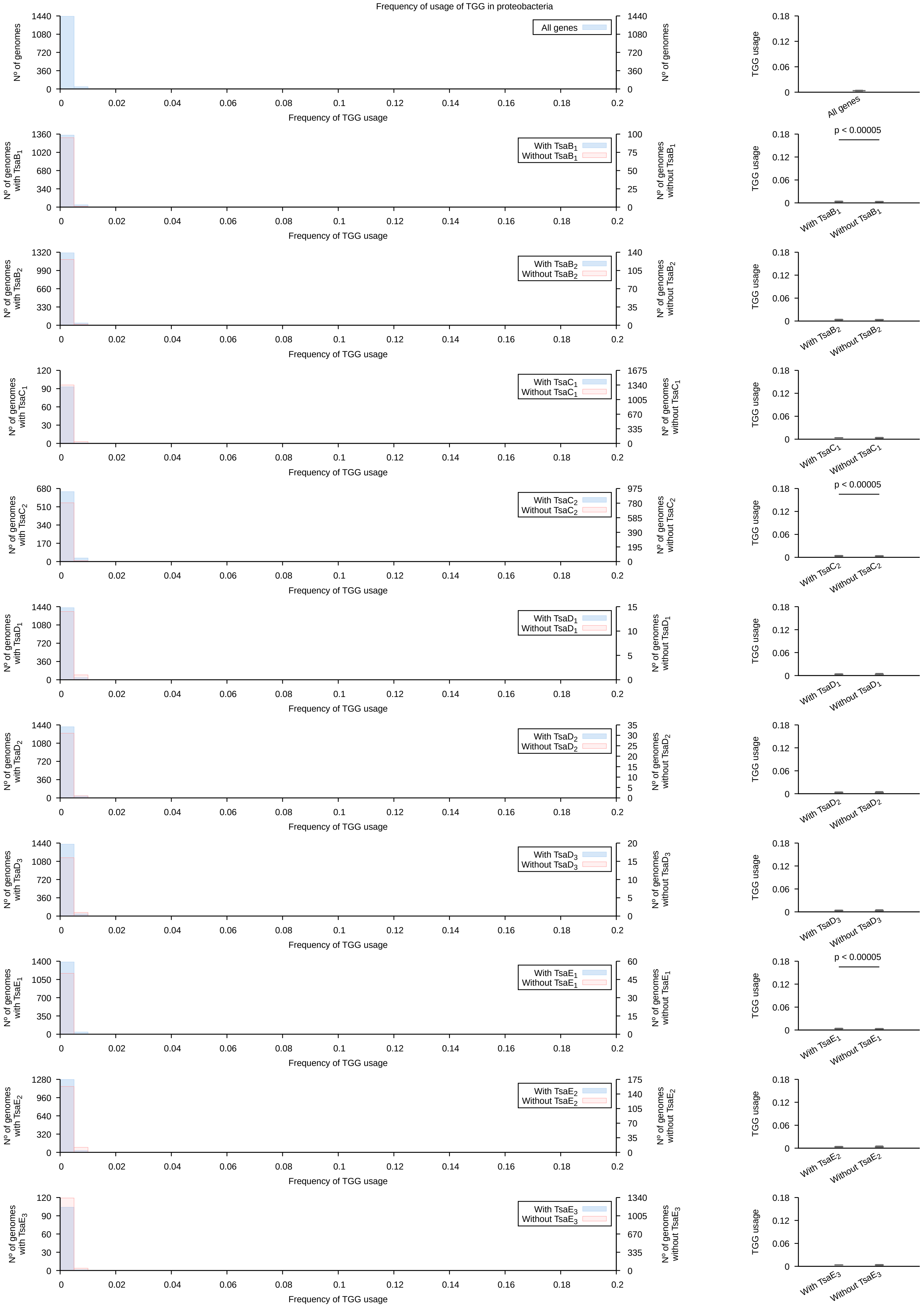
Frequency of usage of TGG in proteobacteria
All genes
Nº of genomes
Frequency of TGG usage
TGG usage
p < 0.00005
With TsaB$_1$
Without TsaB$_1$
Nº of genomes with TsaB$_1$
Nº of genomes without TsaB$_1$
With TsaB$_2$
Without TsaB$_2$
Nº of genomes with TsaB$_2$
Nº of genomes without TsaB$_2$
With TsaC$_1$
Without TsaC$_1$
Nº of genomes with TsaC$_1$
Nº of genomes without TsaC$_1$
With TsaC$_2$
Without TsaC$_2$
Nº of genomes with TsaC$_2$
Nº of genomes without TsaC$_2$
With TsaD$_1$
Without TsaD$_1$
Nº of genomes with TsaD$_1$
Nº of genomes without TsaD$_1$
With TsaD$_2$
Without TsaD$_2$
Nº of genomes with TsaD$_2$
Nº of genomes without TsaD$_2$
With TsaD$_3$
Without TsaD$_3$
Nº of genomes with TsaD$_3$
Nº of genomes without TsaD$_3$
With TsaE$_1$
Without TsaE$_1$
Nº of genomes with TsaE$_1$
Nº of genomes without TsaE$_1$
With TsaE$_2$
Without TsaE$_2$
Nº of genomes with TsaE$_2$
Nº of genomes without TsaE$_2$
With TsaE$_3$
Without TsaE$_3$
Nº of genomes with TsaE$_3$
Nº of genomes without TsaE$_3$

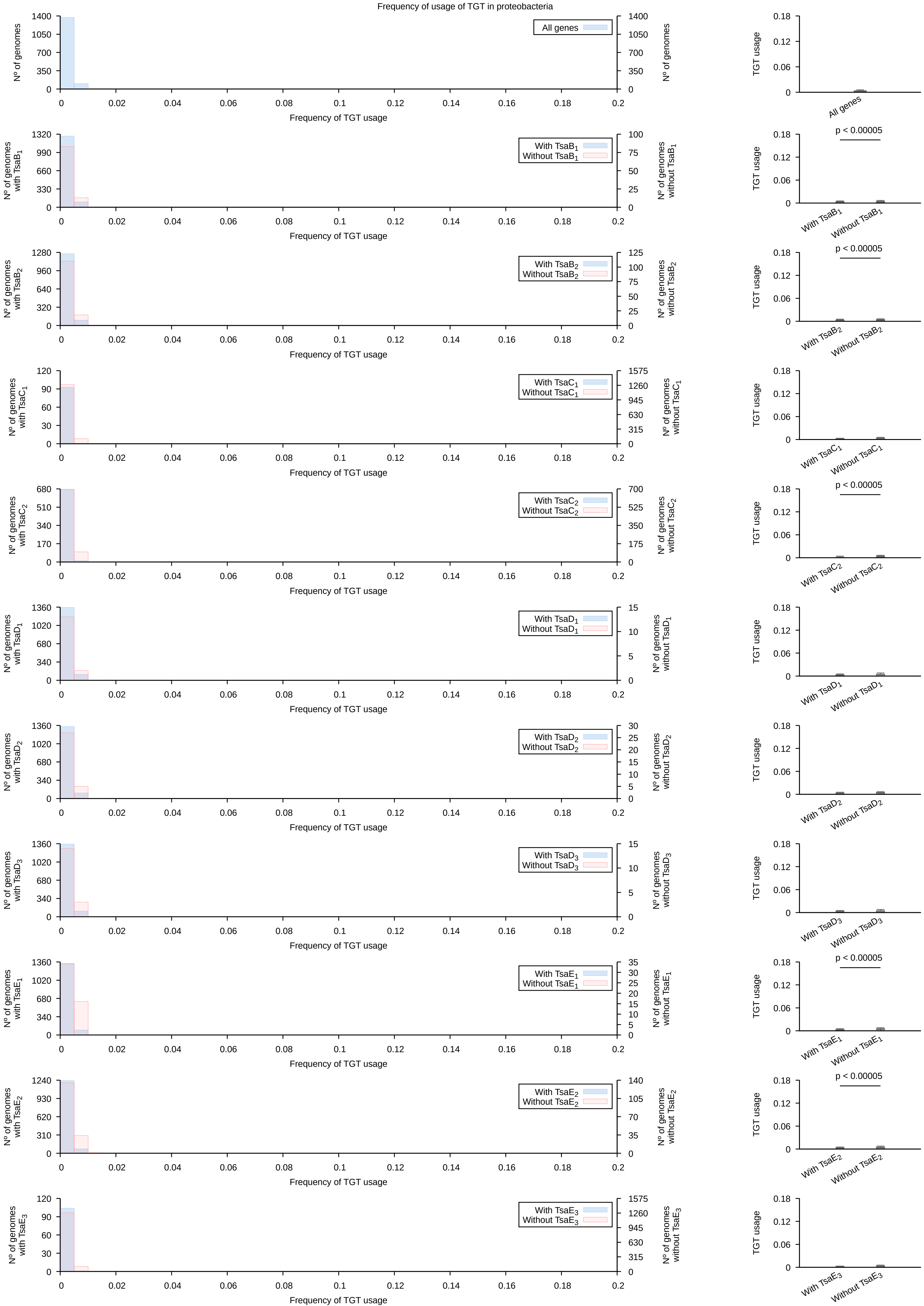
Frequency of usage of TGT in proteobacteria
All genes
Nº of genomes
Frequency of TGT usage
TGT usage
With TsaB1
Without TsaB1
p < 0.00005
With TsaB2
Without TsaB2
With TsaC1
Without TsaC1
With TsaC2
Without TsaC2
With TsaD1
Without TsaD1
With TsaD2
Without TsaD2
With TsaD3
Without TsaD3
With TsaE1
Without TsaE1
With TsaE2
Without TsaE2
With TsaE3
Without TsaE3

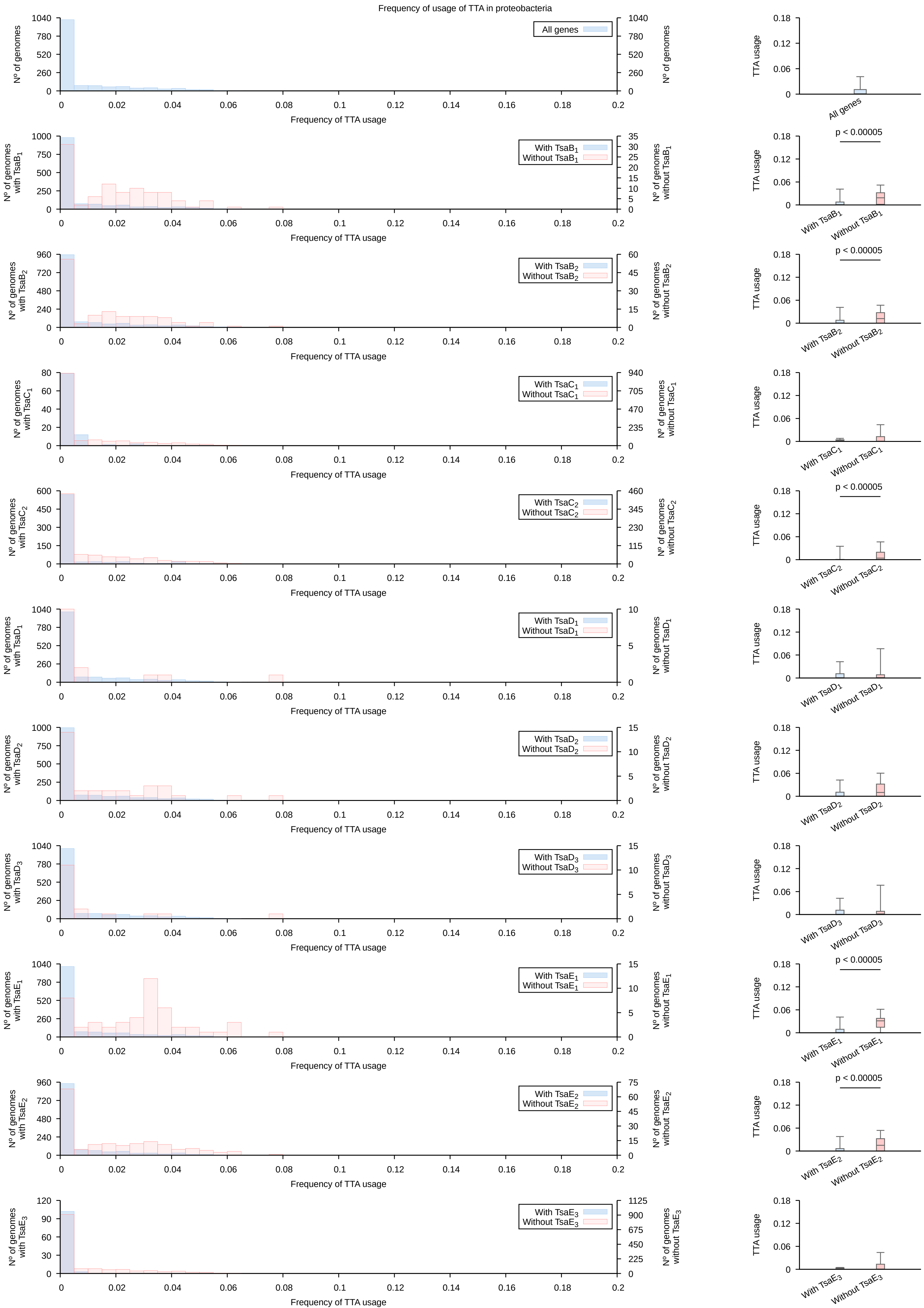

Frequency of usage of TTA in proteobacteria
All genes
Nº of genomes
Frequency of TTA usage
TTA usage
All genes
With $TsaB_1$
Without $TsaB_1$
Nº of genomes with $TsaB_1$
Nº of genomes without $TsaB_1$
p < 0.00005
With $TsaB_2$
Without $TsaB_2$
Nº of genomes with $TsaB_2$
Nº of genomes without $TsaB_2$
p < 0.00005
With $TsaC_1$
Without $TsaC_1$
Nº of genomes with $TsaC_1$
Nº of genomes without $TsaC_1$
With $TsaC_2$
Without $TsaC_2$
Nº of genomes with $TsaC_2$
Nº of genomes without $TsaC_2$
p < 0.00005
With $TsaD_1$
Without $TsaD_1$
Nº of genomes with $TsaD_1$
Nº of genomes without $TsaD_1$
With $TsaD_2$
Without $TsaD_2$
Nº of genomes with $TsaD_2$
Nº of genomes without $TsaD_2$
With $TsaD_3$
Without $TsaD_3$
Nº of genomes with $TsaD_3$
Nº of genomes without $TsaD_3$
With $TsaE_1$
Without $TsaE_1$
Nº of genomes with $TsaE_1$
Nº of genomes without $TsaE_1$
p < 0.00005
With $TsaE_2$
Without $TsaE_2$
Nº of genomes with $TsaE_2$
Nº of genomes without $TsaE_2$
p < 0.00005
With $TsaE_3$
Without $TsaE_3$
Nº of genomes with $TsaE_3$
Nº of genomes without $TsaE_3$

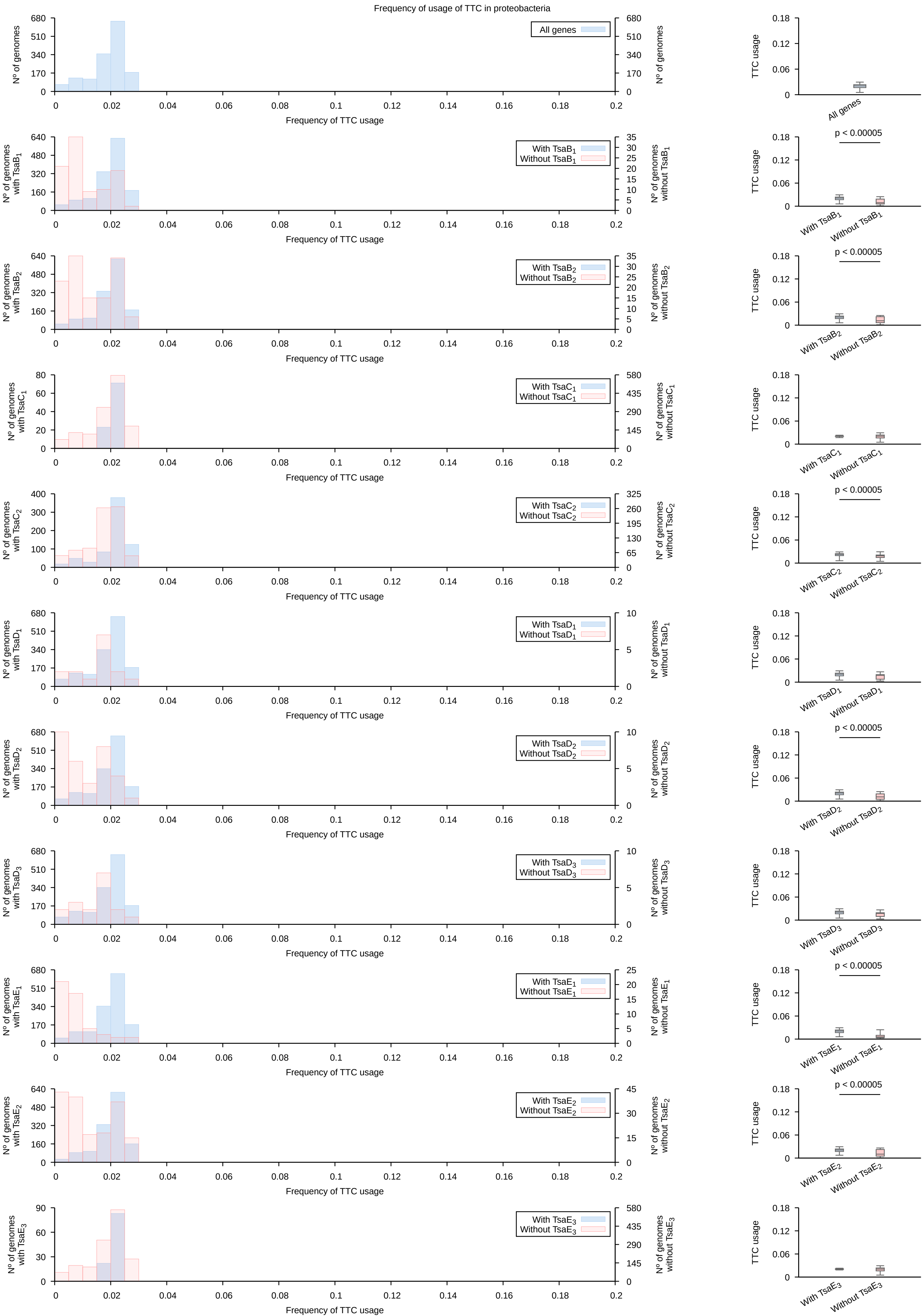

Frequency of usage of TTC in proteobacteria
All genes
Nº of genomes
Frequency of TTC usage
TTC usage
With TsaB1
Without TsaB1
Nº of genomes with TsaB1
Nº of genomes without TsaB1
p < 0.00005
With TsaB2
Without TsaB2
Nº of genomes with TsaB2
Nº of genomes without TsaB2
p < 0.00005
With TsaC1
Without TsaC1
Nº of genomes with TsaC1
Nº of genomes without TsaC1
With TsaC2
Without TsaC2
Nº of genomes with TsaC2
Nº of genomes without TsaC2
p < 0.00005
With TsaD1
Without TsaD1
Nº of genomes with TsaD1
Nº of genomes without TsaD1
With TsaD2
Without TsaD2
Nº of genomes with TsaD2
Nº of genomes without TsaD2
p < 0.00005
With TsaD3
Without TsaD3
Nº of genomes with TsaD3
Nº of genomes without TsaD3
With TsaE1
Without TsaE1
Nº of genomes with TsaE1
Nº of genomes without TsaE1
p < 0.00005
With TsaE2
Without TsaE2
Nº of genomes with TsaE2
Nº of genomes without TsaE2
p < 0.00005
With TsaE3
Without TsaE3
Nº of genomes with TsaE3
Nº of genomes without TsaE3

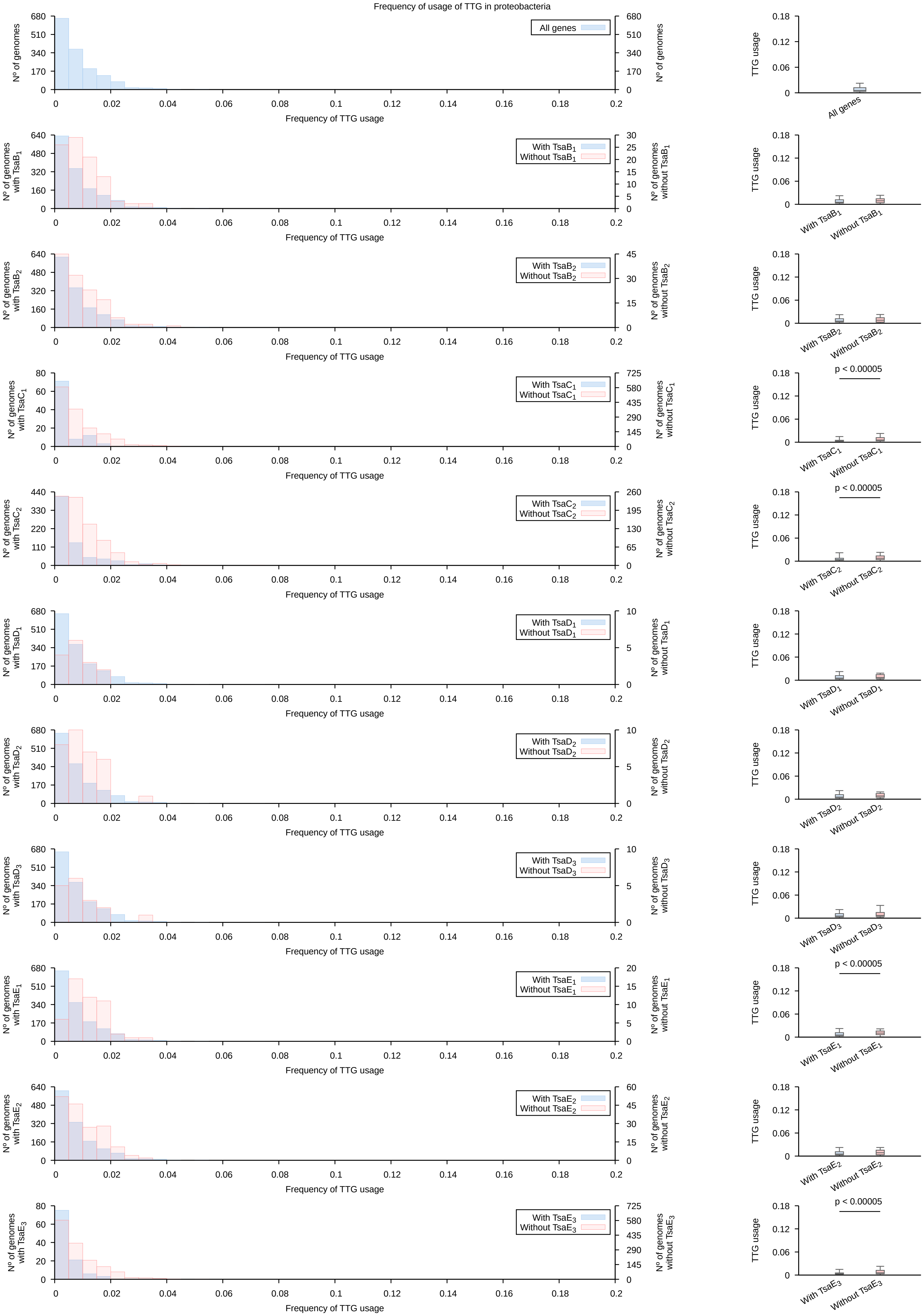
Frequency of usage of TTG in proteobacteria
Nº of genomes
All genes
TTG usage
Frequency of TTG usage
Nº of genomes with TsaB1
Nº of genomes without TsaB1
With TsaB1
Without TsaB1
Nº of genomes with TsaB2
Nº of genomes without TsaB2
With TsaB2
Without TsaB2
Nº of genomes with TsaC1
Nº of genomes without TsaC1
With TsaC1
Without TsaC1
p < 0.00005
Nº of genomes with TsaC2
Nº of genomes without TsaC2
With TsaC2
Without TsaC2
p < 0.00005
Nº of genomes with TsaD1
Nº of genomes without TsaD1
With TsaD1
Without TsaD1
Nº of genomes with TsaD2
Nº of genomes without TsaD2
With TsaD2
Without TsaD2
Nº of genomes with TsaD3
Nº of genomes without TsaD3
With TsaD3
Without TsaD3
Nº of genomes with TsaE1
Nº of genomes without TsaE1
With TsaE1
Without TsaE1
p < 0.00005
Nº of genomes with TsaE2
Nº of genomes without TsaE2
With TsaE2
Without TsaE2
Nº of genomes with TsaE3
Nº of genomes without TsaE3
With TsaE3
Without TsaE3
p < 0.00005

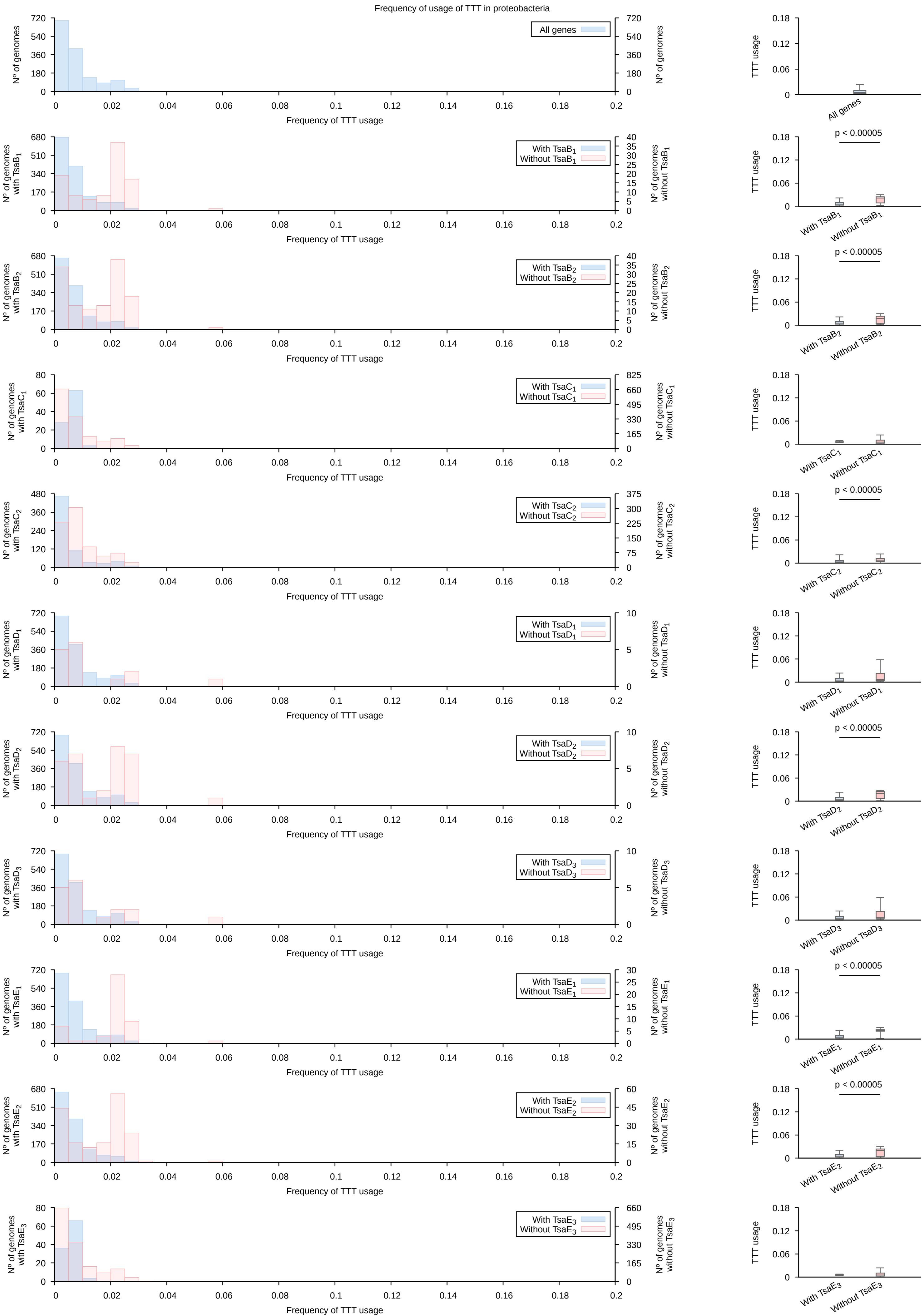
Frequency of usage of TTT in proteobacteria
All genes
Nº of genomes
Frequency of TTT usage
TTT usage
With TsaB$_1$
Without TsaB$_1$
Nº of genomes with TsaB$_1$
Nº of genomes without TsaB$_1$
p < 0.00005
With TsaB$_2$
Without TsaB$_2$
Nº of genomes with TsaB$_2$
Nº of genomes without TsaB$_2$
p < 0.00005
With TsaC$_1$
Without TsaC$_1$
Nº of genomes with TsaC$_1$
Nº of genomes without TsaC$_1$
With TsaC$_2$
Without TsaC$_2$
Nº of genomes with TsaC$_2$
Nº of genomes without TsaC$_2$
p < 0.00005
With TsaD$_1$
Without TsaD$_1$
Nº of genomes with TsaD$_1$
Nº of genomes without TsaD$_1$
With TsaD$_2$
Without TsaD$_2$
Nº of genomes with TsaD$_2$
Nº of genomes without TsaD$_2$
p < 0.00005
With TsaD$_3$
Without TsaD$_3$
Nº of genomes with TsaD$_3$
Nº of genomes without TsaD$_3$
With TsaE$_1$
Without TsaE$_1$
Nº of genomes with TsaE$_1$
Nº of genomes without TsaE$_1$
p < 0.00005
With TsaE$_2$
Without TsaE$_2$
Nº of genomes with TsaE$_2$
Nº of genomes without TsaE$_2$
p < 0.00005
With TsaE$_3$
Without TsaE$_3$
Nº of genomes with TsaE$_3$
Nº of genomes without TsaE$_3$
