# Supplementary material for: Impact of the chemical modification of tRNAs anticodon loop on the variability and evolution of codon usage in proteobacteria
Source: Front Microbiol. 2024 Aug 5;15:1412318. doi: 10.3389/fmicb.2024.1412318 (PMC11332805; doi:10.3389/fmicb.2024.1412318)
Supplement: Supplementary file 1 [file Data_Sheet_1.zip › Supp_figures/Fig_S21.pdf]

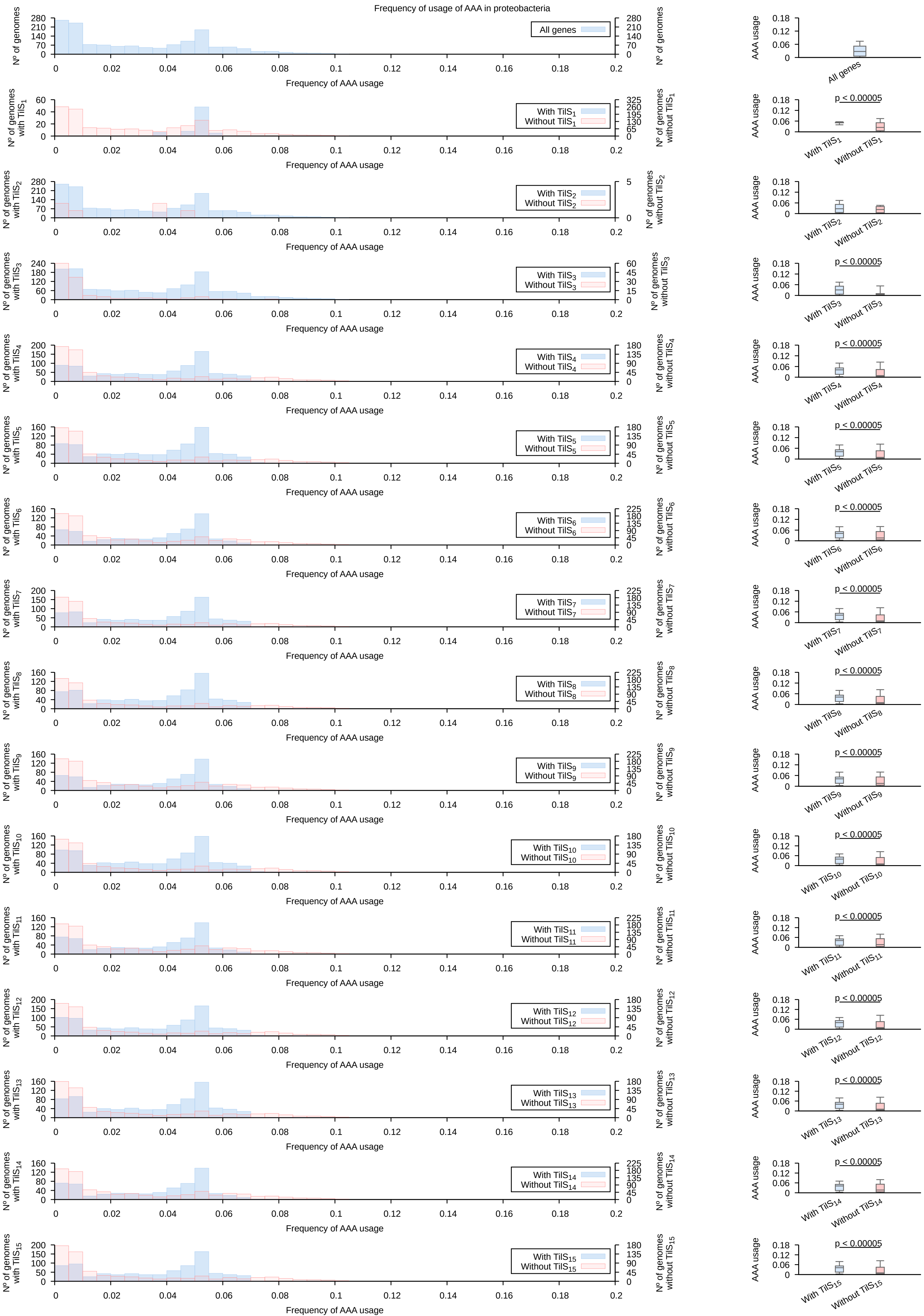

Frequency of usage of AAA in proteobacteria
Nº of genomes
All genes
Frequency of AAA usage
AAA usage
p < 0.00005
Nº of genomes with TilS$_1$
Nº of genomes without TilS$_1$
With TilS$_1$
Without TilS$_1$
Nº of genomes with TilS$_2$
Nº of genomes without TilS$_2$
With TilS$_2$
Without TilS$_2$
Nº of genomes with TilS$_3$
Nº of genomes without TilS$_3$
With TilS$_3$
Without TilS$_3$
Nº of genomes with TilS$_4$
Nº of genomes without TilS$_4$
With TilS$_4$
Without TilS$_4$
Nº of genomes with TilS$_5$
Nº of genomes without TilS$_5$
With TilS$_5$
Without TilS$_5$
Nº of genomes with TilS$_6$
Nº of genomes without TilS$_6$
With TilS$_6$
Without TilS$_6$
Nº of genomes with TilS$_7$
Nº of genomes without TilS$_7$
With TilS$_7$
Without TilS$_7$
Nº of genomes with TilS$_8$
Nº of genomes without TilS$_8$
With TilS$_8$
Without TilS$_8$
Nº of genomes with TilS$_9$
Nº of genomes without TilS$_9$
With TilS$_9$
Without TilS$_9$
Nº of genomes with TilS$_{10}$
Nº of genomes without TilS$_{10}$
With TilS$_{10}$
Without TilS$_{10}$
Nº of genomes with TilS$_{11}$
Nº of genomes without TilS$_{11}$
With TilS$_{11}$
Without TilS$_{11}$
Nº of genomes with TilS$_{12}$
Nº of genomes without TilS$_{12}$
With TilS$_{12}$
Without TilS$_{12}$
Nº of genomes with TilS$_{13}$
Nº of genomes without TilS$_{13}$
With TilS$_{13}$
Without TilS$_{13}$
Nº of genomes with TilS$_{14}$
Nº of genomes without TilS$_{14}$
With TilS$_{14}$
Without TilS$_{14}$
Nº of genomes with TilS$_{15}$
Nº of genomes without TilS$_{15}$
With TilS$_{15}$
Without TilS$_{15}$

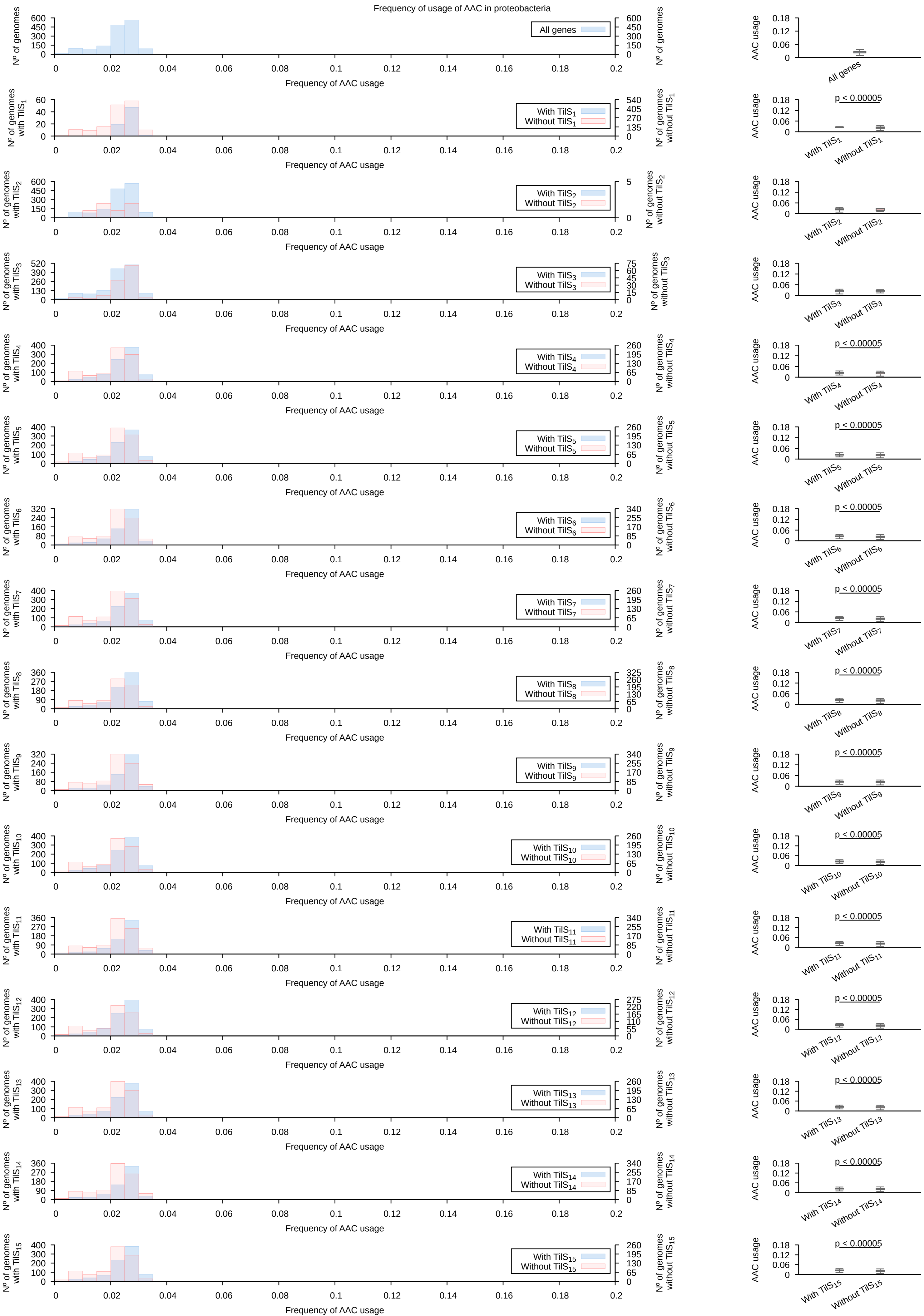
Frequency of usage of AAC in proteobacteria
Nº of genomes
All genes
Frequency of AAC usage
AAC usage
With TilS1
Without TilS1
Nº of genomes with TilS1
Nº of genomes without TilS1
With TilS2
Without TilS2
With TilS3
Without TilS3
With TilS4
Without TilS4
With TilS5
Without TilS5
With TilS6
Without TilS6
With TilS7
Without TilS7
With TilS8
Without TilS8
With TilS9
Without TilS9
With TilS10
Without TilS10
With TilS11
Without TilS11
With TilS12
Without TilS12
With TilS13
Without TilS13
With TilS14
Without TilS14
With TilS15
Without TilS15
p < 0.00005

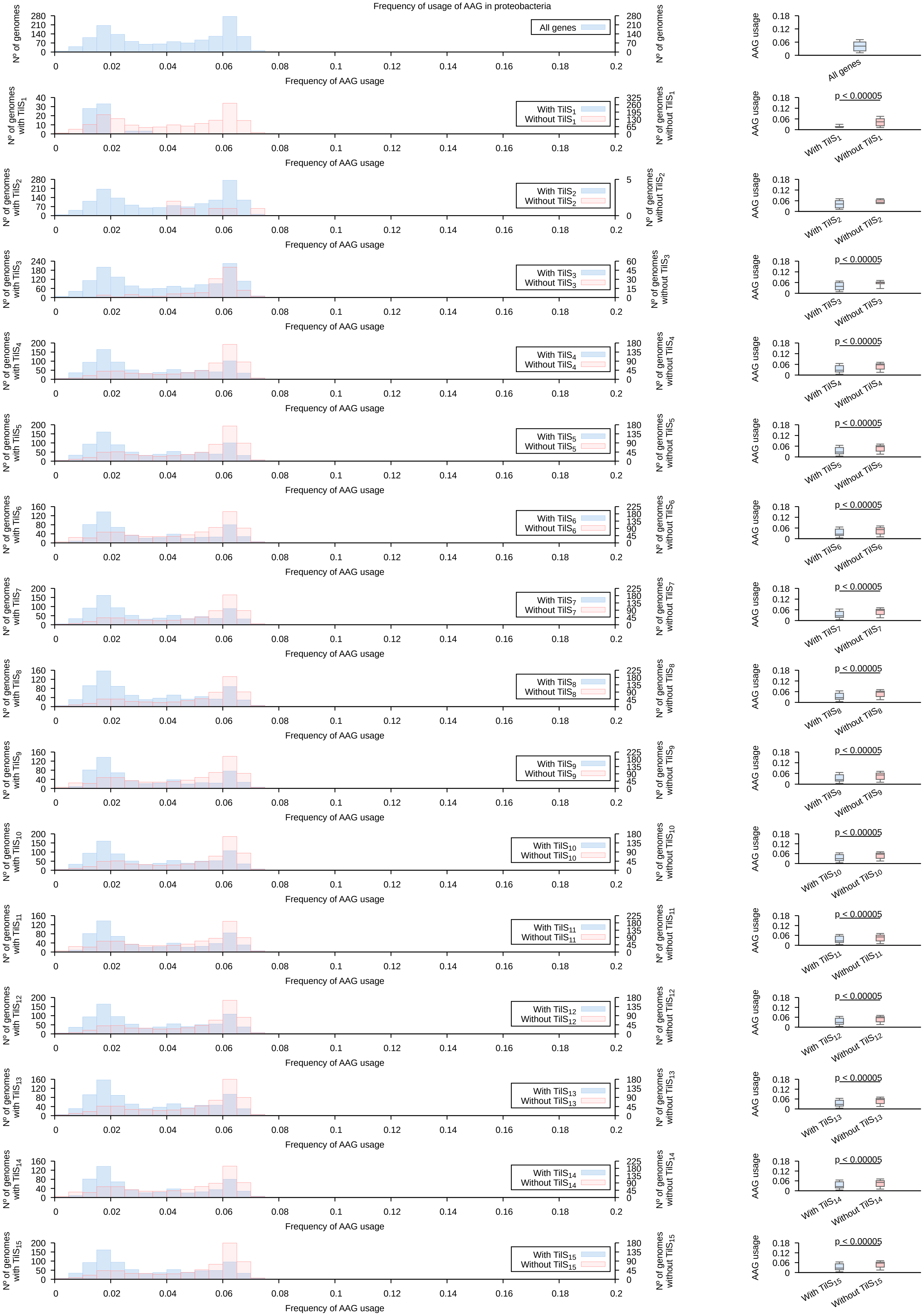

Frequency of usage of AAG in proteobacteria
Nº of genomes
All genes
Frequency of AAG usage
AAG usage
All genes
Nº of genomes with TilS$_1$
Nº of genomes without TilS$_1$
With TilS$_1$
Without TilS$_1$
p < 0.00005
Nº of genomes with TilS$_2$
Nº of genomes without TilS$_2$
With TilS$_2$
Without TilS$_2$
Nº of genomes with TilS$_3$
Nº of genomes without TilS$_3$
With TilS$_3$
Without TilS$_3$
p < 0.00005
Nº of genomes with TilS$_4$
Nº of genomes without TilS$_4$
With TilS$_4$
Without TilS$_4$
p < 0.00005
Nº of genomes with TilS$_5$
Nº of genomes without TilS$_5$
With TilS$_5$
Without TilS$_5$
p < 0.00005
Nº of genomes with TilS$_6$
Nº of genomes without TilS$_6$
With TilS$_6$
Without TilS$_6$
p < 0.00005
Nº of genomes with TilS$_7$
Nº of genomes without TilS$_7$
With TilS$_7$
Without TilS$_7$
p < 0.00005
Nº of genomes with TilS$_8$
Nº of genomes without TilS$_8$
With TilS$_8$
Without TilS$_8$
p < 0.00005
Nº of genomes with TilS$_9$
Nº of genomes without TilS$_9$
With TilS$_9$
Without TilS$_9$
p < 0.00005
Nº of genomes with TilS$_{10}$
Nº of genomes without TilS$_{10}$
With TilS$_{10}$
Without TilS$_{10}$
p < 0.00005
Nº of genomes with TilS$_{11}$
Nº of genomes without TilS$_{11}$
With TilS$_{11}$
Without TilS$_{11}$
p < 0.00005
Nº of genomes with TilS$_{12}$
Nº of genomes without TilS$_{12}$
With TilS$_{12}$
Without TilS$_{12}$
p < 0.00005
Nº of genomes with TilS$_{13}$
Nº of genomes without TilS$_{13}$
With TilS$_{13}$
Without TilS$_{13}$
p < 0.00005
Nº of genomes with TilS$_{14}$
Nº of genomes without TilS$_{14}$
With TilS$_{14}$
Without TilS$_{14}$
p < 0.00005
Nº of genomes with TilS$_{15}$
Nº of genomes without TilS$_{15}$
With TilS$_{15}$
Without TilS$_{15}$
p < 0.00005

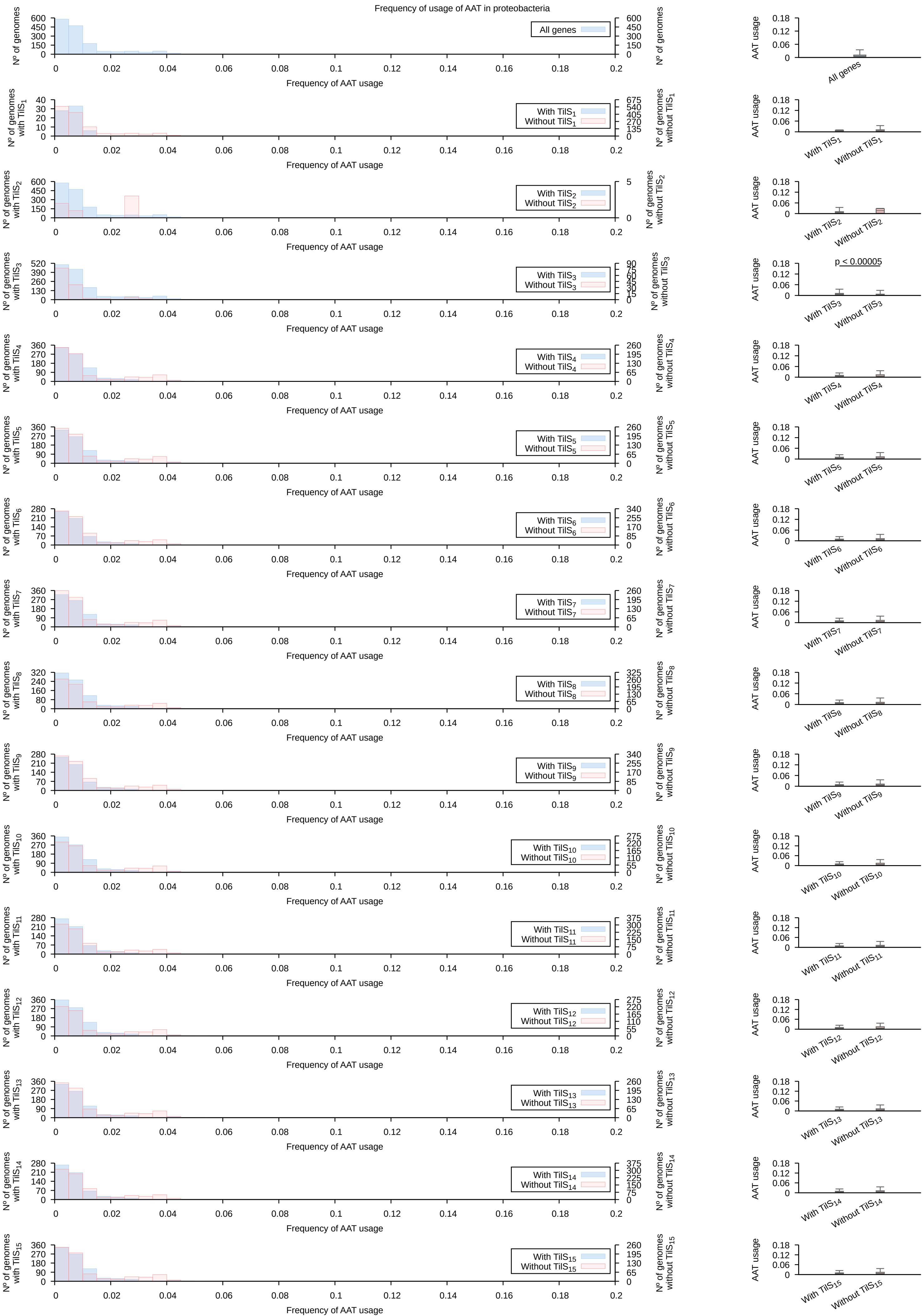

Frequency of usage of AAT in proteobacteria
Nº of genomes
All genes
Frequency of AAT usage
AAT usage
All genes
Nº of genomes with TilS$_1$
Nº of genomes without TilS$_1$
With TilS$_1$
Without TilS$_1$
Nº of genomes with TilS$_2$
Nº of genomes without TilS$_2$
With TilS$_2$
Without TilS$_2$
Nº of genomes with TilS$_3$
Nº of genomes without TilS$_3$
With TilS$_3$
Without TilS$_3$
p < 0.00005
Nº of genomes with TilS$_4$
Nº of genomes without TilS$_4$
With TilS$_4$
Without TilS$_4$
Nº of genomes with TilS$_5$
Nº of genomes without TilS$_5$
With TilS$_5$
Without TilS$_5$
Nº of genomes with TilS$_6$
Nº of genomes without TilS$_6$
With TilS$_6$
Without TilS$_6$
Nº of genomes with TilS$_7$
Nº of genomes without TilS$_7$
With TilS$_7$
Without TilS$_7$
Nº of genomes with TilS$_8$
Nº of genomes without TilS$_8$
With TilS$_8$
Without TilS$_8$
Nº of genomes with TilS$_9$
Nº of genomes without TilS$_9$
With TilS$_9$
Without TilS$_9$
Nº of genomes with TilS$_{10}$
Nº of genomes without TilS$_{10}$
With TilS$_{10}$
Without TilS$_{10}$
Nº of genomes with TilS$_{11}$
Nº of genomes without TilS$_{11}$
With TilS$_{11}$
Without TilS$_{11}$
Nº of genomes with TilS$_{12}$
Nº of genomes without TilS$_{12}$
With TilS$_{12}$
Without TilS$_{12}$
Nº of genomes with TilS$_{13}$
Nº of genomes without TilS$_{13}$
With TilS$_{13}$
Without TilS$_{13}$
Nº of genomes with TilS$_{14}$
Nº of genomes without TilS$_{14}$
With TilS$_{14}$
Without TilS$_{14}$
Nº of genomes with TilS$_{15}$
Nº of genomes without TilS$_{15}$
With TilS$_{15}$
Without TilS$_{15}$

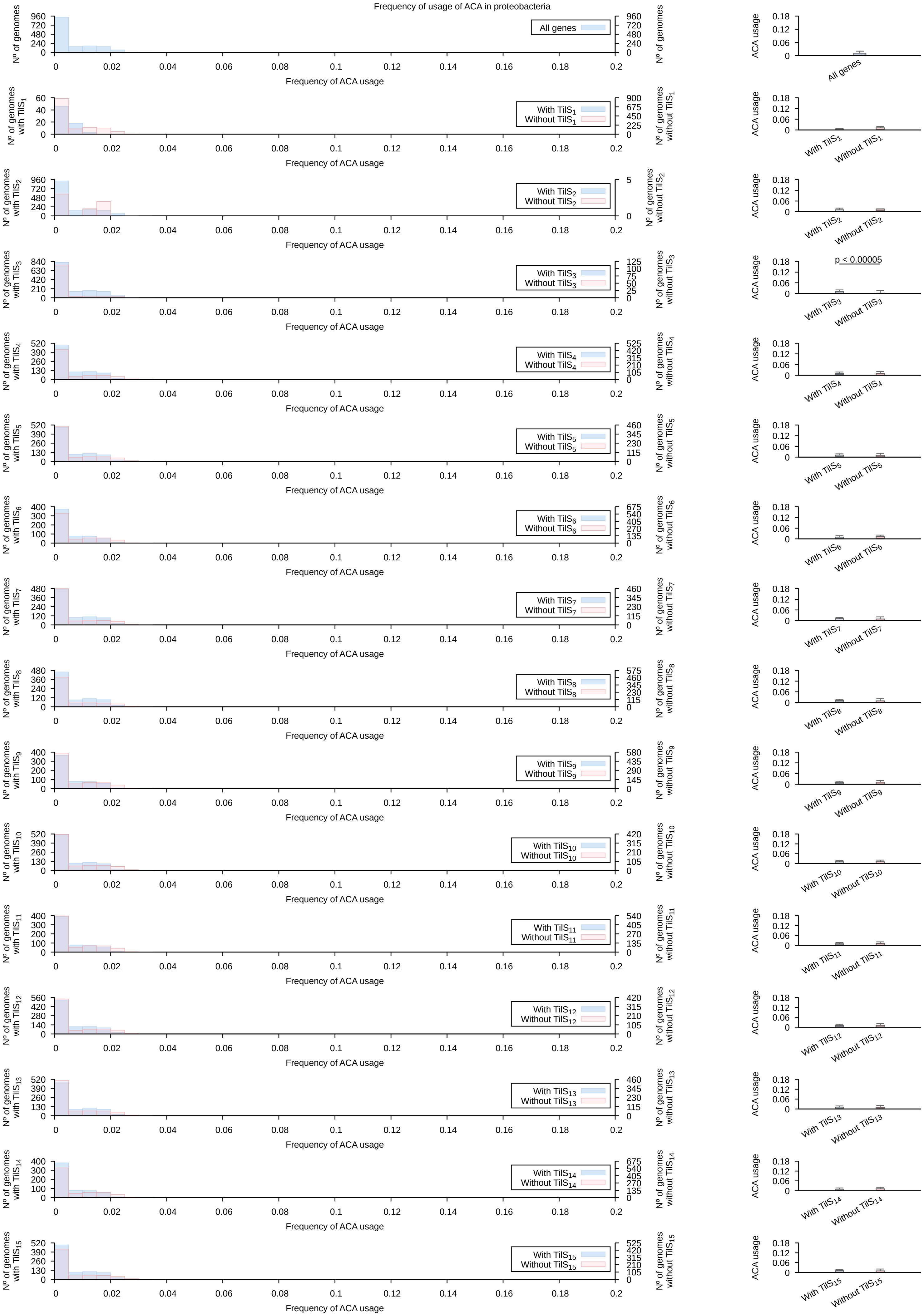

Frequency of usage of ACA in proteobacteria
Nº of genomes
All genes
Frequency of ACA usage
ACA usage
Nº of genomes with TilS1
Nº of genomes without TilS1
With TilS1
Without TilS1
Nº of genomes with TilS2
Nº of genomes without TilS2
With TilS2
Without TilS2
Nº of genomes with TilS3
Nº of genomes without TilS3
With TilS3
Without TilS3
p < 0.00005
Nº of genomes with TilS4
Nº of genomes without TilS4
With TilS4
Without TilS4
Nº of genomes with TilS5
Nº of genomes without TilS5
With TilS5
Without TilS5
Nº of genomes with TilS6
Nº of genomes without TilS6
With TilS6
Without TilS6
Nº of genomes with TilS7
Nº of genomes without TilS7
With TilS7
Without TilS7
Nº of genomes with TilS8
Nº of genomes without TilS8
With TilS8
Without TilS8
Nº of genomes with TilS9
Nº of genomes without TilS9
With TilS9
Without TilS9
Nº of genomes with TilS10
Nº of genomes without TilS10
With TilS10
Without TilS10
Nº of genomes with TilS11
Nº of genomes without TilS11
With TilS11
Without TilS11
Nº of genomes with TilS12
Nº of genomes without TilS12
With TilS12
Without TilS12
Nº of genomes with TilS13
Nº of genomes without TilS13
With TilS13
Without TilS13
Nº of genomes with TilS14
Nº of genomes without TilS14
With TilS14
Without TilS14
Nº of genomes with TilS15
Nº of genomes without TilS15
With TilS15
Without TilS15

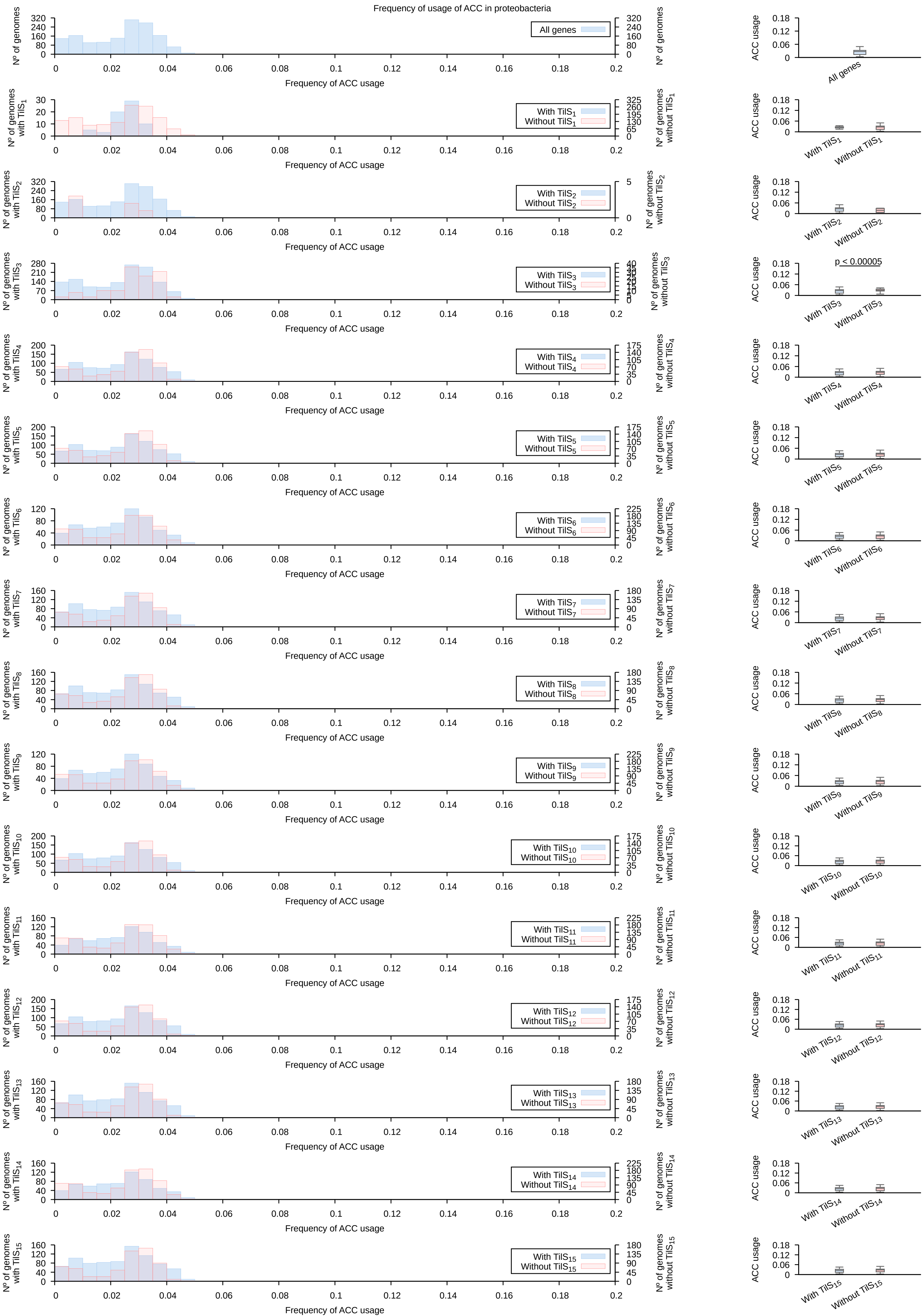

Frequency of usage of ACC in proteobacteria
Nº of genomes
All genes
Frequency of ACC usage
ACC usage
Nº of genomes with TilS$_1$
Nº of genomes without TilS$_1$
With TilS$_1$
Without TilS$_1$
Nº of genomes with TilS$_2$
Nº of genomes without TilS$_2$
With TilS$_2$
Without TilS$_2$
Nº of genomes with TilS$_3$
Nº of genomes without TilS$_3$
With TilS$_3$
Without TilS$_3$
p < 0.00005
Nº of genomes with TilS$_4$
Nº of genomes without TilS$_4$
With TilS$_4$
Without TilS$_4$
Nº of genomes with TilS$_5$
Nº of genomes without TilS$_5$
With TilS$_5$
Without TilS$_5$
Nº of genomes with TilS$_6$
Nº of genomes without TilS$_6$
With TilS$_6$
Without TilS$_6$
Nº of genomes with TilS$_7$
Nº of genomes without TilS$_7$
With TilS$_7$
Without TilS$_7$
Nº of genomes with TilS$_8$
Nº of genomes without TilS$_8$
With TilS$_8$
Without TilS$_8$
Nº of genomes with TilS$_9$
Nº of genomes without TilS$_9$
With TilS$_9$
Without TilS$_9$
Nº of genomes with TilS$_{10}$
Nº of genomes without TilS$_{10}$
With TilS$_{10}$
Without TilS$_{10}$
Nº of genomes with TilS$_{11}$
Nº of genomes without TilS$_{11}$
With TilS$_{11}$
Without TilS$_{11}$
Nº of genomes with TilS$_{12}$
Nº of genomes without TilS$_{12}$
With TilS$_{12}$
Without TilS$_{12}$
Nº of genomes with TilS$_{13}$
Nº of genomes without TilS$_{13}$
With TilS$_{13}$
Without TilS$_{13}$
Nº of genomes with TilS$_{14}$
Nº of genomes without TilS$_{14}$
With TilS$_{14}$
Without TilS$_{14}$
Nº of genomes with TilS$_{15}$
Nº of genomes without TilS$_{15}$
With TilS$_{15}$
Without TilS$_{15}$

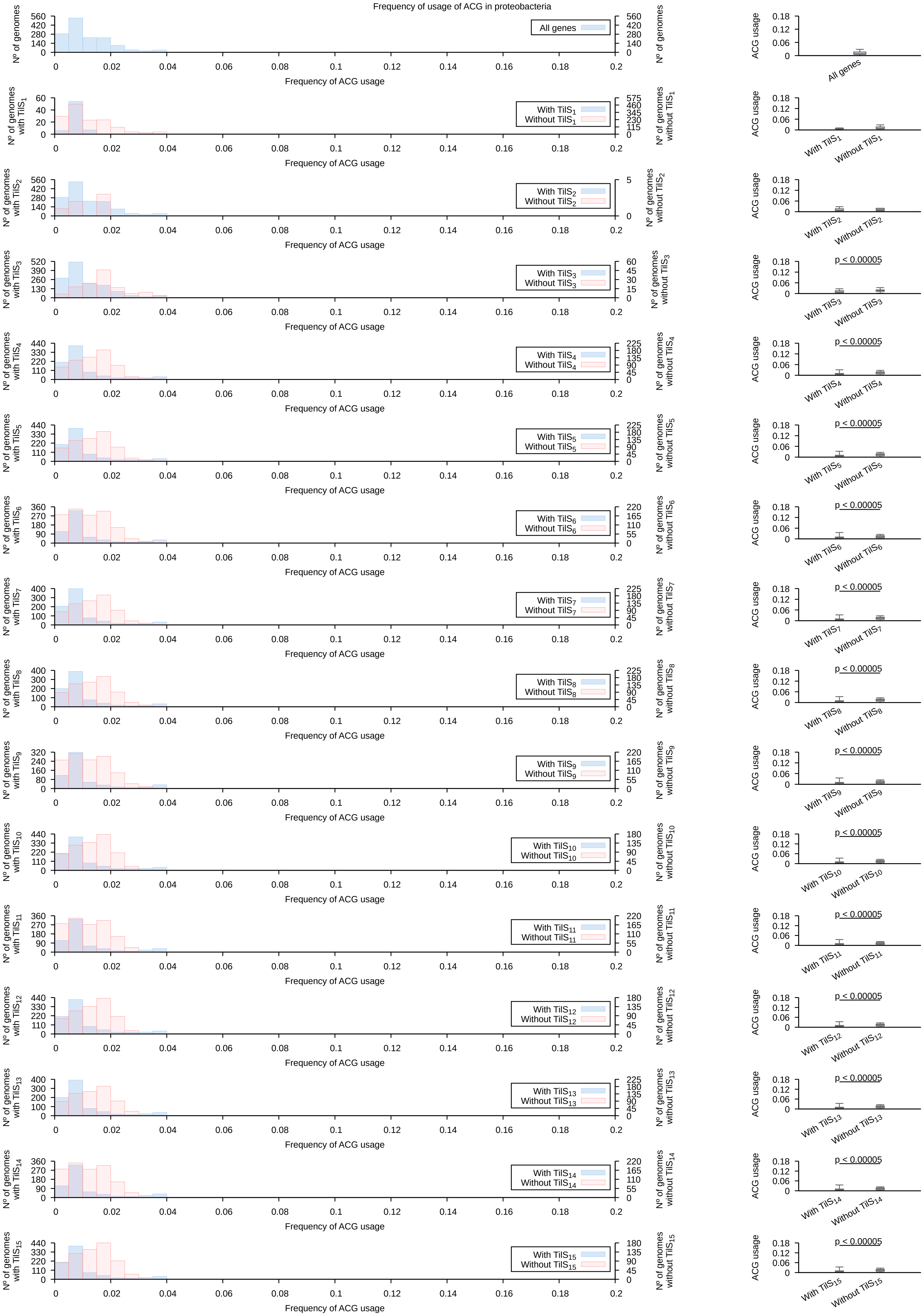

Frequency of usage of ACG in proteobacteria
Frequency of ACG usage
Nº of genomes
ACG usage
All genes
With TilS
Without TilS
p < 0.00005

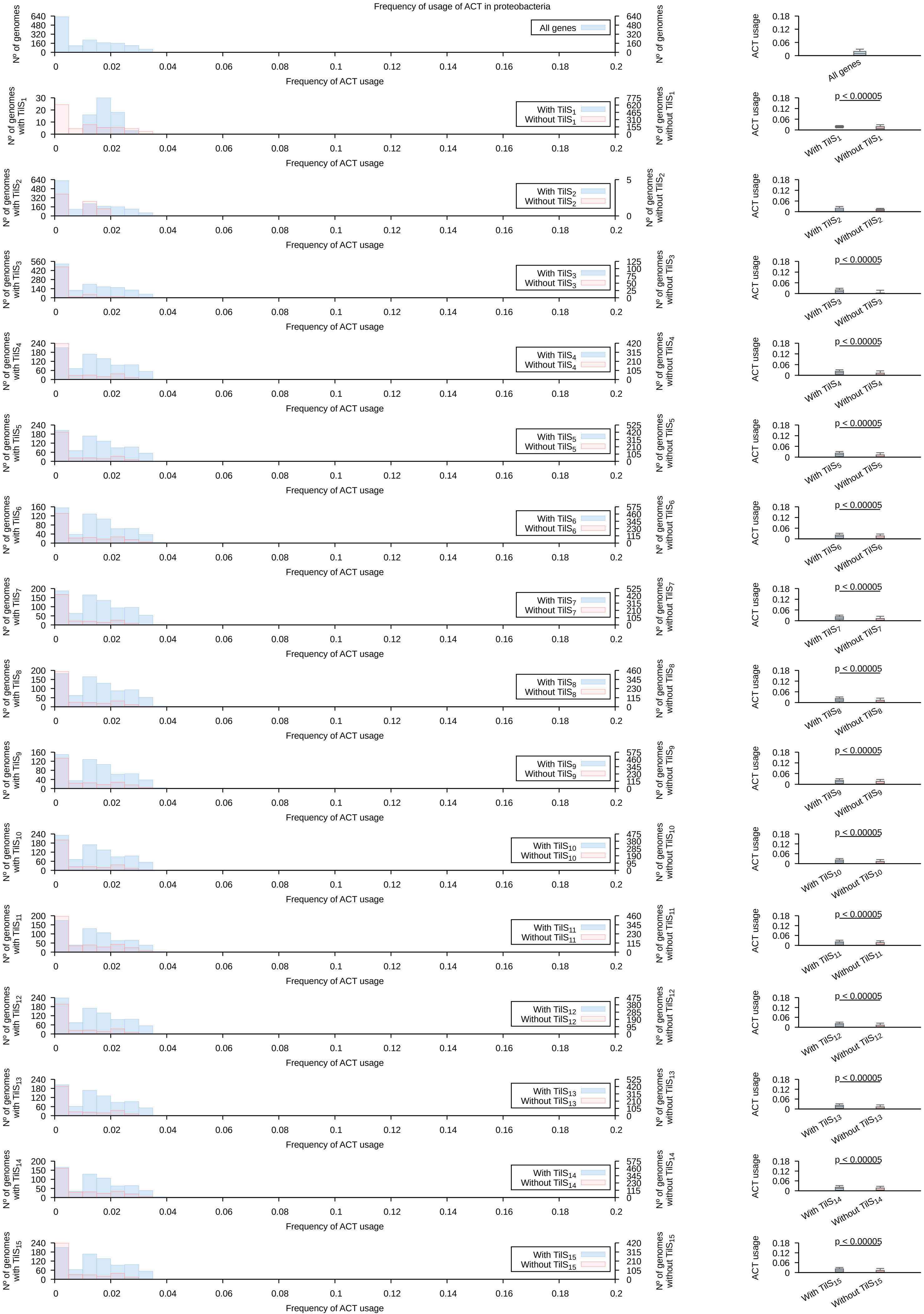
Frequency of usage of ACT in proteobacteria
Nº of genomes
All genes
Frequency of ACT usage
ACT usage
Nº of genomes with TilS1
Nº of genomes without TilS1
With TilS1
Without TilS1
p < 0.00005
Nº of genomes with TilS2
Nº of genomes without TilS2
With TilS2
Without TilS2
Nº of genomes with TilS3
Nº of genomes without TilS3
With TilS3
Without TilS3
Nº of genomes with TilS4
Nº of genomes without TilS4
With TilS4
Without TilS4
Nº of genomes with TilS5
Nº of genomes without TilS5
With TilS5
Without TilS5
Nº of genomes with TilS6
Nº of genomes without TilS6
With TilS6
Without TilS6
Nº of genomes with TilS7
Nº of genomes without TilS7
With TilS7
Without TilS7
Nº of genomes with TilS8
Nº of genomes without TilS8
With TilS8
Without TilS8
Nº of genomes with TilS9
Nº of genomes without TilS9
With TilS9
Without TilS9
Nº of genomes with TilS10
Nº of genomes without TilS10
With TilS10
Without TilS10
Nº of genomes with TilS11
Nº of genomes without TilS11
With TilS11
Without TilS11
Nº of genomes with TilS12
Nº of genomes without TilS12
With TilS12
Without TilS12
Nº of genomes with TilS13
Nº of genomes without TilS13
With TilS13
Without TilS13
Nº of genomes with TilS14
Nº of genomes without TilS14
With TilS14
Without TilS14
Nº of genomes with TilS15
Nº of genomes without TilS15
With TilS15
Without TilS15

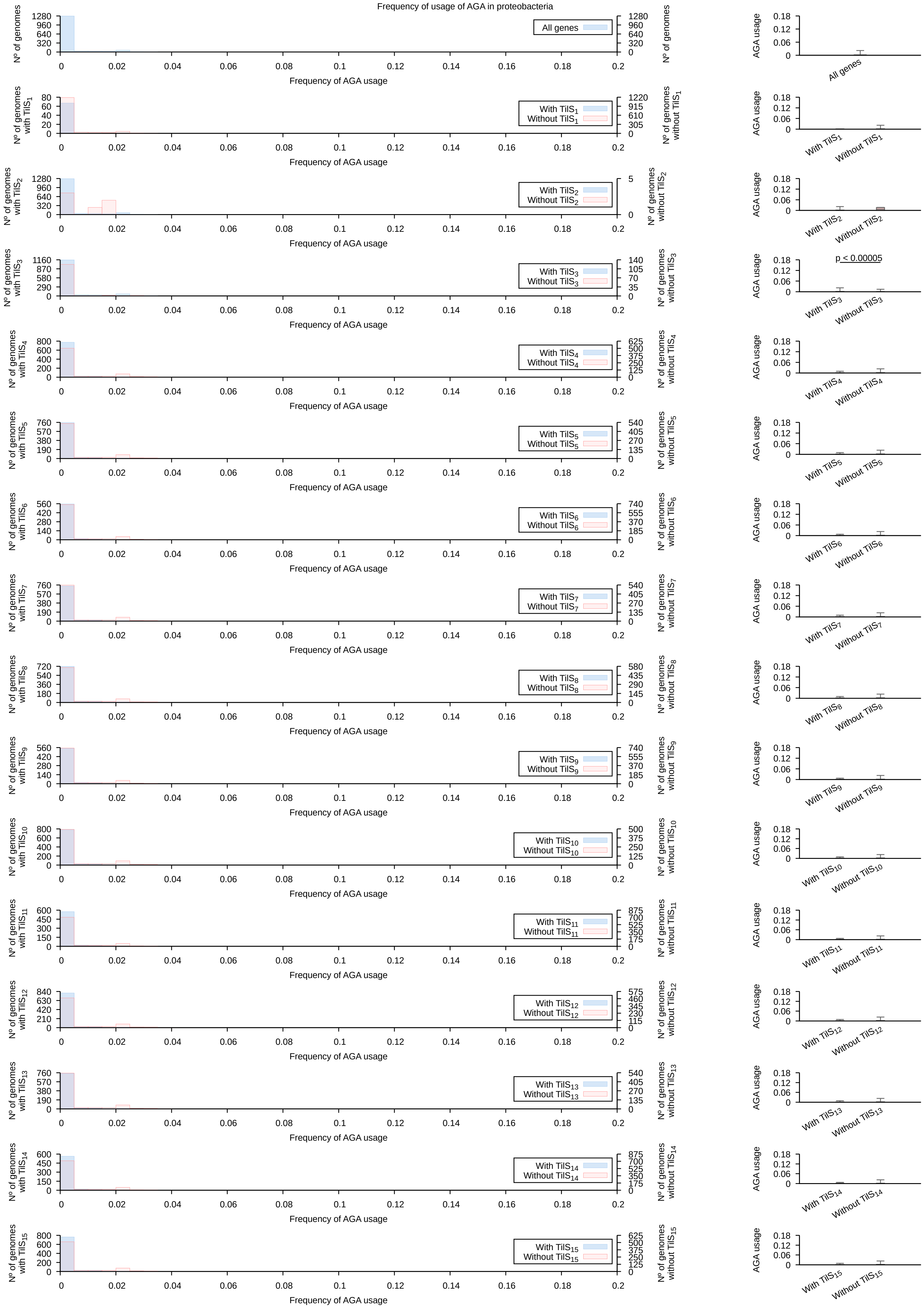
Frequency of usage of AGA in proteobacteria
Nº of genomes
All genes
Frequency of AGA usage
AGA usage
Nº of genomes with TilS$_1$
Nº of genomes without TilS$_1$
With TilS$_1$
Without TilS$_1$
Nº of genomes with TilS$_2$
Nº of genomes without TilS$_2$
With TilS$_2$
Without TilS$_2$
Nº of genomes with TilS$_3$
Nº of genomes without TilS$_3$
With TilS$_3$
Without TilS$_3$
p < 0.00005
Nº of genomes with TilS$_4$
Nº of genomes without TilS$_4$
With TilS$_4$
Without TilS$_4$
Nº of genomes with TilS$_5$
Nº of genomes without TilS$_5$
With TilS$_5$
Without TilS$_5$
Nº of genomes with TilS$_6$
Nº of genomes without TilS$_6$
With TilS$_6$
Without TilS$_6$
Nº of genomes with TilS$_7$
Nº of genomes without TilS$_7$
With TilS$_7$
Without TilS$_7$
Nº of genomes with TilS$_8$
Nº of genomes without TilS$_8$
With TilS$_8$
Without TilS$_8$
Nº of genomes with TilS$_9$
Nº of genomes without TilS$_9$
With TilS$_9$
Without TilS$_9$
Nº of genomes with TilS$_{10}$
Nº of genomes without TilS$_{10}$
With TilS$_{10}$
Without TilS$_{10}$
Nº of genomes with TilS$_{11}$
Nº of genomes without TilS$_{11}$
With TilS$_{11}$
Without TilS$_{11}$
Nº of genomes with TilS$_{12}$
Nº of genomes without TilS$_{12}$
With TilS$_{12}$
Without TilS$_{12}$
Nº of genomes with TilS$_{13}$
Nº of genomes without TilS$_{13}$
With TilS$_{13}$
Without TilS$_{13}$
Nº of genomes with TilS$_{14}$
Nº of genomes without TilS$_{14}$
With TilS$_{14}$
Without TilS$_{14}$
Nº of genomes with TilS$_{15}$
Nº of genomes without TilS$_{15}$
With TilS$_{15}$
Without TilS$_{15}$

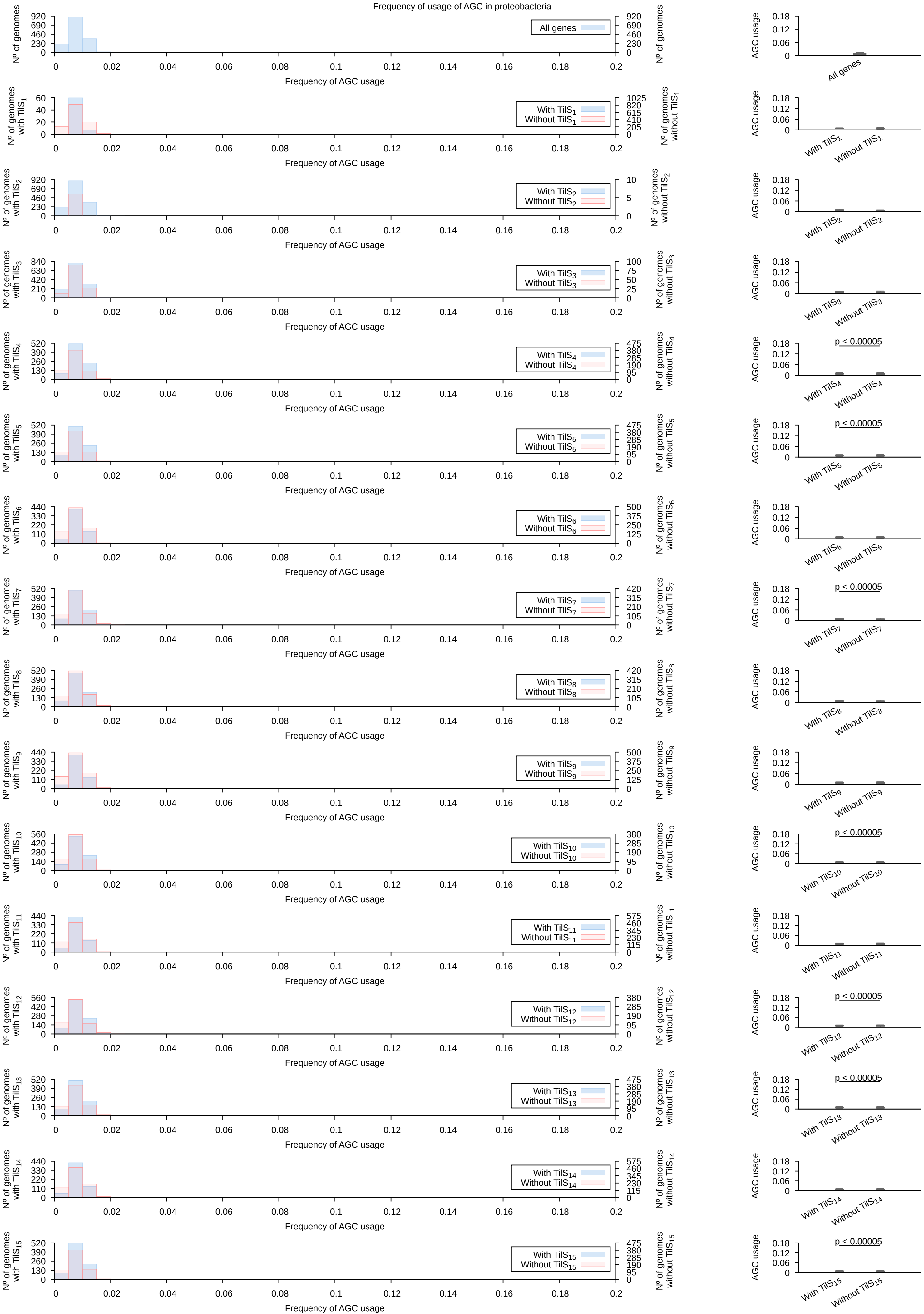
Frequency of usage of AGC in proteobacteria
Nº of genomes
All genes
Frequency of AGC usage
AGC usage
With TilS1
Without TilS1
Nº of genomes with TilS1
Nº of genomes without TilS1
With TilS2
Without TilS2
With TilS3
Without TilS3
With TilS4
Without TilS4
p < 0.00005
With TilS5
Without TilS5
p < 0.00005
With TilS6
Without TilS6
With TilS7
Without TilS7
p < 0.00005
With TilS8
Without TilS8
With TilS9
Without TilS9
With TilS10
Without TilS10
p < 0.00005
With TilS11
Without TilS11
With TilS12
Without TilS12
p < 0.00005
With TilS13
Without TilS13
p < 0.00005
With TilS14
Without TilS14
With TilS15
Without TilS15
p < 0.00005

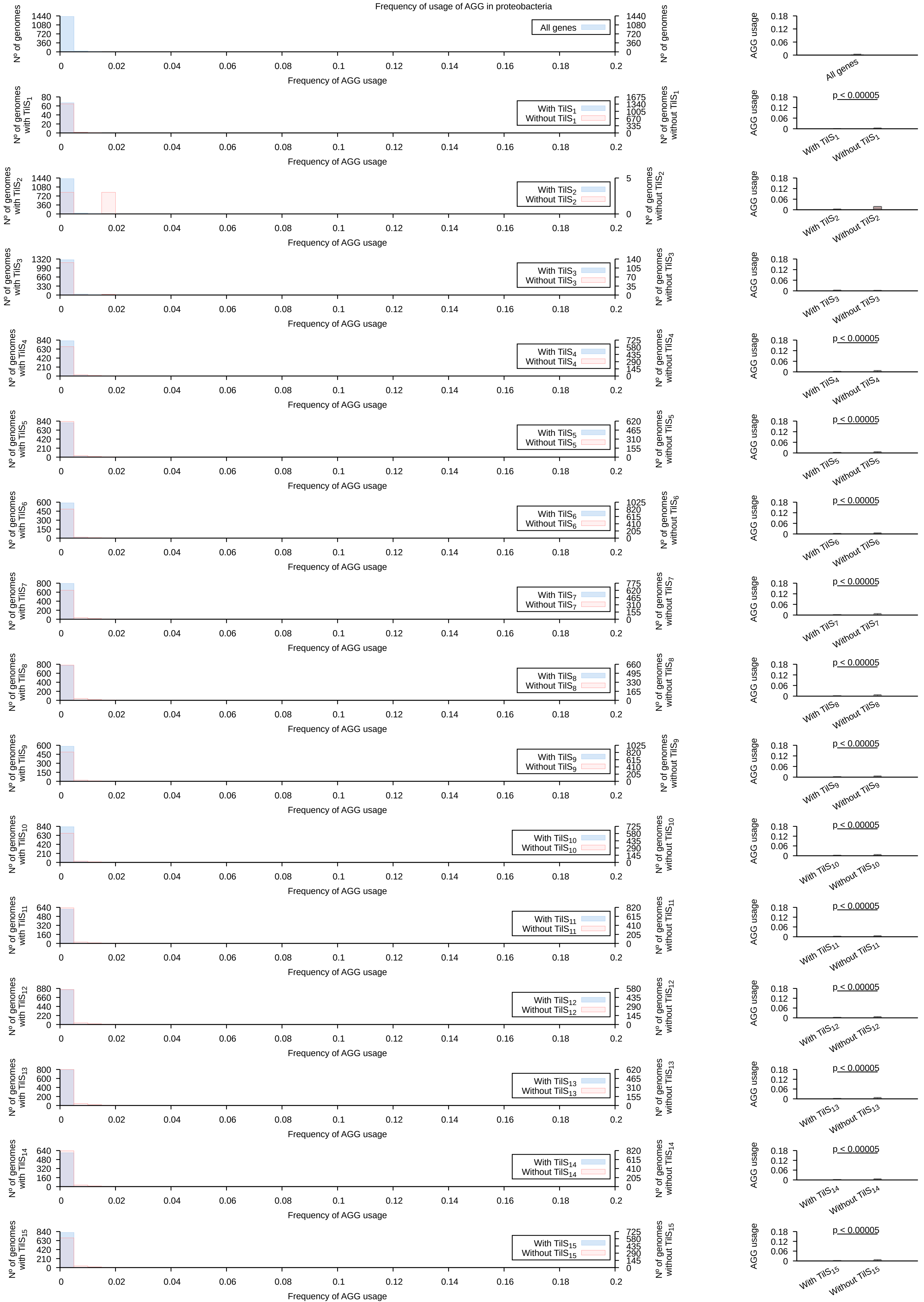

Frequency of usage of AGG in proteobacteria
Nº of genomes
1440 1080 720 360 0
All genes
0 0.02 0.04 0.06 0.08 0.1 0.12 0.14 0.16 0.18 0.2
Frequency of AGG usage
AGG usage
0.18 0.12 0.06 0
All genes
Nº of genomes with TilS1
80 60 40 20 0
With TilS1
Without TilS1
1675 1340 1005 670 335 0
Nº of genomes without TilS1
p < 0.00005
Nº of genomes with TilS2
1440 1080 720 360 0
With TilS2
Without TilS2
5 0
Nº of genomes without TilS2
Nº of genomes with TilS3
1320 990 660 330 0
With TilS3
Without TilS3
140 105 70 35 0
Nº of genomes without TilS3
Nº of genomes with TilS4
840 630 420 210 0
With TilS4
Without TilS4
725 580 435 290 145 0
Nº of genomes without TilS4
p < 0.00005
Nº of genomes with TilS5
840 630 420 210 0
With TilS5
Without TilS5
620 465 310 155 0
Nº of genomes without TilS5
p < 0.00005
Nº of genomes with TilS6
600 450 300 150 0
With TilS6
Without TilS6
1025 820 615 410 205 0
Nº of genomes without TilS6
p < 0.00005
Nº of genomes with TilS7
800 600 400 200 0
With TilS7
Without TilS7
775 620 465 310 155 0
Nº of genomes without TilS7
p < 0.00005
Nº of genomes with TilS8
800 600 400 200 0
With TilS8
Without TilS8
660 495 330 165 0
Nº of genomes without TilS8
p < 0.00005
Nº of genomes with TilS9
600 450 300 150 0
With TilS9
Without TilS9
1025 820 615 410 205 0
Nº of genomes without TilS9
p < 0.00005
Nº of genomes with TilS10
840 630 420 210 0
With TilS10
Without TilS10
725 580 435 290 145 0
Nº of genomes without TilS10
p < 0.00005
Nº of genomes with TilS11
640 480 320 160 0
With TilS11
Without TilS11
820 615 410 205 0
Nº of genomes without TilS11
p < 0.00005
Nº of genomes with TilS12
880 660 440 220 0
With TilS12
Without TilS12
580 435 290 145 0
Nº of genomes without TilS12
p < 0.00005
Nº of genomes with TilS13
800 600 400 200 0
With TilS13
Without TilS13
620 465 310 155 0
Nº of genomes without TilS13
p < 0.00005
Nº of genomes with TilS14
640 480 320 160 0
With TilS14
Without TilS14
820 615 410 205 0
Nº of genomes without TilS14
p < 0.00005
Nº of genomes with TilS15
840 630 420 210 0
With TilS15
Without TilS15
725 580 435 290 145 0
Nº of genomes without TilS15
p < 0.00005

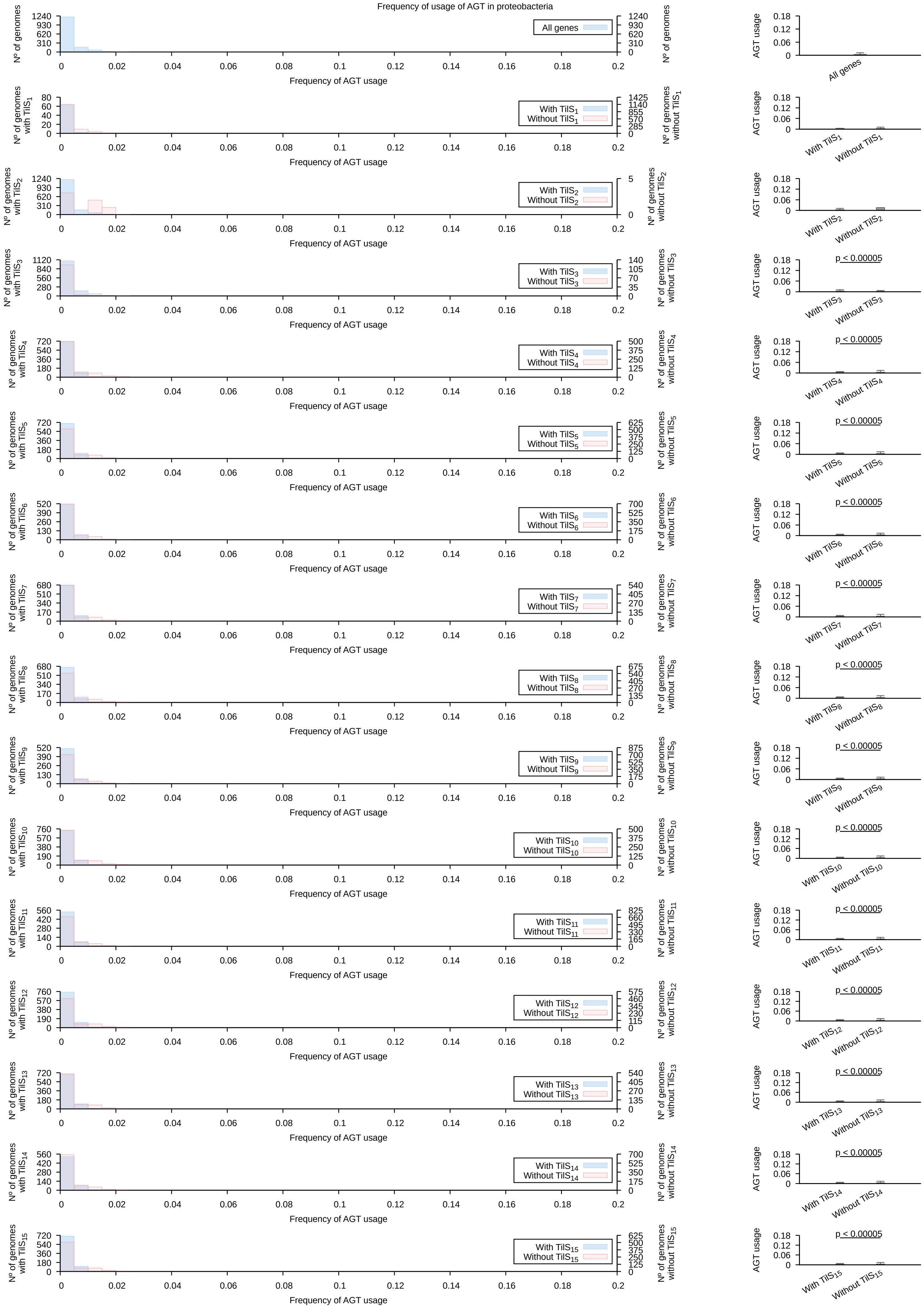

Frequency of usage of AGT in proteobacteria
Nº of genomes
All genes
Frequency of AGT usage
AGT usage
All genes
Nº of genomes with TilS$_1$
Nº of genomes without TilS$_1$
With TilS$_1$
Without TilS$_1$
Nº of genomes with TilS$_2$
Nº of genomes without TilS$_2$
With TilS$_2$
Without TilS$_2$
Nº of genomes with TilS$_3$
Nº of genomes without TilS$_3$
With TilS$_3$
Without TilS$_3$
p < 0.00005
Nº of genomes with TilS$_4$
Nº of genomes without TilS$_4$
With TilS$_4$
Without TilS$_4$
p < 0.00005
Nº of genomes with TilS$_5$
Nº of genomes without TilS$_5$
With TilS$_5$
Without TilS$_5$
p < 0.00005
Nº of genomes with TilS$_6$
Nº of genomes without TilS$_6$
With TilS$_6$
Without TilS$_6$
p < 0.00005
Nº of genomes with TilS$_7$
Nº of genomes without TilS$_7$
With TilS$_7$
Without TilS$_7$
p < 0.00005
Nº of genomes with TilS$_8$
Nº of genomes without TilS$_8$
With TilS$_8$
Without TilS$_8$
p < 0.00005
Nº of genomes with TilS$_9$
Nº of genomes without TilS$_9$
With TilS$_9$
Without TilS$_9$
p < 0.00005
Nº of genomes with TilS$_{10}$
Nº of genomes without TilS$_{10}$
With TilS$_{10}$
Without TilS$_{10}$
p < 0.00005
Nº of genomes with TilS$_{11}$
Nº of genomes without TilS$_{11}$
With TilS$_{11}$
Without TilS$_{11}$
p < 0.00005
Nº of genomes with TilS$_{12}$
Nº of genomes without TilS$_{12}$
With TilS$_{12}$
Without TilS$_{12}$
p < 0.00005
Nº of genomes with TilS$_{13}$
Nº of genomes without TilS$_{13}$
With TilS$_{13}$
Without TilS$_{13}$
p < 0.00005
Nº of genomes with TilS$_{14}$
Nº of genomes without TilS$_{14}$
With TilS$_{14}$
Without TilS$_{14}$
p < 0.00005
Nº of genomes with TilS$_{15}$
Nº of genomes without TilS$_{15}$
With TilS$_{15}$
Without TilS$_{15}$
p < 0.00005

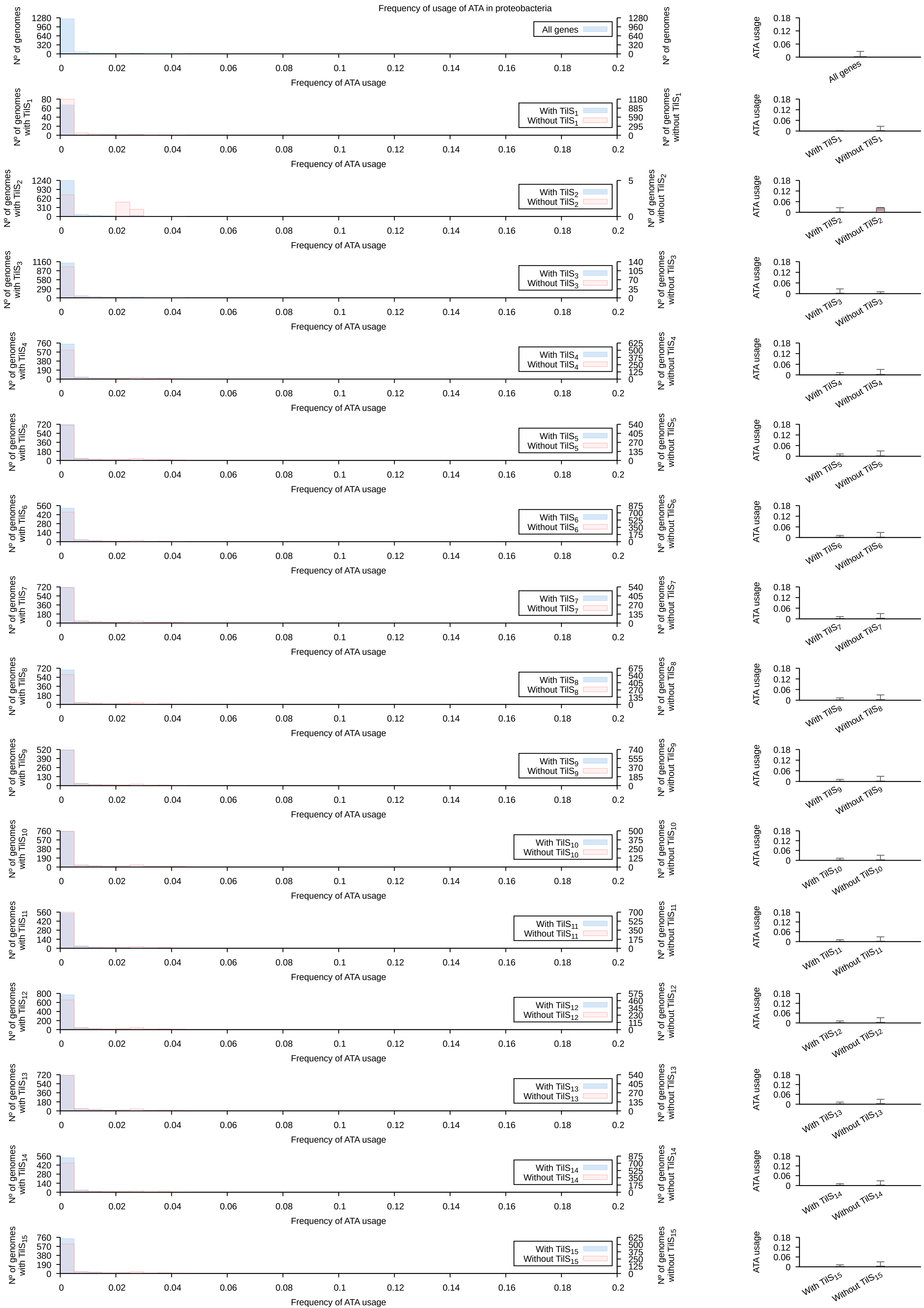

Frequency of usage of ATA in proteobacteria
Nº of genomes
All genes
Frequency of ATA usage
0 0.02 0.04 0.06 0.08 0.1 0.12 0.14 0.16 0.18 0.2
ATA usage
0 0.06 0.12 0.18
Nº of genomes with TilS$_1$
Nº of genomes without TilS$_1$
With TilS$_1$
Without TilS$_1$
Nº of genomes with TilS$_2$
Nº of genomes without TilS$_2$
With TilS$_2$
Without TilS$_2$
Nº of genomes with TilS$_3$
Nº of genomes without TilS$_3$
With TilS$_3$
Without TilS$_3$
Nº of genomes with TilS$_4$
Nº of genomes without TilS$_4$
With TilS$_4$
Without TilS$_4$
Nº of genomes with TilS$_5$
Nº of genomes without TilS$_5$
With TilS$_5$
Without TilS$_5$
Nº of genomes with TilS$_6$
Nº of genomes without TilS$_6$
With TilS$_6$
Without TilS$_6$
Nº of genomes with TilS$_7$
Nº of genomes without TilS$_7$
With TilS$_7$
Without TilS$_7$
Nº of genomes with TilS$_8$
Nº of genomes without TilS$_8$
With TilS$_8$
Without TilS$_8$
Nº of genomes with TilS$_9$
Nº of genomes without TilS$_9$
With TilS$_9$
Without TilS$_9$
Nº of genomes with TilS$_{10}$
Nº of genomes without TilS$_{10}$
With TilS$_{10}$
Without TilS$_{10}$
Nº of genomes with TilS$_{11}$
Nº of genomes without TilS$_{11}$
With TilS$_{11}$
Without TilS$_{11}$
Nº of genomes with TilS$_{12}$
Nº of genomes without TilS$_{12}$
With TilS$_{12}$
Without TilS$_{12}$
Nº of genomes with TilS$_{13}$
Nº of genomes without TilS$_{13}$
With TilS$_{13}$
Without TilS$_{13}$
Nº of genomes with TilS$_{14}$
Nº of genomes without TilS$_{14}$
With TilS$_{14}$
Without TilS$_{14}$
Nº of genomes with TilS$_{15}$
Nº of genomes without TilS$_{15}$
With TilS$_{15}$
Without TilS$_{15}$

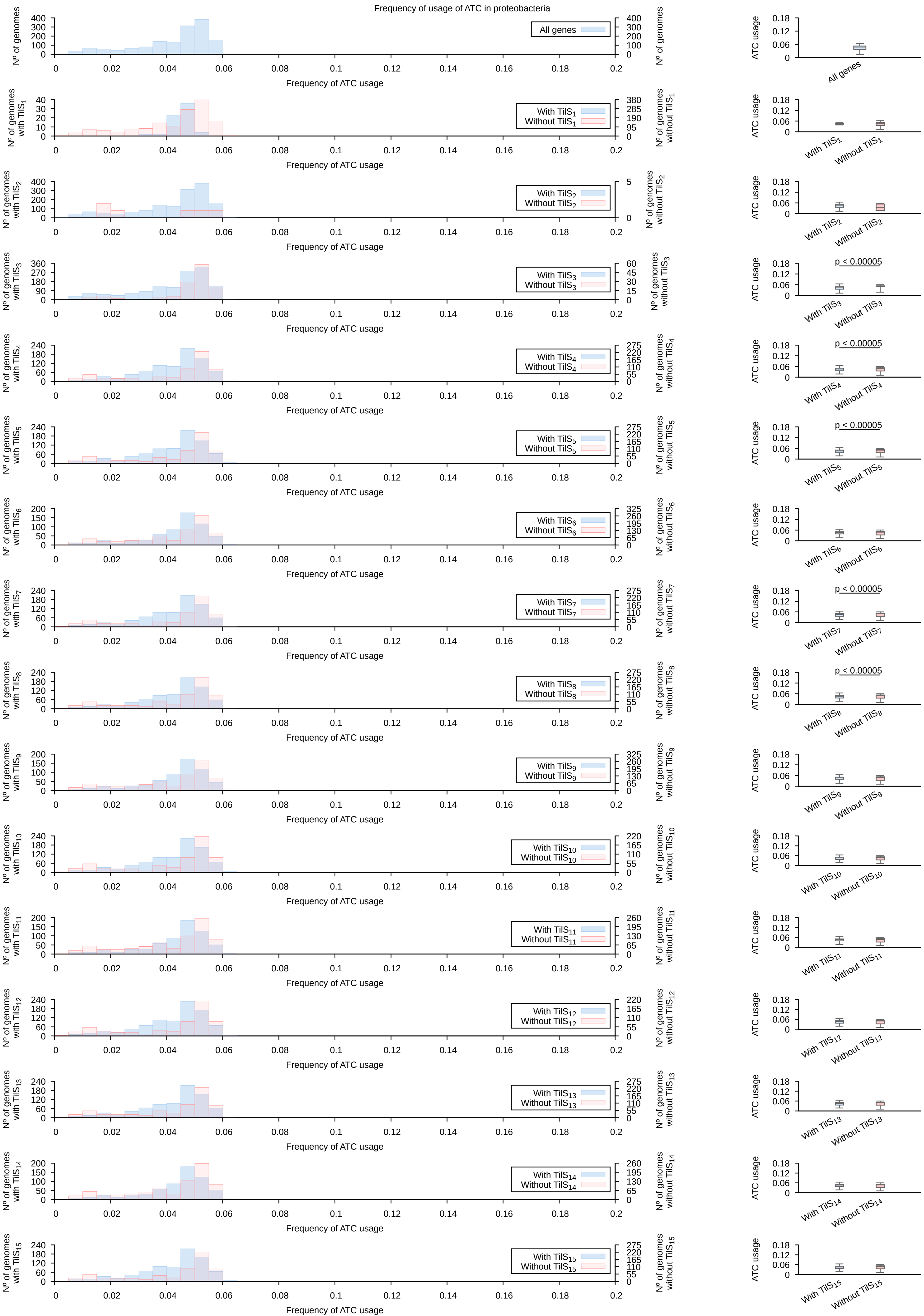

Frequency of usage of ATC in proteobacteria
Nº of genomes
All genes
Frequency of ATC usage
ATC usage
Nº of genomes with TilS$_1$
Nº of genomes without TilS$_1$
With TilS$_1$
Without TilS$_1$
Nº of genomes with TilS$_2$
Nº of genomes without TilS$_2$
With TilS$_2$
Without TilS$_2$
Nº of genomes with TilS$_3$
Nº of genomes without TilS$_3$
With TilS$_3$
Without TilS$_3$
$p < 0.00005$
Nº of genomes with TilS$_4$
Nº of genomes without TilS$_4$
With TilS$_4$
Without TilS$_4$
$p < 0.00005$
Nº of genomes with TilS$_5$
Nº of genomes without TilS$_5$
With TilS$_5$
Without TilS$_5$
$p < 0.00005$
Nº of genomes with TilS$_6$
Nº of genomes without TilS$_6$
With TilS$_6$
Without TilS$_6$
Nº of genomes with TilS$_7$
Nº of genomes without TilS$_7$
With TilS$_7$
Without TilS$_7$
$p < 0.00005$
Nº of genomes with TilS$_8$
Nº of genomes without TilS$_8$
With TilS$_8$
Without TilS$_8$
$p < 0.00005$
Nº of genomes with TilS$_9$
Nº of genomes without TilS$_9$
With TilS$_9$
Without TilS$_9$
Nº of genomes with TilS$_{10}$
Nº of genomes without TilS$_{10}$
With TilS$_{10}$
Without TilS$_{10}$
Nº of genomes with TilS$_{11}$
Nº of genomes without TilS$_{11}$
With TilS$_{11}$
Without TilS$_{11}$
Nº of genomes with TilS$_{12}$
Nº of genomes without TilS$_{12}$
With TilS$_{12}$
Without TilS$_{12}$
Nº of genomes with TilS$_{13}$
Nº of genomes without TilS$_{13}$
With TilS$_{13}$
Without TilS$_{13}$
Nº of genomes with TilS$_{14}$
Nº of genomes without TilS$_{14}$
With TilS$_{14}$
Without TilS$_{14}$
Nº of genomes with TilS$_{15}$
Nº of genomes without TilS$_{15}$
With TilS$_{15}$
Without TilS$_{15}$

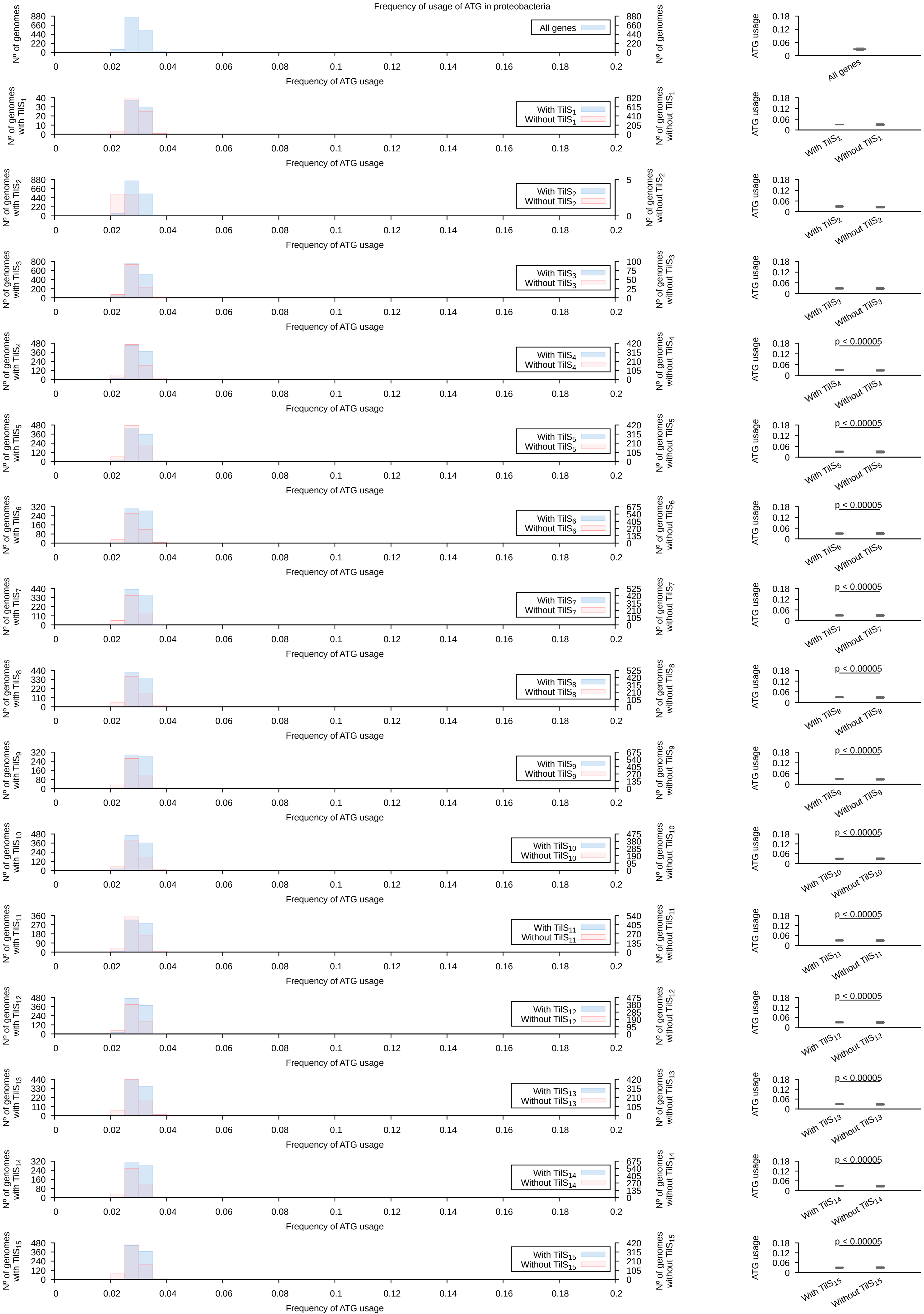

Frequency of usage of ATG in proteobacteria
Nº of genomes
All genes
Frequency of ATG usage
ATG usage
Nº of genomes with TilS$_1$
Nº of genomes without TilS$_1$
With TilS$_1$
Without TilS$_1$
Nº of genomes with TilS$_2$
Nº of genomes without TilS$_2$
With TilS$_2$
Without TilS$_2$
Nº of genomes with TilS$_3$
Nº of genomes without TilS$_3$
With TilS$_3$
Without TilS$_3$
Nº of genomes with TilS$_4$
Nº of genomes without TilS$_4$
With TilS$_4$
Without TilS$_4$
p < 0.00005
Nº of genomes with TilS$_5$
Nº of genomes without TilS$_5$
With TilS$_5$
Without TilS$_5$
p < 0.00005
Nº of genomes with TilS$_6$
Nº of genomes without TilS$_6$
With TilS$_6$
Without TilS$_6$
p < 0.00005
Nº of genomes with TilS$_7$
Nº of genomes without TilS$_7$
With TilS$_7$
Without TilS$_7$
p < 0.00005
Nº of genomes with TilS$_8$
Nº of genomes without TilS$_8$
With TilS$_8$
Without TilS$_8$
p < 0.00005
Nº of genomes with TilS$_9$
Nº of genomes without TilS$_9$
With TilS$_9$
Without TilS$_9$
p < 0.00005
Nº of genomes with TilS$_{10}$
Nº of genomes without TilS$_{10}$
With TilS$_{10}$
Without TilS$_{10}$
p < 0.00005
Nº of genomes with TilS$_{11}$
Nº of genomes without TilS$_{11}$
With TilS$_{11}$
Without TilS$_{11}$
p < 0.00005
Nº of genomes with TilS$_{12}$
Nº of genomes without TilS$_{12}$
With TilS$_{12}$
Without TilS$_{12}$
p < 0.00005
Nº of genomes with TilS$_{13}$
Nº of genomes without TilS$_{13}$
With TilS$_{13}$
Without TilS$_{13}$
p < 0.00005
Nº of genomes with TilS$_{14}$
Nº of genomes without TilS$_{14}$
With TilS$_{14}$
Without TilS$_{14}$
p < 0.00005
Nº of genomes with TilS$_{15}$
Nº of genomes without TilS$_{15}$
With TilS$_{15}$
Without TilS$_{15}$
p < 0.00005

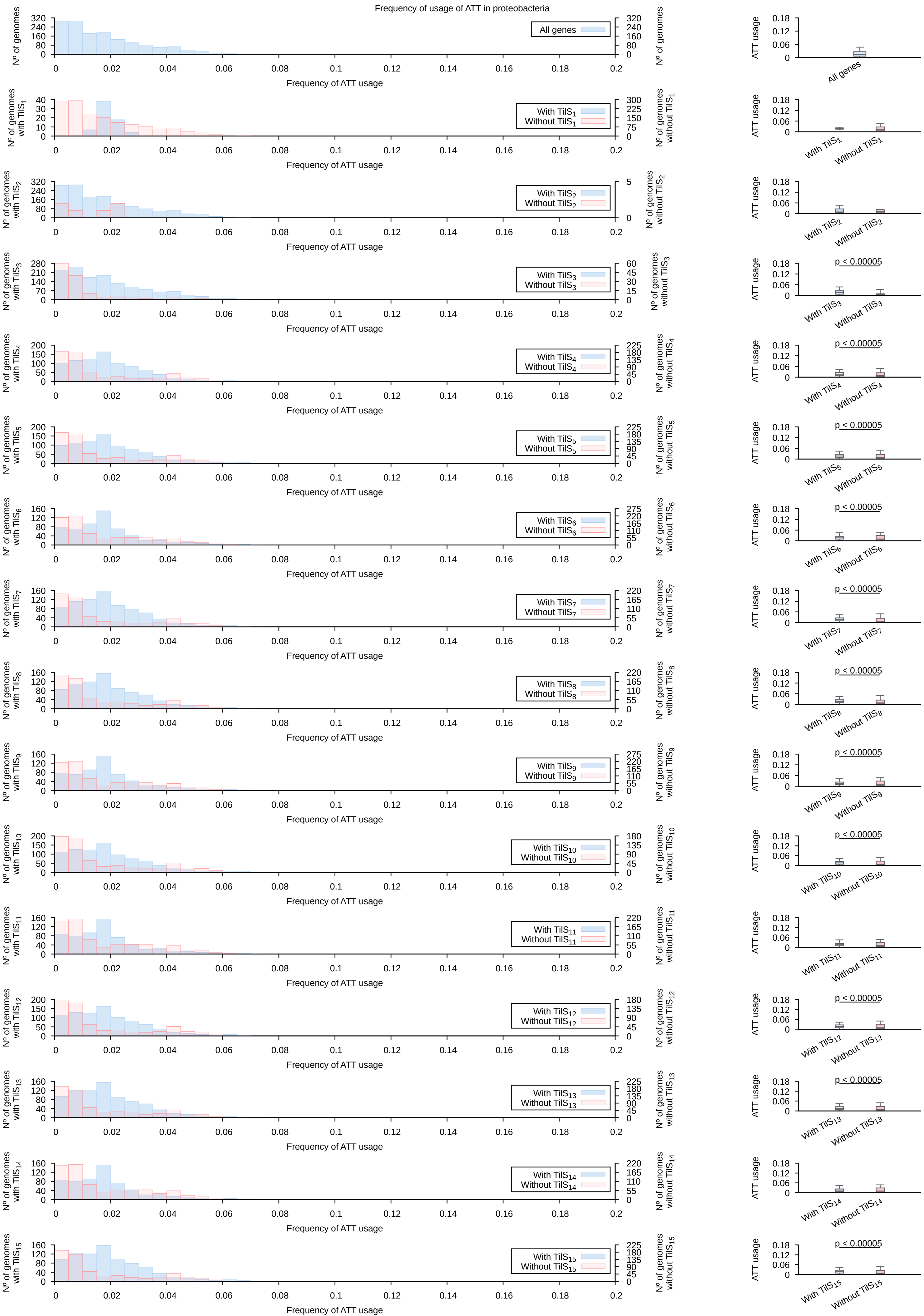

Frequency of usage of ATT in proteobacteria
Nº of genomes
All genes
Frequency of ATT usage
ATT usage
All genes
Nº of genomes with TilS1
Nº of genomes without TilS1
With TilS1
Without TilS1
Nº of genomes with TilS2
Nº of genomes without TilS2
With TilS2
Without TilS2
Nº of genomes with TilS3
Nº of genomes without TilS3
With TilS3
Without TilS3
p < 0.00005
Nº of genomes with TilS4
Nº of genomes without TilS4
With TilS4
Without TilS4
p < 0.00005
Nº of genomes with TilS5
Nº of genomes without TilS5
With TilS5
Without TilS5
p < 0.00005
Nº of genomes with TilS6
Nº of genomes without TilS6
With TilS6
Without TilS6
p < 0.00005
Nº of genomes with TilS7
Nº of genomes without TilS7
With TilS7
Without TilS7
p < 0.00005
Nº of genomes with TilS8
Nº of genomes without TilS8
With TilS8
Without TilS8
p < 0.00005
Nº of genomes with TilS9
Nº of genomes without TilS9
With TilS9
Without TilS9
p < 0.00005
Nº of genomes with TilS10
Nº of genomes without TilS10
With TilS10
Without TilS10
p < 0.00005
Nº of genomes with TilS11
Nº of genomes without TilS11
With TilS11
Without TilS11
Nº of genomes with TilS12
Nº of genomes without TilS12
With TilS12
Without TilS12
p < 0.00005
Nº of genomes with TilS13
Nº of genomes without TilS13
With TilS13
Without TilS13
p < 0.00005
Nº of genomes with TilS14
Nº of genomes without TilS14
With TilS14
Without TilS14
Nº of genomes with TilS15
Nº of genomes without TilS15
With TilS15
Without TilS15
p < 0.00005

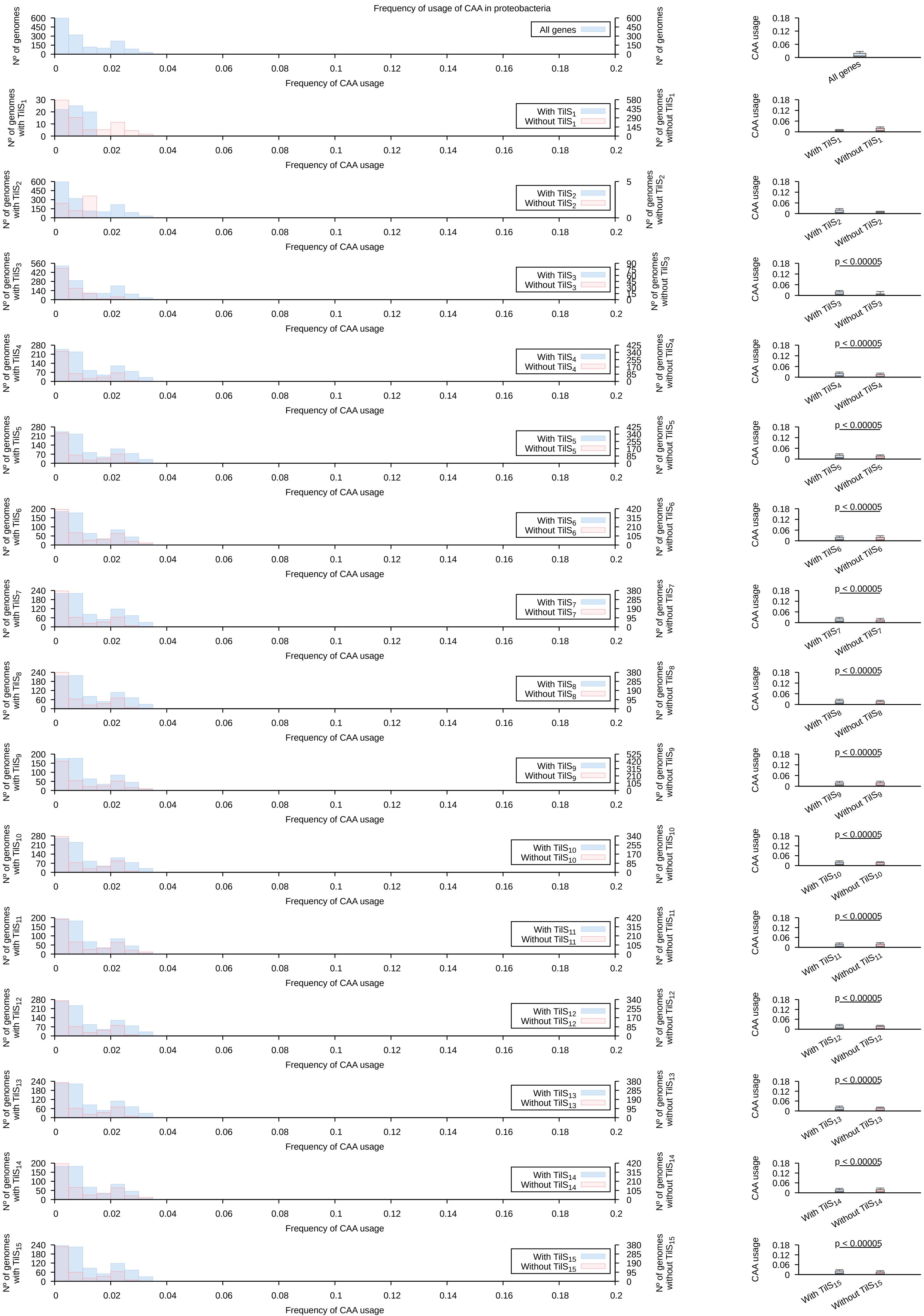
Frequency of usage of CAA in proteobacteria
Nº of genomes
All genes
Frequency of CAA usage
CAA usage
Nº of genomes with TilS1
Nº of genomes without TilS1
With TilS1
Without TilS1
Nº of genomes with TilS2
Nº of genomes without TilS2
With TilS2
Without TilS2
Nº of genomes with TilS3
Nº of genomes without TilS3
With TilS3
Without TilS3
p < 0.00005
Nº of genomes with TilS4
Nº of genomes without TilS4
With TilS4
Without TilS4
p < 0.00005
Nº of genomes with TilS5
Nº of genomes without TilS5
With TilS5
Without TilS5
p < 0.00005
Nº of genomes with TilS6
Nº of genomes without TilS6
With TilS6
Without TilS6
p < 0.00005
Nº of genomes with TilS7
Nº of genomes without TilS7
With TilS7
Without TilS7
p < 0.00005
Nº of genomes with TilS8
Nº of genomes without TilS8
With TilS8
Without TilS8
p < 0.00005
Nº of genomes with TilS9
Nº of genomes without TilS9
With TilS9
Without TilS9
p < 0.00005
Nº of genomes with TilS10
Nº of genomes without TilS10
With TilS10
Without TilS10
p < 0.00005
Nº of genomes with TilS11
Nº of genomes without TilS11
With TilS11
Without TilS11
p < 0.00005
Nº of genomes with TilS12
Nº of genomes without TilS12
With TilS12
Without TilS12
p < 0.00005
Nº of genomes with TilS13
Nº of genomes without TilS13
With TilS13
Without TilS13
p < 0.00005
Nº of genomes with TilS14
Nº of genomes without TilS14
With TilS14
Without TilS14
p < 0.00005
Nº of genomes with TilS15
Nº of genomes without TilS15
With TilS15
Without TilS15
p < 0.00005

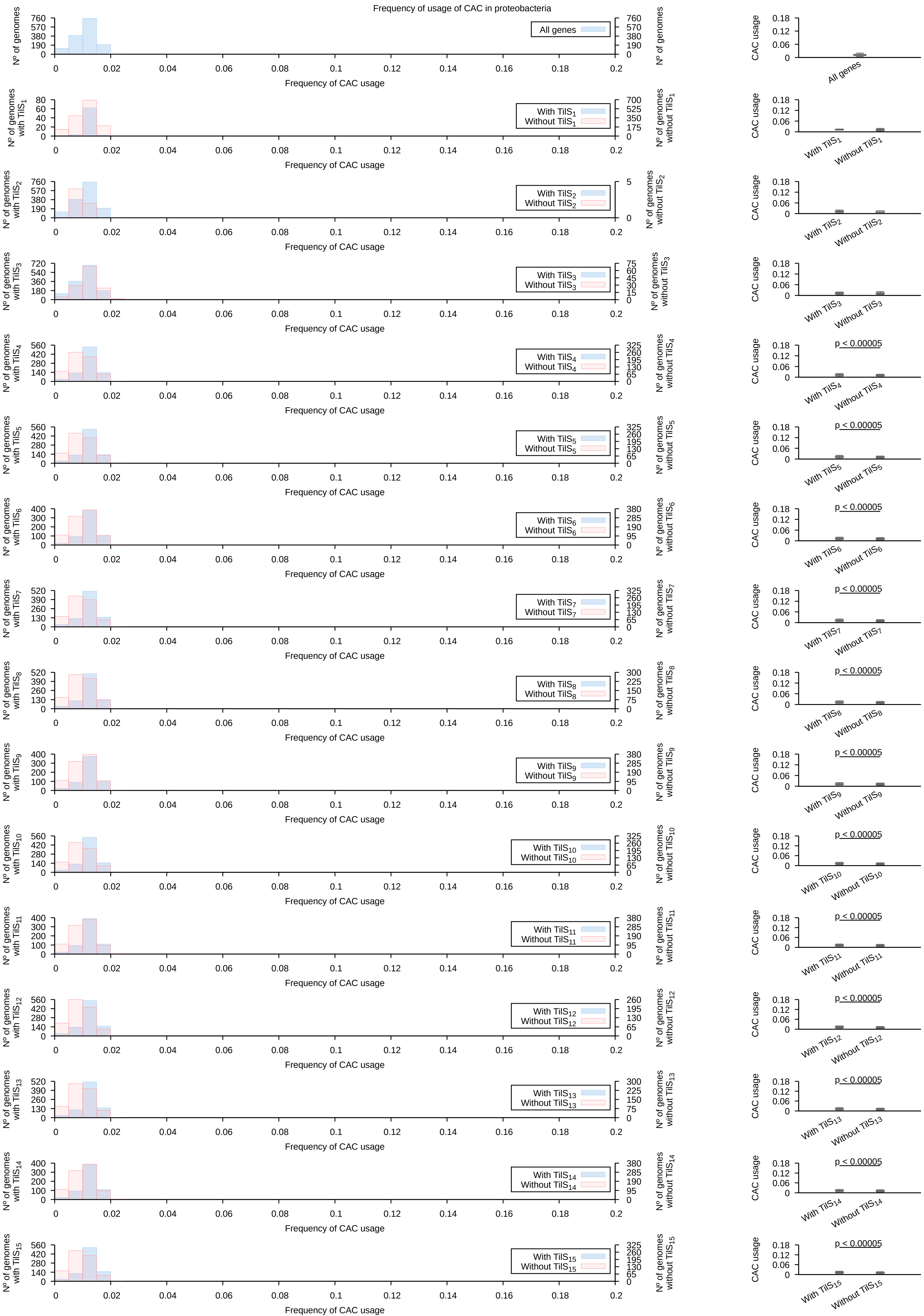

Frequency of usage of CAC in proteobacteria
Nº of genomes
All genes
Frequency of CAC usage
CAC usage
0 0.02 0.04 0.06 0.08 0.1 0.12 0.14 0.16 0.18 0.2
0 0.06 0.12 0.18
Nº of genomes with $TilS_1$
Nº of genomes without $TilS_1$
With $TilS_1$
Without $TilS_1$
Nº of genomes with $TilS_2$
Nº of genomes without $TilS_2$
With $TilS_2$
Without $TilS_2$
Nº of genomes with $TilS_3$
Nº of genomes without $TilS_3$
With $TilS_3$
Without $TilS_3$
Nº of genomes with $TilS_4$
Nº of genomes without $TilS_4$
With $TilS_4$
Without $TilS_4$
p < 0.00005
Nº of genomes with $TilS_5$
Nº of genomes without $TilS_5$
With $TilS_5$
Without $TilS_5$
p < 0.00005
Nº of genomes with $TilS_6$
Nº of genomes without $TilS_6$
With $TilS_6$
Without $TilS_6$
p < 0.00005
Nº of genomes with $TilS_7$
Nº of genomes without $TilS_7$
With $TilS_7$
Without $TilS_7$
p < 0.00005
Nº of genomes with $TilS_8$
Nº of genomes without $TilS_8$
With $TilS_8$
Without $TilS_8$
p < 0.00005
Nº of genomes with $TilS_9$
Nº of genomes without $TilS_9$
With $TilS_9$
Without $TilS_9$
p < 0.00005
Nº of genomes with $TilS_{10}$
Nº of genomes without $TilS_{10}$
With $TilS_{10}$
Without $TilS_{10}$
p < 0.00005
Nº of genomes with $TilS_{11}$
Nº of genomes without $TilS_{11}$
With $TilS_{11}$
Without $TilS_{11}$
p < 0.00005
Nº of genomes with $TilS_{12}$
Nº of genomes without $TilS_{12}$
With $TilS_{12}$
Without $TilS_{12}$
p < 0.00005
Nº of genomes with $TilS_{13}$
Nº of genomes without $TilS_{13}$
With $TilS_{13}$
Without $TilS_{13}$
p < 0.00005
Nº of genomes with $TilS_{14}$
Nº of genomes without $TilS_{14}$
With $TilS_{14}$
Without $TilS_{14}$
p < 0.00005
Nº of genomes with $TilS_{15}$
Nº of genomes without $TilS_{15}$
With $TilS_{15}$
Without $TilS_{15}$
p < 0.00005

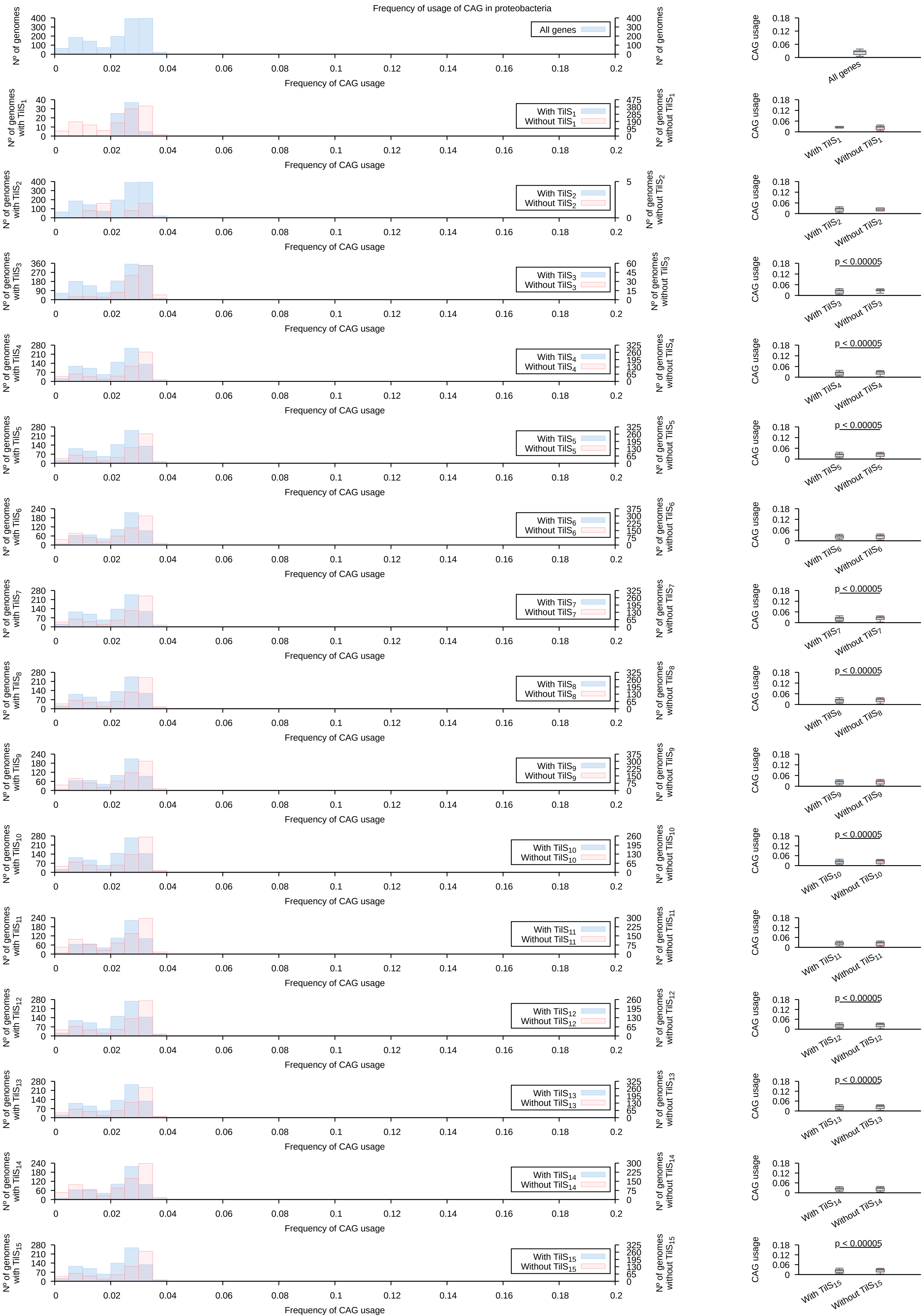

Frequency of usage of CAG in proteobacteria
Nº of genomes
Frequency of CAG usage
All genes
CAG usage
Nº of genomes with TilS1
Nº of genomes without TilS1
With TilS1
Without TilS1
With TilS2
Without TilS2
With TilS3
Without TilS3
With TilS4
Without TilS4
With TilS5
Without TilS5
With TilS6
Without TilS6
With TilS7
Without TilS7
With TilS8
Without TilS8
With TilS9
Without TilS9
With TilS10
Without TilS10
With TilS11
Without TilS11
With TilS12
Without TilS12
With TilS13
Without TilS13
With TilS14
Without TilS14
With TilS15
Without TilS15
p < 0.00005

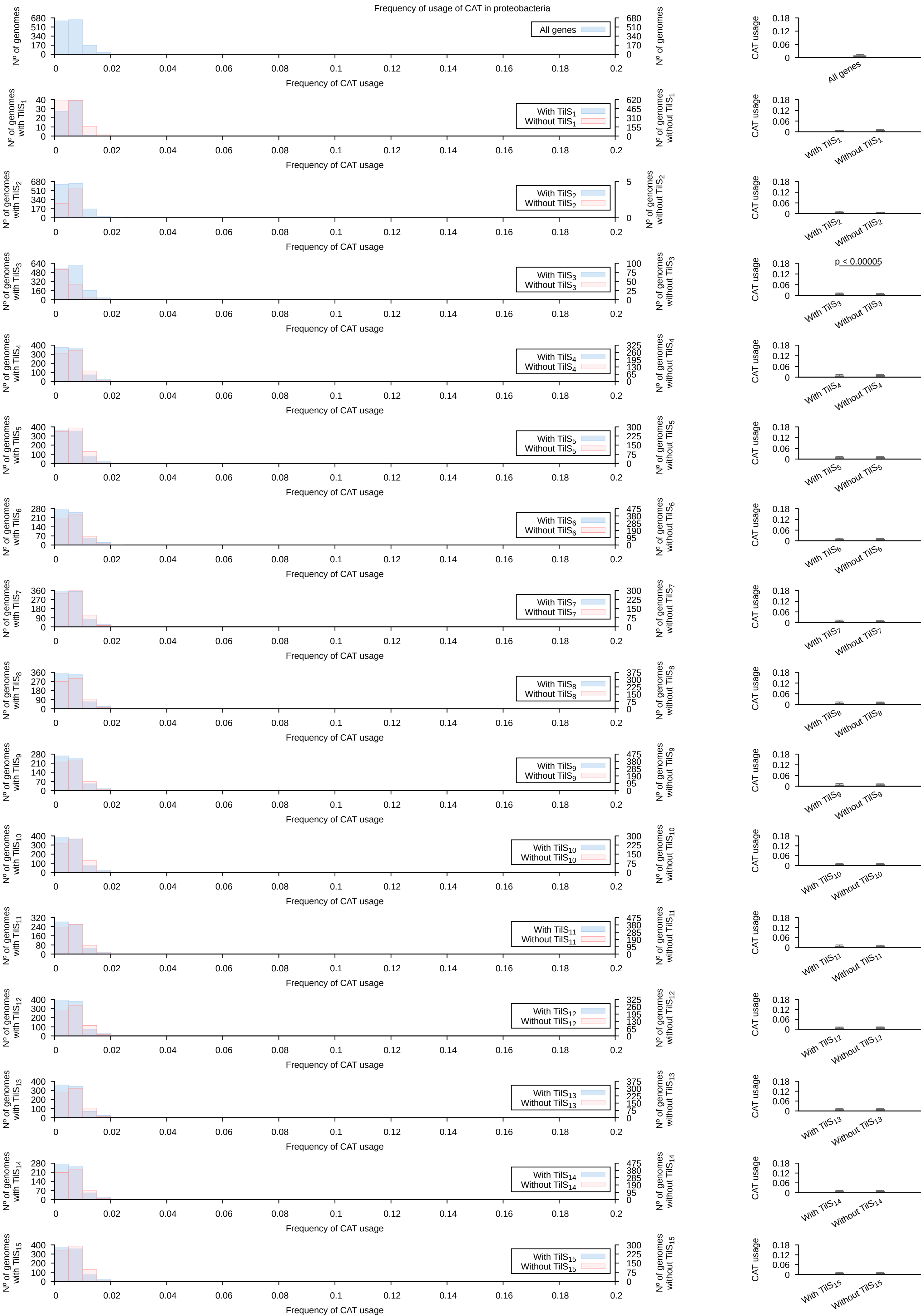

Frequency of usage of CAT in proteobacteria
Nº of genomes
All genes
Frequency of CAT usage
CAT usage
All genes
Nº of genomes with $TilS_1$
Nº of genomes without $TilS_1$
With $TilS_1$
Without $TilS_1$
Nº of genomes with $TilS_2$
Nº of genomes without $TilS_2$
With $TilS_2$
Without $TilS_2$
Nº of genomes with $TilS_3$
Nº of genomes without $TilS_3$
With $TilS_3$
Without $TilS_3$
$p < 0.00005$
Nº of genomes with $TilS_4$
Nº of genomes without $TilS_4$
With $TilS_4$
Without $TilS_4$
Nº of genomes with $TilS_5$
Nº of genomes without $TilS_5$
With $TilS_5$
Without $TilS_5$
Nº of genomes with $TilS_6$
Nº of genomes without $TilS_6$
With $TilS_6$
Without $TilS_6$
Nº of genomes with $TilS_7$
Nº of genomes without $TilS_7$
With $TilS_7$
Without $TilS_7$
Nº of genomes with $TilS_8$
Nº of genomes without $TilS_8$
With $TilS_8$
Without $TilS_8$
Nº of genomes with $TilS_9$
Nº of genomes without $TilS_9$
With $TilS_9$
Without $TilS_9$
Nº of genomes with $TilS_{10}$
Nº of genomes without $TilS_{10}$
With $TilS_{10}$
Without $TilS_{10}$
Nº of genomes with $TilS_{11}$
Nº of genomes without $TilS_{11}$
With $TilS_{11}$
Without $TilS_{11}$
Nº of genomes with $TilS_{12}$
Nº of genomes without $TilS_{12}$
With $TilS_{12}$
Without $TilS_{12}$
Nº of genomes with $TilS_{13}$
Nº of genomes without $TilS_{13}$
With $TilS_{13}$
Without $TilS_{13}$
Nº of genomes with $TilS_{14}$
Nº of genomes without $TilS_{14}$
With $TilS_{14}$
Without $TilS_{14}$
Nº of genomes with $TilS_{15}$
Nº of genomes without $TilS_{15}$
With $TilS_{15}$
Without $TilS_{15}$

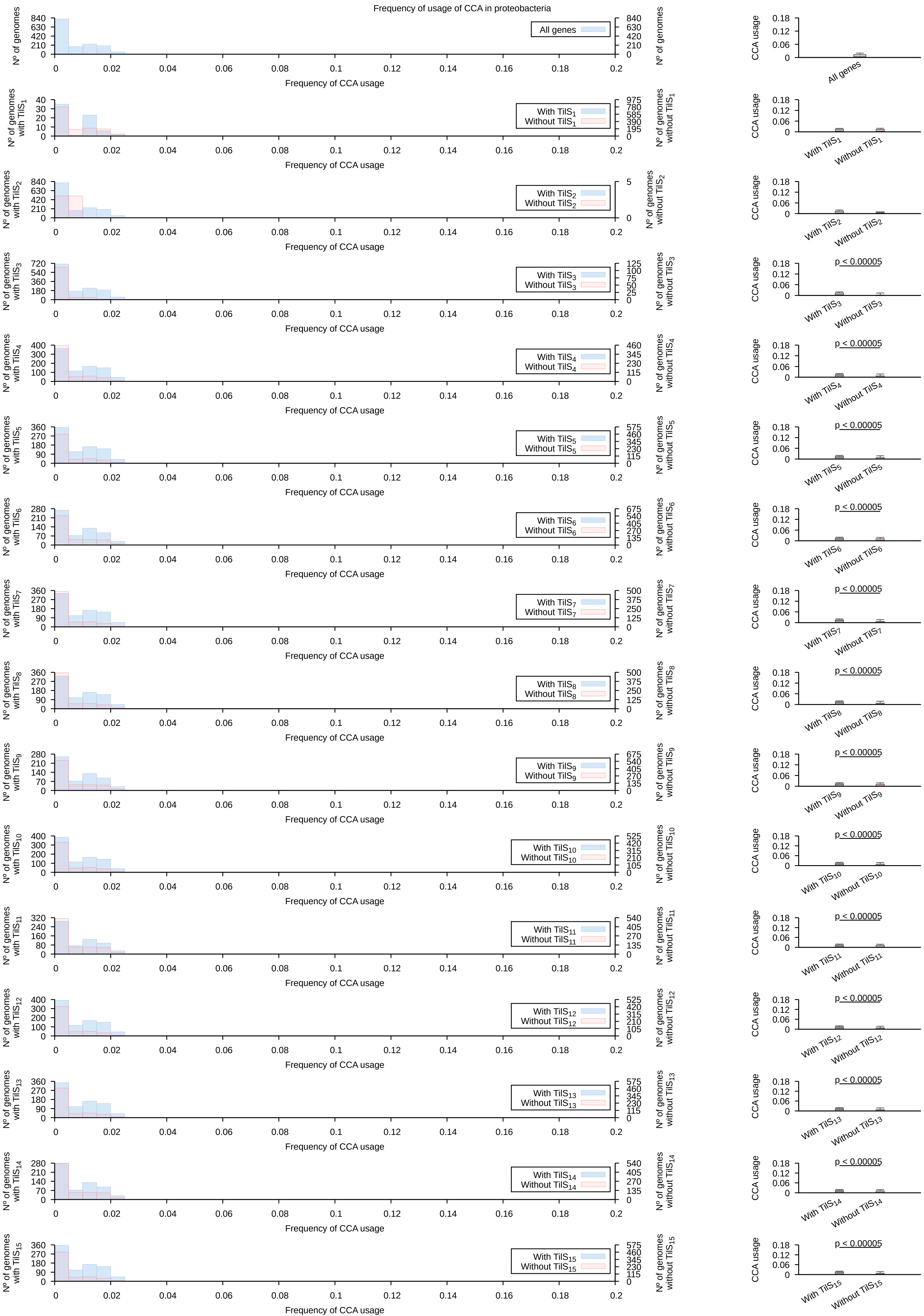

Frequency of usage of CCA in proteobacteria
Nº of genomes
All genes
Frequency of CCA usage
CCA usage
Nº of genomes with TilS1
Nº of genomes without TilS1
With TilS1
Without TilS1
Nº of genomes with TilS2
Nº of genomes without TilS2
With TilS2
Without TilS2
p < 0.00005
With TilS3
Without TilS3
With TilS4
Without TilS4
With TilS5
Without TilS5
With TilS6
Without TilS6
With TilS7
Without TilS7
With TilS8
Without TilS8
With TilS9
Without TilS9
With TilS10
Without TilS10
With TilS11
Without TilS11
With TilS12
Without TilS12
With TilS13
Without TilS13
With TilS14
Without TilS14
With TilS15
Without TilS15

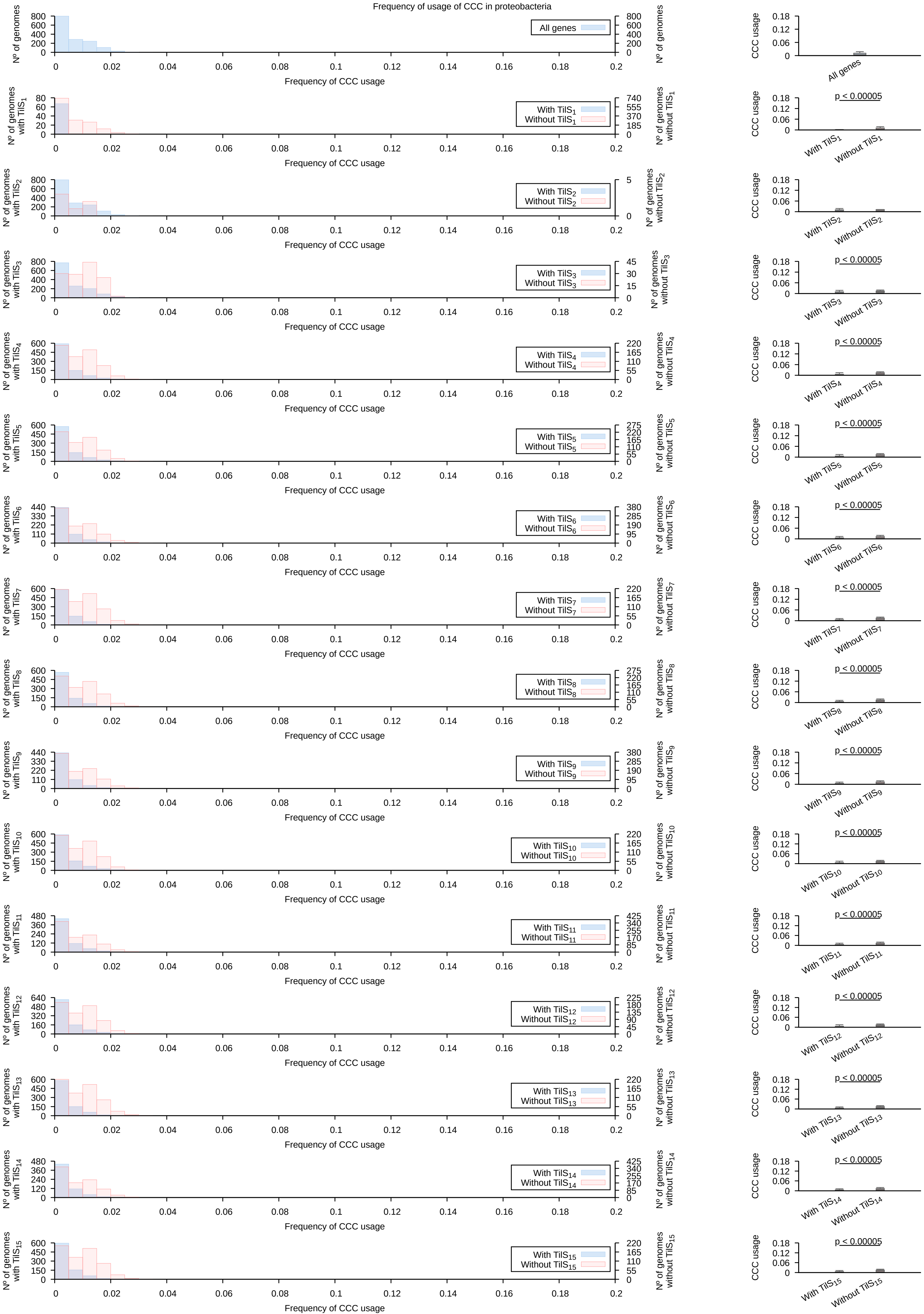
Frequency of usage of CCC in proteobacteria
Nº of genomes
All genes
Frequency of CCC usage
CCC usage
Nº of genomes with TilS$_1$
With TilS$_1$
Without TilS$_1$
Nº of genomes without TilS$_1$
p < 0.00005
Nº of genomes with TilS$_2$
With TilS$_2$
Without TilS$_2$
Nº of genomes without TilS$_2$
Nº of genomes with TilS$_3$
With TilS$_3$
Without TilS$_3$
Nº of genomes without TilS$_3$
Nº of genomes with TilS$_4$
With TilS$_4$
Without TilS$_4$
Nº of genomes without TilS$_4$
Nº of genomes with TilS$_5$
With TilS$_5$
Without TilS$_5$
Nº of genomes without TilS$_5$
Nº of genomes with TilS$_6$
With TilS$_6$
Without TilS$_6$
Nº of genomes without TilS$_6$
Nº of genomes with TilS$_7$
With TilS$_7$
Without TilS$_7$
Nº of genomes without TilS$_7$
Nº of genomes with TilS$_8$
With TilS$_8$
Without TilS$_8$
Nº of genomes without TilS$_8$
Nº of genomes with TilS$_9$
With TilS$_9$
Without TilS$_9$
Nº of genomes without TilS$_9$
Nº of genomes with TilS$_{10}$
With TilS$_{10}$
Without TilS$_{10}$
Nº of genomes without TilS$_{10}$
Nº of genomes with TilS$_{11}$
With TilS$_{11}$
Without TilS$_{11}$
Nº of genomes without TilS$_{11}$
Nº of genomes with TilS$_{12}$
With TilS$_{12}$
Without TilS$_{12}$
Nº of genomes without TilS$_{12}$
Nº of genomes with TilS$_{13}$
With TilS$_{13}$
Without TilS$_{13}$
Nº of genomes without TilS$_{13}$
Nº of genomes with TilS$_{14}$
With TilS$_{14}$
Without TilS$_{14}$
Nº of genomes without TilS$_{14}$
Nº of genomes with TilS$_{15}$
With TilS$_{15}$
Without TilS$_{15}$
Nº of genomes without TilS$_{15}$

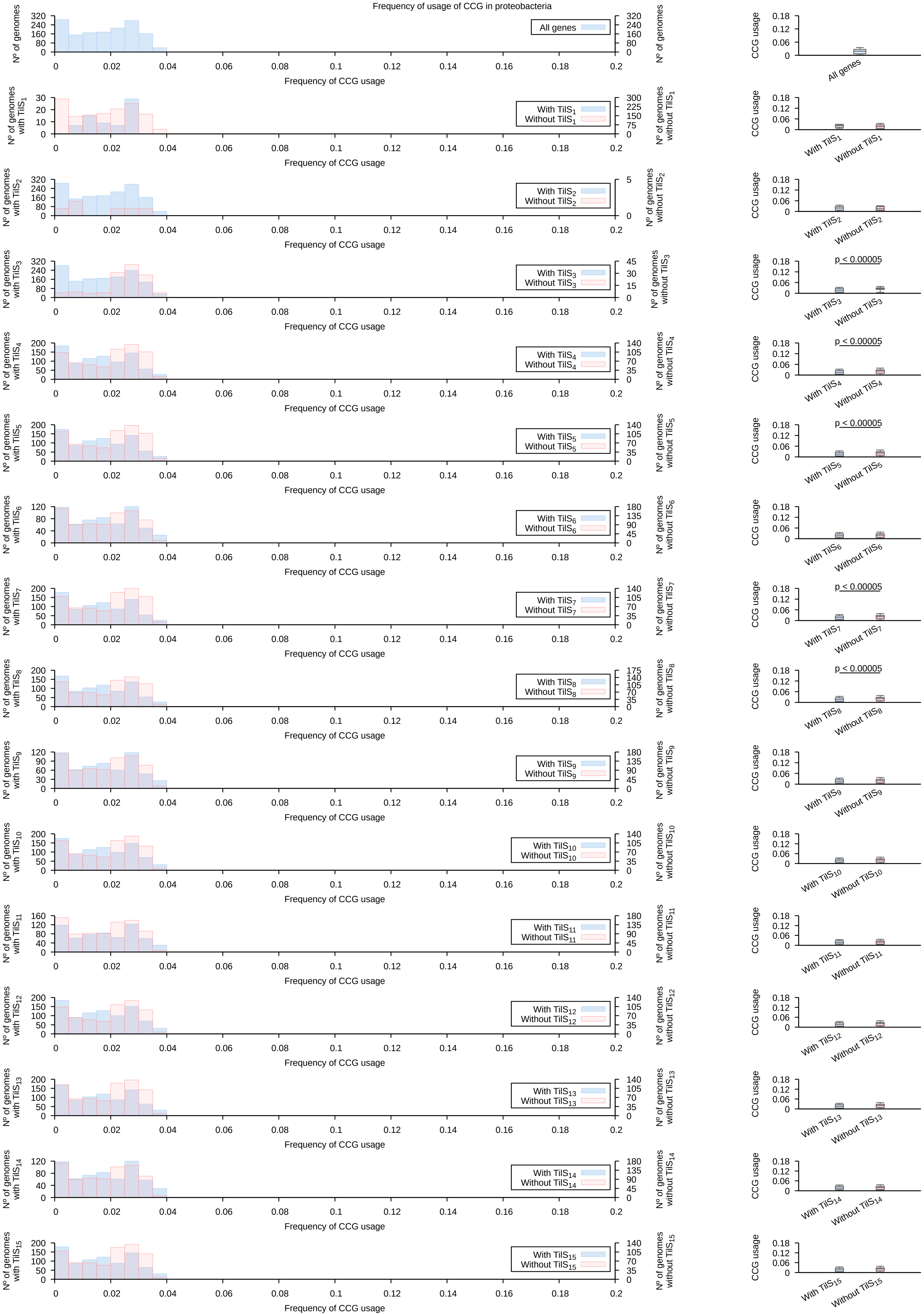
Frequency of usage of CCG in proteobacteria
Nº of genomes
All genes
Frequency of CCG usage
CCG usage
All genes
Nº of genomes with TilS$_1$
With TilS$_1$
Without TilS$_1$
Nº of genomes without TilS$_1$
Nº of genomes with TilS$_2$
With TilS$_2$
Without TilS$_2$
Nº of genomes without TilS$_2$
Nº of genomes with TilS$_3$
With TilS$_3$
Without TilS$_3$
Nº of genomes without TilS$_3$
p < 0.00005
Nº of genomes with TilS$_4$
With TilS$_4$
Without TilS$_4$
Nº of genomes without TilS$_4$
p < 0.00005
Nº of genomes with TilS$_5$
With TilS$_5$
Without TilS$_5$
Nº of genomes without TilS$_5$
p < 0.00005
Nº of genomes with TilS$_6$
With TilS$_6$
Without TilS$_6$
Nº of genomes without TilS$_6$
Nº of genomes with TilS$_7$
With TilS$_7$
Without TilS$_7$
Nº of genomes without TilS$_7$
p < 0.00005
Nº of genomes with TilS$_8$
With TilS$_8$
Without TilS$_8$
Nº of genomes without TilS$_8$
p < 0.00005
Nº of genomes with TilS$_9$
With TilS$_9$
Without TilS$_9$
Nº of genomes without TilS$_9$
Nº of genomes with TilS$_{10}$
With TilS$_{10}$
Without TilS$_{10}$
Nº of genomes without TilS$_{10}$
Nº of genomes with TilS$_{11}$
With TilS$_{11}$
Without TilS$_{11}$
Nº of genomes without TilS$_{11}$
Nº of genomes with TilS$_{12}$
With TilS$_{12}$
Without TilS$_{12}$
Nº of genomes without TilS$_{12}$
Nº of genomes with TilS$_{13}$
With TilS$_{13}$
Without TilS$_{13}$
Nº of genomes without TilS$_{13}$
Nº of genomes with TilS$_{14}$
With TilS$_{14}$
Without TilS$_{14}$
Nº of genomes without TilS$_{14}$
Nº of genomes with TilS$_{15}$
With TilS$_{15}$
Without TilS$_{15}$
Nº of genomes without TilS$_{15}$
Frequency of CCG usage

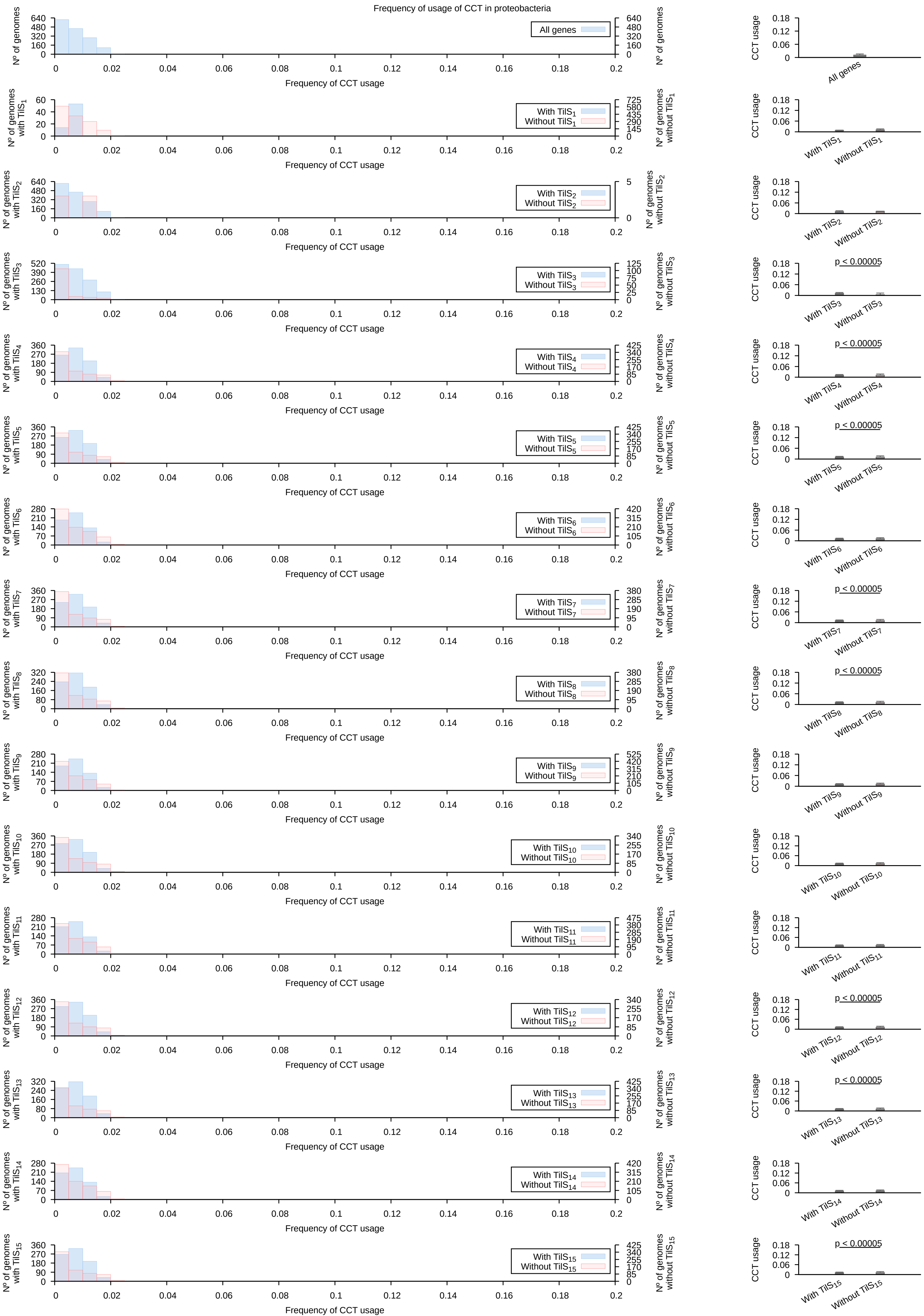

Frequency of usage of CCT in proteobacteria
Nº of genomes
All genes
Frequency of CCT usage
CCT usage
Nº of genomes with TilS$_1$
Nº of genomes without TilS$_1$
With TilS$_1$
Without TilS$_1$
Nº of genomes with TilS$_2$
Nº of genomes without TilS$_2$
With TilS$_2$
Without TilS$_2$
Nº of genomes with TilS$_3$
Nº of genomes without TilS$_3$
With TilS$_3$
Without TilS$_3$
p < 0.00005
Nº of genomes with TilS$_4$
Nº of genomes without TilS$_4$
With TilS$_4$
Without TilS$_4$
p < 0.00005
Nº of genomes with TilS$_5$
Nº of genomes without TilS$_5$
With TilS$_5$
Without TilS$_5$
p < 0.00005
Nº of genomes with TilS$_6$
Nº of genomes without TilS$_6$
With TilS$_6$
Without TilS$_6$
Nº of genomes with TilS$_7$
Nº of genomes without TilS$_7$
With TilS$_7$
Without TilS$_7$
p < 0.00005
Nº of genomes with TilS$_8$
Nº of genomes without TilS$_8$
With TilS$_8$
Without TilS$_8$
p < 0.00005
Nº of genomes with TilS$_9$
Nº of genomes without TilS$_9$
With TilS$_9$
Without TilS$_9$
Nº of genomes with TilS$_{10}$
Nº of genomes without TilS$_{10}$
With TilS$_{10}$
Without TilS$_{10}$
Nº of genomes with TilS$_{11}$
Nº of genomes without TilS$_{11}$
With TilS$_{11}$
Without TilS$_{11}$
Nº of genomes with TilS$_{12}$
Nº of genomes without TilS$_{12}$
With TilS$_{12}$
Without TilS$_{12}$
p < 0.00005
Nº of genomes with TilS$_{13}$
Nº of genomes without TilS$_{13}$
With TilS$_{13}$
Without TilS$_{13}$
p < 0.00005
Nº of genomes with TilS$_{14}$
Nº of genomes without TilS$_{14}$
With TilS$_{14}$
Without TilS$_{14}$
Nº of genomes with TilS$_{15}$
Nº of genomes without TilS$_{15}$
With TilS$_{15}$
Without TilS$_{15}$
p < 0.00005

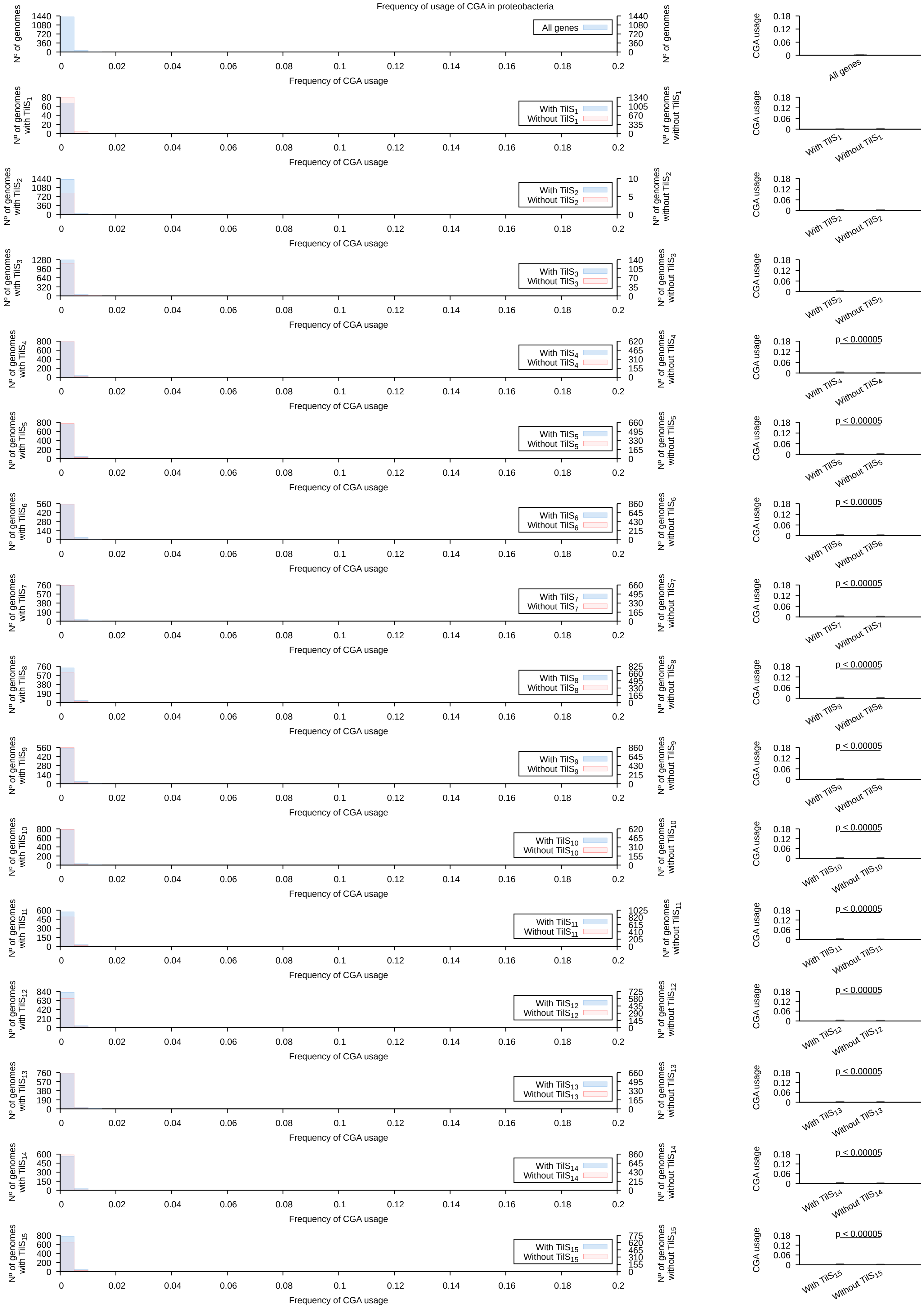
Frequency of usage of CGA in proteobacteria

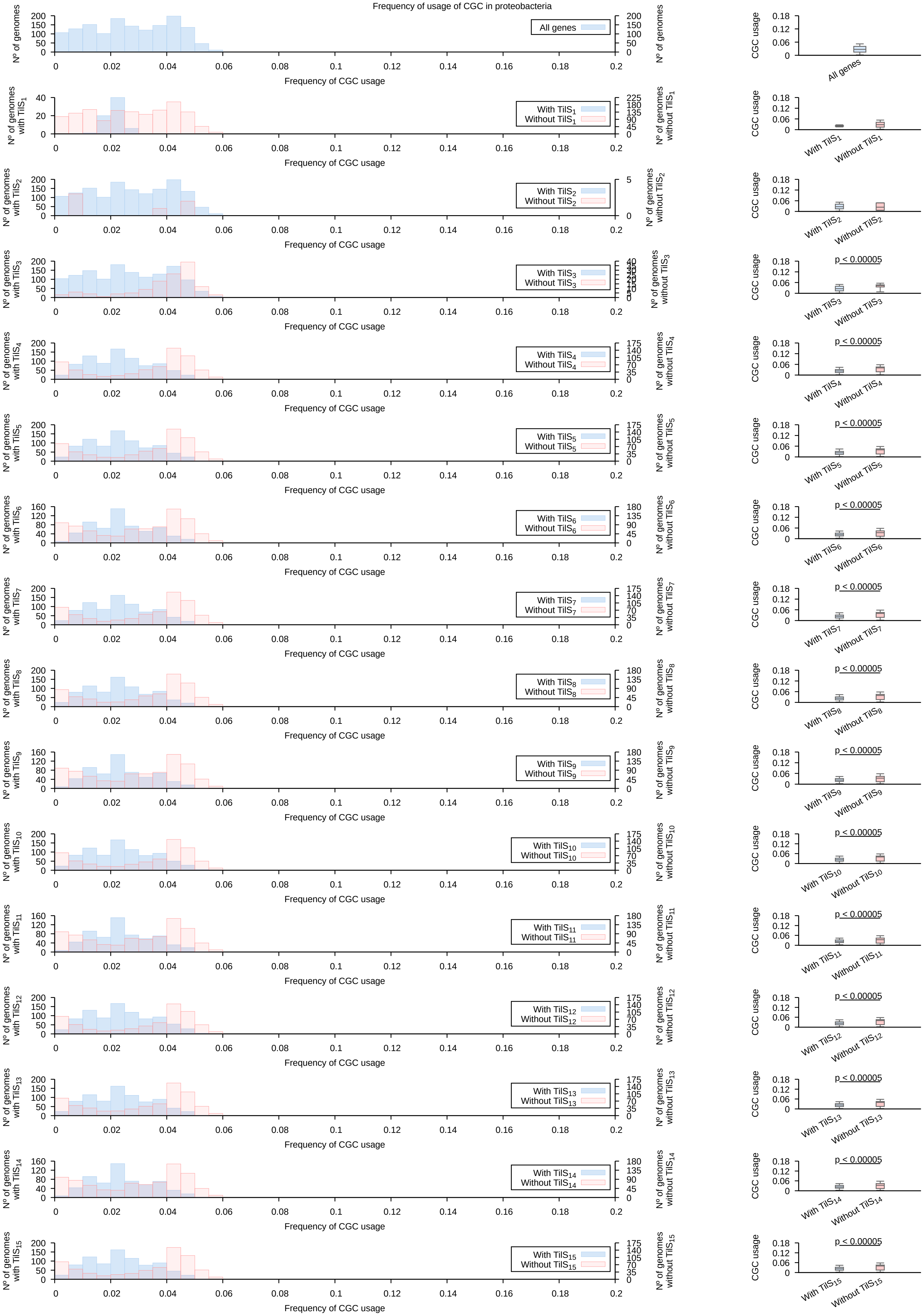
Frequency of usage of CGC in proteobacteria
Nº of genomes
All genes
Frequency of CGC usage
CGC usage
Nº of genomes with TilS$_1$
Nº of genomes without TilS$_1$
With TilS$_1$
Without TilS$_1$
Nº of genomes with TilS$_2$
Nº of genomes without TilS$_2$
With TilS$_2$
Without TilS$_2$
Nº of genomes with TilS$_3$
Nº of genomes without TilS$_3$
With TilS$_3$
Without TilS$_3$
p < 0.00005
Nº of genomes with TilS$_4$
Nº of genomes without TilS$_4$
With TilS$_4$
Without TilS$_4$
Nº of genomes with TilS$_5$
Nº of genomes without TilS$_5$
With TilS$_5$
Without TilS$_5$
Nº of genomes with TilS$_6$
Nº of genomes without TilS$_6$
With TilS$_6$
Without TilS$_6$
Nº of genomes with TilS$_7$
Nº of genomes without TilS$_7$
With TilS$_7$
Without TilS$_7$
Nº of genomes with TilS$_8$
Nº of genomes without TilS$_8$
With TilS$_8$
Without TilS$_8$
Nº of genomes with TilS$_9$
Nº of genomes without TilS$_9$
With TilS$_9$
Without TilS$_9$
Nº of genomes with TilS$_{10}$
Nº of genomes without TilS$_{10}$
With TilS$_{10}$
Without TilS$_{10}$
Nº of genomes with TilS$_{11}$
Nº of genomes without TilS$_{11}$
With TilS$_{11}$
Without TilS$_{11}$
Nº of genomes with TilS$_{12}$
Nº of genomes without TilS$_{12}$
With TilS$_{12}$
Without TilS$_{12}$
Nº of genomes with TilS$_{13}$
Nº of genomes without TilS$_{13}$
With TilS$_{13}$
Without TilS$_{13}$
Nº of genomes with TilS$_{14}$
Nº of genomes without TilS$_{14}$
With TilS$_{14}$
Without TilS$_{14}$
Nº of genomes with TilS$_{15}$
Nº of genomes without TilS$_{15}$
With TilS$_{15}$
Without TilS$_{15}$

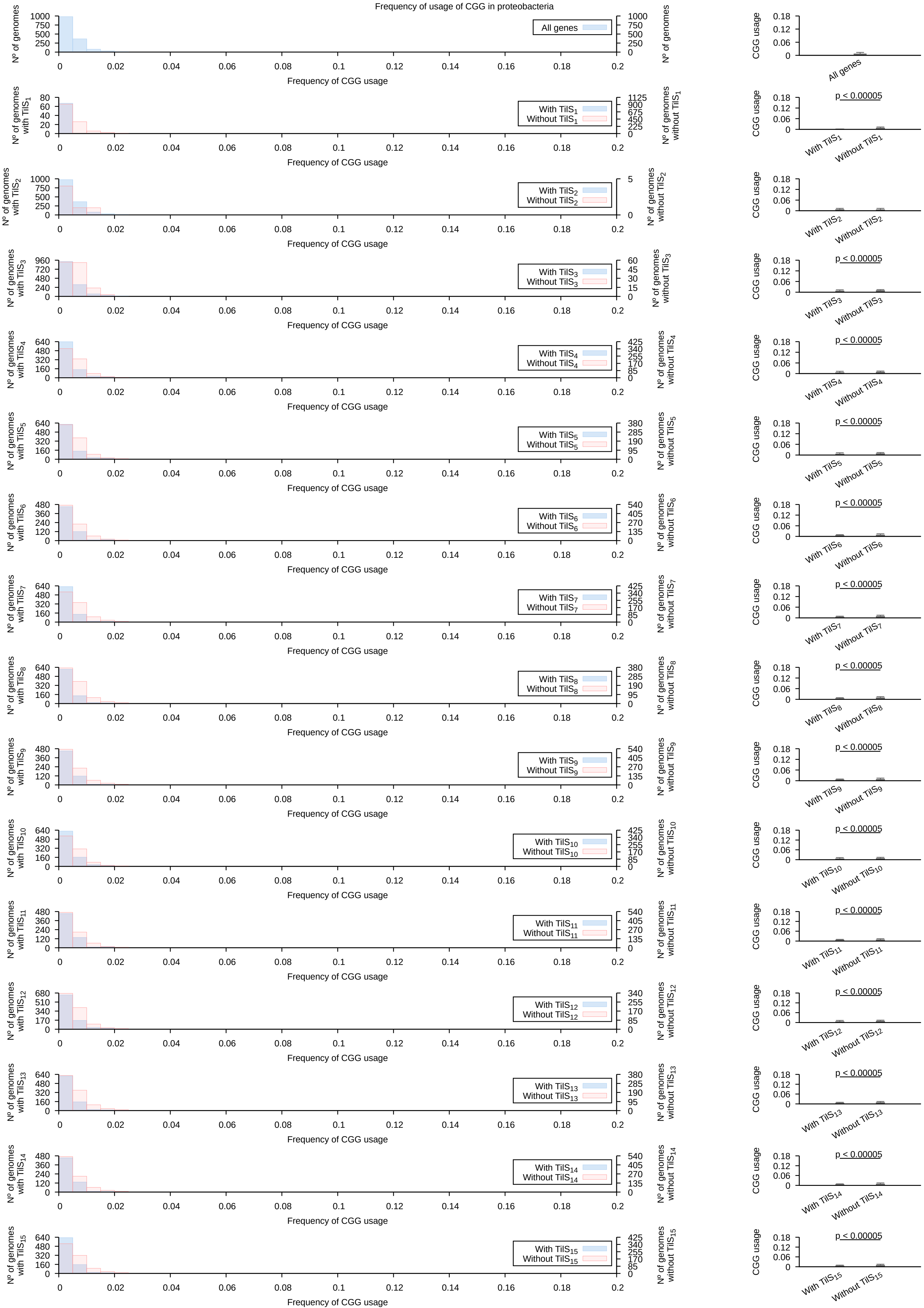

Frequency of usage of CGG in proteobacteria
Nº of genomes
All genes
Frequency of CGG usage
CGG usage
All genes
Nº of genomes with TilS$_1$
Nº of genomes without TilS$_1$
With TilS$_1$
Without TilS$_1$
p < 0.00005
Nº of genomes with TilS$_2$
Nº of genomes without TilS$_2$
With TilS$_2$
Without TilS$_2$
Nº of genomes with TilS$_3$
Nº of genomes without TilS$_3$
With TilS$_3$
Without TilS$_3$
p < 0.00005
Nº of genomes with TilS$_4$
Nº of genomes without TilS$_4$
With TilS$_4$
Without TilS$_4$
p < 0.00005
Nº of genomes with TilS$_5$
Nº of genomes without TilS$_5$
With TilS$_5$
Without TilS$_5$
p < 0.00005
Nº of genomes with TilS$_6$
Nº of genomes without TilS$_6$
With TilS$_6$
Without TilS$_6$
p < 0.00005
Nº of genomes with TilS$_7$
Nº of genomes without TilS$_7$
With TilS$_7$
Without TilS$_7$
p < 0.00005
Nº of genomes with TilS$_8$
Nº of genomes without TilS$_8$
With TilS$_8$
Without TilS$_8$
p < 0.00005
Nº of genomes with TilS$_9$
Nº of genomes without TilS$_9$
With TilS$_9$
Without TilS$_9$
p < 0.00005
Nº of genomes with TilS$_{10}$
Nº of genomes without TilS$_{10}$
With TilS$_{10}$
Without TilS$_{10}$
p < 0.00005
Nº of genomes with TilS$_{11}$
Nº of genomes without TilS$_{11}$
With TilS$_{11}$
Without TilS$_{11}$
p < 0.00005
Nº of genomes with TilS$_{12}$
Nº of genomes without TilS$_{12}$
With TilS$_{12}$
Without TilS$_{12}$
p < 0.00005
Nº of genomes with TilS$_{13}$
Nº of genomes without TilS$_{13}$
With TilS$_{13}$
Without TilS$_{13}$
p < 0.00005
Nº of genomes with TilS$_{14}$
Nº of genomes without TilS$_{14}$
With TilS$_{14}$
Without TilS$_{14}$
p < 0.00005
Nº of genomes with TilS$_{15}$
Nº of genomes without TilS$_{15}$
With TilS$_{15}$
Without TilS$_{15}$
p < 0.00005

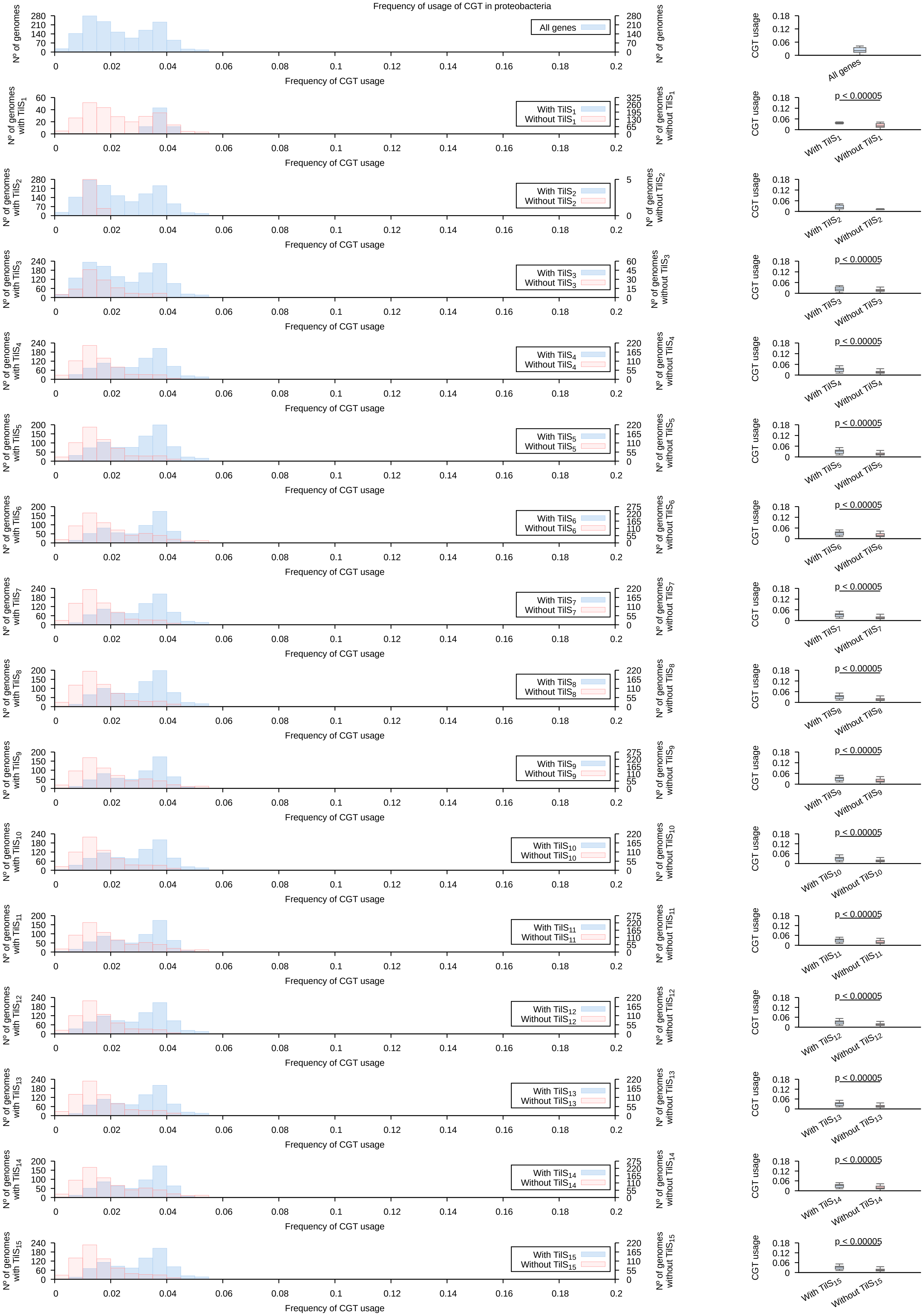

Frequency of usage of CGT in proteobacteria
Nº of genomes
All genes
Frequency of CGT usage
CGT usage
Nº of genomes with TilS$_1$
Nº of genomes without TilS$_1$
With TilS$_1$
Without TilS$_1$
p < 0.00005
Nº of genomes with TilS$_2$
Nº of genomes without TilS$_2$
With TilS$_2$
Without TilS$_2$
Nº of genomes with TilS$_3$
Nº of genomes without TilS$_3$
With TilS$_3$
Without TilS$_3$
Nº of genomes with TilS$_4$
Nº of genomes without TilS$_4$
With TilS$_4$
Without TilS$_4$
Nº of genomes with TilS$_5$
Nº of genomes without TilS$_5$
With TilS$_5$
Without TilS$_5$
Nº of genomes with TilS$_6$
Nº of genomes without TilS$_6$
With TilS$_6$
Without TilS$_6$
Nº of genomes with TilS$_7$
Nº of genomes without TilS$_7$
With TilS$_7$
Without TilS$_7$
Nº of genomes with TilS$_8$
Nº of genomes without TilS$_8$
With TilS$_8$
Without TilS$_8$
Nº of genomes with TilS$_9$
Nº of genomes without TilS$_9$
With TilS$_9$
Without TilS$_9$
Nº of genomes with TilS$_{10}$
Nº of genomes without TilS$_{10}$
With TilS$_{10}$
Without TilS$_{10}$
Nº of genomes with TilS$_{11}$
Nº of genomes without TilS$_{11}$
With TilS$_{11}$
Without TilS$_{11}$
Nº of genomes with TilS$_{12}$
Nº of genomes without TilS$_{12}$
With TilS$_{12}$
Without TilS$_{12}$
Nº of genomes with TilS$_{13}$
Nº of genomes without TilS$_{13}$
With TilS$_{13}$
Without TilS$_{13}$
Nº of genomes with TilS$_{14}$
Nº of genomes without TilS$_{14}$
With TilS$_{14}$
Without TilS$_{14}$
Nº of genomes with TilS$_{15}$
Nº of genomes without TilS$_{15}$
With TilS$_{15}$
Without TilS$_{15}$

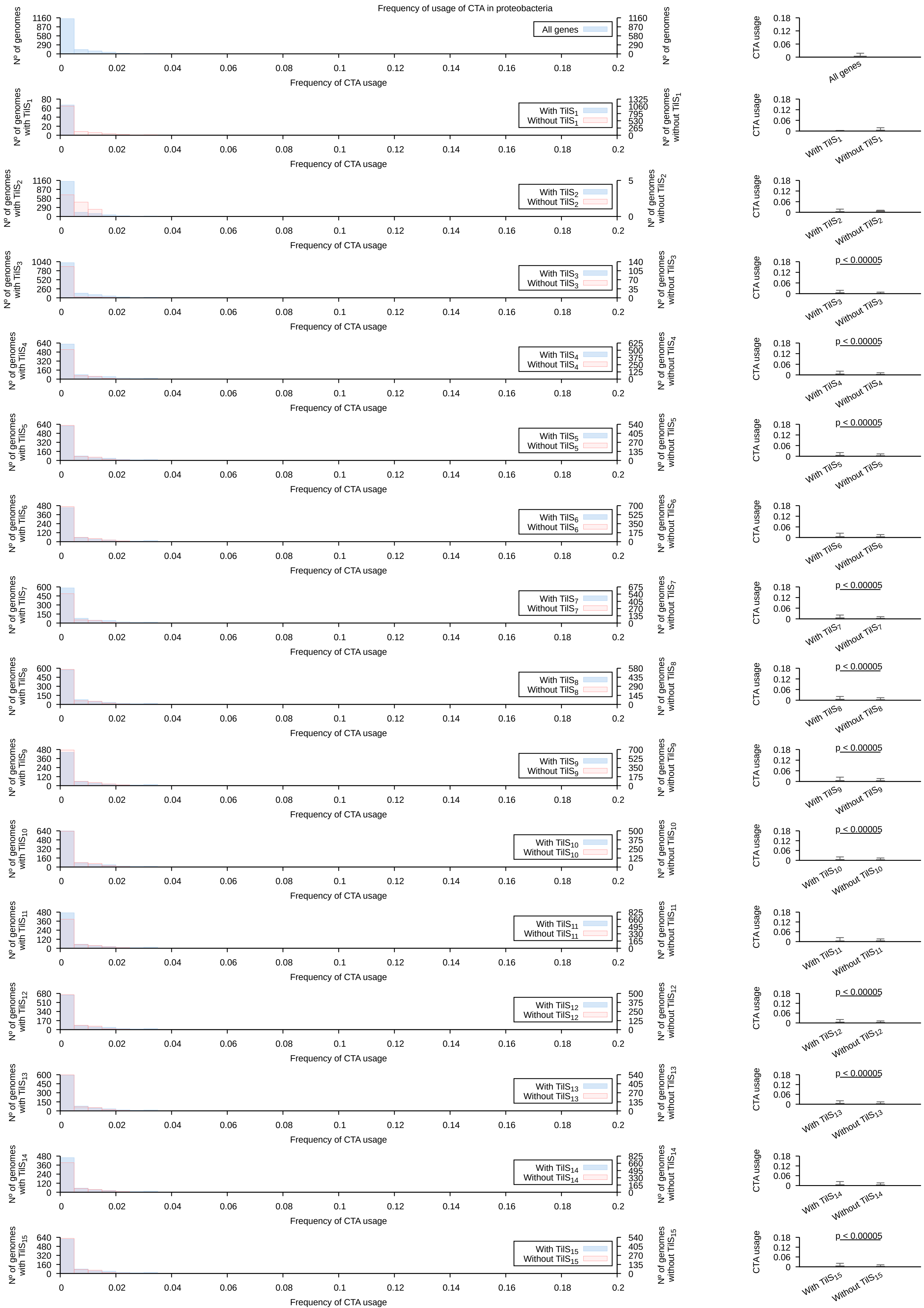

Frequency of usage of CTA in proteobacteria
Nº of genomes
All genes
Frequency of CTA usage
CTA usage
Nº of genomes with TilS$_1$
Nº of genomes without TilS$_1$
With TilS$_1$
Without TilS$_1$
Nº of genomes with TilS$_2$
Nº of genomes without TilS$_2$
With TilS$_2$
Without TilS$_2$
Nº of genomes with TilS$_3$
Nº of genomes without TilS$_3$
With TilS$_3$
Without TilS$_3$
p < 0.00005
Nº of genomes with TilS$_4$
Nº of genomes without TilS$_4$
With TilS$_4$
Without TilS$_4$
p < 0.00005
Nº of genomes with TilS$_5$
Nº of genomes without TilS$_5$
With TilS$_5$
Without TilS$_5$
p < 0.00005
Nº of genomes with TilS$_6$
Nº of genomes without TilS$_6$
With TilS$_6$
Without TilS$_6$
Nº of genomes with TilS$_7$
Nº of genomes without TilS$_7$
With TilS$_7$
Without TilS$_7$
p < 0.00005
Nº of genomes with TilS$_8$
Nº of genomes without TilS$_8$
With TilS$_8$
Without TilS$_8$
p < 0.00005
Nº of genomes with TilS$_9$
Nº of genomes without TilS$_9$
With TilS$_9$
Without TilS$_9$
p < 0.00005
Nº of genomes with TilS$_{10}$
Nº of genomes without TilS$_{10}$
With TilS$_{10}$
Without TilS$_{10}$
p < 0.00005
Nº of genomes with TilS$_{11}$
Nº of genomes without TilS$_{11}$
With TilS$_{11}$
Without TilS$_{11}$
Nº of genomes with TilS$_{12}$
Nº of genomes without TilS$_{12}$
With TilS$_{12}$
Without TilS$_{12}$
p < 0.00005
Nº of genomes with TilS$_{13}$
Nº of genomes without TilS$_{13}$
With TilS$_{13}$
Without TilS$_{13}$
p < 0.00005
Nº of genomes with TilS$_{14}$
Nº of genomes without TilS$_{14}$
With TilS$_{14}$
Without TilS$_{14}$
Nº of genomes with TilS$_{15}$
Nº of genomes without TilS$_{15}$
With TilS$_{15}$
Without TilS$_{15}$
p < 0.00005

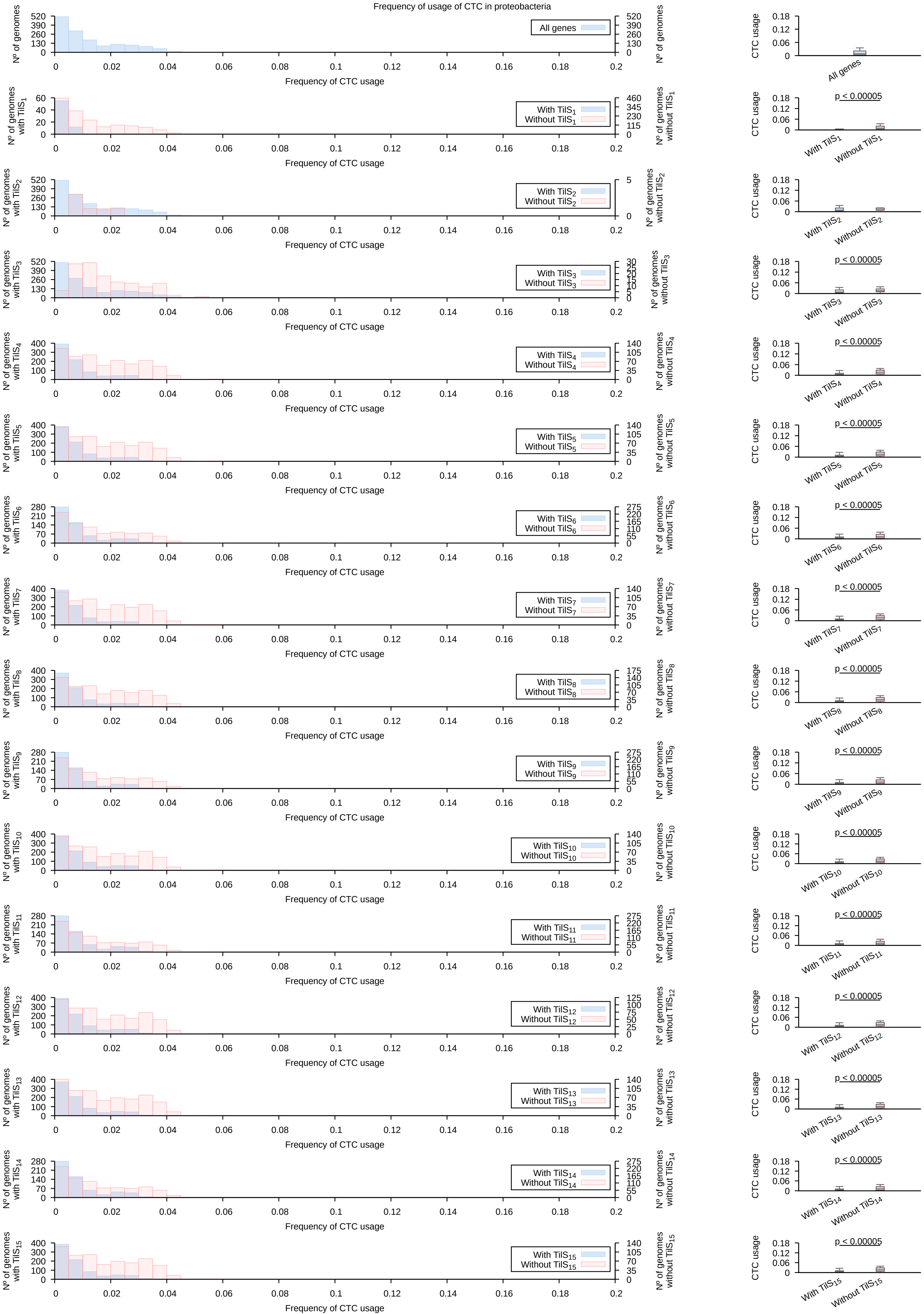

Frequency of usage of CTC in proteobacteria
Nº of genomes
All genes
Frequency of CTC usage
CTC usage
0
0.02
0.04
0.06
0.08
0.1
0.12
0.14
0.16
0.18
0.2
0.06
0.12
0.18
520
390
260
130
All genes
Nº of genomes with TilS$_1$
Nº of genomes without TilS$_1$
With TilS$_1$
Without TilS$_1$
p < 0.00005
Nº of genomes with TilS$_2$
Nº of genomes without TilS$_2$
With TilS$_2$
Without TilS$_2$
Nº of genomes with TilS$_3$
Nº of genomes without TilS$_3$
With TilS$_3$
Without TilS$_3$
Nº of genomes with TilS$_4$
Nº of genomes without TilS$_4$
With TilS$_4$
Without TilS$_4$
Nº of genomes with TilS$_5$
Nº of genomes without TilS$_5$
With TilS$_5$
Without TilS$_5$
Nº of genomes with TilS$_6$
Nº of genomes without TilS$_6$
With TilS$_6$
Without TilS$_6$
Nº of genomes with TilS$_7$
Nº of genomes without TilS$_7$
With TilS$_7$
Without TilS$_7$
Nº of genomes with TilS$_8$
Nº of genomes without TilS$_8$
With TilS$_8$
Without TilS$_8$
Nº of genomes with TilS$_9$
Nº of genomes without TilS$_9$
With TilS$_9$
Without TilS$_9$
Nº of genomes with TilS$_{10}$
Nº of genomes without TilS$_{10}$
With TilS$_{10}$
Without TilS$_{10}$
Nº of genomes with TilS$_{11}$
Nº of genomes without TilS$_{11}$
With TilS$_{11}$
Without TilS$_{11}$
Nº of genomes with TilS$_{12}$
Nº of genomes without TilS$_{12}$
With TilS$_{12}$
Without TilS$_{12}$
Nº of genomes with TilS$_{13}$
Nº of genomes without TilS$_{13}$
With TilS$_{13}$
Without TilS$_{13}$
Nº of genomes with TilS$_{14}$
Nº of genomes without TilS$_{14}$
With TilS$_{14}$
Without TilS$_{14}$
Nº of genomes with TilS$_{15}$
Nº of genomes without TilS$_{15}$
With TilS$_{15}$
Without TilS$_{15}$

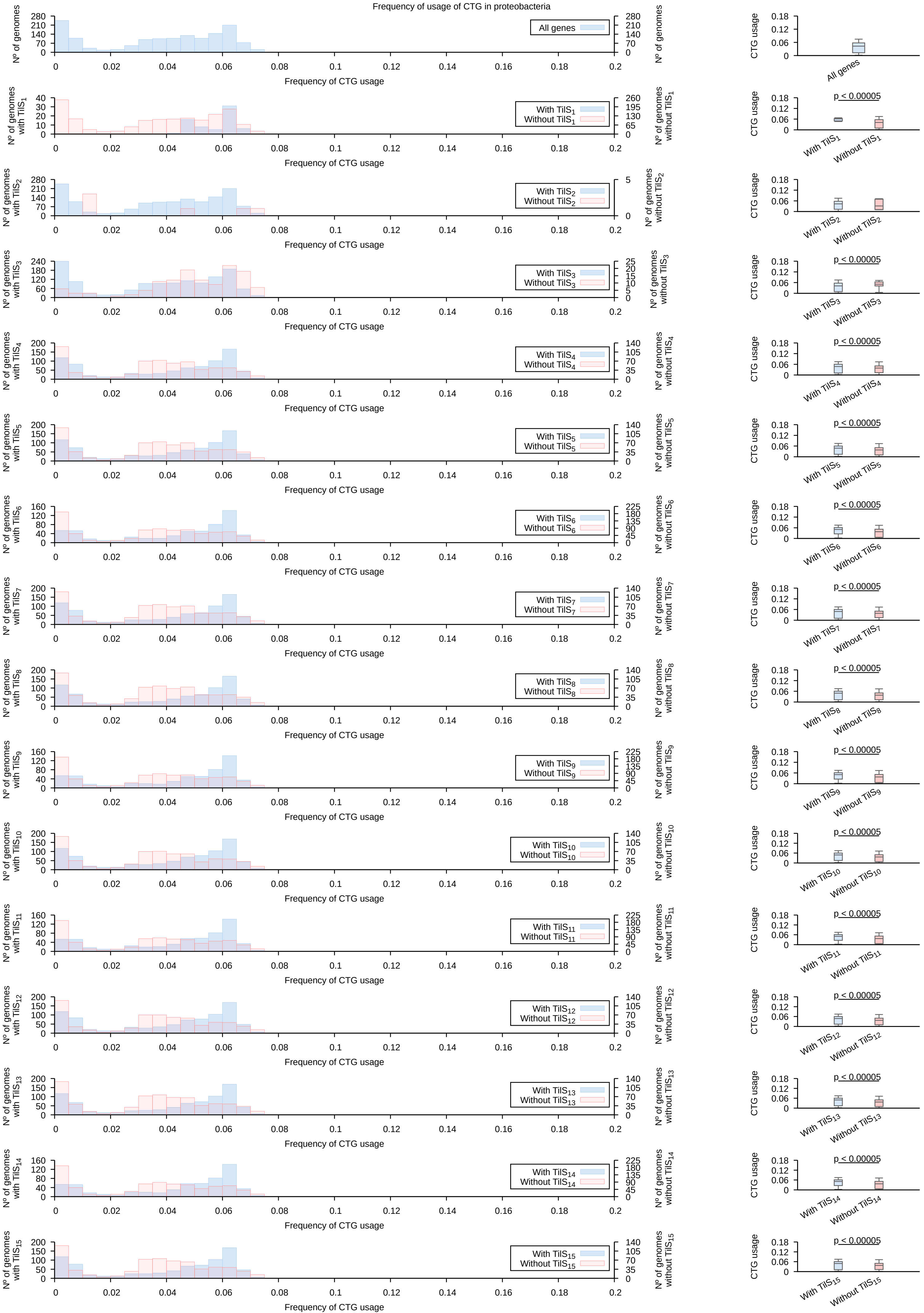

Frequency of usage of CTG in proteobacteria
Nº of genomes
All genes
Frequency of CTG usage
CTG usage
Nº of genomes with TilS$_1$
Nº of genomes without TilS$_1$
With TilS$_1$
Without TilS$_1$
p < 0.00005
Nº of genomes with TilS$_2$
Nº of genomes without TilS$_2$
With TilS$_2$
Without TilS$_2$
Nº of genomes with TilS$_3$
Nº of genomes without TilS$_3$
With TilS$_3$
Without TilS$_3$
Nº of genomes with TilS$_4$
Nº of genomes without TilS$_4$
With TilS$_4$
Without TilS$_4$
Nº of genomes with TilS$_5$
Nº of genomes without TilS$_5$
With TilS$_5$
Without TilS$_5$
Nº of genomes with TilS$_6$
Nº of genomes without TilS$_6$
With TilS$_6$
Without TilS$_6$
Nº of genomes with TilS$_7$
Nº of genomes without TilS$_7$
With TilS$_7$
Without TilS$_7$
Nº of genomes with TilS$_8$
Nº of genomes without TilS$_8$
With TilS$_8$
Without TilS$_8$
Nº of genomes with TilS$_9$
Nº of genomes without TilS$_9$
With TilS$_9$
Without TilS$_9$
Nº of genomes with TilS$_{10}$
Nº of genomes without TilS$_{10}$
With TilS$_{10}$
Without TilS$_{10}$
Nº of genomes with TilS$_{11}$
Nº of genomes without TilS$_{11}$
With TilS$_{11}$
Without TilS$_{11}$
Nº of genomes with TilS$_{12}$
Nº of genomes without TilS$_{12}$
With TilS$_{12}$
Without TilS$_{12}$
Nº of genomes with TilS$_{13}$
Nº of genomes without TilS$_{13}$
With TilS$_{13}$
Without TilS$_{13}$
Nº of genomes with TilS$_{14}$
Nº of genomes without TilS$_{14}$
With TilS$_{14}$
Without TilS$_{14}$
Nº of genomes with TilS$_{15}$
Nº of genomes without TilS$_{15}$
With TilS$_{15}$
Without TilS$_{15}$

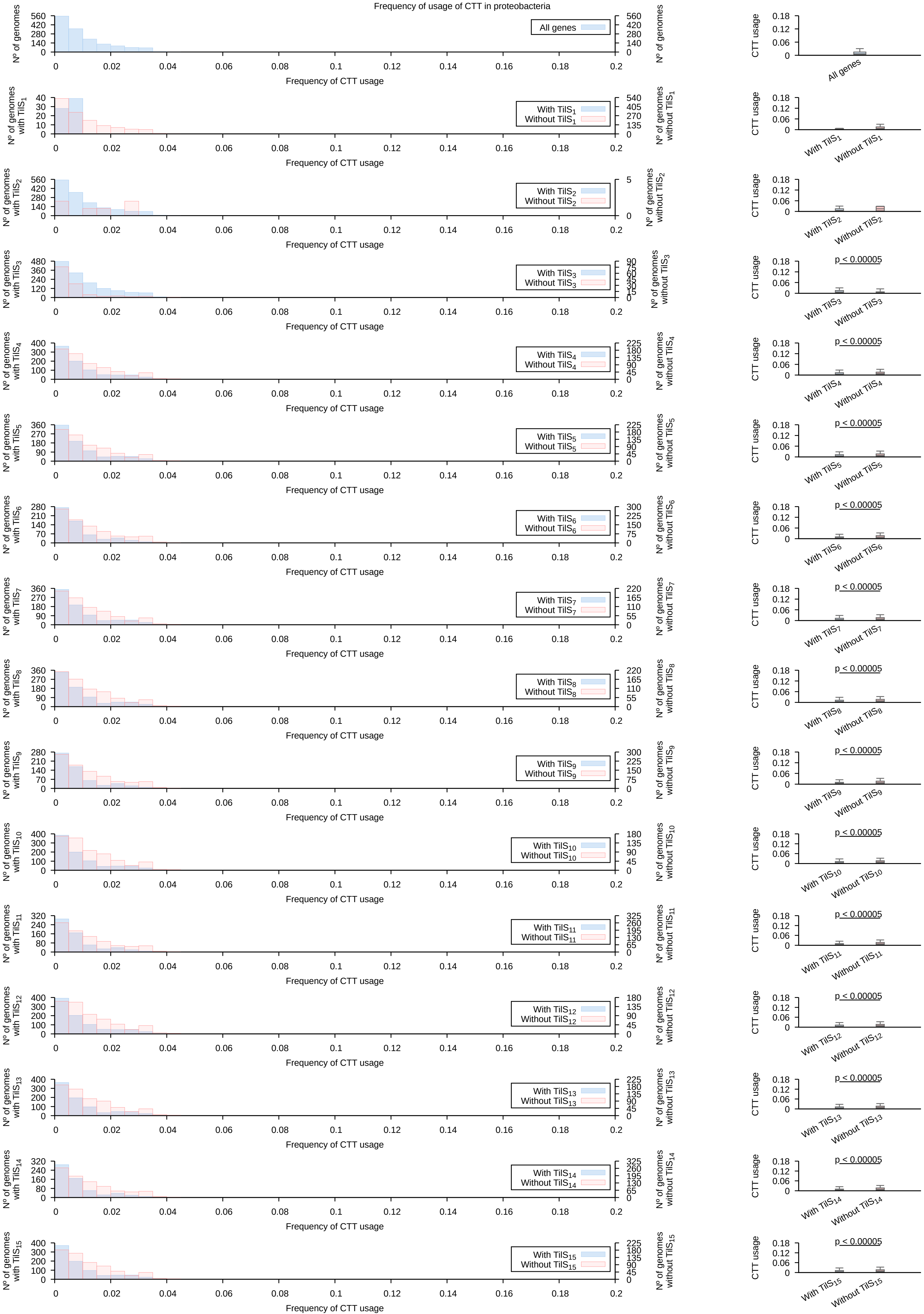
Frequency of usage of CTT in proteobacteria
Nº of genomes
All genes
Frequency of CTT usage
CTT usage
Nº of genomes with TilS1
Nº of genomes without TilS1
With TilS1
Without TilS1
Nº of genomes with TilS2
Nº of genomes without TilS2
With TilS2
Without TilS2
Nº of genomes with TilS3
Nº of genomes without TilS3
With TilS3
Without TilS3
p < 0.00005
Nº of genomes with TilS4
Nº of genomes without TilS4
With TilS4
Without TilS4
p < 0.00005
Nº of genomes with TilS5
Nº of genomes without TilS5
With TilS5
Without TilS5
p < 0.00005
Nº of genomes with TilS6
Nº of genomes without TilS6
With TilS6
Without TilS6
p < 0.00005
Nº of genomes with TilS7
Nº of genomes without TilS7
With TilS7
Without TilS7
p < 0.00005
Nº of genomes with TilS8
Nº of genomes without TilS8
With TilS8
Without TilS8
p < 0.00005
Nº of genomes with TilS9
Nº of genomes without TilS9
With TilS9
Without TilS9
p < 0.00005
Nº of genomes with TilS10
Nº of genomes without TilS10
With TilS10
Without TilS10
p < 0.00005
Nº of genomes with TilS11
Nº of genomes without TilS11
With TilS11
Without TilS11
p < 0.00005
Nº of genomes with TilS12
Nº of genomes without TilS12
With TilS12
Without TilS12
p < 0.00005
Nº of genomes with TilS13
Nº of genomes without TilS13
With TilS13
Without TilS13
p < 0.00005
Nº of genomes with TilS14
Nº of genomes without TilS14
With TilS14
Without TilS14
p < 0.00005
Nº of genomes with TilS15
Nº of genomes without TilS15
With TilS15
Without TilS15
p < 0.00005

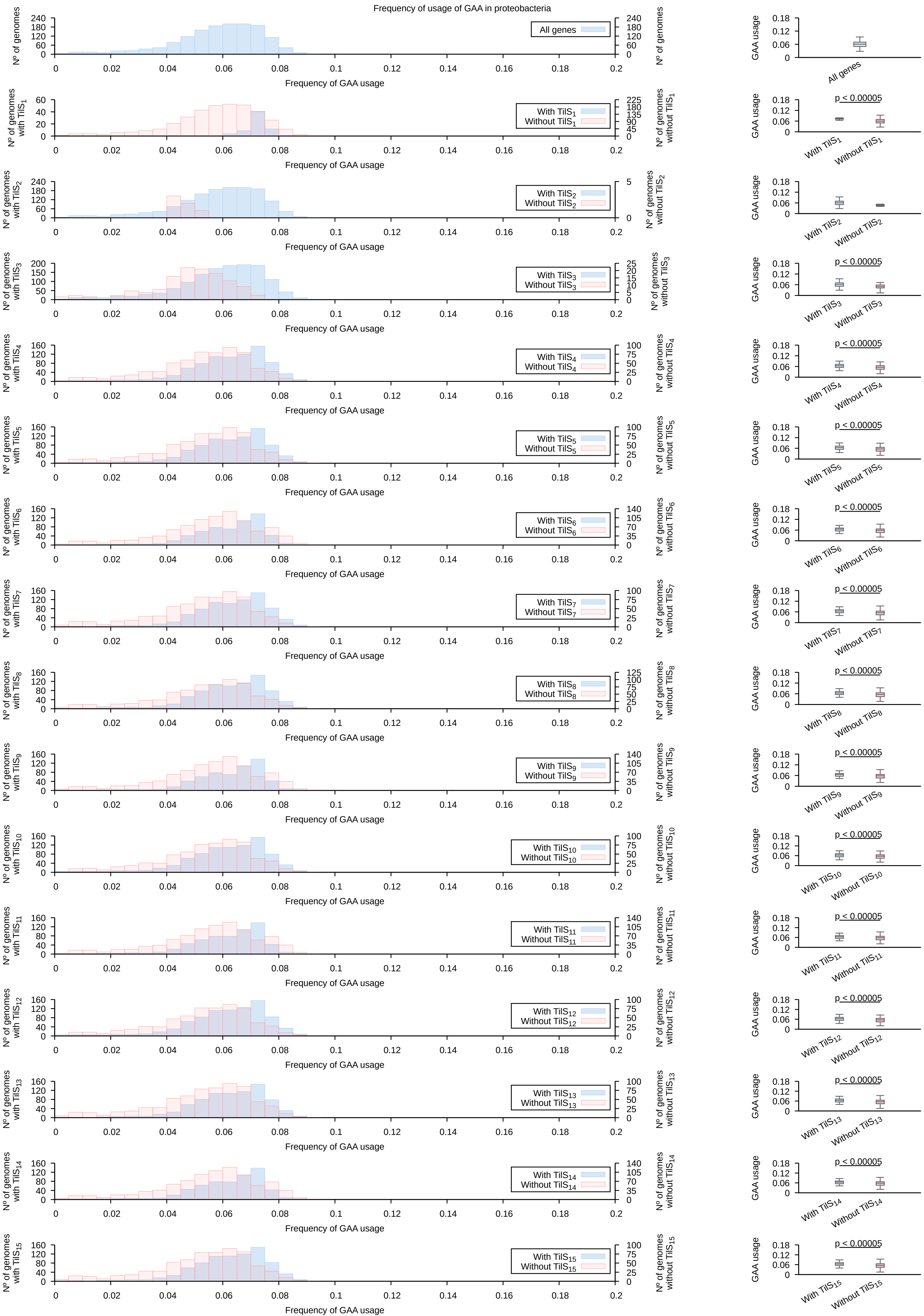
Frequency of usage of GAA in proteobacteria
Nº of genomes
All genes
Frequency of GAA usage
GAA usage
p < 0.00005
With TilS1
Without TilS1
With TilS2
Without TilS2
With TilS3
Without TilS3
With TilS4
Without TilS4
With TilS5
Without TilS5
With TilS6
Without TilS6
With TilS7
Without TilS7
With TilS8
Without TilS8
With TilS9
Without TilS9
With TilS10
Without TilS10
With TilS11
Without TilS11
With TilS12
Without TilS12
With TilS13
Without TilS13
With TilS14
Without TilS14
With TilS15
Without TilS15

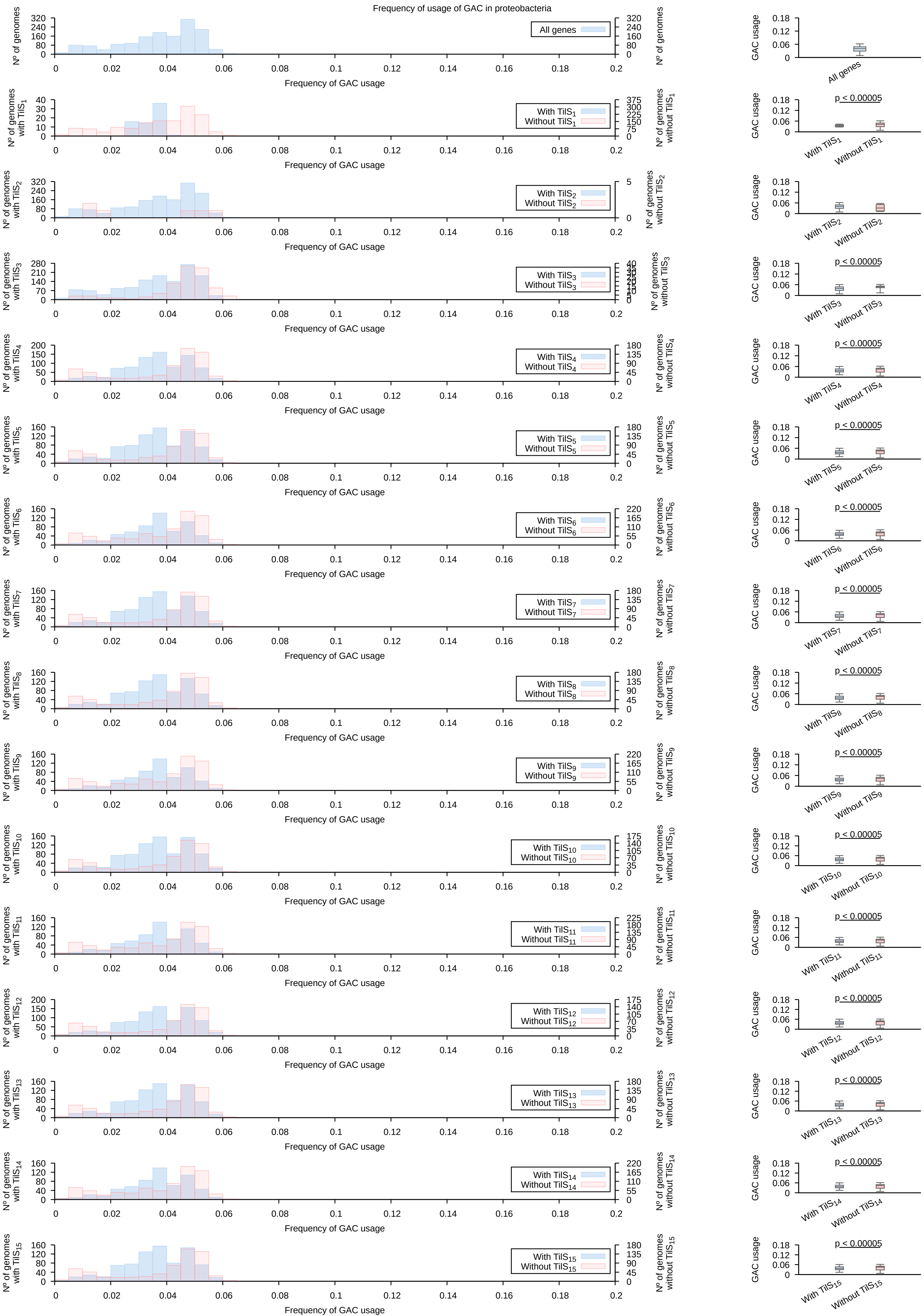

Frequency of usage of GAC in proteobacteria
Nº of genomes
All genes
Frequency of GAC usage
GAC usage
All genes
Nº of genomes with $TilS_1$
Nº of genomes without $TilS_1$
With $TilS_1$
Without $TilS_1$
p < 0.00005
Nº of genomes with $TilS_2$
Nº of genomes without $TilS_2$
With $TilS_2$
Without $TilS_2$
Nº of genomes with $TilS_3$
Nº of genomes without $TilS_3$
With $TilS_3$
Without $TilS_3$
p < 0.00005
Nº of genomes with $TilS_4$
Nº of genomes without $TilS_4$
With $TilS_4$
Without $TilS_4$
p < 0.00005
Nº of genomes with $TilS_5$
Nº of genomes without $TilS_5$
With $TilS_5$
Without $TilS_5$
p < 0.00005
Nº of genomes with $TilS_6$
Nº of genomes without $TilS_6$
With $TilS_6$
Without $TilS_6$
p < 0.00005
Nº of genomes with $TilS_7$
Nº of genomes without $TilS_7$
With $TilS_7$
Without $TilS_7$
p < 0.00005
Nº of genomes with $TilS_8$
Nº of genomes without $TilS_8$
With $TilS_8$
Without $TilS_8$
p < 0.00005
Nº of genomes with $TilS_9$
Nº of genomes without $TilS_9$
With $TilS_9$
Without $TilS_9$
p < 0.00005
Nº of genomes with $TilS_{10}$
Nº of genomes without $TilS_{10}$
With $TilS_{10}$
Without $TilS_{10}$
p < 0.00005
Nº of genomes with $TilS_{11}$
Nº of genomes without $TilS_{11}$
With $TilS_{11}$
Without $TilS_{11}$
p < 0.00005
Nº of genomes with $TilS_{12}$
Nº of genomes without $TilS_{12}$
With $TilS_{12}$
Without $TilS_{12}$
p < 0.00005
Nº of genomes with $TilS_{13}$
Nº of genomes without $TilS_{13}$
With $TilS_{13}$
Without $TilS_{13}$
p < 0.00005
Nº of genomes with $TilS_{14}$
Nº of genomes without $TilS_{14}$
With $TilS_{14}$
Without $TilS_{14}$
p < 0.00005
Nº of genomes with $TilS_{15}$
Nº of genomes without $TilS_{15}$
With $TilS_{15}$
Without $TilS_{15}$
p < 0.00005

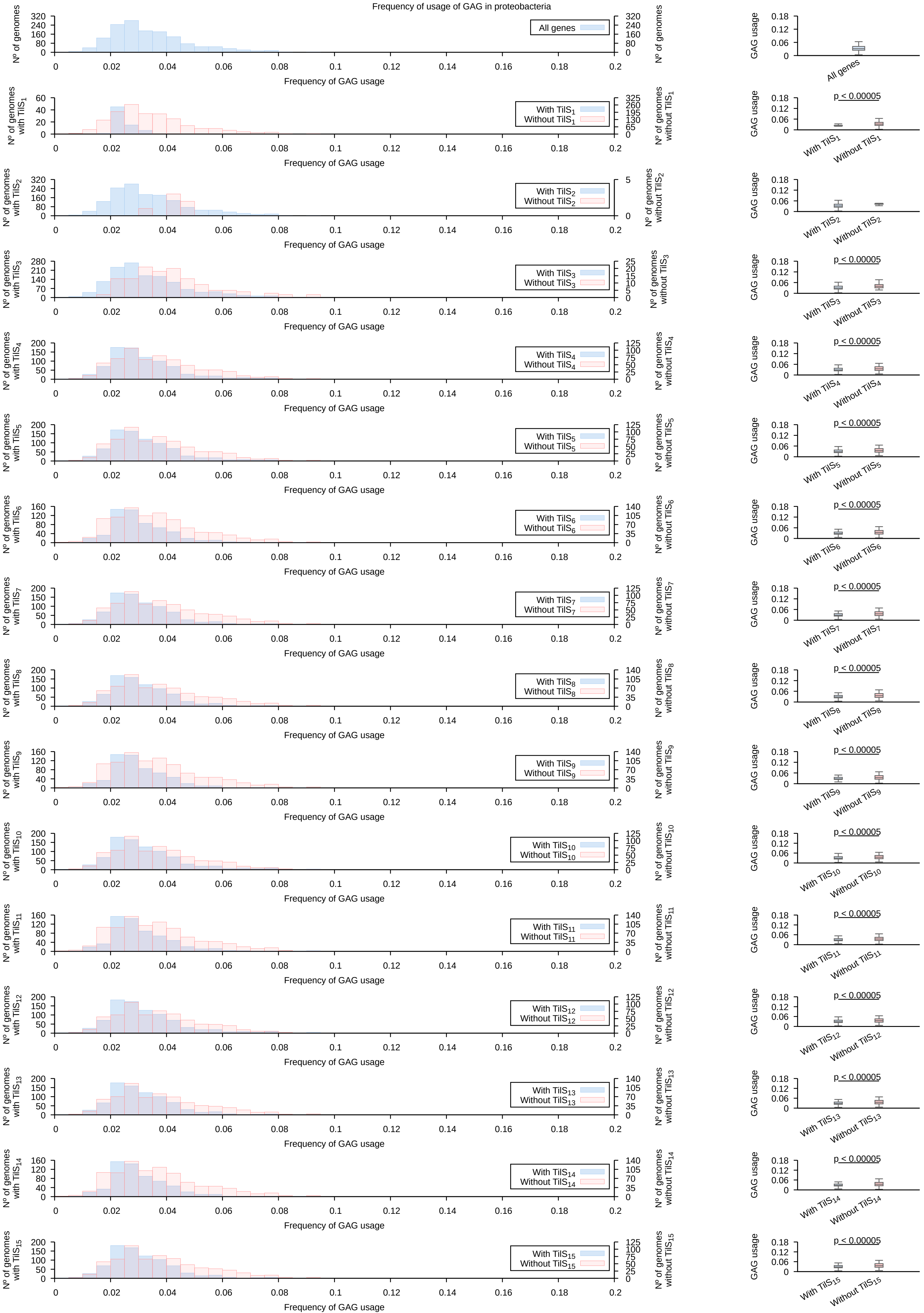

Frequency of usage of GAG in proteobacteria
Nº of genomes
All genes
Frequency of GAG usage
GAG usage
0
0.02
0.04
0.06
0.08
0.1
0.12
0.14
0.16
0.18
0.2
Nº of genomes with TilS$_1$
With TilS$_1$
Without TilS$_1$
Nº of genomes without TilS$_1$
p < 0.00005
Nº of genomes with TilS$_2$
With TilS$_2$
Without TilS$_2$
Nº of genomes without TilS$_2$
Nº of genomes with TilS$_3$
With TilS$_3$
Without TilS$_3$
Nº of genomes without TilS$_3$
Nº of genomes with TilS$_4$
With TilS$_4$
Without TilS$_4$
Nº of genomes without TilS$_4$
Nº of genomes with TilS$_5$
With TilS$_5$
Without TilS$_5$
Nº of genomes without TilS$_5$
Nº of genomes with TilS$_6$
With TilS$_6$
Without TilS$_6$
Nº of genomes without TilS$_6$
Nº of genomes with TilS$_7$
With TilS$_7$
Without TilS$_7$
Nº of genomes without TilS$_7$
Nº of genomes with TilS$_8$
With TilS$_8$
Without TilS$_8$
Nº of genomes without TilS$_8$
Nº of genomes with TilS$_9$
With TilS$_9$
Without TilS$_9$
Nº of genomes without TilS$_9$
Nº of genomes with TilS$_{10}$
With TilS$_{10}$
Without TilS$_{10}$
Nº of genomes without TilS$_{10}$
Nº of genomes with TilS$_{11}$
With TilS$_{11}$
Without TilS$_{11}$
Nº of genomes without TilS$_{11}$
Nº of genomes with TilS$_{12}$
With TilS$_{12}$
Without TilS$_{12}$
Nº of genomes without TilS$_{12}$
Nº of genomes with TilS$_{13}$
With TilS$_{13}$
Without TilS$_{13}$
Nº of genomes without TilS$_{13}$
Nº of genomes with TilS$_{14}$
With TilS$_{14}$
Without TilS$_{14}$
Nº of genomes without TilS$_{14}$
Nº of genomes with TilS$_{15}$
With TilS$_{15}$
Without TilS$_{15}$
Nº of genomes without TilS$_{15}$

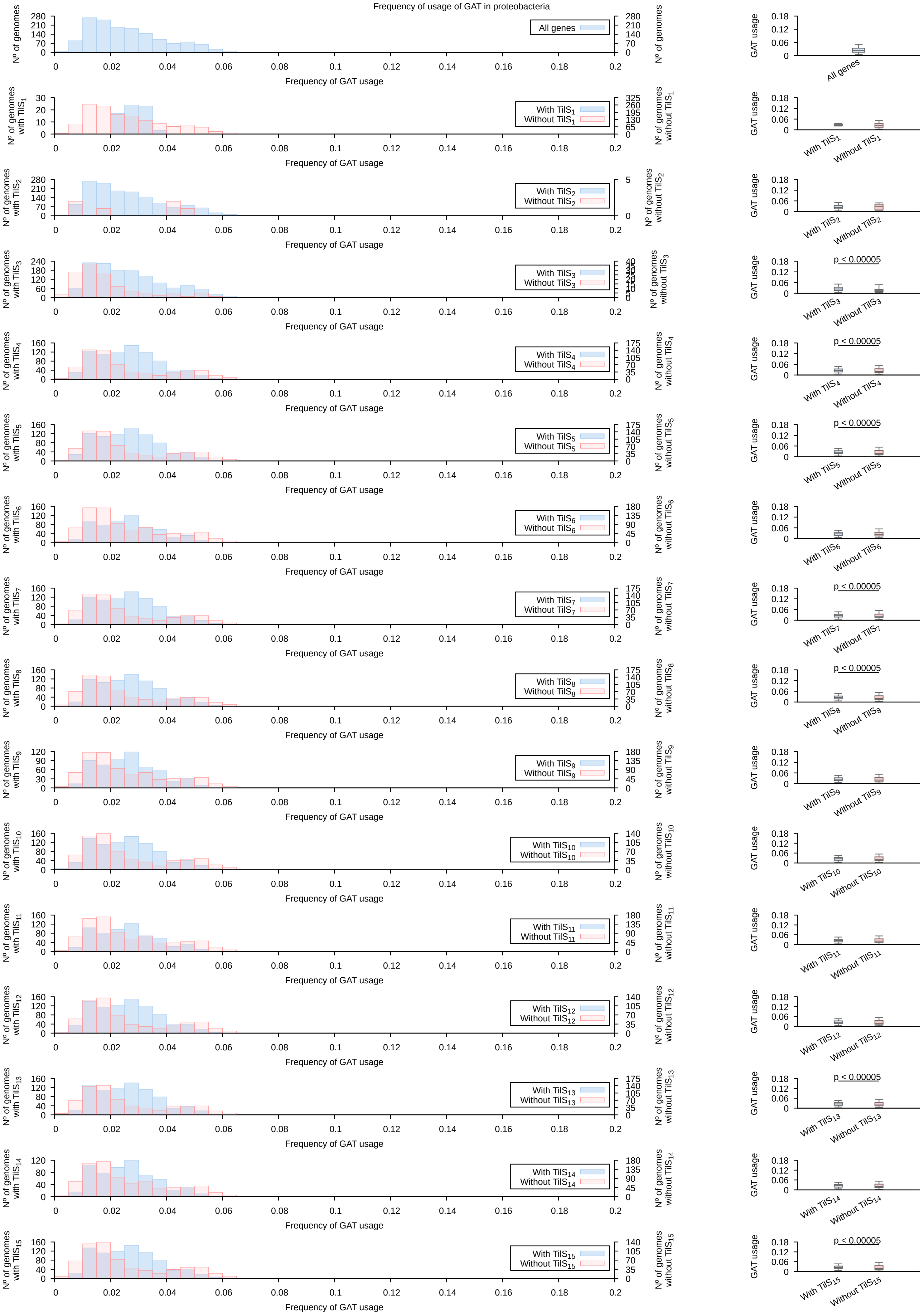
Frequency of usage of GAT in proteobacteria
Nº of genomes
All genes
Frequency of GAT usage
GAT usage
All genes
Nº of genomes with TilS$_1$
Nº of genomes without TilS$_1$
With TilS$_1$
Without TilS$_1$
Nº of genomes with TilS$_2$
Nº of genomes without TilS$_2$
With TilS$_2$
Without TilS$_2$
Nº of genomes with TilS$_3$
Nº of genomes without TilS$_3$
With TilS$_3$
Without TilS$_3$
p < 0.00005
Nº of genomes with TilS$_4$
Nº of genomes without TilS$_4$
With TilS$_4$
Without TilS$_4$
p < 0.00005
Nº of genomes with TilS$_5$
Nº of genomes without TilS$_5$
With TilS$_5$
Without TilS$_5$
p < 0.00005
Nº of genomes with TilS$_6$
Nº of genomes without TilS$_6$
With TilS$_6$
Without TilS$_6$
Nº of genomes with TilS$_7$
Nº of genomes without TilS$_7$
With TilS$_7$
Without TilS$_7$
p < 0.00005
Nº of genomes with TilS$_8$
Nº of genomes without TilS$_8$
With TilS$_8$
Without TilS$_8$
p < 0.00005
Nº of genomes with TilS$_9$
Nº of genomes without TilS$_9$
With TilS$_9$
Without TilS$_9$
Nº of genomes with TilS$_{10}$
Nº of genomes without TilS$_{10}$
With TilS$_{10}$
Without TilS$_{10}$
Nº of genomes with TilS$_{11}$
Nº of genomes without TilS$_{11}$
With TilS$_{11}$
Without TilS$_{11}$
Nº of genomes with TilS$_{12}$
Nº of genomes without TilS$_{12}$
With TilS$_{12}$
Without TilS$_{12}$
Nº of genomes with TilS$_{13}$
Nº of genomes without TilS$_{13}$
With TilS$_{13}$
Without TilS$_{13}$
p < 0.00005
Nº of genomes with TilS$_{14}$
Nº of genomes without TilS$_{14}$
With TilS$_{14}$
Without TilS$_{14}$
Nº of genomes with TilS$_{15}$
Nº of genomes without TilS$_{15}$
With TilS$_{15}$
Without TilS$_{15}$
p < 0.00005

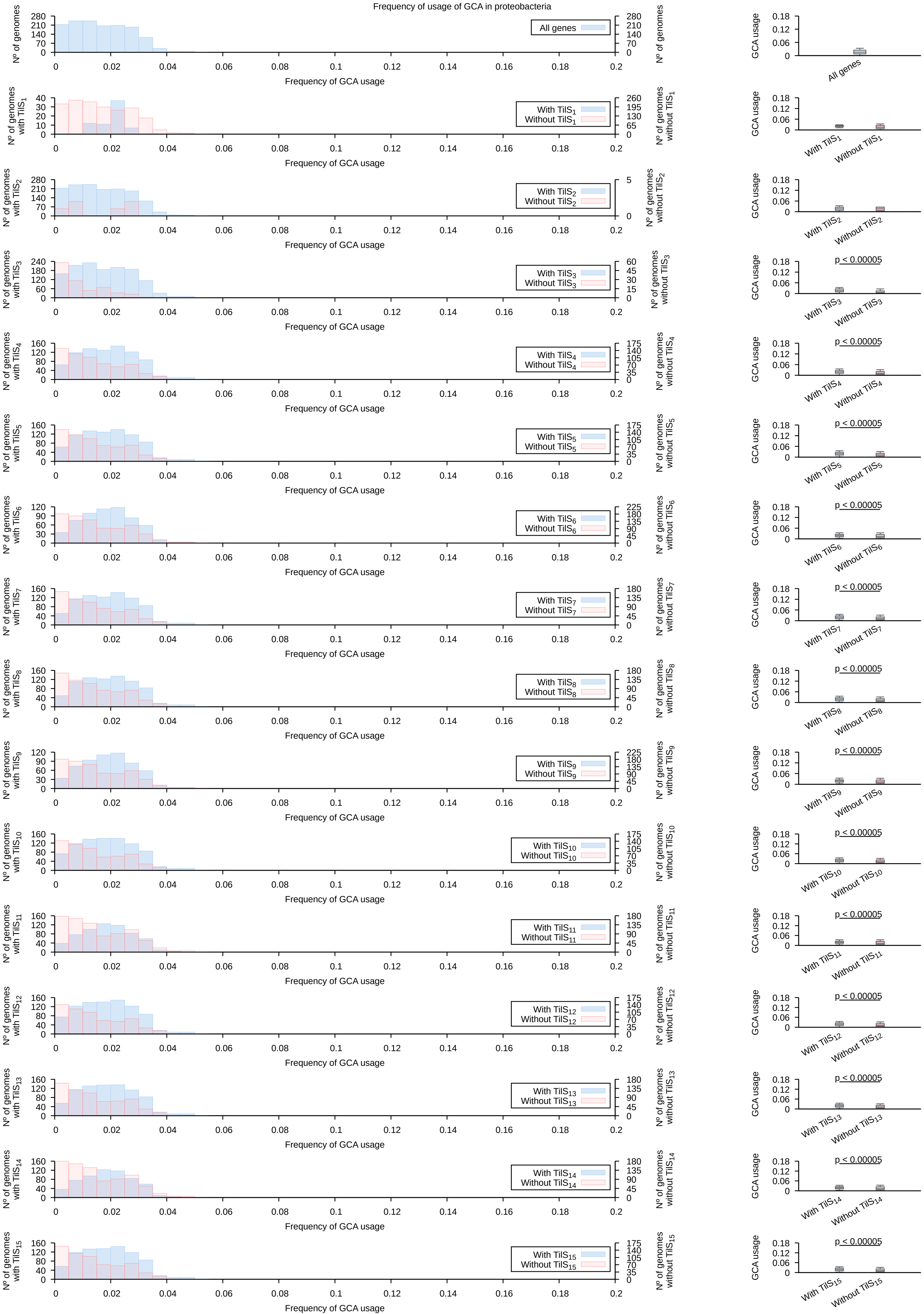

Frequency of usage of GCA in proteobacteria
Nº of genomes
All genes
Frequency of GCA usage
GCA usage
Nº of genomes with TilS$_1$
Nº of genomes without TilS$_1$
With TilS$_1$
Without TilS$_1$
Nº of genomes with TilS$_2$
Nº of genomes without TilS$_2$
With TilS$_2$
Without TilS$_2$
Nº of genomes with TilS$_3$
Nº of genomes without TilS$_3$
With TilS$_3$
Without TilS$_3$
p < 0.00005
Nº of genomes with TilS$_4$
Nº of genomes without TilS$_4$
With TilS$_4$
Without TilS$_4$
p < 0.00005
Nº of genomes with TilS$_5$
Nº of genomes without TilS$_5$
With TilS$_5$
Without TilS$_5$
p < 0.00005
Nº of genomes with TilS$_6$
Nº of genomes without TilS$_6$
With TilS$_6$
Without TilS$_6$
p < 0.00005
Nº of genomes with TilS$_7$
Nº of genomes without TilS$_7$
With TilS$_7$
Without TilS$_7$
p < 0.00005
Nº of genomes with TilS$_8$
Nº of genomes without TilS$_8$
With TilS$_8$
Without TilS$_8$
p < 0.00005
Nº of genomes with TilS$_9$
Nº of genomes without TilS$_9$
With TilS$_9$
Without TilS$_9$
p < 0.00005
Nº of genomes with TilS$_{10}$
Nº of genomes without TilS$_{10}$
With TilS$_{10}$
Without TilS$_{10}$
p < 0.00005
Nº of genomes with TilS$_{11}$
Nº of genomes without TilS$_{11}$
With TilS$_{11}$
Without TilS$_{11}$
p < 0.00005
Nº of genomes with TilS$_{12}$
Nº of genomes without TilS$_{12}$
With TilS$_{12}$
Without TilS$_{12}$
p < 0.00005
Nº of genomes with TilS$_{13}$
Nº of genomes without TilS$_{13}$
With TilS$_{13}$
Without TilS$_{13}$
p < 0.00005
Nº of genomes with TilS$_{14}$
Nº of genomes without TilS$_{14}$
With TilS$_{14}$
Without TilS$_{14}$
p < 0.00005
Nº of genomes with TilS$_{15}$
Nº of genomes without TilS$_{15}$
With TilS$_{15}$
Without TilS$_{15}$
p < 0.00005

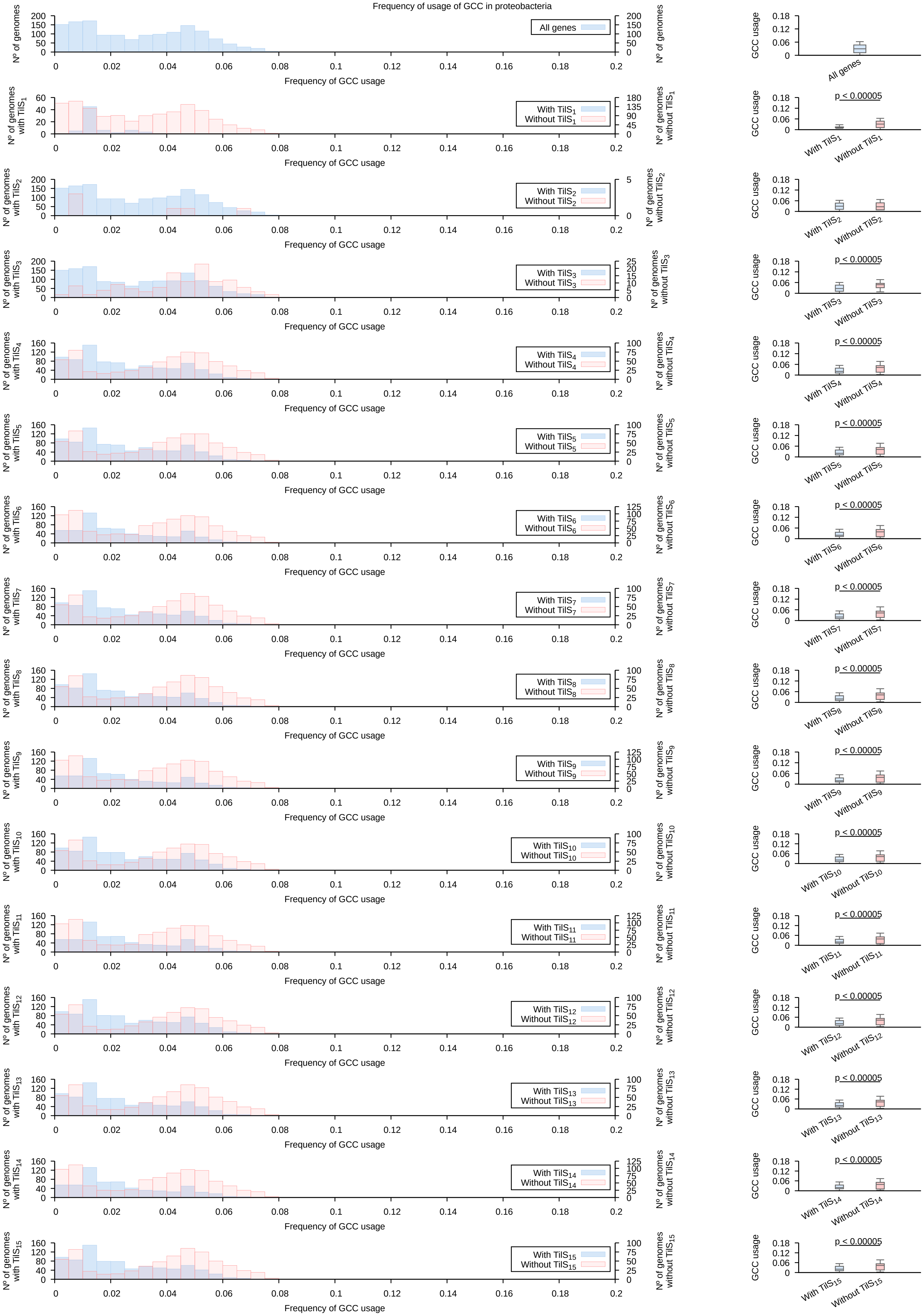

Frequency of usage of GCC in proteobacteria
Nº of genomes
All genes
Frequency of GCC usage
GCC usage
p < 0.00005
Nº of genomes with TilS$_1$
Nº of genomes without TilS$_1$
With TilS$_1$
Without TilS$_1$
Nº of genomes with TilS$_2$
Nº of genomes without TilS$_2$
With TilS$_2$
Without TilS$_2$
Nº of genomes with TilS$_3$
Nº of genomes without TilS$_3$
With TilS$_3$
Without TilS$_3$
Nº of genomes with TilS$_4$
Nº of genomes without TilS$_4$
With TilS$_4$
Without TilS$_4$
Nº of genomes with TilS$_5$
Nº of genomes without TilS$_5$
With TilS$_5$
Without TilS$_5$
Nº of genomes with TilS$_6$
Nº of genomes without TilS$_6$
With TilS$_6$
Without TilS$_6$
Nº of genomes with TilS$_7$
Nº of genomes without TilS$_7$
With TilS$_7$
Without TilS$_7$
Nº of genomes with TilS$_8$
Nº of genomes without TilS$_8$
With TilS$_8$
Without TilS$_8$
Nº of genomes with TilS$_9$
Nº of genomes without TilS$_9$
With TilS$_9$
Without TilS$_9$
Nº of genomes with TilS$_{10}$
Nº of genomes without TilS$_{10}$
With TilS$_{10}$
Without TilS$_{10}$
Nº of genomes with TilS$_{11}$
Nº of genomes without TilS$_{11}$
With TilS$_{11}$
Without TilS$_{11}$
Nº of genomes with TilS$_{12}$
Nº of genomes without TilS$_{12}$
With TilS$_{12}$
Without TilS$_{12}$
Nº of genomes with TilS$_{13}$
Nº of genomes without TilS$_{13}$
With TilS$_{13}$
Without TilS$_{13}$
Nº of genomes with TilS$_{14}$
Nº of genomes without TilS$_{14}$
With TilS$_{14}$
Without TilS$_{14}$
Nº of genomes with TilS$_{15}$
Nº of genomes without TilS$_{15}$
With TilS$_{15}$
Without TilS$_{15}$

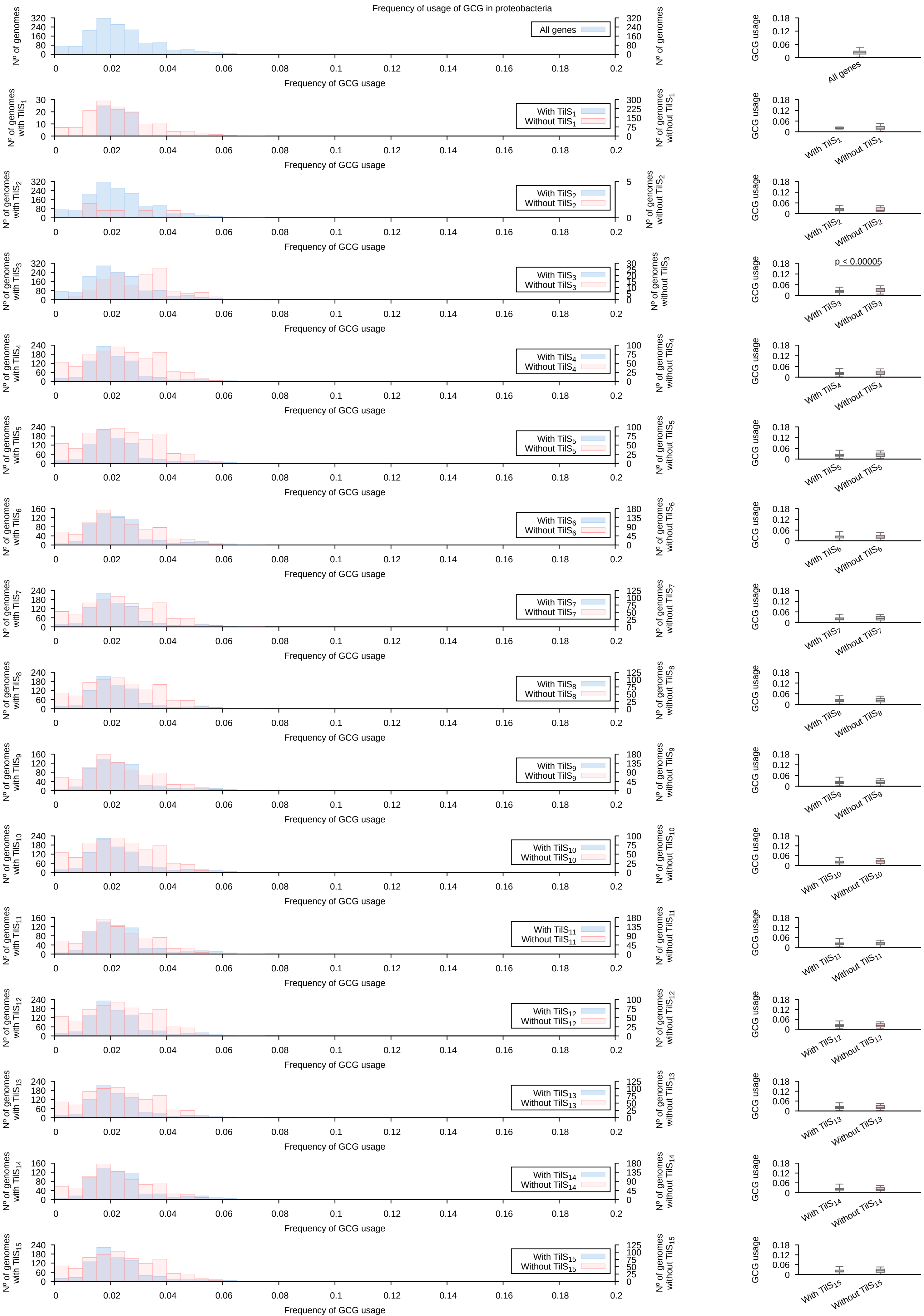

Frequency of usage of GCG in proteobacteria
Nº of genomes
All genes
Frequency of GCG usage
GCG usage
Nº of genomes with TilS$_1$
Nº of genomes without TilS$_1$
With TilS$_1$
Without TilS$_1$
Nº of genomes with TilS$_2$
Nº of genomes without TilS$_2$
With TilS$_2$
Without TilS$_2$
Nº of genomes with TilS$_3$
Nº of genomes without TilS$_3$
With TilS$_3$
Without TilS$_3$
p < 0.00005
Nº of genomes with TilS$_4$
Nº of genomes without TilS$_4$
With TilS$_4$
Without TilS$_4$
Nº of genomes with TilS$_5$
Nº of genomes without TilS$_5$
With TilS$_5$
Without TilS$_5$
Nº of genomes with TilS$_6$
Nº of genomes without TilS$_6$
With TilS$_6$
Without TilS$_6$
Nº of genomes with TilS$_7$
Nº of genomes without TilS$_7$
With TilS$_7$
Without TilS$_7$
Nº of genomes with TilS$_8$
Nº of genomes without TilS$_8$
With TilS$_8$
Without TilS$_8$
Nº of genomes with TilS$_9$
Nº of genomes without TilS$_9$
With TilS$_9$
Without TilS$_9$
Nº of genomes with TilS$_{10}$
Nº of genomes without TilS$_{10}$
With TilS$_{10}$
Without TilS$_{10}$
Nº of genomes with TilS$_{11}$
Nº of genomes without TilS$_{11}$
With TilS$_{11}$
Without TilS$_{11}$
Nº of genomes with TilS$_{12}$
Nº of genomes without TilS$_{12}$
With TilS$_{12}$
Without TilS$_{12}$
Nº of genomes with TilS$_{13}$
Nº of genomes without TilS$_{13}$
With TilS$_{13}$
Without TilS$_{13}$
Nº of genomes with TilS$_{14}$
Nº of genomes without TilS$_{14}$
With TilS$_{14}$
Without TilS$_{14}$
Nº of genomes with TilS$_{15}$
Nº of genomes without TilS$_{15}$
With TilS$_{15}$
Without TilS$_{15}$

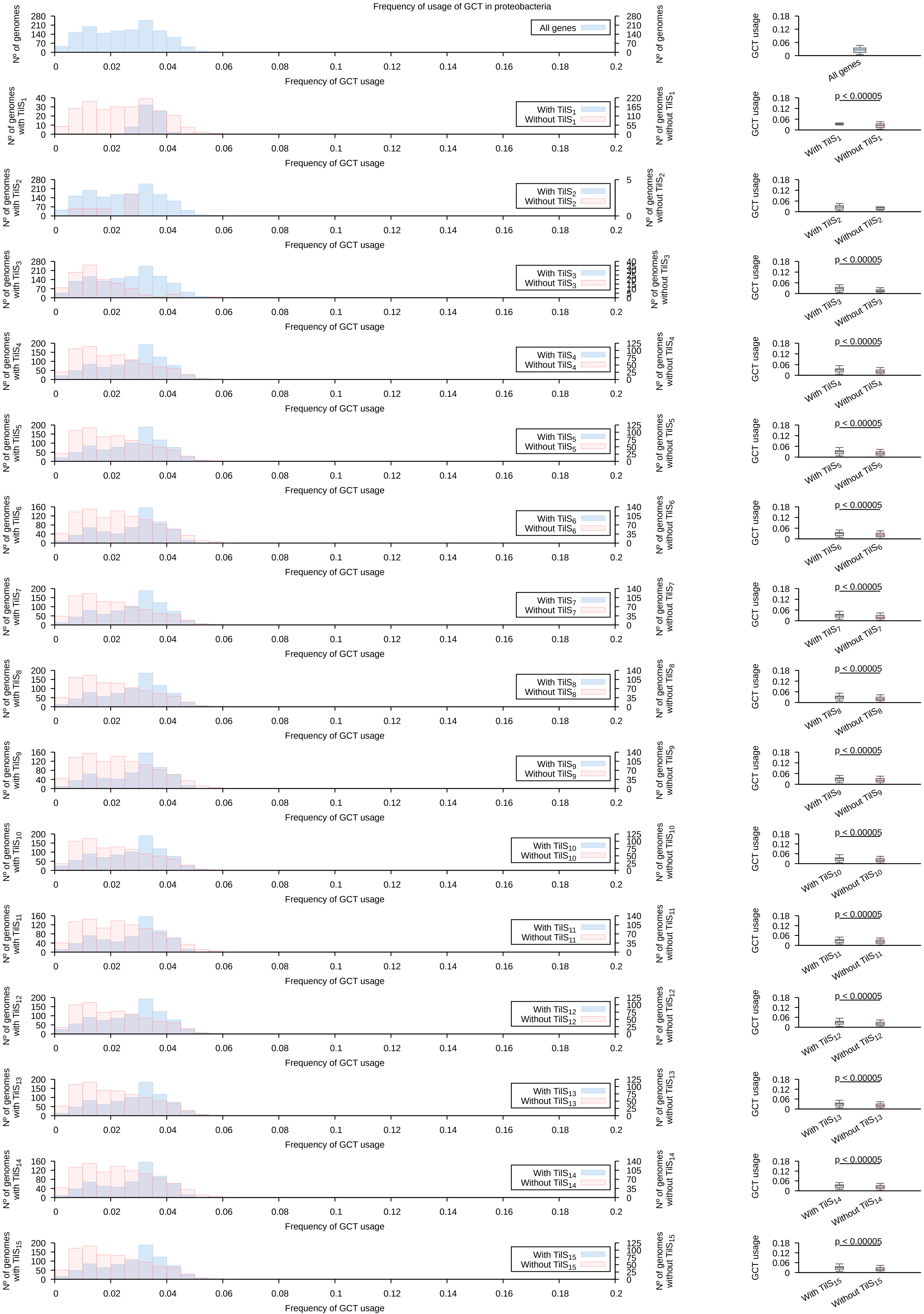

Frequency of usage of GCT in proteobacteria
Nº of genomes
All genes
Frequency of GCT usage
GCT usage
Nº of genomes with TilS$_1$
Nº of genomes without TilS$_1$
With TilS$_1$
Without TilS$_1$
p < 0.00005
Nº of genomes with TilS$_2$
Nº of genomes without TilS$_2$
With TilS$_2$
Without TilS$_2$
Nº of genomes with TilS$_3$
Nº of genomes without TilS$_3$
With TilS$_3$
Without TilS$_3$
p < 0.00005
Nº of genomes with TilS$_4$
Nº of genomes without TilS$_4$
With TilS$_4$
Without TilS$_4$
p < 0.00005
Nº of genomes with TilS$_5$
Nº of genomes without TilS$_5$
With TilS$_5$
Without TilS$_5$
p < 0.00005
Nº of genomes with TilS$_6$
Nº of genomes without TilS$_6$
With TilS$_6$
Without TilS$_6$
p < 0.00005
Nº of genomes with TilS$_7$
Nº of genomes without TilS$_7$
With TilS$_7$
Without TilS$_7$
p < 0.00005
Nº of genomes with TilS$_8$
Nº of genomes without TilS$_8$
With TilS$_8$
Without TilS$_8$
p < 0.00005
Nº of genomes with TilS$_9$
Nº of genomes without TilS$_9$
With TilS$_9$
Without TilS$_9$
p < 0.00005
Nº of genomes with TilS$_{10}$
Nº of genomes without TilS$_{10}$
With TilS$_{10}$
Without TilS$_{10}$
p < 0.00005
Nº of genomes with TilS$_{11}$
Nº of genomes without TilS$_{11}$
With TilS$_{11}$
Without TilS$_{11}$
p < 0.00005
Nº of genomes with TilS$_{12}$
Nº of genomes without TilS$_{12}$
With TilS$_{12}$
Without TilS$_{12}$
p < 0.00005
Nº of genomes with TilS$_{13}$
Nº of genomes without TilS$_{13}$
With TilS$_{13}$
Without TilS$_{13}$
p < 0.00005
Nº of genomes with TilS$_{14}$
Nº of genomes without TilS$_{14}$
With TilS$_{14}$
Without TilS$_{14}$
p < 0.00005
Nº of genomes with TilS$_{15}$
Nº of genomes without TilS$_{15}$
With TilS$_{15}$
Without TilS$_{15}$
p < 0.00005

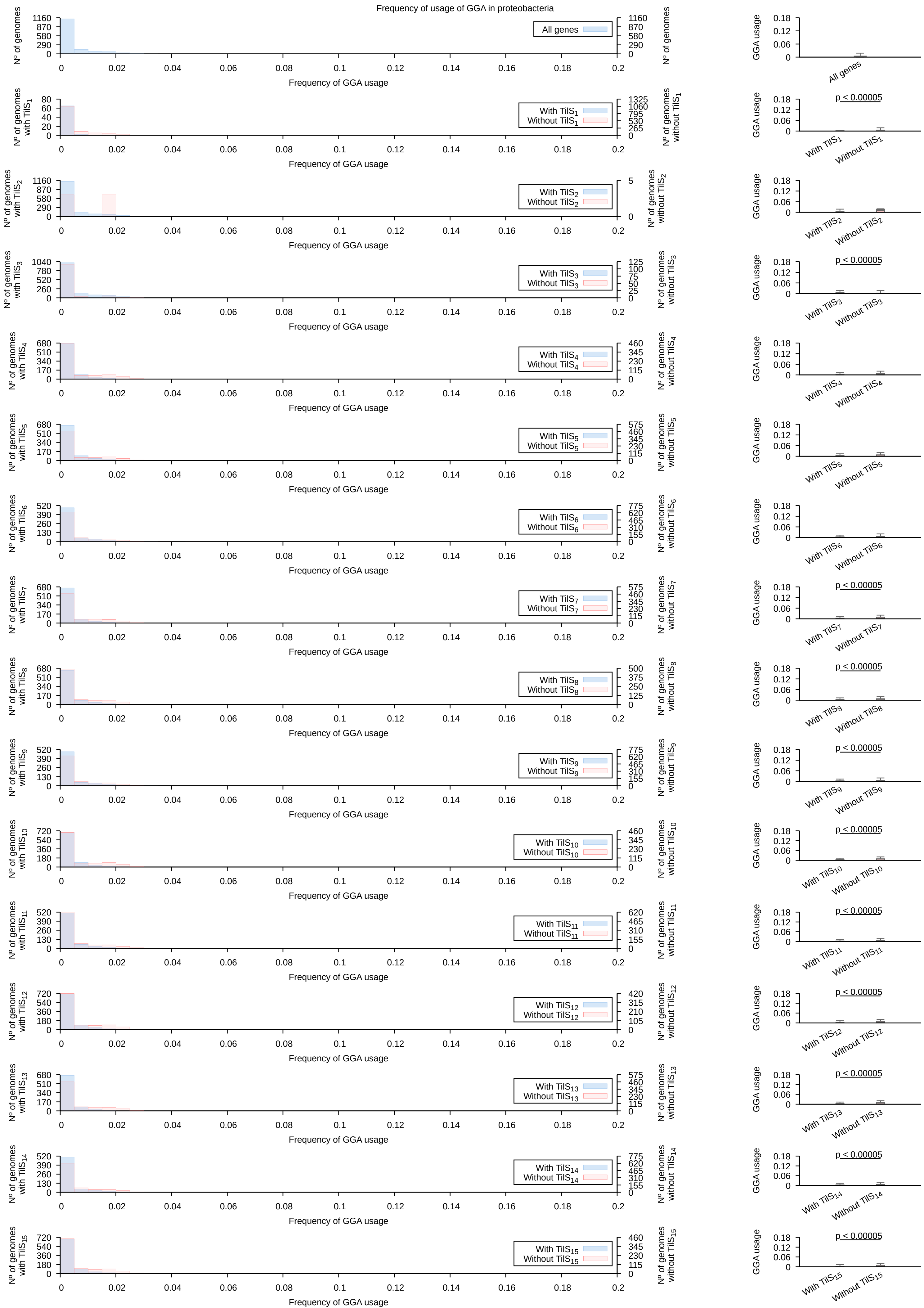

Frequency of usage of GGA in proteobacteria
All genes
Nº of genomes
Frequency of GGA usage
GGA usage
With TilS$_1$
Without TilS$_1$
Nº of genomes with TilS$_1$
Nº of genomes without TilS$_1$
p < 0.00005
With TilS$_2$
Without TilS$_2$
Nº of genomes with TilS$_2$
Nº of genomes without TilS$_2$
With TilS$_3$
Without TilS$_3$
Nº of genomes with TilS$_3$
Nº of genomes without TilS$_3$
p < 0.00005
With TilS$_4$
Without TilS$_4$
Nº of genomes with TilS$_4$
Nº of genomes without TilS$_4$
With TilS$_5$
Without TilS$_5$
Nº of genomes with TilS$_5$
Nº of genomes without TilS$_5$
With TilS$_6$
Without TilS$_6$
Nº of genomes with TilS$_6$
Nº of genomes without TilS$_6$
With TilS$_7$
Without TilS$_7$
Nº of genomes with TilS$_7$
Nº of genomes without TilS$_7$
p < 0.00005
With TilS$_8$
Without TilS$_8$
Nº of genomes with TilS$_8$
Nº of genomes without TilS$_8$
p < 0.00005
With TilS$_9$
Without TilS$_9$
Nº of genomes with TilS$_9$
Nº of genomes without TilS$_9$
p < 0.00005
With TilS$_{10}$
Without TilS$_{10}$
Nº of genomes with TilS$_{10}$
Nº of genomes without TilS$_{10}$
p < 0.00005
With TilS$_{11}$
Without TilS$_{11}$
Nº of genomes with TilS$_{11}$
Nº of genomes without TilS$_{11}$
p < 0.00005
With TilS$_{12}$
Without TilS$_{12}$
Nº of genomes with TilS$_{12}$
Nº of genomes without TilS$_{12}$
p < 0.00005
With TilS$_{13}$
Without TilS$_{13}$
Nº of genomes with TilS$_{13}$
Nº of genomes without TilS$_{13}$
p < 0.00005
With TilS$_{14}$
Without TilS$_{14}$
Nº of genomes with TilS$_{14}$
Nº of genomes without TilS$_{14}$
p < 0.00005
With TilS$_{15}$
Without TilS$_{15}$
Nº of genomes with TilS$_{15}$
Nº of genomes without TilS$_{15}$
p < 0.00005

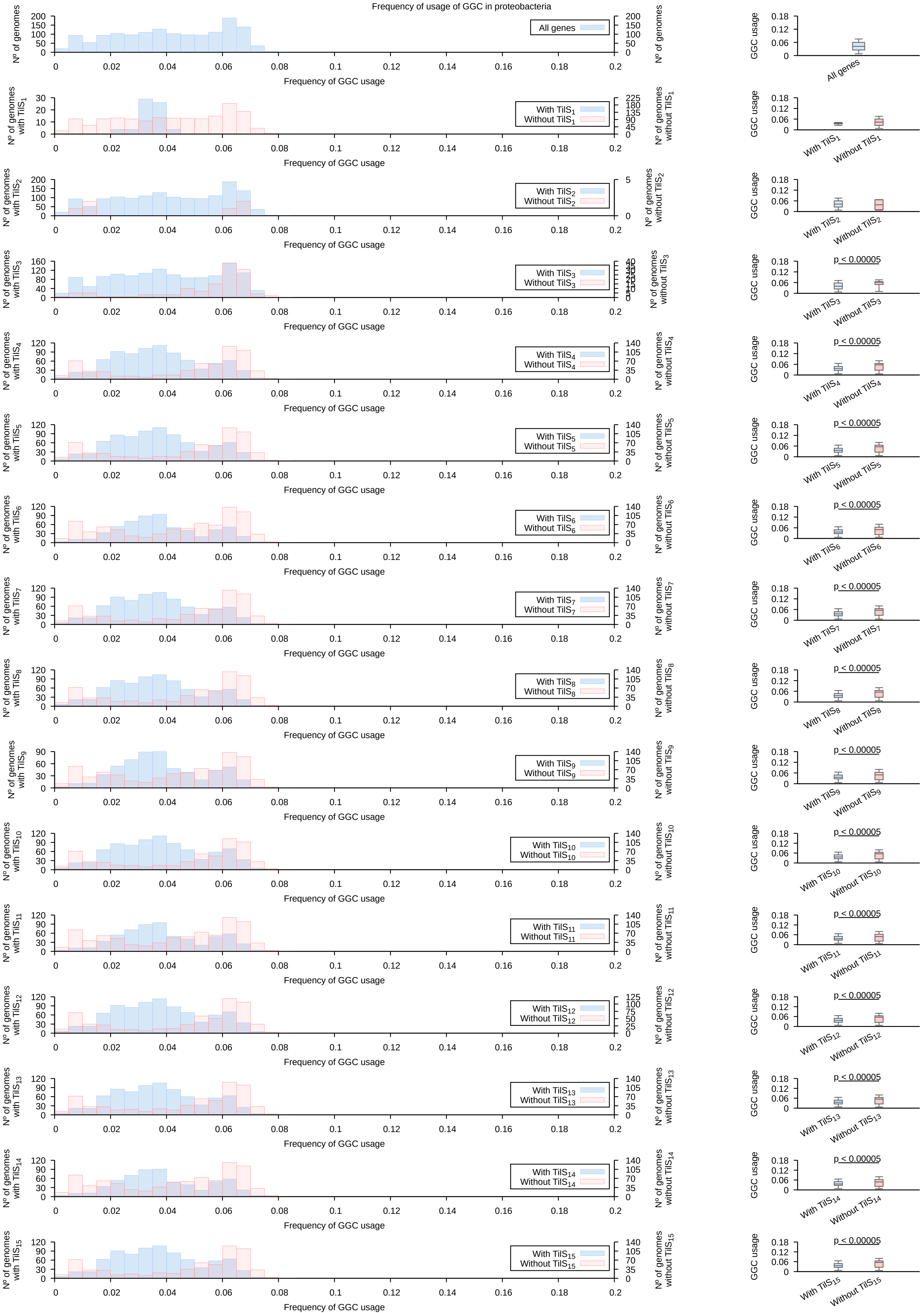
Frequency of usage of GGC in proteobacteria
Nº of genomes
All genes
GGC usage
Frequency of GGC usage
Nº of genomes with TilS$_1$
Nº of genomes without TilS$_1$
With TilS$_1$
Without TilS$_1$
Nº of genomes with TilS$_2$
Nº of genomes without TilS$_2$
With TilS$_2$
Without TilS$_2$
Nº of genomes with TilS$_3$
Nº of genomes without TilS$_3$
With TilS$_3$
Without TilS$_3$
p < 0.00005
Nº of genomes with TilS$_4$
Nº of genomes without TilS$_4$
With TilS$_4$
Without TilS$_4$
p < 0.00005
Nº of genomes with TilS$_5$
Nº of genomes without TilS$_5$
With TilS$_5$
Without TilS$_5$
p < 0.00005
Nº of genomes with TilS$_6$
Nº of genomes without TilS$_6$
With TilS$_6$
Without TilS$_6$
p < 0.00005
Nº of genomes with TilS$_7$
Nº of genomes without TilS$_7$
With TilS$_7$
Without TilS$_7$
p < 0.00005
Nº of genomes with TilS$_8$
Nº of genomes without TilS$_8$
With TilS$_8$
Without TilS$_8$
p < 0.00005
Nº of genomes with TilS$_9$
Nº of genomes without TilS$_9$
With TilS$_9$
Without TilS$_9$
p < 0.00005
Nº of genomes with TilS$_{10}$
Nº of genomes without TilS$_{10}$
With TilS$_{10}$
Without TilS$_{10}$
p < 0.00005
Nº of genomes with TilS$_{11}$
Nº of genomes without TilS$_{11}$
With TilS$_{11}$
Without TilS$_{11}$
p < 0.00005
Nº of genomes with TilS$_{12}$
Nº of genomes without TilS$_{12}$
With TilS$_{12}$
Without TilS$_{12}$
p < 0.00005
Nº of genomes with TilS$_{13}$
Nº of genomes without TilS$_{13}$
With TilS$_{13}$
Without TilS$_{13}$
p < 0.00005
Nº of genomes with TilS$_{14}$
Nº of genomes without TilS$_{14}$
With TilS$_{14}$
Without TilS$_{14}$
p < 0.00005
Nº of genomes with TilS$_{15}$
Nº of genomes without TilS$_{15}$
With TilS$_{15}$
Without TilS$_{15}$
p < 0.00005

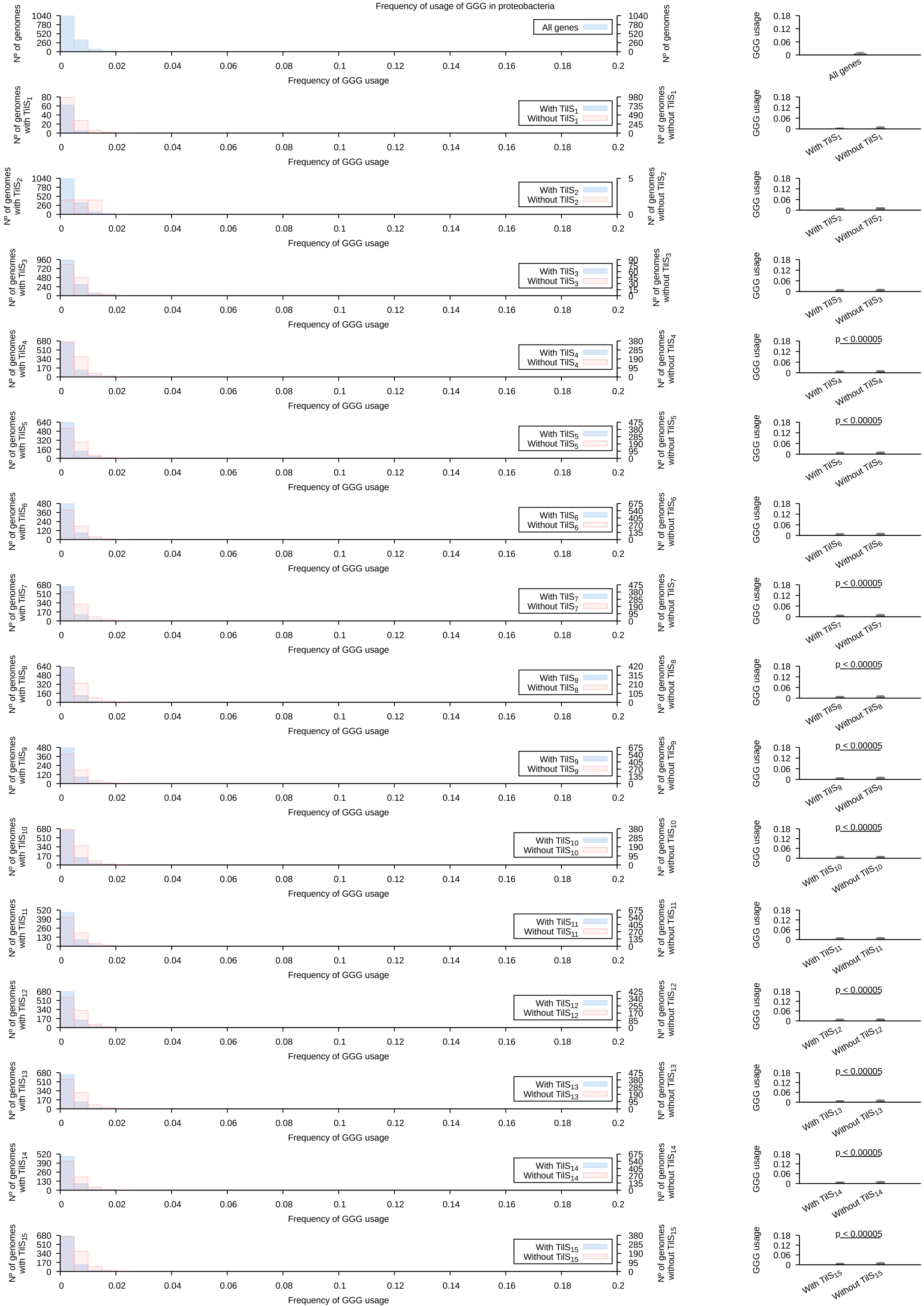
Frequency of usage of GGG in proteobacteria
Nº of genomes
Frequency of GGG usage
All genes
GGG usage
With TilS1
Without TilS1
Nº of genomes with TilS1
Nº of genomes without TilS1
With TilS2
Without TilS2
With TilS3
Without TilS3
With TilS4
Without TilS4
p < 0.00005
With TilS5
Without TilS5
With TilS6
Without TilS6
With TilS7
Without TilS7
With TilS8
Without TilS8
With TilS9
Without TilS9
With TilS10
Without TilS10
With TilS11
Without TilS11
With TilS12
Without TilS12
With TilS13
Without TilS13
With TilS14
Without TilS14
With TilS15
Without TilS15

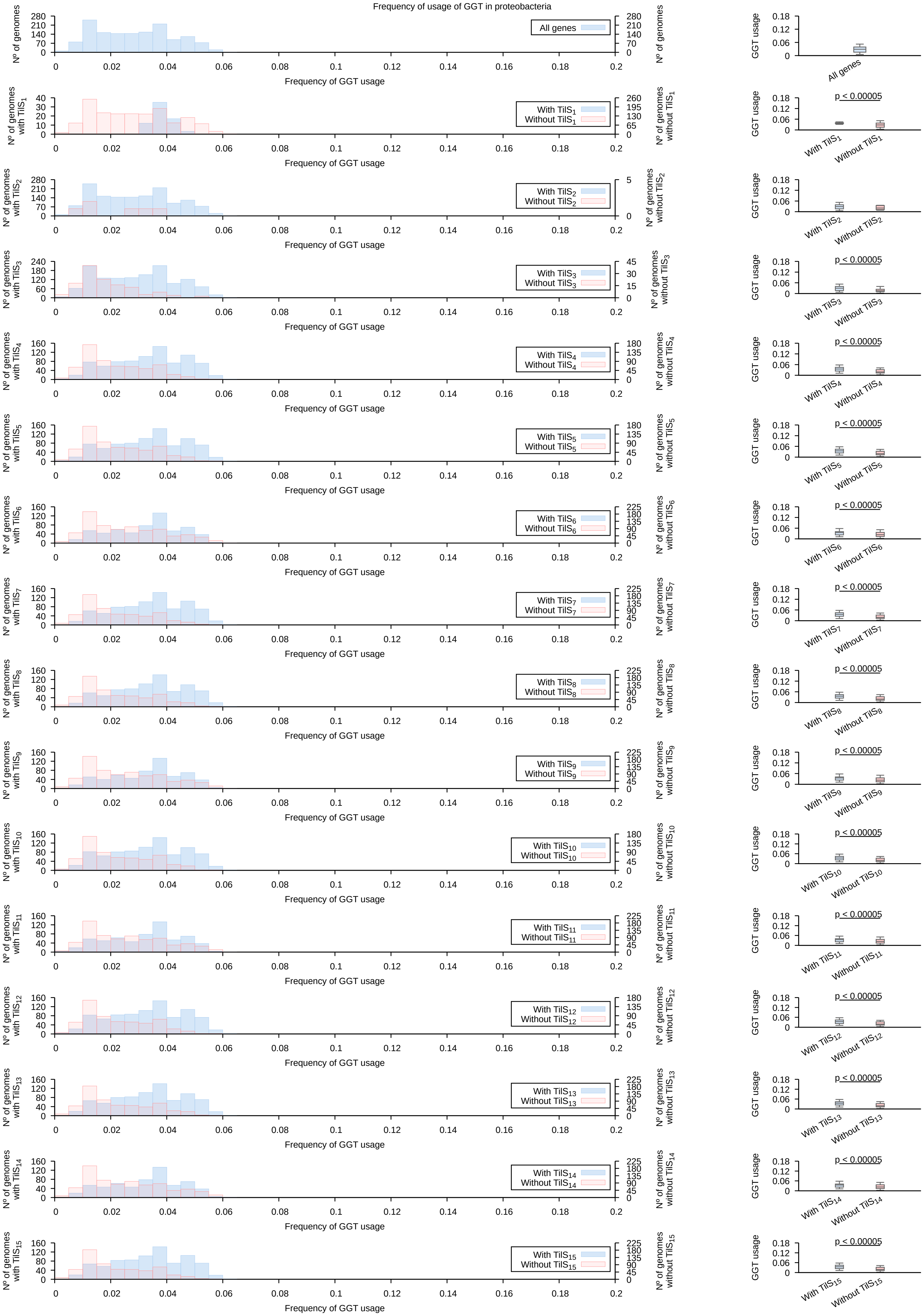

Frequency of usage of GGT in proteobacteria
Nº of genomes
All genes
Frequency of GGT usage
GGT usage
Nº of genomes with TilS1
Nº of genomes without TilS1
With TilS1
Without TilS1
p < 0.00005
Nº of genomes with TilS2
Nº of genomes without TilS2
With TilS2
Without TilS2
Nº of genomes with TilS3
Nº of genomes without TilS3
With TilS3
Without TilS3
Nº of genomes with TilS4
Nº of genomes without TilS4
With TilS4
Without TilS4
Nº of genomes with TilS5
Nº of genomes without TilS5
With TilS5
Without TilS5
Nº of genomes with TilS6
Nº of genomes without TilS6
With TilS6
Without TilS6
Nº of genomes with TilS7
Nº of genomes without TilS7
With TilS7
Without TilS7
Nº of genomes with TilS8
Nº of genomes without TilS8
With TilS8
Without TilS8
Nº of genomes with TilS9
Nº of genomes without TilS9
With TilS9
Without TilS9
Nº of genomes with TilS10
Nº of genomes without TilS10
With TilS10
Without TilS10
Nº of genomes with TilS11
Nº of genomes without TilS11
With TilS11
Without TilS11
Nº of genomes with TilS12
Nº of genomes without TilS12
With TilS12
Without TilS12
Nº of genomes with TilS13
Nº of genomes without TilS13
With TilS13
Without TilS13
Nº of genomes with TilS14
Nº of genomes without TilS14
With TilS14
Without TilS14
Nº of genomes with TilS15
Nº of genomes without TilS15
With TilS15
Without TilS15

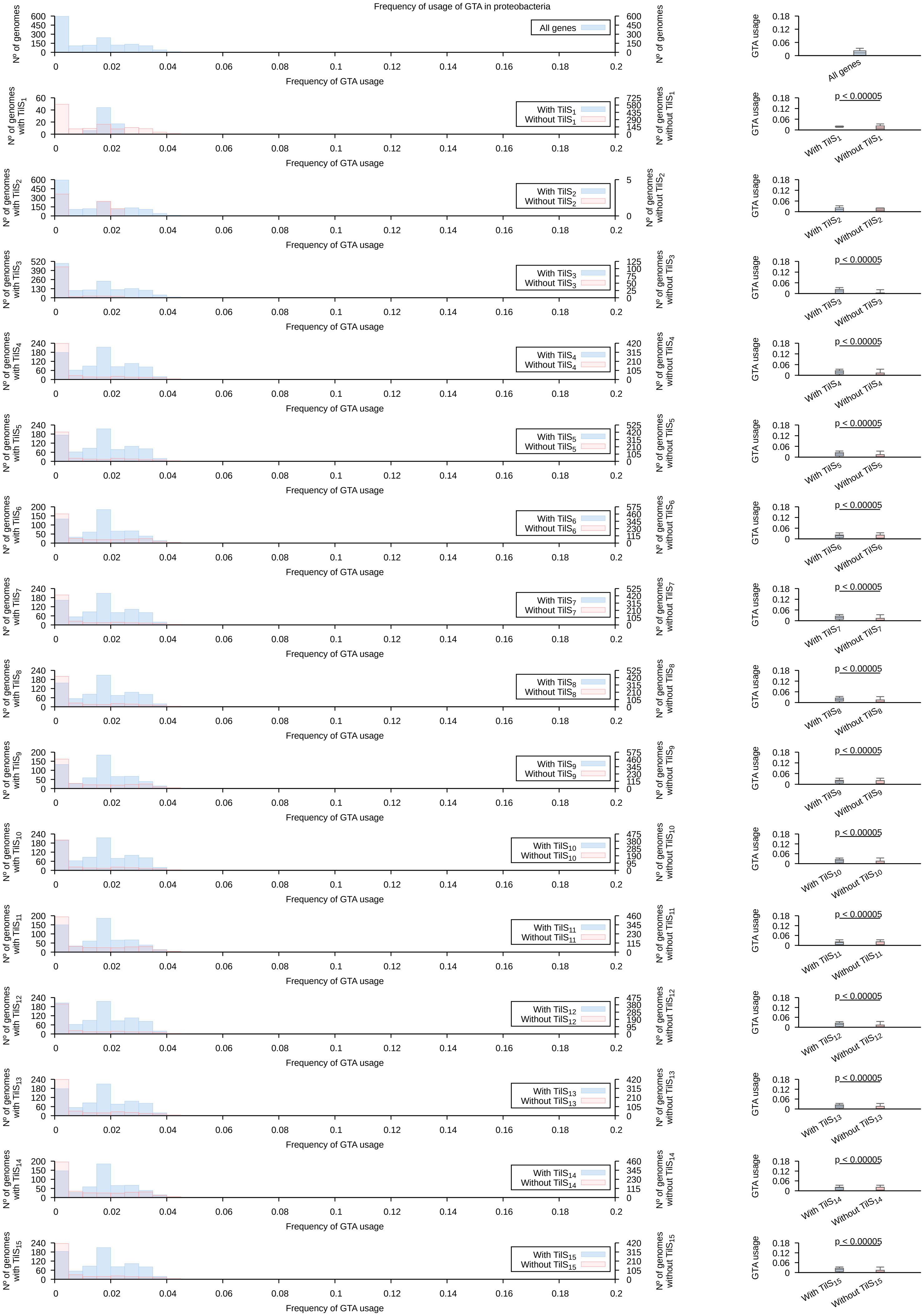
Frequency of usage of GTA in proteobacteria
Nº of genomes
Frequency of GTA usage
GTA usage
All genes
With TilS$_1$
Without TilS$_1$
With TilS$_2$
Without TilS$_2$
With TilS$_3$
Without TilS$_3$
With TilS$_4$
Without TilS$_4$
With TilS$_5$
Without TilS$_5$
With TilS$_6$
Without TilS$_6$
With TilS$_7$
Without TilS$_7$
With TilS$_8$
Without TilS$_8$
With TilS$_9$
Without TilS$_9$
With TilS$_{10}$
Without TilS$_{10}$
With TilS$_{11}$
Without TilS$_{11}$
With TilS$_{12}$
Without TilS$_{12}$
With TilS$_{13}$
Without TilS$_{13}$
With TilS$_{14}$
Without TilS$_{14}$
With TilS$_{15}$
Without TilS$_{15}$
$p < 0.00005$

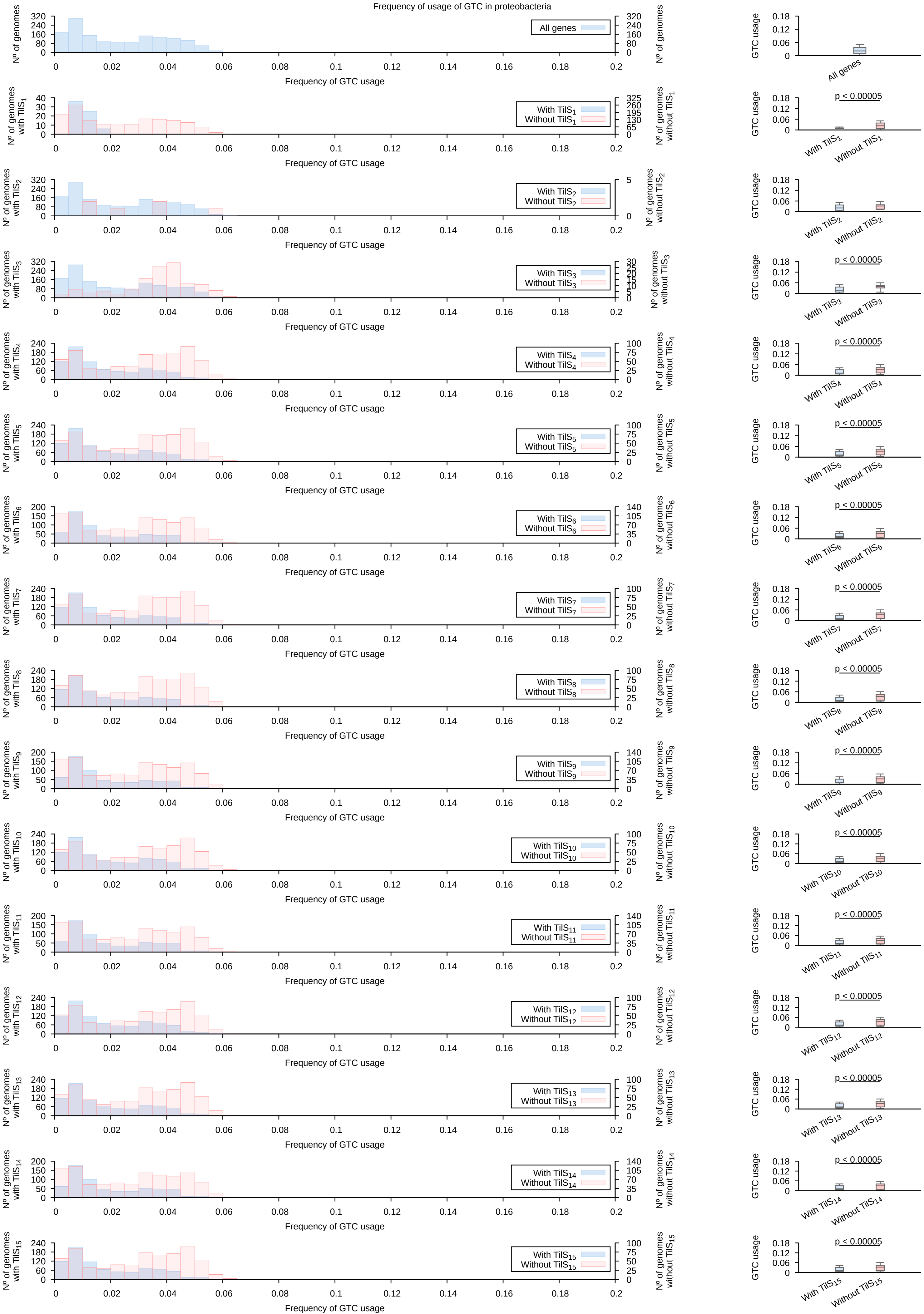

Frequency of usage of GTC in proteobacteria
Nº of genomes
All genes
Frequency of GTC usage
GTC usage
Nº of genomes with TilS1
Nº of genomes without TilS1
With TilS1
Without TilS1
p < 0.00005
Nº of genomes with TilS2
Nº of genomes without TilS2
With TilS2
Without TilS2
Nº of genomes with TilS3
Nº of genomes without TilS3
With TilS3
Without TilS3
p < 0.00005
Nº of genomes with TilS4
Nº of genomes without TilS4
With TilS4
Without TilS4
p < 0.00005
Nº of genomes with TilS5
Nº of genomes without TilS5
With TilS5
Without TilS5
p < 0.00005
Nº of genomes with TilS6
Nº of genomes without TilS6
With TilS6
Without TilS6
p < 0.00005
Nº of genomes with TilS7
Nº of genomes without TilS7
With TilS7
Without TilS7
p < 0.00005
Nº of genomes with TilS8
Nº of genomes without TilS8
With TilS8
Without TilS8
p < 0.00005
Nº of genomes with TilS9
Nº of genomes without TilS9
With TilS9
Without TilS9
p < 0.00005
Nº of genomes with TilS10
Nº of genomes without TilS10
With TilS10
Without TilS10
p < 0.00005
Nº of genomes with TilS11
Nº of genomes without TilS11
With TilS11
Without TilS11
p < 0.00005
Nº of genomes with TilS12
Nº of genomes without TilS12
With TilS12
Without TilS12
p < 0.00005
Nº of genomes with TilS13
Nº of genomes without TilS13
With TilS13
Without TilS13
p < 0.00005
Nº of genomes with TilS14
Nº of genomes without TilS14
With TilS14
Without TilS14
p < 0.00005
Nº of genomes with TilS15
Nº of genomes without TilS15
With TilS15
Without TilS15
p < 0.00005

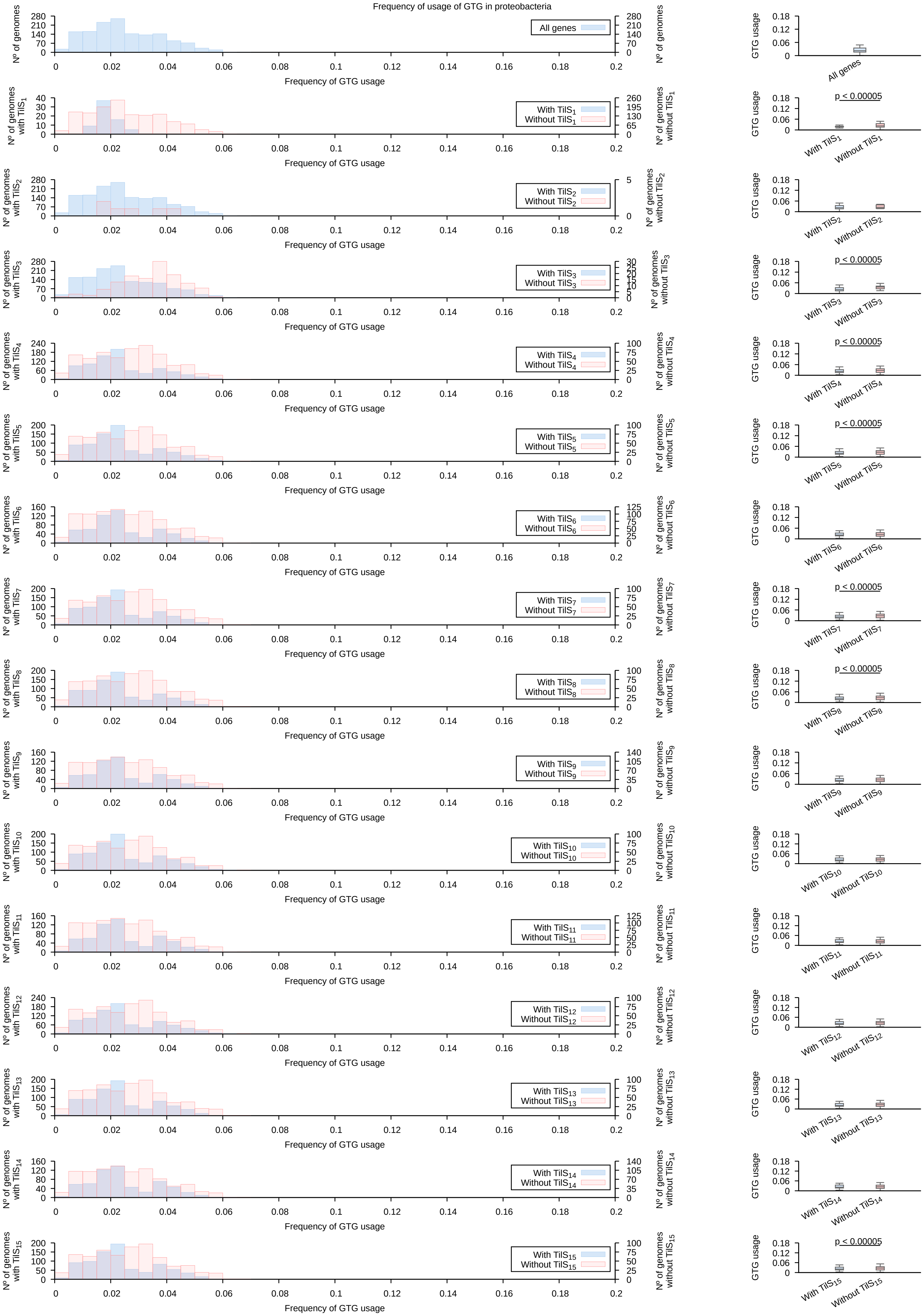
Frequency of usage of GTG in proteobacteria
Nº of genomes
All genes
Frequency of GTG usage
GTG usage
Nº of genomes with TilS1
Nº of genomes without TilS1
With TilS1
Without TilS1
p < 0.00005
Nº of genomes with TilS2
Nº of genomes without TilS2
With TilS2
Without TilS2
Nº of genomes with TilS3
Nº of genomes without TilS3
With TilS3
Without TilS3
p < 0.00005
Nº of genomes with TilS4
Nº of genomes without TilS4
With TilS4
Without TilS4
p < 0.00005
Nº of genomes with TilS5
Nº of genomes without TilS5
With TilS5
Without TilS5
p < 0.00005
Nº of genomes with TilS6
Nº of genomes without TilS6
With TilS6
Without TilS6
Nº of genomes with TilS7
Nº of genomes without TilS7
With TilS7
Without TilS7
p < 0.00005
Nº of genomes with TilS8
Nº of genomes without TilS8
With TilS8
Without TilS8
p < 0.00005
Nº of genomes with TilS9
Nº of genomes without TilS9
With TilS9
Without TilS9
Nº of genomes with TilS10
Nº of genomes without TilS10
With TilS10
Without TilS10
Nº of genomes with TilS11
Nº of genomes without TilS11
With TilS11
Without TilS11
Nº of genomes with TilS12
Nº of genomes without TilS12
With TilS12
Without TilS12
Nº of genomes with TilS13
Nº of genomes without TilS13
With TilS13
Without TilS13
Nº of genomes with TilS14
Nº of genomes without TilS14
With TilS14
Without TilS14
Nº of genomes with TilS15
Nº of genomes without TilS15
With TilS15
Without TilS15
p < 0.00005

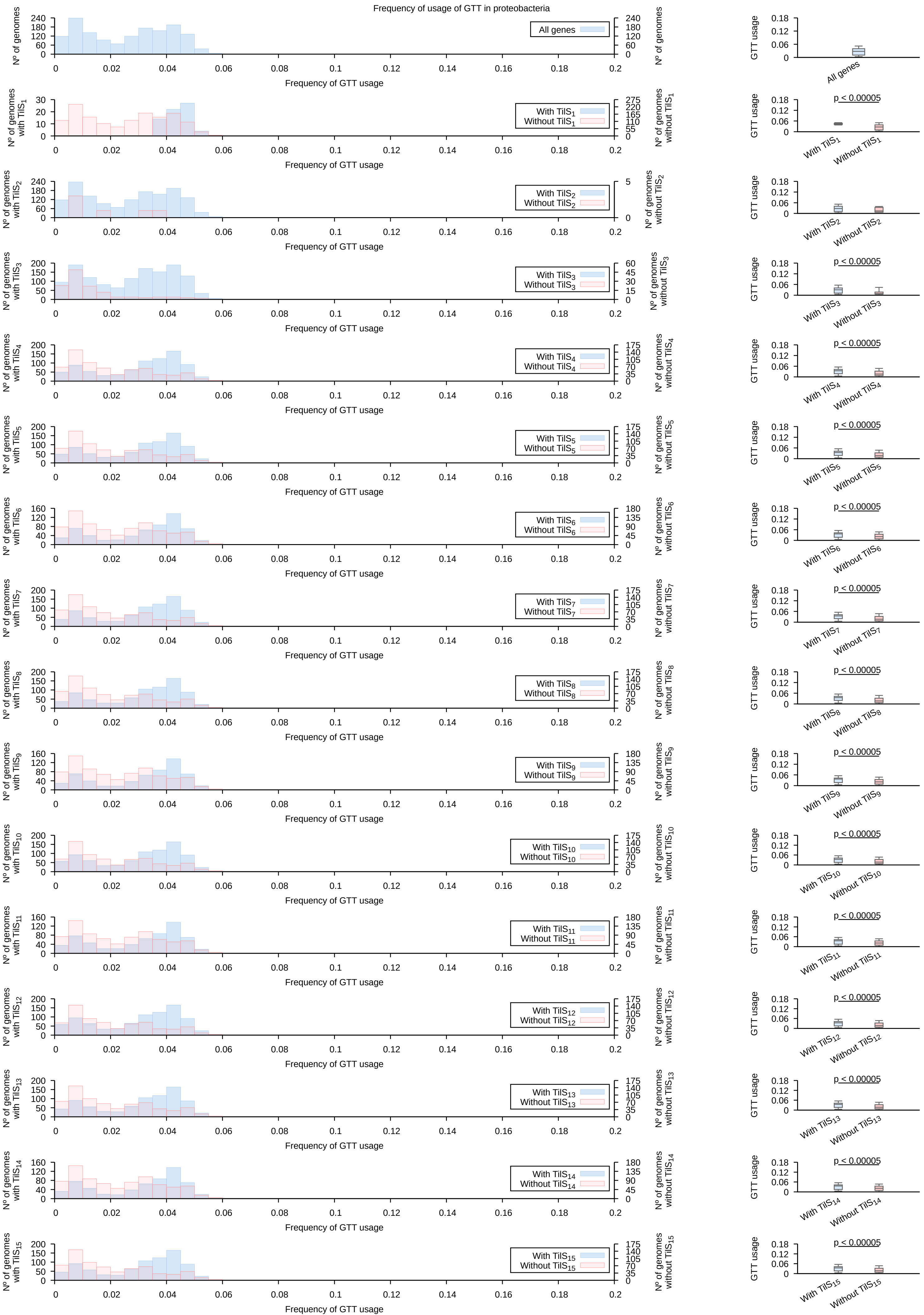
Frequency of usage of GTT in proteobacteria
Nº of genomes
All genes
Frequency of GTT usage
GTT usage
Nº of genomes with TilS$_1$
Nº of genomes without TilS$_1$
With TilS$_1$
Without TilS$_1$
p < 0.00005
With TilS$_2$
Without TilS$_2$
With TilS$_3$
Without TilS$_3$
With TilS$_4$
Without TilS$_4$
With TilS$_5$
Without TilS$_5$
With TilS$_6$
Without TilS$_6$
With TilS$_7$
Without TilS$_7$
With TilS$_8$
Without TilS$_8$
With TilS$_9$
Without TilS$_9$
With TilS$_{10}$
Without TilS$_{10}$
With TilS$_{11}$
Without TilS$_{11}$
With TilS$_{12}$
Without TilS$_{12}$
With TilS$_{13}$
Without TilS$_{13}$
With TilS$_{14}$
Without TilS$_{14}$
With TilS$_{15}$
Without TilS$_{15}$

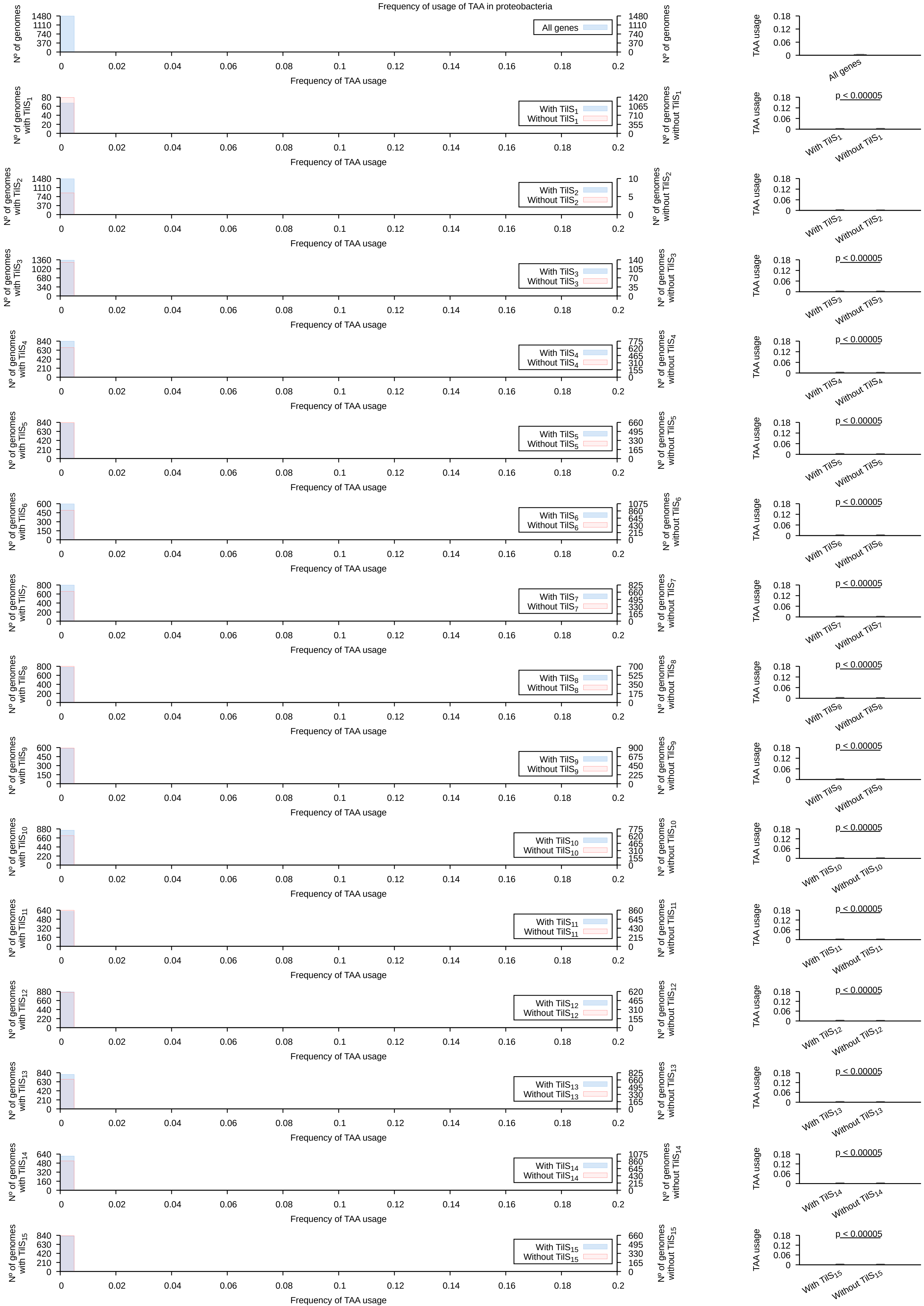

Frequency of usage of TAA in proteobacteria
Nº of genomes
All genes
Frequency of TAA usage
TAA usage
Nº of genomes with TilS1
Nº of genomes without TilS1
With TilS1
Without TilS1
p < 0.00005
With TilS2
Without TilS2
With TilS3
Without TilS3
With TilS4
Without TilS4
With TilS5
Without TilS5
With TilS6
Without TilS6
With TilS7
Without TilS7
With TilS8
Without TilS8
With TilS9
Without TilS9
With TilS10
Without TilS10
With TilS11
Without TilS11
With TilS12
Without TilS12
With TilS13
Without TilS13
With TilS14
Without TilS14
With TilS15
Without TilS15

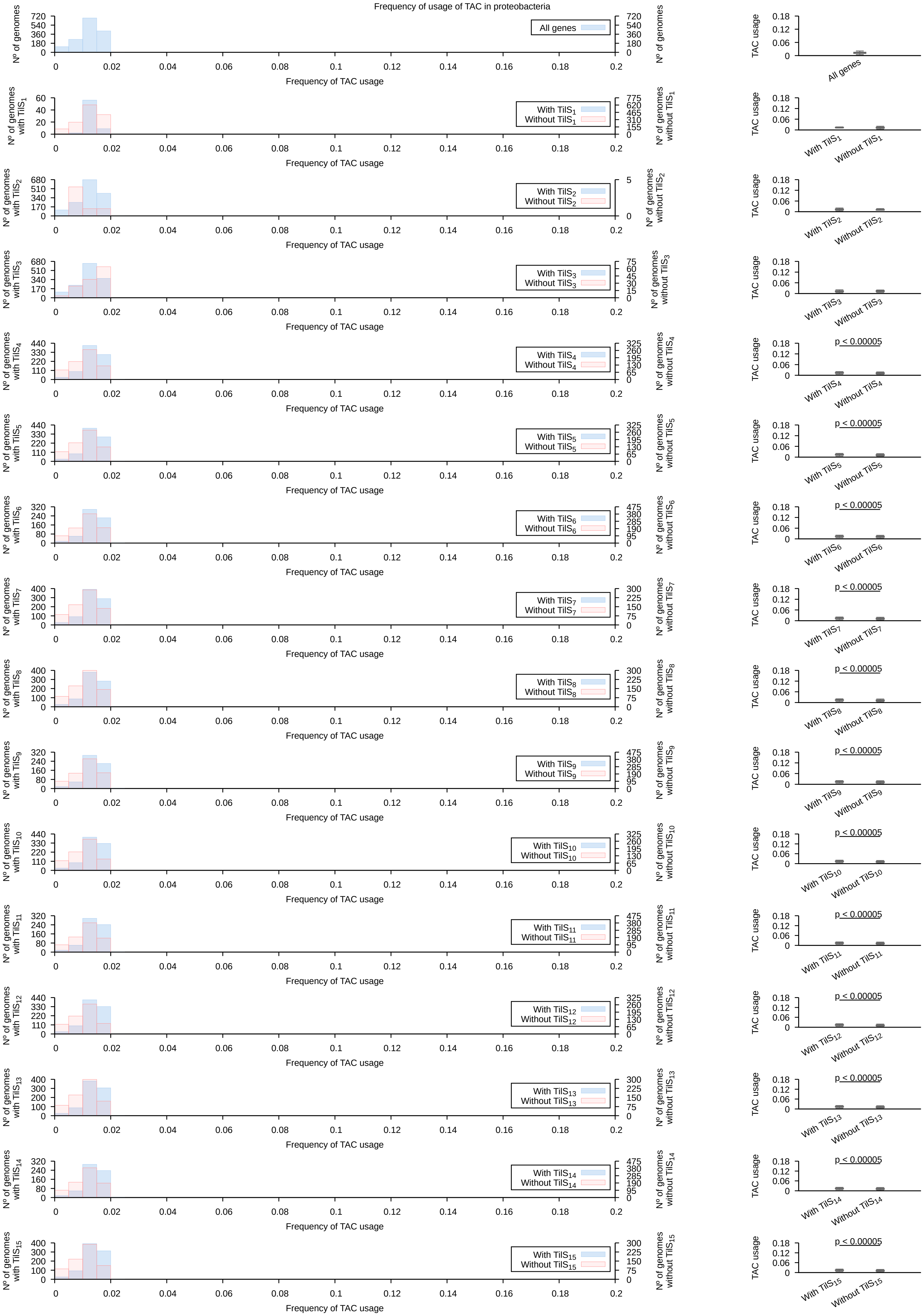

Frequency of usage of TAC in proteobacteria
Nº of genomes
All genes
Frequency of TAC usage
TAC usage
0 0.02 0.04 0.06 0.08 0.1 0.12 0.14 0.16 0.18 0.2
0 0.06 0.12 0.18
Nº of genomes with TilS$_1$
Nº of genomes without TilS$_1$
With TilS$_1$
Without TilS$_1$
Nº of genomes with TilS$_2$
Nº of genomes without TilS$_2$
With TilS$_2$
Without TilS$_2$
Nº of genomes with TilS$_3$
Nº of genomes without TilS$_3$
With TilS$_3$
Without TilS$_3$
Nº of genomes with TilS$_4$
Nº of genomes without TilS$_4$
With TilS$_4$
Without TilS$_4$
p < 0.00005
Nº of genomes with TilS$_5$
Nº of genomes without TilS$_5$
With TilS$_5$
Without TilS$_5$
p < 0.00005
Nº of genomes with TilS$_6$
Nº of genomes without TilS$_6$
With TilS$_6$
Without TilS$_6$
p < 0.00005
Nº of genomes with TilS$_7$
Nº of genomes without TilS$_7$
With TilS$_7$
Without TilS$_7$
p < 0.00005
Nº of genomes with TilS$_8$
Nº of genomes without TilS$_8$
With TilS$_8$
Without TilS$_8$
p < 0.00005
Nº of genomes with TilS$_9$
Nº of genomes without TilS$_9$
With TilS$_9$
Without TilS$_9$
p < 0.00005
Nº of genomes with TilS$_{10}$
Nº of genomes without TilS$_{10}$
With TilS$_{10}$
Without TilS$_{10}$
p < 0.00005
Nº of genomes with TilS$_{11}$
Nº of genomes without TilS$_{11}$
With TilS$_{11}$
Without TilS$_{11}$
p < 0.00005
Nº of genomes with TilS$_{12}$
Nº of genomes without TilS$_{12}$
With TilS$_{12}$
Without TilS$_{12}$
p < 0.00005
Nº of genomes with TilS$_{13}$
Nº of genomes without TilS$_{13}$
With TilS$_{13}$
Without TilS$_{13}$
p < 0.00005
Nº of genomes with TilS$_{14}$
Nº of genomes without TilS$_{14}$
With TilS$_{14}$
Without TilS$_{14}$
p < 0.00005
Nº of genomes with TilS$_{15}$
Nº of genomes without TilS$_{15}$
With TilS$_{15}$
Without TilS$_{15}$
p < 0.00005

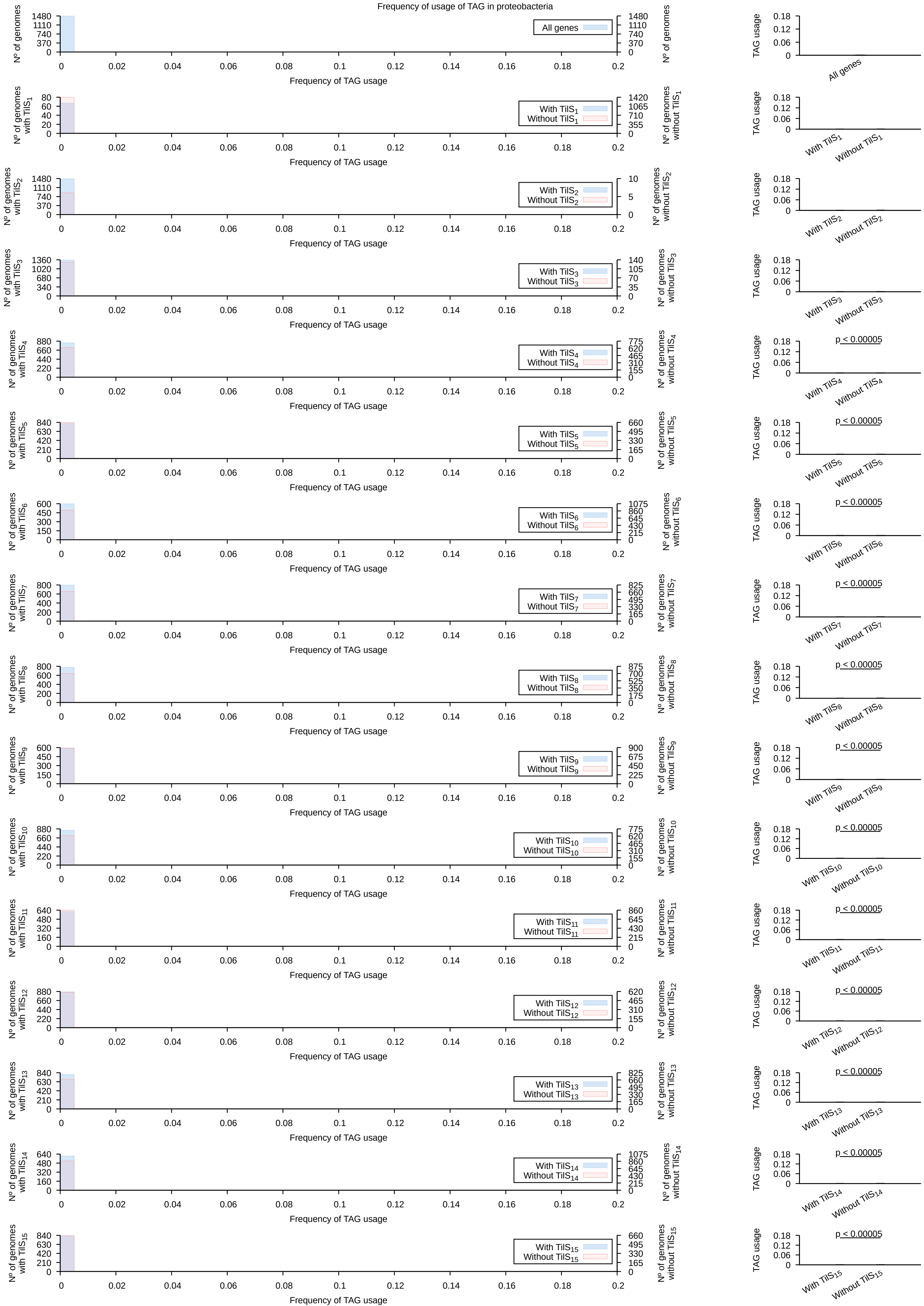

Frequency of usage of TAG in proteobacteria
Nº of genomes
All genes
Frequency of TAG usage
TAG usage
Nº of genomes with TilS1
Nº of genomes without TilS1
With TilS1
Without TilS1
Nº of genomes with TilS2
Nº of genomes without TilS2
With TilS2
Without TilS2
Nº of genomes with TilS3
Nº of genomes without TilS3
With TilS3
Without TilS3
Nº of genomes with TilS4
Nº of genomes without TilS4
With TilS4
Without TilS4
p < 0.00005
Nº of genomes with TilS5
Nº of genomes without TilS5
With TilS5
Without TilS5
Nº of genomes with TilS6
Nº of genomes without TilS6
With TilS6
Without TilS6
Nº of genomes with TilS7
Nº of genomes without TilS7
With TilS7
Without TilS7
Nº of genomes with TilS8
Nº of genomes without TilS8
With TilS8
Without TilS8
Nº of genomes with TilS9
Nº of genomes without TilS9
With TilS9
Without TilS9
Nº of genomes with TilS10
Nº of genomes without TilS10
With TilS10
Without TilS10
Nº of genomes with TilS11
Nº of genomes without TilS11
With TilS11
Without TilS11
Nº of genomes with TilS12
Nº of genomes without TilS12
With TilS12
Without TilS12
Nº of genomes with TilS13
Nº of genomes without TilS13
With TilS13
Without TilS13
Nº of genomes with TilS14
Nº of genomes without TilS14
With TilS14
Without TilS14
Nº of genomes with TilS15
Nº of genomes without TilS15
With TilS15
Without TilS15

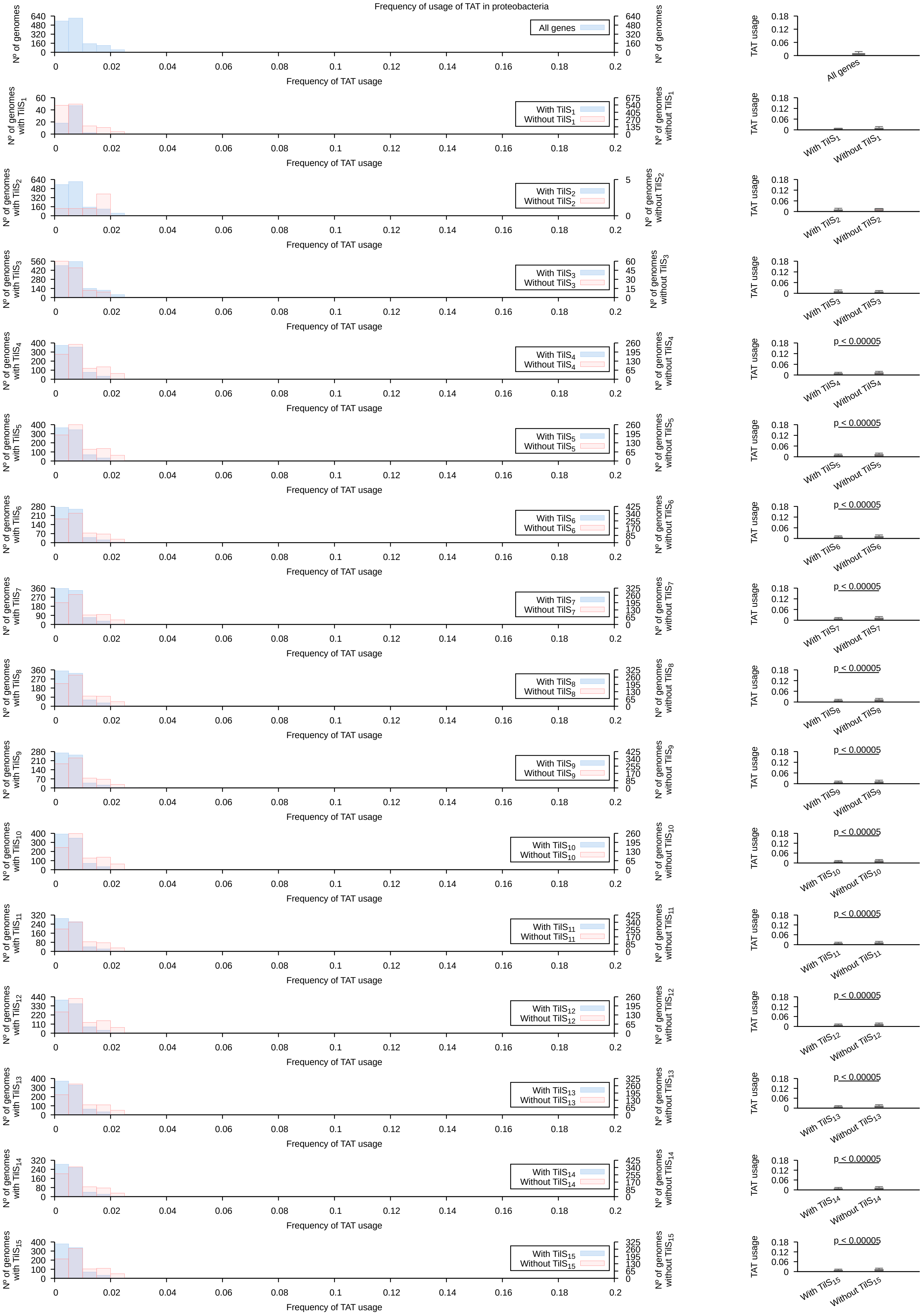
Frequency of usage of TAT in proteobacteria
Nº of genomes
All genes
Frequency of TAT usage
TAT usage
All genes
Nº of genomes with TilS$_1$
Nº of genomes without TilS$_1$
With TilS$_1$
Without TilS$_1$
Nº of genomes with TilS$_2$
Nº of genomes without TilS$_2$
With TilS$_2$
Without TilS$_2$
Nº of genomes with TilS$_3$
Nº of genomes without TilS$_3$
With TilS$_3$
Without TilS$_3$
Nº of genomes with TilS$_4$
Nº of genomes without TilS$_4$
With TilS$_4$
Without TilS$_4$
p < 0.00005
Nº of genomes with TilS$_5$
Nº of genomes without TilS$_5$
With TilS$_5$
Without TilS$_5$
p < 0.00005
Nº of genomes with TilS$_6$
Nº of genomes without TilS$_6$
With TilS$_6$
Without TilS$_6$
p < 0.00005
Nº of genomes with TilS$_7$
Nº of genomes without TilS$_7$
With TilS$_7$
Without TilS$_7$
p < 0.00005
Nº of genomes with TilS$_8$
Nº of genomes without TilS$_8$
With TilS$_8$
Without TilS$_8$
p < 0.00005
Nº of genomes with TilS$_9$
Nº of genomes without TilS$_9$
With TilS$_9$
Without TilS$_9$
p < 0.00005
Nº of genomes with TilS$_{10}$
Nº of genomes without TilS$_{10}$
With TilS$_{10}$
Without TilS$_{10}$
p < 0.00005
Nº of genomes with TilS$_{11}$
Nº of genomes without TilS$_{11}$
With TilS$_{11}$
Without TilS$_{11}$
p < 0.00005
Nº of genomes with TilS$_{12}$
Nº of genomes without TilS$_{12}$
With TilS$_{12}$
Without TilS$_{12}$
p < 0.00005
Nº of genomes with TilS$_{13}$
Nº of genomes without TilS$_{13}$
With TilS$_{13}$
Without TilS$_{13}$
p < 0.00005
Nº of genomes with TilS$_{14}$
Nº of genomes without TilS$_{14}$
With TilS$_{14}$
Without TilS$_{14}$
p < 0.00005
Nº of genomes with TilS$_{15}$
Nº of genomes without TilS$_{15}$
With TilS$_{15}$
Without TilS$_{15}$
p < 0.00005

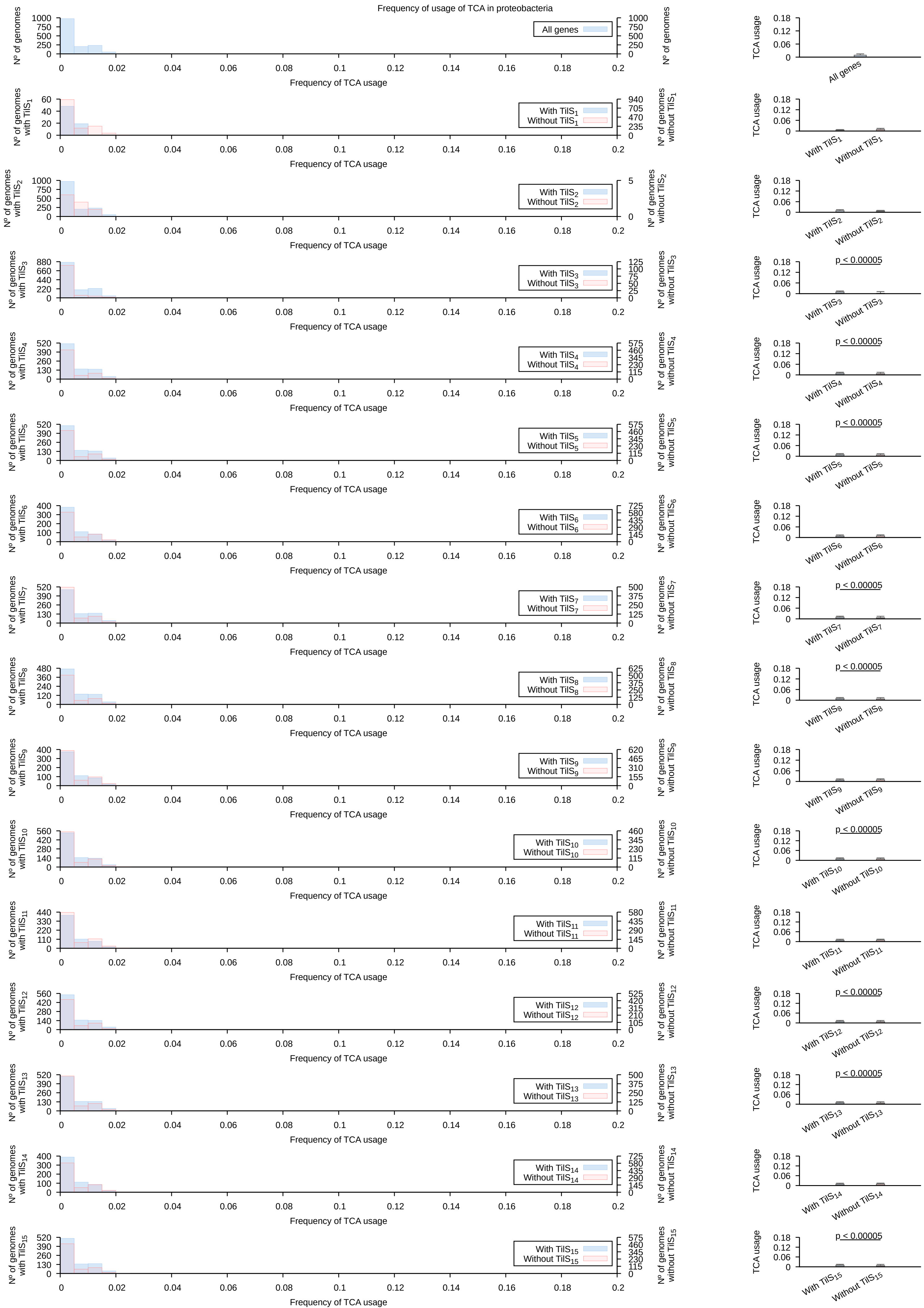

Frequency of usage of TCA in proteobacteria
Nº of genomes
Frequency of TCA usage
All genes
TCA usage
With $TilS_1$
Without $TilS_1$
Nº of genomes with $TilS_1$
Nº of genomes without $TilS_1$
With $TilS_2$
Without $TilS_2$
Nº of genomes with $TilS_2$
Nº of genomes without $TilS_2$
With $TilS_3$
Without $TilS_3$
Nº of genomes with $TilS_3$
Nº of genomes without $TilS_3$
p < 0.00005
With $TilS_4$
Without $TilS_4$
Nº of genomes with $TilS_4$
Nº of genomes without $TilS_4$
p < 0.00005
With $TilS_5$
Without $TilS_5$
Nº of genomes with $TilS_5$
Nº of genomes without $TilS_5$
p < 0.00005
With $TilS_6$
Without $TilS_6$
Nº of genomes with $TilS_6$
Nº of genomes without $TilS_6$
With $TilS_7$
Without $TilS_7$
Nº of genomes with $TilS_7$
Nº of genomes without $TilS_7$
p < 0.00005
With $TilS_8$
Without $TilS_8$
Nº of genomes with $TilS_8$
Nº of genomes without $TilS_8$
p < 0.00005
With $TilS_9$
Without $TilS_9$
Nº of genomes with $TilS_9$
Nº of genomes without $TilS_9$
With $TilS_{10}$
Without $TilS_{10}$
Nº of genomes with $TilS_{10}$
Nº of genomes without $TilS_{10}$
p < 0.00005
With $TilS_{11}$
Without $TilS_{11}$
Nº of genomes with $TilS_{11}$
Nº of genomes without $TilS_{11}$
With $TilS_{12}$
Without $TilS_{12}$
Nº of genomes with $TilS_{12}$
Nº of genomes without $TilS_{12}$
p < 0.00005
With $TilS_{13}$
Without $TilS_{13}$
Nº of genomes with $TilS_{13}$
Nº of genomes without $TilS_{13}$
p < 0.00005
With $TilS_{14}$
Without $TilS_{14}$
Nº of genomes with $TilS_{14}$
Nº of genomes without $TilS_{14}$
With $TilS_{15}$
Without $TilS_{15}$
Nº of genomes with $TilS_{15}$
Nº of genomes without $TilS_{15}$
p < 0.00005

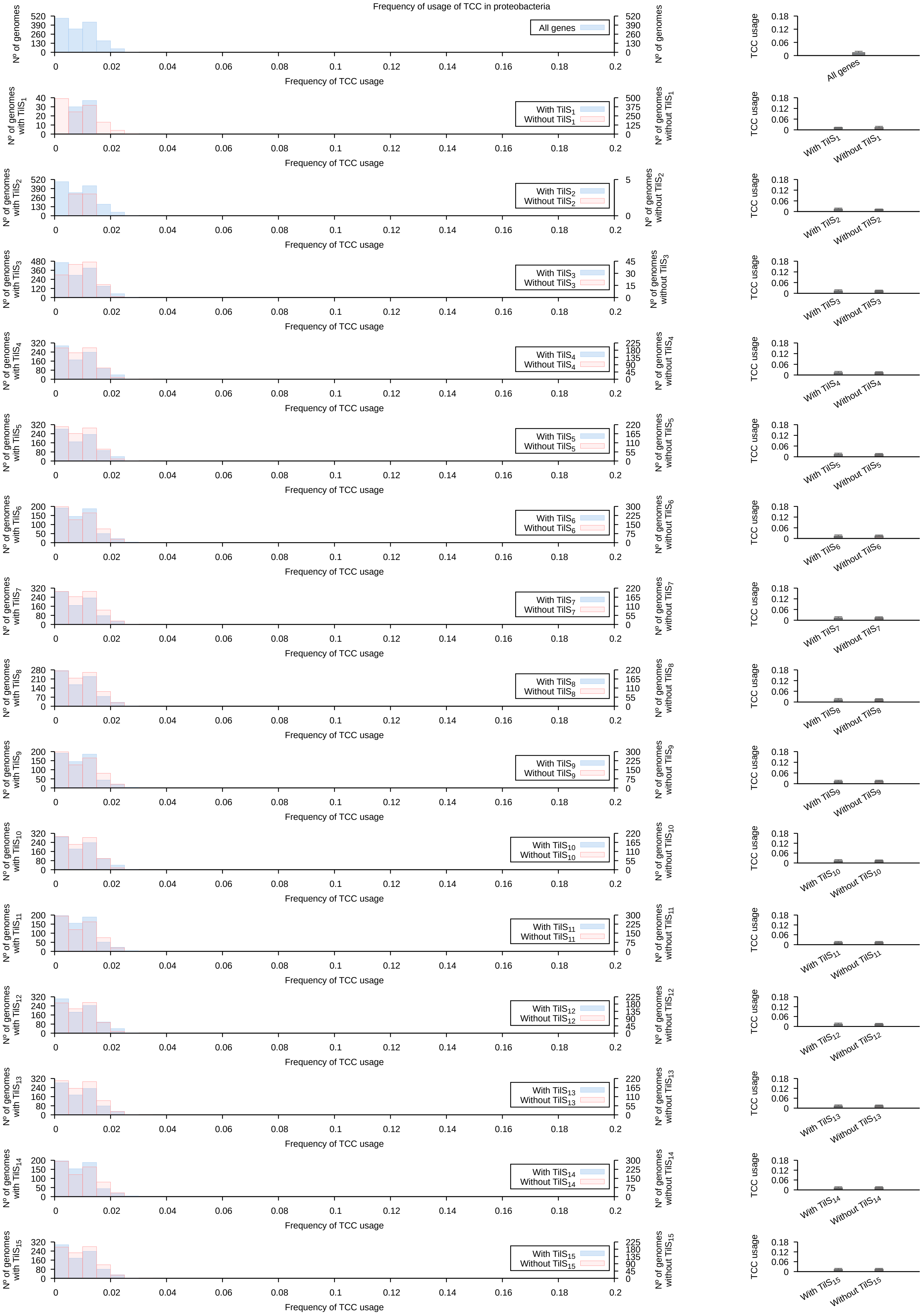
Frequency of usage of TCC in proteobacteria
Nº of genomes
All genes
Frequency of TCC usage
TCC usage
All genes
Nº of genomes with TilS$_1$
With TilS$_1$
Without TilS$_1$
Nº of genomes without TilS$_1$
Nº of genomes with TilS$_2$
With TilS$_2$
Without TilS$_2$
Nº of genomes without TilS$_2$
Nº of genomes with TilS$_3$
With TilS$_3$
Without TilS$_3$
Nº of genomes without TilS$_3$
Nº of genomes with TilS$_4$
With TilS$_4$
Without TilS$_4$
Nº of genomes without TilS$_4$
Nº of genomes with TilS$_5$
With TilS$_5$
Without TilS$_5$
Nº of genomes without TilS$_5$
Nº of genomes with TilS$_6$
With TilS$_6$
Without TilS$_6$
Nº of genomes without TilS$_6$
Nº of genomes with TilS$_7$
With TilS$_7$
Without TilS$_7$
Nº of genomes without TilS$_7$
Nº of genomes with TilS$_8$
With TilS$_8$
Without TilS$_8$
Nº of genomes without TilS$_8$
Nº of genomes with TilS$_9$
With TilS$_9$
Without TilS$_9$
Nº of genomes without TilS$_9$
Nº of genomes with TilS$_{10}$
With TilS$_{10}$
Without TilS$_{10}$
Nº of genomes without TilS$_{10}$
Nº of genomes with TilS$_{11}$
With TilS$_{11}$
Without TilS$_{11}$
Nº of genomes without TilS$_{11}$
Nº of genomes with TilS$_{12}$
With TilS$_{12}$
Without TilS$_{12}$
Nº of genomes without TilS$_{12}$
Nº of genomes with TilS$_{13}$
With TilS$_{13}$
Without TilS$_{13}$
Nº of genomes without TilS$_{13}$
Nº of genomes with TilS$_{14}$
With TilS$_{14}$
Without TilS$_{14}$
Nº of genomes without TilS$_{14}$
Nº of genomes with TilS$_{15}$
With TilS$_{15}$
Without TilS$_{15}$
Nº of genomes without TilS$_{15}$

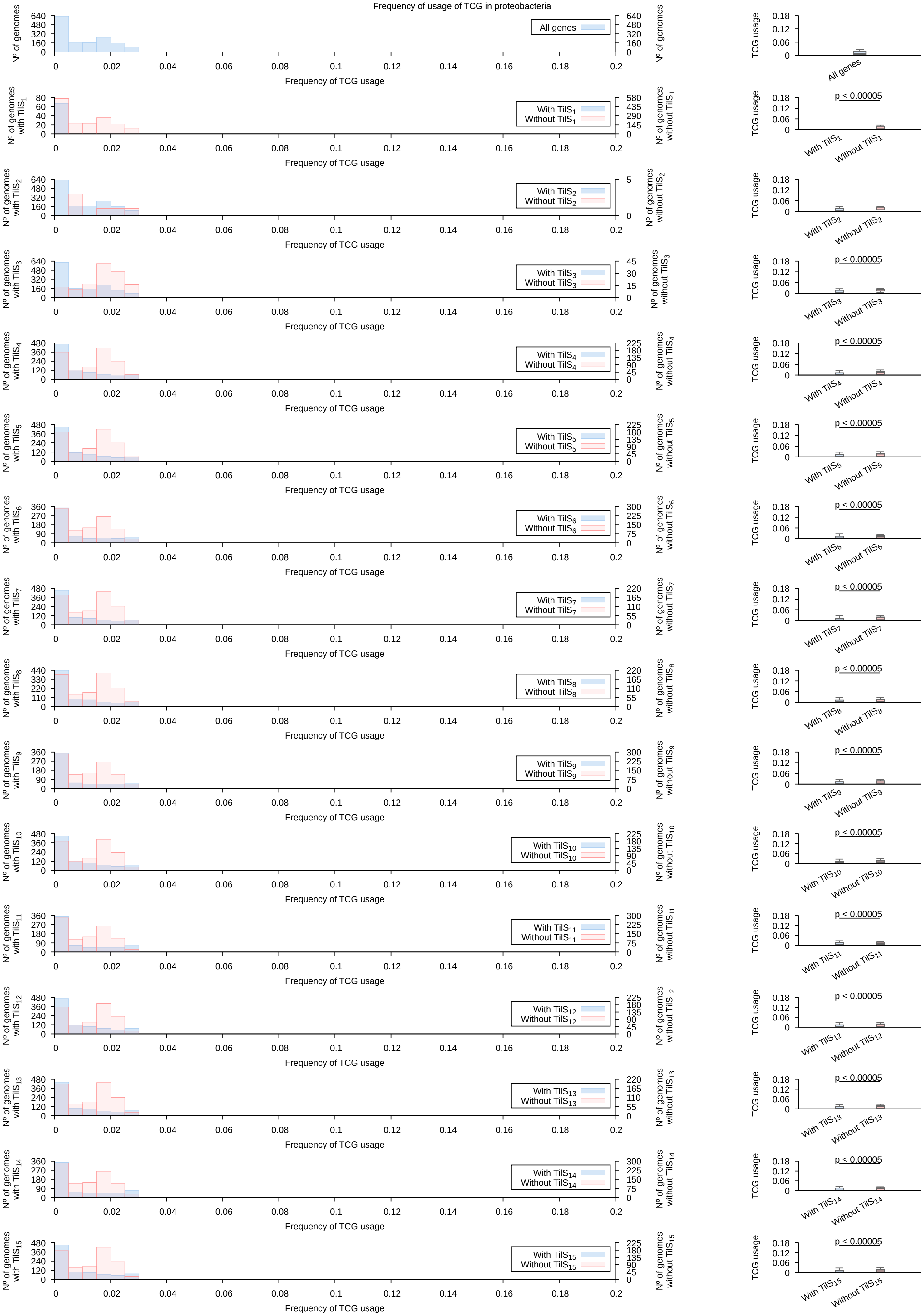

Frequency of usage of TCG in proteobacteria
Nº of genomes
All genes
Frequency of TCG usage
TCG usage
With TilS$_1$
Without TilS$_1$
p < 0.00005
With TilS$_2$
Without TilS$_2$
With TilS$_3$
Without TilS$_3$
With TilS$_4$
Without TilS$_4$
With TilS$_5$
Without TilS$_5$
With TilS$_6$
Without TilS$_6$
With TilS$_7$
Without TilS$_7$
With TilS$_8$
Without TilS$_8$
With TilS$_9$
Without TilS$_9$
With TilS$_{10}$
Without TilS$_{10}$
With TilS$_{11}$
Without TilS$_{11}$
With TilS$_{12}$
Without TilS$_{12}$
With TilS$_{13}$
Without TilS$_{13}$
With TilS$_{14}$
Without TilS$_{14}$
With TilS$_{15}$
Without TilS$_{15}$

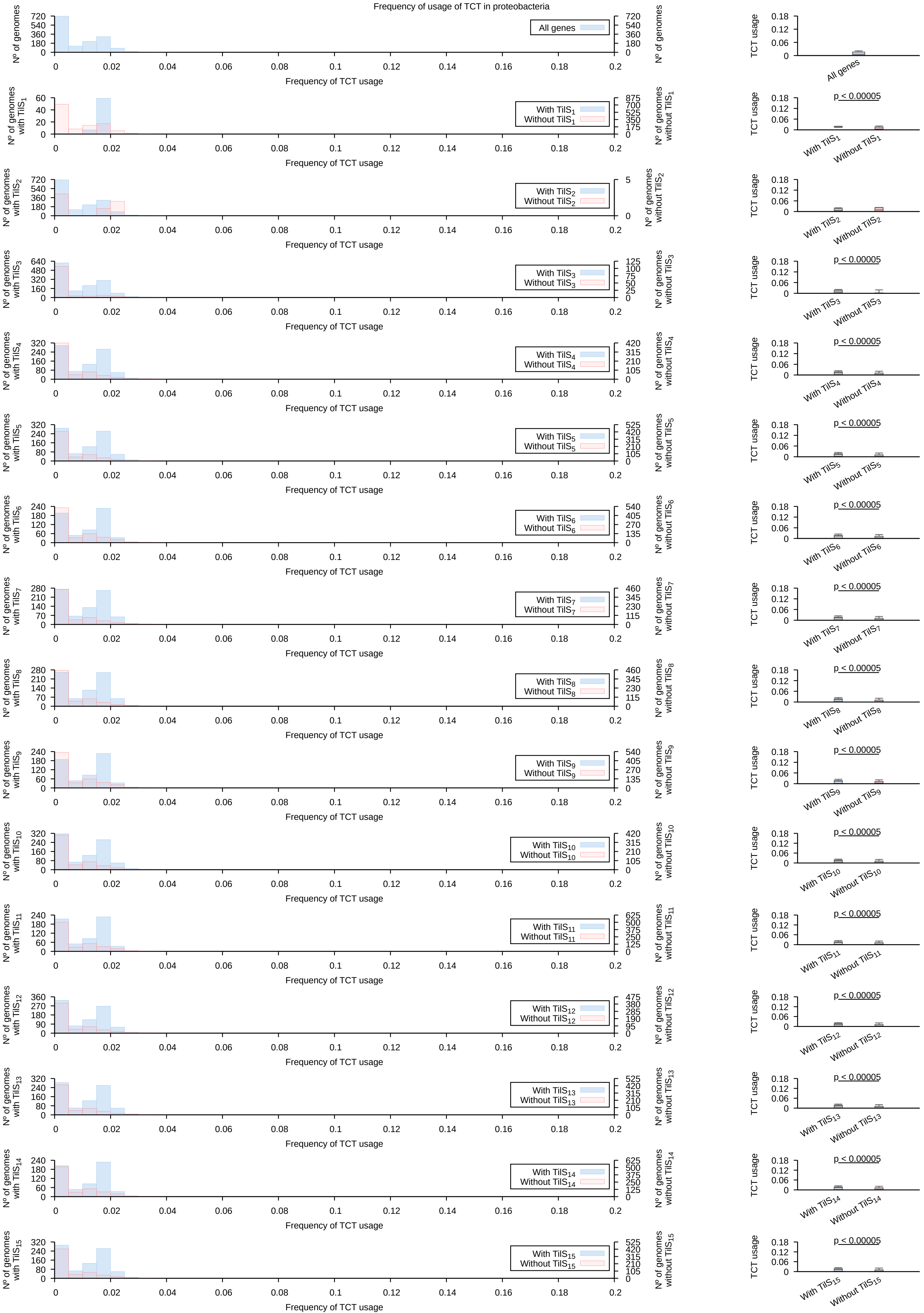
Frequency of usage of TCT in proteobacteria
Nº of genomes
All genes
Frequency of TCT usage
TCT usage
Nº of genomes with $TilS_1$
Nº of genomes without $TilS_1$
With $TilS_1$
Without $TilS_1$
p < 0.00005
Nº of genomes with $TilS_2$
Nº of genomes without $TilS_2$
With $TilS_2$
Without $TilS_2$
Nº of genomes with $TilS_3$
Nº of genomes without $TilS_3$
With $TilS_3$
Without $TilS_3$
Nº of genomes with $TilS_4$
Nº of genomes without $TilS_4$
With $TilS_4$
Without $TilS_4$
Nº of genomes with $TilS_5$
Nº of genomes without $TilS_5$
With $TilS_5$
Without $TilS_5$
Nº of genomes with $TilS_6$
Nº of genomes without $TilS_6$
With $TilS_6$
Without $TilS_6$
Nº of genomes with $TilS_7$
Nº of genomes without $TilS_7$
With $TilS_7$
Without $TilS_7$
Nº of genomes with $TilS_8$
Nº of genomes without $TilS_8$
With $TilS_8$
Without $TilS_8$
Nº of genomes with $TilS_9$
Nº of genomes without $TilS_9$
With $TilS_9$
Without $TilS_9$
Nº of genomes with $TilS_{10}$
Nº of genomes without $TilS_{10}$
With $TilS_{10}$
Without $TilS_{10}$
Nº of genomes with $TilS_{11}$
Nº of genomes without $TilS_{11}$
With $TilS_{11}$
Without $TilS_{11}$
Nº of genomes with $TilS_{12}$
Nº of genomes without $TilS_{12}$
With $TilS_{12}$
Without $TilS_{12}$
Nº of genomes with $TilS_{13}$
Nº of genomes without $TilS_{13}$
With $TilS_{13}$
Without $TilS_{13}$
Nº of genomes with $TilS_{14}$
Nº of genomes without $TilS_{14}$
With $TilS_{14}$
Without $TilS_{14}$
Nº of genomes with $TilS_{15}$
Nº of genomes without $TilS_{15}$
With $TilS_{15}$
Without $TilS_{15}$

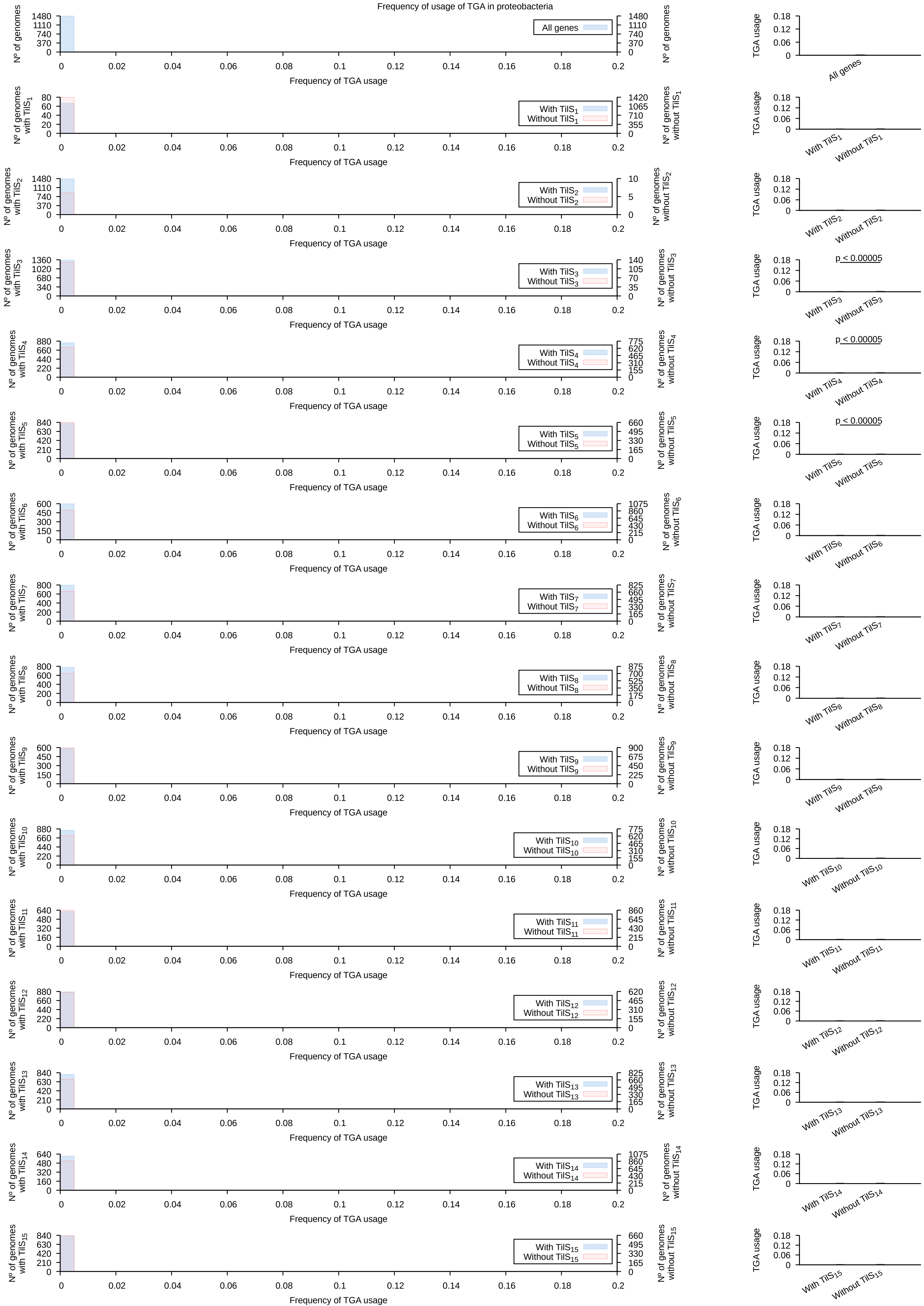
Frequency of usage of TGA in proteobacteria
Nº of genomes
All genes
Frequency of TGA usage
TGA usage
With TilS$_1$
Without TilS$_1$
Nº of genomes with TilS$_1$
Nº of genomes without TilS$_1$
With TilS$_2$
Without TilS$_2$
Nº of genomes with TilS$_2$
Nº of genomes without TilS$_2$
With TilS$_3$
Without TilS$_3$
Nº of genomes with TilS$_3$
Nº of genomes without TilS$_3$
$p < 0.00005$
With TilS$_4$
Without TilS$_4$
Nº of genomes with TilS$_4$
Nº of genomes without TilS$_4$
$p < 0.00005$
With TilS$_5$
Without TilS$_5$
Nº of genomes with TilS$_5$
Nº of genomes without TilS$_5$
$p < 0.00005$
With TilS$_6$
Without TilS$_6$
Nº of genomes with TilS$_6$
Nº of genomes without TilS$_6$
With TilS$_7$
Without TilS$_7$
Nº of genomes with TilS$_7$
Nº of genomes without TilS$_7$
With TilS$_8$
Without TilS$_8$
Nº of genomes with TilS$_8$
Nº of genomes without TilS$_8$
With TilS$_9$
Without TilS$_9$
Nº of genomes with TilS$_9$
Nº of genomes without TilS$_9$
With TilS$_{10}$
Without TilS$_{10}$
Nº of genomes with TilS$_{10}$
Nº of genomes without TilS$_{10}$
With TilS$_{11}$
Without TilS$_{11}$
Nº of genomes with TilS$_{11}$
Nº of genomes without TilS$_{11}$
With TilS$_{12}$
Without TilS$_{12}$
Nº of genomes with TilS$_{12}$
Nº of genomes without TilS$_{12}$
With TilS$_{13}$
Without TilS$_{13}$
Nº of genomes with TilS$_{13}$
Nº of genomes without TilS$_{13}$
With TilS$_{14}$
Without TilS$_{14}$
Nº of genomes with TilS$_{14}$
Nº of genomes without TilS$_{14}$
With TilS$_{15}$
Without TilS$_{15}$
Nº of genomes with TilS$_{15}$
Nº of genomes without TilS$_{15}$

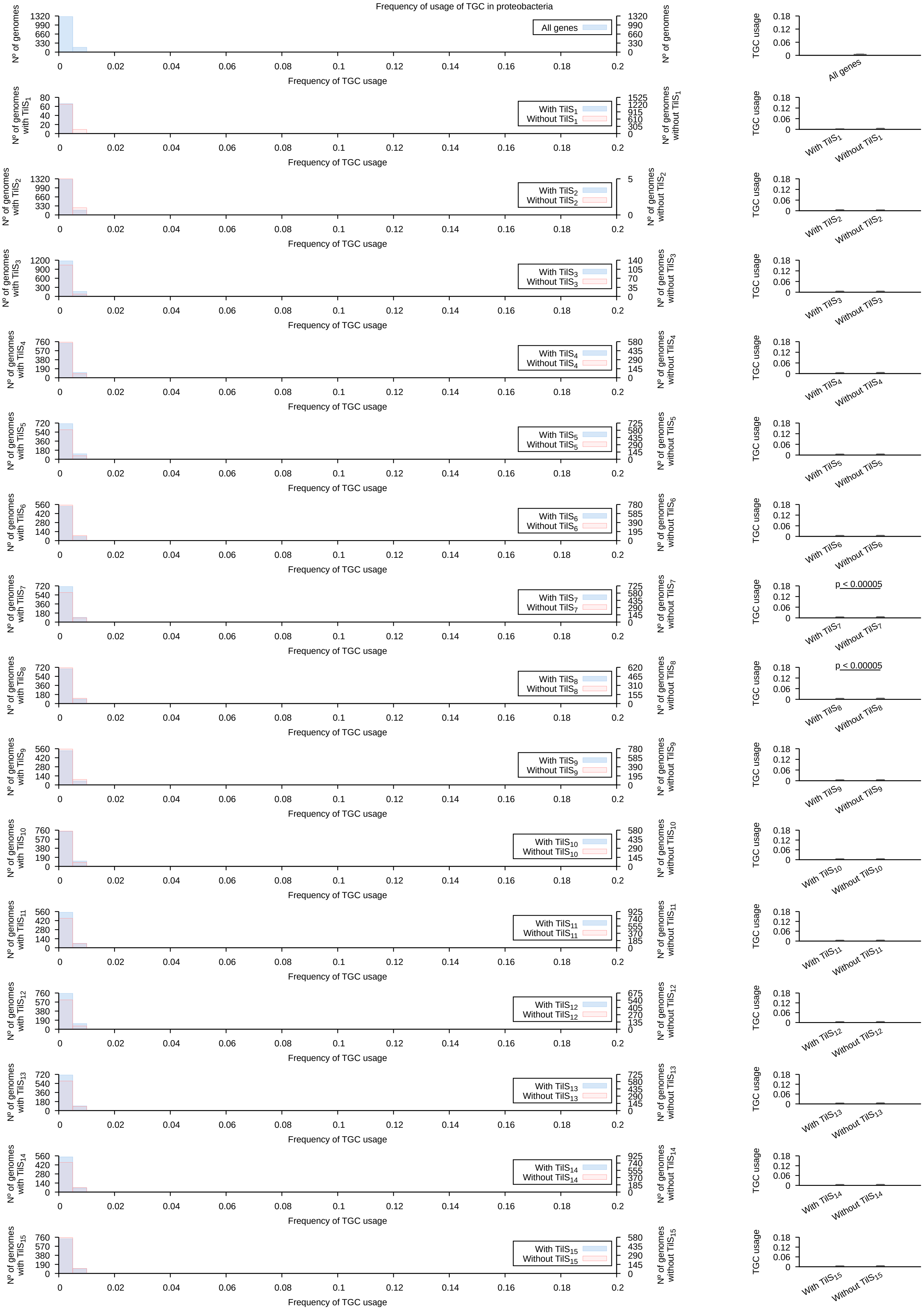

Frequency of usage of TGC in proteobacteria
Nº of genomes
All genes
Frequency of TGC usage
TGC usage
Nº of genomes with TilS$_1$
Nº of genomes without TilS$_1$
With TilS$_1$
Without TilS$_1$
Nº of genomes with TilS$_2$
Nº of genomes without TilS$_2$
With TilS$_2$
Without TilS$_2$
Nº of genomes with TilS$_3$
Nº of genomes without TilS$_3$
With TilS$_3$
Without TilS$_3$
Nº of genomes with TilS$_4$
Nº of genomes without TilS$_4$
With TilS$_4$
Without TilS$_4$
Nº of genomes with TilS$_5$
Nº of genomes without TilS$_5$
With TilS$_5$
Without TilS$_5$
Nº of genomes with TilS$_6$
Nº of genomes without TilS$_6$
With TilS$_6$
Without TilS$_6$
Nº of genomes with TilS$_7$
Nº of genomes without TilS$_7$
With TilS$_7$
Without TilS$_7$
$p < 0.00005$
Nº of genomes with TilS$_8$
Nº of genomes without TilS$_8$
With TilS$_8$
Without TilS$_8$
$p < 0.00005$
Nº of genomes with TilS$_9$
Nº of genomes without TilS$_9$
With TilS$_9$
Without TilS$_9$
Nº of genomes with TilS$_{10}$
Nº of genomes without TilS$_{10}$
With TilS$_{10}$
Without TilS$_{10}$
Nº of genomes with TilS$_{11}$
Nº of genomes without TilS$_{11}$
With TilS$_{11}$
Without TilS$_{11}$
Nº of genomes with TilS$_{12}$
Nº of genomes without TilS$_{12}$
With TilS$_{12}$
Without TilS$_{12}$
Nº of genomes with TilS$_{13}$
Nº of genomes without TilS$_{13}$
With TilS$_{13}$
Without TilS$_{13}$
Nº of genomes with TilS$_{14}$
Nº of genomes without TilS$_{14}$
With TilS$_{14}$
Without TilS$_{14}$
Nº of genomes with TilS$_{15}$
Nº of genomes without TilS$_{15}$
With TilS$_{15}$
Without TilS$_{15}$

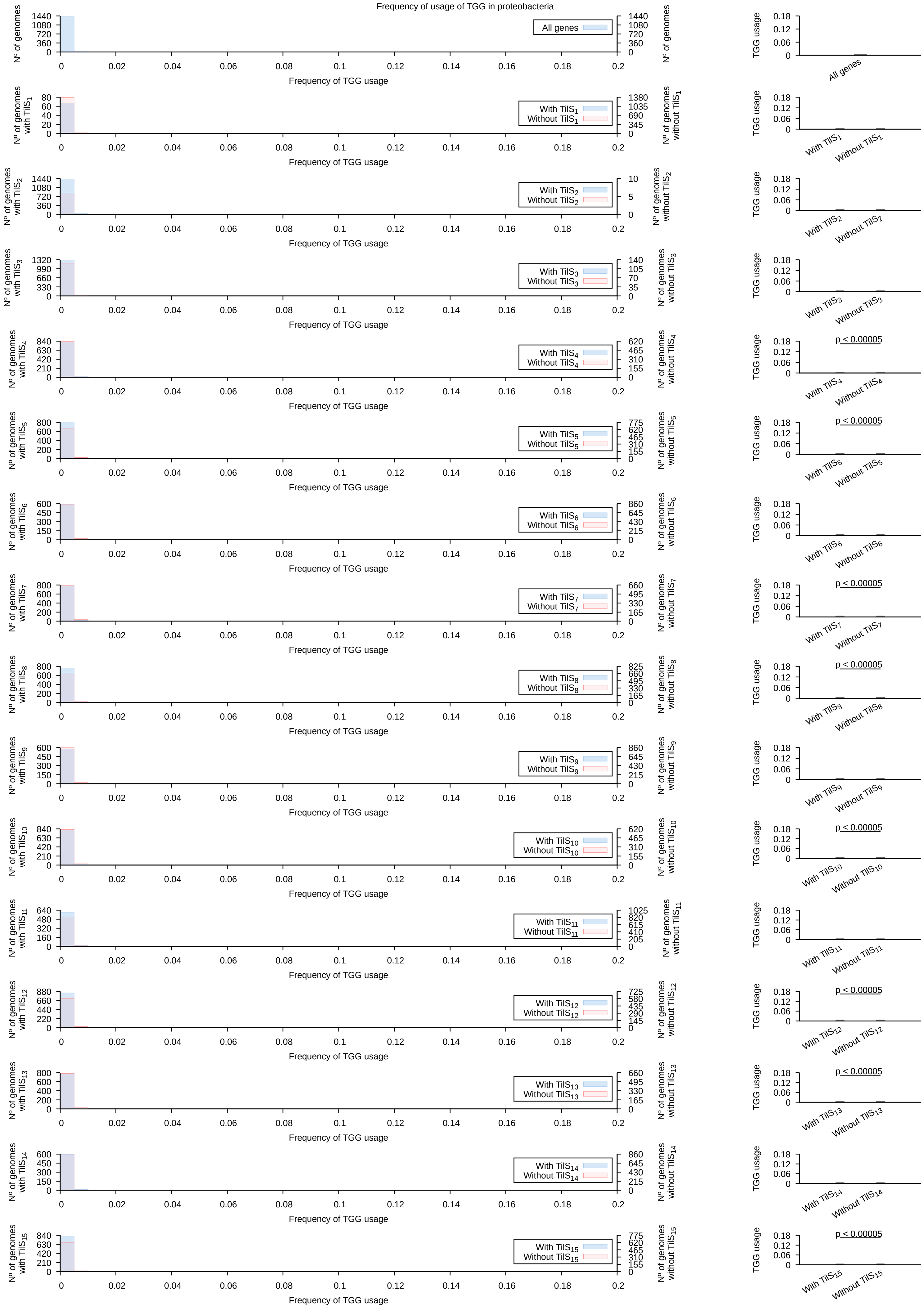

Frequency of usage of TGG in proteobacteria
Nº of genomes
Frequency of TGG usage
All genes
TGG usage
With TilS
Without TilS
p < 0.00005

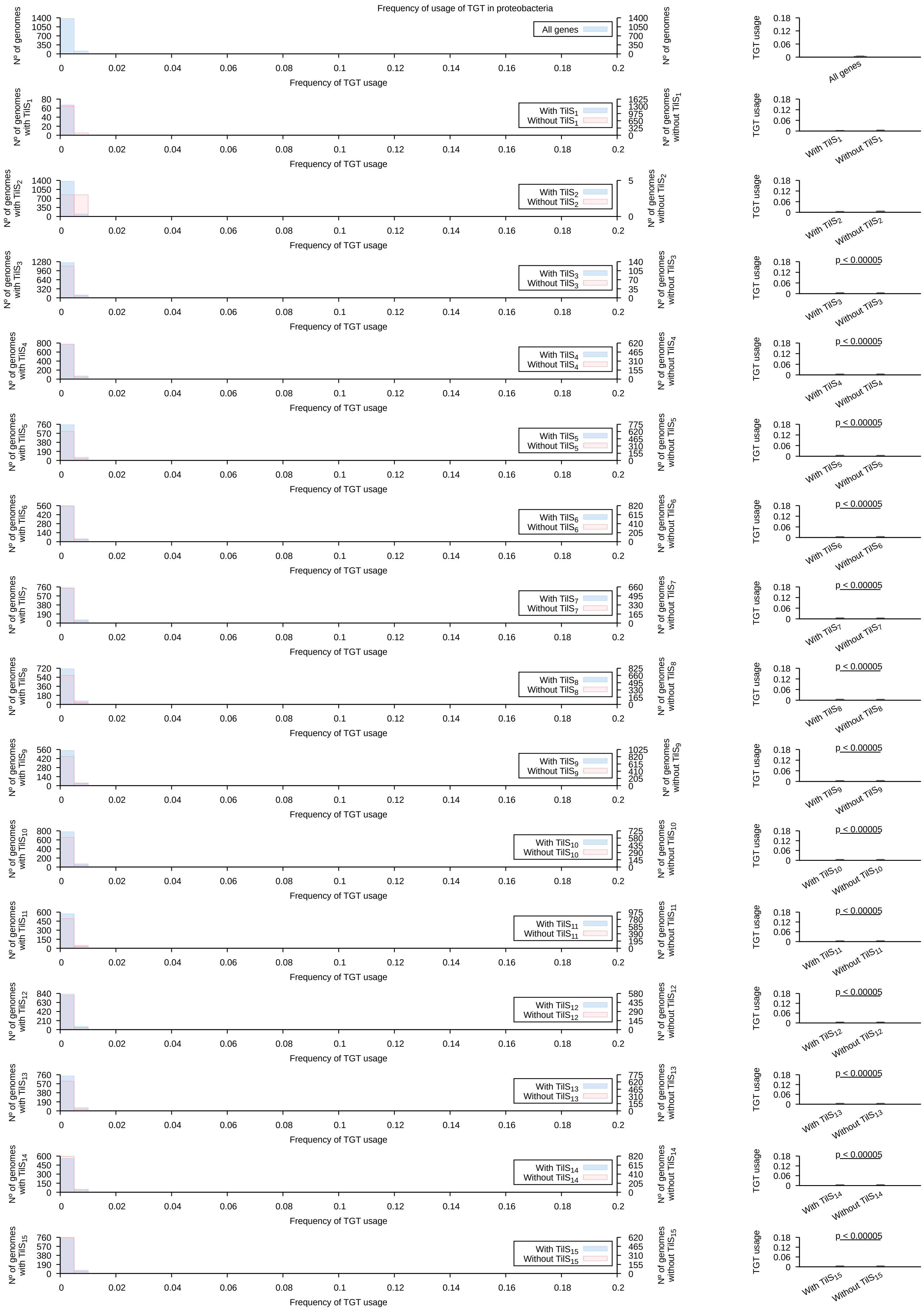

Frequency of usage of TGT in proteobacteria
Nº of genomes
All genes
Frequency of TGT usage
TGT usage
With TilS1
Without TilS1
With TilS2
Without TilS2
With TilS3
Without TilS3
With TilS4
Without TilS4
With TilS5
Without TilS5
With TilS6
Without TilS6
With TilS7
Without TilS7
With TilS8
Without TilS8
With TilS9
Without TilS9
With TilS10
Without TilS10
With TilS11
Without TilS11
With TilS12
Without TilS12
With TilS13
Without TilS13
With TilS14
Without TilS14
With TilS15
Without TilS15
p < 0.00005

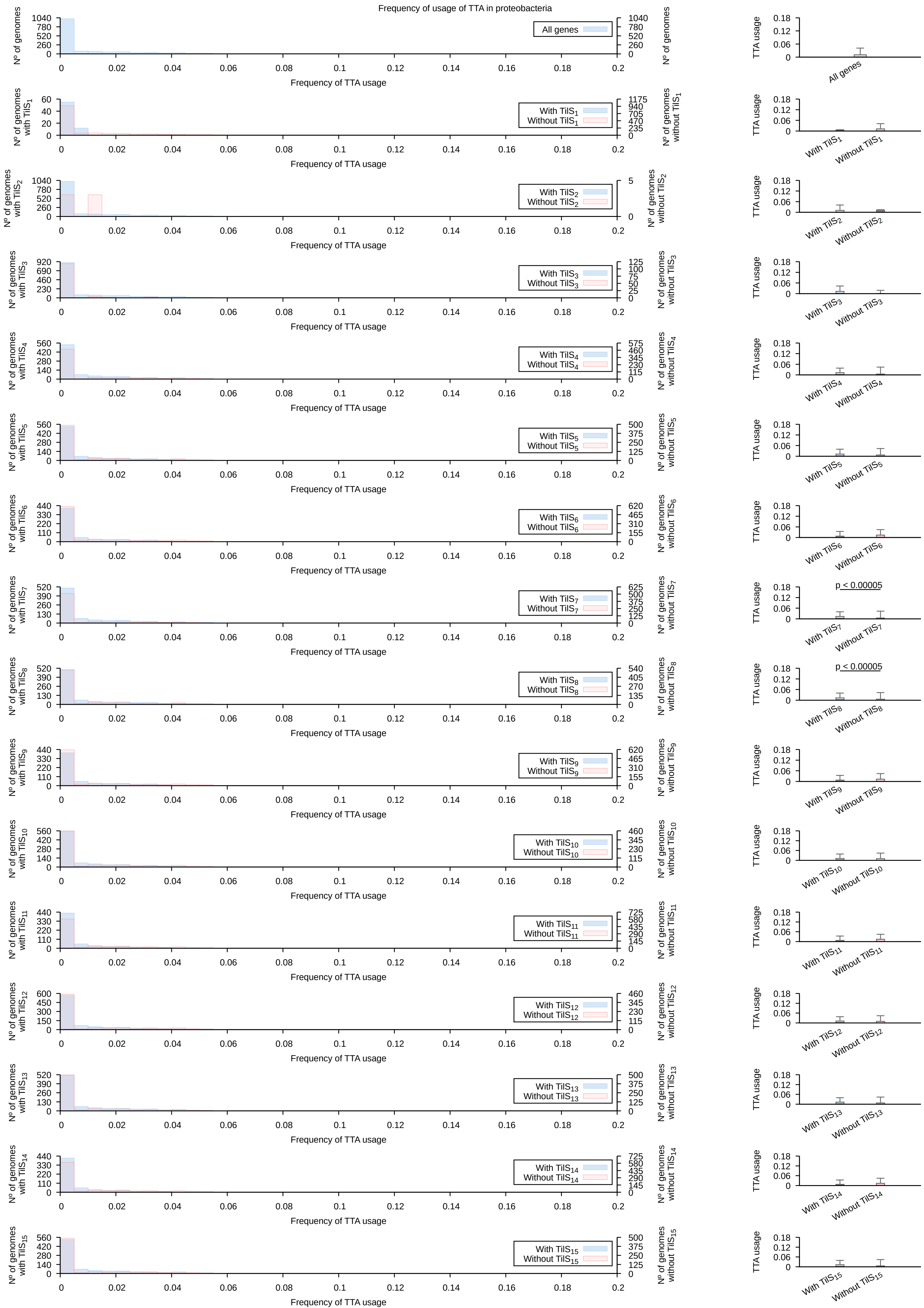

Frequency of usage of TTA in proteobacteria
Nº of genomes
Frequency of TTA usage
TTA usage
All genes
With $TilS_1$
Without $TilS_1$
With $TilS_2$
Without $TilS_2$
With $TilS_3$
Without $TilS_3$
With $TilS_4$
Without $TilS_4$
With $TilS_5$
Without $TilS_5$
With $TilS_6$
Without $TilS_6$
With $TilS_7$
Without $TilS_7$
p < 0.00005
With $TilS_8$
Without $TilS_8$
p < 0.00005
With $TilS_9$
Without $TilS_9$
With $TilS_{10}$
Without $TilS_{10}$
With $TilS_{11}$
Without $TilS_{11}$
With $TilS_{12}$
Without $TilS_{12}$
With $TilS_{13}$
Without $TilS_{13}$
With $TilS_{14}$
Without $TilS_{14}$
With $TilS_{15}$
Without $TilS_{15}$

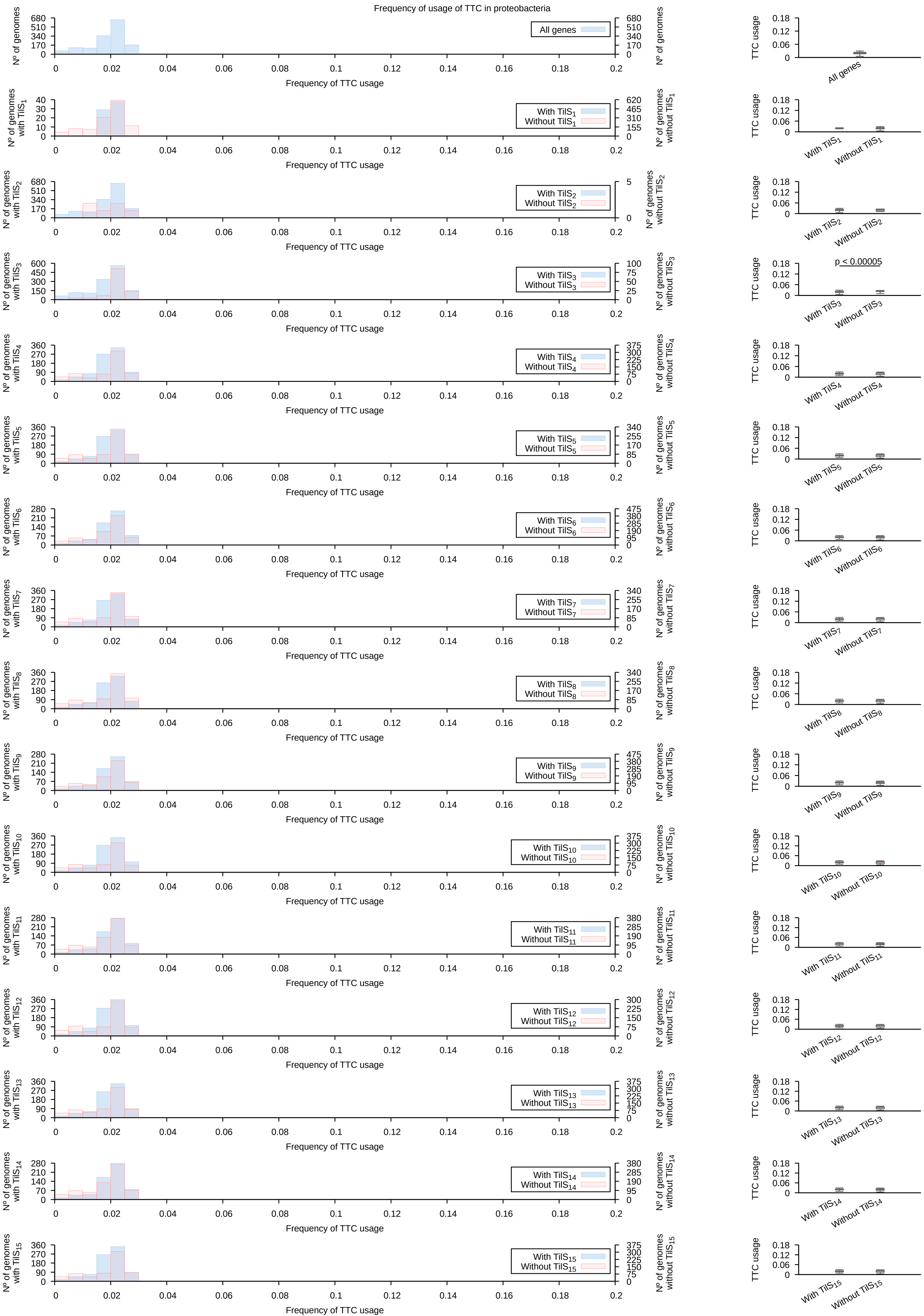

Frequency of usage of TTC in proteobacteria
Nº of genomes
All genes
Frequency of TTC usage
TTC usage
Nº of genomes with TilS1
Nº of genomes without TilS1
With TilS1
Without TilS1
Nº of genomes with TilS2
Nº of genomes without TilS2
With TilS2
Without TilS2
Nº of genomes with TilS3
Nº of genomes without TilS3
With TilS3
Without TilS3
p < 0.00005
Nº of genomes with TilS4
Nº of genomes without TilS4
With TilS4
Without TilS4
Nº of genomes with TilS5
Nº of genomes without TilS5
With TilS5
Without TilS5
Nº of genomes with TilS6
Nº of genomes without TilS6
With TilS6
Without TilS6
Nº of genomes with TilS7
Nº of genomes without TilS7
With TilS7
Without TilS7
Nº of genomes with TilS8
Nº of genomes without TilS8
With TilS8
Without TilS8
Nº of genomes with TilS9
Nº of genomes without TilS9
With TilS9
Without TilS9
Nº of genomes with TilS10
Nº of genomes without TilS10
With TilS10
Without TilS10
Nº of genomes with TilS11
Nº of genomes without TilS11
With TilS11
Without TilS11
Nº of genomes with TilS12
Nº of genomes without TilS12
With TilS12
Without TilS12
Nº of genomes with TilS13
Nº of genomes without TilS13
With TilS13
Without TilS13
Nº of genomes with TilS14
Nº of genomes without TilS14
With TilS14
Without TilS14
Nº of genomes with TilS15
Nº of genomes without TilS15
With TilS15
Without TilS15

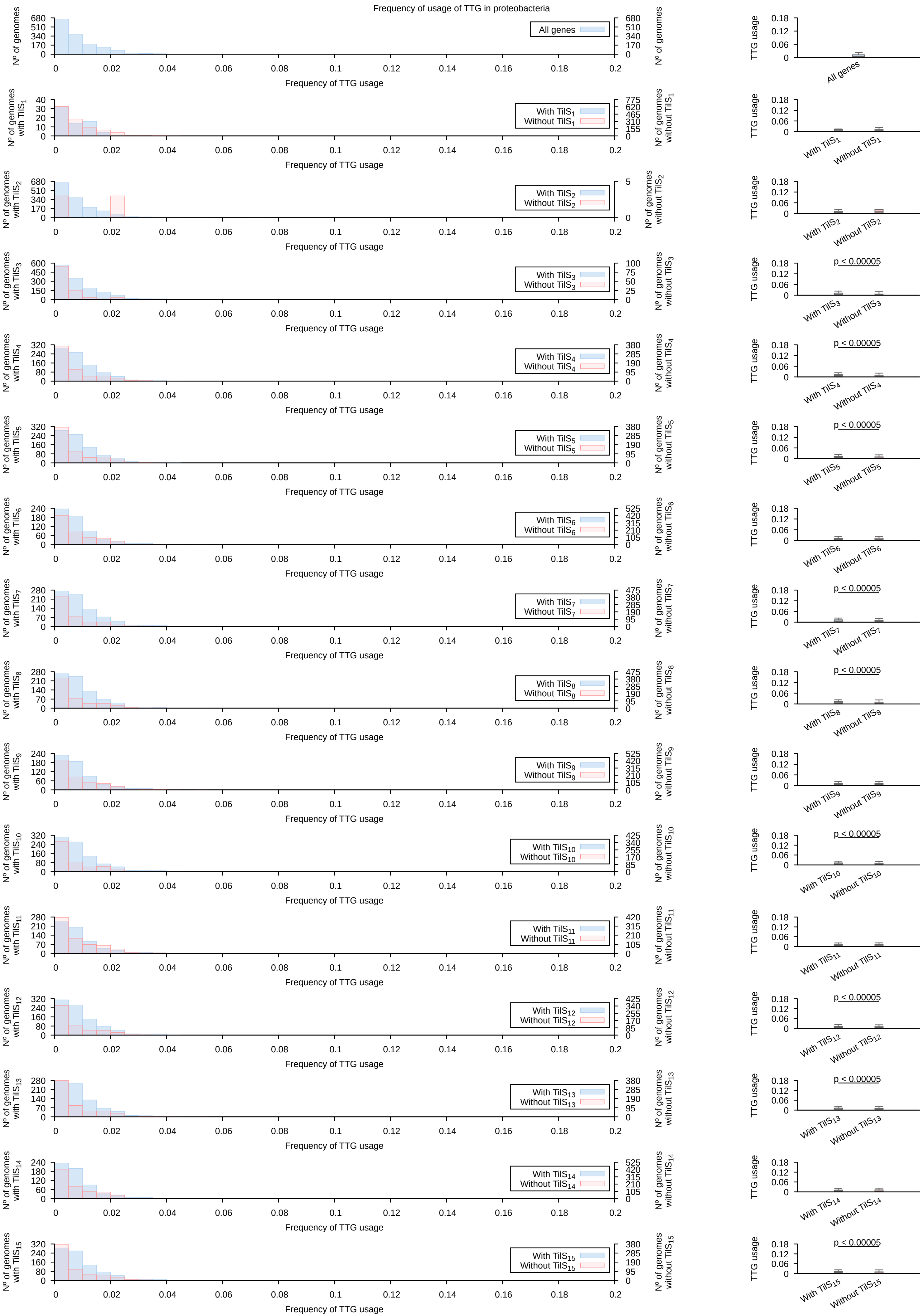

Frequency of usage of TTG in proteobacteria
Nº of genomes
All genes
Frequency of TTG usage
TTG usage
With TilS1
Without TilS1
With TilS2
Without TilS2
With TilS3
Without TilS3
p < 0.00005
With TilS4
Without TilS4
p < 0.00005
With TilS5
Without TilS5
p < 0.00005
With TilS6
Without TilS6
With TilS7
Without TilS7
p < 0.00005
With TilS8
Without TilS8
p < 0.00005
With TilS9
Without TilS9
With TilS10
Without TilS10
p < 0.00005
With TilS11
Without TilS11
With TilS12
Without TilS12
p < 0.00005
With TilS13
Without TilS13
p < 0.00005
With TilS14
Without TilS14
With TilS15
Without TilS15
p < 0.00005

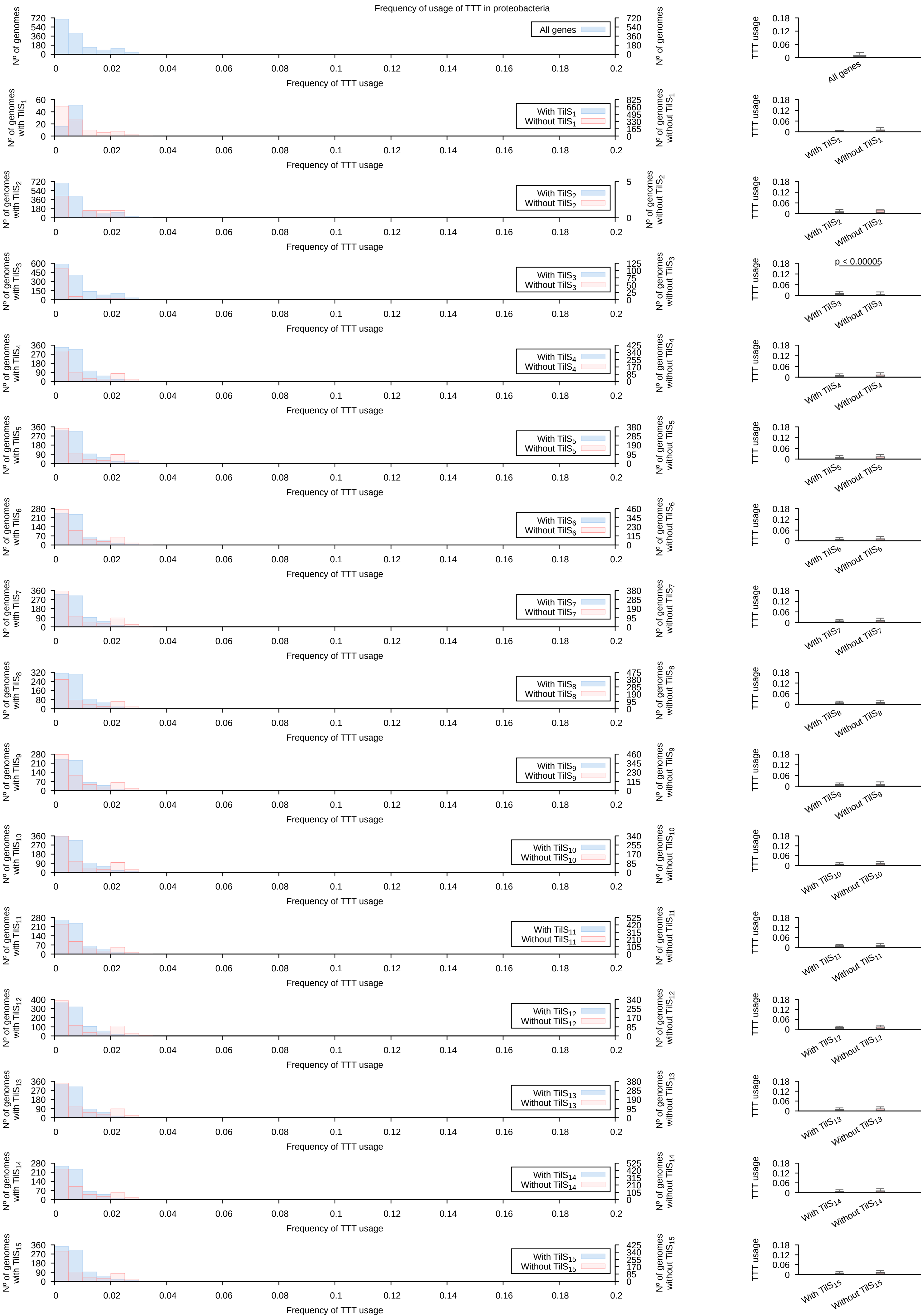

Frequency of usage of TTT in proteobacteria
Nº of genomes
All genes
Frequency of TTT usage
TTT usage
Nº of genomes with TilS$_1$
Nº of genomes without TilS$_1$
With TilS$_1$
Without TilS$_1$
Nº of genomes with TilS$_2$
Nº of genomes without TilS$_2$
With TilS$_2$
Without TilS$_2$
Nº of genomes with TilS$_3$
Nº of genomes without TilS$_3$
With TilS$_3$
Without TilS$_3$
p < 0.00005
Nº of genomes with TilS$_4$
Nº of genomes without TilS$_4$
With TilS$_4$
Without TilS$_4$
Nº of genomes with TilS$_5$
Nº of genomes without TilS$_5$
With TilS$_5$
Without TilS$_5$
Nº of genomes with TilS$_6$
Nº of genomes without TilS$_6$
With TilS$_6$
Without TilS$_6$
Nº of genomes with TilS$_7$
Nº of genomes without TilS$_7$
With TilS$_7$
Without TilS$_7$
Nº of genomes with TilS$_8$
Nº of genomes without TilS$_8$
With TilS$_8$
Without TilS$_8$
Nº of genomes with TilS$_9$
Nº of genomes without TilS$_9$
With TilS$_9$
Without TilS$_9$
Nº of genomes with TilS$_{10}$
Nº of genomes without TilS$_{10}$
With TilS$_{10}$
Without TilS$_{10}$
Nº of genomes with TilS$_{11}$
Nº of genomes without TilS$_{11}$
With TilS$_{11}$
Without TilS$_{11}$
Nº of genomes with TilS$_{12}$
Nº of genomes without TilS$_{12}$
With TilS$_{12}$
Without TilS$_{12}$
Nº of genomes with TilS$_{13}$
Nº of genomes without TilS$_{13}$
With TilS$_{13}$
Without TilS$_{13}$
Nº of genomes with TilS$_{14}$
Nº of genomes without TilS$_{14}$
With TilS$_{14}$
Without TilS$_{14}$
Nº of genomes with TilS$_{15}$
Nº of genomes without TilS$_{15}$
With TilS$_{15}$
Without TilS$_{15}$
