# Supplementary material for: Impact of the chemical modification of tRNAs anticodon loop on the variability and evolution of codon usage in proteobacteria
Source: Front Microbiol. 2024 Aug 5;15:1412318. doi: 10.3389/fmicb.2024.1412318 (PMC11332805; doi:10.3389/fmicb.2024.1412318)
Supplement: Supplementary file 1 [file Data_Sheet_1.zip › Supp_figures/Fig_S19.pdf]

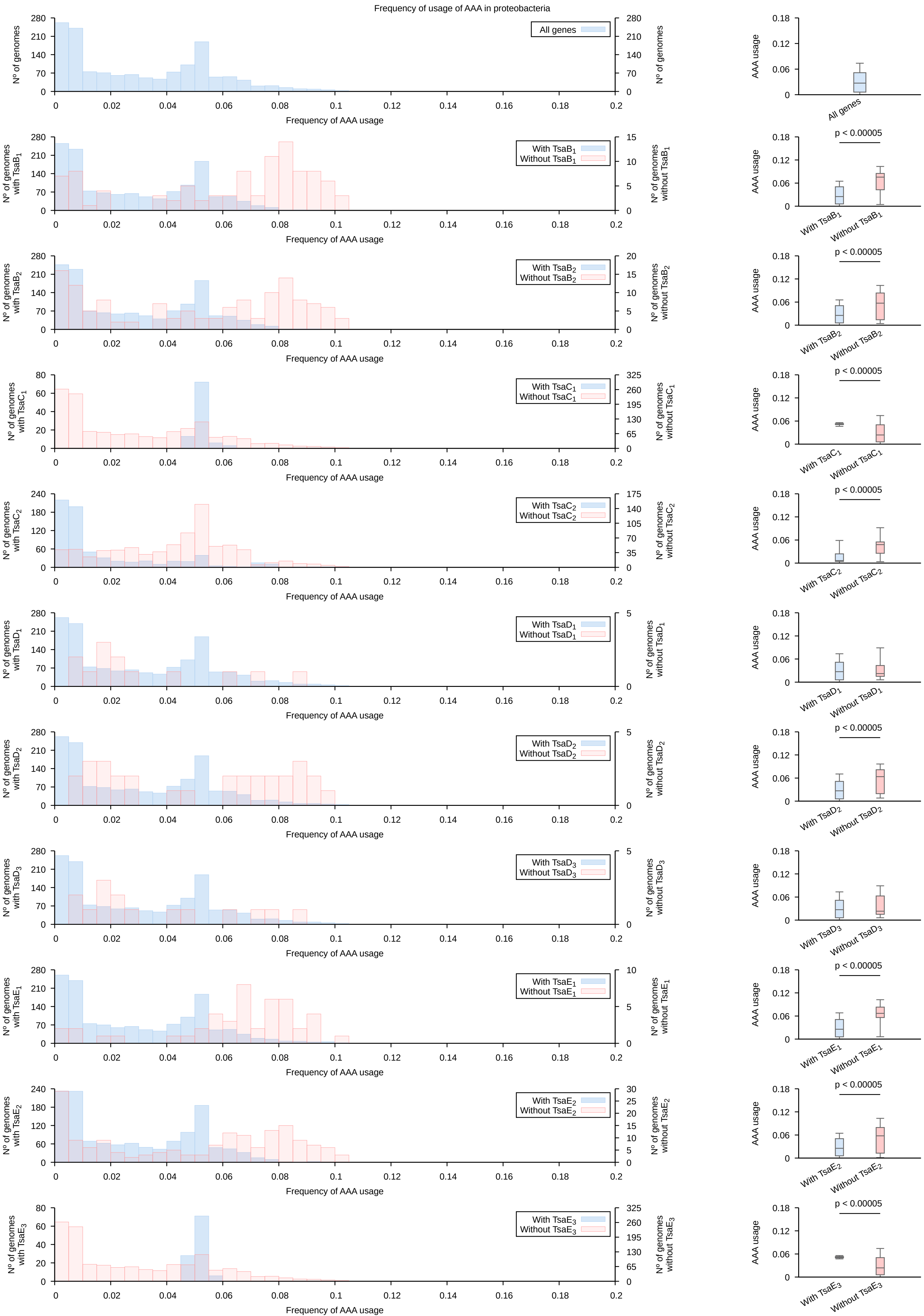

Frequency of usage of AAA in proteobacteria
All genes
Nº of genomes
Frequency of AAA usage
AAA usage
With TsaB1
Without TsaB1
Nº of genomes with TsaB1
Nº of genomes without TsaB1
p < 0.00005
With TsaB2
Without TsaB2
Nº of genomes with TsaB2
Nº of genomes without TsaB2
p < 0.00005
With TsaC1
Without TsaC1
Nº of genomes with TsaC1
Nº of genomes without TsaC1
p < 0.00005
With TsaC2
Without TsaC2
Nº of genomes with TsaC2
Nº of genomes without TsaC2
p < 0.00005
With TsaD1
Without TsaD1
Nº of genomes with TsaD1
Nº of genomes without TsaD1
With TsaD2
Without TsaD2
Nº of genomes with TsaD2
Nº of genomes without TsaD2
p < 0.00005
With TsaD3
Without TsaD3
Nº of genomes with TsaD3
Nº of genomes without TsaD3
With TsaE1
Without TsaE1
Nº of genomes with TsaE1
Nº of genomes without TsaE1
p < 0.00005
With TsaE2
Without TsaE2
Nº of genomes with TsaE2
Nº of genomes without TsaE2
p < 0.00005
With TsaE3
Without TsaE3
Nº of genomes with TsaE3
Nº of genomes without TsaE3
p < 0.00005

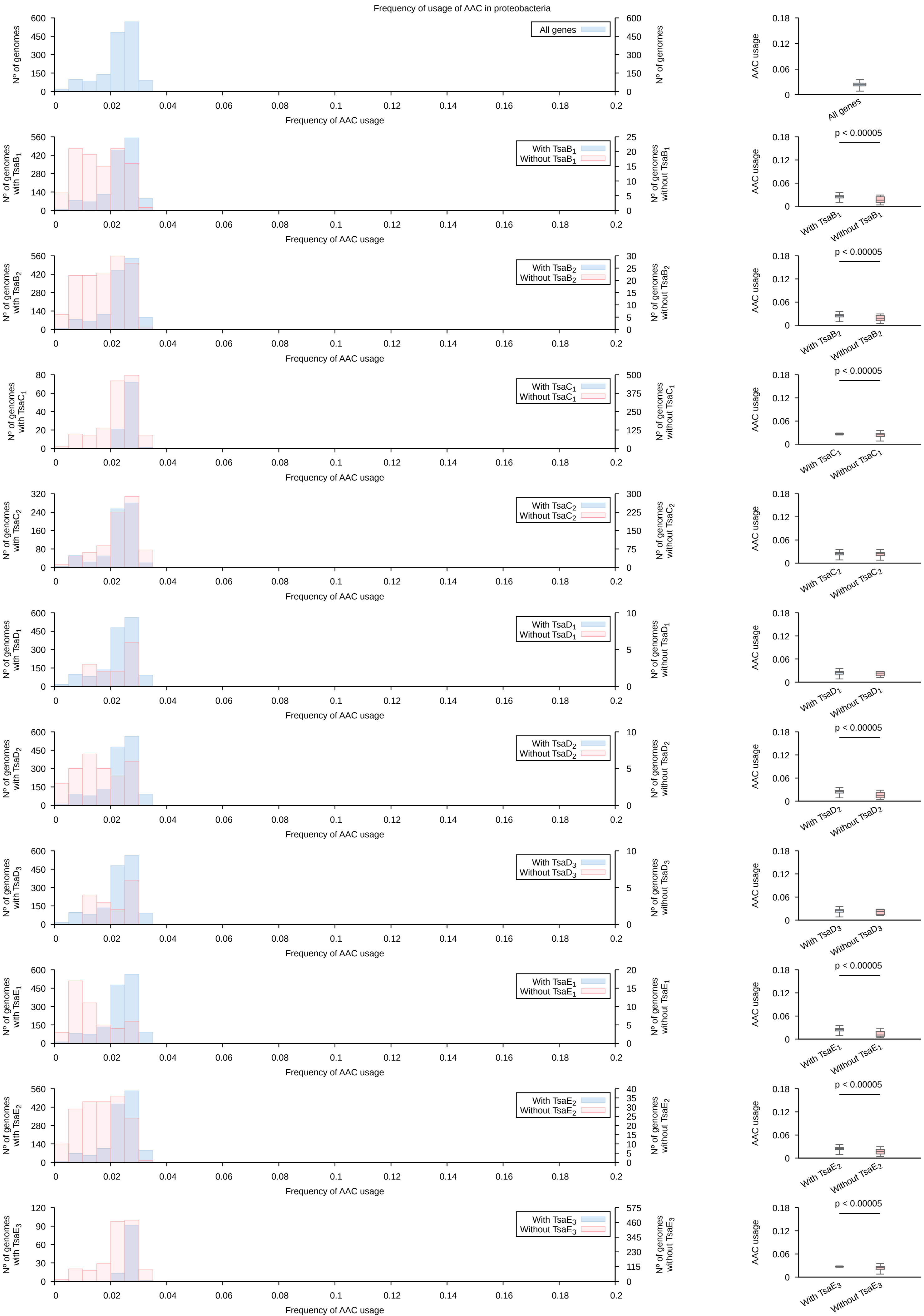

Frequency of usage of AAC in proteobacteria
All genes
Nº of genomes
Frequency of AAC usage
AAC usage
With TsaB1
Without TsaB1
Nº of genomes with TsaB1
Nº of genomes without TsaB1
With TsaB2
Without TsaB2
Nº of genomes with TsaB2
Nº of genomes without TsaB2
With TsaC1
Without TsaC1
Nº of genomes with TsaC1
Nº of genomes without TsaC1
With TsaC2
Without TsaC2
Nº of genomes with TsaC2
Nº of genomes without TsaC2
With TsaD1
Without TsaD1
Nº of genomes with TsaD1
Nº of genomes without TsaD1
With TsaD2
Without TsaD2
Nº of genomes with TsaD2
Nº of genomes without TsaD2
With TsaD3
Without TsaD3
Nº of genomes with TsaD3
Nº of genomes without TsaD3
With TsaE1
Without TsaE1
Nº of genomes with TsaE1
Nº of genomes without TsaE1
With TsaE2
Without TsaE2
Nº of genomes with TsaE2
Nº of genomes without TsaE2
With TsaE3
Without TsaE3
Nº of genomes with TsaE3
Nº of genomes without TsaE3
p < 0.00005

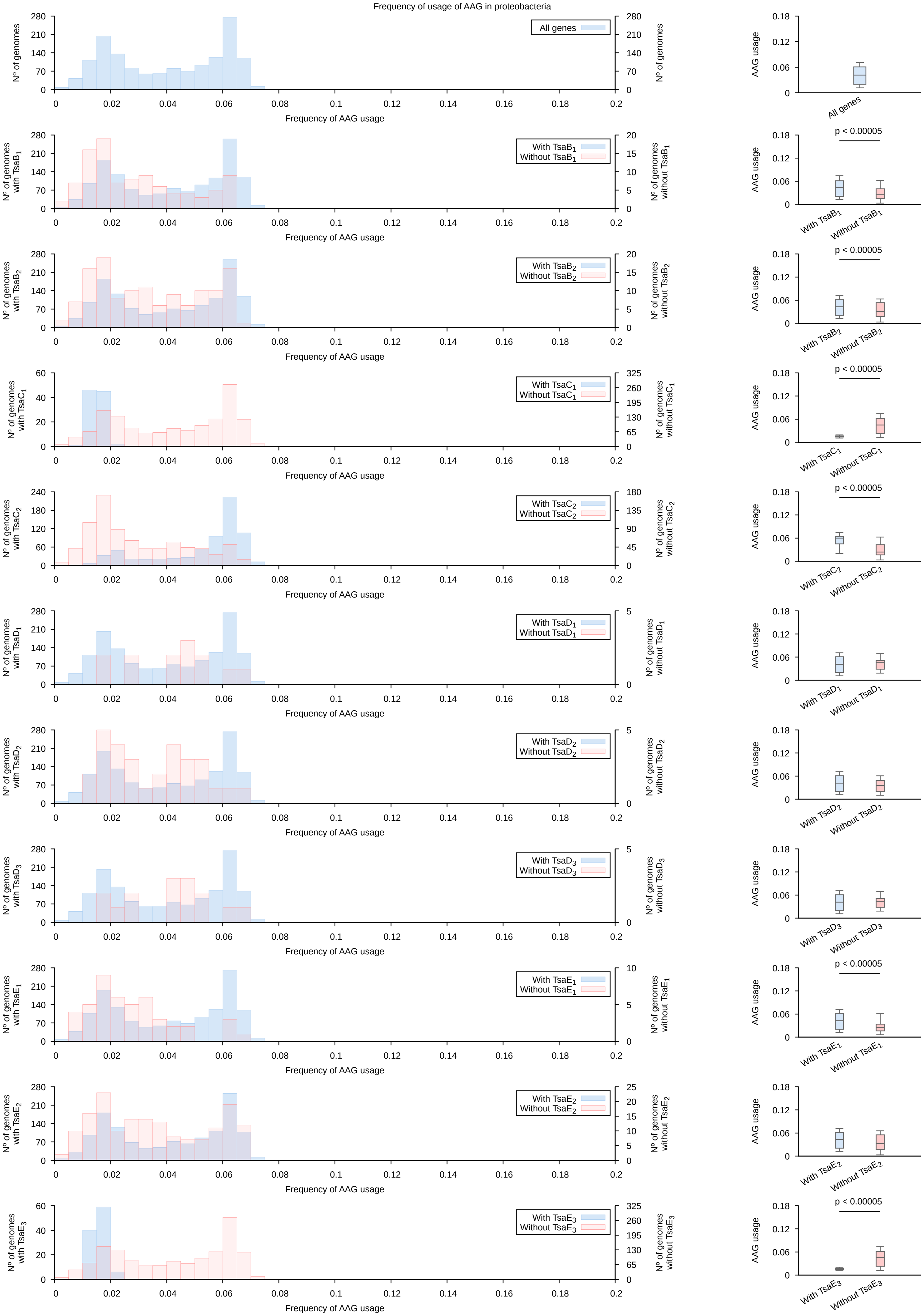

Frequency of usage of AAG in proteobacteria
All genes
Nº of genomes
Frequency of AAG usage
AAG usage
With TsaB$_1$
Without TsaB$_1$
Nº of genomes with TsaB$_1$
Nº of genomes without TsaB$_1$
p < 0.00005
With TsaB$_2$
Without TsaB$_2$
Nº of genomes with TsaB$_2$
Nº of genomes without TsaB$_2$
p < 0.00005
With TsaC$_1$
Without TsaC$_1$
Nº of genomes with TsaC$_1$
Nº of genomes without TsaC$_1$
p < 0.00005
With TsaC$_2$
Without TsaC$_2$
Nº of genomes with TsaC$_2$
Nº of genomes without TsaC$_2$
p < 0.00005
With TsaD$_1$
Without TsaD$_1$
Nº of genomes with TsaD$_1$
Nº of genomes without TsaD$_1$
With TsaD$_2$
Without TsaD$_2$
Nº of genomes with TsaD$_2$
Nº of genomes without TsaD$_2$
With TsaD$_3$
Without TsaD$_3$
Nº of genomes with TsaD$_3$
Nº of genomes without TsaD$_3$
With TsaE$_1$
Without TsaE$_1$
Nº of genomes with TsaE$_1$
Nº of genomes without TsaE$_1$
p < 0.00005
With TsaE$_2$
Without TsaE$_2$
Nº of genomes with TsaE$_2$
Nº of genomes without TsaE$_2$
With TsaE$_3$
Without TsaE$_3$
Nº of genomes with TsaE$_3$
Nº of genomes without TsaE$_3$
p < 0.00005

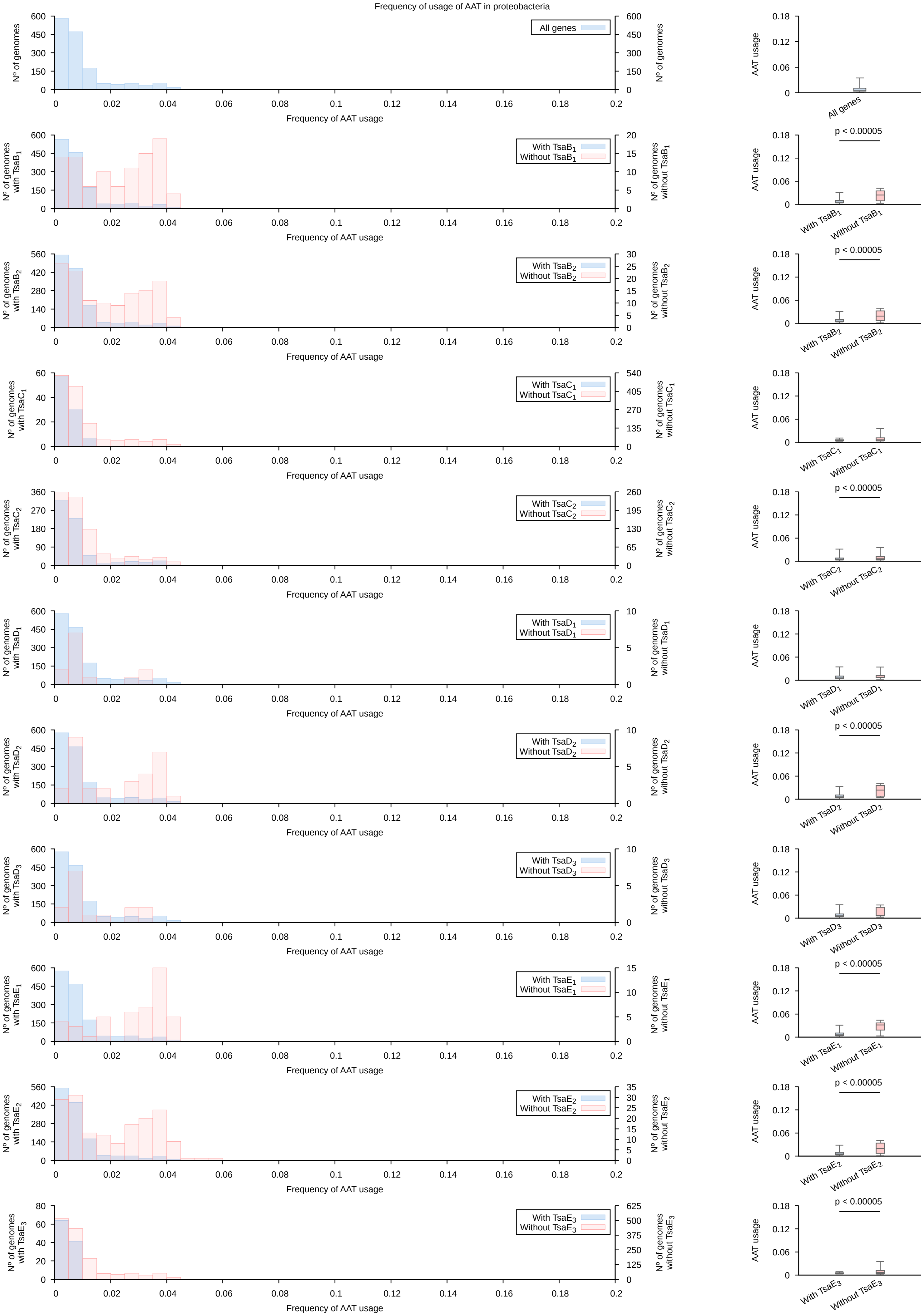
Frequency of usage of AAT in proteobacteria
Nº of genomes
Frequency of AAT usage
All genes
AAT usage
Nº of genomes with TsaB$_1$
Nº of genomes without TsaB$_1$
With TsaB$_1$
Without TsaB$_1$
p < 0.00005
Nº of genomes with TsaB$_2$
Nº of genomes without TsaB$_2$
With TsaB$_2$
Without TsaB$_2$
p < 0.00005
Nº of genomes with TsaC$_1$
Nº of genomes without TsaC$_1$
With TsaC$_1$
Without TsaC$_1$
Nº of genomes with TsaC$_2$
Nº of genomes without TsaC$_2$
With TsaC$_2$
Without TsaC$_2$
p < 0.00005
Nº of genomes with TsaD$_1$
Nº of genomes without TsaD$_1$
With TsaD$_1$
Without TsaD$_1$
Nº of genomes with TsaD$_2$
Nº of genomes without TsaD$_2$
With TsaD$_2$
Without TsaD$_2$
p < 0.00005
Nº of genomes with TsaD$_3$
Nº of genomes without TsaD$_3$
With TsaD$_3$
Without TsaD$_3$
Nº of genomes with TsaE$_1$
Nº of genomes without TsaE$_1$
With TsaE$_1$
Without TsaE$_1$
p < 0.00005
Nº of genomes with TsaE$_2$
Nº of genomes without TsaE$_2$
With TsaE$_2$
Without TsaE$_2$
p < 0.00005
Nº of genomes with TsaE$_3$
Nº of genomes without TsaE$_3$
With TsaE$_3$
Without TsaE$_3$
p < 0.00005

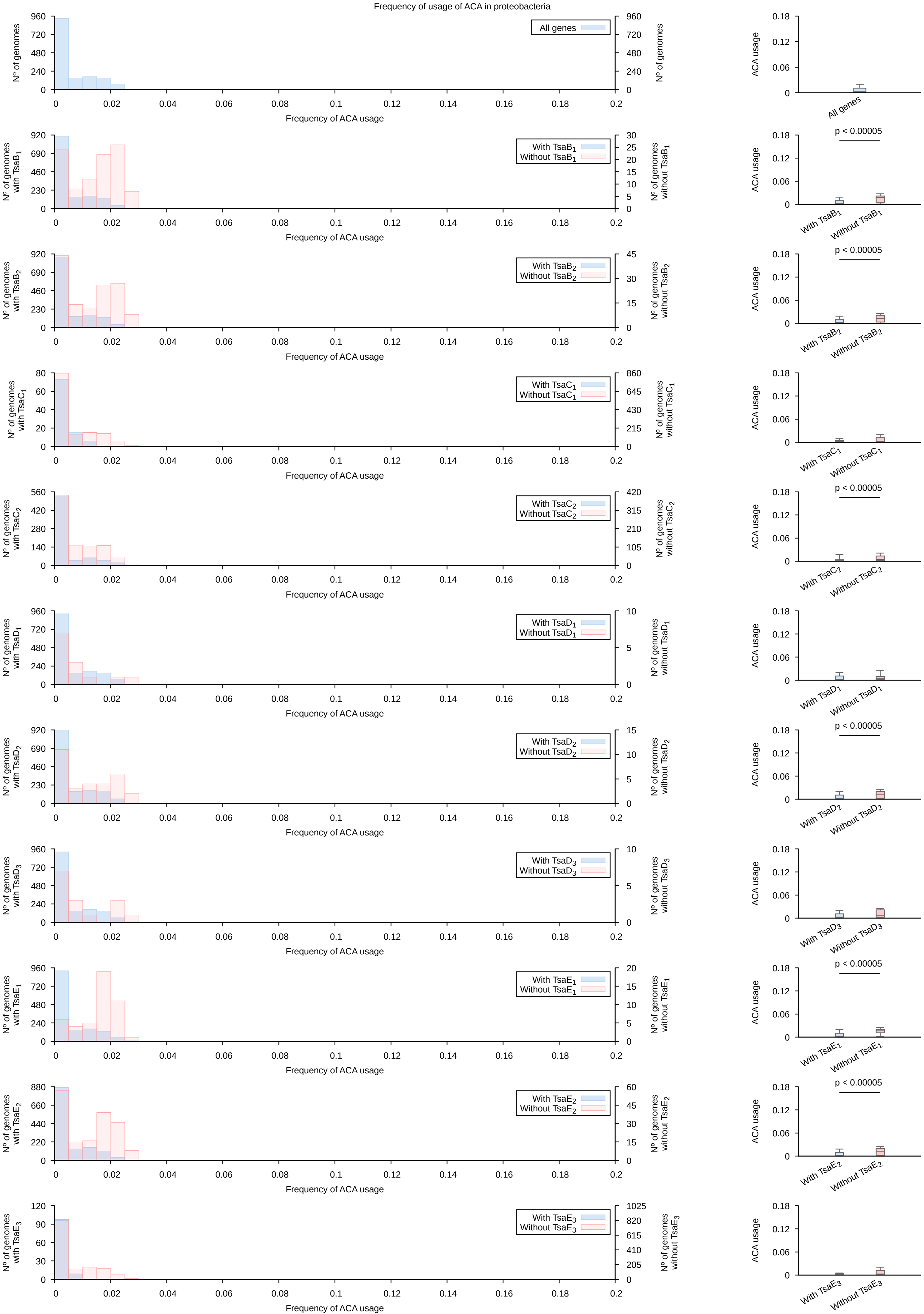
Frequency of usage of ACA in proteobacteria
Nº of genomes
Frequency of ACA usage
ACA usage
All genes
Nº of genomes with $TsaB_1$
Nº of genomes without $TsaB_1$
With $TsaB_1$
Without $TsaB_1$
p < 0.00005
Nº of genomes with $TsaB_2$
Nº of genomes without $TsaB_2$
With $TsaB_2$
Without $TsaB_2$
p < 0.00005
Nº of genomes with $TsaC_1$
Nº of genomes without $TsaC_1$
With $TsaC_1$
Without $TsaC_1$
Nº of genomes with $TsaC_2$
Nº of genomes without $TsaC_2$
With $TsaC_2$
Without $TsaC_2$
p < 0.00005
Nº of genomes with $TsaD_1$
Nº of genomes without $TsaD_1$
With $TsaD_1$
Without $TsaD_1$
Nº of genomes with $TsaD_2$
Nº of genomes without $TsaD_2$
With $TsaD_2$
Without $TsaD_2$
p < 0.00005
Nº of genomes with $TsaD_3$
Nº of genomes without $TsaD_3$
With $TsaD_3$
Without $TsaD_3$
Nº of genomes with $TsaE_1$
Nº of genomes without $TsaE_1$
With $TsaE_1$
Without $TsaE_1$
p < 0.00005
Nº of genomes with $TsaE_2$
Nº of genomes without $TsaE_2$
With $TsaE_2$
Without $TsaE_2$
p < 0.00005
Nº of genomes with $TsaE_3$
Nº of genomes without $TsaE_3$
With $TsaE_3$
Without $TsaE_3$

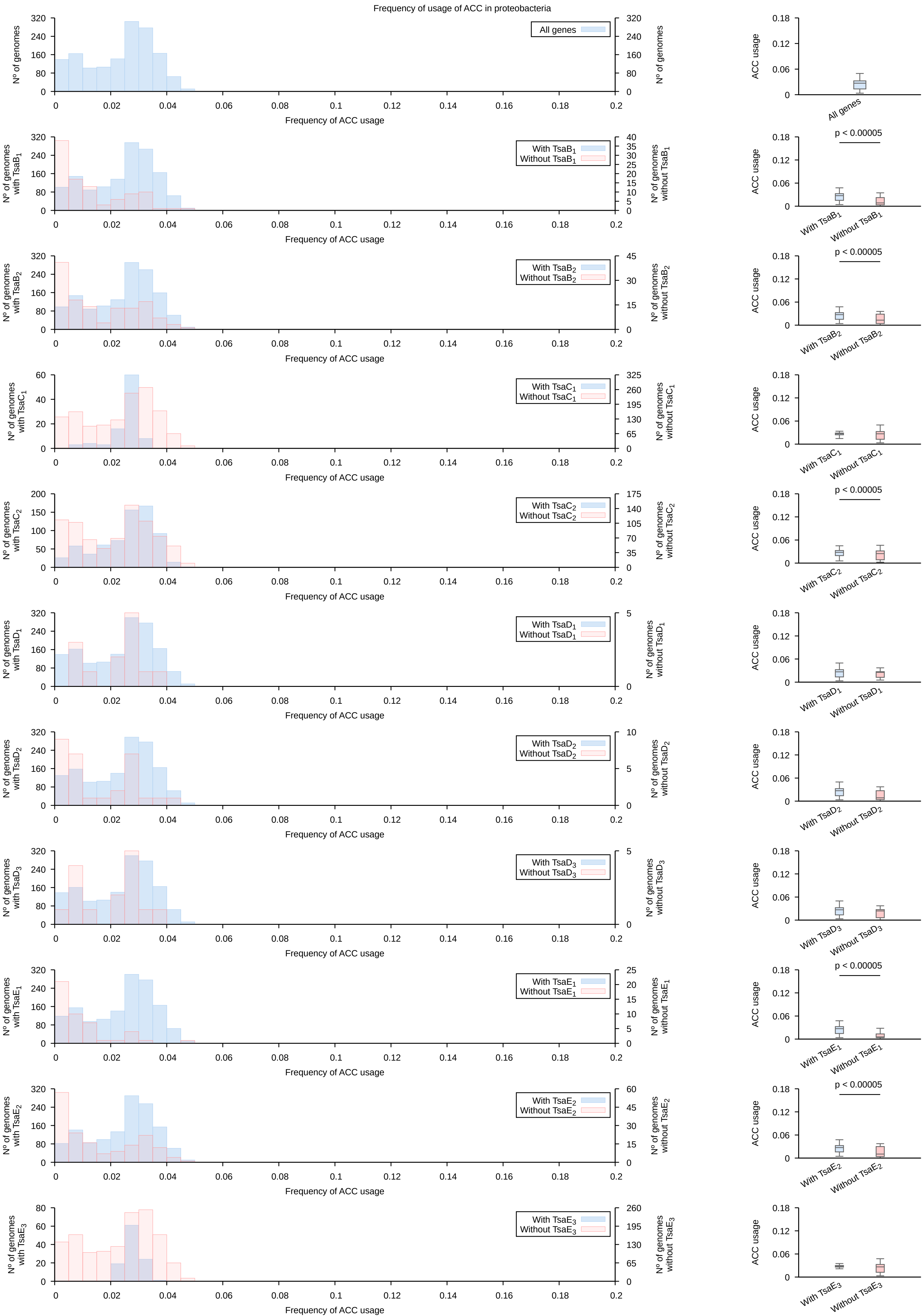
Frequency of usage of ACC in proteobacteria
Nº of genomes
Frequency of ACC usage
All genes
ACC usage
Nº of genomes with $TsaB_1$
Nº of genomes without $TsaB_1$
With $TsaB_1$
Without $TsaB_1$
p < 0.00005
Nº of genomes with $TsaB_2$
Nº of genomes without $TsaB_2$
With $TsaB_2$
Without $TsaB_2$
p < 0.00005
Nº of genomes with $TsaC_1$
Nº of genomes without $TsaC_1$
With $TsaC_1$
Without $TsaC_1$
Nº of genomes with $TsaC_2$
Nº of genomes without $TsaC_2$
With $TsaC_2$
Without $TsaC_2$
p < 0.00005
Nº of genomes with $TsaD_1$
Nº of genomes without $TsaD_1$
With $TsaD_1$
Without $TsaD_1$
Nº of genomes with $TsaD_2$
Nº of genomes without $TsaD_2$
With $TsaD_2$
Without $TsaD_2$
Nº of genomes with $TsaD_3$
Nº of genomes without $TsaD_3$
With $TsaD_3$
Without $TsaD_3$
Nº of genomes with $TsaE_1$
Nº of genomes without $TsaE_1$
With $TsaE_1$
Without $TsaE_1$
p < 0.00005
Nº of genomes with $TsaE_2$
Nº of genomes without $TsaE_2$
With $TsaE_2$
Without $TsaE_2$
p < 0.00005
Nº of genomes with $TsaE_3$
Nº of genomes without $TsaE_3$
With $TsaE_3$
Without $TsaE_3$

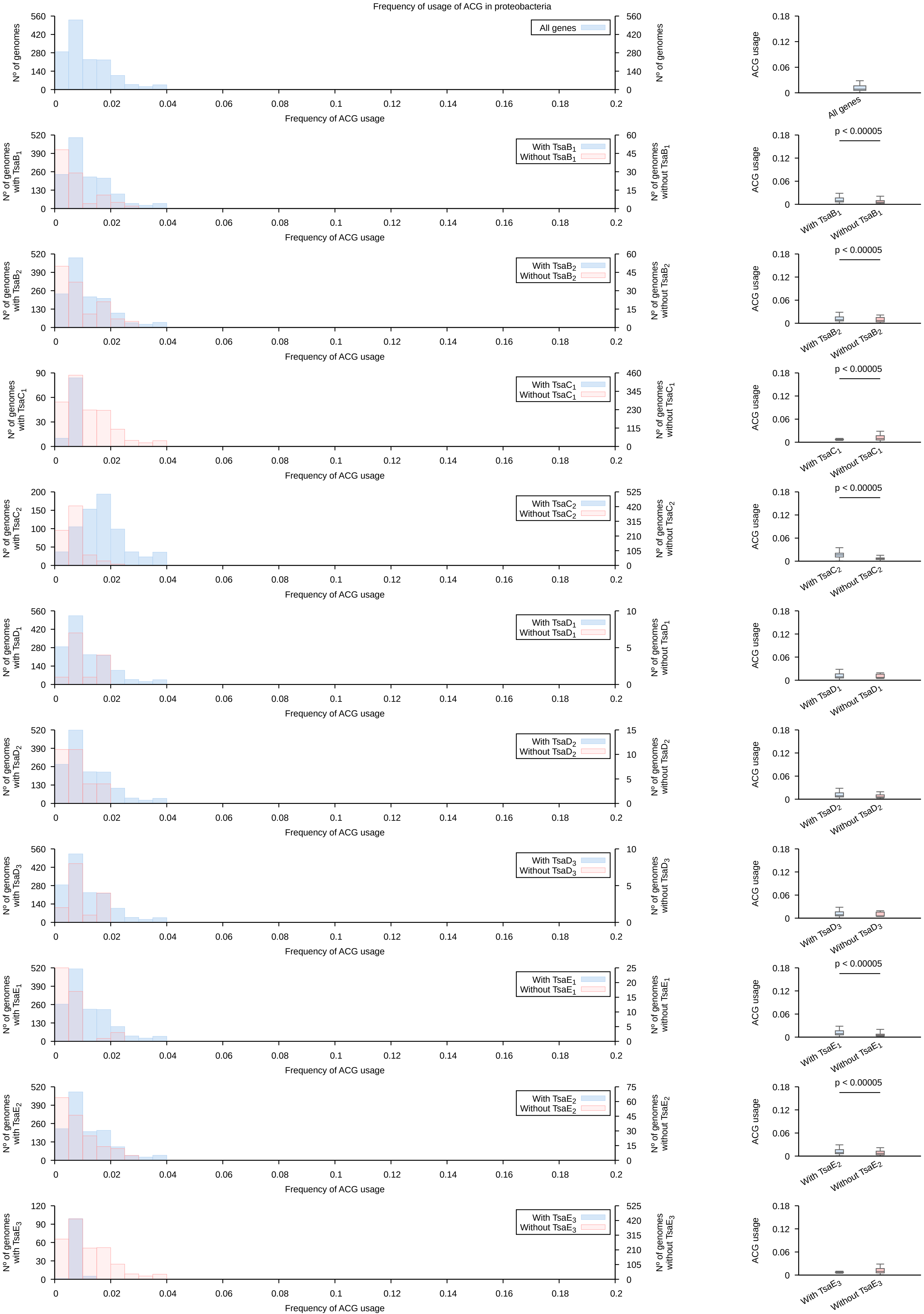
Frequency of usage of ACG in proteobacteria
Nº of genomes
All genes
Frequency of ACG usage
ACG usage
Nº of genomes with TsaB1
Nº of genomes without TsaB1
With TsaB1
Without TsaB1
p < 0.00005
Nº of genomes with TsaB2
Nº of genomes without TsaB2
With TsaB2
Without TsaB2
p < 0.00005
Nº of genomes with TsaC1
Nº of genomes without TsaC1
With TsaC1
Without TsaC1
p < 0.00005
Nº of genomes with TsaC2
Nº of genomes without TsaC2
With TsaC2
Without TsaC2
p < 0.00005
Nº of genomes with TsaD1
Nº of genomes without TsaD1
With TsaD1
Without TsaD1
Nº of genomes with TsaD2
Nº of genomes without TsaD2
With TsaD2
Without TsaD2
Nº of genomes with TsaD3
Nº of genomes without TsaD3
With TsaD3
Without TsaD3
Nº of genomes with TsaE1
Nº of genomes without TsaE1
With TsaE1
Without TsaE1
p < 0.00005
Nº of genomes with TsaE2
Nº of genomes without TsaE2
With TsaE2
Without TsaE2
p < 0.00005
Nº of genomes with TsaE3
Nº of genomes without TsaE3
With TsaE3
Without TsaE3

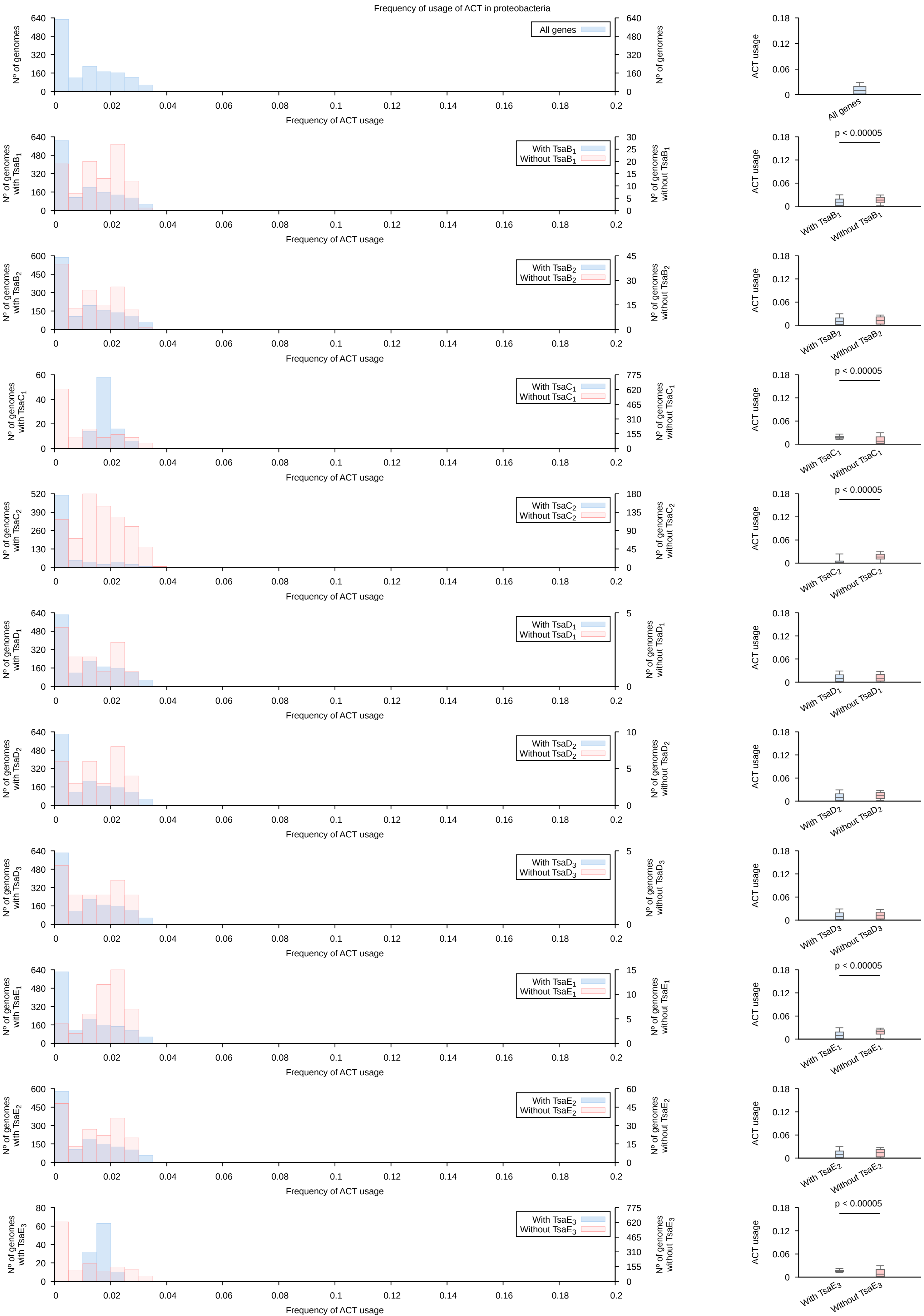
Frequency of usage of ACT in proteobacteria
Nº of genomes
All genes
Frequency of ACT usage
ACT usage
Nº of genomes with TsaB1
Nº of genomes without TsaB1
With TsaB1
Without TsaB1
p < 0.00005
Nº of genomes with TsaB2
Nº of genomes without TsaB2
With TsaB2
Without TsaB2
Nº of genomes with TsaC1
Nº of genomes without TsaC1
With TsaC1
Without TsaC1
p < 0.00005
Nº of genomes with TsaC2
Nº of genomes without TsaC2
With TsaC2
Without TsaC2
p < 0.00005
Nº of genomes with TsaD1
Nº of genomes without TsaD1
With TsaD1
Without TsaD1
Nº of genomes with TsaD2
Nº of genomes without TsaD2
With TsaD2
Without TsaD2
Nº of genomes with TsaD3
Nº of genomes without TsaD3
With TsaD3
Without TsaD3
Nº of genomes with TsaE1
Nº of genomes without TsaE1
With TsaE1
Without TsaE1
p < 0.00005
Nº of genomes with TsaE2
Nº of genomes without TsaE2
With TsaE2
Without TsaE2
Nº of genomes with TsaE3
Nº of genomes without TsaE3
With TsaE3
Without TsaE3
p < 0.00005

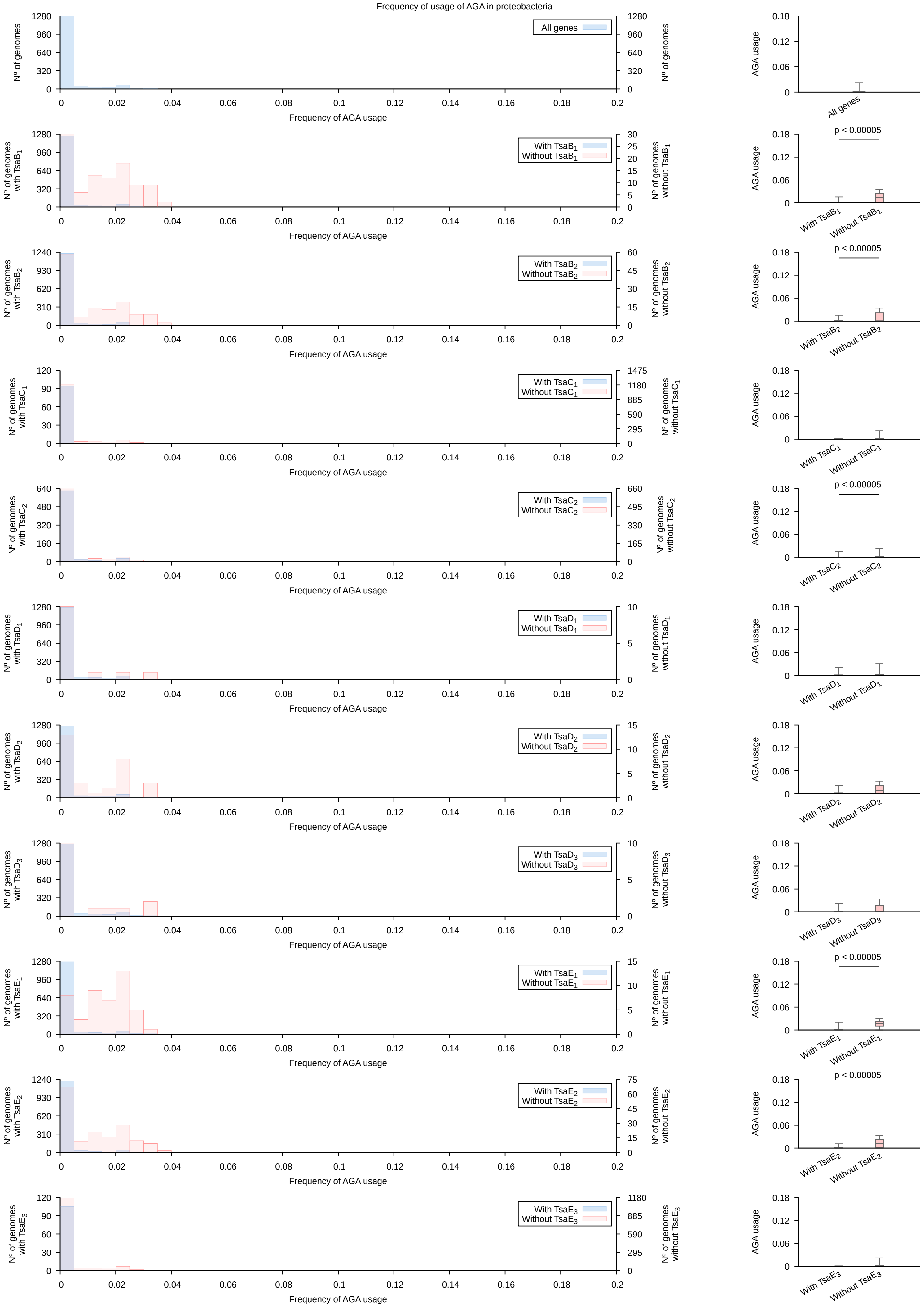
Frequency of usage of AGA in proteobacteria
All genes
Nº of genomes
AGA usage
Frequency of AGA usage
With TsaB1
Without TsaB1
Nº of genomes with TsaB1
Nº of genomes without TsaB1
p < 0.00005
With TsaB2
Without TsaB2
Nº of genomes with TsaB2
Nº of genomes without TsaB2
p < 0.00005
With TsaC1
Without TsaC1
Nº of genomes with TsaC1
Nº of genomes without TsaC1
With TsaC2
Without TsaC2
Nº of genomes with TsaC2
Nº of genomes without TsaC2
p < 0.00005
With TsaD1
Without TsaD1
Nº of genomes with TsaD1
Nº of genomes without TsaD1
With TsaD2
Without TsaD2
Nº of genomes with TsaD2
Nº of genomes without TsaD2
With TsaD3
Without TsaD3
Nº of genomes with TsaD3
Nº of genomes without TsaD3
With TsaE1
Without TsaE1
Nº of genomes with TsaE1
Nº of genomes without TsaE1
p < 0.00005
With TsaE2
Without TsaE2
Nº of genomes with TsaE2
Nº of genomes without TsaE2
p < 0.00005
With TsaE3
Without TsaE3
Nº of genomes with TsaE3
Nº of genomes without TsaE3

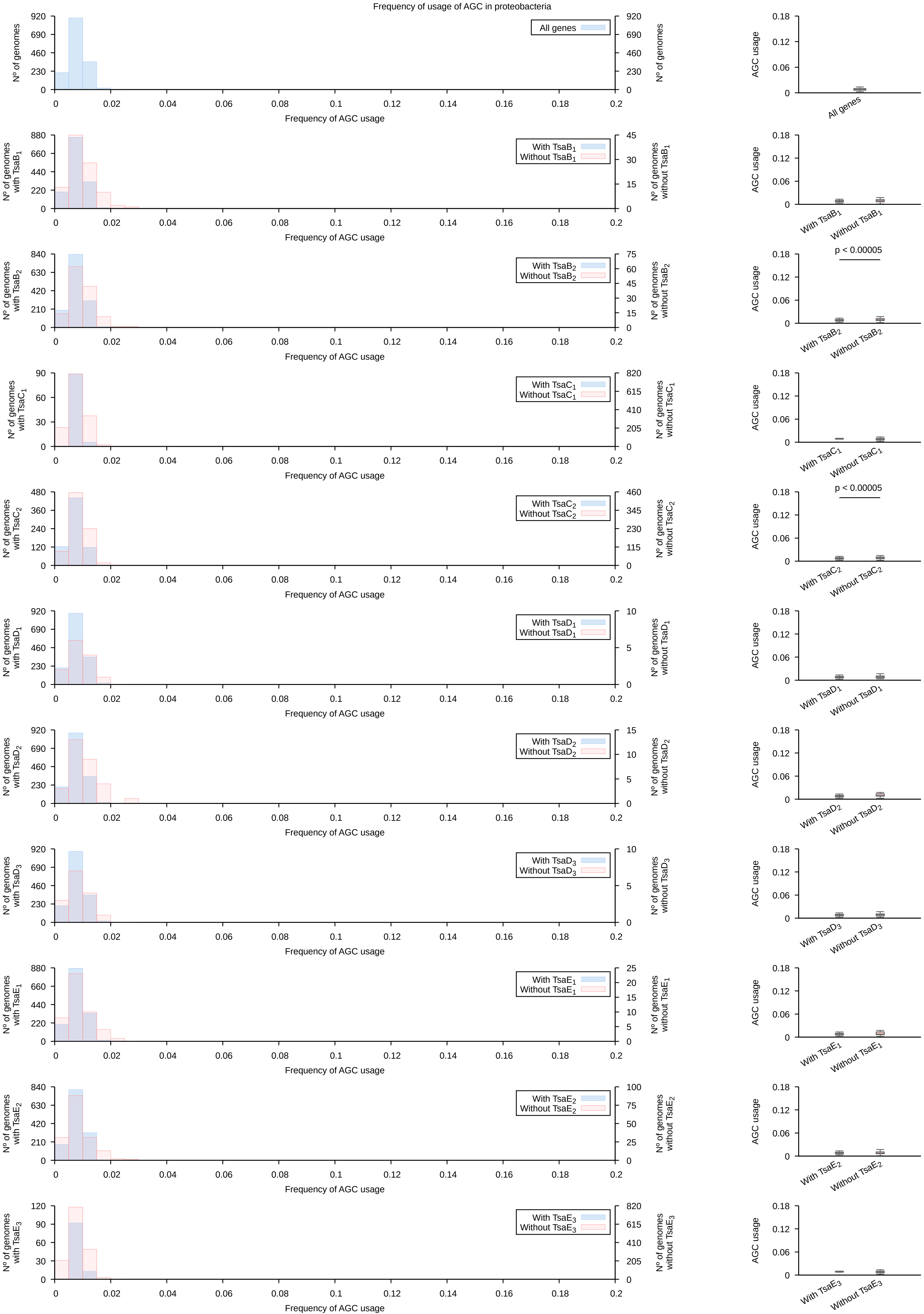

Frequency of usage of AGC in proteobacteria
All genes
Nº of genomes
Frequency of AGC usage
AGC usage
With TsaB1
Without TsaB1
Nº of genomes with TsaB1
Nº of genomes without TsaB1
With TsaB2
Without TsaB2
Nº of genomes with TsaB2
Nº of genomes without TsaB2
p < 0.00005
With TsaC1
Without TsaC1
Nº of genomes with TsaC1
Nº of genomes without TsaC1
With TsaC2
Without TsaC2
Nº of genomes with TsaC2
Nº of genomes without TsaC2
p < 0.00005
With TsaD1
Without TsaD1
Nº of genomes with TsaD1
Nº of genomes without TsaD1
With TsaD2
Without TsaD2
Nº of genomes with TsaD2
Nº of genomes without TsaD2
With TsaD3
Without TsaD3
Nº of genomes with TsaD3
Nº of genomes without TsaD3
With TsaE1
Without TsaE1
Nº of genomes with TsaE1
Nº of genomes without TsaE1
With TsaE2
Without TsaE2
Nº of genomes with TsaE2
Nº of genomes without TsaE2
With TsaE3
Without TsaE3
Nº of genomes with TsaE3
Nº of genomes without TsaE3

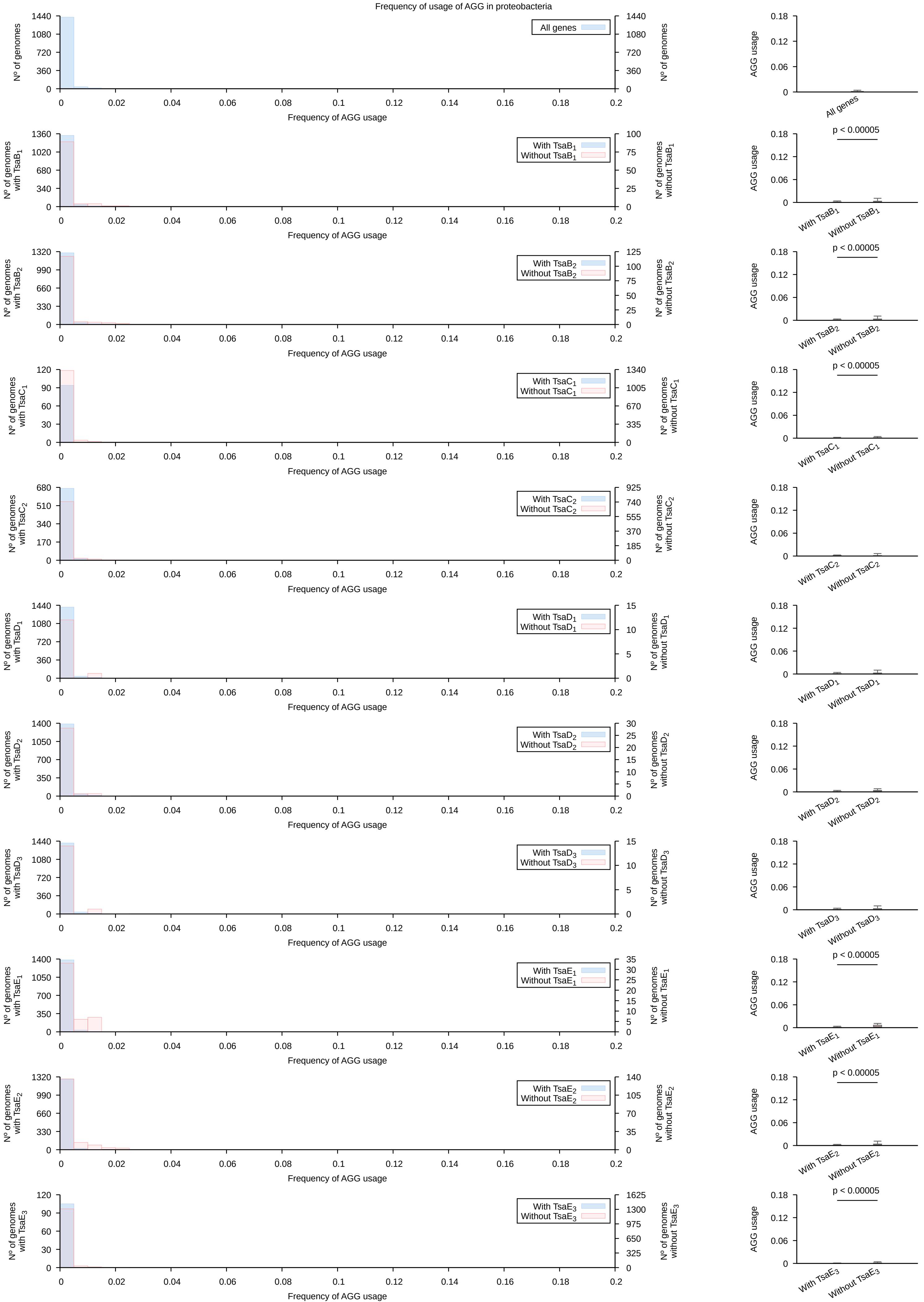
Frequency of usage of AGG in proteobacteria
All genes
Nº of genomes
Frequency of AGG usage
AGG usage
With TsaB1
Without TsaB1
Nº of genomes with TsaB1
Nº of genomes without TsaB1
p < 0.00005
With TsaB2
Without TsaB2
Nº of genomes with TsaB2
Nº of genomes without TsaB2
With TsaC1
Without TsaC1
Nº of genomes with TsaC1
Nº of genomes without TsaC1
With TsaC2
Without TsaC2
Nº of genomes with TsaC2
Nº of genomes without TsaC2
With TsaD1
Without TsaD1
Nº of genomes with TsaD1
Nº of genomes without TsaD1
With TsaD2
Without TsaD2
Nº of genomes with TsaD2
Nº of genomes without TsaD2
With TsaD3
Without TsaD3
Nº of genomes with TsaD3
Nº of genomes without TsaD3
With TsaE1
Without TsaE1
Nº of genomes with TsaE1
Nº of genomes without TsaE1
With TsaE2
Without TsaE2
Nº of genomes with TsaE2
Nº of genomes without TsaE2
With TsaE3
Without TsaE3
Nº of genomes with TsaE3
Nº of genomes without TsaE3

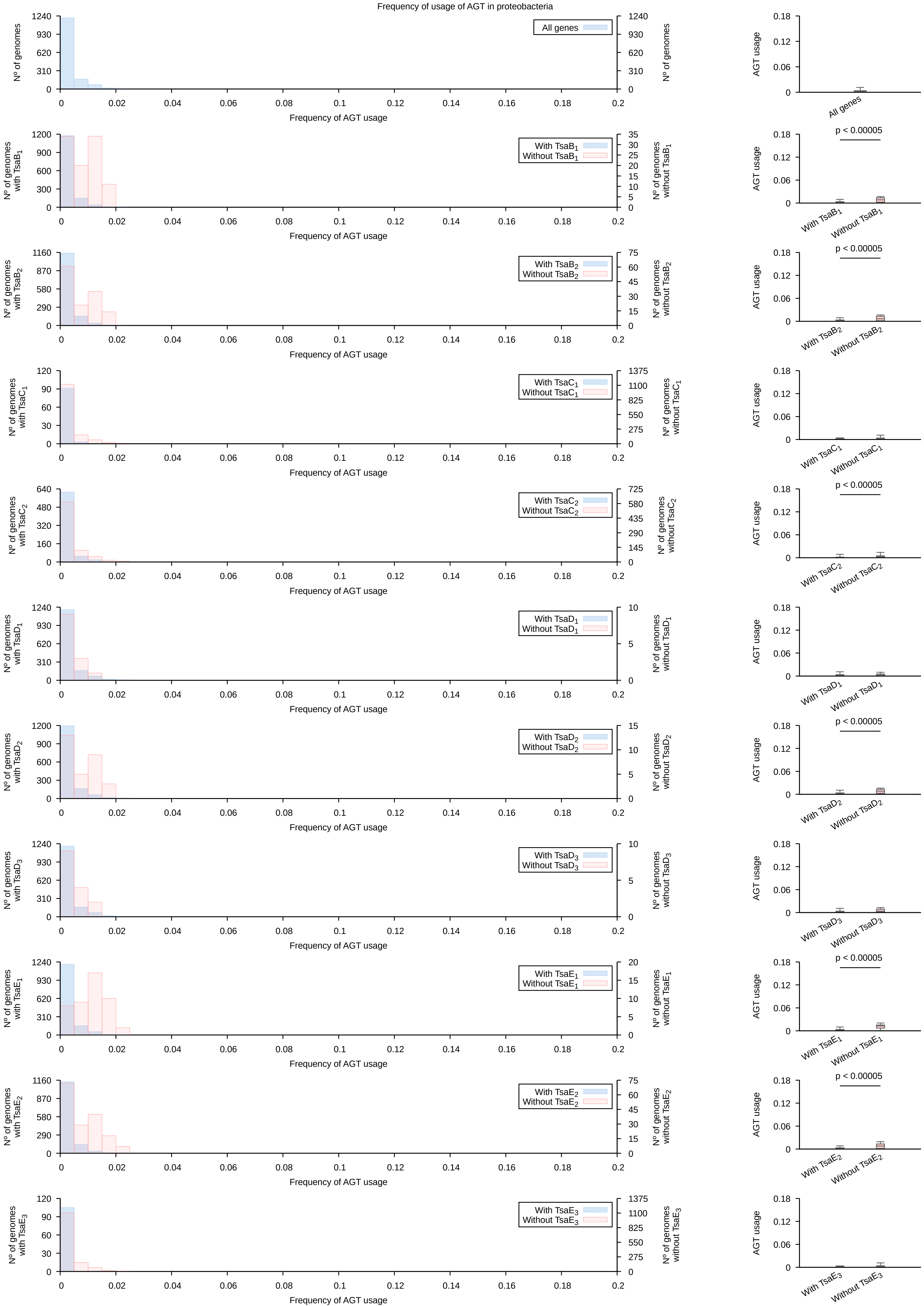
Frequency of usage of AGT in proteobacteria
Nº of genomes
Frequency of AGT usage
AGT usage
All genes
With TsaB1
Without TsaB1
With TsaB2
Without TsaB2
With TsaC1
Without TsaC1
With TsaC2
Without TsaC2
With TsaD1
Without TsaD1
With TsaD2
Without TsaD2
With TsaD3
Without TsaD3
With TsaE1
Without TsaE1
With TsaE2
Without TsaE2
With TsaE3
Without TsaE3
p < 0.00005

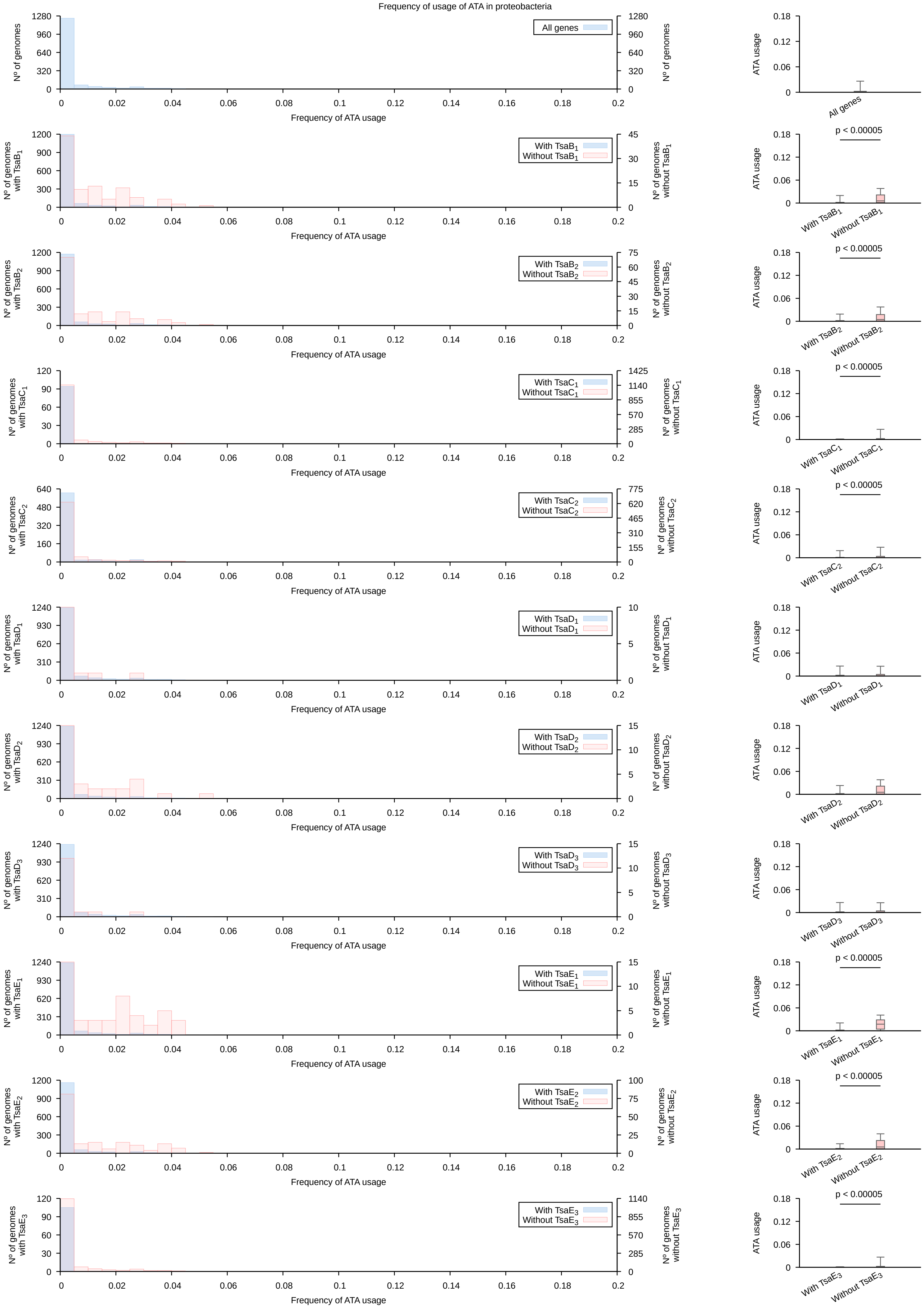

Frequency of usage of ATA in proteobacteria
Nº of genomes
Frequency of ATA usage
All genes
ATA usage
With TsaB1
Without TsaB1
Nº of genomes with TsaB1
Nº of genomes without TsaB1
p < 0.00005
With TsaB2
Without TsaB2
Nº of genomes with TsaB2
Nº of genomes without TsaB2
With TsaC1
Without TsaC1
Nº of genomes with TsaC1
Nº of genomes without TsaC1
With TsaC2
Without TsaC2
Nº of genomes with TsaC2
Nº of genomes without TsaC2
With TsaD1
Without TsaD1
Nº of genomes with TsaD1
Nº of genomes without TsaD1
With TsaD2
Without TsaD2
Nº of genomes with TsaD2
Nº of genomes without TsaD2
With TsaD3
Without TsaD3
Nº of genomes with TsaD3
Nº of genomes without TsaD3
With TsaE1
Without TsaE1
Nº of genomes with TsaE1
Nº of genomes without TsaE1
With TsaE2
Without TsaE2
Nº of genomes with TsaE2
Nº of genomes without TsaE2
With TsaE3
Without TsaE3
Nº of genomes with TsaE3
Nº of genomes without TsaE3

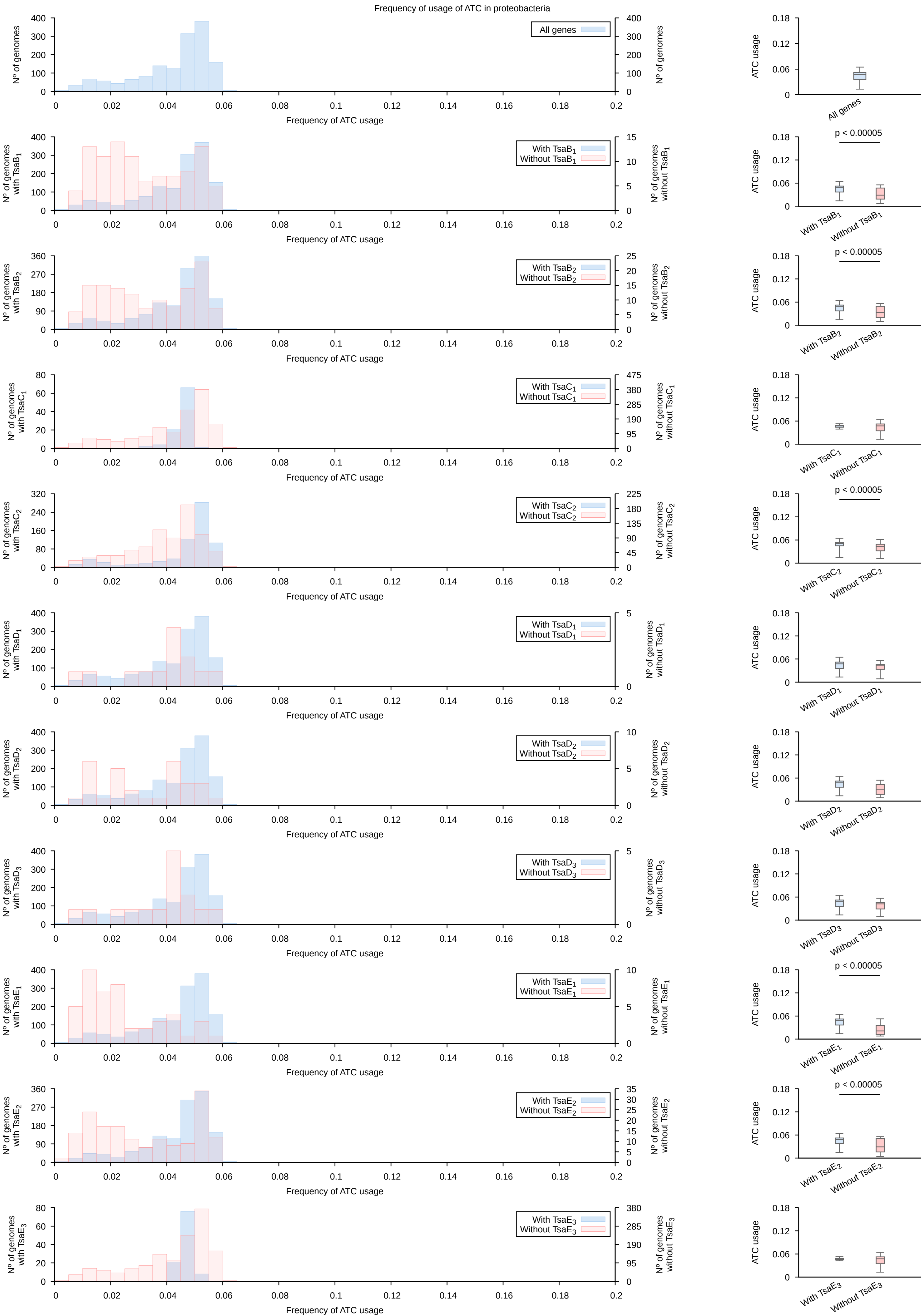

Frequency of usage of ATC in proteobacteria
Nº of genomes
All genes
Frequency of ATC usage
ATC usage
Nº of genomes with TsaB$_1$
Nº of genomes without TsaB$_1$
With TsaB$_1$
Without TsaB$_1$
p < 0.00005
Nº of genomes with TsaB$_2$
Nº of genomes without TsaB$_2$
With TsaB$_2$
Without TsaB$_2$
p < 0.00005
Nº of genomes with TsaC$_1$
Nº of genomes without TsaC$_1$
With TsaC$_1$
Without TsaC$_1$
Nº of genomes with TsaC$_2$
Nº of genomes without TsaC$_2$
With TsaC$_2$
Without TsaC$_2$
p < 0.00005
Nº of genomes with TsaD$_1$
Nº of genomes without TsaD$_1$
With TsaD$_1$
Without TsaD$_1$
Nº of genomes with TsaD$_2$
Nº of genomes without TsaD$_2$
With TsaD$_2$
Without TsaD$_2$
Nº of genomes with TsaD$_3$
Nº of genomes without TsaD$_3$
With TsaD$_3$
Without TsaD$_3$
Nº of genomes with TsaE$_1$
Nº of genomes without TsaE$_1$
With TsaE$_1$
Without TsaE$_1$
p < 0.00005
Nº of genomes with TsaE$_2$
Nº of genomes without TsaE$_2$
With TsaE$_2$
Without TsaE$_2$
p < 0.00005
Nº of genomes with TsaE$_3$
Nº of genomes without TsaE$_3$
With TsaE$_3$
Without TsaE$_3$

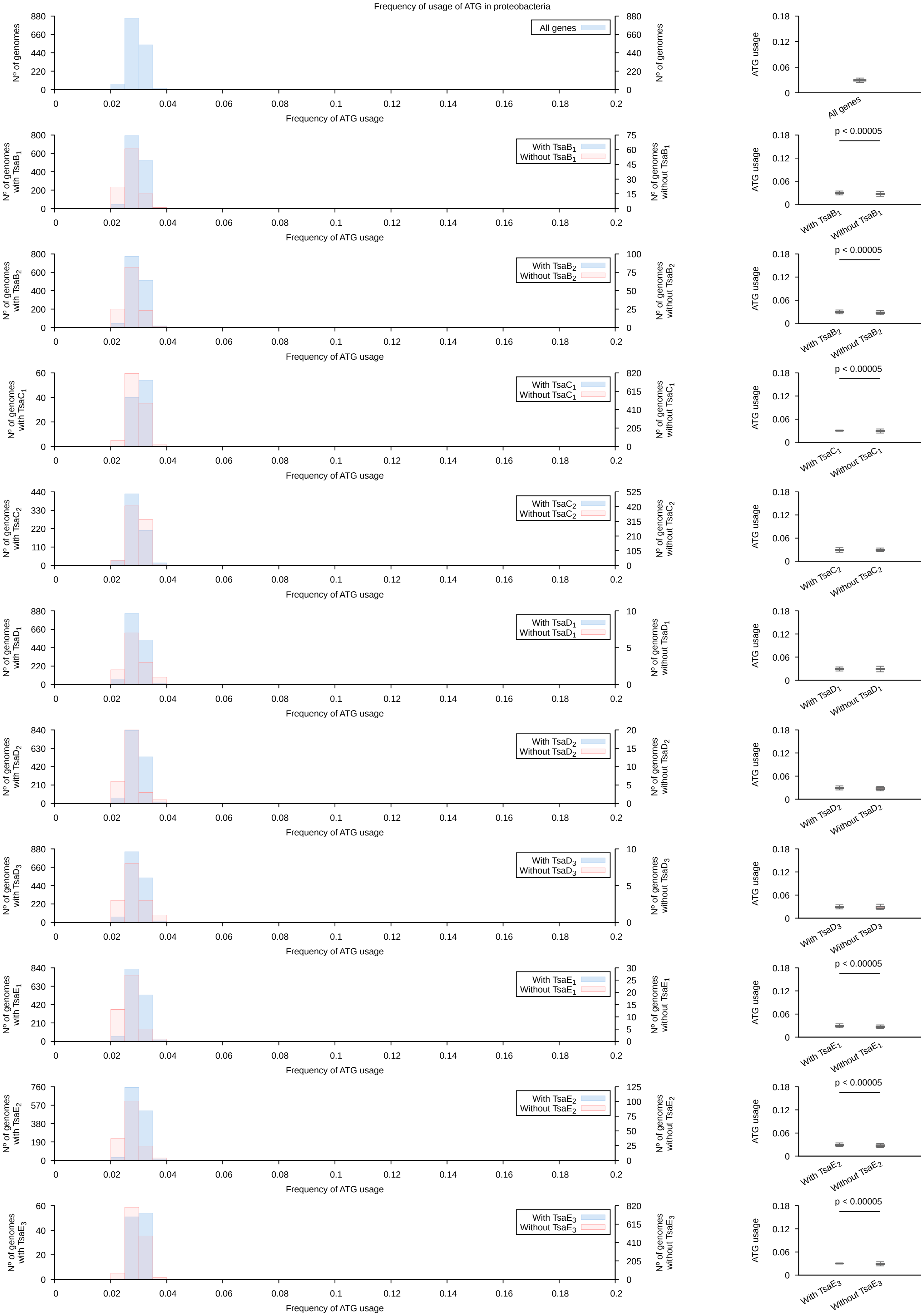

Frequency of usage of ATG in proteobacteria
Nº of genomes
All genes
Frequency of ATG usage
ATG usage
p < 0.00005
With TsaB1
Without TsaB1
With TsaB2
Without TsaB2
With TsaC1
Without TsaC1
With TsaC2
Without TsaC2
With TsaD1
Without TsaD1
With TsaD2
Without TsaD2
With TsaD3
Without TsaD3
With TsaE1
Without TsaE1
With TsaE2
Without TsaE2
With TsaE3
Without TsaE3

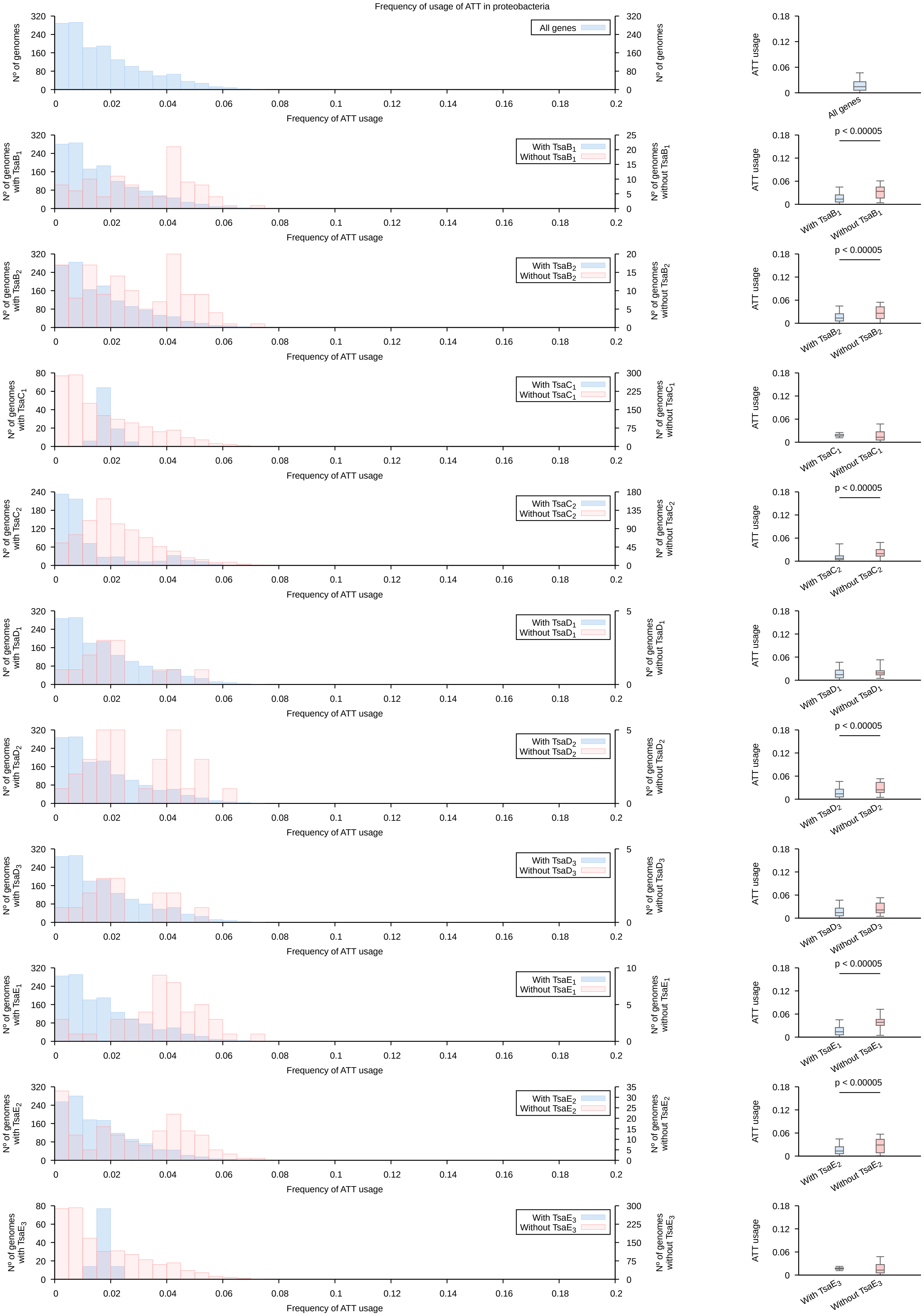

Frequency of usage of ATT in proteobacteria
All genes
Nº of genomes
Frequency of ATT usage
ATT usage
All genes
With $TsaB_1$
Without $TsaB_1$
Nº of genomes with $TsaB_1$
Nº of genomes without $TsaB_1$
p < 0.00005
With $TsaB_2$
Without $TsaB_2$
Nº of genomes with $TsaB_2$
Nº of genomes without $TsaB_2$
p < 0.00005
With $TsaC_1$
Without $TsaC_1$
Nº of genomes with $TsaC_1$
Nº of genomes without $TsaC_1$
With $TsaC_2$
Without $TsaC_2$
Nº of genomes with $TsaC_2$
Nº of genomes without $TsaC_2$
p < 0.00005
With $TsaD_1$
Without $TsaD_1$
Nº of genomes with $TsaD_1$
Nº of genomes without $TsaD_1$
With $TsaD_2$
Without $TsaD_2$
Nº of genomes with $TsaD_2$
Nº of genomes without $TsaD_2$
p < 0.00005
With $TsaD_3$
Without $TsaD_3$
Nº of genomes with $TsaD_3$
Nº of genomes without $TsaD_3$
With $TsaE_1$
Without $TsaE_1$
Nº of genomes with $TsaE_1$
Nº of genomes without $TsaE_1$
p < 0.00005
With $TsaE_2$
Without $TsaE_2$
Nº of genomes with $TsaE_2$
Nº of genomes without $TsaE_2$
p < 0.00005
With $TsaE_3$
Without $TsaE_3$
Nº of genomes with $TsaE_3$
Nº of genomes without $TsaE_3$

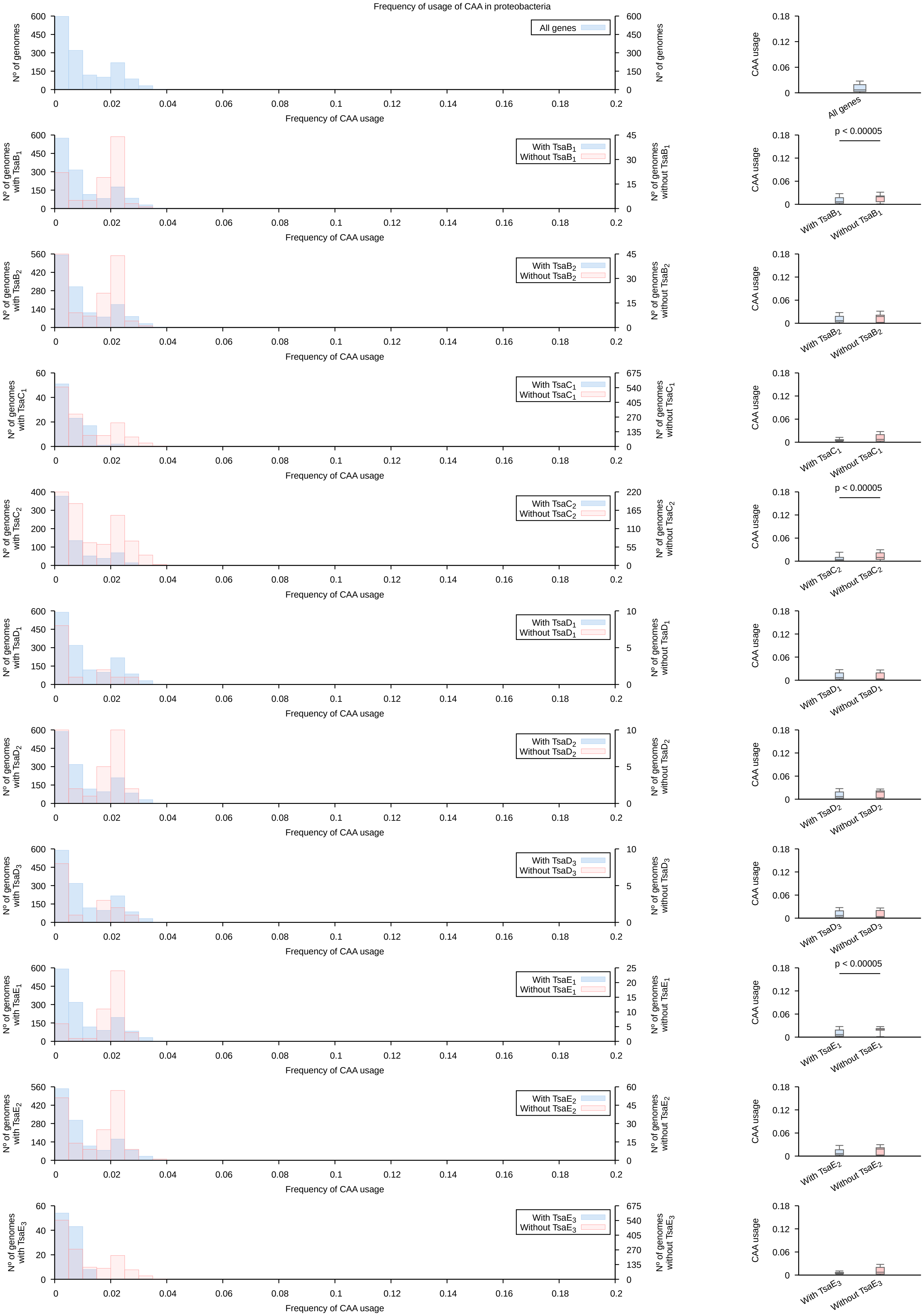

Frequency of usage of CAA in proteobacteria
All genes
Nº of genomes
Frequency of CAA usage
CAA usage
With TsaB$_1$
Without TsaB$_1$
Nº of genomes with TsaB$_1$
Nº of genomes without TsaB$_1$
p < 0.00005
With TsaB$_2$
Without TsaB$_2$
Nº of genomes with TsaB$_2$
Nº of genomes without TsaB$_2$
With TsaC$_1$
Without TsaC$_1$
Nº of genomes with TsaC$_1$
Nº of genomes without TsaC$_1$
With TsaC$_2$
Without TsaC$_2$
Nº of genomes with TsaC$_2$
Nº of genomes without TsaC$_2$
p < 0.00005
With TsaD$_1$
Without TsaD$_1$
Nº of genomes with TsaD$_1$
Nº of genomes without TsaD$_1$
With TsaD$_2$
Without TsaD$_2$
Nº of genomes with TsaD$_2$
Nº of genomes without TsaD$_2$
With TsaD$_3$
Without TsaD$_3$
Nº of genomes with TsaD$_3$
Nº of genomes without TsaD$_3$
With TsaE$_1$
Without TsaE$_1$
Nº of genomes with TsaE$_1$
Nº of genomes without TsaE$_1$
p < 0.00005
With TsaE$_2$
Without TsaE$_2$
Nº of genomes with TsaE$_2$
Nº of genomes without TsaE$_2$
With TsaE$_3$
Without TsaE$_3$
Nº of genomes with TsaE$_3$
Nº of genomes without TsaE$_3$

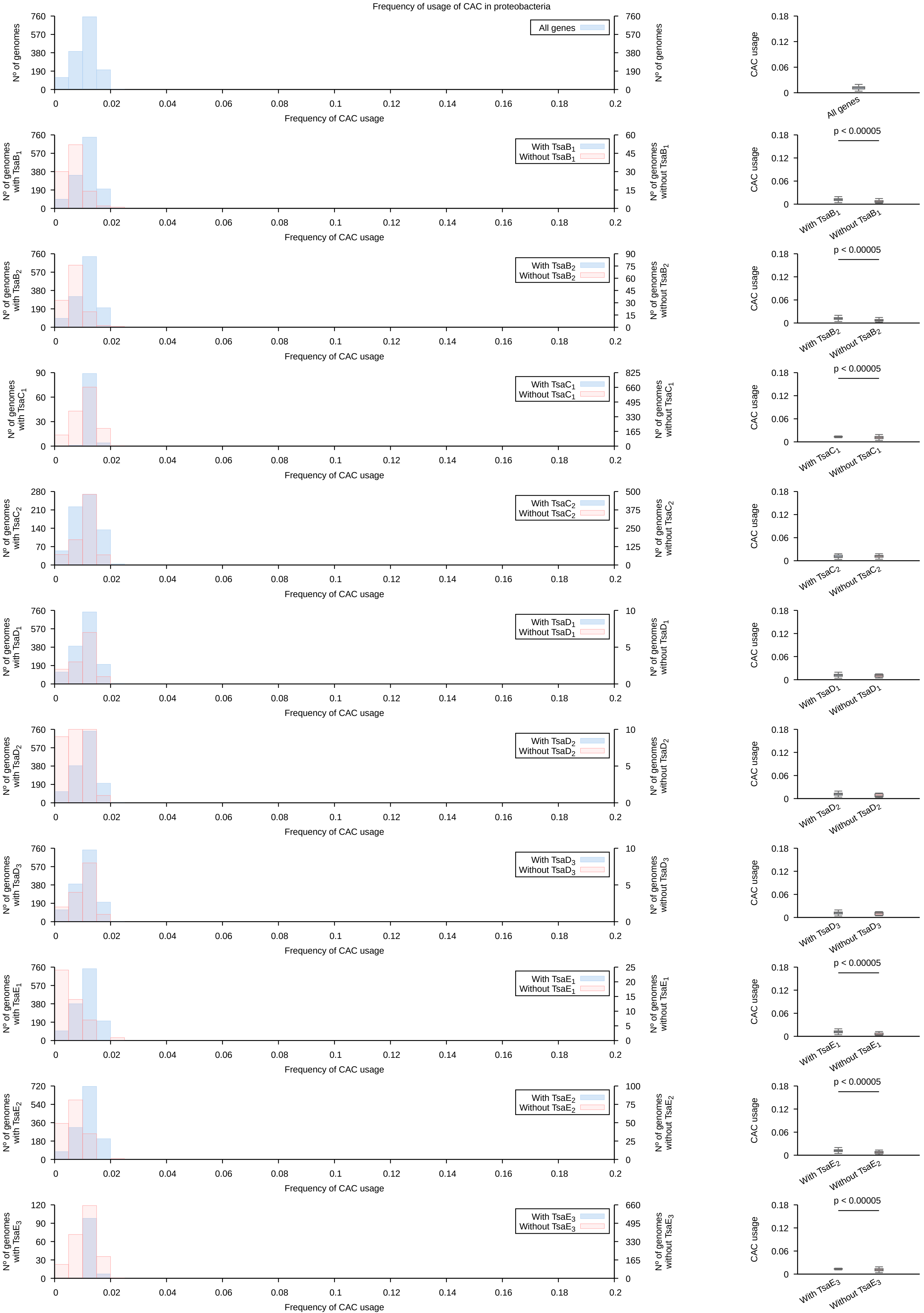
Frequency of usage of CAC in proteobacteria
Nº of genomes
All genes
Frequency of CAC usage
CAC usage
Nº of genomes with $TsaB_1$
Nº of genomes without $TsaB_1$
With $TsaB_1$
Without $TsaB_1$
$p < 0.00005$
Nº of genomes with $TsaB_2$
Nº of genomes without $TsaB_2$
With $TsaB_2$
Without $TsaB_2$
Nº of genomes with $TsaC_1$
Nº of genomes without $TsaC_1$
With $TsaC_1$
Without $TsaC_1$
Nº of genomes with $TsaC_2$
Nº of genomes without $TsaC_2$
With $TsaC_2$
Without $TsaC_2$
Nº of genomes with $TsaD_1$
Nº of genomes without $TsaD_1$
With $TsaD_1$
Without $TsaD_1$
Nº of genomes with $TsaD_2$
Nº of genomes without $TsaD_2$
With $TsaD_2$
Without $TsaD_2$
Nº of genomes with $TsaD_3$
Nº of genomes without $TsaD_3$
With $TsaD_3$
Without $TsaD_3$
Nº of genomes with $TsaE_1$
Nº of genomes without $TsaE_1$
With $TsaE_1$
Without $TsaE_1$
Nº of genomes with $TsaE_2$
Nº of genomes without $TsaE_2$
With $TsaE_2$
Without $TsaE_2$
Nº of genomes with $TsaE_3$
Nº of genomes without $TsaE_3$
With $TsaE_3$
Without $TsaE_3$

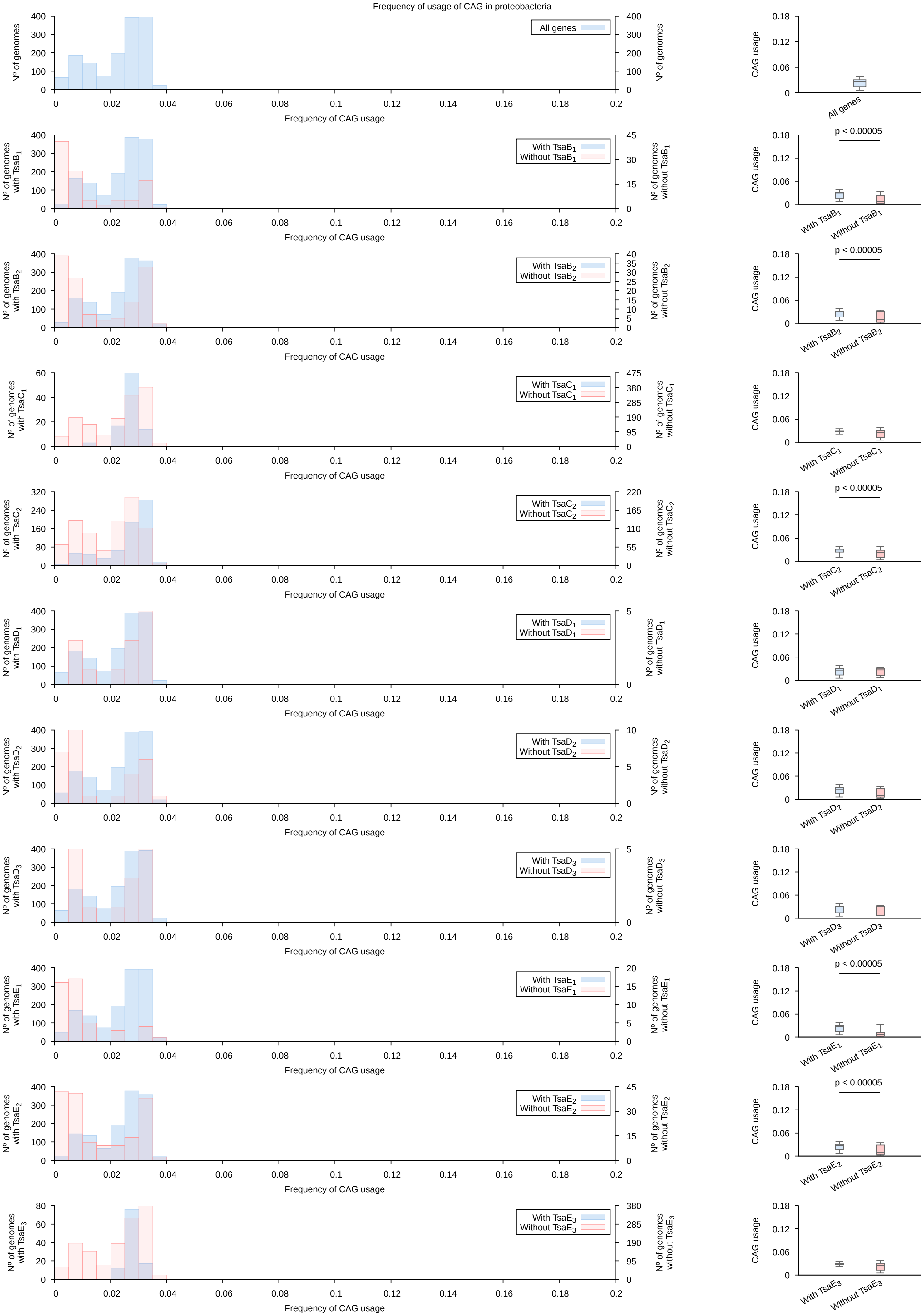
Frequency of usage of CAG in proteobacteria
Nº of genomes
All genes
Frequency of CAG usage
CAG usage
Nº of genomes with TsaB1
Nº of genomes without TsaB1
With TsaB1
Without TsaB1
p < 0.00005
Nº of genomes with TsaB2
Nº of genomes without TsaB2
With TsaB2
Without TsaB2
p < 0.00005
Nº of genomes with TsaC1
Nº of genomes without TsaC1
With TsaC1
Without TsaC1
Nº of genomes with TsaC2
Nº of genomes without TsaC2
With TsaC2
Without TsaC2
p < 0.00005
Nº of genomes with TsaD1
Nº of genomes without TsaD1
With TsaD1
Without TsaD1
Nº of genomes with TsaD2
Nº of genomes without TsaD2
With TsaD2
Without TsaD2
Nº of genomes with TsaD3
Nº of genomes without TsaD3
With TsaD3
Without TsaD3
Nº of genomes with TsaE1
Nº of genomes without TsaE1
With TsaE1
Without TsaE1
p < 0.00005
Nº of genomes with TsaE2
Nº of genomes without TsaE2
With TsaE2
Without TsaE2
p < 0.00005
Nº of genomes with TsaE3
Nº of genomes without TsaE3
With TsaE3
Without TsaE3

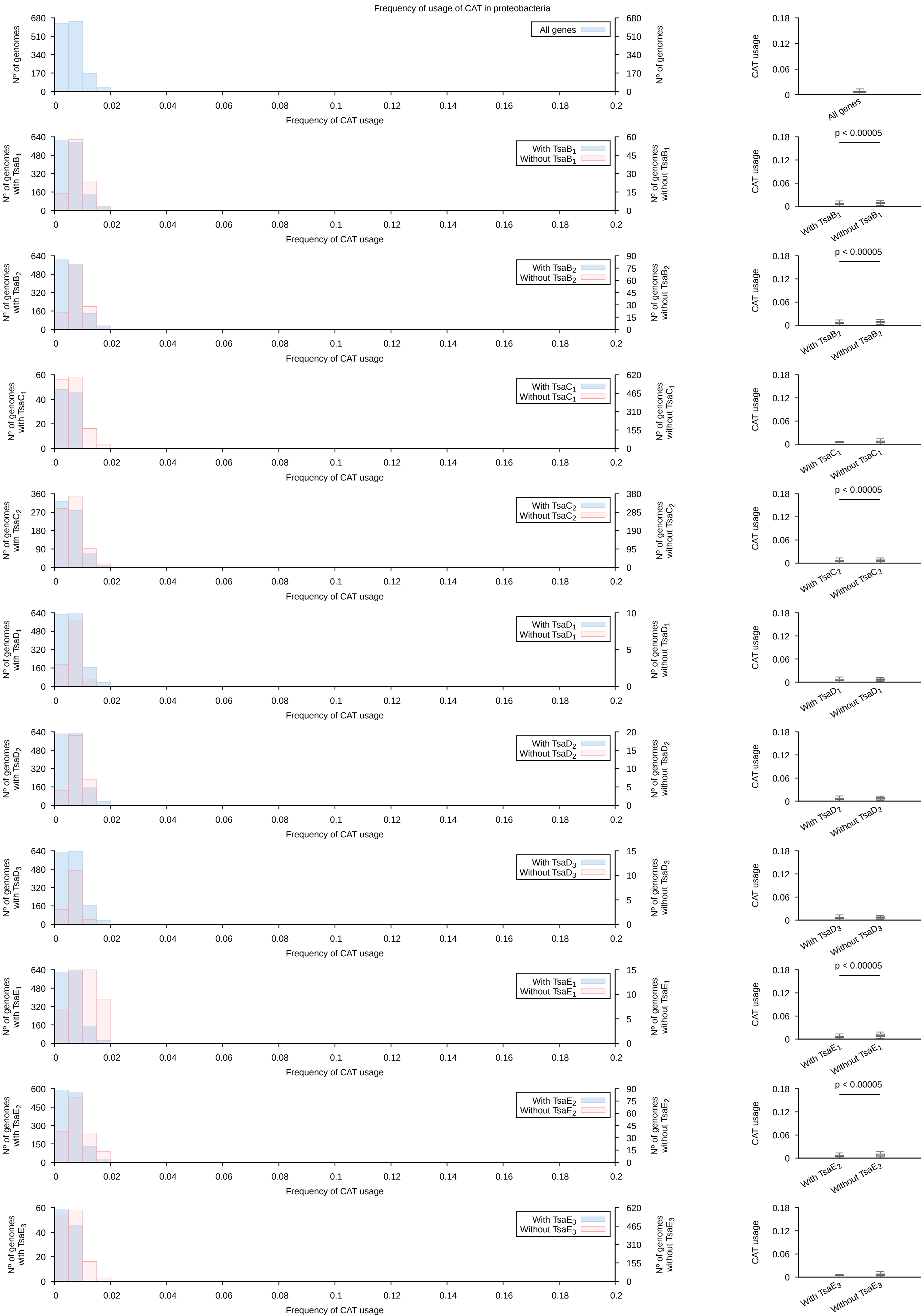

Frequency of usage of CAT in proteobacteria
All genes
With TsaB1
Without TsaB1
With TsaB2
Without TsaB2
With TsaC1
Without TsaC1
With TsaC2
Without TsaC2
With TsaD1
Without TsaD1
With TsaD2
Without TsaD2
With TsaD3
Without TsaD3
With TsaE1
Without TsaE1
With TsaE2
Without TsaE2
With TsaE3
Without TsaE3
Nº of genomes
Nº of genomes with TsaB1
Nº of genomes without TsaB1
Nº of genomes with TsaB2
Nº of genomes without TsaB2
Nº of genomes with TsaC1
Nº of genomes without TsaC1
Nº of genomes with TsaC2
Nº of genomes without TsaC2
Nº of genomes with TsaD1
Nº of genomes without TsaD1
Nº of genomes with TsaD2
Nº of genomes without TsaD2
Nº of genomes with TsaD3
Nº of genomes without TsaD3
Nº of genomes with TsaE1
Nº of genomes without TsaE1
Nº of genomes with TsaE2
Nº of genomes without TsaE2
Nº of genomes with TsaE3
Nº of genomes without TsaE3
Frequency of CAT usage
CAT usage
p < 0.00005

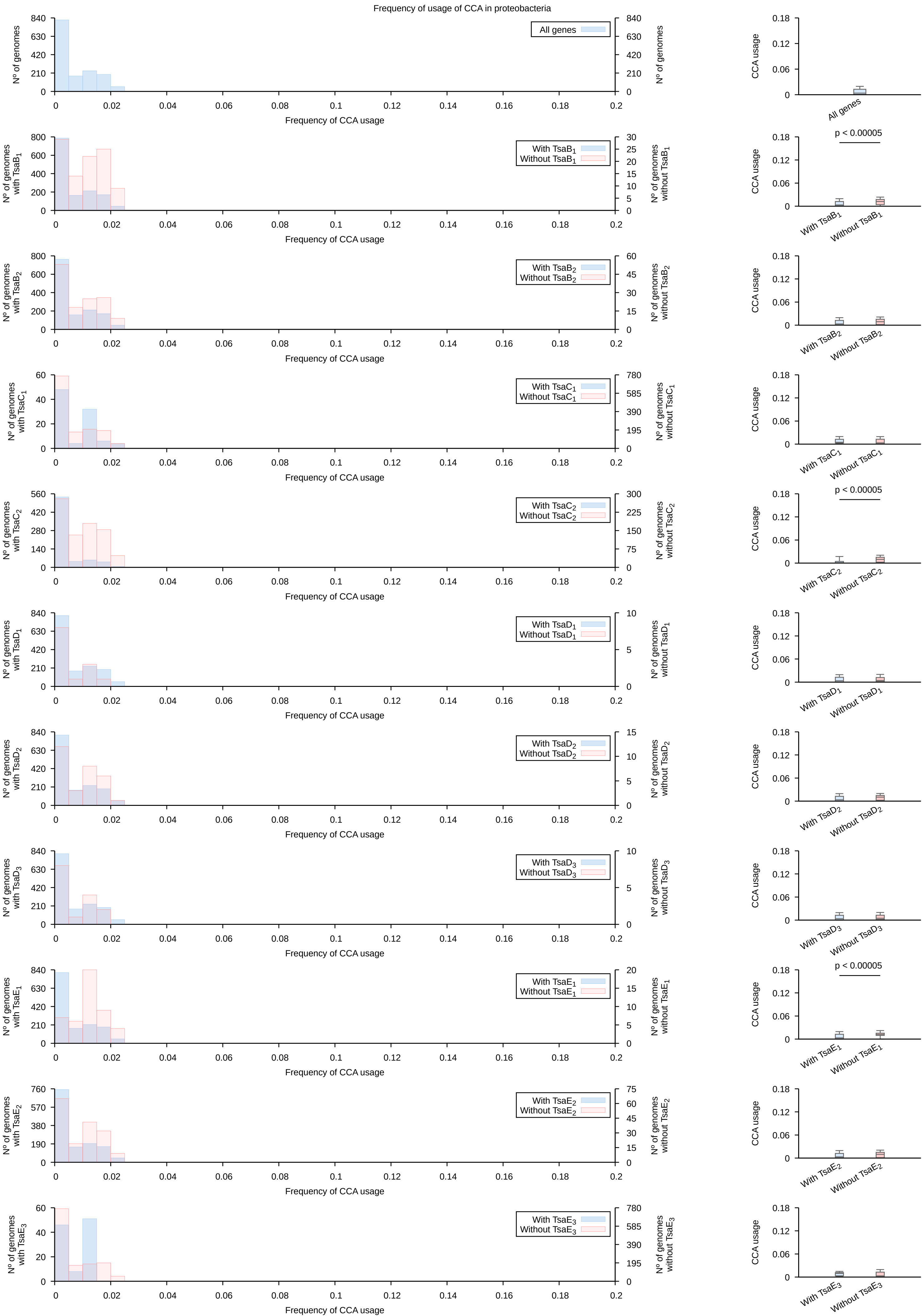

Frequency of usage of CCA in proteobacteria
Nº of genomes
Frequency of CCA usage
All genes
CCA usage
With TsaB$_1$
Without TsaB$_1$
Nº of genomes with TsaB$_1$
Nº of genomes without TsaB$_1$
p < 0.00005
With TsaB$_2$
Without TsaB$_2$
Nº of genomes with TsaB$_2$
Nº of genomes without TsaB$_2$
With TsaC$_1$
Without TsaC$_1$
Nº of genomes with TsaC$_1$
Nº of genomes without TsaC$_1$
With TsaC$_2$
Without TsaC$_2$
Nº of genomes with TsaC$_2$
Nº of genomes without TsaC$_2$
p < 0.00005
With TsaD$_1$
Without TsaD$_1$
Nº of genomes with TsaD$_1$
Nº of genomes without TsaD$_1$
With TsaD$_2$
Without TsaD$_2$
Nº of genomes with TsaD$_2$
Nº of genomes without TsaD$_2$
With TsaD$_3$
Without TsaD$_3$
Nº of genomes with TsaD$_3$
Nº of genomes without TsaD$_3$
With TsaE$_1$
Without TsaE$_1$
Nº of genomes with TsaE$_1$
Nº of genomes without TsaE$_1$
p < 0.00005
With TsaE$_2$
Without TsaE$_2$
Nº of genomes with TsaE$_2$
Nº of genomes without TsaE$_2$
With TsaE$_3$
Without TsaE$_3$
Nº of genomes with TsaE$_3$
Nº of genomes without TsaE$_3$
0 0.02 0.04 0.06 0.08 0.1 0.12 0.14 0.16 0.18 0.2
0 0.06 0.12 0.18

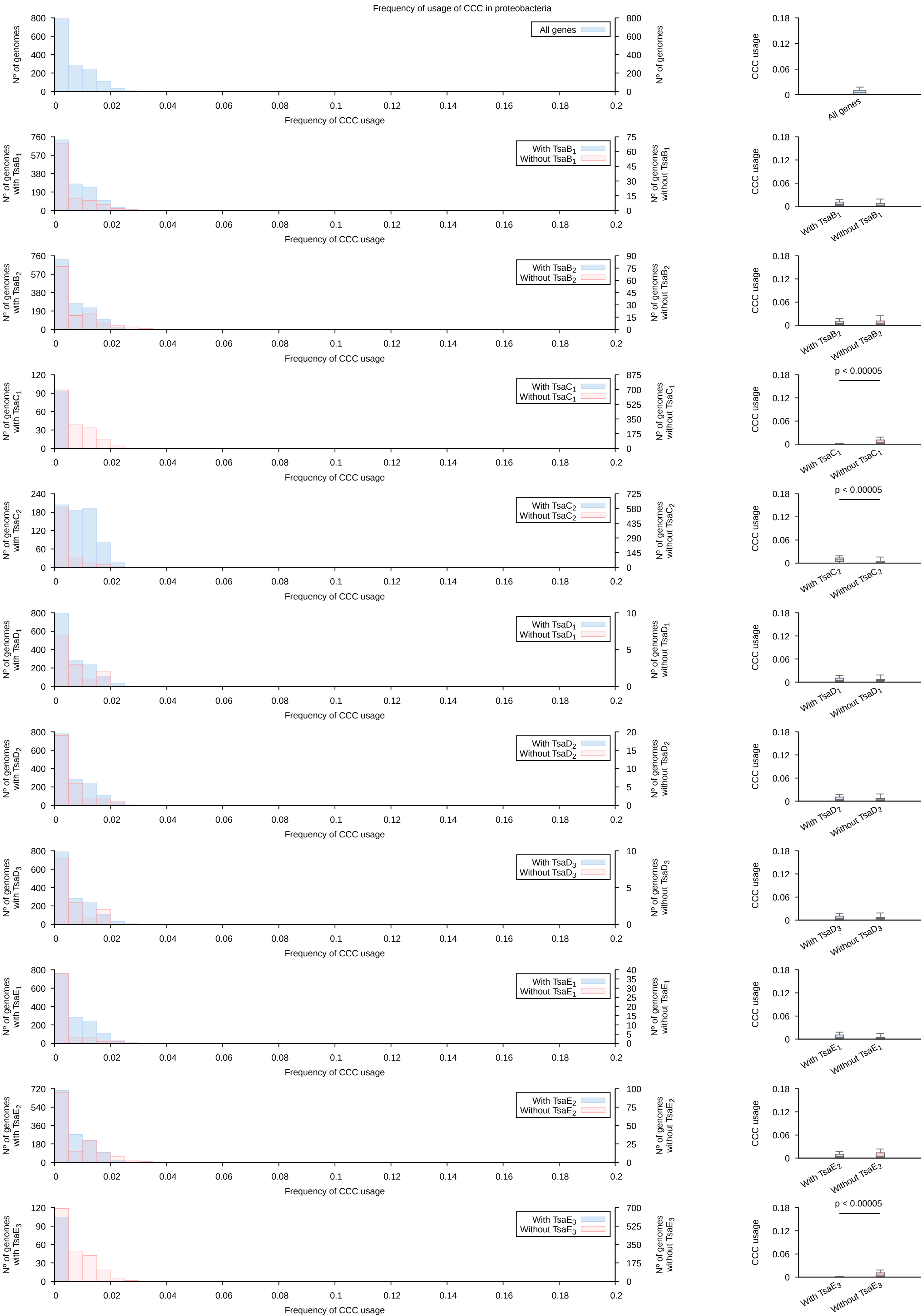

Frequency of usage of CCC in proteobacteria
All genes
Nº of genomes
Frequency of CCC usage
CCC usage
With TsaB1
Without TsaB1
Nº of genomes with TsaB1
Nº of genomes without TsaB1
With TsaB2
Without TsaB2
Nº of genomes with TsaB2
Nº of genomes without TsaB2
With TsaC1
Without TsaC1
Nº of genomes with TsaC1
Nº of genomes without TsaC1
p < 0.00005
With TsaC2
Without TsaC2
Nº of genomes with TsaC2
Nº of genomes without TsaC2
p < 0.00005
With TsaD1
Without TsaD1
Nº of genomes with TsaD1
Nº of genomes without TsaD1
With TsaD2
Without TsaD2
Nº of genomes with TsaD2
Nº of genomes without TsaD2
With TsaD3
Without TsaD3
Nº of genomes with TsaD3
Nº of genomes without TsaD3
With TsaE1
Without TsaE1
Nº of genomes with TsaE1
Nº of genomes without TsaE1
With TsaE2
Without TsaE2
Nº of genomes with TsaE2
Nº of genomes without TsaE2
With TsaE3
Without TsaE3
Nº of genomes with TsaE3
Nº of genomes without TsaE3
p < 0.00005

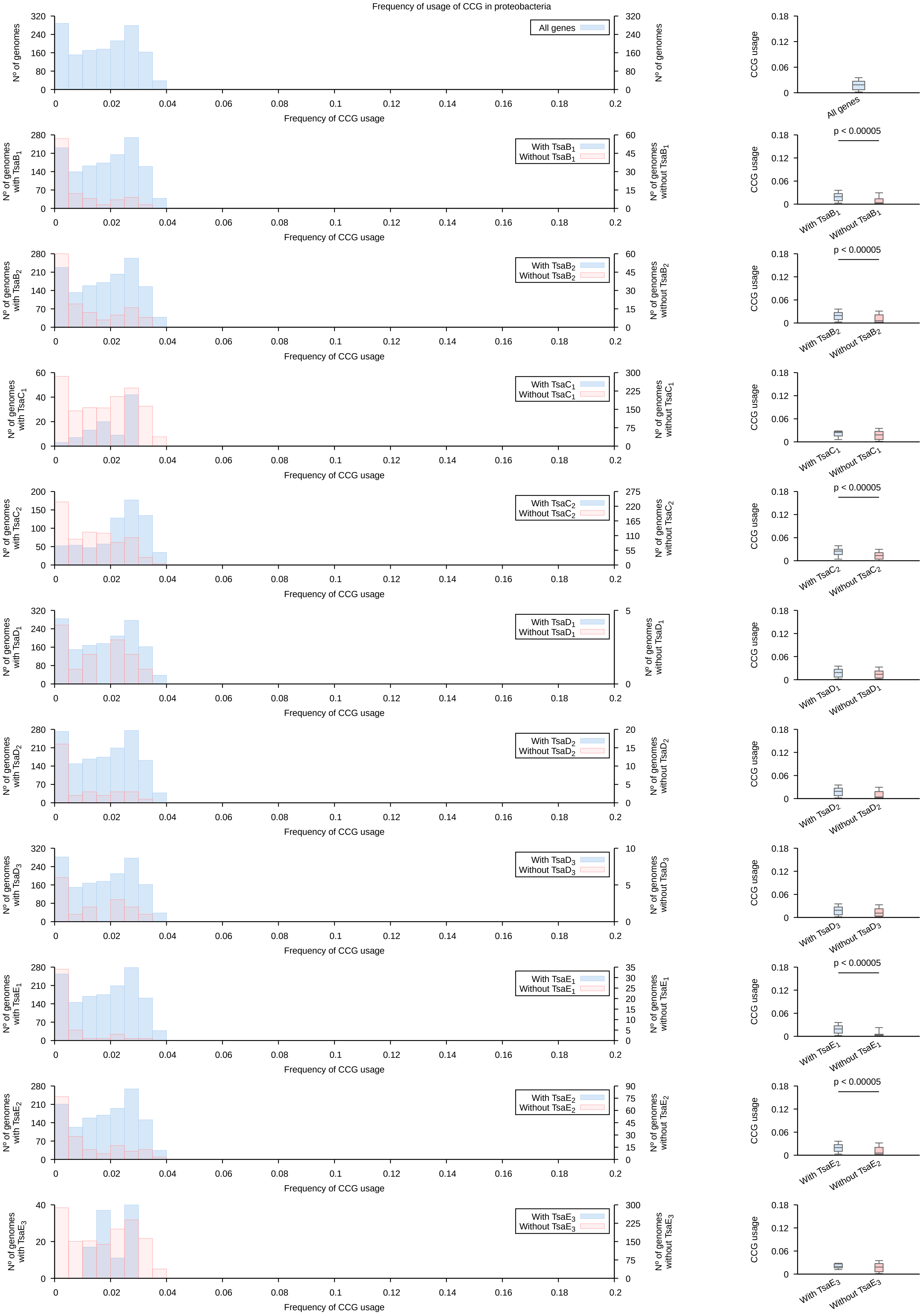

Frequency of usage of CCG in proteobacteria
All genes
Nº of genomes
CCG usage
Frequency of CCG usage
With TsaB1
Without TsaB1
Nº of genomes with TsaB1
Nº of genomes without TsaB1
p < 0.00005
With TsaB2
Without TsaB2
Nº of genomes with TsaB2
Nº of genomes without TsaB2
p < 0.00005
With TsaC1
Without TsaC1
Nº of genomes with TsaC1
Nº of genomes without TsaC1
With TsaC2
Without TsaC2
Nº of genomes with TsaC2
Nº of genomes without TsaC2
p < 0.00005
With TsaD1
Without TsaD1
Nº of genomes with TsaD1
Nº of genomes without TsaD1
With TsaD2
Without TsaD2
Nº of genomes with TsaD2
Nº of genomes without TsaD2
With TsaD3
Without TsaD3
Nº of genomes with TsaD3
Nº of genomes without TsaD3
With TsaE1
Without TsaE1
Nº of genomes with TsaE1
Nº of genomes without TsaE1
p < 0.00005
With TsaE2
Without TsaE2
Nº of genomes with TsaE2
Nº of genomes without TsaE2
p < 0.00005
With TsaE3
Without TsaE3
Nº of genomes with TsaE3
Nº of genomes without TsaE3

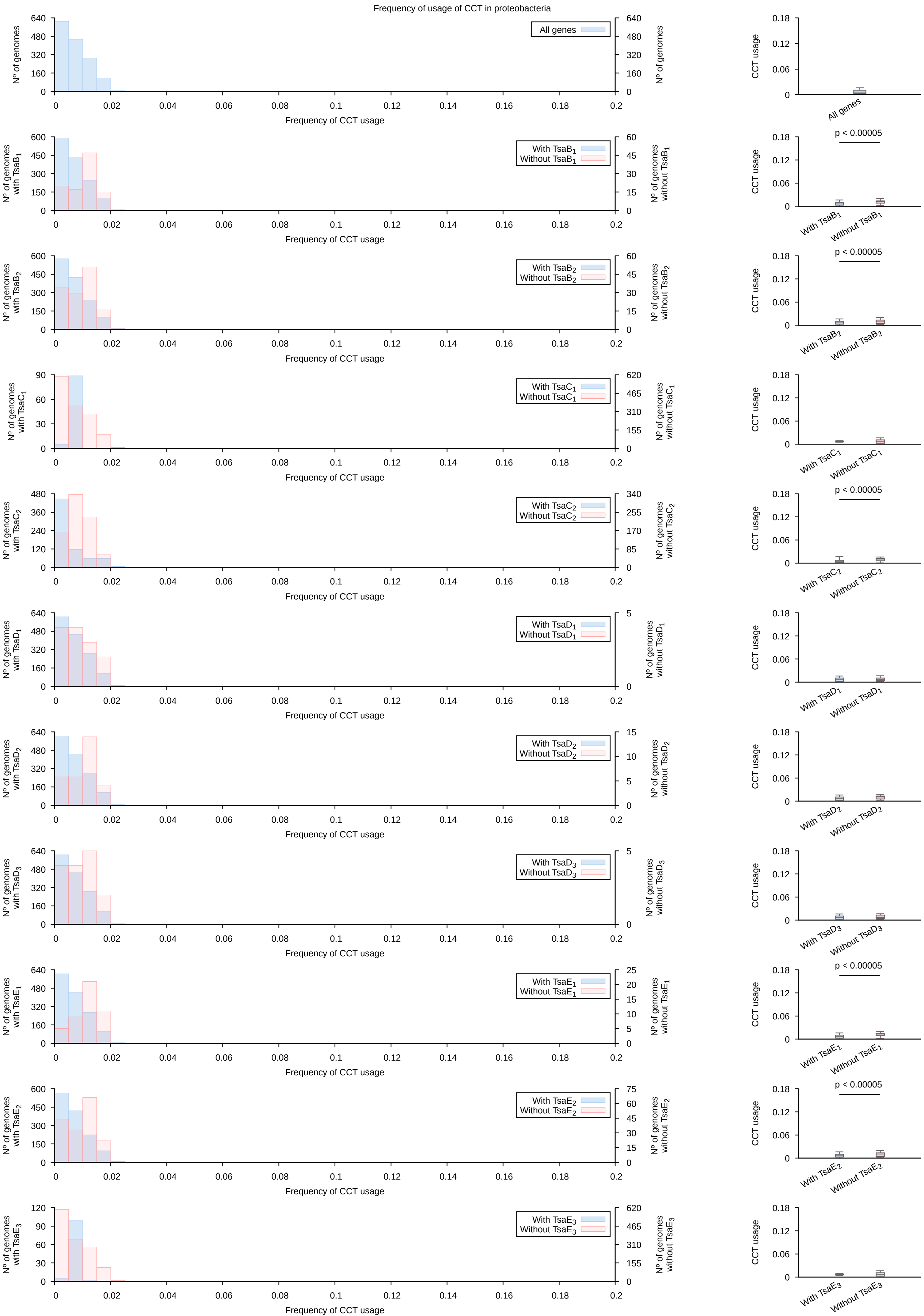

Frequency of usage of CCT in proteobacteria
All genes
Nº of genomes
Frequency of CCT usage
CCT usage
With $TsaB_1$
Without $TsaB_1$
p < 0.00005
With $TsaB_2$
Without $TsaB_2$
p < 0.00005
With $TsaC_1$
Without $TsaC_1$
With $TsaC_2$
Without $TsaC_2$
p < 0.00005
With $TsaD_1$
Without $TsaD_1$
With $TsaD_2$
Without $TsaD_2$
With $TsaD_3$
Without $TsaD_3$
With $TsaE_1$
Without $TsaE_1$
p < 0.00005
With $TsaE_2$
Without $TsaE_2$
p < 0.00005
With $TsaE_3$
Without $TsaE_3$

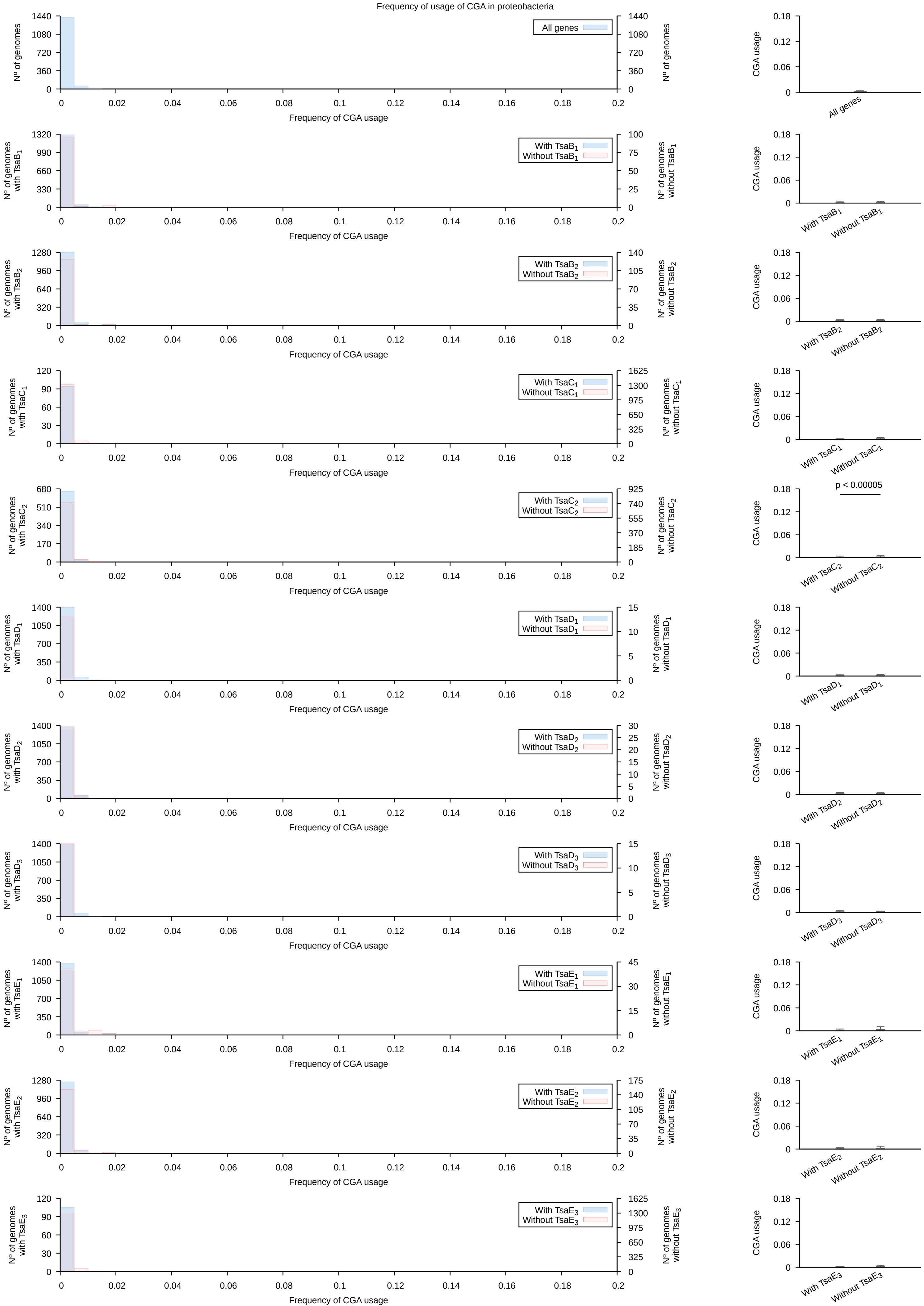
Frequency of usage of CGA in proteobacteria
All genes
With TsaB₁
Without TsaB₁
With TsaB₂
Without TsaB₂
With TsaC₁
Without TsaC₁
With TsaC₂
Without TsaC₂
p < 0.00005
With TsaD₁
Without TsaD₁
With TsaD₂
Without TsaD₂
With TsaD₃
Without TsaD₃
With TsaE₁
Without TsaE₁
With TsaE₂
Without TsaE₂
With TsaE₃
Without TsaE₃
Nº of genomes
CGA usage
Frequency of CGA usage

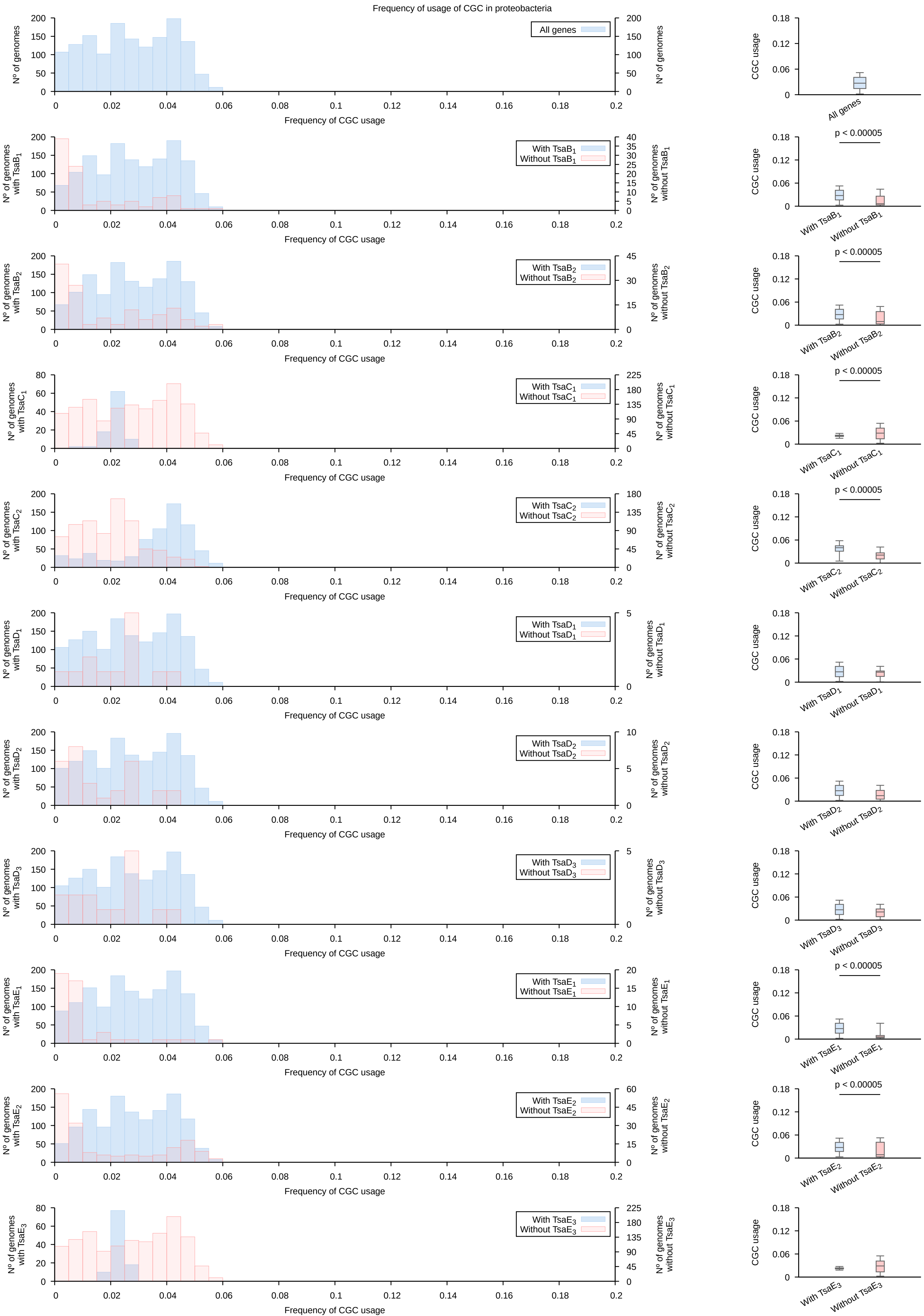
Frequency of usage of CGC in proteobacteria
All genes
Nº of genomes
Frequency of CGC usage
CGC usage
With TsaB1
Without TsaB1
Nº of genomes with TsaB1
Nº of genomes without TsaB1
p < 0.00005
With TsaB2
Without TsaB2
Nº of genomes with TsaB2
Nº of genomes without TsaB2
p < 0.00005
With TsaC1
Without TsaC1
Nº of genomes with TsaC1
Nº of genomes without TsaC1
p < 0.00005
With TsaC2
Without TsaC2
Nº of genomes with TsaC2
Nº of genomes without TsaC2
p < 0.00005
With TsaD1
Without TsaD1
Nº of genomes with TsaD1
Nº of genomes without TsaD1
With TsaD2
Without TsaD2
Nº of genomes with TsaD2
Nº of genomes without TsaD2
With TsaD3
Without TsaD3
Nº of genomes with TsaD3
Nº of genomes without TsaD3
With TsaE1
Without TsaE1
Nº of genomes with TsaE1
Nº of genomes without TsaE1
p < 0.00005
With TsaE2
Without TsaE2
Nº of genomes with TsaE2
Nº of genomes without TsaE2
p < 0.00005
With TsaE3
Without TsaE3
Nº of genomes with TsaE3
Nº of genomes without TsaE3

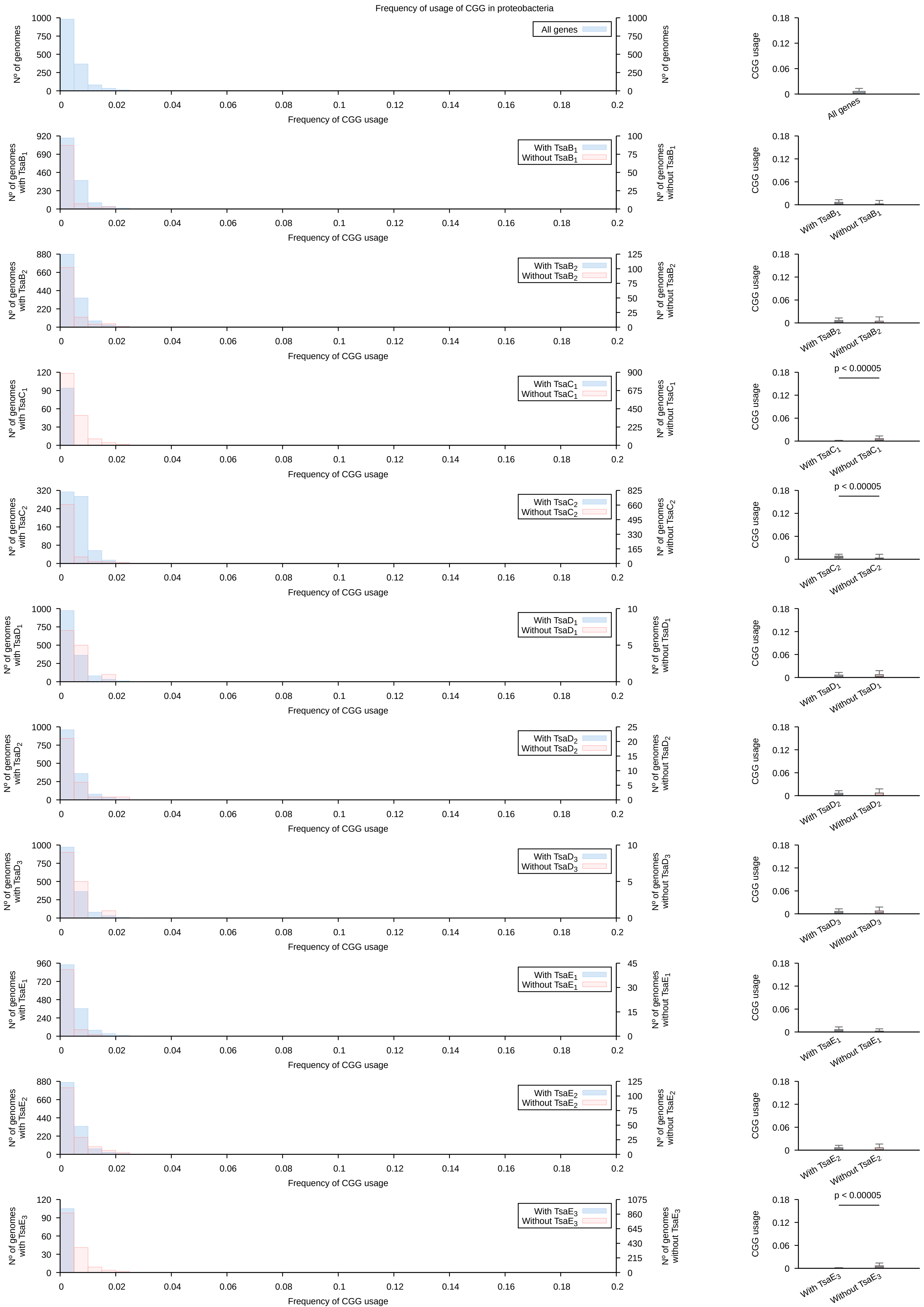
Frequency of usage of CGG in proteobacteria
All genes
Nº of genomes
Frequency of CGG usage
CGG usage
With TsaB$_1$
Without TsaB$_1$
Nº of genomes with TsaB$_1$
Nº of genomes without TsaB$_1$
With TsaB$_2$
Without TsaB$_2$
Nº of genomes with TsaB$_2$
Nº of genomes without TsaB$_2$
With TsaC$_1$
Without TsaC$_1$
Nº of genomes with TsaC$_1$
Nº of genomes without TsaC$_1$
p < 0.00005
With TsaC$_2$
Without TsaC$_2$
Nº of genomes with TsaC$_2$
Nº of genomes without TsaC$_2$
p < 0.00005
With TsaD$_1$
Without TsaD$_1$
Nº of genomes with TsaD$_1$
Nº of genomes without TsaD$_1$
With TsaD$_2$
Without TsaD$_2$
Nº of genomes with TsaD$_2$
Nº of genomes without TsaD$_2$
With TsaD$_3$
Without TsaD$_3$
Nº of genomes with TsaD$_3$
Nº of genomes without TsaD$_3$
With TsaE$_1$
Without TsaE$_1$
Nº of genomes with TsaE$_1$
Nº of genomes without TsaE$_1$
With TsaE$_2$
Without TsaE$_2$
Nº of genomes with TsaE$_2$
Nº of genomes without TsaE$_2$
With TsaE$_3$
Without TsaE$_3$
Nº of genomes with TsaE$_3$
Nº of genomes without TsaE$_3$
p < 0.00005

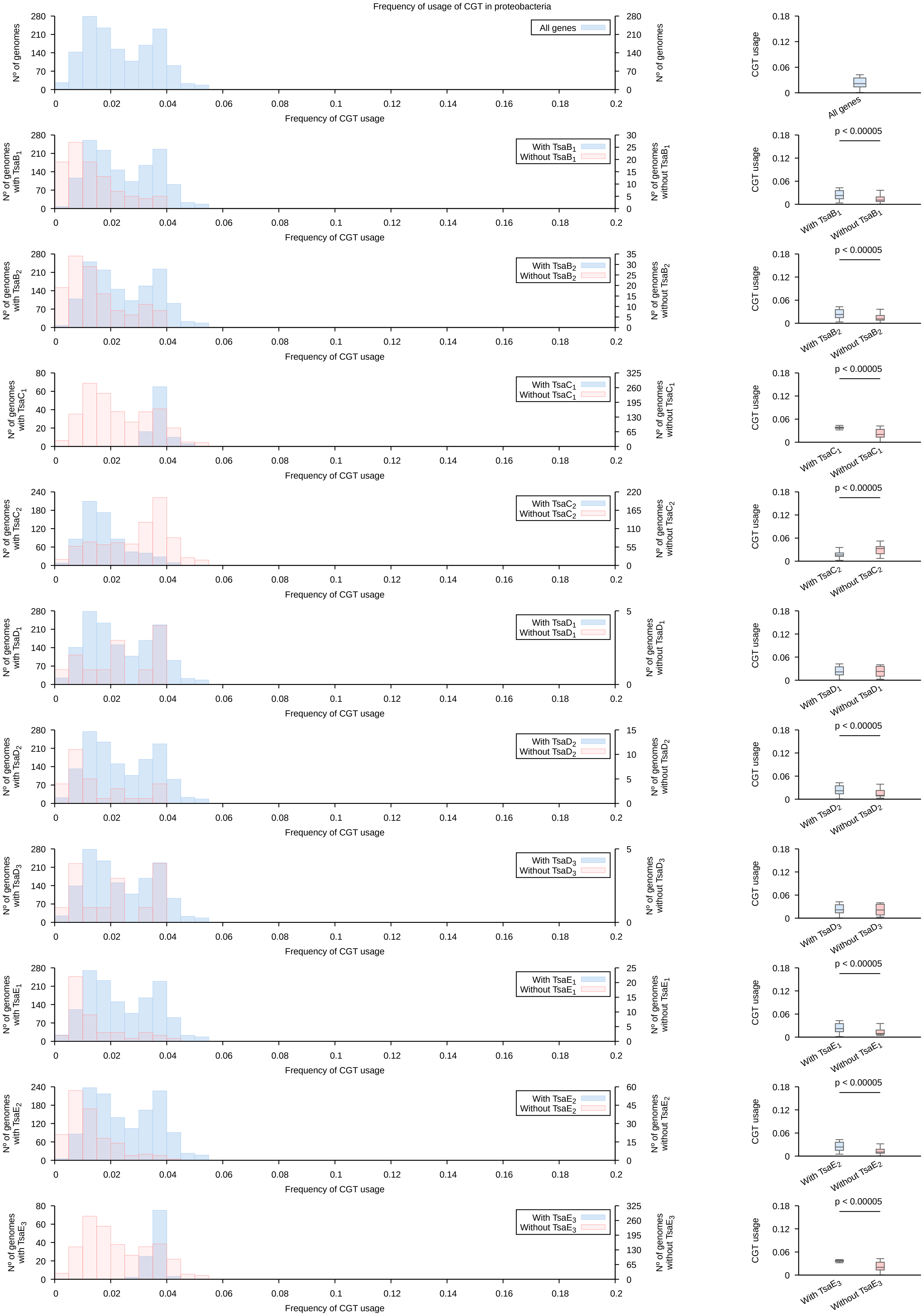

Frequency of usage of CGT in proteobacteria
Nº of genomes
Frequency of CGT usage
CGT usage
All genes
Nº of genomes with TsaB1
Nº of genomes without TsaB1
With TsaB1
Without TsaB1
p < 0.00005
Nº of genomes with TsaB2
Nº of genomes without TsaB2
With TsaB2
Without TsaB2
p < 0.00005
Nº of genomes with TsaC1
Nº of genomes without TsaC1
With TsaC1
Without TsaC1
p < 0.00005
Nº of genomes with TsaC2
Nº of genomes without TsaC2
With TsaC2
Without TsaC2
p < 0.00005
Nº of genomes with TsaD1
Nº of genomes without TsaD1
With TsaD1
Without TsaD1
Nº of genomes with TsaD2
Nº of genomes without TsaD2
With TsaD2
Without TsaD2
p < 0.00005
Nº of genomes with TsaD3
Nº of genomes without TsaD3
With TsaD3
Without TsaD3
Nº of genomes with TsaE1
Nº of genomes without TsaE1
With TsaE1
Without TsaE1
p < 0.00005
Nº of genomes with TsaE2
Nº of genomes without TsaE2
With TsaE2
Without TsaE2
p < 0.00005
Nº of genomes with TsaE3
Nº of genomes without TsaE3
With TsaE3
Without TsaE3
p < 0.00005

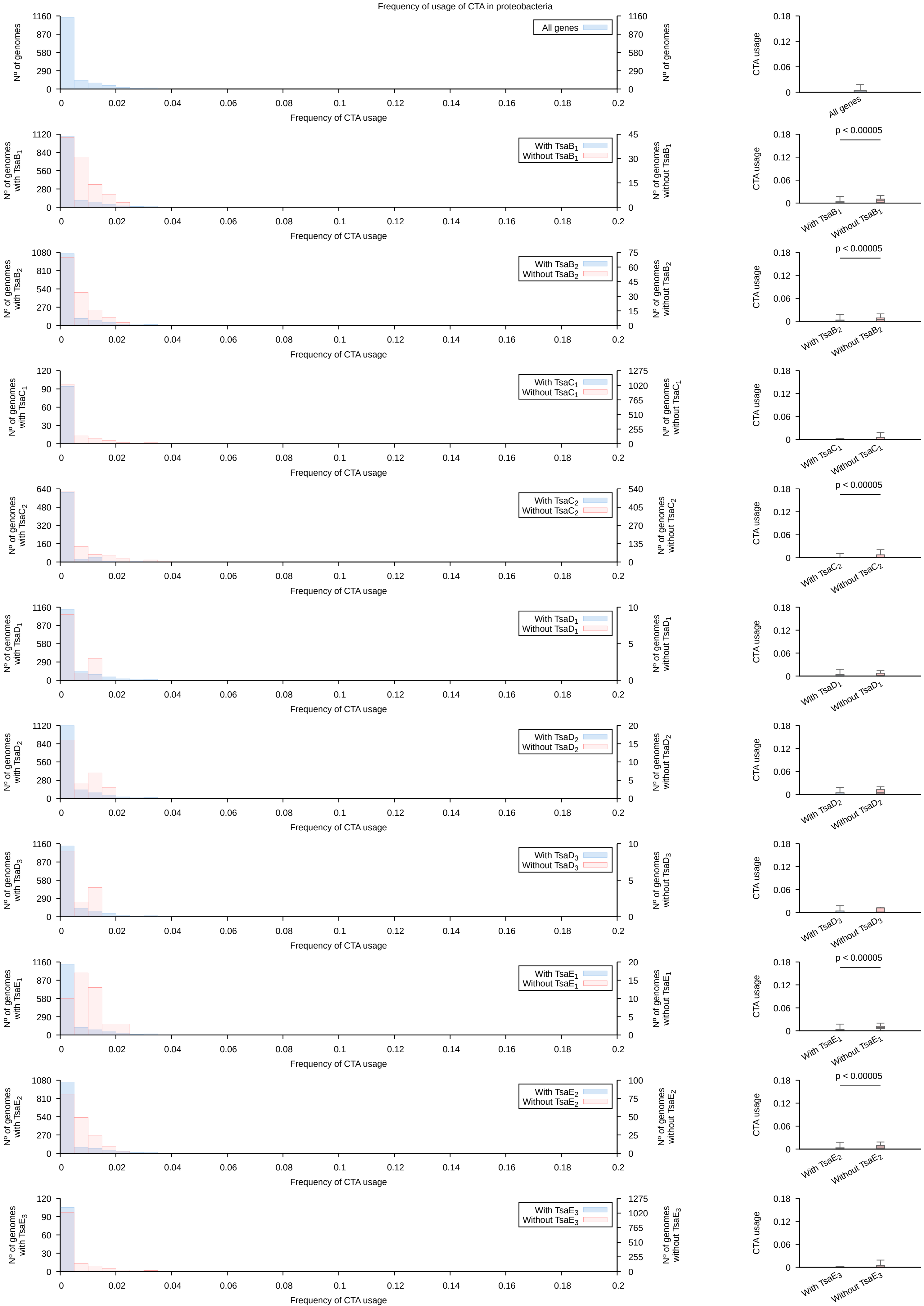

Frequency of usage of CTA in proteobacteria
All genes
With TsaB$_1$
Without TsaB$_1$
With TsaB$_2$
Without TsaB$_2$
With TsaC$_1$
Without TsaC$_1$
With TsaC$_2$
Without TsaC$_2$
With TsaD$_1$
Without TsaD$_1$
With TsaD$_2$
Without TsaD$_2$
With TsaD$_3$
Without TsaD$_3$
With TsaE$_1$
Without TsaE$_1$
With TsaE$_2$
Without TsaE$_2$
With TsaE$_3$
Without TsaE$_3$
Frequency of CTA usage
Nº of genomes
CTA usage
p < 0.00005

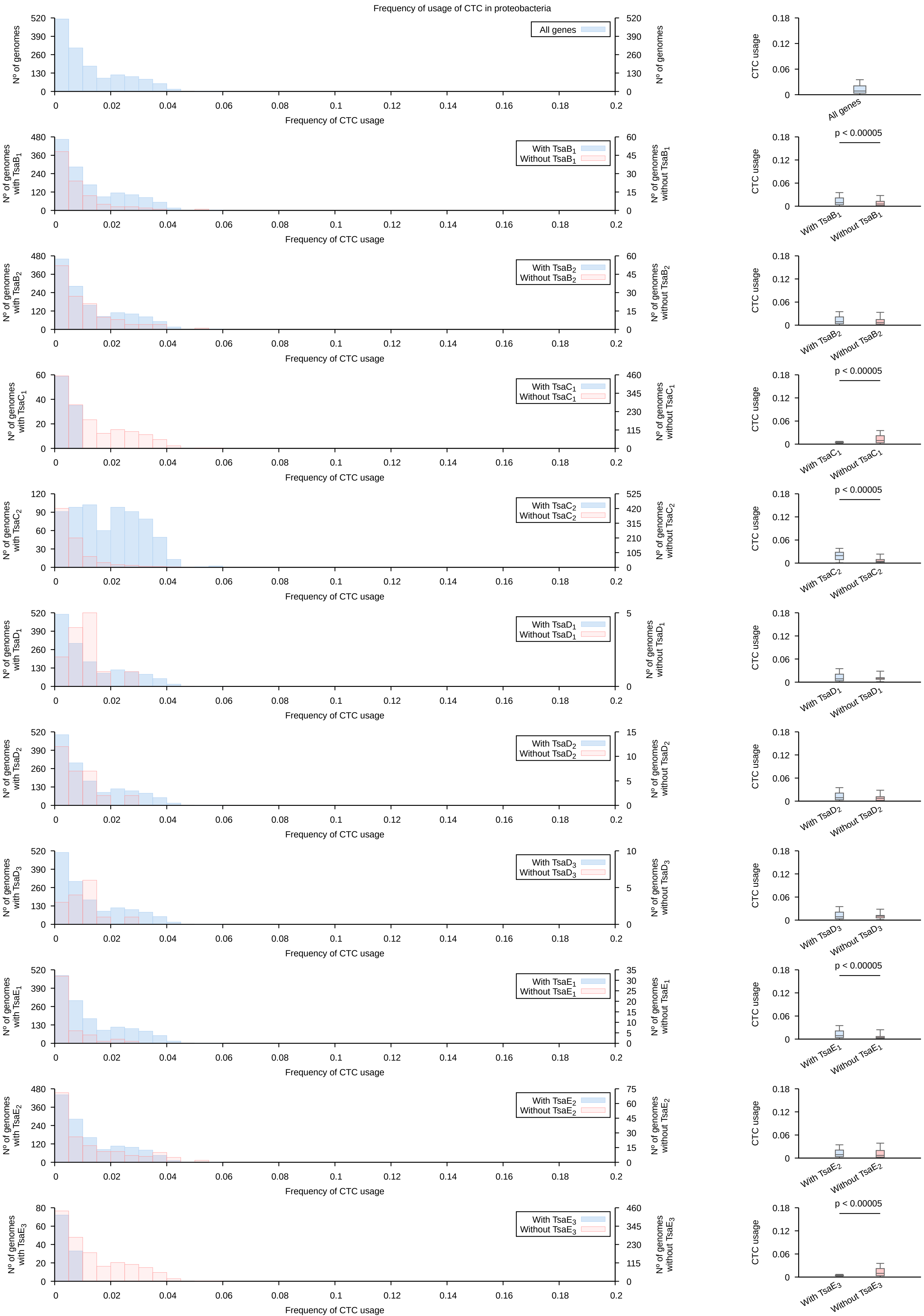

Frequency of usage of CTC in proteobacteria
All genes
Nº of genomes
Frequency of CTC usage
CTC usage
With TsaB1
Without TsaB1
Nº of genomes with TsaB1
Nº of genomes without TsaB1
p < 0.00005
With TsaB2
Without TsaB2
Nº of genomes with TsaB2
Nº of genomes without TsaB2
With TsaC1
Without TsaC1
Nº of genomes with TsaC1
Nº of genomes without TsaC1
p < 0.00005
With TsaC2
Without TsaC2
Nº of genomes with TsaC2
Nº of genomes without TsaC2
p < 0.00005
With TsaD1
Without TsaD1
Nº of genomes with TsaD1
Nº of genomes without TsaD1
With TsaD2
Without TsaD2
Nº of genomes with TsaD2
Nº of genomes without TsaD2
With TsaD3
Without TsaD3
Nº of genomes with TsaD3
Nº of genomes without TsaD3
With TsaE1
Without TsaE1
Nº of genomes with TsaE1
Nº of genomes without TsaE1
p < 0.00005
With TsaE2
Without TsaE2
Nº of genomes with TsaE2
Nº of genomes without TsaE2
With TsaE3
Without TsaE3
Nº of genomes with TsaE3
Nº of genomes without TsaE3
p < 0.00005

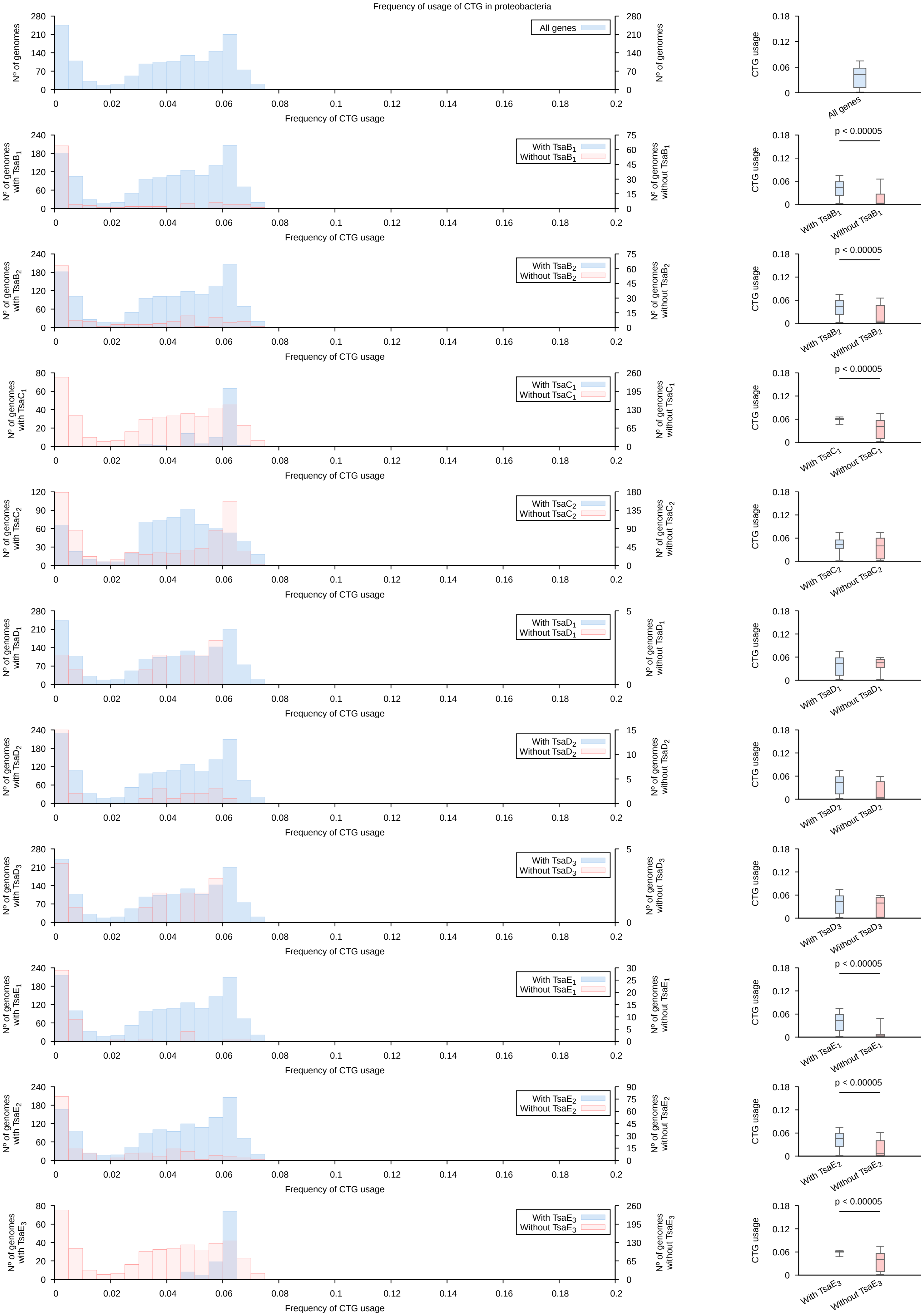
Frequency of usage of CTG in proteobacteria
All genes
Nº of genomes
Frequency of CTG usage
CTG usage
With TsaB1
Without TsaB1
Nº of genomes with TsaB1
Nº of genomes without TsaB1
p < 0.00005
With TsaB2
Without TsaB2
Nº of genomes with TsaB2
Nº of genomes without TsaB2
p < 0.00005
With TsaC1
Without TsaC1
Nº of genomes with TsaC1
Nº of genomes without TsaC1
p < 0.00005
With TsaC2
Without TsaC2
Nº of genomes with TsaC2
Nº of genomes without TsaC2
With TsaD1
Without TsaD1
Nº of genomes with TsaD1
Nº of genomes without TsaD1
With TsaD2
Without TsaD2
Nº of genomes with TsaD2
Nº of genomes without TsaD2
With TsaD3
Without TsaD3
Nº of genomes with TsaD3
Nº of genomes without TsaD3
With TsaE1
Without TsaE1
Nº of genomes with TsaE1
Nº of genomes without TsaE1
p < 0.00005
With TsaE2
Without TsaE2
Nº of genomes with TsaE2
Nº of genomes without TsaE2
p < 0.00005
With TsaE3
Without TsaE3
Nº of genomes with TsaE3
Nº of genomes without TsaE3
p < 0.00005

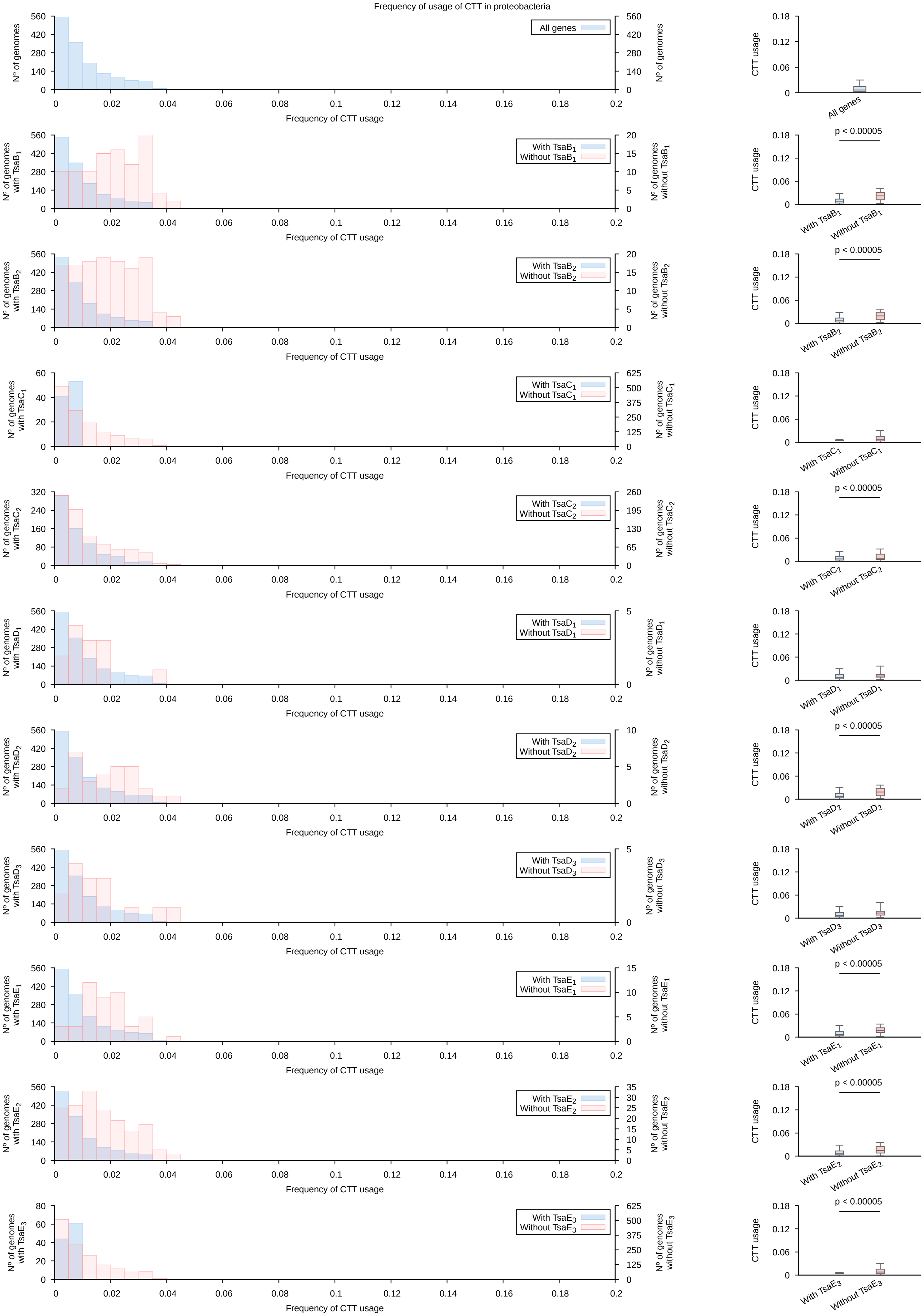
Frequency of usage of CTT in proteobacteria
All genes
Nº of genomes
Frequency of CTT usage
CTT usage
With TsaB1
Without TsaB1
Nº of genomes with TsaB1
Nº of genomes without TsaB1
With TsaB2
Without TsaB2
Nº of genomes with TsaB2
Nº of genomes without TsaB2
With TsaC1
Without TsaC1
Nº of genomes with TsaC1
Nº of genomes without TsaC1
With TsaC2
Without TsaC2
Nº of genomes with TsaC2
Nº of genomes without TsaC2
With TsaD1
Without TsaD1
Nº of genomes with TsaD1
Nº of genomes without TsaD1
With TsaD2
Without TsaD2
Nº of genomes with TsaD2
Nº of genomes without TsaD2
With TsaD3
Without TsaD3
Nº of genomes with TsaD3
Nº of genomes without TsaD3
With TsaE1
Without TsaE1
Nº of genomes with TsaE1
Nº of genomes without TsaE1
With TsaE2
Without TsaE2
Nº of genomes with TsaE2
Nº of genomes without TsaE2
With TsaE3
Without TsaE3
Nº of genomes with TsaE3
Nº of genomes without TsaE3
p < 0.00005

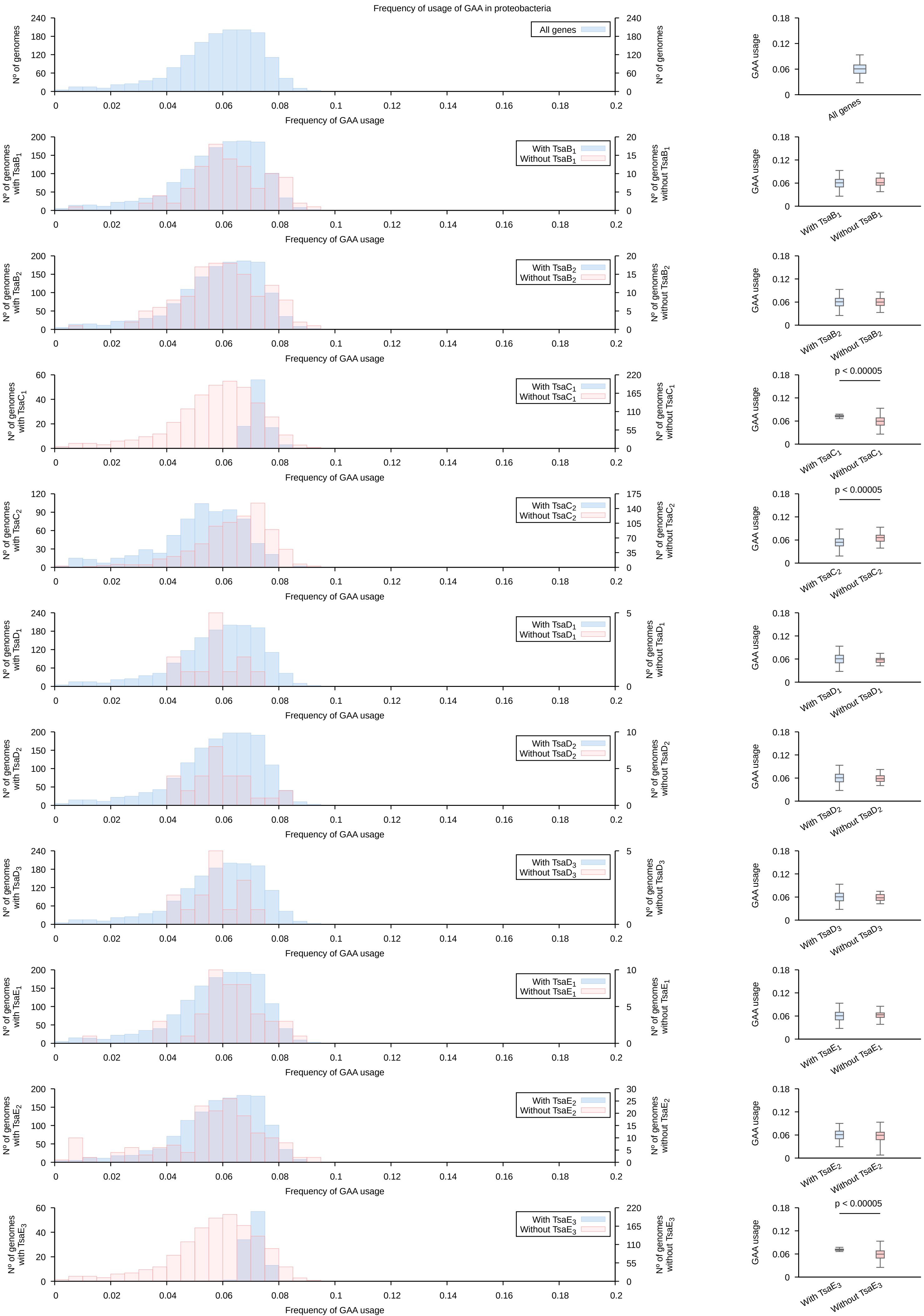
Frequency of usage of GAA in proteobacteria
All genes
With TsaB1
Without TsaB1
With TsaB2
Without TsaB2
With TsaC1
Without TsaC1
With TsaC2
Without TsaC2
With TsaD1
Without TsaD1
With TsaD2
Without TsaD2
With TsaD3
Without TsaD3
With TsaE1
Without TsaE1
With TsaE2
Without TsaE2
With TsaE3
Without TsaE3
Frequency of GAA usage
Nº of genomes
GAA usage
p < 0.00005

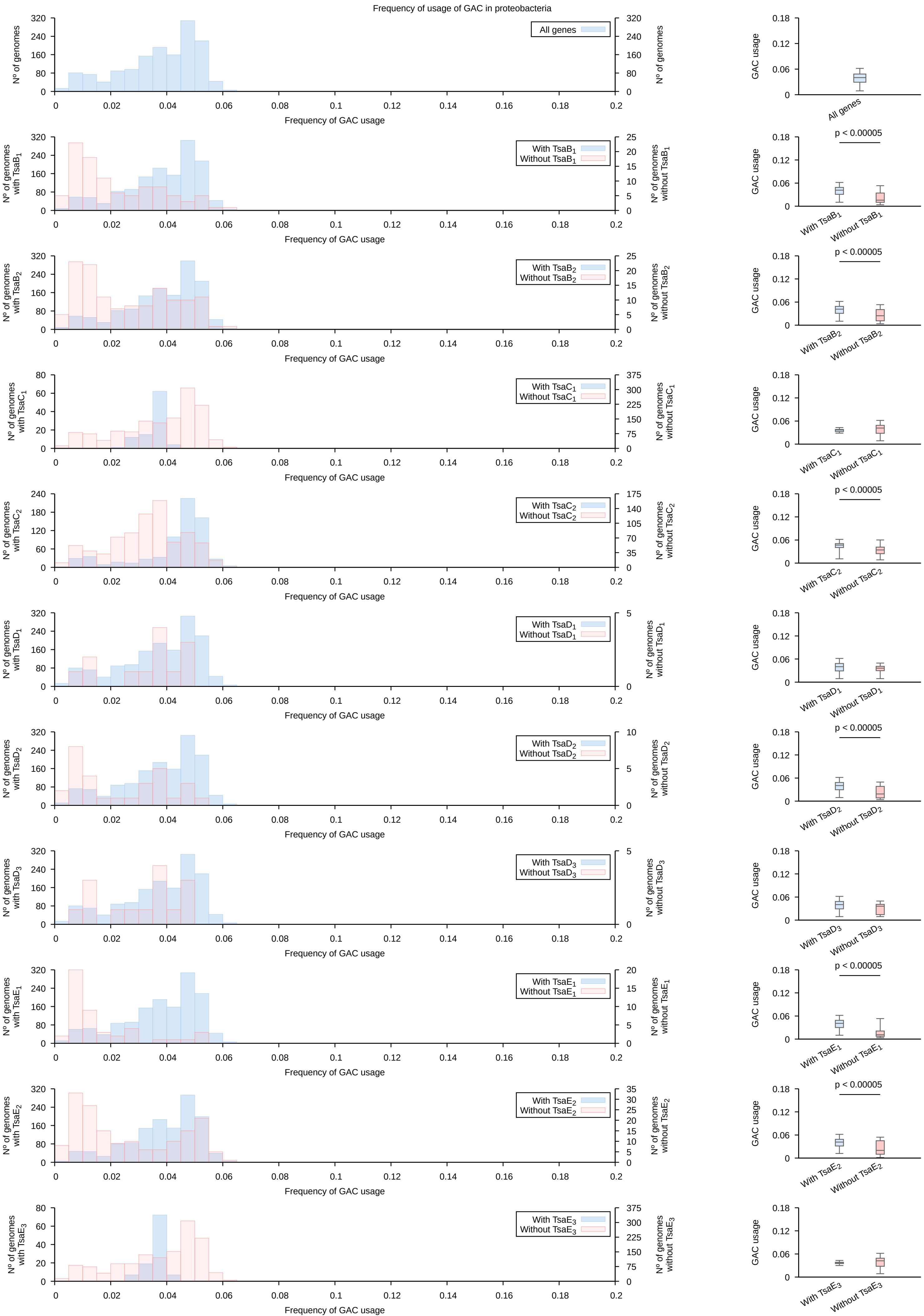

Frequency of usage of GAC in proteobacteria
Nº of genomes
All genes
Frequency of GAC usage
GAC usage
p < 0.00005
Nº of genomes with TsaB1
Nº of genomes without TsaB1
With TsaB1
Without TsaB1
Nº of genomes with TsaB2
Nº of genomes without TsaB2
With TsaB2
Without TsaB2
Nº of genomes with TsaC1
Nº of genomes without TsaC1
With TsaC1
Without TsaC1
Nº of genomes with TsaC2
Nº of genomes without TsaC2
With TsaC2
Without TsaC2
Nº of genomes with TsaD1
Nº of genomes without TsaD1
With TsaD1
Without TsaD1
Nº of genomes with TsaD2
Nº of genomes without TsaD2
With TsaD2
Without TsaD2
Nº of genomes with TsaD3
Nº of genomes without TsaD3
With TsaD3
Without TsaD3
Nº of genomes with TsaE1
Nº of genomes without TsaE1
With TsaE1
Without TsaE1
Nº of genomes with TsaE2
Nº of genomes without TsaE2
With TsaE2
Without TsaE2
Nº of genomes with TsaE3
Nº of genomes without TsaE3
With TsaE3
Without TsaE3

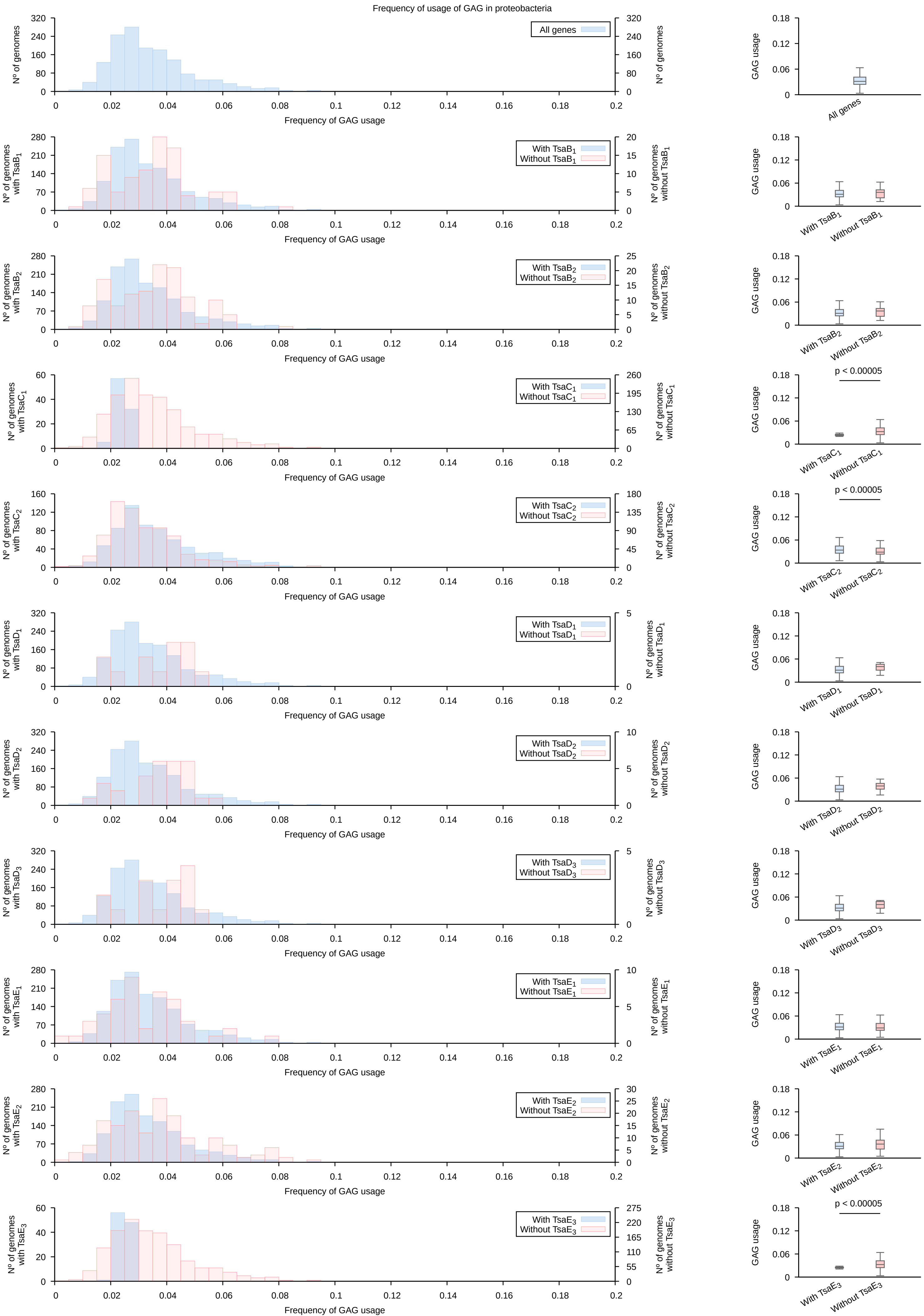

Frequency of usage of GAG in proteobacteria
All genes
Nº of genomes
Frequency of GAG usage
GAG usage
With TsaB$_1$
Without TsaB$_1$
Nº of genomes with TsaB$_1$
Nº of genomes without TsaB$_1$
With TsaB$_2$
Without TsaB$_2$
Nº of genomes with TsaB$_2$
Nº of genomes without TsaB$_2$
With TsaC$_1$
Without TsaC$_1$
Nº of genomes with TsaC$_1$
Nº of genomes without TsaC$_1$
p < 0.00005
With TsaC$_2$
Without TsaC$_2$
Nº of genomes with TsaC$_2$
Nº of genomes without TsaC$_2$
p < 0.00005
With TsaD$_1$
Without TsaD$_1$
Nº of genomes with TsaD$_1$
Nº of genomes without TsaD$_1$
With TsaD$_2$
Without TsaD$_2$
Nº of genomes with TsaD$_2$
Nº of genomes without TsaD$_2$
With TsaD$_3$
Without TsaD$_3$
Nº of genomes with TsaD$_3$
Nº of genomes without TsaD$_3$
With TsaE$_1$
Without TsaE$_1$
Nº of genomes with TsaE$_1$
Nº of genomes without TsaE$_1$
With TsaE$_2$
Without TsaE$_2$
Nº of genomes with TsaE$_2$
Nº of genomes without TsaE$_2$
With TsaE$_3$
Without TsaE$_3$
Nº of genomes with TsaE$_3$
Nº of genomes without TsaE$_3$
p < 0.00005

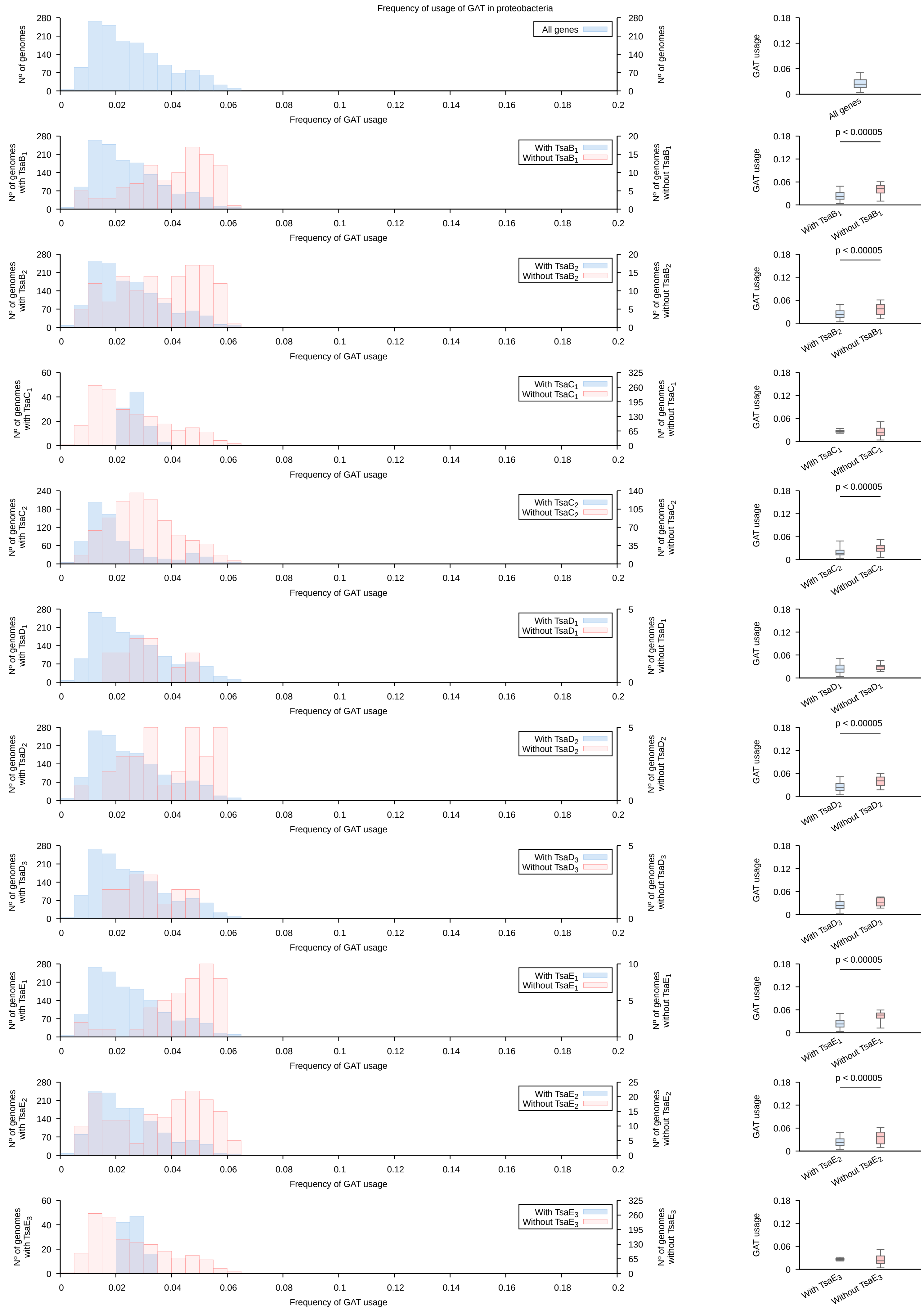

Frequency of usage of GAT in proteobacteria
Nº of genomes
All genes
Frequency of GAT usage
GAT usage
All genes
Nº of genomes with $TsaB_1$
Nº of genomes without $TsaB_1$
With $TsaB_1$
Without $TsaB_1$
p < 0.00005
Nº of genomes with $TsaB_2$
Nº of genomes without $TsaB_2$
With $TsaB_2$
Without $TsaB_2$
p < 0.00005
Nº of genomes with $TsaC_1$
Nº of genomes without $TsaC_1$
With $TsaC_1$
Without $TsaC_1$
Nº of genomes with $TsaC_2$
Nº of genomes without $TsaC_2$
With $TsaC_2$
Without $TsaC_2$
p < 0.00005
Nº of genomes with $TsaD_1$
Nº of genomes without $TsaD_1$
With $TsaD_1$
Without $TsaD_1$
Nº of genomes with $TsaD_2$
Nº of genomes without $TsaD_2$
With $TsaD_2$
Without $TsaD_2$
p < 0.00005
Nº of genomes with $TsaD_3$
Nº of genomes without $TsaD_3$
With $TsaD_3$
Without $TsaD_3$
Nº of genomes with $TsaE_1$
Nº of genomes without $TsaE_1$
With $TsaE_1$
Without $TsaE_1$
p < 0.00005
Nº of genomes with $TsaE_2$
Nº of genomes without $TsaE_2$
With $TsaE_2$
Without $TsaE_2$
p < 0.00005
Nº of genomes with $TsaE_3$
Nº of genomes without $TsaE_3$
With $TsaE_3$
Without $TsaE_3$

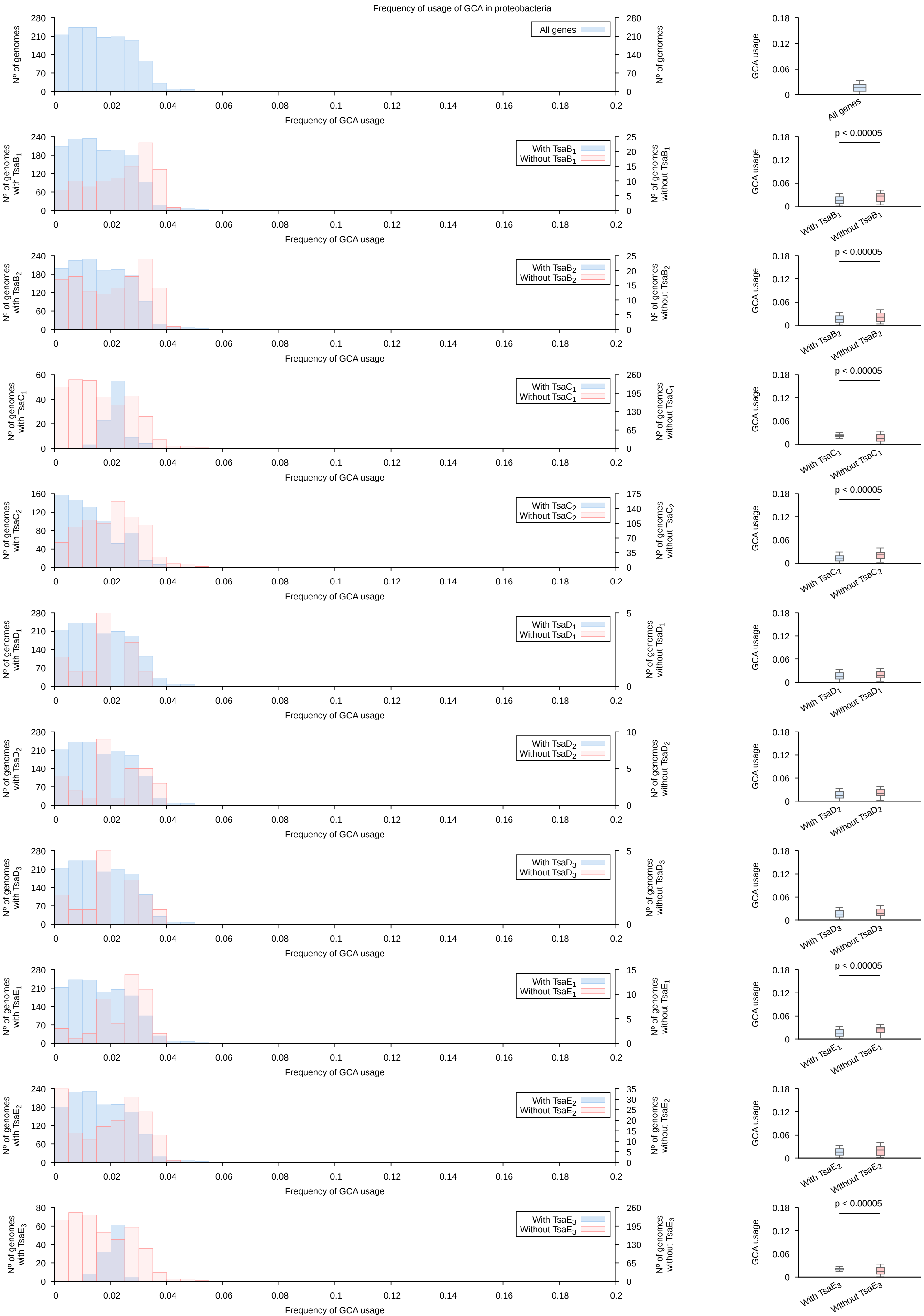

Frequency of usage of GCA in proteobacteria
Nº of genomes
All genes
Frequency of GCA usage
GCA usage
All genes
Nº of genomes with TsaB1
Nº of genomes without TsaB1
With TsaB1
Without TsaB1
p < 0.00005
Nº of genomes with TsaB2
Nº of genomes without TsaB2
With TsaB2
Without TsaB2
p < 0.00005
Nº of genomes with TsaC1
Nº of genomes without TsaC1
With TsaC1
Without TsaC1
p < 0.00005
Nº of genomes with TsaC2
Nº of genomes without TsaC2
With TsaC2
Without TsaC2
p < 0.00005
Nº of genomes with TsaD1
Nº of genomes without TsaD1
With TsaD1
Without TsaD1
Nº of genomes with TsaD2
Nº of genomes without TsaD2
With TsaD2
Without TsaD2
Nº of genomes with TsaD3
Nº of genomes without TsaD3
With TsaD3
Without TsaD3
Nº of genomes with TsaE1
Nº of genomes without TsaE1
With TsaE1
Without TsaE1
p < 0.00005
Nº of genomes with TsaE2
Nº of genomes without TsaE2
With TsaE2
Without TsaE2
Nº of genomes with TsaE3
Nº of genomes without TsaE3
With TsaE3
Without TsaE3
p < 0.00005

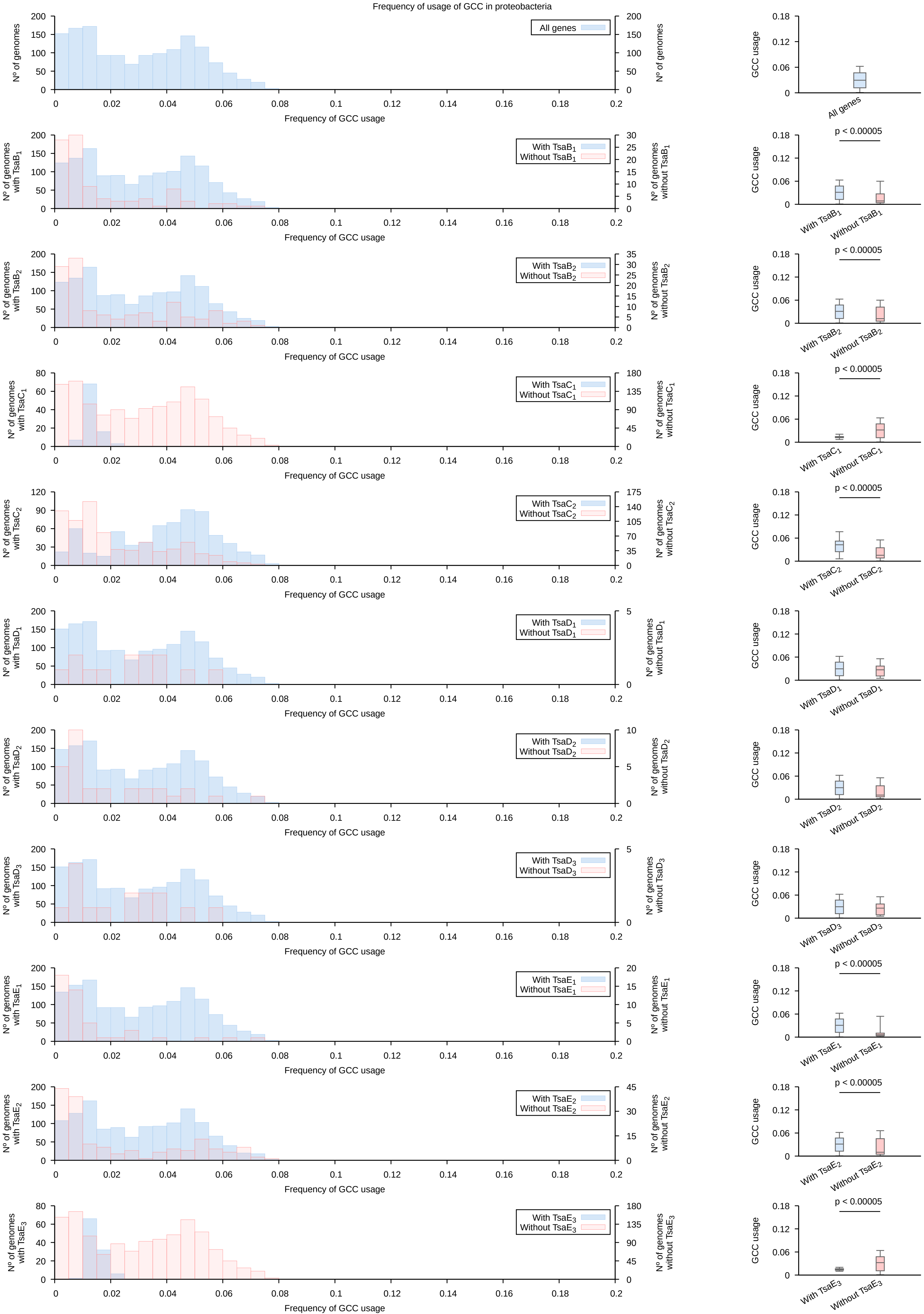
Frequency of usage of GCC in proteobacteria
All genes
Nº of genomes
Frequency of GCC usage
GCC usage
With TsaB1
Without TsaB1
Nº of genomes with TsaB1
Nº of genomes without TsaB1
p < 0.00005
With TsaB2
Without TsaB2
Nº of genomes with TsaB2
Nº of genomes without TsaB2
p < 0.00005
With TsaC1
Without TsaC1
Nº of genomes with TsaC1
Nº of genomes without TsaC1
p < 0.00005
With TsaC2
Without TsaC2
Nº of genomes with TsaC2
Nº of genomes without TsaC2
p < 0.00005
With TsaD1
Without TsaD1
Nº of genomes with TsaD1
Nº of genomes without TsaD1
With TsaD2
Without TsaD2
Nº of genomes with TsaD2
Nº of genomes without TsaD2
With TsaD3
Without TsaD3
Nº of genomes with TsaD3
Nº of genomes without TsaD3
With TsaE1
Without TsaE1
Nº of genomes with TsaE1
Nº of genomes without TsaE1
p < 0.00005
With TsaE2
Without TsaE2
Nº of genomes with TsaE2
Nº of genomes without TsaE2
p < 0.00005
With TsaE3
Without TsaE3
Nº of genomes with TsaE3
Nº of genomes without TsaE3
p < 0.00005

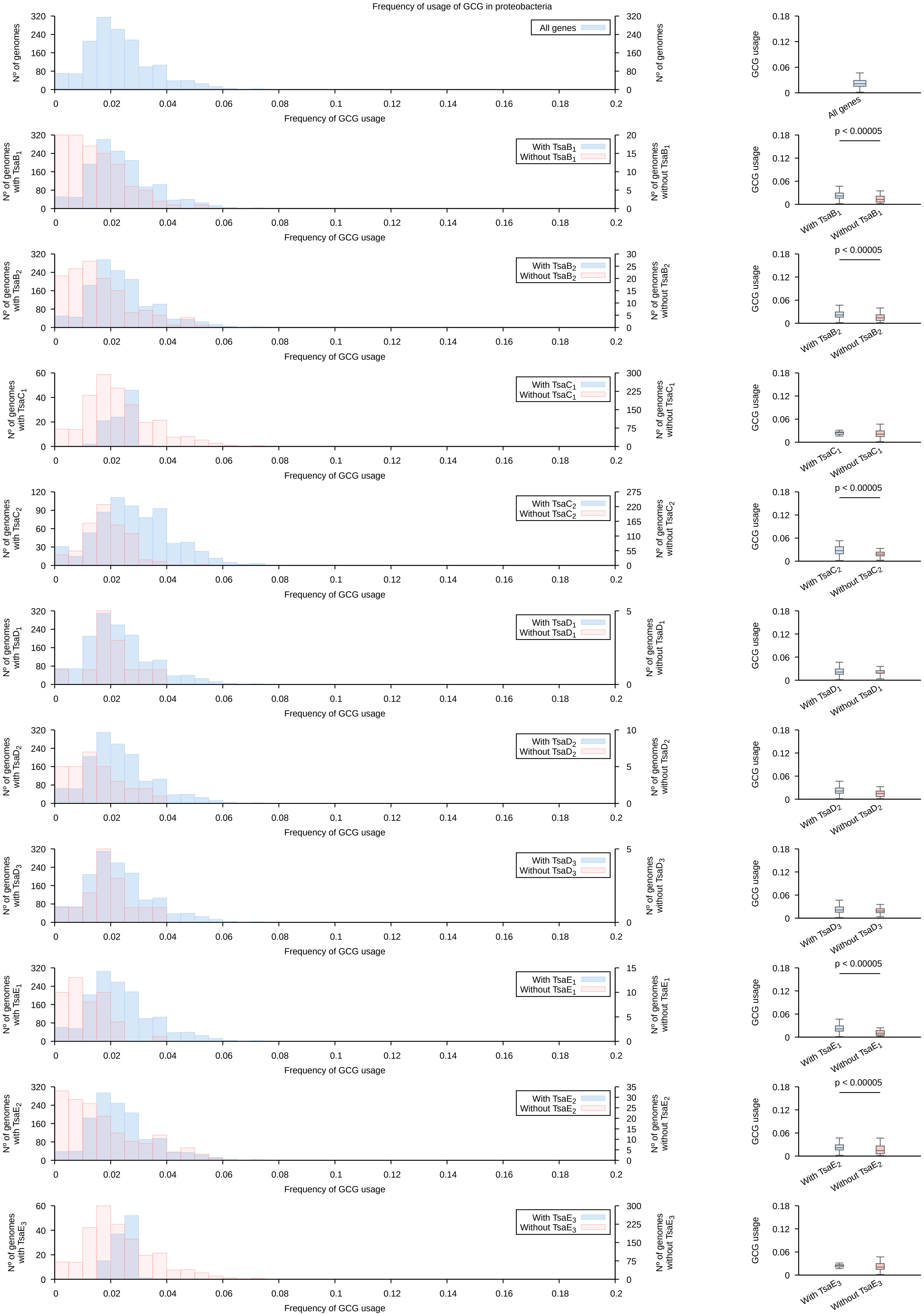
Frequency of usage of GCG in proteobacteria
Nº of genomes
All genes
Frequency of GCG usage
GCG usage
Nº of genomes with TsaB1
Nº of genomes without TsaB1
With TsaB1
Without TsaB1
p < 0.00005
Nº of genomes with TsaB2
Nº of genomes without TsaB2
With TsaB2
Without TsaB2
p < 0.00005
Nº of genomes with TsaC1
Nº of genomes without TsaC1
With TsaC1
Without TsaC1
Nº of genomes with TsaC2
Nº of genomes without TsaC2
With TsaC2
Without TsaC2
p < 0.00005
Nº of genomes with TsaD1
Nº of genomes without TsaD1
With TsaD1
Without TsaD1
Nº of genomes with TsaD2
Nº of genomes without TsaD2
With TsaD2
Without TsaD2
Nº of genomes with TsaD3
Nº of genomes without TsaD3
With TsaD3
Without TsaD3
Nº of genomes with TsaE1
Nº of genomes without TsaE1
With TsaE1
Without TsaE1
p < 0.00005
Nº of genomes with TsaE2
Nº of genomes without TsaE2
With TsaE2
Without TsaE2
p < 0.00005
Nº of genomes with TsaE3
Nº of genomes without TsaE3
With TsaE3
Without TsaE3

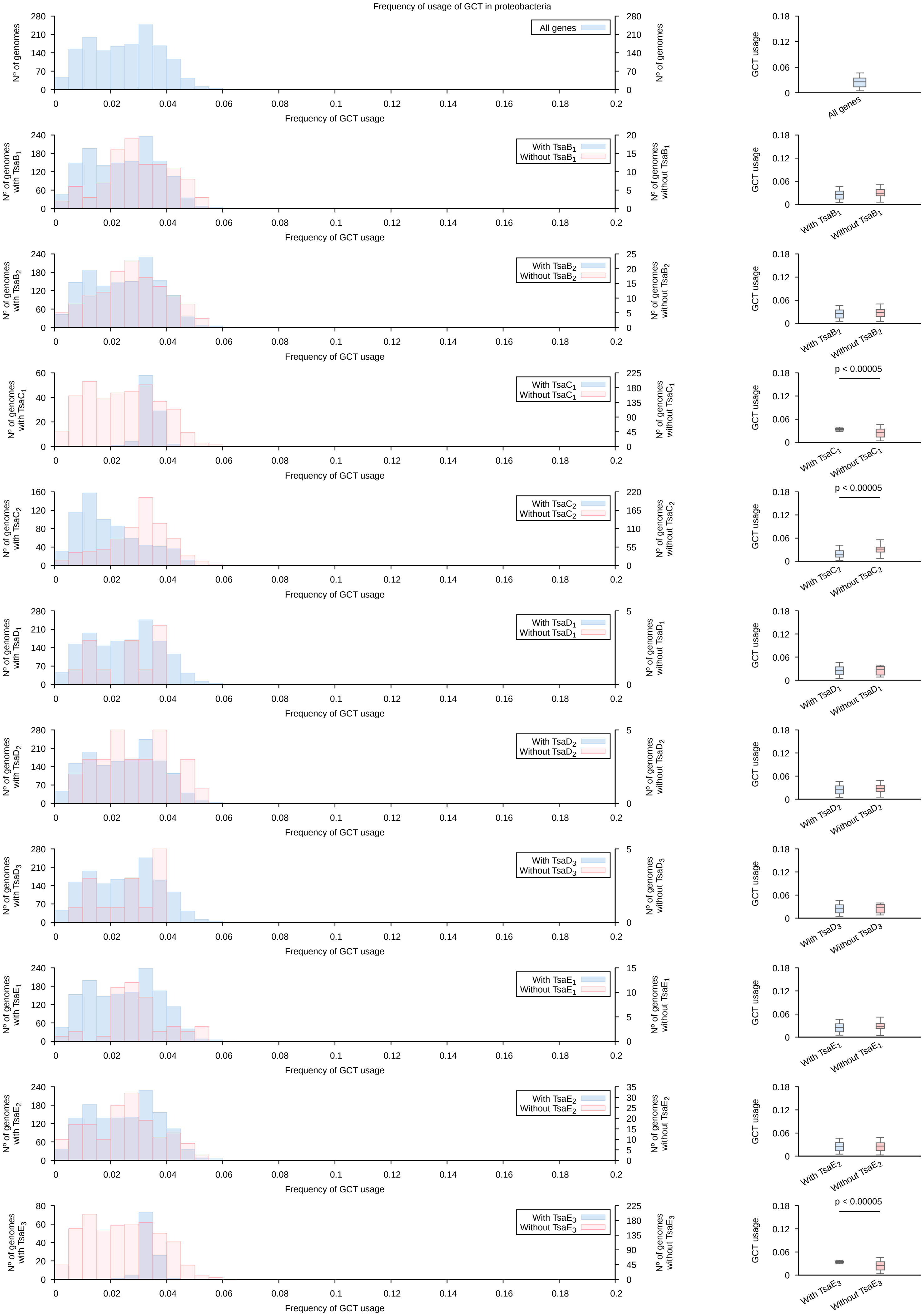

Frequency of usage of GCT in proteobacteria
Nº of genomes
All genes
GCT usage
Frequency of GCT usage
Nº of genomes with $TsaB_1$
Nº of genomes without $TsaB_1$
With $TsaB_1$
Without $TsaB_1$
Nº of genomes with $TsaB_2$
Nº of genomes without $TsaB_2$
With $TsaB_2$
Without $TsaB_2$
Nº of genomes with $TsaC_1$
Nº of genomes without $TsaC_1$
With $TsaC_1$
Without $TsaC_1$
$p < 0.00005$
Nº of genomes with $TsaC_2$
Nº of genomes without $TsaC_2$
With $TsaC_2$
Without $TsaC_2$
$p < 0.00005$
Nº of genomes with $TsaD_1$
Nº of genomes without $TsaD_1$
With $TsaD_1$
Without $TsaD_1$
Nº of genomes with $TsaD_2$
Nº of genomes without $TsaD_2$
With $TsaD_2$
Without $TsaD_2$
Nº of genomes with $TsaD_3$
Nº of genomes without $TsaD_3$
With $TsaD_3$
Without $TsaD_3$
Nº of genomes with $TsaE_1$
Nº of genomes without $TsaE_1$
With $TsaE_1$
Without $TsaE_1$
Nº of genomes with $TsaE_2$
Nº of genomes without $TsaE_2$
With $TsaE_2$
Without $TsaE_2$
Nº of genomes with $TsaE_3$
Nº of genomes without $TsaE_3$
With $TsaE_3$
Without $TsaE_3$
$p < 0.00005$

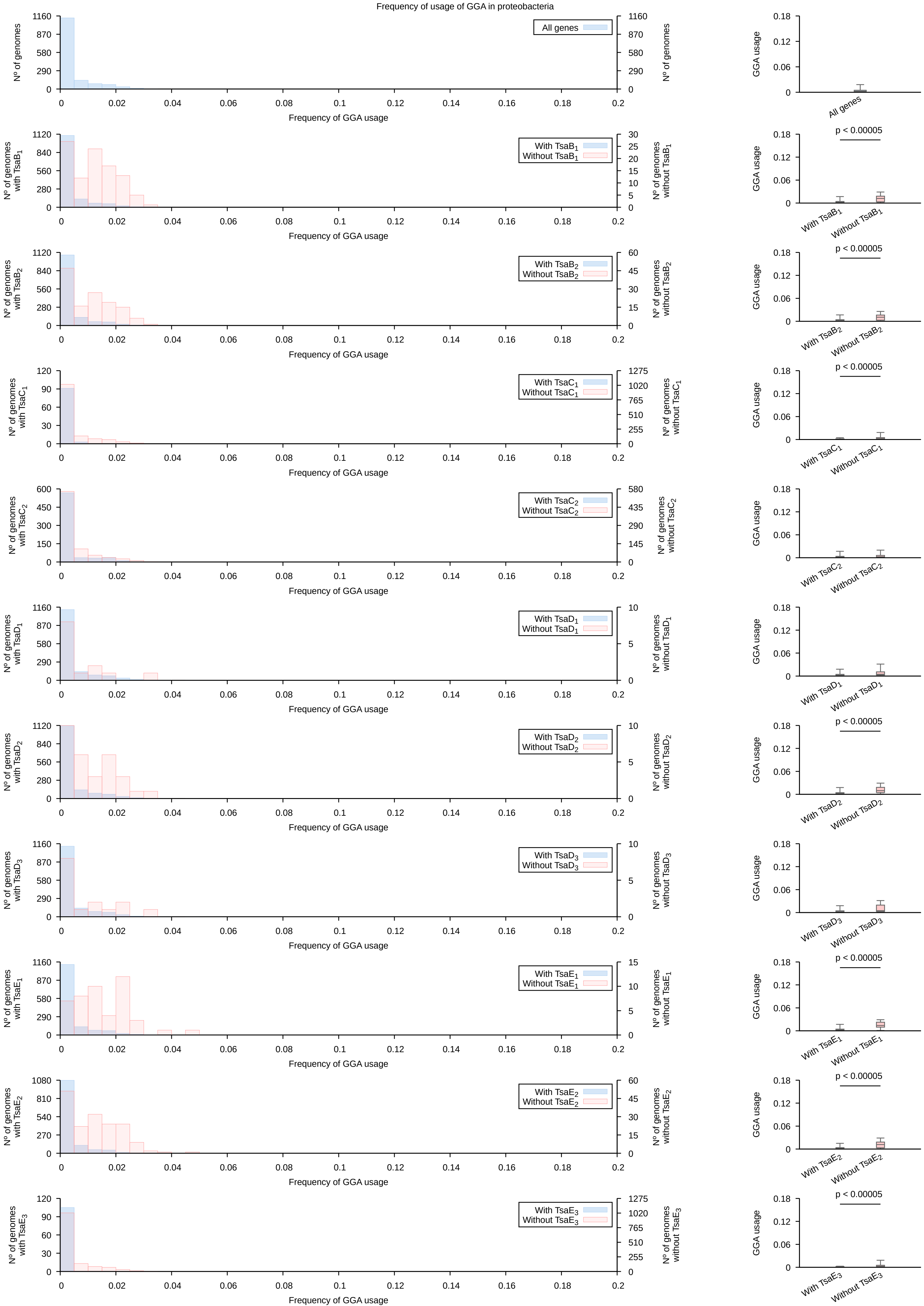

Frequency of usage of GGA in proteobacteria
All genes
Nº of genomes
Frequency of GGA usage
GGA usage
With TsaB1
Without TsaB1
Nº of genomes with TsaB1
Nº of genomes without TsaB1
p < 0.00005
With TsaB2
Without TsaB2
Nº of genomes with TsaB2
Nº of genomes without TsaB2
With TsaC1
Without TsaC1
Nº of genomes with TsaC1
Nº of genomes without TsaC1
With TsaC2
Without TsaC2
Nº of genomes with TsaC2
Nº of genomes without TsaC2
With TsaD1
Without TsaD1
Nº of genomes with TsaD1
Nº of genomes without TsaD1
With TsaD2
Without TsaD2
Nº of genomes with TsaD2
Nº of genomes without TsaD2
With TsaD3
Without TsaD3
Nº of genomes with TsaD3
Nº of genomes without TsaD3
With TsaE1
Without TsaE1
Nº of genomes with TsaE1
Nº of genomes without TsaE1
With TsaE2
Without TsaE2
Nº of genomes with TsaE2
Nº of genomes without TsaE2
With TsaE3
Without TsaE3
Nº of genomes with TsaE3
Nº of genomes without TsaE3

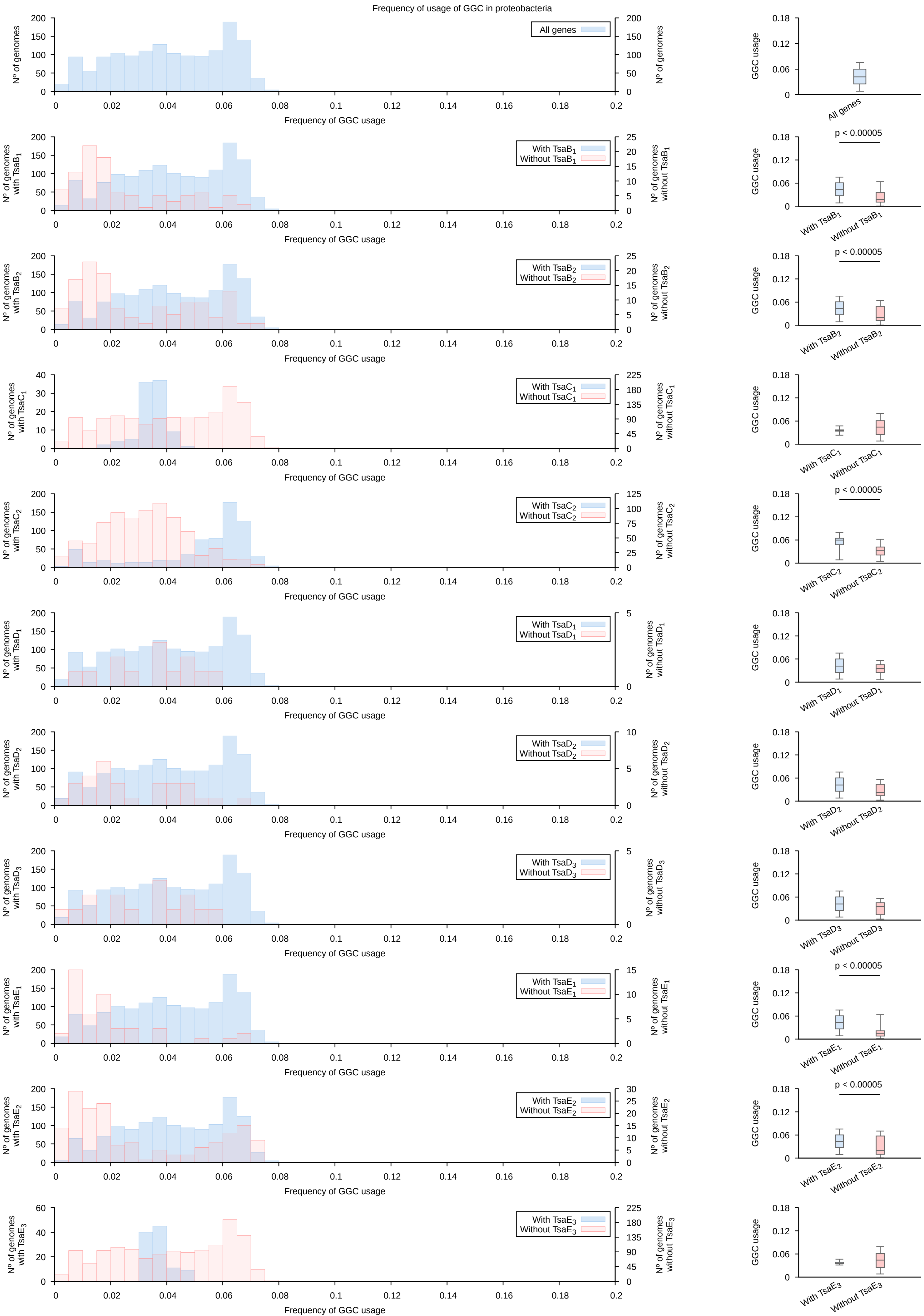
Frequency of usage of GGC in proteobacteria
Nº of genomes
All genes
GGC usage
Frequency of GGC usage
Nº of genomes with TsaB$_1$
Nº of genomes without TsaB$_1$
With TsaB$_1$
Without TsaB$_1$
p < 0.00005
Nº of genomes with TsaB$_2$
Nº of genomes without TsaB$_2$
With TsaB$_2$
Without TsaB$_2$
p < 0.00005
Nº of genomes with TsaC$_1$
Nº of genomes without TsaC$_1$
With TsaC$_1$
Without TsaC$_1$
Nº of genomes with TsaC$_2$
Nº of genomes without TsaC$_2$
With TsaC$_2$
Without TsaC$_2$
p < 0.00005
Nº of genomes with TsaD$_1$
Nº of genomes without TsaD$_1$
With TsaD$_1$
Without TsaD$_1$
Nº of genomes with TsaD$_2$
Nº of genomes without TsaD$_2$
With TsaD$_2$
Without TsaD$_2$
Nº of genomes with TsaD$_3$
Nº of genomes without TsaD$_3$
With TsaD$_3$
Without TsaD$_3$
Nº of genomes with TsaE$_1$
Nº of genomes without TsaE$_1$
With TsaE$_1$
Without TsaE$_1$
p < 0.00005
Nº of genomes with TsaE$_2$
Nº of genomes without TsaE$_2$
With TsaE$_2$
Without TsaE$_2$
p < 0.00005
Nº of genomes with TsaE$_3$
Nº of genomes without TsaE$_3$
With TsaE$_3$
Without TsaE$_3$

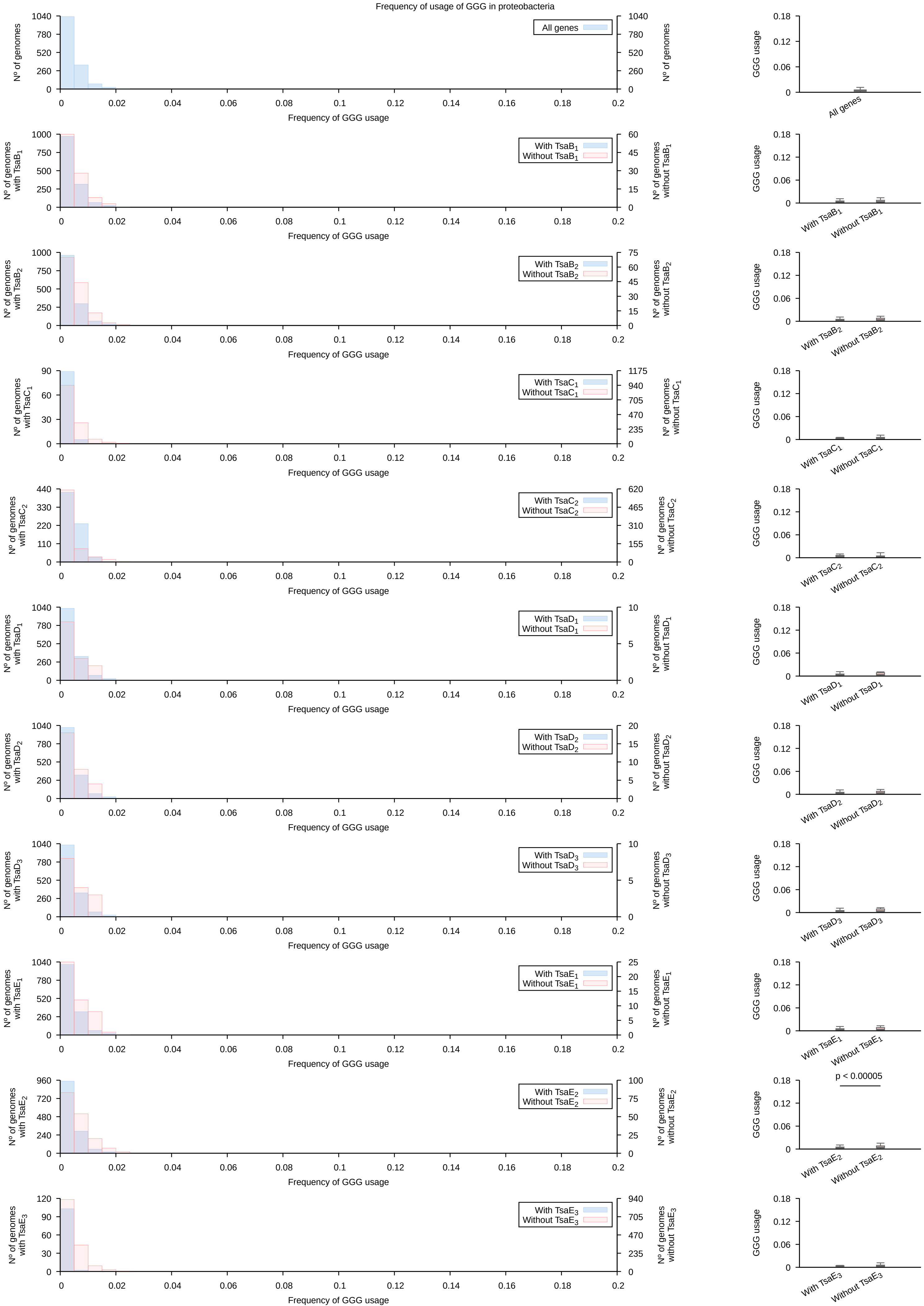
Frequency of usage of GGG in proteobacteria
Nº of genomes
Frequency of GGG usage
GGG usage
All genes
With TsaB1
Without TsaB1
With TsaB2
Without TsaB2
With TsaC1
Without TsaC1
With TsaC2
Without TsaC2
With TsaD1
Without TsaD1
With TsaD2
Without TsaD2
With TsaD3
Without TsaD3
With TsaE1
Without TsaE1
With TsaE2
Without TsaE2
p < 0.00005
With TsaE3
Without TsaE3

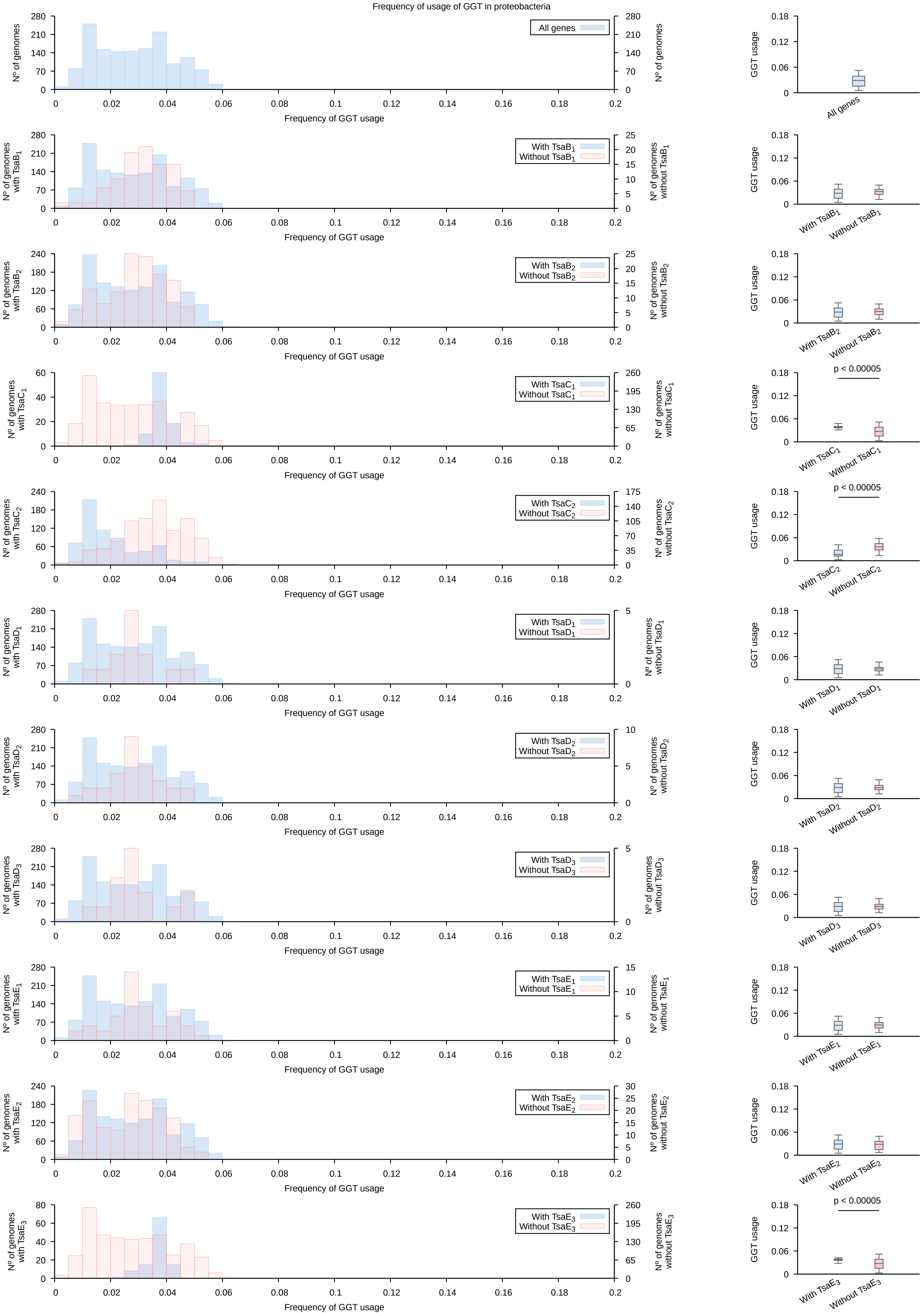
Frequency of usage of GGT in proteobacteria
Nº of genomes
Frequency of GGT usage
All genes
GGT usage
With TsaB1
Without TsaB1
Nº of genomes with TsaB1
Nº of genomes without TsaB1
With TsaB2
Without TsaB2
Nº of genomes with TsaB2
Nº of genomes without TsaB2
With TsaC1
Without TsaC1
Nº of genomes with TsaC1
Nº of genomes without TsaC1
p < 0.00005
With TsaC2
Without TsaC2
Nº of genomes with TsaC2
Nº of genomes without TsaC2
p < 0.00005
With TsaD1
Without TsaD1
Nº of genomes with TsaD1
Nº of genomes without TsaD1
With TsaD2
Without TsaD2
Nº of genomes with TsaD2
Nº of genomes without TsaD2
With TsaD3
Without TsaD3
Nº of genomes with TsaD3
Nº of genomes without TsaD3
With TsaE1
Without TsaE1
Nº of genomes with TsaE1
Nº of genomes without TsaE1
With TsaE2
Without TsaE2
Nº of genomes with TsaE2
Nº of genomes without TsaE2
With TsaE3
Without TsaE3
Nº of genomes with TsaE3
Nº of genomes without TsaE3
p < 0.00005

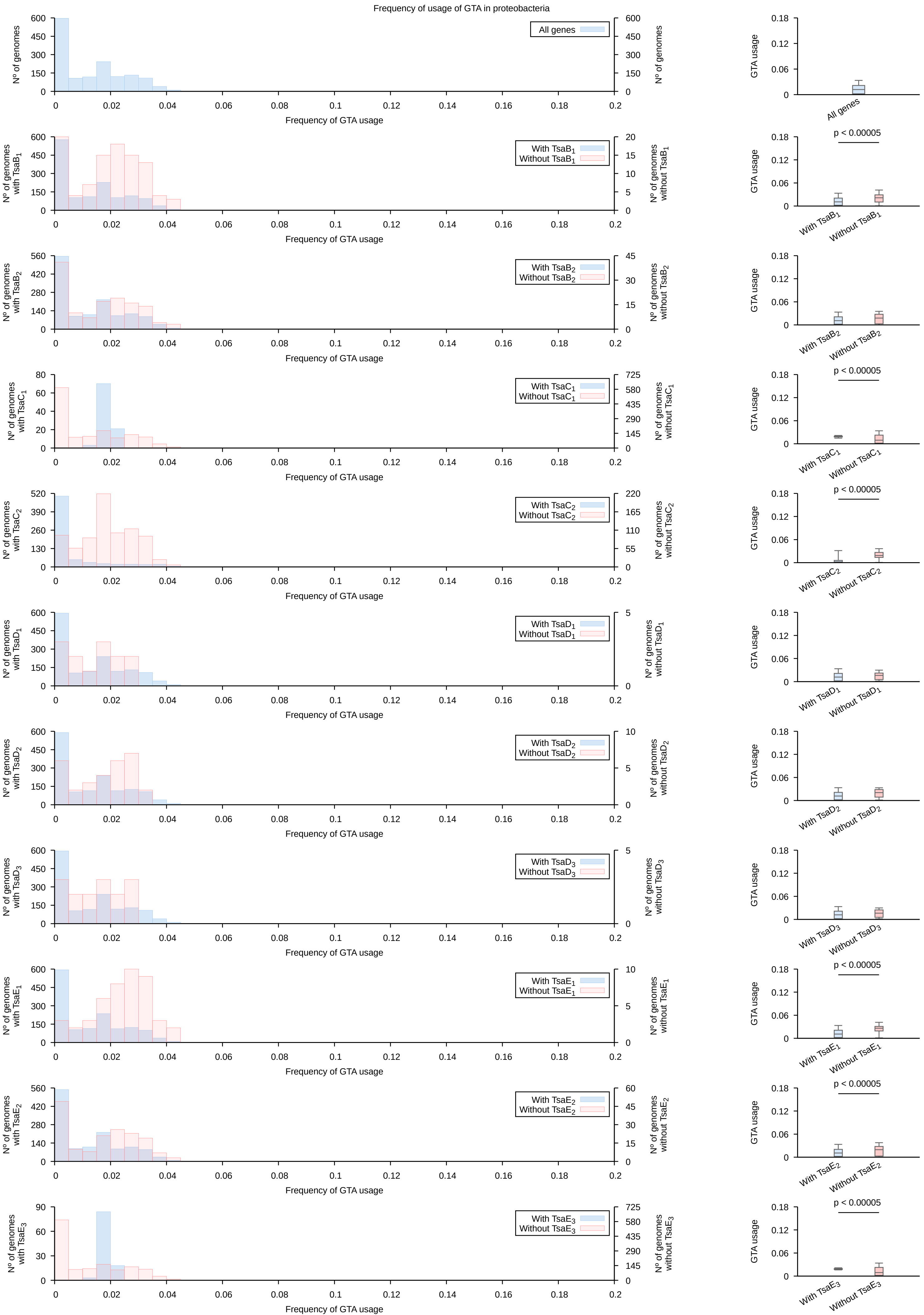

Frequency of usage of GTA in proteobacteria
All genes
Nº of genomes
Frequency of GTA usage
GTA usage
With TsaB1
Without TsaB1
Nº of genomes with TsaB1
Nº of genomes without TsaB1
p < 0.00005
With TsaB2
Without TsaB2
Nº of genomes with TsaB2
Nº of genomes without TsaB2
With TsaC1
Without TsaC1
Nº of genomes with TsaC1
Nº of genomes without TsaC1
p < 0.00005
With TsaC2
Without TsaC2
Nº of genomes with TsaC2
Nº of genomes without TsaC2
p < 0.00005
With TsaD1
Without TsaD1
Nº of genomes with TsaD1
Nº of genomes without TsaD1
With TsaD2
Without TsaD2
Nº of genomes with TsaD2
Nº of genomes without TsaD2
With TsaD3
Without TsaD3
Nº of genomes with TsaD3
Nº of genomes without TsaD3
With TsaE1
Without TsaE1
Nº of genomes with TsaE1
Nº of genomes without TsaE1
p < 0.00005
With TsaE2
Without TsaE2
Nº of genomes with TsaE2
Nº of genomes without TsaE2
p < 0.00005
With TsaE3
Without TsaE3
Nº of genomes with TsaE3
Nº of genomes without TsaE3
p < 0.00005

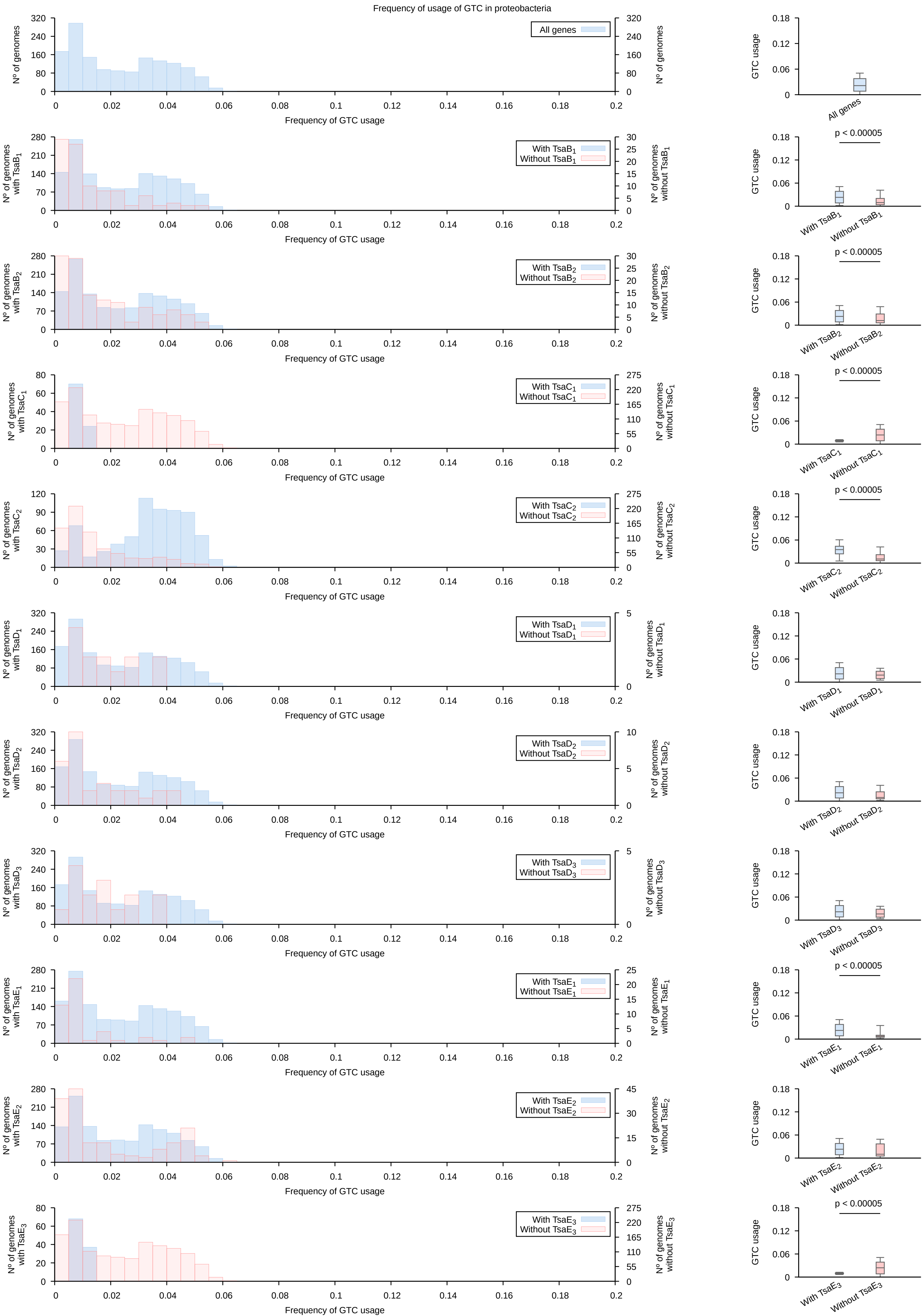
Frequency of usage of GTC in proteobacteria
All genes
Nº of genomes
Frequency of GTC usage
GTC usage
With $TsaB_1$
Without $TsaB_1$
Nº of genomes with $TsaB_1$
Nº of genomes without $TsaB_1$
p < 0.00005
With $TsaB_2$
Without $TsaB_2$
Nº of genomes with $TsaB_2$
Nº of genomes without $TsaB_2$
p < 0.00005
With $TsaC_1$
Without $TsaC_1$
Nº of genomes with $TsaC_1$
Nº of genomes without $TsaC_1$
p < 0.00005
With $TsaC_2$
Without $TsaC_2$
Nº of genomes with $TsaC_2$
Nº of genomes without $TsaC_2$
p < 0.00005
With $TsaD_1$
Without $TsaD_1$
Nº of genomes with $TsaD_1$
Nº of genomes without $TsaD_1$
With $TsaD_2$
Without $TsaD_2$
Nº of genomes with $TsaD_2$
Nº of genomes without $TsaD_2$
With $TsaD_3$
Without $TsaD_3$
Nº of genomes with $TsaD_3$
Nº of genomes without $TsaD_3$
With $TsaE_1$
Without $TsaE_1$
Nº of genomes with $TsaE_1$
Nº of genomes without $TsaE_1$
p < 0.00005
With $TsaE_2$
Without $TsaE_2$
Nº of genomes with $TsaE_2$
Nº of genomes without $TsaE_2$
With $TsaE_3$
Without $TsaE_3$
Nº of genomes with $TsaE_3$
Nº of genomes without $TsaE_3$
p < 0.00005

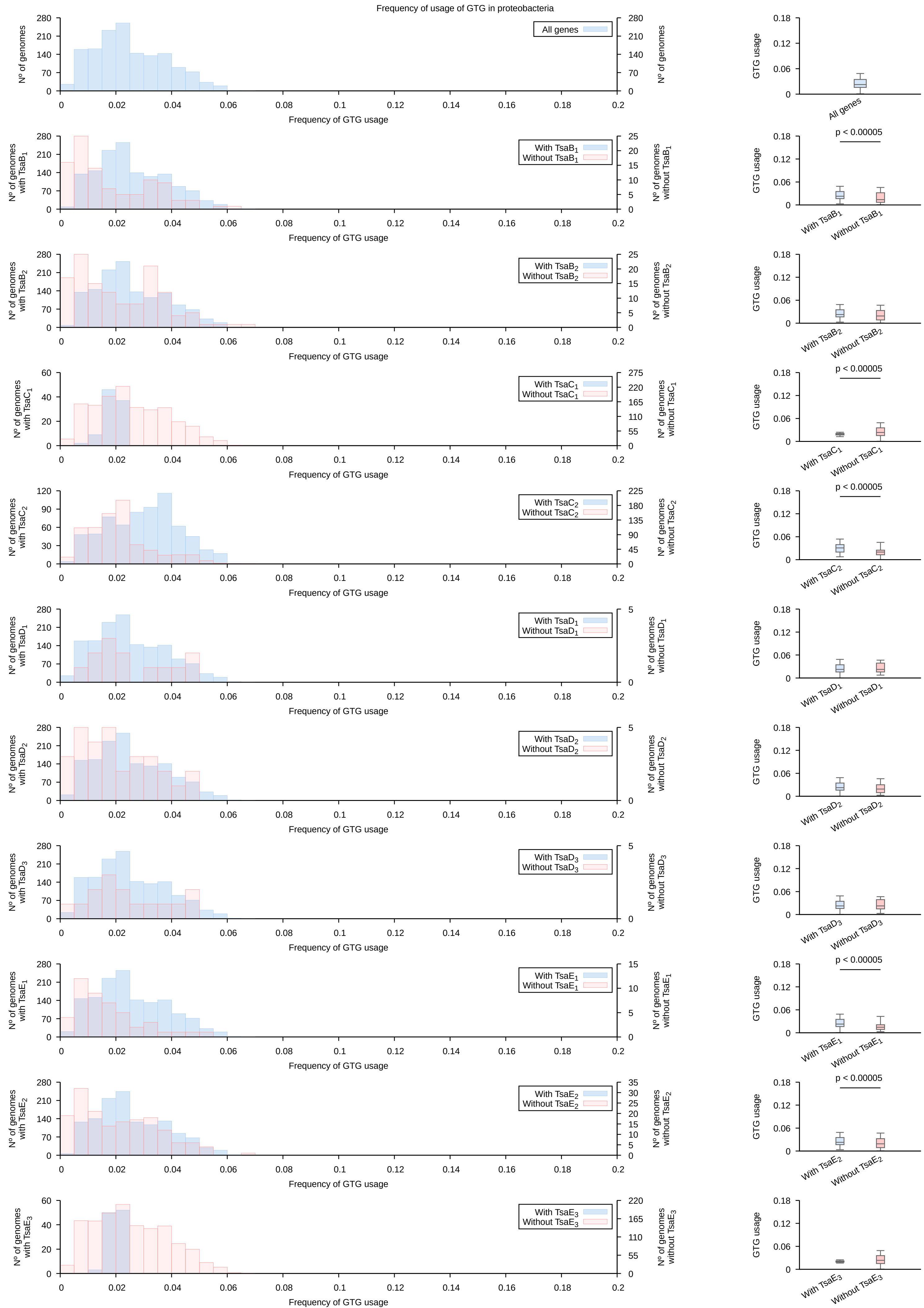

Frequency of usage of GTG in proteobacteria
Nº of genomes
All genes
GTG usage
Frequency of GTG usage
Nº of genomes with TsaB$_1$
Nº of genomes without TsaB$_1$
With TsaB$_1$
Without TsaB$_1$
p < 0.00005
Nº of genomes with TsaB$_2$
Nº of genomes without TsaB$_2$
With TsaB$_2$
Without TsaB$_2$
Nº of genomes with TsaC$_1$
Nº of genomes without TsaC$_1$
With TsaC$_1$
Without TsaC$_1$
p < 0.00005
Nº of genomes with TsaC$_2$
Nº of genomes without TsaC$_2$
With TsaC$_2$
Without TsaC$_2$
p < 0.00005
Nº of genomes with TsaD$_1$
Nº of genomes without TsaD$_1$
With TsaD$_1$
Without TsaD$_1$
Nº of genomes with TsaD$_2$
Nº of genomes without TsaD$_2$
With TsaD$_2$
Without TsaD$_2$
Nº of genomes with TsaD$_3$
Nº of genomes without TsaD$_3$
With TsaD$_3$
Without TsaD$_3$
Nº of genomes with TsaE$_1$
Nº of genomes without TsaE$_1$
With TsaE$_1$
Without TsaE$_1$
p < 0.00005
Nº of genomes with TsaE$_2$
Nº of genomes without TsaE$_2$
With TsaE$_2$
Without TsaE$_2$
p < 0.00005
Nº of genomes with TsaE$_3$
Nº of genomes without TsaE$_3$
With TsaE$_3$
Without TsaE$_3$

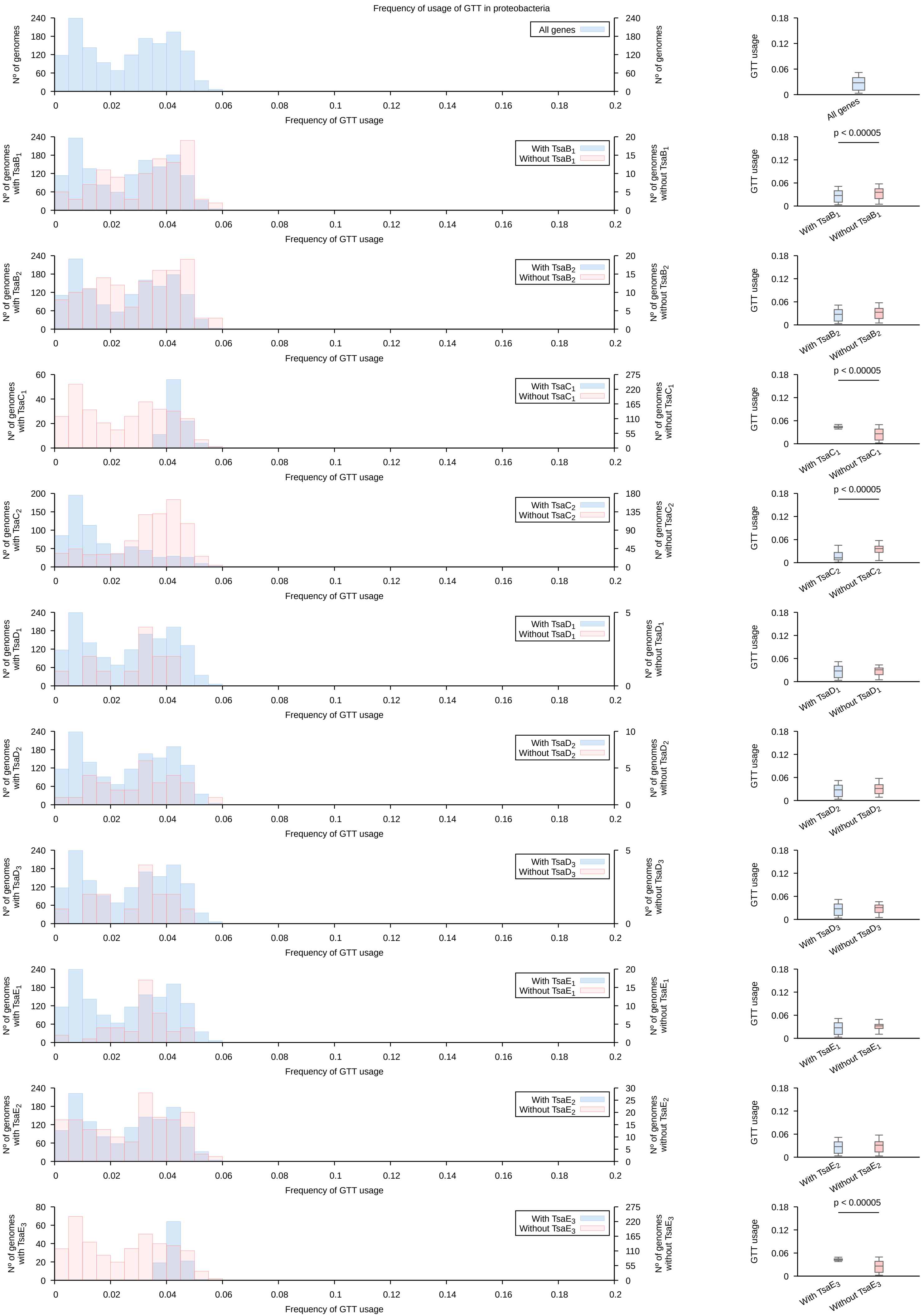
Frequency of usage of GTT in proteobacteria
Nº of genomes
All genes
GTT usage
Frequency of GTT usage
Nº of genomes with TsaB$_1$
Nº of genomes without TsaB$_1$
With TsaB$_1$
Without TsaB$_1$
p < 0.00005
Nº of genomes with TsaB$_2$
Nº of genomes without TsaB$_2$
With TsaB$_2$
Without TsaB$_2$
Nº of genomes with TsaC$_1$
Nº of genomes without TsaC$_1$
With TsaC$_1$
Without TsaC$_1$
p < 0.00005
Nº of genomes with TsaC$_2$
Nº of genomes without TsaC$_2$
With TsaC$_2$
Without TsaC$_2$
p < 0.00005
Nº of genomes with TsaD$_1$
Nº of genomes without TsaD$_1$
With TsaD$_1$
Without TsaD$_1$
Nº of genomes with TsaD$_2$
Nº of genomes without TsaD$_2$
With TsaD$_2$
Without TsaD$_2$
Nº of genomes with TsaD$_3$
Nº of genomes without TsaD$_3$
With TsaD$_3$
Without TsaD$_3$
Nº of genomes with TsaE$_1$
Nº of genomes without TsaE$_1$
With TsaE$_1$
Without TsaE$_1$
Nº of genomes with TsaE$_2$
Nº of genomes without TsaE$_2$
With TsaE$_2$
Without TsaE$_2$
Nº of genomes with TsaE$_3$
Nº of genomes without TsaE$_3$
With TsaE$_3$
Without TsaE$_3$
p < 0.00005

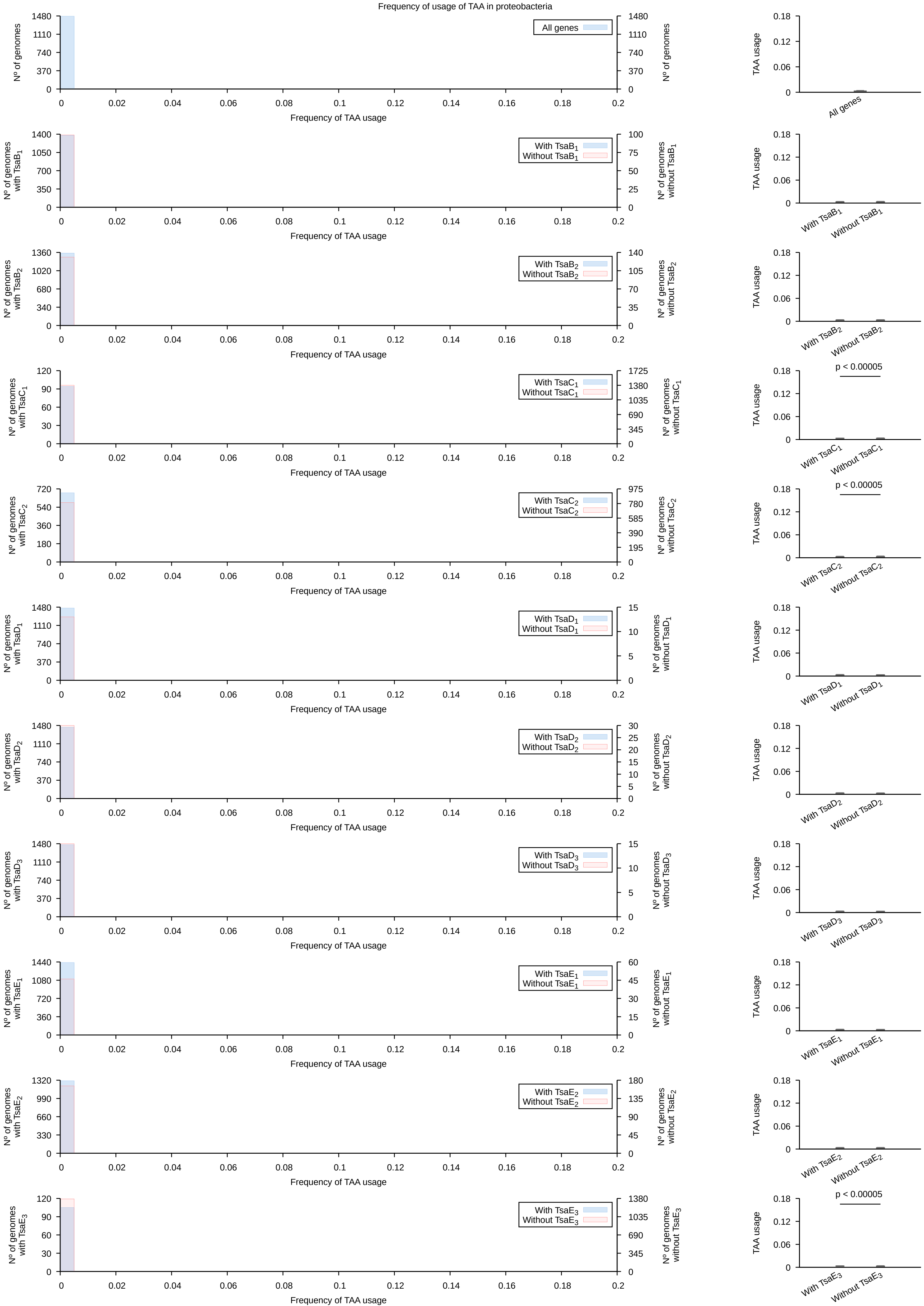
Frequency of usage of TAA in proteobacteria
Nº of genomes
Frequency of TAA usage
TAA usage
All genes
Nº of genomes with TsaB$_1$
Nº of genomes without TsaB$_1$
With TsaB$_1$
Without TsaB$_1$
Nº of genomes with TsaB$_2$
Nº of genomes without TsaB$_2$
With TsaB$_2$
Without TsaB$_2$
Nº of genomes with TsaC$_1$
Nº of genomes without TsaC$_1$
With TsaC$_1$
Without TsaC$_1$
p < 0.00005
Nº of genomes with TsaC$_2$
Nº of genomes without TsaC$_2$
With TsaC$_2$
Without TsaC$_2$
p < 0.00005
Nº of genomes with TsaD$_1$
Nº of genomes without TsaD$_1$
With TsaD$_1$
Without TsaD$_1$
Nº of genomes with TsaD$_2$
Nº of genomes without TsaD$_2$
With TsaD$_2$
Without TsaD$_2$
Nº of genomes with TsaD$_3$
Nº of genomes without TsaD$_3$
With TsaD$_3$
Without TsaD$_3$
Nº of genomes with TsaE$_1$
Nº of genomes without TsaE$_1$
With TsaE$_1$
Without TsaE$_1$
Nº of genomes with TsaE$_2$
Nº of genomes without TsaE$_2$
With TsaE$_2$
Without TsaE$_2$
Nº of genomes with TsaE$_3$
Nº of genomes without TsaE$_3$
With TsaE$_3$
Without TsaE$_3$
p < 0.00005

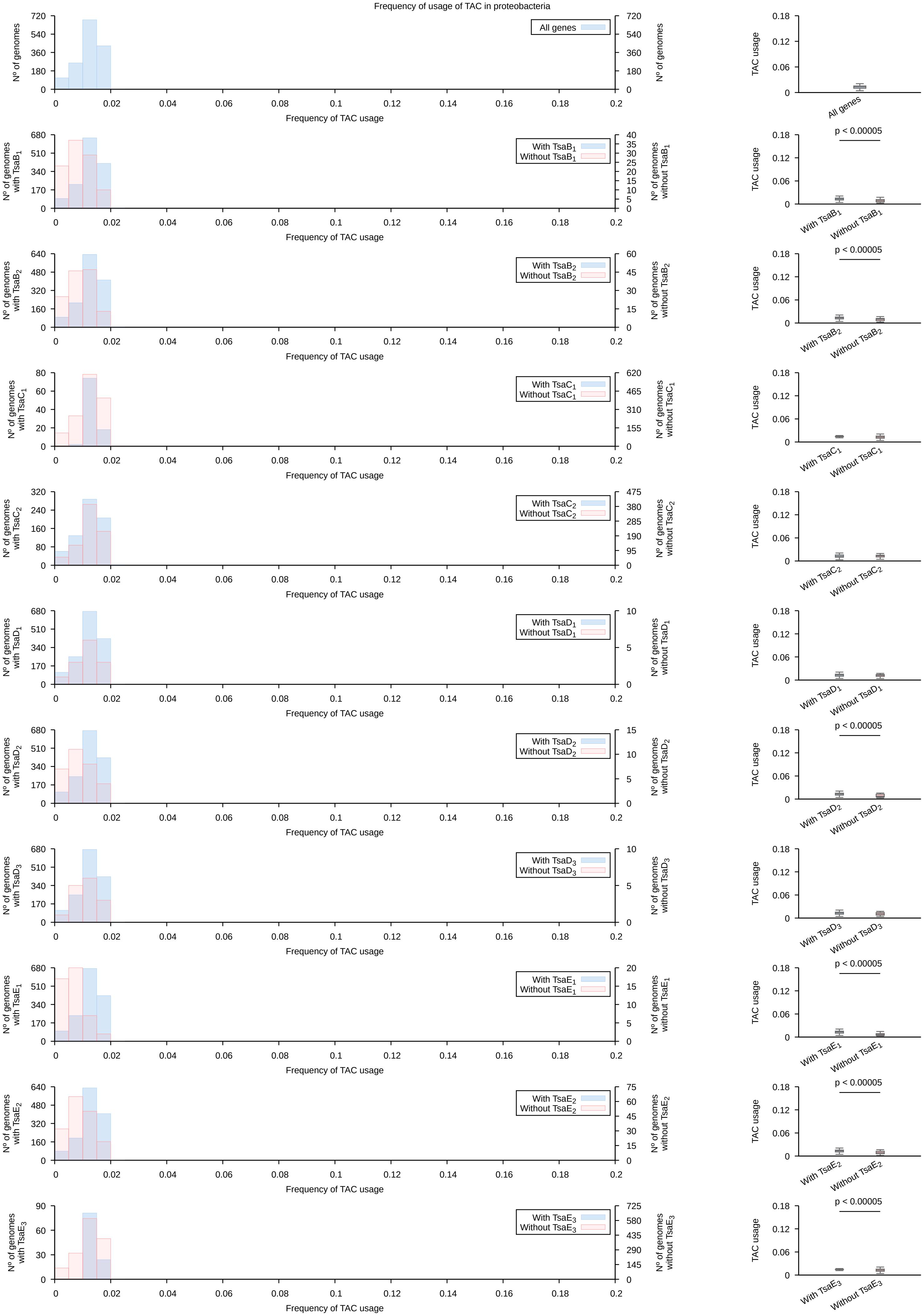
Frequency of usage of TAC in proteobacteria
All genes
With $TsaB_1$
Without $TsaB_1$
With $TsaB_2$
Without $TsaB_2$
With $TsaC_1$
Without $TsaC_1$
With $TsaC_2$
Without $TsaC_2$
With $TsaD_1$
Without $TsaD_1$
With $TsaD_2$
Without $TsaD_2$
With $TsaD_3$
Without $TsaD_3$
With $TsaE_1$
Without $TsaE_1$
With $TsaE_2$
Without $TsaE_2$
With $TsaE_3$
Without $TsaE_3$
Frequency of TAC usage
Nº of genomes
TAC usage
p < 0.00005

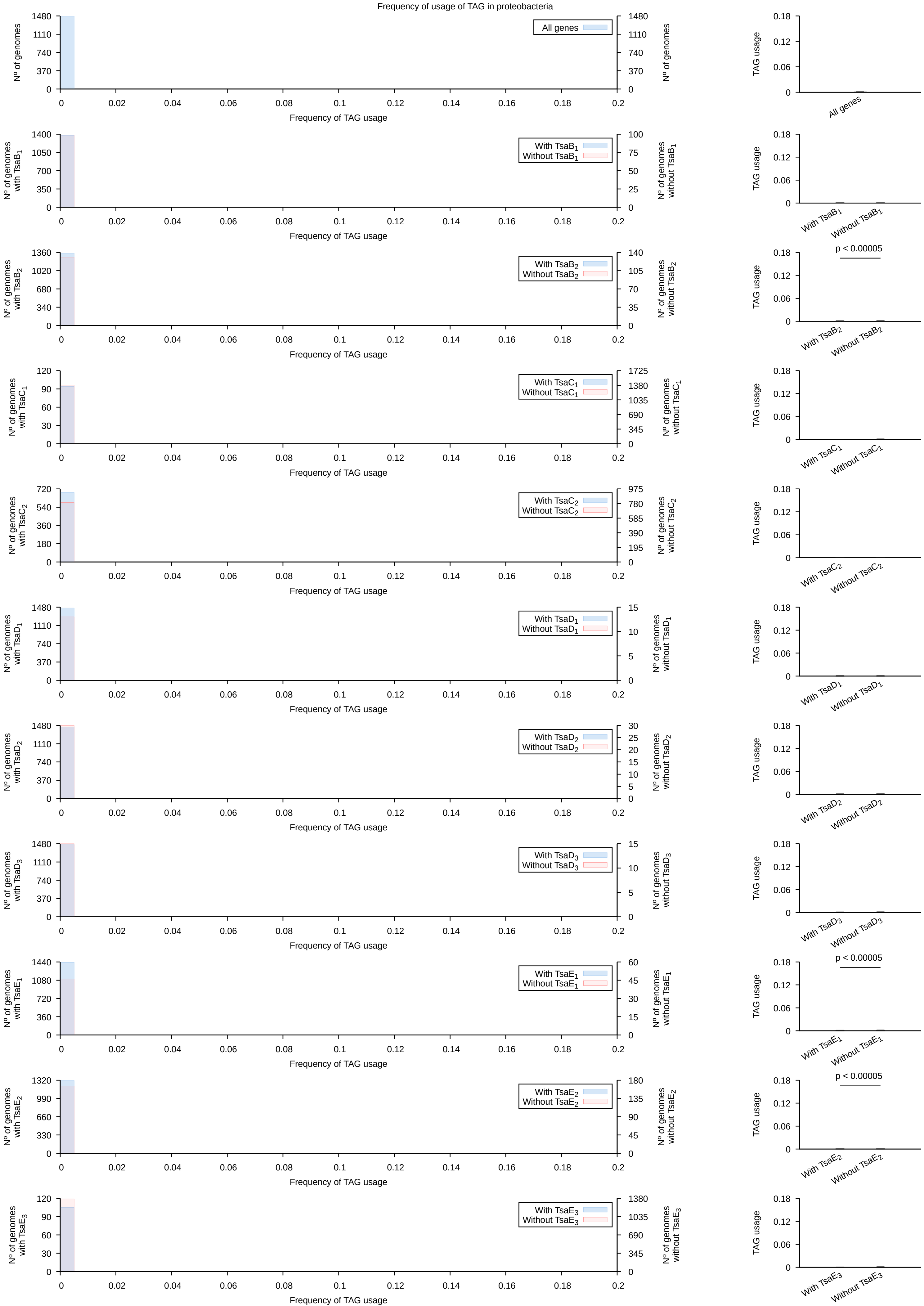

Frequency of usage of TAG in proteobacteria
Nº of genomes
All genes
Frequency of TAG usage
TAG usage
Nº of genomes with TsaB$_1$
Nº of genomes without TsaB$_1$
With TsaB$_1$
Without TsaB$_1$
Nº of genomes with TsaB$_2$
Nº of genomes without TsaB$_2$
With TsaB$_2$
Without TsaB$_2$
p < 0.00005
Nº of genomes with TsaC$_1$
Nº of genomes without TsaC$_1$
With TsaC$_1$
Without TsaC$_1$
Nº of genomes with TsaC$_2$
Nº of genomes without TsaC$_2$
With TsaC$_2$
Without TsaC$_2$
Nº of genomes with TsaD$_1$
Nº of genomes without TsaD$_1$
With TsaD$_1$
Without TsaD$_1$
Nº of genomes with TsaD$_2$
Nº of genomes without TsaD$_2$
With TsaD$_2$
Without TsaD$_2$
Nº of genomes with TsaD$_3$
Nº of genomes without TsaD$_3$
With TsaD$_3$
Without TsaD$_3$
Nº of genomes with TsaE$_1$
Nº of genomes without TsaE$_1$
With TsaE$_1$
Without TsaE$_1$
p < 0.00005
Nº of genomes with TsaE$_2$
Nº of genomes without TsaE$_2$
With TsaE$_2$
Without TsaE$_2$
p < 0.00005
Nº of genomes with TsaE$_3$
Nº of genomes without TsaE$_3$
With TsaE$_3$
Without TsaE$_3$

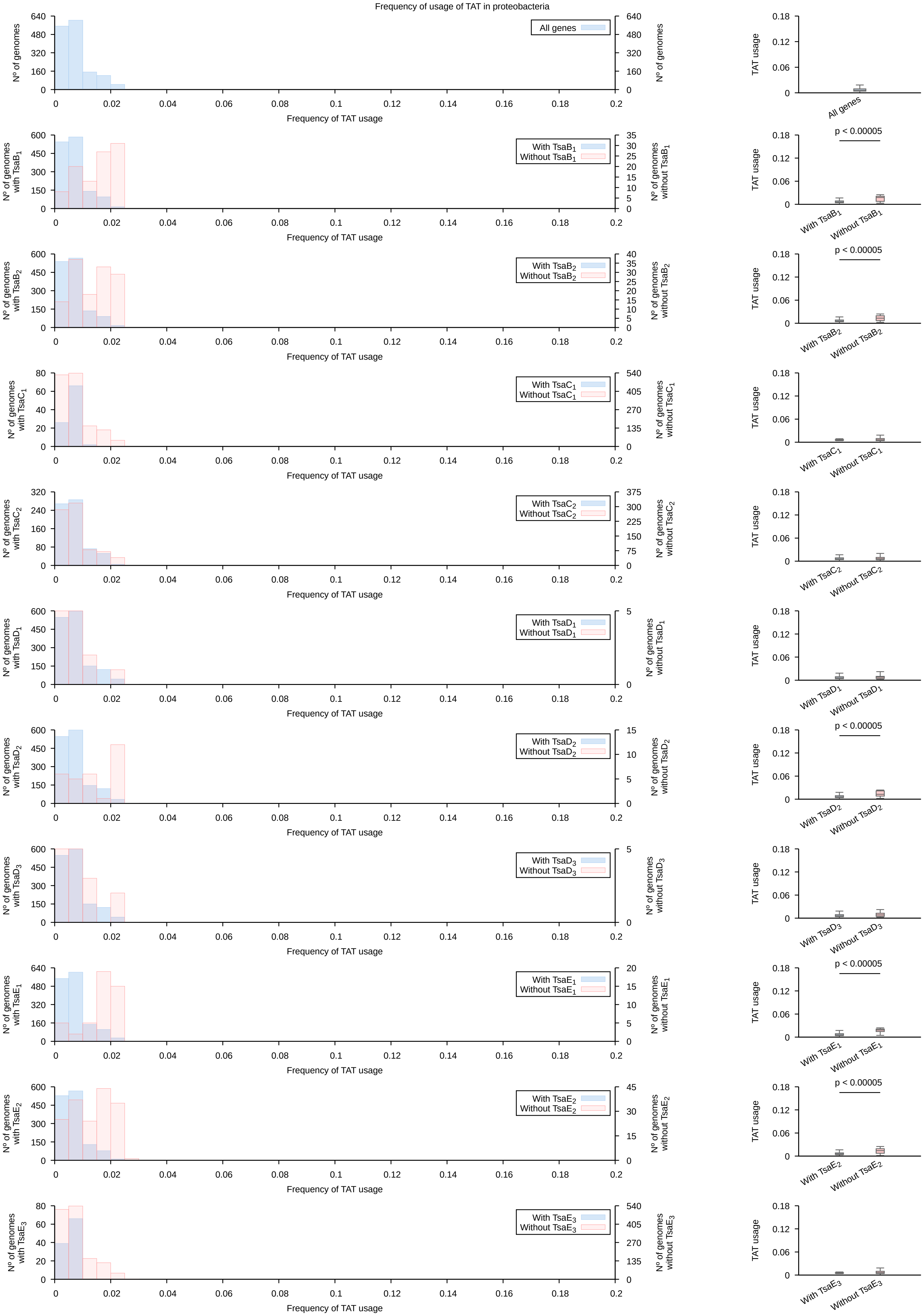

Frequency of usage of TAT in proteobacteria
Nº of genomes
All genes
Frequency of TAT usage
TAT usage
Nº of genomes with TsaB1
Nº of genomes without TsaB1
With TsaB1
Without TsaB1
p < 0.00005
Nº of genomes with TsaB2
Nº of genomes without TsaB2
With TsaB2
Without TsaB2
p < 0.00005
Nº of genomes with TsaC1
Nº of genomes without TsaC1
With TsaC1
Without TsaC1
Nº of genomes with TsaC2
Nº of genomes without TsaC2
With TsaC2
Without TsaC2
Nº of genomes with TsaD1
Nº of genomes without TsaD1
With TsaD1
Without TsaD1
Nº of genomes with TsaD2
Nº of genomes without TsaD2
With TsaD2
Without TsaD2
p < 0.00005
Nº of genomes with TsaD3
Nº of genomes without TsaD3
With TsaD3
Without TsaD3
Nº of genomes with TsaE1
Nº of genomes without TsaE1
With TsaE1
Without TsaE1
p < 0.00005
Nº of genomes with TsaE2
Nº of genomes without TsaE2
With TsaE2
Without TsaE2
p < 0.00005
Nº of genomes with TsaE3
Nº of genomes without TsaE3
With TsaE3
Without TsaE3

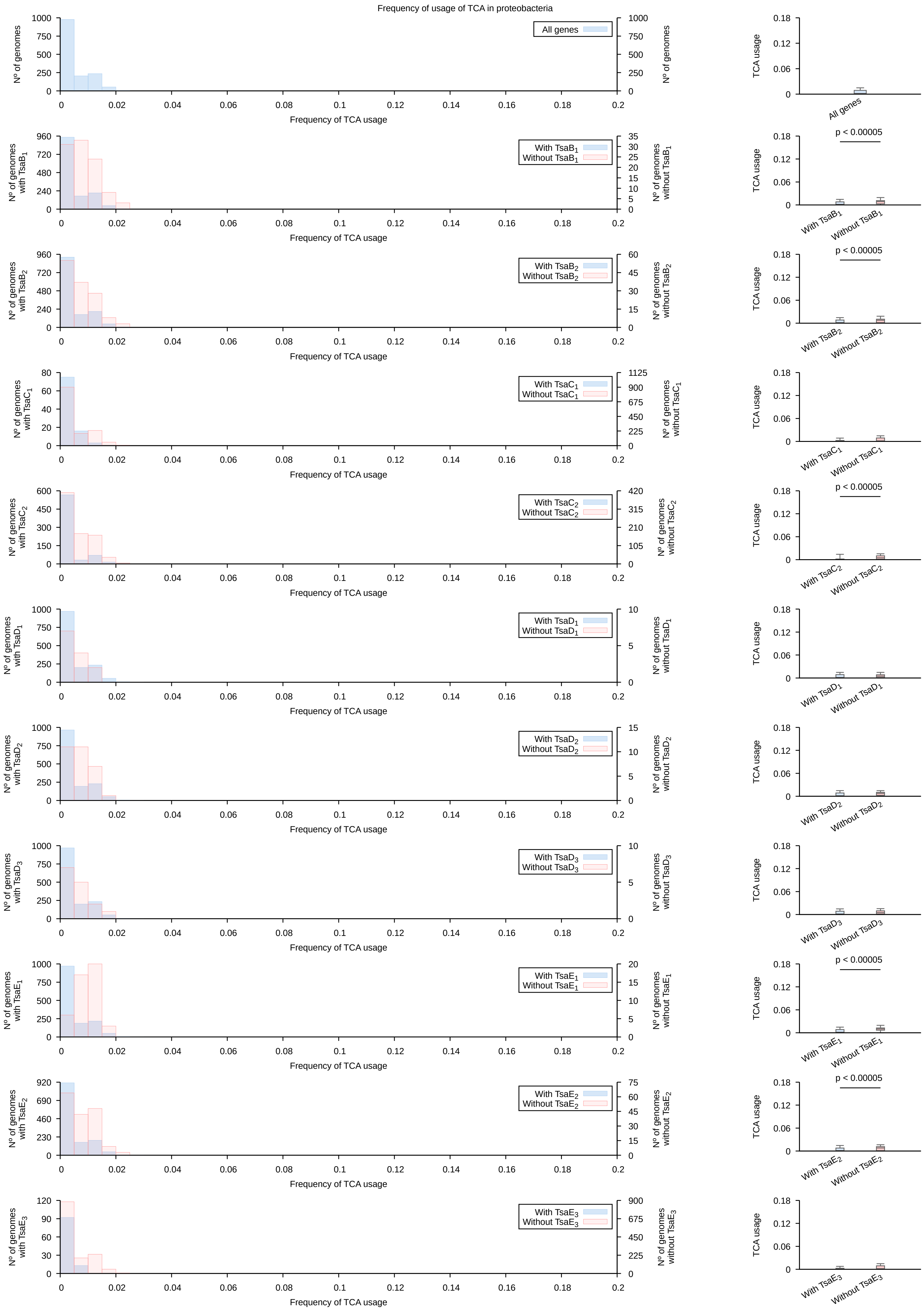

Frequency of usage of TCA in proteobacteria
All genes
Nº of genomes
Frequency of TCA usage
TCA usage
With TsaB1
Without TsaB1
Nº of genomes with TsaB1
Nº of genomes without TsaB1
With TsaB2
Without TsaB2
Nº of genomes with TsaB2
Nº of genomes without TsaB2
With TsaC1
Without TsaC1
Nº of genomes with TsaC1
Nº of genomes without TsaC1
With TsaC2
Without TsaC2
Nº of genomes with TsaC2
Nº of genomes without TsaC2
With TsaD1
Without TsaD1
Nº of genomes with TsaD1
Nº of genomes without TsaD1
With TsaD2
Without TsaD2
Nº of genomes with TsaD2
Nº of genomes without TsaD2
With TsaD3
Without TsaD3
Nº of genomes with TsaD3
Nº of genomes without TsaD3
With TsaE1
Without TsaE1
Nº of genomes with TsaE1
Nº of genomes without TsaE1
With TsaE2
Without TsaE2
Nº of genomes with TsaE2
Nº of genomes without TsaE2
With TsaE3
Without TsaE3
Nº of genomes with TsaE3
Nº of genomes without TsaE3
p < 0.00005

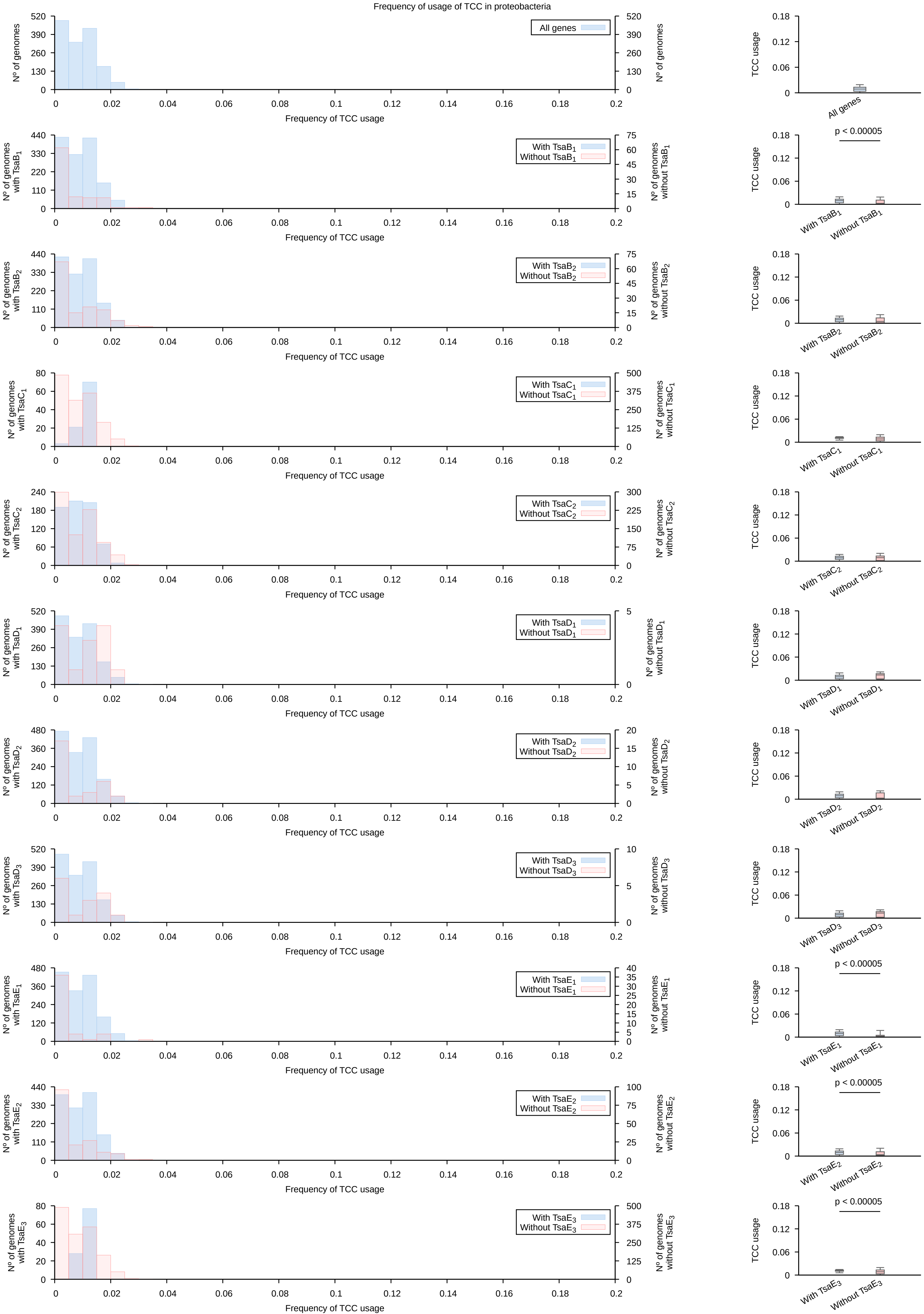
Frequency of usage of TCC in proteobacteria
Nº of genomes
All genes
Frequency of TCC usage
TCC usage
Nº of genomes with TsaB1
Nº of genomes without TsaB1
With TsaB1
Without TsaB1
p < 0.00005
Nº of genomes with TsaB2
Nº of genomes without TsaB2
With TsaB2
Without TsaB2
Nº of genomes with TsaC1
Nº of genomes without TsaC1
With TsaC1
Without TsaC1
Nº of genomes with TsaC2
Nº of genomes without TsaC2
With TsaC2
Without TsaC2
Nº of genomes with TsaD1
Nº of genomes without TsaD1
With TsaD1
Without TsaD1
Nº of genomes with TsaD2
Nº of genomes without TsaD2
With TsaD2
Without TsaD2
Nº of genomes with TsaD3
Nº of genomes without TsaD3
With TsaD3
Without TsaD3
Nº of genomes with TsaE1
Nº of genomes without TsaE1
With TsaE1
Without TsaE1
p < 0.00005
Nº of genomes with TsaE2
Nº of genomes without TsaE2
With TsaE2
Without TsaE2
p < 0.00005
Nº of genomes with TsaE3
Nº of genomes without TsaE3
With TsaE3
Without TsaE3
p < 0.00005

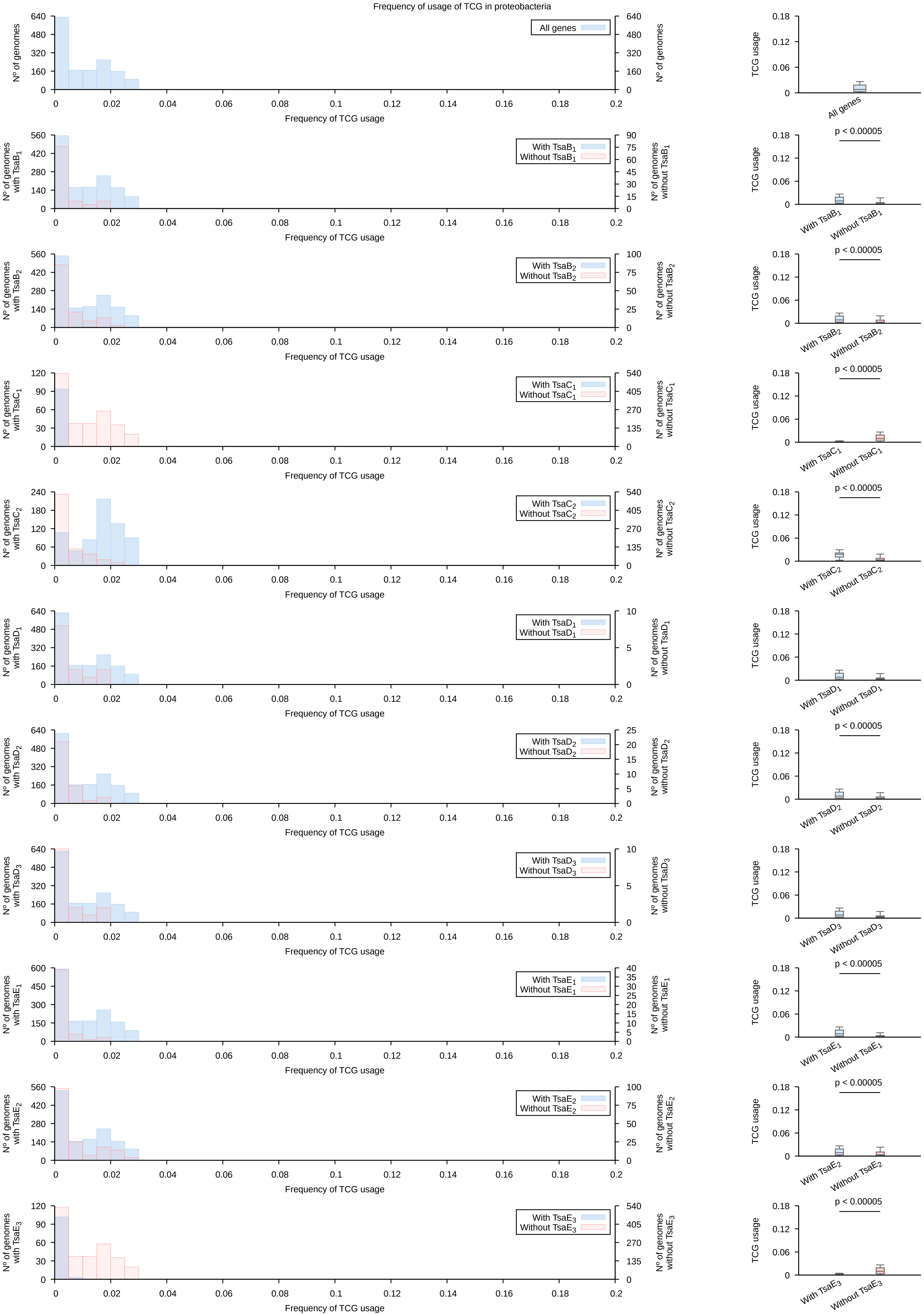

Frequency of usage of TCG in proteobacteria
All genes
Nº of genomes
Frequency of TCG usage
TCG usage
With TsaB1
Without TsaB1
Nº of genomes with TsaB1
Nº of genomes without TsaB1
p < 0.00005
With TsaB2
Without TsaB2
Nº of genomes with TsaB2
Nº of genomes without TsaB2
p < 0.00005
With TsaC1
Without TsaC1
Nº of genomes with TsaC1
Nº of genomes without TsaC1
p < 0.00005
With TsaC2
Without TsaC2
Nº of genomes with TsaC2
Nº of genomes without TsaC2
p < 0.00005
With TsaD1
Without TsaD1
Nº of genomes with TsaD1
Nº of genomes without TsaD1
With TsaD2
Without TsaD2
Nº of genomes with TsaD2
Nº of genomes without TsaD2
p < 0.00005
With TsaD3
Without TsaD3
Nº of genomes with TsaD3
Nº of genomes without TsaD3
With TsaE1
Without TsaE1
Nº of genomes with TsaE1
Nº of genomes without TsaE1
p < 0.00005
With TsaE2
Without TsaE2
Nº of genomes with TsaE2
Nº of genomes without TsaE2
p < 0.00005
With TsaE3
Without TsaE3
Nº of genomes with TsaE3
Nº of genomes without TsaE3
p < 0.00005

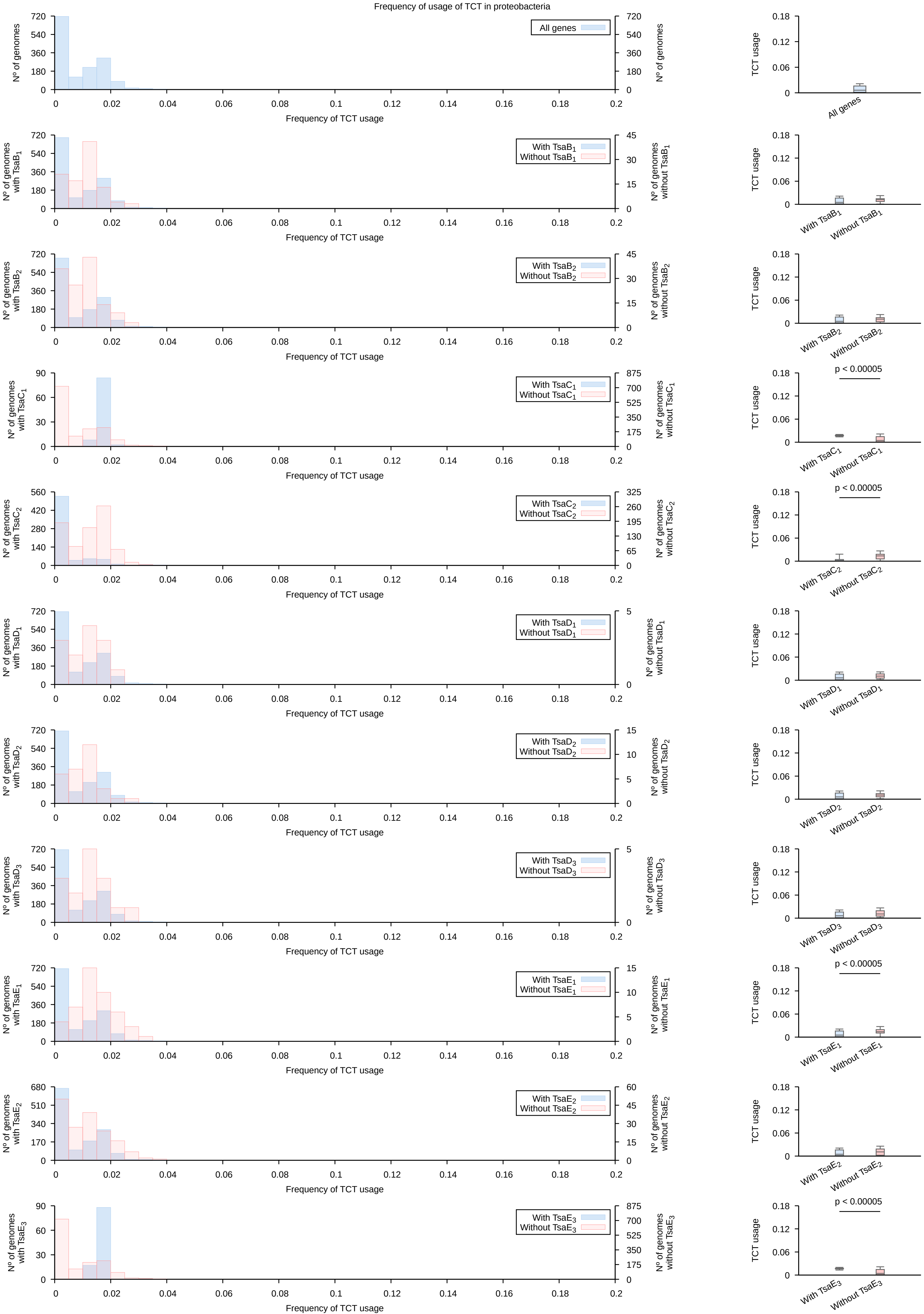

Frequency of usage of TCT in proteobacteria
Nº of genomes
All genes
Frequency of TCT usage
TCT usage
Nº of genomes with TsaB$_1$
Nº of genomes without TsaB$_1$
With TsaB$_1$
Without TsaB$_1$
Nº of genomes with TsaB$_2$
Nº of genomes without TsaB$_2$
With TsaB$_2$
Without TsaB$_2$
Nº of genomes with TsaC$_1$
Nº of genomes without TsaC$_1$
With TsaC$_1$
Without TsaC$_1$
p < 0.00005
Nº of genomes with TsaC$_2$
Nº of genomes without TsaC$_2$
With TsaC$_2$
Without TsaC$_2$
p < 0.00005
Nº of genomes with TsaD$_1$
Nº of genomes without TsaD$_1$
With TsaD$_1$
Without TsaD$_1$
Nº of genomes with TsaD$_2$
Nº of genomes without TsaD$_2$
With TsaD$_2$
Without TsaD$_2$
Nº of genomes with TsaD$_3$
Nº of genomes without TsaD$_3$
With TsaD$_3$
Without TsaD$_3$
Nº of genomes with TsaE$_1$
Nº of genomes without TsaE$_1$
With TsaE$_1$
Without TsaE$_1$
p < 0.00005
Nº of genomes with TsaE$_2$
Nº of genomes without TsaE$_2$
With TsaE$_2$
Without TsaE$_2$
Nº of genomes with TsaE$_3$
Nº of genomes without TsaE$_3$
With TsaE$_3$
Without TsaE$_3$
p < 0.00005

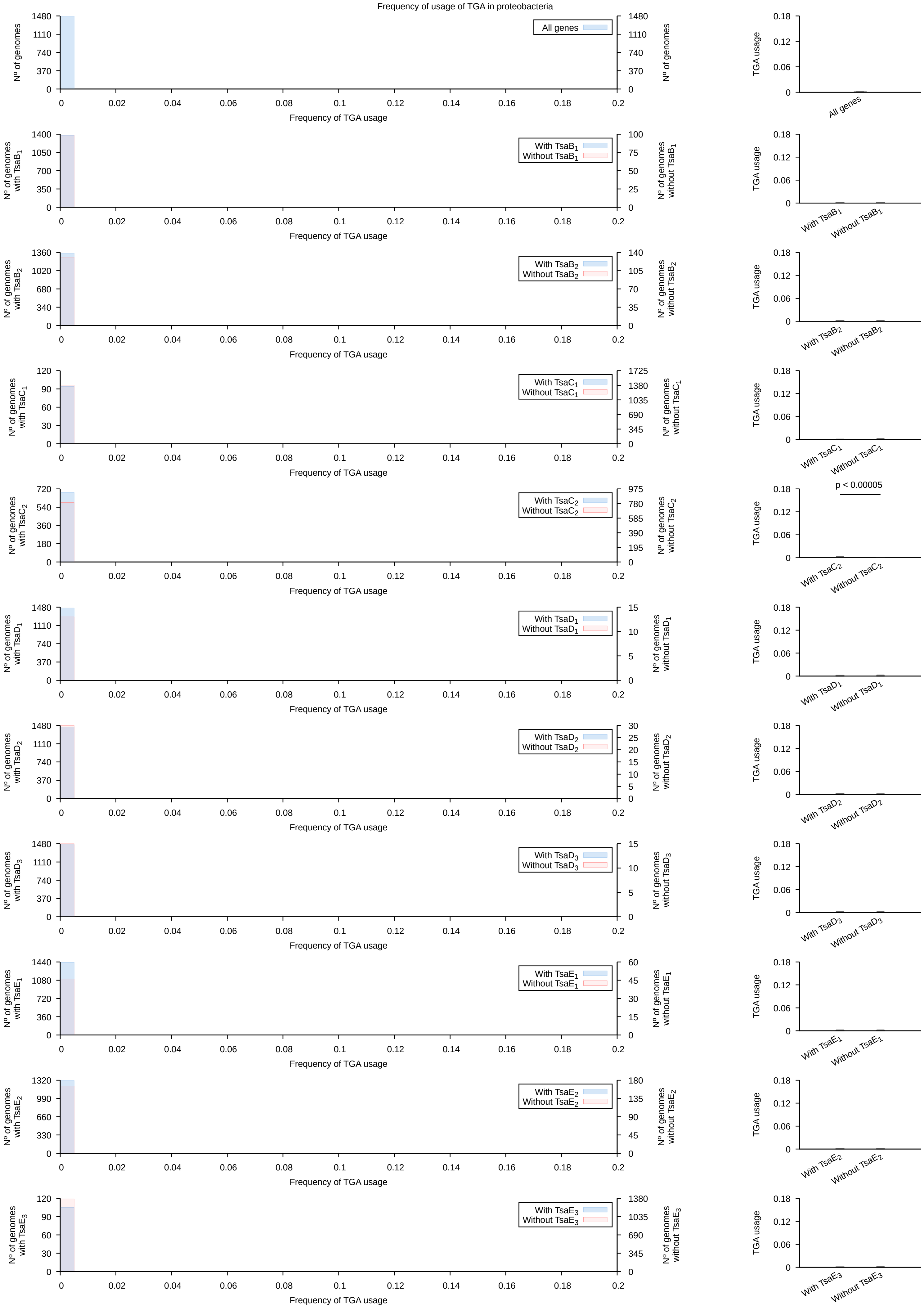

Frequency of usage of TGA in proteobacteria
Nº of genomes
All genes
Frequency of TGA usage
TGA usage
Nº of genomes with TsaB1
Nº of genomes without TsaB1
With TsaB1
Without TsaB1
Nº of genomes with TsaB2
Nº of genomes without TsaB2
With TsaB2
Without TsaB2
Nº of genomes with TsaC1
Nº of genomes without TsaC1
With TsaC1
Without TsaC1
Nº of genomes with TsaC2
Nº of genomes without TsaC2
With TsaC2
Without TsaC2
p < 0.00005
Nº of genomes with TsaD1
Nº of genomes without TsaD1
With TsaD1
Without TsaD1
Nº of genomes with TsaD2
Nº of genomes without TsaD2
With TsaD2
Without TsaD2
Nº of genomes with TsaD3
Nº of genomes without TsaD3
With TsaD3
Without TsaD3
Nº of genomes with TsaE1
Nº of genomes without TsaE1
With TsaE1
Without TsaE1
Nº of genomes with TsaE2
Nº of genomes without TsaE2
With TsaE2
Without TsaE2
Nº of genomes with TsaE3
Nº of genomes without TsaE3
With TsaE3
Without TsaE3

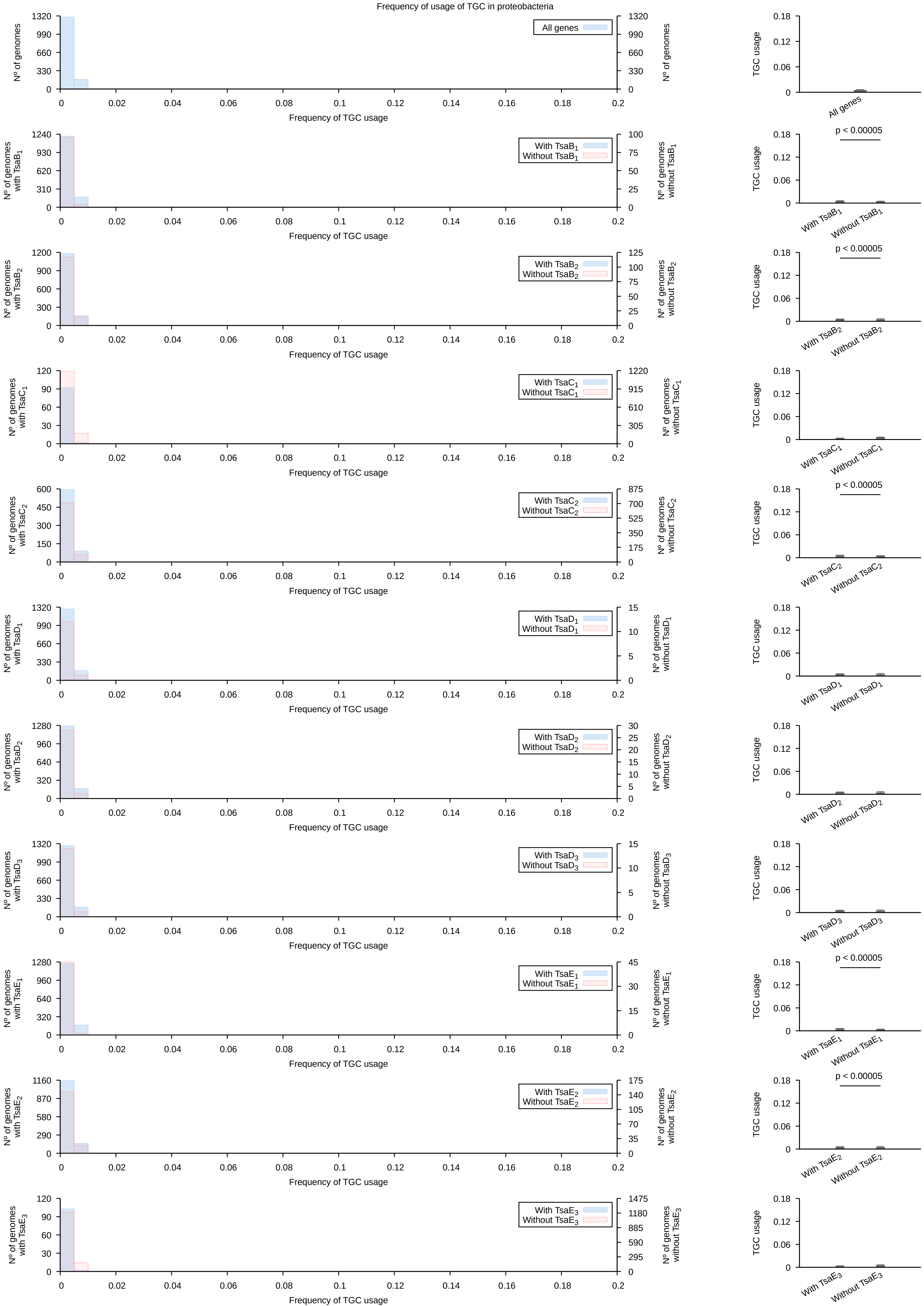

Frequency of usage of TGC in proteobacteria
All genes
Nº of genomes
Frequency of TGC usage
TGC usage
0 0.02 0.04 0.06 0.08 0.1 0.12 0.14 0.16 0.18 0.2
0 0.06 0.12 0.18
With TsaB$_1$
Without TsaB$_1$
Nº of genomes with TsaB$_1$
Nº of genomes without TsaB$_1$
p < 0.00005
With TsaB$_2$
Without TsaB$_2$
Nº of genomes with TsaB$_2$
Nº of genomes without TsaB$_2$
p < 0.00005
With TsaC$_1$
Without TsaC$_1$
Nº of genomes with TsaC$_1$
Nº of genomes without TsaC$_1$
With TsaC$_2$
Without TsaC$_2$
Nº of genomes with TsaC$_2$
Nº of genomes without TsaC$_2$
p < 0.00005
With TsaD$_1$
Without TsaD$_1$
Nº of genomes with TsaD$_1$
Nº of genomes without TsaD$_1$
With TsaD$_2$
Without TsaD$_2$
Nº of genomes with TsaD$_2$
Nº of genomes without TsaD$_2$
With TsaD$_3$
Without TsaD$_3$
Nº of genomes with TsaD$_3$
Nº of genomes without TsaD$_3$
With TsaE$_1$
Without TsaE$_1$
Nº of genomes with TsaE$_1$
Nº of genomes without TsaE$_1$
p < 0.00005
With TsaE$_2$
Without TsaE$_2$
Nº of genomes with TsaE$_2$
Nº of genomes without TsaE$_2$
p < 0.00005
With TsaE$_3$
Without TsaE$_3$
Nº of genomes with TsaE$_3$
Nº of genomes without TsaE$_3$

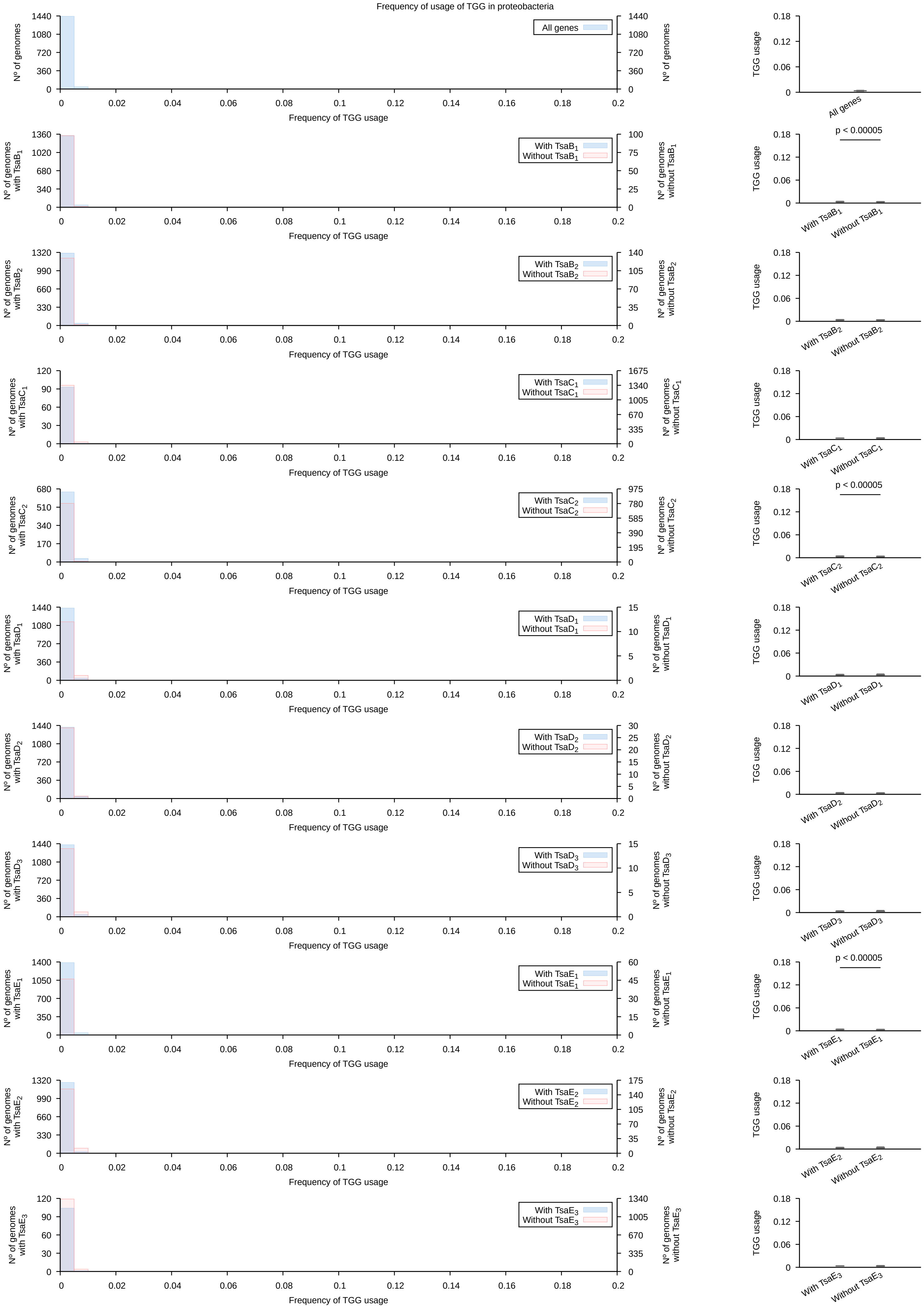

Frequency of usage of TGG in proteobacteria
All genes
Nº of genomes
Frequency of TGG usage
TGG usage
With TsaB1
Without TsaB1
Nº of genomes with TsaB1
Nº of genomes without TsaB1
p < 0.00005
With TsaB2
Without TsaB2
Nº of genomes with TsaB2
Nº of genomes without TsaB2
With TsaC1
Without TsaC1
Nº of genomes with TsaC1
Nº of genomes without TsaC1
With TsaC2
Without TsaC2
Nº of genomes with TsaC2
Nº of genomes without TsaC2
p < 0.00005
With TsaD1
Without TsaD1
Nº of genomes with TsaD1
Nº of genomes without TsaD1
With TsaD2
Without TsaD2
Nº of genomes with TsaD2
Nº of genomes without TsaD2
With TsaD3
Without TsaD3
Nº of genomes with TsaD3
Nº of genomes without TsaD3
With TsaE1
Without TsaE1
Nº of genomes with TsaE1
Nº of genomes without TsaE1
p < 0.00005
With TsaE2
Without TsaE2
Nº of genomes with TsaE2
Nº of genomes without TsaE2
With TsaE3
Without TsaE3
Nº of genomes with TsaE3
Nº of genomes without TsaE3

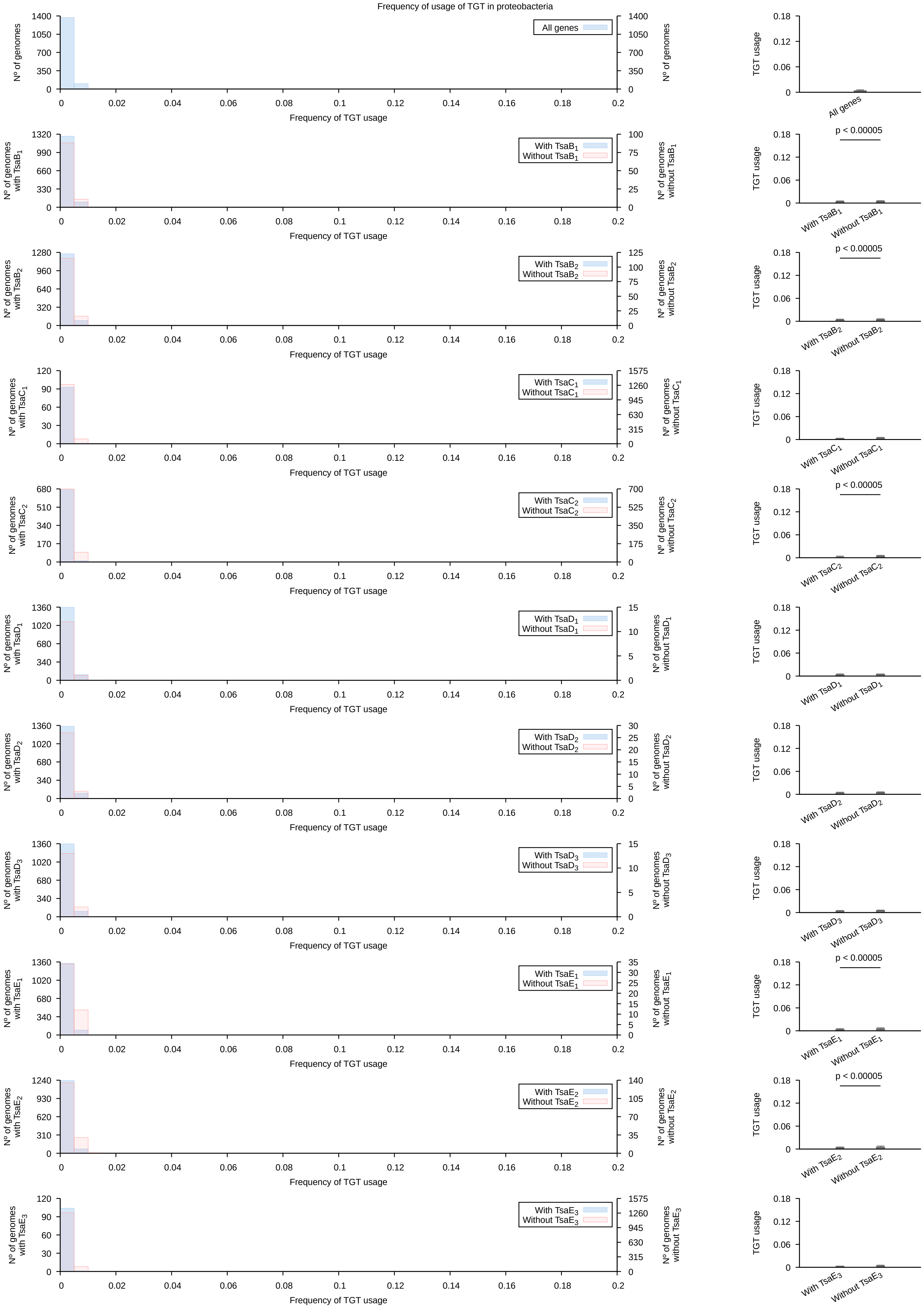
Frequency of usage of TGT in proteobacteria
All genes
Nº of genomes
Frequency of TGT usage
TGT usage
With TsaB$_1$
Without TsaB$_1$
Nº of genomes with TsaB$_1$
Nº of genomes without TsaB$_1$
p < 0.00005
With TsaB$_2$
Without TsaB$_2$
Nº of genomes with TsaB$_2$
Nº of genomes without TsaB$_2$
p < 0.00005
With TsaC$_1$
Without TsaC$_1$
Nº of genomes with TsaC$_1$
Nº of genomes without TsaC$_1$
With TsaC$_2$
Without TsaC$_2$
Nº of genomes with TsaC$_2$
Nº of genomes without TsaC$_2$
p < 0.00005
With TsaD$_1$
Without TsaD$_1$
Nº of genomes with TsaD$_1$
Nº of genomes without TsaD$_1$
With TsaD$_2$
Without TsaD$_2$
Nº of genomes with TsaD$_2$
Nº of genomes without TsaD$_2$
With TsaD$_3$
Without TsaD$_3$
Nº of genomes with TsaD$_3$
Nº of genomes without TsaD$_3$
With TsaE$_1$
Without TsaE$_1$
Nº of genomes with TsaE$_1$
Nº of genomes without TsaE$_1$
p < 0.00005
With TsaE$_2$
Without TsaE$_2$
Nº of genomes with TsaE$_2$
Nº of genomes without TsaE$_2$
p < 0.00005
With TsaE$_3$
Without TsaE$_3$
Nº of genomes with TsaE$_3$
Nº of genomes without TsaE$_3$

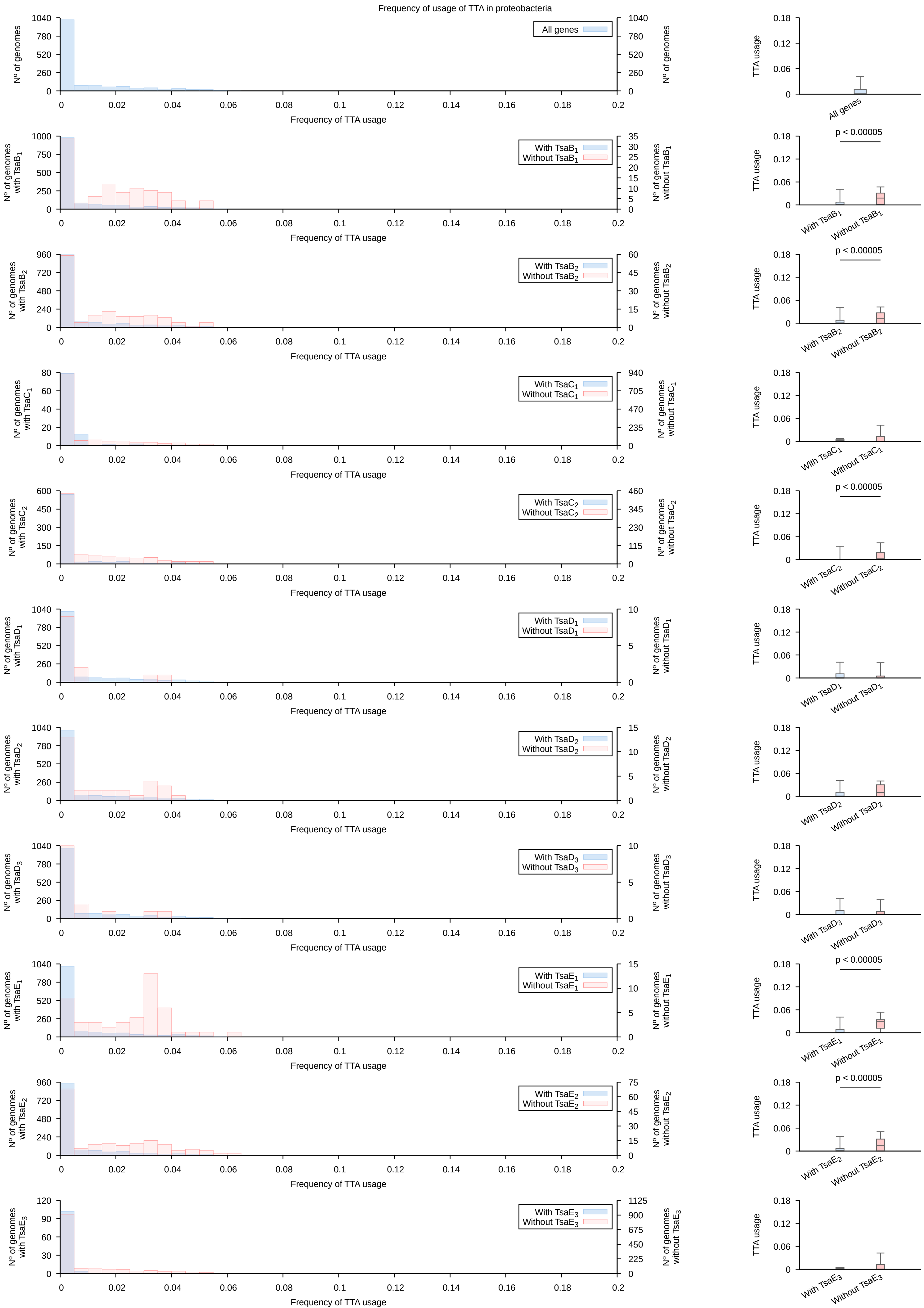
Frequency of usage of TTA in proteobacteria
Nº of genomes
Frequency of TTA usage
TTA usage
All genes
With TsaB$_1$
Without TsaB$_1$
Nº of genomes with TsaB$_1$
Nº of genomes without TsaB$_1$
p < 0.00005
With TsaB$_2$
Without TsaB$_2$
Nº of genomes with TsaB$_2$
Nº of genomes without TsaB$_2$
p < 0.00005
With TsaC$_1$
Without TsaC$_1$
Nº of genomes with TsaC$_1$
Nº of genomes without TsaC$_1$
With TsaC$_2$
Without TsaC$_2$
Nº of genomes with TsaC$_2$
Nº of genomes without TsaC$_2$
p < 0.00005
With TsaD$_1$
Without TsaD$_1$
Nº of genomes with TsaD$_1$
Nº of genomes without TsaD$_1$
With TsaD$_2$
Without TsaD$_2$
Nº of genomes with TsaD$_2$
Nº of genomes without TsaD$_2$
With TsaD$_3$
Without TsaD$_3$
Nº of genomes with TsaD$_3$
Nº of genomes without TsaD$_3$
With TsaE$_1$
Without TsaE$_1$
Nº of genomes with TsaE$_1$
Nº of genomes without TsaE$_1$
p < 0.00005
With TsaE$_2$
Without TsaE$_2$
Nº of genomes with TsaE$_2$
Nº of genomes without TsaE$_2$
p < 0.00005
With TsaE$_3$
Without TsaE$_3$
Nº of genomes with TsaE$_3$
Nº of genomes without TsaE$_3$

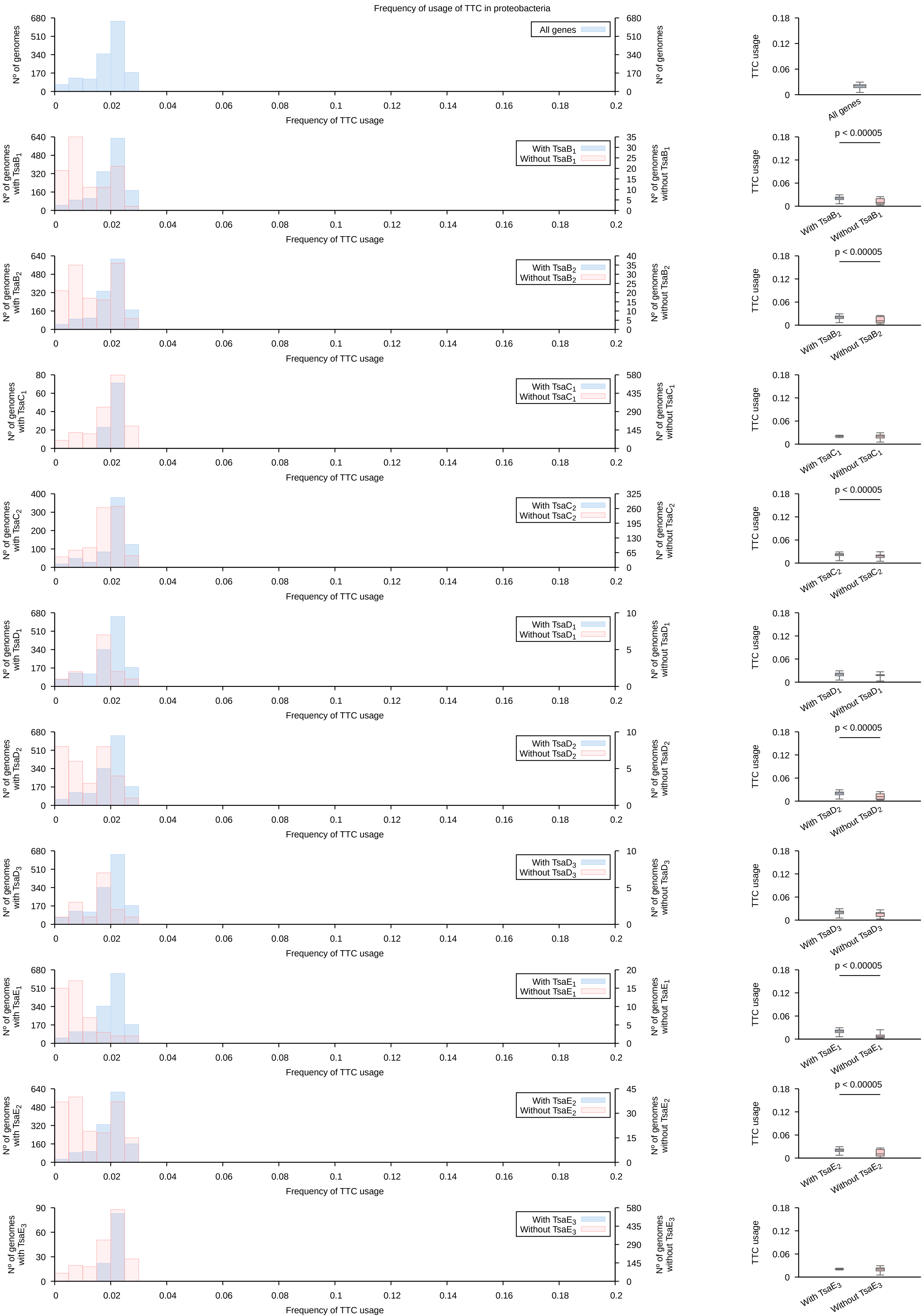
Frequency of usage of TTC in proteobacteria
All genes
With TsaB1
Without TsaB1
With TsaB2
Without TsaB2
With TsaC1
Without TsaC1
With TsaC2
Without TsaC2
With TsaD1
Without TsaD1
With TsaD2
Without TsaD2
With TsaD3
Without TsaD3
With TsaE1
Without TsaE1
With TsaE2
Without TsaE2
With TsaE3
Without TsaE3
Nº of genomes
Frequency of TTC usage
TTC usage
p < 0.00005

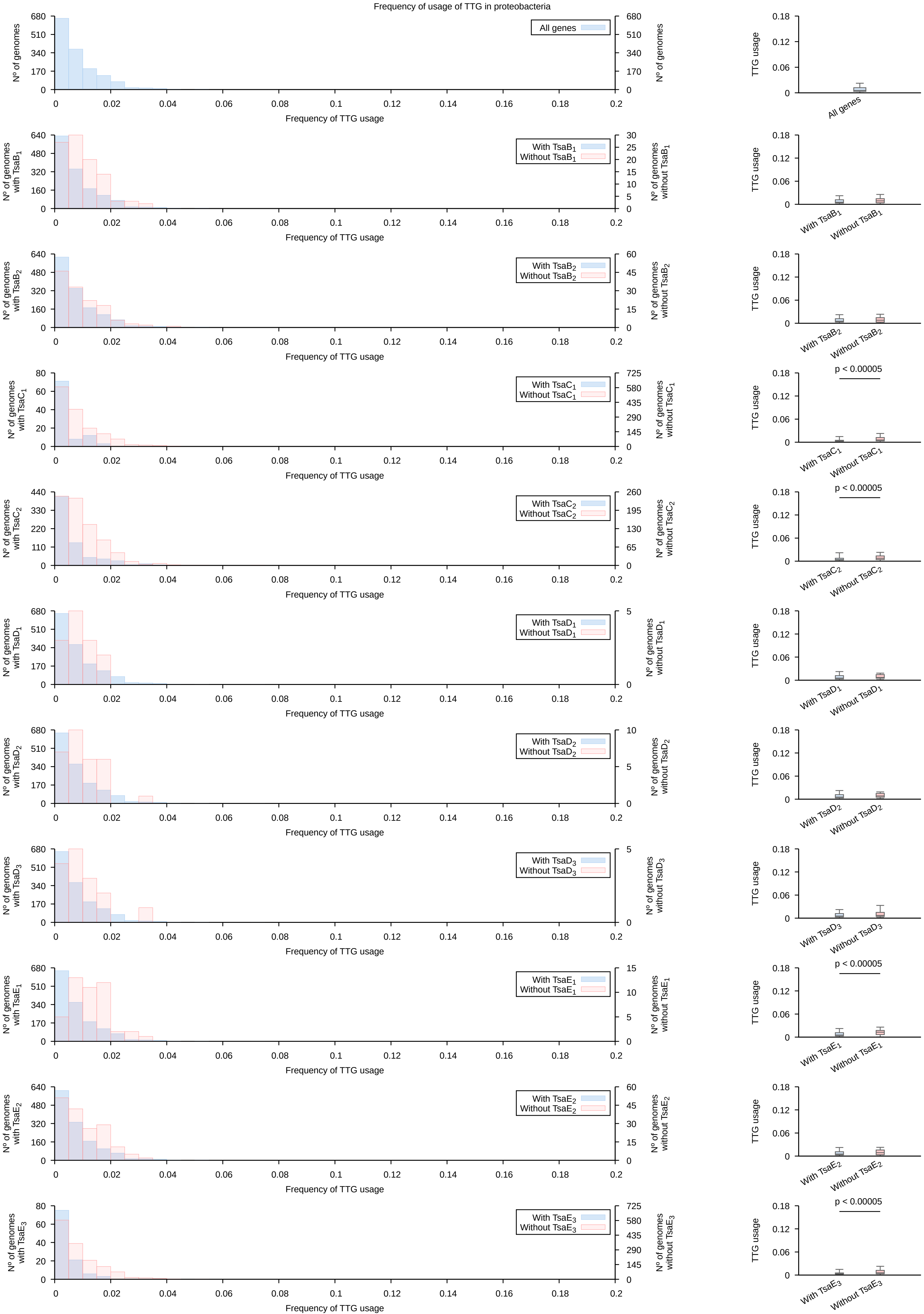
Frequency of usage of TTG in proteobacteria
Nº of genomes
All genes
Frequency of TTG usage
TTG usage
0 0.02 0.04 0.06 0.08 0.1 0.12 0.14 0.16 0.18 0.2
0 0.06 0.12 0.18
Nº of genomes with TsaB$_1$
Nº of genomes without TsaB$_1$
With TsaB$_1$
Without TsaB$_1$
Nº of genomes with TsaB$_2$
Nº of genomes without TsaB$_2$
With TsaB$_2$
Without TsaB$_2$
Nº of genomes with TsaC$_1$
Nº of genomes without TsaC$_1$
With TsaC$_1$
Without TsaC$_1$
p < 0.00005
Nº of genomes with TsaC$_2$
Nº of genomes without TsaC$_2$
With TsaC$_2$
Without TsaC$_2$
p < 0.00005
Nº of genomes with TsaD$_1$
Nº of genomes without TsaD$_1$
With TsaD$_1$
Without TsaD$_1$
Nº of genomes with TsaD$_2$
Nº of genomes without TsaD$_2$
With TsaD$_2$
Without TsaD$_2$
Nº of genomes with TsaD$_3$
Nº of genomes without TsaD$_3$
With TsaD$_3$
Without TsaD$_3$
Nº of genomes with TsaE$_1$
Nº of genomes without TsaE$_1$
With TsaE$_1$
Without TsaE$_1$
p < 0.00005
Nº of genomes with TsaE$_2$
Nº of genomes without TsaE$_2$
With TsaE$_2$
Without TsaE$_2$
Nº of genomes with TsaE$_3$
Nº of genomes without TsaE$_3$
With TsaE$_3$
Without TsaE$_3$
p < 0.00005

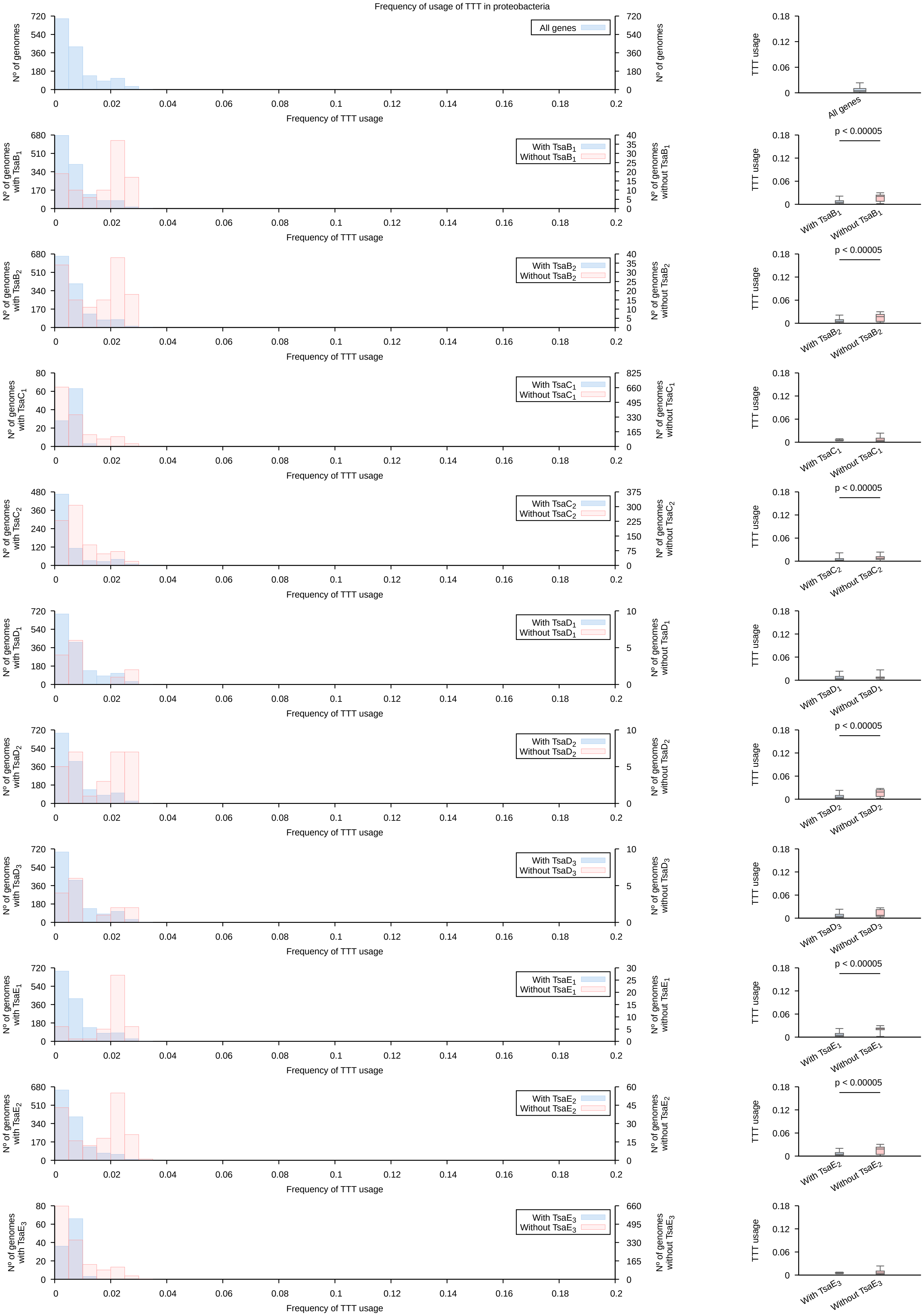

Frequency of usage of TTT in proteobacteria
Nº of genomes
All genes
Frequency of TTT usage
TTT usage
p < 0.00005
Nº of genomes with TsaB1
Nº of genomes without TsaB1
With TsaB1
Without TsaB1
Nº of genomes with TsaB2
Nº of genomes without TsaB2
With TsaB2
Without TsaB2
Nº of genomes with TsaC1
Nº of genomes without TsaC1
With TsaC1
Without TsaC1
Nº of genomes with TsaC2
Nº of genomes without TsaC2
With TsaC2
Without TsaC2
Nº of genomes with TsaD1
Nº of genomes without TsaD1
With TsaD1
Without TsaD1
Nº of genomes with TsaD2
Nº of genomes without TsaD2
With TsaD2
Without TsaD2
Nº of genomes with TsaD3
Nº of genomes without TsaD3
With TsaD3
Without TsaD3
Nº of genomes with TsaE1
Nº of genomes without TsaE1
With TsaE1
Without TsaE1
Nº of genomes with TsaE2
Nº of genomes without TsaE2
With TsaE2
Without TsaE2
Nº of genomes with TsaE3
Nº of genomes without TsaE3
With TsaE3
Without TsaE3
