# Supplementary material for: Impact of the chemical modification of tRNAs anticodon loop on the variability and evolution of codon usage in proteobacteria
Source: Front Microbiol. 2024 Aug 5;15:1412318. doi: 10.3389/fmicb.2024.1412318 (PMC11332805; doi:10.3389/fmicb.2024.1412318)
Supplement: Supplementary file 1 [file Data_Sheet_1.zip › Supp_figures/Fig_S20.pdf]

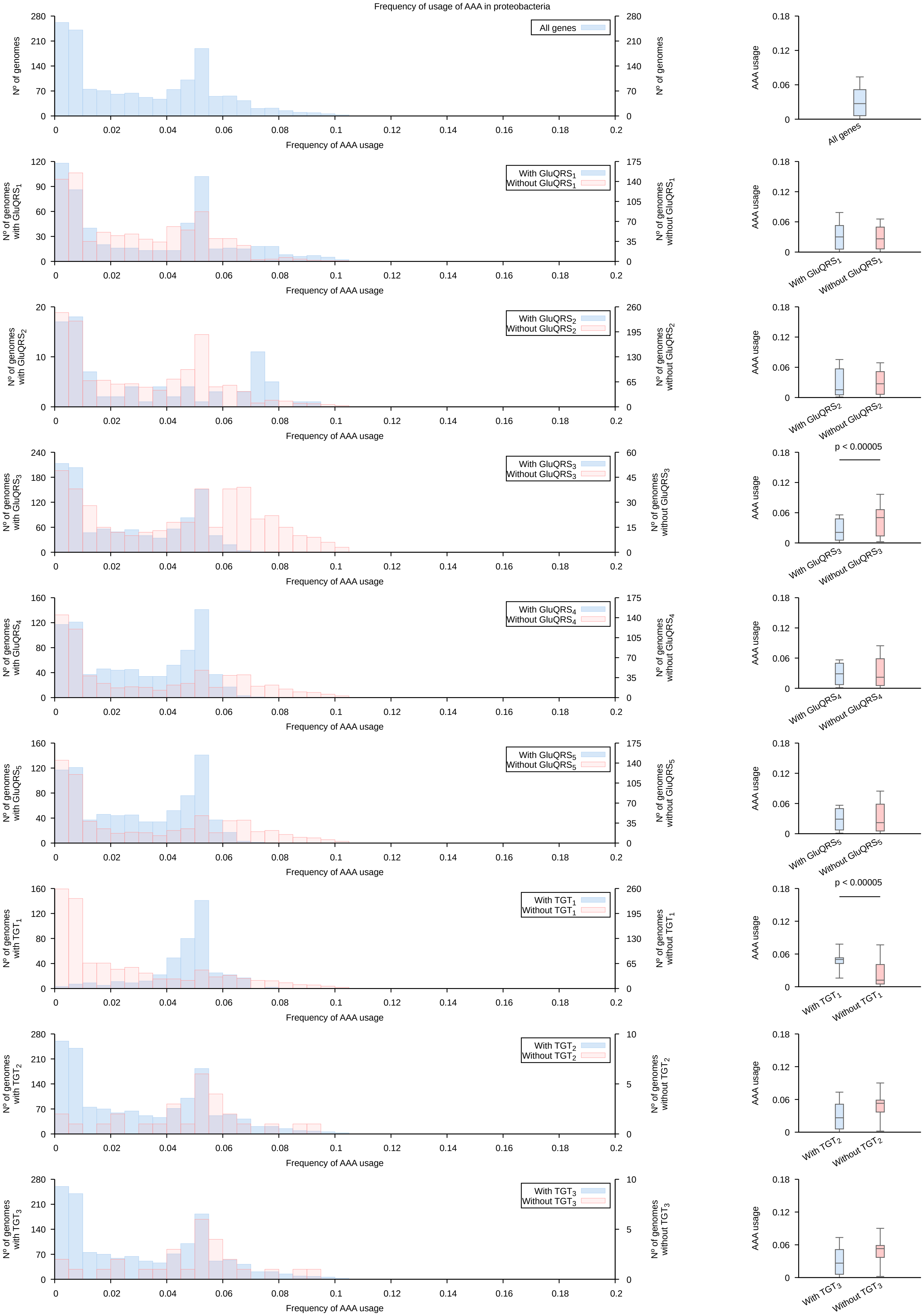

Frequency of usage of AAA in proteobacteria
All genes
Nº of genomes
Frequency of AAA usage
AAA usage
With GluQRS1
Without GluQRS1
Nº of genomes with GluQRS1
Nº of genomes without GluQRS1
With GluQRS2
Without GluQRS2
Nº of genomes with GluQRS2
Nº of genomes without GluQRS2
With GluQRS3
Without GluQRS3
Nº of genomes with GluQRS3
Nº of genomes without GluQRS3
p < 0.00005
With GluQRS4
Without GluQRS4
Nº of genomes with GluQRS4
Nº of genomes without GluQRS4
With GluQRS5
Without GluQRS5
Nº of genomes with GluQRS5
Nº of genomes without GluQRS5
With TGT1
Without TGT1
Nº of genomes with TGT1
Nº of genomes without TGT1
p < 0.00005
With TGT2
Without TGT2
Nº of genomes with TGT2
Nº of genomes without TGT2
With TGT3
Without TGT3
Nº of genomes with TGT3
Nº of genomes without TGT3

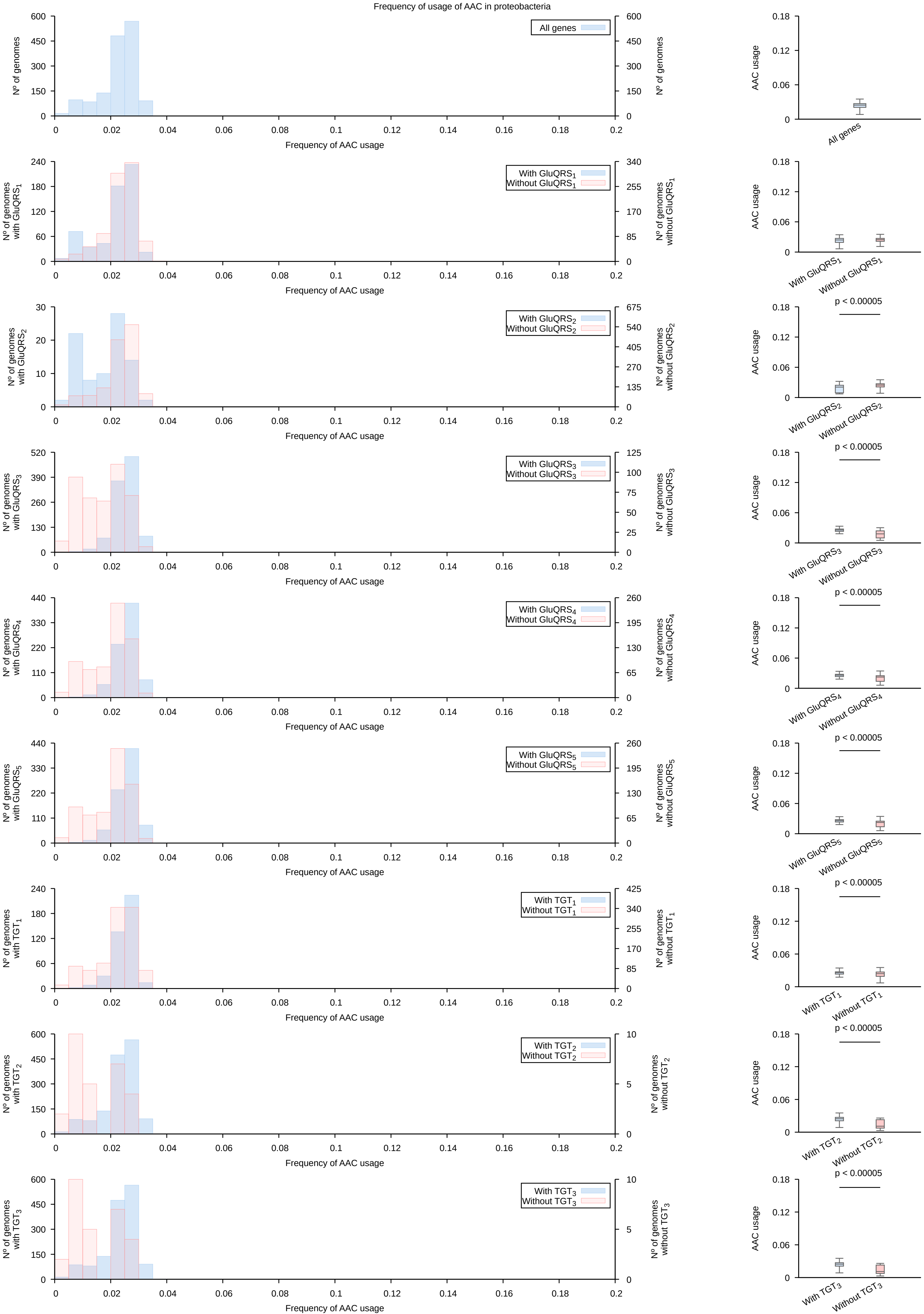
Frequency of usage of AAC in proteobacteria
All genes
Nº of genomes
Frequency of AAC usage
AAC usage
With GluQRS$_1$
Without GluQRS$_1$
Nº of genomes with GluQRS$_1$
Nº of genomes without GluQRS$_1$
With GluQRS$_2$
Without GluQRS$_2$
Nº of genomes with GluQRS$_2$
Nº of genomes without GluQRS$_2$
p < 0.00005
With GluQRS$_3$
Without GluQRS$_3$
Nº of genomes with GluQRS$_3$
Nº of genomes without GluQRS$_3$
p < 0.00005
With GluQRS$_4$
Without GluQRS$_4$
Nº of genomes with GluQRS$_4$
Nº of genomes without GluQRS$_4$
p < 0.00005
With GluQRS$_5$
Without GluQRS$_5$
Nº of genomes with GluQRS$_5$
Nº of genomes without GluQRS$_5$
p < 0.00005
With TGT$_1$
Without TGT$_1$
Nº of genomes with TGT$_1$
Nº of genomes without TGT$_1$
p < 0.00005
With TGT$_2$
Without TGT$_2$
Nº of genomes with TGT$_2$
Nº of genomes without TGT$_2$
p < 0.00005
With TGT$_3$
Without TGT$_3$
Nº of genomes with TGT$_3$
Nº of genomes without TGT$_3$
p < 0.00005

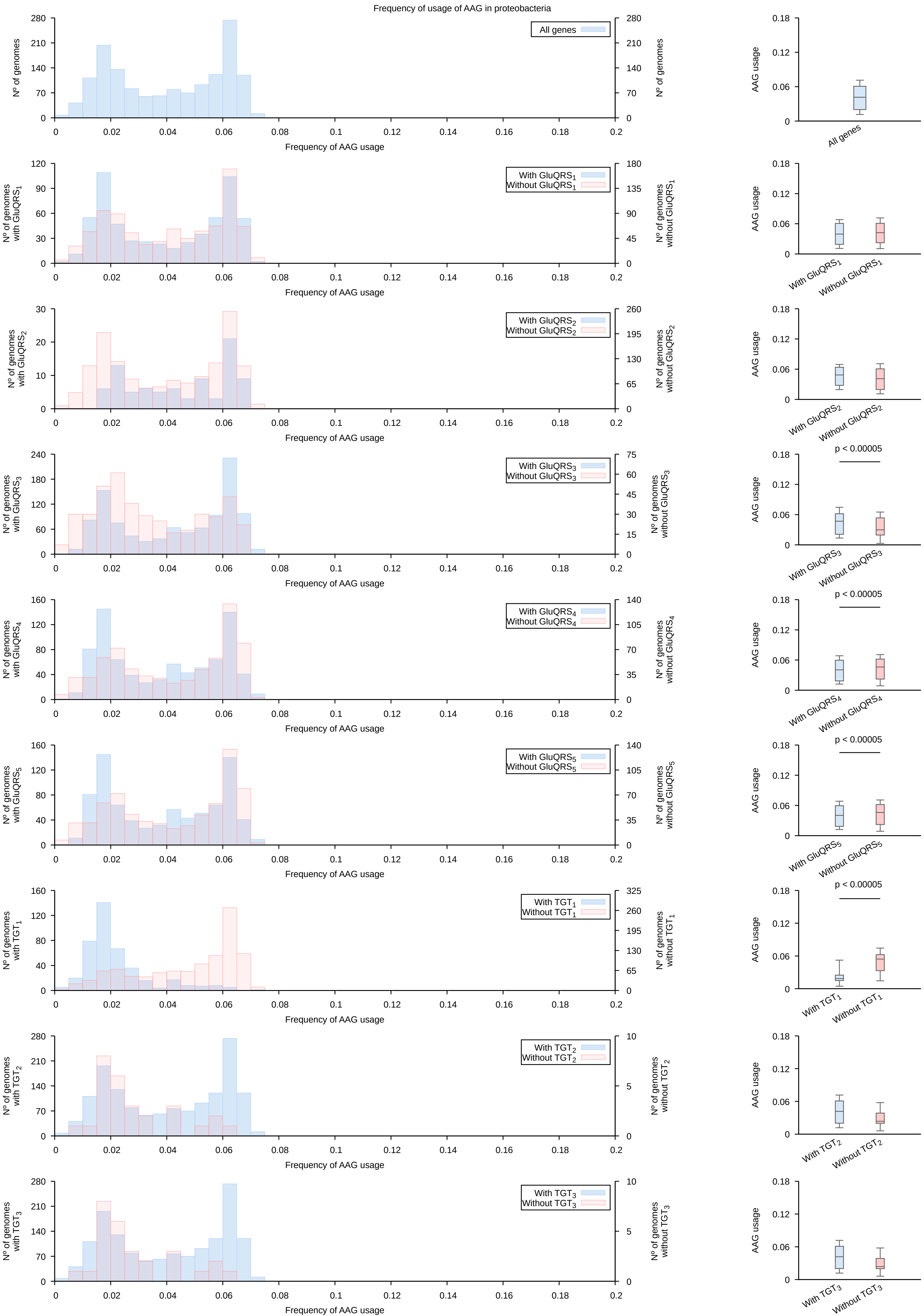

Frequency of usage of AAG in proteobacteria
Nº of genomes
All genes
AAG usage
Frequency of AAG usage
Nº of genomes with GluQRS$_1$
Nº of genomes without GluQRS$_1$
With GluQRS$_1$
Without GluQRS$_1$
Nº of genomes with GluQRS$_2$
Nº of genomes without GluQRS$_2$
With GluQRS$_2$
Without GluQRS$_2$
Nº of genomes with GluQRS$_3$
Nº of genomes without GluQRS$_3$
With GluQRS$_3$
Without GluQRS$_3$
$p < 0.00005$
Nº of genomes with GluQRS$_4$
Nº of genomes without GluQRS$_4$
With GluQRS$_4$
Without GluQRS$_4$
$p < 0.00005$
Nº of genomes with GluQRS$_5$
Nº of genomes without GluQRS$_5$
With GluQRS$_5$
Without GluQRS$_5$
$p < 0.00005$
Nº of genomes with TGT$_1$
Nº of genomes without TGT$_1$
With TGT$_1$
Without TGT$_1$
$p < 0.00005$
Nº of genomes with TGT$_2$
Nº of genomes without TGT$_2$
With TGT$_2$
Without TGT$_2$
Nº of genomes with TGT$_3$
Nº of genomes without TGT$_3$
With TGT$_3$
Without TGT$_3$

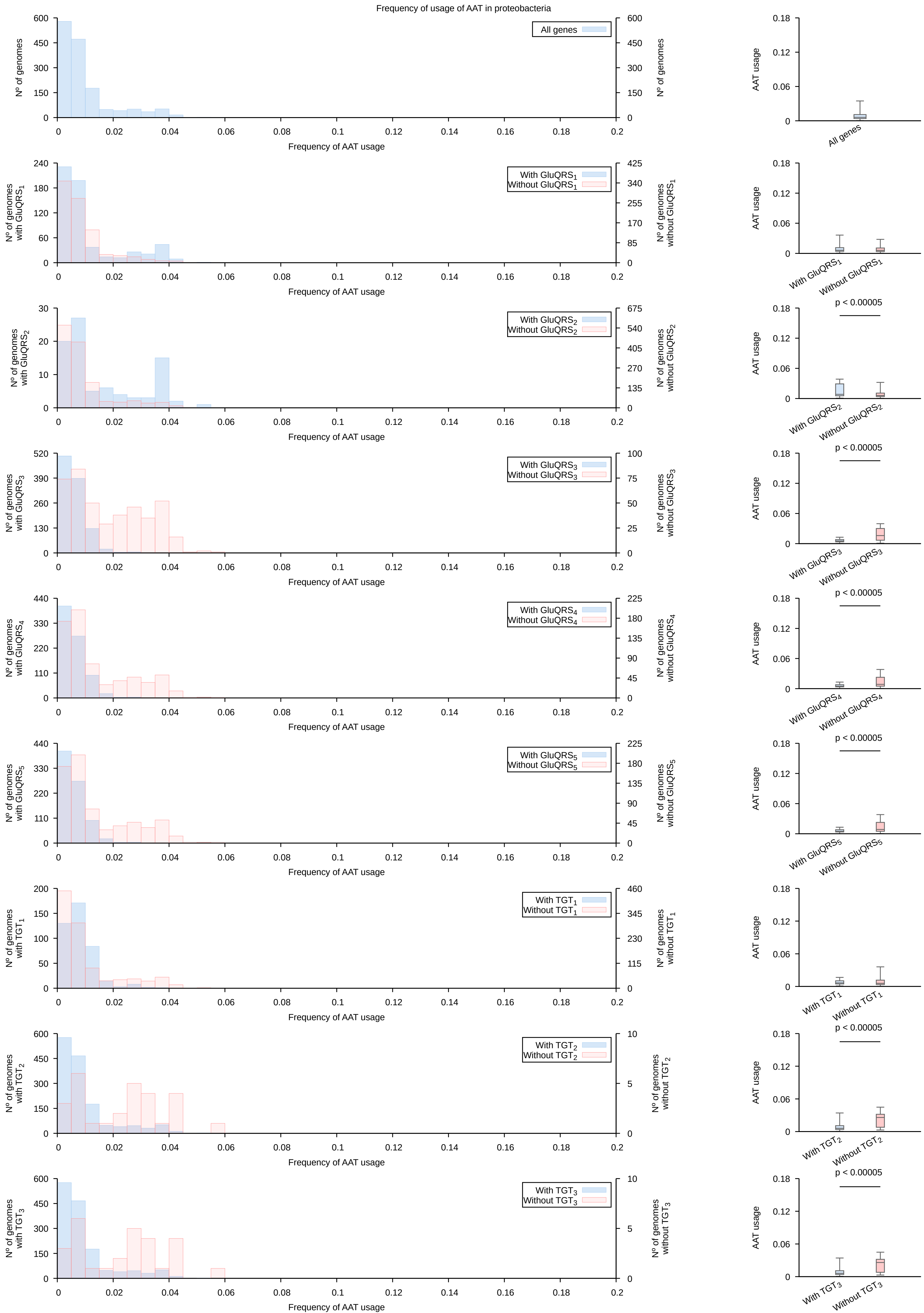
Frequency of usage of AAT in proteobacteria
All genes
Nº of genomes
Frequency of AAT usage
AAT usage
With GluQRS$_1$
Without GluQRS$_1$
Nº of genomes with GluQRS$_1$
Nº of genomes without GluQRS$_1$
With GluQRS$_2$
Without GluQRS$_2$
Nº of genomes with GluQRS$_2$
Nº of genomes without GluQRS$_2$
p < 0.00005
With GluQRS$_3$
Without GluQRS$_3$
Nº of genomes with GluQRS$_3$
Nº of genomes without GluQRS$_3$
p < 0.00005
With GluQRS$_4$
Without GluQRS$_4$
Nº of genomes with GluQRS$_4$
Nº of genomes without GluQRS$_4$
p < 0.00005
With GluQRS$_5$
Without GluQRS$_5$
Nº of genomes with GluQRS$_5$
Nº of genomes without GluQRS$_5$
p < 0.00005
With TGT$_1$
Without TGT$_1$
Nº of genomes with TGT$_1$
Nº of genomes without TGT$_1$
With TGT$_2$
Without TGT$_2$
Nº of genomes with TGT$_2$
Nº of genomes without TGT$_2$
p < 0.00005
With TGT$_3$
Without TGT$_3$
Nº of genomes with TGT$_3$
Nº of genomes without TGT$_3$
p < 0.00005

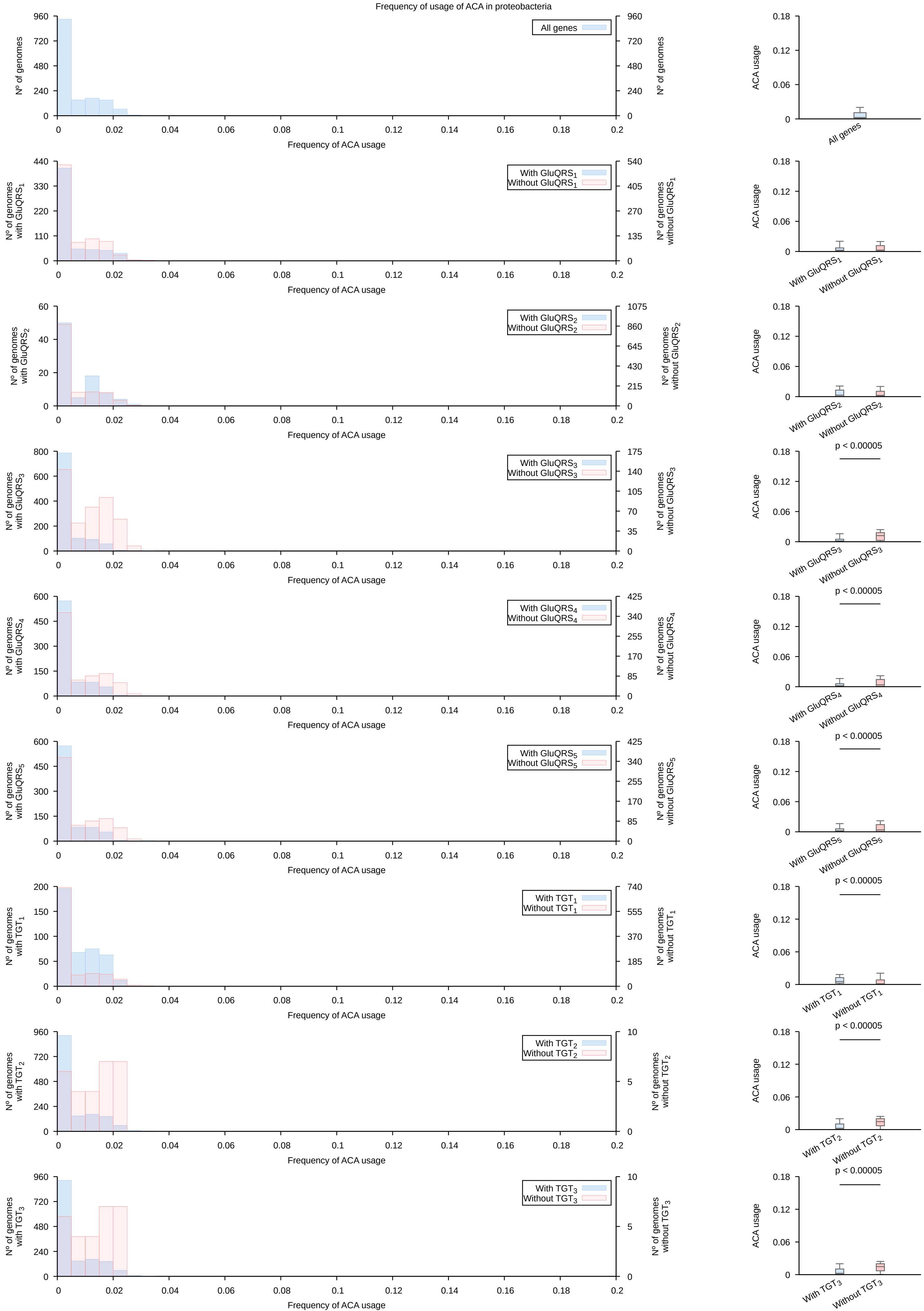

Frequency of usage of ACA in proteobacteria
All genes
Nº of genomes
Frequency of ACA usage
ACA usage
With GluQRS$_1$
Without GluQRS$_1$
Nº of genomes with GluQRS$_1$
Nº of genomes without GluQRS$_1$
With GluQRS$_2$
Without GluQRS$_2$
Nº of genomes with GluQRS$_2$
Nº of genomes without GluQRS$_2$
With GluQRS$_3$
Without GluQRS$_3$
Nº of genomes with GluQRS$_3$
Nº of genomes without GluQRS$_3$
p < 0.00005
With GluQRS$_4$
Without GluQRS$_4$
Nº of genomes with GluQRS$_4$
Nº of genomes without GluQRS$_4$
p < 0.00005
With GluQRS$_5$
Without GluQRS$_5$
Nº of genomes with GluQRS$_5$
Nº of genomes without GluQRS$_5$
p < 0.00005
With TGT$_1$
Without TGT$_1$
Nº of genomes with TGT$_1$
Nº of genomes without TGT$_1$
p < 0.00005
With TGT$_2$
Without TGT$_2$
Nº of genomes with TGT$_2$
Nº of genomes without TGT$_2$
p < 0.00005
With TGT$_3$
Without TGT$_3$
Nº of genomes with TGT$_3$
Nº of genomes without TGT$_3$
p < 0.00005

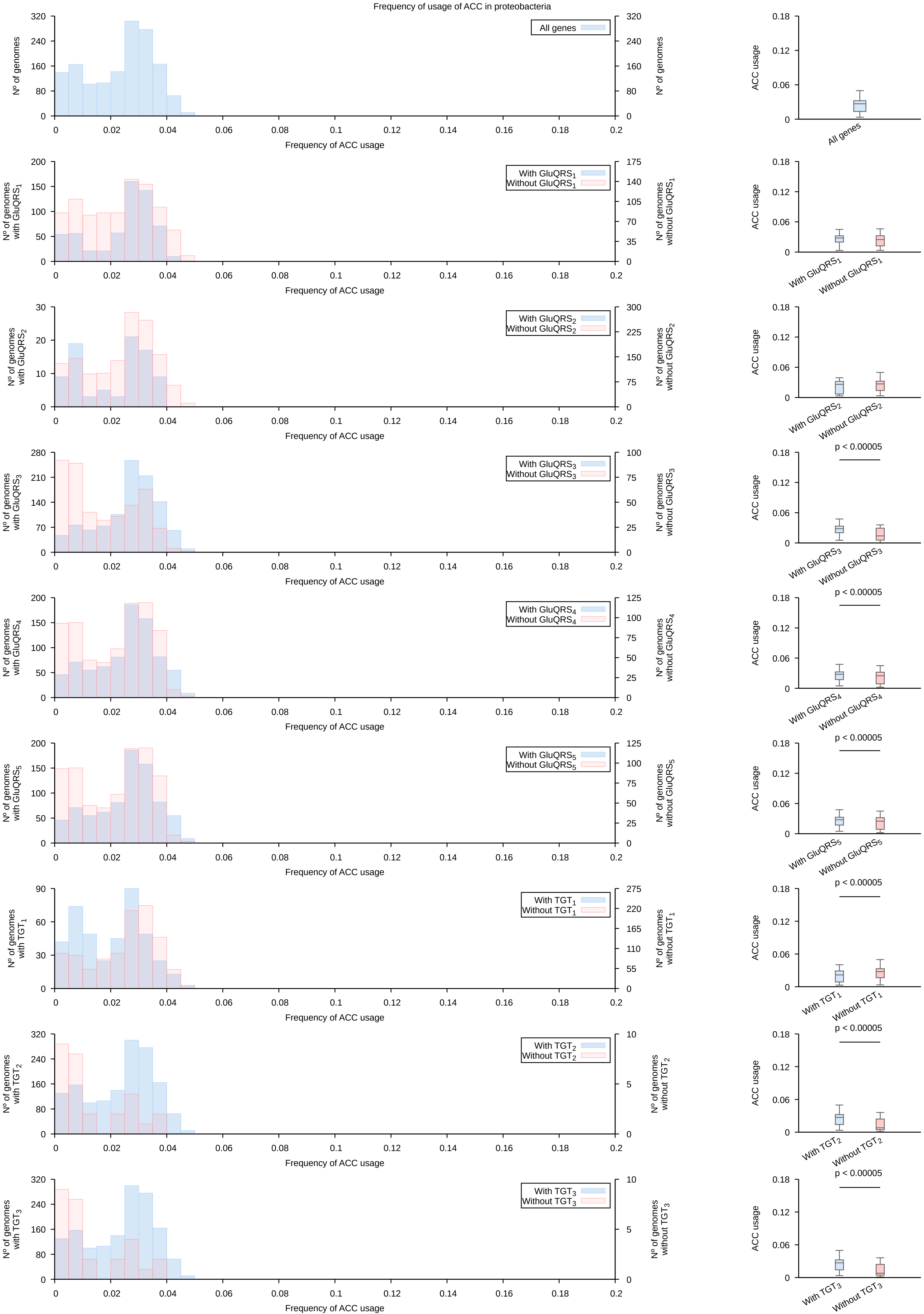

Frequency of usage of ACC in proteobacteria
All genes
Nº of genomes
Frequency of ACC usage
ACC usage
With GluQRS$_1$
Without GluQRS$_1$
Nº of genomes with GluQRS$_1$
Nº of genomes without GluQRS$_1$
With GluQRS$_2$
Without GluQRS$_2$
Nº of genomes with GluQRS$_2$
Nº of genomes without GluQRS$_2$
With GluQRS$_3$
Without GluQRS$_3$
Nº of genomes with GluQRS$_3$
Nº of genomes without GluQRS$_3$
p < 0.00005
With GluQRS$_4$
Without GluQRS$_4$
Nº of genomes with GluQRS$_4$
Nº of genomes without GluQRS$_4$
p < 0.00005
With GluQRS$_5$
Without GluQRS$_5$
Nº of genomes with GluQRS$_5$
Nº of genomes without GluQRS$_5$
p < 0.00005
With TGT$_1$
Without TGT$_1$
Nº of genomes with TGT$_1$
Nº of genomes without TGT$_1$
p < 0.00005
With TGT$_2$
Without TGT$_2$
Nº of genomes with TGT$_2$
Nº of genomes without TGT$_2$
p < 0.00005
With TGT$_3$
Without TGT$_3$
Nº of genomes with TGT$_3$
Nº of genomes without TGT$_3$
p < 0.00005

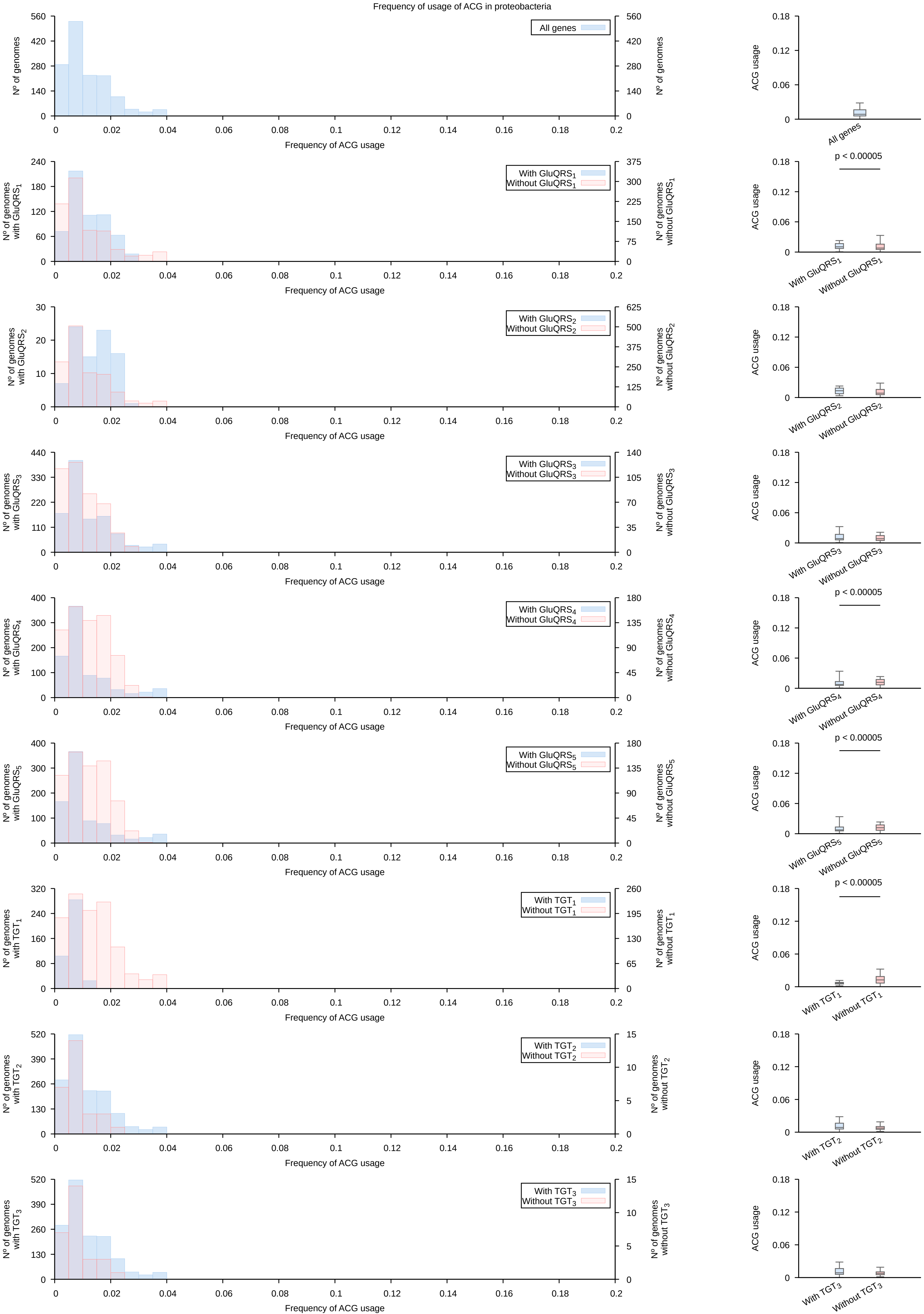
Frequency of usage of ACG in proteobacteria
All genes
Nº of genomes
Frequency of ACG usage
ACG usage
With GluQRS$_1$
Without GluQRS$_1$
Nº of genomes with GluQRS$_1$
Nº of genomes without GluQRS$_1$
p < 0.00005
With GluQRS$_2$
Without GluQRS$_2$
Nº of genomes with GluQRS$_2$
Nº of genomes without GluQRS$_2$
With GluQRS$_3$
Without GluQRS$_3$
Nº of genomes with GluQRS$_3$
Nº of genomes without GluQRS$_3$
With GluQRS$_4$
Without GluQRS$_4$
Nº of genomes with GluQRS$_4$
Nº of genomes without GluQRS$_4$
p < 0.00005
With GluQRS$_5$
Without GluQRS$_5$
Nº of genomes with GluQRS$_5$
Nº of genomes without GluQRS$_5$
p < 0.00005
With TGT$_1$
Without TGT$_1$
Nº of genomes with TGT$_1$
Nº of genomes without TGT$_1$
p < 0.00005
With TGT$_2$
Without TGT$_2$
Nº of genomes with TGT$_2$
Nº of genomes without TGT$_2$
With TGT$_3$
Without TGT$_3$
Nº of genomes with TGT$_3$
Nº of genomes without TGT$_3$

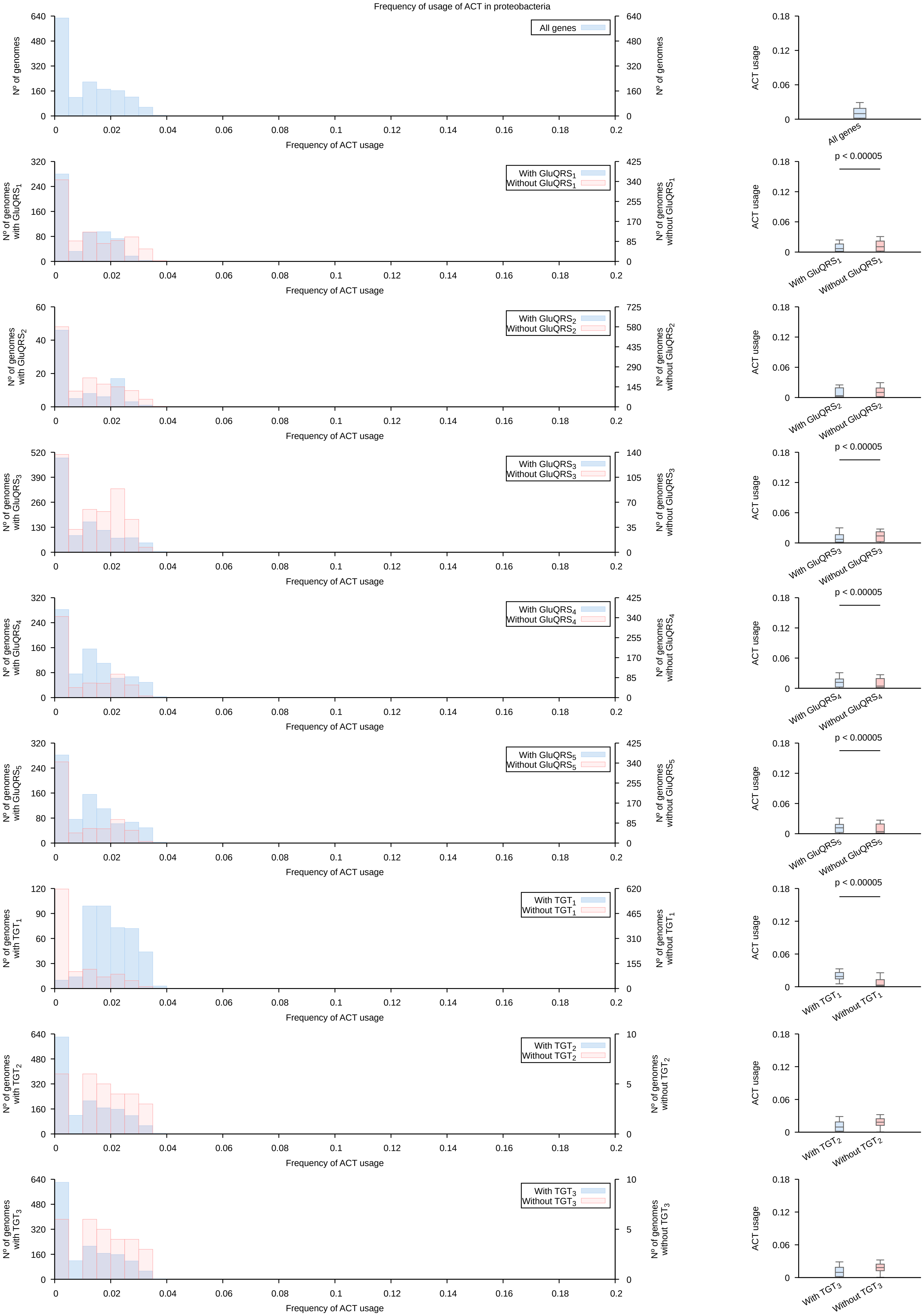

Frequency of usage of ACT in proteobacteria
All genes
With GluQRS1
Without GluQRS1
With GluQRS2
Without GluQRS2
With GluQRS3
Without GluQRS3
With GluQRS4
Without GluQRS4
With GluQRS5
Without GluQRS5
With TGT1
Without TGT1
With TGT2
Without TGT2
With TGT3
Without TGT3
Frequency of ACT usage
Nº of genomes
ACT usage
p < 0.00005

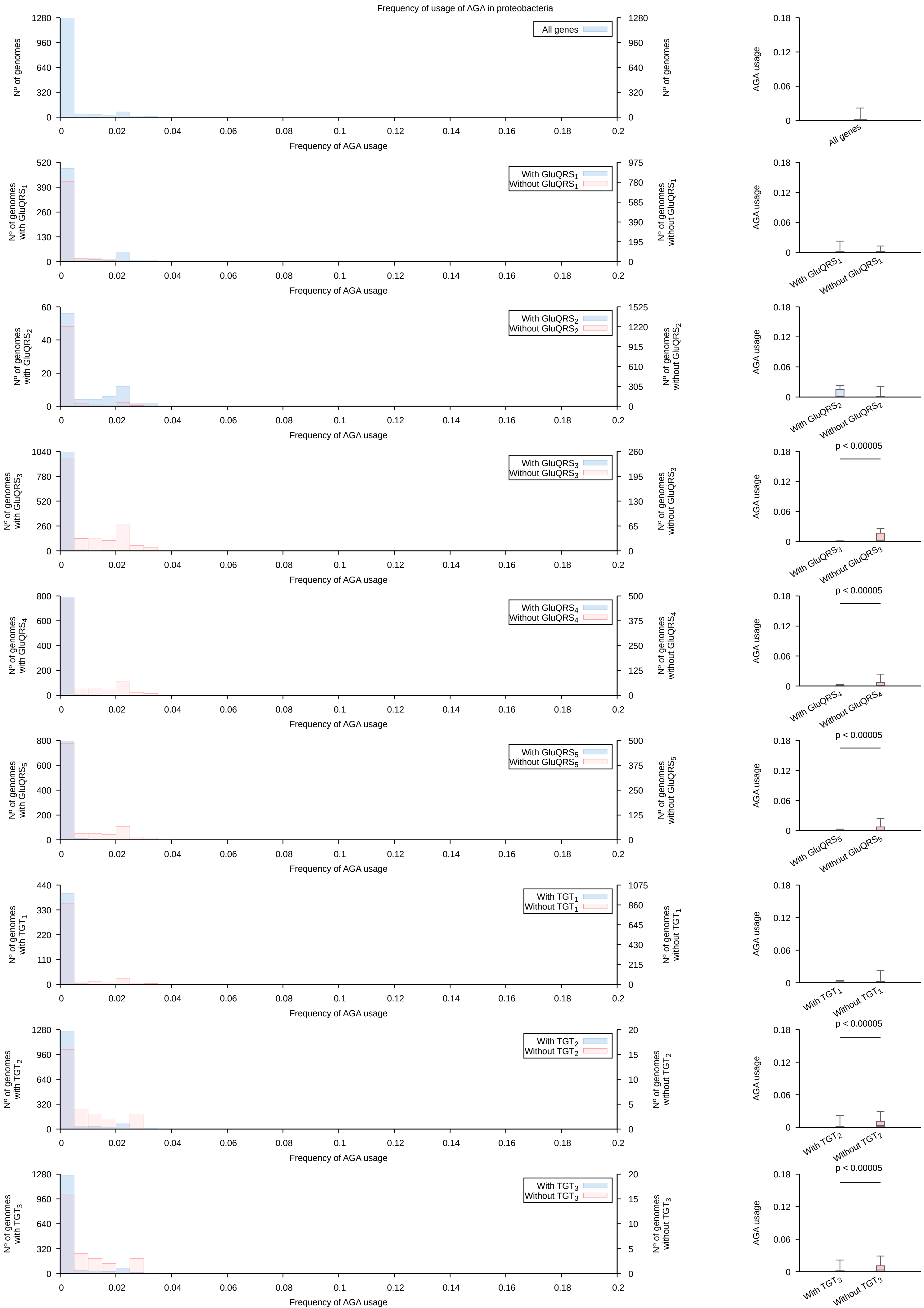
Frequency of usage of AGA in proteobacteria
All genes
Nº of genomes
Frequency of AGA usage
AGA usage
With GluQRS$_1$
Without GluQRS$_1$
Nº of genomes with GluQRS$_1$
Nº of genomes without GluQRS$_1$
With GluQRS$_2$
Without GluQRS$_2$
Nº of genomes with GluQRS$_2$
Nº of genomes without GluQRS$_2$
With GluQRS$_3$
Without GluQRS$_3$
Nº of genomes with GluQRS$_3$
Nº of genomes without GluQRS$_3$
p < 0.00005
With GluQRS$_4$
Without GluQRS$_4$
Nº of genomes with GluQRS$_4$
Nº of genomes without GluQRS$_4$
p < 0.00005
With GluQRS$_5$
Without GluQRS$_5$
Nº of genomes with GluQRS$_5$
Nº of genomes without GluQRS$_5$
p < 0.00005
With TGT$_1$
Without TGT$_1$
Nº of genomes with TGT$_1$
Nº of genomes without TGT$_1$
With TGT$_2$
Without TGT$_2$
Nº of genomes with TGT$_2$
Nº of genomes without TGT$_2$
p < 0.00005
With TGT$_3$
Without TGT$_3$
Nº of genomes with TGT$_3$
Nº of genomes without TGT$_3$
p < 0.00005

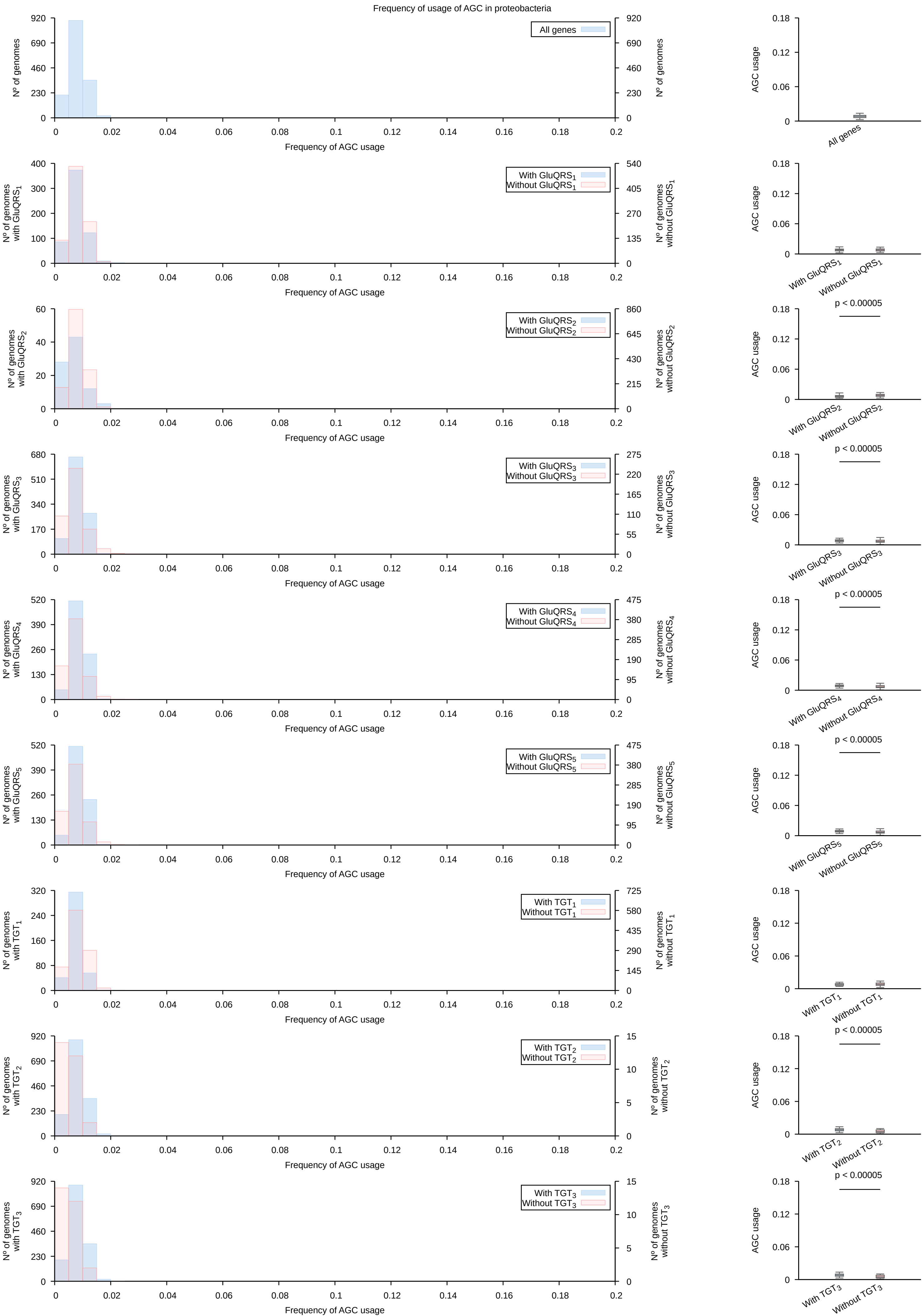

Frequency of usage of AGC in proteobacteria
All genes
With GluQRS1
Without GluQRS1
With GluQRS2
Without GluQRS2
With GluQRS3
Without GluQRS3
With GluQRS4
Without GluQRS4
With GluQRS5
Without GluQRS5
With TGT1
Without TGT1
With TGT2
Without TGT2
With TGT3
Without TGT3
Nº of genomes
Nº of genomes with GluQRS1
Nº of genomes without GluQRS1
Nº of genomes with GluQRS2
Nº of genomes without GluQRS2
Nº of genomes with GluQRS3
Nº of genomes without GluQRS3
Nº of genomes with GluQRS4
Nº of genomes without GluQRS4
Nº of genomes with GluQRS5
Nº of genomes without GluQRS5
Nº of genomes with TGT1
Nº of genomes without TGT1
Nº of genomes with TGT2
Nº of genomes without TGT2
Nº of genomes with TGT3
Nº of genomes without TGT3
Frequency of AGC usage
AGC usage
p < 0.00005

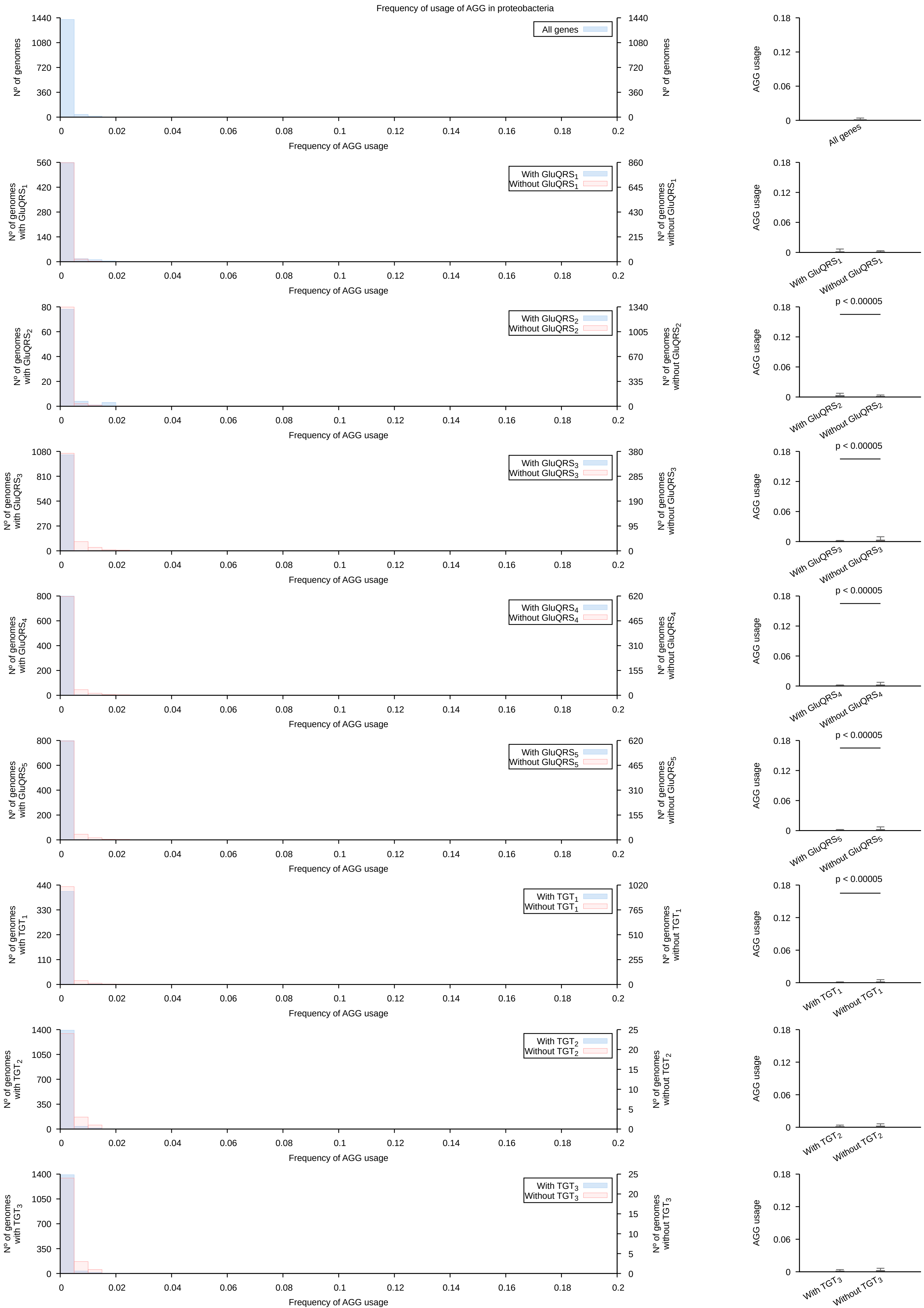
Frequency of usage of AGG in proteobacteria
All genes
With GluQRS1
Without GluQRS1
With GluQRS2
Without GluQRS2
With GluQRS3
Without GluQRS3
With GluQRS4
Without GluQRS4
With GluQRS5
Without GluQRS5
With TGT1
Without TGT1
With TGT2
Without TGT2
With TGT3
Without TGT3
Nº of genomes
Frequency of AGG usage
AGG usage
p < 0.00005

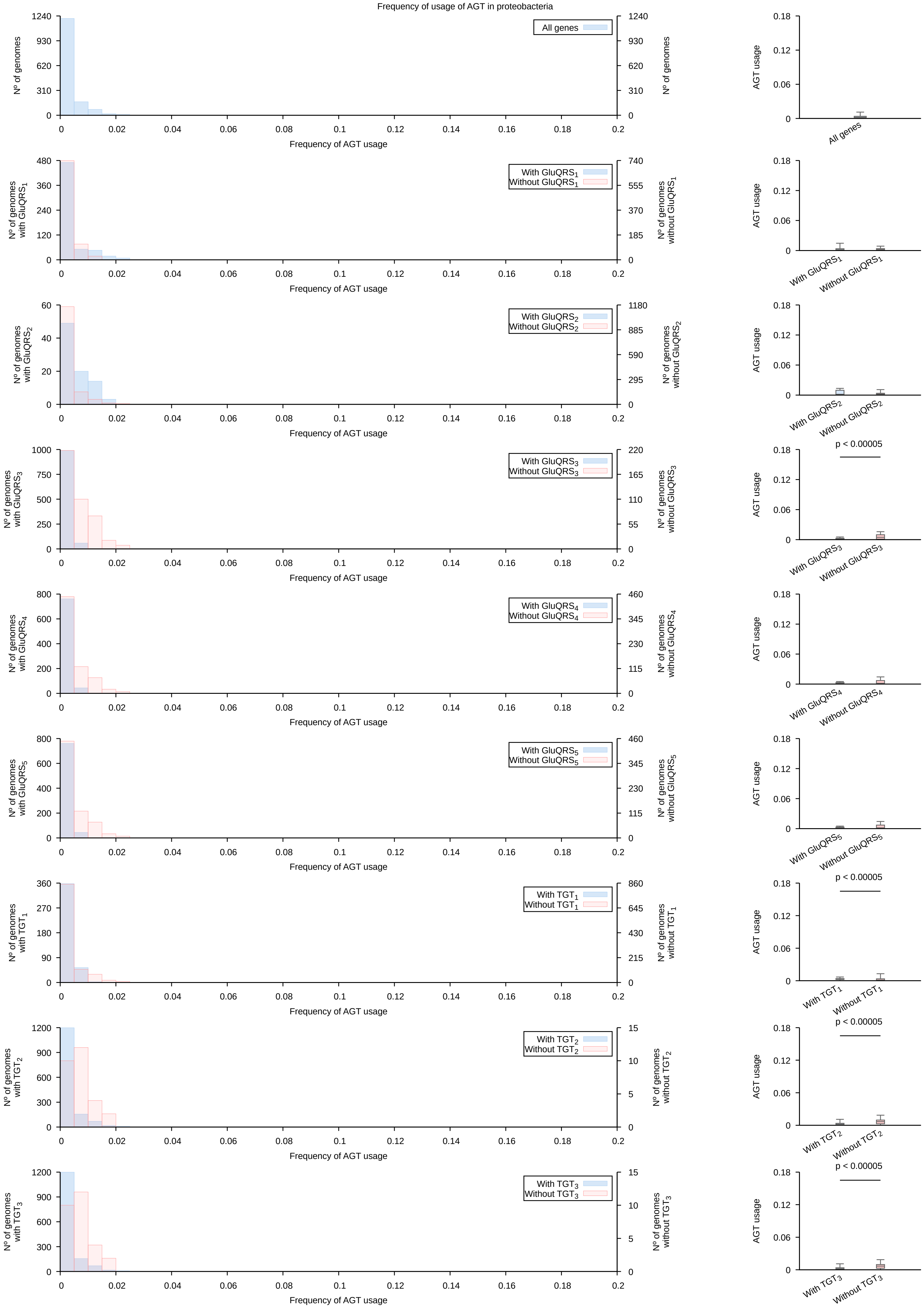
Frequency of usage of AGT in proteobacteria
All genes
With GluQRS$_1$
Without GluQRS$_1$
With GluQRS$_2$
Without GluQRS$_2$
With GluQRS$_3$
Without GluQRS$_3$
With GluQRS$_4$
Without GluQRS$_4$
With GluQRS$_5$
Without GluQRS$_5$
With TGT$_1$
Without TGT$_1$
With TGT$_2$
Without TGT$_2$
With TGT$_3$
Without TGT$_3$
Nº of genomes
Nº of genomes with GluQRS$_1$
Nº of genomes without GluQRS$_1$
Nº of genomes with GluQRS$_2$
Nº of genomes without GluQRS$_2$
Nº of genomes with GluQRS$_3$
Nº of genomes without GluQRS$_3$
Nº of genomes with GluQRS$_4$
Nº of genomes without GluQRS$_4$
Nº of genomes with GluQRS$_5$
Nº of genomes without GluQRS$_5$
Nº of genomes with TGT$_1$
Nº of genomes without TGT$_1$
Nº of genomes with TGT$_2$
Nº of genomes without TGT$_2$
Nº of genomes with TGT$_3$
Nº of genomes without TGT$_3$
Frequency of AGT usage
AGT usage
p < 0.00005

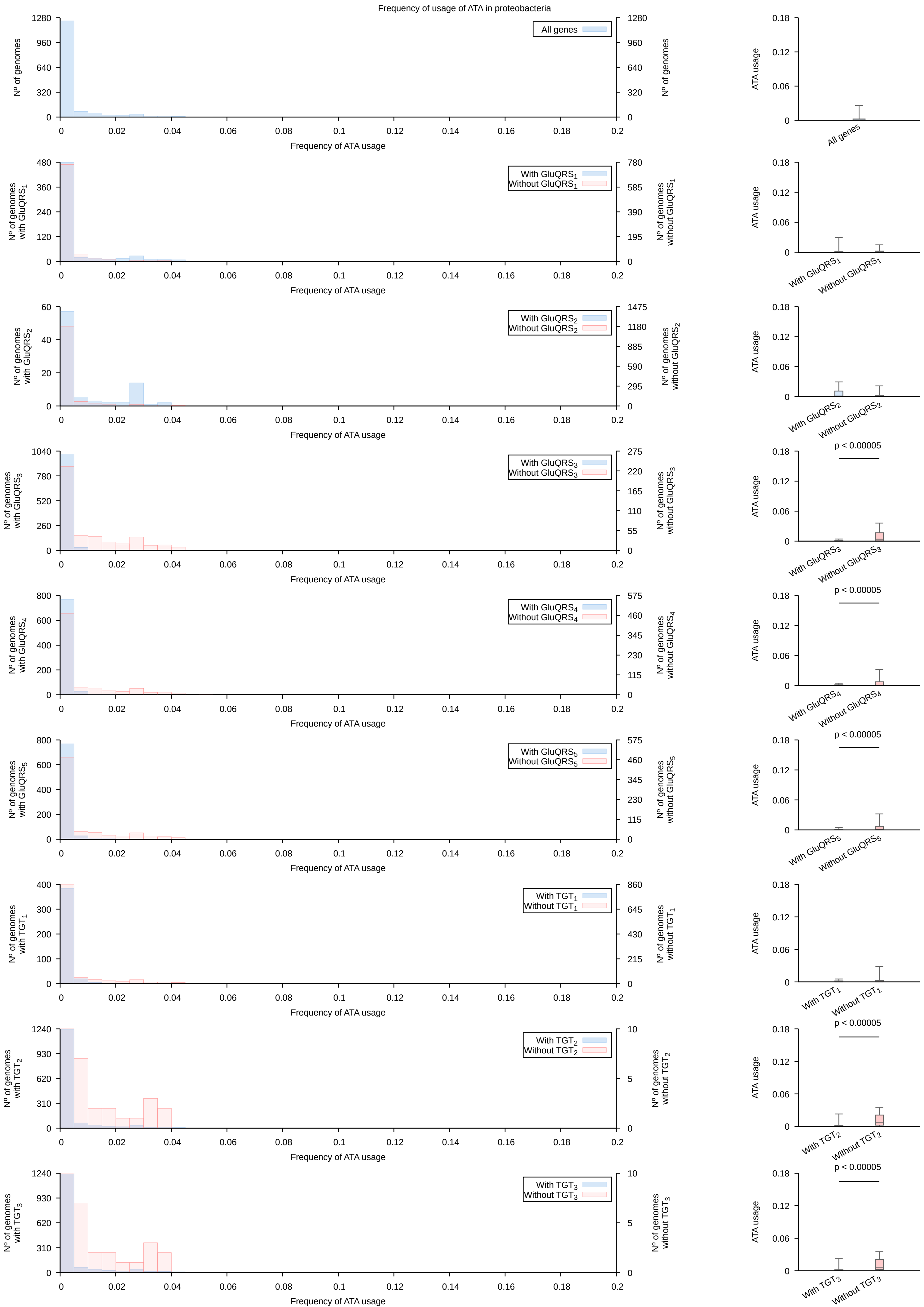
Frequency of usage of ATA in proteobacteria
All genes
Nº of genomes
Frequency of ATA usage
ATA usage
With GluQRS$_1$
Without GluQRS$_1$
Nº of genomes with GluQRS$_1$
Nº of genomes without GluQRS$_1$
With GluQRS$_2$
Without GluQRS$_2$
Nº of genomes with GluQRS$_2$
Nº of genomes without GluQRS$_2$
With GluQRS$_3$
Without GluQRS$_3$
Nº of genomes with GluQRS$_3$
Nº of genomes without GluQRS$_3$
p < 0.00005
With GluQRS$_4$
Without GluQRS$_4$
Nº of genomes with GluQRS$_4$
Nº of genomes without GluQRS$_4$
p < 0.00005
With GluQRS$_5$
Without GluQRS$_5$
Nº of genomes with GluQRS$_5$
Nº of genomes without GluQRS$_5$
p < 0.00005
With TGT$_1$
Without TGT$_1$
Nº of genomes with TGT$_1$
Nº of genomes without TGT$_1$
With TGT$_2$
Without TGT$_2$
Nº of genomes with TGT$_2$
Nº of genomes without TGT$_2$
p < 0.00005
With TGT$_3$
Without TGT$_3$
Nº of genomes with TGT$_3$
Nº of genomes without TGT$_3$
p < 0.00005

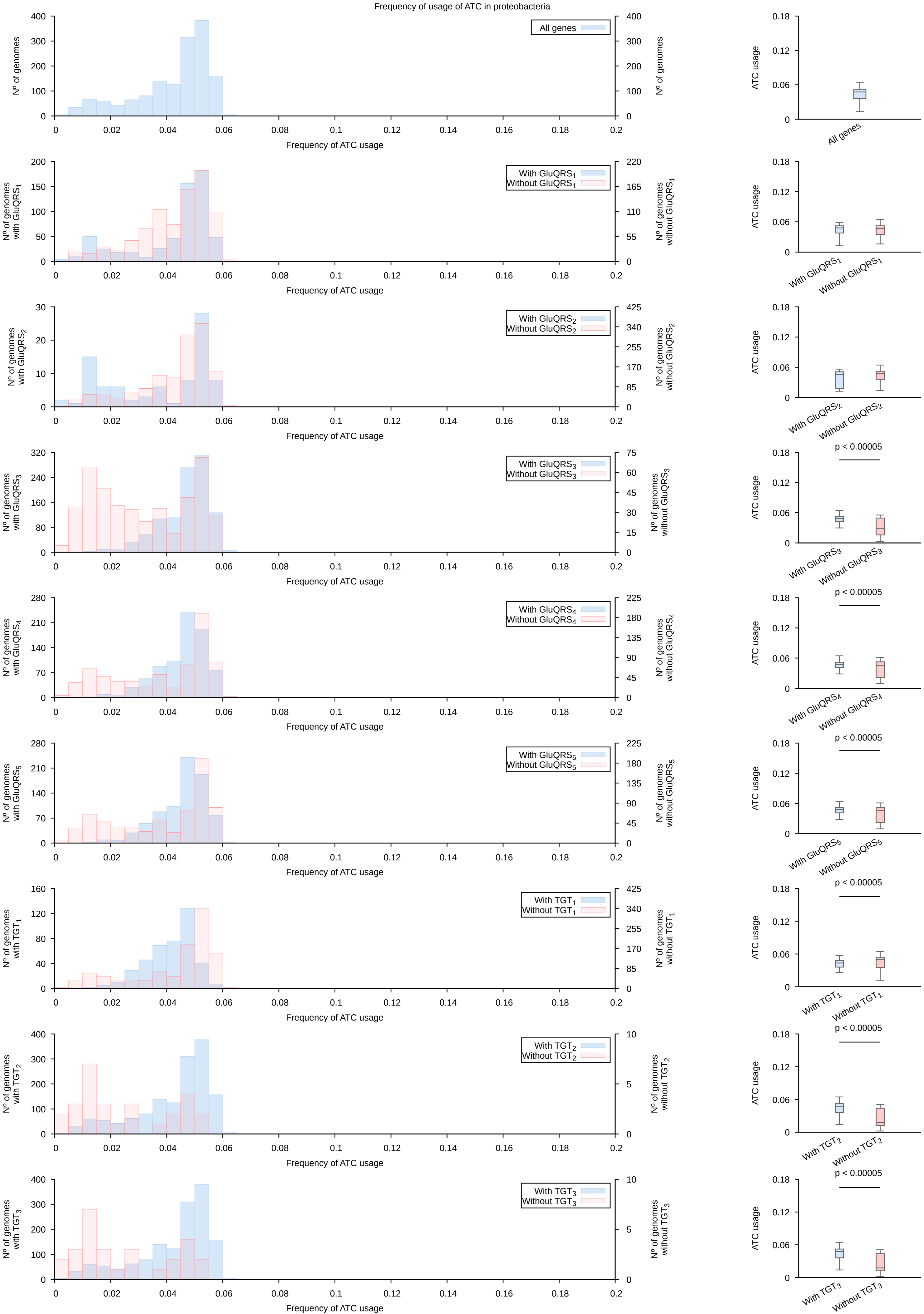

Frequency of usage of ATC in proteobacteria
All genes
Nº of genomes
Frequency of ATC usage
ATC usage
With GluQRS1
Without GluQRS1
Nº of genomes with GluQRS1
Nº of genomes without GluQRS1
With GluQRS2
Without GluQRS2
Nº of genomes with GluQRS2
Nº of genomes without GluQRS2
p < 0.00005
With GluQRS3
Without GluQRS3
Nº of genomes with GluQRS3
Nº of genomes without GluQRS3
With GluQRS4
Without GluQRS4
Nº of genomes with GluQRS4
Nº of genomes without GluQRS4
With GluQRS5
Without GluQRS5
Nº of genomes with GluQRS5
Nº of genomes without GluQRS5
With TGT1
Without TGT1
Nº of genomes with TGT1
Nº of genomes without TGT1
With TGT2
Without TGT2
Nº of genomes with TGT2
Nº of genomes without TGT2
With TGT3
Without TGT3
Nº of genomes with TGT3
Nº of genomes without TGT3

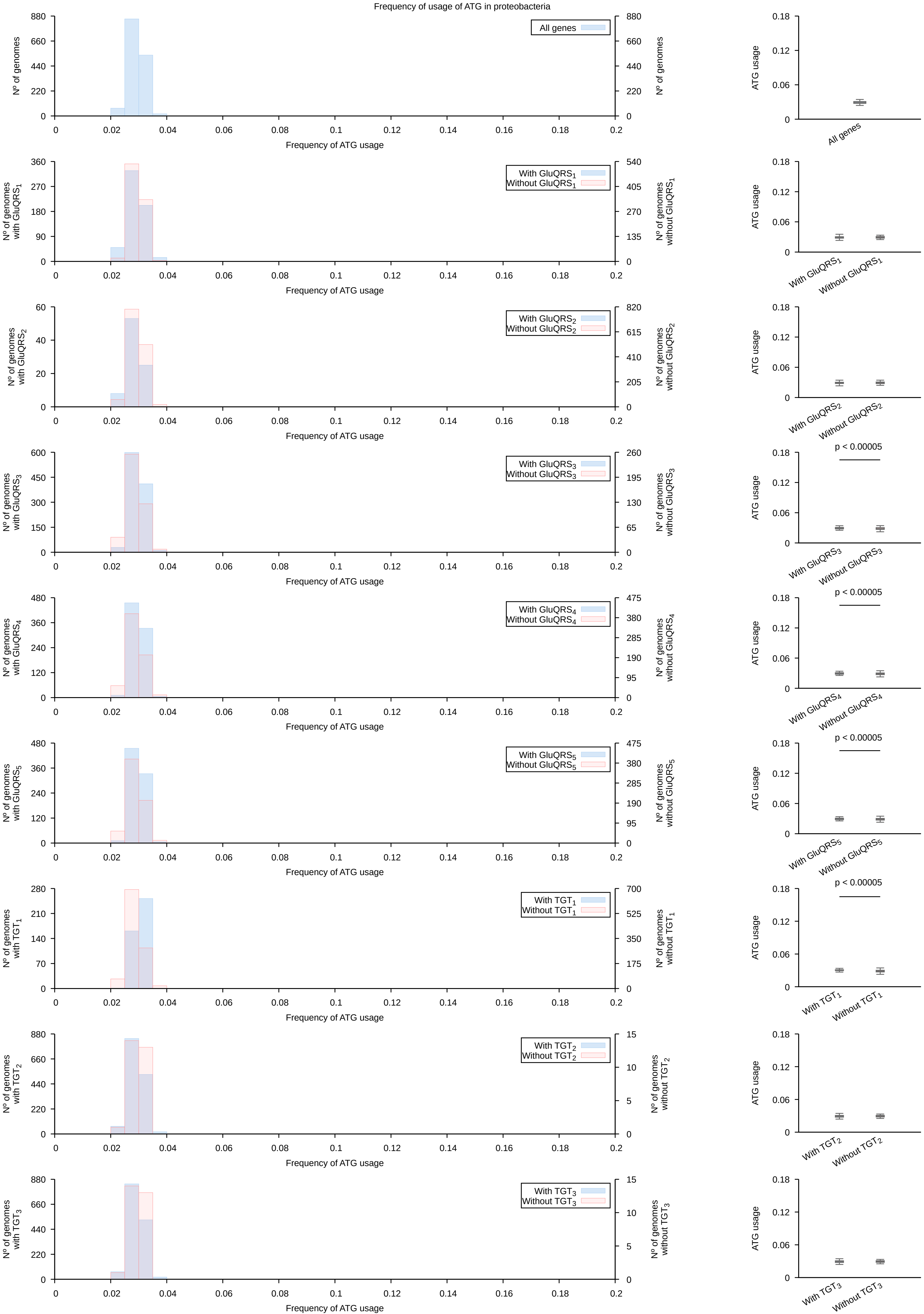

Frequency of usage of ATG in proteobacteria
Nº of genomes
All genes
Frequency of ATG usage
ATG usage
Nº of genomes with GluQRS$_1$
Nº of genomes without GluQRS$_1$
With GluQRS$_1$
Without GluQRS$_1$
Nº of genomes with GluQRS$_2$
Nº of genomes without GluQRS$_2$
With GluQRS$_2$
Without GluQRS$_2$
Nº of genomes with GluQRS$_3$
Nº of genomes without GluQRS$_3$
With GluQRS$_3$
Without GluQRS$_3$
p < 0.00005
Nº of genomes with GluQRS$_4$
Nº of genomes without GluQRS$_4$
With GluQRS$_4$
Without GluQRS$_4$
p < 0.00005
Nº of genomes with GluQRS$_5$
Nº of genomes without GluQRS$_5$
With GluQRS$_5$
Without GluQRS$_5$
p < 0.00005
Nº of genomes with TGT$_1$
Nº of genomes without TGT$_1$
With TGT$_1$
Without TGT$_1$
p < 0.00005
Nº of genomes with TGT$_2$
Nº of genomes without TGT$_2$
With TGT$_2$
Without TGT$_2$
Nº of genomes with TGT$_3$
Nº of genomes without TGT$_3$
With TGT$_3$
Without TGT$_3$

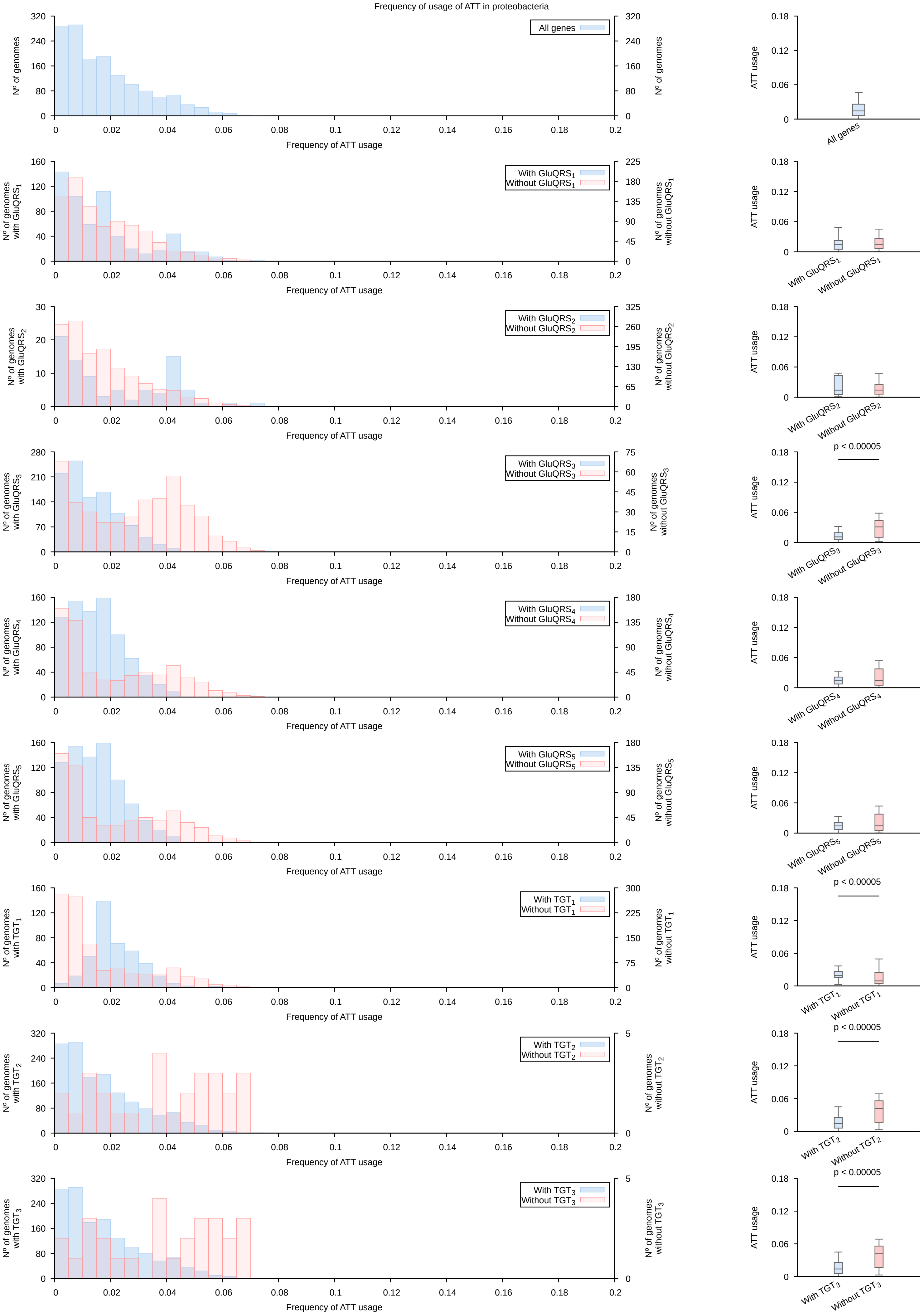

Frequency of usage of ATT in proteobacteria
All genes
Nº of genomes
Frequency of ATT usage
ATT usage
With GluQRS$_1$
Without GluQRS$_1$
Nº of genomes with GluQRS$_1$
Nº of genomes without GluQRS$_1$
With GluQRS$_2$
Without GluQRS$_2$
Nº of genomes with GluQRS$_2$
Nº of genomes without GluQRS$_2$
With GluQRS$_3$
Without GluQRS$_3$
Nº of genomes with GluQRS$_3$
Nº of genomes without GluQRS$_3$
p < 0.00005
With GluQRS$_4$
Without GluQRS$_4$
Nº of genomes with GluQRS$_4$
Nº of genomes without GluQRS$_4$
With GluQRS$_5$
Without GluQRS$_5$
Nº of genomes with GluQRS$_5$
Nº of genomes without GluQRS$_5$
With TGT$_1$
Without TGT$_1$
Nº of genomes with TGT$_1$
Nº of genomes without TGT$_1$
p < 0.00005
With TGT$_2$
Without TGT$_2$
Nº of genomes with TGT$_2$
Nº of genomes without TGT$_2$
p < 0.00005
With TGT$_3$
Without TGT$_3$
Nº of genomes with TGT$_3$
Nº of genomes without TGT$_3$
p < 0.00005

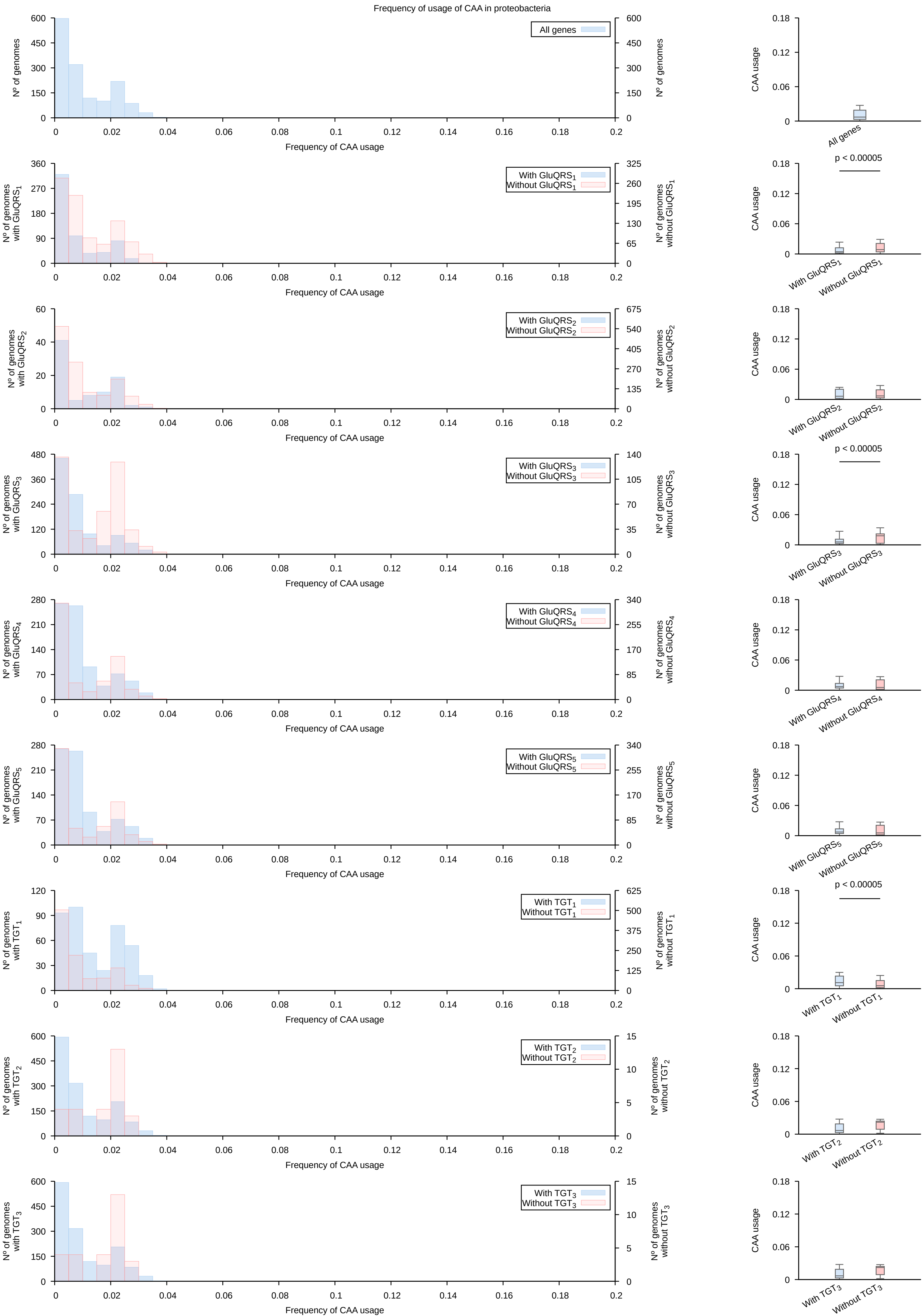
Frequency of usage of CAA in proteobacteria
All genes
Nº of genomes
Frequency of CAA usage
CAA usage
With GluQRS$_1$
Without GluQRS$_1$
Nº of genomes with GluQRS$_1$
Nº of genomes without GluQRS$_1$
p < 0.00005
With GluQRS$_2$
Without GluQRS$_2$
Nº of genomes with GluQRS$_2$
Nº of genomes without GluQRS$_2$
With GluQRS$_3$
Without GluQRS$_3$
Nº of genomes with GluQRS$_3$
Nº of genomes without GluQRS$_3$
p < 0.00005
With GluQRS$_4$
Without GluQRS$_4$
Nº of genomes with GluQRS$_4$
Nº of genomes without GluQRS$_4$
With GluQRS$_5$
Without GluQRS$_5$
Nº of genomes with GluQRS$_5$
Nº of genomes without GluQRS$_5$
With TGT$_1$
Without TGT$_1$
Nº of genomes with TGT$_1$
Nº of genomes without TGT$_1$
p < 0.00005
With TGT$_2$
Without TGT$_2$
Nº of genomes with TGT$_2$
Nº of genomes without TGT$_2$
With TGT$_3$
Without TGT$_3$
Nº of genomes with TGT$_3$
Nº of genomes without TGT$_3$

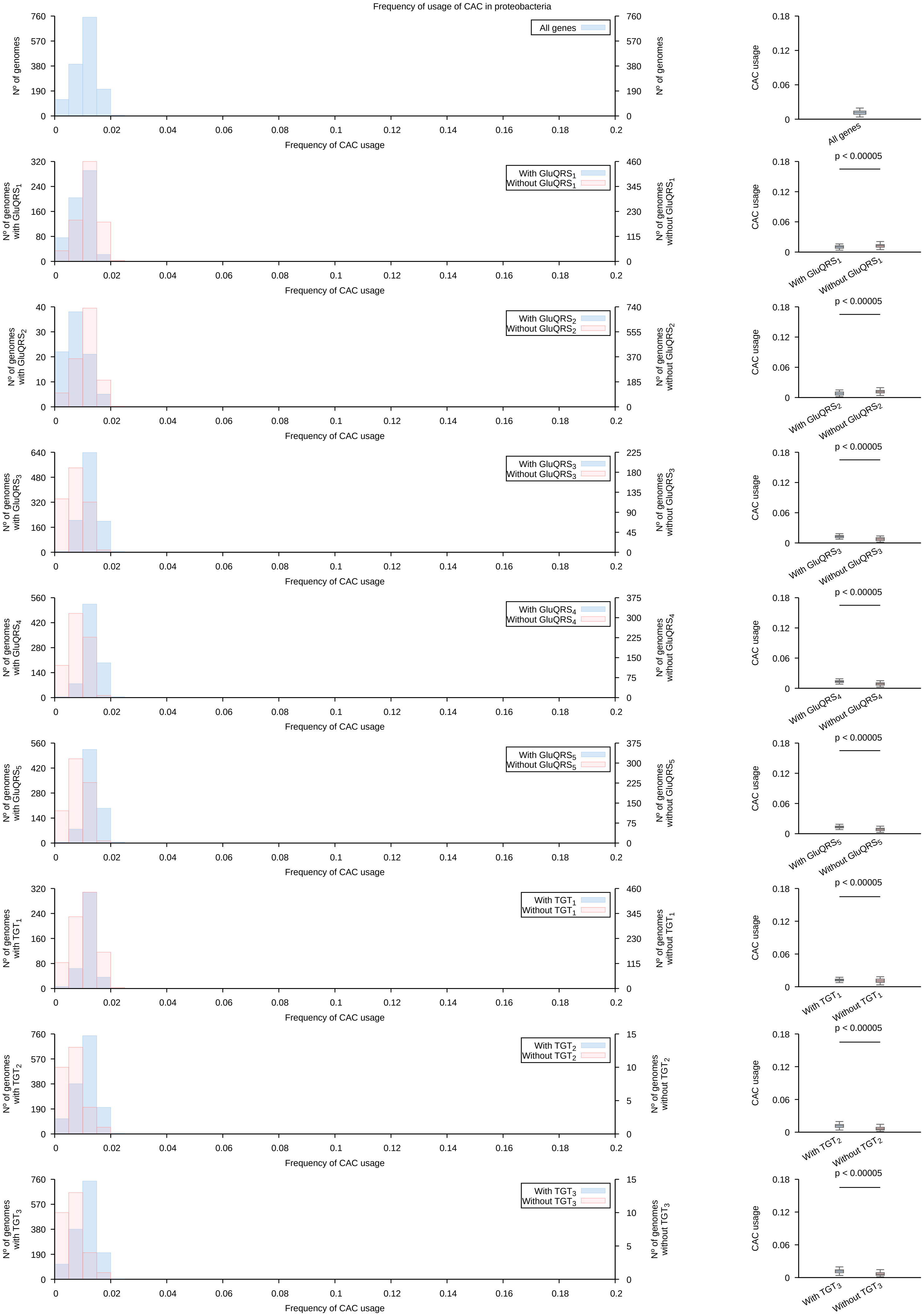
Frequency of usage of CAC in proteobacteria
Nº of genomes
Frequency of CAC usage
CAC usage
All genes
Nº of genomes with GluQRS$_1$
Nº of genomes without GluQRS$_1$
With GluQRS$_1$
Without GluQRS$_1$
p < 0.00005
Nº of genomes with GluQRS$_2$
Nº of genomes without GluQRS$_2$
With GluQRS$_2$
Without GluQRS$_2$
p < 0.00005
Nº of genomes with GluQRS$_3$
Nº of genomes without GluQRS$_3$
With GluQRS$_3$
Without GluQRS$_3$
p < 0.00005
Nº of genomes with GluQRS$_4$
Nº of genomes without GluQRS$_4$
With GluQRS$_4$
Without GluQRS$_4$
p < 0.00005
Nº of genomes with GluQRS$_5$
Nº of genomes without GluQRS$_5$
With GluQRS$_5$
Without GluQRS$_5$
p < 0.00005
Nº of genomes with TGT$_1$
Nº of genomes without TGT$_1$
With TGT$_1$
Without TGT$_1$
p < 0.00005
Nº of genomes with TGT$_2$
Nº of genomes without TGT$_2$
With TGT$_2$
Without TGT$_2$
p < 0.00005
Nº of genomes with TGT$_3$
Nº of genomes without TGT$_3$
With TGT$_3$
Without TGT$_3$
p < 0.00005

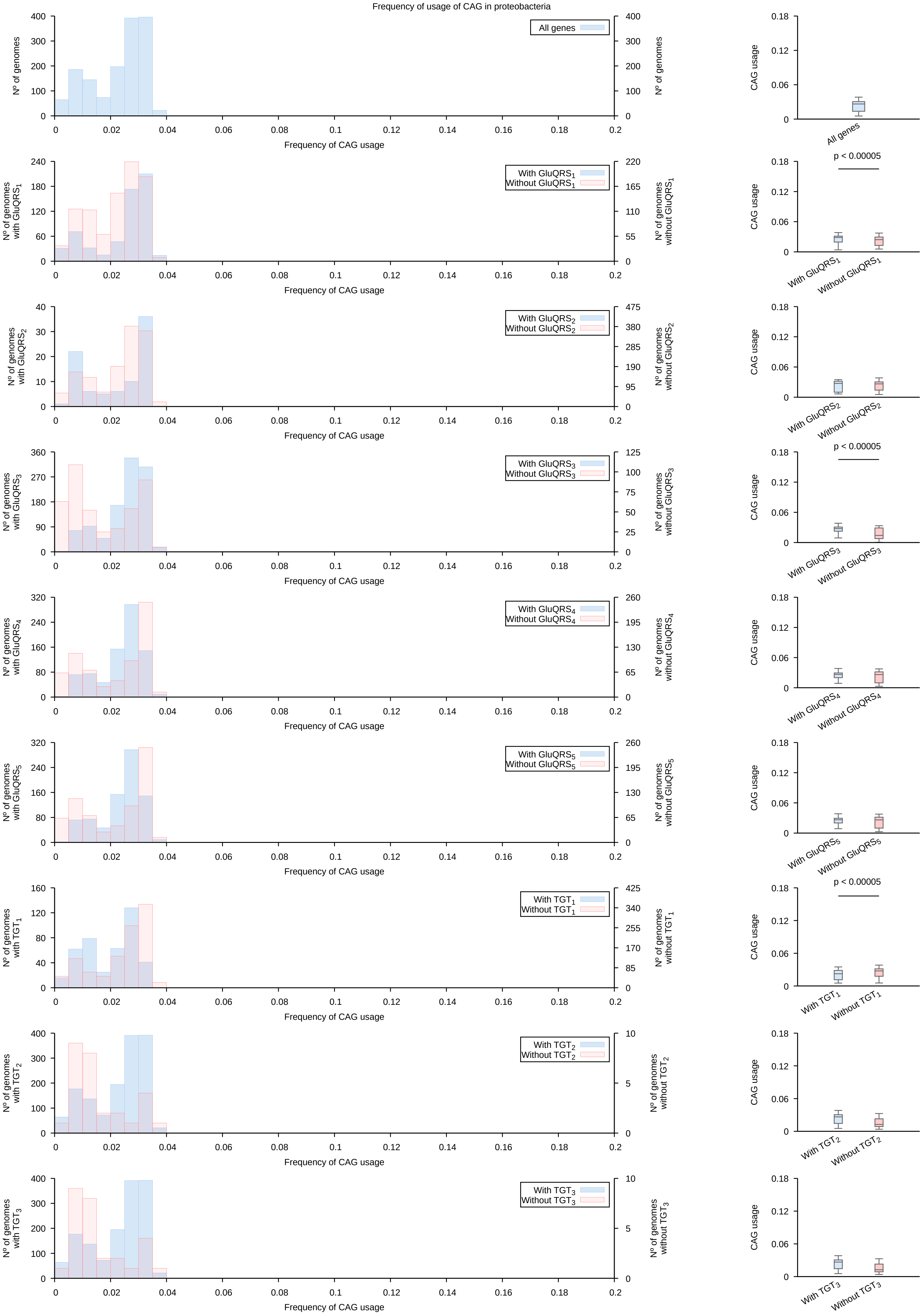
Frequency of usage of CAG in proteobacteria
All genes
Nº of genomes
Frequency of CAG usage
CAG usage
With GluQRS$_1$
Without GluQRS$_1$
Nº of genomes with GluQRS$_1$
Nº of genomes without GluQRS$_1$
p < 0.00005
With GluQRS$_2$
Without GluQRS$_2$
Nº of genomes with GluQRS$_2$
Nº of genomes without GluQRS$_2$
With GluQRS$_3$
Without GluQRS$_3$
Nº of genomes with GluQRS$_3$
Nº of genomes without GluQRS$_3$
p < 0.00005
With GluQRS$_4$
Without GluQRS$_4$
Nº of genomes with GluQRS$_4$
Nº of genomes without GluQRS$_4$
With GluQRS$_5$
Without GluQRS$_5$
Nº of genomes with GluQRS$_5$
Nº of genomes without GluQRS$_5$
With TGT$_1$
Without TGT$_1$
Nº of genomes with TGT$_1$
Nº of genomes without TGT$_1$
p < 0.00005
With TGT$_2$
Without TGT$_2$
Nº of genomes with TGT$_2$
Nº of genomes without TGT$_2$
With TGT$_3$
Without TGT$_3$
Nº of genomes with TGT$_3$
Nº of genomes without TGT$_3$

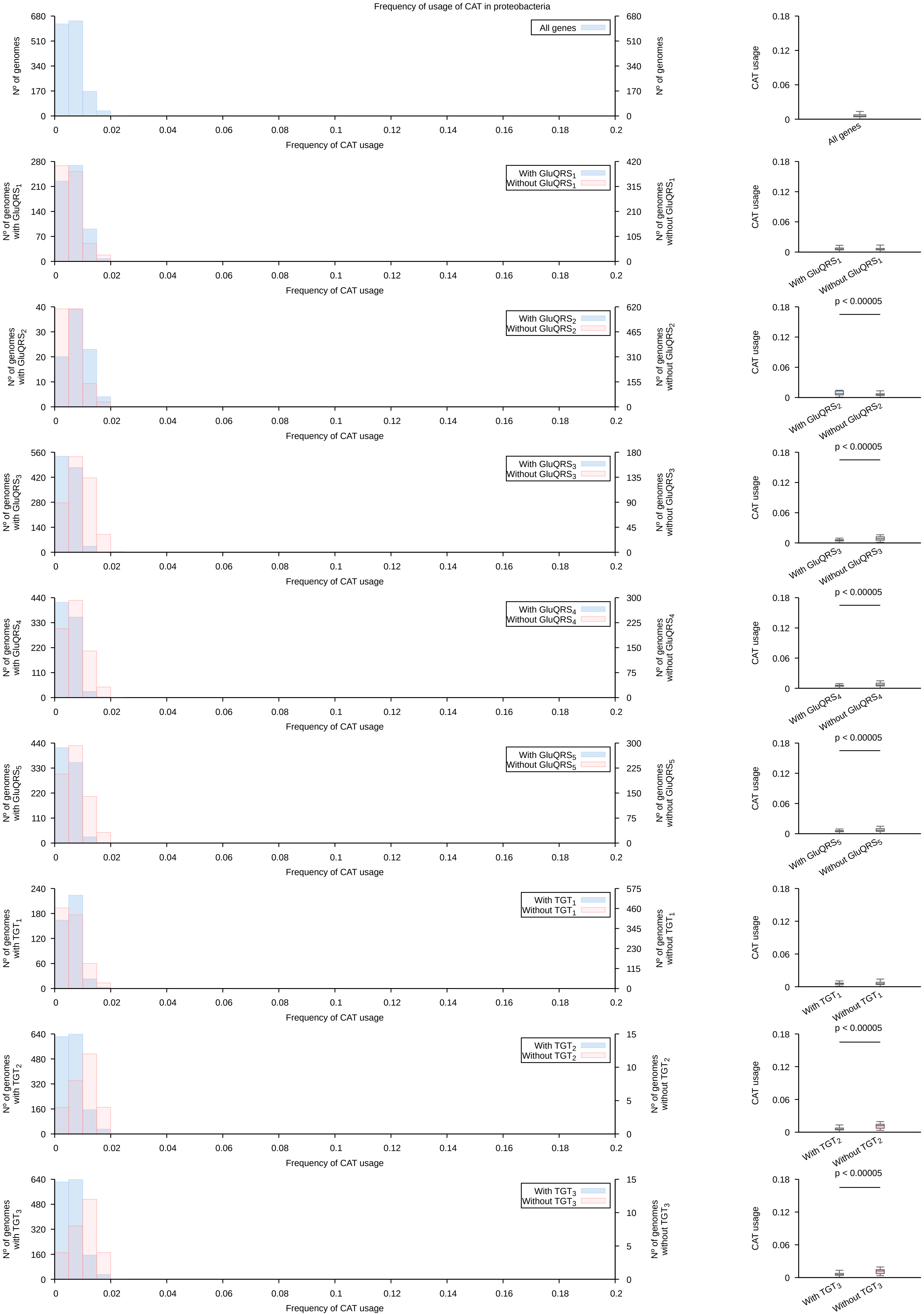
Frequency of usage of CAT in proteobacteria
Frequency of CAT usage
Nº of genomes
CAT usage
All genes
Nº of genomes with GluQRS$_1$
Nº of genomes without GluQRS$_1$
With GluQRS$_1$
Without GluQRS$_1$
Nº of genomes with GluQRS$_2$
Nº of genomes without GluQRS$_2$
With GluQRS$_2$
Without GluQRS$_2$
p < 0.00005
Nº of genomes with GluQRS$_3$
Nº of genomes without GluQRS$_3$
With GluQRS$_3$
Without GluQRS$_3$
p < 0.00005
Nº of genomes with GluQRS$_4$
Nº of genomes without GluQRS$_4$
With GluQRS$_4$
Without GluQRS$_4$
p < 0.00005
Nº of genomes with GluQRS$_5$
Nº of genomes without GluQRS$_5$
With GluQRS$_5$
Without GluQRS$_5$
p < 0.00005
Nº of genomes with TGT$_1$
Nº of genomes without TGT$_1$
With TGT$_1$
Without TGT$_1$
Nº of genomes with TGT$_2$
Nº of genomes without TGT$_2$
With TGT$_2$
Without TGT$_2$
p < 0.00005
Nº of genomes with TGT$_3$
Nº of genomes without TGT$_3$
With TGT$_3$
Without TGT$_3$
p < 0.00005

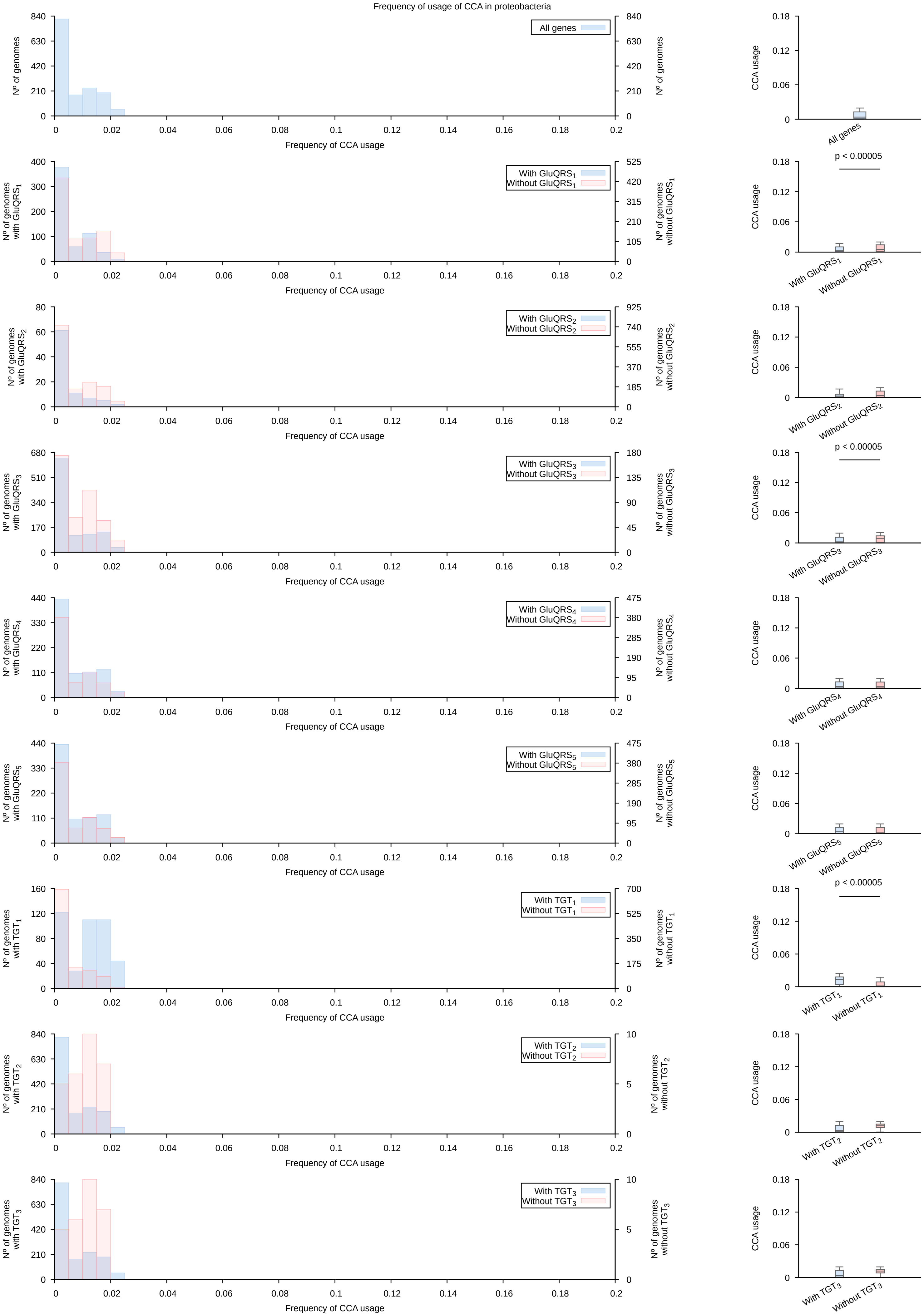
Frequency of usage of CCA in proteobacteria
Nº of genomes
Frequency of CCA usage
All genes
CCA usage
p < 0.00005
Nº of genomes with $GluQRS_1$
Nº of genomes without $GluQRS_1$
With $GluQRS_1$
Without $GluQRS_1$
Nº of genomes with $GluQRS_2$
Nº of genomes without $GluQRS_2$
With $GluQRS_2$
Without $GluQRS_2$
Nº of genomes with $GluQRS_3$
Nº of genomes without $GluQRS_3$
With $GluQRS_3$
Without $GluQRS_3$
Nº of genomes with $GluQRS_4$
Nº of genomes without $GluQRS_4$
With $GluQRS_4$
Without $GluQRS_4$
Nº of genomes with $GluQRS_5$
Nº of genomes without $GluQRS_5$
With $GluQRS_5$
Without $GluQRS_5$
Nº of genomes with TGT_1
Nº of genomes without TGT_1
With TGT_1
Without TGT_1
Nº of genomes with TGT_2
Nº of genomes without TGT_2
With TGT_2
Without TGT_2
Nº of genomes with TGT_3
Nº of genomes without TGT_3
With TGT_3
Without TGT_3

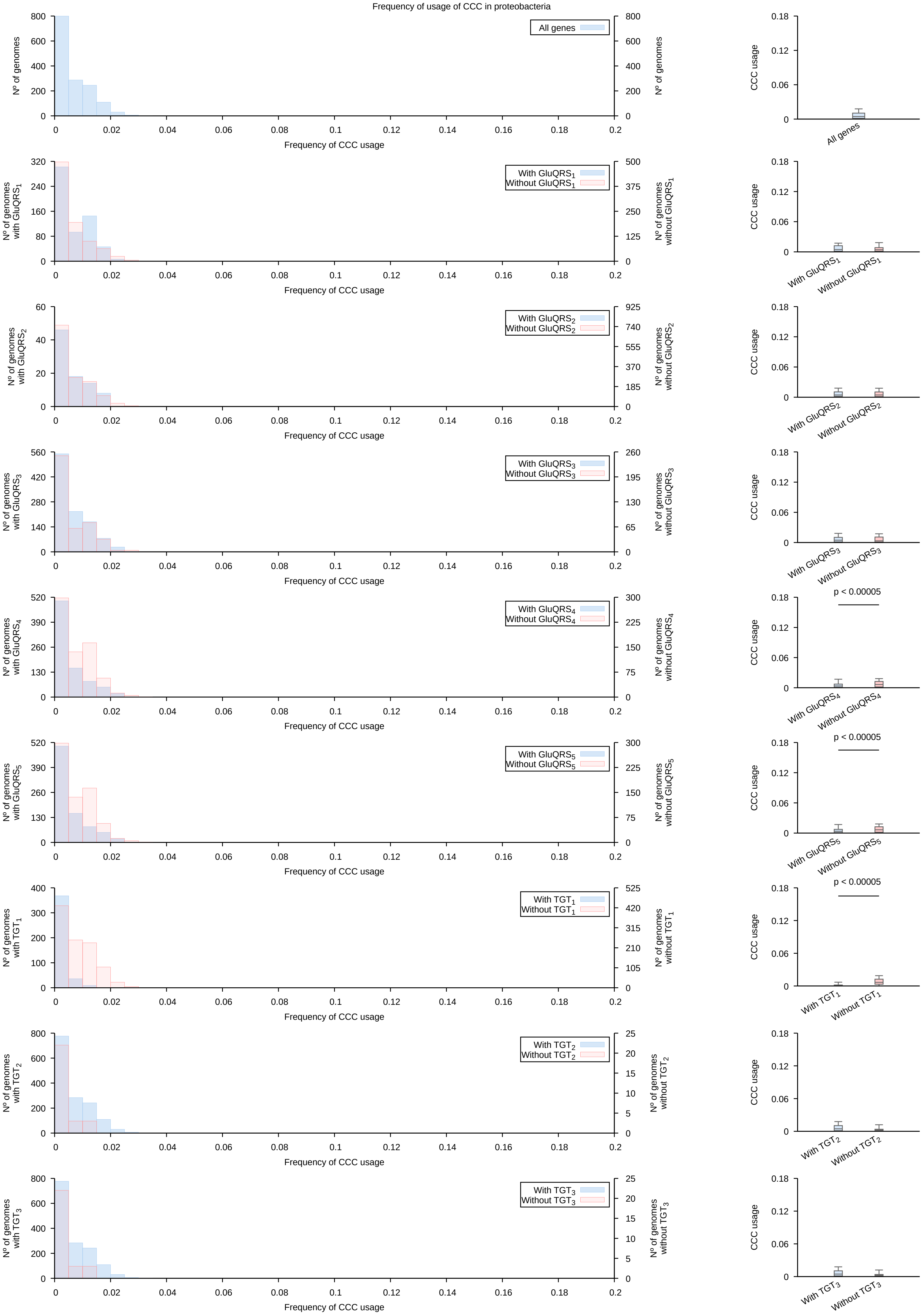
Frequency of usage of CCC in proteobacteria
All genes
With GluQRS$_1$
Without GluQRS$_1$
With GluQRS$_2$
Without GluQRS$_2$
With GluQRS$_3$
Without GluQRS$_3$
With GluQRS$_4$
Without GluQRS$_4$
With GluQRS$_5$
Without GluQRS$_5$
With TGT$_1$
Without TGT$_1$
With TGT$_2$
Without TGT$_2$
With TGT$_3$
Without TGT$_3$
Nº of genomes
Nº of genomes with GluQRS$_1$
Nº of genomes without GluQRS$_1$
Nº of genomes with GluQRS$_2$
Nº of genomes without GluQRS$_2$
Nº of genomes with GluQRS$_3$
Nº of genomes without GluQRS$_3$
Nº of genomes with GluQRS$_4$
Nº of genomes without GluQRS$_4$
Nº of genomes with GluQRS$_5$
Nº of genomes without GluQRS$_5$
Nº of genomes with TGT$_1$
Nº of genomes without TGT$_1$
Nº of genomes with TGT$_2$
Nº of genomes without TGT$_2$
Nº of genomes with TGT$_3$
Nº of genomes without TGT$_3$
Frequency of CCC usage
CCC usage
p < 0.00005

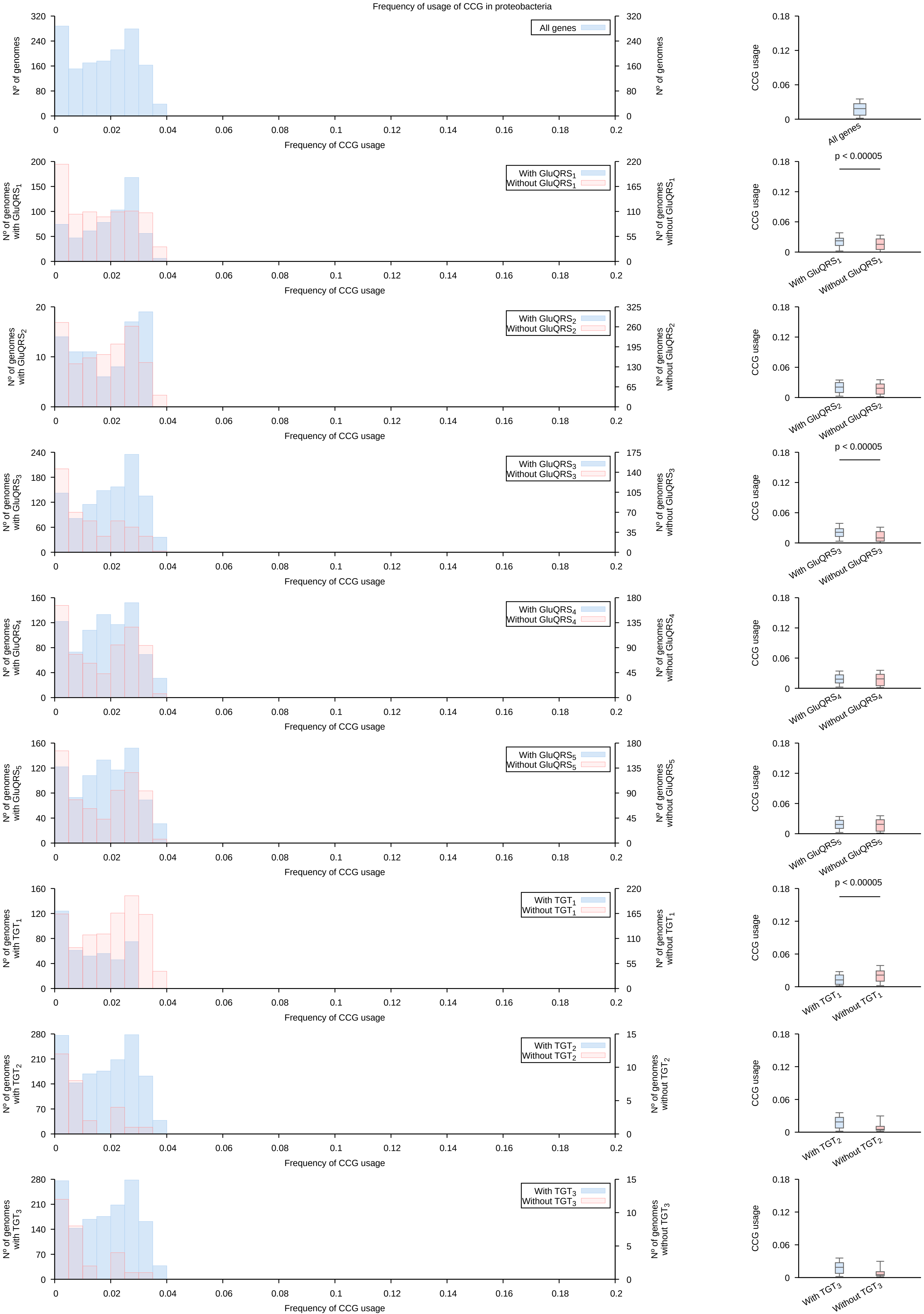
Frequency of usage of CCG in proteobacteria
All genes
Nº of genomes
Frequency of CCG usage
CCG usage
With GluQRS$_1$
Without GluQRS$_1$
Nº of genomes with GluQRS$_1$
Nº of genomes without GluQRS$_1$
p < 0.00005
With GluQRS$_2$
Without GluQRS$_2$
Nº of genomes with GluQRS$_2$
Nº of genomes without GluQRS$_2$
With GluQRS$_3$
Without GluQRS$_3$
Nº of genomes with GluQRS$_3$
Nº of genomes without GluQRS$_3$
p < 0.00005
With GluQRS$_4$
Without GluQRS$_4$
Nº of genomes with GluQRS$_4$
Nº of genomes without GluQRS$_4$
With GluQRS$_5$
Without GluQRS$_5$
Nº of genomes with GluQRS$_5$
Nº of genomes without GluQRS$_5$
With TGT$_1$
Without TGT$_1$
Nº of genomes with TGT$_1$
Nº of genomes without TGT$_1$
p < 0.00005
With TGT$_2$
Without TGT$_2$
Nº of genomes with TGT$_2$
Nº of genomes without TGT$_2$
With TGT$_3$
Without TGT$_3$
Nº of genomes with TGT$_3$
Nº of genomes without TGT$_3$

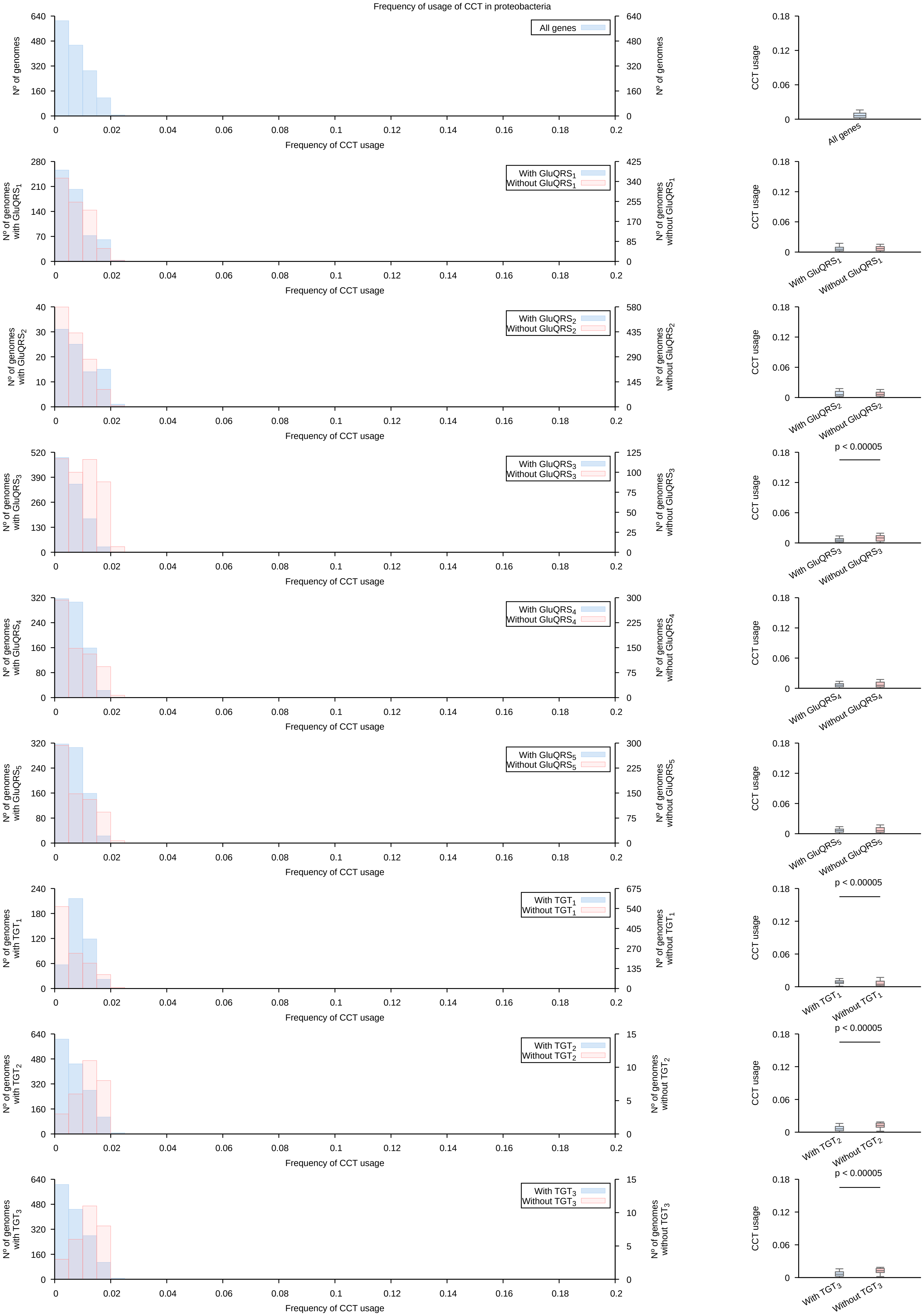
Frequency of usage of CCT in proteobacteria
All genes
With GluQRS$_1$
Without GluQRS$_1$
With GluQRS$_2$
Without GluQRS$_2$
With GluQRS$_3$
Without GluQRS$_3$
With GluQRS$_4$
Without GluQRS$_4$
With GluQRS$_5$
Without GluQRS$_5$
With TGT$_1$
Without TGT$_1$
With TGT$_2$
Without TGT$_2$
With TGT$_3$
Without TGT$_3$
Nº of genomes
Nº of genomes with GluQRS$_1$
Nº of genomes without GluQRS$_1$
Nº of genomes with GluQRS$_2$
Nº of genomes without GluQRS$_2$
Nº of genomes with GluQRS$_3$
Nº of genomes without GluQRS$_3$
Nº of genomes with GluQRS$_4$
Nº of genomes without GluQRS$_4$
Nº of genomes with GluQRS$_5$
Nº of genomes without GluQRS$_5$
Nº of genomes with TGT$_1$
Nº of genomes without TGT$_1$
Nº of genomes with TGT$_2$
Nº of genomes without TGT$_2$
Nº of genomes with TGT$_3$
Nº of genomes without TGT$_3$
Frequency of CCT usage
CCT usage
p < 0.00005

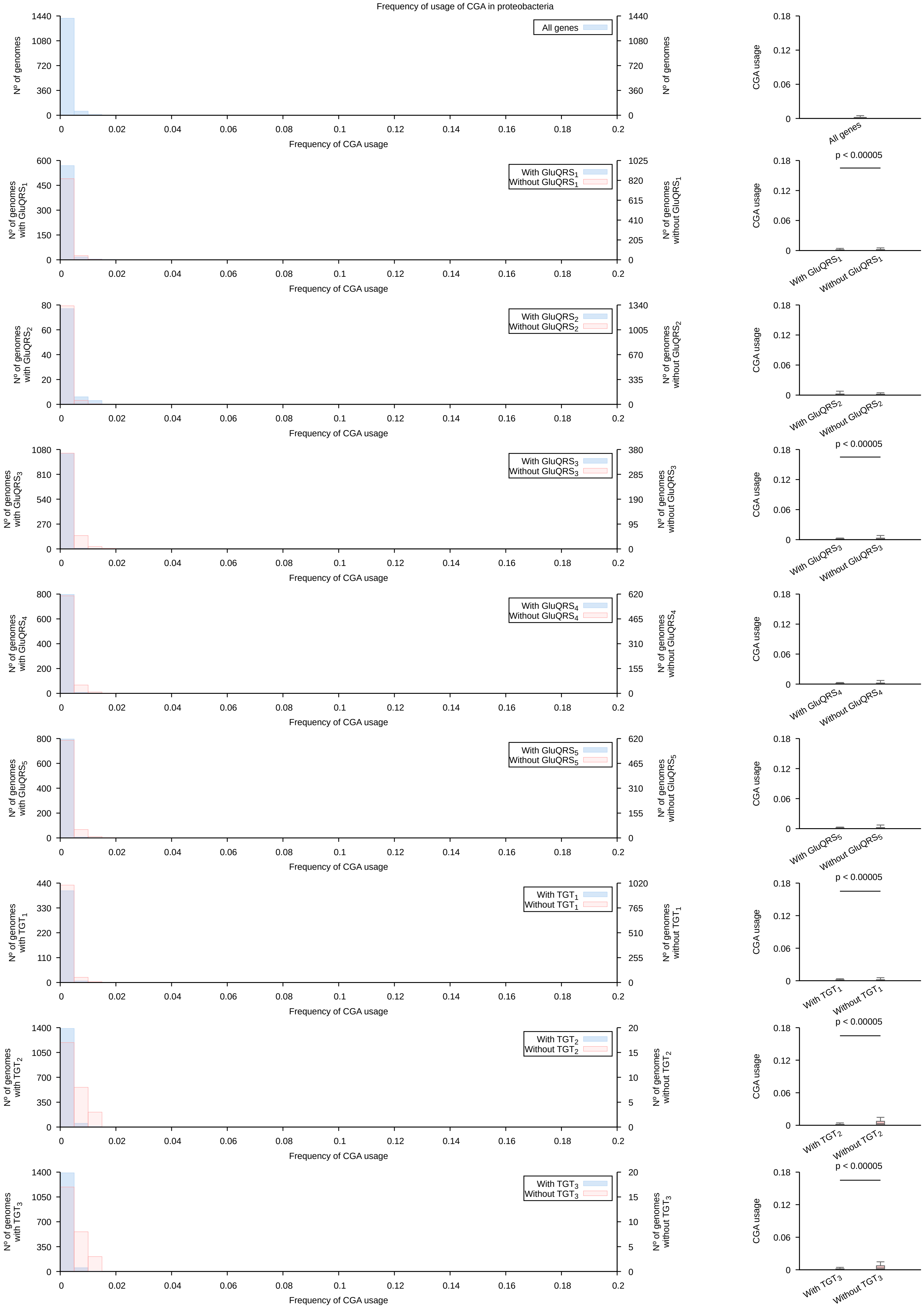

Frequency of usage of CGA in proteobacteria
All genes
Nº of genomes
Frequency of CGA usage
CGA usage
With GluQRS$_1$
Without GluQRS$_1$
Nº of genomes with GluQRS$_1$
Nº of genomes without GluQRS$_1$
p < 0.00005
With GluQRS$_2$
Without GluQRS$_2$
Nº of genomes with GluQRS$_2$
Nº of genomes without GluQRS$_2$
With GluQRS$_3$
Without GluQRS$_3$
Nº of genomes with GluQRS$_3$
Nº of genomes without GluQRS$_3$
p < 0.00005
With GluQRS$_4$
Without GluQRS$_4$
Nº of genomes with GluQRS$_4$
Nº of genomes without GluQRS$_4$
With GluQRS$_5$
Without GluQRS$_5$
Nº of genomes with GluQRS$_5$
Nº of genomes without GluQRS$_5$
With TGT$_1$
Without TGT$_1$
Nº of genomes with TGT$_1$
Nº of genomes without TGT$_1$
p < 0.00005
With TGT$_2$
Without TGT$_2$
Nº of genomes with TGT$_2$
Nº of genomes without TGT$_2$
p < 0.00005
With TGT$_3$
Without TGT$_3$
Nº of genomes with TGT$_3$
Nº of genomes without TGT$_3$
p < 0.00005

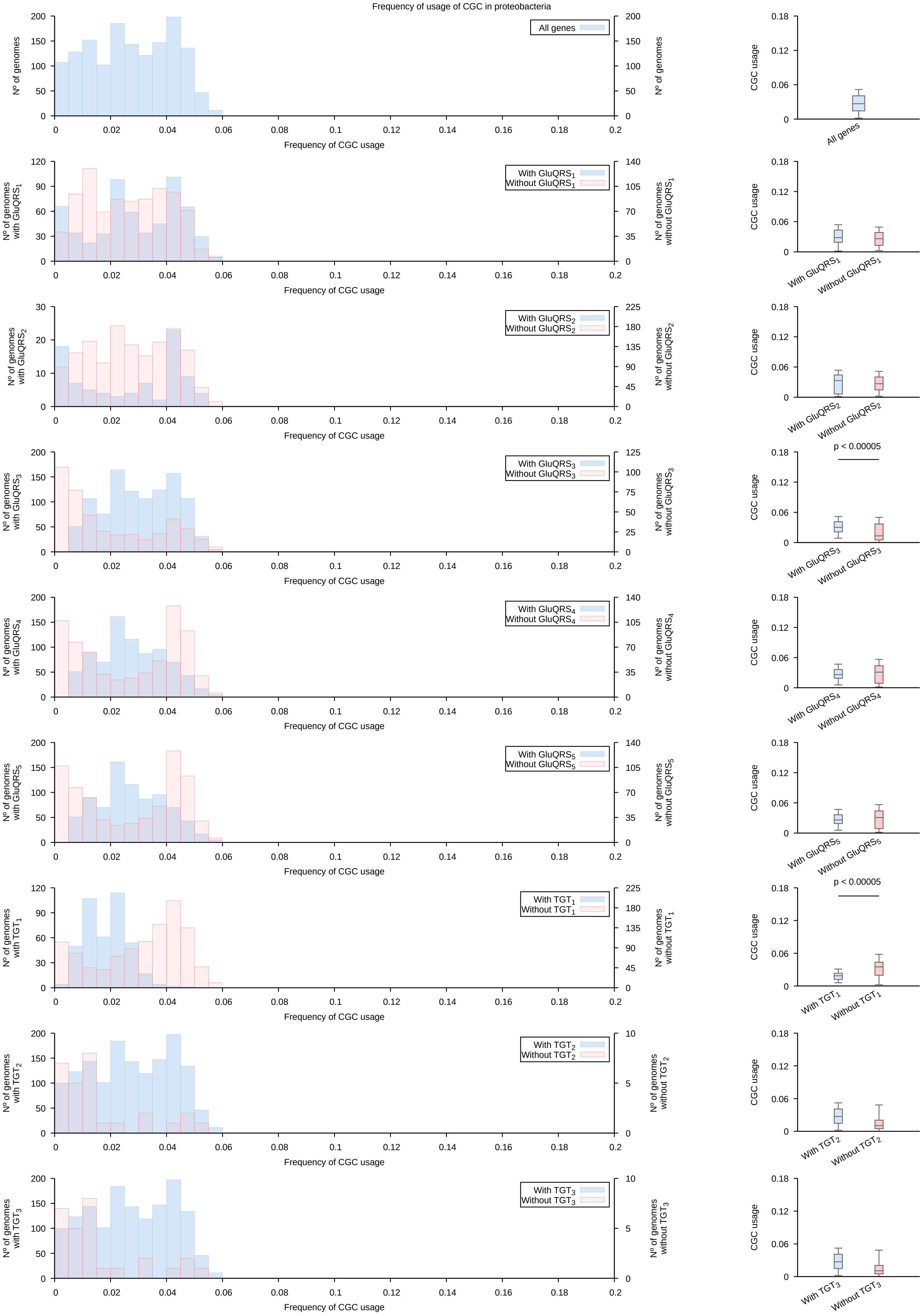

Frequency of usage of CGC in proteobacteria
All genes
With GluQRS1
Without GluQRS1
With GluQRS2
Without GluQRS2
With GluQRS3
Without GluQRS3
With GluQRS4
Without GluQRS4
With GluQRS5
Without GluQRS5
With TGT1
Without TGT1
With TGT2
Without TGT2
With TGT3
Without TGT3
Nº of genomes
Nº of genomes with GluQRS1
Nº of genomes without GluQRS1
Nº of genomes with GluQRS2
Nº of genomes without GluQRS2
Nº of genomes with GluQRS3
Nº of genomes without GluQRS3
Nº of genomes with GluQRS4
Nº of genomes without GluQRS4
Nº of genomes with GluQRS5
Nº of genomes without GluQRS5
Nº of genomes with TGT1
Nº of genomes without TGT1
Nº of genomes with TGT2
Nº of genomes without TGT2
Nº of genomes with TGT3
Nº of genomes without TGT3
Frequency of CGC usage
CGC usage
p < 0.00005
p < 0.00005

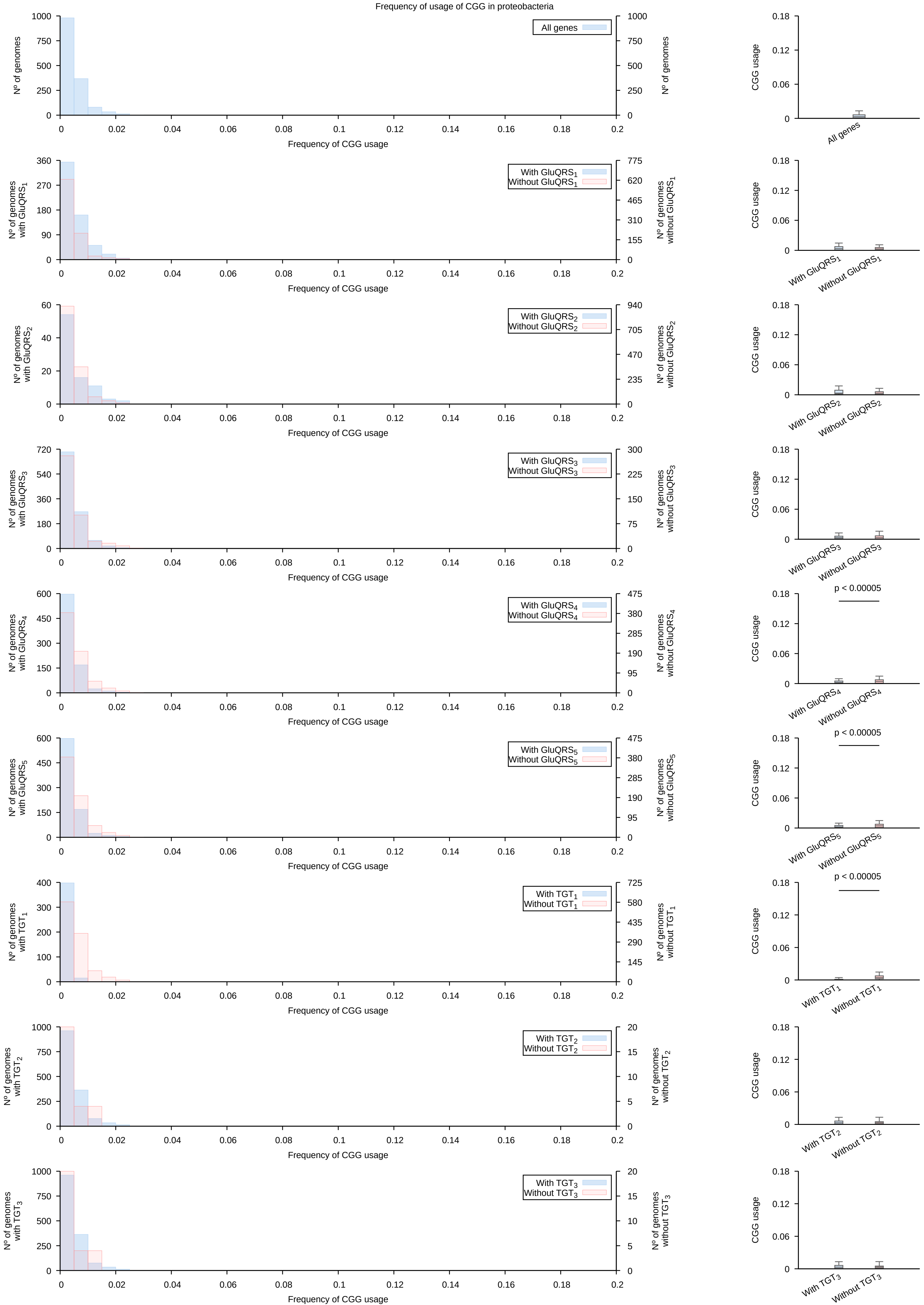

Frequency of usage of CGG in proteobacteria
All genes
Nº of genomes
Frequency of CGG usage
CGG usage
With GluQRS1
Without GluQRS1
Nº of genomes with GluQRS1
Nº of genomes without GluQRS1
With GluQRS2
Without GluQRS2
Nº of genomes with GluQRS2
Nº of genomes without GluQRS2
With GluQRS3
Without GluQRS3
Nº of genomes with GluQRS3
Nº of genomes without GluQRS3
With GluQRS4
Without GluQRS4
Nº of genomes with GluQRS4
Nº of genomes without GluQRS4
p < 0.00005
With GluQRS5
Without GluQRS5
Nº of genomes with GluQRS5
Nº of genomes without GluQRS5
p < 0.00005
With TGT1
Without TGT1
Nº of genomes with TGT1
Nº of genomes without TGT1
p < 0.00005
With TGT2
Without TGT2
Nº of genomes with TGT2
Nº of genomes without TGT2
With TGT3
Without TGT3
Nº of genomes with TGT3
Nº of genomes without TGT3

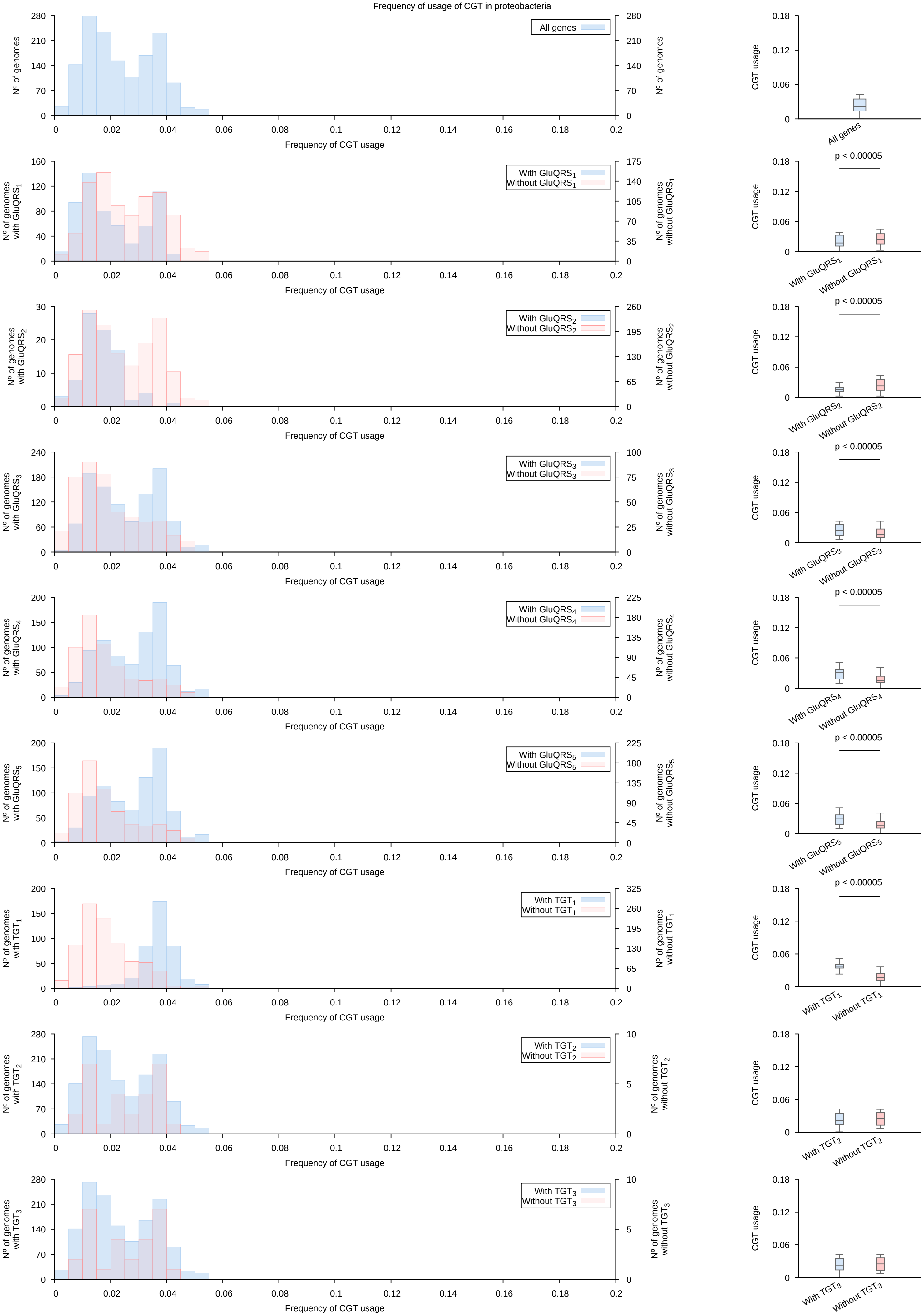

Frequency of usage of CGT in proteobacteria
All genes
With $GluQRS_1$
Without $GluQRS_1$
With $GluQRS_2$
Without $GluQRS_2$
With $GluQRS_3$
Without $GluQRS_3$
With $GluQRS_4$
Without $GluQRS_4$
With $GluQRS_5$
Without $GluQRS_5$
With TGT_1
Without TGT_1
With TGT_2
Without TGT_2
With TGT_3
Without TGT_3
Nº of genomes
Frequency of CGT usage
CGT usage
$p < 0.00005$

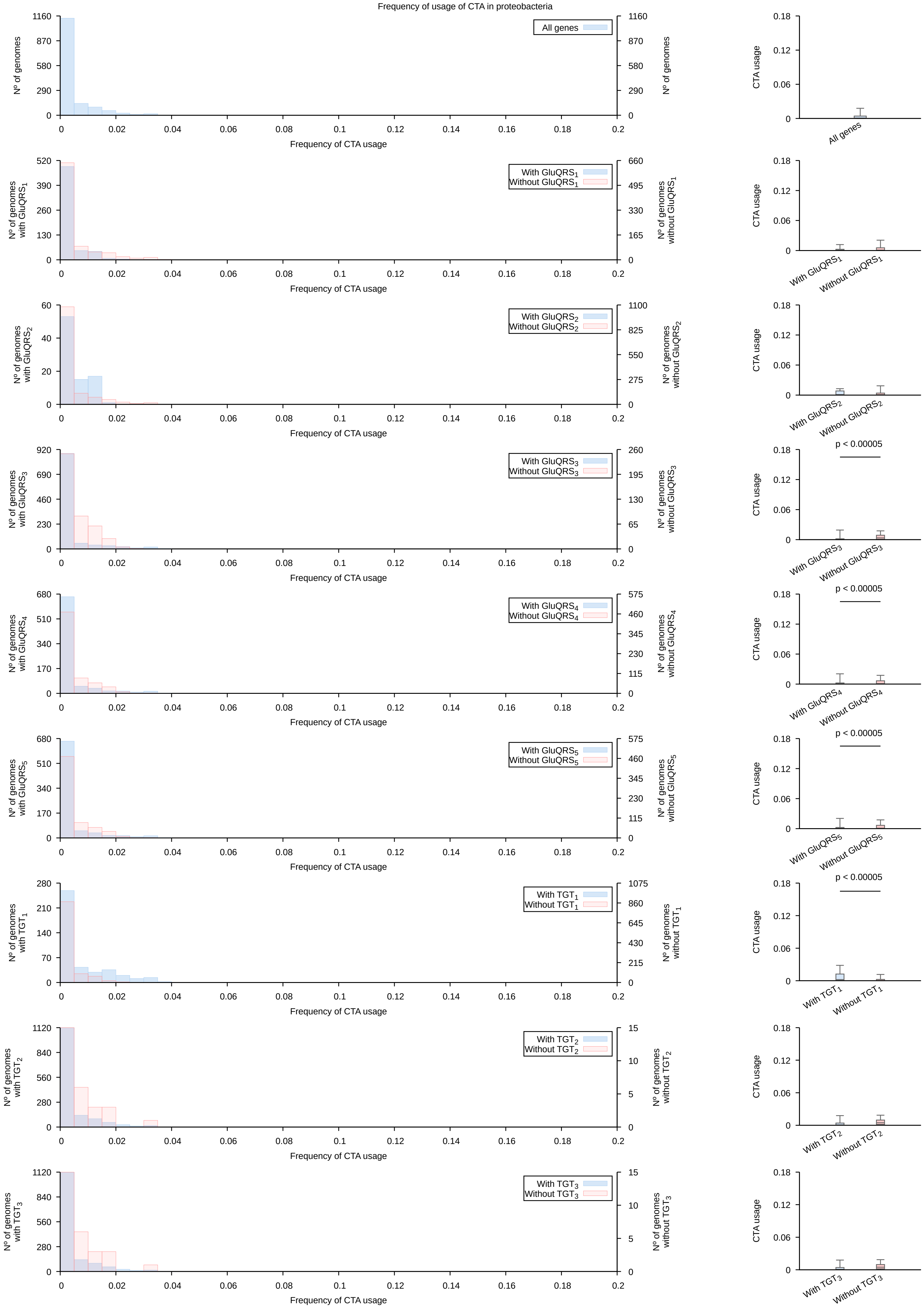
Frequency of usage of CTA in proteobacteria
All genes
With GluQRS$_1$
Without GluQRS$_1$
With GluQRS$_2$
Without GluQRS$_2$
With GluQRS$_3$
Without GluQRS$_3$
With GluQRS$_4$
Without GluQRS$_4$
With GluQRS$_5$
Without GluQRS$_5$
With TGT$_1$
Without TGT$_1$
With TGT$_2$
Without TGT$_2$
With TGT$_3$
Without TGT$_3$
Nº of genomes
Nº of genomes with GluQRS$_1$
Nº of genomes without GluQRS$_1$
Nº of genomes with GluQRS$_2$
Nº of genomes without GluQRS$_2$
Nº of genomes with GluQRS$_3$
Nº of genomes without GluQRS$_3$
Nº of genomes with GluQRS$_4$
Nº of genomes without GluQRS$_4$
Nº of genomes with GluQRS$_5$
Nº of genomes without GluQRS$_5$
Nº of genomes with TGT$_1$
Nº of genomes without TGT$_1$
Nº of genomes with TGT$_2$
Nº of genomes without TGT$_2$
Nº of genomes with TGT$_3$
Nº of genomes without TGT$_3$
Frequency of CTA usage
CTA usage
p < 0.00005

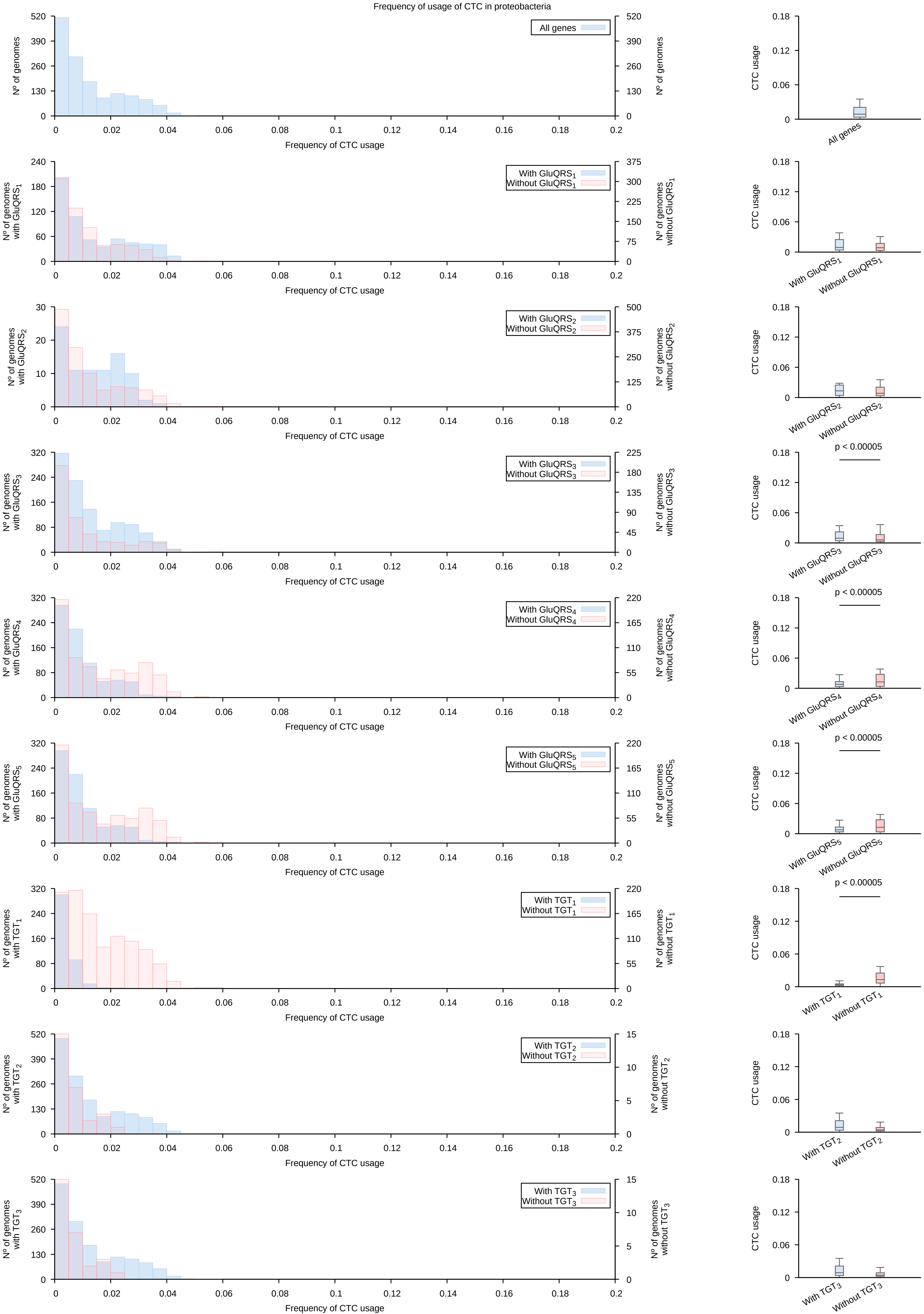
Frequency of usage of CTC in proteobacteria
Nº of genomes
Frequency of CTC usage
CTC usage
All genes
With GluQRS1
Without GluQRS1
With GluQRS2
Without GluQRS2
With GluQRS3
Without GluQRS3
With GluQRS4
Without GluQRS4
With GluQRS5
Without GluQRS5
With TGT1
Without TGT1
With TGT2
Without TGT2
With TGT3
Without TGT3
p < 0.00005

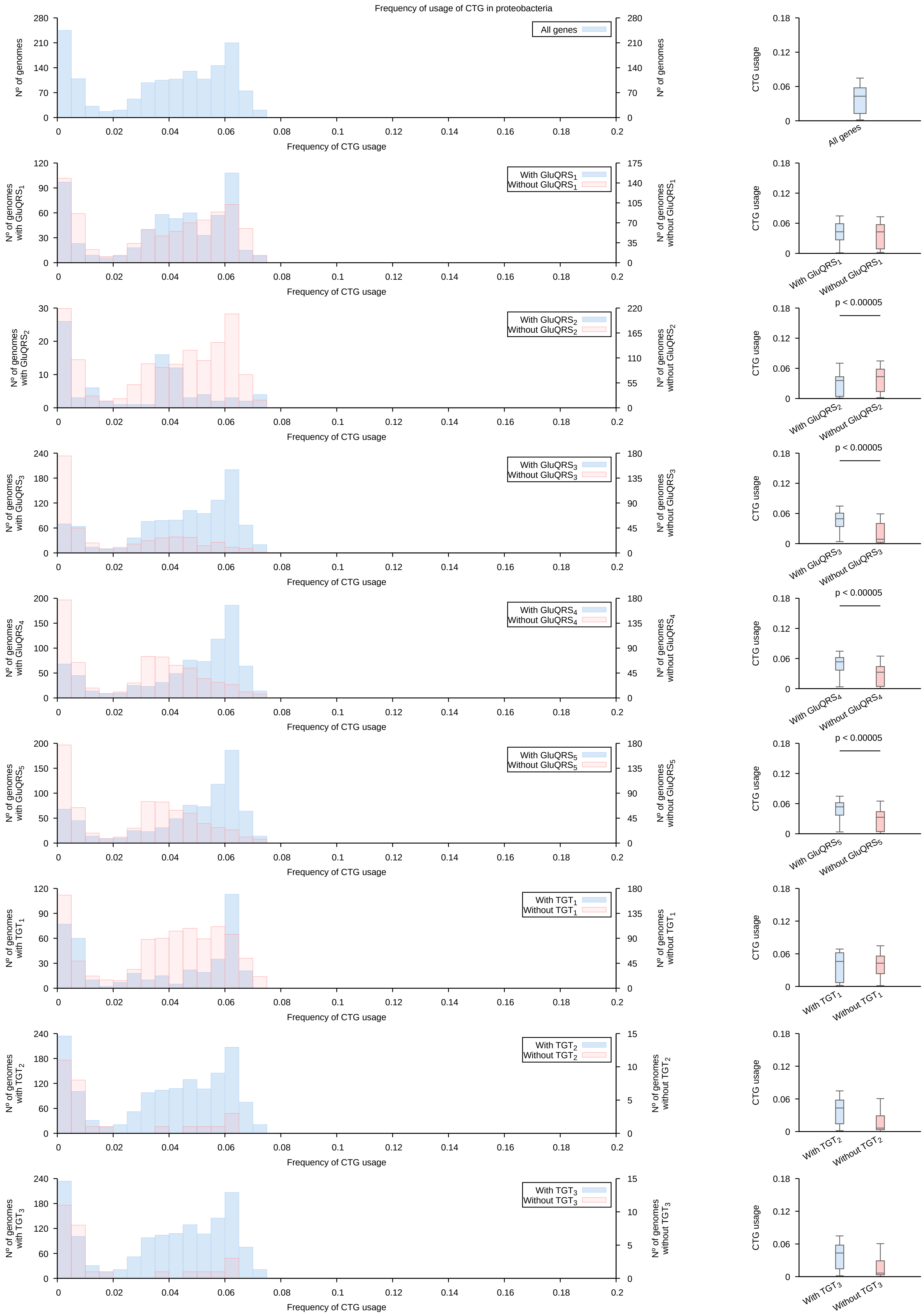

Frequency of usage of CTG in proteobacteria
All genes
Nº of genomes
Frequency of CTG usage
CTG usage
With GluQRS$_1$
Without GluQRS$_1$
Nº of genomes with GluQRS$_1$
Nº of genomes without GluQRS$_1$
With GluQRS$_2$
Without GluQRS$_2$
Nº of genomes with GluQRS$_2$
Nº of genomes without GluQRS$_2$
p < 0.00005
With GluQRS$_3$
Without GluQRS$_3$
Nº of genomes with GluQRS$_3$
Nº of genomes without GluQRS$_3$
p < 0.00005
With GluQRS$_4$
Without GluQRS$_4$
Nº of genomes with GluQRS$_4$
Nº of genomes without GluQRS$_4$
p < 0.00005
With GluQRS$_5$
Without GluQRS$_5$
Nº of genomes with GluQRS$_5$
Nº of genomes without GluQRS$_5$
p < 0.00005
With TGT$_1$
Without TGT$_1$
Nº of genomes with TGT$_1$
Nº of genomes without TGT$_1$
With TGT$_2$
Without TGT$_2$
Nº of genomes with TGT$_2$
Nº of genomes without TGT$_2$
With TGT$_3$
Without TGT$_3$
Nº of genomes with TGT$_3$
Nº of genomes without TGT$_3$

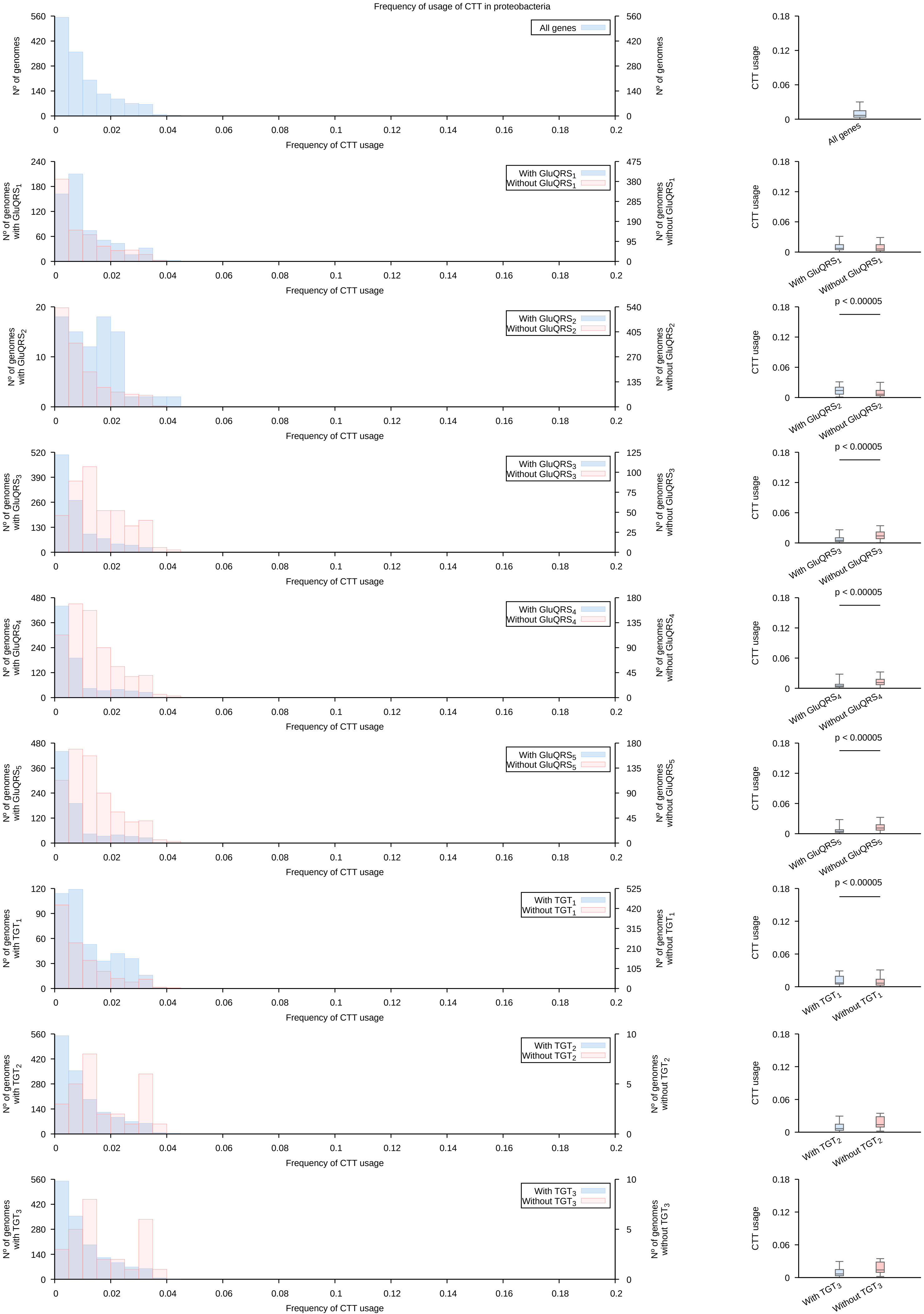
Frequency of usage of CTT in proteobacteria
Nº of genomes
Frequency of CTT usage
All genes
CTT usage
Nº of genomes with GluQRS1
Nº of genomes without GluQRS1
With GluQRS1
Without GluQRS1
Nº of genomes with GluQRS2
Nº of genomes without GluQRS2
With GluQRS2
Without GluQRS2
p < 0.00005
Nº of genomes with GluQRS3
Nº of genomes without GluQRS3
With GluQRS3
Without GluQRS3
p < 0.00005
Nº of genomes with GluQRS4
Nº of genomes without GluQRS4
With GluQRS4
Without GluQRS4
p < 0.00005
Nº of genomes with GluQRS5
Nº of genomes without GluQRS5
With GluQRS5
Without GluQRS5
p < 0.00005
Nº of genomes with TGT1
Nº of genomes without TGT1
With TGT1
Without TGT1
p < 0.00005
Nº of genomes with TGT2
Nº of genomes without TGT2
With TGT2
Without TGT2
Nº of genomes with TGT3
Nº of genomes without TGT3
With TGT3
Without TGT3

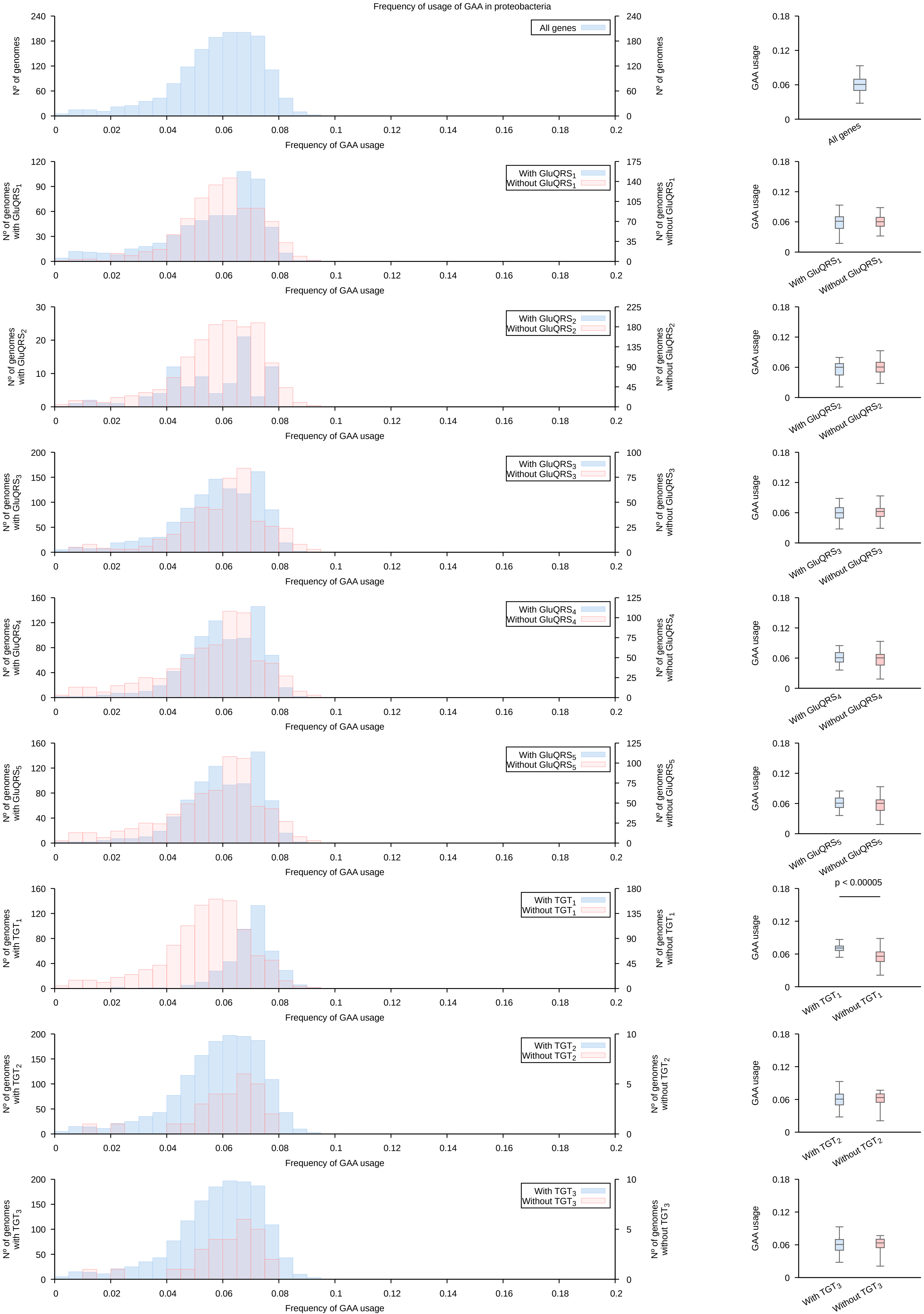
Frequency of usage of GAA in proteobacteria
Nº of genomes
Frequency of GAA usage
All genes
GAA usage
Nº of genomes with GluQRS$_1$
Nº of genomes without GluQRS$_1$
With GluQRS$_1$
Without GluQRS$_1$
Nº of genomes with GluQRS$_2$
Nº of genomes without GluQRS$_2$
With GluQRS$_2$
Without GluQRS$_2$
Nº of genomes with GluQRS$_3$
Nº of genomes without GluQRS$_3$
With GluQRS$_3$
Without GluQRS$_3$
Nº of genomes with GluQRS$_4$
Nº of genomes without GluQRS$_4$
With GluQRS$_4$
Without GluQRS$_4$
Nº of genomes with GluQRS$_5$
Nº of genomes without GluQRS$_5$
With GluQRS$_5$
Without GluQRS$_5$
Nº of genomes with TGT$_1$
Nº of genomes without TGT$_1$
With TGT$_1$
Without TGT$_1$
p < 0.00005
Nº of genomes with TGT$_2$
Nº of genomes without TGT$_2$
With TGT$_2$
Without TGT$_2$
Nº of genomes with TGT$_3$
Nº of genomes without TGT$_3$
With TGT$_3$
Without TGT$_3$

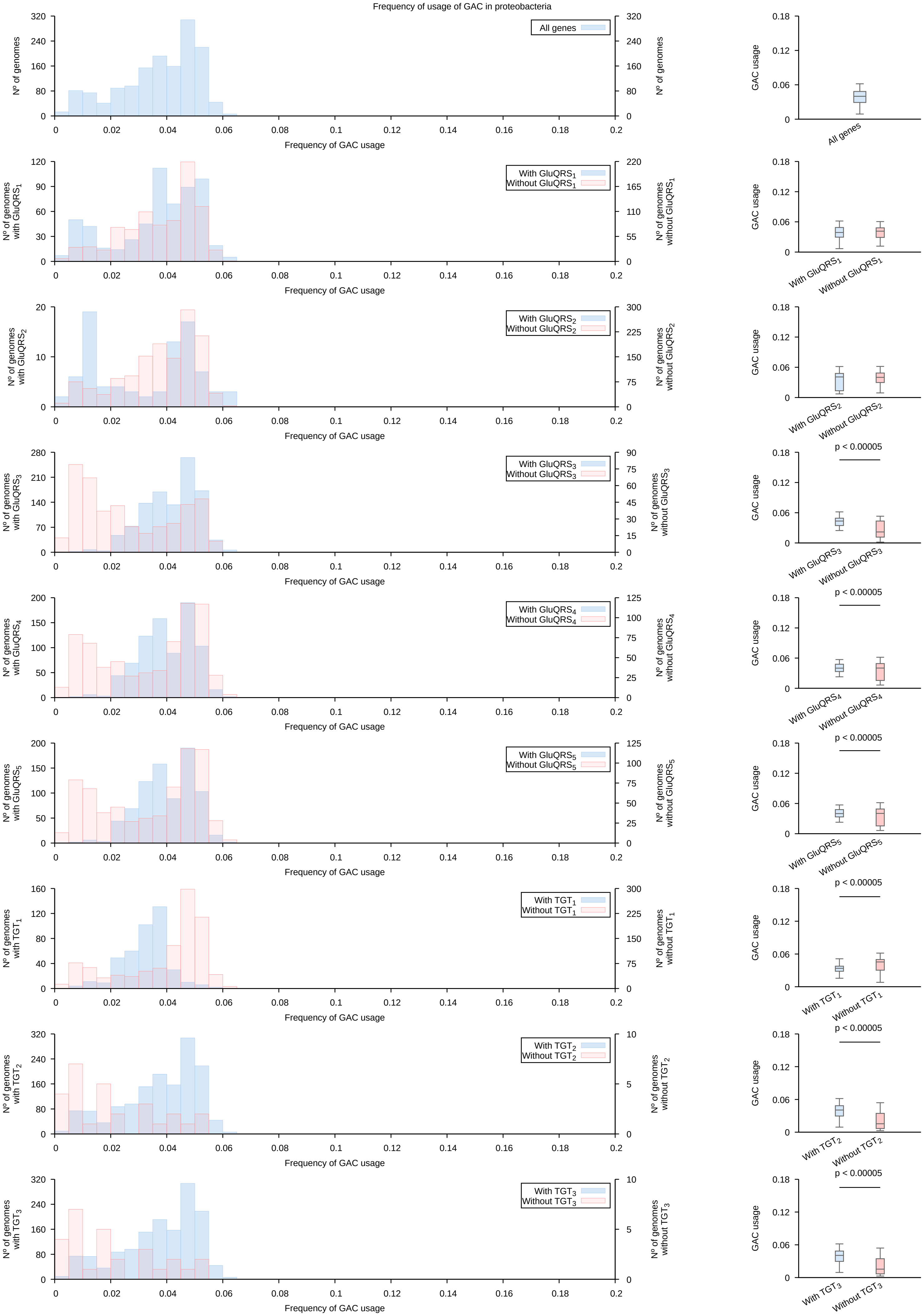
Frequency of usage of GAC in proteobacteria
All genes
With GluQRS$_1$
Without GluQRS$_1$
With GluQRS$_2$
Without GluQRS$_2$
With GluQRS$_3$
Without GluQRS$_3$
With GluQRS$_4$
Without GluQRS$_4$
With GluQRS$_5$
Without GluQRS$_5$
With TGT$_1$
Without TGT$_1$
With TGT$_2$
Without TGT$_2$
With TGT$_3$
Without TGT$_3$
Nº of genomes
Frequency of GAC usage
GAC usage
p < 0.00005

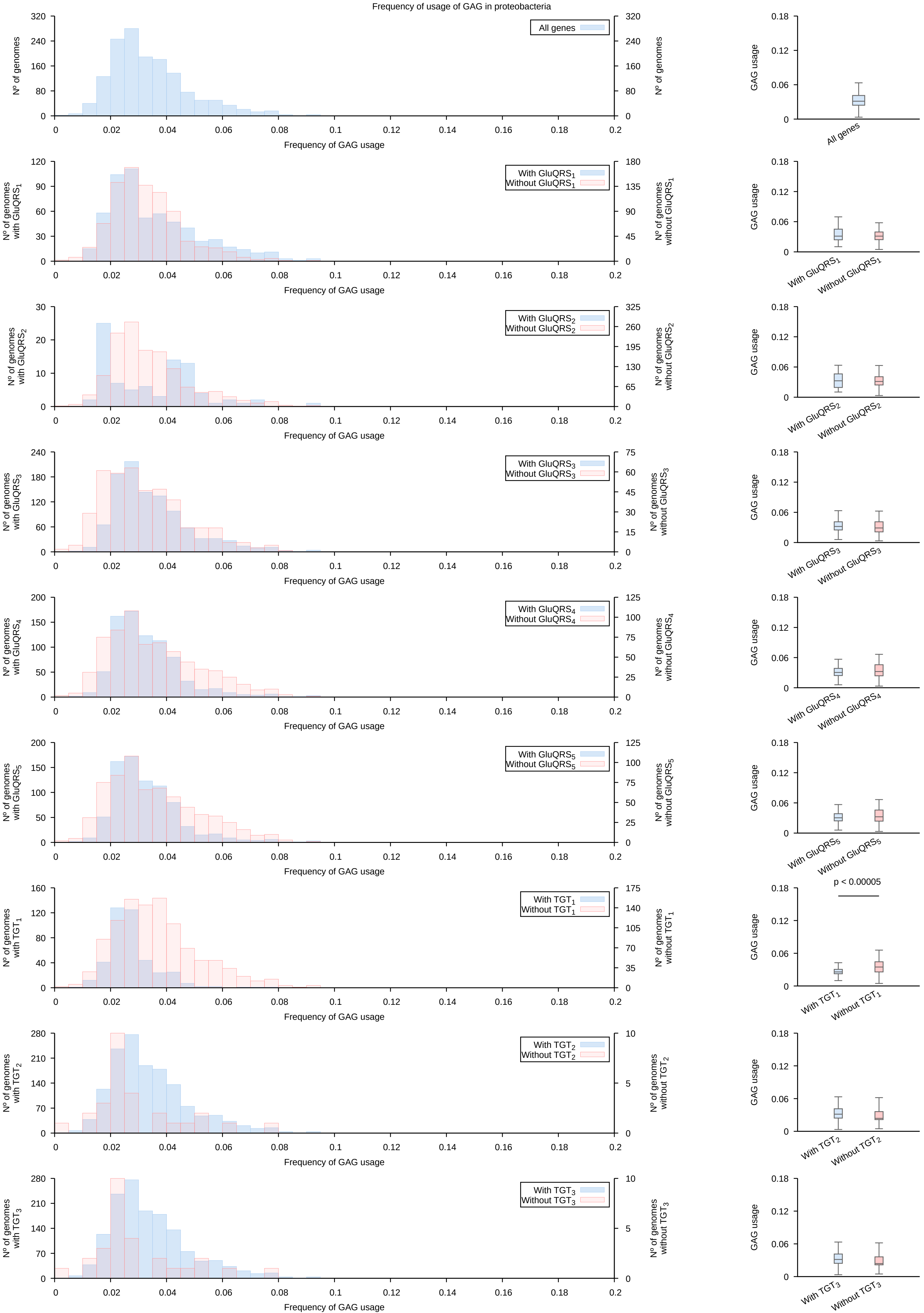
Frequency of usage of GAG in proteobacteria
Nº of genomes
Frequency of GAG usage
GAG usage
All genes
Nº of genomes with GluQRS$_1$
Nº of genomes without GluQRS$_1$
With GluQRS$_1$
Without GluQRS$_1$
Nº of genomes with GluQRS$_2$
Nº of genomes without GluQRS$_2$
With GluQRS$_2$
Without GluQRS$_2$
Nº of genomes with GluQRS$_3$
Nº of genomes without GluQRS$_3$
With GluQRS$_3$
Without GluQRS$_3$
Nº of genomes with GluQRS$_4$
Nº of genomes without GluQRS$_4$
With GluQRS$_4$
Without GluQRS$_4$
Nº of genomes with GluQRS$_5$
Nº of genomes without GluQRS$_5$
With GluQRS$_5$
Without GluQRS$_5$
Nº of genomes with TGT$_1$
Nº of genomes without TGT$_1$
With TGT$_1$
Without TGT$_1$
p < 0.00005
Nº of genomes with TGT$_2$
Nº of genomes without TGT$_2$
With TGT$_2$
Without TGT$_2$
Nº of genomes with TGT$_3$
Nº of genomes without TGT$_3$
With TGT$_3$
Without TGT$_3$

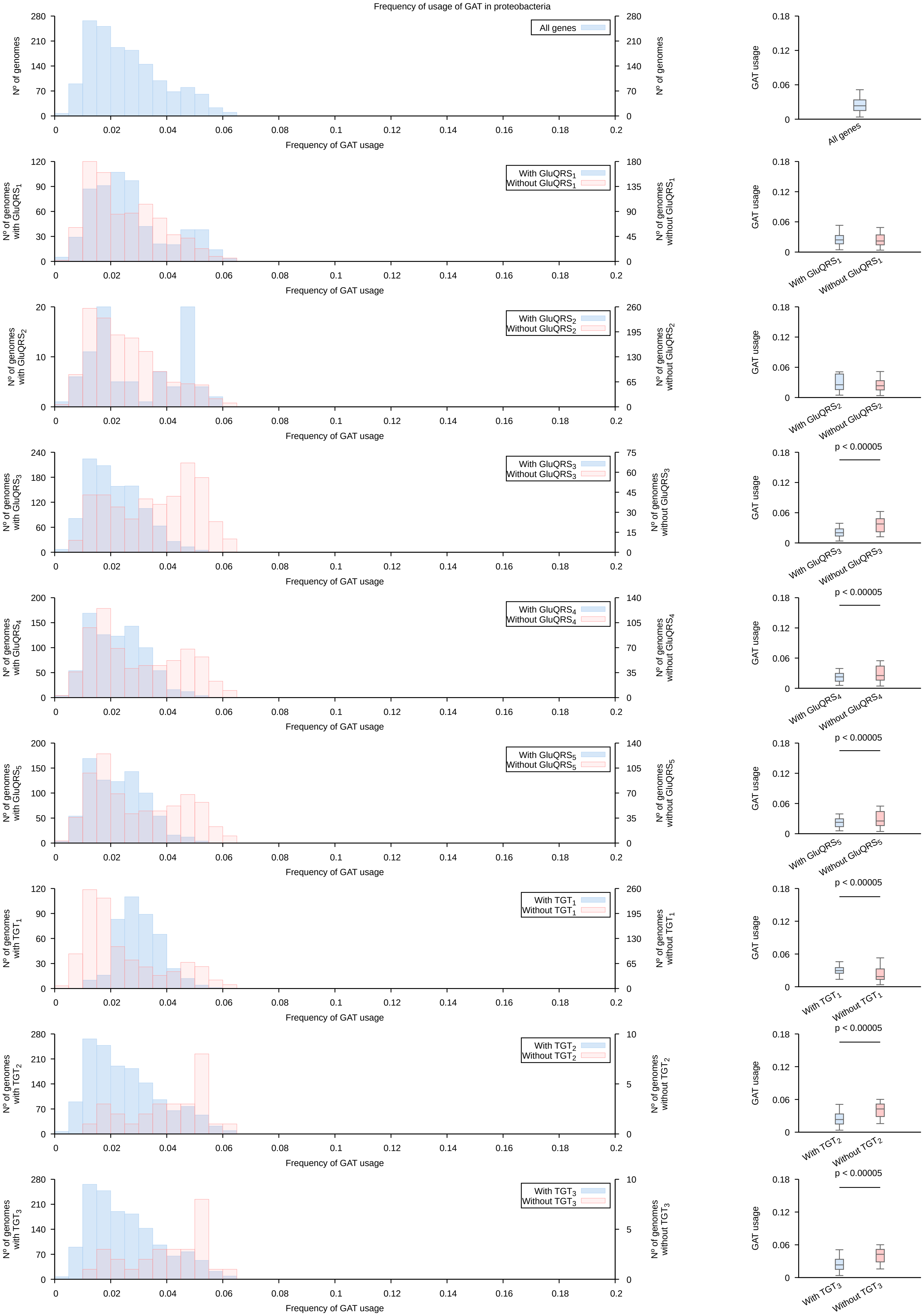

Frequency of usage of GAT in proteobacteria
Nº of genomes
All genes
Frequency of GAT usage
GAT usage
Nº of genomes with GluQRS$_1$
Nº of genomes without GluQRS$_1$
With GluQRS$_1$
Without GluQRS$_1$
Nº of genomes with GluQRS$_2$
Nº of genomes without GluQRS$_2$
With GluQRS$_2$
Without GluQRS$_2$
Nº of genomes with GluQRS$_3$
Nº of genomes without GluQRS$_3$
With GluQRS$_3$
Without GluQRS$_3$
p < 0.00005
Nº of genomes with GluQRS$_4$
Nº of genomes without GluQRS$_4$
With GluQRS$_4$
Without GluQRS$_4$
p < 0.00005
Nº of genomes with GluQRS$_5$
Nº of genomes without GluQRS$_5$
With GluQRS$_5$
Without GluQRS$_5$
p < 0.00005
Nº of genomes with TGT$_1$
Nº of genomes without TGT$_1$
With TGT$_1$
Without TGT$_1$
p < 0.00005
Nº of genomes with TGT$_2$
Nº of genomes without TGT$_2$
With TGT$_2$
Without TGT$_2$
p < 0.00005
Nº of genomes with TGT$_3$
Nº of genomes without TGT$_3$
With TGT$_3$
Without TGT$_3$
p < 0.00005

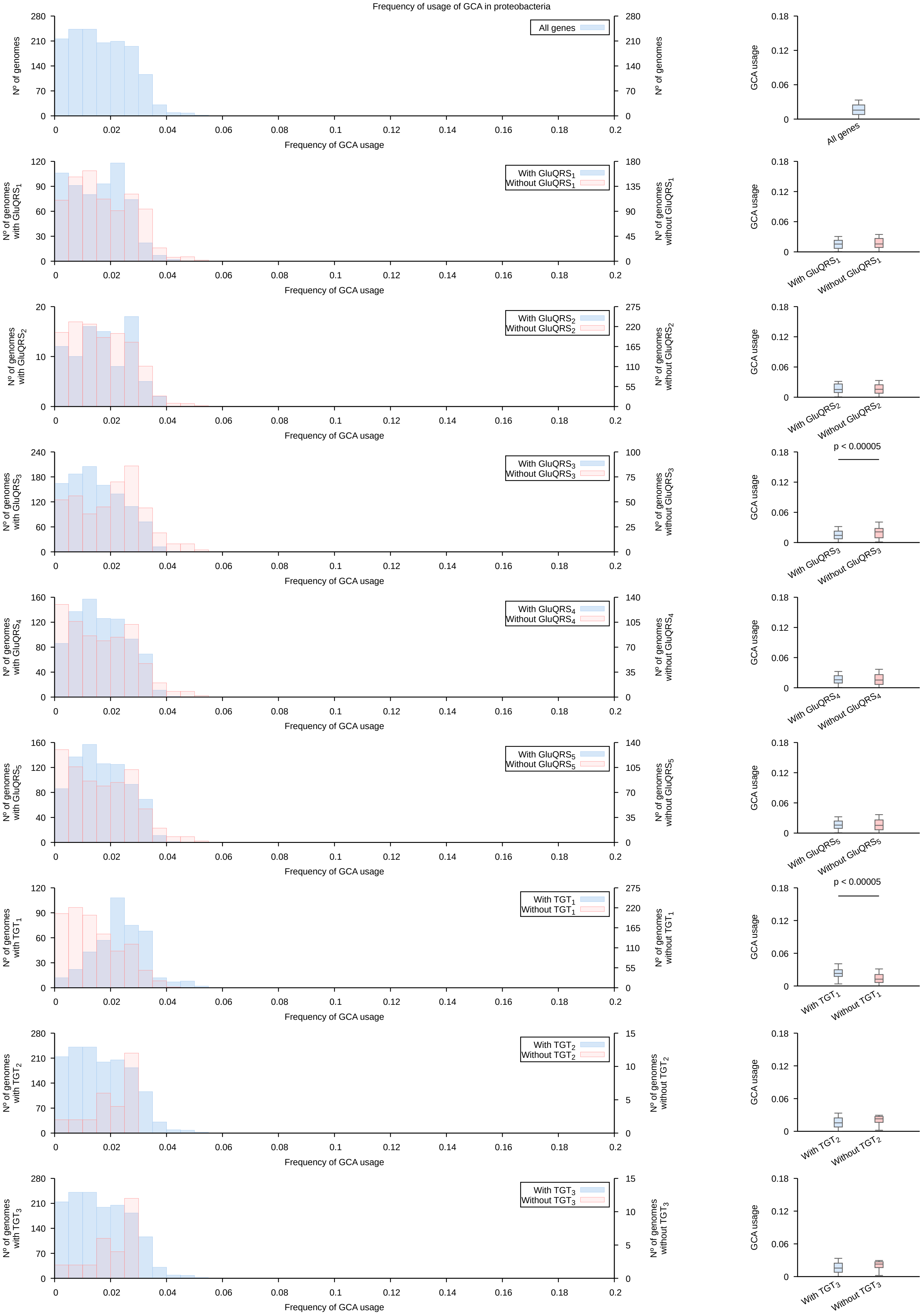

Frequency of usage of GCA in proteobacteria
Nº of genomes
All genes
Frequency of GCA usage
GCA usage
Nº of genomes with GluQRS$_1$
Nº of genomes without GluQRS$_1$
With GluQRS$_1$
Without GluQRS$_1$
Nº of genomes with GluQRS$_2$
Nº of genomes without GluQRS$_2$
With GluQRS$_2$
Without GluQRS$_2$
Nº of genomes with GluQRS$_3$
Nº of genomes without GluQRS$_3$
With GluQRS$_3$
Without GluQRS$_3$
p < 0.00005
Nº of genomes with GluQRS$_4$
Nº of genomes without GluQRS$_4$
With GluQRS$_4$
Without GluQRS$_4$
Nº of genomes with GluQRS$_5$
Nº of genomes without GluQRS$_5$
With GluQRS$_5$
Without GluQRS$_5$
Nº of genomes with TGT$_1$
Nº of genomes without TGT$_1$
With TGT$_1$
Without TGT$_1$
p < 0.00005
Nº of genomes with TGT$_2$
Nº of genomes without TGT$_2$
With TGT$_2$
Without TGT$_2$
Nº of genomes with TGT$_3$
Nº of genomes without TGT$_3$
With TGT$_3$
Without TGT$_3$

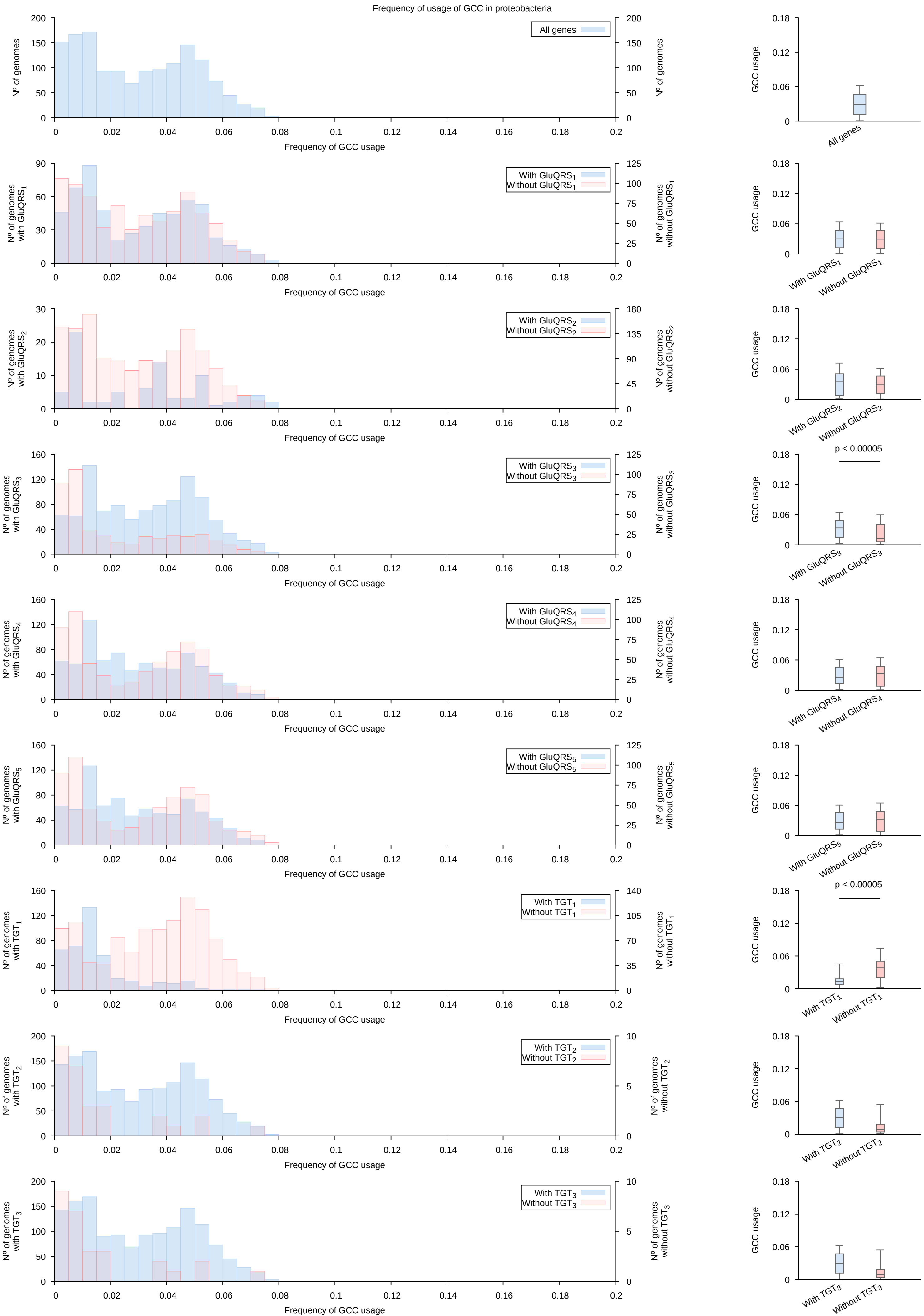

Frequency of usage of GCC in proteobacteria
All genes
With GluQRS$_1$
Without GluQRS$_1$
With GluQRS$_2$
Without GluQRS$_2$
With GluQRS$_3$
Without GluQRS$_3$
With GluQRS$_4$
Without GluQRS$_4$
With GluQRS$_5$
Without GluQRS$_5$
With TGT$_1$
Without TGT$_1$
With TGT$_2$
Without TGT$_2$
With TGT$_3$
Without TGT$_3$
Nº of genomes
Nº of genomes with GluQRS$_1$
Nº of genomes without GluQRS$_1$
Nº of genomes with GluQRS$_2$
Nº of genomes without GluQRS$_2$
Nº of genomes with GluQRS$_3$
Nº of genomes without GluQRS$_3$
Nº of genomes with GluQRS$_4$
Nº of genomes without GluQRS$_4$
Nº of genomes with GluQRS$_5$
Nº of genomes without GluQRS$_5$
Nº of genomes with TGT$_1$
Nº of genomes without TGT$_1$
Nº of genomes with TGT$_2$
Nº of genomes without TGT$_2$
Nº of genomes with TGT$_3$
Nº of genomes without TGT$_3$
Frequency of GCC usage
GCC usage
p < 0.00005
p < 0.00005

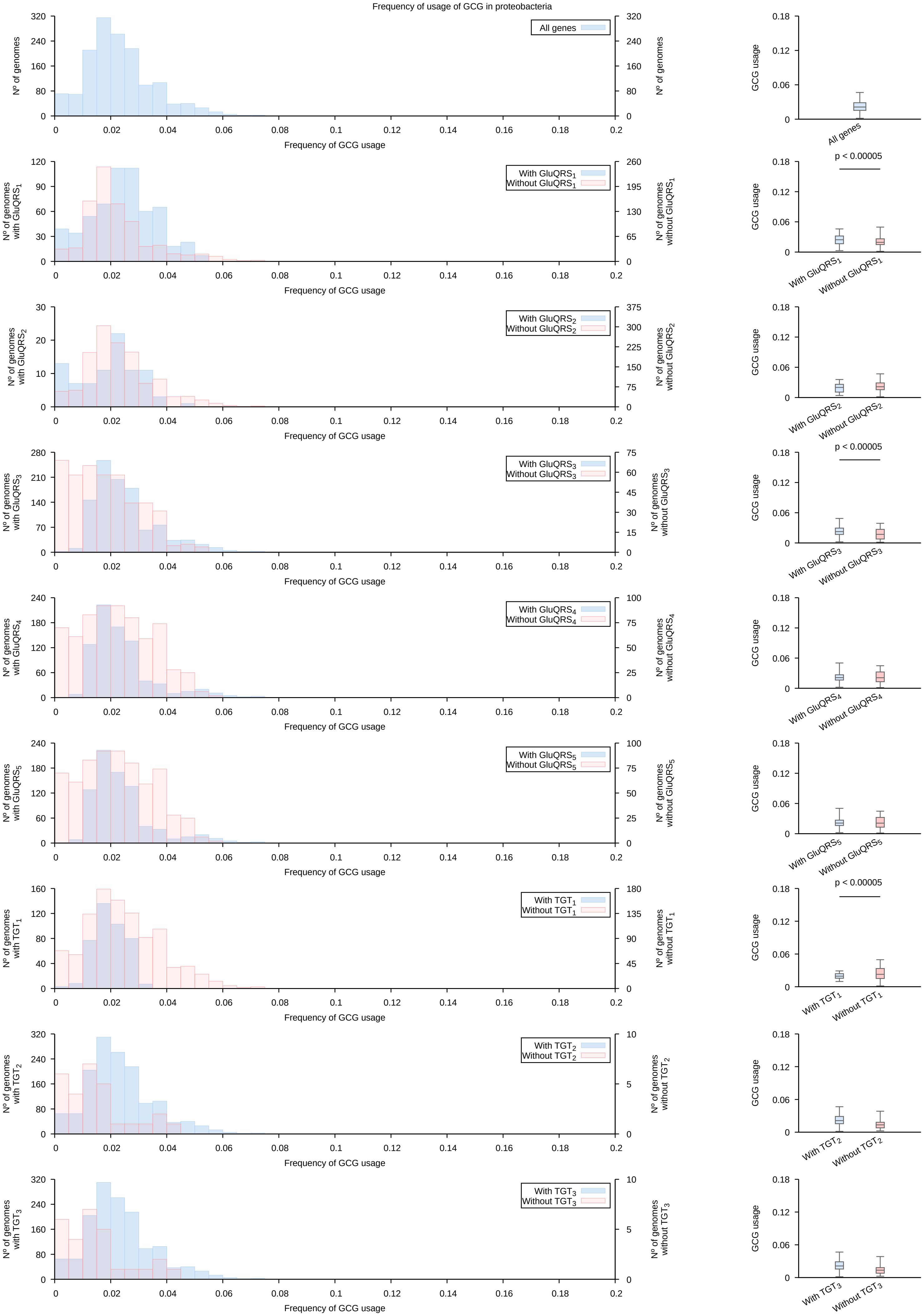
Frequency of usage of GCG in proteobacteria
All genes
Nº of genomes
Frequency of GCG usage
GCG usage
With GluQRS1
Without GluQRS1
Nº of genomes with GluQRS1
Nº of genomes without GluQRS1
p < 0.00005
With GluQRS2
Without GluQRS2
Nº of genomes with GluQRS2
Nº of genomes without GluQRS2
With GluQRS3
Without GluQRS3
Nº of genomes with GluQRS3
Nº of genomes without GluQRS3
p < 0.00005
With GluQRS4
Without GluQRS4
Nº of genomes with GluQRS4
Nº of genomes without GluQRS4
With GluQRS5
Without GluQRS5
Nº of genomes with GluQRS5
Nº of genomes without GluQRS5
With TGT1
Without TGT1
Nº of genomes with TGT1
Nº of genomes without TGT1
p < 0.00005
With TGT2
Without TGT2
Nº of genomes with TGT2
Nº of genomes without TGT2
With TGT3
Without TGT3
Nº of genomes with TGT3
Nº of genomes without TGT3

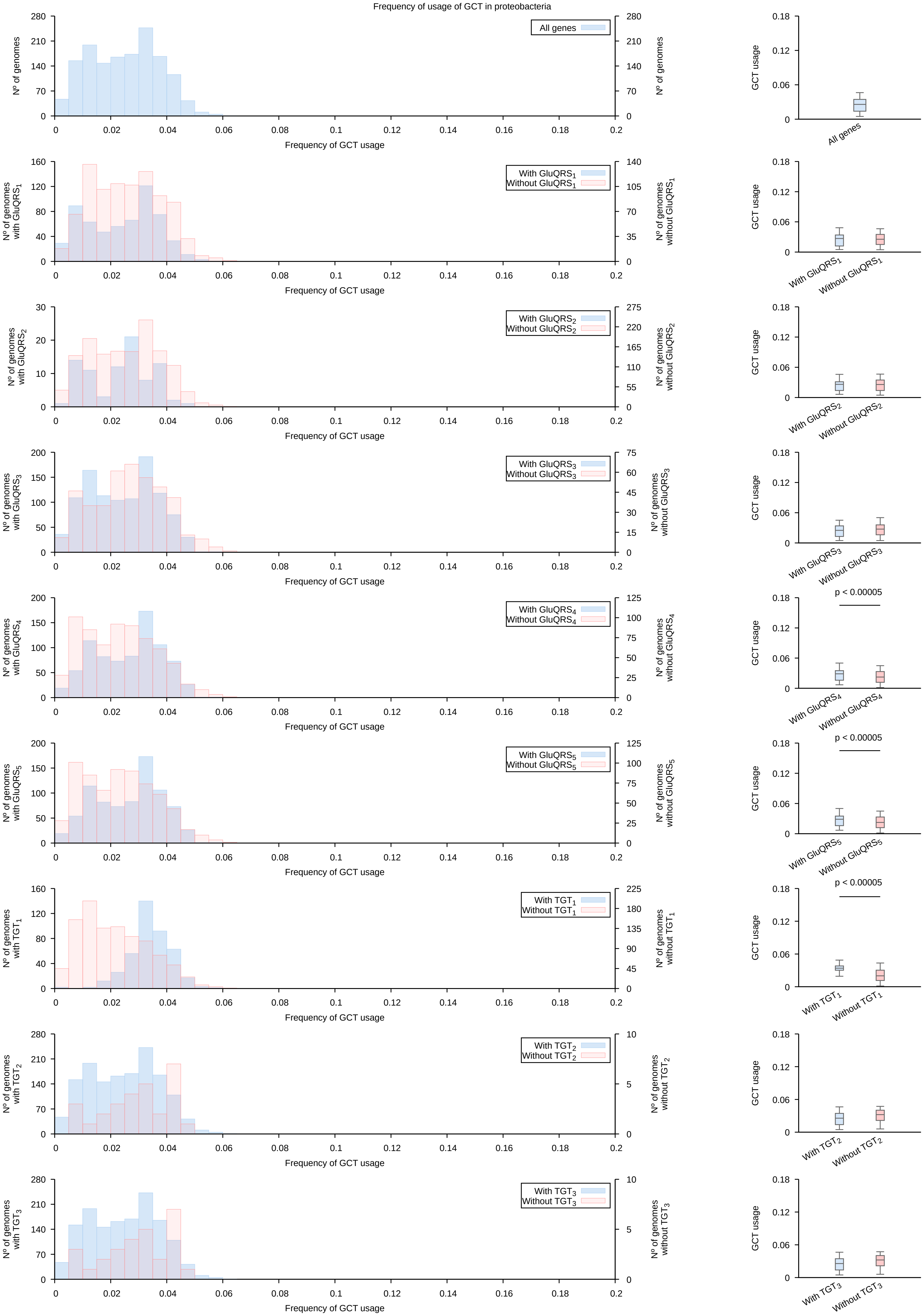
Frequency of usage of GCT in proteobacteria
All genes
Nº of genomes
Frequency of GCT usage
GCT usage
With GluQRS$_1$
Without GluQRS$_1$
Nº of genomes with GluQRS$_1$
Nº of genomes without GluQRS$_1$
With GluQRS$_2$
Without GluQRS$_2$
Nº of genomes with GluQRS$_2$
Nº of genomes without GluQRS$_2$
With GluQRS$_3$
Without GluQRS$_3$
Nº of genomes with GluQRS$_3$
Nº of genomes without GluQRS$_3$
With GluQRS$_4$
Without GluQRS$_4$
Nº of genomes with GluQRS$_4$
Nº of genomes without GluQRS$_4$
p < 0.00005
With GluQRS$_5$
Without GluQRS$_5$
Nº of genomes with GluQRS$_5$
Nº of genomes without GluQRS$_5$
p < 0.00005
With TGT$_1$
Without TGT$_1$
Nº of genomes with TGT$_1$
Nº of genomes without TGT$_1$
p < 0.00005
With TGT$_2$
Without TGT$_2$
Nº of genomes with TGT$_2$
Nº of genomes without TGT$_2$
With TGT$_3$
Without TGT$_3$
Nº of genomes with TGT$_3$
Nº of genomes without TGT$_3$

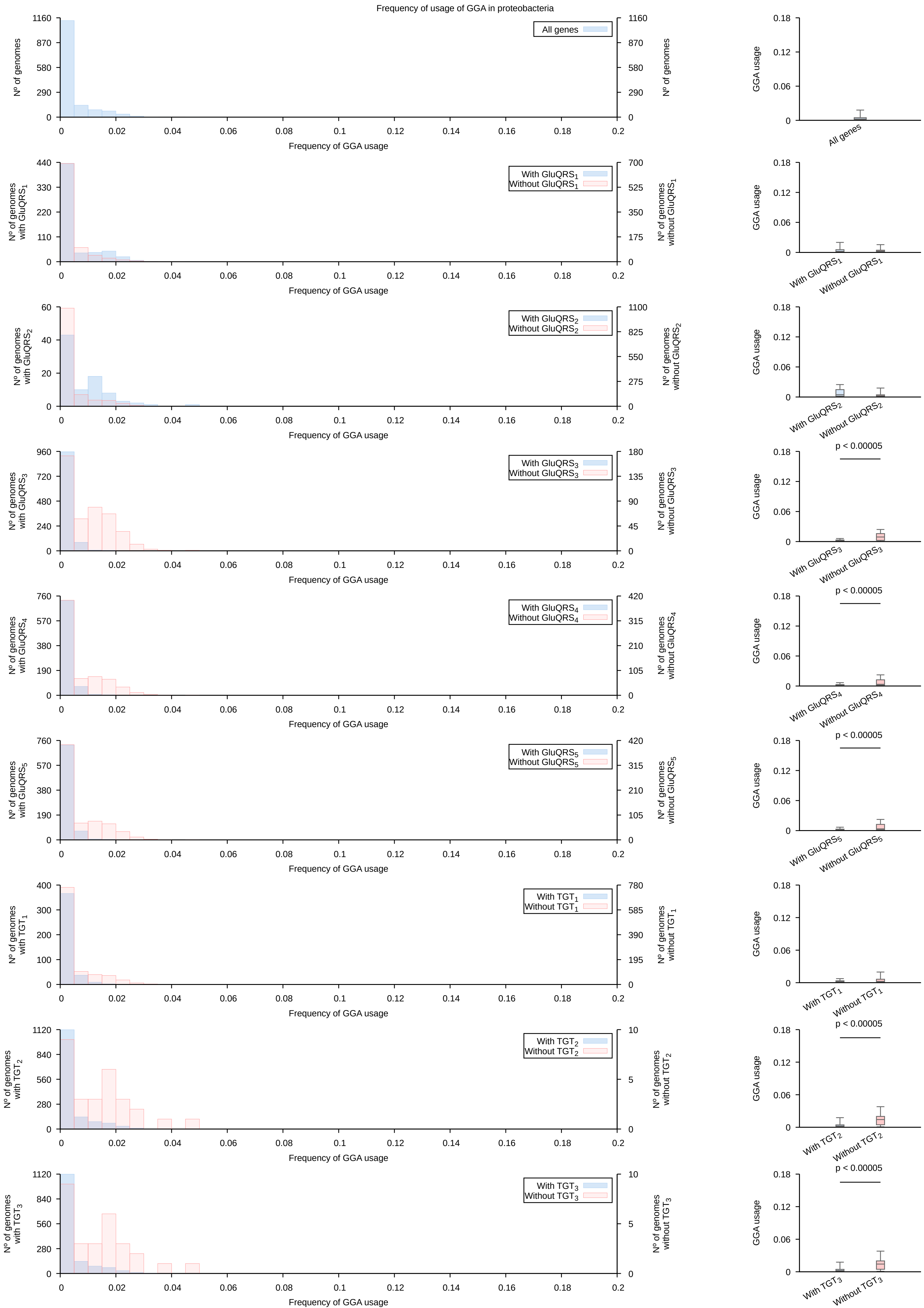
Frequency of usage of GGA in proteobacteria
All genes
With GluQRS$_1$
Without GluQRS$_1$
With GluQRS$_2$
Without GluQRS$_2$
With GluQRS$_3$
Without GluQRS$_3$
With GluQRS$_4$
Without GluQRS$_4$
With GluQRS$_5$
Without GluQRS$_5$
With TGT$_1$
Without TGT$_1$
With TGT$_2$
Without TGT$_2$
With TGT$_3$
Without TGT$_3$
Nº of genomes
Nº of genomes with GluQRS$_1$
Nº of genomes without GluQRS$_1$
Nº of genomes with GluQRS$_2$
Nº of genomes without GluQRS$_2$
Nº of genomes with GluQRS$_3$
Nº of genomes without GluQRS$_3$
Nº of genomes with GluQRS$_4$
Nº of genomes without GluQRS$_4$
Nº of genomes with GluQRS$_5$
Nº of genomes without GluQRS$_5$
Nº of genomes with TGT$_1$
Nº of genomes without TGT$_1$
Nº of genomes with TGT$_2$
Nº of genomes without TGT$_2$
Nº of genomes with TGT$_3$
Nº of genomes without TGT$_3$
Frequency of GGA usage
GGA usage
p < 0.00005

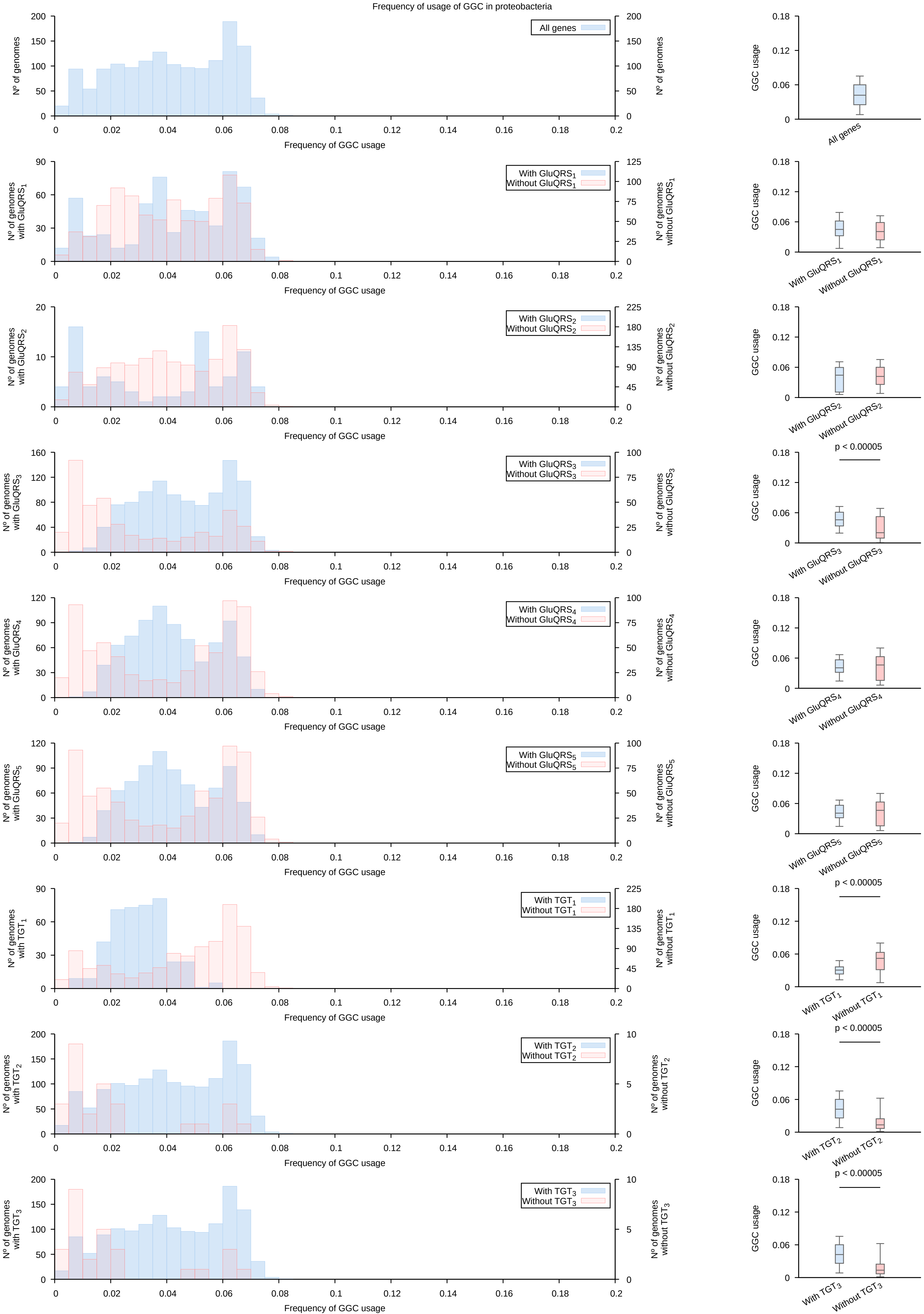

Frequency of usage of GGC in proteobacteria
All genes
With GluQRS1
Without GluQRS1
With GluQRS2
Without GluQRS2
With GluQRS3
Without GluQRS3
With GluQRS4
Without GluQRS4
With GluQRS5
Without GluQRS5
With TGT1
Without TGT1
With TGT2
Without TGT2
With TGT3
Without TGT3
Nº of genomes
Frequency of GGC usage
GGC usage
p < 0.00005

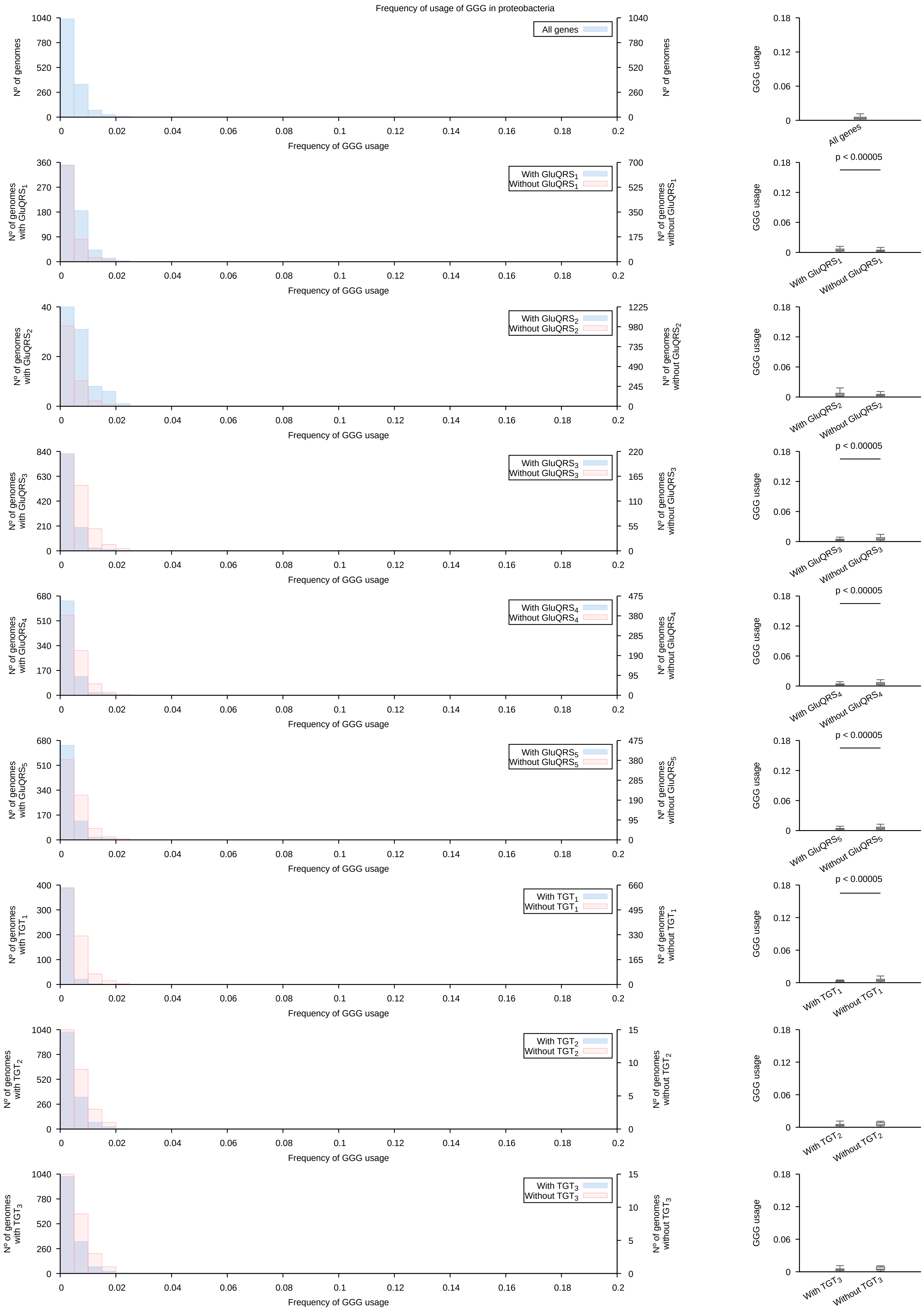
Frequency of usage of GGG in proteobacteria
All genes
With GluQRS$_1$
Without GluQRS$_1$
With GluQRS$_2$
Without GluQRS$_2$
With GluQRS$_3$
Without GluQRS$_3$
With GluQRS$_4$
Without GluQRS$_4$
With GluQRS$_5$
Without GluQRS$_5$
With TGT$_1$
Without TGT$_1$
With TGT$_2$
Without TGT$_2$
With TGT$_3$
Without TGT$_3$
Nº of genomes
Nº of genomes with GluQRS$_1$
Nº of genomes without GluQRS$_1$
Nº of genomes with GluQRS$_2$
Nº of genomes without GluQRS$_2$
Nº of genomes with GluQRS$_3$
Nº of genomes without GluQRS$_3$
Nº of genomes with GluQRS$_4$
Nº of genomes without GluQRS$_4$
Nº of genomes with GluQRS$_5$
Nº of genomes without GluQRS$_5$
Nº of genomes with TGT$_1$
Nº of genomes without TGT$_1$
Nº of genomes with TGT$_2$
Nº of genomes without TGT$_2$
Nº of genomes with TGT$_3$
Nº of genomes without TGT$_3$
Frequency of GGG usage
GGG usage
p < 0.00005

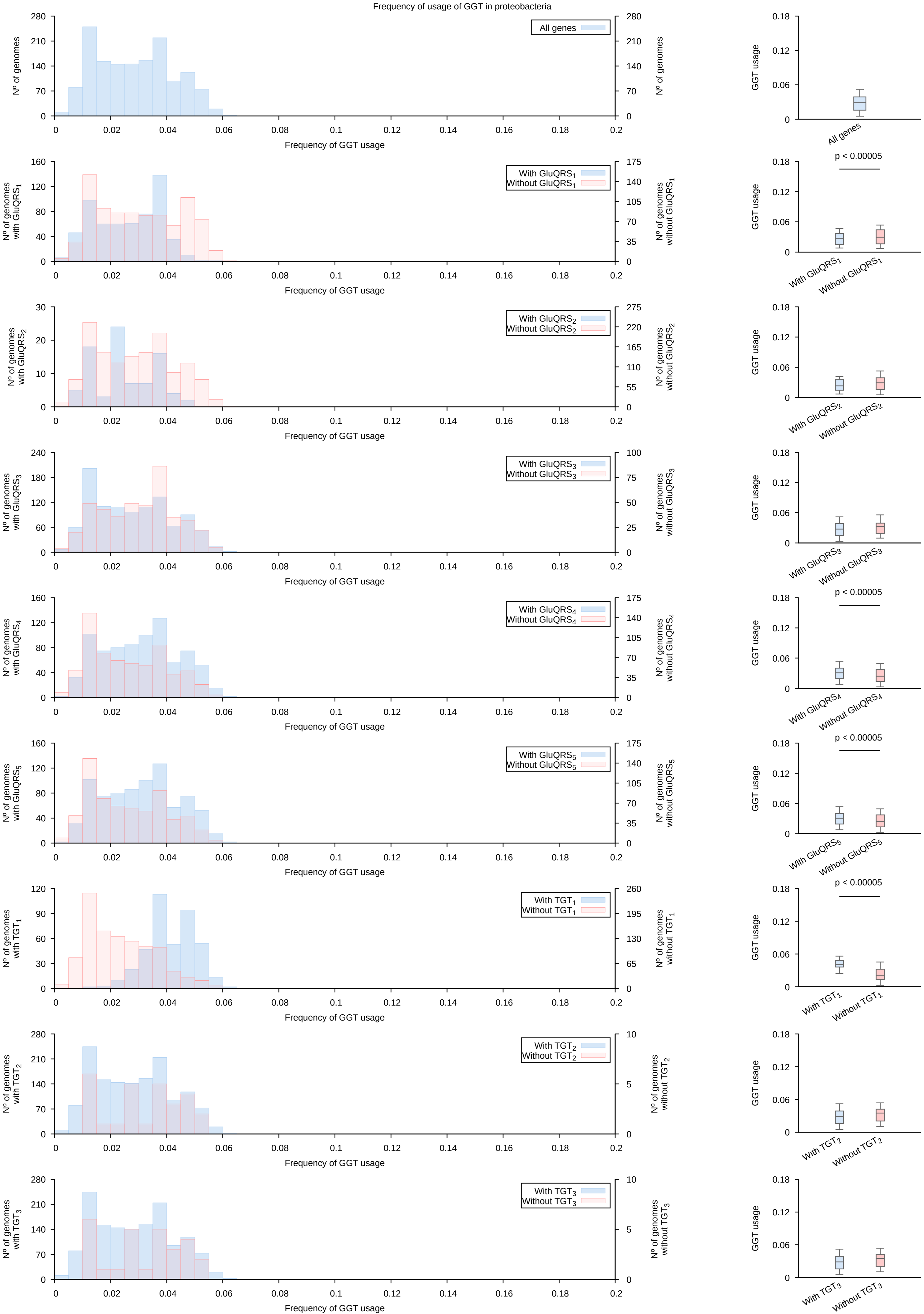
Frequency of usage of GGT in proteobacteria
All genes
Nº of genomes
Frequency of GGT usage
GGT usage
With GluQRS$_1$
Without GluQRS$_1$
Nº of genomes with GluQRS$_1$
Nº of genomes without GluQRS$_1$
p < 0.00005
With GluQRS$_2$
Without GluQRS$_2$
Nº of genomes with GluQRS$_2$
Nº of genomes without GluQRS$_2$
With GluQRS$_3$
Without GluQRS$_3$
Nº of genomes with GluQRS$_3$
Nº of genomes without GluQRS$_3$
With GluQRS$_4$
Without GluQRS$_4$
Nº of genomes with GluQRS$_4$
Nº of genomes without GluQRS$_4$
p < 0.00005
With GluQRS$_5$
Without GluQRS$_5$
Nº of genomes with GluQRS$_5$
Nº of genomes without GluQRS$_5$
p < 0.00005
With TGT$_1$
Without TGT$_1$
Nº of genomes with TGT$_1$
Nº of genomes without TGT$_1$
p < 0.00005
With TGT$_2$
Without TGT$_2$
Nº of genomes with TGT$_2$
Nº of genomes without TGT$_2$
With TGT$_3$
Without TGT$_3$
Nº of genomes with TGT$_3$
Nº of genomes without TGT$_3$

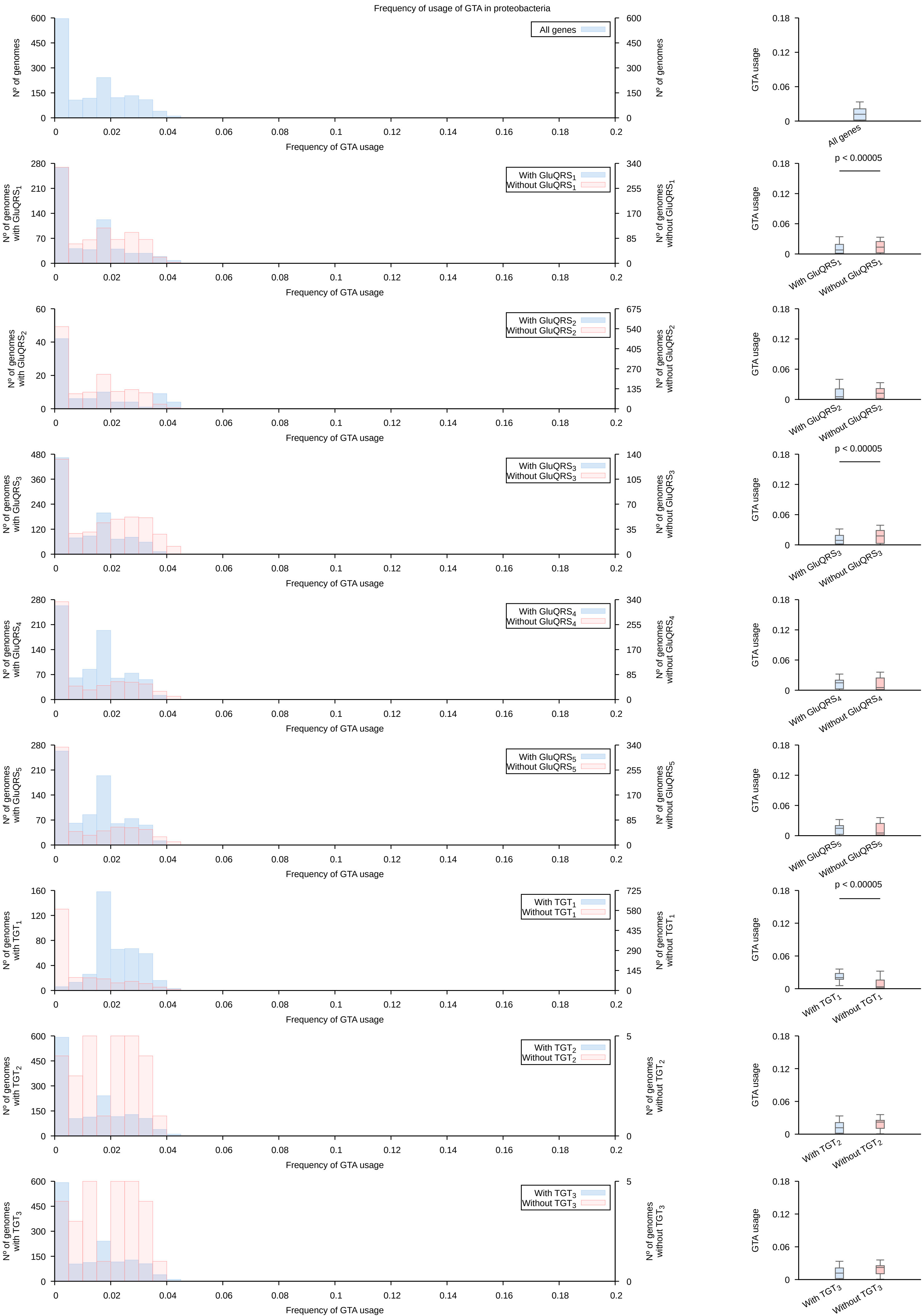
Frequency of usage of GTA in proteobacteria
All genes
With GluQRS$_1$
Without GluQRS$_1$
With GluQRS$_2$
Without GluQRS$_2$
With GluQRS$_3$
Without GluQRS$_3$
With GluQRS$_4$
Without GluQRS$_4$
With GluQRS$_5$
Without GluQRS$_5$
With TGT$_1$
Without TGT$_1$
With TGT$_2$
Without TGT$_2$
With TGT$_3$
Without TGT$_3$
Nº of genomes
Frequency of GTA usage
GTA usage
p < 0.00005

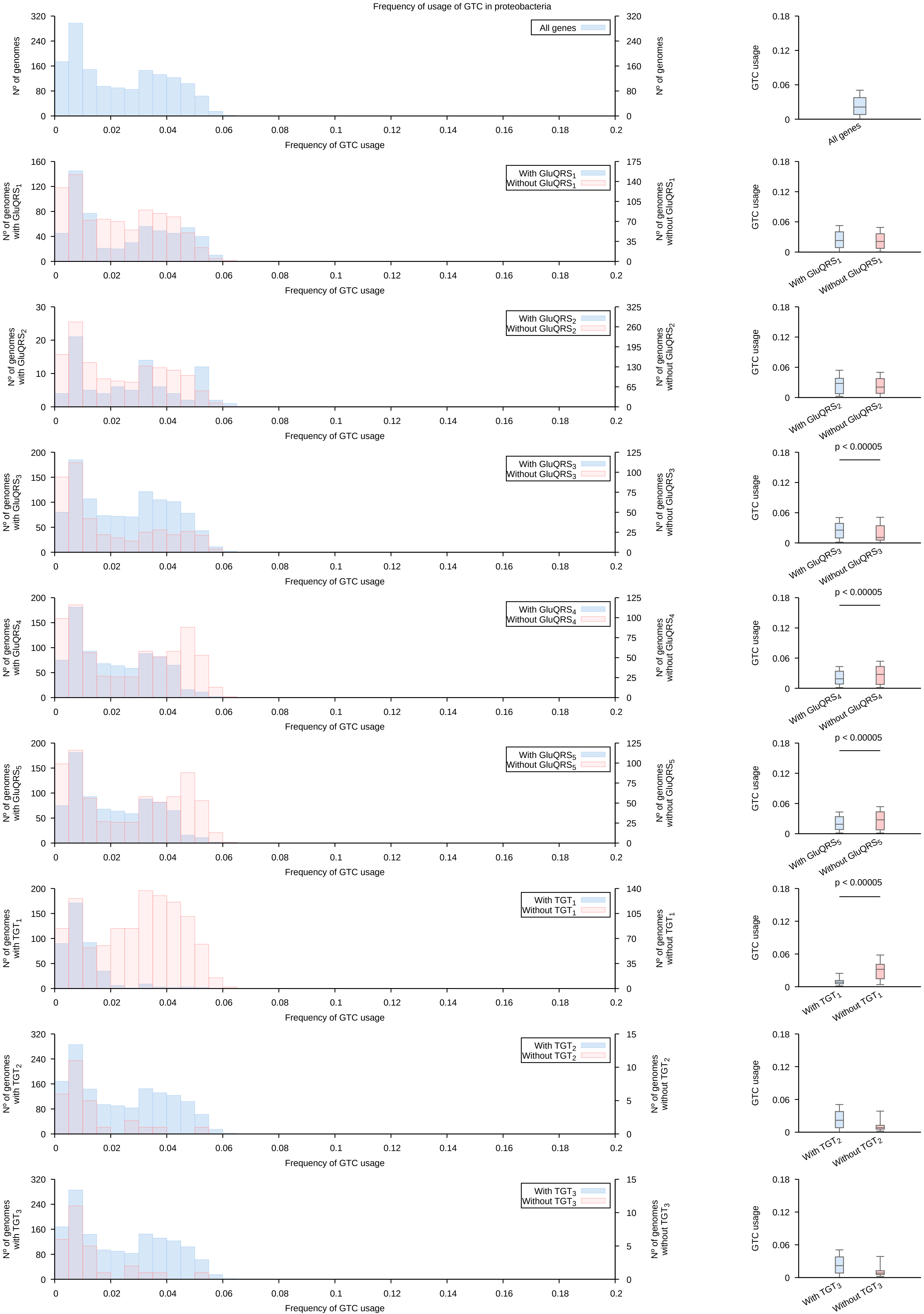

Frequency of usage of GTC in proteobacteria
All genes
With GluQRS$_1$
Without GluQRS$_1$
With GluQRS$_2$
Without GluQRS$_2$
With GluQRS$_3$
Without GluQRS$_3$
With GluQRS$_4$
Without GluQRS$_4$
With GluQRS$_5$
Without GluQRS$_5$
With TGT$_1$
Without TGT$_1$
With TGT$_2$
Without TGT$_2$
With TGT$_3$
Without TGT$_3$
Nº of genomes
Frequency of GTC usage
GTC usage
p < 0.00005

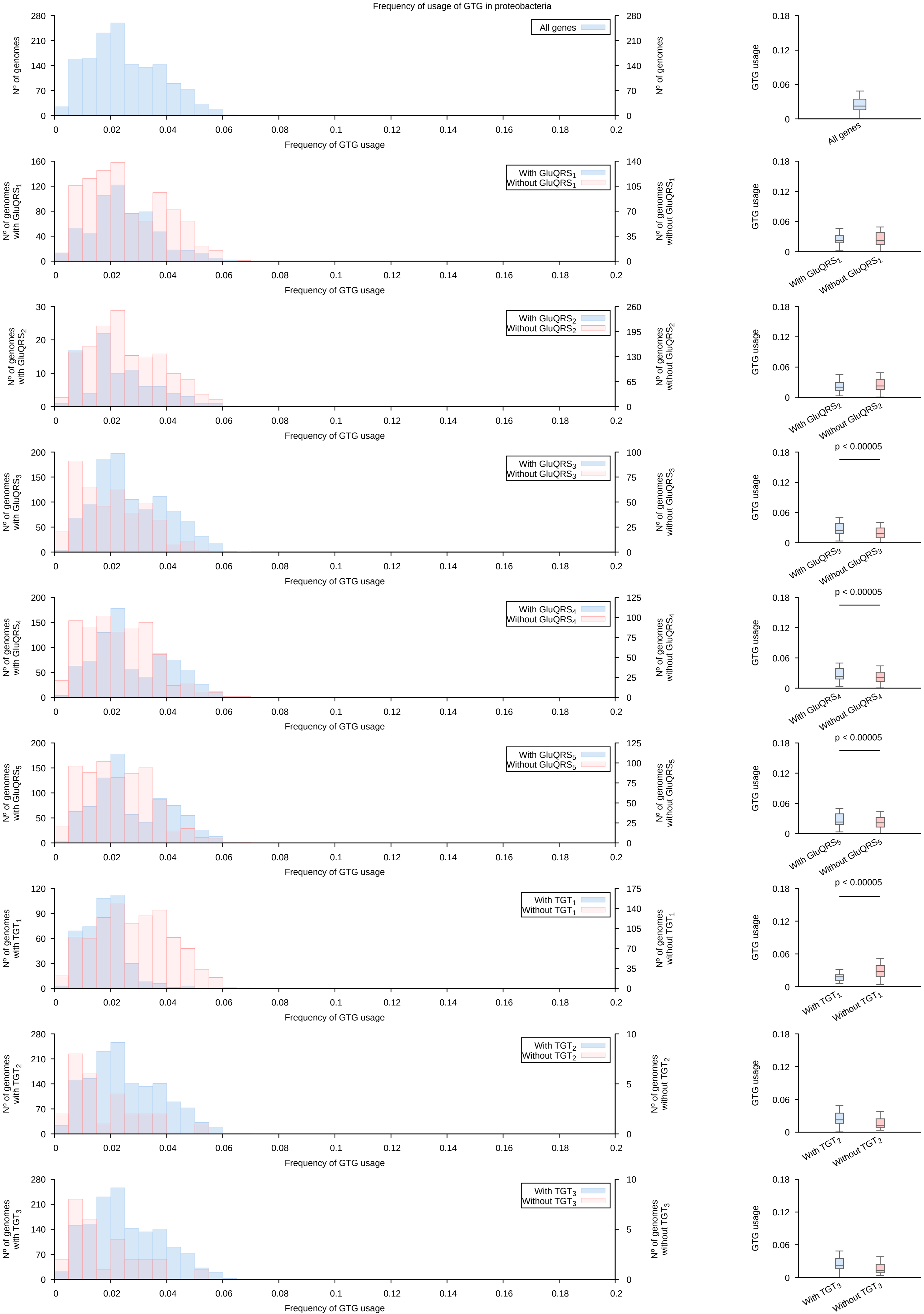

Frequency of usage of GTG in proteobacteria
All genes
With GluQRS1
Without GluQRS1
With GluQRS2
Without GluQRS2
With GluQRS3
Without GluQRS3
With GluQRS4
Without GluQRS4
With GluQRS5
Without GluQRS5
With TGT1
Without TGT1
With TGT2
Without TGT2
With TGT3
Without TGT3
Nº of genomes
Frequency of GTG usage
GTG usage
p < 0.00005

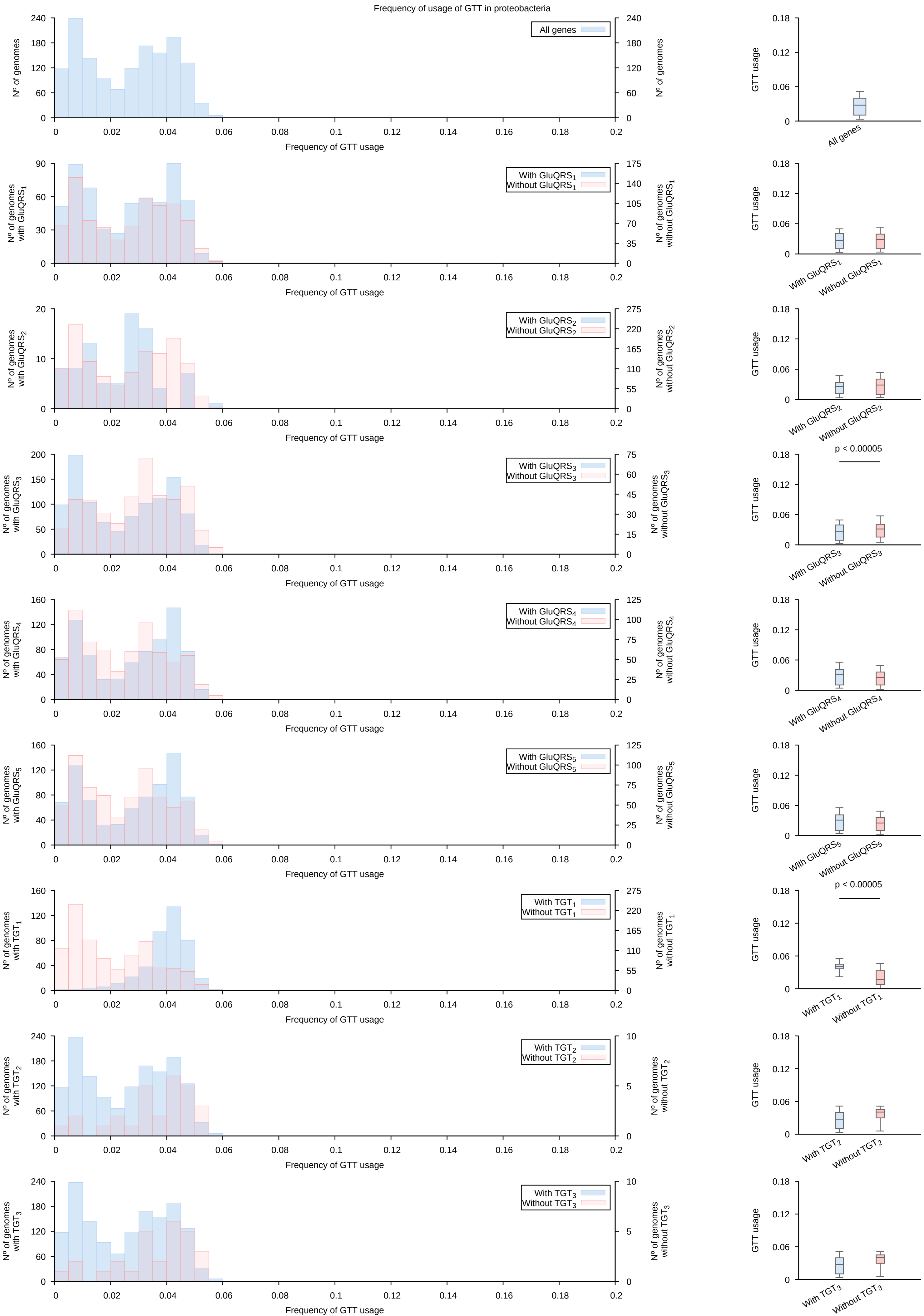
Frequency of usage of GTT in proteobacteria
Nº of genomes
Frequency of GTT usage
All genes
GTT usage
Nº of genomes with GluQRS$_1$
Nº of genomes without GluQRS$_1$
With GluQRS$_1$
Without GluQRS$_1$
Nº of genomes with GluQRS$_2$
Nº of genomes without GluQRS$_2$
With GluQRS$_2$
Without GluQRS$_2$
Nº of genomes with GluQRS$_3$
Nº of genomes without GluQRS$_3$
With GluQRS$_3$
Without GluQRS$_3$
p < 0.00005
Nº of genomes with GluQRS$_4$
Nº of genomes without GluQRS$_4$
With GluQRS$_4$
Without GluQRS$_4$
Nº of genomes with GluQRS$_5$
Nº of genomes without GluQRS$_5$
With GluQRS$_5$
Without GluQRS$_5$
Nº of genomes with TGT$_1$
Nº of genomes without TGT$_1$
With TGT$_1$
Without TGT$_1$
p < 0.00005
Nº of genomes with TGT$_2$
Nº of genomes without TGT$_2$
With TGT$_2$
Without TGT$_2$
Nº of genomes with TGT$_3$
Nº of genomes without TGT$_3$
With TGT$_3$
Without TGT$_3$

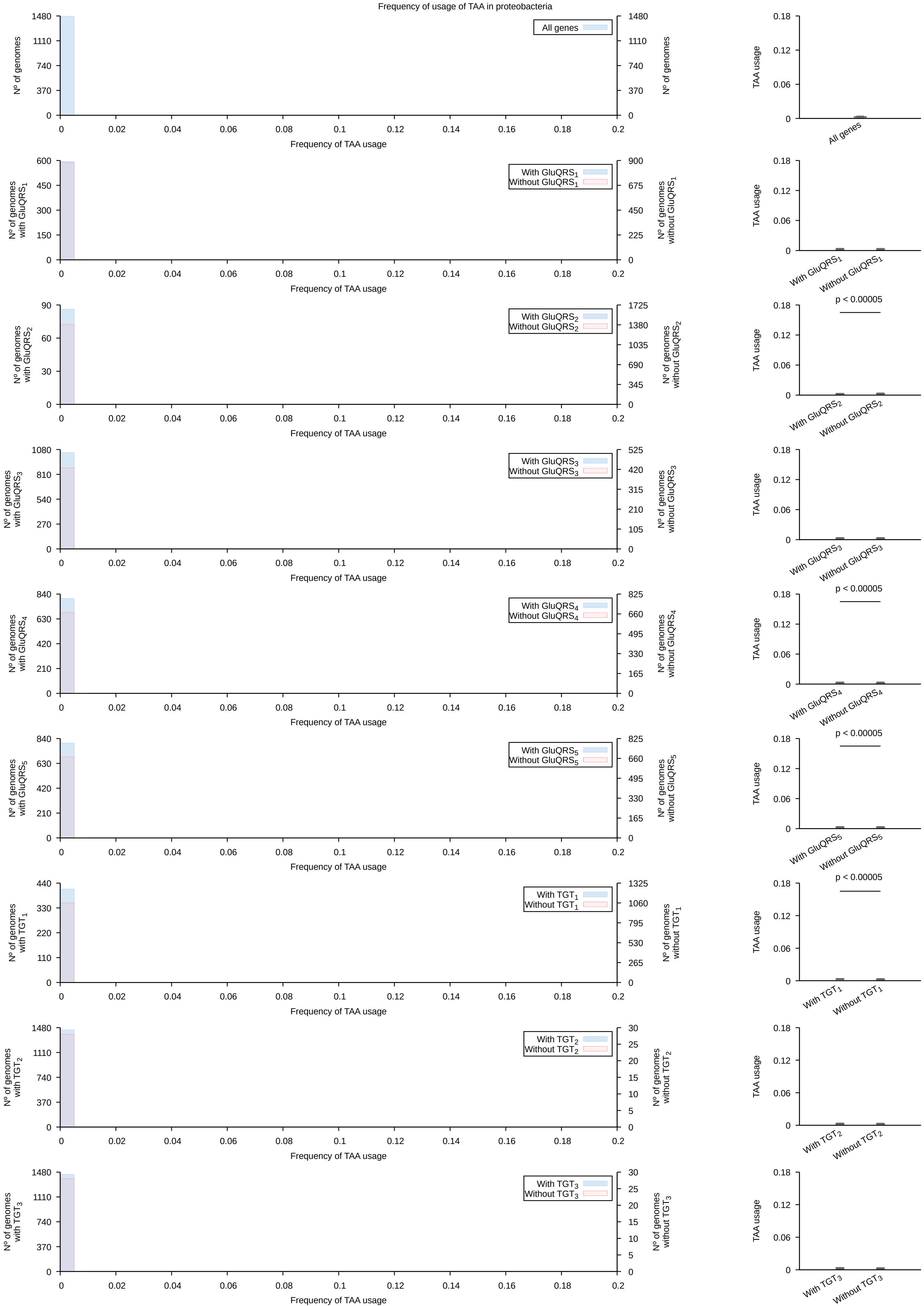
Frequency of usage of TAA in proteobacteria
All genes
With GluQRS$_1$
Without GluQRS$_1$
With GluQRS$_2$
Without GluQRS$_2$
With GluQRS$_3$
Without GluQRS$_3$
With GluQRS$_4$
Without GluQRS$_4$
With GluQRS$_5$
Without GluQRS$_5$
With TGT$_1$
Without TGT$_1$
With TGT$_2$
Without TGT$_2$
With TGT$_3$
Without TGT$_3$
Nº of genomes
Frequency of TAA usage
TAA usage
p < 0.00005

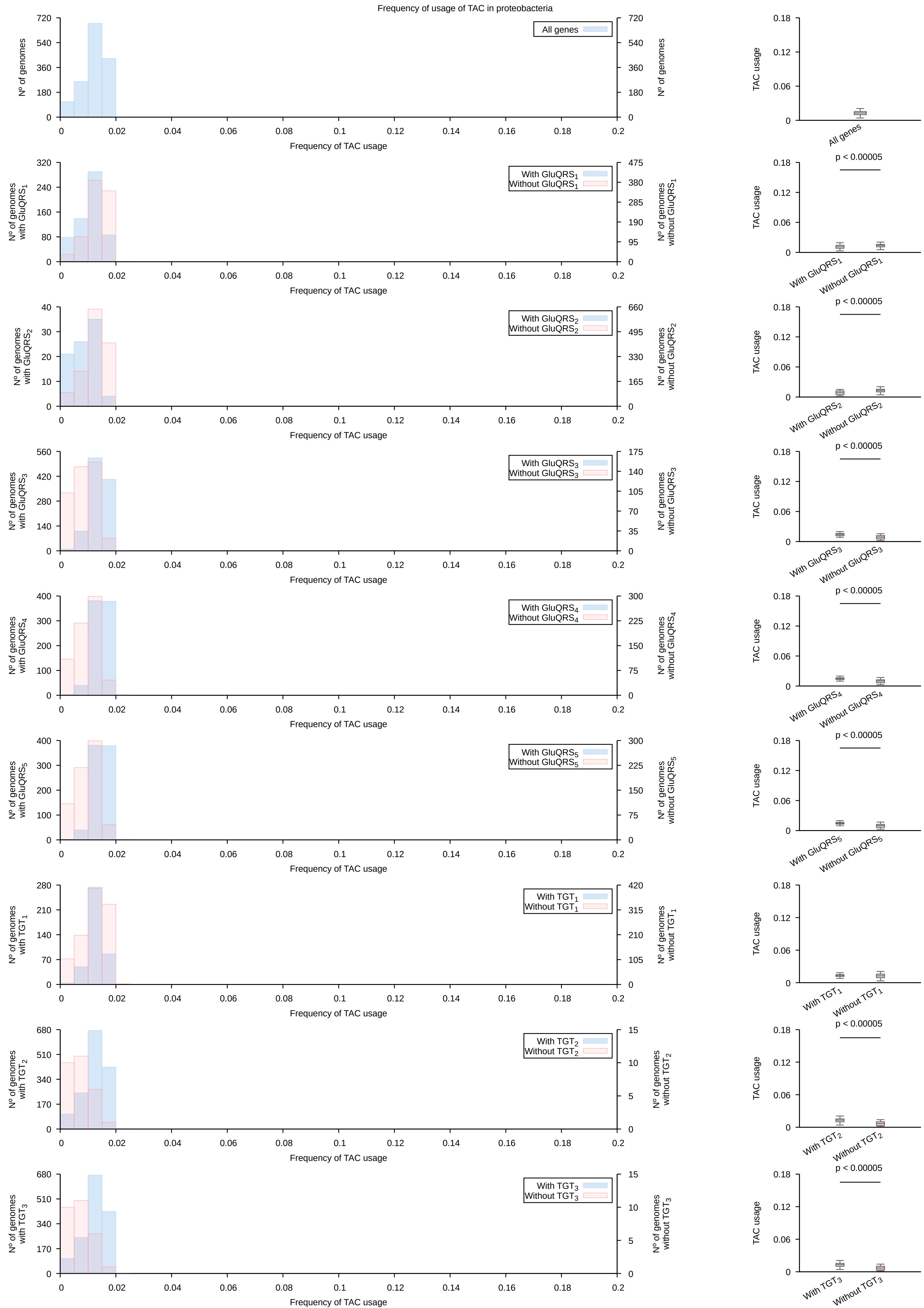
Frequency of usage of TAC in proteobacteria
All genes
Nº of genomes
Frequency of TAC usage
TAC usage
With GluQRS$_1$
Without GluQRS$_1$
Nº of genomes with GluQRS$_1$
Nº of genomes without GluQRS$_1$
p < 0.00005
With GluQRS$_2$
Without GluQRS$_2$
Nº of genomes with GluQRS$_2$
Nº of genomes without GluQRS$_2$
p < 0.00005
With GluQRS$_3$
Without GluQRS$_3$
Nº of genomes with GluQRS$_3$
Nº of genomes without GluQRS$_3$
p < 0.00005
With GluQRS$_4$
Without GluQRS$_4$
Nº of genomes with GluQRS$_4$
Nº of genomes without GluQRS$_4$
p < 0.00005
With GluQRS$_5$
Without GluQRS$_5$
Nº of genomes with GluQRS$_5$
Nº of genomes without GluQRS$_5$
p < 0.00005
With TGT$_1$
Without TGT$_1$
Nº of genomes with TGT$_1$
Nº of genomes without TGT$_1$
With TGT$_2$
Without TGT$_2$
Nº of genomes with TGT$_2$
Nº of genomes without TGT$_2$
p < 0.00005
With TGT$_3$
Without TGT$_3$
Nº of genomes with TGT$_3$
Nº of genomes without TGT$_3$
p < 0.00005

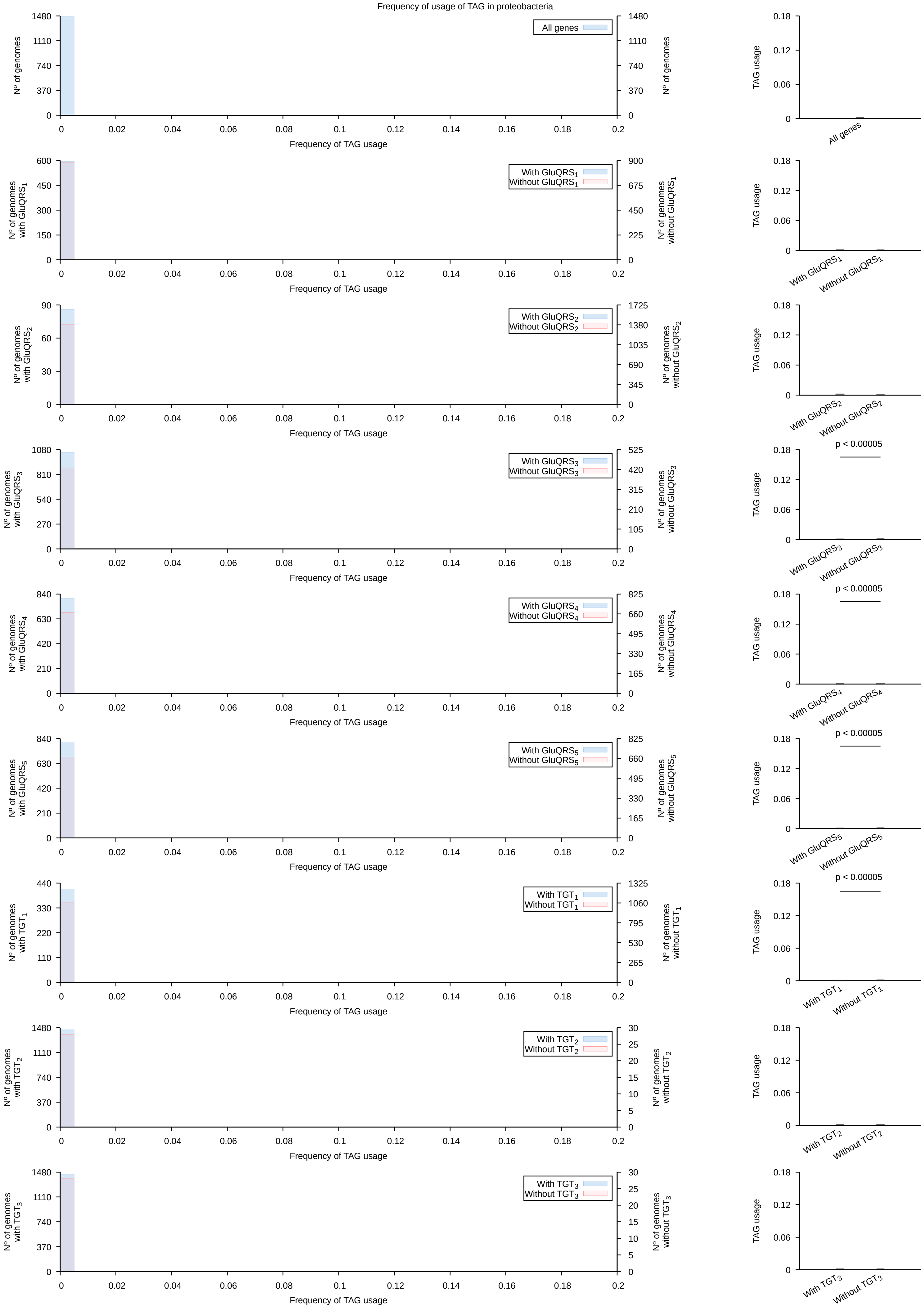
Frequency of usage of TAG in proteobacteria
Nº of genomes
Frequency of TAG usage
TAG usage
All genes
With GluQRS1
Without GluQRS1
With GluQRS2
Without GluQRS2
With GluQRS3
Without GluQRS3
With GluQRS4
Without GluQRS4
With GluQRS5
Without GluQRS5
With TGT1
Without TGT1
With TGT2
Without TGT2
With TGT3
Without TGT3
p < 0.00005

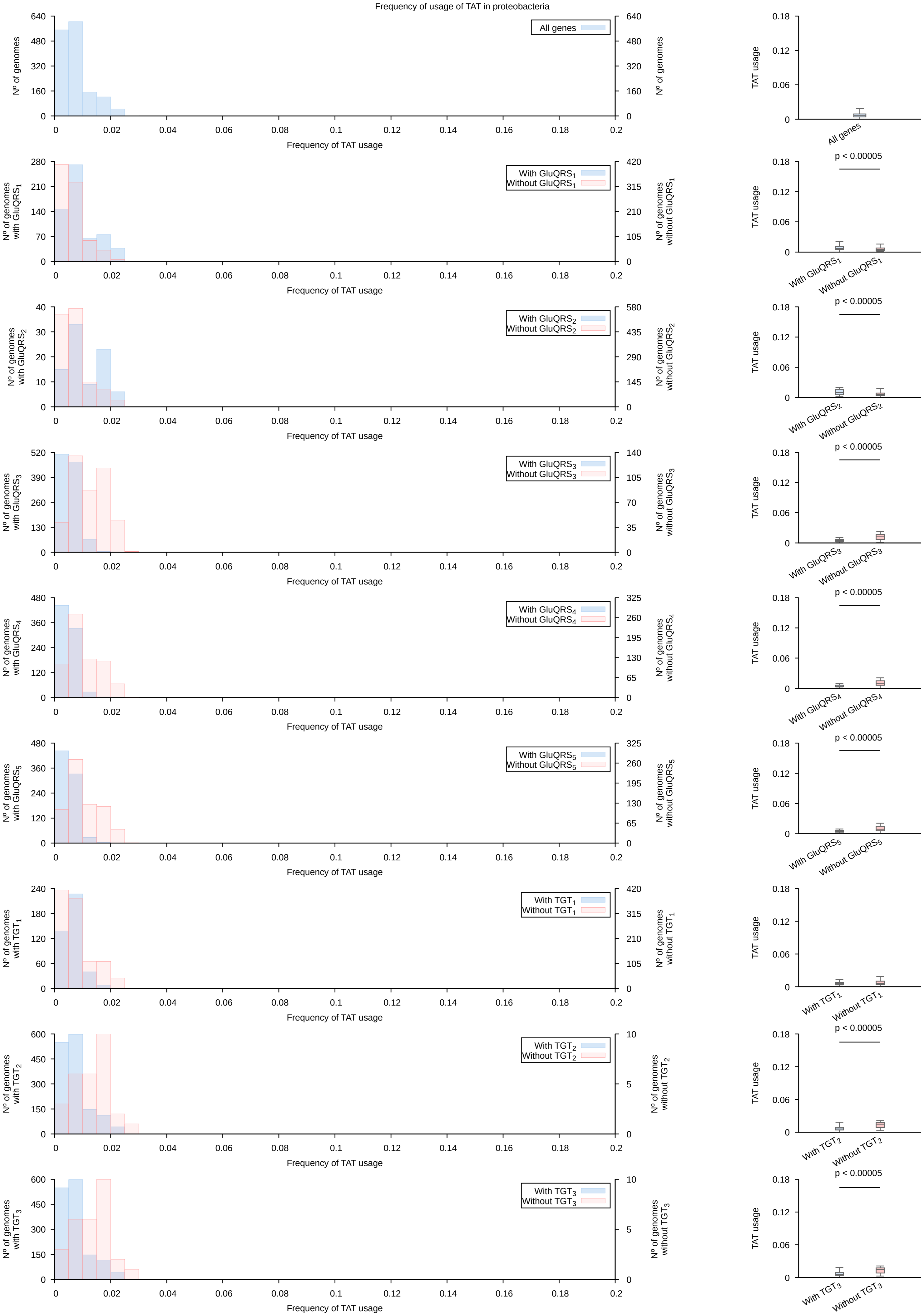
Frequency of usage of TAT in proteobacteria
All genes
Nº of genomes
Frequency of TAT usage
TAT usage
With GluQRS$_1$
Without GluQRS$_1$
Nº of genomes with GluQRS$_1$
Nº of genomes without GluQRS$_1$
p < 0.00005
With GluQRS$_2$
Without GluQRS$_2$
Nº of genomes with GluQRS$_2$
Nº of genomes without GluQRS$_2$
With GluQRS$_3$
Without GluQRS$_3$
Nº of genomes with GluQRS$_3$
Nº of genomes without GluQRS$_3$
With GluQRS$_4$
Without GluQRS$_4$
Nº of genomes with GluQRS$_4$
Nº of genomes without GluQRS$_4$
With GluQRS$_5$
Without GluQRS$_5$
Nº of genomes with GluQRS$_5$
Nº of genomes without GluQRS$_5$
With TGT$_1$
Without TGT$_1$
Nº of genomes with TGT$_1$
Nº of genomes without TGT$_1$
With TGT$_2$
Without TGT$_2$
Nº of genomes with TGT$_2$
Nº of genomes without TGT$_2$
With TGT$_3$
Without TGT$_3$
Nº of genomes with TGT$_3$
Nº of genomes without TGT$_3$

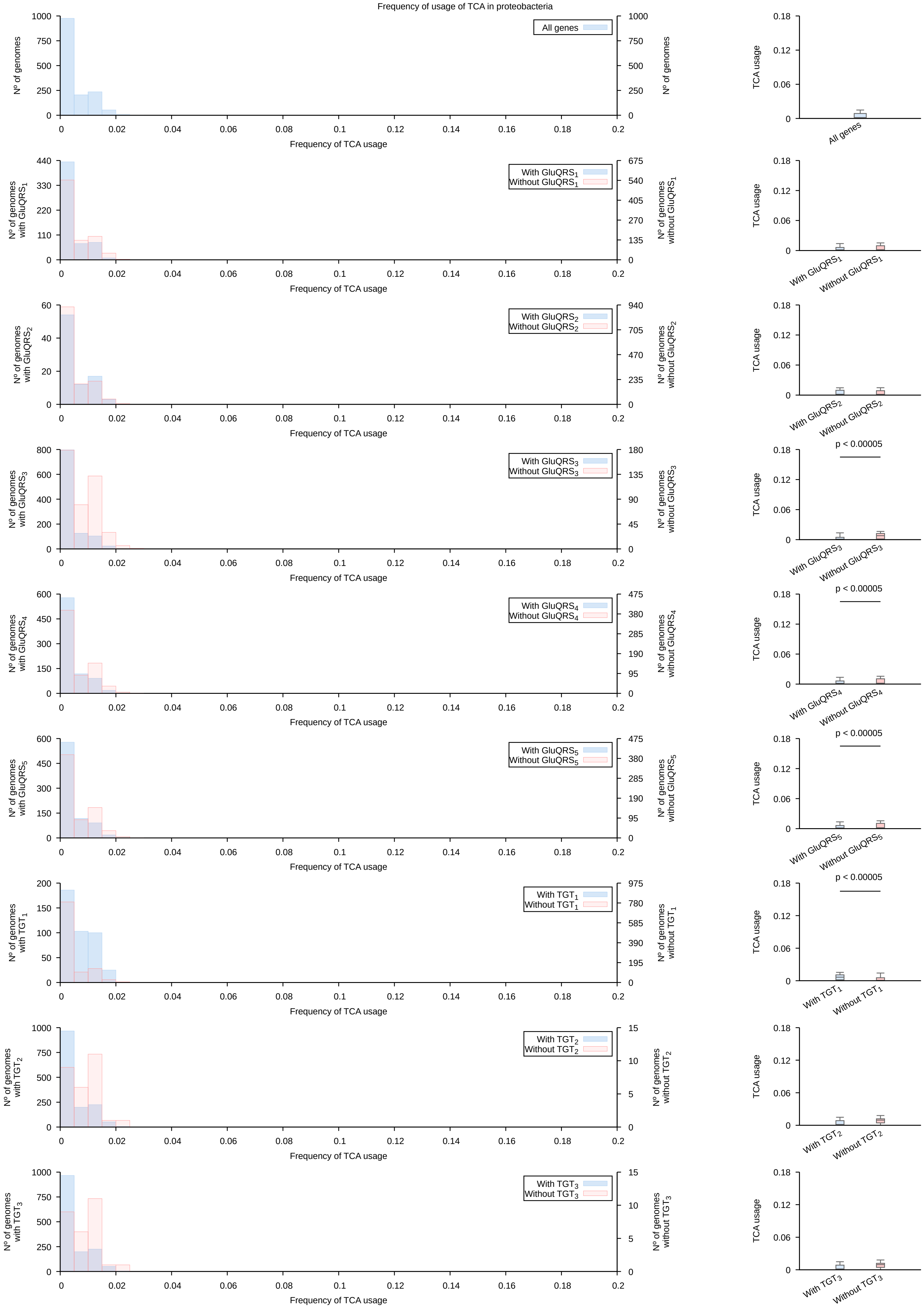

Frequency of usage of TCA in proteobacteria
All genes
Nº of genomes
Frequency of TCA usage
TCA usage
With GluQRS$_1$
Without GluQRS$_1$
Nº of genomes with GluQRS$_1$
Nº of genomes without GluQRS$_1$
With GluQRS$_2$
Without GluQRS$_2$
Nº of genomes with GluQRS$_2$
Nº of genomes without GluQRS$_2$
With GluQRS$_3$
Without GluQRS$_3$
Nº of genomes with GluQRS$_3$
Nº of genomes without GluQRS$_3$
p < 0.00005
With GluQRS$_4$
Without GluQRS$_4$
Nº of genomes with GluQRS$_4$
Nº of genomes without GluQRS$_4$
p < 0.00005
With GluQRS$_5$
Without GluQRS$_5$
Nº of genomes with GluQRS$_5$
Nº of genomes without GluQRS$_5$
p < 0.00005
With TGT$_1$
Without TGT$_1$
Nº of genomes with TGT$_1$
Nº of genomes without TGT$_1$
p < 0.00005
With TGT$_2$
Without TGT$_2$
Nº of genomes with TGT$_2$
Nº of genomes without TGT$_2$
With TGT$_3$
Without TGT$_3$
Nº of genomes with TGT$_3$
Nº of genomes without TGT$_3$

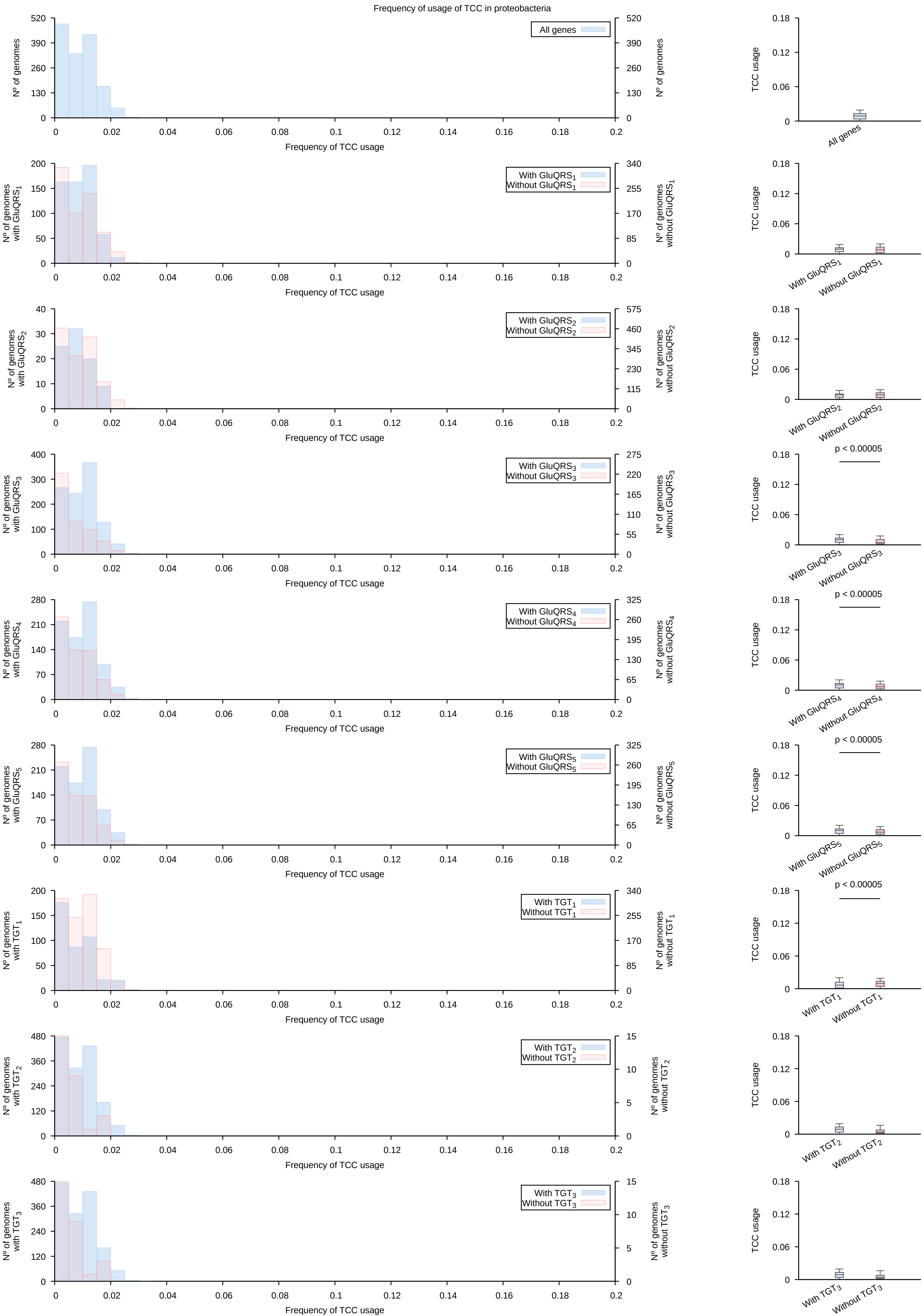
Frequency of usage of TCC in proteobacteria
Nº of genomes
Frequency of TCC usage
TCC usage
All genes
Nº of genomes with $GluQRS_1$
Nº of genomes without $GluQRS_1$
With $GluQRS_1$
Without $GluQRS_1$
Nº of genomes with $GluQRS_2$
Nº of genomes without $GluQRS_2$
With $GluQRS_2$
Without $GluQRS_2$
Nº of genomes with $GluQRS_3$
Nº of genomes without $GluQRS_3$
With $GluQRS_3$
Without $GluQRS_3$
Nº of genomes with $GluQRS_4$
Nº of genomes without $GluQRS_4$
With $GluQRS_4$
Without $GluQRS_4$
Nº of genomes with $GluQRS_5$
Nº of genomes without $GluQRS_5$
With $GluQRS_5$
Without $GluQRS_5$
Nº of genomes with TGT_1
Nº of genomes without TGT_1
With TGT_1
Without TGT_1
Nº of genomes with TGT_2
Nº of genomes without TGT_2
With TGT_2
Without TGT_2
Nº of genomes with TGT_3
Nº of genomes without TGT_3
With TGT_3
Without TGT_3
$p < 0.00005$
0 0.02 0.04 0.06 0.08 0.1 0.12 0.14 0.16 0.18 0.2
0 0.06 0.12 0.18

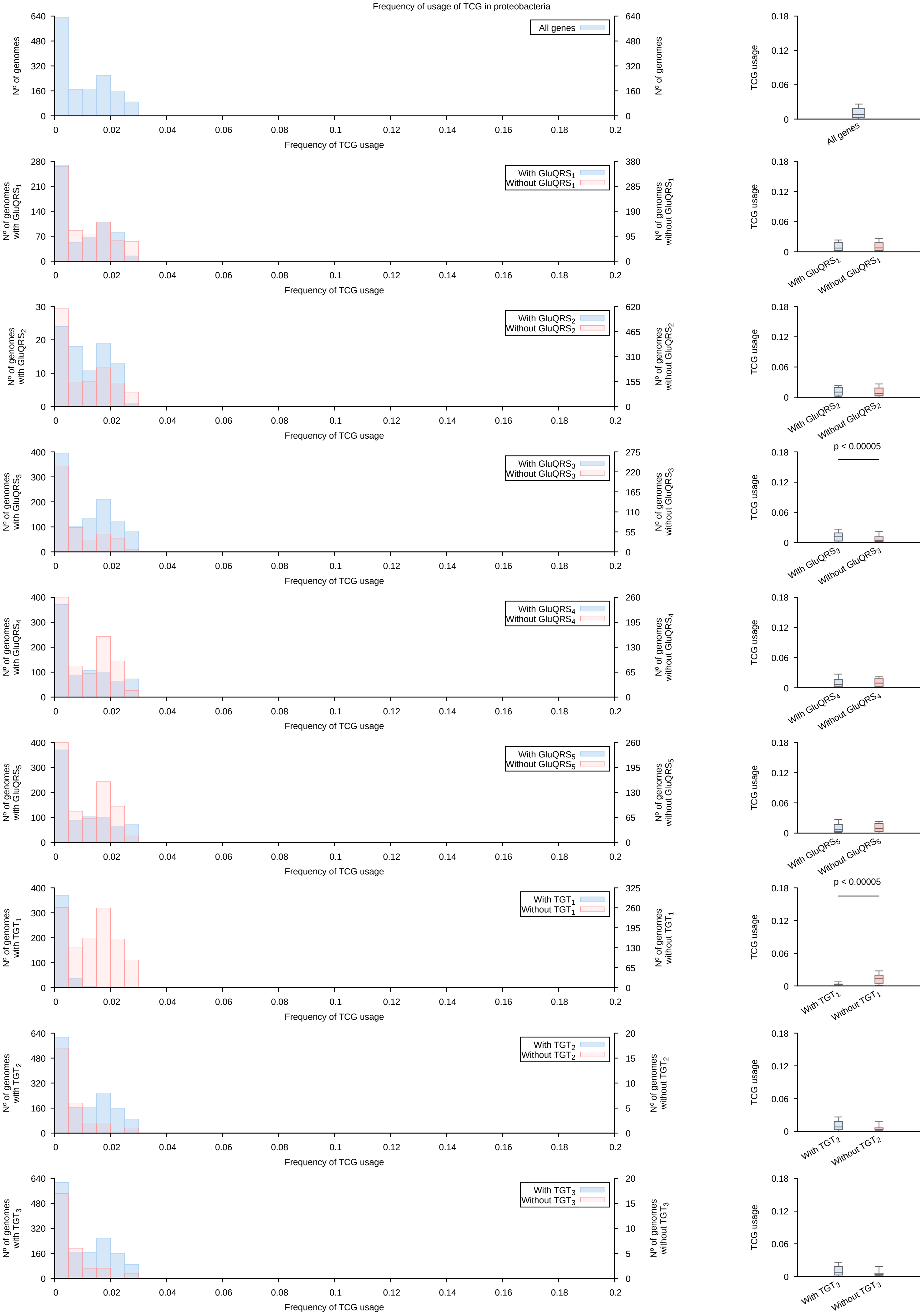

Frequency of usage of TCG in proteobacteria
All genes
Nº of genomes
Frequency of TCG usage
TCG usage
All genes
With GluQRS$_1$
Without GluQRS$_1$
Nº of genomes with GluQRS$_1$
Nº of genomes without GluQRS$_1$
With GluQRS$_2$
Without GluQRS$_2$
Nº of genomes with GluQRS$_2$
Nº of genomes without GluQRS$_2$
With GluQRS$_3$
Without GluQRS$_3$
Nº of genomes with GluQRS$_3$
Nº of genomes without GluQRS$_3$
p < 0.00005
With GluQRS$_4$
Without GluQRS$_4$
Nº of genomes with GluQRS$_4$
Nº of genomes without GluQRS$_4$
With GluQRS$_5$
Without GluQRS$_5$
Nº of genomes with GluQRS$_5$
Nº of genomes without GluQRS$_5$
With TGT$_1$
Without TGT$_1$
Nº of genomes with TGT$_1$
Nº of genomes without TGT$_1$
p < 0.00005
With TGT$_2$
Without TGT$_2$
Nº of genomes with TGT$_2$
Nº of genomes without TGT$_2$
With TGT$_3$
Without TGT$_3$
Nº of genomes with TGT$_3$
Nº of genomes without TGT$_3$

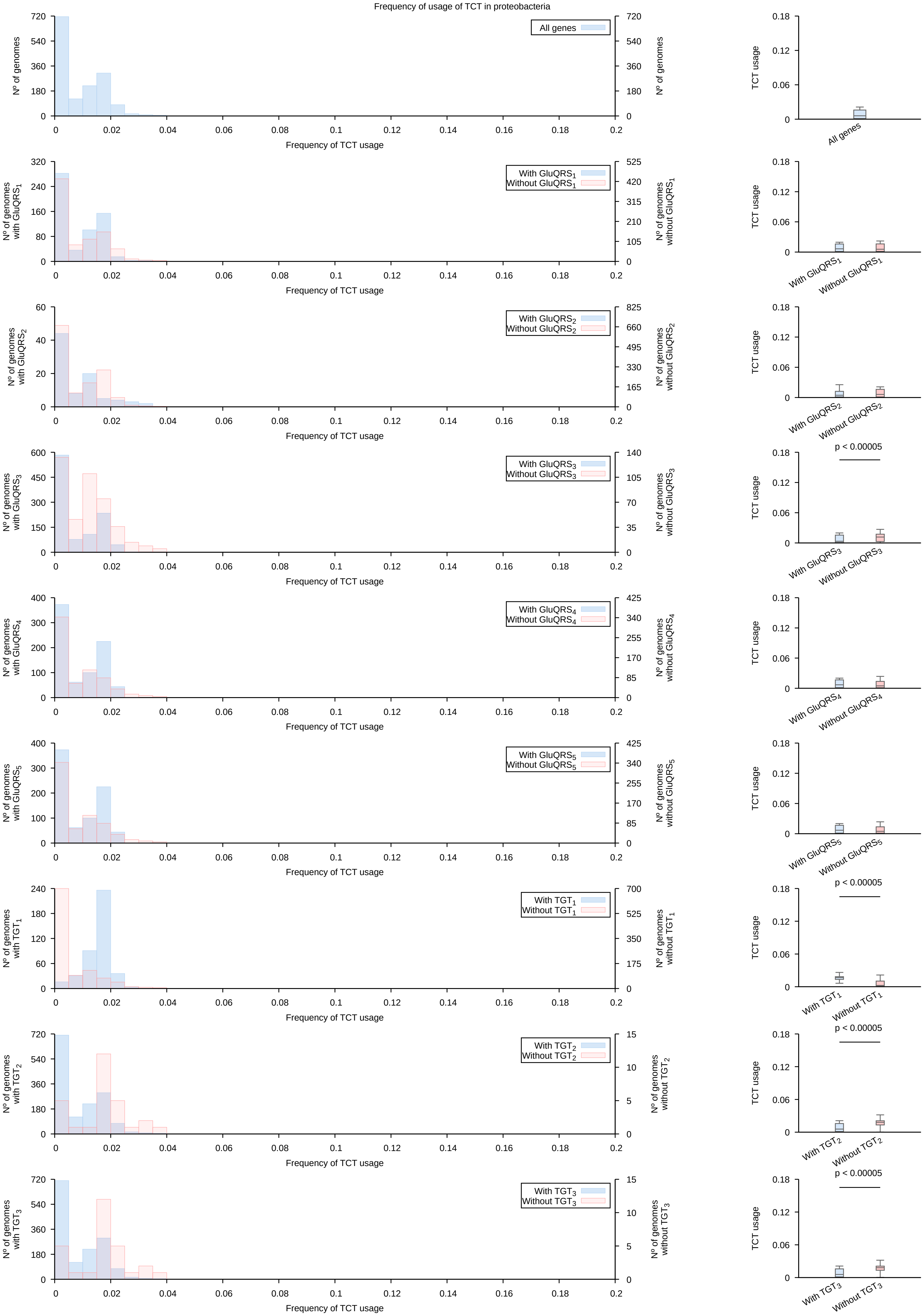

Frequency of usage of TCT in proteobacteria
Nº of genomes
Frequency of TCT usage
TCT usage
All genes
Nº of genomes with GluQRS$_1$
Nº of genomes without GluQRS$_1$
With GluQRS$_1$
Without GluQRS$_1$
Nº of genomes with GluQRS$_2$
Nº of genomes without GluQRS$_2$
With GluQRS$_2$
Without GluQRS$_2$
Nº of genomes with GluQRS$_3$
Nº of genomes without GluQRS$_3$
With GluQRS$_3$
Without GluQRS$_3$
p < 0.00005
Nº of genomes with GluQRS$_4$
Nº of genomes without GluQRS$_4$
With GluQRS$_4$
Without GluQRS$_4$
Nº of genomes with GluQRS$_5$
Nº of genomes without GluQRS$_5$
With GluQRS$_5$
Without GluQRS$_5$
Nº of genomes with TGT$_1$
Nº of genomes without TGT$_1$
With TGT$_1$
Without TGT$_1$
p < 0.00005
Nº of genomes with TGT$_2$
Nº of genomes without TGT$_2$
With TGT$_2$
Without TGT$_2$
p < 0.00005
Nº of genomes with TGT$_3$
Nº of genomes without TGT$_3$
With TGT$_3$
Without TGT$_3$
p < 0.00005

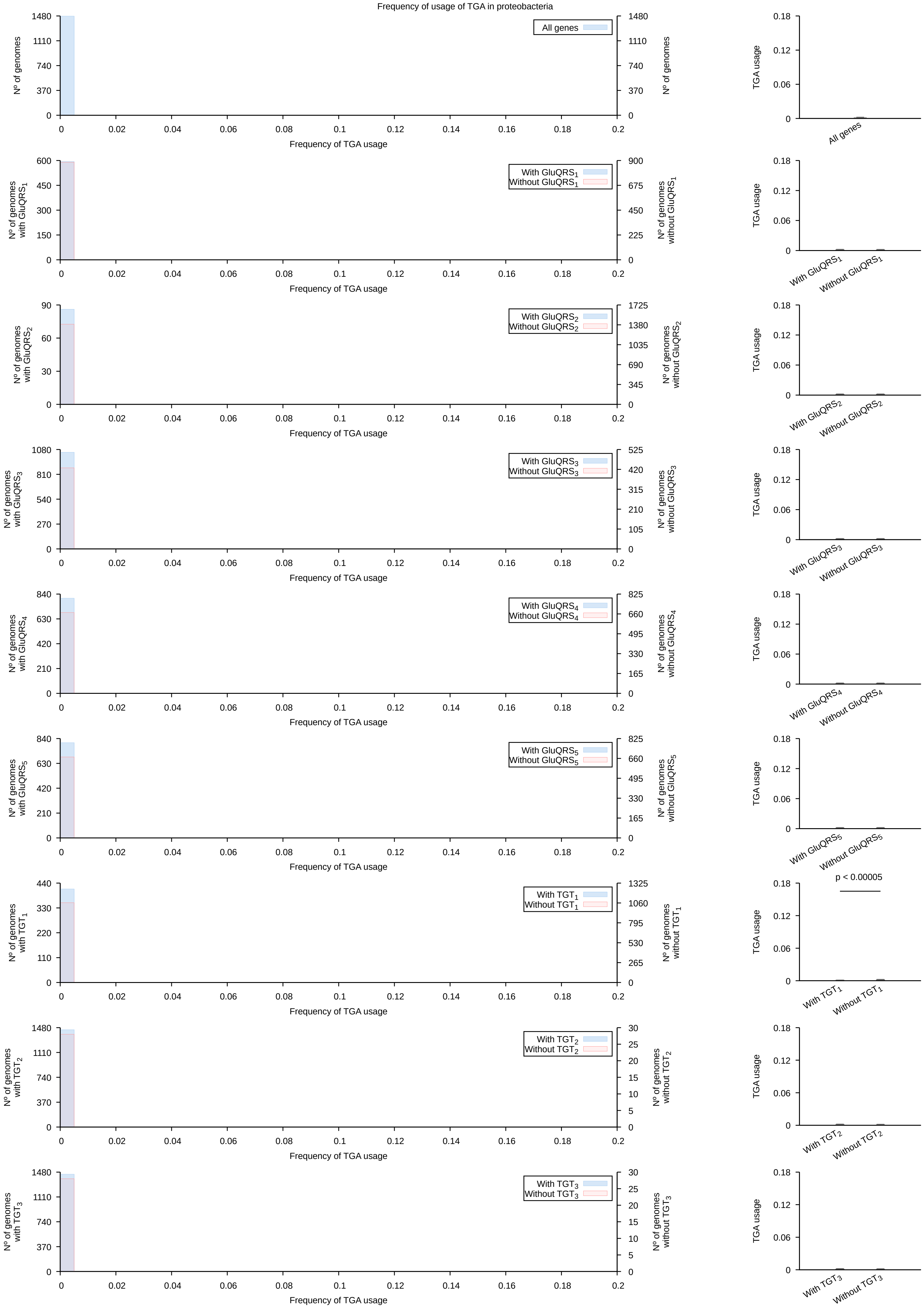
Frequency of usage of TGA in proteobacteria
All genes
With GluQRS1
Without GluQRS1
With GluQRS2
Without GluQRS2
With GluQRS3
Without GluQRS3
With GluQRS4
Without GluQRS4
With GluQRS5
Without GluQRS5
With TGT1
Without TGT1
With TGT2
Without TGT2
With TGT3
Without TGT3
Nº of genomes
Frequency of TGA usage
TGA usage
p < 0.00005

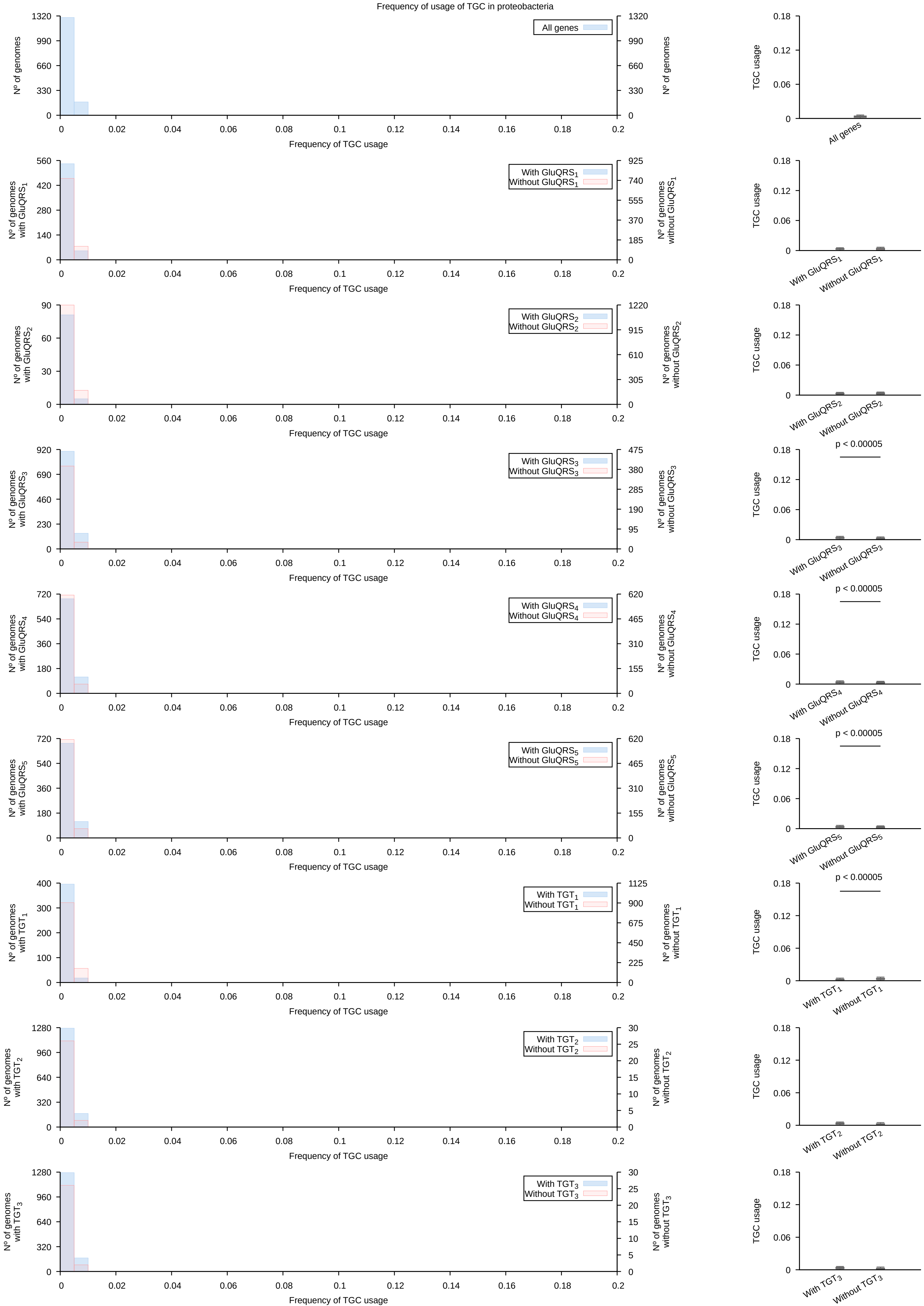
Frequency of usage of TGC in proteobacteria
All genes
With GluQRS1
Without GluQRS1
With GluQRS2
Without GluQRS2
With GluQRS3
Without GluQRS3
With GluQRS4
Without GluQRS4
With GluQRS5
Without GluQRS5
With TGT1
Without TGT1
With TGT2
Without TGT2
With TGT3
Without TGT3
Nº of genomes
Frequency of TGC usage
TGC usage
p < 0.00005

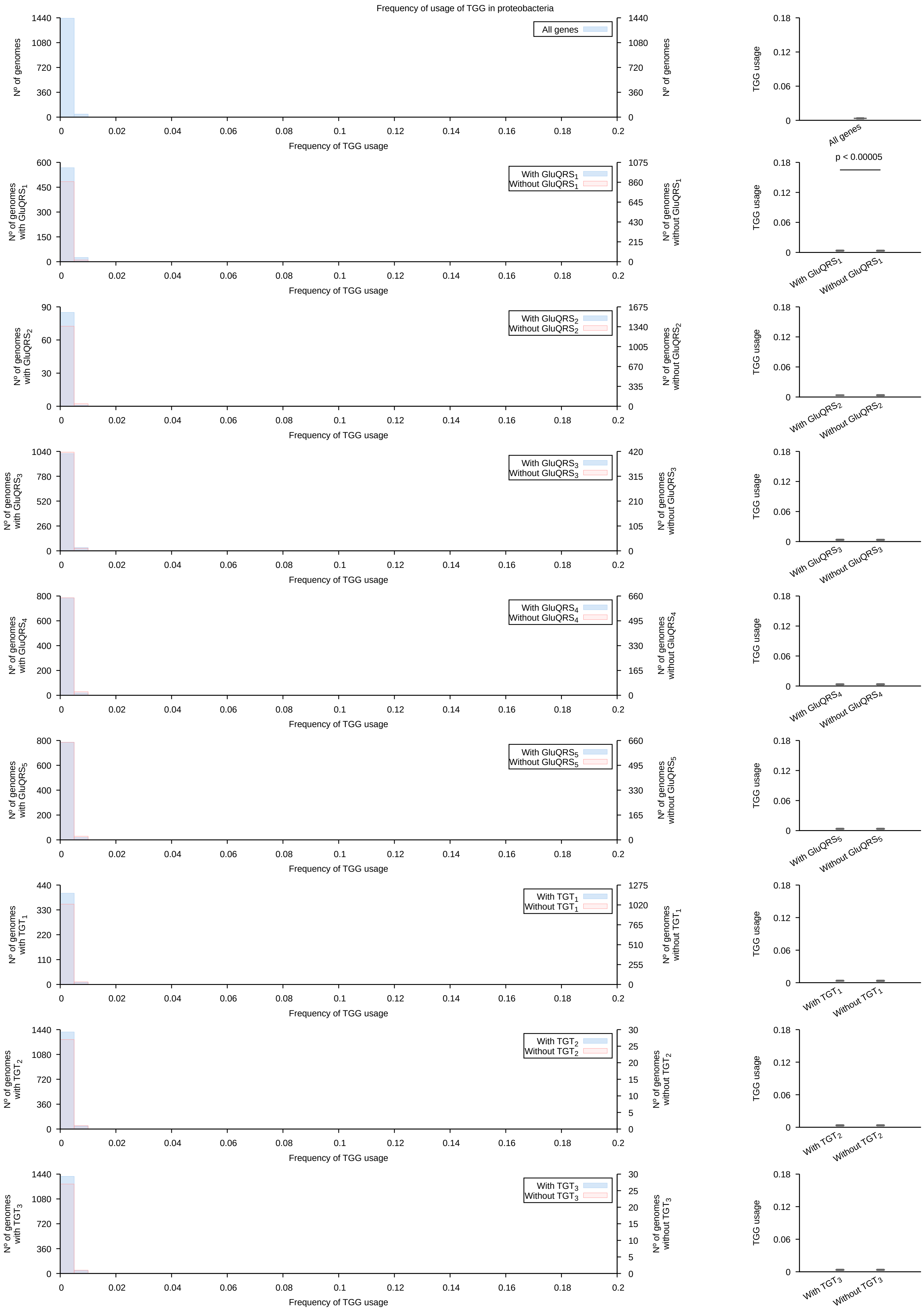

Frequency of usage of TGG in proteobacteria
All genes
Nº of genomes
Frequency of TGG usage
TGG usage
With GluQRS$_1$
Without GluQRS$_1$
Nº of genomes with GluQRS$_1$
Nº of genomes without GluQRS$_1$
p < 0.00005
With GluQRS$_2$
Without GluQRS$_2$
Nº of genomes with GluQRS$_2$
Nº of genomes without GluQRS$_2$
With GluQRS$_3$
Without GluQRS$_3$
Nº of genomes with GluQRS$_3$
Nº of genomes without GluQRS$_3$
With GluQRS$_4$
Without GluQRS$_4$
Nº of genomes with GluQRS$_4$
Nº of genomes without GluQRS$_4$
With GluQRS$_5$
Without GluQRS$_5$
Nº of genomes with GluQRS$_5$
Nº of genomes without GluQRS$_5$
With TGT$_1$
Without TGT$_1$
Nº of genomes with TGT$_1$
Nº of genomes without TGT$_1$
With TGT$_2$
Without TGT$_2$
Nº of genomes with TGT$_2$
Nº of genomes without TGT$_2$
With TGT$_3$
Without TGT$_3$
Nº of genomes with TGT$_3$
Nº of genomes without TGT$_3$

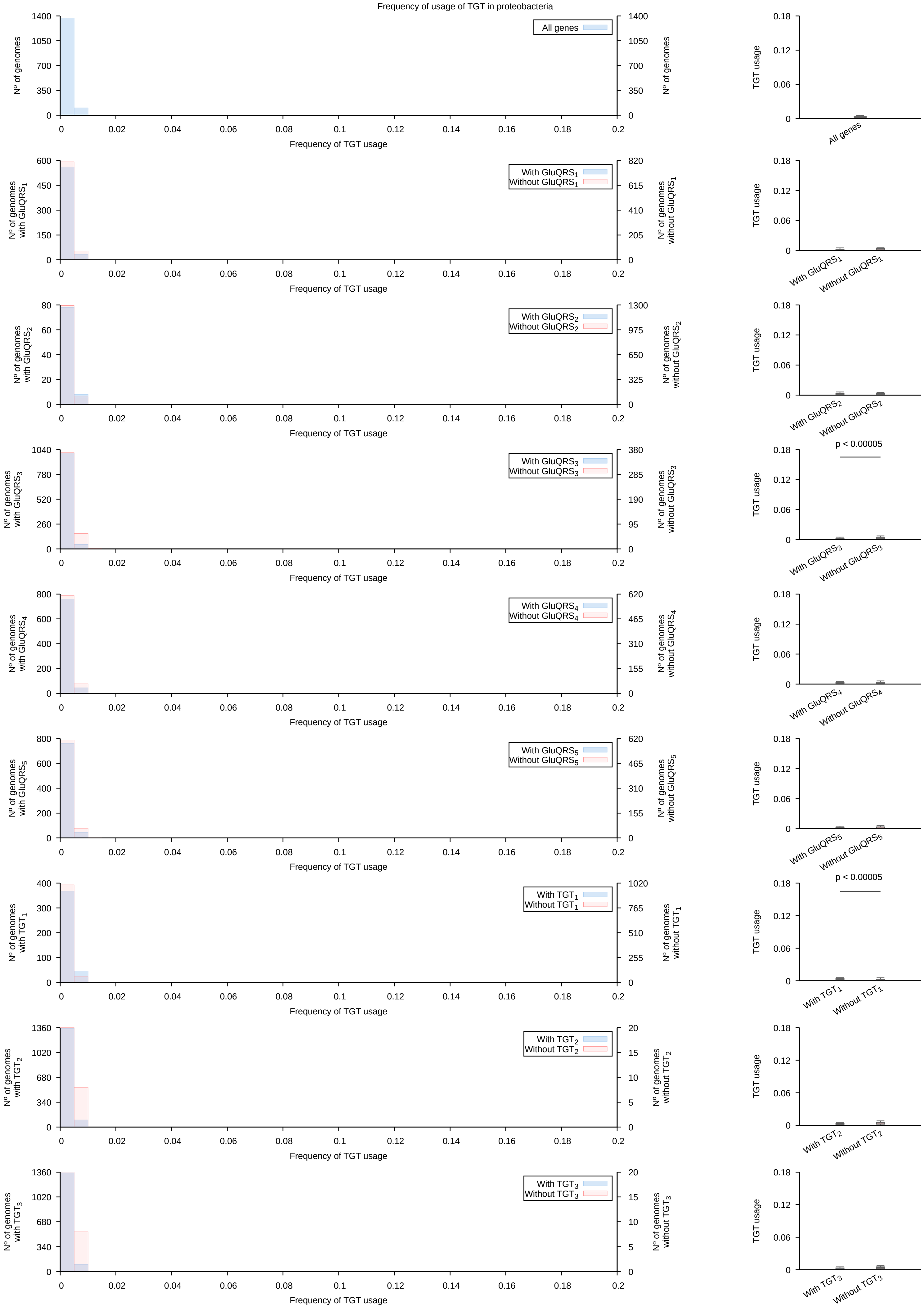
Frequency of usage of TGT in proteobacteria
All genes
With GluQRS1
Without GluQRS1
With GluQRS2
Without GluQRS2
With GluQRS3
Without GluQRS3
With GluQRS4
Without GluQRS4
With GluQRS5
Without GluQRS5
With TGT1
Without TGT1
With TGT2
Without TGT2
With TGT3
Without TGT3
Frequency of TGT usage
Nº of genomes
TGT usage
p < 0.00005

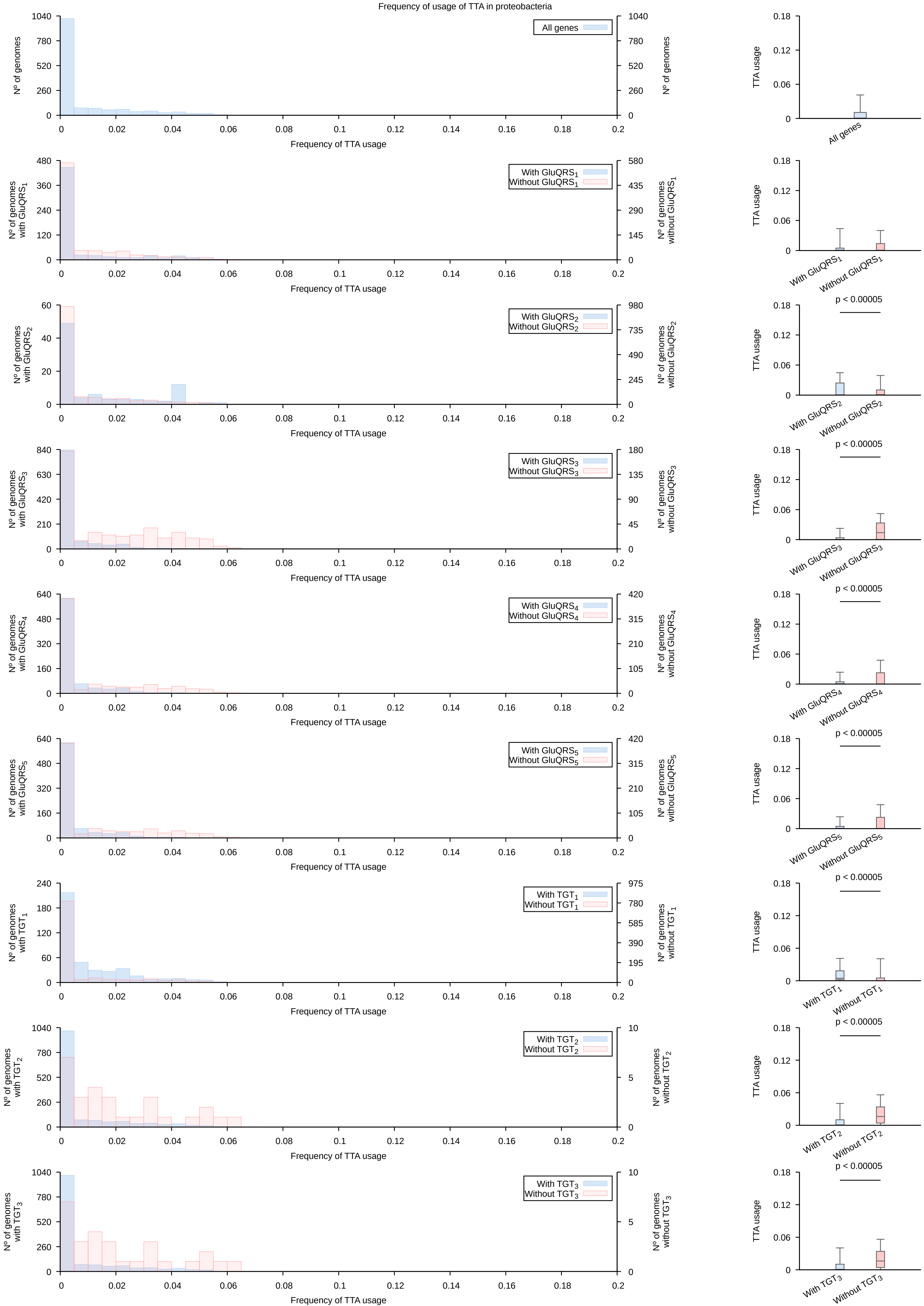
Frequency of usage of TTA in proteobacteria
All genes
Nº of genomes
Frequency of TTA usage
TTA usage
With GluQRS$_1$
Without GluQRS$_1$
Nº of genomes with GluQRS$_1$
Nº of genomes without GluQRS$_1$
With GluQRS$_2$
Without GluQRS$_2$
Nº of genomes with GluQRS$_2$
Nº of genomes without GluQRS$_2$
With GluQRS$_3$
Without GluQRS$_3$
Nº of genomes with GluQRS$_3$
Nº of genomes without GluQRS$_3$
With GluQRS$_4$
Without GluQRS$_4$
Nº of genomes with GluQRS$_4$
Nº of genomes without GluQRS$_4$
With GluQRS$_5$
Without GluQRS$_5$
Nº of genomes with GluQRS$_5$
Nº of genomes without GluQRS$_5$
With TGT$_1$
Without TGT$_1$
Nº of genomes with TGT$_1$
Nº of genomes without TGT$_1$
With TGT$_2$
Without TGT$_2$
Nº of genomes with TGT$_2$
Nº of genomes without TGT$_2$
With TGT$_3$
Without TGT$_3$
Nº of genomes with TGT$_3$
Nº of genomes without TGT$_3$
$p < 0.00005$

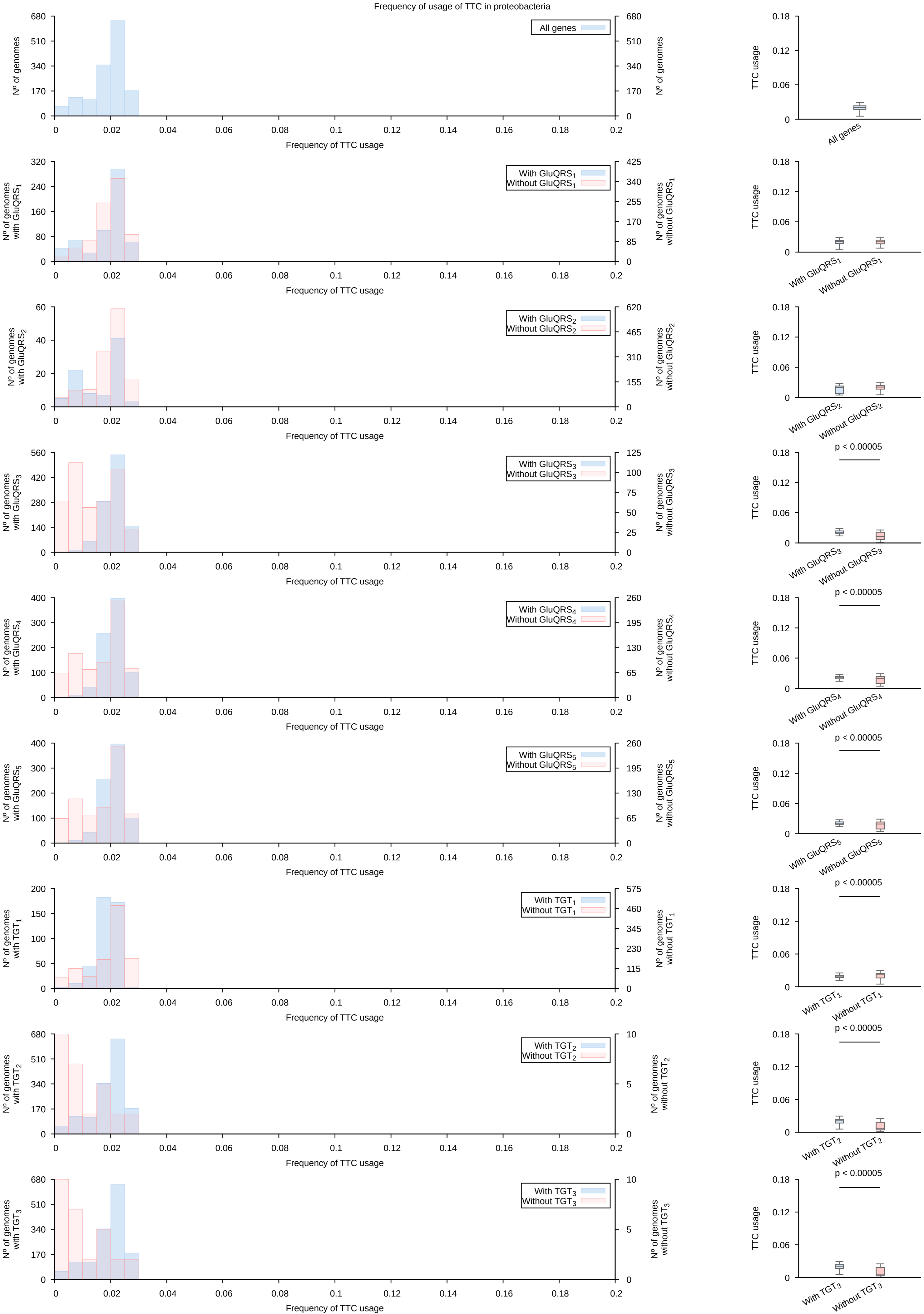
Frequency of usage of TTC in proteobacteria
All genes
With GluQRS1
Without GluQRS1
With GluQRS2
Without GluQRS2
With GluQRS3
Without GluQRS3
With GluQRS4
Without GluQRS4
With GluQRS5
Without GluQRS5
With TGT1
Without TGT1
With TGT2
Without TGT2
With TGT3
Without TGT3
Nº of genomes
Frequency of TTC usage
TTC usage
p < 0.00005

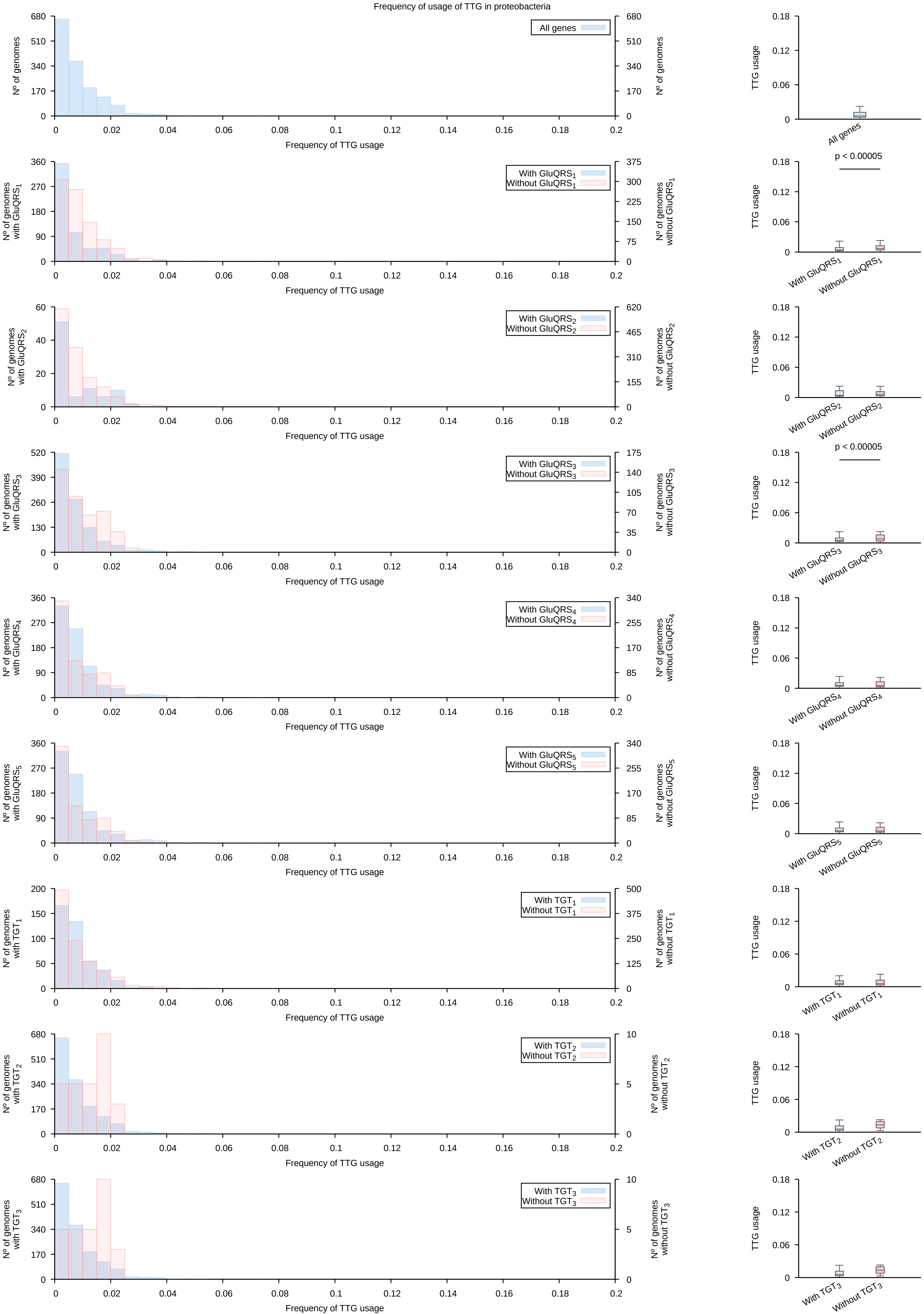

Frequency of usage of TTG in proteobacteria
All genes
Nº of genomes
Frequency of TTG usage
TTG usage
With GluQRS$_1$
Without GluQRS$_1$
Nº of genomes with GluQRS$_1$
Nº of genomes without GluQRS$_1$
p < 0.00005
With GluQRS$_2$
Without GluQRS$_2$
Nº of genomes with GluQRS$_2$
Nº of genomes without GluQRS$_2$
With GluQRS$_3$
Without GluQRS$_3$
Nº of genomes with GluQRS$_3$
Nº of genomes without GluQRS$_3$
p < 0.00005
With GluQRS$_4$
Without GluQRS$_4$
Nº of genomes with GluQRS$_4$
Nº of genomes without GluQRS$_4$
With GluQRS$_5$
Without GluQRS$_5$
Nº of genomes with GluQRS$_5$
Nº of genomes without GluQRS$_5$
With TGT$_1$
Without TGT$_1$
Nº of genomes with TGT$_1$
Nº of genomes without TGT$_1$
With TGT$_2$
Without TGT$_2$
Nº of genomes with TGT$_2$
Nº of genomes without TGT$_2$
With TGT$_3$
Without TGT$_3$
Nº of genomes with TGT$_3$
Nº of genomes without TGT$_3$

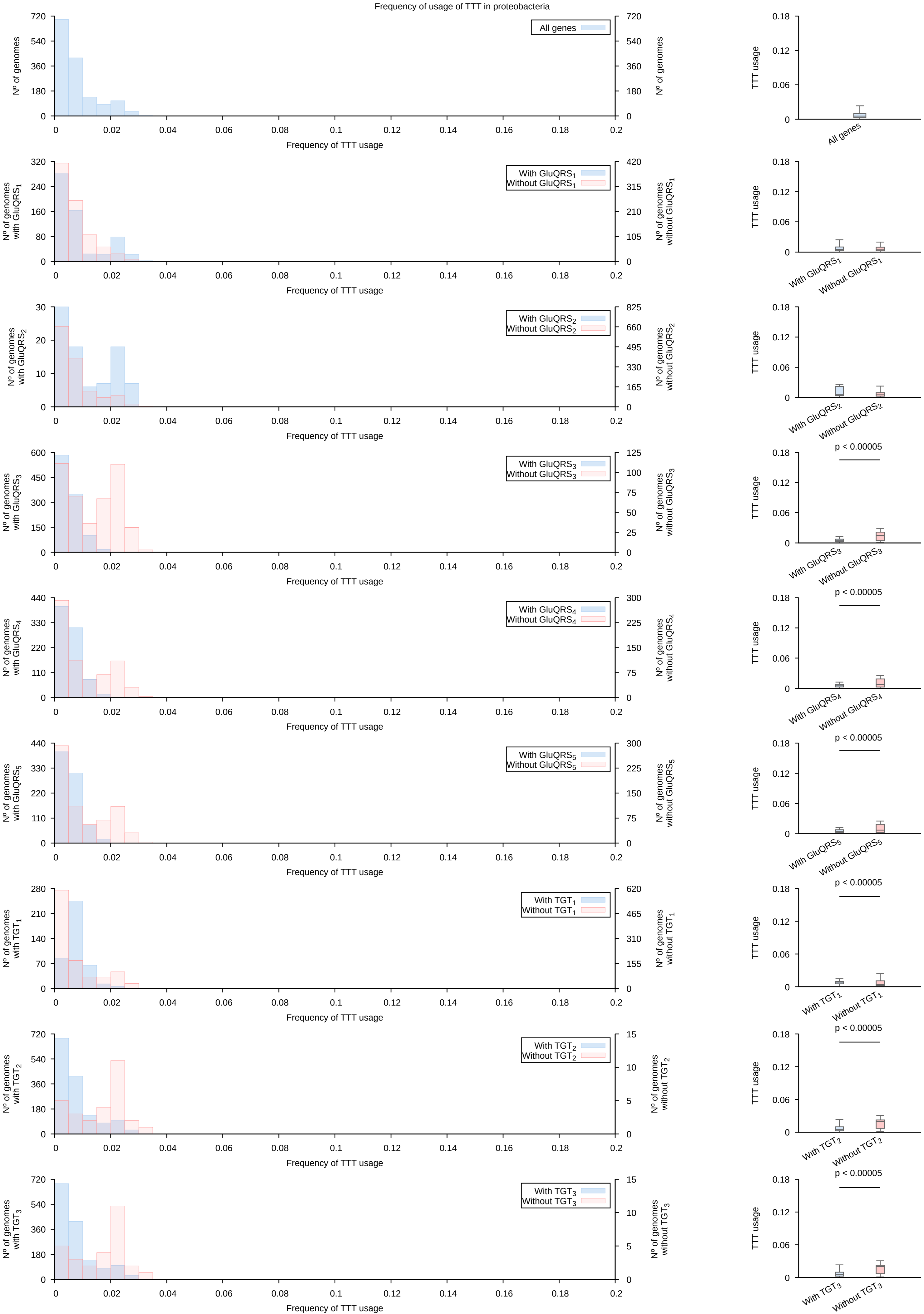
Frequency of usage of TTT in proteobacteria
All genes
With GluQRS$_1$
Without GluQRS$_1$
With GluQRS$_2$
Without GluQRS$_2$
With GluQRS$_3$
Without GluQRS$_3$
With GluQRS$_4$
Without GluQRS$_4$
With GluQRS$_5$
Without GluQRS$_5$
With TGT$_1$
Without TGT$_1$
With TGT$_2$
Without TGT$_2$
With TGT$_3$
Without TGT$_3$
Nº of genomes
Nº of genomes with GluQRS$_1$
Nº of genomes without GluQRS$_1$
Nº of genomes with GluQRS$_2$
Nº of genomes without GluQRS$_2$
Nº of genomes with GluQRS$_3$
Nº of genomes without GluQRS$_3$
Nº of genomes with GluQRS$_4$
Nº of genomes without GluQRS$_4$
Nº of genomes with GluQRS$_5$
Nº of genomes without GluQRS$_5$
Nº of genomes with TGT$_1$
Nº of genomes without TGT$_1$
Nº of genomes with TGT$_2$
Nº of genomes without TGT$_2$
Nº of genomes with TGT$_3$
Nº of genomes without TGT$_3$
Frequency of TTT usage
TTT usage
p < 0.00005
0 0.02 0.04 0.06 0.08 0.1 0.12 0.14 0.16 0.18 0.2
0 0.06 0.12 0.18
