# Supplementary material for: Impact of the chemical modification of tRNAs anticodon loop on the variability and evolution of codon usage in proteobacteria
Source: Front Microbiol. 2024 Aug 5;15:1412318. doi: 10.3389/fmicb.2024.1412318 (PMC11332805; doi:10.3389/fmicb.2024.1412318)
Supplement: Supplementary file 1 [file Data_Sheet_1.zip › Supp_figures/Fig_S24A.pdf]

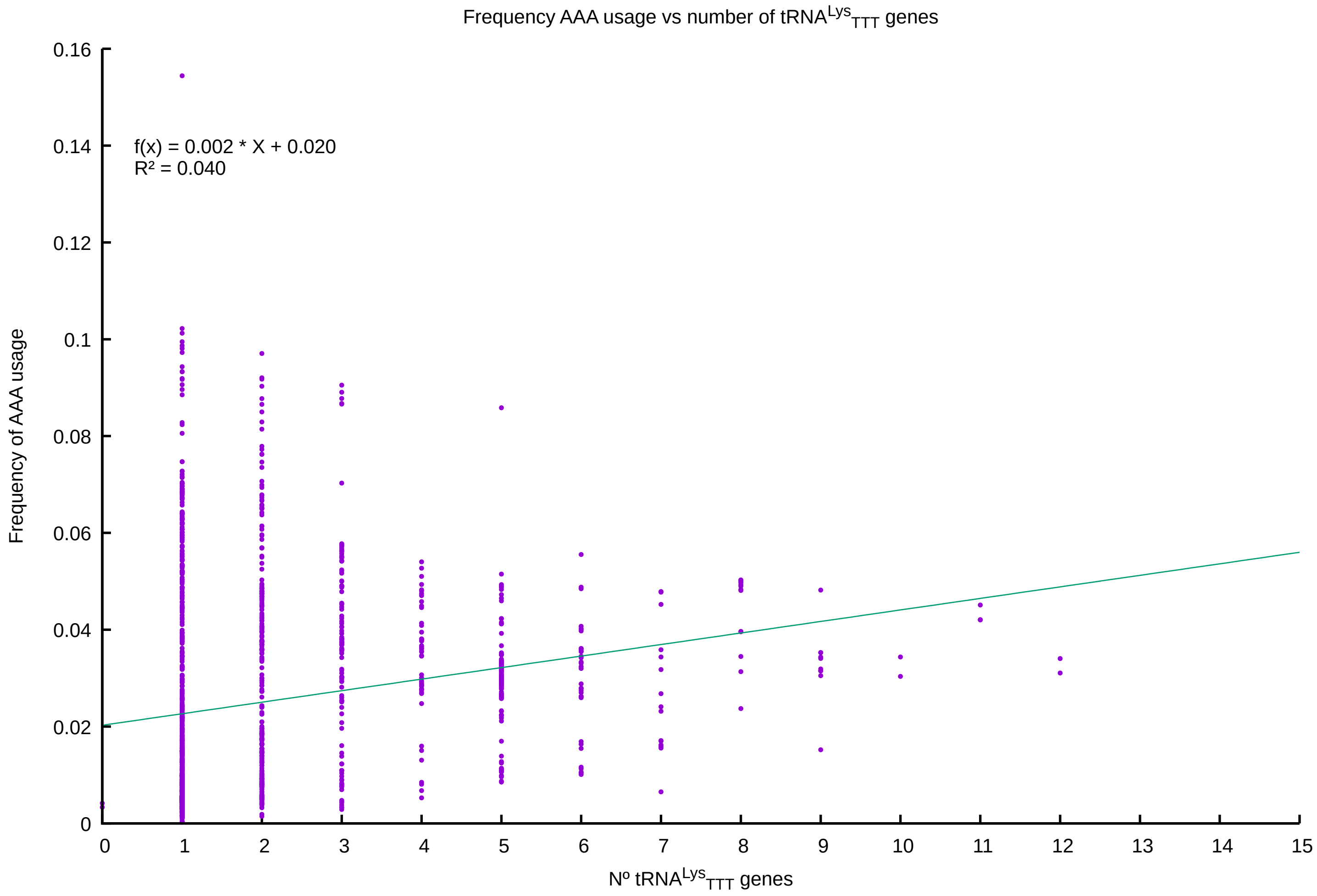

Frequency AAA usage vs number of tRNALysTTT genes
f(x) = 0.002 * X + 0.020
R² = 0.040
Frequency of AAA usage
0.16
0.14
0.12
0.1
0.08
0.06
0.04
0.02
0
0 1 2 3 4 5 6 7 8 9 10 11 12 13 14 15
Nº tRNALysTTT genes

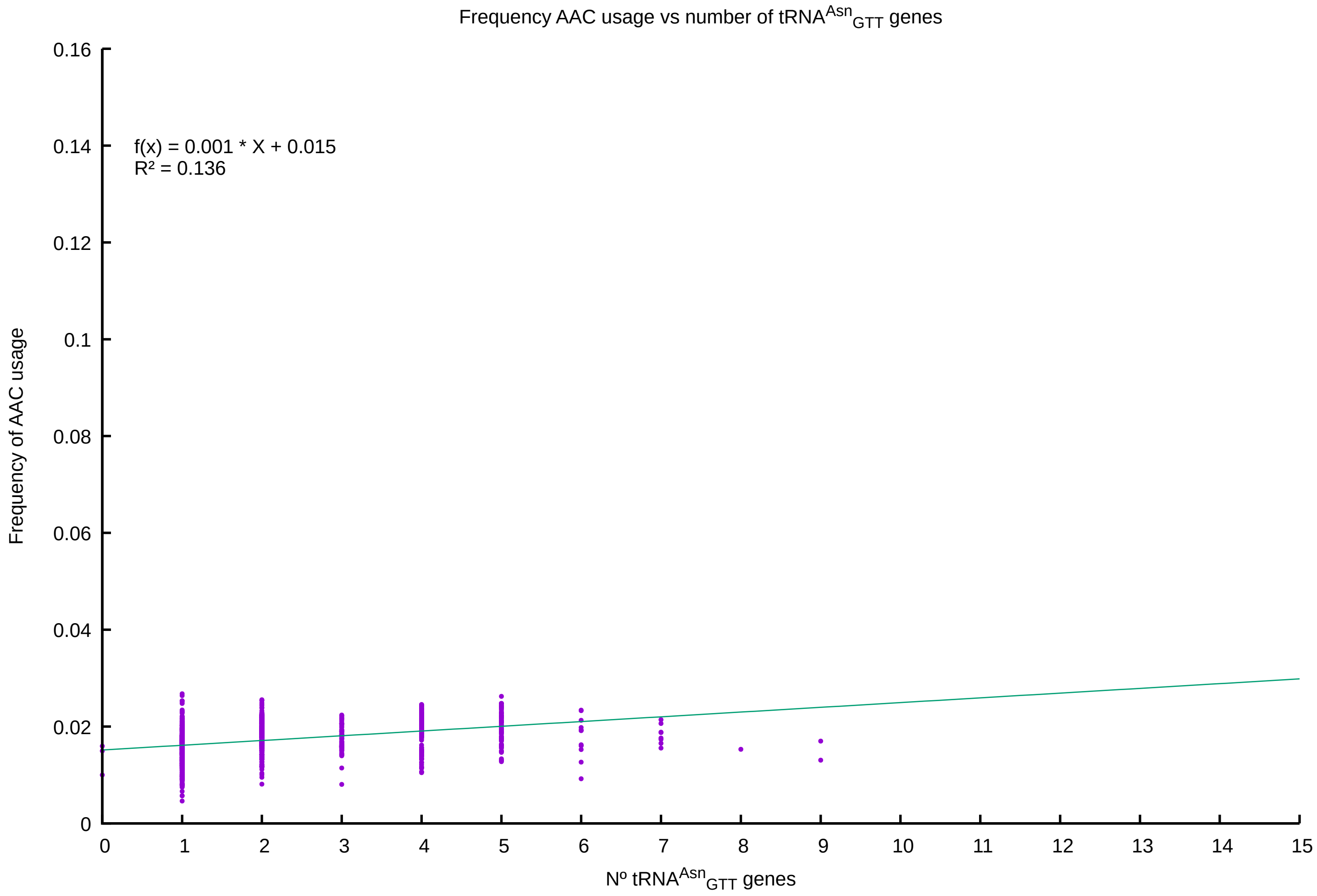

Frequency AAC usage vs number of tRNA^Asn_GTT genes
f(x) = 0.001 * X + 0.015
R² = 0.136
Frequency of AAC usage
0
0.02
0.04
0.06
0.08
0.1
0.12
0.14
0.16
0
1
2
3
4
5
6
7
8
9
10
11
12
13
14
15
Nº tRNA^Asn_GTT genes

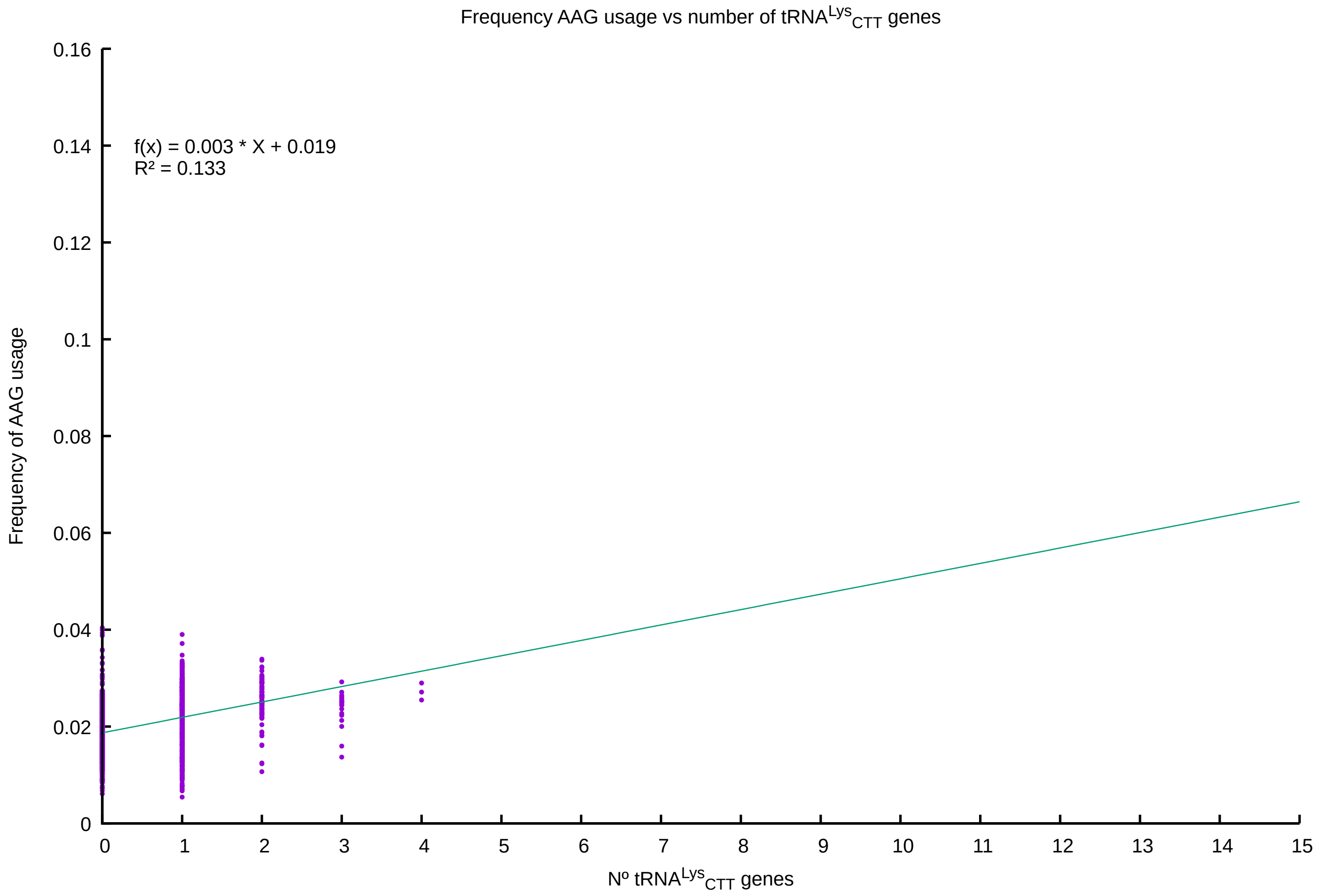

Frequency AAG usage vs number of tRNA^Lys_CTT genes
f(x) = 0.003 * X + 0.019
R² = 0.133
Frequency of AAG usage
0.16
0.14
0.12
0.1
0.08
0.06
0.04
0.02
0
0 1 2 3 4 5 6 7 8 9 10 11 12 13 14 15
Nº tRNA^Lys_CTT genes

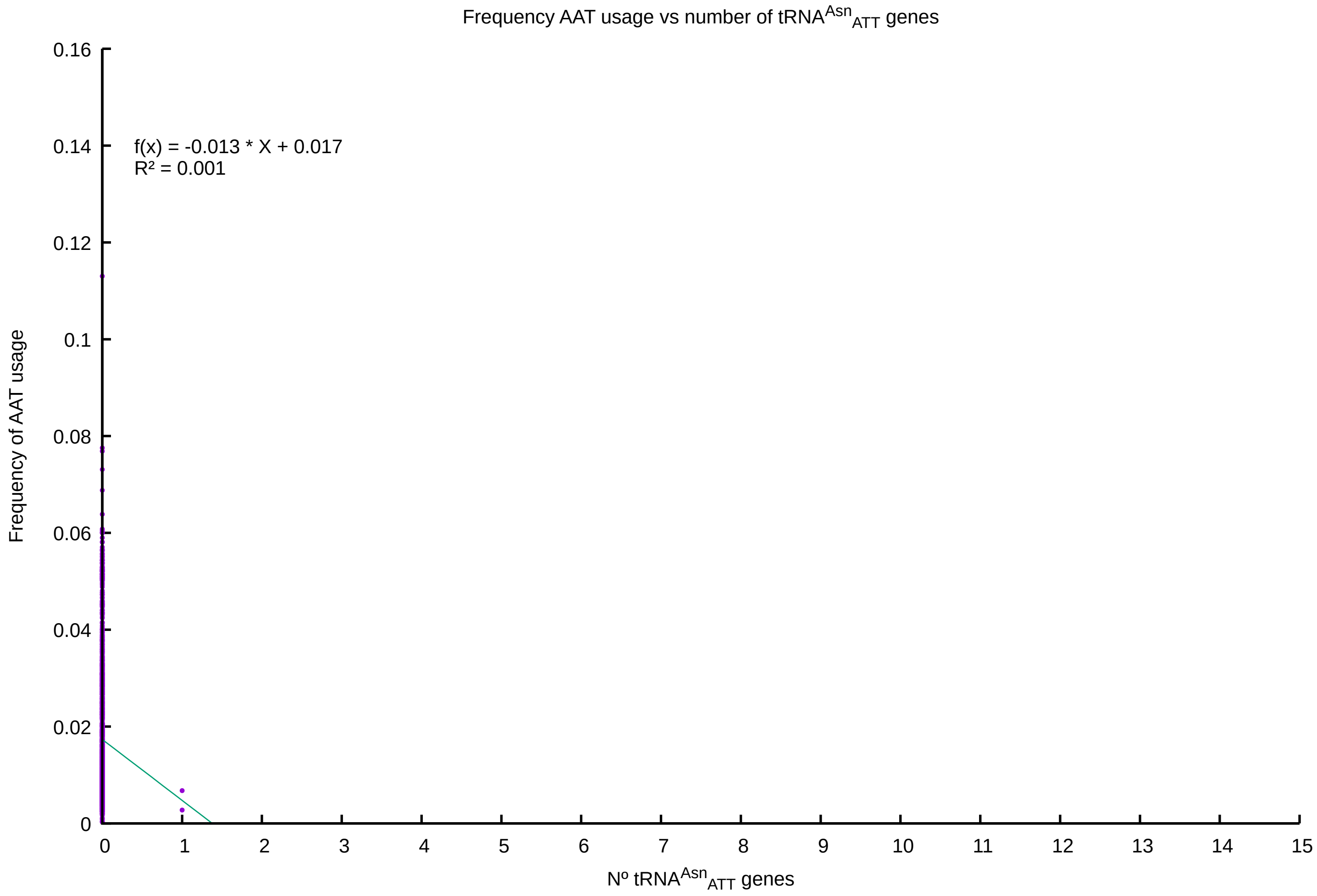

Frequency AAT usage vs number of tRNA^Asn_ATT genes
f(x) = -0.013 * X + 0.017
R² = 0.001
Frequency of AAT usage
0.16
0.14
0.12
0.1
0.08
0.06
0.04
0.02
0
0 1 2 3 4 5 6 7 8 9 10 11 12 13 14 15
Nº tRNA^Asn_ATT genes

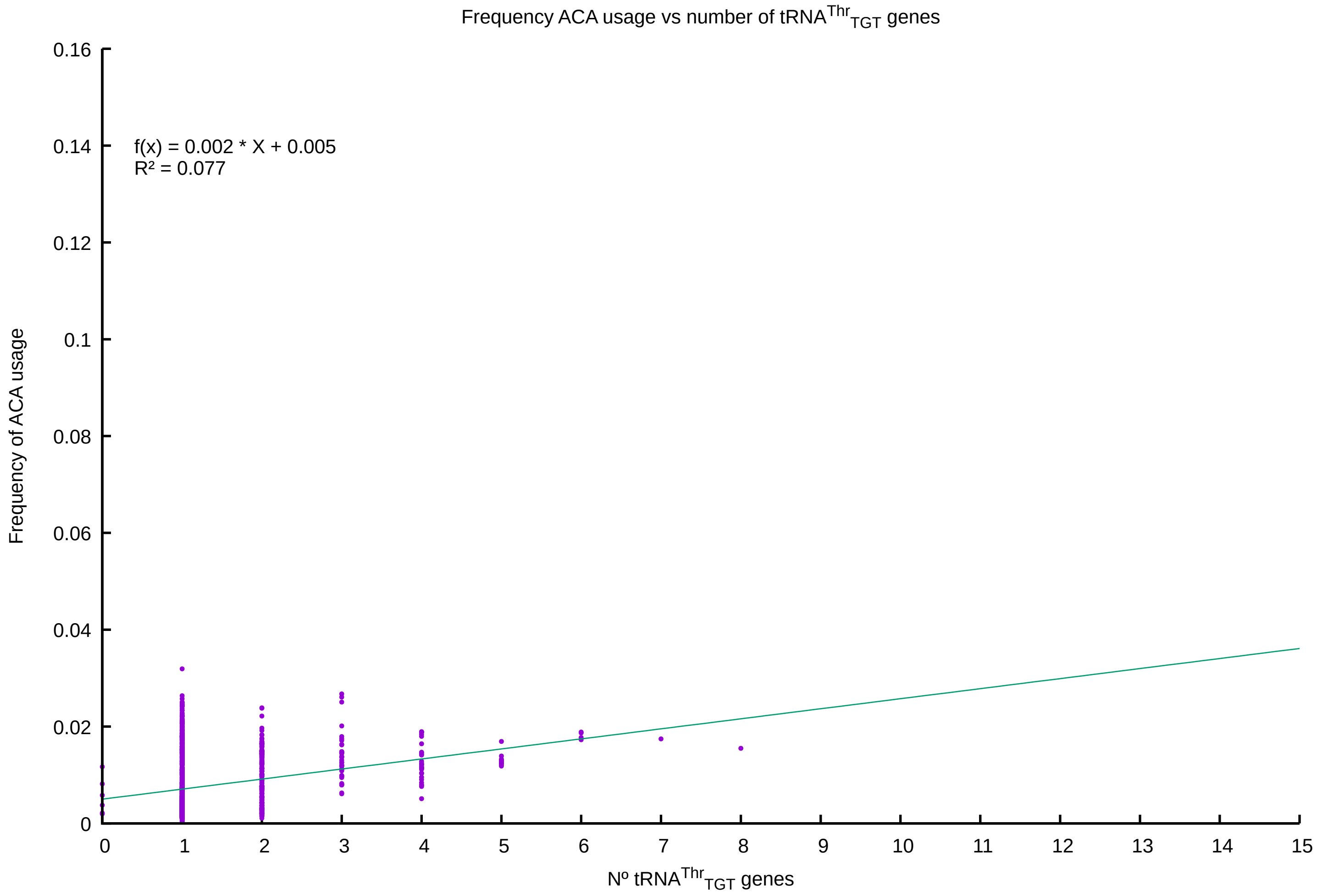

Frequency ACA usage vs number of tRNA$^{Thr}_{TGT}$ genes
f(x) = 0.002 * X + 0.005
R^2 = 0.077
Frequency of ACA usage
0.16
0.14
0.12
0.1
0.08
0.06
0.04
0.02
0
0 1 2 3 4 5 6 7 8 9 10 11 12 13 14 15
Nº tRNA$^{Thr}_{TGT}$ genes

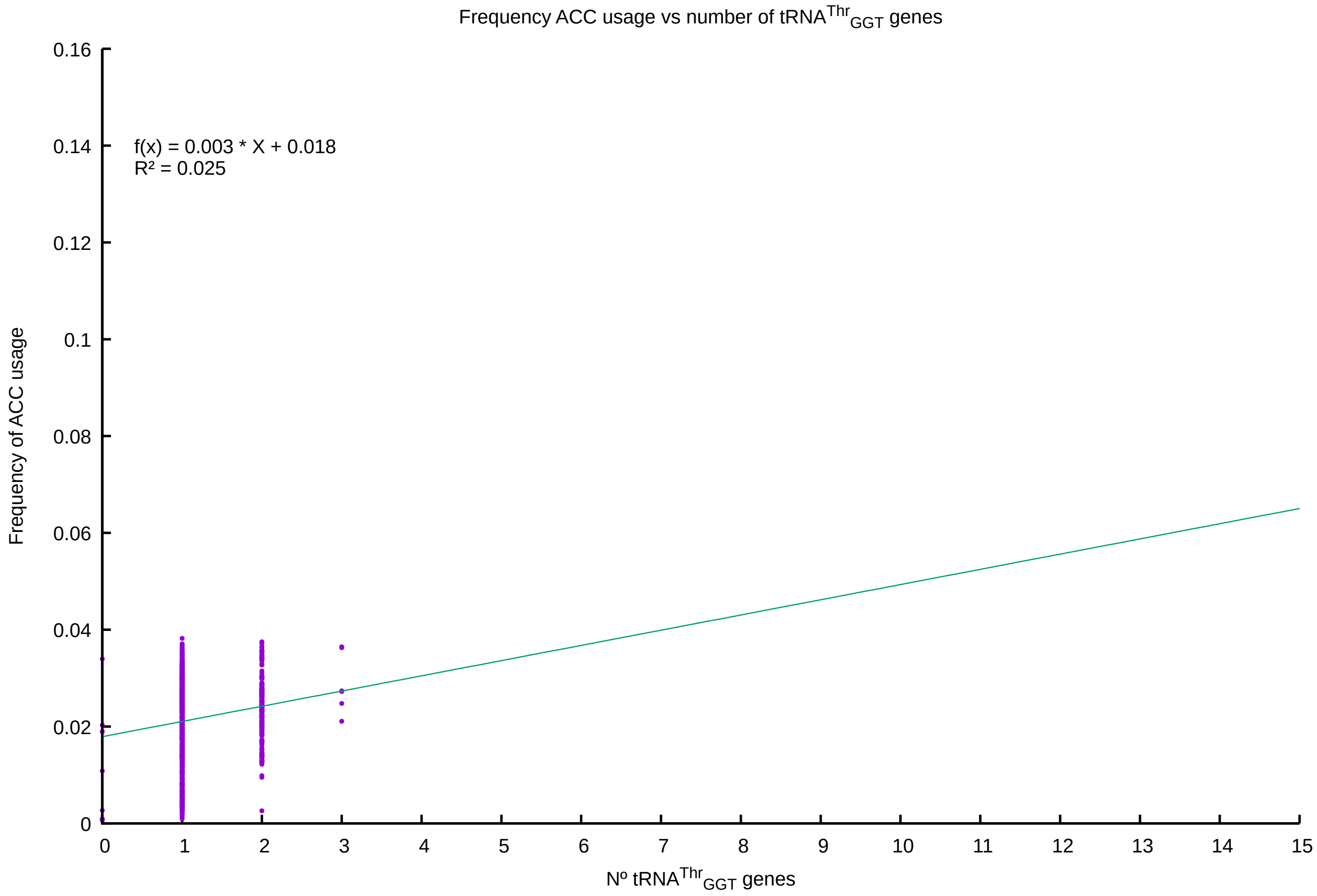

Frequency ACC usage vs number of tRNAThrGGT genes
f(x) = 0.003 * X + 0.018
R² = 0.025
Frequency of ACC usage
0.16
0.14
0.12
0.1
0.08
0.06
0.04
0.02
0
0 1 2 3 4 5 6 7 8 9 10 11 12 13 14 15
Nº tRNAThrGGT genes

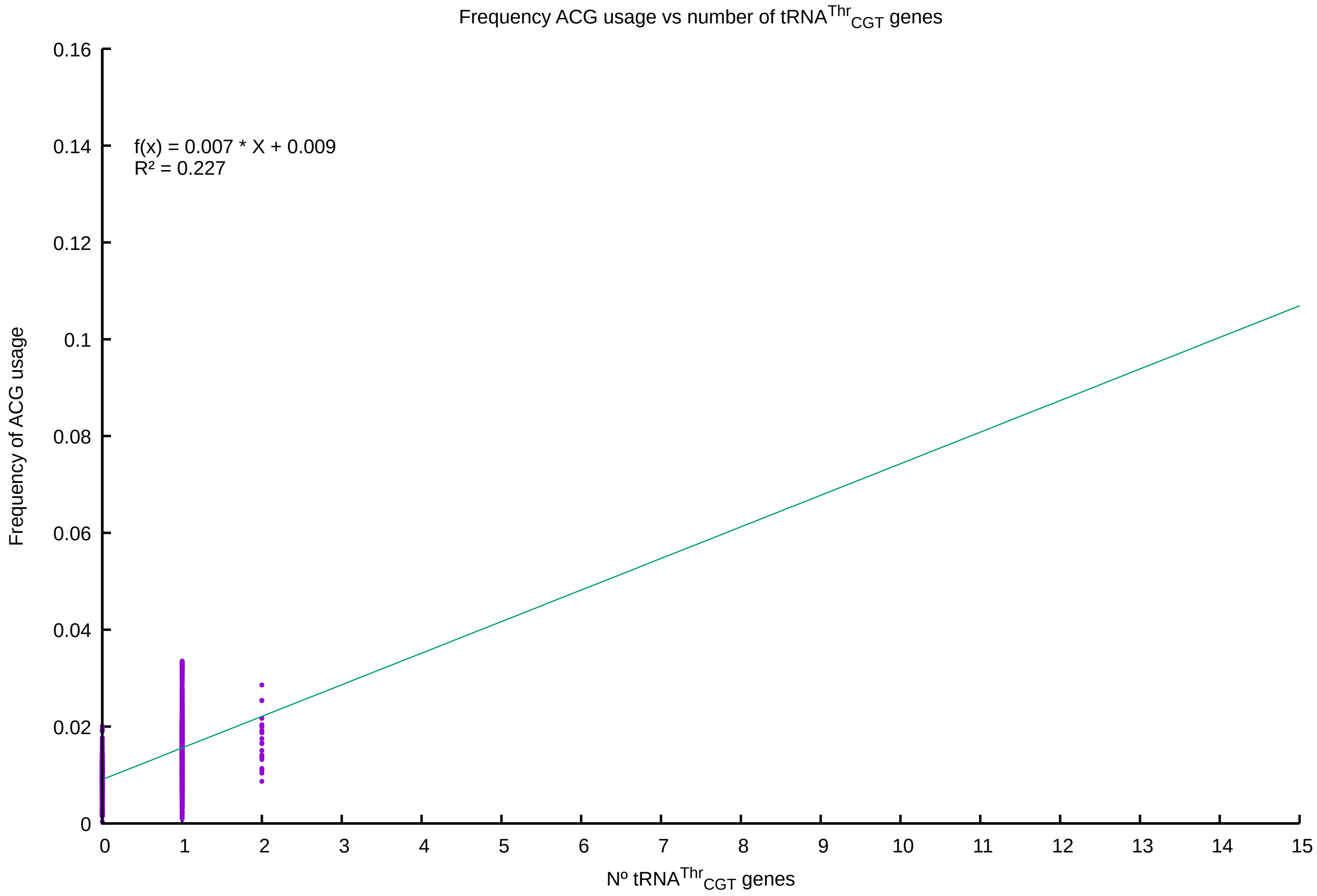

Frequency ACG usage vs number of tRNA^Thr_CGT genes
f(x) = 0.007 * X + 0.009
R² = 0.227
Frequency of ACG usage
0.16
0.14
0.12
0.1
0.08
0.06
0.04
0.02
0
0 1 2 3 4 5 6 7 8 9 10 11 12 13 14 15
Nº tRNA^Thr_CGT genes

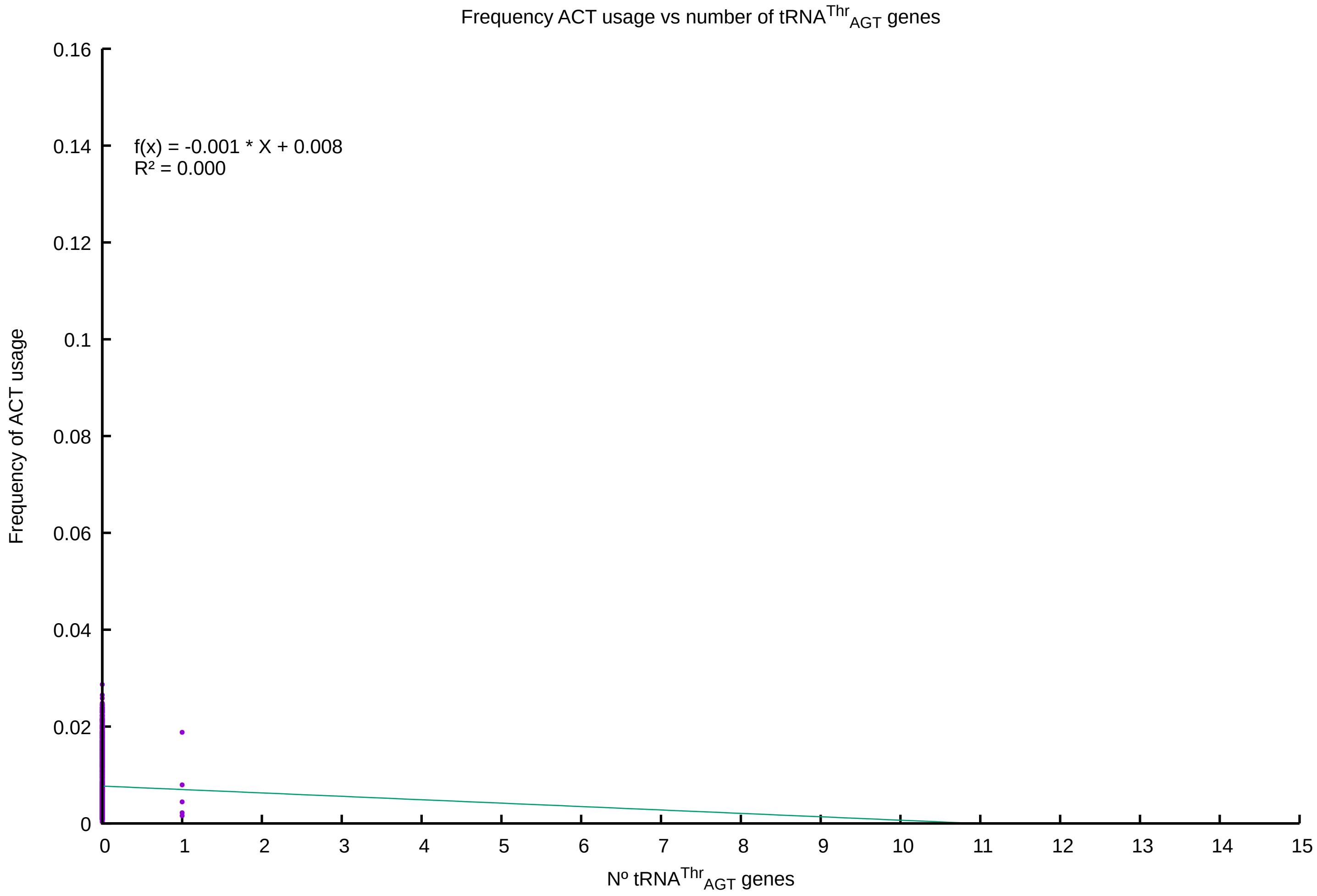
Frequency ACT usage vs number of tRNAThrAGT genes
f(x) = -0.001 * X + 0.008
R² = 0.000
Frequency of ACT usage
Nº tRNAThrAGT genes
0
0.02
0.04
0.06
0.08
0.1
0.12
0.14
0.16
0
1
2
3
4
5
6
7
8
9
10
11
12
13
14
15

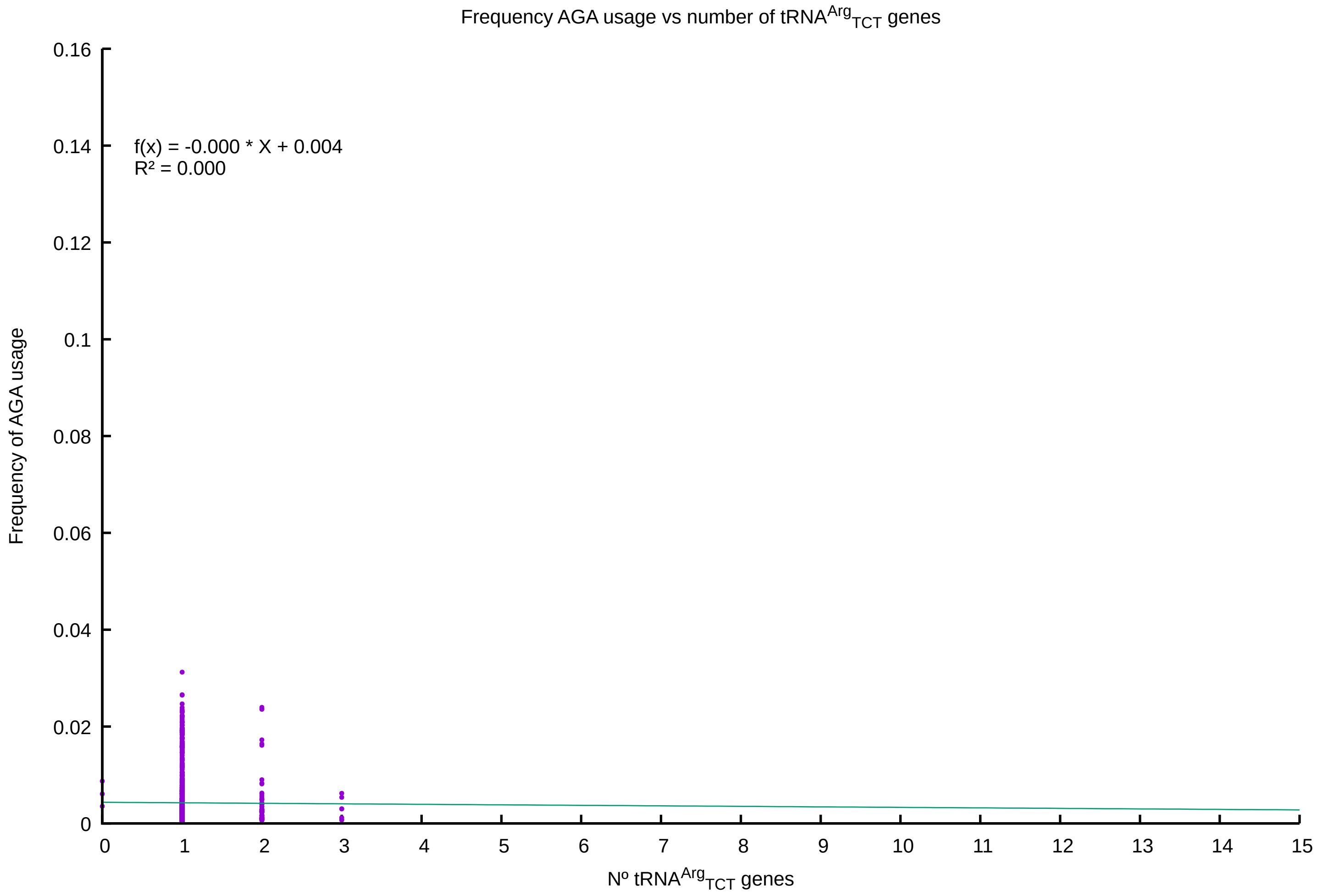

Frequency AGA usage vs number of tRNA^Arg_TCT genes
f(x) = -0.000 * X + 0.004
R² = 0.000
Frequency of AGA usage
0.16
0.14
0.12
0.1
0.08
0.06
0.04
0.02
0
0 1 2 3 4 5 6 7 8 9 10 11 12 13 14 15
Nº tRNA^Arg_TCT genes

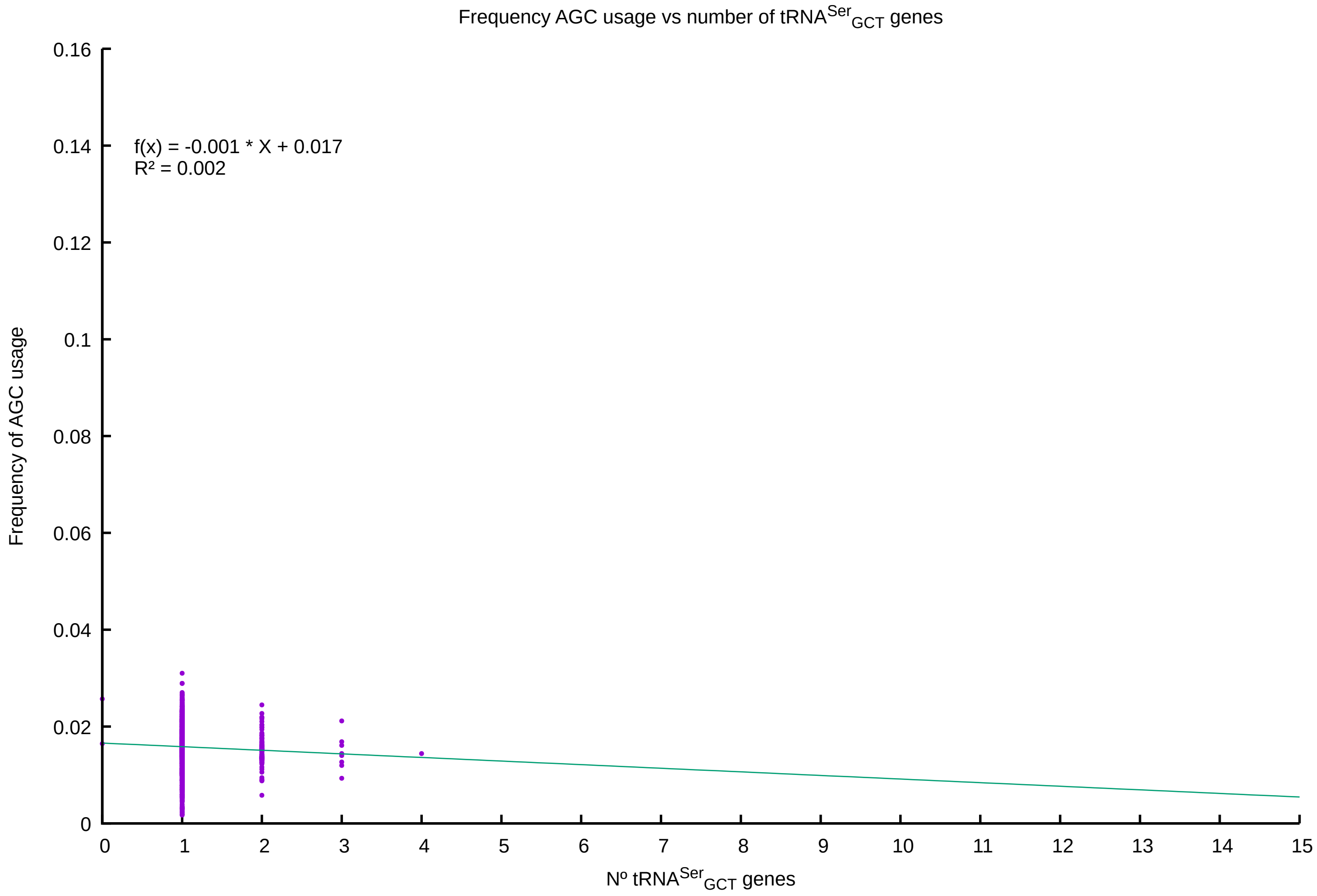
Frequency AGC usage vs number of tRNA^Ser_GCT genes
f(x) = -0.001 * X + 0.017
R² = 0.002
Frequency of AGC usage
0.16
0.14
0.12
0.1
0.08
0.06
0.04
0.02
0
0 1 2 3 4 5 6 7 8 9 10 11 12 13 14 15
Nº tRNA^Ser_GCT genes

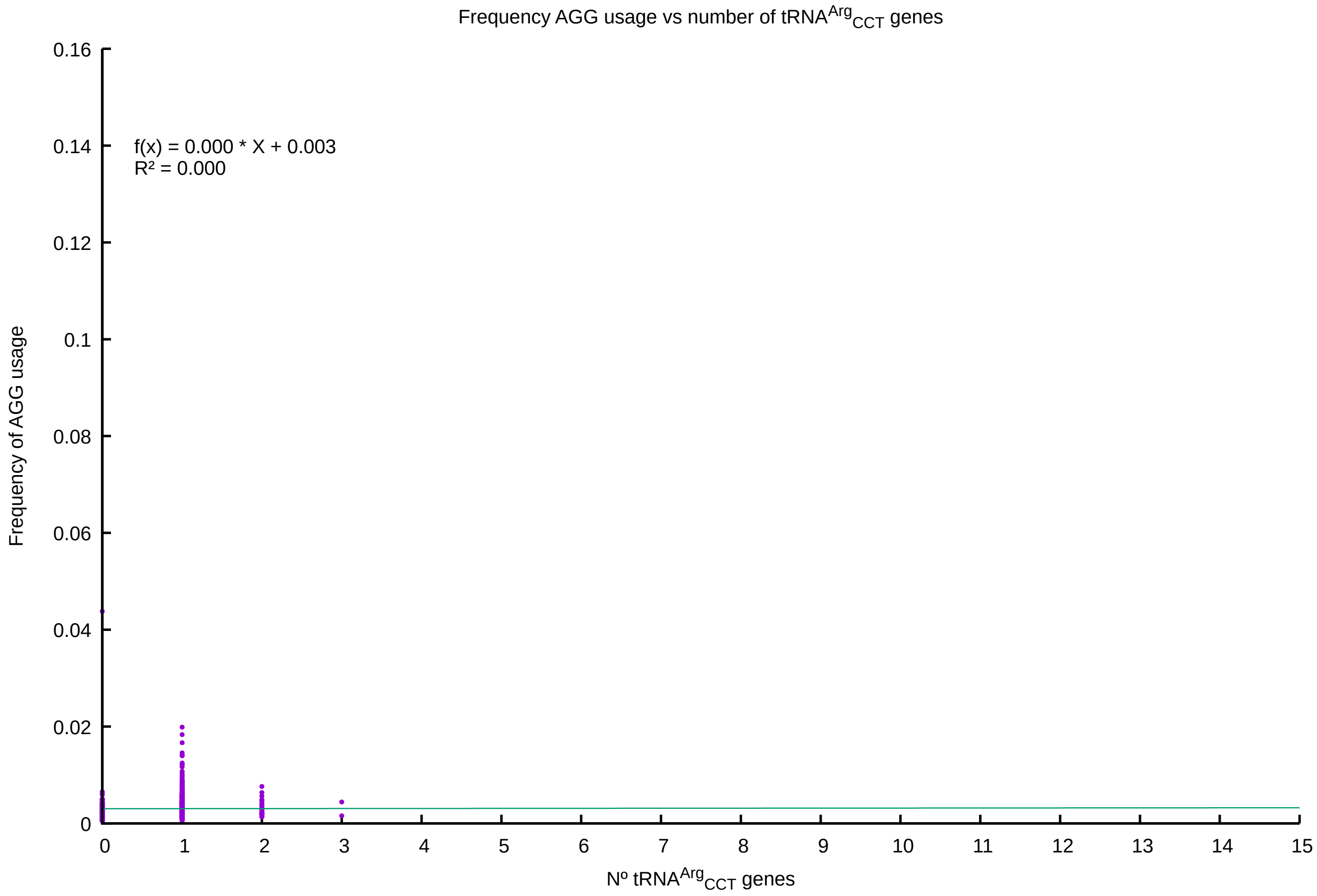

Frequency AGG usage vs number of tRNA^Arg_CCT genes
f(x) = 0.000 * X + 0.003
R² = 0.000
Frequency of AGG usage
0.16
0.14
0.12
0.1
0.08
0.06
0.04
0.02
0
0 1 2 3 4 5 6 7 8 9 10 11 12 13 14 15
Nº tRNA^Arg_CCT genes

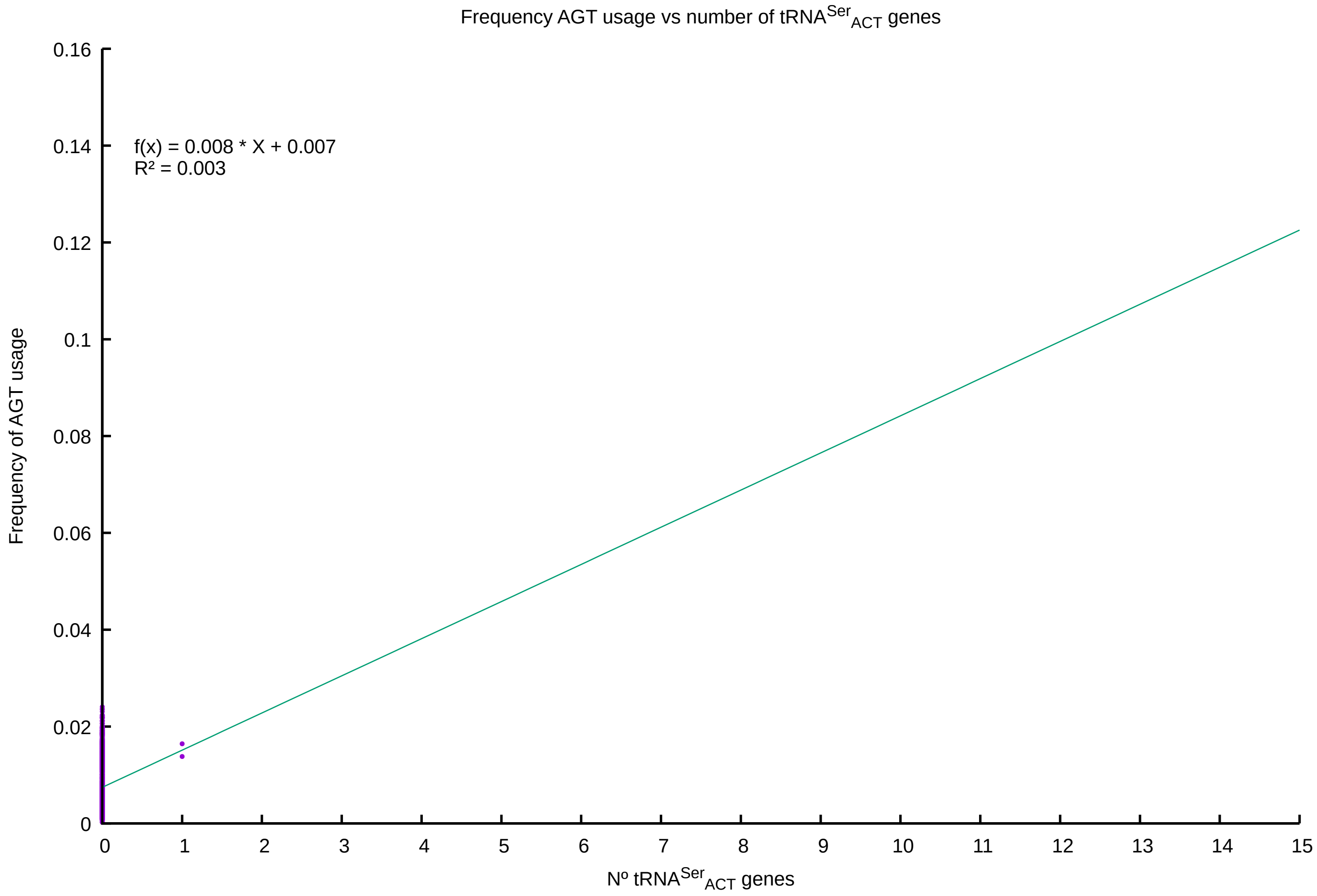
Frequency AGT usage vs number of tRNA^Ser_ACT genes
f(x) = 0.008 * X + 0.007
R² = 0.003
Frequency of AGT usage
0.16
0.14
0.12
0.1
0.08
0.06
0.04
0.02
0
0 1 2 3 4 5 6 7 8 9 10 11 12 13 14 15
Nº tRNA^Ser_ACT genes

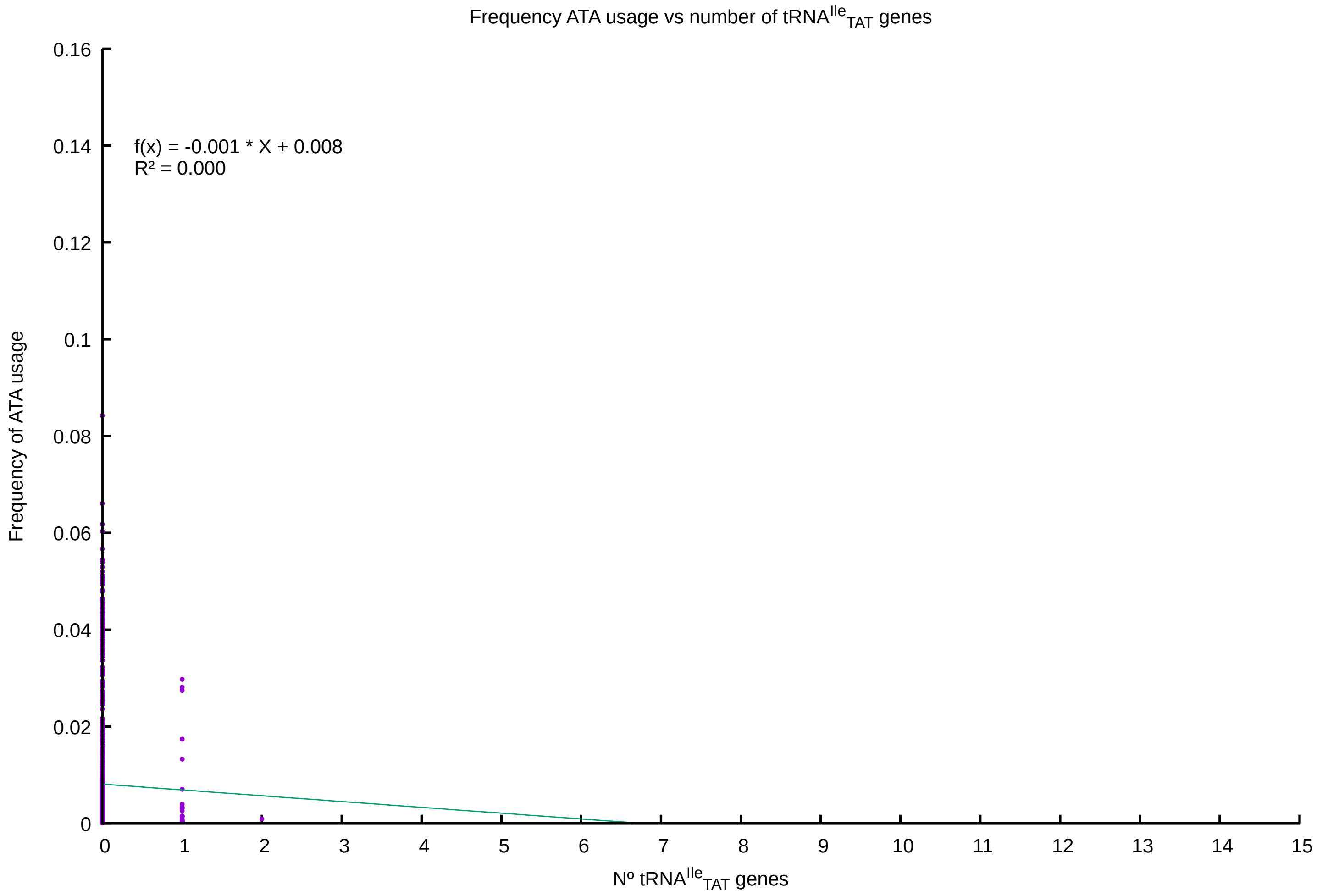
Frequency ATA usage vs number of tRNA^Ile_TAT genes
f(x) = -0.001 * X + 0.008
R² = 0.000
Frequency of ATA usage
0.16
0.14
0.12
0.1
0.08
0.06
0.04
0.02
0
0 1 2 3 4 5 6 7 8 9 10 11 12 13 14 15
Nº tRNA^Ile_TAT genes

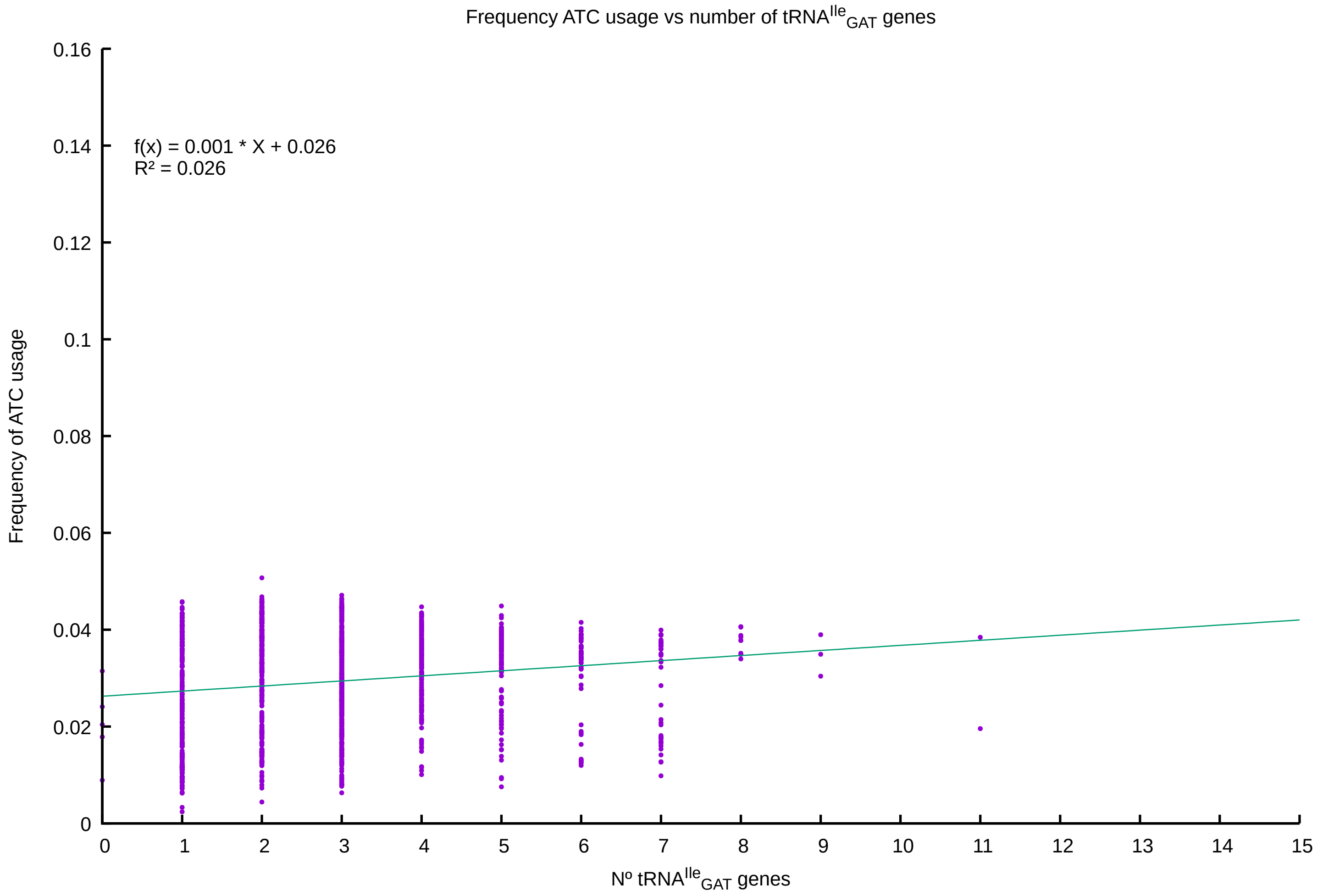

Frequency ATC usage vs number of tRNA^Ile_GAT genes
f(x) = 0.001 * X + 0.026
R² = 0.026
Frequency of ATC usage
0.16
0.14
0.12
0.1
0.08
0.06
0.04
0.02
0
0 1 2 3 4 5 6 7 8 9 10 11 12 13 14 15
Nº tRNA^Ile_GAT genes

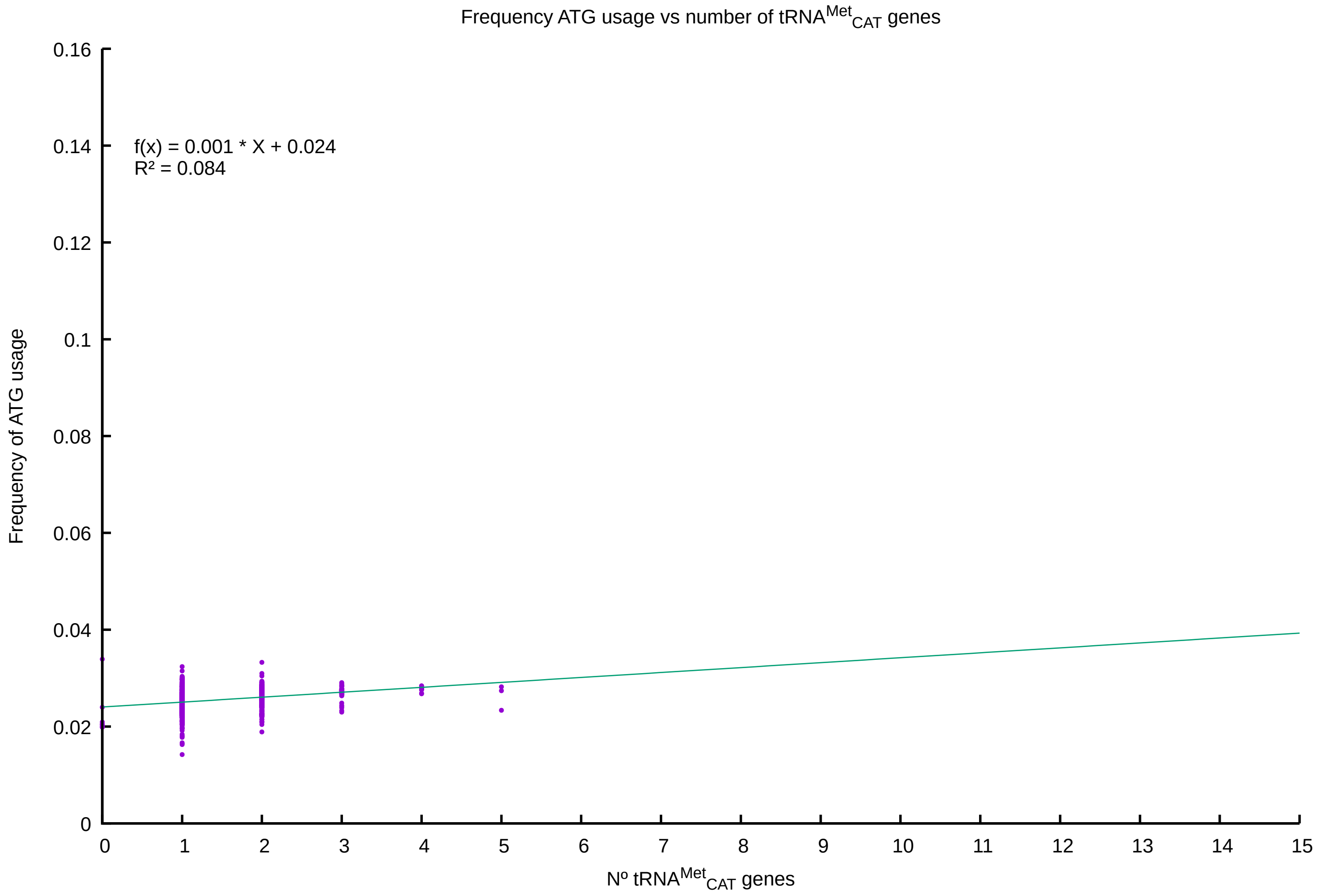
Frequency ATG usage vs number of tRNAMetCAT genes
f(x) = 0.001 * X + 0.024
R² = 0.084
Frequency of ATG usage
0.16
0.14
0.12
0.1
0.08
0.06
0.04
0.02
0
0 1 2 3 4 5 6 7 8 9 10 11 12 13 14 15
Nº tRNAMetCAT genes

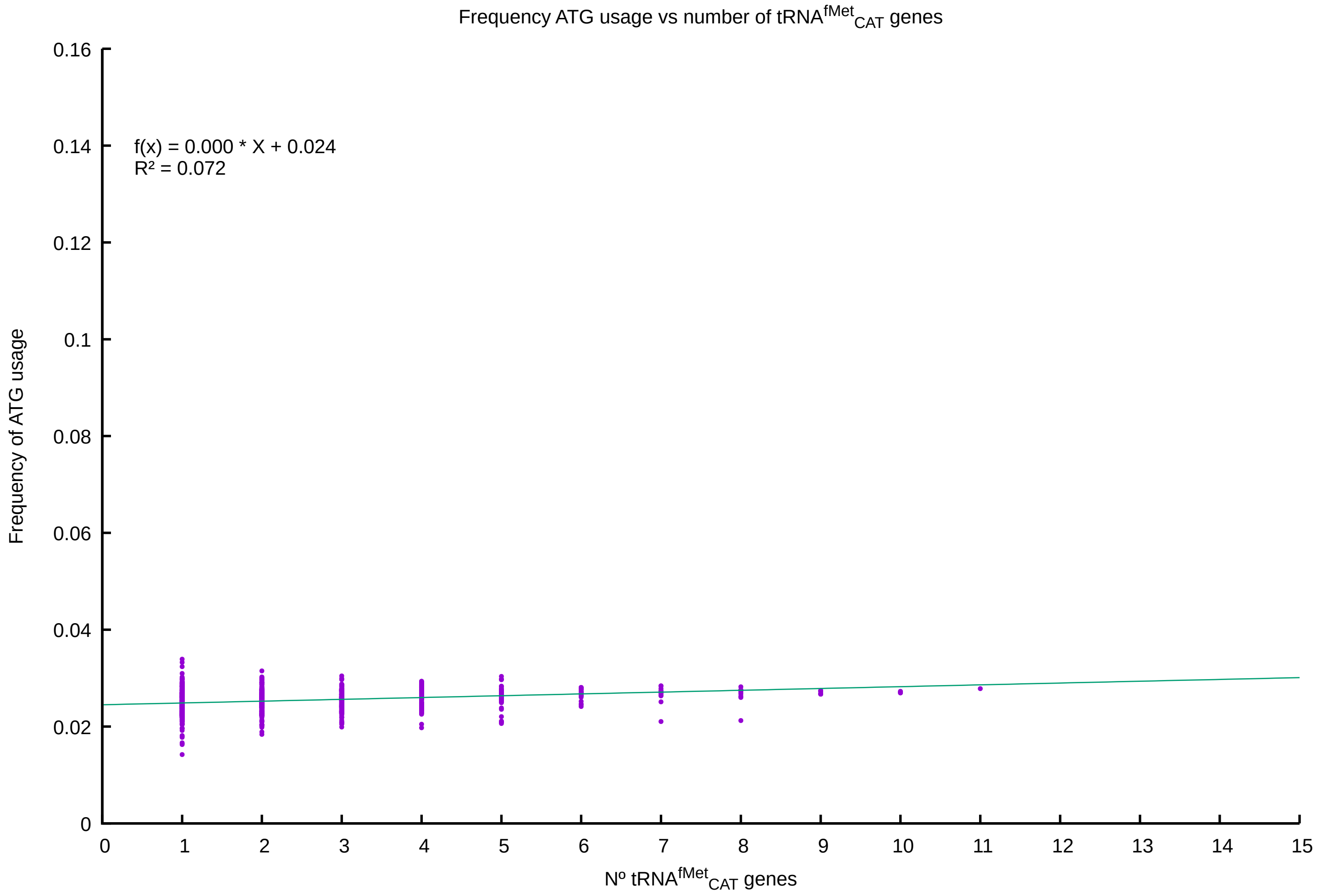
Frequency ATG usage vs number of tRNA fMet CAT genes
f(x) = 0.000 * X + 0.024
R² = 0.072
Frequency of ATG usage
0.16
0.14
0.12
0.1
0.08
0.06
0.04
0.02
0
0 1 2 3 4 5 6 7 8 9 10 11 12 13 14 15
Nº tRNA fMet CAT genes

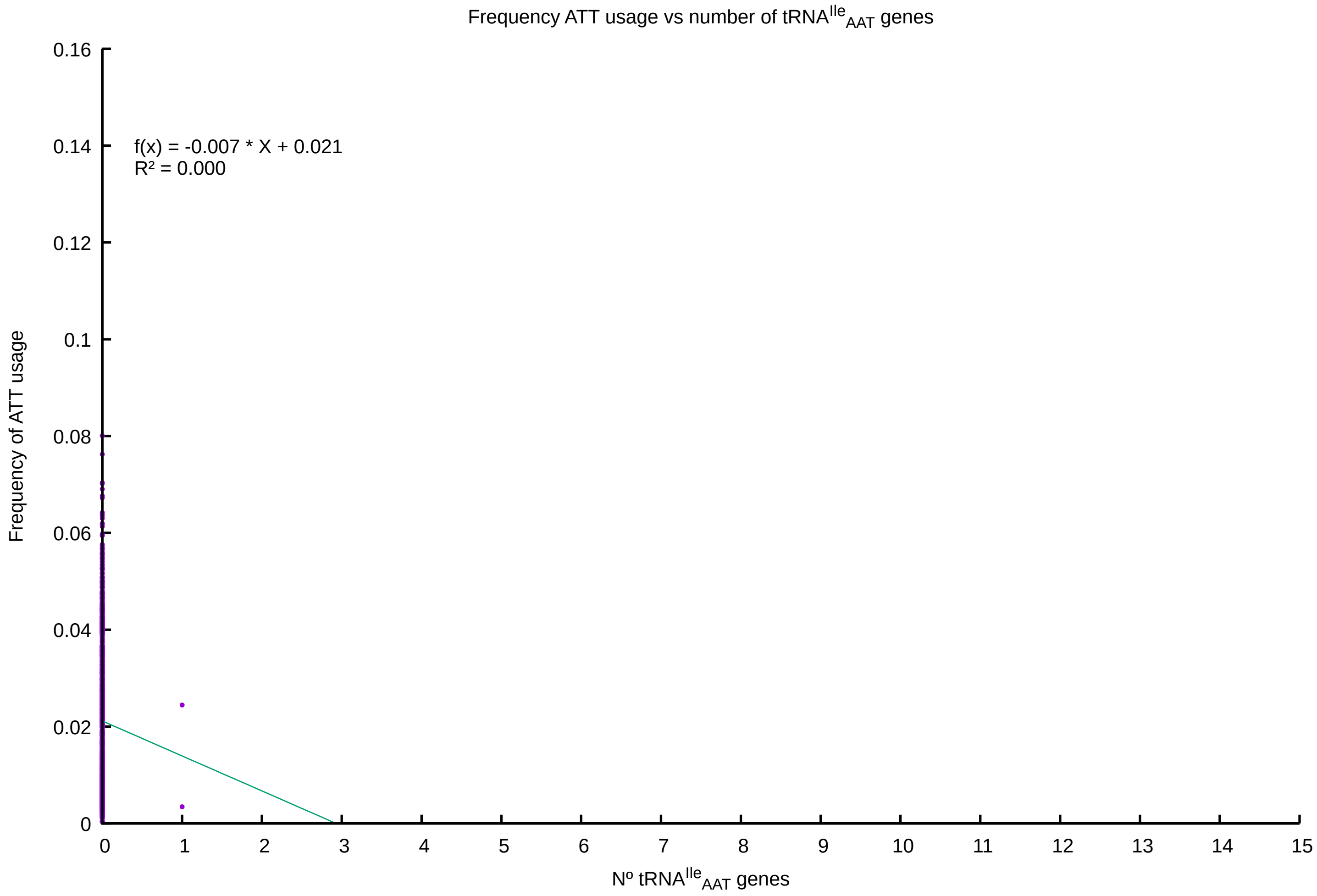
Frequency ATT usage vs number of tRNA^Ile_AAT genes
f(x) = -0.007 * X + 0.021
R² = 0.000
Frequency of ATT usage
0.16
0.14
0.12
0.1
0.08
0.06
0.04
0.02
0
0 1 2 3 4 5 6 7 8 9 10 11 12 13 14 15
Nº tRNA^Ile_AAT genes

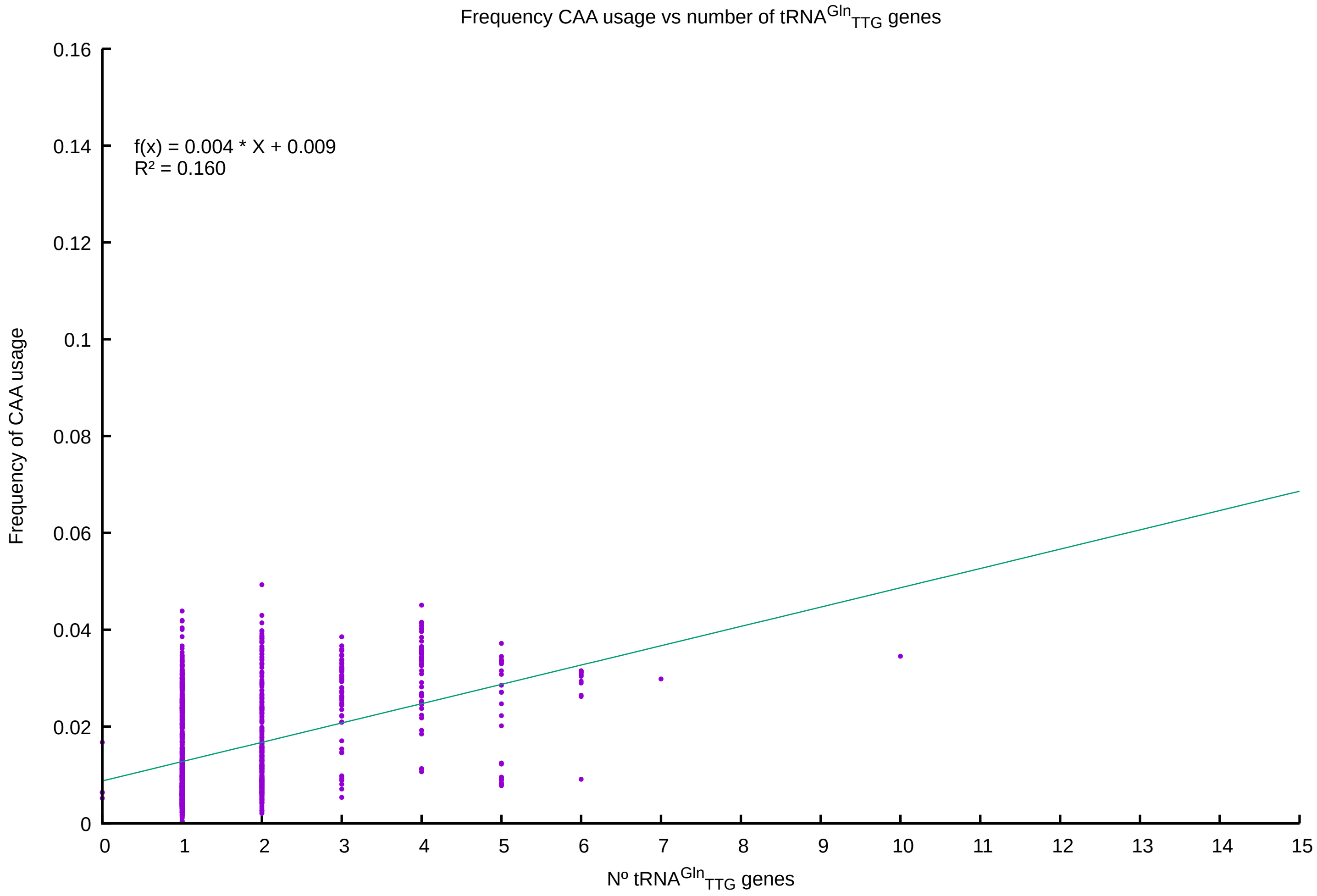
Frequency CAA usage vs number of tRNA^Gln_TTG genes
f(x) = 0.004 * X + 0.009
R² = 0.160
Frequency of CAA usage
0
0.02
0.04
0.06
0.08
0.1
0.12
0.14
0.16
0
1
2
3
4
5
6
7
8
9
10
11
12
13
14
15
Nº tRNA^Gln_TTG genes

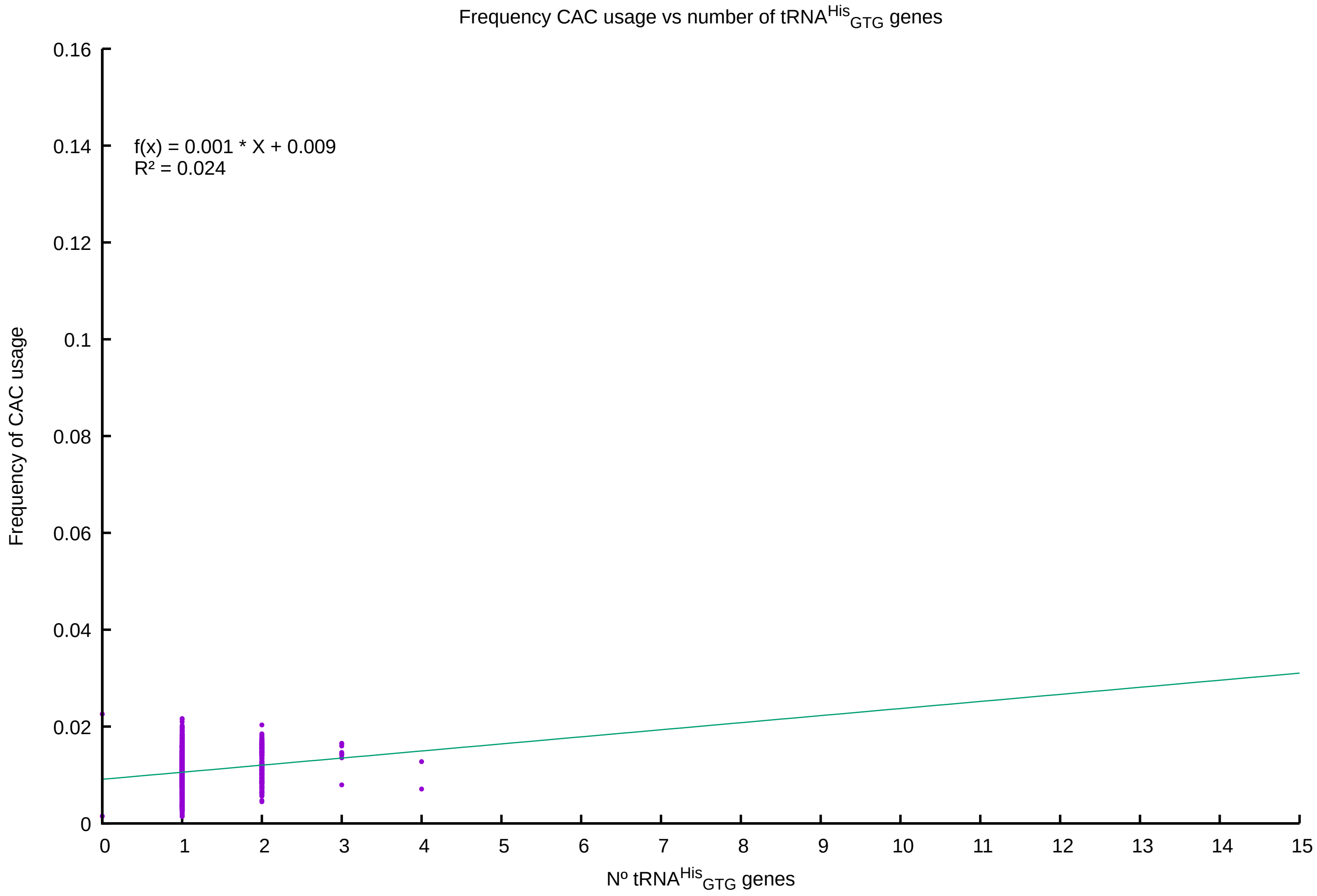

Frequency CAC usage vs number of tRNA$^{His}_{GTG}$ genes
f(x) = 0.001 * X + 0.009
R^2 = 0.024
Frequency of CAC usage
0.16
0.14
0.12
0.1
0.08
0.06
0.04
0.02
0
0 1 2 3 4 5 6 7 8 9 10 11 12 13 14 15
Nº tRNA$^{His}_{GTG}$ genes

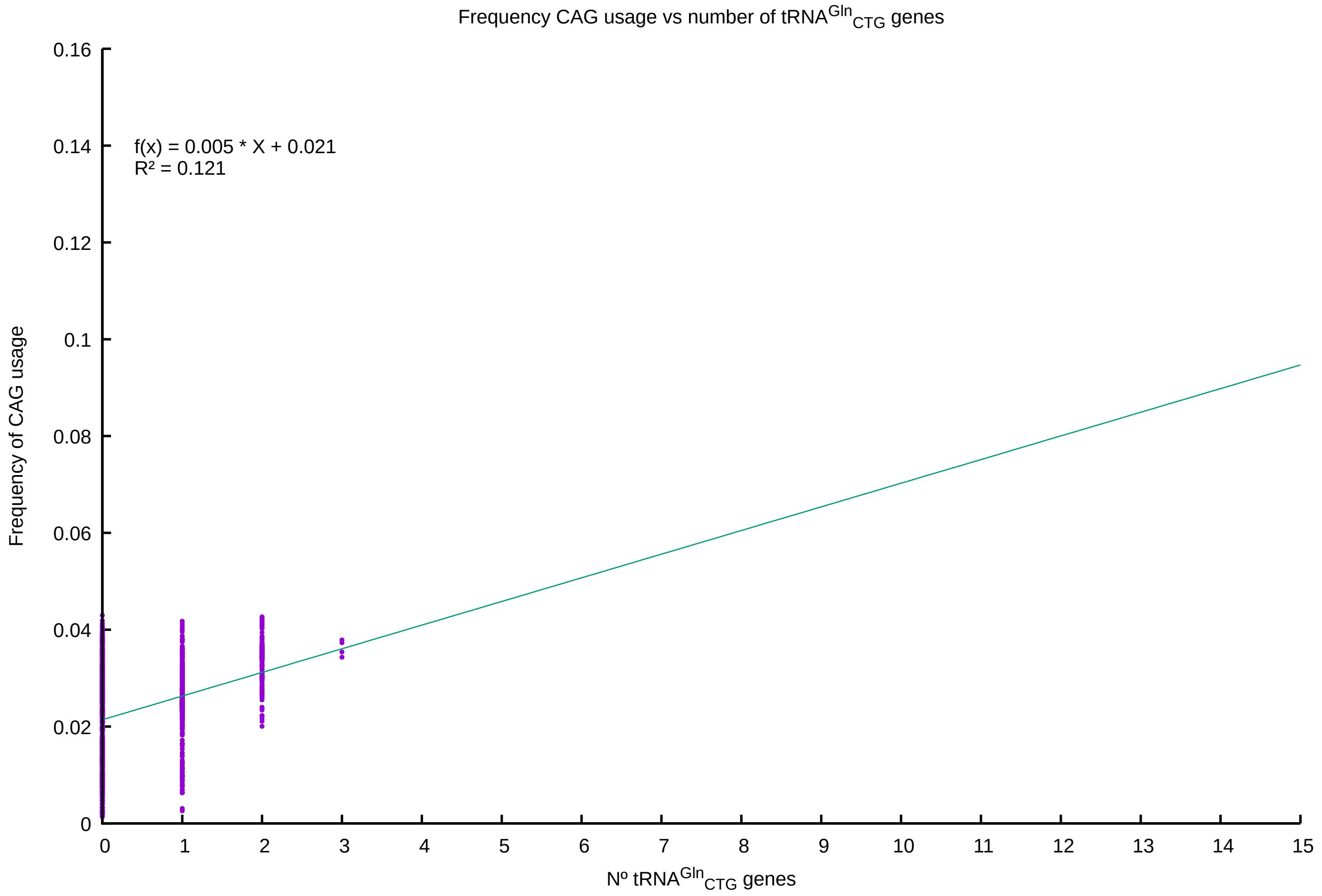
Frequency CAG usage vs number of tRNA^Gln_CTG genes
f(x) = 0.005 * X + 0.021
R² = 0.121
Frequency of CAG usage
0
0.02
0.04
0.06
0.08
0.1
0.12
0.14
0.16
0
1
2
3
4
5
6
7
8
9
10
11
12
13
14
15
Nº tRNA^Gln_CTG genes

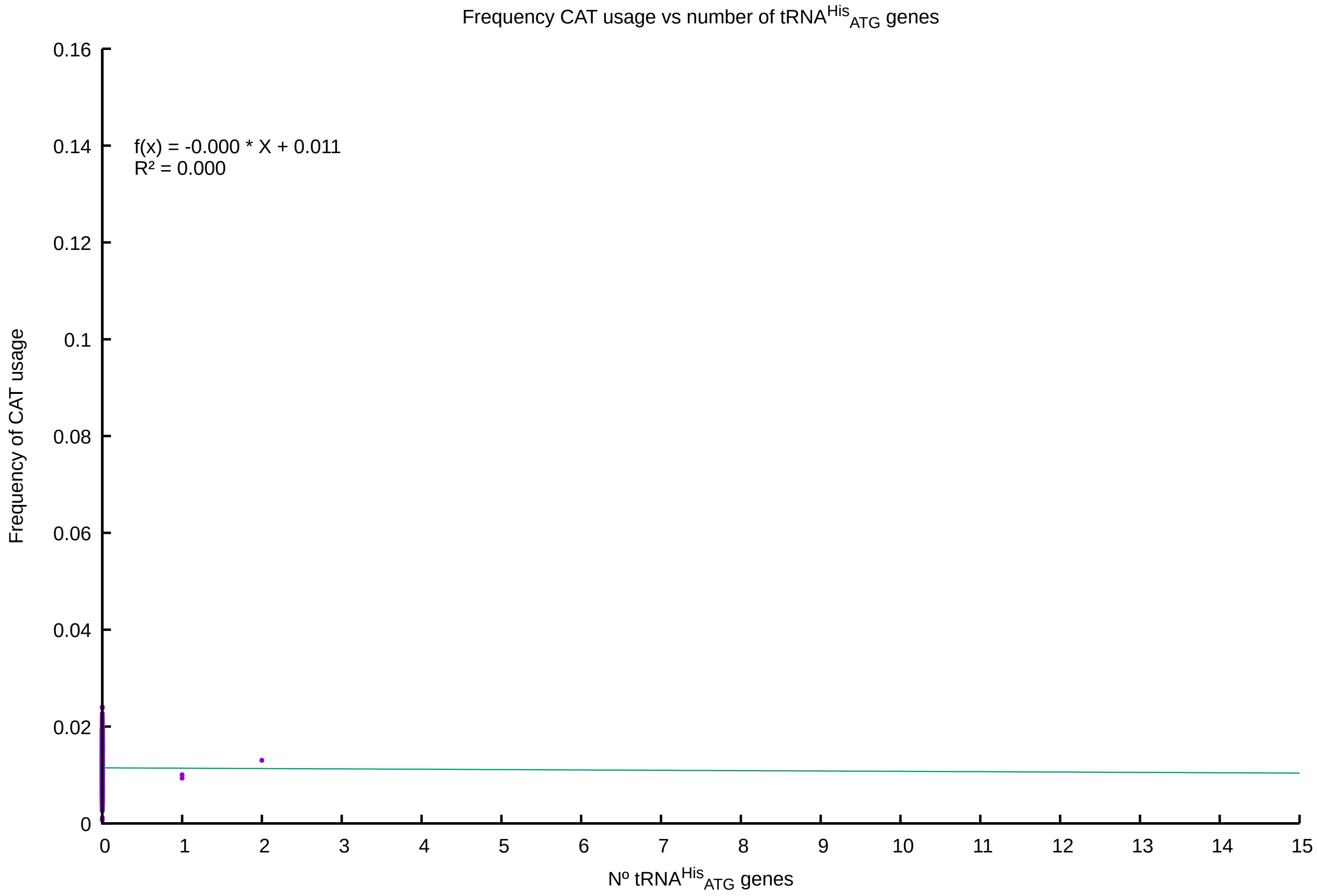

Frequency CAT usage vs number of tRNA$^{His}_{ATG}$ genes
f(x) = -0.000 * X + 0.011
R² = 0.000
Frequency of CAT usage
0.16
0.14
0.12
0.1
0.08
0.06
0.04
0.02
0
0 1 2 3 4 5 6 7 8 9 10 11 12 13 14 15
Nº tRNA$^{His}_{ATG}$ genes

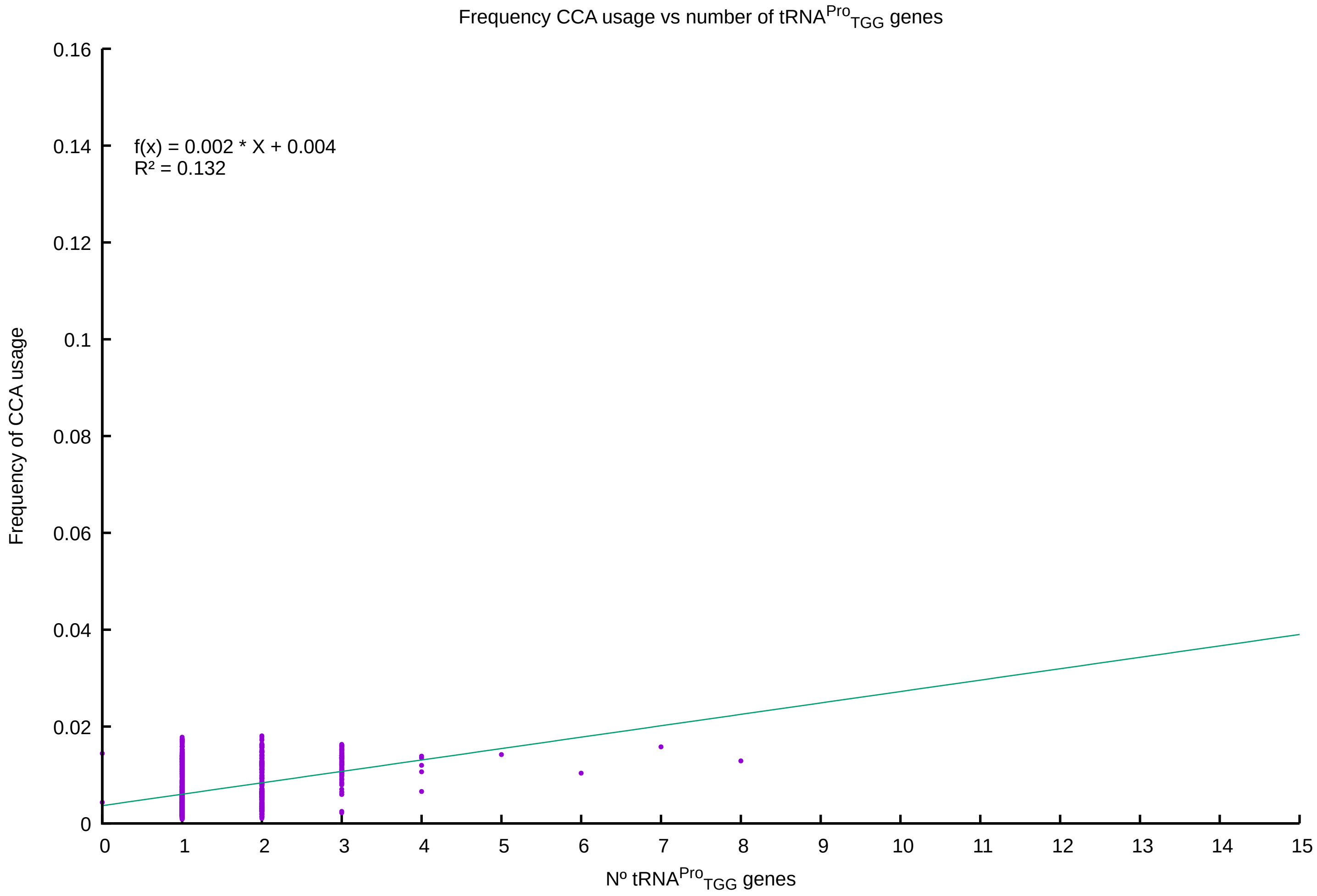
Frequency CCA usage vs number of tRNAPro TGG genes
f(x) = 0.002 * X + 0.004
R² = 0.132
Frequency of CCA usage
0.16
0.14
0.12
0.1
0.08
0.06
0.04
0.02
0
0 1 2 3 4 5 6 7 8 9 10 11 12 13 14 15
Nº tRNAPro TGG genes

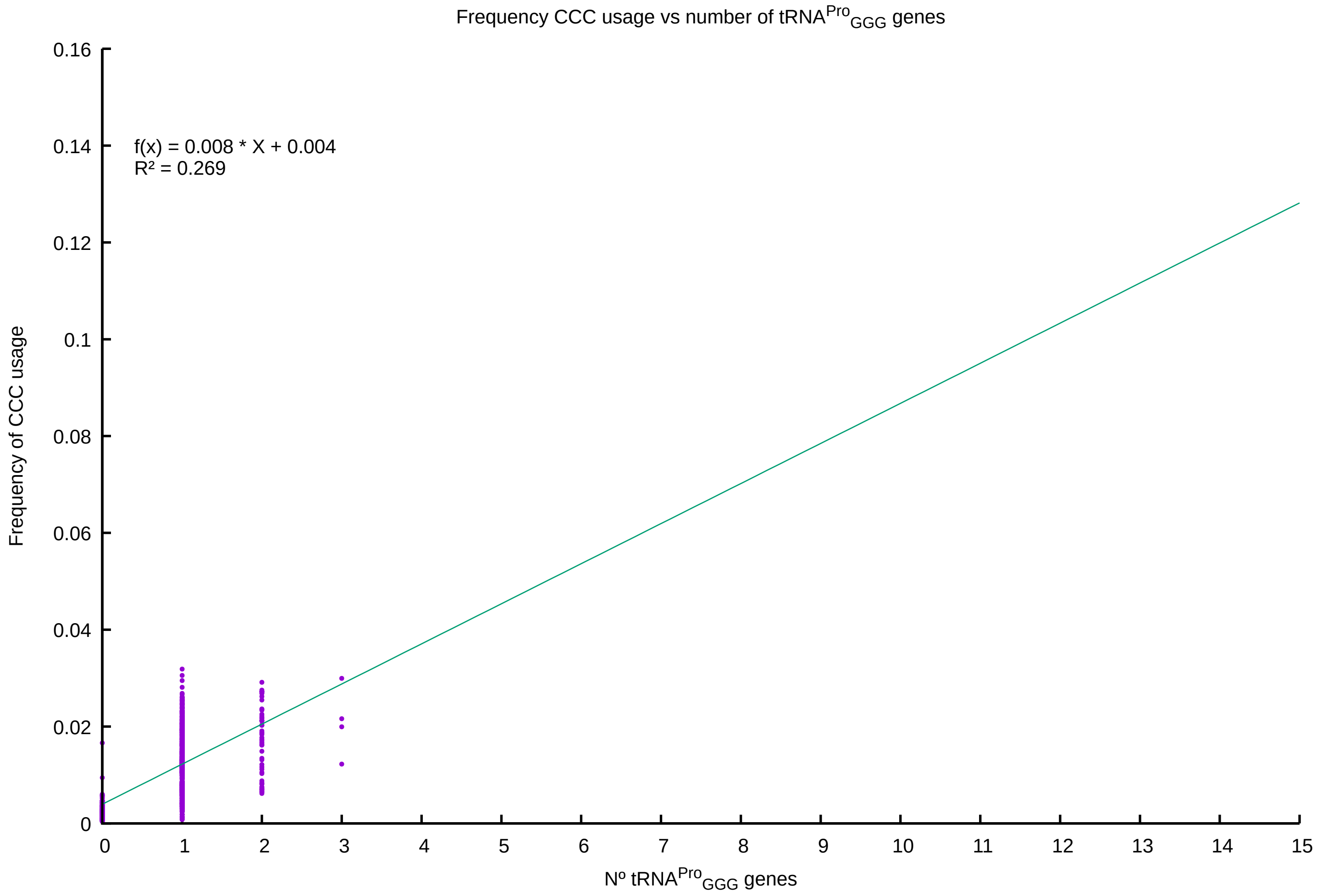

Frequency CCC usage vs number of tRNAProGGG genes
f(x) = 0.008 * X + 0.004
R² = 0.269
Frequency of CCC usage
0.16
0.14
0.12
0.1
0.08
0.06
0.04
0.02
0
0 1 2 3 4 5 6 7 8 9 10 11 12 13 14 15
Nº tRNAProGGG genes

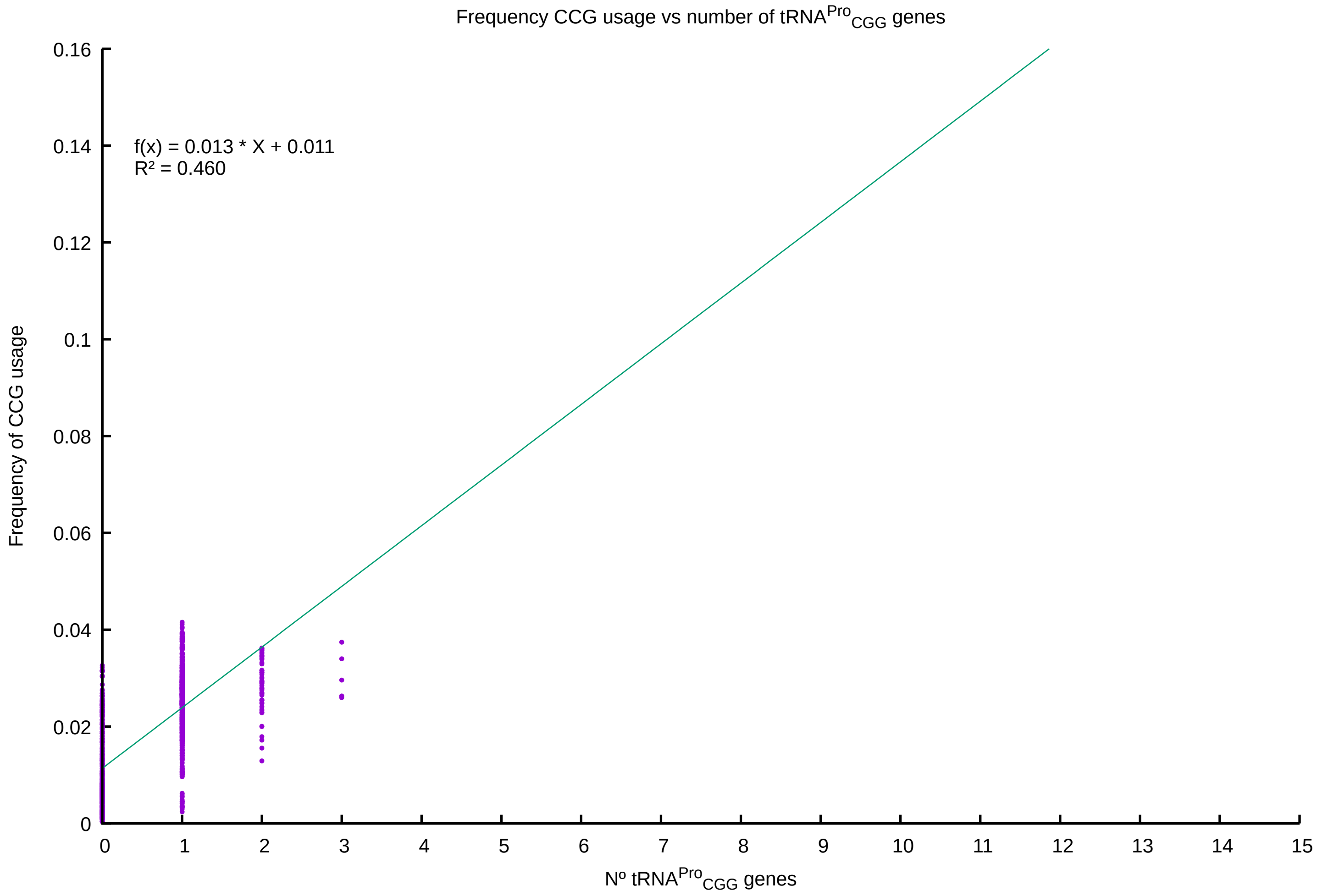

Frequency CCG usage vs number of tRNAProCGG genes
f(x) = 0.013 * X + 0.011
R² = 0.460
Frequency of CCG usage
0.16
0.14
0.12
0.1
0.08
0.06
0.04
0.02
0
0 1 2 3 4 5 6 7 8 9 10 11 12 13 14 15
Nº tRNAProCGG genes

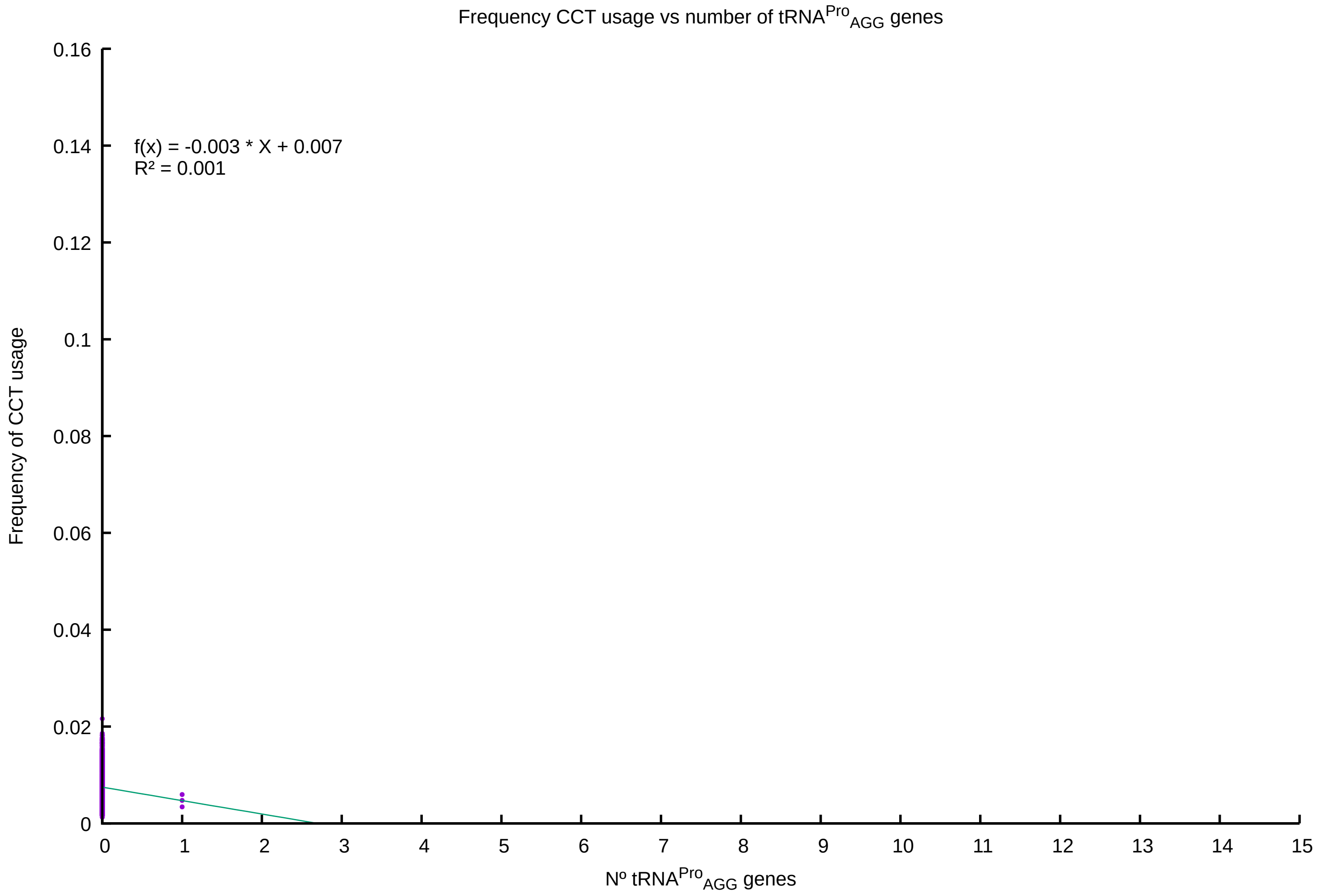

Frequency CCT usage vs number of tRNA^Pro_AGG genes
f(x) = -0.003 * X + 0.007
R² = 0.001
Frequency of CCT usage
0.16
0.14
0.12
0.1
0.08
0.06
0.04
0.02
0
0 1 2 3 4 5 6 7 8 9 10 11 12 13 14 15
Nº tRNA^Pro_AGG genes

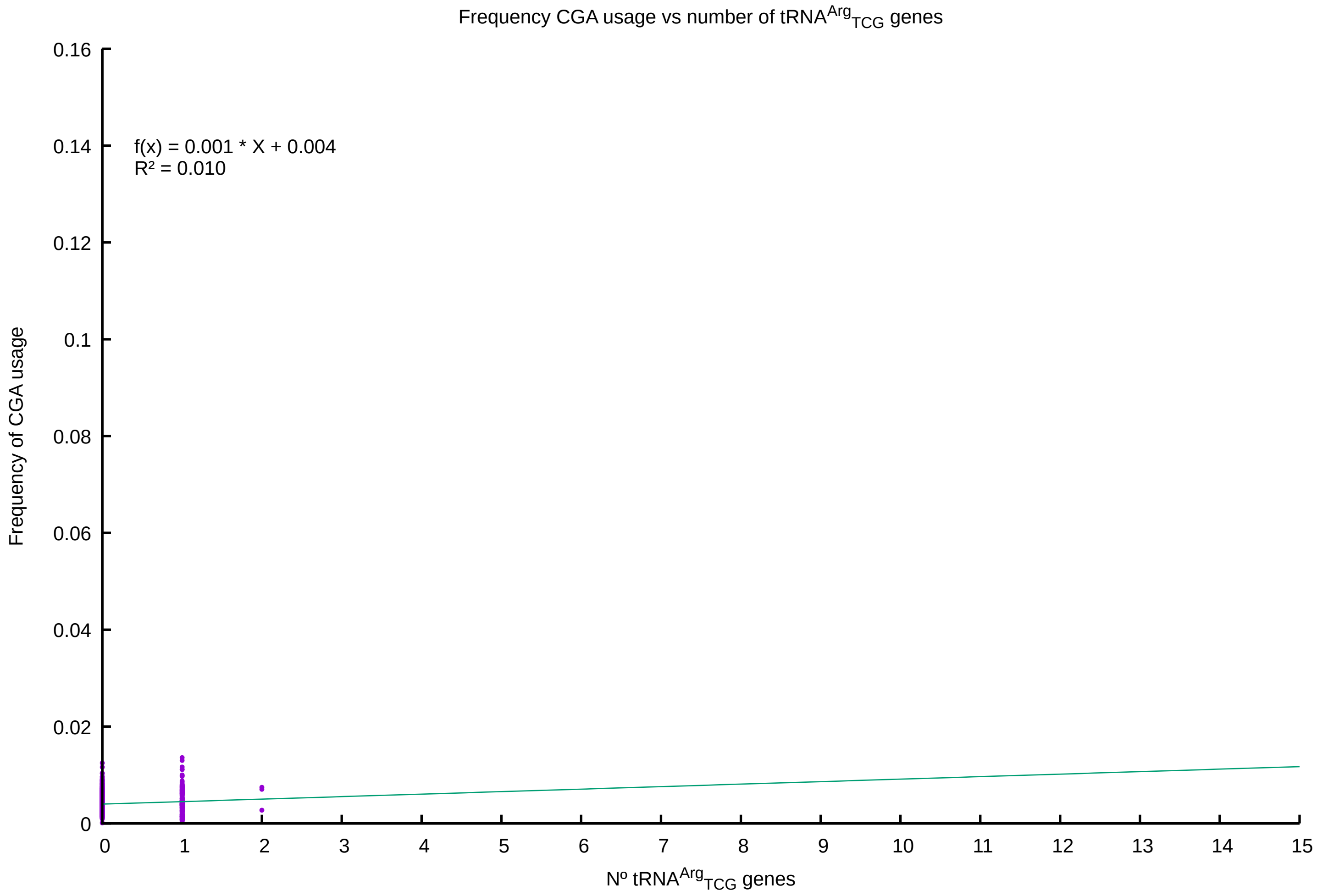
Frequency CGA usage vs number of tRNA^Arg_TCG genes
f(x) = 0.001 * X + 0.004
R² = 0.010
Frequency of CGA usage
0.16
0.14
0.12
0.1
0.08
0.06
0.04
0.02
0
0 1 2 3 4 5 6 7 8 9 10 11 12 13 14 15
Nº tRNA^Arg_TCG genes

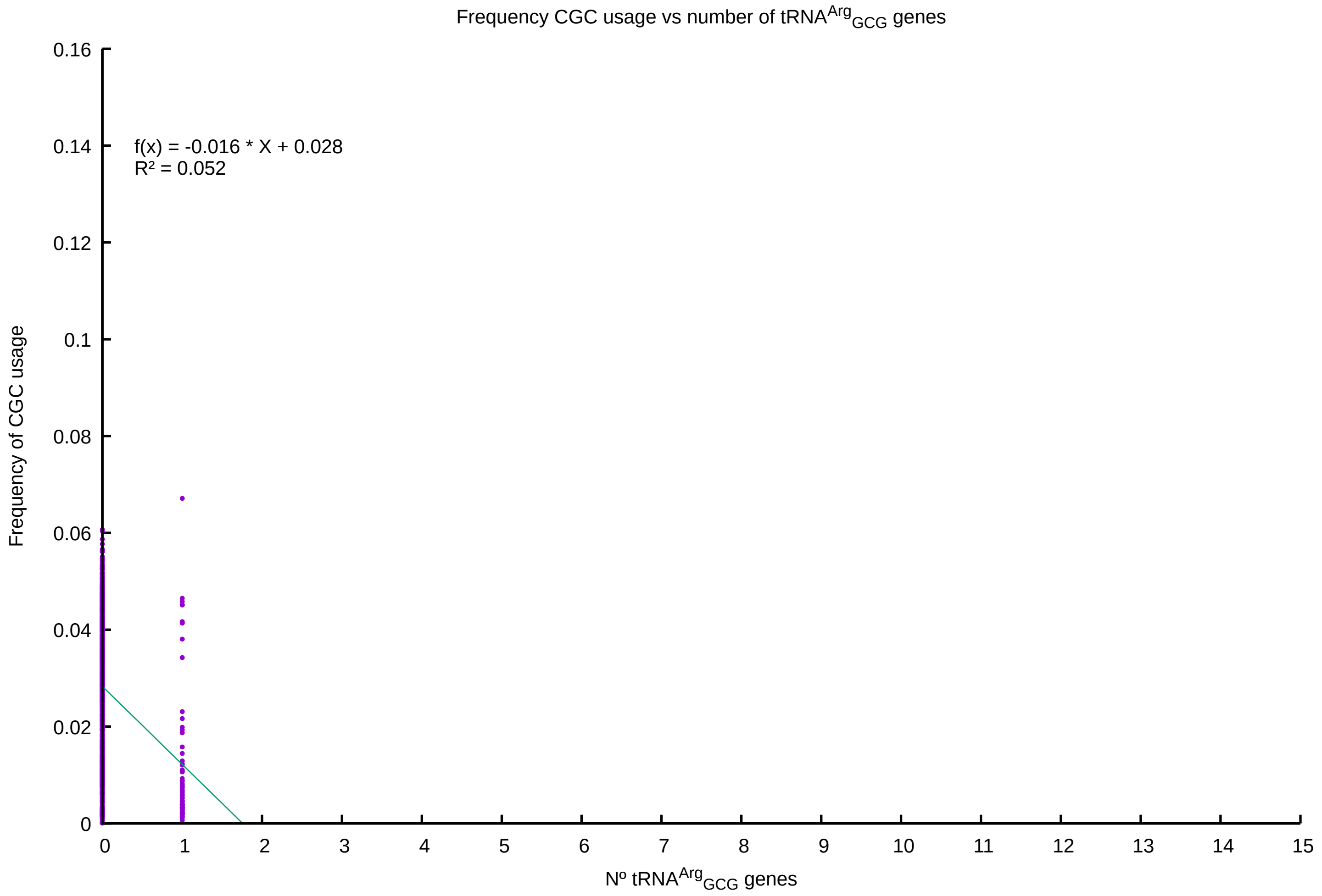
Frequency CGC usage vs number of tRNA^Arg_GCG genes
f(x) = -0.016 * X + 0.028
R² = 0.052
Frequency of CGC usage
0.16
0.14
0.12
0.1
0.08
0.06
0.04
0.02
0
0 1 2 3 4 5 6 7 8 9 10 11 12 13 14 15
Nº tRNA^Arg_GCG genes

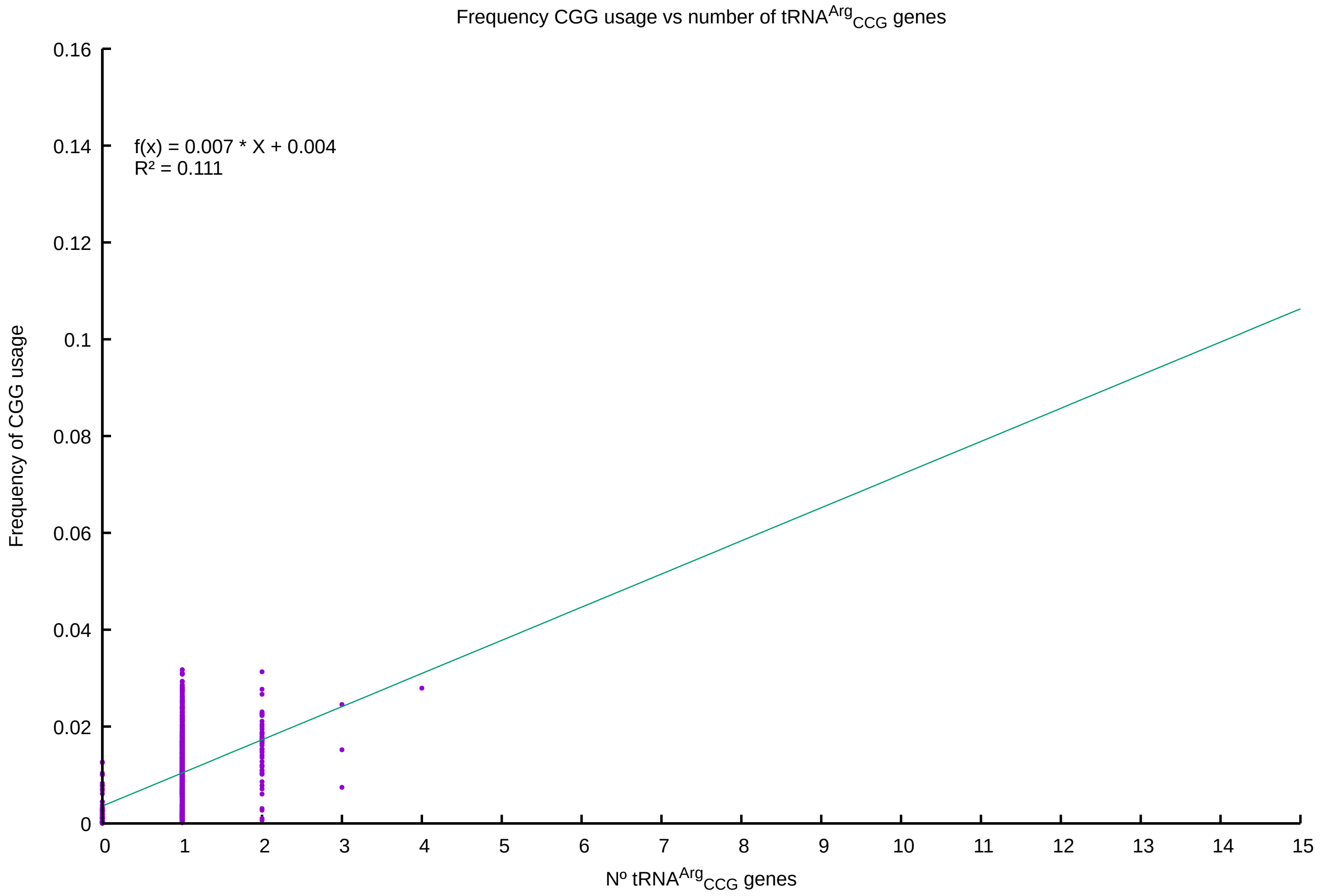
Frequency CGG usage vs number of tRNA^Arg_CCG genes
f(x) = 0.007 * X + 0.004
R² = 0.111
Frequency of CGG usage
0.16
0.14
0.12
0.1
0.08
0.06
0.04
0.02
0
0 1 2 3 4 5 6 7 8 9 10 11 12 13 14 15
Nº tRNA^Arg_CCG genes

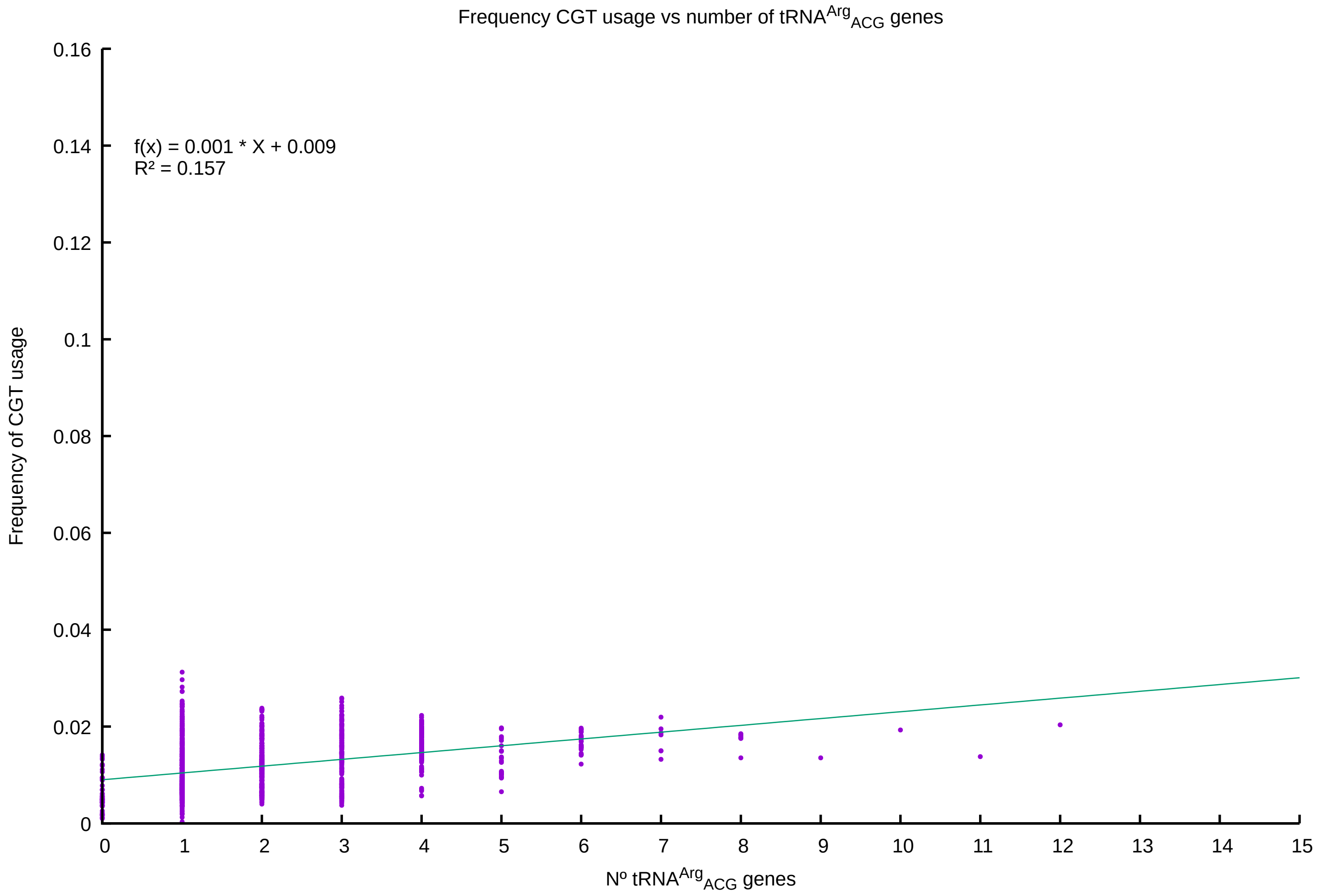

Frequency CGT usage vs number of tRNA^Arg_ACG genes
f(x) = 0.001 * X + 0.009
R² = 0.157
Frequency of CGT usage
0.16
0.14
0.12
0.1
0.08
0.06
0.04
0.02
0
0 1 2 3 4 5 6 7 8 9 10 11 12 13 14 15
Nº tRNA^Arg_ACG genes

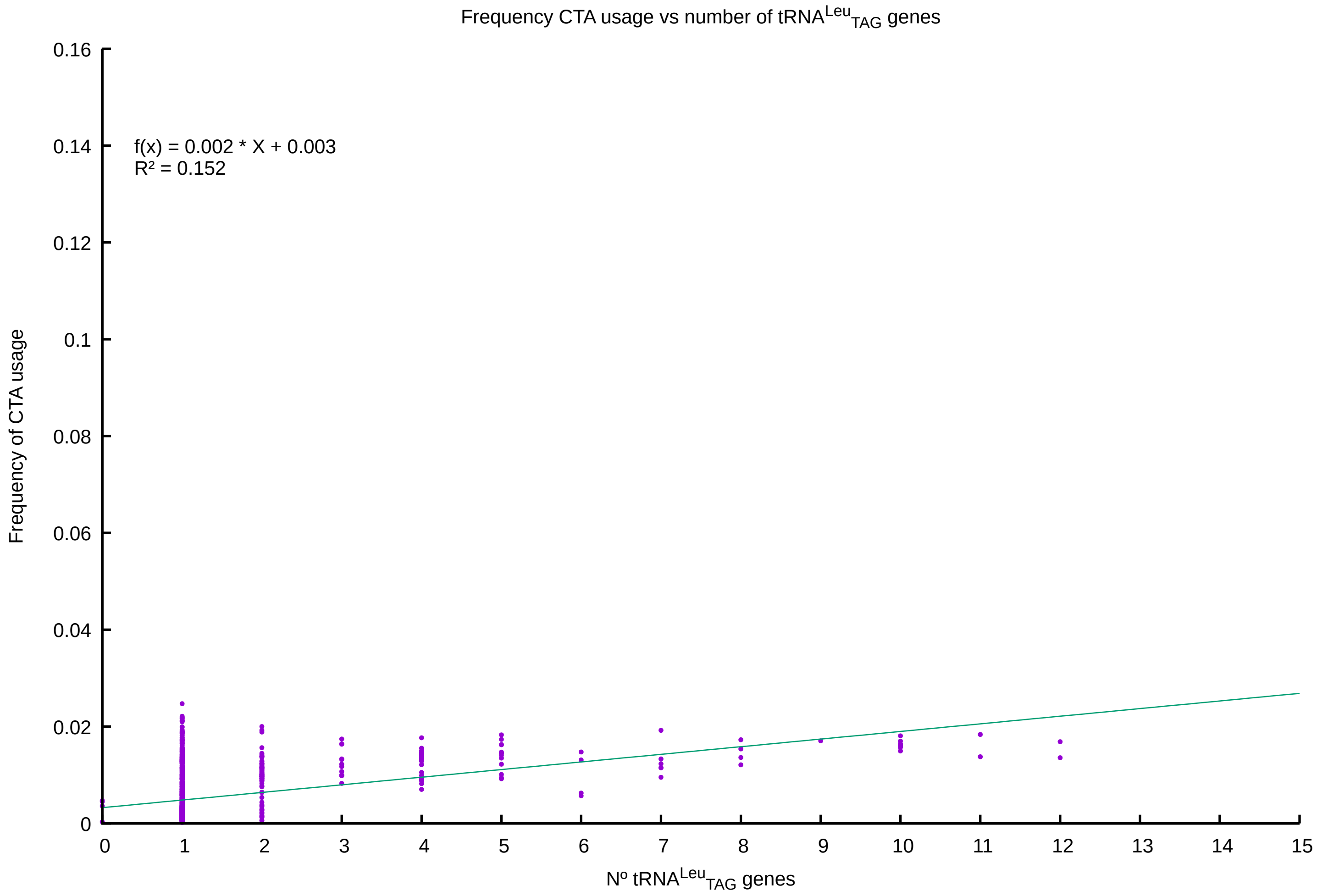

Frequency CTA usage vs number of tRNA Leu TAG genes
f(x) = 0.002 * X + 0.003
R² = 0.152
Frequency of CTA usage
0.16
0.14
0.12
0.1
0.08
0.06
0.04
0.02
0
0 1 2 3 4 5 6 7 8 9 10 11 12 13 14 15
Nº tRNA Leu TAG genes

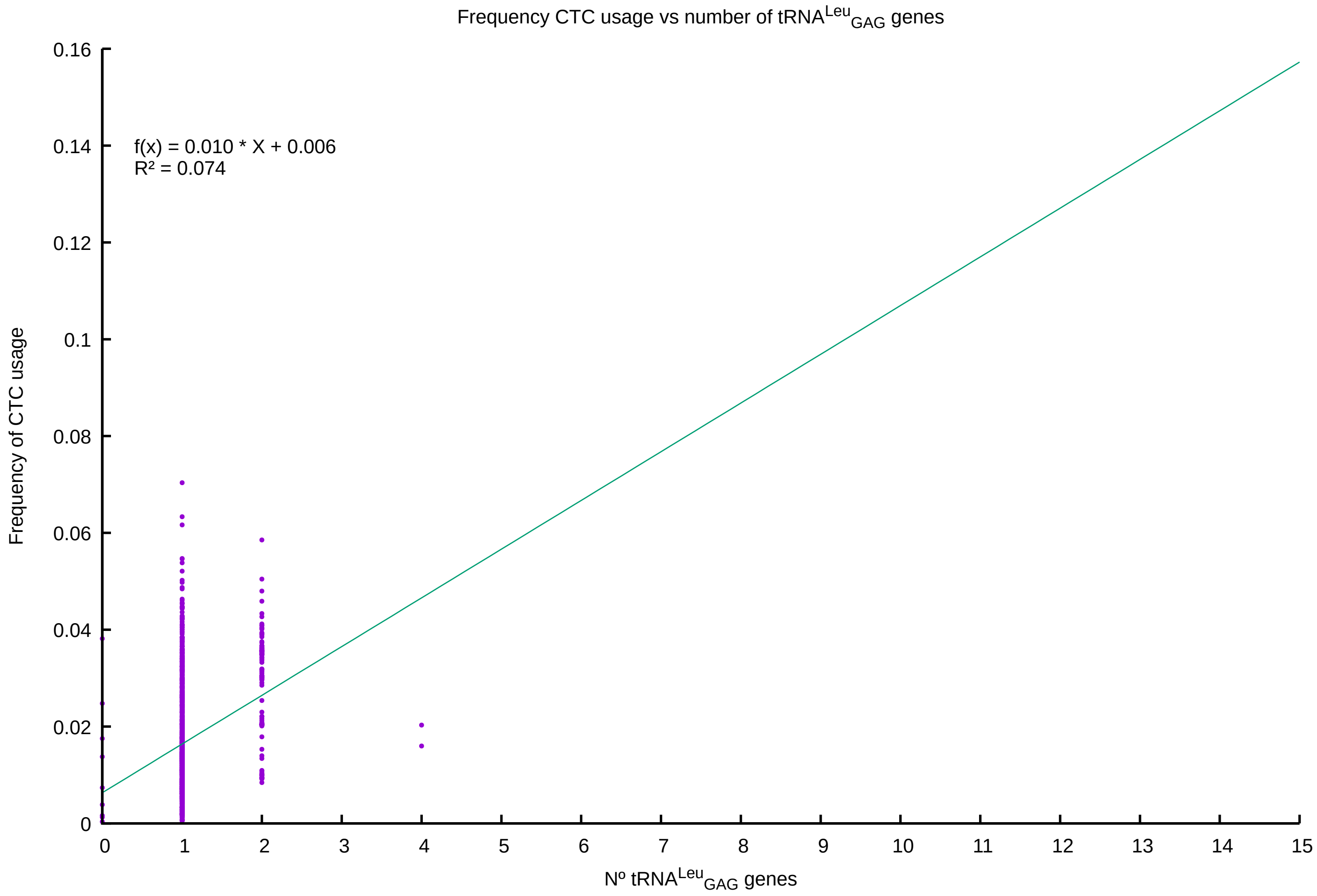
Frequency CTC usage vs number of tRNALeuGAG genes
f(x) = 0.010 * X + 0.006
R² = 0.074
Frequency of CTC usage
Nº tRNALeuGAG genes
0.16
0.14
0.12
0.1
0.08
0.06
0.04
0.02
0
0 1 2 3 4 5 6 7 8 9 10 11 12 13 14 15

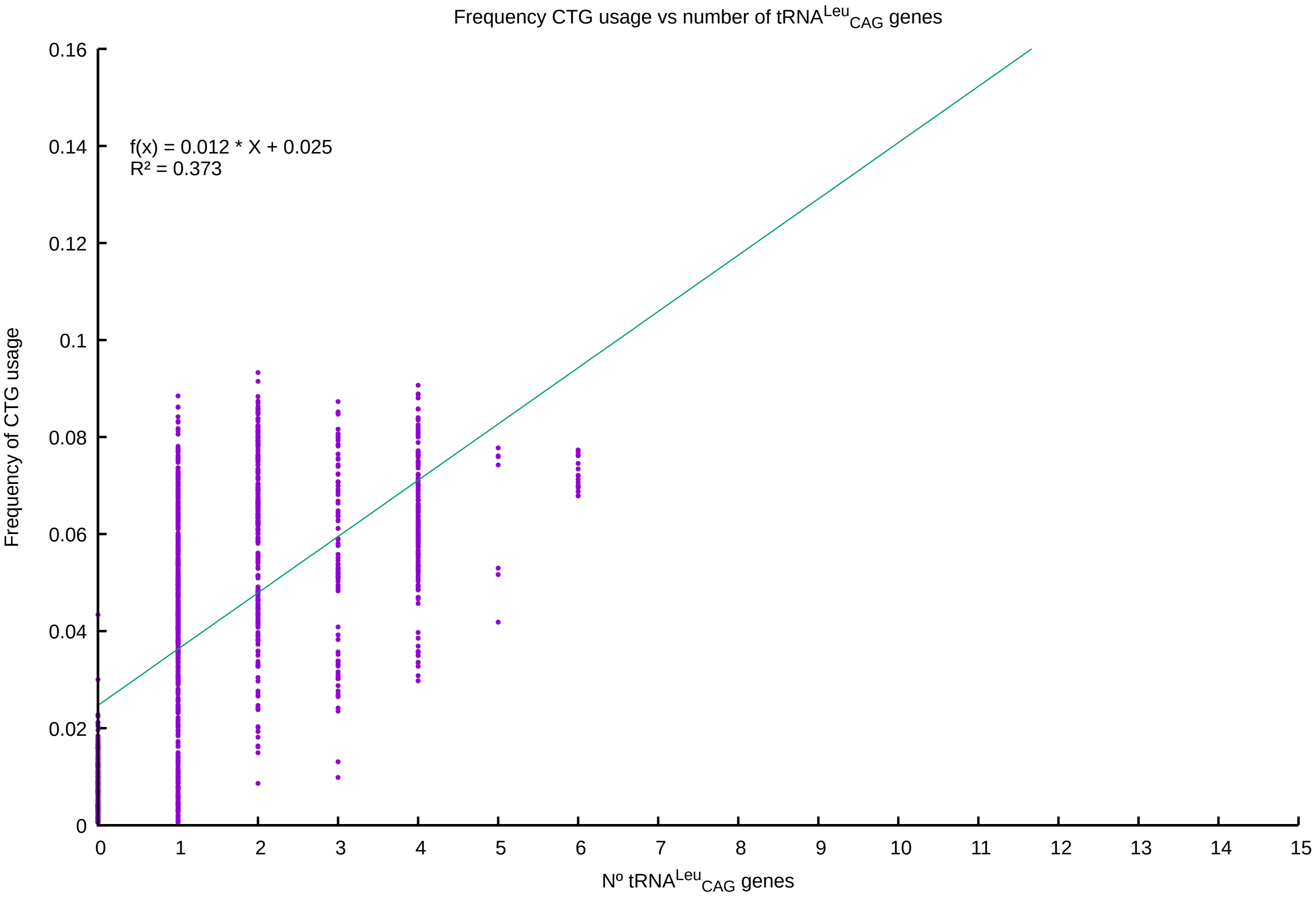

Frequency CTG usage vs number of tRNA$^{Leu}_{CAG}$ genes
f(x) = 0.012 * X + 0.025
R^2 = 0.373
Frequency of CTG usage
0.16
0.14
0.12
0.1
0.08
0.06
0.04
0.02
0
0 1 2 3 4 5 6 7 8 9 10 11 12 13 14 15
Nº tRNA$^{Leu}_{CAG}$ genes

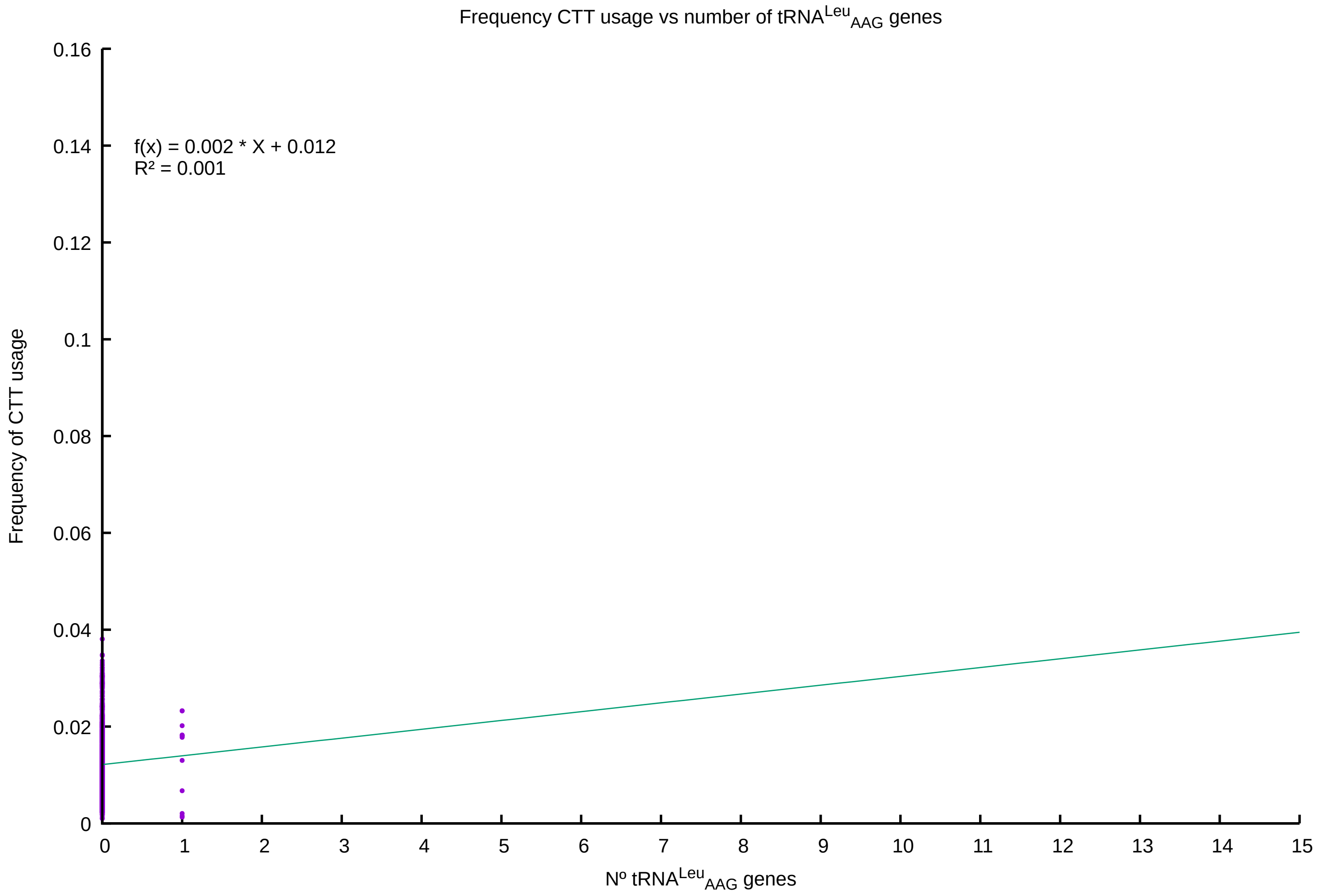

Frequency CTT usage vs number of tRNALeuAAG genes
f(x) = 0.002 * X + 0.012
R² = 0.001
Frequency of CTT usage
0.16
0.14
0.12
0.1
0.08
0.06
0.04
0.02
0
0 1 2 3 4 5 6 7 8 9 10 11 12 13 14 15
Nº tRNALeuAAG genes

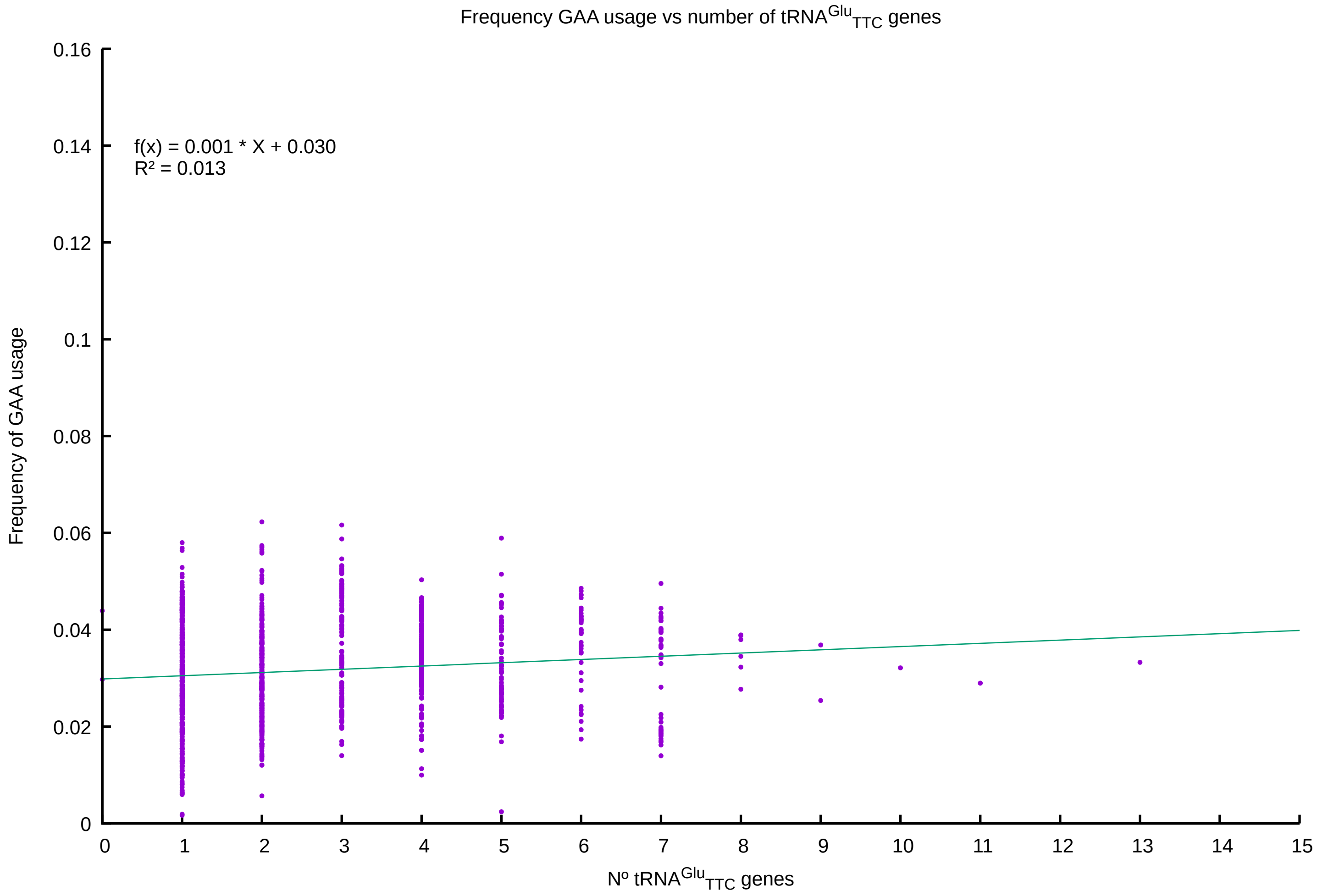

Frequency GAA usage vs number of tRNAGluTTC genes
f(x) = 0.001 * X + 0.030
R² = 0.013
Frequency of GAA usage
Nº tRNAGluTTC genes
0 0.02 0.04 0.06 0.08 0.1 0.12 0.14 0.16
0 1 2 3 4 5 6 7 8 9 10 11 12 13 14 15

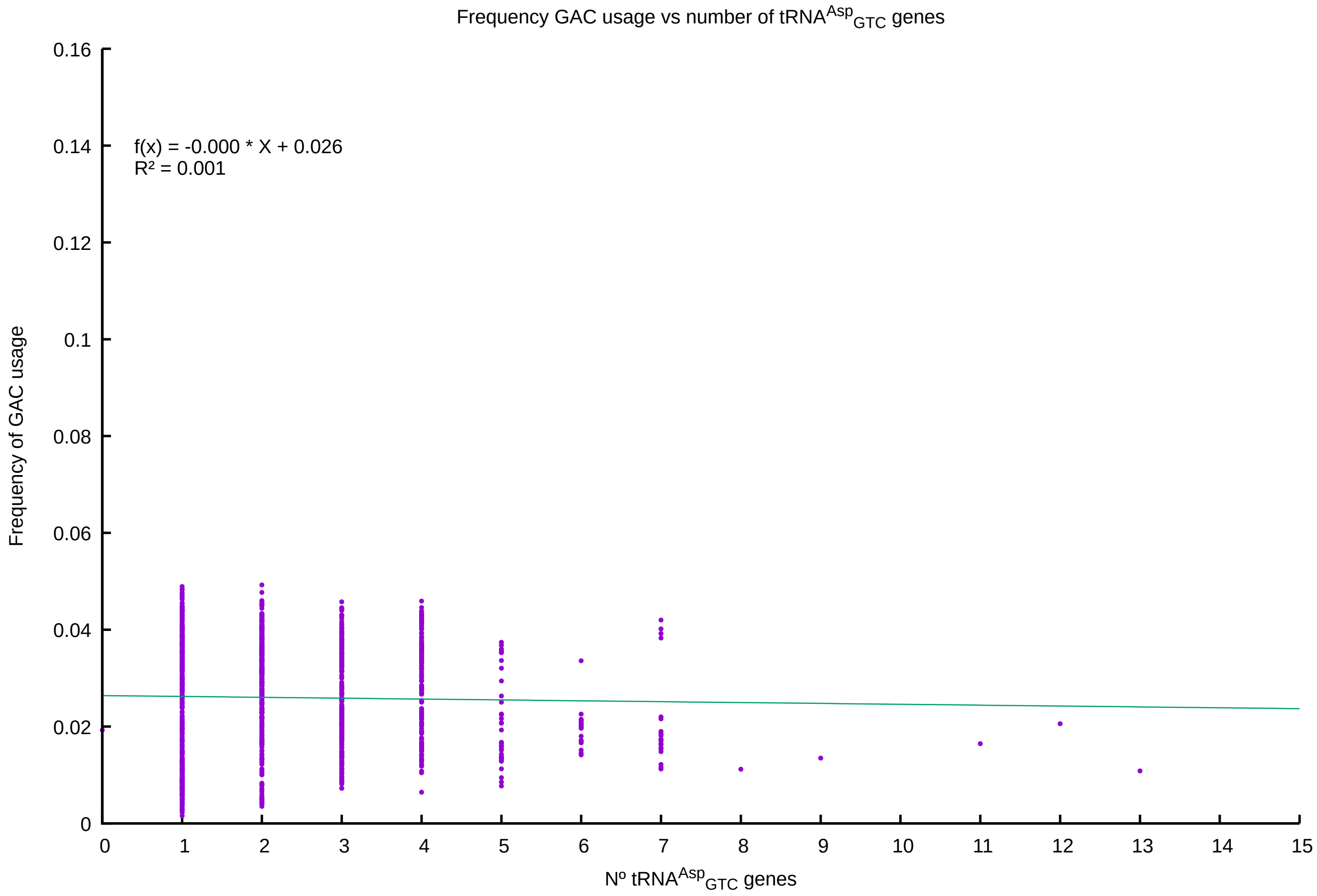
Frequency GAC usage vs number of tRNAAspGTC genes
f(x) = -0.000 * X + 0.026
R² = 0.001
Frequency of GAC usage
0.16
0.14
0.12
0.1
0.08
0.06
0.04
0.02
0
0 1 2 3 4 5 6 7 8 9 10 11 12 13 14 15
Nº tRNAAspGTC genes

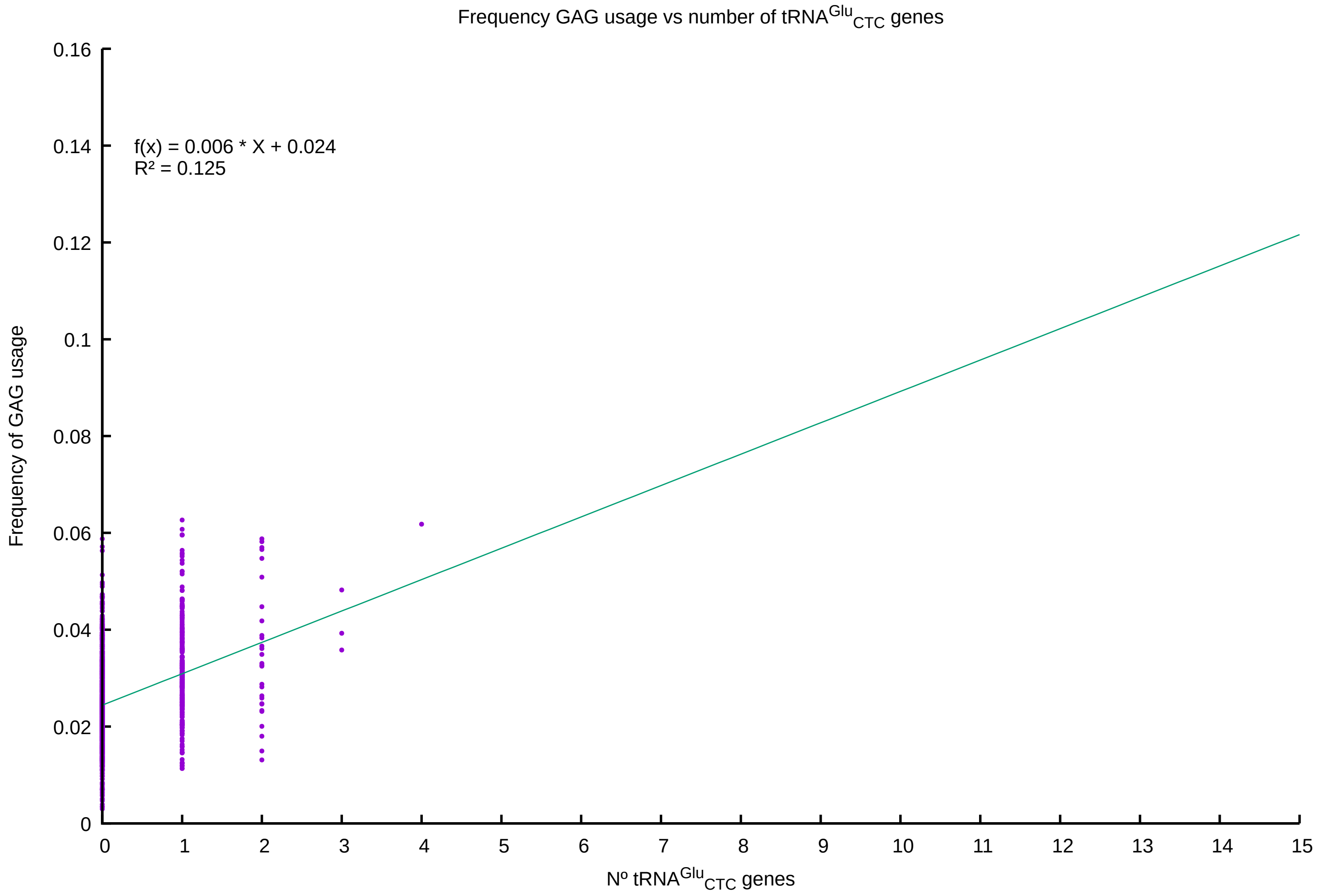
Frequency GAG usage vs number of tRNA$^{Glu}_{CTC}$ genes
f(x) = 0.006 * X + 0.024
R^2 = 0.125
Frequency of GAG usage
Nº tRNA$^{Glu}_{CTC}$ genes
0.16
0.14
0.12
0.1
0.08
0.06
0.04
0.02
0
0 1 2 3 4 5 6 7 8 9 10 11 12 13 14 15

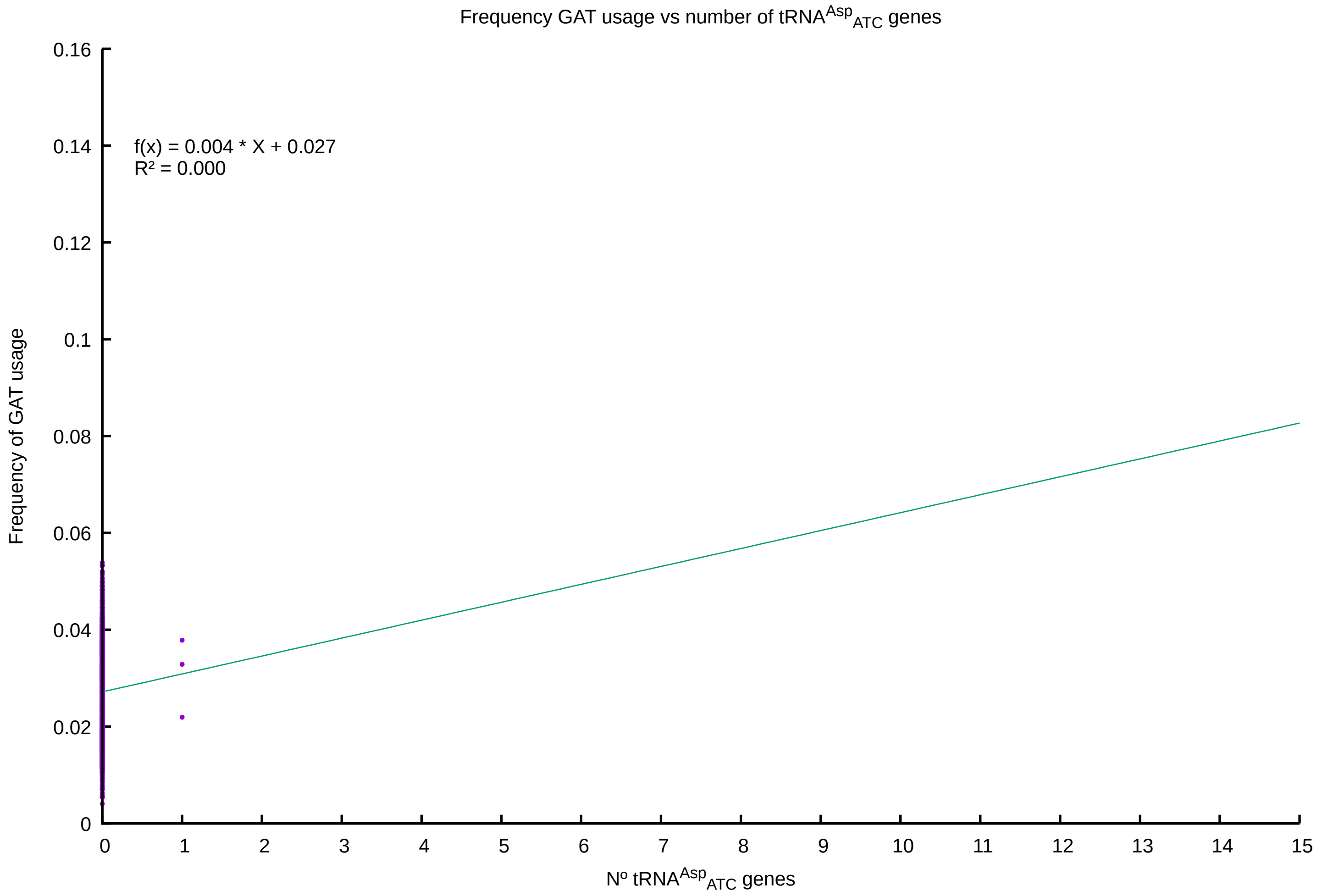

Frequency GAT usage vs number of tRNA$^{Asp}_{ATC}$ genes
f(x) = 0.004 * X + 0.027
R^2 = 0.000
Frequency of GAT usage
0.16
0.14
0.12
0.1
0.08
0.06
0.04
0.02
0
0 1 2 3 4 5 6 7 8 9 10 11 12 13 14 15
Nº tRNA$^{Asp}_{ATC}$ genes

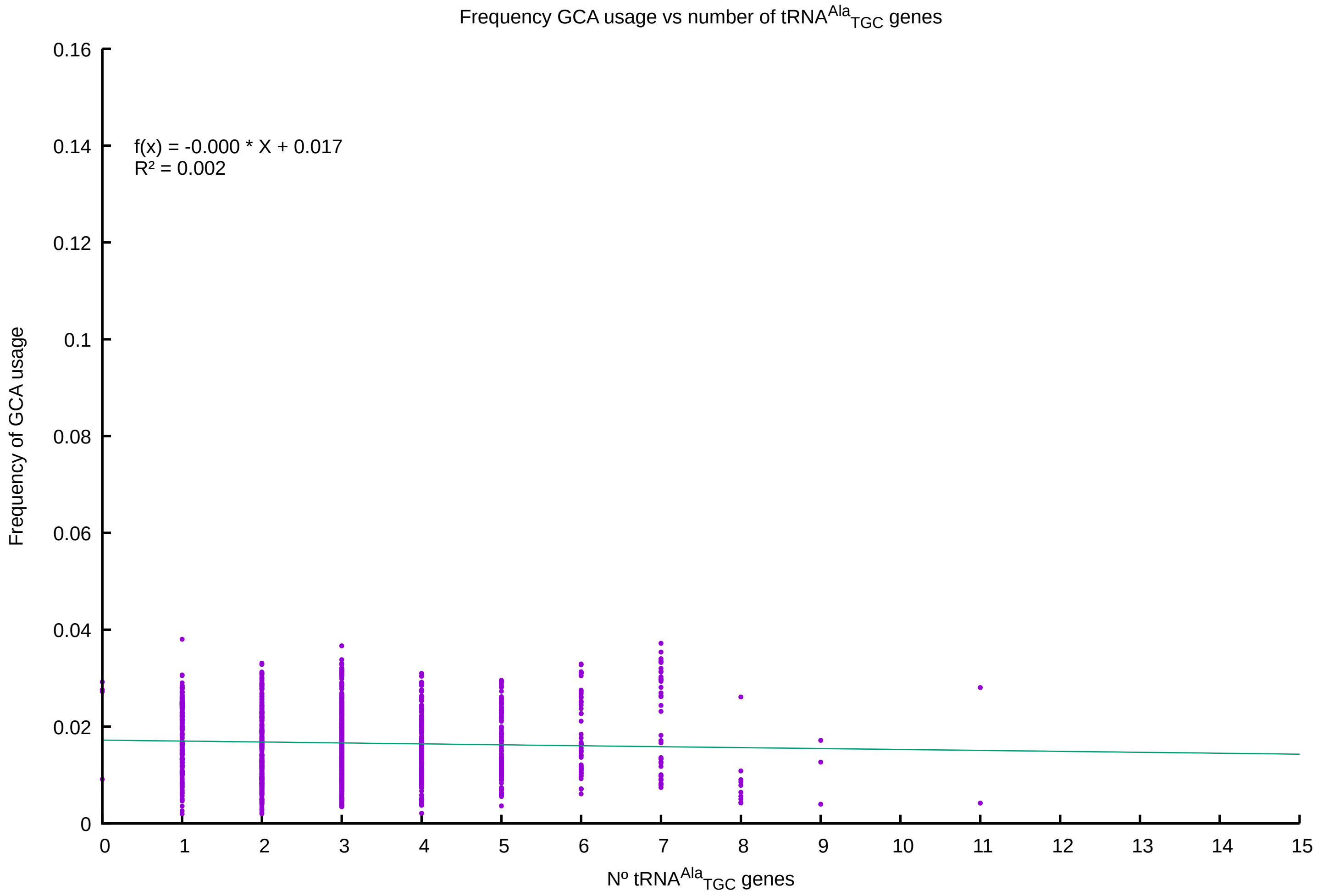

Frequency GCA usage vs number of tRNA^Ala_TGC genes
f(x) = -0.000 * X + 0.017
R² = 0.002
Frequency of GCA usage
0.16
0.14
0.12
0.1
0.08
0.06
0.04
0.02
0
0 1 2 3 4 5 6 7 8 9 10 11 12 13 14 15
Nº tRNA^Ala_TGC genes

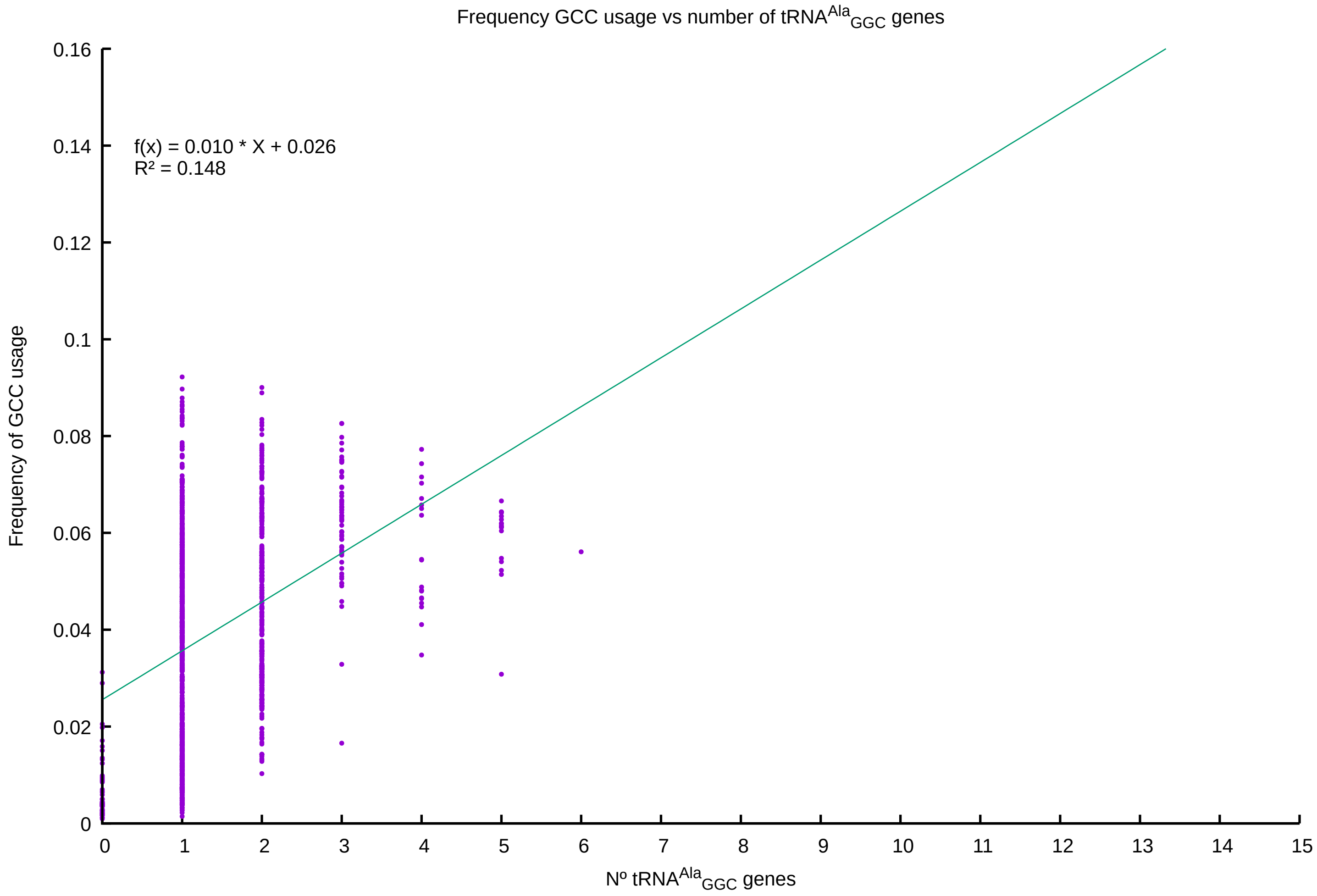

Frequency GCC usage vs number of tRNA^Ala_GGC genes
f(x) = 0.010 * X + 0.026
R² = 0.148
Frequency of GCC usage
Nº tRNA^Ala_GGC genes
0
0.02
0.04
0.06
0.08
0.1
0.12
0.14
0.16
0
1
2
3
4
5
6
7
8
9
10
11
12
13
14
15

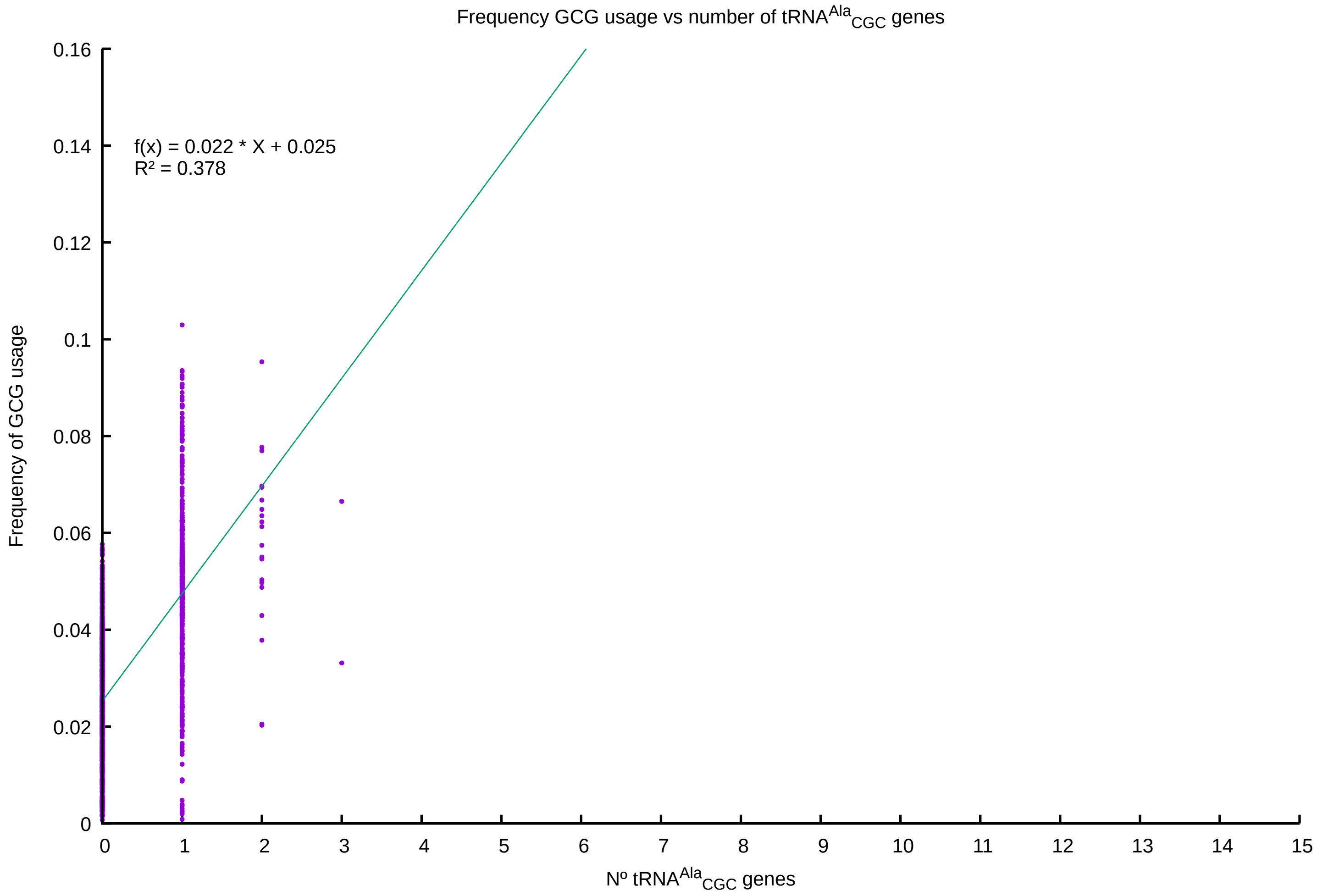
Frequency GCG usage vs number of tRNA$^{Ala}_{CGC}$ genes
f(x) = 0.022 * X + 0.025
R^2 = 0.378
Frequency of GCG usage
0
0.02
0.04
0.06
0.08
0.1
0.12
0.14
0.16
0
1
2
3
4
5
6
7
8
9
10
11
12
13
14
15
Nº tRNA$^{Ala}_{CGC}$ genes

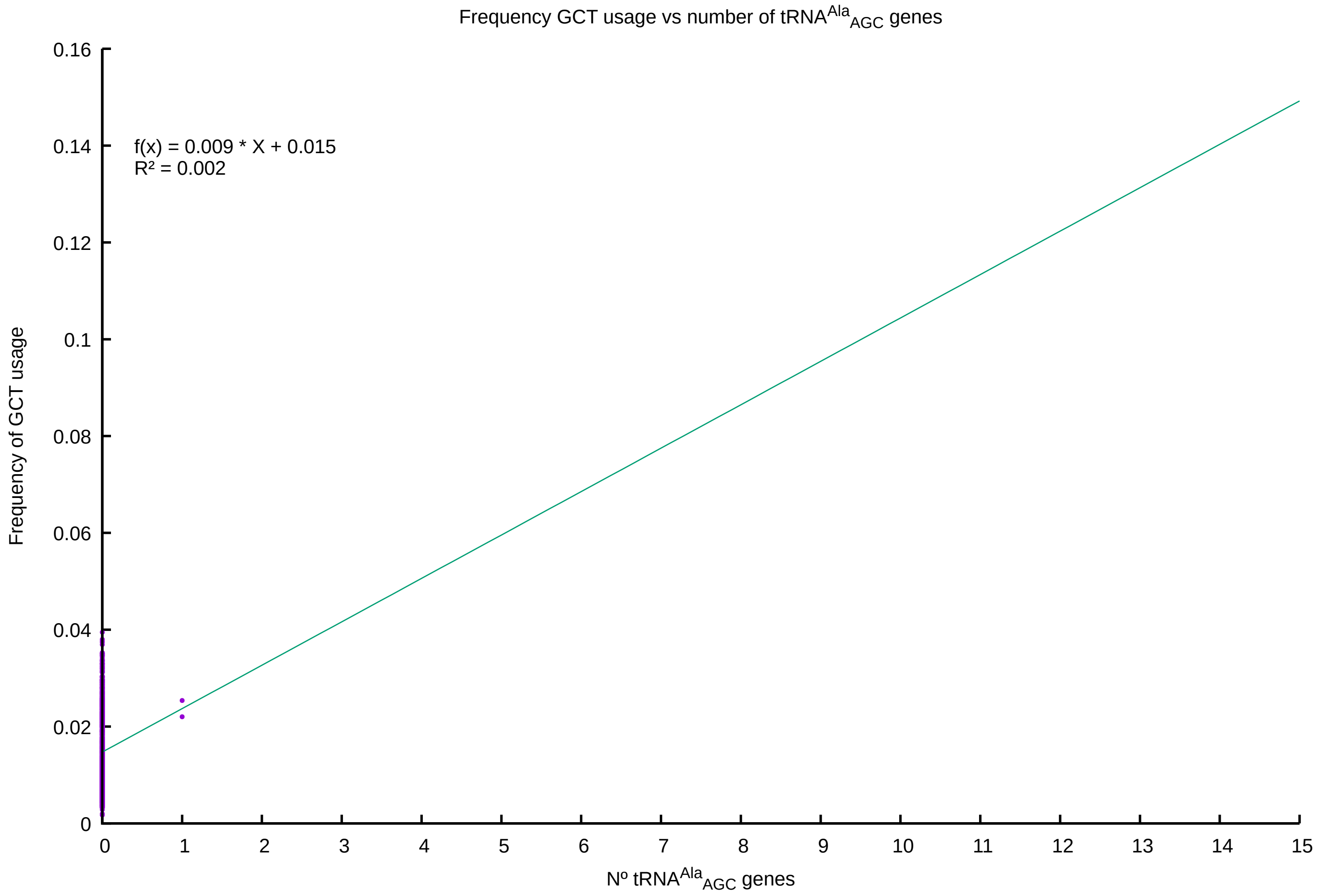
Frequency GCT usage vs number of tRNA^Ala_AGC genes
f(x) = 0.009 * X + 0.015
R² = 0.002
Frequency of GCT usage
0.16
0.14
0.12
0.1
0.08
0.06
0.04
0.02
0
0 1 2 3 4 5 6 7 8 9 10 11 12 13 14 15
Nº tRNA^Ala_AGC genes

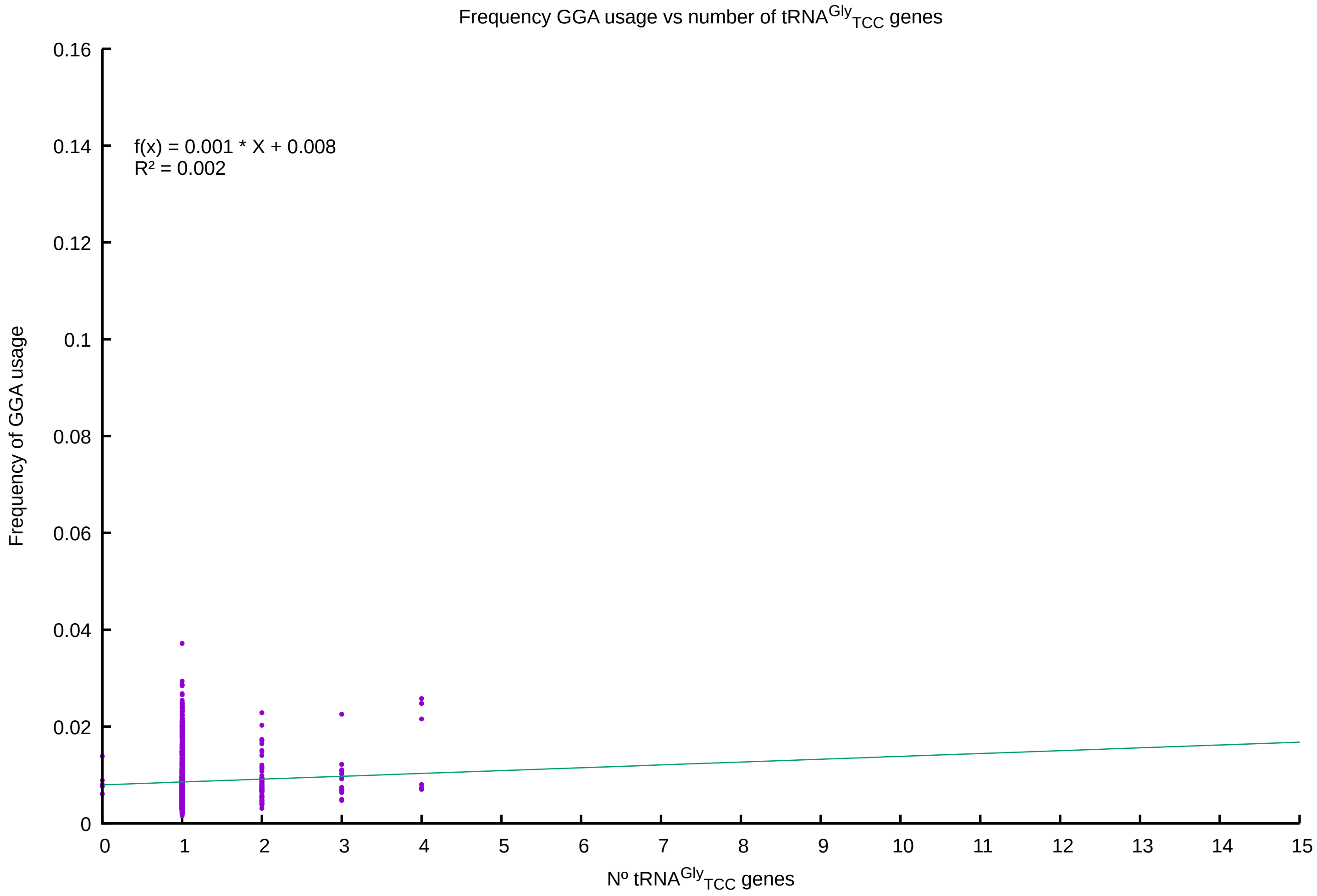

Frequency GGA usage vs number of tRNAGlyTCC genes
f(x) = 0.001 * X + 0.008
R² = 0.002
Frequency of GGA usage
0.16
0.14
0.12
0.1
0.08
0.06
0.04
0.02
0
0
1
2
3
4
5
6
7
8
9
10
11
12
13
14
15
Nº tRNAGlyTCC genes

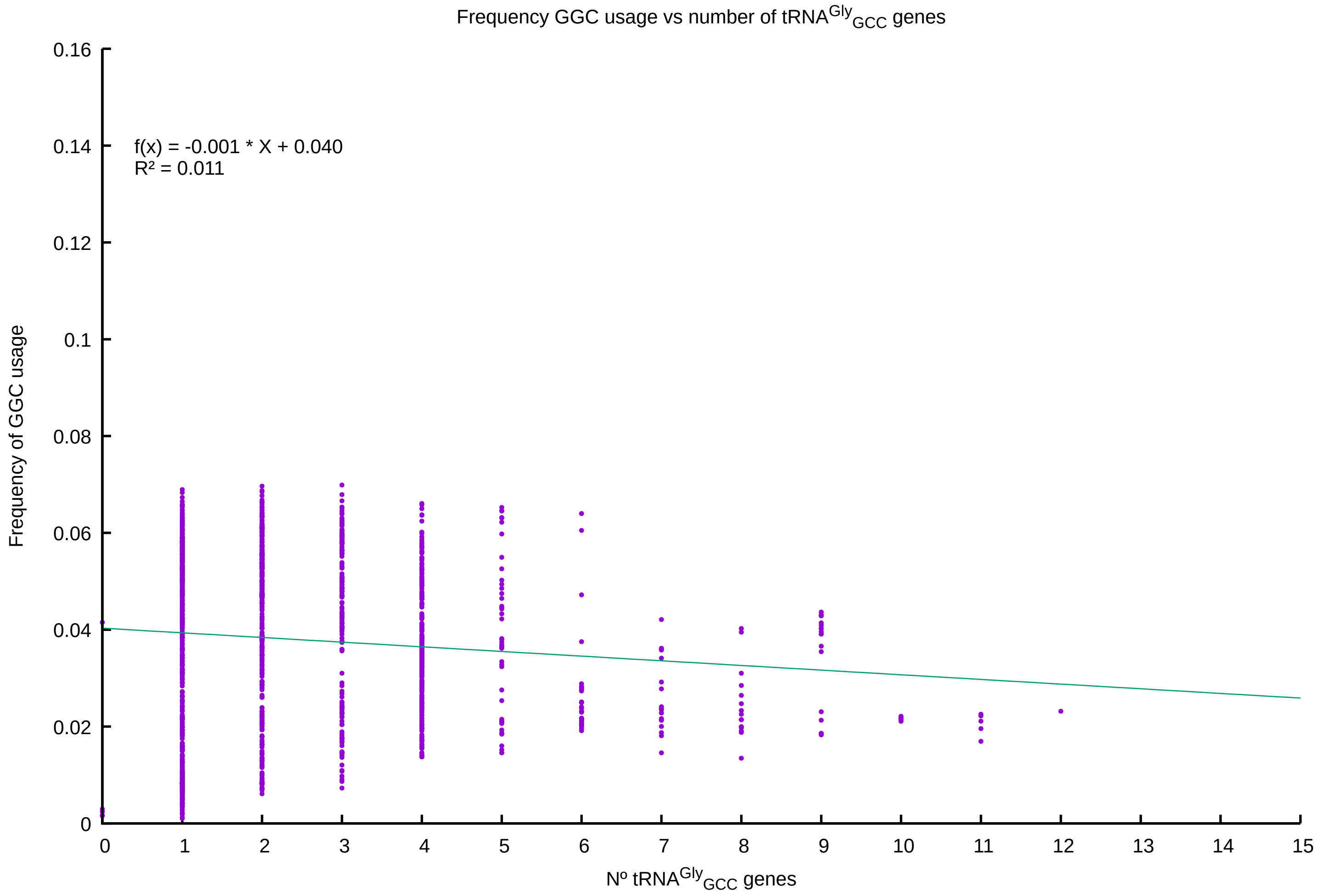
Frequency GGC usage vs number of tRNAGlyGCC genes
f(x) = -0.001 * X + 0.040
R² = 0.011
Frequency of GGC usage
Nº tRNAGlyGCC genes
0
0.02
0.04
0.06
0.08
0.1
0.12
0.14
0.16
0
1
2
3
4
5
6
7
8
9
10
11
12
13
14
15

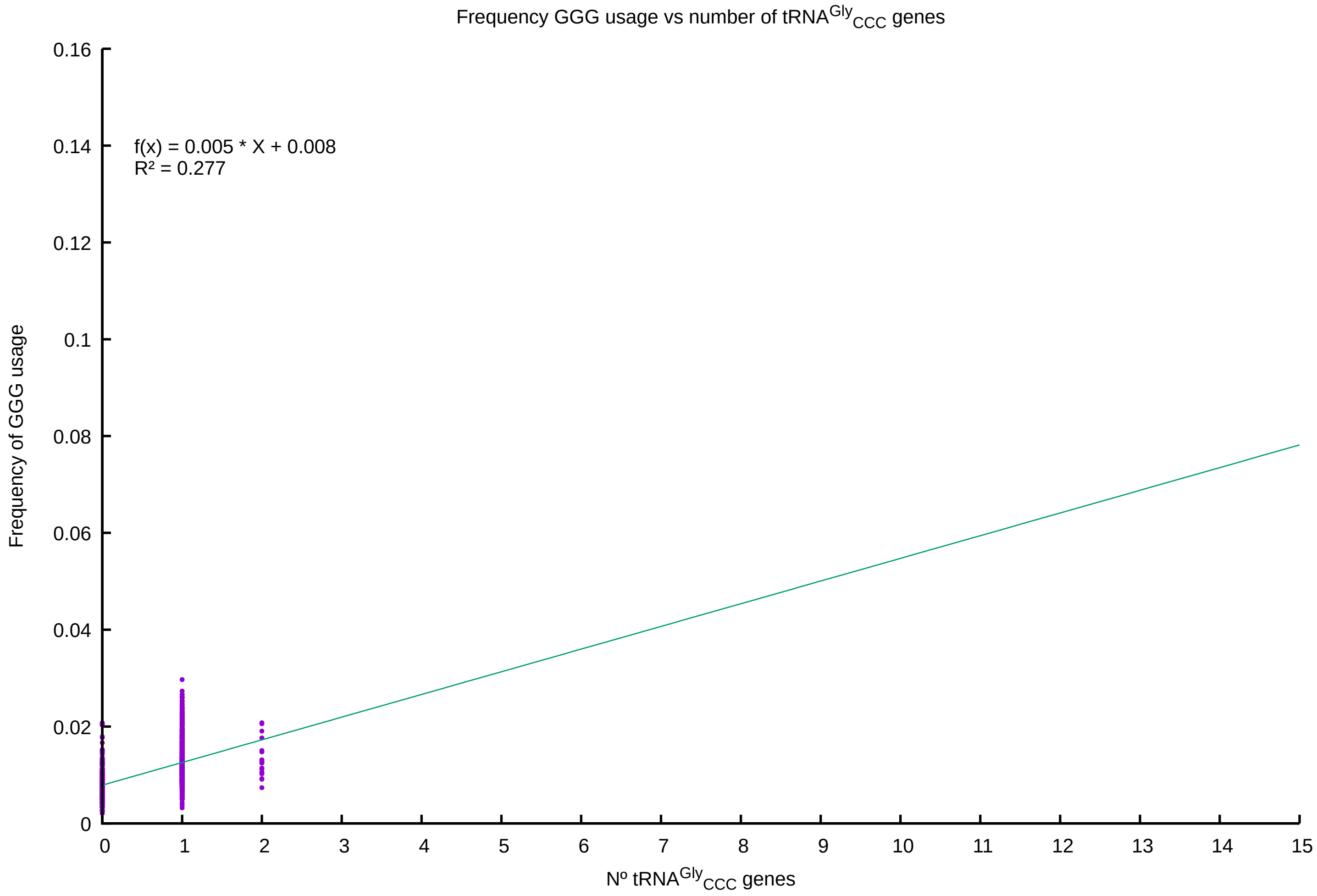
Frequency GGG usage vs number of tRNA$^{Gly}_{CCC}$ genes
f(x) = 0.005 * X + 0.008
R^2 = 0.277
Frequency of GGG usage
0.16
0.14
0.12
0.1
0.08
0.06
0.04
0.02
0
0 1 2 3 4 5 6 7 8 9 10 11 12 13 14 15
Nº tRNA$^{Gly}_{CCC}$ genes

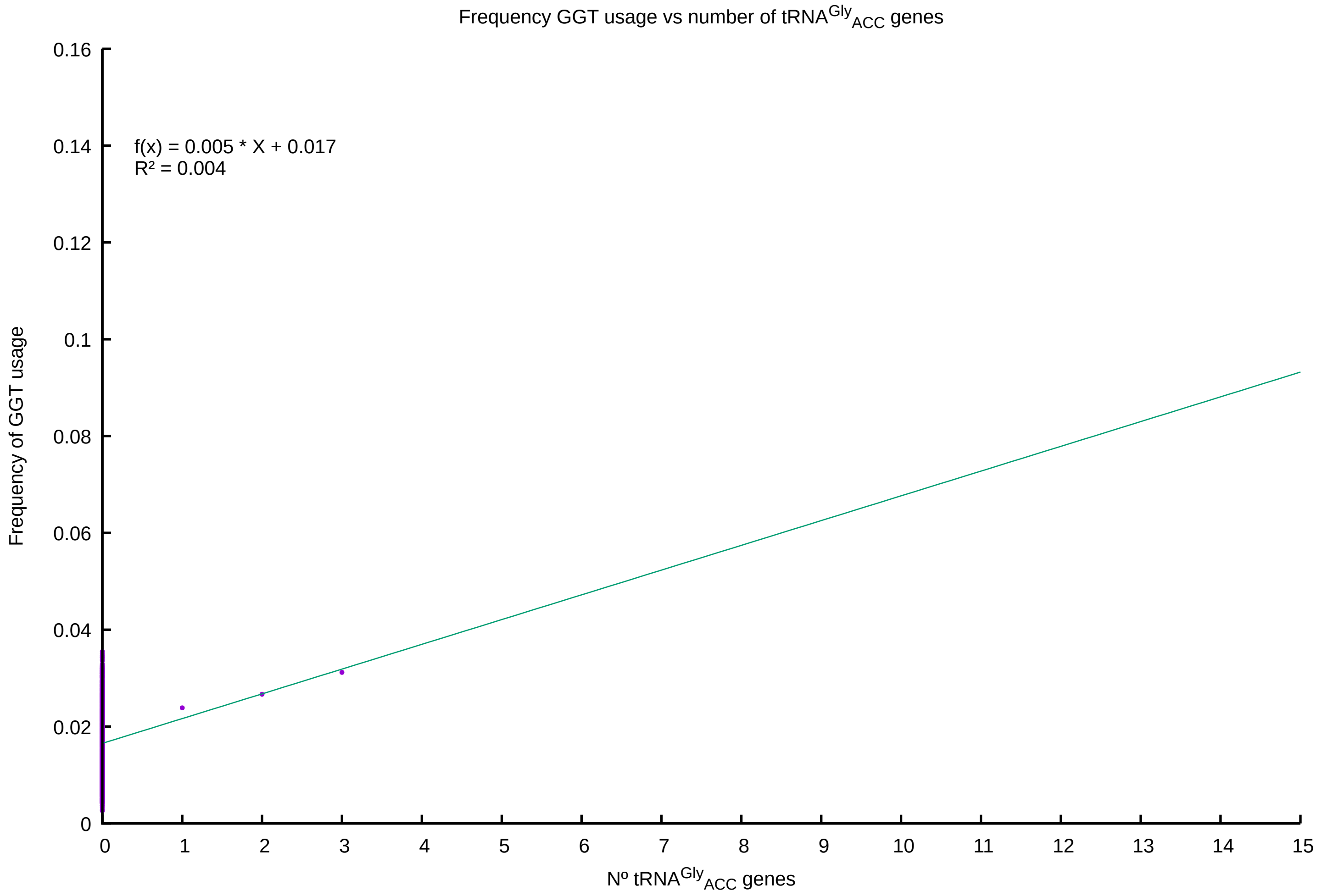

Frequency GGT usage vs number of tRNA^Gly_ACC genes
f(x) = 0.005 * X + 0.017
R² = 0.004
Frequency of GGT usage
0.16
0.14
0.12
0.1
0.08
0.06
0.04
0.02
0
0 1 2 3 4 5 6 7 8 9 10 11 12 13 14 15
Nº tRNA^Gly_ACC genes

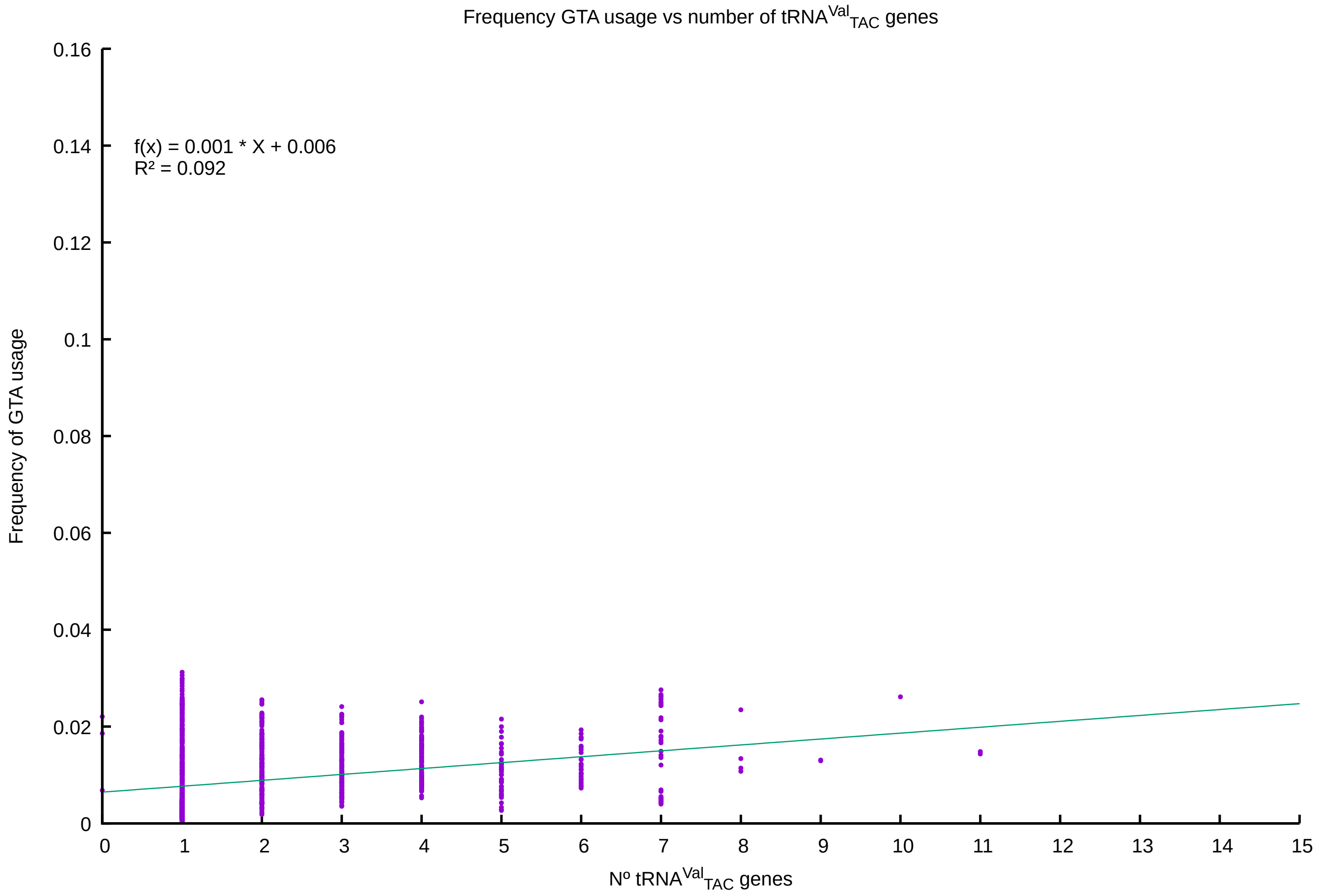
Frequency GTA usage vs number of tRNA Val TAC genes
f(x) = 0.001 * X + 0.006
R² = 0.092
Frequency of GTA usage
0.16
0.14
0.12
0.1
0.08
0.06
0.04
0.02
0
0 1 2 3 4 5 6 7 8 9 10 11 12 13 14 15
Nº tRNA Val TAC genes

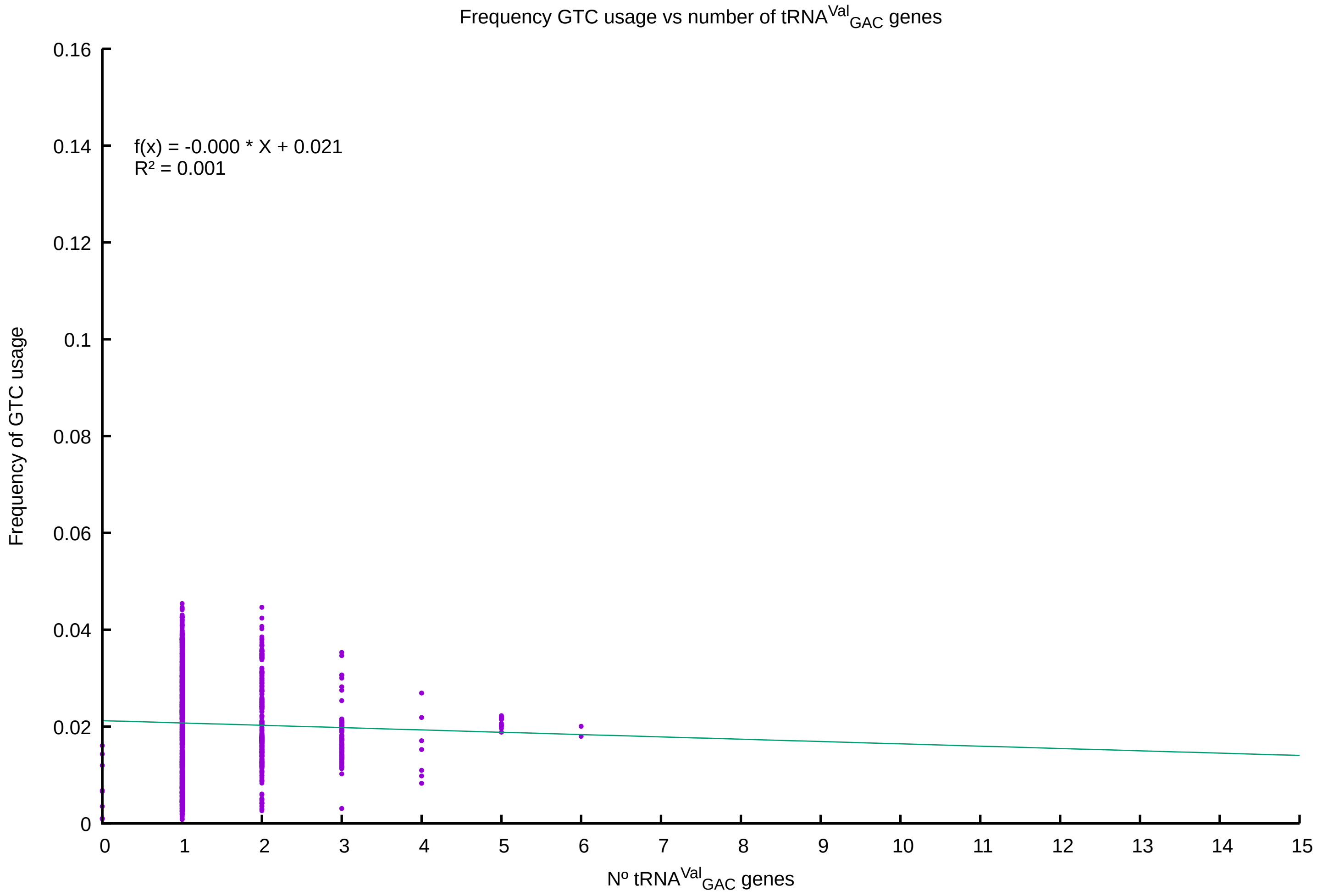
Frequency GTC usage vs number of tRNA^Val_GAC genes
f(x) = -0.000 * X + 0.021
R² = 0.001
Frequency of GTC usage
0.16
0.14
0.12
0.1
0.08
0.06
0.04
0.02
0
0 1 2 3 4 5 6 7 8 9 10 11 12 13 14 15
Nº tRNA^Val_GAC genes

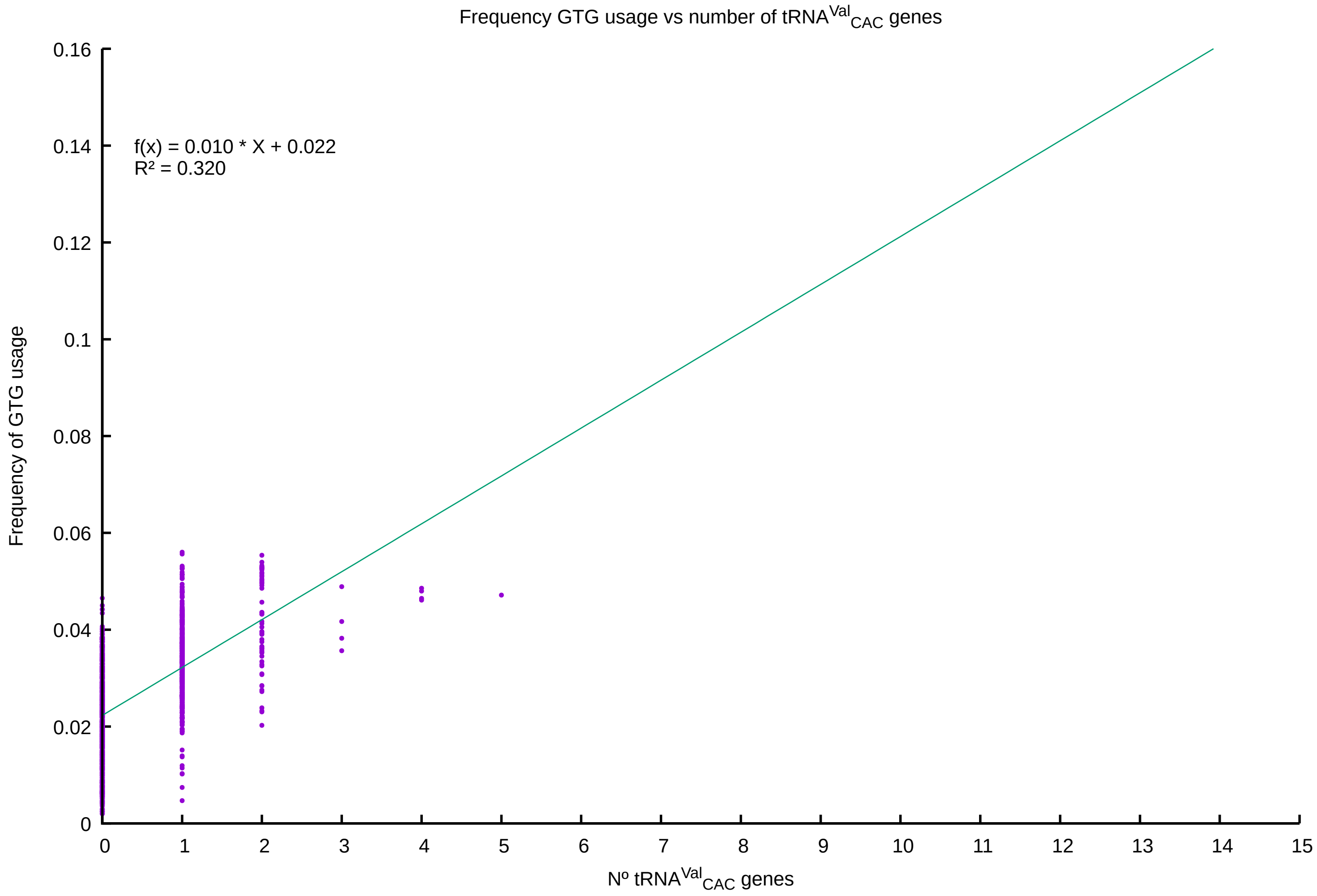

Frequency GTG usage vs number of tRNAValCAC genes
f(x) = 0.010 * X + 0.022
R² = 0.320
Frequency of GTG usage
0.16
0.14
0.12
0.1
0.08
0.06
0.04
0.02
0
0 1 2 3 4 5 6 7 8 9 10 11 12 13 14 15
Nº tRNAValCAC genes

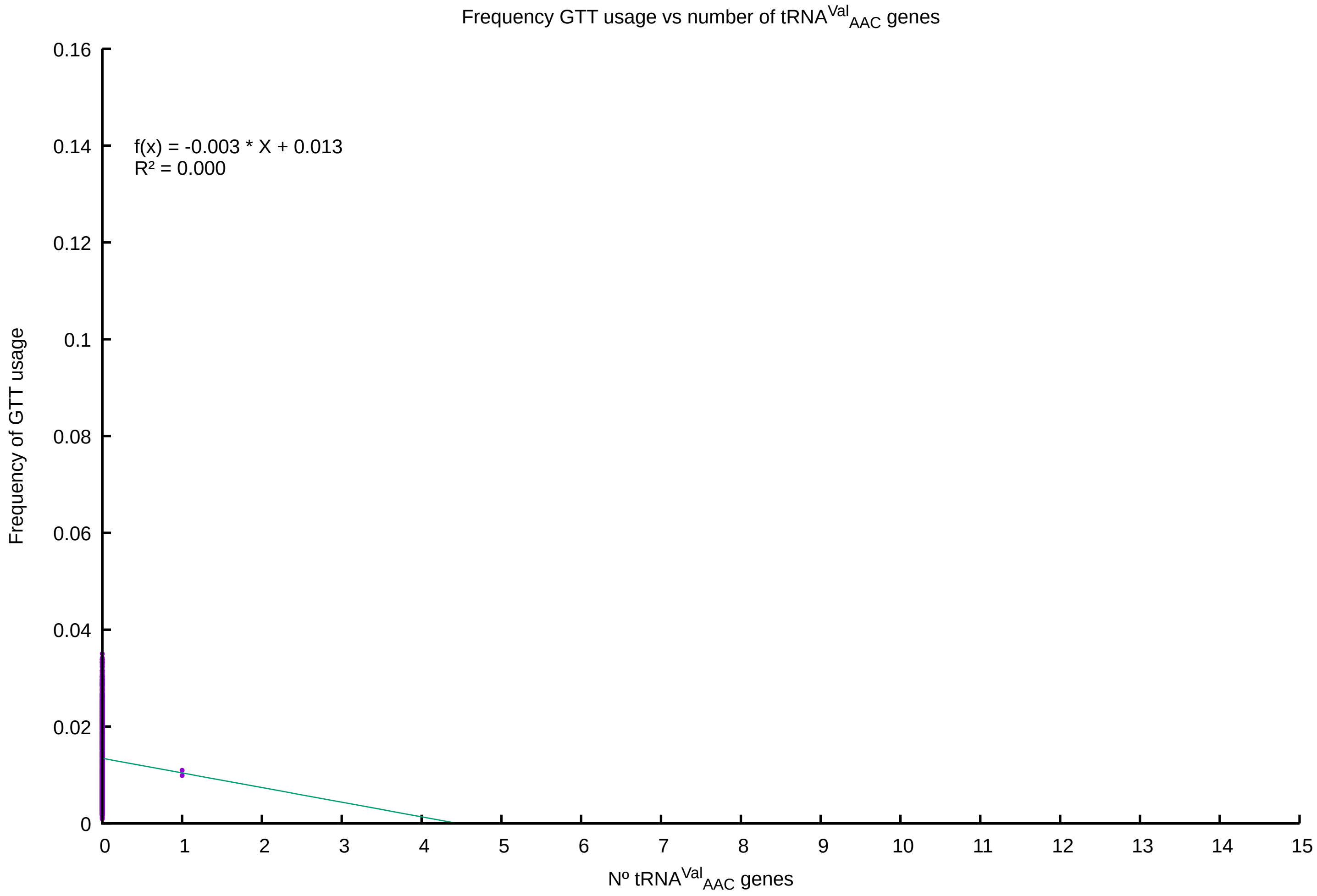

Frequency GTT usage vs number of tRNAValAAC genes
f(x) = -0.003 * X + 0.013
R² = 0.000
Frequency of GTT usage
0.16
0.14
0.12
0.1
0.08
0.06
0.04
0.02
0
0
1
2
3
4
5
6
7
8
9
10
11
12
13
14
15
Nº tRNAValAAC genes

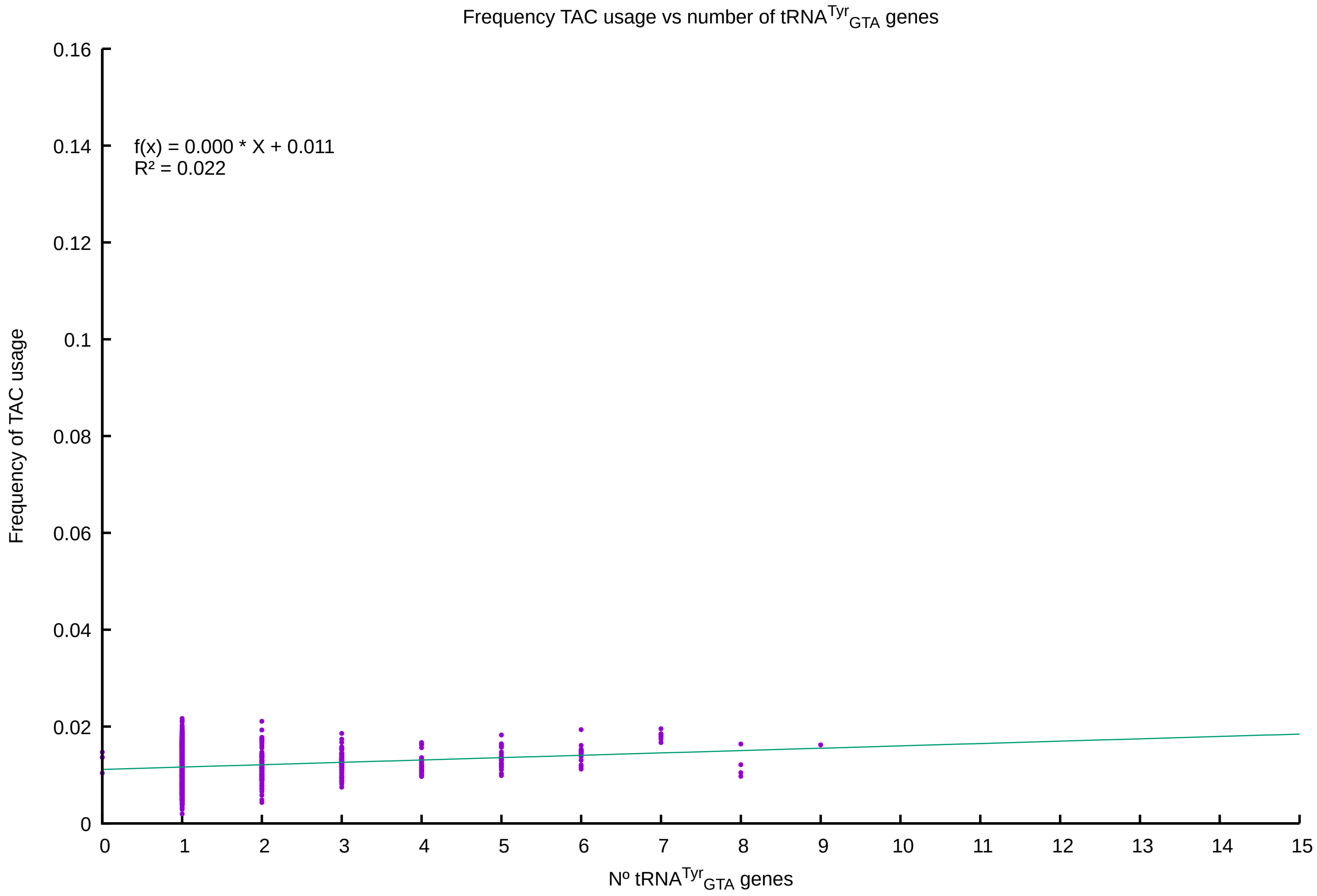
Frequency TAC usage vs number of tRNATyrGTA genes
f(x) = 0.000 * X + 0.011
R² = 0.022
Frequency of TAC usage
0.16
0.14
0.12
0.1
0.08
0.06
0.04
0.02
0
0 1 2 3 4 5 6 7 8 9 10 11 12 13 14 15
Nº tRNATyrGTA genes

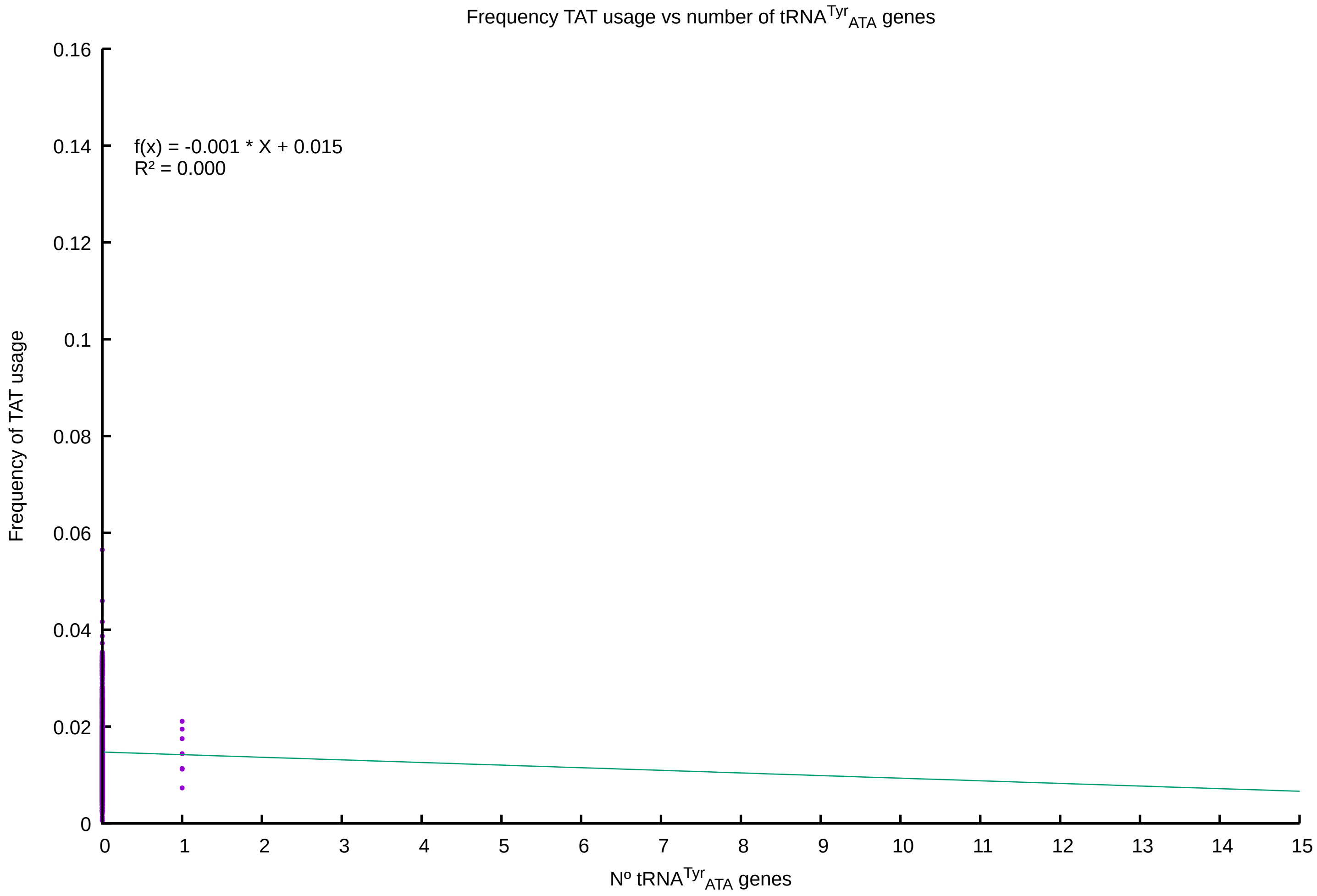

Frequency TAT usage vs number of tRNA Tyr ATA genes
f(x) = -0.001 * X + 0.015
R² = 0.000
Frequency of TAT usage
0.16
0.14
0.12
0.1
0.08
0.06
0.04
0.02
0
0 1 2 3 4 5 6 7 8 9 10 11 12 13 14 15
Nº tRNA Tyr ATA genes

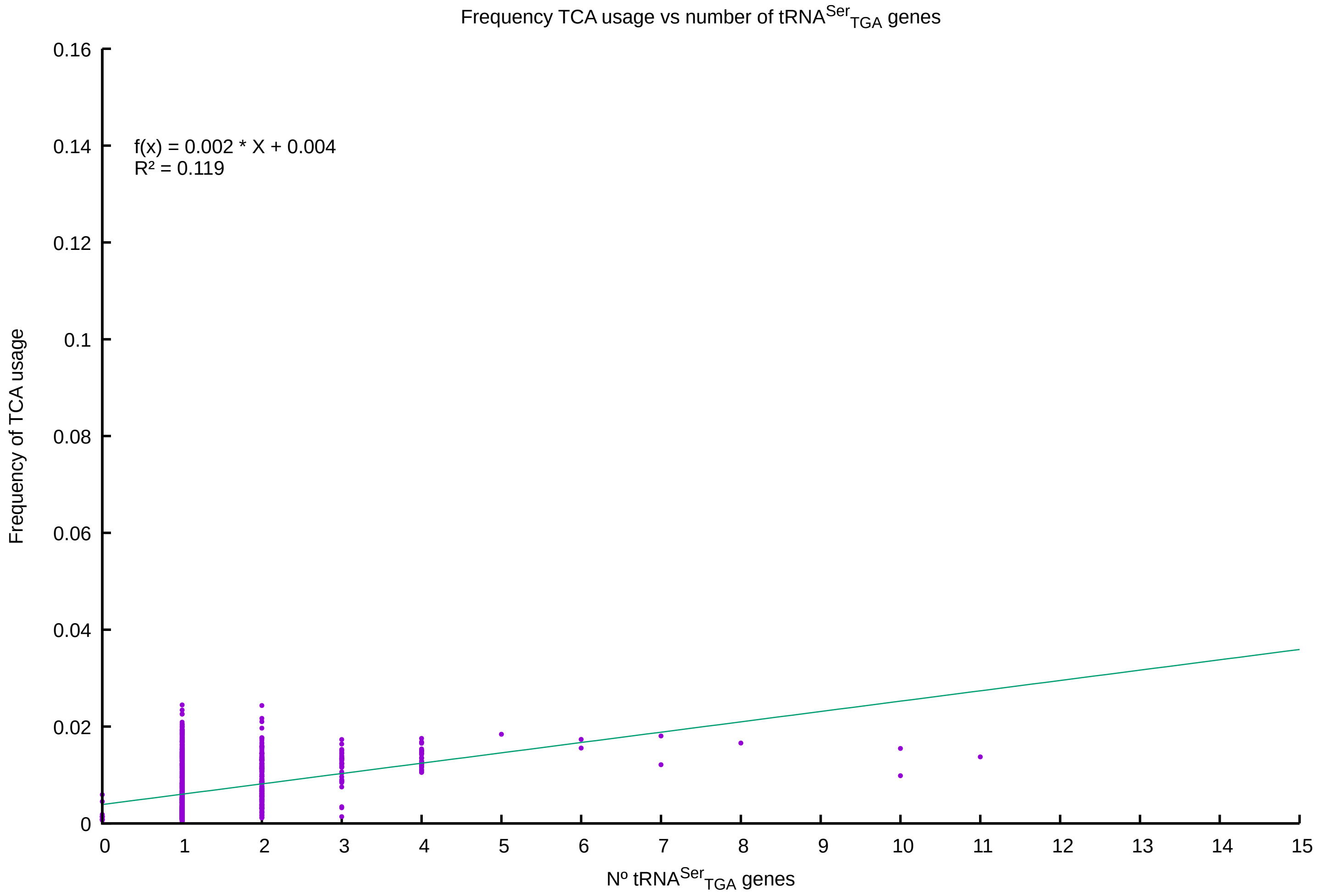
Frequency TCA usage vs number of tRNA^Ser_TGA genes
f(x) = 0.002 * X + 0.004
R² = 0.119
Frequency of TCA usage
0.16
0.14
0.12
0.1
0.08
0.06
0.04
0.02
0
0 1 2 3 4 5 6 7 8 9 10 11 12 13 14 15
Nº tRNA^Ser_TGA genes

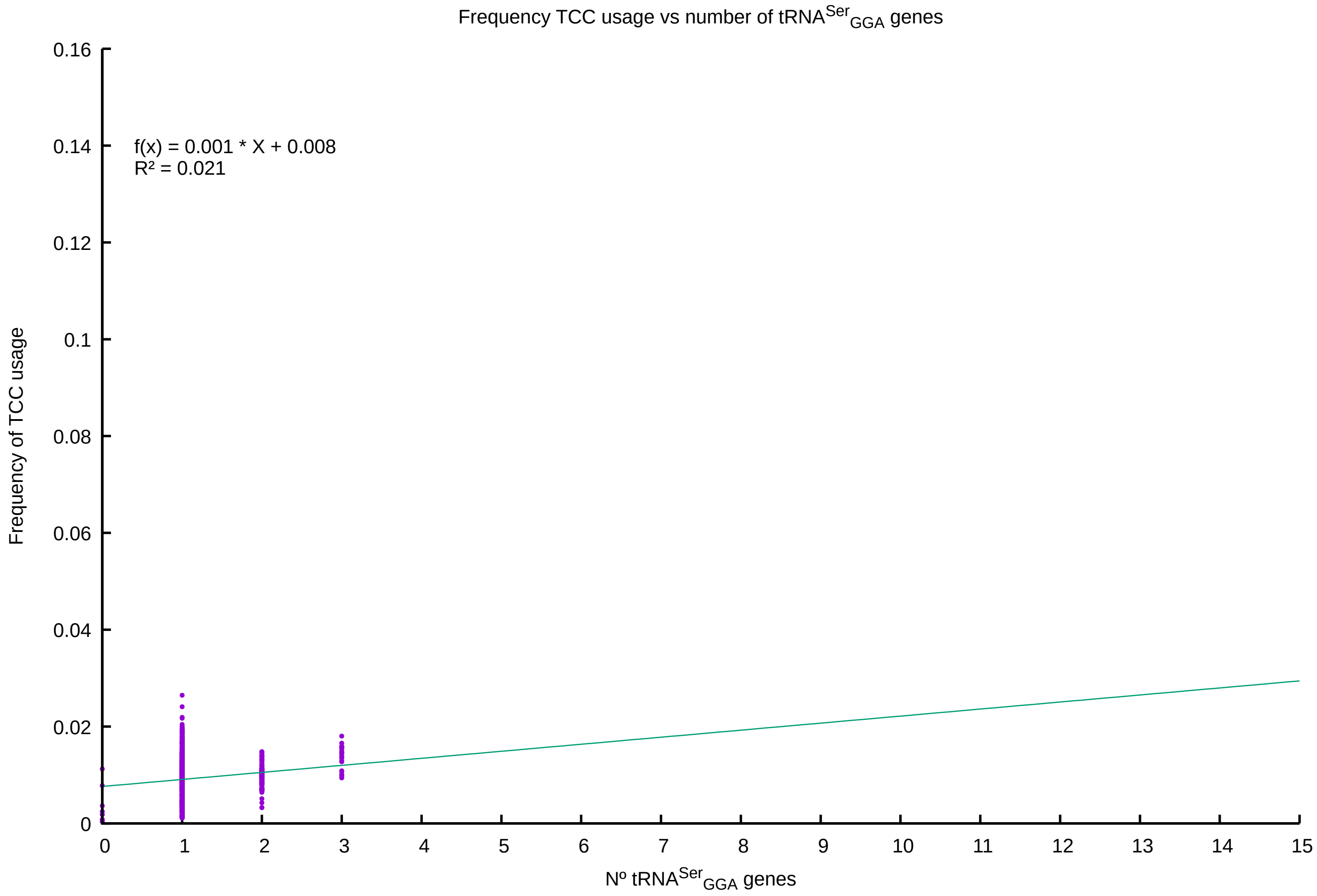

Frequency TCC usage vs number of tRNA$^{Ser}_{GGA}$ genes
f(x) = 0.001 * X + 0.008
R² = 0.021
Frequency of TCC usage
0.16
0.14
0.12
0.1
0.08
0.06
0.04
0.02
0
0 1 2 3 4 5 6 7 8 9 10 11 12 13 14 15
Nº tRNA$^{Ser}_{GGA}$ genes

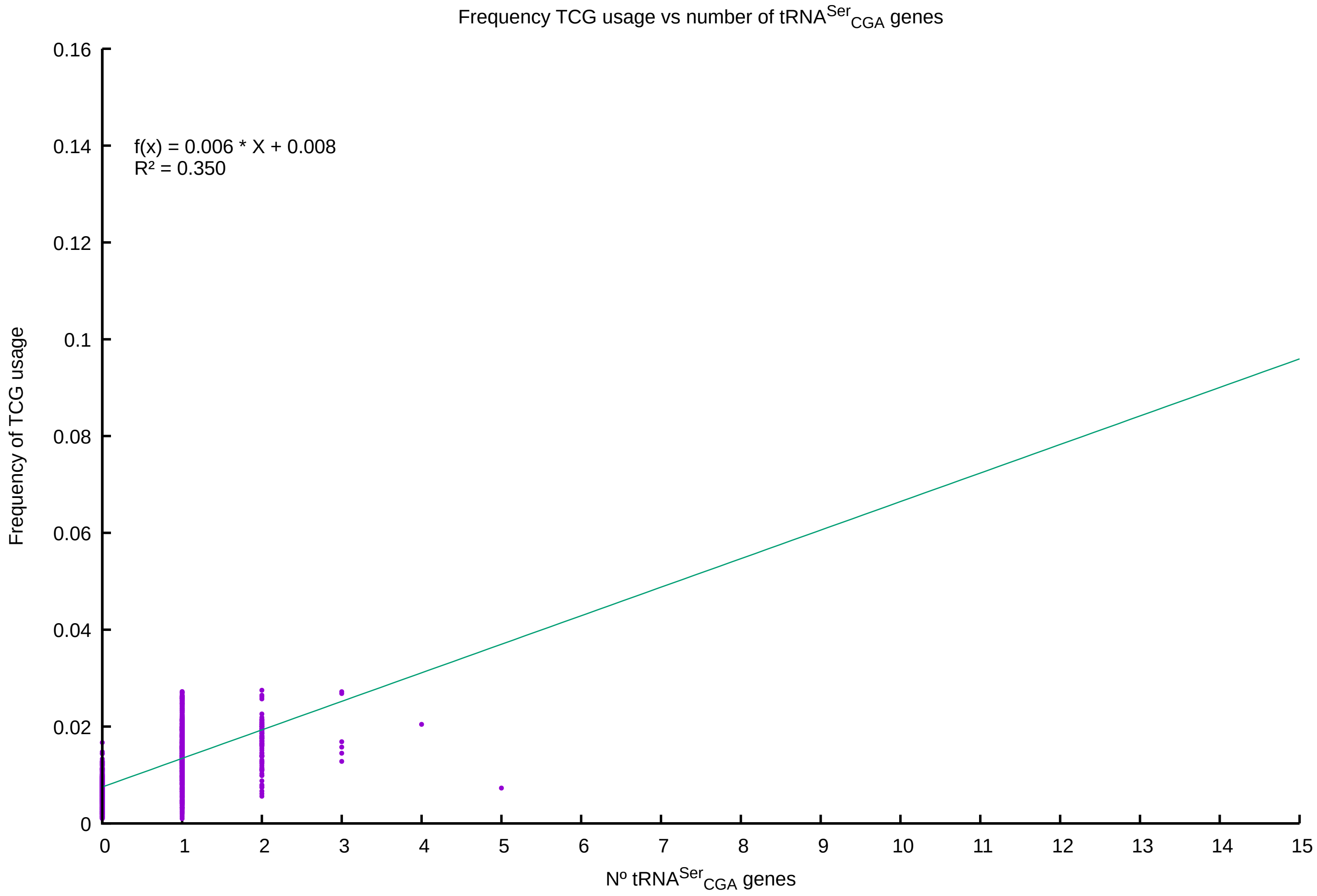

Frequency TCG usage vs number of tRNA$^{Ser}_{CGA}$ genes
f(x) = 0.006 * X + 0.008
R^2 = 0.350
Frequency of TCG usage
0.16
0.14
0.12
0.1
0.08
0.06
0.04
0.02
0
0 1 2 3 4 5 6 7 8 9 10 11 12 13 14 15
Nº tRNA$^{Ser}_{CGA}$ genes

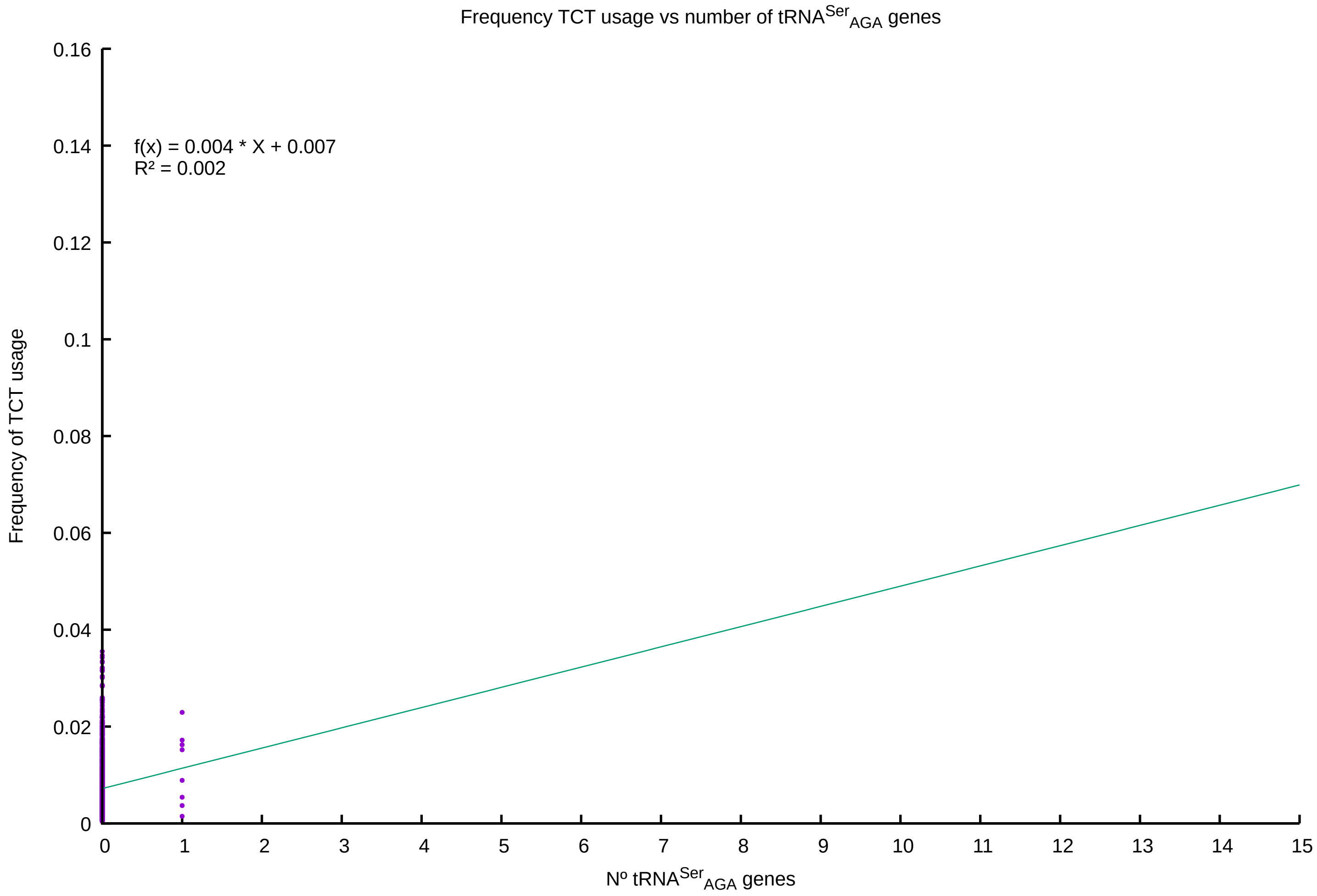
Frequency TCT usage vs number of tRNASerAGA genes
f(x) = 0.004 * X + 0.007
R² = 0.002
Frequency of TCT usage
0.16
0.14
0.12
0.1
0.08
0.06
0.04
0.02
0
0 1 2 3 4 5 6 7 8 9 10 11 12 13 14 15
Nº tRNASerAGA genes

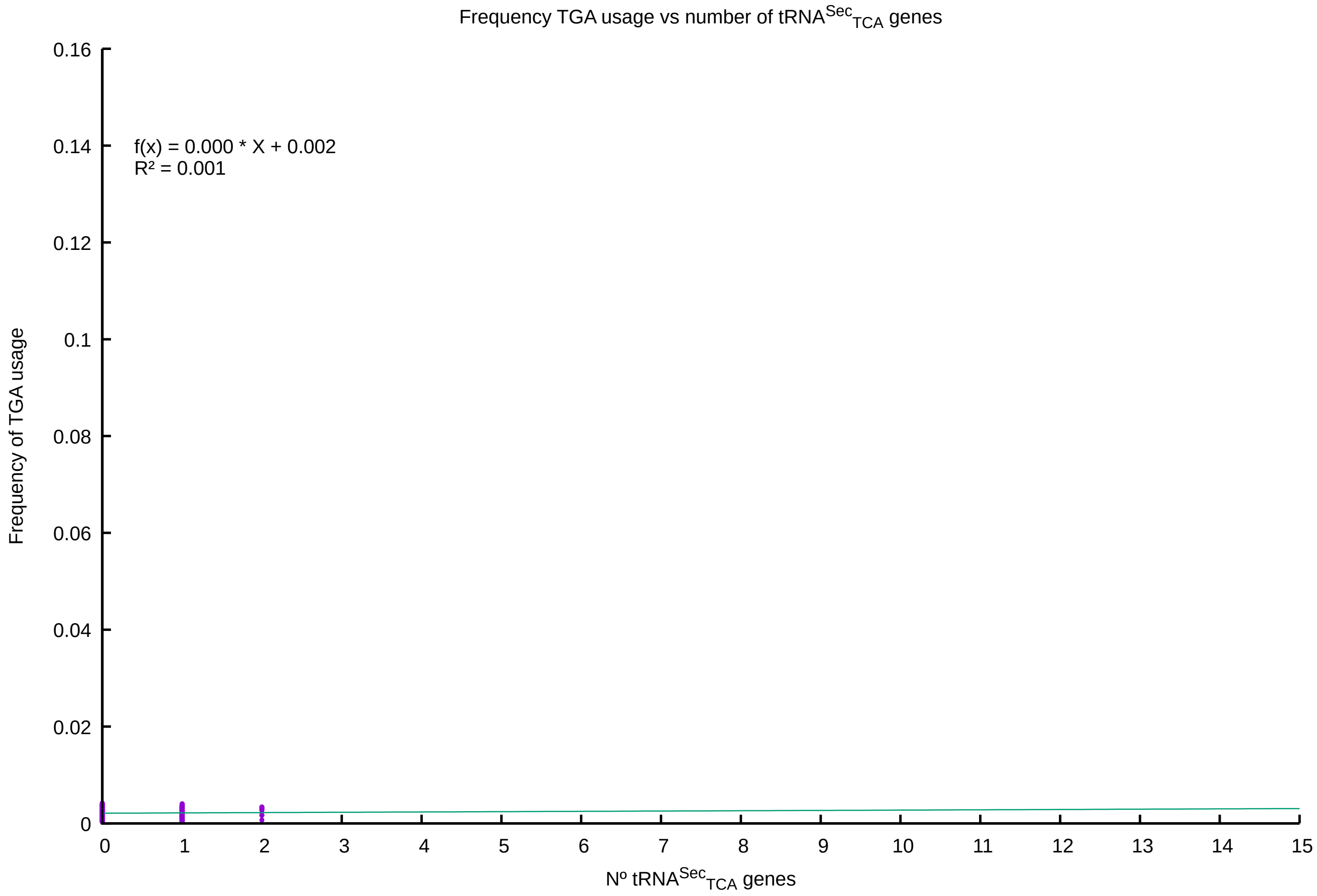
Frequency TGA usage vs number of tRNA^Sec_TCA genes
f(x) = 0.000 * X + 0.002
R² = 0.001
Frequency of TGA usage
0.16
0.14
0.12
0.1
0.08
0.06
0.04
0.02
0
0 1 2 3 4 5 6 7 8 9 10 11 12 13 14 15
Nº tRNA^Sec_TCA genes

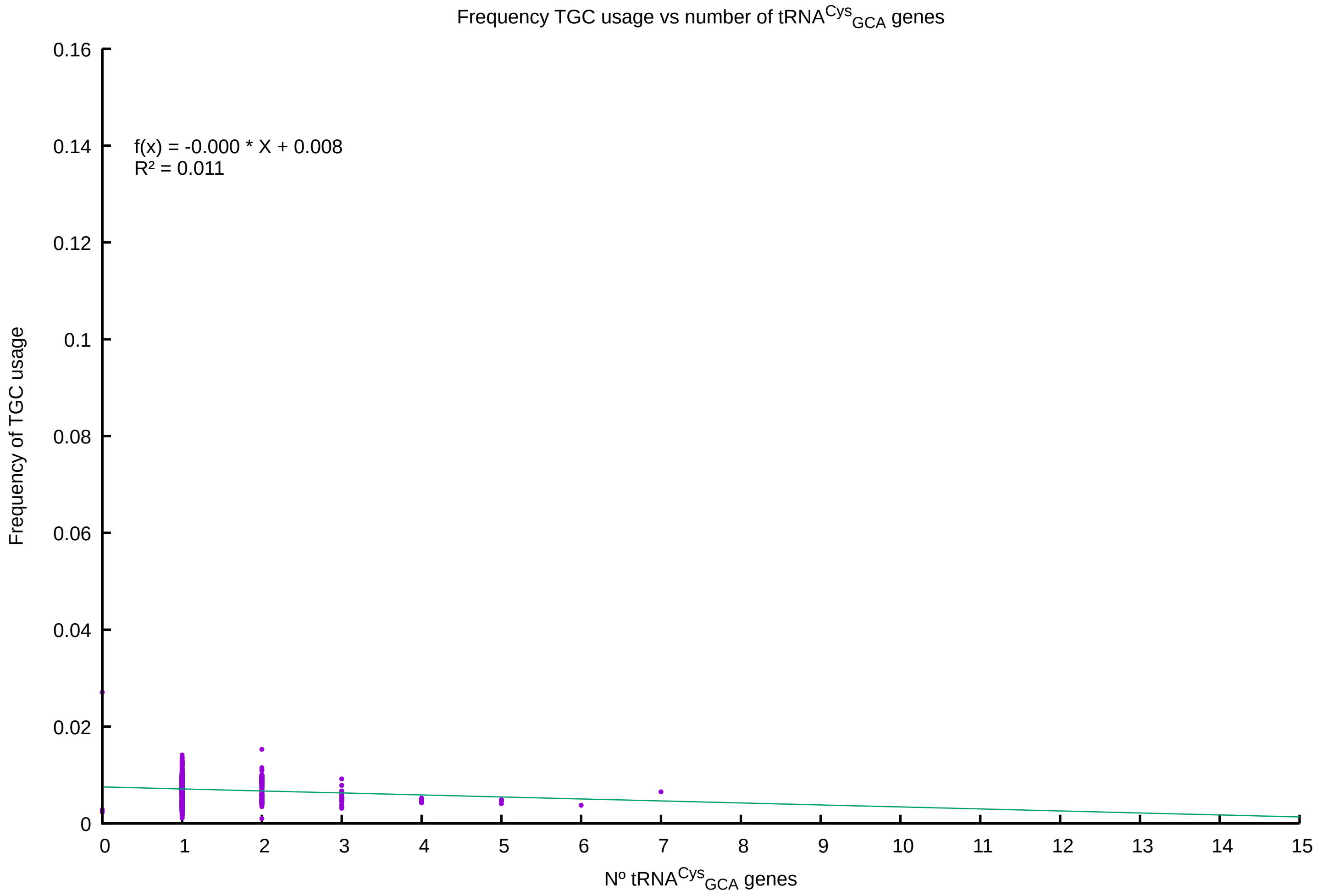

Frequency TGC usage vs number of tRNA$^{Cys}_{GCA}$ genes
f(x) = -0.000 * X + 0.008
R^2 = 0.011
Frequency of TGC usage
0.16
0.14
0.12
0.1
0.08
0.06
0.04
0.02
0
0 1 2 3 4 5 6 7 8 9 10 11 12 13 14 15
Nº tRNA$^{Cys}_{GCA}$ genes

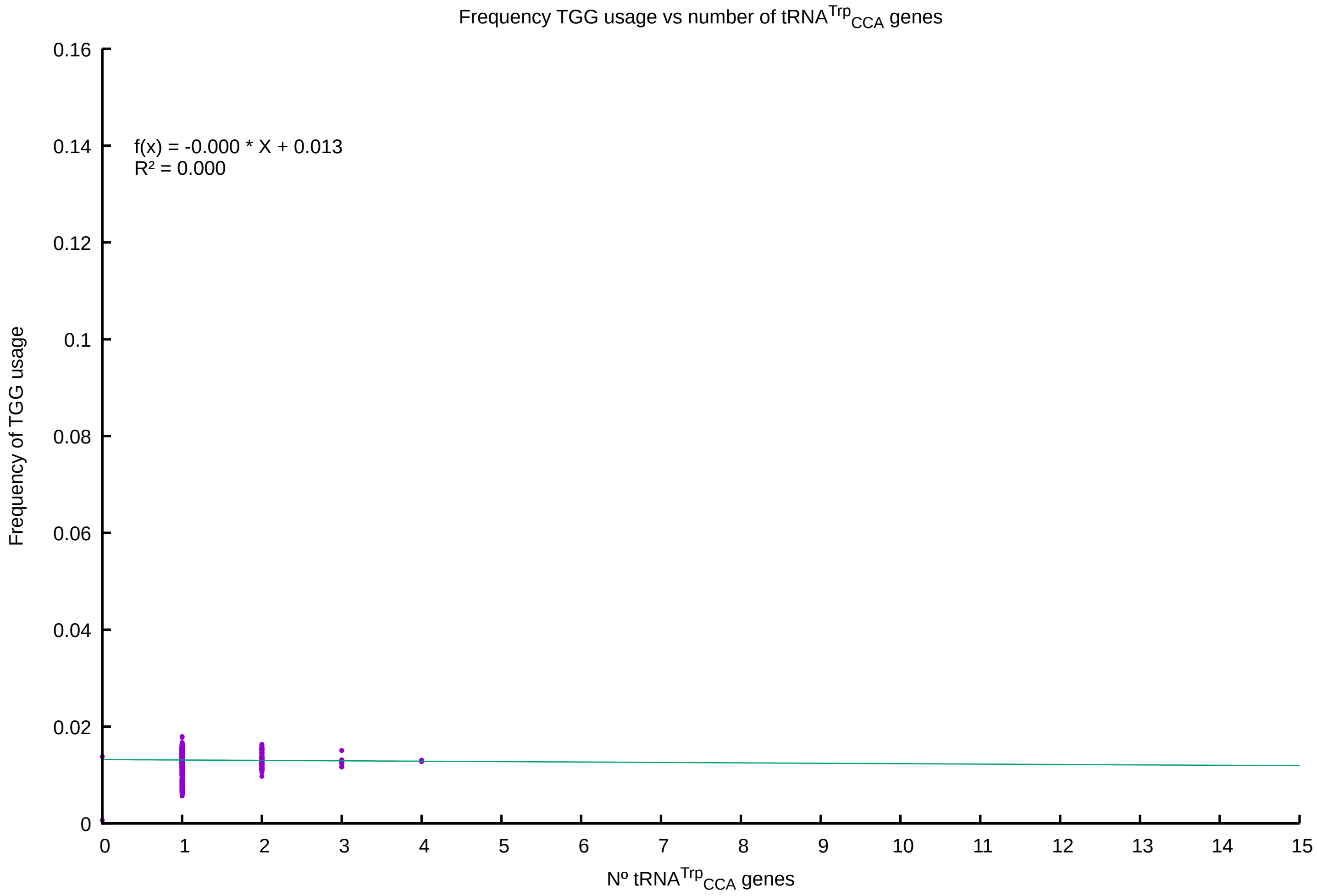

Frequency TGG usage vs number of tRNA^Trp_CCA genes
f(x) = -0.000 * X + 0.013
R² = 0.000
Frequency of TGG usage
0.16
0.14
0.12
0.1
0.08
0.06
0.04
0.02
0
0 1 2 3 4 5 6 7 8 9 10 11 12 13 14 15
Nº tRNA^Trp_CCA genes

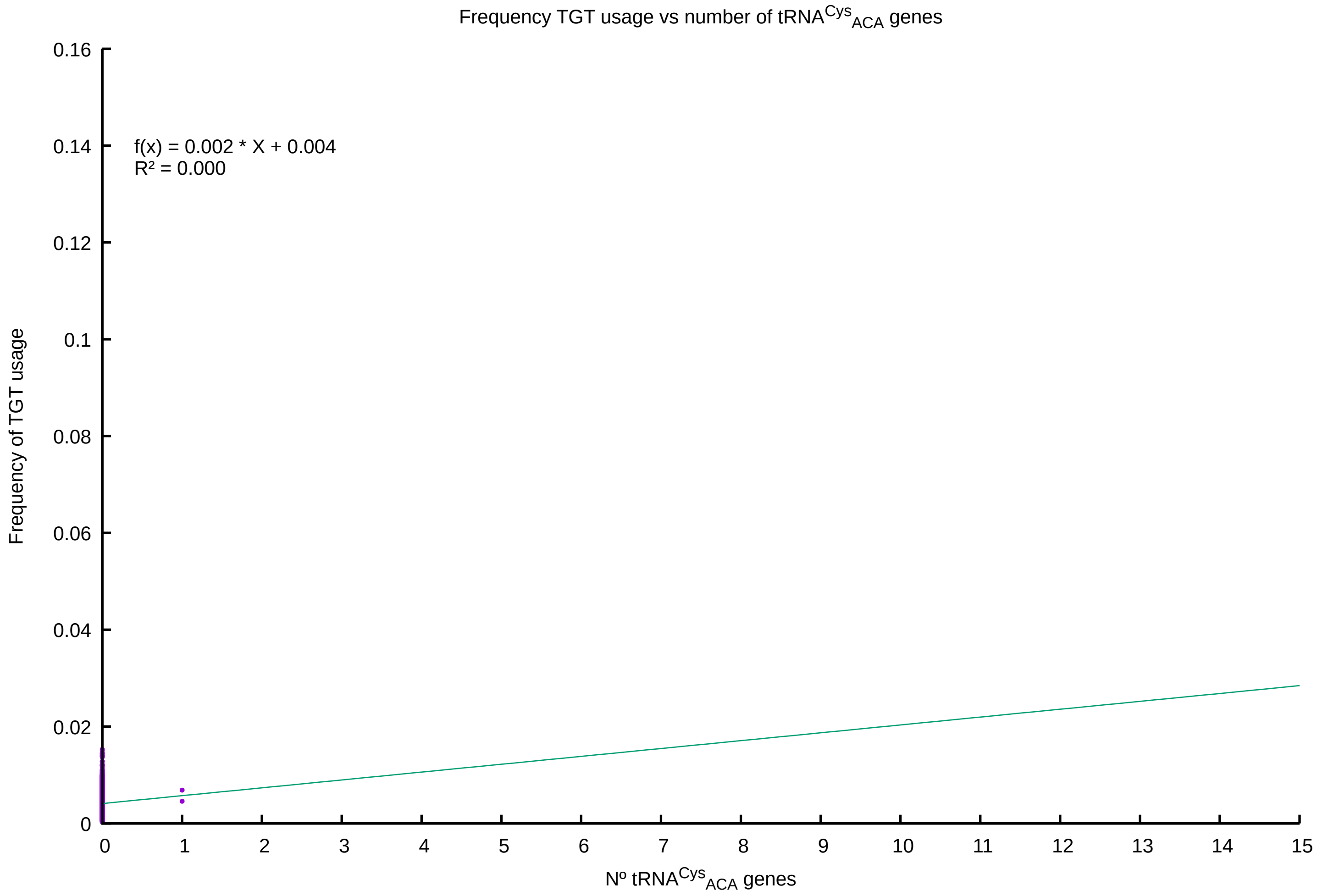

Frequency TGT usage vs number of tRNA^Cys_ACA genes
f(x) = 0.002 * X + 0.004
R² = 0.000
Frequency of TGT usage
0.16
0.14
0.12
0.1
0.08
0.06
0.04
0.02
0
0 1 2 3 4 5 6 7 8 9 10 11 12 13 14 15
Nº tRNA^Cys_ACA genes

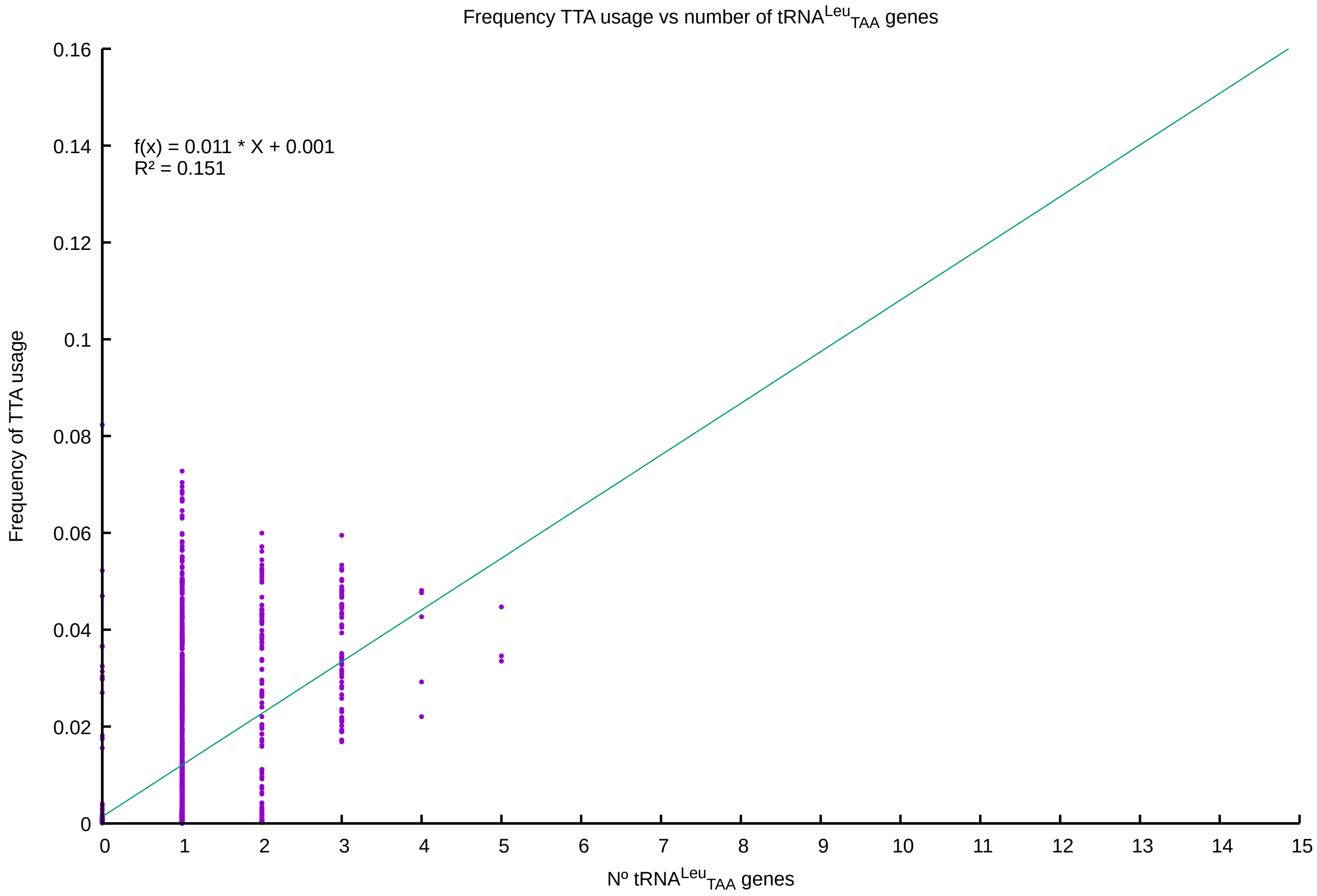

Frequency TTA usage vs number of tRNALeuTAA genes
f(x) = 0.011 * X + 0.001
R² = 0.151
Frequency of TTA usage
0.16
0.14
0.12
0.1
0.08
0.06
0.04
0.02
0
0 1 2 3 4 5 6 7 8 9 10 11 12 13 14 15
Nº tRNALeuTAA genes

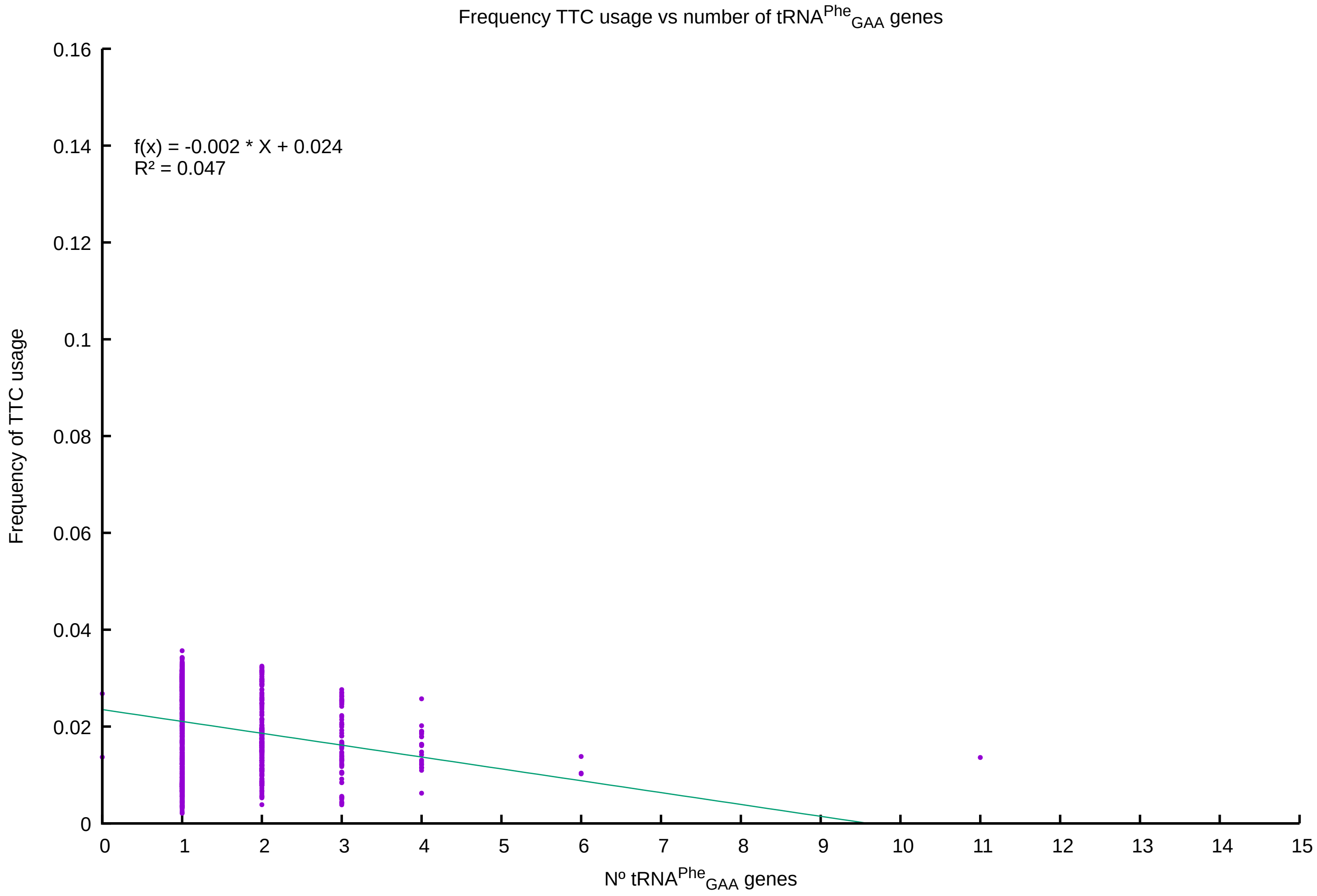

Frequency TTC usage vs number of tRNAPheGAA genes
f(x) = -0.002 * X + 0.024
R² = 0.047
Frequency of TTC usage
0.16
0.14
0.12
0.1
0.08
0.06
0.04
0.02
0
0 1 2 3 4 5 6 7 8 9 10 11 12 13 14 15
Nº tRNAPheGAA genes

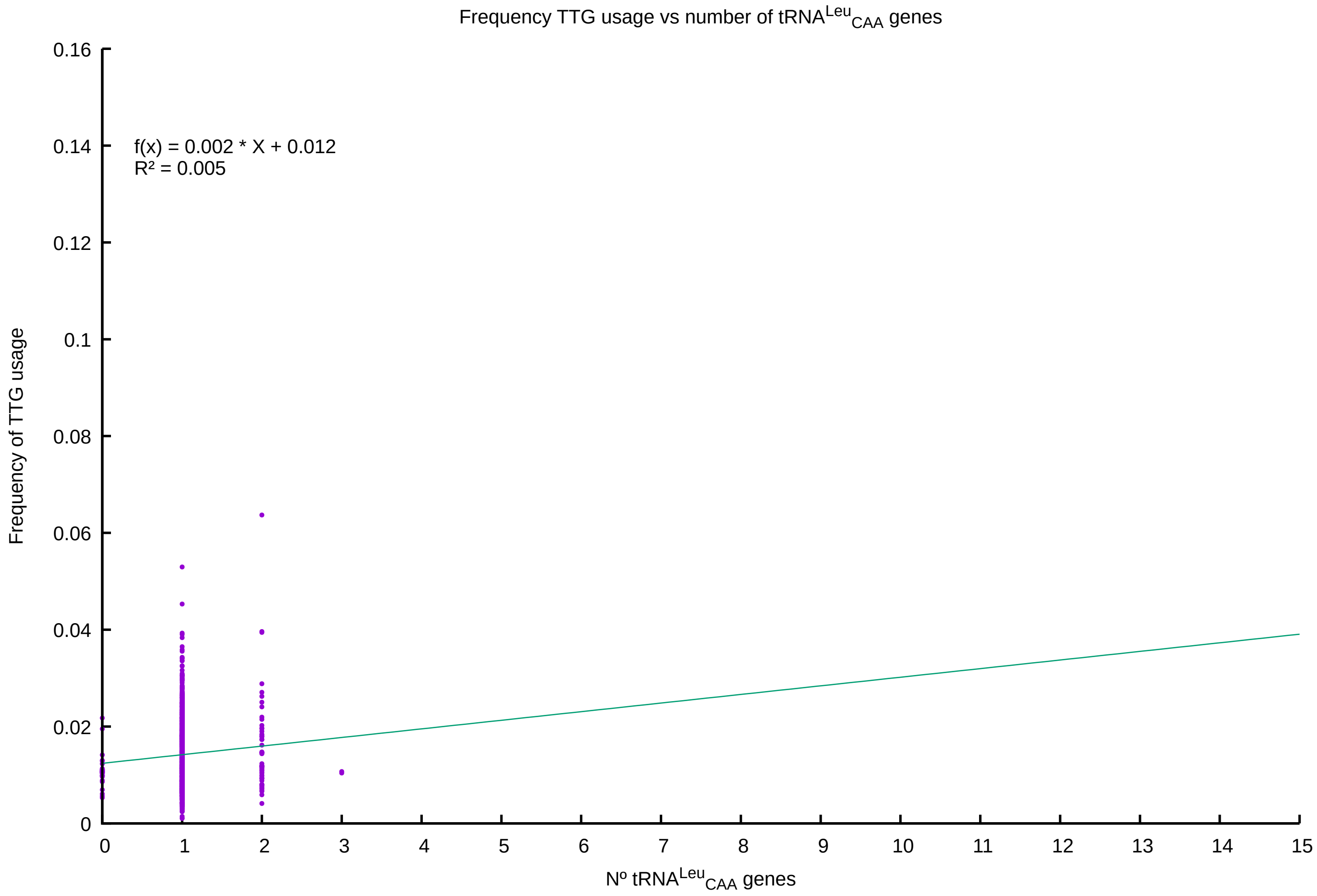

Frequency TTG usage vs number of tRNA$^{Leu}_{CAA}$ genes
f(x) = 0.002 * X + 0.012
R^2 = 0.005
Frequency of TTG usage
0.16
0.14
0.12
0.1
0.08
0.06
0.04
0.02
0
0 1 2 3 4 5 6 7 8 9 10 11 12 13 14 15
Nº tRNA$^{Leu}_{CAA}$ genes

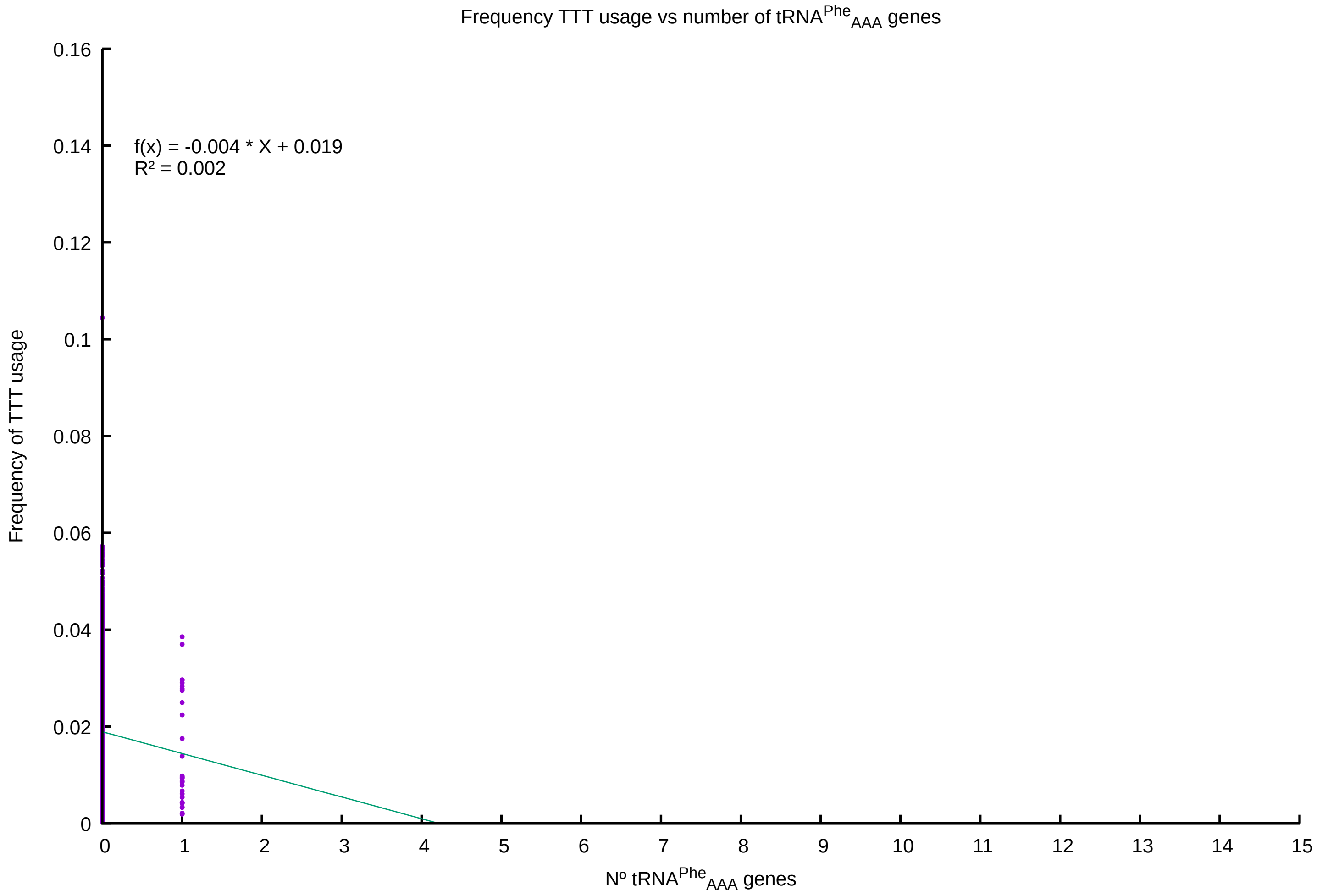

Frequency TTT usage vs number of tRNA^Phe_AAA genes
f(x) = -0.004 * X + 0.019
R² = 0.002
Frequency of TTT usage
Nº tRNA^Phe_AAA genes
0.16
0.14
0.12
0.1
0.08
0.06
0.04
0.02
0
0 1 2 3 4 5 6 7 8 9 10 11 12 13 14 15
