# Supplementary material for: Impact of the chemical modification of tRNAs anticodon loop on the variability and evolution of codon usage in proteobacteria
Source: Front Microbiol. 2024 Aug 5;15:1412318. doi: 10.3389/fmicb.2024.1412318 (PMC11332805; doi:10.3389/fmicb.2024.1412318)
Supplement: Supplementary file 1 [file Data_Sheet_1.zip › Supp_figures/Fig_S24B.pdf]

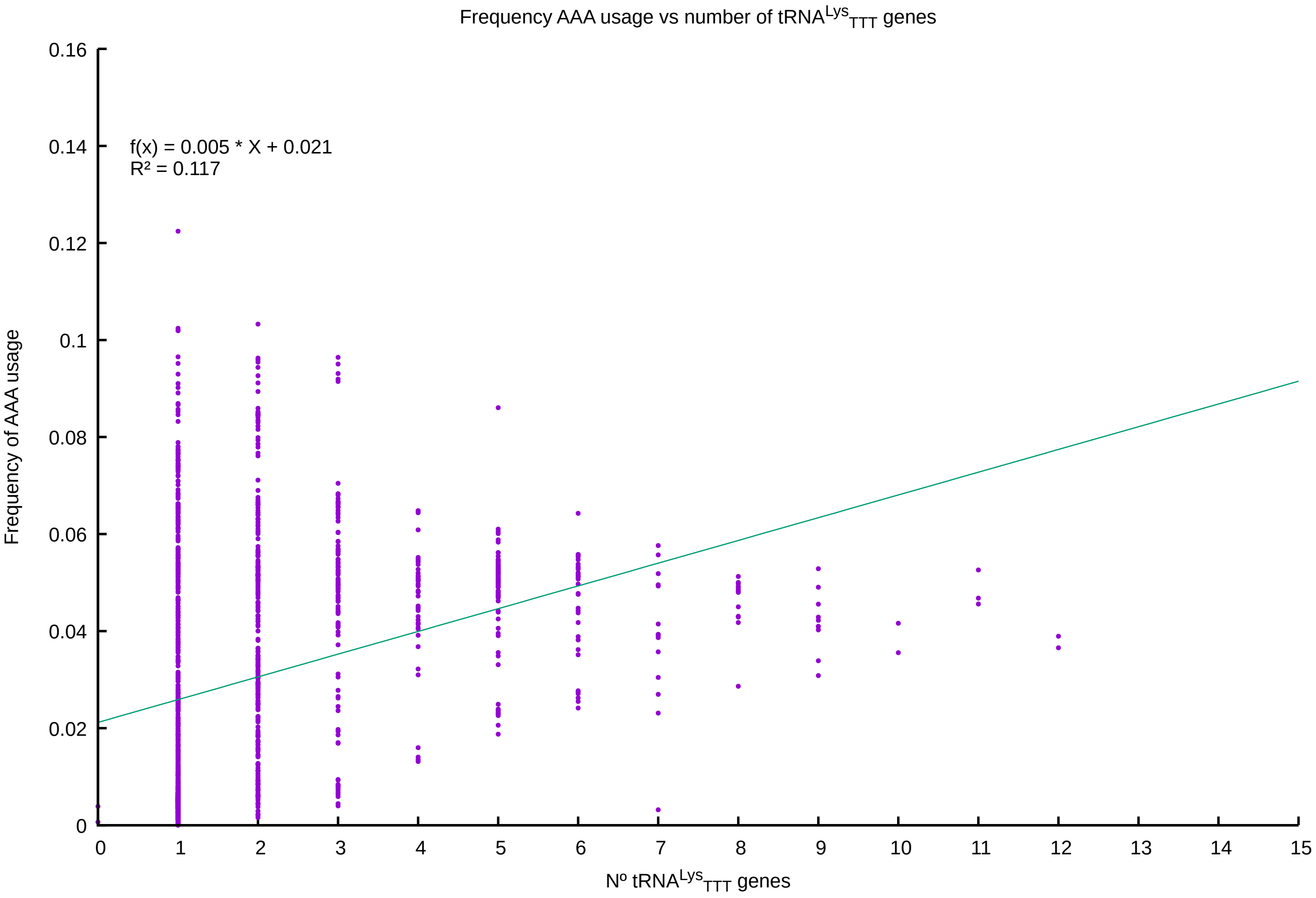
Frequency AAA usage vs number of tRNA^Lys_TTT genes
f(x) = 0.005 * X + 0.021
R² = 0.117
Frequency of AAA usage
0.16
0.14
0.12
0.1
0.08
0.06
0.04
0.02
0
0 1 2 3 4 5 6 7 8 9 10 11 12 13 14 15
Nº tRNA^Lys_TTT genes

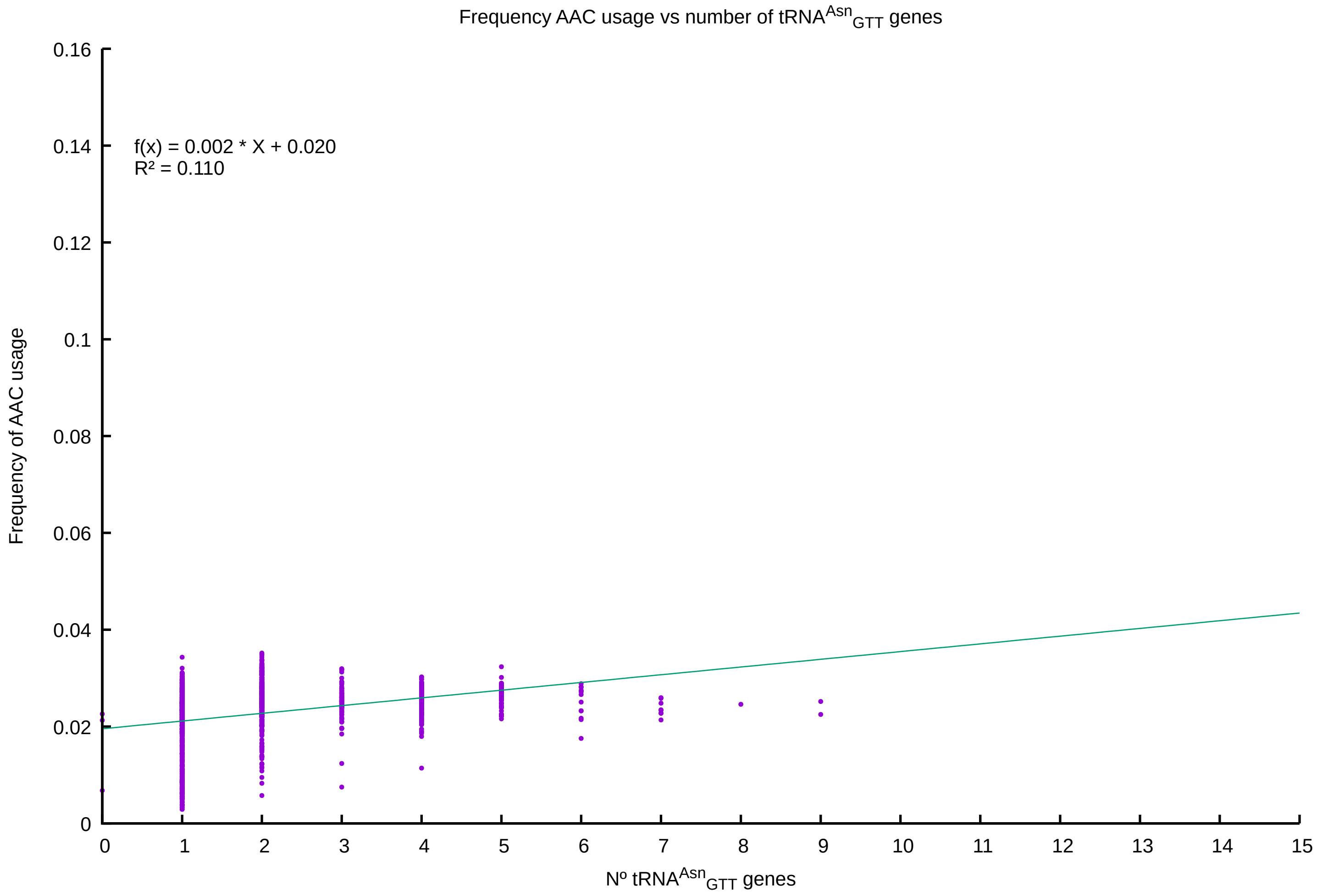
Frequency AAC usage vs number of tRNA^Asn_GTT genes
f(x) = 0.002 * X + 0.020
R² = 0.110
Frequency of AAC usage
0.16
0.14
0.12
0.1
0.08
0.06
0.04
0.02
0
0 1 2 3 4 5 6 7 8 9 10 11 12 13 14 15
Nº tRNA^Asn_GTT genes

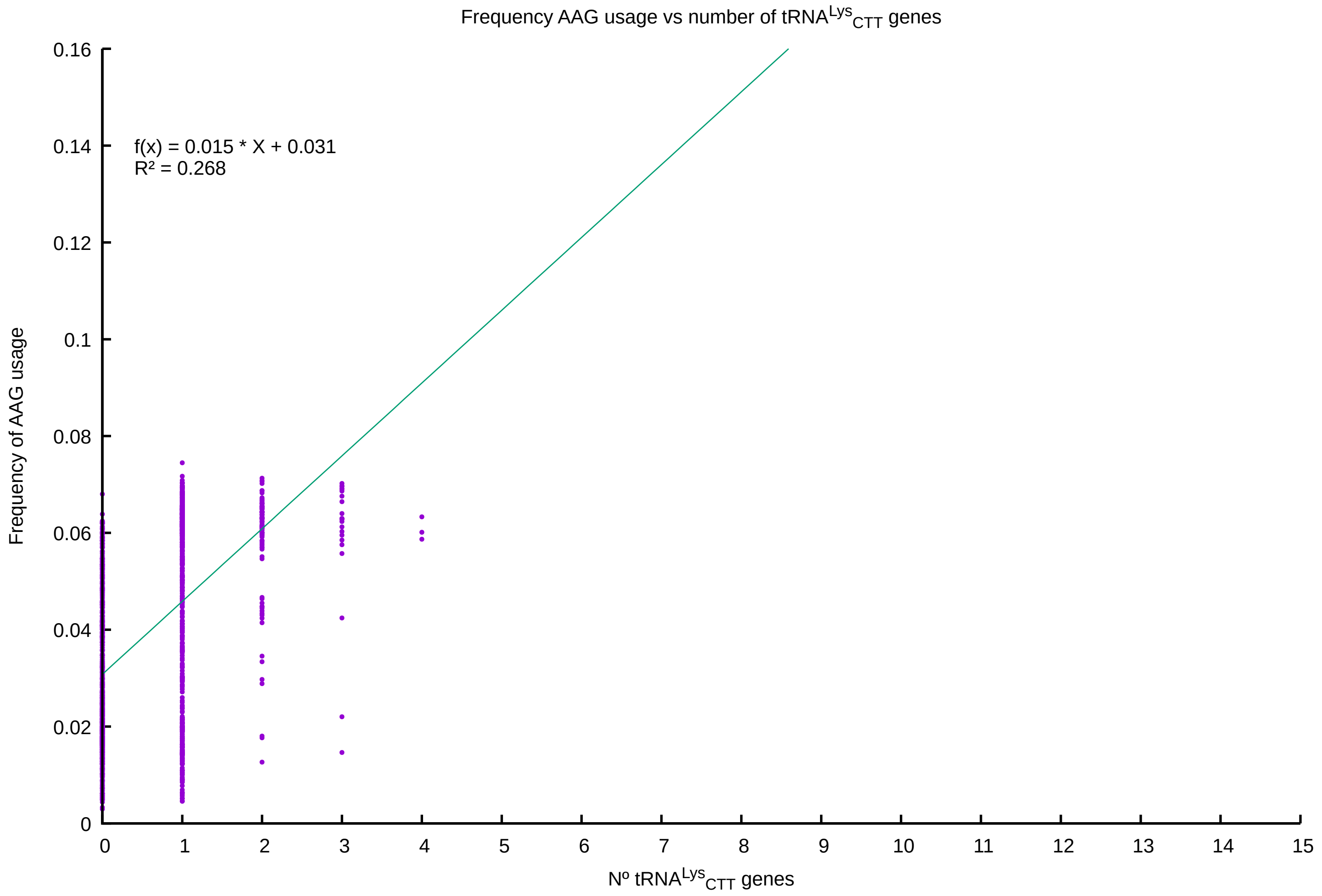

Frequency AAG usage vs number of tRNA$^{Lys}_{CTT}$ genes
f(x) = 0.015 * X + 0.031
R² = 0.268
Frequency of AAG usage
Nº tRNA$^{Lys}_{CTT}$ genes
0.16
0.14
0.12
0.1
0.08
0.06
0.04
0.02
0
0 1 2 3 4 5 6 7 8 9 10 11 12 13 14 15

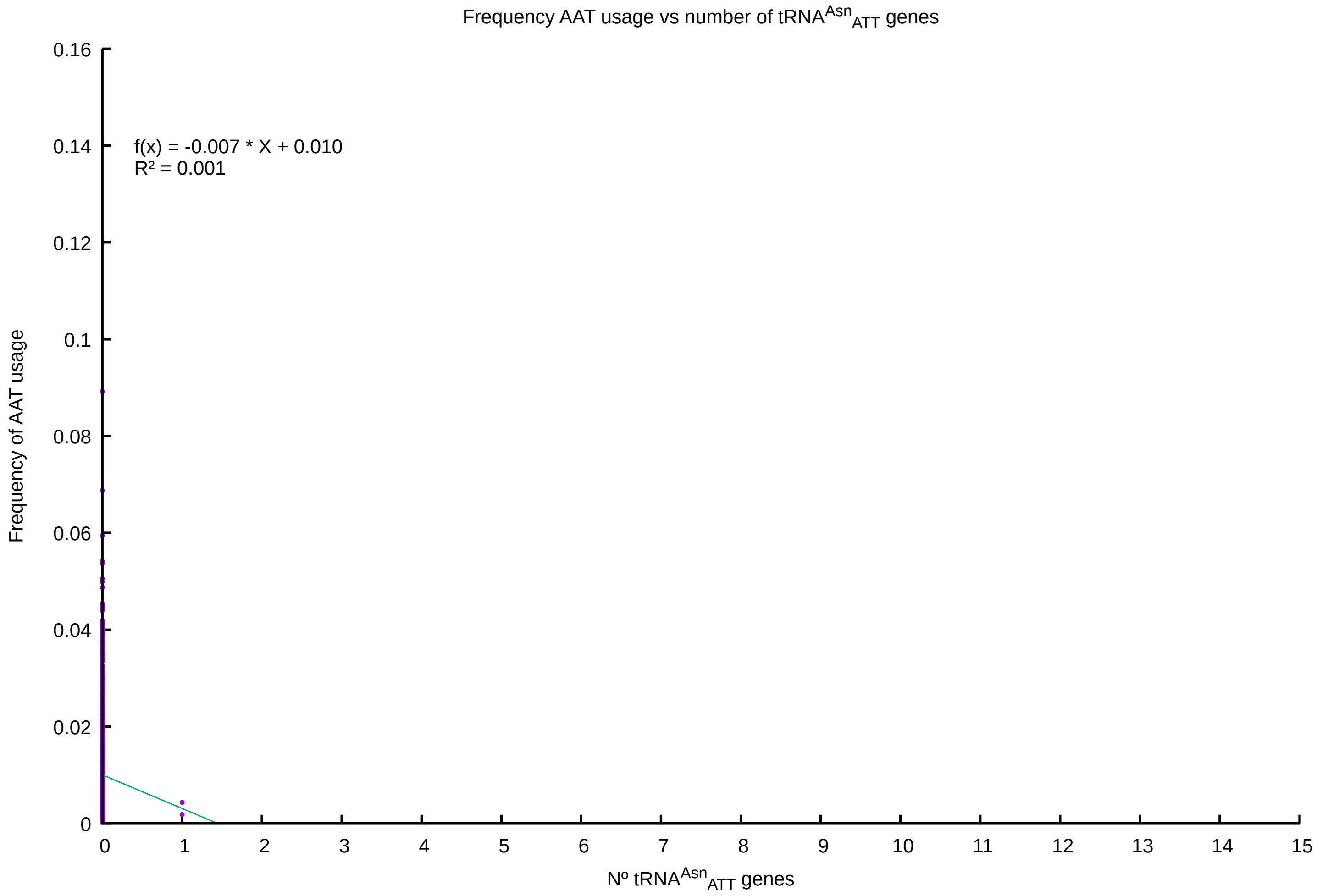

Frequency AAT usage vs number of tRNA^Asn_ATT genes
f(x) = -0.007 * X + 0.010
R² = 0.001
Frequency of AAT usage
0.16
0.14
0.12
0.1
0.08
0.06
0.04
0.02
0
0 1 2 3 4 5 6 7 8 9 10 11 12 13 14 15
Nº tRNA^Asn_ATT genes

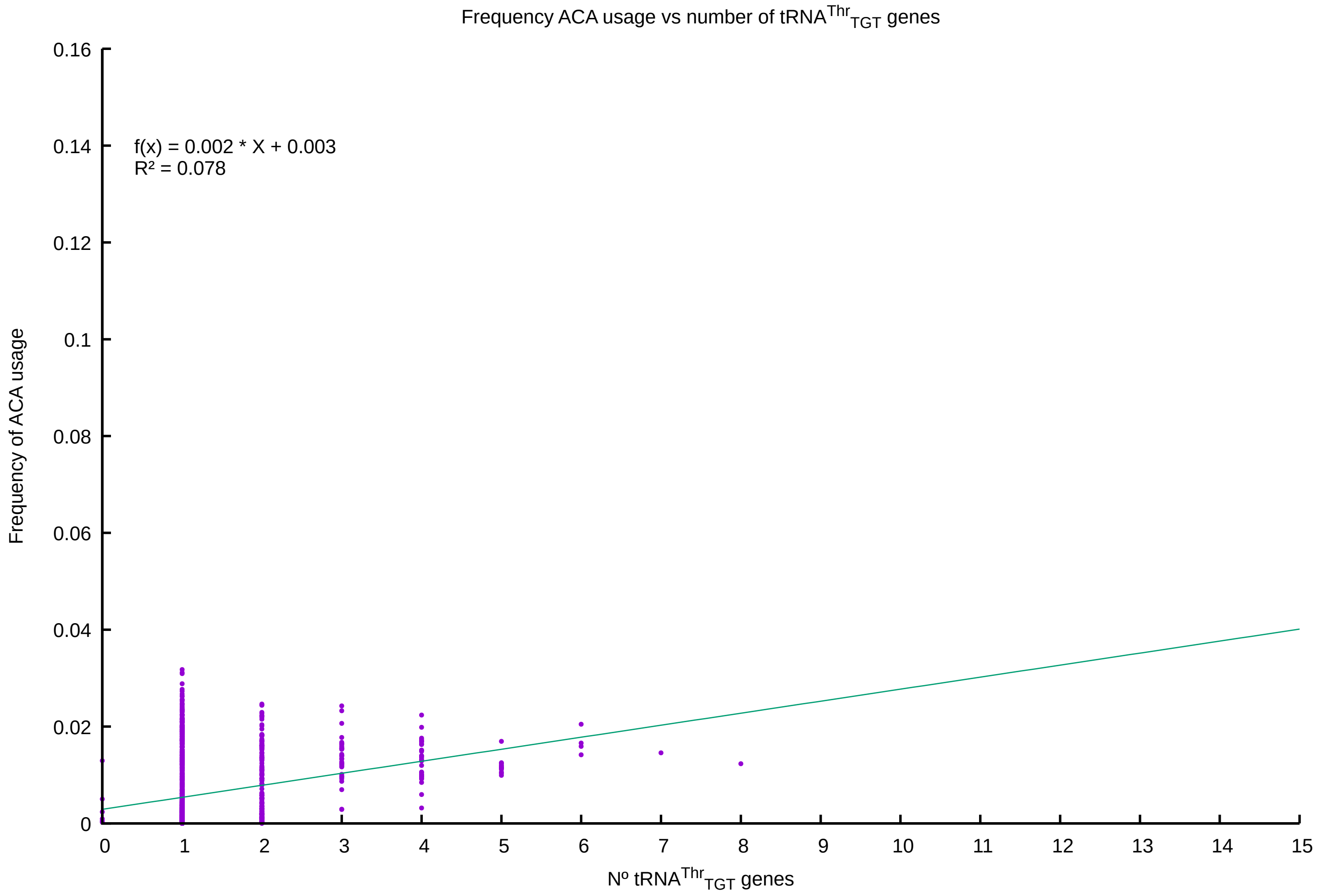

Frequency ACA usage vs number of tRNAThrTGT genes
f(x) = 0.002 * X + 0.003
R² = 0.078
Frequency of ACA usage
0.16
0.14
0.12
0.1
0.08
0.06
0.04
0.02
0
0 1 2 3 4 5 6 7 8 9 10 11 12 13 14 15
Nº tRNAThrTGT genes

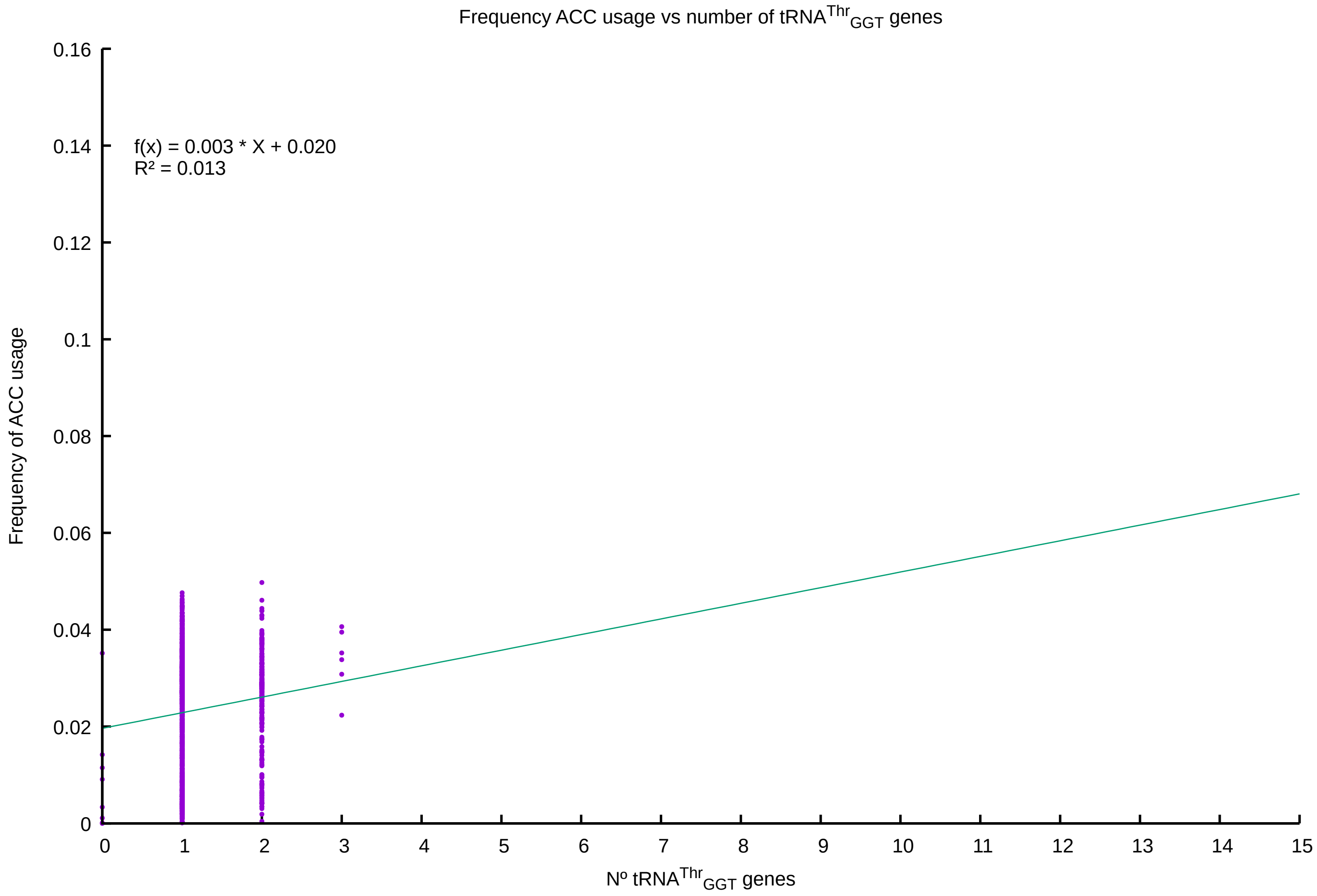
Frequency ACC usage vs number of tRNA^Thr_GGT genes
f(x) = 0.003 * X + 0.020
R² = 0.013
Frequency of ACC usage
Nº tRNA^Thr_GGT genes
0.16
0.14
0.12
0.1
0.08
0.06
0.04
0.02
0
0 1 2 3 4 5 6 7 8 9 10 11 12 13 14 15

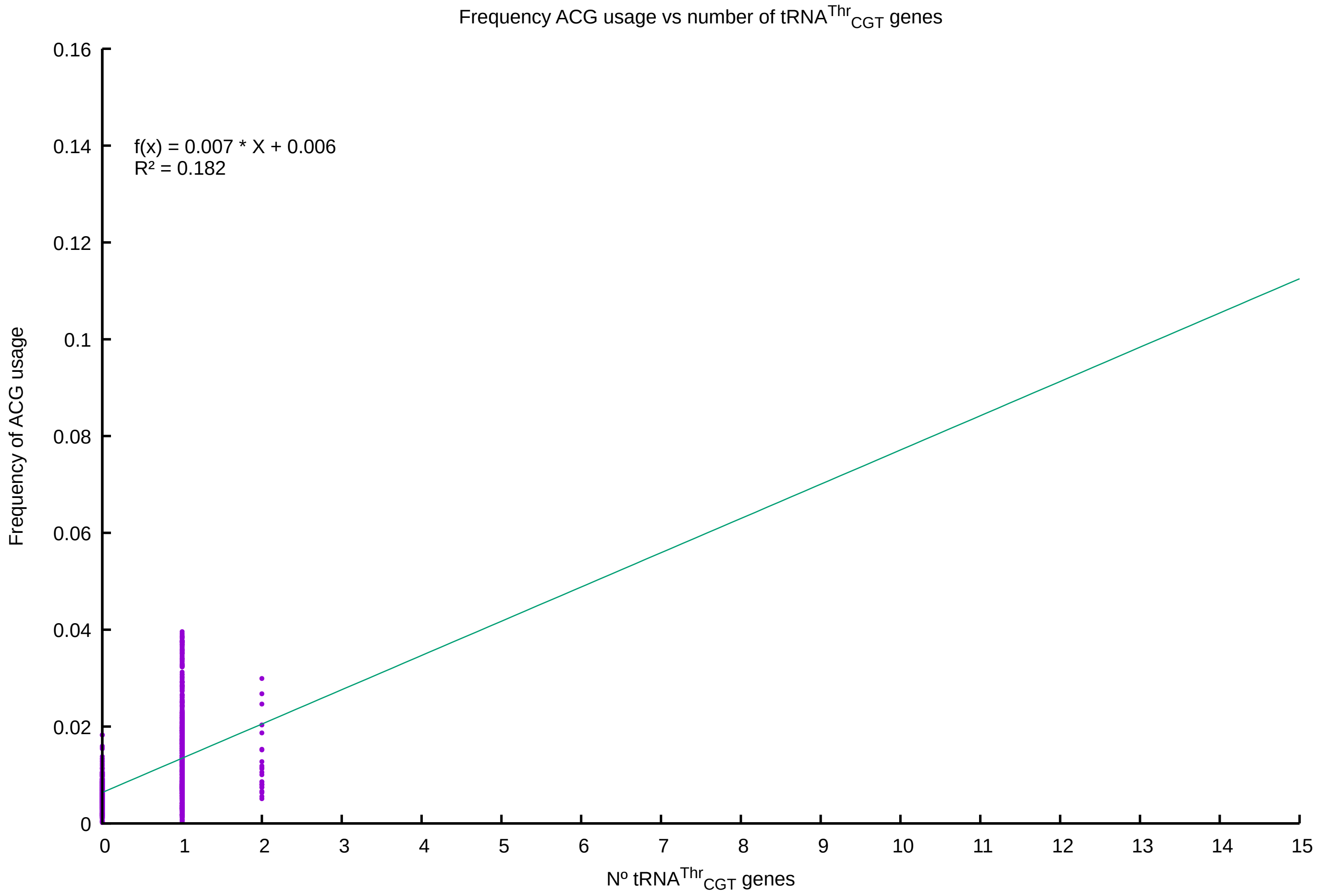

Frequency ACG usage vs number of tRNAThrCGT genes
f(x) = 0.007 * X + 0.006
R² = 0.182
Frequency of ACG usage
0
0.02
0.04
0.06
0.08
0.1
0.12
0.14
0.16
0 1 2 3 4 5 6 7 8 9 10 11 12 13 14 15
Nº tRNAThrCGT genes

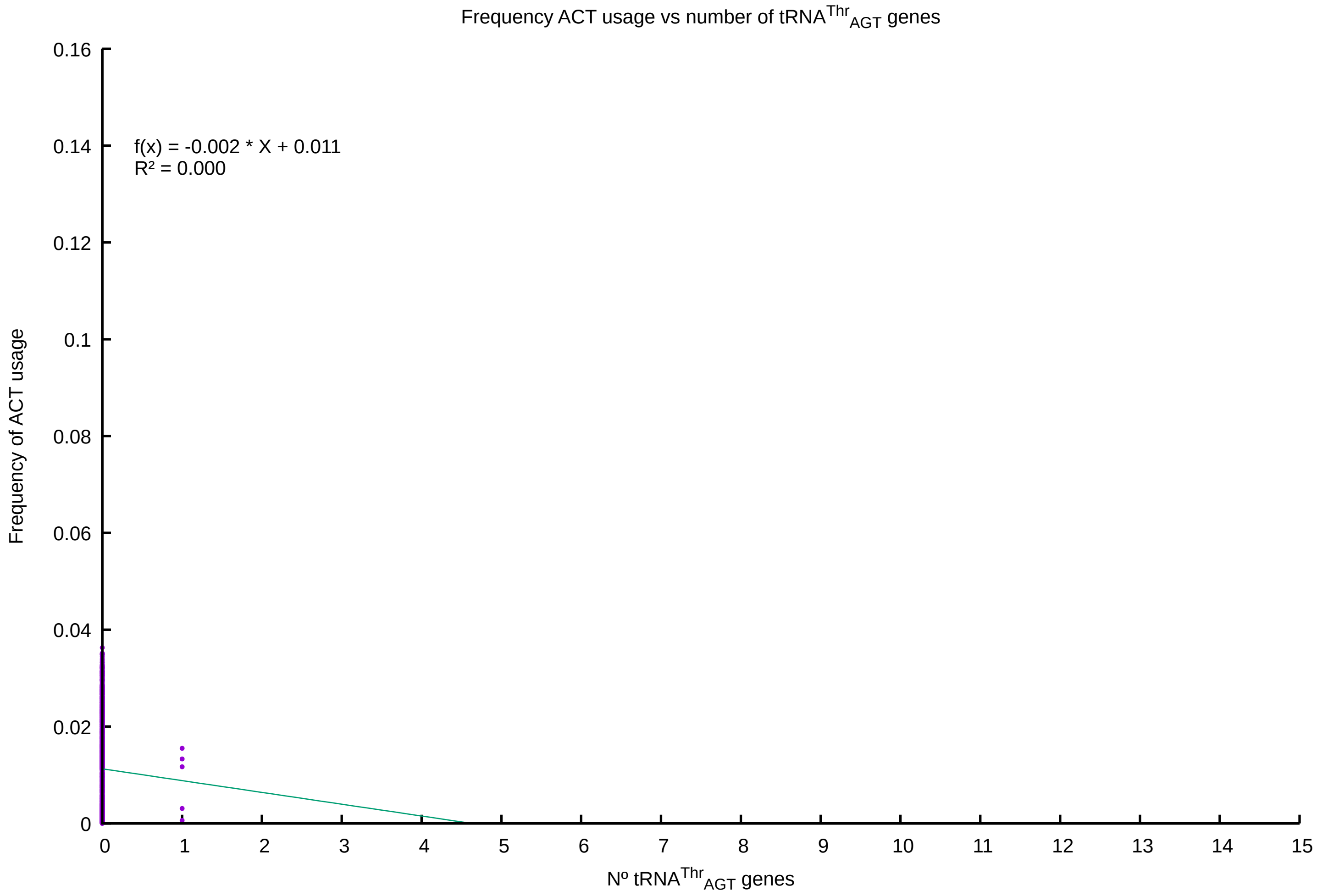
Frequency ACT usage vs number of tRNAThrAGT genes
f(x) = -0.002 * X + 0.011
R² = 0.000
Frequency of ACT usage
0.16
0.14
0.12
0.1
0.08
0.06
0.04
0.02
0
0 1 2 3 4 5 6 7 8 9 10 11 12 13 14 15
Nº tRNAThrAGT genes

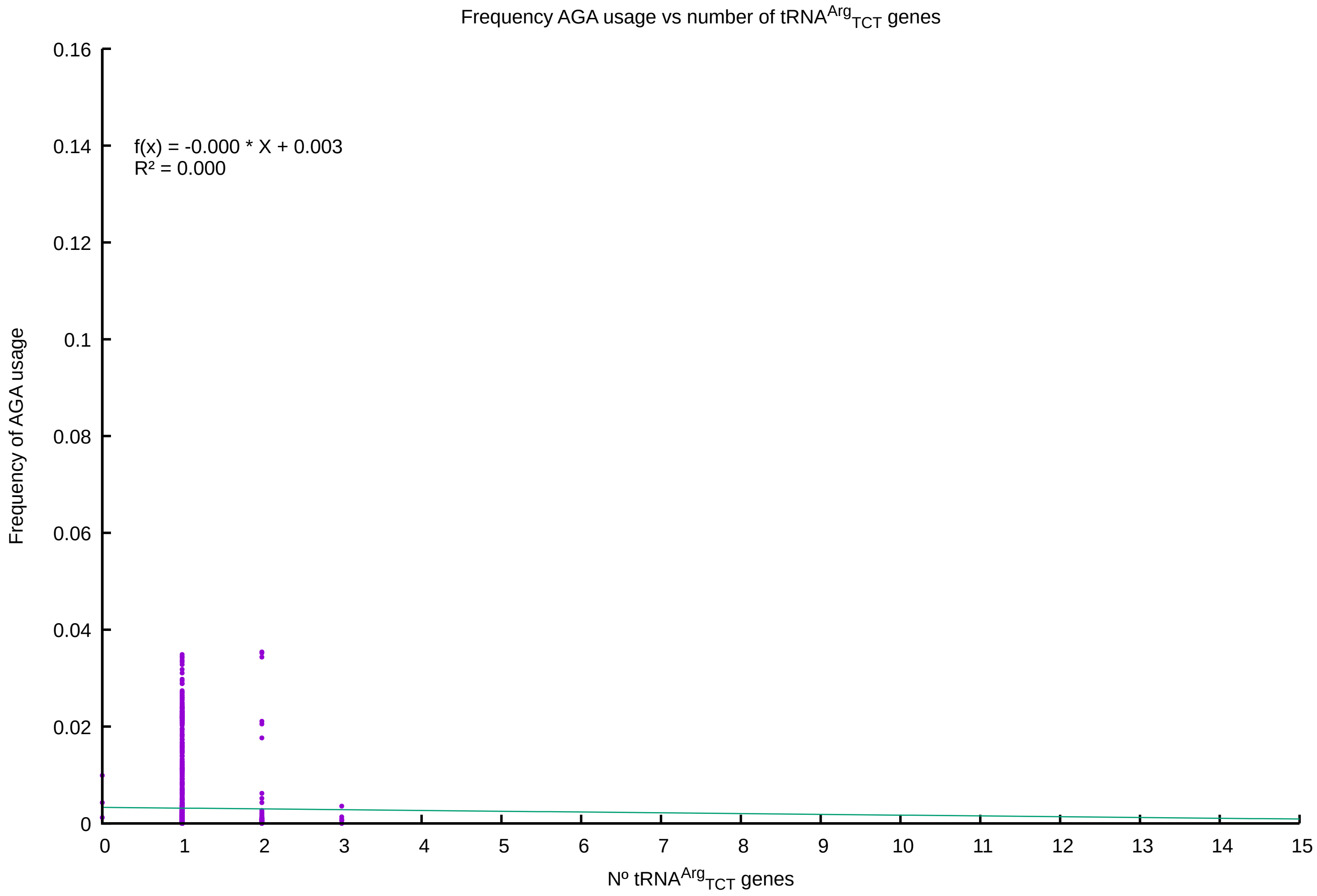

Frequency AGA usage vs number of tRNA^Arg_TCT genes
f(x) = -0.000 * X + 0.003
R² = 0.000
Frequency of AGA usage
0.16
0.14
0.12
0.1
0.08
0.06
0.04
0.02
0
0 1 2 3 4 5 6 7 8 9 10 11 12 13 14 15
Nº tRNA^Arg_TCT genes

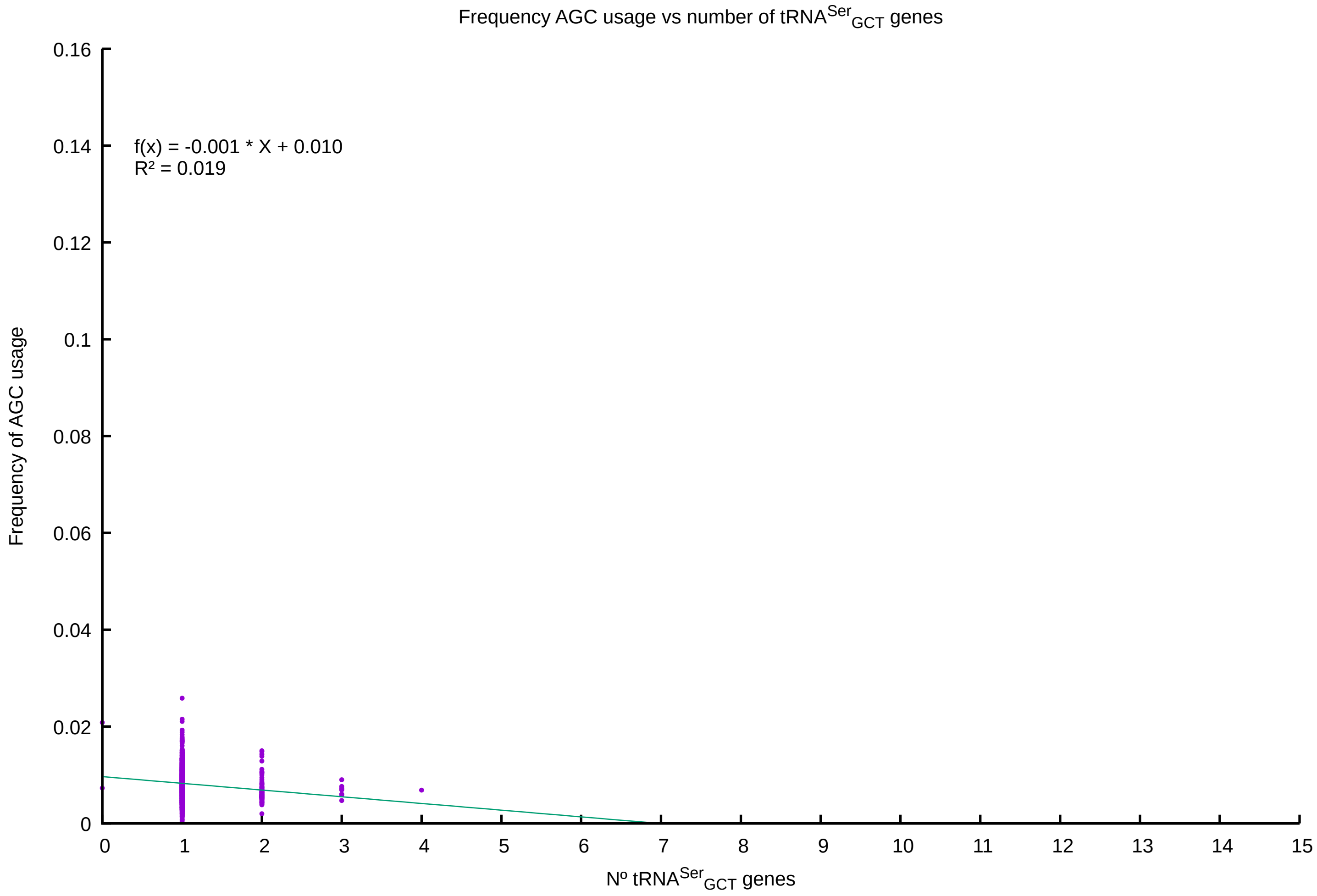

Frequency AGC usage vs number of tRNA^Ser_GCT genes
f(x) = -0.001 * X + 0.010
R² = 0.019
Frequency of AGC usage
0.16
0.14
0.12
0.1
0.08
0.06
0.04
0.02
0
0 1 2 3 4 5 6 7 8 9 10 11 12 13 14 15
Nº tRNA^Ser_GCT genes

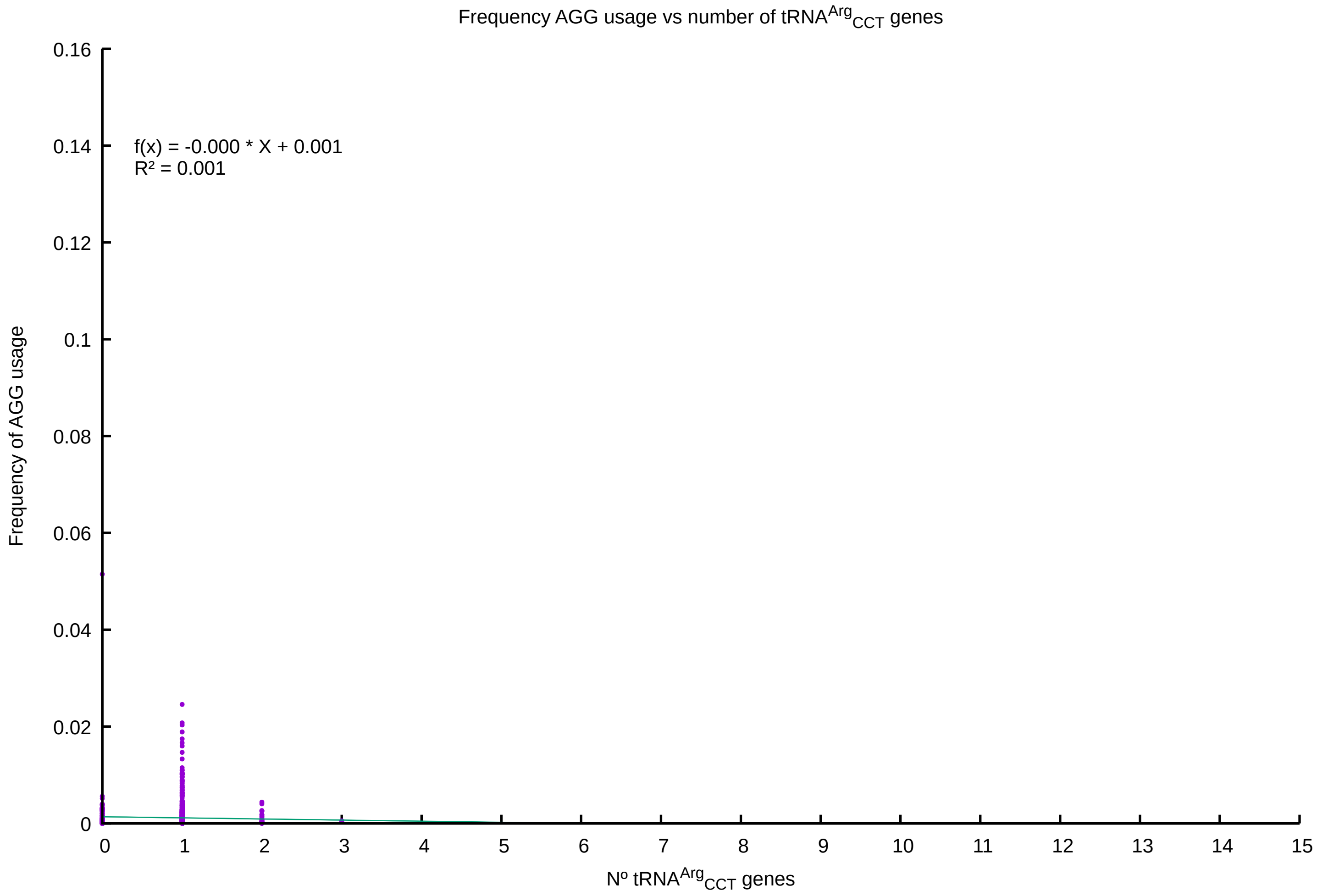

Frequency AGG usage vs number of tRNA^Arg_CCT genes
f(x) = -0.000 * X + 0.001
R² = 0.001
Frequency of AGG usage
0.16
0.14
0.12
0.1
0.08
0.06
0.04
0.02
0
0 1 2 3 4 5 6 7 8 9 10 11 12 13 14 15
Nº tRNA^Arg_CCT genes

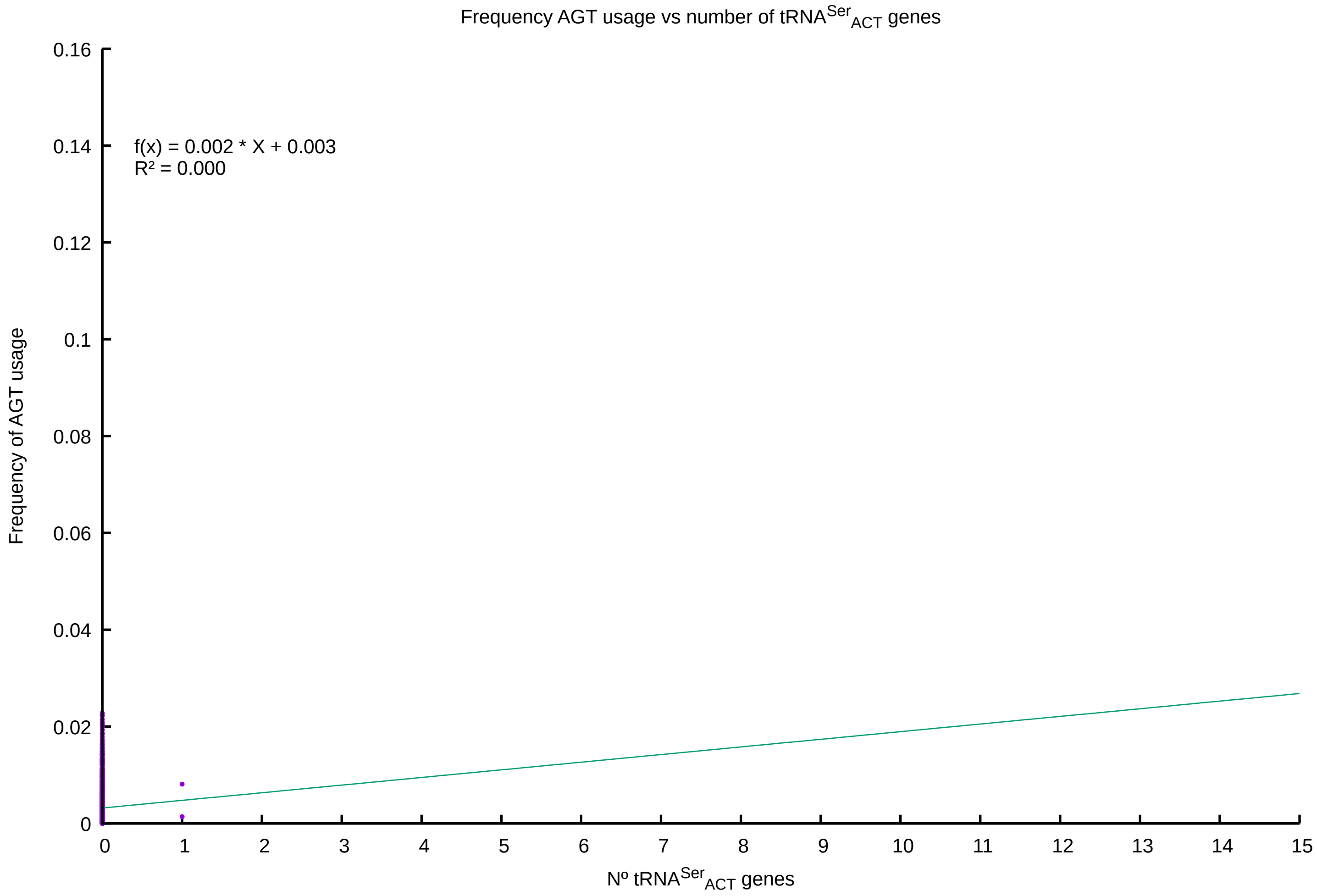
Frequency AGT usage vs number of tRNA^Ser_ACT genes
f(x) = 0.002 * X + 0.003
R² = 0.000
Frequency of AGT usage
Nº tRNA^Ser_ACT genes
0.16
0.14
0.12
0.1
0.08
0.06
0.04
0.02
0
0
1
2
3
4
5
6
7
8
9
10
11
12
13
14
15

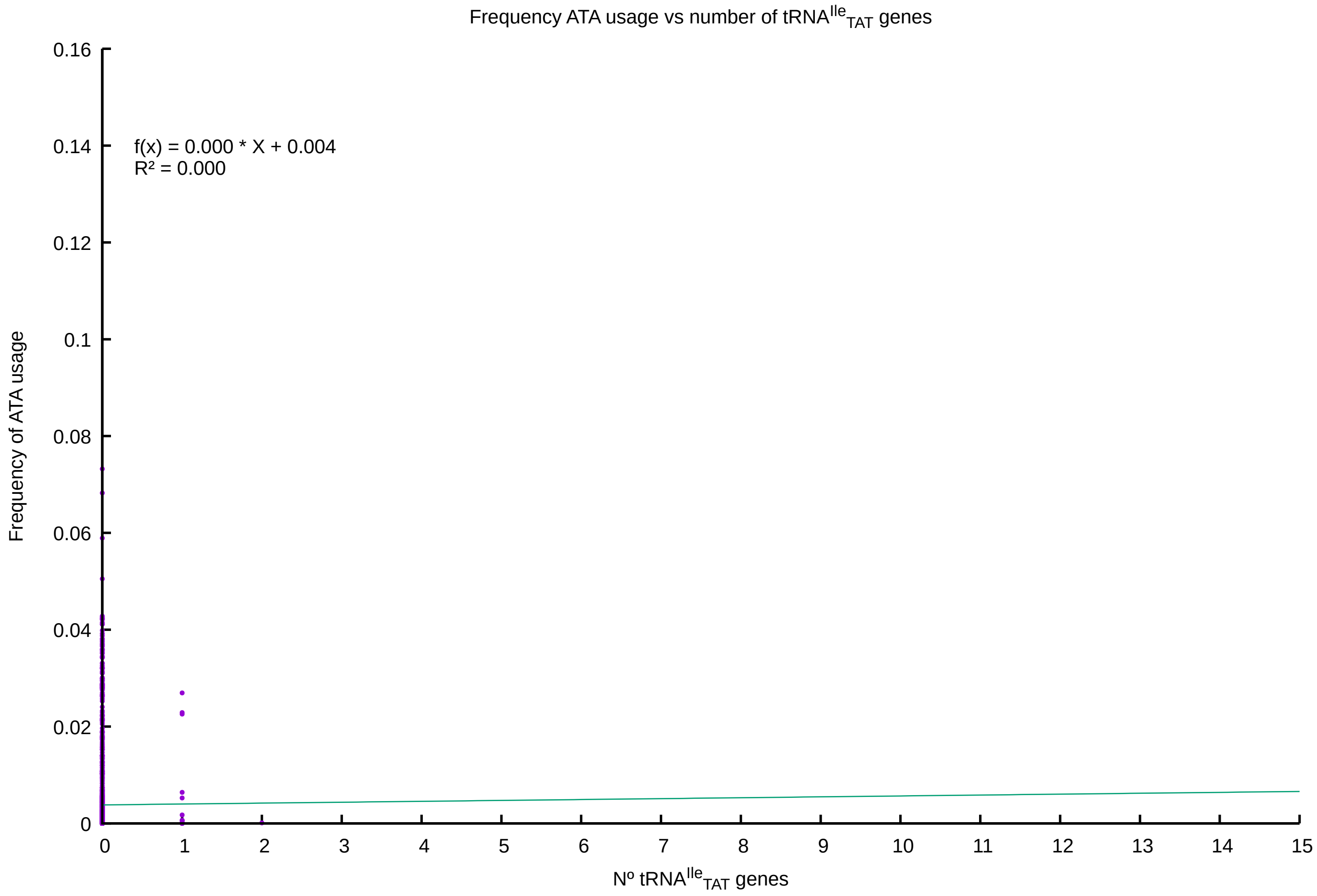
Frequency ATA usage vs number of tRNA^Ile_TAT genes
f(x) = 0.000 * X + 0.004
R² = 0.000
Frequency of ATA usage
0.16
0.14
0.12
0.1
0.08
0.06
0.04
0.02
0
0 1 2 3 4 5 6 7 8 9 10 11 12 13 14 15
Nº tRNA^Ile_TAT genes

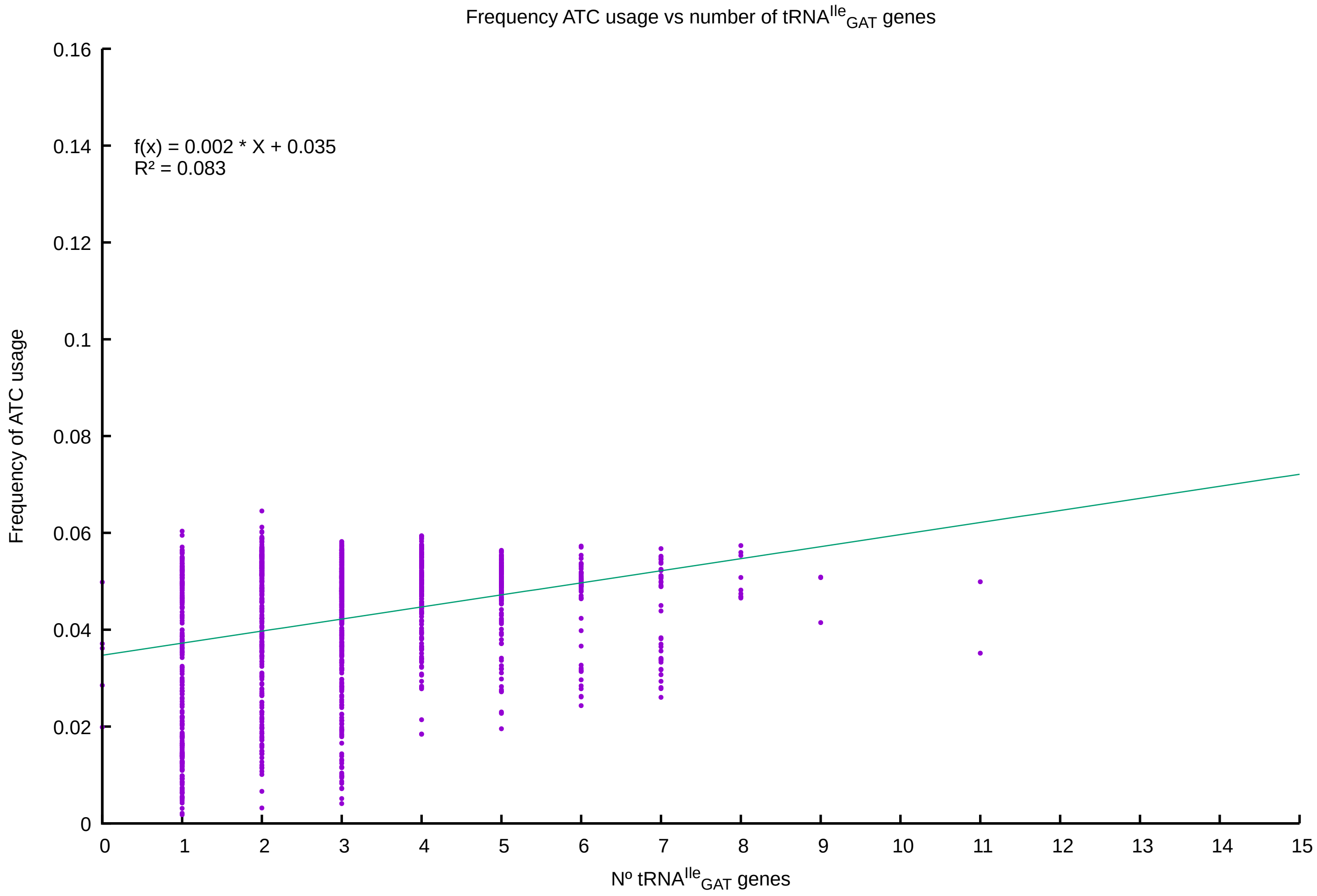

Frequency ATC usage vs number of tRNA^Ile_GAT genes
f(x) = 0.002 * X + 0.035
R² = 0.083
Frequency of ATC usage
0.16
0.14
0.12
0.1
0.08
0.06
0.04
0.02
0
0 1 2 3 4 5 6 7 8 9 10 11 12 13 14 15
Nº tRNA^Ile_GAT genes

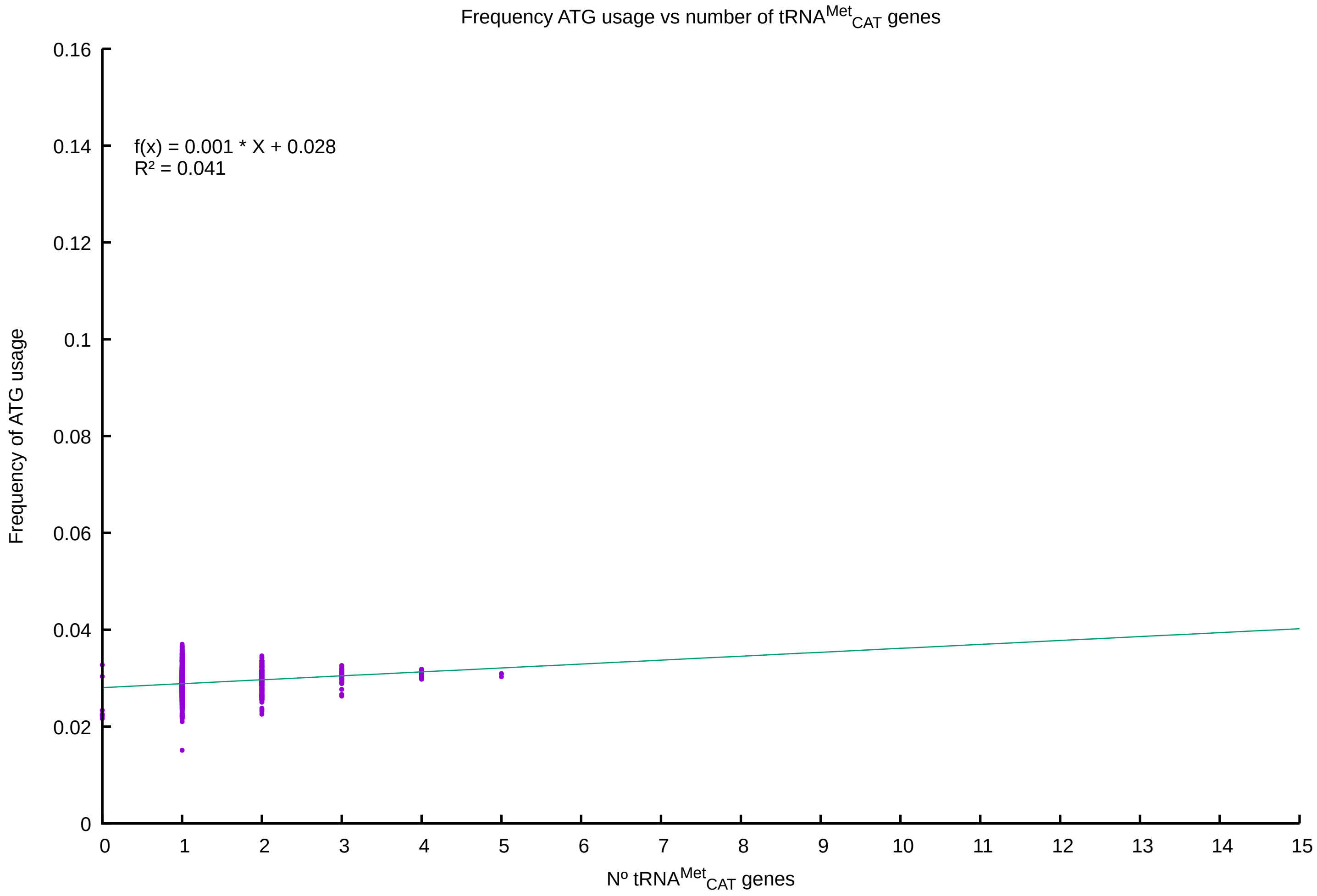

Frequency ATG usage vs number of tRNA^Met_CAT genes
f(x) = 0.001 * X + 0.028
R² = 0.041
Frequency of ATG usage
0.16
0.14
0.12
0.1
0.08
0.06
0.04
0.02
0
0 1 2 3 4 5 6 7 8 9 10 11 12 13 14 15
Nº tRNA^Met_CAT genes

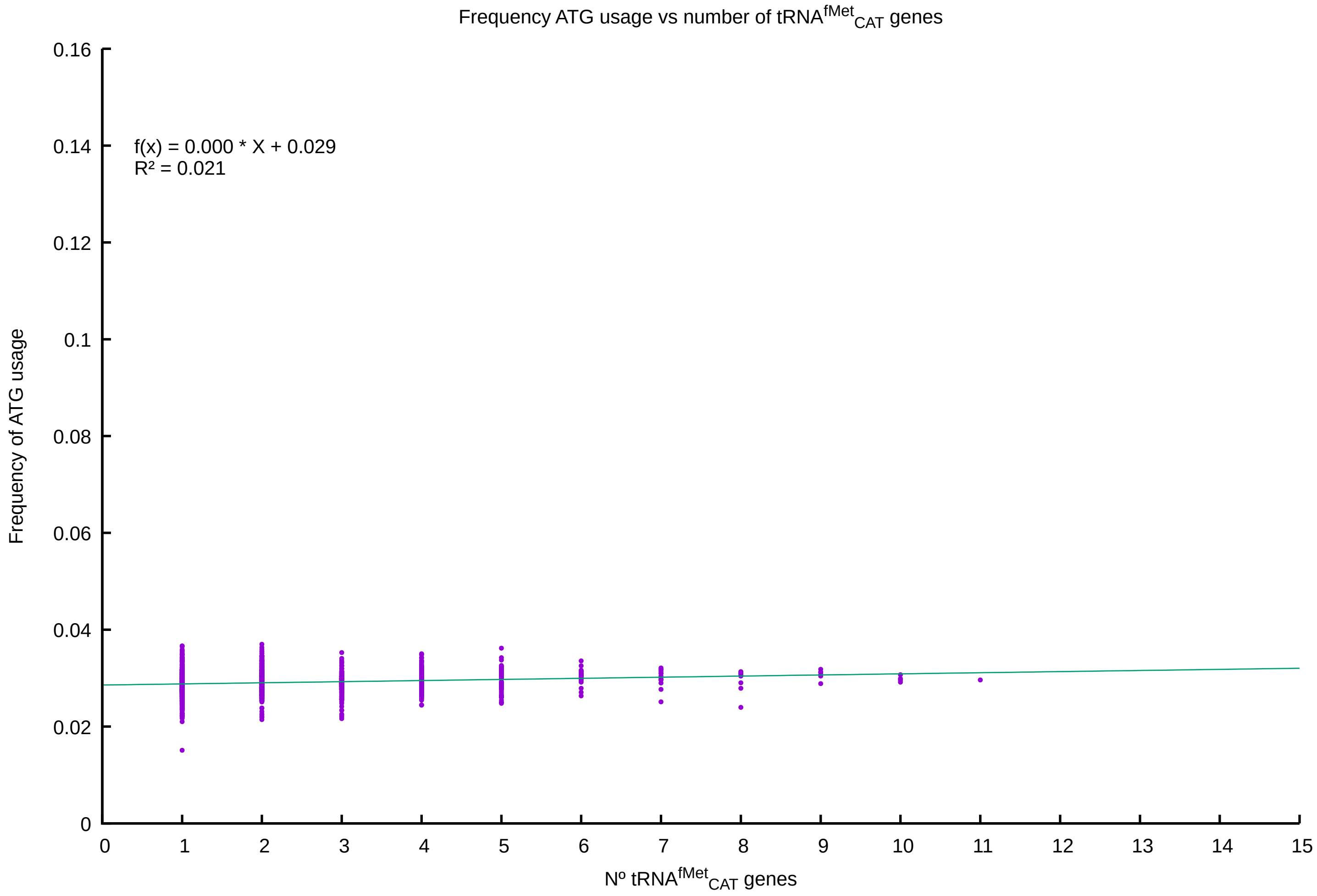

Frequency ATG usage vs number of tRNAfMetCAT genes
f(x) = 0.000 * X + 0.029
R² = 0.021
Frequency of ATG usage
0.16
0.14
0.12
0.1
0.08
0.06
0.04
0.02
0
0 1 2 3 4 5 6 7 8 9 10 11 12 13 14 15
Nº tRNAfMetCAT genes

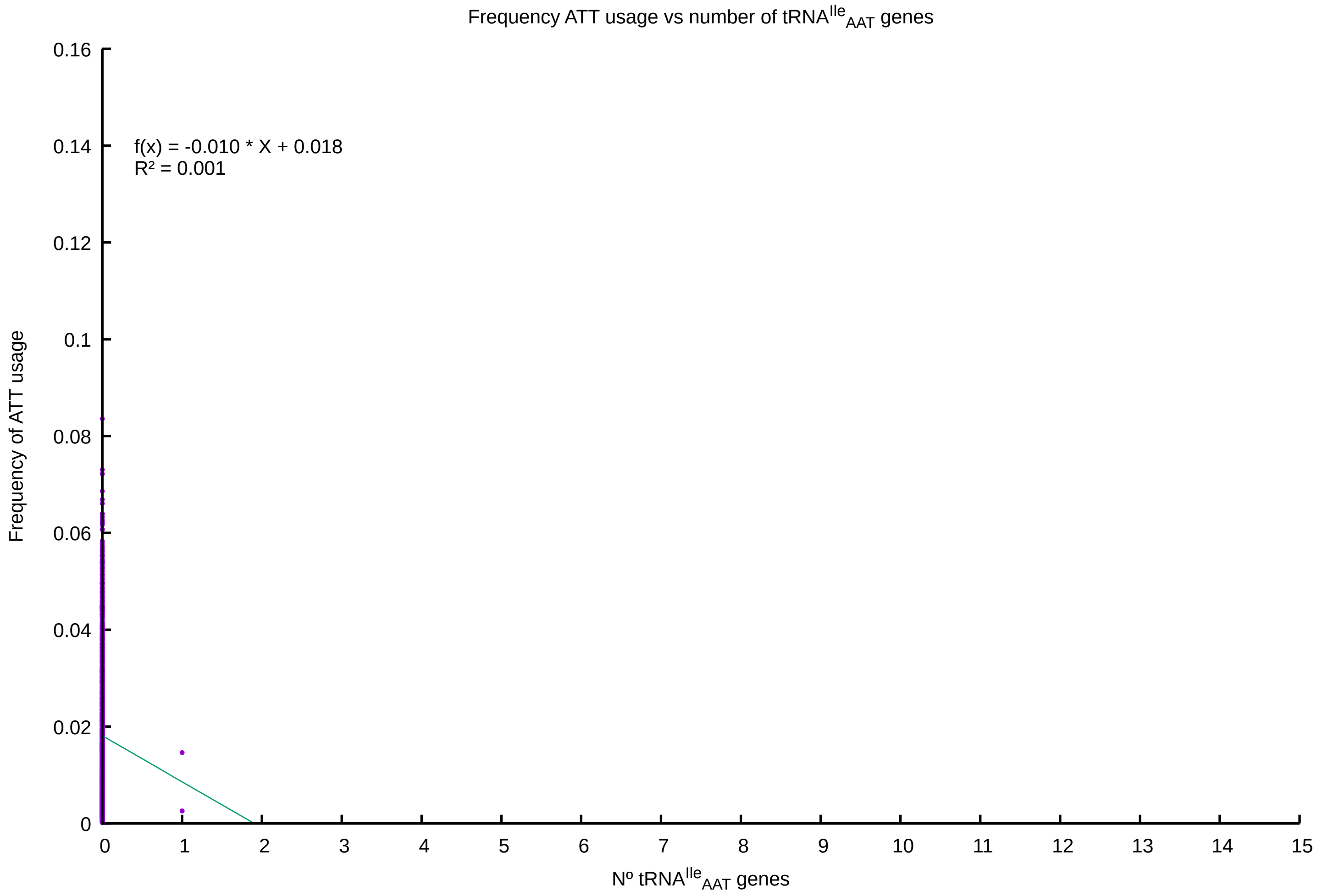

Frequency ATT usage vs number of tRNAIleAAT genes
f(x) = -0.010 * X + 0.018
R² = 0.001
Frequency of ATT usage
0.16
0.14
0.12
0.1
0.08
0.06
0.04
0.02
0
0 1 2 3 4 5 6 7 8 9 10 11 12 13 14 15
Nº tRNAIleAAT genes

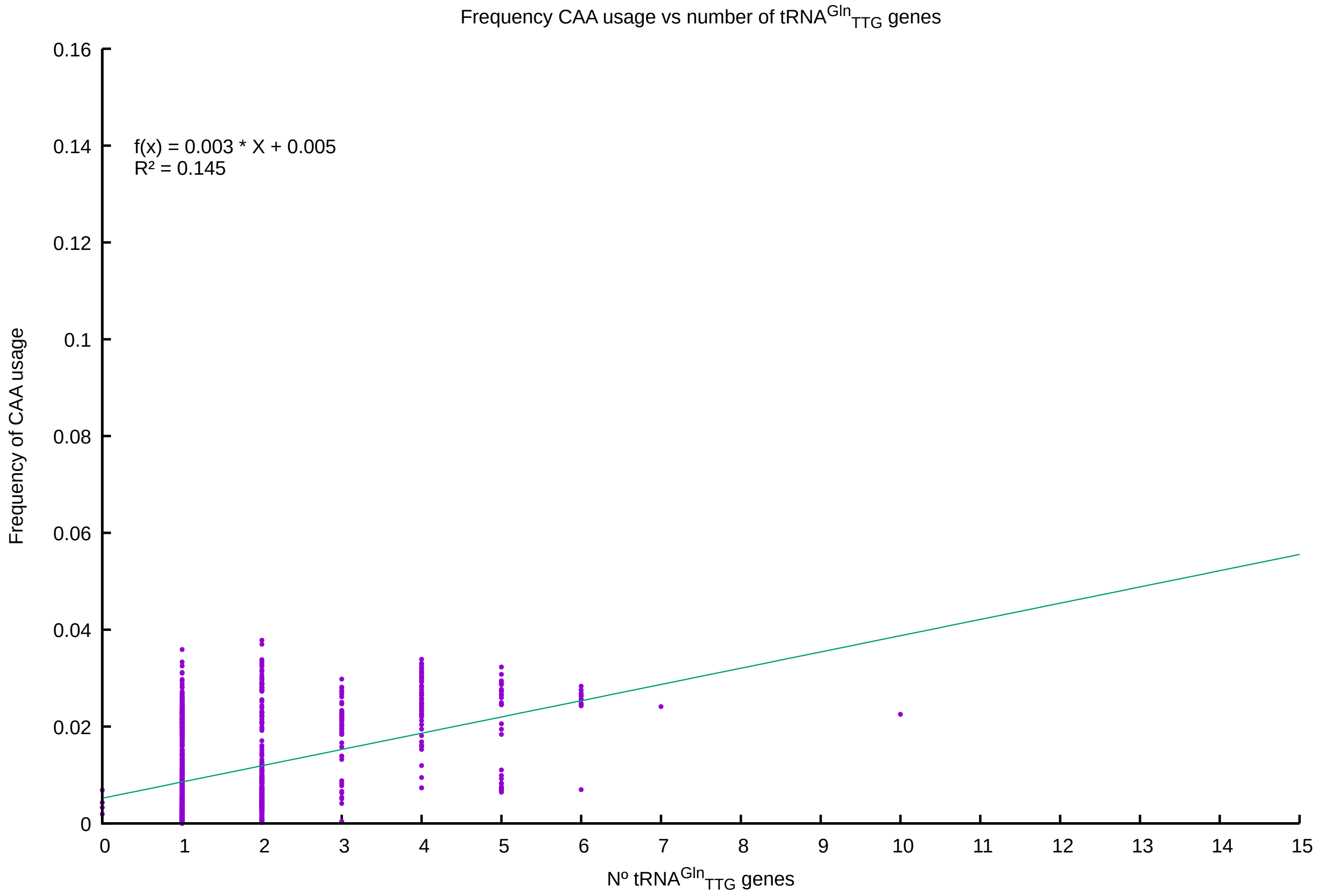
Frequency CAA usage vs number of tRNA^Gln_TTG genes
f(x) = 0.003 * X + 0.005
R² = 0.145
Frequency of CAA usage
0.16
0.14
0.12
0.1
0.08
0.06
0.04
0.02
0
0 1 2 3 4 5 6 7 8 9 10 11 12 13 14 15
Nº tRNA^Gln_TTG genes

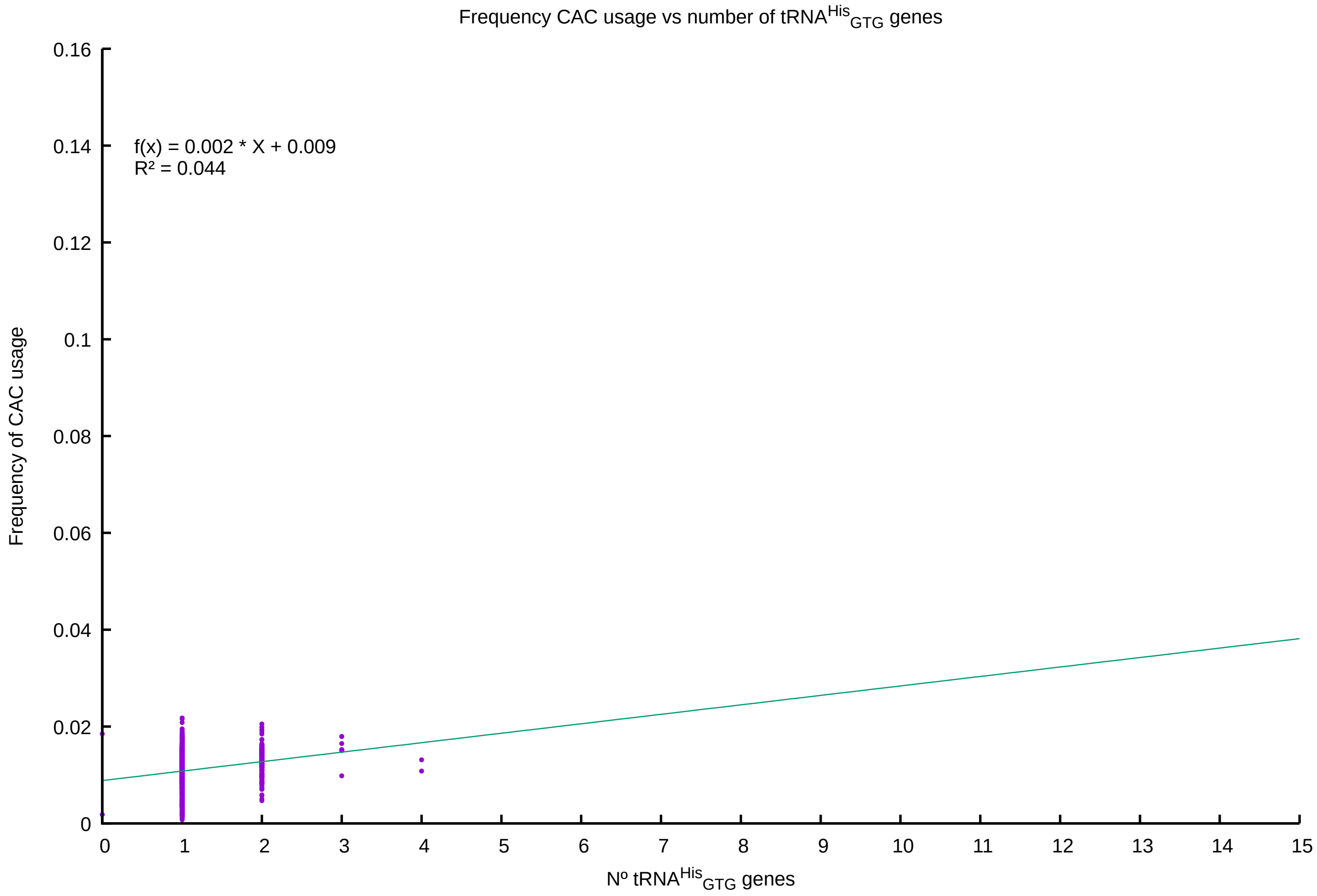

Frequency CAC usage vs number of tRNA^His_GTG genes
f(x) = 0.002 * X + 0.009
R² = 0.044
Frequency of CAC usage
0.16
0.14
0.12
0.1
0.08
0.06
0.04
0.02
0
0 1 2 3 4 5 6 7 8 9 10 11 12 13 14 15
Nº tRNA^His_GTG genes

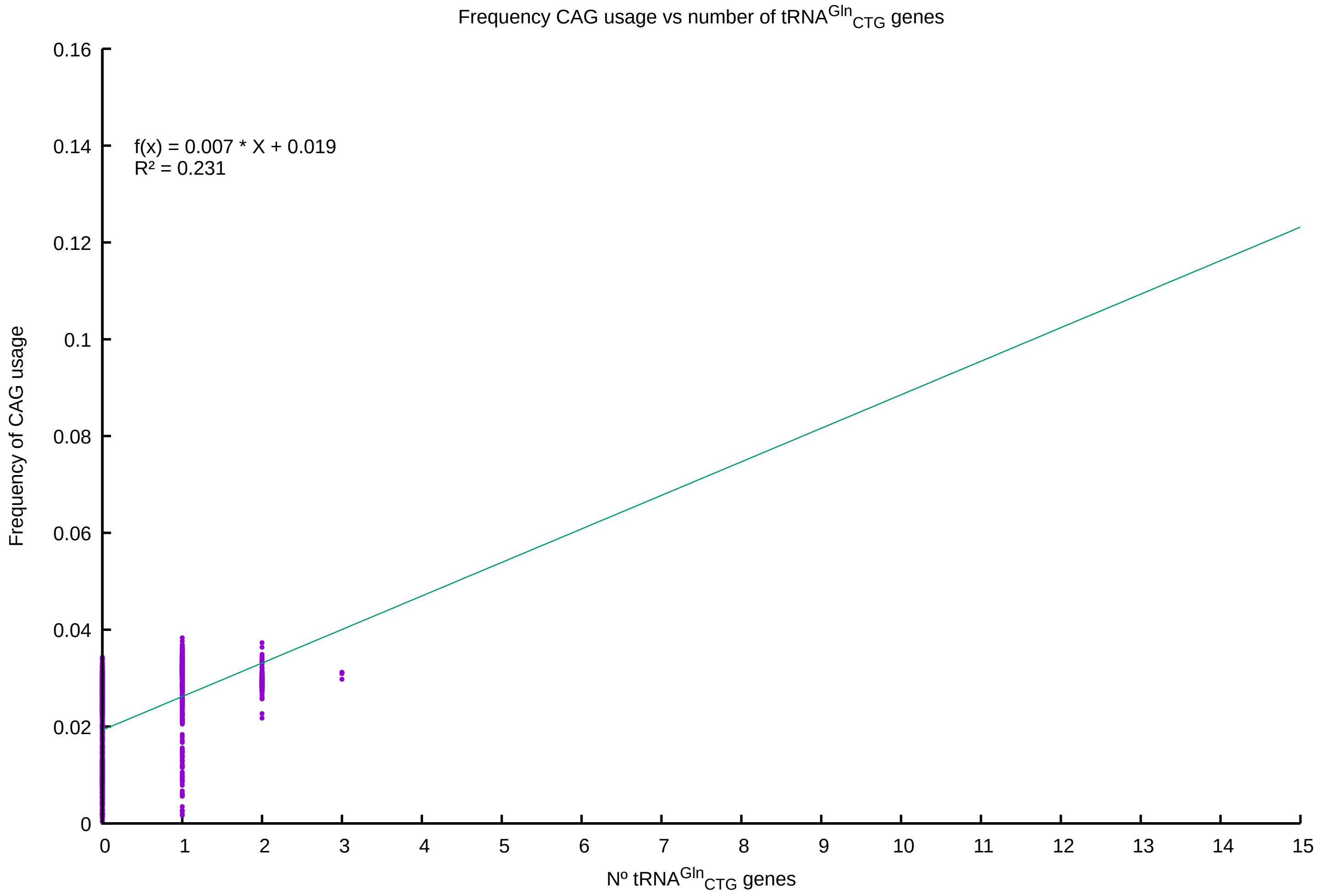

Frequency CAG usage vs number of tRNAGlnCTG genes
f(x) = 0.007 * X + 0.019
R² = 0.231
Frequency of CAG usage
0.16
0.14
0.12
0.1
0.08
0.06
0.04
0.02
0
0 1 2 3 4 5 6 7 8 9 10 11 12 13 14 15
Nº tRNAGlnCTG genes

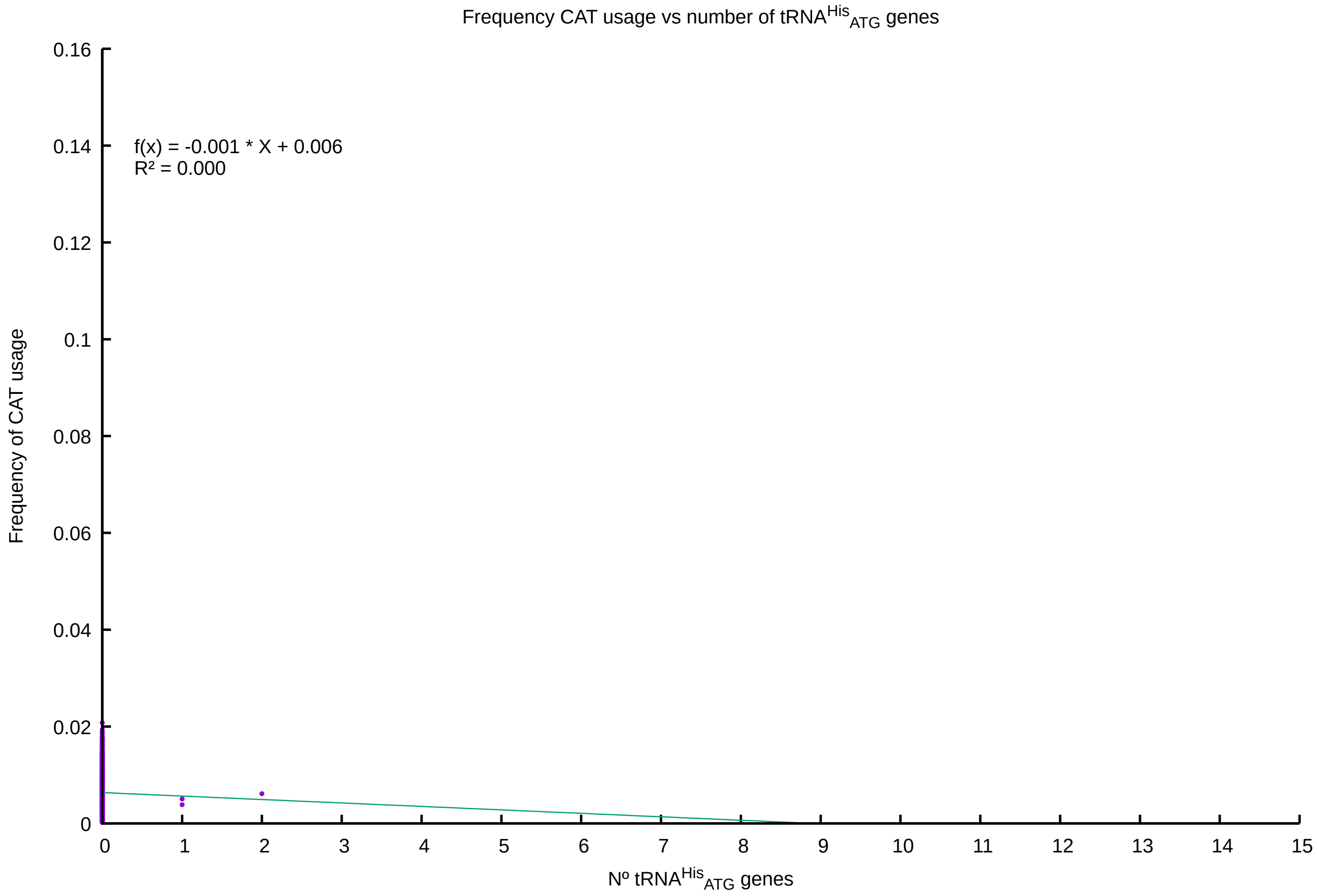

Frequency CAT usage vs number of tRNA$^{His}_{ATG}$ genes
f(x) = -0.001 * X + 0.006
R^2 = 0.000
Frequency of CAT usage
0.16
0.14
0.12
0.1
0.08
0.06
0.04
0.02
0
0 1 2 3 4 5 6 7 8 9 10 11 12 13 14 15
Nº tRNA$^{His}_{ATG}$ genes

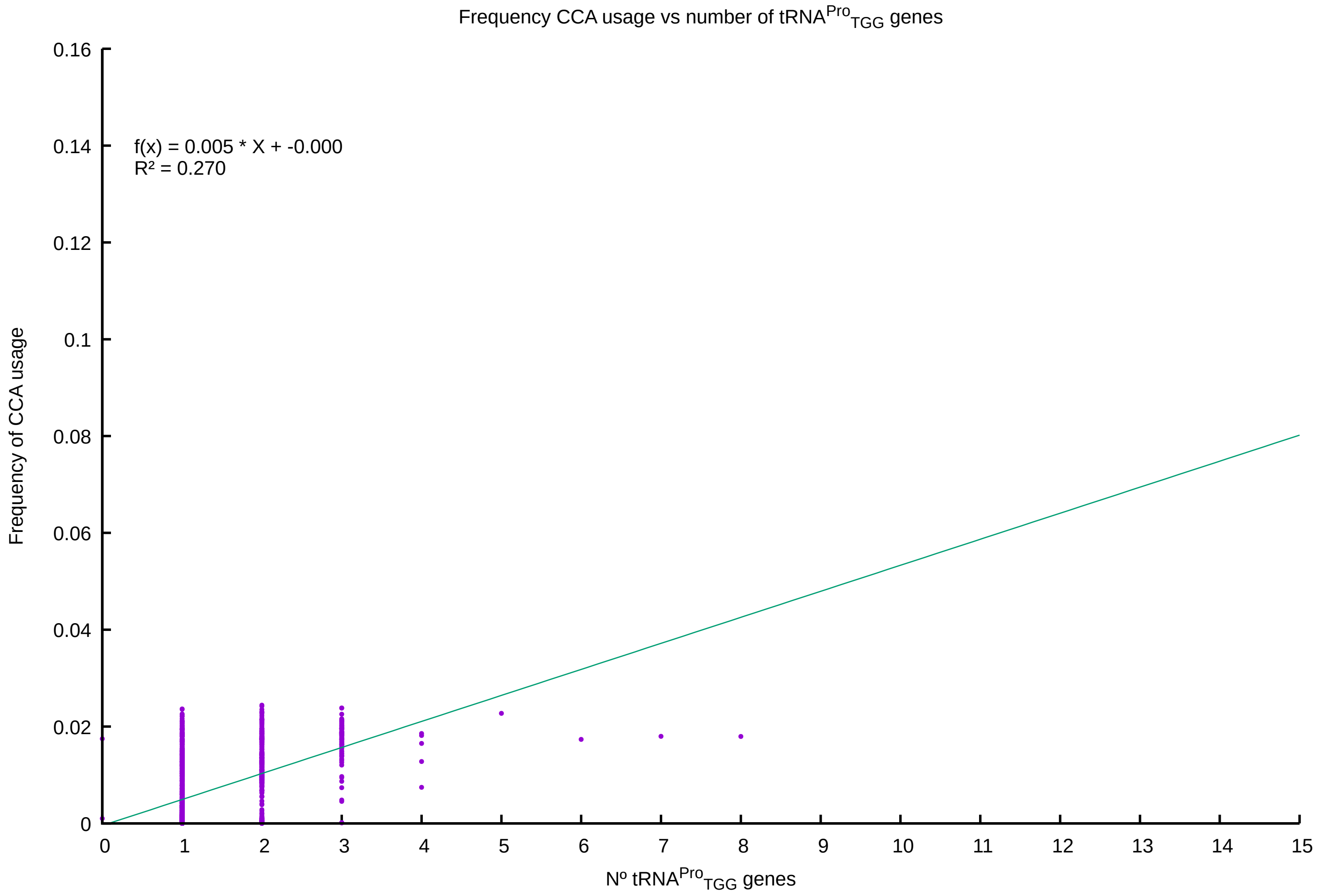

Frequency CCA usage vs number of tRNA^Pro_TGG genes
f(x) = 0.005 * X + -0.000
R² = 0.270
Frequency of CCA usage
0.16
0.14
0.12
0.1
0.08
0.06
0.04
0.02
0
0 1 2 3 4 5 6 7 8 9 10 11 12 13 14 15
Nº tRNA^Pro_TGG genes

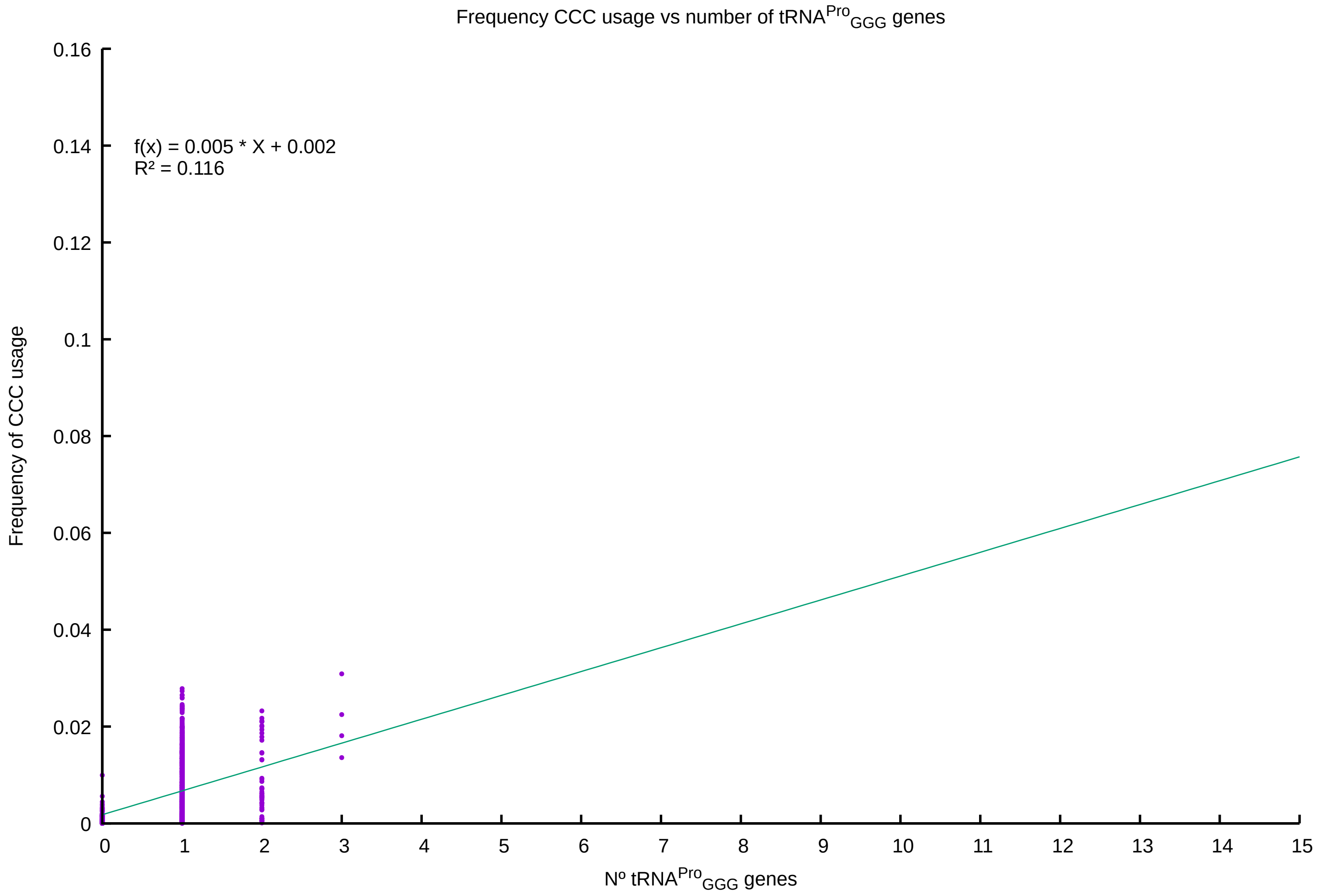

Frequency CCC usage vs number of tRNAProGGG genes
Frequency of CCC usage
f(x) = 0.005 * X + 0.002
R² = 0.116
0.16
0.14
0.12
0.1
0.08
0.06
0.04
0.02
0
0 1 2 3 4 5 6 7 8 9 10 11 12 13 14 15
Nº tRNAProGGG genes

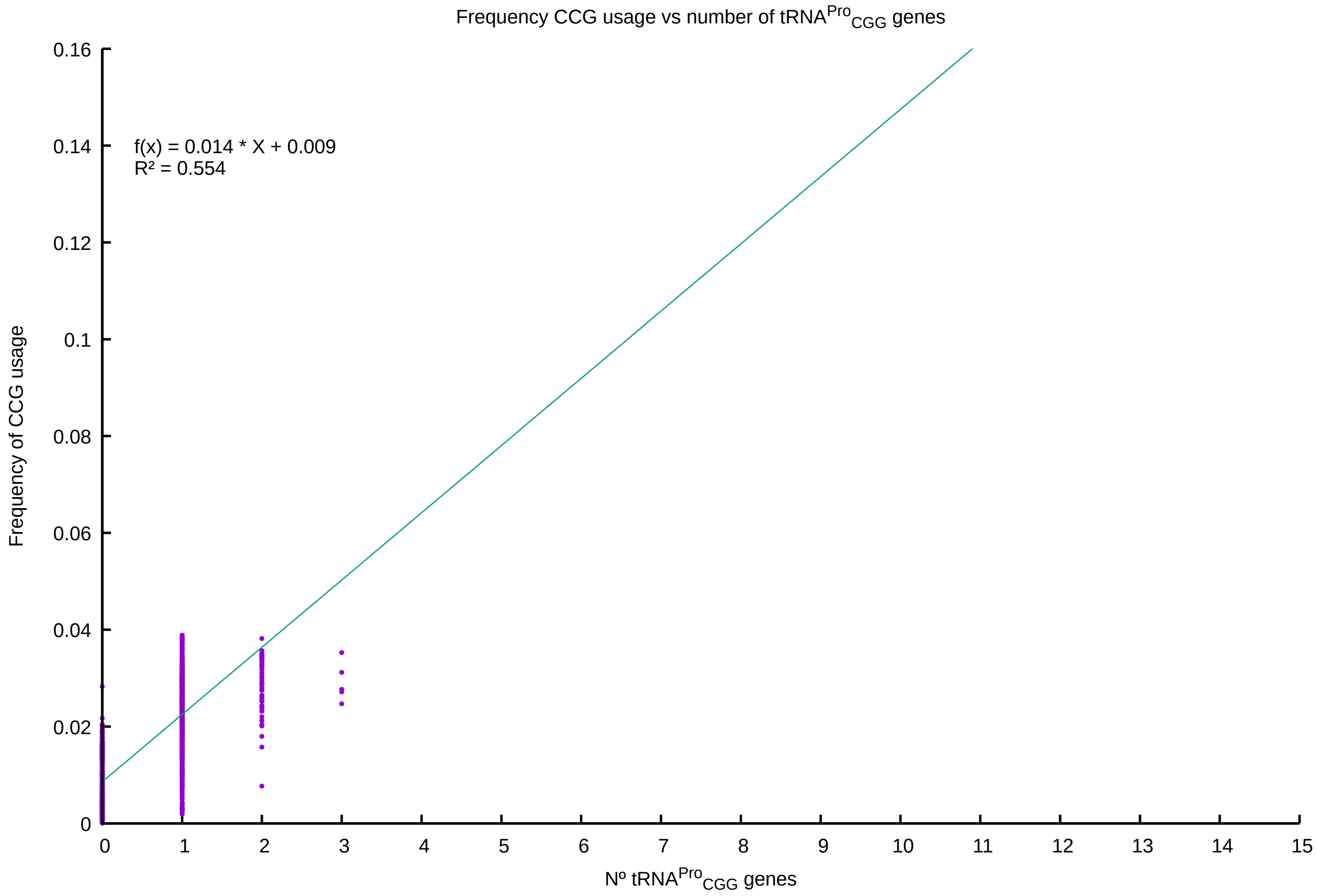

Frequency CCG usage vs number of tRNA^Pro_CGG genes
f(x) = 0.014 * X + 0.009
R² = 0.554
Frequency of CCG usage
0.16
0.14
0.12
0.1
0.08
0.06
0.04
0.02
0
0 1 2 3 4 5 6 7 8 9 10 11 12 13 14 15
Nº tRNA^Pro_CGG genes

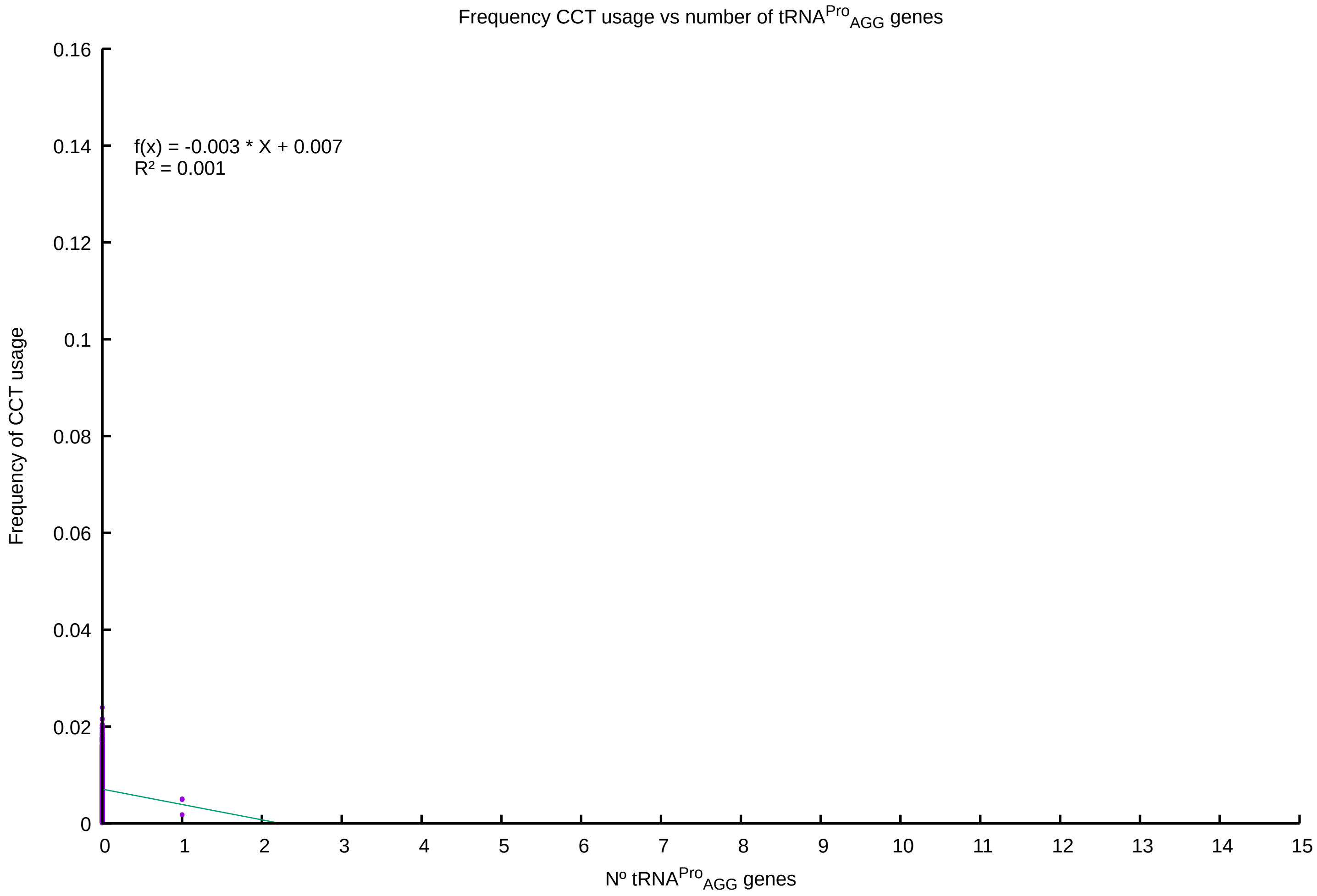

Frequency CCT usage vs number of tRNA^Pro_AGG genes
f(x) = -0.003 * X + 0.007
R² = 0.001
Frequency of CCT usage
0.16
0.14
0.12
0.1
0.08
0.06
0.04
0.02
0
0 1 2 3 4 5 6 7 8 9 10 11 12 13 14 15
Nº tRNA^Pro_AGG genes

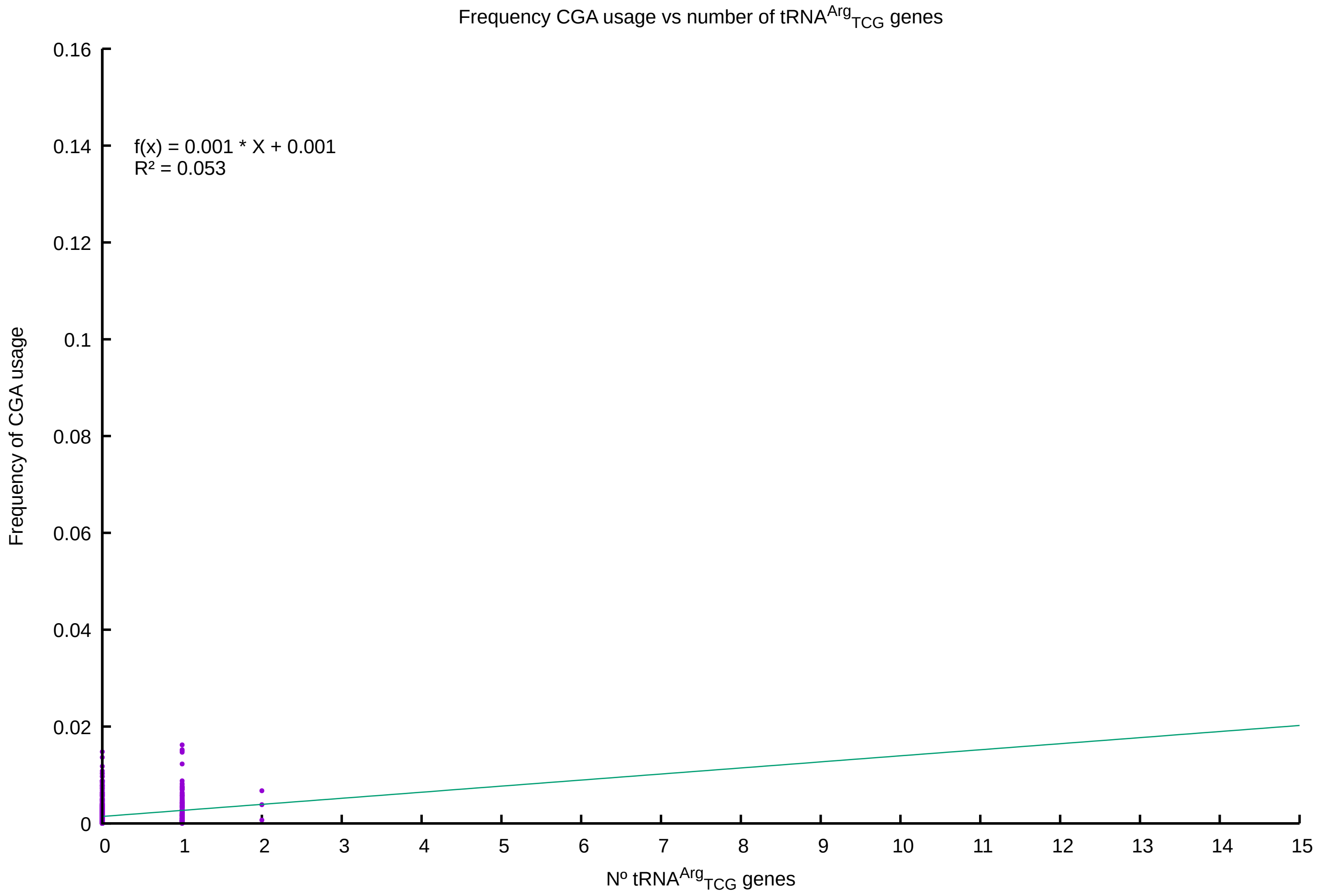

Frequency CGA usage vs number of tRNA^Arg_TCG genes
f(x) = 0.001 * X + 0.001
R² = 0.053
Frequency of CGA usage
0.16
0.14
0.12
0.1
0.08
0.06
0.04
0.02
0
0 1 2 3 4 5 6 7 8 9 10 11 12 13 14 15
Nº tRNA^Arg_TCG genes

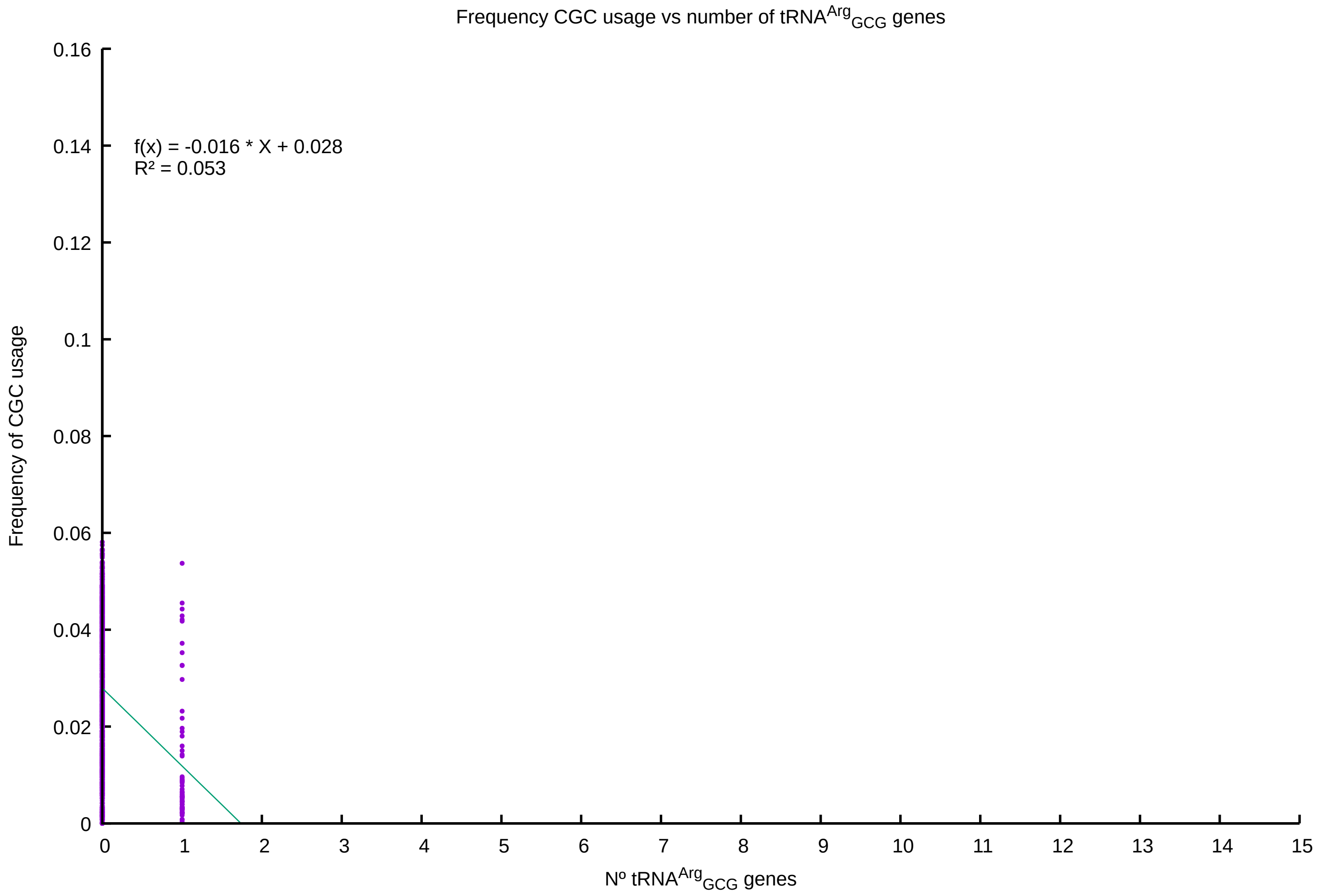
Frequency CGC usage vs number of tRNAArgGCG genes
f(x) = -0.016 * X + 0.028
R² = 0.053
Frequency of CGC usage
0.16
0.14
0.12
0.1
0.08
0.06
0.04
0.02
0
0 1 2 3 4 5 6 7 8 9 10 11 12 13 14 15
Nº tRNAArgGCG genes

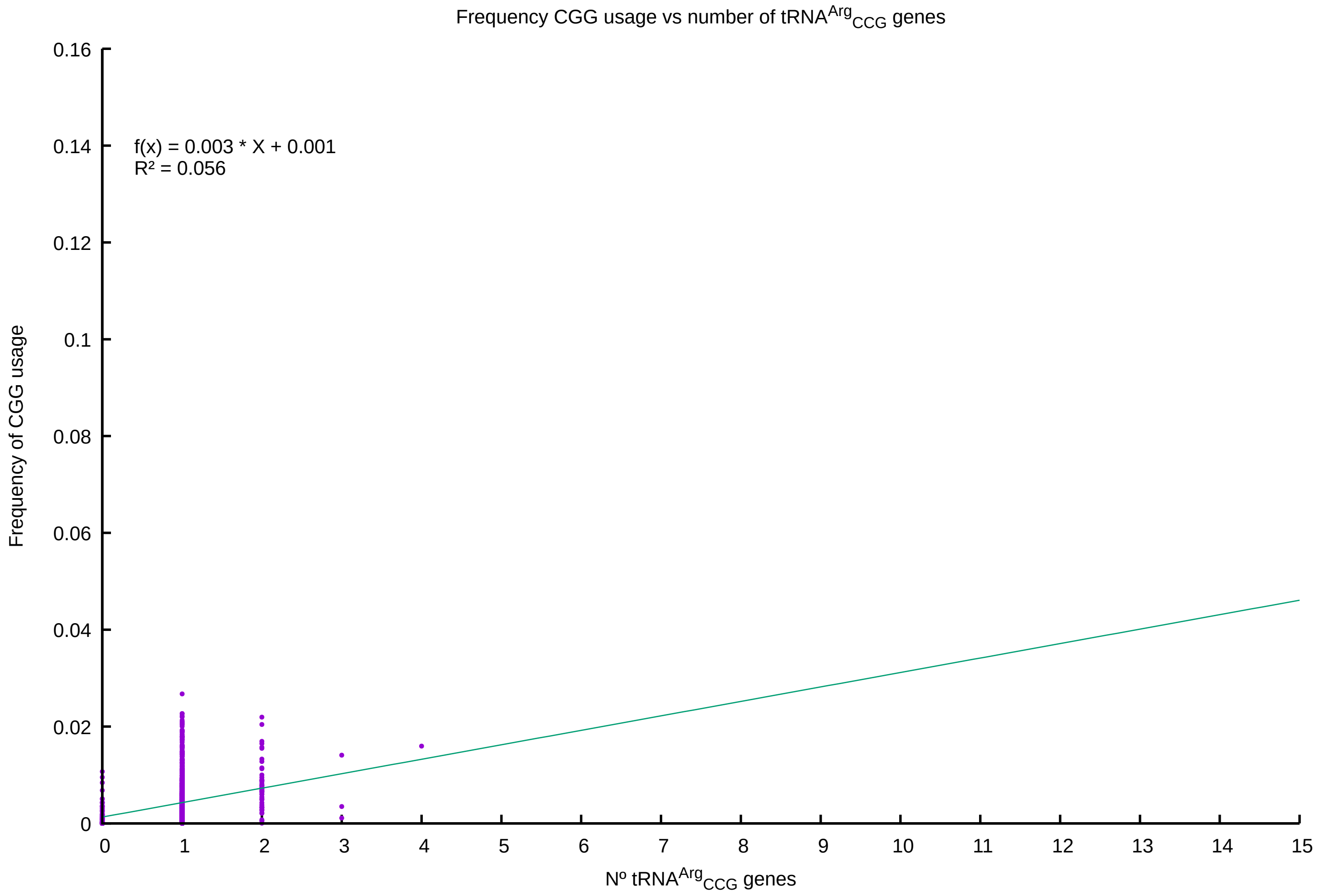

Frequency CGG usage vs number of tRNAArgCCG genes
f(x) = 0.003 * X + 0.001
R² = 0.056
Frequency of CGG usage
0.16
0.14
0.12
0.1
0.08
0.06
0.04
0.02
0
0 1 2 3 4 5 6 7 8 9 10 11 12 13 14 15
Nº tRNAArgCCG genes

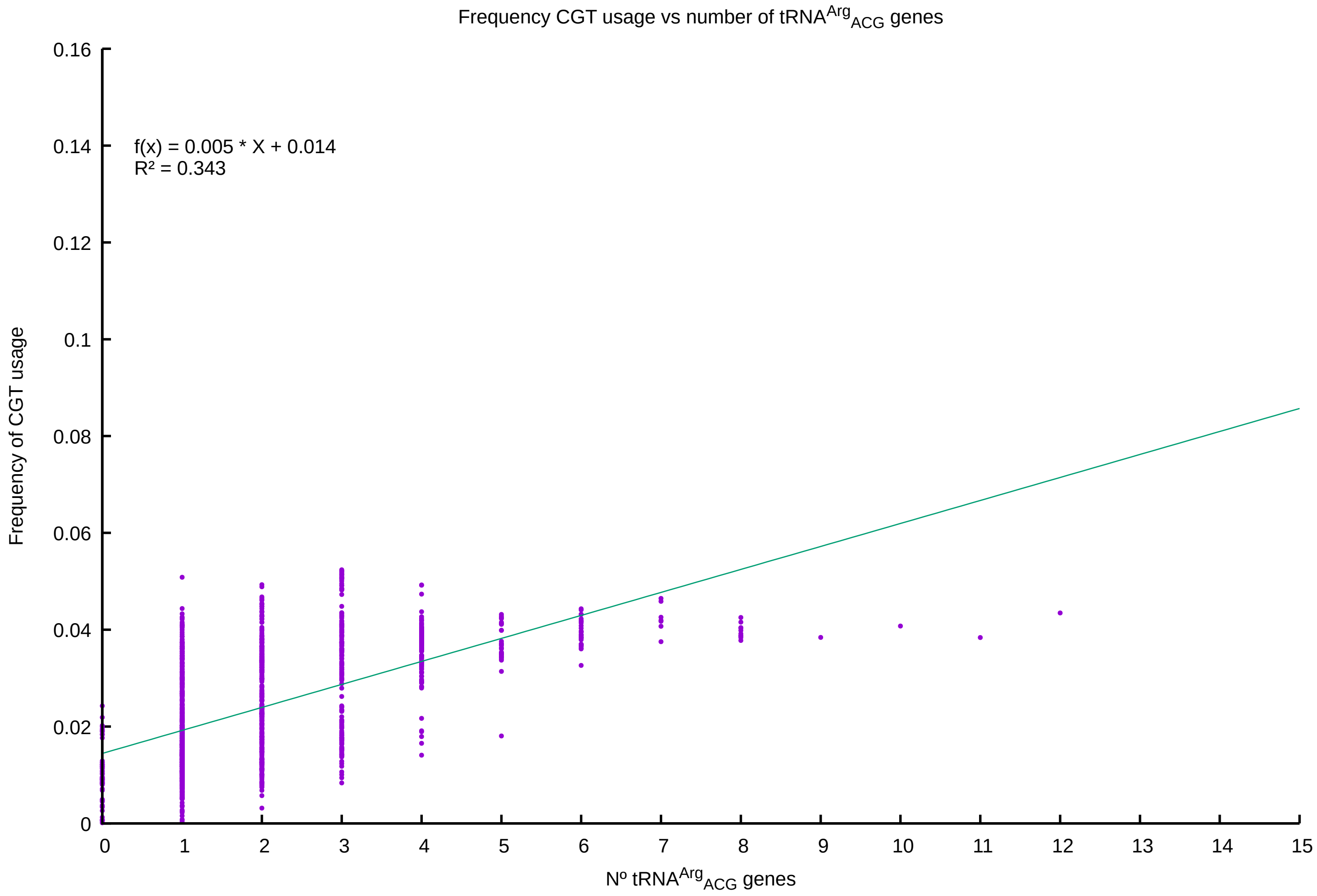
Frequency CGT usage vs number of tRNA^Arg_ACG genes
f(x) = 0.005 * X + 0.014
R² = 0.343
Frequency of CGT usage
0.16
0.14
0.12
0.1
0.08
0.06
0.04
0.02
0
0 1 2 3 4 5 6 7 8 9 10 11 12 13 14 15
Nº tRNA^Arg_ACG genes

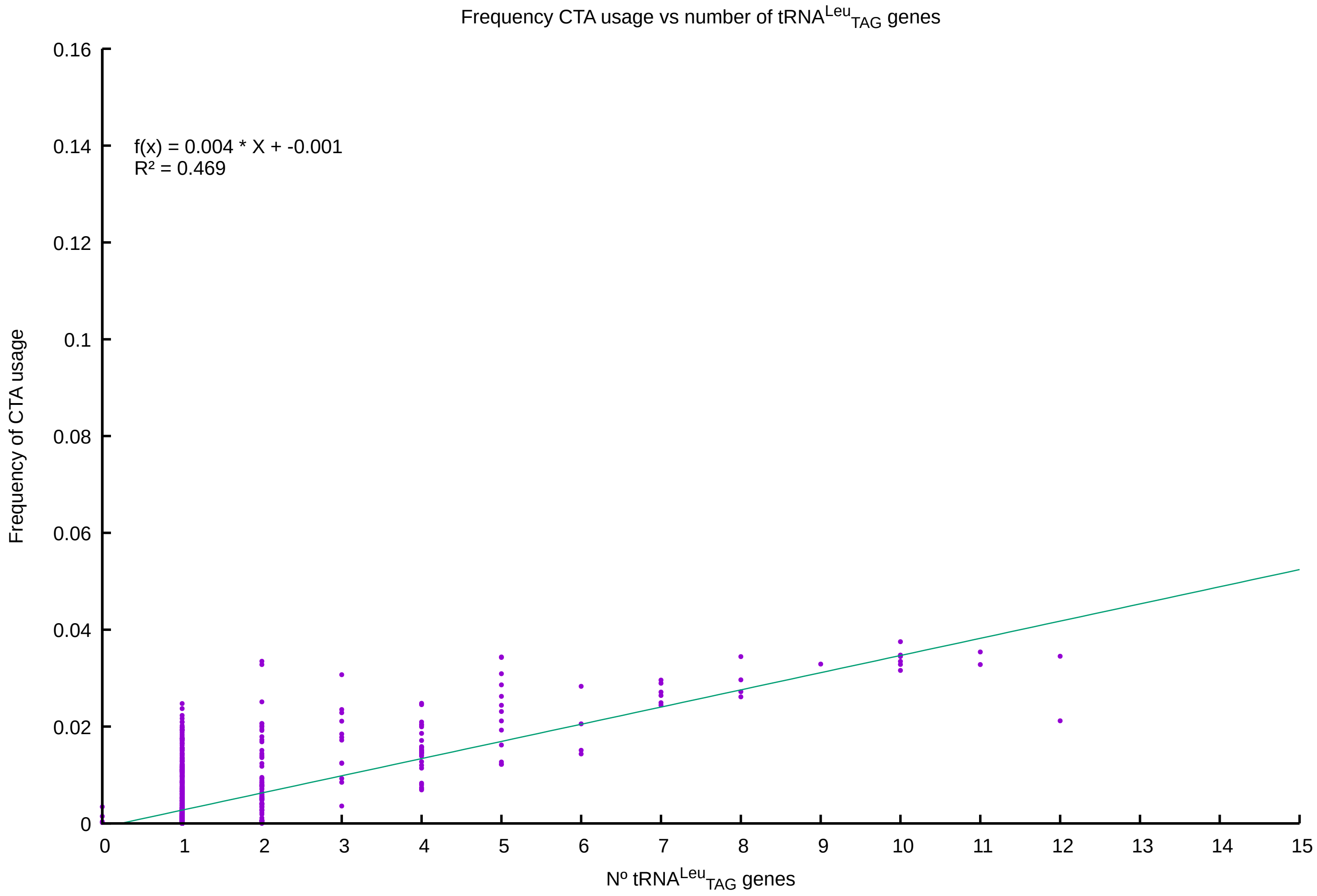

Frequency CTA usage vs number of tRNALeuTAG genes
f(x) = 0.004 * X + -0.001
R² = 0.469
Frequency of CTA usage
0.16
0.14
0.12
0.1
0.08
0.06
0.04
0.02
0
0 1 2 3 4 5 6 7 8 9 10 11 12 13 14 15
Nº tRNALeuTAG genes

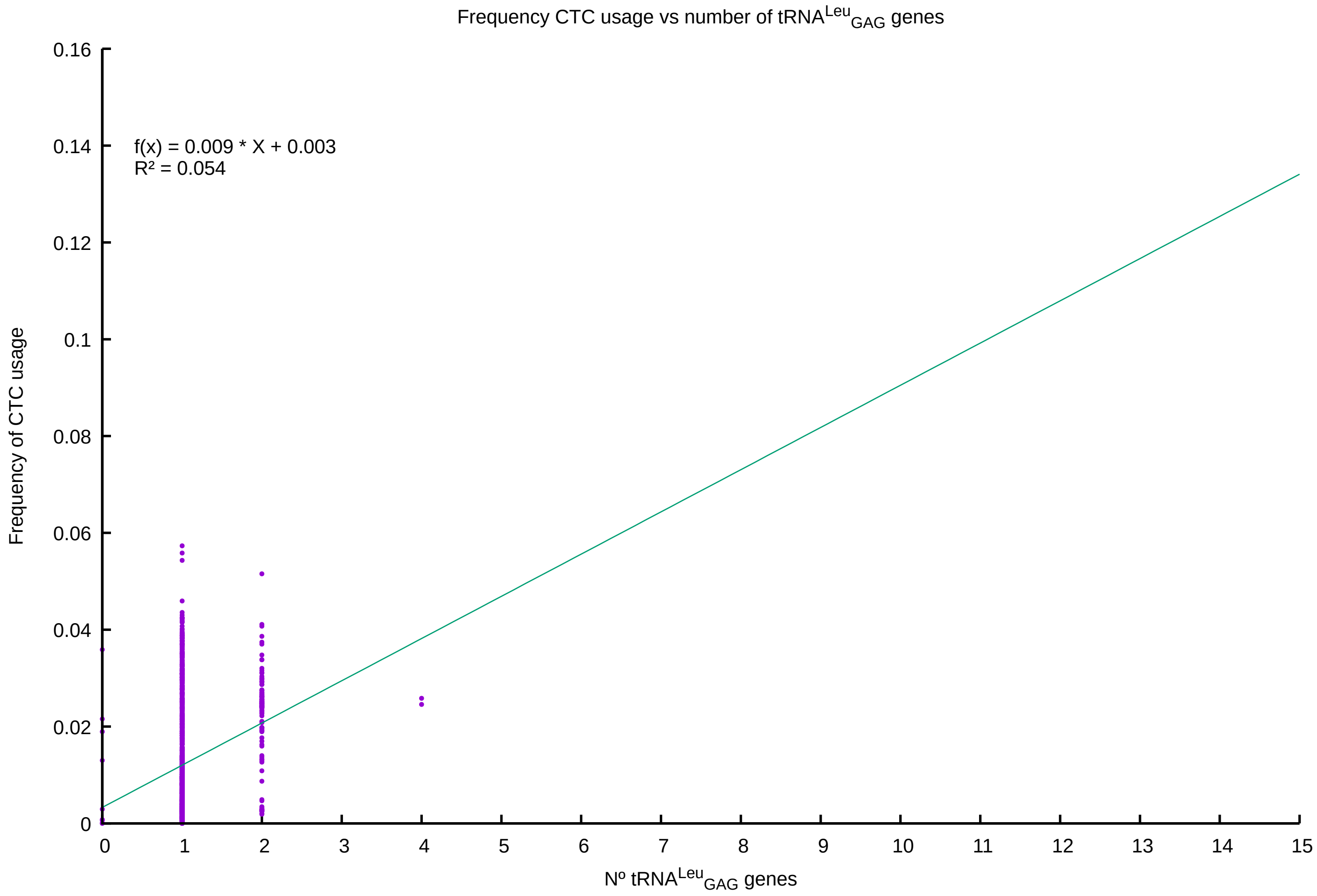

Frequency CTC usage vs number of tRNA^Leu_GAG genes
f(x) = 0.009 * X + 0.003
R² = 0.054
Frequency of CTC usage
0.16
0.14
0.12
0.1
0.08
0.06
0.04
0.02
0
0 1 2 3 4 5 6 7 8 9 10 11 12 13 14 15
Nº tRNA^Leu_GAG genes

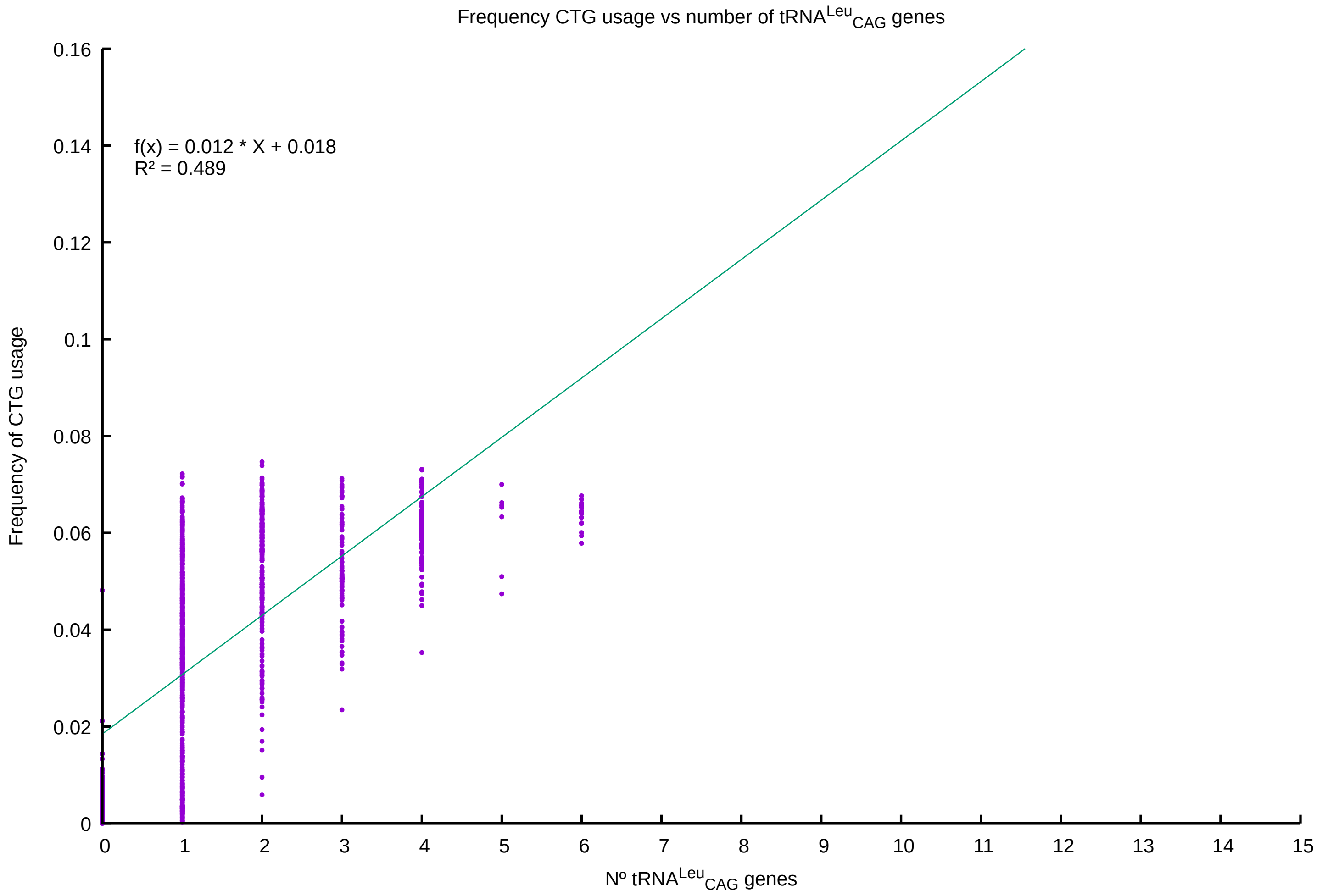

Frequency CTG usage vs number of tRNALeuCAG genes
f(x) = 0.012 * X + 0.018
R² = 0.489
Frequency of CTG usage
0
0.02
0.04
0.06
0.08
0.1
0.12
0.14
0.16
0
1
2
3
4
5
6
7
8
9
10
11
12
13
14
15
Nº tRNALeuCAG genes

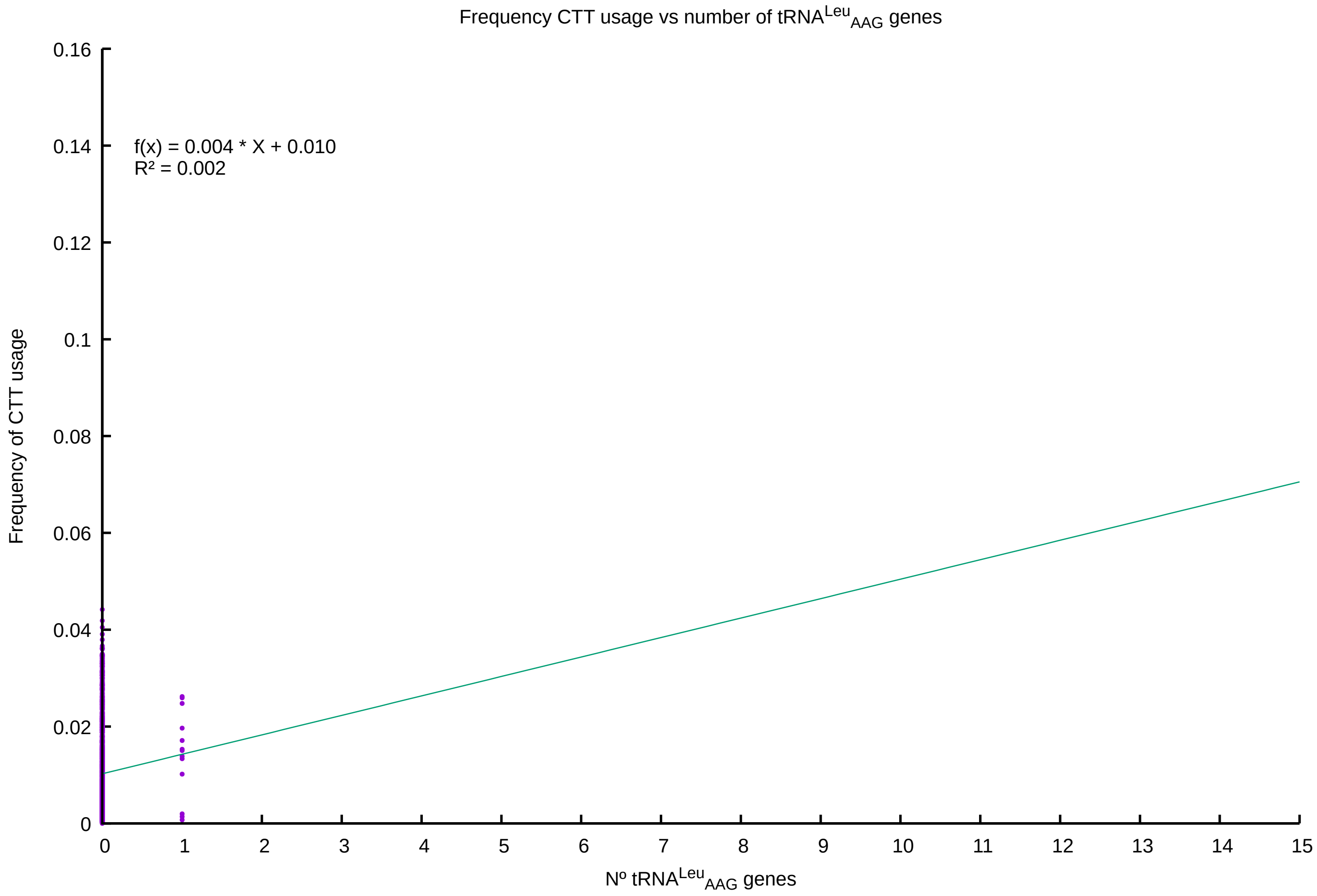

Frequency CTT usage vs number of tRNALeuAAG genes
f(x) = 0.004 * X + 0.010
R² = 0.002
Frequency of CTT usage
0.16
0.14
0.12
0.1
0.08
0.06
0.04
0.02
0
0 1 2 3 4 5 6 7 8 9 10 11 12 13 14 15
Nº tRNALeuAAG genes

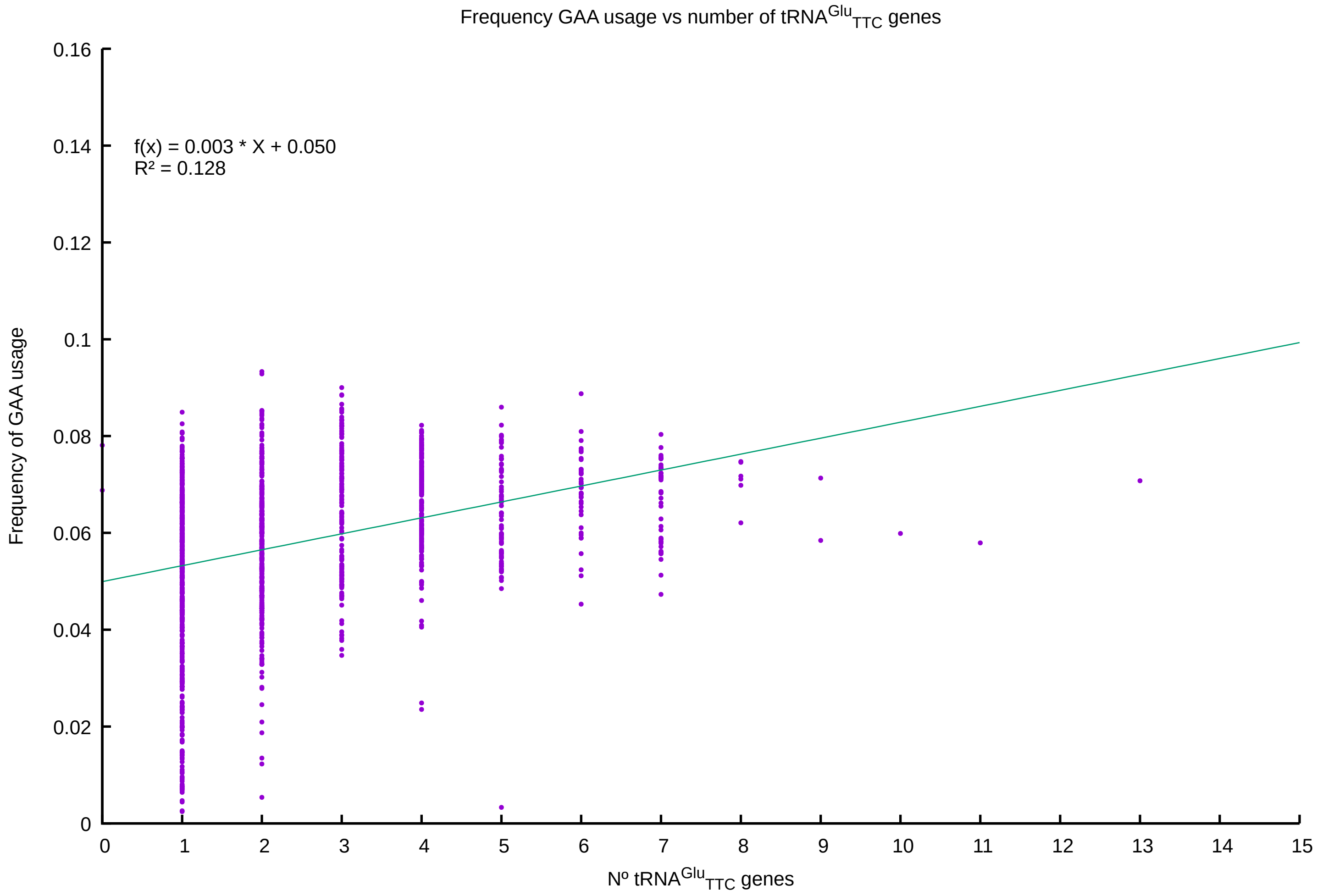

Frequency GAA usage vs number of tRNA^Glu_TTC genes
f(x) = 0.003 * X + 0.050
R² = 0.128
Frequency of GAA usage
0.16
0.14
0.12
0.1
0.08
0.06
0.04
0.02
0
0 1 2 3 4 5 6 7 8 9 10 11 12 13 14 15
Nº tRNA^Glu_TTC genes

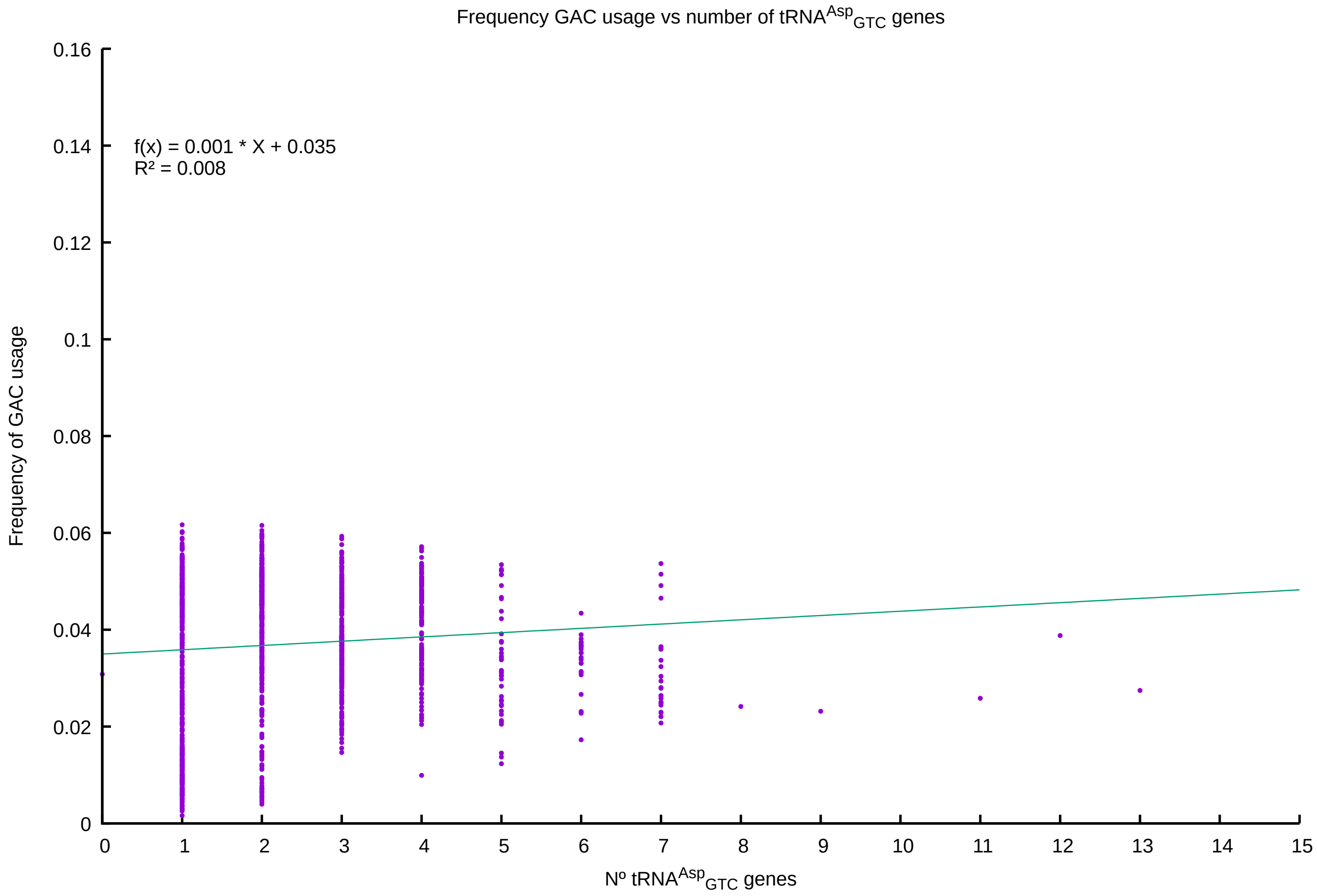
Frequency GAC usage vs number of tRNA^Asp_GTC genes
f(x) = 0.001 * X + 0.035
R² = 0.008
Frequency of GAC usage
0
0.02
0.04
0.06
0.08
0.1
0.12
0.14
0.16
0
1
2
3
4
5
6
7
8
9
10
11
12
13
14
15
Nº tRNA^Asp_GTC genes

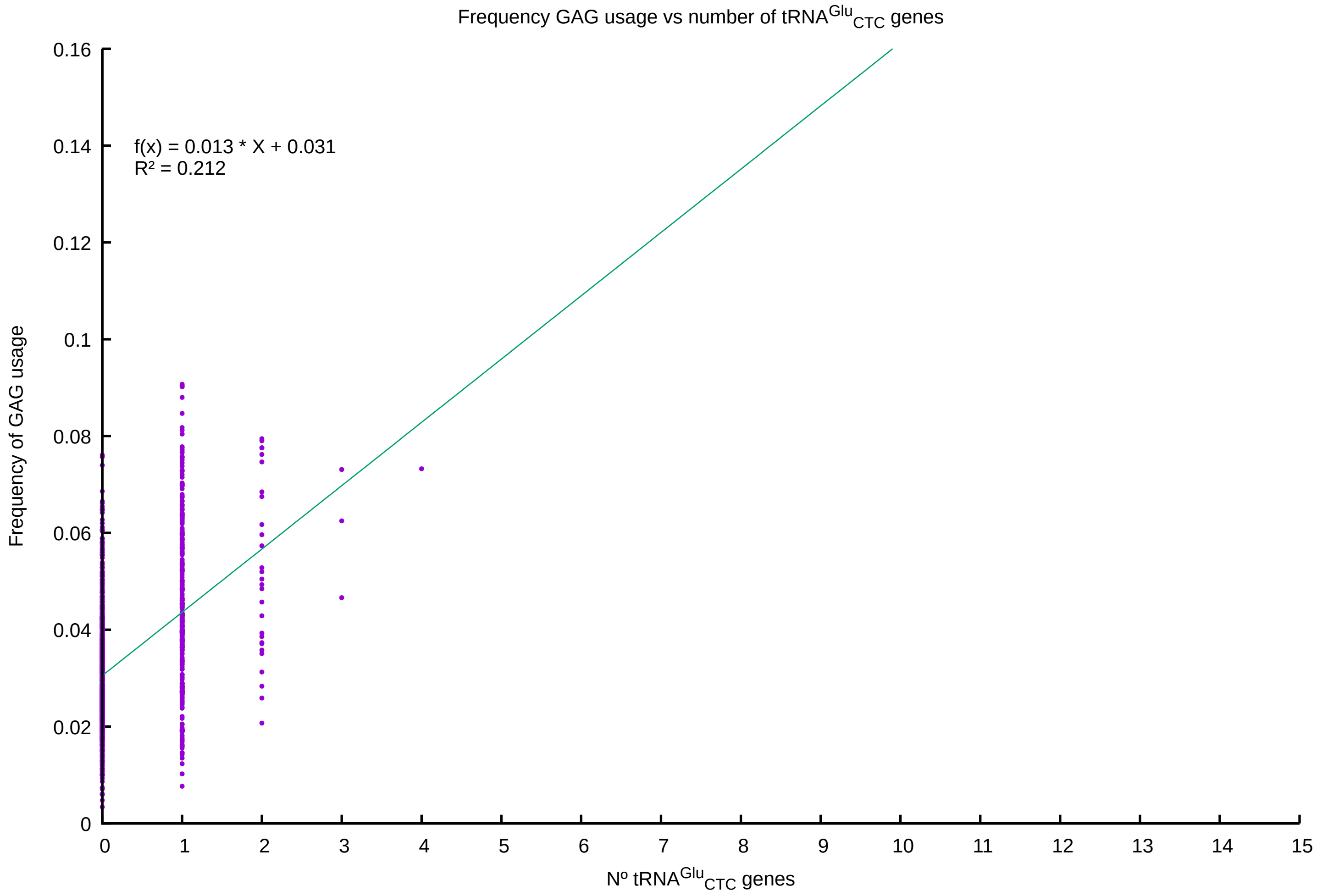
Frequency GAG usage vs number of tRNA^Glu_CTC genes
f(x) = 0.013 * X + 0.031
R² = 0.212
Frequency of GAG usage
0.16
0.14
0.12
0.1
0.08
0.06
0.04
0.02
0
0 1 2 3 4 5 6 7 8 9 10 11 12 13 14 15
Nº tRNA^Glu_CTC genes

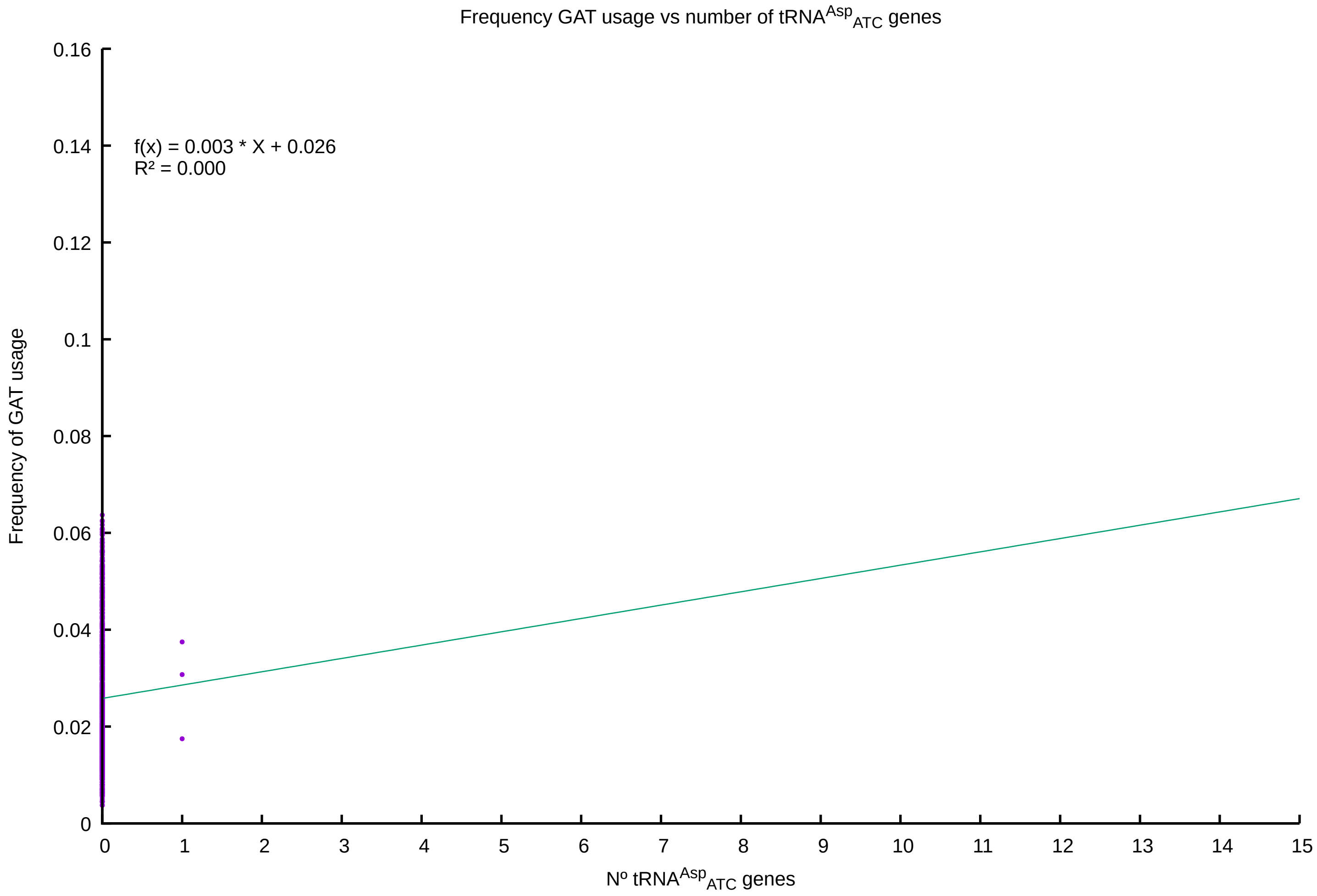

Frequency GAT usage vs number of tRNA^Asp_ATC genes
f(x) = 0.003 * X + 0.026
R² = 0.000
Frequency of GAT usage
Nº tRNA^Asp_ATC genes
0.16
0.14
0.12
0.1
0.08
0.06
0.04
0.02
0
0 1 2 3 4 5 6 7 8 9 10 11 12 13 14 15

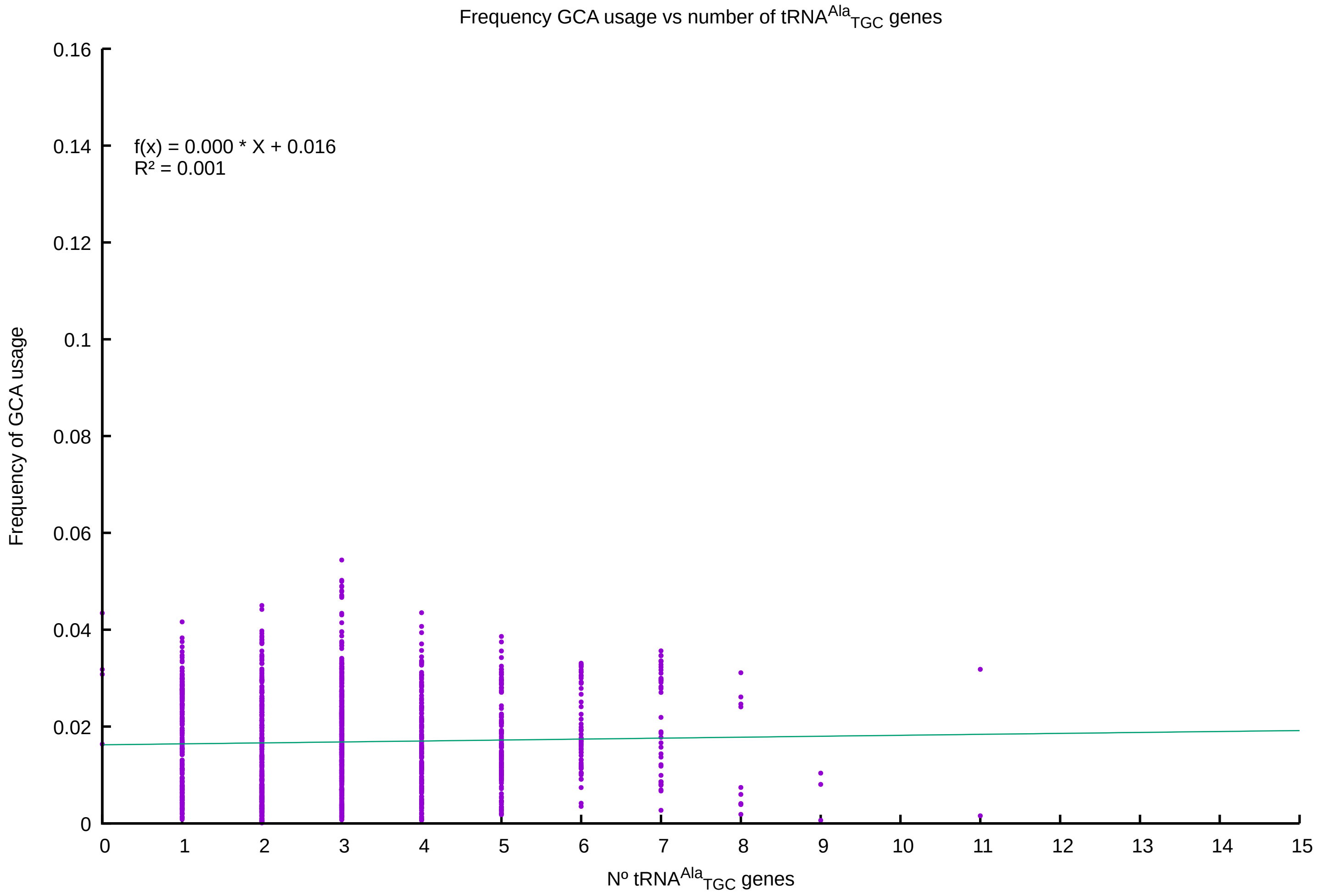

Frequency GCA usage vs number of $tRNA^{Ala}_{TGC}$ genes
f(x) = 0.000 * X + 0.016
R^2 = 0.001
Frequency of GCA usage
0.16
0.14
0.12
0.1
0.08
0.06
0.04
0.02
0
0 1 2 3 4 5 6 7 8 9 10 11 12 13 14 15
Nº $tRNA^{Ala}_{TGC}$ genes

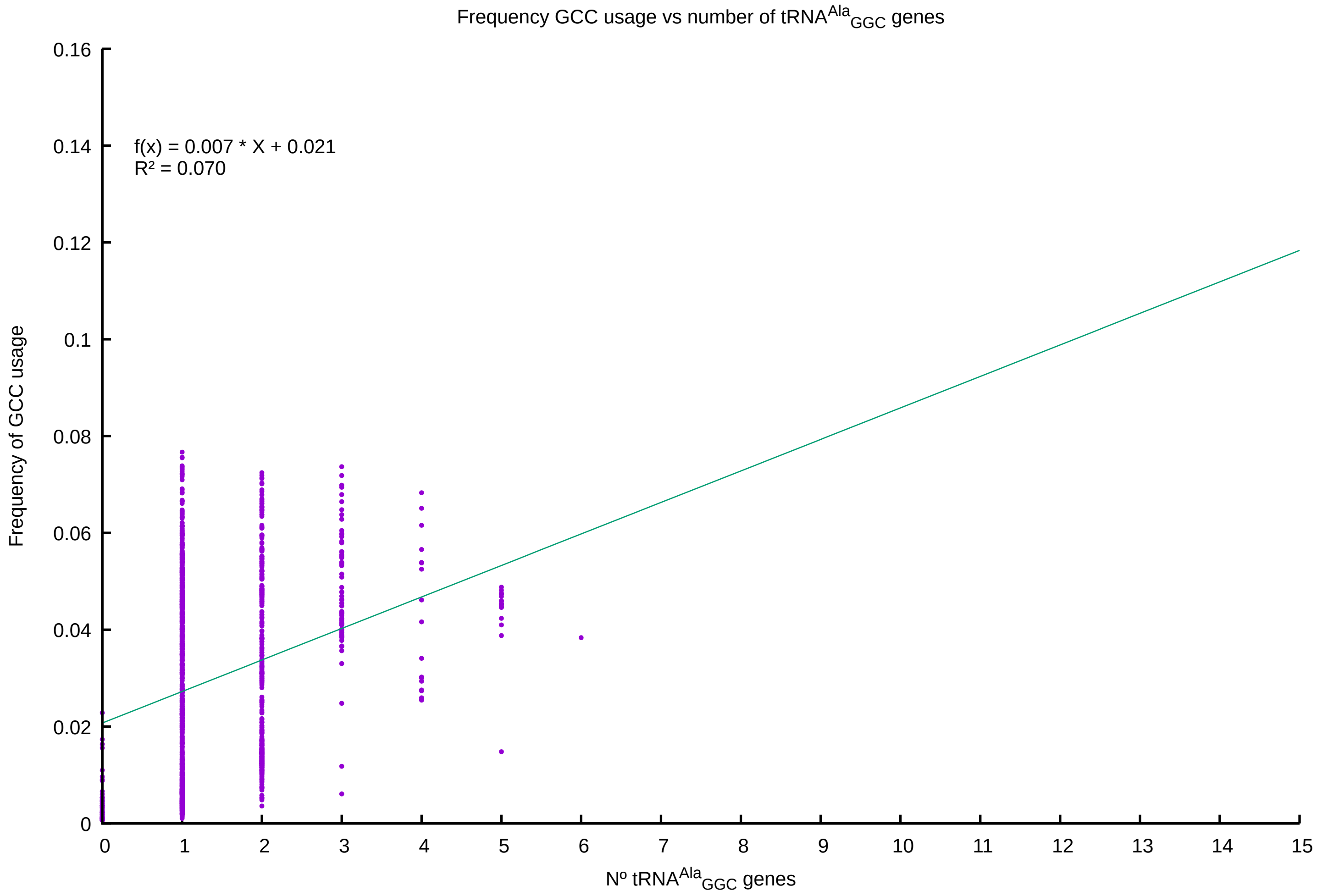

Frequency GCC usage vs number of tRNA^Ala_GGC genes
f(x) = 0.007 * X + 0.021
R² = 0.070
Frequency of GCC usage
0.16
0.14
0.12
0.1
0.08
0.06
0.04
0.02
0
0 1 2 3 4 5 6 7 8 9 10 11 12 13 14 15
Nº tRNA^Ala_GGC genes

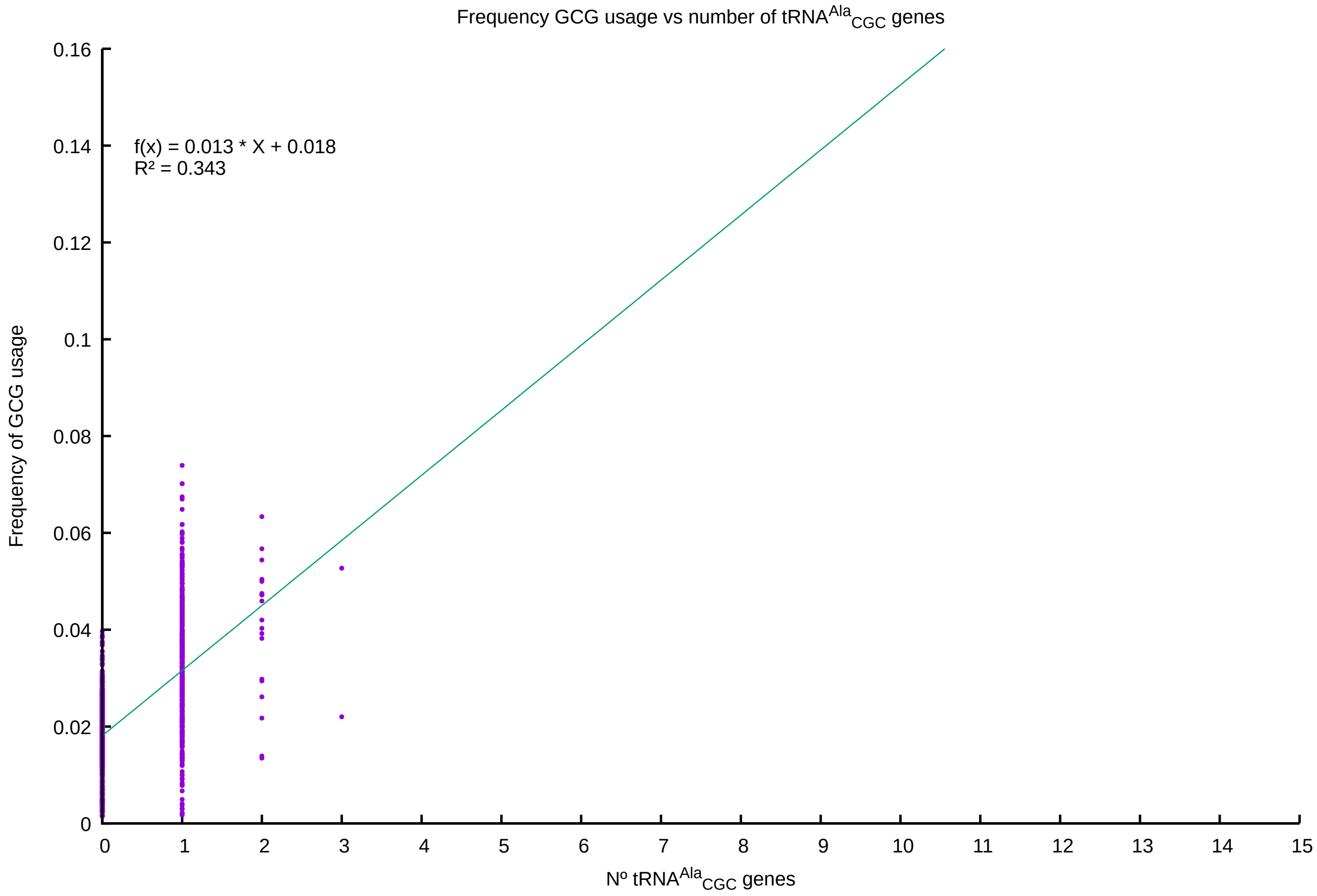
Frequency GCG usage vs number of tRNA^Ala_CGC genes
f(x) = 0.013 * X + 0.018
R² = 0.343
Frequency of GCG usage
0.16
0.14
0.12
0.1
0.08
0.06
0.04
0.02
0
0 1 2 3 4 5 6 7 8 9 10 11 12 13 14 15
Nº tRNA^Ala_CGC genes

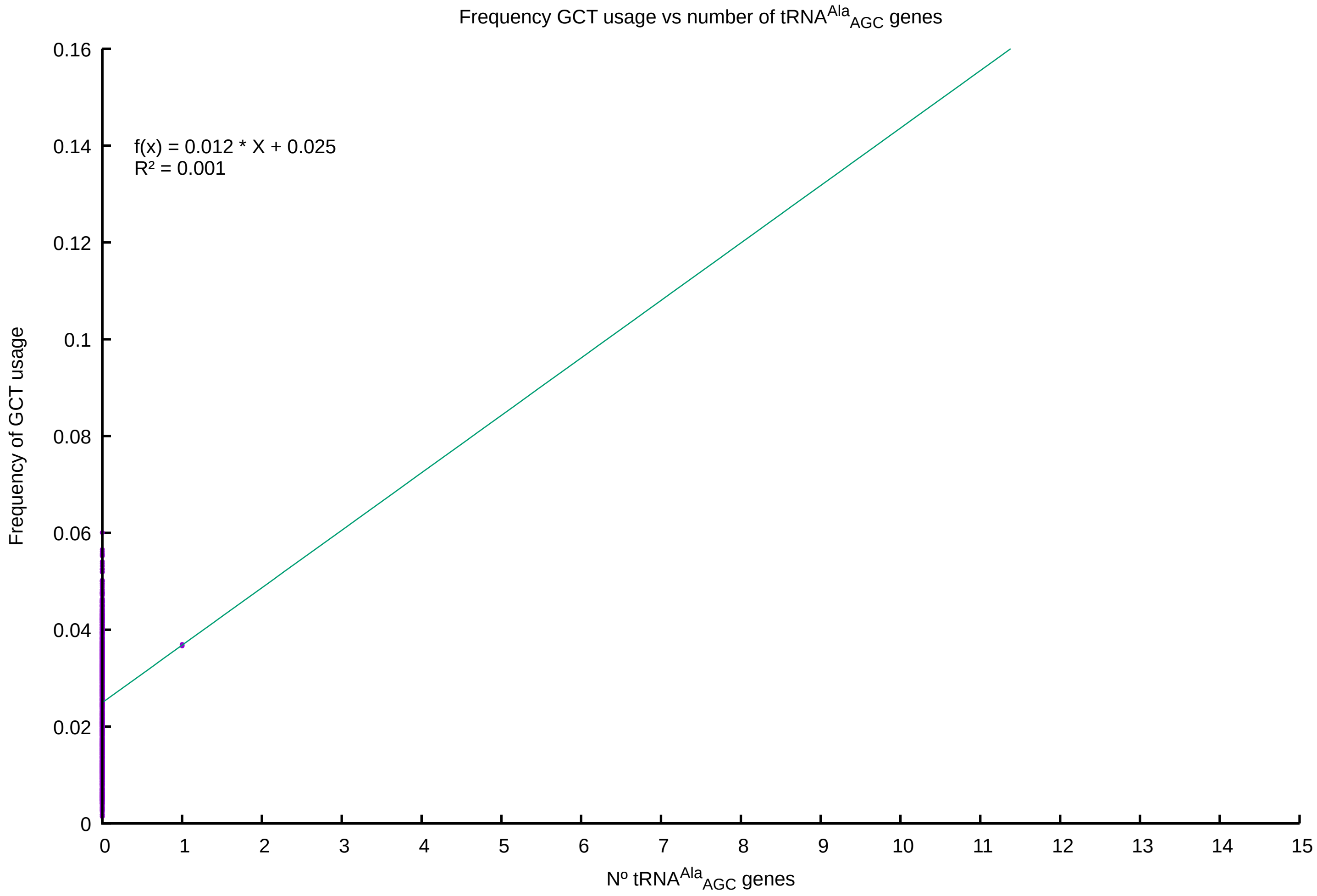

Frequency GCT usage vs number of tRNA^Ala_AGC genes
f(x) = 0.012 * X + 0.025
R² = 0.001
Frequency of GCT usage
0.16
0.14
0.12
0.1
0.08
0.06
0.04
0.02
0
0 1 2 3 4 5 6 7 8 9 10 11 12 13 14 15
Nº tRNA^Ala_AGC genes

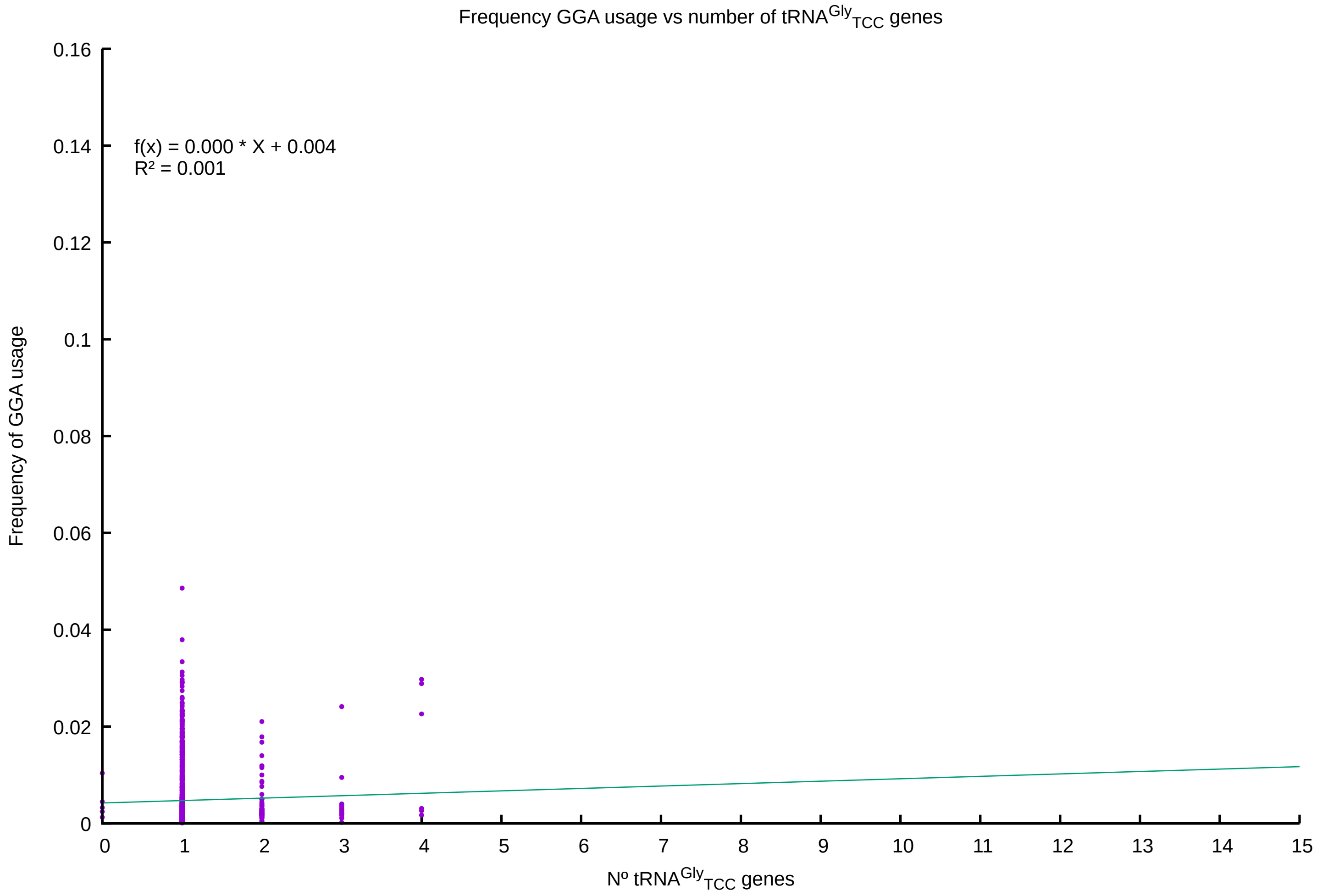

Frequency GGA usage vs number of tRNA^Gly_TCC genes
f(x) = 0.000 * X + 0.004
R² = 0.001
Frequency of GGA usage
Nº tRNA^Gly_TCC genes
0
0.02
0.04
0.06
0.08
0.1
0.12
0.14
0.16
0 1 2 3 4 5 6 7 8 9 10 11 12 13 14 15

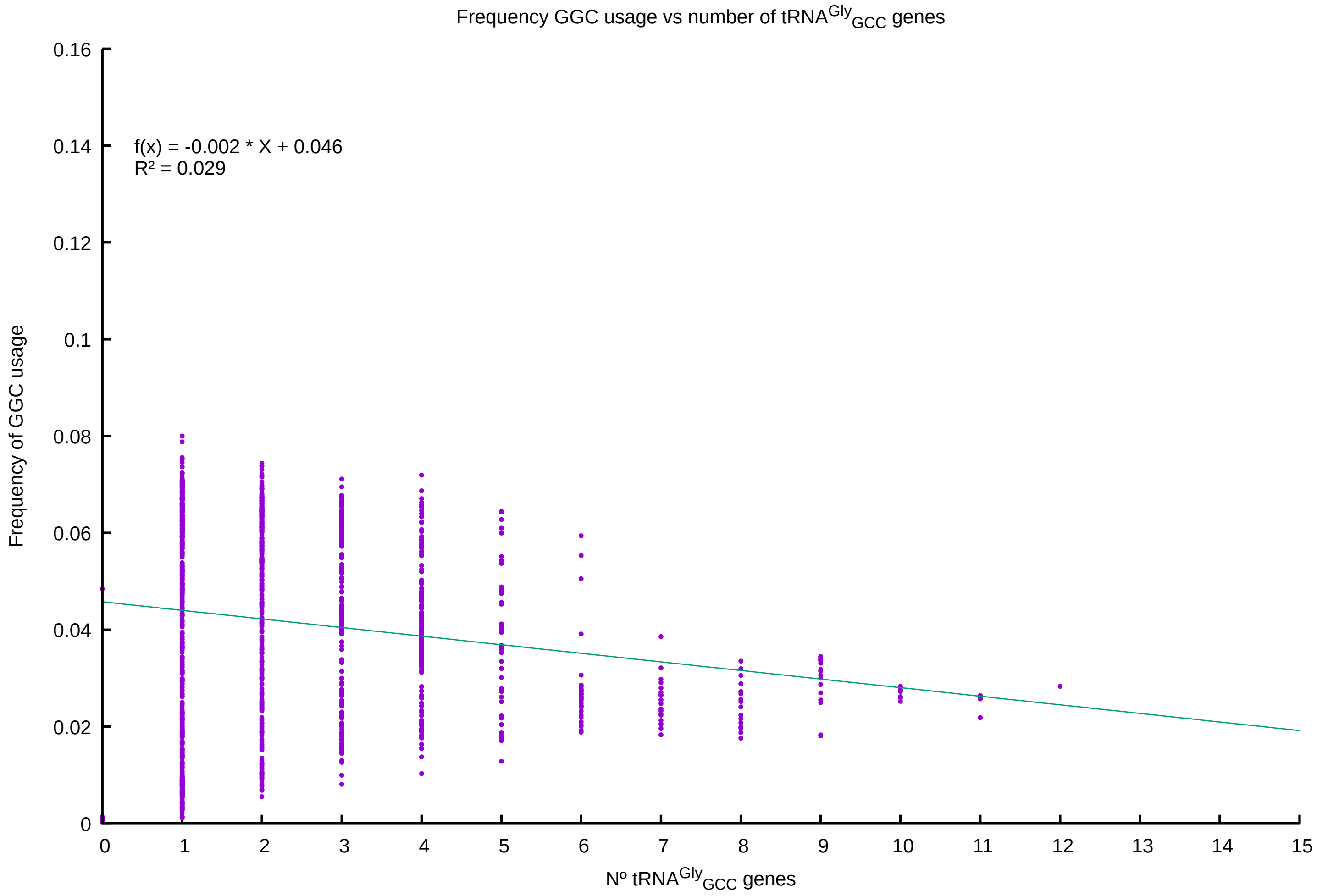

Frequency GGC usage vs number of tRNAGlyGCC genes
f(x) = -0.002 * X + 0.046
R² = 0.029
Frequency of GGC usage
0.16
0.14
0.12
0.1
0.08
0.06
0.04
0.02
0
0 1 2 3 4 5 6 7 8 9 10 11 12 13 14 15
Nº tRNAGlyGCC genes

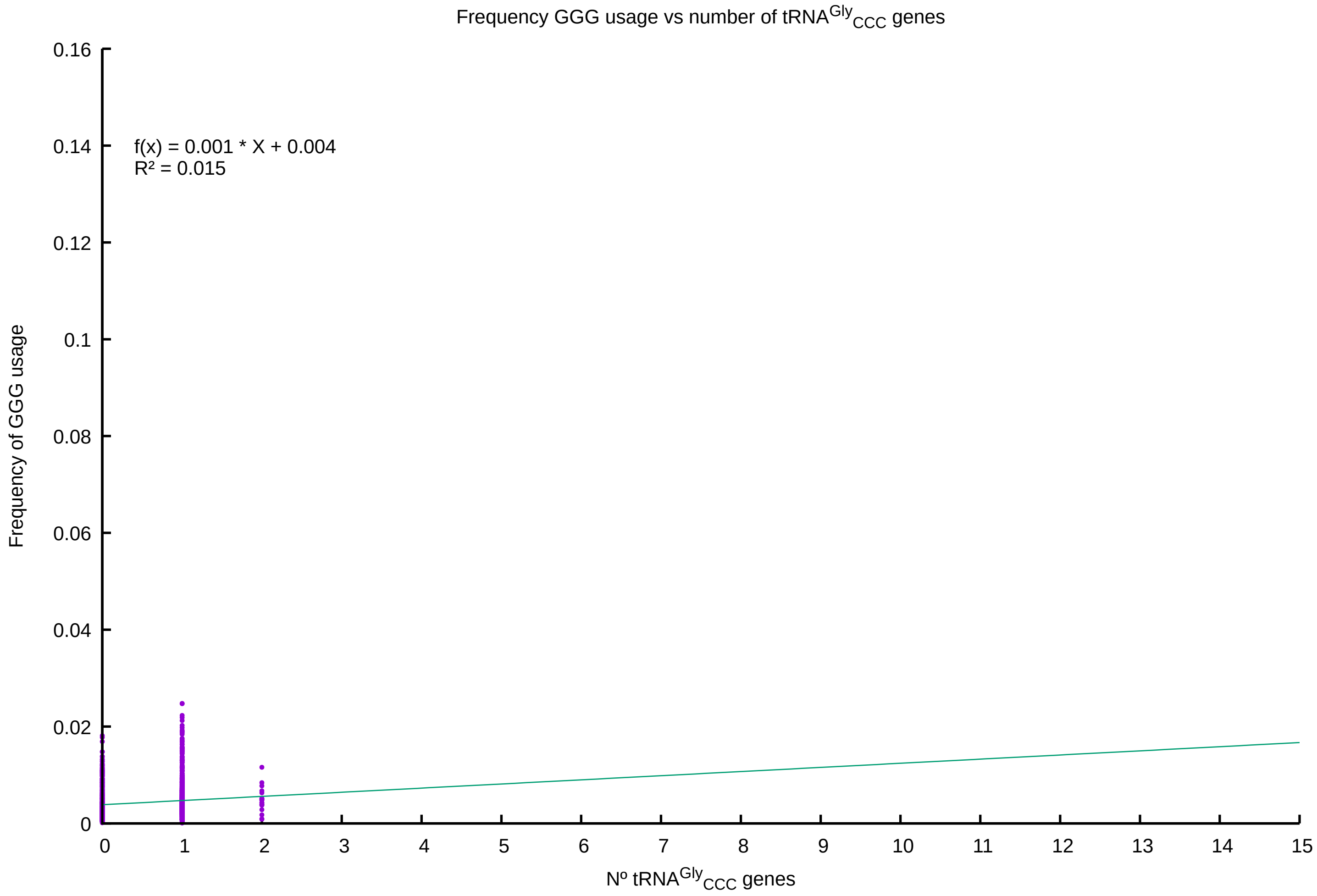
Frequency GGG usage vs number of tRNAGlyCCC genes
f(x) = 0.001 * X + 0.004
R² = 0.015
Frequency of GGG usage
0.16
0.14
0.12
0.1
0.08
0.06
0.04
0.02
0
0 1 2 3 4 5 6 7 8 9 10 11 12 13 14 15
Nº tRNAGlyCCC genes

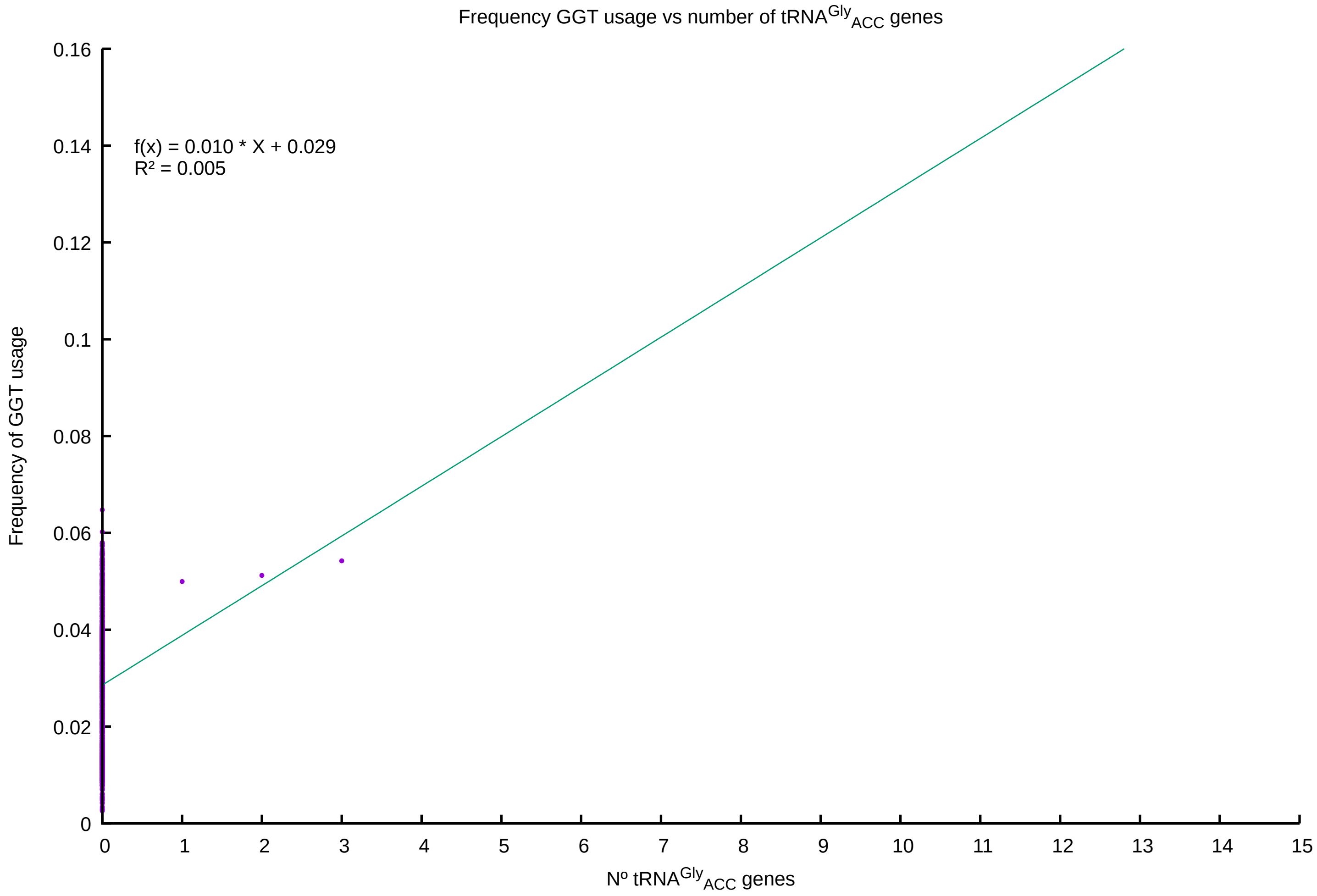

Frequency GGT usage vs number of tRNA^Gly_ACC genes
f(x) = 0.010 * X + 0.029
R² = 0.005
Frequency of GGT usage
Nº tRNA^Gly_ACC genes
0
0.02
0.04
0.06
0.08
0.1
0.12
0.14
0.16
0 1 2 3 4 5 6 7 8 9 10 11 12 13 14 15

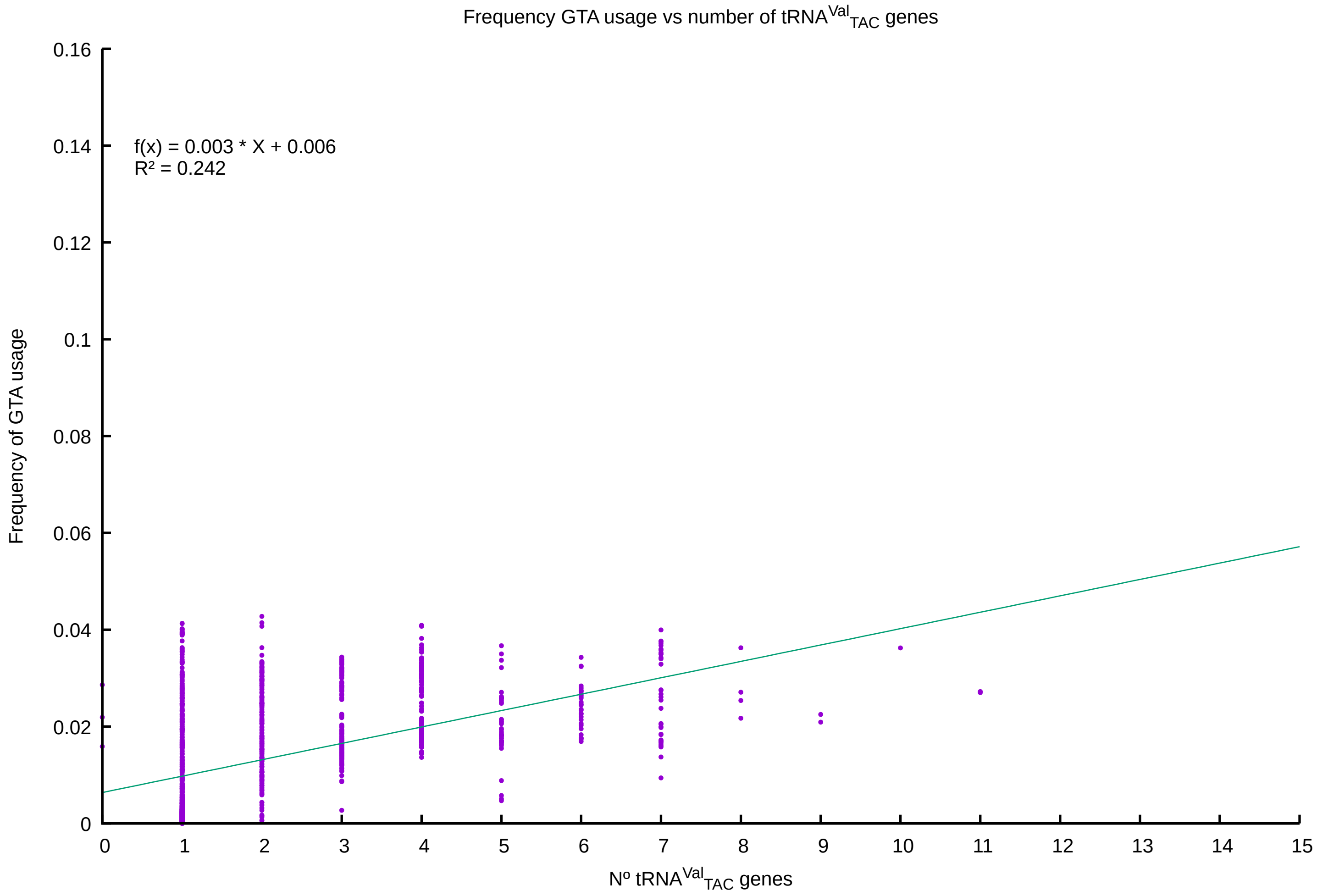
Frequency GTA usage vs number of tRNA^Val_TAC genes
f(x) = 0.003 * X + 0.006
R² = 0.242
Frequency of GTA usage
0.16
0.14
0.12
0.1
0.08
0.06
0.04
0.02
0
0 1 2 3 4 5 6 7 8 9 10 11 12 13 14 15
Nº tRNA^Val_TAC genes

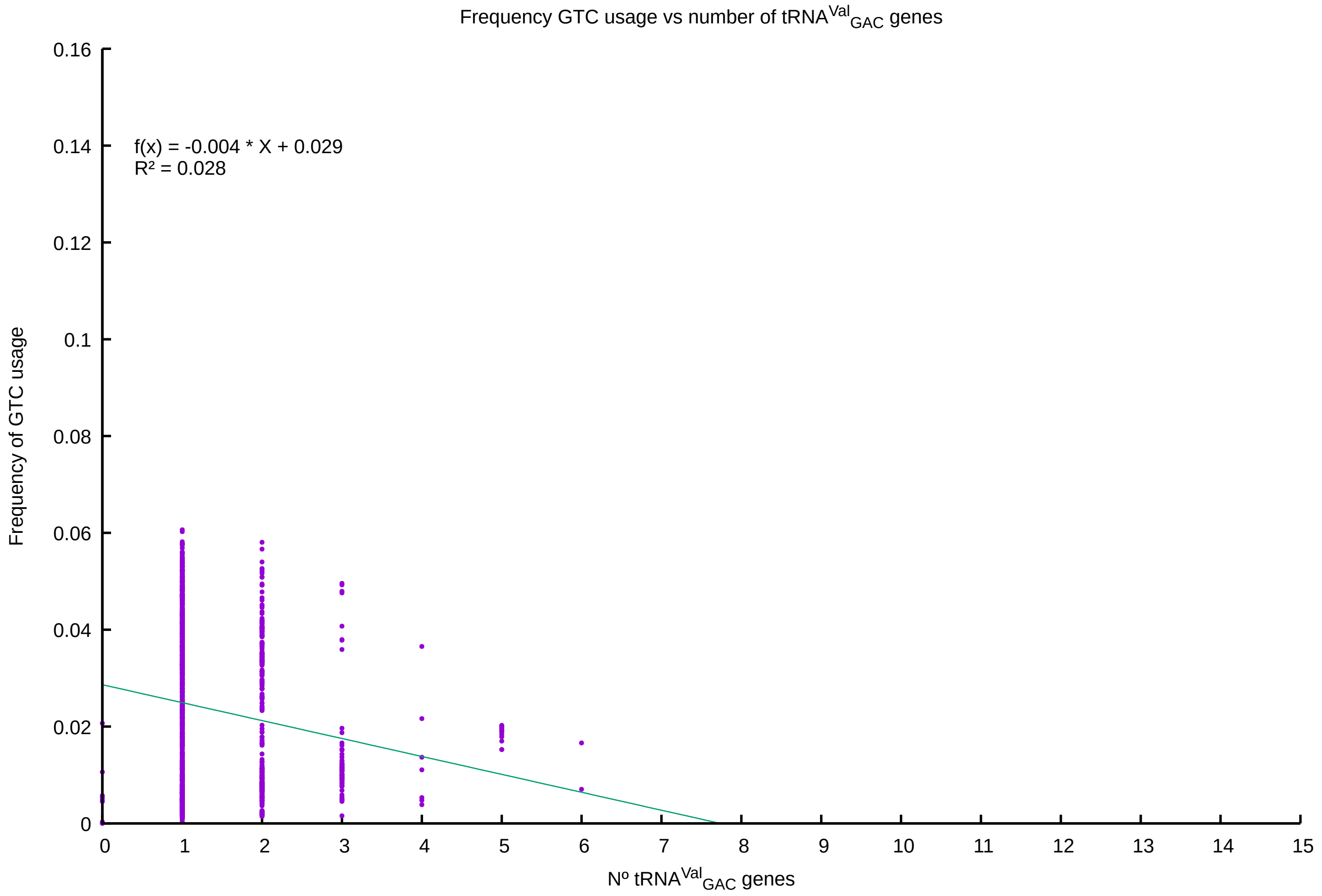
Frequency GTC usage vs number of tRNAValGAC genes
f(x) = -0.004 * X + 0.029
R² = 0.028
Frequency of GTC usage
Nº tRNAValGAC genes
0
0.02
0.04
0.06
0.08
0.1
0.12
0.14
0.16
0
1
2
3
4
5
6
7
8
9
10
11
12
13
14
15

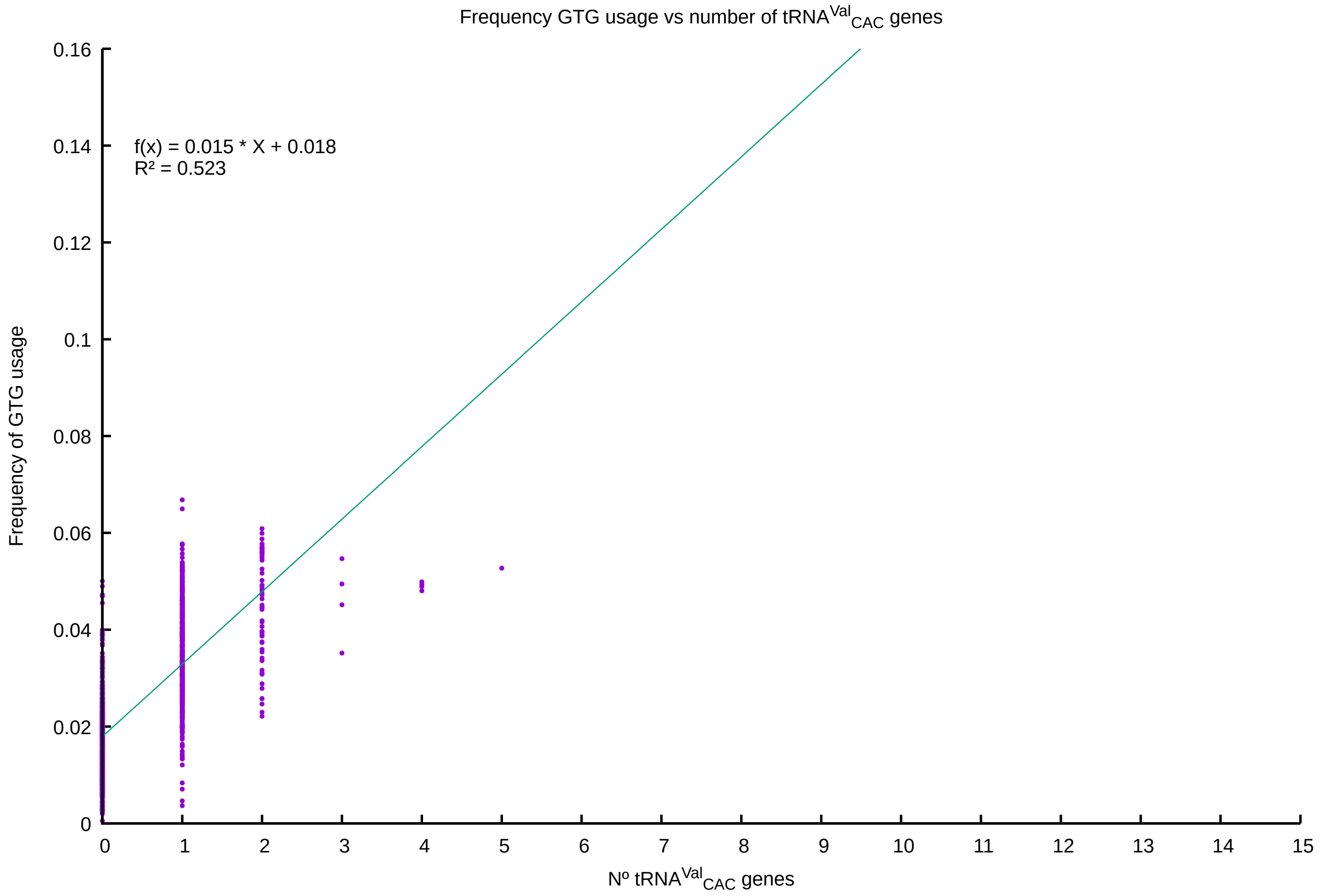
Frequency GTG usage vs number of tRNA Val CAC genes
f(x) = 0.015 * X + 0.018
R² = 0.523
Frequency of GTG usage
0.16
0.14
0.12
0.1
0.08
0.06
0.04
0.02
0
0 1 2 3 4 5 6 7 8 9 10 11 12 13 14 15
Nº tRNA Val CAC genes

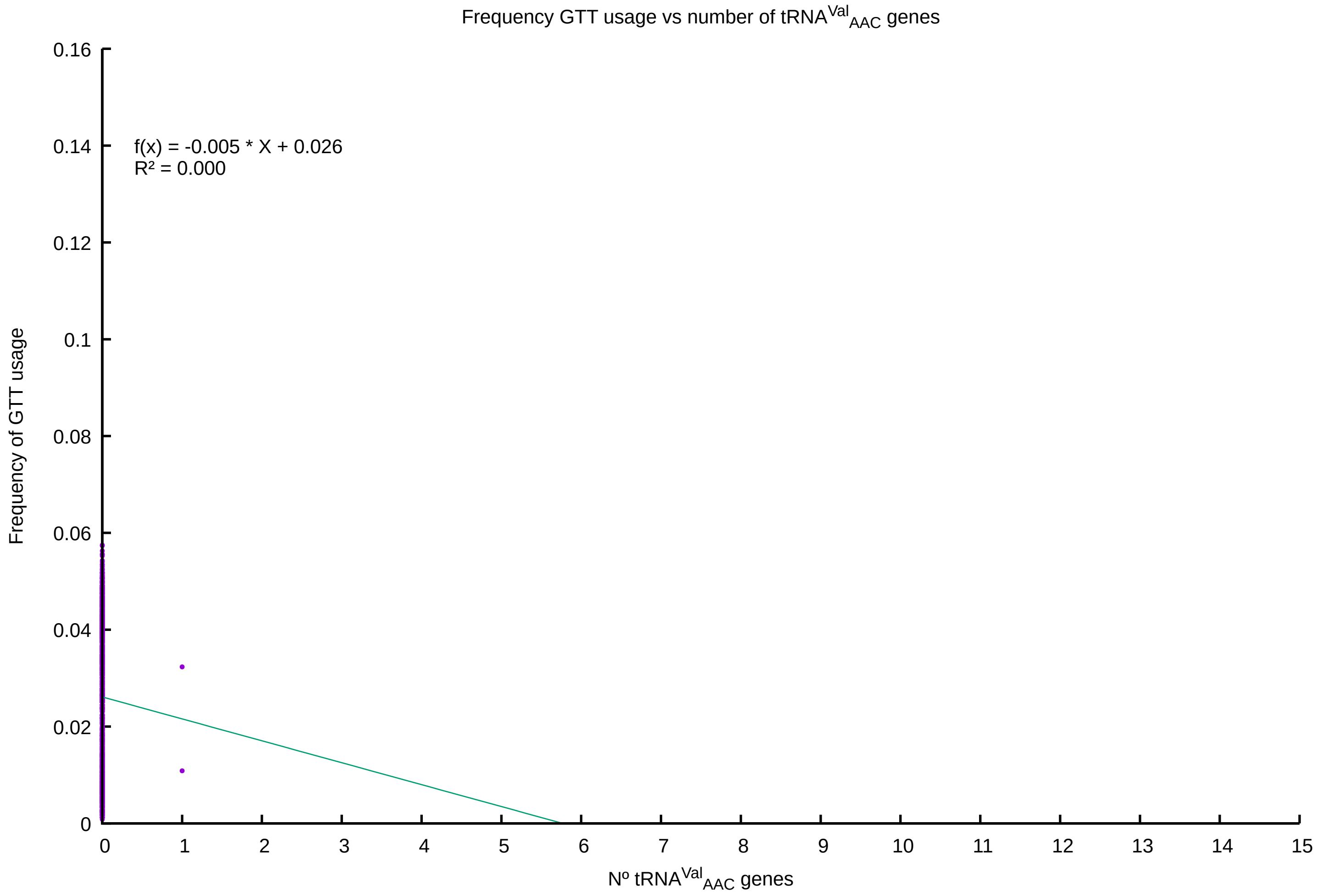
Frequency GTT usage vs number of tRNA^Val_AAC genes
f(x) = -0.005 * X + 0.026
R² = 0.000
Frequency of GTT usage
Nº tRNA^Val_AAC genes
0
0.02
0.04
0.06
0.08
0.1
0.12
0.14
0.16
0 1 2 3 4 5 6 7 8 9 10 11 12 13 14 15

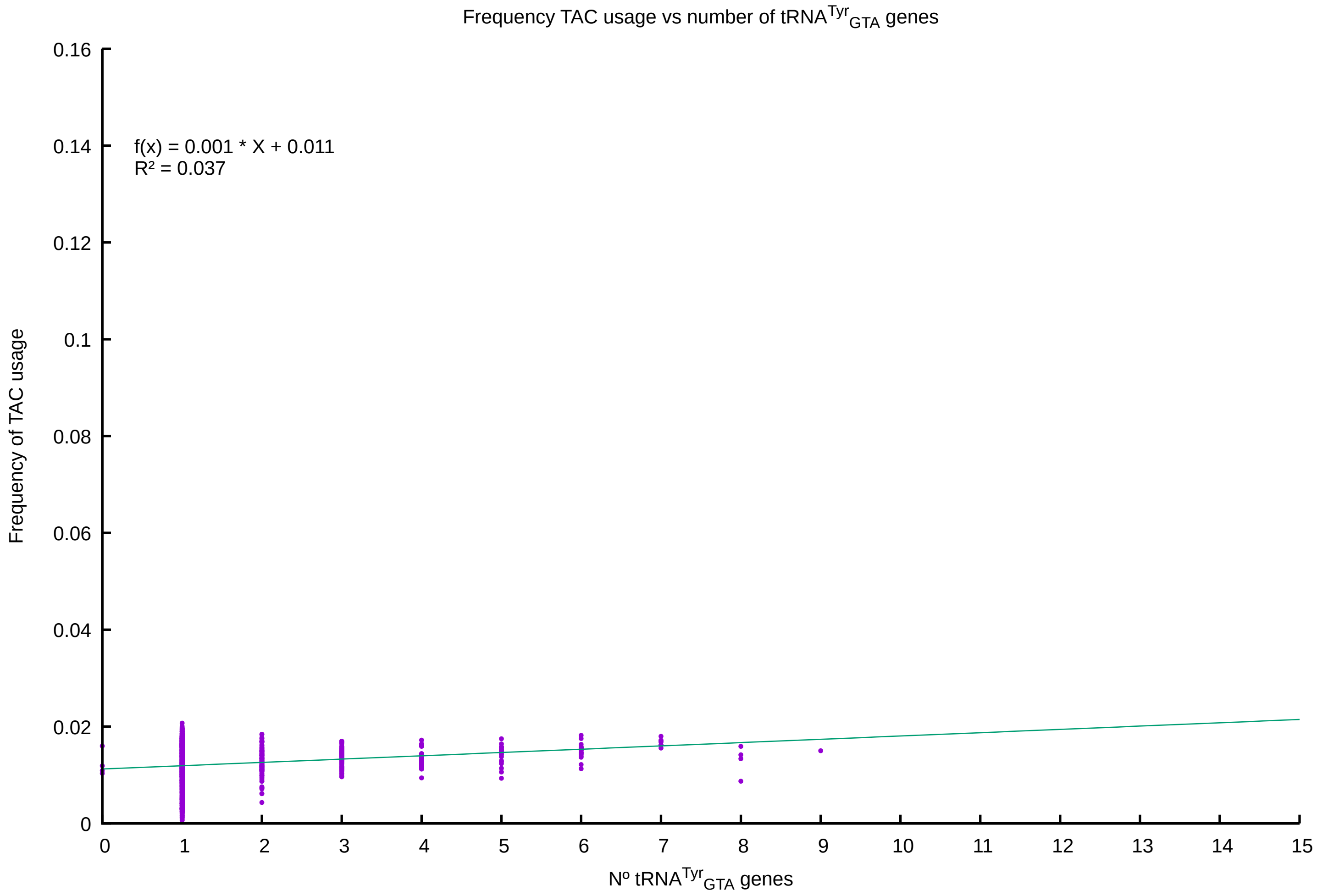

Frequency TAC usage vs number of tRNA^Tyr_GTA genes
f(x) = 0.001 * X + 0.011
R² = 0.037
Frequency of TAC usage
0.16
0.14
0.12
0.1
0.08
0.06
0.04
0.02
0
0 1 2 3 4 5 6 7 8 9 10 11 12 13 14 15
Nº tRNA^Tyr_GTA genes

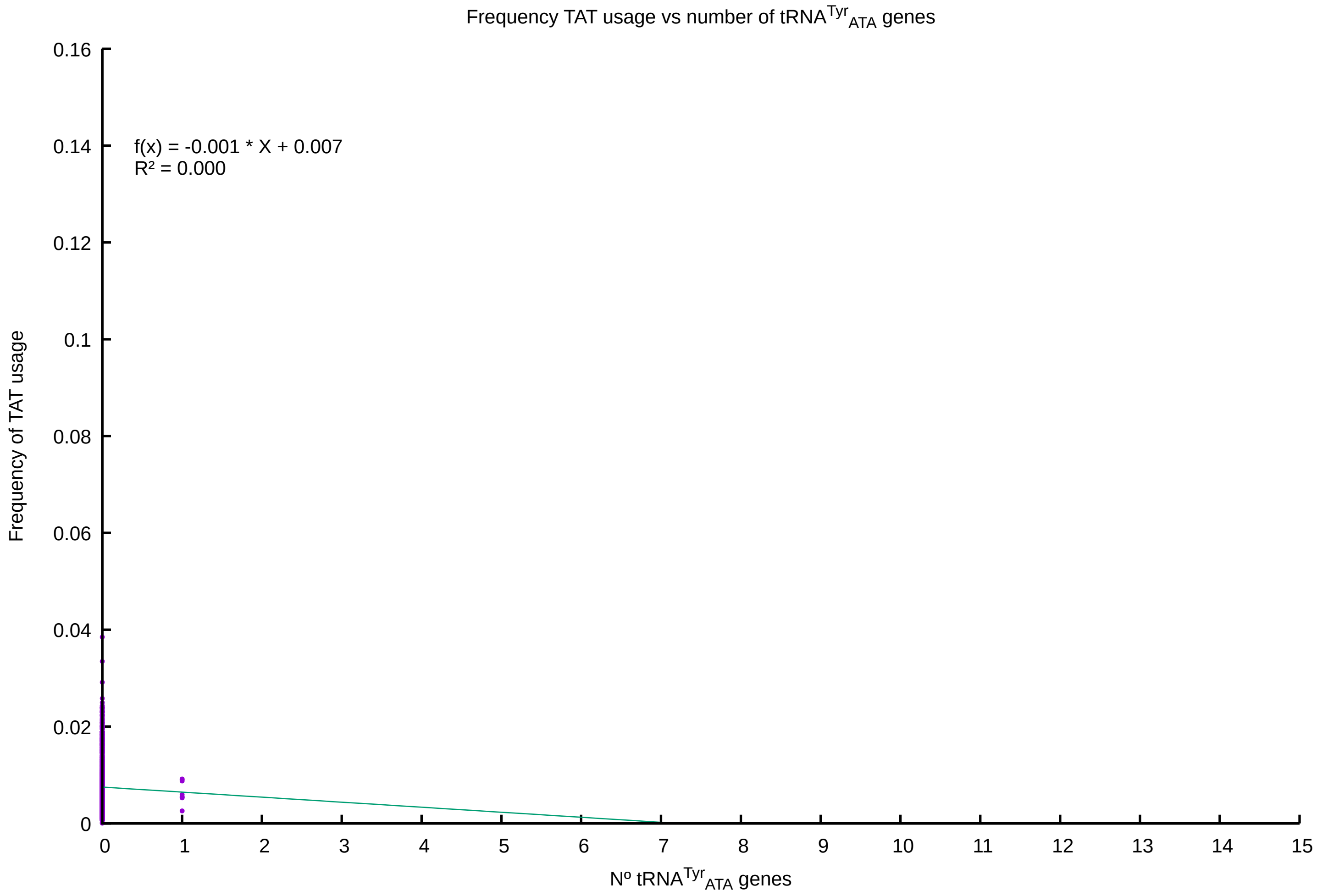

Frequency TAT usage vs number of tRNA$^{Tyr}_{ATA}$ genes
f(x) = -0.001 * X + 0.007
R² = 0.000
Frequency of TAT usage
0.16
0.14
0.12
0.1
0.08
0.06
0.04
0.02
0
0
1
2
3
4
5
6
7
8
9
10
11
12
13
14
15
Nº tRNA$^{Tyr}_{ATA}$ genes

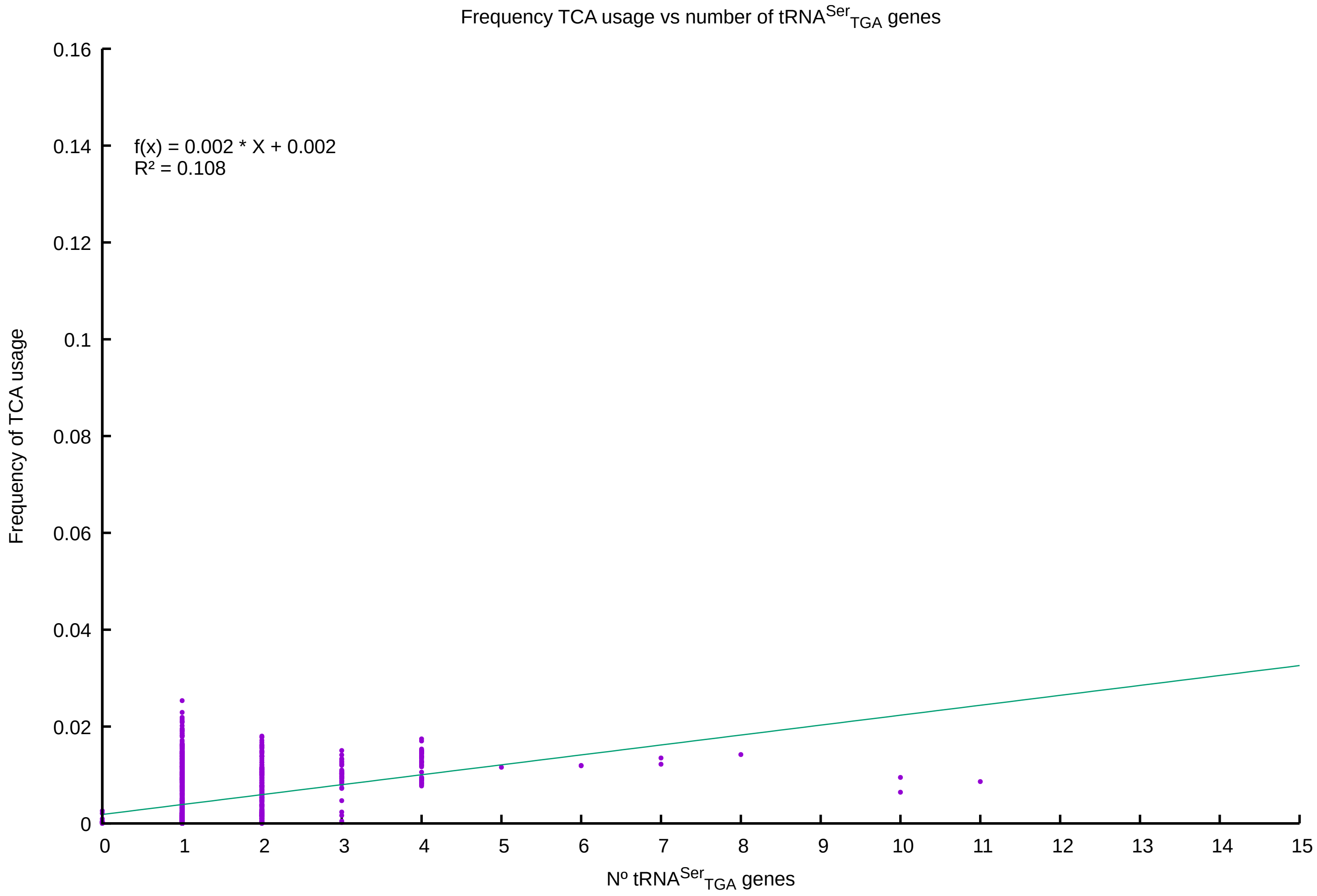

Frequency TCA usage vs number of tRNASerTGA genes
f(x) = 0.002 * X + 0.002
R² = 0.108
Frequency of TCA usage
0.16
0.14
0.12
0.1
0.08
0.06
0.04
0.02
0
0 1 2 3 4 5 6 7 8 9 10 11 12 13 14 15
Nº tRNASerTGA genes

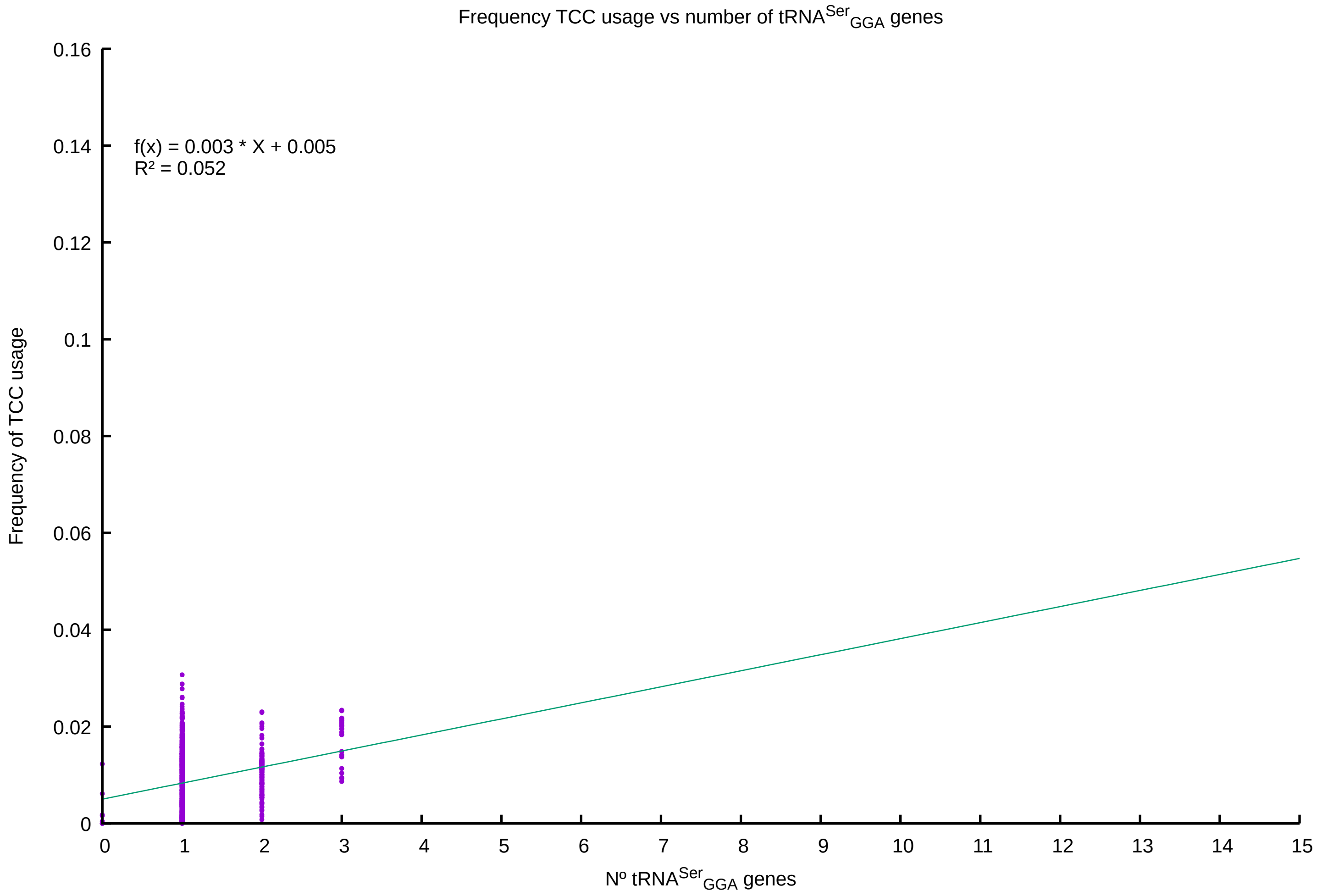

Frequency TCC usage vs number of tRNA$^{Ser}_{GGA}$ genes
f(x) = 0.003 * X + 0.005
R² = 0.052
Frequency of TCC usage
0.16
0.14
0.12
0.1
0.08
0.06
0.04
0.02
0
0 1 2 3 4 5 6 7 8 9 10 11 12 13 14 15
Nº tRNA$^{Ser}_{GGA}$ genes

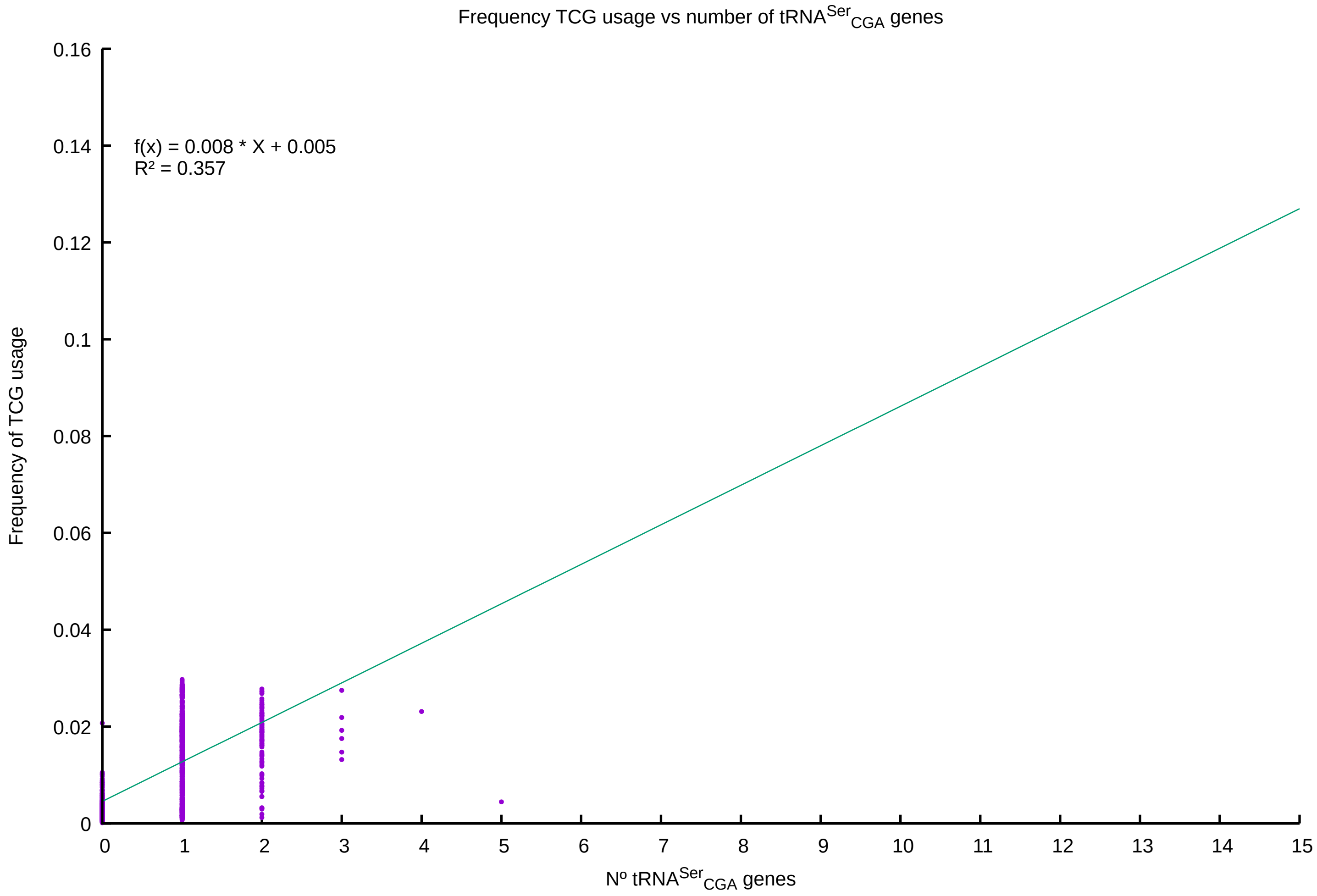

Frequency TCG usage vs number of tRNA^Ser_CGA genes
f(x) = 0.008 * X + 0.005
R² = 0.357
Frequency of TCG usage
0.16
0.14
0.12
0.1
0.08
0.06
0.04
0.02
0
0 1 2 3 4 5 6 7 8 9 10 11 12 13 14 15
Nº tRNA^Ser_CGA genes

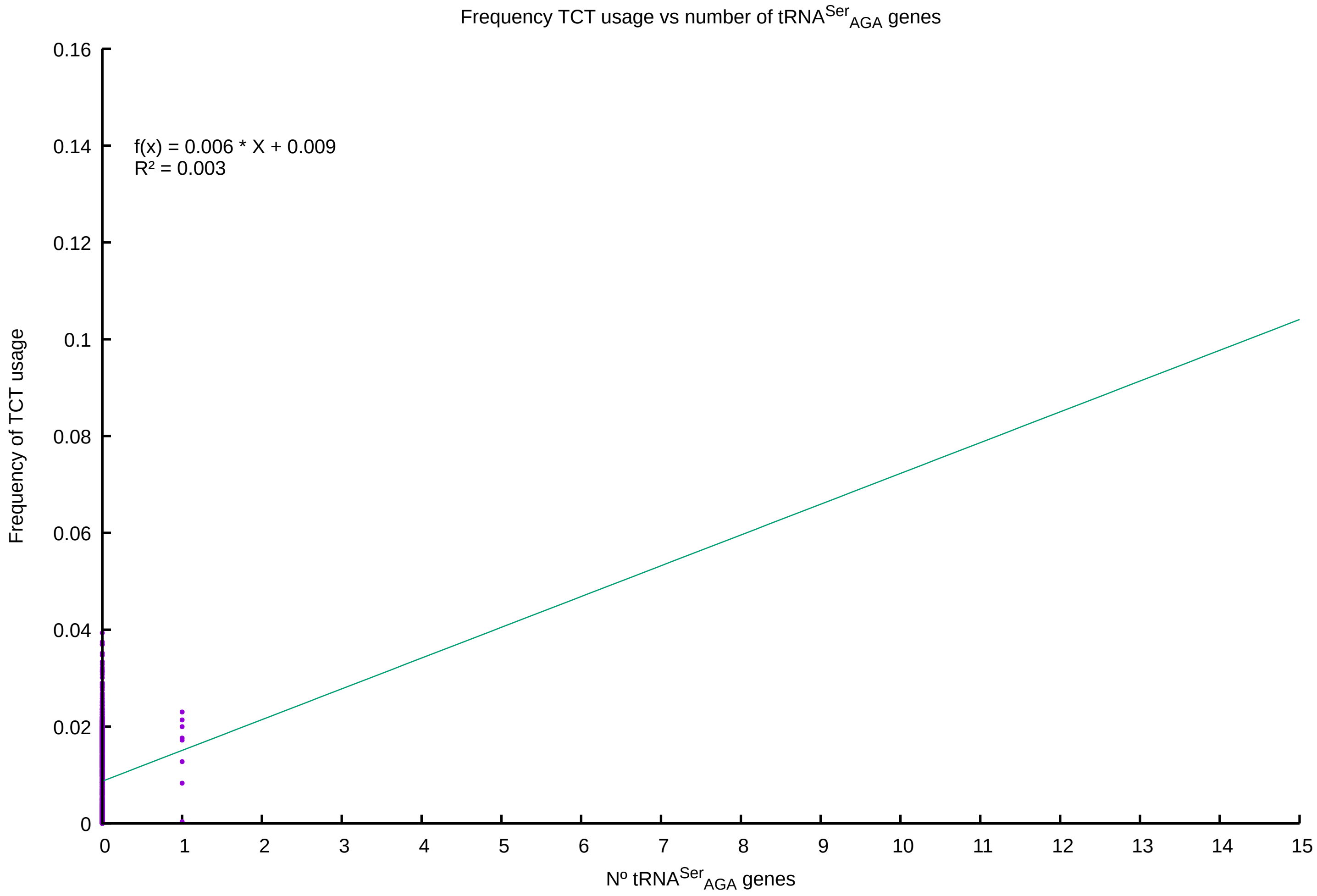

Frequency TCT usage vs number of tRNASerAGA genes
f(x) = 0.006 * X + 0.009
R² = 0.003
Frequency of TCT usage
0.16
0.14
0.12
0.1
0.08
0.06
0.04
0.02
0
0 1 2 3 4 5 6 7 8 9 10 11 12 13 14 15
Nº tRNASerAGA genes

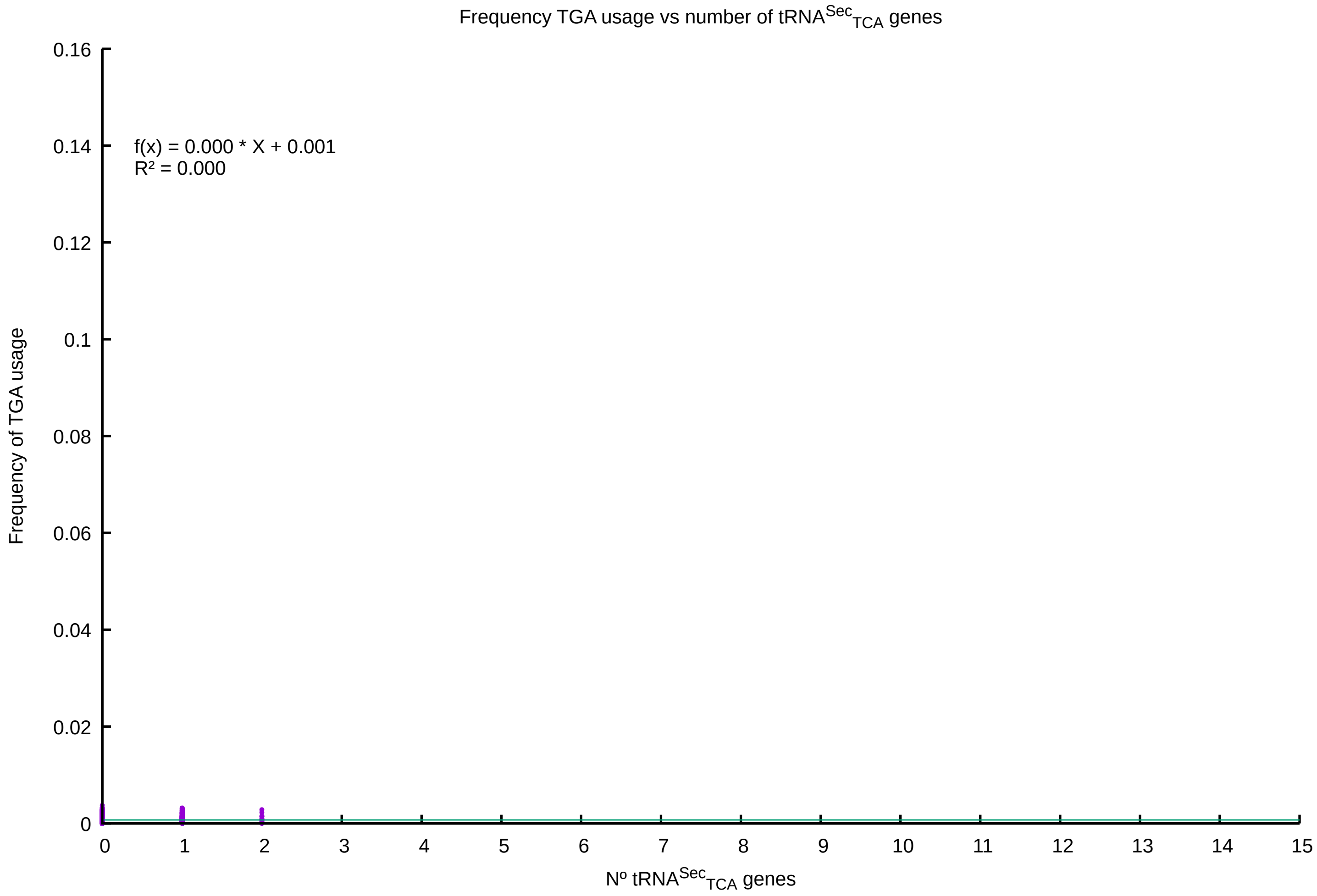
Frequency TGA usage vs number of tRNASecTCA genes
f(x) = 0.000 * X + 0.001
R² = 0.000
Frequency of TGA usage
0.16
0.14
0.12
0.1
0.08
0.06
0.04
0.02
0
0 1 2 3 4 5 6 7 8 9 10 11 12 13 14 15
Nº tRNASecTCA genes

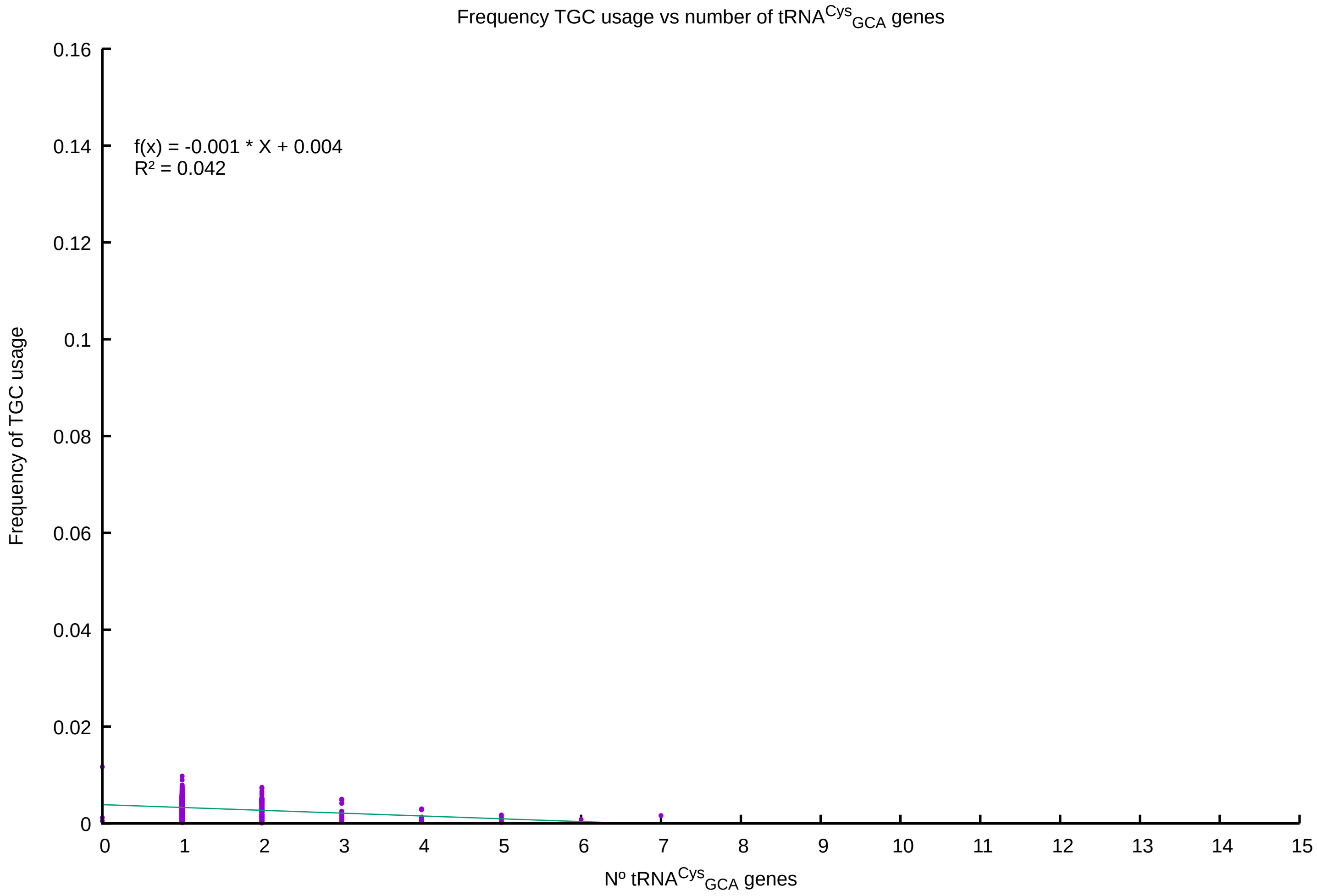
Frequency TGC usage vs number of tRNACysGCA genes
f(x) = -0.001 * X + 0.004
R² = 0.042
Frequency of TGC usage
0.16
0.14
0.12
0.1
0.08
0.06
0.04
0.02
0
0 1 2 3 4 5 6 7 8 9 10 11 12 13 14 15
Nº tRNACysGCA genes

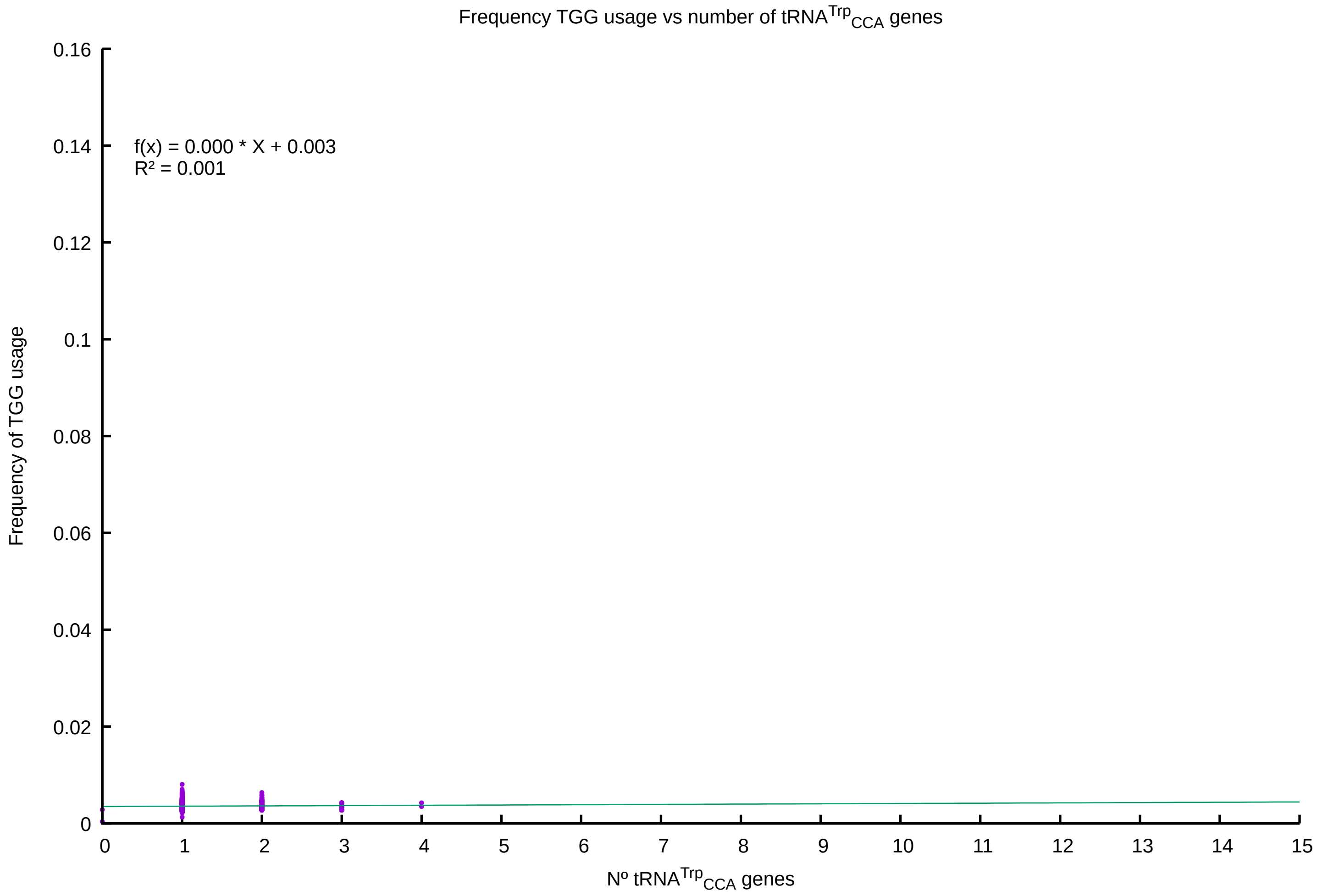
Frequency TGG usage vs number of tRNA^Trp_CCA genes
f(x) = 0.000 * X + 0.003
R² = 0.001
Frequency of TGG usage
0.16
0.14
0.12
0.1
0.08
0.06
0.04
0.02
0
0 1 2 3 4 5 6 7 8 9 10 11 12 13 14 15
Nº tRNA^Trp_CCA genes

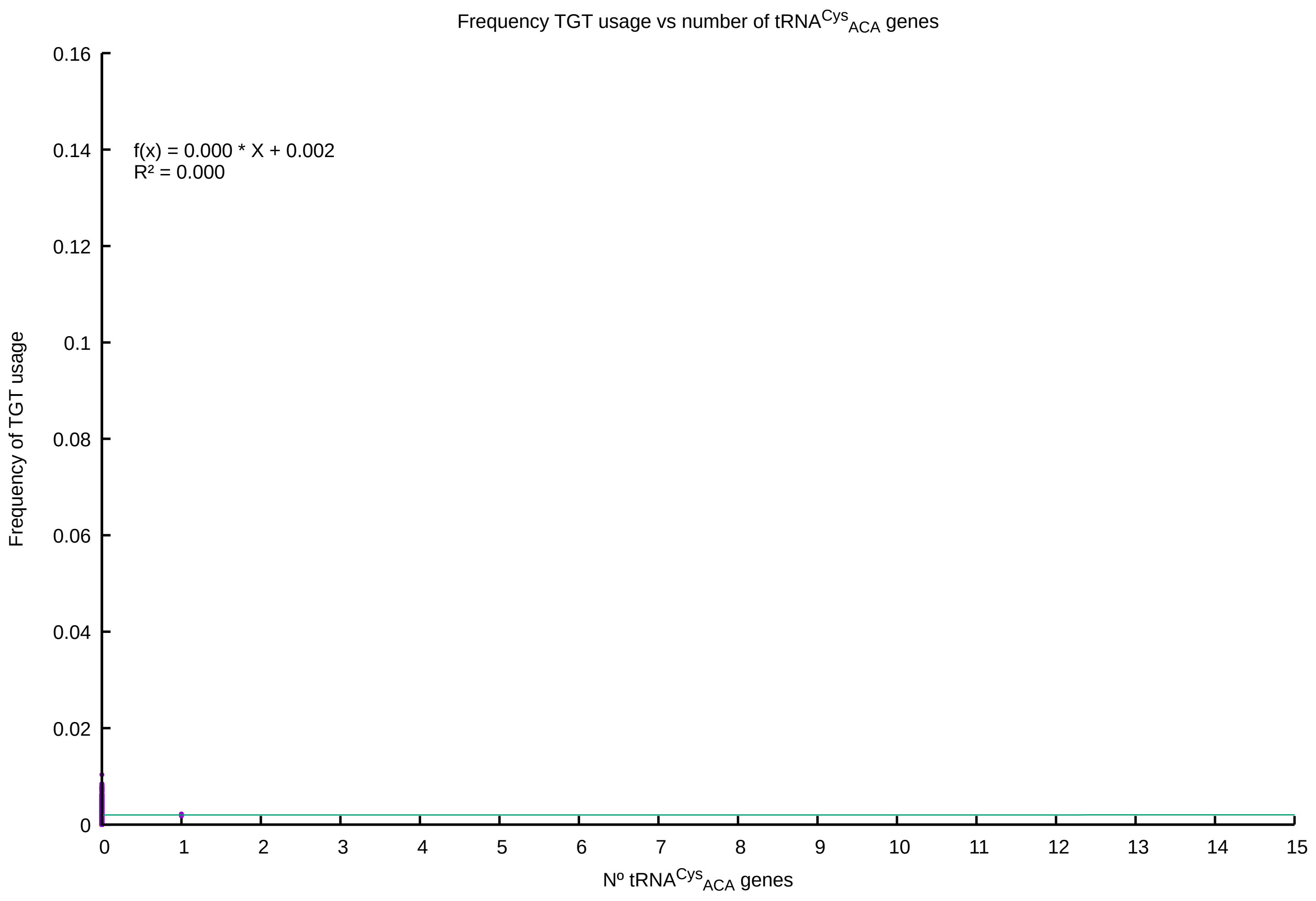

Frequency TGT usage vs number of tRNA$^{Cys}_{ACA}$ genes
f(x) = 0.000 * X + 0.002
R² = 0.000
Frequency of TGT usage
0.16
0.14
0.12
0.1
0.08
0.06
0.04
0.02
0
0 1 2 3 4 5 6 7 8 9 10 11 12 13 14 15
Nº tRNA$^{Cys}_{ACA}$ genes

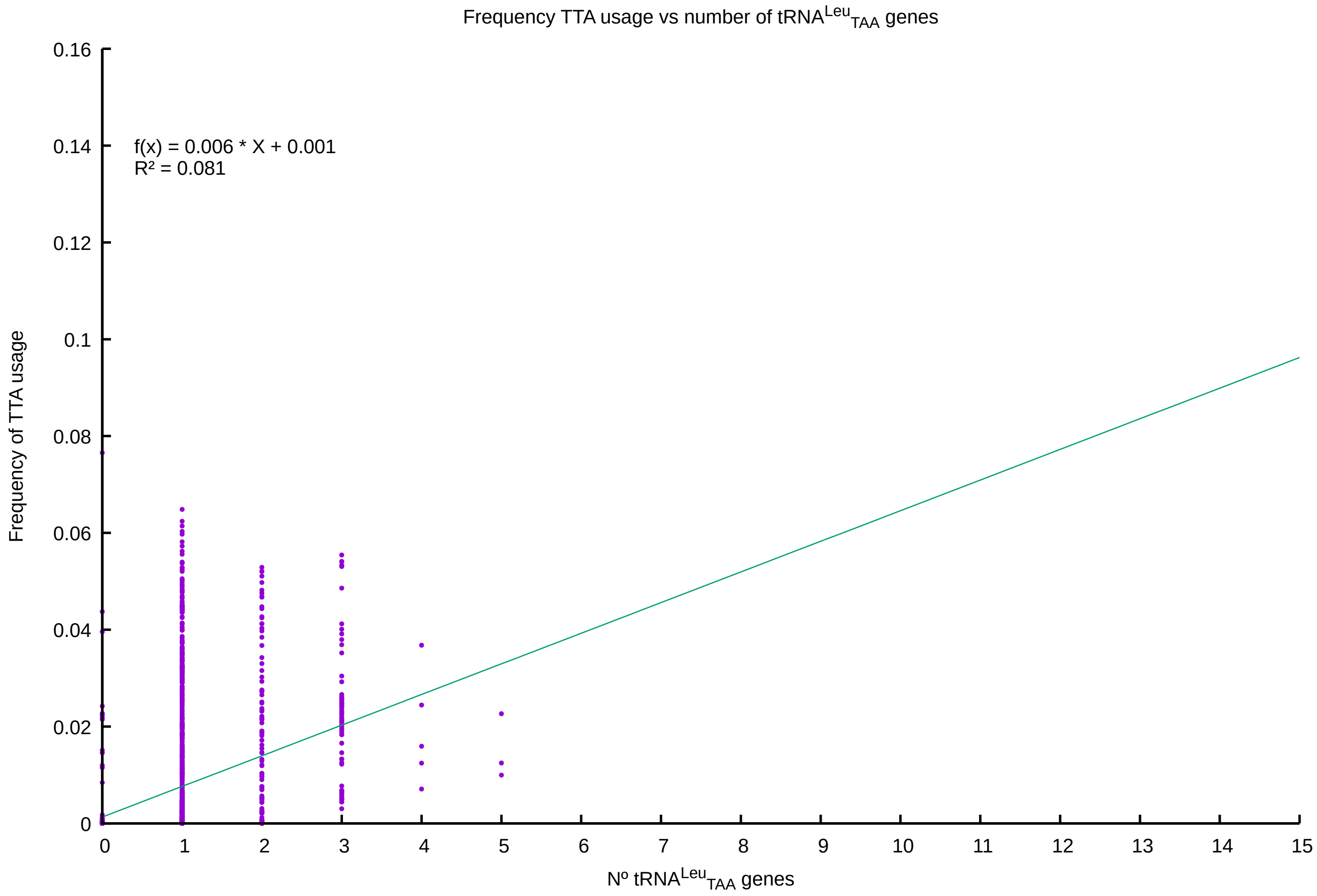

Frequency TTA usage vs number of tRNA^Leu_TAA genes
f(x) = 0.006 * X + 0.001
R² = 0.081
0.16
0.14
0.12
0.1
0.08
0.06
0.04
0.02
0
Frequency of TTA usage
0 1 2 3 4 5 6 7 8 9 10 11 12 13 14 15
Nº tRNA^Leu_TAA genes

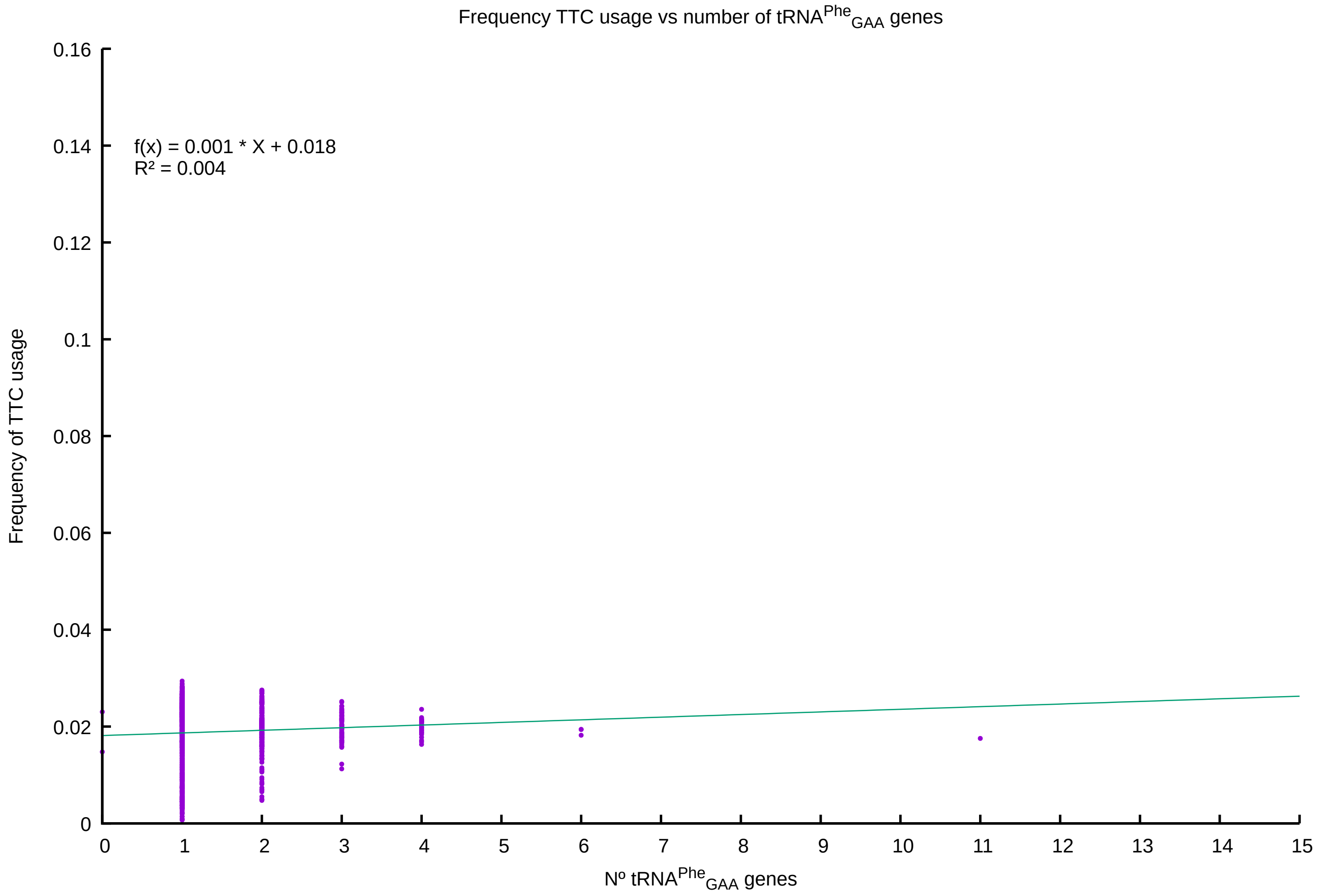

Frequency TTC usage vs number of tRNA^Phe_GAA genes
f(x) = 0.001 * X + 0.018
R² = 0.004
Frequency of TTC usage
0.16
0.14
0.12
0.1
0.08
0.06
0.04
0.02
0
0 1 2 3 4 5 6 7 8 9 10 11 12 13 14 15
Nº tRNA^Phe_GAA genes

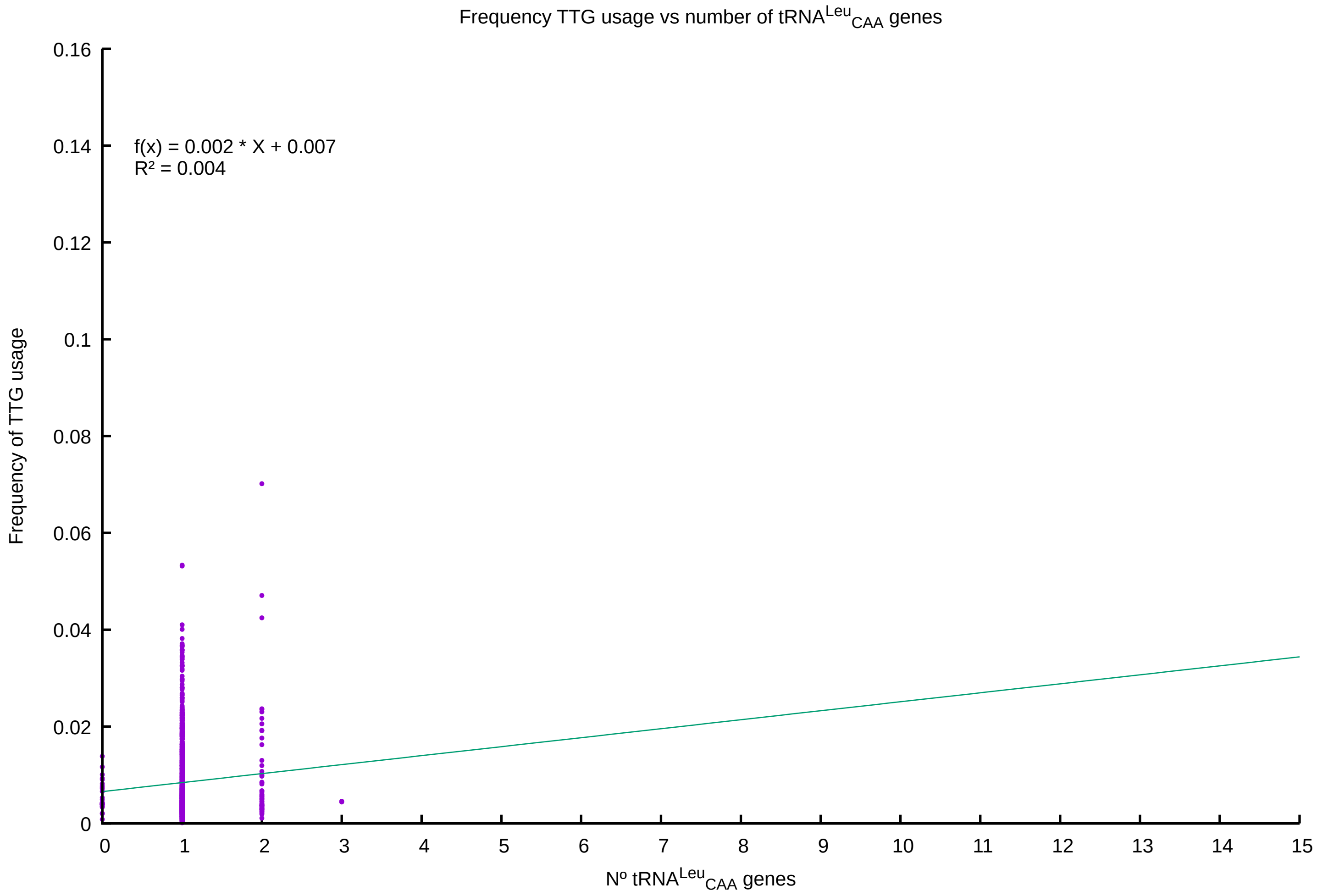
Frequency TTG usage vs number of tRNALeuCAA genes
f(x) = 0.002 * X + 0.007
R² = 0.004
Frequency of TTG usage
0.16
0.14
0.12
0.1
0.08
0.06
0.04
0.02
0
0 1 2 3 4 5 6 7 8 9 10 11 12 13 14 15
Nº tRNALeuCAA genes

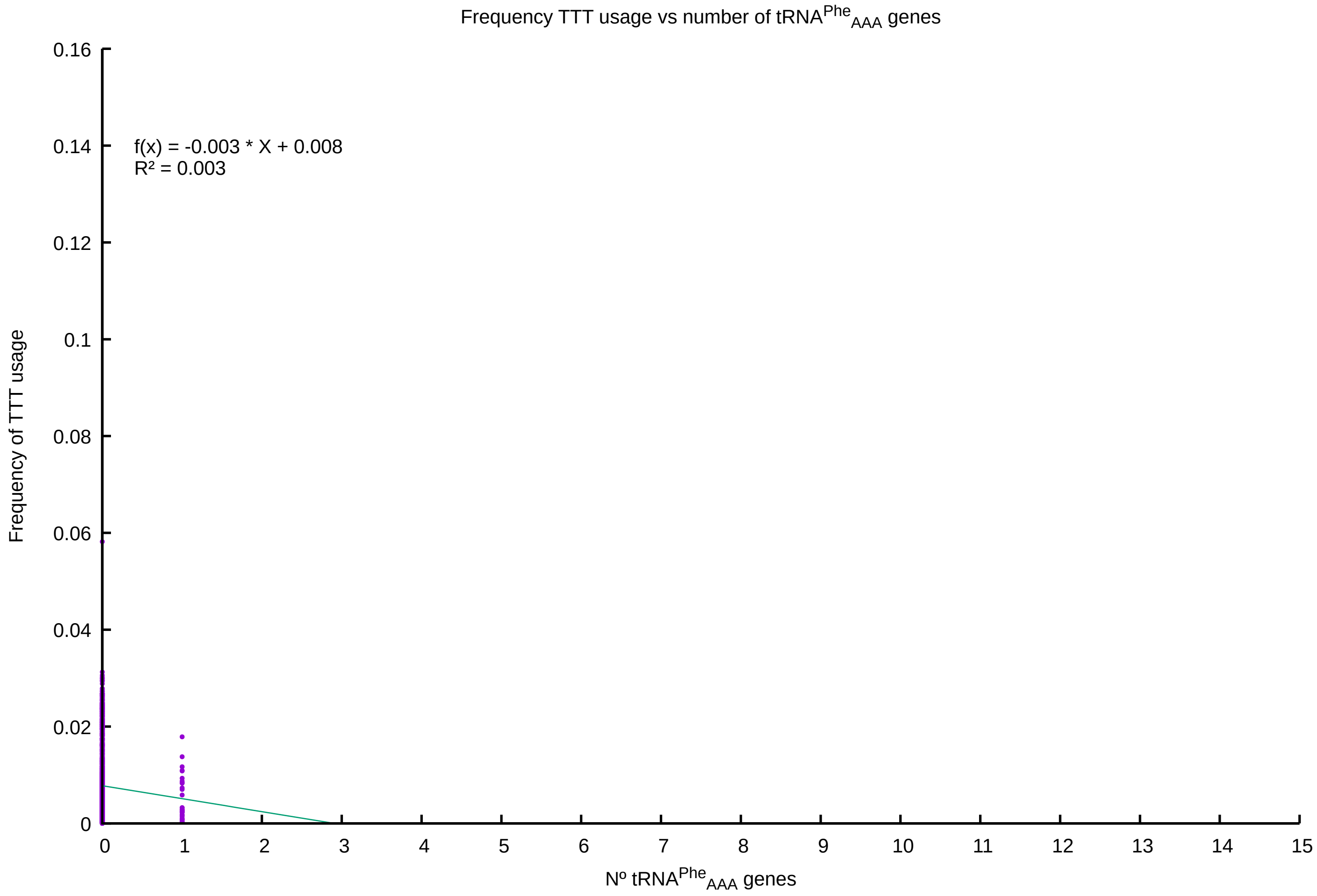

Frequency TTT usage vs number of tRNA^Phe_AAA genes
f(x) = -0.003 * X + 0.008
R² = 0.003
Frequency of TTT usage
0.16
0.14
0.12
0.1
0.08
0.06
0.04
0.02
0
0 1 2 3 4 5 6 7 8 9 10 11 12 13 14 15
Nº tRNA^Phe_AAA genes
