# Supplementary material for: Impact of the chemical modification of tRNAs anticodon loop on the variability and evolution of codon usage in proteobacteria
Source: Front Microbiol. 2024 Aug 5;15:1412318. doi: 10.3389/fmicb.2024.1412318 (PMC11332805; doi:10.3389/fmicb.2024.1412318)
Supplement: Supplementary file 1 [file Data_Sheet_1.zip › Supp_figures/Fig_S23.pdf]

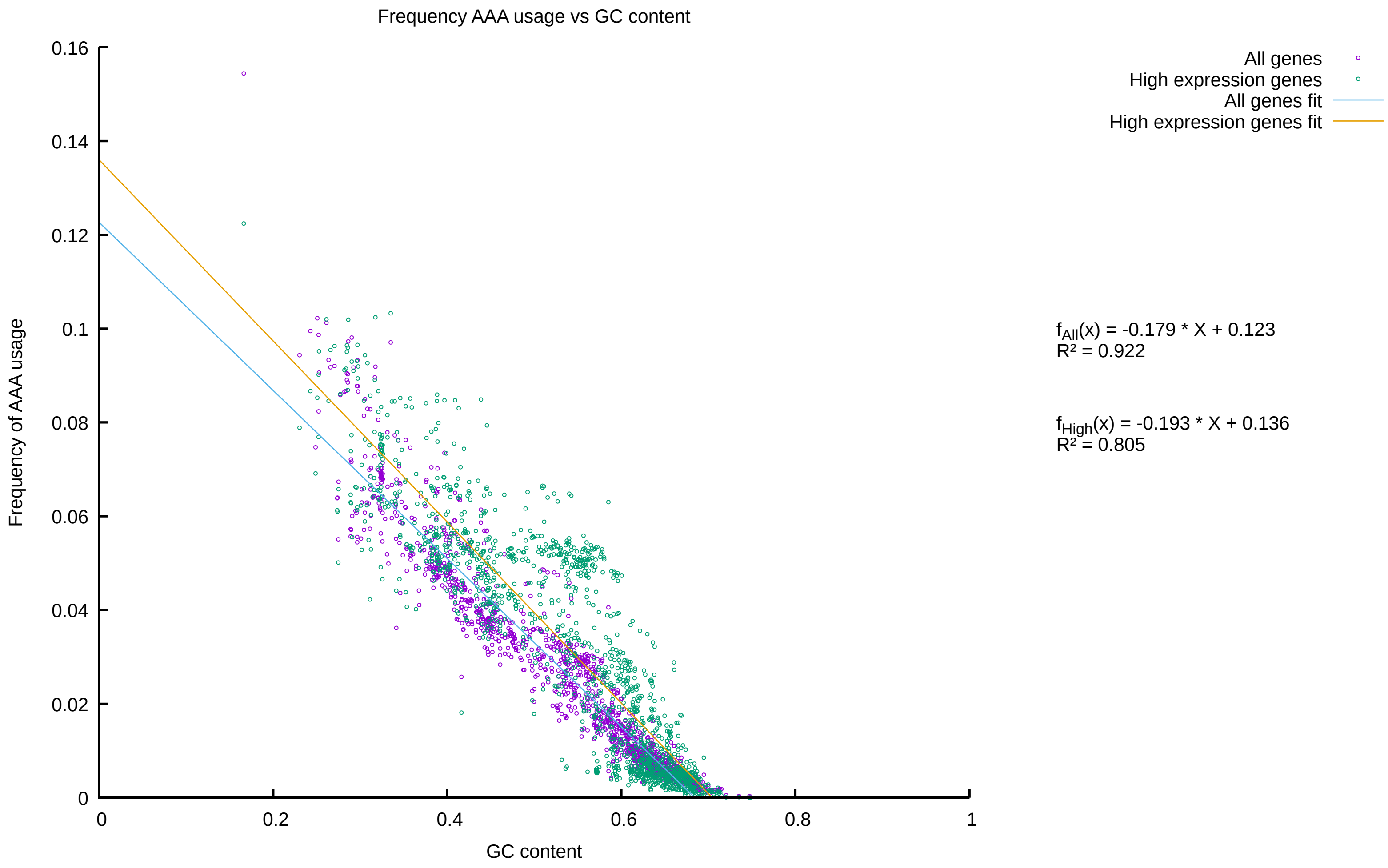

Frequency AAA usage vs GC content
Frequency of AAA usage
GC content
0.16
0.14
0.12
0.1
0.08
0.06
0.04
0.02
0
0
0.2
0.4
0.6
0.8
1
All genes
High expression genes
All genes fit
High expression genes fit
$f_{All}(x) = -0.179 * X + 0.123$
$R^2 = 0.922$
$f_{High}(x) = -0.193 * X + 0.136$
$R^2 = 0.805$

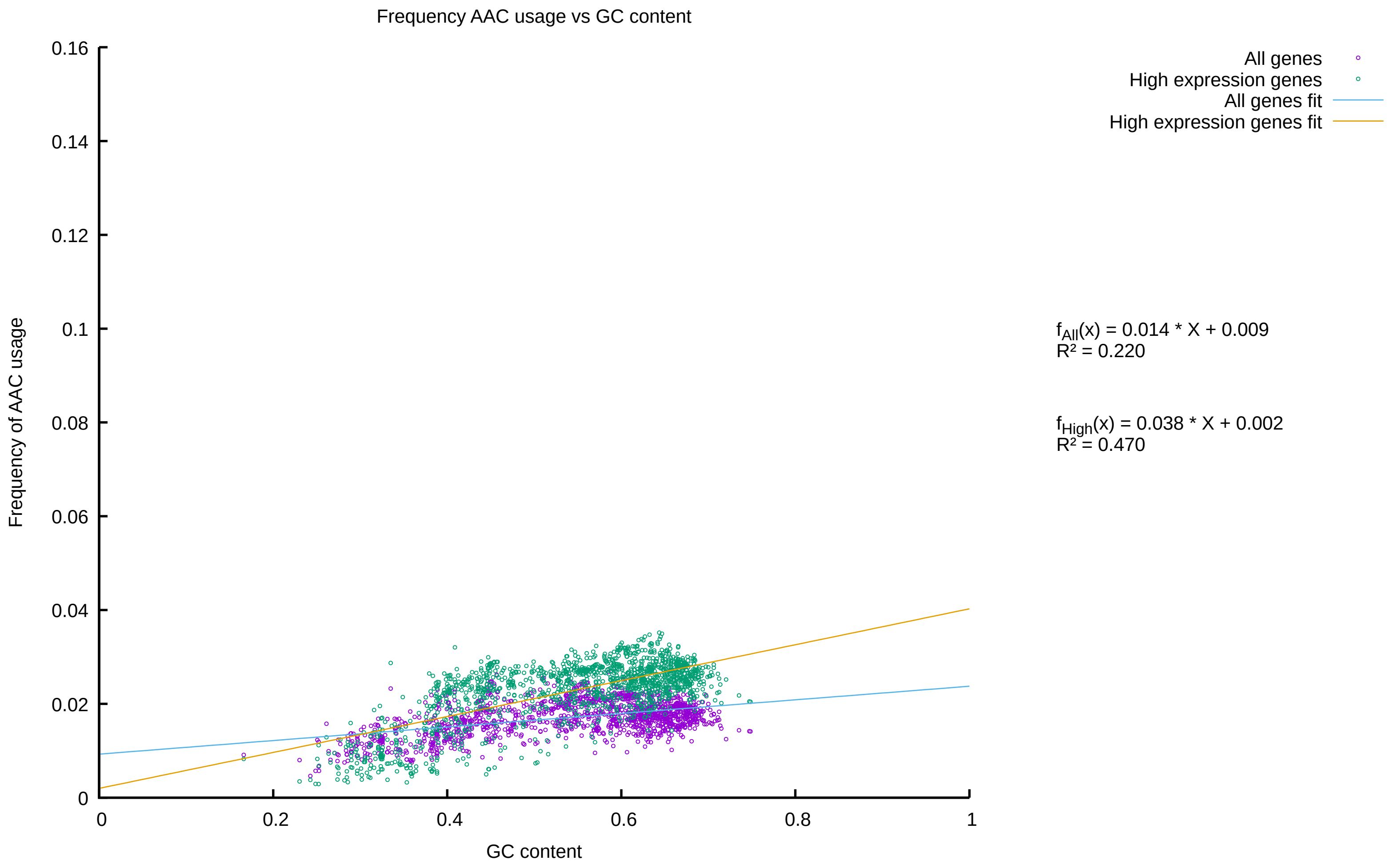
Frequency AAC usage vs GC content
0.16
0.14
0.12
0.1
0.08
0.06
0.04
0.02
0
Frequency of AAC usage
0
0.2
0.4
0.6
0.8
1
GC content
All genes
High expression genes
All genes fit
High expression genes fit
$f_{All}(x) = 0.014 * X + 0.009$
$R^2 = 0.220$
$f_{High}(x) = 0.038 * X + 0.002$
$R^2 = 0.470$

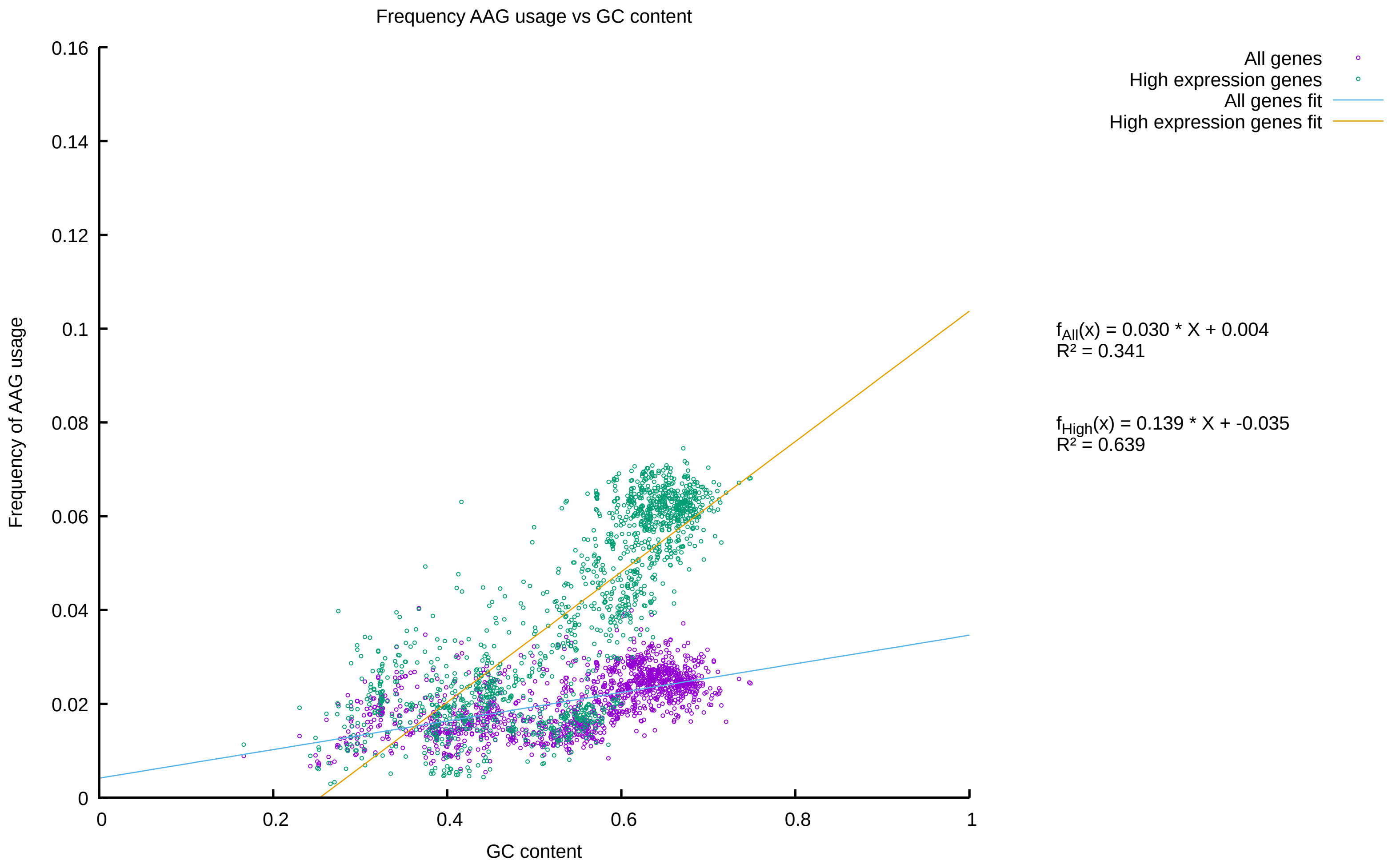

$f_{All}(x) = 0.030 * X + 0.004$
$R^2 = 0.341$

$f_{High}(x) = 0.139 * X + -0.035$
$R^2 = 0.639$

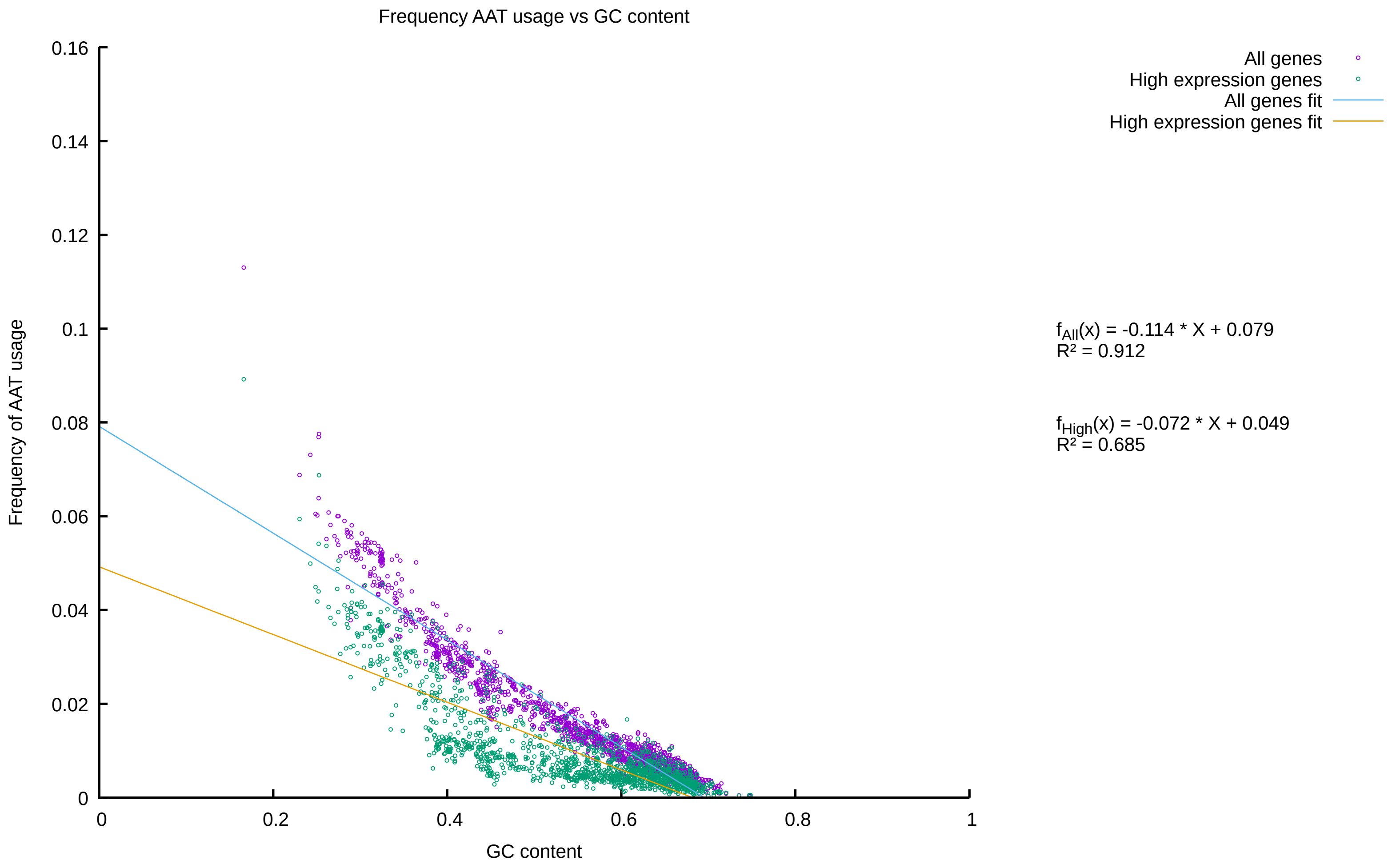

$f_{All}(x) = -0.114 * X + 0.079$
$R^2 = 0.912$

$f_{High}(x) = -0.072 * X + 0.049$
$R^2 = 0.685$

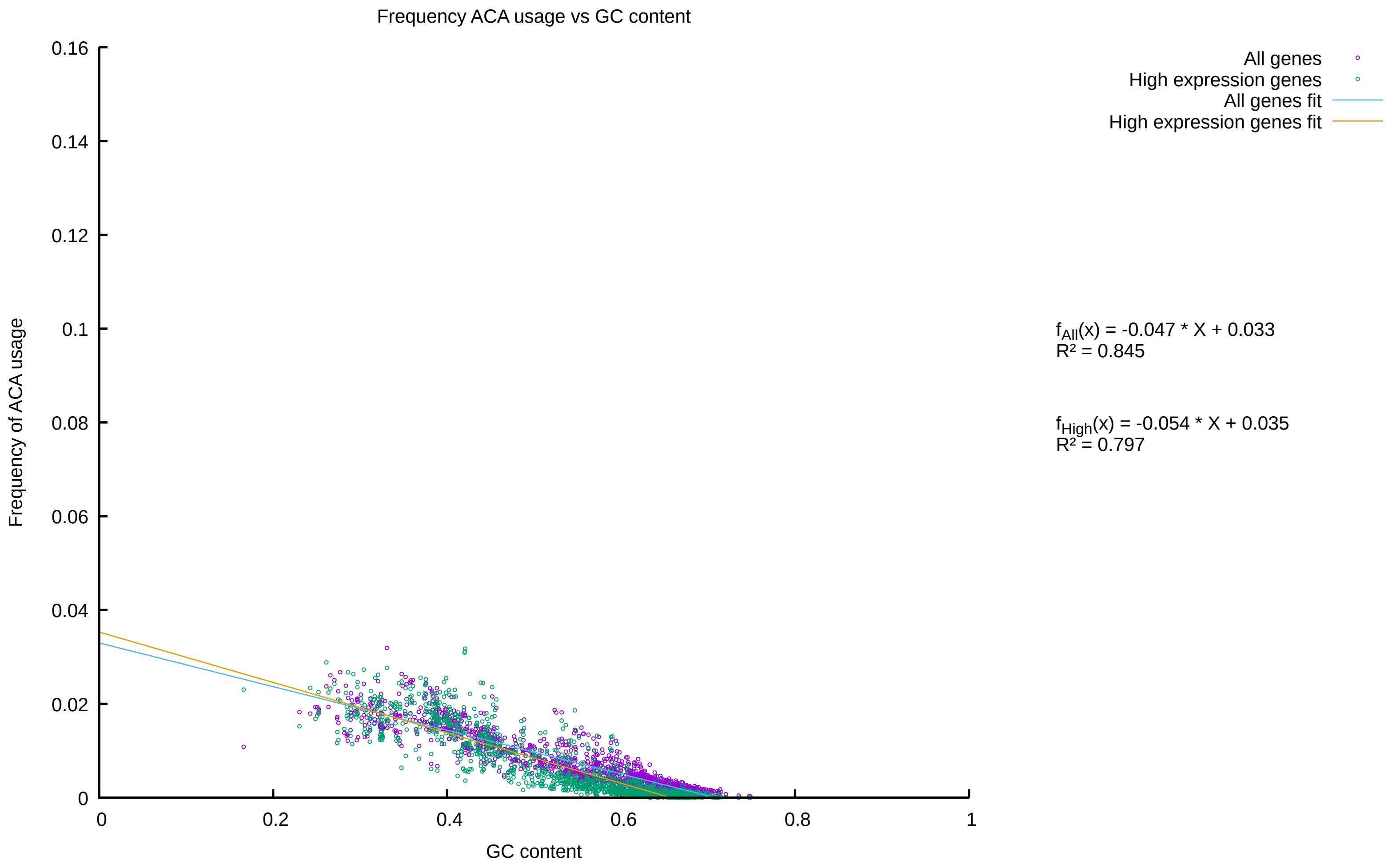

Frequency ACA usage vs GC content
All genes
High expression genes
All genes fit
High expression genes fit
$f_{All}(x) = -0.047 * X + 0.033$
$R^2 = 0.845$
$f_{High}(x) = -0.054 * X + 0.035$
$R^2 = 0.797$
Frequency of ACA usage
GC content
0.16
0.14
0.12
0.1
0.08
0.06
0.04
0.02
0
0.2
0.4
0.6
0.8
1

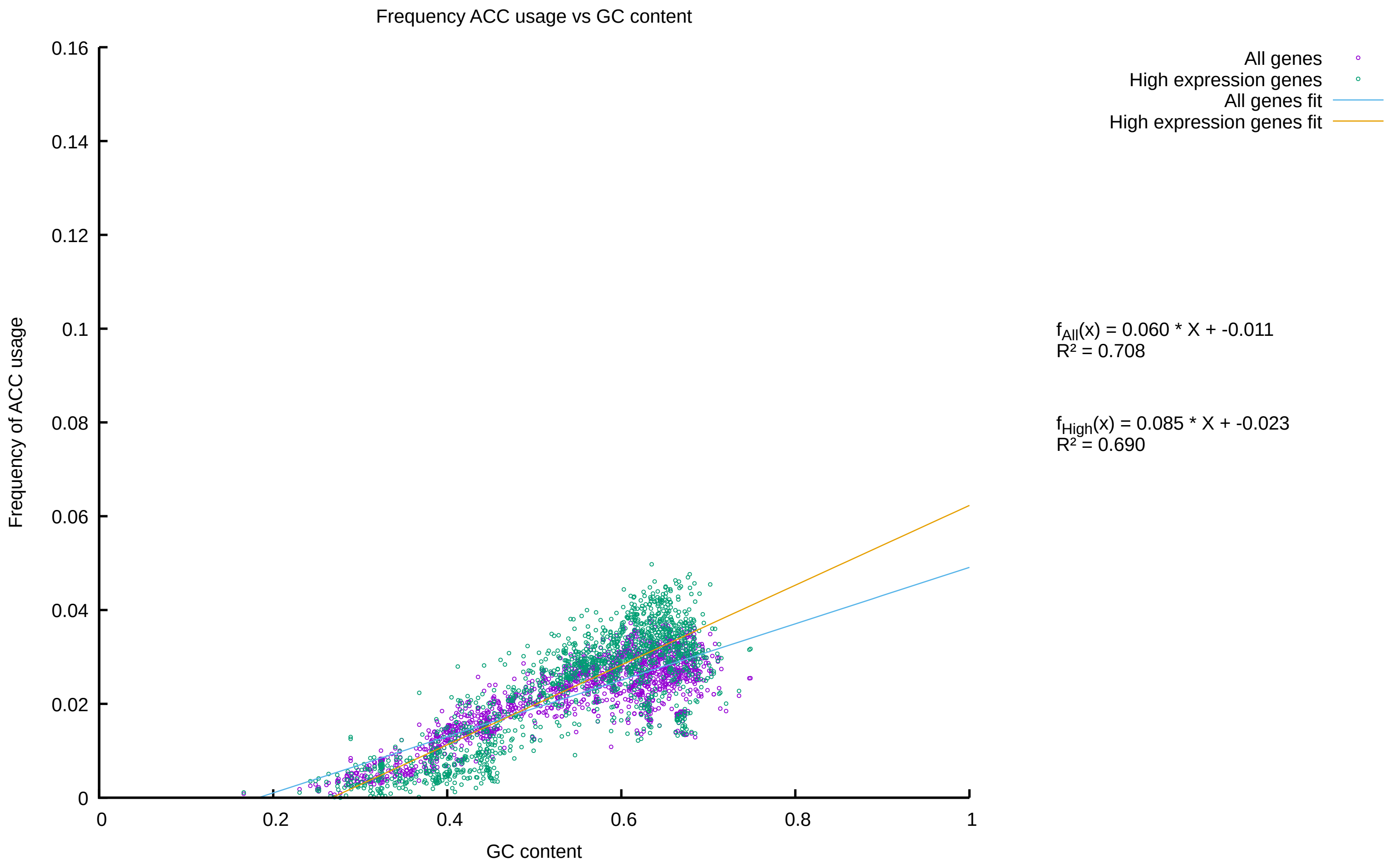

$f_{All}(x) = 0.060 * X + -0.011$
$R^2 = 0.708$

$f_{High}(x) = 0.085 * X + -0.023$
$R^2 = 0.690$

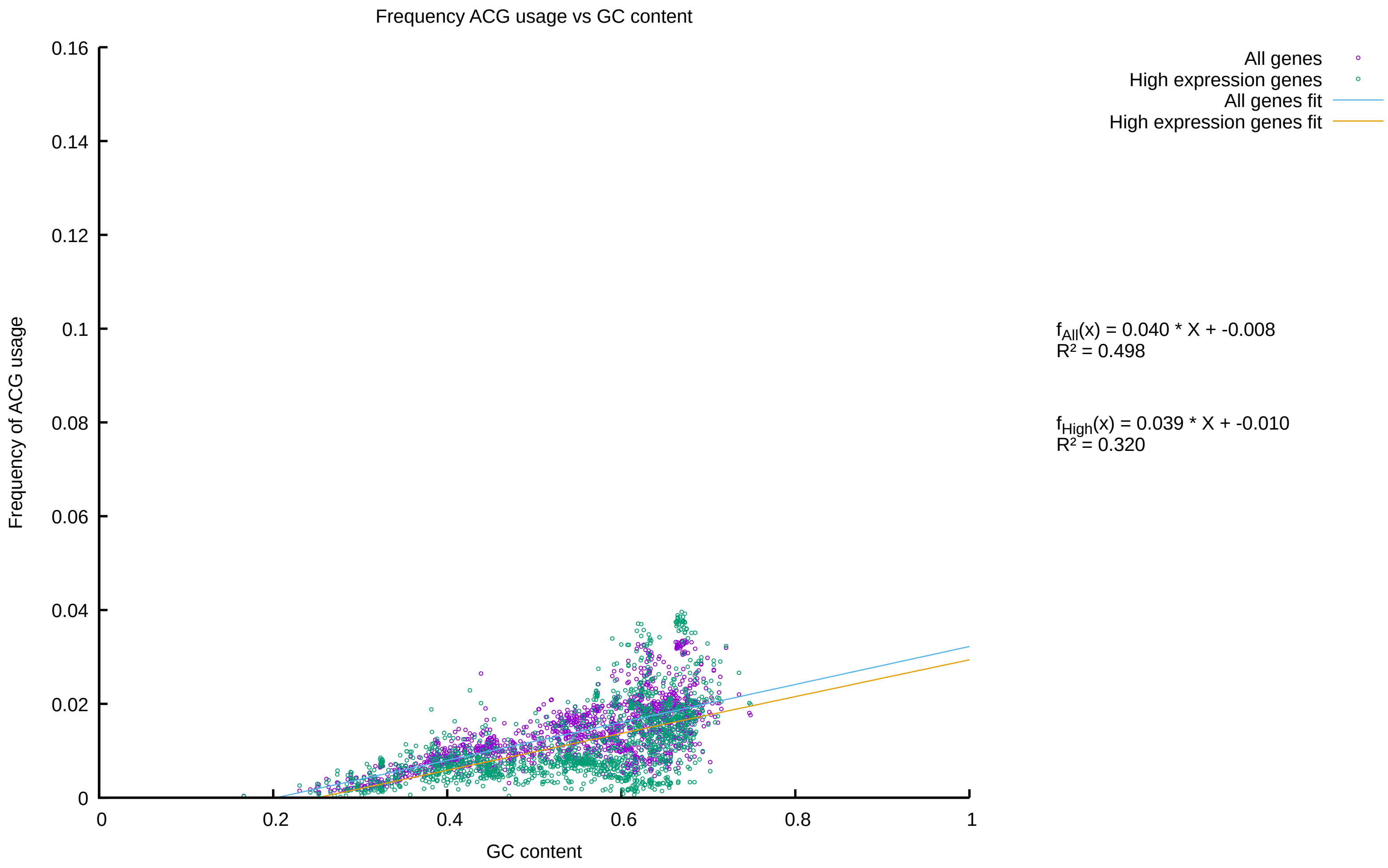

Frequency ACG usage vs GC content
0.16
0.14
0.12
0.1
0.08
0.06
0.04
0.02
0
Frequency of ACG usage
0
0.2
0.4
0.6
0.8
1
GC content
All genes
High expression genes
All genes fit
High expression genes fit
$f_{All}(x) = 0.040 * X + -0.008$
$R^2 = 0.498$
$f_{High}(x) = 0.039 * X + -0.010$
$R^2 = 0.320$

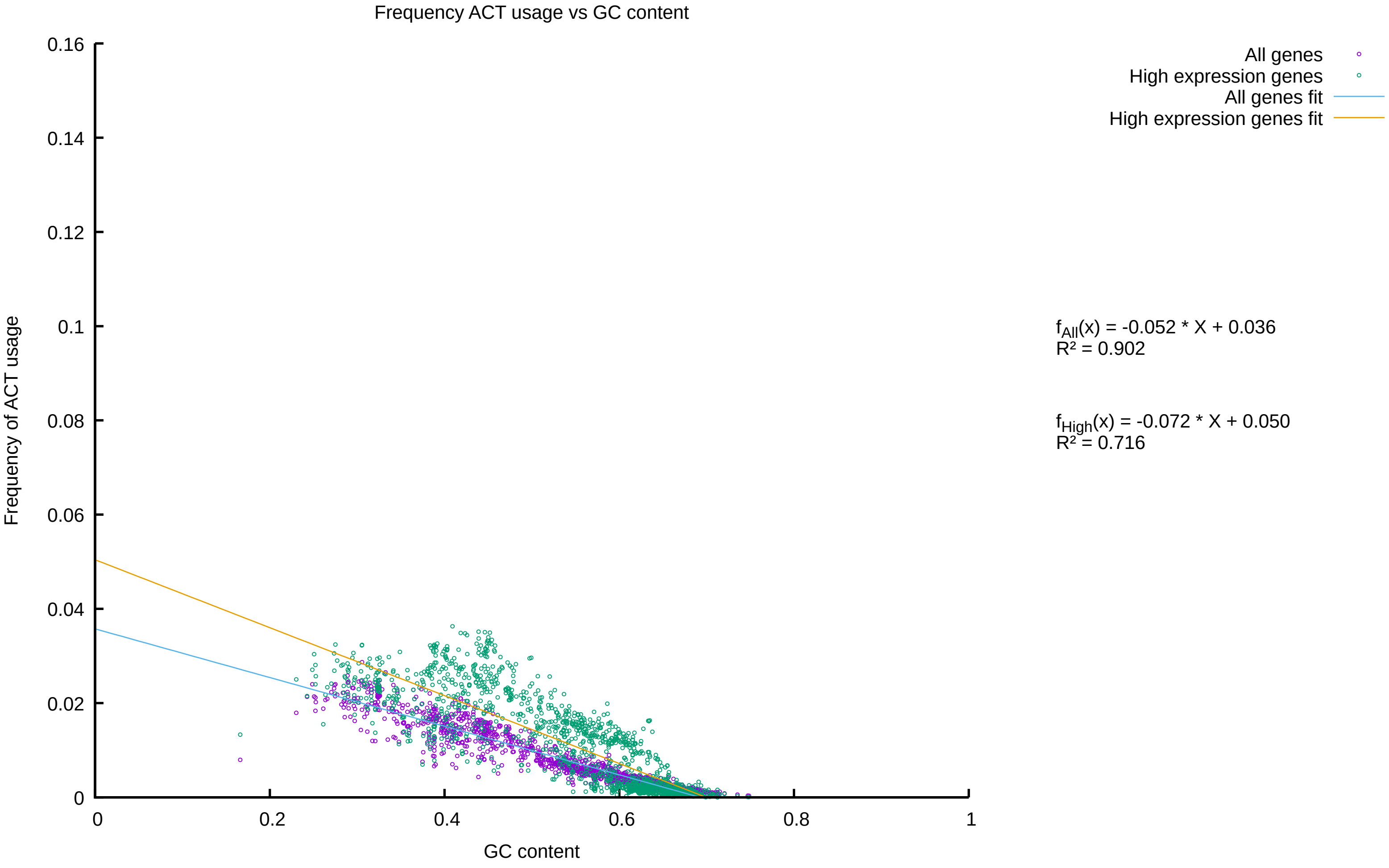
Frequency ACT usage vs GC content
Frequency of ACT usage
GC content
0
0.02
0.04
0.06
0.08
0.1
0.12
0.14
0.16
0.2
0.4
0.6
0.8
1
All genes
High expression genes
All genes fit
High expression genes fit
f_All(x) = -0.052 * X + 0.036
R² = 0.902
f_High(x) = -0.072 * X + 0.050
R² = 0.716

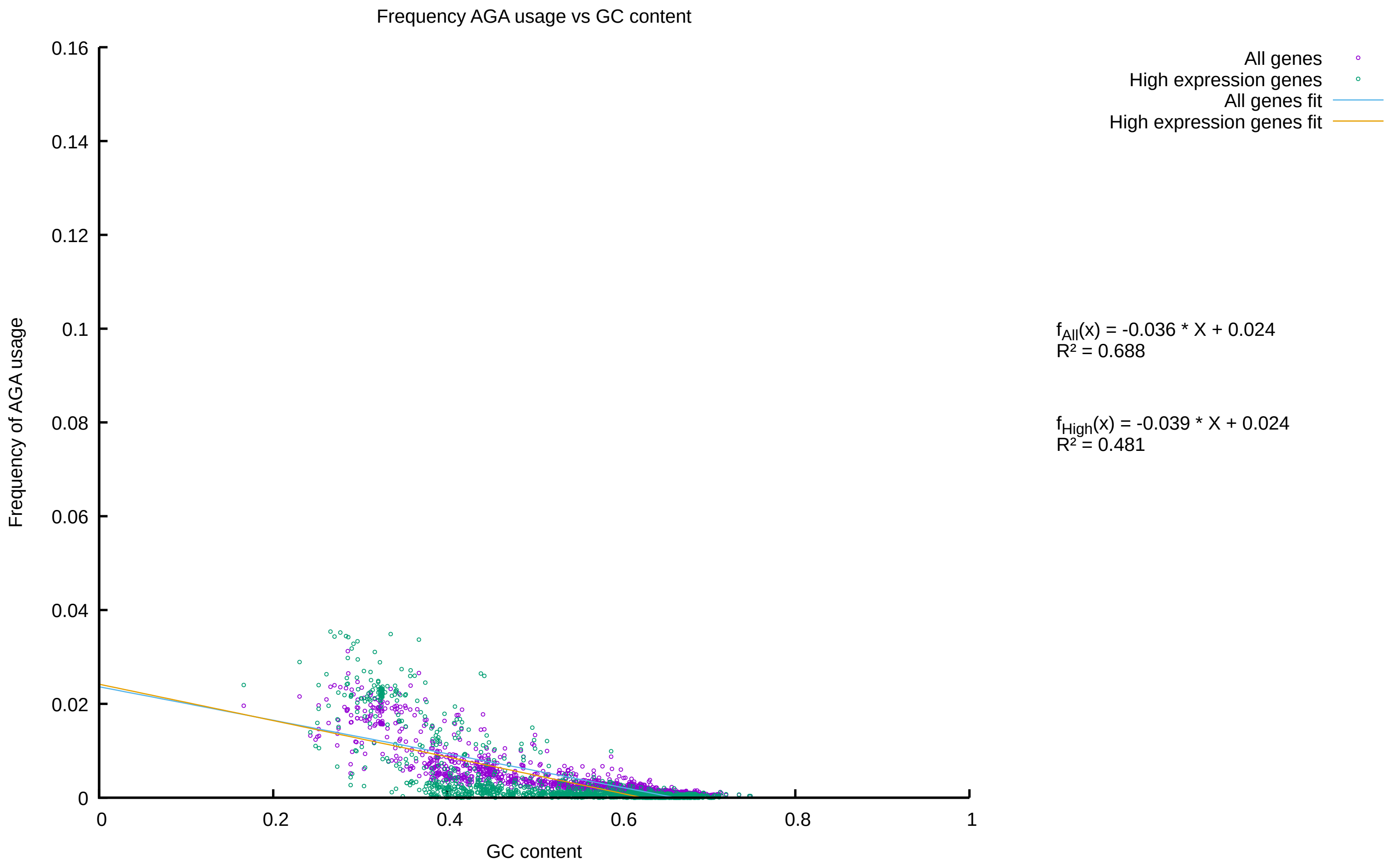

$f_{All}(x) = -0.036 * X + 0.024$
$R^2 = 0.688$

$f_{High}(x) = -0.039 * X + 0.024$
$R^2 = 0.481$

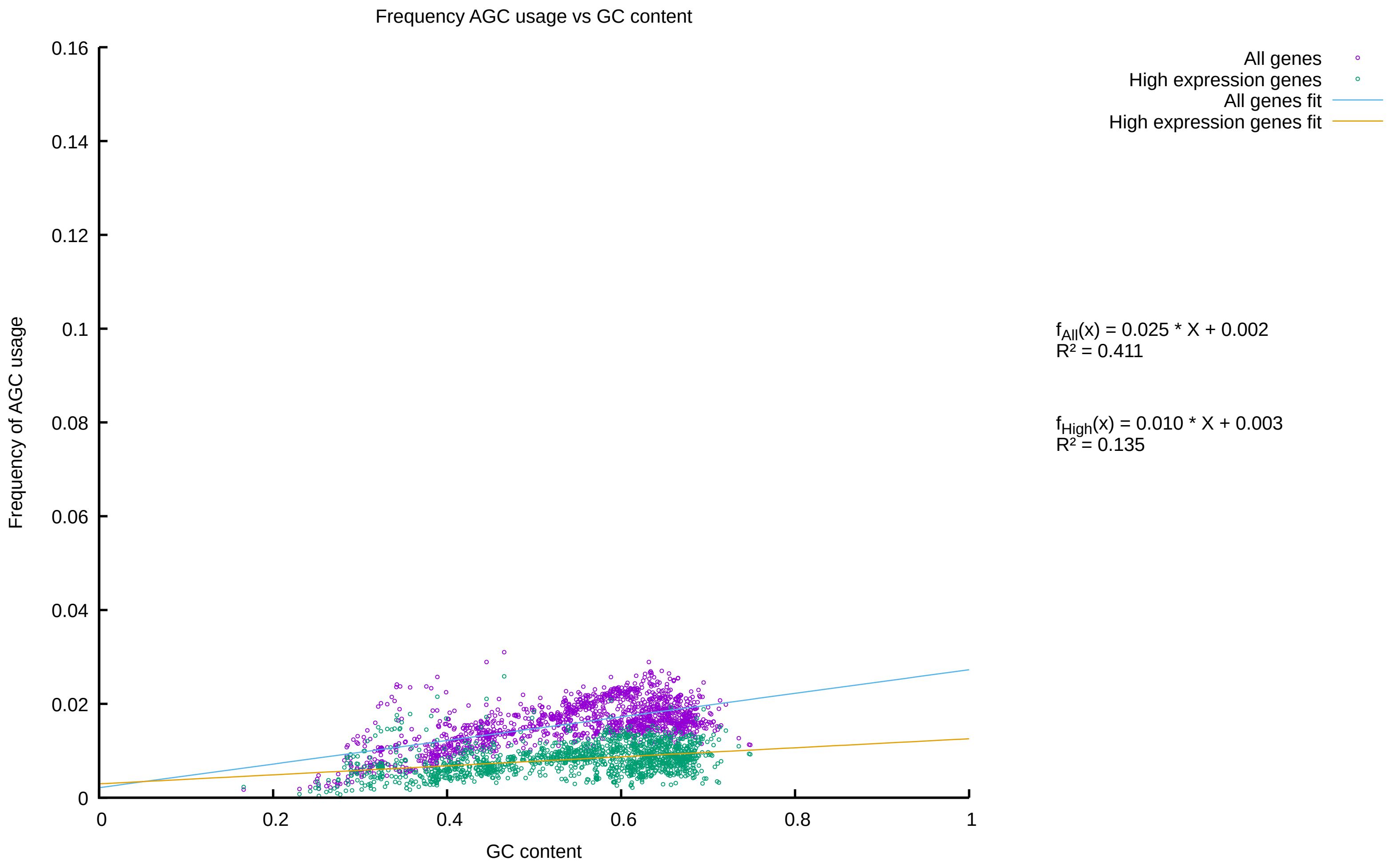
Frequency AGC usage vs GC content
0.16
0.14
0.12
0.1
0.08
0.06
0.04
0.02
0
Frequency of AGC usage
0
0.2
0.4
0.6
0.8
1
GC content
All genes
High expression genes
All genes fit
High expression genes fit
$f_{All}(x) = 0.025 * X + 0.002$
$R^2 = 0.411$
$f_{High}(x) = 0.010 * X + 0.003$
$R^2 = 0.135$

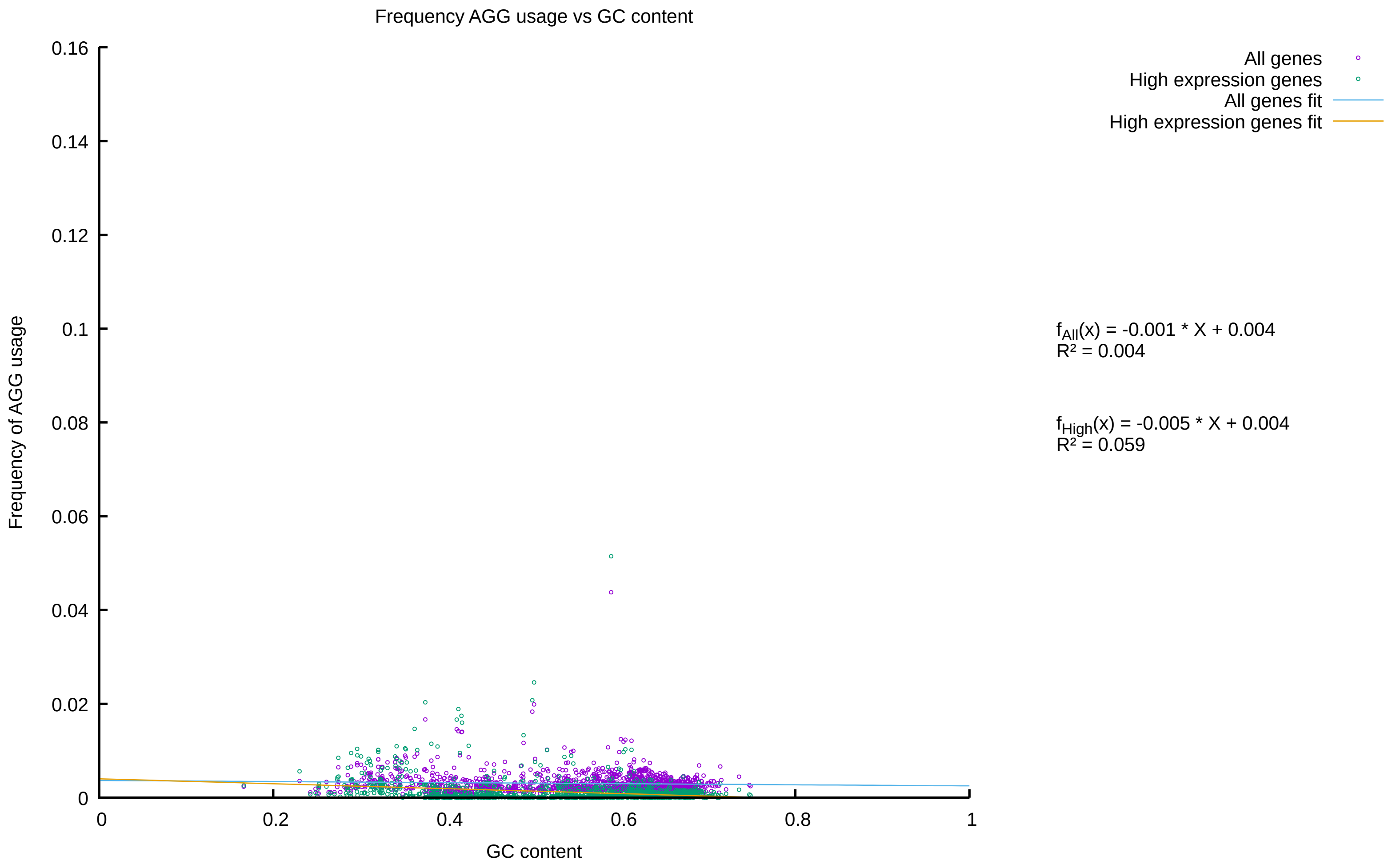

Frequency AGG usage vs GC content
Frequency of AGG usage
GC content
0.16
0.14
0.12
0.1
0.08
0.06
0.04
0.02
0
0
0.2
0.4
0.6
0.8
1
All genes
High expression genes
All genes fit
High expression genes fit
$f_{All}(x) = -0.001 * X + 0.004$
$R^2 = 0.004$
$f_{High}(x) = -0.005 * X + 0.004$
$R^2 = 0.059$

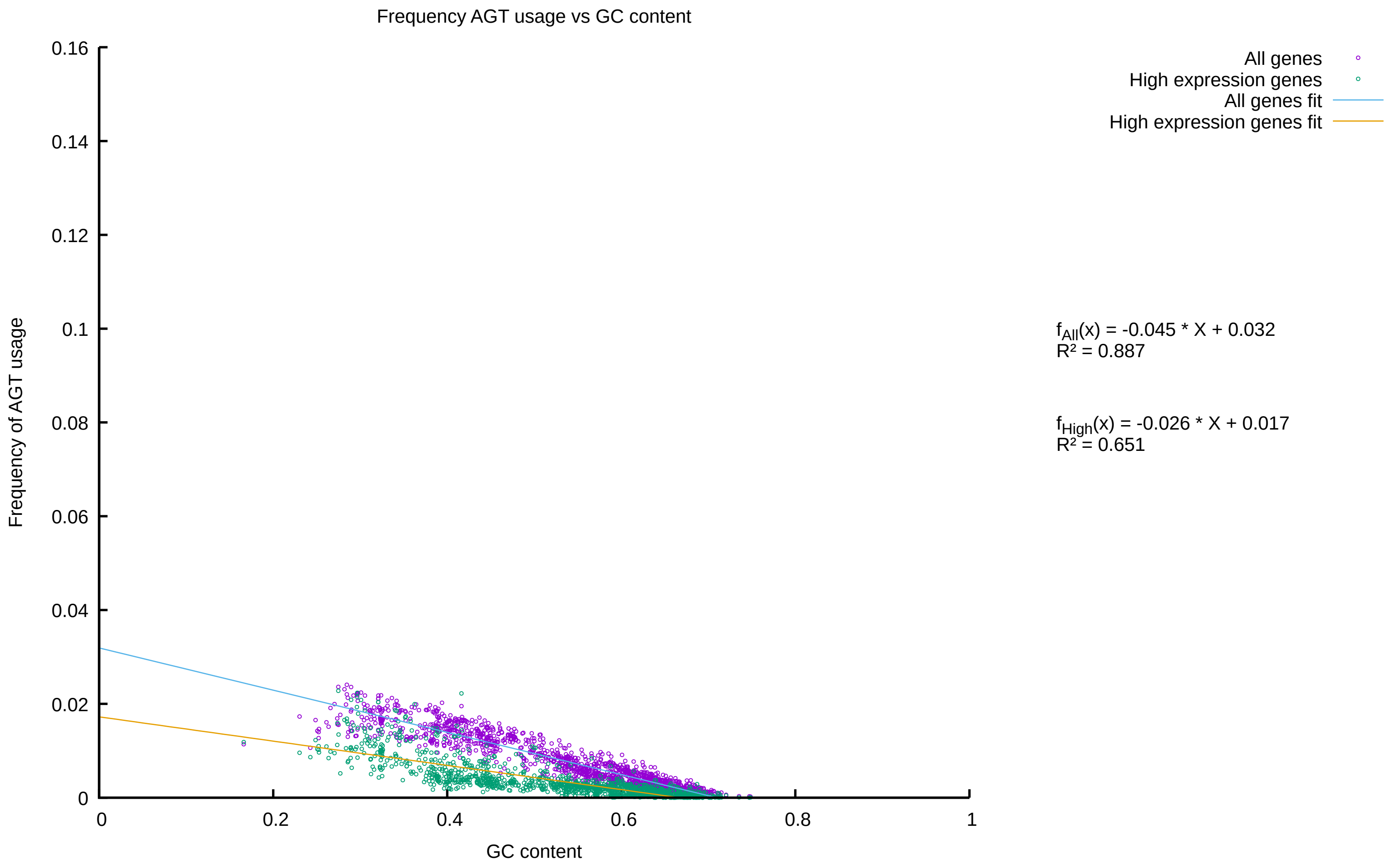
Frequency AGT usage vs GC content
0.16
0.14
0.12
0.1
0.08
0.06
0.04
0.02
0
Frequency of AGT usage
0
0.2
0.4
0.6
0.8
1
GC content
All genes
High expression genes
All genes fit
High expression genes fit
$f_{All}(x) = -0.045 * X + 0.032$
$R^2 = 0.887$
$f_{High}(x) = -0.026 * X + 0.017$
$R^2 = 0.651$

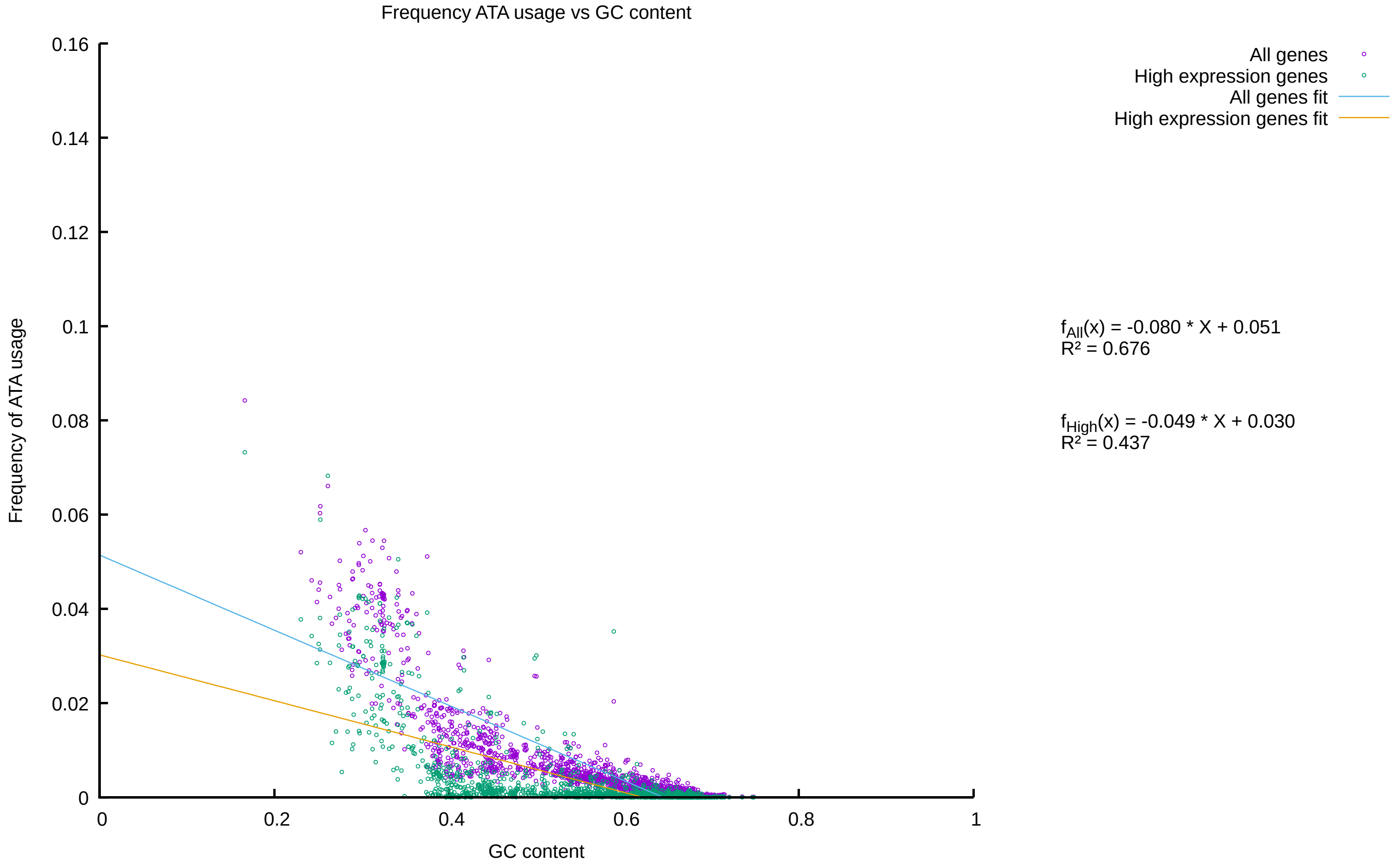
Frequency ATA usage vs GC content
Frequency of ATA usage
GC content
0
0.02
0.04
0.06
0.08
0.1
0.12
0.14
0.16
0
0.2
0.4
0.6
0.8
1
All genes
High expression genes
All genes fit
High expression genes fit
$f_{All}(x) = -0.080 * X + 0.051$
$R^2 = 0.676$
$f_{High}(x) = -0.049 * X + 0.030$
$R^2 = 0.437$

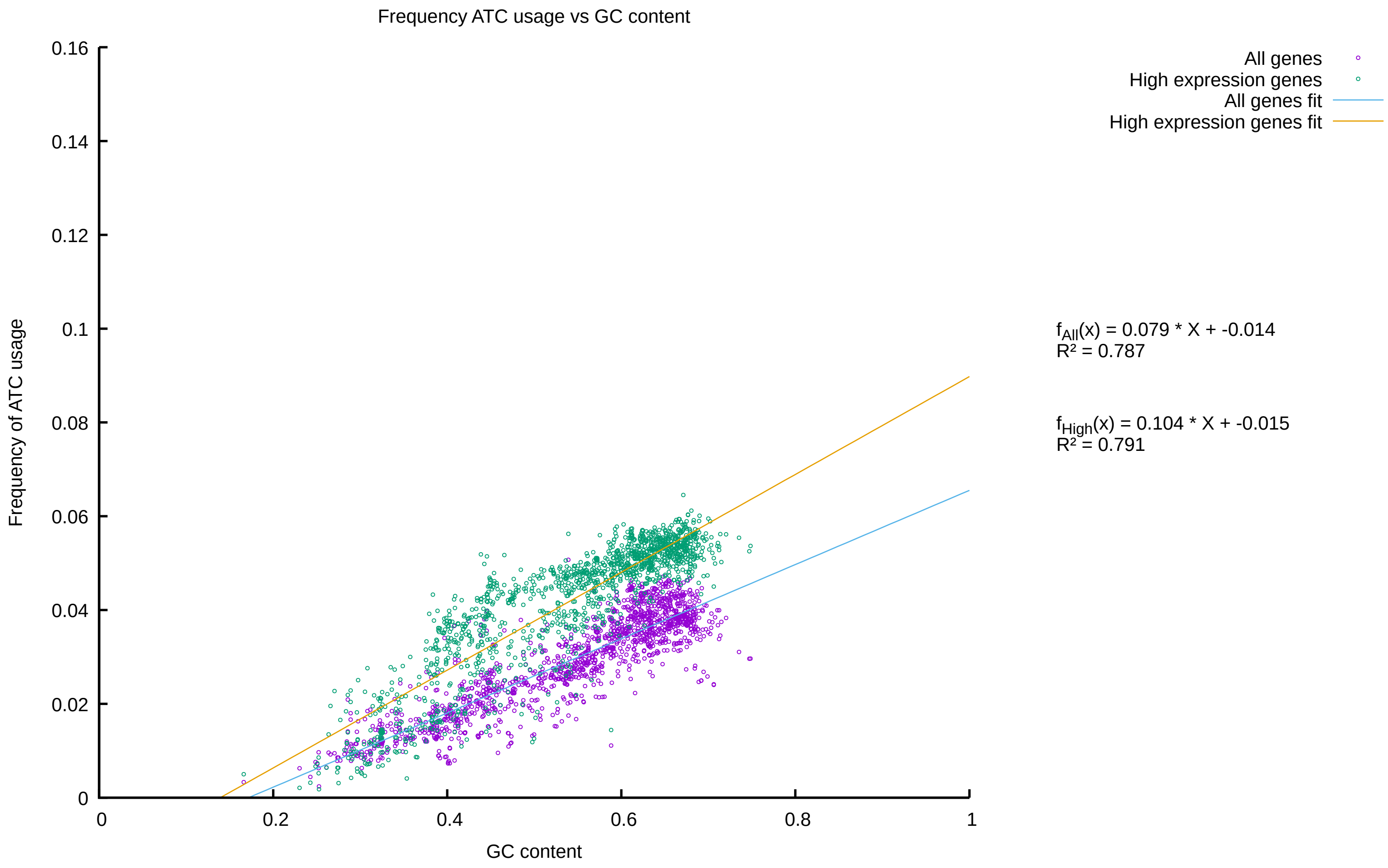

Frequency ATC usage vs GC content
0.16
0.14
0.12
0.1
0.08
0.06
0.04
0.02
0
0
0.2
0.4
0.6
0.8
1
Frequency of ATC usage
GC content
All genes
High expression genes
All genes fit
High expression genes fit
$f_{All}(x)$ = 0.079 * X + -0.014
R^2 = 0.787
$f_{High}(x)$ = 0.104 * X + -0.015
R^2 = 0.791

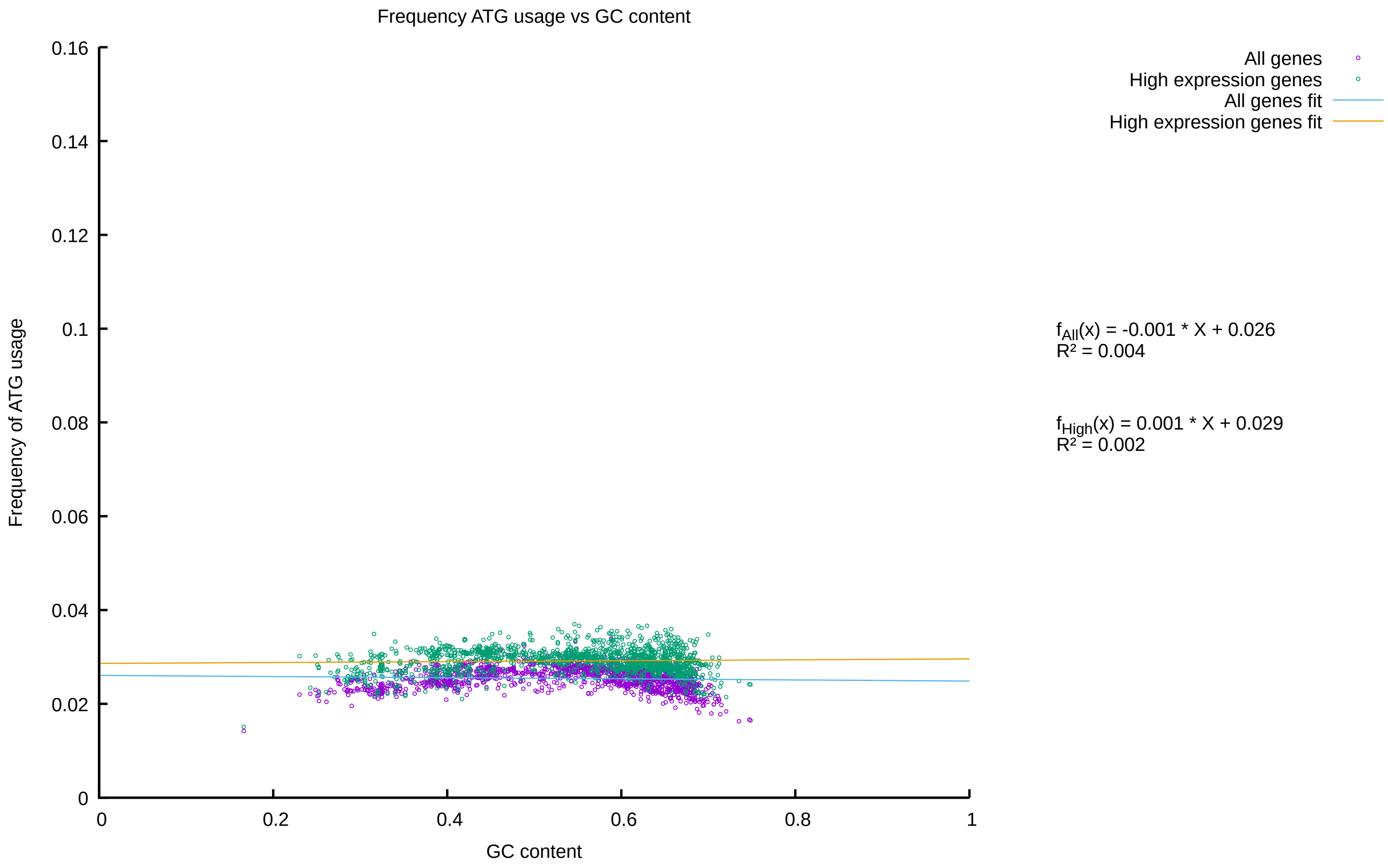

Frequency ATG usage vs GC content
Frequency of ATG usage
GC content
0.16
0.14
0.12
0.1
0.08
0.06
0.04
0.02
0
0
0.2
0.4
0.6
0.8
1
All genes
High expression genes
All genes fit
High expression genes fit
$f_{All}(x) = -0.001 * X + 0.026$
$R^2 = 0.004$
$f_{High}(x) = 0.001 * X + 0.029$
$R^2 = 0.002$

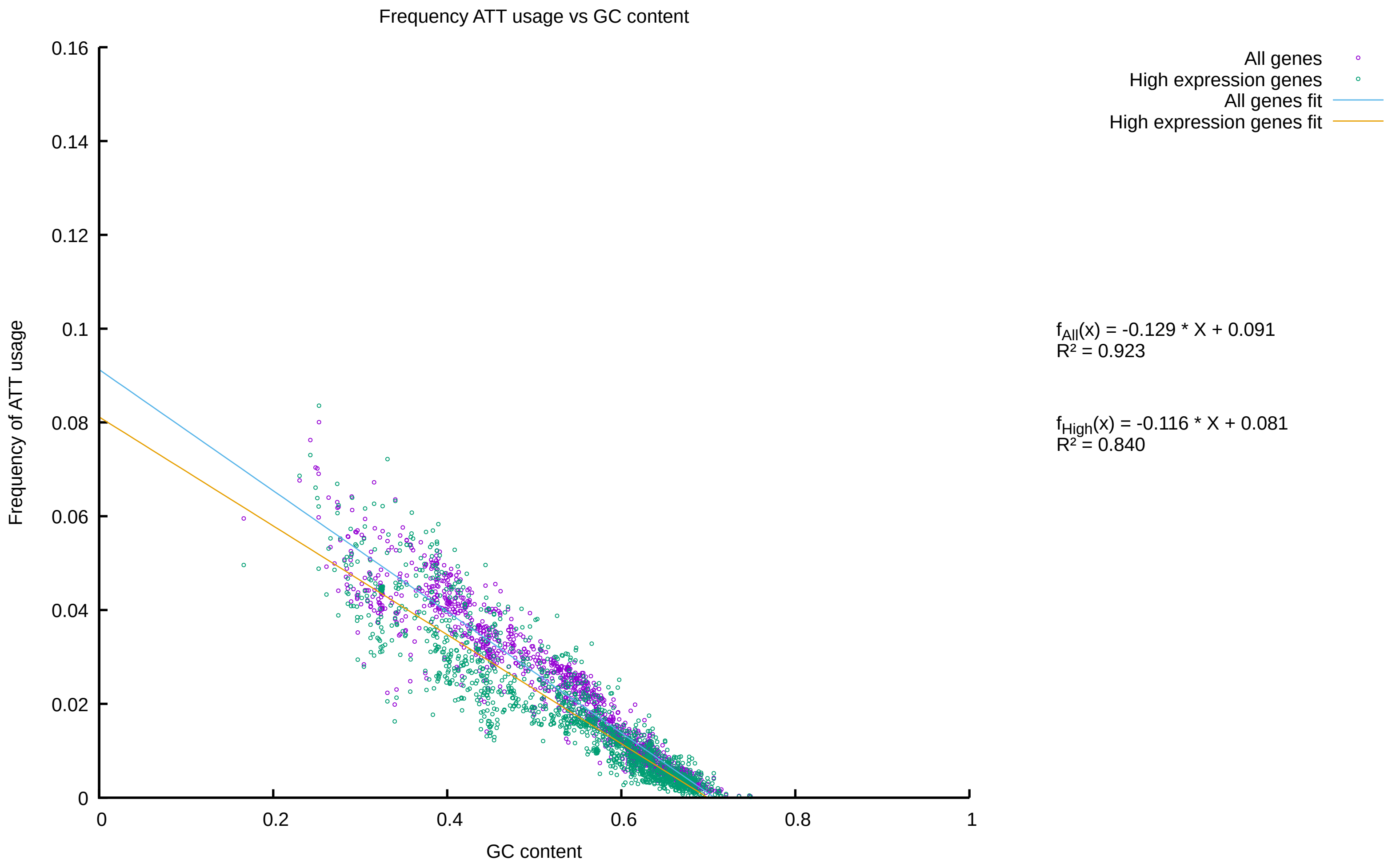

Frequency ATT usage vs GC content
0.16
0.14
0.12
0.1
0.08
0.06
0.04
0.02
0
Frequency of ATT usage
0
0.2
0.4
0.6
0.8
1
GC content
All genes
High expression genes
All genes fit
High expression genes fit
$f_{All}(x) = -0.129 * X + 0.091$
$R^2 = 0.923$
$f_{High}(x) = -0.116 * X + 0.081$
$R^2 = 0.840$

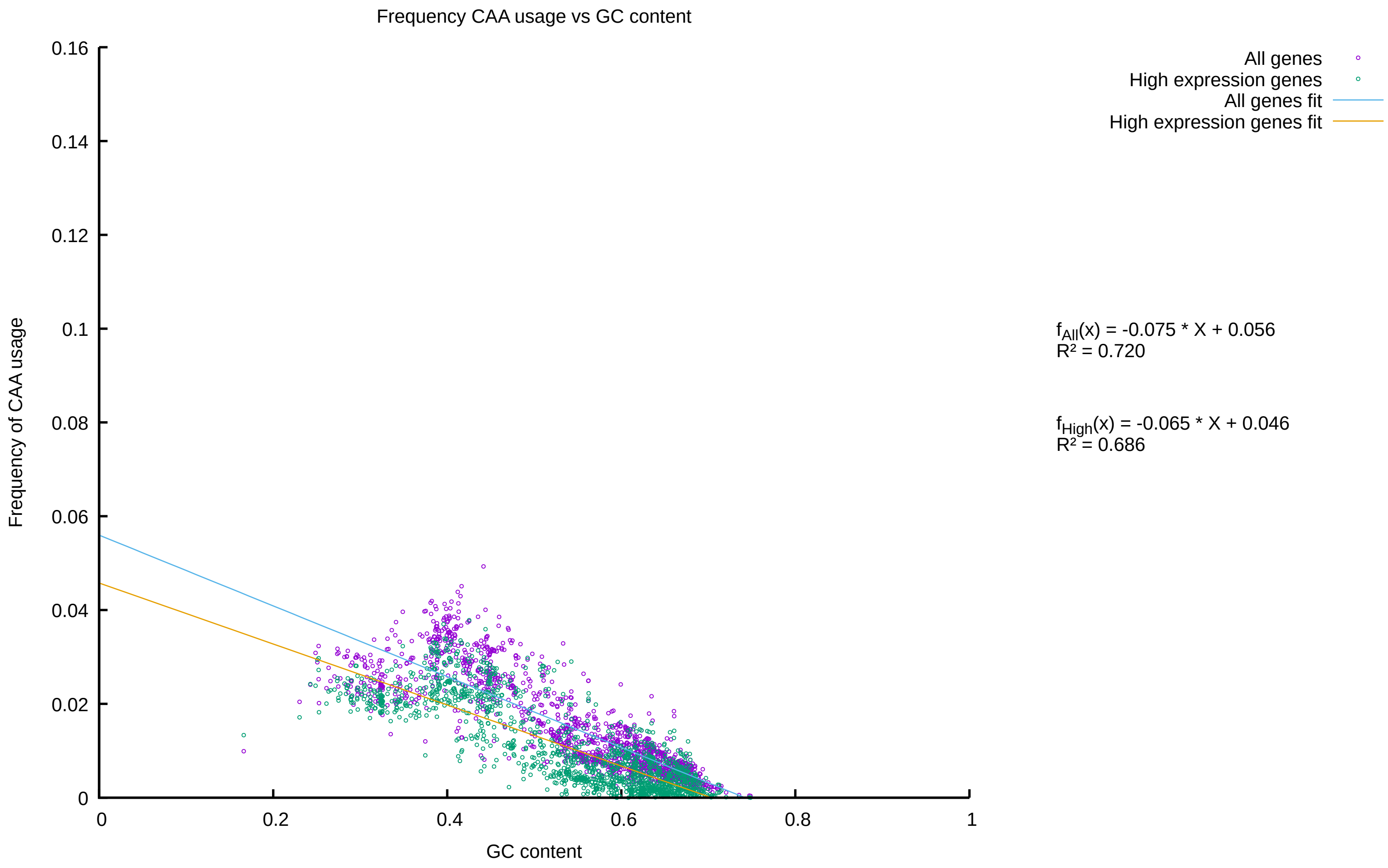
Frequency CAA usage vs GC content
Frequency of CAA usage
GC content
0.16
0.14
0.12
0.1
0.08
0.06
0.04
0.02
0
0
0.2
0.4
0.6
0.8
1
All genes
High expression genes
All genes fit
High expression genes fit
$f_{All}(x) = -0.075 * X + 0.056$
$R^2 = 0.720$
$f_{High}(x) = -0.065 * X + 0.046$
$R^2 = 0.686$

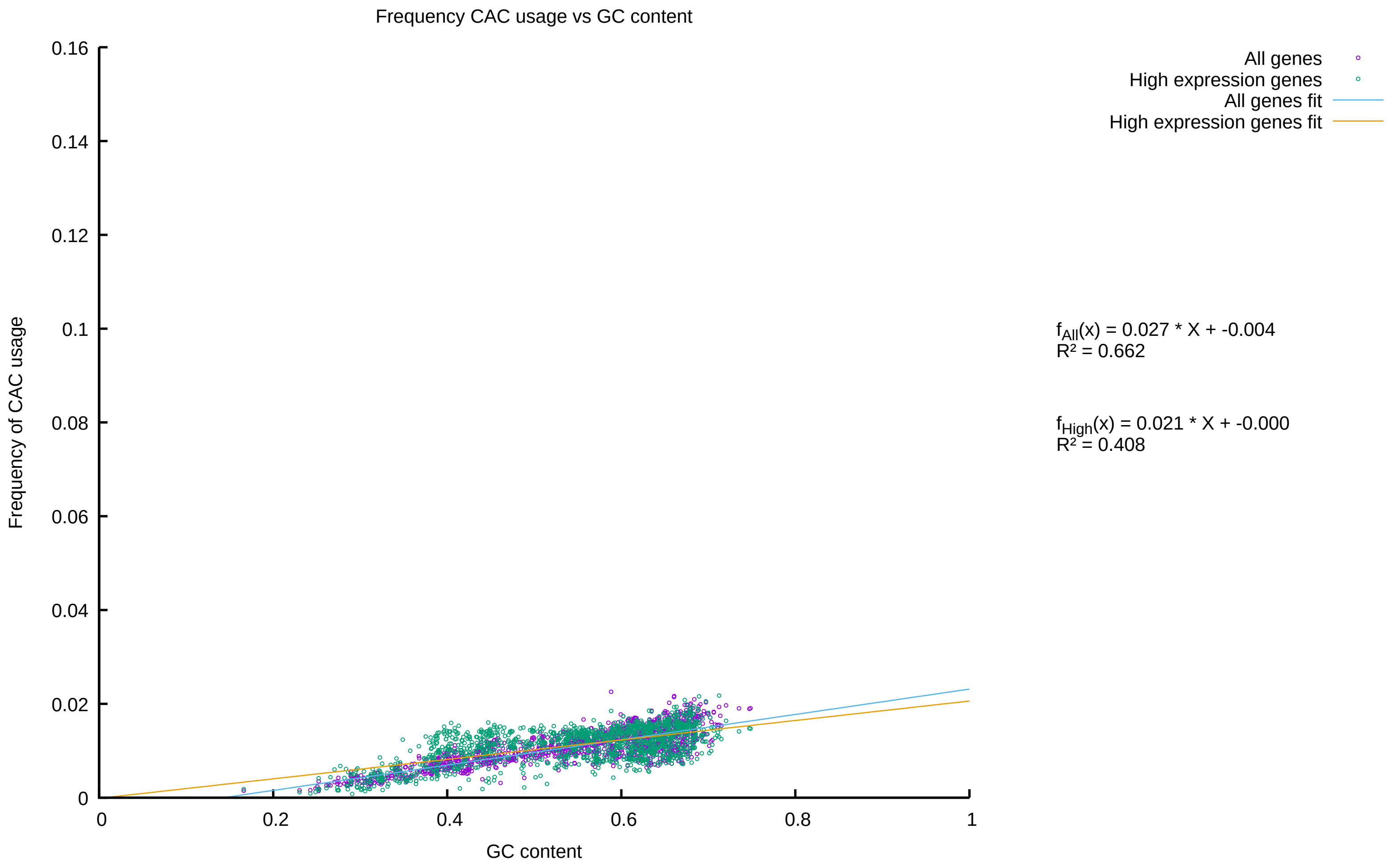
Frequency CAC usage vs GC content
Frequency of CAC usage
GC content
0.16
0.14
0.12
0.1
0.08
0.06
0.04
0.02
0
0
0.2
0.4
0.6
0.8
1
All genes
High expression genes
All genes fit
High expression genes fit
$f_{All}(x) = 0.027 * X + -0.004$
$R^2 = 0.662$
$f_{High}(x) = 0.021 * X + -0.000$
$R^2 = 0.408$

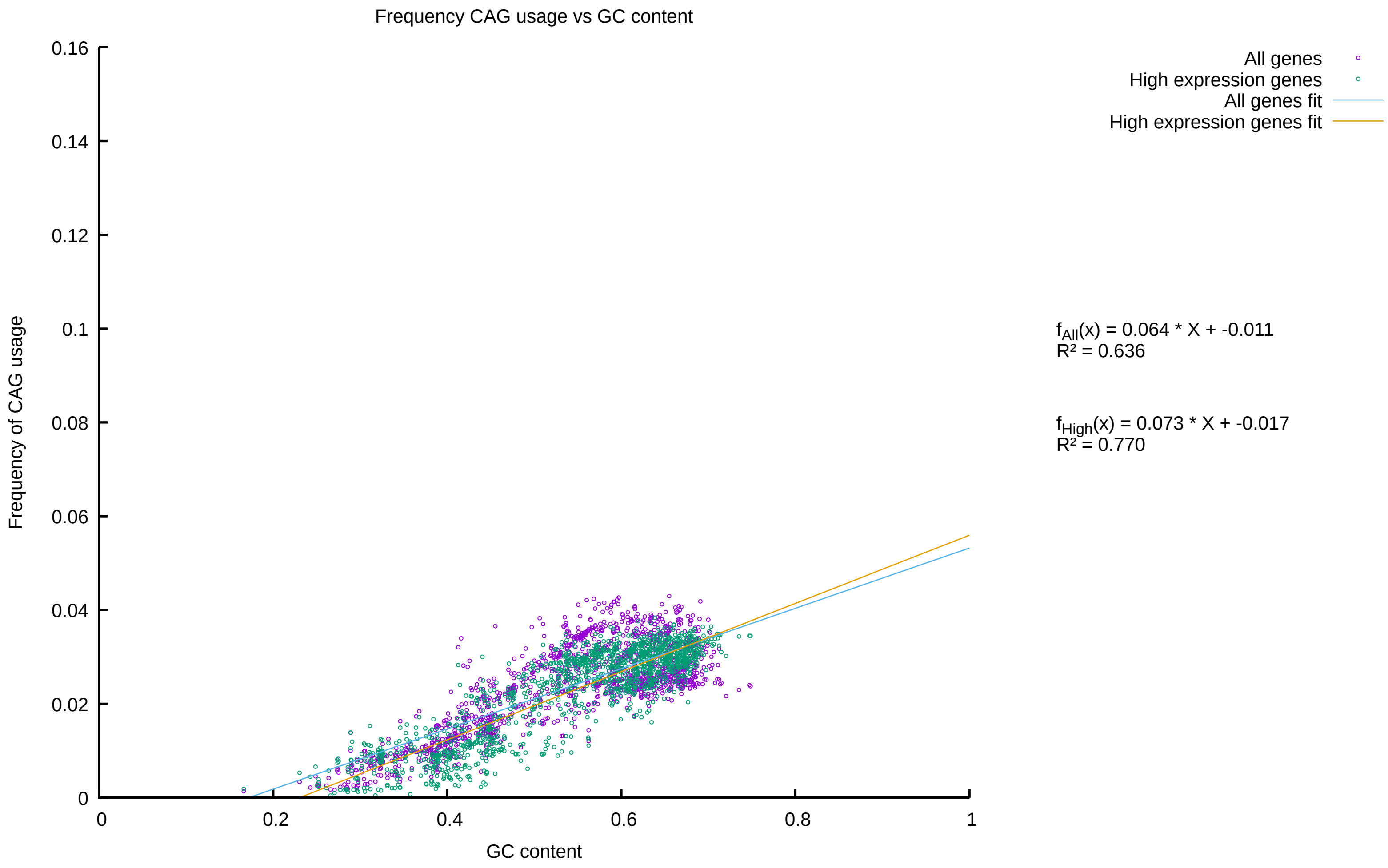

Frequency CAG usage vs GC content
Frequency of CAG usage
0.16
0.14
0.12
0.1
0.08
0.06
0.04
0.02
0
0
0.2
0.4
0.6
0.8
1
GC content
All genes
High expression genes
All genes fit
High expression genes fit
$f_{All}(x) = 0.064 * X + -0.011$
$R^2 = 0.636$
$f_{High}(x) = 0.073 * X + -0.017$
$R^2 = 0.770$

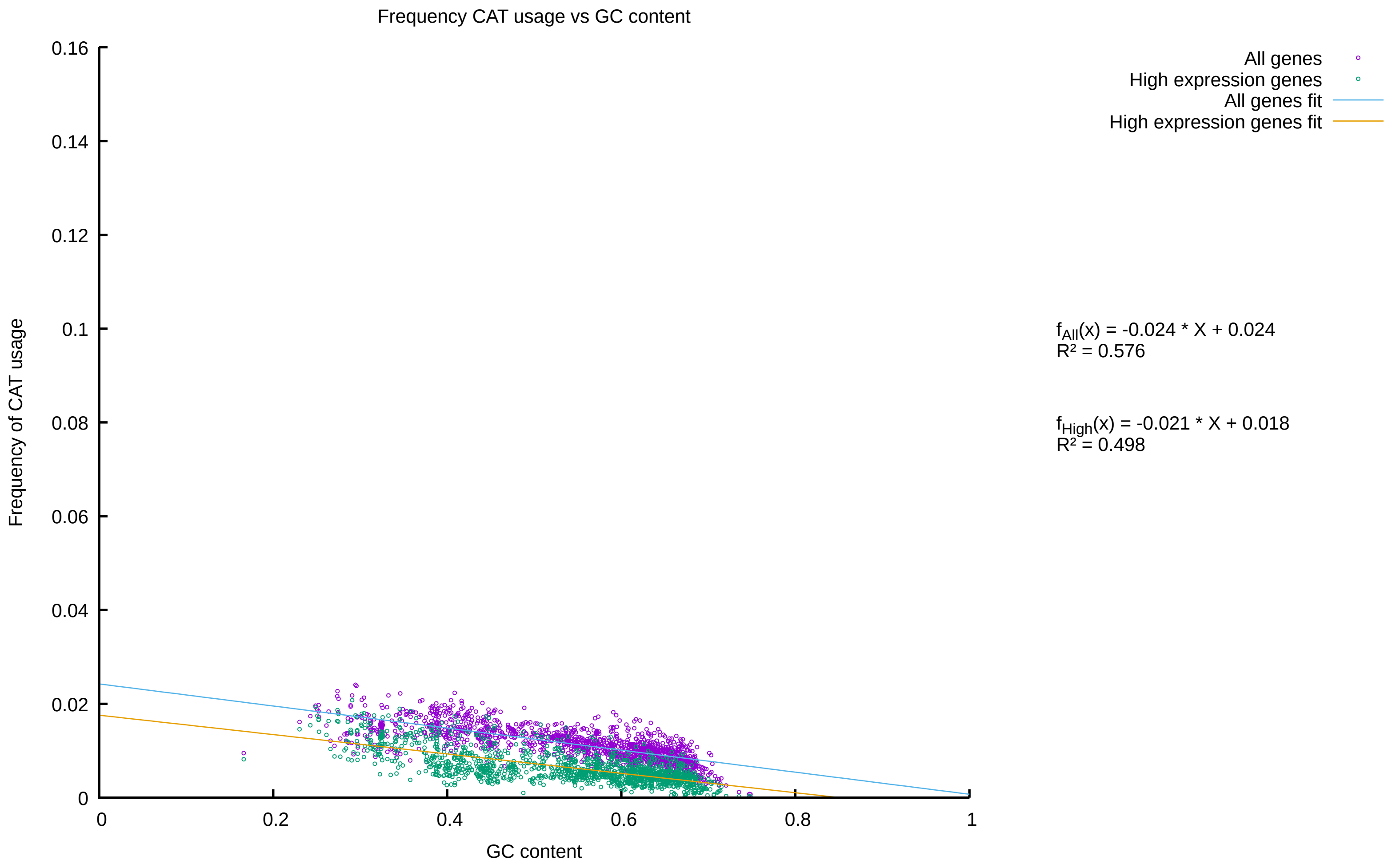
Frequency CAT usage vs GC content
Frequency of CAT usage
GC content
0.16
0.14
0.12
0.1
0.08
0.06
0.04
0.02
0
0
0.2
0.4
0.6
0.8
1
All genes
High expression genes
All genes fit
High expression genes fit
f_All(x) = -0.024 * X + 0.024
R² = 0.576
f_High(x) = -0.021 * X + 0.018
R² = 0.498

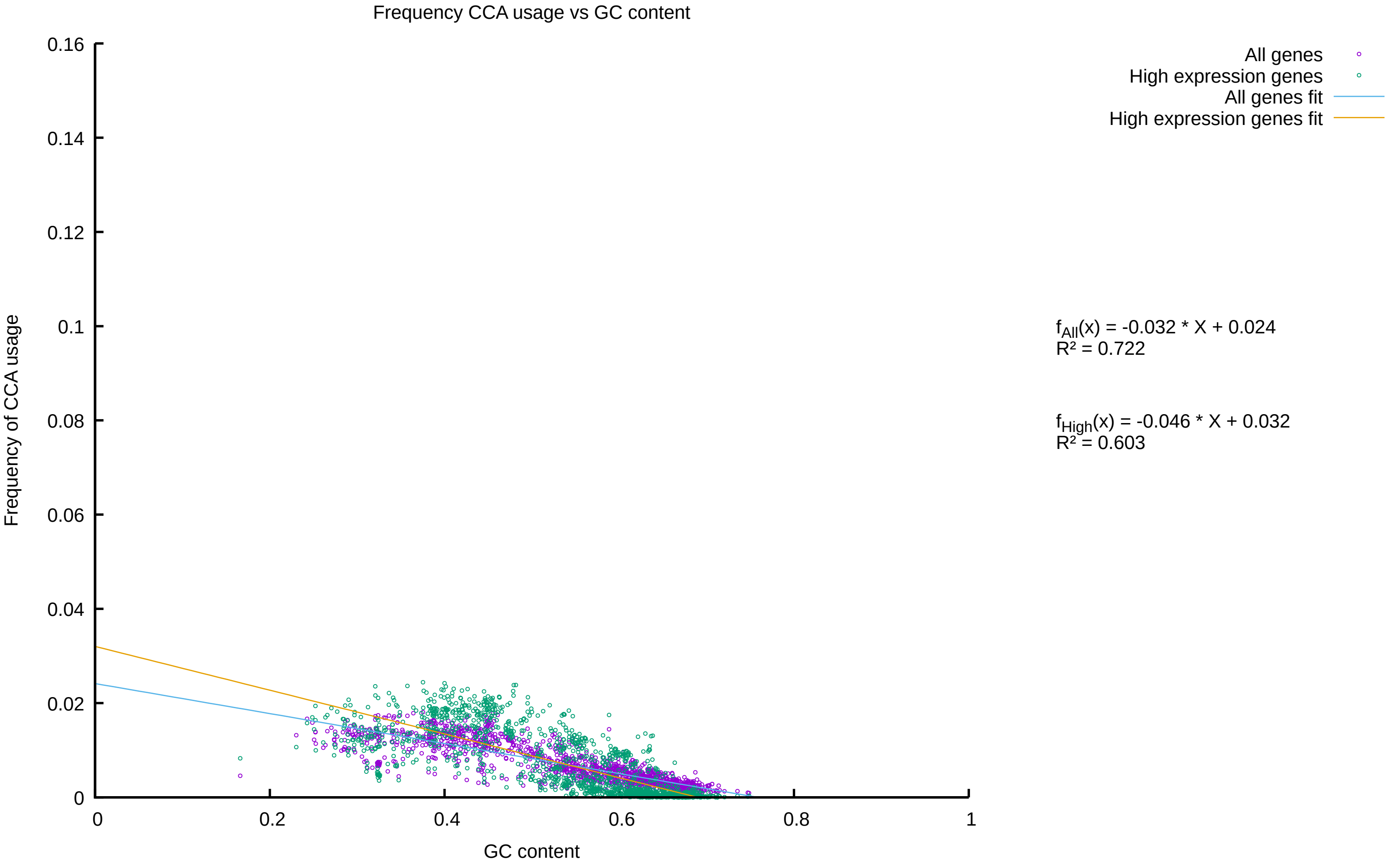

$f_{All}(x) = -0.032 * X + 0.024$
$R^2 = 0.722$

$f_{High}(x) = -0.046 * X + 0.032$
$R^2 = 0.603$

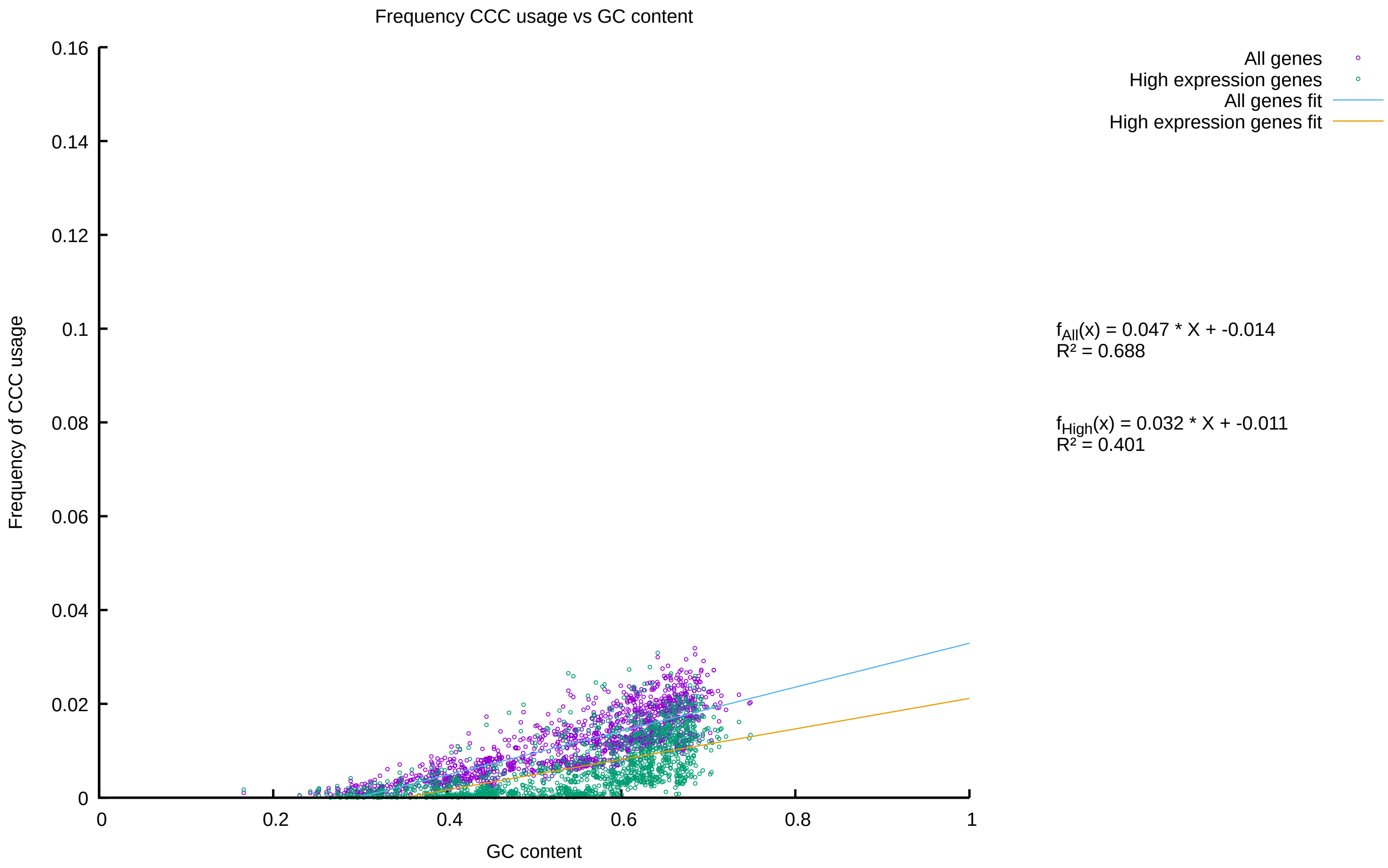

Frequency CCC usage vs GC content
All genes
High expression genes
All genes fit
High expression genes fit
f_All(x) = 0.047 * X + -0.014
R² = 0.688
f_High(x) = 0.032 * X + -0.011
R² = 0.401
Frequency of CCC usage
GC content
0.16
0.14
0.12
0.1
0.08
0.06
0.04
0.02
0
0.2
0.4
0.6
0.8
1

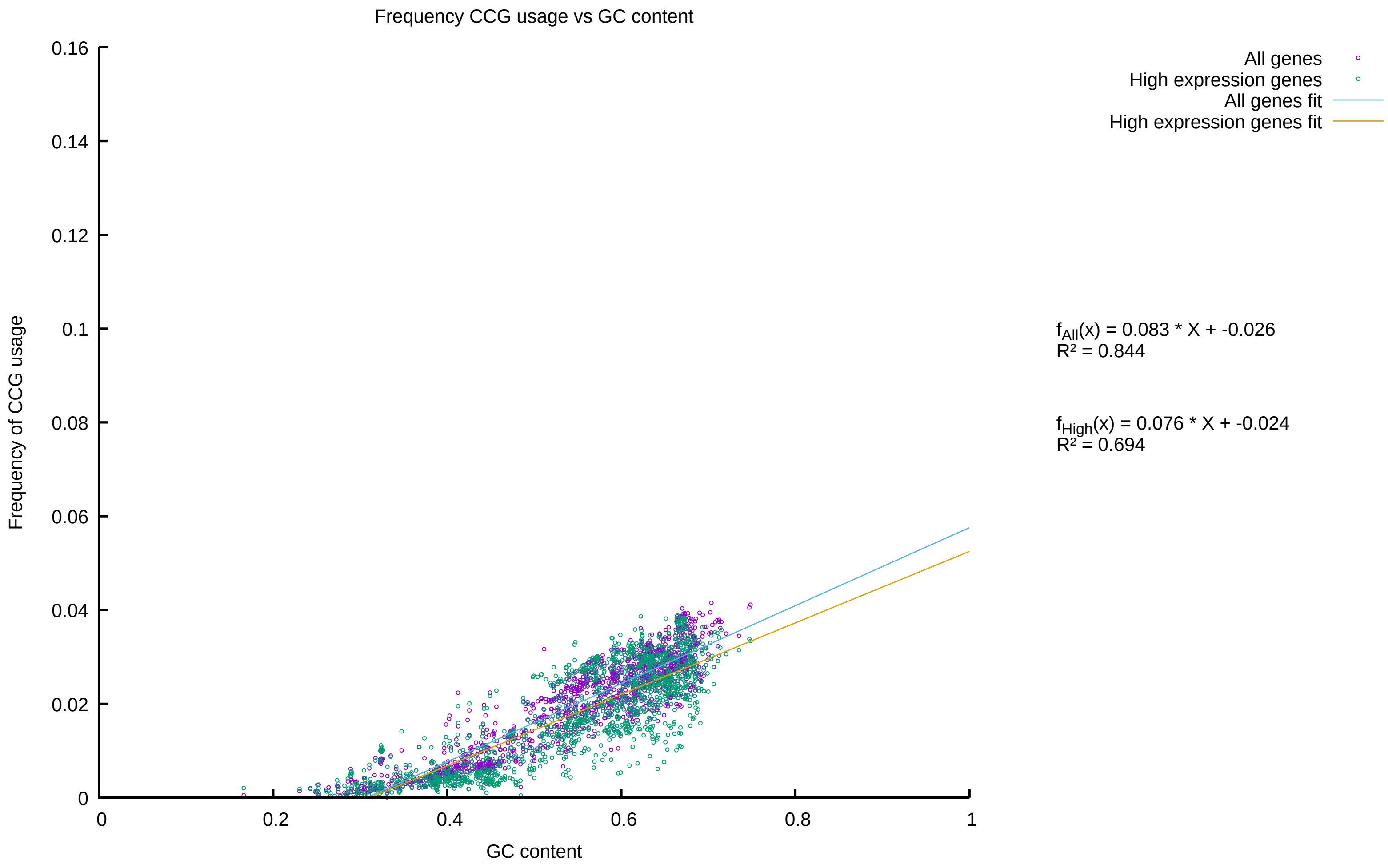

Frequency CCG usage vs GC content
0.16
0.14
0.12
0.1
0.08
0.06
0.04
0.02
0
Frequency of CCG usage
0
0.2
0.4
0.6
0.8
1
GC content
All genes
High expression genes
All genes fit
High expression genes fit
$f_{All}(x) = 0.083 * X + -0.026$
$R^2 = 0.844$
$f_{High}(x) = 0.076 * X + -0.024$
$R^2 = 0.694$

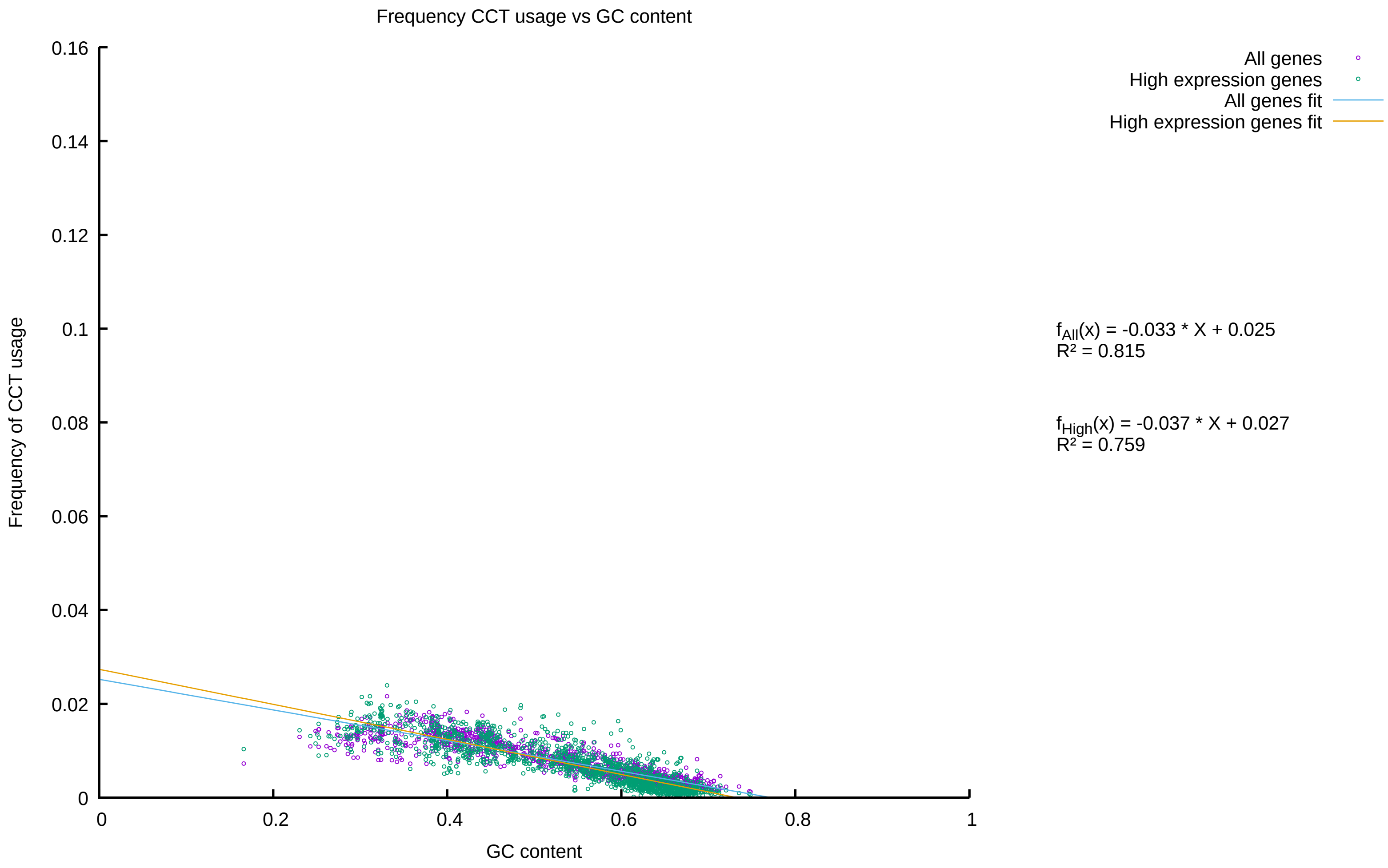
Frequency CCT usage vs GC content
0.16
0.14
0.12
0.1
0.08
0.06
0.04
0.02
0
0.2
0.4
0.6
0.8
1
Frequency of CCT usage
GC content
All genes
High expression genes
All genes fit
High expression genes fit
$f_{All}(x) = -0.033 * X + 0.025$
$R^2 = 0.815$
$f_{High}(x) = -0.037 * X + 0.027$
$R^2 = 0.759$

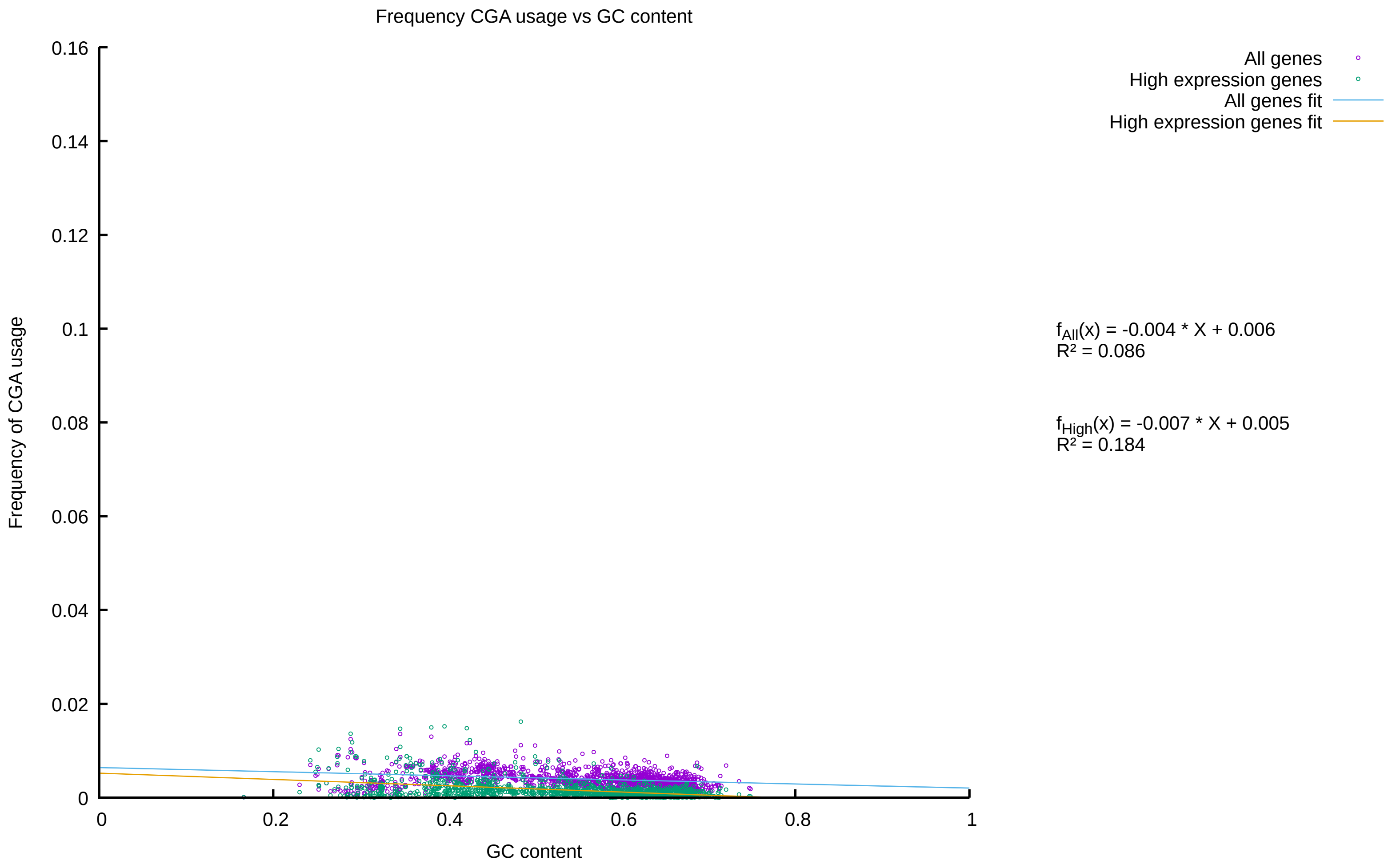
Frequency CGA usage vs GC content
Frequency of CGA usage
GC content
0.16
0.14
0.12
0.1
0.08
0.06
0.04
0.02
0
0
0.2
0.4
0.6
0.8
1
All genes
High expression genes
All genes fit
High expression genes fit
f_All(x) = -0.004 * X + 0.006
R² = 0.086
f_High(x) = -0.007 * X + 0.005
R² = 0.184

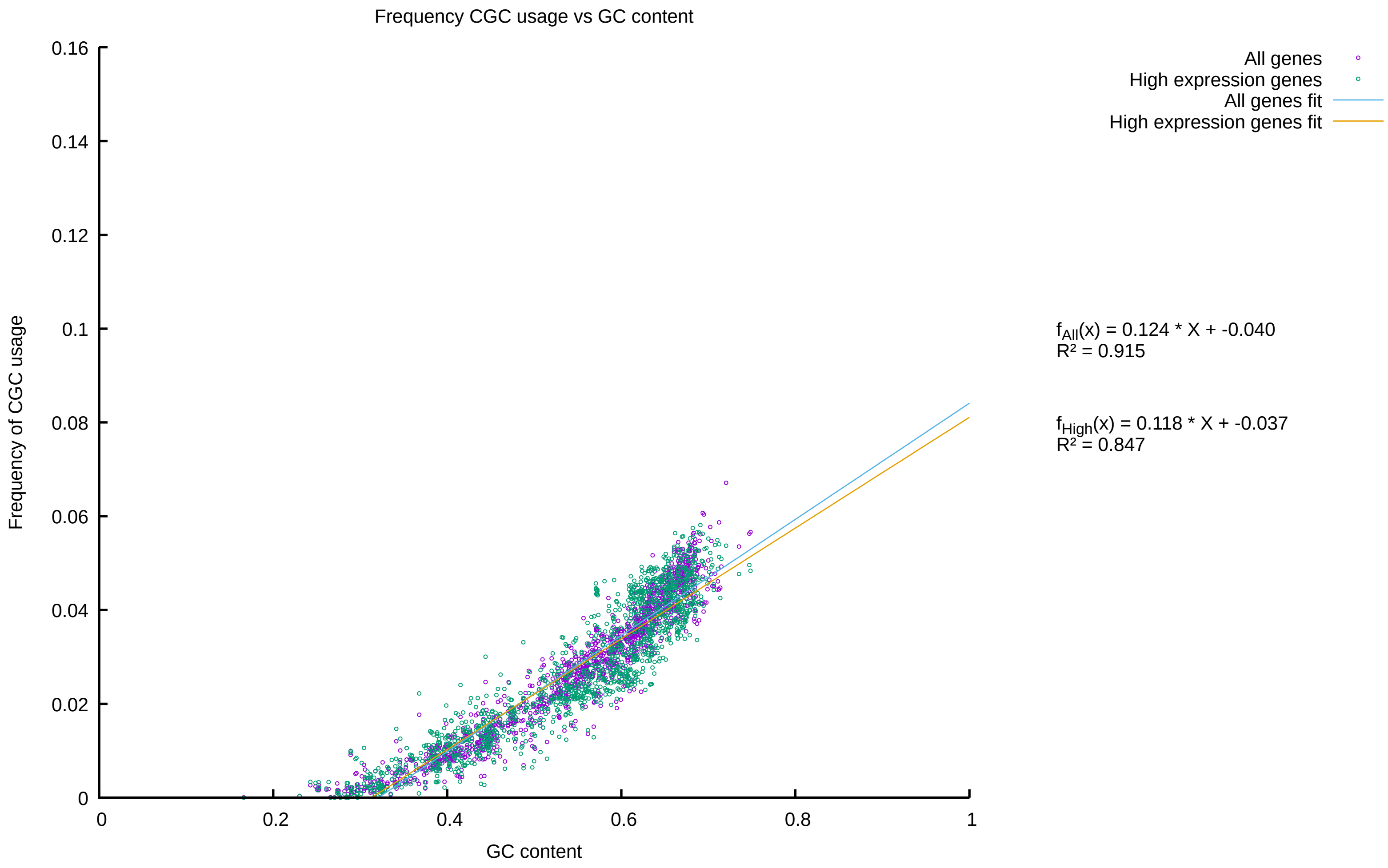

Frequency CGC usage vs GC content
0.16
0.14
0.12
0.1
0.08
0.06
0.04
0.02
0
Frequency of CGC usage
0
0.2
0.4
0.6
0.8
1
GC content
All genes
High expression genes
All genes fit
High expression genes fit
$f_{All}(x) = 0.124 * X + -0.040$
$R^2 = 0.915$
$f_{High}(x) = 0.118 * X + -0.037$
$R^2 = 0.847$

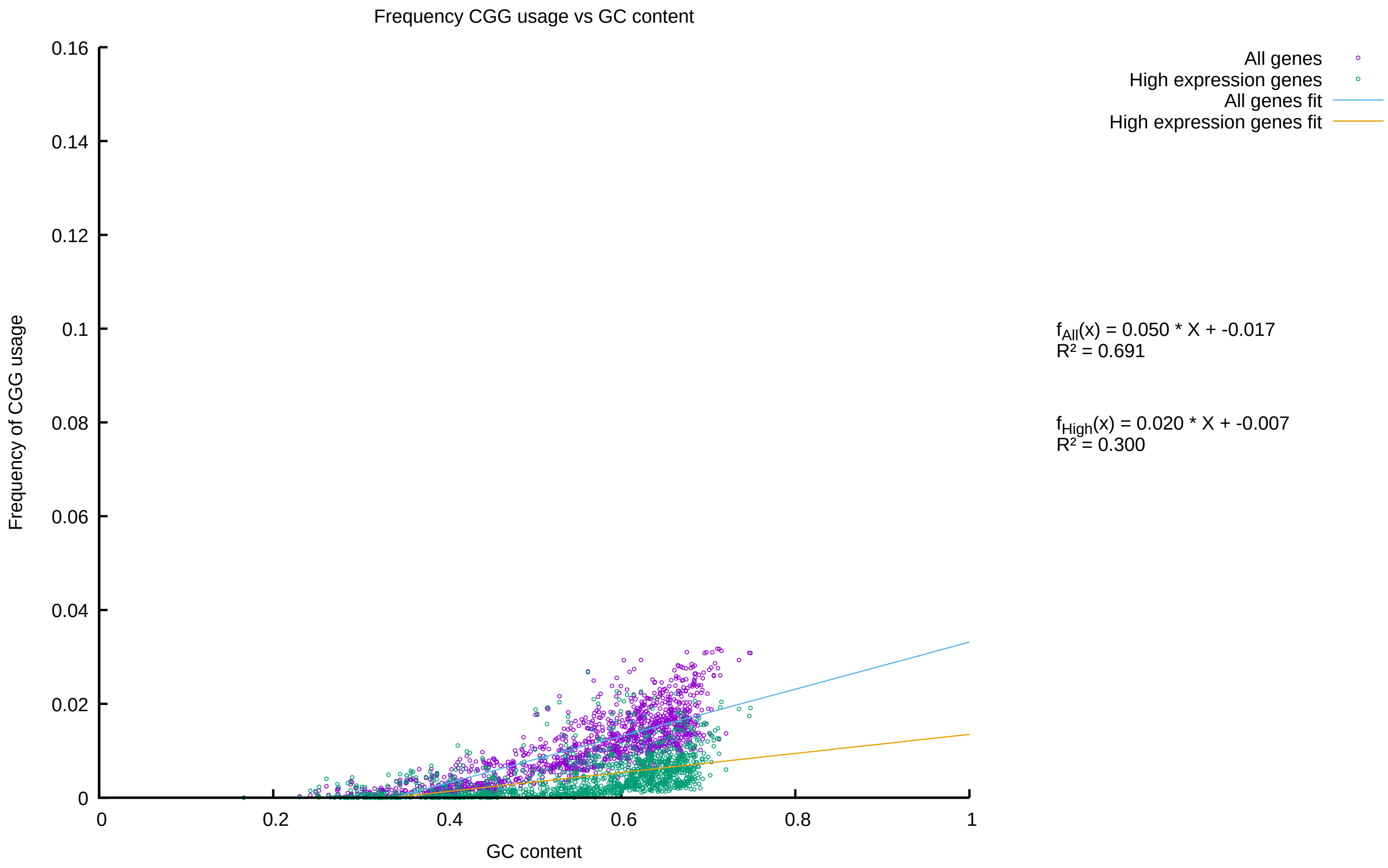

$f_{All}(x) = 0.050 * X + -0.017$
$R^2 = 0.691$

$f_{High}(x) = 0.020 * X + -0.007$
$R^2 = 0.300$

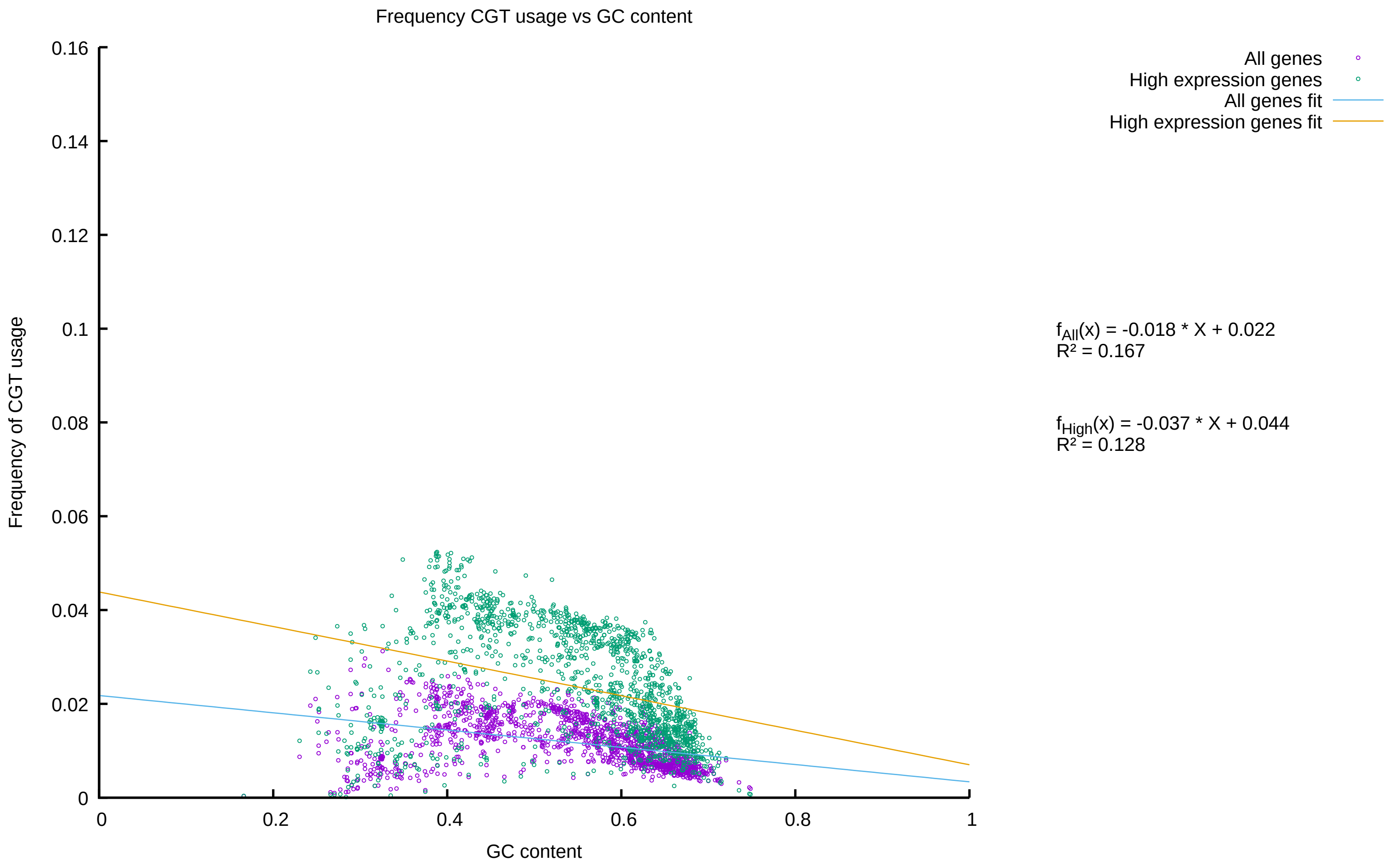

$f_{All}(x) = -0.018 * X + 0.022$
$R^2 = 0.167$

$f_{High}(x) = -0.037 * X + 0.044$
$R^2 = 0.128$

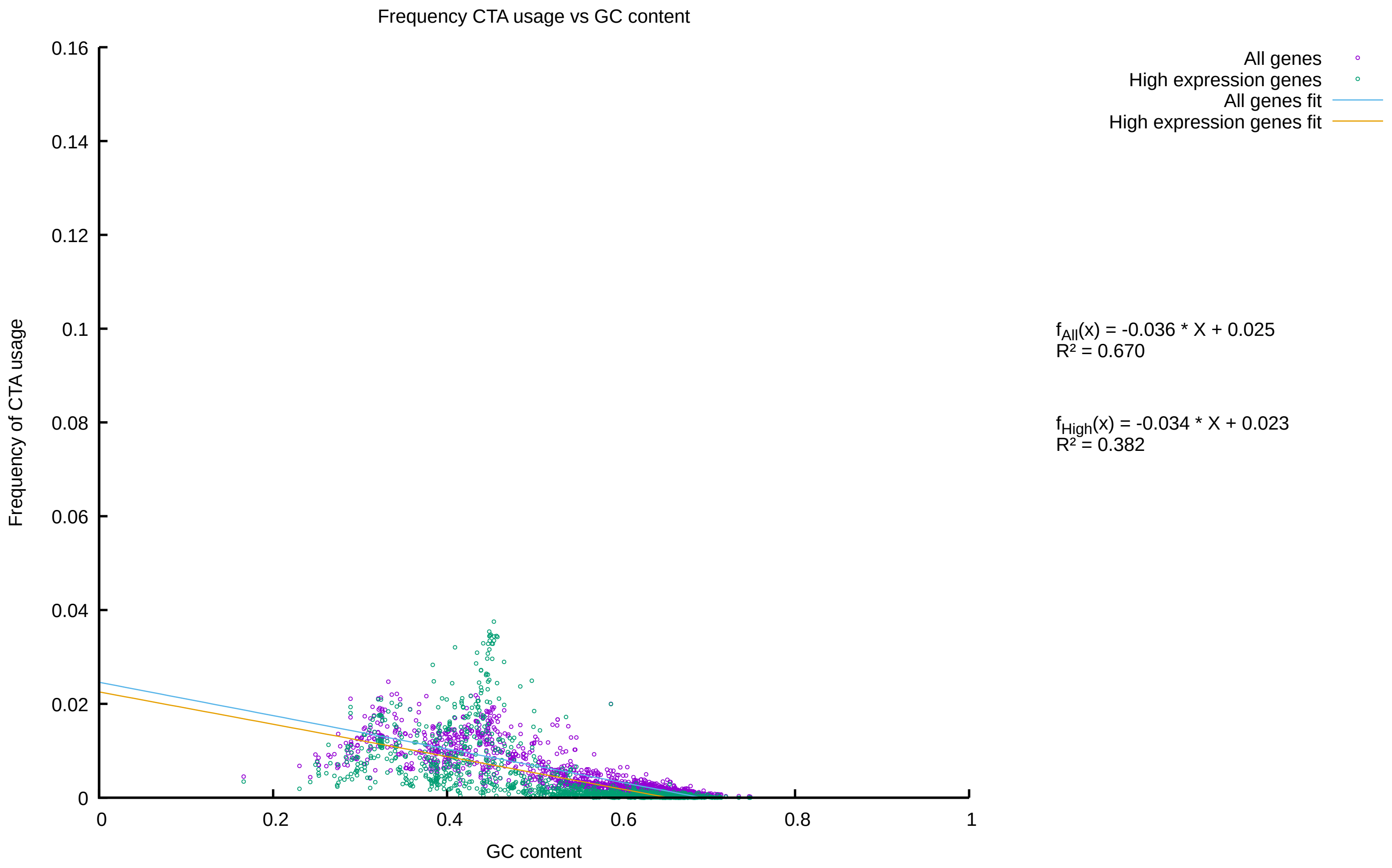

Frequency CTA usage vs GC content
0.16
0.14
0.12
0.1
0.08
0.06
0.04
0.02
0
Frequency of CTA usage
0
0.2
0.4
0.6
0.8
1
GC content
All genes
High expression genes
All genes fit
High expression genes fit
f_All(x) = -0.036 * X + 0.025
R² = 0.670
f_High(x) = -0.034 * X + 0.023
R² = 0.382

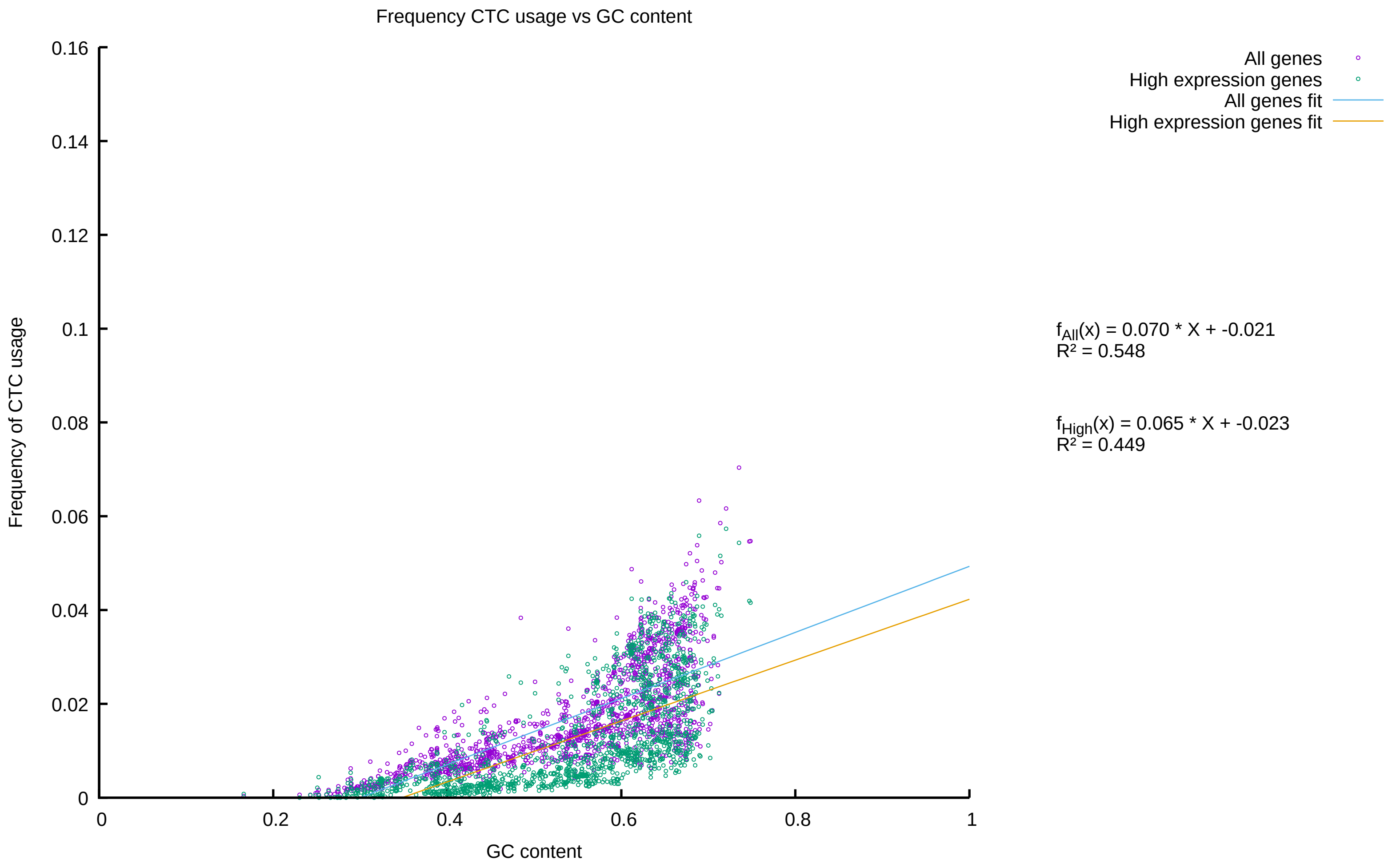
Frequency CTC usage vs GC content
Frequency of CTC usage
GC content
0.16
0.14
0.12
0.1
0.08
0.06
0.04
0.02
0
0
0.2
0.4
0.6
0.8
1
All genes
High expression genes
All genes fit
High expression genes fit
$f_{All}(x) = 0.070 * X + -0.021$
$R^2 = 0.548$
$f_{High}(x) = 0.065 * X + -0.023$
$R^2 = 0.449$

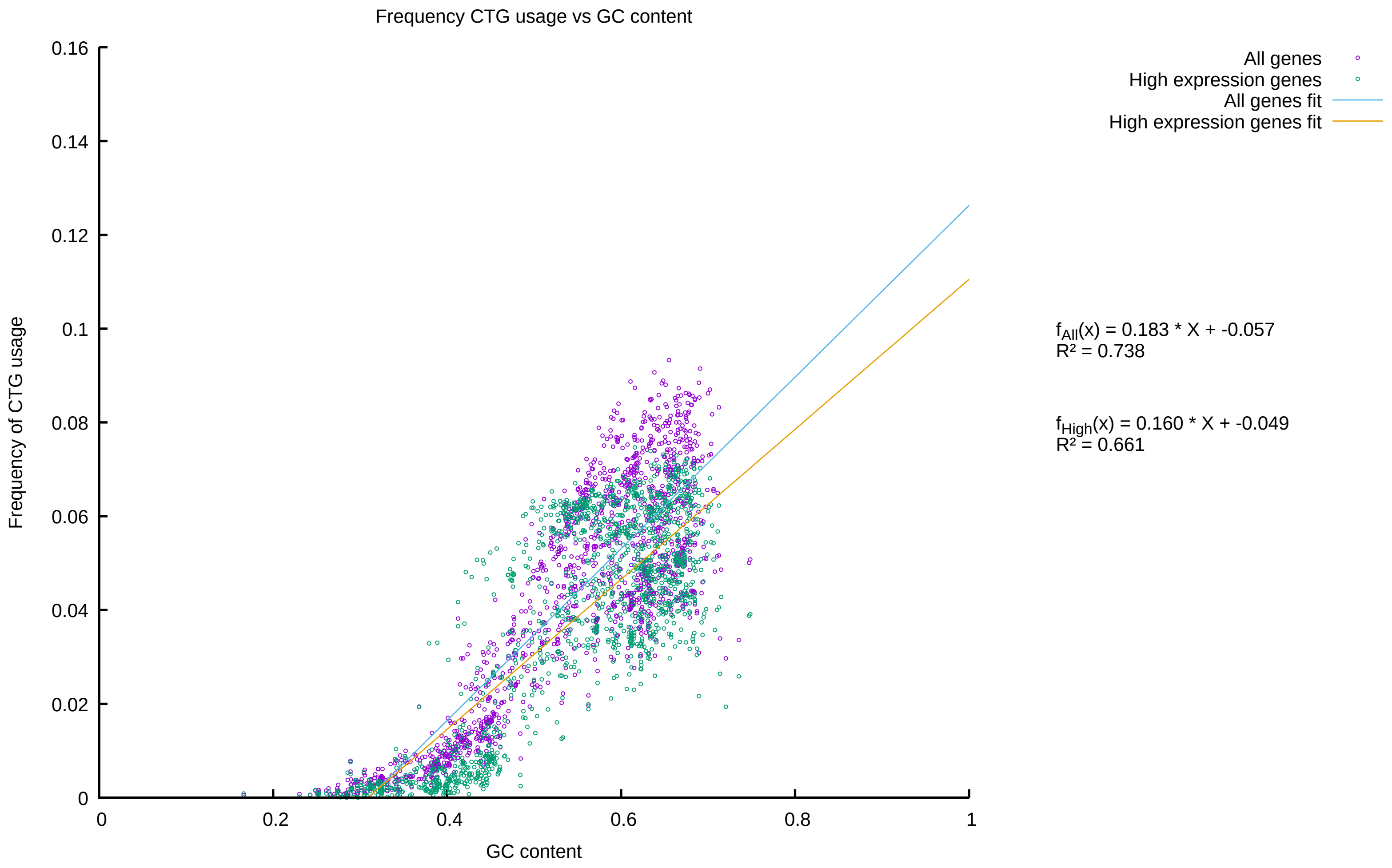

Frequency CTG usage vs GC content
All genes
High expression genes
All genes fit
High expression genes fit
$f_{All}(x) = 0.183 * X + -0.057$
$R^2 = 0.738$
$f_{High}(x) = 0.160 * X + -0.049$
$R^2 = 0.661$
Frequency of CTG usage
GC content
0.16
0.14
0.12
0.1
0.08
0.06
0.04
0.02
0
0
0.2
0.4
0.6
0.8
1

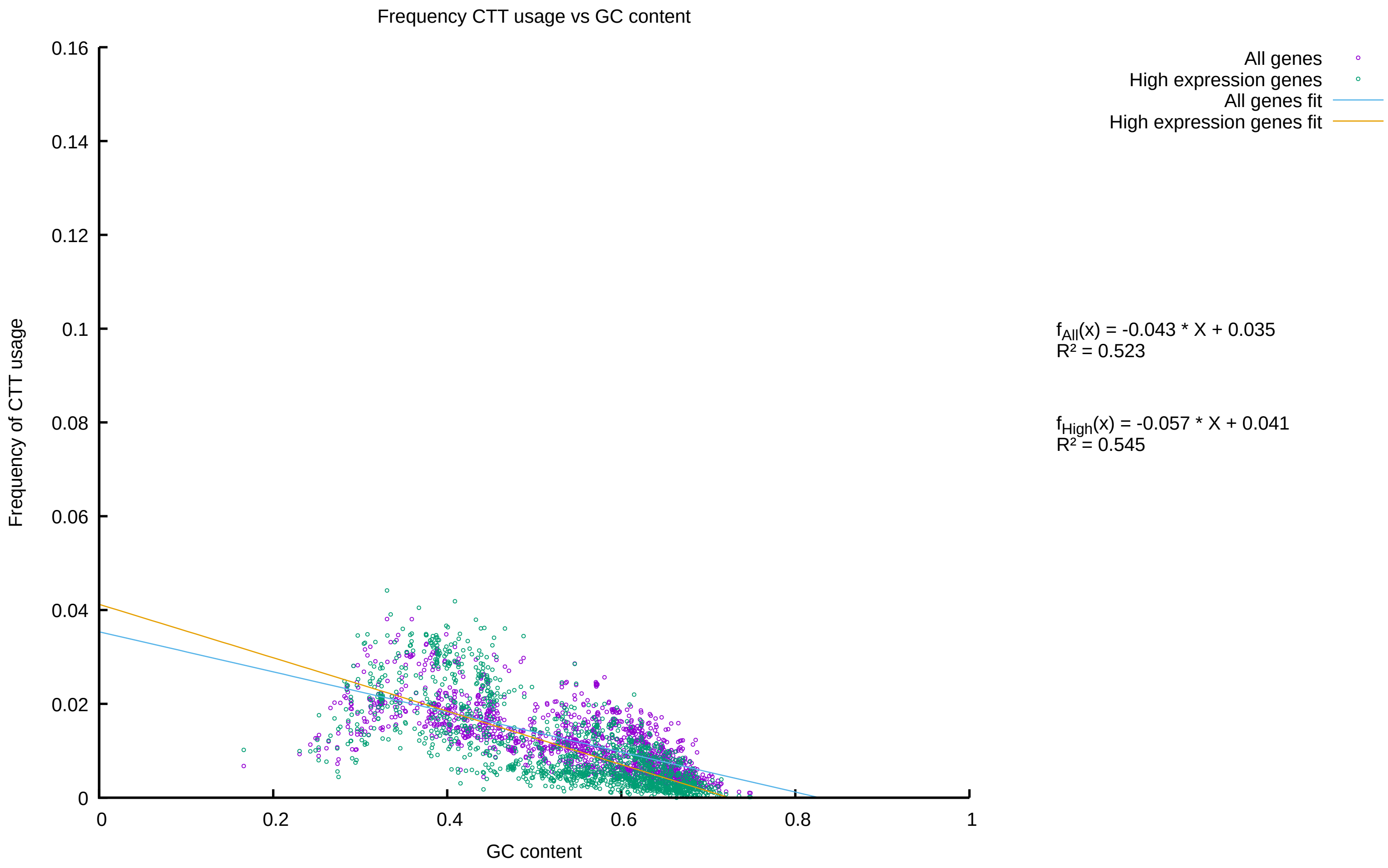
Frequency CTT usage vs GC content
Frequency of CTT usage
GC content
0.16
0.14
0.12
0.1
0.08
0.06
0.04
0.02
0
0
0.2
0.4
0.6
0.8
1
All genes
High expression genes
All genes fit
High expression genes fit
$f_{All}(x) = -0.043 * X + 0.035$
$R^2 = 0.523$
$f_{High}(x) = -0.057 * X + 0.041$
$R^2 = 0.545$

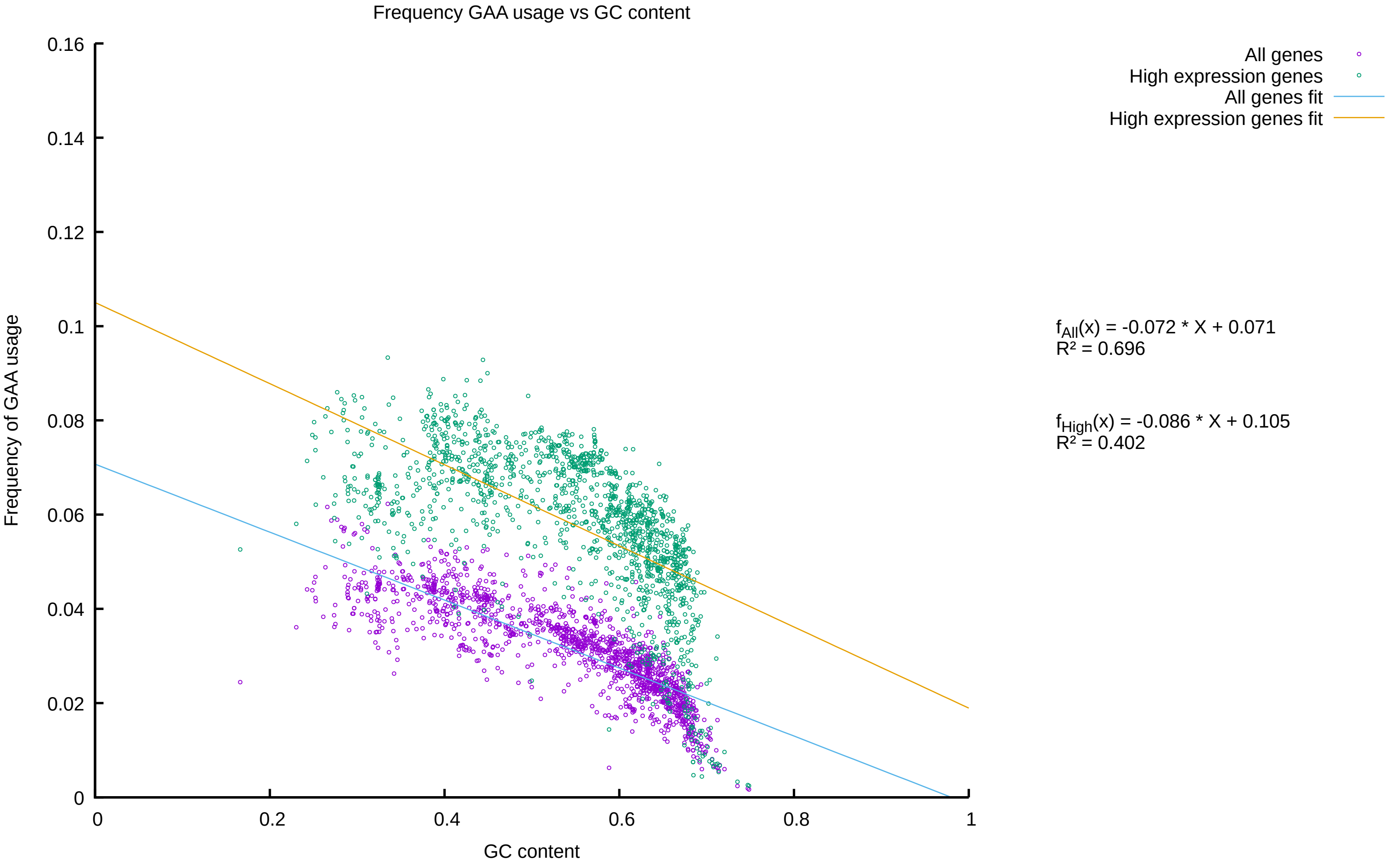

$f_{All}(x) = -0.072 * X + 0.071$
$R^2 = 0.696$

$f_{High}(x) = -0.086 * X + 0.105$
$R^2 = 0.402$

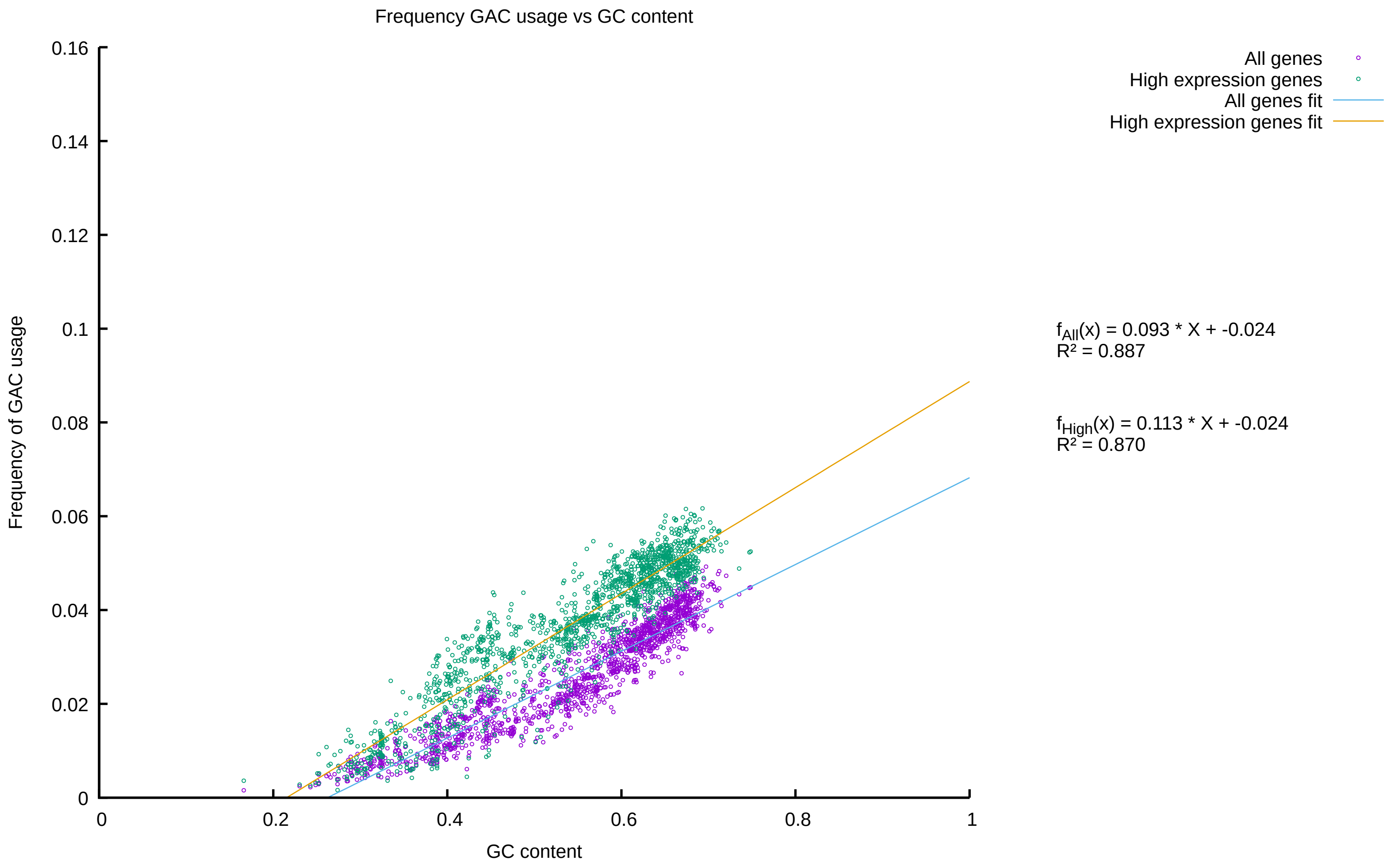
Frequency GAC usage vs GC content
All genes
High expression genes
All genes fit
High expression genes fit
$f_{All}(x) = 0.093 * X + -0.024$
$R^2 = 0.887$
$f_{High}(x) = 0.113 * X + -0.024$
$R^2 = 0.870$
Frequency of GAC usage
0.16
0.14
0.12
0.1
0.08
0.06
0.04
0.02
0
GC content
0
0.2
0.4
0.6
0.8
1

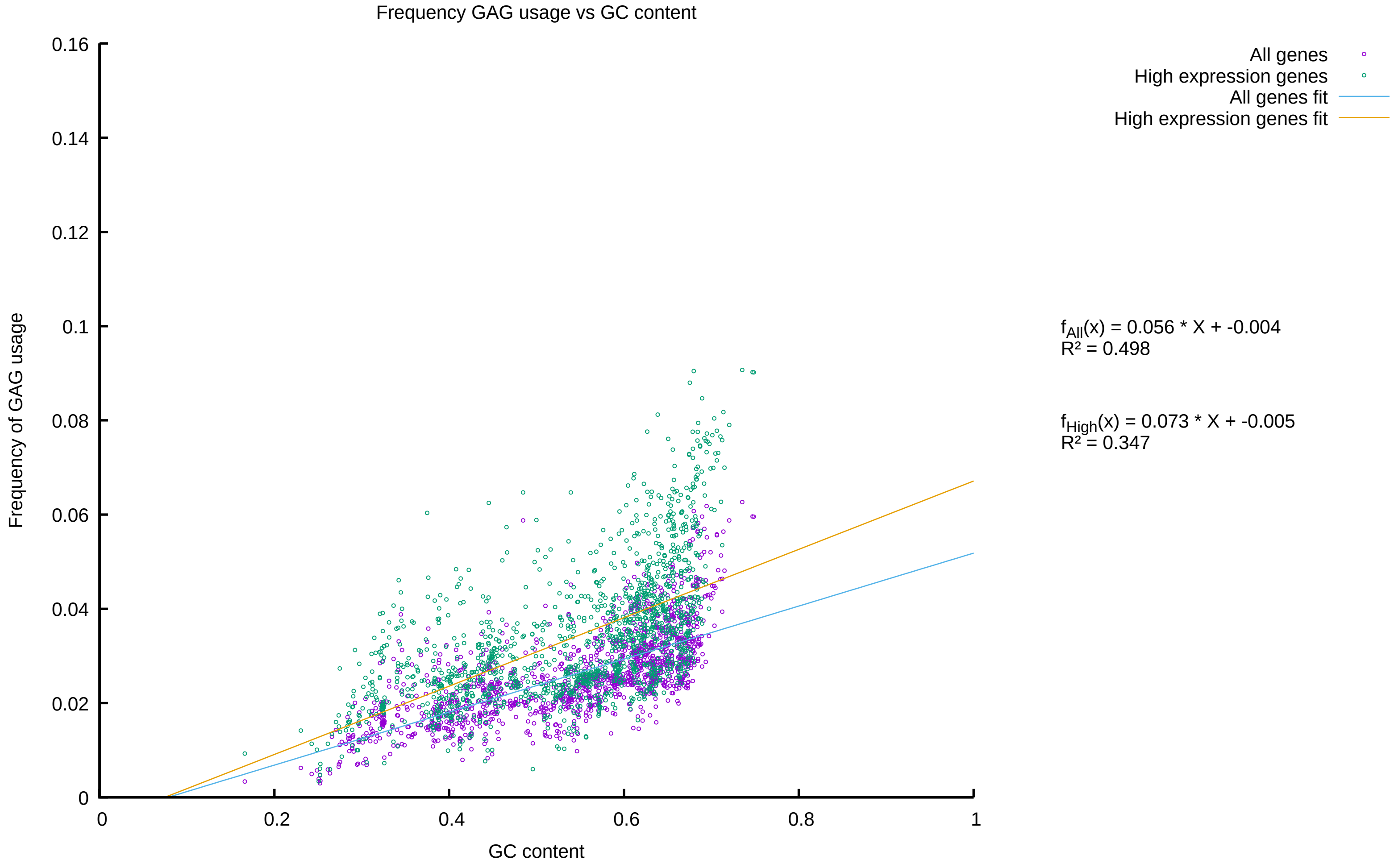

$f_{All}(x) = 0.056 * X + -0.004$
$R^2 = 0.498$

$f_{High}(x) = 0.073 * X + -0.005$
$R^2 = 0.347$

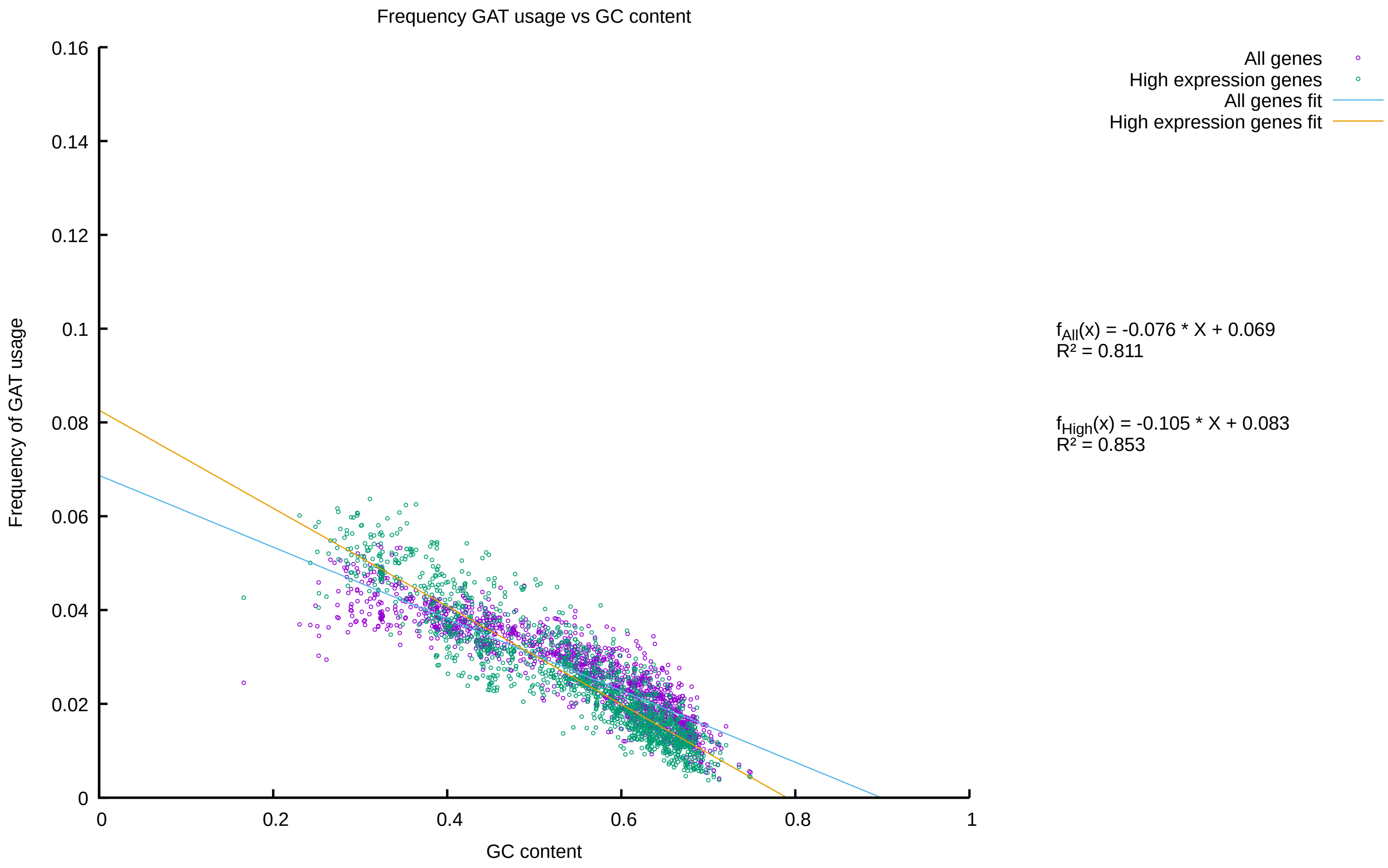

Frequency GAT usage vs GC content
Frequency of GAT usage
GC content
0.16
0.14
0.12
0.1
0.08
0.06
0.04
0.02
0
0.2
0.4
0.6
0.8
1
All genes
High expression genes
All genes fit
High expression genes fit
$f_{All}(x) = -0.076 * X + 0.069$
$R^2 = 0.811$
$f_{High}(x) = -0.105 * X + 0.083$
$R^2 = 0.853$

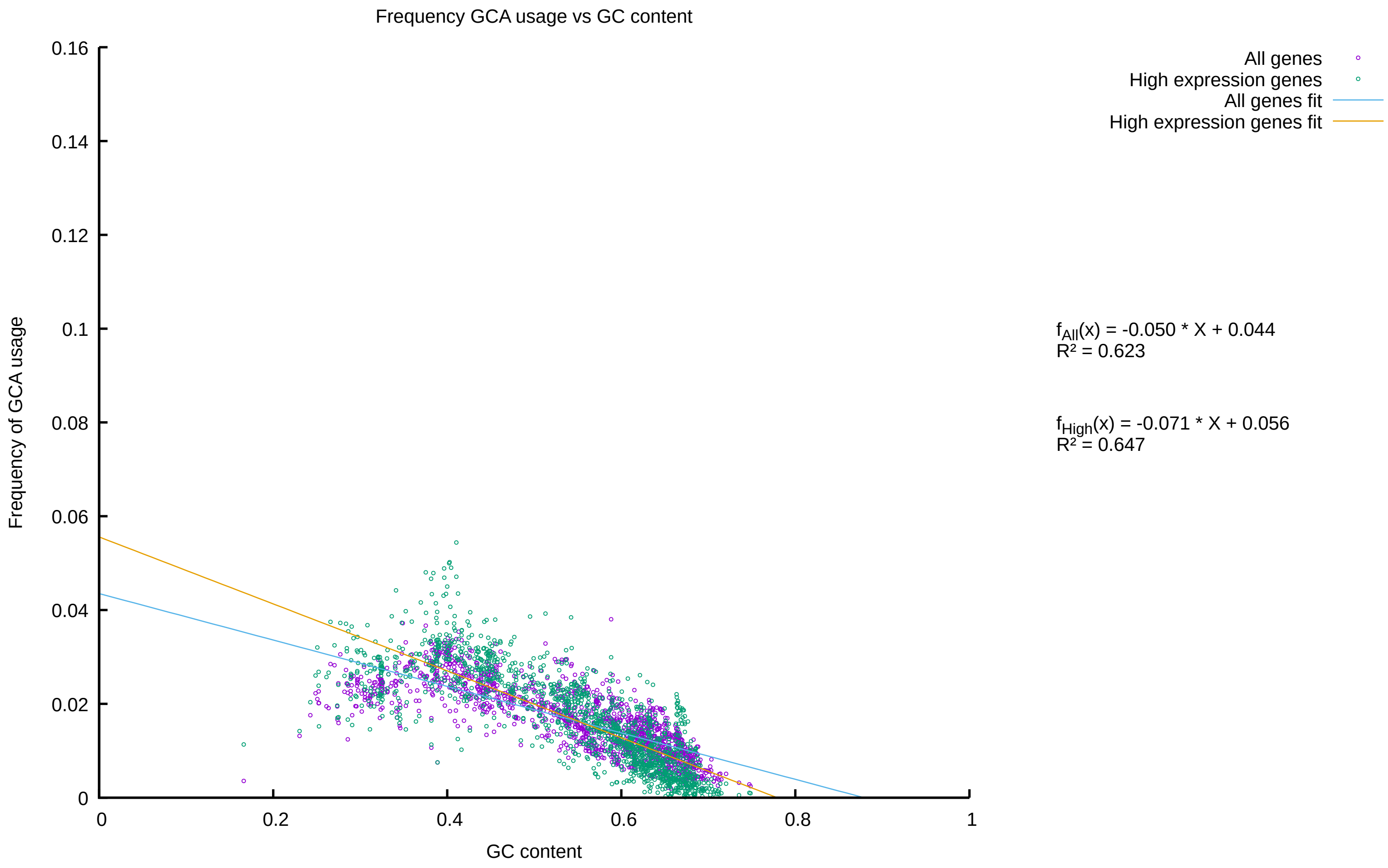

$f_{All}(x) = -0.050 * X + 0.044$
$R^2 = 0.623$

$f_{High}(x) = -0.071 * X + 0.056$
$R^2 = 0.647$

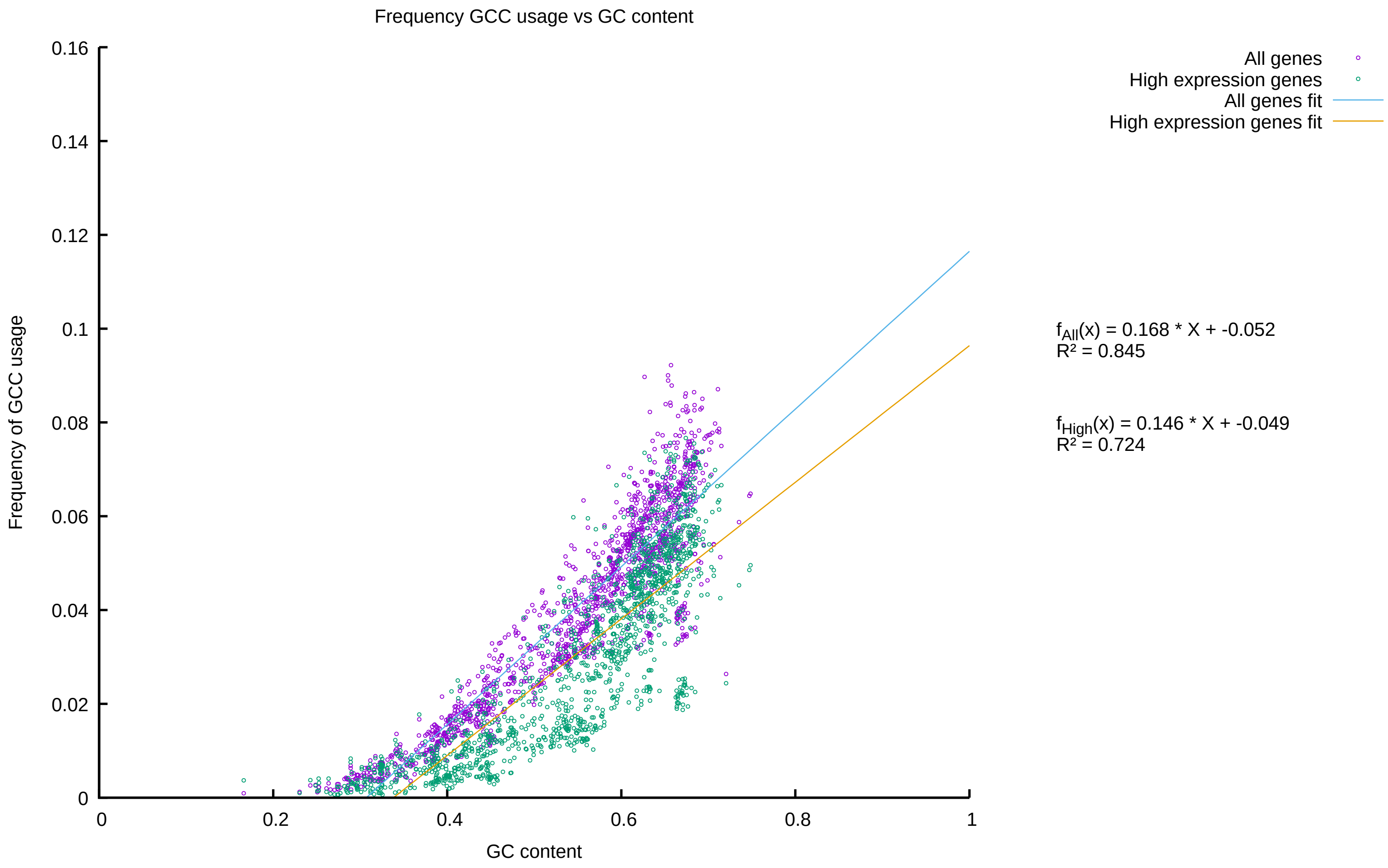

Frequency GCC usage vs GC content
Frequency of GCC usage
GC content
0
0.02
0.04
0.06
0.08
0.1
0.12
0.14
0.16
0
0.2
0.4
0.6
0.8
1
All genes
High expression genes
All genes fit
High expression genes fit
$f_{All}(x) = 0.168 * X + -0.052$
$R^2 = 0.845$
$f_{High}(x) = 0.146 * X + -0.049$
$R^2 = 0.724$

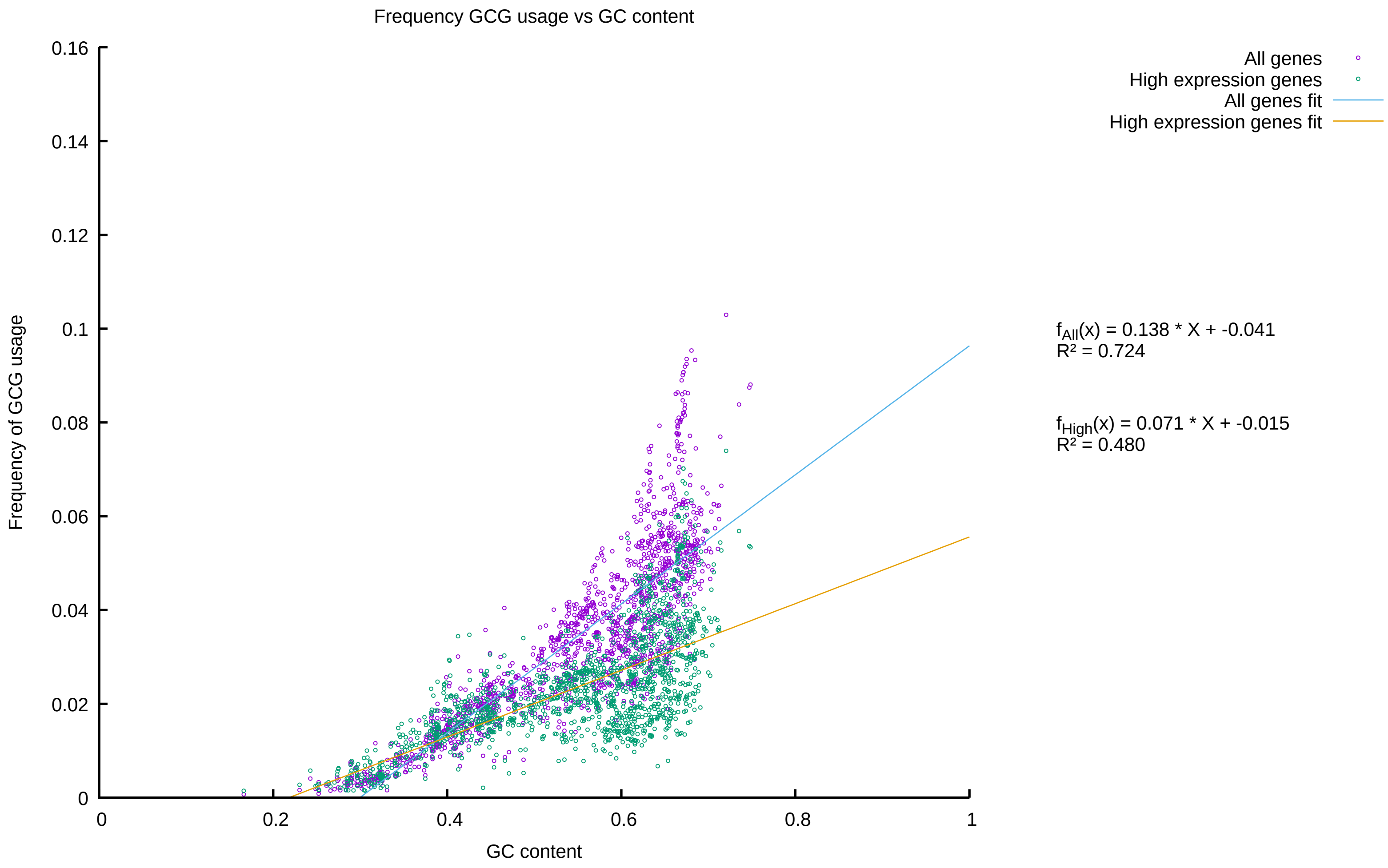

Frequency GCG usage vs GC content
Frequency of GCG usage
GC content
0.16
0.14
0.12
0.1
0.08
0.06
0.04
0.02
0
0
0.2
0.4
0.6
0.8
1
All genes
High expression genes
All genes fit
High expression genes fit
$f_{All}(x) = 0.138 * X + -0.041$
$R^2 = 0.724$
$f_{High}(x) = 0.071 * X + -0.015$
$R^2 = 0.480$

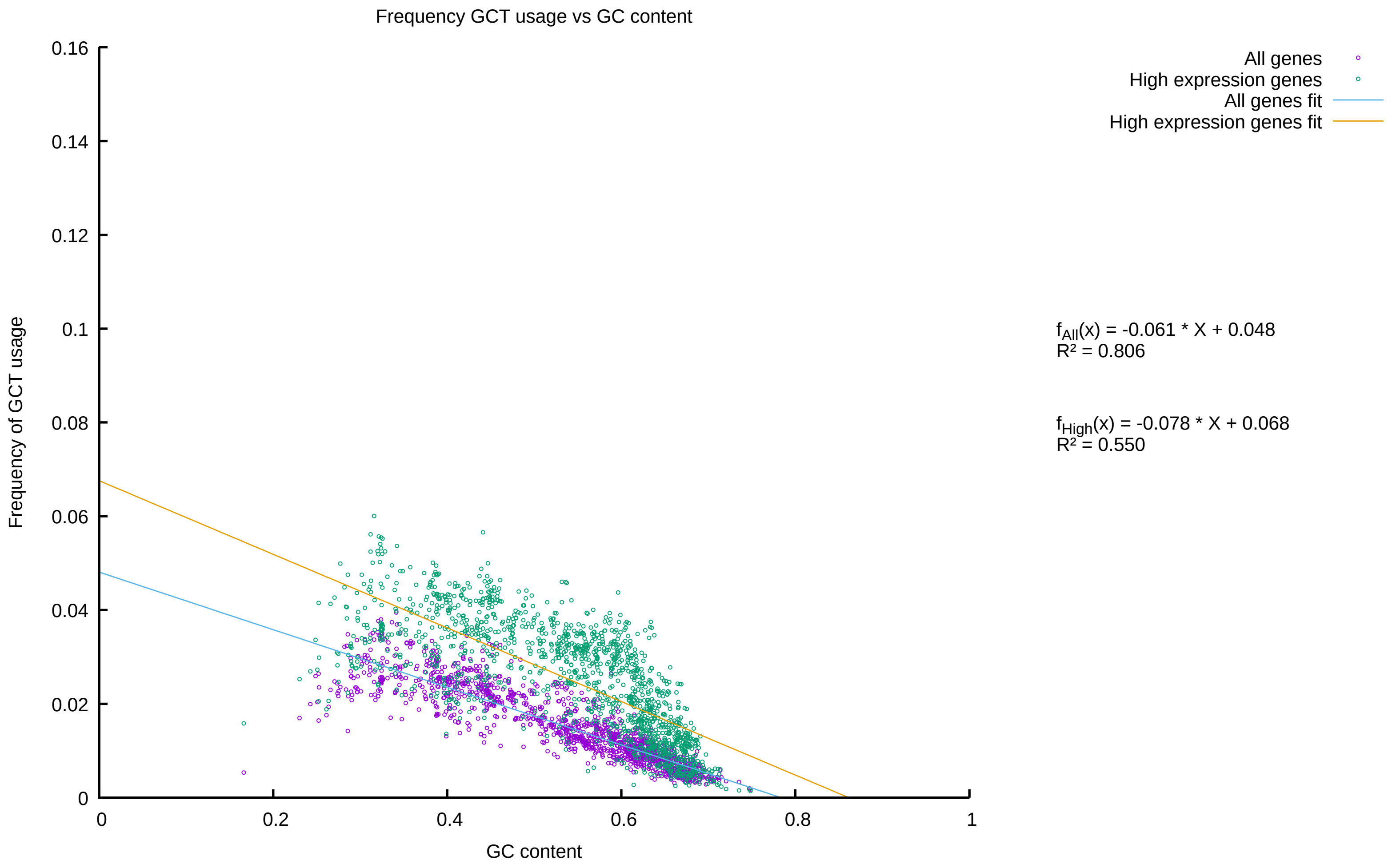
Frequency GCT usage vs GC content
0.16
0.14
0.12
0.1
0.08
0.06
0.04
0.02
0
Frequency of GCT usage
0
0.2
0.4
0.6
0.8
1
GC content
All genes
High expression genes
All genes fit
High expression genes fit
fAll(x) = -0.061 * X + 0.048
R² = 0.806
fHigh(x) = -0.078 * X + 0.068
R² = 0.550

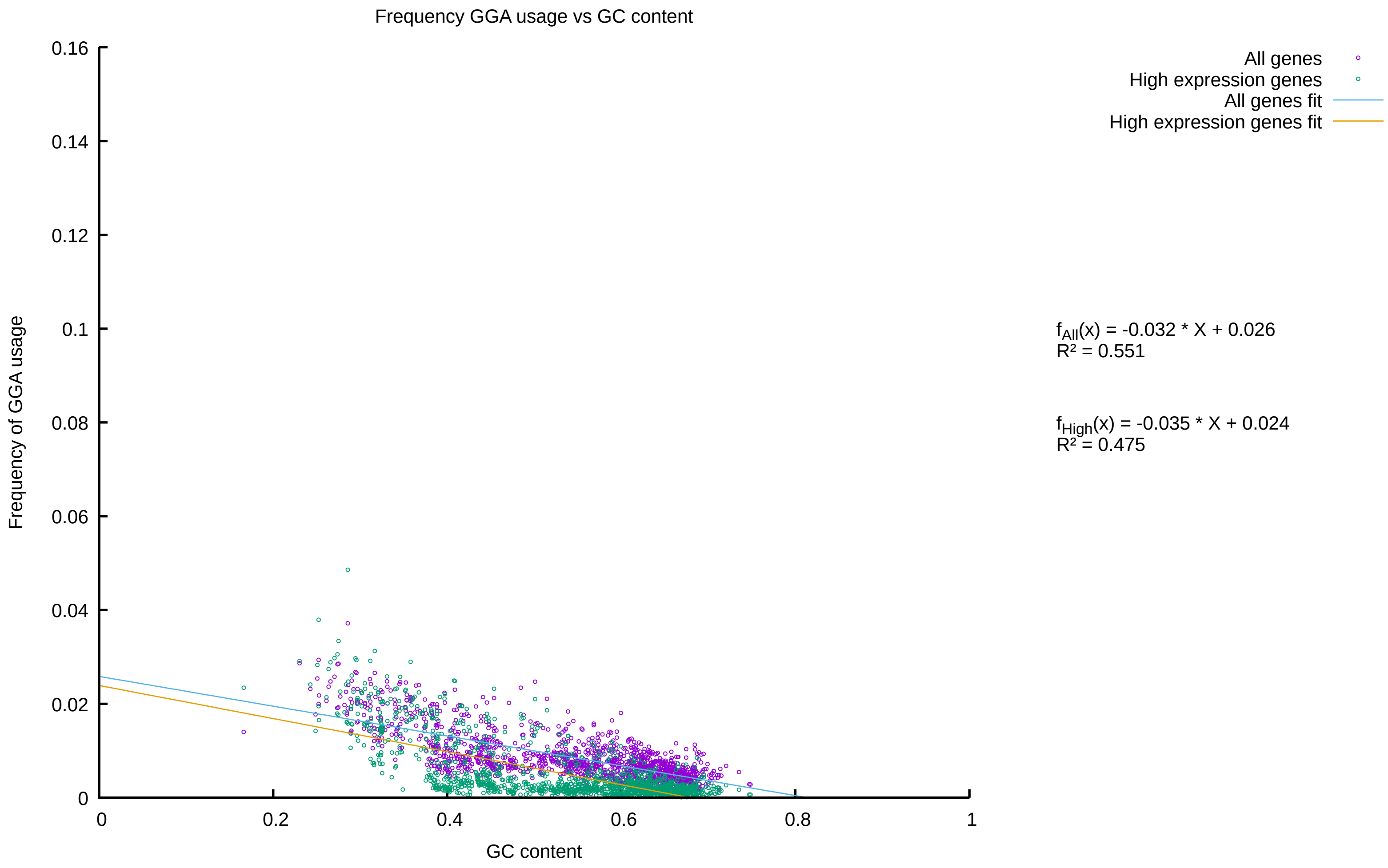

Frequency GGA usage vs GC content
Frequency of GGA usage
GC content
0.16
0.14
0.12
0.1
0.08
0.06
0.04
0.02
0
0
0.2
0.4
0.6
0.8
1
All genes
High expression genes
All genes fit
High expression genes fit
$f_{All}(x) = -0.032 * X + 0.026$
$R^2 = 0.551$
$f_{High}(x) = -0.035 * X + 0.024$
$R^2 = 0.475$

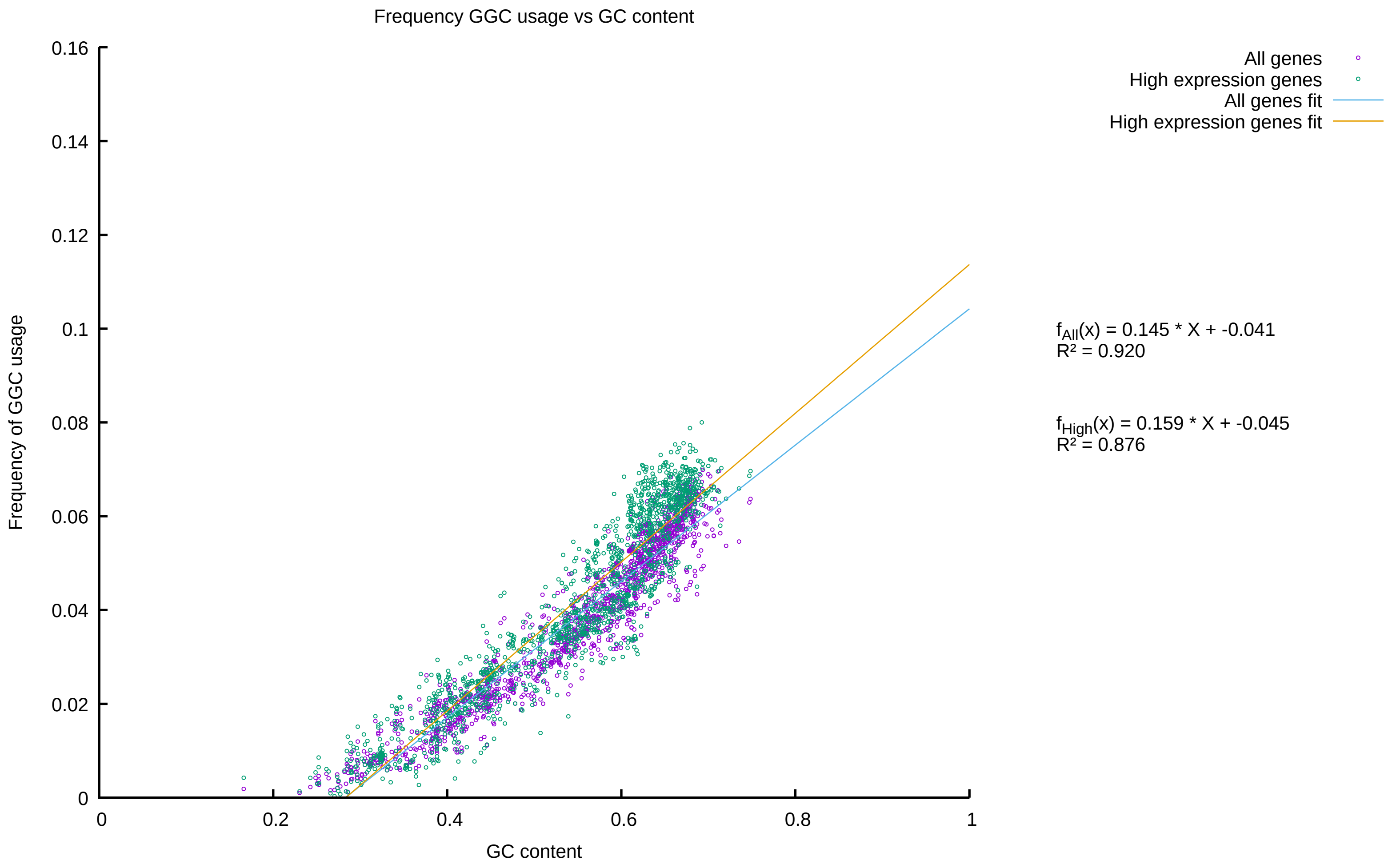

Frequency GGC usage vs GC content
All genes
High expression genes
All genes fit
High expression genes fit
$f_{All}(x) = 0.145 * X + -0.041$
$R^2 = 0.920$
$f_{High}(x) = 0.159 * X + -0.045$
$R^2 = 0.876$
Frequency of GGC usage
GC content
0.16
0.14
0.12
0.1
0.08
0.06
0.04
0.02
0
0
0.2
0.4
0.6
0.8
1

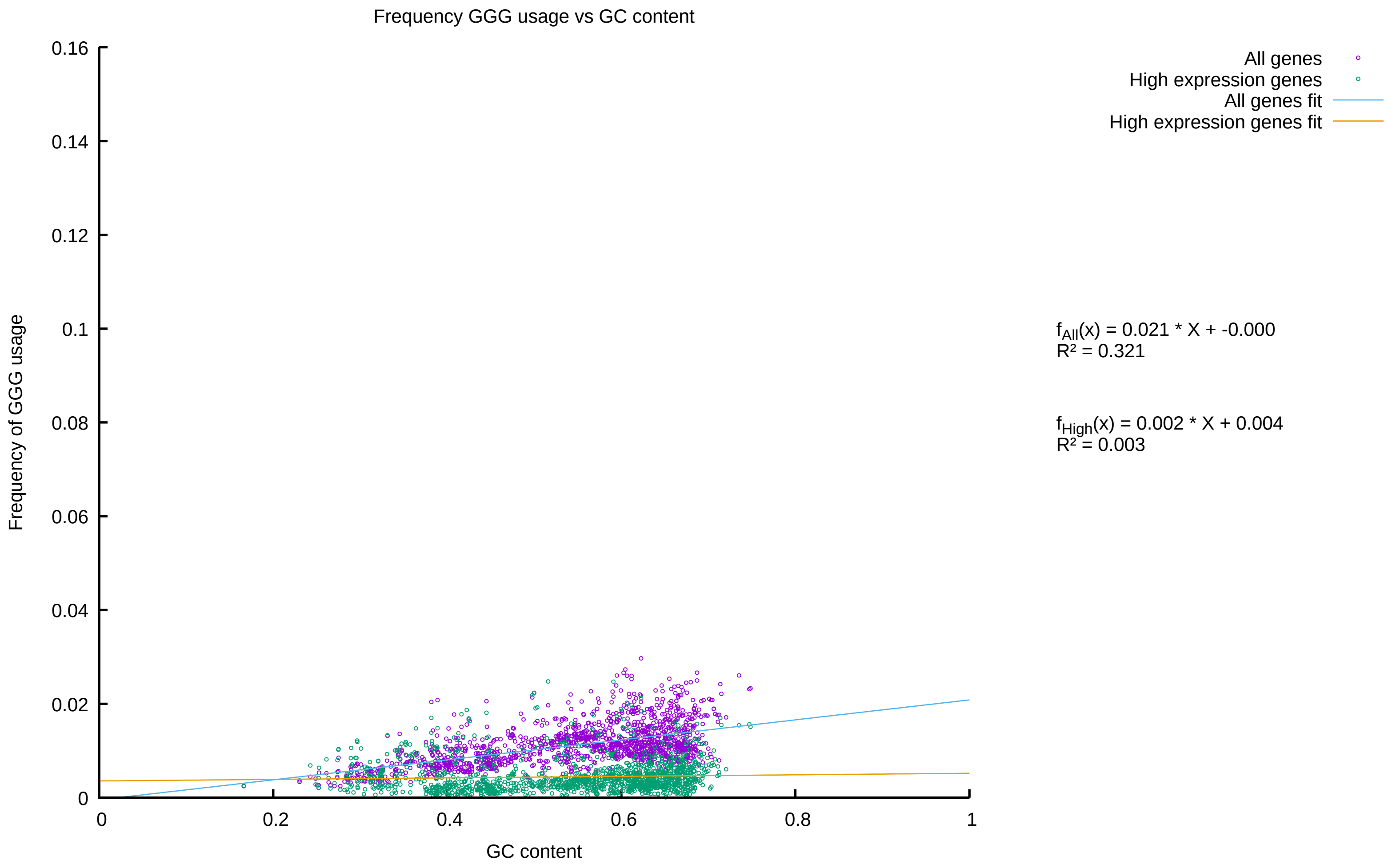

Frequency GGG usage vs GC content
Frequency of GGG usage
GC content
0.16
0.14
0.12
0.1
0.08
0.06
0.04
0.02
0
0
0.2
0.4
0.6
0.8
1
All genes
High expression genes
All genes fit
High expression genes fit
$f_{All}(x) = 0.021 * X + -0.000$
$R^2 = 0.321$
$f_{High}(x) = 0.002 * X + 0.004$
$R^2 = 0.003$

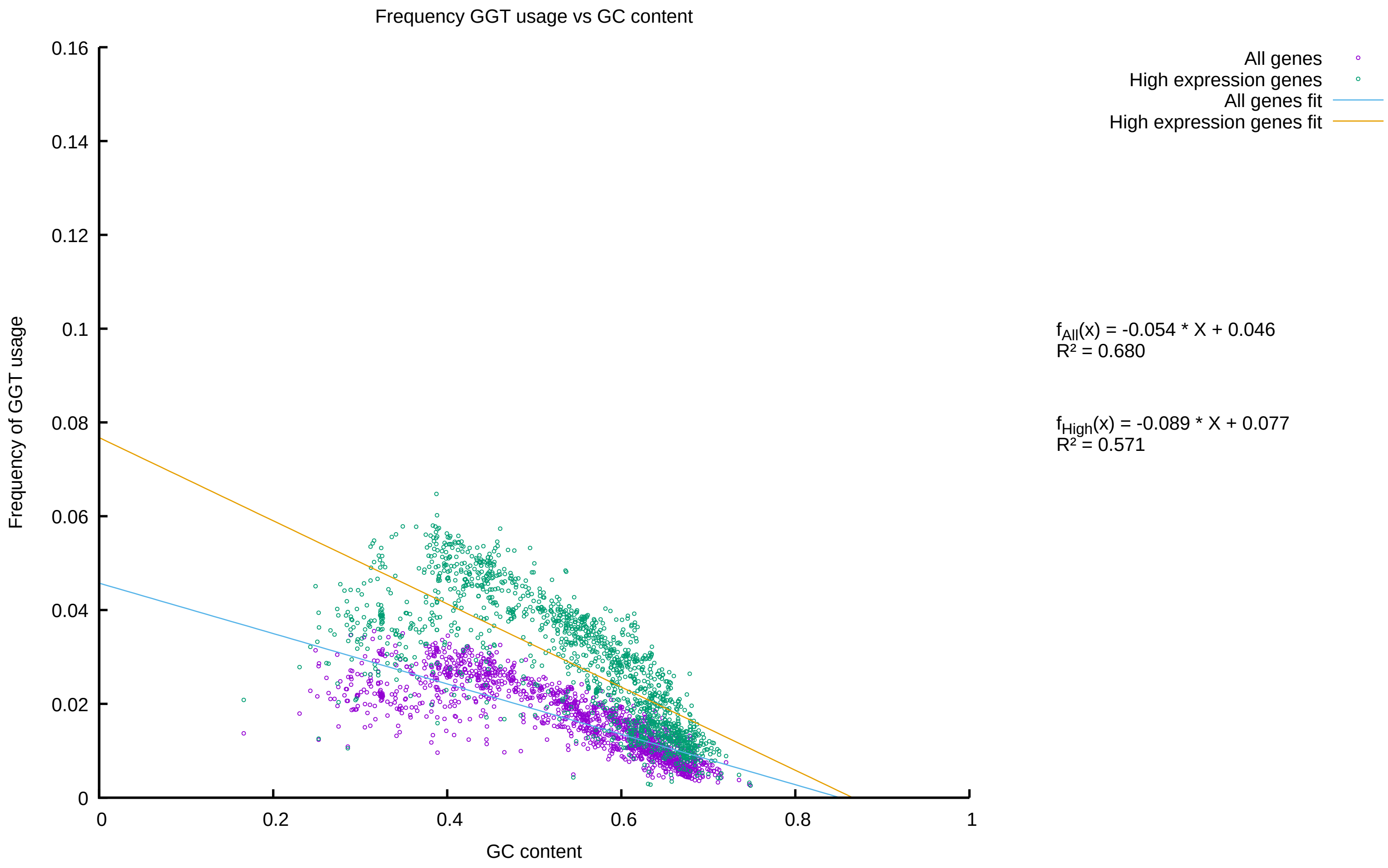
Frequency GGT usage vs GC content
0.16
0.14
0.12
0.1
0.08
0.06
0.04
0.02
0
0
0.2
0.4
0.6
0.8
1
Frequency of GGT usage
GC content
All genes
High expression genes
All genes fit
High expression genes fit
$f_{All}(x) = -0.054 * X + 0.046$
$R^2 = 0.680$
$f_{High}(x) = -0.089 * X + 0.077$
$R^2 = 0.571$

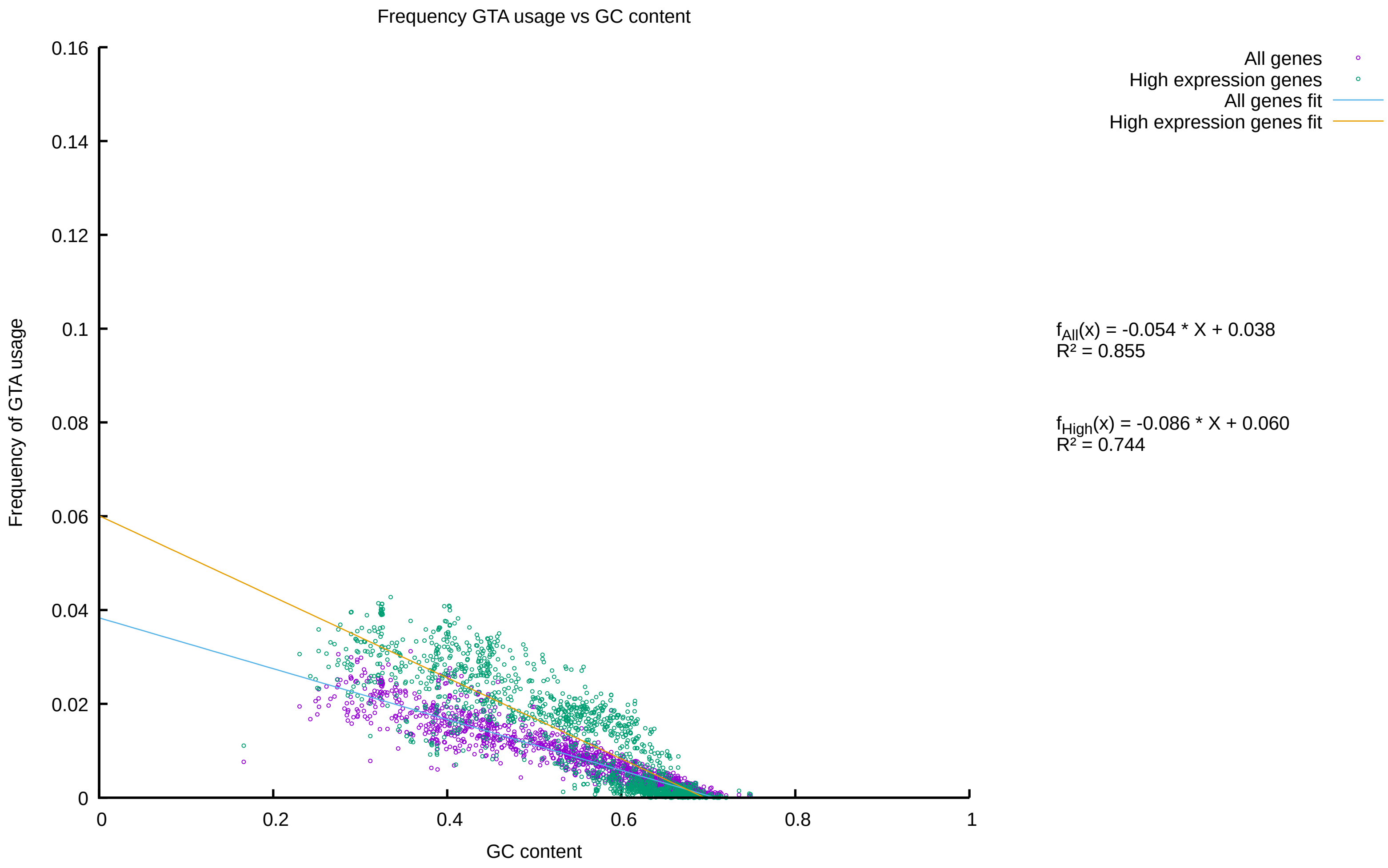
Frequency GTA usage vs GC content
Frequency of GTA usage
GC content
0.16
0.14
0.12
0.1
0.08
0.06
0.04
0.02
0
0
0.2
0.4
0.6
0.8
1
All genes
High expression genes
All genes fit
High expression genes fit
$f_{All}(x) = -0.054 * X + 0.038$
$R^2 = 0.855$
$f_{High}(x) = -0.086 * X + 0.060$
$R^2 = 0.744$

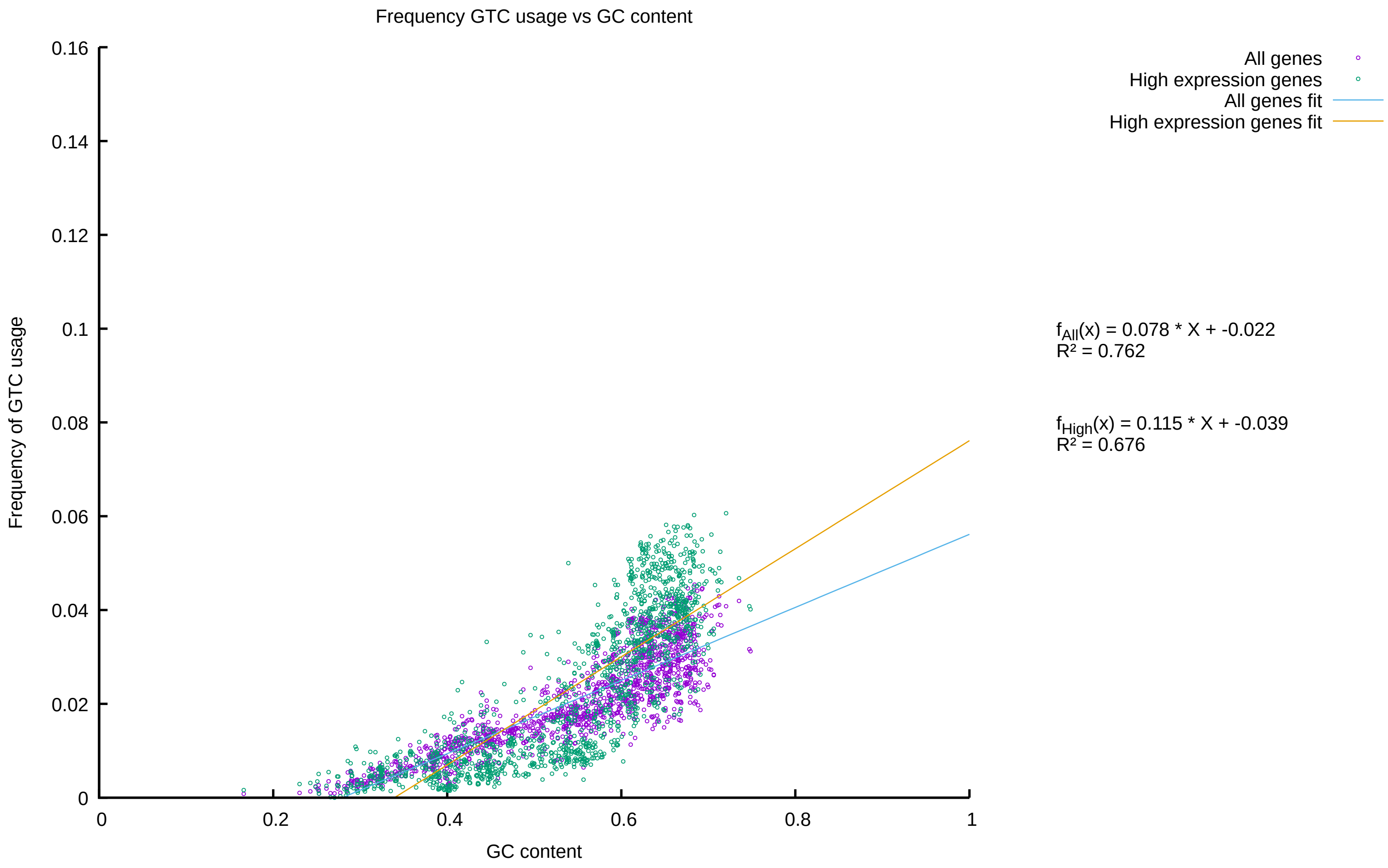
Frequency GTC usage vs GC content
Frequency of GTC usage
GC content
0.16
0.14
0.12
0.1
0.08
0.06
0.04
0.02
0
0
0.2
0.4
0.6
0.8
1
All genes
High expression genes
All genes fit
High expression genes fit
$f_{All}(x) = 0.078 * X + -0.022$
$R^2 = 0.762$
$f_{High}(x) = 0.115 * X + -0.039$
$R^2 = 0.676$

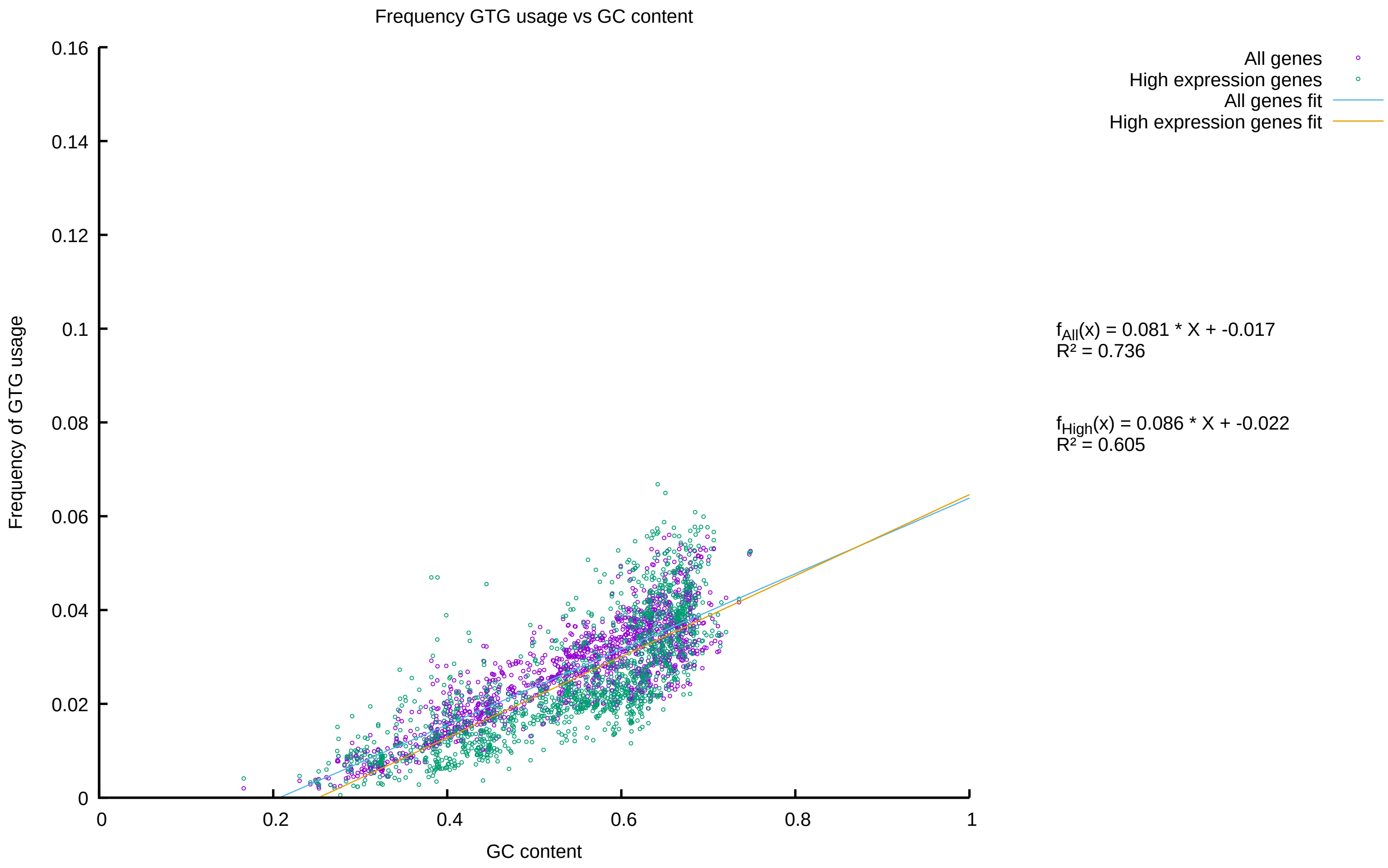
Frequency GTG usage vs GC content
0.16
0.14
0.12
0.1
0.08
0.06
0.04
0.02
0
Frequency of GTG usage
0
0.2
0.4
0.6
0.8
1
GC content
All genes
High expression genes
All genes fit
High expression genes fit
$f_{All}(x) = 0.081 * X + -0.017$
$R^2 = 0.736$
$f_{High}(x) = 0.086 * X + -0.022$
$R^2 = 0.605$

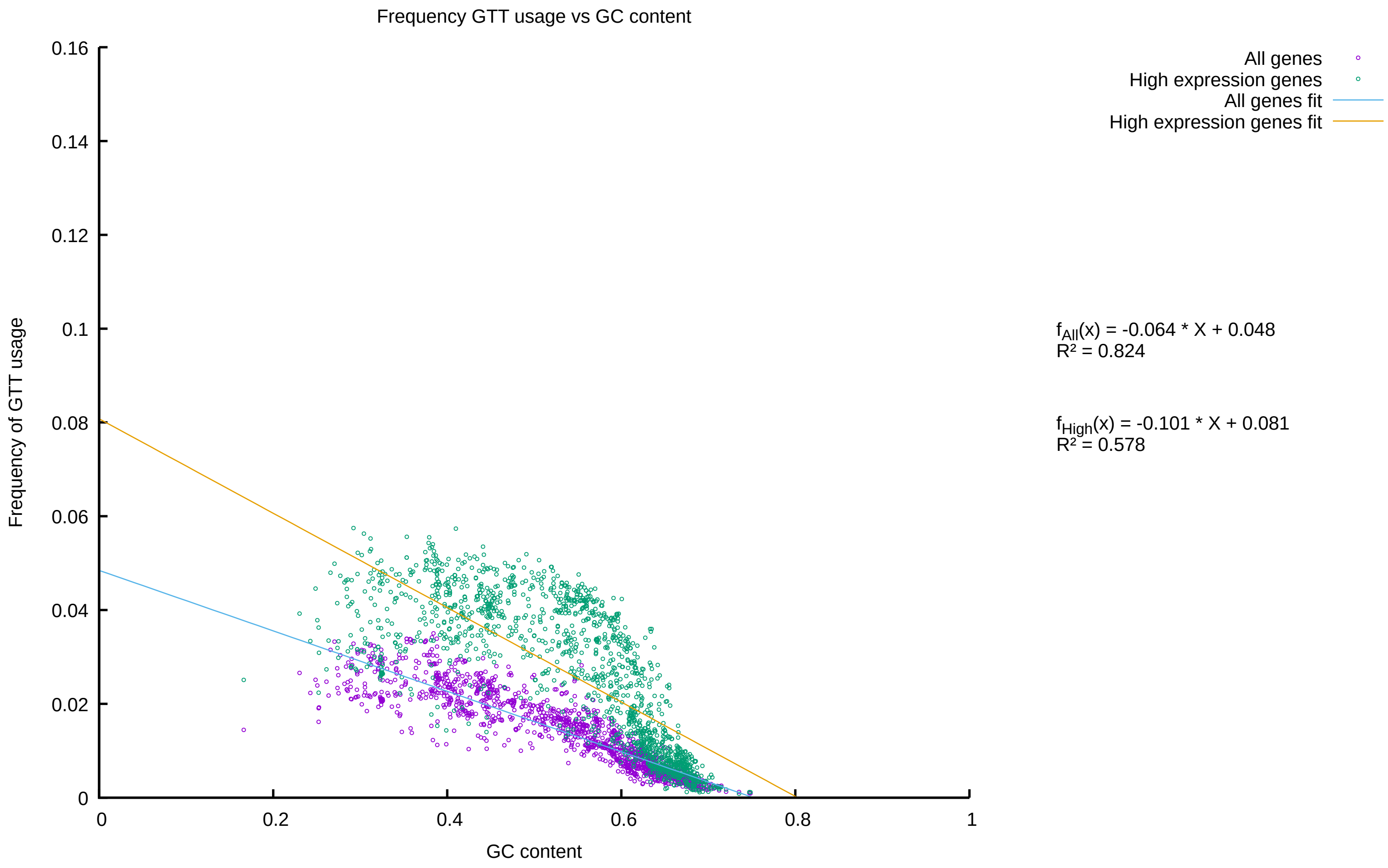

$f_{All}(x) = -0.064 * X + 0.048$
$R^2 = 0.824$

$f_{High}(x) = -0.101 * X + 0.081$
$R^2 = 0.578$

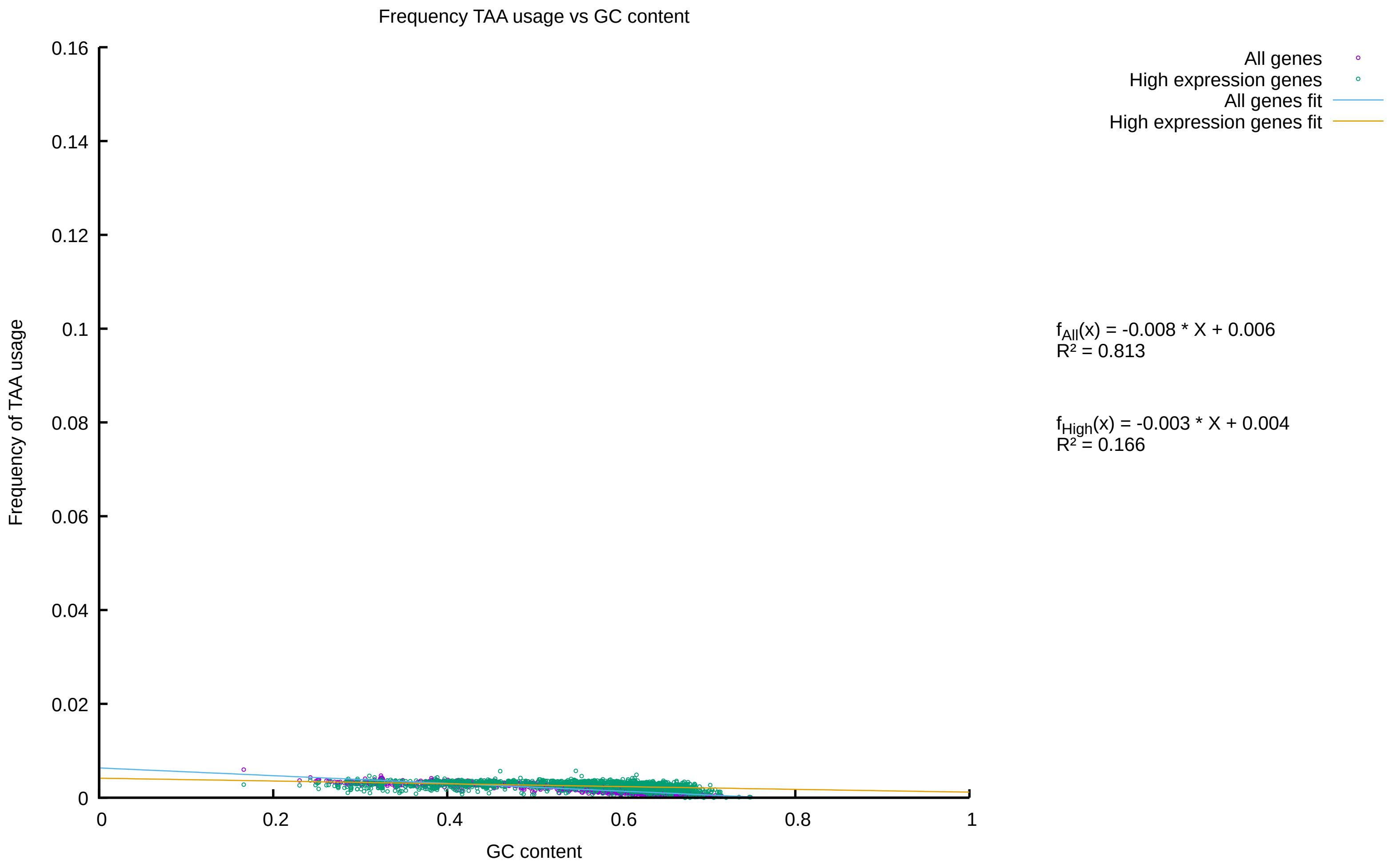

Frequency TAA usage vs GC content
All genes
High expression genes
All genes fit
High expression genes fit
$f_{All}(x) = -0.008 * X + 0.006$
$R^2 = 0.813$
$f_{High}(x) = -0.003 * X + 0.004$
$R^2 = 0.166$
Frequency of TAA usage
GC content
0.16
0.14
0.12
0.1
0.08
0.06
0.04
0.02
0
0.2
0.4
0.6
0.8
1

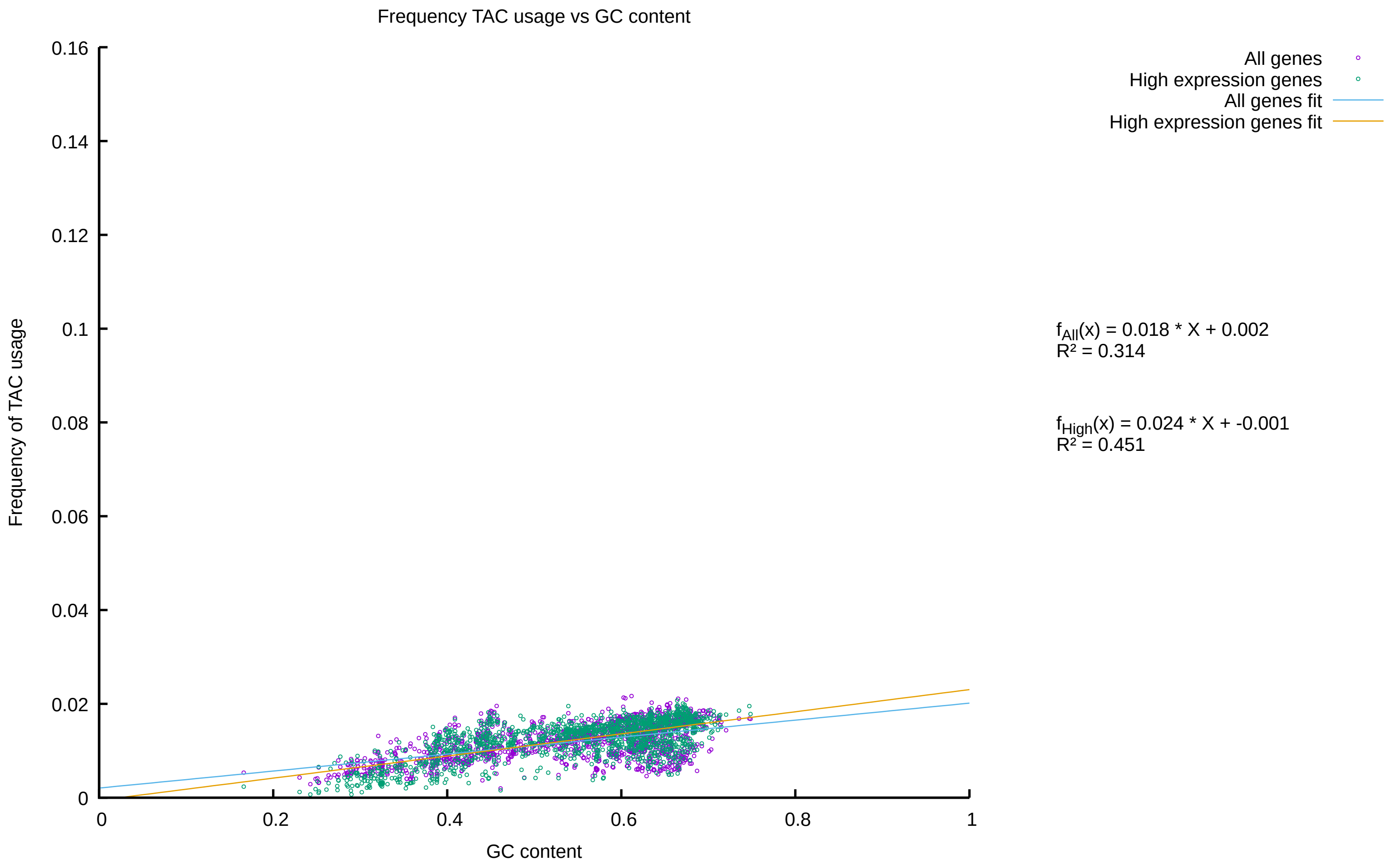

$f_{All}(x) = 0.018 * X + 0.002$
$R^2 = 0.314$

$f_{High}(x) = 0.024 * X + -0.001$
$R^2 = 0.451$

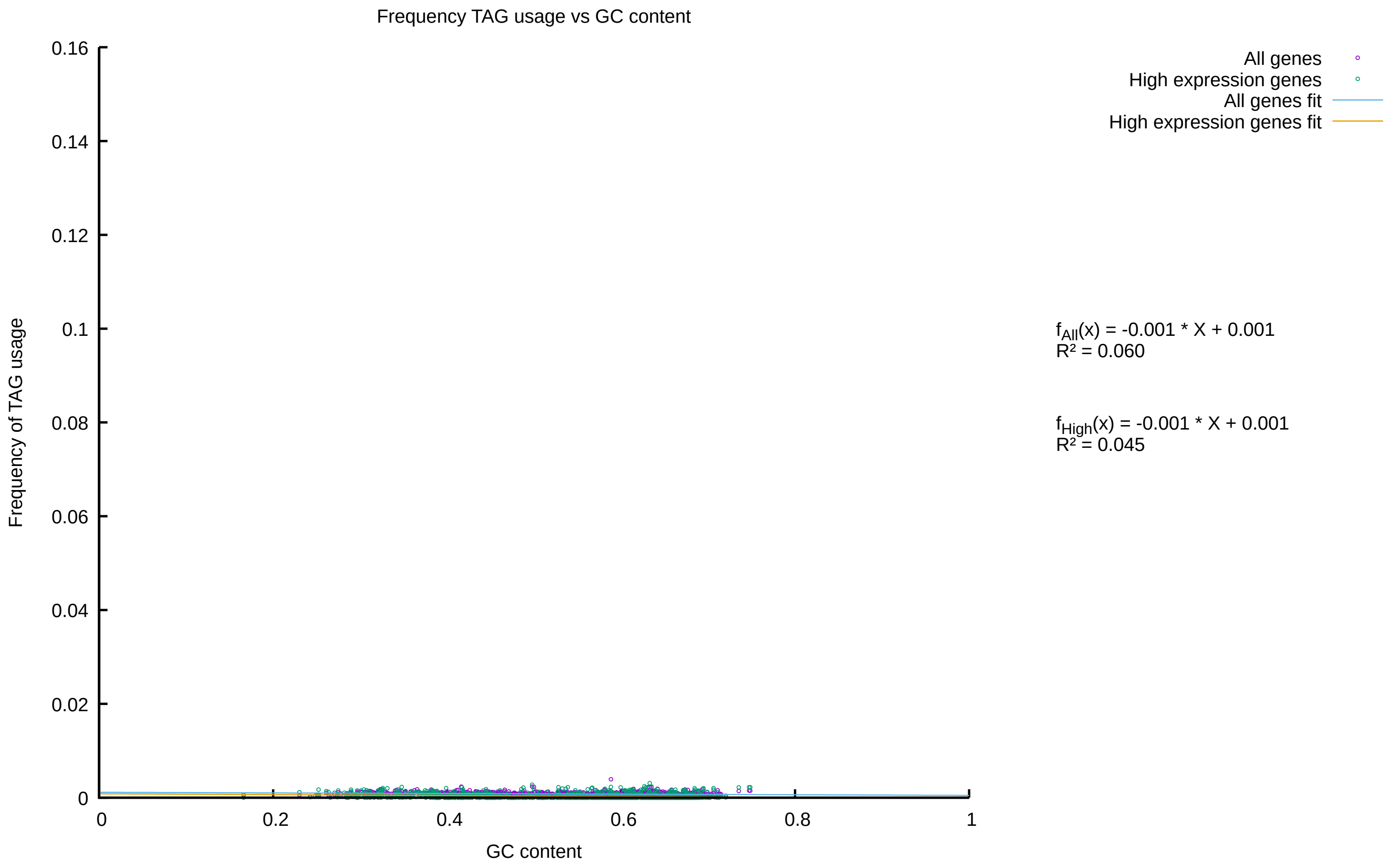

Frequency TAG usage vs GC content
Frequency of TAG usage
GC content
0.16
0.14
0.12
0.1
0.08
0.06
0.04
0.02
0
0
0.2
0.4
0.6
0.8
1
All genes
High expression genes
All genes fit
High expression genes fit
f_All(x) = -0.001 * X + 0.001
R² = 0.060
f_High(x) = -0.001 * X + 0.001
R² = 0.045

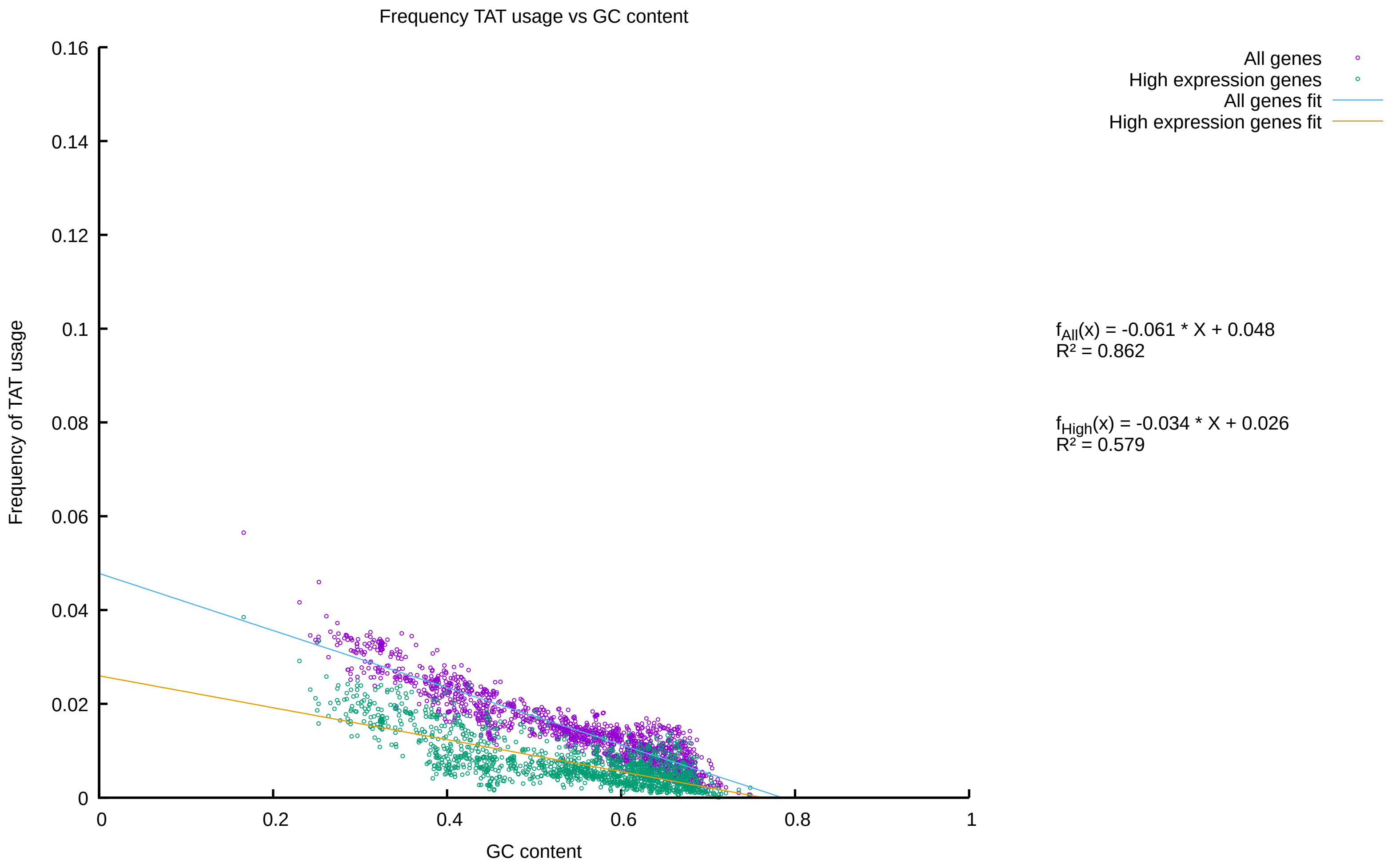

Frequency TAT usage vs GC content
Frequency of TAT usage
GC content
0.16
0.14
0.12
0.1
0.08
0.06
0.04
0.02
0
0
0.2
0.4
0.6
0.8
1
All genes
High expression genes
All genes fit
High expression genes fit
$f_{All}(x) = -0.061 * X + 0.048$
$R^2 = 0.862$
$f_{High}(x) = -0.034 * X + 0.026$
$R^2 = 0.579$

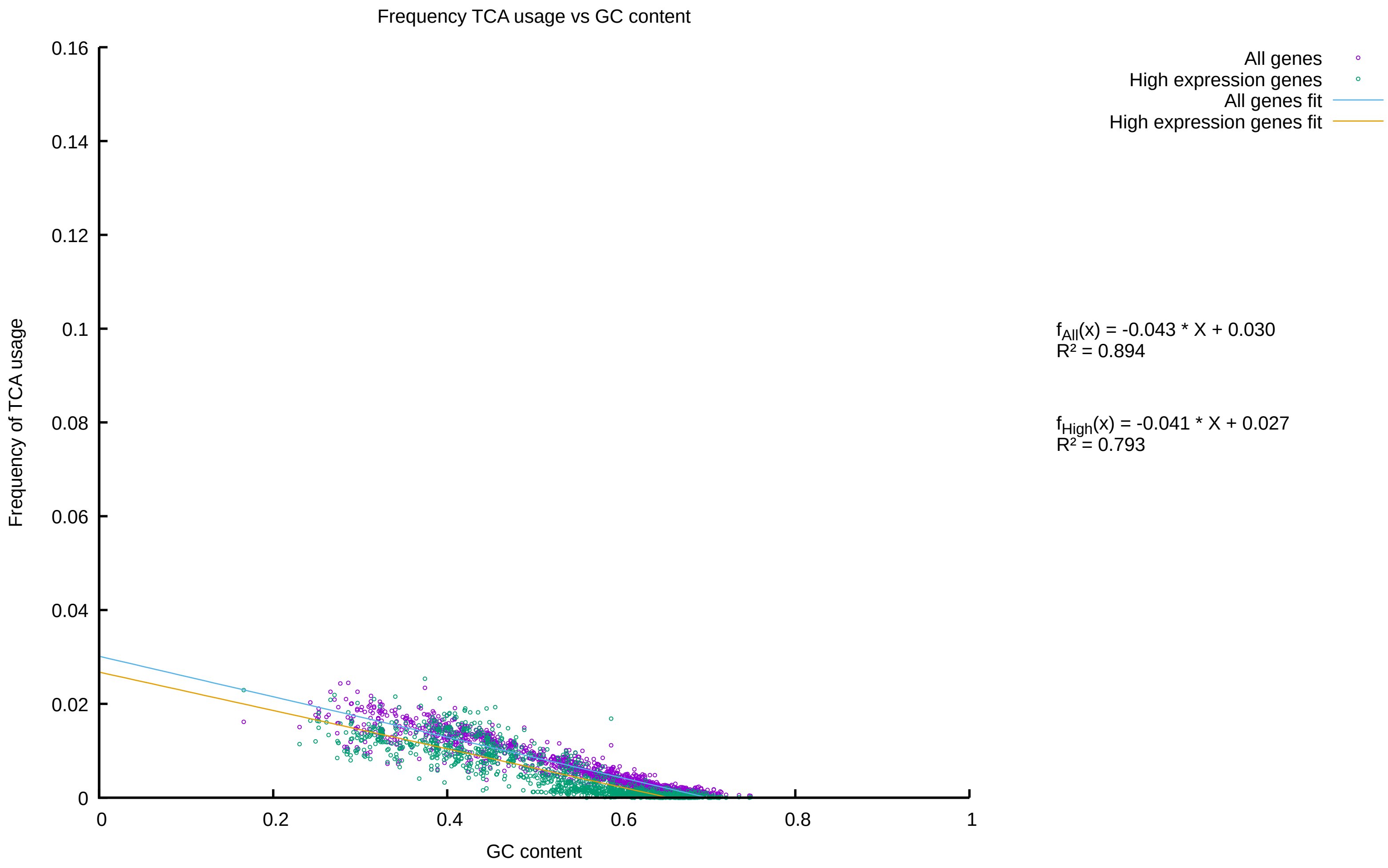

Frequency TCA usage vs GC content
0.16
0.14
0.12
0.1
0.08
0.06
0.04
0.02
0
0
0.2
0.4
0.6
0.8
1
Frequency of TCA usage
GC content
All genes
High expression genes
All genes fit
High expression genes fit
$f_{All}(x) = -0.043 * X + 0.030$
$R^2 = 0.894$
$f_{High}(x) = -0.041 * X + 0.027$
$R^2 = 0.793$

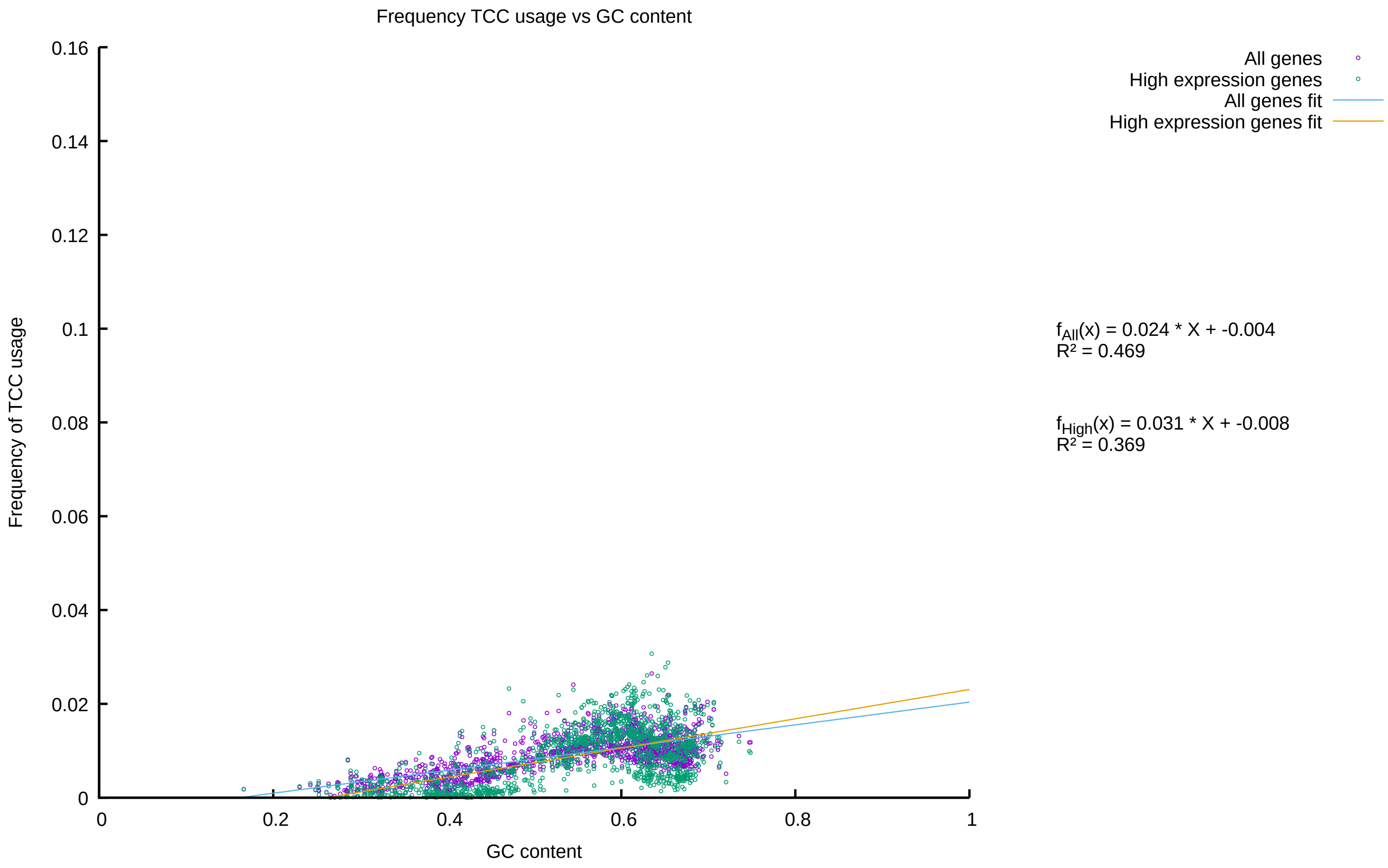

Frequency TCC usage vs GC content
0.16
0.14
0.12
0.1
0.08
0.06
0.04
0.02
0
Frequency of TCC usage
0
0.2
0.4
0.6
0.8
1
GC content
All genes
High expression genes
All genes fit
High expression genes fit
$f_{All}(x) = 0.024 * X + -0.004$
$R^2 = 0.469$
$f_{High}(x) = 0.031 * X + -0.008$
$R^2 = 0.369$

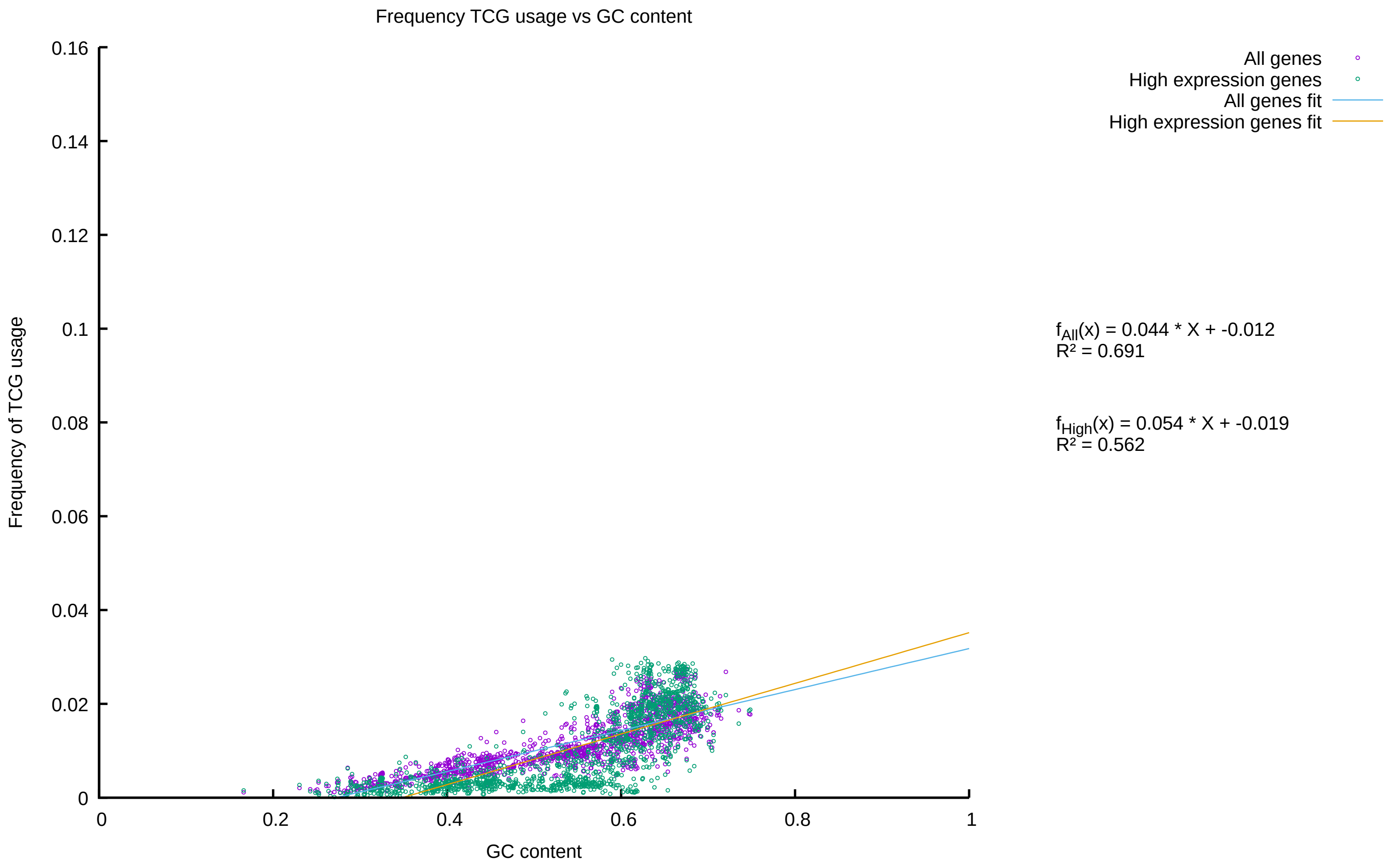
Frequency TCG usage vs GC content
0.16
0.14
0.12
0.1
0.08
0.06
0.04
0.02
0
Frequency of TCG usage
0
0.2
0.4
0.6
0.8
1
GC content
All genes
High expression genes
All genes fit
High expression genes fit
f_All(x) = 0.044 * X + -0.012
R² = 0.691
f_High(x) = 0.054 * X + -0.019
R² = 0.562

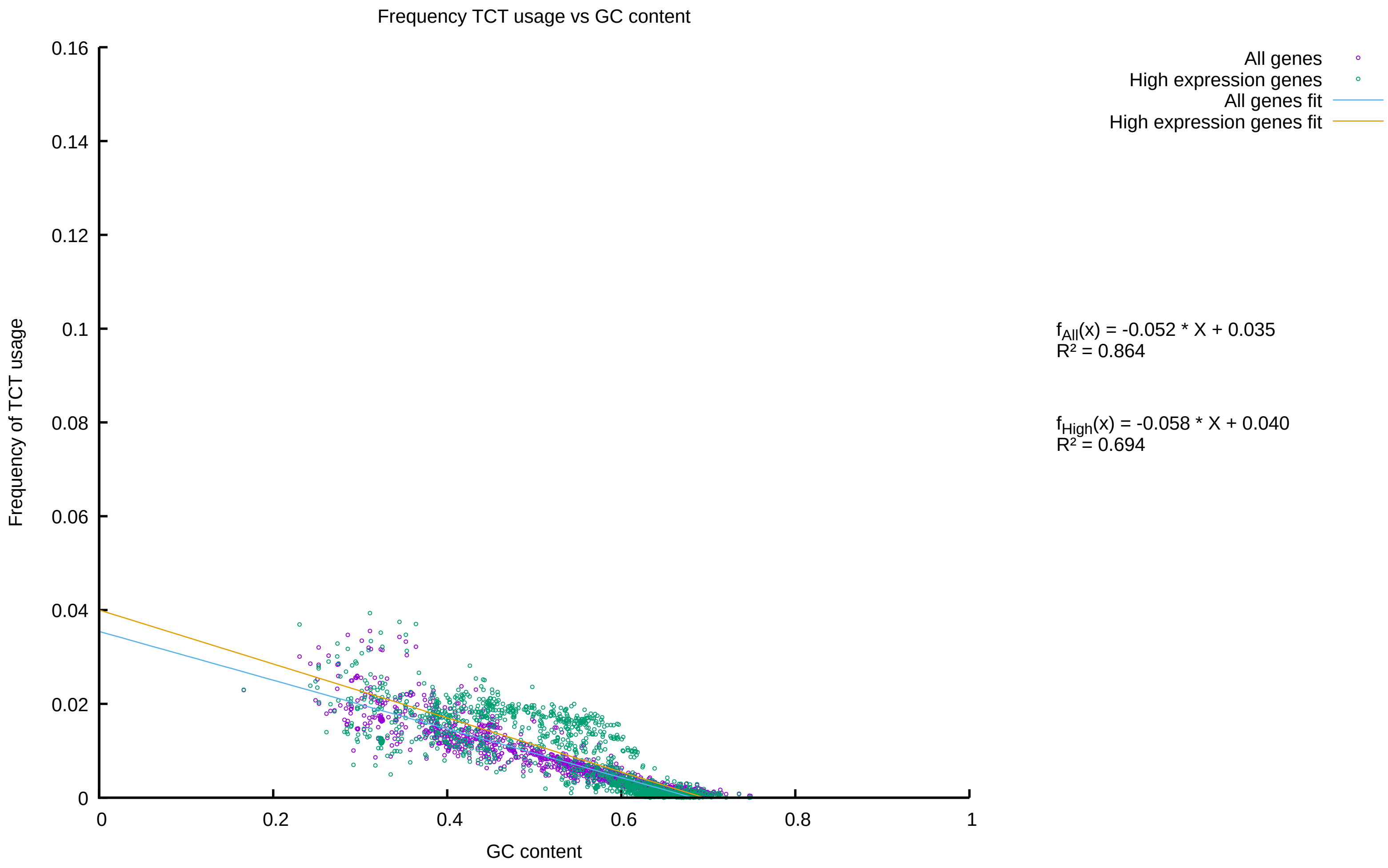

Frequency TCT usage vs GC content
Frequency of TCT usage
GC content
0
0.02
0.04
0.06
0.08
0.1
0.12
0.14
0.16
0
0.2
0.4
0.6
0.8
1
All genes
High expression genes
All genes fit
High expression genes fit
f_All(x) = -0.052 * X + 0.035
R² = 0.864
f_High(x) = -0.058 * X + 0.040
R² = 0.694

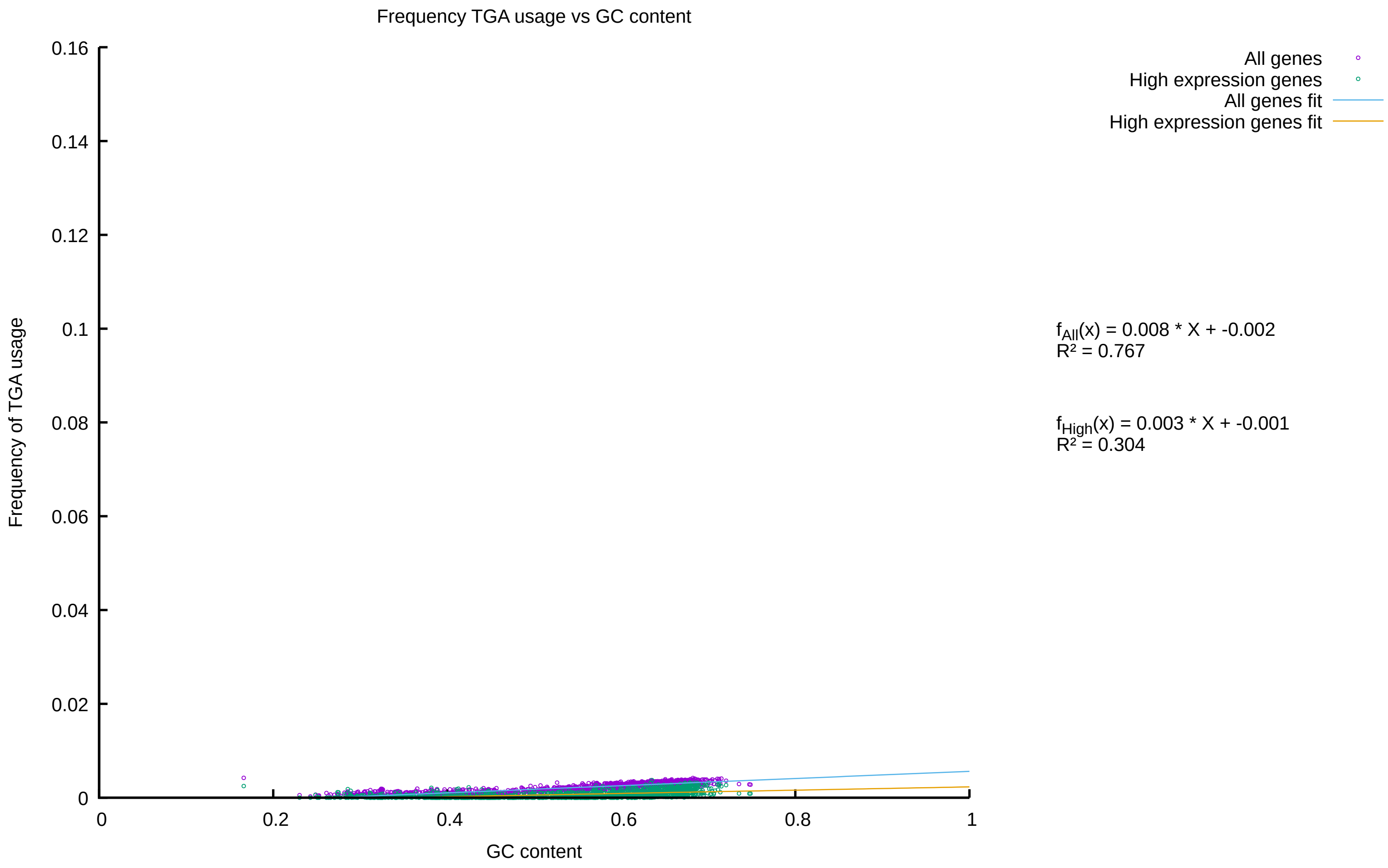
Frequency TGA usage vs GC content
Frequency of TGA usage
GC content
0.16
0.14
0.12
0.1
0.08
0.06
0.04
0.02
0
0
0.2
0.4
0.6
0.8
1
All genes
High expression genes
All genes fit
High expression genes fit
$f_{All}(x) = 0.008 * X + -0.002$
$R^2 = 0.767$
$f_{High}(x) = 0.003 * X + -0.001$
$R^2 = 0.304$

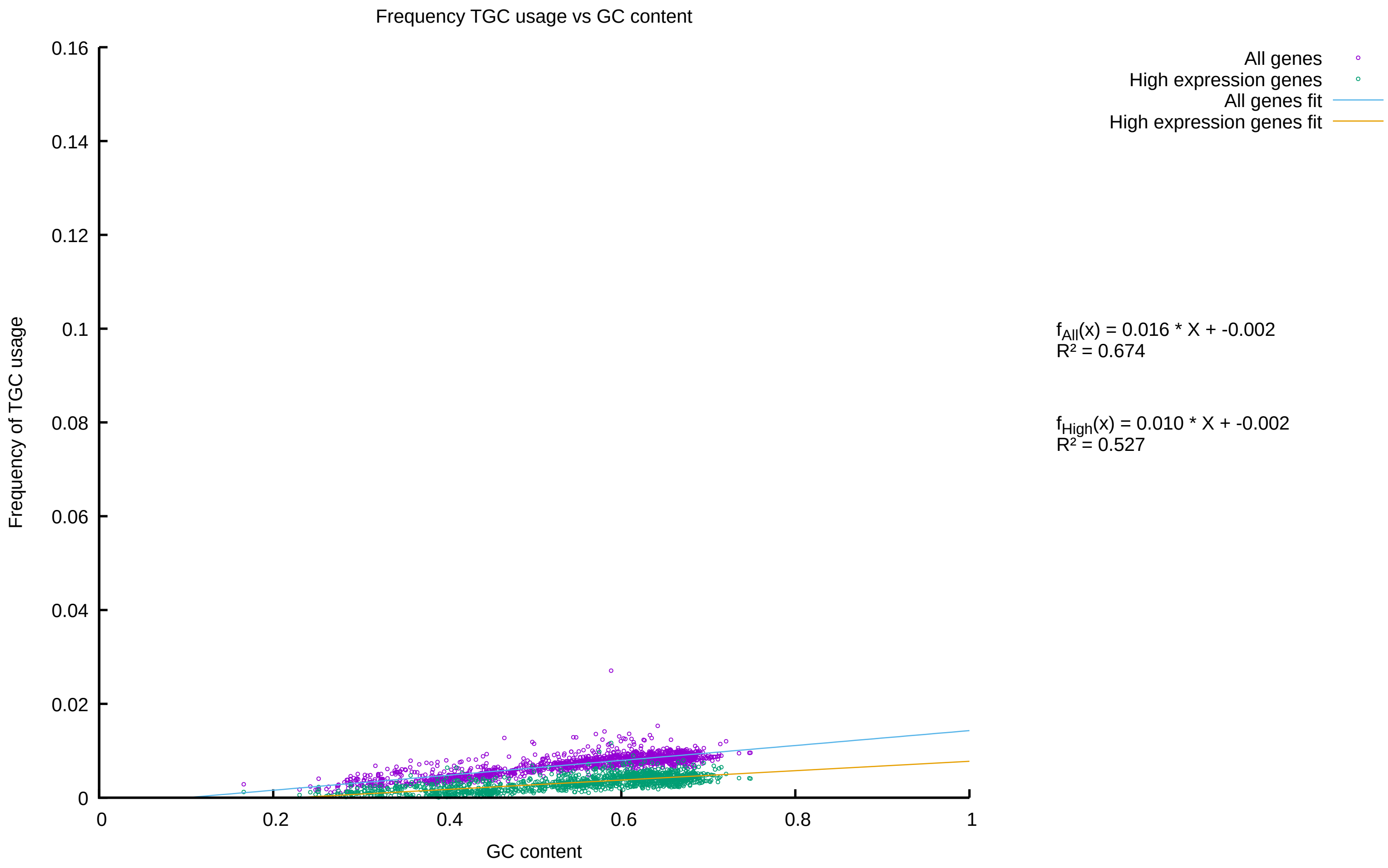
Frequency TGC usage vs GC content
Frequency of TGC usage
GC content
0.16
0.14
0.12
0.1
0.08
0.06
0.04
0.02
0
0
0.2
0.4
0.6
0.8
1
All genes
High expression genes
All genes fit
High expression genes fit
$f_{All}(x) = 0.016 * X + -0.002$
$R^2 = 0.674$
$f_{High}(x) = 0.010 * X + -0.002$
$R^2 = 0.527$

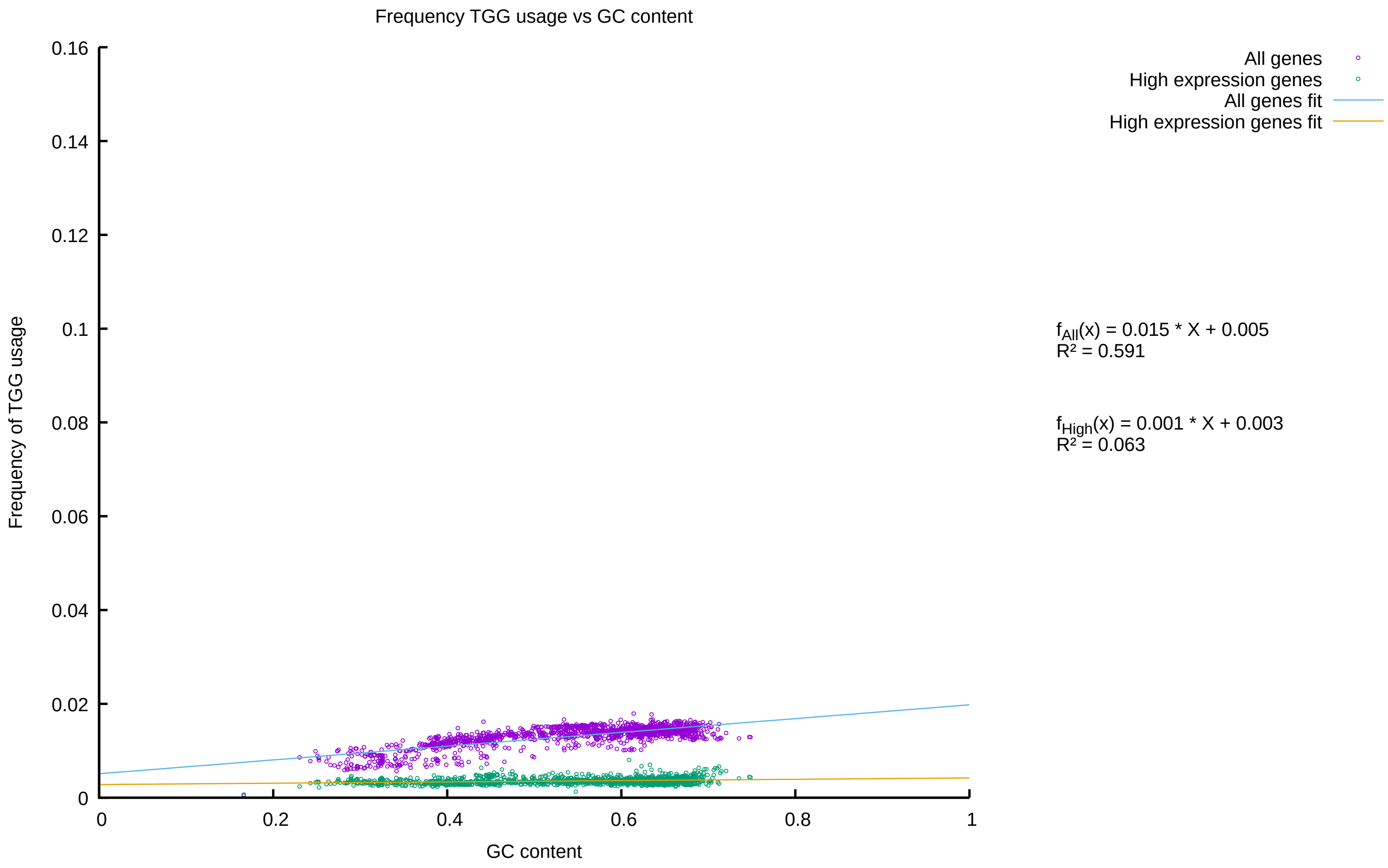

Frequency TGG usage vs GC content
Frequency of TGG usage
GC content
0.16
0.14
0.12
0.1
0.08
0.06
0.04
0.02
0
0
0.2
0.4
0.6
0.8
1
All genes
High expression genes
All genes fit
High expression genes fit
$f_{All}(x) = 0.015 * X + 0.005$
$R^2 = 0.591$
$f_{High}(x) = 0.001 * X + 0.003$
$R^2 = 0.063$

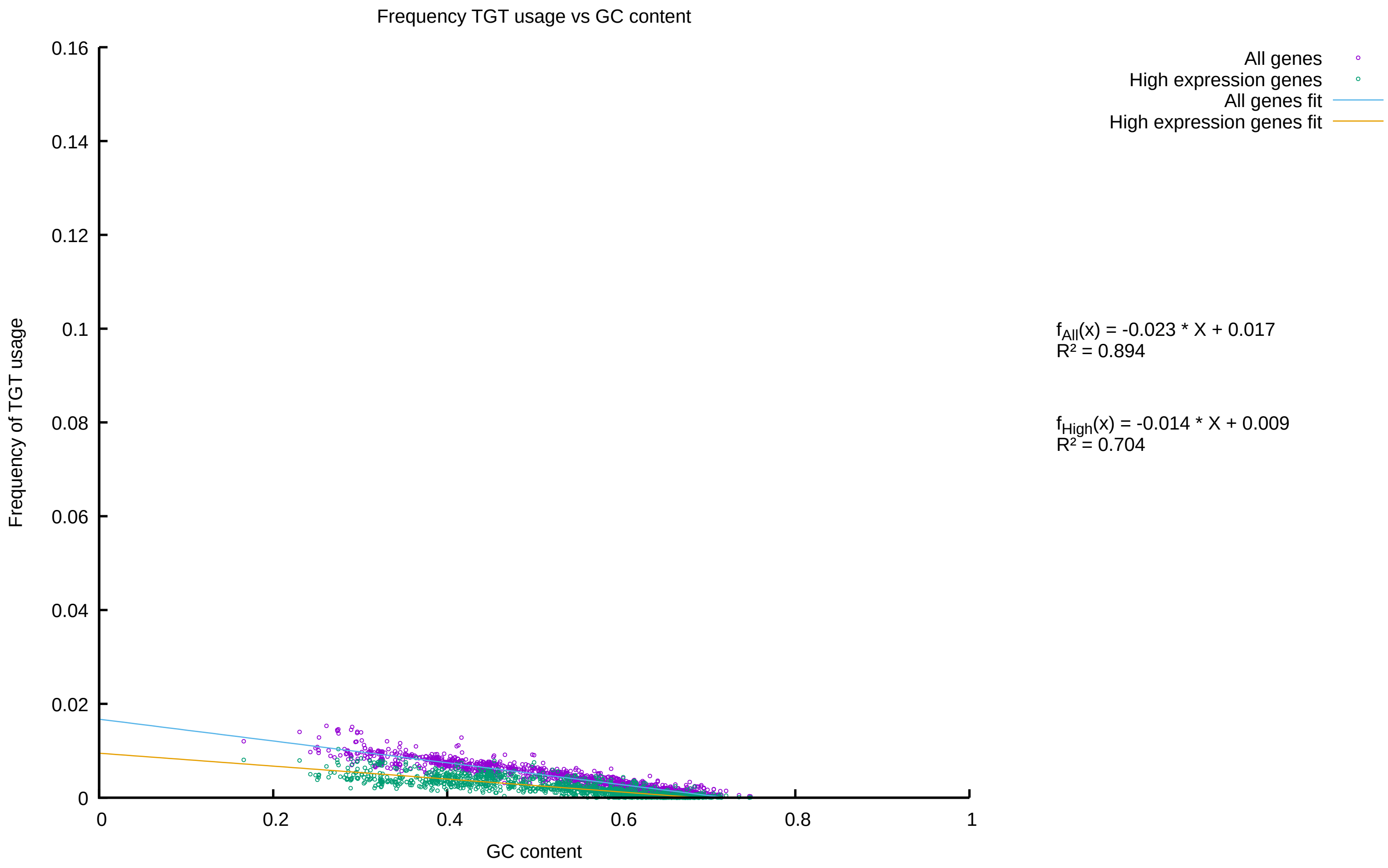
Frequency TGT usage vs GC content
Frequency of TGT usage
GC content
0
0.02
0.04
0.06
0.08
0.1
0.12
0.14
0.16
0
0.2
0.4
0.6
0.8
1
All genes
High expression genes
All genes fit
High expression genes fit
$f_{All}(x) = -0.023 * X + 0.017$
$R^2 = 0.894$
$f_{High}(x) = -0.014 * X + 0.009$
$R^2 = 0.704$

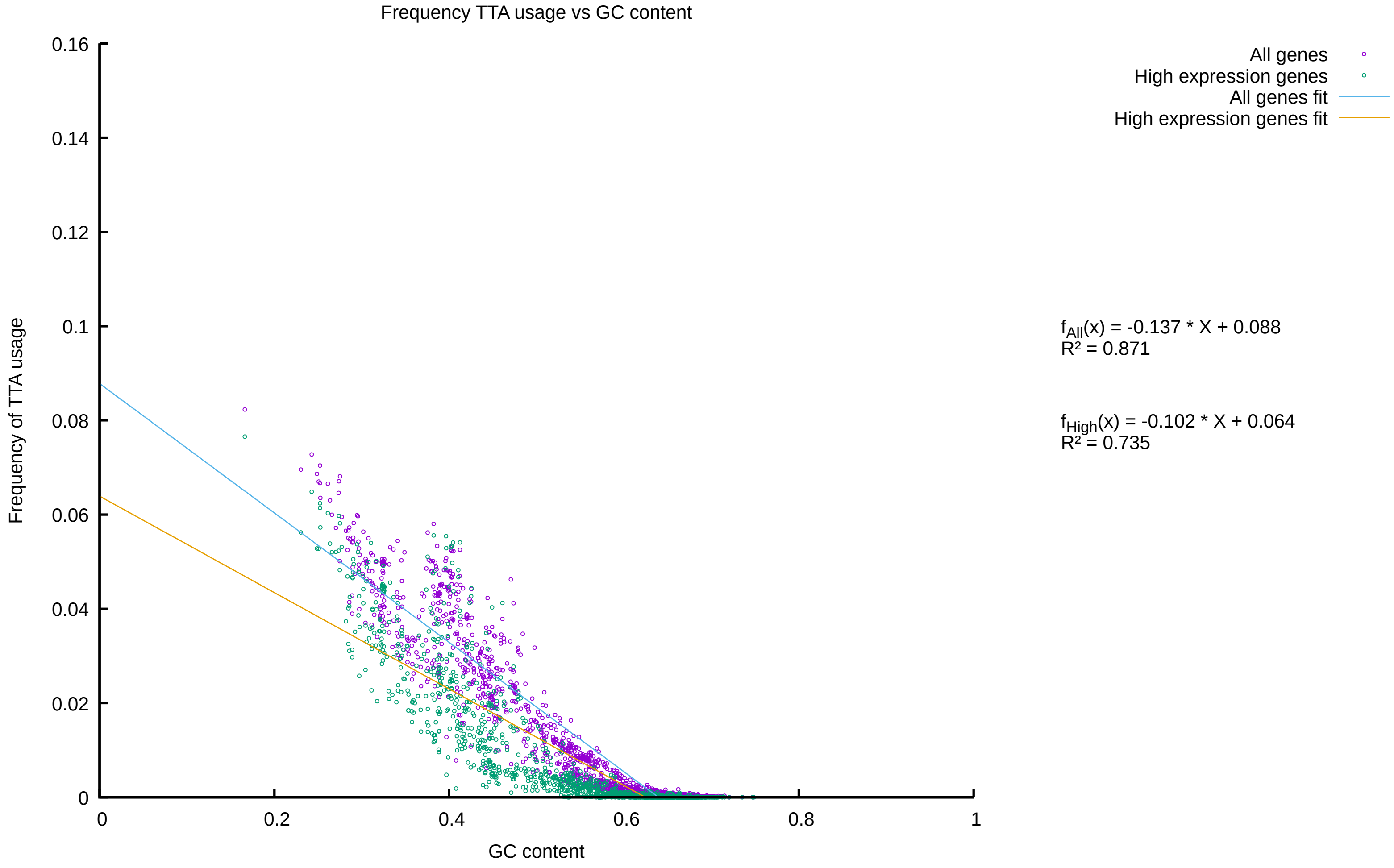
Frequency TTA usage vs GC content
Frequency of TTA usage
GC content
0.16
0.14
0.12
0.1
0.08
0.06
0.04
0.02
0
0
0.2
0.4
0.6
0.8
1
All genes
High expression genes
All genes fit
High expression genes fit
$f_{All}(x) = -0.137 * X + 0.088$
$R^2 = 0.871$
$f_{High}(x) = -0.102 * X + 0.064$
$R^2 = 0.735$

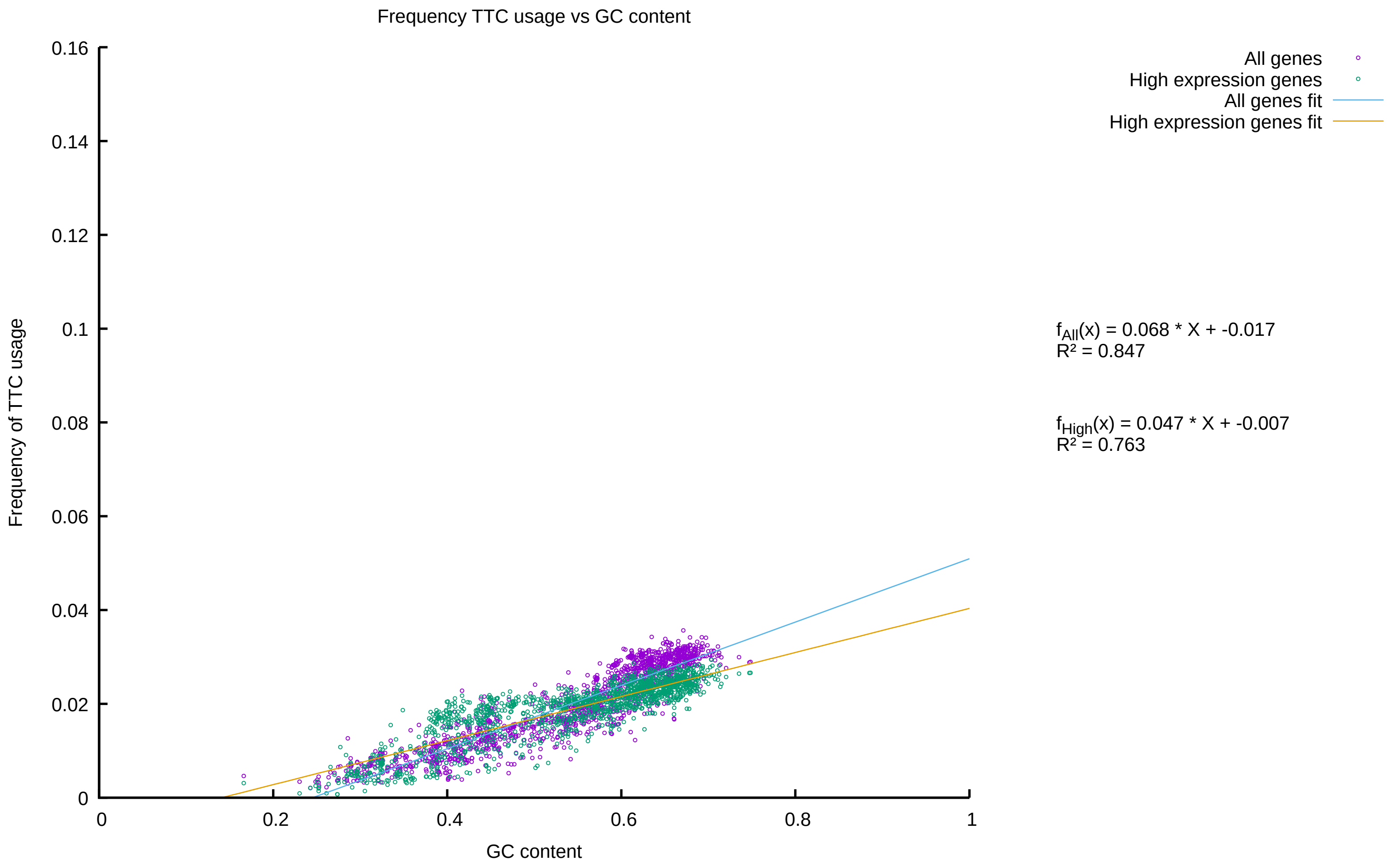

Frequency TTC usage vs GC content
0.16
0.14
0.12
0.1
0.08
0.06
0.04
0.02
0
0
0.2
0.4
0.6
0.8
1
Frequency of TTC usage
GC content
All genes
High expression genes
All genes fit
High expression genes fit
$f_{All}(x) = 0.068 * X + -0.017$
$R^2 = 0.847$
$f_{High}(x) = 0.047 * X + -0.007$
$R^2 = 0.763$

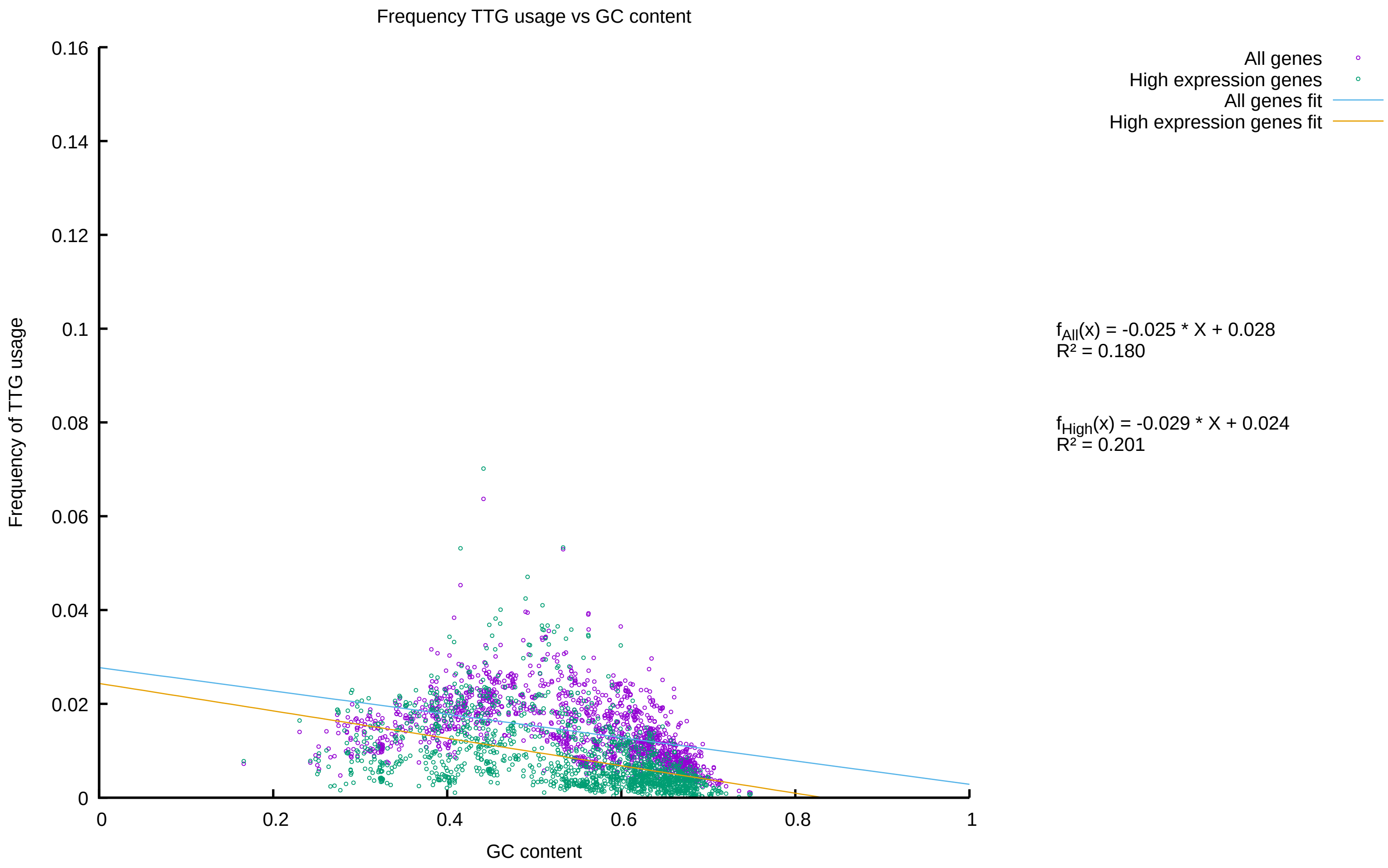
Frequency TTG usage vs GC content
All genes
High expression genes
All genes fit
High expression genes fit
fAll(x) = -0.025 * X + 0.028
R² = 0.180
fHigh(x) = -0.029 * X + 0.024
R² = 0.201
Frequency of TTG usage
GC content
0
0.02
0.04
0.06
0.08
0.1
0.12
0.14
0.16
0
0.2
0.4
0.6
0.8
1

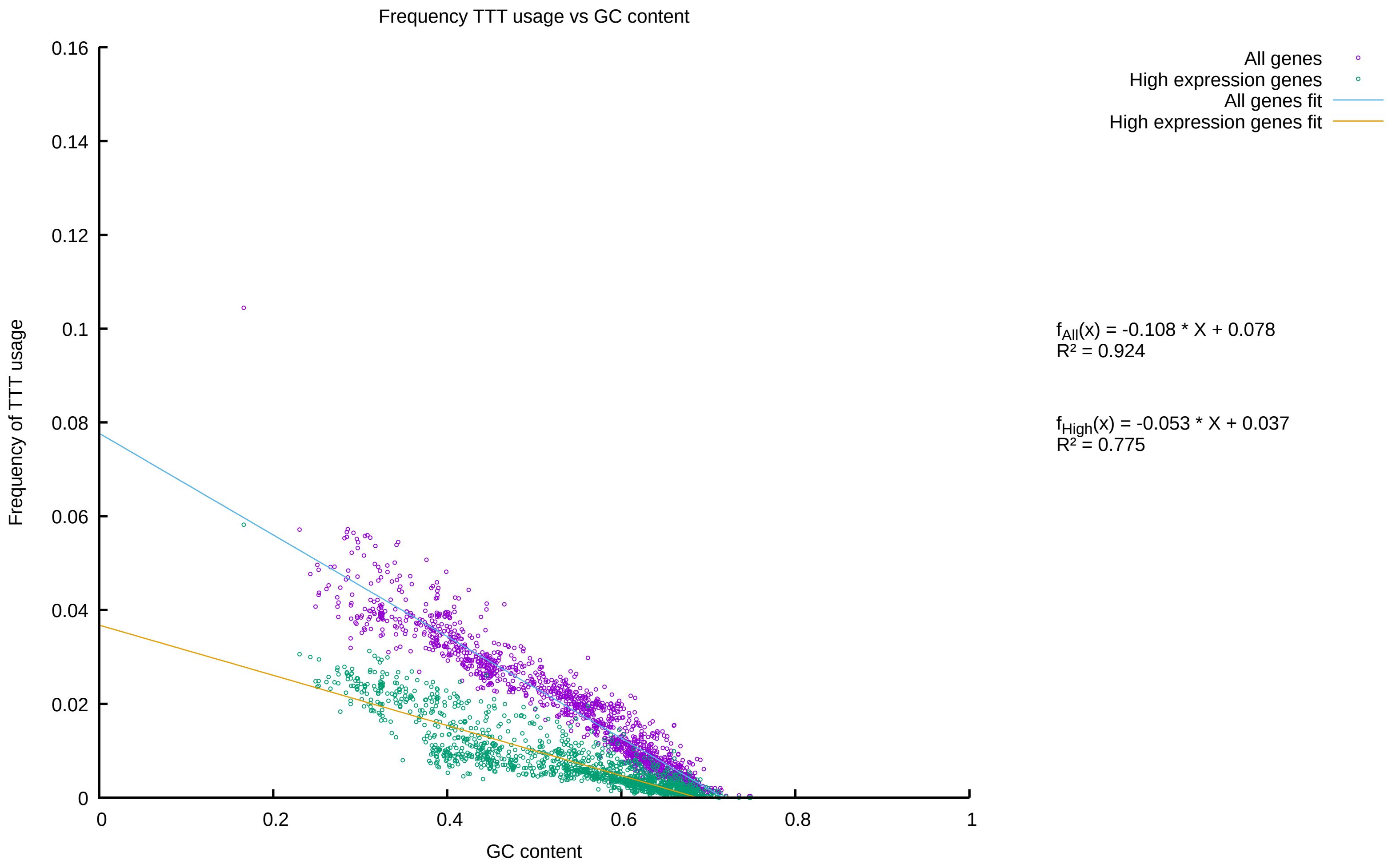
Frequency TTT usage vs GC content
All genes
High expression genes
All genes fit
High expression genes fit
f_All(x) = -0.108 * X + 0.078
R² = 0.924
f_High(x) = -0.053 * X + 0.037
R² = 0.775
Frequency of TTT usage
GC content
0.16
0.14
0.12
0.1
0.08
0.06
0.04
0.02
0
0.2
0.4
0.6
0.8
1
