# Supplementary material for: Impact of the chemical modification of tRNAs anticodon loop on the variability and evolution of codon usage in proteobacteria
Source: Front Microbiol. 2024 Aug 5;15:1412318. doi: 10.3389/fmicb.2024.1412318 (PMC11332805; doi:10.3389/fmicb.2024.1412318)
Supplement: Supplementary file 1 [file Data_Sheet_1.zip › Supp_figures/Fig_S4.pdf]

A)

2nd base

1st base	T			C			A			G			3rd base
T	TTT	0.0339	Phe	TCT	0.0152	Ser	TAT	0.0190	Tyr	TGT	0.0070	Cys	T
	TTC	0.0225		TCC	0.0108		TAC	0.0100		TGC	0.0056		C
	TTA	0.0423	Leu	TCA	0.0133		TAA	0.0027	Stop	TGA	0.0027	Stop	A
	TTG	0.0165		TCG	0.0156		TAG	0.0007		TGG	0.0053	Trp	G
C	CTT	0.0167	Leu	CCT	0.0110	Pro	CAT	0.0091	His	CGT	0.0135	Arg	T
	CTC	0.0287		CCC	0.0177		CAC	0.0103		CGC	0.0401		C
	CTA	0.0125		CCA	0.0114		CAA	0.0266	Gln	CGA	0.0043		A
	CTG	0.0677		CCG	0.0273		CAG	0.0259		CGG	0.0179		G
A	ATT	0.0395	Ile	ACT	0.0161	Thr	AAT	0.0320	Asn	AGT	0.0142	Ser	T
	ATC	0.0280		ACC	0.0229		AAC	0.0094		AGC	0.0116		C
	ATA	0.0187		ACA	0.0148		AAA	0.0519	Lys	AGA	0.0098	Arg	A
	ATG	0.0055	Met	ACG	0.0153		AAG	0.0156		AGG	0.0037		G
G	GTT	0.0212	Val	GCT	0.0204	Ala	GAT	0.0253	Asp	GGT	0.0202	Gly	T
	GTC	0.0287		GCC	0.0570		GAC	0.0310		GGC	0.0474		C
	GTA	0.0172		GCA	0.0188		GAA	0.0257	Glu	GGA	0.0119		A
	GTG	0.0286		GCG	0.0475		GAG	0.0231		GGG	0.0112		G

B)

2nd base

1st base	T			C			A			G			3rd base
T	TTT	0.0188	Phe	TCT	0.0188	Ser	TAT	0.0135	Tyr	TGT	0.0046	Cys	T
	TTC	0.0174		TCC	0.0154		TAC	0.0110		TGC	0.0041		C
	TTA	0.0313	Leu	TCA	0.0132		TAA	0.0022	Stop	TGA	0.0018	Stop	A
	TTG	0.0170		TCG	0.0205		TAG	0.0011		TGG	0.0015	Trp	G
C	CTT	0.0228	Leu	CCT	0.0131	Pro	CAT	0.0083	His	CGT	0.0301	Arg	T
	CTC	0.0286		CCC	0.0146		CAC	0.0098		CGC	0.0399		C
	CTA	0.0121		CCA	0.0178		CAA	0.0229	Gln	CGA	0.0030		A
	CTG	0.0608		CCG	0.0278		CAG	0.0246		CGG	0.0092		G
A	ATT	0.0375	Ile	ACT	0.0252	Thr	AAT	0.0243	Asn	AGT	0.0080	Ser	T
	ATC	0.0367		ACC	0.0322		AAC	0.0170		AGC	0.0077		C
	ATA	0.0120		ACA	0.0171		AAA	0.0607	Lys	AGA	0.0113	Arg	A
	ATG	0.0060	Met	ACG	0.0185		AAG	0.0502		AGG	0.0027		G
G	GTT	0.0398	Val	GCT	0.0328	Ala	GAT	0.0351	Asp	GGT	0.0364	Gly	T
	GTC	0.0423		GCC	0.0512		GAC	0.0387		GGC	0.0535		C
	GTA	0.0300		GCA	0.0269		GAA	0.0381	Glu	GGA	0.0135		A
	GTG	0.0346		GCG	0.0289		GAG	0.0346		GGG	0.0072		G
