# Supplementary material for: Impact of the chemical modification of tRNAs anticodon loop on the variability and evolution of codon usage in proteobacteria
Source: Front Microbiol. 2024 Aug 5;15:1412318. doi: 10.3389/fmicb.2024.1412318 (PMC11332805; doi:10.3389/fmicb.2024.1412318)
Supplement: Supplementary file 1 [file Data_Sheet_1.zip › Supp_figures/Fig_S1.pdf]

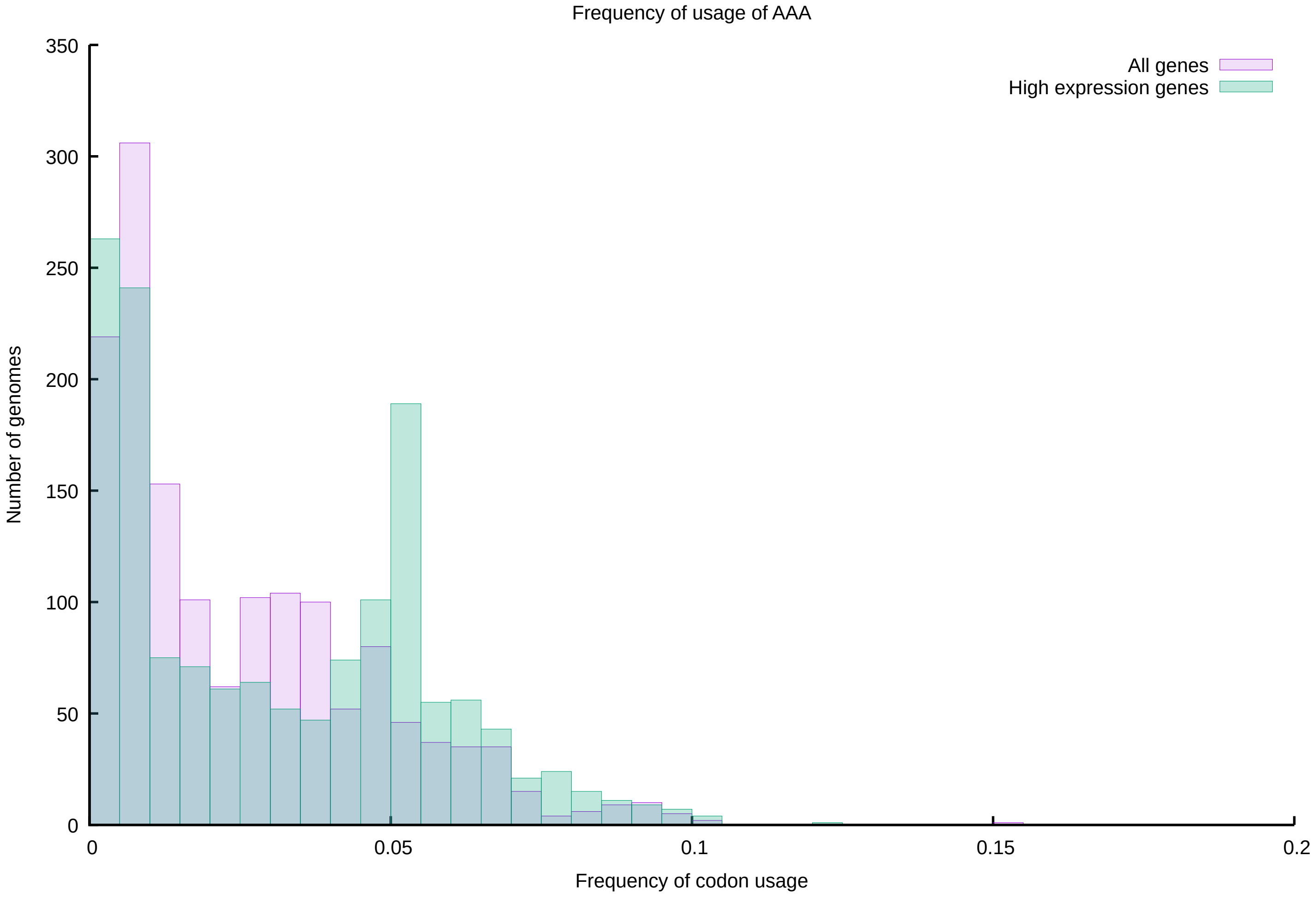

Frequency of usage of AAA
All genes
High expression genes
Number of genomes
350
300
250
200
150
100
50
0
0
0.05
0.1
0.15
0.2
Frequency of codon usage

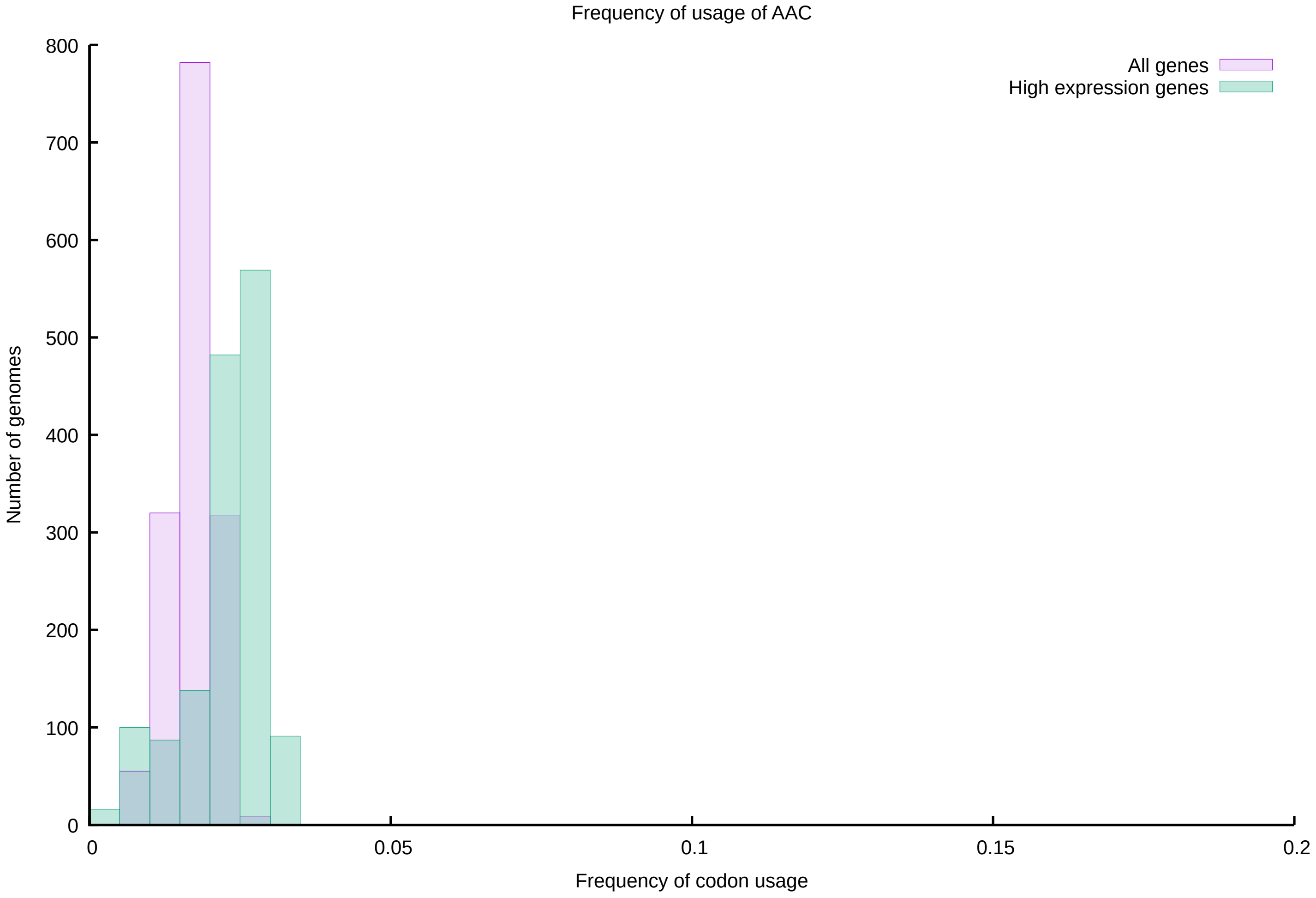
Frequency of usage of AAC
All genes
High expression genes
Number of genomes
Frequency of codon usage
0
100
200
300
400
500
600
700
800
0.05
0.1
0.15
0.2

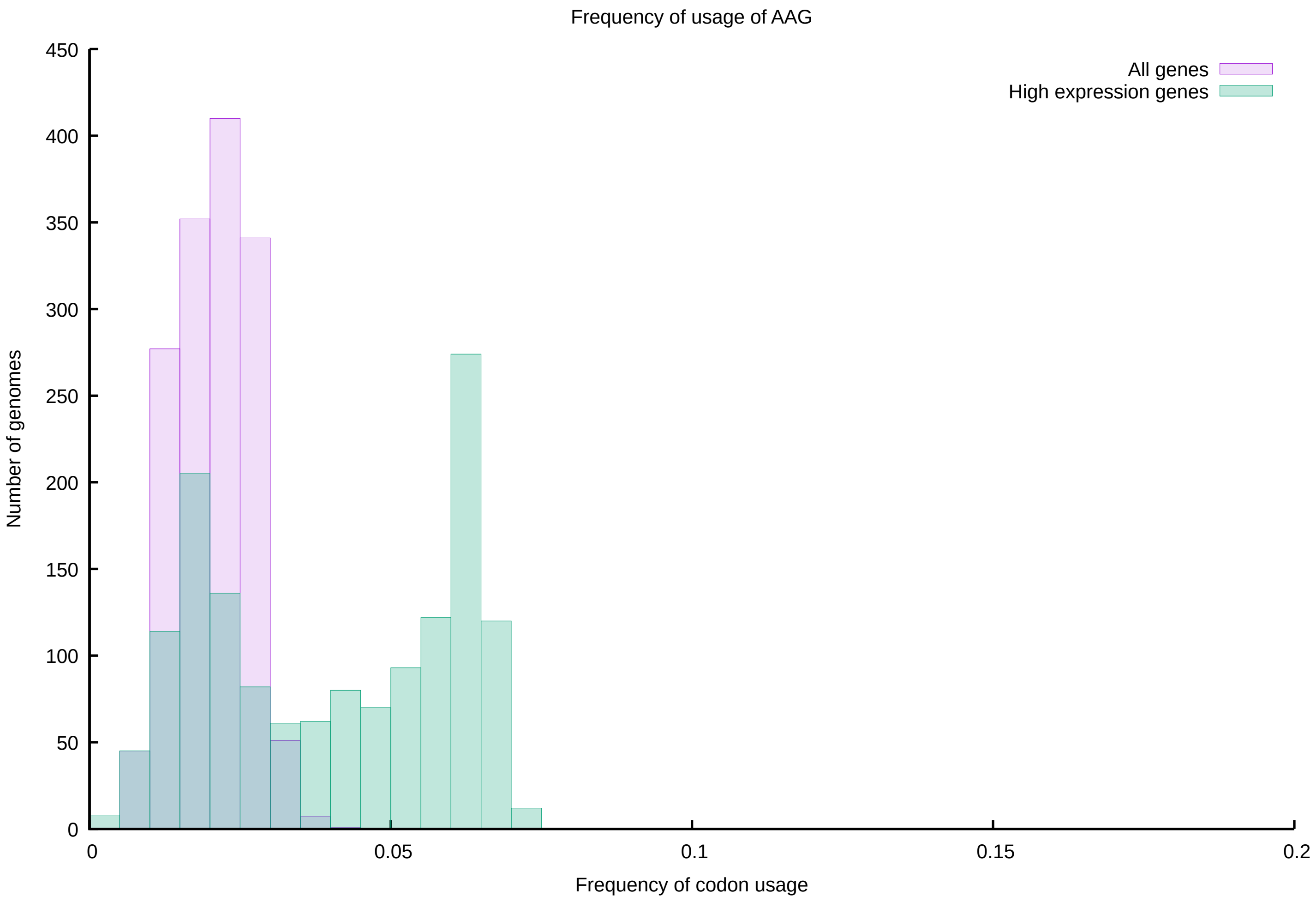
Frequency of usage of AAG
All genes
High expression genes
Number of genomes
0
50
100
150
200
250
300
350
400
450
0
0.05
0.1
0.15
0.2
Frequency of codon usage

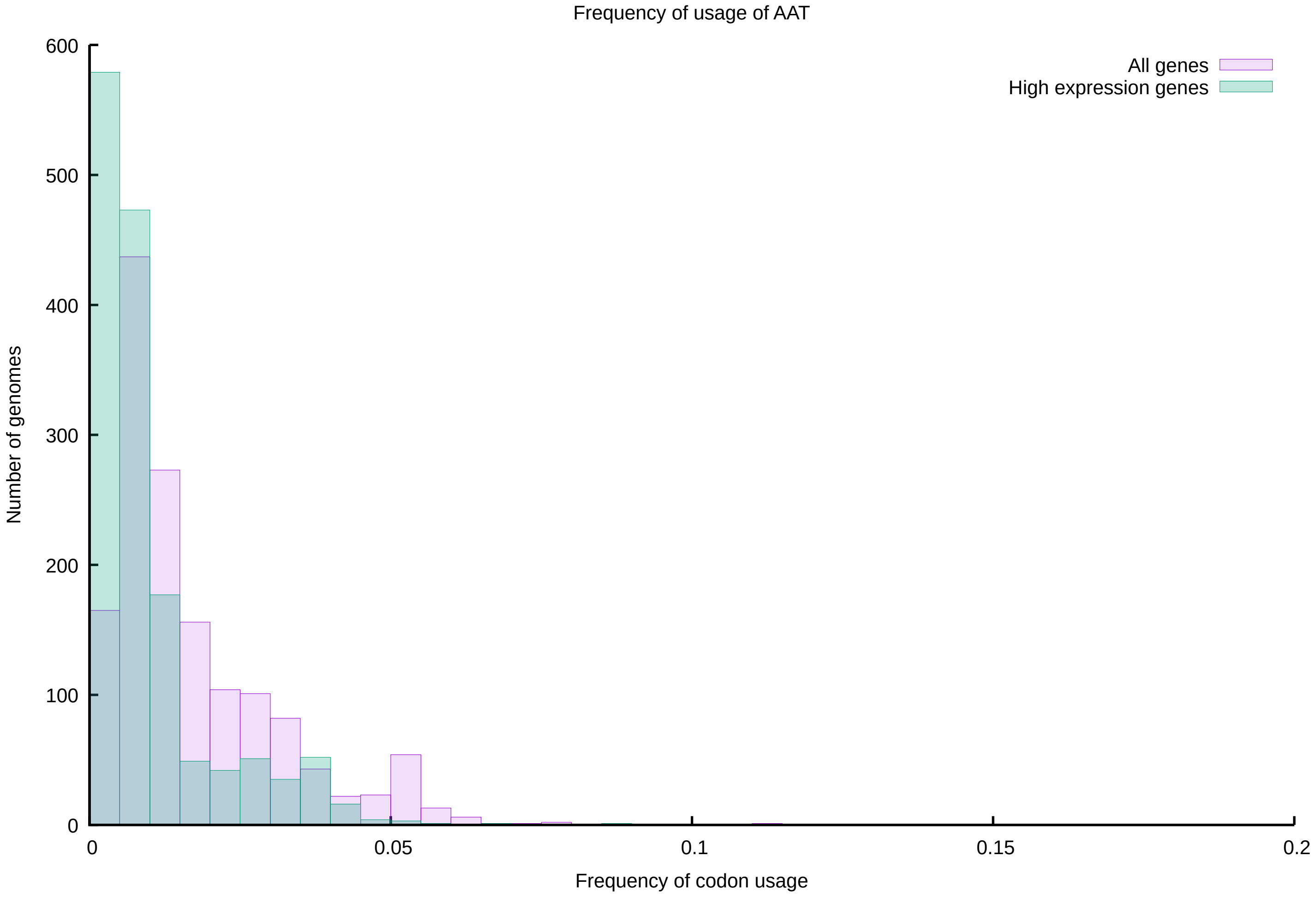
Frequency of usage of AAT
All genes
High expression genes
Number of genomes
600
500
400
300
200
100
0
0
0.05
0.1
0.15
0.2
Frequency of codon usage

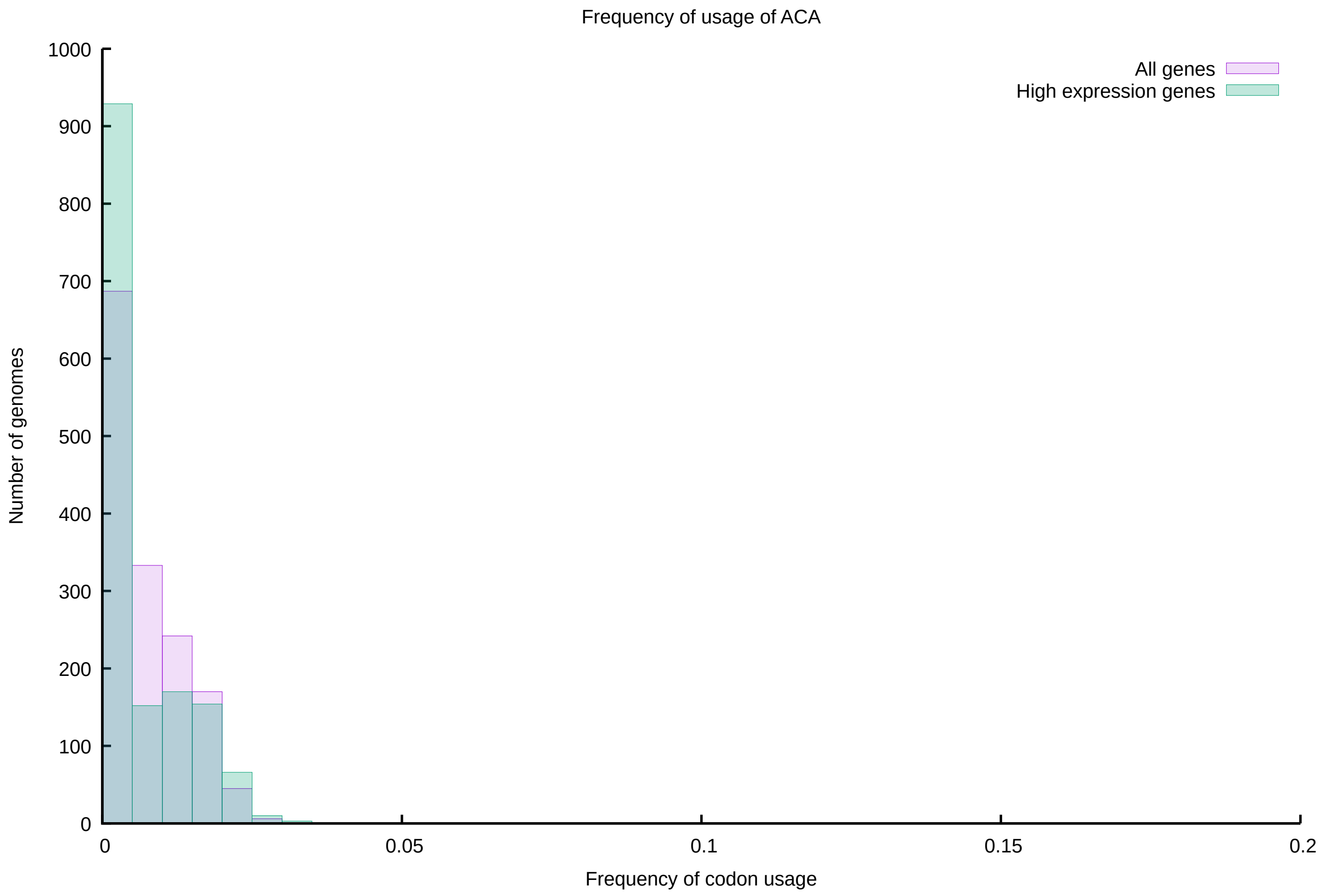
Frequency of usage of ACA
All genes
High expression genes
Number of genomes
0
100
200
300
400
500
600
700
800
900
1000
0
0.05
0.1
0.15
0.2
Frequency of codon usage

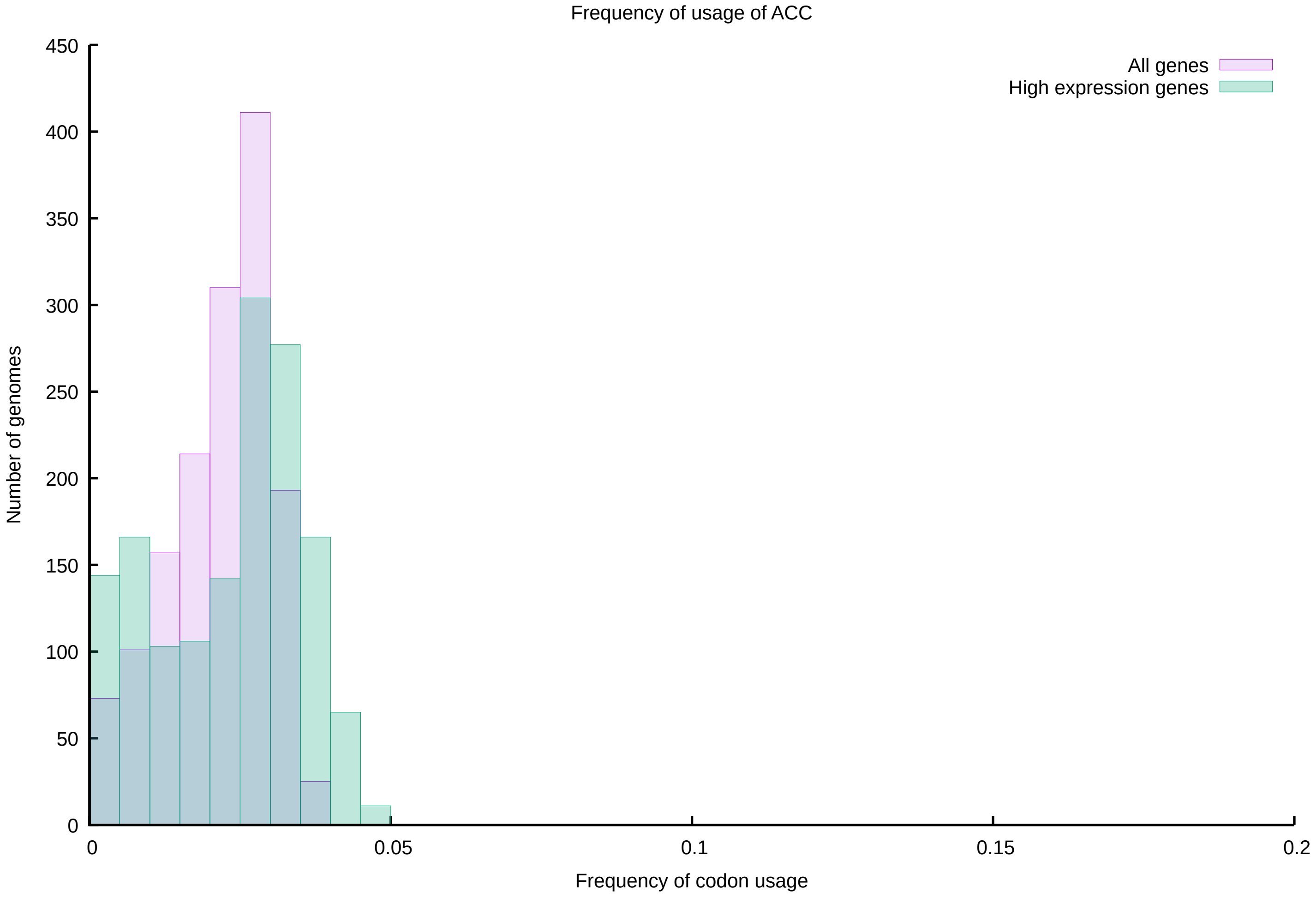
Frequency of usage of ACC
All genes
High expression genes
Number of genomes
Frequency of codon usage
0
50
100
150
200
250
300
350
400
450
0
0.05
0.1
0.15
0.2

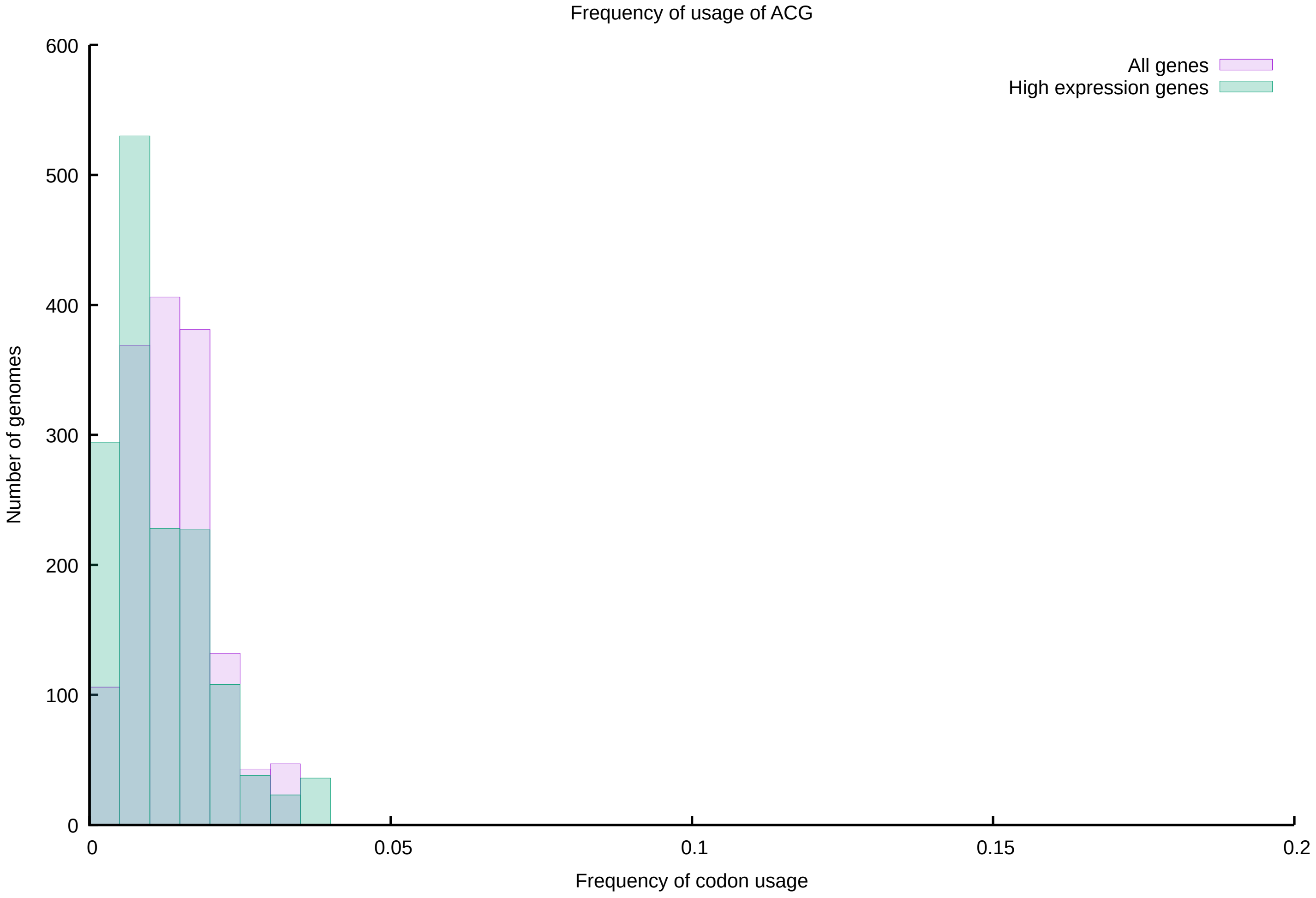
Frequency of usage of ACG
All genes
High expression genes
Number of genomes
Frequency of codon usage
0
100
200
300
400
500
600
0
0.05
0.1
0.15
0.2

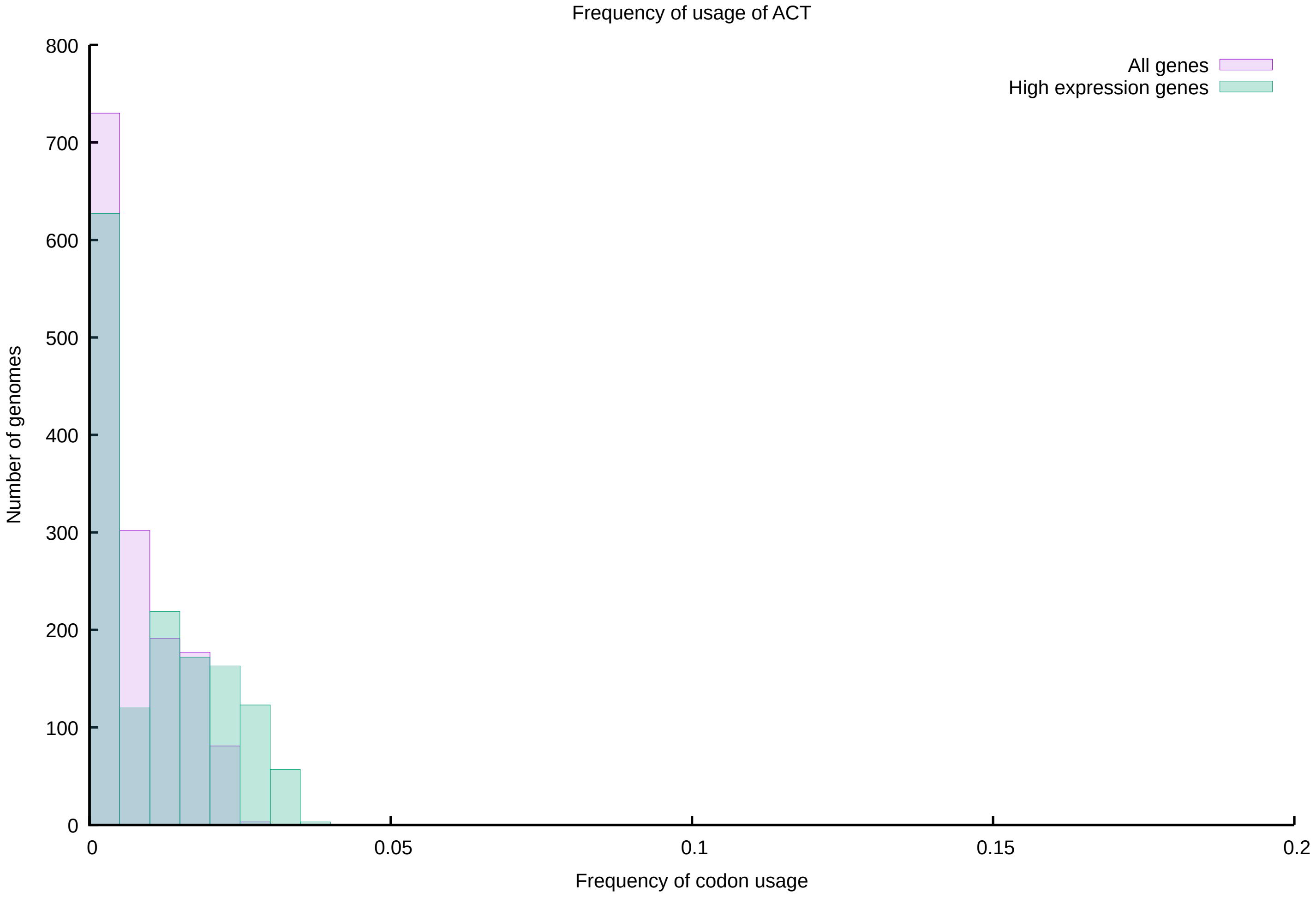

Frequency of usage of ACT
All genes
High expression genes
Number of genomes
800
700
600
500
400
300
200
100
0
0
0.05
0.1
0.15
0.2
Frequency of codon usage

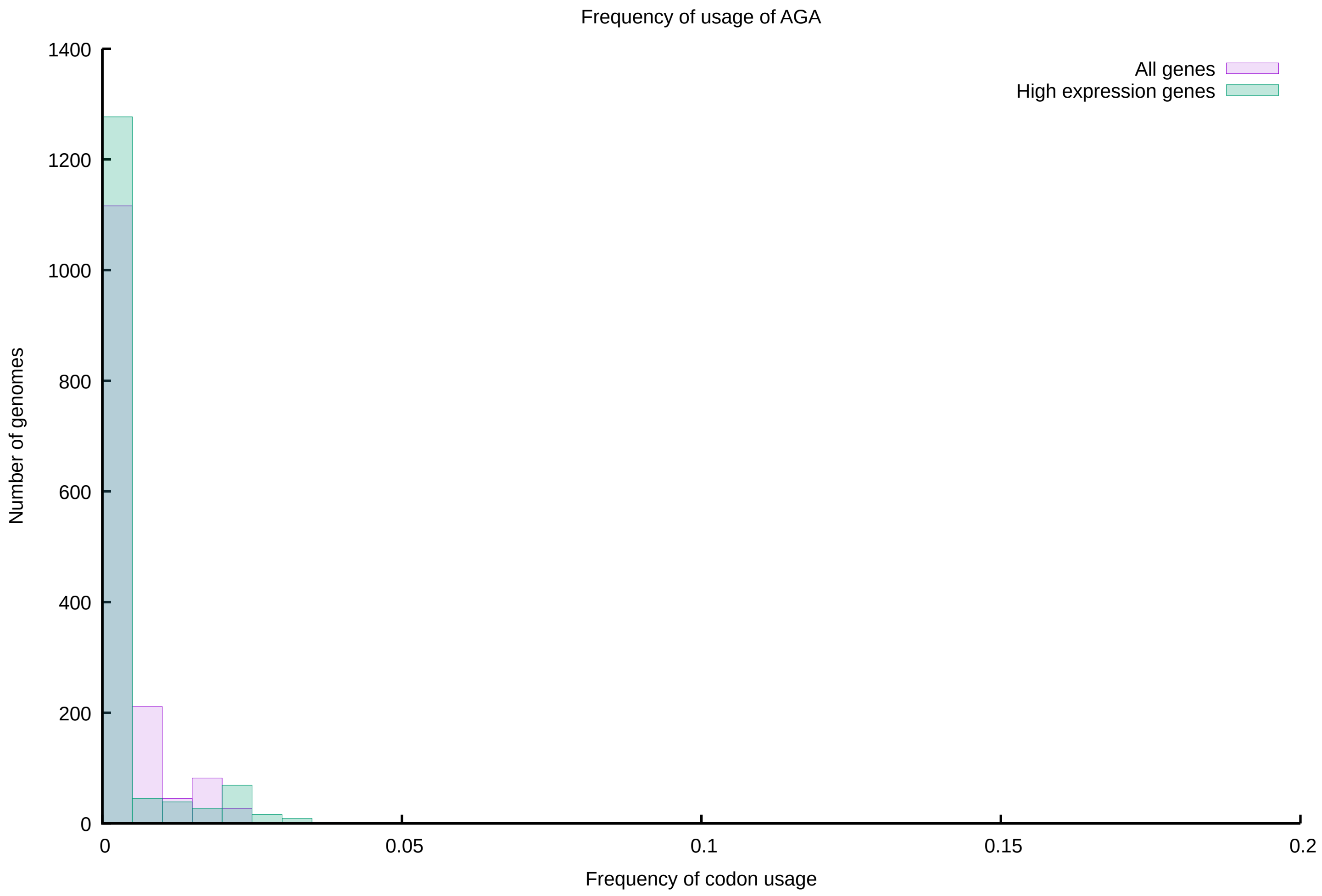

Frequency of usage of AGA
All genes
High expression genes
Number of genomes
0
200
400
600
800
1000
1200
1400
0
0.05
0.1
0.15
0.2
Frequency of codon usage

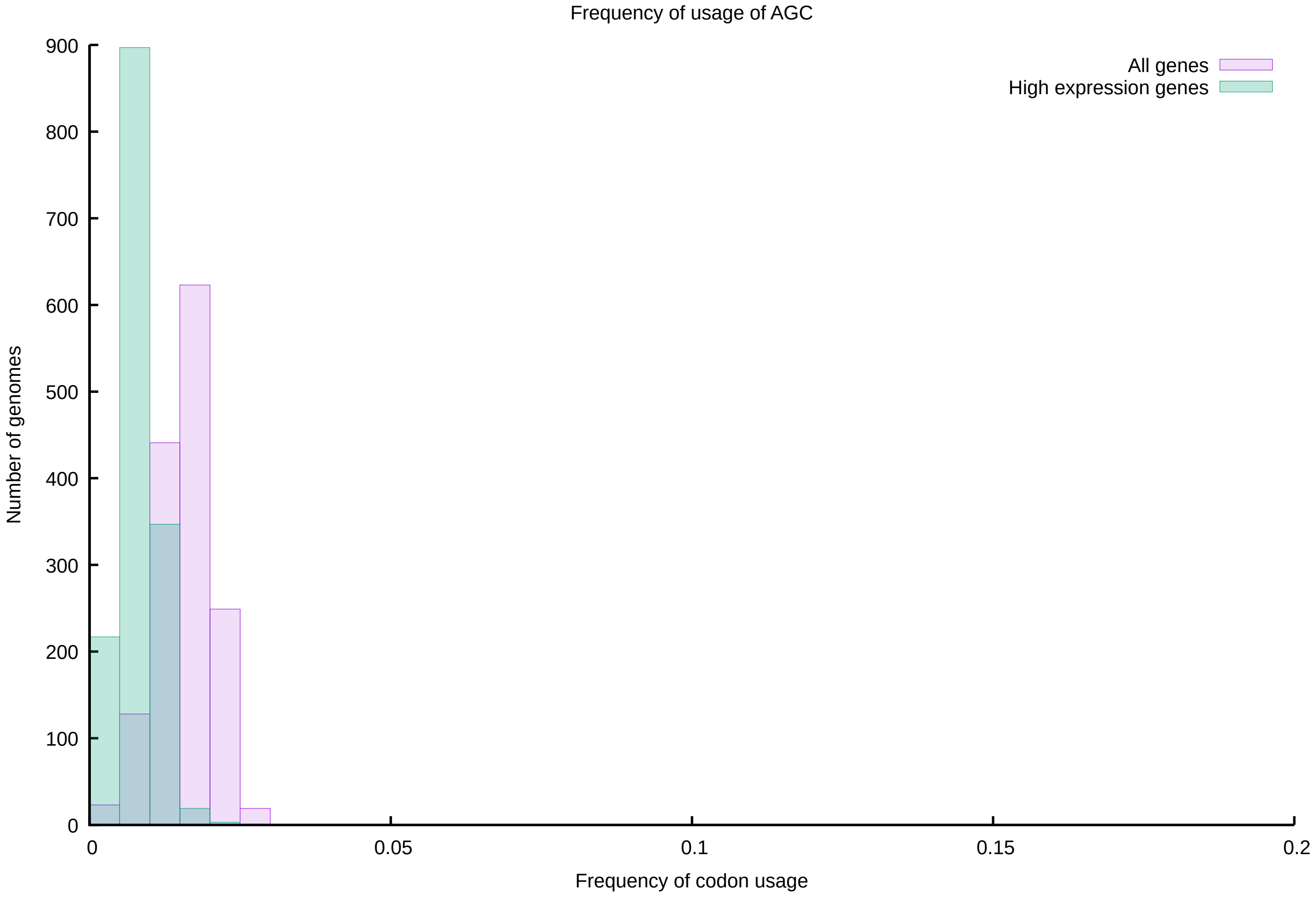

Frequency of usage of AGC
All genes
High expression genes
Number of genomes
0
100
200
300
400
500
600
700
800
900
0
0.05
0.1
0.15
0.2
Frequency of codon usage

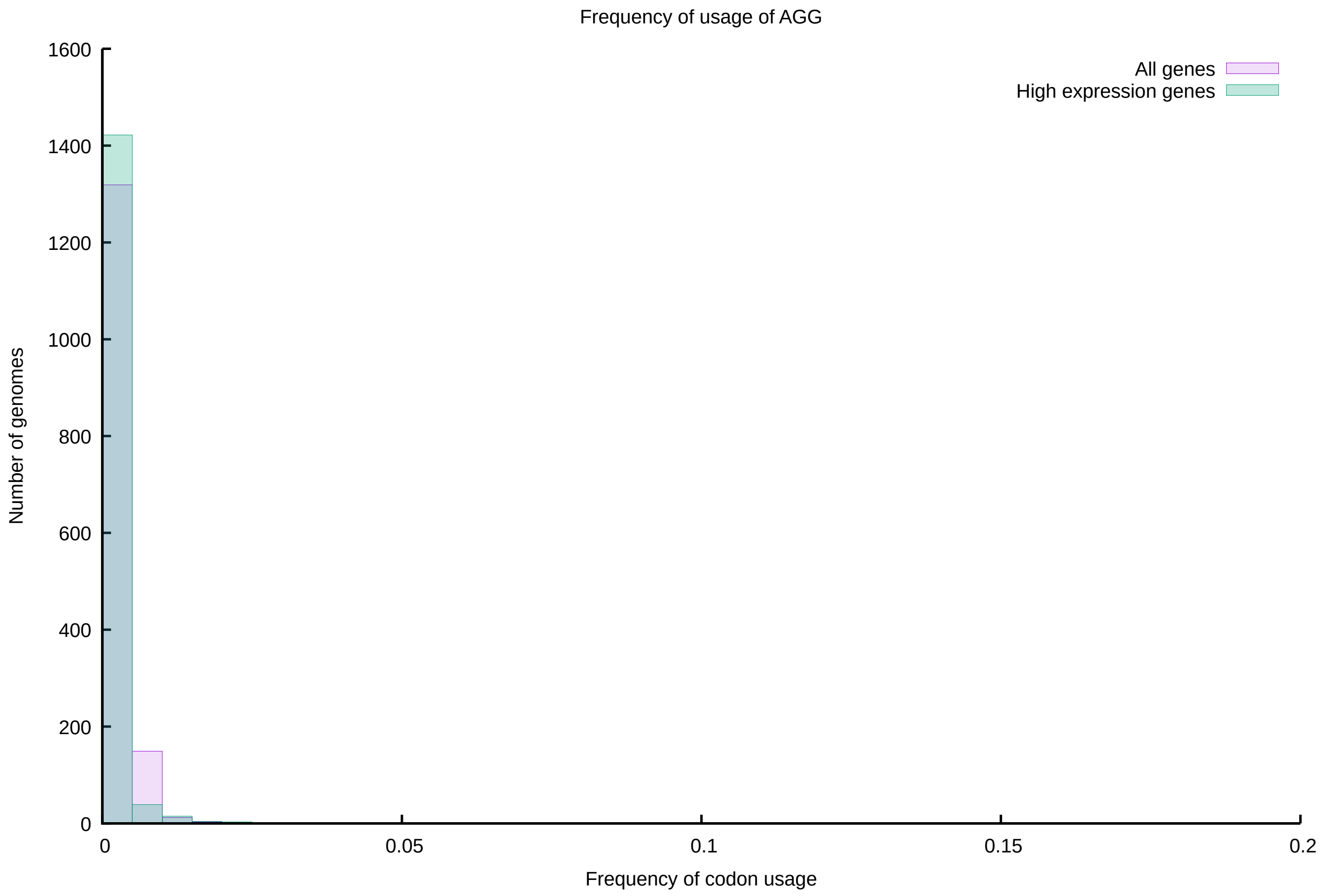

Frequency of usage of AGG
All genes
High expression genes
Number of genomes
1600
1400
1200
1000
800
600
400
200
0
0
0.05
0.1
0.15
0.2
Frequency of codon usage

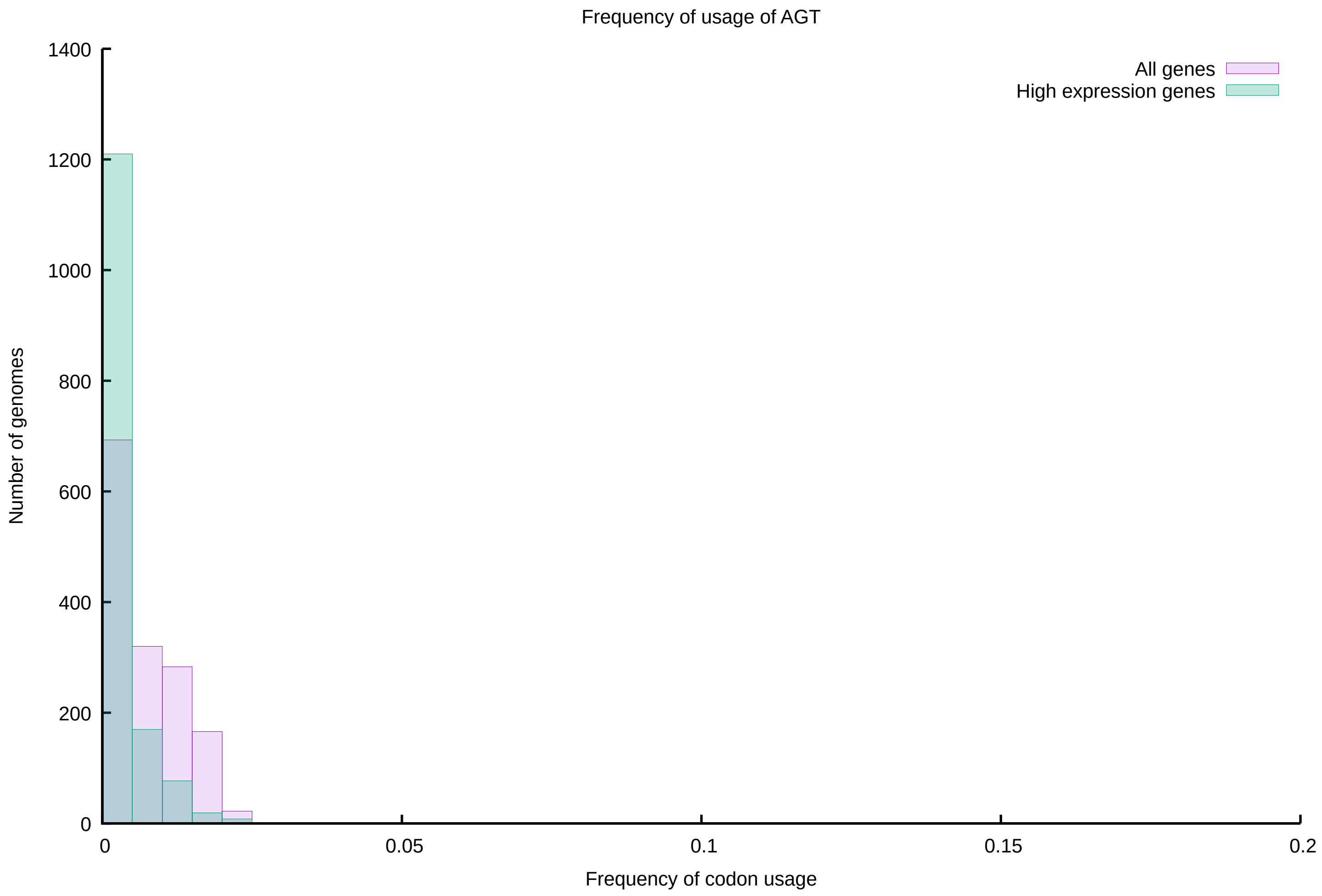
Frequency of usage of AGT
All genes
High expression genes
Number of genomes
0
200
400
600
800
1000
1200
1400
0
0.05
0.1
0.15
0.2
Frequency of codon usage

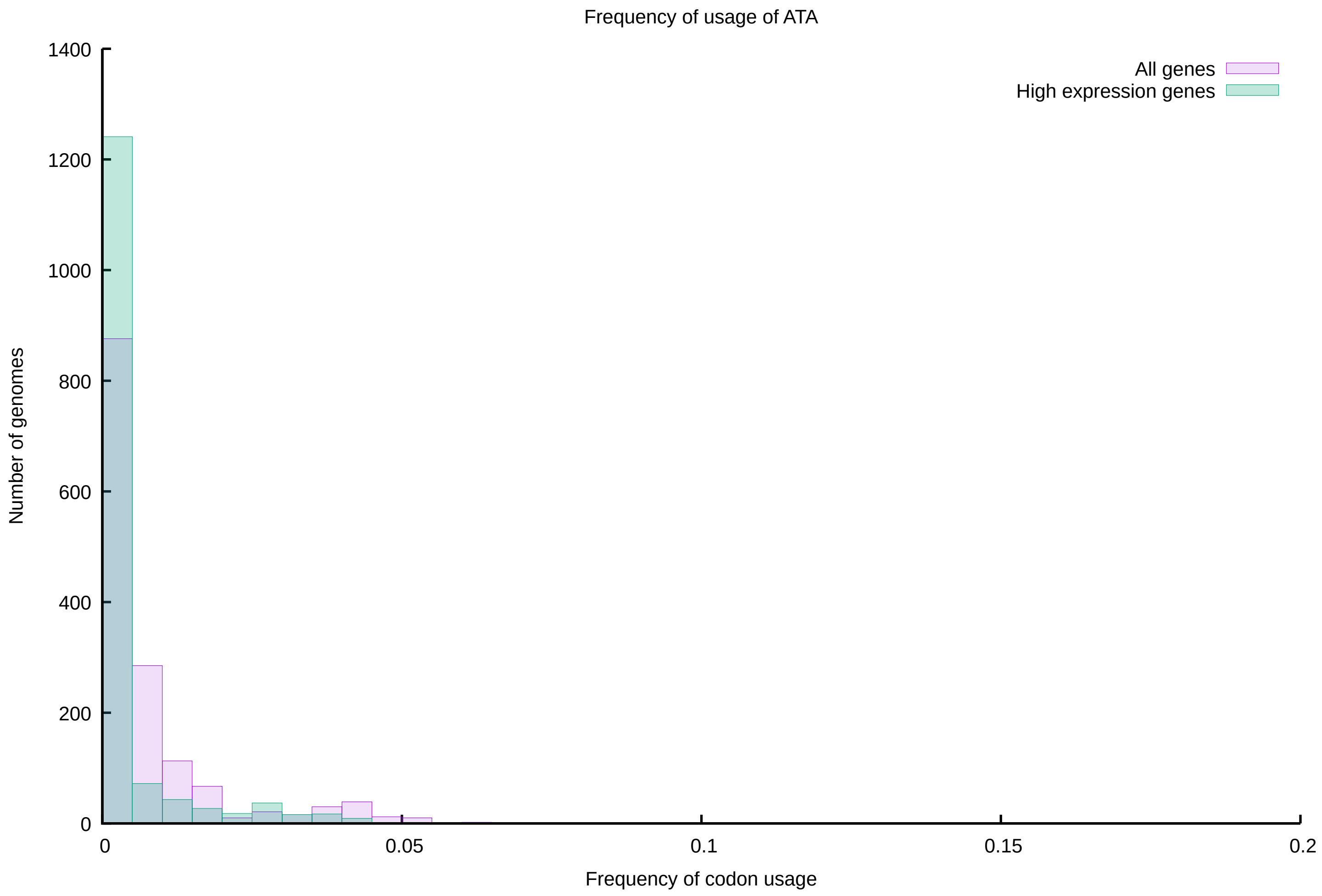

Frequency of usage of ATA
All genes
High expression genes
Number of genomes
Frequency of codon usage
0
200
400
600
800
1000
1200
1400
0
0.05
0.1
0.15
0.2

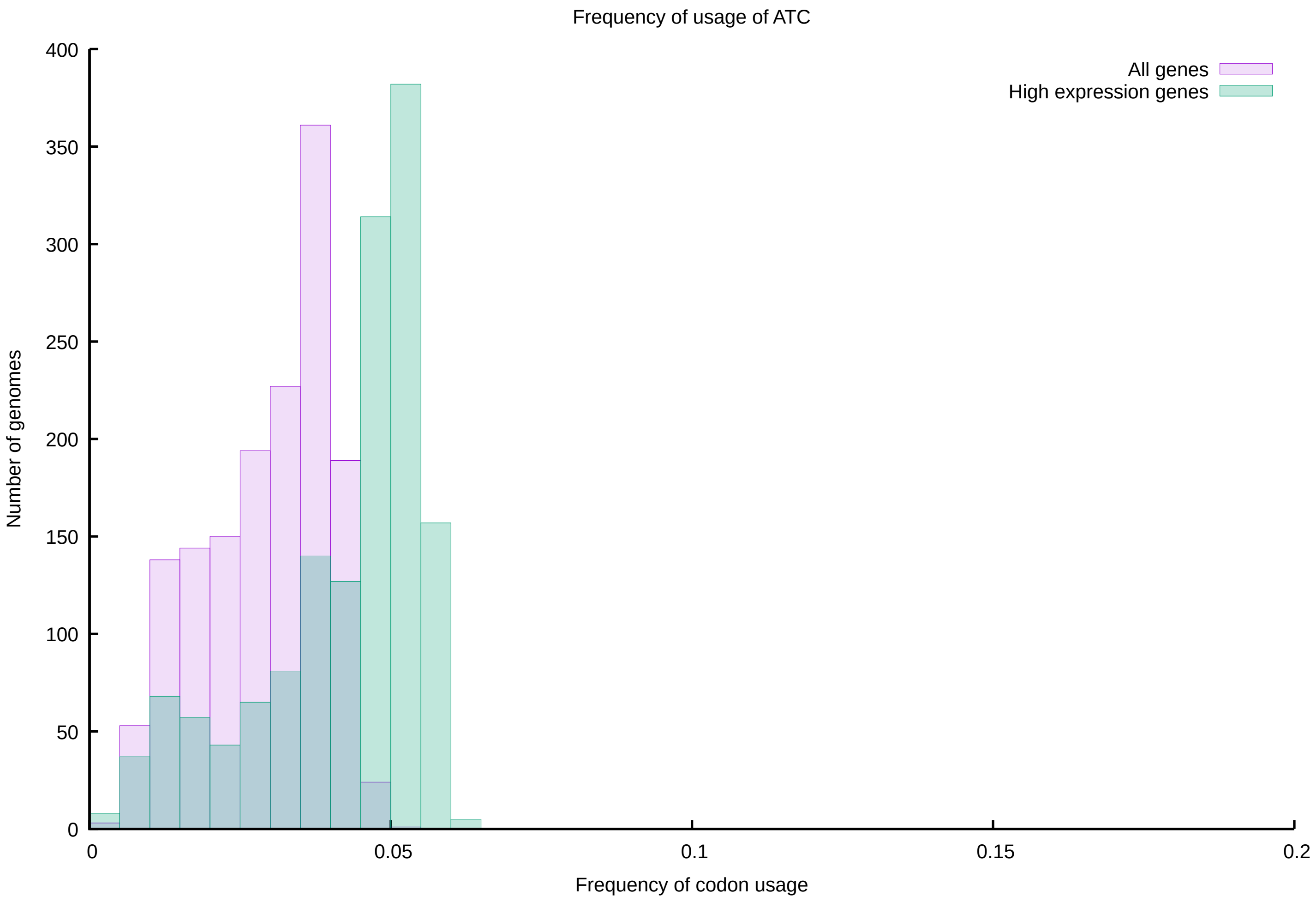

Frequency of usage of ATC
All genes
High expression genes
Number of genomes
Frequency of codon usage
0
50
100
150
200
250
300
350
400
0
0.05
0.1
0.15
0.2

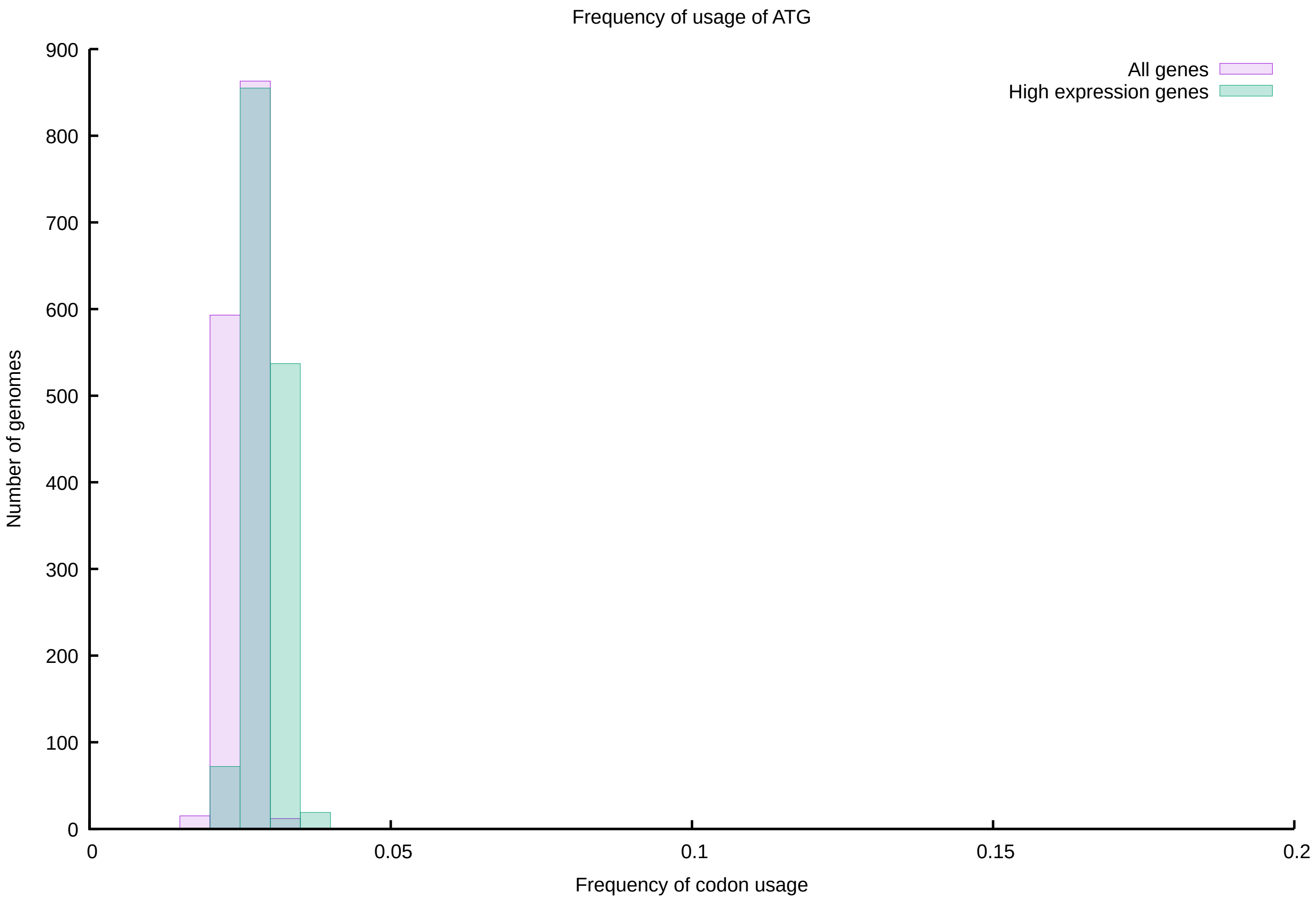
Frequency of usage of ATG
All genes
High expression genes
900
800
700
600
500
400
300
200
100
0
Number of genomes
0
0.05
0.1
0.15
0.2
Frequency of codon usage

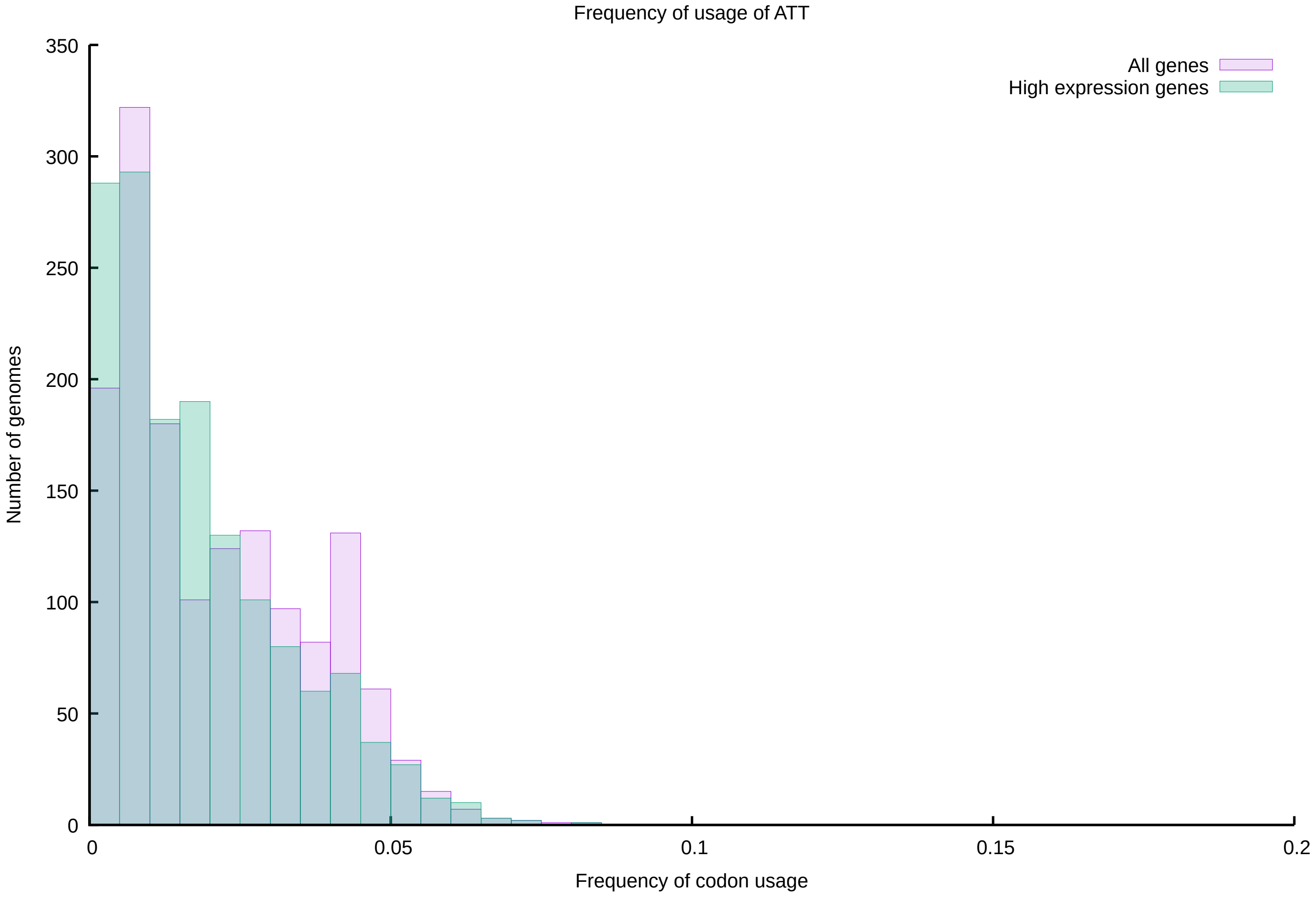

Frequency of usage of ATT
All genes
High expression genes
Number of genomes
Frequency of codon usage
0
50
100
150
200
250
300
350
0
0.05
0.1
0.15
0.2

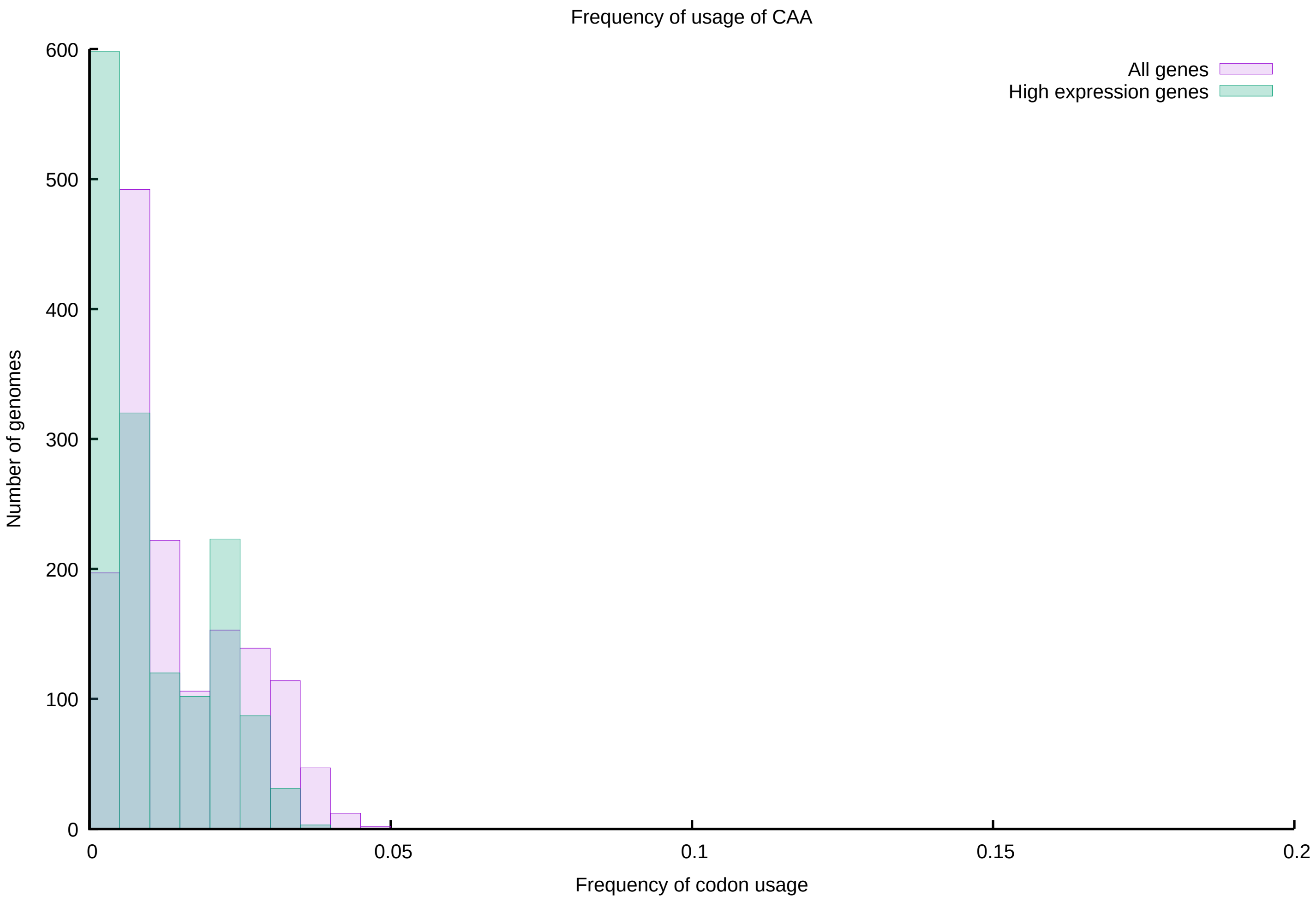

Frequency of usage of CAA
All genes
High expression genes
Number of genomes
Frequency of codon usage
0
100
200
300
400
500
600
0.05
0.1
0.15
0.2

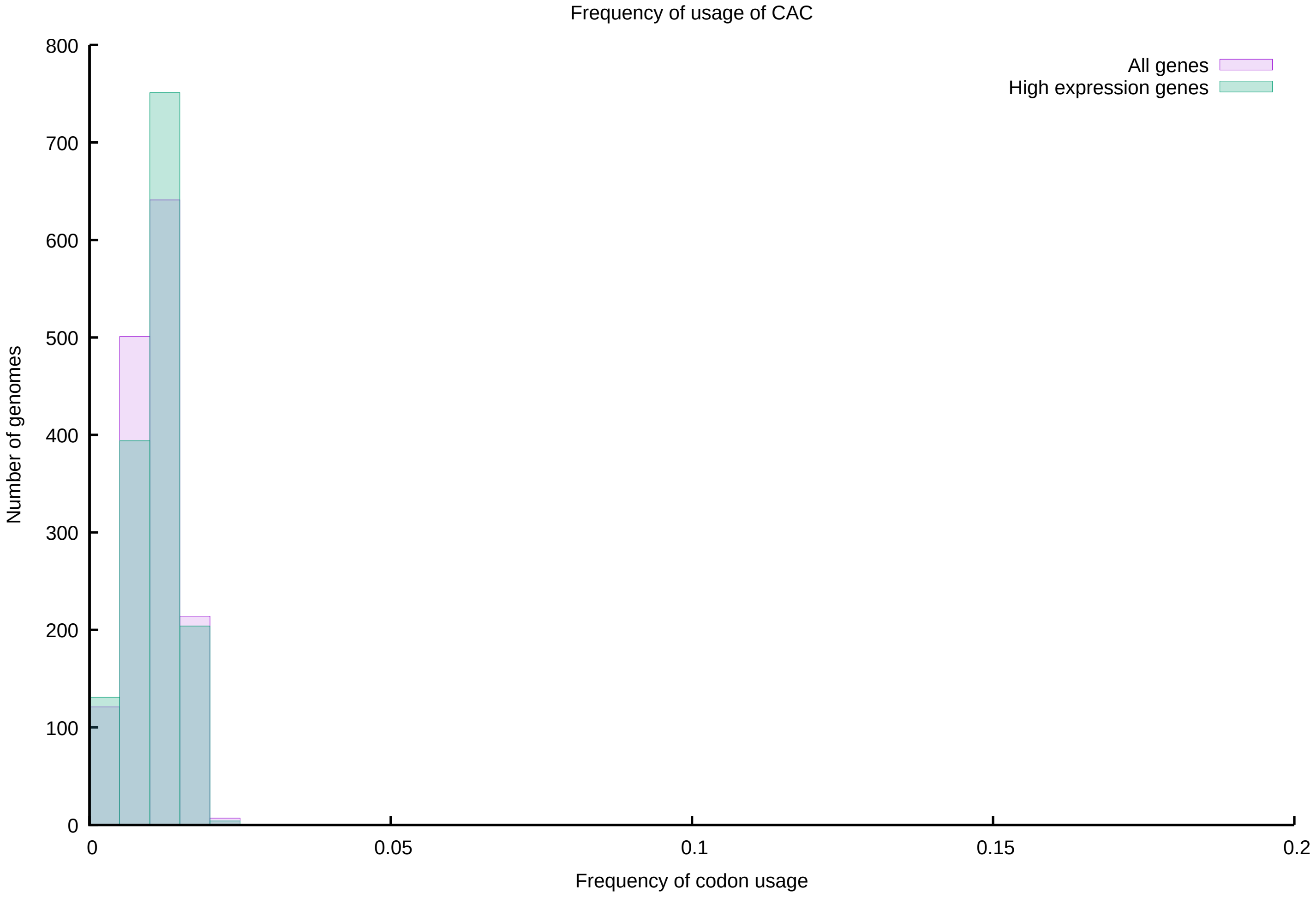
Frequency of usage of CAC
All genes
High expression genes
Number of genomes
800
700
600
500
400
300
200
100
0
0
0.05
0.1
0.15
0.2
Frequency of codon usage

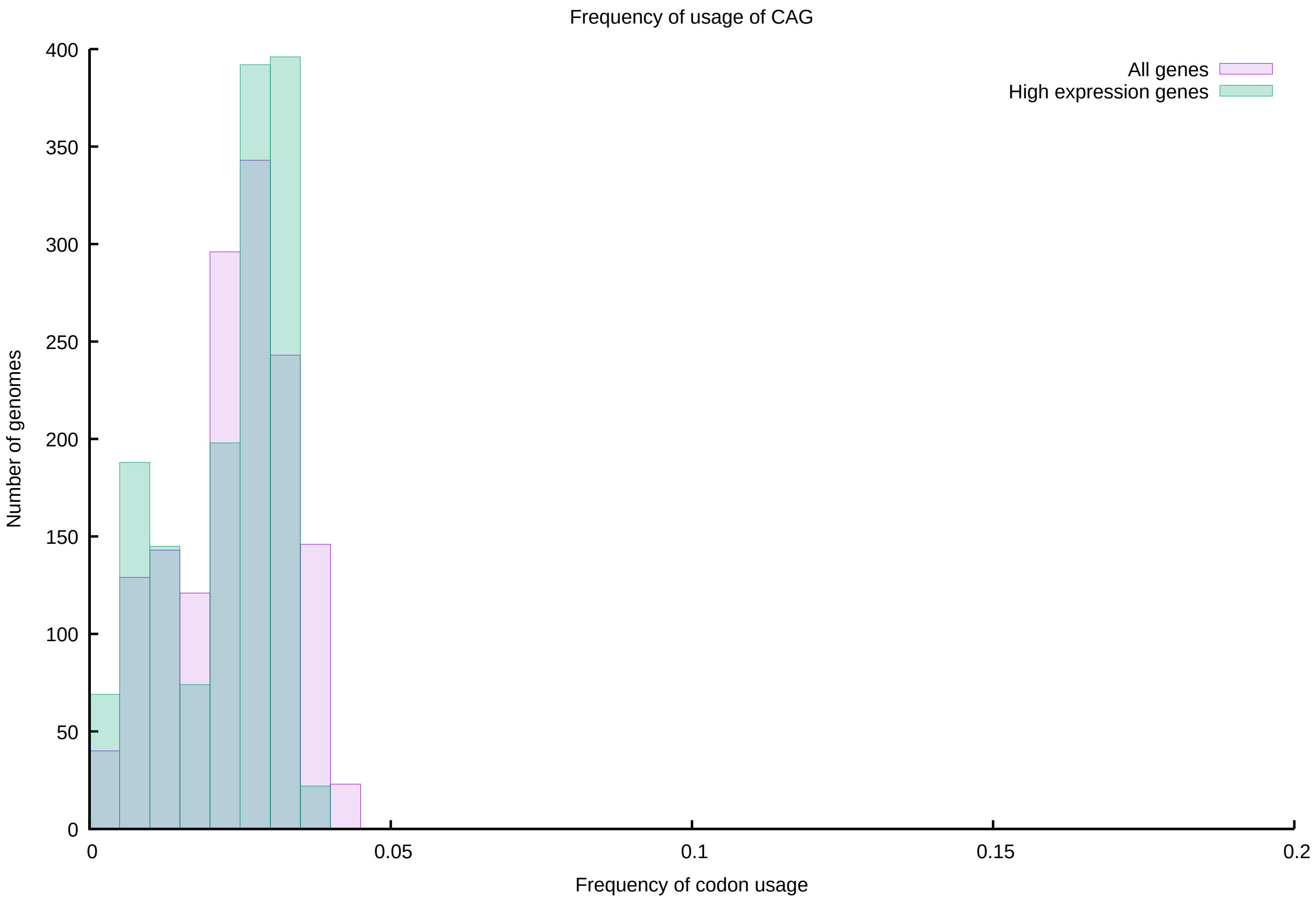

Frequency of usage of CAG
All genes
High expression genes
Number of genomes
Frequency of codon usage
0
50
100
150
200
250
300
350
400
0
0.05
0.1
0.15
0.2

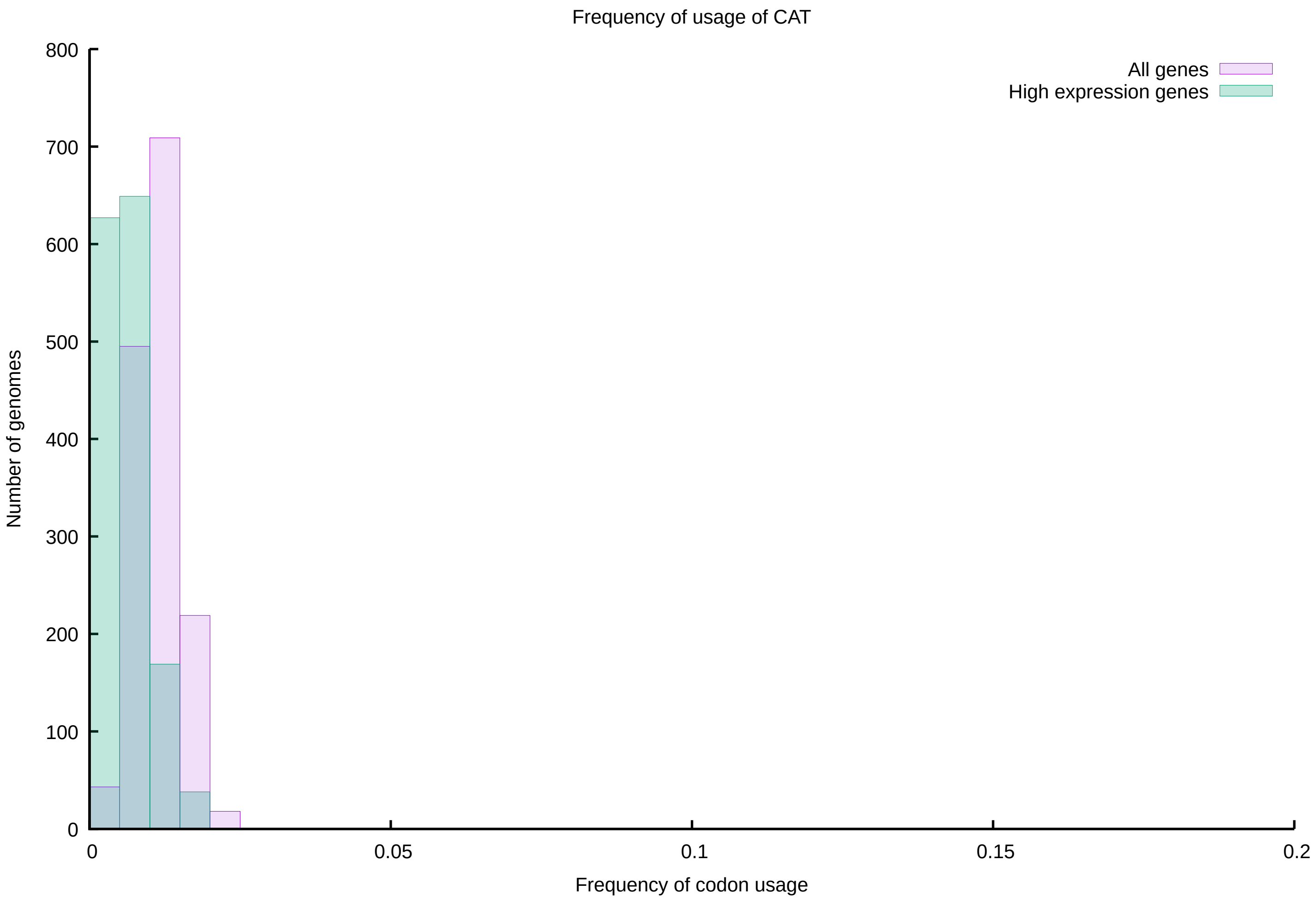

Frequency of usage of CAT
All genes
High expression genes
Number of genomes
0
100
200
300
400
500
600
700
800
0
0.05
0.1
0.15
0.2
Frequency of codon usage

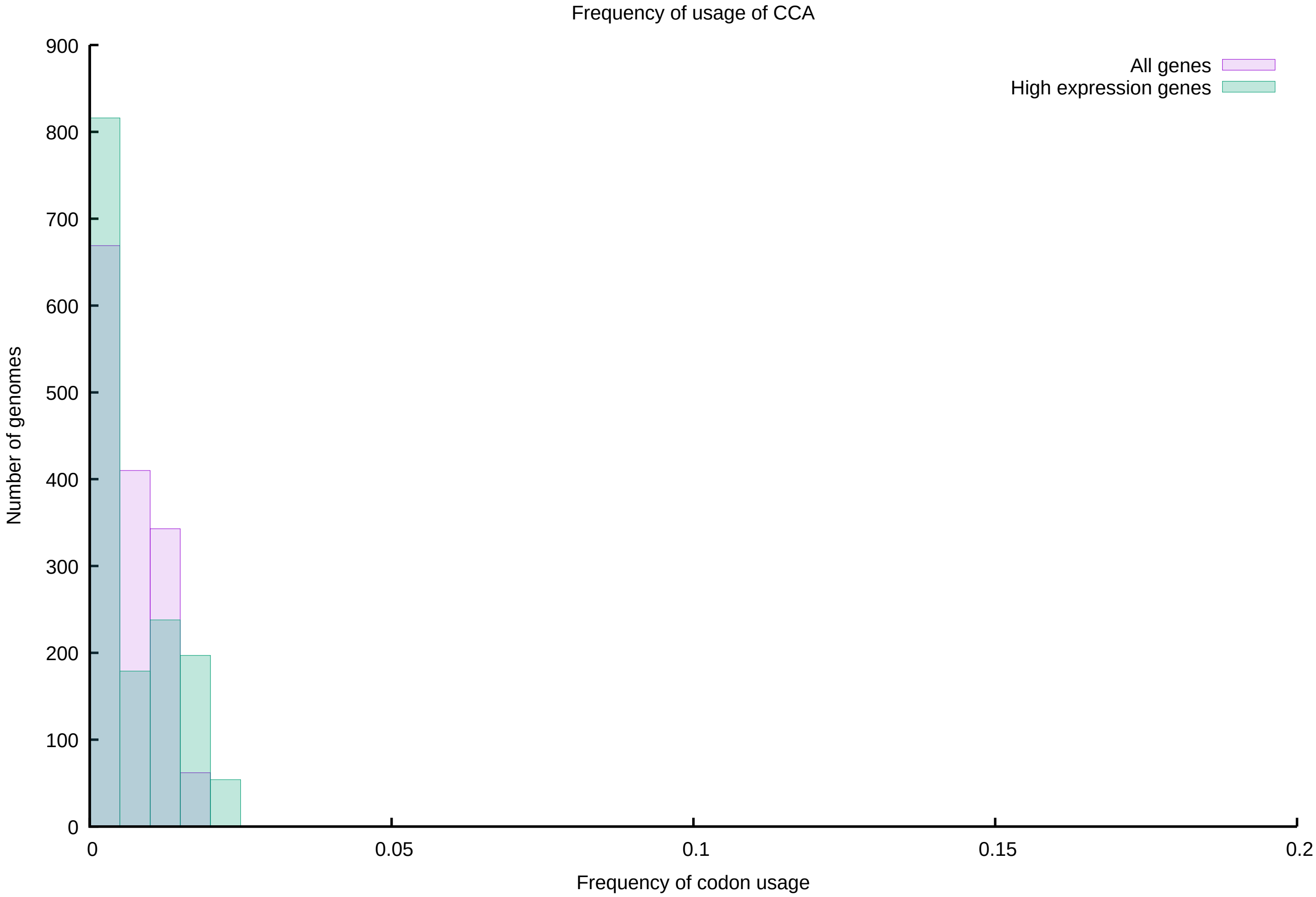
Frequency of usage of CCA
All genes
High expression genes
Number of genomes
0
100
200
300
400
500
600
700
800
900
0
0.05
0.1
0.15
0.2
Frequency of codon usage

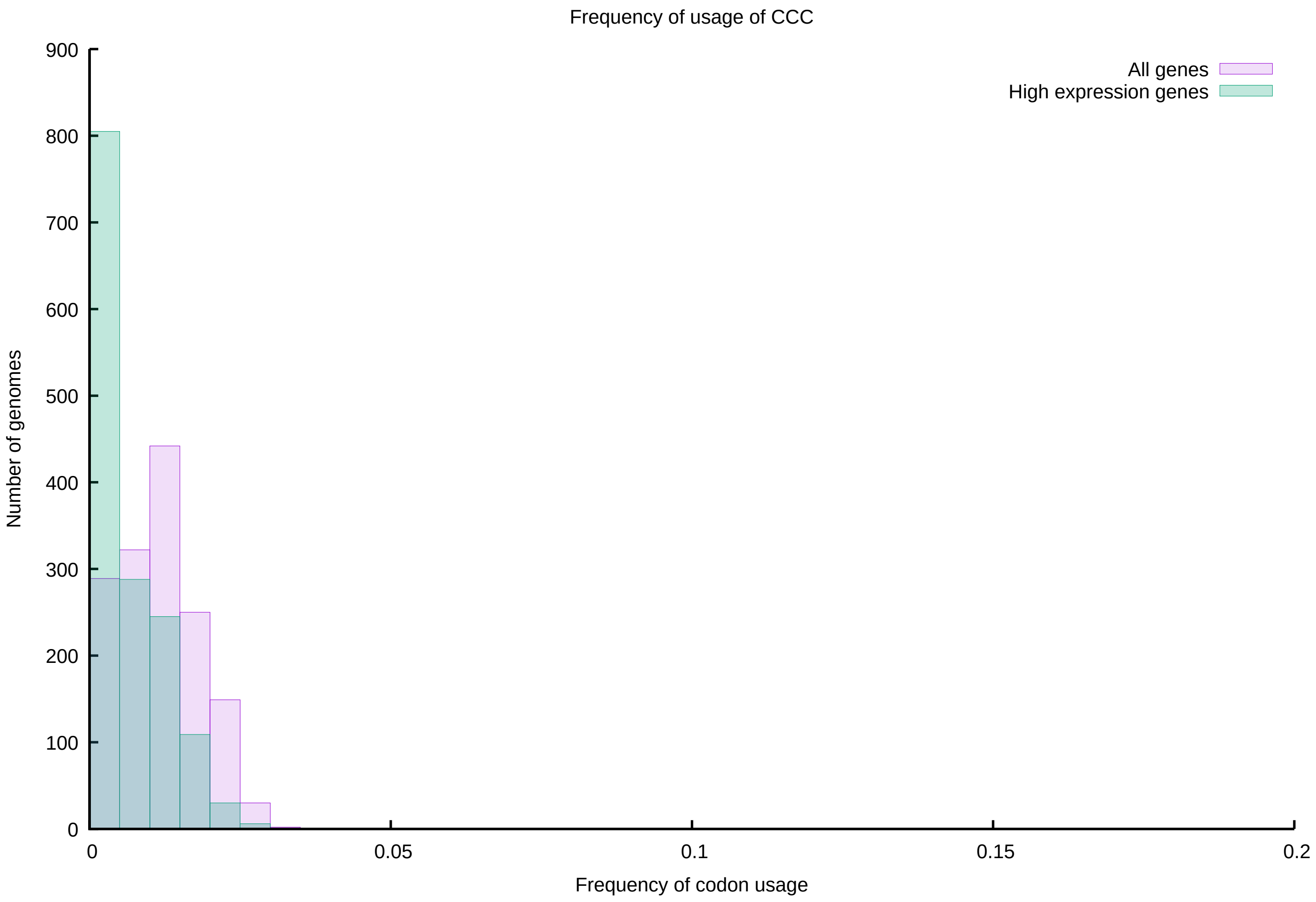

Frequency of usage of CCC
All genes
High expression genes
Number of genomes
900
800
700
600
500
400
300
200
100
0
0
0.05
0.1
0.15
0.2
Frequency of codon usage

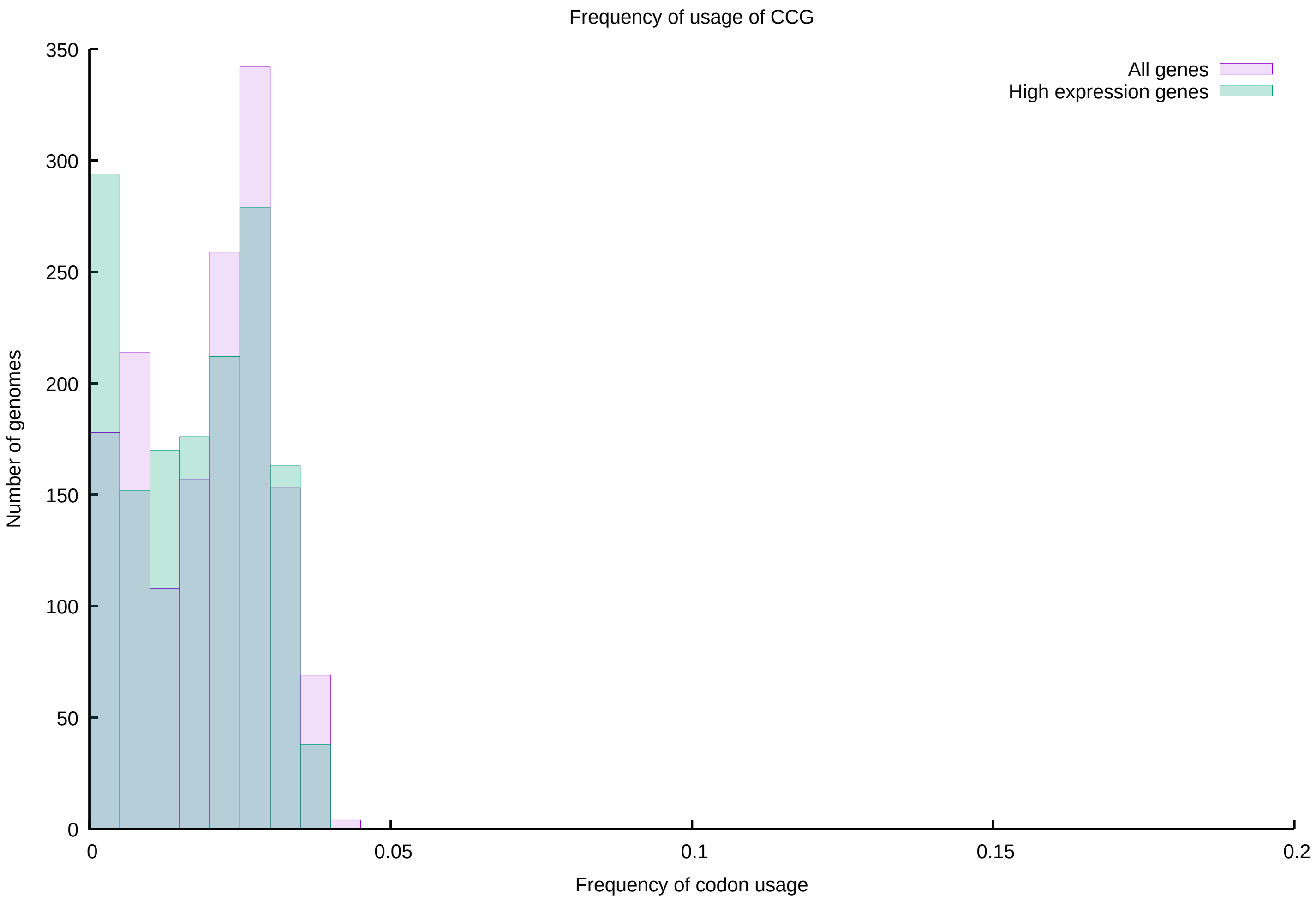

Frequency of usage of CCG
All genes
High expression genes
Number of genomes
350
300
250
200
150
100
50
0
0
0.05
0.1
0.15
0.2
Frequency of codon usage

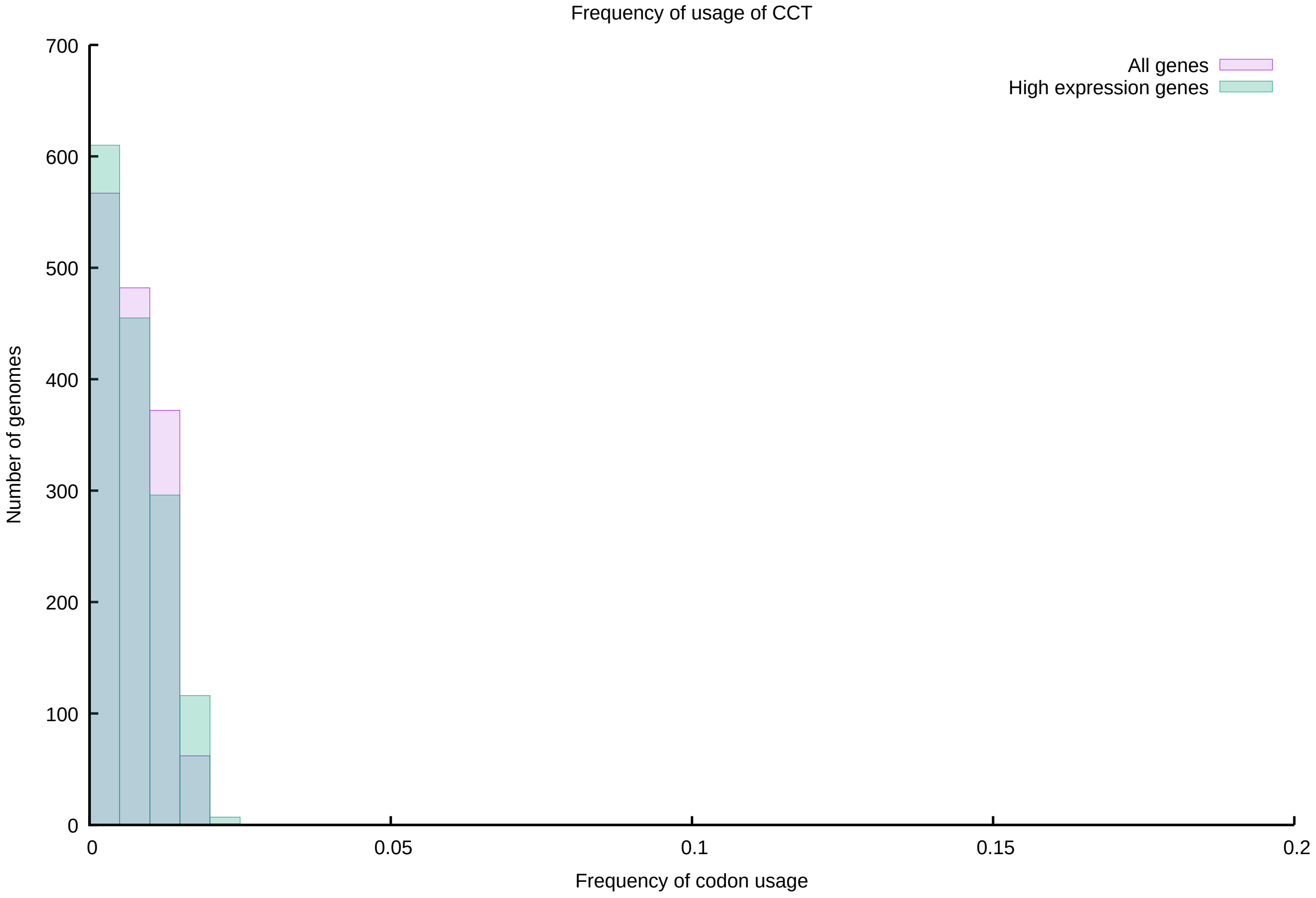

Frequency of usage of CCT
All genes
High expression genes
Number of genomes
0
100
200
300
400
500
600
700
0
0.05
0.1
0.15
0.2
Frequency of codon usage

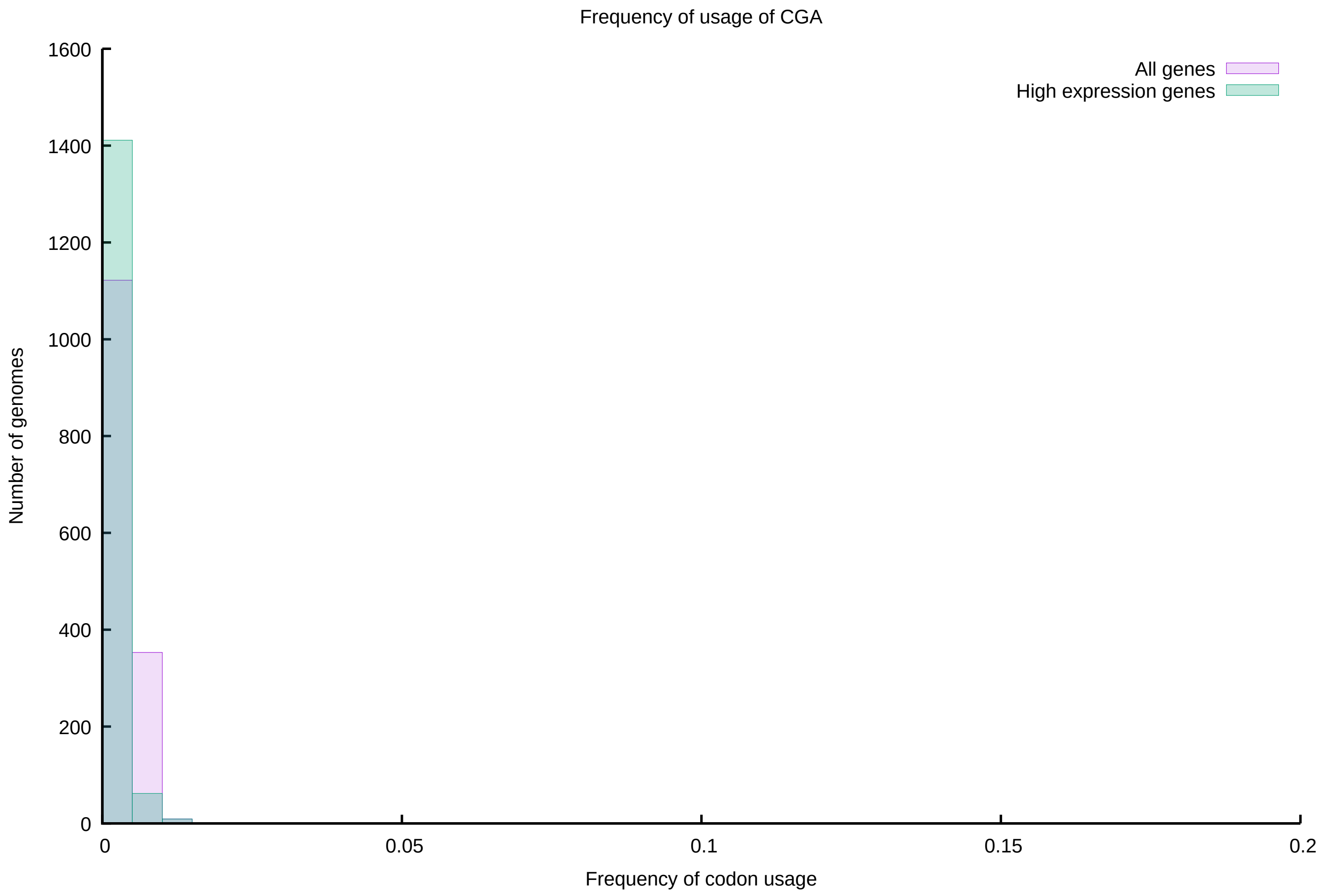

Frequency of usage of CGA
All genes
High expression genes
Number of genomes
1600
1400
1200
1000
800
600
400
200
0
0
0.05
0.1
0.15
0.2
Frequency of codon usage

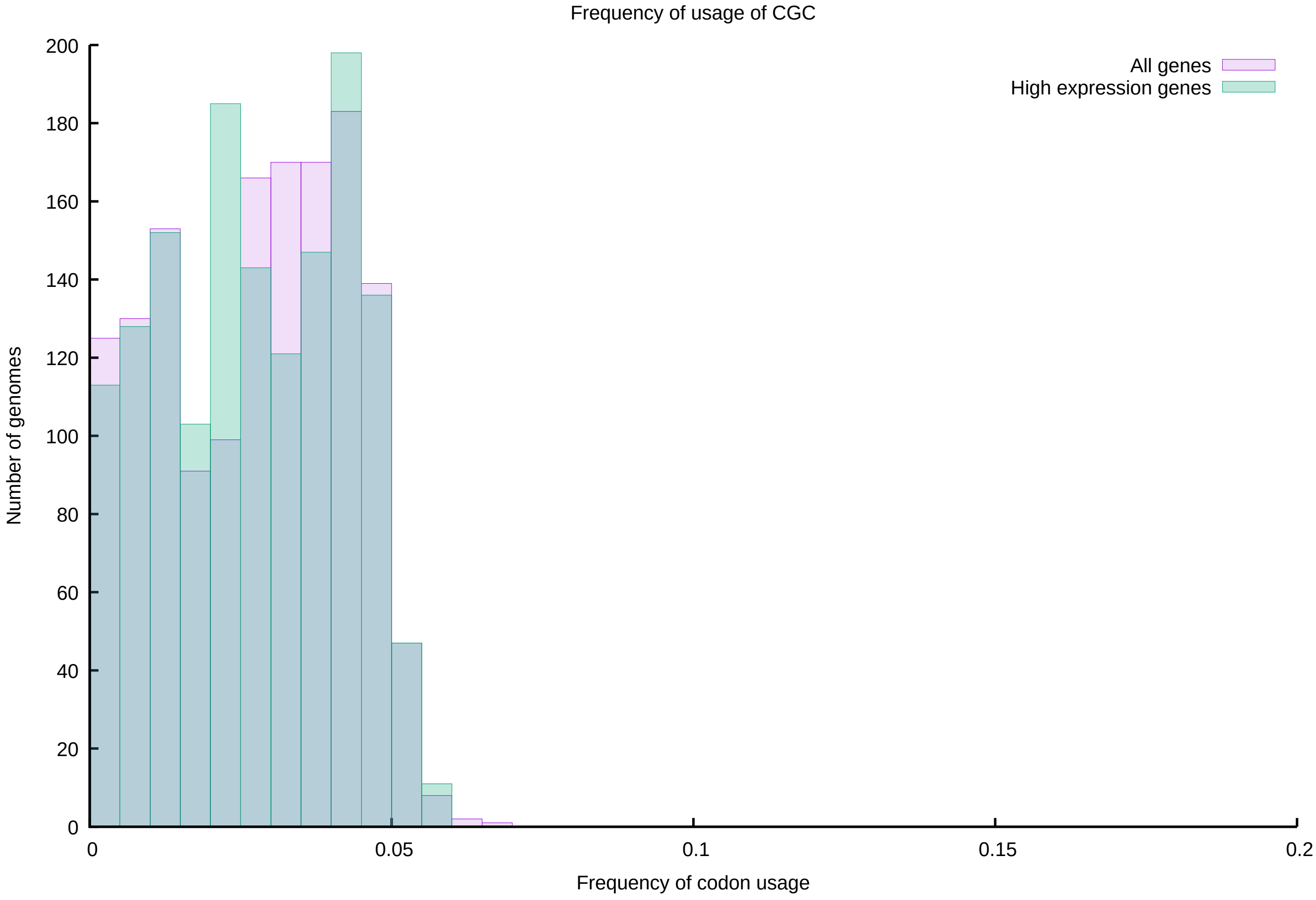

Frequency of usage of CGC
All genes
High expression genes
Number of genomes
Frequency of codon usage
0
20
40
60
80
100
120
140
160
180
200
0
0.05
0.1
0.15
0.2

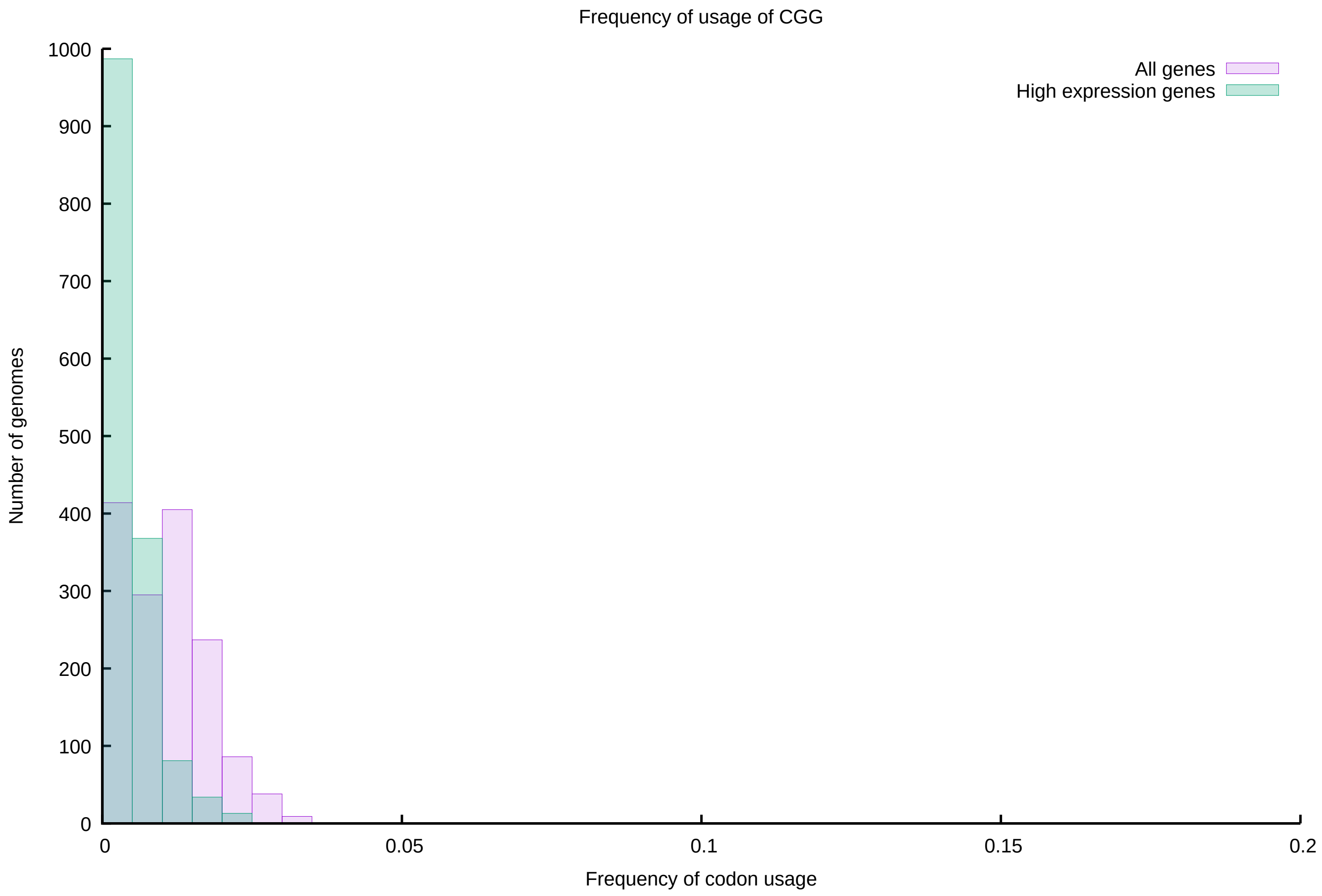

Frequency of usage of CGG
All genes
High expression genes
Number of genomes
1000
900
800
700
600
500
400
300
200
100
0
0
0.05
0.1
0.15
0.2
Frequency of codon usage

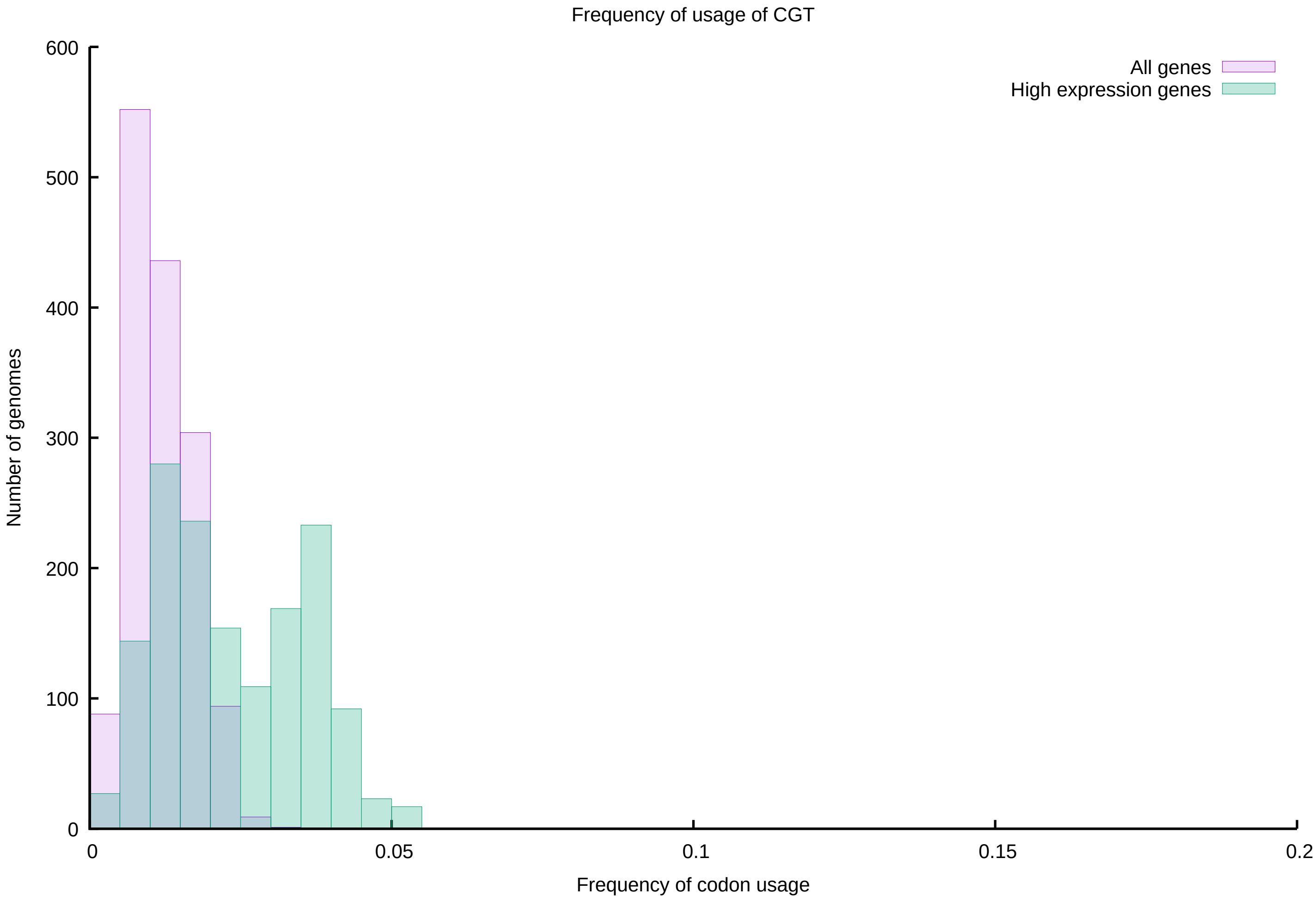
Frequency of usage of CGT
All genes
High expression genes
Number of genomes
Frequency of codon usage
0
100
200
300
400
500
600
0
0.05
0.1
0.15
0.2

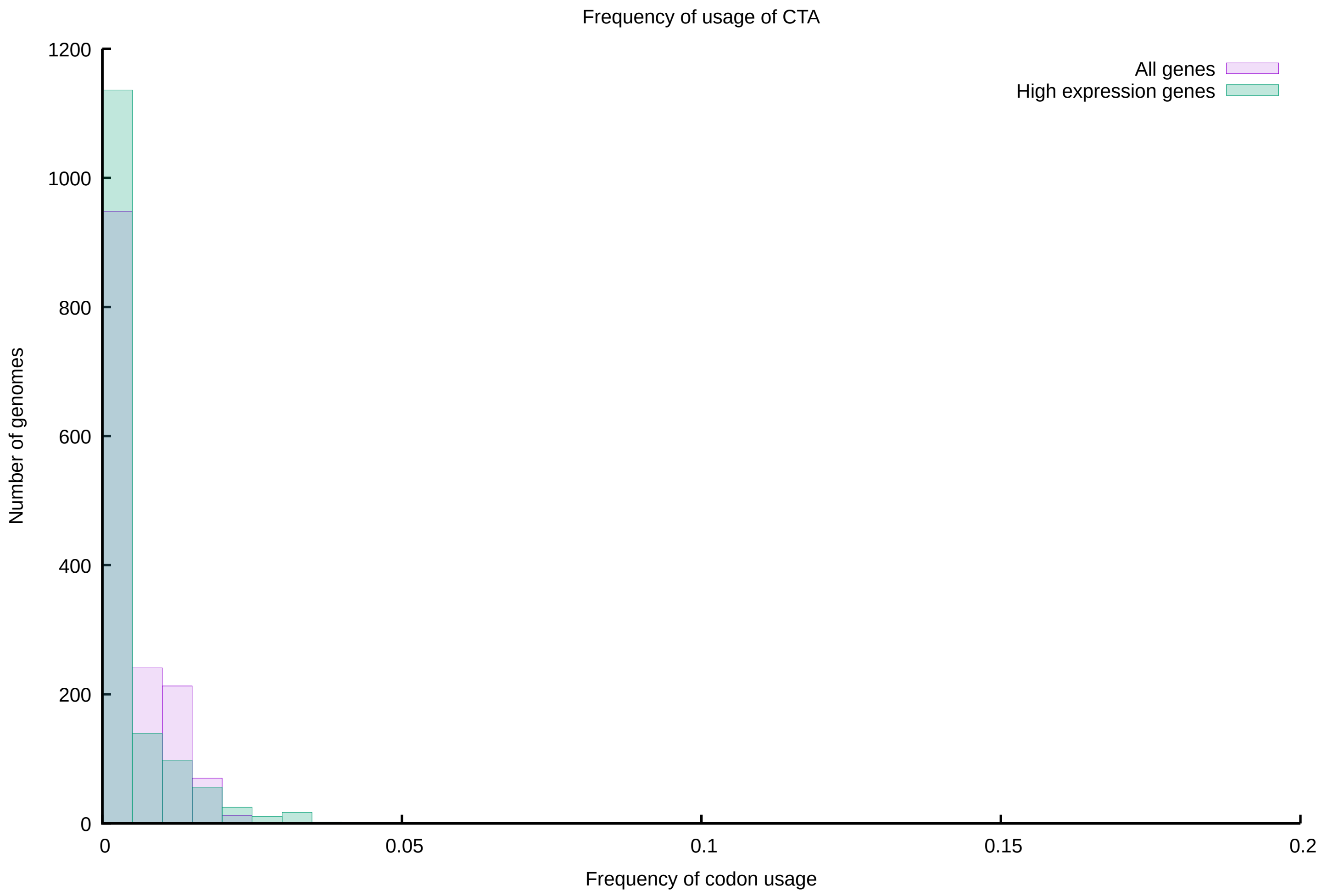

Frequency of usage of CTA
All genes
High expression genes
Number of genomes
1200
1000
800
600
400
200
0
0
0.05
0.1
0.15
0.2
Frequency of codon usage

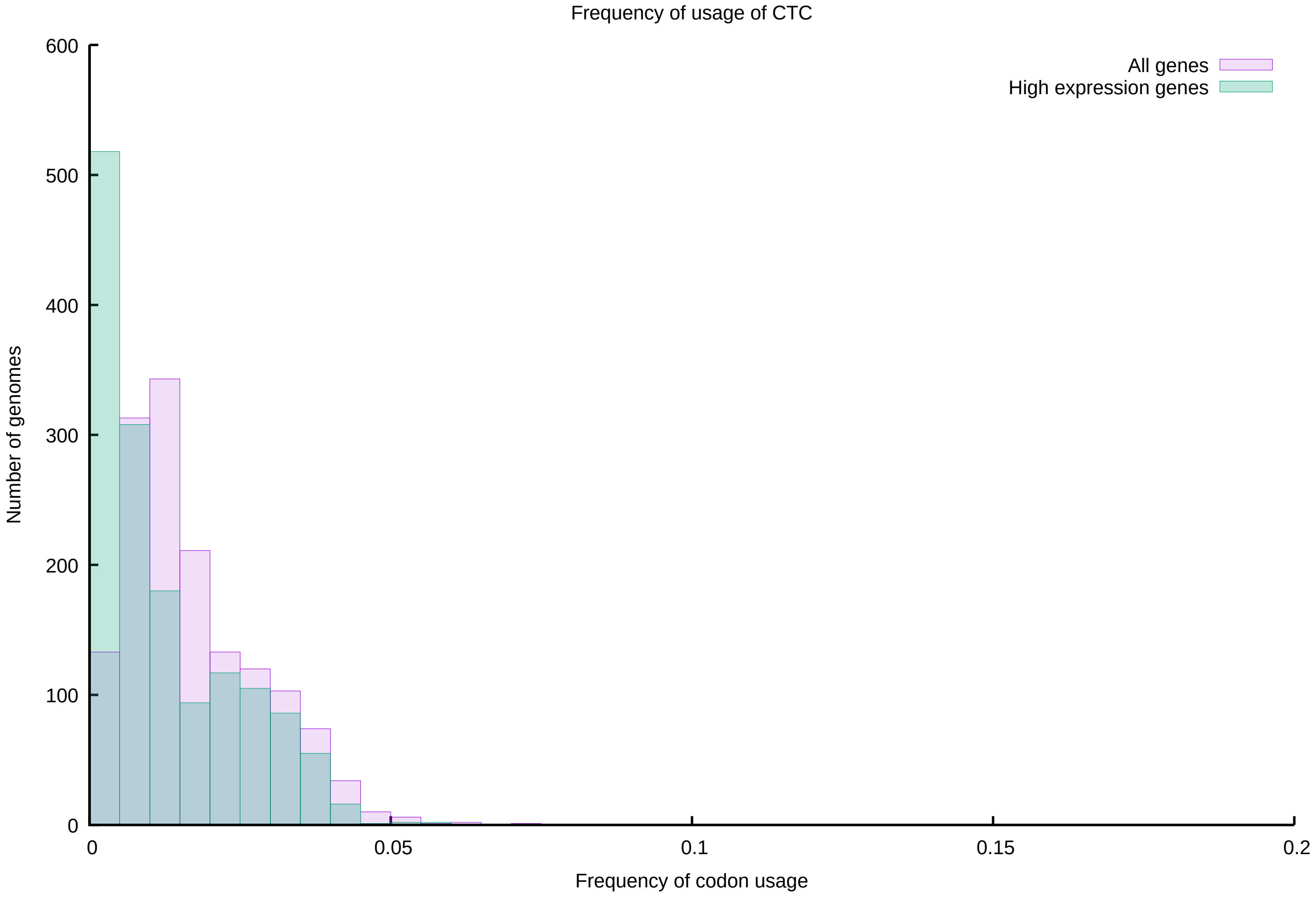

Frequency of usage of CTC
All genes
High expression genes
Number of genomes
600
500
400
300
200
100
0
0
0.05
0.1
0.15
0.2
Frequency of codon usage

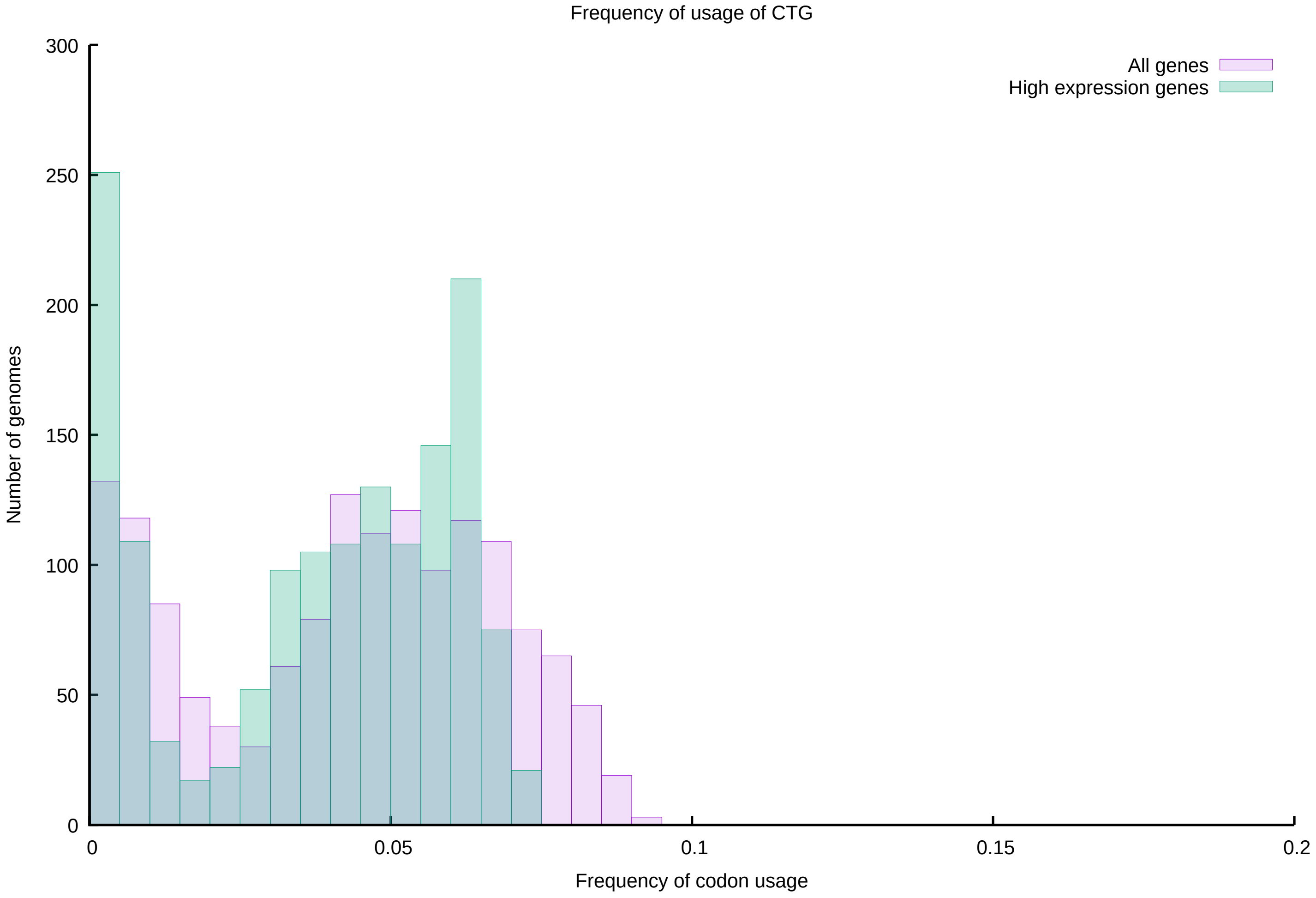
Frequency of usage of CTG
All genes
High expression genes
Number of genomes
300
250
200
150
100
50
0
0
0.05
0.1
0.15
0.2
Frequency of codon usage

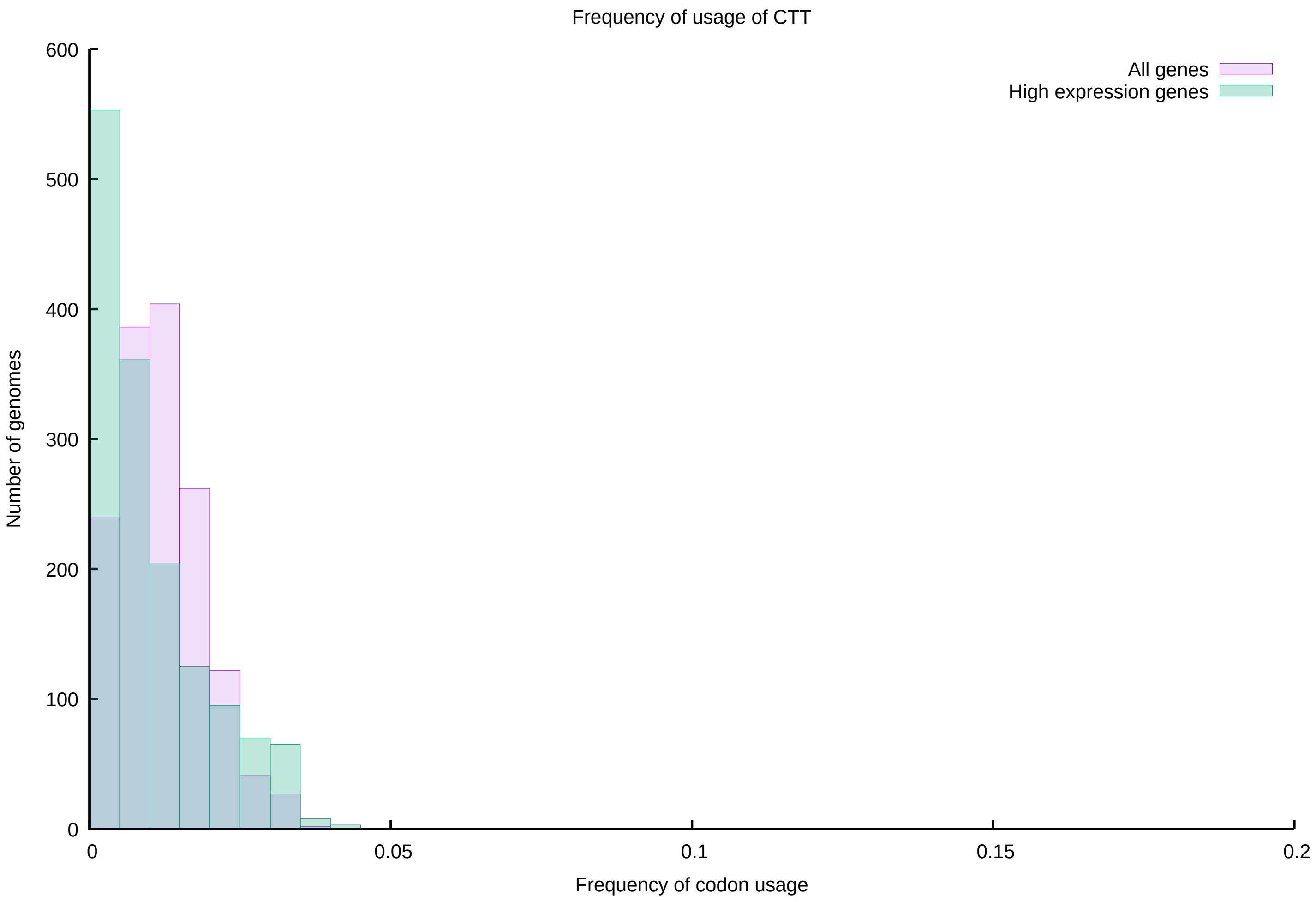
Frequency of usage of CTT
All genes
High expression genes
Number of genomes
Frequency of codon usage
0
100
200
300
400
500
600
0
0.05
0.1
0.15
0.2

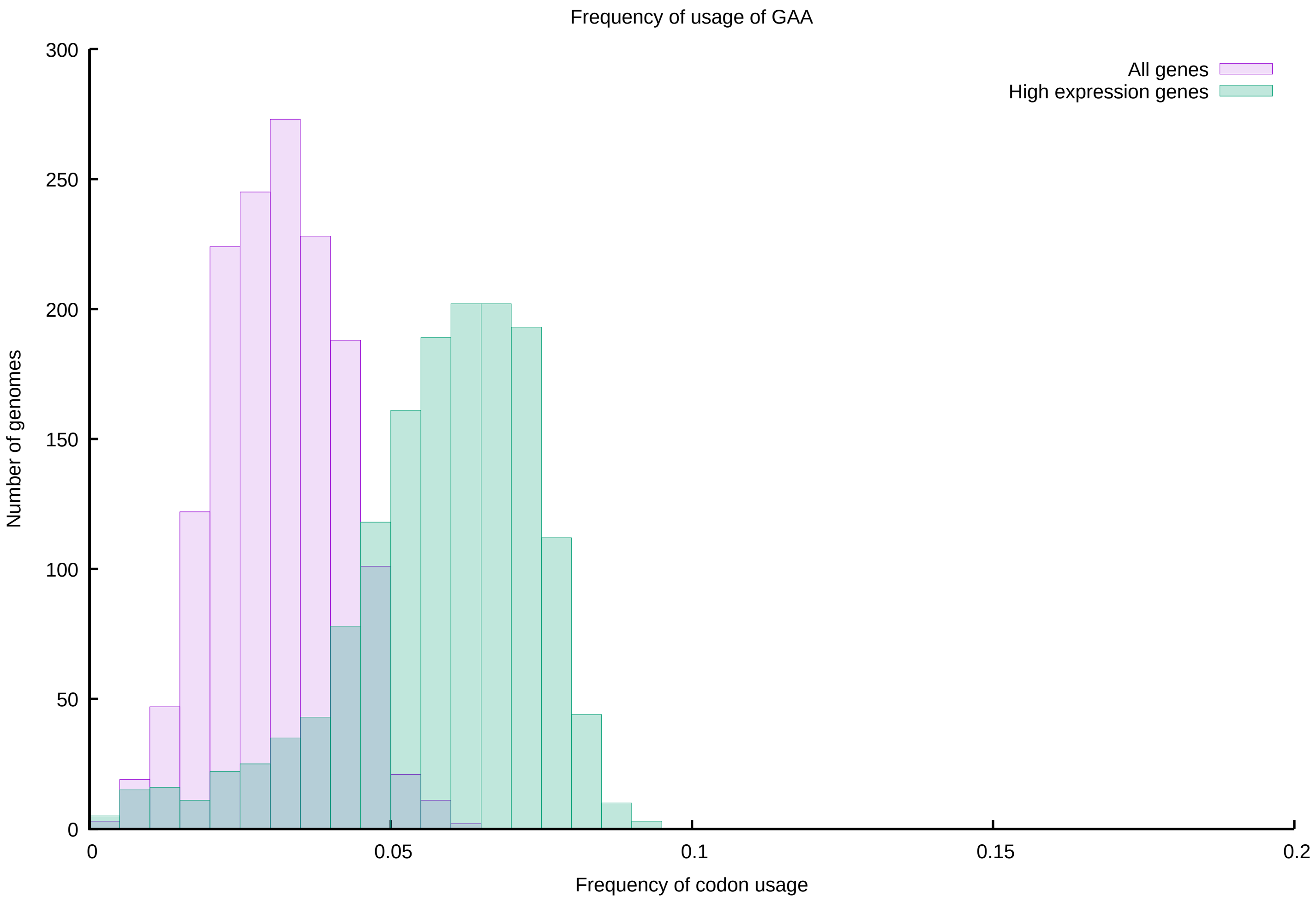
Frequency of usage of GAA
All genes
High expression genes
Number of genomes
0
50
100
150
200
250
300
0
0.05
0.1
0.15
0.2
Frequency of codon usage

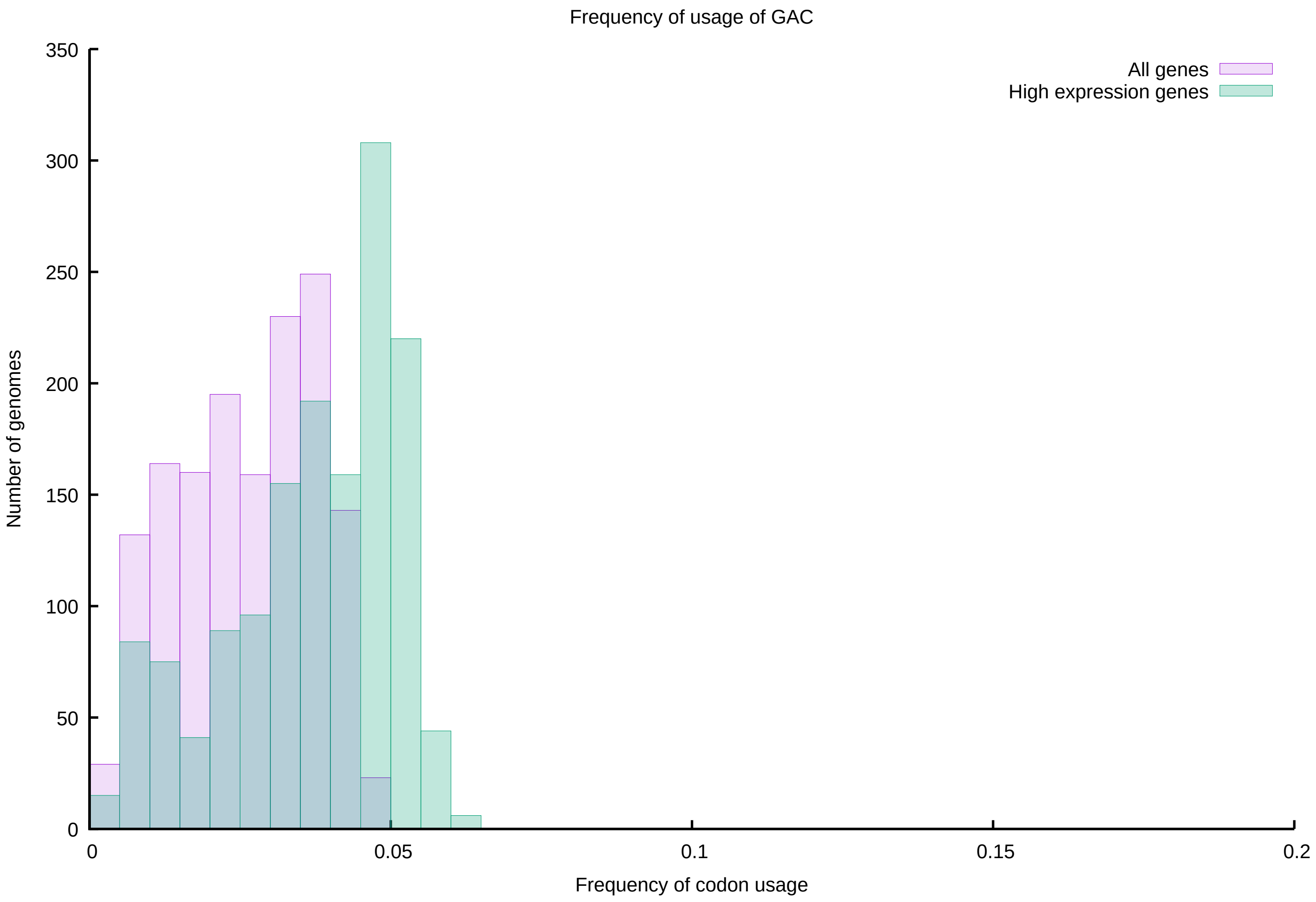
Frequency of usage of GAC
All genes
High expression genes
Number of genomes
Frequency of codon usage
0
50
100
150
200
250
300
350
0
0.05
0.1
0.15
0.2

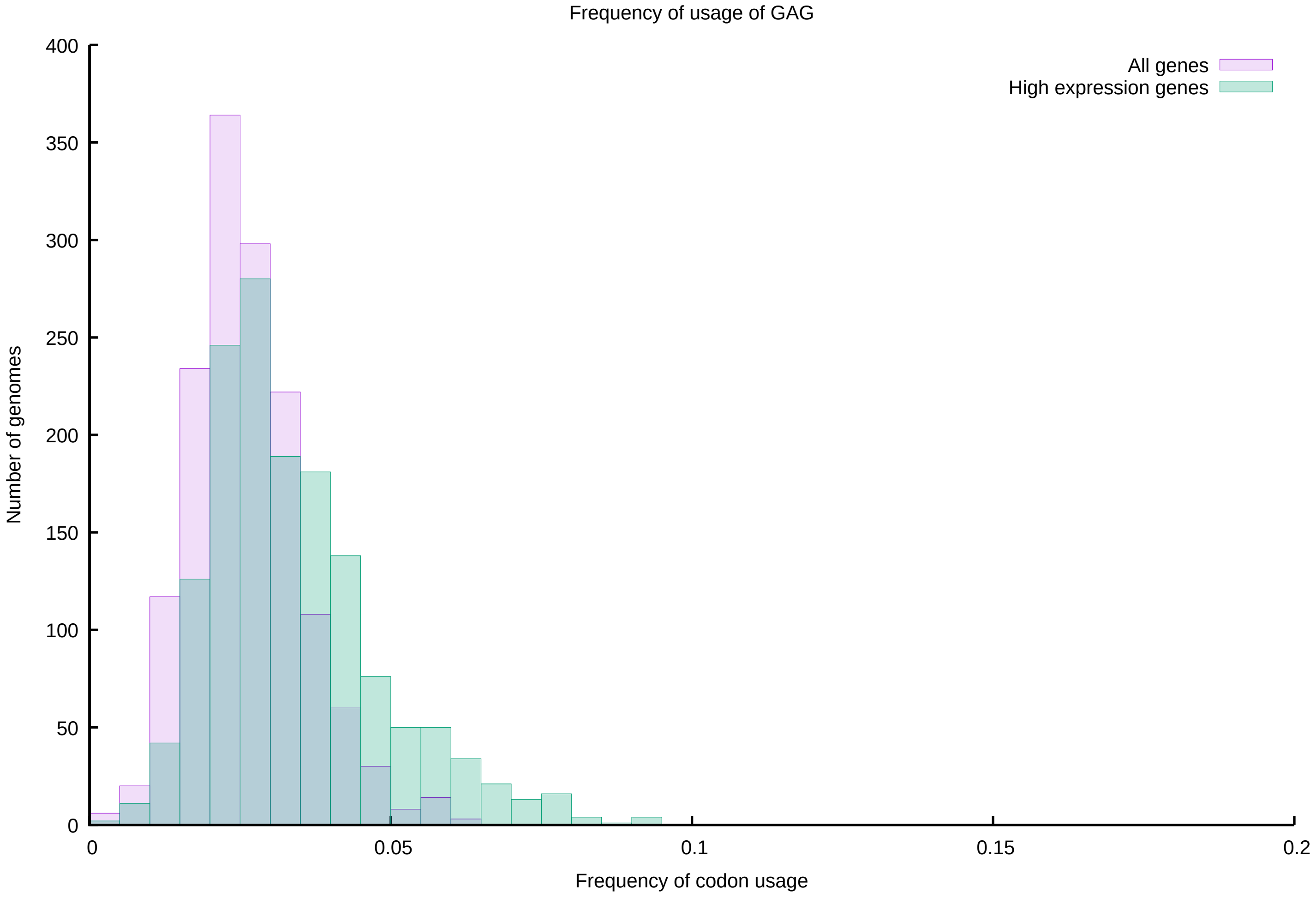
Frequency of usage of GAG
All genes
High expression genes
Number of genomes
Frequency of codon usage
0
50
100
150
200
250
300
350
400
0
0.05
0.1
0.15
0.2

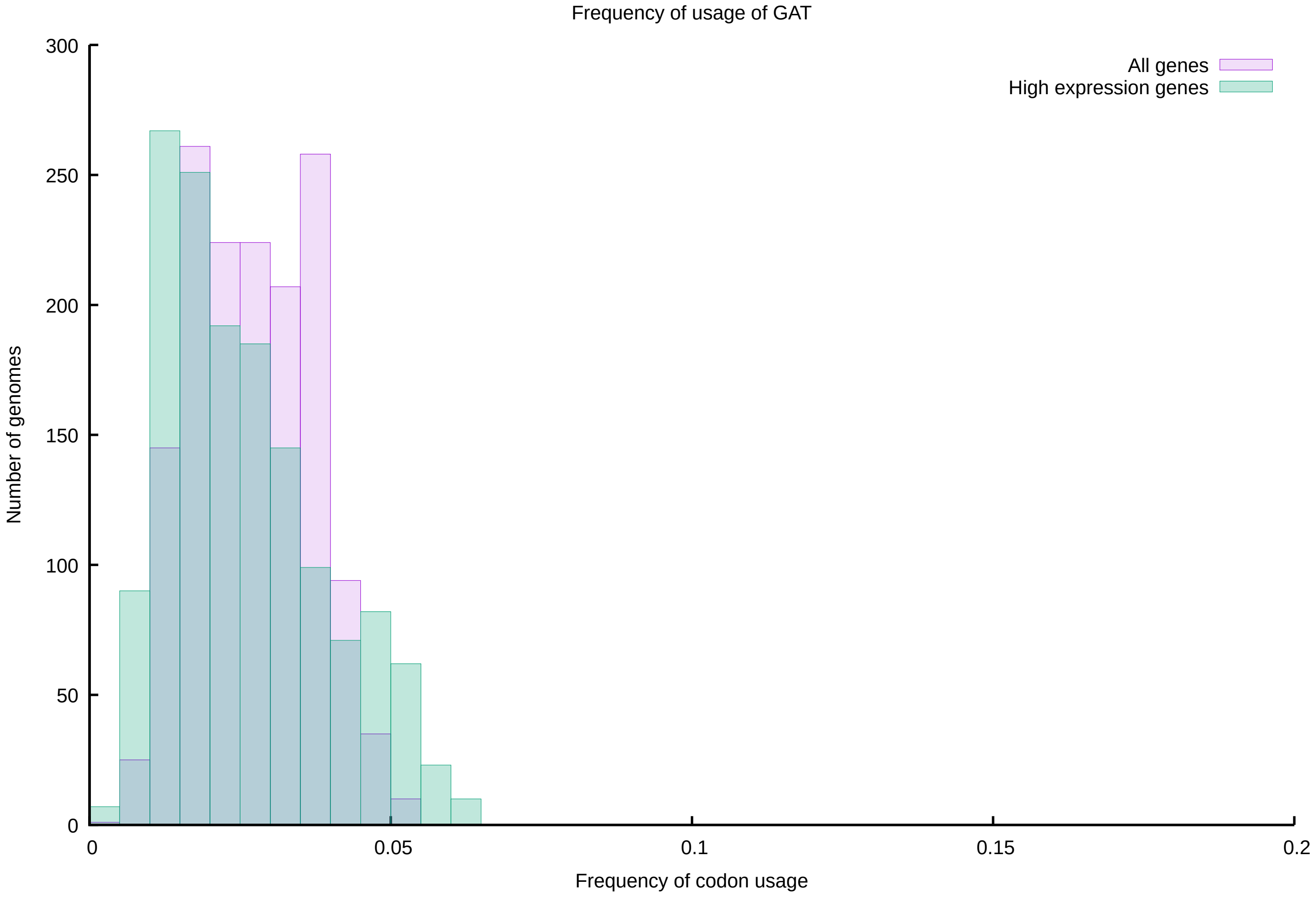
Frequency of usage of GAT
All genes
High expression genes
Number of genomes
Frequency of codon usage
0
50
100
150
200
250
300
0
0.05
0.1
0.15
0.2

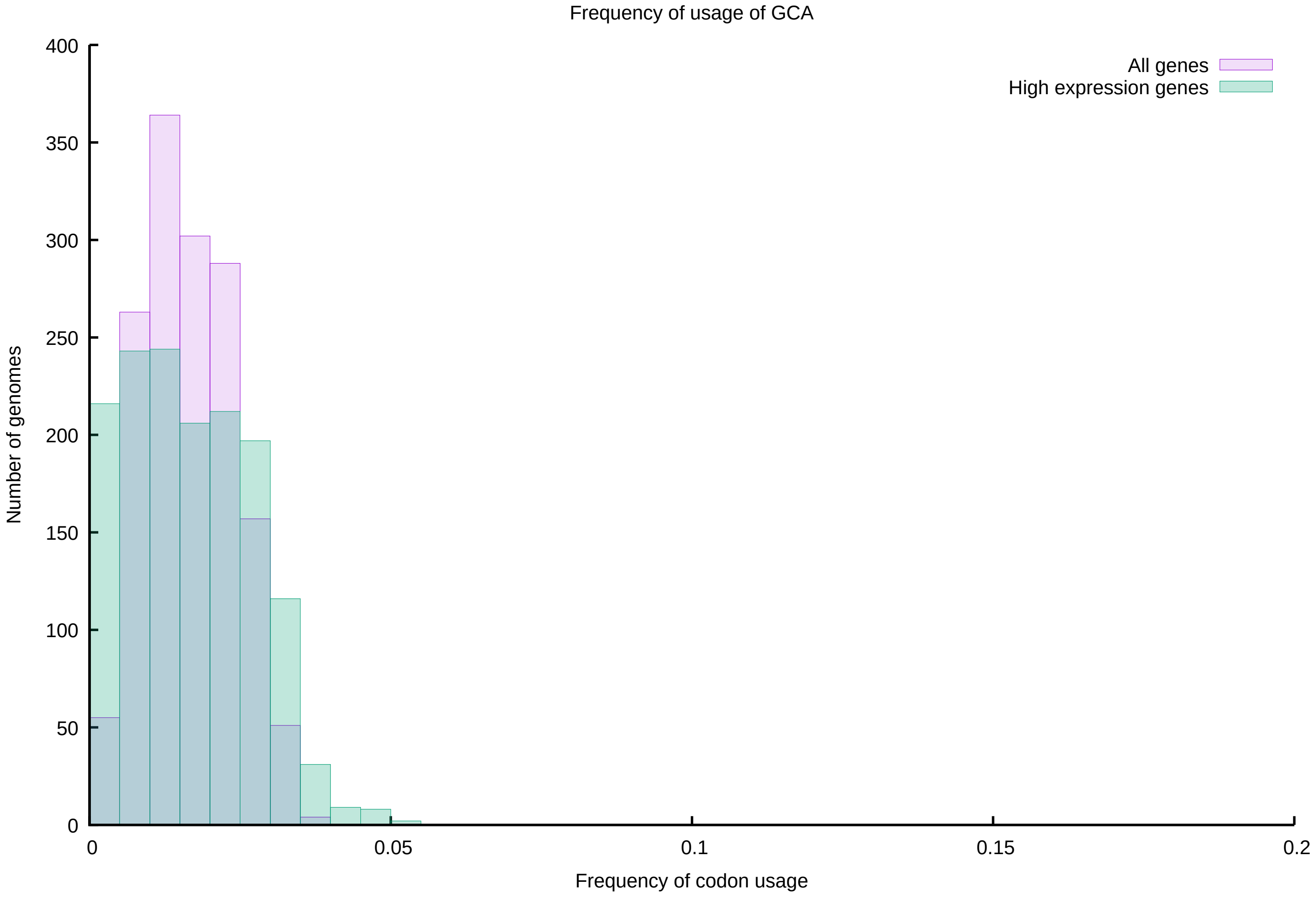

Frequency of usage of GCA
All genes
High expression genes
Number of genomes
400
350
300
250
200
150
100
50
0
0
0.05
0.1
0.15
0.2
Frequency of codon usage

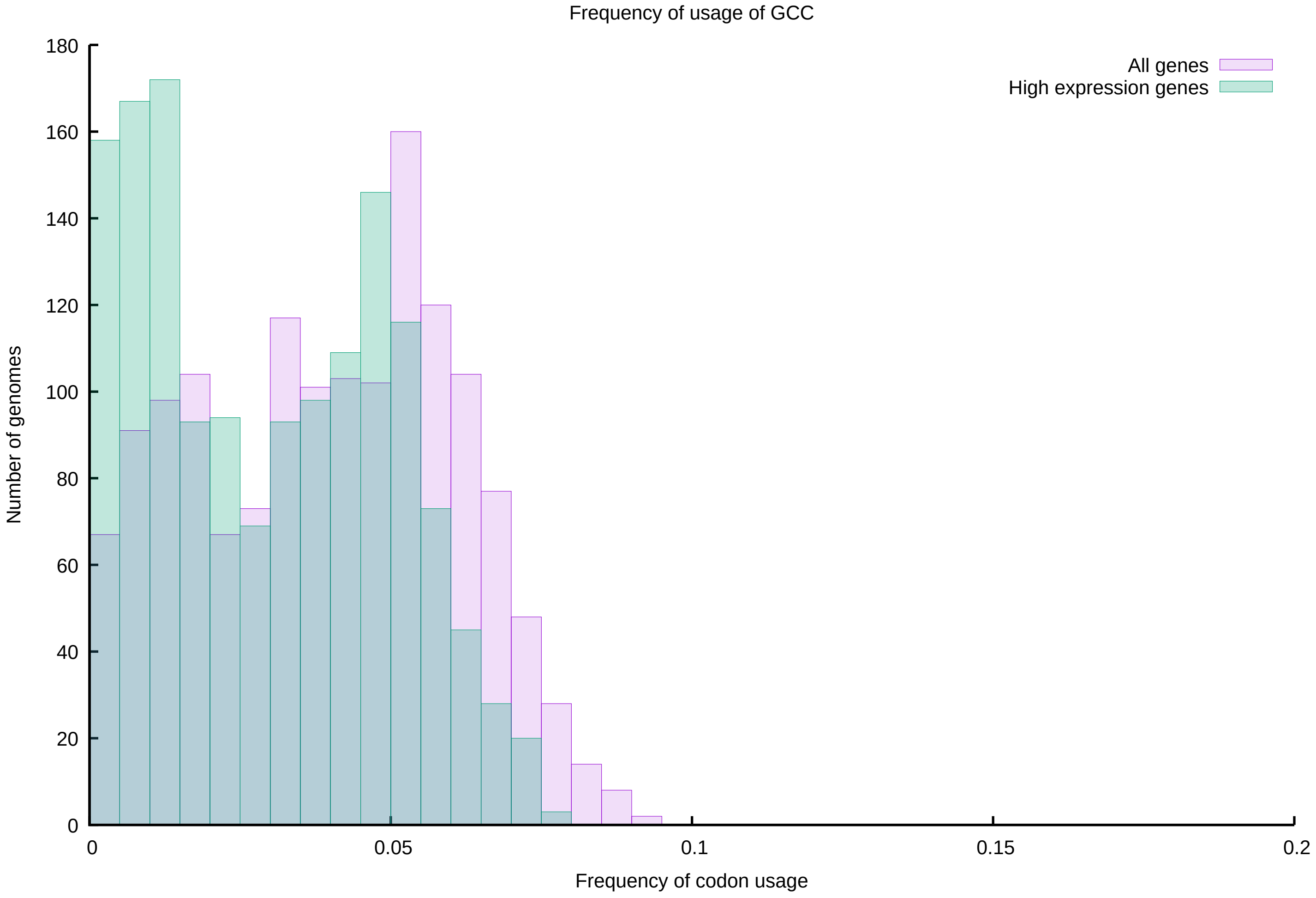

Frequency of usage of GCC
All genes
High expression genes
Number of genomes
Frequency of codon usage
0
20
40
60
80
100
120
140
160
180
0
0.05
0.1
0.15
0.2

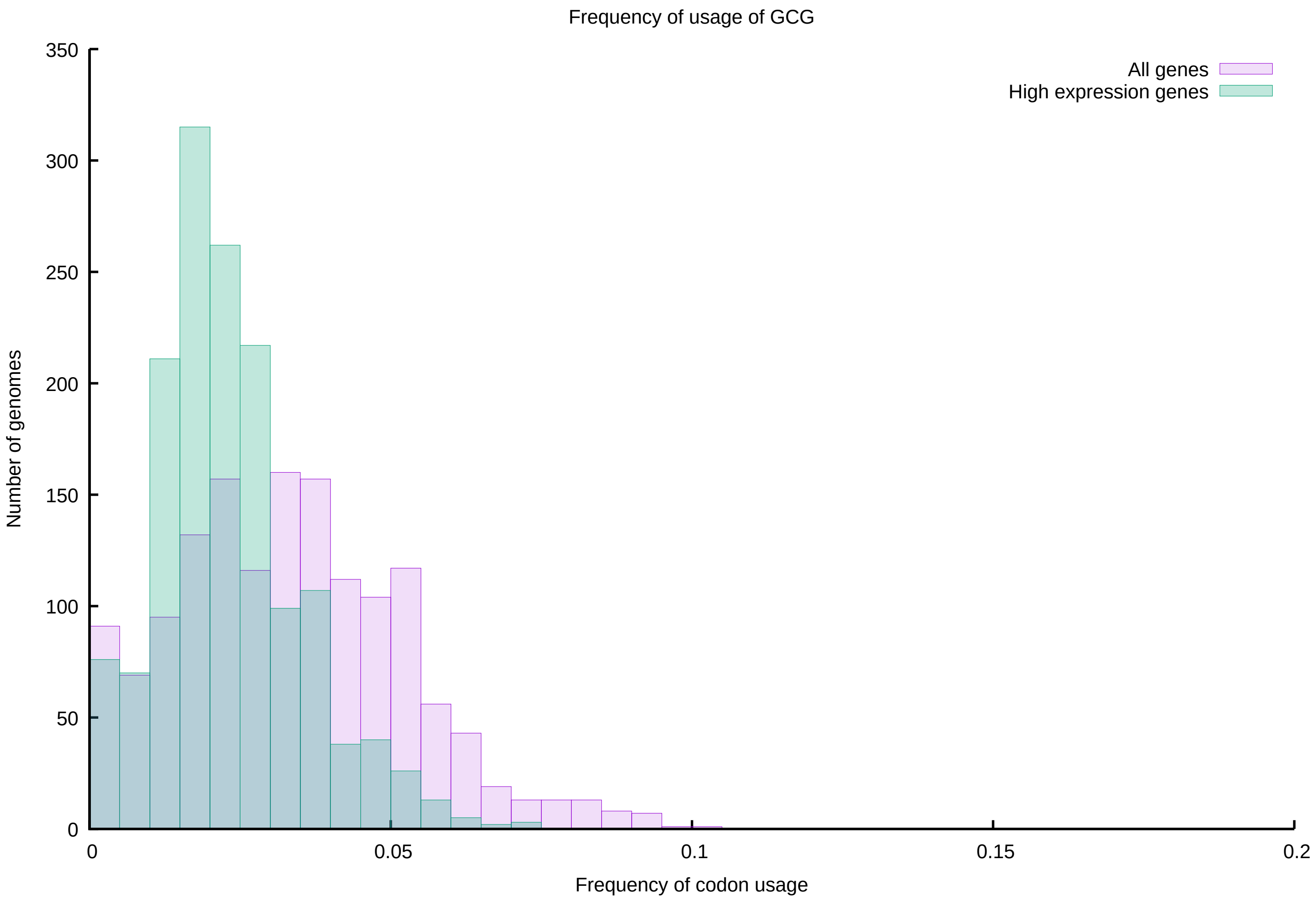
Frequency of usage of GCG
All genes
High expression genes
Number of genomes
350
300
250
200
150
100
50
0
0
0.05
0.1
0.15
0.2
Frequency of codon usage

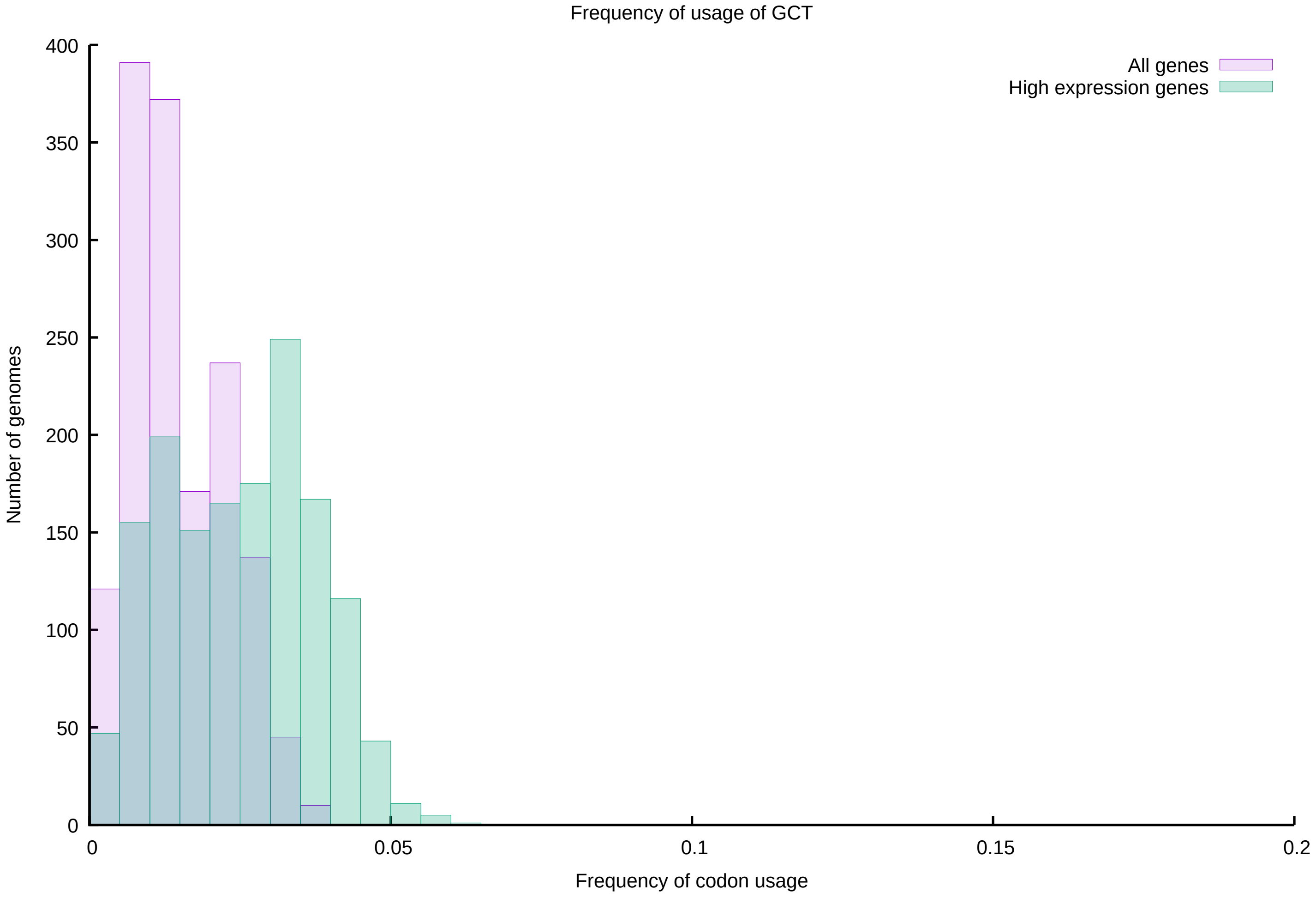
Frequency of usage of GCT
All genes
High expression genes
Number of genomes
400
350
300
250
200
150
100
50
0
0
0.05
0.1
0.15
0.2
Frequency of codon usage

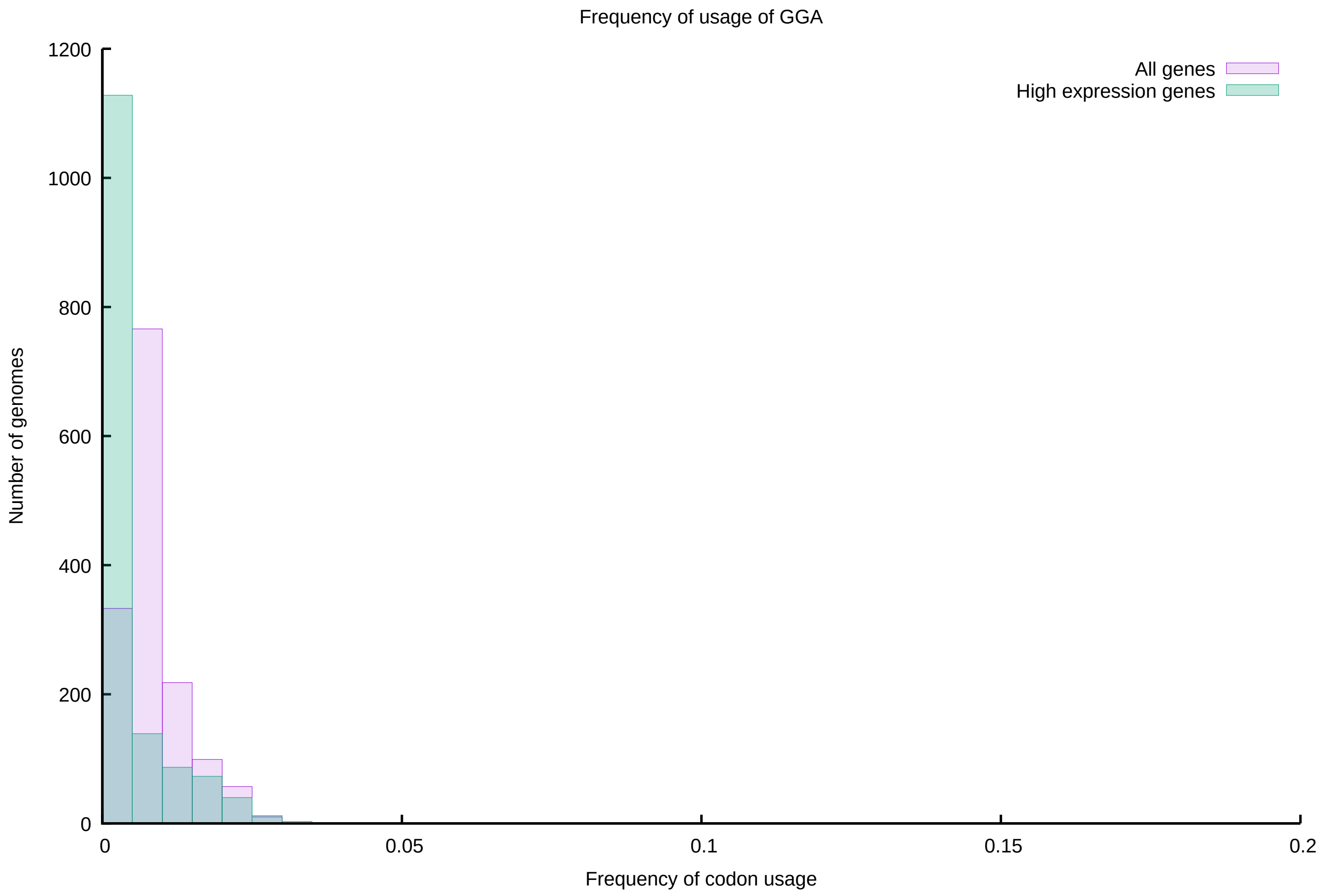

Frequency of usage of GGA
All genes
High expression genes
Number of genomes
Frequency of codon usage
0
200
400
600
800
1000
1200
0
0.05
0.1
0.15
0.2

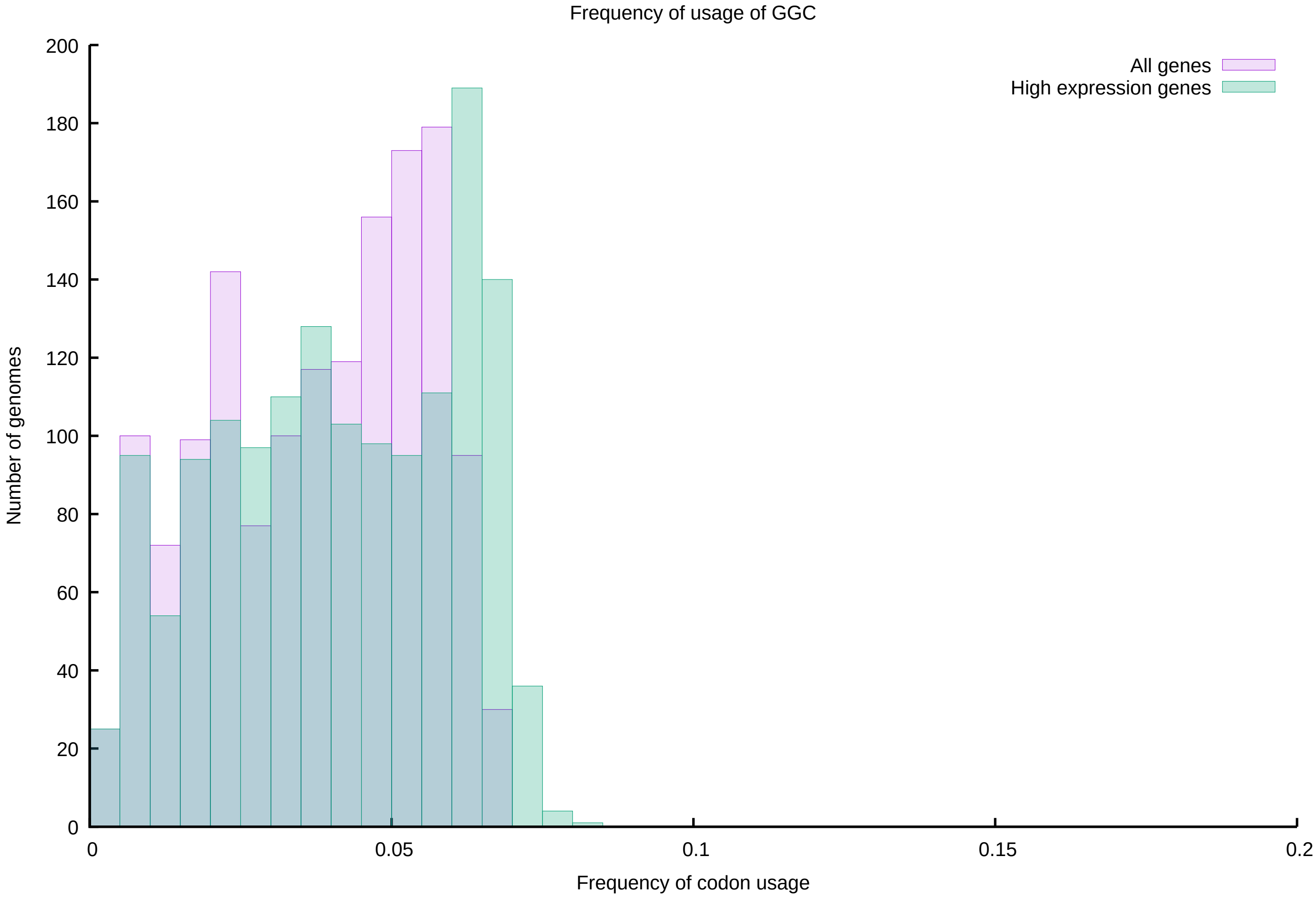

Frequency of usage of GGC
All genes
High expression genes
Number of genomes
Frequency of codon usage
0
20
40
60
80
100
120
140
160
180
200
0
0.05
0.1
0.15
0.2

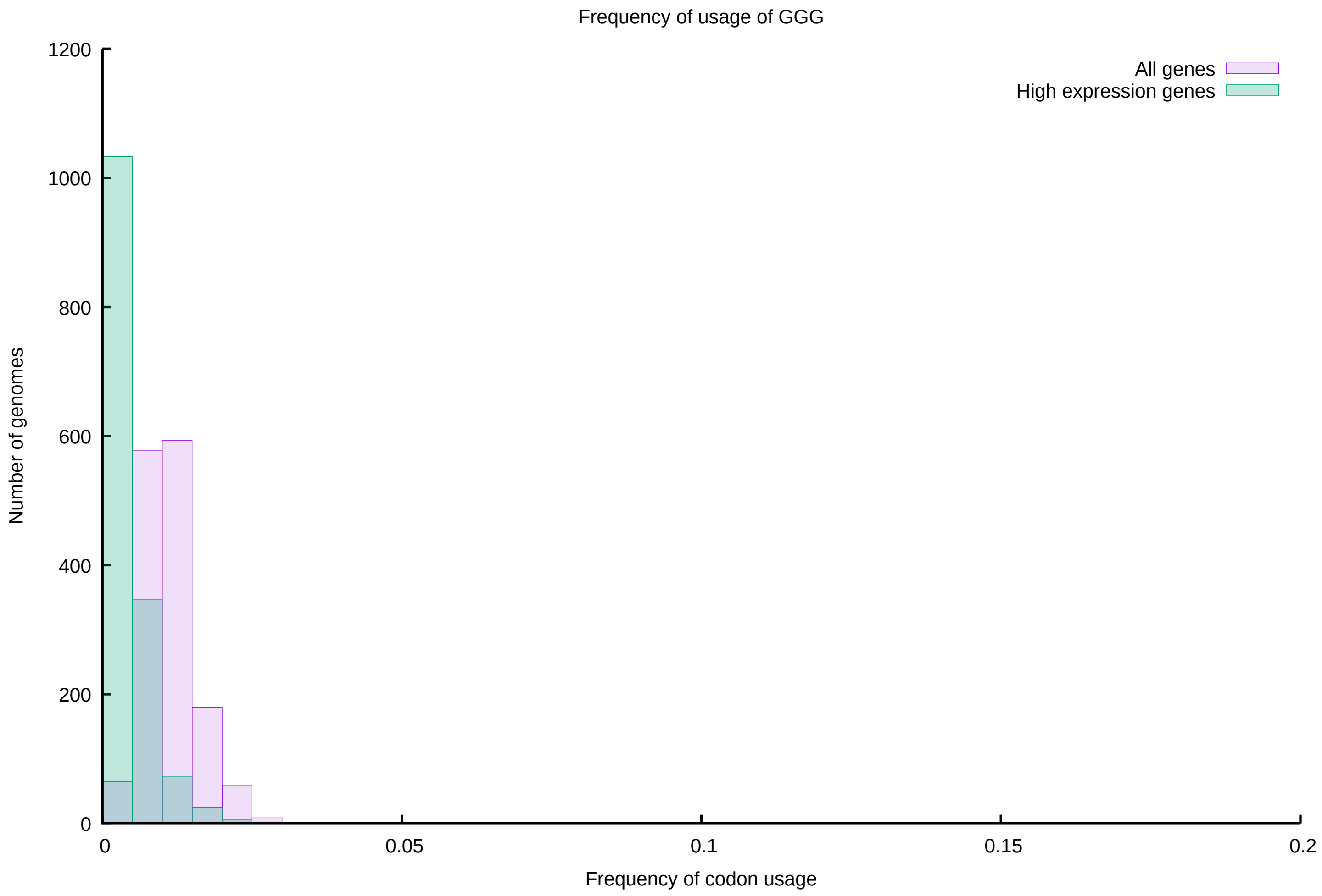
Frequency of usage of GGG
All genes
High expression genes
Number of genomes
1200
1000
800
600
400
200
0
0
0.05
0.1
0.15
0.2
Frequency of codon usage

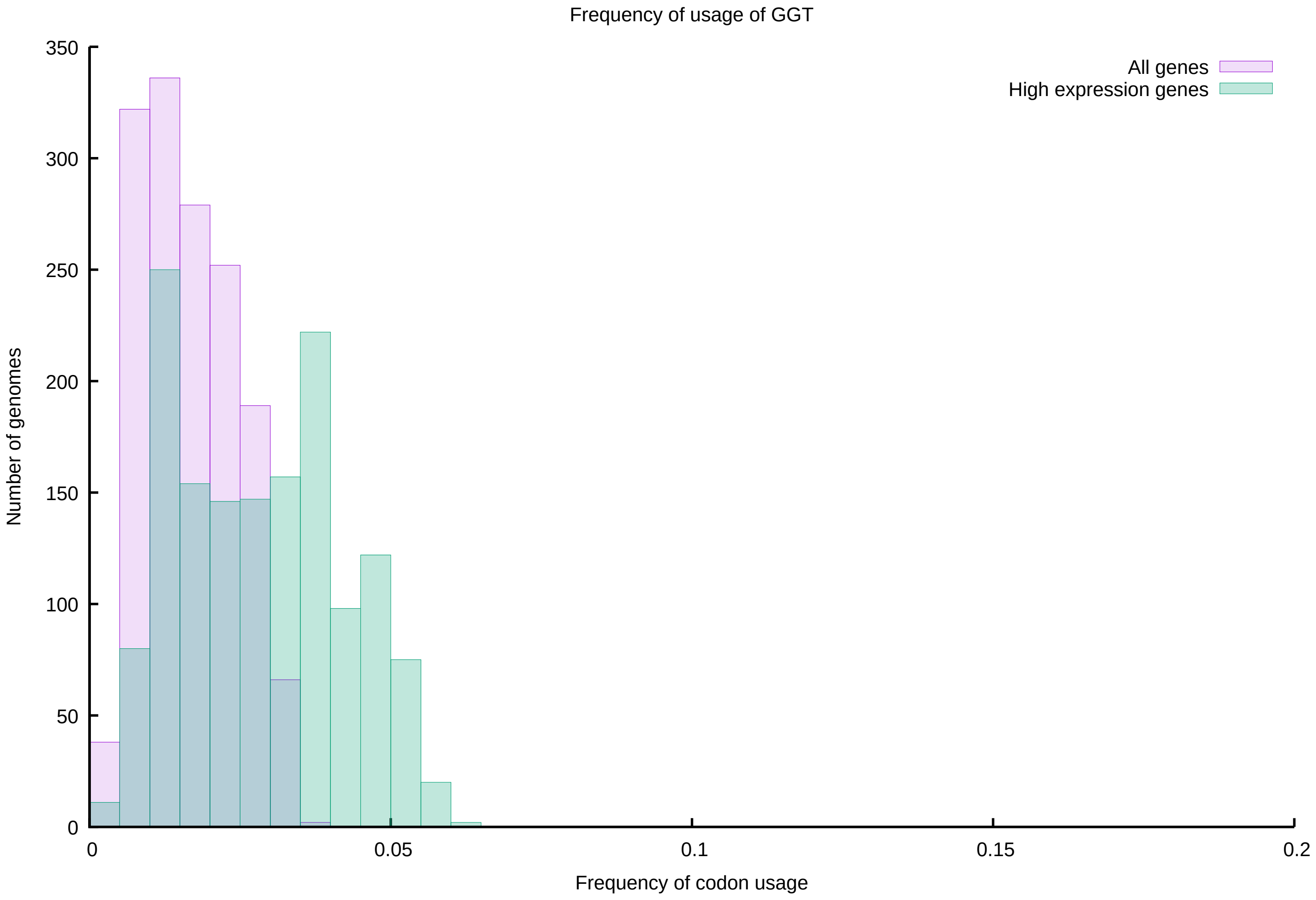
Frequency of usage of GGT
All genes
High expression genes
Number of genomes
0
50
100
150
200
250
300
350
0
0.05
0.1
0.15
0.2
Frequency of codon usage

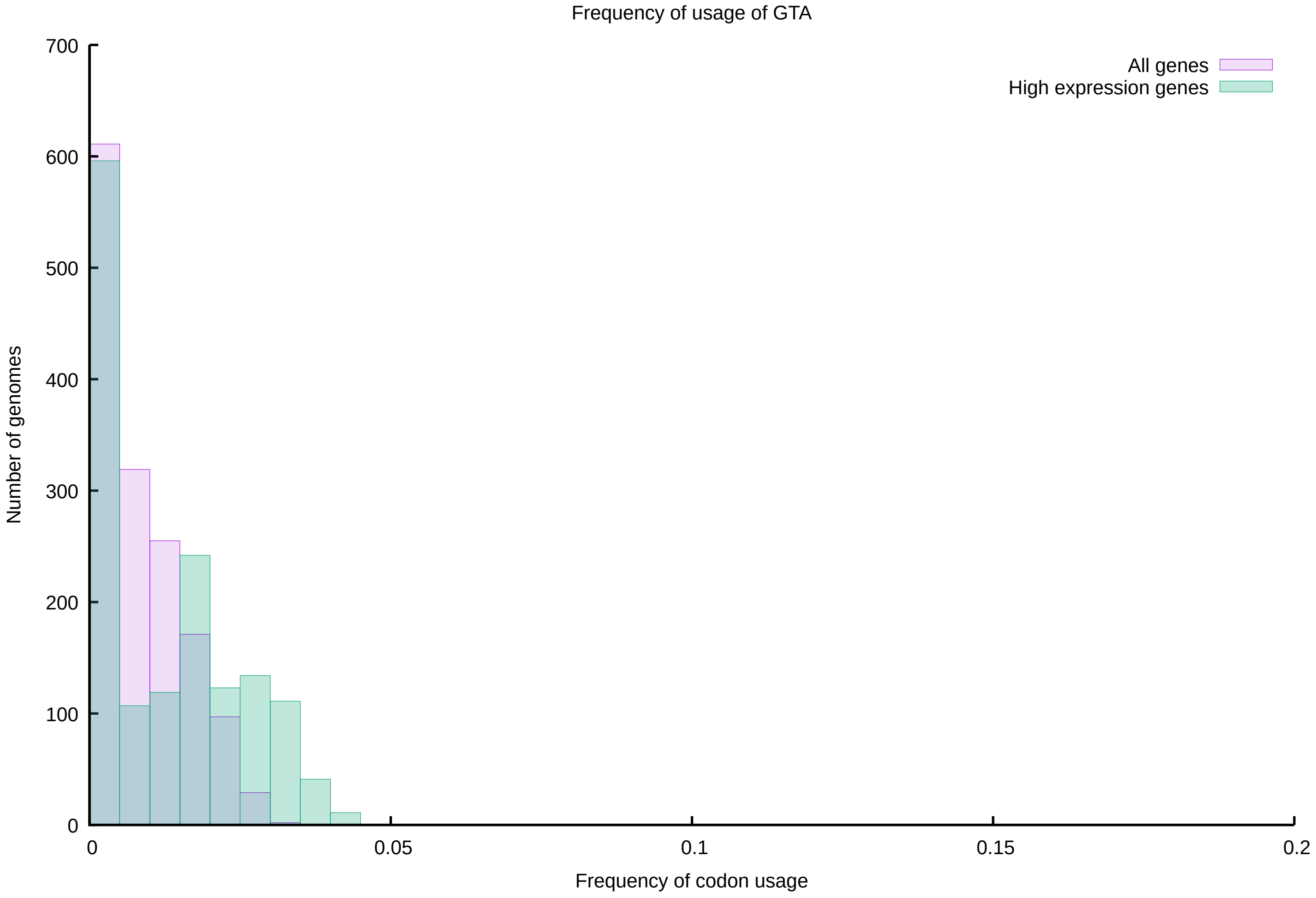

Frequency of usage of GTA
All genes
High expression genes
Number of genomes
0
100
200
300
400
500
600
700
0
0.05
0.1
0.15
0.2
Frequency of codon usage

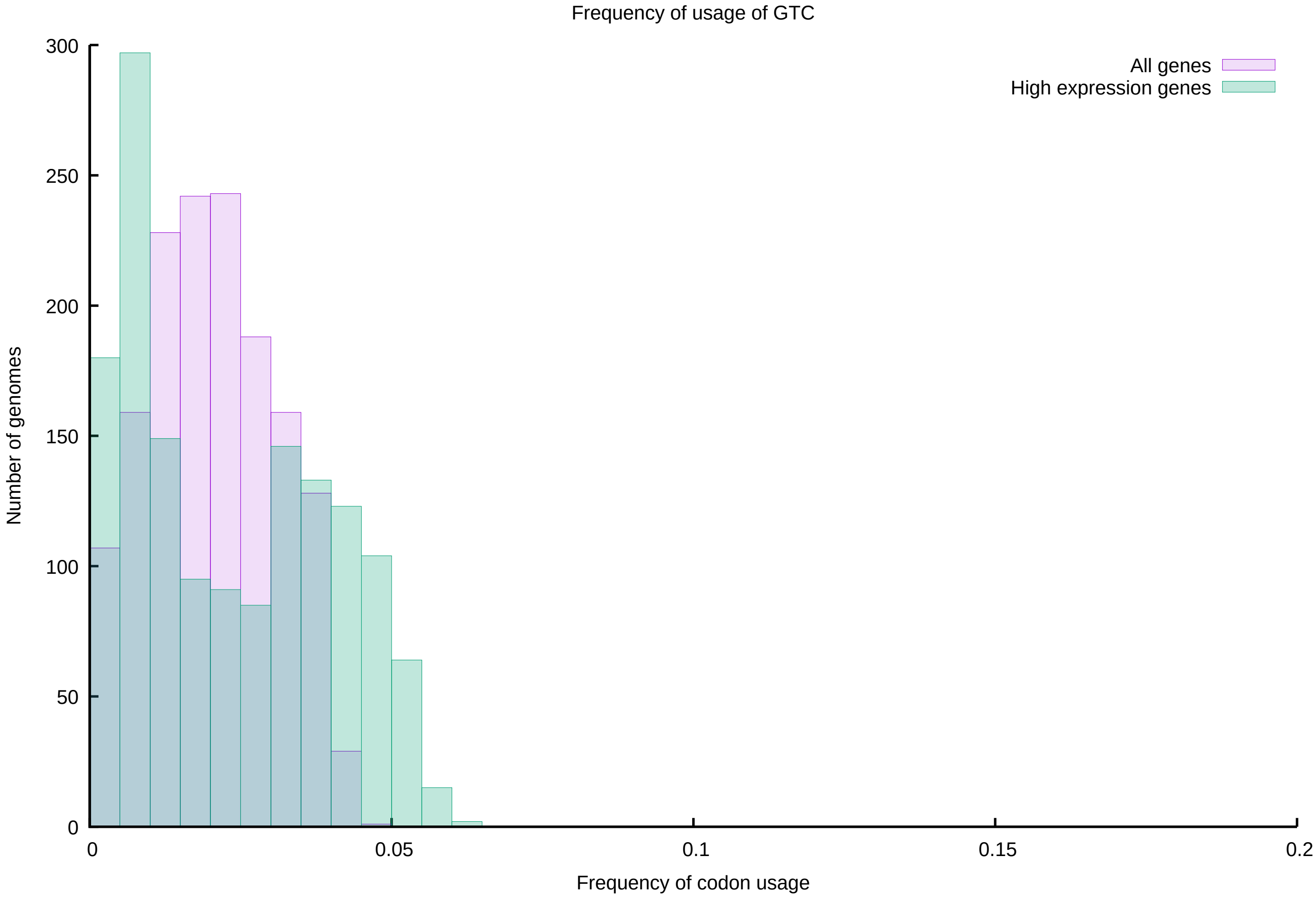
Frequency of usage of GTC
All genes
High expression genes
Number of genomes
Frequency of codon usage
300
250
200
150
100
50
0
0
0.05
0.1
0.15
0.2

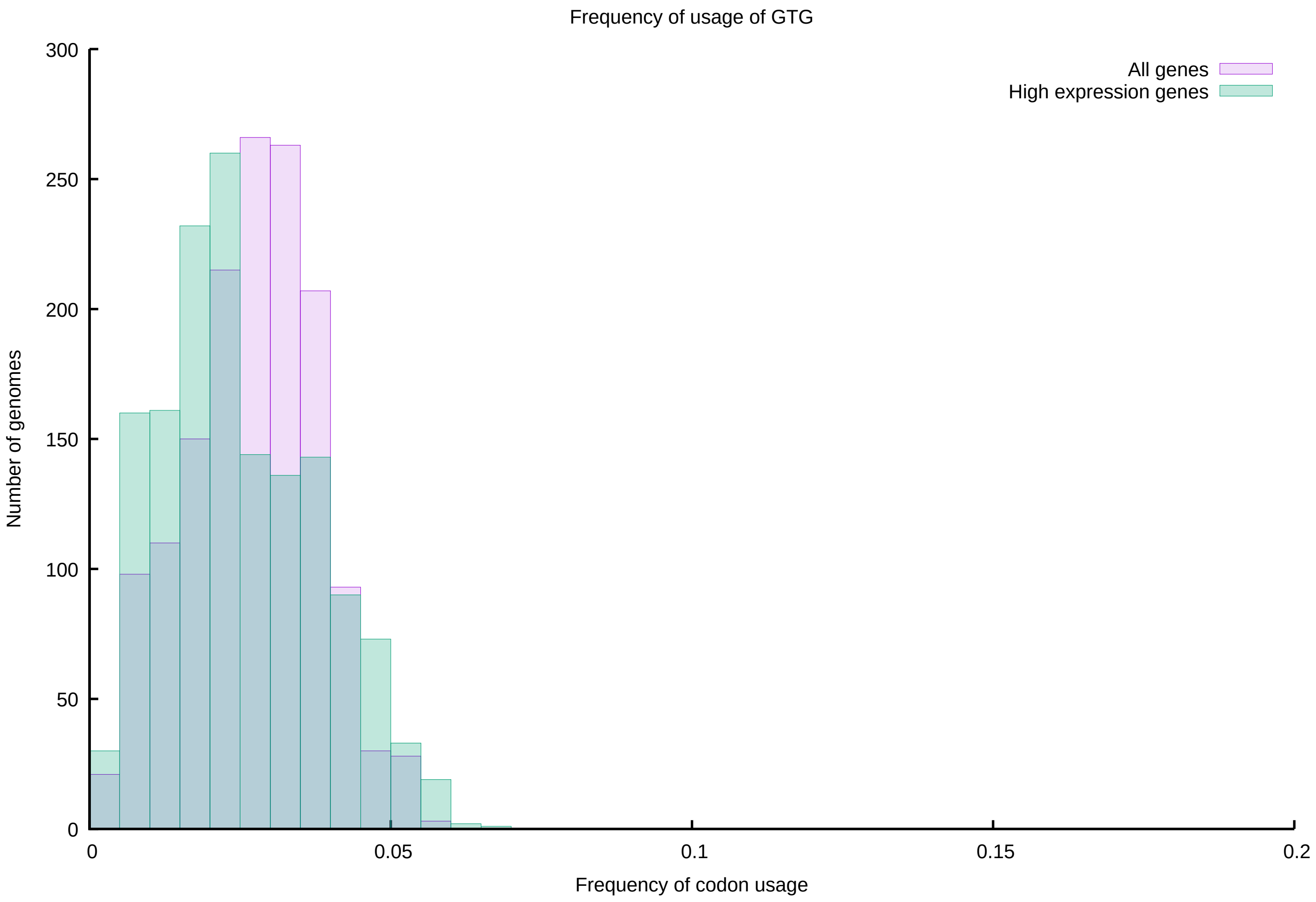

Frequency of usage of GTG
All genes
High expression genes
Number of genomes
Frequency of codon usage
300
250
200
150
100
50
0
0
0.05
0.1
0.15
0.2

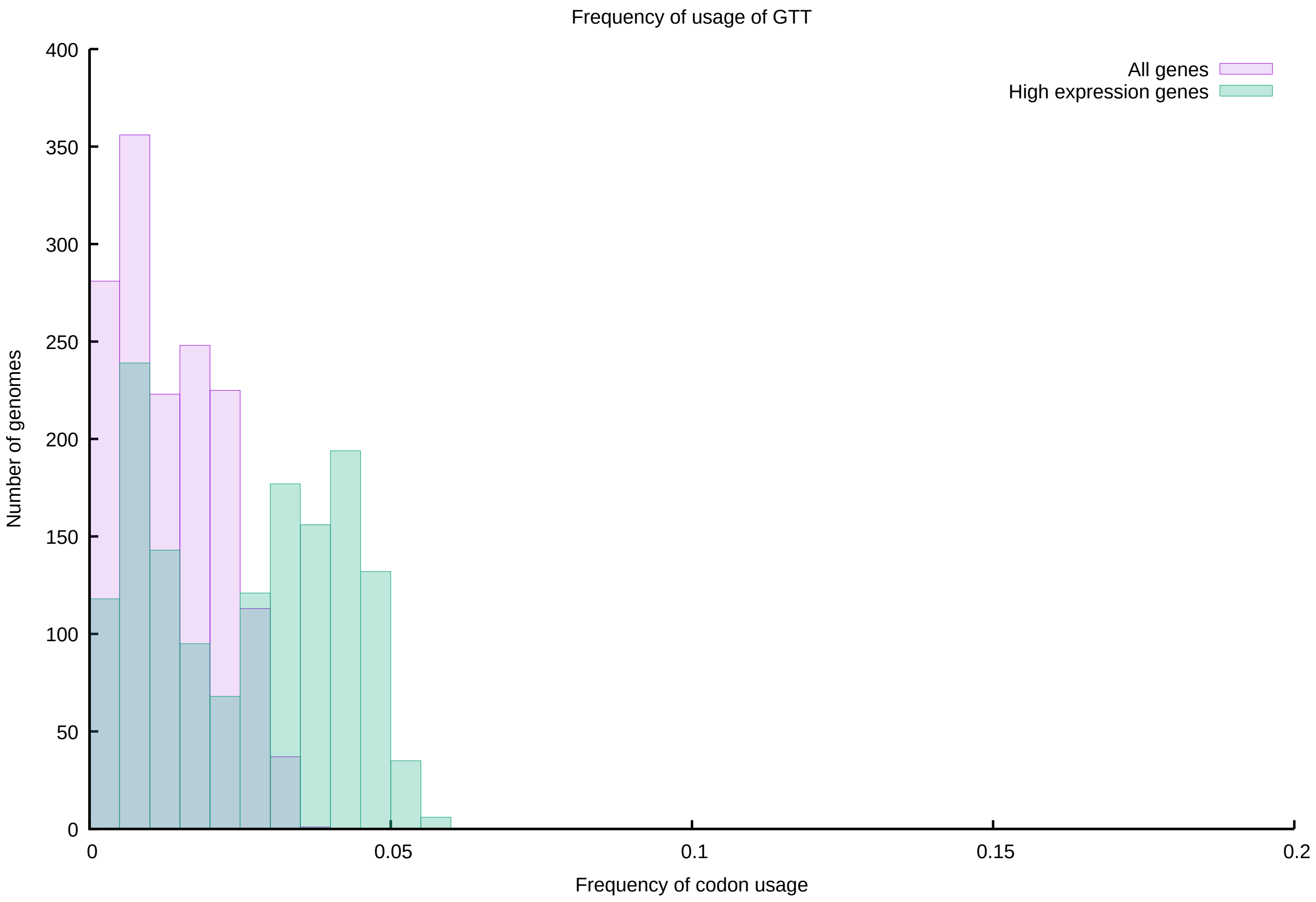
Frequency of usage of GTT
All genes
High expression genes
Number of genomes
400
350
300
250
200
150
100
50
0
0
0.05
0.1
0.15
0.2
Frequency of codon usage

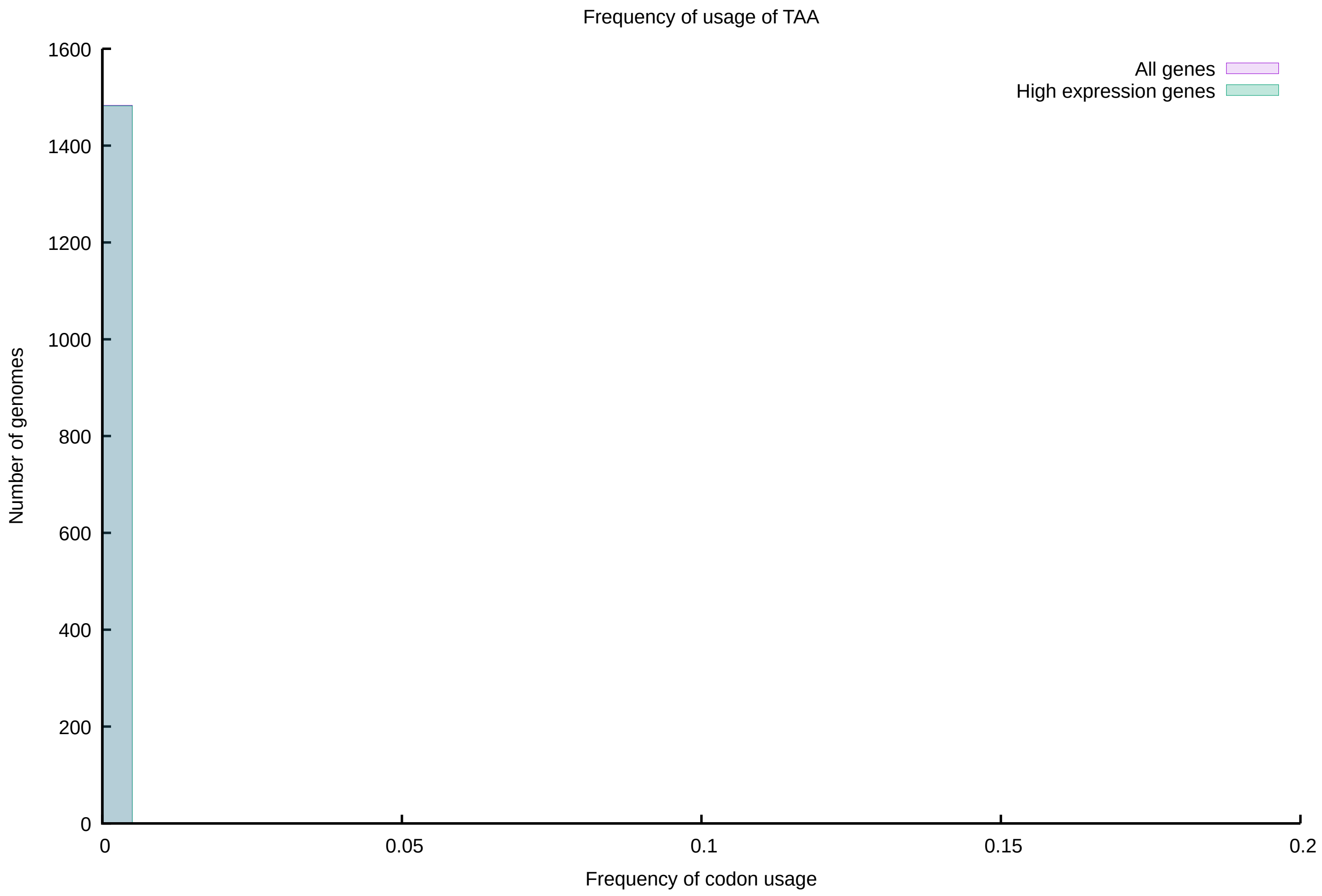

Frequency of usage of TAA
All genes
High expression genes
Number of genomes
Frequency of codon usage
0
200
400
600
800
1000
1200
1400
1600
0.05
0.1
0.15
0.2

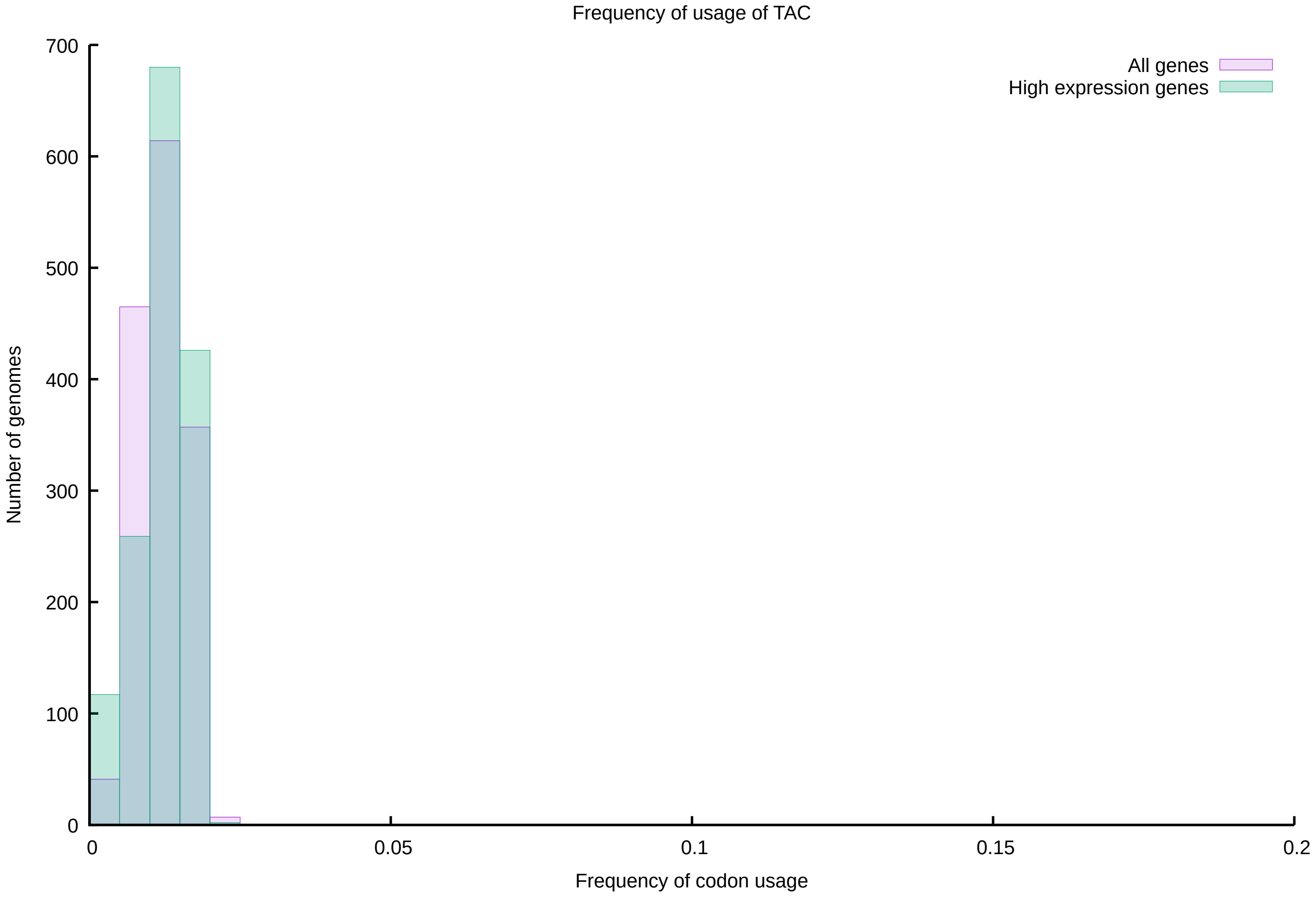

Frequency of usage of TAC
All genes
High expression genes
Number of genomes
Frequency of codon usage
0
100
200
300
400
500
600
700
0
0.05
0.1
0.15
0.2

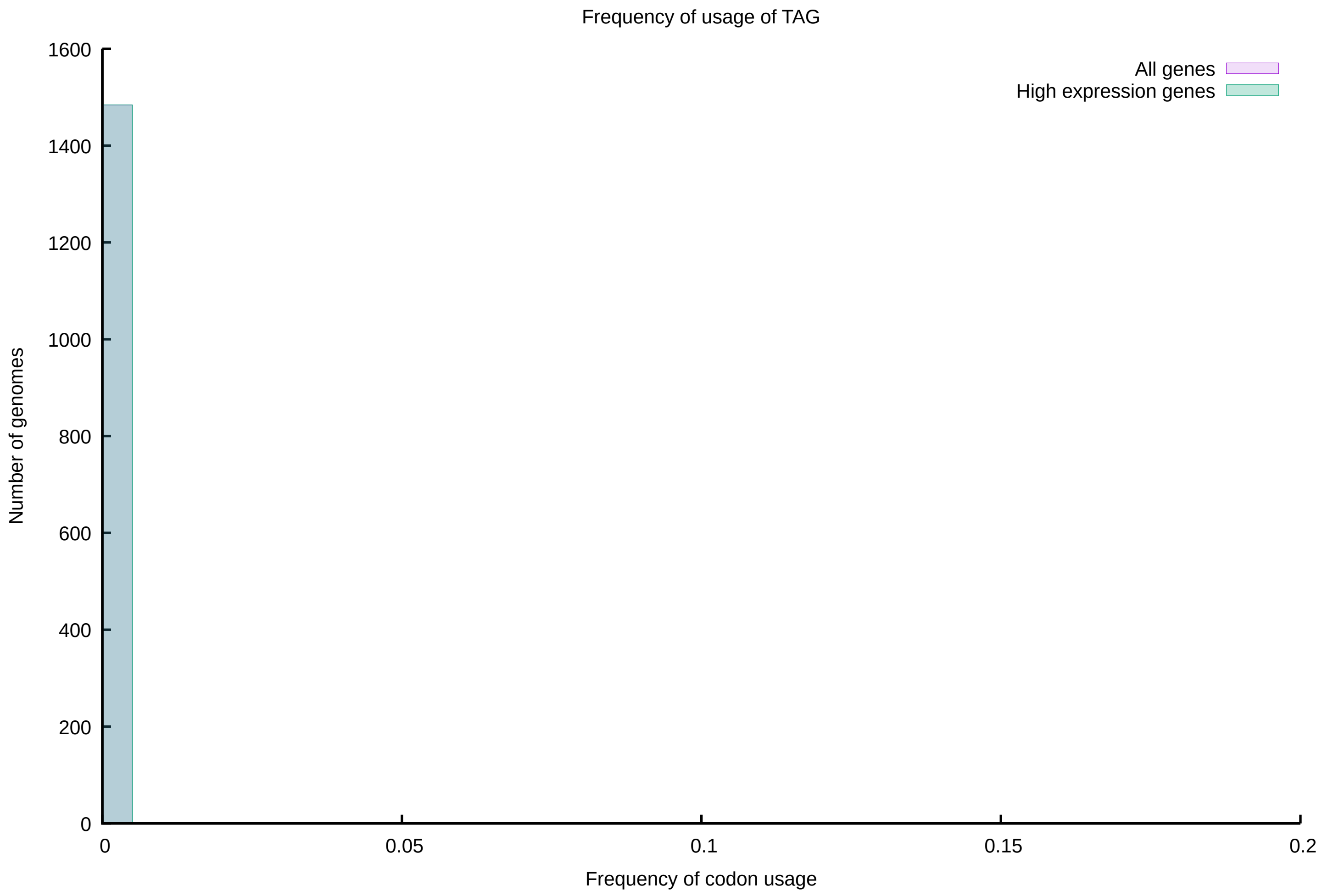
Frequency of usage of TAG
All genes
High expression genes
Number of genomes
1600
1400
1200
1000
800
600
400
200
0
0
0.05
0.1
0.15
0.2
Frequency of codon usage

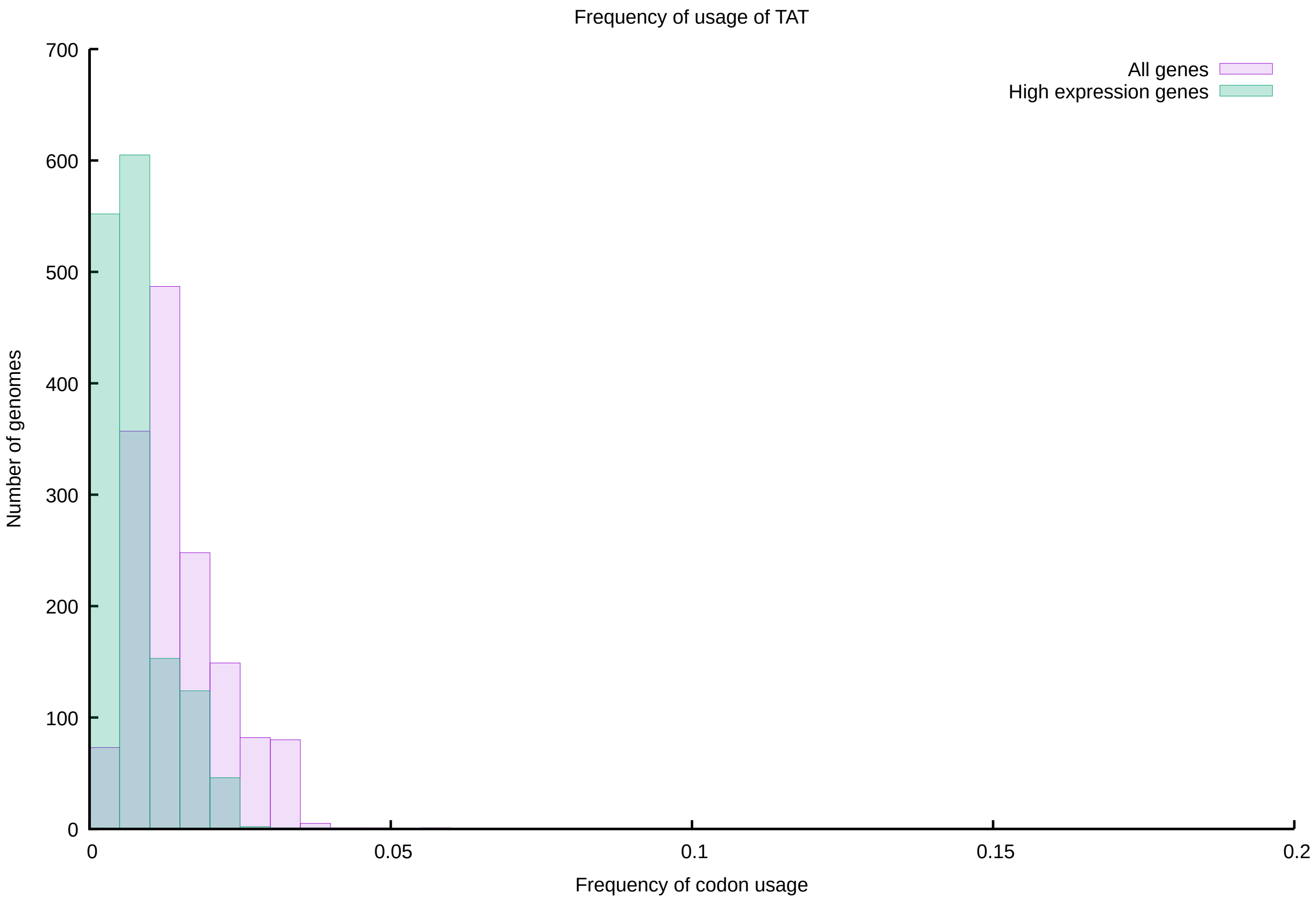
Frequency of usage of TAT
All genes
High expression genes
Number of genomes
0
100
200
300
400
500
600
700
0
0.05
0.1
0.15
0.2
Frequency of codon usage

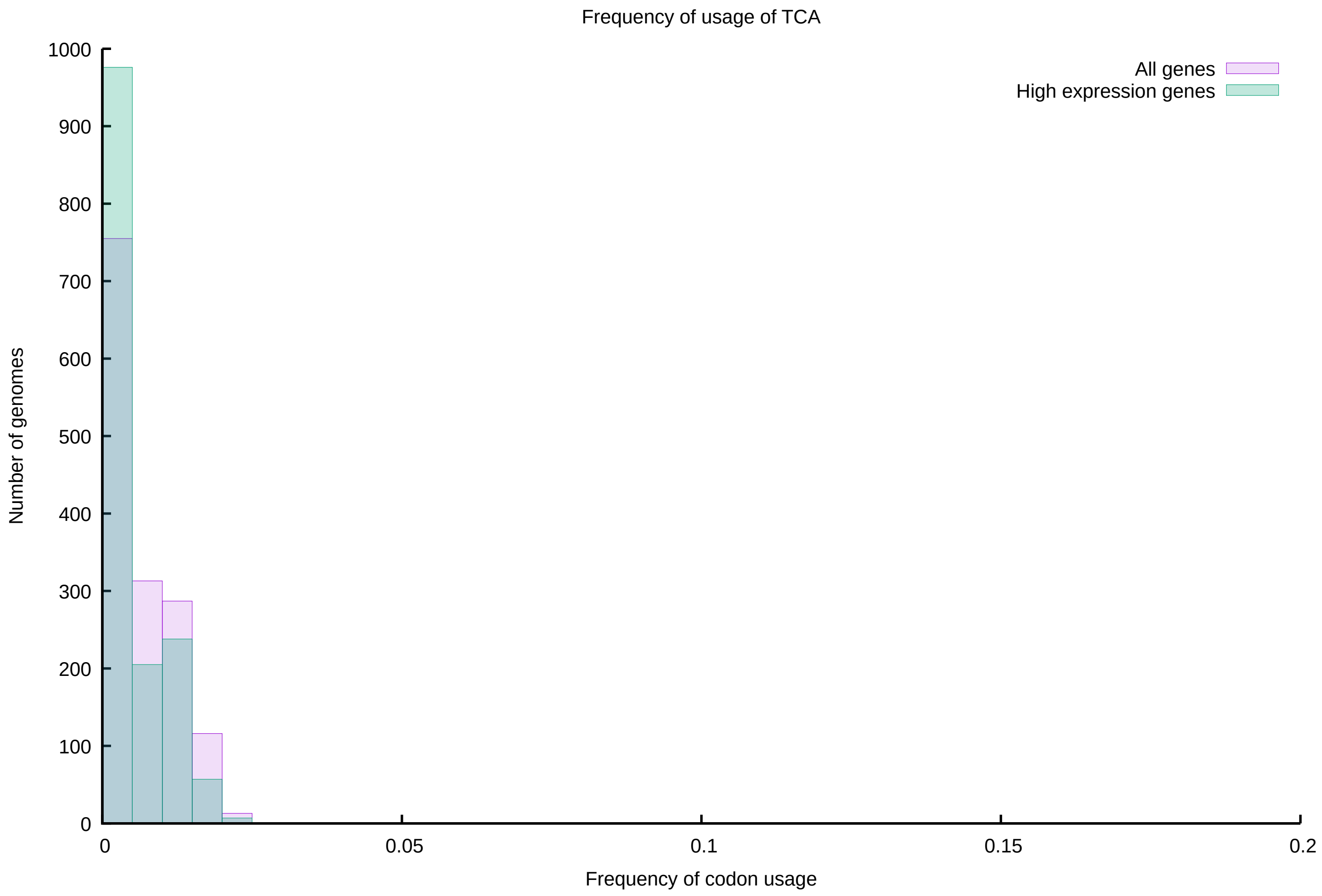
Frequency of usage of TCA
All genes
High expression genes
Number of genomes
Frequency of codon usage
0
100
200
300
400
500
600
700
800
900
1000
0
0.05
0.1
0.15
0.2

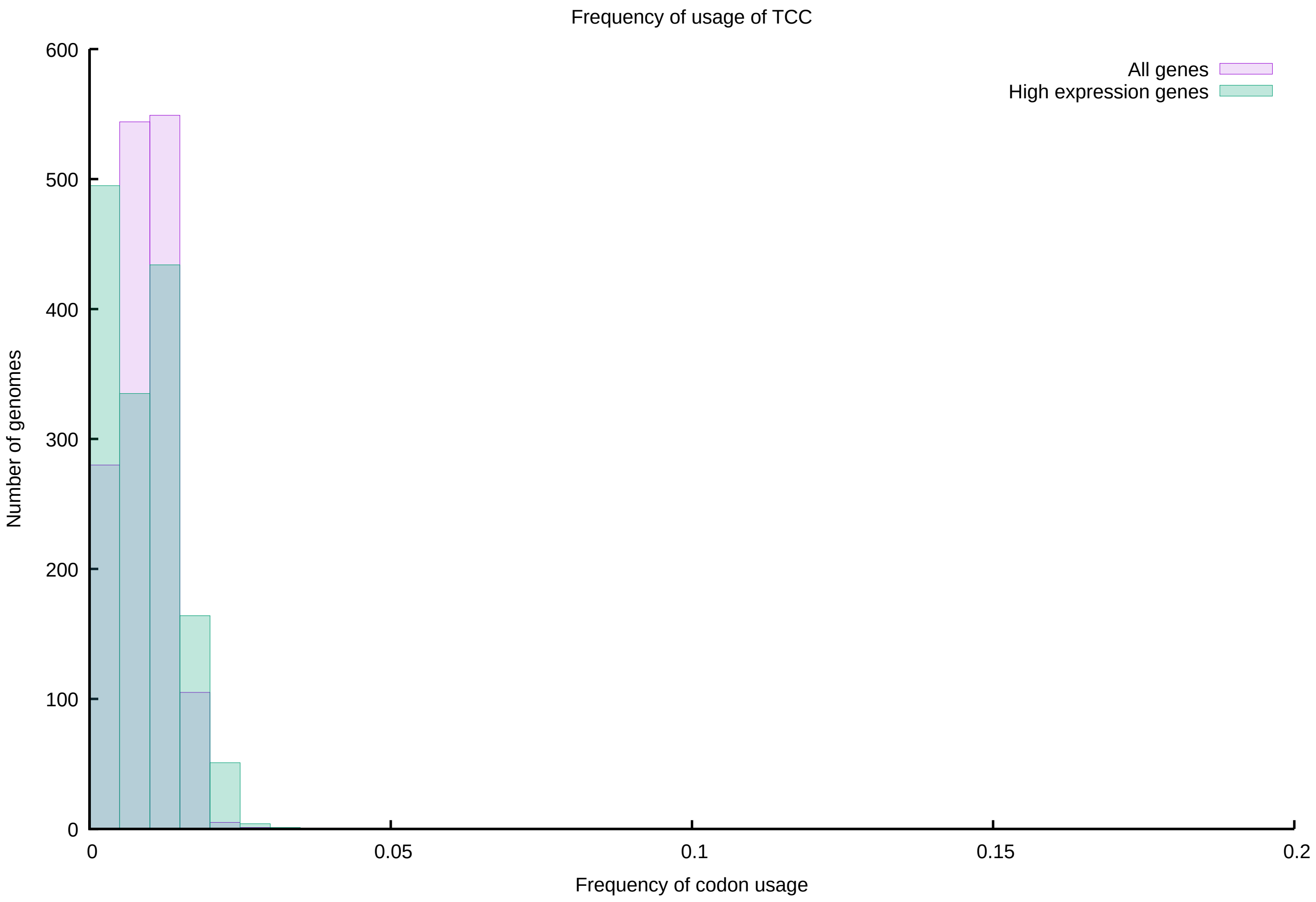

Frequency of usage of TCC
All genes
High expression genes
Number of genomes
0
100
200
300
400
500
600
0
0.05
0.1
0.15
0.2
Frequency of codon usage

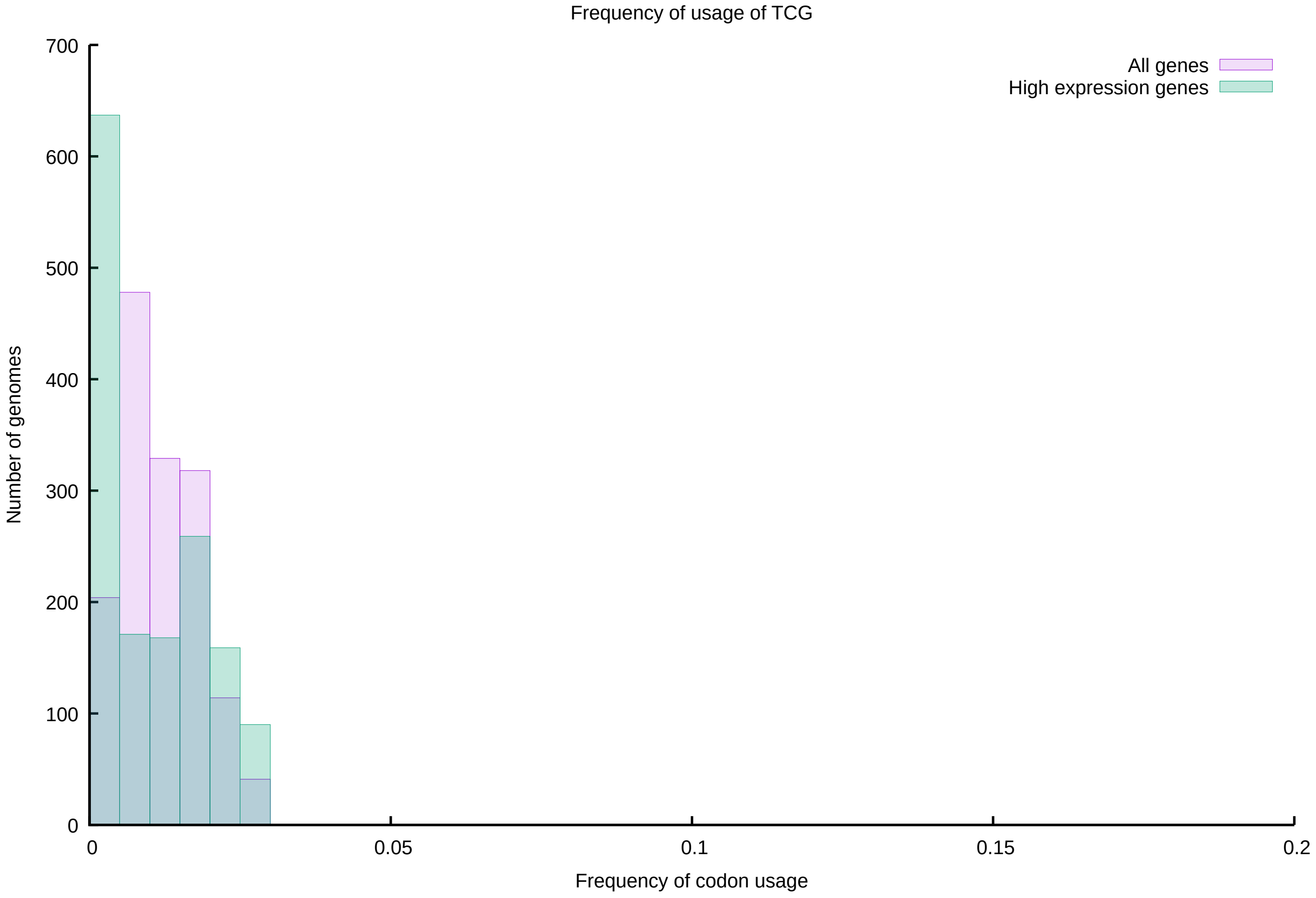
Frequency of usage of TCG
All genes
High expression genes
Number of genomes
0
100
200
300
400
500
600
700
0
0.05
0.1
0.15
0.2
Frequency of codon usage

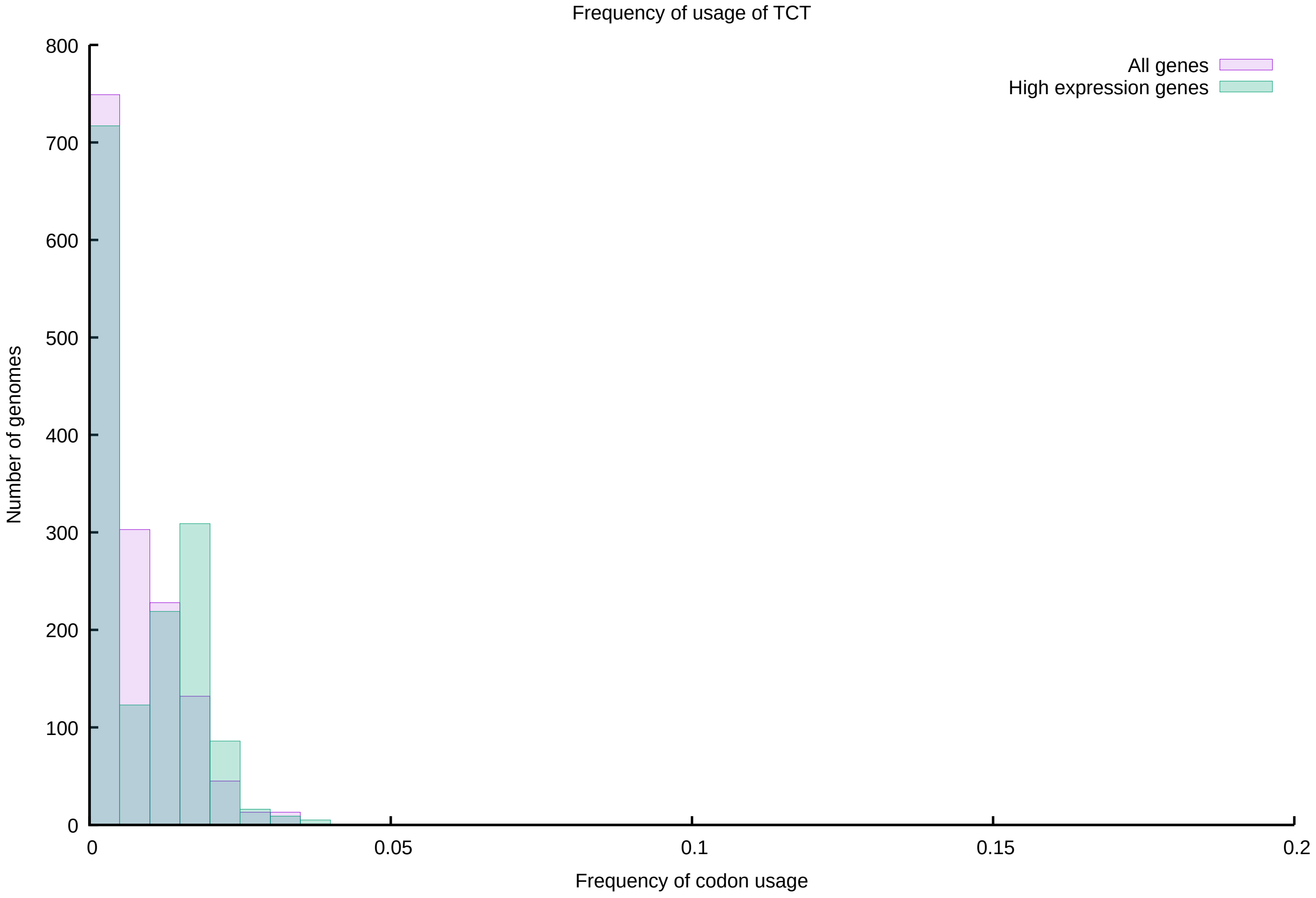

Frequency of usage of TCT
All genes
High expression genes
Number of genomes
Frequency of codon usage
0
100
200
300
400
500
600
700
800
0
0.05
0.1
0.15
0.2

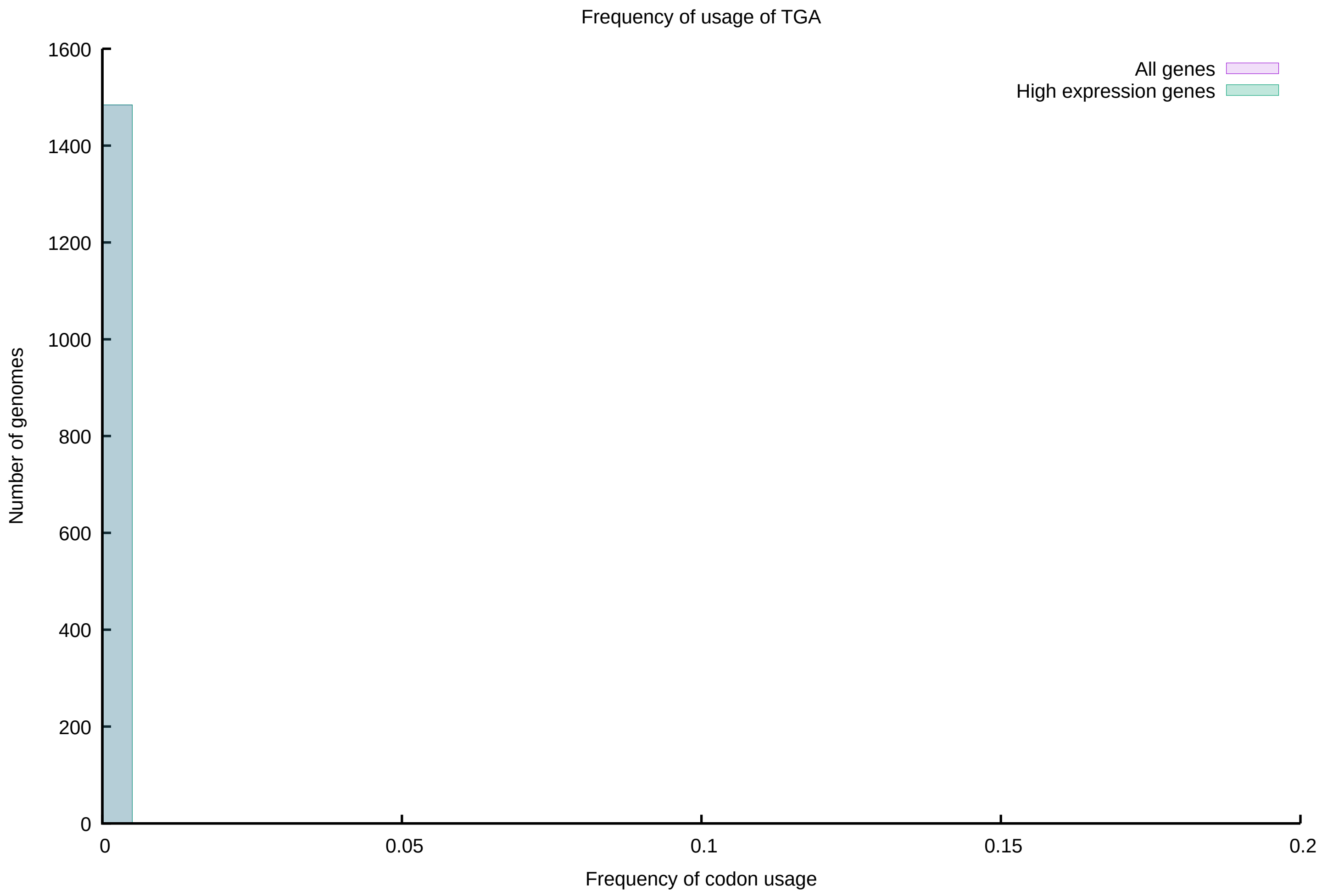
Frequency of usage of TGA
All genes
High expression genes
Number of genomes
Frequency of codon usage
0
200
400
600
800
1000
1200
1400
1600
0
0.05
0.1
0.15
0.2

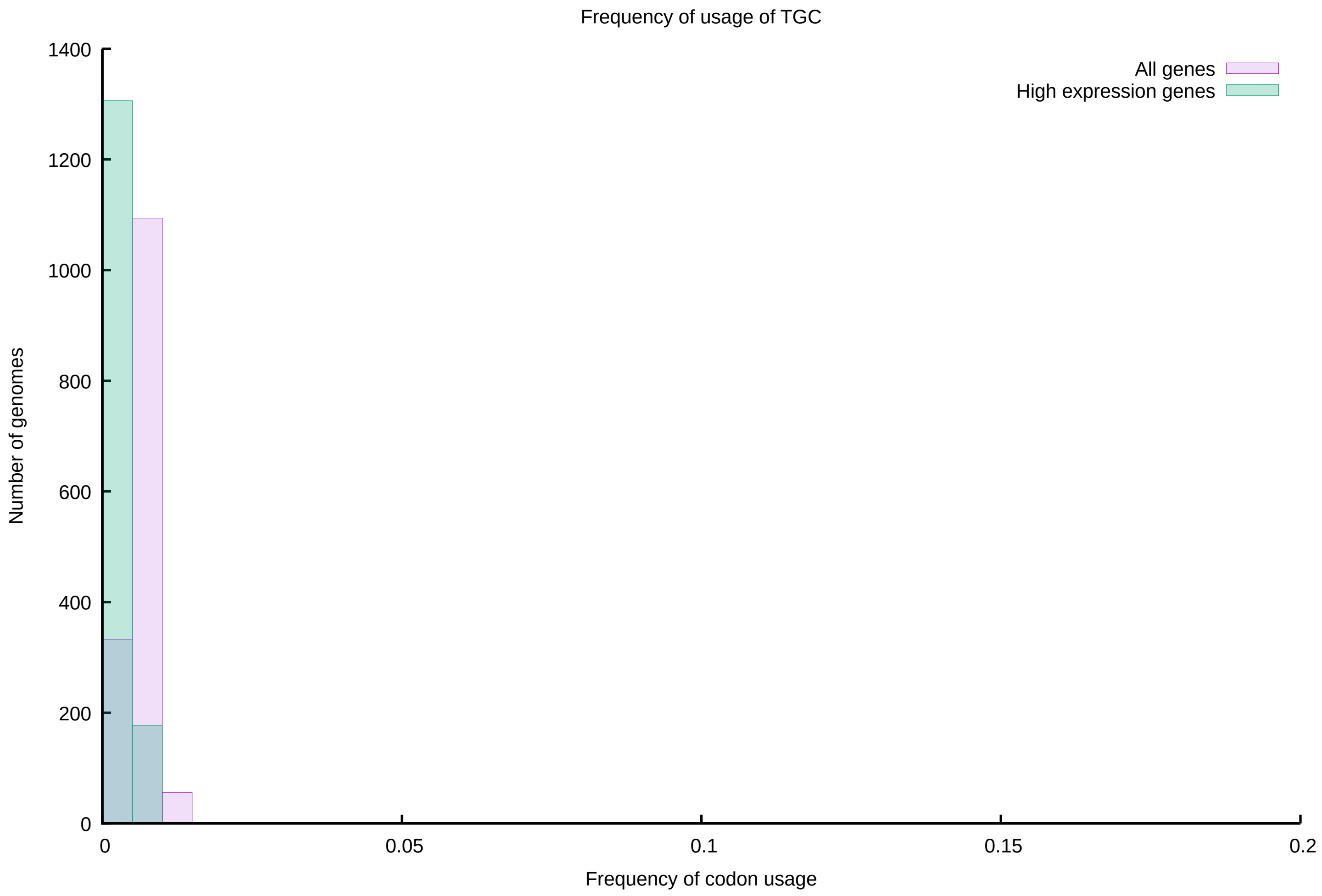
Frequency of usage of TGC
All genes
High expression genes
Number of genomes
Frequency of codon usage
0
200
400
600
800
1000
1200
1400
0
0.05
0.1
0.15
0.2

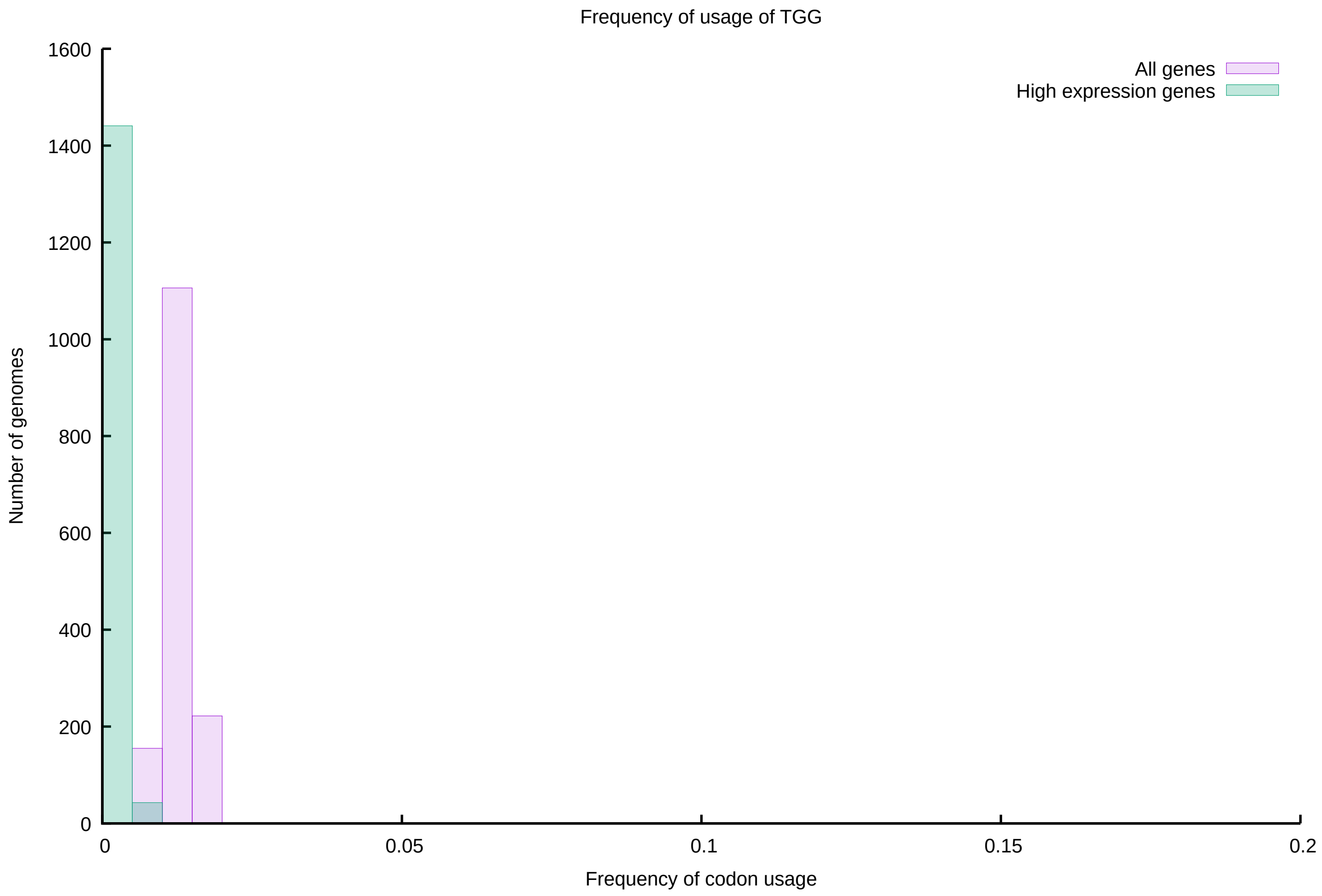

Frequency of usage of TGG
All genes
High expression genes
Number of genomes
Frequency of codon usage
0
200
400
600
800
1000
1200
1400
1600
0.05
0.1
0.15
0.2

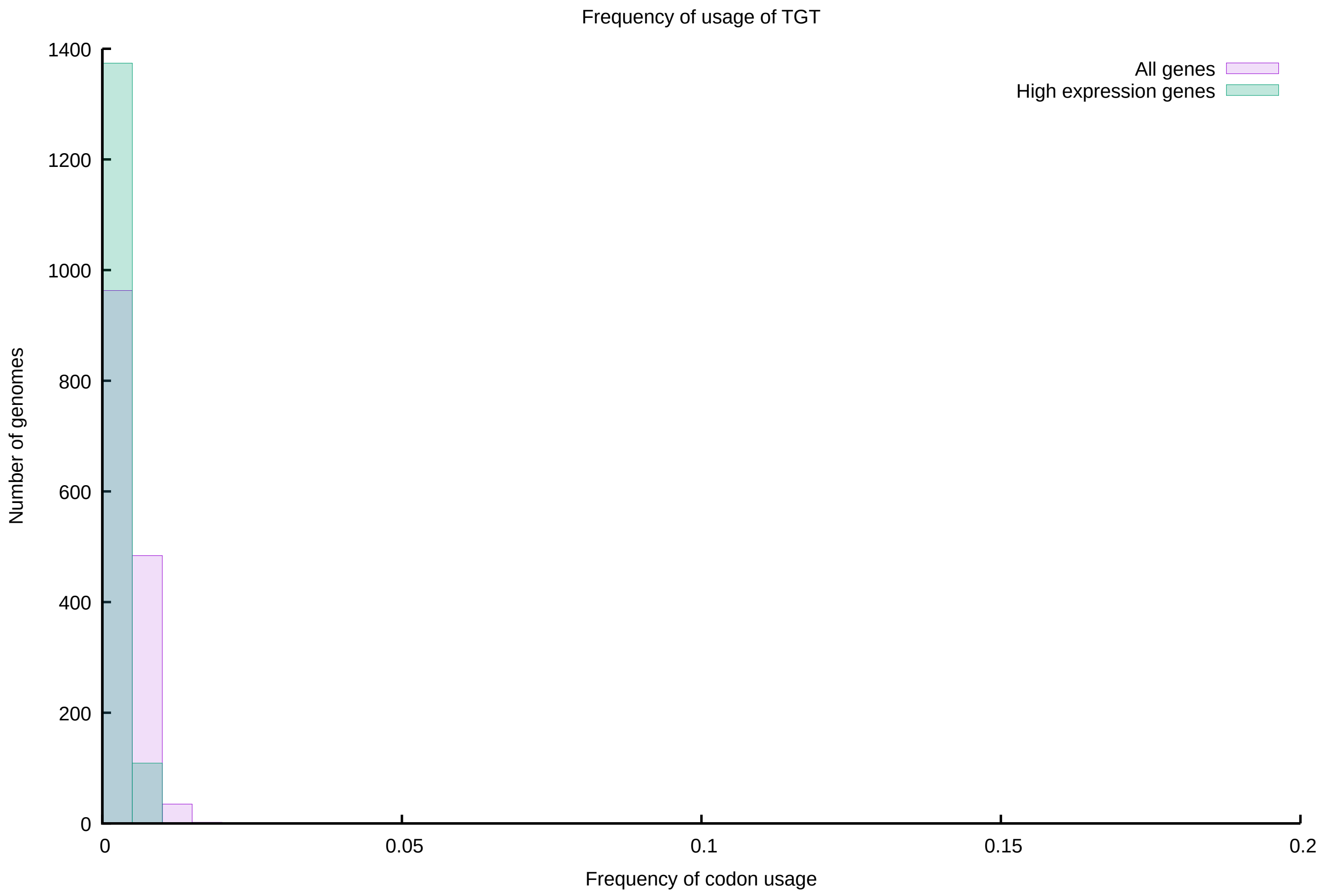

Frequency of usage of TGT
All genes
High expression genes
Number of genomes
1400
1200
1000
800
600
400
200
0
0
0.05
0.1
0.15
0.2
Frequency of codon usage

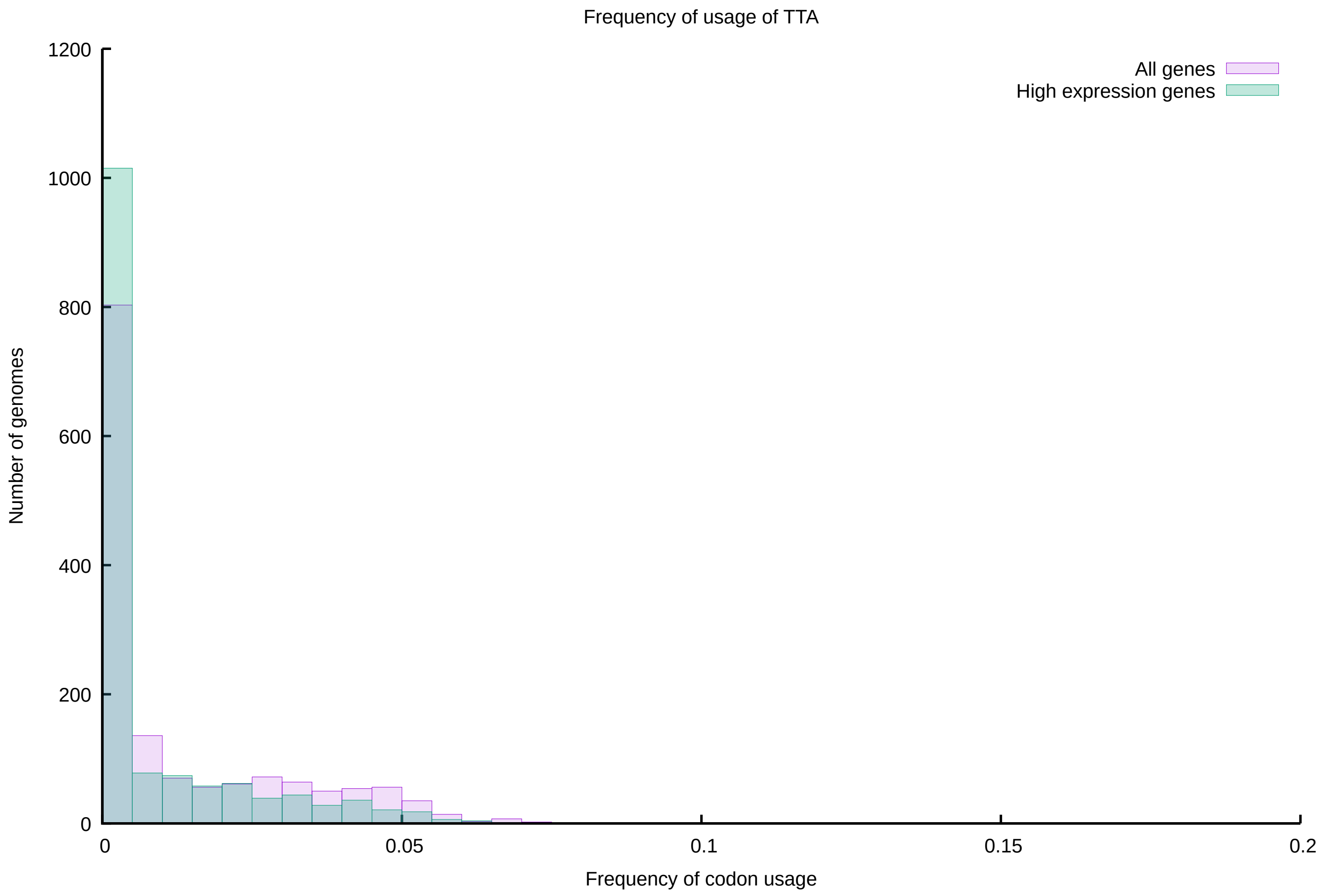

Frequency of usage of TTA
All genes
High expression genes
Number of genomes
Frequency of codon usage
0
200
400
600
800
1000
1200
0
0.05
0.1
0.15
0.2

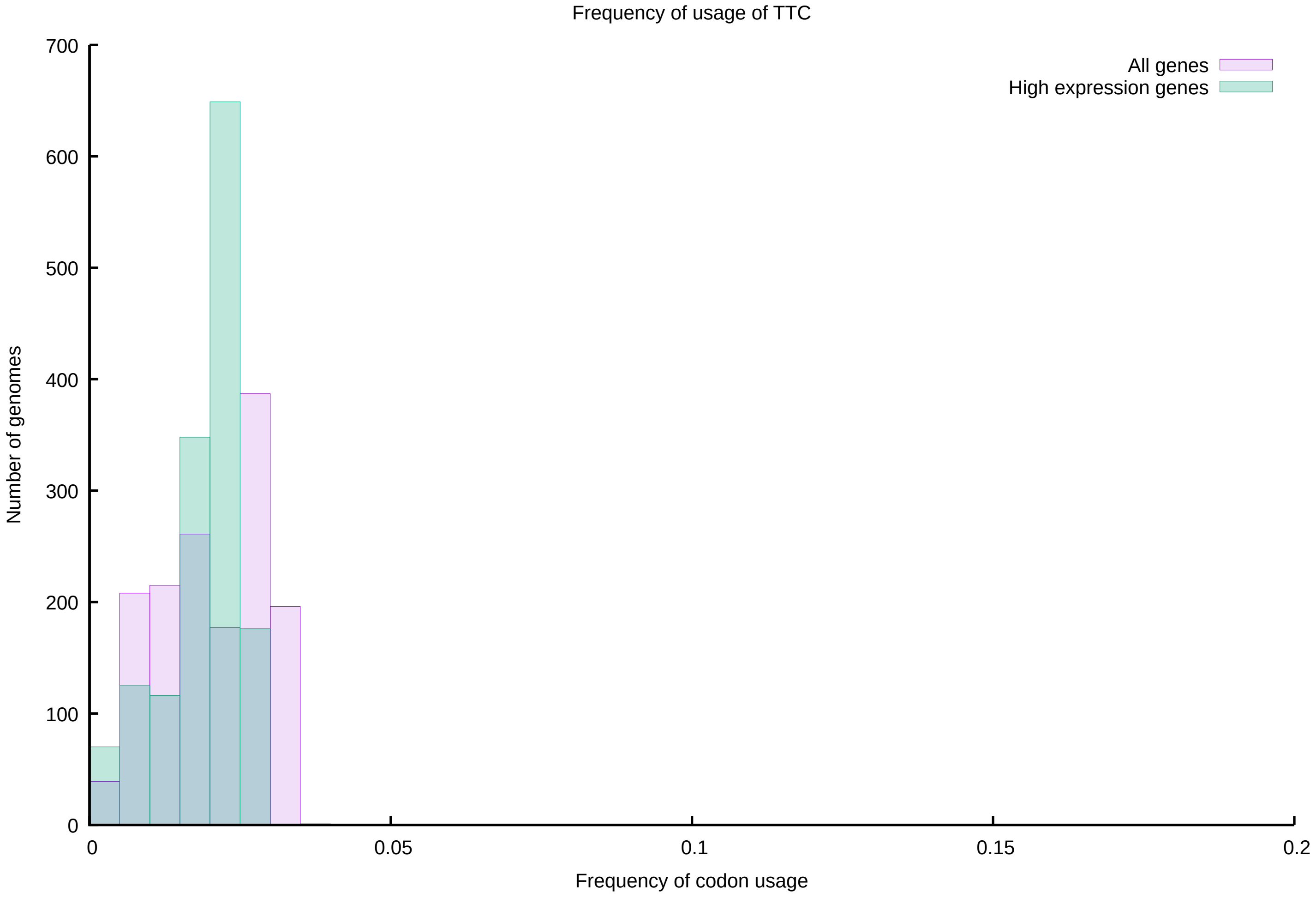

Frequency of usage of TTC
All genes
High expression genes
Number of genomes
700
600
500
400
300
200
100
0
0
0.05
0.1
0.15
0.2
Frequency of codon usage

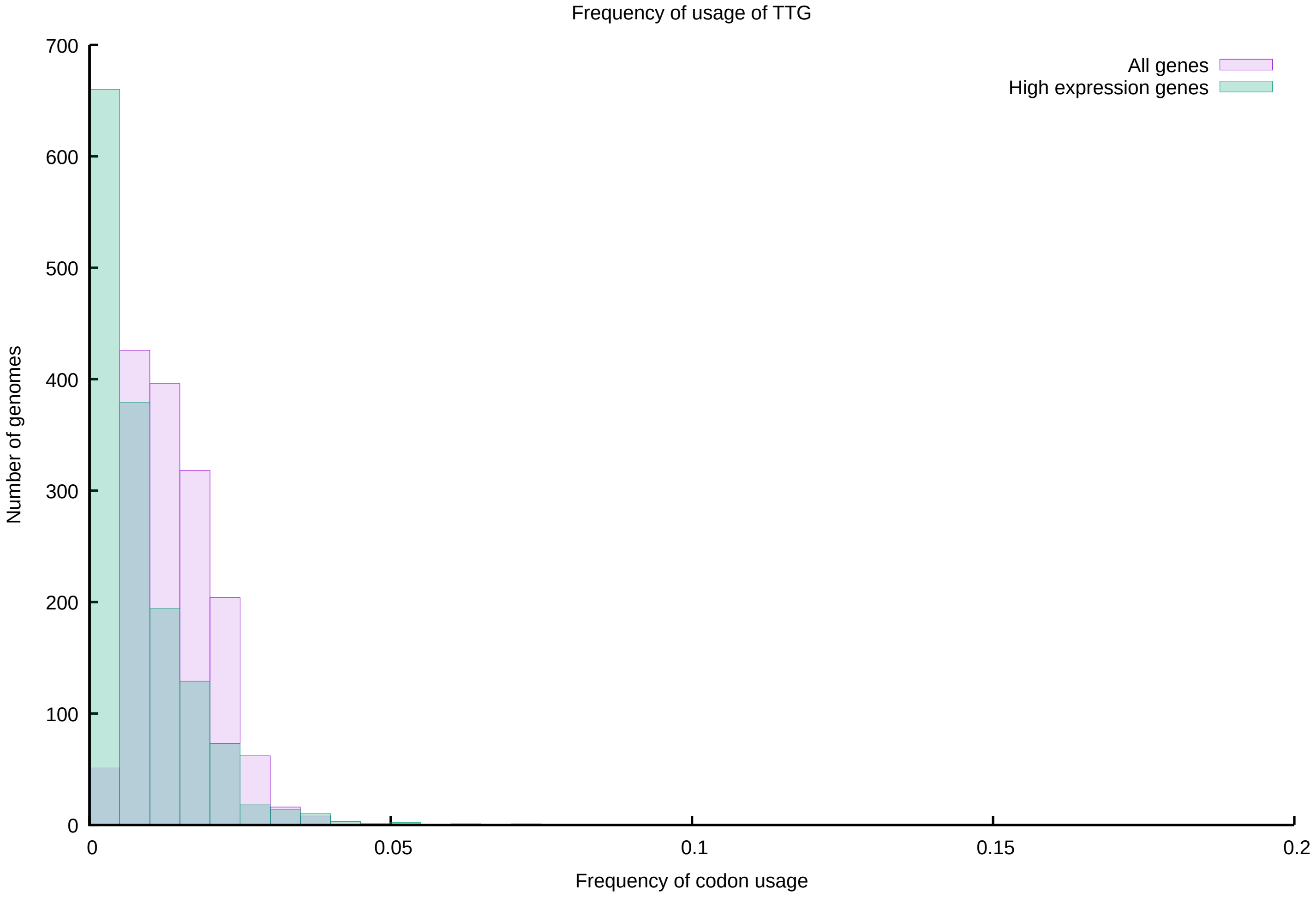

Frequency of usage of TTG
All genes
High expression genes
Number of genomes
700
600
500
400
300
200
100
0
0
0.05
0.1
0.15
0.2
Frequency of codon usage

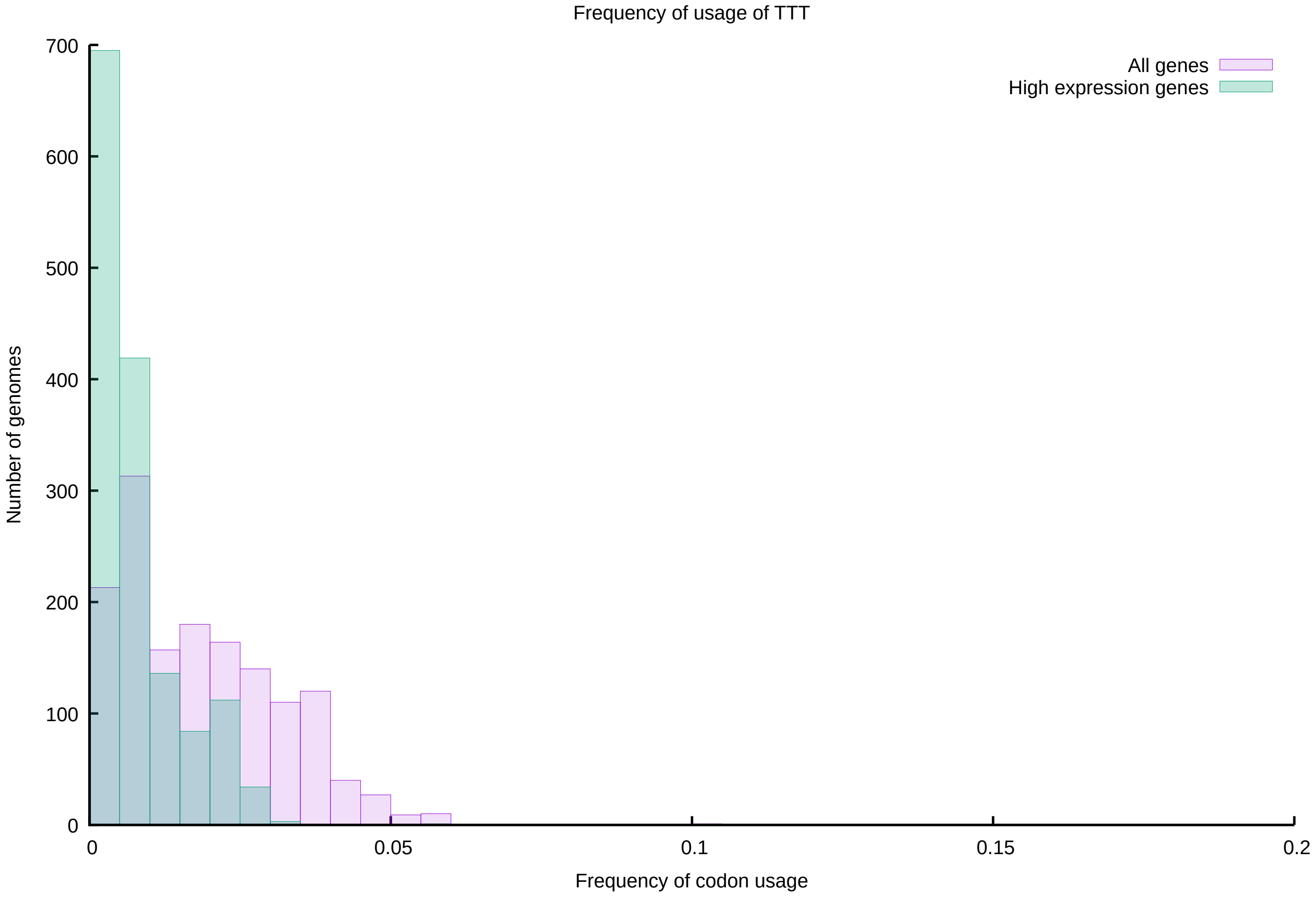

Frequency of usage of TTT
All genes
High expression genes
Number of genomes
0
100
200
300
400
500
600
700
0
0.05
0.1
0.15
0.2
Frequency of codon usage
