# Supplementary material for: Impact of the chemical modification of tRNAs anticodon loop on the variability and evolution of codon usage in proteobacteria
Source: Front Microbiol. 2024 Aug 5;15:1412318. doi: 10.3389/fmicb.2024.1412318 (PMC11332805; doi:10.3389/fmicb.2024.1412318)
Supplement: Supplementary file 1 [file Data_Sheet_1.zip › Supp_figures/Fig_S5.pdf]

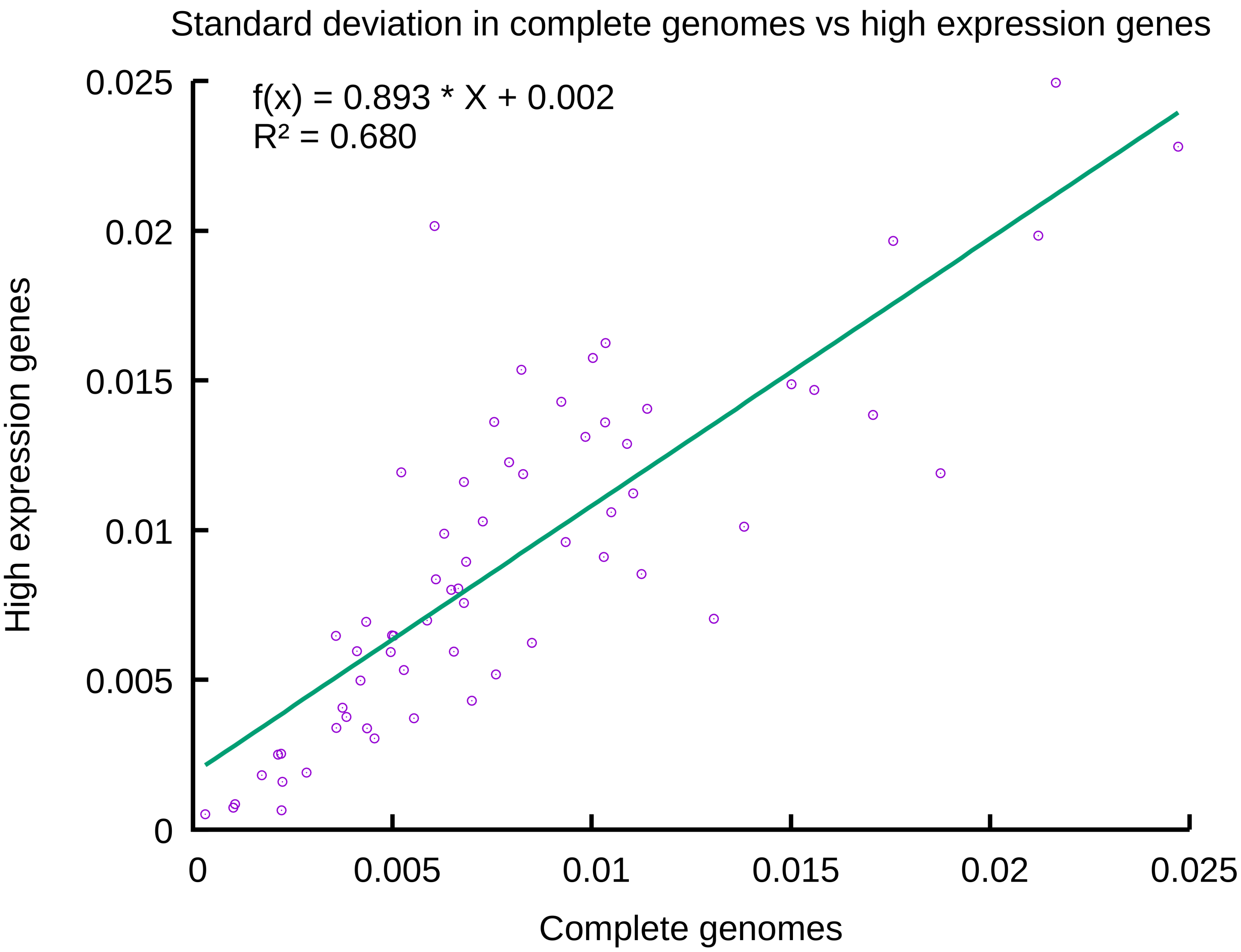

Standard deviation in complete genomes vs high expression genes
f(x) = 0.893 * X + 0.002
R² = 0.680
High expression genes
Complete genomes
0
0.005
0.01
0.015
0.02
0.025

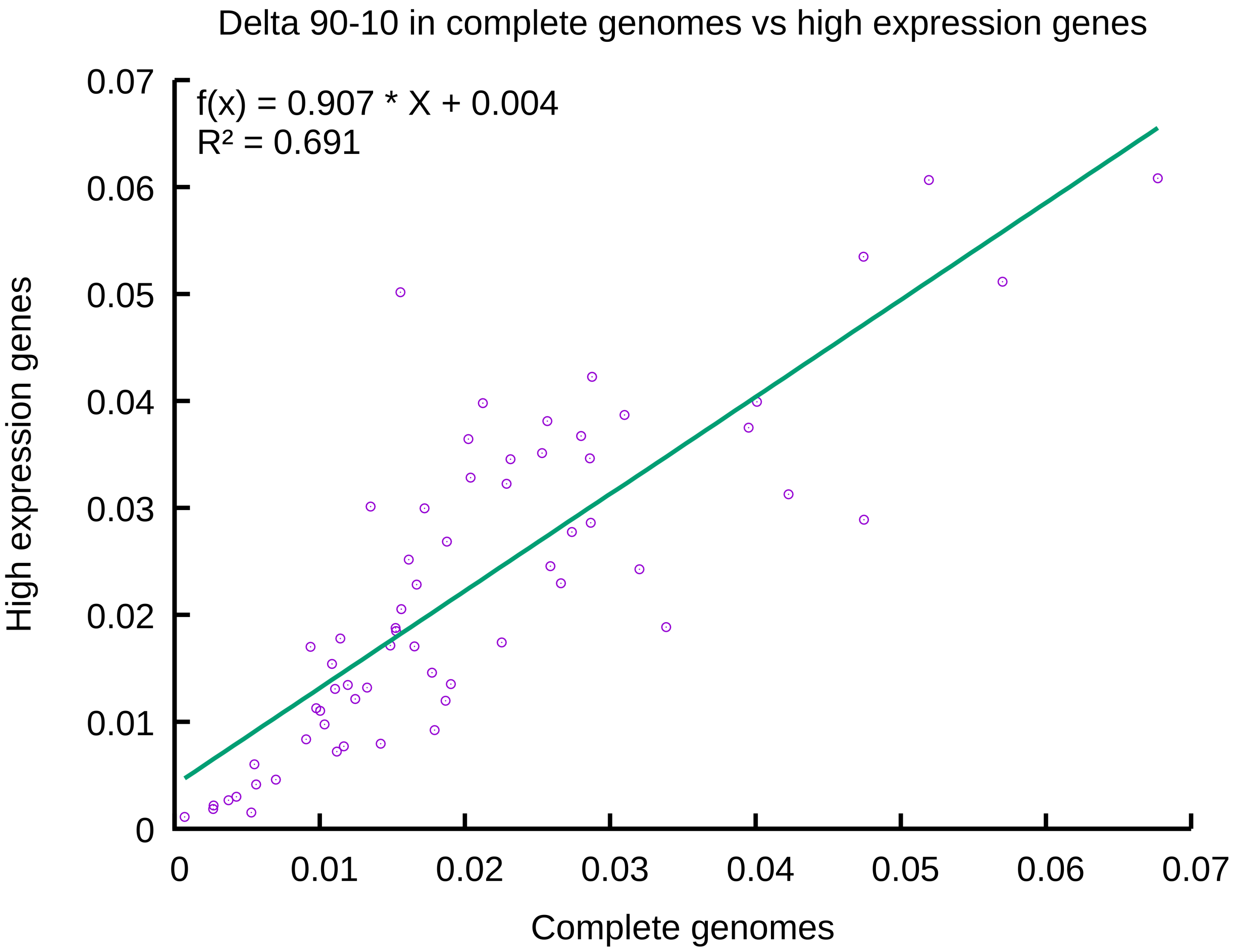

Delta 90-10 in complete genomes vs high expression genes
f(x) = 0.907 * X + 0.004
R² = 0.691
High expression genes
Complete genomes
0
0.01
0.02
0.03
0.04
0.05
0.06
0.07

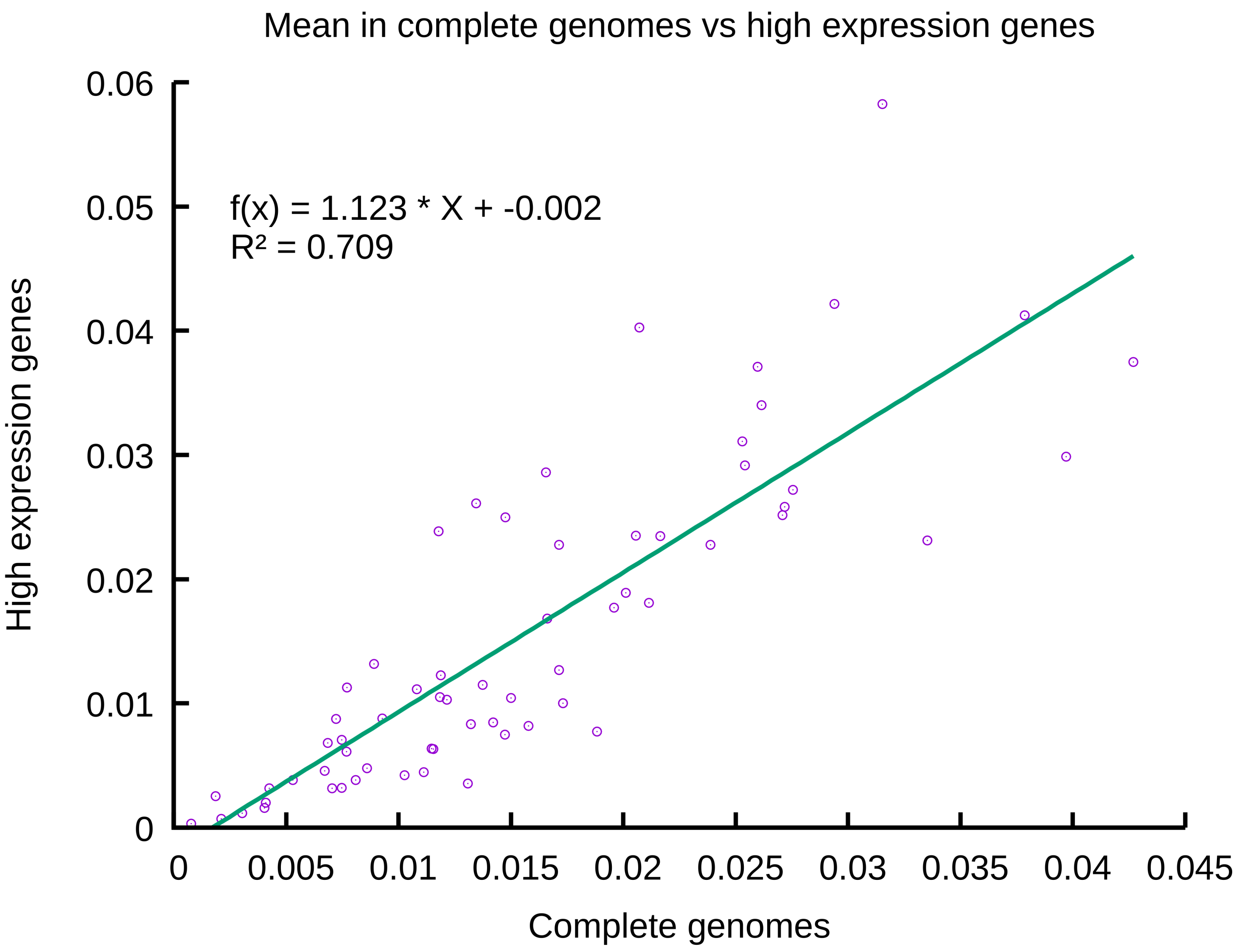
Mean in complete genomes vs high expression genes
f(x) = 1.123 * X + -0.002
R² = 0.709
High expression genes
0.06
0.05
0.04
0.03
0.02
0.01
0
0
0.005
0.01
0.015
0.02
0.025
0.03
0.035
0.04
0.045
Complete genomes
