# Supplementary material for: Impact of the chemical modification of tRNAs anticodon loop on the variability and evolution of codon usage in proteobacteria
Source: Front Microbiol. 2024 Aug 5;15:1412318. doi: 10.3389/fmicb.2024.1412318 (PMC11332805; doi:10.3389/fmicb.2024.1412318)
Supplement: Supplementary file 1 [file Data_Sheet_1.zip › Supp_figures/Fig_S9.pdf]

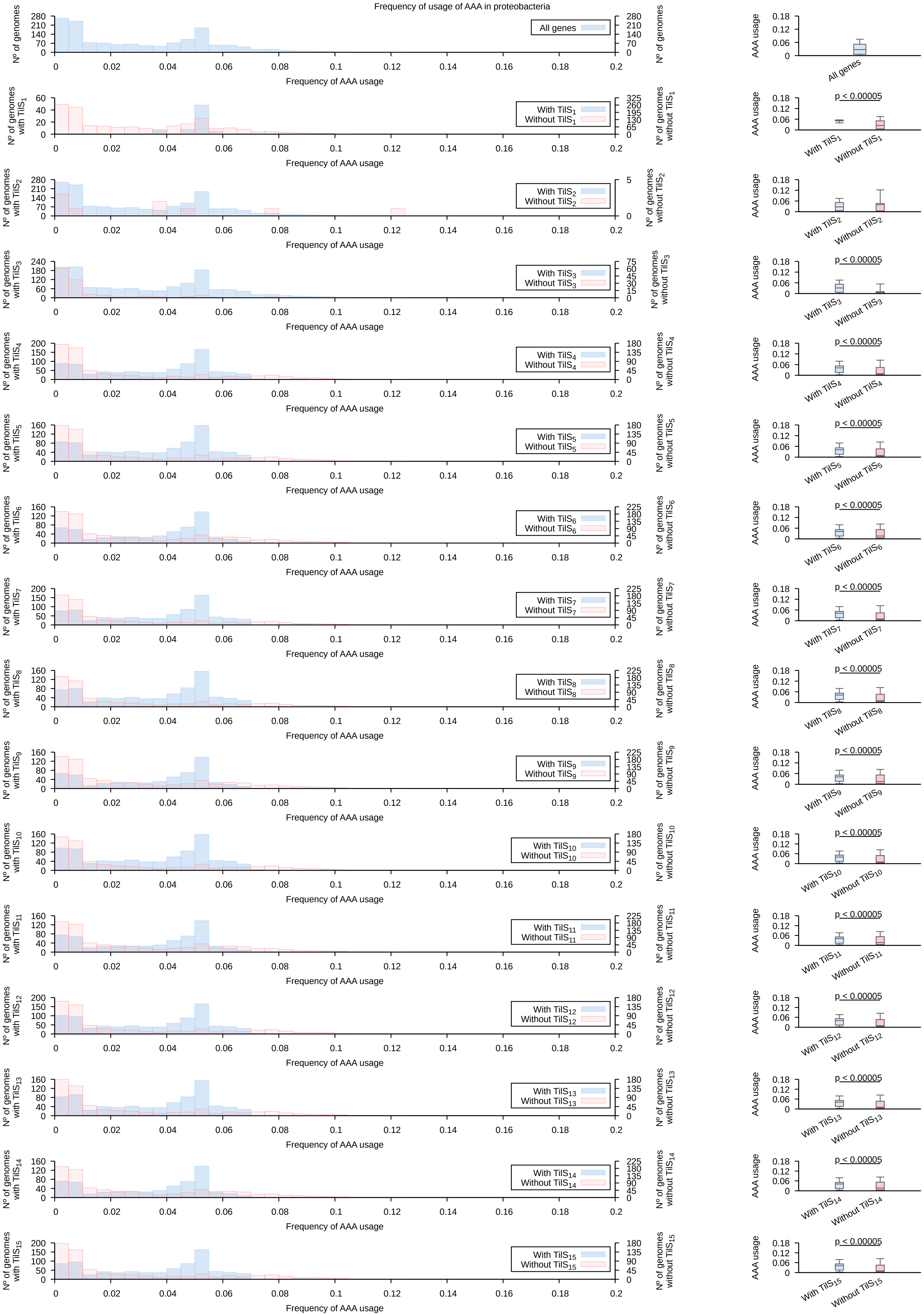

Frequency of usage of AAA in proteobacteria
Nº of genomes
All genes
Frequency of AAA usage
AAA usage
0 0.02 0.04 0.06 0.08 0.1 0.12 0.14 0.16 0.18 0.2
0 0.06 0.12 0.18
Nº of genomes with TilS$_1$
Nº of genomes without TilS$_1$
With TilS$_1$
Without TilS$_1$
p < 0.00005
Nº of genomes with TilS$_2$
Nº of genomes without TilS$_2$
With TilS$_2$
Without TilS$_2$
Nº of genomes with TilS$_3$
Nº of genomes without TilS$_3$
With TilS$_3$
Without TilS$_3$
p < 0.00005
Nº of genomes with TilS$_4$
Nº of genomes without TilS$_4$
With TilS$_4$
Without TilS$_4$
p < 0.00005
Nº of genomes with TilS$_5$
Nº of genomes without TilS$_5$
With TilS$_5$
Without TilS$_5$
p < 0.00005
Nº of genomes with TilS$_6$
Nº of genomes without TilS$_6$
With TilS$_6$
Without TilS$_6$
p < 0.00005
Nº of genomes with TilS$_7$
Nº of genomes without TilS$_7$
With TilS$_7$
Without TilS$_7$
p < 0.00005
Nº of genomes with TilS$_8$
Nº of genomes without TilS$_8$
With TilS$_8$
Without TilS$_8$
p < 0.00005
Nº of genomes with TilS$_9$
Nº of genomes without TilS$_9$
With TilS$_9$
Without TilS$_9$
p < 0.00005
Nº of genomes with TilS$_{10}$
Nº of genomes without TilS$_{10}$
With TilS$_{10}$
Without TilS$_{10}$
p < 0.00005
Nº of genomes with TilS$_{11}$
Nº of genomes without TilS$_{11}$
With TilS$_{11}$
Without TilS$_{11}$
p < 0.00005
Nº of genomes with TilS$_{12}$
Nº of genomes without TilS$_{12}$
With TilS$_{12}$
Without TilS$_{12}$
p < 0.00005
Nº of genomes with TilS$_{13}$
Nº of genomes without TilS$_{13}$
With TilS$_{13}$
Without TilS$_{13}$
p < 0.00005
Nº of genomes with TilS$_{14}$
Nº of genomes without TilS$_{14}$
With TilS$_{14}$
Without TilS$_{14}$
p < 0.00005
Nº of genomes with TilS$_{15}$
Nº of genomes without TilS$_{15}$
With TilS$_{15}$
Without TilS$_{15}$
p < 0.00005

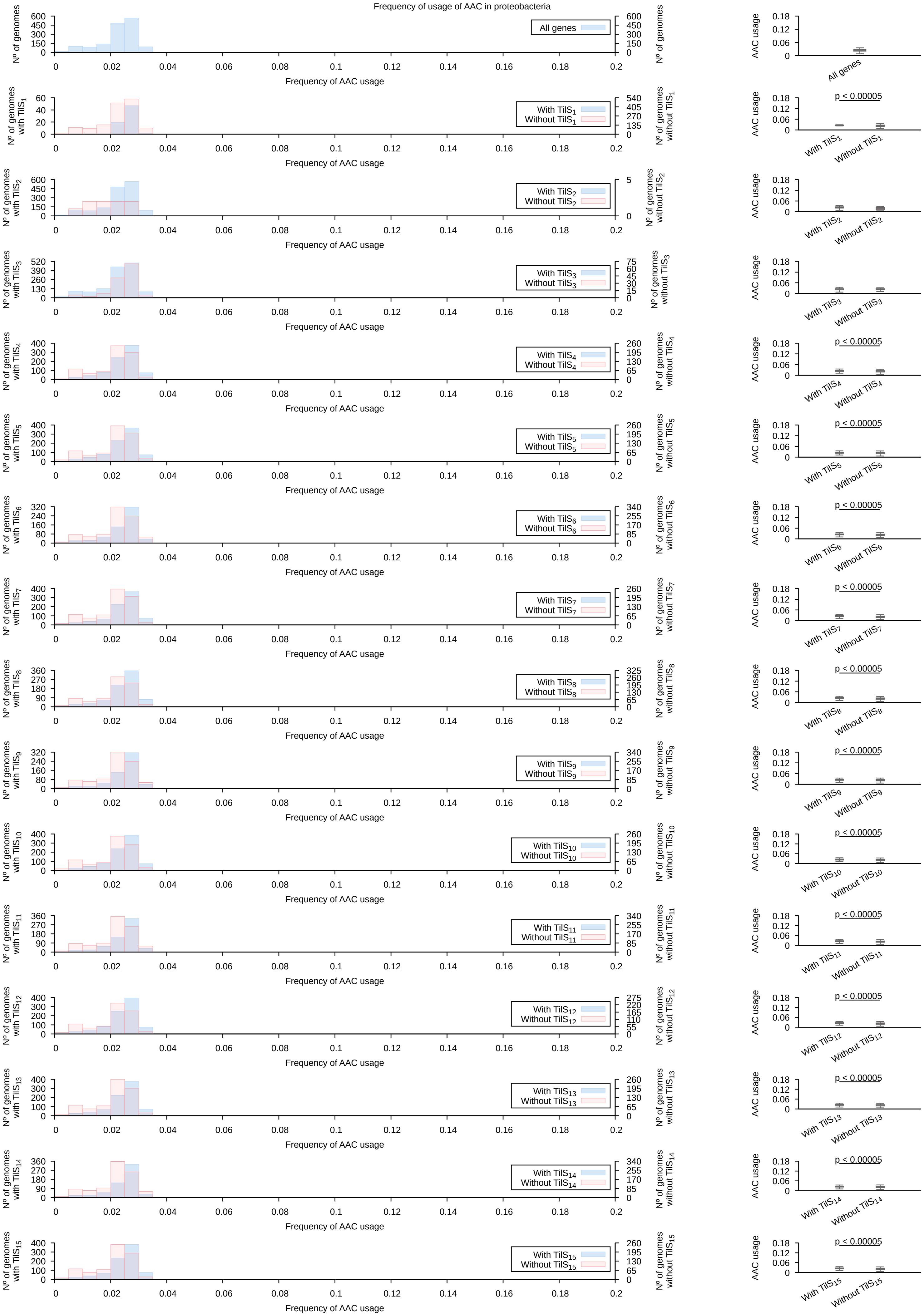

Frequency of usage of AAC in proteobacteria
Nº of genomes
All genes
Frequency of AAC usage
AAC usage
p < 0.00005
Nº of genomes with $TilS_1$
Nº of genomes without $TilS_1$
With $TilS_1$
Without $TilS_1$
Nº of genomes with $TilS_2$
Nº of genomes without $TilS_2$
With $TilS_2$
Without $TilS_2$
Nº of genomes with $TilS_3$
Nº of genomes without $TilS_3$
With $TilS_3$
Without $TilS_3$
Nº of genomes with $TilS_4$
Nº of genomes without $TilS_4$
With $TilS_4$
Without $TilS_4$
Nº of genomes with $TilS_5$
Nº of genomes without $TilS_5$
With $TilS_5$
Without $TilS_5$
Nº of genomes with $TilS_6$
Nº of genomes without $TilS_6$
With $TilS_6$
Without $TilS_6$
Nº of genomes with $TilS_7$
Nº of genomes without $TilS_7$
With $TilS_7$
Without $TilS_7$
Nº of genomes with $TilS_8$
Nº of genomes without $TilS_8$
With $TilS_8$
Without $TilS_8$
Nº of genomes with $TilS_9$
Nº of genomes without $TilS_9$
With $TilS_9$
Without $TilS_9$
Nº of genomes with $TilS_{10}$
Nº of genomes without $TilS_{10}$
With $TilS_{10}$
Without $TilS_{10}$
Nº of genomes with $TilS_{11}$
Nº of genomes without $TilS_{11}$
With $TilS_{11}$
Without $TilS_{11}$
Nº of genomes with $TilS_{12}$
Nº of genomes without $TilS_{12}$
With $TilS_{12}$
Without $TilS_{12}$
Nº of genomes with $TilS_{13}$
Nº of genomes without $TilS_{13}$
With $TilS_{13}$
Without $TilS_{13}$
Nº of genomes with $TilS_{14}$
Nº of genomes without $TilS_{14}$
With $TilS_{14}$
Without $TilS_{14}$
Nº of genomes with $TilS_{15}$
Nº of genomes without $TilS_{15}$
With $TilS_{15}$
Without $TilS_{15}$

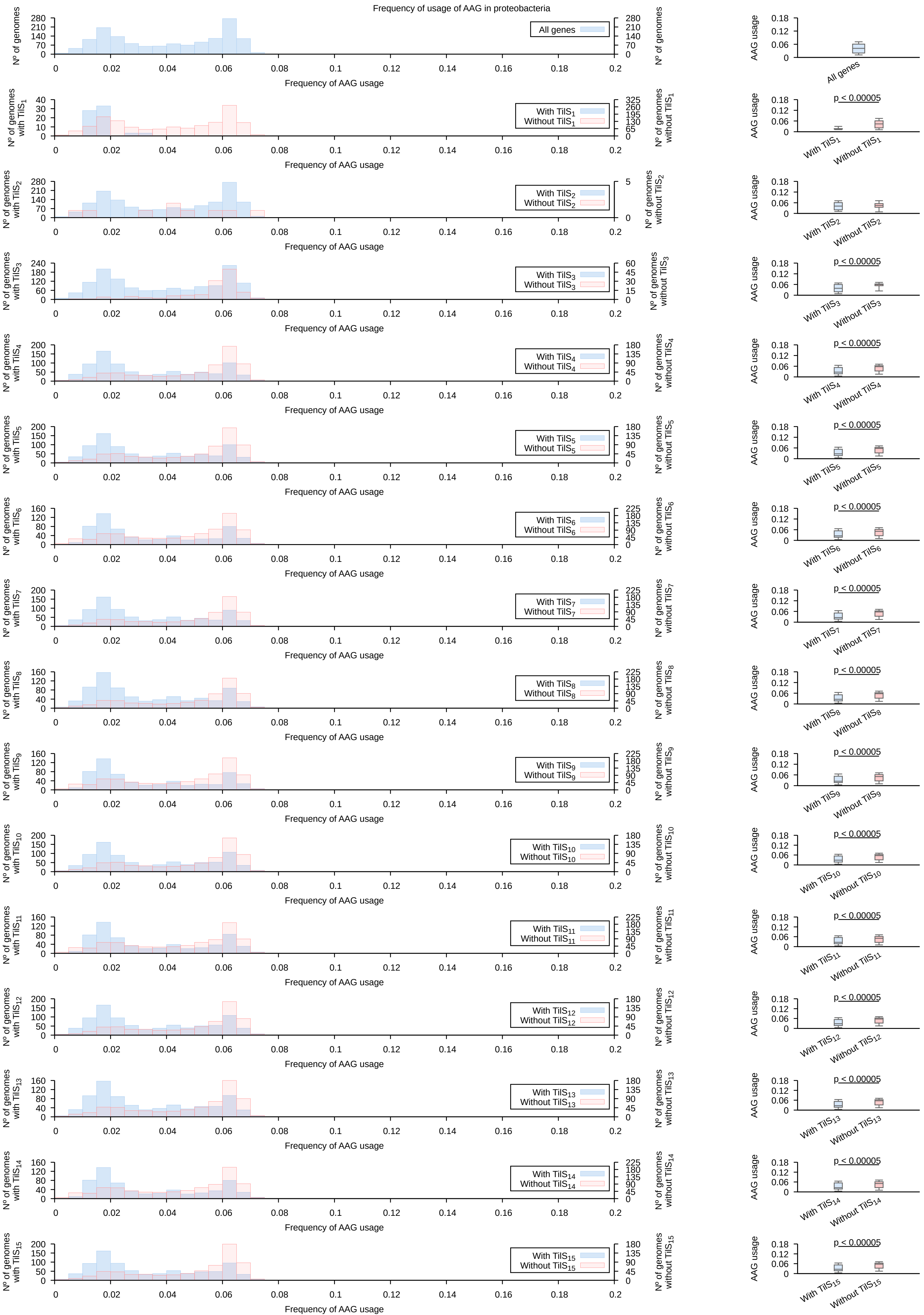

Frequency of usage of AAG in proteobacteria
Nº of genomes
All genes
Frequency of AAG usage
AAG usage
All genes
Nº of genomes with $TilS_1$
Nº of genomes without $TilS_1$
With $TilS_1$
Without $TilS_1$
p < 0.00005
Nº of genomes with $TilS_2$
Nº of genomes without $TilS_2$
With $TilS_2$
Without $TilS_2$
Nº of genomes with $TilS_3$
Nº of genomes without $TilS_3$
With $TilS_3$
Without $TilS_3$
p < 0.00005
Nº of genomes with $TilS_4$
Nº of genomes without $TilS_4$
With $TilS_4$
Without $TilS_4$
p < 0.00005
Nº of genomes with $TilS_5$
Nº of genomes without $TilS_5$
With $TilS_5$
Without $TilS_5$
p < 0.00005
Nº of genomes with $TilS_6$
Nº of genomes without $TilS_6$
With $TilS_6$
Without $TilS_6$
p < 0.00005
Nº of genomes with $TilS_7$
Nº of genomes without $TilS_7$
With $TilS_7$
Without $TilS_7$
p < 0.00005
Nº of genomes with $TilS_8$
Nº of genomes without $TilS_8$
With $TilS_8$
Without $TilS_8$
p < 0.00005
Nº of genomes with $TilS_9$
Nº of genomes without $TilS_9$
With $TilS_9$
Without $TilS_9$
p < 0.00005
Nº of genomes with $TilS_{10}$
Nº of genomes without $TilS_{10}$
With $TilS_{10}$
Without $TilS_{10}$
p < 0.00005
Nº of genomes with $TilS_{11}$
Nº of genomes without $TilS_{11}$
With $TilS_{11}$
Without $TilS_{11}$
p < 0.00005
Nº of genomes with $TilS_{12}$
Nº of genomes without $TilS_{12}$
With $TilS_{12}$
Without $TilS_{12}$
p < 0.00005
Nº of genomes with $TilS_{13}$
Nº of genomes without $TilS_{13}$
With $TilS_{13}$
Without $TilS_{13}$
p < 0.00005
Nº of genomes with $TilS_{14}$
Nº of genomes without $TilS_{14}$
With $TilS_{14}$
Without $TilS_{14}$
p < 0.00005
Nº of genomes with $TilS_{15}$
Nº of genomes without $TilS_{15}$
With $TilS_{15}$
Without $TilS_{15}$
p < 0.00005

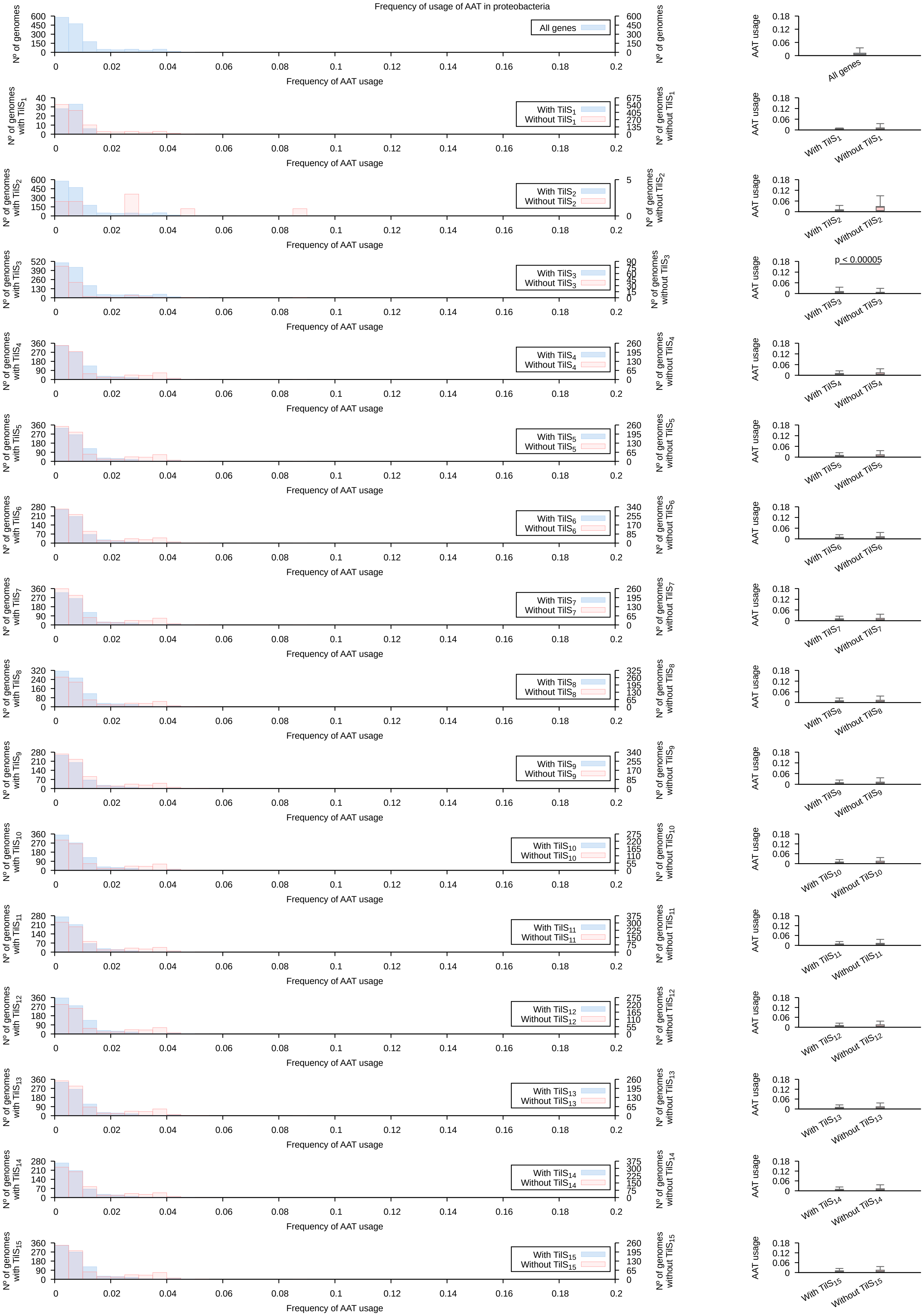
Frequency of usage of AAT in proteobacteria
Nº of genomes
All genes
Frequency of AAT usage
AAT usage
Nº of genomes with TilS$_1$
Nº of genomes without TilS$_1$
With TilS$_1$
Without TilS$_1$
Nº of genomes with TilS$_2$
Nº of genomes without TilS$_2$
With TilS$_2$
Without TilS$_2$
Nº of genomes with TilS$_3$
Nº of genomes without TilS$_3$
With TilS$_3$
Without TilS$_3$
p < 0.00005
Nº of genomes with TilS$_4$
Nº of genomes without TilS$_4$
With TilS$_4$
Without TilS$_4$
Nº of genomes with TilS$_5$
Nº of genomes without TilS$_5$
With TilS$_5$
Without TilS$_5$
Nº of genomes with TilS$_6$
Nº of genomes without TilS$_6$
With TilS$_6$
Without TilS$_6$
Nº of genomes with TilS$_7$
Nº of genomes without TilS$_7$
With TilS$_7$
Without TilS$_7$
Nº of genomes with TilS$_8$
Nº of genomes without TilS$_8$
With TilS$_8$
Without TilS$_8$
Nº of genomes with TilS$_9$
Nº of genomes without TilS$_9$
With TilS$_9$
Without TilS$_9$
Nº of genomes with TilS$_{10}$
Nº of genomes without TilS$_{10}$
With TilS$_{10}$
Without TilS$_{10}$
Nº of genomes with TilS$_{11}$
Nº of genomes without TilS$_{11}$
With TilS$_{11}$
Without TilS$_{11}$
Nº of genomes with TilS$_{12}$
Nº of genomes without TilS$_{12}$
With TilS$_{12}$
Without TilS$_{12}$
Nº of genomes with TilS$_{13}$
Nº of genomes without TilS$_{13}$
With TilS$_{13}$
Without TilS$_{13}$
Nº of genomes with TilS$_{14}$
Nº of genomes without TilS$_{14}$
With TilS$_{14}$
Without TilS$_{14}$
Nº of genomes with TilS$_{15}$
Nº of genomes without TilS$_{15}$
With TilS$_{15}$
Without TilS$_{15}$

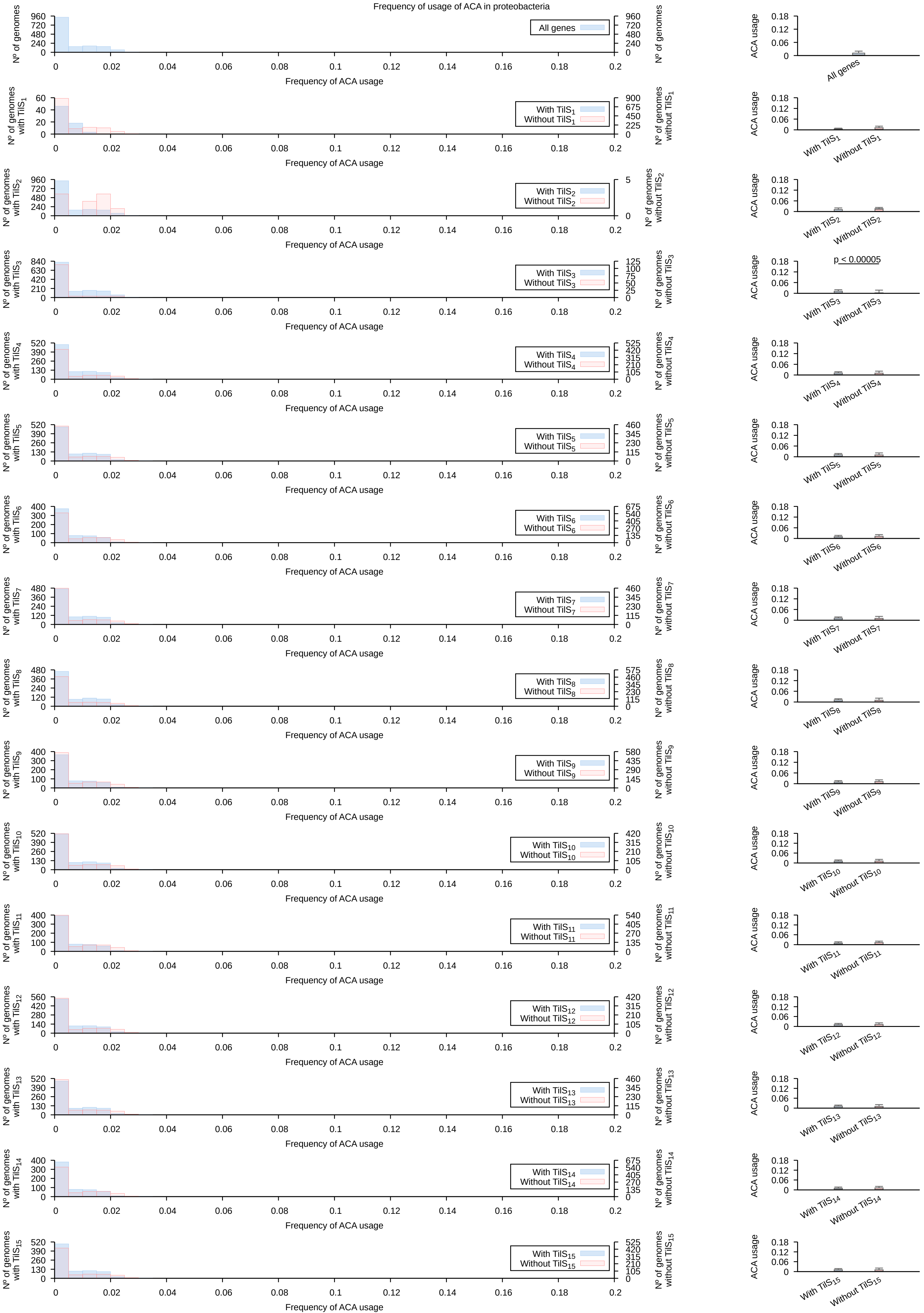

Frequency of usage of ACA in proteobacteria
Nº of genomes
All genes
Frequency of ACA usage
ACA usage
Nº of genomes with TilS1
With TilS1
Without TilS1
Nº of genomes without TilS1
Nº of genomes with TilS2
With TilS2
Without TilS2
Nº of genomes without TilS2
Nº of genomes with TilS3
With TilS3
Without TilS3
Nº of genomes without TilS3
p < 0.00005
Nº of genomes with TilS4
With TilS4
Without TilS4
Nº of genomes without TilS4
Nº of genomes with TilS5
With TilS5
Without TilS5
Nº of genomes without TilS5
Nº of genomes with TilS6
With TilS6
Without TilS6
Nº of genomes without TilS6
Nº of genomes with TilS7
With TilS7
Without TilS7
Nº of genomes without TilS7
Nº of genomes with TilS8
With TilS8
Without TilS8
Nº of genomes without TilS8
Nº of genomes with TilS9
With TilS9
Without TilS9
Nº of genomes without TilS9
Nº of genomes with TilS10
With TilS10
Without TilS10
Nº of genomes without TilS10
Nº of genomes with TilS11
With TilS11
Without TilS11
Nº of genomes without TilS11
Nº of genomes with TilS12
With TilS12
Without TilS12
Nº of genomes without TilS12
Nº of genomes with TilS13
With TilS13
Without TilS13
Nº of genomes without TilS13
Nº of genomes with TilS14
With TilS14
Without TilS14
Nº of genomes without TilS14
Nº of genomes with TilS15
With TilS15
Without TilS15
Nº of genomes without TilS15

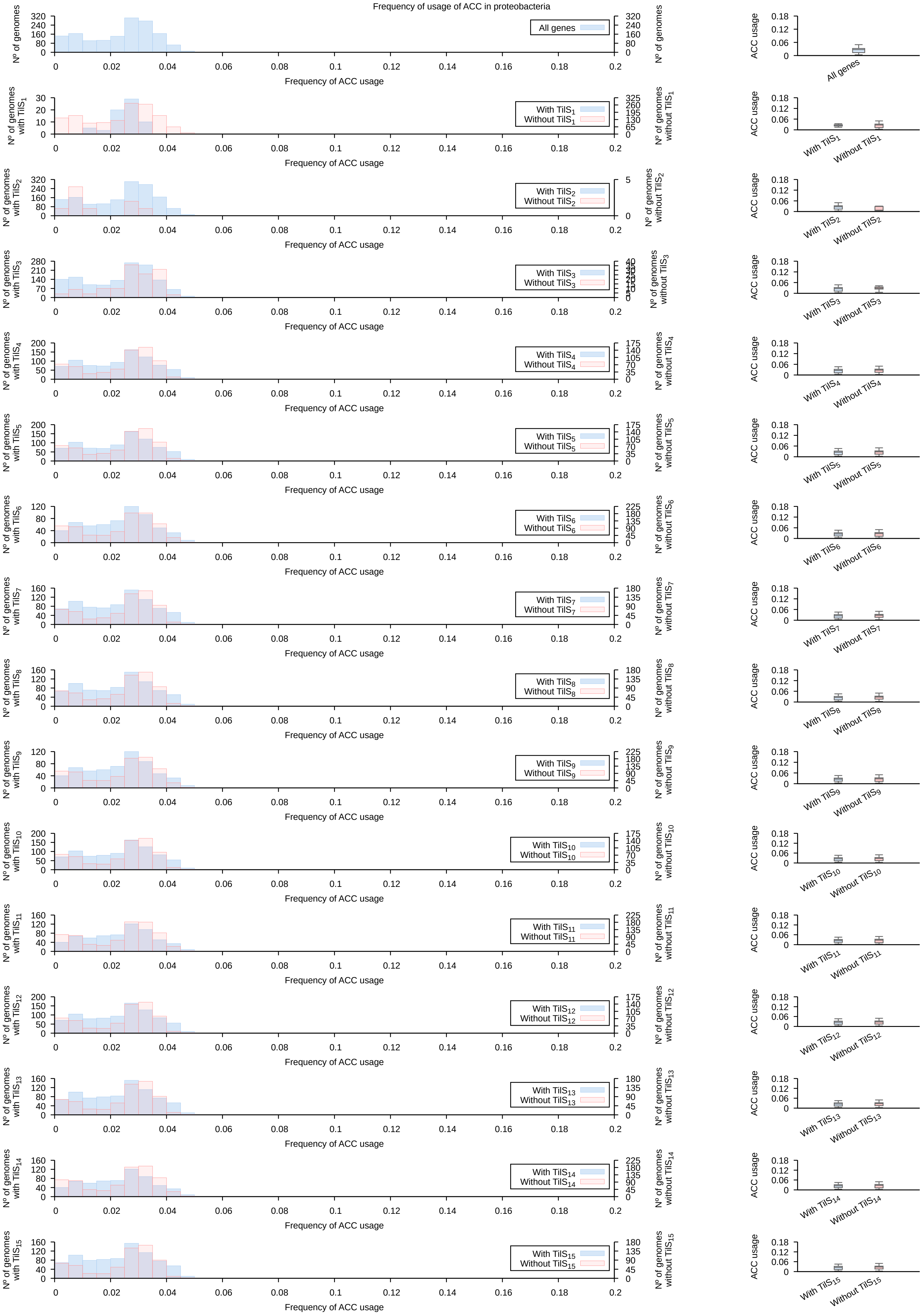

Frequency of usage of ACC in proteobacteria
Nº of genomes
All genes
Frequency of ACC usage
ACC usage
Nº of genomes with $TilS_1$
Nº of genomes without $TilS_1$
With $TilS_1$
Without $TilS_1$
Nº of genomes with $TilS_2$
Nº of genomes without $TilS_2$
With $TilS_2$
Without $TilS_2$
Nº of genomes with $TilS_3$
Nº of genomes without $TilS_3$
With $TilS_3$
Without $TilS_3$
Nº of genomes with $TilS_4$
Nº of genomes without $TilS_4$
With $TilS_4$
Without $TilS_4$
Nº of genomes with $TilS_5$
Nº of genomes without $TilS_5$
With $TilS_5$
Without $TilS_5$
Nº of genomes with $TilS_6$
Nº of genomes without $TilS_6$
With $TilS_6$
Without $TilS_6$
Nº of genomes with $TilS_7$
Nº of genomes without $TilS_7$
With $TilS_7$
Without $TilS_7$
Nº of genomes with $TilS_8$
Nº of genomes without $TilS_8$
With $TilS_8$
Without $TilS_8$
Nº of genomes with $TilS_9$
Nº of genomes without $TilS_9$
With $TilS_9$
Without $TilS_9$
Nº of genomes with $TilS_{10}$
Nº of genomes without $TilS_{10}$
With $TilS_{10}$
Without $TilS_{10}$
Nº of genomes with $TilS_{11}$
Nº of genomes without $TilS_{11}$
With $TilS_{11}$
Without $TilS_{11}$
Nº of genomes with $TilS_{12}$
Nº of genomes without $TilS_{12}$
With $TilS_{12}$
Without $TilS_{12}$
Nº of genomes with $TilS_{13}$
Nº of genomes without $TilS_{13}$
With $TilS_{13}$
Without $TilS_{13}$
Nº of genomes with $TilS_{14}$
Nº of genomes without $TilS_{14}$
With $TilS_{14}$
Without $TilS_{14}$
Nº of genomes with $TilS_{15}$
Nº of genomes without $TilS_{15}$
With $TilS_{15}$
Without $TilS_{15}$
0 0.02 0.04 0.06 0.08 0.1 0.12 0.14 0.16 0.18 0.2
0 0.06 0.12 0.18

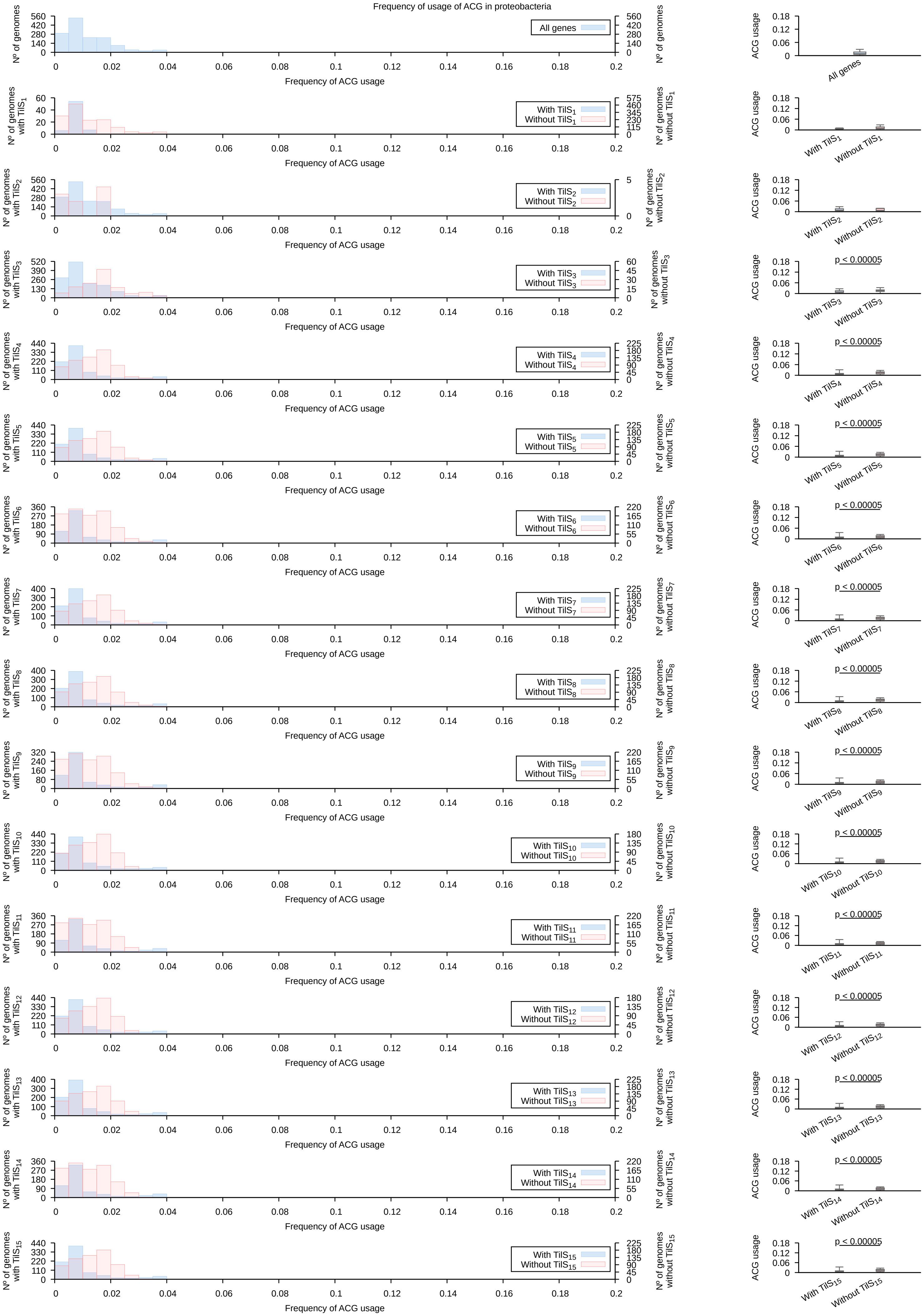
Frequency of usage of ACG in proteobacteria
Nº of genomes
560 420 280 140 0
All genes
0 0.02 0.04 0.06 0.08 0.1 0.12 0.14 0.16 0.18 0.2
Frequency of ACG usage
ACG usage
0.18 0.12 0.06 0
All genes
Nº of genomes with TilS$_1$
60 40 20 0
With TilS$_1$
Without TilS$_1$
575 460 345 230 115 0
Nº of genomes without TilS$_1$
Frequency of ACG usage
With TilS$_1$
Without TilS$_1$
Nº of genomes with TilS$_2$
560 420 280 140 0
With TilS$_2$
Without TilS$_2$
5 0
Nº of genomes without TilS$_2$
Frequency of ACG usage
With TilS$_2$
Without TilS$_2$
Nº of genomes with TilS$_3$
520 390 260 130 0
With TilS$_3$
Without TilS$_3$
60 45 30 15 0
Nº of genomes without TilS$_3$
Frequency of ACG usage
p < 0.00005
With TilS$_3$
Without TilS$_3$
Nº of genomes with TilS$_4$
440 330 220 110 0
With TilS$_4$
Without TilS$_4$
225 180 135 90 45 0
Nº of genomes without TilS$_4$
Frequency of ACG usage
p < 0.00005
With TilS$_4$
Without TilS$_4$
Nº of genomes with TilS$_5$
440 330 220 110 0
With TilS$_5$
Without TilS$_5$
225 180 135 90 45 0
Nº of genomes without TilS$_5$
Frequency of ACG usage
p < 0.00005
With TilS$_5$
Without TilS$_5$
Nº of genomes with TilS$_6$
360 270 180 90 0
With TilS$_6$
Without TilS$_6$
220 165 110 55 0
Nº of genomes without TilS$_6$
Frequency of ACG usage
p < 0.00005
With TilS$_6$
Without TilS$_6$
Nº of genomes with TilS$_7$
400 300 200 100 0
With TilS$_7$
Without TilS$_7$
225 180 135 90 45 0
Nº of genomes without TilS$_7$
Frequency of ACG usage
p < 0.00005
With TilS$_7$
Without TilS$_7$
Nº of genomes with TilS$_8$
400 300 200 100 0
With TilS$_8$
Without TilS$_8$
225 180 135 90 45 0
Nº of genomes without TilS$_8$
Frequency of ACG usage
p < 0.00005
With TilS$_8$
Without TilS$_8$
Nº of genomes with TilS$_9$
320 240 160 80 0
With TilS$_9$
Without TilS$_9$
220 165 110 55 0
Nº of genomes without TilS$_9$
Frequency of ACG usage
p < 0.00005
With TilS$_9$
Without TilS$_9$
Nº of genomes with TilS$_{10}$
440 330 220 110 0
With TilS$_{10}$
Without TilS$_{10}$
180 135 90 45 0
Nº of genomes without TilS$_{10}$
Frequency of ACG usage
p < 0.00005
With TilS$_{10}$
Without TilS$_{10}$
Nº of genomes with TilS$_{11}$
360 270 180 90 0
With TilS$_{11}$
Without TilS$_{11}$
220 165 110 55 0
Nº of genomes without TilS$_{11}$
Frequency of ACG usage
p < 0.00005
With TilS$_{11}$
Without TilS$_{11}$
Nº of genomes with TilS$_{12}$
440 330 220 110 0
With TilS$_{12}$
Without TilS$_{12}$
180 135 90 45 0
Nº of genomes without TilS$_{12}$
Frequency of ACG usage
p < 0.00005
With TilS$_{12}$
Without TilS$_{12}$
Nº of genomes with TilS$_{13}$
400 300 200 100 0
With TilS$_{13}$
Without TilS$_{13}$
225 180 135 90 45 0
Nº of genomes without TilS$_{13}$
Frequency of ACG usage
p < 0.00005
With TilS$_{13}$
Without TilS$_{13}$
Nº of genomes with TilS$_{14}$
360 270 180 90 0
With TilS$_{14}$
Without TilS$_{14}$
220 165 110 55 0
Nº of genomes without TilS$_{14}$
Frequency of ACG usage
p < 0.00005
With TilS$_{14}$
Without TilS$_{14}$
Nº of genomes with TilS$_{15}$
440 330 220 110 0
With TilS$_{15}$
Without TilS$_{15}$
225 180 135 90 45 0
Nº of genomes without TilS$_{15}$
Frequency of ACG usage
p < 0.00005
With TilS$_{15}$
Without TilS$_{15}$

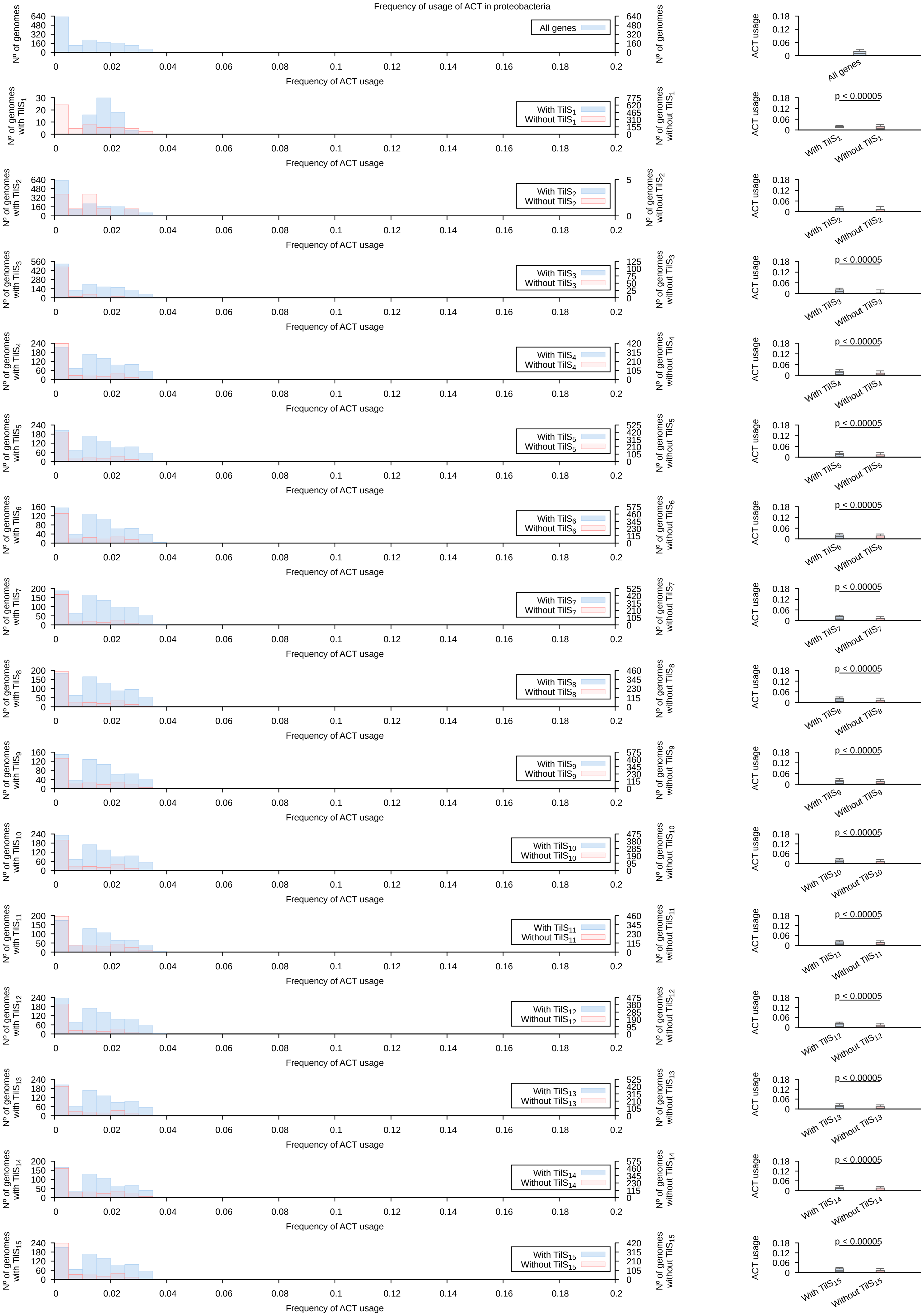

Frequency of usage of ACT in proteobacteria
Nº of genomes
All genes
Frequency of ACT usage
ACT usage
p < 0.00005
Nº of genomes with $TilS_1$
Nº of genomes without $TilS_1$
With $TilS_1$
Without $TilS_1$
Nº of genomes with $TilS_2$
Nº of genomes without $TilS_2$
With $TilS_2$
Without $TilS_2$
Nº of genomes with $TilS_3$
Nº of genomes without $TilS_3$
With $TilS_3$
Without $TilS_3$
Nº of genomes with $TilS_4$
Nº of genomes without $TilS_4$
With $TilS_4$
Without $TilS_4$
Nº of genomes with $TilS_5$
Nº of genomes without $TilS_5$
With $TilS_5$
Without $TilS_5$
Nº of genomes with $TilS_6$
Nº of genomes without $TilS_6$
With $TilS_6$
Without $TilS_6$
Nº of genomes with $TilS_7$
Nº of genomes without $TilS_7$
With $TilS_7$
Without $TilS_7$
Nº of genomes with $TilS_8$
Nº of genomes without $TilS_8$
With $TilS_8$
Without $TilS_8$
Nº of genomes with $TilS_9$
Nº of genomes without $TilS_9$
With $TilS_9$
Without $TilS_9$
Nº of genomes with $TilS_{10}$
Nº of genomes without $TilS_{10}$
With $TilS_{10}$
Without $TilS_{10}$
Nº of genomes with $TilS_{11}$
Nº of genomes without $TilS_{11}$
With $TilS_{11}$
Without $TilS_{11}$
Nº of genomes with $TilS_{12}$
Nº of genomes without $TilS_{12}$
With $TilS_{12}$
Without $TilS_{12}$
Nº of genomes with $TilS_{13}$
Nº of genomes without $TilS_{13}$
With $TilS_{13}$
Without $TilS_{13}$
Nº of genomes with $TilS_{14}$
Nº of genomes without $TilS_{14}$
With $TilS_{14}$
Without $TilS_{14}$
Nº of genomes with $TilS_{15}$
Nº of genomes without $TilS_{15}$
With $TilS_{15}$
Without $TilS_{15}$

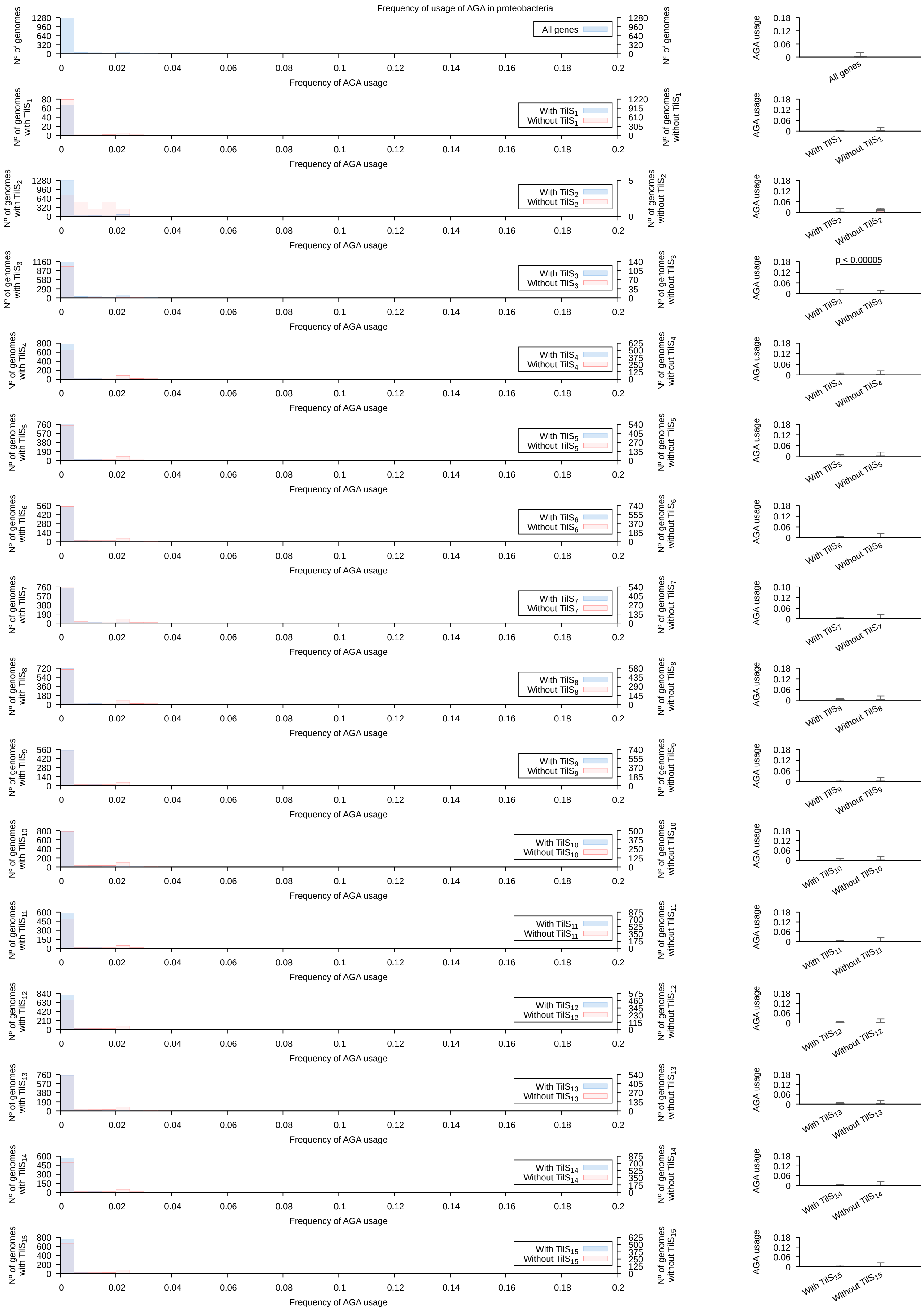

Frequency of usage of AGA in proteobacteria
Nº of genomes
All genes
Frequency of AGA usage
AGA usage
Nº of genomes with TilS$_1$
Nº of genomes without TilS$_1$
With TilS$_1$
Without TilS$_1$
Nº of genomes with TilS$_2$
Nº of genomes without TilS$_2$
With TilS$_2$
Without TilS$_2$
Nº of genomes with TilS$_3$
Nº of genomes without TilS$_3$
With TilS$_3$
Without TilS$_3$
p < 0.00005
Nº of genomes with TilS$_4$
Nº of genomes without TilS$_4$
With TilS$_4$
Without TilS$_4$
Nº of genomes with TilS$_5$
Nº of genomes without TilS$_5$
With TilS$_5$
Without TilS$_5$
Nº of genomes with TilS$_6$
Nº of genomes without TilS$_6$
With TilS$_6$
Without TilS$_6$
Nº of genomes with TilS$_7$
Nº of genomes without TilS$_7$
With TilS$_7$
Without TilS$_7$
Nº of genomes with TilS$_8$
Nº of genomes without TilS$_8$
With TilS$_8$
Without TilS$_8$
Nº of genomes with TilS$_9$
Nº of genomes without TilS$_9$
With TilS$_9$
Without TilS$_9$
Nº of genomes with TilS$_{10}$
Nº of genomes without TilS$_{10}$
With TilS$_{10}$
Without TilS$_{10}$
Nº of genomes with TilS$_{11}$
Nº of genomes without TilS$_{11}$
With TilS$_{11}$
Without TilS$_{11}$
Nº of genomes with TilS$_{12}$
Nº of genomes without TilS$_{12}$
With TilS$_{12}$
Without TilS$_{12}$
Nº of genomes with TilS$_{13}$
Nº of genomes without TilS$_{13}$
With TilS$_{13}$
Without TilS$_{13}$
Nº of genomes with TilS$_{14}$
Nº of genomes without TilS$_{14}$
With TilS$_{14}$
Without TilS$_{14}$
Nº of genomes with TilS$_{15}$
Nº of genomes without TilS$_{15}$
With TilS$_{15}$
Without TilS$_{15}$

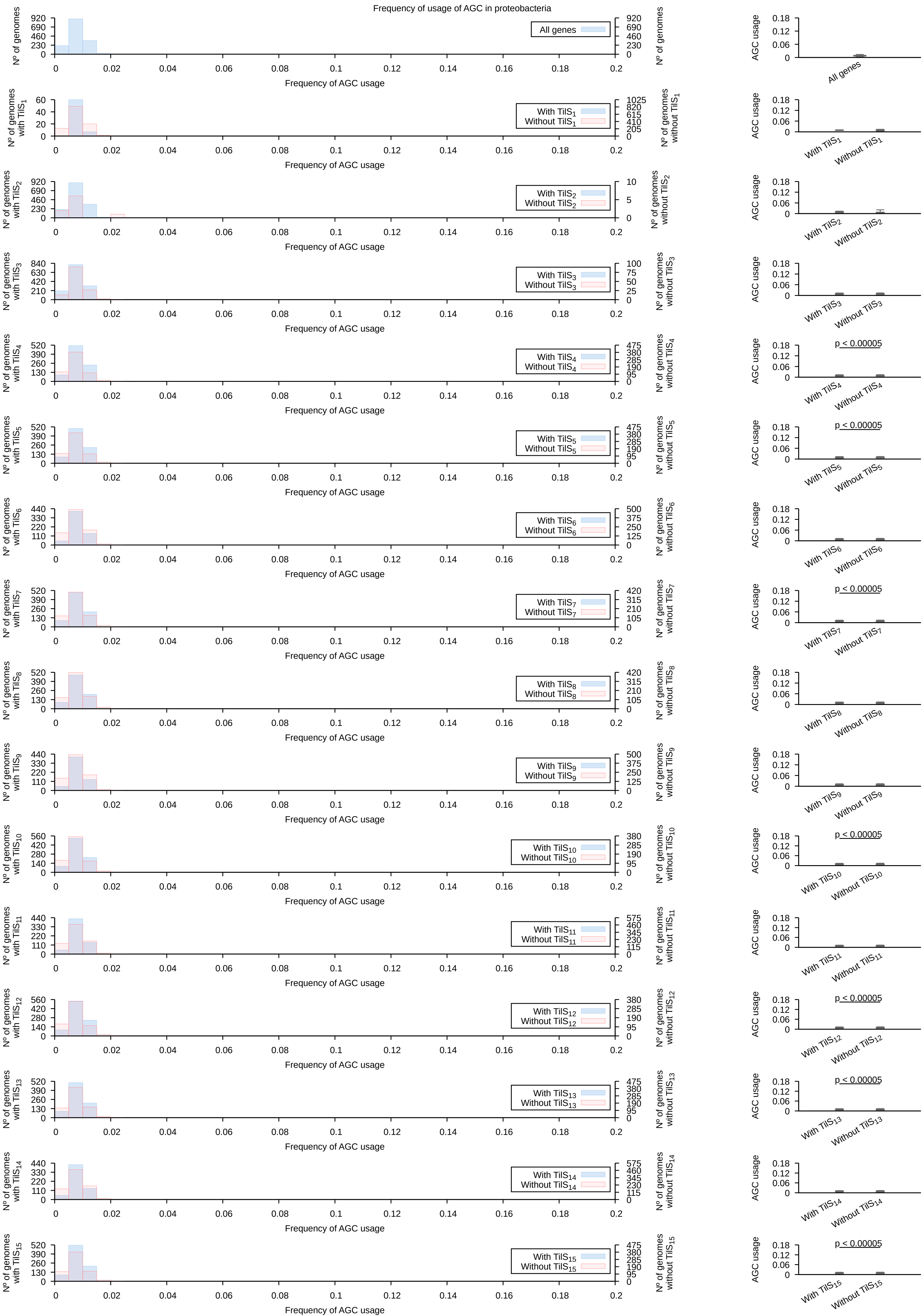

Frequency of usage of AGC in proteobacteria
Nº of genomes
All genes
Frequency of AGC usage
AGC usage
All genes
Nº of genomes with TilS1
With TilS1
Without TilS1
Nº of genomes without TilS1
Nº of genomes with TilS2
With TilS2
Without TilS2
Nº of genomes without TilS2
Nº of genomes with TilS3
With TilS3
Without TilS3
Nº of genomes without TilS3
Nº of genomes with TilS4
With TilS4
Without TilS4
Nº of genomes without TilS4
p < 0.00005
Nº of genomes with TilS5
With TilS5
Without TilS5
Nº of genomes without TilS5
p < 0.00005
Nº of genomes with TilS6
With TilS6
Without TilS6
Nº of genomes without TilS6
Nº of genomes with TilS7
With TilS7
Without TilS7
Nº of genomes without TilS7
p < 0.00005
Nº of genomes with TilS8
With TilS8
Without TilS8
Nº of genomes without TilS8
Nº of genomes with TilS9
With TilS9
Without TilS9
Nº of genomes without TilS9
Nº of genomes with TilS10
With TilS10
Without TilS10
Nº of genomes without TilS10
p < 0.00005
Nº of genomes with TilS11
With TilS11
Without TilS11
Nº of genomes without TilS11
Nº of genomes with TilS12
With TilS12
Without TilS12
Nº of genomes without TilS12
p < 0.00005
Nº of genomes with TilS13
With TilS13
Without TilS13
Nº of genomes without TilS13
p < 0.00005
Nº of genomes with TilS14
With TilS14
Without TilS14
Nº of genomes without TilS14
Nº of genomes with TilS15
With TilS15
Without TilS15
Nº of genomes without TilS15
p < 0.00005

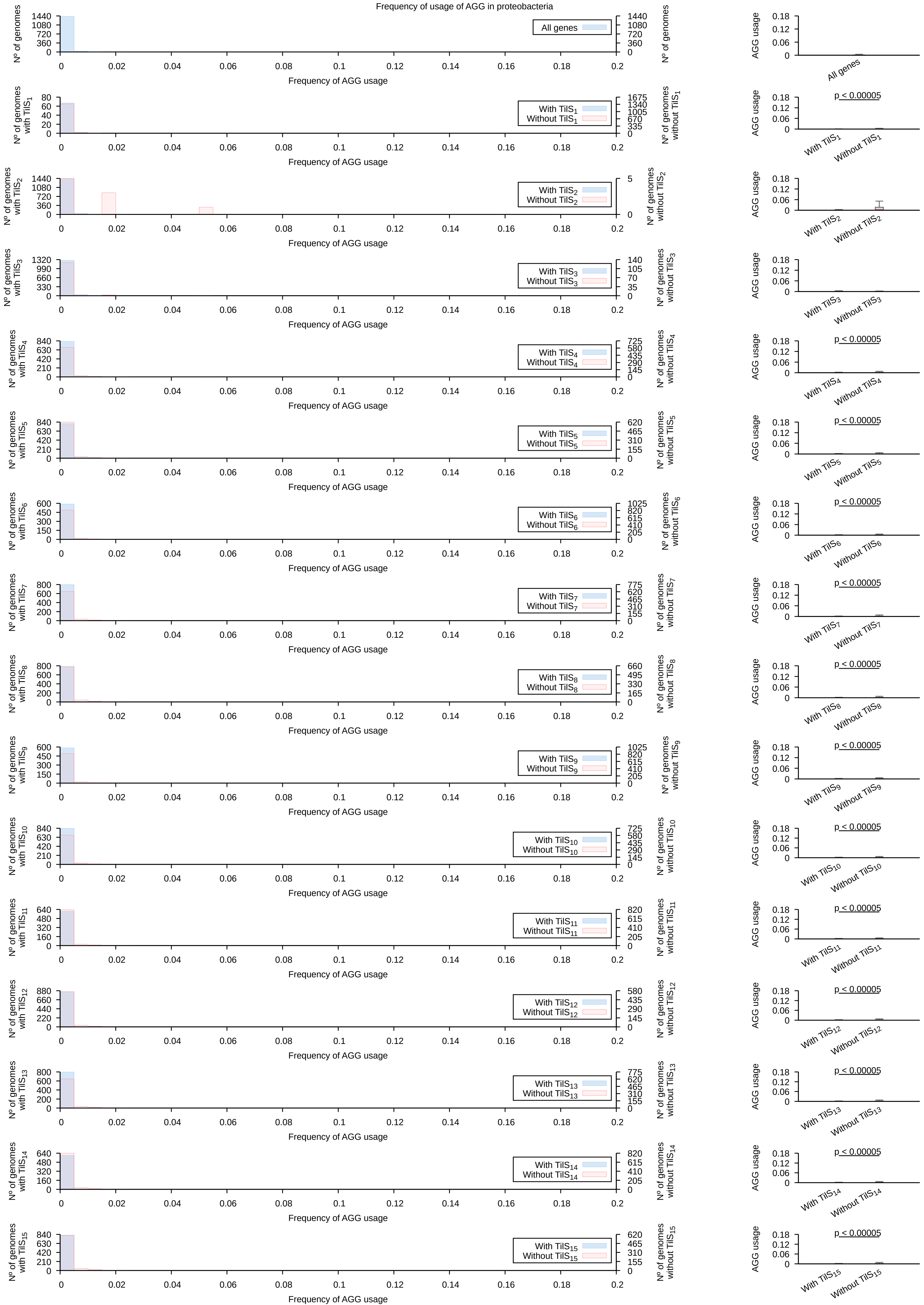

Frequency of usage of AGG in proteobacteria
Nº of genomes
All genes
Frequency of AGG usage
AGG usage
With TilS1
Without TilS1
Nº of genomes with TilS1
Nº of genomes without TilS1
p < 0.00005
With TilS2
Without TilS2
Nº of genomes with TilS2
Nº of genomes without TilS2
With TilS3
Without TilS3
Nº of genomes with TilS3
Nº of genomes without TilS3
With TilS4
Without TilS4
With TilS5
Without TilS5
With TilS6
Without TilS6
With TilS7
Without TilS7
With TilS8
Without TilS8
With TilS9
Without TilS9
With TilS10
Without TilS10
With TilS11
Without TilS11
With TilS12
Without TilS12
With TilS13
Without TilS13
With TilS14
Without TilS14
With TilS15
Without TilS15

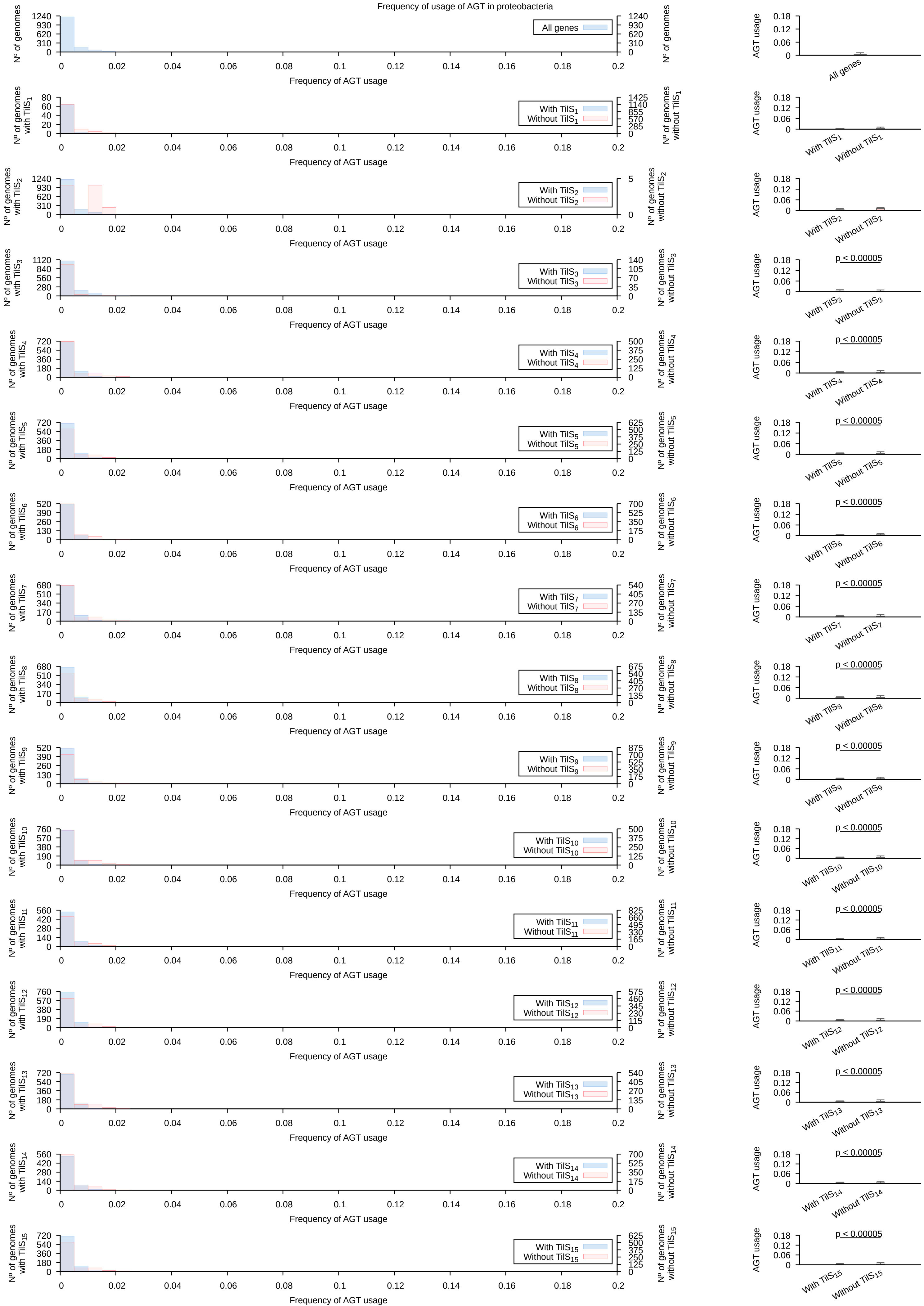

Frequency of usage of AGT in proteobacteria
Nº of genomes
All genes
Frequency of AGT usage
AGT usage
Nº of genomes with TilS$_1$
Nº of genomes without TilS$_1$
With TilS$_1$
Without TilS$_1$
Nº of genomes with TilS$_2$
Nº of genomes without TilS$_2$
With TilS$_2$
Without TilS$_2$
Nº of genomes with TilS$_3$
Nº of genomes without TilS$_3$
With TilS$_3$
Without TilS$_3$
p < 0.00005
Nº of genomes with TilS$_4$
Nº of genomes without TilS$_4$
With TilS$_4$
Without TilS$_4$
p < 0.00005
Nº of genomes with TilS$_5$
Nº of genomes without TilS$_5$
With TilS$_5$
Without TilS$_5$
p < 0.00005
Nº of genomes with TilS$_6$
Nº of genomes without TilS$_6$
With TilS$_6$
Without TilS$_6$
p < 0.00005
Nº of genomes with TilS$_7$
Nº of genomes without TilS$_7$
With TilS$_7$
Without TilS$_7$
p < 0.00005
Nº of genomes with TilS$_8$
Nº of genomes without TilS$_8$
With TilS$_8$
Without TilS$_8$
p < 0.00005
Nº of genomes with TilS$_9$
Nº of genomes without TilS$_9$
With TilS$_9$
Without TilS$_9$
p < 0.00005
Nº of genomes with TilS$_{10}$
Nº of genomes without TilS$_{10}$
With TilS$_{10}$
Without TilS$_{10}$
p < 0.00005
Nº of genomes with TilS$_{11}$
Nº of genomes without TilS$_{11}$
With TilS$_{11}$
Without TilS$_{11}$
p < 0.00005
Nº of genomes with TilS$_{12}$
Nº of genomes without TilS$_{12}$
With TilS$_{12}$
Without TilS$_{12}$
p < 0.00005
Nº of genomes with TilS$_{13}$
Nº of genomes without TilS$_{13}$
With TilS$_{13}$
Without TilS$_{13}$
p < 0.00005
Nº of genomes with TilS$_{14}$
Nº of genomes without TilS$_{14}$
With TilS$_{14}$
Without TilS$_{14}$
p < 0.00005
Nº of genomes with TilS$_{15}$
Nº of genomes without TilS$_{15}$
With TilS$_{15}$
Without TilS$_{15}$
p < 0.00005

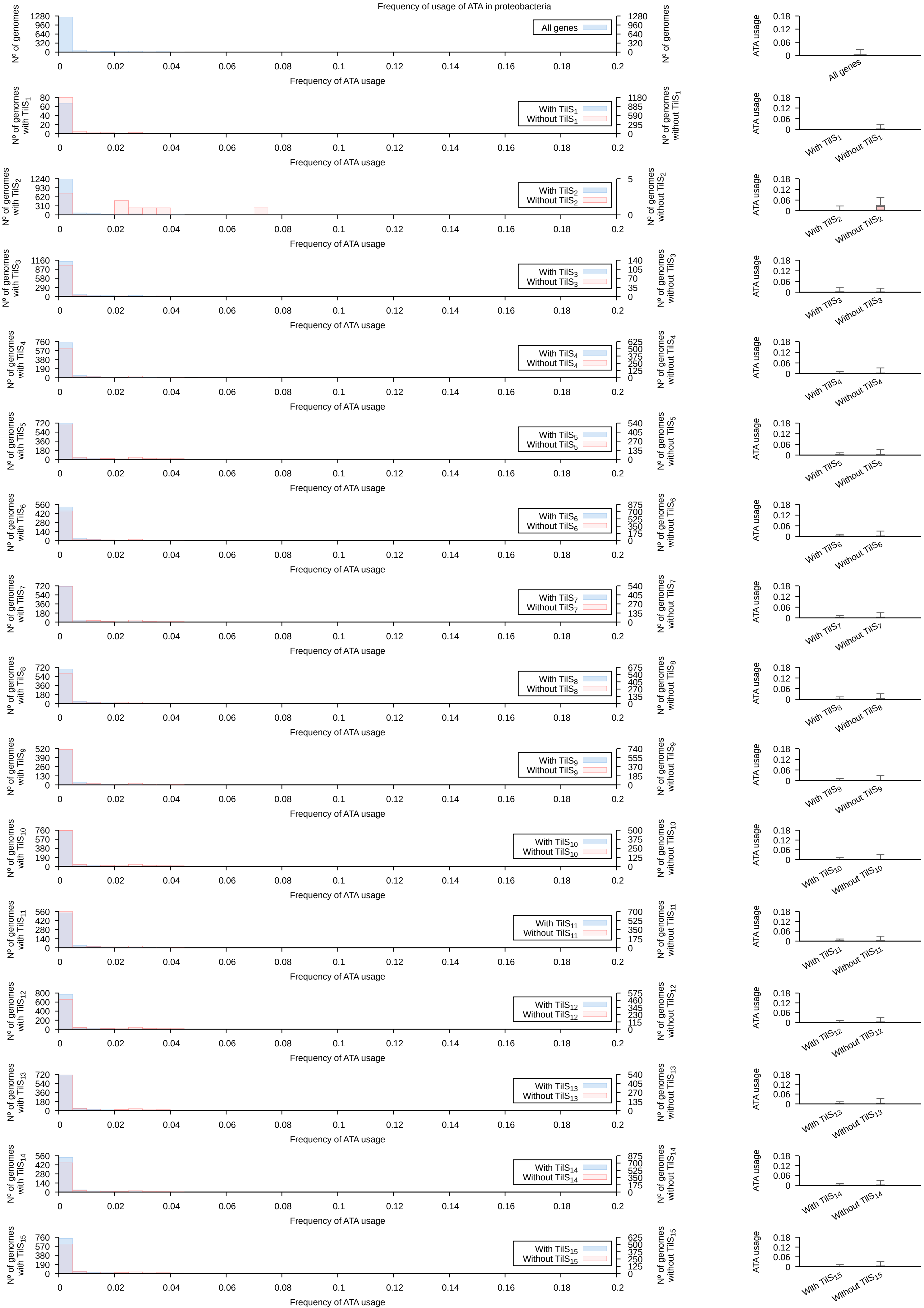

Frequency of usage of ATA in proteobacteria
All genes
Nº of genomes
Frequency of ATA usage
ATA usage
Nº of genomes with TilS$_1$
Nº of genomes without TilS$_1$
With TilS$_1$
Without TilS$_1$
Nº of genomes with TilS$_2$
Nº of genomes without TilS$_2$
With TilS$_2$
Without TilS$_2$
Nº of genomes with TilS$_3$
Nº of genomes without TilS$_3$
With TilS$_3$
Without TilS$_3$
Nº of genomes with TilS$_4$
Nº of genomes without TilS$_4$
With TilS$_4$
Without TilS$_4$
Nº of genomes with TilS$_5$
Nº of genomes without TilS$_5$
With TilS$_5$
Without TilS$_5$
Nº of genomes with TilS$_6$
Nº of genomes without TilS$_6$
With TilS$_6$
Without TilS$_6$
Nº of genomes with TilS$_7$
Nº of genomes without TilS$_7$
With TilS$_7$
Without TilS$_7$
Nº of genomes with TilS$_8$
Nº of genomes without TilS$_8$
With TilS$_8$
Without TilS$_8$
Nº of genomes with TilS$_9$
Nº of genomes without TilS$_9$
With TilS$_9$
Without TilS$_9$
Nº of genomes with TilS$_{10}$
Nº of genomes without TilS$_{10}$
With TilS$_{10}$
Without TilS$_{10}$
Nº of genomes with TilS$_{11}$
Nº of genomes without TilS$_{11}$
With TilS$_{11}$
Without TilS$_{11}$
Nº of genomes with TilS$_{12}$
Nº of genomes without TilS$_{12}$
With TilS$_{12}$
Without TilS$_{12}$
Nº of genomes with TilS$_{13}$
Nº of genomes without TilS$_{13}$
With TilS$_{13}$
Without TilS$_{13}$
Nº of genomes with TilS$_{14}$
Nº of genomes without TilS$_{14}$
With TilS$_{14}$
Without TilS$_{14}$
Nº of genomes with TilS$_{15}$
Nº of genomes without TilS$_{15}$
With TilS$_{15}$
Without TilS$_{15}$

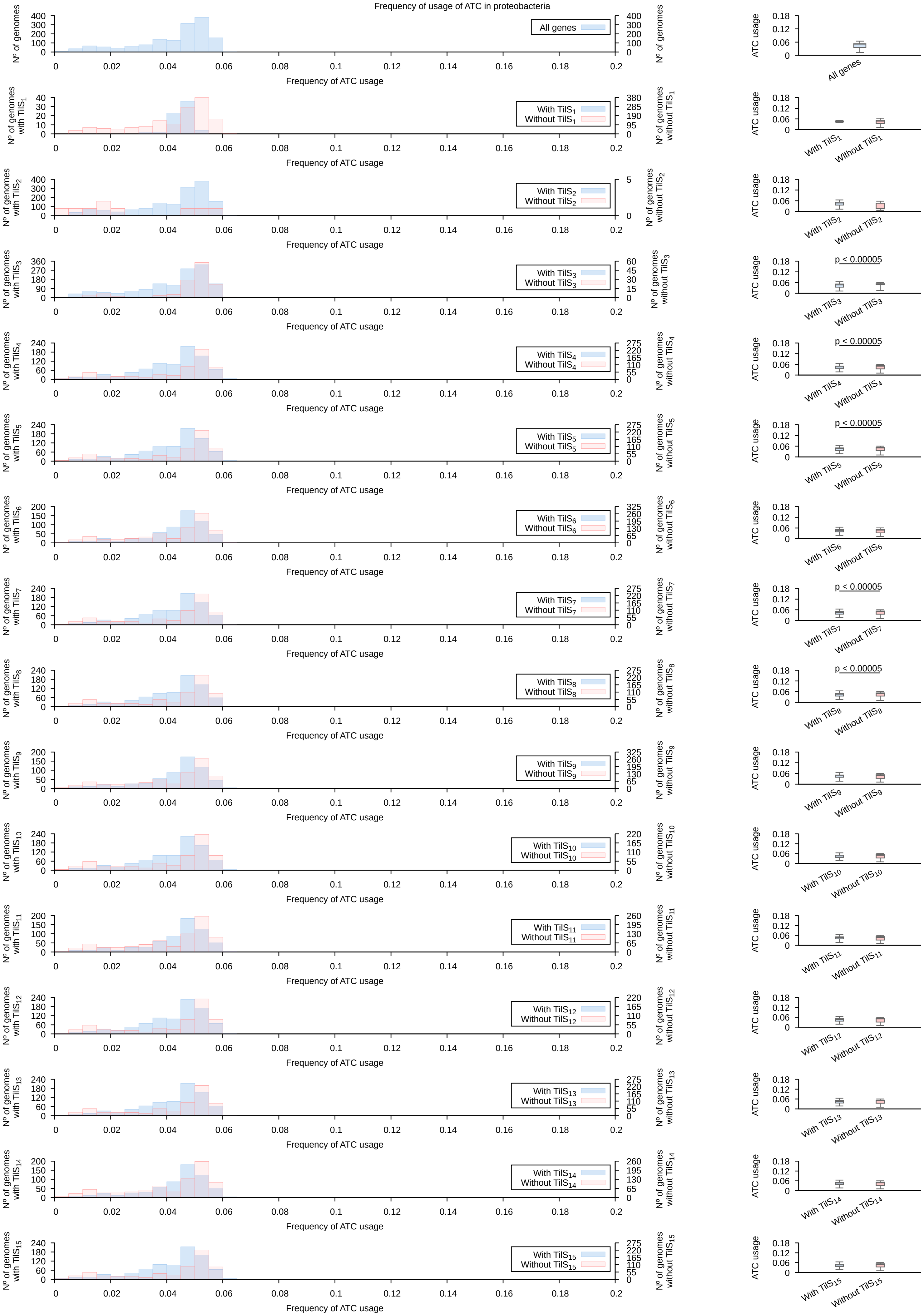
Frequency of usage of ATC in proteobacteria
Nº of genomes
All genes
Frequency of ATC usage
ATC usage
Nº of genomes with TilS1
Nº of genomes without TilS1
With TilS1
Without TilS1
p < 0.00005

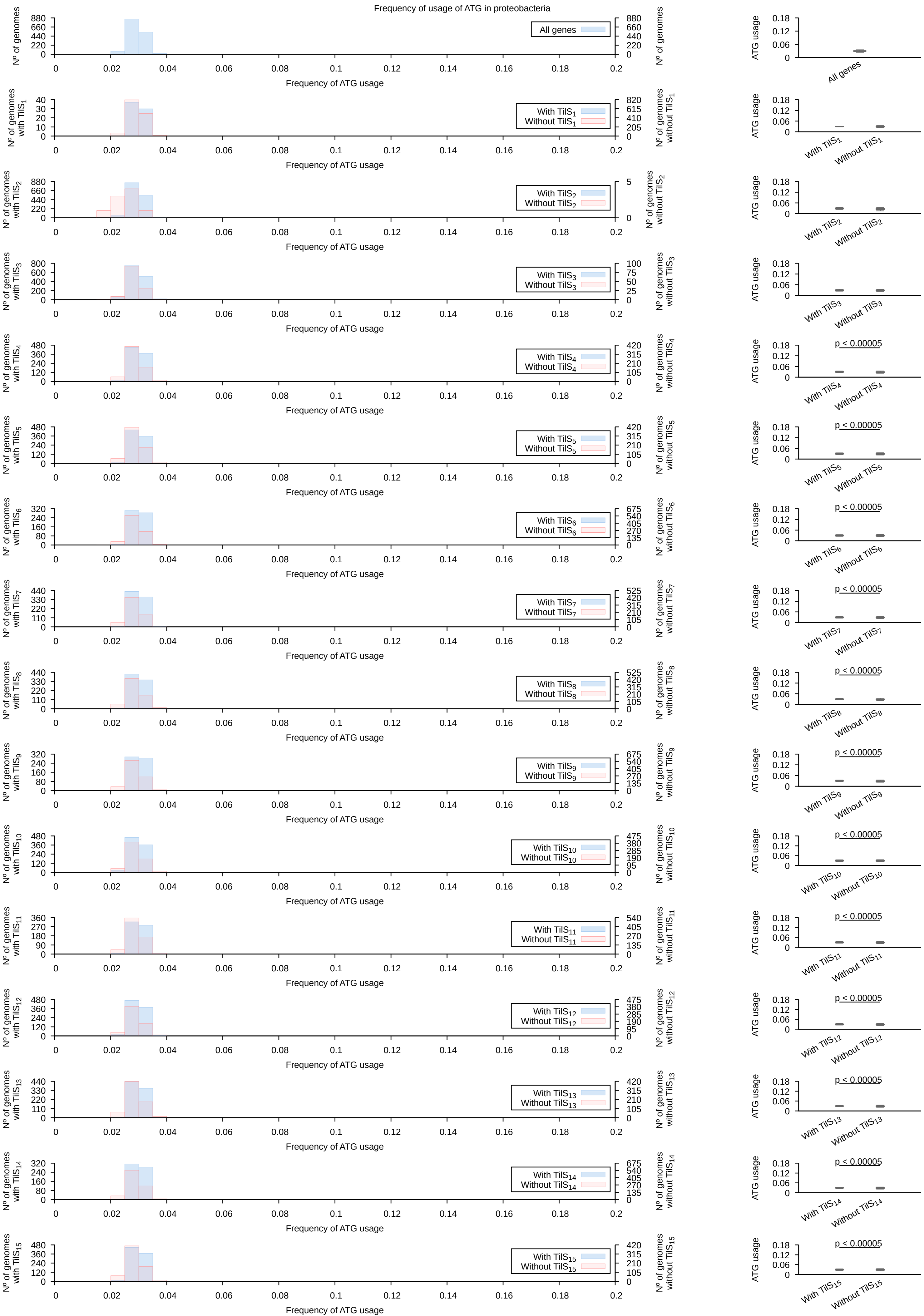

Frequency of usage of ATG in proteobacteria
Nº of genomes
All genes
Frequency of ATG usage
ATG usage
Nº of genomes with TilS$_1$
Nº of genomes without TilS$_1$
With TilS$_1$
Without TilS$_1$
Nº of genomes with TilS$_2$
Nº of genomes without TilS$_2$
With TilS$_2$
Without TilS$_2$
Nº of genomes with TilS$_3$
Nº of genomes without TilS$_3$
With TilS$_3$
Without TilS$_3$
Nº of genomes with TilS$_4$
Nº of genomes without TilS$_4$
With TilS$_4$
Without TilS$_4$
$p < 0.00005$
Nº of genomes with TilS$_5$
Nº of genomes without TilS$_5$
With TilS$_5$
Without TilS$_5$
Nº of genomes with TilS$_6$
Nº of genomes without TilS$_6$
With TilS$_6$
Without TilS$_6$
Nº of genomes with TilS$_7$
Nº of genomes without TilS$_7$
With TilS$_7$
Without TilS$_7$
Nº of genomes with TilS$_8$
Nº of genomes without TilS$_8$
With TilS$_8$
Without TilS$_8$
Nº of genomes with TilS$_9$
Nº of genomes without TilS$_9$
With TilS$_9$
Without TilS$_9$
Nº of genomes with TilS$_{10}$
Nº of genomes without TilS$_{10}$
With TilS$_{10}$
Without TilS$_{10}$
Nº of genomes with TilS$_{11}$
Nº of genomes without TilS$_{11}$
With TilS$_{11}$
Without TilS$_{11}$
Nº of genomes with TilS$_{12}$
Nº of genomes without TilS$_{12}$
With TilS$_{12}$
Without TilS$_{12}$
Nº of genomes with TilS$_{13}$
Nº of genomes without TilS$_{13}$
With TilS$_{13}$
Without TilS$_{13}$
Nº of genomes with TilS$_{14}$
Nº of genomes without TilS$_{14}$
With TilS$_{14}$
Without TilS$_{14}$
Nº of genomes with TilS$_{15}$
Nº of genomes without TilS$_{15}$
With TilS$_{15}$
Without TilS$_{15}$

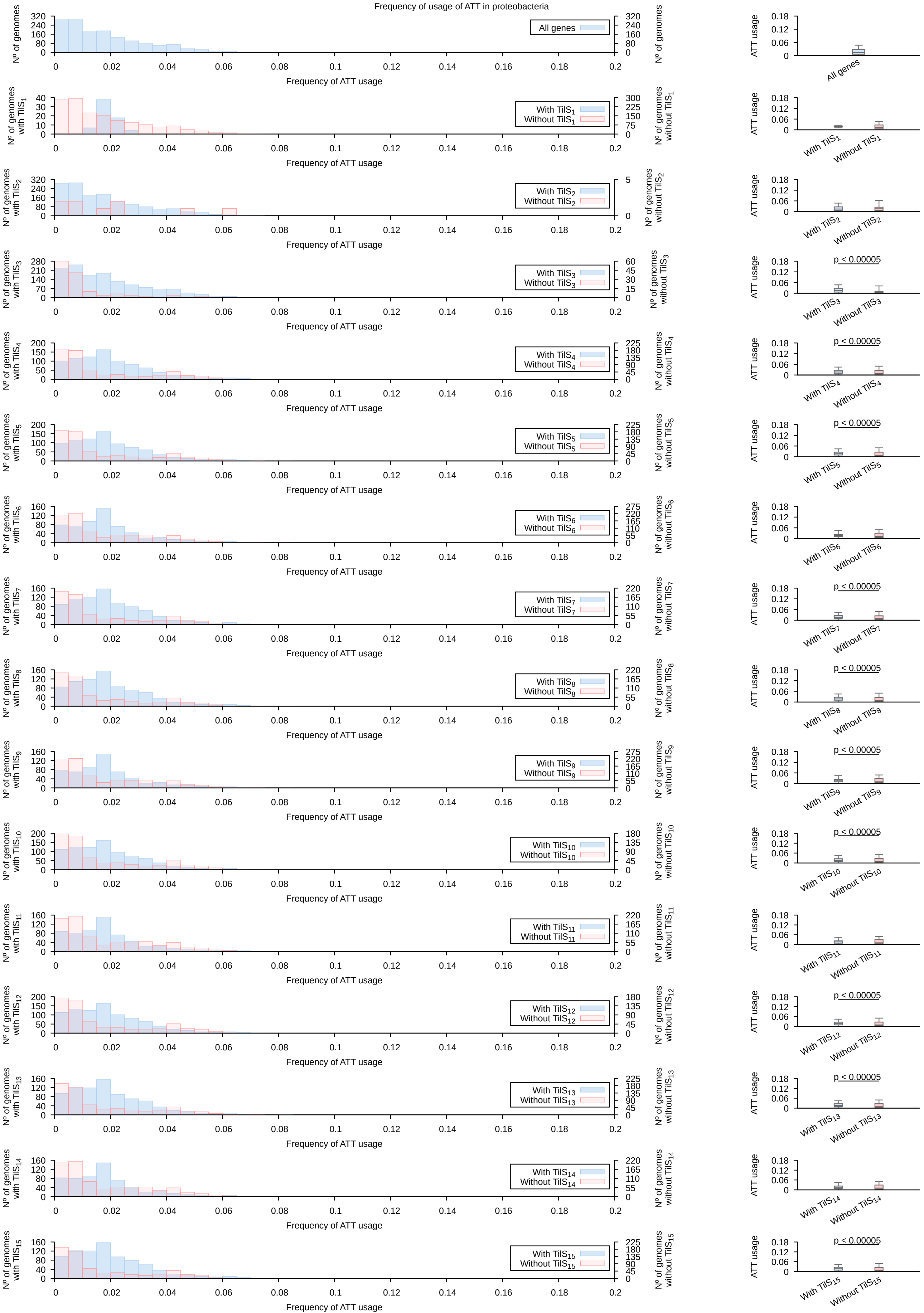

Frequency of usage of ATT in proteobacteria
Nº of genomes
All genes
Frequency of ATT usage
ATT usage
Nº of genomes with TilS1
Nº of genomes without TilS1
With TilS1
Without TilS1
Nº of genomes with TilS2
Nº of genomes without TilS2
With TilS2
Without TilS2
Nº of genomes with TilS3
Nº of genomes without TilS3
With TilS3
Without TilS3
p < 0.00005
Nº of genomes with TilS4
Nº of genomes without TilS4
With TilS4
Without TilS4
p < 0.00005
Nº of genomes with TilS5
Nº of genomes without TilS5
With TilS5
Without TilS5
p < 0.00005
Nº of genomes with TilS6
Nº of genomes without TilS6
With TilS6
Without TilS6
Nº of genomes with TilS7
Nº of genomes without TilS7
With TilS7
Without TilS7
p < 0.00005
Nº of genomes with TilS8
Nº of genomes without TilS8
With TilS8
Without TilS8
p < 0.00005
Nº of genomes with TilS9
Nº of genomes without TilS9
With TilS9
Without TilS9
p < 0.00005
Nº of genomes with TilS10
Nº of genomes without TilS10
With TilS10
Without TilS10
p < 0.00005
Nº of genomes with TilS11
Nº of genomes without TilS11
With TilS11
Without TilS11
Nº of genomes with TilS12
Nº of genomes without TilS12
With TilS12
Without TilS12
p < 0.00005
Nº of genomes with TilS13
Nº of genomes without TilS13
With TilS13
Without TilS13
p < 0.00005
Nº of genomes with TilS14
Nº of genomes without TilS14
With TilS14
Without TilS14
Nº of genomes with TilS15
Nº of genomes without TilS15
With TilS15
Without TilS15
p < 0.00005

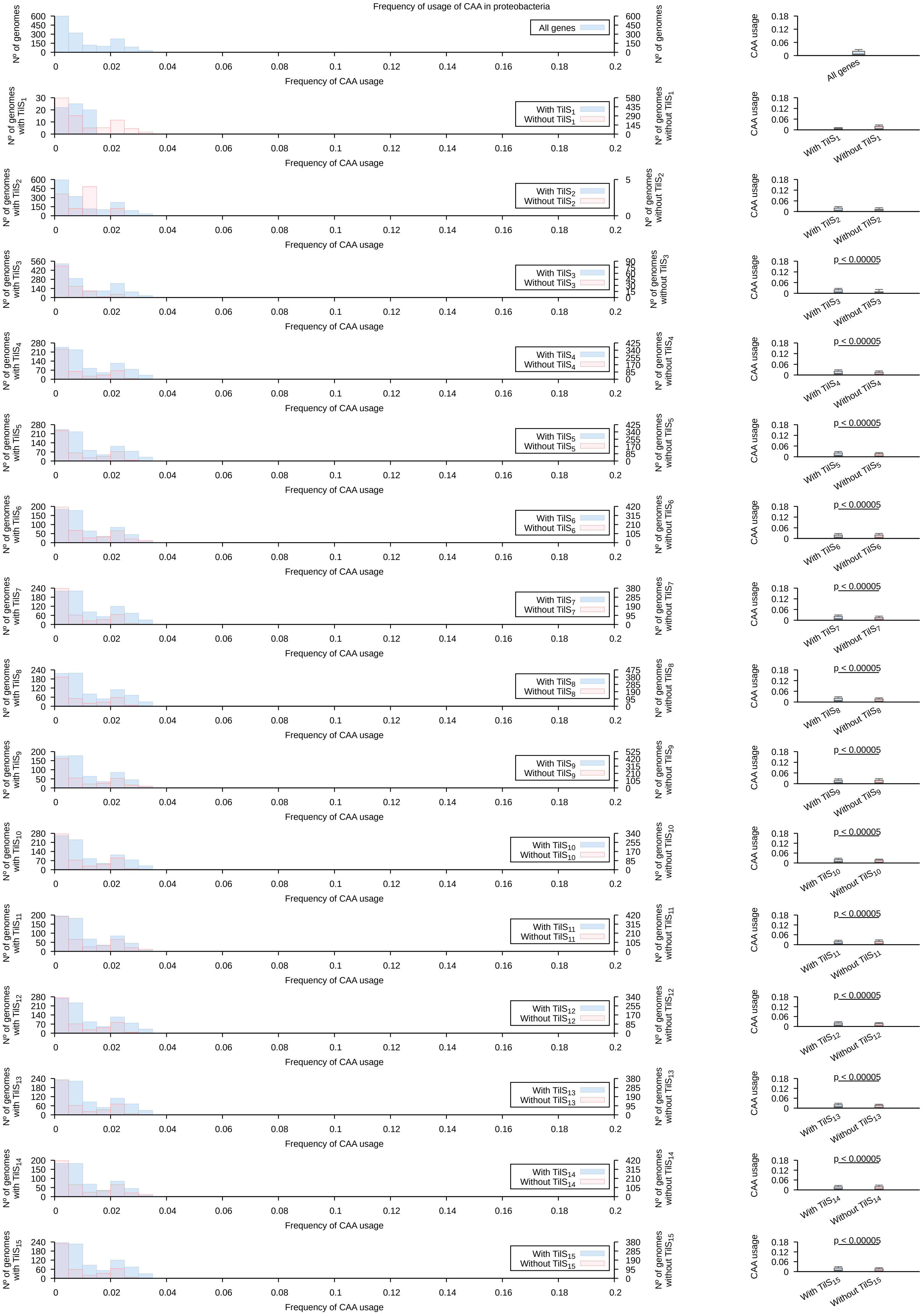
Frequency of usage of CAA in proteobacteria
Nº of genomes
All genes
Frequency of CAA usage
CAA usage
With TilS1
Without TilS1
Nº of genomes with TilS
Nº of genomes without TilS
p < 0.00005
0
0.02
0.04
0.06
0.08
0.1
0.12
0.14
0.16
0.18
0.2

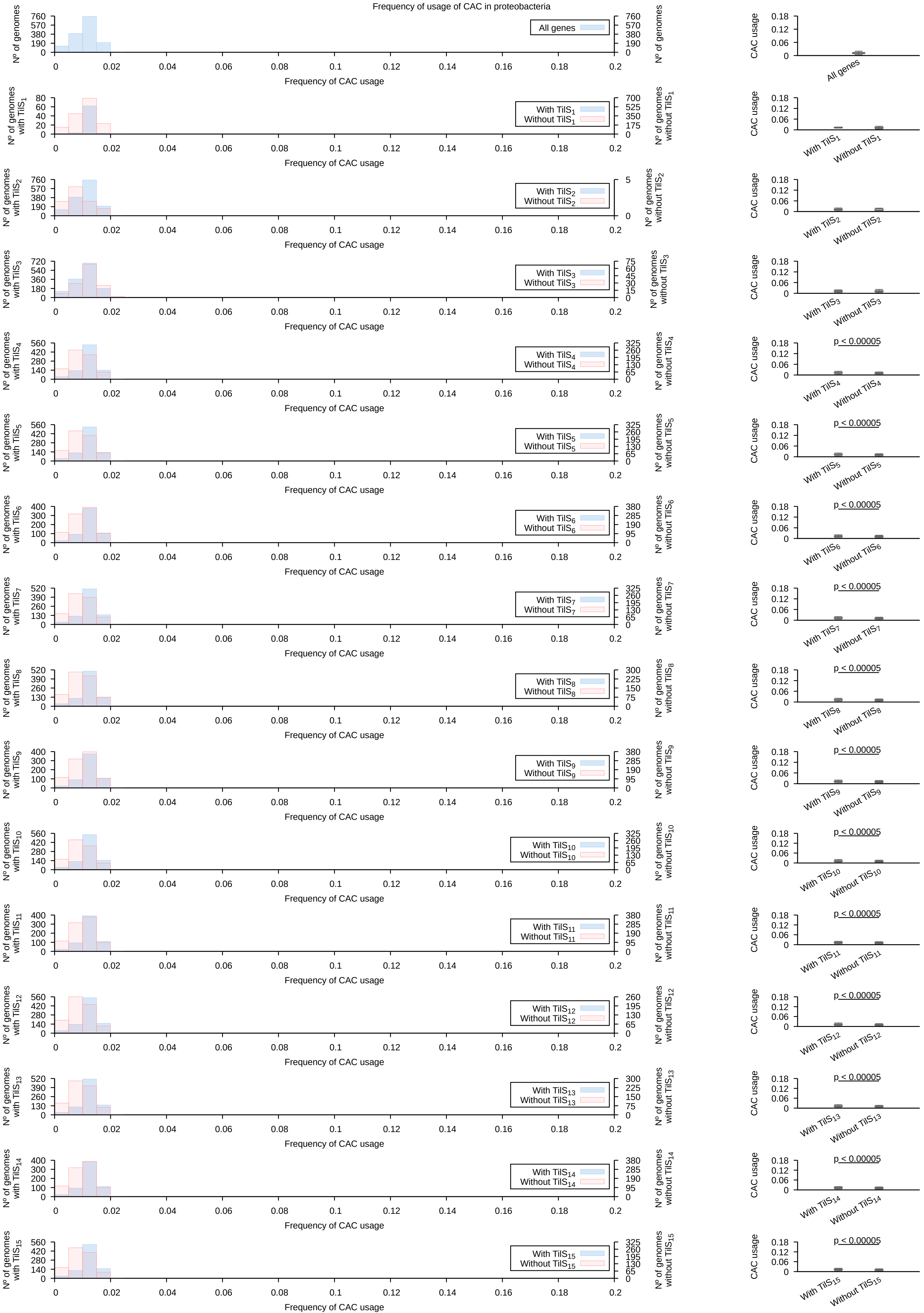

Frequency of usage of CAC in proteobacteria
Nº of genomes
All genes
Frequency of CAC usage
CAC usage
Nº of genomes with TilS$_1$
Nº of genomes without TilS$_1$
With TilS$_1$
Without TilS$_1$
Nº of genomes with TilS$_2$
Nº of genomes without TilS$_2$
With TilS$_2$
Without TilS$_2$
Nº of genomes with TilS$_3$
Nº of genomes without TilS$_3$
With TilS$_3$
Without TilS$_3$
Nº of genomes with TilS$_4$
Nº of genomes without TilS$_4$
With TilS$_4$
Without TilS$_4$
Nº of genomes with TilS$_5$
Nº of genomes without TilS$_5$
With TilS$_5$
Without TilS$_5$
Nº of genomes with TilS$_6$
Nº of genomes without TilS$_6$
With TilS$_6$
Without TilS$_6$
Nº of genomes with TilS$_7$
Nº of genomes without TilS$_7$
With TilS$_7$
Without TilS$_7$
Nº of genomes with TilS$_8$
Nº of genomes without TilS$_8$
With TilS$_8$
Without TilS$_8$
Nº of genomes with TilS$_9$
Nº of genomes without TilS$_9$
With TilS$_9$
Without TilS$_9$
Nº of genomes with TilS$_{10}$
Nº of genomes without TilS$_{10}$
With TilS$_{10}$
Without TilS$_{10}$
Nº of genomes with TilS$_{11}$
Nº of genomes without TilS$_{11}$
With TilS$_{11}$
Without TilS$_{11}$
Nº of genomes with TilS$_{12}$
Nº of genomes without TilS$_{12}$
With TilS$_{12}$
Without TilS$_{12}$
Nº of genomes with TilS$_{13}$
Nº of genomes without TilS$_{13}$
With TilS$_{13}$
Without TilS$_{13}$
Nº of genomes with TilS$_{14}$
Nº of genomes without TilS$_{14}$
With TilS$_{14}$
Without TilS$_{14}$
Nº of genomes with TilS$_{15}$
Nº of genomes without TilS$_{15}$
With TilS$_{15}$
Without TilS$_{15}$
p < 0.00005

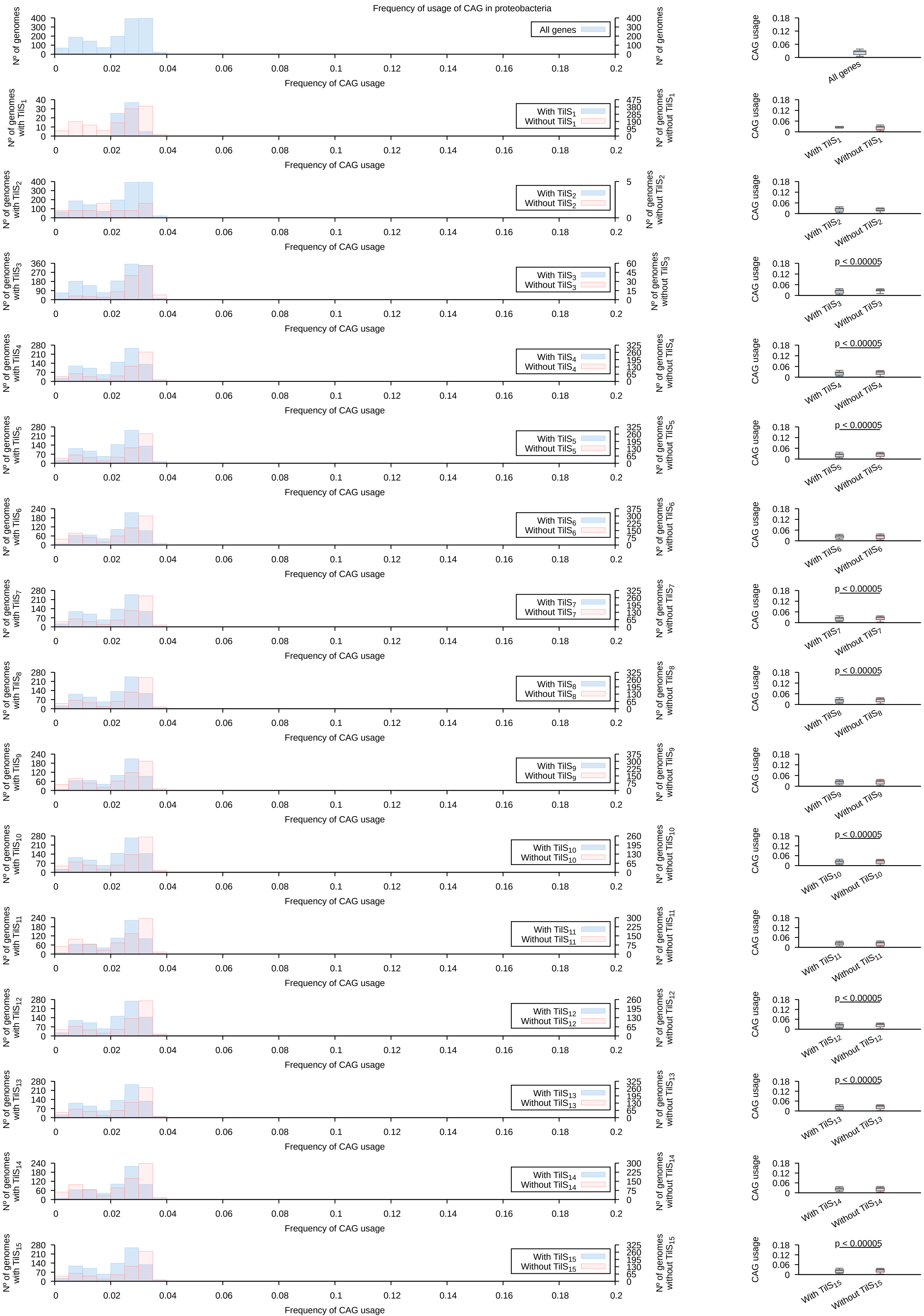

Frequency of usage of CAG in proteobacteria
Nº of genomes
All genes
Frequency of CAG usage
CAG usage
Nº of genomes with TilS1
Nº of genomes without TilS1
With TilS1
Without TilS1
Nº of genomes with TilS2
Nº of genomes without TilS2
With TilS2
Without TilS2
Nº of genomes with TilS3
Nº of genomes without TilS3
With TilS3
Without TilS3
p < 0.00005
Nº of genomes with TilS4
Nº of genomes without TilS4
With TilS4
Without TilS4
p < 0.00005
Nº of genomes with TilS5
Nº of genomes without TilS5
With TilS5
Without TilS5
p < 0.00005
Nº of genomes with TilS6
Nº of genomes without TilS6
With TilS6
Without TilS6
Nº of genomes with TilS7
Nº of genomes without TilS7
With TilS7
Without TilS7
p < 0.00005
Nº of genomes with TilS8
Nº of genomes without TilS8
With TilS8
Without TilS8
p < 0.00005
Nº of genomes with TilS9
Nº of genomes without TilS9
With TilS9
Without TilS9
Nº of genomes with TilS10
Nº of genomes without TilS10
With TilS10
Without TilS10
p < 0.00005
Nº of genomes with TilS11
Nº of genomes without TilS11
With TilS11
Without TilS11
Nº of genomes with TilS12
Nº of genomes without TilS12
With TilS12
Without TilS12
p < 0.00005
Nº of genomes with TilS13
Nº of genomes without TilS13
With TilS13
Without TilS13
p < 0.00005
Nº of genomes with TilS14
Nº of genomes without TilS14
With TilS14
Without TilS14
Nº of genomes with TilS15
Nº of genomes without TilS15
With TilS15
Without TilS15
p < 0.00005

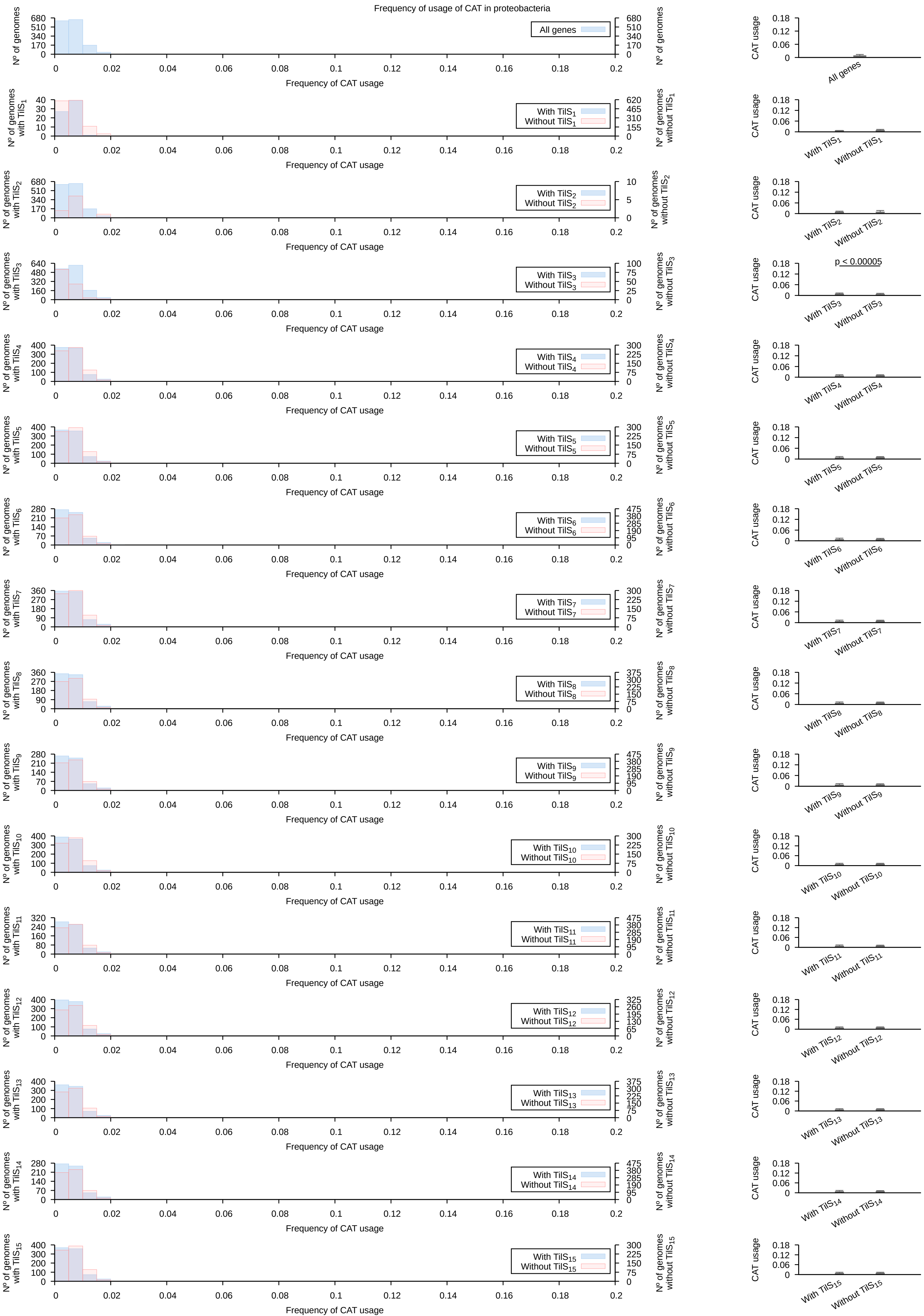

Frequency of usage of CAT in proteobacteria
Nº of genomes
680 510 340 170 0
All genes
0 0.02 0.04 0.06 0.08 0.1 0.12 0.14 0.16 0.18 0.2
Frequency of CAT usage
CAT usage
0.18 0.12 0.06 0
All genes
Nº of genomes with $TilS_1$
40 30 20 10 0
Nº of genomes without $TilS_1$
620 465 310 155 0
With $TilS_1$
Without $TilS_1$
Nº of genomes with $TilS_2$
680 510 340 170 0
Nº of genomes without $TilS_2$
10 5 0
With $TilS_2$
Without $TilS_2$
Nº of genomes with $TilS_3$
640 480 320 160 0
Nº of genomes without $TilS_3$
100 75 50 25 0
With $TilS_3$
Without $TilS_3$
p < 0.00005
Nº of genomes with $TilS_4$
400 300 200 100 0
Nº of genomes without $TilS_4$
300 225 150 75 0
With $TilS_4$
Without $TilS_4$
Nº of genomes with $TilS_5$
400 300 200 100 0
Nº of genomes without $TilS_5$
300 225 150 75 0
With $TilS_5$
Without $TilS_5$
Nº of genomes with $TilS_6$
280 210 140 70 0
Nº of genomes without $TilS_6$
475 380 285 190 95 0
With $TilS_6$
Without $TilS_6$
Nº of genomes with $TilS_7$
360 270 180 90 0
Nº of genomes without $TilS_7$
300 225 150 75 0
With $TilS_7$
Without $TilS_7$
Nº of genomes with $TilS_8$
360 270 180 90 0
Nº of genomes without $TilS_8$
375 300 225 150 75 0
With $TilS_8$
Without $TilS_8$
Nº of genomes with $TilS_9$
280 210 140 70 0
Nº of genomes without $TilS_9$
475 380 285 190 95 0
With $TilS_9$
Without $TilS_9$
Nº of genomes with $TilS_{10}$
400 300 200 100 0
Nº of genomes without $TilS_{10}$
300 225 150 75 0
With $TilS_{10}$
Without $TilS_{10}$
Nº of genomes with $TilS_{11}$
320 240 160 80 0
Nº of genomes without $TilS_{11}$
475 380 285 190 95 0
With $TilS_{11}$
Without $TilS_{11}$
Nº of genomes with $TilS_{12}$
400 300 200 100 0
Nº of genomes without $TilS_{12}$
325 260 195 130 65 0
With $TilS_{12}$
Without $TilS_{12}$
Nº of genomes with $TilS_{13}$
400 300 200 100 0
Nº of genomes without $TilS_{13}$
375 300 225 150 75 0
With $TilS_{13}$
Without $TilS_{13}$
Nº of genomes with $TilS_{14}$
280 210 140 70 0
Nº of genomes without $TilS_{14}$
475 380 285 190 95 0
With $TilS_{14}$
Without $TilS_{14}$
Nº of genomes with $TilS_{15}$
400 300 200 100 0
Nº of genomes without $TilS_{15}$
300 225 150 75 0
With $TilS_{15}$
Without $TilS_{15}$

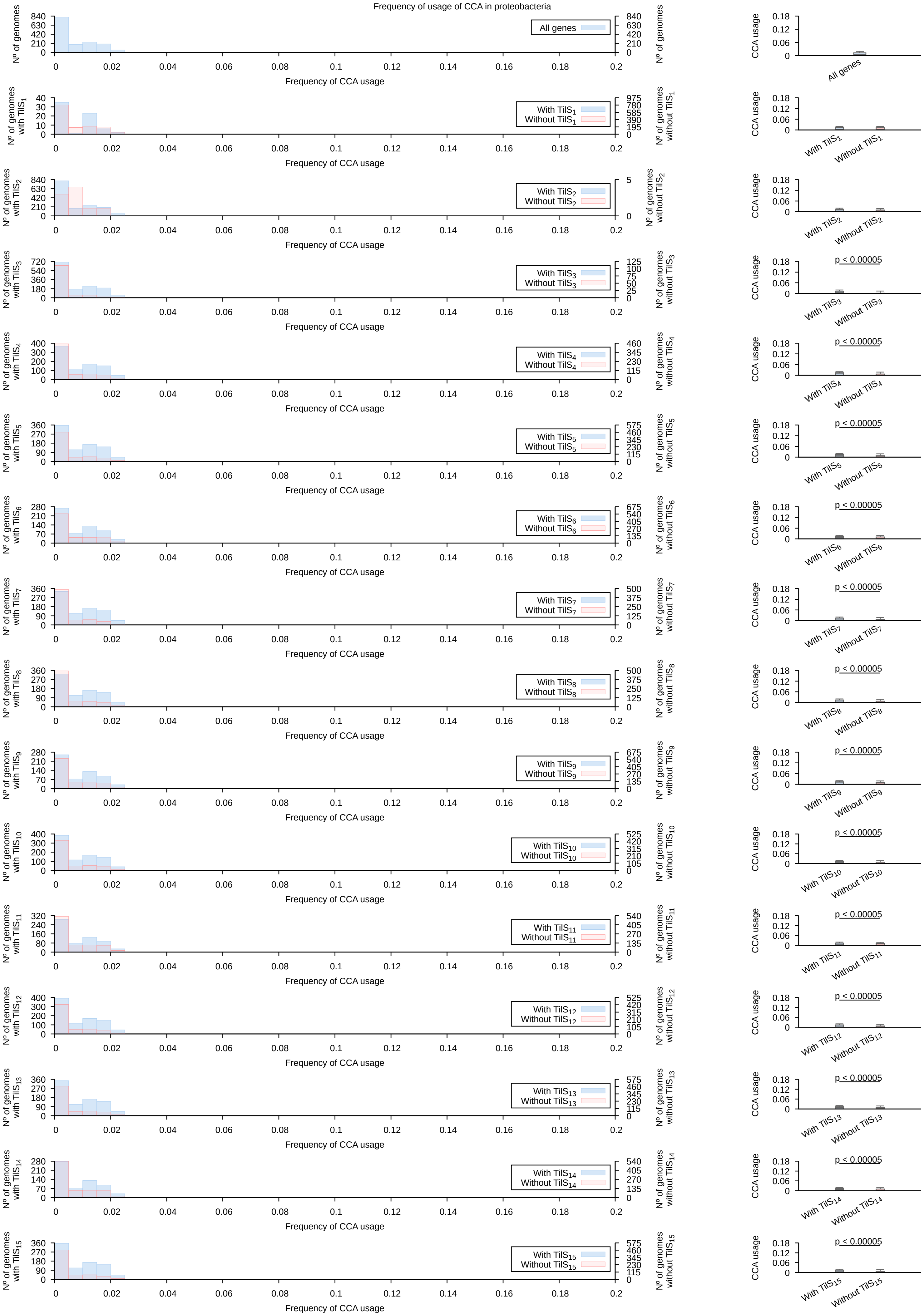

Frequency of usage of CCA in proteobacteria
Nº of genomes
Frequency of CCA usage
CCA usage
All genes
With TilS1
Without TilS1
Nº of genomes with TilS1
Nº of genomes without TilS1
With TilS2
Without TilS2
With TilS3
Without TilS3
p < 0.00005
With TilS4
Without TilS4
With TilS5
Without TilS5
With TilS6
Without TilS6
With TilS7
Without TilS7
With TilS8
Without TilS8
With TilS9
Without TilS9
With TilS10
Without TilS10
With TilS11
Without TilS11
With TilS12
Without TilS12
With TilS13
Without TilS13
With TilS14
Without TilS14
With TilS15
Without TilS15

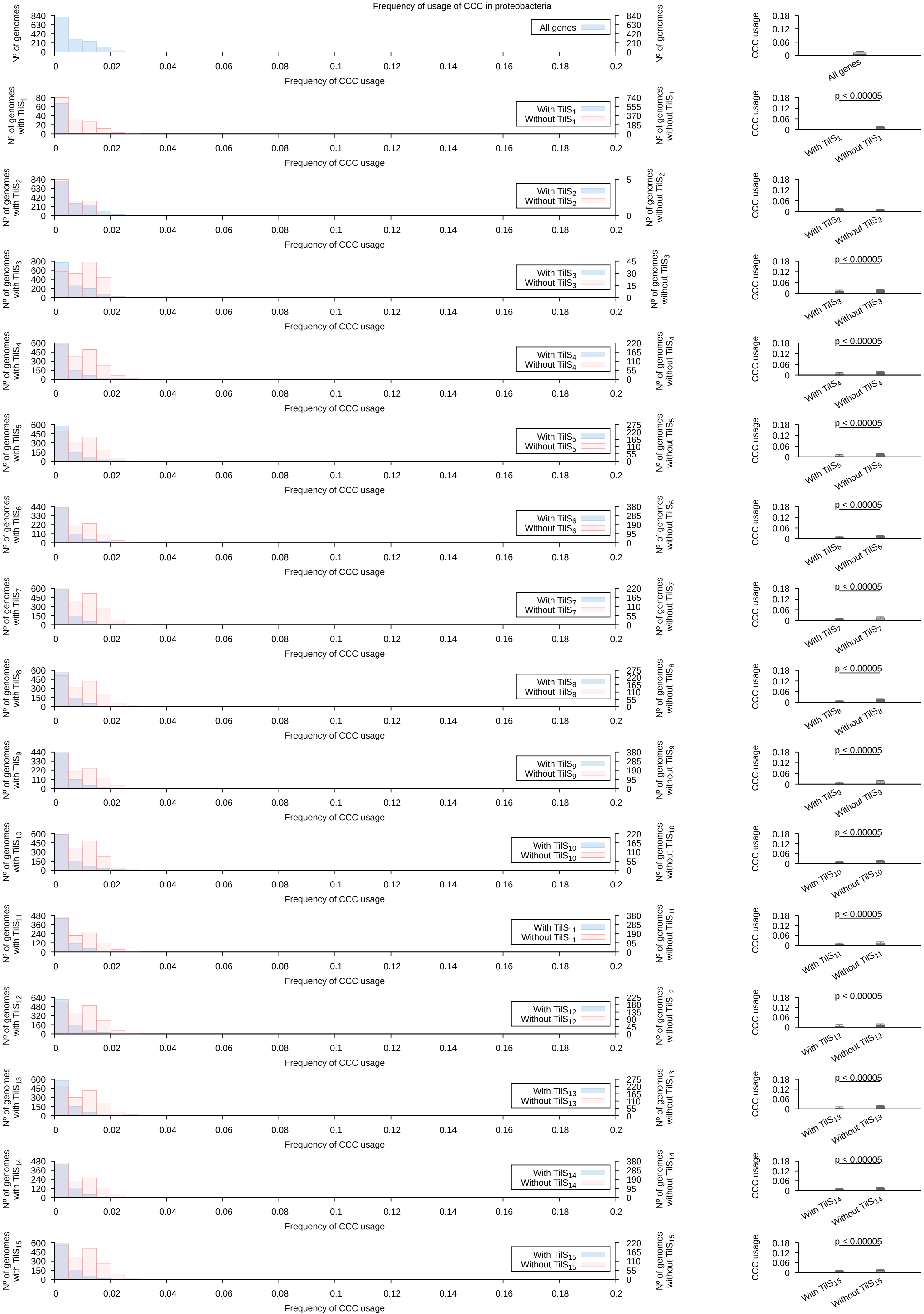

Frequency of usage of CCC in proteobacteria
Nº of genomes
All genes
Frequency of CCC usage
CCC usage
All genes
Nº of genomes with TilS$_1$
Nº of genomes without TilS$_1$
With TilS$_1$
Without TilS$_1$
p < 0.00005
Nº of genomes with TilS$_2$
Nº of genomes without TilS$_2$
With TilS$_2$
Without TilS$_2$
Nº of genomes with TilS$_3$
Nº of genomes without TilS$_3$
With TilS$_3$
Without TilS$_3$
p < 0.00005
Nº of genomes with TilS$_4$
Nº of genomes without TilS$_4$
With TilS$_4$
Without TilS$_4$
p < 0.00005
Nº of genomes with TilS$_5$
Nº of genomes without TilS$_5$
With TilS$_5$
Without TilS$_5$
p < 0.00005
Nº of genomes with TilS$_6$
Nº of genomes without TilS$_6$
With TilS$_6$
Without TilS$_6$
p < 0.00005
Nº of genomes with TilS$_7$
Nº of genomes without TilS$_7$
With TilS$_7$
Without TilS$_7$
p < 0.00005
Nº of genomes with TilS$_8$
Nº of genomes without TilS$_8$
With TilS$_8$
Without TilS$_8$
p < 0.00005
Nº of genomes with TilS$_9$
Nº of genomes without TilS$_9$
With TilS$_9$
Without TilS$_9$
p < 0.00005
Nº of genomes with TilS$_{10}$
Nº of genomes without TilS$_{10}$
With TilS$_{10}$
Without TilS$_{10}$
p < 0.00005
Nº of genomes with TilS$_{11}$
Nº of genomes without TilS$_{11}$
With TilS$_{11}$
Without TilS$_{11}$
p < 0.00005
Nº of genomes with TilS$_{12}$
Nº of genomes without TilS$_{12}$
With TilS$_{12}$
Without TilS$_{12}$
p < 0.00005
Nº of genomes with TilS$_{13}$
Nº of genomes without TilS$_{13}$
With TilS$_{13}$
Without TilS$_{13}$
p < 0.00005
Nº of genomes with TilS$_{14}$
Nº of genomes without TilS$_{14}$
With TilS$_{14}$
Without TilS$_{14}$
p < 0.00005
Nº of genomes with TilS$_{15}$
Nº of genomes without TilS$_{15}$
With TilS$_{15}$
Without TilS$_{15}$
p < 0.00005

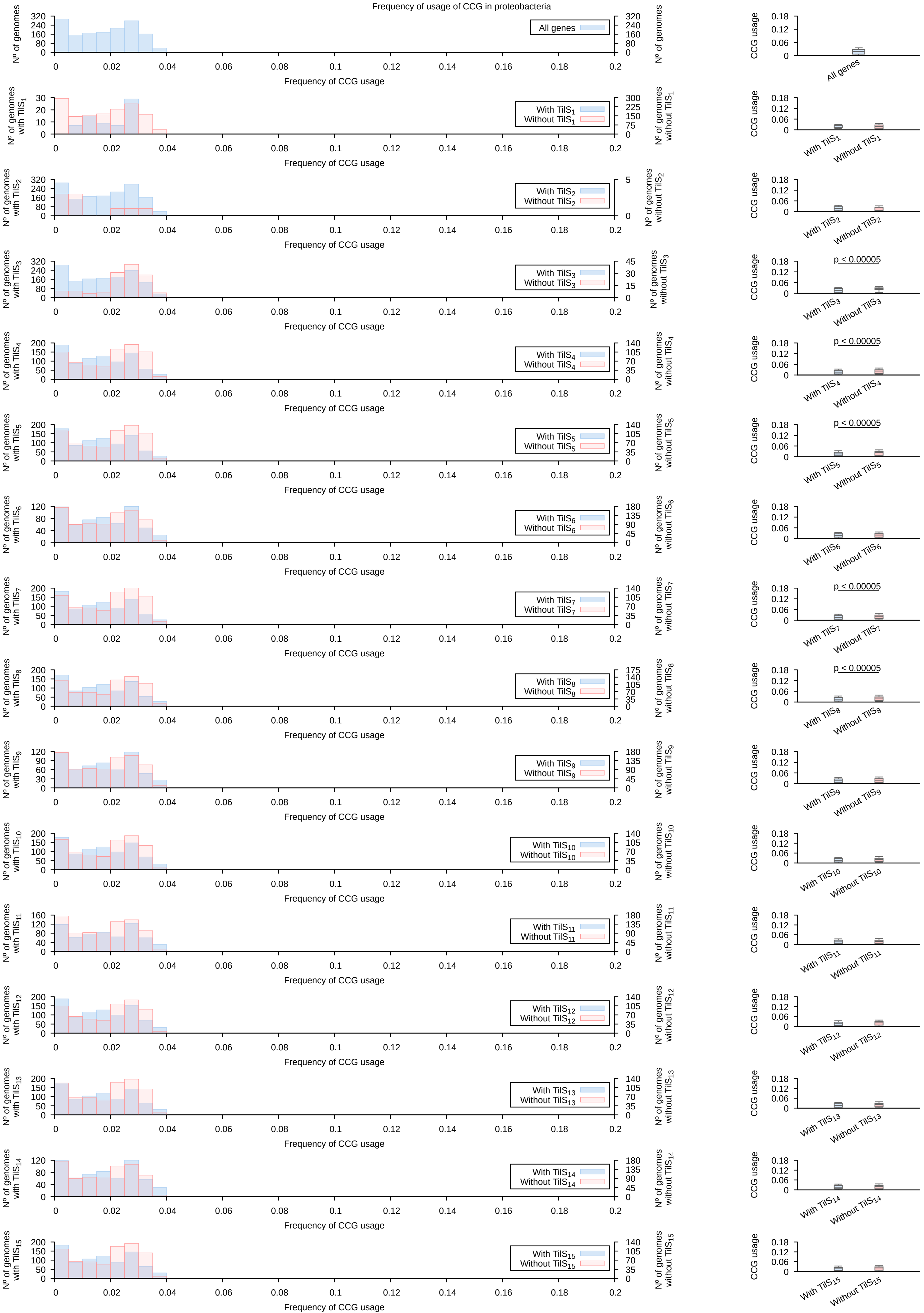
Frequency of usage of CCG in proteobacteria
Nº of genomes
All genes
Frequency of CCG usage
CCG usage
p < 0.00005
Nº of genomes with TilS1 … Nº of genomes with TilS15
Nº of genomes without TilS1 … Nº of genomes without TilS15
With TilS1 … With TilS15
Without TilS1 … Without TilS15
0 0.02 0.04 0.06 0.08 0.1 0.12 0.14 0.16 0.18 0.2
0 0.06 0.12 0.18

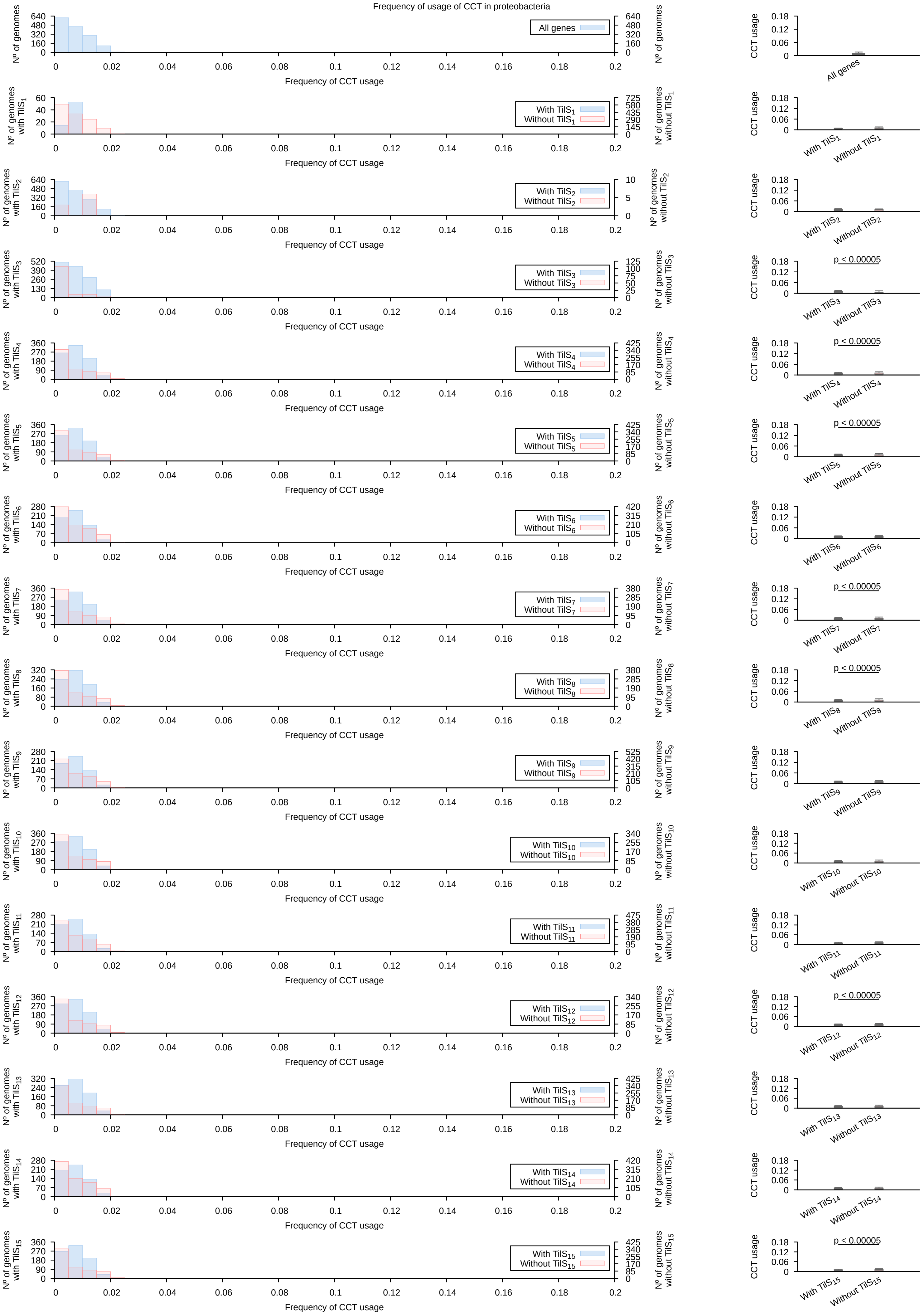
Frequency of usage of CCT in proteobacteria
Nº of genomes
All genes
Frequency of CCT usage
CCT usage
Nº of genomes with TilS1
Nº of genomes without TilS1
With TilS1
Without TilS1
Nº of genomes with TilS2
Nº of genomes without TilS2
With TilS2
Without TilS2
Nº of genomes with TilS3
Nº of genomes without TilS3
With TilS3
Without TilS3
p < 0.00005
Nº of genomes with TilS4
Nº of genomes without TilS4
With TilS4
Without TilS4
Nº of genomes with TilS5
Nº of genomes without TilS5
With TilS5
Without TilS5
Nº of genomes with TilS6
Nº of genomes without TilS6
With TilS6
Without TilS6
Nº of genomes with TilS7
Nº of genomes without TilS7
With TilS7
Without TilS7
Nº of genomes with TilS8
Nº of genomes without TilS8
With TilS8
Without TilS8
Nº of genomes with TilS9
Nº of genomes without TilS9
With TilS9
Without TilS9
Nº of genomes with TilS10
Nº of genomes without TilS10
With TilS10
Without TilS10
Nº of genomes with TilS11
Nº of genomes without TilS11
With TilS11
Without TilS11
Nº of genomes with TilS12
Nº of genomes without TilS12
With TilS12
Without TilS12
Nº of genomes with TilS13
Nº of genomes without TilS13
With TilS13
Without TilS13
Nº of genomes with TilS14
Nº of genomes without TilS14
With TilS14
Without TilS14
Nº of genomes with TilS15
Nº of genomes without TilS15
With TilS15
Without TilS15

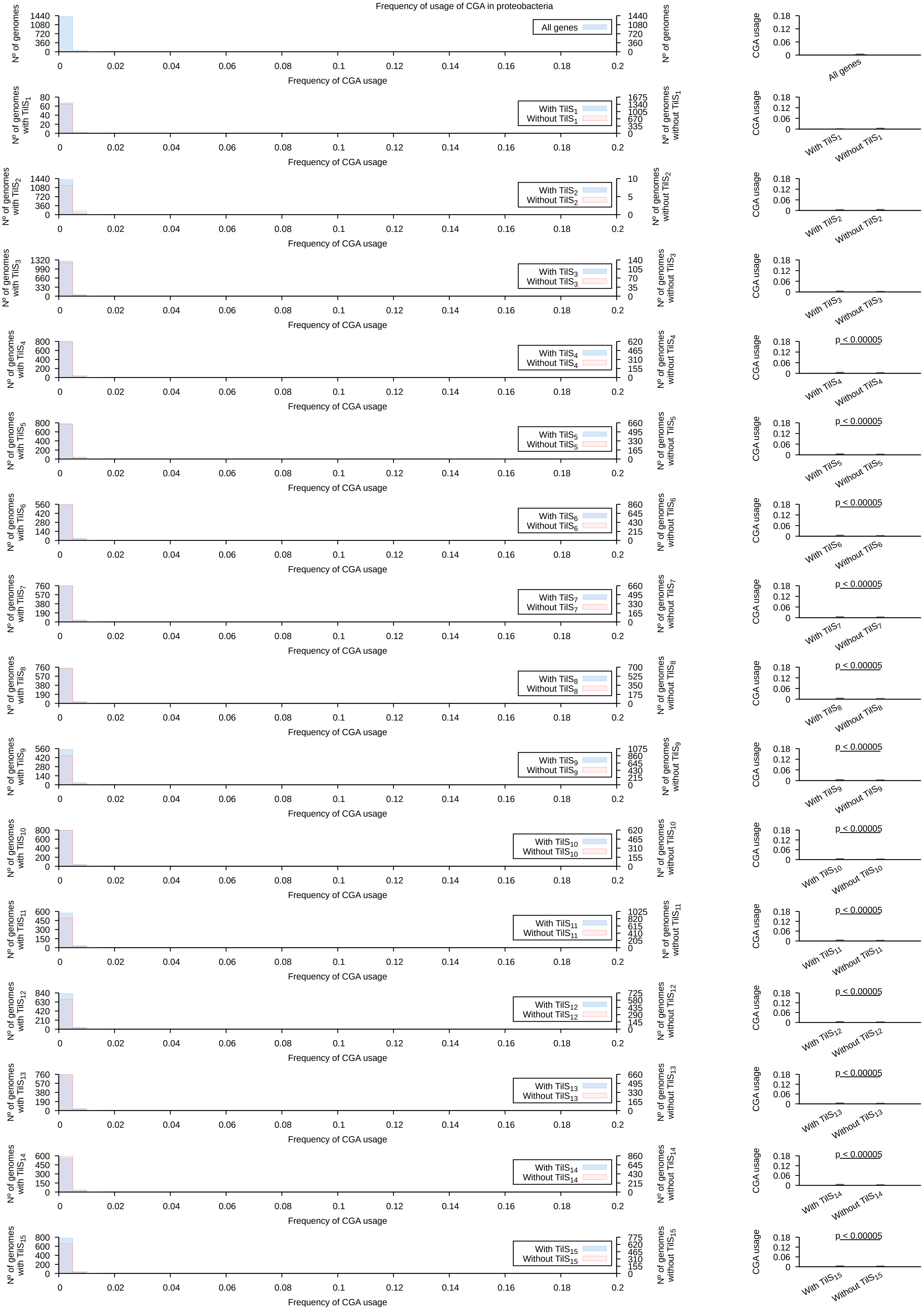

Frequency of usage of CGA in proteobacteria
Nº of genomes
All genes
Frequency of CGA usage
CGA usage
Nº of genomes with TilS1
With TilS1
Without TilS1
Nº of genomes without TilS1
Nº of genomes with TilS2
With TilS2
Without TilS2
Nº of genomes without TilS2
Nº of genomes with TilS3
With TilS3
Without TilS3
Nº of genomes without TilS3
Nº of genomes with TilS4
With TilS4
Without TilS4
Nº of genomes without TilS4
p < 0.00005
Nº of genomes with TilS5
With TilS5
Without TilS5
Nº of genomes without TilS5
Nº of genomes with TilS6
With TilS6
Without TilS6
Nº of genomes without TilS6
Nº of genomes with TilS7
With TilS7
Without TilS7
Nº of genomes without TilS7
Nº of genomes with TilS8
With TilS8
Without TilS8
Nº of genomes without TilS8
Nº of genomes with TilS9
With TilS9
Without TilS9
Nº of genomes without TilS9
Nº of genomes with TilS10
With TilS10
Without TilS10
Nº of genomes without TilS10
Nº of genomes with TilS11
With TilS11
Without TilS11
Nº of genomes without TilS11
Nº of genomes with TilS12
With TilS12
Without TilS12
Nº of genomes without TilS12
Nº of genomes with TilS13
With TilS13
Without TilS13
Nº of genomes without TilS13
Nº of genomes with TilS14
With TilS14
Without TilS14
Nº of genomes without TilS14
Nº of genomes with TilS15
With TilS15
Without TilS15
Nº of genomes without TilS15

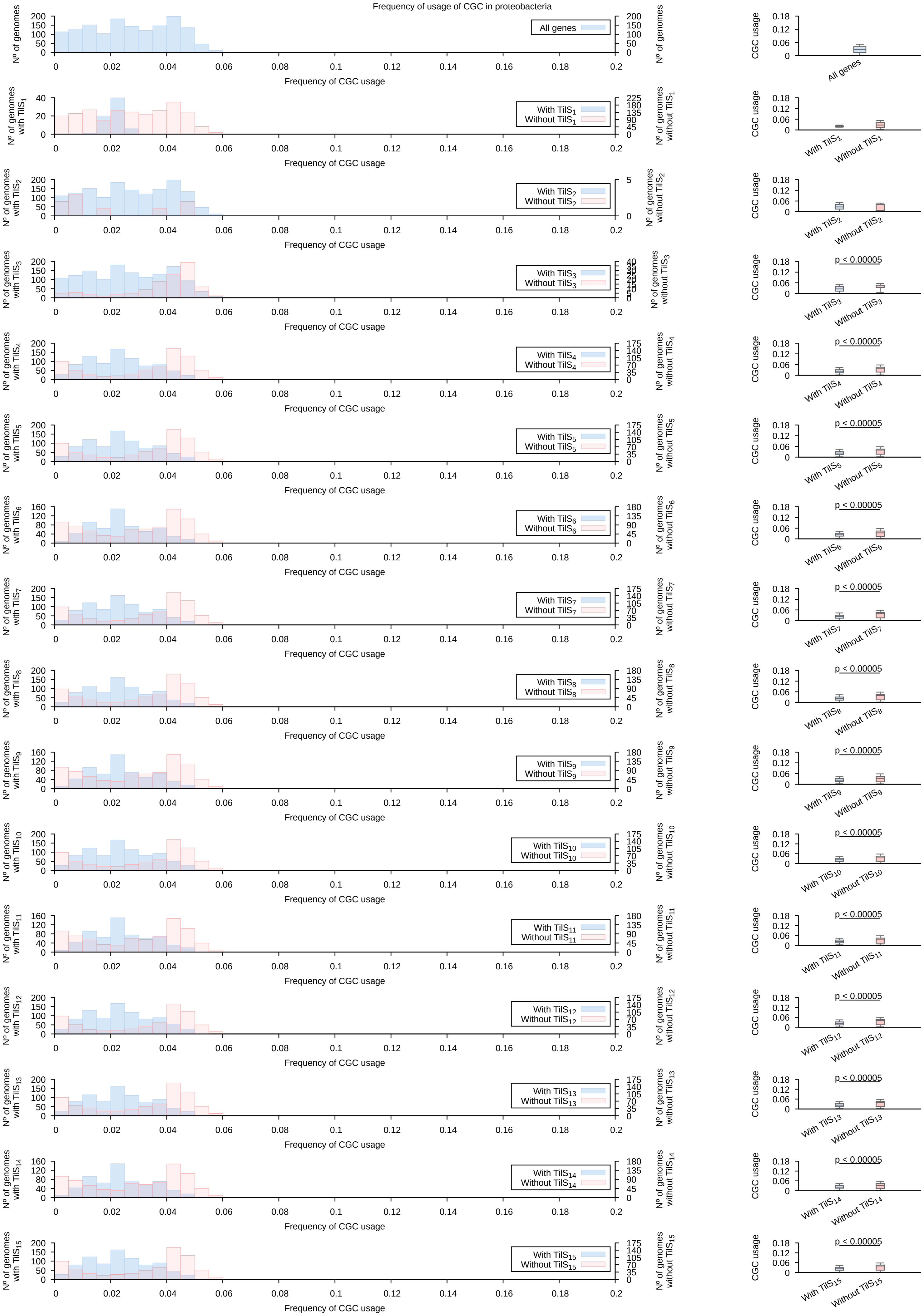

Frequency of usage of CGC in proteobacteria
Nº of genomes
All genes
Frequency of CGC usage
CGC usage
With TilS1
Without TilS1
With TilS2
Without TilS2
With TilS3
Without TilS3
With TilS4
Without TilS4
With TilS5
Without TilS5
With TilS6
Without TilS6
With TilS7
Without TilS7
With TilS8
Without TilS8
With TilS9
Without TilS9
With TilS10
Without TilS10
With TilS11
Without TilS11
With TilS12
Without TilS12
With TilS13
Without TilS13
With TilS14
Without TilS14
With TilS15
Without TilS15
p < 0.00005

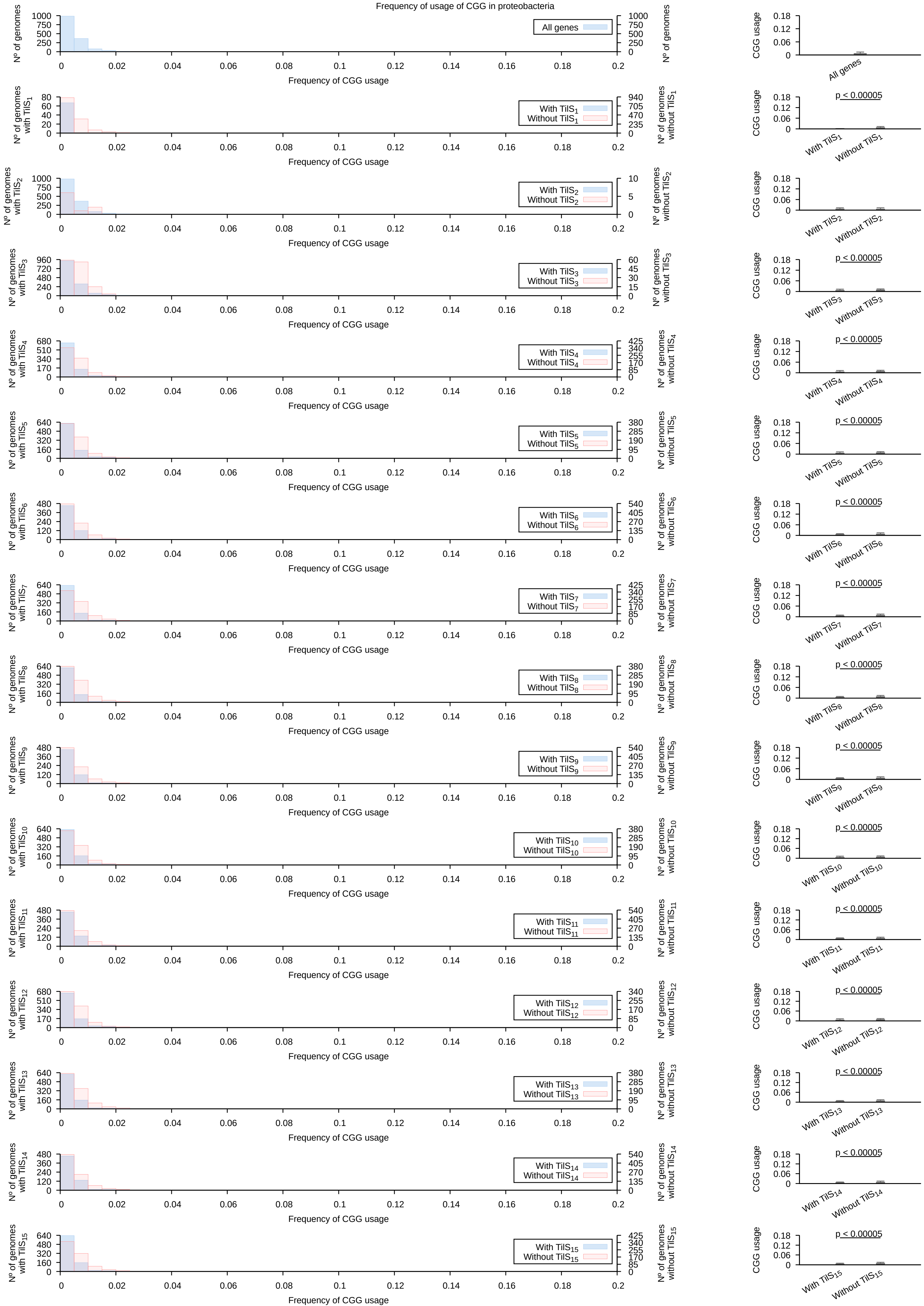

Frequency of usage of CGG in proteobacteria
Nº of genomes
All genes
Frequency of CGG usage
CGG usage
Nº of genomes with TilS1
Nº of genomes without TilS1
With TilS1
Without TilS1
p < 0.00005
Nº of genomes with TilS2
Nº of genomes without TilS2
With TilS2
Without TilS2
Nº of genomes with TilS3
Nº of genomes without TilS3
With TilS3
Without TilS3
Nº of genomes with TilS4
Nº of genomes without TilS4
With TilS4
Without TilS4
Nº of genomes with TilS5
Nº of genomes without TilS5
With TilS5
Without TilS5
Nº of genomes with TilS6
Nº of genomes without TilS6
With TilS6
Without TilS6
Nº of genomes with TilS7
Nº of genomes without TilS7
With TilS7
Without TilS7
Nº of genomes with TilS8
Nº of genomes without TilS8
With TilS8
Without TilS8
Nº of genomes with TilS9
Nº of genomes without TilS9
With TilS9
Without TilS9
Nº of genomes with TilS10
Nº of genomes without TilS10
With TilS10
Without TilS10
Nº of genomes with TilS11
Nº of genomes without TilS11
With TilS11
Without TilS11
Nº of genomes with TilS12
Nº of genomes without TilS12
With TilS12
Without TilS12
Nº of genomes with TilS13
Nº of genomes without TilS13
With TilS13
Without TilS13
Nº of genomes with TilS14
Nº of genomes without TilS14
With TilS14
Without TilS14
Nº of genomes with TilS15
Nº of genomes without TilS15
With TilS15
Without TilS15

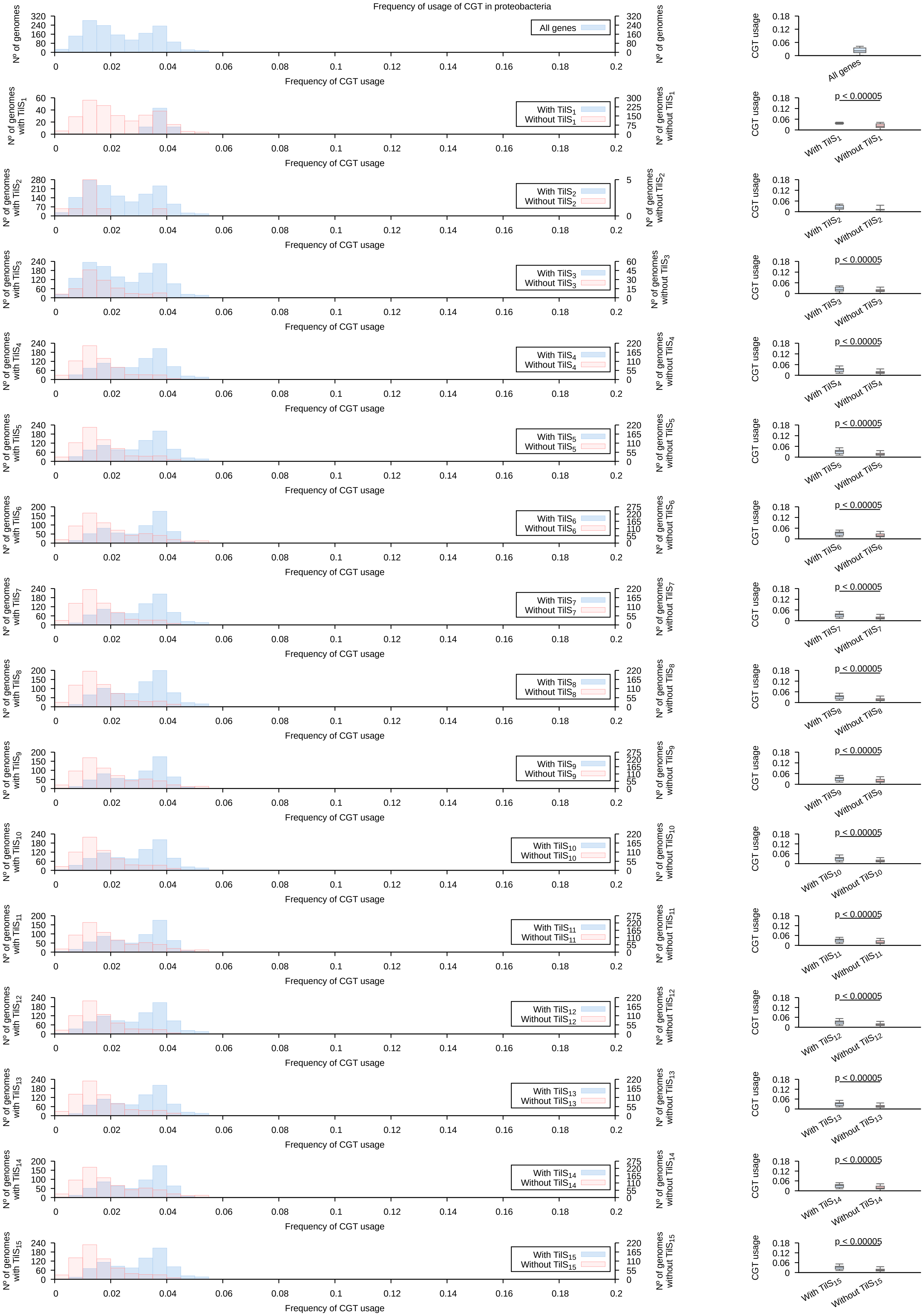
Frequency of usage of CGT in proteobacteria
Nº of genomes
All genes
Frequency of CGT usage
CGT usage
Nº of genomes with TilS$_1$
Nº of genomes without TilS$_1$
With TilS$_1$
Without TilS$_1$
p < 0.00005
Nº of genomes with TilS$_2$
Nº of genomes without TilS$_2$
With TilS$_2$
Without TilS$_2$
Nº of genomes with TilS$_3$
Nº of genomes without TilS$_3$
With TilS$_3$
Without TilS$_3$
Nº of genomes with TilS$_4$
Nº of genomes without TilS$_4$
With TilS$_4$
Without TilS$_4$
Nº of genomes with TilS$_5$
Nº of genomes without TilS$_5$
With TilS$_5$
Without TilS$_5$
Nº of genomes with TilS$_6$
Nº of genomes without TilS$_6$
With TilS$_6$
Without TilS$_6$
Nº of genomes with TilS$_7$
Nº of genomes without TilS$_7$
With TilS$_7$
Without TilS$_7$
Nº of genomes with TilS$_8$
Nº of genomes without TilS$_8$
With TilS$_8$
Without TilS$_8$
Nº of genomes with TilS$_9$
Nº of genomes without TilS$_9$
With TilS$_9$
Without TilS$_9$
Nº of genomes with TilS$_{10}$
Nº of genomes without TilS$_{10}$
With TilS$_{10}$
Without TilS$_{10}$
Nº of genomes with TilS$_{11}$
Nº of genomes without TilS$_{11}$
With TilS$_{11}$
Without TilS$_{11}$
Nº of genomes with TilS$_{12}$
Nº of genomes without TilS$_{12}$
With TilS$_{12}$
Without TilS$_{12}$
Nº of genomes with TilS$_{13}$
Nº of genomes without TilS$_{13}$
With TilS$_{13}$
Without TilS$_{13}$
Nº of genomes with TilS$_{14}$
Nº of genomes without TilS$_{14}$
With TilS$_{14}$
Without TilS$_{14}$
Nº of genomes with TilS$_{15}$
Nº of genomes without TilS$_{15}$
With TilS$_{15}$
Without TilS$_{15}$

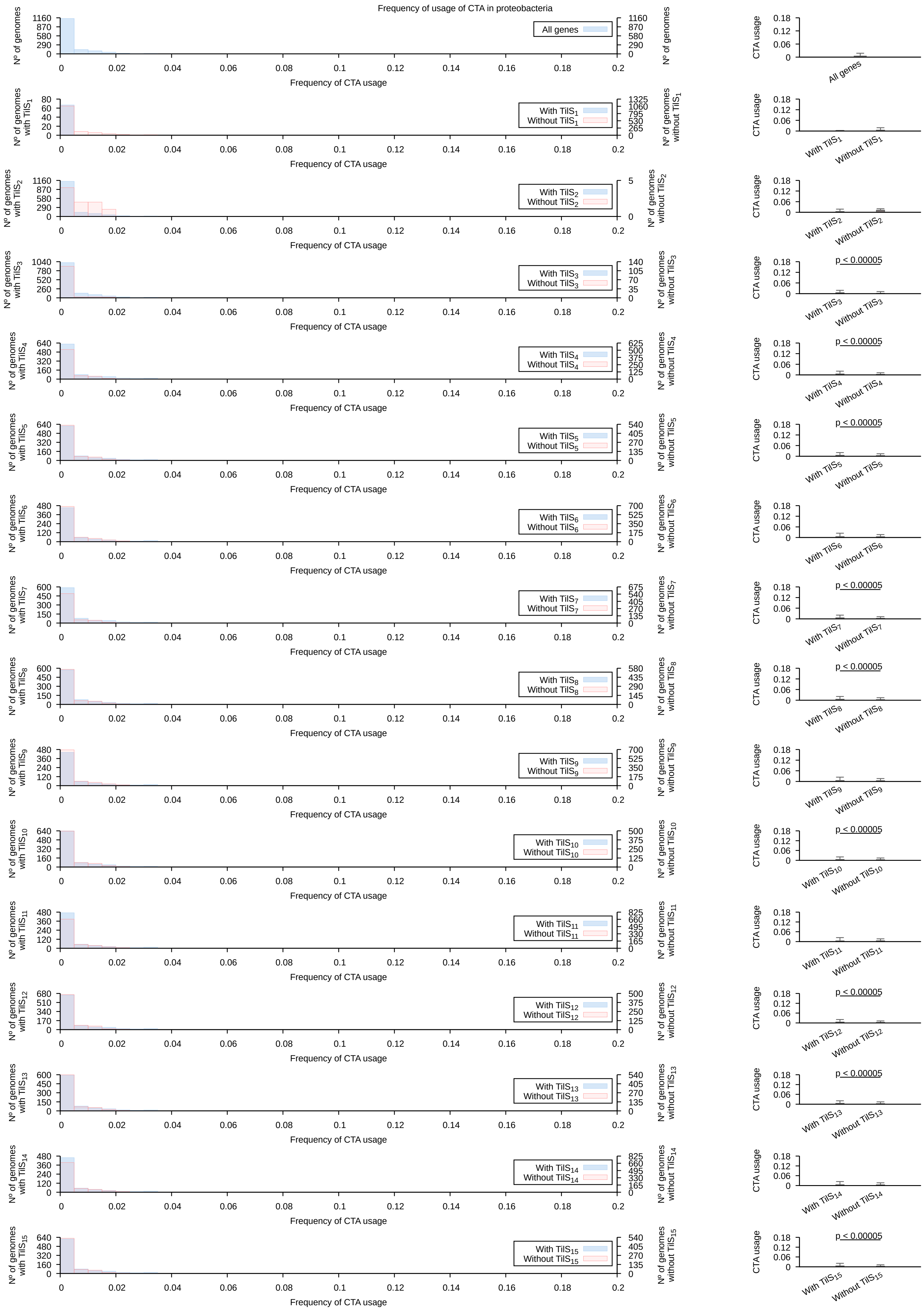
Frequency of usage of CTA in proteobacteria
Nº of genomes
Frequency of CTA usage
CTA usage
All genes
With TilS1
Without TilS1
Nº of genomes with TilS1
Nº of genomes without TilS1
With TilS2
Without TilS2
With TilS3
Without TilS3
p < 0.00005
With TilS4
Without TilS4
p < 0.00005
With TilS5
Without TilS5
p < 0.00005
With TilS6
Without TilS6
With TilS7
Without TilS7
p < 0.00005
With TilS8
Without TilS8
p < 0.00005
With TilS9
Without TilS9
With TilS10
Without TilS10
p < 0.00005
With TilS11
Without TilS11
With TilS12
Without TilS12
p < 0.00005
With TilS13
Without TilS13
p < 0.00005
With TilS14
Without TilS14
With TilS15
Without TilS15
p < 0.00005

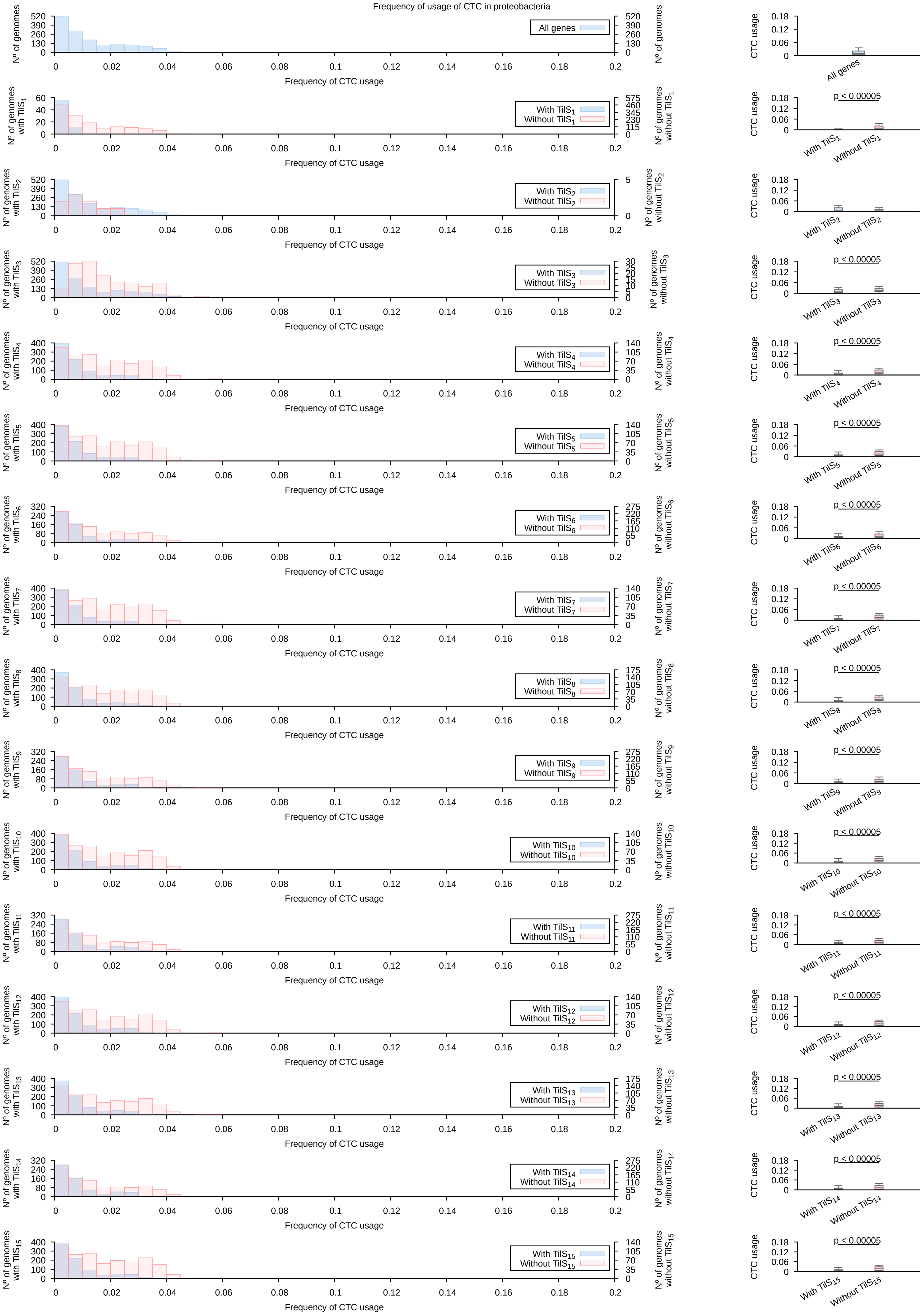

Frequency of usage of CTC in proteobacteria
Nº of genomes
All genes
Frequency of CTC usage
CTC usage
Nº of genomes with TilS1
Nº of genomes without TilS1
With TilS1
Without TilS1
p < 0.00005
Nº of genomes with TilS2
Nº of genomes without TilS2
With TilS2
Without TilS2
Nº of genomes with TilS3
Nº of genomes without TilS3
With TilS3
Without TilS3
p < 0.00005
Nº of genomes with TilS4
Nº of genomes without TilS4
With TilS4
Without TilS4
p < 0.00005
Nº of genomes with TilS5
Nº of genomes without TilS5
With TilS5
Without TilS5
p < 0.00005
Nº of genomes with TilS6
Nº of genomes without TilS6
With TilS6
Without TilS6
p < 0.00005
Nº of genomes with TilS7
Nº of genomes without TilS7
With TilS7
Without TilS7
p < 0.00005
Nº of genomes with TilS8
Nº of genomes without TilS8
With TilS8
Without TilS8
p < 0.00005
Nº of genomes with TilS9
Nº of genomes without TilS9
With TilS9
Without TilS9
p < 0.00005
Nº of genomes with TilS10
Nº of genomes without TilS10
With TilS10
Without TilS10
p < 0.00005
Nº of genomes with TilS11
Nº of genomes without TilS11
With TilS11
Without TilS11
p < 0.00005
Nº of genomes with TilS12
Nº of genomes without TilS12
With TilS12
Without TilS12
p < 0.00005
Nº of genomes with TilS13
Nº of genomes without TilS13
With TilS13
Without TilS13
p < 0.00005
Nº of genomes with TilS14
Nº of genomes without TilS14
With TilS14
Without TilS14
p < 0.00005
Nº of genomes with TilS15
Nº of genomes without TilS15
With TilS15
Without TilS15
p < 0.00005

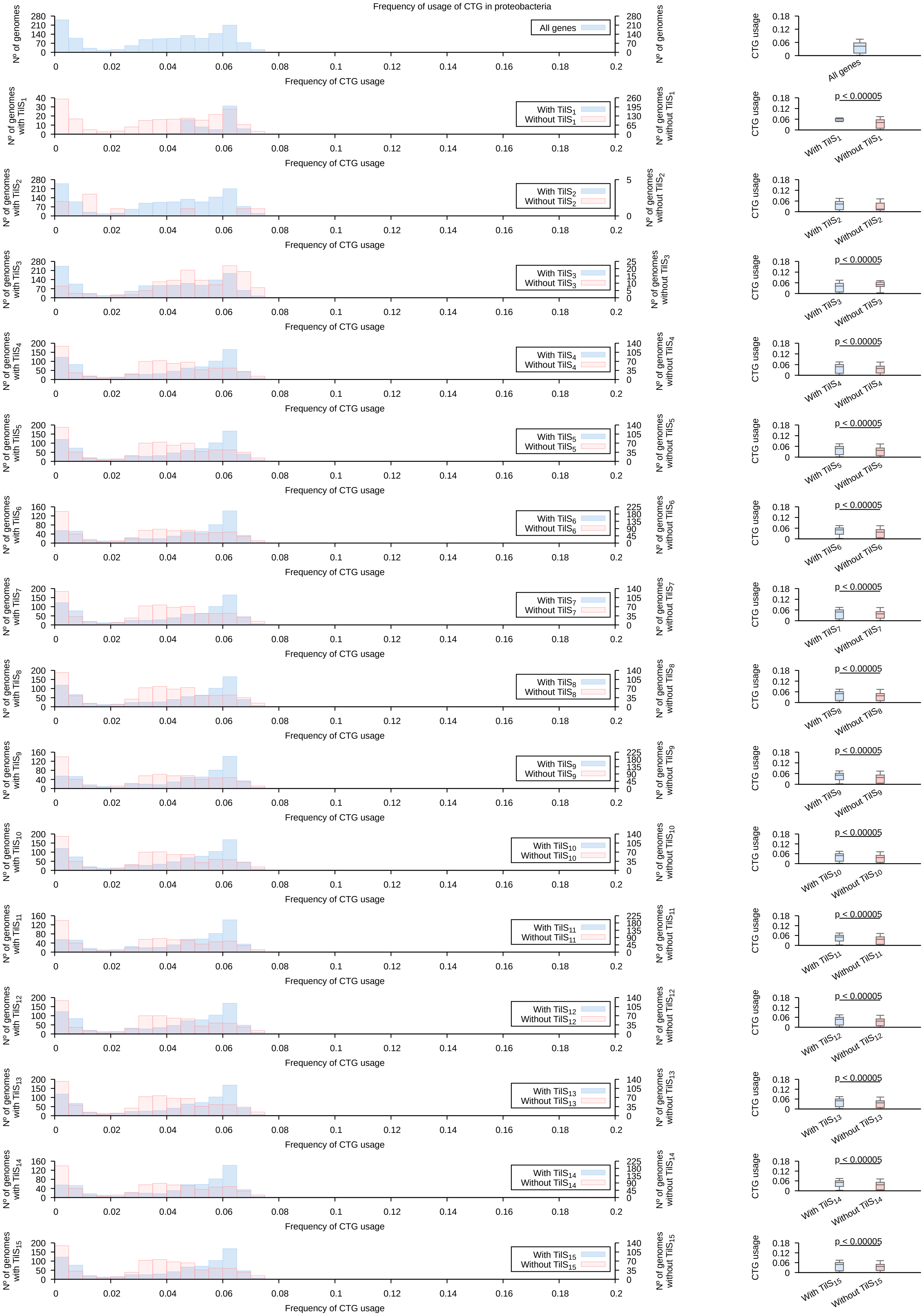

Frequency of usage of CTG in proteobacteria
All genes
Nº of genomes
Frequency of CTG usage
CTG usage
With TilS
Without TilS
Nº of genomes with TilS
Nº of genomes without TilS
p < 0.00005

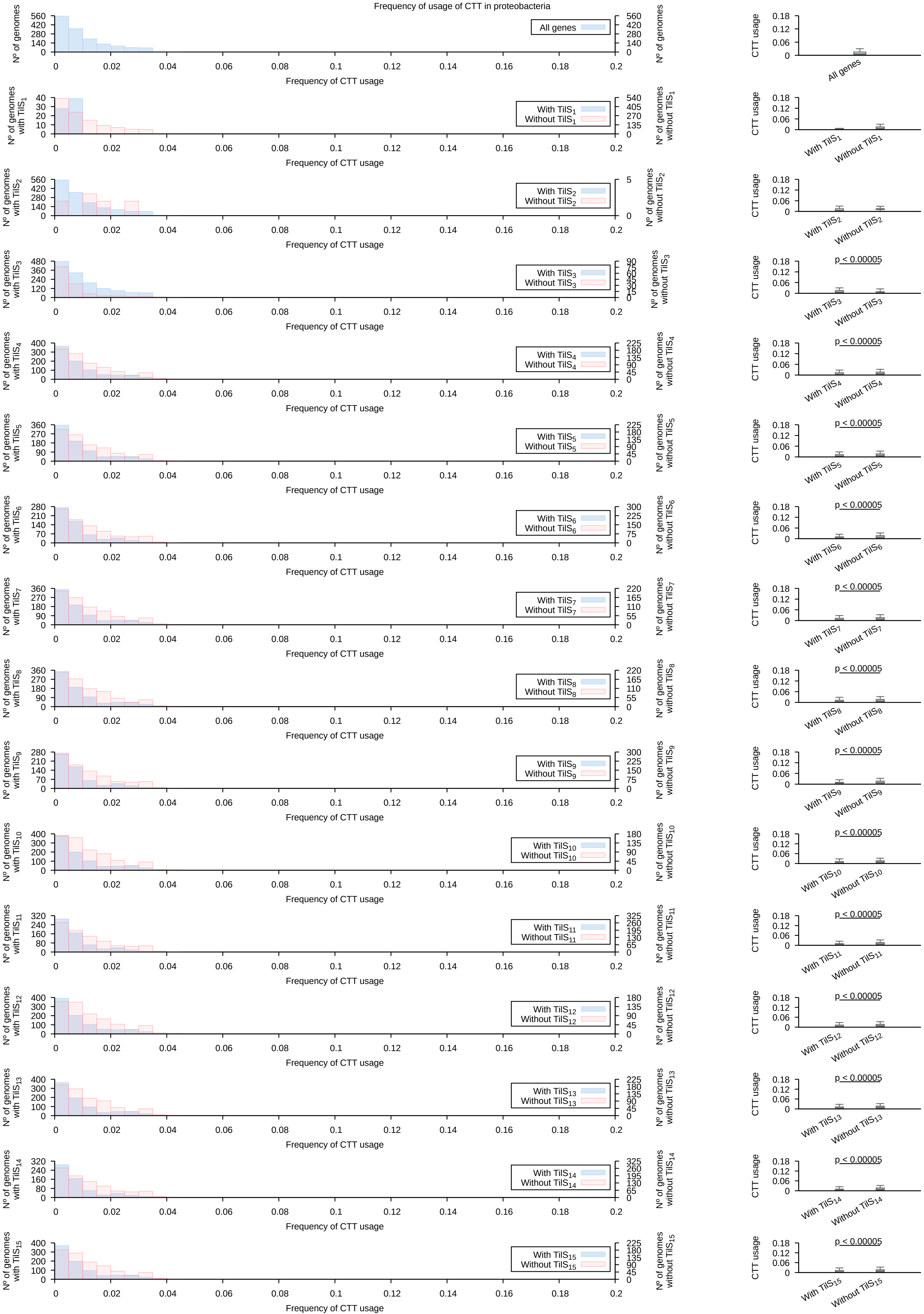

Frequency of usage of CTT in proteobacteria
Nº of genomes
Frequency of CTT usage
All genes
CTT usage
Nº of genomes with TilS1
Nº of genomes without TilS1
With TilS1
Without TilS1
Nº of genomes with TilS2
Nº of genomes without TilS2
With TilS2
Without TilS2
Nº of genomes with TilS3
Nº of genomes without TilS3
With TilS3
Without TilS3
p < 0.00005
Nº of genomes with TilS4
Nº of genomes without TilS4
With TilS4
Without TilS4
p < 0.00005
Nº of genomes with TilS5
Nº of genomes without TilS5
With TilS5
Without TilS5
p < 0.00005
Nº of genomes with TilS6
Nº of genomes without TilS6
With TilS6
Without TilS6
p < 0.00005
Nº of genomes with TilS7
Nº of genomes without TilS7
With TilS7
Without TilS7
p < 0.00005
Nº of genomes with TilS8
Nº of genomes without TilS8
With TilS8
Without TilS8
p < 0.00005
Nº of genomes with TilS9
Nº of genomes without TilS9
With TilS9
Without TilS9
p < 0.00005
Nº of genomes with TilS10
Nº of genomes without TilS10
With TilS10
Without TilS10
p < 0.00005
Nº of genomes with TilS11
Nº of genomes without TilS11
With TilS11
Without TilS11
p < 0.00005
Nº of genomes with TilS12
Nº of genomes without TilS12
With TilS12
Without TilS12
p < 0.00005
Nº of genomes with TilS13
Nº of genomes without TilS13
With TilS13
Without TilS13
p < 0.00005
Nº of genomes with TilS14
Nº of genomes without TilS14
With TilS14
Without TilS14
p < 0.00005
Nº of genomes with TilS15
Nº of genomes without TilS15
With TilS15
Without TilS15
p < 0.00005

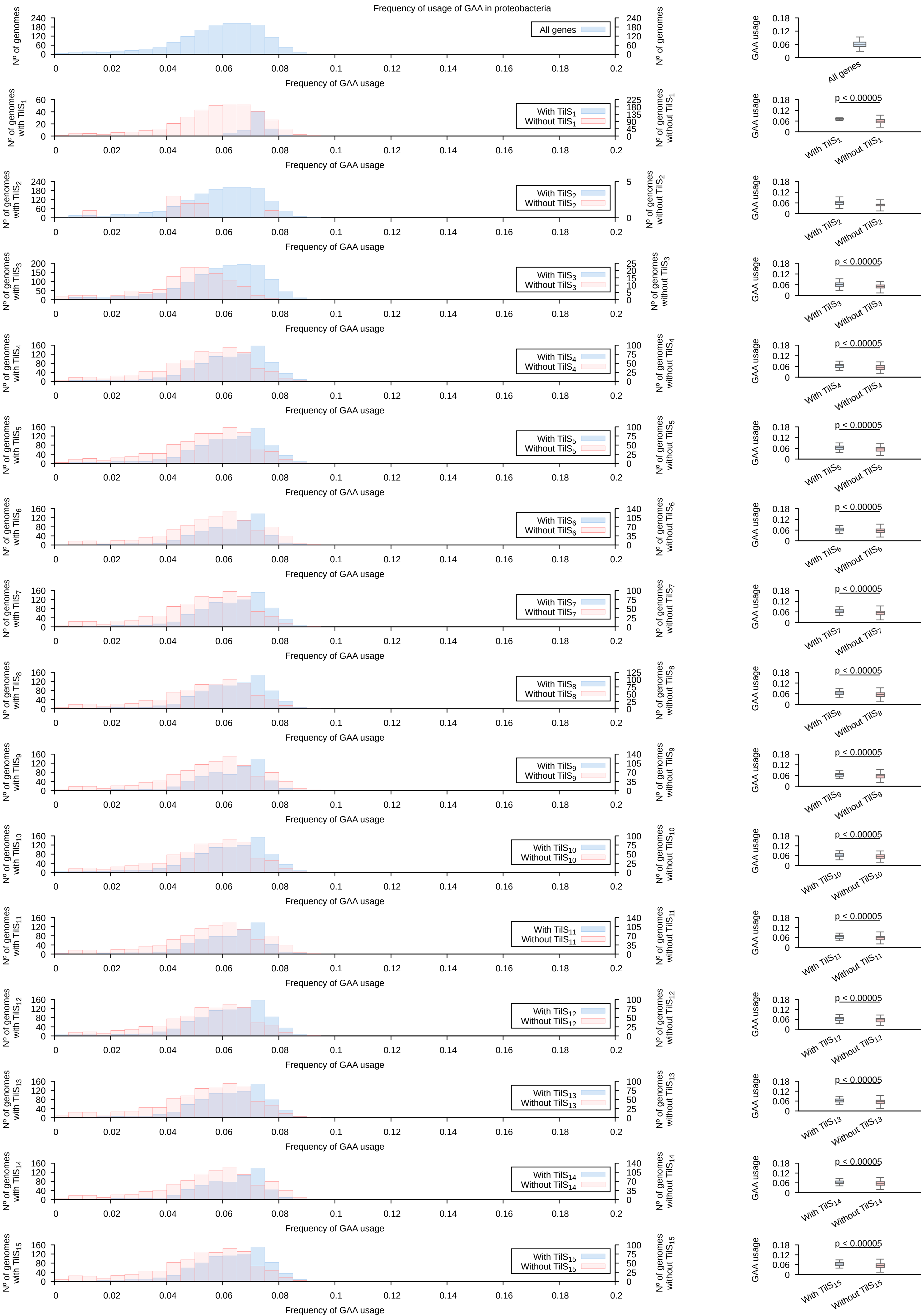

Frequency of usage of GAA in proteobacteria
Nº of genomes
All genes
Frequency of GAA usage
GAA usage
All genes
Nº of genomes with TilS$_1$
Nº of genomes without TilS$_1$
With TilS$_1$
Without TilS$_1$
p < 0.00005
Nº of genomes with TilS$_2$
Nº of genomes without TilS$_2$
With TilS$_2$
Without TilS$_2$
Nº of genomes with TilS$_3$
Nº of genomes without TilS$_3$
With TilS$_3$
Without TilS$_3$
p < 0.00005
Nº of genomes with TilS$_4$
Nº of genomes without TilS$_4$
With TilS$_4$
Without TilS$_4$
p < 0.00005
Nº of genomes with TilS$_5$
Nº of genomes without TilS$_5$
With TilS$_5$
Without TilS$_5$
p < 0.00005
Nº of genomes with TilS$_6$
Nº of genomes without TilS$_6$
With TilS$_6$
Without TilS$_6$
p < 0.00005
Nº of genomes with TilS$_7$
Nº of genomes without TilS$_7$
With TilS$_7$
Without TilS$_7$
p < 0.00005
Nº of genomes with TilS$_8$
Nº of genomes without TilS$_8$
With TilS$_8$
Without TilS$_8$
p < 0.00005
Nº of genomes with TilS$_9$
Nº of genomes without TilS$_9$
With TilS$_9$
Without TilS$_9$
p < 0.00005
Nº of genomes with TilS$_{10}$
Nº of genomes without TilS$_{10}$
With TilS$_{10}$
Without TilS$_{10}$
p < 0.00005
Nº of genomes with TilS$_{11}$
Nº of genomes without TilS$_{11}$
With TilS$_{11}$
Without TilS$_{11}$
p < 0.00005
Nº of genomes with TilS$_{12}$
Nº of genomes without TilS$_{12}$
With TilS$_{12}$
Without TilS$_{12}$
p < 0.00005
Nº of genomes with TilS$_{13}$
Nº of genomes without TilS$_{13}$
With TilS$_{13}$
Without TilS$_{13}$
p < 0.00005
Nº of genomes with TilS$_{14}$
Nº of genomes without TilS$_{14}$
With TilS$_{14}$
Without TilS$_{14}$
p < 0.00005
Nº of genomes with TilS$_{15}$
Nº of genomes without TilS$_{15}$
With TilS$_{15}$
Without TilS$_{15}$
p < 0.00005

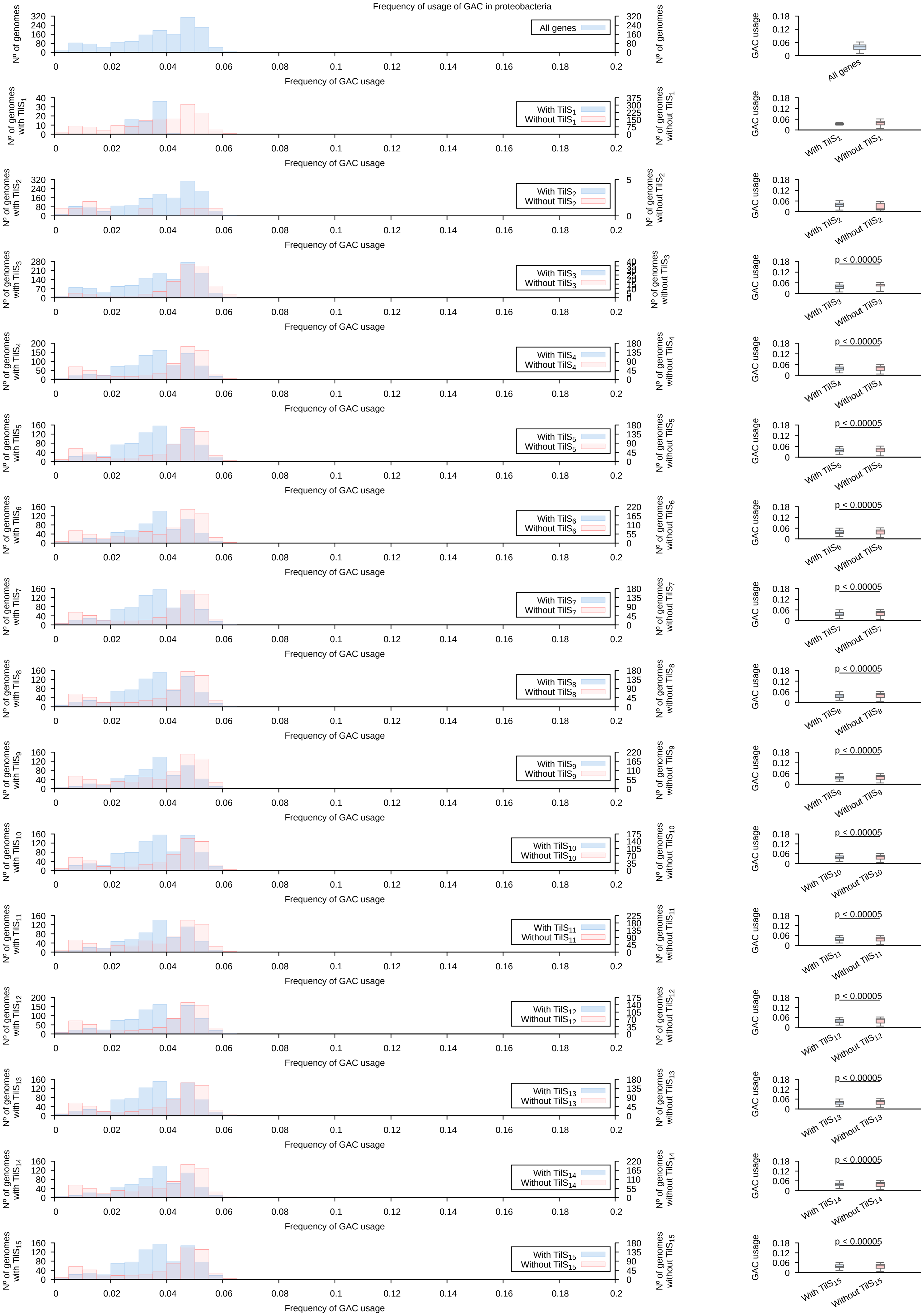

Frequency of usage of GAC in proteobacteria
Nº of genomes
All genes
Frequency of GAC usage
GAC usage
All genes
Nº of genomes with TilS$_1$
With TilS$_1$
Without TilS$_1$
Nº of genomes without TilS$_1$
Nº of genomes with TilS$_2$
With TilS$_2$
Without TilS$_2$
Nº of genomes without TilS$_2$
Nº of genomes with TilS$_3$
With TilS$_3$
Without TilS$_3$
Nº of genomes without TilS$_3$
p < 0.00005
Nº of genomes with TilS$_4$
With TilS$_4$
Without TilS$_4$
Nº of genomes without TilS$_4$
p < 0.00005
Nº of genomes with TilS$_5$
With TilS$_5$
Without TilS$_5$
Nº of genomes without TilS$_5$
p < 0.00005
Nº of genomes with TilS$_6$
With TilS$_6$
Without TilS$_6$
Nº of genomes without TilS$_6$
p < 0.00005
Nº of genomes with TilS$_7$
With TilS$_7$
Without TilS$_7$
Nº of genomes without TilS$_7$
p < 0.00005
Nº of genomes with TilS$_8$
With TilS$_8$
Without TilS$_8$
Nº of genomes without TilS$_8$
p < 0.00005
Nº of genomes with TilS$_9$
With TilS$_9$
Without TilS$_9$
Nº of genomes without TilS$_9$
p < 0.00005
Nº of genomes with TilS$_{10}$
With TilS$_{10}$
Without TilS$_{10}$
Nº of genomes without TilS$_{10}$
p < 0.00005
Nº of genomes with TilS$_{11}$
With TilS$_{11}$
Without TilS$_{11}$
Nº of genomes without TilS$_{11}$
p < 0.00005
Nº of genomes with TilS$_{12}$
With TilS$_{12}$
Without TilS$_{12}$
Nº of genomes without TilS$_{12}$
p < 0.00005
Nº of genomes with TilS$_{13}$
With TilS$_{13}$
Without TilS$_{13}$
Nº of genomes without TilS$_{13}$
p < 0.00005
Nº of genomes with TilS$_{14}$
With TilS$_{14}$
Without TilS$_{14}$
Nº of genomes without TilS$_{14}$
p < 0.00005
Nº of genomes with TilS$_{15}$
With TilS$_{15}$
Without TilS$_{15}$
Nº of genomes without TilS$_{15}$
p < 0.00005

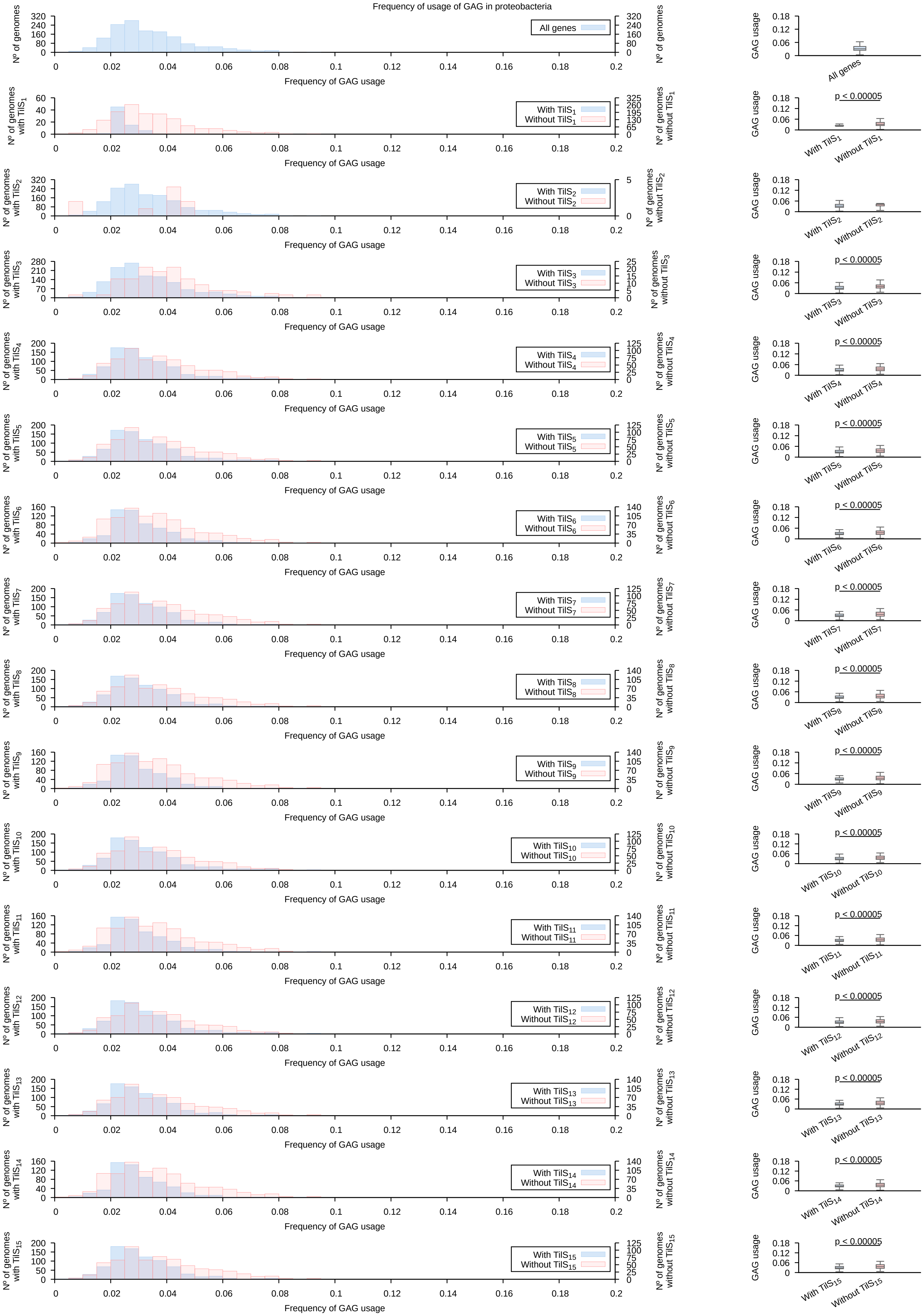

Frequency of usage of GAG in proteobacteria
Nº of genomes
All genes
Frequency of GAG usage
GAG usage
Nº of genomes with TilS$_1$
Nº of genomes without TilS$_1$
With TilS$_1$
Without TilS$_1$
p < 0.00005
Nº of genomes with TilS$_2$
Nº of genomes without TilS$_2$
With TilS$_2$
Without TilS$_2$
Nº of genomes with TilS$_3$
Nº of genomes without TilS$_3$
With TilS$_3$
Without TilS$_3$
p < 0.00005
Nº of genomes with TilS$_4$
Nº of genomes without TilS$_4$
With TilS$_4$
Without TilS$_4$
p < 0.00005
Nº of genomes with TilS$_5$
Nº of genomes without TilS$_5$
With TilS$_5$
Without TilS$_5$
p < 0.00005
Nº of genomes with TilS$_6$
Nº of genomes without TilS$_6$
With TilS$_6$
Without TilS$_6$
p < 0.00005
Nº of genomes with TilS$_7$
Nº of genomes without TilS$_7$
With TilS$_7$
Without TilS$_7$
p < 0.00005
Nº of genomes with TilS$_8$
Nº of genomes without TilS$_8$
With TilS$_8$
Without TilS$_8$
p < 0.00005
Nº of genomes with TilS$_9$
Nº of genomes without TilS$_9$
With TilS$_9$
Without TilS$_9$
p < 0.00005
Nº of genomes with TilS$_{10}$
Nº of genomes without TilS$_{10}$
With TilS$_{10}$
Without TilS$_{10}$
p < 0.00005
Nº of genomes with TilS$_{11}$
Nº of genomes without TilS$_{11}$
With TilS$_{11}$
Without TilS$_{11}$
p < 0.00005
Nº of genomes with TilS$_{12}$
Nº of genomes without TilS$_{12}$
With TilS$_{12}$
Without TilS$_{12}$
p < 0.00005
Nº of genomes with TilS$_{13}$
Nº of genomes without TilS$_{13}$
With TilS$_{13}$
Without TilS$_{13}$
p < 0.00005
Nº of genomes with TilS$_{14}$
Nº of genomes without TilS$_{14}$
With TilS$_{14}$
Without TilS$_{14}$
p < 0.00005
Nº of genomes with TilS$_{15}$
Nº of genomes without TilS$_{15}$
With TilS$_{15}$
Without TilS$_{15}$
p < 0.00005

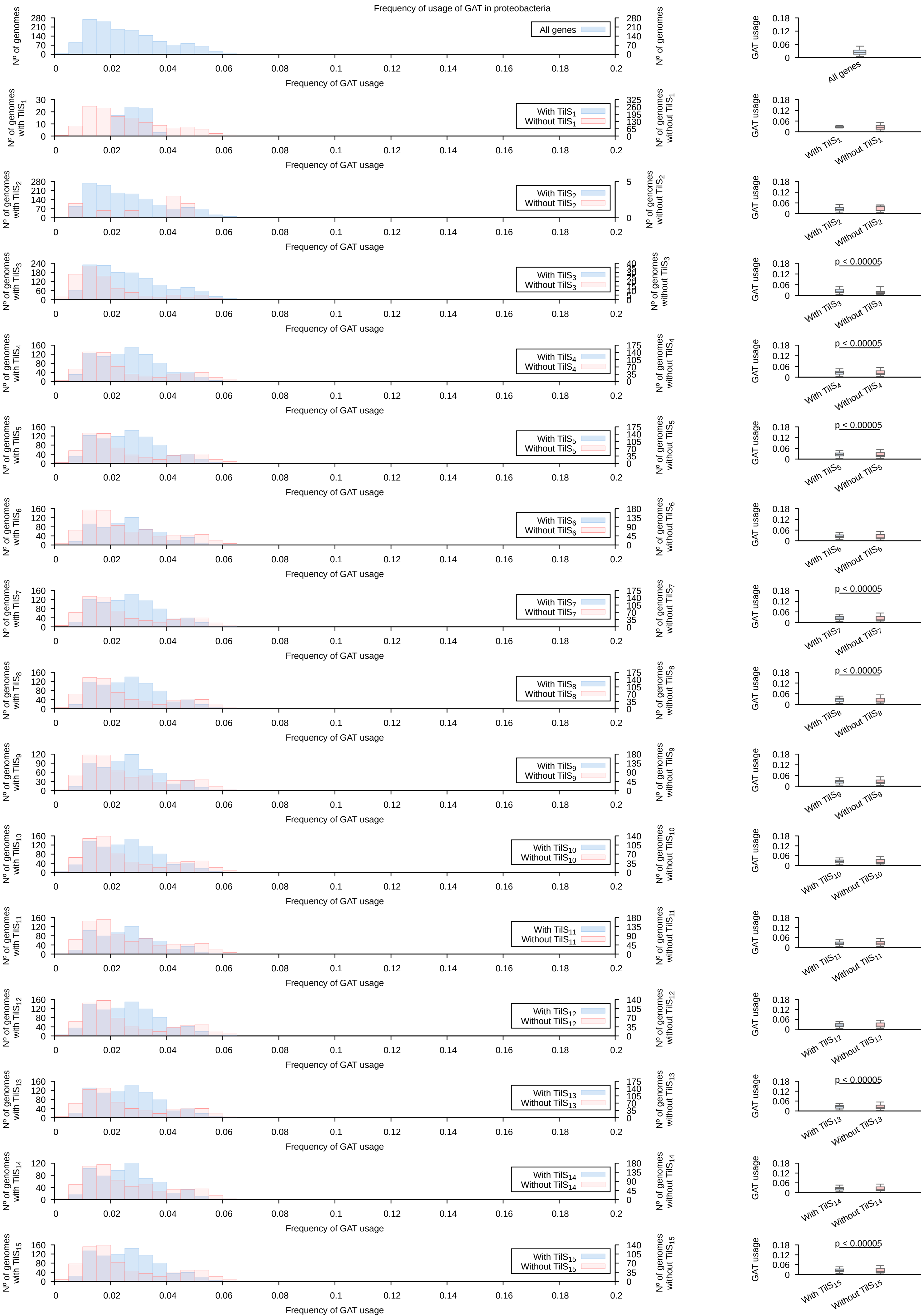

Frequency of usage of GAT in proteobacteria
Nº of genomes
All genes
Frequency of GAT usage
GAT usage
With TilS$_1$
Without TilS$_1$
Nº of genomes with TilS$_1$
Nº of genomes without TilS$_1$
With TilS$_2$
Without TilS$_2$
Nº of genomes with TilS$_2$
Nº of genomes without TilS$_2$
With TilS$_3$
Without TilS$_3$
Nº of genomes with TilS$_3$
Nº of genomes without TilS$_3$
p < 0.00005
With TilS$_4$
Without TilS$_4$
Nº of genomes with TilS$_4$
Nº of genomes without TilS$_4$
p < 0.00005
With TilS$_5$
Without TilS$_5$
Nº of genomes with TilS$_5$
Nº of genomes without TilS$_5$
p < 0.00005
With TilS$_6$
Without TilS$_6$
Nº of genomes with TilS$_6$
Nº of genomes without TilS$_6$
With TilS$_7$
Without TilS$_7$
Nº of genomes with TilS$_7$
Nº of genomes without TilS$_7$
p < 0.00005
With TilS$_8$
Without TilS$_8$
Nº of genomes with TilS$_8$
Nº of genomes without TilS$_8$
p < 0.00005
With TilS$_9$
Without TilS$_9$
Nº of genomes with TilS$_9$
Nº of genomes without TilS$_9$
With TilS$_{10}$
Without TilS$_{10}$
Nº of genomes with TilS$_{10}$
Nº of genomes without TilS$_{10}$
With TilS$_{11}$
Without TilS$_{11}$
Nº of genomes with TilS$_{11}$
Nº of genomes without TilS$_{11}$
With TilS$_{12}$
Without TilS$_{12}$
Nº of genomes with TilS$_{12}$
Nº of genomes without TilS$_{12}$
With TilS$_{13}$
Without TilS$_{13}$
Nº of genomes with TilS$_{13}$
Nº of genomes without TilS$_{13}$
p < 0.00005
With TilS$_{14}$
Without TilS$_{14}$
Nº of genomes with TilS$_{14}$
Nº of genomes without TilS$_{14}$
With TilS$_{15}$
Without TilS$_{15}$
Nº of genomes with TilS$_{15}$
Nº of genomes without TilS$_{15}$
p < 0.00005

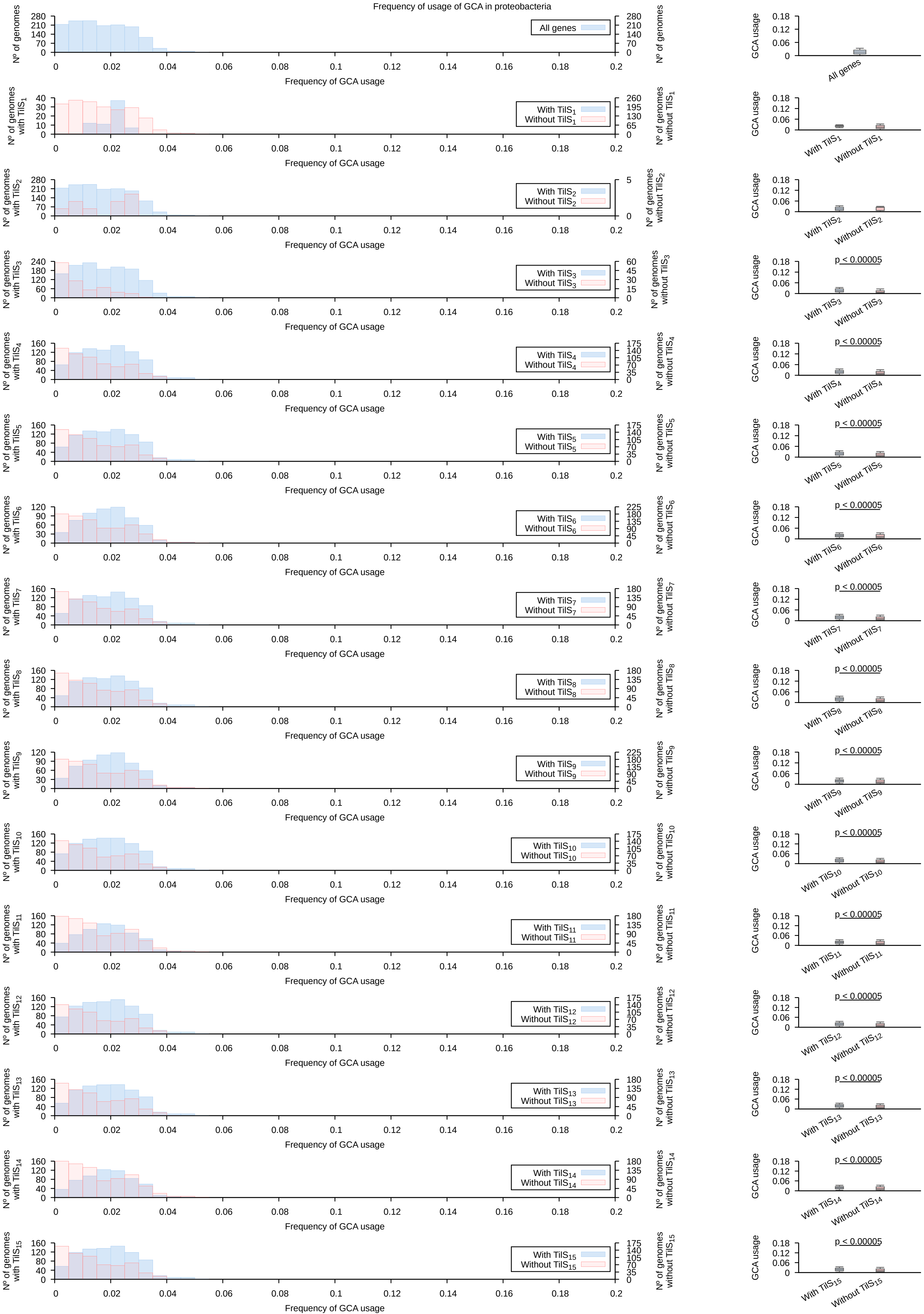

Frequency of usage of GCA in proteobacteria
Nº of genomes
All genes
Frequency of GCA usage
GCA usage
Nº of genomes with TilS1
Nº of genomes without TilS1
With TilS1
Without TilS1
Nº of genomes with TilS2
Nº of genomes without TilS2
With TilS2
Without TilS2
Nº of genomes with TilS3
Nº of genomes without TilS3
With TilS3
Without TilS3
p < 0.00005
Nº of genomes with TilS4
Nº of genomes without TilS4
With TilS4
Without TilS4
Nº of genomes with TilS5
Nº of genomes without TilS5
With TilS5
Without TilS5
Nº of genomes with TilS6
Nº of genomes without TilS6
With TilS6
Without TilS6
Nº of genomes with TilS7
Nº of genomes without TilS7
With TilS7
Without TilS7
Nº of genomes with TilS8
Nº of genomes without TilS8
With TilS8
Without TilS8
Nº of genomes with TilS9
Nº of genomes without TilS9
With TilS9
Without TilS9
Nº of genomes with TilS10
Nº of genomes without TilS10
With TilS10
Without TilS10
Nº of genomes with TilS11
Nº of genomes without TilS11
With TilS11
Without TilS11
Nº of genomes with TilS12
Nº of genomes without TilS12
With TilS12
Without TilS12
Nº of genomes with TilS13
Nº of genomes without TilS13
With TilS13
Without TilS13
Nº of genomes with TilS14
Nº of genomes without TilS14
With TilS14
Without TilS14
Nº of genomes with TilS15
Nº of genomes without TilS15
With TilS15
Without TilS15

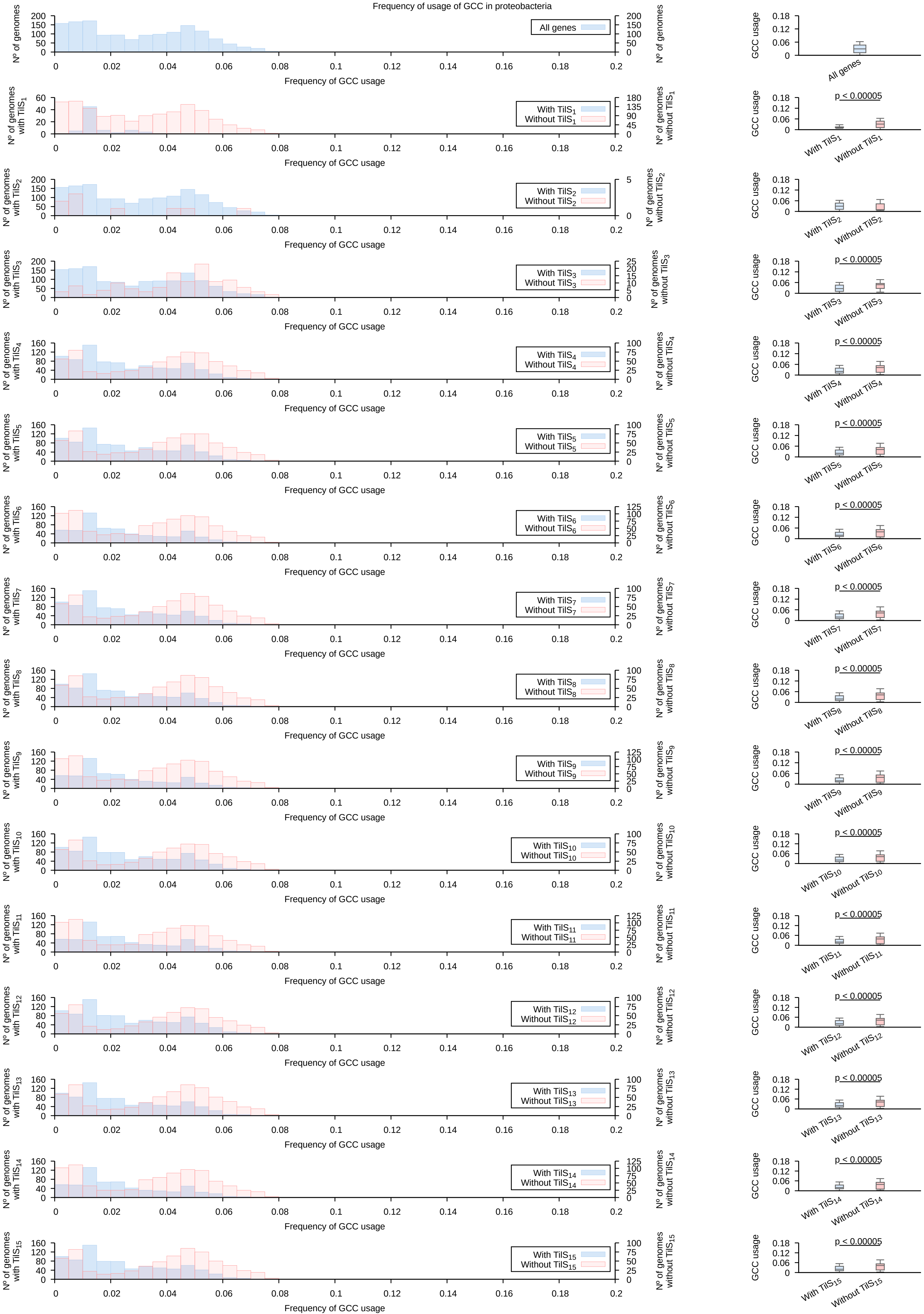

Frequency of usage of GCC in proteobacteria
Nº of genomes
All genes
Frequency of GCC usage
GCC usage
0.18
0.12
0.06
0
0
0.02
0.04
0.06
0.08
0.1
0.12
0.14
0.16
0.18
0.2
Nº of genomes with TilS1
Nº of genomes without TilS1
With TilS1
Without TilS1
p < 0.00005
Nº of genomes with TilS2
Nº of genomes without TilS2
With TilS2
Without TilS2
Nº of genomes with TilS3
Nº of genomes without TilS3
With TilS3
Without TilS3
Nº of genomes with TilS4
Nº of genomes without TilS4
With TilS4
Without TilS4
Nº of genomes with TilS5
Nº of genomes without TilS5
With TilS5
Without TilS5
Nº of genomes with TilS6
Nº of genomes without TilS6
With TilS6
Without TilS6
Nº of genomes with TilS7
Nº of genomes without TilS7
With TilS7
Without TilS7
Nº of genomes with TilS8
Nº of genomes without TilS8
With TilS8
Without TilS8
Nº of genomes with TilS9
Nº of genomes without TilS9
With TilS9
Without TilS9
Nº of genomes with TilS10
Nº of genomes without TilS10
With TilS10
Without TilS10
Nº of genomes with TilS11
Nº of genomes without TilS11
With TilS11
Without TilS11
Nº of genomes with TilS12
Nº of genomes without TilS12
With TilS12
Without TilS12
Nº of genomes with TilS13
Nº of genomes without TilS13
With TilS13
Without TilS13
Nº of genomes with TilS14
Nº of genomes without TilS14
With TilS14
Without TilS14
Nº of genomes with TilS15
Nº of genomes without TilS15
With TilS15
Without TilS15

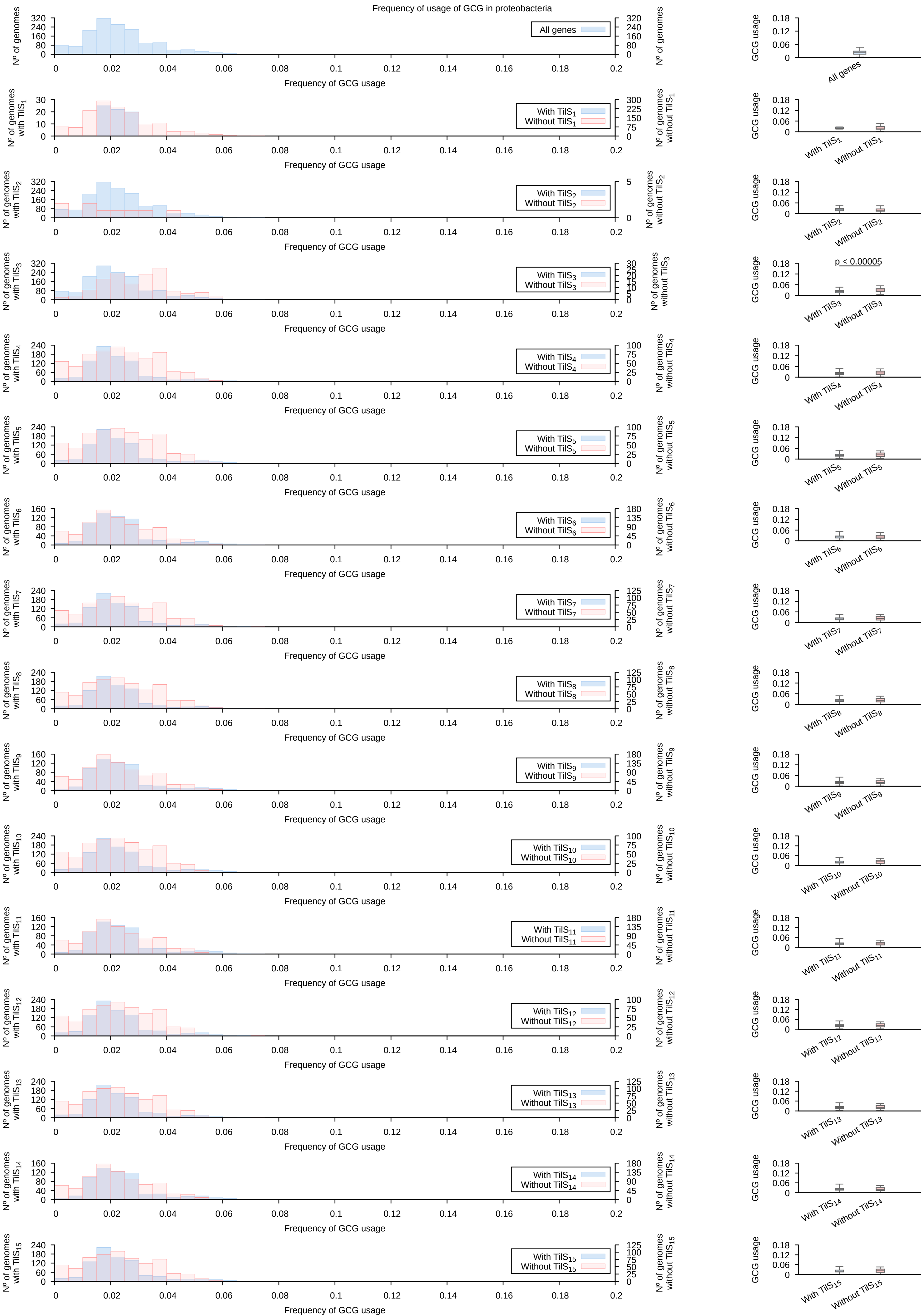

Frequency of usage of GCG in proteobacteria
Nº of genomes
All genes
Frequency of GCG usage
GCG usage
Nº of genomes with TilS$_1$
Nº of genomes without TilS$_1$
With TilS$_1$
Without TilS$_1$
Nº of genomes with TilS$_2$
Nº of genomes without TilS$_2$
With TilS$_2$
Without TilS$_2$
Nº of genomes with TilS$_3$
Nº of genomes without TilS$_3$
With TilS$_3$
Without TilS$_3$
$p < 0.00005$
Nº of genomes with TilS$_4$
Nº of genomes without TilS$_4$
With TilS$_4$
Without TilS$_4$
Nº of genomes with TilS$_5$
Nº of genomes without TilS$_5$
With TilS$_5$
Without TilS$_5$
Nº of genomes with TilS$_6$
Nº of genomes without TilS$_6$
With TilS$_6$
Without TilS$_6$
Nº of genomes with TilS$_7$
Nº of genomes without TilS$_7$
With TilS$_7$
Without TilS$_7$
Nº of genomes with TilS$_8$
Nº of genomes without TilS$_8$
With TilS$_8$
Without TilS$_8$
Nº of genomes with TilS$_9$
Nº of genomes without TilS$_9$
With TilS$_9$
Without TilS$_9$
Nº of genomes with TilS$_{10}$
Nº of genomes without TilS$_{10}$
With TilS$_{10}$
Without TilS$_{10}$
Nº of genomes with TilS$_{11}$
Nº of genomes without TilS$_{11}$
With TilS$_{11}$
Without TilS$_{11}$
Nº of genomes with TilS$_{12}$
Nº of genomes without TilS$_{12}$
With TilS$_{12}$
Without TilS$_{12}$
Nº of genomes with TilS$_{13}$
Nº of genomes without TilS$_{13}$
With TilS$_{13}$
Without TilS$_{13}$
Nº of genomes with TilS$_{14}$
Nº of genomes without TilS$_{14}$
With TilS$_{14}$
Without TilS$_{14}$
Nº of genomes with TilS$_{15}$
Nº of genomes without TilS$_{15}$
With TilS$_{15}$
Without TilS$_{15}$

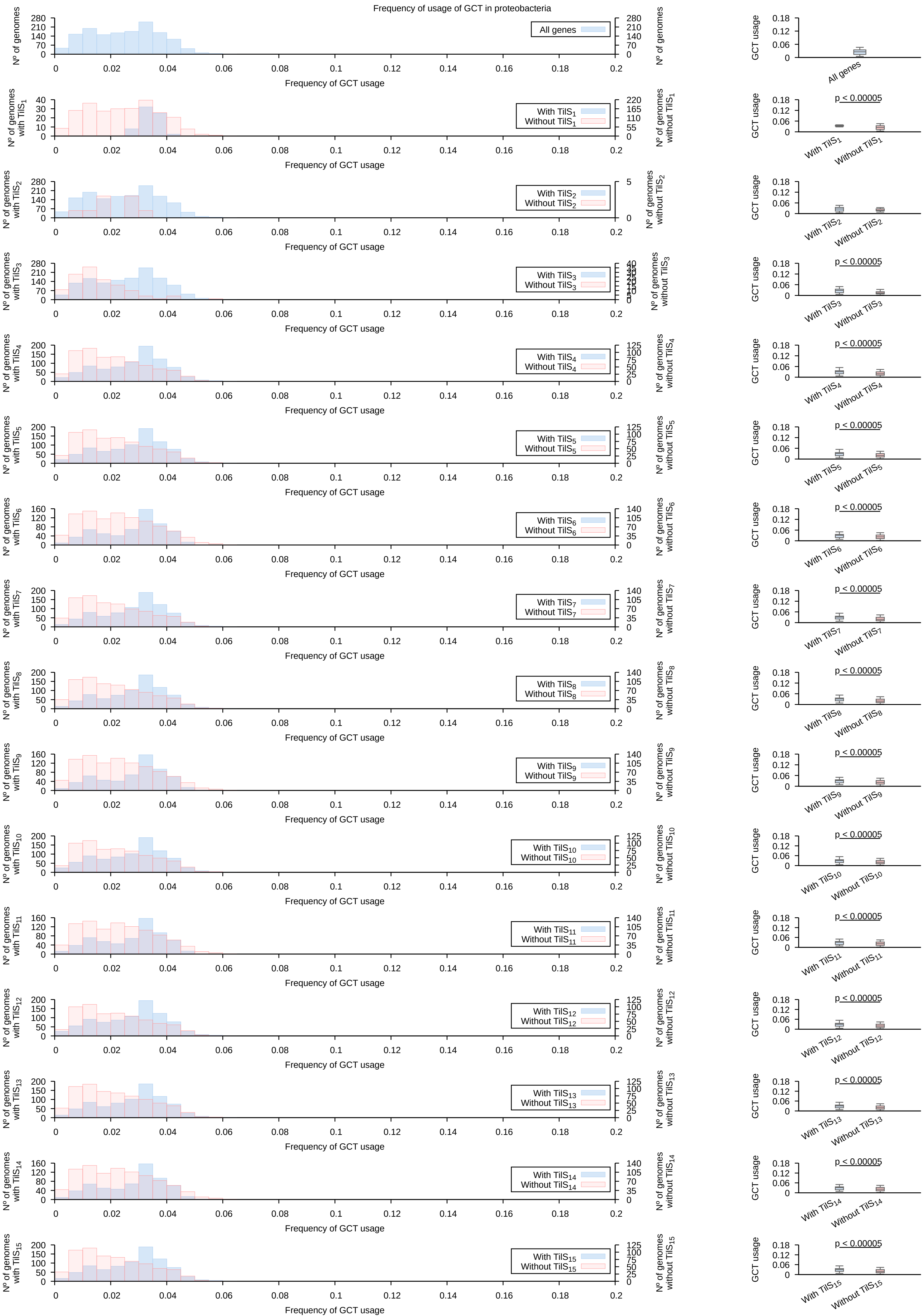

Frequency of usage of GCT in proteobacteria
Nº of genomes
All genes
Frequency of GCT usage
GCT usage
All genes
Nº of genomes with TilS$_1$
With TilS$_1$
Without TilS$_1$
Nº of genomes without TilS$_1$
p < 0.00005
Nº of genomes with TilS$_2$
With TilS$_2$
Without TilS$_2$
Nº of genomes without TilS$_2$
Nº of genomes with TilS$_3$
With TilS$_3$
Without TilS$_3$
Nº of genomes without TilS$_3$
p < 0.00005
Nº of genomes with TilS$_4$
With TilS$_4$
Without TilS$_4$
Nº of genomes without TilS$_4$
p < 0.00005
Nº of genomes with TilS$_5$
With TilS$_5$
Without TilS$_5$
Nº of genomes without TilS$_5$
p < 0.00005
Nº of genomes with TilS$_6$
With TilS$_6$
Without TilS$_6$
Nº of genomes without TilS$_6$
p < 0.00005
Nº of genomes with TilS$_7$
With TilS$_7$
Without TilS$_7$
Nº of genomes without TilS$_7$
p < 0.00005
Nº of genomes with TilS$_8$
With TilS$_8$
Without TilS$_8$
Nº of genomes without TilS$_8$
p < 0.00005
Nº of genomes with TilS$_9$
With TilS$_9$
Without TilS$_9$
Nº of genomes without TilS$_9$
p < 0.00005
Nº of genomes with TilS$_{10}$
With TilS$_{10}$
Without TilS$_{10}$
Nº of genomes without TilS$_{10}$
p < 0.00005
Nº of genomes with TilS$_{11}$
With TilS$_{11}$
Without TilS$_{11}$
Nº of genomes without TilS$_{11}$
p < 0.00005
Nº of genomes with TilS$_{12}$
With TilS$_{12}$
Without TilS$_{12}$
Nº of genomes without TilS$_{12}$
p < 0.00005
Nº of genomes with TilS$_{13}$
With TilS$_{13}$
Without TilS$_{13}$
Nº of genomes without TilS$_{13}$
p < 0.00005
Nº of genomes with TilS$_{14}$
With TilS$_{14}$
Without TilS$_{14}$
Nº of genomes without TilS$_{14}$
p < 0.00005
Nº of genomes with TilS$_{15}$
With TilS$_{15}$
Without TilS$_{15}$
Nº of genomes without TilS$_{15}$
p < 0.00005

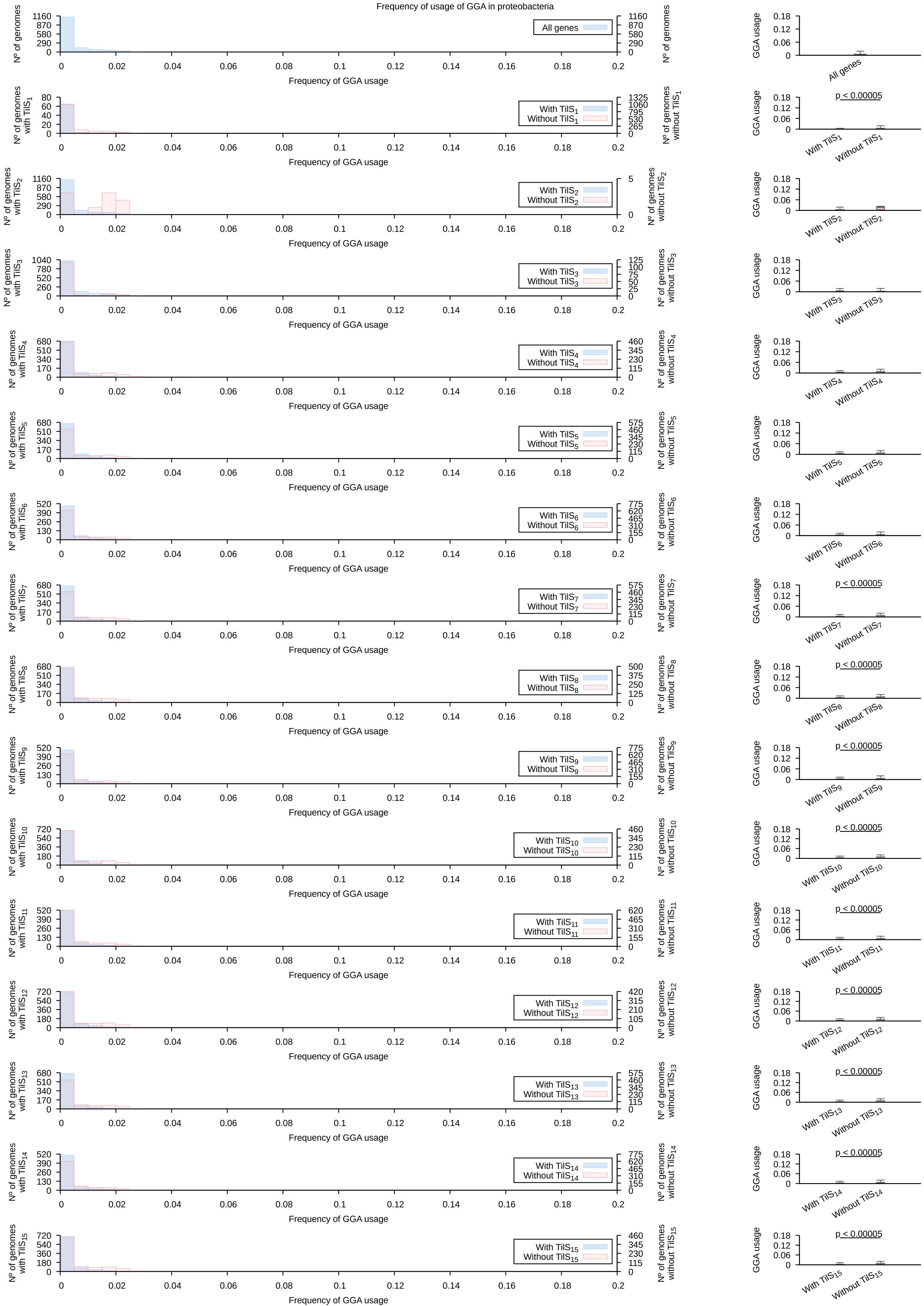

Frequency of usage of GGA in proteobacteria
Nº of genomes
All genes
Frequency of GGA usage
GGA usage
All genes
Nº of genomes with TilS$_1$
Nº of genomes without TilS$_1$
With TilS$_1$
Without TilS$_1$
p < 0.00005
Nº of genomes with TilS$_2$
Nº of genomes without TilS$_2$
With TilS$_2$
Without TilS$_2$
Nº of genomes with TilS$_3$
Nº of genomes without TilS$_3$
With TilS$_3$
Without TilS$_3$
Nº of genomes with TilS$_4$
Nº of genomes without TilS$_4$
With TilS$_4$
Without TilS$_4$
Nº of genomes with TilS$_5$
Nº of genomes without TilS$_5$
With TilS$_5$
Without TilS$_5$
Nº of genomes with TilS$_6$
Nº of genomes without TilS$_6$
With TilS$_6$
Without TilS$_6$
Nº of genomes with TilS$_7$
Nº of genomes without TilS$_7$
With TilS$_7$
Without TilS$_7$
p < 0.00005
Nº of genomes with TilS$_8$
Nº of genomes without TilS$_8$
With TilS$_8$
Without TilS$_8$
p < 0.00005
Nº of genomes with TilS$_9$
Nº of genomes without TilS$_9$
With TilS$_9$
Without TilS$_9$
p < 0.00005
Nº of genomes with TilS$_{10}$
Nº of genomes without TilS$_{10}$
With TilS$_{10}$
Without TilS$_{10}$
p < 0.00005
Nº of genomes with TilS$_{11}$
Nº of genomes without TilS$_{11}$
With TilS$_{11}$
Without TilS$_{11}$
p < 0.00005
Nº of genomes with TilS$_{12}$
Nº of genomes without TilS$_{12}$
With TilS$_{12}$
Without TilS$_{12}$
p < 0.00005
Nº of genomes with TilS$_{13}$
Nº of genomes without TilS$_{13}$
With TilS$_{13}$
Without TilS$_{13}$
p < 0.00005
Nº of genomes with TilS$_{14}$
Nº of genomes without TilS$_{14}$
With TilS$_{14}$
Without TilS$_{14}$
p < 0.00005
Nº of genomes with TilS$_{15}$
Nº of genomes without TilS$_{15}$
With TilS$_{15}$
Without TilS$_{15}$
p < 0.00005

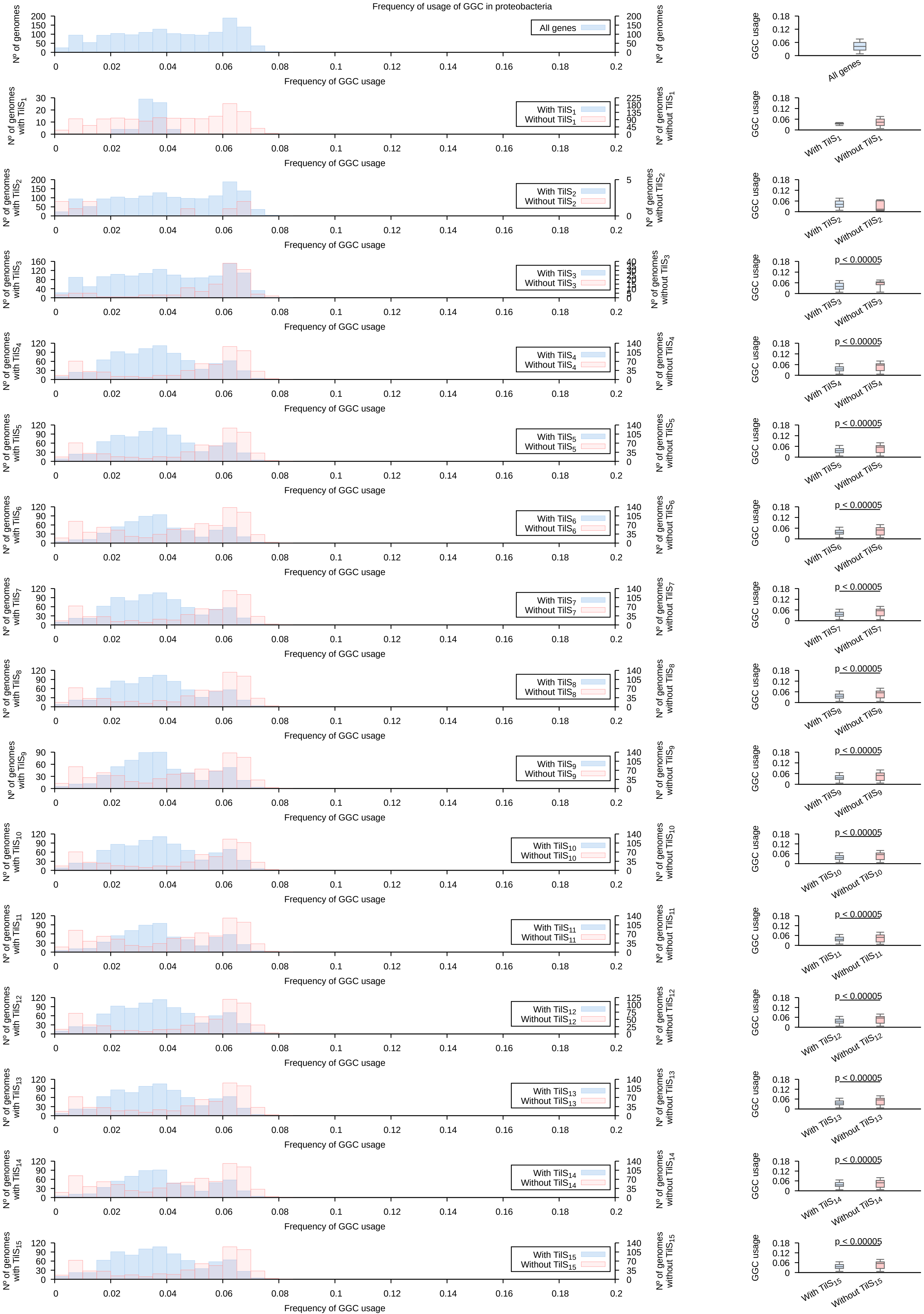
Frequency of usage of GGC in proteobacteria
Nº of genomes
All genes
Frequency of GGC usage
GGC usage
Nº of genomes with TilS$_1$
Nº of genomes without TilS$_1$
With TilS$_1$
Without TilS$_1$
Nº of genomes with TilS$_2$
Nº of genomes without TilS$_2$
With TilS$_2$
Without TilS$_2$
Nº of genomes with TilS$_3$
Nº of genomes without TilS$_3$
With TilS$_3$
Without TilS$_3$
$p < 0.00005$
Nº of genomes with TilS$_4$
Nº of genomes without TilS$_4$
With TilS$_4$
Without TilS$_4$
Nº of genomes with TilS$_5$
Nº of genomes without TilS$_5$
With TilS$_5$
Without TilS$_5$
Nº of genomes with TilS$_6$
Nº of genomes without TilS$_6$
With TilS$_6$
Without TilS$_6$
Nº of genomes with TilS$_7$
Nº of genomes without TilS$_7$
With TilS$_7$
Without TilS$_7$
Nº of genomes with TilS$_8$
Nº of genomes without TilS$_8$
With TilS$_8$
Without TilS$_8$
Nº of genomes with TilS$_9$
Nº of genomes without TilS$_9$
With TilS$_9$
Without TilS$_9$
Nº of genomes with TilS$_{10}$
Nº of genomes without TilS$_{10}$
With TilS$_{10}$
Without TilS$_{10}$
Nº of genomes with TilS$_{11}$
Nº of genomes without TilS$_{11}$
With TilS$_{11}$
Without TilS$_{11}$
Nº of genomes with TilS$_{12}$
Nº of genomes without TilS$_{12}$
With TilS$_{12}$
Without TilS$_{12}$
Nº of genomes with TilS$_{13}$
Nº of genomes without TilS$_{13}$
With TilS$_{13}$
Without TilS$_{13}$
Nº of genomes with TilS$_{14}$
Nº of genomes without TilS$_{14}$
With TilS$_{14}$
Without TilS$_{14}$
Nº of genomes with TilS$_{15}$
Nº of genomes without TilS$_{15}$
With TilS$_{15}$
Without TilS$_{15}$
0 0.02 0.04 0.06 0.08 0.1 0.12 0.14 0.16 0.18 0.2
0 0.06 0.12 0.18

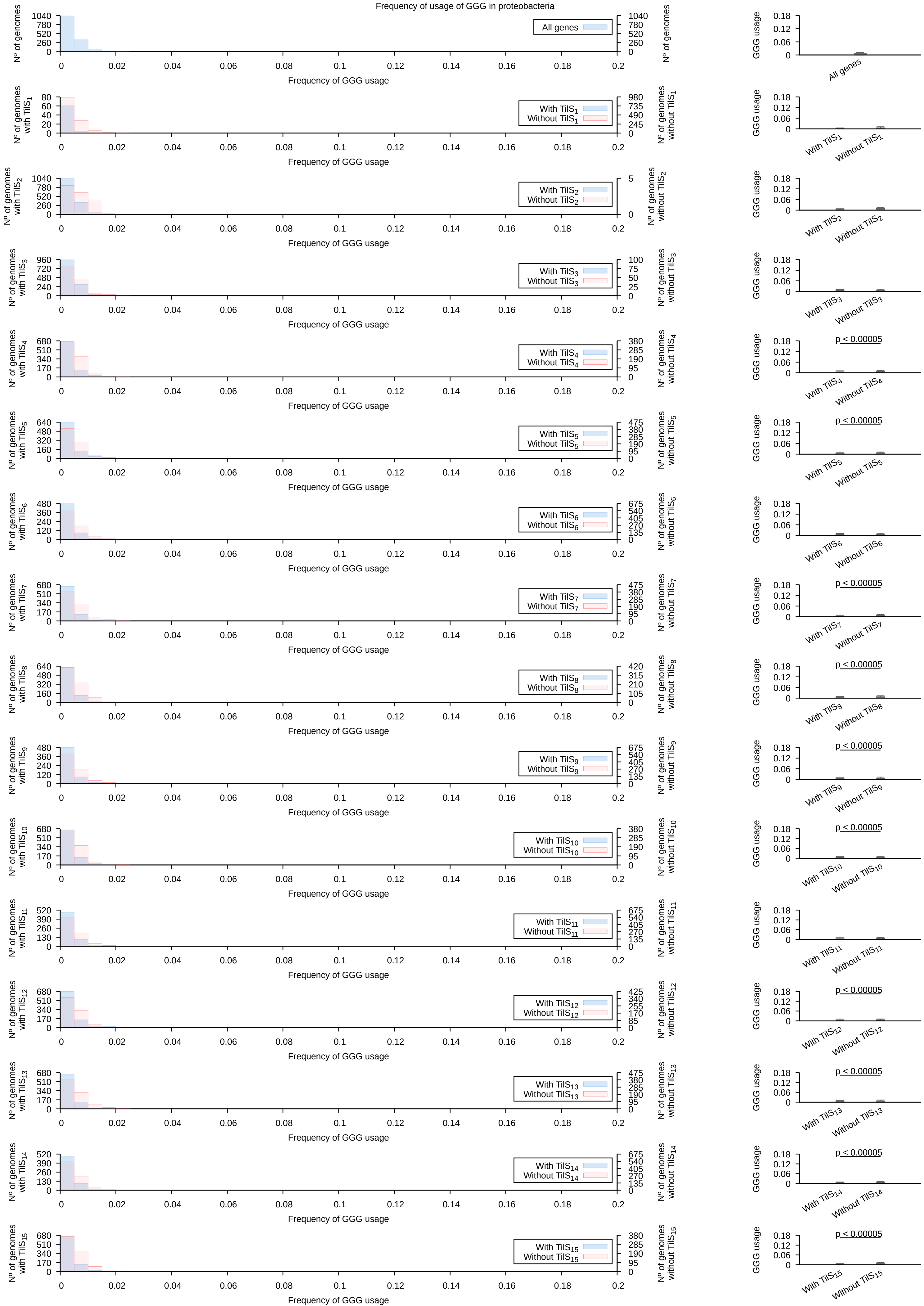
Frequency of usage of GGG in proteobacteria
Nº of genomes
All genes
Frequency of GGG usage
GGG usage
Nº of genomes with TilS1
Nº of genomes without TilS1
With TilS1
Without TilS1
Nº of genomes with TilS2
Nº of genomes without TilS2
With TilS2
Without TilS2
Nº of genomes with TilS3
Nº of genomes without TilS3
With TilS3
Without TilS3
Nº of genomes with TilS4
Nº of genomes without TilS4
With TilS4
Without TilS4
p < 0.00005
Nº of genomes with TilS5
Nº of genomes without TilS5
With TilS5
Without TilS5
p < 0.00005
Nº of genomes with TilS6
Nº of genomes without TilS6
With TilS6
Without TilS6
Nº of genomes with TilS7
Nº of genomes without TilS7
With TilS7
Without TilS7
p < 0.00005
Nº of genomes with TilS8
Nº of genomes without TilS8
With TilS8
Without TilS8
p < 0.00005
Nº of genomes with TilS9
Nº of genomes without TilS9
With TilS9
Without TilS9
p < 0.00005
Nº of genomes with TilS10
Nº of genomes without TilS10
With TilS10
Without TilS10
p < 0.00005
Nº of genomes with TilS11
Nº of genomes without TilS11
With TilS11
Without TilS11
Nº of genomes with TilS12
Nº of genomes without TilS12
With TilS12
Without TilS12
p < 0.00005
Nº of genomes with TilS13
Nº of genomes without TilS13
With TilS13
Without TilS13
p < 0.00005
Nº of genomes with TilS14
Nº of genomes without TilS14
With TilS14
Without TilS14
p < 0.00005
Nº of genomes with TilS15
Nº of genomes without TilS15
With TilS15
Without TilS15
p < 0.00005

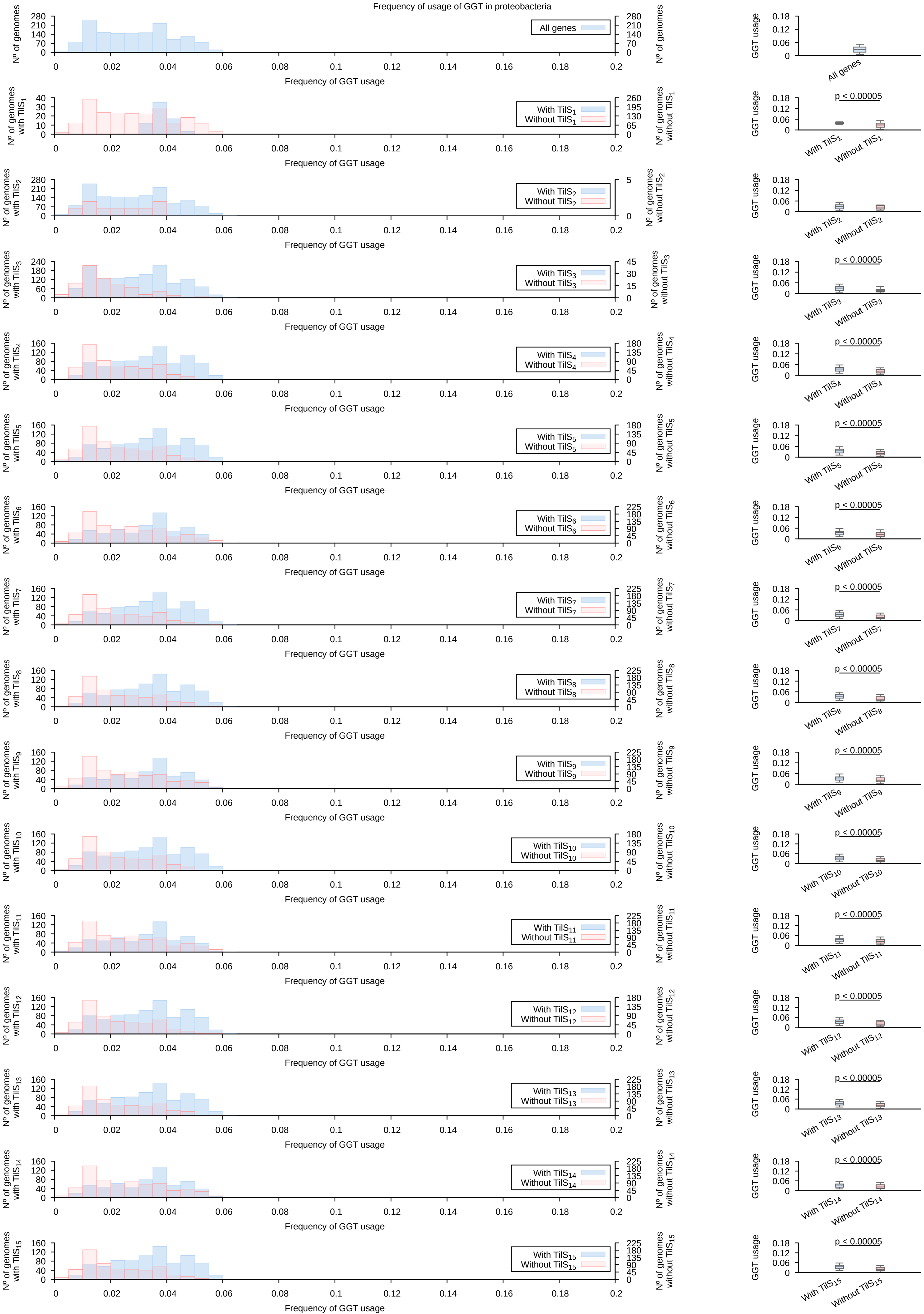

Frequency of usage of GGT in proteobacteria
Nº of genomes
All genes
Frequency of GGT usage
GGT usage
Nº of genomes with TilS
Nº of genomes without TilS
With TilS
Without TilS
p < 0.00005

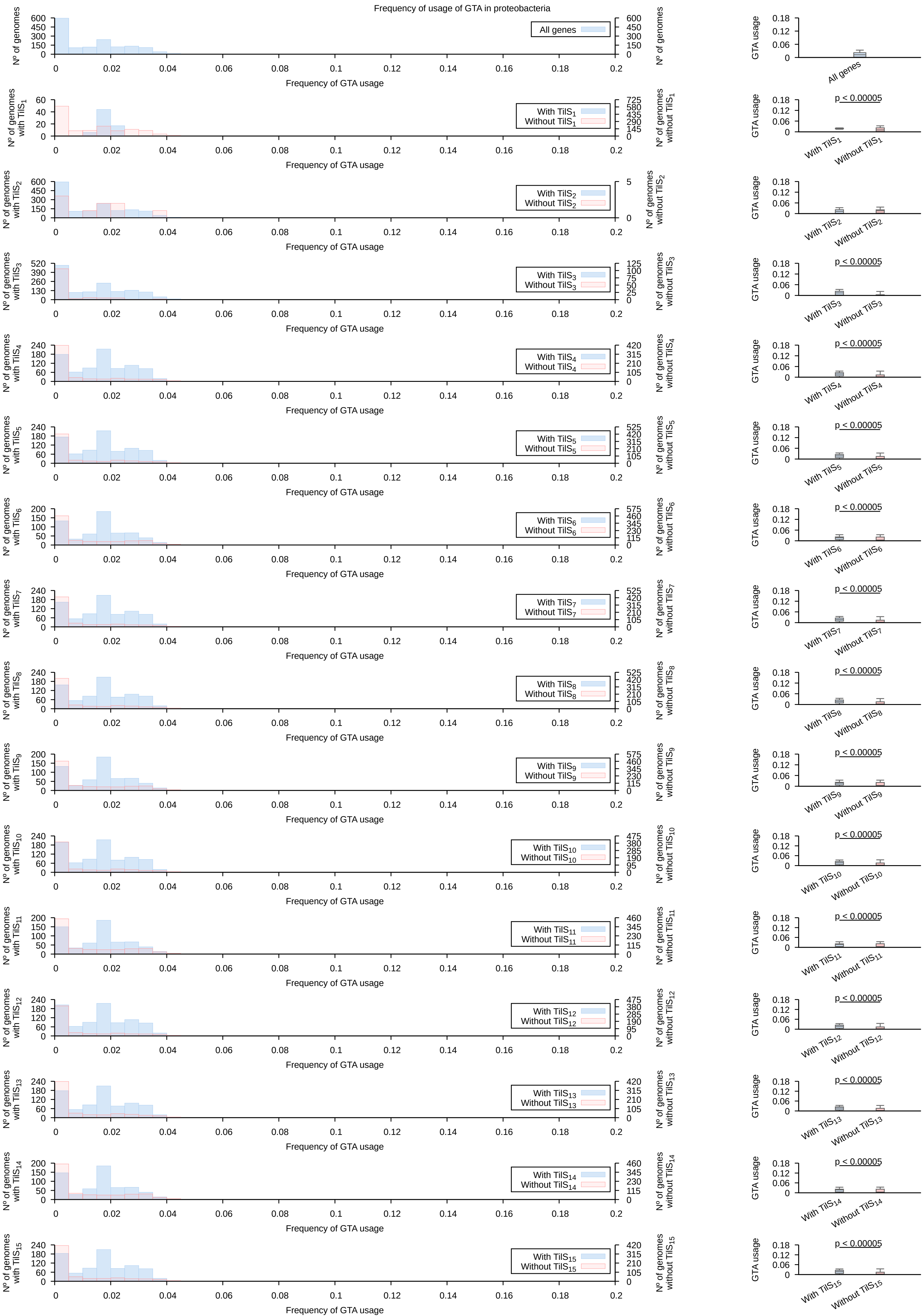

Frequency of usage of GTA in proteobacteria
Nº of genomes
All genes
Frequency of GTA usage
GTA usage
p < 0.00005
With TilS1
Without TilS1
With TilS15
Without TilS15

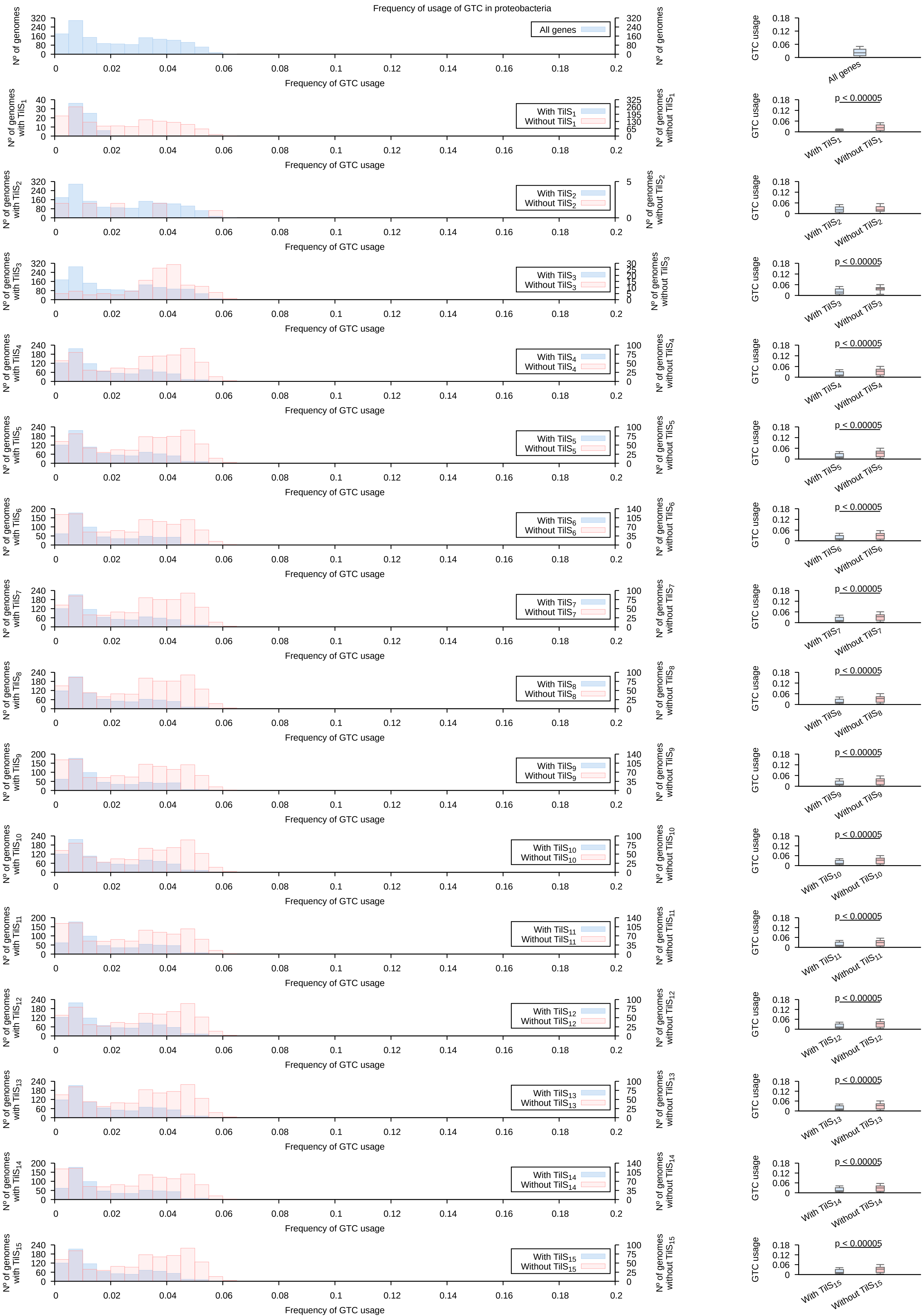

Frequency of usage of GTC in proteobacteria
Nº of genomes
All genes
Frequency of GTC usage
GTC usage
Nº of genomes with TilS$_1$
Nº of genomes without TilS$_1$
With TilS$_1$
Without TilS$_1$
p < 0.00005
Nº of genomes with TilS$_2$
Nº of genomes without TilS$_2$
With TilS$_2$
Without TilS$_2$
Nº of genomes with TilS$_3$
Nº of genomes without TilS$_3$
With TilS$_3$
Without TilS$_3$
p < 0.00005
Nº of genomes with TilS$_4$
Nº of genomes without TilS$_4$
With TilS$_4$
Without TilS$_4$
p < 0.00005
Nº of genomes with TilS$_5$
Nº of genomes without TilS$_5$
With TilS$_5$
Without TilS$_5$
p < 0.00005
Nº of genomes with TilS$_6$
Nº of genomes without TilS$_6$
With TilS$_6$
Without TilS$_6$
p < 0.00005
Nº of genomes with TilS$_7$
Nº of genomes without TilS$_7$
With TilS$_7$
Without TilS$_7$
p < 0.00005
Nº of genomes with TilS$_8$
Nº of genomes without TilS$_8$
With TilS$_8$
Without TilS$_8$
p < 0.00005
Nº of genomes with TilS$_9$
Nº of genomes without TilS$_9$
With TilS$_9$
Without TilS$_9$
p < 0.00005
Nº of genomes with TilS$_{10}$
Nº of genomes without TilS$_{10}$
With TilS$_{10}$
Without TilS$_{10}$
p < 0.00005
Nº of genomes with TilS$_{11}$
Nº of genomes without TilS$_{11}$
With TilS$_{11}$
Without TilS$_{11}$
p < 0.00005
Nº of genomes with TilS$_{12}$
Nº of genomes without TilS$_{12}$
With TilS$_{12}$
Without TilS$_{12}$
p < 0.00005
Nº of genomes with TilS$_{13}$
Nº of genomes without TilS$_{13}$
With TilS$_{13}$
Without TilS$_{13}$
p < 0.00005
Nº of genomes with TilS$_{14}$
Nº of genomes without TilS$_{14}$
With TilS$_{14}$
Without TilS$_{14}$
p < 0.00005
Nº of genomes with TilS$_{15}$
Nº of genomes without TilS$_{15}$
With TilS$_{15}$
Without TilS$_{15}$
p < 0.00005

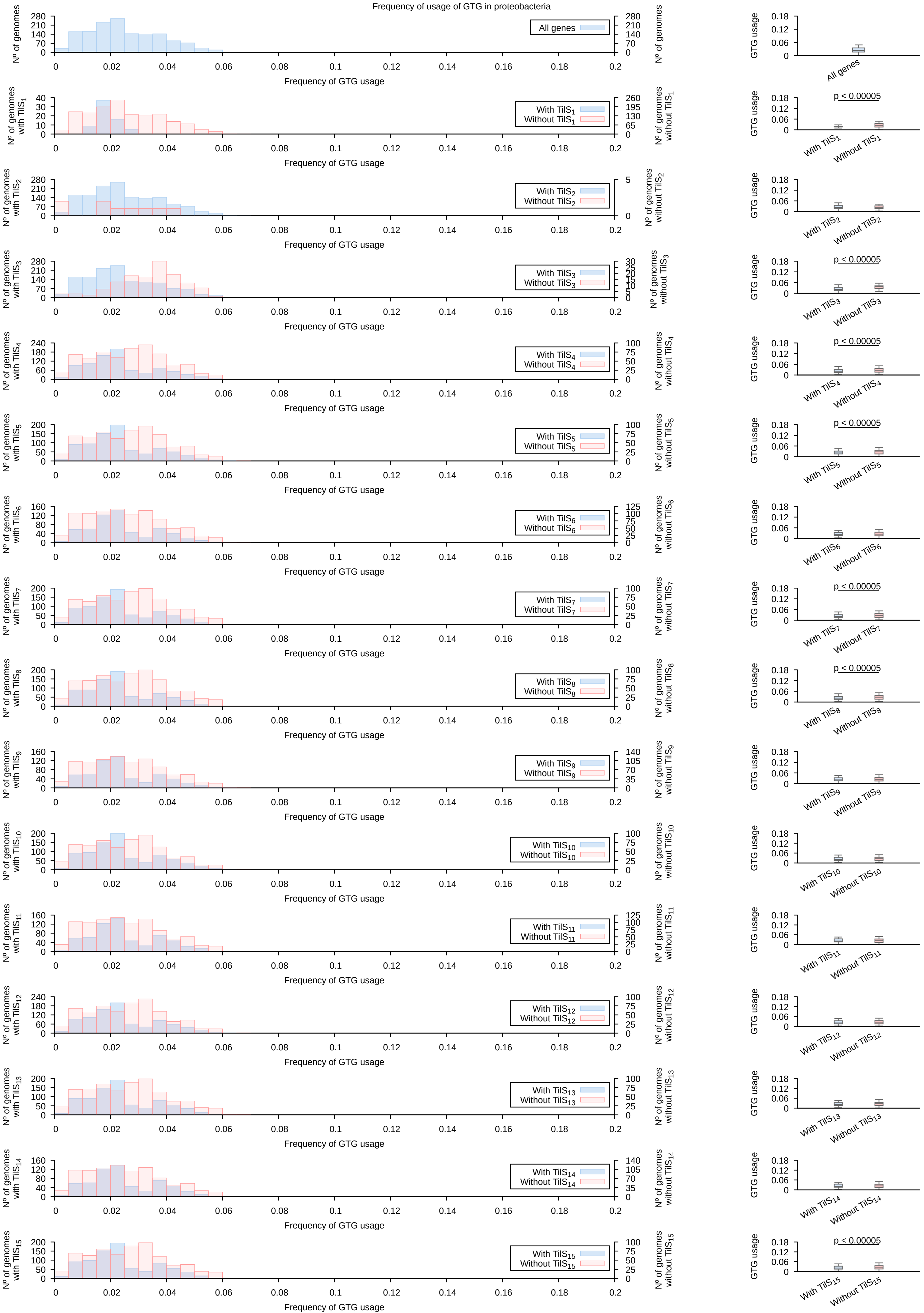

Frequency of usage of GTG in proteobacteria
Nº of genomes
All genes
Frequency of GTG usage
0 0.02 0.04 0.06 0.08 0.1 0.12 0.14 0.16 0.18 0.2
GTG usage
0.18 0.12 0.06 0
All genes
Nº of genomes with TilS1
Nº of genomes without TilS1
With TilS1
Without TilS1
p < 0.00005
Nº of genomes with TilS2
Nº of genomes without TilS2
With TilS2
Without TilS2
Nº of genomes with TilS3
Nº of genomes without TilS3
With TilS3
Without TilS3
p < 0.00005
Nº of genomes with TilS4
Nº of genomes without TilS4
With TilS4
Without TilS4
p < 0.00005
Nº of genomes with TilS5
Nº of genomes without TilS5
With TilS5
Without TilS5
p < 0.00005
Nº of genomes with TilS6
Nº of genomes without TilS6
With TilS6
Without TilS6
Nº of genomes with TilS7
Nº of genomes without TilS7
With TilS7
Without TilS7
p < 0.00005
Nº of genomes with TilS8
Nº of genomes without TilS8
With TilS8
Without TilS8
p < 0.00005
Nº of genomes with TilS9
Nº of genomes without TilS9
With TilS9
Without TilS9
Nº of genomes with TilS10
Nº of genomes without TilS10
With TilS10
Without TilS10
Nº of genomes with TilS11
Nº of genomes without TilS11
With TilS11
Without TilS11
Nº of genomes with TilS12
Nº of genomes without TilS12
With TilS12
Without TilS12
Nº of genomes with TilS13
Nº of genomes without TilS13
With TilS13
Without TilS13
Nº of genomes with TilS14
Nº of genomes without TilS14
With TilS14
Without TilS14
Nº of genomes with TilS15
Nº of genomes without TilS15
With TilS15
Without TilS15
p < 0.00005

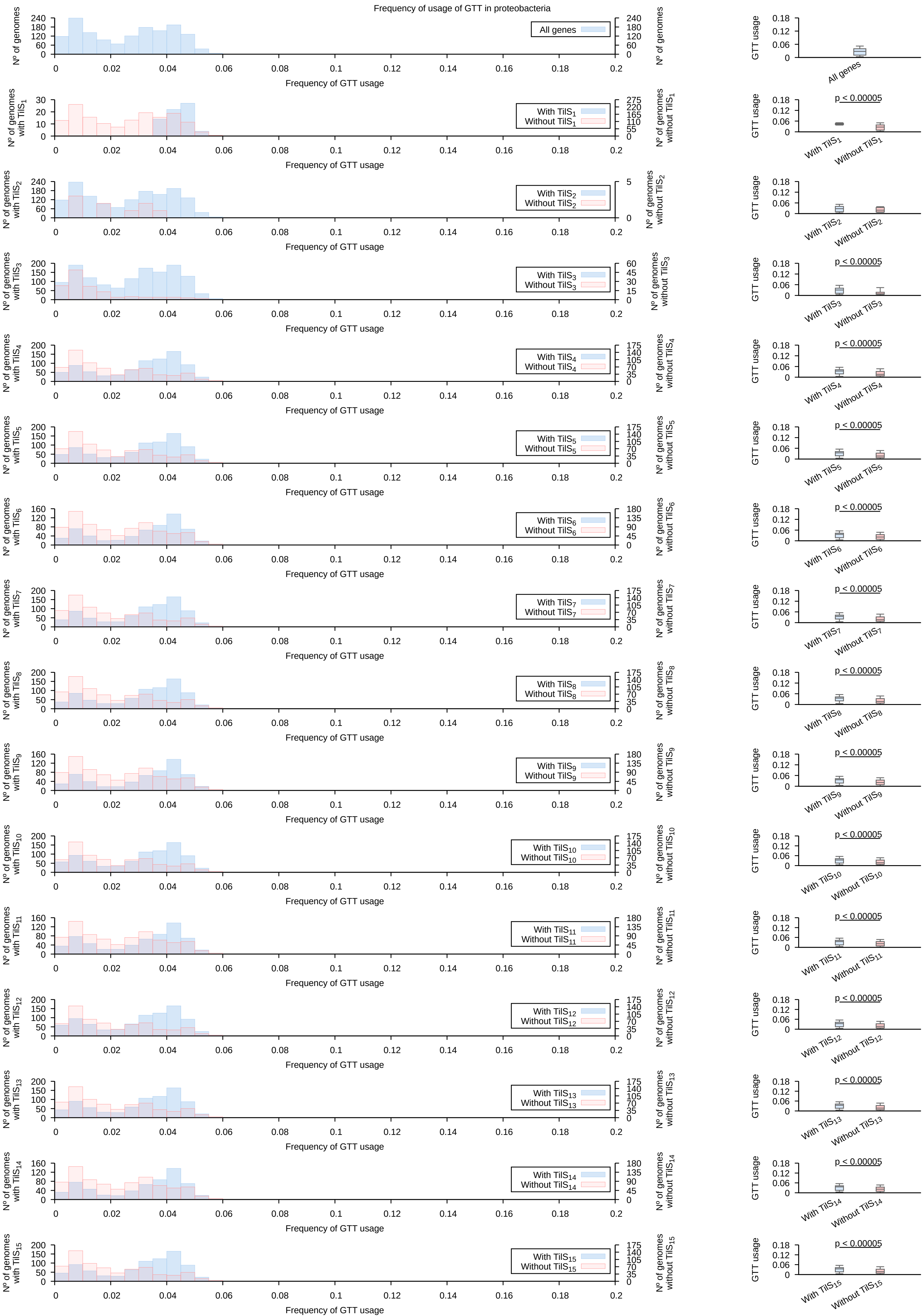

Frequency of usage of GTT in proteobacteria
Nº of genomes
All genes
Frequency of GTT usage
GTT usage
All genes
Nº of genomes with TilS$_1$
Nº of genomes without TilS$_1$
With TilS$_1$
Without TilS$_1$
p < 0.00005
Nº of genomes with TilS$_2$
Nº of genomes without TilS$_2$
With TilS$_2$
Without TilS$_2$
Nº of genomes with TilS$_3$
Nº of genomes without TilS$_3$
With TilS$_3$
Without TilS$_3$
p < 0.00005
Nº of genomes with TilS$_4$
Nº of genomes without TilS$_4$
With TilS$_4$
Without TilS$_4$
p < 0.00005
Nº of genomes with TilS$_5$
Nº of genomes without TilS$_5$
With TilS$_5$
Without TilS$_5$
p < 0.00005
Nº of genomes with TilS$_6$
Nº of genomes without TilS$_6$
With TilS$_6$
Without TilS$_6$
p < 0.00005
Nº of genomes with TilS$_7$
Nº of genomes without TilS$_7$
With TilS$_7$
Without TilS$_7$
p < 0.00005
Nº of genomes with TilS$_8$
Nº of genomes without TilS$_8$
With TilS$_8$
Without TilS$_8$
p < 0.00005
Nº of genomes with TilS$_9$
Nº of genomes without TilS$_9$
With TilS$_9$
Without TilS$_9$
p < 0.00005
Nº of genomes with TilS$_{10}$
Nº of genomes without TilS$_{10}$
With TilS$_{10}$
Without TilS$_{10}$
p < 0.00005
Nº of genomes with TilS$_{11}$
Nº of genomes without TilS$_{11}$
With TilS$_{11}$
Without TilS$_{11}$
p < 0.00005
Nº of genomes with TilS$_{12}$
Nº of genomes without TilS$_{12}$
With TilS$_{12}$
Without TilS$_{12}$
p < 0.00005
Nº of genomes with TilS$_{13}$
Nº of genomes without TilS$_{13}$
With TilS$_{13}$
Without TilS$_{13}$
p < 0.00005
Nº of genomes with TilS$_{14}$
Nº of genomes without TilS$_{14}$
With TilS$_{14}$
Without TilS$_{14}$
p < 0.00005
Nº of genomes with TilS$_{15}$
Nº of genomes without TilS$_{15}$
With TilS$_{15}$
Without TilS$_{15}$
p < 0.00005

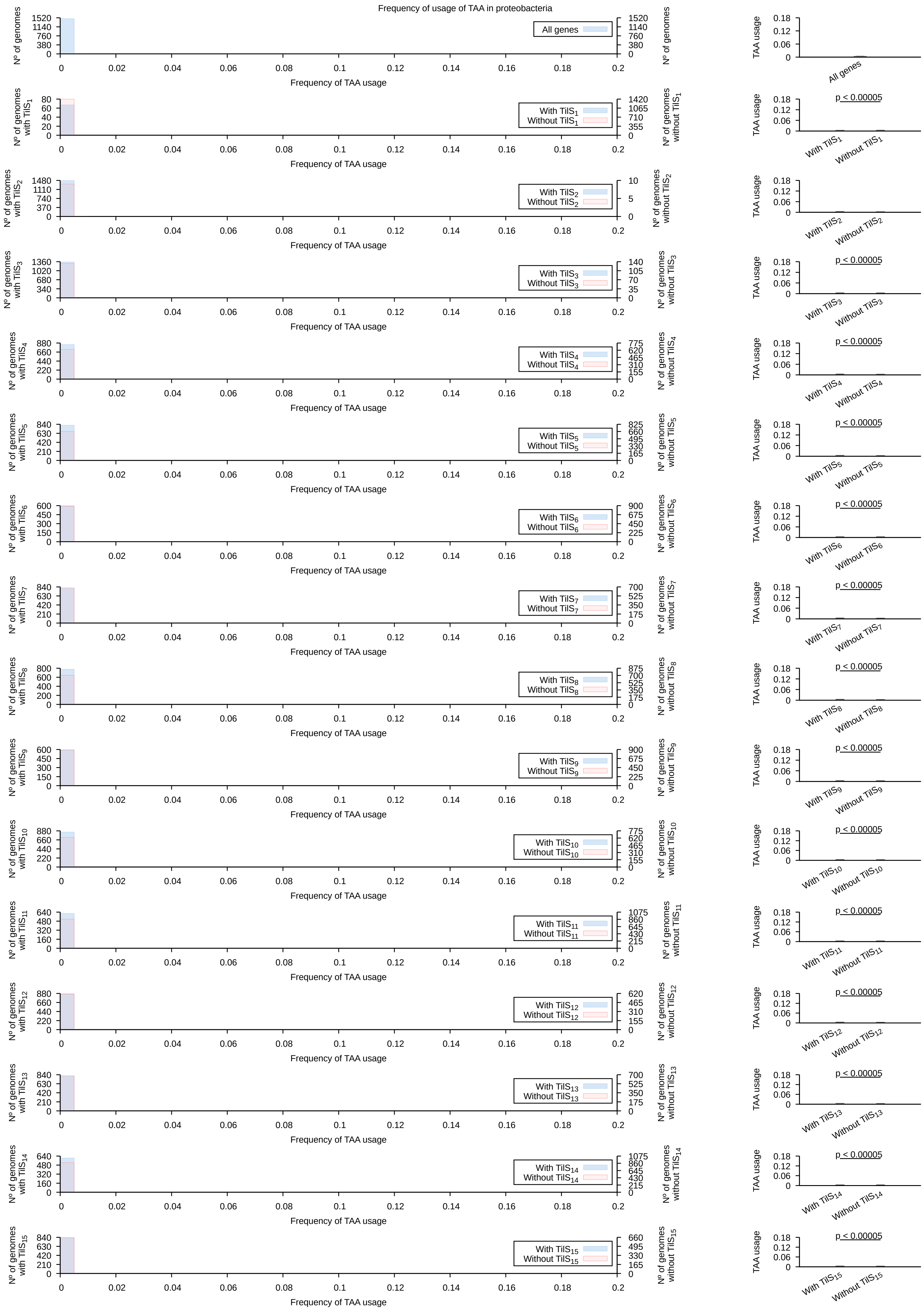

Frequency of usage of TAA in proteobacteria
Nº of genomes
All genes
Frequency of TAA usage
0
0.02
0.04
0.06
0.08
0.1
0.12
0.14
0.16
0.18
0.2
1520
1140
760
380
TAA usage
0.18
0.12
0.06
Nº of genomes with TilS$_1$
Nº of genomes without TilS$_1$
With TilS$_1$
Without TilS$_1$
p < 0.00005
80
60
40
20
1420
1065
710
355
Nº of genomes with TilS$_2$
Nº of genomes without TilS$_2$
With TilS$_2$
Without TilS$_2$
1480
1110
740
370
10
5
Nº of genomes with TilS$_3$
Nº of genomes without TilS$_3$
With TilS$_3$
Without TilS$_3$
1360
1020
680
340
140
105
70
35
Nº of genomes with TilS$_4$
Nº of genomes without TilS$_4$
With TilS$_4$
Without TilS$_4$
880
660
440
220
775
620
465
310
155
Nº of genomes with TilS$_5$
Nº of genomes without TilS$_5$
With TilS$_5$
Without TilS$_5$
840
630
420
210
825
660
495
330
165
Nº of genomes with TilS$_6$
Nº of genomes without TilS$_6$
With TilS$_6$
Without TilS$_6$
600
450
300
150
900
675
450
225
Nº of genomes with TilS$_7$
Nº of genomes without TilS$_7$
With TilS$_7$
Without TilS$_7$
840
630
420
210
700
525
350
175
Nº of genomes with TilS$_8$
Nº of genomes without TilS$_8$
With TilS$_8$
Without TilS$_8$
800
600
400
200
875
700
525
350
175
Nº of genomes with TilS$_9$
Nº of genomes without TilS$_9$
With TilS$_9$
Without TilS$_9$
600
450
300
150
900
675
450
225
Nº of genomes with TilS$_{10}$
Nº of genomes without TilS$_{10}$
With TilS$_{10}$
Without TilS$_{10}$
880
660
440
220
775
620
465
310
155
Nº of genomes with TilS$_{11}$
Nº of genomes without TilS$_{11}$
With TilS$_{11}$
Without TilS$_{11}$
640
480
320
160
1075
860
645
430
215
Nº of genomes with TilS$_{12}$
Nº of genomes without TilS$_{12}$
With TilS$_{12}$
Without TilS$_{12}$
880
660
440
220
620
465
310
155
Nº of genomes with TilS$_{13}$
Nº of genomes without TilS$_{13}$
With TilS$_{13}$
Without TilS$_{13}$
840
630
420
210
700
525
350
175
Nº of genomes with TilS$_{14}$
Nº of genomes without TilS$_{14}$
With TilS$_{14}$
Without TilS$_{14}$
640
480
320
160
1075
860
645
430
215
Nº of genomes with TilS$_{15}$
Nº of genomes without TilS$_{15}$
With TilS$_{15}$
Without TilS$_{15}$
840
630
420
210
660
495
330
165

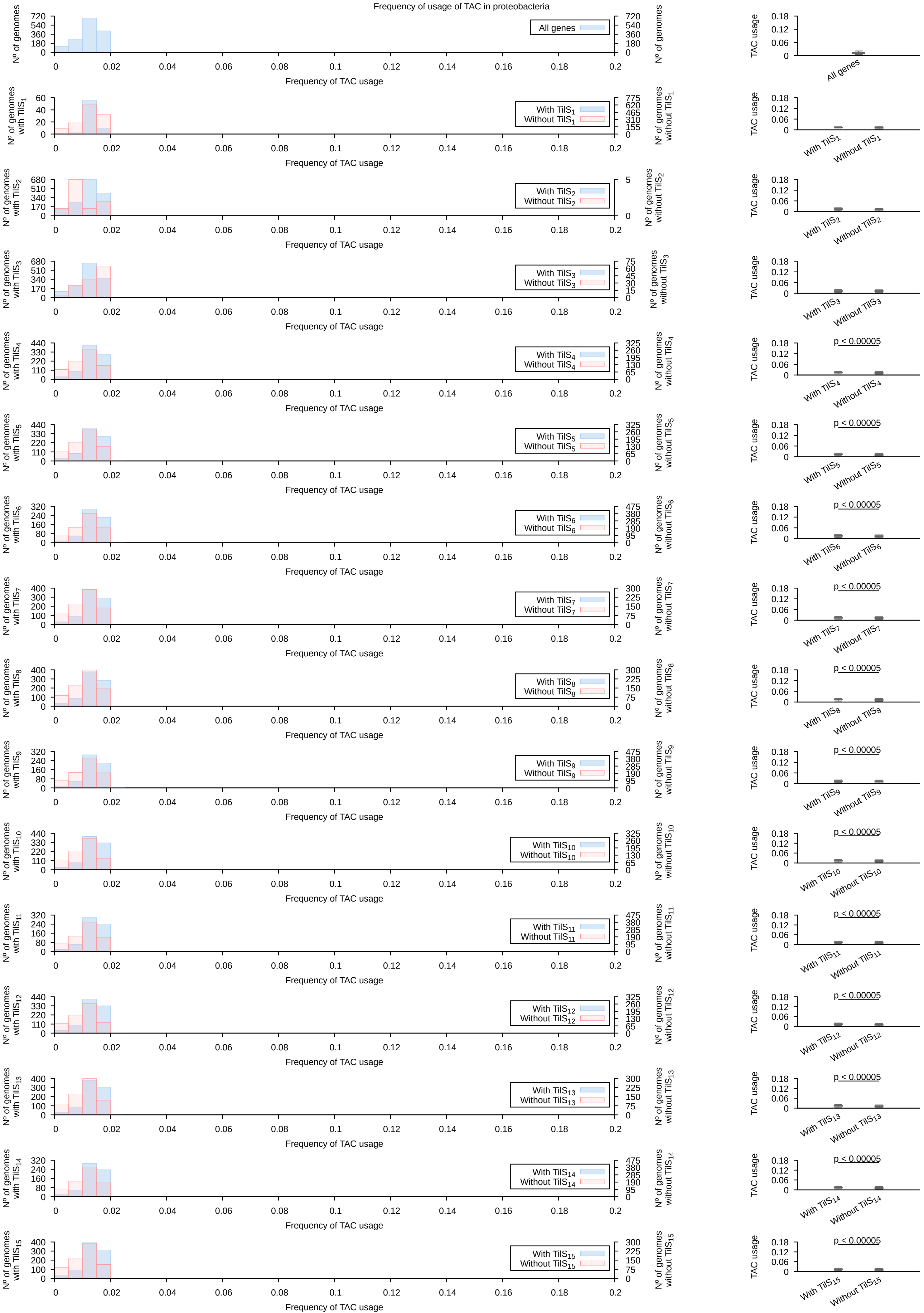

Frequency of usage of TAC in proteobacteria
Nº of genomes
All genes
Frequency of TAC usage
TAC usage
0
0.02
0.04
0.06
0.08
0.1
0.12
0.14
0.16
0.18
0.2
Nº of genomes with TilS1
Nº of genomes without TilS1
With TilS1
Without TilS1
Nº of genomes with TilS2
Nº of genomes without TilS2
With TilS2
Without TilS2
Nº of genomes with TilS3
Nº of genomes without TilS3
With TilS3
Without TilS3
Nº of genomes with TilS4
Nº of genomes without TilS4
With TilS4
Without TilS4
p < 0.00005
Nº of genomes with TilS5
Nº of genomes without TilS5
With TilS5
Without TilS5
p < 0.00005
Nº of genomes with TilS6
Nº of genomes without TilS6
With TilS6
Without TilS6
p < 0.00005
Nº of genomes with TilS7
Nº of genomes without TilS7
With TilS7
Without TilS7
p < 0.00005
Nº of genomes with TilS8
Nº of genomes without TilS8
With TilS8
Without TilS8
p < 0.00005
Nº of genomes with TilS9
Nº of genomes without TilS9
With TilS9
Without TilS9
p < 0.00005
Nº of genomes with TilS10
Nº of genomes without TilS10
With TilS10
Without TilS10
p < 0.00005
Nº of genomes with TilS11
Nº of genomes without TilS11
With TilS11
Without TilS11
p < 0.00005
Nº of genomes with TilS12
Nº of genomes without TilS12
With TilS12
Without TilS12
p < 0.00005
Nº of genomes with TilS13
Nº of genomes without TilS13
With TilS13
Without TilS13
p < 0.00005
Nº of genomes with TilS14
Nº of genomes without TilS14
With TilS14
Without TilS14
p < 0.00005
Nº of genomes with TilS15
Nº of genomes without TilS15
With TilS15
Without TilS15
p < 0.00005

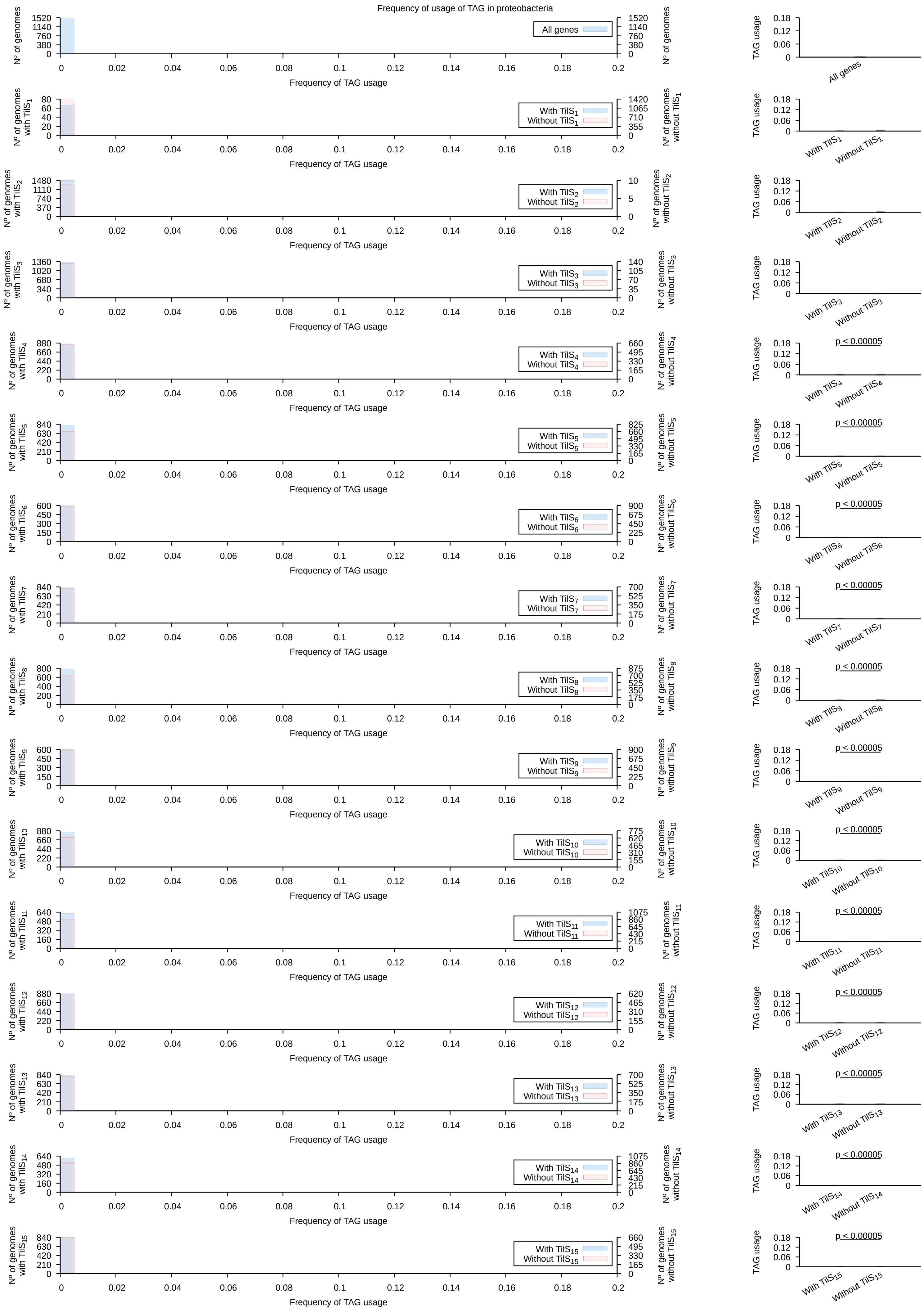

Frequency of usage of TAG in proteobacteria
Nº of genomes
1520 1140 760 380 0
All genes
0 0.02 0.04 0.06 0.08 0.1 0.12 0.14 0.16 0.18 0.2
Frequency of TAG usage
TAG usage
0.18 0.12 0.06 0
All genes
Nº of genomes with TilS1
80 60 40 20 0
With TilS1
Without TilS1
1420 1065 710 355 0
Nº of genomes without TilS1
Nº of genomes with TilS2
1480 1110 740 370 0
With TilS2
Without TilS2
10 5 0
Nº of genomes without TilS2
Nº of genomes with TilS3
1360 1020 680 340 0
With TilS3
Without TilS3
140 105 70 35 0
Nº of genomes without TilS3
Nº of genomes with TilS4
880 660 440 220 0
With TilS4
Without TilS4
660 495 330 165 0
Nº of genomes without TilS4
p < 0.00005
Nº of genomes with TilS5
840 630 420 210 0
With TilS5
Without TilS5
825 660 495 330 165 0
Nº of genomes without TilS5
Nº of genomes with TilS6
600 450 300 150 0
With TilS6
Without TilS6
900 675 450 225 0
Nº of genomes without TilS6
Nº of genomes with TilS7
840 630 420 210 0
With TilS7
Without TilS7
700 525 350 175 0
Nº of genomes without TilS7
Nº of genomes with TilS8
800 600 400 200 0
With TilS8
Without TilS8
875 700 525 350 175 0
Nº of genomes without TilS8
Nº of genomes with TilS9
600 450 300 150 0
With TilS9
Without TilS9
900 675 450 225 0
Nº of genomes without TilS9
Nº of genomes with TilS10
880 660 440 220 0
With TilS10
Without TilS10
775 620 465 310 155 0
Nº of genomes without TilS10
Nº of genomes with TilS11
640 480 320 160 0
With TilS11
Without TilS11
1075 860 645 430 215 0
Nº of genomes without TilS11
Nº of genomes with TilS12
880 660 440 220 0
With TilS12
Without TilS12
620 465 310 155 0
Nº of genomes without TilS12
Nº of genomes with TilS13
840 630 420 210 0
With TilS13
Without TilS13
700 525 350 175 0
Nº of genomes without TilS13
Nº of genomes with TilS14
640 480 320 160 0
With TilS14
Without TilS14
1075 860 645 430 215 0
Nº of genomes without TilS14
Nº of genomes with TilS15
840 630 420 210 0
With TilS15
Without TilS15
660 495 330 165 0
Nº of genomes without TilS15

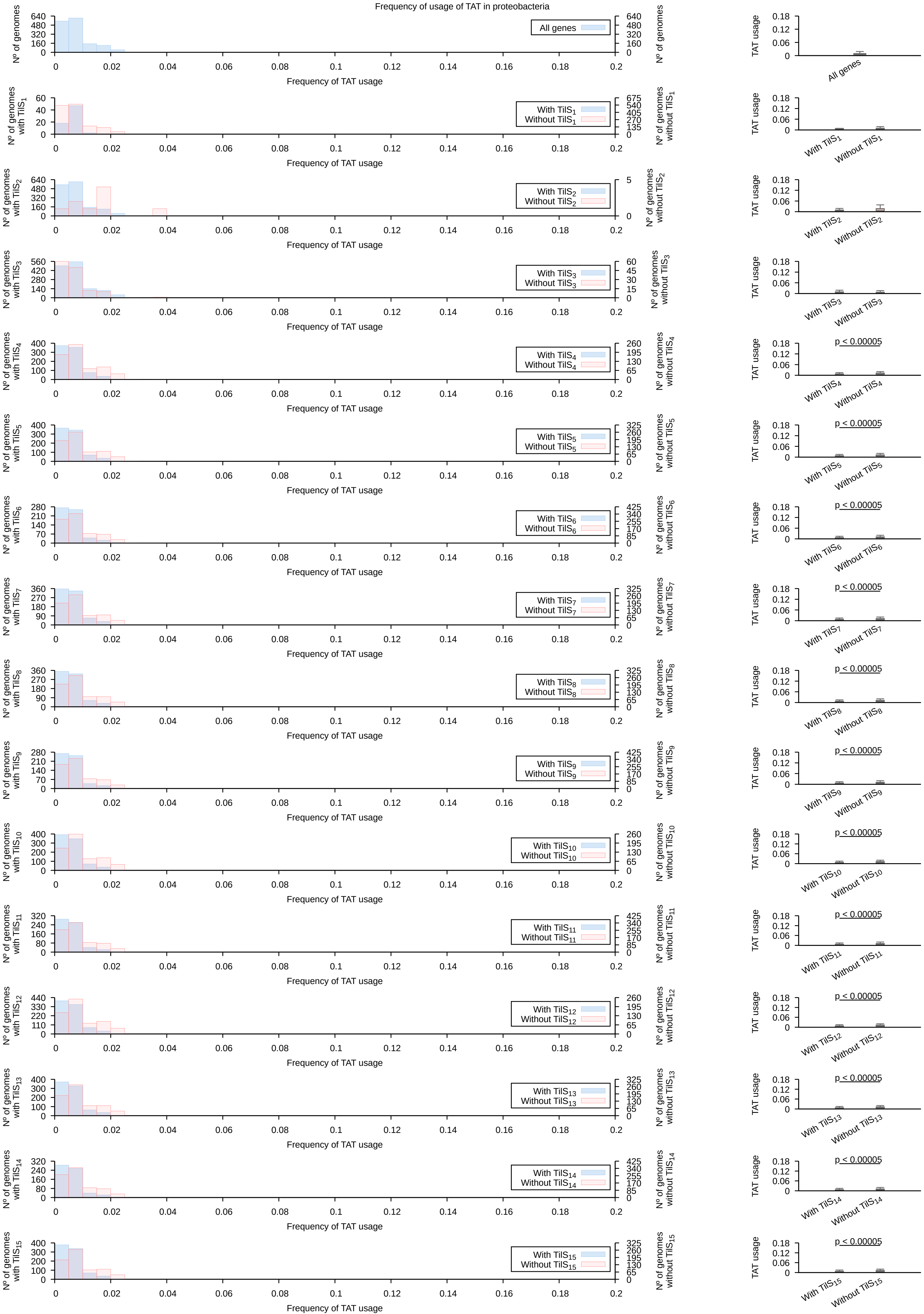

Frequency of usage of TAT in proteobacteria
All genes
Nº of genomes
Frequency of TAT usage
TAT usage
With TilS$_1$
Without TilS$_1$
Nº of genomes with TilS$_1$
Nº of genomes without TilS$_1$
With TilS$_2$
Without TilS$_2$
With TilS$_3$
Without TilS$_3$
With TilS$_4$
Without TilS$_4$
With TilS$_5$
Without TilS$_5$
With TilS$_6$
Without TilS$_6$
With TilS$_7$
Without TilS$_7$
With TilS$_8$
Without TilS$_8$
With TilS$_9$
Without TilS$_9$
With TilS$_{10}$
Without TilS$_{10}$
With TilS$_{11}$
Without TilS$_{11}$
With TilS$_{12}$
Without TilS$_{12}$
With TilS$_{13}$
Without TilS$_{13}$
With TilS$_{14}$
Without TilS$_{14}$
With TilS$_{15}$
Without TilS$_{15}$
p < 0.00005

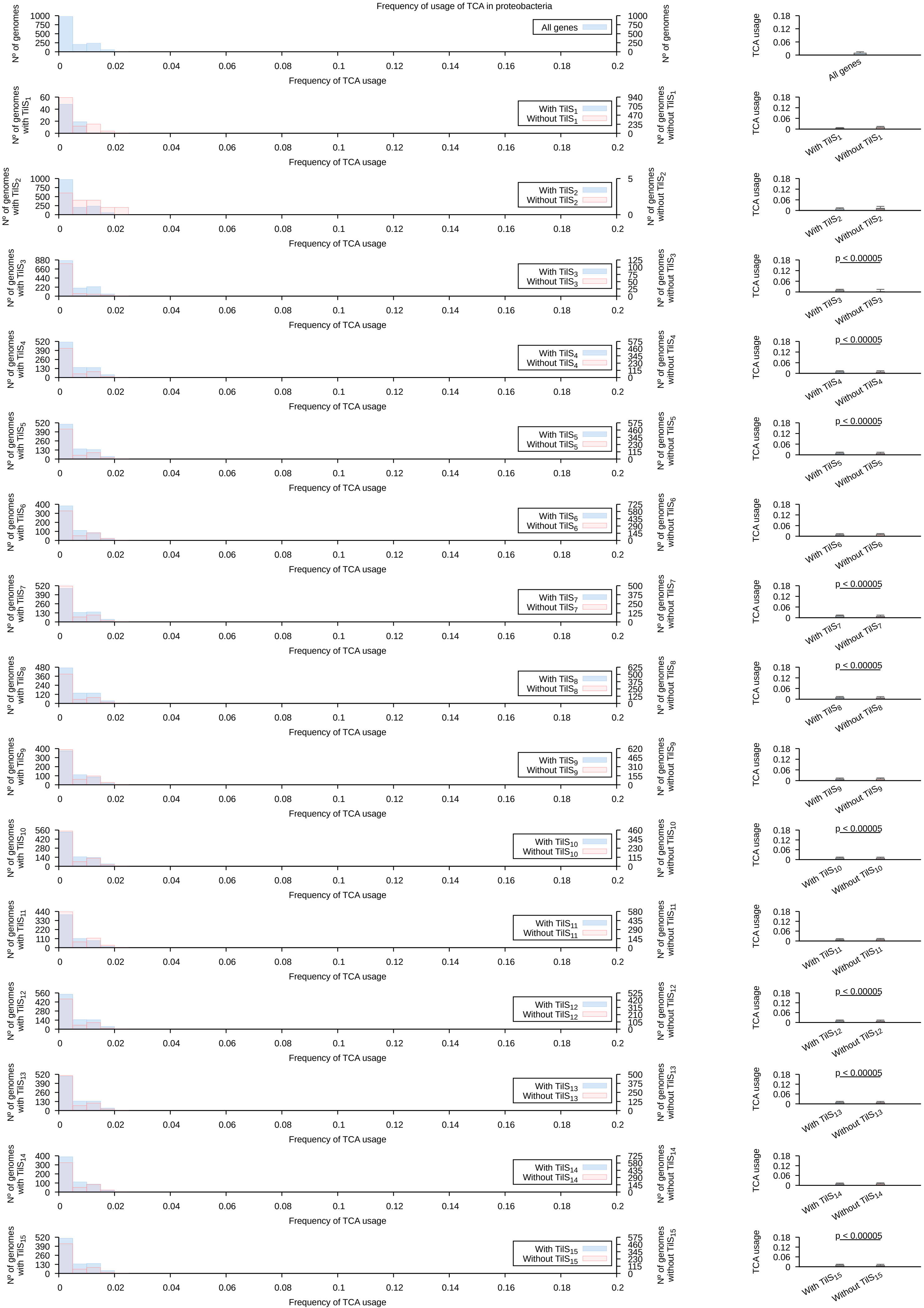

Frequency of usage of TCA in proteobacteria
Nº of genomes
1000
750
500
250
0
All genes
TCA usage
0.18
0.12
0.06
0
All genes
Frequency of TCA usage
0
0.02
0.04
0.06
0.08
0.1
0.12
0.14
0.16
0.18
0.2
Nº of genomes with TilS$_1$
60
40
20
0
With TilS$_1$
Without TilS$_1$
940
705
470
235
0
Nº of genomes without TilS$_1$
Nº of genomes with TilS$_2$
With TilS$_2$
Without TilS$_2$
5
Nº of genomes without TilS$_2$
Nº of genomes with TilS$_3$
880
660
440
220
With TilS$_3$
Without TilS$_3$
125
100
75
50
25
Nº of genomes without TilS$_3$
p < 0.00005
Nº of genomes with TilS$_4$
520
390
260
130
With TilS$_4$
Without TilS$_4$
575
460
345
230
115
Nº of genomes without TilS$_4$
Nº of genomes with TilS$_5$
With TilS$_5$
Without TilS$_5$
Nº of genomes without TilS$_5$
Nº of genomes with TilS$_6$
400
300
200
100
With TilS$_6$
Without TilS$_6$
725
580
435
290
145
Nº of genomes without TilS$_6$
Nº of genomes with TilS$_7$
With TilS$_7$
Without TilS$_7$
500
375
250
125
Nº of genomes without TilS$_7$
Nº of genomes with TilS$_8$
480
360
240
120
With TilS$_8$
Without TilS$_8$
625
500
375
250
125
Nº of genomes without TilS$_8$
Nº of genomes with TilS$_9$
With TilS$_9$
Without TilS$_9$
620
465
310
155
Nº of genomes without TilS$_9$
Nº of genomes with TilS$_{10}$
560
420
280
140
With TilS$_{10}$
Without TilS$_{10}$
460
345
230
115
Nº of genomes without TilS$_{10}$
Nº of genomes with TilS$_{11}$
440
330
220
110
With TilS$_{11}$
Without TilS$_{11}$
580
435
290
145
Nº of genomes without TilS$_{11}$
Nº of genomes with TilS$_{12}$
With TilS$_{12}$
Without TilS$_{12}$
525
420
315
210
105
Nº of genomes without TilS$_{12}$
Nº of genomes with TilS$_{13}$
With TilS$_{13}$
Without TilS$_{13}$
Nº of genomes without TilS$_{13}$
Nº of genomes with TilS$_{14}$
With TilS$_{14}$
Without TilS$_{14}$
Nº of genomes without TilS$_{14}$
Nº of genomes with TilS$_{15}$
With TilS$_{15}$
Without TilS$_{15}$
Nº of genomes without TilS$_{15}$

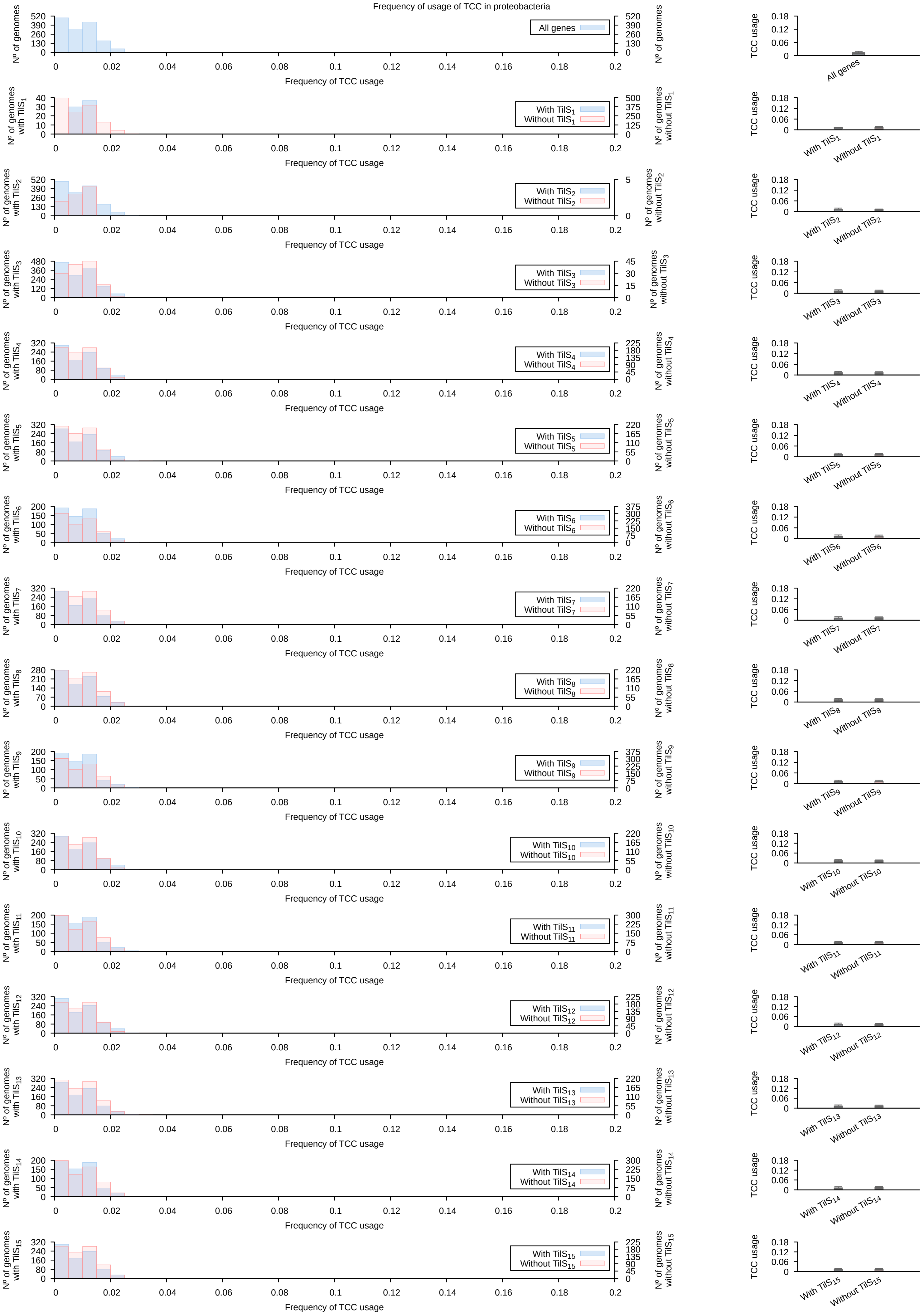
Frequency of usage of TCC in proteobacteria
Nº of genomes
All genes
Frequency of TCC usage
TCC usage
Nº of genomes with TilS$_1$
Nº of genomes without TilS$_1$
With TilS$_1$
Without TilS$_1$
Nº of genomes with TilS$_2$
Nº of genomes without TilS$_2$
With TilS$_2$
Without TilS$_2$
Nº of genomes with TilS$_3$
Nº of genomes without TilS$_3$
With TilS$_3$
Without TilS$_3$
Nº of genomes with TilS$_4$
Nº of genomes without TilS$_4$
With TilS$_4$
Without TilS$_4$
Nº of genomes with TilS$_5$
Nº of genomes without TilS$_5$
With TilS$_5$
Without TilS$_5$
Nº of genomes with TilS$_6$
Nº of genomes without TilS$_6$
With TilS$_6$
Without TilS$_6$
Nº of genomes with TilS$_7$
Nº of genomes without TilS$_7$
With TilS$_7$
Without TilS$_7$
Nº of genomes with TilS$_8$
Nº of genomes without TilS$_8$
With TilS$_8$
Without TilS$_8$
Nº of genomes with TilS$_9$
Nº of genomes without TilS$_9$
With TilS$_9$
Without TilS$_9$
Nº of genomes with TilS$_{10}$
Nº of genomes without TilS$_{10}$
With TilS$_{10}$
Without TilS$_{10}$
Nº of genomes with TilS$_{11}$
Nº of genomes without TilS$_{11}$
With TilS$_{11}$
Without TilS$_{11}$
Nº of genomes with TilS$_{12}$
Nº of genomes without TilS$_{12}$
With TilS$_{12}$
Without TilS$_{12}$
Nº of genomes with TilS$_{13}$
Nº of genomes without TilS$_{13}$
With TilS$_{13}$
Without TilS$_{13}$
Nº of genomes with TilS$_{14}$
Nº of genomes without TilS$_{14}$
With TilS$_{14}$
Without TilS$_{14}$
Nº of genomes with TilS$_{15}$
Nº of genomes without TilS$_{15}$
With TilS$_{15}$
Without TilS$_{15}$

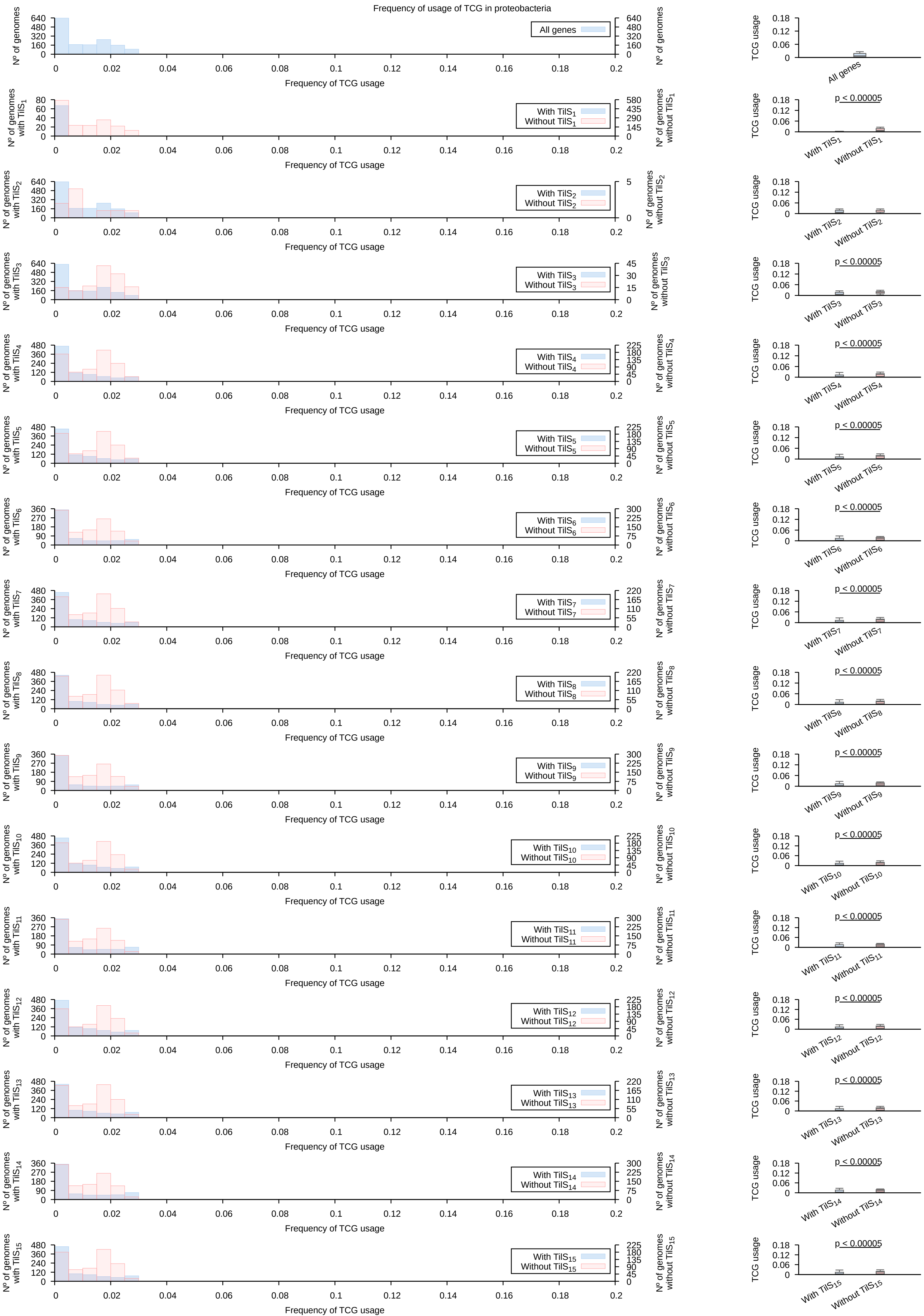
Frequency of usage of TCG in proteobacteria
Nº of genomes
All genes
Frequency of TCG usage
TCG usage
All genes
Nº of genomes with TilS$_1$
With TilS$_1$
Without TilS$_1$
Nº of genomes without TilS$_1$
p < 0.00005
Nº of genomes with TilS$_2$
With TilS$_2$
Without TilS$_2$
Nº of genomes without TilS$_2$
Nº of genomes with TilS$_3$
With TilS$_3$
Without TilS$_3$
Nº of genomes without TilS$_3$
p < 0.00005
Nº of genomes with TilS$_4$
With TilS$_4$
Without TilS$_4$
Nº of genomes without TilS$_4$
p < 0.00005
Nº of genomes with TilS$_5$
With TilS$_5$
Without TilS$_5$
Nº of genomes without TilS$_5$
p < 0.00005
Nº of genomes with TilS$_6$
With TilS$_6$
Without TilS$_6$
Nº of genomes without TilS$_6$
p < 0.00005
Nº of genomes with TilS$_7$
With TilS$_7$
Without TilS$_7$
Nº of genomes without TilS$_7$
p < 0.00005
Nº of genomes with TilS$_8$
With TilS$_8$
Without TilS$_8$
Nº of genomes without TilS$_8$
p < 0.00005
Nº of genomes with TilS$_9$
With TilS$_9$
Without TilS$_9$
Nº of genomes without TilS$_9$
p < 0.00005
Nº of genomes with TilS$_{10}$
With TilS$_{10}$
Without TilS$_{10}$
Nº of genomes without TilS$_{10}$
p < 0.00005
Nº of genomes with TilS$_{11}$
With TilS$_{11}$
Without TilS$_{11}$
Nº of genomes without TilS$_{11}$
p < 0.00005
Nº of genomes with TilS$_{12}$
With TilS$_{12}$
Without TilS$_{12}$
Nº of genomes without TilS$_{12}$
p < 0.00005
Nº of genomes with TilS$_{13}$
With TilS$_{13}$
Without TilS$_{13}$
Nº of genomes without TilS$_{13}$
p < 0.00005
Nº of genomes with TilS$_{14}$
With TilS$_{14}$
Without TilS$_{14}$
Nº of genomes without TilS$_{14}$
p < 0.00005
Nº of genomes with TilS$_{15}$
With TilS$_{15}$
Without TilS$_{15}$
Nº of genomes without TilS$_{15}$
p < 0.00005

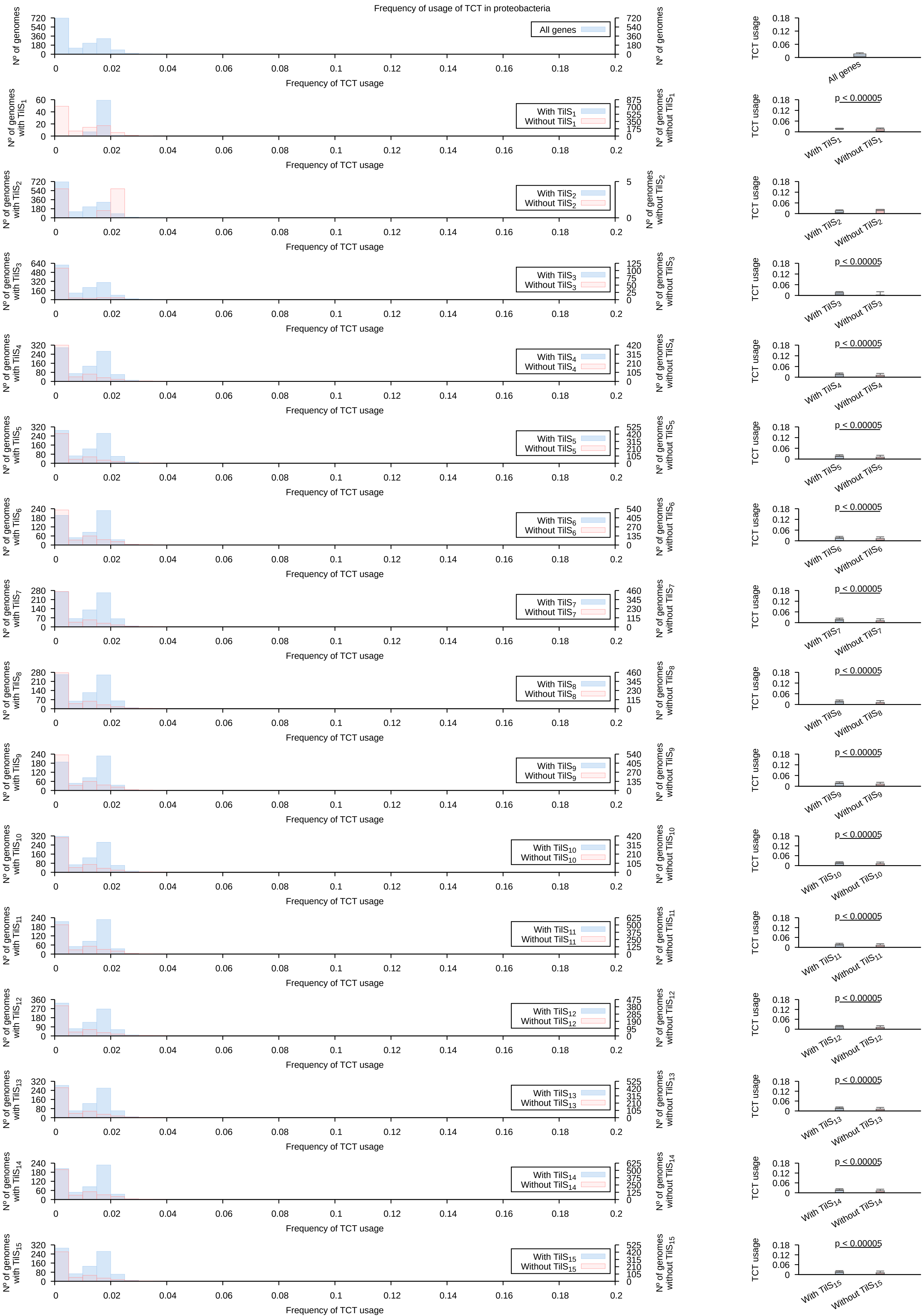
Frequency of usage of TCT in proteobacteria
Nº of genomes
Frequency of TCT usage
All genes
TCT usage
With TilS$_1$
Without TilS$_1$
Nº of genomes with TilS$_1$
Nº of genomes without TilS$_1$
p < 0.00005
With TilS$_2$
Without TilS$_2$
Nº of genomes with TilS$_2$
Nº of genomes without TilS$_2$
With TilS$_3$
Without TilS$_3$
With TilS$_4$
Without TilS$_4$
With TilS$_5$
Without TilS$_5$
With TilS$_6$
Without TilS$_6$
With TilS$_7$
Without TilS$_7$
With TilS$_8$
Without TilS$_8$
With TilS$_9$
Without TilS$_9$
With TilS$_{10}$
Without TilS$_{10}$
With TilS$_{11}$
Without TilS$_{11}$
With TilS$_{12}$
Without TilS$_{12}$
With TilS$_{13}$
Without TilS$_{13}$
With TilS$_{14}$
Without TilS$_{14}$
With TilS$_{15}$
Without TilS$_{15}$

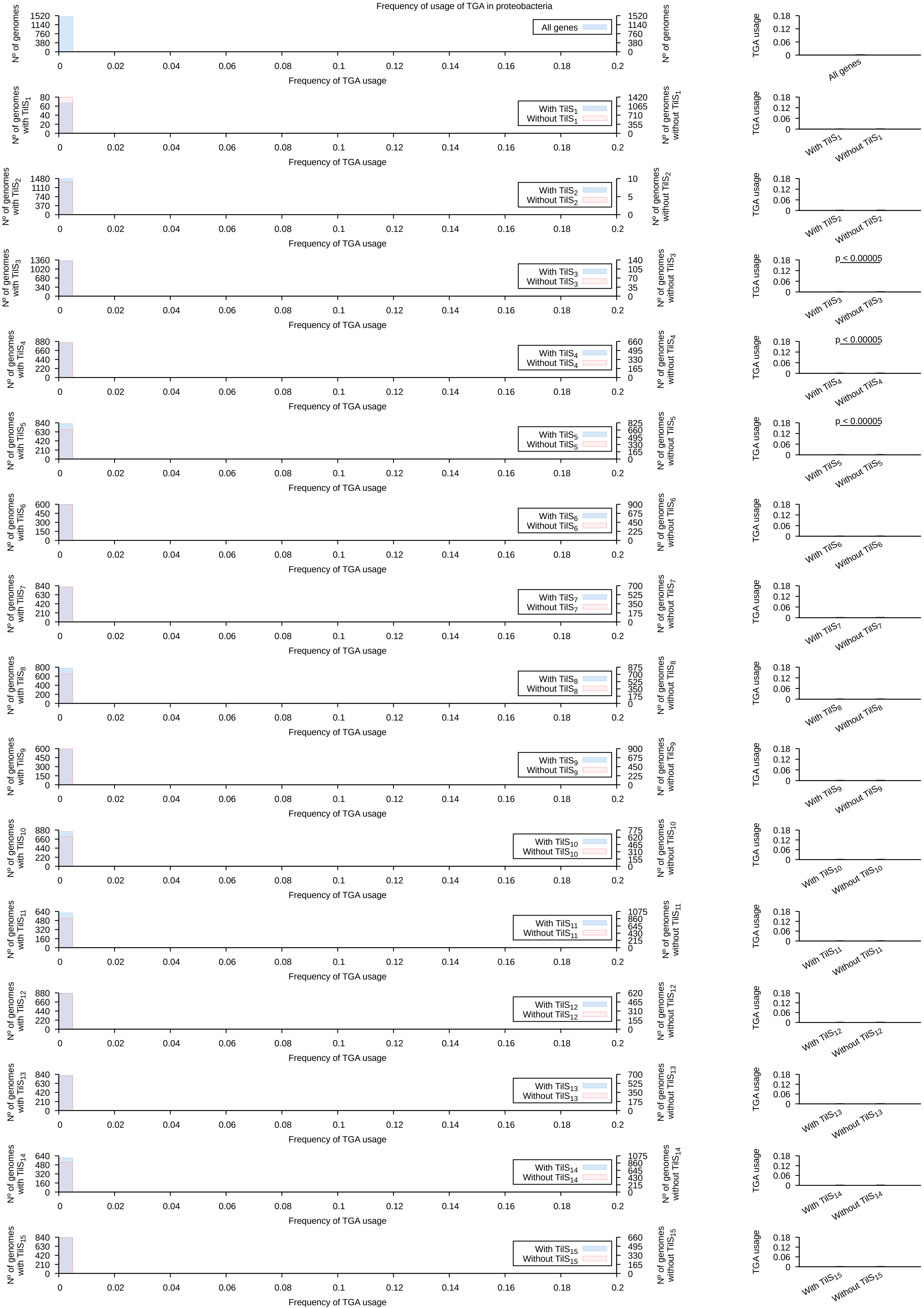

Frequency of usage of TGA in proteobacteria
Nº of genomes
All genes
Frequency of TGA usage
TGA usage
Nº of genomes with $TilS_1$
Nº of genomes without $TilS_1$
With $TilS_1$
Without $TilS_1$
Nº of genomes with $TilS_2$
Nº of genomes without $TilS_2$
With $TilS_2$
Without $TilS_2$
Nº of genomes with $TilS_3$
Nº of genomes without $TilS_3$
With $TilS_3$
Without $TilS_3$
$p < 0.00005$
Nº of genomes with $TilS_4$
Nº of genomes without $TilS_4$
With $TilS_4$
Without $TilS_4$
$p < 0.00005$
Nº of genomes with $TilS_5$
Nº of genomes without $TilS_5$
With $TilS_5$
Without $TilS_5$
$p < 0.00005$
Nº of genomes with $TilS_6$
Nº of genomes without $TilS_6$
With $TilS_6$
Without $TilS_6$
Nº of genomes with $TilS_7$
Nº of genomes without $TilS_7$
With $TilS_7$
Without $TilS_7$
Nº of genomes with $TilS_8$
Nº of genomes without $TilS_8$
With $TilS_8$
Without $TilS_8$
Nº of genomes with $TilS_9$
Nº of genomes without $TilS_9$
With $TilS_9$
Without $TilS_9$
Nº of genomes with $TilS_{10}$
Nº of genomes without $TilS_{10}$
With $TilS_{10}$
Without $TilS_{10}$
Nº of genomes with $TilS_{11}$
Nº of genomes without $TilS_{11}$
With $TilS_{11}$
Without $TilS_{11}$
Nº of genomes with $TilS_{12}$
Nº of genomes without $TilS_{12}$
With $TilS_{12}$
Without $TilS_{12}$
Nº of genomes with $TilS_{13}$
Nº of genomes without $TilS_{13}$
With $TilS_{13}$
Without $TilS_{13}$
Nº of genomes with $TilS_{14}$
Nº of genomes without $TilS_{14}$
With $TilS_{14}$
Without $TilS_{14}$
Nº of genomes with $TilS_{15}$
Nº of genomes without $TilS_{15}$
With $TilS_{15}$
Without $TilS_{15}$

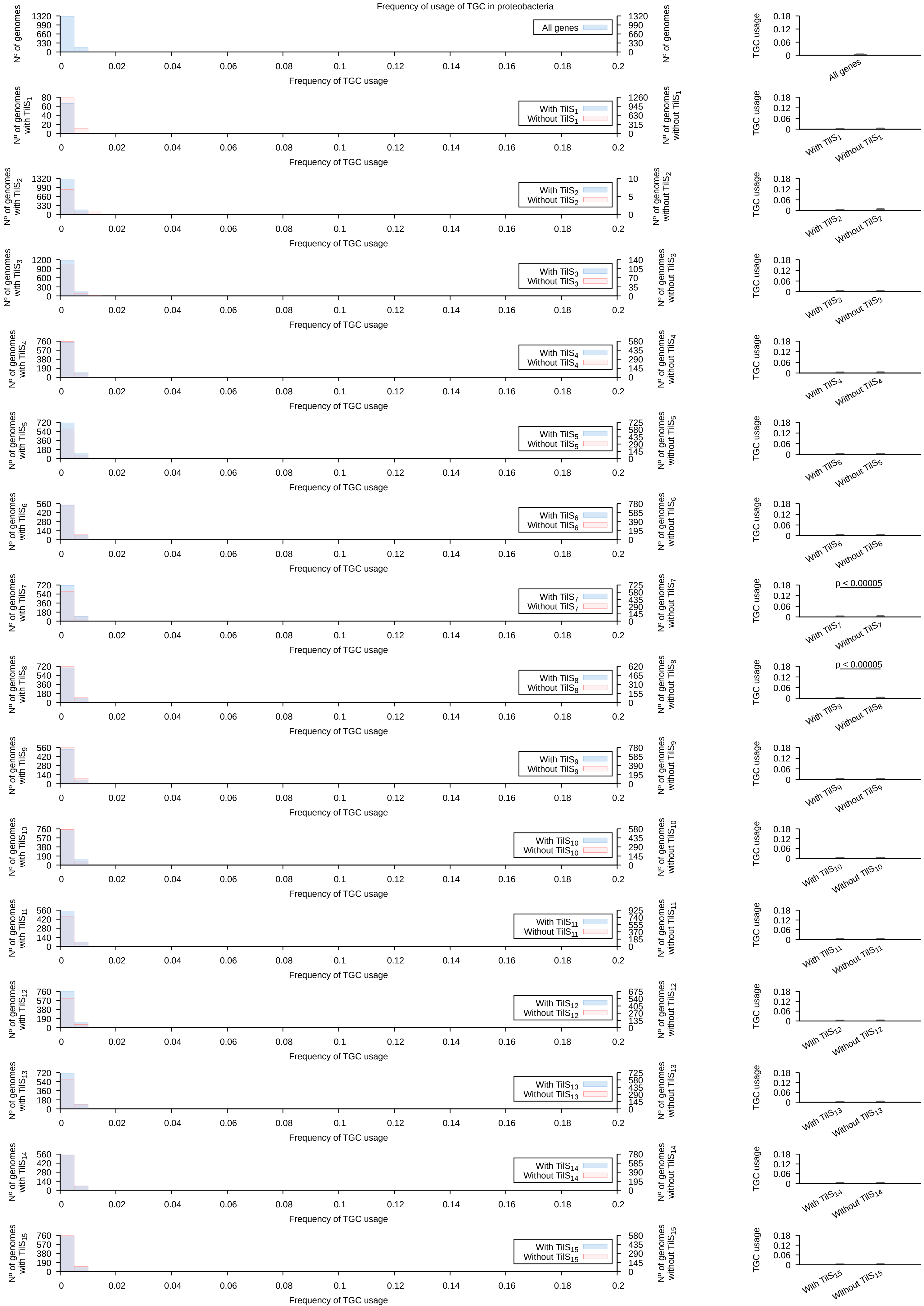

Frequency of usage of TGC in proteobacteria
Nº of genomes
1320 990 660 330 0
All genes
Frequency of TGC usage
0 0.02 0.04 0.06 0.08 0.1 0.12 0.14 0.16 0.18 0.2
TGC usage
0.18 0.12 0.06 0
All genes
Nº of genomes with TilS$_1$
80 60 40 20 0
With TilS$_1$
Without TilS$_1$
1260 945 630 315 0
Nº of genomes without TilS$_1$
Nº of genomes with TilS$_2$
1320 990 660 330 0
With TilS$_2$
Without TilS$_2$
10 5 0
Nº of genomes without TilS$_2$
Nº of genomes with TilS$_3$
1200 900 600 300 0
With TilS$_3$
Without TilS$_3$
140 105 70 35 0
Nº of genomes without TilS$_3$
Nº of genomes with TilS$_4$
760 570 380 190 0
With TilS$_4$
Without TilS$_4$
580 435 290 145 0
Nº of genomes without TilS$_4$
Nº of genomes with TilS$_5$
720 540 360 180 0
With TilS$_5$
Without TilS$_5$
725 580 435 290 145 0
Nº of genomes without TilS$_5$
Nº of genomes with TilS$_6$
560 420 280 140 0
With TilS$_6$
Without TilS$_6$
780 585 390 195 0
Nº of genomes without TilS$_6$
Nº of genomes with TilS$_7$
720 540 360 180 0
With TilS$_7$
Without TilS$_7$
725 580 435 290 145 0
Nº of genomes without TilS$_7$
p < 0.00005
Nº of genomes with TilS$_8$
720 540 360 180 0
With TilS$_8$
Without TilS$_8$
620 465 310 155 0
Nº of genomes without TilS$_8$
p < 0.00005
Nº of genomes with TilS$_9$
560 420 280 140 0
With TilS$_9$
Without TilS$_9$
780 585 390 195 0
Nº of genomes without TilS$_9$
Nº of genomes with TilS$_{10}$
760 570 380 190 0
With TilS$_{10}$
Without TilS$_{10}$
580 435 290 145 0
Nº of genomes without TilS$_{10}$
Nº of genomes with TilS$_{11}$
560 420 280 140 0
With TilS$_{11}$
Without TilS$_{11}$
925 740 555 370 185 0
Nº of genomes without TilS$_{11}$
Nº of genomes with TilS$_{12}$
760 570 380 190 0
With TilS$_{12}$
Without TilS$_{12}$
675 540 405 270 135 0
Nº of genomes without TilS$_{12}$
Nº of genomes with TilS$_{13}$
720 540 360 180 0
With TilS$_{13}$
Without TilS$_{13}$
725 580 435 290 145 0
Nº of genomes without TilS$_{13}$
Nº of genomes with TilS$_{14}$
560 420 280 140 0
With TilS$_{14}$
Without TilS$_{14}$
780 585 390 195 0
Nº of genomes without TilS$_{14}$
Nº of genomes with TilS$_{15}$
760 570 380 190 0
With TilS$_{15}$
Without TilS$_{15}$
580 435 290 145 0
Nº of genomes without TilS$_{15}$

Frequency of usage of TGG in proteobacteria

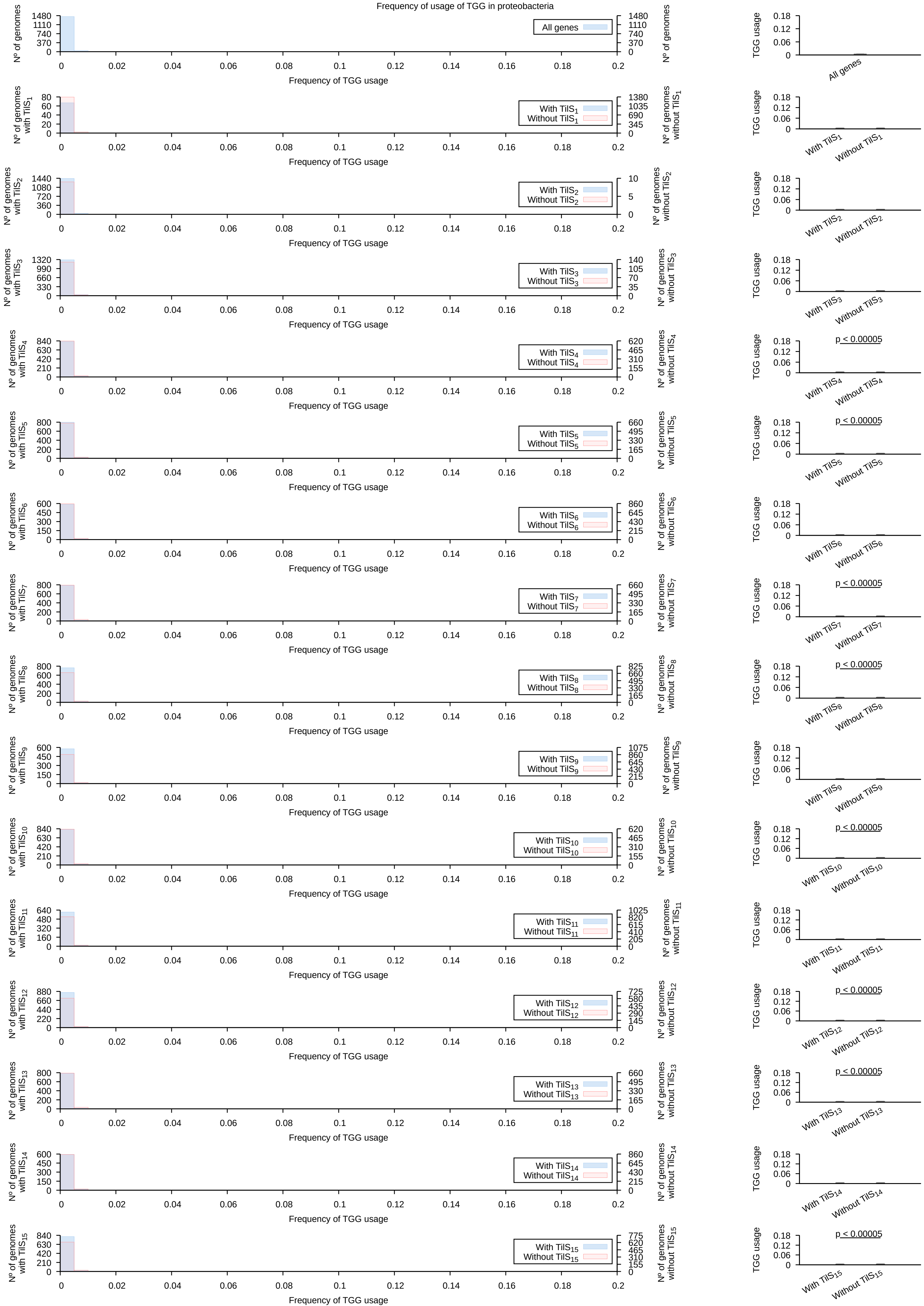

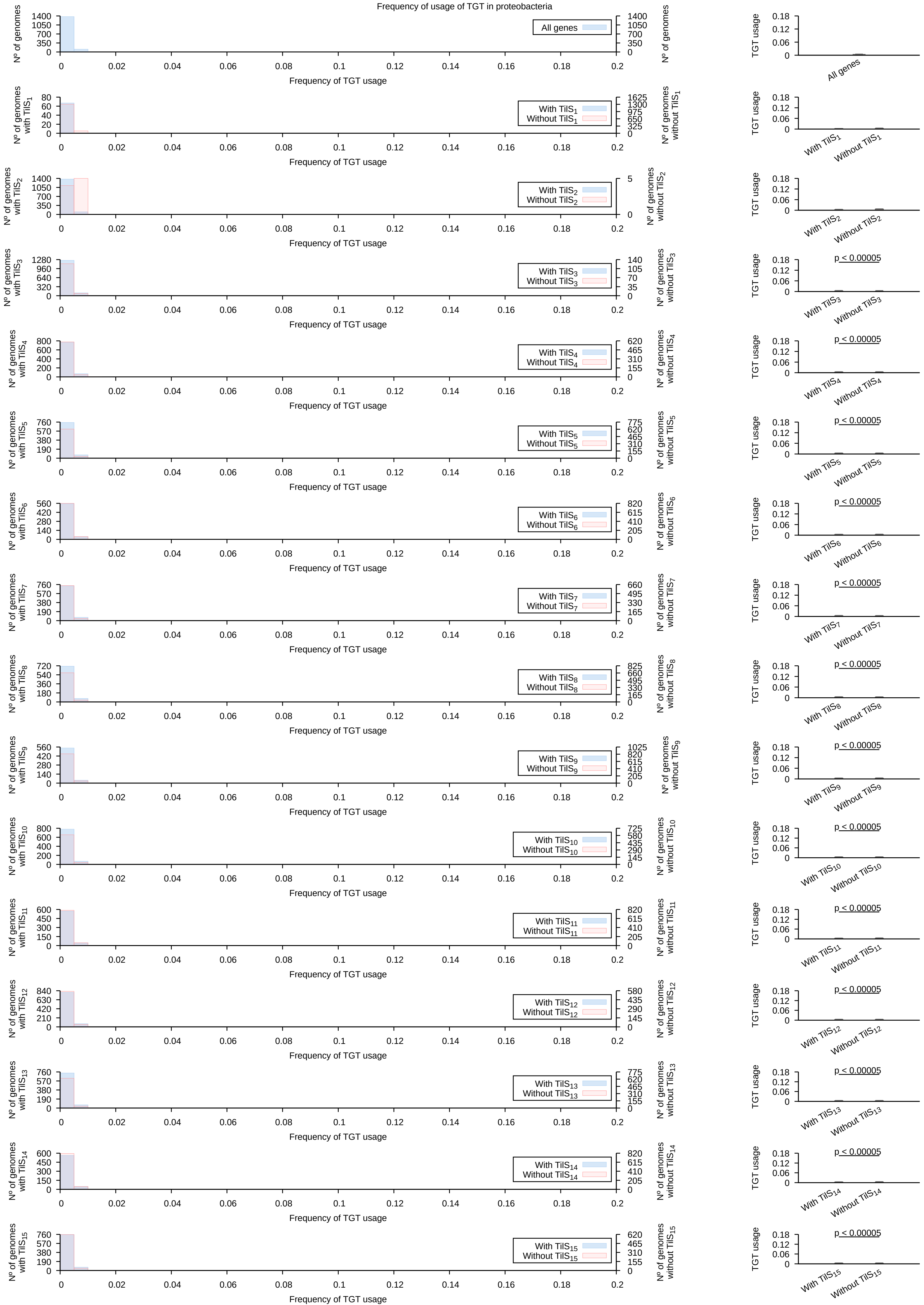

Frequency of usage of TGT in proteobacteria
Nº of genomes
Frequency of TGT usage
All genes
TGT usage
Nº of genomes with TilS1
Nº of genomes without TilS1
With TilS1
Without TilS1
Nº of genomes with TilS2
Nº of genomes without TilS2
With TilS2
Without TilS2
Nº of genomes with TilS3
Nº of genomes without TilS3
With TilS3
Without TilS3
p < 0.00005
Nº of genomes with TilS4
Nº of genomes without TilS4
With TilS4
Without TilS4
Nº of genomes with TilS5
Nº of genomes without TilS5
With TilS5
Without TilS5
Nº of genomes with TilS6
Nº of genomes without TilS6
With TilS6
Without TilS6
Nº of genomes with TilS7
Nº of genomes without TilS7
With TilS7
Without TilS7
Nº of genomes with TilS8
Nº of genomes without TilS8
With TilS8
Without TilS8
Nº of genomes with TilS9
Nº of genomes without TilS9
With TilS9
Without TilS9
Nº of genomes with TilS10
Nº of genomes without TilS10
With TilS10
Without TilS10
Nº of genomes with TilS11
Nº of genomes without TilS11
With TilS11
Without TilS11
Nº of genomes with TilS12
Nº of genomes without TilS12
With TilS12
Without TilS12
Nº of genomes with TilS13
Nº of genomes without TilS13
With TilS13
Without TilS13
Nº of genomes with TilS14
Nº of genomes without TilS14
With TilS14
Without TilS14
Nº of genomes with TilS15
Nº of genomes without TilS15
With TilS15
Without TilS15

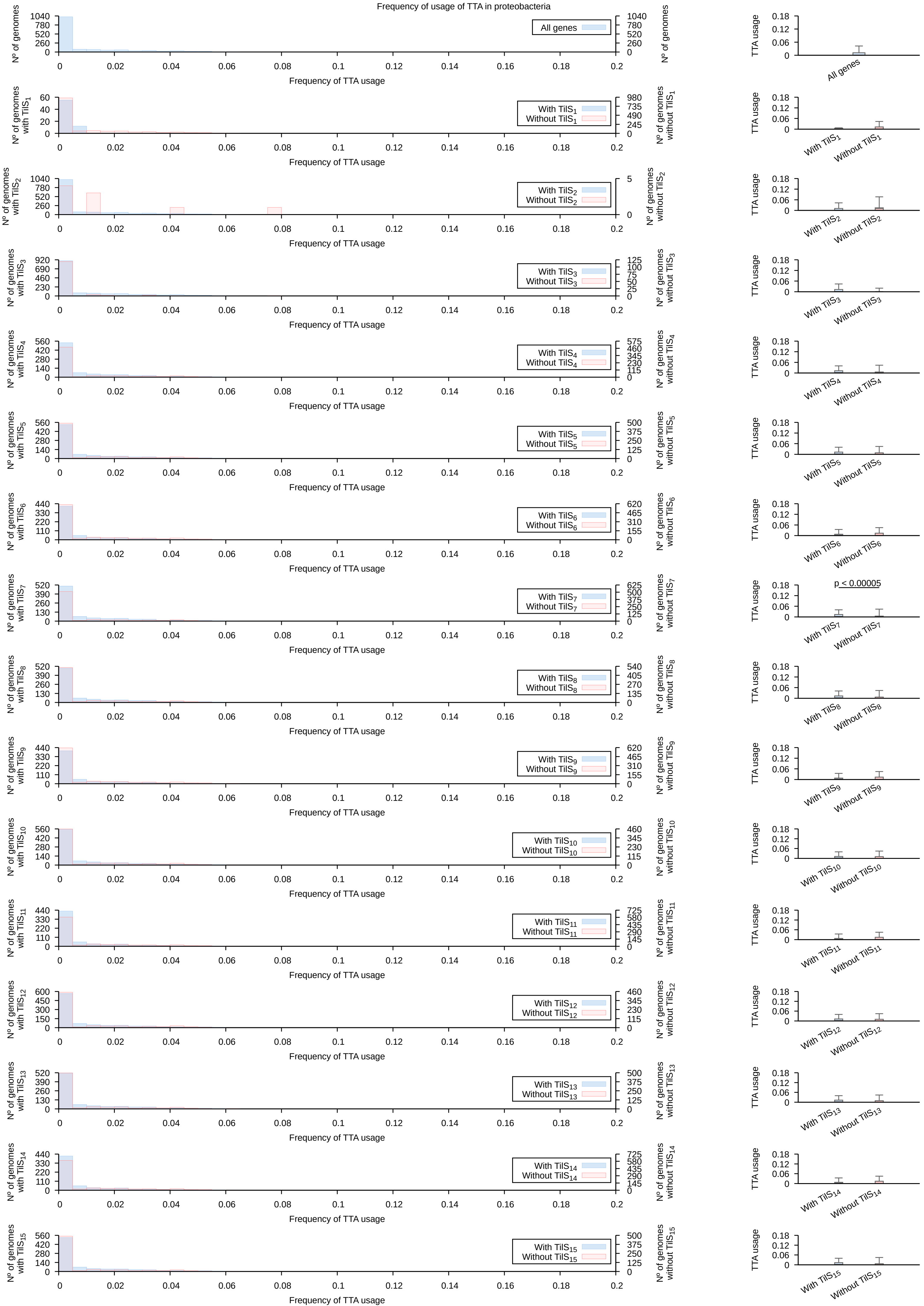

Frequency of usage of TTA in proteobacteria
Nº of genomes
All genes
Frequency of TTA usage
TTA usage
With TilS1
Without TilS1
Nº of genomes with TilS1
Nº of genomes without TilS1
With TilS2
Without TilS2
With TilS3
Without TilS3
With TilS4
Without TilS4
With TilS5
Without TilS5
With TilS6
Without TilS6
With TilS7
Without TilS7
p < 0.00005
With TilS8
Without TilS8
With TilS9
Without TilS9
With TilS10
Without TilS10
With TilS11
Without TilS11
With TilS12
Without TilS12
With TilS13
Without TilS13
With TilS14
Without TilS14
With TilS15
Without TilS15

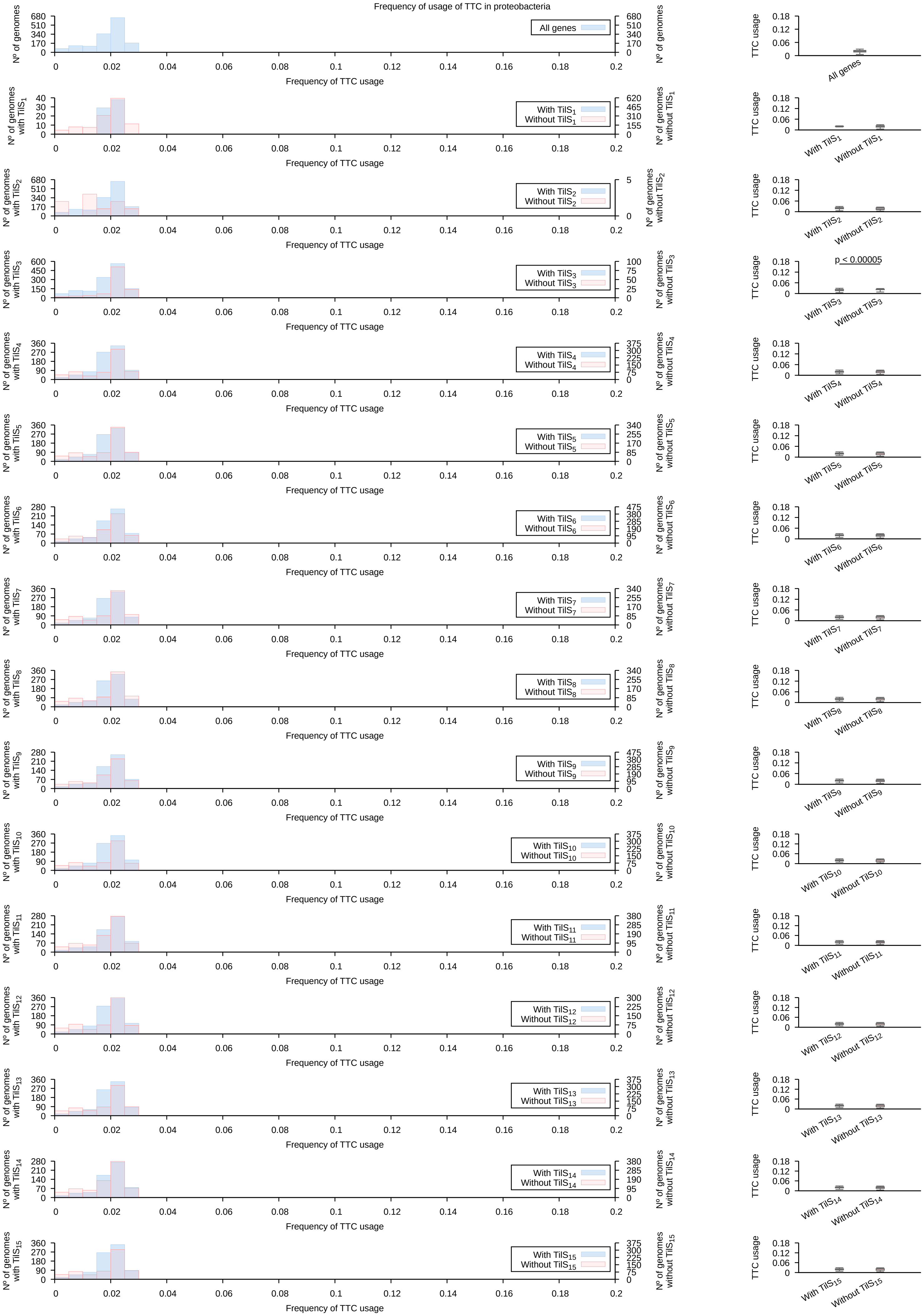
Frequency of usage of TTC in proteobacteria
Nº of genomes
All genes
Frequency of TTC usage
TTC usage
Nº of genomes with $TilS_1$
Nº of genomes without $TilS_1$
With $TilS_1$
Without $TilS_1$
Nº of genomes with $TilS_2$
Nº of genomes without $TilS_2$
With $TilS_2$
Without $TilS_2$
Nº of genomes with $TilS_3$
Nº of genomes without $TilS_3$
With $TilS_3$
Without $TilS_3$
p < 0.00005
Nº of genomes with $TilS_4$
Nº of genomes without $TilS_4$
With $TilS_4$
Without $TilS_4$
Nº of genomes with $TilS_5$
Nº of genomes without $TilS_5$
With $TilS_5$
Without $TilS_5$
Nº of genomes with $TilS_6$
Nº of genomes without $TilS_6$
With $TilS_6$
Without $TilS_6$
Nº of genomes with $TilS_7$
Nº of genomes without $TilS_7$
With $TilS_7$
Without $TilS_7$
Nº of genomes with $TilS_8$
Nº of genomes without $TilS_8$
With $TilS_8$
Without $TilS_8$
Nº of genomes with $TilS_9$
Nº of genomes without $TilS_9$
With $TilS_9$
Without $TilS_9$
Nº of genomes with $TilS_{10}$
Nº of genomes without $TilS_{10}$
With $TilS_{10}$
Without $TilS_{10}$
Nº of genomes with $TilS_{11}$
Nº of genomes without $TilS_{11}$
With $TilS_{11}$
Without $TilS_{11}$
Nº of genomes with $TilS_{12}$
Nº of genomes without $TilS_{12}$
With $TilS_{12}$
Without $TilS_{12}$
Nº of genomes with $TilS_{13}$
Nº of genomes without $TilS_{13}$
With $TilS_{13}$
Without $TilS_{13}$
Nº of genomes with $TilS_{14}$
Nº of genomes without $TilS_{14}$
With $TilS_{14}$
Without $TilS_{14}$
Nº of genomes with $TilS_{15}$
Nº of genomes without $TilS_{15}$
With $TilS_{15}$
Without $TilS_{15}$

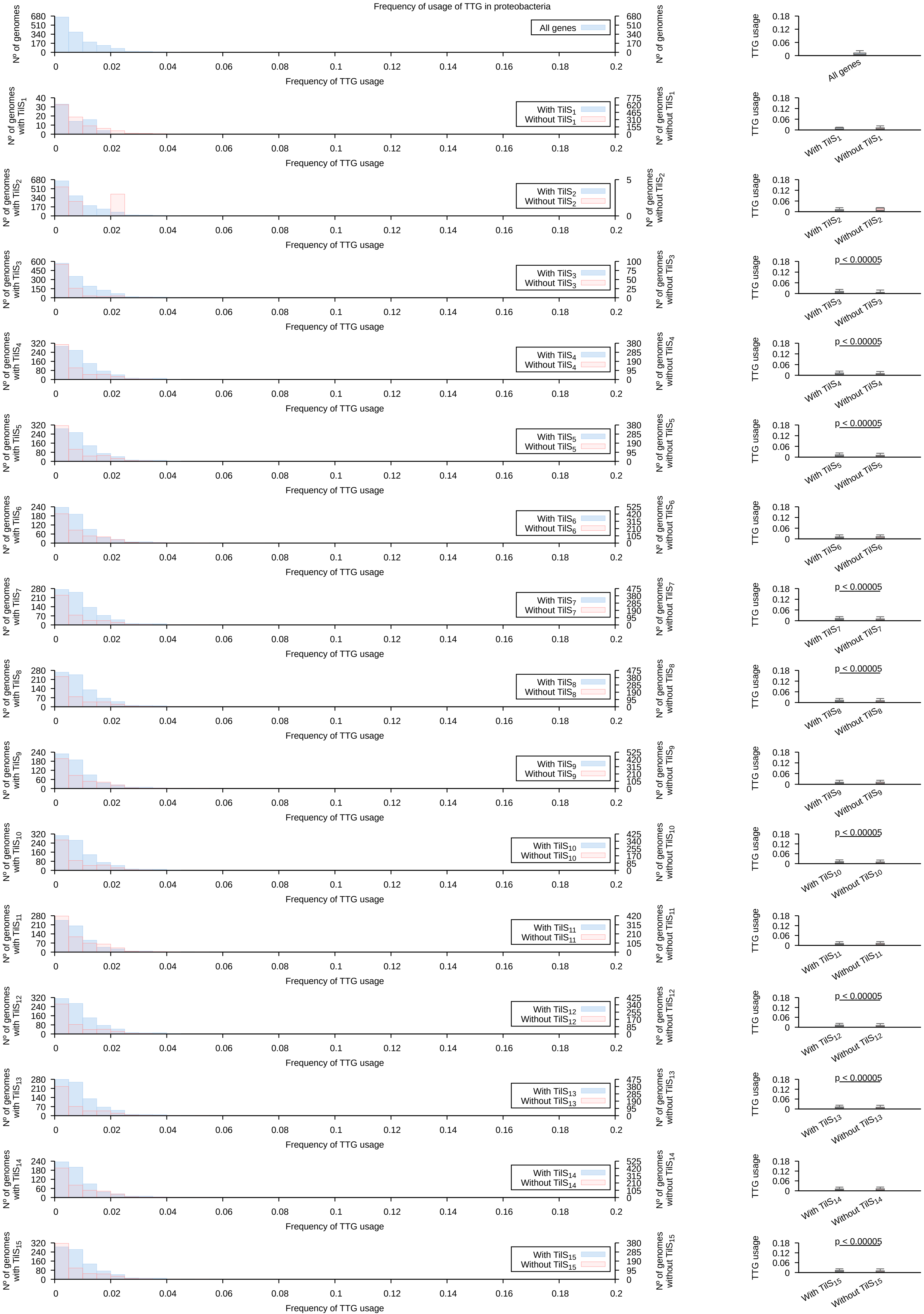

Frequency of usage of TTG in proteobacteria
Nº of genomes
All genes
Frequency of TTG usage
TTG usage
All genes
Nº of genomes with $TilS_1$
Nº of genomes without $TilS_1$
With $TilS_1$
Without $TilS_1$
Nº of genomes with $TilS_2$
Nº of genomes without $TilS_2$
With $TilS_2$
Without $TilS_2$
Nº of genomes with $TilS_3$
Nº of genomes without $TilS_3$
With $TilS_3$
Without $TilS_3$
p < 0.00005
Nº of genomes with $TilS_4$
Nº of genomes without $TilS_4$
With $TilS_4$
Without $TilS_4$
p < 0.00005
Nº of genomes with $TilS_5$
Nº of genomes without $TilS_5$
With $TilS_5$
Without $TilS_5$
p < 0.00005
Nº of genomes with $TilS_6$
Nº of genomes without $TilS_6$
With $TilS_6$
Without $TilS_6$
Nº of genomes with $TilS_7$
Nº of genomes without $TilS_7$
With $TilS_7$
Without $TilS_7$
p < 0.00005
Nº of genomes with $TilS_8$
Nº of genomes without $TilS_8$
With $TilS_8$
Without $TilS_8$
p < 0.00005
Nº of genomes with $TilS_9$
Nº of genomes without $TilS_9$
With $TilS_9$
Without $TilS_9$
Nº of genomes with $TilS_{10}$
Nº of genomes without $TilS_{10}$
With $TilS_{10}$
Without $TilS_{10}$
p < 0.00005
Nº of genomes with $TilS_{11}$
Nº of genomes without $TilS_{11}$
With $TilS_{11}$
Without $TilS_{11}$
Nº of genomes with $TilS_{12}$
Nº of genomes without $TilS_{12}$
With $TilS_{12}$
Without $TilS_{12}$
p < 0.00005
Nº of genomes with $TilS_{13}$
Nº of genomes without $TilS_{13}$
With $TilS_{13}$
Without $TilS_{13}$
p < 0.00005
Nº of genomes with $TilS_{14}$
Nº of genomes without $TilS_{14}$
With $TilS_{14}$
Without $TilS_{14}$
Nº of genomes with $TilS_{15}$
Nº of genomes without $TilS_{15}$
With $TilS_{15}$
Without $TilS_{15}$
p < 0.00005

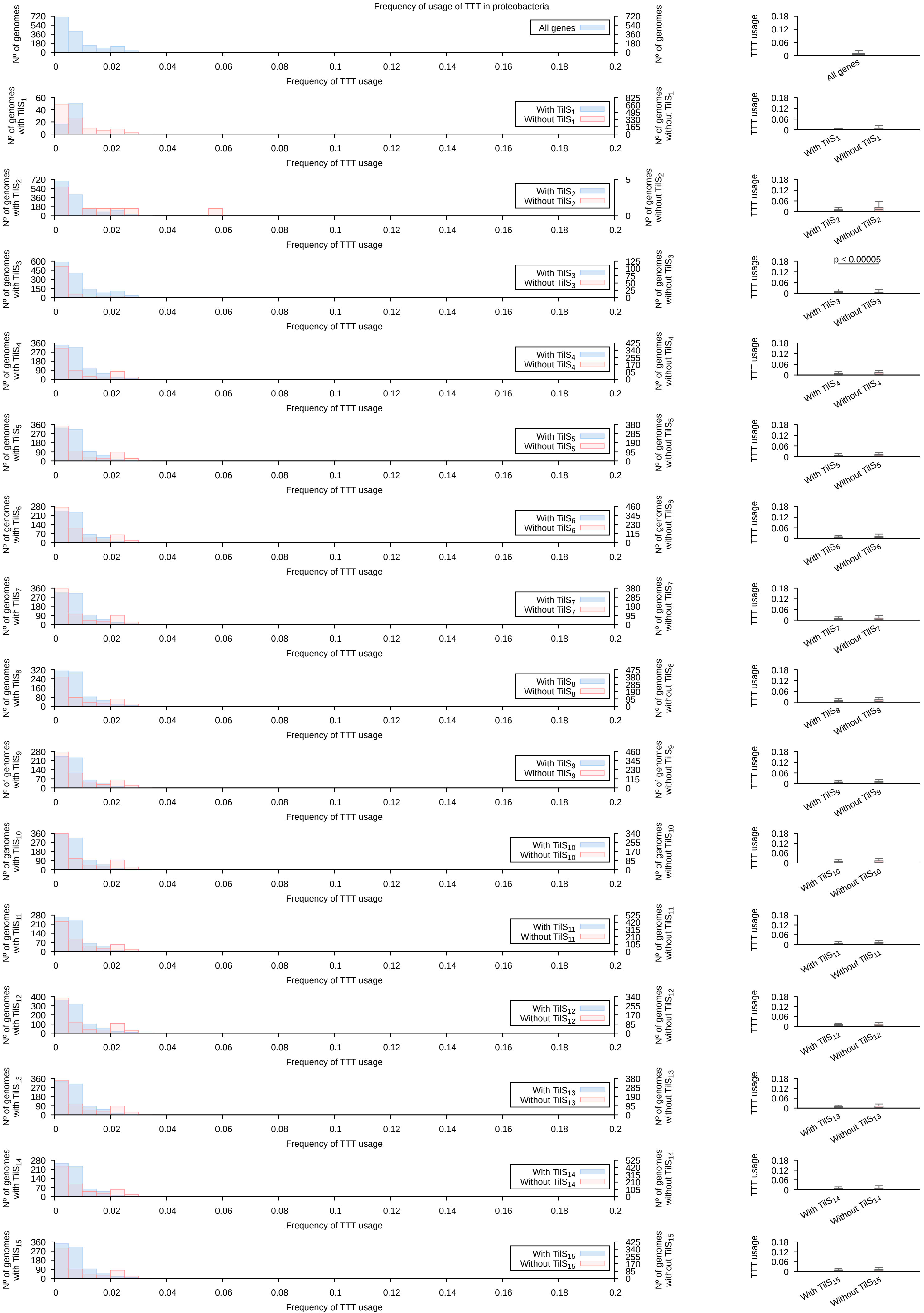

Frequency of usage of TTT in proteobacteria
Nº of genomes
All genes
Frequency of TTT usage
TTT usage
p < 0.00005
Nº of genomes with TilS$_1$
With TilS$_1$
Without TilS$_1$
Nº of genomes without TilS$_1$
Nº of genomes with TilS$_2$
With TilS$_2$
Without TilS$_2$
Nº of genomes without TilS$_2$
Nº of genomes with TilS$_3$
With TilS$_3$
Without TilS$_3$
Nº of genomes without TilS$_3$
Nº of genomes with TilS$_4$
With TilS$_4$
Without TilS$_4$
Nº of genomes without TilS$_4$
Nº of genomes with TilS$_5$
With TilS$_5$
Without TilS$_5$
Nº of genomes without TilS$_5$
Nº of genomes with TilS$_6$
With TilS$_6$
Without TilS$_6$
Nº of genomes without TilS$_6$
Nº of genomes with TilS$_7$
With TilS$_7$
Without TilS$_7$
Nº of genomes without TilS$_7$
Nº of genomes with TilS$_8$
With TilS$_8$
Without TilS$_8$
Nº of genomes without TilS$_8$
Nº of genomes with TilS$_9$
With TilS$_9$
Without TilS$_9$
Nº of genomes without TilS$_9$
Nº of genomes with TilS$_{10}$
With TilS$_{10}$
Without TilS$_{10}$
Nº of genomes without TilS$_{10}$
Nº of genomes with TilS$_{11}$
With TilS$_{11}$
Without TilS$_{11}$
Nº of genomes without TilS$_{11}$
Nº of genomes with TilS$_{12}$
With TilS$_{12}$
Without TilS$_{12}$
Nº of genomes without TilS$_{12}$
Nº of genomes with TilS$_{13}$
With TilS$_{13}$
Without TilS$_{13}$
Nº of genomes without TilS$_{13}$
Nº of genomes with TilS$_{14}$
With TilS$_{14}$
Without TilS$_{14}$
Nº of genomes without TilS$_{14}$
Nº of genomes with TilS$_{15}$
With TilS$_{15}$
Without TilS$_{15}$
Nº of genomes without TilS$_{15}$
