# Supplementary material for: Impact of the chemical modification of tRNAs anticodon loop on the variability and evolution of codon usage in proteobacteria
Source: Front Microbiol. 2024 Aug 5;15:1412318. doi: 10.3389/fmicb.2024.1412318 (PMC11332805; doi:10.3389/fmicb.2024.1412318)
Supplement: Supplementary file 1 [file Data_Sheet_1.zip › Supp_figures/Fig_S10.pdf]

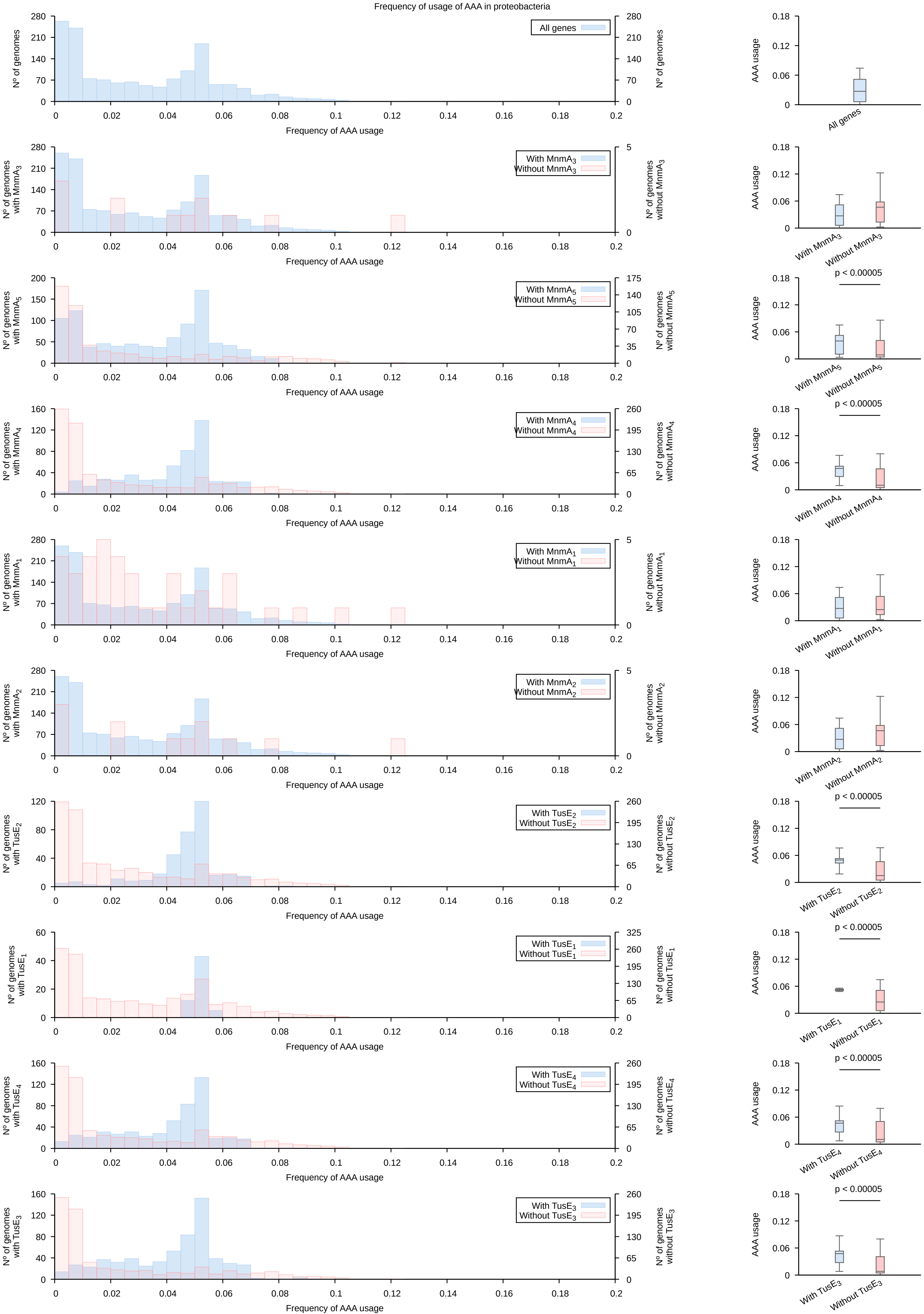

Frequency of usage of AAA in proteobacteria
Nº of genomes
Frequency of AAA usage
All genes
AAA usage
Nº of genomes with MnmA$_3$
Nº of genomes without MnmA$_3$
With MnmA$_3$
Without MnmA$_3$
Nº of genomes with MnmA$_5$
Nº of genomes without MnmA$_5$
With MnmA$_5$
Without MnmA$_5$
p < 0.00005
Nº of genomes with MnmA$_4$
Nº of genomes without MnmA$_4$
With MnmA$_4$
Without MnmA$_4$
p < 0.00005
Nº of genomes with MnmA$_1$
Nº of genomes without MnmA$_1$
With MnmA$_1$
Without MnmA$_1$
Nº of genomes with MnmA$_2$
Nº of genomes without MnmA$_2$
With MnmA$_2$
Without MnmA$_2$
Nº of genomes with TusE$_2$
Nº of genomes without TusE$_2$
With TusE$_2$
Without TusE$_2$
p < 0.00005
Nº of genomes with TusE$_1$
Nº of genomes without TusE$_1$
With TusE$_1$
Without TusE$_1$
p < 0.00005
Nº of genomes with TusE$_4$
Nº of genomes without TusE$_4$
With TusE$_4$
Without TusE$_4$
p < 0.00005
Nº of genomes with TusE$_3$
Nº of genomes without TusE$_3$
With TusE$_3$
Without TusE$_3$
p < 0.00005

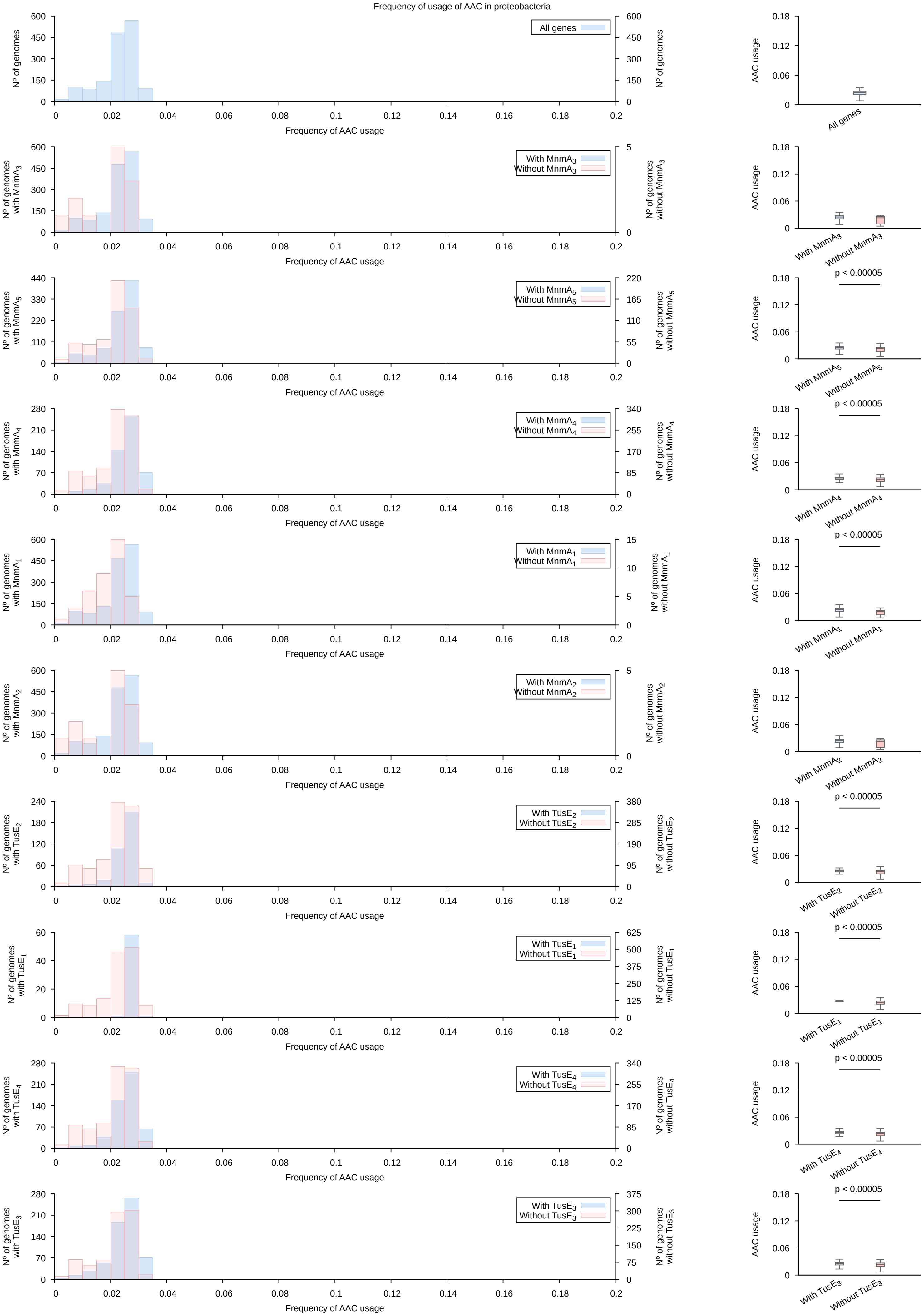

Frequency of usage of AAC in proteobacteria
Nº of genomes
Frequency of AAC usage
AAC usage
All genes
Nº of genomes with MnmA3
Nº of genomes without MnmA3
With MnmA3
Without MnmA3
Nº of genomes with MnmA5
Nº of genomes without MnmA5
With MnmA5
Without MnmA5
Nº of genomes with MnmA4
Nº of genomes without MnmA4
With MnmA4
Without MnmA4
Nº of genomes with MnmA1
Nº of genomes without MnmA1
With MnmA1
Without MnmA1
Nº of genomes with MnmA2
Nº of genomes without MnmA2
With MnmA2
Without MnmA2
Nº of genomes with TusE2
Nº of genomes without TusE2
With TusE2
Without TusE2
Nº of genomes with TusE1
Nº of genomes without TusE1
With TusE1
Without TusE1
Nº of genomes with TusE4
Nº of genomes without TusE4
With TusE4
Without TusE4
Nº of genomes with TusE3
Nº of genomes without TusE3
With TusE3
Without TusE3
p < 0.00005

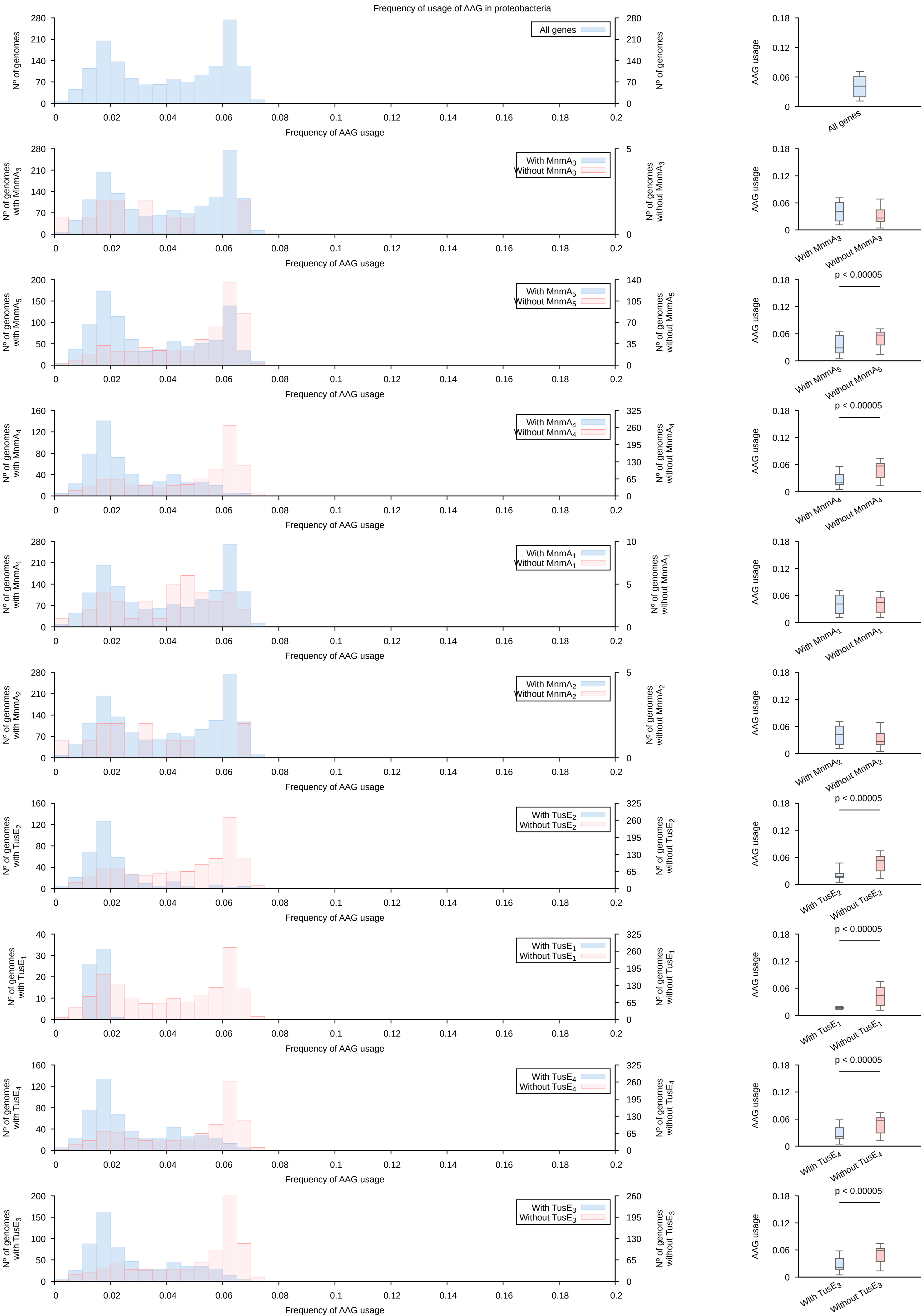

Frequency of usage of AAG in proteobacteria
Nº of genomes
Frequency of AAG usage
All genes
AAG usage
All genes
Nº of genomes with MnmA$_3$
Nº of genomes without MnmA$_3$
With MnmA$_3$
Without MnmA$_3$
Nº of genomes with MnmA$_5$
Nº of genomes without MnmA$_5$
With MnmA$_5$
Without MnmA$_5$
p < 0.00005
Nº of genomes with MnmA$_4$
Nº of genomes without MnmA$_4$
With MnmA$_4$
Without MnmA$_4$
p < 0.00005
Nº of genomes with MnmA$_1$
Nº of genomes without MnmA$_1$
With MnmA$_1$
Without MnmA$_1$
Nº of genomes with MnmA$_2$
Nº of genomes without MnmA$_2$
With MnmA$_2$
Without MnmA$_2$
Nº of genomes with TusE$_2$
Nº of genomes without TusE$_2$
With TusE$_2$
Without TusE$_2$
p < 0.00005
Nº of genomes with TusE$_1$
Nº of genomes without TusE$_1$
With TusE$_1$
Without TusE$_1$
p < 0.00005
Nº of genomes with TusE$_4$
Nº of genomes without TusE$_4$
With TusE$_4$
Without TusE$_4$
p < 0.00005
Nº of genomes with TusE$_3$
Nº of genomes without TusE$_3$
With TusE$_3$
Without TusE$_3$
p < 0.00005

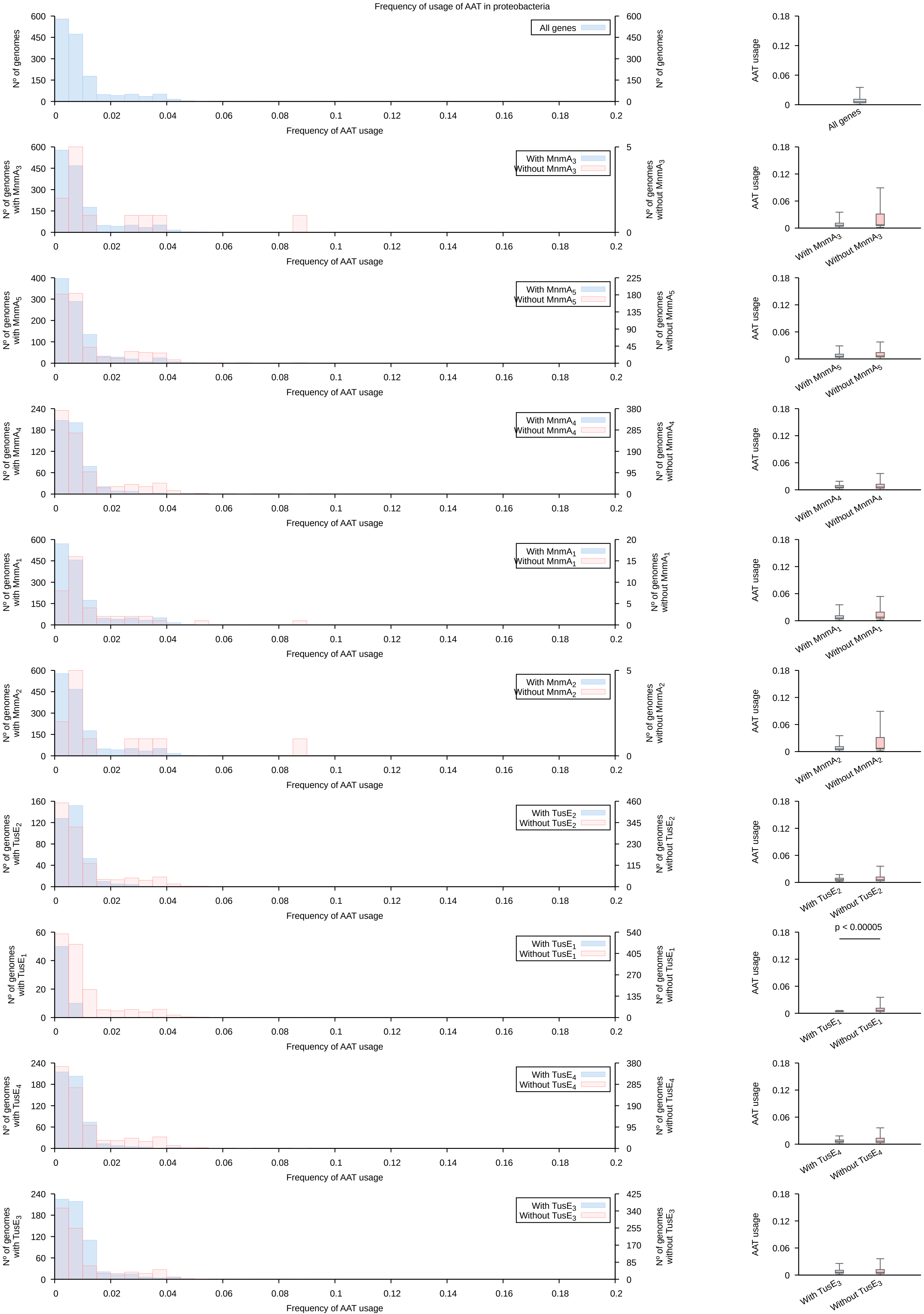
Frequency of usage of AAT in proteobacteria
All genes
Nº of genomes
Frequency of AAT usage
AAT usage
With MnmA$_3$
Without MnmA$_3$
Nº of genomes with MnmA$_3$
Nº of genomes without MnmA$_3$
With MnmA$_5$
Without MnmA$_5$
Nº of genomes with MnmA$_5$
Nº of genomes without MnmA$_5$
With MnmA$_4$
Without MnmA$_4$
Nº of genomes with MnmA$_4$
Nº of genomes without MnmA$_4$
With MnmA$_1$
Without MnmA$_1$
Nº of genomes with MnmA$_1$
Nº of genomes without MnmA$_1$
With MnmA$_2$
Without MnmA$_2$
Nº of genomes with MnmA$_2$
Nº of genomes without MnmA$_2$
With TusE$_2$
Without TusE$_2$
Nº of genomes with TusE$_2$
Nº of genomes without TusE$_2$
With TusE$_1$
Without TusE$_1$
Nº of genomes with TusE$_1$
Nº of genomes without TusE$_1$
p < 0.00005
With TusE$_4$
Without TusE$_4$
Nº of genomes with TusE$_4$
Nº of genomes without TusE$_4$
With TusE$_3$
Without TusE$_3$
Nº of genomes with TusE$_3$
Nº of genomes without TusE$_3$

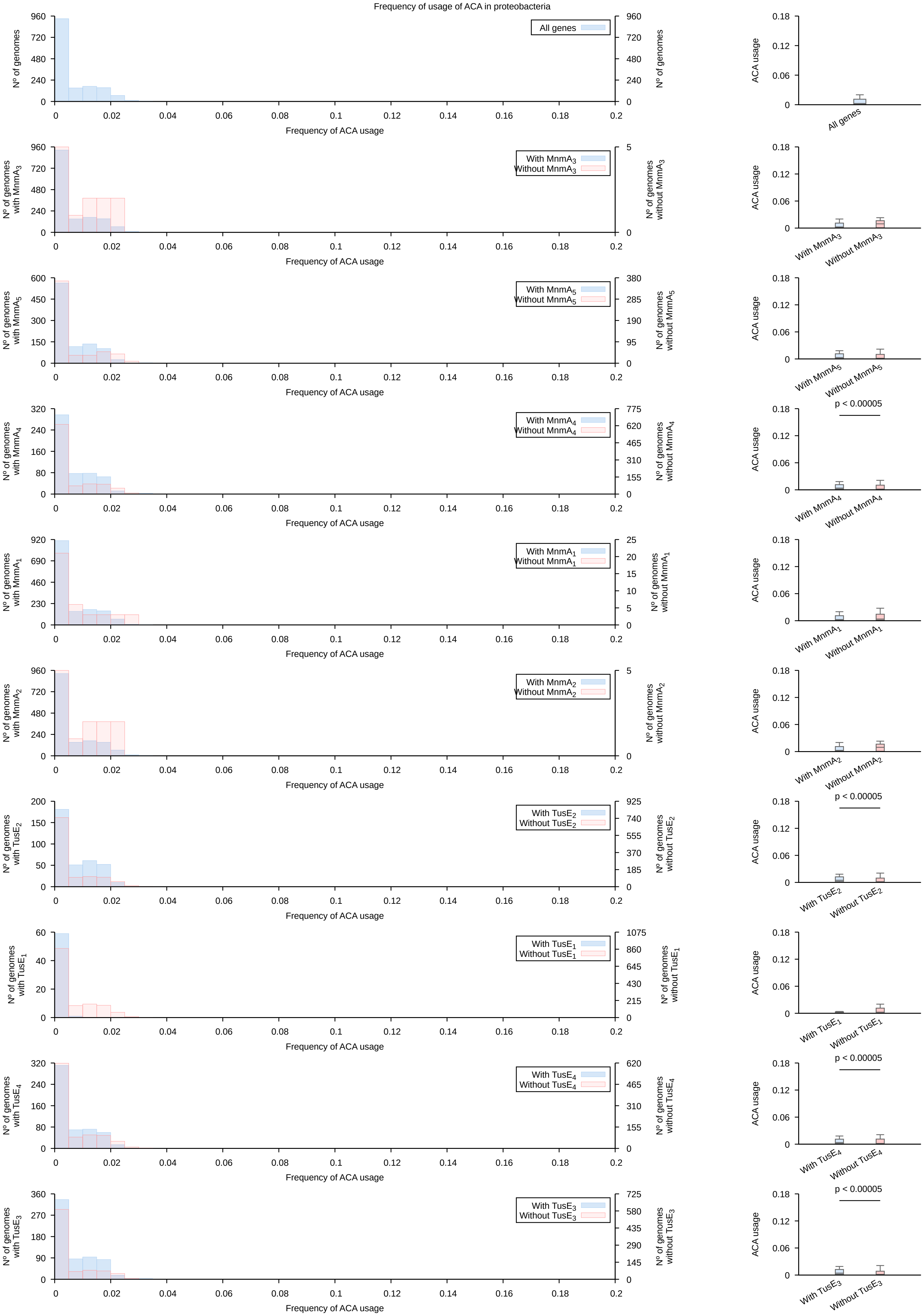
Frequency of usage of ACA in proteobacteria
All genes
Nº of genomes
Frequency of ACA usage
ACA usage
With MnmA$_3$
Without MnmA$_3$
Nº of genomes with MnmA$_3$
Nº of genomes without MnmA$_3$
With MnmA$_5$
Without MnmA$_5$
Nº of genomes with MnmA$_5$
Nº of genomes without MnmA$_5$
With MnmA$_4$
Without MnmA$_4$
Nº of genomes with MnmA$_4$
Nº of genomes without MnmA$_4$
p < 0.00005
With MnmA$_1$
Without MnmA$_1$
Nº of genomes with MnmA$_1$
Nº of genomes without MnmA$_1$
With MnmA$_2$
Without MnmA$_2$
Nº of genomes with MnmA$_2$
Nº of genomes without MnmA$_2$
With TusE$_2$
Without TusE$_2$
Nº of genomes with TusE$_2$
Nº of genomes without TusE$_2$
p < 0.00005
With TusE$_1$
Without TusE$_1$
Nº of genomes with TusE$_1$
Nº of genomes without TusE$_1$
With TusE$_4$
Without TusE$_4$
Nº of genomes with TusE$_4$
Nº of genomes without TusE$_4$
p < 0.00005
With TusE$_3$
Without TusE$_3$
Nº of genomes with TusE$_3$
Nº of genomes without TusE$_3$
p < 0.00005

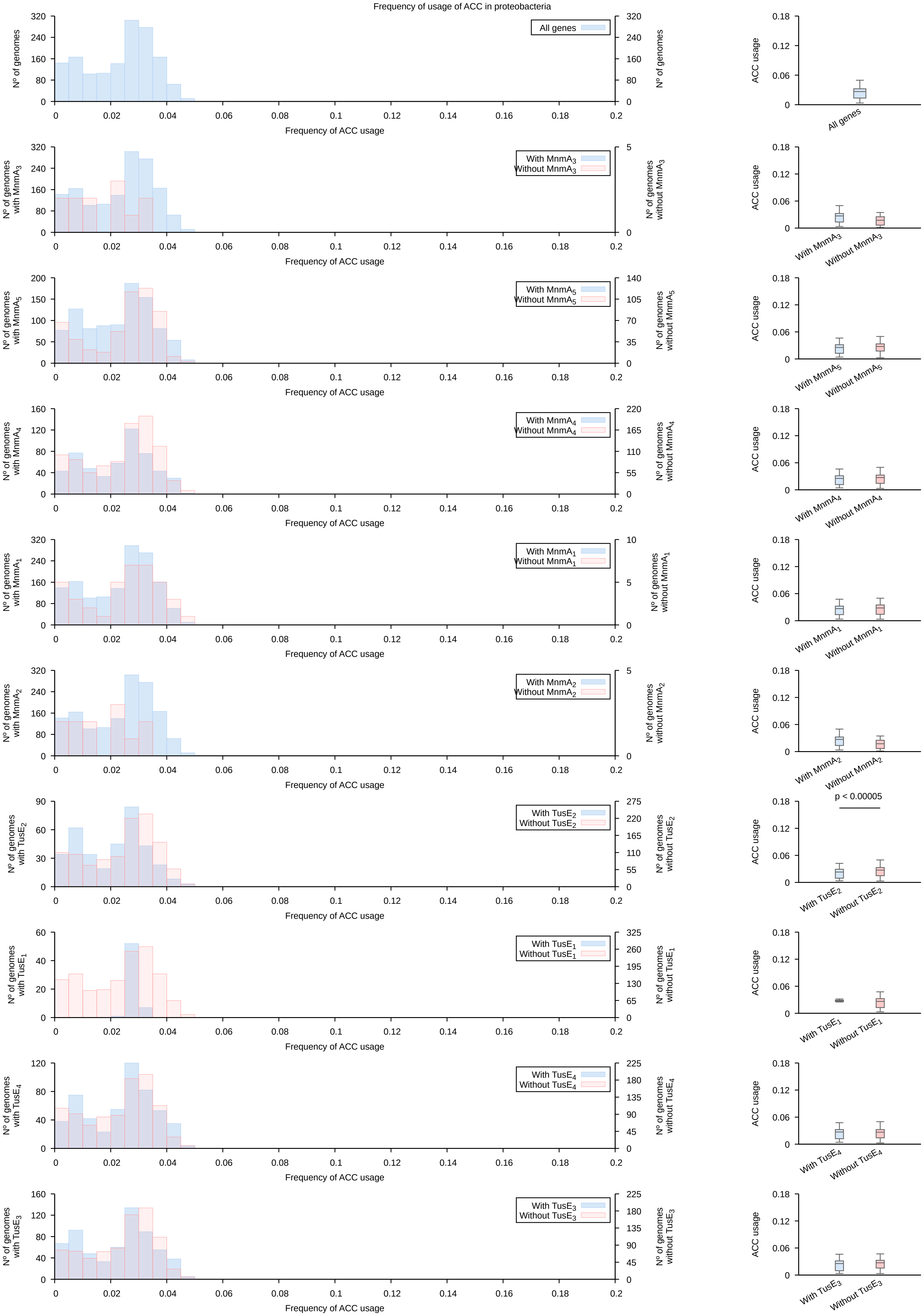

Frequency of usage of ACC in proteobacteria
Nº of genomes
All genes
Frequency of ACC usage
ACC usage
All genes
Nº of genomes with $MnmA_3$
With $MnmA_3$
Without $MnmA_3$
Nº of genomes without $MnmA_3$
Nº of genomes with $MnmA_5$
With $MnmA_5$
Without $MnmA_5$
Nº of genomes without $MnmA_5$
Nº of genomes with $MnmA_4$
With $MnmA_4$
Without $MnmA_4$
Nº of genomes without $MnmA_4$
Nº of genomes with $MnmA_1$
With $MnmA_1$
Without $MnmA_1$
Nº of genomes without $MnmA_1$
Nº of genomes with $MnmA_2$
With $MnmA_2$
Without $MnmA_2$
Nº of genomes without $MnmA_2$
Nº of genomes with $TusE_2$
With $TusE_2$
Without $TusE_2$
Nº of genomes without $TusE_2$
p < 0.00005
Nº of genomes with $TusE_1$
With $TusE_1$
Without $TusE_1$
Nº of genomes without $TusE_1$
Nº of genomes with $TusE_4$
With $TusE_4$
Without $TusE_4$
Nº of genomes without $TusE_4$
Nº of genomes with $TusE_3$
With $TusE_3$
Without $TusE_3$
Nº of genomes without $TusE_3$

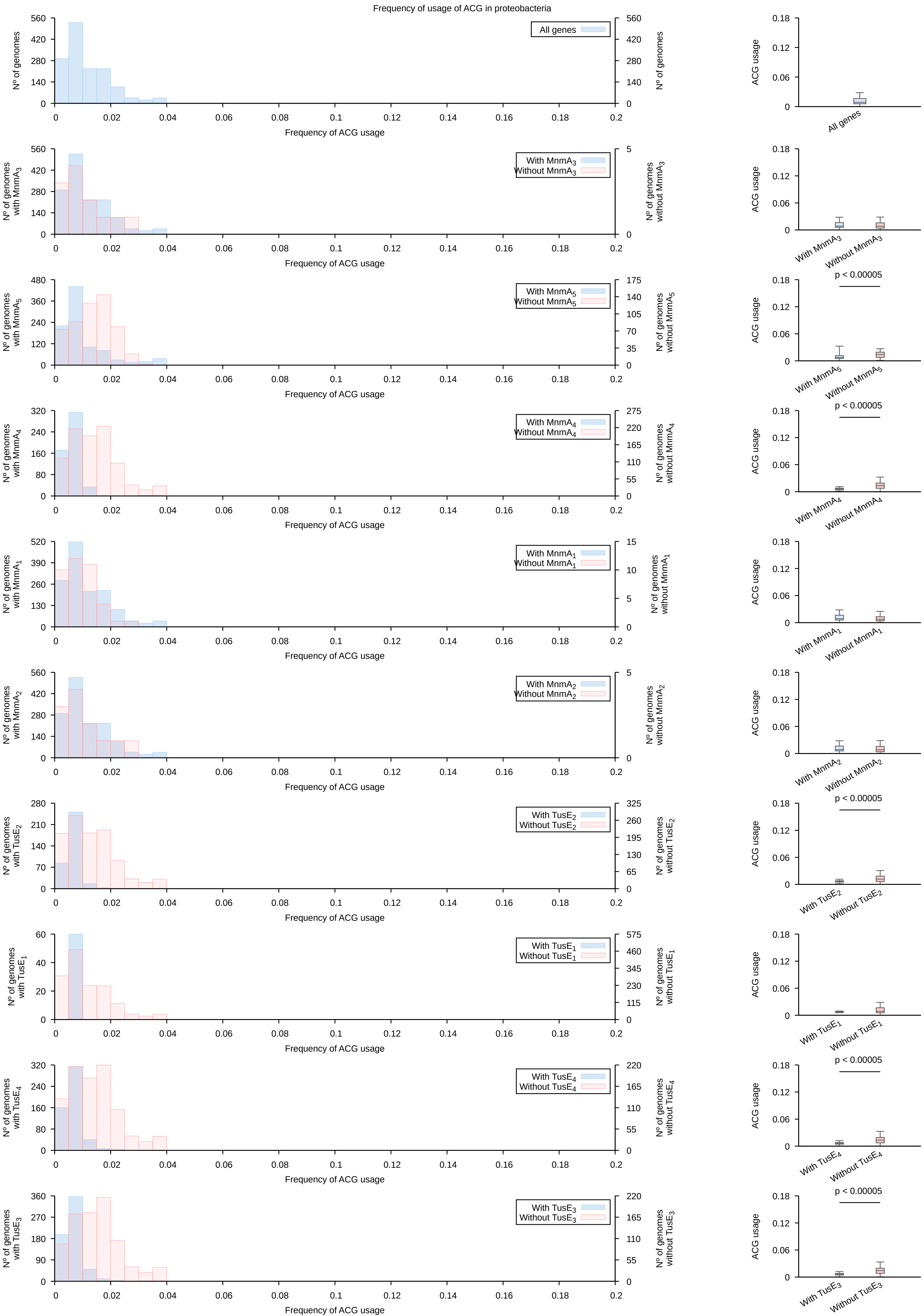

Frequency of usage of ACG in proteobacteria
All genes
Nº of genomes
Frequency of ACG usage
ACG usage
With MnmA$_3$
Without MnmA$_3$
Nº of genomes with MnmA$_3$
Nº of genomes without MnmA$_3$
With MnmA$_5$
Without MnmA$_5$
Nº of genomes with MnmA$_5$
Nº of genomes without MnmA$_5$
p < 0.00005
With MnmA$_4$
Without MnmA$_4$
Nº of genomes with MnmA$_4$
Nº of genomes without MnmA$_4$
p < 0.00005
With MnmA$_1$
Without MnmA$_1$
Nº of genomes with MnmA$_1$
Nº of genomes without MnmA$_1$
With MnmA$_2$
Without MnmA$_2$
Nº of genomes with MnmA$_2$
Nº of genomes without MnmA$_2$
With TusE$_2$
Without TusE$_2$
Nº of genomes with TusE$_2$
Nº of genomes without TusE$_2$
p < 0.00005
With TusE$_1$
Without TusE$_1$
Nº of genomes with TusE$_1$
Nº of genomes without TusE$_1$
With TusE$_4$
Without TusE$_4$
Nº of genomes with TusE$_4$
Nº of genomes without TusE$_4$
p < 0.00005
With TusE$_3$
Without TusE$_3$
Nº of genomes with TusE$_3$
Nº of genomes without TusE$_3$
p < 0.00005

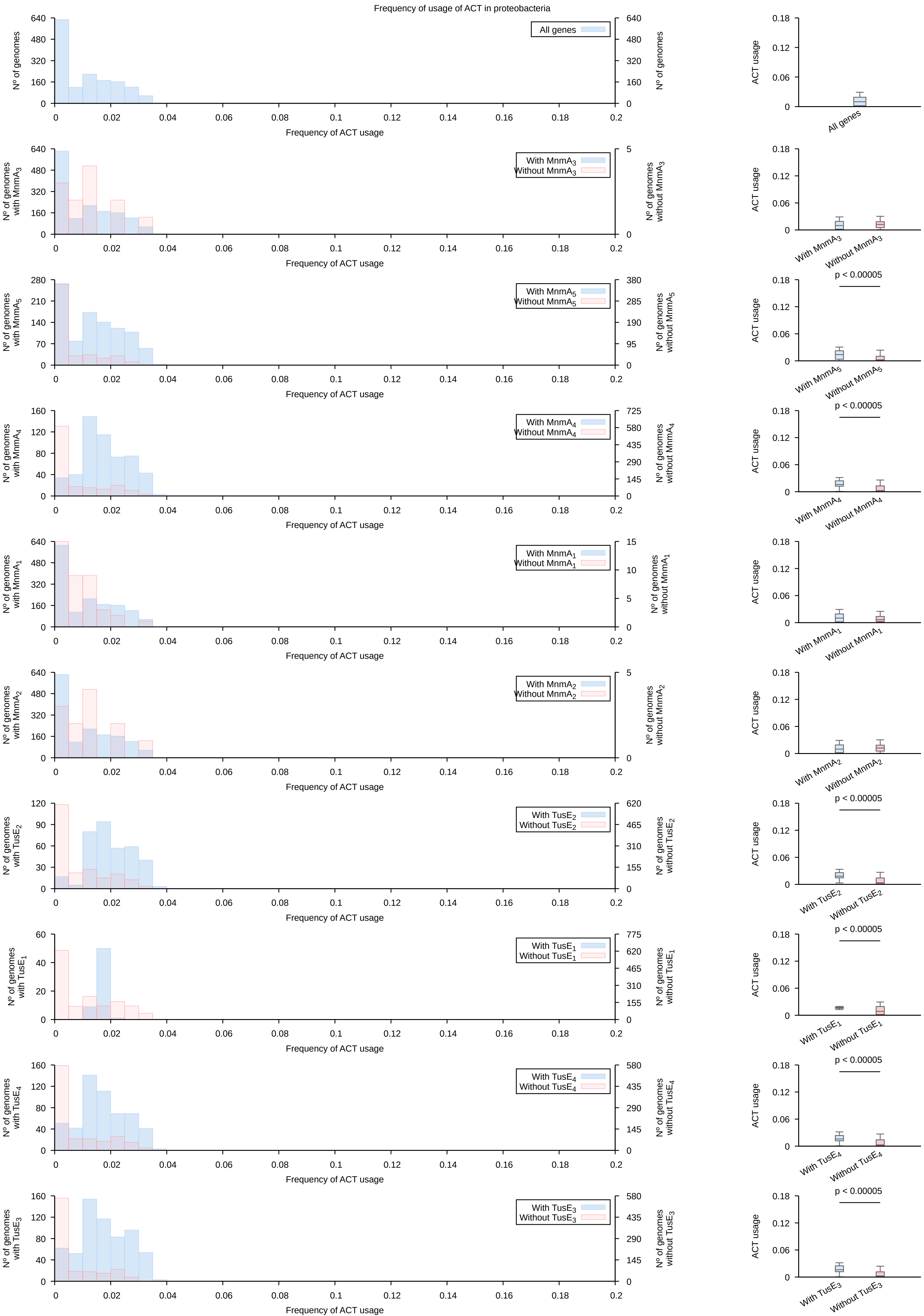

Frequency of usage of ACT in proteobacteria
All genes
Nº of genomes
Frequency of ACT usage
ACT usage
With MnmA$_3$
Without MnmA$_3$
Nº of genomes with MnmA$_3$
Nº of genomes without MnmA$_3$
With MnmA$_5$
Without MnmA$_5$
Nº of genomes with MnmA$_5$
Nº of genomes without MnmA$_5$
p < 0.00005
With MnmA$_4$
Without MnmA$_4$
Nº of genomes with MnmA$_4$
Nº of genomes without MnmA$_4$
p < 0.00005
With MnmA$_1$
Without MnmA$_1$
Nº of genomes with MnmA$_1$
Nº of genomes without MnmA$_1$
With MnmA$_2$
Without MnmA$_2$
Nº of genomes with MnmA$_2$
Nº of genomes without MnmA$_2$
With TusE$_2$
Without TusE$_2$
Nº of genomes with TusE$_2$
Nº of genomes without TusE$_2$
p < 0.00005
With TusE$_1$
Without TusE$_1$
Nº of genomes with TusE$_1$
Nº of genomes without TusE$_1$
p < 0.00005
With TusE$_4$
Without TusE$_4$
Nº of genomes with TusE$_4$
Nº of genomes without TusE$_4$
p < 0.00005
With TusE$_3$
Without TusE$_3$
Nº of genomes with TusE$_3$
Nº of genomes without TusE$_3$
p < 0.00005

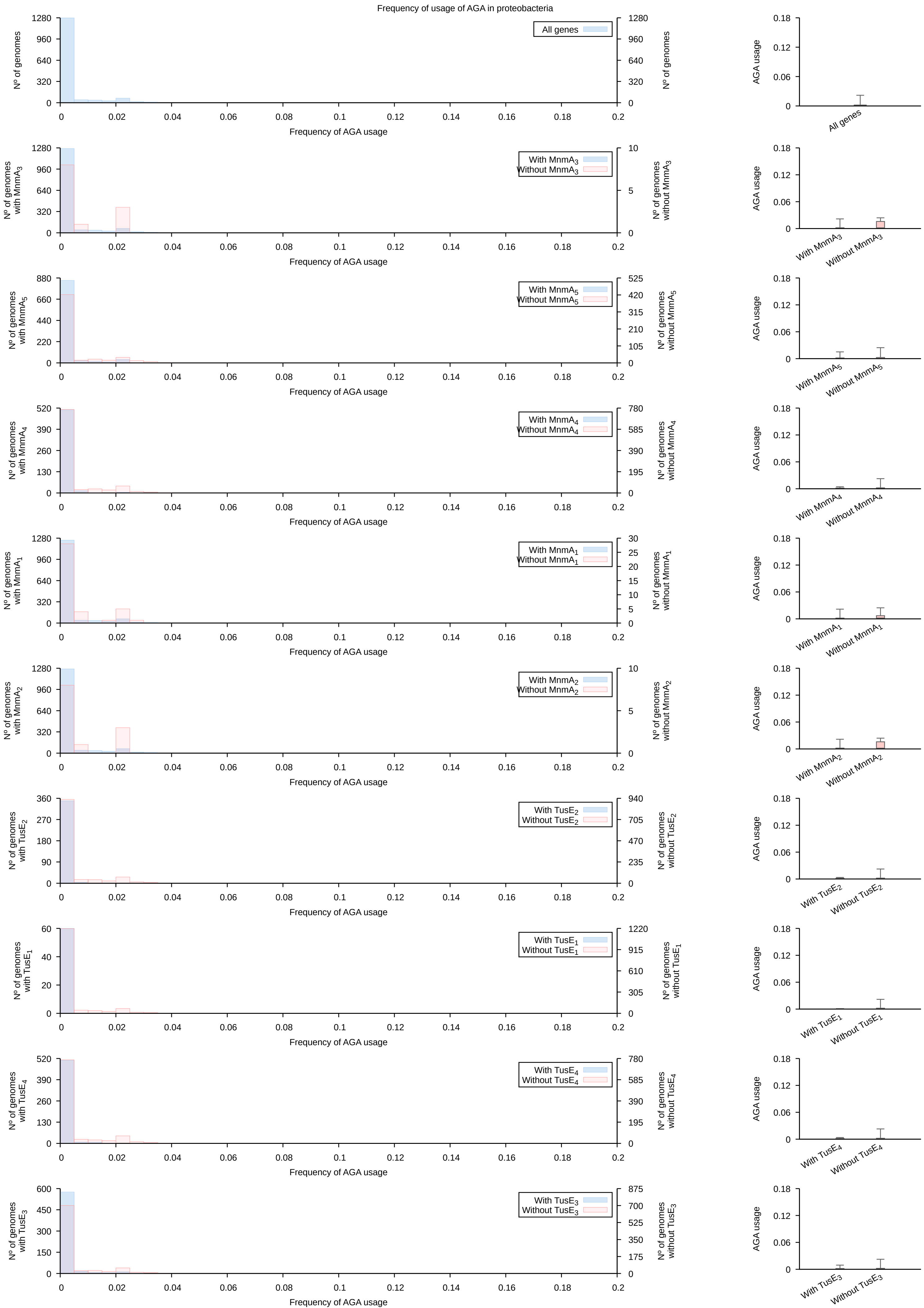
Frequency of usage of AGA in proteobacteria
All genes
Nº of genomes
Frequency of AGA usage
AGA usage
With $MnmA_3$
Without $MnmA_3$
Nº of genomes with $MnmA_3$
Nº of genomes without $MnmA_3$
With $MnmA_5$
Without $MnmA_5$
Nº of genomes with $MnmA_5$
Nº of genomes without $MnmA_5$
With $MnmA_4$
Without $MnmA_4$
Nº of genomes with $MnmA_4$
Nº of genomes without $MnmA_4$
With $MnmA_1$
Without $MnmA_1$
Nº of genomes with $MnmA_1$
Nº of genomes without $MnmA_1$
With $MnmA_2$
Without $MnmA_2$
Nº of genomes with $MnmA_2$
Nº of genomes without $MnmA_2$
With $TusE_2$
Without $TusE_2$
Nº of genomes with $TusE_2$
Nº of genomes without $TusE_2$
With $TusE_1$
Without $TusE_1$
Nº of genomes with $TusE_1$
Nº of genomes without $TusE_1$
With $TusE_4$
Without $TusE_4$
Nº of genomes with $TusE_4$
Nº of genomes without $TusE_4$
With $TusE_3$
Without $TusE_3$
Nº of genomes with $TusE_3$
Nº of genomes without $TusE_3$

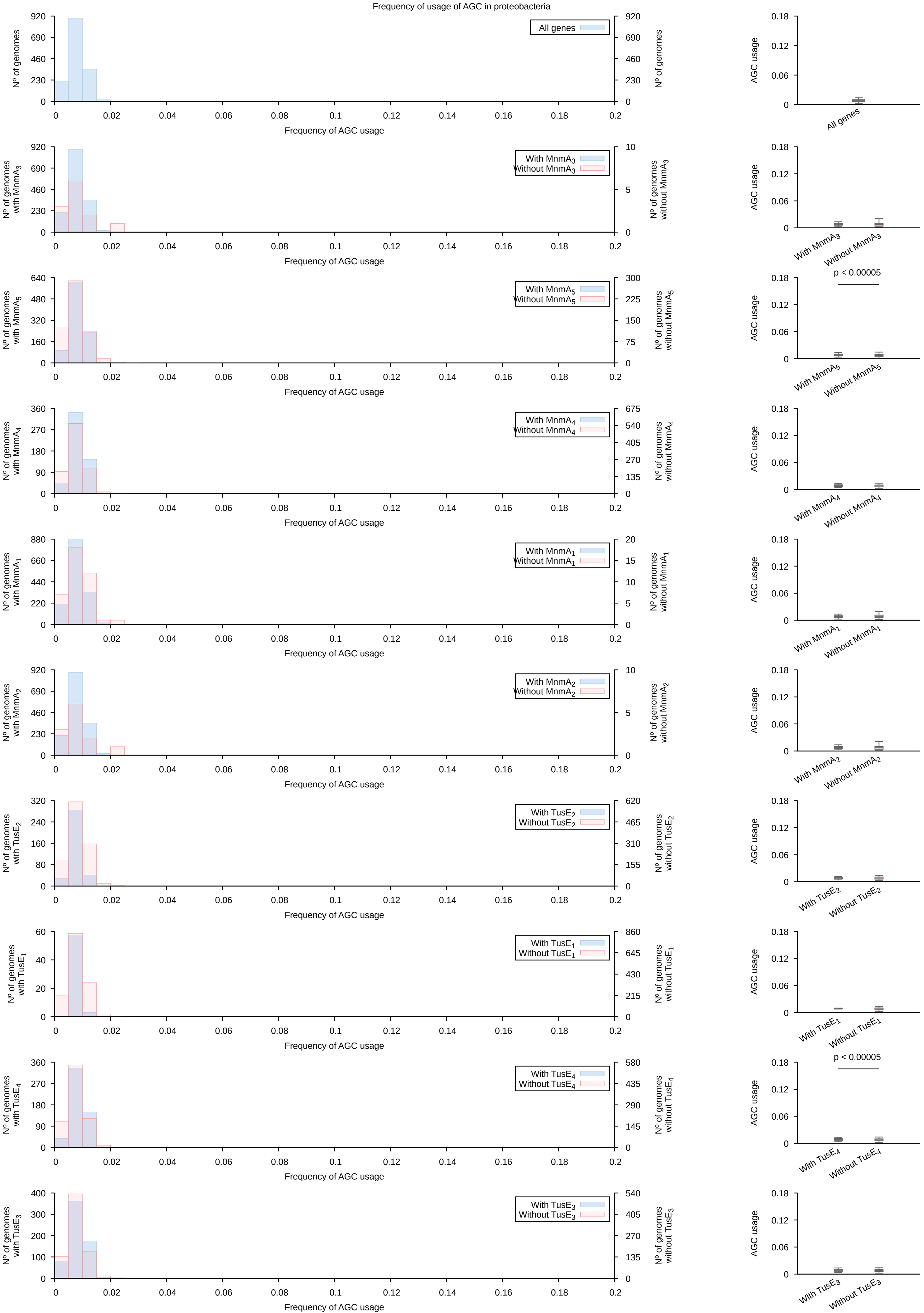
Frequency of usage of AGC in proteobacteria
Nº of genomes
All genes
Frequency of AGC usage
AGC usage
Nº of genomes with $MnmA_3$
Nº of genomes without $MnmA_3$
With $MnmA_3$
Without $MnmA_3$
Nº of genomes with $MnmA_5$
Nº of genomes without $MnmA_5$
With $MnmA_5$
Without $MnmA_5$
p < 0.00005
Nº of genomes with $MnmA_4$
Nº of genomes without $MnmA_4$
With $MnmA_4$
Without $MnmA_4$
Nº of genomes with $MnmA_1$
Nº of genomes without $MnmA_1$
With $MnmA_1$
Without $MnmA_1$
Nº of genomes with $MnmA_2$
Nº of genomes without $MnmA_2$
With $MnmA_2$
Without $MnmA_2$
Nº of genomes with $TusE_2$
Nº of genomes without $TusE_2$
With $TusE_2$
Without $TusE_2$
Nº of genomes with $TusE_1$
Nº of genomes without $TusE_1$
With $TusE_1$
Without $TusE_1$
Nº of genomes with $TusE_4$
Nº of genomes without $TusE_4$
With $TusE_4$
Without $TusE_4$
p < 0.00005
Nº of genomes with $TusE_3$
Nº of genomes without $TusE_3$
With $TusE_3$
Without $TusE_3$

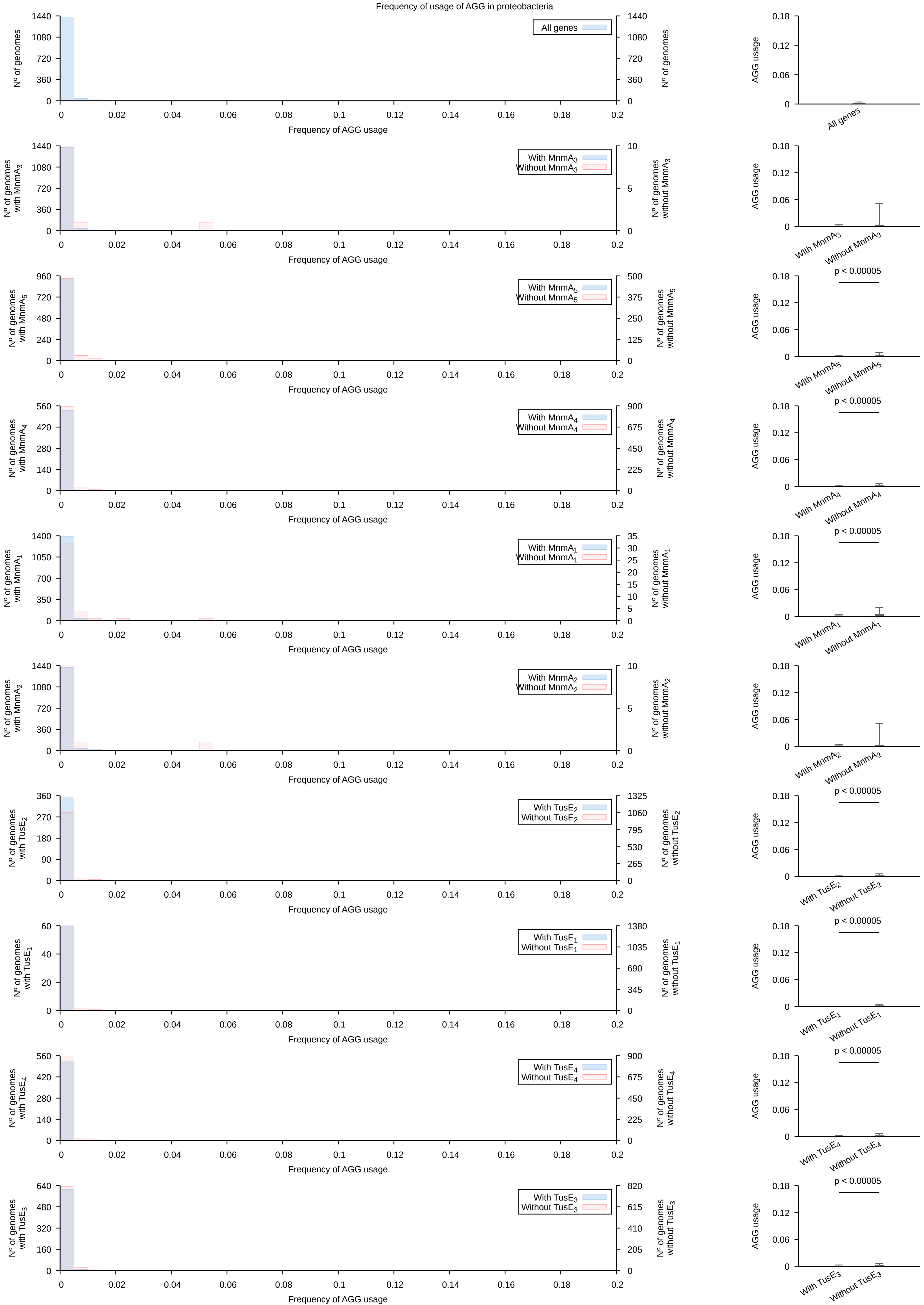

Frequency of usage of AGG in proteobacteria
Nº of genomes
All genes
Frequency of AGG usage
AGG usage
Nº of genomes with MnmA$_3$
Nº of genomes without MnmA$_3$
With MnmA$_3$
Without MnmA$_3$
Nº of genomes with MnmA$_5$
Nº of genomes without MnmA$_5$
With MnmA$_5$
Without MnmA$_5$
p < 0.00005
Nº of genomes with MnmA$_4$
Nº of genomes without MnmA$_4$
With MnmA$_4$
Without MnmA$_4$
p < 0.00005
Nº of genomes with MnmA$_1$
Nº of genomes without MnmA$_1$
With MnmA$_1$
Without MnmA$_1$
p < 0.00005
Nº of genomes with MnmA$_2$
Nº of genomes without MnmA$_2$
With MnmA$_2$
Without MnmA$_2$
Nº of genomes with TusE$_2$
Nº of genomes without TusE$_2$
With TusE$_2$
Without TusE$_2$
p < 0.00005
Nº of genomes with TusE$_1$
Nº of genomes without TusE$_1$
With TusE$_1$
Without TusE$_1$
p < 0.00005
Nº of genomes with TusE$_4$
Nº of genomes without TusE$_4$
With TusE$_4$
Without TusE$_4$
p < 0.00005
Nº of genomes with TusE$_3$
Nº of genomes without TusE$_3$
With TusE$_3$
Without TusE$_3$
p < 0.00005

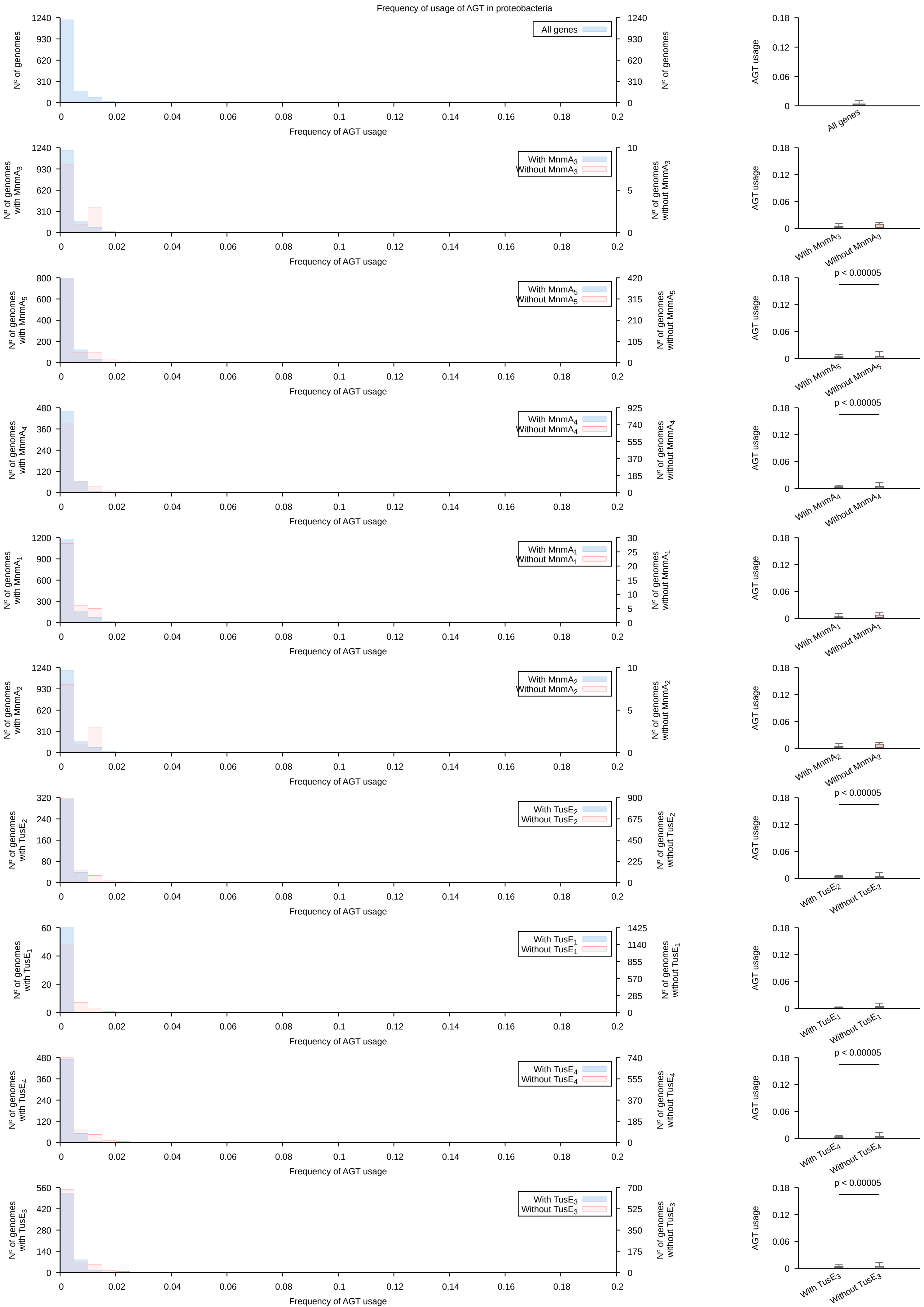

Frequency of usage of AGT in proteobacteria
Nº of genomes
Frequency of AGT usage
All genes
AGT usage
Nº of genomes with $MnmA_3$
Nº of genomes without $MnmA_3$
With $MnmA_3$
Without $MnmA_3$
Nº of genomes with $MnmA_5$
Nº of genomes without $MnmA_5$
With $MnmA_5$
Without $MnmA_5$
p < 0.00005
Nº of genomes with $MnmA_4$
Nº of genomes without $MnmA_4$
With $MnmA_4$
Without $MnmA_4$
p < 0.00005
Nº of genomes with $MnmA_1$
Nº of genomes without $MnmA_1$
With $MnmA_1$
Without $MnmA_1$
Nº of genomes with $MnmA_2$
Nº of genomes without $MnmA_2$
With $MnmA_2$
Without $MnmA_2$
Nº of genomes with $TusE_2$
Nº of genomes without $TusE_2$
With $TusE_2$
Without $TusE_2$
p < 0.00005
Nº of genomes with $TusE_1$
Nº of genomes without $TusE_1$
With $TusE_1$
Without $TusE_1$
Nº of genomes with $TusE_4$
Nº of genomes without $TusE_4$
With $TusE_4$
Without $TusE_4$
p < 0.00005
Nº of genomes with $TusE_3$
Nº of genomes without $TusE_3$
With $TusE_3$
Without $TusE_3$
p < 0.00005

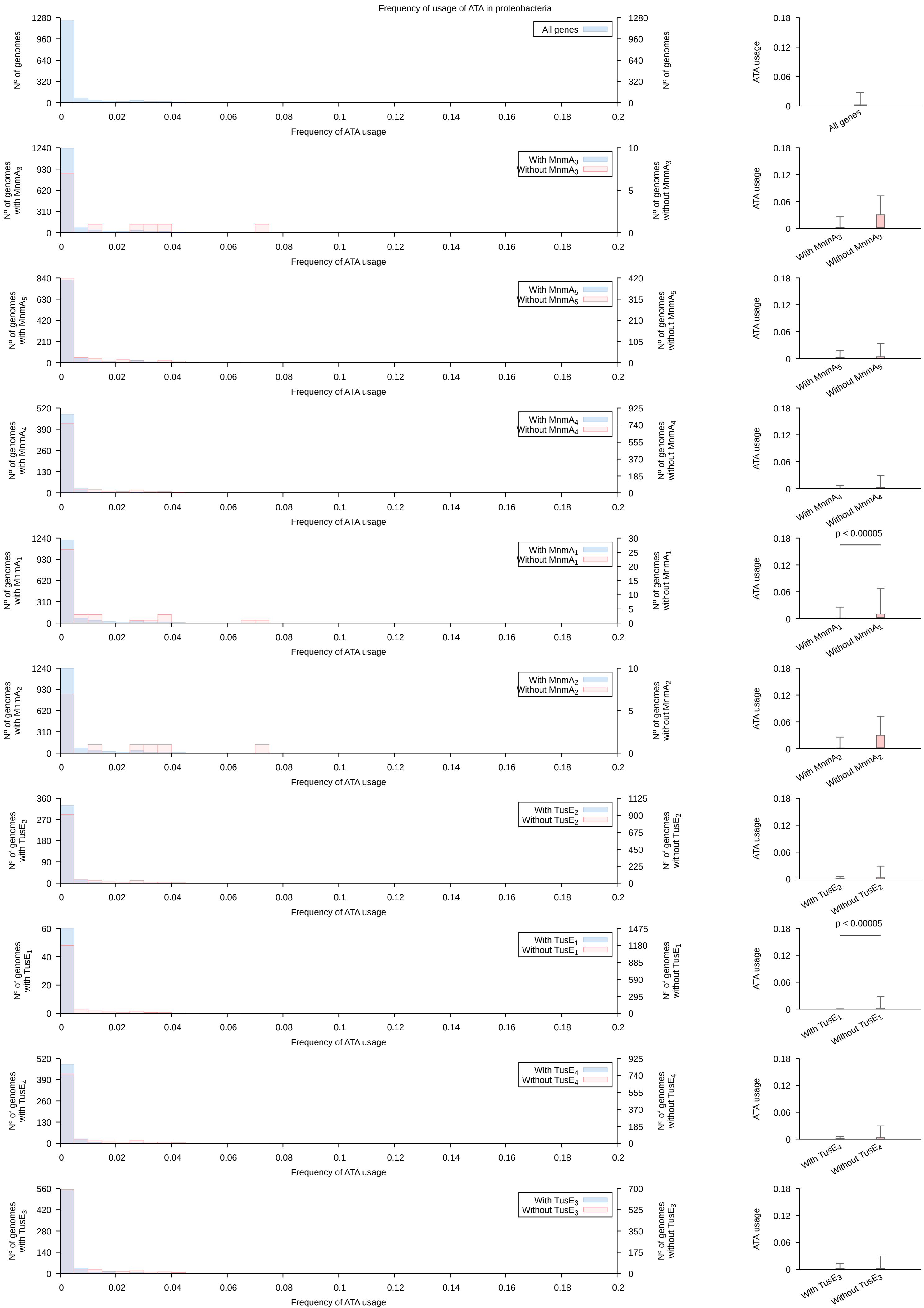
Frequency of usage of ATA in proteobacteria
All genes
Nº of genomes
ATA usage
Frequency of ATA usage
With MnmA3
Without MnmA3
With MnmA5
Without MnmA5
With MnmA4
Without MnmA4
With MnmA1
Without MnmA1
p < 0.00005
With MnmA2
Without MnmA2
With TusE2
Without TusE2
With TusE1
Without TusE1
p < 0.00005
With TusE4
Without TusE4
With TusE3
Without TusE3

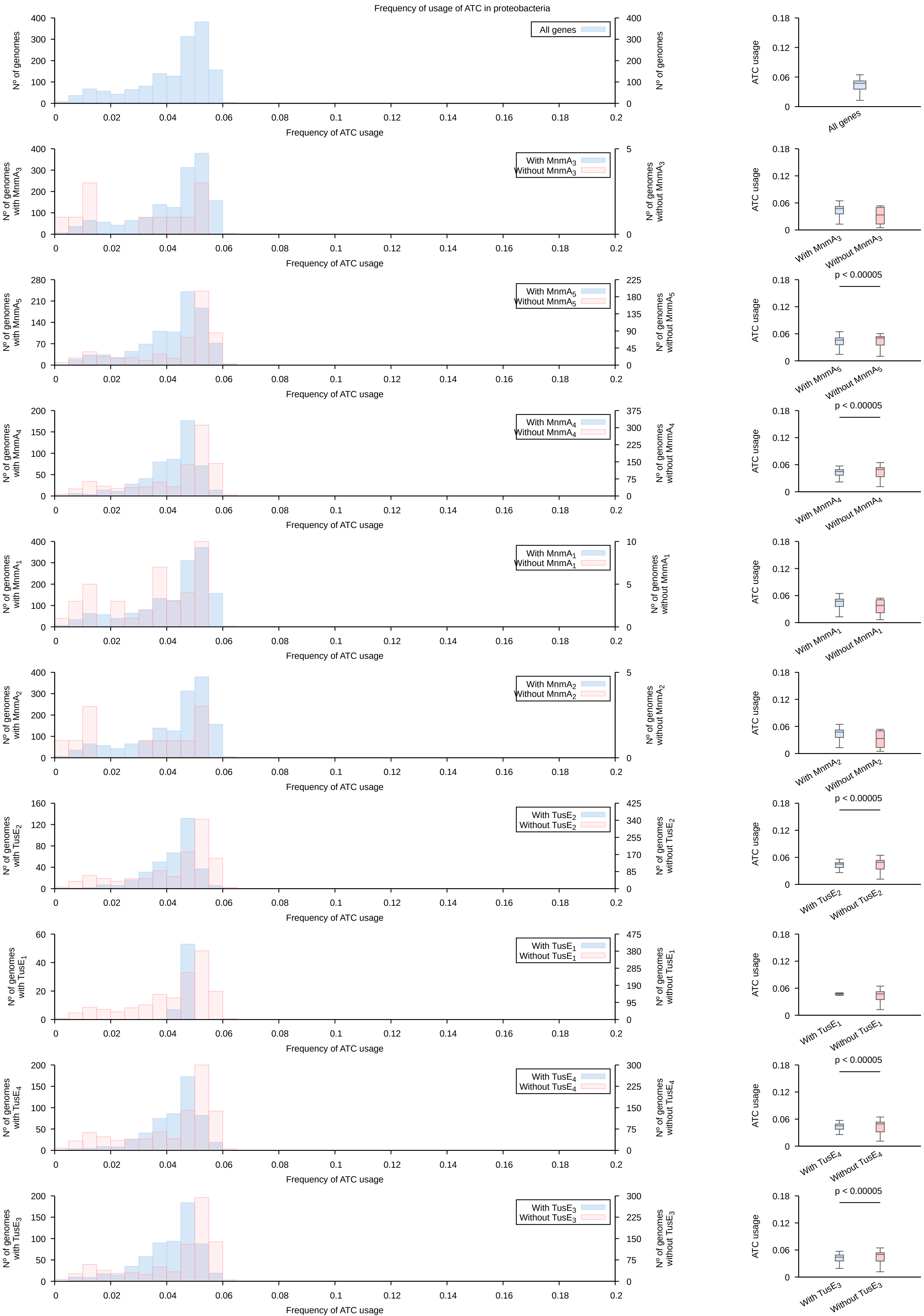
Frequency of usage of ATC in proteobacteria
All genes
With MnmA3
Without MnmA3
With MnmA5
Without MnmA5
With MnmA4
Without MnmA4
With MnmA1
Without MnmA1
With MnmA2
Without MnmA2
With TusE2
Without TusE2
With TusE1
Without TusE1
With TusE4
Without TusE4
With TusE3
Without TusE3
Nº of genomes
Frequency of ATC usage
ATC usage
p < 0.00005

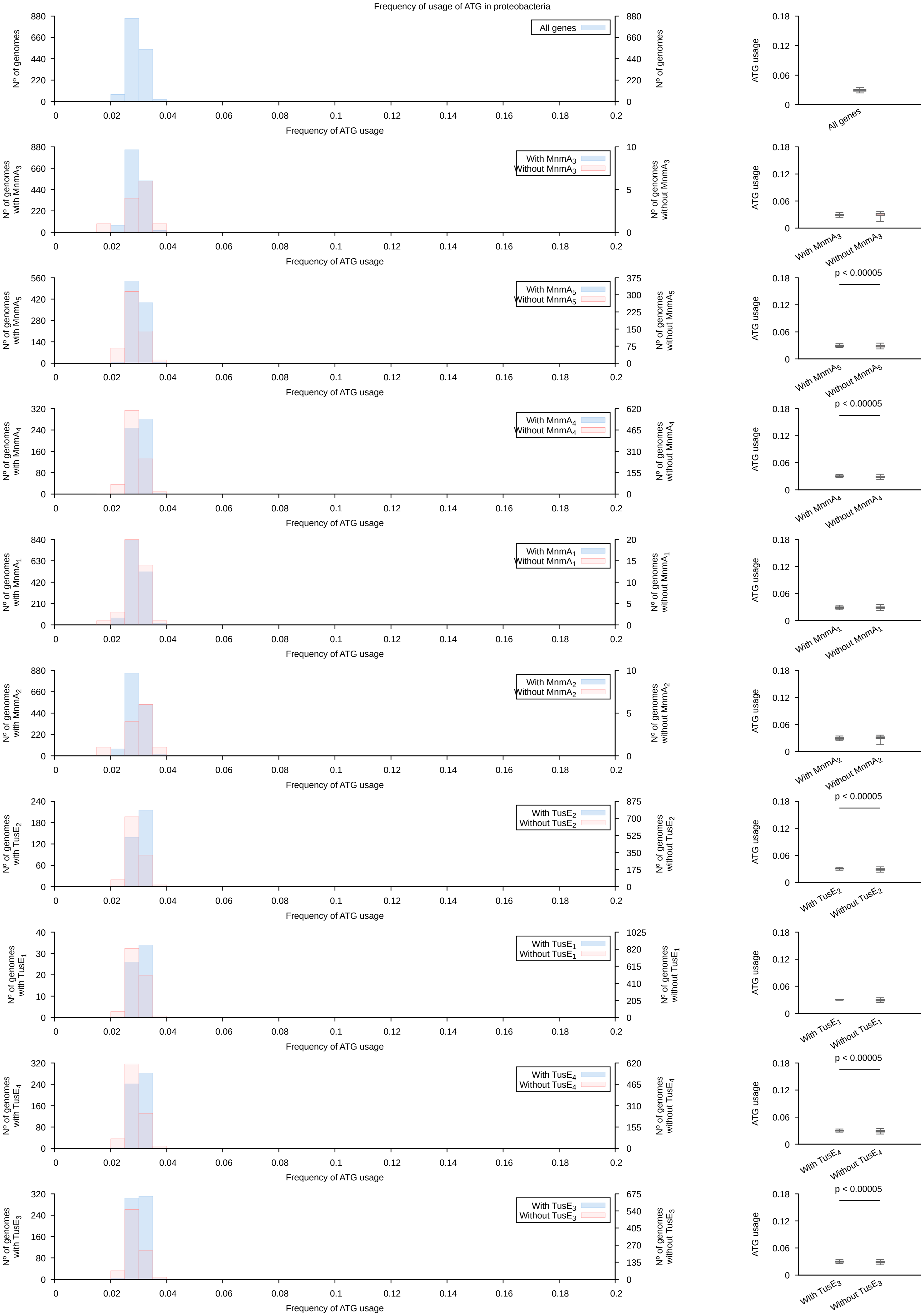
Frequency of usage of ATG in proteobacteria
All genes
Nº of genomes
Frequency of ATG usage
ATG usage
With MnmA$_3$
Without MnmA$_3$
Nº of genomes with MnmA$_3$
Nº of genomes without MnmA$_3$
With MnmA$_5$
Without MnmA$_5$
Nº of genomes with MnmA$_5$
Nº of genomes without MnmA$_5$
p < 0.00005
With MnmA$_4$
Without MnmA$_4$
Nº of genomes with MnmA$_4$
Nº of genomes without MnmA$_4$
p < 0.00005
With MnmA$_1$
Without MnmA$_1$
Nº of genomes with MnmA$_1$
Nº of genomes without MnmA$_1$
With MnmA$_2$
Without MnmA$_2$
Nº of genomes with MnmA$_2$
Nº of genomes without MnmA$_2$
With TusE$_2$
Without TusE$_2$
Nº of genomes with TusE$_2$
Nº of genomes without TusE$_2$
p < 0.00005
With TusE$_1$
Without TusE$_1$
Nº of genomes with TusE$_1$
Nº of genomes without TusE$_1$
With TusE$_4$
Without TusE$_4$
Nº of genomes with TusE$_4$
Nº of genomes without TusE$_4$
p < 0.00005
With TusE$_3$
Without TusE$_3$
Nº of genomes with TusE$_3$
Nº of genomes without TusE$_3$
p < 0.00005

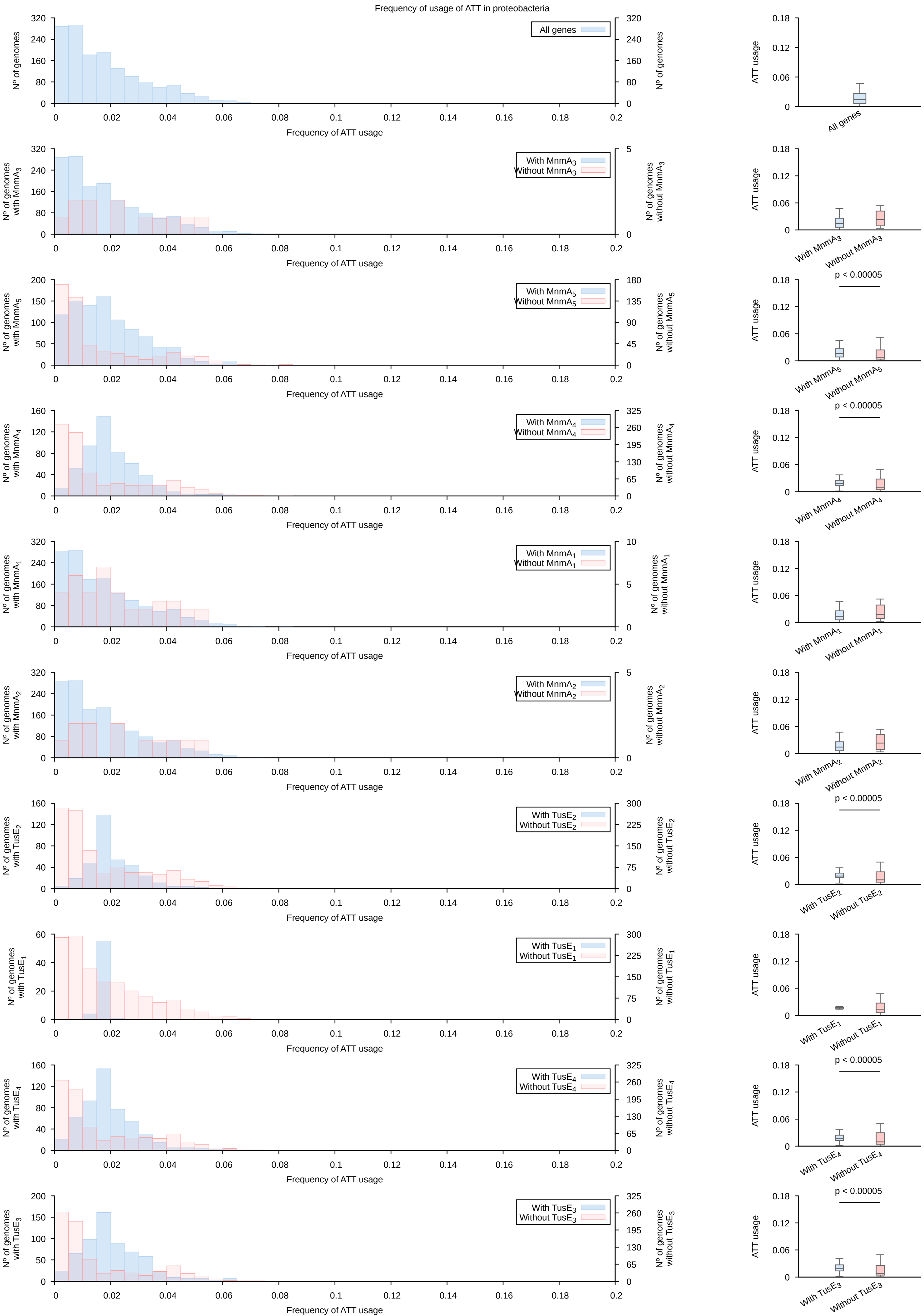
Frequency of usage of ATT in proteobacteria
Nº of genomes
All genes
Frequency of ATT usage
ATT usage
All genes
Nº of genomes with MnmA$_3$
Nº of genomes without MnmA$_3$
With MnmA$_3$
Without MnmA$_3$
Nº of genomes with MnmA$_5$
Nº of genomes without MnmA$_5$
With MnmA$_5$
Without MnmA$_5$
p < 0.00005
Nº of genomes with MnmA$_4$
Nº of genomes without MnmA$_4$
With MnmA$_4$
Without MnmA$_4$
p < 0.00005
Nº of genomes with MnmA$_1$
Nº of genomes without MnmA$_1$
With MnmA$_1$
Without MnmA$_1$
Nº of genomes with MnmA$_2$
Nº of genomes without MnmA$_2$
With MnmA$_2$
Without MnmA$_2$
Nº of genomes with TusE$_2$
Nº of genomes without TusE$_2$
With TusE$_2$
Without TusE$_2$
p < 0.00005
Nº of genomes with TusE$_1$
Nº of genomes without TusE$_1$
With TusE$_1$
Without TusE$_1$
Nº of genomes with TusE$_4$
Nº of genomes without TusE$_4$
With TusE$_4$
Without TusE$_4$
p < 0.00005
Nº of genomes with TusE$_3$
Nº of genomes without TusE$_3$
With TusE$_3$
Without TusE$_3$
p < 0.00005

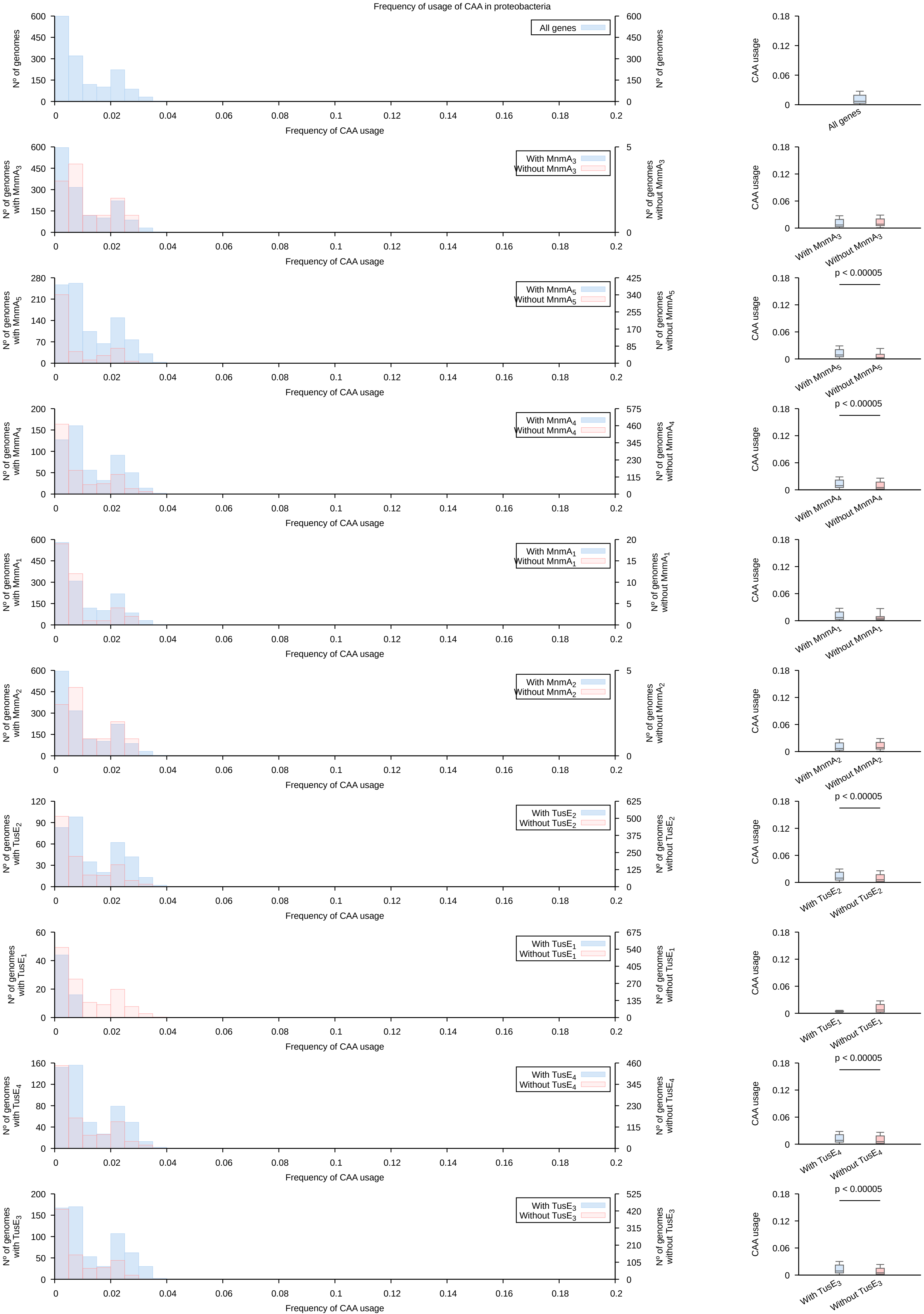
Frequency of usage of CAA in proteobacteria
All genes
Nº of genomes
Frequency of CAA usage
CAA usage
With MnmA$_3$
Without MnmA$_3$
Nº of genomes with MnmA$_3$
Nº of genomes without MnmA$_3$
With MnmA$_5$
Without MnmA$_5$
Nº of genomes with MnmA$_5$
Nº of genomes without MnmA$_5$
p < 0.00005
With MnmA$_4$
Without MnmA$_4$
Nº of genomes with MnmA$_4$
Nº of genomes without MnmA$_4$
p < 0.00005
With MnmA$_1$
Without MnmA$_1$
Nº of genomes with MnmA$_1$
Nº of genomes without MnmA$_1$
With MnmA$_2$
Without MnmA$_2$
Nº of genomes with MnmA$_2$
Nº of genomes without MnmA$_2$
With TusE$_2$
Without TusE$_2$
Nº of genomes with TusE$_2$
Nº of genomes without TusE$_2$
p < 0.00005
With TusE$_1$
Without TusE$_1$
Nº of genomes with TusE$_1$
Nº of genomes without TusE$_1$
With TusE$_4$
Without TusE$_4$
Nº of genomes with TusE$_4$
Nº of genomes without TusE$_4$
p < 0.00005
With TusE$_3$
Without TusE$_3$
Nº of genomes with TusE$_3$
Nº of genomes without TusE$_3$
p < 0.00005

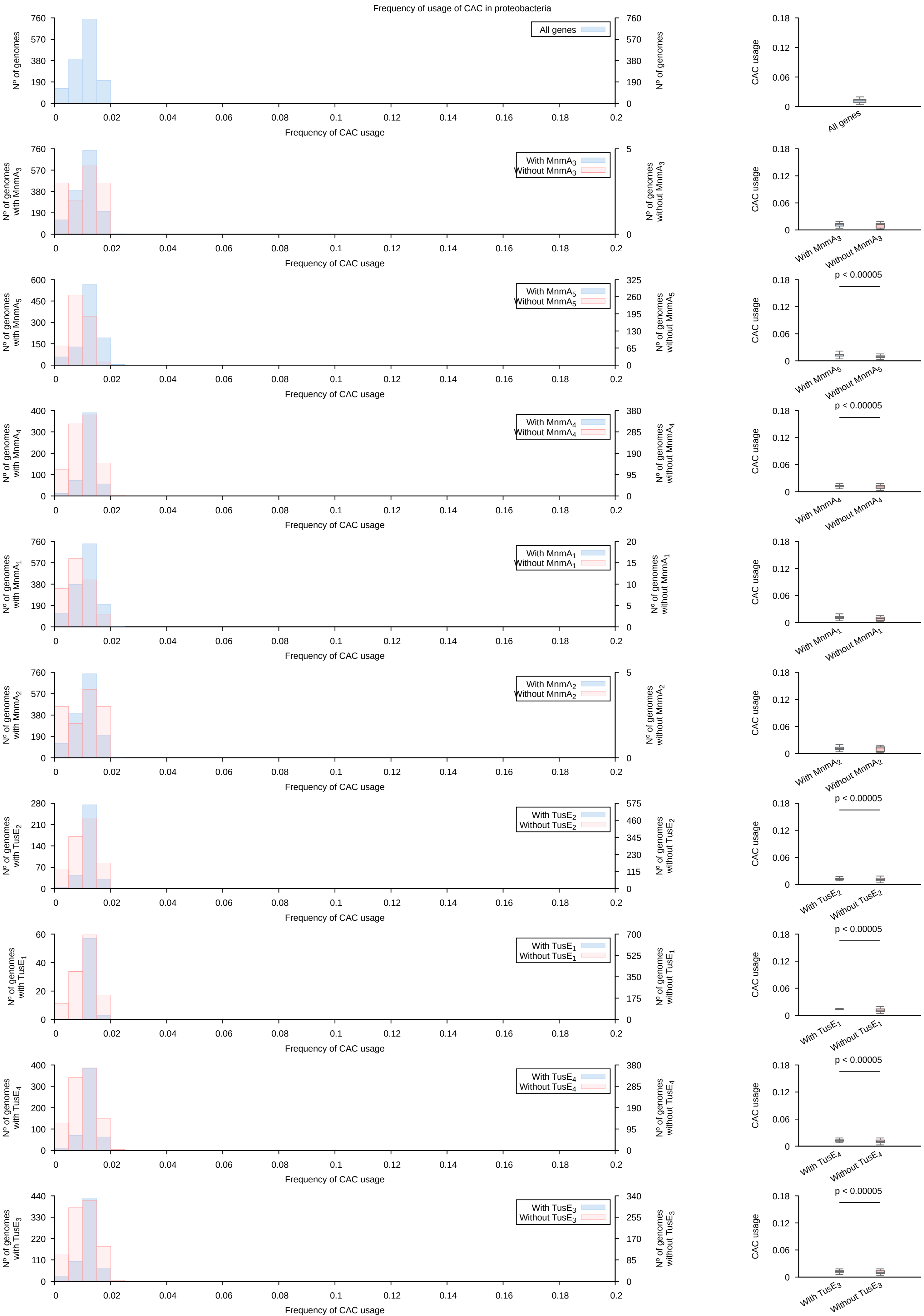

Frequency of usage of CAC in proteobacteria
All genes
Nº of genomes
Frequency of CAC usage
CAC usage
With MnmA3
Without MnmA3
Nº of genomes with MnmA3
Nº of genomes without MnmA3
With MnmA5
Without MnmA5
Nº of genomes with MnmA5
Nº of genomes without MnmA5
p < 0.00005
With MnmA4
Without MnmA4
Nº of genomes with MnmA4
Nº of genomes without MnmA4
p < 0.00005
With MnmA1
Without MnmA1
Nº of genomes with MnmA1
Nº of genomes without MnmA1
With MnmA2
Without MnmA2
Nº of genomes with MnmA2
Nº of genomes without MnmA2
With TusE2
Without TusE2
Nº of genomes with TusE2
Nº of genomes without TusE2
p < 0.00005
With TusE1
Without TusE1
Nº of genomes with TusE1
Nº of genomes without TusE1
p < 0.00005
With TusE4
Without TusE4
Nº of genomes with TusE4
Nº of genomes without TusE4
p < 0.00005
With TusE3
Without TusE3
Nº of genomes with TusE3
Nº of genomes without TusE3
p < 0.00005

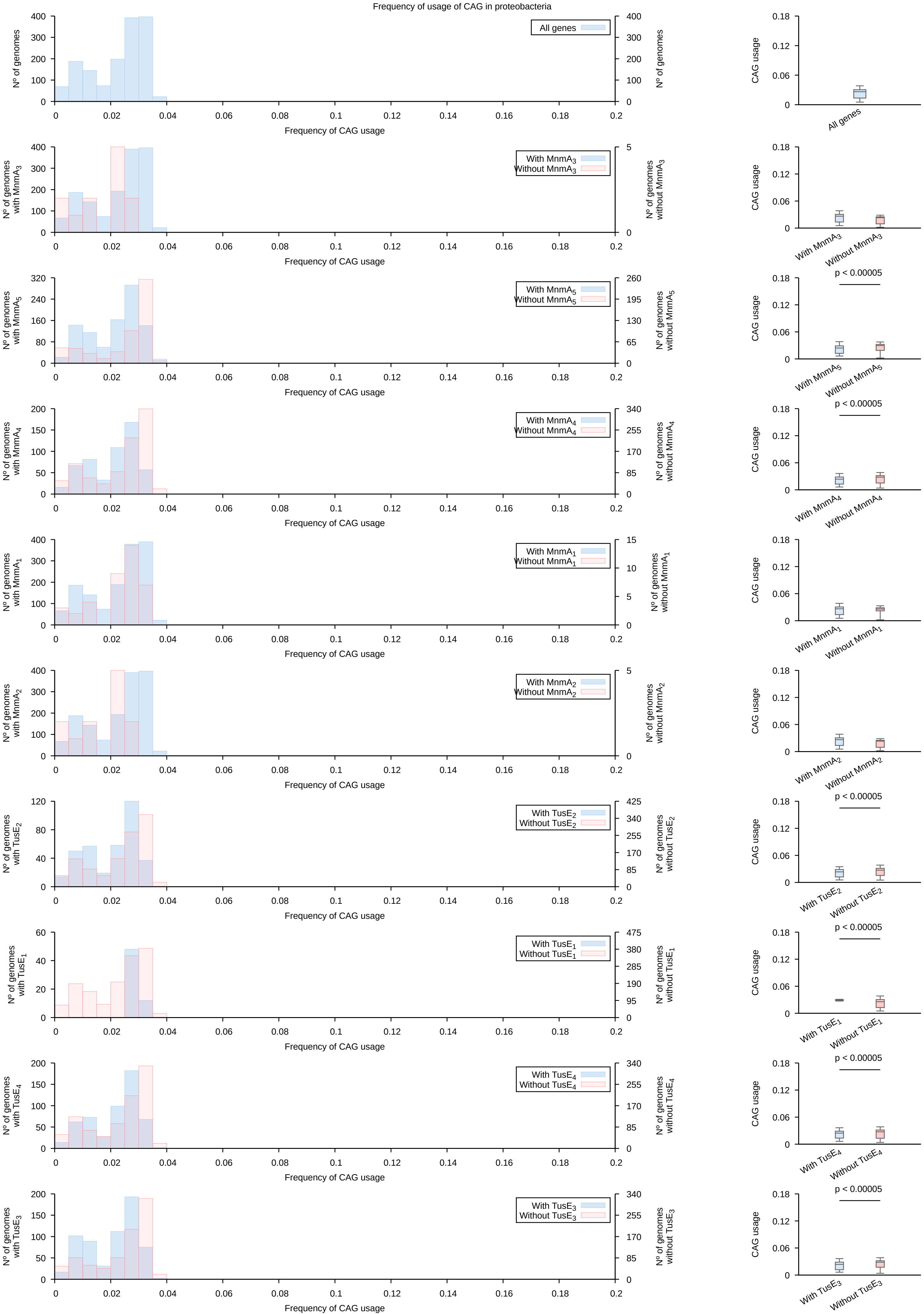

Frequency of usage of CAG in proteobacteria
Nº of genomes
All genes
Frequency of CAG usage
CAG usage
All genes
Nº of genomes with MnmA$_3$
Nº of genomes without MnmA$_3$
With MnmA$_3$
Without MnmA$_3$
Nº of genomes with MnmA$_5$
Nº of genomes without MnmA$_5$
With MnmA$_5$
Without MnmA$_5$
p < 0.00005
Nº of genomes with MnmA$_4$
Nº of genomes without MnmA$_4$
With MnmA$_4$
Without MnmA$_4$
p < 0.00005
Nº of genomes with MnmA$_1$
Nº of genomes without MnmA$_1$
With MnmA$_1$
Without MnmA$_1$
Nº of genomes with MnmA$_2$
Nº of genomes without MnmA$_2$
With MnmA$_2$
Without MnmA$_2$
Nº of genomes with TusE$_2$
Nº of genomes without TusE$_2$
With TusE$_2$
Without TusE$_2$
p < 0.00005
Nº of genomes with TusE$_1$
Nº of genomes without TusE$_1$
With TusE$_1$
Without TusE$_1$
p < 0.00005
Nº of genomes with TusE$_4$
Nº of genomes without TusE$_4$
With TusE$_4$
Without TusE$_4$
p < 0.00005
Nº of genomes with TusE$_3$
Nº of genomes without TusE$_3$
With TusE$_3$
Without TusE$_3$
p < 0.00005

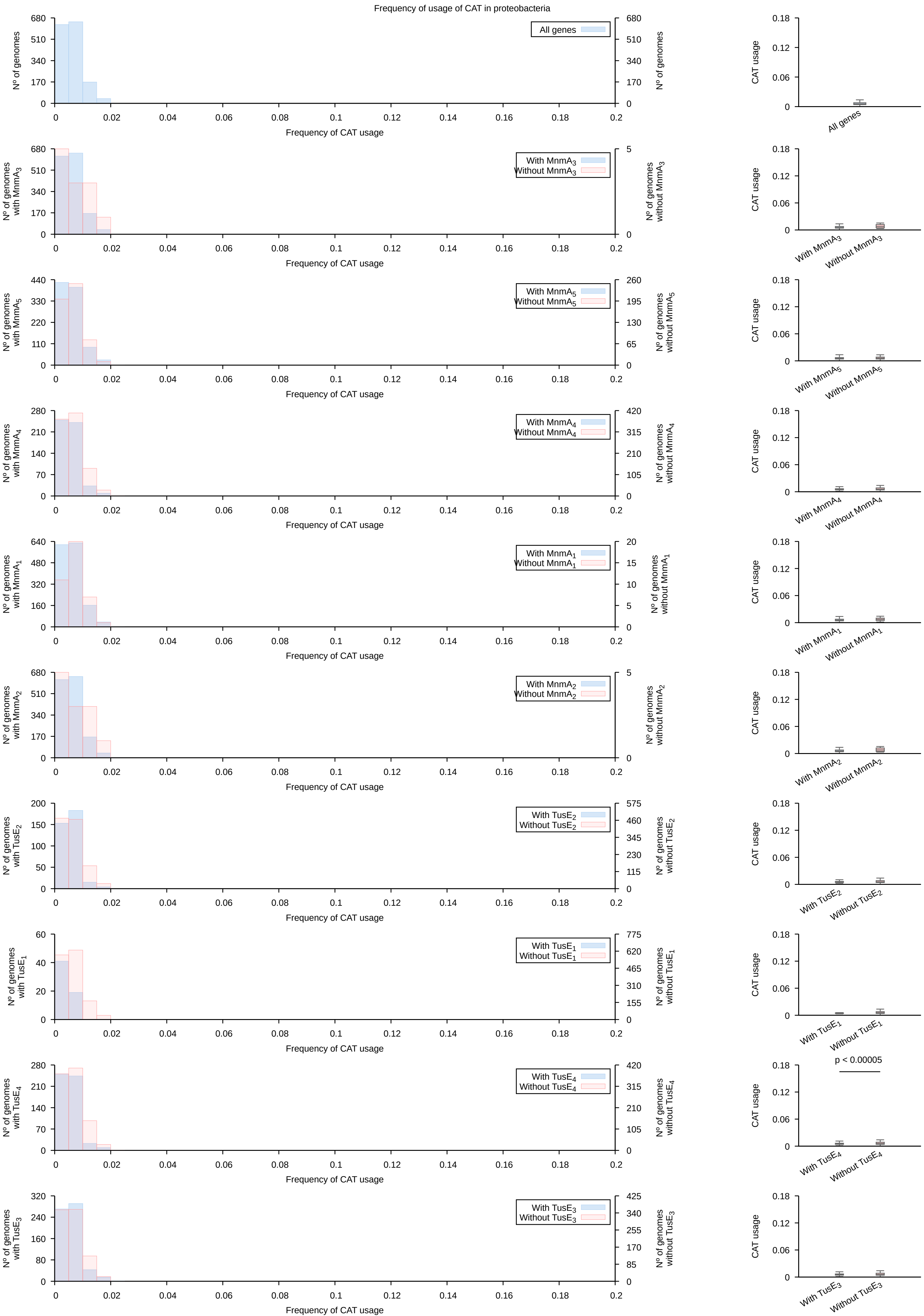

Frequency of usage of CAT in proteobacteria
Nº of genomes
Frequency of CAT usage
CAT usage
All genes
With MnmA3
Without MnmA3
Nº of genomes with MnmA3
Nº of genomes without MnmA3
With MnmA5
Without MnmA5
Nº of genomes with MnmA5
Nº of genomes without MnmA5
With MnmA4
Without MnmA4
Nº of genomes with MnmA4
Nº of genomes without MnmA4
With MnmA1
Without MnmA1
Nº of genomes with MnmA1
Nº of genomes without MnmA1
With MnmA2
Without MnmA2
Nº of genomes with MnmA2
Nº of genomes without MnmA2
With TusE2
Without TusE2
Nº of genomes with TusE2
Nº of genomes without TusE2
With TusE1
Without TusE1
Nº of genomes with TusE1
Nº of genomes without TusE1
With TusE4
Without TusE4
Nº of genomes with TusE4
Nº of genomes without TusE4
p < 0.00005
With TusE3
Without TusE3
Nº of genomes with TusE3
Nº of genomes without TusE3

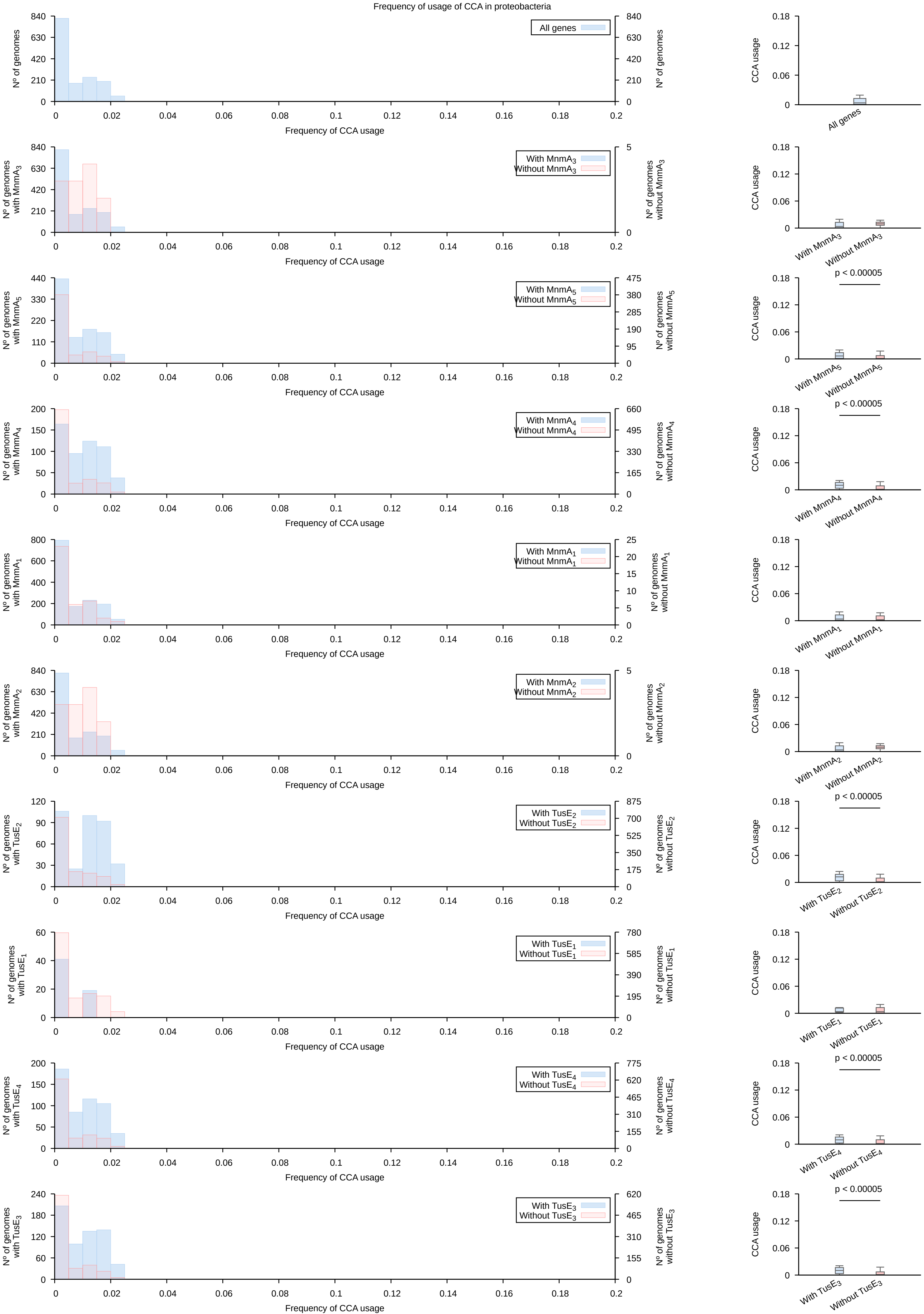

Frequency of usage of CCA in proteobacteria
All genes
Nº of genomes
Frequency of CCA usage
CCA usage
With MnmA$_3$
Without MnmA$_3$
Nº of genomes with MnmA$_3$
Nº of genomes without MnmA$_3$
With MnmA$_5$
Without MnmA$_5$
Nº of genomes with MnmA$_5$
Nº of genomes without MnmA$_5$
p < 0.00005
With MnmA$_4$
Without MnmA$_4$
Nº of genomes with MnmA$_4$
Nº of genomes without MnmA$_4$
p < 0.00005
With MnmA$_1$
Without MnmA$_1$
Nº of genomes with MnmA$_1$
Nº of genomes without MnmA$_1$
With MnmA$_2$
Without MnmA$_2$
Nº of genomes with MnmA$_2$
Nº of genomes without MnmA$_2$
With TusE$_2$
Without TusE$_2$
Nº of genomes with TusE$_2$
Nº of genomes without TusE$_2$
p < 0.00005
With TusE$_1$
Without TusE$_1$
Nº of genomes with TusE$_1$
Nº of genomes without TusE$_1$
With TusE$_4$
Without TusE$_4$
Nº of genomes with TusE$_4$
Nº of genomes without TusE$_4$
p < 0.00005
With TusE$_3$
Without TusE$_3$
Nº of genomes with TusE$_3$
Nº of genomes without TusE$_3$
p < 0.00005

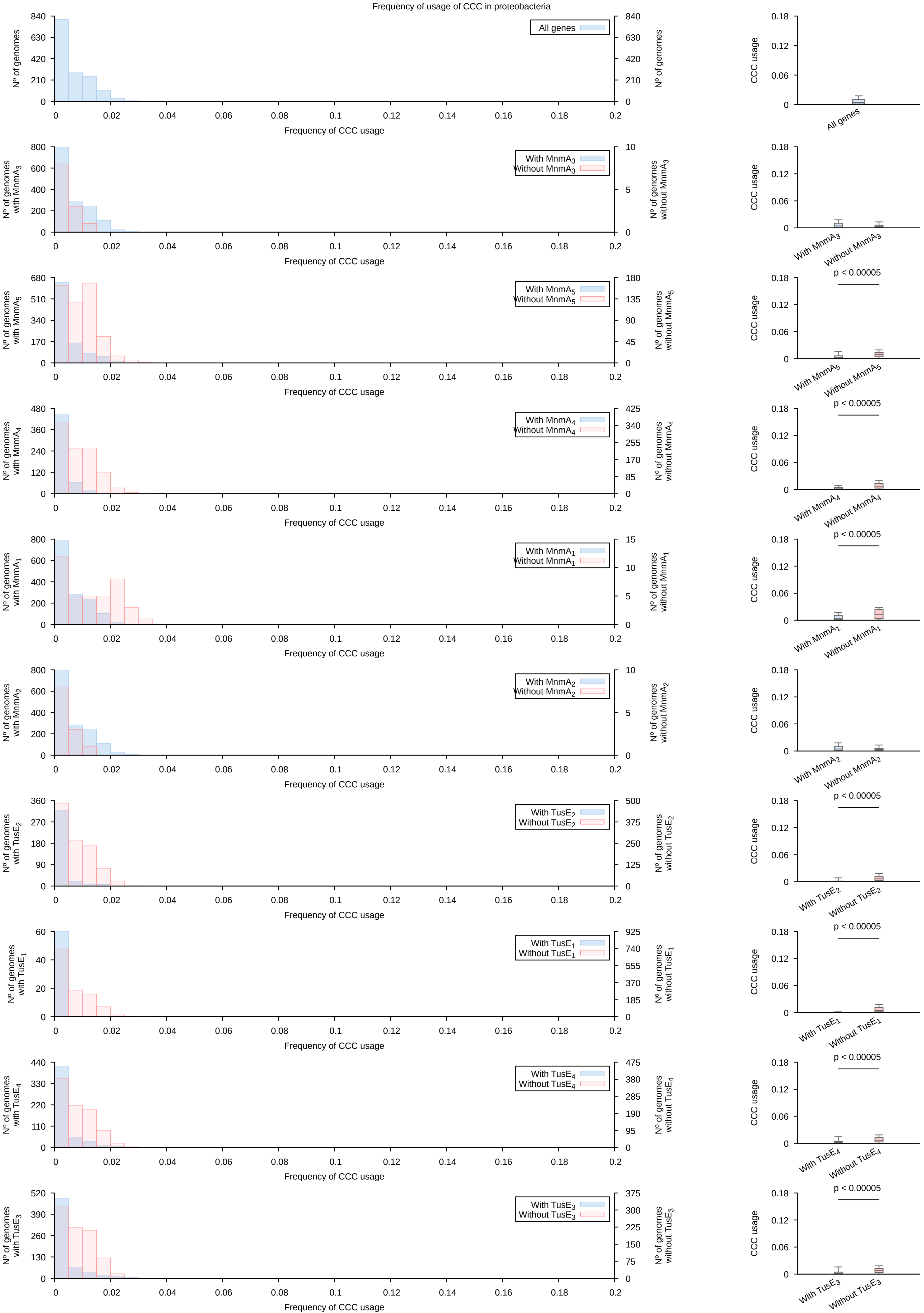

Frequency of usage of CCC in proteobacteria
All genes
Nº of genomes
Frequency of CCC usage
CCC usage
With $MnmA_3$
Without $MnmA_3$
Nº of genomes with $MnmA_3$
Nº of genomes without $MnmA_3$
With $MnmA_5$
Without $MnmA_5$
Nº of genomes with $MnmA_5$
Nº of genomes without $MnmA_5$
p < 0.00005
With $MnmA_4$
Without $MnmA_4$
Nº of genomes with $MnmA_4$
Nº of genomes without $MnmA_4$
p < 0.00005
With $MnmA_1$
Without $MnmA_1$
Nº of genomes with $MnmA_1$
Nº of genomes without $MnmA_1$
p < 0.00005
With $MnmA_2$
Without $MnmA_2$
Nº of genomes with $MnmA_2$
Nº of genomes without $MnmA_2$
With $TusE_2$
Without $TusE_2$
Nº of genomes with $TusE_2$
Nº of genomes without $TusE_2$
p < 0.00005
With $TusE_1$
Without $TusE_1$
Nº of genomes with $TusE_1$
Nº of genomes without $TusE_1$
p < 0.00005
With $TusE_4$
Without $TusE_4$
Nº of genomes with $TusE_4$
Nº of genomes without $TusE_4$
p < 0.00005
With $TusE_3$
Without $TusE_3$
Nº of genomes with $TusE_3$
Nº of genomes without $TusE_3$
p < 0.00005

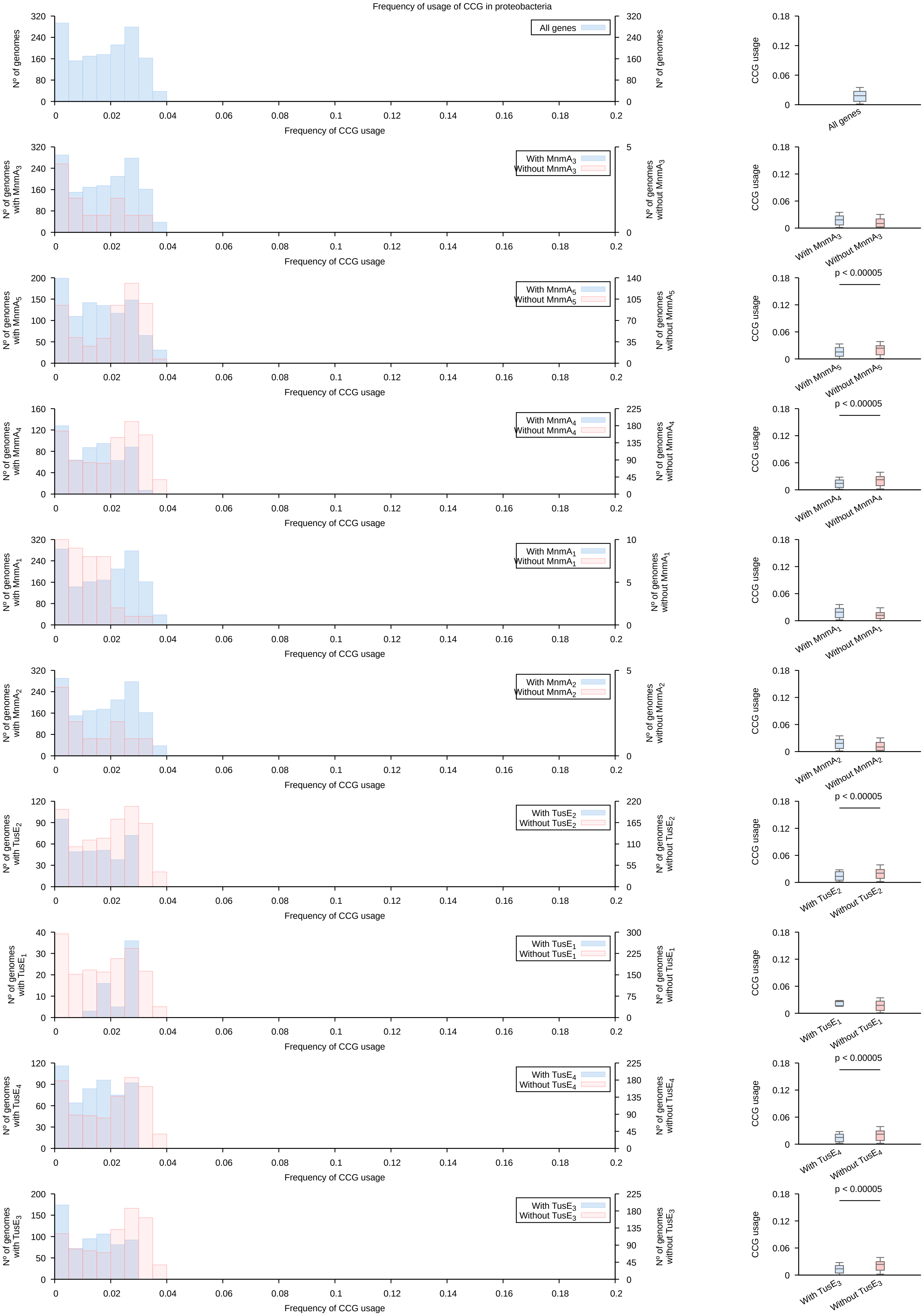

Frequency of usage of CCG in proteobacteria
Nº of genomes
Frequency of CCG usage
CCG usage
All genes
With MnmA3
Without MnmA3
With MnmA5
Without MnmA5
With MnmA4
Without MnmA4
With MnmA1
Without MnmA1
With MnmA2
Without MnmA2
With TusE2
Without TusE2
With TusE1
Without TusE1
With TusE4
Without TusE4
With TusE3
Without TusE3
p < 0.00005

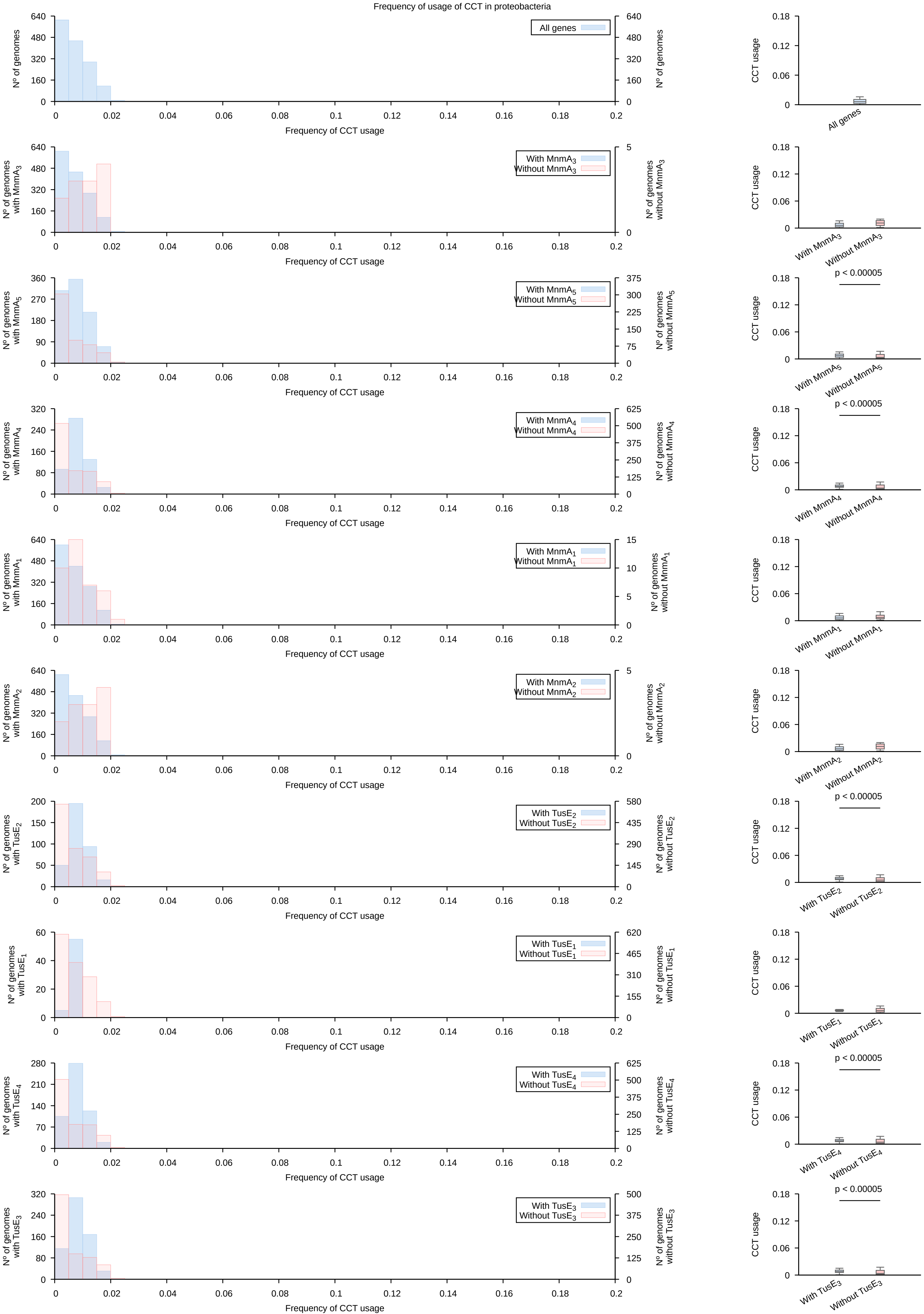
Frequency of usage of CCT in proteobacteria
All genes
Nº of genomes
Frequency of CCT usage
CCT usage
With MnmA3
Without MnmA3
Nº of genomes with MnmA3
Nº of genomes without MnmA3
With MnmA5
Without MnmA5
Nº of genomes with MnmA5
Nº of genomes without MnmA5
p < 0.00005
With MnmA4
Without MnmA4
Nº of genomes with MnmA4
Nº of genomes without MnmA4
p < 0.00005
With MnmA1
Without MnmA1
Nº of genomes with MnmA1
Nº of genomes without MnmA1
With MnmA2
Without MnmA2
Nº of genomes with MnmA2
Nº of genomes without MnmA2
With TusE2
Without TusE2
Nº of genomes with TusE2
Nº of genomes without TusE2
p < 0.00005
With TusE1
Without TusE1
Nº of genomes with TusE1
Nº of genomes without TusE1
With TusE4
Without TusE4
Nº of genomes with TusE4
Nº of genomes without TusE4
p < 0.00005
With TusE3
Without TusE3
Nº of genomes with TusE3
Nº of genomes without TusE3
p < 0.00005

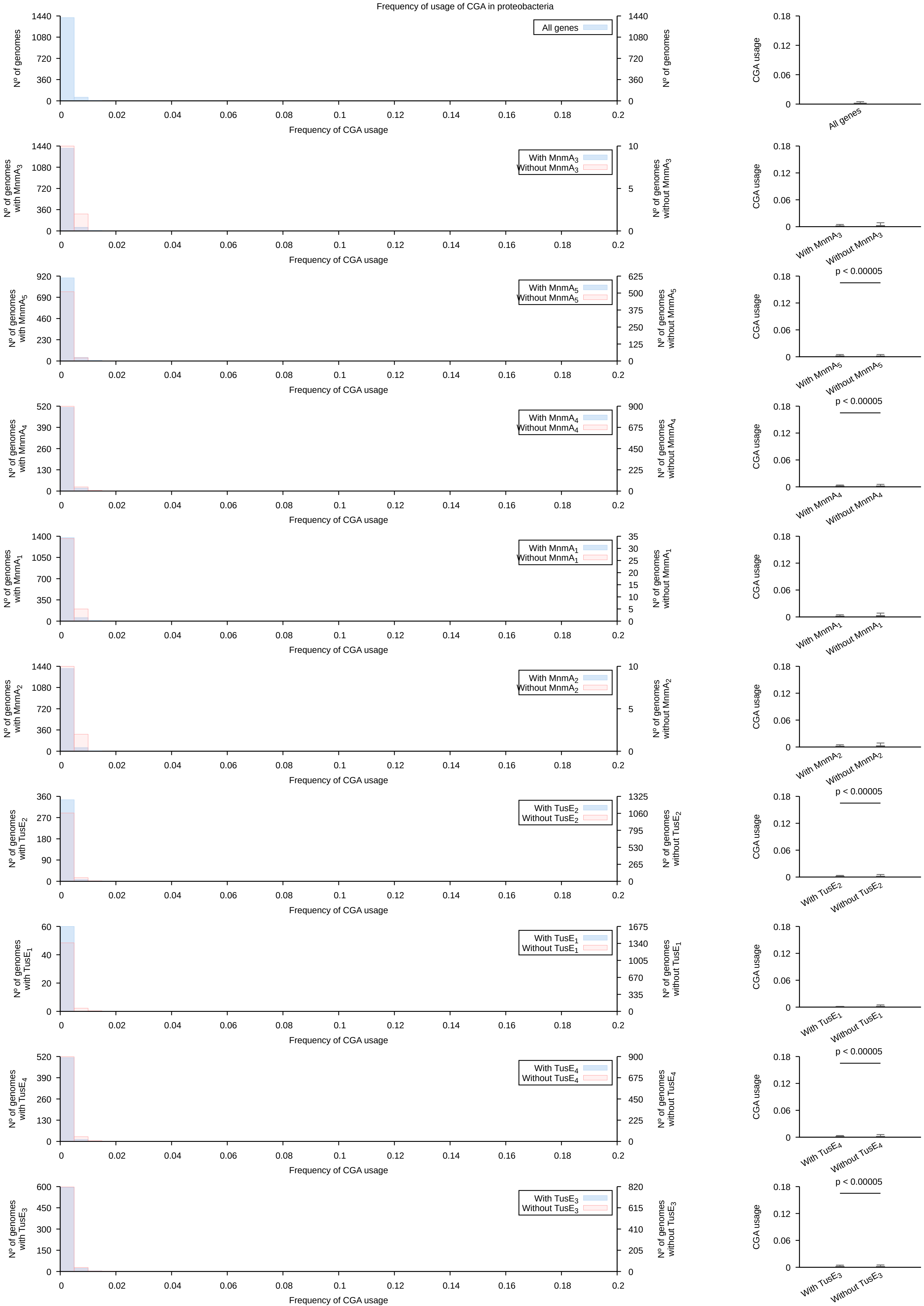
Frequency of usage of CGA in proteobacteria
Nº of genomes
Frequency of CGA usage
CGA usage
All genes
With MnmA$_3$
Without MnmA$_3$
Nº of genomes with MnmA$_3$
Nº of genomes without MnmA$_3$
With MnmA$_5$
Without MnmA$_5$
Nº of genomes with MnmA$_5$
Nº of genomes without MnmA$_5$
p < 0.00005
With MnmA$_4$
Without MnmA$_4$
Nº of genomes with MnmA$_4$
Nº of genomes without MnmA$_4$
p < 0.00005
With MnmA$_1$
Without MnmA$_1$
Nº of genomes with MnmA$_1$
Nº of genomes without MnmA$_1$
With MnmA$_2$
Without MnmA$_2$
Nº of genomes with MnmA$_2$
Nº of genomes without MnmA$_2$
With TusE$_2$
Without TusE$_2$
Nº of genomes with TusE$_2$
Nº of genomes without TusE$_2$
p < 0.00005
With TusE$_1$
Without TusE$_1$
Nº of genomes with TusE$_1$
Nº of genomes without TusE$_1$
With TusE$_4$
Without TusE$_4$
Nº of genomes with TusE$_4$
Nº of genomes without TusE$_4$
p < 0.00005
With TusE$_3$
Without TusE$_3$
Nº of genomes with TusE$_3$
Nº of genomes without TusE$_3$
p < 0.00005

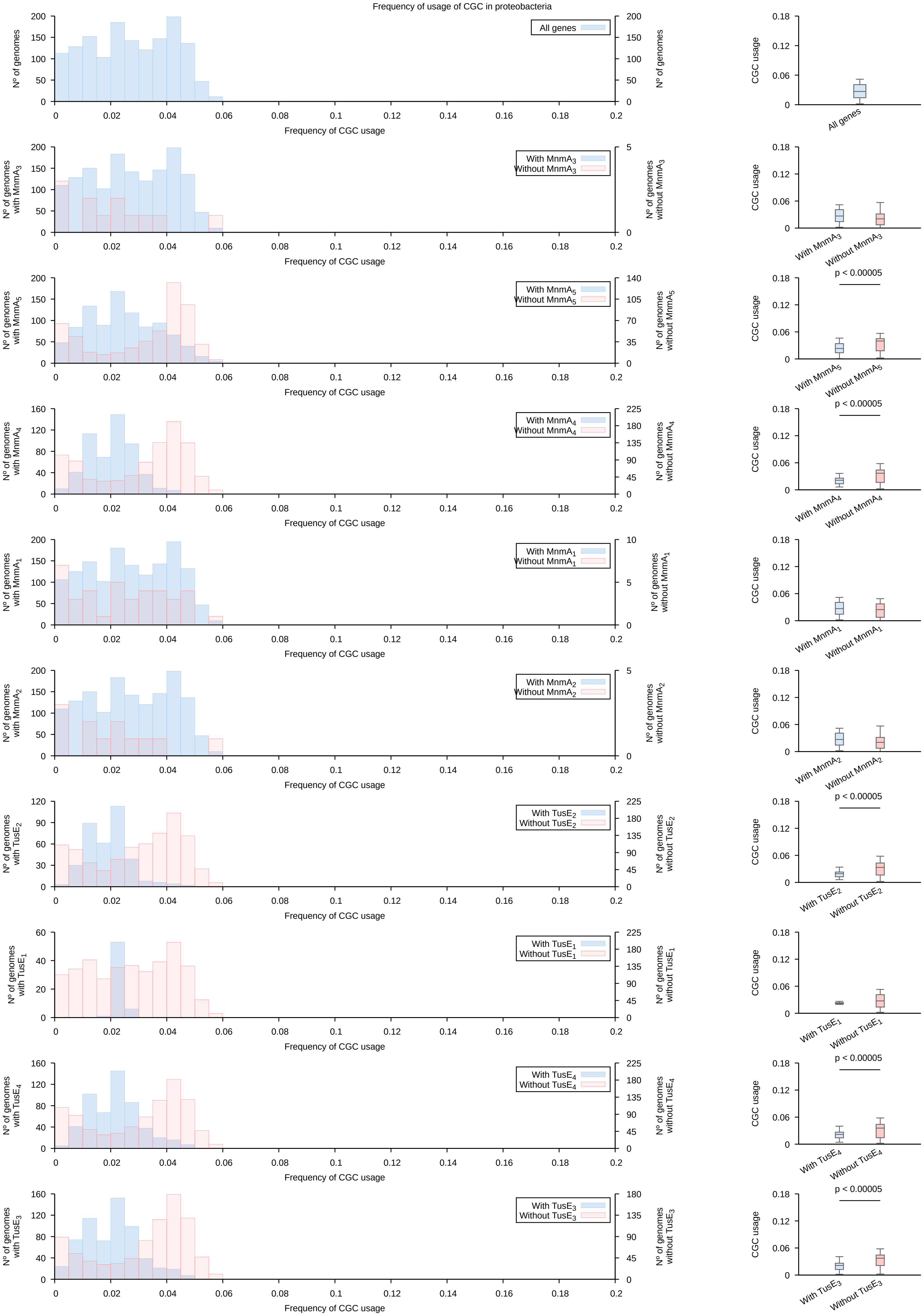
Frequency of usage of CGC in proteobacteria
Nº of genomes
Frequency of CGC usage
CGC usage
All genes
With MnmA3
Without MnmA3
With MnmA5
Without MnmA5
With MnmA4
Without MnmA4
With MnmA1
Without MnmA1
With MnmA2
Without MnmA2
With TusE2
Without TusE2
With TusE1
Without TusE1
With TusE4
Without TusE4
With TusE3
Without TusE3
Nº of genomes with MnmA3
Nº of genomes without MnmA3
p < 0.00005

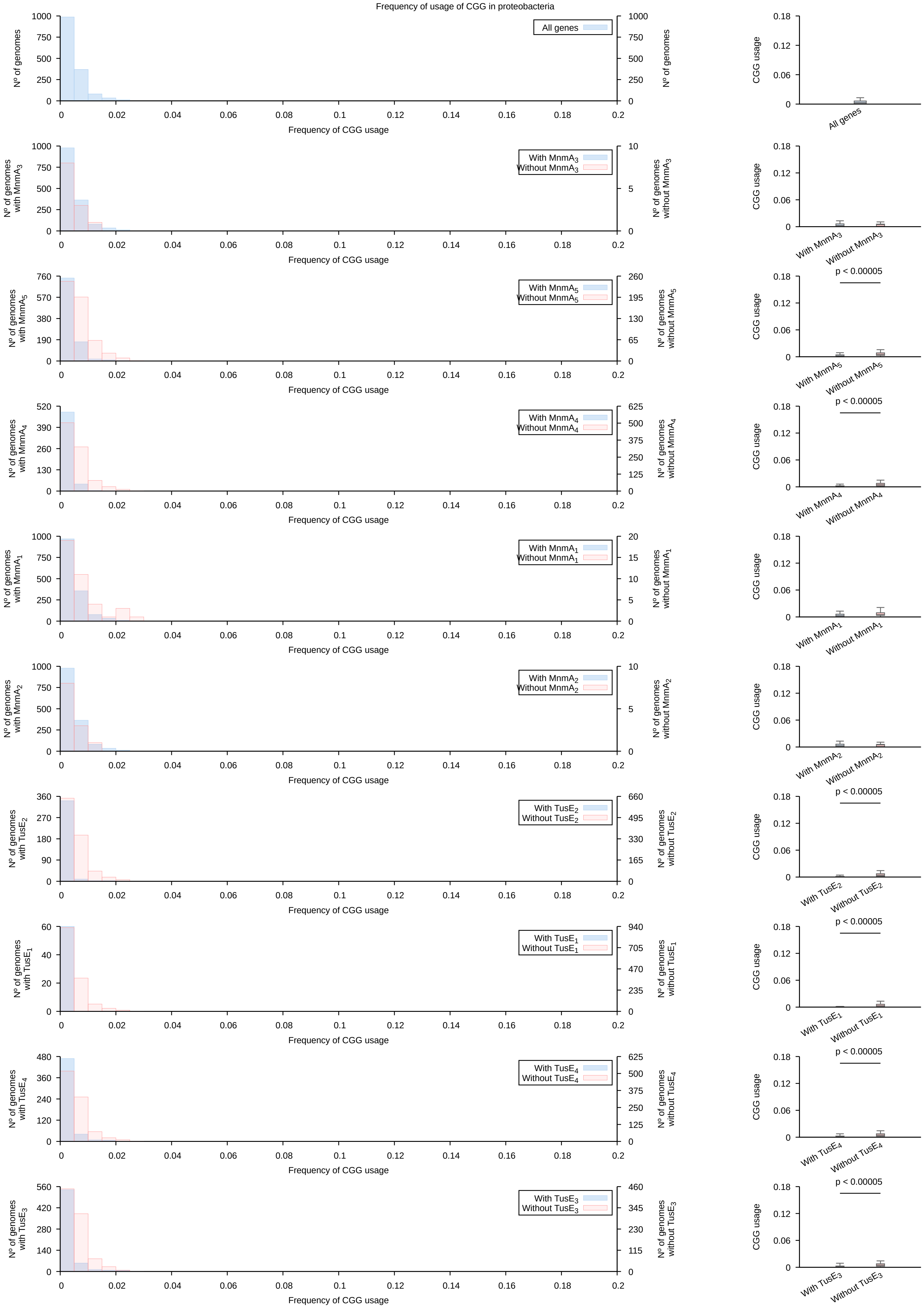

Frequency of usage of CGG in proteobacteria
Nº of genomes
All genes
Frequency of CGG usage
CGG usage
Nº of genomes with MnmA$_3$
Nº of genomes without MnmA$_3$
With MnmA$_3$
Without MnmA$_3$
Nº of genomes with MnmA$_5$
Nº of genomes without MnmA$_5$
With MnmA$_5$
Without MnmA$_5$
p < 0.00005
Nº of genomes with MnmA$_4$
Nº of genomes without MnmA$_4$
With MnmA$_4$
Without MnmA$_4$
p < 0.00005
Nº of genomes with MnmA$_1$
Nº of genomes without MnmA$_1$
With MnmA$_1$
Without MnmA$_1$
Nº of genomes with MnmA$_2$
Nº of genomes without MnmA$_2$
With MnmA$_2$
Without MnmA$_2$
Nº of genomes with TusE$_2$
Nº of genomes without TusE$_2$
With TusE$_2$
Without TusE$_2$
p < 0.00005
Nº of genomes with TusE$_1$
Nº of genomes without TusE$_1$
With TusE$_1$
Without TusE$_1$
p < 0.00005
Nº of genomes with TusE$_4$
Nº of genomes without TusE$_4$
With TusE$_4$
Without TusE$_4$
p < 0.00005
Nº of genomes with TusE$_3$
Nº of genomes without TusE$_3$
With TusE$_3$
Without TusE$_3$
p < 0.00005

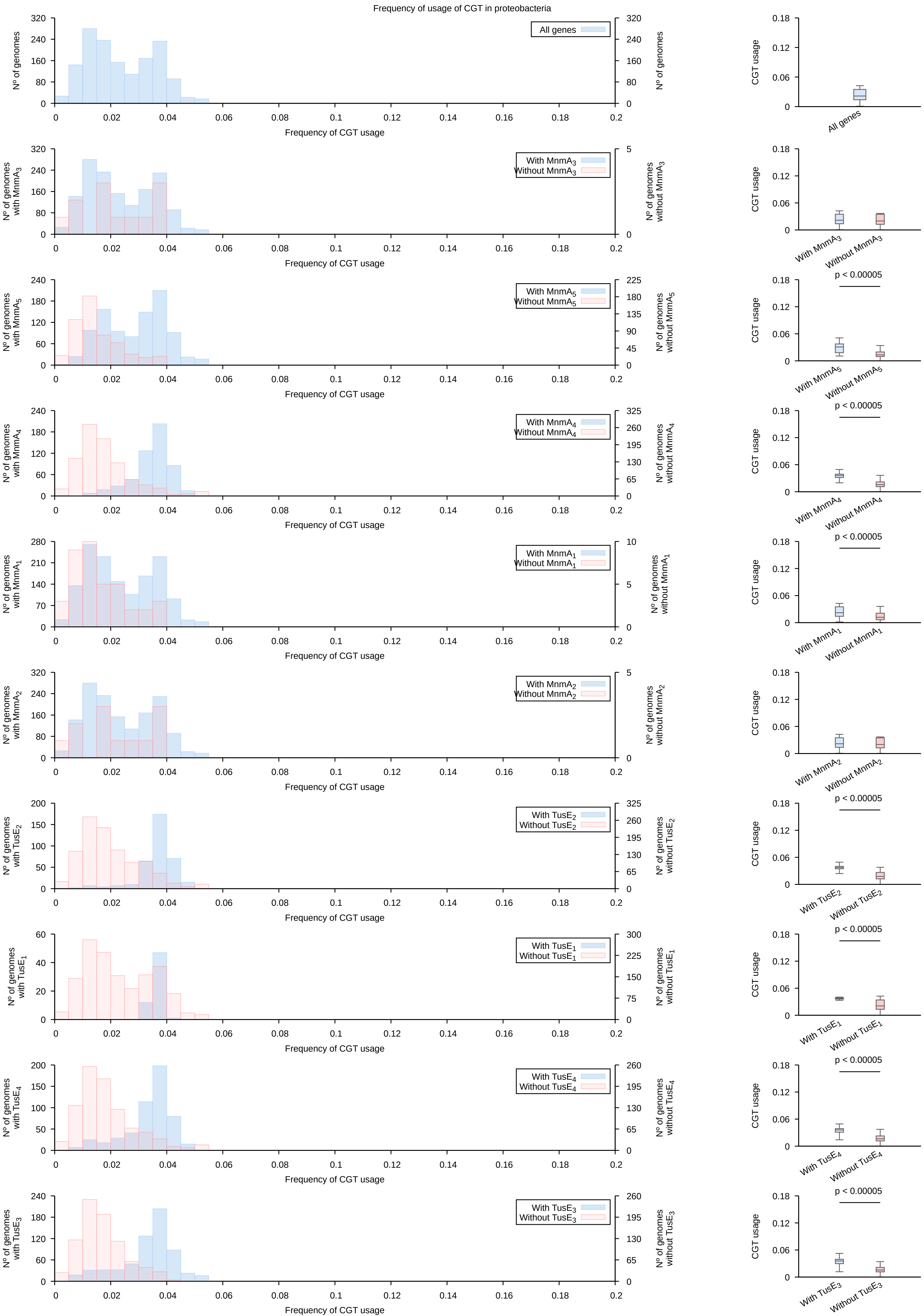
Frequency of usage of CGT in proteobacteria
Nº of genomes
All genes
Frequency of CGT usage
CGT usage
Nº of genomes with MnmA$_3$
With MnmA$_3$
Without MnmA$_3$
Nº of genomes without MnmA$_3$
Nº of genomes with MnmA$_5$
With MnmA$_5$
Without MnmA$_5$
Nº of genomes without MnmA$_5$
p < 0.00005
Nº of genomes with MnmA$_4$
With MnmA$_4$
Without MnmA$_4$
Nº of genomes without MnmA$_4$
p < 0.00005
Nº of genomes with MnmA$_1$
With MnmA$_1$
Without MnmA$_1$
Nº of genomes without MnmA$_1$
p < 0.00005
Nº of genomes with MnmA$_2$
With MnmA$_2$
Without MnmA$_2$
Nº of genomes without MnmA$_2$
Nº of genomes with TusE$_2$
With TusE$_2$
Without TusE$_2$
Nº of genomes without TusE$_2$
p < 0.00005
Nº of genomes with TusE$_1$
With TusE$_1$
Without TusE$_1$
Nº of genomes without TusE$_1$
p < 0.00005
Nº of genomes with TusE$_4$
With TusE$_4$
Without TusE$_4$
Nº of genomes without TusE$_4$
p < 0.00005
Nº of genomes with TusE$_3$
With TusE$_3$
Without TusE$_3$
Nº of genomes without TusE$_3$
p < 0.00005

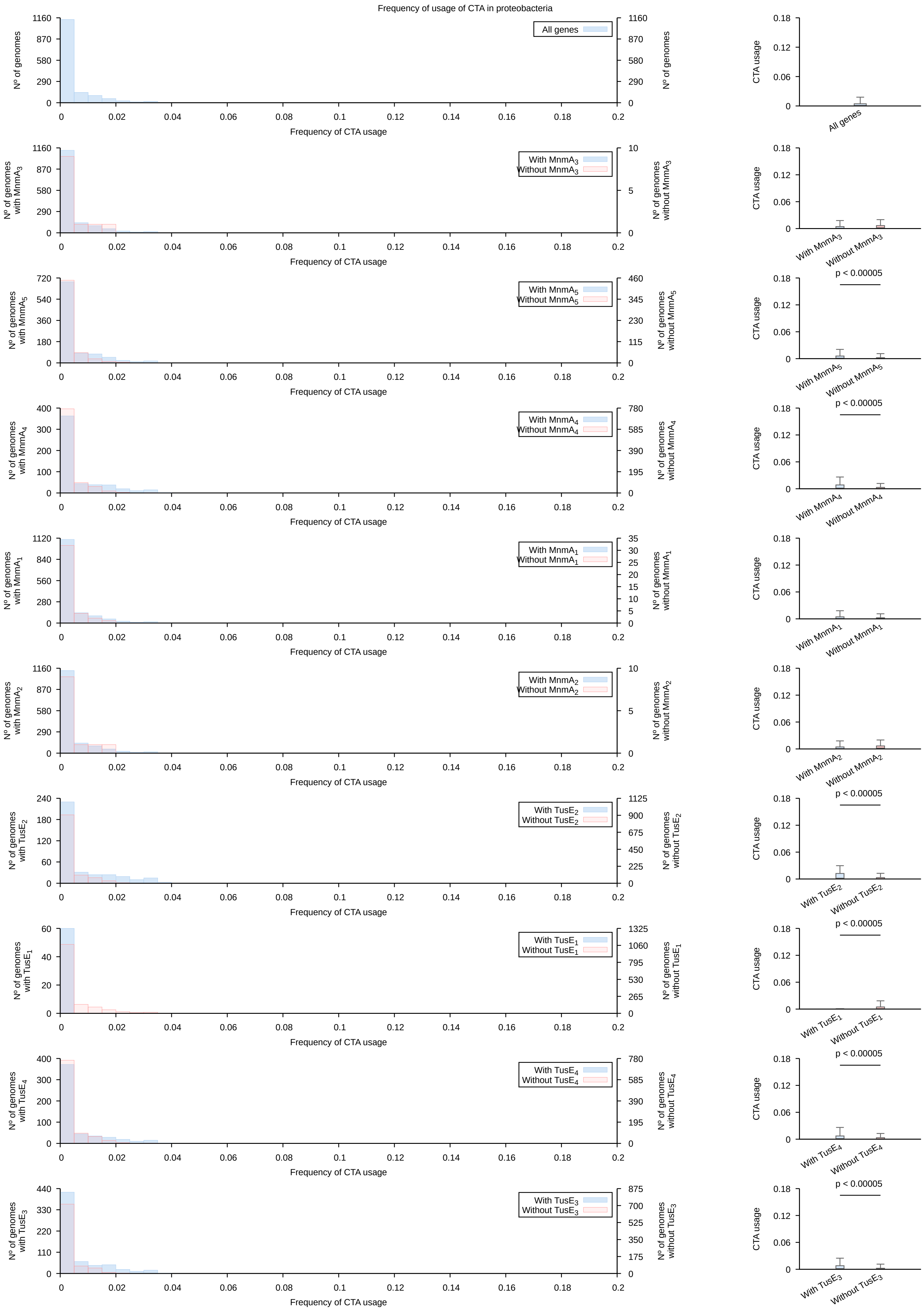

Frequency of usage of CTA in proteobacteria
All genes
Nº of genomes
Frequency of CTA usage
CTA usage
With MnmA$_3$
Without MnmA$_3$
Nº of genomes with MnmA$_3$
Nº of genomes without MnmA$_3$
With MnmA$_5$
Without MnmA$_5$
Nº of genomes with MnmA$_5$
Nº of genomes without MnmA$_5$
p < 0.00005
With MnmA$_4$
Without MnmA$_4$
Nº of genomes with MnmA$_4$
Nº of genomes without MnmA$_4$
p < 0.00005
With MnmA$_1$
Without MnmA$_1$
Nº of genomes with MnmA$_1$
Nº of genomes without MnmA$_1$
With MnmA$_2$
Without MnmA$_2$
Nº of genomes with MnmA$_2$
Nº of genomes without MnmA$_2$
With TusE$_2$
Without TusE$_2$
Nº of genomes with TusE$_2$
Nº of genomes without TusE$_2$
p < 0.00005
With TusE$_1$
Without TusE$_1$
Nº of genomes with TusE$_1$
Nº of genomes without TusE$_1$
p < 0.00005
With TusE$_4$
Without TusE$_4$
Nº of genomes with TusE$_4$
Nº of genomes without TusE$_4$
p < 0.00005
With TusE$_3$
Without TusE$_3$
Nº of genomes with TusE$_3$
Nº of genomes without TusE$_3$
p < 0.00005

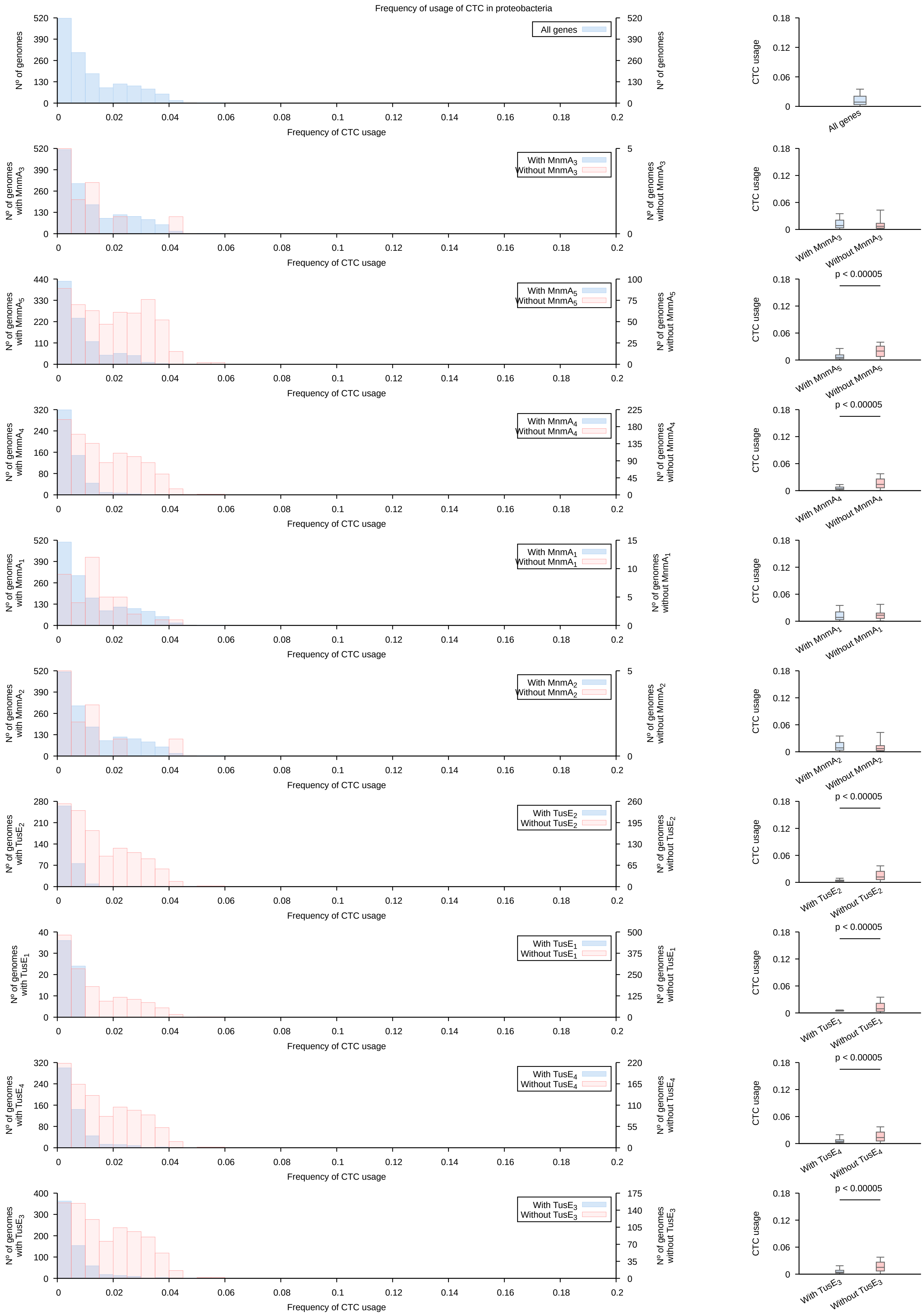
Frequency of usage of CTC in proteobacteria
Nº of genomes
Frequency of CTC usage
CTC usage
All genes
Nº of genomes with MnmA3
Nº of genomes without MnmA3
With MnmA3
Without MnmA3
Nº of genomes with MnmA5
Nº of genomes without MnmA5
With MnmA5
Without MnmA5
p < 0.00005
Nº of genomes with MnmA4
Nº of genomes without MnmA4
With MnmA4
Without MnmA4
p < 0.00005
Nº of genomes with MnmA1
Nº of genomes without MnmA1
With MnmA1
Without MnmA1
Nº of genomes with MnmA2
Nº of genomes without MnmA2
With MnmA2
Without MnmA2
Nº of genomes with TusE2
Nº of genomes without TusE2
With TusE2
Without TusE2
p < 0.00005
Nº of genomes with TusE1
Nº of genomes without TusE1
With TusE1
Without TusE1
p < 0.00005
Nº of genomes with TusE4
Nº of genomes without TusE4
With TusE4
Without TusE4
p < 0.00005
Nº of genomes with TusE3
Nº of genomes without TusE3
With TusE3
Without TusE3
p < 0.00005
0
0.02
0.04
0.06
0.08
0.1
0.12
0.14
0.16
0.18
0.2

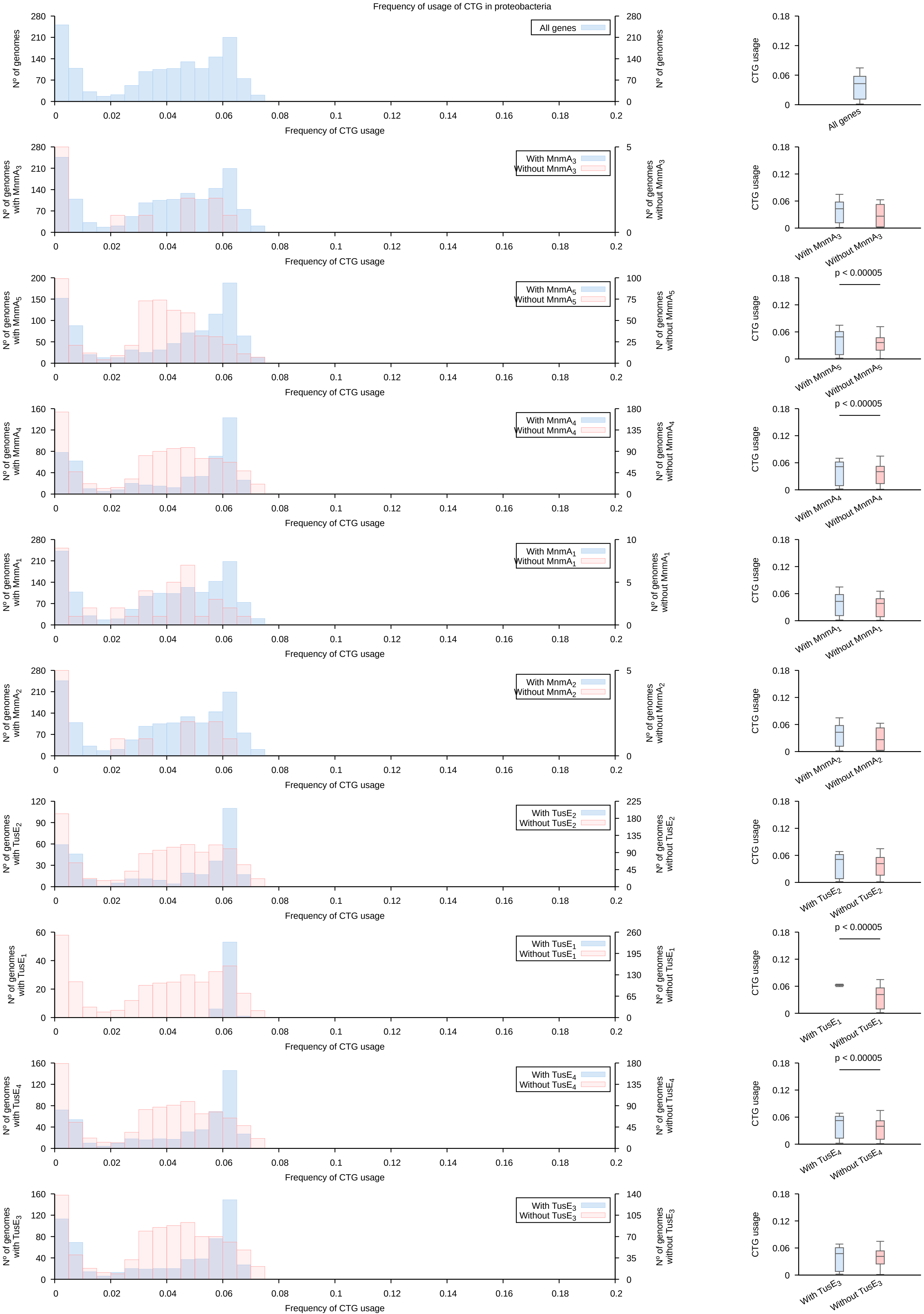

Frequency of usage of CTG in proteobacteria
All genes
Nº of genomes
Frequency of CTG usage
CTG usage
With $MnmA_3$
Without $MnmA_3$
Nº of genomes with $MnmA_3$
Nº of genomes without $MnmA_3$
With $MnmA_5$
Without $MnmA_5$
Nº of genomes with $MnmA_5$
Nº of genomes without $MnmA_5$
p < 0.00005
With $MnmA_4$
Without $MnmA_4$
Nº of genomes with $MnmA_4$
Nº of genomes without $MnmA_4$
p < 0.00005
With $MnmA_1$
Without $MnmA_1$
Nº of genomes with $MnmA_1$
Nº of genomes without $MnmA_1$
With $MnmA_2$
Without $MnmA_2$
Nº of genomes with $MnmA_2$
Nº of genomes without $MnmA_2$
With $TusE_2$
Without $TusE_2$
Nº of genomes with $TusE_2$
Nº of genomes without $TusE_2$
With $TusE_1$
Without $TusE_1$
Nº of genomes with $TusE_1$
Nº of genomes without $TusE_1$
p < 0.00005
With $TusE_4$
Without $TusE_4$
Nº of genomes with $TusE_4$
Nº of genomes without $TusE_4$
p < 0.00005
With $TusE_3$
Without $TusE_3$
Nº of genomes with $TusE_3$
Nº of genomes without $TusE_3$
0 0.02 0.04 0.06 0.08 0.1 0.12 0.14 0.16 0.18 0.2
0 0.06 0.12 0.18

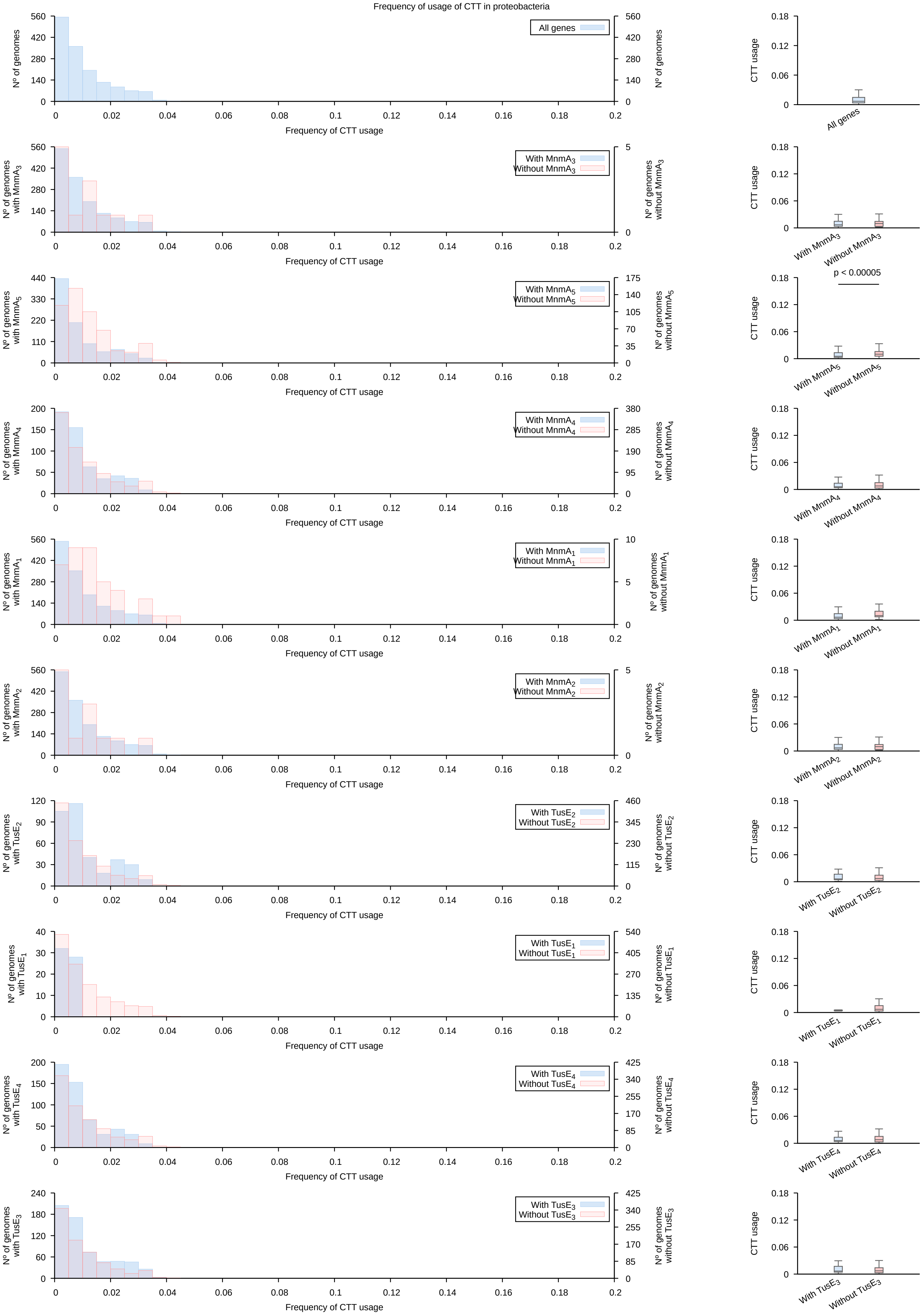

Frequency of usage of CTT in proteobacteria
Nº of genomes
All genes
Frequency of CTT usage
CTT usage
Nº of genomes with MnmA3
Nº of genomes without MnmA3
With MnmA3
Without MnmA3
Nº of genomes with MnmA5
Nº of genomes without MnmA5
With MnmA5
Without MnmA5
p < 0.00005
Nº of genomes with MnmA4
Nº of genomes without MnmA4
With MnmA4
Without MnmA4
Nº of genomes with MnmA1
Nº of genomes without MnmA1
With MnmA1
Without MnmA1
Nº of genomes with MnmA2
Nº of genomes without MnmA2
With MnmA2
Without MnmA2
Nº of genomes with TusE2
Nº of genomes without TusE2
With TusE2
Without TusE2
Nº of genomes with TusE1
Nº of genomes without TusE1
With TusE1
Without TusE1
Nº of genomes with TusE4
Nº of genomes without TusE4
With TusE4
Without TusE4
Nº of genomes with TusE3
Nº of genomes without TusE3
With TusE3
Without TusE3

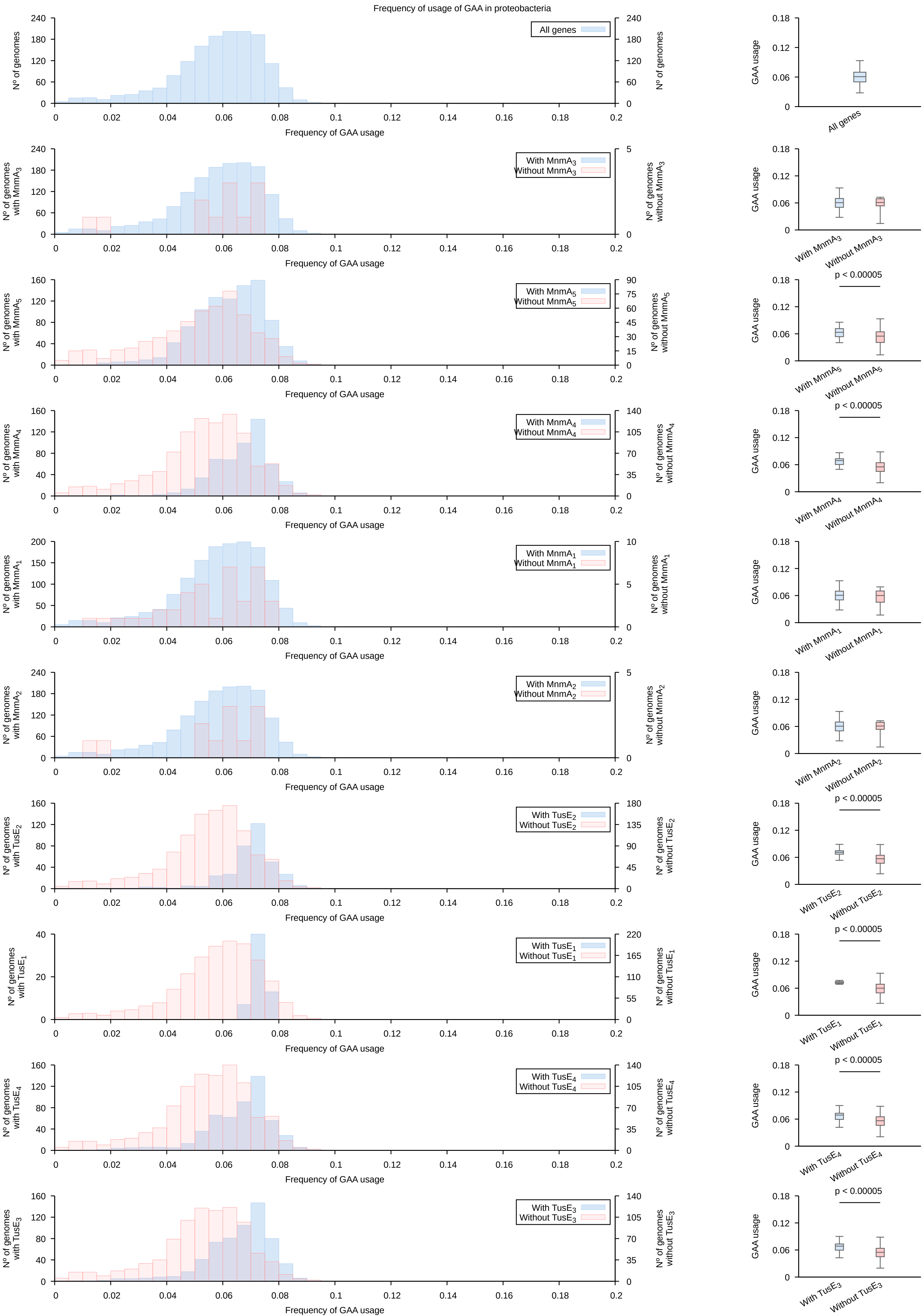
Frequency of usage of GAA in proteobacteria
All genes
Nº of genomes
Frequency of GAA usage
GAA usage
With MnmA$_3$
Without MnmA$_3$
Nº of genomes with MnmA$_3$
Nº of genomes without MnmA$_3$
With MnmA$_5$
Without MnmA$_5$
Nº of genomes with MnmA$_5$
Nº of genomes without MnmA$_5$
p < 0.00005
With MnmA$_4$
Without MnmA$_4$
Nº of genomes with MnmA$_4$
Nº of genomes without MnmA$_4$
With MnmA$_1$
Without MnmA$_1$
Nº of genomes with MnmA$_1$
Nº of genomes without MnmA$_1$
With MnmA$_2$
Without MnmA$_2$
Nº of genomes with MnmA$_2$
Nº of genomes without MnmA$_2$
With TusE$_2$
Without TusE$_2$
Nº of genomes with TusE$_2$
Nº of genomes without TusE$_2$
With TusE$_1$
Without TusE$_1$
Nº of genomes with TusE$_1$
Nº of genomes without TusE$_1$
With TusE$_4$
Without TusE$_4$
Nº of genomes with TusE$_4$
Nº of genomes without TusE$_4$
With TusE$_3$
Without TusE$_3$
Nº of genomes with TusE$_3$
Nº of genomes without TusE$_3$

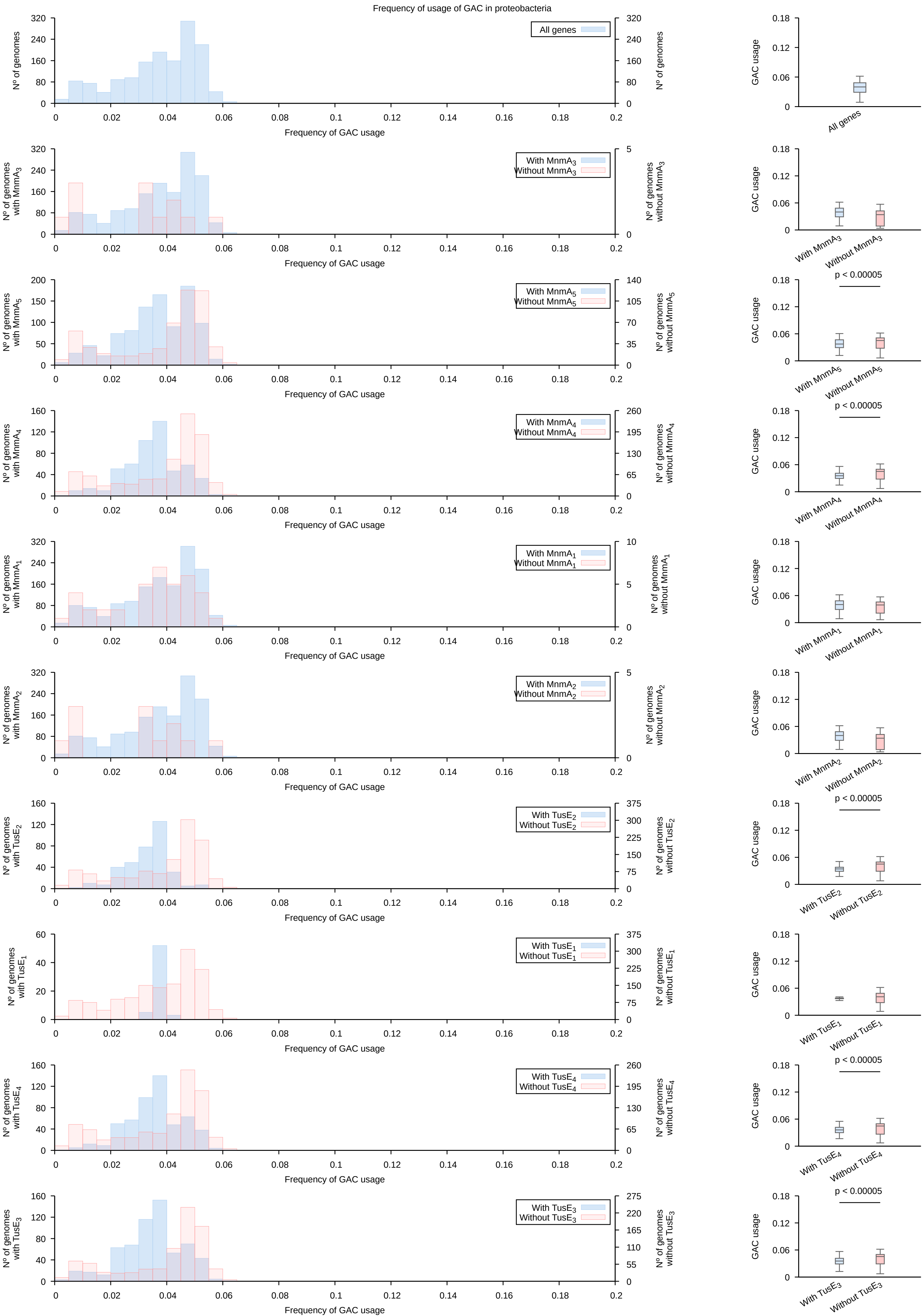

Frequency of usage of GAC in proteobacteria
Nº of genomes
All genes
Frequency of GAC usage
GAC usage
All genes
Nº of genomes with MnmA$_3$
Nº of genomes without MnmA$_3$
With MnmA$_3$
Without MnmA$_3$
Nº of genomes with MnmA$_5$
Nº of genomes without MnmA$_5$
With MnmA$_5$
Without MnmA$_5$
p < 0.00005
Nº of genomes with MnmA$_4$
Nº of genomes without MnmA$_4$
With MnmA$_4$
Without MnmA$_4$
p < 0.00005
Nº of genomes with MnmA$_1$
Nº of genomes without MnmA$_1$
With MnmA$_1$
Without MnmA$_1$
Nº of genomes with MnmA$_2$
Nº of genomes without MnmA$_2$
With MnmA$_2$
Without MnmA$_2$
Nº of genomes with TusE$_2$
Nº of genomes without TusE$_2$
With TusE$_2$
Without TusE$_2$
p < 0.00005
Nº of genomes with TusE$_1$
Nº of genomes without TusE$_1$
With TusE$_1$
Without TusE$_1$
Nº of genomes with TusE$_4$
Nº of genomes without TusE$_4$
With TusE$_4$
Without TusE$_4$
p < 0.00005
Nº of genomes with TusE$_3$
Nº of genomes without TusE$_3$
With TusE$_3$
Without TusE$_3$
p < 0.00005

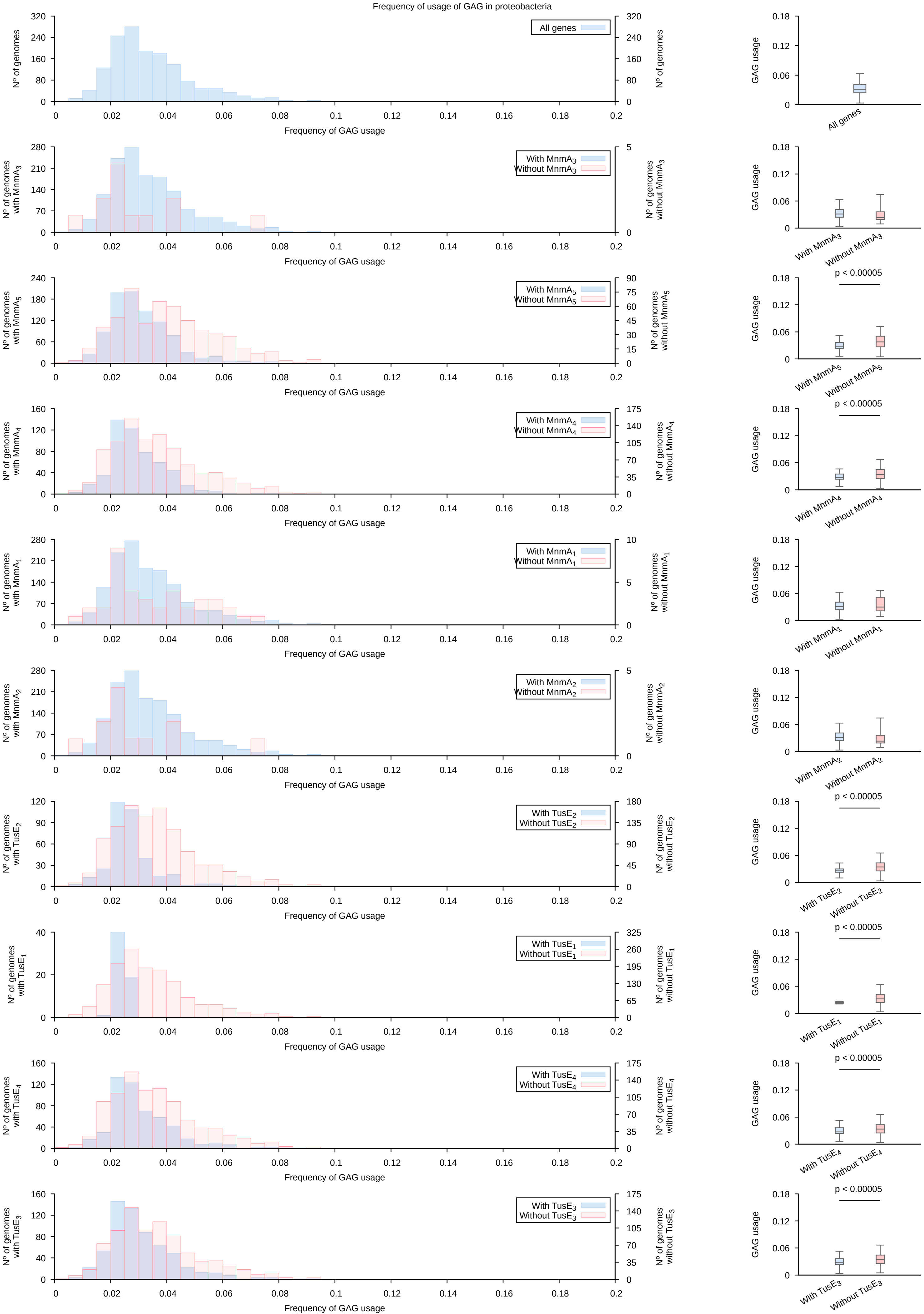
Frequency of usage of GAG in proteobacteria
All genes
With MnmA3
Without MnmA3
With MnmA5
Without MnmA5
With MnmA4
Without MnmA4
With MnmA1
Without MnmA1
With MnmA2
Without MnmA2
With TusE2
Without TusE2
With TusE1
Without TusE1
With TusE4
Without TusE4
With TusE3
Without TusE3
Nº of genomes
Frequency of GAG usage
GAG usage
p < 0.00005

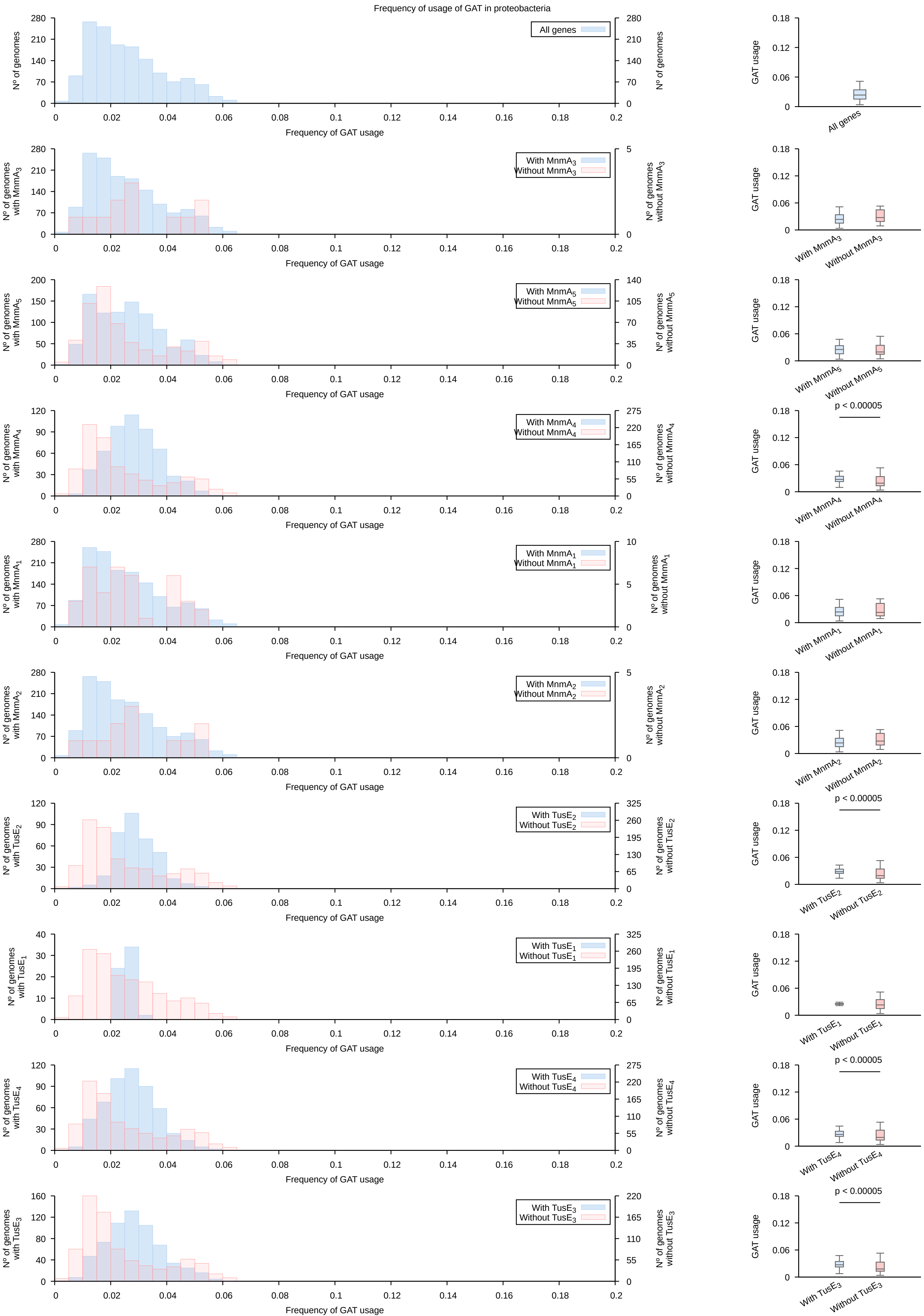
Frequency of usage of GAT in proteobacteria
Nº of genomes
All genes
Frequency of GAT usage
GAT usage
With MnmA$_3$
Without MnmA$_3$
Nº of genomes with MnmA$_3$
Nº of genomes without MnmA$_3$
With MnmA$_5$
Without MnmA$_5$
Nº of genomes with MnmA$_5$
Nº of genomes without MnmA$_5$
p < 0.00005
With MnmA$_4$
Without MnmA$_4$
Nº of genomes with MnmA$_4$
Nº of genomes without MnmA$_4$
With MnmA$_1$
Without MnmA$_1$
Nº of genomes with MnmA$_1$
Nº of genomes without MnmA$_1$
With MnmA$_2$
Without MnmA$_2$
Nº of genomes with MnmA$_2$
Nº of genomes without MnmA$_2$
p < 0.00005
With TusE$_2$
Without TusE$_2$
Nº of genomes with TusE$_2$
Nº of genomes without TusE$_2$
With TusE$_1$
Without TusE$_1$
Nº of genomes with TusE$_1$
Nº of genomes without TusE$_1$
p < 0.00005
With TusE$_4$
Without TusE$_4$
Nº of genomes with TusE$_4$
Nº of genomes without TusE$_4$
p < 0.00005
With TusE$_3$
Without TusE$_3$
Nº of genomes with TusE$_3$
Nº of genomes without TusE$_3$

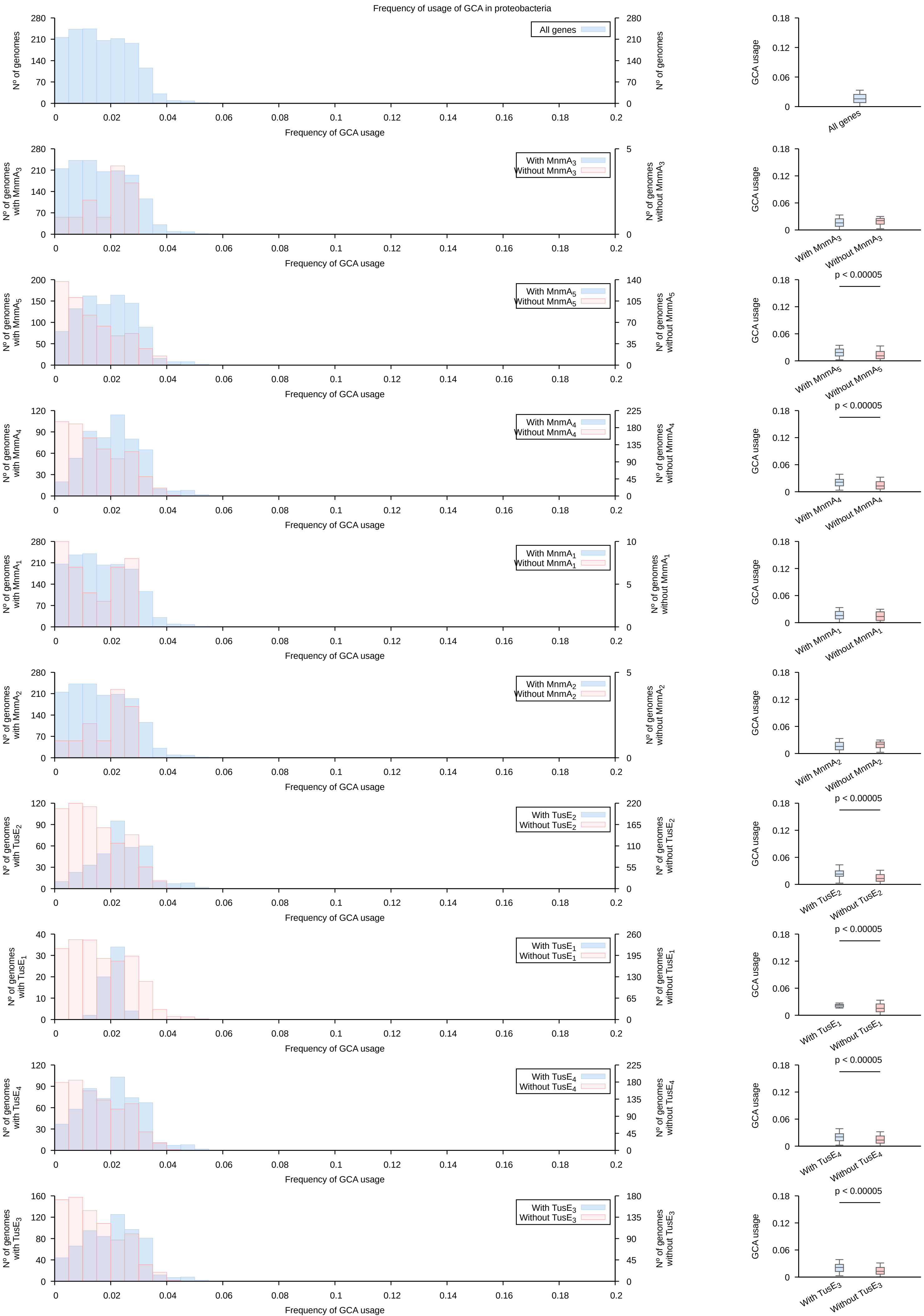

Frequency of usage of GCA in proteobacteria
Nº of genomes
All genes
Frequency of GCA usage
GCA usage
Nº of genomes with MnmA$_3$
Nº of genomes without MnmA$_3$
With MnmA$_3$
Without MnmA$_3$
Nº of genomes with MnmA$_5$
Nº of genomes without MnmA$_5$
With MnmA$_5$
Without MnmA$_5$
p < 0.00005
Nº of genomes with MnmA$_4$
Nº of genomes without MnmA$_4$
With MnmA$_4$
Without MnmA$_4$
p < 0.00005
Nº of genomes with MnmA$_1$
Nº of genomes without MnmA$_1$
With MnmA$_1$
Without MnmA$_1$
Nº of genomes with MnmA$_2$
Nº of genomes without MnmA$_2$
With MnmA$_2$
Without MnmA$_2$
Nº of genomes with TusE$_2$
Nº of genomes without TusE$_2$
With TusE$_2$
Without TusE$_2$
p < 0.00005
Nº of genomes with TusE$_1$
Nº of genomes without TusE$_1$
With TusE$_1$
Without TusE$_1$
p < 0.00005
Nº of genomes with TusE$_4$
Nº of genomes without TusE$_4$
With TusE$_4$
Without TusE$_4$
p < 0.00005
Nº of genomes with TusE$_3$
Nº of genomes without TusE$_3$
With TusE$_3$
Without TusE$_3$
p < 0.00005

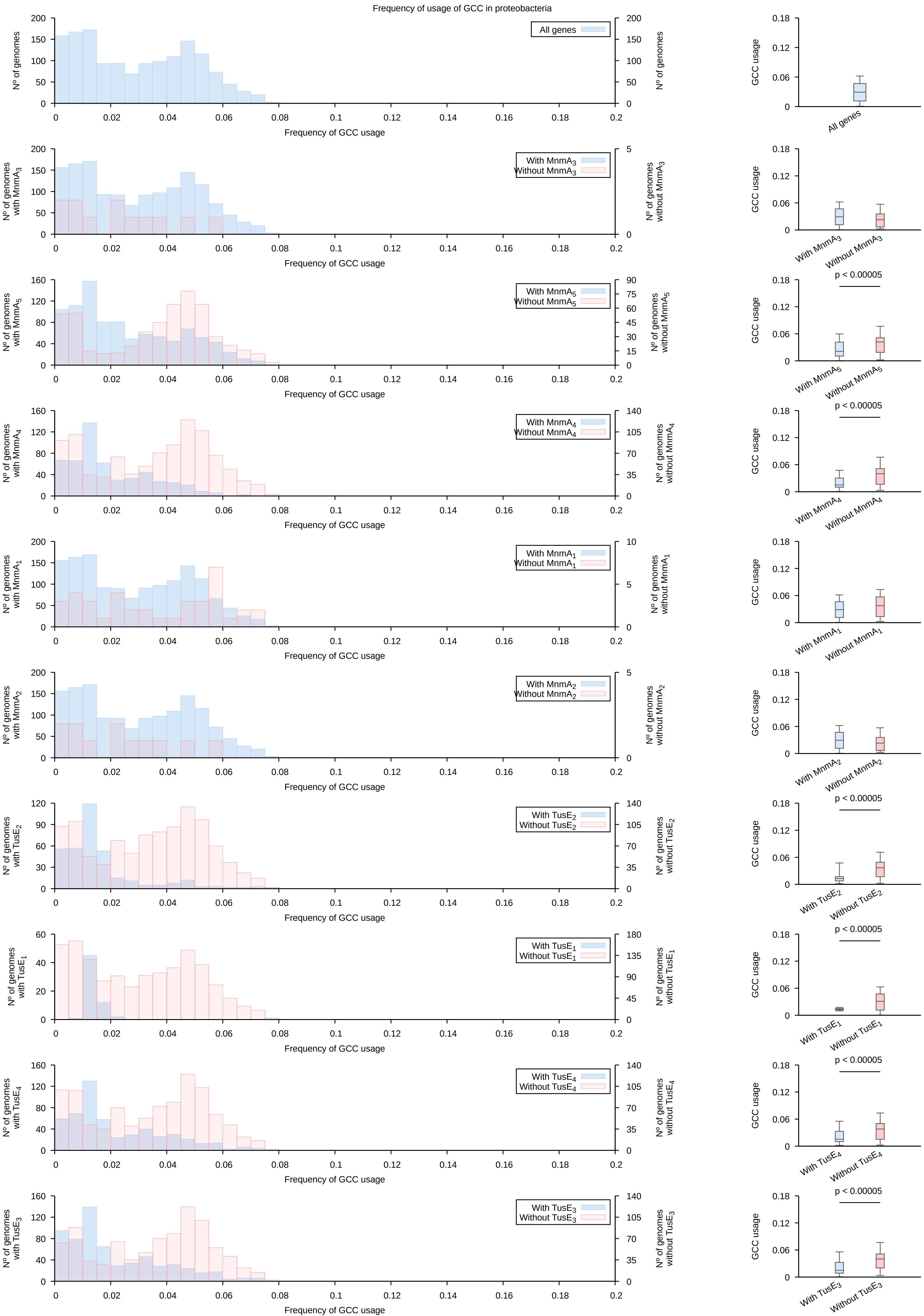

Frequency of usage of GCC in proteobacteria
All genes
Nº of genomes
Frequency of GCC usage
GCC usage
With MnmA$_3$
Without MnmA$_3$
Nº of genomes with MnmA$_3$
Nº of genomes without MnmA$_3$
With MnmA$_5$
Without MnmA$_5$
Nº of genomes with MnmA$_5$
Nº of genomes without MnmA$_5$
p < 0.00005
With MnmA$_4$
Without MnmA$_4$
Nº of genomes with MnmA$_4$
Nº of genomes without MnmA$_4$
p < 0.00005
With MnmA$_1$
Without MnmA$_1$
Nº of genomes with MnmA$_1$
Nº of genomes without MnmA$_1$
With MnmA$_2$
Without MnmA$_2$
Nº of genomes with MnmA$_2$
Nº of genomes without MnmA$_2$
With TusE$_2$
Without TusE$_2$
Nº of genomes with TusE$_2$
Nº of genomes without TusE$_2$
p < 0.00005
With TusE$_1$
Without TusE$_1$
Nº of genomes with TusE$_1$
Nº of genomes without TusE$_1$
p < 0.00005
With TusE$_4$
Without TusE$_4$
Nº of genomes with TusE$_4$
Nº of genomes without TusE$_4$
p < 0.00005
With TusE$_3$
Without TusE$_3$
Nº of genomes with TusE$_3$
Nº of genomes without TusE$_3$
p < 0.00005

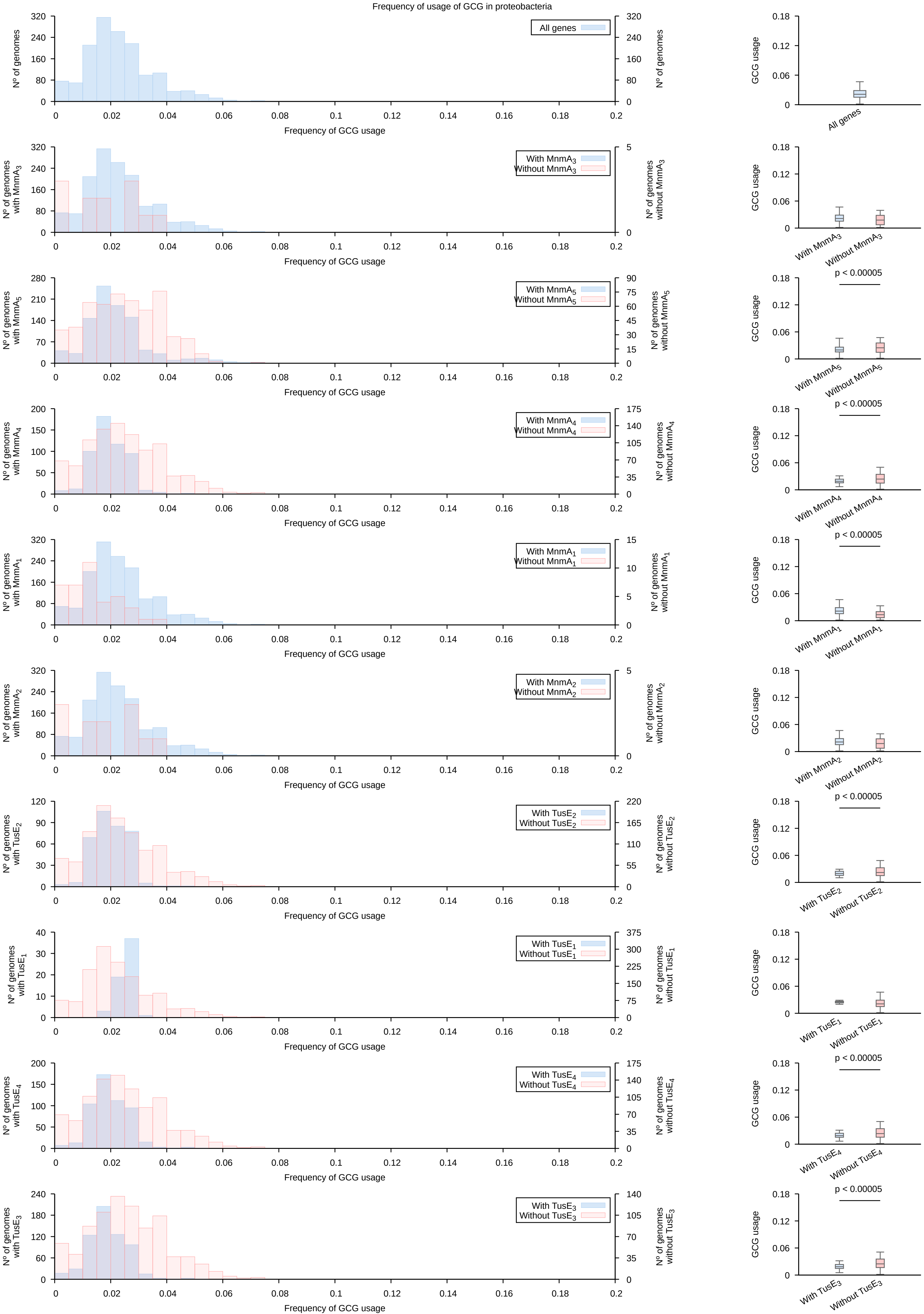

Frequency of usage of GCG in proteobacteria
Nº of genomes
All genes
Frequency of GCG usage
GCG usage
Nº of genomes with MnmA3
Nº of genomes without MnmA3
With MnmA3
Without MnmA3
Nº of genomes with MnmA5
Nº of genomes without MnmA5
With MnmA5
Without MnmA5
p < 0.00005
Nº of genomes with MnmA4
Nº of genomes without MnmA4
With MnmA4
Without MnmA4
p < 0.00005
Nº of genomes with MnmA1
Nº of genomes without MnmA1
With MnmA1
Without MnmA1
p < 0.00005
Nº of genomes with MnmA2
Nº of genomes without MnmA2
With MnmA2
Without MnmA2
Nº of genomes with TusE2
Nº of genomes without TusE2
With TusE2
Without TusE2
p < 0.00005
Nº of genomes with TusE1
Nº of genomes without TusE1
With TusE1
Without TusE1
Nº of genomes with TusE4
Nº of genomes without TusE4
With TusE4
Without TusE4
p < 0.00005
Nº of genomes with TusE3
Nº of genomes without TusE3
With TusE3
Without TusE3
p < 0.00005

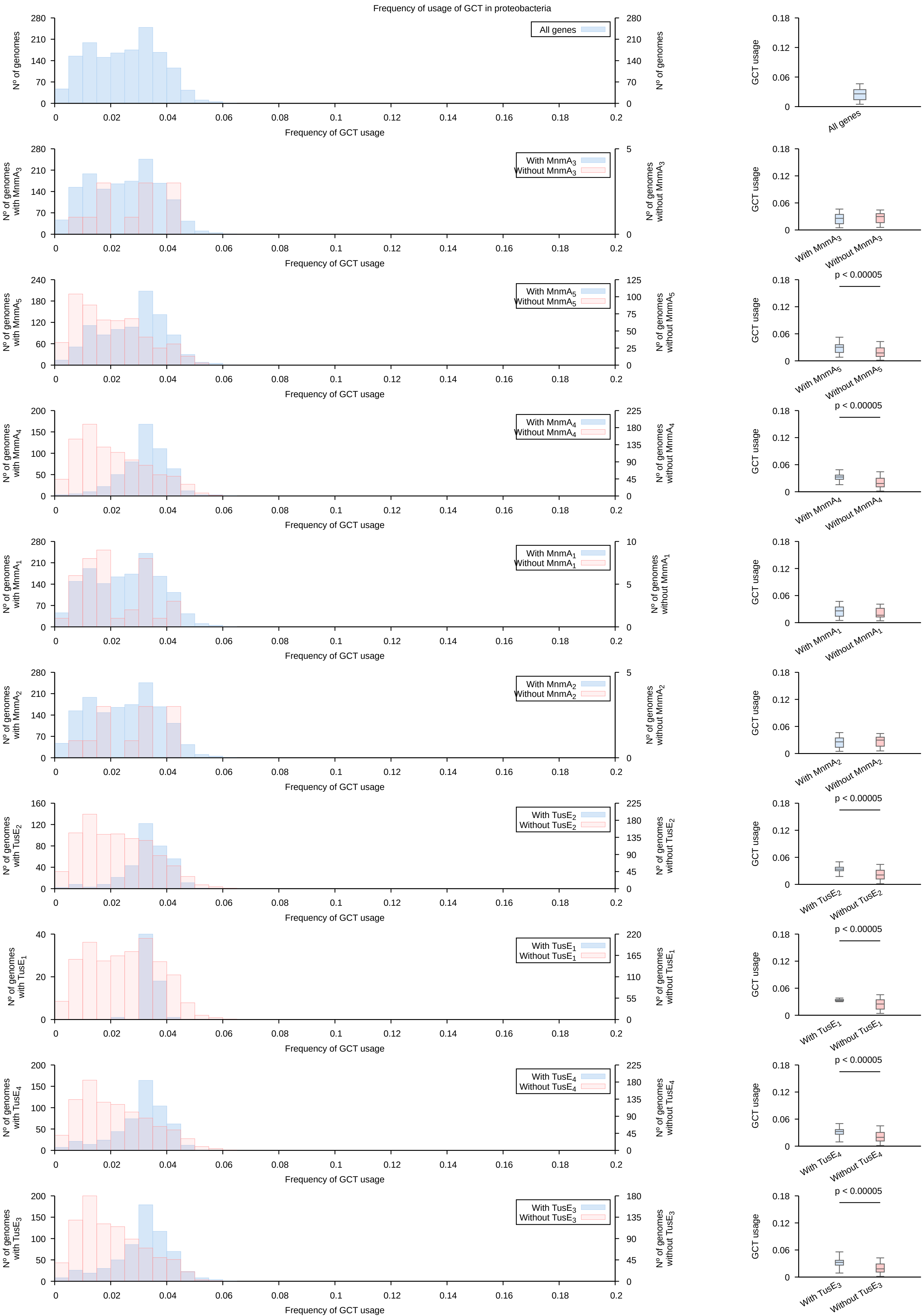
Frequency of usage of GCT in proteobacteria
All genes
Nº of genomes
Frequency of GCT usage
GCT usage
With MnmA3
Without MnmA3
Nº of genomes with MnmA3
Nº of genomes without MnmA3
With MnmA5
Without MnmA5
Nº of genomes with MnmA5
Nº of genomes without MnmA5
p < 0.00005
With MnmA4
Without MnmA4
Nº of genomes with MnmA4
Nº of genomes without MnmA4
p < 0.00005
With MnmA1
Without MnmA1
Nº of genomes with MnmA1
Nº of genomes without MnmA1
With MnmA2
Without MnmA2
Nº of genomes with MnmA2
Nº of genomes without MnmA2
With TusE2
Without TusE2
Nº of genomes with TusE2
Nº of genomes without TusE2
p < 0.00005
With TusE1
Without TusE1
Nº of genomes with TusE1
Nº of genomes without TusE1
p < 0.00005
With TusE4
Without TusE4
Nº of genomes with TusE4
Nº of genomes without TusE4
p < 0.00005
With TusE3
Without TusE3
Nº of genomes with TusE3
Nº of genomes without TusE3
p < 0.00005

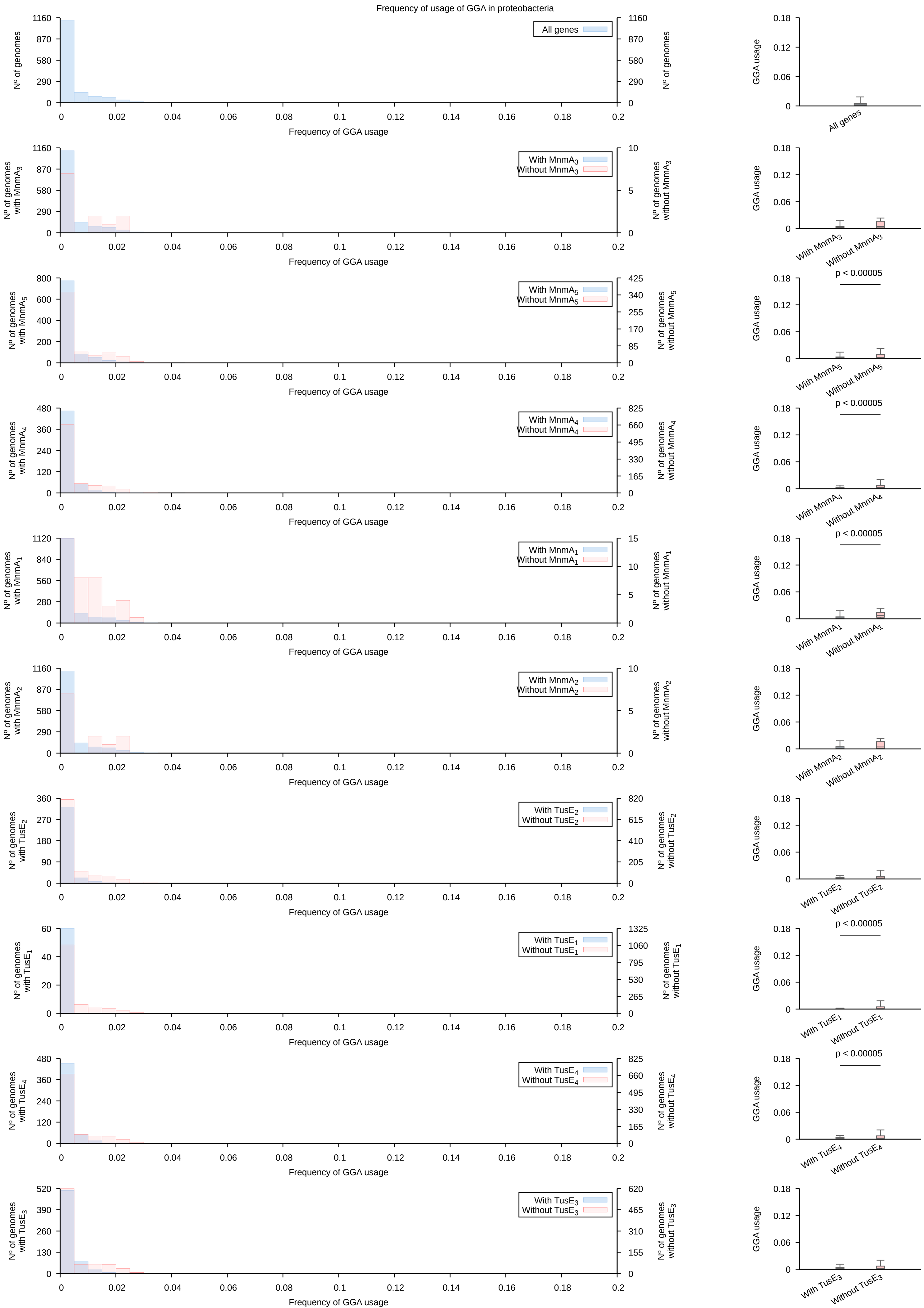

Frequency of usage of GGA in proteobacteria
Nº of genomes
All genes
Frequency of GGA usage
GGA usage
All genes
Nº of genomes with MnmA3
Nº of genomes without MnmA3
With MnmA3
Without MnmA3
Nº of genomes with MnmA5
Nº of genomes without MnmA5
With MnmA5
Without MnmA5
p < 0.00005
Nº of genomes with MnmA4
Nº of genomes without MnmA4
With MnmA4
Without MnmA4
p < 0.00005
Nº of genomes with MnmA1
Nº of genomes without MnmA1
With MnmA1
Without MnmA1
p < 0.00005
Nº of genomes with MnmA2
Nº of genomes without MnmA2
With MnmA2
Without MnmA2
Nº of genomes with TusE2
Nº of genomes without TusE2
With TusE2
Without TusE2
Nº of genomes with TusE1
Nº of genomes without TusE1
With TusE1
Without TusE1
p < 0.00005
Nº of genomes with TusE4
Nº of genomes without TusE4
With TusE4
Without TusE4
p < 0.00005
Nº of genomes with TusE3
Nº of genomes without TusE3
With TusE3
Without TusE3

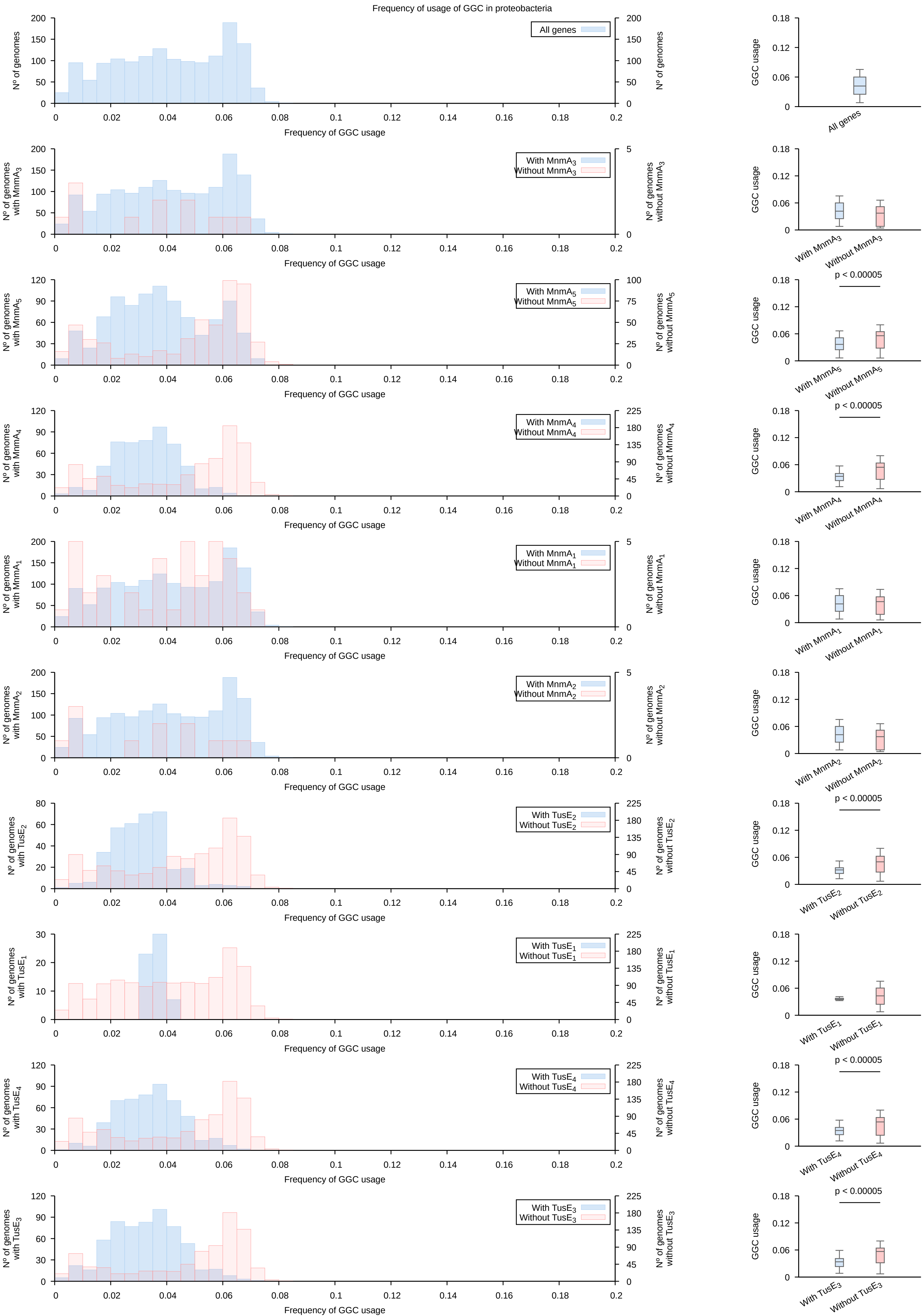
Frequency of usage of GGC in proteobacteria
Nº of genomes
All genes
Frequency of GGC usage
GGC usage
All genes
Nº of genomes with MnmA$_3$
Nº of genomes without MnmA$_3$
With MnmA$_3$
Without MnmA$_3$
Nº of genomes with MnmA$_5$
Nº of genomes without MnmA$_5$
With MnmA$_5$
Without MnmA$_5$
p < 0.00005
Nº of genomes with MnmA$_4$
Nº of genomes without MnmA$_4$
With MnmA$_4$
Without MnmA$_4$
p < 0.00005
Nº of genomes with MnmA$_1$
Nº of genomes without MnmA$_1$
With MnmA$_1$
Without MnmA$_1$
Nº of genomes with MnmA$_2$
Nº of genomes without MnmA$_2$
With MnmA$_2$
Without MnmA$_2$
Nº of genomes with TusE$_2$
Nº of genomes without TusE$_2$
With TusE$_2$
Without TusE$_2$
p < 0.00005
Nº of genomes with TusE$_1$
Nº of genomes without TusE$_1$
With TusE$_1$
Without TusE$_1$
Nº of genomes with TusE$_4$
Nº of genomes without TusE$_4$
With TusE$_4$
Without TusE$_4$
p < 0.00005
Nº of genomes with TusE$_3$
Nº of genomes without TusE$_3$
With TusE$_3$
Without TusE$_3$
p < 0.00005

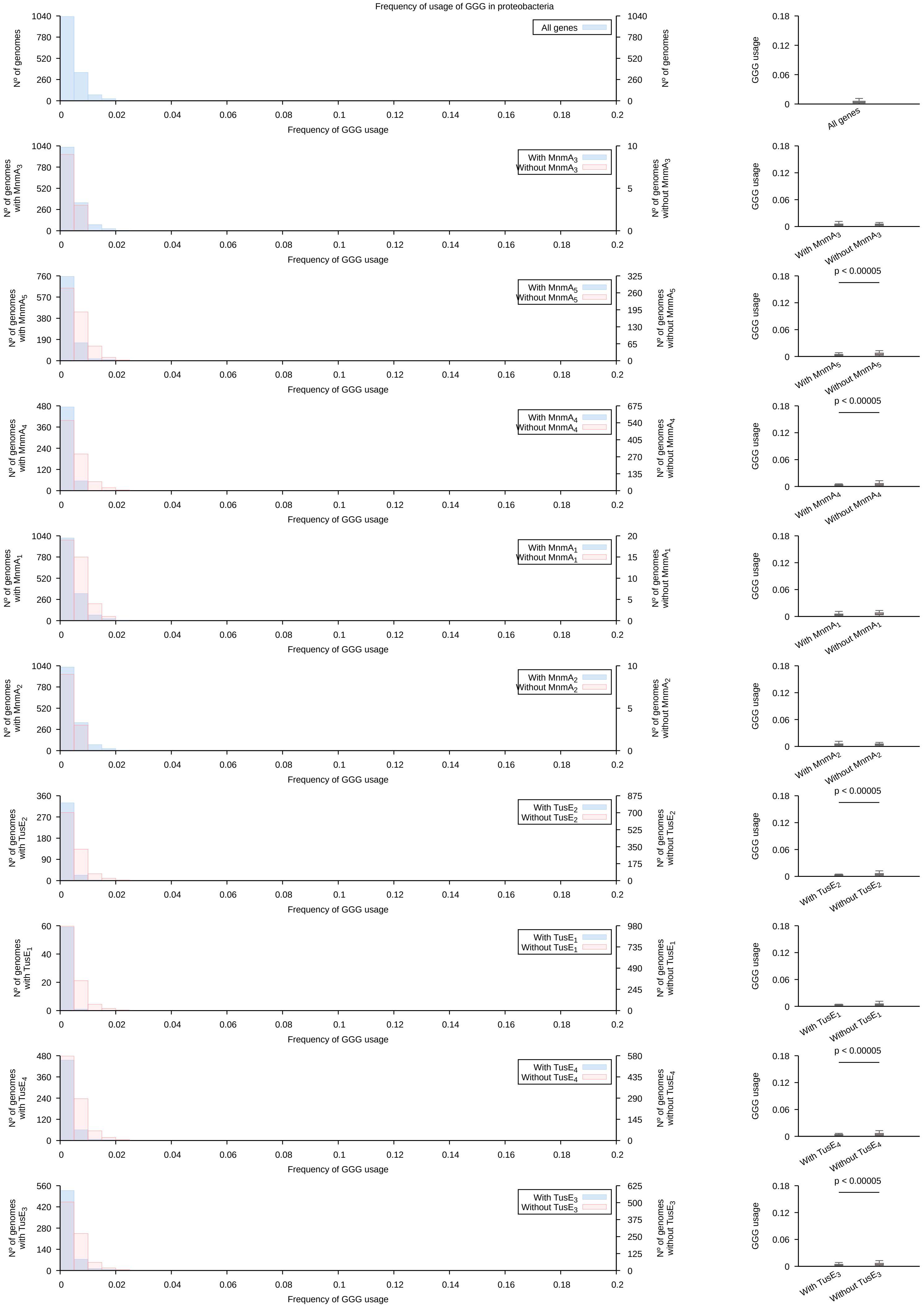
Frequency of usage of GGG in proteobacteria
Nº of genomes
All genes
Frequency of GGG usage
GGG usage
Nº of genomes with MnmA$_3$
With MnmA$_3$
Without MnmA$_3$
Nº of genomes without MnmA$_3$
Nº of genomes with MnmA$_5$
With MnmA$_5$
Without MnmA$_5$
Nº of genomes without MnmA$_5$
p < 0.00005
Nº of genomes with MnmA$_4$
With MnmA$_4$
Without MnmA$_4$
Nº of genomes without MnmA$_4$
p < 0.00005
Nº of genomes with MnmA$_1$
With MnmA$_1$
Without MnmA$_1$
Nº of genomes without MnmA$_1$
Nº of genomes with MnmA$_2$
With MnmA$_2$
Without MnmA$_2$
Nº of genomes without MnmA$_2$
Nº of genomes with TusE$_2$
With TusE$_2$
Without TusE$_2$
Nº of genomes without TusE$_2$
p < 0.00005
Nº of genomes with TusE$_1$
With TusE$_1$
Without TusE$_1$
Nº of genomes without TusE$_1$
Nº of genomes with TusE$_4$
With TusE$_4$
Without TusE$_4$
Nº of genomes without TusE$_4$
p < 0.00005
Nº of genomes with TusE$_3$
With TusE$_3$
Without TusE$_3$
Nº of genomes without TusE$_3$
p < 0.00005

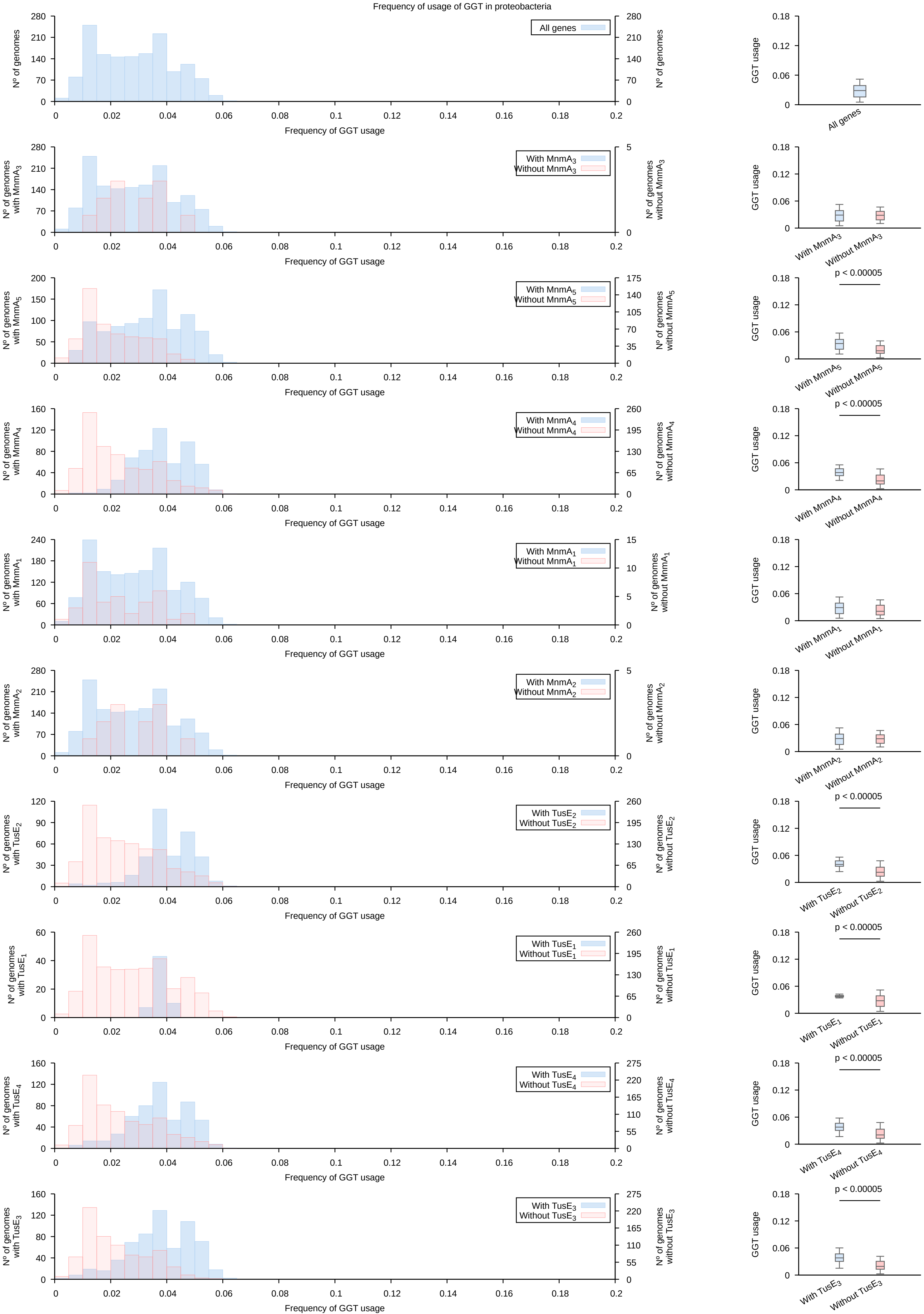

Frequency of usage of GGT in proteobacteria
Nº of genomes
Frequency of GGT usage
GGT usage
All genes
Nº of genomes with MnmA$_3$
Nº of genomes without MnmA$_3$
With MnmA$_3$
Without MnmA$_3$
Nº of genomes with MnmA$_5$
Nº of genomes without MnmA$_5$
With MnmA$_5$
Without MnmA$_5$
p < 0.00005
Nº of genomes with MnmA$_4$
Nº of genomes without MnmA$_4$
With MnmA$_4$
Without MnmA$_4$
p < 0.00005
Nº of genomes with MnmA$_1$
Nº of genomes without MnmA$_1$
With MnmA$_1$
Without MnmA$_1$
Nº of genomes with MnmA$_2$
Nº of genomes without MnmA$_2$
With MnmA$_2$
Without MnmA$_2$
Nº of genomes with TusE$_2$
Nº of genomes without TusE$_2$
With TusE$_2$
Without TusE$_2$
p < 0.00005
Nº of genomes with TusE$_1$
Nº of genomes without TusE$_1$
With TusE$_1$
Without TusE$_1$
p < 0.00005
Nº of genomes with TusE$_4$
Nº of genomes without TusE$_4$
With TusE$_4$
Without TusE$_4$
p < 0.00005
Nº of genomes with TusE$_3$
Nº of genomes without TusE$_3$
With TusE$_3$
Without TusE$_3$
p < 0.00005

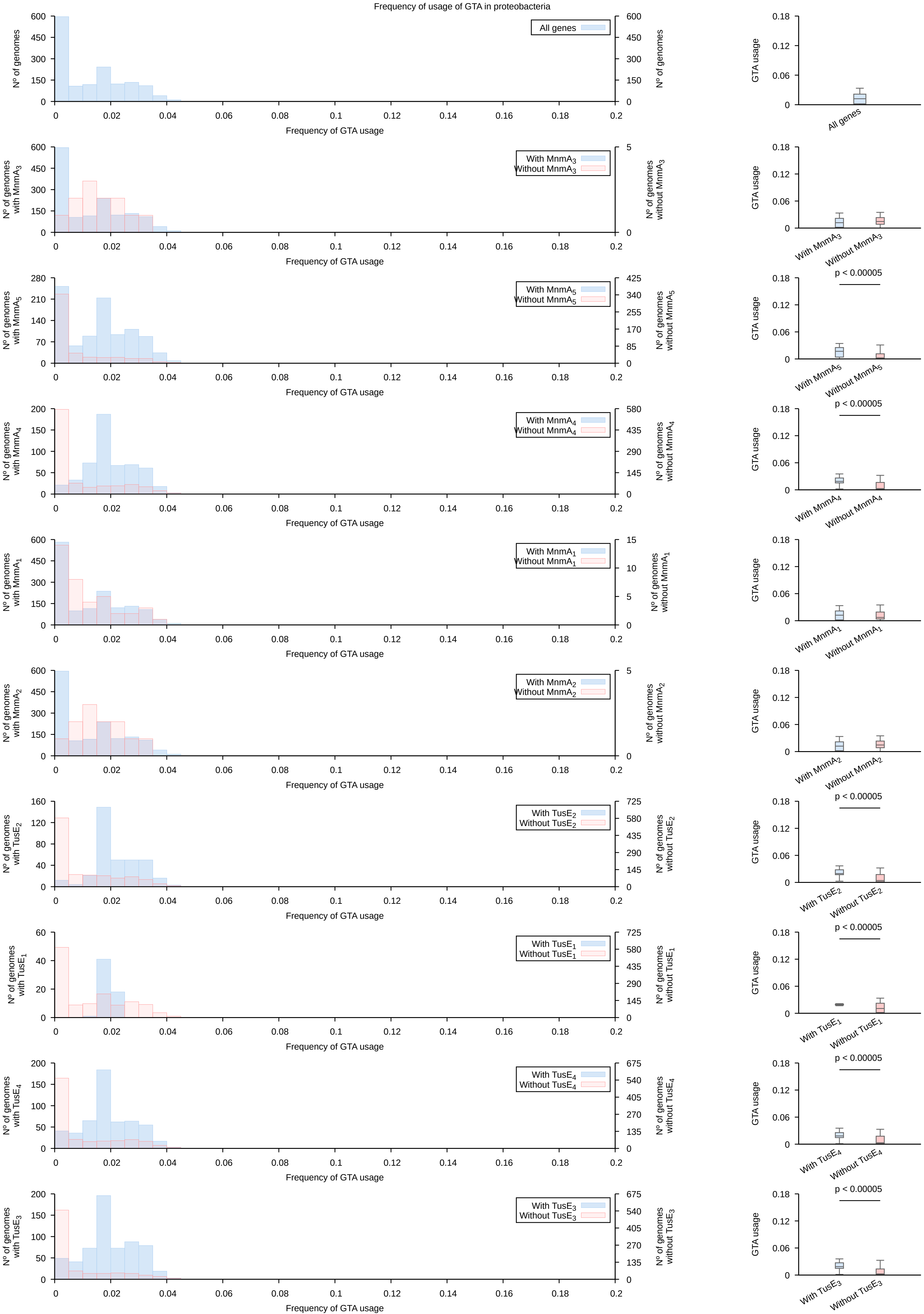
Frequency of usage of GTA in proteobacteria
Nº of genomes
All genes
Frequency of GTA usage
GTA usage
Nº of genomes with MnmA$_3$
Nº of genomes without MnmA$_3$
With MnmA$_3$
Without MnmA$_3$
Nº of genomes with MnmA$_5$
Nº of genomes without MnmA$_5$
With MnmA$_5$
Without MnmA$_5$
p < 0.00005
Nº of genomes with MnmA$_4$
Nº of genomes without MnmA$_4$
With MnmA$_4$
Without MnmA$_4$
p < 0.00005
Nº of genomes with MnmA$_1$
Nº of genomes without MnmA$_1$
With MnmA$_1$
Without MnmA$_1$
Nº of genomes with MnmA$_2$
Nº of genomes without MnmA$_2$
With MnmA$_2$
Without MnmA$_2$
Nº of genomes with TusE$_2$
Nº of genomes without TusE$_2$
With TusE$_2$
Without TusE$_2$
p < 0.00005
Nº of genomes with TusE$_1$
Nº of genomes without TusE$_1$
With TusE$_1$
Without TusE$_1$
p < 0.00005
Nº of genomes with TusE$_4$
Nº of genomes without TusE$_4$
With TusE$_4$
Without TusE$_4$
p < 0.00005
Nº of genomes with TusE$_3$
Nº of genomes without TusE$_3$
With TusE$_3$
Without TusE$_3$
p < 0.00005

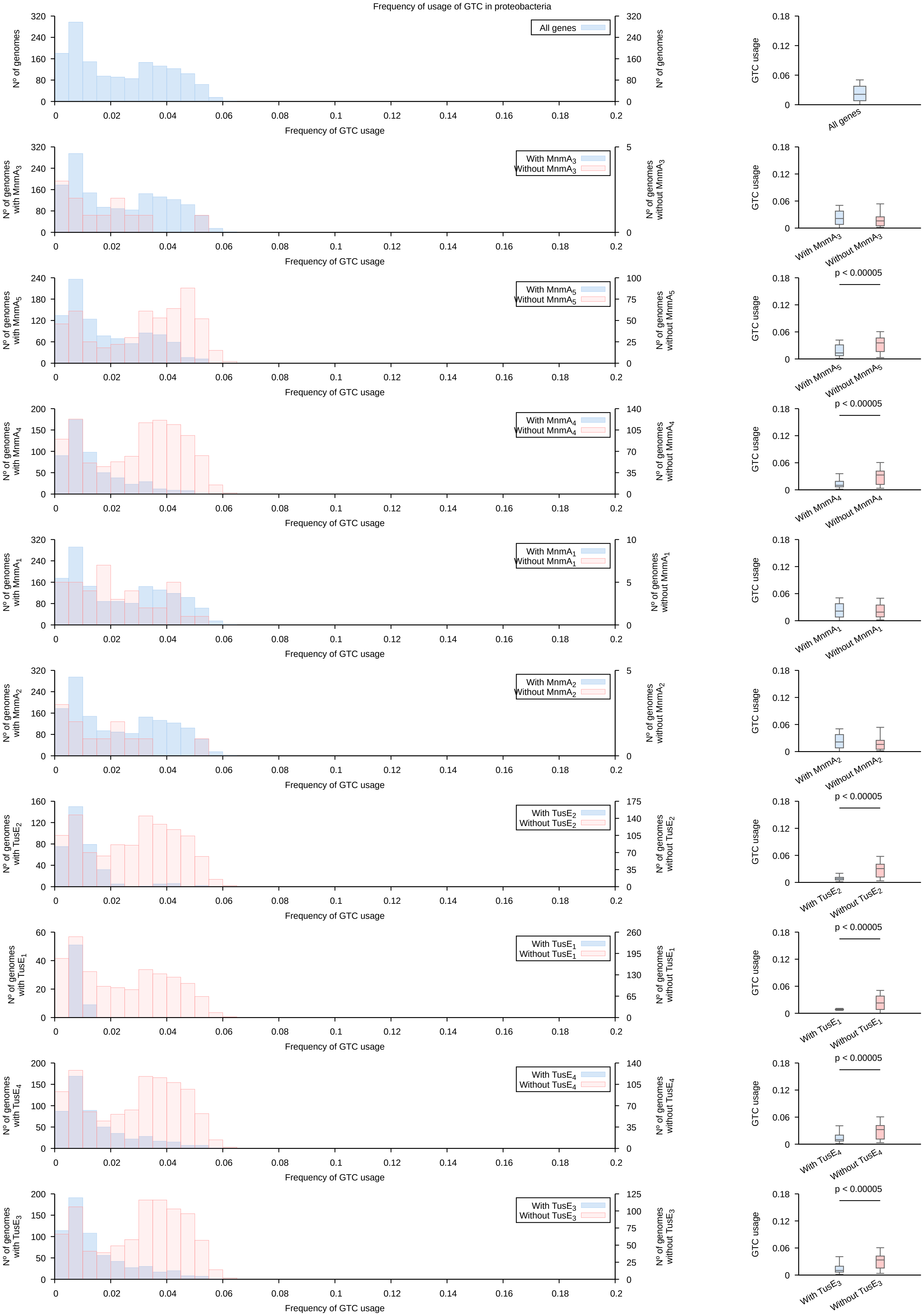
Frequency of usage of GTC in proteobacteria
All genes
Nº of genomes
Frequency of GTC usage
GTC usage
With MnmA$_3$
Without MnmA$_3$
Nº of genomes with MnmA$_3$
Nº of genomes without MnmA$_3$
With MnmA$_5$
Without MnmA$_5$
Nº of genomes with MnmA$_5$
Nº of genomes without MnmA$_5$
p < 0.00005
With MnmA$_4$
Without MnmA$_4$
Nº of genomes with MnmA$_4$
Nº of genomes without MnmA$_4$
p < 0.00005
With MnmA$_1$
Without MnmA$_1$
Nº of genomes with MnmA$_1$
Nº of genomes without MnmA$_1$
With MnmA$_2$
Without MnmA$_2$
Nº of genomes with MnmA$_2$
Nº of genomes without MnmA$_2$
With TusE$_2$
Without TusE$_2$
Nº of genomes with TusE$_2$
Nº of genomes without TusE$_2$
p < 0.00005
With TusE$_1$
Without TusE$_1$
Nº of genomes with TusE$_1$
Nº of genomes without TusE$_1$
p < 0.00005
With TusE$_4$
Without TusE$_4$
Nº of genomes with TusE$_4$
Nº of genomes without TusE$_4$
p < 0.00005
With TusE$_3$
Without TusE$_3$
Nº of genomes with TusE$_3$
Nº of genomes without TusE$_3$
p < 0.00005

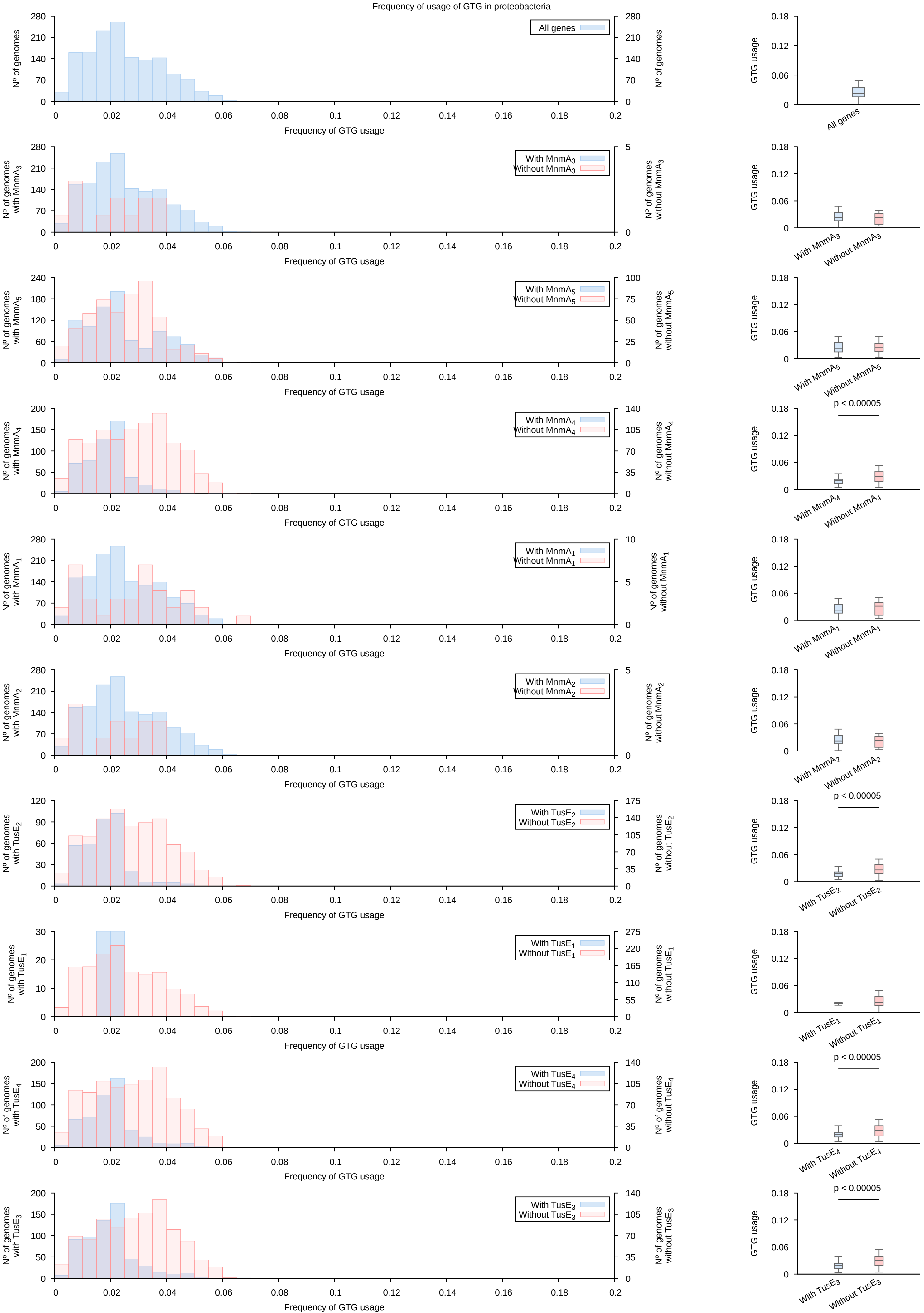
Frequency of usage of GTG in proteobacteria
All genes
Nº of genomes
GTG usage
Frequency of GTG usage
With MnmA$_3$
Without MnmA$_3$
Nº of genomes with MnmA$_3$
Nº of genomes without MnmA$_3$
With MnmA$_5$
Without MnmA$_5$
Nº of genomes with MnmA$_5$
Nº of genomes without MnmA$_5$
p < 0.00005
With MnmA$_4$
Without MnmA$_4$
Nº of genomes with MnmA$_4$
Nº of genomes without MnmA$_4$
With MnmA$_1$
Without MnmA$_1$
Nº of genomes with MnmA$_1$
Nº of genomes without MnmA$_1$
With MnmA$_2$
Without MnmA$_2$
Nº of genomes with MnmA$_2$
Nº of genomes without MnmA$_2$
p < 0.00005
With TusE$_2$
Without TusE$_2$
Nº of genomes with TusE$_2$
Nº of genomes without TusE$_2$
With TusE$_1$
Without TusE$_1$
Nº of genomes with TusE$_1$
Nº of genomes without TusE$_1$
p < 0.00005
With TusE$_4$
Without TusE$_4$
Nº of genomes with TusE$_4$
Nº of genomes without TusE$_4$
p < 0.00005
With TusE$_3$
Without TusE$_3$
Nº of genomes with TusE$_3$
Nº of genomes without TusE$_3$

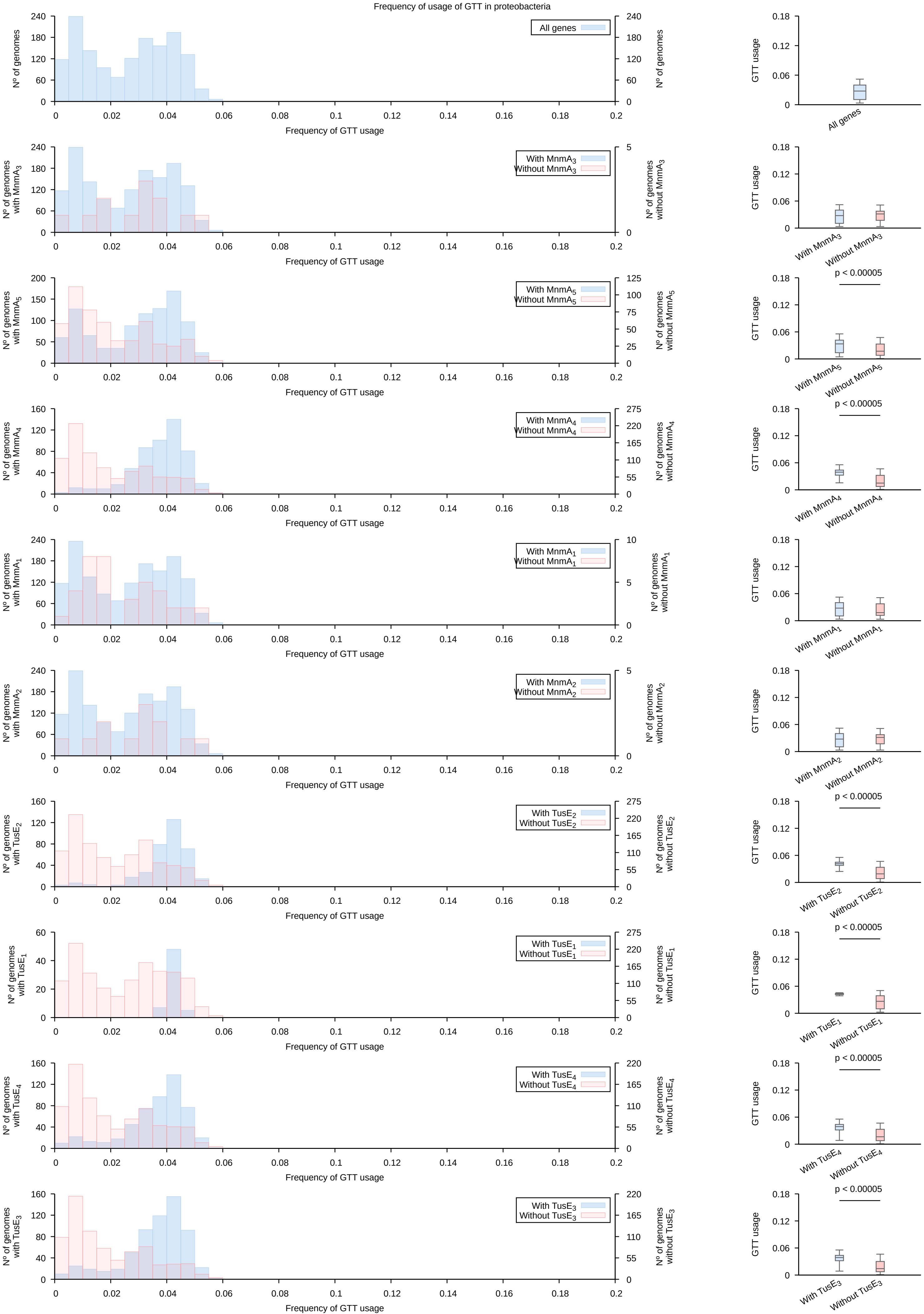

Frequency of usage of GTT in proteobacteria
All genes
With MnmA$_3$
Without MnmA$_3$
With MnmA$_5$
Without MnmA$_5$
With MnmA$_4$
Without MnmA$_4$
With MnmA$_1$
Without MnmA$_1$
With MnmA$_2$
Without MnmA$_2$
With TusE$_2$
Without TusE$_2$
With TusE$_1$
Without TusE$_1$
With TusE$_4$
Without TusE$_4$
With TusE$_3$
Without TusE$_3$
Nº of genomes
Nº of genomes with MnmA$_3$
Nº of genomes without MnmA$_3$
Nº of genomes with MnmA$_5$
Nº of genomes without MnmA$_5$
Nº of genomes with MnmA$_4$
Nº of genomes without MnmA$_4$
Nº of genomes with MnmA$_1$
Nº of genomes without MnmA$_1$
Nº of genomes with MnmA$_2$
Nº of genomes without MnmA$_2$
Nº of genomes with TusE$_2$
Nº of genomes without TusE$_2$
Nº of genomes with TusE$_1$
Nº of genomes without TusE$_1$
Nº of genomes with TusE$_4$
Nº of genomes without TusE$_4$
Nº of genomes with TusE$_3$
Nº of genomes without TusE$_3$
Frequency of GTT usage
GTT usage
p < 0.00005

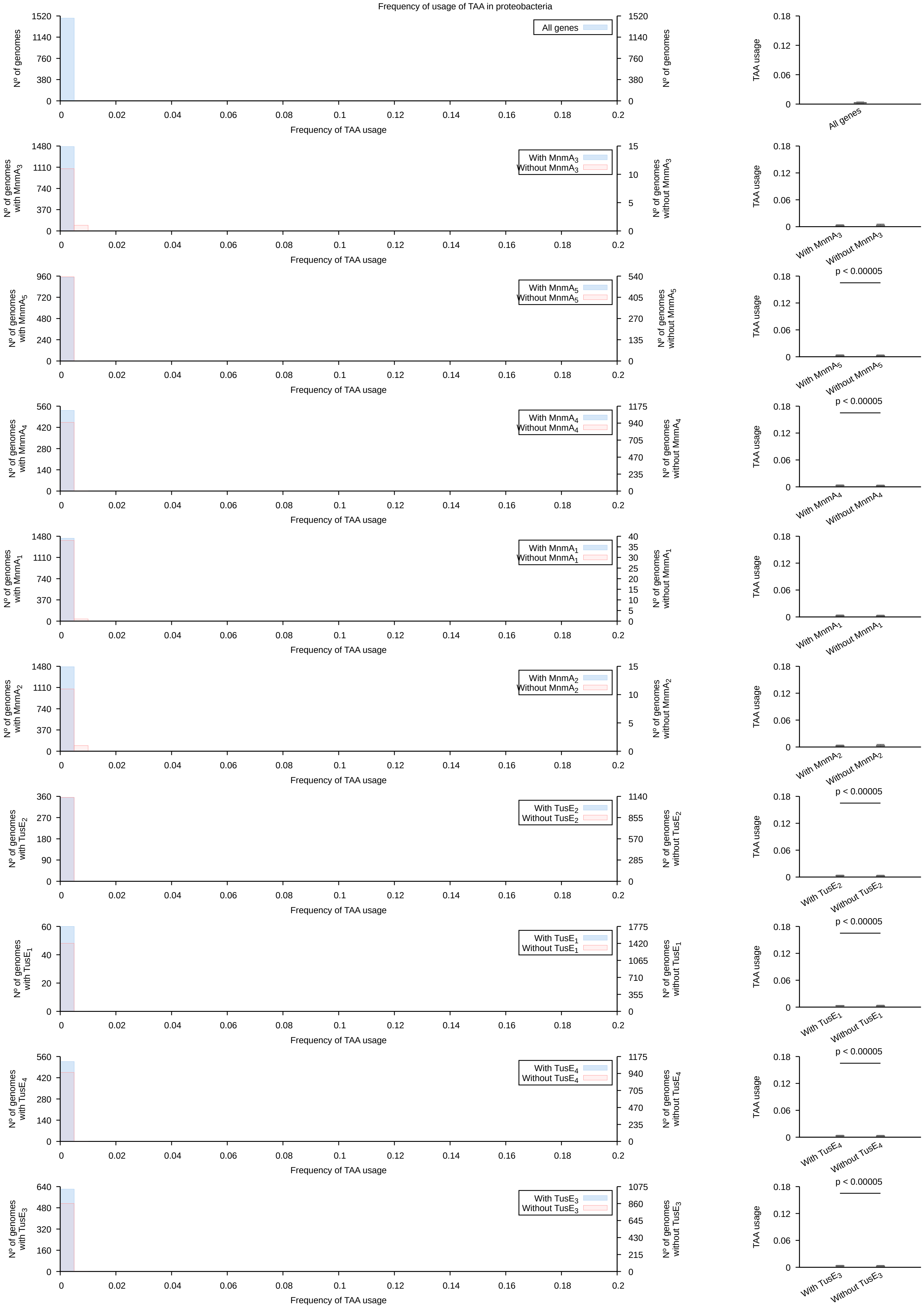

Frequency of usage of TAA in proteobacteria
All genes
With MnmA$_3$
Without MnmA$_3$
With MnmA$_5$
Without MnmA$_5$
With MnmA$_4$
Without MnmA$_4$
With MnmA$_1$
Without MnmA$_1$
With MnmA$_2$
Without MnmA$_2$
With TusE$_2$
Without TusE$_2$
With TusE$_1$
Without TusE$_1$
With TusE$_4$
Without TusE$_4$
With TusE$_3$
Without TusE$_3$
Nº of genomes
Frequency of TAA usage
TAA usage
p < 0.00005

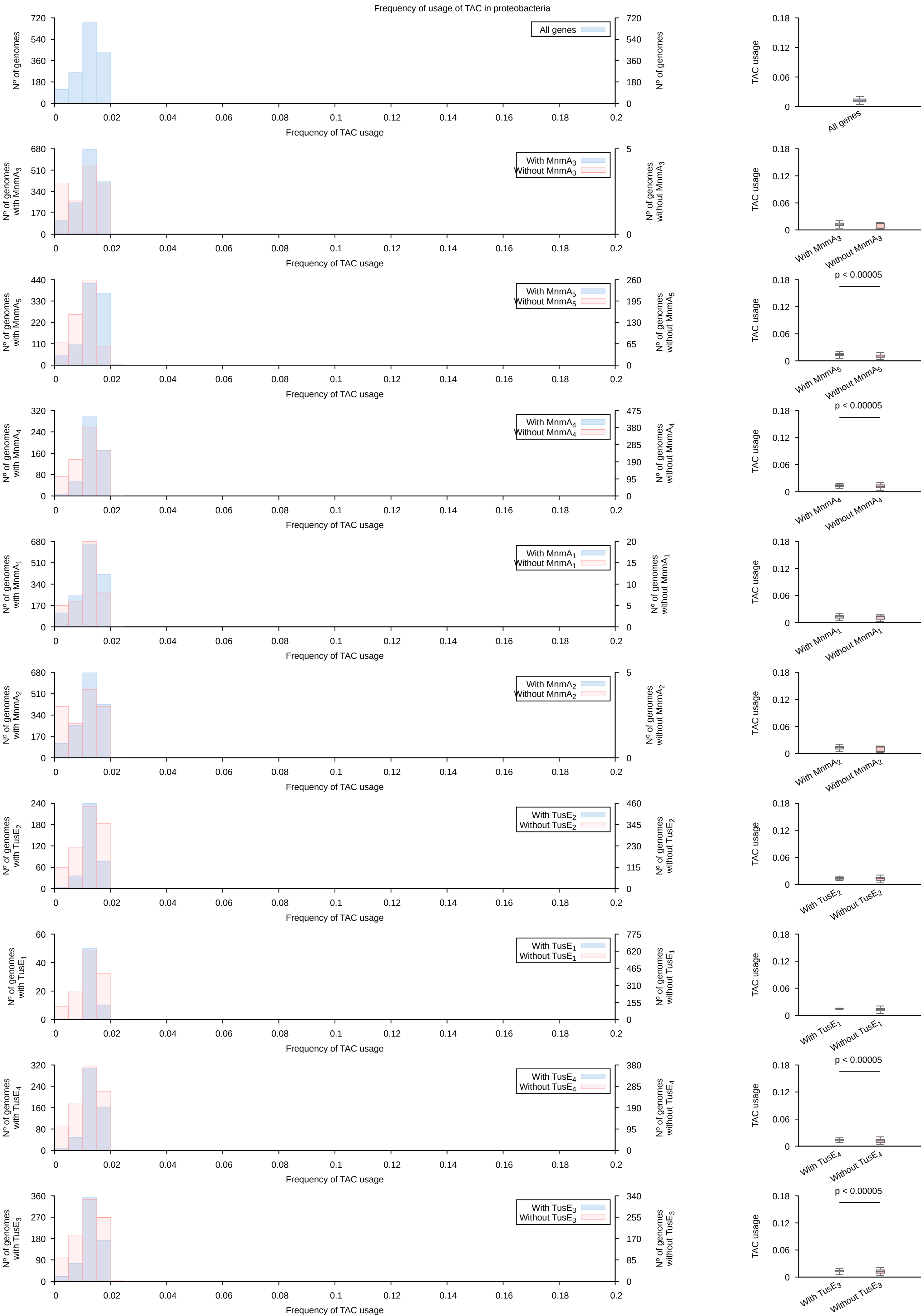
Frequency of usage of TAC in proteobacteria
Nº of genomes
Frequency of TAC usage
TAC usage
All genes
With MnmA$_3$
Without MnmA$_3$
Nº of genomes with MnmA$_3$
Nº of genomes without MnmA$_3$
With MnmA$_5$
Without MnmA$_5$
Nº of genomes with MnmA$_5$
Nº of genomes without MnmA$_5$
p < 0.00005
With MnmA$_4$
Without MnmA$_4$
Nº of genomes with MnmA$_4$
Nº of genomes without MnmA$_4$
p < 0.00005
With MnmA$_1$
Without MnmA$_1$
Nº of genomes with MnmA$_1$
Nº of genomes without MnmA$_1$
With MnmA$_2$
Without MnmA$_2$
Nº of genomes with MnmA$_2$
Nº of genomes without MnmA$_2$
With TusE$_2$
Without TusE$_2$
Nº of genomes with TusE$_2$
Nº of genomes without TusE$_2$
With TusE$_1$
Without TusE$_1$
Nº of genomes with TusE$_1$
Nº of genomes without TusE$_1$
With TusE$_4$
Without TusE$_4$
Nº of genomes with TusE$_4$
Nº of genomes without TusE$_4$
p < 0.00005
With TusE$_3$
Without TusE$_3$
Nº of genomes with TusE$_3$
Nº of genomes without TusE$_3$
p < 0.00005

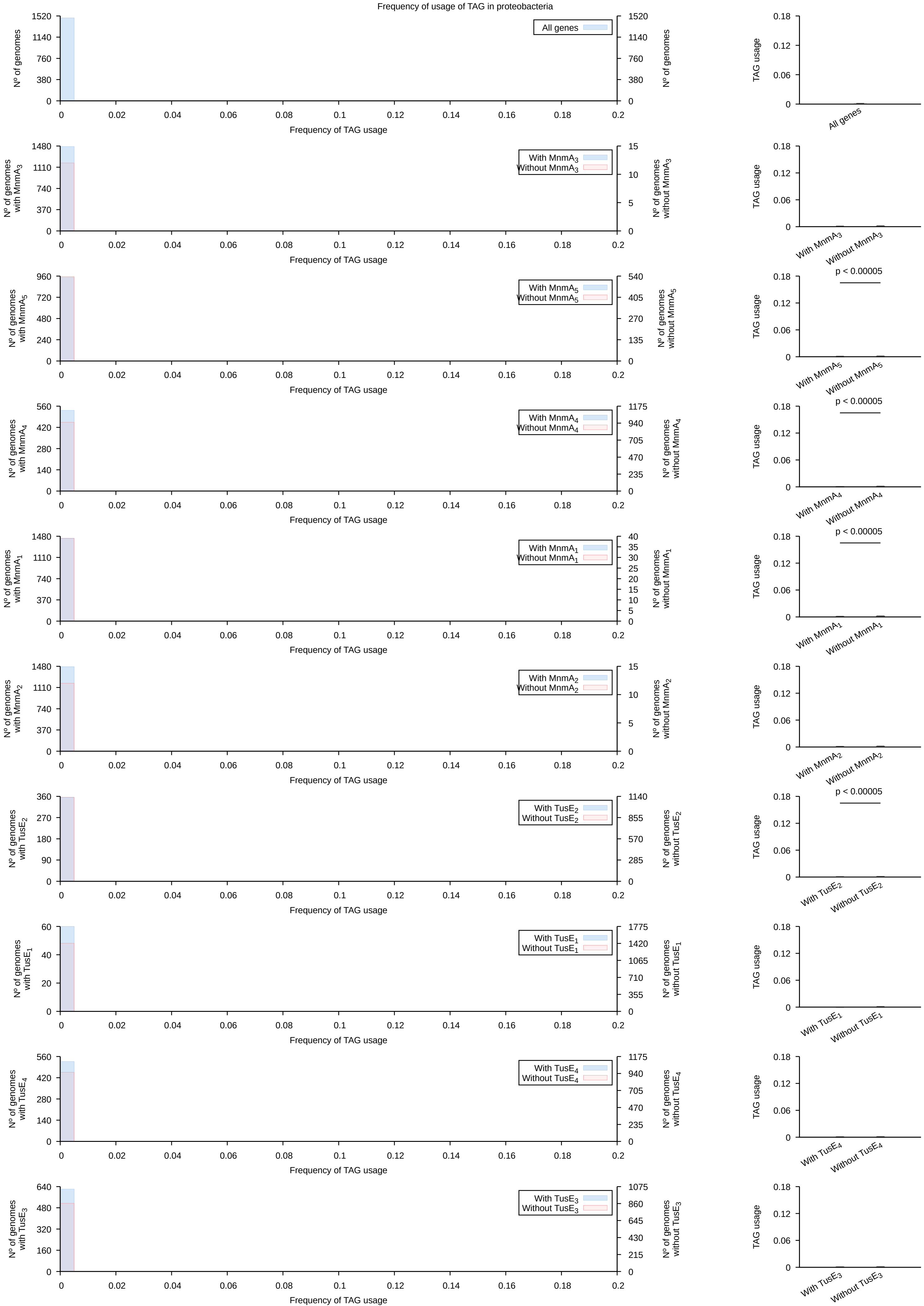

Frequency of usage of TAG in proteobacteria
All genes
With MnmA3
Without MnmA3
With MnmA5
Without MnmA5
With MnmA4
Without MnmA4
With MnmA1
Without MnmA1
With MnmA2
Without MnmA2
With TusE2
Without TusE2
With TusE1
Without TusE1
With TusE4
Without TusE4
With TusE3
Without TusE3
Frequency of TAG usage
Nº of genomes
TAG usage
p < 0.00005

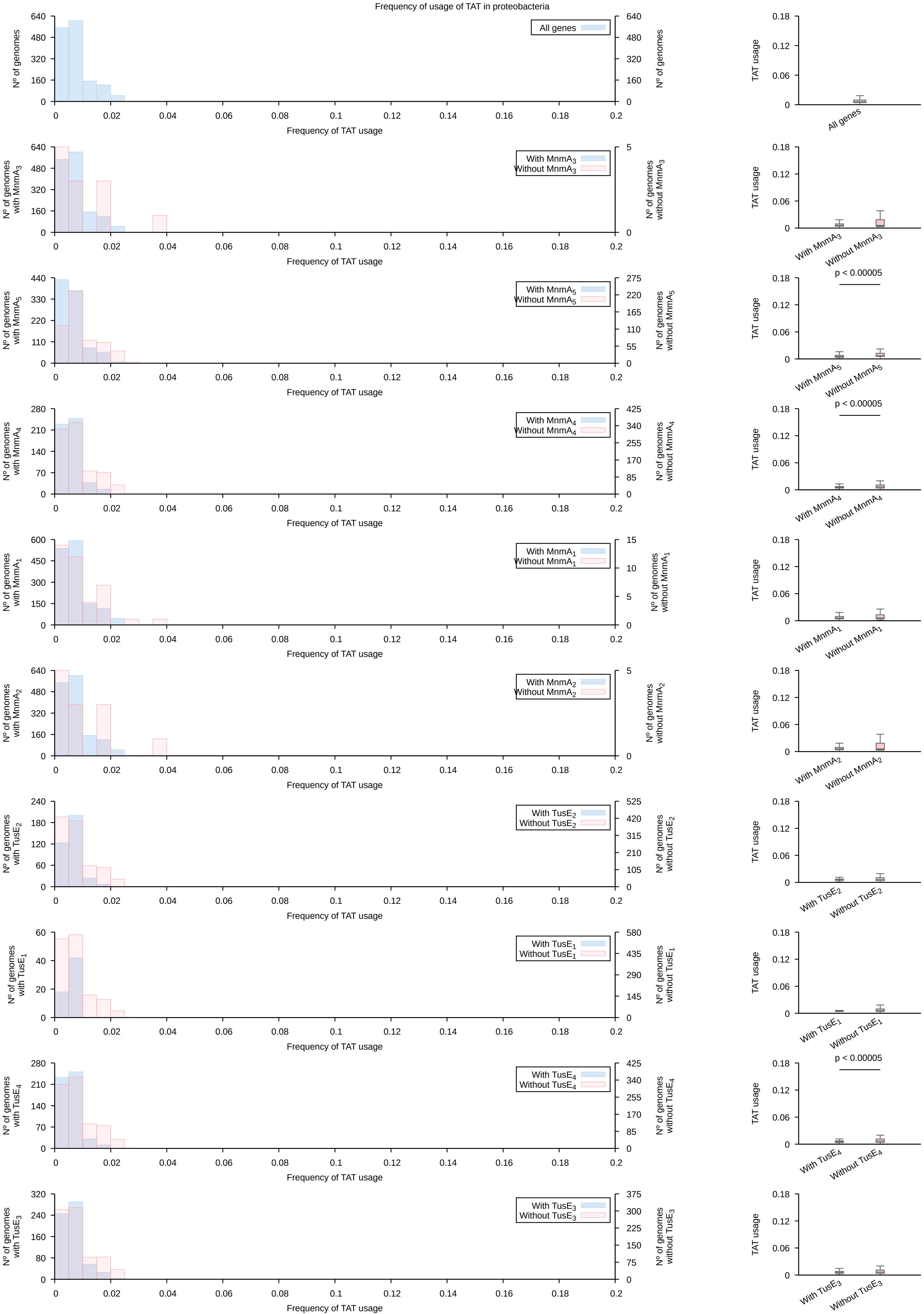
Frequency of usage of TAT in proteobacteria
Nº of genomes
Frequency of TAT usage
TAT usage
All genes
Nº of genomes with MnmA$_3$
Nº of genomes without MnmA$_3$
With MnmA$_3$
Without MnmA$_3$
Nº of genomes with MnmA$_5$
Nº of genomes without MnmA$_5$
With MnmA$_5$
Without MnmA$_5$
p < 0.00005
Nº of genomes with MnmA$_4$
Nº of genomes without MnmA$_4$
With MnmA$_4$
Without MnmA$_4$
p < 0.00005
Nº of genomes with MnmA$_1$
Nº of genomes without MnmA$_1$
With MnmA$_1$
Without MnmA$_1$
Nº of genomes with MnmA$_2$
Nº of genomes without MnmA$_2$
With MnmA$_2$
Without MnmA$_2$
Nº of genomes with TusE$_2$
Nº of genomes without TusE$_2$
With TusE$_2$
Without TusE$_2$
Nº of genomes with TusE$_1$
Nº of genomes without TusE$_1$
With TusE$_1$
Without TusE$_1$
Nº of genomes with TusE$_4$
Nº of genomes without TusE$_4$
With TusE$_4$
Without TusE$_4$
p < 0.00005
Nº of genomes with TusE$_3$
Nº of genomes without TusE$_3$
With TusE$_3$
Without TusE$_3$

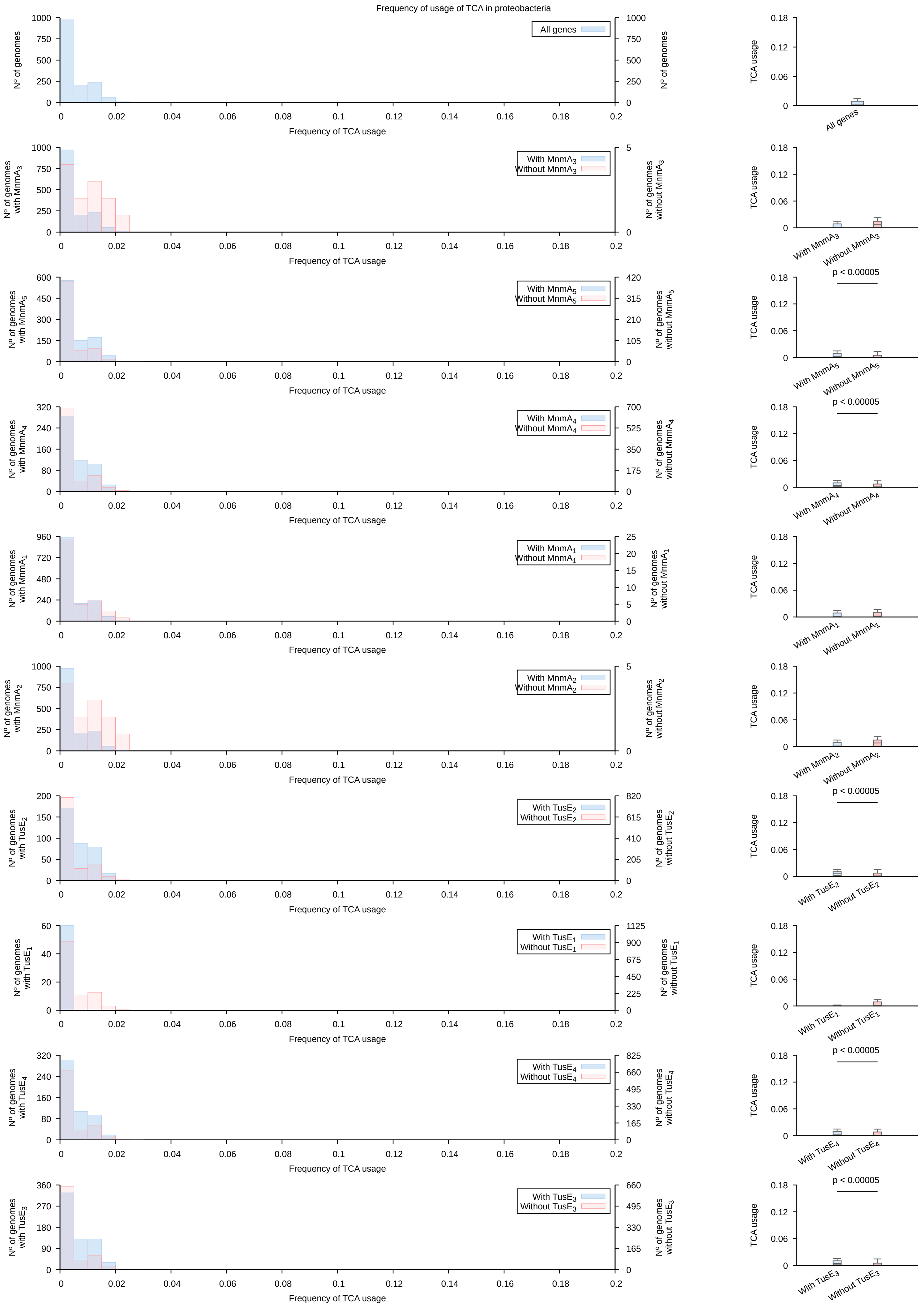
Frequency of usage of TCA in proteobacteria
All genes
Nº of genomes
Frequency of TCA usage
TCA usage
With MnmA$_3$
Without MnmA$_3$
Nº of genomes with MnmA$_3$
Nº of genomes without MnmA$_3$
With MnmA$_5$
Without MnmA$_5$
Nº of genomes with MnmA$_5$
Nº of genomes without MnmA$_5$
p < 0.00005
With MnmA$_4$
Without MnmA$_4$
Nº of genomes with MnmA$_4$
Nº of genomes without MnmA$_4$
p < 0.00005
With MnmA$_1$
Without MnmA$_1$
Nº of genomes with MnmA$_1$
Nº of genomes without MnmA$_1$
With MnmA$_2$
Without MnmA$_2$
Nº of genomes with MnmA$_2$
Nº of genomes without MnmA$_2$
With TusE$_2$
Without TusE$_2$
Nº of genomes with TusE$_2$
Nº of genomes without TusE$_2$
p < 0.00005
With TusE$_1$
Without TusE$_1$
Nº of genomes with TusE$_1$
Nº of genomes without TusE$_1$
With TusE$_4$
Without TusE$_4$
Nº of genomes with TusE$_4$
Nº of genomes without TusE$_4$
p < 0.00005
With TusE$_3$
Without TusE$_3$
Nº of genomes with TusE$_3$
Nº of genomes without TusE$_3$
p < 0.00005

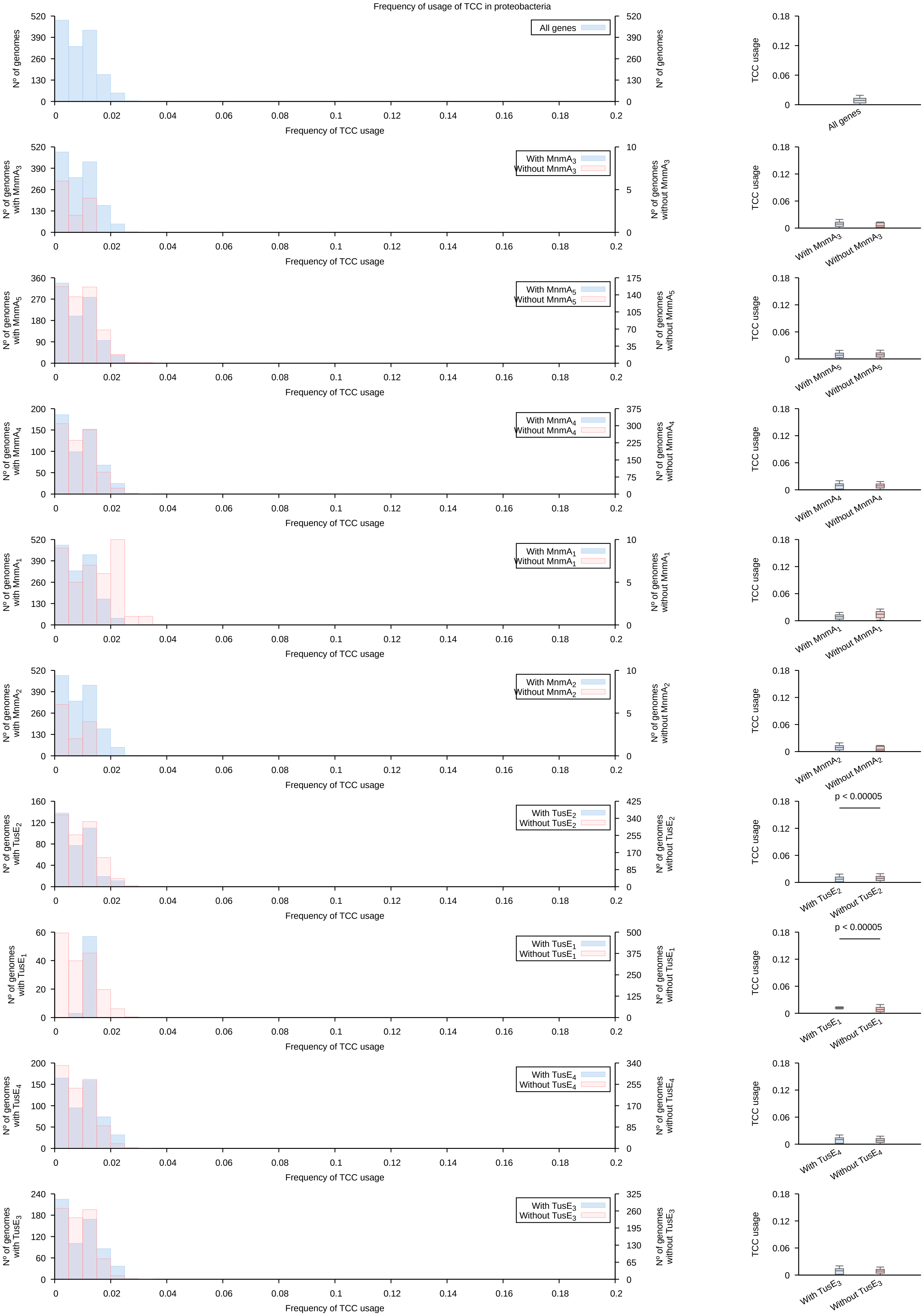

Frequency of usage of TCC in proteobacteria
Nº of genomes
All genes
Frequency of TCC usage
TCC usage
Nº of genomes with MnmA3
Nº of genomes without MnmA3
With MnmA3
Without MnmA3
Nº of genomes with MnmA5
Nº of genomes without MnmA5
With MnmA5
Without MnmA5
Nº of genomes with MnmA4
Nº of genomes without MnmA4
With MnmA4
Without MnmA4
Nº of genomes with MnmA1
Nº of genomes without MnmA1
With MnmA1
Without MnmA1
Nº of genomes with MnmA2
Nº of genomes without MnmA2
With MnmA2
Without MnmA2
Nº of genomes with TusE2
Nº of genomes without TusE2
With TusE2
Without TusE2
p < 0.00005
Nº of genomes with TusE1
Nº of genomes without TusE1
With TusE1
Without TusE1
p < 0.00005
Nº of genomes with TusE4
Nº of genomes without TusE4
With TusE4
Without TusE4
Nº of genomes with TusE3
Nº of genomes without TusE3
With TusE3
Without TusE3

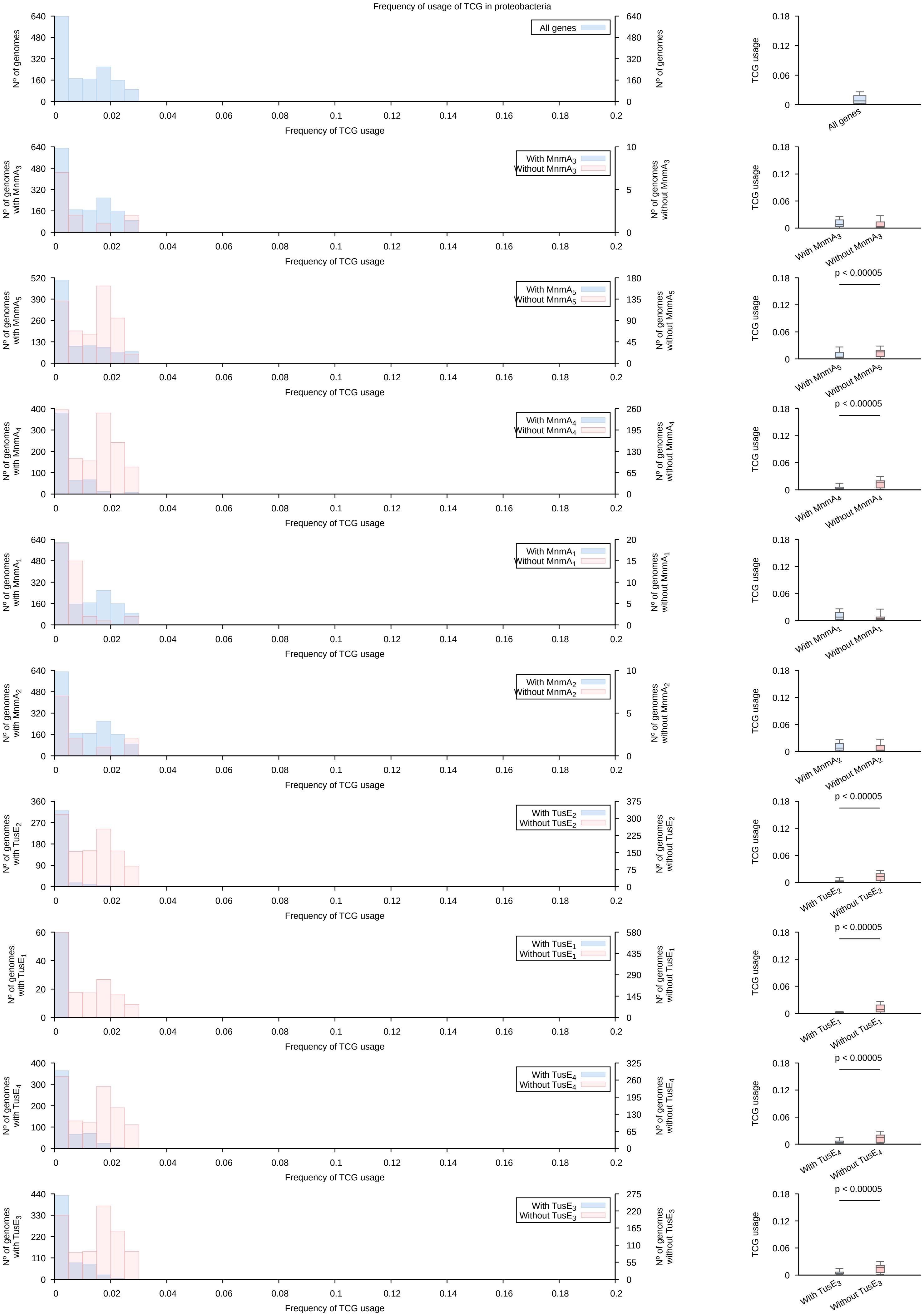

Frequency of usage of TCG in proteobacteria
All genes
With MnmA$_3$
Without MnmA$_3$
With MnmA$_5$
Without MnmA$_5$
With MnmA$_4$
Without MnmA$_4$
With MnmA$_1$
Without MnmA$_1$
With MnmA$_2$
Without MnmA$_2$
With TusE$_2$
Without TusE$_2$
With TusE$_1$
Without TusE$_1$
With TusE$_4$
Without TusE$_4$
With TusE$_3$
Without TusE$_3$
Nº of genomes
Frequency of TCG usage
TCG usage
p < 0.00005

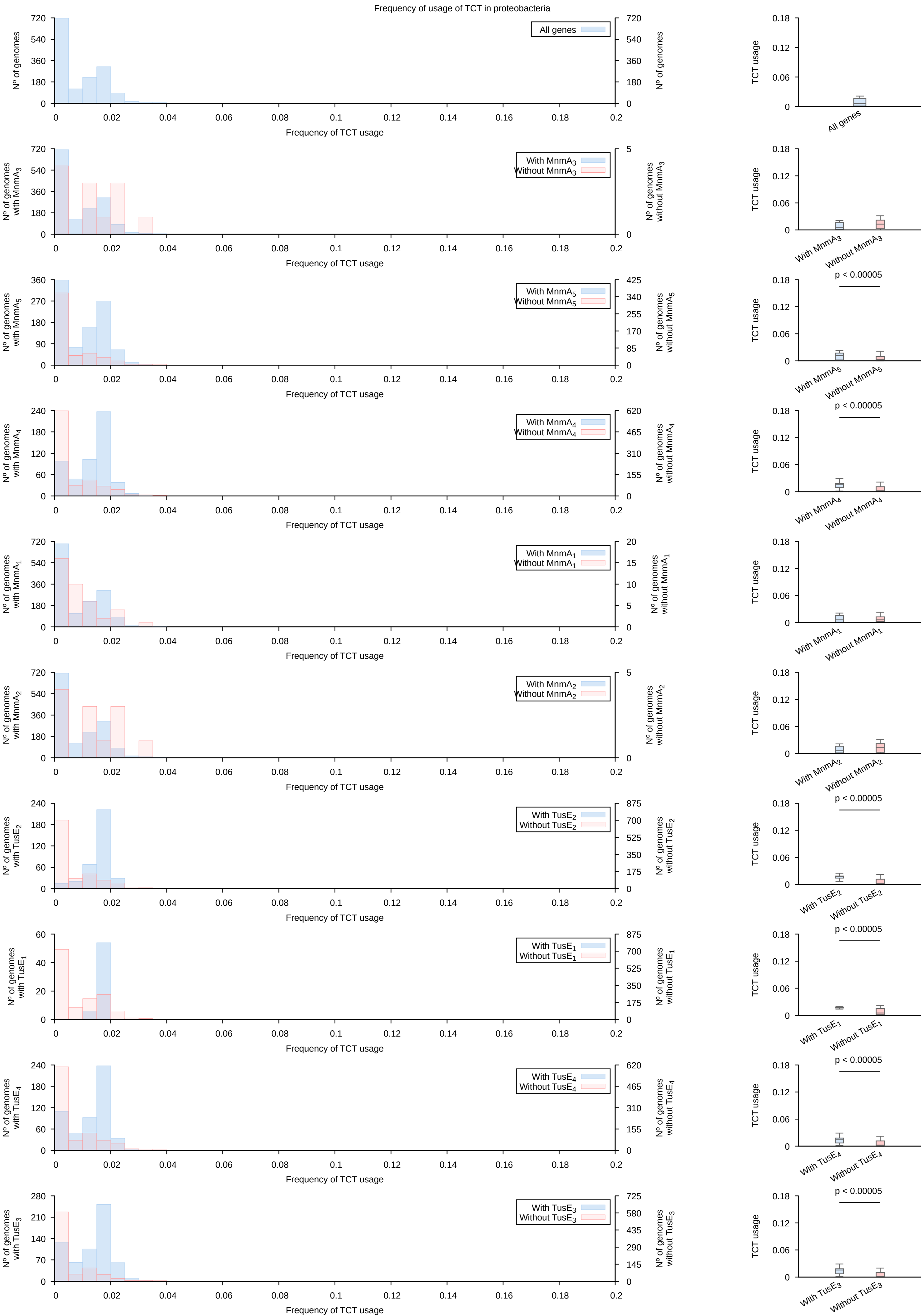

Frequency of usage of TCT in proteobacteria
Nº of genomes
Frequency of TCT usage
TCT usage
All genes
With $MnmA_3$
Without $MnmA_3$
Nº of genomes with $MnmA_3$
Nº of genomes without $MnmA_3$
With $MnmA_5$
Without $MnmA_5$
Nº of genomes with $MnmA_5$
Nº of genomes without $MnmA_5$
p < 0.00005
With $MnmA_4$
Without $MnmA_4$
Nº of genomes with $MnmA_4$
Nº of genomes without $MnmA_4$
p < 0.00005
With $MnmA_1$
Without $MnmA_1$
Nº of genomes with $MnmA_1$
Nº of genomes without $MnmA_1$
With $MnmA_2$
Without $MnmA_2$
Nº of genomes with $MnmA_2$
Nº of genomes without $MnmA_2$
With $TusE_2$
Without $TusE_2$
Nº of genomes with $TusE_2$
Nº of genomes without $TusE_2$
p < 0.00005
With $TusE_1$
Without $TusE_1$
Nº of genomes with $TusE_1$
Nº of genomes without $TusE_1$
p < 0.00005
With $TusE_4$
Without $TusE_4$
Nº of genomes with $TusE_4$
Nº of genomes without $TusE_4$
p < 0.00005
With $TusE_3$
Without $TusE_3$
Nº of genomes with $TusE_3$
Nº of genomes without $TusE_3$
p < 0.00005

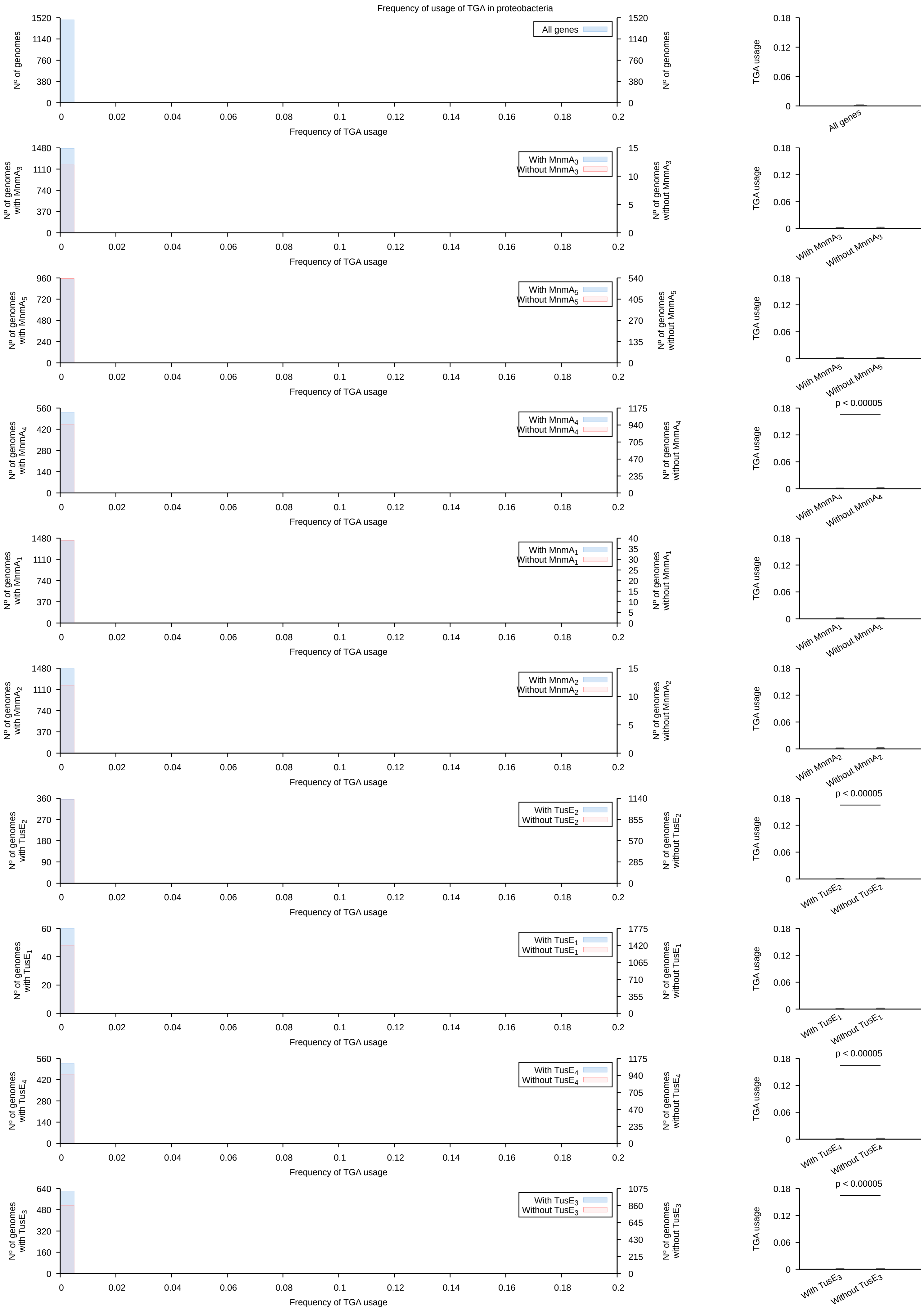
Frequency of usage of TGA in proteobacteria
All genes
Nº of genomes
Frequency of TGA usage
TGA usage
With MnmA$_3$
Without MnmA$_3$
Nº of genomes with MnmA$_3$
Nº of genomes without MnmA$_3$
With MnmA$_5$
Without MnmA$_5$
Nº of genomes with MnmA$_5$
Nº of genomes without MnmA$_5$
With MnmA$_4$
Without MnmA$_4$
Nº of genomes with MnmA$_4$
Nº of genomes without MnmA$_4$
p < 0.00005
With MnmA$_1$
Without MnmA$_1$
Nº of genomes with MnmA$_1$
Nº of genomes without MnmA$_1$
With MnmA$_2$
Without MnmA$_2$
Nº of genomes with MnmA$_2$
Nº of genomes without MnmA$_2$
With TusE$_2$
Without TusE$_2$
Nº of genomes with TusE$_2$
Nº of genomes without TusE$_2$
p < 0.00005
With TusE$_1$
Without TusE$_1$
Nº of genomes with TusE$_1$
Nº of genomes without TusE$_1$
With TusE$_4$
Without TusE$_4$
Nº of genomes with TusE$_4$
Nº of genomes without TusE$_4$
p < 0.00005
With TusE$_3$
Without TusE$_3$
Nº of genomes with TusE$_3$
Nº of genomes without TusE$_3$
p < 0.00005

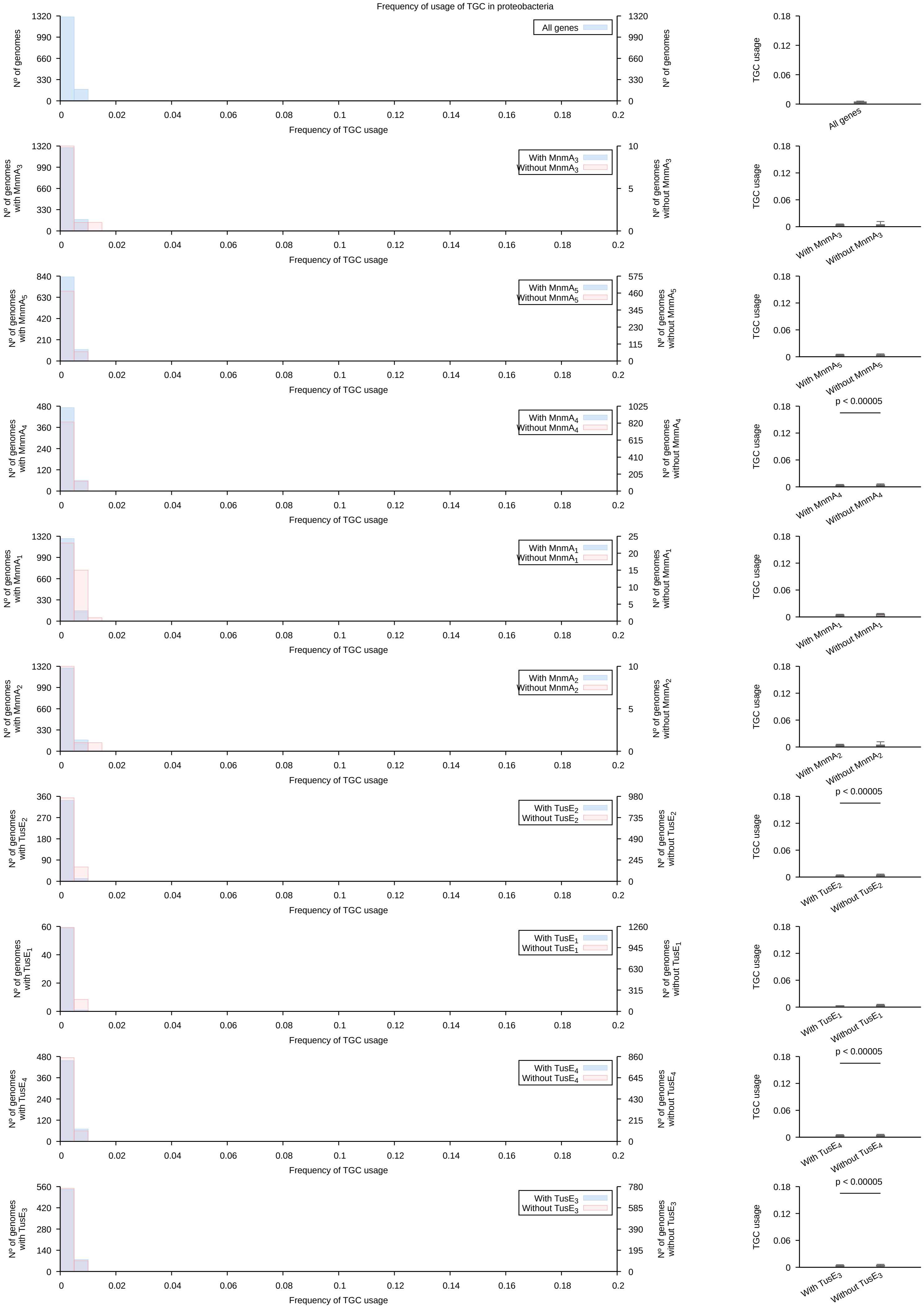
Frequency of usage of TGC in proteobacteria
Nº of genomes
Frequency of TGC usage
TGC usage
All genes
With MnmA$_3$
Without MnmA$_3$
Nº of genomes with MnmA$_3$
Nº of genomes without MnmA$_3$
With MnmA$_5$
Without MnmA$_5$
Nº of genomes with MnmA$_5$
Nº of genomes without MnmA$_5$
With MnmA$_4$
Without MnmA$_4$
Nº of genomes with MnmA$_4$
Nº of genomes without MnmA$_4$
p < 0.00005
With MnmA$_1$
Without MnmA$_1$
Nº of genomes with MnmA$_1$
Nº of genomes without MnmA$_1$
With MnmA$_2$
Without MnmA$_2$
Nº of genomes with MnmA$_2$
Nº of genomes without MnmA$_2$
With TusE$_2$
Without TusE$_2$
Nº of genomes with TusE$_2$
Nº of genomes without TusE$_2$
p < 0.00005
With TusE$_1$
Without TusE$_1$
Nº of genomes with TusE$_1$
Nº of genomes without TusE$_1$
With TusE$_4$
Without TusE$_4$
Nº of genomes with TusE$_4$
Nº of genomes without TusE$_4$
p < 0.00005
With TusE$_3$
Without TusE$_3$
Nº of genomes with TusE$_3$
Nº of genomes without TusE$_3$
p < 0.00005

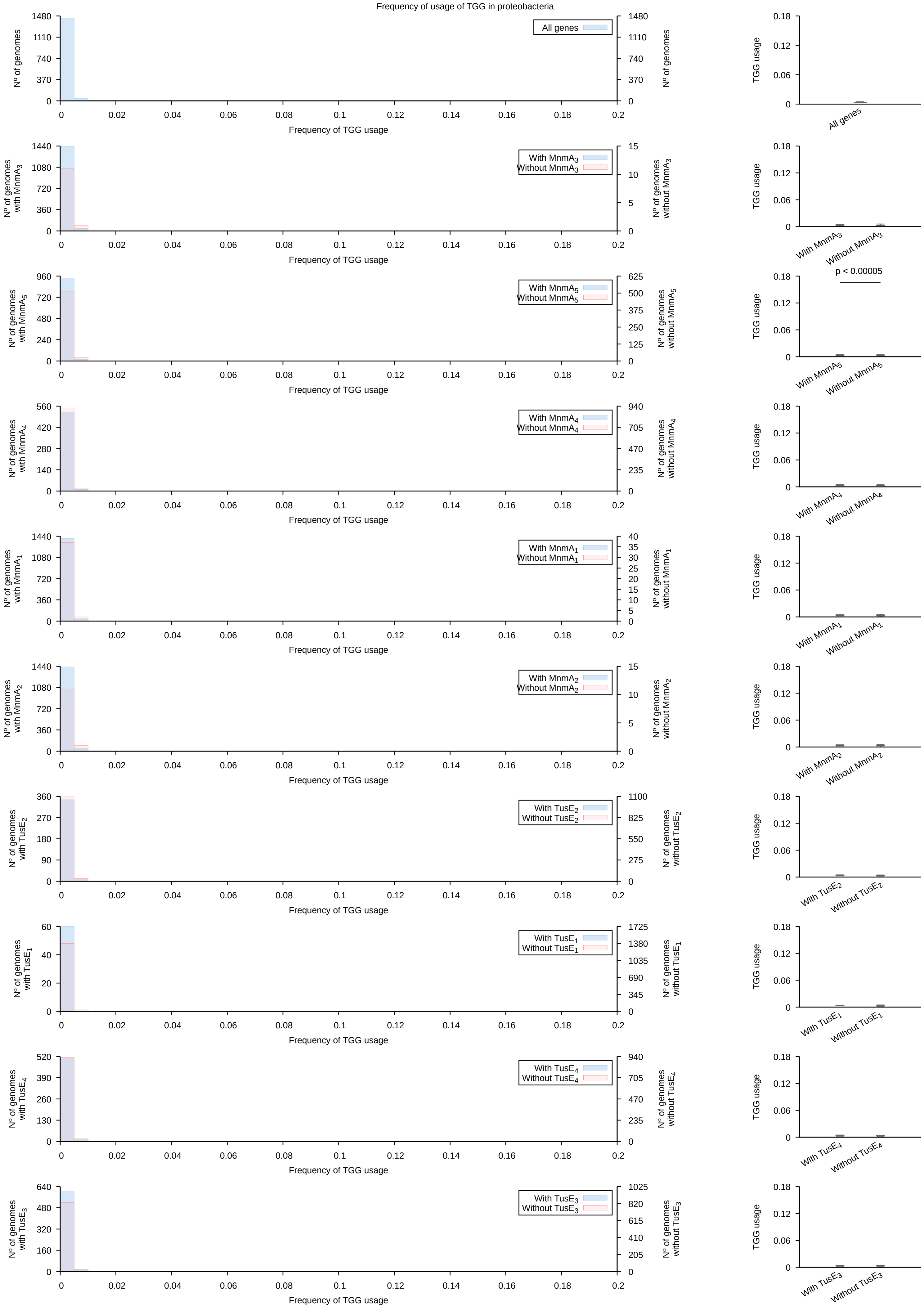

Frequency of usage of TGG in proteobacteria
All genes
With MnmA$_3$
Without MnmA$_3$
With MnmA$_5$
Without MnmA$_5$
p < 0.00005
With MnmA$_4$
Without MnmA$_4$
With MnmA$_1$
Without MnmA$_1$
With MnmA$_2$
Without MnmA$_2$
With TusE$_2$
Without TusE$_2$
With TusE$_1$
Without TusE$_1$
With TusE$_4$
Without TusE$_4$
With TusE$_3$
Without TusE$_3$
Nº of genomes
Frequency of TGG usage
TGG usage

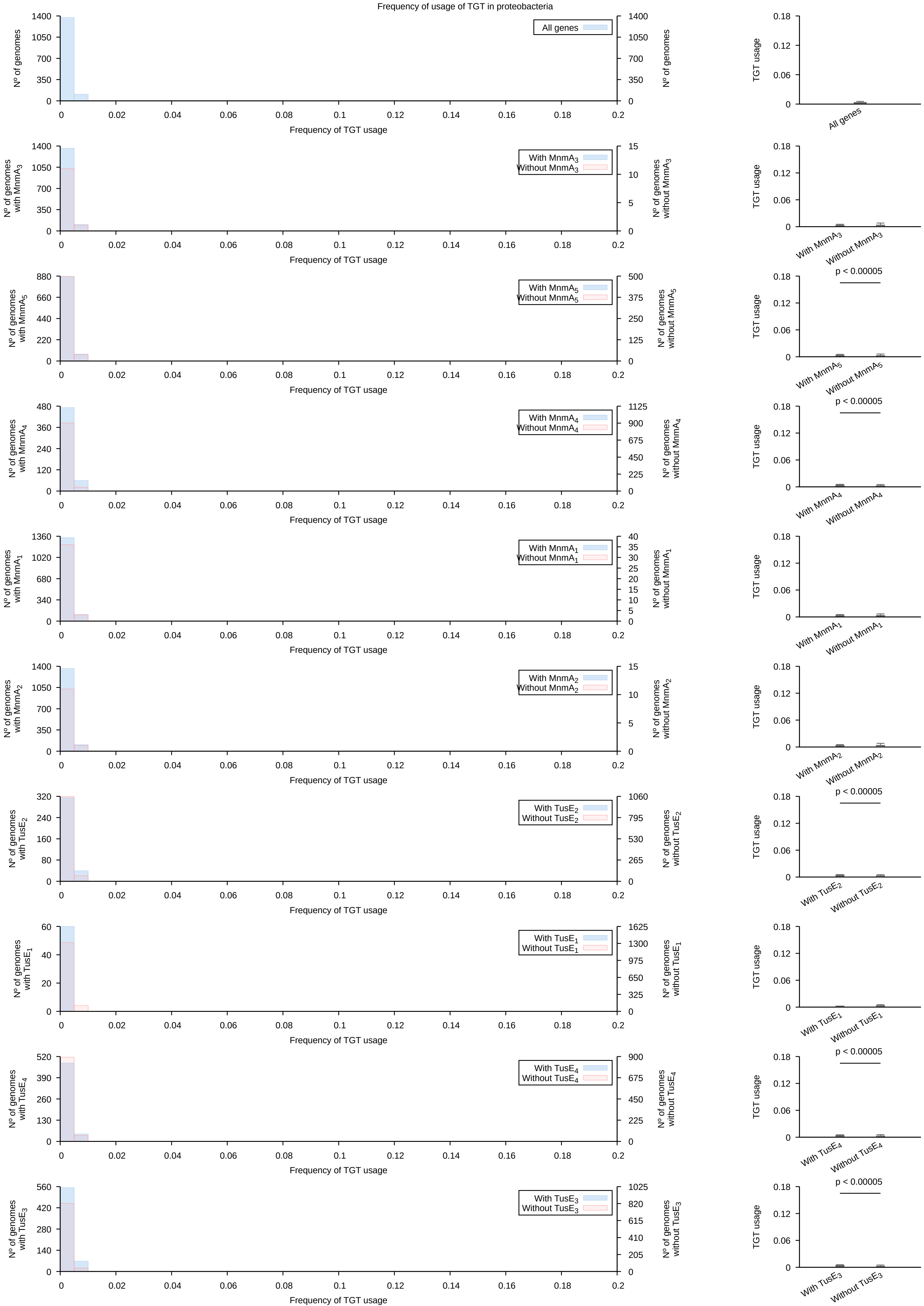

Frequency of usage of TGT in proteobacteria
All genes
With MnmA$_3$
Without MnmA$_3$
With MnmA$_5$
Without MnmA$_5$
With MnmA$_4$
Without MnmA$_4$
With MnmA$_1$
Without MnmA$_1$
With MnmA$_2$
Without MnmA$_2$
With TusE$_2$
Without TusE$_2$
With TusE$_1$
Without TusE$_1$
With TusE$_4$
Without TusE$_4$
With TusE$_3$
Without TusE$_3$
Nº of genomes
Frequency of TGT usage
TGT usage
p < 0.00005

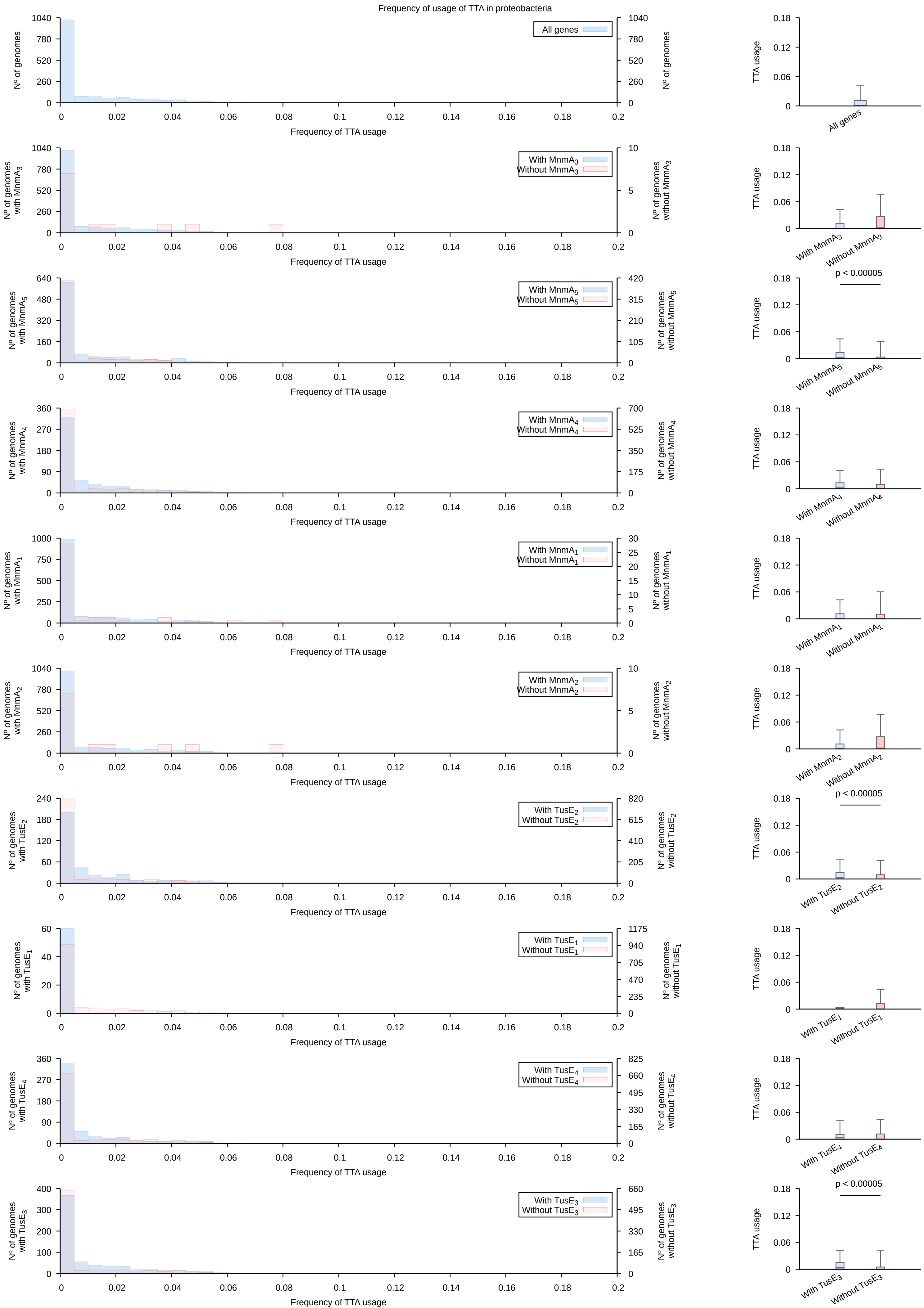

Frequency of usage of TTA in proteobacteria
All genes
Nº of genomes
Frequency of TTA usage
TTA usage
All genes
With $MnmA_3$
Without $MnmA_3$
Nº of genomes with $MnmA_3$
Nº of genomes without $MnmA_3$
With $MnmA_5$
Without $MnmA_5$
Nº of genomes with $MnmA_5$
Nº of genomes without $MnmA_5$
p < 0.00005
With $MnmA_4$
Without $MnmA_4$
Nº of genomes with $MnmA_4$
Nº of genomes without $MnmA_4$
With $MnmA_1$
Without $MnmA_1$
Nº of genomes with $MnmA_1$
Nº of genomes without $MnmA_1$
With $MnmA_2$
Without $MnmA_2$
Nº of genomes with $MnmA_2$
Nº of genomes without $MnmA_2$
With $TusE_2$
Without $TusE_2$
Nº of genomes with $TusE_2$
Nº of genomes without $TusE_2$
p < 0.00005
With $TusE_1$
Without $TusE_1$
Nº of genomes with $TusE_1$
Nº of genomes without $TusE_1$
With $TusE_4$
Without $TusE_4$
Nº of genomes with $TusE_4$
Nº of genomes without $TusE_4$
With $TusE_3$
Without $TusE_3$
Nº of genomes with $TusE_3$
Nº of genomes without $TusE_3$
p < 0.00005

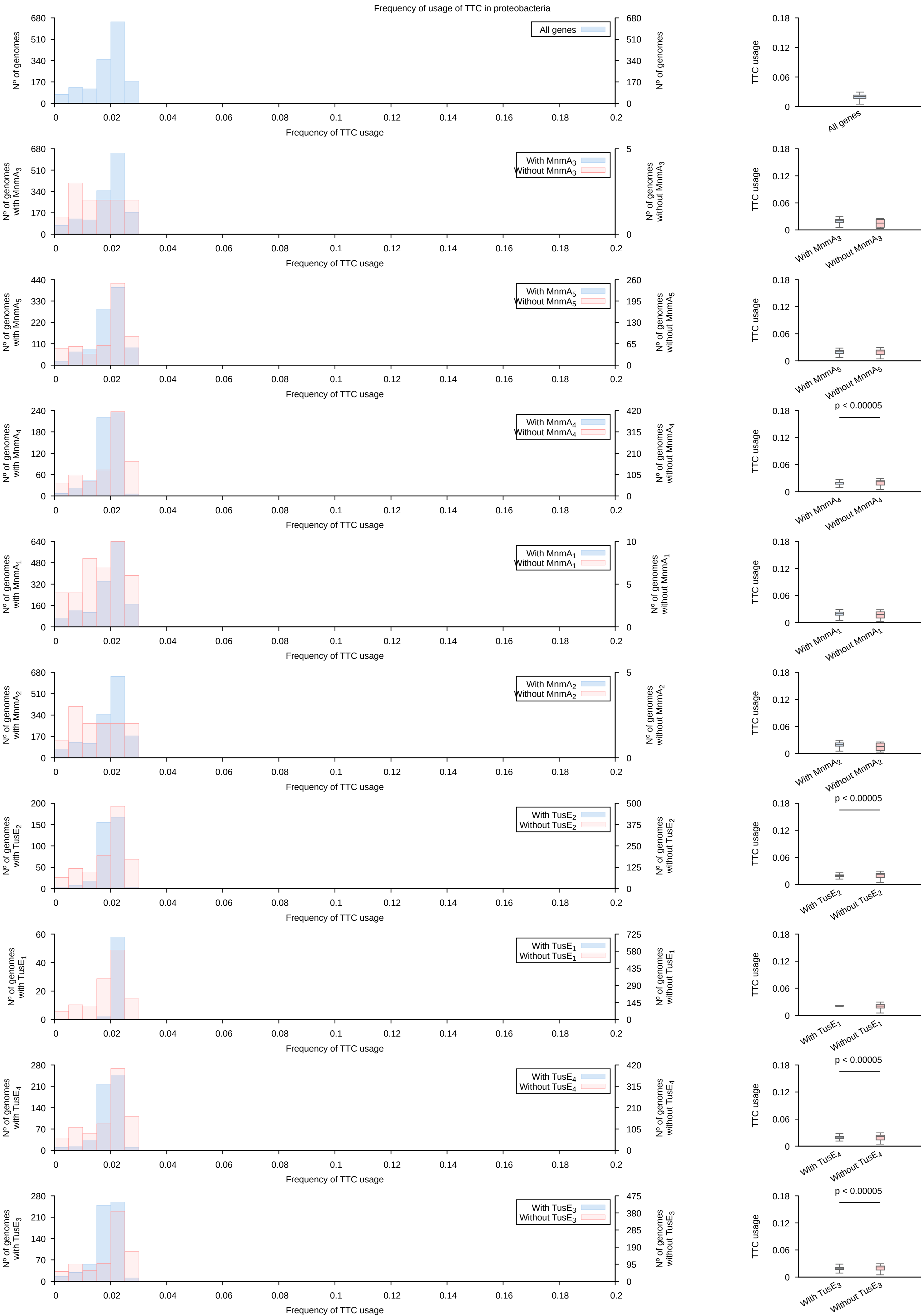

Frequency of usage of TTC in proteobacteria
All genes
Nº of genomes
Frequency of TTC usage
TTC usage
0
0.02
0.04
0.06
0.08
0.1
0.12
0.14
0.16
0.18
0.2
0.06
0.12
0.18
With MnmA3
Without MnmA3
Nº of genomes with MnmA3
Nº of genomes without MnmA3
With MnmA5
Without MnmA5
Nº of genomes with MnmA5
Nº of genomes without MnmA5
With MnmA4
Without MnmA4
Nº of genomes with MnmA4
Nº of genomes without MnmA4
p < 0.00005
With MnmA1
Without MnmA1
Nº of genomes with MnmA1
Nº of genomes without MnmA1
With MnmA2
Without MnmA2
Nº of genomes with MnmA2
Nº of genomes without MnmA2
With TusE2
Without TusE2
Nº of genomes with TusE2
Nº of genomes without TusE2
p < 0.00005
With TusE1
Without TusE1
Nº of genomes with TusE1
Nº of genomes without TusE1
With TusE4
Without TusE4
Nº of genomes with TusE4
Nº of genomes without TusE4
p < 0.00005
With TusE3
Without TusE3
Nº of genomes with TusE3
Nº of genomes without TusE3
p < 0.00005

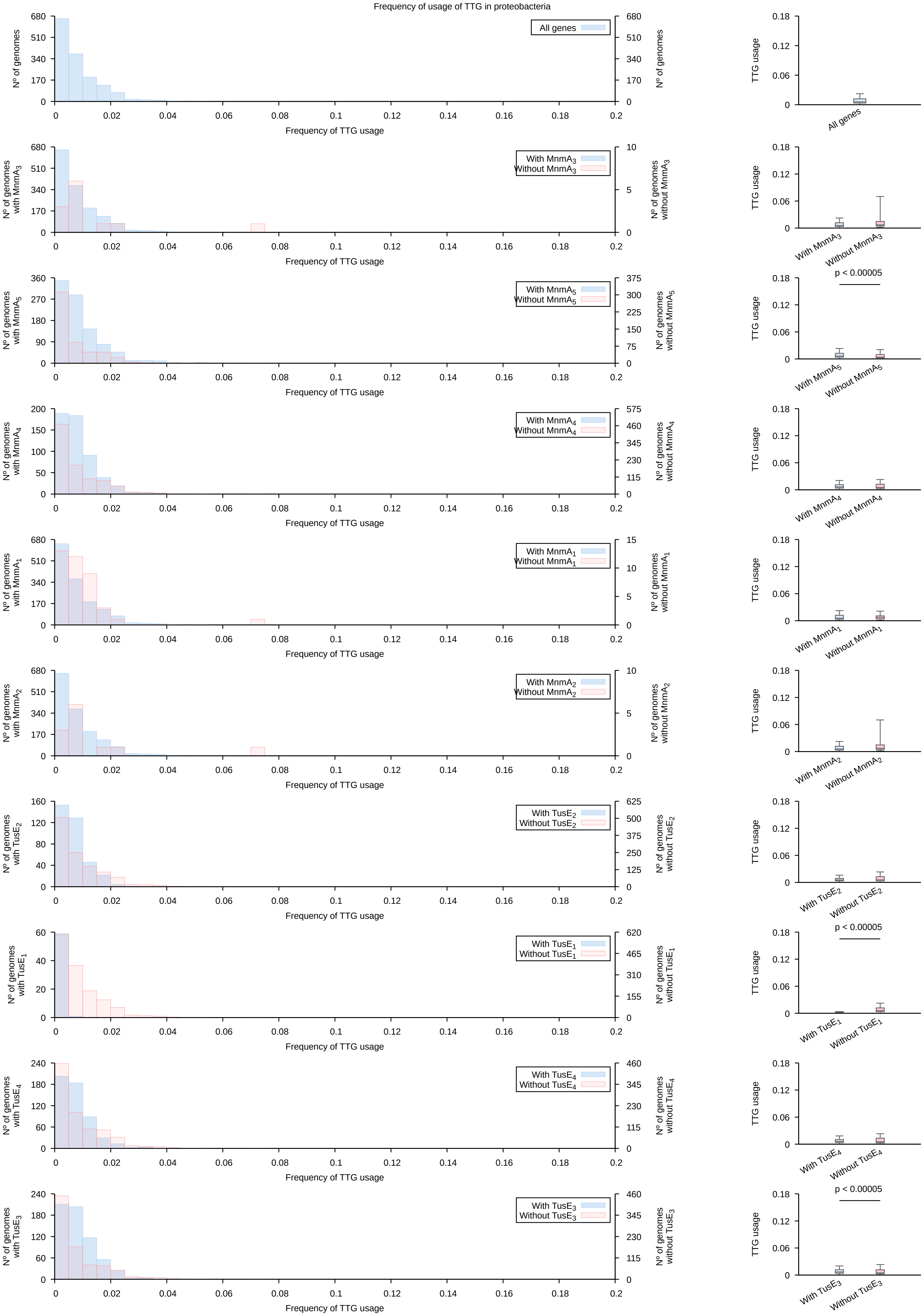

Frequency of usage of TTG in proteobacteria
All genes
Nº of genomes
Frequency of TTG usage
TTG usage
With MnmA$_3$
Without MnmA$_3$
Nº of genomes with MnmA$_3$
Nº of genomes without MnmA$_3$
With MnmA$_5$
Without MnmA$_5$
Nº of genomes with MnmA$_5$
Nº of genomes without MnmA$_5$
p < 0.00005
With MnmA$_4$
Without MnmA$_4$
Nº of genomes with MnmA$_4$
Nº of genomes without MnmA$_4$
With MnmA$_1$
Without MnmA$_1$
Nº of genomes with MnmA$_1$
Nº of genomes without MnmA$_1$
With MnmA$_2$
Without MnmA$_2$
Nº of genomes with MnmA$_2$
Nº of genomes without MnmA$_2$
With TusE$_2$
Without TusE$_2$
Nº of genomes with TusE$_2$
Nº of genomes without TusE$_2$
With TusE$_1$
Without TusE$_1$
Nº of genomes with TusE$_1$
Nº of genomes without TusE$_1$
p < 0.00005
With TusE$_4$
Without TusE$_4$
Nº of genomes with TusE$_4$
Nº of genomes without TusE$_4$
With TusE$_3$
Without TusE$_3$
Nº of genomes with TusE$_3$
Nº of genomes without TusE$_3$
p < 0.00005

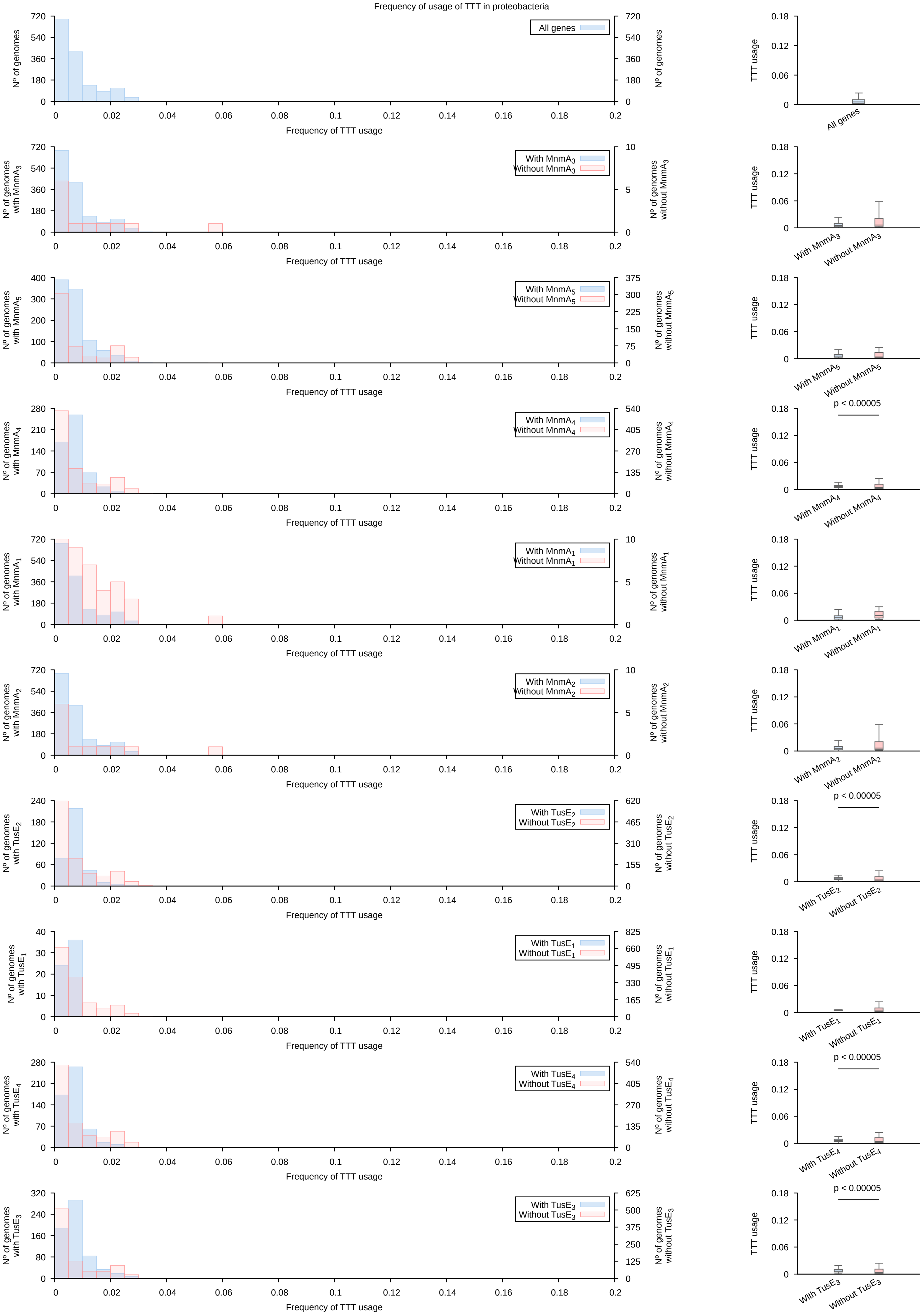
Frequency of usage of TTT in proteobacteria
Nº of genomes
All genes
Frequency of TTT usage
TTT usage
Nº of genomes with $MnmA_3$
Nº of genomes without $MnmA_3$
With $MnmA_3$
Without $MnmA_3$
Nº of genomes with $MnmA_5$
Nº of genomes without $MnmA_5$
With $MnmA_5$
Without $MnmA_5$
Nº of genomes with $MnmA_4$
Nº of genomes without $MnmA_4$
With $MnmA_4$
Without $MnmA_4$
$p < 0.00005$
Nº of genomes with $MnmA_1$
Nº of genomes without $MnmA_1$
With $MnmA_1$
Without $MnmA_1$
Nº of genomes with $MnmA_2$
Nº of genomes without $MnmA_2$
With $MnmA_2$
Without $MnmA_2$
Nº of genomes with $TusE_2$
Nº of genomes without $TusE_2$
With $TusE_2$
Without $TusE_2$
$p < 0.00005$
Nº of genomes with $TusE_1$
Nº of genomes without $TusE_1$
With $TusE_1$
Without $TusE_1$
Nº of genomes with $TusE_4$
Nº of genomes without $TusE_4$
With $TusE_4$
Without $TusE_4$
$p < 0.00005$
Nº of genomes with $TusE_3$
Nº of genomes without $TusE_3$
With $TusE_3$
Without $TusE_3$
$p < 0.00005$
