# Supplementary material for: Impact of the chemical modification of tRNAs anticodon loop on the variability and evolution of codon usage in proteobacteria
Source: Front Microbiol. 2024 Aug 5;15:1412318. doi: 10.3389/fmicb.2024.1412318 (PMC11332805; doi:10.3389/fmicb.2024.1412318)
Supplement: Supplementary file 1 [file Data_Sheet_1.zip › Supp_figures/Fig_S13.pdf]

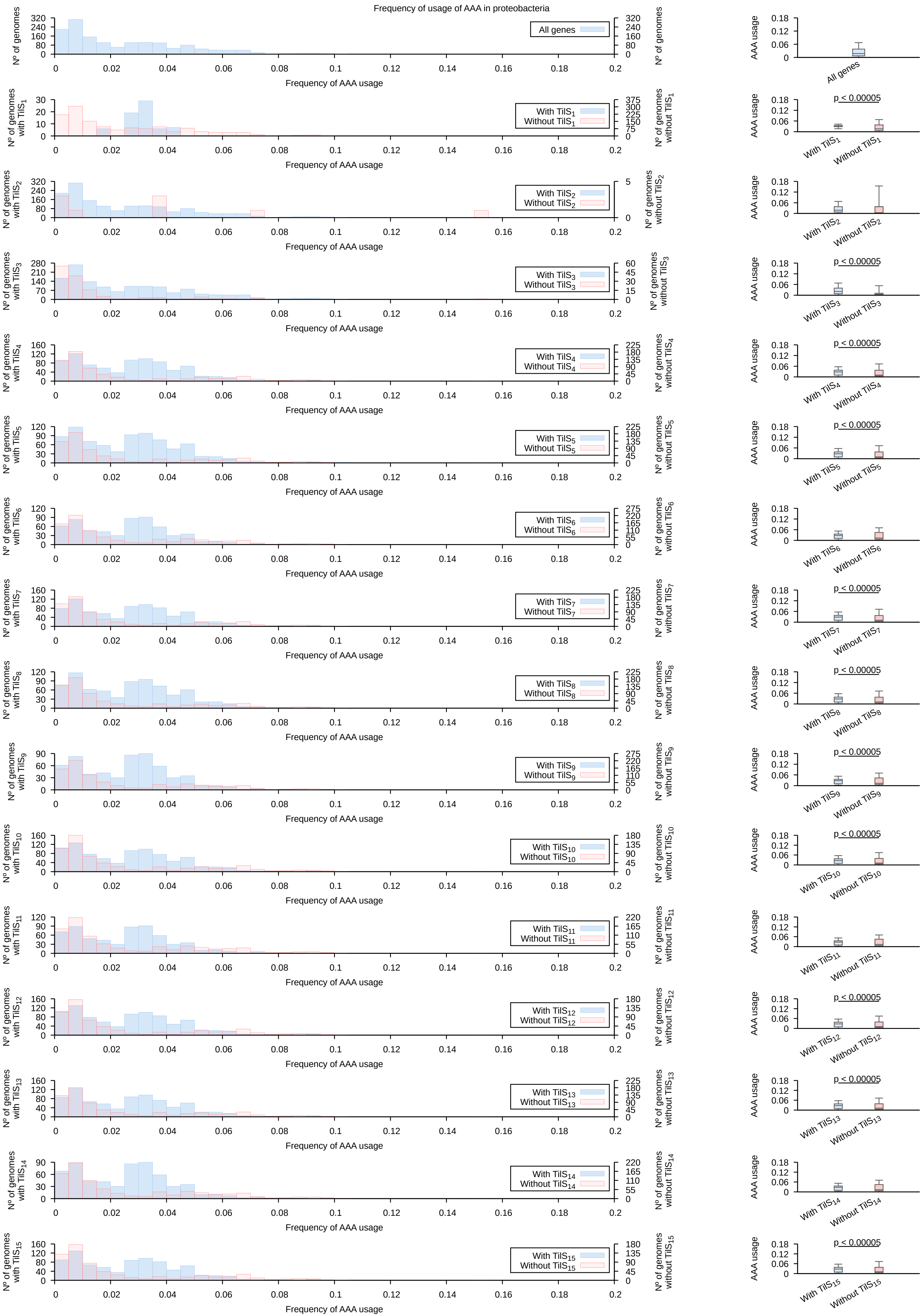
Frequency of usage of AAA in proteobacteria
Nº of genomes
All genes
Frequency of AAA usage
AAA usage
Nº of genomes with TilS1
Nº of genomes without TilS1
With TilS1
Without TilS1
p < 0.00005
Nº of genomes with TilS2
Nº of genomes without TilS2
With TilS2
Without TilS2
Nº of genomes with TilS3
Nº of genomes without TilS3
With TilS3
Without TilS3
Nº of genomes with TilS4
Nº of genomes without TilS4
With TilS4
Without TilS4
Nº of genomes with TilS5
Nº of genomes without TilS5
With TilS5
Without TilS5
Nº of genomes with TilS6
Nº of genomes without TilS6
With TilS6
Without TilS6
Nº of genomes with TilS7
Nº of genomes without TilS7
With TilS7
Without TilS7
Nº of genomes with TilS8
Nº of genomes without TilS8
With TilS8
Without TilS8
Nº of genomes with TilS9
Nº of genomes without TilS9
With TilS9
Without TilS9
Nº of genomes with TilS10
Nº of genomes without TilS10
With TilS10
Without TilS10
Nº of genomes with TilS11
Nº of genomes without TilS11
With TilS11
Without TilS11
Nº of genomes with TilS12
Nº of genomes without TilS12
With TilS12
Without TilS12
Nº of genomes with TilS13
Nº of genomes without TilS13
With TilS13
Without TilS13
Nº of genomes with TilS14
Nº of genomes without TilS14
With TilS14
Without TilS14
Nº of genomes with TilS15
Nº of genomes without TilS15
With TilS15
Without TilS15

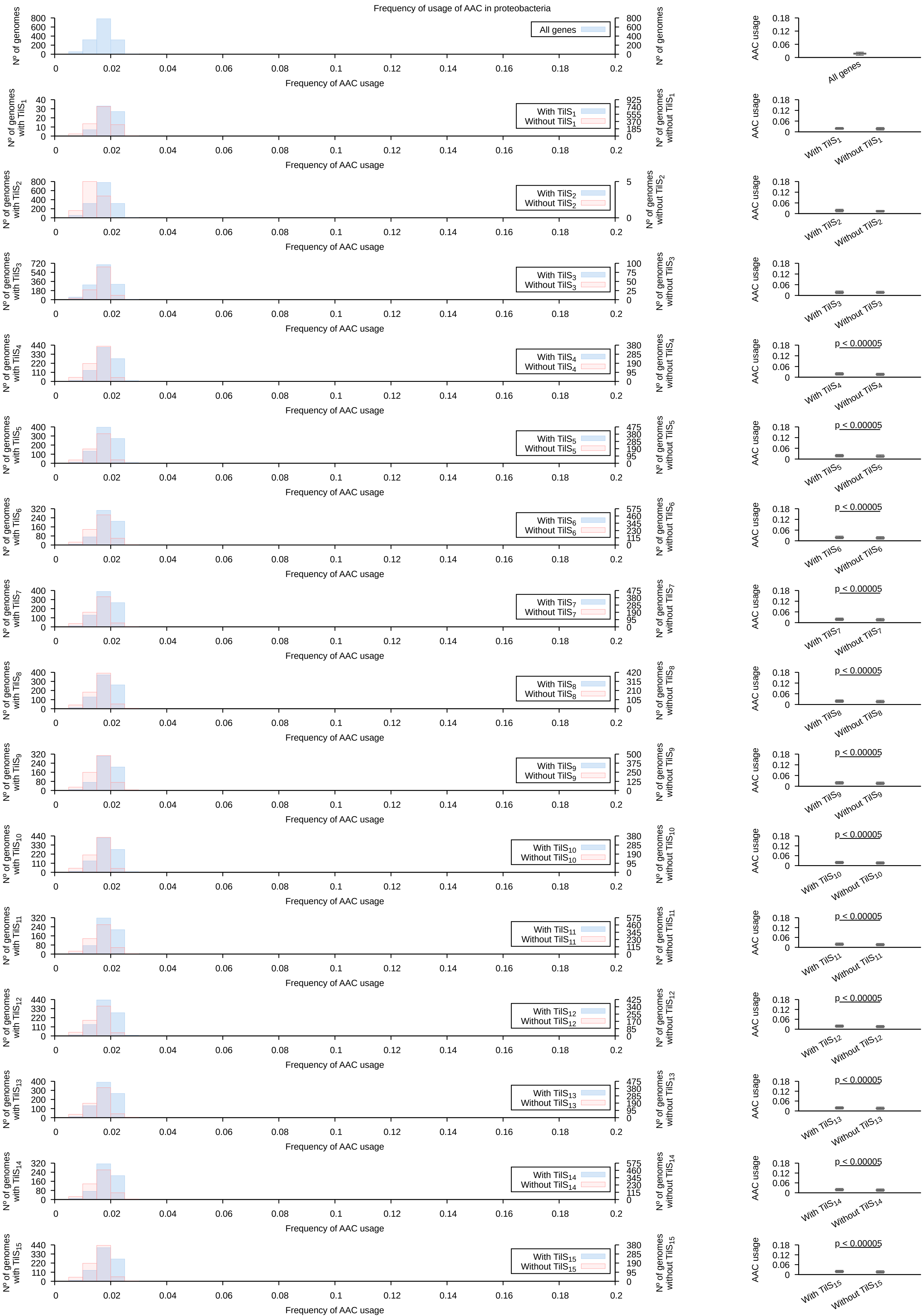

Frequency of usage of AAC in proteobacteria
Nº of genomes
All genes
Frequency of AAC usage
AAC usage
0 0.02 0.04 0.06 0.08 0.1 0.12 0.14 0.16 0.18 0.2
0 0.06 0.12 0.18
Nº of genomes with $TilS_1$
Nº of genomes without $TilS_1$
With $TilS_1$
Without $TilS_1$
Nº of genomes with $TilS_2$
Nº of genomes without $TilS_2$
With $TilS_2$
Without $TilS_2$
Nº of genomes with $TilS_3$
Nº of genomes without $TilS_3$
With $TilS_3$
Without $TilS_3$
Nº of genomes with $TilS_4$
Nº of genomes without $TilS_4$
With $TilS_4$
Without $TilS_4$
p < 0.00005
Nº of genomes with $TilS_5$
Nº of genomes without $TilS_5$
With $TilS_5$
Without $TilS_5$
p < 0.00005
Nº of genomes with $TilS_6$
Nº of genomes without $TilS_6$
With $TilS_6$
Without $TilS_6$
p < 0.00005
Nº of genomes with $TilS_7$
Nº of genomes without $TilS_7$
With $TilS_7$
Without $TilS_7$
p < 0.00005
Nº of genomes with $TilS_8$
Nº of genomes without $TilS_8$
With $TilS_8$
Without $TilS_8$
p < 0.00005
Nº of genomes with $TilS_9$
Nº of genomes without $TilS_9$
With $TilS_9$
Without $TilS_9$
p < 0.00005
Nº of genomes with $TilS_{10}$
Nº of genomes without $TilS_{10}$
With $TilS_{10}$
Without $TilS_{10}$
p < 0.00005
Nº of genomes with $TilS_{11}$
Nº of genomes without $TilS_{11}$
With $TilS_{11}$
Without $TilS_{11}$
p < 0.00005
Nº of genomes with $TilS_{12}$
Nº of genomes without $TilS_{12}$
With $TilS_{12}$
Without $TilS_{12}$
p < 0.00005
Nº of genomes with $TilS_{13}$
Nº of genomes without $TilS_{13}$
With $TilS_{13}$
Without $TilS_{13}$
p < 0.00005
Nº of genomes with $TilS_{14}$
Nº of genomes without $TilS_{14}$
With $TilS_{14}$
Without $TilS_{14}$
p < 0.00005
Nº of genomes with $TilS_{15}$
Nº of genomes without $TilS_{15}$
With $TilS_{15}$
Without $TilS_{15}$
p < 0.00005

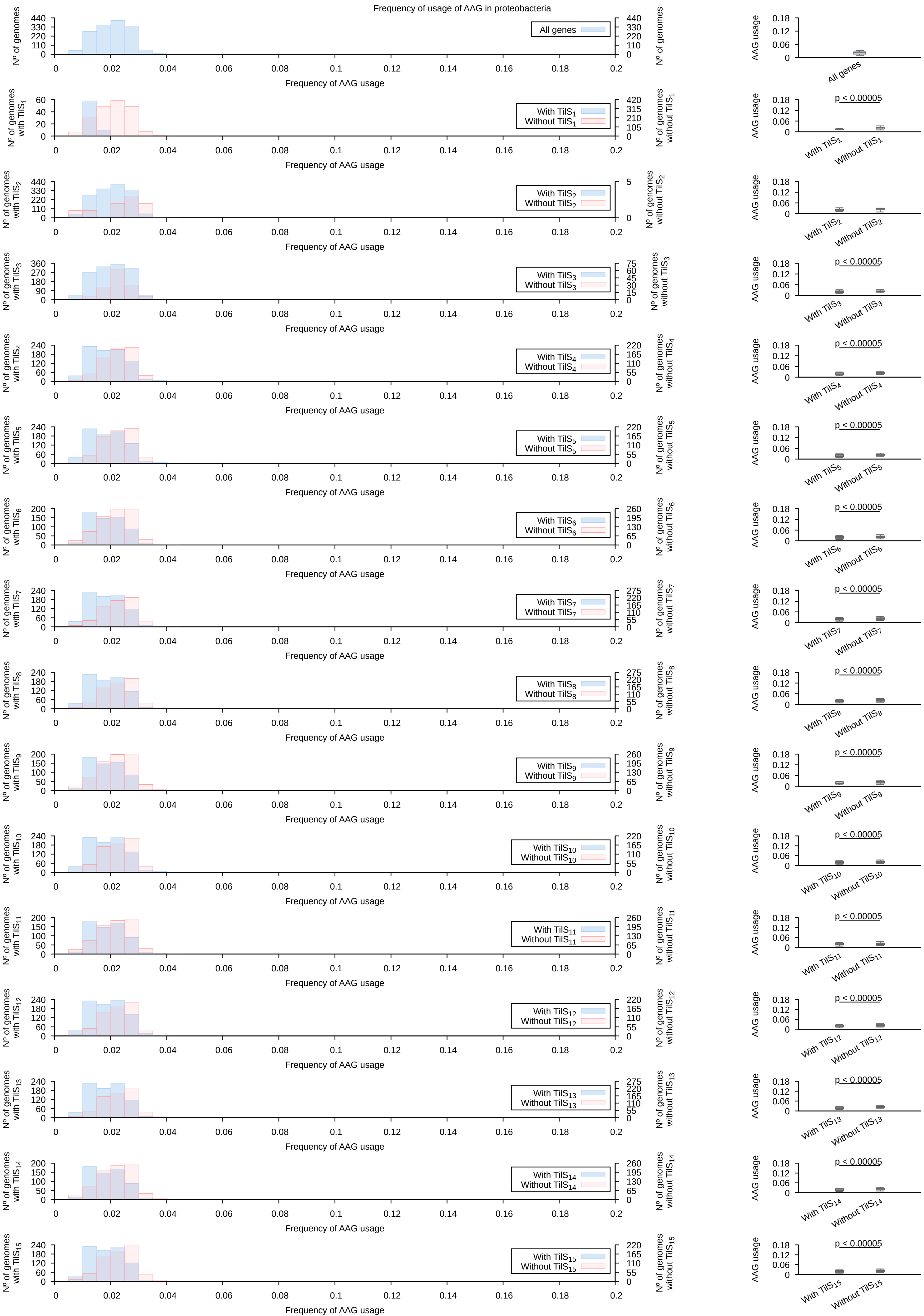

Frequency of usage of AAG in proteobacteria
Nº of genomes
All genes
Frequency of AAG usage
AAG usage
p < 0.00005
With TilS$_1$
Without TilS$_1$
With TilS$_2$
Without TilS$_2$
With TilS$_3$
Without TilS$_3$
With TilS$_4$
Without TilS$_4$
With TilS$_5$
Without TilS$_5$
With TilS$_6$
Without TilS$_6$
With TilS$_7$
Without TilS$_7$
With TilS$_8$
Without TilS$_8$
With TilS$_9$
Without TilS$_9$
With TilS$_{10}$
Without TilS$_{10}$
With TilS$_{11}$
Without TilS$_{11}$
With TilS$_{12}$
Without TilS$_{12}$
With TilS$_{13}$
Without TilS$_{13}$
With TilS$_{14}$
Without TilS$_{14}$
With TilS$_{15}$
Without TilS$_{15}$

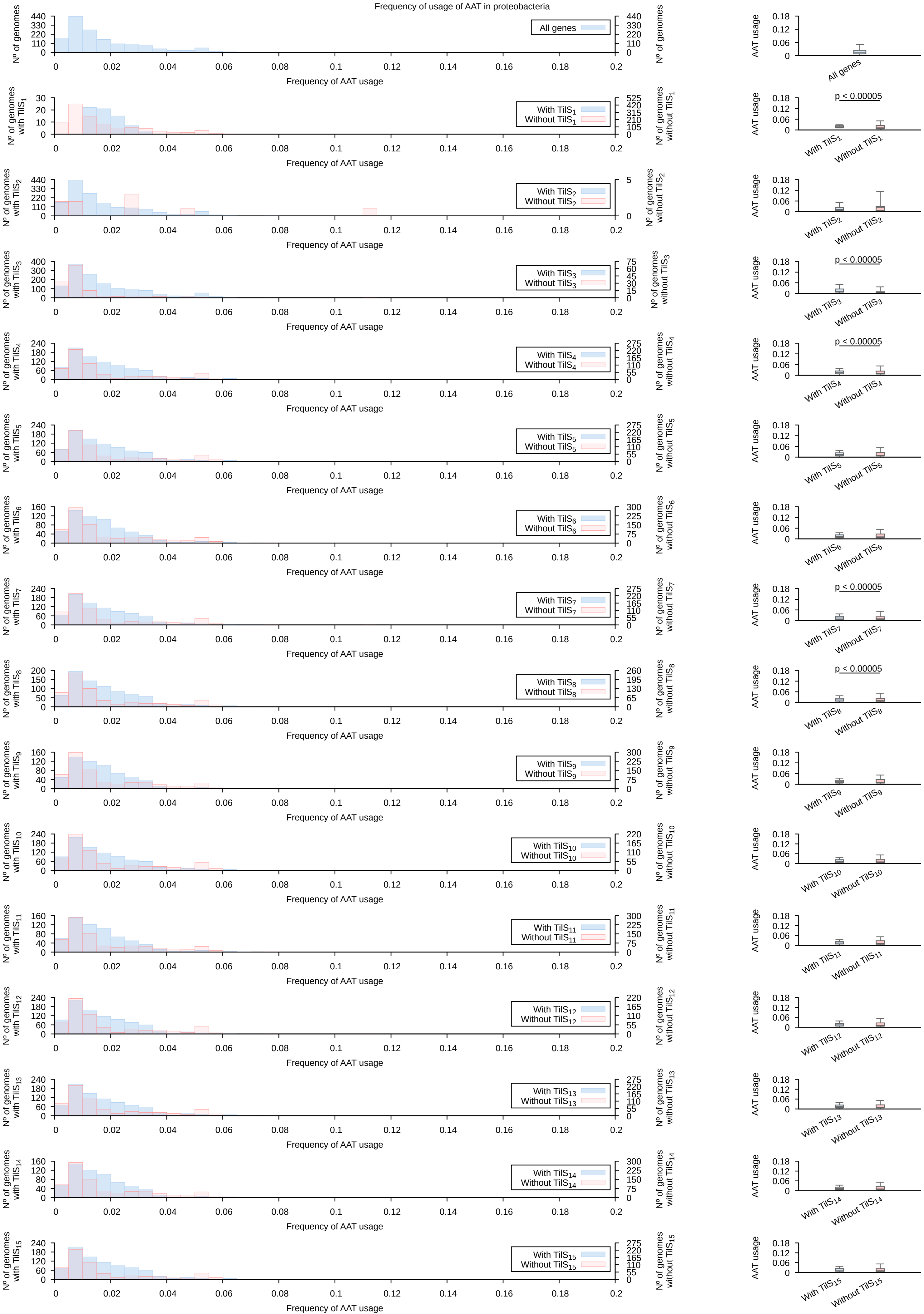
Frequency of usage of AAT in proteobacteria
Nº of genomes
All genes
Frequency of AAT usage
AAT usage
Nº of genomes with TilS1
Nº of genomes without TilS1
With TilS1
Without TilS1
p < 0.00005
Nº of genomes with TilS2
Nº of genomes without TilS2
With TilS2
Without TilS2
Nº of genomes with TilS3
Nº of genomes without TilS3
With TilS3
Without TilS3
p < 0.00005
Nº of genomes with TilS4
Nº of genomes without TilS4
With TilS4
Without TilS4
p < 0.00005
Nº of genomes with TilS5
Nº of genomes without TilS5
With TilS5
Without TilS5
Nº of genomes with TilS6
Nº of genomes without TilS6
With TilS6
Without TilS6
Nº of genomes with TilS7
Nº of genomes without TilS7
With TilS7
Without TilS7
p < 0.00005
Nº of genomes with TilS8
Nº of genomes without TilS8
With TilS8
Without TilS8
p < 0.00005
Nº of genomes with TilS9
Nº of genomes without TilS9
With TilS9
Without TilS9
Nº of genomes with TilS10
Nº of genomes without TilS10
With TilS10
Without TilS10
Nº of genomes with TilS11
Nº of genomes without TilS11
With TilS11
Without TilS11
Nº of genomes with TilS12
Nº of genomes without TilS12
With TilS12
Without TilS12
Nº of genomes with TilS13
Nº of genomes without TilS13
With TilS13
Without TilS13
Nº of genomes with TilS14
Nº of genomes without TilS14
With TilS14
Without TilS14
Nº of genomes with TilS15
Nº of genomes without TilS15
With TilS15
Without TilS15

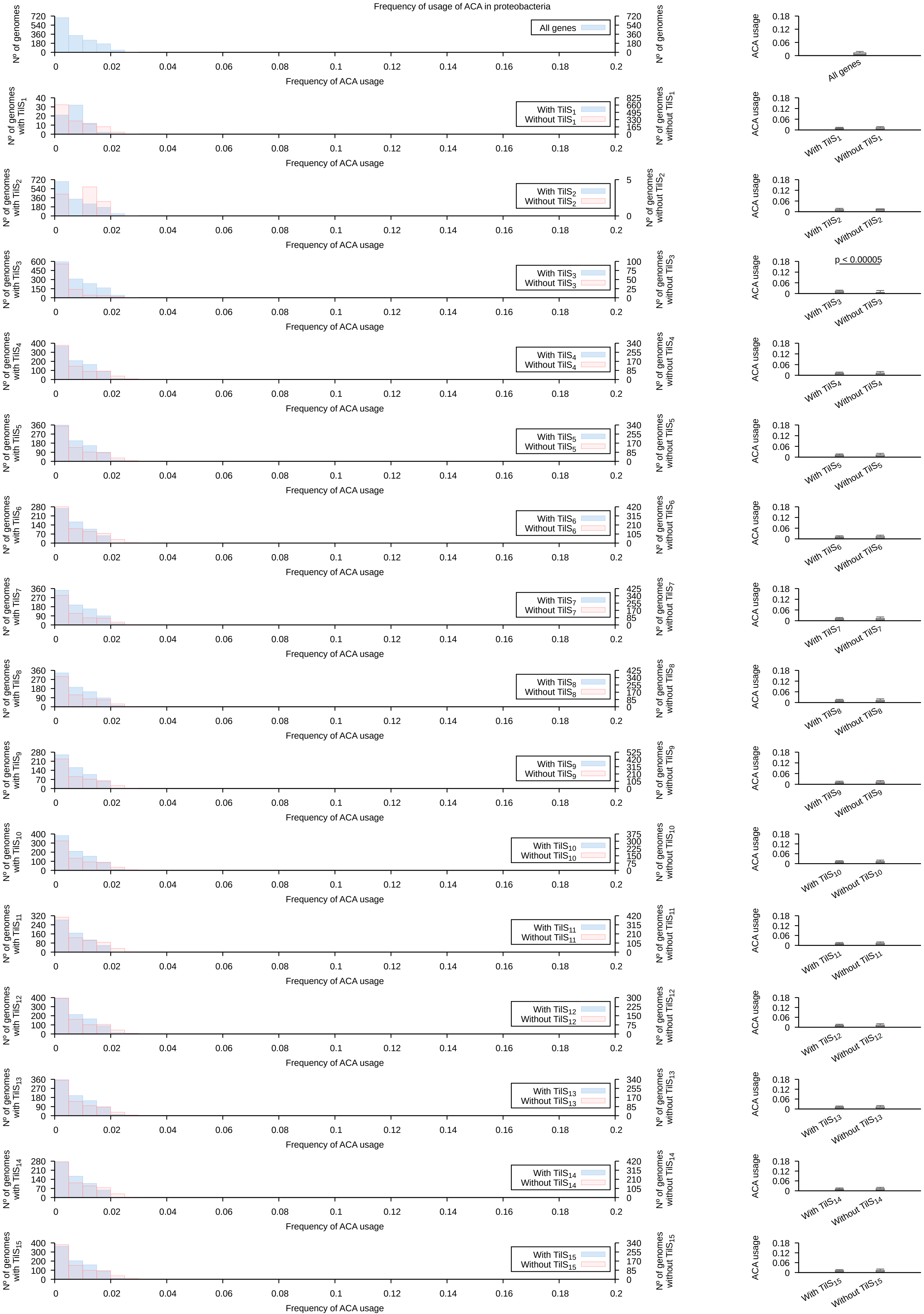
Frequency of usage of ACA in proteobacteria
Nº of genomes
All genes
Frequency of ACA usage
ACA usage
Nº of genomes with TilS$_1$
Nº of genomes without TilS$_1$
With TilS$_1$
Without TilS$_1$
Nº of genomes with TilS$_2$
Nº of genomes without TilS$_2$
With TilS$_2$
Without TilS$_2$
Nº of genomes with TilS$_3$
Nº of genomes without TilS$_3$
With TilS$_3$
Without TilS$_3$
p < 0.00005
Nº of genomes with TilS$_4$
Nº of genomes without TilS$_4$
With TilS$_4$
Without TilS$_4$
Nº of genomes with TilS$_5$
Nº of genomes without TilS$_5$
With TilS$_5$
Without TilS$_5$
Nº of genomes with TilS$_6$
Nº of genomes without TilS$_6$
With TilS$_6$
Without TilS$_6$
Nº of genomes with TilS$_7$
Nº of genomes without TilS$_7$
With TilS$_7$
Without TilS$_7$
Nº of genomes with TilS$_8$
Nº of genomes without TilS$_8$
With TilS$_8$
Without TilS$_8$
Nº of genomes with TilS$_9$
Nº of genomes without TilS$_9$
With TilS$_9$
Without TilS$_9$
Nº of genomes with TilS$_{10}$
Nº of genomes without TilS$_{10}$
With TilS$_{10}$
Without TilS$_{10}$
Nº of genomes with TilS$_{11}$
Nº of genomes without TilS$_{11}$
With TilS$_{11}$
Without TilS$_{11}$
Nº of genomes with TilS$_{12}$
Nº of genomes without TilS$_{12}$
With TilS$_{12}$
Without TilS$_{12}$
Nº of genomes with TilS$_{13}$
Nº of genomes without TilS$_{13}$
With TilS$_{13}$
Without TilS$_{13}$
Nº of genomes with TilS$_{14}$
Nº of genomes without TilS$_{14}$
With TilS$_{14}$
Without TilS$_{14}$
Nº of genomes with TilS$_{15}$
Nº of genomes without TilS$_{15}$
With TilS$_{15}$
Without TilS$_{15}$

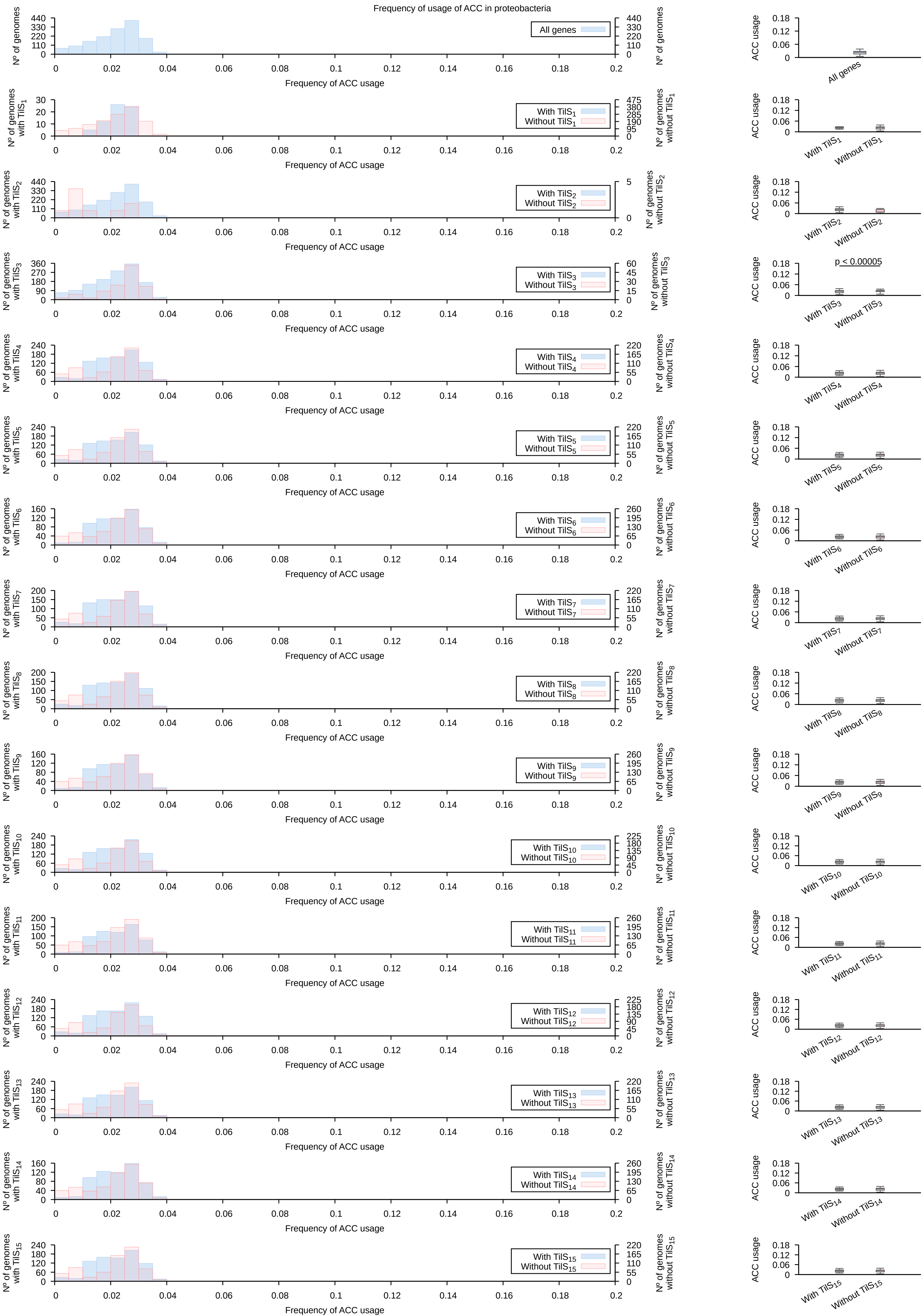

Frequency of usage of ACC in proteobacteria
Nº of genomes
All genes
Frequency of ACC usage
ACC usage
Nº of genomes with TilS1
Nº of genomes without TilS1
With TilS1
Without TilS1
Nº of genomes with TilS2
Nº of genomes without TilS2
With TilS2
Without TilS2
Nº of genomes with TilS3
Nº of genomes without TilS3
With TilS3
Without TilS3
p < 0.00005
Nº of genomes with TilS4
Nº of genomes without TilS4
With TilS4
Without TilS4
Nº of genomes with TilS5
Nº of genomes without TilS5
With TilS5
Without TilS5
Nº of genomes with TilS6
Nº of genomes without TilS6
With TilS6
Without TilS6
Nº of genomes with TilS7
Nº of genomes without TilS7
With TilS7
Without TilS7
Nº of genomes with TilS8
Nº of genomes without TilS8
With TilS8
Without TilS8
Nº of genomes with TilS9
Nº of genomes without TilS9
With TilS9
Without TilS9
Nº of genomes with TilS10
Nº of genomes without TilS10
With TilS10
Without TilS10
Nº of genomes with TilS11
Nº of genomes without TilS11
With TilS11
Without TilS11
Nº of genomes with TilS12
Nº of genomes without TilS12
With TilS12
Without TilS12
Nº of genomes with TilS13
Nº of genomes without TilS13
With TilS13
Without TilS13
Nº of genomes with TilS14
Nº of genomes without TilS14
With TilS14
Without TilS14
Nº of genomes with TilS15
Nº of genomes without TilS15
With TilS15
Without TilS15

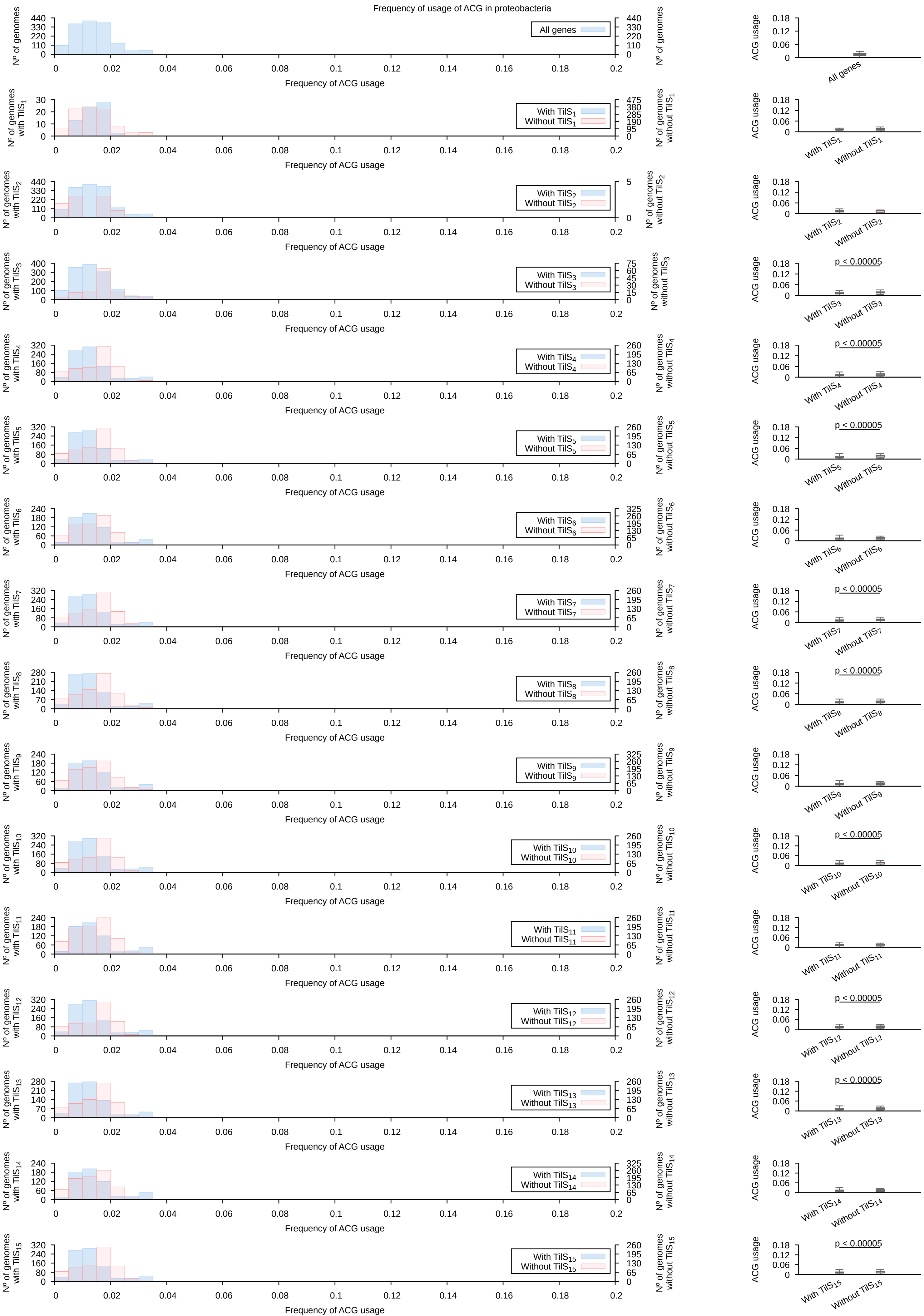

Frequency of usage of ACG in proteobacteria
Nº of genomes
All genes
Frequency of ACG usage
ACG usage
Nº of genomes with TilS$_1$
With TilS$_1$
Without TilS$_1$
Nº of genomes without TilS$_1$
Nº of genomes with TilS$_2$
With TilS$_2$
Without TilS$_2$
Nº of genomes without TilS$_2$
Nº of genomes with TilS$_3$
With TilS$_3$
Without TilS$_3$
Nº of genomes without TilS$_3$
p < 0.00005
Nº of genomes with TilS$_4$
With TilS$_4$
Without TilS$_4$
Nº of genomes without TilS$_4$
p < 0.00005
Nº of genomes with TilS$_5$
With TilS$_5$
Without TilS$_5$
Nº of genomes without TilS$_5$
p < 0.00005
Nº of genomes with TilS$_6$
With TilS$_6$
Without TilS$_6$
Nº of genomes without TilS$_6$
Nº of genomes with TilS$_7$
With TilS$_7$
Without TilS$_7$
Nº of genomes without TilS$_7$
p < 0.00005
Nº of genomes with TilS$_8$
With TilS$_8$
Without TilS$_8$
Nº of genomes without TilS$_8$
p < 0.00005
Nº of genomes with TilS$_9$
With TilS$_9$
Without TilS$_9$
Nº of genomes without TilS$_9$
Nº of genomes with TilS$_{10}$
With TilS$_{10}$
Without TilS$_{10}$
Nº of genomes without TilS$_{10}$
p < 0.00005
Nº of genomes with TilS$_{11}$
With TilS$_{11}$
Without TilS$_{11}$
Nº of genomes without TilS$_{11}$
Nº of genomes with TilS$_{12}$
With TilS$_{12}$
Without TilS$_{12}$
Nº of genomes without TilS$_{12}$
p < 0.00005
Nº of genomes with TilS$_{13}$
With TilS$_{13}$
Without TilS$_{13}$
Nº of genomes without TilS$_{13}$
p < 0.00005
Nº of genomes with TilS$_{14}$
With TilS$_{14}$
Without TilS$_{14}$
Nº of genomes without TilS$_{14}$
Nº of genomes with TilS$_{15}$
With TilS$_{15}$
Without TilS$_{15}$
Nº of genomes without TilS$_{15}$
p < 0.00005

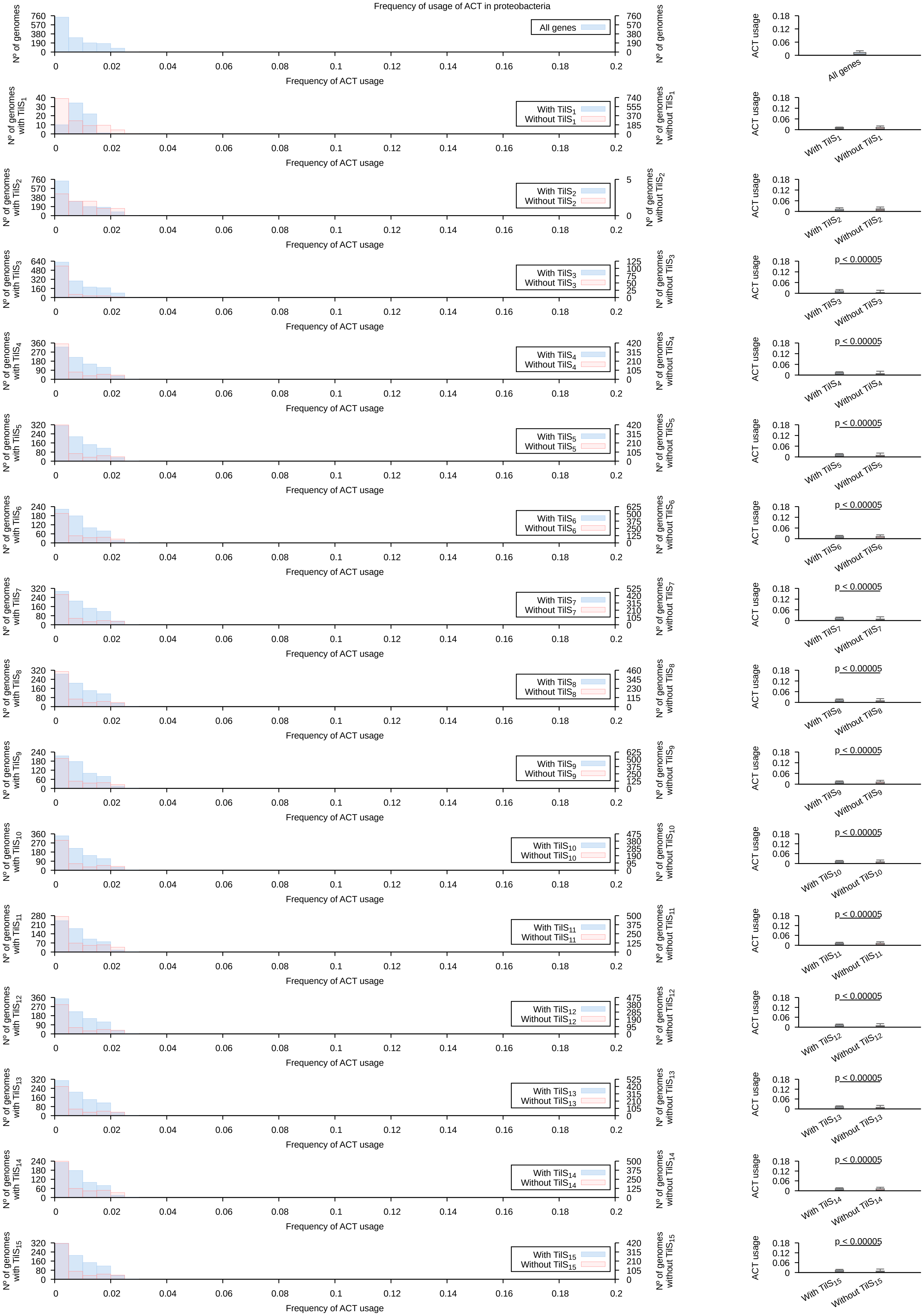
Frequency of usage of ACT in proteobacteria
Nº of genomes
All genes
Frequency of ACT usage
ACT usage
Nº of genomes with TilS$_1$
Nº of genomes without TilS$_1$
With TilS$_1$
Without TilS$_1$
Nº of genomes with TilS$_2$
Nº of genomes without TilS$_2$
With TilS$_2$
Without TilS$_2$
Nº of genomes with TilS$_3$
Nº of genomes without TilS$_3$
With TilS$_3$
Without TilS$_3$
Nº of genomes with TilS$_4$
Nº of genomes without TilS$_4$
With TilS$_4$
Without TilS$_4$
Nº of genomes with TilS$_5$
Nº of genomes without TilS$_5$
With TilS$_5$
Without TilS$_5$
Nº of genomes with TilS$_6$
Nº of genomes without TilS$_6$
With TilS$_6$
Without TilS$_6$
Nº of genomes with TilS$_7$
Nº of genomes without TilS$_7$
With TilS$_7$
Without TilS$_7$
Nº of genomes with TilS$_8$
Nº of genomes without TilS$_8$
With TilS$_8$
Without TilS$_8$
Nº of genomes with TilS$_9$
Nº of genomes without TilS$_9$
With TilS$_9$
Without TilS$_9$
Nº of genomes with TilS$_{10}$
Nº of genomes without TilS$_{10}$
With TilS$_{10}$
Without TilS$_{10}$
Nº of genomes with TilS$_{11}$
Nº of genomes without TilS$_{11}$
With TilS$_{11}$
Without TilS$_{11}$
Nº of genomes with TilS$_{12}$
Nº of genomes without TilS$_{12}$
With TilS$_{12}$
Without TilS$_{12}$
Nº of genomes with TilS$_{13}$
Nº of genomes without TilS$_{13}$
With TilS$_{13}$
Without TilS$_{13}$
Nº of genomes with TilS$_{14}$
Nº of genomes without TilS$_{14}$
With TilS$_{14}$
Without TilS$_{14}$
Nº of genomes with TilS$_{15}$
Nº of genomes without TilS$_{15}$
With TilS$_{15}$
Without TilS$_{15}$
$p < 0.00005$

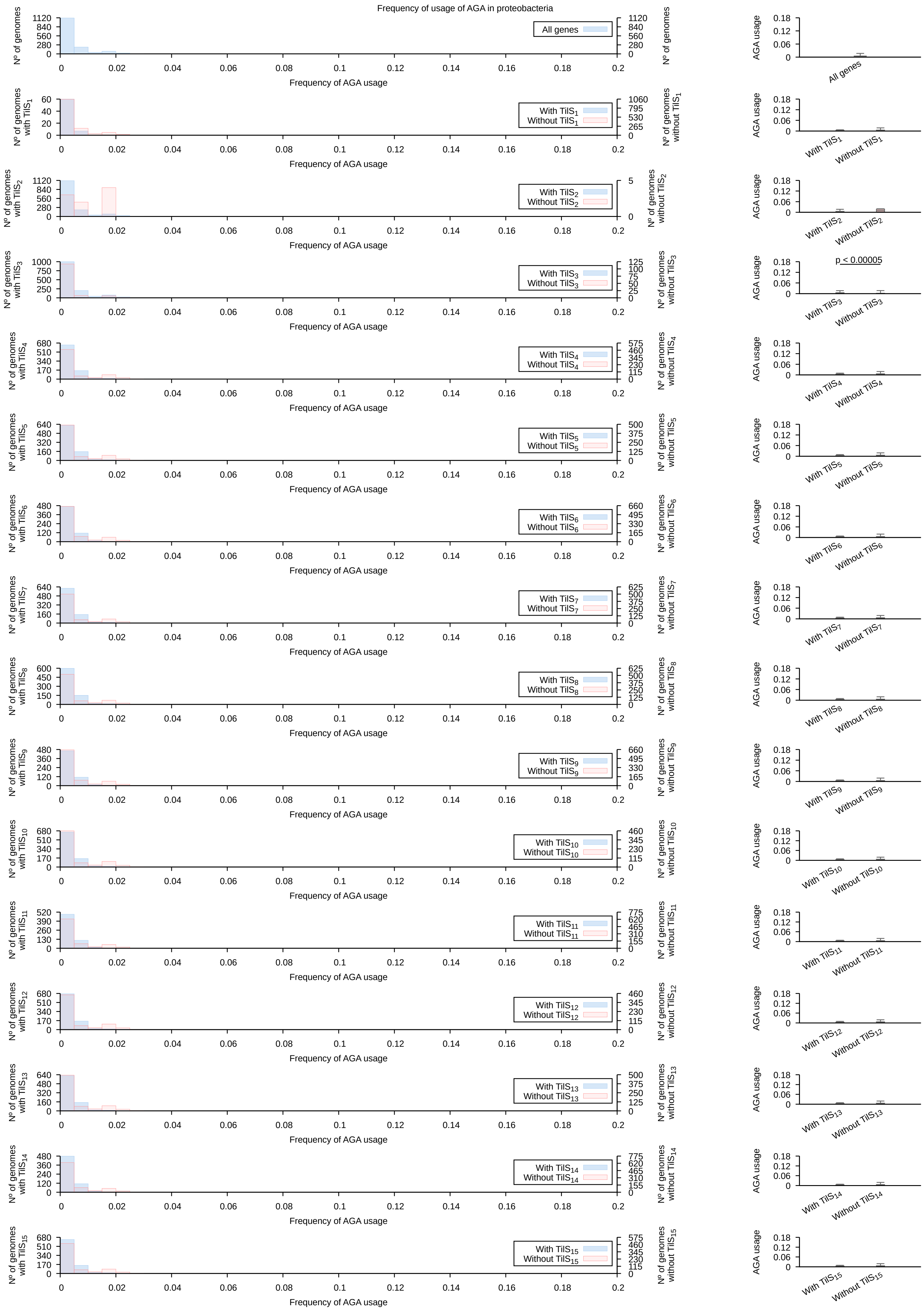

Frequency of usage of AGA in proteobacteria
All genes
Nº of genomes
Frequency of AGA usage
AGA usage
0
0.02
0.04
0.06
0.08
0.1
0.12
0.14
0.16
0.18
0.2
0.06
0.12
0.18
1120
840
560
280
With TilS1
Without TilS1
Nº of genomes with TilS1
Nº of genomes without TilS1
With TilS2
Without TilS2
With TilS3
Without TilS3
p < 0.00005
With TilS4
Without TilS4
With TilS5
Without TilS5
With TilS6
Without TilS6
With TilS7
Without TilS7
With TilS8
Without TilS8
With TilS9
Without TilS9
With TilS10
Without TilS10
With TilS11
Without TilS11
With TilS12
Without TilS12
With TilS13
Without TilS13
With TilS14
Without TilS14
With TilS15
Without TilS15

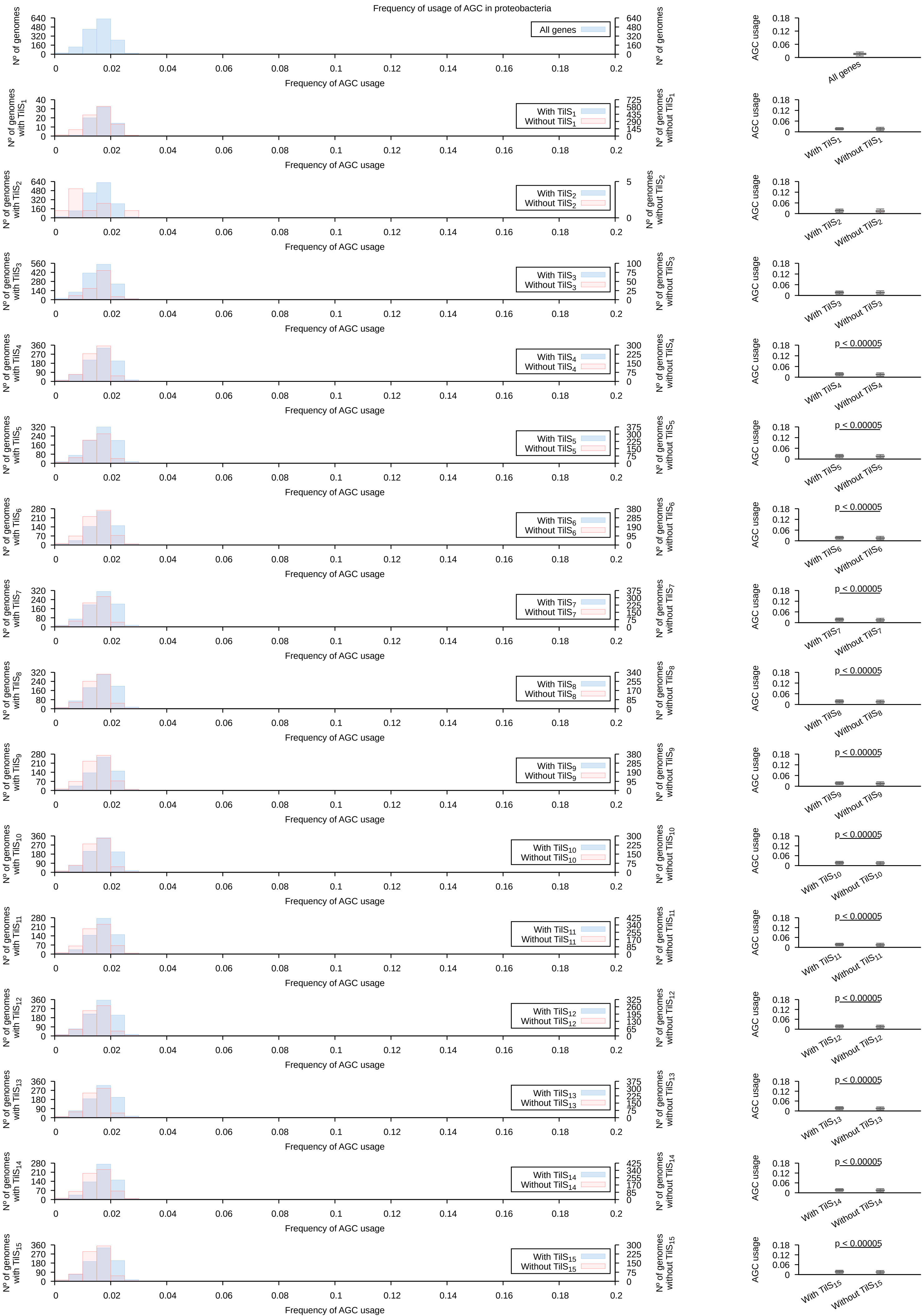

Frequency of usage of AGC in proteobacteria
Nº of genomes
All genes
Frequency of AGC usage
AGC usage
Nº of genomes with $TilS_1$
Nº of genomes without $TilS_1$
With $TilS_1$
Without $TilS_1$
Nº of genomes with $TilS_2$
Nº of genomes without $TilS_2$
With $TilS_2$
Without $TilS_2$
Nº of genomes with $TilS_3$
Nº of genomes without $TilS_3$
With $TilS_3$
Without $TilS_3$
Nº of genomes with $TilS_4$
Nº of genomes without $TilS_4$
With $TilS_4$
Without $TilS_4$
p < 0.00005
Nº of genomes with $TilS_5$
Nº of genomes without $TilS_5$
With $TilS_5$
Without $TilS_5$
p < 0.00005
Nº of genomes with $TilS_6$
Nº of genomes without $TilS_6$
With $TilS_6$
Without $TilS_6$
p < 0.00005
Nº of genomes with $TilS_7$
Nº of genomes without $TilS_7$
With $TilS_7$
Without $TilS_7$
p < 0.00005
Nº of genomes with $TilS_8$
Nº of genomes without $TilS_8$
With $TilS_8$
Without $TilS_8$
p < 0.00005
Nº of genomes with $TilS_9$
Nº of genomes without $TilS_9$
With $TilS_9$
Without $TilS_9$
p < 0.00005
Nº of genomes with $TilS_{10}$
Nº of genomes without $TilS_{10}$
With $TilS_{10}$
Without $TilS_{10}$
p < 0.00005
Nº of genomes with $TilS_{11}$
Nº of genomes without $TilS_{11}$
With $TilS_{11}$
Without $TilS_{11}$
p < 0.00005
Nº of genomes with $TilS_{12}$
Nº of genomes without $TilS_{12}$
With $TilS_{12}$
Without $TilS_{12}$
p < 0.00005
Nº of genomes with $TilS_{13}$
Nº of genomes without $TilS_{13}$
With $TilS_{13}$
Without $TilS_{13}$
p < 0.00005
Nº of genomes with $TilS_{14}$
Nº of genomes without $TilS_{14}$
With $TilS_{14}$
Without $TilS_{14}$
p < 0.00005
Nº of genomes with $TilS_{15}$
Nº of genomes without $TilS_{15}$
With $TilS_{15}$
Without $TilS_{15}$
p < 0.00005

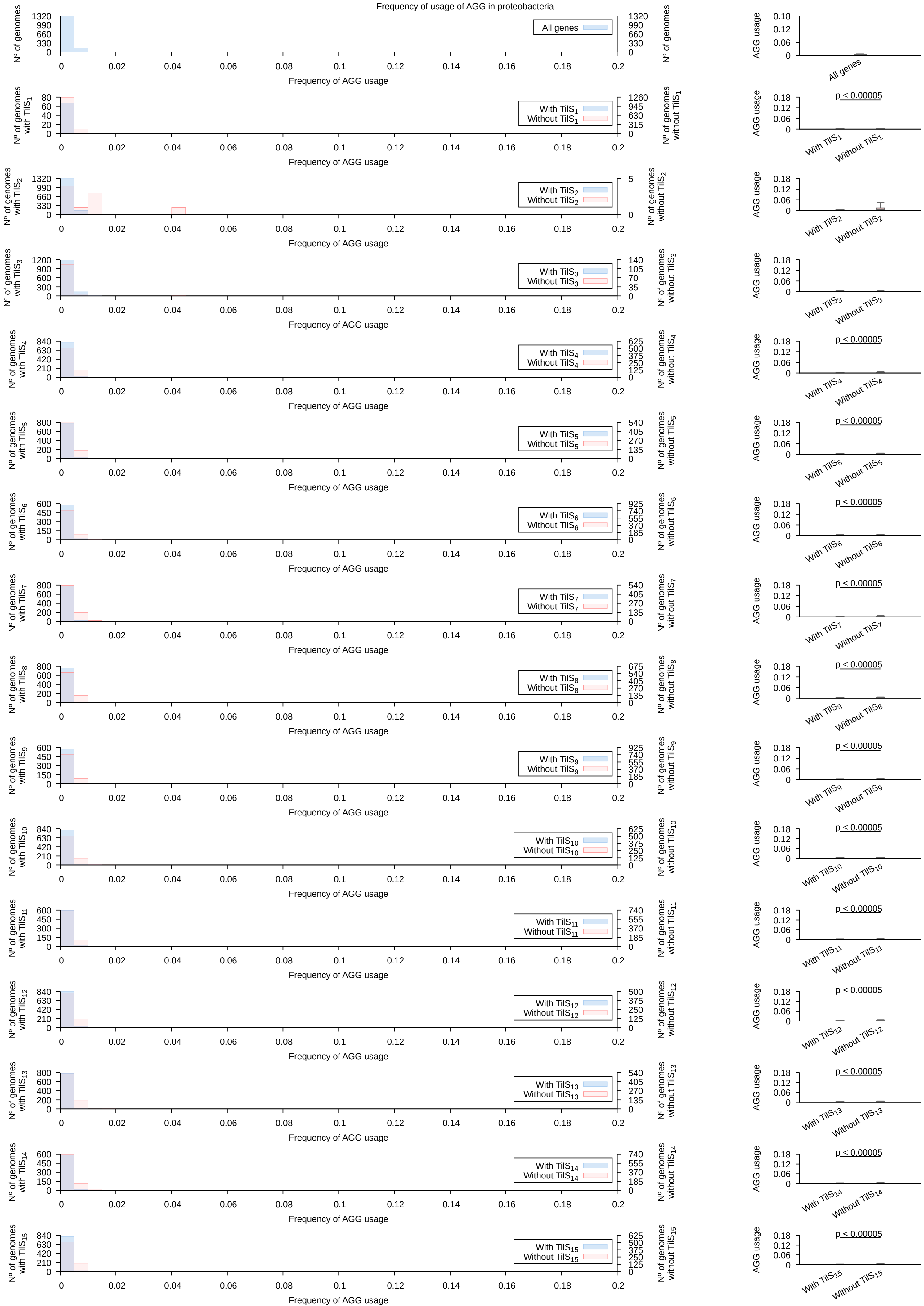

Frequency of usage of AGG in proteobacteria
Nº of genomes
All genes
Frequency of AGG usage
AGG usage
All genes
Nº of genomes with $TilS_1$
With $TilS_1$
Without $TilS_1$
Nº of genomes without $TilS_1$
p < 0.00005
Nº of genomes with $TilS_2$
With $TilS_2$
Without $TilS_2$
Nº of genomes without $TilS_2$
Nº of genomes with $TilS_3$
With $TilS_3$
Without $TilS_3$
Nº of genomes without $TilS_3$
Nº of genomes with $TilS_4$
With $TilS_4$
Without $TilS_4$
Nº of genomes without $TilS_4$
p < 0.00005
Nº of genomes with $TilS_5$
With $TilS_5$
Without $TilS_5$
Nº of genomes without $TilS_5$
p < 0.00005
Nº of genomes with $TilS_6$
With $TilS_6$
Without $TilS_6$
Nº of genomes without $TilS_6$
p < 0.00005
Nº of genomes with $TilS_7$
With $TilS_7$
Without $TilS_7$
Nº of genomes without $TilS_7$
p < 0.00005
Nº of genomes with $TilS_8$
With $TilS_8$
Without $TilS_8$
Nº of genomes without $TilS_8$
p < 0.00005
Nº of genomes with $TilS_9$
With $TilS_9$
Without $TilS_9$
Nº of genomes without $TilS_9$
p < 0.00005
Nº of genomes with $TilS_{10}$
With $TilS_{10}$
Without $TilS_{10}$
Nº of genomes without $TilS_{10}$
p < 0.00005
Nº of genomes with $TilS_{11}$
With $TilS_{11}$
Without $TilS_{11}$
Nº of genomes without $TilS_{11}$
p < 0.00005
Nº of genomes with $TilS_{12}$
With $TilS_{12}$
Without $TilS_{12}$
Nº of genomes without $TilS_{12}$
p < 0.00005
Nº of genomes with $TilS_{13}$
With $TilS_{13}$
Without $TilS_{13}$
Nº of genomes without $TilS_{13}$
p < 0.00005
Nº of genomes with $TilS_{14}$
With $TilS_{14}$
Without $TilS_{14}$
Nº of genomes without $TilS_{14}$
p < 0.00005
Nº of genomes with $TilS_{15}$
With $TilS_{15}$
Without $TilS_{15}$
Nº of genomes without $TilS_{15}$
p < 0.00005

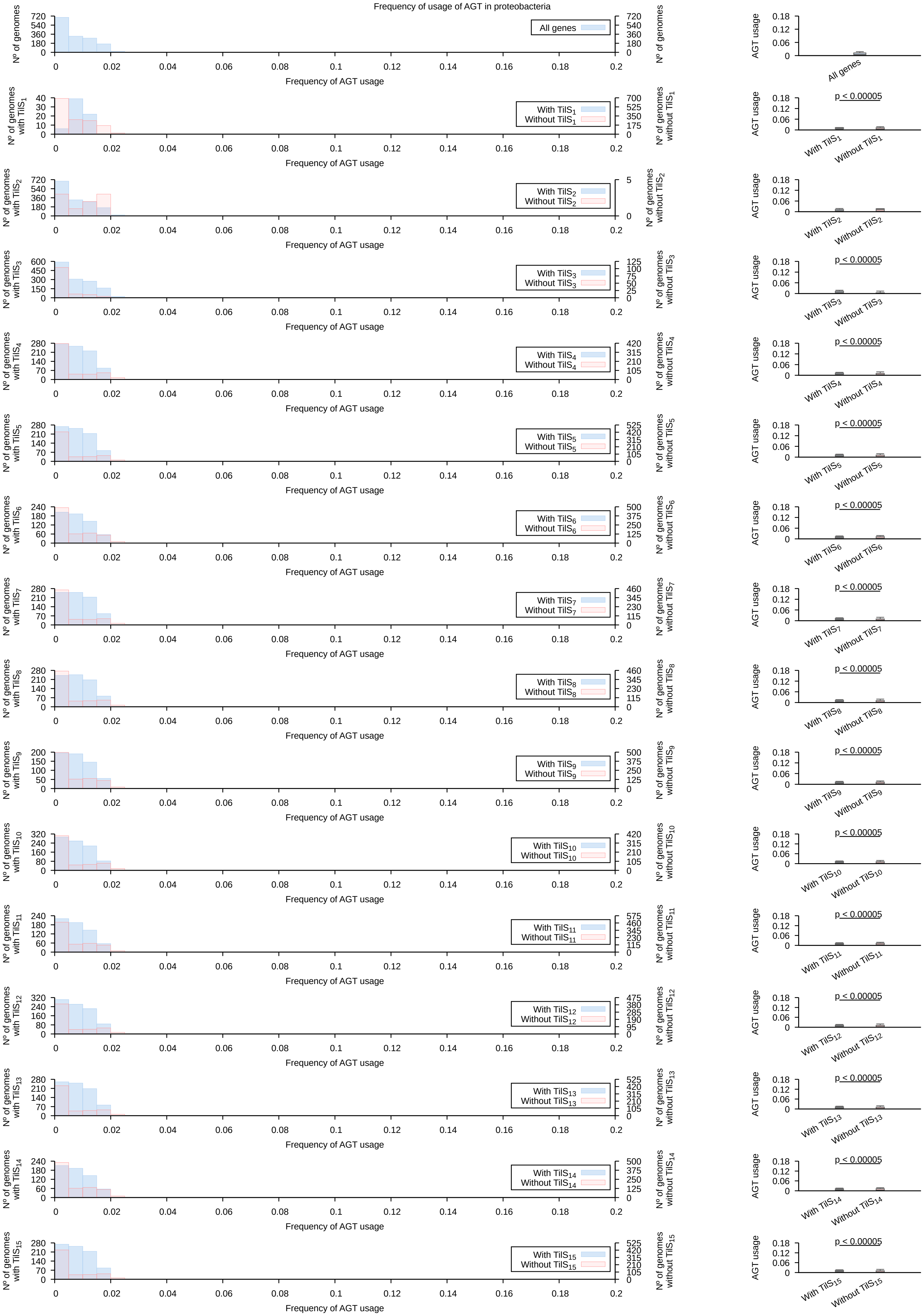
Frequency of usage of AGT in proteobacteria
Nº of genomes
All genes
Frequency of AGT usage
AGT usage
p < 0.00005
Nº of genomes with TilS1
Nº of genomes without TilS1
With TilS1
Without TilS1
With TilS2
Without TilS2
With TilS3
Without TilS3
With TilS4
Without TilS4
With TilS5
Without TilS5
With TilS6
Without TilS6
With TilS7
Without TilS7
With TilS8
Without TilS8
With TilS9
Without TilS9
With TilS10
Without TilS10
With TilS11
Without TilS11
With TilS12
Without TilS12
With TilS13
Without TilS13
With TilS14
Without TilS14
With TilS15
Without TilS15

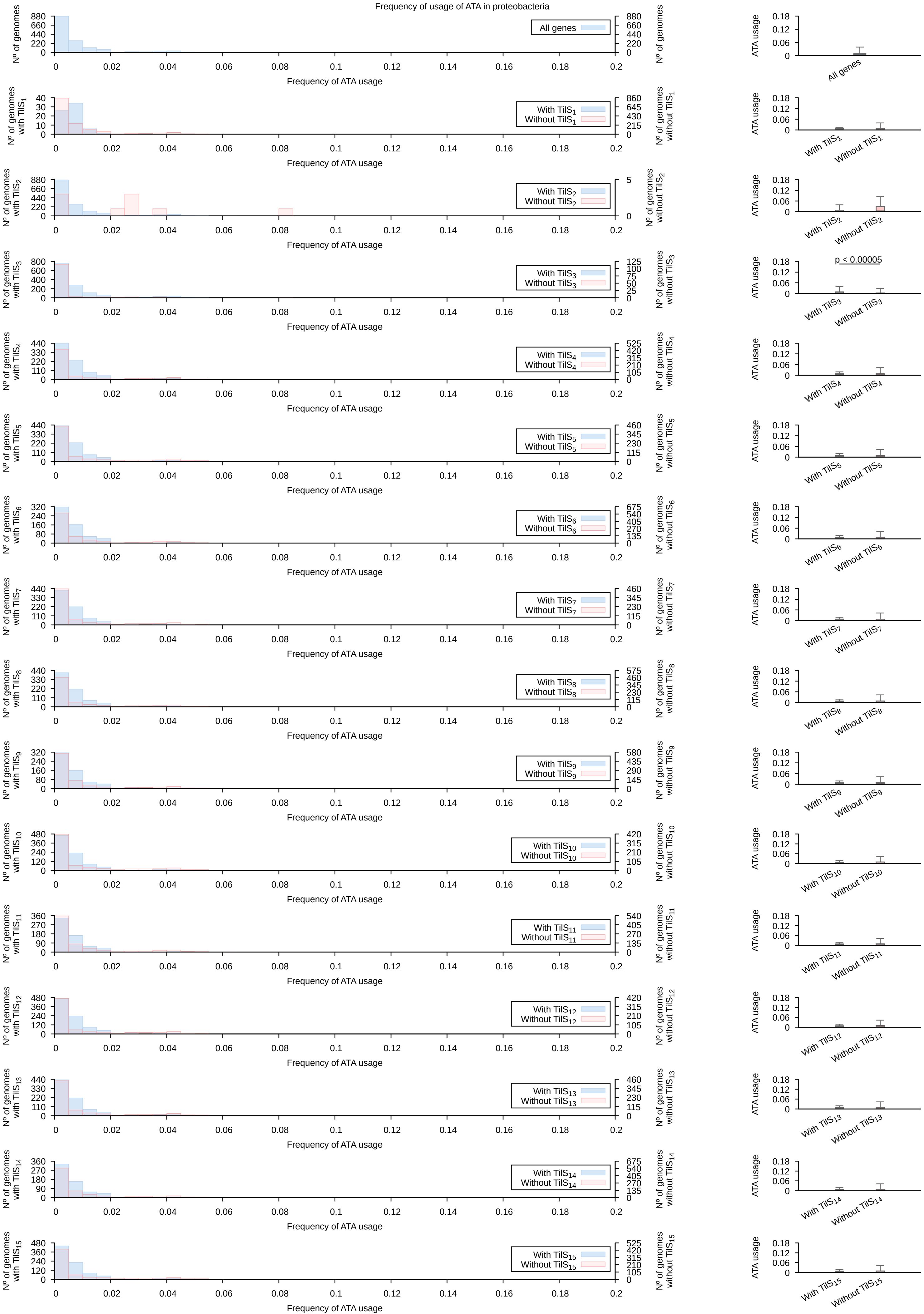
Frequency of usage of ATA in proteobacteria
Nº of genomes
All genes
Frequency of ATA usage
ATA usage
Nº of genomes with TilS$_1$
With TilS$_1$
Without TilS$_1$
Nº of genomes without TilS$_1$
Nº of genomes with TilS$_2$
With TilS$_2$
Without TilS$_2$
Nº of genomes without TilS$_2$
Nº of genomes with TilS$_3$
With TilS$_3$
Without TilS$_3$
Nº of genomes without TilS$_3$
$p < 0.00005$
Nº of genomes with TilS$_4$
With TilS$_4$
Without TilS$_4$
Nº of genomes without TilS$_4$
Nº of genomes with TilS$_5$
With TilS$_5$
Without TilS$_5$
Nº of genomes without TilS$_5$
Nº of genomes with TilS$_6$
With TilS$_6$
Without TilS$_6$
Nº of genomes without TilS$_6$
Nº of genomes with TilS$_7$
With TilS$_7$
Without TilS$_7$
Nº of genomes without TilS$_7$
Nº of genomes with TilS$_8$
With TilS$_8$
Without TilS$_8$
Nº of genomes without TilS$_8$
Nº of genomes with TilS$_9$
With TilS$_9$
Without TilS$_9$
Nº of genomes without TilS$_9$
Nº of genomes with TilS$_{10}$
With TilS$_{10}$
Without TilS$_{10}$
Nº of genomes without TilS$_{10}$
Nº of genomes with TilS$_{11}$
With TilS$_{11}$
Without TilS$_{11}$
Nº of genomes without TilS$_{11}$
Nº of genomes with TilS$_{12}$
With TilS$_{12}$
Without TilS$_{12}$
Nº of genomes without TilS$_{12}$
Nº of genomes with TilS$_{13}$
With TilS$_{13}$
Without TilS$_{13}$
Nº of genomes without TilS$_{13}$
Nº of genomes with TilS$_{14}$
With TilS$_{14}$
Without TilS$_{14}$
Nº of genomes without TilS$_{14}$
Nº of genomes with TilS$_{15}$
With TilS$_{15}$
Without TilS$_{15}$
Nº of genomes without TilS$_{15}$

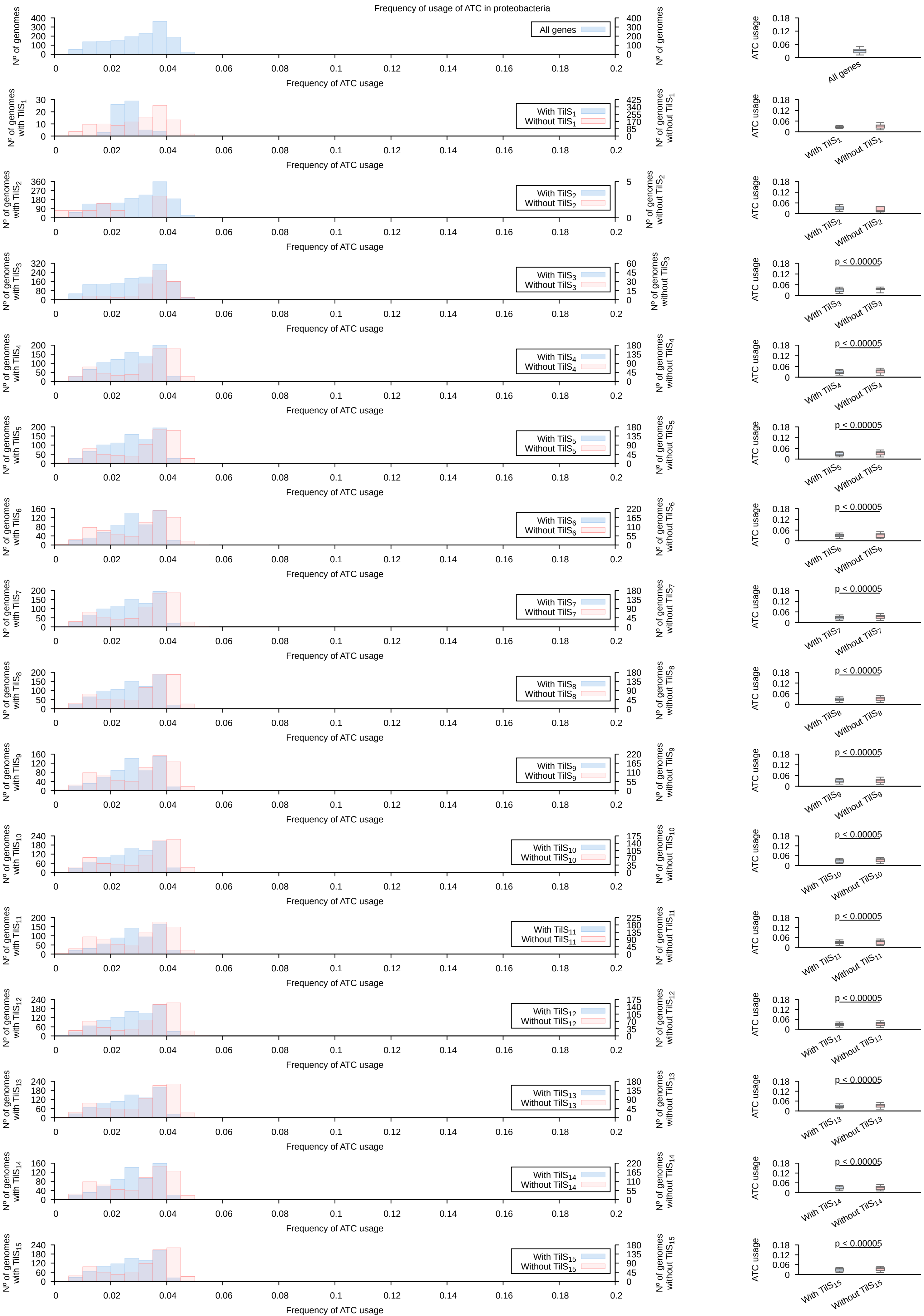

Frequency of usage of ATC in proteobacteria
Nº of genomes
All genes
Frequency of ATC usage
ATC usage
Nº of genomes with TilS$_1$
Nº of genomes without TilS$_1$
With TilS$_1$
Without TilS$_1$
Nº of genomes with TilS$_2$
Nº of genomes without TilS$_2$
With TilS$_2$
Without TilS$_2$
Nº of genomes with TilS$_3$
Nº of genomes without TilS$_3$
With TilS$_3$
Without TilS$_3$
p < 0.00005
Nº of genomes with TilS$_4$
Nº of genomes without TilS$_4$
With TilS$_4$
Without TilS$_4$
Nº of genomes with TilS$_5$
Nº of genomes without TilS$_5$
With TilS$_5$
Without TilS$_5$
Nº of genomes with TilS$_6$
Nº of genomes without TilS$_6$
With TilS$_6$
Without TilS$_6$
Nº of genomes with TilS$_7$
Nº of genomes without TilS$_7$
With TilS$_7$
Without TilS$_7$
Nº of genomes with TilS$_8$
Nº of genomes without TilS$_8$
With TilS$_8$
Without TilS$_8$
Nº of genomes with TilS$_9$
Nº of genomes without TilS$_9$
With TilS$_9$
Without TilS$_9$
Nº of genomes with TilS$_{10}$
Nº of genomes without TilS$_{10}$
With TilS$_{10}$
Without TilS$_{10}$
Nº of genomes with TilS$_{11}$
Nº of genomes without TilS$_{11}$
With TilS$_{11}$
Without TilS$_{11}$
Nº of genomes with TilS$_{12}$
Nº of genomes without TilS$_{12}$
With TilS$_{12}$
Without TilS$_{12}$
Nº of genomes with TilS$_{13}$
Nº of genomes without TilS$_{13}$
With TilS$_{13}$
Without TilS$_{13}$
Nº of genomes with TilS$_{14}$
Nº of genomes without TilS$_{14}$
With TilS$_{14}$
Without TilS$_{14}$
Nº of genomes with TilS$_{15}$
Nº of genomes without TilS$_{15}$
With TilS$_{15}$
Without TilS$_{15}$

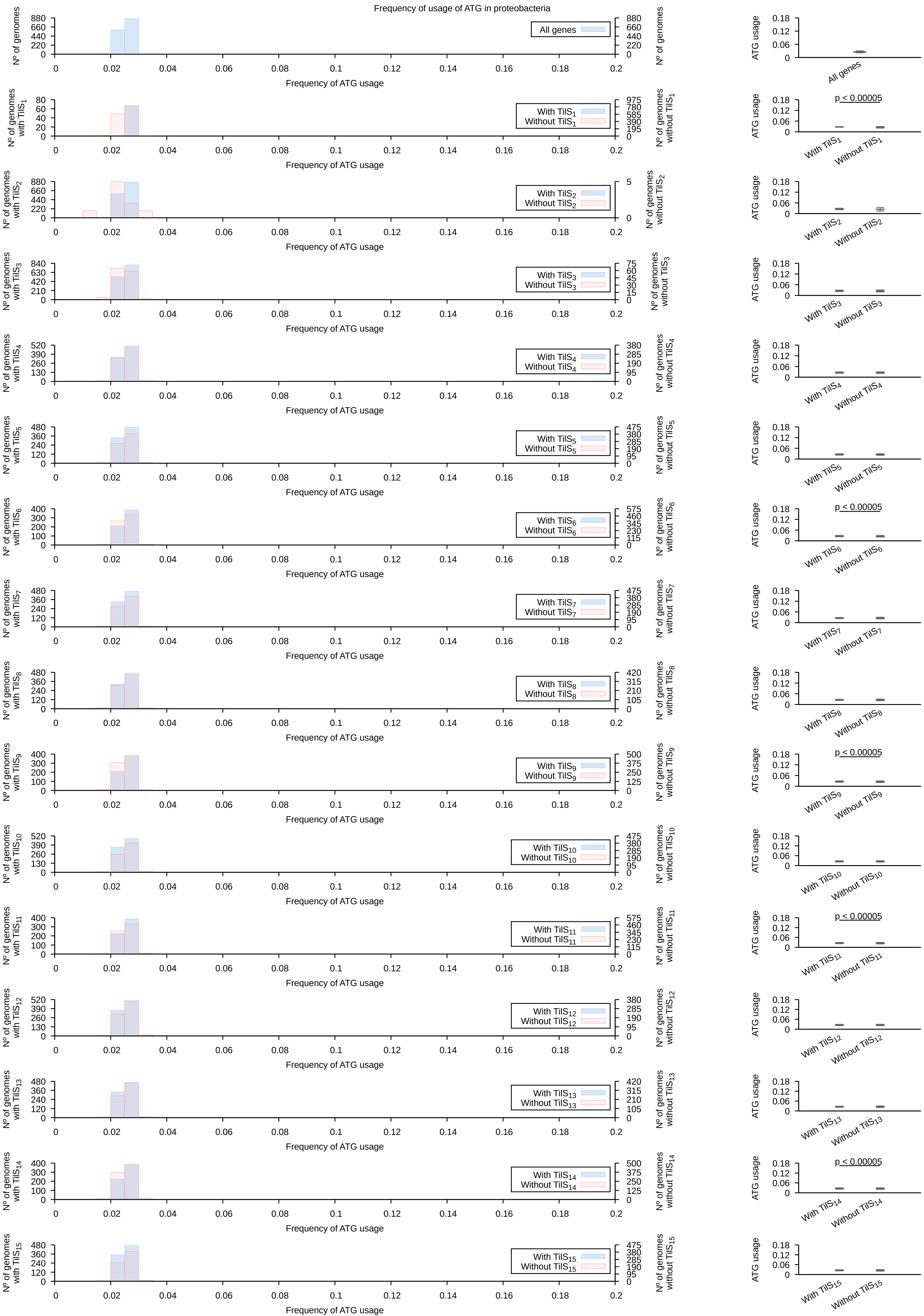

Frequency of usage of ATG in proteobacteria
Nº of genomes
All genes
Frequency of ATG usage
ATG usage
Nº of genomes with TilS$_1$
Nº of genomes without TilS$_1$
With TilS$_1$
Without TilS$_1$
p < 0.00005
Nº of genomes with TilS$_2$
Nº of genomes without TilS$_2$
With TilS$_2$
Without TilS$_2$
Nº of genomes with TilS$_3$
Nº of genomes without TilS$_3$
With TilS$_3$
Without TilS$_3$
Nº of genomes with TilS$_4$
Nº of genomes without TilS$_4$
With TilS$_4$
Without TilS$_4$
Nº of genomes with TilS$_5$
Nº of genomes without TilS$_5$
With TilS$_5$
Without TilS$_5$
Nº of genomes with TilS$_6$
Nº of genomes without TilS$_6$
With TilS$_6$
Without TilS$_6$
p < 0.00005
Nº of genomes with TilS$_7$
Nº of genomes without TilS$_7$
With TilS$_7$
Without TilS$_7$
Nº of genomes with TilS$_8$
Nº of genomes without TilS$_8$
With TilS$_8$
Without TilS$_8$
Nº of genomes with TilS$_9$
Nº of genomes without TilS$_9$
With TilS$_9$
Without TilS$_9$
p < 0.00005
Nº of genomes with TilS$_{10}$
Nº of genomes without TilS$_{10}$
With TilS$_{10}$
Without TilS$_{10}$
Nº of genomes with TilS$_{11}$
Nº of genomes without TilS$_{11}$
With TilS$_{11}$
Without TilS$_{11}$
p < 0.00005
Nº of genomes with TilS$_{12}$
Nº of genomes without TilS$_{12}$
With TilS$_{12}$
Without TilS$_{12}$
Nº of genomes with TilS$_{13}$
Nº of genomes without TilS$_{13}$
With TilS$_{13}$
Without TilS$_{13}$
Nº of genomes with TilS$_{14}$
Nº of genomes without TilS$_{14}$
With TilS$_{14}$
Without TilS$_{14}$
p < 0.00005
Nº of genomes with TilS$_{15}$
Nº of genomes without TilS$_{15}$
With TilS$_{15}$
Without TilS$_{15}$

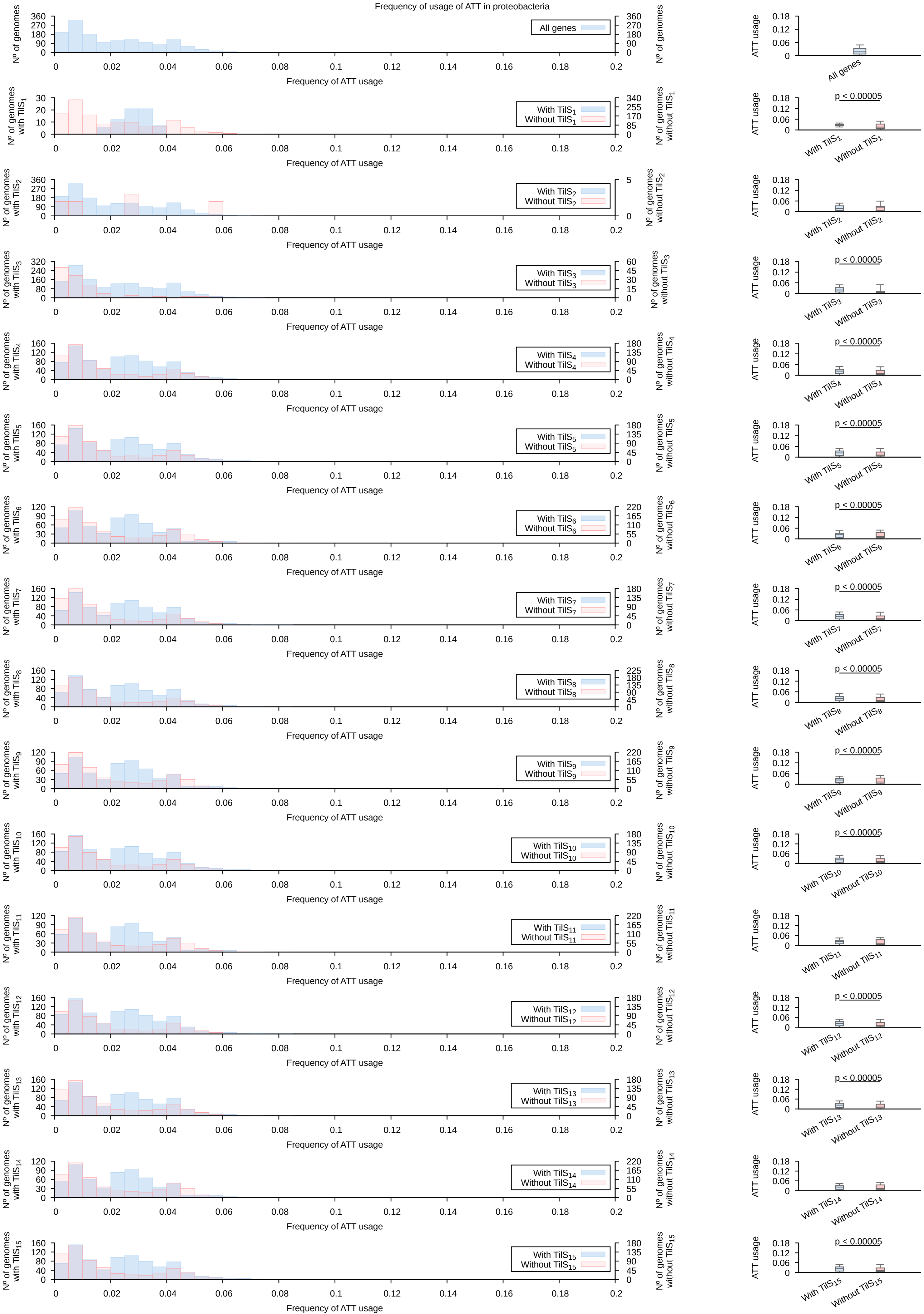

Frequency of usage of ATT in proteobacteria
Nº of genomes
All genes
Frequency of ATT usage
ATT usage
All genes
Nº of genomes with TilS1
With TilS1
Without TilS1
Nº of genomes without TilS1
p < 0.00005
Nº of genomes with TilS2
With TilS2
Without TilS2
Nº of genomes without TilS2
Nº of genomes with TilS3
With TilS3
Without TilS3
Nº of genomes without TilS3
p < 0.00005
Nº of genomes with TilS4
With TilS4
Without TilS4
Nº of genomes without TilS4
p < 0.00005
Nº of genomes with TilS5
With TilS5
Without TilS5
Nº of genomes without TilS5
p < 0.00005
Nº of genomes with TilS6
With TilS6
Without TilS6
Nº of genomes without TilS6
p < 0.00005
Nº of genomes with TilS7
With TilS7
Without TilS7
Nº of genomes without TilS7
p < 0.00005
Nº of genomes with TilS8
With TilS8
Without TilS8
Nº of genomes without TilS8
p < 0.00005
Nº of genomes with TilS9
With TilS9
Without TilS9
Nº of genomes without TilS9
p < 0.00005
Nº of genomes with TilS10
With TilS10
Without TilS10
Nº of genomes without TilS10
p < 0.00005
Nº of genomes with TilS11
With TilS11
Without TilS11
Nº of genomes without TilS11
Nº of genomes with TilS12
With TilS12
Without TilS12
Nº of genomes without TilS12
p < 0.00005
Nº of genomes with TilS13
With TilS13
Without TilS13
Nº of genomes without TilS13
p < 0.00005
Nº of genomes with TilS14
With TilS14
Without TilS14
Nº of genomes without TilS14
Nº of genomes with TilS15
With TilS15
Without TilS15
Nº of genomes without TilS15
p < 0.00005

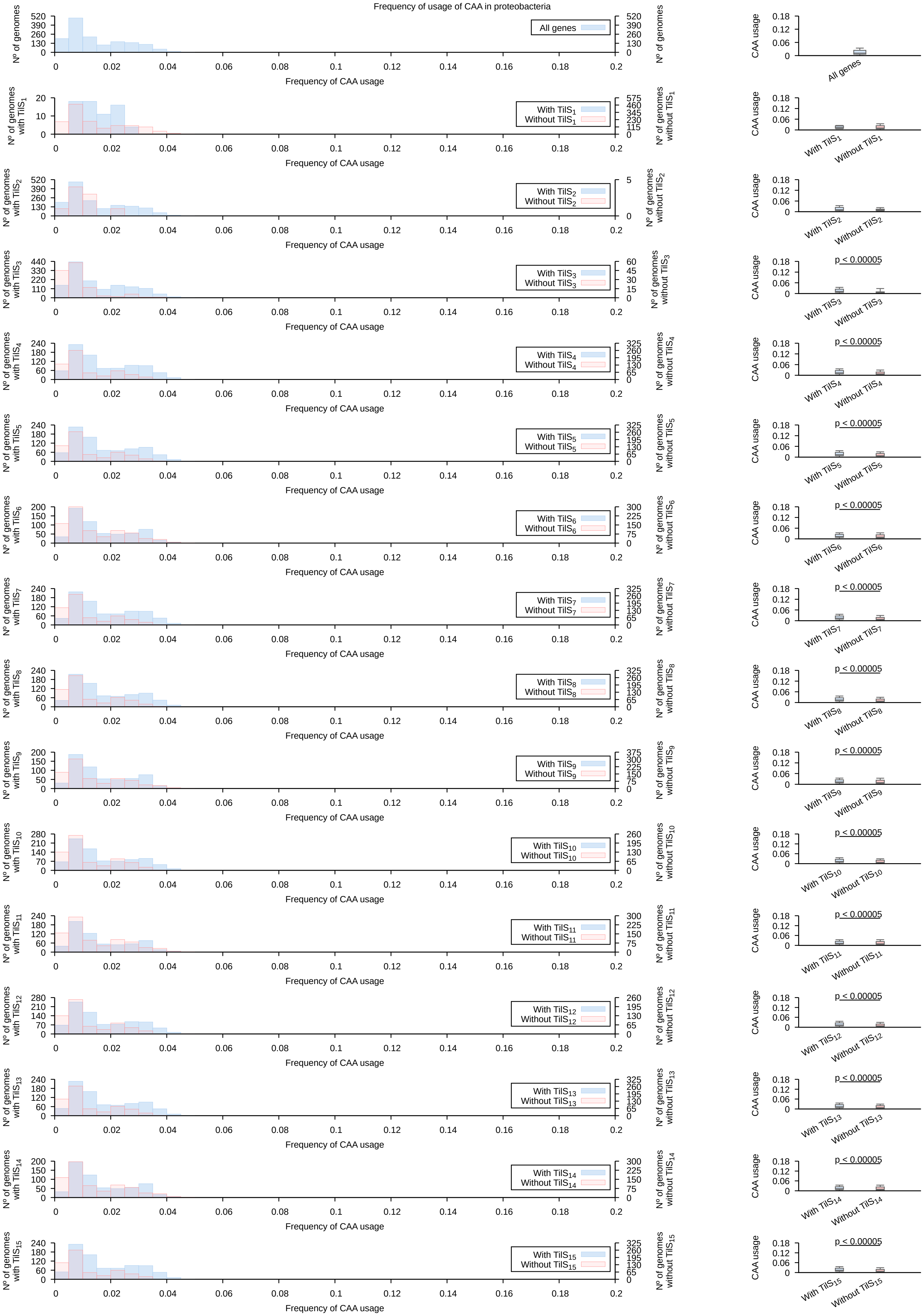

Frequency of usage of CAA in proteobacteria
Nº of genomes
All genes
Frequency of CAA usage
CAA usage
All genes
Nº of genomes with $TilS_1$
With $TilS_1$
Without $TilS_1$
Nº of genomes without $TilS_1$
Nº of genomes with $TilS_2$
With $TilS_2$
Without $TilS_2$
Nº of genomes without $TilS_2$
Nº of genomes with $TilS_3$
With $TilS_3$
Without $TilS_3$
Nº of genomes without $TilS_3$
p < 0.00005
Nº of genomes with $TilS_4$
With $TilS_4$
Without $TilS_4$
Nº of genomes without $TilS_4$
p < 0.00005
Nº of genomes with $TilS_5$
With $TilS_5$
Without $TilS_5$
Nº of genomes without $TilS_5$
p < 0.00005
Nº of genomes with $TilS_6$
With $TilS_6$
Without $TilS_6$
Nº of genomes without $TilS_6$
p < 0.00005
Nº of genomes with $TilS_7$
With $TilS_7$
Without $TilS_7$
Nº of genomes without $TilS_7$
p < 0.00005
Nº of genomes with $TilS_8$
With $TilS_8$
Without $TilS_8$
Nº of genomes without $TilS_8$
p < 0.00005
Nº of genomes with $TilS_9$
With $TilS_9$
Without $TilS_9$
Nº of genomes without $TilS_9$
p < 0.00005
Nº of genomes with $TilS_{10}$
With $TilS_{10}$
Without $TilS_{10}$
Nº of genomes without $TilS_{10}$
p < 0.00005
Nº of genomes with $TilS_{11}$
With $TilS_{11}$
Without $TilS_{11}$
Nº of genomes without $TilS_{11}$
p < 0.00005
Nº of genomes with $TilS_{12}$
With $TilS_{12}$
Without $TilS_{12}$
Nº of genomes without $TilS_{12}$
p < 0.00005
Nº of genomes with $TilS_{13}$
With $TilS_{13}$
Without $TilS_{13}$
Nº of genomes without $TilS_{13}$
p < 0.00005
Nº of genomes with $TilS_{14}$
With $TilS_{14}$
Without $TilS_{14}$
Nº of genomes without $TilS_{14}$
p < 0.00005
Nº of genomes with $TilS_{15}$
With $TilS_{15}$
Without $TilS_{15}$
Nº of genomes without $TilS_{15}$
p < 0.00005

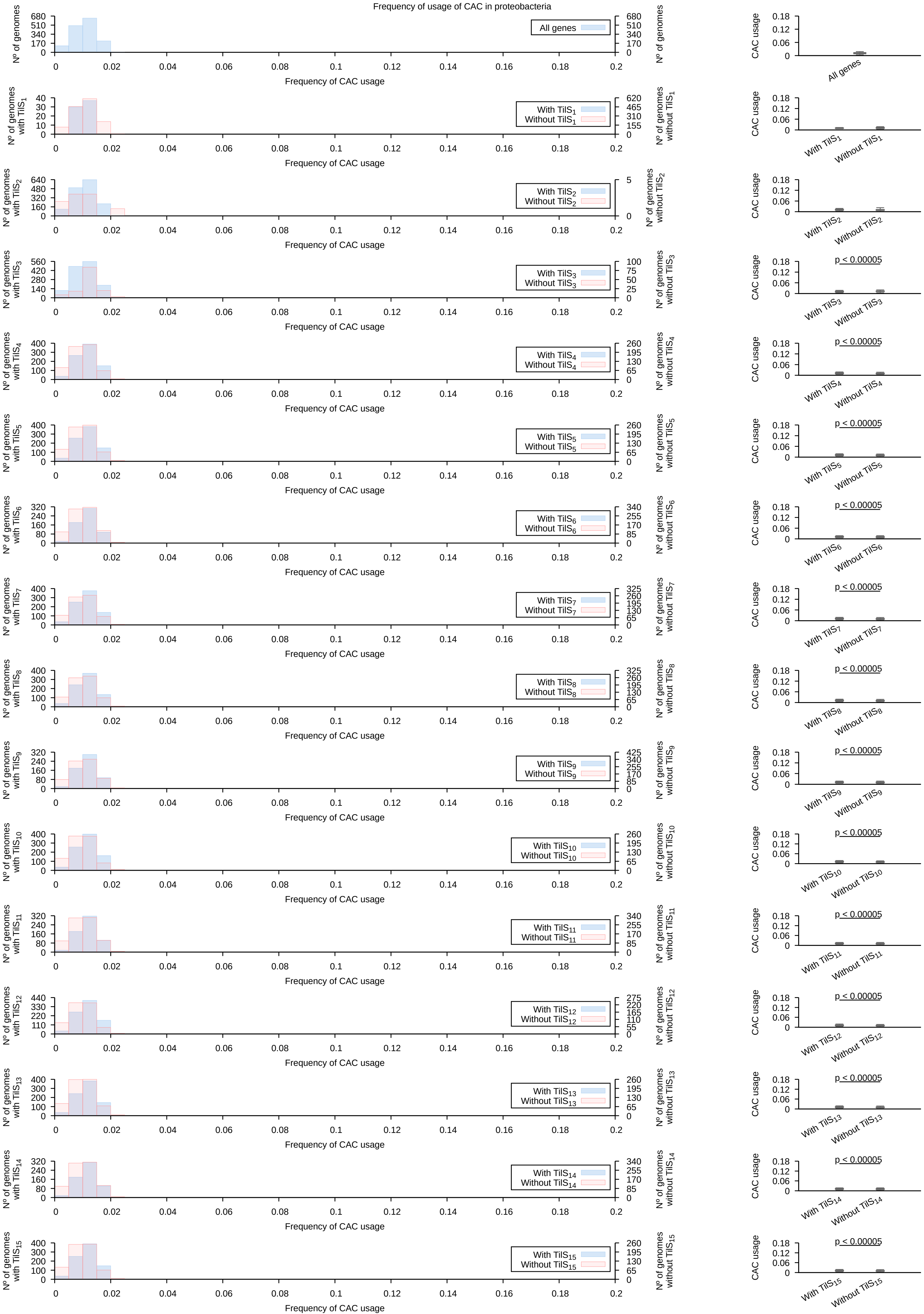

Frequency of usage of CAC in proteobacteria
Nº of genomes
All genes
Frequency of CAC usage
CAC usage
Nº of genomes with TilS$_1$
Nº of genomes without TilS$_1$
With TilS$_1$
Without TilS$_1$
Nº of genomes with TilS$_2$
Nº of genomes without TilS$_2$
With TilS$_2$
Without TilS$_2$
Nº of genomes with TilS$_3$
Nº of genomes without TilS$_3$
With TilS$_3$
Without TilS$_3$
Nº of genomes with TilS$_4$
Nº of genomes without TilS$_4$
With TilS$_4$
Without TilS$_4$
Nº of genomes with TilS$_5$
Nº of genomes without TilS$_5$
With TilS$_5$
Without TilS$_5$
Nº of genomes with TilS$_6$
Nº of genomes without TilS$_6$
With TilS$_6$
Without TilS$_6$
Nº of genomes with TilS$_7$
Nº of genomes without TilS$_7$
With TilS$_7$
Without TilS$_7$
Nº of genomes with TilS$_8$
Nº of genomes without TilS$_8$
With TilS$_8$
Without TilS$_8$
Nº of genomes with TilS$_9$
Nº of genomes without TilS$_9$
With TilS$_9$
Without TilS$_9$
Nº of genomes with TilS$_{10}$
Nº of genomes without TilS$_{10}$
With TilS$_{10}$
Without TilS$_{10}$
Nº of genomes with TilS$_{11}$
Nº of genomes without TilS$_{11}$
With TilS$_{11}$
Without TilS$_{11}$
Nº of genomes with TilS$_{12}$
Nº of genomes without TilS$_{12}$
With TilS$_{12}$
Without TilS$_{12}$
Nº of genomes with TilS$_{13}$
Nº of genomes without TilS$_{13}$
With TilS$_{13}$
Without TilS$_{13}$
Nº of genomes with TilS$_{14}$
Nº of genomes without TilS$_{14}$
With TilS$_{14}$
Without TilS$_{14}$
Nº of genomes with TilS$_{15}$
Nº of genomes without TilS$_{15}$
With TilS$_{15}$
Without TilS$_{15}$
$p < 0.00005$

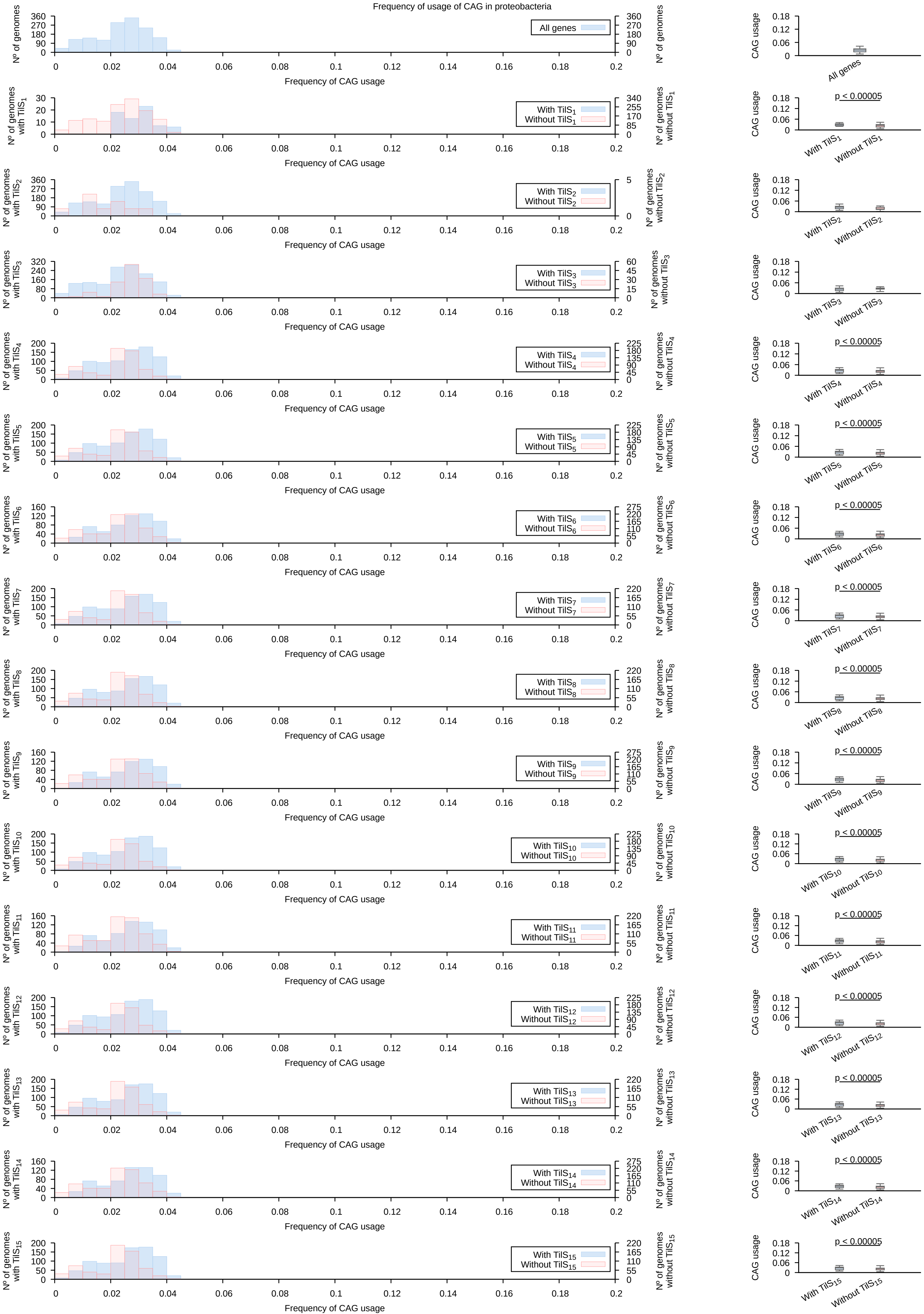
Frequency of usage of CAG in proteobacteria
Nº of genomes
All genes
Frequency of CAG usage
CAG usage
Nº of genomes with $TilS_1$
Nº of genomes without $TilS_1$
With $TilS_1$
Without $TilS_1$
p < 0.00005
Nº of genomes with $TilS_2$
Nº of genomes without $TilS_2$
With $TilS_2$
Without $TilS_2$
Nº of genomes with $TilS_3$
Nº of genomes without $TilS_3$
With $TilS_3$
Without $TilS_3$
Nº of genomes with $TilS_4$
Nº of genomes without $TilS_4$
With $TilS_4$
Without $TilS_4$
p < 0.00005
Nº of genomes with $TilS_5$
Nº of genomes without $TilS_5$
With $TilS_5$
Without $TilS_5$
p < 0.00005
Nº of genomes with $TilS_6$
Nº of genomes without $TilS_6$
With $TilS_6$
Without $TilS_6$
p < 0.00005
Nº of genomes with $TilS_7$
Nº of genomes without $TilS_7$
With $TilS_7$
Without $TilS_7$
p < 0.00005
Nº of genomes with $TilS_8$
Nº of genomes without $TilS_8$
With $TilS_8$
Without $TilS_8$
p < 0.00005
Nº of genomes with $TilS_9$
Nº of genomes without $TilS_9$
With $TilS_9$
Without $TilS_9$
p < 0.00005
Nº of genomes with $TilS_{10}$
Nº of genomes without $TilS_{10}$
With $TilS_{10}$
Without $TilS_{10}$
p < 0.00005
Nº of genomes with $TilS_{11}$
Nº of genomes without $TilS_{11}$
With $TilS_{11}$
Without $TilS_{11}$
p < 0.00005
Nº of genomes with $TilS_{12}$
Nº of genomes without $TilS_{12}$
With $TilS_{12}$
Without $TilS_{12}$
p < 0.00005
Nº of genomes with $TilS_{13}$
Nº of genomes without $TilS_{13}$
With $TilS_{13}$
Without $TilS_{13}$
p < 0.00005
Nº of genomes with $TilS_{14}$
Nº of genomes without $TilS_{14}$
With $TilS_{14}$
Without $TilS_{14}$
p < 0.00005
Nº of genomes with $TilS_{15}$
Nº of genomes without $TilS_{15}$
With $TilS_{15}$
Without $TilS_{15}$
p < 0.00005

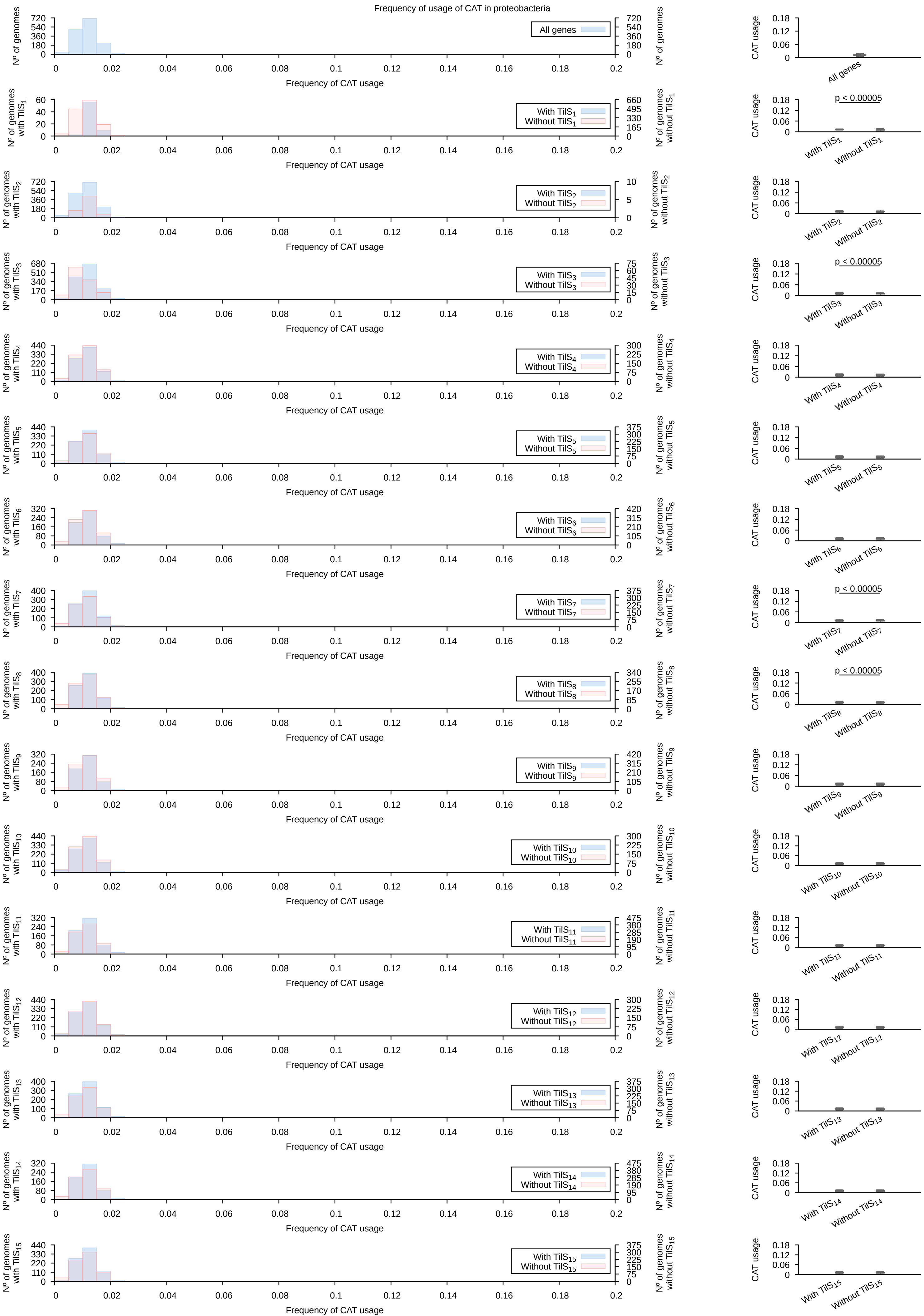

Frequency of usage of CAT in proteobacteria
Nº of genomes
All genes
Frequency of CAT usage
CAT usage
All genes
Nº of genomes with TilS$_1$
Nº of genomes without TilS$_1$
With TilS$_1$
Without TilS$_1$
p < 0.00005
Nº of genomes with TilS$_2$
Nº of genomes without TilS$_2$
With TilS$_2$
Without TilS$_2$
Nº of genomes with TilS$_3$
Nº of genomes without TilS$_3$
With TilS$_3$
Without TilS$_3$
p < 0.00005
Nº of genomes with TilS$_4$
Nº of genomes without TilS$_4$
With TilS$_4$
Without TilS$_4$
Nº of genomes with TilS$_5$
Nº of genomes without TilS$_5$
With TilS$_5$
Without TilS$_5$
Nº of genomes with TilS$_6$
Nº of genomes without TilS$_6$
With TilS$_6$
Without TilS$_6$
Nº of genomes with TilS$_7$
Nº of genomes without TilS$_7$
With TilS$_7$
Without TilS$_7$
p < 0.00005
Nº of genomes with TilS$_8$
Nº of genomes without TilS$_8$
With TilS$_8$
Without TilS$_8$
p < 0.00005
Nº of genomes with TilS$_9$
Nº of genomes without TilS$_9$
With TilS$_9$
Without TilS$_9$
Nº of genomes with TilS$_{10}$
Nº of genomes without TilS$_{10}$
With TilS$_{10}$
Without TilS$_{10}$
Nº of genomes with TilS$_{11}$
Nº of genomes without TilS$_{11}$
With TilS$_{11}$
Without TilS$_{11}$
Nº of genomes with TilS$_{12}$
Nº of genomes without TilS$_{12}$
With TilS$_{12}$
Without TilS$_{12}$
Nº of genomes with TilS$_{13}$
Nº of genomes without TilS$_{13}$
With TilS$_{13}$
Without TilS$_{13}$
Nº of genomes with TilS$_{14}$
Nº of genomes without TilS$_{14}$
With TilS$_{14}$
Without TilS$_{14}$
Nº of genomes with TilS$_{15}$
Nº of genomes without TilS$_{15}$
With TilS$_{15}$
Without TilS$_{15}$

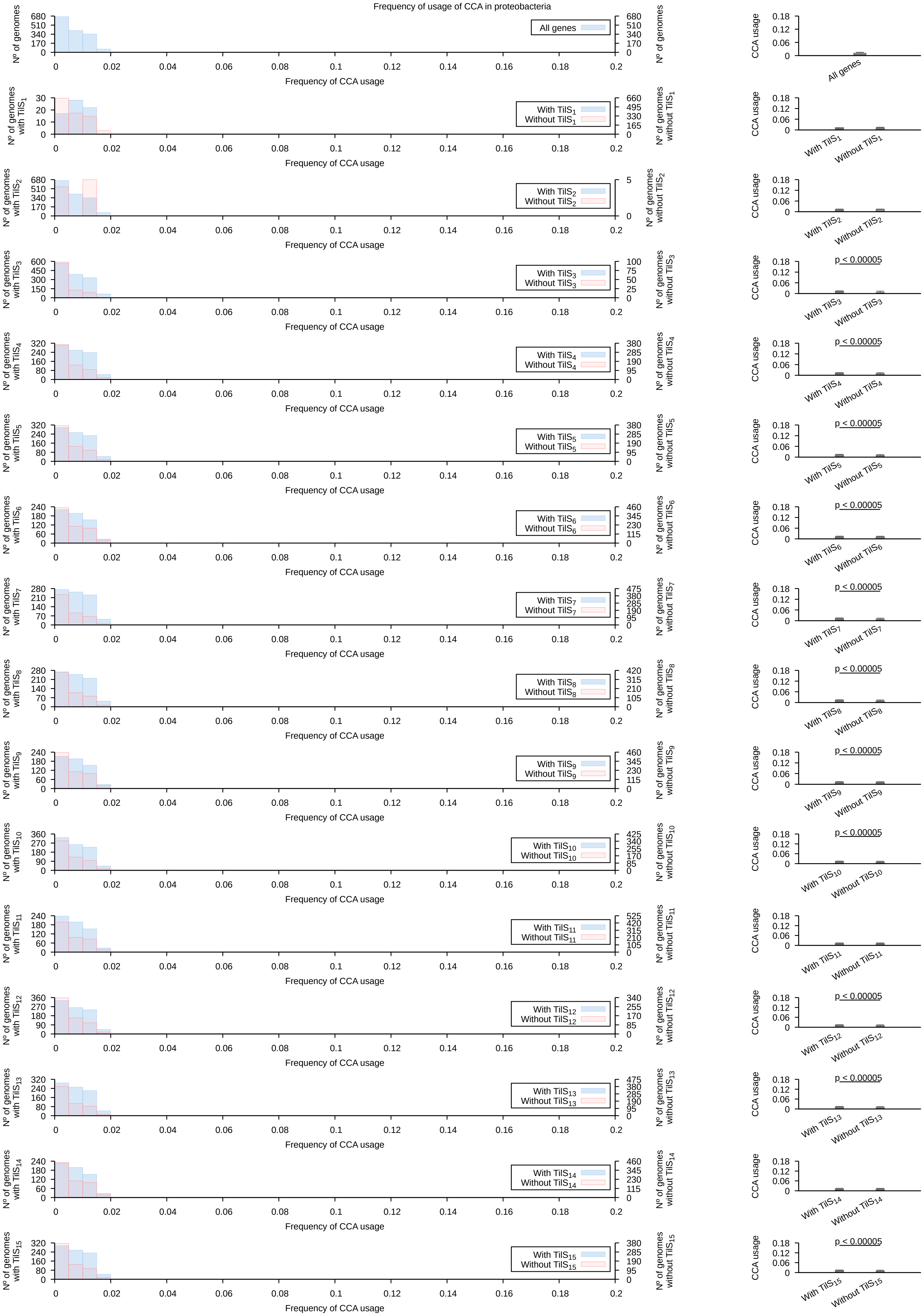

Frequency of usage of CCA in proteobacteria
Nº of genomes
Frequency of CCA usage
All genes
CCA usage
Nº of genomes with TilS$_1$
Nº of genomes without TilS$_1$
With TilS$_1$
Without TilS$_1$
Nº of genomes with TilS$_2$
Nº of genomes without TilS$_2$
With TilS$_2$
Without TilS$_2$
Nº of genomes with TilS$_3$
Nº of genomes without TilS$_3$
With TilS$_3$
Without TilS$_3$
p < 0.00005
Nº of genomes with TilS$_4$
Nº of genomes without TilS$_4$
With TilS$_4$
Without TilS$_4$
p < 0.00005
Nº of genomes with TilS$_5$
Nº of genomes without TilS$_5$
With TilS$_5$
Without TilS$_5$
p < 0.00005
Nº of genomes with TilS$_6$
Nº of genomes without TilS$_6$
With TilS$_6$
Without TilS$_6$
p < 0.00005
Nº of genomes with TilS$_7$
Nº of genomes without TilS$_7$
With TilS$_7$
Without TilS$_7$
p < 0.00005
Nº of genomes with TilS$_8$
Nº of genomes without TilS$_8$
With TilS$_8$
Without TilS$_8$
p < 0.00005
Nº of genomes with TilS$_9$
Nº of genomes without TilS$_9$
With TilS$_9$
Without TilS$_9$
p < 0.00005
Nº of genomes with TilS$_{10}$
Nº of genomes without TilS$_{10}$
With TilS$_{10}$
Without TilS$_{10}$
p < 0.00005
Nº of genomes with TilS$_{11}$
Nº of genomes without TilS$_{11}$
With TilS$_{11}$
Without TilS$_{11}$
Nº of genomes with TilS$_{12}$
Nº of genomes without TilS$_{12}$
With TilS$_{12}$
Without TilS$_{12}$
p < 0.00005
Nº of genomes with TilS$_{13}$
Nº of genomes without TilS$_{13}$
With TilS$_{13}$
Without TilS$_{13}$
p < 0.00005
Nº of genomes with TilS$_{14}$
Nº of genomes without TilS$_{14}$
With TilS$_{14}$
Without TilS$_{14}$
Nº of genomes with TilS$_{15}$
Nº of genomes without TilS$_{15}$
With TilS$_{15}$
Without TilS$_{15}$
p < 0.00005

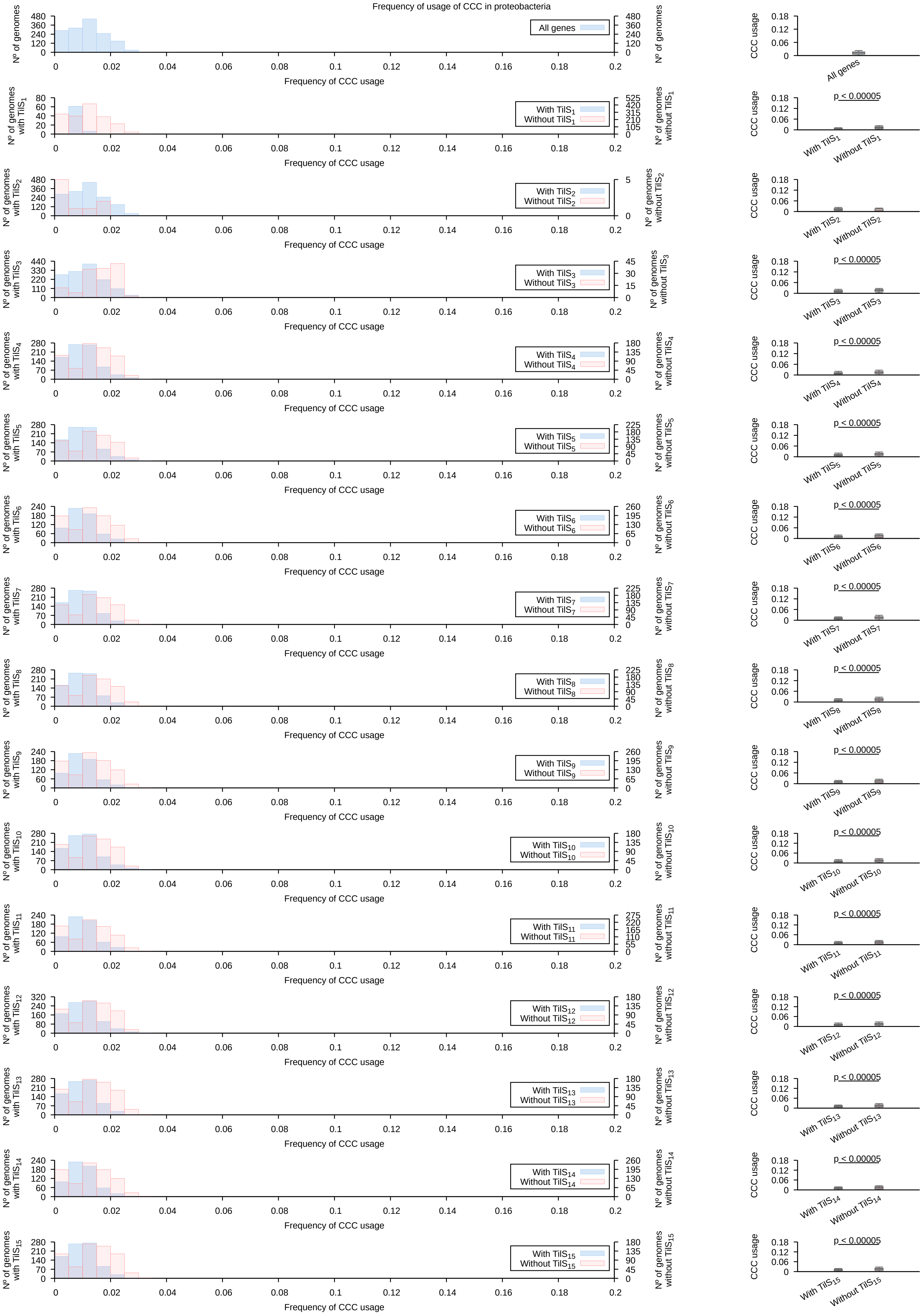

Frequency of usage of CCC in proteobacteria
Nº of genomes
All genes
Frequency of CCC usage
CCC usage
Nº of genomes with TilS$_1$
Nº of genomes without TilS$_1$
With TilS$_1$
Without TilS$_1$
p < 0.00005
Nº of genomes with TilS$_2$
Nº of genomes without TilS$_2$
With TilS$_2$
Without TilS$_2$
Nº of genomes with TilS$_3$
Nº of genomes without TilS$_3$
With TilS$_3$
Without TilS$_3$
p < 0.00005
Nº of genomes with TilS$_4$
Nº of genomes without TilS$_4$
With TilS$_4$
Without TilS$_4$
p < 0.00005
Nº of genomes with TilS$_5$
Nº of genomes without TilS$_5$
With TilS$_5$
Without TilS$_5$
p < 0.00005
Nº of genomes with TilS$_6$
Nº of genomes without TilS$_6$
With TilS$_6$
Without TilS$_6$
p < 0.00005
Nº of genomes with TilS$_7$
Nº of genomes without TilS$_7$
With TilS$_7$
Without TilS$_7$
p < 0.00005
Nº of genomes with TilS$_8$
Nº of genomes without TilS$_8$
With TilS$_8$
Without TilS$_8$
p < 0.00005
Nº of genomes with TilS$_9$
Nº of genomes without TilS$_9$
With TilS$_9$
Without TilS$_9$
p < 0.00005
Nº of genomes with TilS$_{10}$
Nº of genomes without TilS$_{10}$
With TilS$_{10}$
Without TilS$_{10}$
p < 0.00005
Nº of genomes with TilS$_{11}$
Nº of genomes without TilS$_{11}$
With TilS$_{11}$
Without TilS$_{11}$
p < 0.00005
Nº of genomes with TilS$_{12}$
Nº of genomes without TilS$_{12}$
With TilS$_{12}$
Without TilS$_{12}$
p < 0.00005
Nº of genomes with TilS$_{13}$
Nº of genomes without TilS$_{13}$
With TilS$_{13}$
Without TilS$_{13}$
p < 0.00005
Nº of genomes with TilS$_{14}$
Nº of genomes without TilS$_{14}$
With TilS$_{14}$
Without TilS$_{14}$
p < 0.00005
Nº of genomes with TilS$_{15}$
Nº of genomes without TilS$_{15}$
With TilS$_{15}$
Without TilS$_{15}$
p < 0.00005

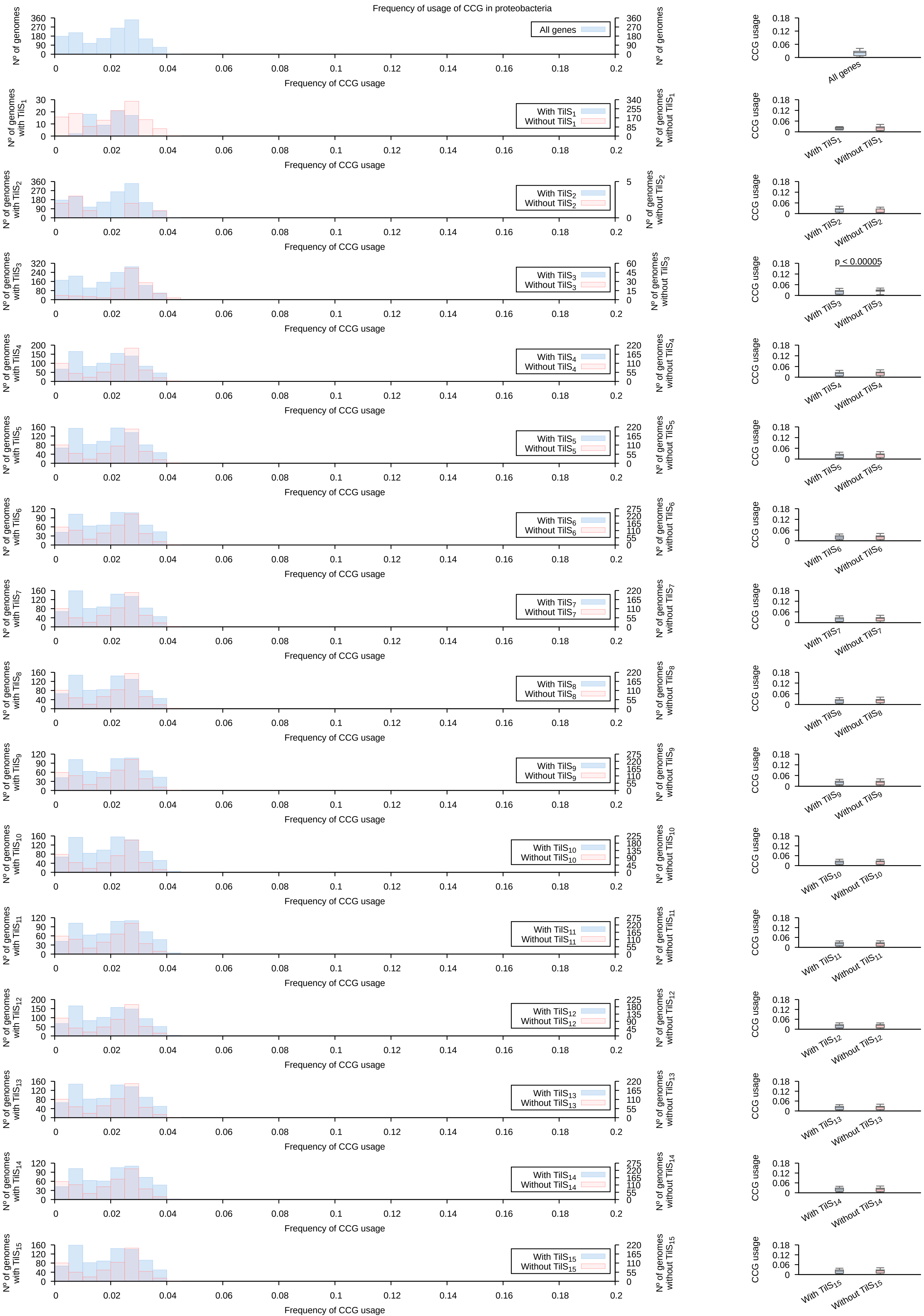

Frequency of usage of CCG in proteobacteria
Nº of genomes
All genes
Frequency of CCG usage
CCG usage
Nº of genomes with TilS$_1$
Nº of genomes without TilS$_1$
With TilS$_1$
Without TilS$_1$
Nº of genomes with TilS$_2$
Nº of genomes without TilS$_2$
With TilS$_2$
Without TilS$_2$
Nº of genomes with TilS$_3$
Nº of genomes without TilS$_3$
With TilS$_3$
Without TilS$_3$
p < 0.00005
Nº of genomes with TilS$_4$
Nº of genomes without TilS$_4$
With TilS$_4$
Without TilS$_4$
Nº of genomes with TilS$_5$
Nº of genomes without TilS$_5$
With TilS$_5$
Without TilS$_5$
Nº of genomes with TilS$_6$
Nº of genomes without TilS$_6$
With TilS$_6$
Without TilS$_6$
Nº of genomes with TilS$_7$
Nº of genomes without TilS$_7$
With TilS$_7$
Without TilS$_7$
Nº of genomes with TilS$_8$
Nº of genomes without TilS$_8$
With TilS$_8$
Without TilS$_8$
Nº of genomes with TilS$_9$
Nº of genomes without TilS$_9$
With TilS$_9$
Without TilS$_9$
Nº of genomes with TilS$_{10}$
Nº of genomes without TilS$_{10}$
With TilS$_{10}$
Without TilS$_{10}$
Nº of genomes with TilS$_{11}$
Nº of genomes without TilS$_{11}$
With TilS$_{11}$
Without TilS$_{11}$
Nº of genomes with TilS$_{12}$
Nº of genomes without TilS$_{12}$
With TilS$_{12}$
Without TilS$_{12}$
Nº of genomes with TilS$_{13}$
Nº of genomes without TilS$_{13}$
With TilS$_{13}$
Without TilS$_{13}$
Nº of genomes with TilS$_{14}$
Nº of genomes without TilS$_{14}$
With TilS$_{14}$
Without TilS$_{14}$
Nº of genomes with TilS$_{15}$
Nº of genomes without TilS$_{15}$
With TilS$_{15}$
Without TilS$_{15}$

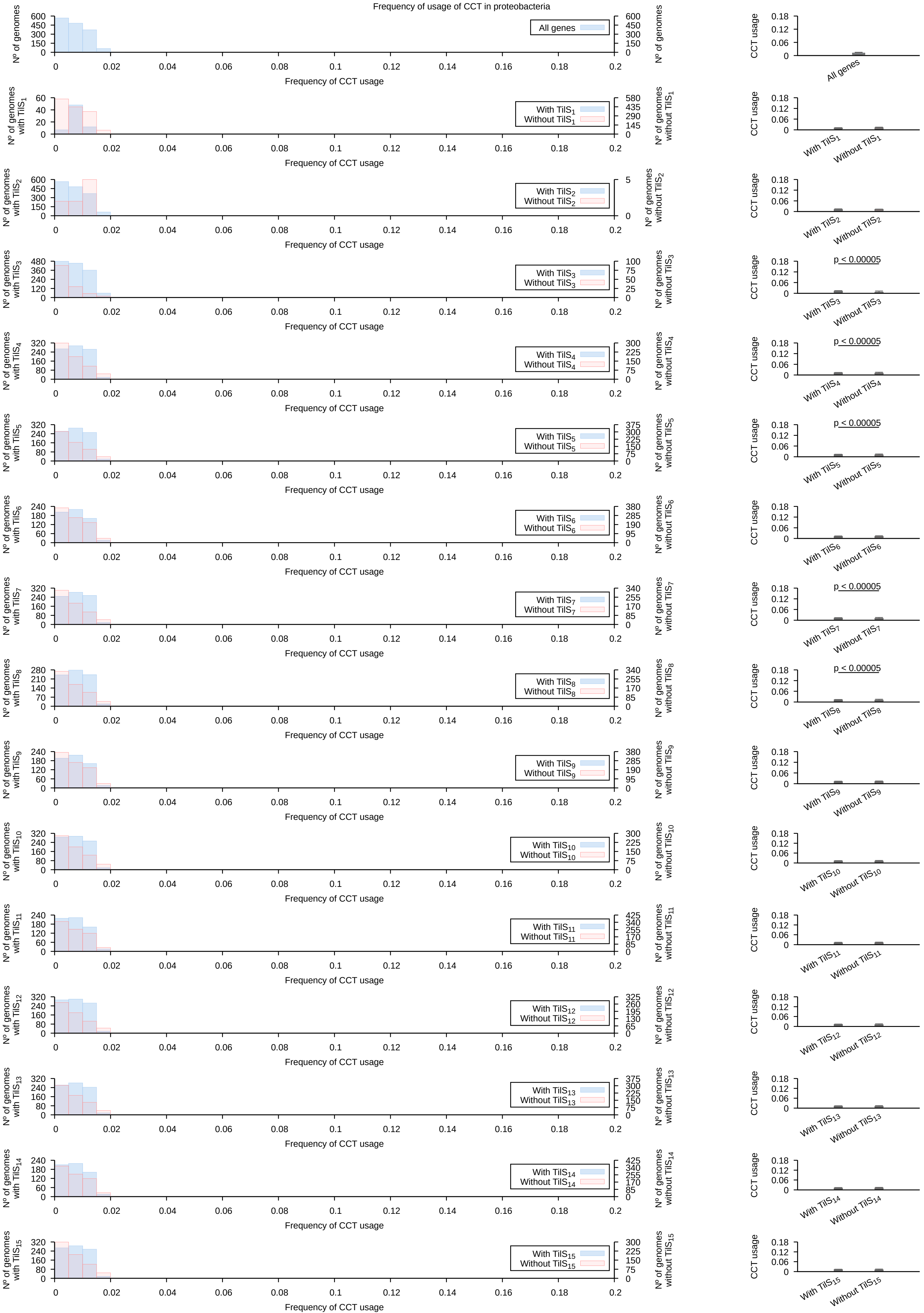
Frequency of usage of CCT in proteobacteria
Nº of genomes
All genes
Frequency of CCT usage
CCT usage
Nº of genomes with TilS$_1$
Nº of genomes without TilS$_1$
With TilS$_1$
Without TilS$_1$
Nº of genomes with TilS$_2$
Nº of genomes without TilS$_2$
With TilS$_2$
Without TilS$_2$
Nº of genomes with TilS$_3$
Nº of genomes without TilS$_3$
With TilS$_3$
Without TilS$_3$
p < 0.00005
Nº of genomes with TilS$_4$
Nº of genomes without TilS$_4$
With TilS$_4$
Without TilS$_4$
p < 0.00005
Nº of genomes with TilS$_5$
Nº of genomes without TilS$_5$
With TilS$_5$
Without TilS$_5$
p < 0.00005
Nº of genomes with TilS$_6$
Nº of genomes without TilS$_6$
With TilS$_6$
Without TilS$_6$
Nº of genomes with TilS$_7$
Nº of genomes without TilS$_7$
With TilS$_7$
Without TilS$_7$
p < 0.00005
Nº of genomes with TilS$_8$
Nº of genomes without TilS$_8$
With TilS$_8$
Without TilS$_8$
p < 0.00005
Nº of genomes with TilS$_9$
Nº of genomes without TilS$_9$
With TilS$_9$
Without TilS$_9$
Nº of genomes with TilS$_{10}$
Nº of genomes without TilS$_{10}$
With TilS$_{10}$
Without TilS$_{10}$
Nº of genomes with TilS$_{11}$
Nº of genomes without TilS$_{11}$
With TilS$_{11}$
Without TilS$_{11}$
Nº of genomes with TilS$_{12}$
Nº of genomes without TilS$_{12}$
With TilS$_{12}$
Without TilS$_{12}$
Nº of genomes with TilS$_{13}$
Nº of genomes without TilS$_{13}$
With TilS$_{13}$
Without TilS$_{13}$
Nº of genomes with TilS$_{14}$
Nº of genomes without TilS$_{14}$
With TilS$_{14}$
Without TilS$_{14}$
Nº of genomes with TilS$_{15}$
Nº of genomes without TilS$_{15}$
With TilS$_{15}$
Without TilS$_{15}$

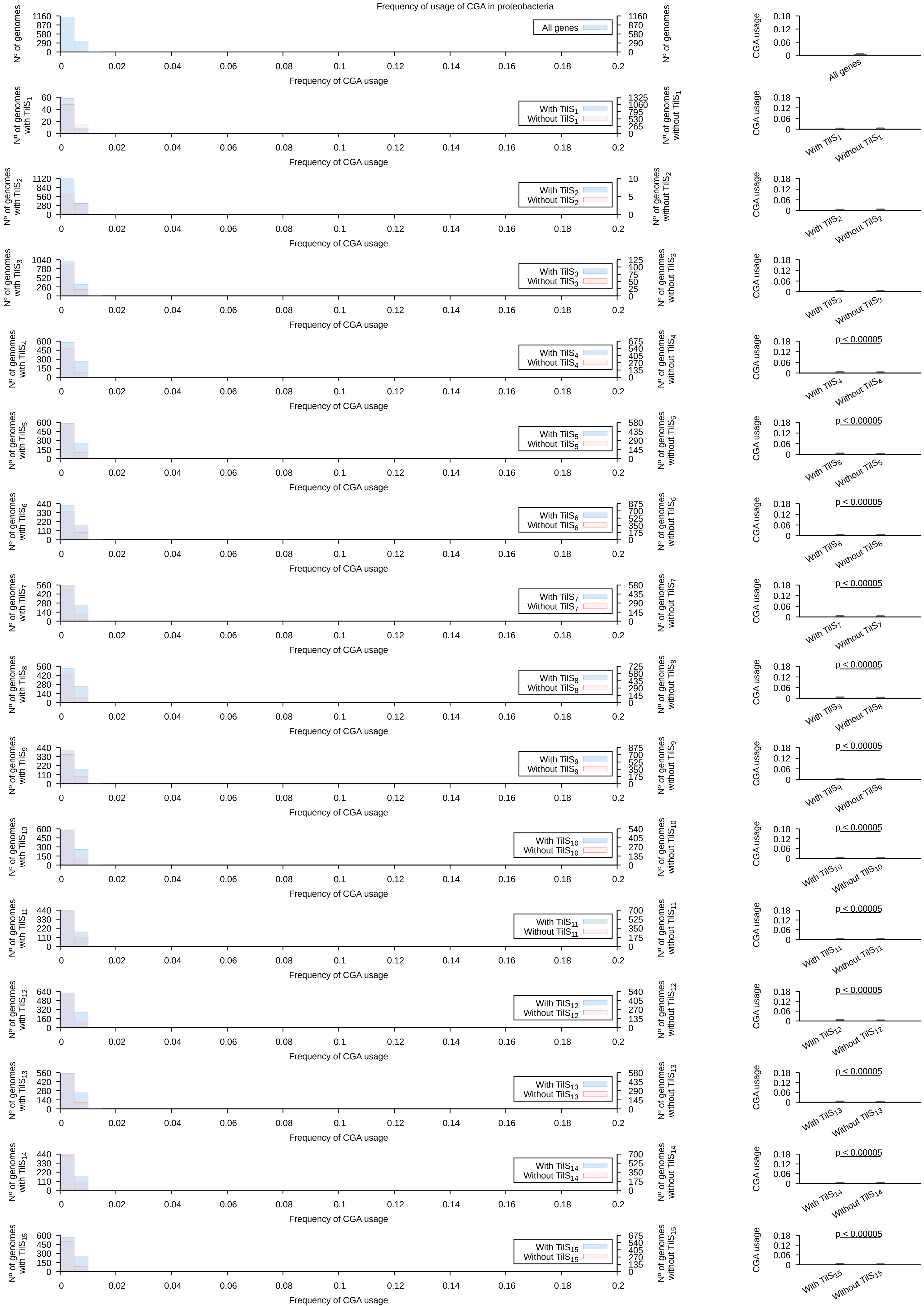

Frequency of usage of CGA in proteobacteria
Nº of genomes
1160 870 580 290 0
All genes
Frequency of CGA usage
0 0.02 0.04 0.06 0.08 0.1 0.12 0.14 0.16 0.18 0.2
CGA usage
0.18 0.12 0.06 0
Nº of genomes with TilS1
60 40 20 0
With TilS1
Without TilS1
1325 1060 795 530 265 0
Nº of genomes without TilS1
Nº of genomes with TilS2
1120 840 560 280 0
With TilS2
Without TilS2
10 5 0
Nº of genomes without TilS2
Nº of genomes with TilS3
1040 780 520 260 0
With TilS3
Without TilS3
125 100 75 50 25 0
Nº of genomes without TilS3
Nº of genomes with TilS4
600 450 300 150 0
With TilS4
Without TilS4
675 540 405 270 135 0
Nº of genomes without TilS4
p < 0.00005
Nº of genomes with TilS5
600 450 300 150 0
With TilS5
Without TilS5
580 435 290 145 0
Nº of genomes without TilS5
p < 0.00005
Nº of genomes with TilS6
440 330 220 110 0
With TilS6
Without TilS6
875 700 525 350 175 0
Nº of genomes without TilS6
p < 0.00005
Nº of genomes with TilS7
560 420 280 140 0
With TilS7
Without TilS7
580 435 290 145 0
Nº of genomes without TilS7
p < 0.00005
Nº of genomes with TilS8
560 420 280 140 0
With TilS8
Without TilS8
725 580 435 290 145 0
Nº of genomes without TilS8
p < 0.00005
Nº of genomes with TilS9
440 330 220 110 0
With TilS9
Without TilS9
875 700 525 350 175 0
Nº of genomes without TilS9
p < 0.00005
Nº of genomes with TilS10
600 450 300 150 0
With TilS10
Without TilS10
540 405 270 135 0
Nº of genomes without TilS10
p < 0.00005
Nº of genomes with TilS11
440 330 220 110 0
With TilS11
Without TilS11
700 525 350 175 0
Nº of genomes without TilS11
p < 0.00005
Nº of genomes with TilS12
640 480 320 160 0
With TilS12
Without TilS12
540 405 270 135 0
Nº of genomes without TilS12
p < 0.00005
Nº of genomes with TilS13
560 420 280 140 0
With TilS13
Without TilS13
580 435 290 145 0
Nº of genomes without TilS13
p < 0.00005
Nº of genomes with TilS14
440 330 220 110 0
With TilS14
Without TilS14
700 525 350 175 0
Nº of genomes without TilS14
p < 0.00005
Nº of genomes with TilS15
600 450 300 150 0
With TilS15
Without TilS15
675 540 405 270 135 0
Nº of genomes without TilS15
p < 0.00005

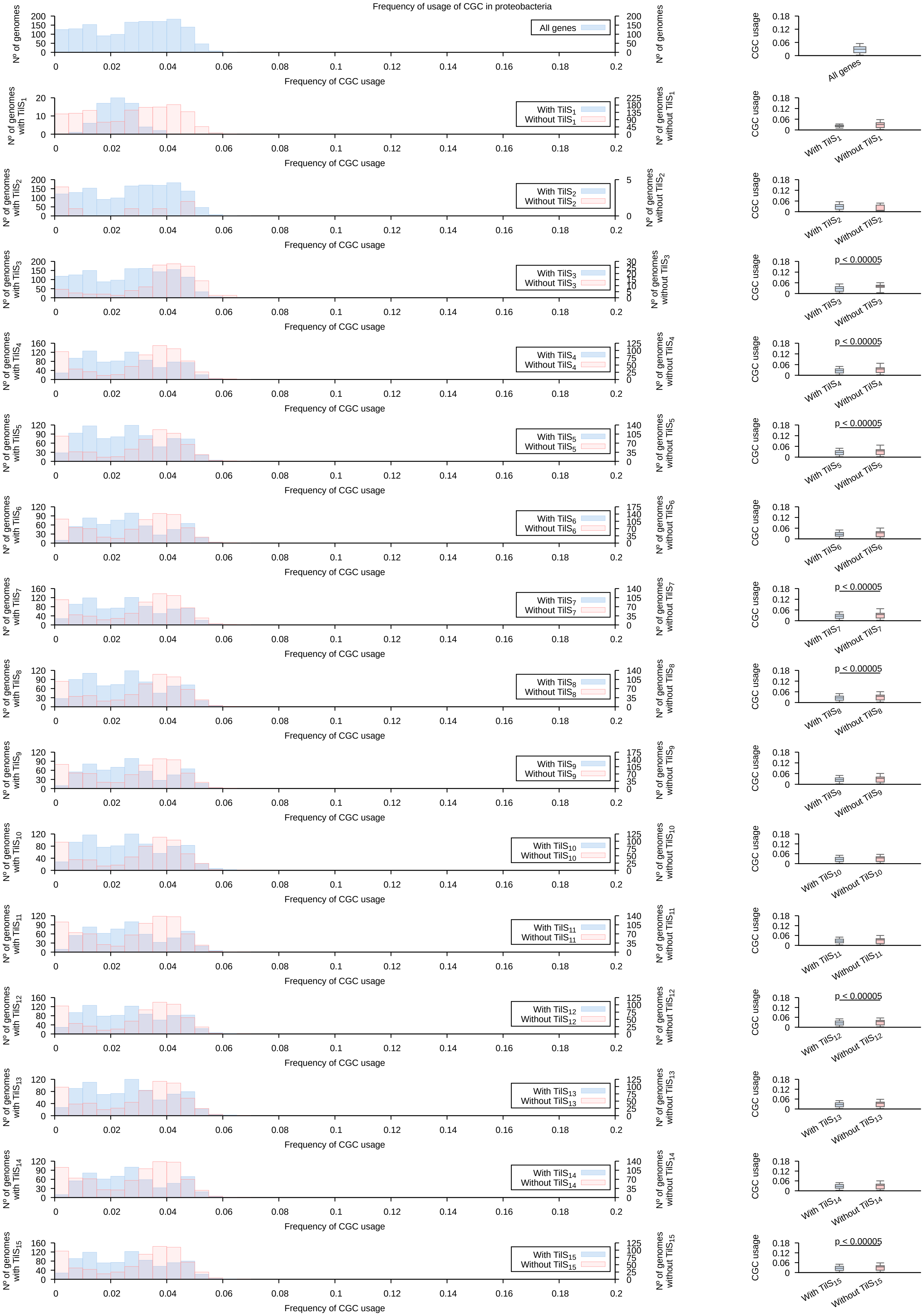

Frequency of usage of CGC in proteobacteria
Nº of genomes
Frequency of CGC usage
CGC usage
All genes
With $TilS_1$
Without $TilS_1$
With $TilS_2$
Without $TilS_2$
With $TilS_3$
Without $TilS_3$
With $TilS_4$
Without $TilS_4$
With $TilS_5$
Without $TilS_5$
With $TilS_6$
Without $TilS_6$
With $TilS_7$
Without $TilS_7$
With $TilS_8$
Without $TilS_8$
With $TilS_9$
Without $TilS_9$
With $TilS_{10}$
Without $TilS_{10}$
With $TilS_{11}$
Without $TilS_{11}$
With $TilS_{12}$
Without $TilS_{12}$
With $TilS_{13}$
Without $TilS_{13}$
With $TilS_{14}$
Without $TilS_{14}$
With $TilS_{15}$
Without $TilS_{15}$
Nº of genomes with $TilS_1$ … $TilS_{15}$
Nº of genomes without $TilS_1$ … $TilS_{15}$
$p < 0.00005$

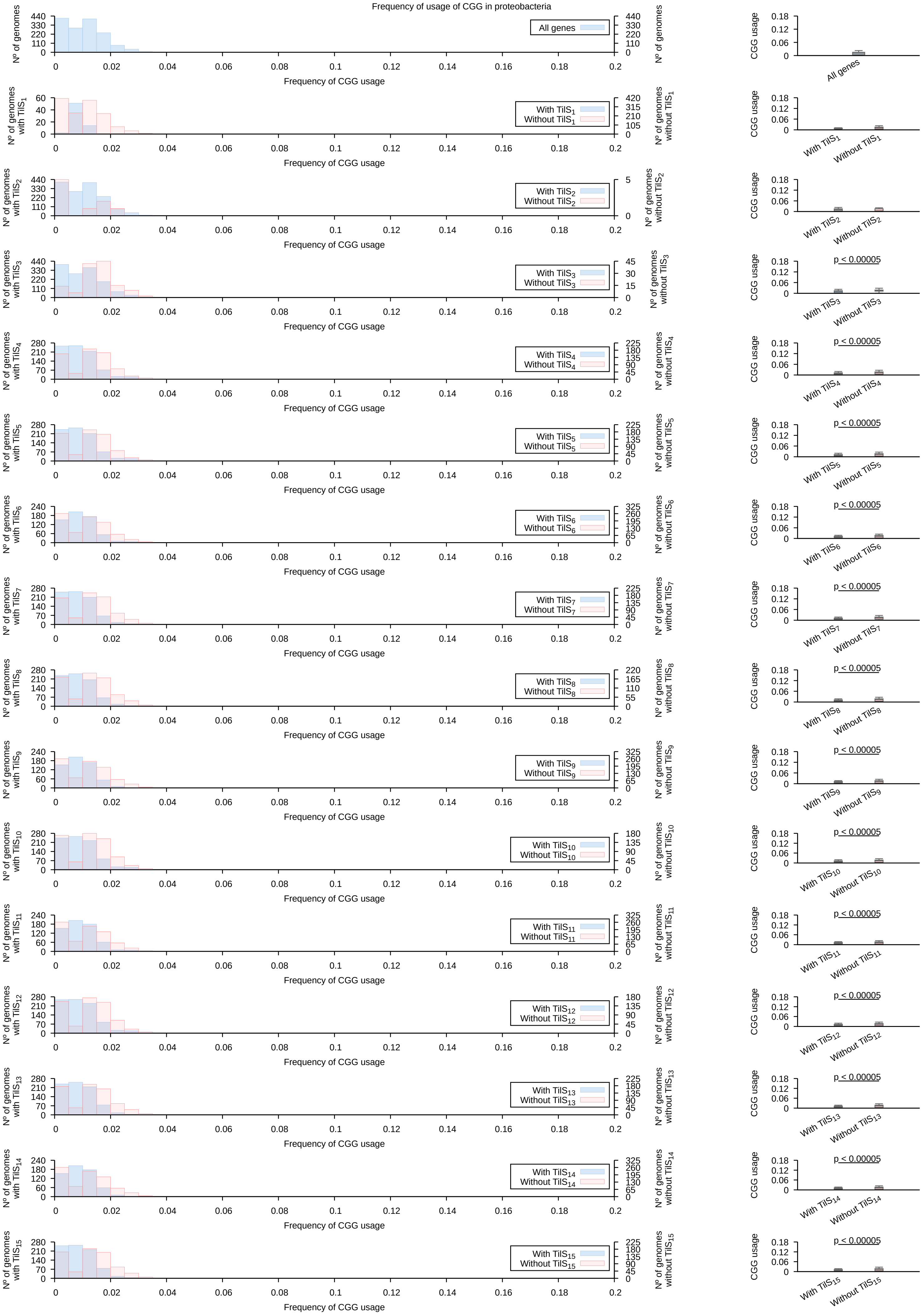

Frequency of usage of CGG in proteobacteria
Nº of genomes
All genes
Frequency of CGG usage
CGG usage
Nº of genomes with TilS$_1$
Nº of genomes without TilS$_1$
With TilS$_1$
Without TilS$_1$
Nº of genomes with TilS$_2$
Nº of genomes without TilS$_2$
With TilS$_2$
Without TilS$_2$
Nº of genomes with TilS$_3$
Nº of genomes without TilS$_3$
With TilS$_3$
Without TilS$_3$
p < 0.00005
Nº of genomes with TilS$_4$
Nº of genomes without TilS$_4$
With TilS$_4$
Without TilS$_4$
p < 0.00005
Nº of genomes with TilS$_5$
Nº of genomes without TilS$_5$
With TilS$_5$
Without TilS$_5$
p < 0.00005
Nº of genomes with TilS$_6$
Nº of genomes without TilS$_6$
With TilS$_6$
Without TilS$_6$
p < 0.00005
Nº of genomes with TilS$_7$
Nº of genomes without TilS$_7$
With TilS$_7$
Without TilS$_7$
p < 0.00005
Nº of genomes with TilS$_8$
Nº of genomes without TilS$_8$
With TilS$_8$
Without TilS$_8$
p < 0.00005
Nº of genomes with TilS$_9$
Nº of genomes without TilS$_9$
With TilS$_9$
Without TilS$_9$
p < 0.00005
Nº of genomes with TilS$_{10}$
Nº of genomes without TilS$_{10}$
With TilS$_{10}$
Without TilS$_{10}$
p < 0.00005
Nº of genomes with TilS$_{11}$
Nº of genomes without TilS$_{11}$
With TilS$_{11}$
Without TilS$_{11}$
p < 0.00005
Nº of genomes with TilS$_{12}$
Nº of genomes without TilS$_{12}$
With TilS$_{12}$
Without TilS$_{12}$
p < 0.00005
Nº of genomes with TilS$_{13}$
Nº of genomes without TilS$_{13}$
With TilS$_{13}$
Without TilS$_{13}$
p < 0.00005
Nº of genomes with TilS$_{14}$
Nº of genomes without TilS$_{14}$
With TilS$_{14}$
Without TilS$_{14}$
p < 0.00005
Nº of genomes with TilS$_{15}$
Nº of genomes without TilS$_{15}$
With TilS$_{15}$
Without TilS$_{15}$
p < 0.00005

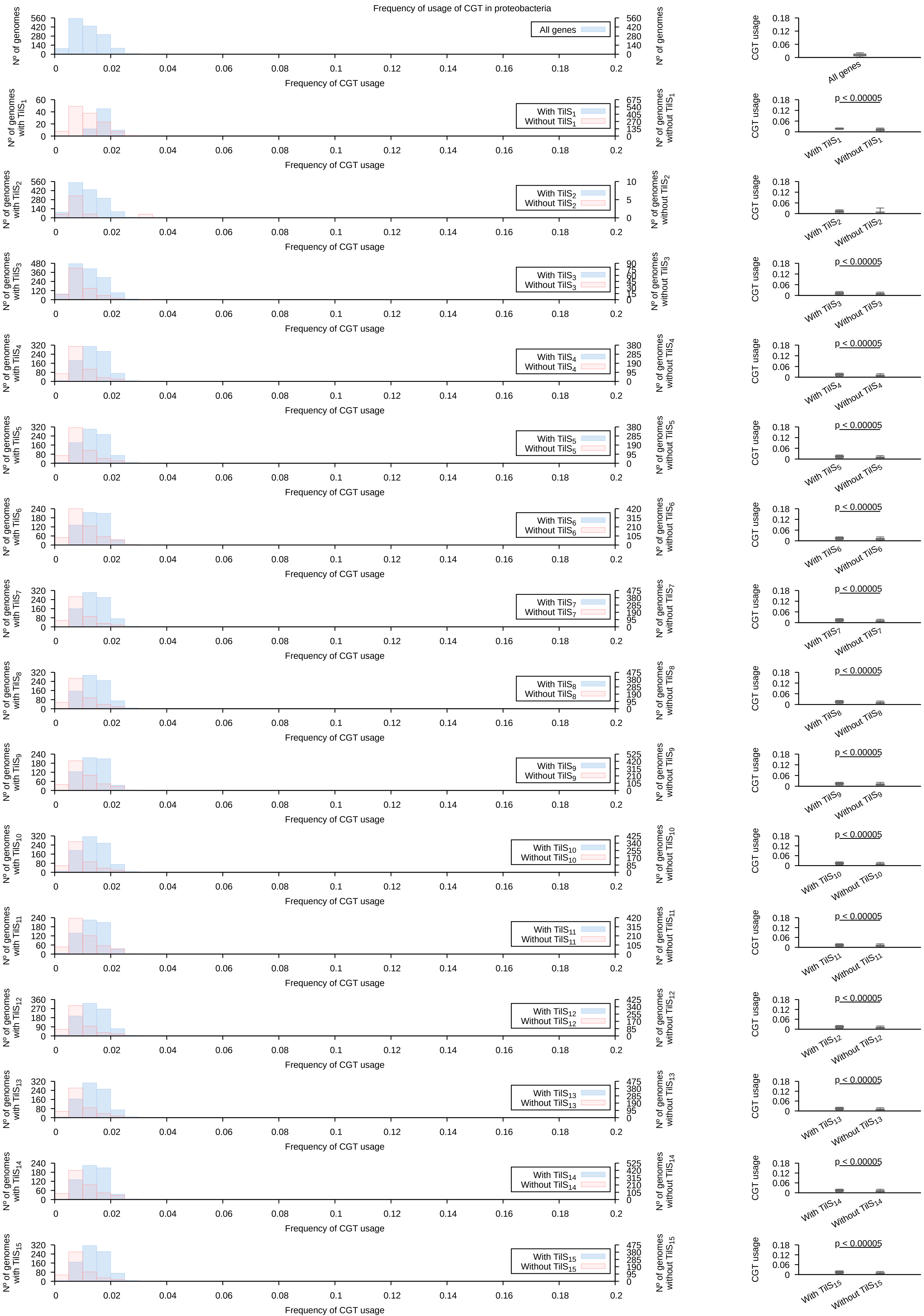

Frequency of usage of CGT in proteobacteria
Nº of genomes
All genes
Frequency of CGT usage
CGT usage
0
0.02
0.04
0.06
0.08
0.1
0.12
0.14
0.16
0.18
0.2
0.18
0.12
0.06
Nº of genomes with TilS$_1$
Nº of genomes without TilS$_1$
With TilS$_1$
Without TilS$_1$
p < 0.00005
Nº of genomes with TilS$_2$
Nº of genomes without TilS$_2$
With TilS$_2$
Without TilS$_2$
Nº of genomes with TilS$_3$
Nº of genomes without TilS$_3$
With TilS$_3$
Without TilS$_3$
p < 0.00005
Nº of genomes with TilS$_4$
Nº of genomes without TilS$_4$
With TilS$_4$
Without TilS$_4$
p < 0.00005
Nº of genomes with TilS$_5$
Nº of genomes without TilS$_5$
With TilS$_5$
Without TilS$_5$
p < 0.00005
Nº of genomes with TilS$_6$
Nº of genomes without TilS$_6$
With TilS$_6$
Without TilS$_6$
p < 0.00005
Nº of genomes with TilS$_7$
Nº of genomes without TilS$_7$
With TilS$_7$
Without TilS$_7$
p < 0.00005
Nº of genomes with TilS$_8$
Nº of genomes without TilS$_8$
With TilS$_8$
Without TilS$_8$
p < 0.00005
Nº of genomes with TilS$_9$
Nº of genomes without TilS$_9$
With TilS$_9$
Without TilS$_9$
p < 0.00005
Nº of genomes with TilS$_{10}$
Nº of genomes without TilS$_{10}$
With TilS$_{10}$
Without TilS$_{10}$
p < 0.00005
Nº of genomes with TilS$_{11}$
Nº of genomes without TilS$_{11}$
With TilS$_{11}$
Without TilS$_{11}$
p < 0.00005
Nº of genomes with TilS$_{12}$
Nº of genomes without TilS$_{12}$
With TilS$_{12}$
Without TilS$_{12}$
p < 0.00005
Nº of genomes with TilS$_{13}$
Nº of genomes without TilS$_{13}$
With TilS$_{13}$
Without TilS$_{13}$
p < 0.00005
Nº of genomes with TilS$_{14}$
Nº of genomes without TilS$_{14}$
With TilS$_{14}$
Without TilS$_{14}$
p < 0.00005
Nº of genomes with TilS$_{15}$
Nº of genomes without TilS$_{15}$
With TilS$_{15}$
Without TilS$_{15}$
p < 0.00005

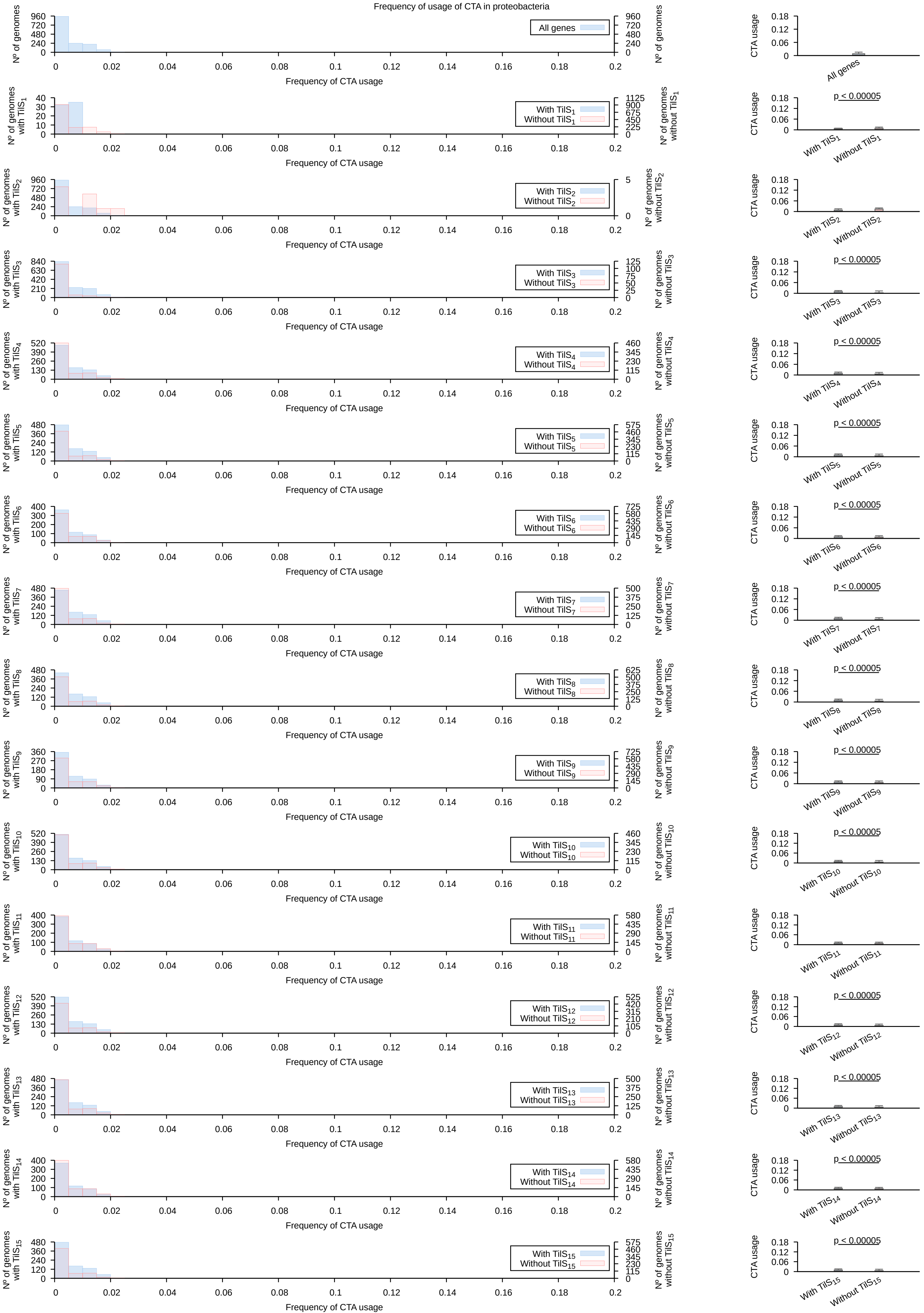

Frequency of usage of CTA in proteobacteria
Nº of genomes
All genes
Frequency of CTA usage
CTA usage
Nº of genomes with $TilS_1$
Nº of genomes without $TilS_1$
With $TilS_1$
Without $TilS_1$
$p < 0.00005$
Nº of genomes with $TilS_2$
Nº of genomes without $TilS_2$
With $TilS_2$
Without $TilS_2$
Nº of genomes with $TilS_3$
Nº of genomes without $TilS_3$
With $TilS_3$
Without $TilS_3$
$p < 0.00005$
Nº of genomes with $TilS_4$
Nº of genomes without $TilS_4$
With $TilS_4$
Without $TilS_4$
$p < 0.00005$
Nº of genomes with $TilS_5$
Nº of genomes without $TilS_5$
With $TilS_5$
Without $TilS_5$
$p < 0.00005$
Nº of genomes with $TilS_6$
Nº of genomes without $TilS_6$
With $TilS_6$
Without $TilS_6$
$p < 0.00005$
Nº of genomes with $TilS_7$
Nº of genomes without $TilS_7$
With $TilS_7$
Without $TilS_7$
$p < 0.00005$
Nº of genomes with $TilS_8$
Nº of genomes without $TilS_8$
With $TilS_8$
Without $TilS_8$
$p < 0.00005$
Nº of genomes with $TilS_9$
Nº of genomes without $TilS_9$
With $TilS_9$
Without $TilS_9$
$p < 0.00005$
Nº of genomes with $TilS_{10}$
Nº of genomes without $TilS_{10}$
With $TilS_{10}$
Without $TilS_{10}$
$p < 0.00005$
Nº of genomes with $TilS_{11}$
Nº of genomes without $TilS_{11}$
With $TilS_{11}$
Without $TilS_{11}$
Nº of genomes with $TilS_{12}$
Nº of genomes without $TilS_{12}$
With $TilS_{12}$
Without $TilS_{12}$
$p < 0.00005$
Nº of genomes with $TilS_{13}$
Nº of genomes without $TilS_{13}$
With $TilS_{13}$
Without $TilS_{13}$
$p < 0.00005$
Nº of genomes with $TilS_{14}$
Nº of genomes without $TilS_{14}$
With $TilS_{14}$
Without $TilS_{14}$
$p < 0.00005$
Nº of genomes with $TilS_{15}$
Nº of genomes without $TilS_{15}$
With $TilS_{15}$
Without $TilS_{15}$
$p < 0.00005$

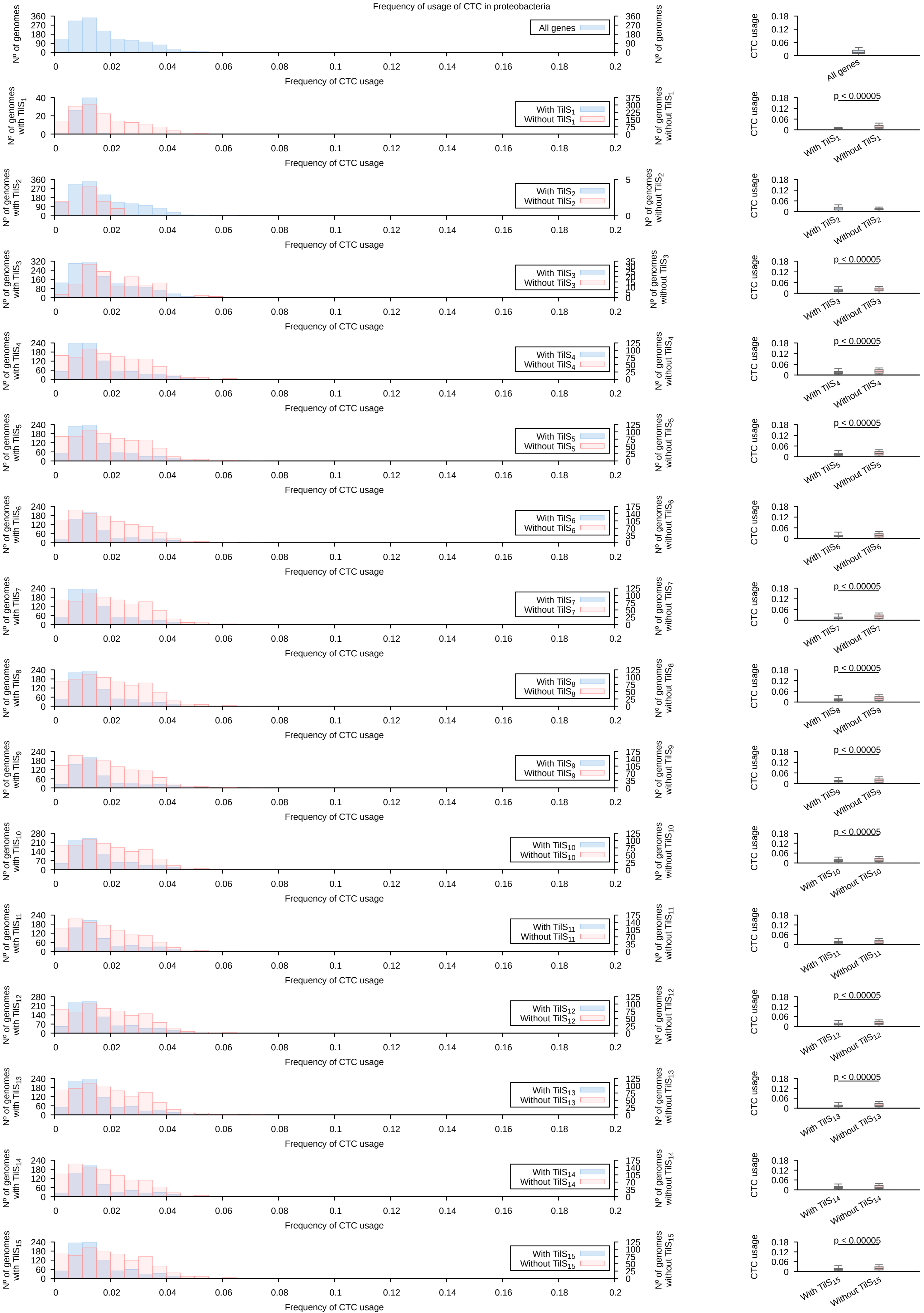

Frequency of usage of CTC in proteobacteria
Nº of genomes
All genes
Frequency of CTC usage
CTC usage
Nº of genomes with TilS1
Nº of genomes without TilS1
With TilS1
Without TilS1
p < 0.00005
Nº of genomes with TilS2
Nº of genomes without TilS2
With TilS2
Without TilS2
Nº of genomes with TilS3
Nº of genomes without TilS3
With TilS3
Without TilS3
p < 0.00005
Nº of genomes with TilS4
Nº of genomes without TilS4
With TilS4
Without TilS4
p < 0.00005
Nº of genomes with TilS5
Nº of genomes without TilS5
With TilS5
Without TilS5
p < 0.00005
Nº of genomes with TilS6
Nº of genomes without TilS6
With TilS6
Without TilS6
Nº of genomes with TilS7
Nº of genomes without TilS7
With TilS7
Without TilS7
p < 0.00005
Nº of genomes with TilS8
Nº of genomes without TilS8
With TilS8
Without TilS8
p < 0.00005
Nº of genomes with TilS9
Nº of genomes without TilS9
With TilS9
Without TilS9
p < 0.00005
Nº of genomes with TilS10
Nº of genomes without TilS10
With TilS10
Without TilS10
p < 0.00005
Nº of genomes with TilS11
Nº of genomes without TilS11
With TilS11
Without TilS11
Nº of genomes with TilS12
Nº of genomes without TilS12
With TilS12
Without TilS12
p < 0.00005
Nº of genomes with TilS13
Nº of genomes without TilS13
With TilS13
Without TilS13
p < 0.00005
Nº of genomes with TilS14
Nº of genomes without TilS14
With TilS14
Without TilS14
Nº of genomes with TilS15
Nº of genomes without TilS15
With TilS15
Without TilS15
p < 0.00005

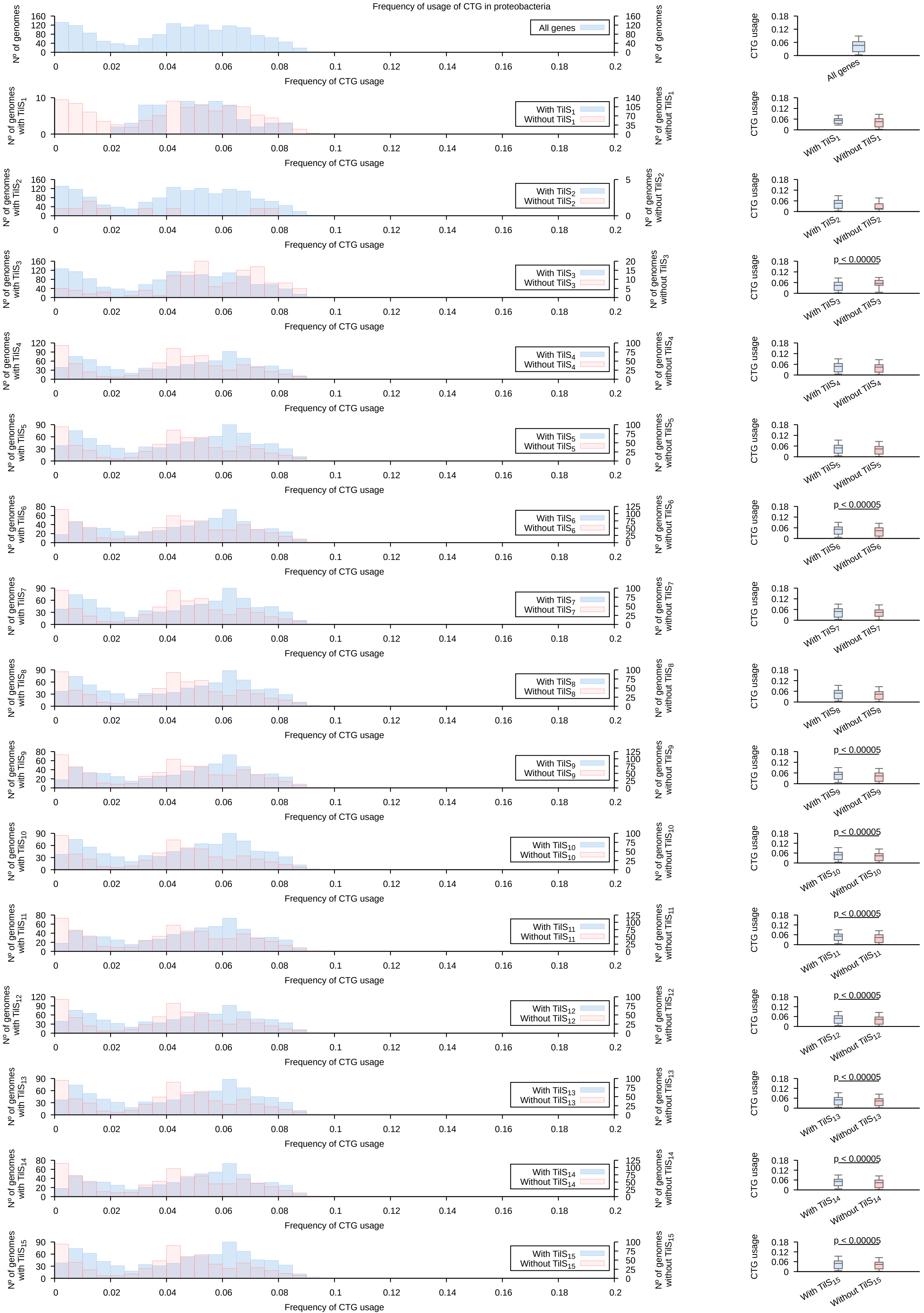
Frequency of usage of CTG in proteobacteria
Nº of genomes
All genes
Frequency of CTG usage
CTG usage
Nº of genomes with TilS$_1$
Nº of genomes without TilS$_1$
With TilS$_1$
Without TilS$_1$
Nº of genomes with TilS$_2$
Nº of genomes without TilS$_2$
With TilS$_2$
Without TilS$_2$
Nº of genomes with TilS$_3$
Nº of genomes without TilS$_3$
With TilS$_3$
Without TilS$_3$
p < 0.00005
Nº of genomes with TilS$_4$
Nº of genomes without TilS$_4$
With TilS$_4$
Without TilS$_4$
Nº of genomes with TilS$_5$
Nº of genomes without TilS$_5$
With TilS$_5$
Without TilS$_5$
Nº of genomes with TilS$_6$
Nº of genomes without TilS$_6$
With TilS$_6$
Without TilS$_6$
p < 0.00005
Nº of genomes with TilS$_7$
Nº of genomes without TilS$_7$
With TilS$_7$
Without TilS$_7$
Nº of genomes with TilS$_8$
Nº of genomes without TilS$_8$
With TilS$_8$
Without TilS$_8$
Nº of genomes with TilS$_9$
Nº of genomes without TilS$_9$
With TilS$_9$
Without TilS$_9$
p < 0.00005
Nº of genomes with TilS$_{10}$
Nº of genomes without TilS$_{10}$
With TilS$_{10}$
Without TilS$_{10}$
p < 0.00005
Nº of genomes with TilS$_{11}$
Nº of genomes without TilS$_{11}$
With TilS$_{11}$
Without TilS$_{11}$
p < 0.00005
Nº of genomes with TilS$_{12}$
Nº of genomes without TilS$_{12}$
With TilS$_{12}$
Without TilS$_{12}$
p < 0.00005
Nº of genomes with TilS$_{13}$
Nº of genomes without TilS$_{13}$
With TilS$_{13}$
Without TilS$_{13}$
p < 0.00005
Nº of genomes with TilS$_{14}$
Nº of genomes without TilS$_{14}$
With TilS$_{14}$
Without TilS$_{14}$
p < 0.00005
Nº of genomes with TilS$_{15}$
Nº of genomes without TilS$_{15}$
With TilS$_{15}$
Without TilS$_{15}$
p < 0.00005

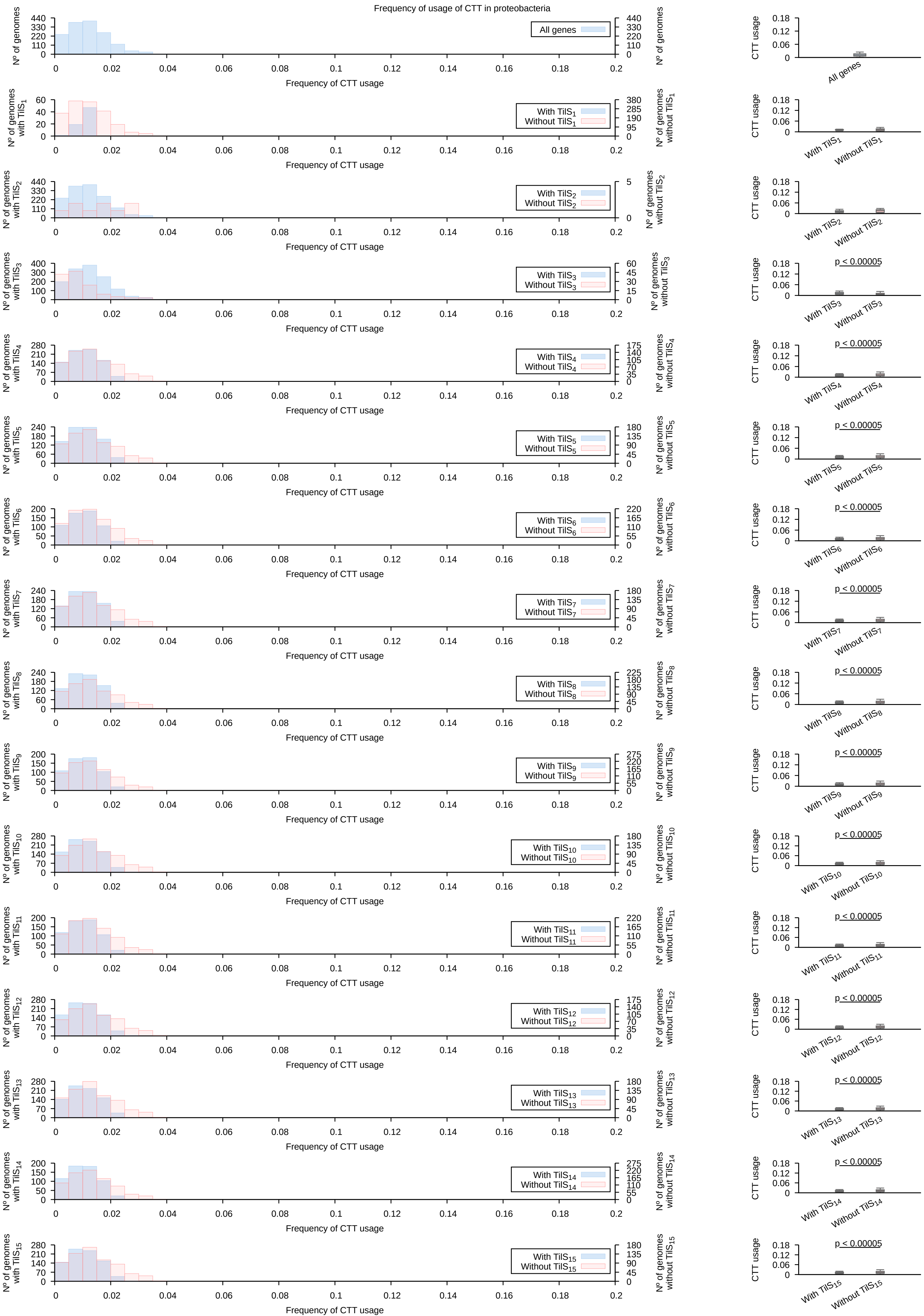

Frequency of usage of CTT in proteobacteria
Nº of genomes
All genes
Frequency of CTT usage
CTT usage
0
0.02
0.04
0.06
0.08
0.1
0.12
0.14
0.16
0.18
0.2
0.06
0.12
0.18
Nº of genomes with TilS$_1$
Nº of genomes without TilS$_1$
With TilS$_1$
Without TilS$_1$
Nº of genomes with TilS$_2$
Nº of genomes without TilS$_2$
With TilS$_2$
Without TilS$_2$
Nº of genomes with TilS$_3$
Nº of genomes without TilS$_3$
With TilS$_3$
Without TilS$_3$
p < 0.00005
Nº of genomes with TilS$_4$
Nº of genomes without TilS$_4$
With TilS$_4$
Without TilS$_4$
p < 0.00005
Nº of genomes with TilS$_5$
Nº of genomes without TilS$_5$
With TilS$_5$
Without TilS$_5$
p < 0.00005
Nº of genomes with TilS$_6$
Nº of genomes without TilS$_6$
With TilS$_6$
Without TilS$_6$
p < 0.00005
Nº of genomes with TilS$_7$
Nº of genomes without TilS$_7$
With TilS$_7$
Without TilS$_7$
p < 0.00005
Nº of genomes with TilS$_8$
Nº of genomes without TilS$_8$
With TilS$_8$
Without TilS$_8$
p < 0.00005
Nº of genomes with TilS$_9$
Nº of genomes without TilS$_9$
With TilS$_9$
Without TilS$_9$
p < 0.00005
Nº of genomes with TilS$_{10}$
Nº of genomes without TilS$_{10}$
With TilS$_{10}$
Without TilS$_{10}$
p < 0.00005
Nº of genomes with TilS$_{11}$
Nº of genomes without TilS$_{11}$
With TilS$_{11}$
Without TilS$_{11}$
p < 0.00005
Nº of genomes with TilS$_{12}$
Nº of genomes without TilS$_{12}$
With TilS$_{12}$
Without TilS$_{12}$
p < 0.00005
Nº of genomes with TilS$_{13}$
Nº of genomes without TilS$_{13}$
With TilS$_{13}$
Without TilS$_{13}$
p < 0.00005
Nº of genomes with TilS$_{14}$
Nº of genomes without TilS$_{14}$
With TilS$_{14}$
Without TilS$_{14}$
p < 0.00005
Nº of genomes with TilS$_{15}$
Nº of genomes without TilS$_{15}$
With TilS$_{15}$
Without TilS$_{15}$
p < 0.00005

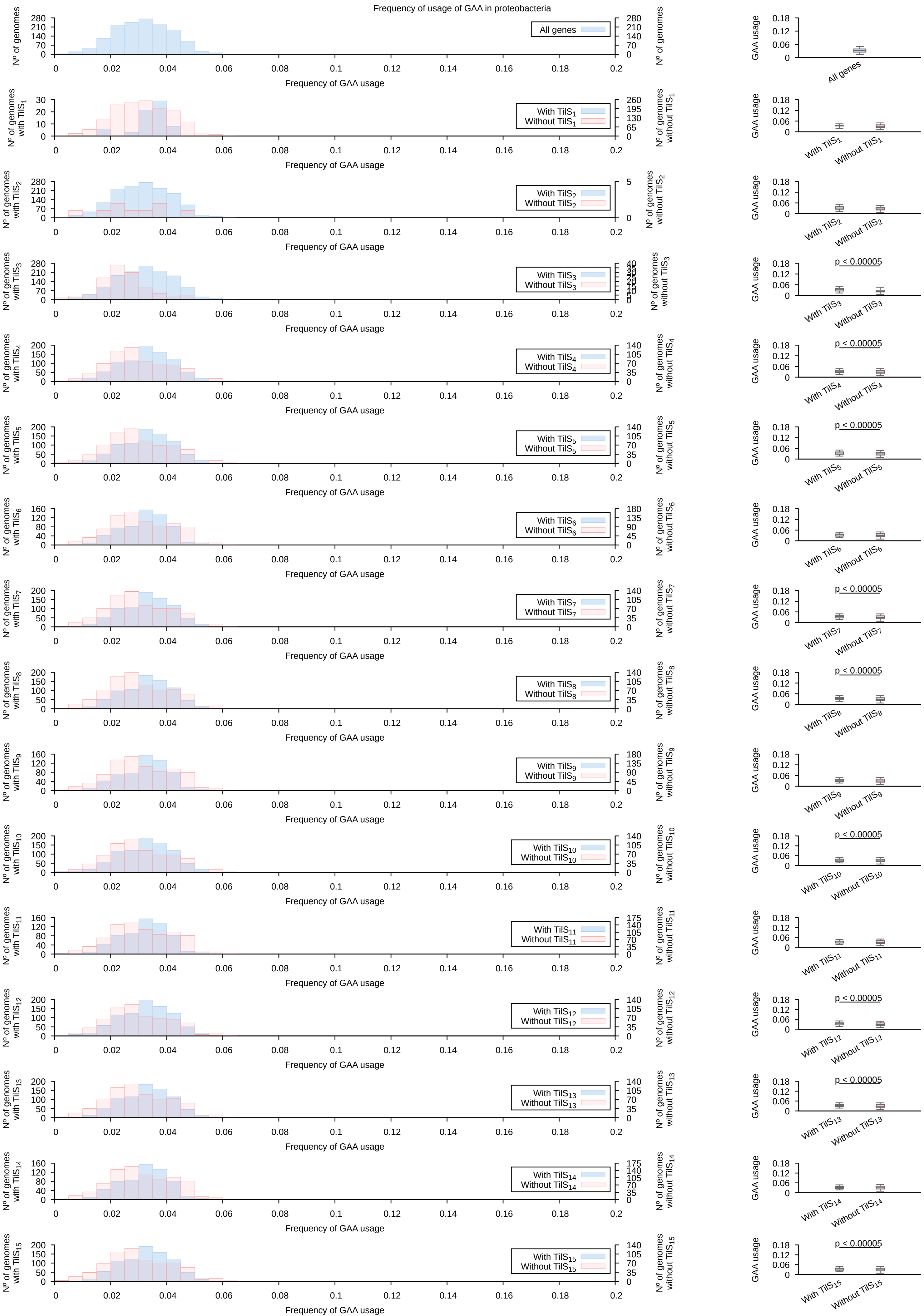
Frequency of usage of GAA in proteobacteria
Nº of genomes
All genes
Frequency of GAA usage
GAA usage
0 0.02 0.04 0.06 0.08 0.1 0.12 0.14 0.16 0.18 0.2
0 0.06 0.12 0.18
Nº of genomes with TilS1
Nº of genomes without TilS1
With TilS1
Without TilS1
Nº of genomes with TilS2
Nº of genomes without TilS2
With TilS2
Without TilS2
Nº of genomes with TilS3
Nº of genomes without TilS3
With TilS3
Without TilS3
p < 0.00005
Nº of genomes with TilS4
Nº of genomes without TilS4
With TilS4
Without TilS4
p < 0.00005
Nº of genomes with TilS5
Nº of genomes without TilS5
With TilS5
Without TilS5
p < 0.00005
Nº of genomes with TilS6
Nº of genomes without TilS6
With TilS6
Without TilS6
Nº of genomes with TilS7
Nº of genomes without TilS7
With TilS7
Without TilS7
p < 0.00005
Nº of genomes with TilS8
Nº of genomes without TilS8
With TilS8
Without TilS8
p < 0.00005
Nº of genomes with TilS9
Nº of genomes without TilS9
With TilS9
Without TilS9
Nº of genomes with TilS10
Nº of genomes without TilS10
With TilS10
Without TilS10
p < 0.00005
Nº of genomes with TilS11
Nº of genomes without TilS11
With TilS11
Without TilS11
Nº of genomes with TilS12
Nº of genomes without TilS12
With TilS12
Without TilS12
p < 0.00005
Nº of genomes with TilS13
Nº of genomes without TilS13
With TilS13
Without TilS13
p < 0.00005
Nº of genomes with TilS14
Nº of genomes without TilS14
With TilS14
Without TilS14
Nº of genomes with TilS15
Nº of genomes without TilS15
With TilS15
Without TilS15
p < 0.00005

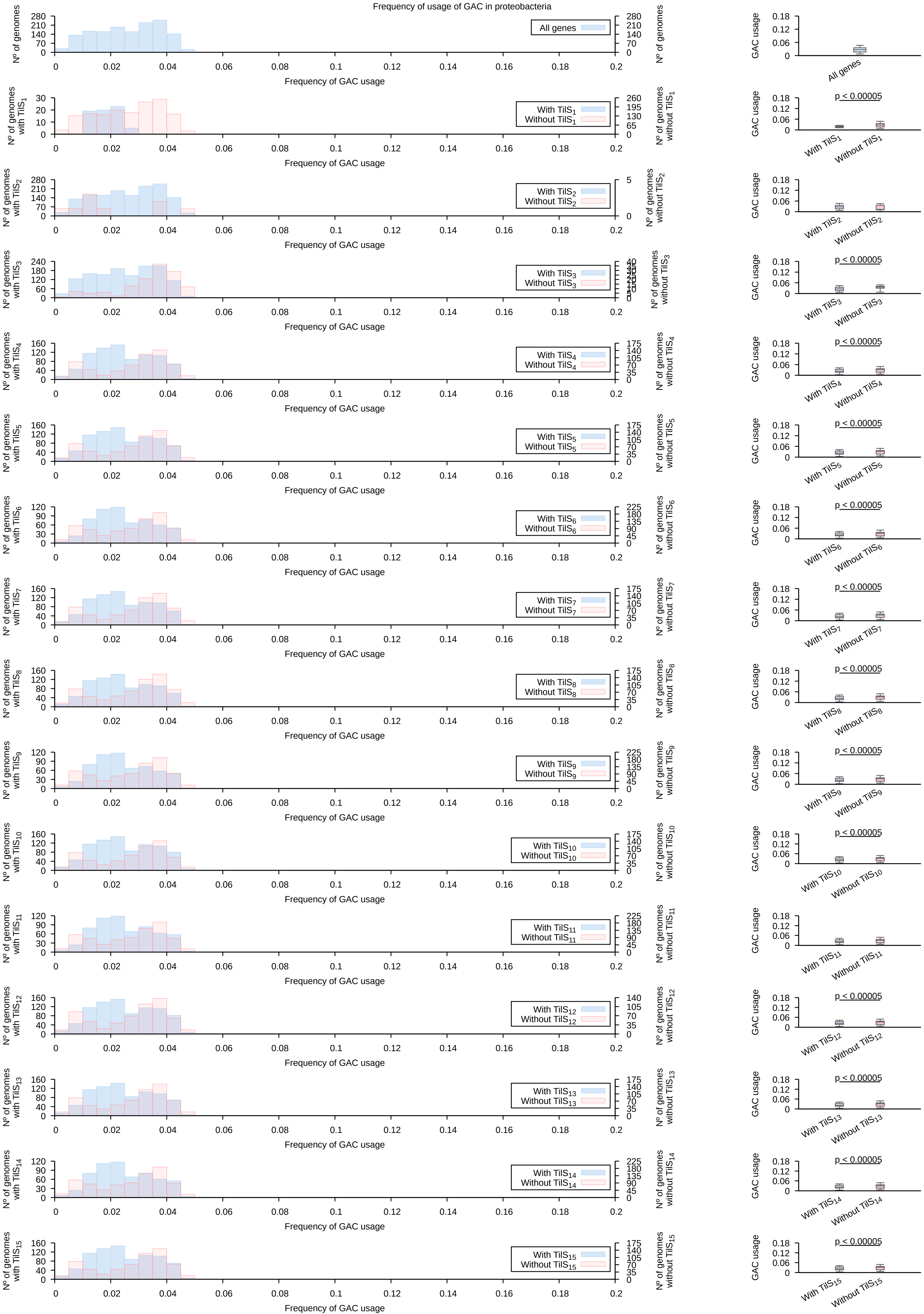

Frequency of usage of GAC in proteobacteria
Nº of genomes
All genes
Frequency of GAC usage
GAC usage
Nº of genomes with TilS1
Nº of genomes without TilS1
With TilS1
Without TilS1
p < 0.00005
Nº of genomes with TilS2
Nº of genomes without TilS2
With TilS2
Without TilS2
Nº of genomes with TilS3
Nº of genomes without TilS3
With TilS3
Without TilS3
p < 0.00005
Nº of genomes with TilS4
Nº of genomes without TilS4
With TilS4
Without TilS4
p < 0.00005
Nº of genomes with TilS5
Nº of genomes without TilS5
With TilS5
Without TilS5
p < 0.00005
Nº of genomes with TilS6
Nº of genomes without TilS6
With TilS6
Without TilS6
p < 0.00005
Nº of genomes with TilS7
Nº of genomes without TilS7
With TilS7
Without TilS7
p < 0.00005
Nº of genomes with TilS8
Nº of genomes without TilS8
With TilS8
Without TilS8
p < 0.00005
Nº of genomes with TilS9
Nº of genomes without TilS9
With TilS9
Without TilS9
p < 0.00005
Nº of genomes with TilS10
Nº of genomes without TilS10
With TilS10
Without TilS10
p < 0.00005
Nº of genomes with TilS11
Nº of genomes without TilS11
With TilS11
Without TilS11
Nº of genomes with TilS12
Nº of genomes without TilS12
With TilS12
Without TilS12
p < 0.00005
Nº of genomes with TilS13
Nº of genomes without TilS13
With TilS13
Without TilS13
p < 0.00005
Nº of genomes with TilS14
Nº of genomes without TilS14
With TilS14
Without TilS14
p < 0.00005
Nº of genomes with TilS15
Nº of genomes without TilS15
With TilS15
Without TilS15
p < 0.00005

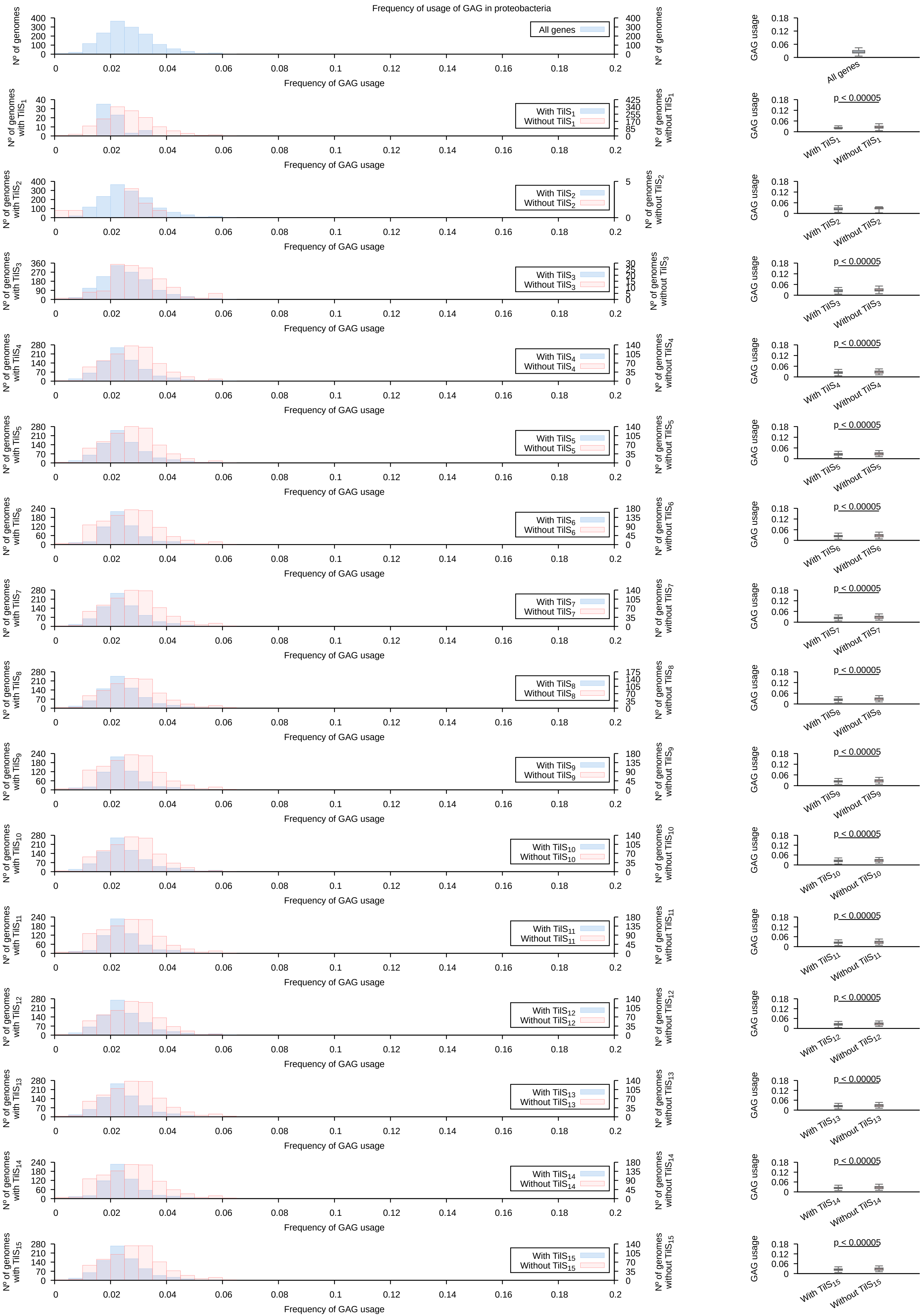
Frequency of usage of GAG in proteobacteria
Nº of genomes
All genes
Frequency of GAG usage
GAG usage
Nº of genomes with $TilS_1$
With $TilS_1$
Without $TilS_1$
Nº of genomes without $TilS_1$
p < 0.00005
Nº of genomes with $TilS_2$
With $TilS_2$
Without $TilS_2$
Nº of genomes without $TilS_2$
Nº of genomes with $TilS_3$
With $TilS_3$
Without $TilS_3$
Nº of genomes without $TilS_3$
Nº of genomes with $TilS_4$
With $TilS_4$
Without $TilS_4$
Nº of genomes without $TilS_4$
Nº of genomes with $TilS_5$
With $TilS_5$
Without $TilS_5$
Nº of genomes without $TilS_5$
Nº of genomes with $TilS_6$
With $TilS_6$
Without $TilS_6$
Nº of genomes without $TilS_6$
Nº of genomes with $TilS_7$
With $TilS_7$
Without $TilS_7$
Nº of genomes without $TilS_7$
Nº of genomes with $TilS_8$
With $TilS_8$
Without $TilS_8$
Nº of genomes without $TilS_8$
Nº of genomes with $TilS_9$
With $TilS_9$
Without $TilS_9$
Nº of genomes without $TilS_9$
Nº of genomes with $TilS_{10}$
With $TilS_{10}$
Without $TilS_{10}$
Nº of genomes without $TilS_{10}$
Nº of genomes with $TilS_{11}$
With $TilS_{11}$
Without $TilS_{11}$
Nº of genomes without $TilS_{11}$
Nº of genomes with $TilS_{12}$
With $TilS_{12}$
Without $TilS_{12}$
Nº of genomes without $TilS_{12}$
Nº of genomes with $TilS_{13}$
With $TilS_{13}$
Without $TilS_{13}$
Nº of genomes without $TilS_{13}$
Nº of genomes with $TilS_{14}$
With $TilS_{14}$
Without $TilS_{14}$
Nº of genomes without $TilS_{14}$
Nº of genomes with $TilS_{15}$
With $TilS_{15}$
Without $TilS_{15}$
Nº of genomes without $TilS_{15}$

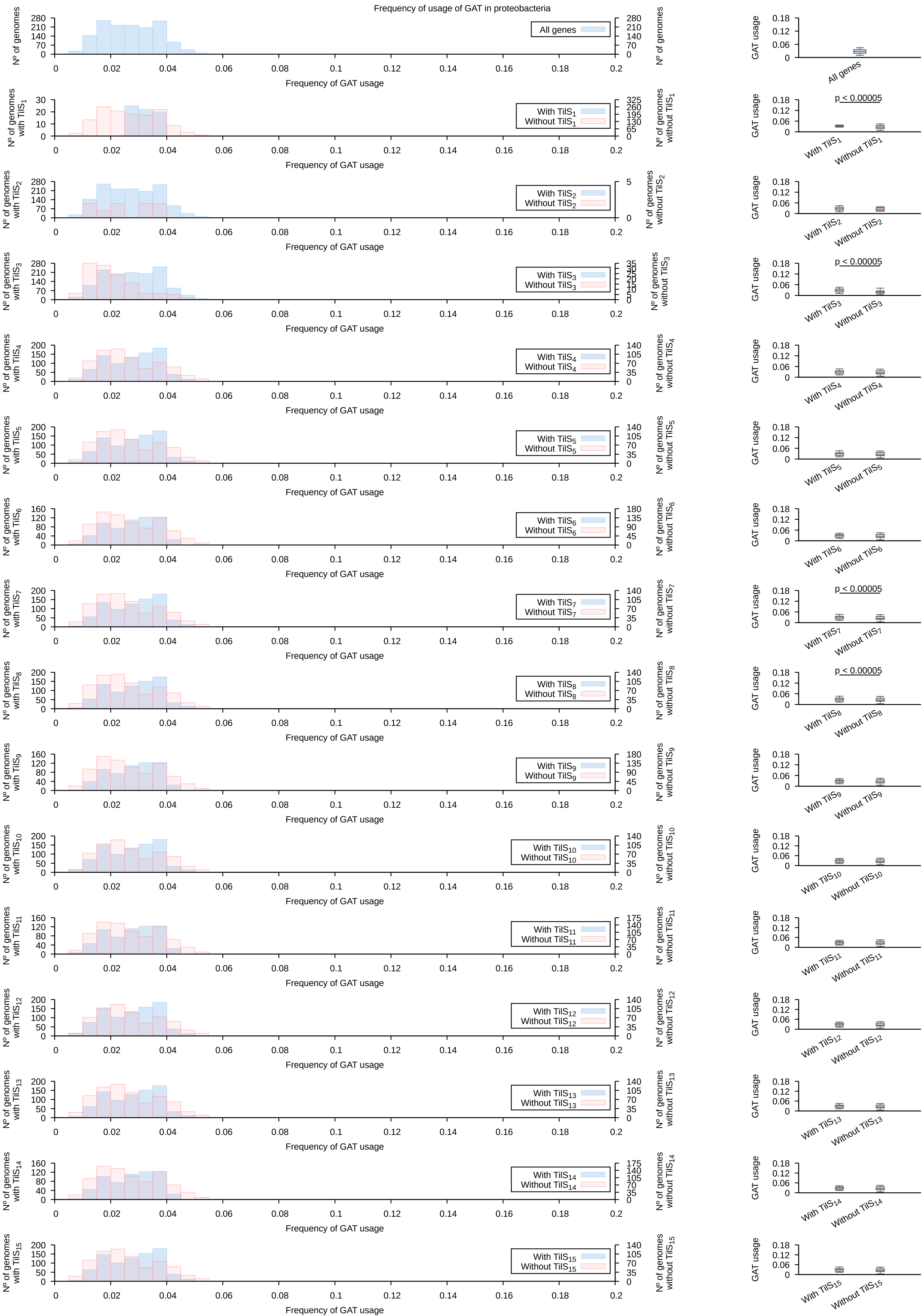

Frequency of usage of GAT in proteobacteria
Nº of genomes
All genes
Frequency of GAT usage
GAT usage
p < 0.00005
Nº of genomes with $TilS_1$
Nº of genomes without $TilS_1$
With $TilS_1$
Without $TilS_1$
With $TilS_2$
Without $TilS_2$
With $TilS_3$
Without $TilS_3$
With $TilS_4$
Without $TilS_4$
With $TilS_5$
Without $TilS_5$
With $TilS_6$
Without $TilS_6$
With $TilS_7$
Without $TilS_7$
With $TilS_8$
Without $TilS_8$
With $TilS_9$
Without $TilS_9$
With $TilS_{10}$
Without $TilS_{10}$
With $TilS_{11}$
Without $TilS_{11}$
With $TilS_{12}$
Without $TilS_{12}$
With $TilS_{13}$
Without $TilS_{13}$
With $TilS_{14}$
Without $TilS_{14}$
With $TilS_{15}$
Without $TilS_{15}$

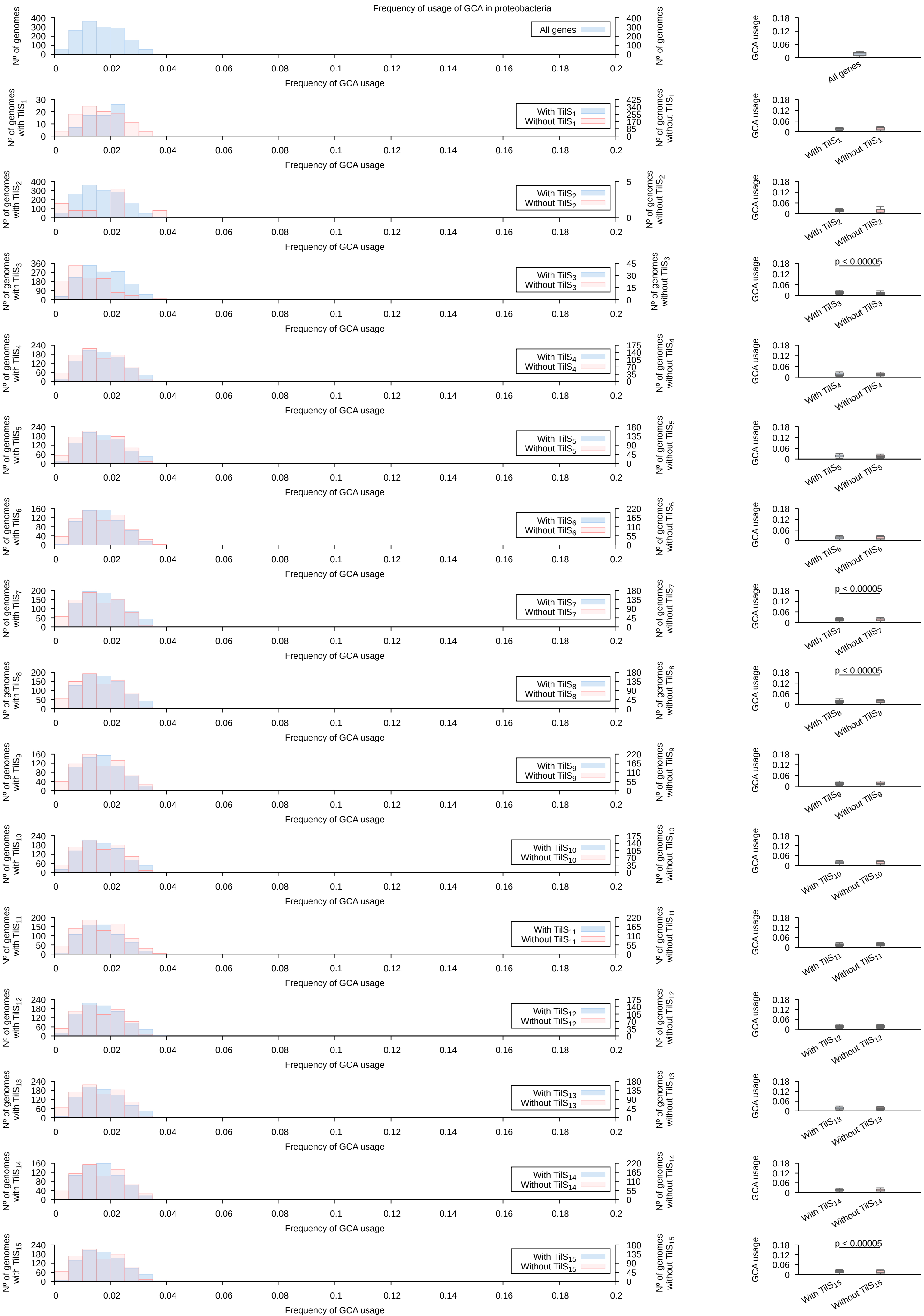

Frequency of usage of GCA in proteobacteria
Nº of genomes
All genes
Frequency of GCA usage
GCA usage
All genes
Nº of genomes with TilS$_1$
With TilS$_1$
Without TilS$_1$
Nº of genomes without TilS$_1$
Nº of genomes with TilS$_2$
With TilS$_2$
Without TilS$_2$
Nº of genomes without TilS$_2$
Nº of genomes with TilS$_3$
With TilS$_3$
Without TilS$_3$
Nº of genomes without TilS$_3$
p < 0.00005
Nº of genomes with TilS$_4$
With TilS$_4$
Without TilS$_4$
Nº of genomes without TilS$_4$
Nº of genomes with TilS$_5$
With TilS$_5$
Without TilS$_5$
Nº of genomes without TilS$_5$
Nº of genomes with TilS$_6$
With TilS$_6$
Without TilS$_6$
Nº of genomes without TilS$_6$
Nº of genomes with TilS$_7$
With TilS$_7$
Without TilS$_7$
Nº of genomes without TilS$_7$
p < 0.00005
Nº of genomes with TilS$_8$
With TilS$_8$
Without TilS$_8$
Nº of genomes without TilS$_8$
p < 0.00005
Nº of genomes with TilS$_9$
With TilS$_9$
Without TilS$_9$
Nº of genomes without TilS$_9$
Nº of genomes with TilS$_{10}$
With TilS$_{10}$
Without TilS$_{10}$
Nº of genomes without TilS$_{10}$
Nº of genomes with TilS$_{11}$
With TilS$_{11}$
Without TilS$_{11}$
Nº of genomes without TilS$_{11}$
Nº of genomes with TilS$_{12}$
With TilS$_{12}$
Without TilS$_{12}$
Nº of genomes without TilS$_{12}$
Nº of genomes with TilS$_{13}$
With TilS$_{13}$
Without TilS$_{13}$
Nº of genomes without TilS$_{13}$
Nº of genomes with TilS$_{14}$
With TilS$_{14}$
Without TilS$_{14}$
Nº of genomes without TilS$_{14}$
Nº of genomes with TilS$_{15}$
With TilS$_{15}$
Without TilS$_{15}$
Nº of genomes without TilS$_{15}$
p < 0.00005

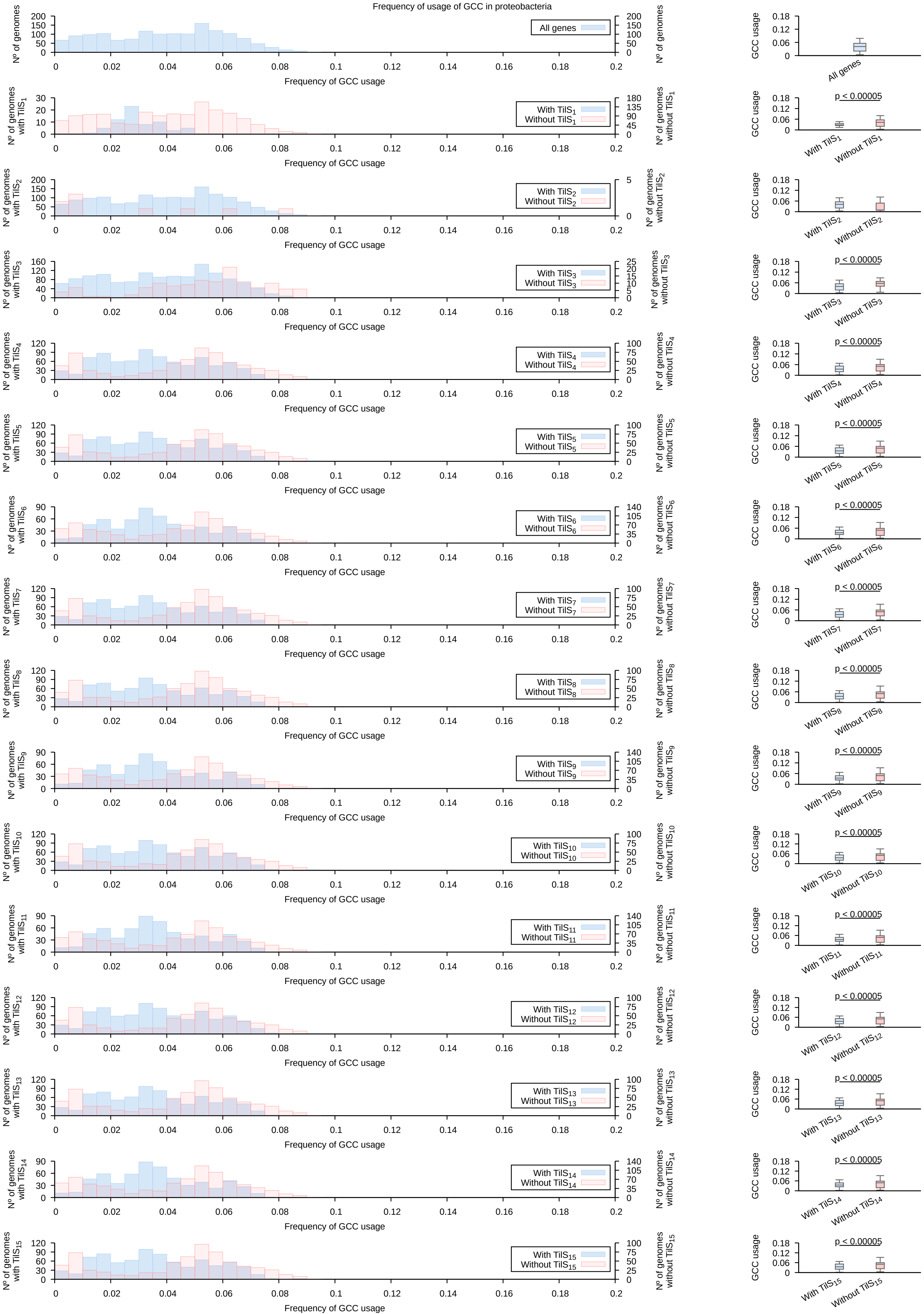

Frequency of usage of GCC in proteobacteria
Nº of genomes
All genes
Frequency of GCC usage
GCC usage
All genes
Nº of genomes with $TilS_1$
Nº of genomes without $TilS_1$
With $TilS_1$
Without $TilS_1$
$p < 0.00005$
Nº of genomes with $TilS_2$
Nº of genomes without $TilS_2$
With $TilS_2$
Without $TilS_2$
Nº of genomes with $TilS_3$
Nº of genomes without $TilS_3$
With $TilS_3$
Without $TilS_3$
$p < 0.00005$
Nº of genomes with $TilS_4$
Nº of genomes without $TilS_4$
With $TilS_4$
Without $TilS_4$
$p < 0.00005$
Nº of genomes with $TilS_5$
Nº of genomes without $TilS_5$
With $TilS_5$
Without $TilS_5$
$p < 0.00005$
Nº of genomes with $TilS_6$
Nº of genomes without $TilS_6$
With $TilS_6$
Without $TilS_6$
$p < 0.00005$
Nº of genomes with $TilS_7$
Nº of genomes without $TilS_7$
With $TilS_7$
Without $TilS_7$
$p < 0.00005$
Nº of genomes with $TilS_8$
Nº of genomes without $TilS_8$
With $TilS_8$
Without $TilS_8$
$p < 0.00005$
Nº of genomes with $TilS_9$
Nº of genomes without $TilS_9$
With $TilS_9$
Without $TilS_9$
$p < 0.00005$
Nº of genomes with $TilS_{10}$
Nº of genomes without $TilS_{10}$
With $TilS_{10}$
Without $TilS_{10}$
$p < 0.00005$
Nº of genomes with $TilS_{11}$
Nº of genomes without $TilS_{11}$
With $TilS_{11}$
Without $TilS_{11}$
$p < 0.00005$
Nº of genomes with $TilS_{12}$
Nº of genomes without $TilS_{12}$
With $TilS_{12}$
Without $TilS_{12}$
$p < 0.00005$
Nº of genomes with $TilS_{13}$
Nº of genomes without $TilS_{13}$
With $TilS_{13}$
Without $TilS_{13}$
$p < 0.00005$
Nº of genomes with $TilS_{14}$
Nº of genomes without $TilS_{14}$
With $TilS_{14}$
Without $TilS_{14}$
$p < 0.00005$
Nº of genomes with $TilS_{15}$
Nº of genomes without $TilS_{15}$
With $TilS_{15}$
Without $TilS_{15}$
$p < 0.00005$

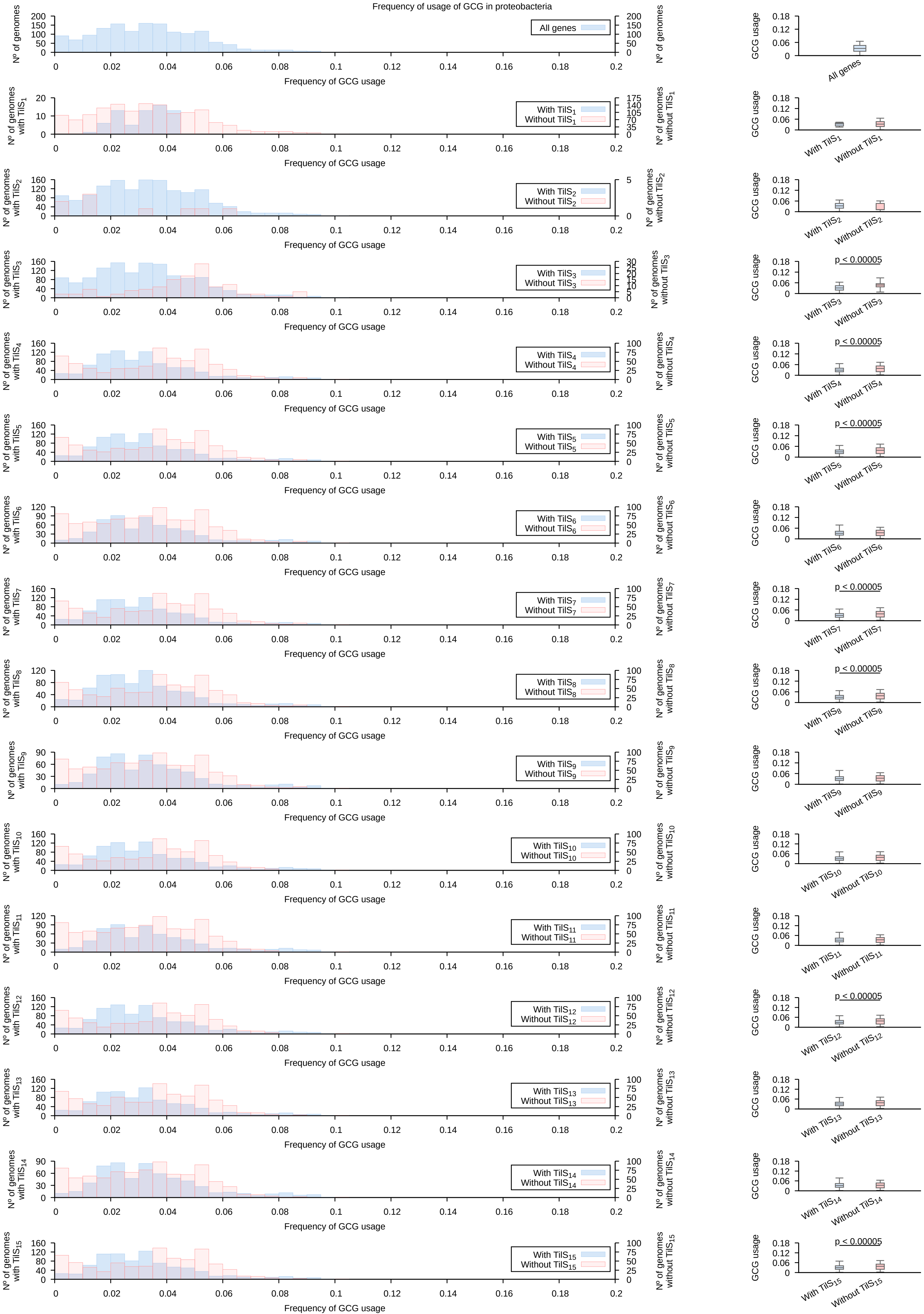
Frequency of usage of GCG in proteobacteria
Nº of genomes
All genes
Frequency of GCG usage
GCG usage
Nº of genomes with TilS$_1$
Nº of genomes without TilS$_1$
With TilS$_1$
Without TilS$_1$
Nº of genomes with TilS$_2$
Nº of genomes without TilS$_2$
With TilS$_2$
Without TilS$_2$
Nº of genomes with TilS$_3$
Nº of genomes without TilS$_3$
With TilS$_3$
Without TilS$_3$
p < 0.00005
Nº of genomes with TilS$_4$
Nº of genomes without TilS$_4$
With TilS$_4$
Without TilS$_4$
p < 0.00005
Nº of genomes with TilS$_5$
Nº of genomes without TilS$_5$
With TilS$_5$
Without TilS$_5$
p < 0.00005
Nº of genomes with TilS$_6$
Nº of genomes without TilS$_6$
With TilS$_6$
Without TilS$_6$
Nº of genomes with TilS$_7$
Nº of genomes without TilS$_7$
With TilS$_7$
Without TilS$_7$
p < 0.00005
Nº of genomes with TilS$_8$
Nº of genomes without TilS$_8$
With TilS$_8$
Without TilS$_8$
p < 0.00005
Nº of genomes with TilS$_9$
Nº of genomes without TilS$_9$
With TilS$_9$
Without TilS$_9$
Nº of genomes with TilS$_{10}$
Nº of genomes without TilS$_{10}$
With TilS$_{10}$
Without TilS$_{10}$
Nº of genomes with TilS$_{11}$
Nº of genomes without TilS$_{11}$
With TilS$_{11}$
Without TilS$_{11}$
Nº of genomes with TilS$_{12}$
Nº of genomes without TilS$_{12}$
With TilS$_{12}$
Without TilS$_{12}$
p < 0.00005
Nº of genomes with TilS$_{13}$
Nº of genomes without TilS$_{13}$
With TilS$_{13}$
Without TilS$_{13}$
Nº of genomes with TilS$_{14}$
Nº of genomes without TilS$_{14}$
With TilS$_{14}$
Without TilS$_{14}$
Nº of genomes with TilS$_{15}$
Nº of genomes without TilS$_{15}$
With TilS$_{15}$
Without TilS$_{15}$
p < 0.00005

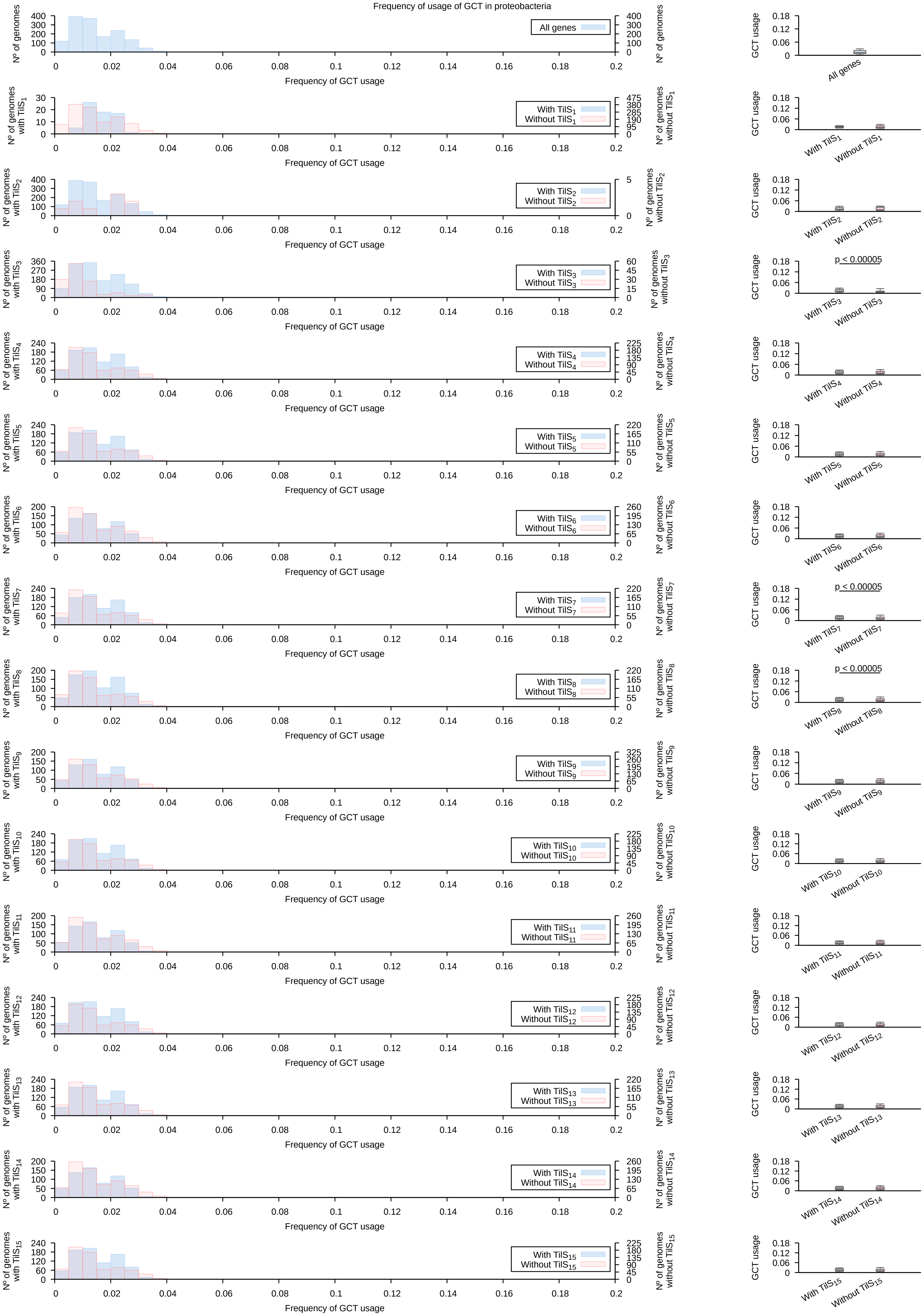
Frequency of usage of GCT in proteobacteria
Nº of genomes
All genes
Frequency of GCT usage
GCT usage
0
0.02
0.04
0.06
0.08
0.1
0.12
0.14
0.16
0.18
0.2
Nº of genomes with TilS1
Nº of genomes without TilS1
With TilS1
Without TilS1
Nº of genomes with TilS2
Nº of genomes without TilS2
With TilS2
Without TilS2
Nº of genomes with TilS3
Nº of genomes without TilS3
With TilS3
Without TilS3
p < 0.00005
Nº of genomes with TilS4
Nº of genomes without TilS4
With TilS4
Without TilS4
Nº of genomes with TilS5
Nº of genomes without TilS5
With TilS5
Without TilS5
Nº of genomes with TilS6
Nº of genomes without TilS6
With TilS6
Without TilS6
Nº of genomes with TilS7
Nº of genomes without TilS7
With TilS7
Without TilS7
p < 0.00005
Nº of genomes with TilS8
Nº of genomes without TilS8
With TilS8
Without TilS8
p < 0.00005
Nº of genomes with TilS9
Nº of genomes without TilS9
With TilS9
Without TilS9
Nº of genomes with TilS10
Nº of genomes without TilS10
With TilS10
Without TilS10
Nº of genomes with TilS11
Nº of genomes without TilS11
With TilS11
Without TilS11
Nº of genomes with TilS12
Nº of genomes without TilS12
With TilS12
Without TilS12
Nº of genomes with TilS13
Nº of genomes without TilS13
With TilS13
Without TilS13
Nº of genomes with TilS14
Nº of genomes without TilS14
With TilS14
Without TilS14
Nº of genomes with TilS15
Nº of genomes without TilS15
With TilS15
Without TilS15

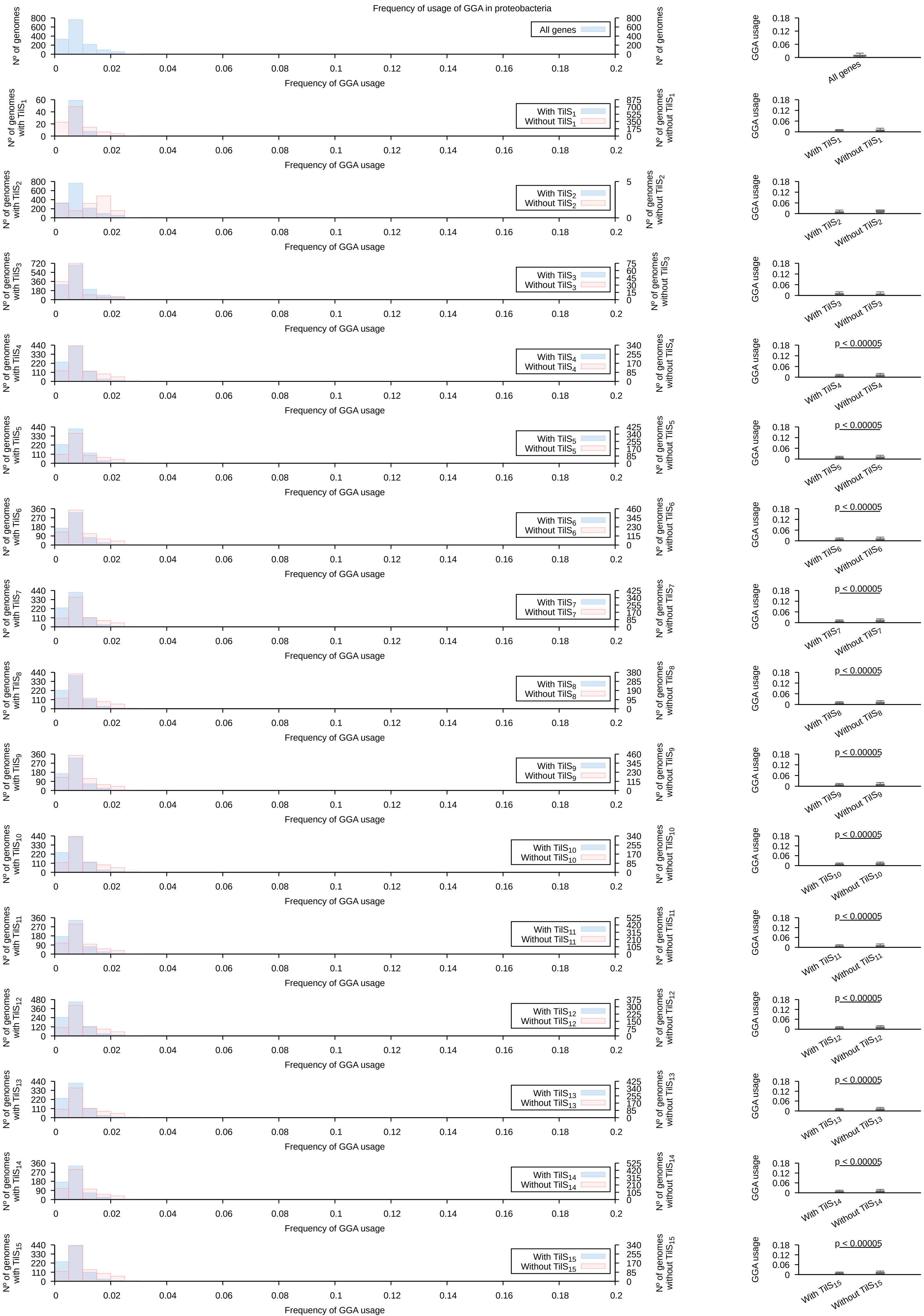

Frequency of usage of GGA in proteobacteria
Nº of genomes
All genes
Frequency of GGA usage
GGA usage
All genes
Nº of genomes with TilS1
With TilS1
Without TilS1
Nº of genomes without TilS1
Nº of genomes with TilS2
With TilS2
Without TilS2
Nº of genomes without TilS2
Nº of genomes with TilS3
With TilS3
Without TilS3
Nº of genomes without TilS3
Nº of genomes with TilS4
With TilS4
Without TilS4
Nº of genomes without TilS4
p < 0.00005
Nº of genomes with TilS5
With TilS5
Without TilS5
Nº of genomes without TilS5
p < 0.00005
Nº of genomes with TilS6
With TilS6
Without TilS6
Nº of genomes without TilS6
p < 0.00005
Nº of genomes with TilS7
With TilS7
Without TilS7
Nº of genomes without TilS7
p < 0.00005
Nº of genomes with TilS8
With TilS8
Without TilS8
Nº of genomes without TilS8
p < 0.00005
Nº of genomes with TilS9
With TilS9
Without TilS9
Nº of genomes without TilS9
p < 0.00005
Nº of genomes with TilS10
With TilS10
Without TilS10
Nº of genomes without TilS10
p < 0.00005
Nº of genomes with TilS11
With TilS11
Without TilS11
Nº of genomes without TilS11
p < 0.00005
Nº of genomes with TilS12
With TilS12
Without TilS12
Nº of genomes without TilS12
p < 0.00005
Nº of genomes with TilS13
With TilS13
Without TilS13
Nº of genomes without TilS13
p < 0.00005
Nº of genomes with TilS14
With TilS14
Without TilS14
Nº of genomes without TilS14
p < 0.00005
Nº of genomes with TilS15
With TilS15
Without TilS15
Nº of genomes without TilS15
p < 0.00005

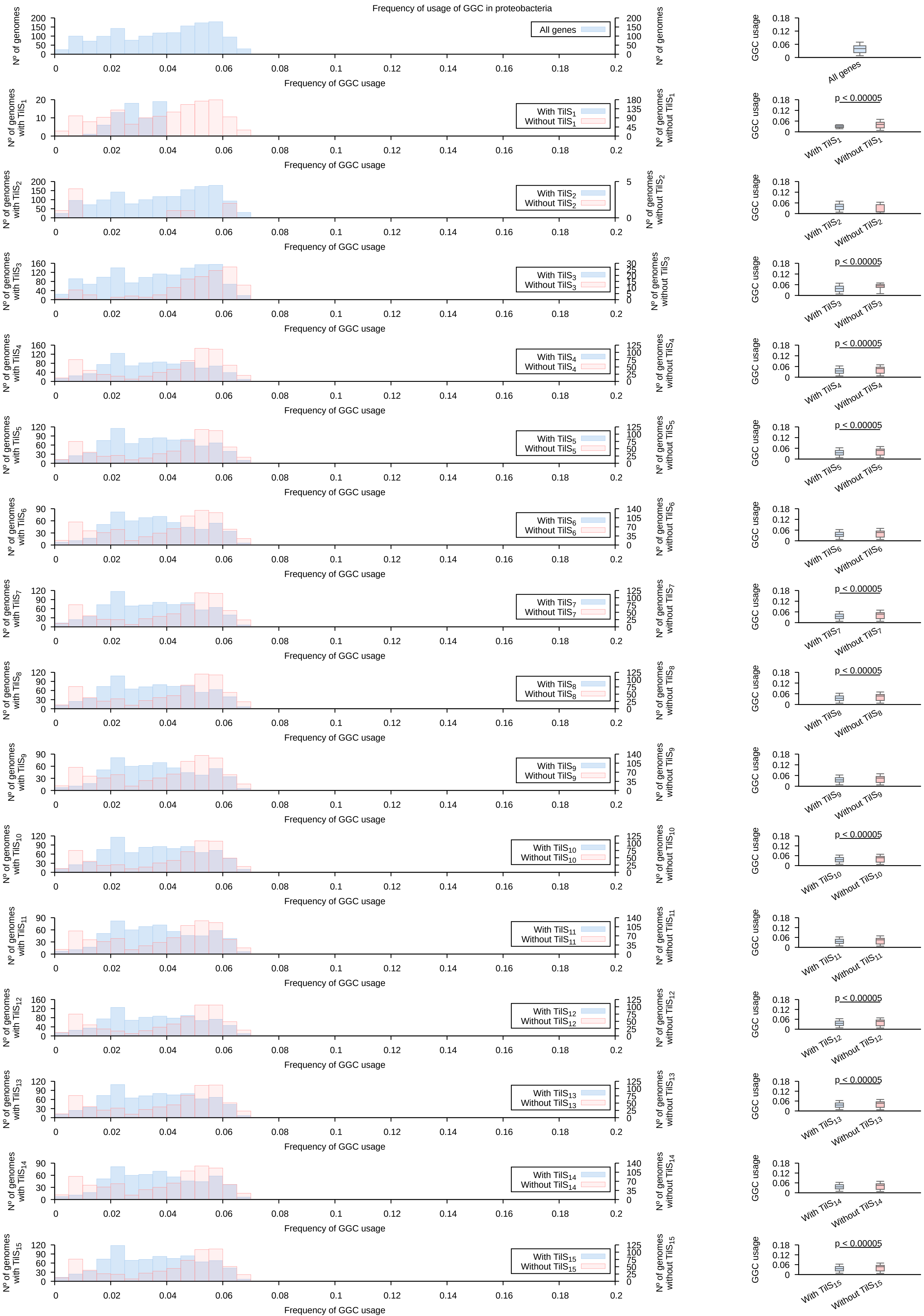

Frequency of usage of GGC in proteobacteria
Nº of genomes
All genes
Frequency of GGC usage
GGC usage
Nº of genomes with TilS1
Nº of genomes without TilS1
With TilS1
Without TilS1
p < 0.00005
Nº of genomes with TilS2
Nº of genomes without TilS2
With TilS2
Without TilS2
Nº of genomes with TilS3
Nº of genomes without TilS3
With TilS3
Without TilS3
p < 0.00005
Nº of genomes with TilS4
Nº of genomes without TilS4
With TilS4
Without TilS4
p < 0.00005
Nº of genomes with TilS5
Nº of genomes without TilS5
With TilS5
Without TilS5
p < 0.00005
Nº of genomes with TilS6
Nº of genomes without TilS6
With TilS6
Without TilS6
Nº of genomes with TilS7
Nº of genomes without TilS7
With TilS7
Without TilS7
p < 0.00005
Nº of genomes with TilS8
Nº of genomes without TilS8
With TilS8
Without TilS8
p < 0.00005
Nº of genomes with TilS9
Nº of genomes without TilS9
With TilS9
Without TilS9
Nº of genomes with TilS10
Nº of genomes without TilS10
With TilS10
Without TilS10
p < 0.00005
Nº of genomes with TilS11
Nº of genomes without TilS11
With TilS11
Without TilS11
Nº of genomes with TilS12
Nº of genomes without TilS12
With TilS12
Without TilS12
p < 0.00005
Nº of genomes with TilS13
Nº of genomes without TilS13
With TilS13
Without TilS13
p < 0.00005
Nº of genomes with TilS14
Nº of genomes without TilS14
With TilS14
Without TilS14
Nº of genomes with TilS15
Nº of genomes without TilS15
With TilS15
Without TilS15
p < 0.00005

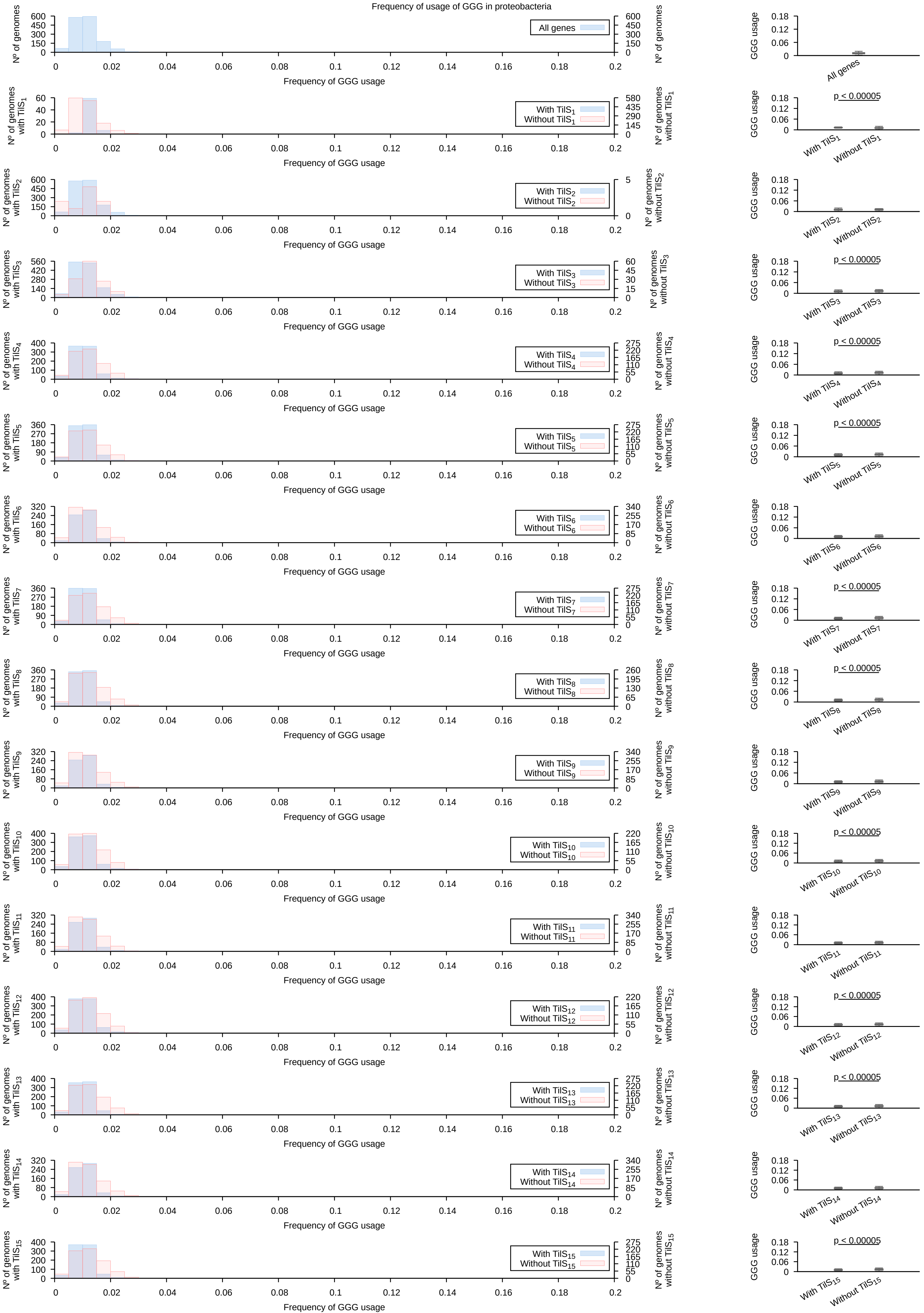

Frequency of usage of GGG in proteobacteria
Nº of genomes
All genes
Frequency of GGG usage
GGG usage
p < 0.00005
Nº of genomes with TilS$_1$
Nº of genomes without TilS$_1$
With TilS$_1$
Without TilS$_1$
Nº of genomes with TilS$_2$
Nº of genomes without TilS$_2$
With TilS$_2$
Without TilS$_2$
Nº of genomes with TilS$_3$
Nº of genomes without TilS$_3$
With TilS$_3$
Without TilS$_3$
Nº of genomes with TilS$_4$
Nº of genomes without TilS$_4$
With TilS$_4$
Without TilS$_4$
Nº of genomes with TilS$_5$
Nº of genomes without TilS$_5$
With TilS$_5$
Without TilS$_5$
Nº of genomes with TilS$_6$
Nº of genomes without TilS$_6$
With TilS$_6$
Without TilS$_6$
Nº of genomes with TilS$_7$
Nº of genomes without TilS$_7$
With TilS$_7$
Without TilS$_7$
Nº of genomes with TilS$_8$
Nº of genomes without TilS$_8$
With TilS$_8$
Without TilS$_8$
Nº of genomes with TilS$_9$
Nº of genomes without TilS$_9$
With TilS$_9$
Without TilS$_9$
Nº of genomes with TilS$_{10}$
Nº of genomes without TilS$_{10}$
With TilS$_{10}$
Without TilS$_{10}$
Nº of genomes with TilS$_{11}$
Nº of genomes without TilS$_{11}$
With TilS$_{11}$
Without TilS$_{11}$
Nº of genomes with TilS$_{12}$
Nº of genomes without TilS$_{12}$
With TilS$_{12}$
Without TilS$_{12}$
Nº of genomes with TilS$_{13}$
Nº of genomes without TilS$_{13}$
With TilS$_{13}$
Without TilS$_{13}$
Nº of genomes with TilS$_{14}$
Nº of genomes without TilS$_{14}$
With TilS$_{14}$
Without TilS$_{14}$
Nº of genomes with TilS$_{15}$
Nº of genomes without TilS$_{15}$
With TilS$_{15}$
Without TilS$_{15}$

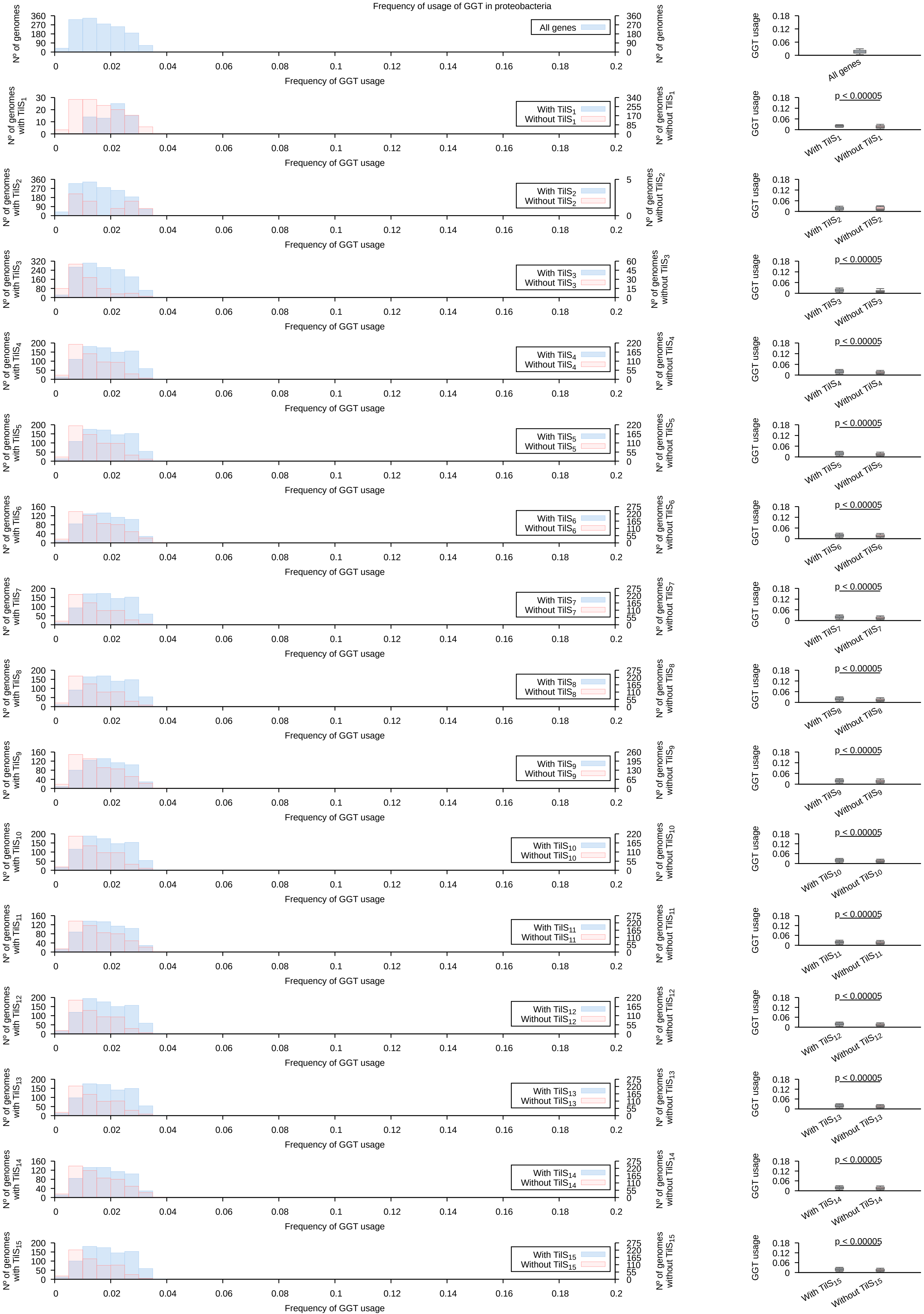

Frequency of usage of GGT in proteobacteria
Nº of genomes
All genes
Frequency of GGT usage
GGT usage
p < 0.00005
Nº of genomes with TilS$_1$
Nº of genomes without TilS$_1$
With TilS$_1$
Without TilS$_1$
Nº of genomes with TilS$_2$
Nº of genomes without TilS$_2$
With TilS$_2$
Without TilS$_2$
Nº of genomes with TilS$_3$
Nº of genomes without TilS$_3$
With TilS$_3$
Without TilS$_3$
Nº of genomes with TilS$_4$
Nº of genomes without TilS$_4$
With TilS$_4$
Without TilS$_4$
Nº of genomes with TilS$_5$
Nº of genomes without TilS$_5$
With TilS$_5$
Without TilS$_5$
Nº of genomes with TilS$_6$
Nº of genomes without TilS$_6$
With TilS$_6$
Without TilS$_6$
Nº of genomes with TilS$_7$
Nº of genomes without TilS$_7$
With TilS$_7$
Without TilS$_7$
Nº of genomes with TilS$_8$
Nº of genomes without TilS$_8$
With TilS$_8$
Without TilS$_8$
Nº of genomes with TilS$_9$
Nº of genomes without TilS$_9$
With TilS$_9$
Without TilS$_9$
Nº of genomes with TilS$_{10}$
Nº of genomes without TilS$_{10}$
With TilS$_{10}$
Without TilS$_{10}$
Nº of genomes with TilS$_{11}$
Nº of genomes without TilS$_{11}$
With TilS$_{11}$
Without TilS$_{11}$
Nº of genomes with TilS$_{12}$
Nº of genomes without TilS$_{12}$
With TilS$_{12}$
Without TilS$_{12}$
Nº of genomes with TilS$_{13}$
Nº of genomes without TilS$_{13}$
With TilS$_{13}$
Without TilS$_{13}$
Nº of genomes with TilS$_{14}$
Nº of genomes without TilS$_{14}$
With TilS$_{14}$
Without TilS$_{14}$
Nº of genomes with TilS$_{15}$
Nº of genomes without TilS$_{15}$
With TilS$_{15}$
Without TilS$_{15}$

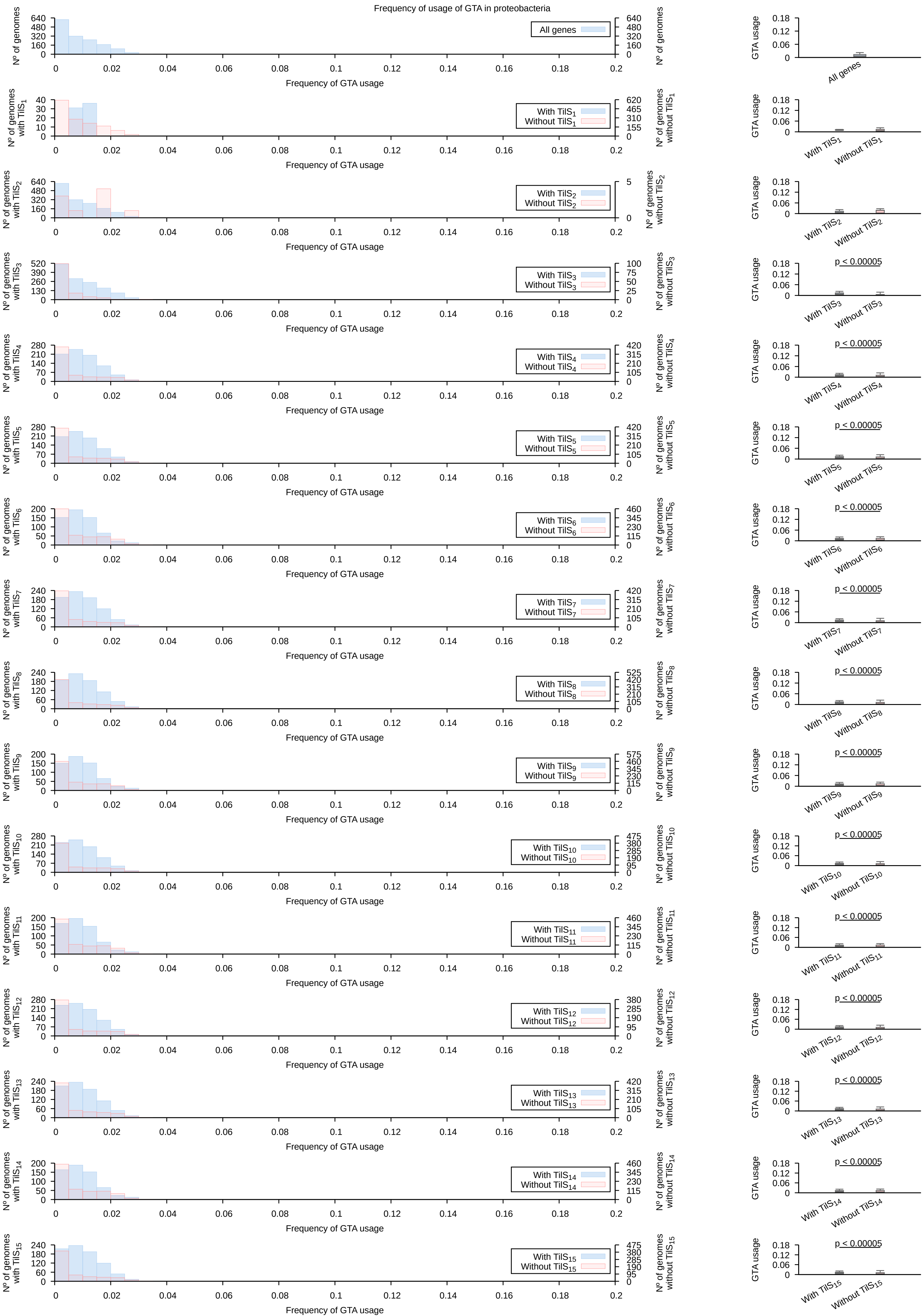
Frequency of usage of GTA in proteobacteria
Nº of genomes
All genes
Frequency of GTA usage
GTA usage
Nº of genomes with TilS$_1$
Nº of genomes without TilS$_1$
With TilS$_1$
Without TilS$_1$
Nº of genomes with TilS$_2$
Nº of genomes without TilS$_2$
With TilS$_2$
Without TilS$_2$
Nº of genomes with TilS$_3$
Nº of genomes without TilS$_3$
With TilS$_3$
Without TilS$_3$
p < 0.00005
Nº of genomes with TilS$_4$
Nº of genomes without TilS$_4$
With TilS$_4$
Without TilS$_4$
Nº of genomes with TilS$_5$
Nº of genomes without TilS$_5$
With TilS$_5$
Without TilS$_5$
Nº of genomes with TilS$_6$
Nº of genomes without TilS$_6$
With TilS$_6$
Without TilS$_6$
Nº of genomes with TilS$_7$
Nº of genomes without TilS$_7$
With TilS$_7$
Without TilS$_7$
Nº of genomes with TilS$_8$
Nº of genomes without TilS$_8$
With TilS$_8$
Without TilS$_8$
Nº of genomes with TilS$_9$
Nº of genomes without TilS$_9$
With TilS$_9$
Without TilS$_9$
Nº of genomes with TilS$_{10}$
Nº of genomes without TilS$_{10}$
With TilS$_{10}$
Without TilS$_{10}$
Nº of genomes with TilS$_{11}$
Nº of genomes without TilS$_{11}$
With TilS$_{11}$
Without TilS$_{11}$
Nº of genomes with TilS$_{12}$
Nº of genomes without TilS$_{12}$
With TilS$_{12}$
Without TilS$_{12}$
Nº of genomes with TilS$_{13}$
Nº of genomes without TilS$_{13}$
With TilS$_{13}$
Without TilS$_{13}$
Nº of genomes with TilS$_{14}$
Nº of genomes without TilS$_{14}$
With TilS$_{14}$
Without TilS$_{14}$
Nº of genomes with TilS$_{15}$
Nº of genomes without TilS$_{15}$
With TilS$_{15}$
Without TilS$_{15}$

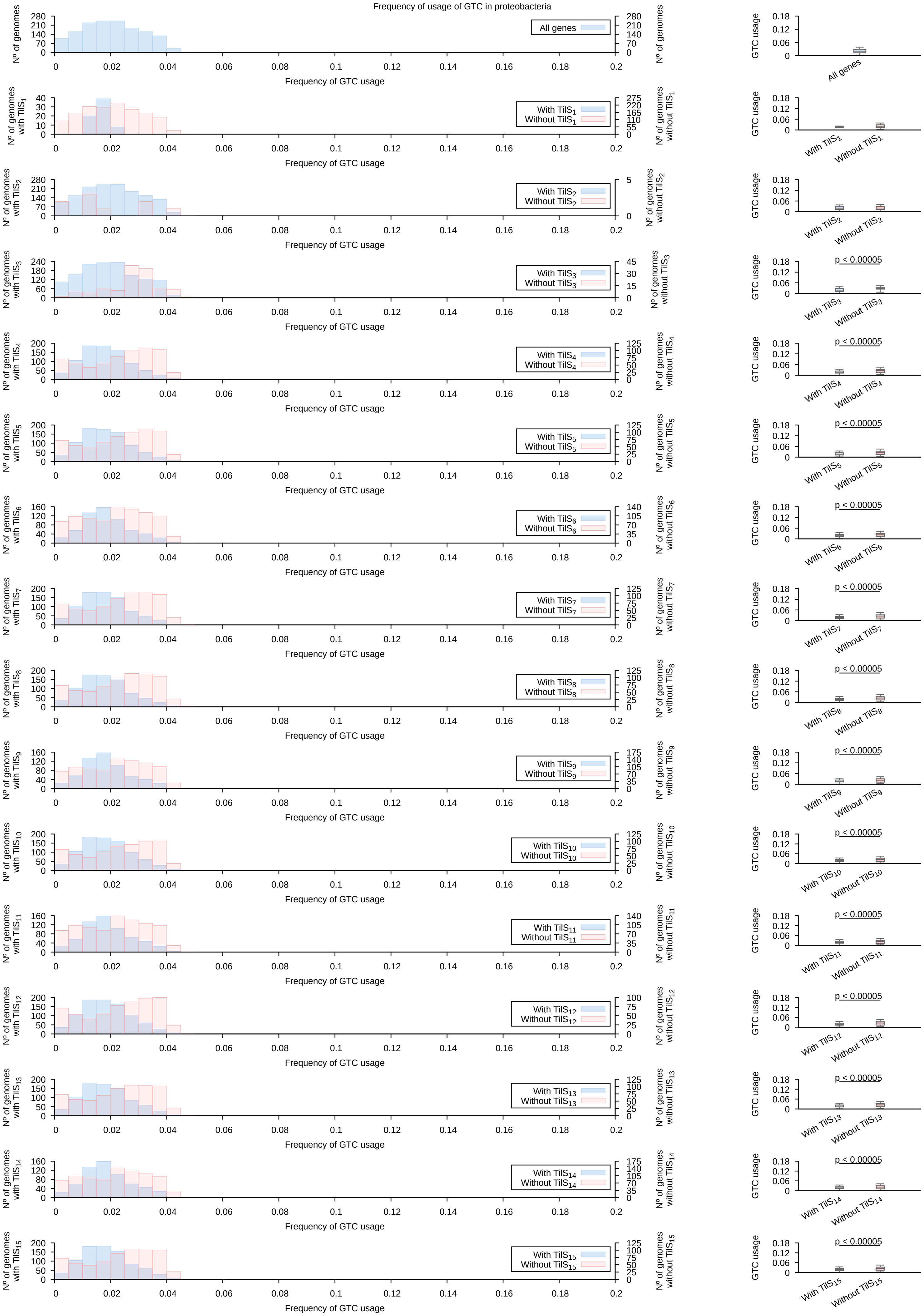

Frequency of usage of GTC in proteobacteria
Nº of genomes
All genes
Frequency of GTC usage
GTC usage
Nº of genomes with TilS$_1$
Nº of genomes without TilS$_1$
With TilS$_1$
Without TilS$_1$
Nº of genomes with TilS$_2$
Nº of genomes without TilS$_2$
With TilS$_2$
Without TilS$_2$
Nº of genomes with TilS$_3$
Nº of genomes without TilS$_3$
With TilS$_3$
Without TilS$_3$
p < 0.00005
Nº of genomes with TilS$_4$
Nº of genomes without TilS$_4$
With TilS$_4$
Without TilS$_4$
Nº of genomes with TilS$_5$
Nº of genomes without TilS$_5$
With TilS$_5$
Without TilS$_5$
Nº of genomes with TilS$_6$
Nº of genomes without TilS$_6$
With TilS$_6$
Without TilS$_6$
Nº of genomes with TilS$_7$
Nº of genomes without TilS$_7$
With TilS$_7$
Without TilS$_7$
Nº of genomes with TilS$_8$
Nº of genomes without TilS$_8$
With TilS$_8$
Without TilS$_8$
Nº of genomes with TilS$_9$
Nº of genomes without TilS$_9$
With TilS$_9$
Without TilS$_9$
Nº of genomes with TilS$_{10}$
Nº of genomes without TilS$_{10}$
With TilS$_{10}$
Without TilS$_{10}$
Nº of genomes with TilS$_{11}$
Nº of genomes without TilS$_{11}$
With TilS$_{11}$
Without TilS$_{11}$
Nº of genomes with TilS$_{12}$
Nº of genomes without TilS$_{12}$
With TilS$_{12}$
Without TilS$_{12}$
Nº of genomes with TilS$_{13}$
Nº of genomes without TilS$_{13}$
With TilS$_{13}$
Without TilS$_{13}$
Nº of genomes with TilS$_{14}$
Nº of genomes without TilS$_{14}$
With TilS$_{14}$
Without TilS$_{14}$
Nº of genomes with TilS$_{15}$
Nº of genomes without TilS$_{15}$
With TilS$_{15}$
Without TilS$_{15}$

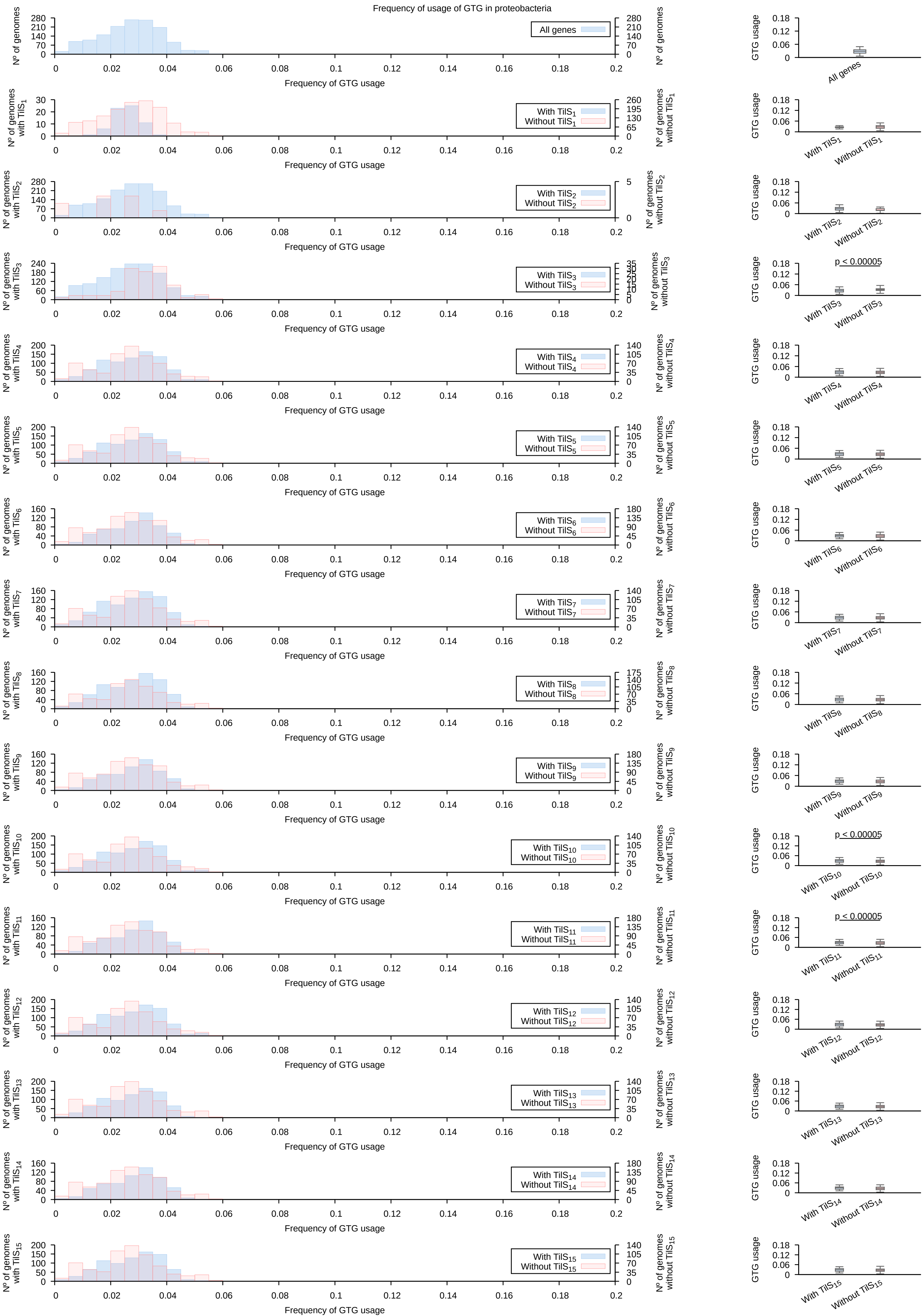
Frequency of usage of GTG in proteobacteria
Nº of genomes
All genes
Frequency of GTG usage
GTG usage
Nº of genomes with $TilS_1$
Nº of genomes without $TilS_1$
With $TilS_1$
Without $TilS_1$
Nº of genomes with $TilS_2$
Nº of genomes without $TilS_2$
With $TilS_2$
Without $TilS_2$
Nº of genomes with $TilS_3$
Nº of genomes without $TilS_3$
With $TilS_3$
Without $TilS_3$
p < 0.00005
Nº of genomes with $TilS_4$
Nº of genomes without $TilS_4$
With $TilS_4$
Without $TilS_4$
Nº of genomes with $TilS_5$
Nº of genomes without $TilS_5$
With $TilS_5$
Without $TilS_5$
Nº of genomes with $TilS_6$
Nº of genomes without $TilS_6$
With $TilS_6$
Without $TilS_6$
Nº of genomes with $TilS_7$
Nº of genomes without $TilS_7$
With $TilS_7$
Without $TilS_7$
Nº of genomes with $TilS_8$
Nº of genomes without $TilS_8$
With $TilS_8$
Without $TilS_8$
Nº of genomes with $TilS_9$
Nº of genomes without $TilS_9$
With $TilS_9$
Without $TilS_9$
Nº of genomes with $TilS_{10}$
Nº of genomes without $TilS_{10}$
With $TilS_{10}$
Without $TilS_{10}$
p < 0.00005
Nº of genomes with $TilS_{11}$
Nº of genomes without $TilS_{11}$
With $TilS_{11}$
Without $TilS_{11}$
p < 0.00005
Nº of genomes with $TilS_{12}$
Nº of genomes without $TilS_{12}$
With $TilS_{12}$
Without $TilS_{12}$
Nº of genomes with $TilS_{13}$
Nº of genomes without $TilS_{13}$
With $TilS_{13}$
Without $TilS_{13}$
Nº of genomes with $TilS_{14}$
Nº of genomes without $TilS_{14}$
With $TilS_{14}$
Without $TilS_{14}$
Nº of genomes with $TilS_{15}$
Nº of genomes without $TilS_{15}$
With $TilS_{15}$
Without $TilS_{15}$

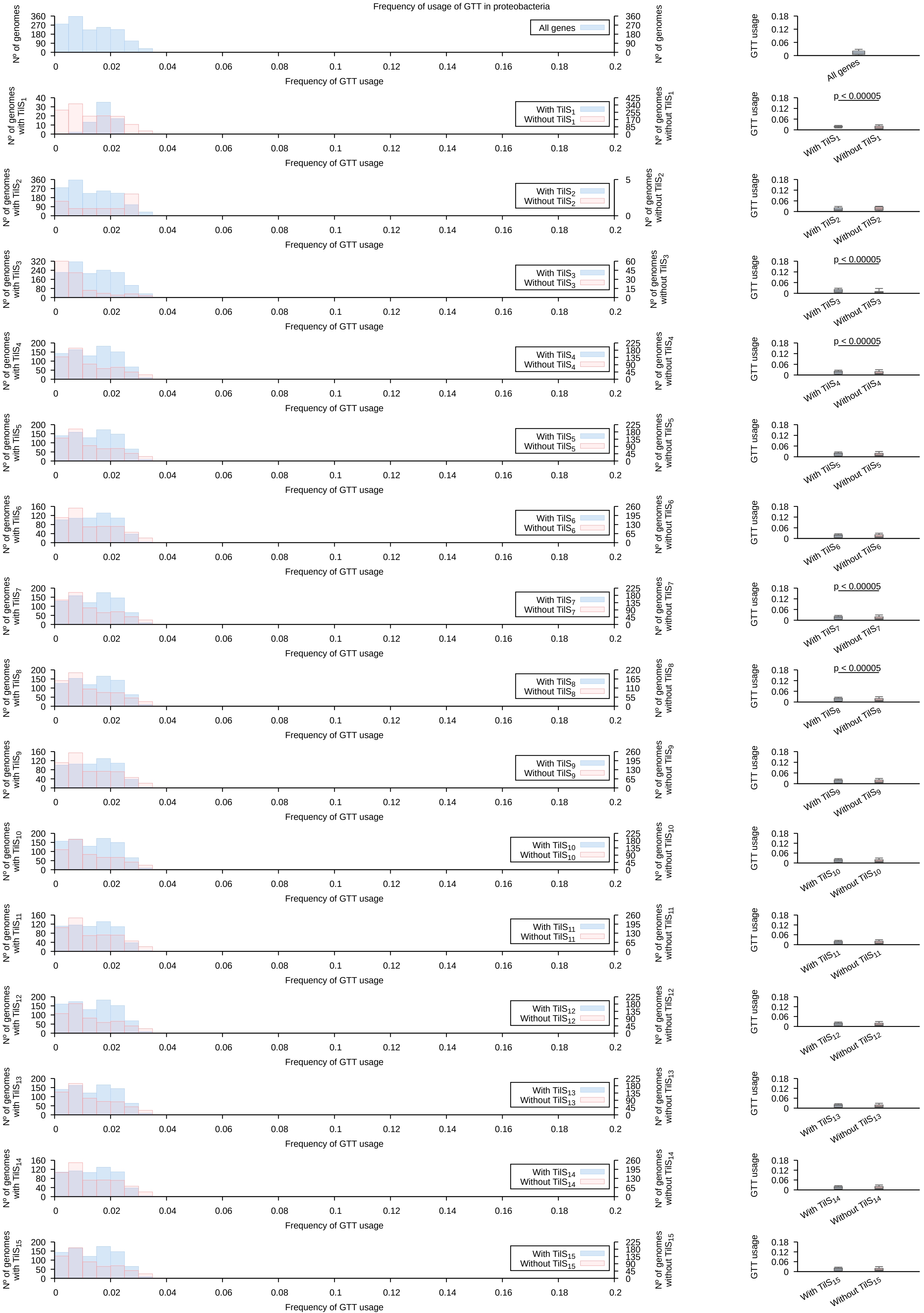

Frequency of usage of GTT in proteobacteria
Nº of genomes
All genes
Frequency of GTT usage
GTT usage
Nº of genomes with TilS1
Nº of genomes without TilS1
With TilS1
Without TilS1
p < 0.00005
Nº of genomes with TilS2
Nº of genomes without TilS2
With TilS2
Without TilS2
Nº of genomes with TilS3
Nº of genomes without TilS3
With TilS3
Without TilS3
p < 0.00005
Nº of genomes with TilS4
Nº of genomes without TilS4
With TilS4
Without TilS4
p < 0.00005
Nº of genomes with TilS5
Nº of genomes without TilS5
With TilS5
Without TilS5
Nº of genomes with TilS6
Nº of genomes without TilS6
With TilS6
Without TilS6
Nº of genomes with TilS7
Nº of genomes without TilS7
With TilS7
Without TilS7
p < 0.00005
Nº of genomes with TilS8
Nº of genomes without TilS8
With TilS8
Without TilS8
p < 0.00005
Nº of genomes with TilS9
Nº of genomes without TilS9
With TilS9
Without TilS9
Nº of genomes with TilS10
Nº of genomes without TilS10
With TilS10
Without TilS10
Nº of genomes with TilS11
Nº of genomes without TilS11
With TilS11
Without TilS11
Nº of genomes with TilS12
Nº of genomes without TilS12
With TilS12
Without TilS12
Nº of genomes with TilS13
Nº of genomes without TilS13
With TilS13
Without TilS13
Nº of genomes with TilS14
Nº of genomes without TilS14
With TilS14
Without TilS14
Nº of genomes with TilS15
Nº of genomes without TilS15
With TilS15
Without TilS15

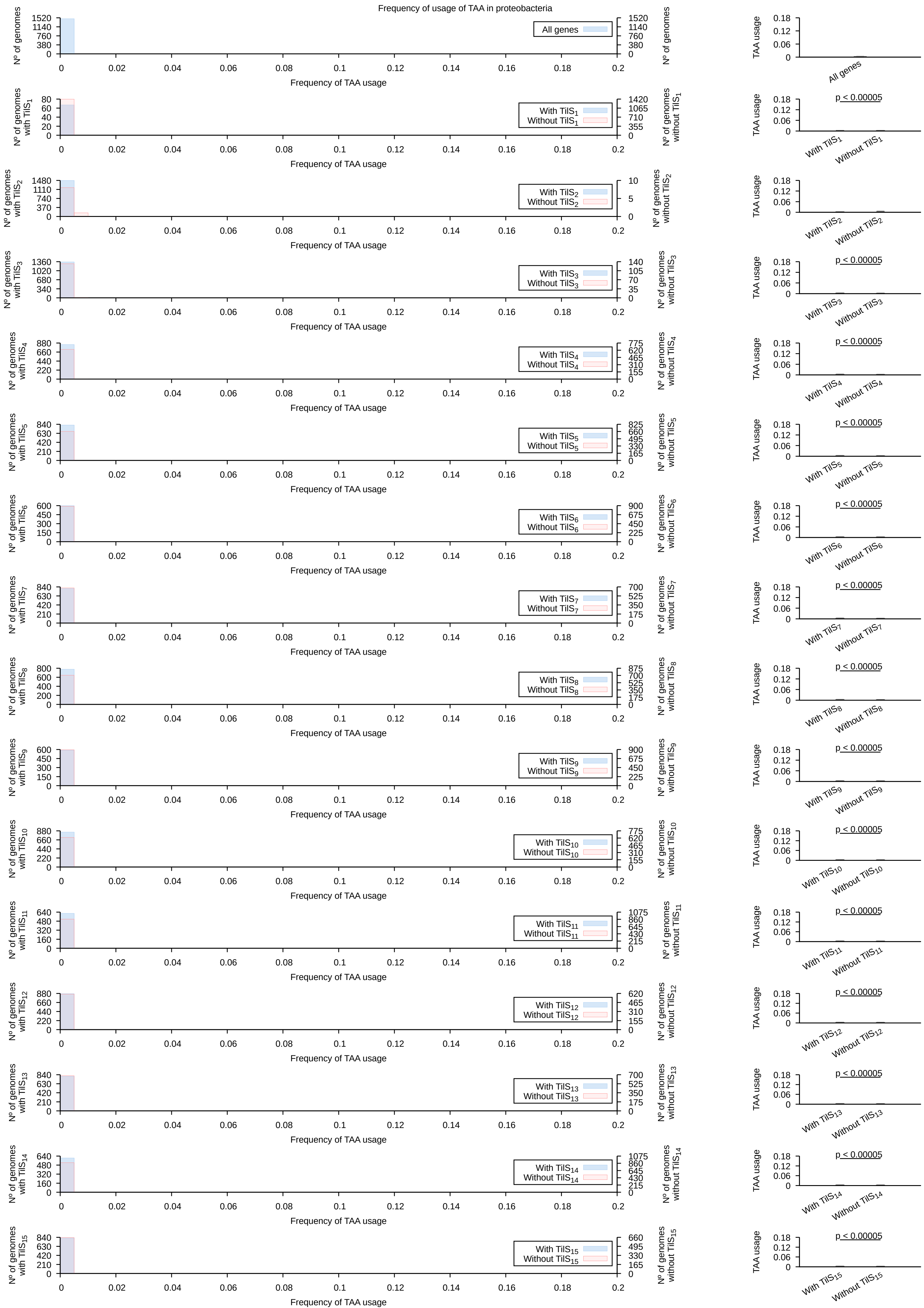

Frequency of usage of TAA in proteobacteria
Nº of genomes
All genes
Frequency of TAA usage
TAA usage
Nº of genomes with TilS1
Nº of genomes without TilS1
With TilS1
Without TilS1
p < 0.00005
Nº of genomes with TilS2
Nº of genomes without TilS2
With TilS2
Without TilS2
Nº of genomes with TilS3
Nº of genomes without TilS3
With TilS3
Without TilS3
p < 0.00005
Nº of genomes with TilS4
Nº of genomes without TilS4
With TilS4
Without TilS4
p < 0.00005
Nº of genomes with TilS5
Nº of genomes without TilS5
With TilS5
Without TilS5
p < 0.00005
Nº of genomes with TilS6
Nº of genomes without TilS6
With TilS6
Without TilS6
p < 0.00005
Nº of genomes with TilS7
Nº of genomes without TilS7
With TilS7
Without TilS7
p < 0.00005
Nº of genomes with TilS8
Nº of genomes without TilS8
With TilS8
Without TilS8
p < 0.00005
Nº of genomes with TilS9
Nº of genomes without TilS9
With TilS9
Without TilS9
p < 0.00005
Nº of genomes with TilS10
Nº of genomes without TilS10
With TilS10
Without TilS10
p < 0.00005
Nº of genomes with TilS11
Nº of genomes without TilS11
With TilS11
Without TilS11
p < 0.00005
Nº of genomes with TilS12
Nº of genomes without TilS12
With TilS12
Without TilS12
p < 0.00005
Nº of genomes with TilS13
Nº of genomes without TilS13
With TilS13
Without TilS13
p < 0.00005
Nº of genomes with TilS14
Nº of genomes without TilS14
With TilS14
Without TilS14
p < 0.00005
Nº of genomes with TilS15
Nº of genomes without TilS15
With TilS15
Without TilS15
p < 0.00005

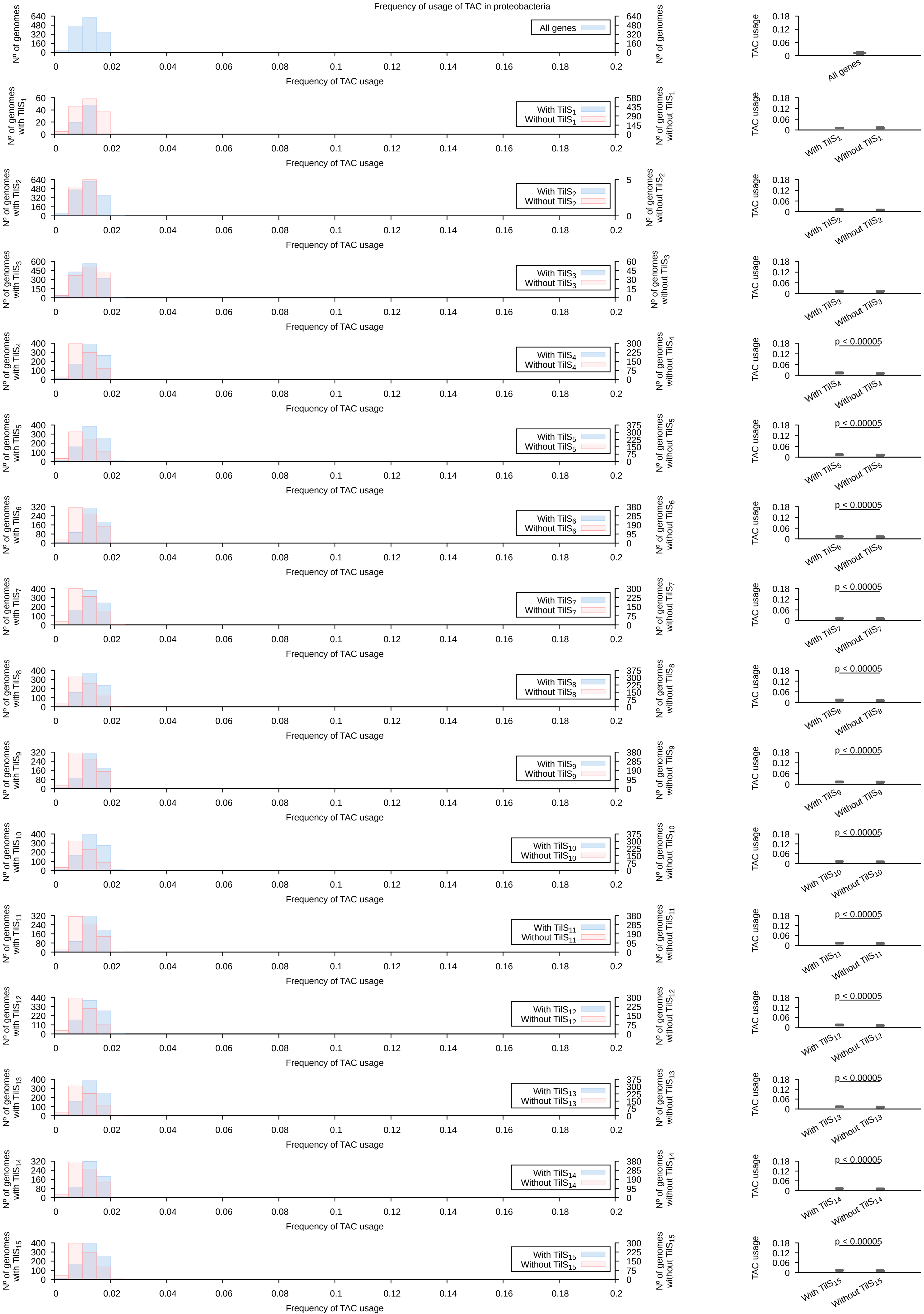

Frequency of usage of TAC in proteobacteria
Nº of genomes
All genes
Frequency of TAC usage
TAC usage
Nº of genomes with TilS$_1$
Nº of genomes without TilS$_1$
With TilS$_1$
Without TilS$_1$
Nº of genomes with TilS$_2$
Nº of genomes without TilS$_2$
With TilS$_2$
Without TilS$_2$
Nº of genomes with TilS$_3$
Nº of genomes without TilS$_3$
With TilS$_3$
Without TilS$_3$
p < 0.00005
With TilS$_4$
Without TilS$_4$
With TilS$_5$
Without TilS$_5$
With TilS$_6$
Without TilS$_6$
With TilS$_7$
Without TilS$_7$
With TilS$_8$
Without TilS$_8$
With TilS$_9$
Without TilS$_9$
With TilS$_{10}$
Without TilS$_{10}$
With TilS$_{11}$
Without TilS$_{11}$
With TilS$_{12}$
Without TilS$_{12}$
With TilS$_{13}$
Without TilS$_{13}$
With TilS$_{14}$
Without TilS$_{14}$
With TilS$_{15}$
Without TilS$_{15}$

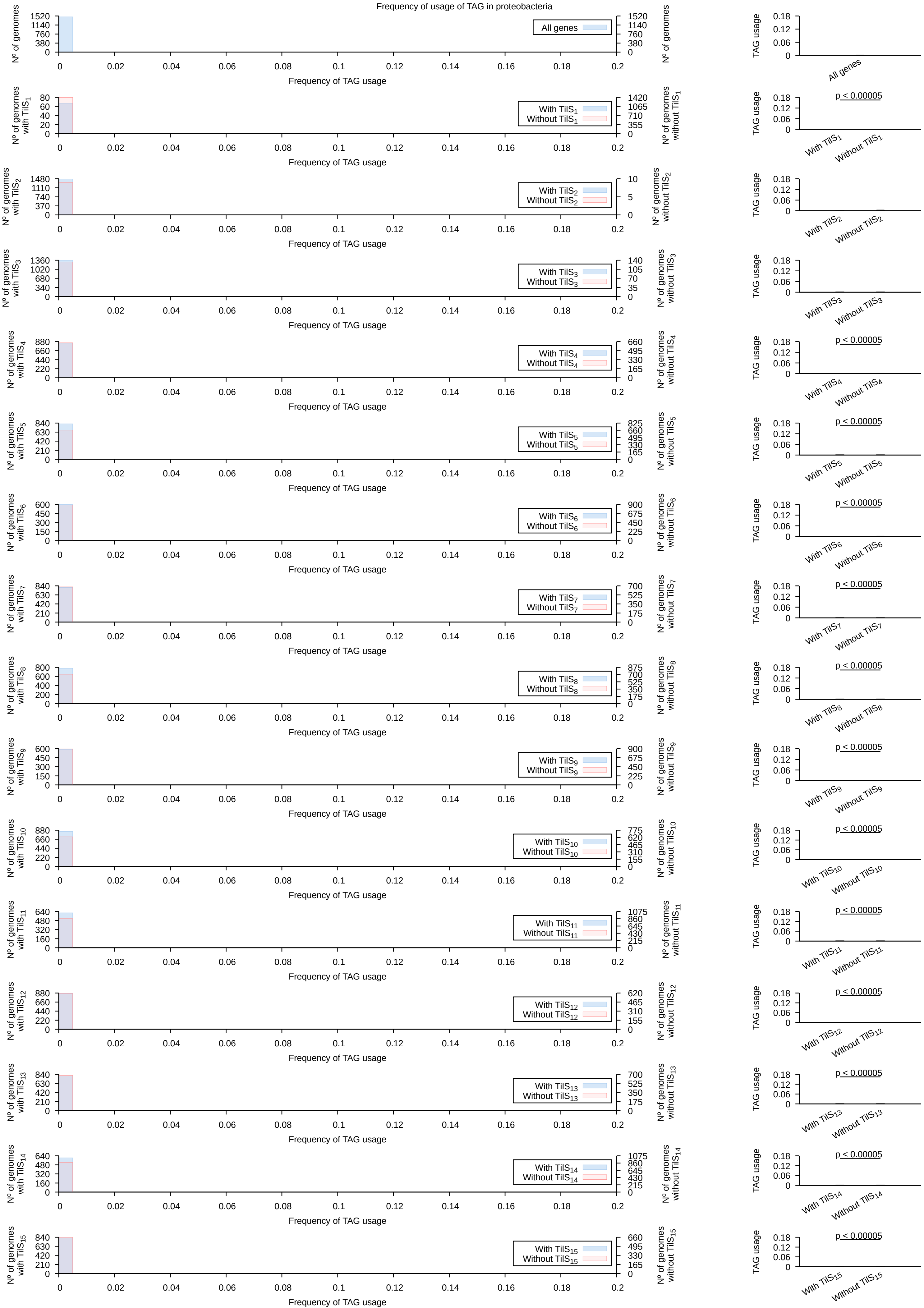

Frequency of usage of TAG in proteobacteria
Nº of genomes
1520 1140 760 380 0
All genes
Nº of genomes
TAG usage
0.18 0.12 0.06 0
All genes
0 0.02 0.04 0.06 0.08 0.1 0.12 0.14 0.16 0.18 0.2
Frequency of TAG usage
Nº of genomes with $TilS_1$
80 60 40 20 0
With $TilS_1$
Without $TilS_1$
1420 1065 710 355 0
Nº of genomes without $TilS_1$
TAG usage
p < 0.00005
With $TilS_1$
Without $TilS_1$
Frequency of TAG usage
Nº of genomes with $TilS_2$
1480 1110 740 370 0
With $TilS_2$
Without $TilS_2$
10 5 0
Nº of genomes without $TilS_2$
TAG usage
With $TilS_2$
Without $TilS_2$
Frequency of TAG usage
Nº of genomes with $TilS_3$
1360 1020 680 340 0
With $TilS_3$
Without $TilS_3$
140 105 70 35 0
Nº of genomes without $TilS_3$
TAG usage
With $TilS_3$
Without $TilS_3$
Frequency of TAG usage
Nº of genomes with $TilS_4$
880 660 440 220 0
With $TilS_4$
Without $TilS_4$
660 495 330 165 0
Nº of genomes without $TilS_4$
TAG usage
p < 0.00005
With $TilS_4$
Without $TilS_4$
Frequency of TAG usage
Nº of genomes with $TilS_5$
840 630 420 210 0
With $TilS_5$
Without $TilS_5$
825 660 495 330 165 0
Nº of genomes without $TilS_5$
TAG usage
p < 0.00005
With $TilS_5$
Without $TilS_5$
Frequency of TAG usage
Nº of genomes with $TilS_6$
600 450 300 150 0
With $TilS_6$
Without $TilS_6$
900 675 450 225 0
Nº of genomes without $TilS_6$
TAG usage
p < 0.00005
With $TilS_6$
Without $TilS_6$
Frequency of TAG usage
Nº of genomes with $TilS_7$
840 630 420 210 0
With $TilS_7$
Without $TilS_7$
700 525 350 175 0
Nº of genomes without $TilS_7$
TAG usage
p < 0.00005
With $TilS_7$
Without $TilS_7$
Frequency of TAG usage
Nº of genomes with $TilS_8$
800 600 400 200 0
With $TilS_8$
Without $TilS_8$
875 700 525 350 175 0
Nº of genomes without $TilS_8$
TAG usage
p < 0.00005
With $TilS_8$
Without $TilS_8$
Frequency of TAG usage
Nº of genomes with $TilS_9$
600 450 300 150 0
With $TilS_9$
Without $TilS_9$
900 675 450 225 0
Nº of genomes without $TilS_9$
TAG usage
p < 0.00005
With $TilS_9$
Without $TilS_9$
Frequency of TAG usage
Nº of genomes with $TilS_{10}$
880 660 440 220 0
With $TilS_{10}$
Without $TilS_{10}$
775 620 465 310 155 0
Nº of genomes without $TilS_{10}$
TAG usage
p < 0.00005
With $TilS_{10}$
Without $TilS_{10}$
Frequency of TAG usage
Nº of genomes with $TilS_{11}$
640 480 320 160 0
With $TilS_{11}$
Without $TilS_{11}$
1075 860 645 430 215 0
Nº of genomes without $TilS_{11}$
TAG usage
p < 0.00005
With $TilS_{11}$
Without $TilS_{11}$
Frequency of TAG usage
Nº of genomes with $TilS_{12}$
880 660 440 220 0
With $TilS_{12}$
Without $TilS_{12}$
620 465 310 155 0
Nº of genomes without $TilS_{12}$
TAG usage
p < 0.00005
With $TilS_{12}$
Without $TilS_{12}$
Frequency of TAG usage
Nº of genomes with $TilS_{13}$
840 630 420 210 0
With $TilS_{13}$
Without $TilS_{13}$
700 525 350 175 0
Nº of genomes without $TilS_{13}$
TAG usage
p < 0.00005
With $TilS_{13}$
Without $TilS_{13}$
Frequency of TAG usage
Nº of genomes with $TilS_{14}$
640 480 320 160 0
With $TilS_{14}$
Without $TilS_{14}$
1075 860 645 430 215 0
Nº of genomes without $TilS_{14}$
TAG usage
p < 0.00005
With $TilS_{14}$
Without $TilS_{14}$
Frequency of TAG usage
Nº of genomes with $TilS_{15}$
840 630 420 210 0
With $TilS_{15}$
Without $TilS_{15}$
660 495 330 165 0
Nº of genomes without $TilS_{15}$
TAG usage
p < 0.00005
With $TilS_{15}$
Without $TilS_{15}$
Frequency of TAG usage

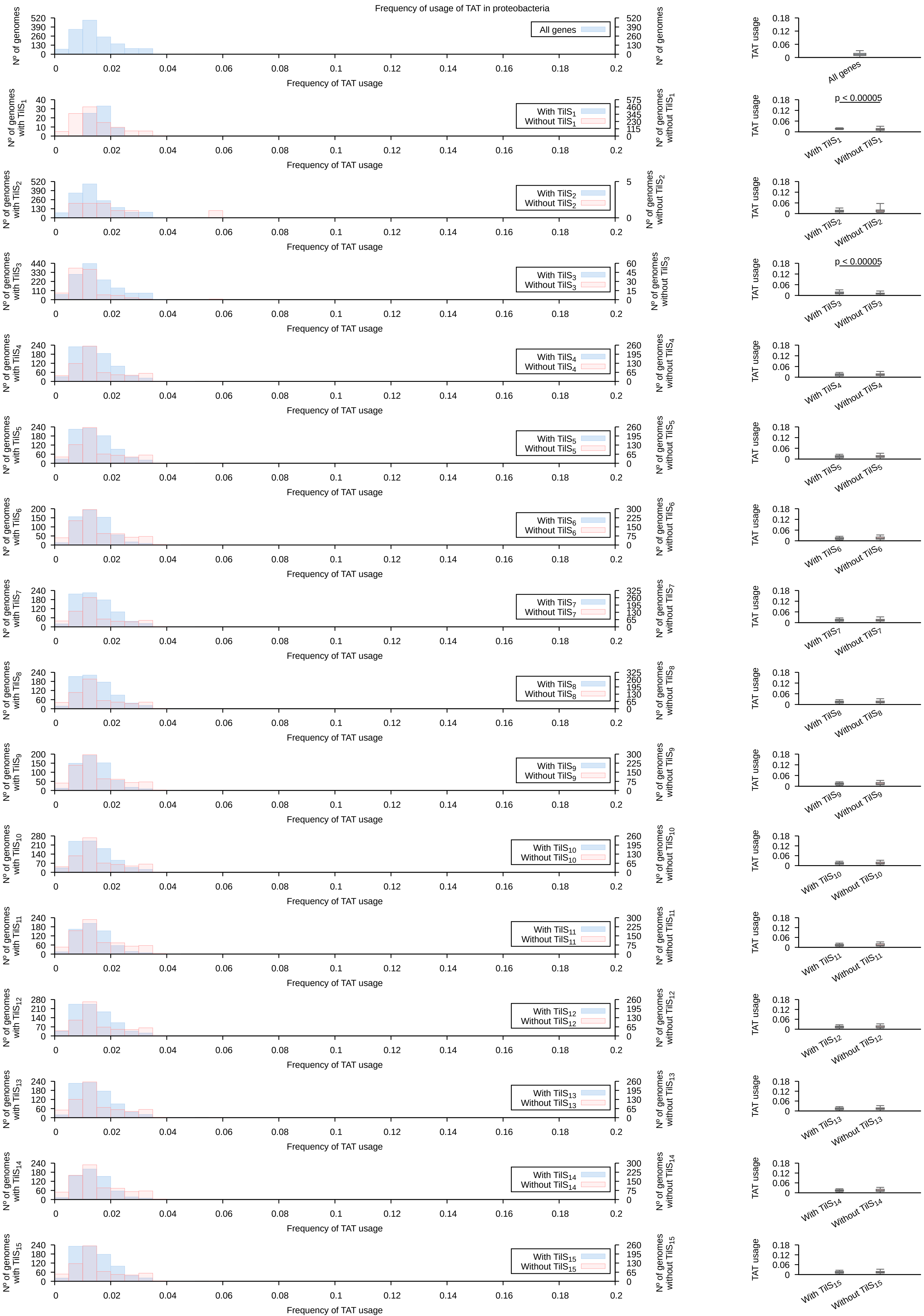

Frequency of usage of TAT in proteobacteria
Nº of genomes
All genes
Frequency of TAT usage
TAT usage
p < 0.00005
Nº of genomes with TilS$_1$
Nº of genomes without TilS$_1$
With TilS$_1$
Without TilS$_1$
Nº of genomes with TilS$_2$
Nº of genomes without TilS$_2$
With TilS$_2$
Without TilS$_2$
Nº of genomes with TilS$_3$
Nº of genomes without TilS$_3$
With TilS$_3$
Without TilS$_3$
Nº of genomes with TilS$_4$
Nº of genomes without TilS$_4$
With TilS$_4$
Without TilS$_4$
Nº of genomes with TilS$_5$
Nº of genomes without TilS$_5$
With TilS$_5$
Without TilS$_5$
Nº of genomes with TilS$_6$
Nº of genomes without TilS$_6$
With TilS$_6$
Without TilS$_6$
Nº of genomes with TilS$_7$
Nº of genomes without TilS$_7$
With TilS$_7$
Without TilS$_7$
Nº of genomes with TilS$_8$
Nº of genomes without TilS$_8$
With TilS$_8$
Without TilS$_8$
Nº of genomes with TilS$_9$
Nº of genomes without TilS$_9$
With TilS$_9$
Without TilS$_9$
Nº of genomes with TilS$_{10}$
Nº of genomes without TilS$_{10}$
With TilS$_{10}$
Without TilS$_{10}$
Nº of genomes with TilS$_{11}$
Nº of genomes without TilS$_{11}$
With TilS$_{11}$
Without TilS$_{11}$
Nº of genomes with TilS$_{12}$
Nº of genomes without TilS$_{12}$
With TilS$_{12}$
Without TilS$_{12}$
Nº of genomes with TilS$_{13}$
Nº of genomes without TilS$_{13}$
With TilS$_{13}$
Without TilS$_{13}$
Nº of genomes with TilS$_{14}$
Nº of genomes without TilS$_{14}$
With TilS$_{14}$
Without TilS$_{14}$
Nº of genomes with TilS$_{15}$
Nº of genomes without TilS$_{15}$
With TilS$_{15}$
Without TilS$_{15}$

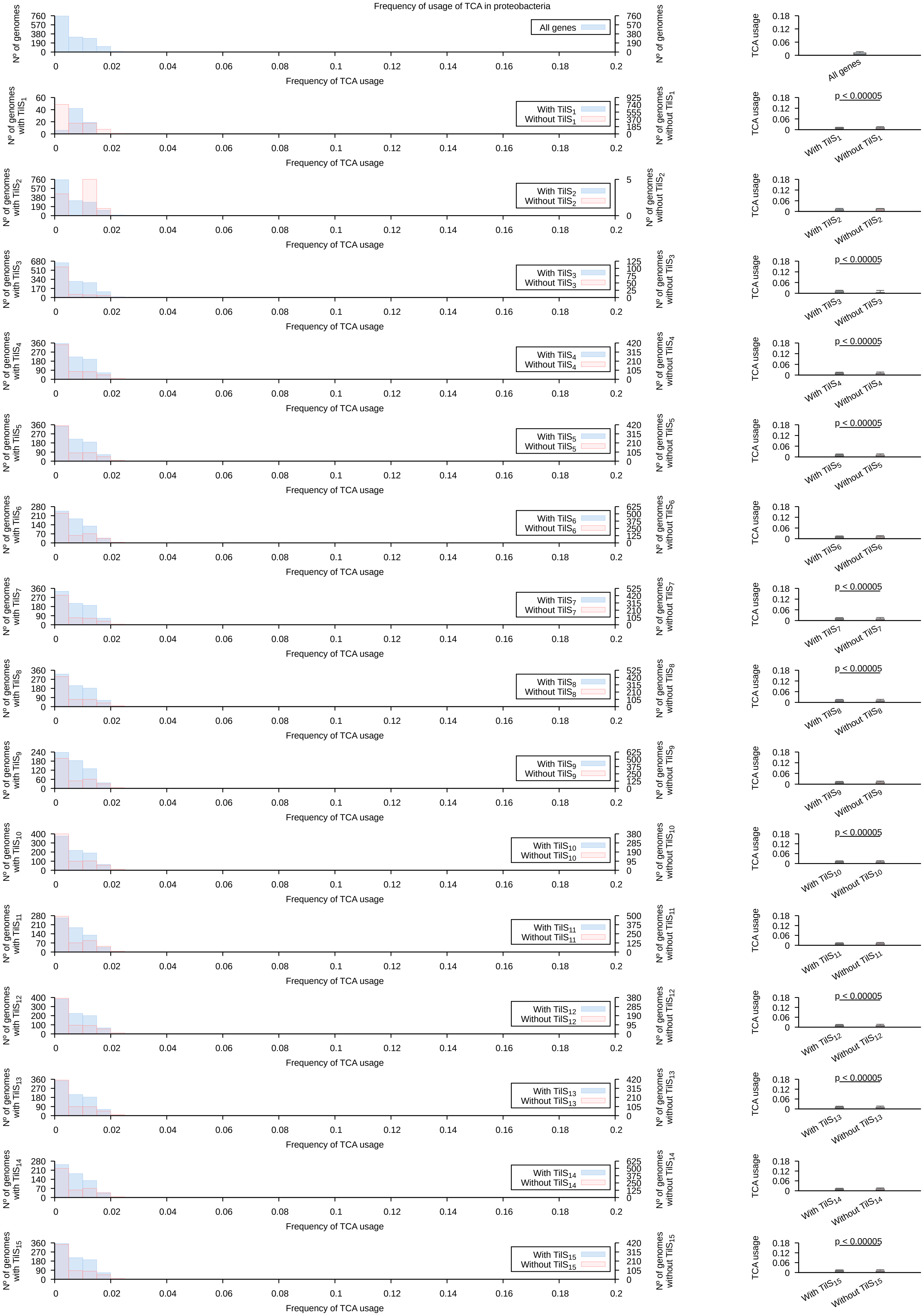

Frequency of usage of TCA in proteobacteria
Nº of genomes
All genes
Frequency of TCA usage
TCA usage
Nº of genomes with TilS$_1$
Nº of genomes without TilS$_1$
With TilS$_1$
Without TilS$_1$
p < 0.00005
Nº of genomes with TilS$_2$
Nº of genomes without TilS$_2$
With TilS$_2$
Without TilS$_2$
Nº of genomes with TilS$_3$
Nº of genomes without TilS$_3$
With TilS$_3$
Without TilS$_3$
p < 0.00005
Nº of genomes with TilS$_4$
Nº of genomes without TilS$_4$
With TilS$_4$
Without TilS$_4$
p < 0.00005
Nº of genomes with TilS$_5$
Nº of genomes without TilS$_5$
With TilS$_5$
Without TilS$_5$
p < 0.00005
Nº of genomes with TilS$_6$
Nº of genomes without TilS$_6$
With TilS$_6$
Without TilS$_6$
Nº of genomes with TilS$_7$
Nº of genomes without TilS$_7$
With TilS$_7$
Without TilS$_7$
p < 0.00005
Nº of genomes with TilS$_8$
Nº of genomes without TilS$_8$
With TilS$_8$
Without TilS$_8$
p < 0.00005
Nº of genomes with TilS$_9$
Nº of genomes without TilS$_9$
With TilS$_9$
Without TilS$_9$
Nº of genomes with TilS$_{10}$
Nº of genomes without TilS$_{10}$
With TilS$_{10}$
Without TilS$_{10}$
p < 0.00005
Nº of genomes with TilS$_{11}$
Nº of genomes without TilS$_{11}$
With TilS$_{11}$
Without TilS$_{11}$
Nº of genomes with TilS$_{12}$
Nº of genomes without TilS$_{12}$
With TilS$_{12}$
Without TilS$_{12}$
p < 0.00005
Nº of genomes with TilS$_{13}$
Nº of genomes without TilS$_{13}$
With TilS$_{13}$
Without TilS$_{13}$
p < 0.00005
Nº of genomes with TilS$_{14}$
Nº of genomes without TilS$_{14}$
With TilS$_{14}$
Without TilS$_{14}$
Nº of genomes with TilS$_{15}$
Nº of genomes without TilS$_{15}$
With TilS$_{15}$
Without TilS$_{15}$
p < 0.00005

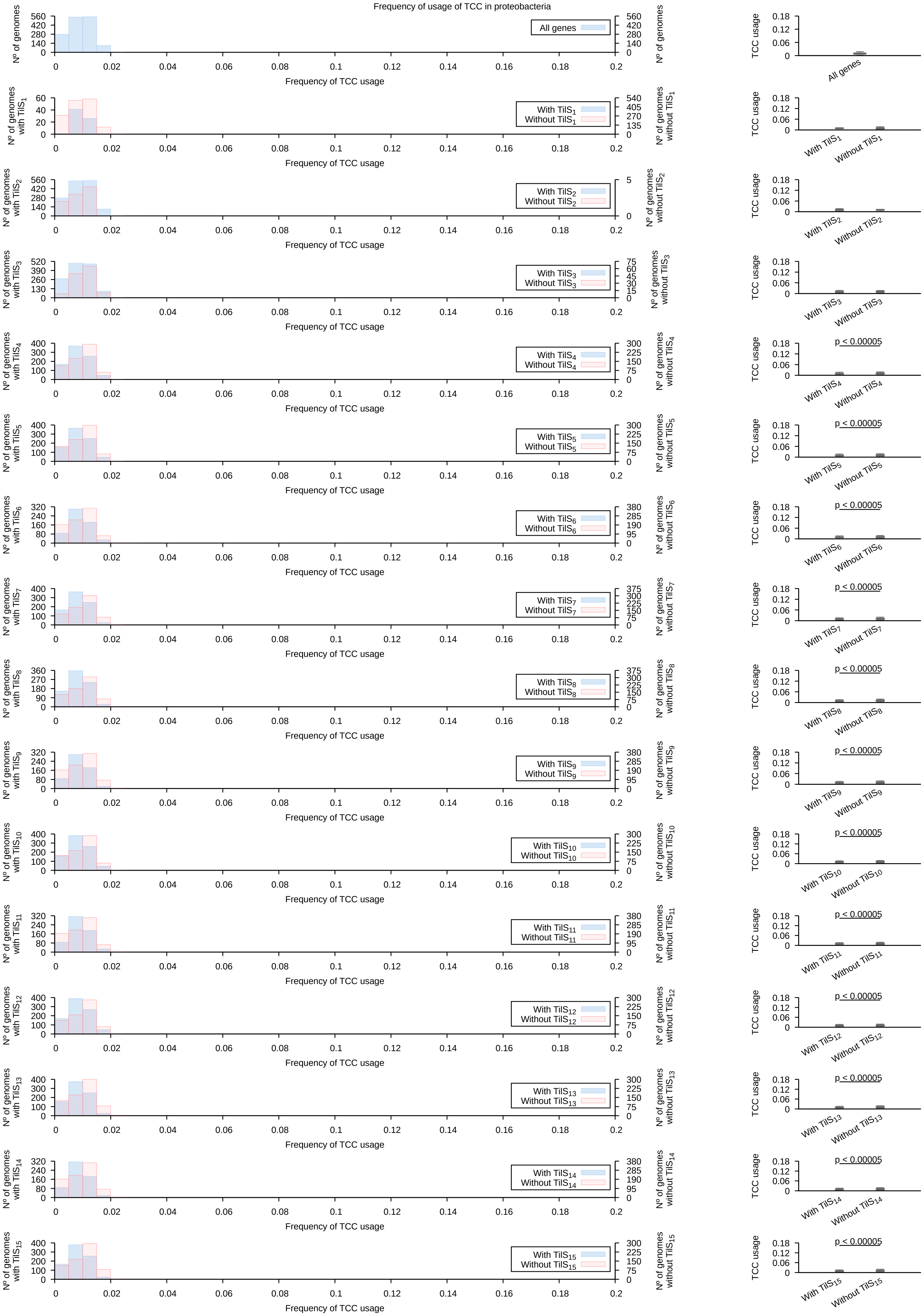

Frequency of usage of TCC in proteobacteria
Nº of genomes
All genes
Frequency of TCC usage
0 0.02 0.04 0.06 0.08 0.1 0.12 0.14 0.16 0.18 0.2
TCC usage
0 0.06 0.12 0.18
Nº of genomes with TilS$_1$
With TilS$_1$
Without TilS$_1$
Nº of genomes without TilS$_1$
Nº of genomes with TilS$_2$
With TilS$_2$
Without TilS$_2$
Nº of genomes without TilS$_2$
Nº of genomes with TilS$_3$
With TilS$_3$
Without TilS$_3$
Nº of genomes without TilS$_3$
Nº of genomes with TilS$_4$
With TilS$_4$
Without TilS$_4$
Nº of genomes without TilS$_4$
p < 0.00005
Nº of genomes with TilS$_5$
With TilS$_5$
Without TilS$_5$
Nº of genomes without TilS$_5$
p < 0.00005
Nº of genomes with TilS$_6$
With TilS$_6$
Without TilS$_6$
Nº of genomes without TilS$_6$
p < 0.00005
Nº of genomes with TilS$_7$
With TilS$_7$
Without TilS$_7$
Nº of genomes without TilS$_7$
p < 0.00005
Nº of genomes with TilS$_8$
With TilS$_8$
Without TilS$_8$
Nº of genomes without TilS$_8$
p < 0.00005
Nº of genomes with TilS$_9$
With TilS$_9$
Without TilS$_9$
Nº of genomes without TilS$_9$
p < 0.00005
Nº of genomes with TilS$_{10}$
With TilS$_{10}$
Without TilS$_{10}$
Nº of genomes without TilS$_{10}$
p < 0.00005
Nº of genomes with TilS$_{11}$
With TilS$_{11}$
Without TilS$_{11}$
Nº of genomes without TilS$_{11}$
p < 0.00005
Nº of genomes with TilS$_{12}$
With TilS$_{12}$
Without TilS$_{12}$
Nº of genomes without TilS$_{12}$
p < 0.00005
Nº of genomes with TilS$_{13}$
With TilS$_{13}$
Without TilS$_{13}$
Nº of genomes without TilS$_{13}$
p < 0.00005
Nº of genomes with TilS$_{14}$
With TilS$_{14}$
Without TilS$_{14}$
Nº of genomes without TilS$_{14}$
p < 0.00005
Nº of genomes with TilS$_{15}$
With TilS$_{15}$
Without TilS$_{15}$
Nº of genomes without TilS$_{15}$
p < 0.00005

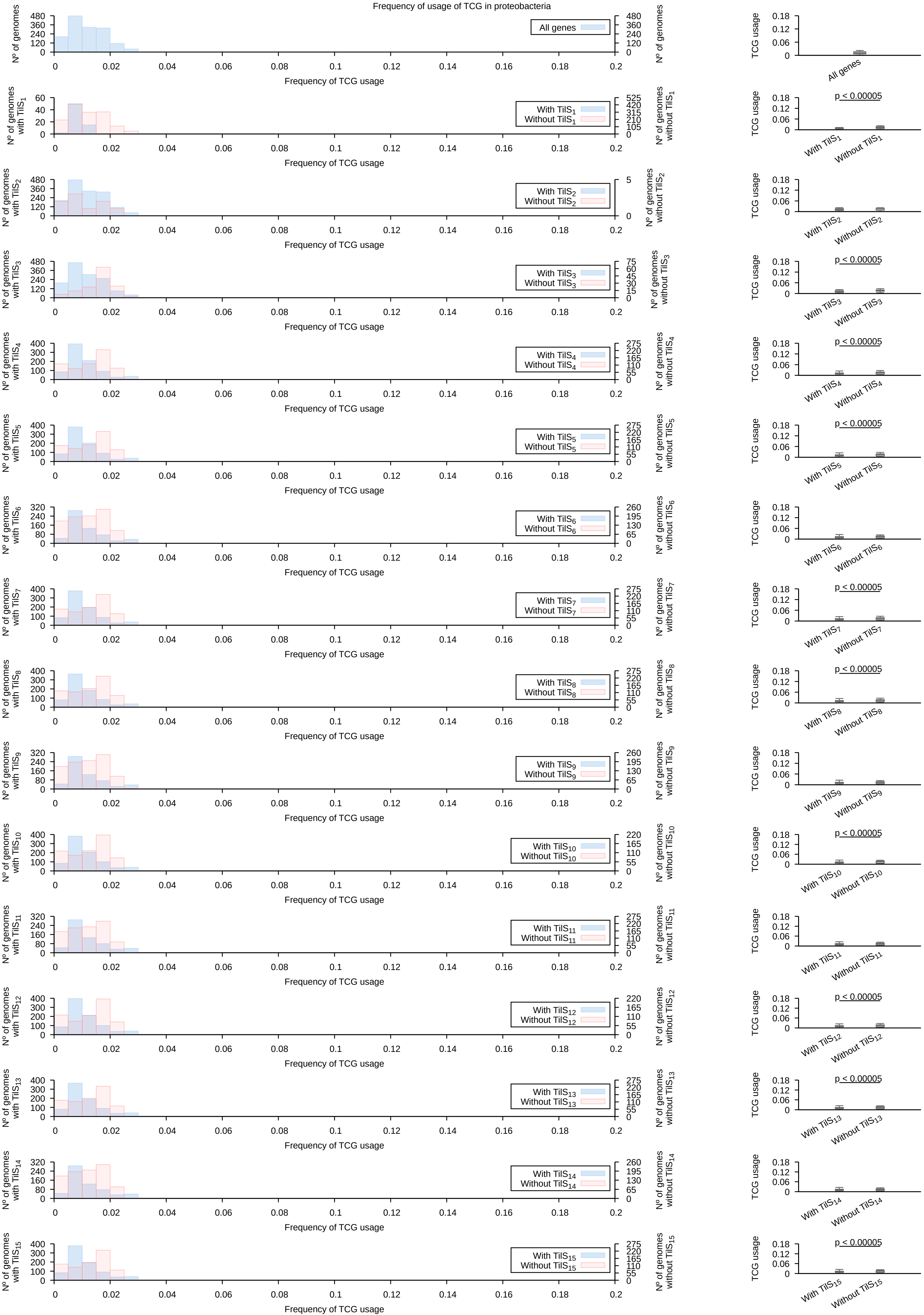

Frequency of usage of TCG in proteobacteria
Nº of genomes
All genes
Frequency of TCG usage
TCG usage
All genes
Nº of genomes with TilS1
Nº of genomes without TilS1
With TilS1
Without TilS1
p < 0.00005
Nº of genomes with TilS2
Nº of genomes without TilS2
With TilS2
Without TilS2
Nº of genomes with TilS3
Nº of genomes without TilS3
With TilS3
Without TilS3
p < 0.00005
Nº of genomes with TilS4
Nº of genomes without TilS4
With TilS4
Without TilS4
p < 0.00005
Nº of genomes with TilS5
Nº of genomes without TilS5
With TilS5
Without TilS5
p < 0.00005
Nº of genomes with TilS6
Nº of genomes without TilS6
With TilS6
Without TilS6
Nº of genomes with TilS7
Nº of genomes without TilS7
With TilS7
Without TilS7
p < 0.00005
Nº of genomes with TilS8
Nº of genomes without TilS8
With TilS8
Without TilS8
p < 0.00005
Nº of genomes with TilS9
Nº of genomes without TilS9
With TilS9
Without TilS9
Nº of genomes with TilS10
Nº of genomes without TilS10
With TilS10
Without TilS10
p < 0.00005
Nº of genomes with TilS11
Nº of genomes without TilS11
With TilS11
Without TilS11
Nº of genomes with TilS12
Nº of genomes without TilS12
With TilS12
Without TilS12
p < 0.00005
Nº of genomes with TilS13
Nº of genomes without TilS13
With TilS13
Without TilS13
p < 0.00005
Nº of genomes with TilS14
Nº of genomes without TilS14
With TilS14
Without TilS14
Nº of genomes with TilS15
Nº of genomes without TilS15
With TilS15
Without TilS15
p < 0.00005

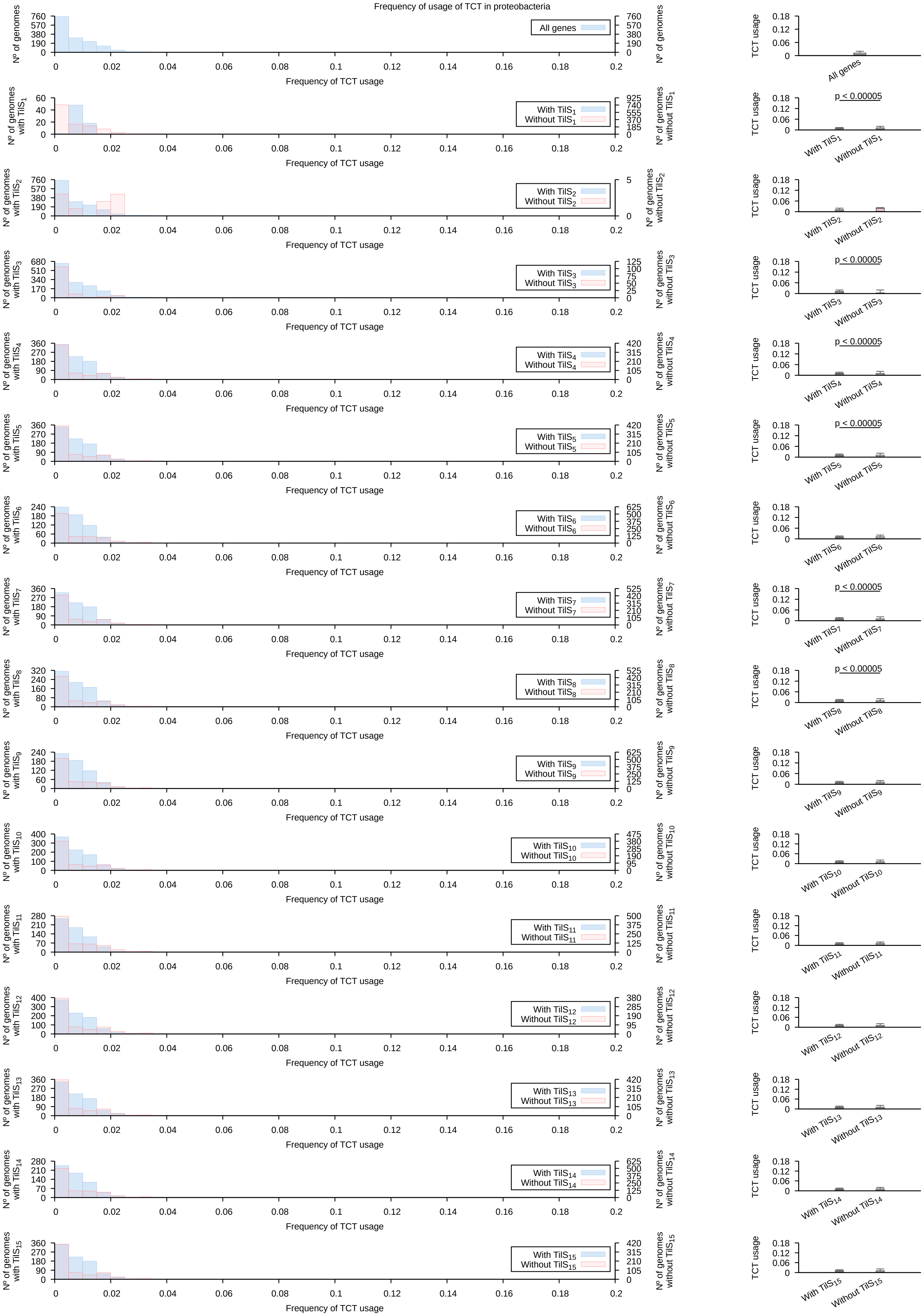

Frequency of usage of TCT in proteobacteria
Nº of genomes
Frequency of TCT usage
All genes
TCT usage
With TilS$_1$
Without TilS$_1$
Nº of genomes with TilS$_1$
Nº of genomes without TilS$_1$
p < 0.00005
With TilS$_2$
Without TilS$_2$
Nº of genomes with TilS$_2$
Nº of genomes without TilS$_2$
With TilS$_3$
Without TilS$_3$
Nº of genomes with TilS$_3$
Nº of genomes without TilS$_3$
p < 0.00005
With TilS$_4$
Without TilS$_4$
Nº of genomes with TilS$_4$
Nº of genomes without TilS$_4$
p < 0.00005
With TilS$_5$
Without TilS$_5$
Nº of genomes with TilS$_5$
Nº of genomes without TilS$_5$
p < 0.00005
With TilS$_6$
Without TilS$_6$
Nº of genomes with TilS$_6$
Nº of genomes without TilS$_6$
With TilS$_7$
Without TilS$_7$
Nº of genomes with TilS$_7$
Nº of genomes without TilS$_7$
p < 0.00005
With TilS$_8$
Without TilS$_8$
Nº of genomes with TilS$_8$
Nº of genomes without TilS$_8$
p < 0.00005
With TilS$_9$
Without TilS$_9$
Nº of genomes with TilS$_9$
Nº of genomes without TilS$_9$
With TilS$_{10}$
Without TilS$_{10}$
Nº of genomes with TilS$_{10}$
Nº of genomes without TilS$_{10}$
With TilS$_{11}$
Without TilS$_{11}$
Nº of genomes with TilS$_{11}$
Nº of genomes without TilS$_{11}$
With TilS$_{12}$
Without TilS$_{12}$
Nº of genomes with TilS$_{12}$
Nº of genomes without TilS$_{12}$
With TilS$_{13}$
Without TilS$_{13}$
Nº of genomes with TilS$_{13}$
Nº of genomes without TilS$_{13}$
With TilS$_{14}$
Without TilS$_{14}$
Nº of genomes with TilS$_{14}$
Nº of genomes without TilS$_{14}$
With TilS$_{15}$
Without TilS$_{15}$
Nº of genomes with TilS$_{15}$
Nº of genomes without TilS$_{15}$

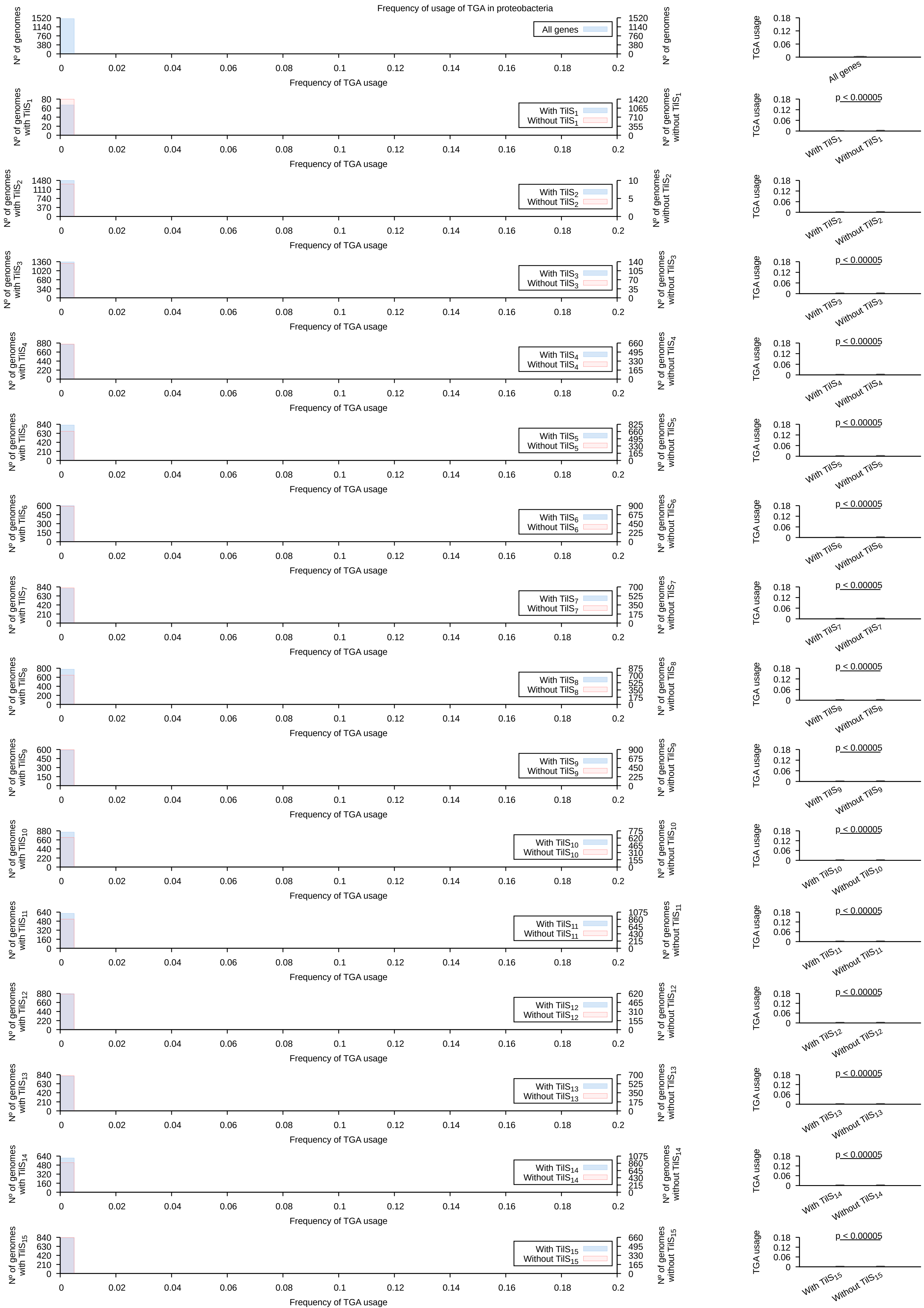
Frequency of usage of TGA in proteobacteria
Nº of genomes
All genes
Frequency of TGA usage
TGA usage
p < 0.00005
Nº of genomes with TilS1 / Nº of genomes without TilS1
With TilS1 / Without TilS1
Nº of genomes with TilS2 / Nº of genomes without TilS2
With TilS2 / Without TilS2
Nº of genomes with TilS3 / Nº of genomes without TilS3
With TilS3 / Without TilS3
Nº of genomes with TilS4 / Nº of genomes without TilS4
With TilS4 / Without TilS4
Nº of genomes with TilS5 / Nº of genomes without TilS5
With TilS5 / Without TilS5
Nº of genomes with TilS6 / Nº of genomes without TilS6
With TilS6 / Without TilS6
Nº of genomes with TilS7 / Nº of genomes without TilS7
With TilS7 / Without TilS7
Nº of genomes with TilS8 / Nº of genomes without TilS8
With TilS8 / Without TilS8
Nº of genomes with TilS9 / Nº of genomes without TilS9
With TilS9 / Without TilS9
Nº of genomes with TilS10 / Nº of genomes without TilS10
With TilS10 / Without TilS10
Nº of genomes with TilS11 / Nº of genomes without TilS11
With TilS11 / Without TilS11
Nº of genomes with TilS12 / Nº of genomes without TilS12
With TilS12 / Without TilS12
Nº of genomes with TilS13 / Nº of genomes without TilS13
With TilS13 / Without TilS13
Nº of genomes with TilS14 / Nº of genomes without TilS14
With TilS14 / Without TilS14
Nº of genomes with TilS15 / Nº of genomes without TilS15
With TilS15 / Without TilS15

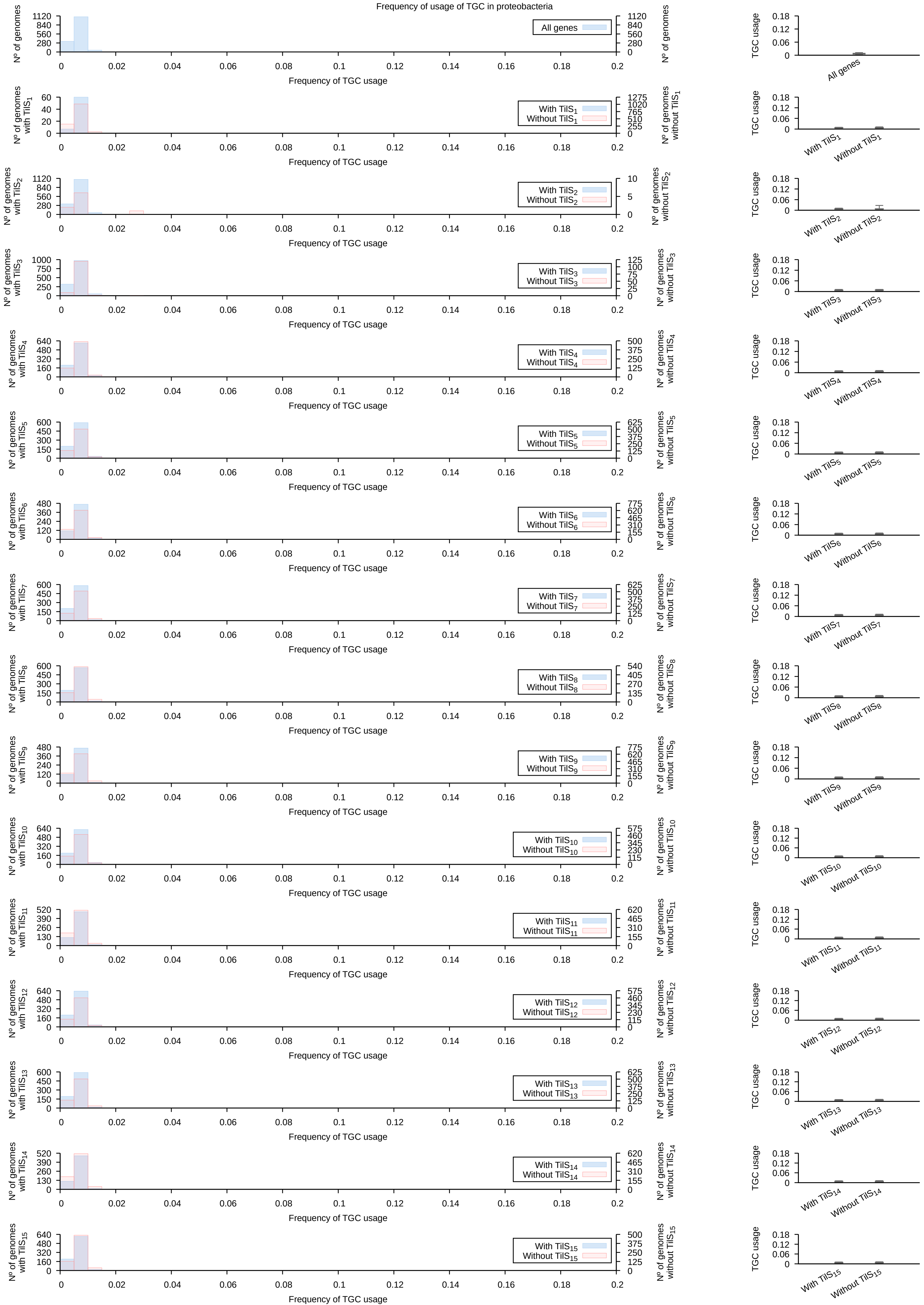

Frequency of usage of TGC in proteobacteria
Nº of genomes
1120 840 560 280 0
All genes
Frequency of TGC usage
0 0.02 0.04 0.06 0.08 0.1 0.12 0.14 0.16 0.18 0.2
TGC usage
0.18 0.12 0.06 0
Nº of genomes with TilS$_1$
60 40 20 0
With TilS$_1$
Without TilS$_1$
1275 1020 765 510 255 0
Nº of genomes without TilS$_1$
Nº of genomes with TilS$_2$
1120 840 560 280 0
With TilS$_2$
Without TilS$_2$
10 5 0
Nº of genomes without TilS$_2$
Nº of genomes with TilS$_3$
1000 750 500 250 0
With TilS$_3$
Without TilS$_3$
125 100 75 50 25 0
Nº of genomes without TilS$_3$
Nº of genomes with TilS$_4$
640 480 320 160 0
With TilS$_4$
Without TilS$_4$
500 375 250 125 0
Nº of genomes without TilS$_4$
Nº of genomes with TilS$_5$
600 450 300 150 0
With TilS$_5$
Without TilS$_5$
625 500 375 250 125 0
Nº of genomes without TilS$_5$
Nº of genomes with TilS$_6$
480 360 240 120 0
With TilS$_6$
Without TilS$_6$
775 620 465 310 155 0
Nº of genomes without TilS$_6$
Nº of genomes with TilS$_7$
600 450 300 150 0
With TilS$_7$
Without TilS$_7$
625 500 375 250 125 0
Nº of genomes without TilS$_7$
Nº of genomes with TilS$_8$
600 450 300 150 0
With TilS$_8$
Without TilS$_8$
540 405 270 135 0
Nº of genomes without TilS$_8$
Nº of genomes with TilS$_9$
480 360 240 120 0
With TilS$_9$
Without TilS$_9$
775 620 465 310 155 0
Nº of genomes without TilS$_9$
Nº of genomes with TilS$_{10}$
640 480 320 160 0
With TilS$_{10}$
Without TilS$_{10}$
575 460 345 230 115 0
Nº of genomes without TilS$_{10}$
Nº of genomes with TilS$_{11}$
520 390 260 130 0
With TilS$_{11}$
Without TilS$_{11}$
620 465 310 155 0
Nº of genomes without TilS$_{11}$
Nº of genomes with TilS$_{12}$
640 480 320 160 0
With TilS$_{12}$
Without TilS$_{12}$
575 460 345 230 115 0
Nº of genomes without TilS$_{12}$
Nº of genomes with TilS$_{13}$
600 450 300 150 0
With TilS$_{13}$
Without TilS$_{13}$
625 500 375 250 125 0
Nº of genomes without TilS$_{13}$
Nº of genomes with TilS$_{14}$
520 390 260 130 0
With TilS$_{14}$
Without TilS$_{14}$
620 465 310 155 0
Nº of genomes without TilS$_{14}$
Nº of genomes with TilS$_{15}$
640 480 320 160 0
With TilS$_{15}$
Without TilS$_{15}$
500 375 250 125 0
Nº of genomes without TilS$_{15}$

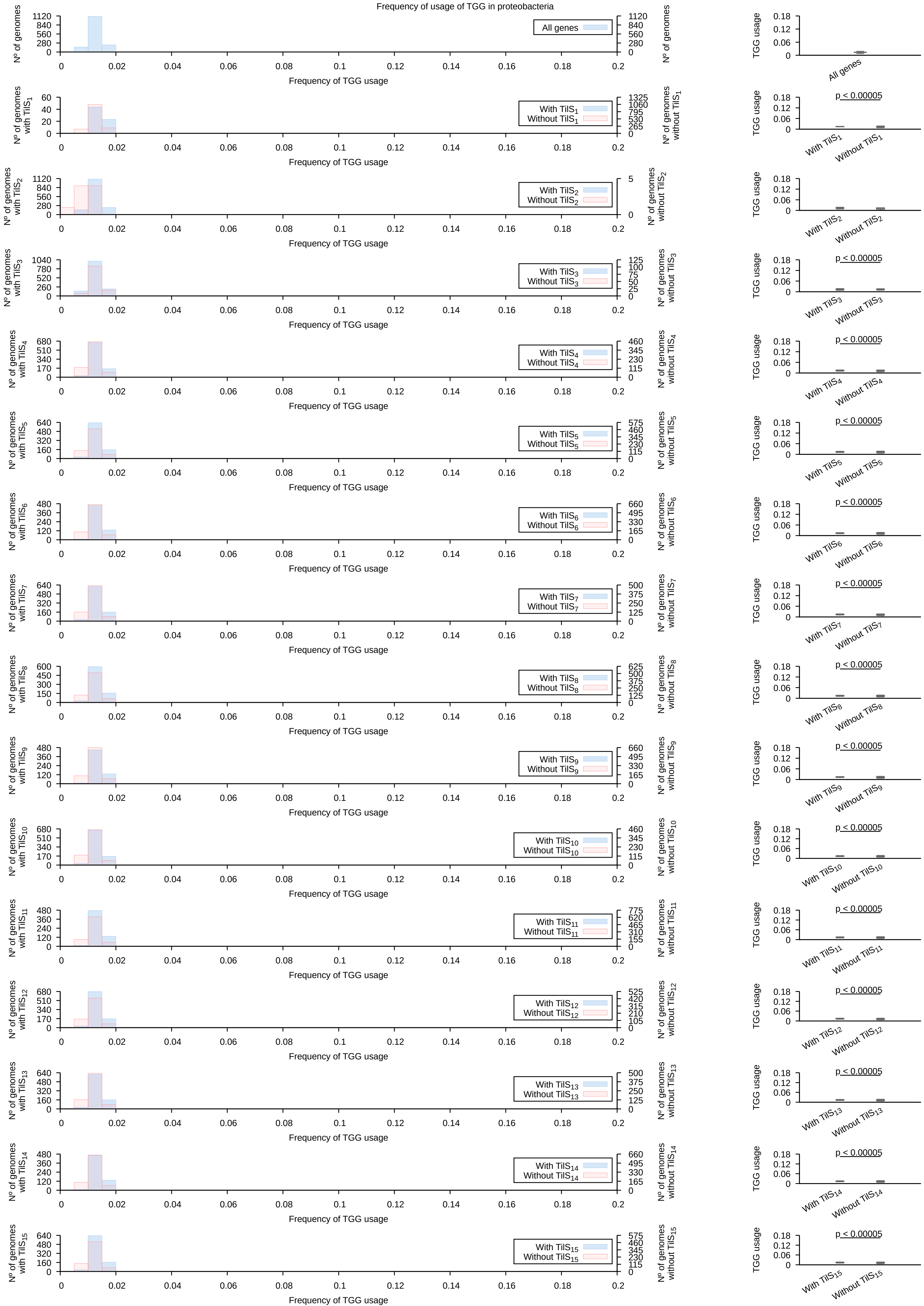

Frequency of usage of TGG in proteobacteria
Nº of genomes
All genes
Frequency of TGG usage
TGG usage
With TilS$_1$
Without TilS$_1$
With TilS$_2$
Without TilS$_2$
With TilS$_3$
Without TilS$_3$
With TilS$_4$
Without TilS$_4$
With TilS$_5$
Without TilS$_5$
With TilS$_6$
Without TilS$_6$
With TilS$_7$
Without TilS$_7$
With TilS$_8$
Without TilS$_8$
With TilS$_9$
Without TilS$_9$
With TilS$_{10}$
Without TilS$_{10}$
With TilS$_{11}$
Without TilS$_{11}$
With TilS$_{12}$
Without TilS$_{12}$
With TilS$_{13}$
Without TilS$_{13}$
With TilS$_{14}$
Without TilS$_{14}$
With TilS$_{15}$
Without TilS$_{15}$
$p < 0.00005$

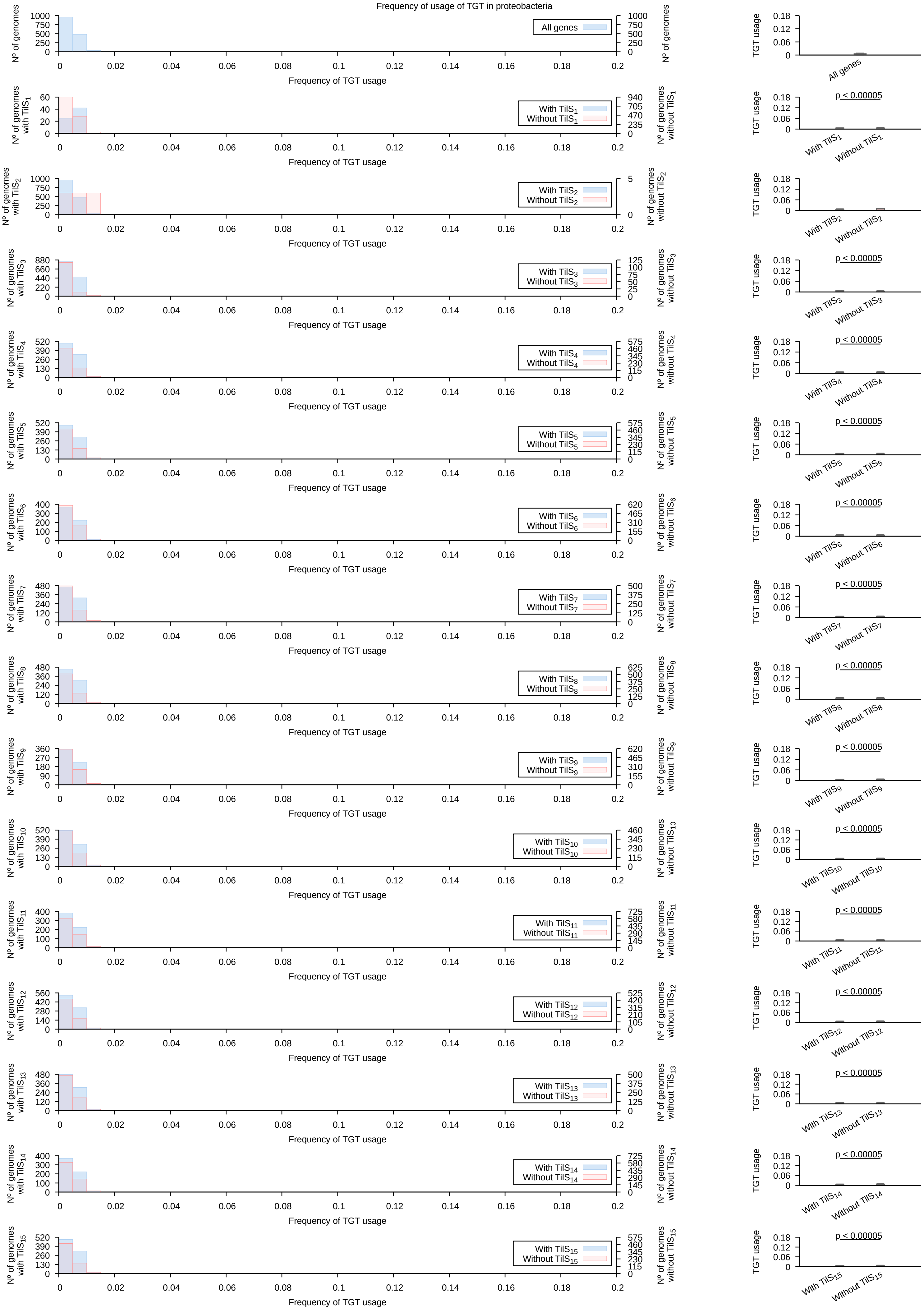
Frequency of usage of TGT in proteobacteria
All genes
Nº of genomes
Frequency of TGT usage
TGT usage
With TilS$_1$
Without TilS$_1$
Nº of genomes with TilS$_1$
Nº of genomes without TilS$_1$
With TilS$_2$
Without TilS$_2$
Nº of genomes with TilS$_2$
Nº of genomes without TilS$_2$
With TilS$_3$
Without TilS$_3$
With TilS$_4$
Without TilS$_4$
With TilS$_5$
Without TilS$_5$
With TilS$_6$
Without TilS$_6$
With TilS$_7$
Without TilS$_7$
With TilS$_8$
Without TilS$_8$
With TilS$_9$
Without TilS$_9$
With TilS$_{10}$
Without TilS$_{10}$
With TilS$_{11}$
Without TilS$_{11}$
With TilS$_{12}$
Without TilS$_{12}$
With TilS$_{13}$
Without TilS$_{13}$
With TilS$_{14}$
Without TilS$_{14}$
With TilS$_{15}$
Without TilS$_{15}$
$p < 0.00005$

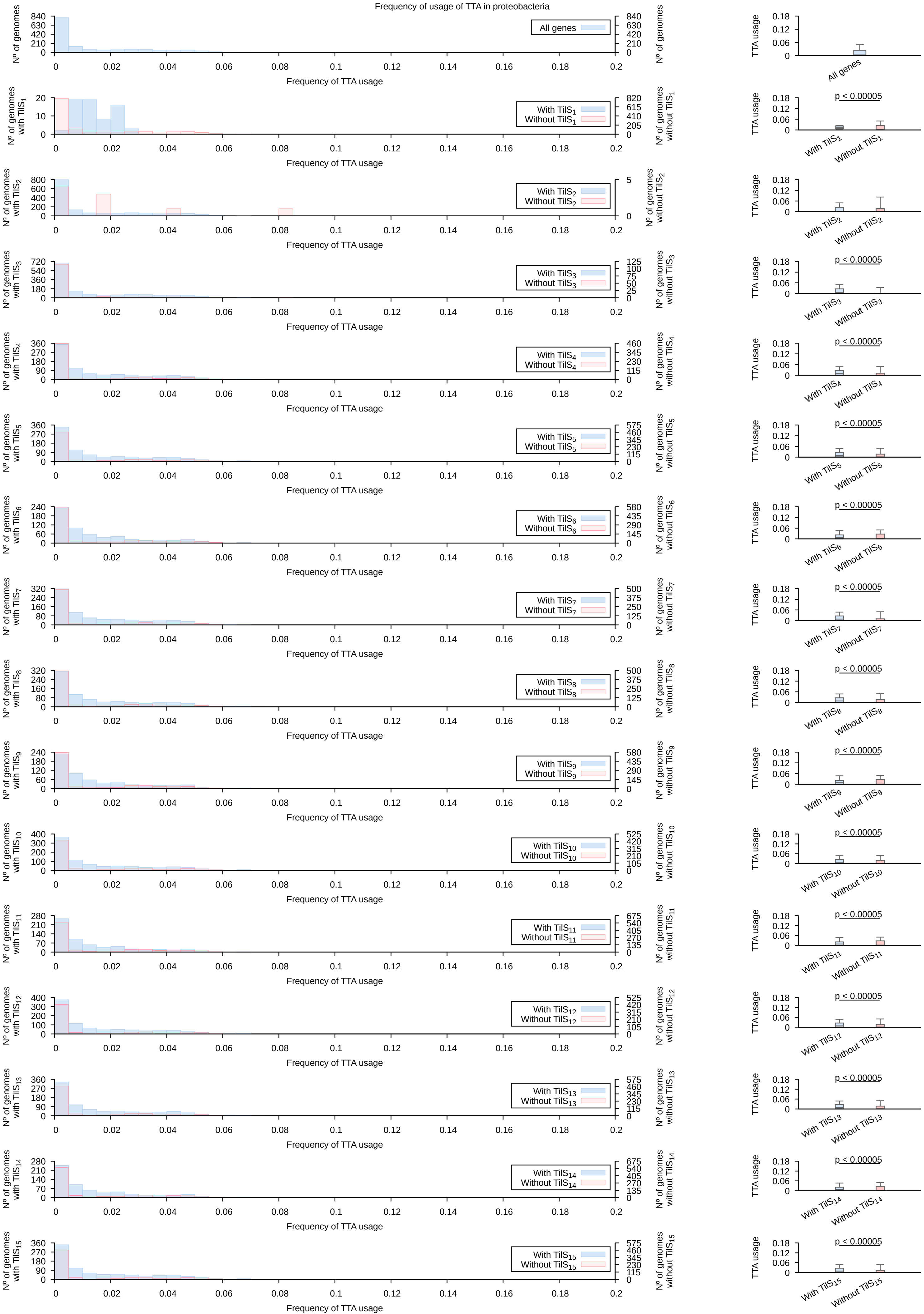

Frequency of usage of TTA in proteobacteria
Nº of genomes
840 630 420 210 0
All genes
0 0.02 0.04 0.06 0.08 0.1 0.12 0.14 0.16 0.18 0.2
Frequency of TTA usage
TTA usage
0.18 0.12 0.06 0
Nº of genomes with TilS$_1$
20 10 0
With TilS$_1$
Without TilS$_1$
820 615 410 205 0
Nº of genomes without TilS$_1$
p < 0.00005
Without TilS$_1$
Nº of genomes with TilS$_2$
800 600 400 200 0
With TilS$_2$
Without TilS$_2$
5 0
Nº of genomes without TilS$_2$
Nº of genomes with TilS$_3$
720 540 360 180 0
With TilS$_3$
Without TilS$_3$
125 100 75 50 25 0
Nº of genomes without TilS$_3$
Nº of genomes with TilS$_4$
360 270 180 90 0
With TilS$_4$
Without TilS$_4$
460 345 230 115 0
Nº of genomes without TilS$_4$
Nº of genomes with TilS$_5$
360 270 180 90 0
With TilS$_5$
Without TilS$_5$
575 460 345 230 115 0
Nº of genomes without TilS$_5$
Nº of genomes with TilS$_6$
240 180 120 60 0
With TilS$_6$
Without TilS$_6$
580 435 290 145 0
Nº of genomes without TilS$_6$
Nº of genomes with TilS$_7$
320 240 160 80 0
With TilS$_7$
Without TilS$_7$
500 375 250 125 0
Nº of genomes without TilS$_7$
Nº of genomes with TilS$_8$
320 240 160 80 0
With TilS$_8$
Without TilS$_8$
500 375 250 125 0
Nº of genomes without TilS$_8$
Nº of genomes with TilS$_9$
240 180 120 60 0
With TilS$_9$
Without TilS$_9$
580 435 290 145 0
Nº of genomes without TilS$_9$
Nº of genomes with TilS$_{10}$
400 300 200 100 0
With TilS$_{10}$
Without TilS$_{10}$
525 420 315 210 105 0
Nº of genomes without TilS$_{10}$
Nº of genomes with TilS$_{11}$
280 210 140 70 0
With TilS$_{11}$
Without TilS$_{11}$
675 540 405 270 135 0
Nº of genomes without TilS$_{11}$
Nº of genomes with TilS$_{12}$
400 300 200 100 0
With TilS$_{12}$
Without TilS$_{12}$
525 420 315 210 105 0
Nº of genomes without TilS$_{12}$
Nº of genomes with TilS$_{13}$
360 270 180 90 0
With TilS$_{13}$
Without TilS$_{13}$
575 460 345 230 115 0
Nº of genomes without TilS$_{13}$
Nº of genomes with TilS$_{14}$
280 210 140 70 0
With TilS$_{14}$
Without TilS$_{14}$
675 540 405 270 135 0
Nº of genomes without TilS$_{14}$
Nº of genomes with TilS$_{15}$
360 270 180 90 0
With TilS$_{15}$
Without TilS$_{15}$
575 460 345 230 115 0
Nº of genomes without TilS$_{15}$

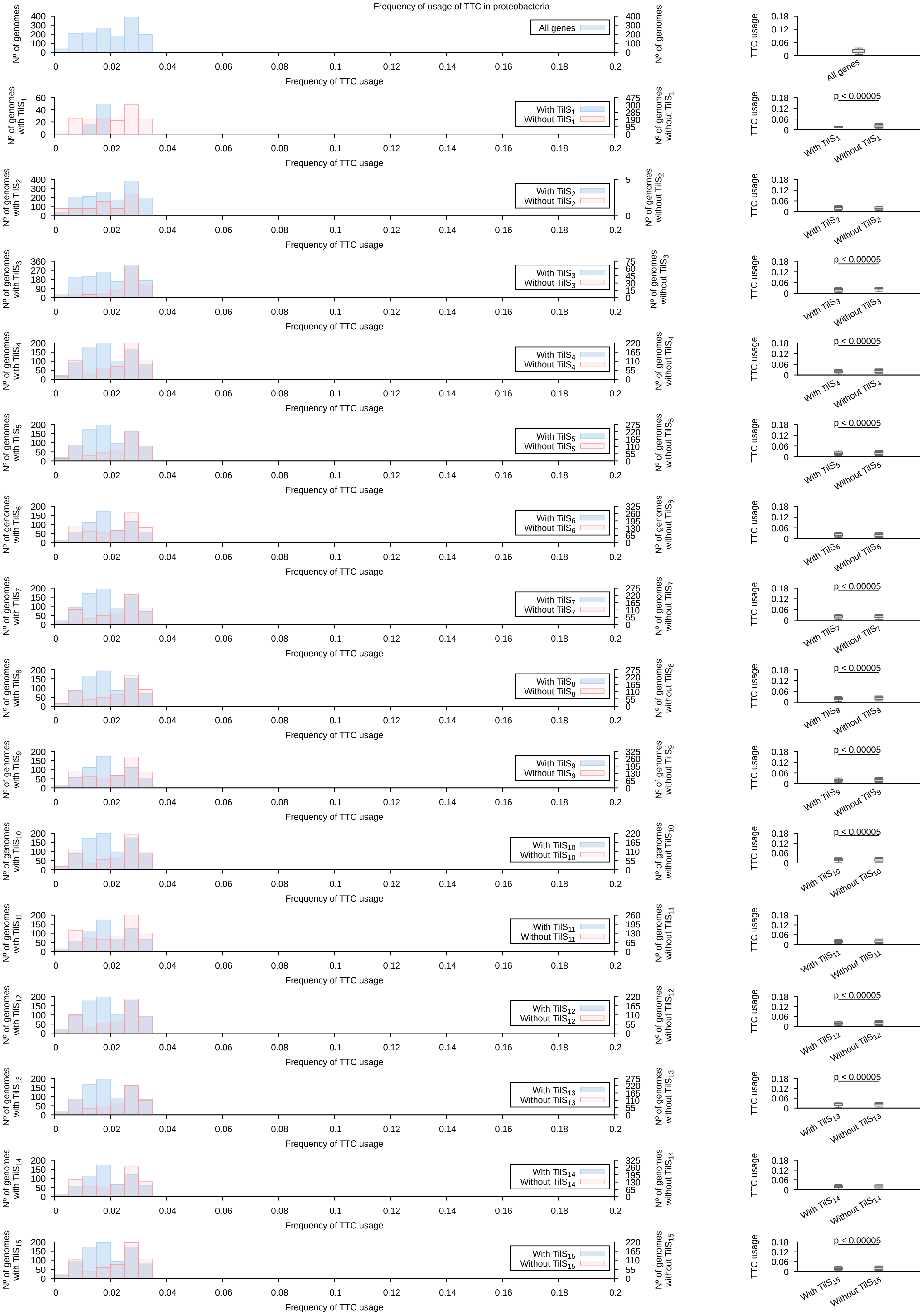
Frequency of usage of TTC in proteobacteria
Nº of genomes
All genes
Frequency of TTC usage
TTC usage
p < 0.00005
Nº of genomes with TilS$_1$
Nº of genomes without TilS$_1$
With TilS$_1$
Without TilS$_1$
Nº of genomes with TilS$_2$
Nº of genomes without TilS$_2$
With TilS$_2$
Without TilS$_2$
Nº of genomes with TilS$_3$
Nº of genomes without TilS$_3$
With TilS$_3$
Without TilS$_3$
Nº of genomes with TilS$_4$
Nº of genomes without TilS$_4$
With TilS$_4$
Without TilS$_4$
Nº of genomes with TilS$_5$
Nº of genomes without TilS$_5$
With TilS$_5$
Without TilS$_5$
Nº of genomes with TilS$_6$
Nº of genomes without TilS$_6$
With TilS$_6$
Without TilS$_6$
Nº of genomes with TilS$_7$
Nº of genomes without TilS$_7$
With TilS$_7$
Without TilS$_7$
Nº of genomes with TilS$_8$
Nº of genomes without TilS$_8$
With TilS$_8$
Without TilS$_8$
Nº of genomes with TilS$_9$
Nº of genomes without TilS$_9$
With TilS$_9$
Without TilS$_9$
Nº of genomes with TilS$_{10}$
Nº of genomes without TilS$_{10}$
With TilS$_{10}$
Without TilS$_{10}$
Nº of genomes with TilS$_{11}$
Nº of genomes without TilS$_{11}$
With TilS$_{11}$
Without TilS$_{11}$
Nº of genomes with TilS$_{12}$
Nº of genomes without TilS$_{12}$
With TilS$_{12}$
Without TilS$_{12}$
Nº of genomes with TilS$_{13}$
Nº of genomes without TilS$_{13}$
With TilS$_{13}$
Without TilS$_{13}$
Nº of genomes with TilS$_{14}$
Nº of genomes without TilS$_{14}$
With TilS$_{14}$
Without TilS$_{14}$
Nº of genomes with TilS$_{15}$
Nº of genomes without TilS$_{15}$
With TilS$_{15}$
Without TilS$_{15}$
0 0.02 0.04 0.06 0.08 0.1 0.12 0.14 0.16 0.18 0.2
0 0.06 0.12 0.18

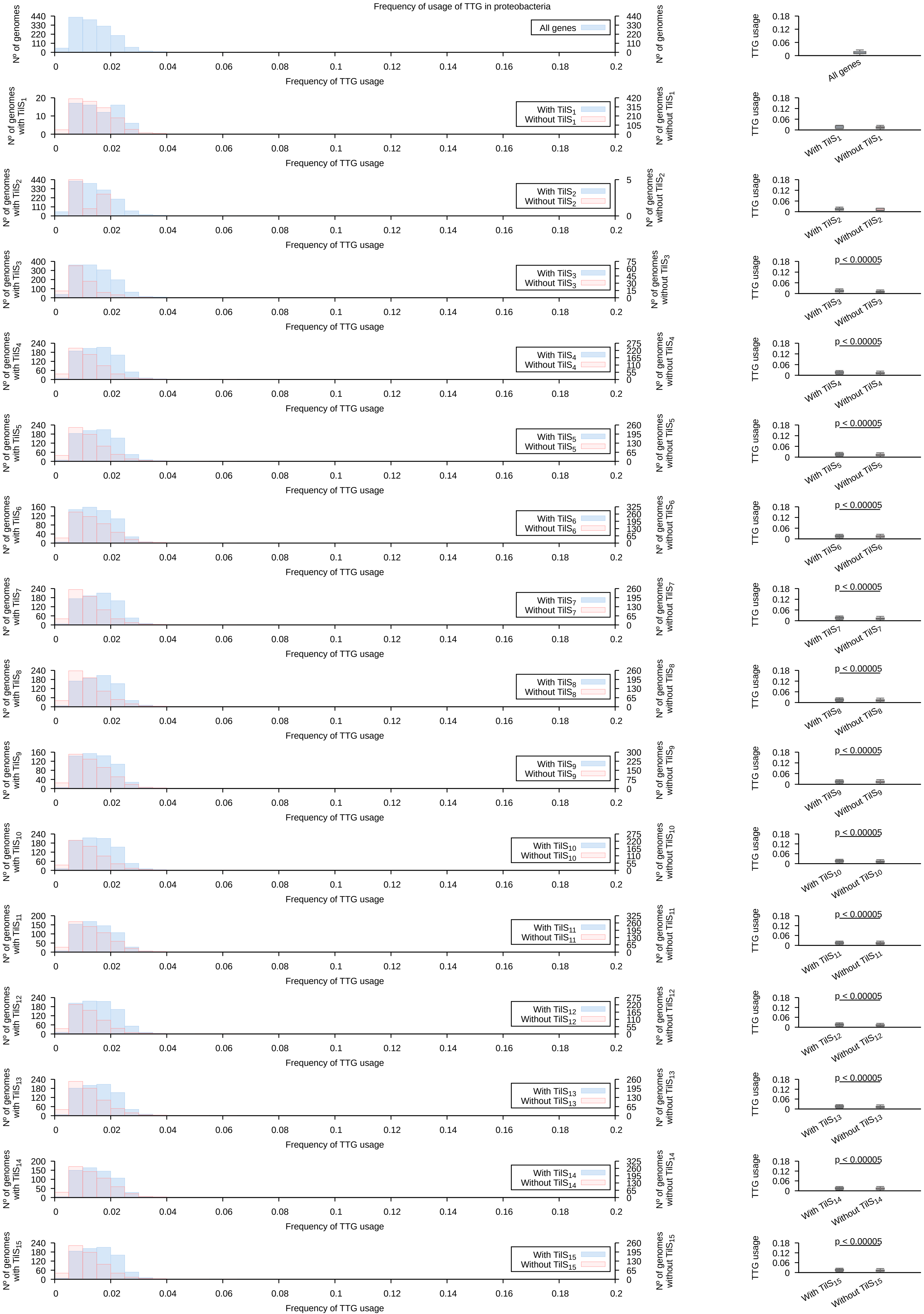

Frequency of usage of TTG in proteobacteria
Nº of genomes
All genes
Frequency of TTG usage
TTG usage
Nº of genomes with TilS1
Nº of genomes without TilS1
With TilS1
Without TilS1
Nº of genomes with TilS2
Nº of genomes without TilS2
With TilS2
Without TilS2
Nº of genomes with TilS3
Nº of genomes without TilS3
With TilS3
Without TilS3
Nº of genomes with TilS4
Nº of genomes without TilS4
With TilS4
Without TilS4
Nº of genomes with TilS5
Nº of genomes without TilS5
With TilS5
Without TilS5
Nº of genomes with TilS6
Nº of genomes without TilS6
With TilS6
Without TilS6
Nº of genomes with TilS7
Nº of genomes without TilS7
With TilS7
Without TilS7
Nº of genomes with TilS8
Nº of genomes without TilS8
With TilS8
Without TilS8
Nº of genomes with TilS9
Nº of genomes without TilS9
With TilS9
Without TilS9
Nº of genomes with TilS10
Nº of genomes without TilS10
With TilS10
Without TilS10
Nº of genomes with TilS11
Nº of genomes without TilS11
With TilS11
Without TilS11
Nº of genomes with TilS12
Nº of genomes without TilS12
With TilS12
Without TilS12
Nº of genomes with TilS13
Nº of genomes without TilS13
With TilS13
Without TilS13
Nº of genomes with TilS14
Nº of genomes without TilS14
With TilS14
Without TilS14
Nº of genomes with TilS15
Nº of genomes without TilS15
With TilS15
Without TilS15
p < 0.00005

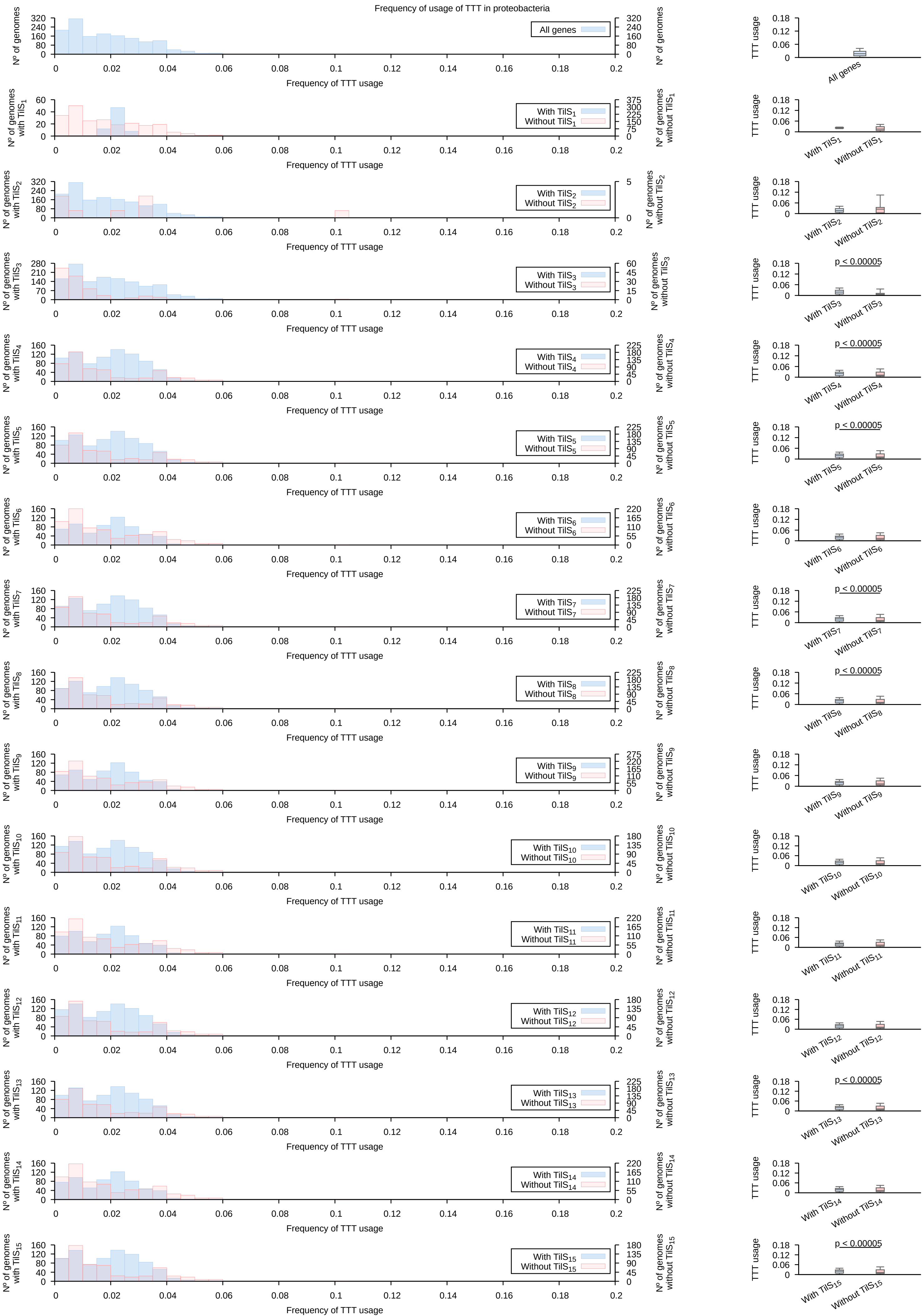
Frequency of usage of TTT in proteobacteria
