# Supplementary material for: Impact of the chemical modification of tRNAs anticodon loop on the variability and evolution of codon usage in proteobacteria
Source: Front Microbiol. 2024 Aug 5;15:1412318. doi: 10.3389/fmicb.2024.1412318 (PMC11332805; doi:10.3389/fmicb.2024.1412318)
Supplement: Supplementary file 1 [file Data_Sheet_1.zip › Supp_figures/Fig_S11.pdf]

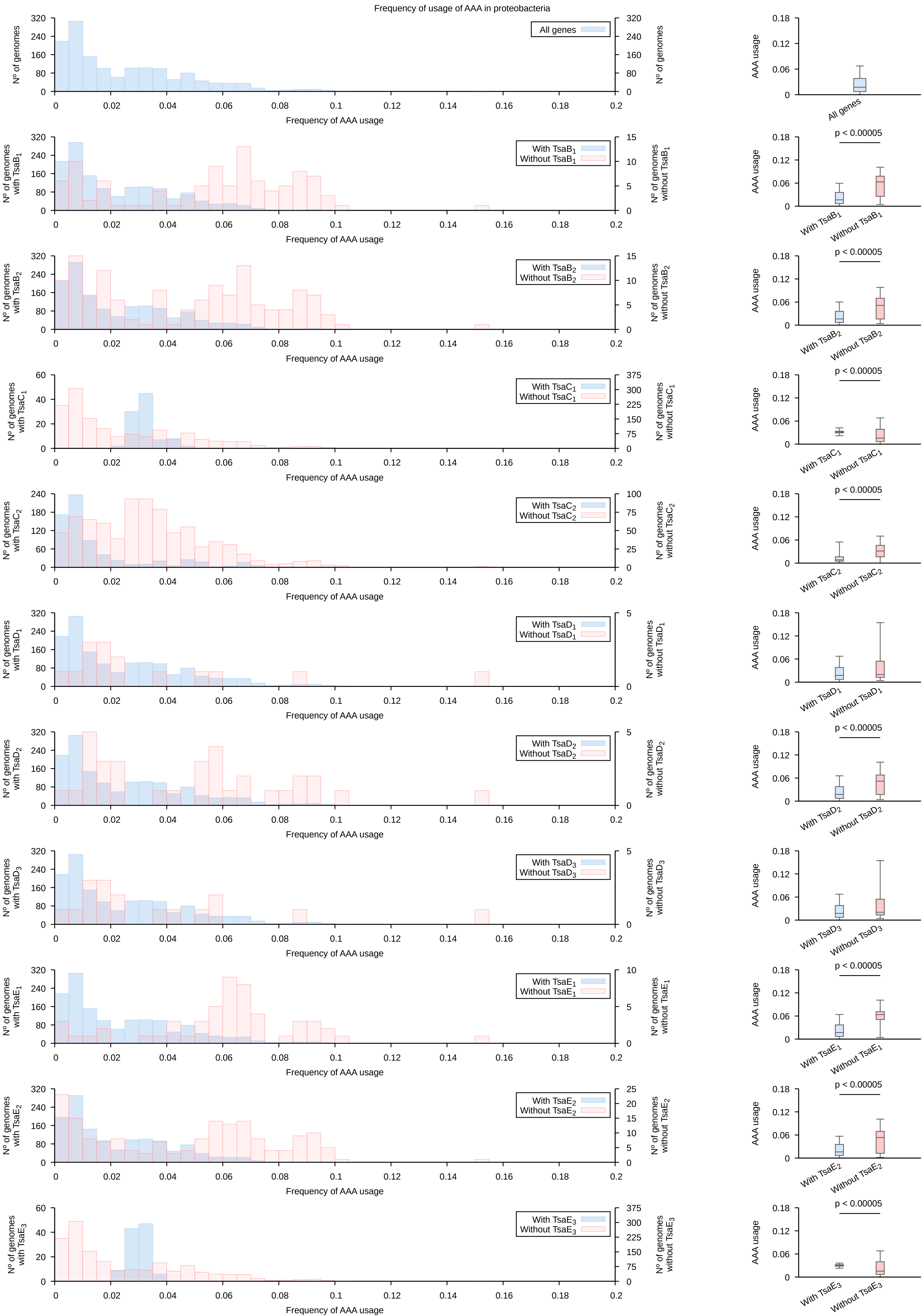

Frequency of usage of AAA in proteobacteria
Nº of genomes
All genes
Frequency of AAA usage
AAA usage
Nº of genomes with TsaB1
Nº of genomes without TsaB1
With TsaB1
Without TsaB1
p < 0.00005
Nº of genomes with TsaB2
Nº of genomes without TsaB2
With TsaB2
Without TsaB2
Nº of genomes with TsaC1
Nº of genomes without TsaC1
With TsaC1
Without TsaC1
Nº of genomes with TsaC2
Nº of genomes without TsaC2
With TsaC2
Without TsaC2
Nº of genomes with TsaD1
Nº of genomes without TsaD1
With TsaD1
Without TsaD1
Nº of genomes with TsaD2
Nº of genomes without TsaD2
With TsaD2
Without TsaD2
Nº of genomes with TsaD3
Nº of genomes without TsaD3
With TsaD3
Without TsaD3
Nº of genomes with TsaE1
Nº of genomes without TsaE1
With TsaE1
Without TsaE1
Nº of genomes with TsaE2
Nº of genomes without TsaE2
With TsaE2
Without TsaE2
Nº of genomes with TsaE3
Nº of genomes without TsaE3
With TsaE3
Without TsaE3

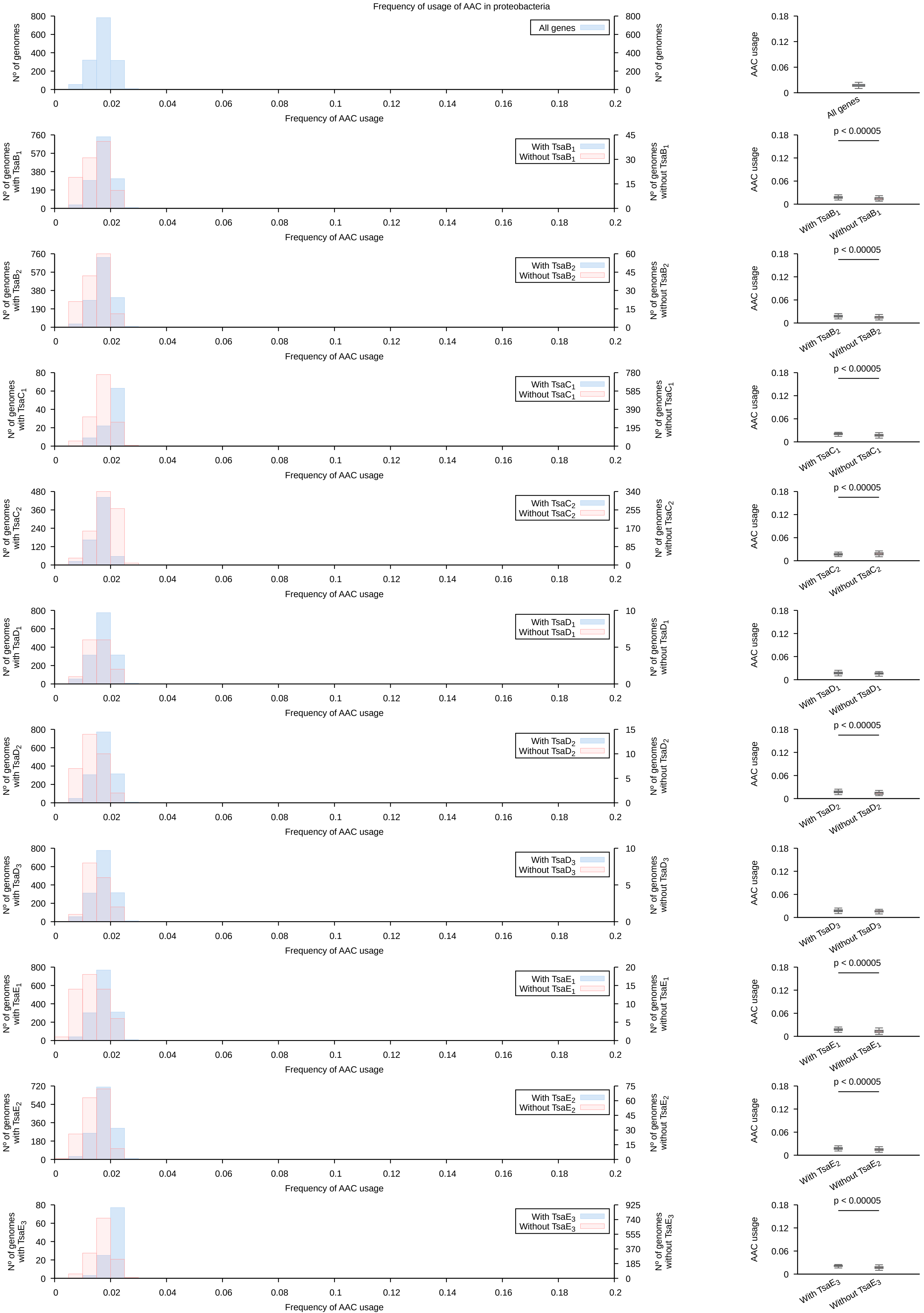

Frequency of usage of AAC in proteobacteria
Nº of genomes
Frequency of AAC usage
AAC usage
All genes
p < 0.00005
With TsaB$_1$
Without TsaB$_1$
Nº of genomes with TsaB$_1$
Nº of genomes without TsaB$_1$
With TsaB$_2$
Without TsaB$_2$
Nº of genomes with TsaB$_2$
Nº of genomes without TsaB$_2$
With TsaC$_1$
Without TsaC$_1$
Nº of genomes with TsaC$_1$
Nº of genomes without TsaC$_1$
With TsaC$_2$
Without TsaC$_2$
Nº of genomes with TsaC$_2$
Nº of genomes without TsaC$_2$
With TsaD$_1$
Without TsaD$_1$
Nº of genomes with TsaD$_1$
Nº of genomes without TsaD$_1$
With TsaD$_2$
Without TsaD$_2$
Nº of genomes with TsaD$_2$
Nº of genomes without TsaD$_2$
With TsaD$_3$
Without TsaD$_3$
Nº of genomes with TsaD$_3$
Nº of genomes without TsaD$_3$
With TsaE$_1$
Without TsaE$_1$
Nº of genomes with TsaE$_1$
Nº of genomes without TsaE$_1$
With TsaE$_2$
Without TsaE$_2$
Nº of genomes with TsaE$_2$
Nº of genomes without TsaE$_2$
With TsaE$_3$
Without TsaE$_3$
Nº of genomes with TsaE$_3$
Nº of genomes without TsaE$_3$

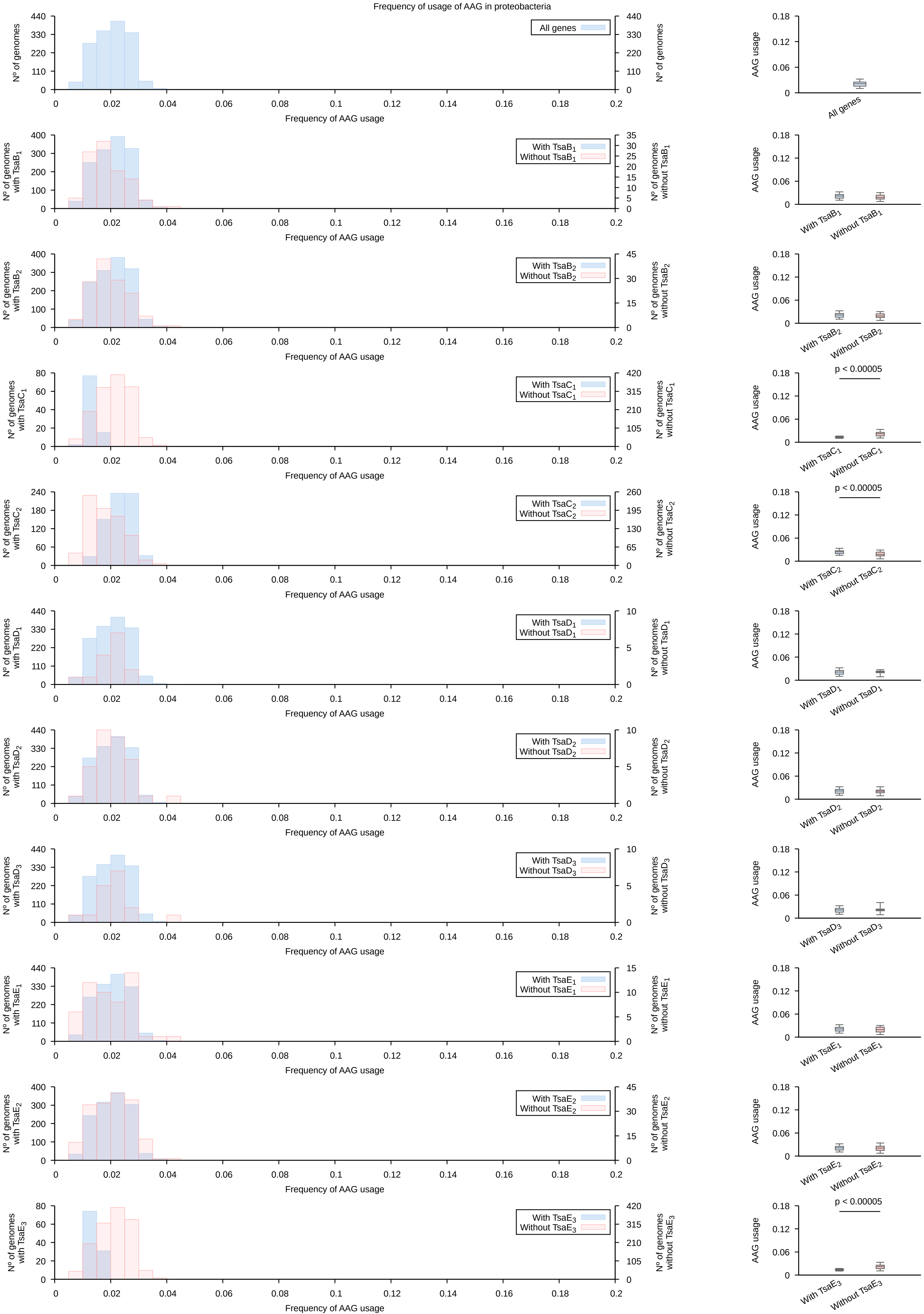

Frequency of usage of AAG in proteobacteria
Nº of genomes
All genes
Frequency of AAG usage
AAG usage
p < 0.00005
With TsaB$_1$
Without TsaB$_1$
Nº of genomes with TsaB$_1$
Nº of genomes without TsaB$_1$
With TsaB$_2$
Without TsaB$_2$
Nº of genomes with TsaB$_2$
Nº of genomes without TsaB$_2$
With TsaC$_1$
Without TsaC$_1$
Nº of genomes with TsaC$_1$
Nº of genomes without TsaC$_1$
With TsaC$_2$
Without TsaC$_2$
Nº of genomes with TsaC$_2$
Nº of genomes without TsaC$_2$
With TsaD$_1$
Without TsaD$_1$
Nº of genomes with TsaD$_1$
Nº of genomes without TsaD$_1$
With TsaD$_2$
Without TsaD$_2$
Nº of genomes with TsaD$_2$
Nº of genomes without TsaD$_2$
With TsaD$_3$
Without TsaD$_3$
Nº of genomes with TsaD$_3$
Nº of genomes without TsaD$_3$
With TsaE$_1$
Without TsaE$_1$
Nº of genomes with TsaE$_1$
Nº of genomes without TsaE$_1$
With TsaE$_2$
Without TsaE$_2$
Nº of genomes with TsaE$_2$
Nº of genomes without TsaE$_2$
With TsaE$_3$
Without TsaE$_3$
Nº of genomes with TsaE$_3$
Nº of genomes without TsaE$_3$

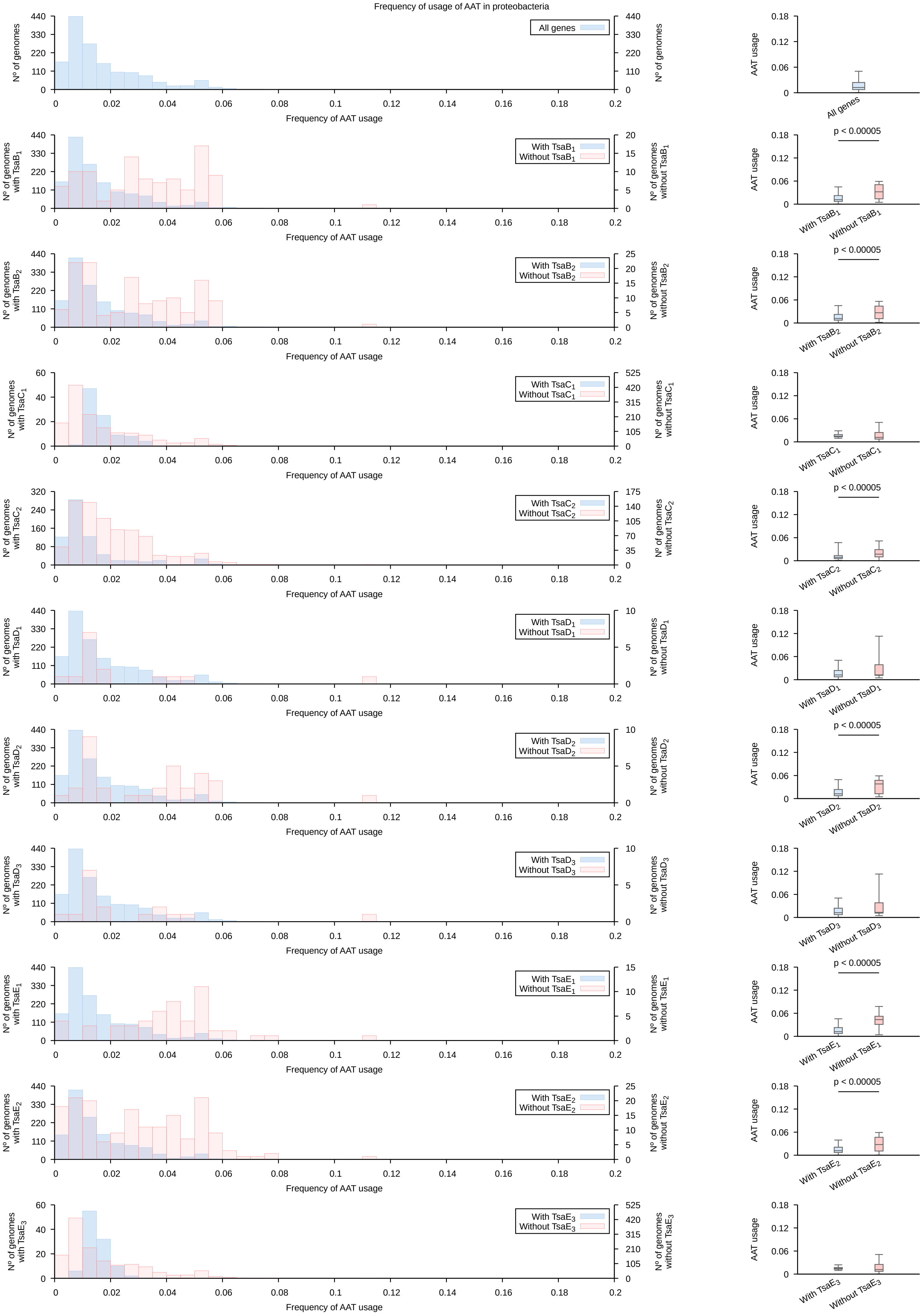
Frequency of usage of AAT in proteobacteria
Nº of genomes
All genes
Frequency of AAT usage
AAT usage
Nº of genomes with TsaB1
Nº of genomes without TsaB1
With TsaB1
Without TsaB1
p < 0.00005
Nº of genomes with TsaB2
Nº of genomes without TsaB2
With TsaB2
Without TsaB2
Nº of genomes with TsaC1
Nº of genomes without TsaC1
With TsaC1
Without TsaC1
Nº of genomes with TsaC2
Nº of genomes without TsaC2
With TsaC2
Without TsaC2
Nº of genomes with TsaD1
Nº of genomes without TsaD1
With TsaD1
Without TsaD1
Nº of genomes with TsaD2
Nº of genomes without TsaD2
With TsaD2
Without TsaD2
Nº of genomes with TsaD3
Nº of genomes without TsaD3
With TsaD3
Without TsaD3
Nº of genomes with TsaE1
Nº of genomes without TsaE1
With TsaE1
Without TsaE1
Nº of genomes with TsaE2
Nº of genomes without TsaE2
With TsaE2
Without TsaE2
Nº of genomes with TsaE3
Nº of genomes without TsaE3
With TsaE3
Without TsaE3

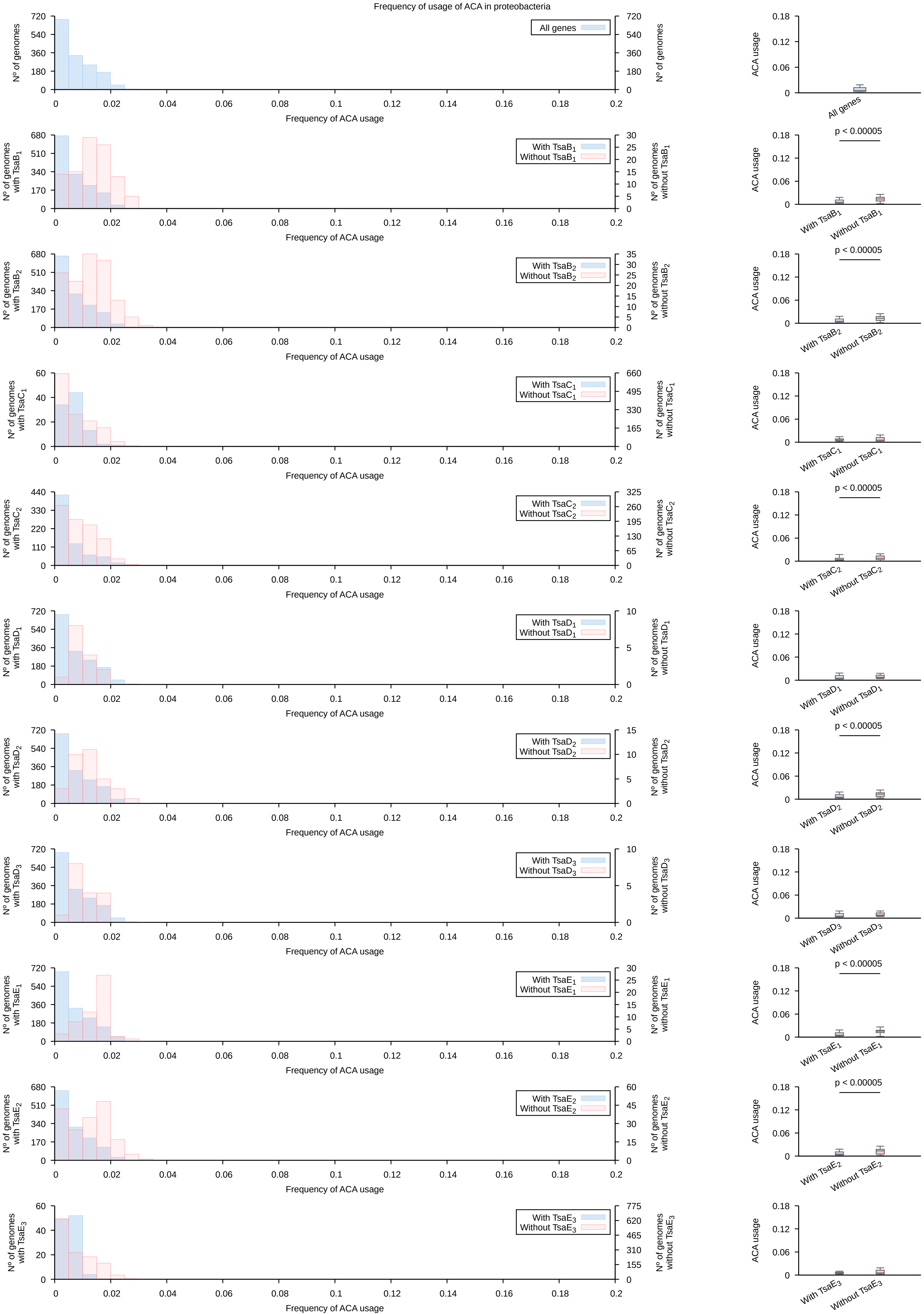

Frequency of usage of ACA in proteobacteria
All genes
Nº of genomes
Frequency of ACA usage
ACA usage
With TsaB$_1$
Without TsaB$_1$
Nº of genomes with TsaB$_1$
Nº of genomes without TsaB$_1$
p < 0.00005
With TsaB$_2$
Without TsaB$_2$
Nº of genomes with TsaB$_2$
Nº of genomes without TsaB$_2$
p < 0.00005
With TsaC$_1$
Without TsaC$_1$
Nº of genomes with TsaC$_1$
Nº of genomes without TsaC$_1$
With TsaC$_2$
Without TsaC$_2$
Nº of genomes with TsaC$_2$
Nº of genomes without TsaC$_2$
p < 0.00005
With TsaD$_1$
Without TsaD$_1$
Nº of genomes with TsaD$_1$
Nº of genomes without TsaD$_1$
With TsaD$_2$
Without TsaD$_2$
Nº of genomes with TsaD$_2$
Nº of genomes without TsaD$_2$
p < 0.00005
With TsaD$_3$
Without TsaD$_3$
Nº of genomes with TsaD$_3$
Nº of genomes without TsaD$_3$
With TsaE$_1$
Without TsaE$_1$
Nº of genomes with TsaE$_1$
Nº of genomes without TsaE$_1$
p < 0.00005
With TsaE$_2$
Without TsaE$_2$
Nº of genomes with TsaE$_2$
Nº of genomes without TsaE$_2$
p < 0.00005
With TsaE$_3$
Without TsaE$_3$
Nº of genomes with TsaE$_3$
Nº of genomes without TsaE$_3$

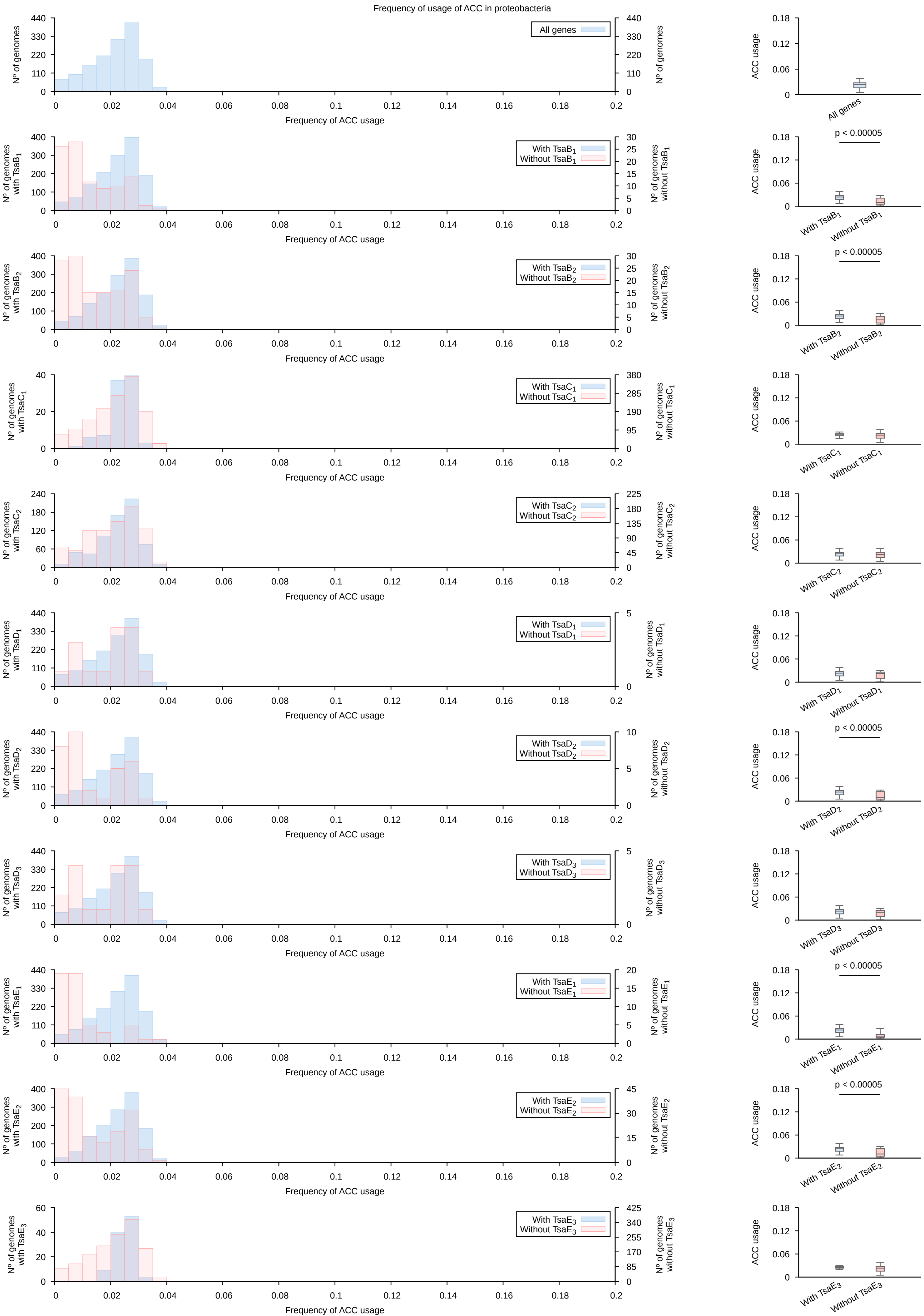
Frequency of usage of ACC in proteobacteria
All genes
Nº of genomes
Frequency of ACC usage
ACC usage
0
0.02
0.04
0.06
0.08
0.1
0.12
0.14
0.16
0.18
0.2
With TsaB1
Without TsaB1
Nº of genomes with TsaB1
Nº of genomes without TsaB1
p < 0.00005
With TsaB2
Without TsaB2
Nº of genomes with TsaB2
Nº of genomes without TsaB2
p < 0.00005
With TsaC1
Without TsaC1
Nº of genomes with TsaC1
Nº of genomes without TsaC1
With TsaC2
Without TsaC2
Nº of genomes with TsaC2
Nº of genomes without TsaC2
With TsaD1
Without TsaD1
Nº of genomes with TsaD1
Nº of genomes without TsaD1
With TsaD2
Without TsaD2
Nº of genomes with TsaD2
Nº of genomes without TsaD2
p < 0.00005
With TsaD3
Without TsaD3
Nº of genomes with TsaD3
Nº of genomes without TsaD3
With TsaE1
Without TsaE1
Nº of genomes with TsaE1
Nº of genomes without TsaE1
p < 0.00005
With TsaE2
Without TsaE2
Nº of genomes with TsaE2
Nº of genomes without TsaE2
p < 0.00005
With TsaE3
Without TsaE3
Nº of genomes with TsaE3
Nº of genomes without TsaE3

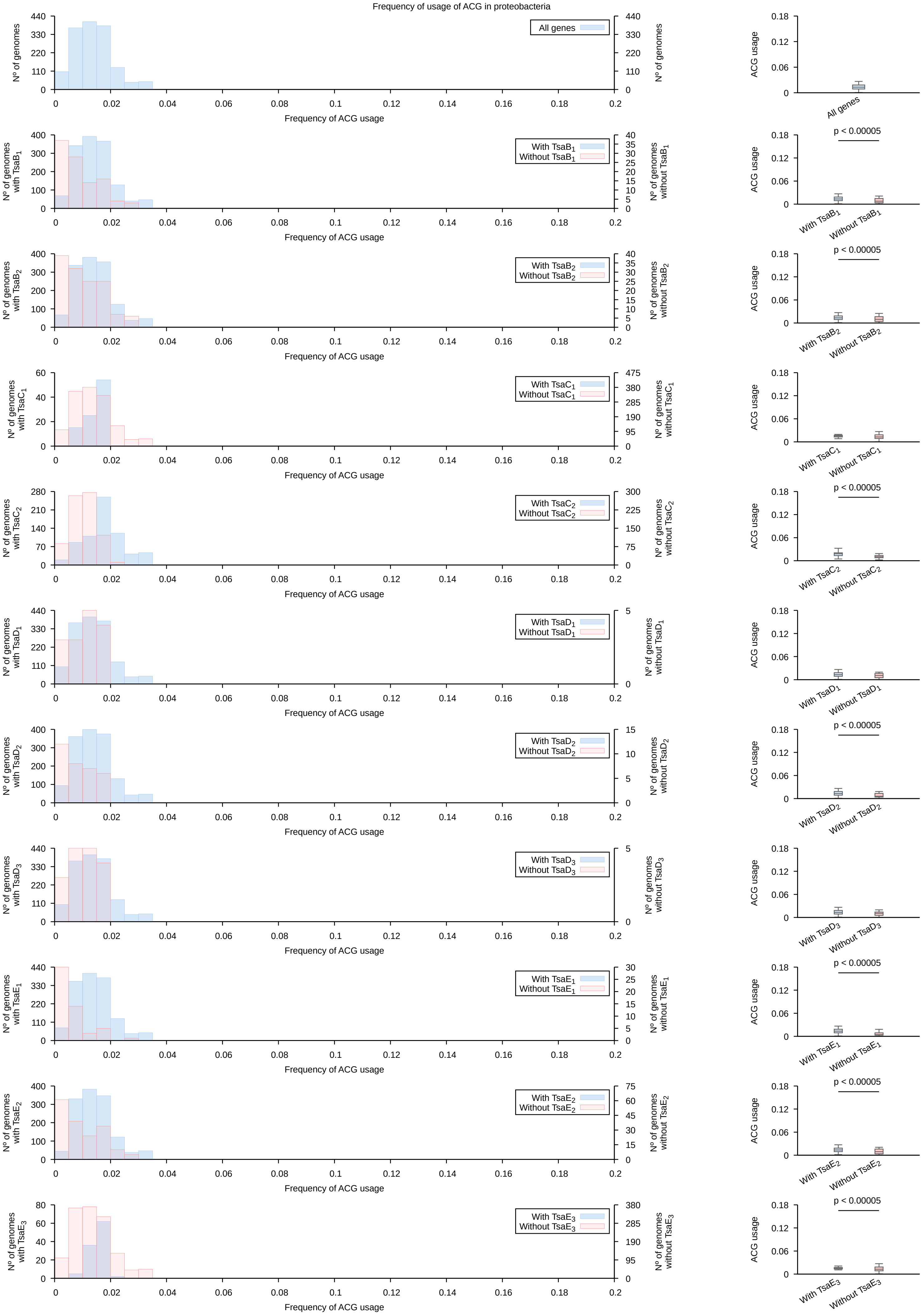

Frequency of usage of ACG in proteobacteria
All genes
Nº of genomes
Frequency of ACG usage
ACG usage
With TsaB$_1$
Without TsaB$_1$
Nº of genomes with TsaB$_1$
Nº of genomes without TsaB$_1$
p < 0.00005
With TsaB$_2$
Without TsaB$_2$
Nº of genomes with TsaB$_2$
Nº of genomes without TsaB$_2$
p < 0.00005
With TsaC$_1$
Without TsaC$_1$
Nº of genomes with TsaC$_1$
Nº of genomes without TsaC$_1$
With TsaC$_2$
Without TsaC$_2$
Nº of genomes with TsaC$_2$
Nº of genomes without TsaC$_2$
p < 0.00005
With TsaD$_1$
Without TsaD$_1$
Nº of genomes with TsaD$_1$
Nº of genomes without TsaD$_1$
With TsaD$_2$
Without TsaD$_2$
Nº of genomes with TsaD$_2$
Nº of genomes without TsaD$_2$
p < 0.00005
With TsaD$_3$
Without TsaD$_3$
Nº of genomes with TsaD$_3$
Nº of genomes without TsaD$_3$
With TsaE$_1$
Without TsaE$_1$
Nº of genomes with TsaE$_1$
Nº of genomes without TsaE$_1$
p < 0.00005
With TsaE$_2$
Without TsaE$_2$
Nº of genomes with TsaE$_2$
Nº of genomes without TsaE$_2$
p < 0.00005
With TsaE$_3$
Without TsaE$_3$
Nº of genomes with TsaE$_3$
Nº of genomes without TsaE$_3$
p < 0.00005

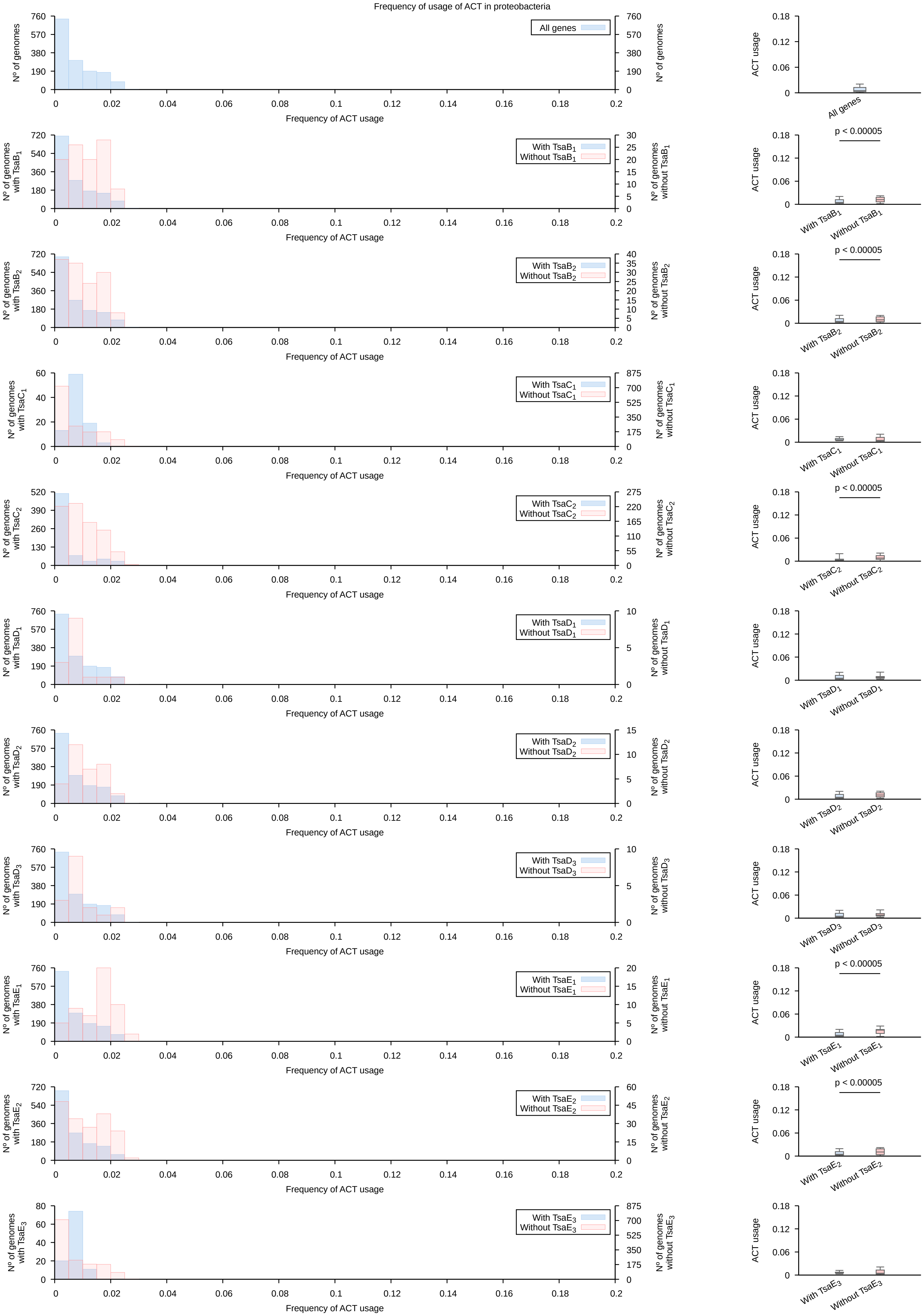

Frequency of usage of ACT in proteobacteria
All genes
Nº of genomes
Frequency of ACT usage
ACT usage
With TsaB1
Without TsaB1
Nº of genomes with TsaB1
Nº of genomes without TsaB1
p < 0.00005
With TsaB2
Without TsaB2
Nº of genomes with TsaB2
Nº of genomes without TsaB2
p < 0.00005
With TsaC1
Without TsaC1
Nº of genomes with TsaC1
Nº of genomes without TsaC1
With TsaC2
Without TsaC2
Nº of genomes with TsaC2
Nº of genomes without TsaC2
p < 0.00005
With TsaD1
Without TsaD1
Nº of genomes with TsaD1
Nº of genomes without TsaD1
With TsaD2
Without TsaD2
Nº of genomes with TsaD2
Nº of genomes without TsaD2
With TsaD3
Without TsaD3
Nº of genomes with TsaD3
Nº of genomes without TsaD3
With TsaE1
Without TsaE1
Nº of genomes with TsaE1
Nº of genomes without TsaE1
p < 0.00005
With TsaE2
Without TsaE2
Nº of genomes with TsaE2
Nº of genomes without TsaE2
p < 0.00005
With TsaE3
Without TsaE3
Nº of genomes with TsaE3
Nº of genomes without TsaE3
0 0.02 0.04 0.06 0.08 0.1 0.12 0.14 0.16 0.18 0.2
0 0.06 0.12 0.18

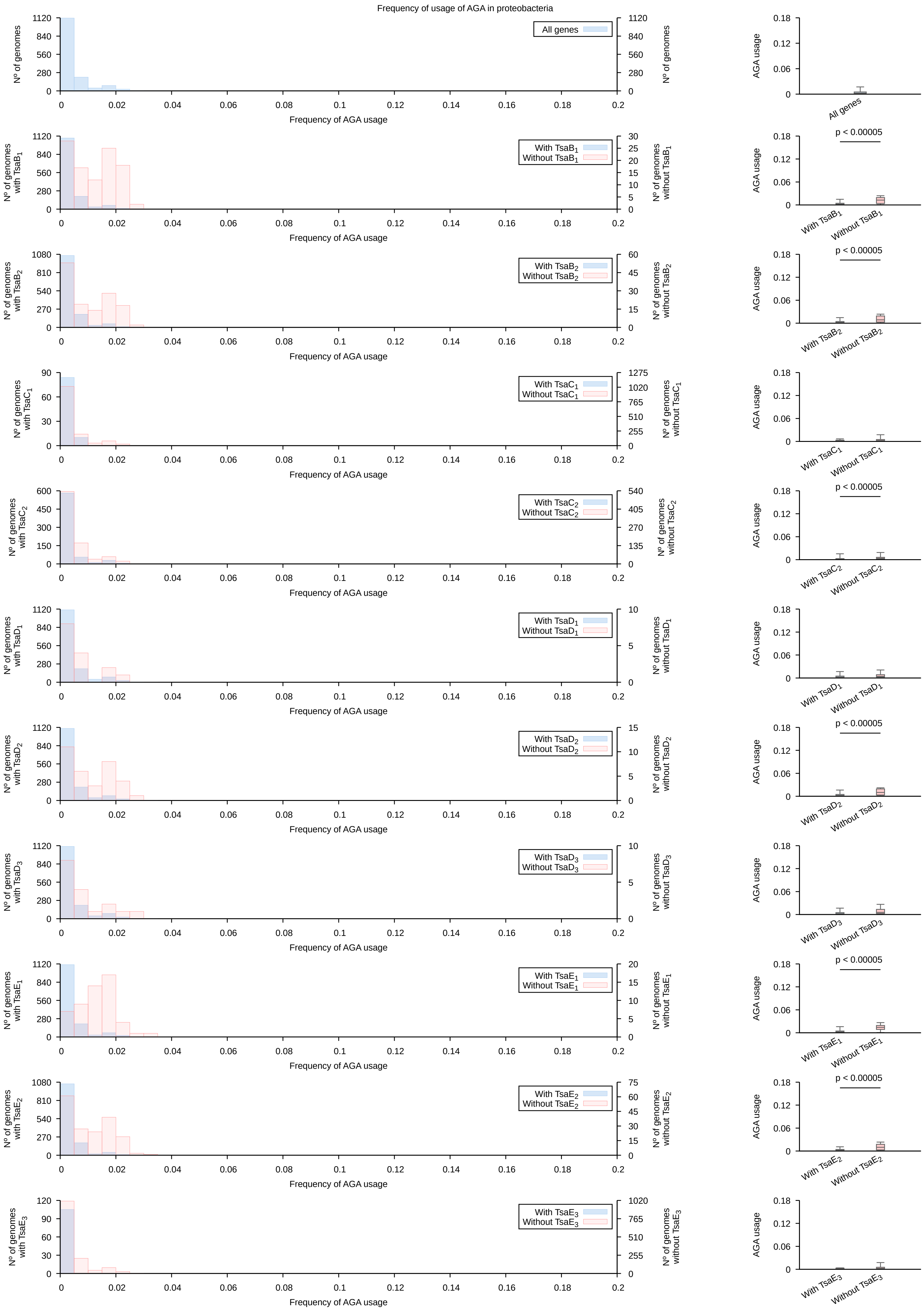
Frequency of usage of AGA in proteobacteria
Nº of genomes
All genes
Frequency of AGA usage
AGA usage
Nº of genomes with $TsaB_1$
Nº of genomes without $TsaB_1$
With $TsaB_1$
Without $TsaB_1$
$p < 0.00005$
Nº of genomes with $TsaB_2$
Nº of genomes without $TsaB_2$
With $TsaB_2$
Without $TsaB_2$
$p < 0.00005$
Nº of genomes with $TsaC_1$
Nº of genomes without $TsaC_1$
With $TsaC_1$
Without $TsaC_1$
Nº of genomes with $TsaC_2$
Nº of genomes without $TsaC_2$
With $TsaC_2$
Without $TsaC_2$
$p < 0.00005$
Nº of genomes with $TsaD_1$
Nº of genomes without $TsaD_1$
With $TsaD_1$
Without $TsaD_1$
Nº of genomes with $TsaD_2$
Nº of genomes without $TsaD_2$
With $TsaD_2$
Without $TsaD_2$
$p < 0.00005$
Nº of genomes with $TsaD_3$
Nº of genomes without $TsaD_3$
With $TsaD_3$
Without $TsaD_3$
Nº of genomes with $TsaE_1$
Nº of genomes without $TsaE_1$
With $TsaE_1$
Without $TsaE_1$
$p < 0.00005$
Nº of genomes with $TsaE_2$
Nº of genomes without $TsaE_2$
With $TsaE_2$
Without $TsaE_2$
$p < 0.00005$
Nº of genomes with $TsaE_3$
Nº of genomes without $TsaE_3$
With $TsaE_3$
Without $TsaE_3$

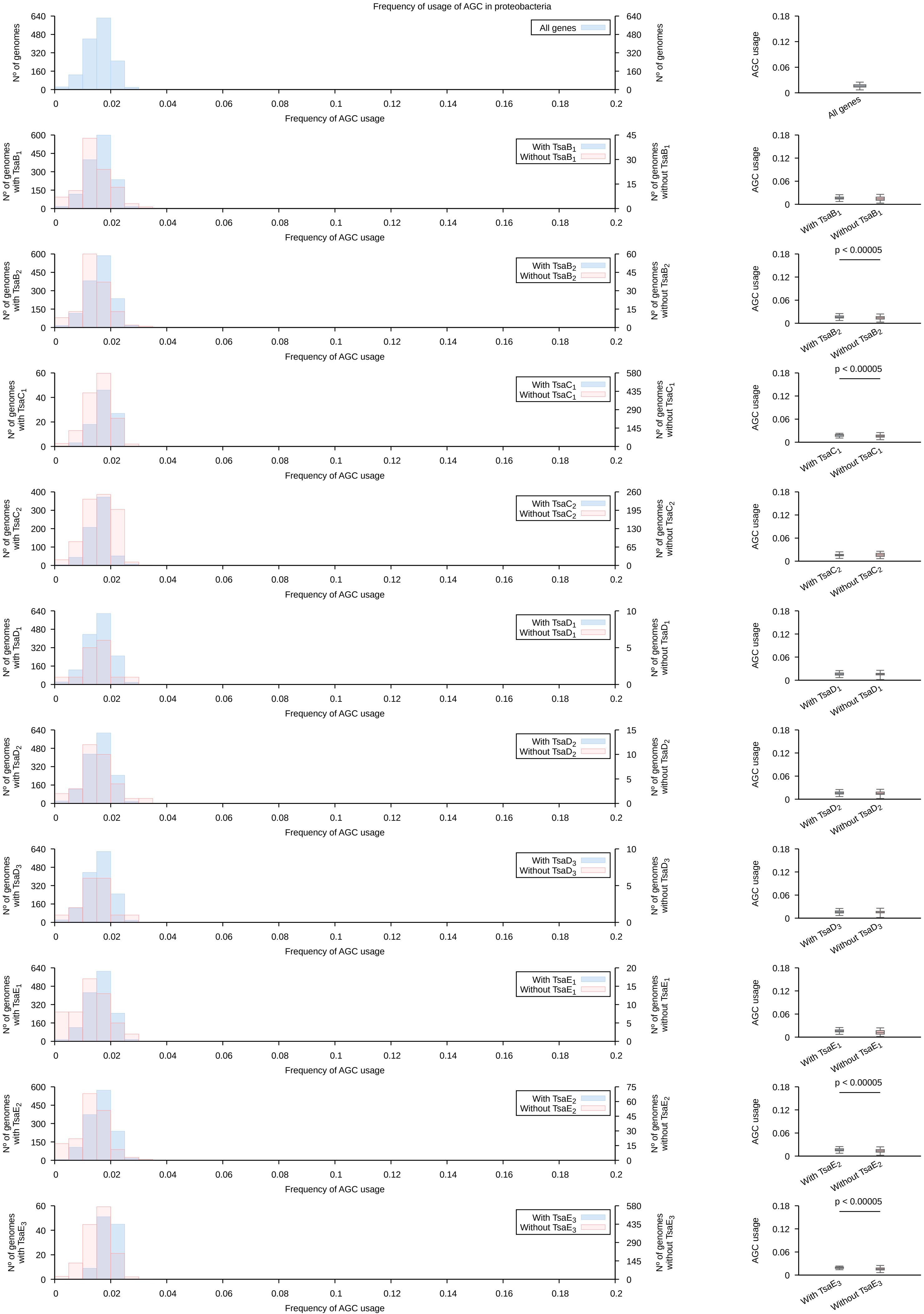

Frequency of usage of AGC in proteobacteria
All genes
Nº of genomes
Frequency of AGC usage
AGC usage
With TsaB$_1$
Without TsaB$_1$
Nº of genomes with TsaB$_1$
Nº of genomes without TsaB$_1$
With TsaB$_2$
Without TsaB$_2$
Nº of genomes with TsaB$_2$
Nº of genomes without TsaB$_2$
p < 0.00005
With TsaC$_1$
Without TsaC$_1$
Nº of genomes with TsaC$_1$
Nº of genomes without TsaC$_1$
p < 0.00005
With TsaC$_2$
Without TsaC$_2$
Nº of genomes with TsaC$_2$
Nº of genomes without TsaC$_2$
With TsaD$_1$
Without TsaD$_1$
Nº of genomes with TsaD$_1$
Nº of genomes without TsaD$_1$
With TsaD$_2$
Without TsaD$_2$
Nº of genomes with TsaD$_2$
Nº of genomes without TsaD$_2$
With TsaD$_3$
Without TsaD$_3$
Nº of genomes with TsaD$_3$
Nº of genomes without TsaD$_3$
With TsaE$_1$
Without TsaE$_1$
Nº of genomes with TsaE$_1$
Nº of genomes without TsaE$_1$
With TsaE$_2$
Without TsaE$_2$
Nº of genomes with TsaE$_2$
Nº of genomes without TsaE$_2$
p < 0.00005
With TsaE$_3$
Without TsaE$_3$
Nº of genomes with TsaE$_3$
Nº of genomes without TsaE$_3$
p < 0.00005

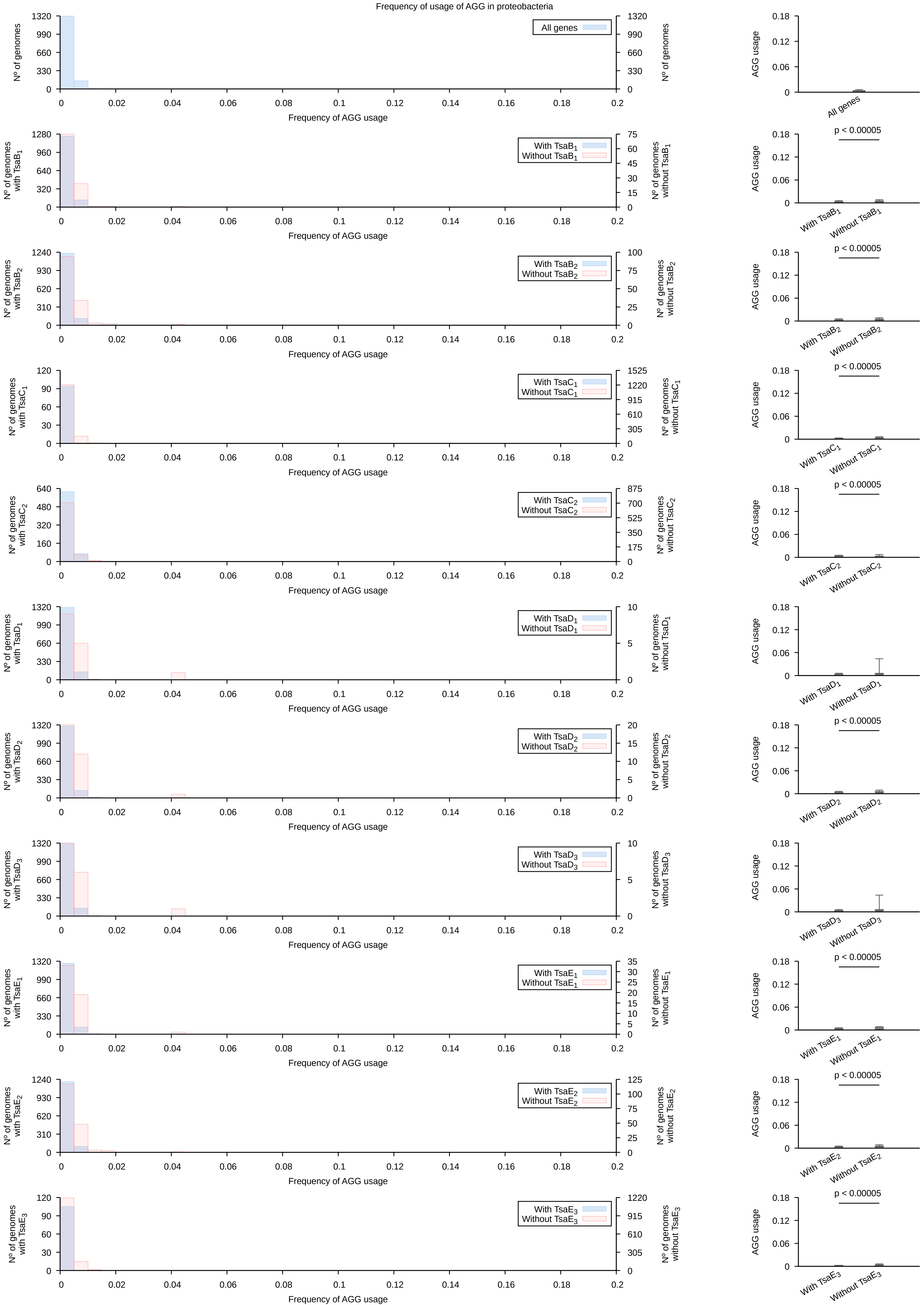
Frequency of usage of AGG in proteobacteria
All genes
Nº of genomes
Frequency of AGG usage
AGG usage
With TsaB$_1$
Without TsaB$_1$
Nº of genomes with TsaB$_1$
Nº of genomes without TsaB$_1$
With TsaB$_2$
Without TsaB$_2$
Nº of genomes with TsaB$_2$
Nº of genomes without TsaB$_2$
With TsaC$_1$
Without TsaC$_1$
Nº of genomes with TsaC$_1$
Nº of genomes without TsaC$_1$
With TsaC$_2$
Without TsaC$_2$
Nº of genomes with TsaC$_2$
Nº of genomes without TsaC$_2$
With TsaD$_1$
Without TsaD$_1$
Nº of genomes with TsaD$_1$
Nº of genomes without TsaD$_1$
With TsaD$_2$
Without TsaD$_2$
Nº of genomes with TsaD$_2$
Nº of genomes without TsaD$_2$
With TsaD$_3$
Without TsaD$_3$
Nº of genomes with TsaD$_3$
Nº of genomes without TsaD$_3$
With TsaE$_1$
Without TsaE$_1$
Nº of genomes with TsaE$_1$
Nº of genomes without TsaE$_1$
With TsaE$_2$
Without TsaE$_2$
Nº of genomes with TsaE$_2$
Nº of genomes without TsaE$_2$
With TsaE$_3$
Without TsaE$_3$
Nº of genomes with TsaE$_3$
Nº of genomes without TsaE$_3$
p < 0.00005

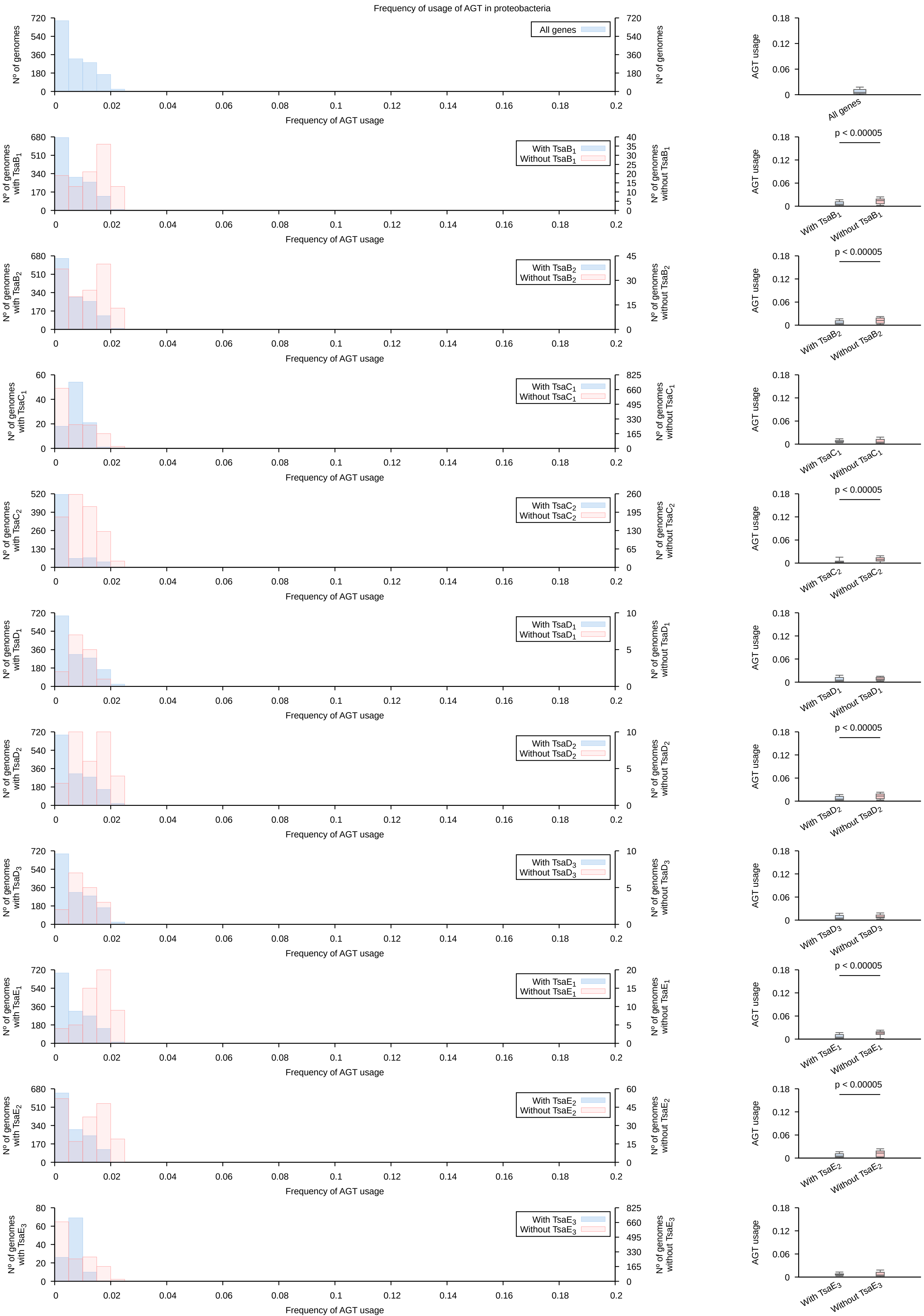

Frequency of usage of AGT in proteobacteria
All genes
Nº of genomes
AGT usage
Frequency of AGT usage
With TsaB$_1$
Without TsaB$_1$
Nº of genomes with TsaB$_1$
Nº of genomes without TsaB$_1$
p < 0.00005
With TsaB$_2$
Without TsaB$_2$
Nº of genomes with TsaB$_2$
Nº of genomes without TsaB$_2$
p < 0.00005
With TsaC$_1$
Without TsaC$_1$
Nº of genomes with TsaC$_1$
Nº of genomes without TsaC$_1$
With TsaC$_2$
Without TsaC$_2$
Nº of genomes with TsaC$_2$
Nº of genomes without TsaC$_2$
p < 0.00005
With TsaD$_1$
Without TsaD$_1$
Nº of genomes with TsaD$_1$
Nº of genomes without TsaD$_1$
With TsaD$_2$
Without TsaD$_2$
Nº of genomes with TsaD$_2$
Nº of genomes without TsaD$_2$
p < 0.00005
With TsaD$_3$
Without TsaD$_3$
Nº of genomes with TsaD$_3$
Nº of genomes without TsaD$_3$
With TsaE$_1$
Without TsaE$_1$
Nº of genomes with TsaE$_1$
Nº of genomes without TsaE$_1$
p < 0.00005
With TsaE$_2$
Without TsaE$_2$
Nº of genomes with TsaE$_2$
Nº of genomes without TsaE$_2$
p < 0.00005
With TsaE$_3$
Without TsaE$_3$
Nº of genomes with TsaE$_3$
Nº of genomes without TsaE$_3$

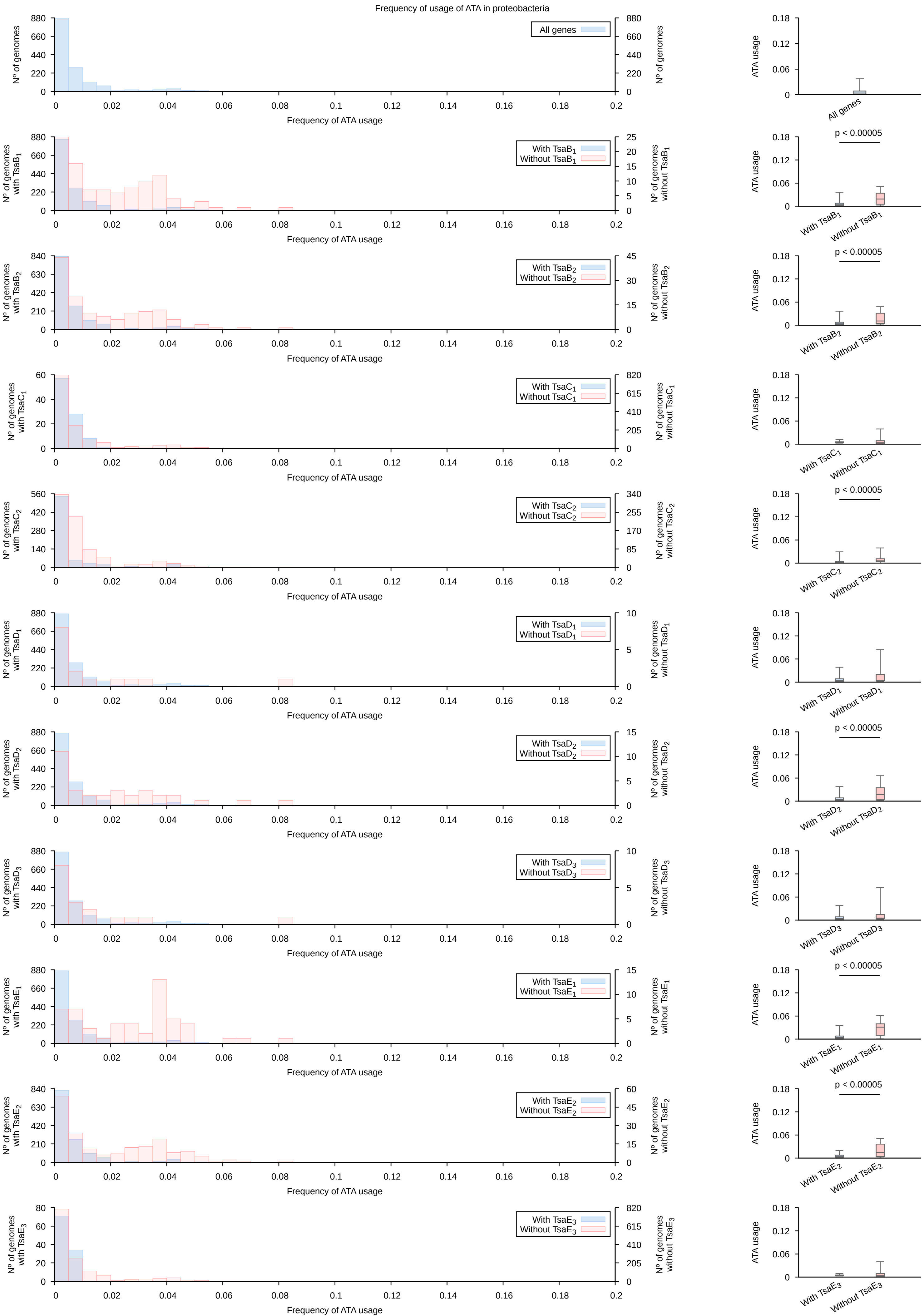
Frequency of usage of ATA in proteobacteria
Nº of genomes
All genes
Frequency of ATA usage
ATA usage
0
0.02
0.04
0.06
0.08
0.1
0.12
0.14
0.16
0.18
0.2
Nº of genomes with TsaB$_1$
Nº of genomes without TsaB$_1$
With TsaB$_1$
Without TsaB$_1$
p < 0.00005
Nº of genomes with TsaB$_2$
Nº of genomes without TsaB$_2$
With TsaB$_2$
Without TsaB$_2$
p < 0.00005
Nº of genomes with TsaC$_1$
Nº of genomes without TsaC$_1$
With TsaC$_1$
Without TsaC$_1$
Nº of genomes with TsaC$_2$
Nº of genomes without TsaC$_2$
With TsaC$_2$
Without TsaC$_2$
p < 0.00005
Nº of genomes with TsaD$_1$
Nº of genomes without TsaD$_1$
With TsaD$_1$
Without TsaD$_1$
Nº of genomes with TsaD$_2$
Nº of genomes without TsaD$_2$
With TsaD$_2$
Without TsaD$_2$
p < 0.00005
Nº of genomes with TsaD$_3$
Nº of genomes without TsaD$_3$
With TsaD$_3$
Without TsaD$_3$
Nº of genomes with TsaE$_1$
Nº of genomes without TsaE$_1$
With TsaE$_1$
Without TsaE$_1$
p < 0.00005
Nº of genomes with TsaE$_2$
Nº of genomes without TsaE$_2$
With TsaE$_2$
Without TsaE$_2$
p < 0.00005
Nº of genomes with TsaE$_3$
Nº of genomes without TsaE$_3$
With TsaE$_3$
Without TsaE$_3$

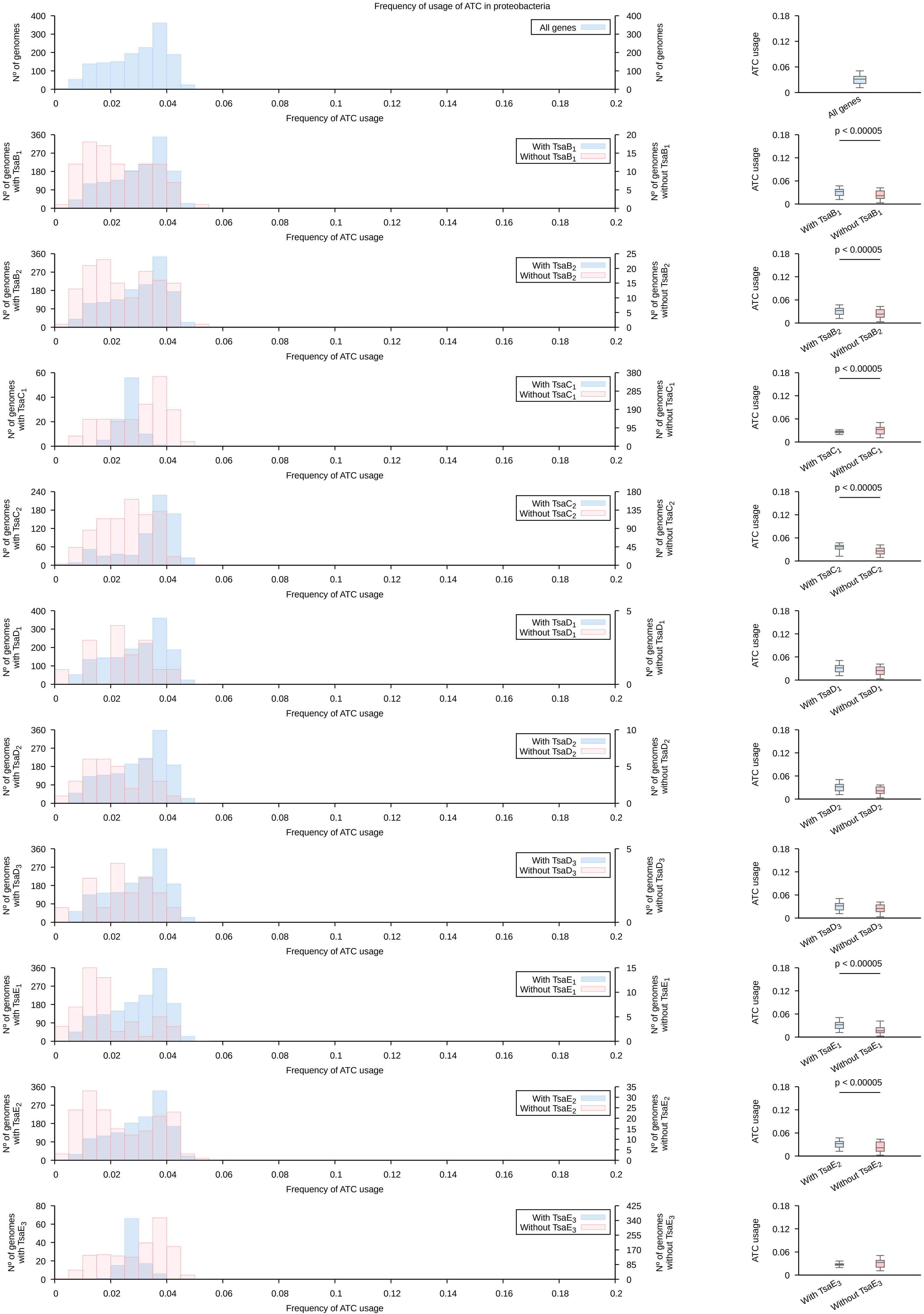

Frequency of usage of ATC in proteobacteria
Nº of genomes
All genes
Frequency of ATC usage
ATC usage
All genes
Nº of genomes with TsaB$_1$
Nº of genomes without TsaB$_1$
With TsaB$_1$
Without TsaB$_1$
p < 0.00005
Nº of genomes with TsaB$_2$
Nº of genomes without TsaB$_2$
With TsaB$_2$
Without TsaB$_2$
p < 0.00005
Nº of genomes with TsaC$_1$
Nº of genomes without TsaC$_1$
With TsaC$_1$
Without TsaC$_1$
p < 0.00005
Nº of genomes with TsaC$_2$
Nº of genomes without TsaC$_2$
With TsaC$_2$
Without TsaC$_2$
p < 0.00005
Nº of genomes with TsaD$_1$
Nº of genomes without TsaD$_1$
With TsaD$_1$
Without TsaD$_1$
Nº of genomes with TsaD$_2$
Nº of genomes without TsaD$_2$
With TsaD$_2$
Without TsaD$_2$
Nº of genomes with TsaD$_3$
Nº of genomes without TsaD$_3$
With TsaD$_3$
Without TsaD$_3$
Nº of genomes with TsaE$_1$
Nº of genomes without TsaE$_1$
With TsaE$_1$
Without TsaE$_1$
p < 0.00005
Nº of genomes with TsaE$_2$
Nº of genomes without TsaE$_2$
With TsaE$_2$
Without TsaE$_2$
p < 0.00005
Nº of genomes with TsaE$_3$
Nº of genomes without TsaE$_3$
With TsaE$_3$
Without TsaE$_3$

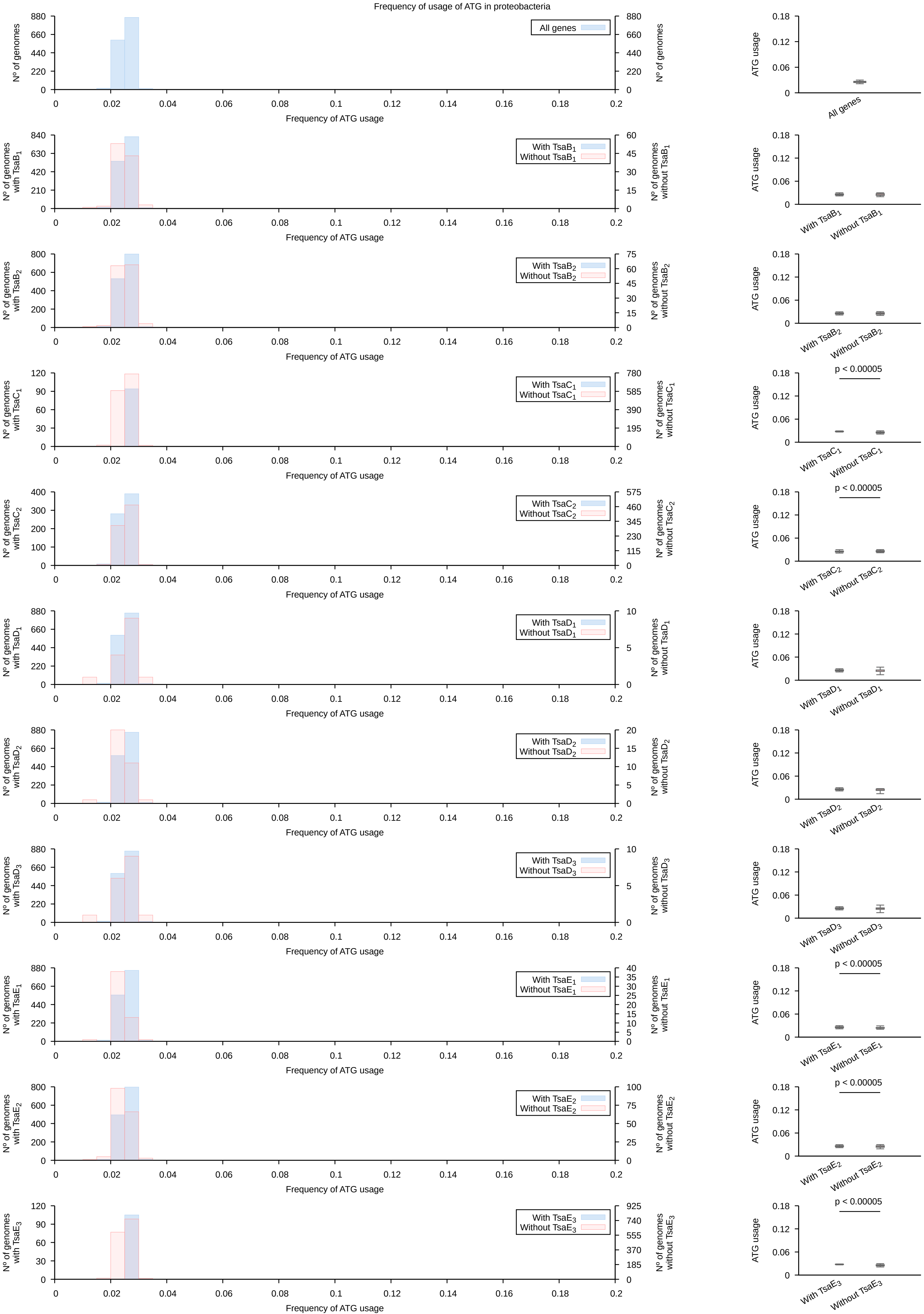

Frequency of usage of ATG in proteobacteria
Nº of genomes
Frequency of ATG usage
All genes
ATG usage
With TsaB1
Without TsaB1
Nº of genomes with TsaB1
Nº of genomes without TsaB1
With TsaB2
Without TsaB2
Nº of genomes with TsaB2
Nº of genomes without TsaB2
With TsaC1
Without TsaC1
Nº of genomes with TsaC1
Nº of genomes without TsaC1
p < 0.00005
With TsaC2
Without TsaC2
Nº of genomes with TsaC2
Nº of genomes without TsaC2
p < 0.00005
With TsaD1
Without TsaD1
Nº of genomes with TsaD1
Nº of genomes without TsaD1
With TsaD2
Without TsaD2
Nº of genomes with TsaD2
Nº of genomes without TsaD2
With TsaD3
Without TsaD3
Nº of genomes with TsaD3
Nº of genomes without TsaD3
With TsaE1
Without TsaE1
Nº of genomes with TsaE1
Nº of genomes without TsaE1
p < 0.00005
With TsaE2
Without TsaE2
Nº of genomes with TsaE2
Nº of genomes without TsaE2
p < 0.00005
With TsaE3
Without TsaE3
Nº of genomes with TsaE3
Nº of genomes without TsaE3
p < 0.00005

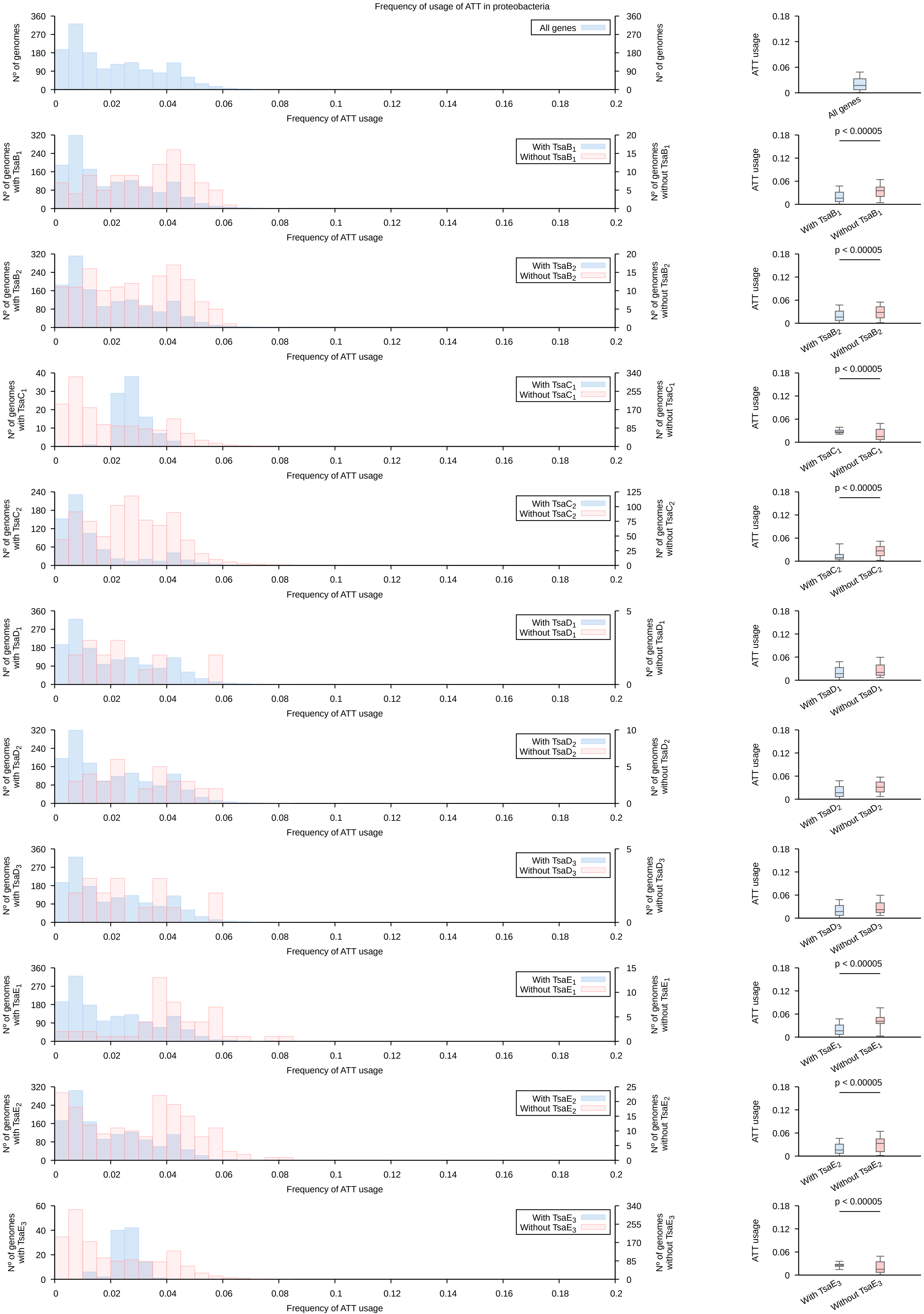
Frequency of usage of ATT in proteobacteria
All genes
Nº of genomes
Frequency of ATT usage
ATT usage
With $TsaB_1$
Without $TsaB_1$
Nº of genomes with $TsaB_1$
Nº of genomes without $TsaB_1$
p < 0.00005
With $TsaB_2$
Without $TsaB_2$
Nº of genomes with $TsaB_2$
Nº of genomes without $TsaB_2$
p < 0.00005
With $TsaC_1$
Without $TsaC_1$
Nº of genomes with $TsaC_1$
Nº of genomes without $TsaC_1$
p < 0.00005
With $TsaC_2$
Without $TsaC_2$
Nº of genomes with $TsaC_2$
Nº of genomes without $TsaC_2$
p < 0.00005
With $TsaD_1$
Without $TsaD_1$
Nº of genomes with $TsaD_1$
Nº of genomes without $TsaD_1$
With $TsaD_2$
Without $TsaD_2$
Nº of genomes with $TsaD_2$
Nº of genomes without $TsaD_2$
With $TsaD_3$
Without $TsaD_3$
Nº of genomes with $TsaD_3$
Nº of genomes without $TsaD_3$
With $TsaE_1$
Without $TsaE_1$
Nº of genomes with $TsaE_1$
Nº of genomes without $TsaE_1$
p < 0.00005
With $TsaE_2$
Without $TsaE_2$
Nº of genomes with $TsaE_2$
Nº of genomes without $TsaE_2$
p < 0.00005
With $TsaE_3$
Without $TsaE_3$
Nº of genomes with $TsaE_3$
Nº of genomes without $TsaE_3$
p < 0.00005

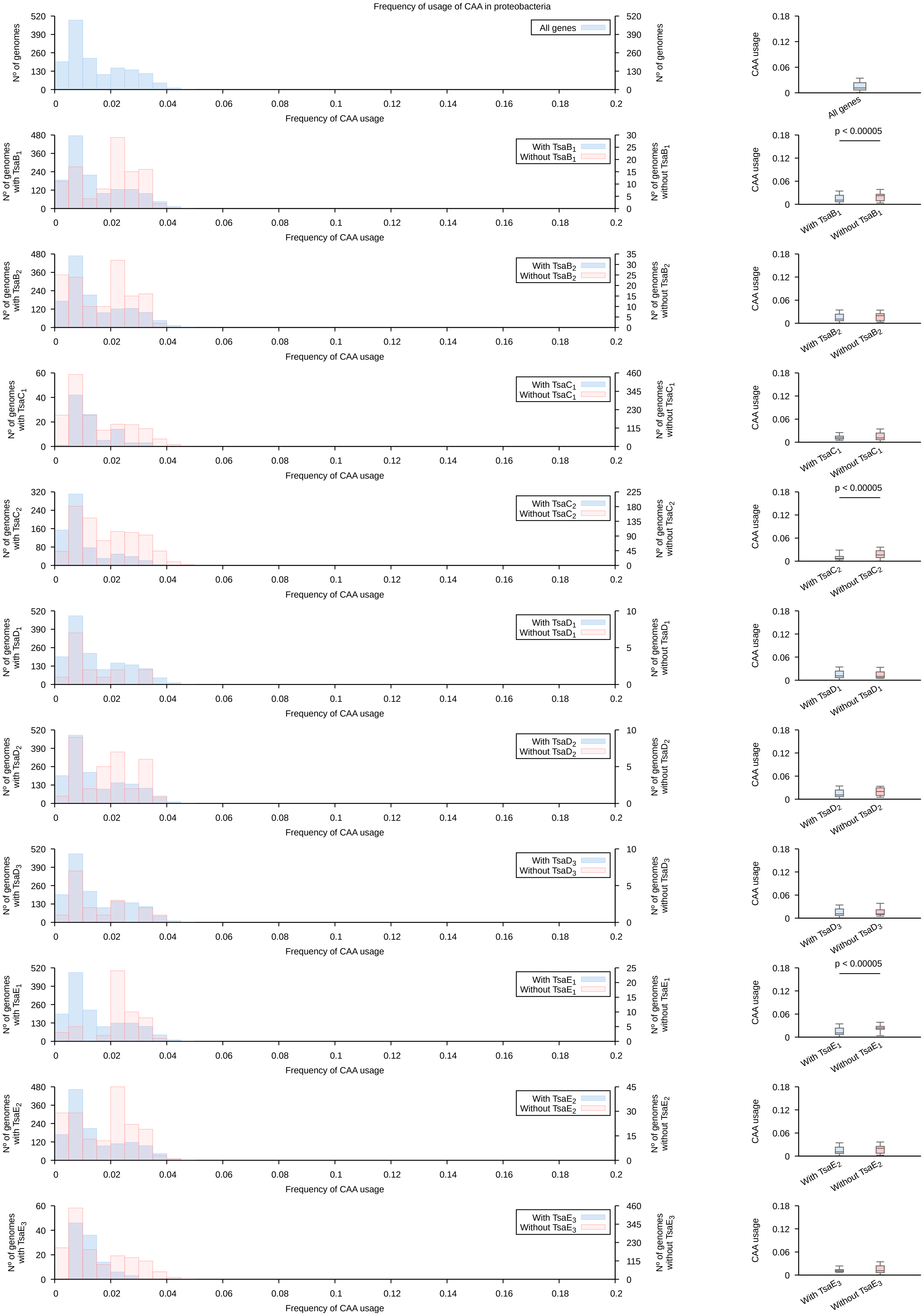
Frequency of usage of CAA in proteobacteria
All genes
Nº of genomes
Frequency of CAA usage
CAA usage
With TsaB1
Without TsaB1
Nº of genomes with TsaB1
Nº of genomes without TsaB1
p < 0.00005
With TsaB2
Without TsaB2
Nº of genomes with TsaB2
Nº of genomes without TsaB2
With TsaC1
Without TsaC1
Nº of genomes with TsaC1
Nº of genomes without TsaC1
With TsaC2
Without TsaC2
Nº of genomes with TsaC2
Nº of genomes without TsaC2
p < 0.00005
With TsaD1
Without TsaD1
Nº of genomes with TsaD1
Nº of genomes without TsaD1
With TsaD2
Without TsaD2
Nº of genomes with TsaD2
Nº of genomes without TsaD2
With TsaD3
Without TsaD3
Nº of genomes with TsaD3
Nº of genomes without TsaD3
With TsaE1
Without TsaE1
Nº of genomes with TsaE1
Nº of genomes without TsaE1
p < 0.00005
With TsaE2
Without TsaE2
Nº of genomes with TsaE2
Nº of genomes without TsaE2
With TsaE3
Without TsaE3
Nº of genomes with TsaE3
Nº of genomes without TsaE3

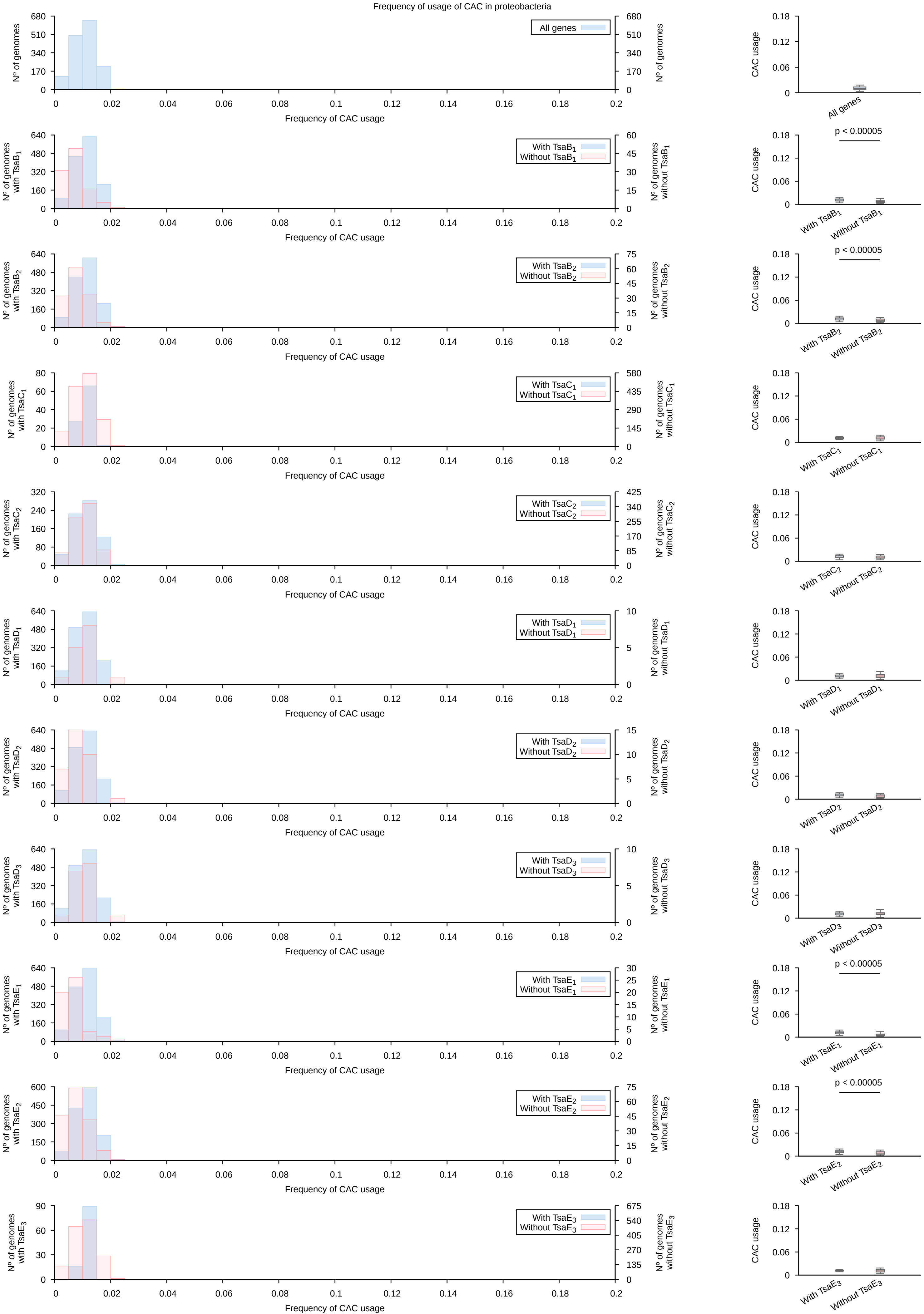
Frequency of usage of CAC in proteobacteria
All genes
With TsaB1
Without TsaB1
With TsaB2
Without TsaB2
With TsaC1
Without TsaC1
With TsaC2
Without TsaC2
With TsaD1
Without TsaD1
With TsaD2
Without TsaD2
With TsaD3
Without TsaD3
With TsaE1
Without TsaE1
With TsaE2
Without TsaE2
With TsaE3
Without TsaE3
Nº of genomes
Frequency of CAC usage
CAC usage
p < 0.00005

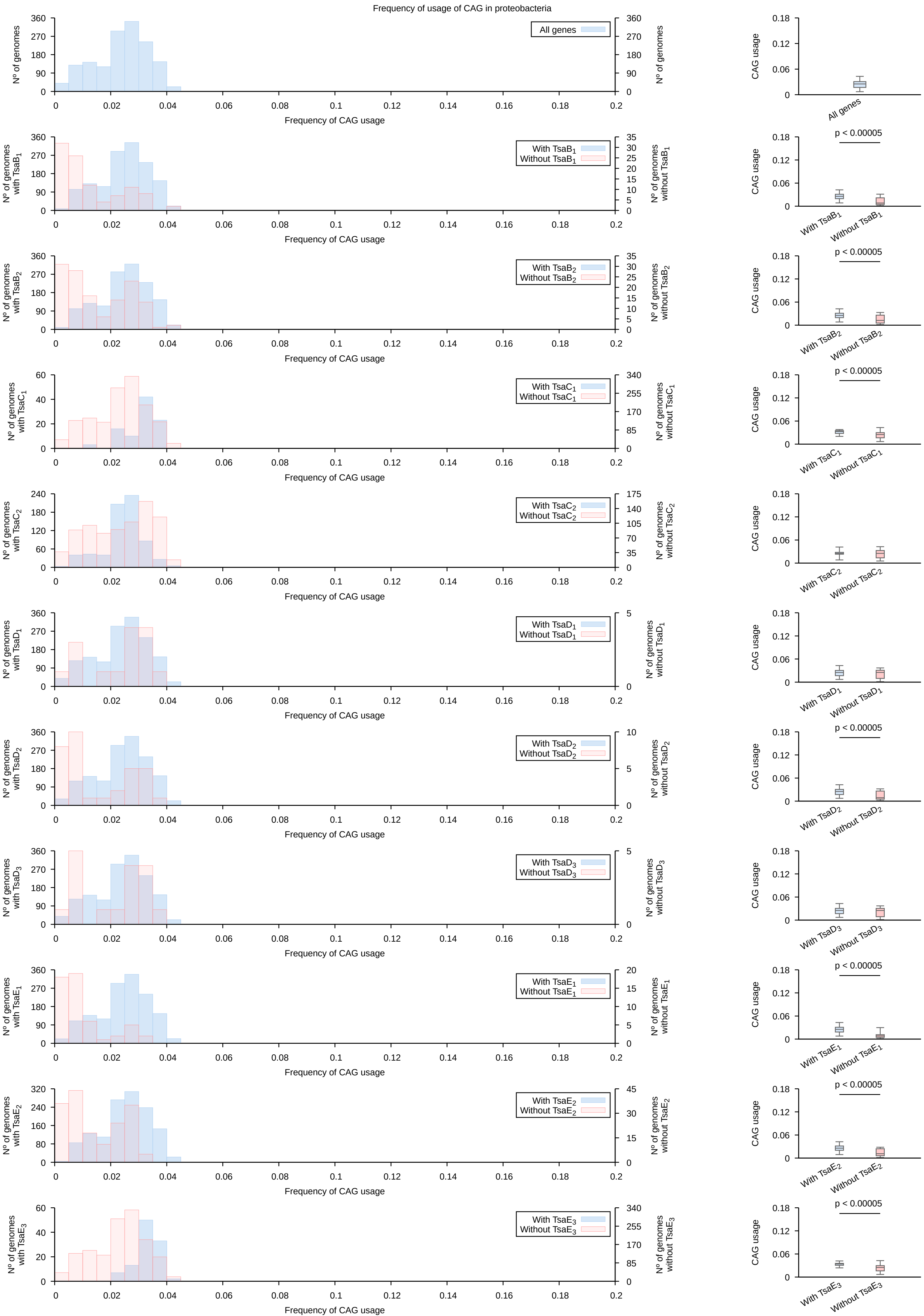

Frequency of usage of CAG in proteobacteria
All genes
Nº of genomes
Frequency of CAG usage
CAG usage
With $TsaB_1$
Without $TsaB_1$
Nº of genomes with $TsaB_1$
Nº of genomes without $TsaB_1$
p < 0.00005
With $TsaB_2$
Without $TsaB_2$
Nº of genomes with $TsaB_2$
Nº of genomes without $TsaB_2$
p < 0.00005
With $TsaC_1$
Without $TsaC_1$
Nº of genomes with $TsaC_1$
Nº of genomes without $TsaC_1$
p < 0.00005
With $TsaC_2$
Without $TsaC_2$
Nº of genomes with $TsaC_2$
Nº of genomes without $TsaC_2$
With $TsaD_1$
Without $TsaD_1$
Nº of genomes with $TsaD_1$
Nº of genomes without $TsaD_1$
With $TsaD_2$
Without $TsaD_2$
Nº of genomes with $TsaD_2$
Nº of genomes without $TsaD_2$
p < 0.00005
With $TsaD_3$
Without $TsaD_3$
Nº of genomes with $TsaD_3$
Nº of genomes without $TsaD_3$
With $TsaE_1$
Without $TsaE_1$
Nº of genomes with $TsaE_1$
Nº of genomes without $TsaE_1$
p < 0.00005
With $TsaE_2$
Without $TsaE_2$
Nº of genomes with $TsaE_2$
Nº of genomes without $TsaE_2$
p < 0.00005
With $TsaE_3$
Without $TsaE_3$
Nº of genomes with $TsaE_3$
Nº of genomes without $TsaE_3$
p < 0.00005

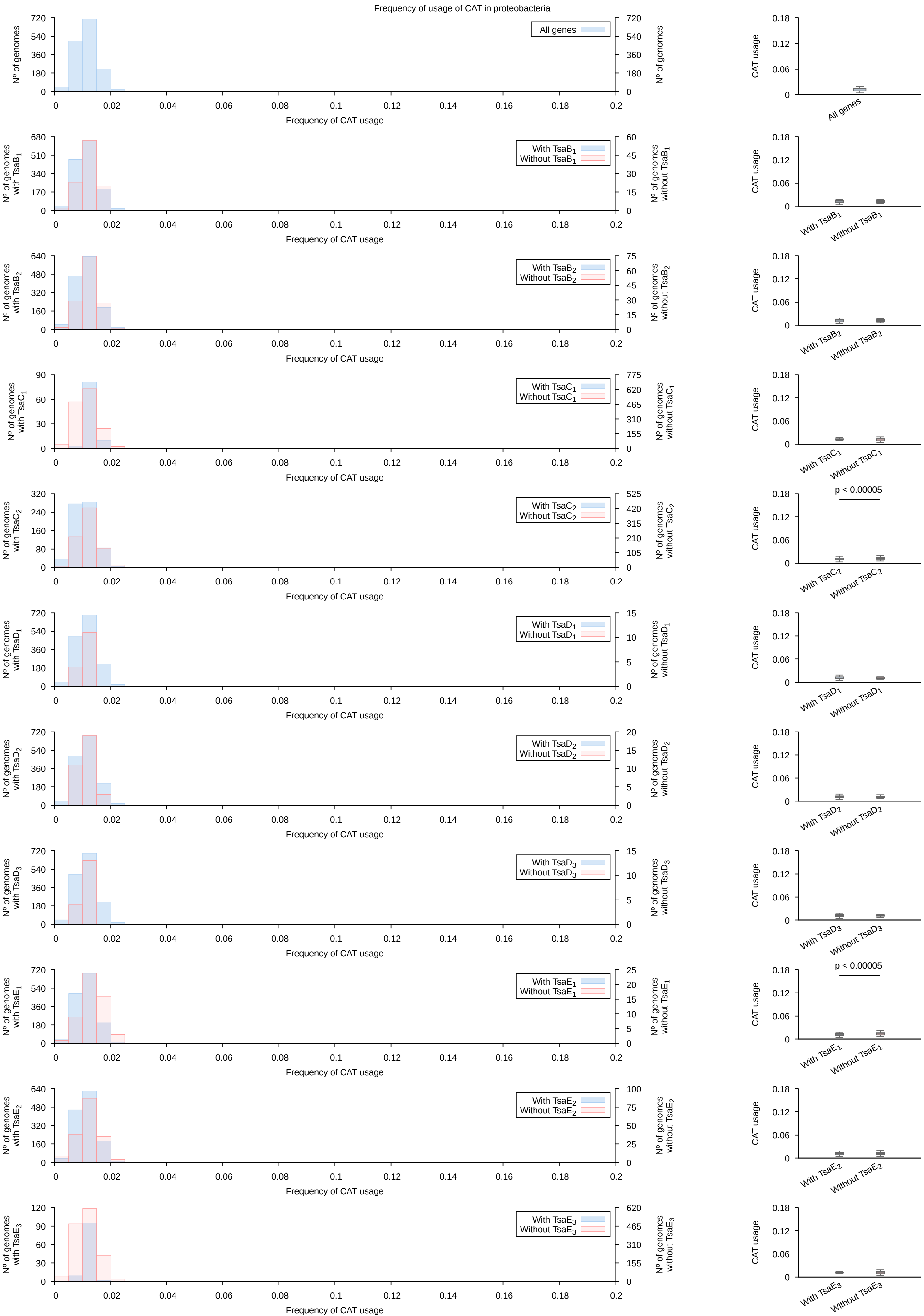
Frequency of usage of CAT in proteobacteria
All genes
Nº of genomes
Frequency of CAT usage
CAT usage
With TsaB$_1$
Without TsaB$_1$
Nº of genomes with TsaB$_1$
Nº of genomes without TsaB$_1$
With TsaB$_2$
Without TsaB$_2$
Nº of genomes with TsaB$_2$
Nº of genomes without TsaB$_2$
With TsaC$_1$
Without TsaC$_1$
Nº of genomes with TsaC$_1$
Nº of genomes without TsaC$_1$
With TsaC$_2$
Without TsaC$_2$
Nº of genomes with TsaC$_2$
Nº of genomes without TsaC$_2$
p < 0.00005
With TsaD$_1$
Without TsaD$_1$
Nº of genomes with TsaD$_1$
Nº of genomes without TsaD$_1$
With TsaD$_2$
Without TsaD$_2$
Nº of genomes with TsaD$_2$
Nº of genomes without TsaD$_2$
With TsaD$_3$
Without TsaD$_3$
Nº of genomes with TsaD$_3$
Nº of genomes without TsaD$_3$
With TsaE$_1$
Without TsaE$_1$
Nº of genomes with TsaE$_1$
Nº of genomes without TsaE$_1$
p < 0.00005
With TsaE$_2$
Without TsaE$_2$
Nº of genomes with TsaE$_2$
Nº of genomes without TsaE$_2$
With TsaE$_3$
Without TsaE$_3$
Nº of genomes with TsaE$_3$
Nº of genomes without TsaE$_3$

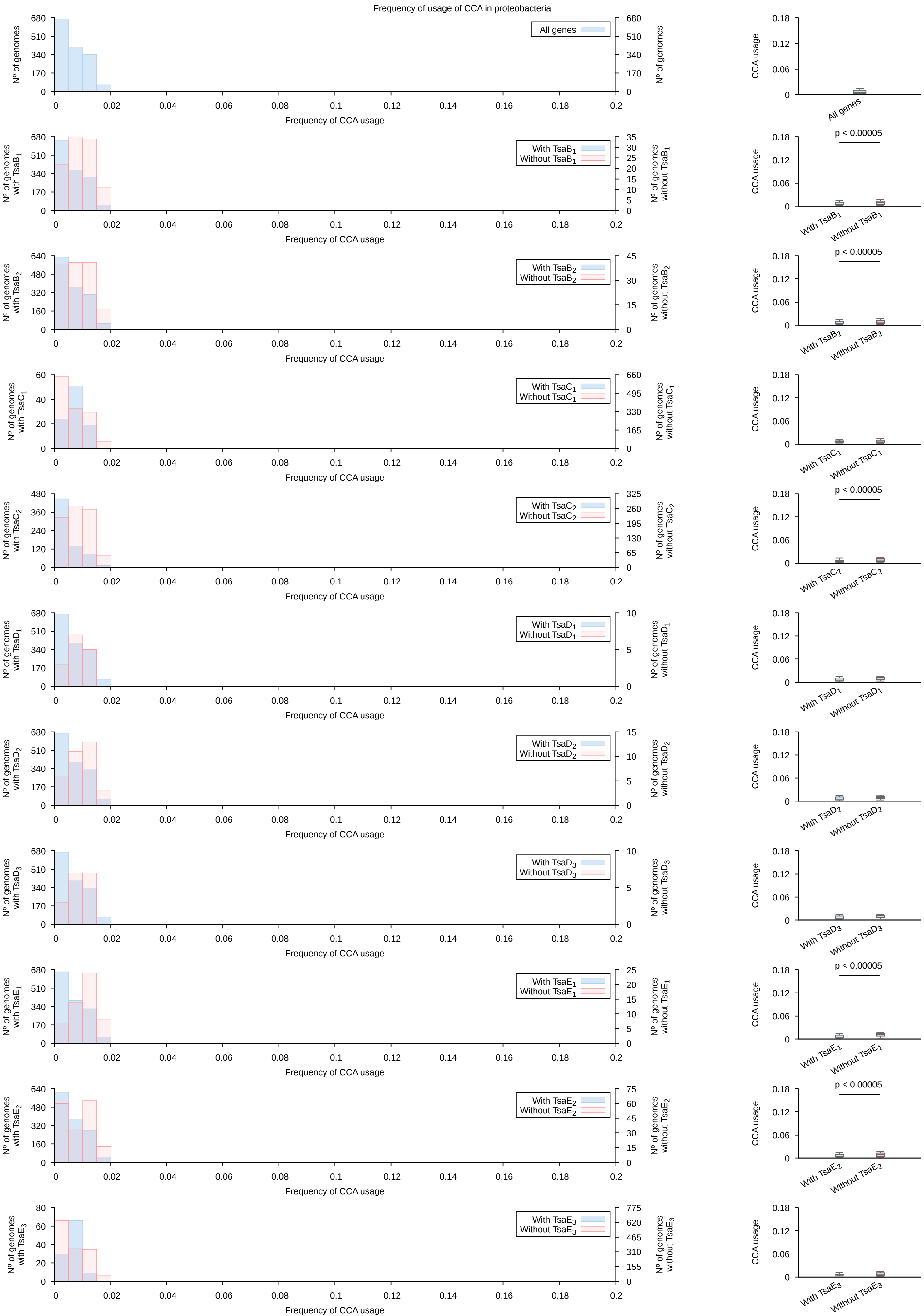

Frequency of usage of CCA in proteobacteria
All genes
Nº of genomes
Frequency of CCA usage
CCA usage
With $TsaB_1$
Without $TsaB_1$
Nº of genomes with $TsaB_1$
Nº of genomes without $TsaB_1$
p < 0.00005
With $TsaB_2$
Without $TsaB_2$
Nº of genomes with $TsaB_2$
Nº of genomes without $TsaB_2$
p < 0.00005
With $TsaC_1$
Without $TsaC_1$
Nº of genomes with $TsaC_1$
Nº of genomes without $TsaC_1$
With $TsaC_2$
Without $TsaC_2$
Nº of genomes with $TsaC_2$
Nº of genomes without $TsaC_2$
p < 0.00005
With $TsaD_1$
Without $TsaD_1$
Nº of genomes with $TsaD_1$
Nº of genomes without $TsaD_1$
With $TsaD_2$
Without $TsaD_2$
Nº of genomes with $TsaD_2$
Nº of genomes without $TsaD_2$
With $TsaD_3$
Without $TsaD_3$
Nº of genomes with $TsaD_3$
Nº of genomes without $TsaD_3$
With $TsaE_1$
Without $TsaE_1$
Nº of genomes with $TsaE_1$
Nº of genomes without $TsaE_1$
p < 0.00005
With $TsaE_2$
Without $TsaE_2$
Nº of genomes with $TsaE_2$
Nº of genomes without $TsaE_2$
p < 0.00005
With $TsaE_3$
Without $TsaE_3$
Nº of genomes with $TsaE_3$
Nº of genomes without $TsaE_3$

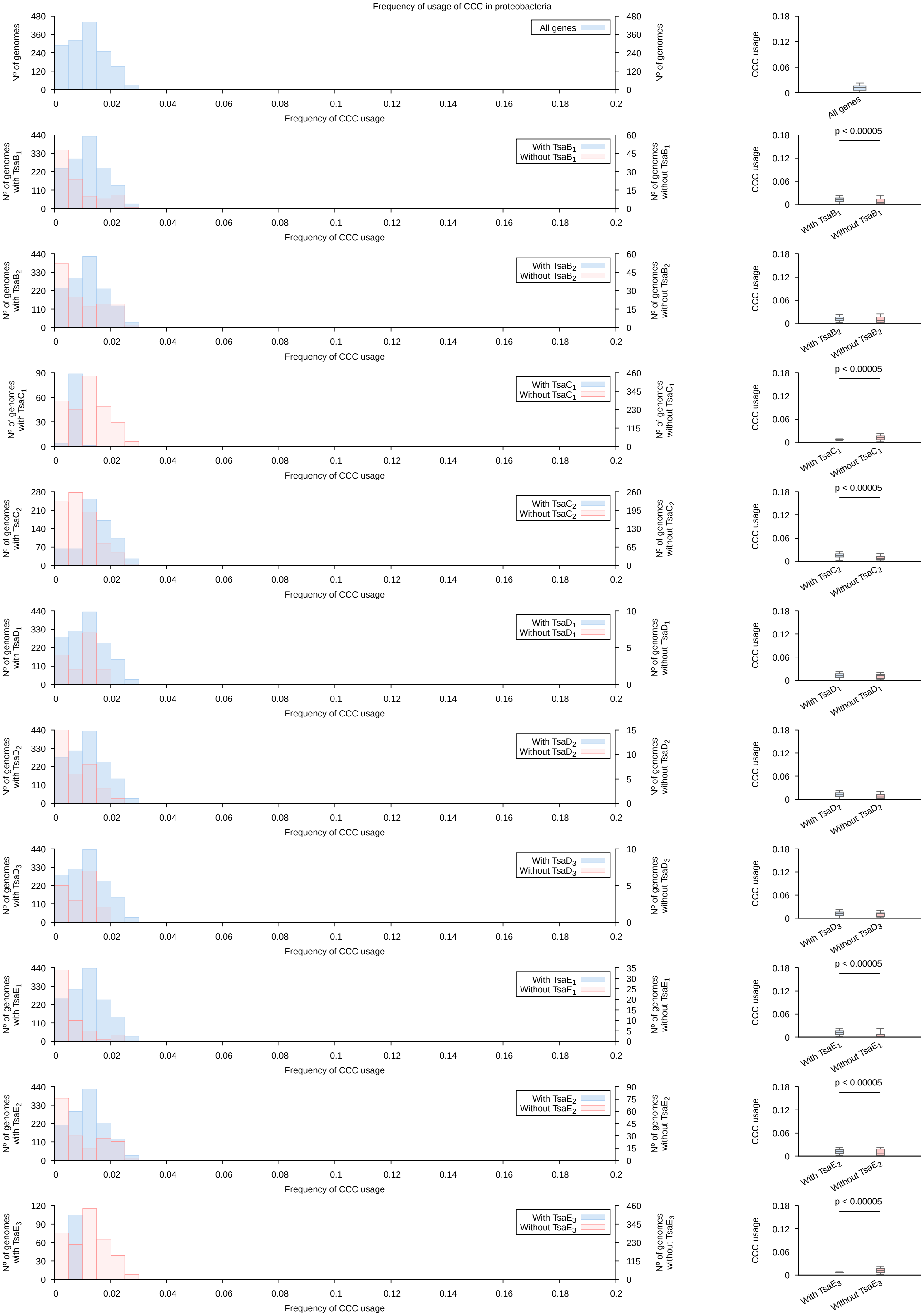
Frequency of usage of CCC in proteobacteria
All genes
Nº of genomes
Frequency of CCC usage
CCC usage
With TsaB1
Without TsaB1
Nº of genomes with TsaB1
Nº of genomes without TsaB1
p < 0.00005
With TsaB2
Without TsaB2
Nº of genomes with TsaB2
Nº of genomes without TsaB2
With TsaC1
Without TsaC1
Nº of genomes with TsaC1
Nº of genomes without TsaC1
p < 0.00005
With TsaC2
Without TsaC2
Nº of genomes with TsaC2
Nº of genomes without TsaC2
p < 0.00005
With TsaD1
Without TsaD1
Nº of genomes with TsaD1
Nº of genomes without TsaD1
With TsaD2
Without TsaD2
Nº of genomes with TsaD2
Nº of genomes without TsaD2
With TsaD3
Without TsaD3
Nº of genomes with TsaD3
Nº of genomes without TsaD3
With TsaE1
Without TsaE1
Nº of genomes with TsaE1
Nº of genomes without TsaE1
p < 0.00005
With TsaE2
Without TsaE2
Nº of genomes with TsaE2
Nº of genomes without TsaE2
p < 0.00005
With TsaE3
Without TsaE3
Nº of genomes with TsaE3
Nº of genomes without TsaE3
p < 0.00005

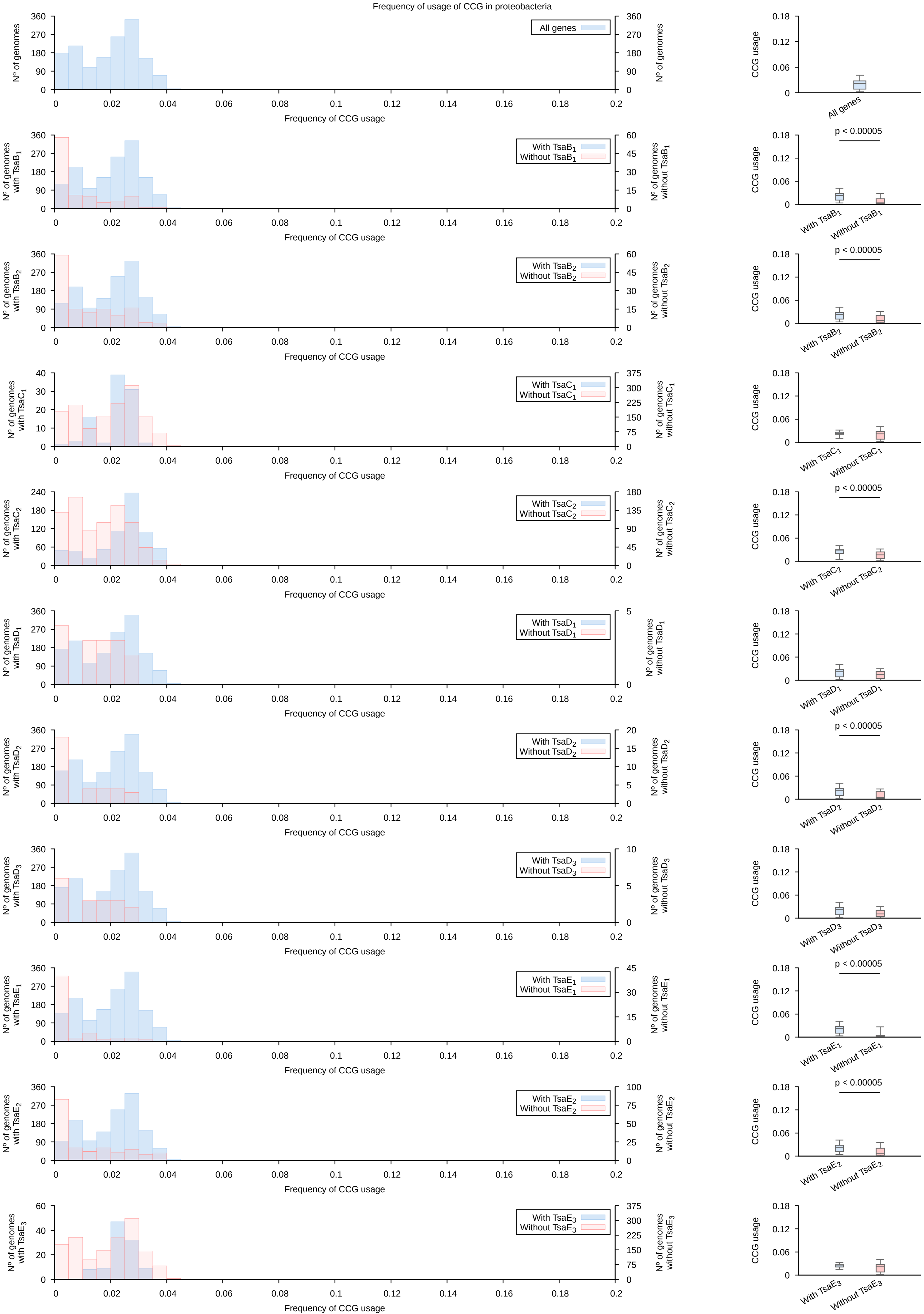

Frequency of usage of CCG in proteobacteria
Nº of genomes
All genes
Frequency of CCG usage
CCG usage
Nº of genomes with TsaB1
Nº of genomes without TsaB1
With TsaB1
Without TsaB1
p < 0.00005
Nº of genomes with TsaB2
Nº of genomes without TsaB2
With TsaB2
Without TsaB2
p < 0.00005
Nº of genomes with TsaC1
Nº of genomes without TsaC1
With TsaC1
Without TsaC1
Nº of genomes with TsaC2
Nº of genomes without TsaC2
With TsaC2
Without TsaC2
p < 0.00005
Nº of genomes with TsaD1
Nº of genomes without TsaD1
With TsaD1
Without TsaD1
Nº of genomes with TsaD2
Nº of genomes without TsaD2
With TsaD2
Without TsaD2
p < 0.00005
Nº of genomes with TsaD3
Nº of genomes without TsaD3
With TsaD3
Without TsaD3
Nº of genomes with TsaE1
Nº of genomes without TsaE1
With TsaE1
Without TsaE1
p < 0.00005
Nº of genomes with TsaE2
Nº of genomes without TsaE2
With TsaE2
Without TsaE2
p < 0.00005
Nº of genomes with TsaE3
Nº of genomes without TsaE3
With TsaE3
Without TsaE3

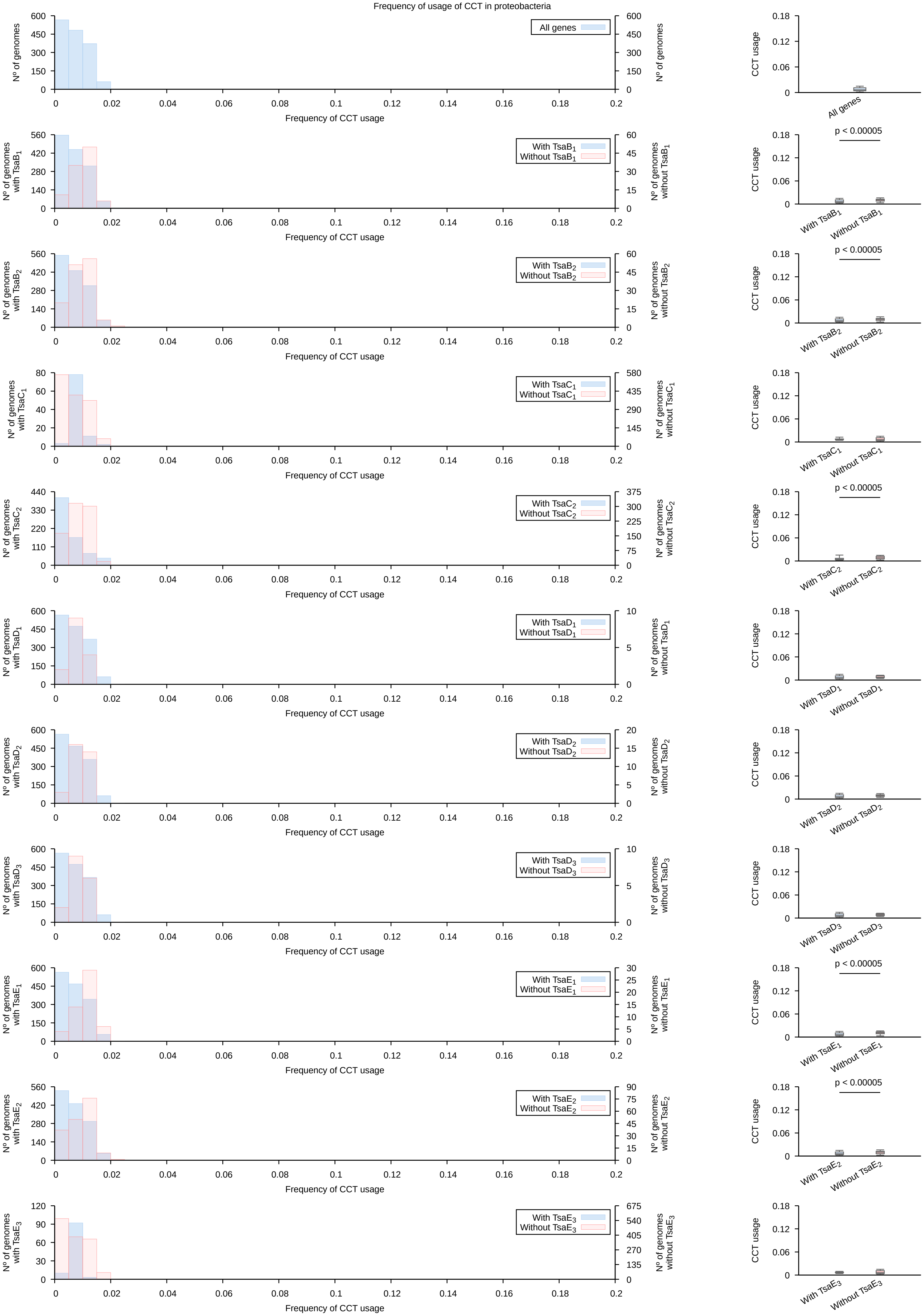
Frequency of usage of CCT in proteobacteria
All genes
With TsaB1
Without TsaB1
With TsaB2
Without TsaB2
With TsaC1
Without TsaC1
With TsaC2
Without TsaC2
With TsaD1
Without TsaD1
With TsaD2
Without TsaD2
With TsaD3
Without TsaD3
With TsaE1
Without TsaE1
With TsaE2
Without TsaE2
With TsaE3
Without TsaE3
Nº of genomes
Nº of genomes with TsaB1
Nº of genomes without TsaB1
Nº of genomes with TsaB2
Nº of genomes without TsaB2
Nº of genomes with TsaC1
Nº of genomes without TsaC1
Nº of genomes with TsaC2
Nº of genomes without TsaC2
Nº of genomes with TsaD1
Nº of genomes without TsaD1
Nº of genomes with TsaD2
Nº of genomes without TsaD2
Nº of genomes with TsaD3
Nº of genomes without TsaD3
Nº of genomes with TsaE1
Nº of genomes without TsaE1
Nº of genomes with TsaE2
Nº of genomes without TsaE2
Nº of genomes with TsaE3
Nº of genomes without TsaE3
Frequency of CCT usage
0 0.02 0.04 0.06 0.08 0.1 0.12 0.14 0.16 0.18 0.2
CCT usage
0 0.06 0.12 0.18
p < 0.00005

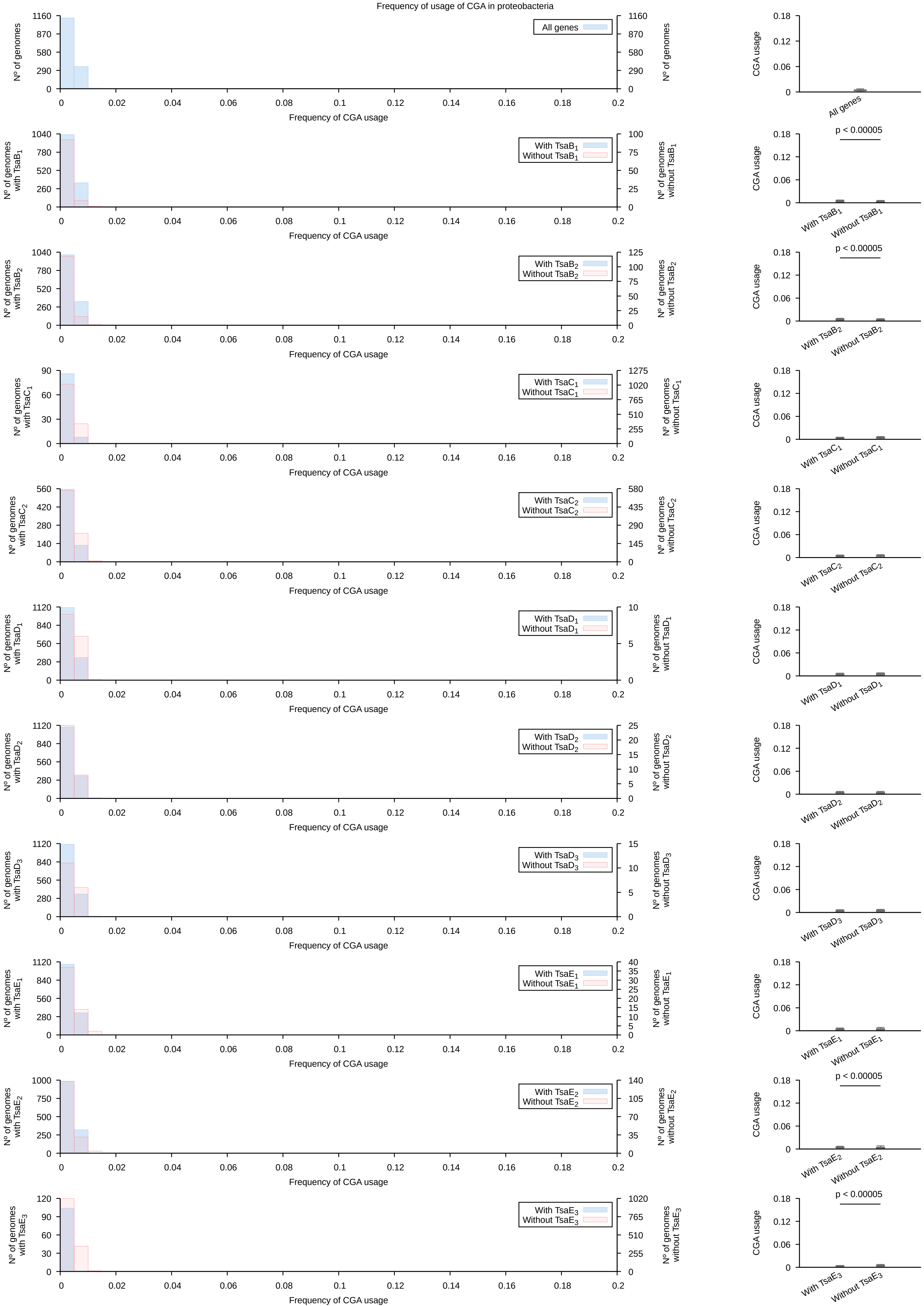
Frequency of usage of CGA in proteobacteria
All genes
With TsaB1
Without TsaB1
With TsaB2
Without TsaB2
With TsaC1
Without TsaC1
With TsaC2
Without TsaC2
With TsaD1
Without TsaD1
With TsaD2
Without TsaD2
With TsaD3
Without TsaD3
With TsaE1
Without TsaE1
With TsaE2
Without TsaE2
With TsaE3
Without TsaE3
Nº of genomes
Frequency of CGA usage
CGA usage
p < 0.00005

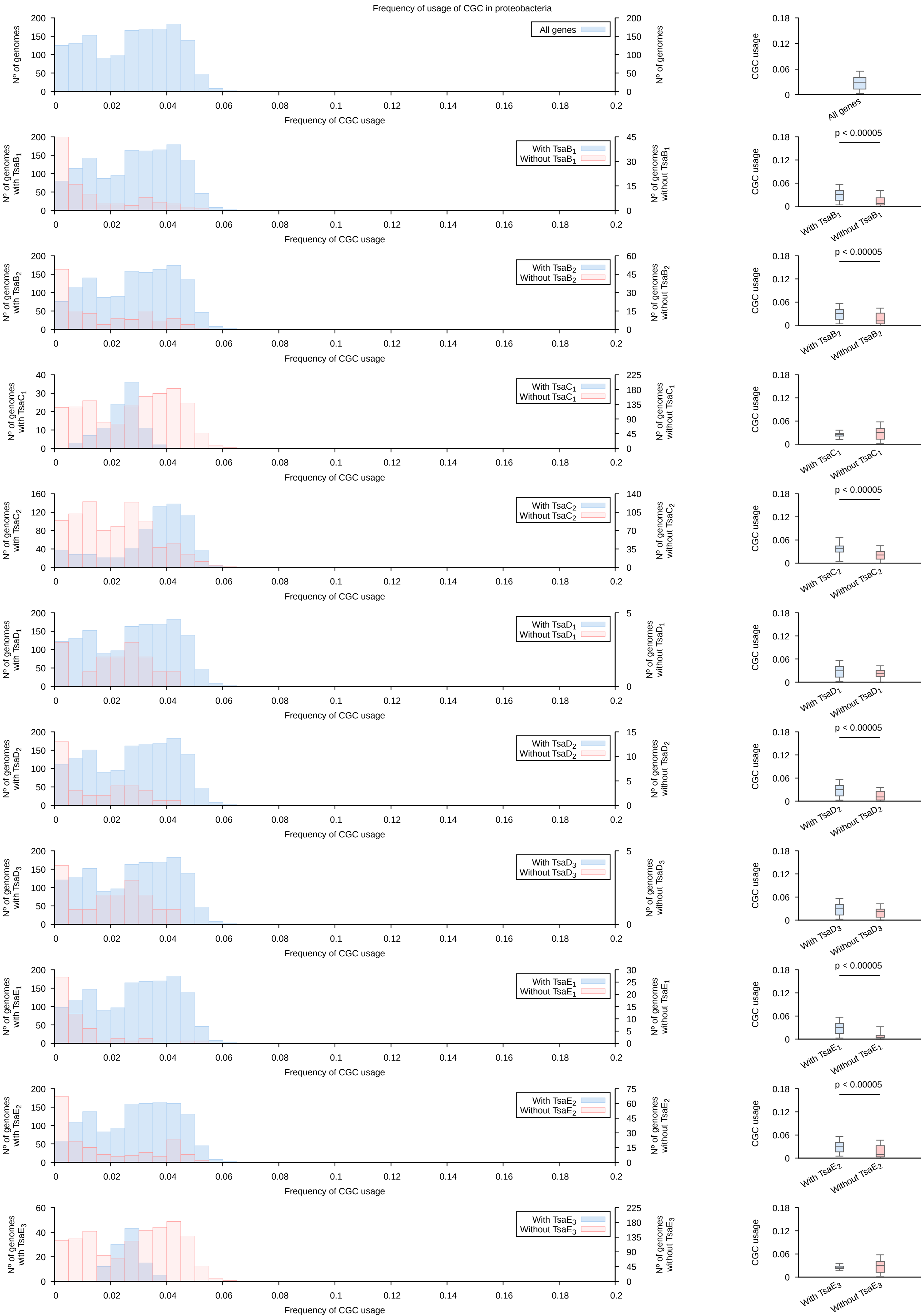
Frequency of usage of CGC in proteobacteria
All genes
Nº of genomes
Frequency of CGC usage
CGC usage
With TsaB$_1$
Without TsaB$_1$
Nº of genomes with TsaB$_1$
Nº of genomes without TsaB$_1$
p < 0.00005
With TsaB$_2$
Without TsaB$_2$
Nº of genomes with TsaB$_2$
Nº of genomes without TsaB$_2$
p < 0.00005
With TsaC$_1$
Without TsaC$_1$
Nº of genomes with TsaC$_1$
Nº of genomes without TsaC$_1$
With TsaC$_2$
Without TsaC$_2$
Nº of genomes with TsaC$_2$
Nº of genomes without TsaC$_2$
p < 0.00005
With TsaD$_1$
Without TsaD$_1$
Nº of genomes with TsaD$_1$
Nº of genomes without TsaD$_1$
With TsaD$_2$
Without TsaD$_2$
Nº of genomes with TsaD$_2$
Nº of genomes without TsaD$_2$
p < 0.00005
With TsaD$_3$
Without TsaD$_3$
Nº of genomes with TsaD$_3$
Nº of genomes without TsaD$_3$
With TsaE$_1$
Without TsaE$_1$
Nº of genomes with TsaE$_1$
Nº of genomes without TsaE$_1$
p < 0.00005
With TsaE$_2$
Without TsaE$_2$
Nº of genomes with TsaE$_2$
Nº of genomes without TsaE$_2$
p < 0.00005
With TsaE$_3$
Without TsaE$_3$
Nº of genomes with TsaE$_3$
Nº of genomes without TsaE$_3$

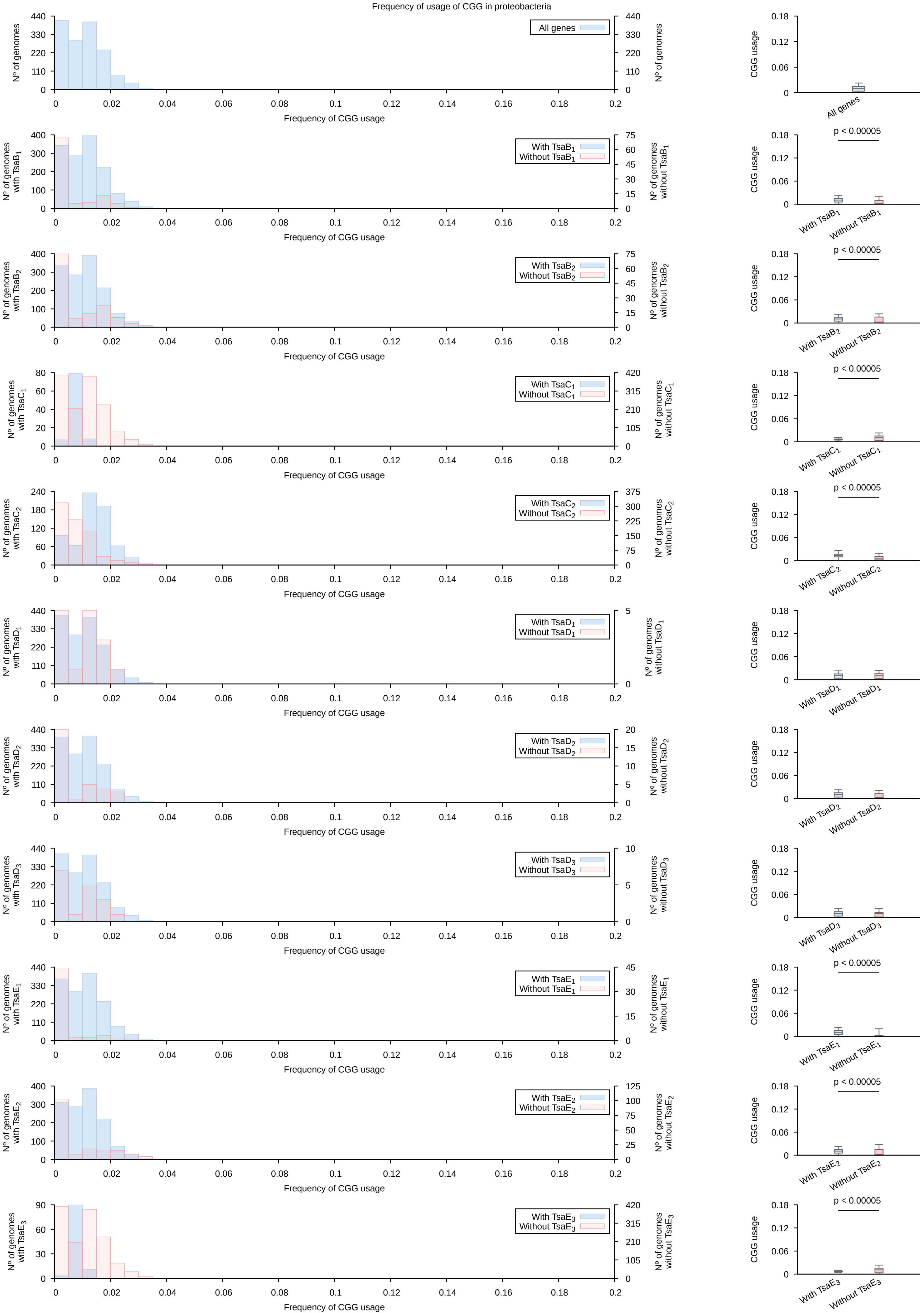

Frequency of usage of CGG in proteobacteria
All genes
Nº of genomes
Frequency of CGG usage
CGG usage
With TsaB1
Without TsaB1
Nº of genomes with TsaB1
Nº of genomes without TsaB1
With TsaB2
Without TsaB2
Nº of genomes with TsaB2
Nº of genomes without TsaB2
With TsaC1
Without TsaC1
Nº of genomes with TsaC1
Nº of genomes without TsaC1
With TsaC2
Without TsaC2
Nº of genomes with TsaC2
Nº of genomes without TsaC2
With TsaD1
Without TsaD1
Nº of genomes with TsaD1
Nº of genomes without TsaD1
With TsaD2
Without TsaD2
Nº of genomes with TsaD2
Nº of genomes without TsaD2
With TsaD3
Without TsaD3
Nº of genomes with TsaD3
Nº of genomes without TsaD3
With TsaE1
Without TsaE1
Nº of genomes with TsaE1
Nº of genomes without TsaE1
With TsaE2
Without TsaE2
Nº of genomes with TsaE2
Nº of genomes without TsaE2
With TsaE3
Without TsaE3
Nº of genomes with TsaE3
Nº of genomes without TsaE3
p < 0.00005

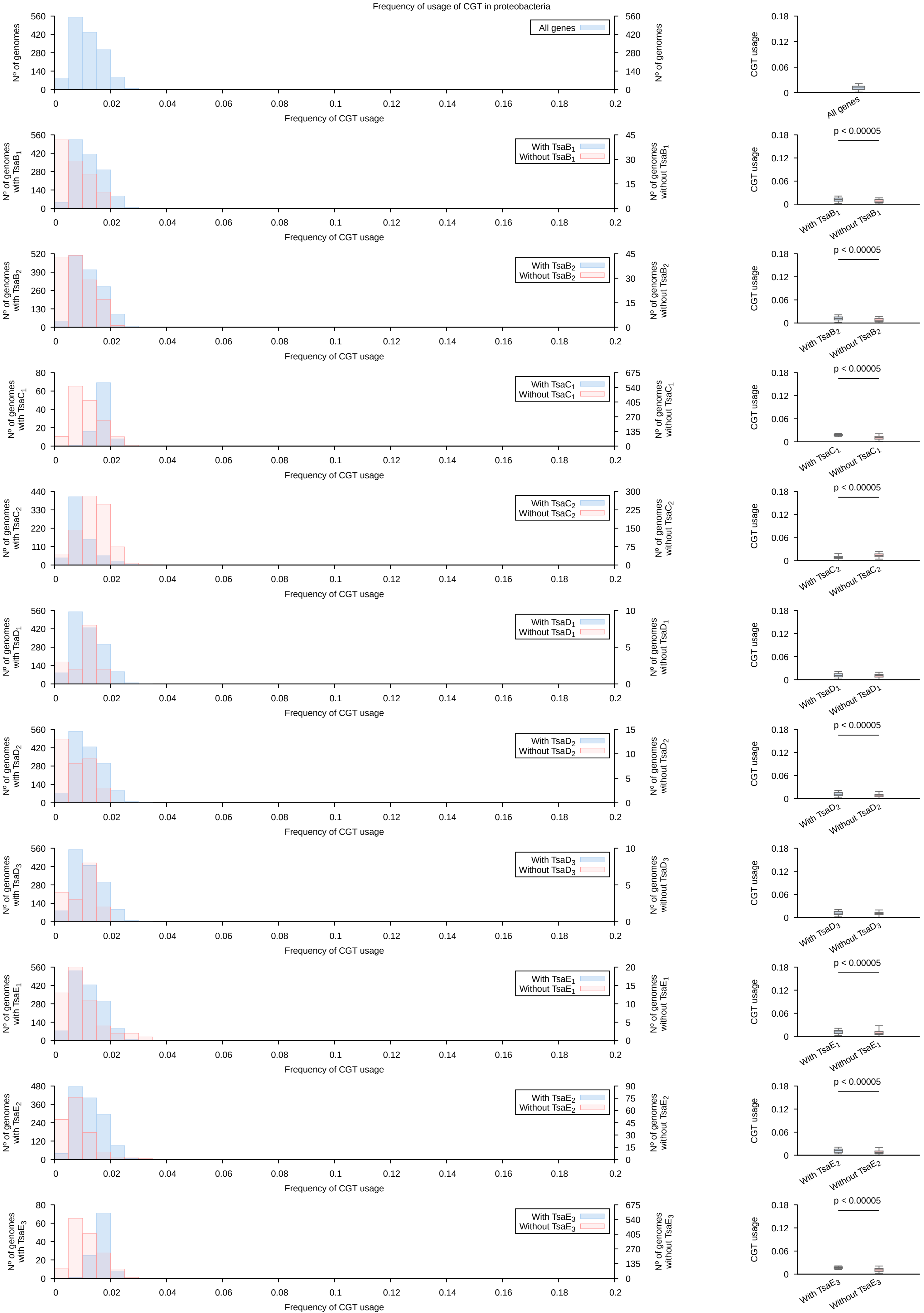
Frequency of usage of CGT in proteobacteria
All genes
Nº of genomes
Frequency of CGT usage
CGT usage
With TsaB1
Without TsaB1
Nº of genomes with TsaB1
Nº of genomes without TsaB1
p < 0.00005
With TsaB2
Without TsaB2
Nº of genomes with TsaB2
Nº of genomes without TsaB2
p < 0.00005
With TsaC1
Without TsaC1
Nº of genomes with TsaC1
Nº of genomes without TsaC1
p < 0.00005
With TsaC2
Without TsaC2
Nº of genomes with TsaC2
Nº of genomes without TsaC2
p < 0.00005
With TsaD1
Without TsaD1
Nº of genomes with TsaD1
Nº of genomes without TsaD1
With TsaD2
Without TsaD2
Nº of genomes with TsaD2
Nº of genomes without TsaD2
p < 0.00005
With TsaD3
Without TsaD3
Nº of genomes with TsaD3
Nº of genomes without TsaD3
With TsaE1
Without TsaE1
Nº of genomes with TsaE1
Nº of genomes without TsaE1
p < 0.00005
With TsaE2
Without TsaE2
Nº of genomes with TsaE2
Nº of genomes without TsaE2
p < 0.00005
With TsaE3
Without TsaE3
Nº of genomes with TsaE3
Nº of genomes without TsaE3
p < 0.00005

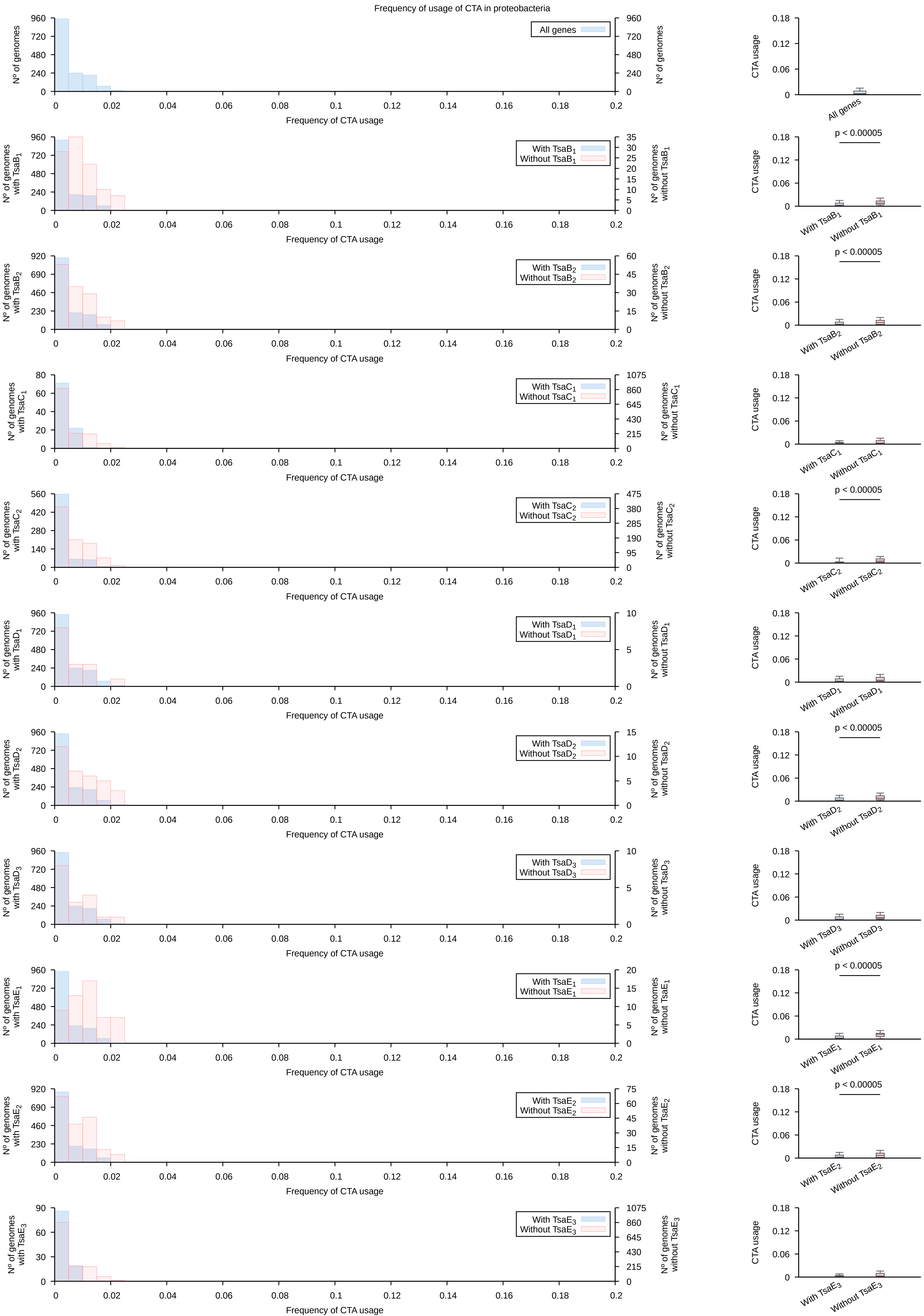
Frequency of usage of CTA in proteobacteria
All genes
Nº of genomes
Frequency of CTA usage
CTA usage
With TsaB$_1$
Without TsaB$_1$
Nº of genomes with TsaB$_1$
Nº of genomes without TsaB$_1$
p < 0.00005
With TsaB$_2$
Without TsaB$_2$
Nº of genomes with TsaB$_2$
Nº of genomes without TsaB$_2$
p < 0.00005
With TsaC$_1$
Without TsaC$_1$
Nº of genomes with TsaC$_1$
Nº of genomes without TsaC$_1$
With TsaC$_2$
Without TsaC$_2$
Nº of genomes with TsaC$_2$
Nº of genomes without TsaC$_2$
p < 0.00005
With TsaD$_1$
Without TsaD$_1$
Nº of genomes with TsaD$_1$
Nº of genomes without TsaD$_1$
With TsaD$_2$
Without TsaD$_2$
Nº of genomes with TsaD$_2$
Nº of genomes without TsaD$_2$
p < 0.00005
With TsaD$_3$
Without TsaD$_3$
Nº of genomes with TsaD$_3$
Nº of genomes without TsaD$_3$
With TsaE$_1$
Without TsaE$_1$
Nº of genomes with TsaE$_1$
Nº of genomes without TsaE$_1$
p < 0.00005
With TsaE$_2$
Without TsaE$_2$
Nº of genomes with TsaE$_2$
Nº of genomes without TsaE$_2$
p < 0.00005
With TsaE$_3$
Without TsaE$_3$
Nº of genomes with TsaE$_3$
Nº of genomes without TsaE$_3$

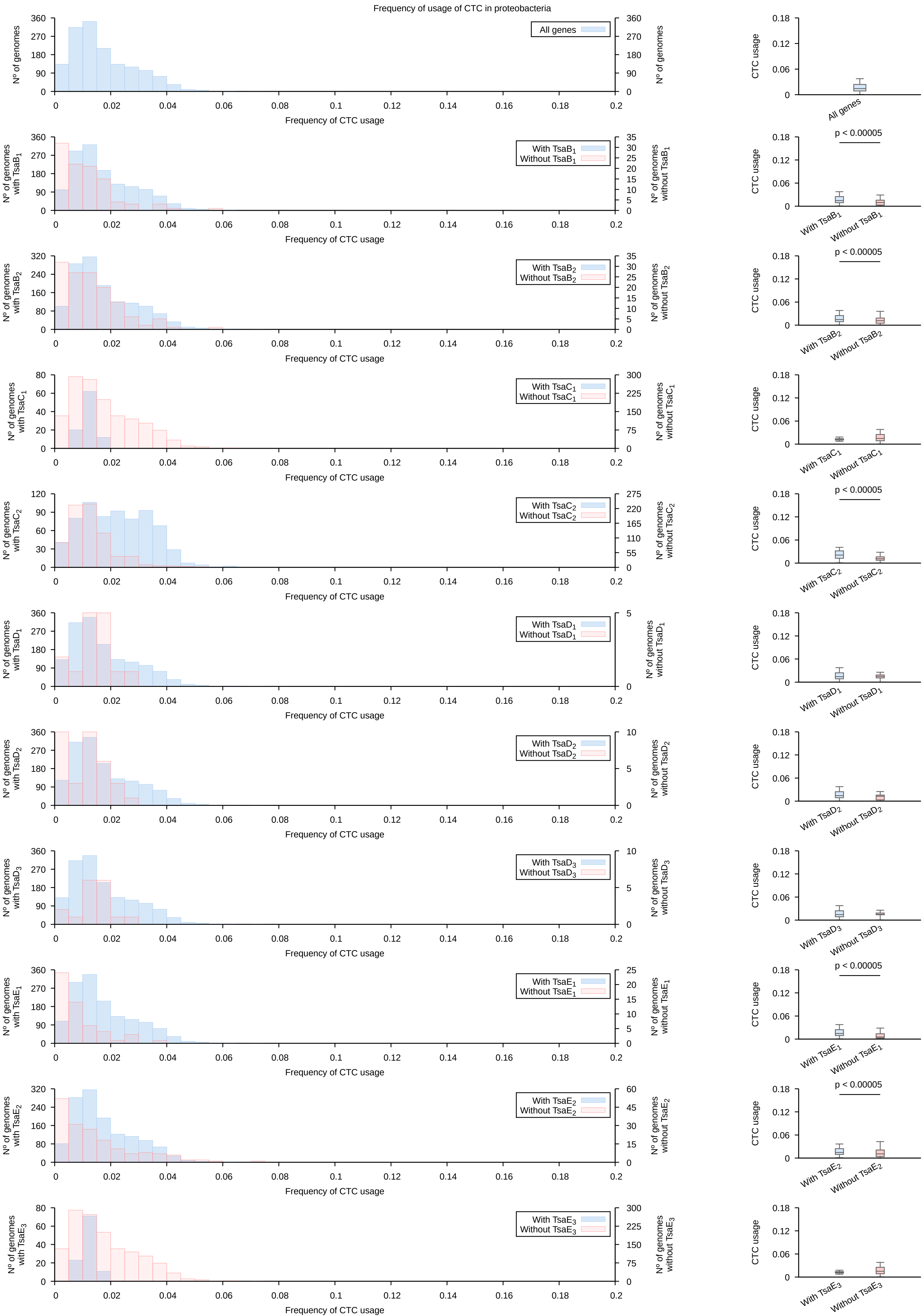
Frequency of usage of CTC in proteobacteria
All genes
With TsaB$_1$
Without TsaB$_1$
With TsaB$_2$
Without TsaB$_2$
With TsaC$_1$
Without TsaC$_1$
With TsaC$_2$
Without TsaC$_2$
With TsaD$_1$
Without TsaD$_1$
With TsaD$_2$
Without TsaD$_2$
With TsaD$_3$
Without TsaD$_3$
With TsaE$_1$
Without TsaE$_1$
With TsaE$_2$
Without TsaE$_2$
With TsaE$_3$
Without TsaE$_3$
Nº of genomes
Nº of genomes with TsaB$_1$
Nº of genomes without TsaB$_1$
Nº of genomes with TsaB$_2$
Nº of genomes without TsaB$_2$
Nº of genomes with TsaC$_1$
Nº of genomes without TsaC$_1$
Nº of genomes with TsaC$_2$
Nº of genomes without TsaC$_2$
Nº of genomes with TsaD$_1$
Nº of genomes without TsaD$_1$
Nº of genomes with TsaD$_2$
Nº of genomes without TsaD$_2$
Nº of genomes with TsaD$_3$
Nº of genomes without TsaD$_3$
Nº of genomes with TsaE$_1$
Nº of genomes without TsaE$_1$
Nº of genomes with TsaE$_2$
Nº of genomes without TsaE$_2$
Nº of genomes with TsaE$_3$
Nº of genomes without TsaE$_3$
Frequency of CTC usage
CTC usage
p < 0.00005

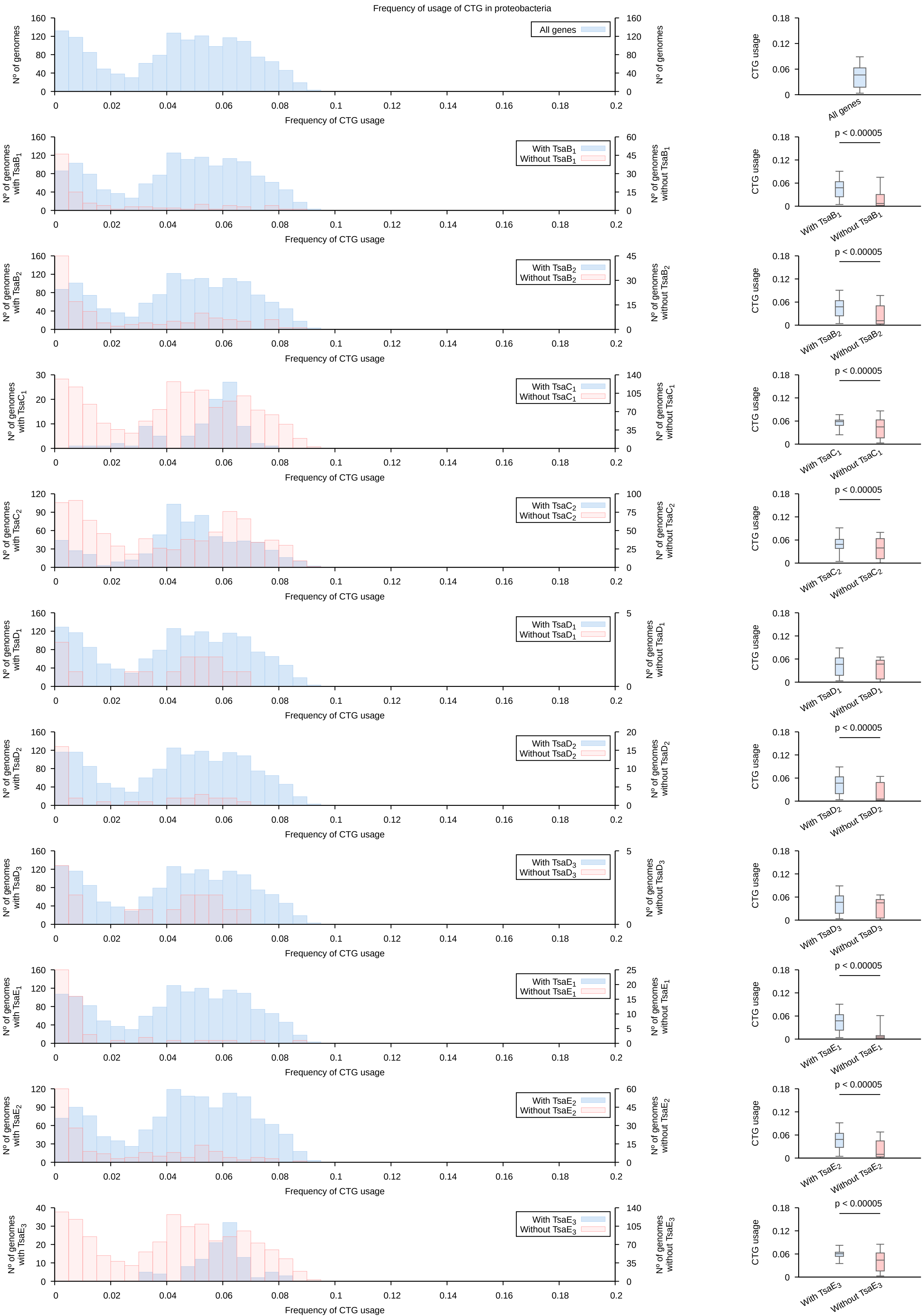

Frequency of usage of CTG in proteobacteria
Nº of genomes
All genes
Frequency of CTG usage
CTG usage
Nº of genomes with TsaB1
Nº of genomes without TsaB1
With TsaB1
Without TsaB1
p < 0.00005
Nº of genomes with TsaB2
Nº of genomes without TsaB2
With TsaB2
Without TsaB2
Nº of genomes with TsaC1
Nº of genomes without TsaC1
With TsaC1
Without TsaC1
Nº of genomes with TsaC2
Nº of genomes without TsaC2
With TsaC2
Without TsaC2
Nº of genomes with TsaD1
Nº of genomes without TsaD1
With TsaD1
Without TsaD1
Nº of genomes with TsaD2
Nº of genomes without TsaD2
With TsaD2
Without TsaD2
Nº of genomes with TsaD3
Nº of genomes without TsaD3
With TsaD3
Without TsaD3
Nº of genomes with TsaE1
Nº of genomes without TsaE1
With TsaE1
Without TsaE1
Nº of genomes with TsaE2
Nº of genomes without TsaE2
With TsaE2
Without TsaE2
Nº of genomes with TsaE3
Nº of genomes without TsaE3
With TsaE3
Without TsaE3

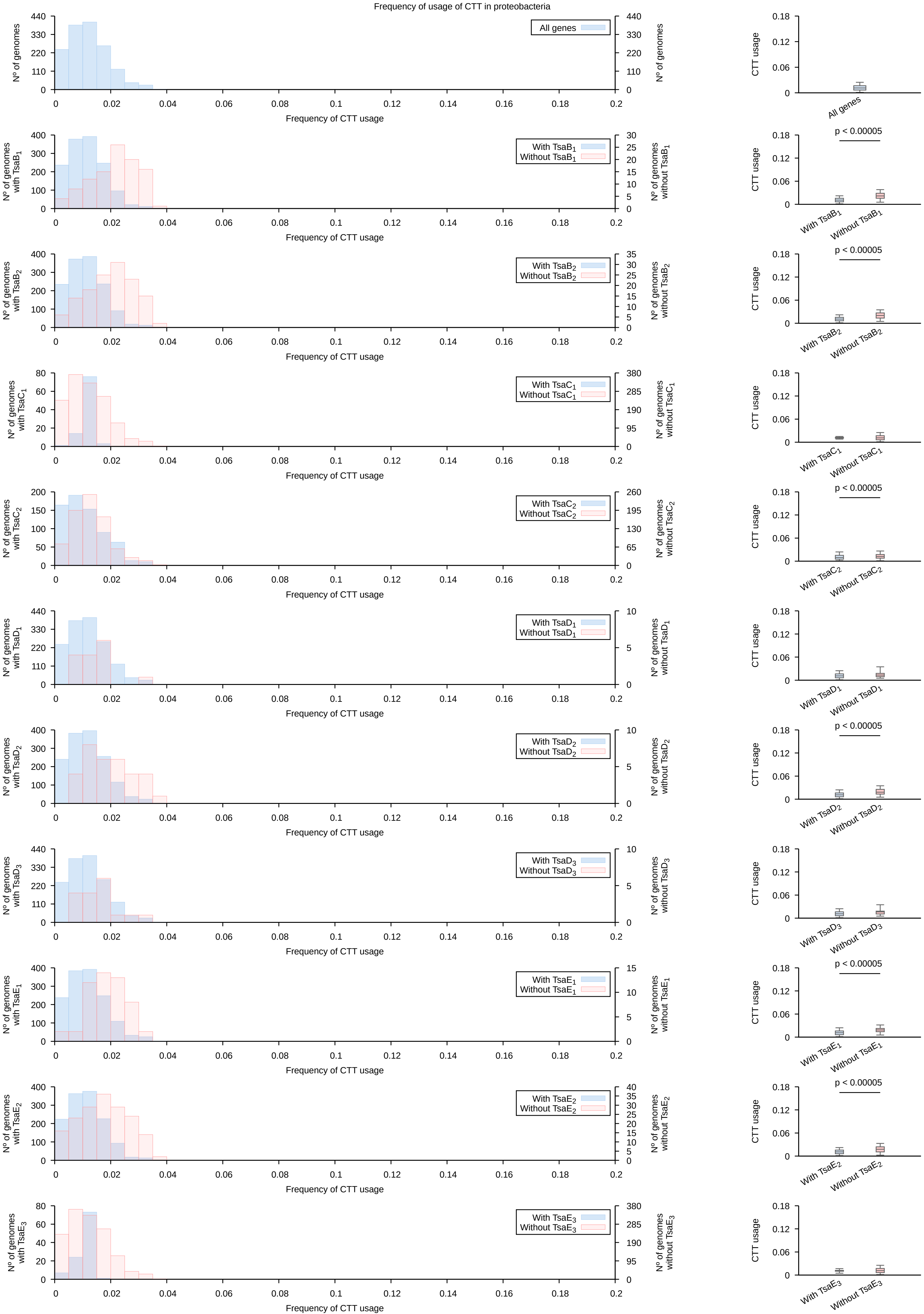
Frequency of usage of CTT in proteobacteria
Nº of genomes
Frequency of CTT usage
CTT usage
All genes
With TsaB1
Without TsaB1
Nº of genomes with TsaB1
Nº of genomes without TsaB1
p < 0.00005
With TsaB2
Without TsaB2
Nº of genomes with TsaB2
Nº of genomes without TsaB2
With TsaC1
Without TsaC1
Nº of genomes with TsaC1
Nº of genomes without TsaC1
With TsaC2
Without TsaC2
Nº of genomes with TsaC2
Nº of genomes without TsaC2
With TsaD1
Without TsaD1
Nº of genomes with TsaD1
Nº of genomes without TsaD1
With TsaD2
Without TsaD2
Nº of genomes with TsaD2
Nº of genomes without TsaD2
With TsaD3
Without TsaD3
Nº of genomes with TsaD3
Nº of genomes without TsaD3
With TsaE1
Without TsaE1
Nº of genomes with TsaE1
Nº of genomes without TsaE1
With TsaE2
Without TsaE2
Nº of genomes with TsaE2
Nº of genomes without TsaE2
With TsaE3
Without TsaE3
Nº of genomes with TsaE3
Nº of genomes without TsaE3

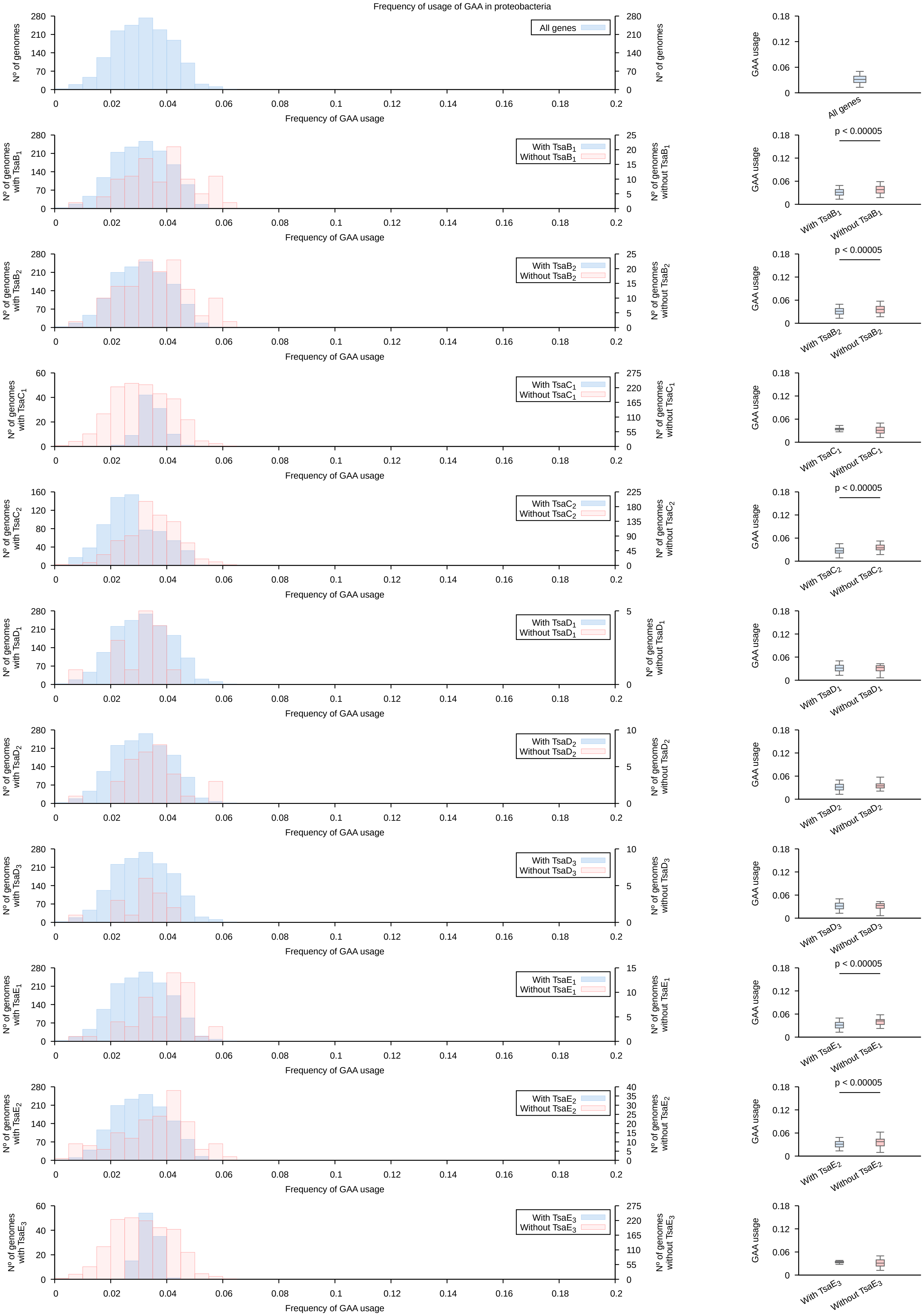
Frequency of usage of GAA in proteobacteria
Nº of genomes
All genes
Frequency of GAA usage
GAA usage
Nº of genomes with $TsaB_1$
Nº of genomes without $TsaB_1$
With $TsaB_1$
Without $TsaB_1$
p < 0.00005
Nº of genomes with $TsaB_2$
Nº of genomes without $TsaB_2$
With $TsaB_2$
Without $TsaB_2$
p < 0.00005
Nº of genomes with $TsaC_1$
Nº of genomes without $TsaC_1$
With $TsaC_1$
Without $TsaC_1$
Nº of genomes with $TsaC_2$
Nº of genomes without $TsaC_2$
With $TsaC_2$
Without $TsaC_2$
p < 0.00005
Nº of genomes with $TsaD_1$
Nº of genomes without $TsaD_1$
With $TsaD_1$
Without $TsaD_1$
Nº of genomes with $TsaD_2$
Nº of genomes without $TsaD_2$
With $TsaD_2$
Without $TsaD_2$
Nº of genomes with $TsaD_3$
Nº of genomes without $TsaD_3$
With $TsaD_3$
Without $TsaD_3$
Nº of genomes with $TsaE_1$
Nº of genomes without $TsaE_1$
With $TsaE_1$
Without $TsaE_1$
p < 0.00005
Nº of genomes with $TsaE_2$
Nº of genomes without $TsaE_2$
With $TsaE_2$
Without $TsaE_2$
p < 0.00005
Nº of genomes with $TsaE_3$
Nº of genomes without $TsaE_3$
With $TsaE_3$
Without $TsaE_3$

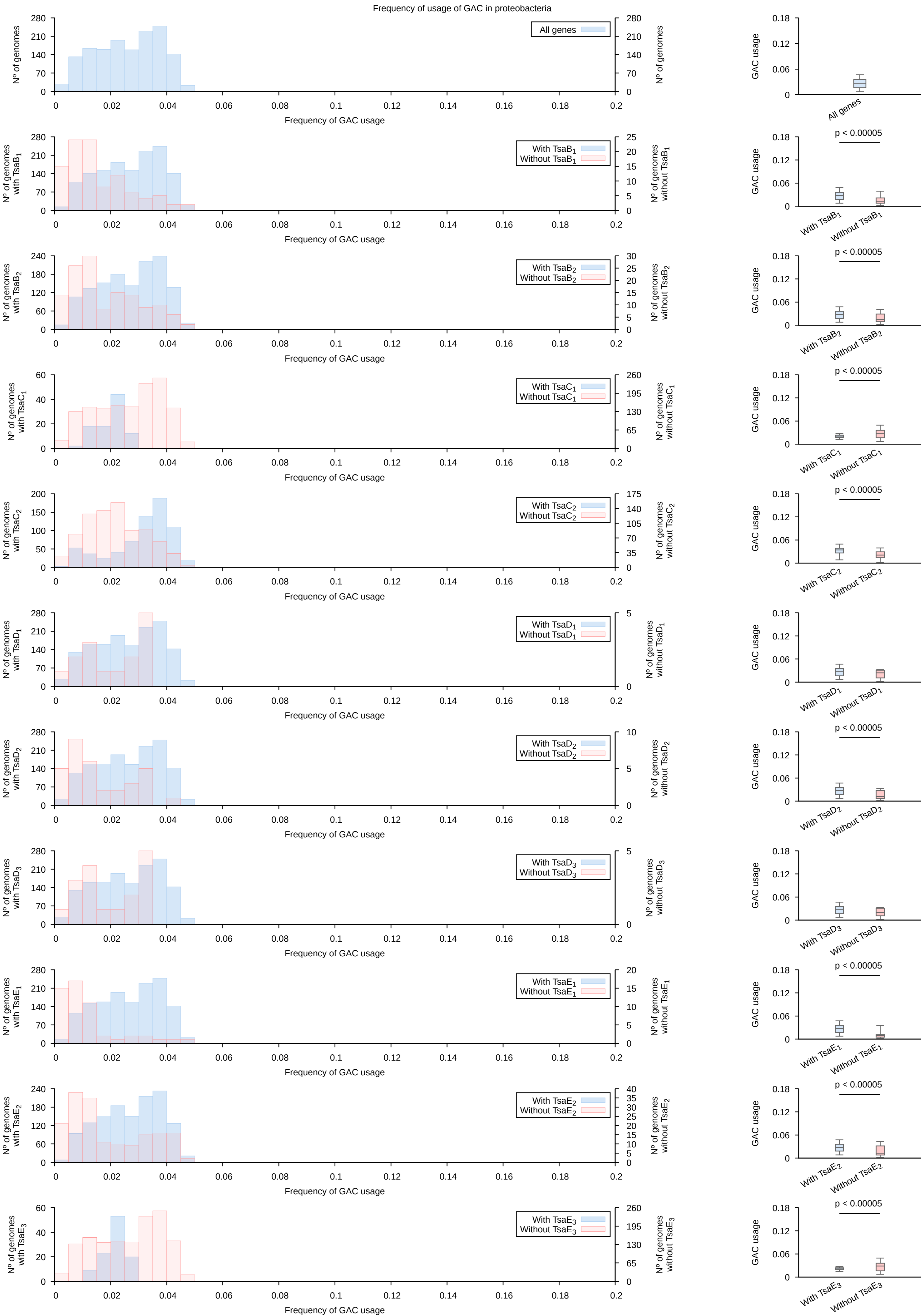
Frequency of usage of GAC in proteobacteria
All genes
Nº of genomes
Frequency of GAC usage
GAC usage
All genes
With TsaB$_1$
Without TsaB$_1$
Nº of genomes with TsaB$_1$
Nº of genomes without TsaB$_1$
p < 0.00005
With TsaB$_2$
Without TsaB$_2$
Nº of genomes with TsaB$_2$
Nº of genomes without TsaB$_2$
p < 0.00005
With TsaC$_1$
Without TsaC$_1$
Nº of genomes with TsaC$_1$
Nº of genomes without TsaC$_1$
p < 0.00005
With TsaC$_2$
Without TsaC$_2$
Nº of genomes with TsaC$_2$
Nº of genomes without TsaC$_2$
p < 0.00005
With TsaD$_1$
Without TsaD$_1$
Nº of genomes with TsaD$_1$
Nº of genomes without TsaD$_1$
With TsaD$_2$
Without TsaD$_2$
Nº of genomes with TsaD$_2$
Nº of genomes without TsaD$_2$
p < 0.00005
With TsaD$_3$
Without TsaD$_3$
Nº of genomes with TsaD$_3$
Nº of genomes without TsaD$_3$
With TsaE$_1$
Without TsaE$_1$
Nº of genomes with TsaE$_1$
Nº of genomes without TsaE$_1$
p < 0.00005
With TsaE$_2$
Without TsaE$_2$
Nº of genomes with TsaE$_2$
Nº of genomes without TsaE$_2$
p < 0.00005
With TsaE$_3$
Without TsaE$_3$
Nº of genomes with TsaE$_3$
Nº of genomes without TsaE$_3$
p < 0.00005
0 0.02 0.04 0.06 0.08 0.1 0.12 0.14 0.16 0.18 0.2
0 0.06 0.12 0.18

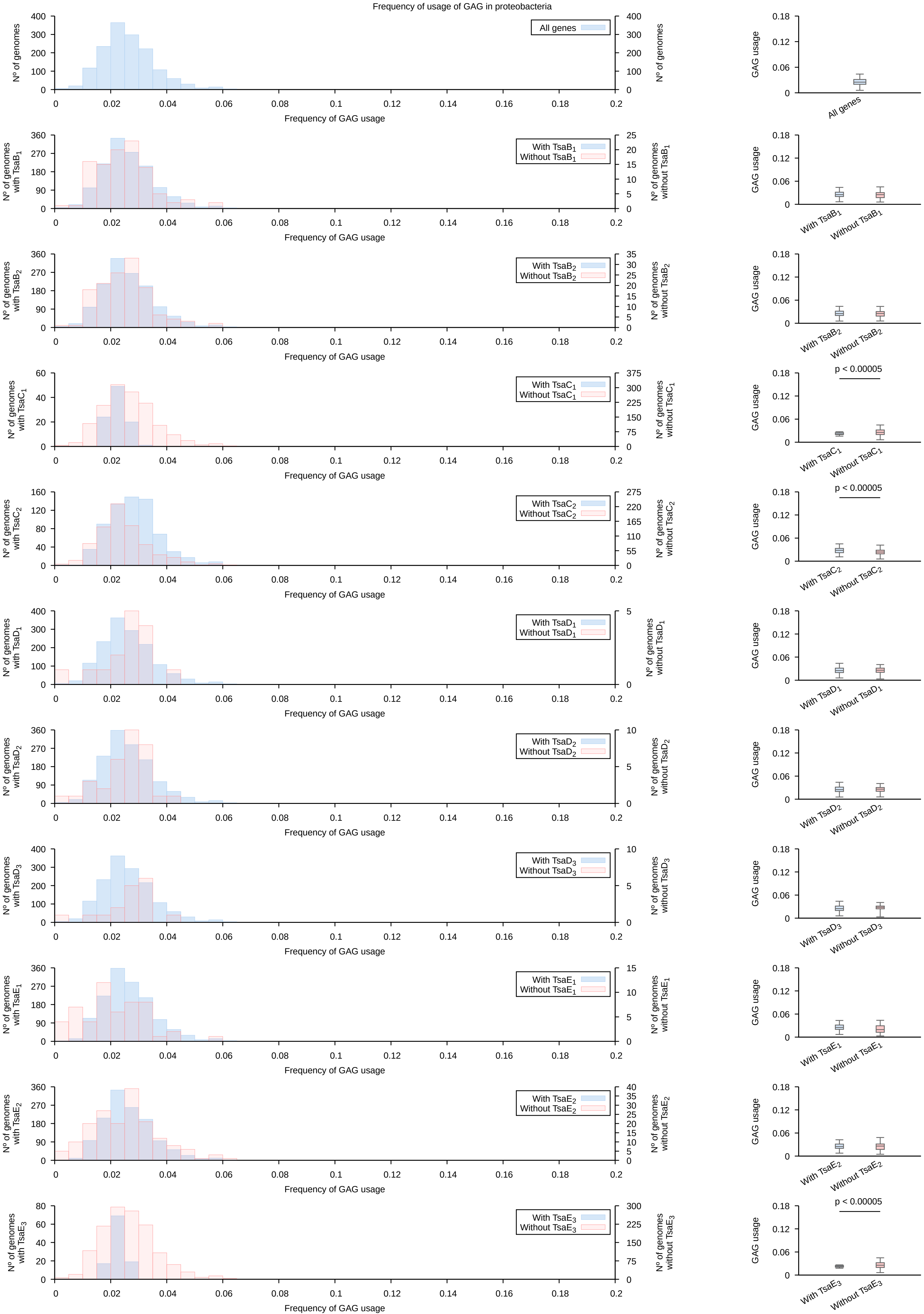
Frequency of usage of GAG in proteobacteria
All genes
Nº of genomes
GAG usage
Frequency of GAG usage
With TsaB$_1$
Without TsaB$_1$
Nº of genomes with TsaB$_1$
Nº of genomes without TsaB$_1$
With TsaB$_2$
Without TsaB$_2$
Nº of genomes with TsaB$_2$
Nº of genomes without TsaB$_2$
With TsaC$_1$
Without TsaC$_1$
Nº of genomes with TsaC$_1$
Nº of genomes without TsaC$_1$
p < 0.00005
With TsaC$_2$
Without TsaC$_2$
Nº of genomes with TsaC$_2$
Nº of genomes without TsaC$_2$
p < 0.00005
With TsaD$_1$
Without TsaD$_1$
Nº of genomes with TsaD$_1$
Nº of genomes without TsaD$_1$
With TsaD$_2$
Without TsaD$_2$
Nº of genomes with TsaD$_2$
Nº of genomes without TsaD$_2$
With TsaD$_3$
Without TsaD$_3$
Nº of genomes with TsaD$_3$
Nº of genomes without TsaD$_3$
With TsaE$_1$
Without TsaE$_1$
Nº of genomes with TsaE$_1$
Nº of genomes without TsaE$_1$
With TsaE$_2$
Without TsaE$_2$
Nº of genomes with TsaE$_2$
Nº of genomes without TsaE$_2$
With TsaE$_3$
Without TsaE$_3$
Nº of genomes with TsaE$_3$
Nº of genomes without TsaE$_3$
p < 0.00005

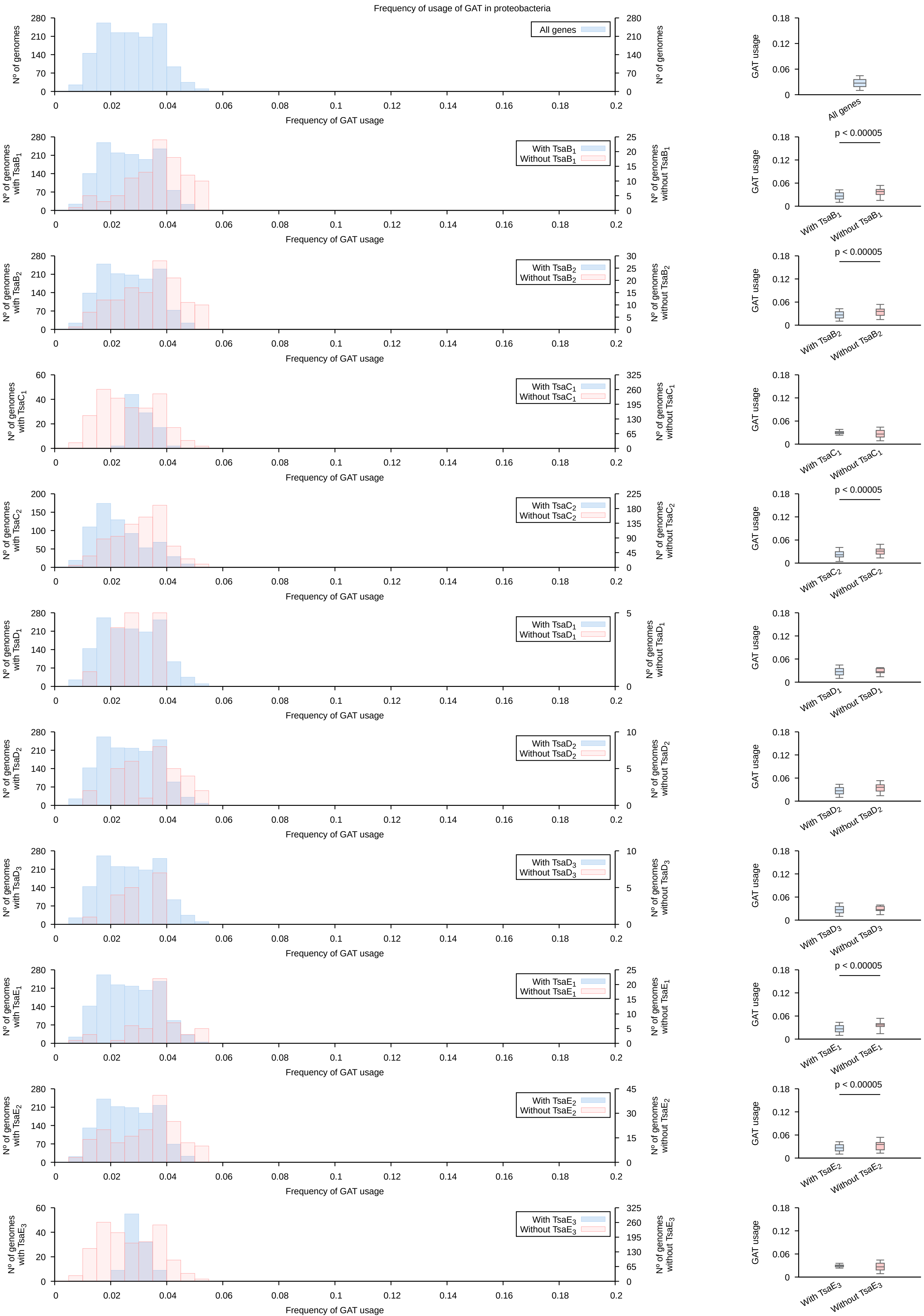

Frequency of usage of GAT in proteobacteria
All genes
Nº of genomes
Frequency of GAT usage
GAT usage
With TsaB1
Without TsaB1
Nº of genomes with TsaB1
Nº of genomes without TsaB1
p < 0.00005
With TsaB2
Without TsaB2
Nº of genomes with TsaB2
Nº of genomes without TsaB2
With TsaC1
Without TsaC1
Nº of genomes with TsaC1
Nº of genomes without TsaC1
With TsaC2
Without TsaC2
Nº of genomes with TsaC2
Nº of genomes without TsaC2
With TsaD1
Without TsaD1
Nº of genomes with TsaD1
Nº of genomes without TsaD1
With TsaD2
Without TsaD2
Nº of genomes with TsaD2
Nº of genomes without TsaD2
With TsaD3
Without TsaD3
Nº of genomes with TsaD3
Nº of genomes without TsaD3
With TsaE1
Without TsaE1
Nº of genomes with TsaE1
Nº of genomes without TsaE1
With TsaE2
Without TsaE2
Nº of genomes with TsaE2
Nº of genomes without TsaE2
With TsaE3
Without TsaE3
Nº of genomes with TsaE3
Nº of genomes without TsaE3
0 0.02 0.04 0.06 0.08 0.1 0.12 0.14 0.16 0.18 0.2
0 0.06 0.12 0.18

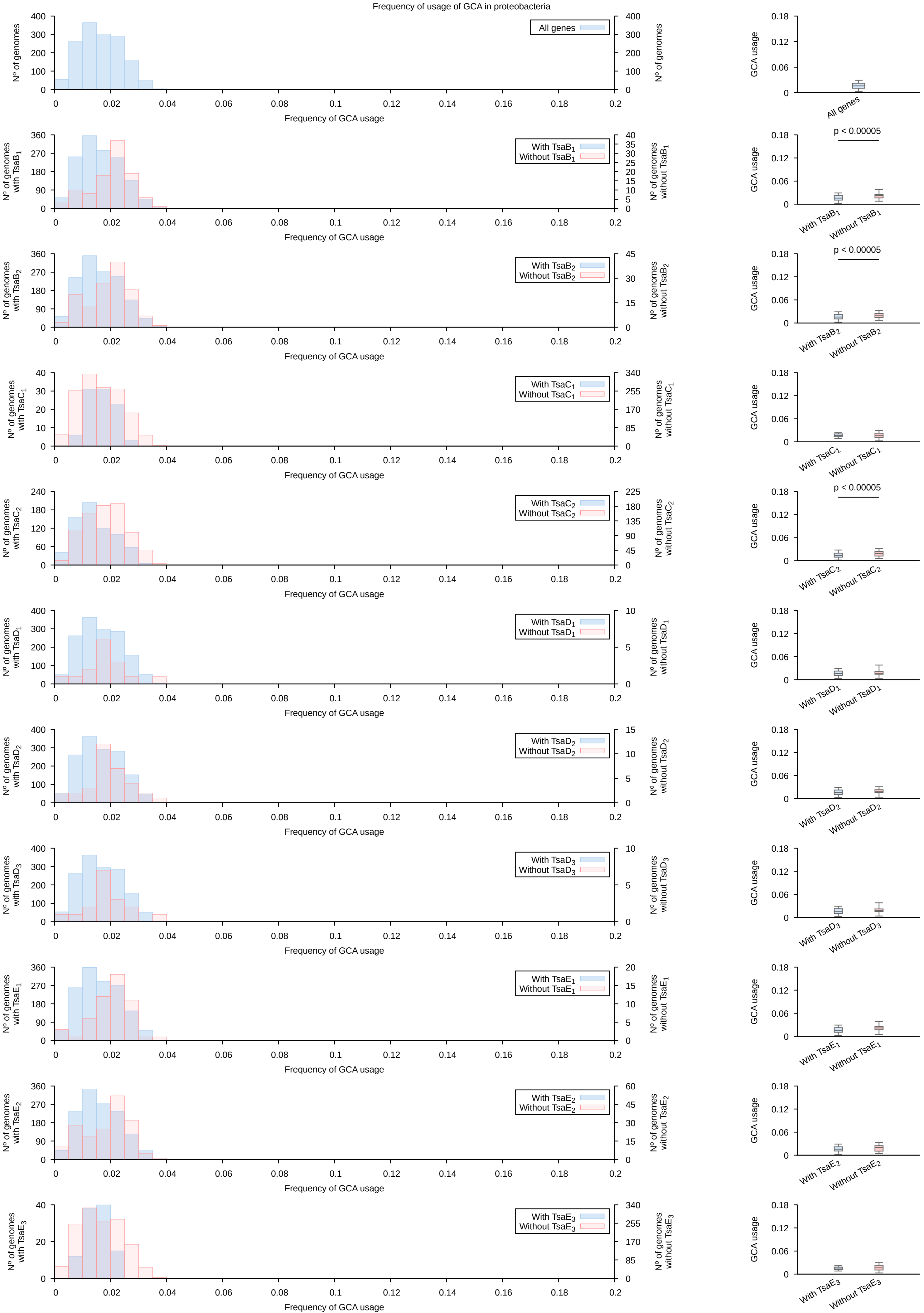

Frequency of usage of GCA in proteobacteria
Nº of genomes
Frequency of GCA usage
All genes
GCA usage
Nº of genomes with $TsaB_1$
Nº of genomes without $TsaB_1$
With $TsaB_1$
Without $TsaB_1$
$p < 0.00005$
Nº of genomes with $TsaB_2$
Nº of genomes without $TsaB_2$
With $TsaB_2$
Without $TsaB_2$
$p < 0.00005$
Nº of genomes with $TsaC_1$
Nº of genomes without $TsaC_1$
With $TsaC_1$
Without $TsaC_1$
Nº of genomes with $TsaC_2$
Nº of genomes without $TsaC_2$
With $TsaC_2$
Without $TsaC_2$
$p < 0.00005$
Nº of genomes with $TsaD_1$
Nº of genomes without $TsaD_1$
With $TsaD_1$
Without $TsaD_1$
Nº of genomes with $TsaD_2$
Nº of genomes without $TsaD_2$
With $TsaD_2$
Without $TsaD_2$
Nº of genomes with $TsaD_3$
Nº of genomes without $TsaD_3$
With $TsaD_3$
Without $TsaD_3$
Nº of genomes with $TsaE_1$
Nº of genomes without $TsaE_1$
With $TsaE_1$
Without $TsaE_1$
Nº of genomes with $TsaE_2$
Nº of genomes without $TsaE_2$
With $TsaE_2$
Without $TsaE_2$
Nº of genomes with $TsaE_3$
Nº of genomes without $TsaE_3$
With $TsaE_3$
Without $TsaE_3$

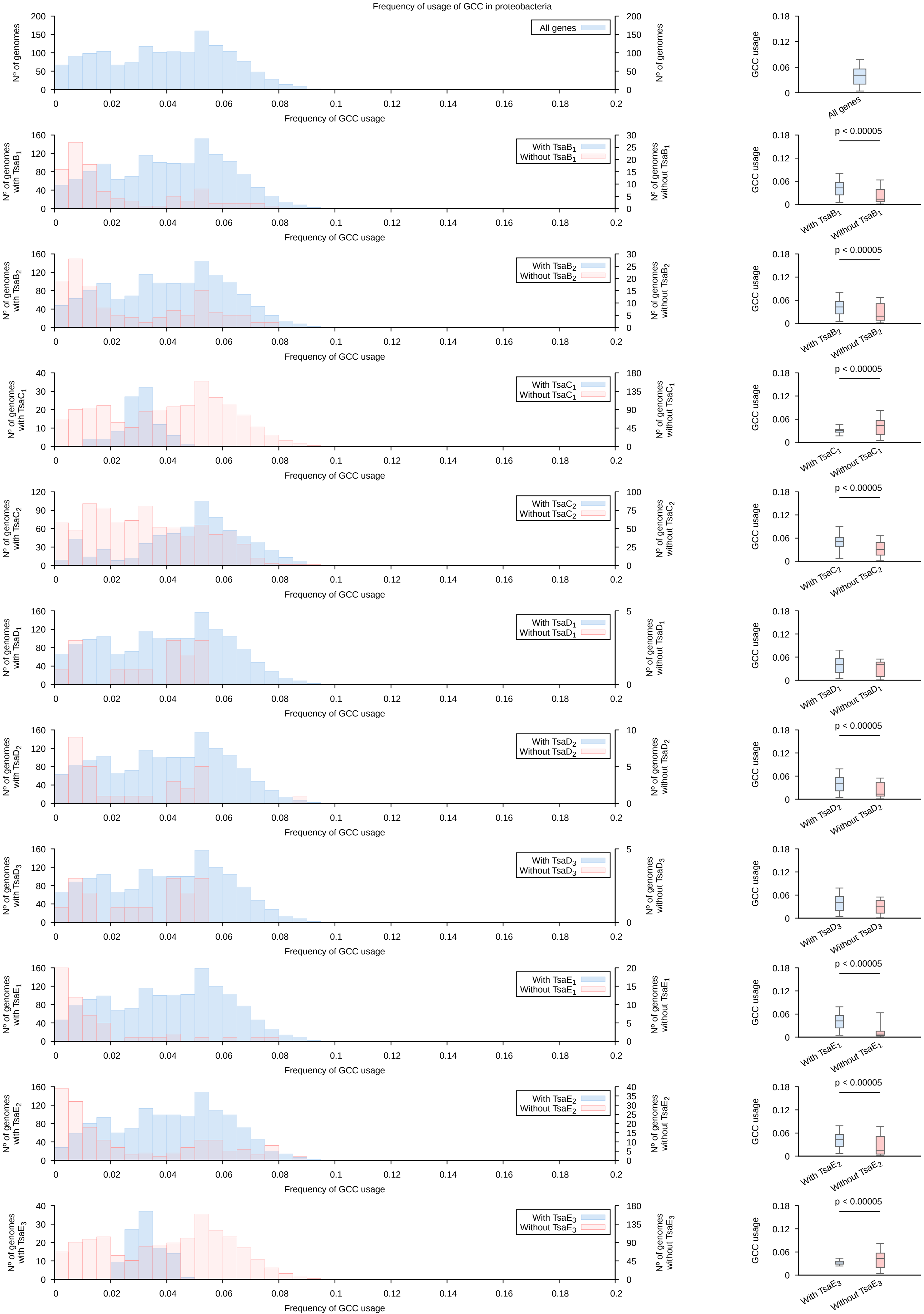

Frequency of usage of GCC in proteobacteria
All genes
Nº of genomes
Frequency of GCC usage
GCC usage
With TsaB$_1$
Without TsaB$_1$
Nº of genomes with TsaB$_1$
Nº of genomes without TsaB$_1$
With TsaB$_2$
Without TsaB$_2$
Nº of genomes with TsaB$_2$
Nº of genomes without TsaB$_2$
With TsaC$_1$
Without TsaC$_1$
Nº of genomes with TsaC$_1$
Nº of genomes without TsaC$_1$
With TsaC$_2$
Without TsaC$_2$
Nº of genomes with TsaC$_2$
Nº of genomes without TsaC$_2$
With TsaD$_1$
Without TsaD$_1$
Nº of genomes with TsaD$_1$
Nº of genomes without TsaD$_1$
With TsaD$_2$
Without TsaD$_2$
Nº of genomes with TsaD$_2$
Nº of genomes without TsaD$_2$
With TsaD$_3$
Without TsaD$_3$
Nº of genomes with TsaD$_3$
Nº of genomes without TsaD$_3$
With TsaE$_1$
Without TsaE$_1$
Nº of genomes with TsaE$_1$
Nº of genomes without TsaE$_1$
With TsaE$_2$
Without TsaE$_2$
Nº of genomes with TsaE$_2$
Nº of genomes without TsaE$_2$
With TsaE$_3$
Without TsaE$_3$
Nº of genomes with TsaE$_3$
Nº of genomes without TsaE$_3$
p < 0.00005

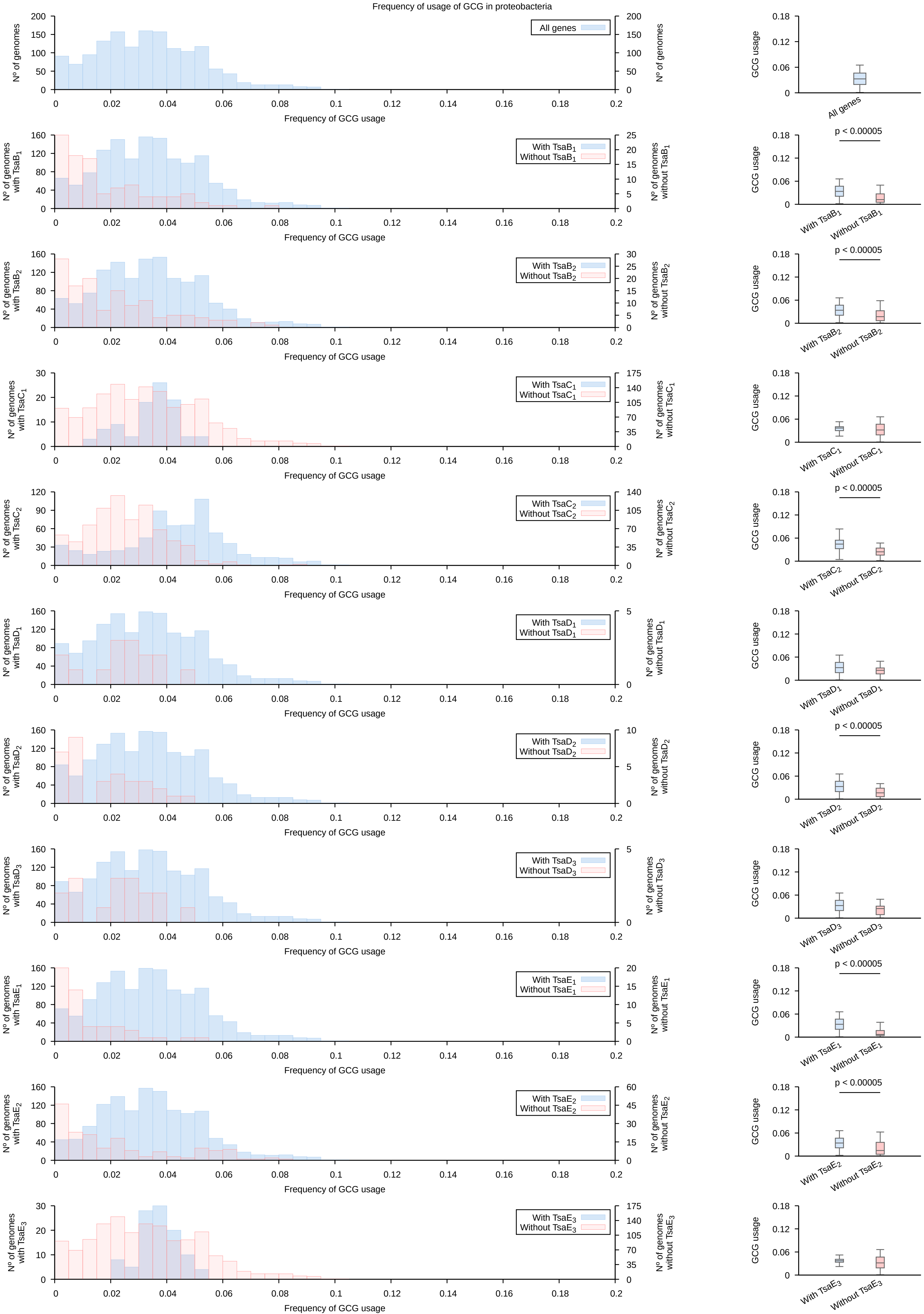
Frequency of usage of GCG in proteobacteria
All genes
With TsaB$_1$
Without TsaB$_1$
With TsaB$_2$
Without TsaB$_2$
With TsaC$_1$
Without TsaC$_1$
With TsaC$_2$
Without TsaC$_2$
With TsaD$_1$
Without TsaD$_1$
With TsaD$_2$
Without TsaD$_2$
With TsaD$_3$
Without TsaD$_3$
With TsaE$_1$
Without TsaE$_1$
With TsaE$_2$
Without TsaE$_2$
With TsaE$_3$
Without TsaE$_3$
Nº of genomes
Frequency of GCG usage
GCG usage
p < 0.00005

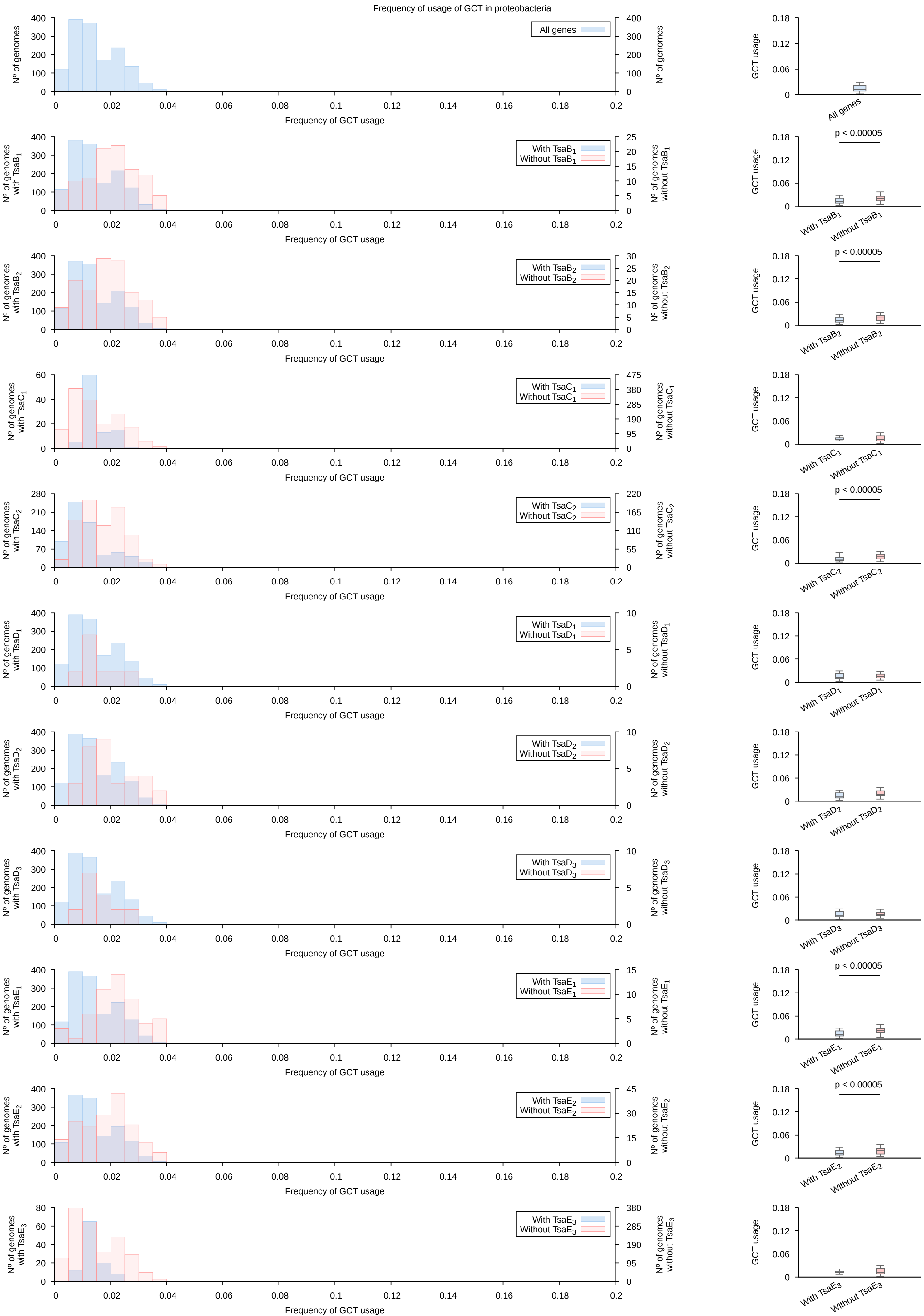
Frequency of usage of GCT in proteobacteria
Nº of genomes
All genes
Frequency of GCT usage
GCT usage
All genes
Nº of genomes with $TsaB_1$
With $TsaB_1$
Without $TsaB_1$
Nº of genomes without $TsaB_1$
$p < 0.00005$
Nº of genomes with $TsaB_2$
With $TsaB_2$
Without $TsaB_2$
Nº of genomes without $TsaB_2$
$p < 0.00005$
Nº of genomes with $TsaC_1$
With $TsaC_1$
Without $TsaC_1$
Nº of genomes without $TsaC_1$
Nº of genomes with $TsaC_2$
With $TsaC_2$
Without $TsaC_2$
Nº of genomes without $TsaC_2$
$p < 0.00005$
Nº of genomes with $TsaD_1$
With $TsaD_1$
Without $TsaD_1$
Nº of genomes without $TsaD_1$
Nº of genomes with $TsaD_2$
With $TsaD_2$
Without $TsaD_2$
Nº of genomes without $TsaD_2$
Nº of genomes with $TsaD_3$
With $TsaD_3$
Without $TsaD_3$
Nº of genomes without $TsaD_3$
Nº of genomes with $TsaE_1$
With $TsaE_1$
Without $TsaE_1$
Nº of genomes without $TsaE_1$
$p < 0.00005$
Nº of genomes with $TsaE_2$
With $TsaE_2$
Without $TsaE_2$
Nº of genomes without $TsaE_2$
$p < 0.00005$
Nº of genomes with $TsaE_3$
With $TsaE_3$
Without $TsaE_3$
Nº of genomes without $TsaE_3$

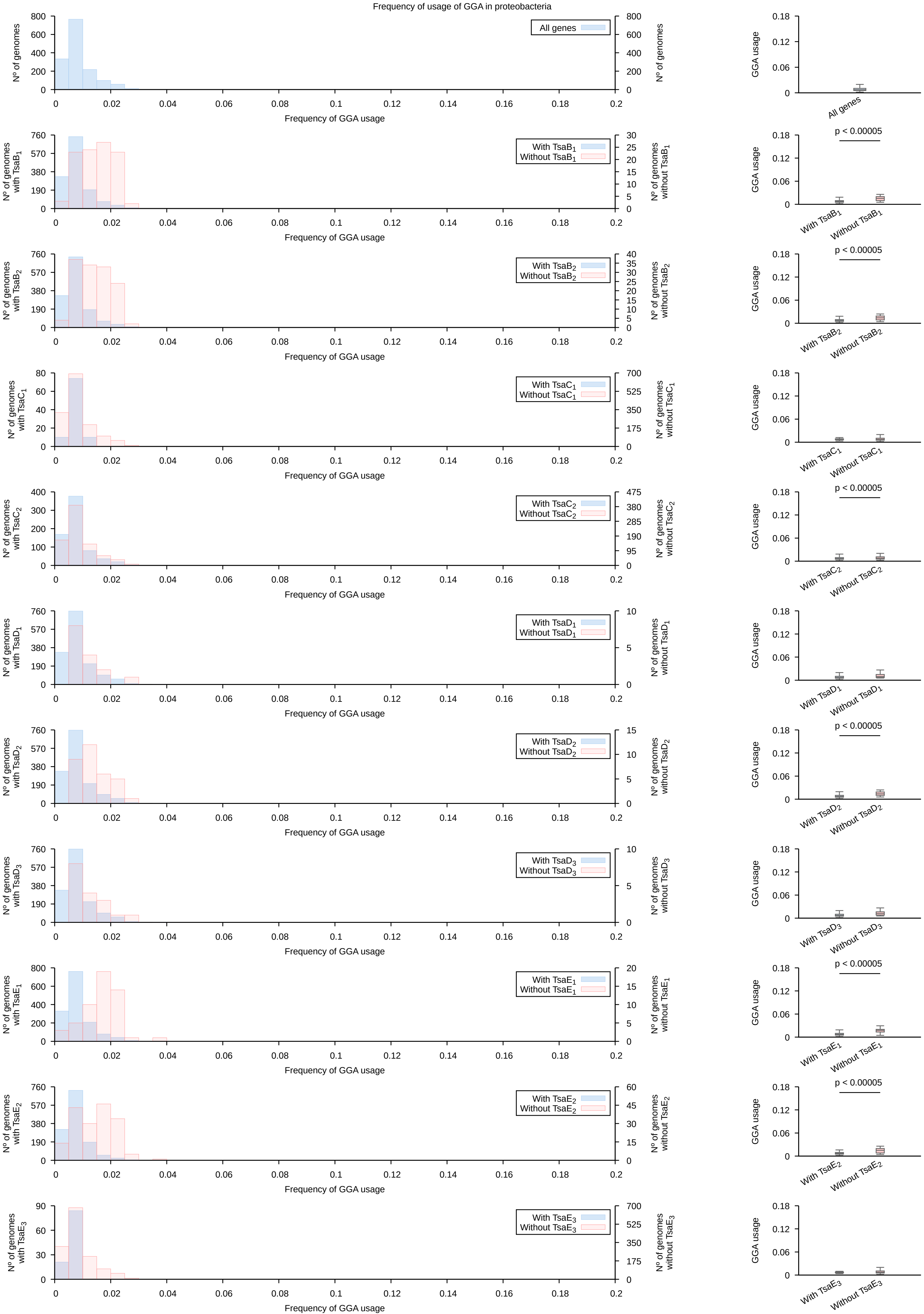
Frequency of usage of GGA in proteobacteria
All genes
Nº of genomes
Frequency of GGA usage
GGA usage
With TsaB$_1$
Without TsaB$_1$
Nº of genomes with TsaB$_1$
Nº of genomes without TsaB$_1$
p < 0.00005
With TsaB$_2$
Without TsaB$_2$
Nº of genomes with TsaB$_2$
Nº of genomes without TsaB$_2$
p < 0.00005
With TsaC$_1$
Without TsaC$_1$
Nº of genomes with TsaC$_1$
Nº of genomes without TsaC$_1$
With TsaC$_2$
Without TsaC$_2$
Nº of genomes with TsaC$_2$
Nº of genomes without TsaC$_2$
p < 0.00005
With TsaD$_1$
Without TsaD$_1$
Nº of genomes with TsaD$_1$
Nº of genomes without TsaD$_1$
With TsaD$_2$
Without TsaD$_2$
Nº of genomes with TsaD$_2$
Nº of genomes without TsaD$_2$
p < 0.00005
With TsaD$_3$
Without TsaD$_3$
Nº of genomes with TsaD$_3$
Nº of genomes without TsaD$_3$
With TsaE$_1$
Without TsaE$_1$
Nº of genomes with TsaE$_1$
Nº of genomes without TsaE$_1$
p < 0.00005
With TsaE$_2$
Without TsaE$_2$
Nº of genomes with TsaE$_2$
Nº of genomes without TsaE$_2$
p < 0.00005
With TsaE$_3$
Without TsaE$_3$
Nº of genomes with TsaE$_3$
Nº of genomes without TsaE$_3$

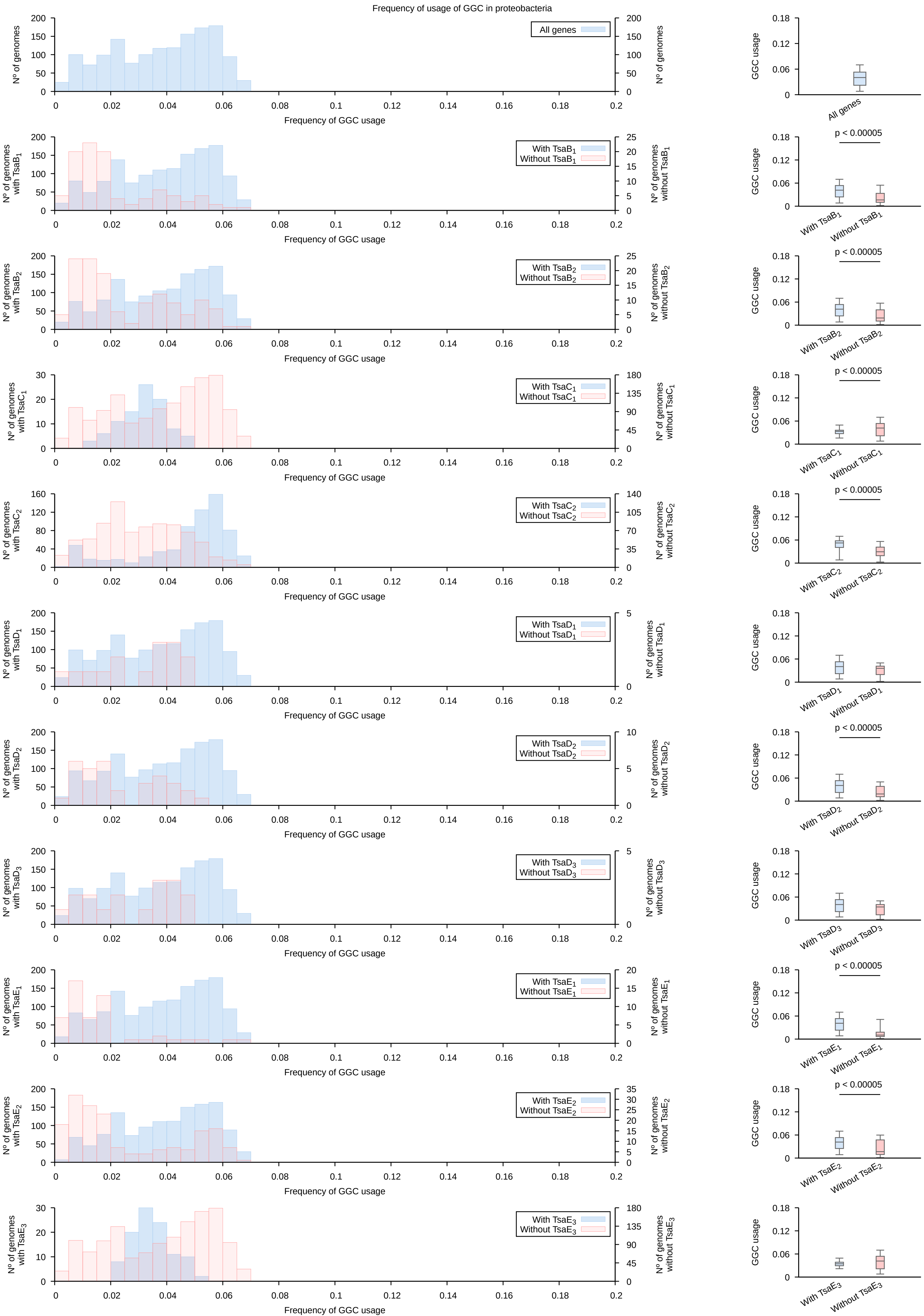
Frequency of usage of GGC in proteobacteria
All genes
Nº of genomes
Frequency of GGC usage
GGC usage
With TsaB1
Without TsaB1
Nº of genomes with TsaB1
Nº of genomes without TsaB1
With TsaB2
Without TsaB2
Nº of genomes with TsaB2
Nº of genomes without TsaB2
With TsaC1
Without TsaC1
Nº of genomes with TsaC1
Nº of genomes without TsaC1
With TsaC2
Without TsaC2
Nº of genomes with TsaC2
Nº of genomes without TsaC2
With TsaD1
Without TsaD1
Nº of genomes with TsaD1
Nº of genomes without TsaD1
With TsaD2
Without TsaD2
Nº of genomes with TsaD2
Nº of genomes without TsaD2
With TsaD3
Without TsaD3
Nº of genomes with TsaD3
Nº of genomes without TsaD3
With TsaE1
Without TsaE1
Nº of genomes with TsaE1
Nº of genomes without TsaE1
With TsaE2
Without TsaE2
Nº of genomes with TsaE2
Nº of genomes without TsaE2
With TsaE3
Without TsaE3
Nº of genomes with TsaE3
Nº of genomes without TsaE3
p < 0.00005
0 0.02 0.04 0.06 0.08 0.1 0.12 0.14 0.16 0.18 0.2
0 0.06 0.12 0.18

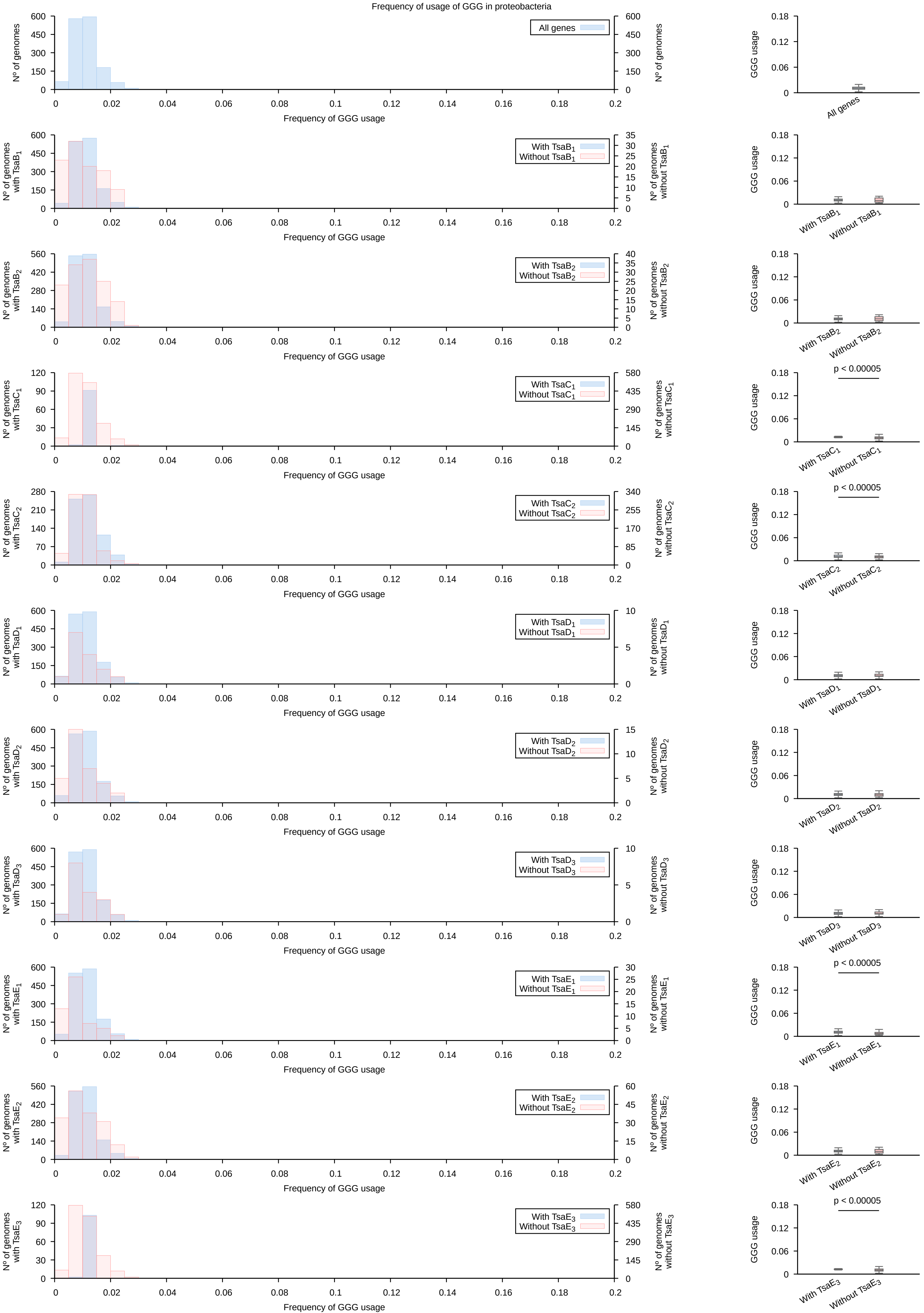
Frequency of usage of GGG in proteobacteria
All genes
Nº of genomes
Frequency of GGG usage
GGG usage
With TsaB1
Without TsaB1
Nº of genomes with TsaB1
Nº of genomes without TsaB1
With TsaB2
Without TsaB2
Nº of genomes with TsaB2
Nº of genomes without TsaB2
With TsaC1
Without TsaC1
Nº of genomes with TsaC1
Nº of genomes without TsaC1
p < 0.00005
With TsaC2
Without TsaC2
Nº of genomes with TsaC2
Nº of genomes without TsaC2
p < 0.00005
With TsaD1
Without TsaD1
Nº of genomes with TsaD1
Nº of genomes without TsaD1
With TsaD2
Without TsaD2
Nº of genomes with TsaD2
Nº of genomes without TsaD2
With TsaD3
Without TsaD3
Nº of genomes with TsaD3
Nº of genomes without TsaD3
With TsaE1
Without TsaE1
Nº of genomes with TsaE1
Nº of genomes without TsaE1
p < 0.00005
With TsaE2
Without TsaE2
Nº of genomes with TsaE2
Nº of genomes without TsaE2
With TsaE3
Without TsaE3
Nº of genomes with TsaE3
Nº of genomes without TsaE3
p < 0.00005

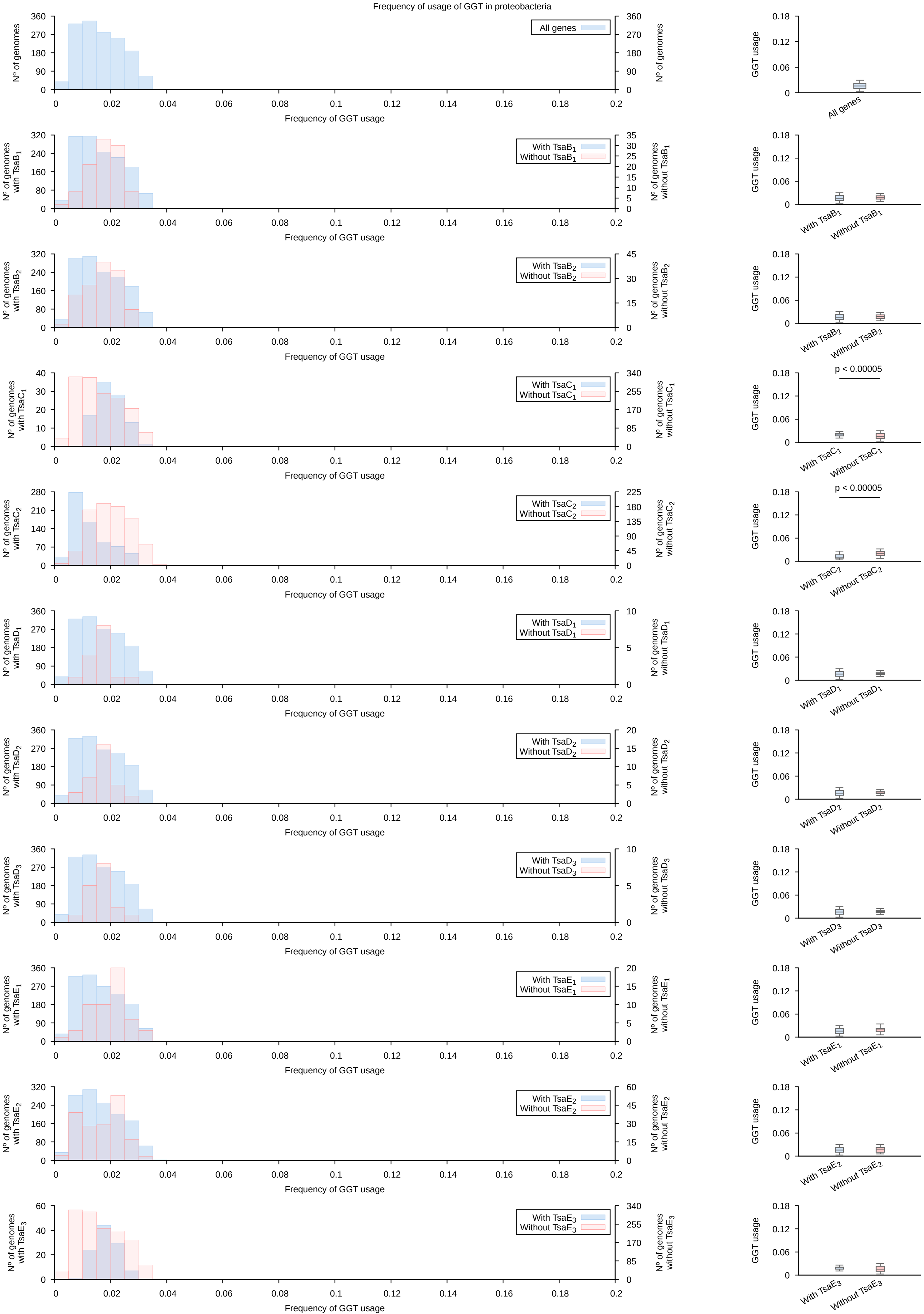
Frequency of usage of GGT in proteobacteria
Nº of genomes
0
90
180
270
360
All genes
0
0.02
0.04
0.06
0.08
0.1
0.12
0.14
0.16
0.18
0.2
Frequency of GGT usage
GGT usage
0
0.06
0.12
0.18
All genes
Nº of genomes with TsaB1
0
80
160
240
320
With TsaB1
Without TsaB1
Nº of genomes without TsaB1
0
5
10
15
20
25
30
35
Frequency of GGT usage
GGT usage
With TsaB1
Without TsaB1
Nº of genomes with TsaB2
With TsaB2
Without TsaB2
Nº of genomes without TsaB2
0
15
30
45
Frequency of GGT usage
GGT usage
With TsaB2
Without TsaB2
Nº of genomes with TsaC1
0
10
20
30
40
With TsaC1
Without TsaC1
Nº of genomes without TsaC1
0
85
170
255
340
Frequency of GGT usage
GGT usage
p < 0.00005
With TsaC1
Without TsaC1
Nº of genomes with TsaC2
0
70
140
210
280
With TsaC2
Without TsaC2
Nº of genomes without TsaC2
0
45
90
135
180
225
Frequency of GGT usage
GGT usage
p < 0.00005
With TsaC2
Without TsaC2
Nº of genomes with TsaD1
0
90
180
270
360
With TsaD1
Without TsaD1
Nº of genomes without TsaD1
0
5
10
Frequency of GGT usage
GGT usage
With TsaD1
Without TsaD1
Nº of genomes with TsaD2
With TsaD2
Without TsaD2
Nº of genomes without TsaD2
0
5
10
15
20
Frequency of GGT usage
GGT usage
With TsaD2
Without TsaD2
Nº of genomes with TsaD3
With TsaD3
Without TsaD3
Nº of genomes without TsaD3
0
5
10
Frequency of GGT usage
GGT usage
With TsaD3
Without TsaD3
Nº of genomes with TsaE1
With TsaE1
Without TsaE1
Nº of genomes without TsaE1
0
5
10
15
20
Frequency of GGT usage
GGT usage
With TsaE1
Without TsaE1
Nº of genomes with TsaE2
0
80
160
240
320
With TsaE2
Without TsaE2
Nº of genomes without TsaE2
0
15
30
45
60
Frequency of GGT usage
GGT usage
With TsaE2
Without TsaE2
Nº of genomes with TsaE3
0
20
40
60
With TsaE3
Without TsaE3
Nº of genomes without TsaE3
0
85
170
255
340
Frequency of GGT usage
GGT usage
With TsaE3
Without TsaE3

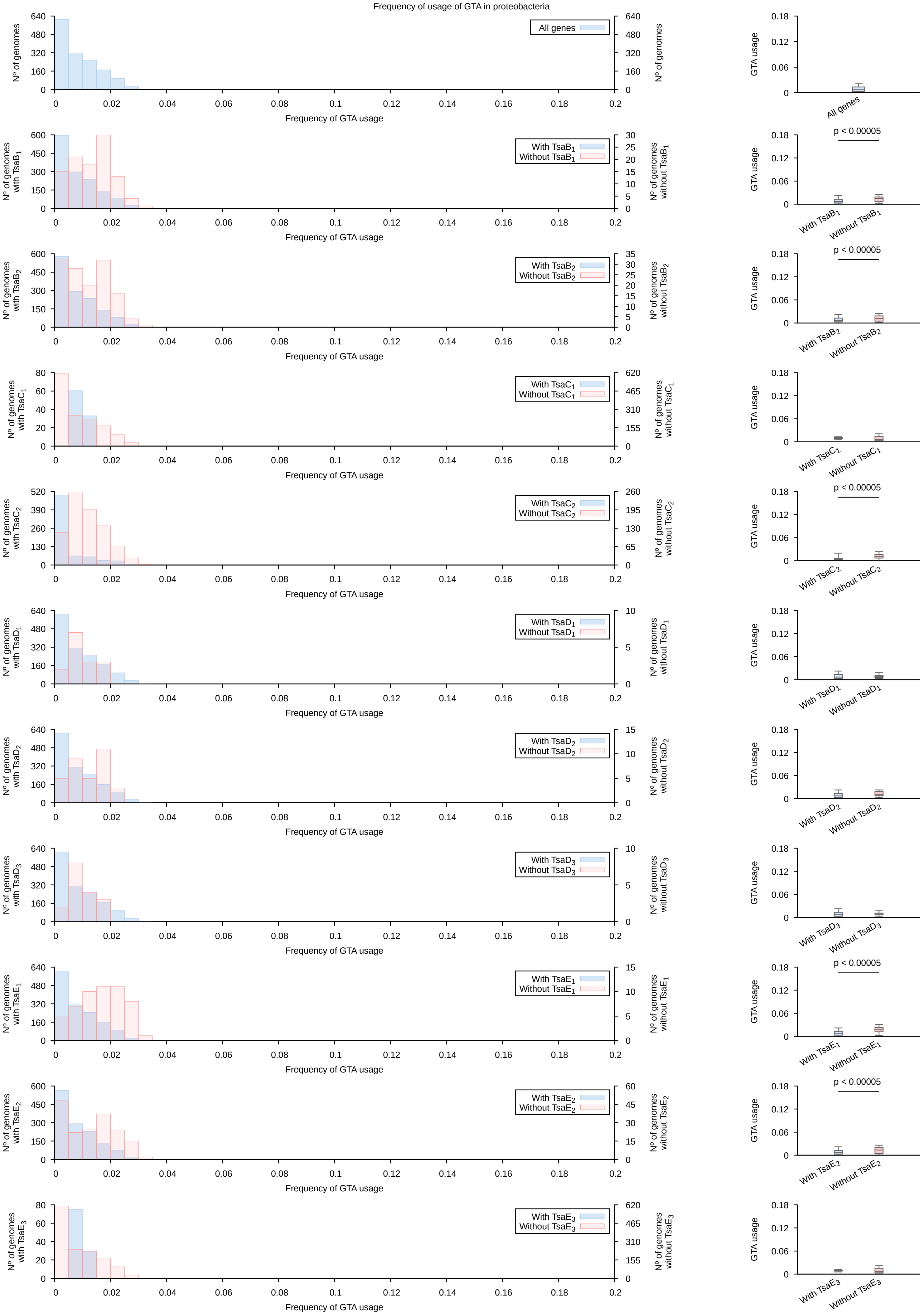

Frequency of usage of GTA in proteobacteria
Nº of genomes
All genes
Frequency of GTA usage
GTA usage
Nº of genomes with TsaB$_1$
Nº of genomes without TsaB$_1$
With TsaB$_1$
Without TsaB$_1$
p < 0.00005
Nº of genomes with TsaB$_2$
Nº of genomes without TsaB$_2$
With TsaB$_2$
Without TsaB$_2$
p < 0.00005
Nº of genomes with TsaC$_1$
Nº of genomes without TsaC$_1$
With TsaC$_1$
Without TsaC$_1$
Nº of genomes with TsaC$_2$
Nº of genomes without TsaC$_2$
With TsaC$_2$
Without TsaC$_2$
p < 0.00005
Nº of genomes with TsaD$_1$
Nº of genomes without TsaD$_1$
With TsaD$_1$
Without TsaD$_1$
Nº of genomes with TsaD$_2$
Nº of genomes without TsaD$_2$
With TsaD$_2$
Without TsaD$_2$
Nº of genomes with TsaD$_3$
Nº of genomes without TsaD$_3$
With TsaD$_3$
Without TsaD$_3$
Nº of genomes with TsaE$_1$
Nº of genomes without TsaE$_1$
With TsaE$_1$
Without TsaE$_1$
p < 0.00005
Nº of genomes with TsaE$_2$
Nº of genomes without TsaE$_2$
With TsaE$_2$
Without TsaE$_2$
p < 0.00005
Nº of genomes with TsaE$_3$
Nº of genomes without TsaE$_3$
With TsaE$_3$
Without TsaE$_3$

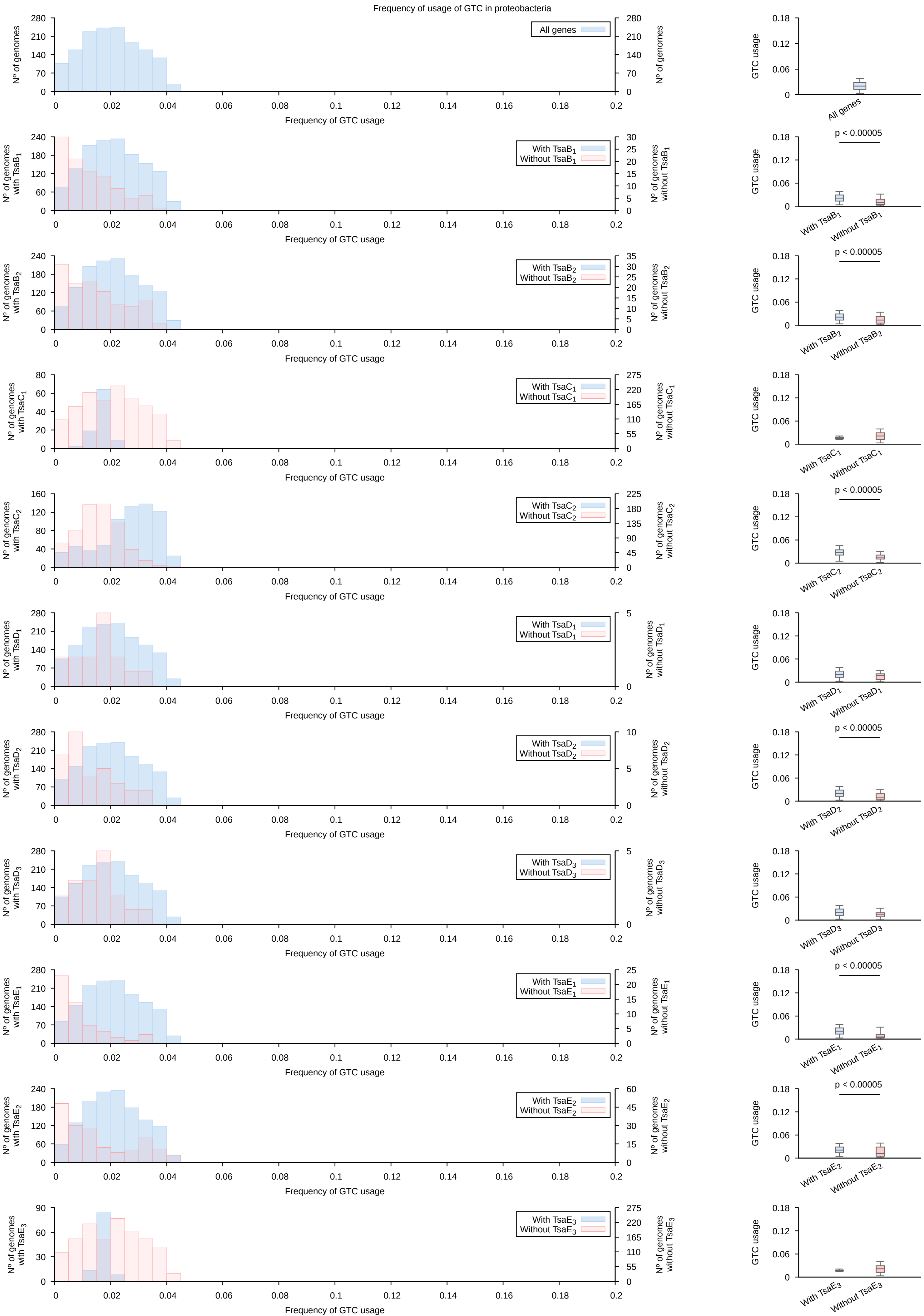
Frequency of usage of GTC in proteobacteria
All genes
Nº of genomes
Frequency of GTC usage
GTC usage
0
0.02
0.04
0.06
0.08
0.1
0.12
0.14
0.16
0.18
0.2
With TsaB$_1$
Without TsaB$_1$
Nº of genomes with TsaB$_1$
Nº of genomes without TsaB$_1$
p < 0.00005
With TsaB$_2$
Without TsaB$_2$
Nº of genomes with TsaB$_2$
Nº of genomes without TsaB$_2$
p < 0.00005
With TsaC$_1$
Without TsaC$_1$
Nº of genomes with TsaC$_1$
Nº of genomes without TsaC$_1$
With TsaC$_2$
Without TsaC$_2$
Nº of genomes with TsaC$_2$
Nº of genomes without TsaC$_2$
p < 0.00005
With TsaD$_1$
Without TsaD$_1$
Nº of genomes with TsaD$_1$
Nº of genomes without TsaD$_1$
With TsaD$_2$
Without TsaD$_2$
Nº of genomes with TsaD$_2$
Nº of genomes without TsaD$_2$
p < 0.00005
With TsaD$_3$
Without TsaD$_3$
Nº of genomes with TsaD$_3$
Nº of genomes without TsaD$_3$
With TsaE$_1$
Without TsaE$_1$
Nº of genomes with TsaE$_1$
Nº of genomes without TsaE$_1$
p < 0.00005
With TsaE$_2$
Without TsaE$_2$
Nº of genomes with TsaE$_2$
Nº of genomes without TsaE$_2$
p < 0.00005
With TsaE$_3$
Without TsaE$_3$
Nº of genomes with TsaE$_3$
Nº of genomes without TsaE$_3$

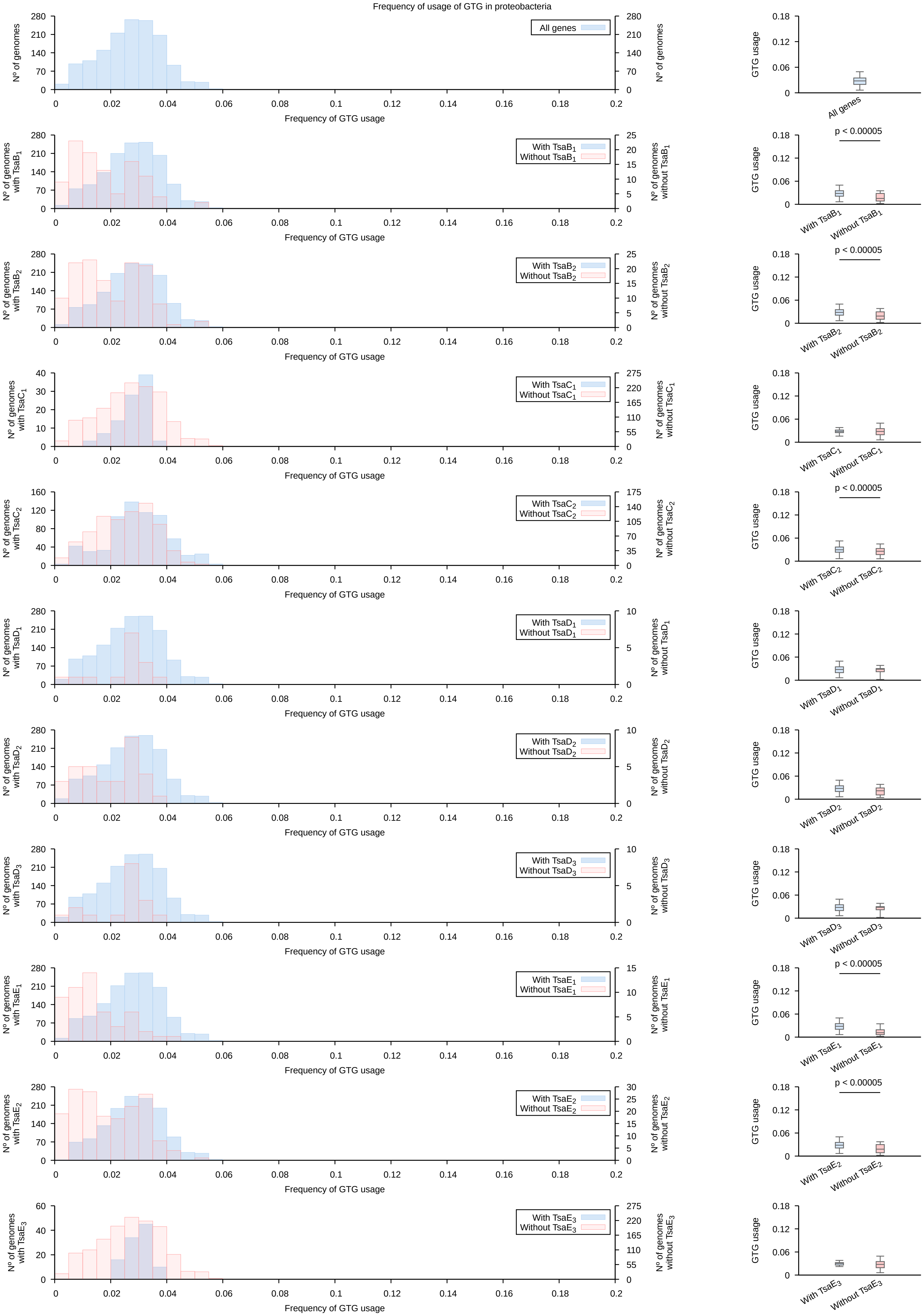

Frequency of usage of GTG in proteobacteria
Nº of genomes
All genes
GTG usage
Frequency of GTG usage
Nº of genomes with TsaB$_1$
Nº of genomes without TsaB$_1$
With TsaB$_1$
Without TsaB$_1$
p < 0.00005
Nº of genomes with TsaB$_2$
Nº of genomes without TsaB$_2$
With TsaB$_2$
Without TsaB$_2$
p < 0.00005
Nº of genomes with TsaC$_1$
Nº of genomes without TsaC$_1$
With TsaC$_1$
Without TsaC$_1$
Nº of genomes with TsaC$_2$
Nº of genomes without TsaC$_2$
With TsaC$_2$
Without TsaC$_2$
p < 0.00005
Nº of genomes with TsaD$_1$
Nº of genomes without TsaD$_1$
With TsaD$_1$
Without TsaD$_1$
Nº of genomes with TsaD$_2$
Nº of genomes without TsaD$_2$
With TsaD$_2$
Without TsaD$_2$
Nº of genomes with TsaD$_3$
Nº of genomes without TsaD$_3$
With TsaD$_3$
Without TsaD$_3$
Nº of genomes with TsaE$_1$
Nº of genomes without TsaE$_1$
With TsaE$_1$
Without TsaE$_1$
p < 0.00005
Nº of genomes with TsaE$_2$
Nº of genomes without TsaE$_2$
With TsaE$_2$
Without TsaE$_2$
p < 0.00005
Nº of genomes with TsaE$_3$
Nº of genomes without TsaE$_3$
With TsaE$_3$
Without TsaE$_3$

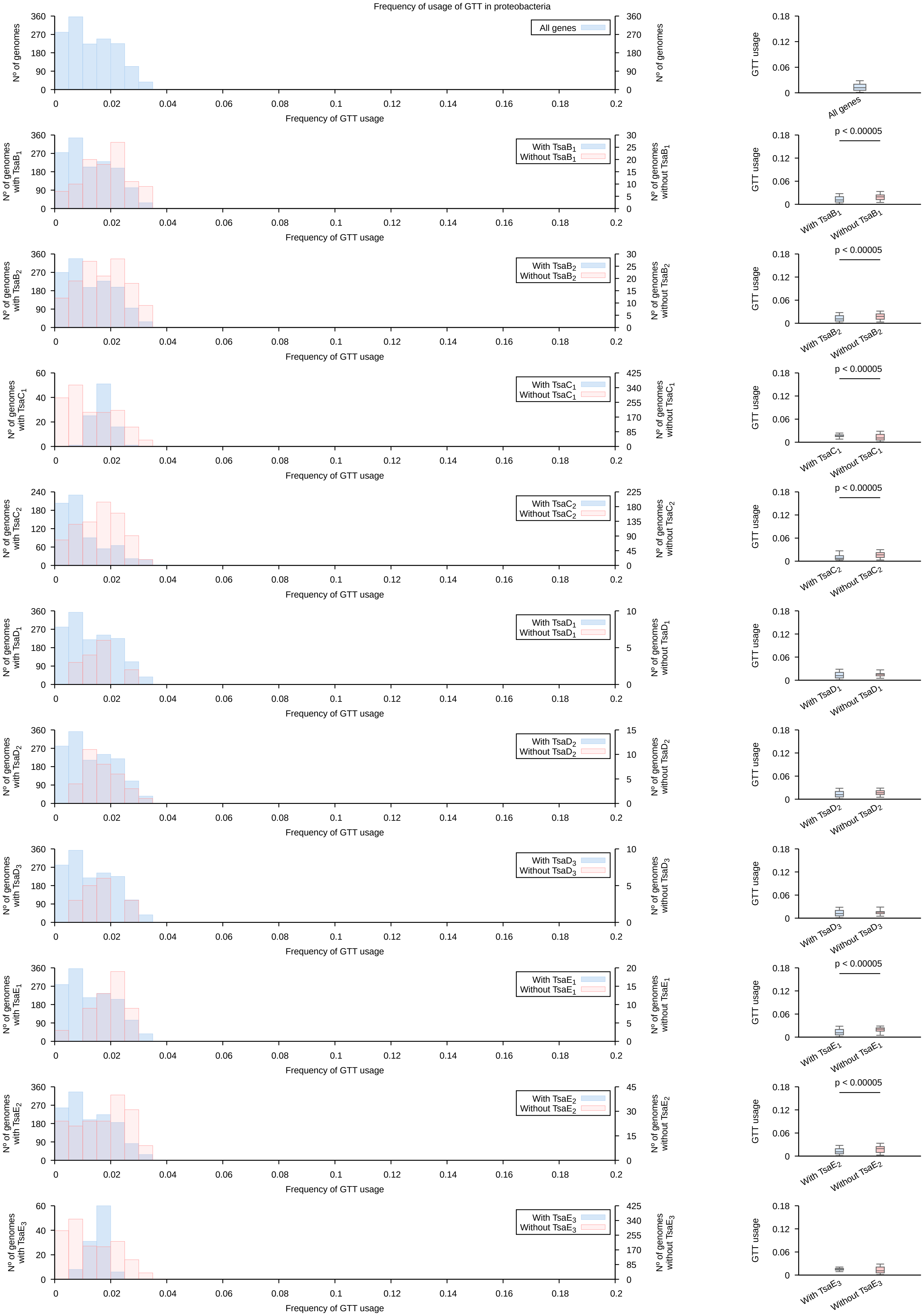
Frequency of usage of GTT in proteobacteria
All genes
With $TsaB_1$ / Without $TsaB_1$
With $TsaB_2$ / Without $TsaB_2$
With $TsaC_1$ / Without $TsaC_1$
With $TsaC_2$ / Without $TsaC_2$
With $TsaD_1$ / Without $TsaD_1$
With $TsaD_2$ / Without $TsaD_2$
With $TsaD_3$ / Without $TsaD_3$
With $TsaE_1$ / Without $TsaE_1$
With $TsaE_2$ / Without $TsaE_2$
With $TsaE_3$ / Without $TsaE_3$
Nº of genomes
Frequency of GTT usage
GTT usage
p < 0.00005

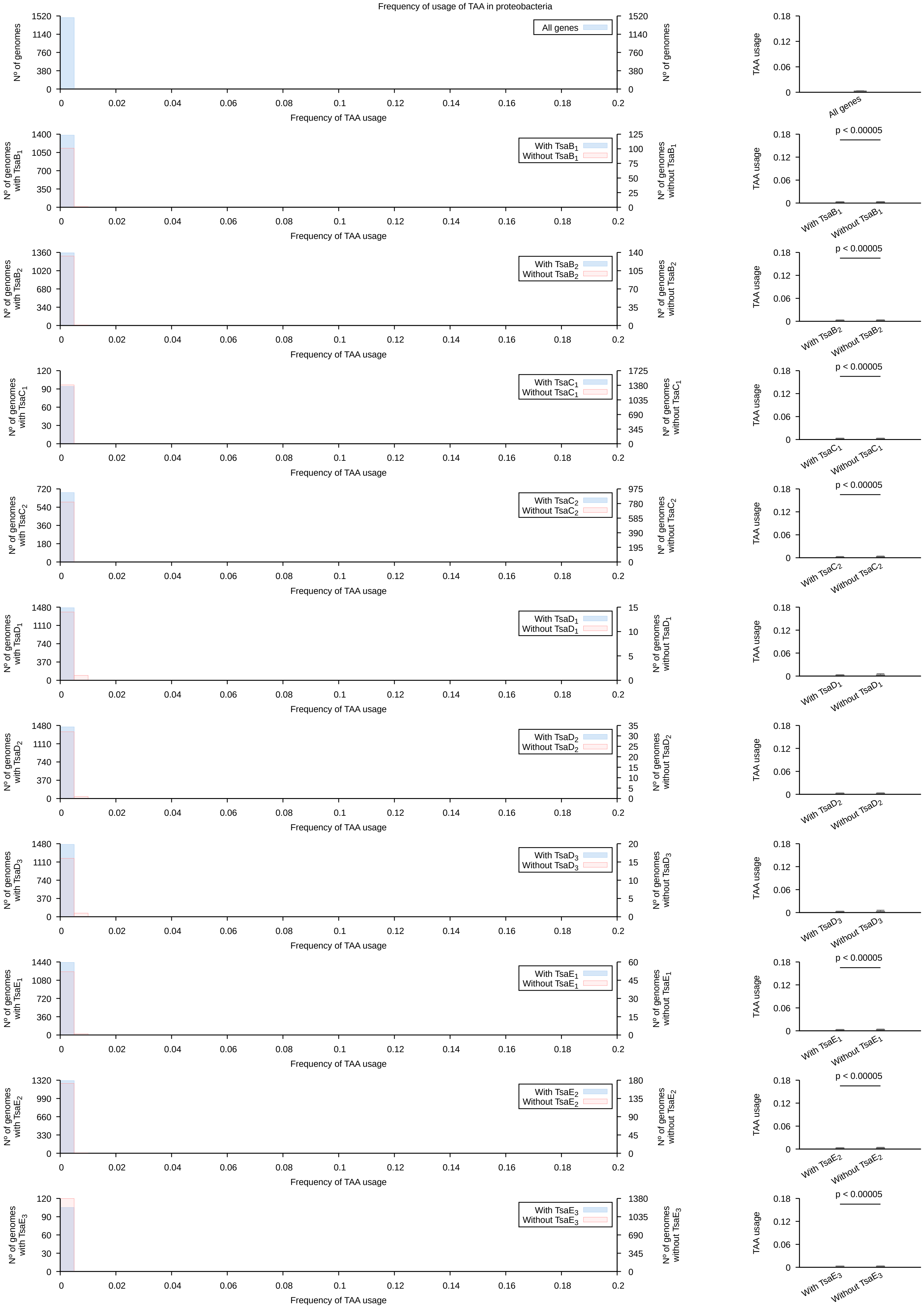
Frequency of usage of TAA in proteobacteria
Nº of genomes
Frequency of TAA usage
All genes
TAA usage
With TsaB$_1$
Without TsaB$_1$
p < 0.00005
With TsaB$_2$
Without TsaB$_2$
p < 0.00005
With TsaC$_1$
Without TsaC$_1$
p < 0.00005
With TsaC$_2$
Without TsaC$_2$
p < 0.00005
With TsaD$_1$
Without TsaD$_1$
With TsaD$_2$
Without TsaD$_2$
With TsaD$_3$
Without TsaD$_3$
With TsaE$_1$
Without TsaE$_1$
p < 0.00005
With TsaE$_2$
Without TsaE$_2$
p < 0.00005
With TsaE$_3$
Without TsaE$_3$
p < 0.00005

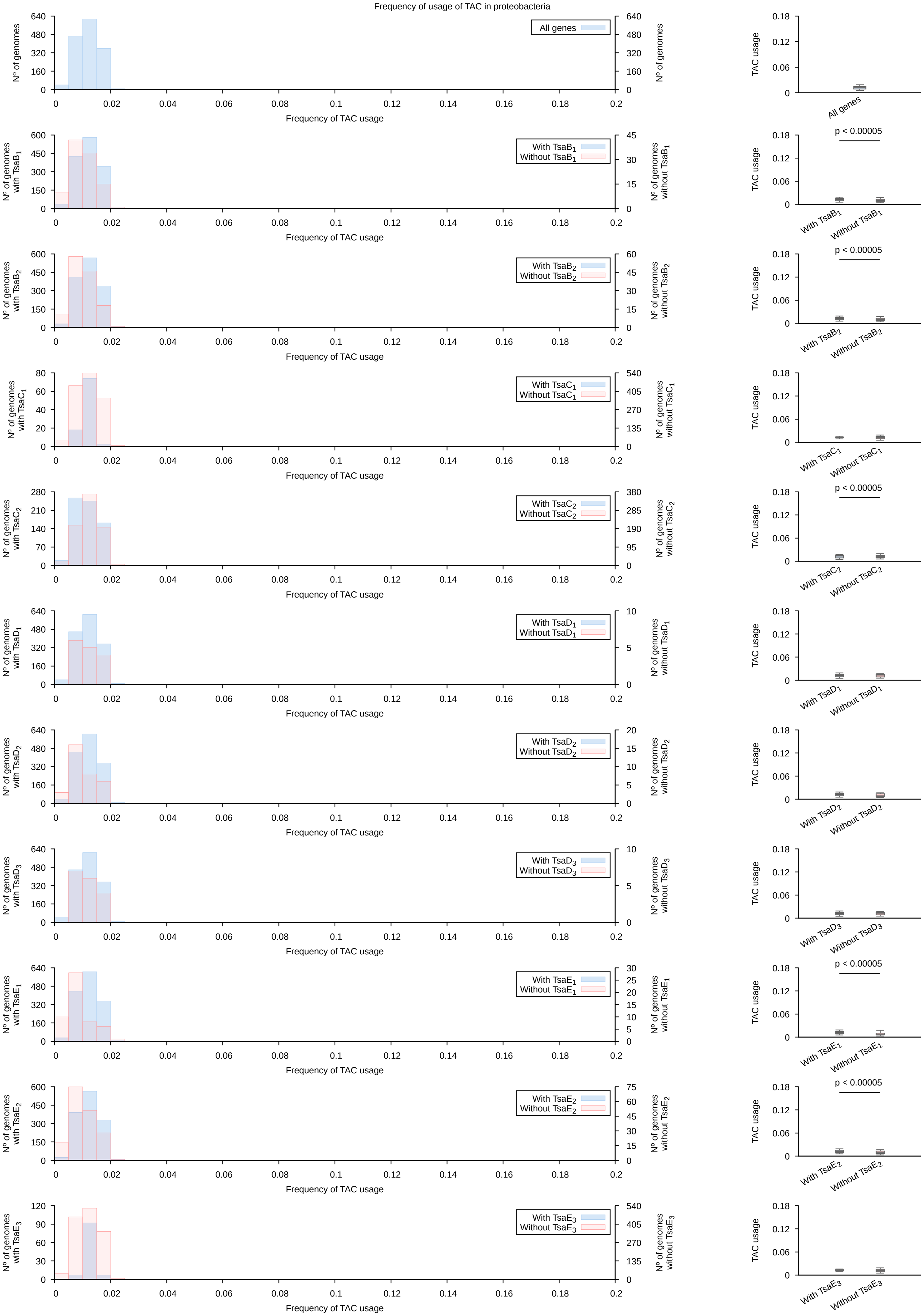
Frequency of usage of TAC in proteobacteria
All genes
Nº of genomes
Frequency of TAC usage
TAC usage
With $TsaB_1$
Without $TsaB_1$
Nº of genomes with $TsaB_1$
Nº of genomes without $TsaB_1$
p < 0.00005
With $TsaB_2$
Without $TsaB_2$
Nº of genomes with $TsaB_2$
Nº of genomes without $TsaB_2$
p < 0.00005
With $TsaC_1$
Without $TsaC_1$
Nº of genomes with $TsaC_1$
Nº of genomes without $TsaC_1$
With $TsaC_2$
Without $TsaC_2$
Nº of genomes with $TsaC_2$
Nº of genomes without $TsaC_2$
p < 0.00005
With $TsaD_1$
Without $TsaD_1$
Nº of genomes with $TsaD_1$
Nº of genomes without $TsaD_1$
With $TsaD_2$
Without $TsaD_2$
Nº of genomes with $TsaD_2$
Nº of genomes without $TsaD_2$
With $TsaD_3$
Without $TsaD_3$
Nº of genomes with $TsaD_3$
Nº of genomes without $TsaD_3$
With $TsaE_1$
Without $TsaE_1$
Nº of genomes with $TsaE_1$
Nº of genomes without $TsaE_1$
p < 0.00005
With $TsaE_2$
Without $TsaE_2$
Nº of genomes with $TsaE_2$
Nº of genomes without $TsaE_2$
p < 0.00005
With $TsaE_3$
Without $TsaE_3$
Nº of genomes with $TsaE_3$
Nº of genomes without $TsaE_3$
0 0.02 0.04 0.06 0.08 0.1 0.12 0.14 0.16 0.18 0.2
0 0.06 0.12 0.18

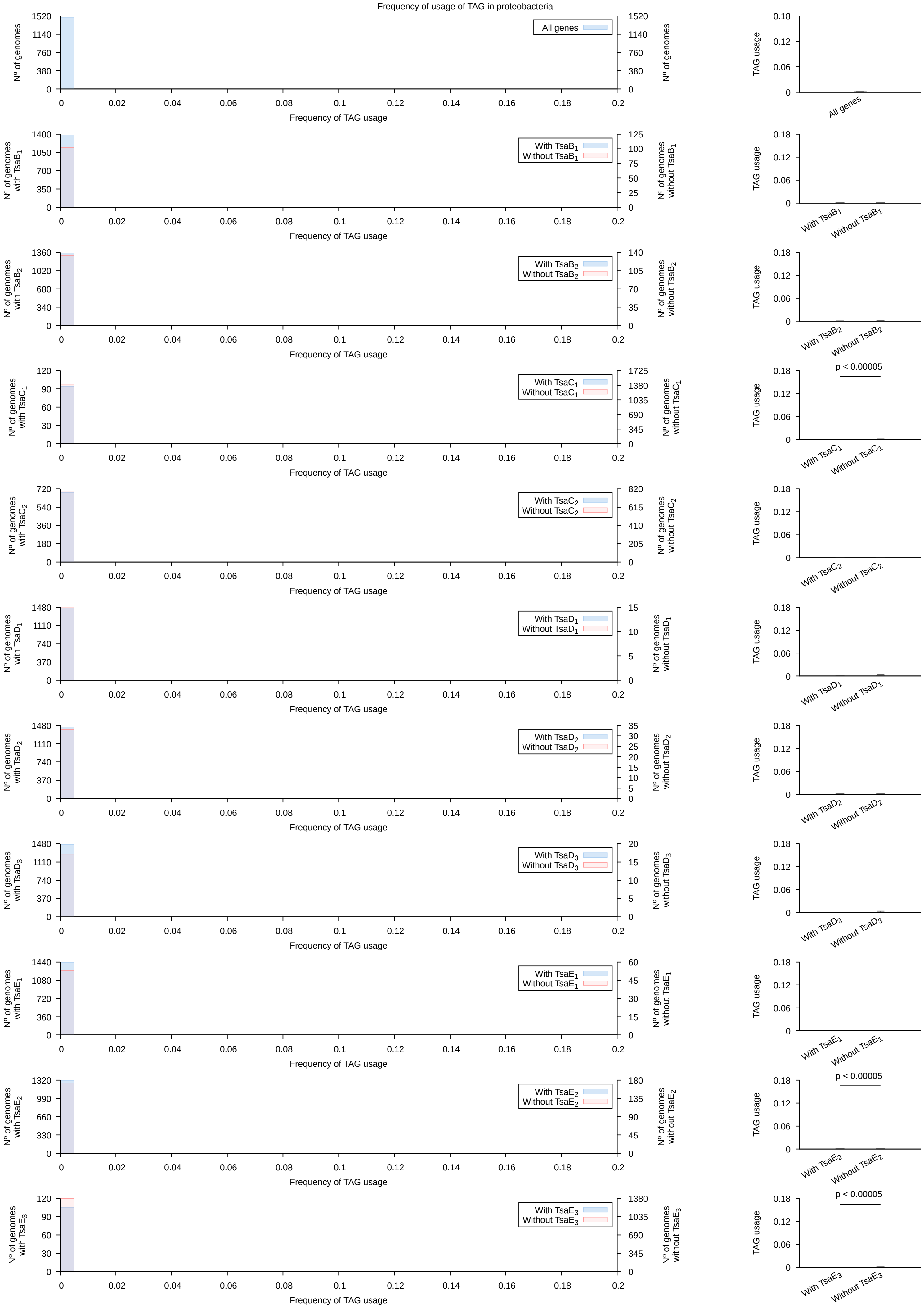

Frequency of usage of TAG in proteobacteria
Nº of genomes
All genes
Frequency of TAG usage
TAG usage
All genes
Nº of genomes with TsaB$_1$
With TsaB$_1$
Without TsaB$_1$
Nº of genomes without TsaB$_1$
Nº of genomes with TsaB$_2$
With TsaB$_2$
Without TsaB$_2$
Nº of genomes without TsaB$_2$
Nº of genomes with TsaC$_1$
With TsaC$_1$
Without TsaC$_1$
Nº of genomes without TsaC$_1$
p < 0.00005
Nº of genomes with TsaC$_2$
With TsaC$_2$
Without TsaC$_2$
Nº of genomes without TsaC$_2$
Nº of genomes with TsaD$_1$
With TsaD$_1$
Without TsaD$_1$
Nº of genomes without TsaD$_1$
Nº of genomes with TsaD$_2$
With TsaD$_2$
Without TsaD$_2$
Nº of genomes without TsaD$_2$
Nº of genomes with TsaD$_3$
With TsaD$_3$
Without TsaD$_3$
Nº of genomes without TsaD$_3$
Nº of genomes with TsaE$_1$
With TsaE$_1$
Without TsaE$_1$
Nº of genomes without TsaE$_1$
Nº of genomes with TsaE$_2$
With TsaE$_2$
Without TsaE$_2$
Nº of genomes without TsaE$_2$
p < 0.00005
Nº of genomes with TsaE$_3$
With TsaE$_3$
Without TsaE$_3$
Nº of genomes without TsaE$_3$
p < 0.00005

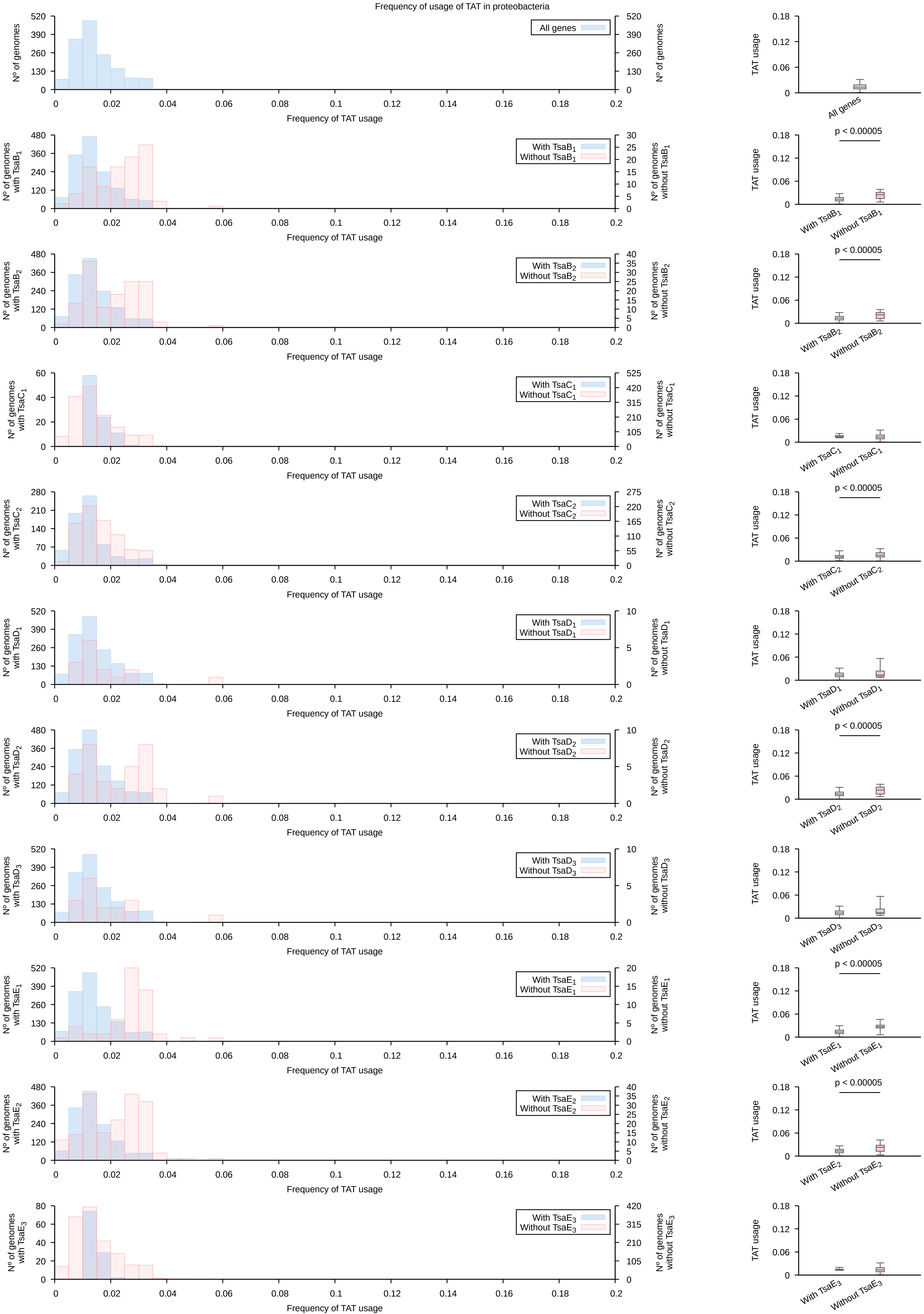

Frequency of usage of TAT in proteobacteria
All genes
Nº of genomes
Frequency of TAT usage
TAT usage
With TsaB1
Without TsaB1
Nº of genomes with TsaB1
Nº of genomes without TsaB1
p < 0.00005
With TsaB2
Without TsaB2
Nº of genomes with TsaB2
Nº of genomes without TsaB2
p < 0.00005
With TsaC1
Without TsaC1
Nº of genomes with TsaC1
Nº of genomes without TsaC1
With TsaC2
Without TsaC2
Nº of genomes with TsaC2
Nº of genomes without TsaC2
p < 0.00005
With TsaD1
Without TsaD1
Nº of genomes with TsaD1
Nº of genomes without TsaD1
With TsaD2
Without TsaD2
Nº of genomes with TsaD2
Nº of genomes without TsaD2
p < 0.00005
With TsaD3
Without TsaD3
Nº of genomes with TsaD3
Nº of genomes without TsaD3
With TsaE1
Without TsaE1
Nº of genomes with TsaE1
Nº of genomes without TsaE1
p < 0.00005
With TsaE2
Without TsaE2
Nº of genomes with TsaE2
Nº of genomes without TsaE2
p < 0.00005
With TsaE3
Without TsaE3
Nº of genomes with TsaE3
Nº of genomes without TsaE3

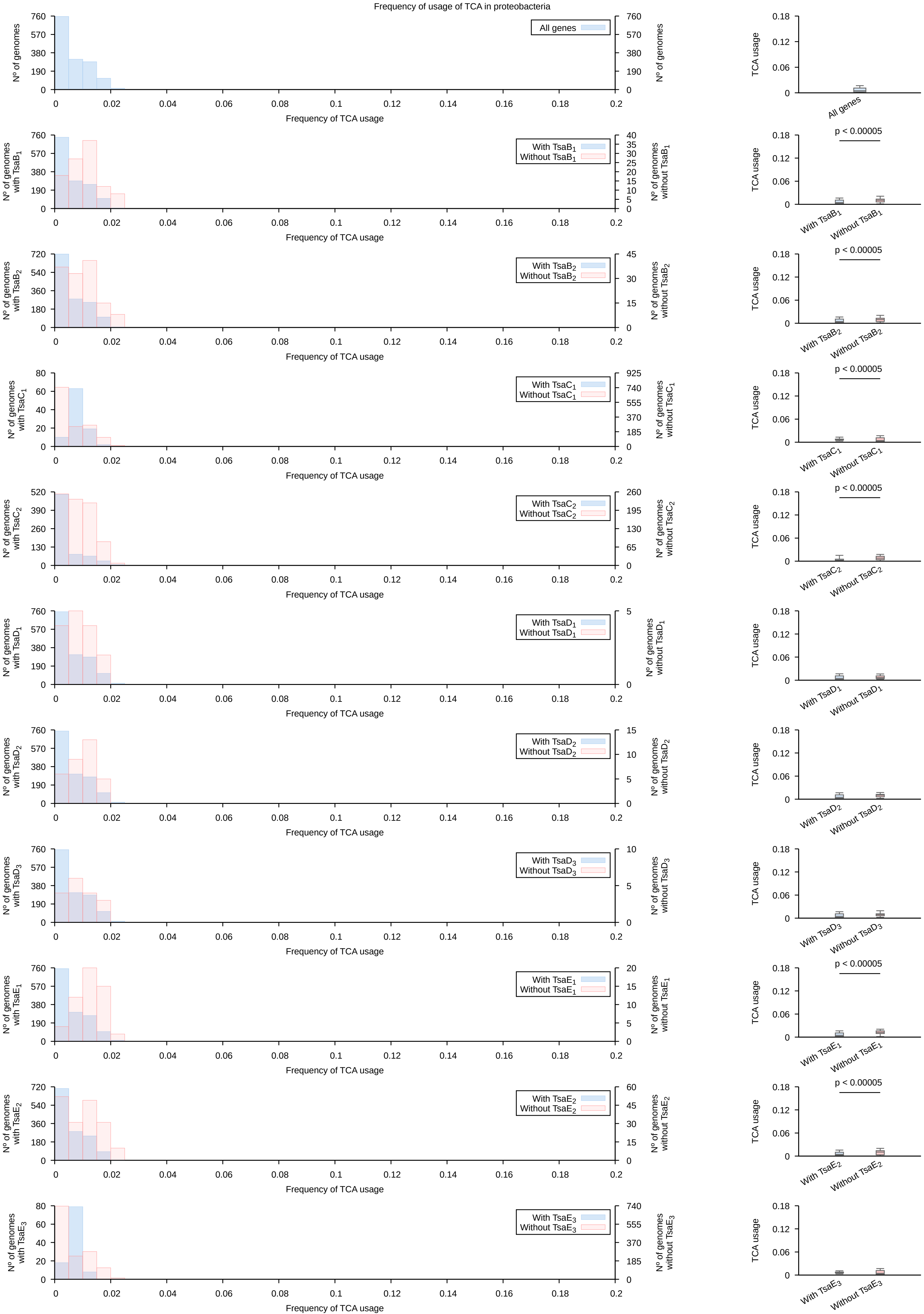

Frequency of usage of TCA in proteobacteria
All genes
Nº of genomes
Frequency of TCA usage
TCA usage
With TsaB1
Without TsaB1
Nº of genomes with TsaB1
Nº of genomes without TsaB1
p < 0.00005
With TsaB2
Without TsaB2
Nº of genomes with TsaB2
Nº of genomes without TsaB2
p < 0.00005
With TsaC1
Without TsaC1
Nº of genomes with TsaC1
Nº of genomes without TsaC1
p < 0.00005
With TsaC2
Without TsaC2
Nº of genomes with TsaC2
Nº of genomes without TsaC2
p < 0.00005
With TsaD1
Without TsaD1
Nº of genomes with TsaD1
Nº of genomes without TsaD1
With TsaD2
Without TsaD2
Nº of genomes with TsaD2
Nº of genomes without TsaD2
With TsaD3
Without TsaD3
Nº of genomes with TsaD3
Nº of genomes without TsaD3
With TsaE1
Without TsaE1
Nº of genomes with TsaE1
Nº of genomes without TsaE1
p < 0.00005
With TsaE2
Without TsaE2
Nº of genomes with TsaE2
Nº of genomes without TsaE2
p < 0.00005
With TsaE3
Without TsaE3
Nº of genomes with TsaE3
Nº of genomes without TsaE3

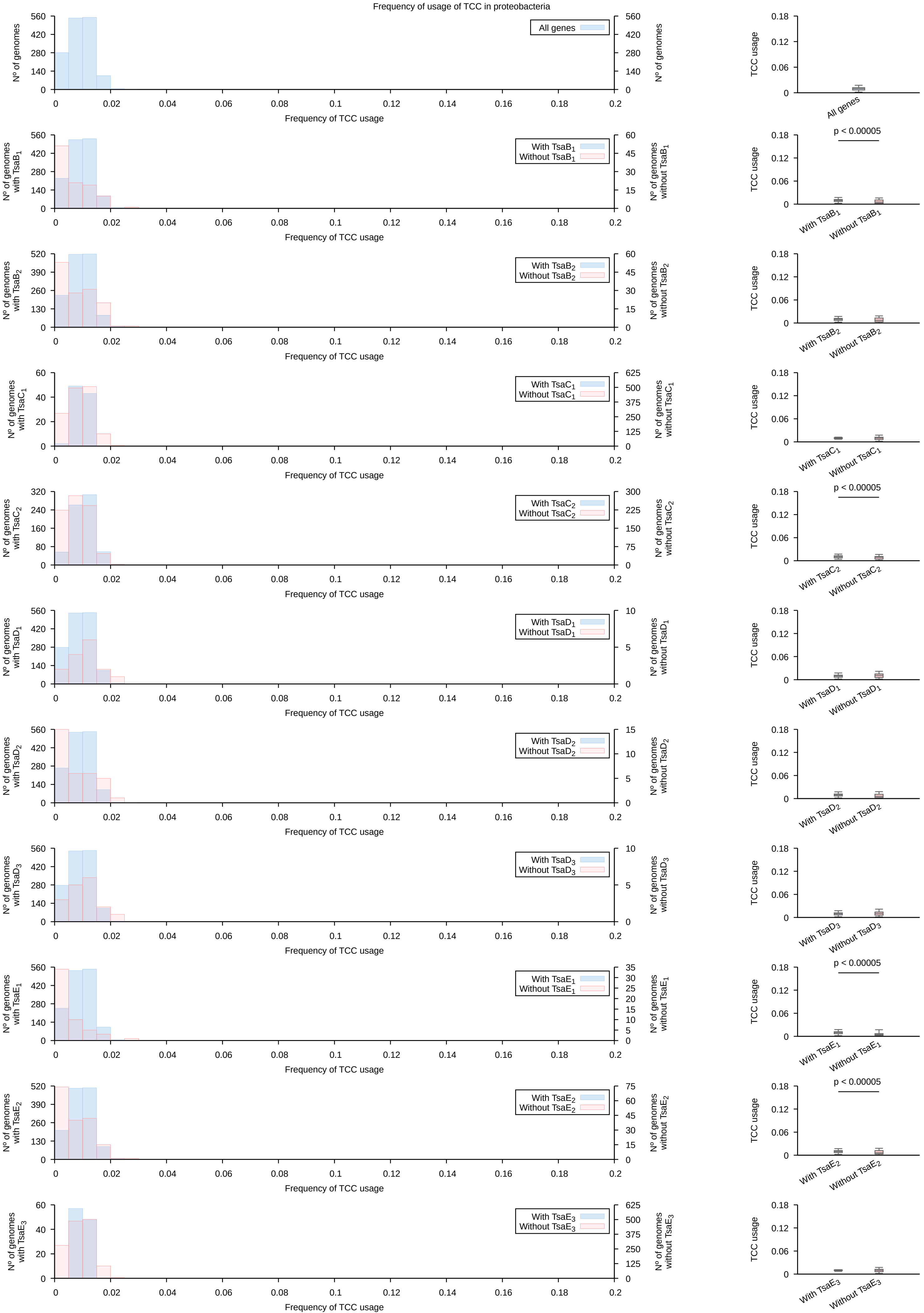

Frequency of usage of TCC in proteobacteria
Nº of genomes
All genes
Frequency of TCC usage
TCC usage
Nº of genomes with TsaB1
Nº of genomes without TsaB1
With TsaB1
Without TsaB1
p < 0.00005
Nº of genomes with TsaB2
Nº of genomes without TsaB2
With TsaB2
Without TsaB2
Nº of genomes with TsaC1
Nº of genomes without TsaC1
With TsaC1
Without TsaC1
Nº of genomes with TsaC2
Nº of genomes without TsaC2
With TsaC2
Without TsaC2
p < 0.00005
Nº of genomes with TsaD1
Nº of genomes without TsaD1
With TsaD1
Without TsaD1
Nº of genomes with TsaD2
Nº of genomes without TsaD2
With TsaD2
Without TsaD2
Nº of genomes with TsaD3
Nº of genomes without TsaD3
With TsaD3
Without TsaD3
Nº of genomes with TsaE1
Nº of genomes without TsaE1
With TsaE1
Without TsaE1
p < 0.00005
Nº of genomes with TsaE2
Nº of genomes without TsaE2
With TsaE2
Without TsaE2
p < 0.00005
Nº of genomes with TsaE3
Nº of genomes without TsaE3
With TsaE3
Without TsaE3

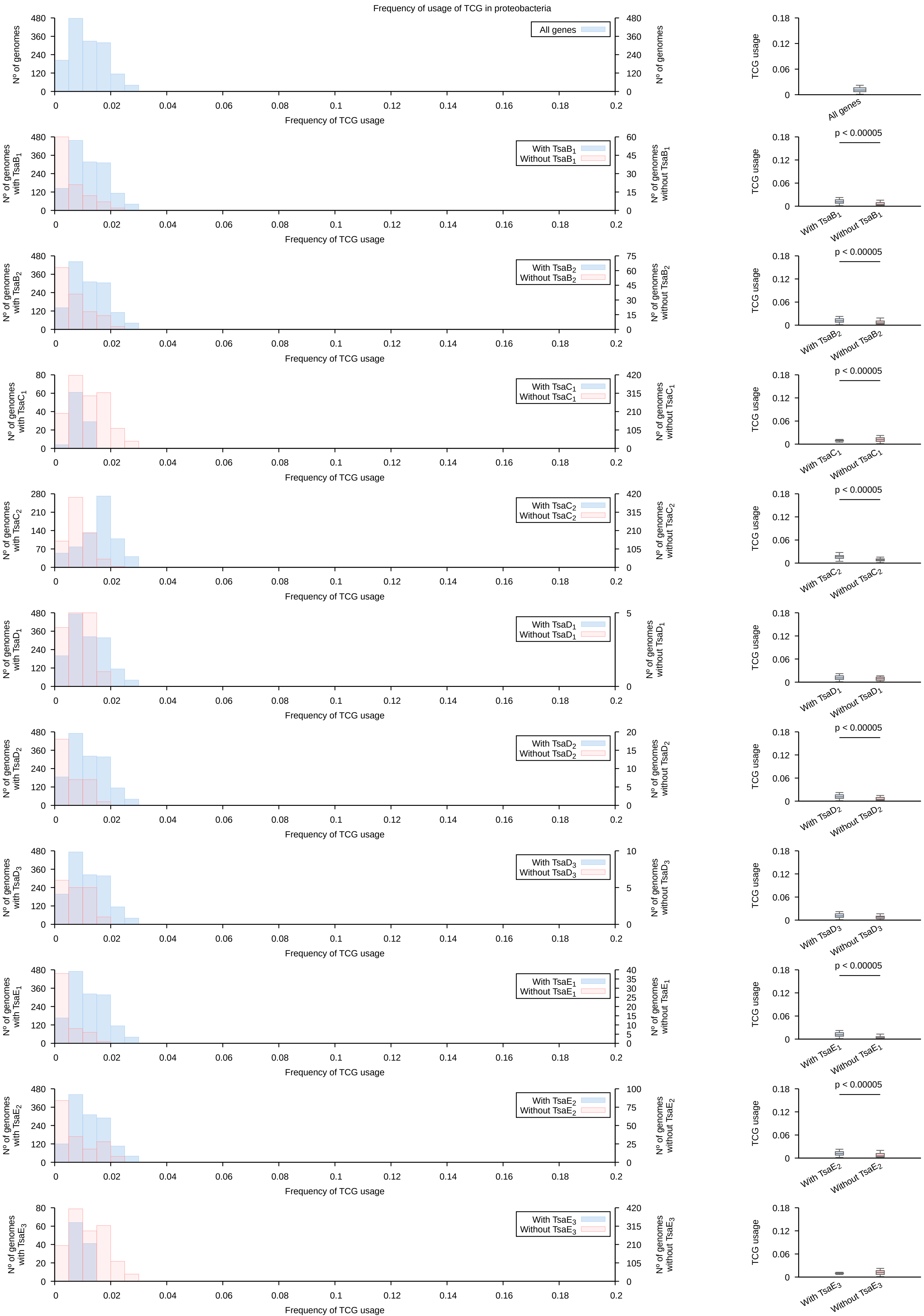

Frequency of usage of TCG in proteobacteria
All genes
Nº of genomes
Frequency of TCG usage
TCG usage
With TsaB1
Without TsaB1
Nº of genomes with TsaB1
Nº of genomes without TsaB1
p < 0.00005
With TsaB2
Without TsaB2
Nº of genomes with TsaB2
Nº of genomes without TsaB2
p < 0.00005
With TsaC1
Without TsaC1
Nº of genomes with TsaC1
Nº of genomes without TsaC1
p < 0.00005
With TsaC2
Without TsaC2
Nº of genomes with TsaC2
Nº of genomes without TsaC2
p < 0.00005
With TsaD1
Without TsaD1
Nº of genomes with TsaD1
Nº of genomes without TsaD1
With TsaD2
Without TsaD2
Nº of genomes with TsaD2
Nº of genomes without TsaD2
p < 0.00005
With TsaD3
Without TsaD3
Nº of genomes with TsaD3
Nº of genomes without TsaD3
With TsaE1
Without TsaE1
Nº of genomes with TsaE1
Nº of genomes without TsaE1
p < 0.00005
With TsaE2
Without TsaE2
Nº of genomes with TsaE2
Nº of genomes without TsaE2
p < 0.00005
With TsaE3
Without TsaE3
Nº of genomes with TsaE3
Nº of genomes without TsaE3

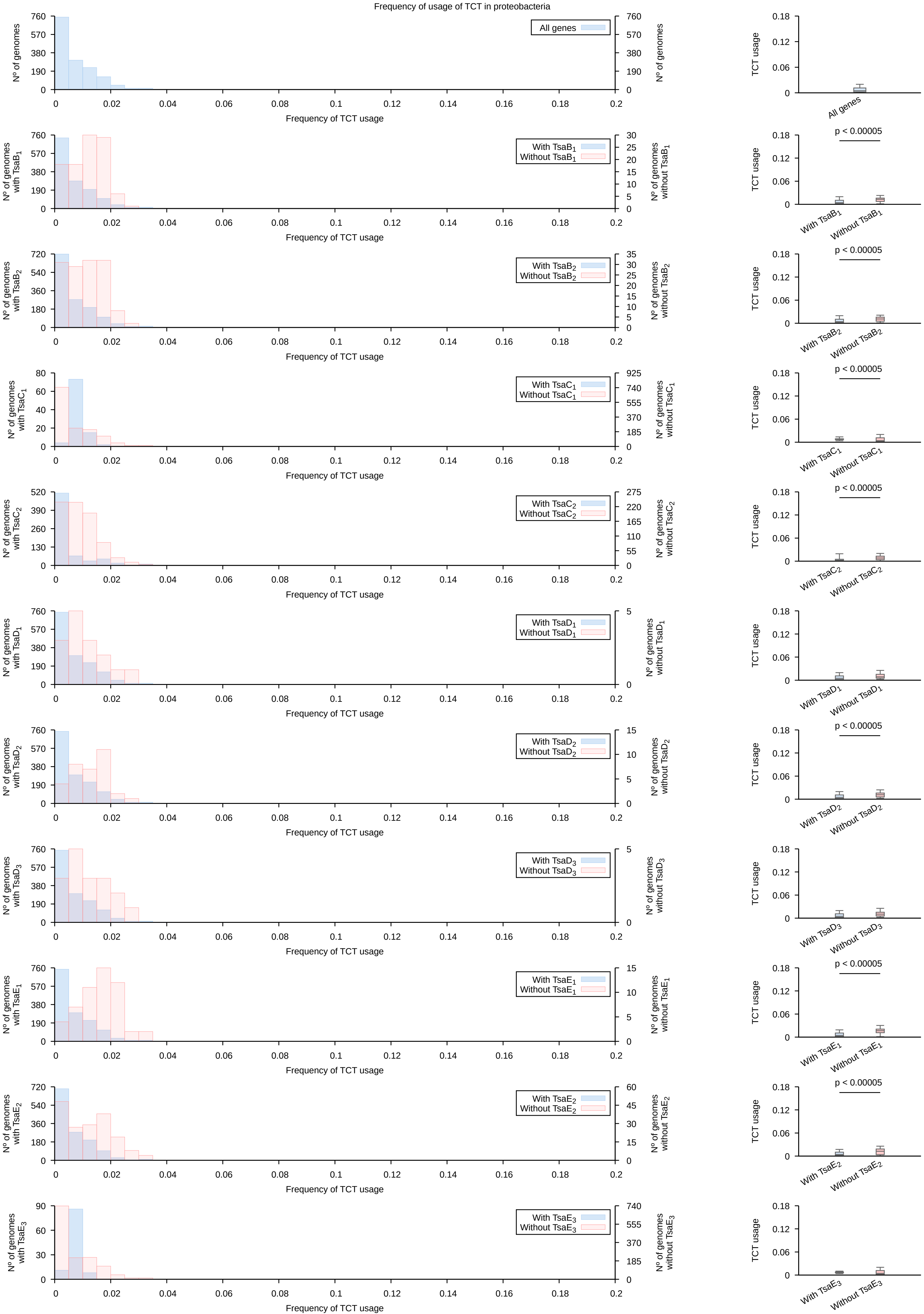
Frequency of usage of TCT in proteobacteria
All genes
With TsaB1
Without TsaB1
With TsaB2
Without TsaB2
With TsaC1
Without TsaC1
With TsaC2
Without TsaC2
With TsaD1
Without TsaD1
With TsaD2
Without TsaD2
With TsaD3
Without TsaD3
With TsaE1
Without TsaE1
With TsaE2
Without TsaE2
With TsaE3
Without TsaE3
Nº of genomes
Frequency of TCT usage
TCT usage
p < 0.00005

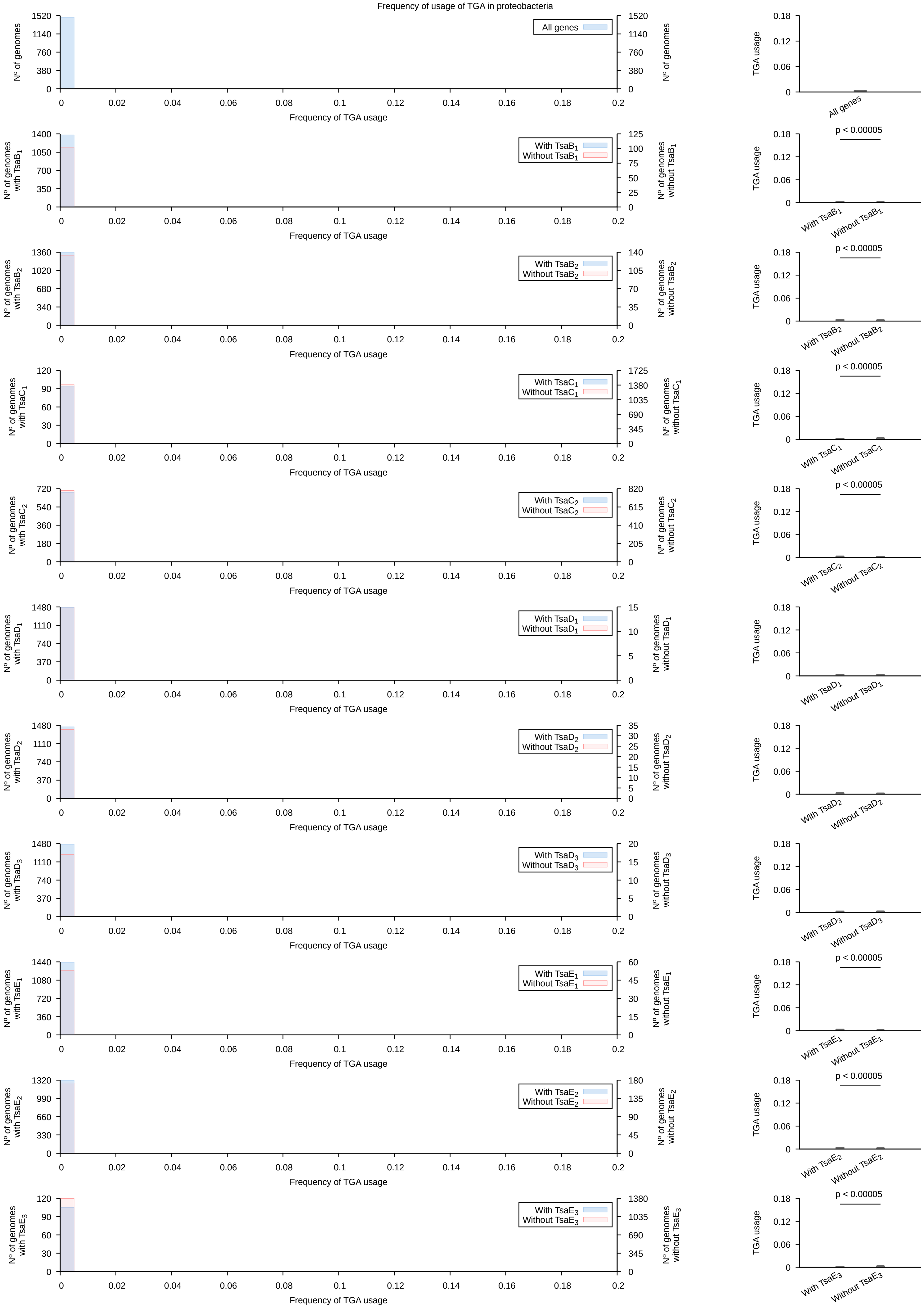
Frequency of usage of TGA in proteobacteria
Nº of genomes
Frequency of TGA usage
All genes
TGA usage
Nº of genomes with TsaB$_1$
Nº of genomes without TsaB$_1$
With TsaB$_1$
Without TsaB$_1$
p < 0.00005
Nº of genomes with TsaB$_2$
Nº of genomes without TsaB$_2$
With TsaB$_2$
Without TsaB$_2$
p < 0.00005
Nº of genomes with TsaC$_1$
Nº of genomes without TsaC$_1$
With TsaC$_1$
Without TsaC$_1$
p < 0.00005
Nº of genomes with TsaC$_2$
Nº of genomes without TsaC$_2$
With TsaC$_2$
Without TsaC$_2$
p < 0.00005
Nº of genomes with TsaD$_1$
Nº of genomes without TsaD$_1$
With TsaD$_1$
Without TsaD$_1$
Nº of genomes with TsaD$_2$
Nº of genomes without TsaD$_2$
With TsaD$_2$
Without TsaD$_2$
Nº of genomes with TsaD$_3$
Nº of genomes without TsaD$_3$
With TsaD$_3$
Without TsaD$_3$
Nº of genomes with TsaE$_1$
Nº of genomes without TsaE$_1$
With TsaE$_1$
Without TsaE$_1$
p < 0.00005
Nº of genomes with TsaE$_2$
Nº of genomes without TsaE$_2$
With TsaE$_2$
Without TsaE$_2$
p < 0.00005
Nº of genomes with TsaE$_3$
Nº of genomes without TsaE$_3$
With TsaE$_3$
Without TsaE$_3$
p < 0.00005

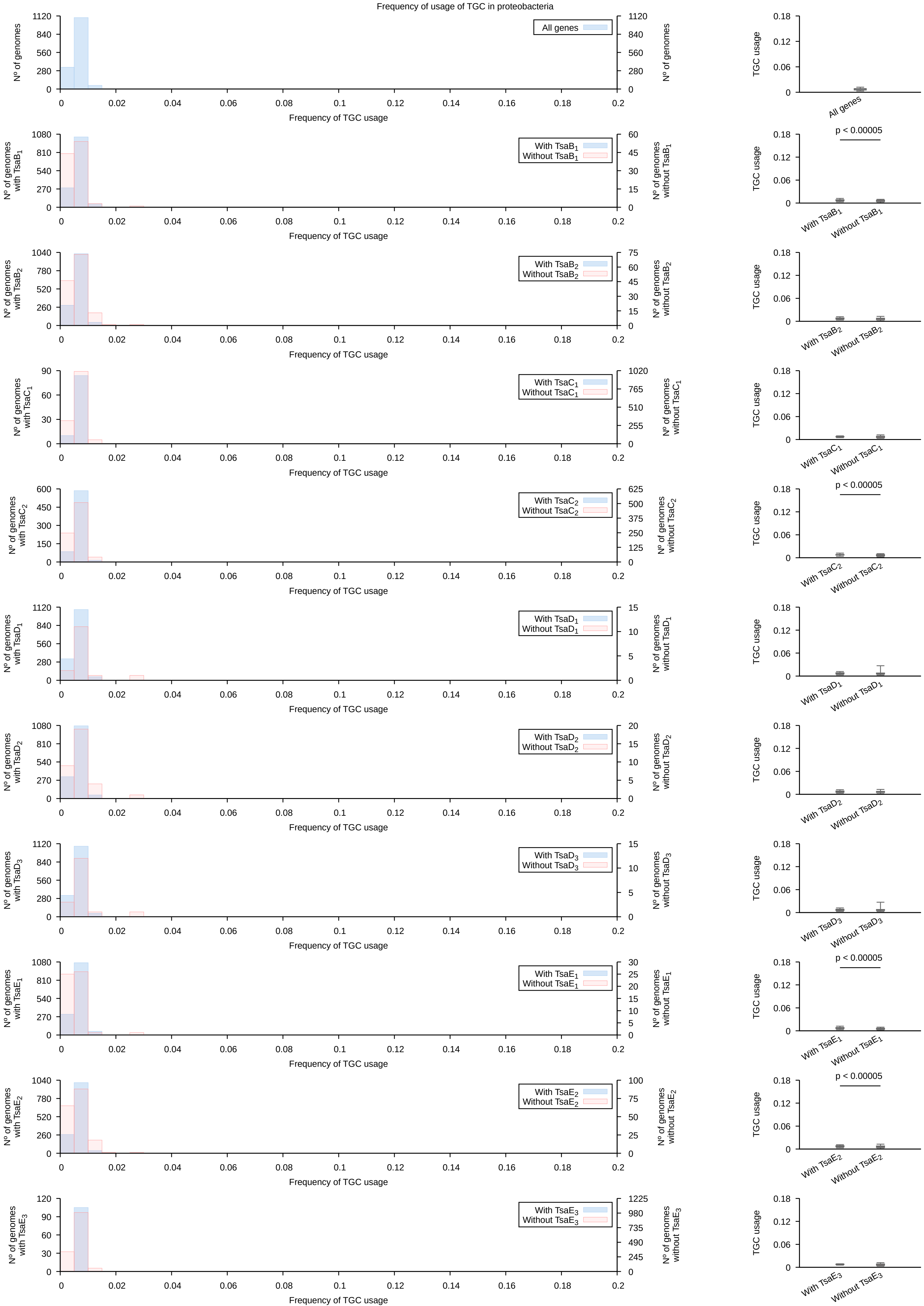
Frequency of usage of TGC in proteobacteria
Nº of genomes
All genes
Frequency of TGC usage
TGC usage
All genes
Nº of genomes with TsaB1
Nº of genomes without TsaB1
With TsaB1
Without TsaB1
p < 0.00005
Nº of genomes with TsaB2
Nº of genomes without TsaB2
With TsaB2
Without TsaB2
Nº of genomes with TsaC1
Nº of genomes without TsaC1
With TsaC1
Without TsaC1
Nº of genomes with TsaC2
Nº of genomes without TsaC2
With TsaC2
Without TsaC2
p < 0.00005
Nº of genomes with TsaD1
Nº of genomes without TsaD1
With TsaD1
Without TsaD1
Nº of genomes with TsaD2
Nº of genomes without TsaD2
With TsaD2
Without TsaD2
Nº of genomes with TsaD3
Nº of genomes without TsaD3
With TsaD3
Without TsaD3
Nº of genomes with TsaE1
Nº of genomes without TsaE1
With TsaE1
Without TsaE1
p < 0.00005
Nº of genomes with TsaE2
Nº of genomes without TsaE2
With TsaE2
Without TsaE2
p < 0.00005
Nº of genomes with TsaE3
Nº of genomes without TsaE3
With TsaE3
Without TsaE3

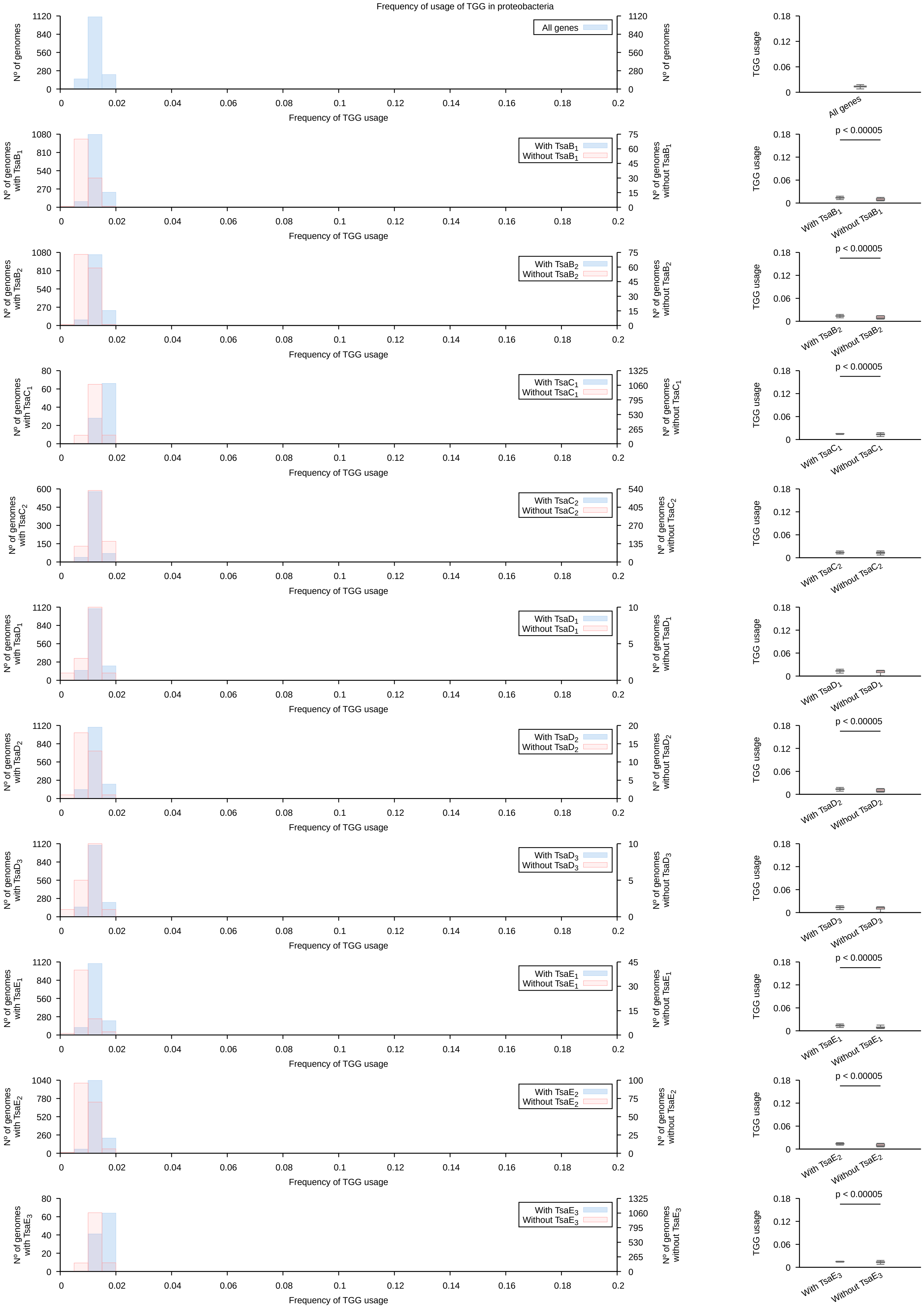

Frequency of usage of TGG in proteobacteria
Nº of genomes
Frequency of TGG usage
TGG usage
All genes
With TsaB$_1$
Without TsaB$_1$
Nº of genomes with TsaB$_1$
Nº of genomes without TsaB$_1$
p < 0.00005
With TsaB$_2$
Without TsaB$_2$
Nº of genomes with TsaB$_2$
Nº of genomes without TsaB$_2$
p < 0.00005
With TsaC$_1$
Without TsaC$_1$
Nº of genomes with TsaC$_1$
Nº of genomes without TsaC$_1$
p < 0.00005
With TsaC$_2$
Without TsaC$_2$
Nº of genomes with TsaC$_2$
Nº of genomes without TsaC$_2$
With TsaD$_1$
Without TsaD$_1$
Nº of genomes with TsaD$_1$
Nº of genomes without TsaD$_1$
With TsaD$_2$
Without TsaD$_2$
Nº of genomes with TsaD$_2$
Nº of genomes without TsaD$_2$
p < 0.00005
With TsaD$_3$
Without TsaD$_3$
Nº of genomes with TsaD$_3$
Nº of genomes without TsaD$_3$
With TsaE$_1$
Without TsaE$_1$
Nº of genomes with TsaE$_1$
Nº of genomes without TsaE$_1$
p < 0.00005
With TsaE$_2$
Without TsaE$_2$
Nº of genomes with TsaE$_2$
Nº of genomes without TsaE$_2$
p < 0.00005
With TsaE$_3$
Without TsaE$_3$
Nº of genomes with TsaE$_3$
Nº of genomes without TsaE$_3$
p < 0.00005

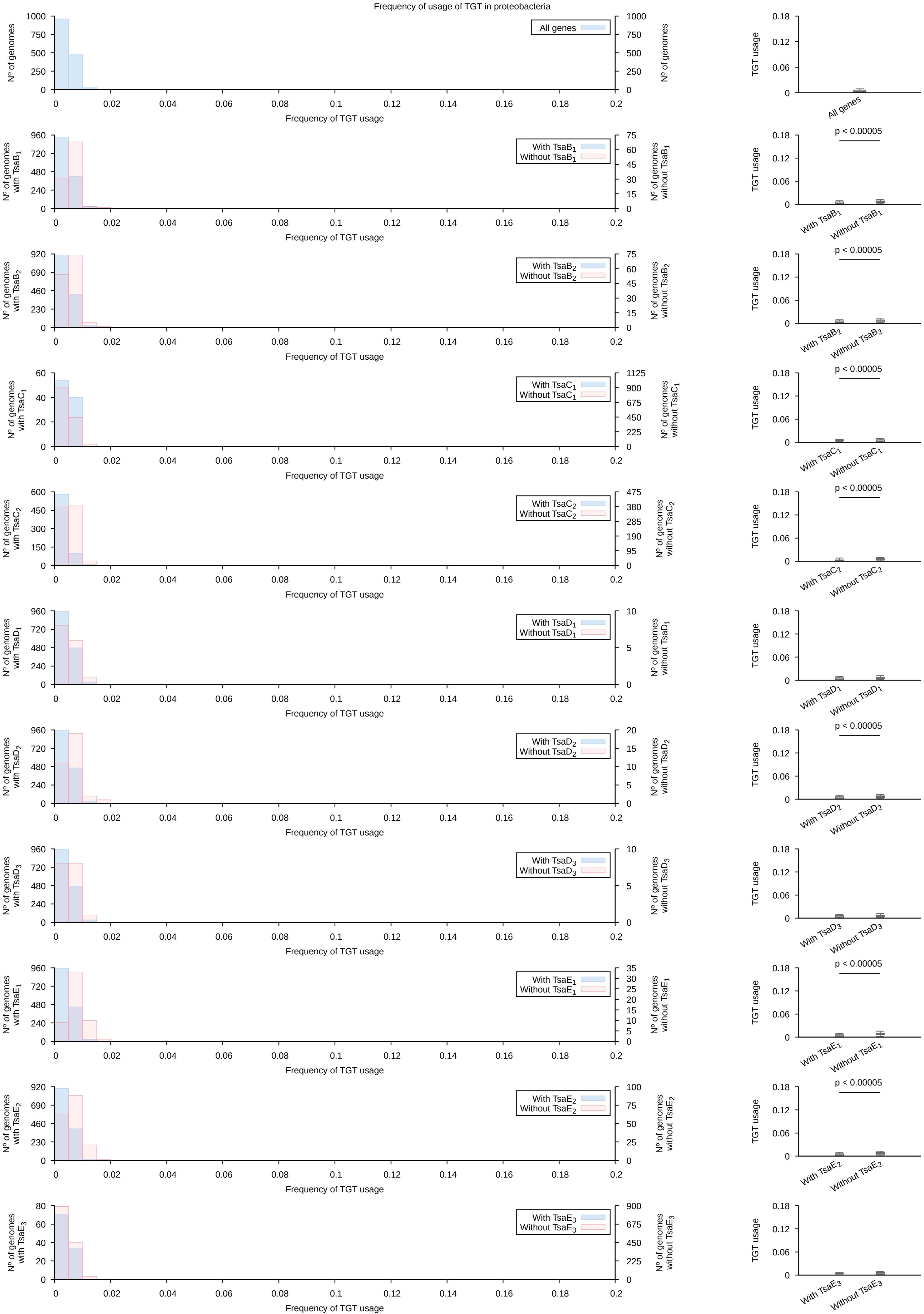

Frequency of usage of TGT in proteobacteria
Nº of genomes
Frequency of TGT usage
TGT usage
All genes
With TsaB1
Without TsaB1
Nº of genomes with TsaB1
Nº of genomes without TsaB1
p < 0.00005
With TsaB2
Without TsaB2
Nº of genomes with TsaB2
Nº of genomes without TsaB2
p < 0.00005
With TsaC1
Without TsaC1
Nº of genomes with TsaC1
Nº of genomes without TsaC1
p < 0.00005
With TsaC2
Without TsaC2
Nº of genomes with TsaC2
Nº of genomes without TsaC2
p < 0.00005
With TsaD1
Without TsaD1
Nº of genomes with TsaD1
Nº of genomes without TsaD1
With TsaD2
Without TsaD2
Nº of genomes with TsaD2
Nº of genomes without TsaD2
p < 0.00005
With TsaD3
Without TsaD3
Nº of genomes with TsaD3
Nº of genomes without TsaD3
With TsaE1
Without TsaE1
Nº of genomes with TsaE1
Nº of genomes without TsaE1
p < 0.00005
With TsaE2
Without TsaE2
Nº of genomes with TsaE2
Nº of genomes without TsaE2
p < 0.00005
With TsaE3
Without TsaE3
Nº of genomes with TsaE3
Nº of genomes without TsaE3

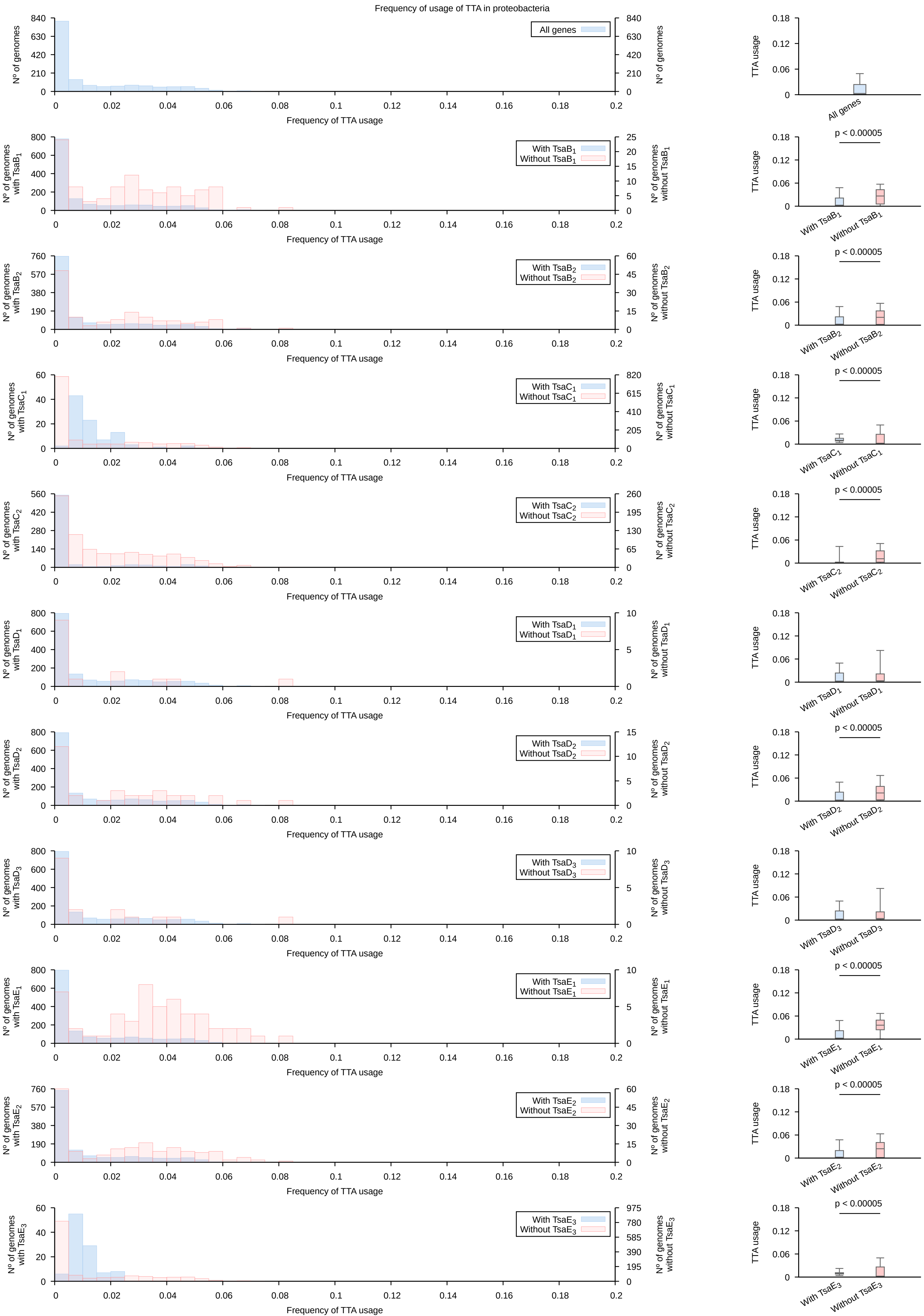

Frequency of usage of TTA in proteobacteria
Nº of genomes
All genes
Frequency of TTA usage
TTA usage
Nº of genomes with TsaB1
Nº of genomes without TsaB1
With TsaB1
Without TsaB1
p < 0.00005
Nº of genomes with TsaB2
Nº of genomes without TsaB2
With TsaB2
Without TsaB2
Nº of genomes with TsaC1
Nº of genomes without TsaC1
With TsaC1
Without TsaC1
Nº of genomes with TsaC2
Nº of genomes without TsaC2
With TsaC2
Without TsaC2
Nº of genomes with TsaD1
Nº of genomes without TsaD1
With TsaD1
Without TsaD1
Nº of genomes with TsaD2
Nº of genomes without TsaD2
With TsaD2
Without TsaD2
Nº of genomes with TsaD3
Nº of genomes without TsaD3
With TsaD3
Without TsaD3
Nº of genomes with TsaE1
Nº of genomes without TsaE1
With TsaE1
Without TsaE1
Nº of genomes with TsaE2
Nº of genomes without TsaE2
With TsaE2
Without TsaE2
Nº of genomes with TsaE3
Nº of genomes without TsaE3
With TsaE3
Without TsaE3

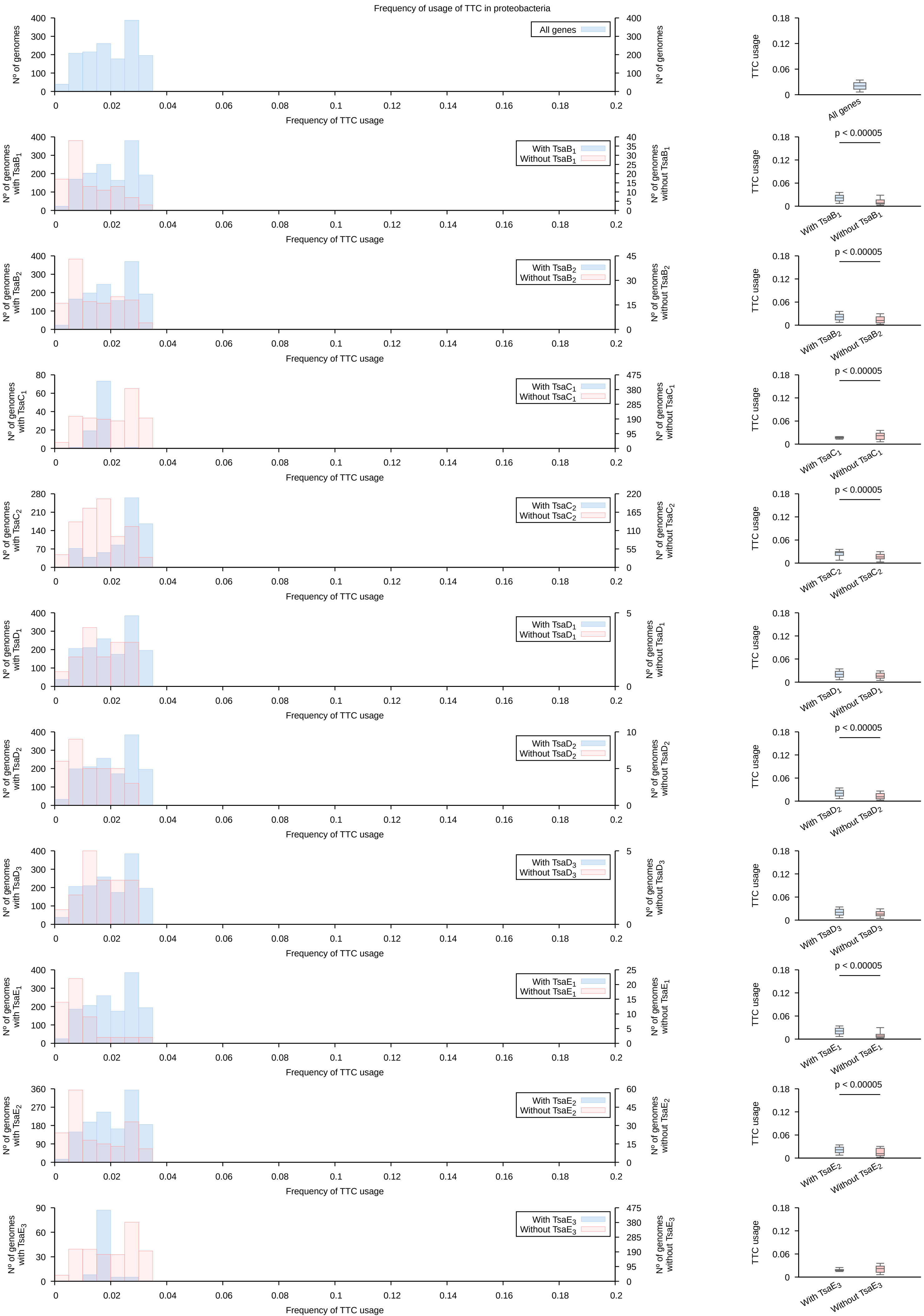

Frequency of usage of TTC in proteobacteria
All genes
Nº of genomes
Frequency of TTC usage
TTC usage
With TsaB1
Without TsaB1
Nº of genomes with TsaB1
Nº of genomes without TsaB1
p < 0.00005
With TsaB2
Without TsaB2
Nº of genomes with TsaB2
Nº of genomes without TsaB2
p < 0.00005
With TsaC1
Without TsaC1
Nº of genomes with TsaC1
Nº of genomes without TsaC1
p < 0.00005
With TsaC2
Without TsaC2
Nº of genomes with TsaC2
Nº of genomes without TsaC2
p < 0.00005
With TsaD1
Without TsaD1
Nº of genomes with TsaD1
Nº of genomes without TsaD1
With TsaD2
Without TsaD2
Nº of genomes with TsaD2
Nº of genomes without TsaD2
p < 0.00005
With TsaD3
Without TsaD3
Nº of genomes with TsaD3
Nº of genomes without TsaD3
With TsaE1
Without TsaE1
Nº of genomes with TsaE1
Nº of genomes without TsaE1
p < 0.00005
With TsaE2
Without TsaE2
Nº of genomes with TsaE2
Nº of genomes without TsaE2
p < 0.00005
With TsaE3
Without TsaE3
Nº of genomes with TsaE3
Nº of genomes without TsaE3

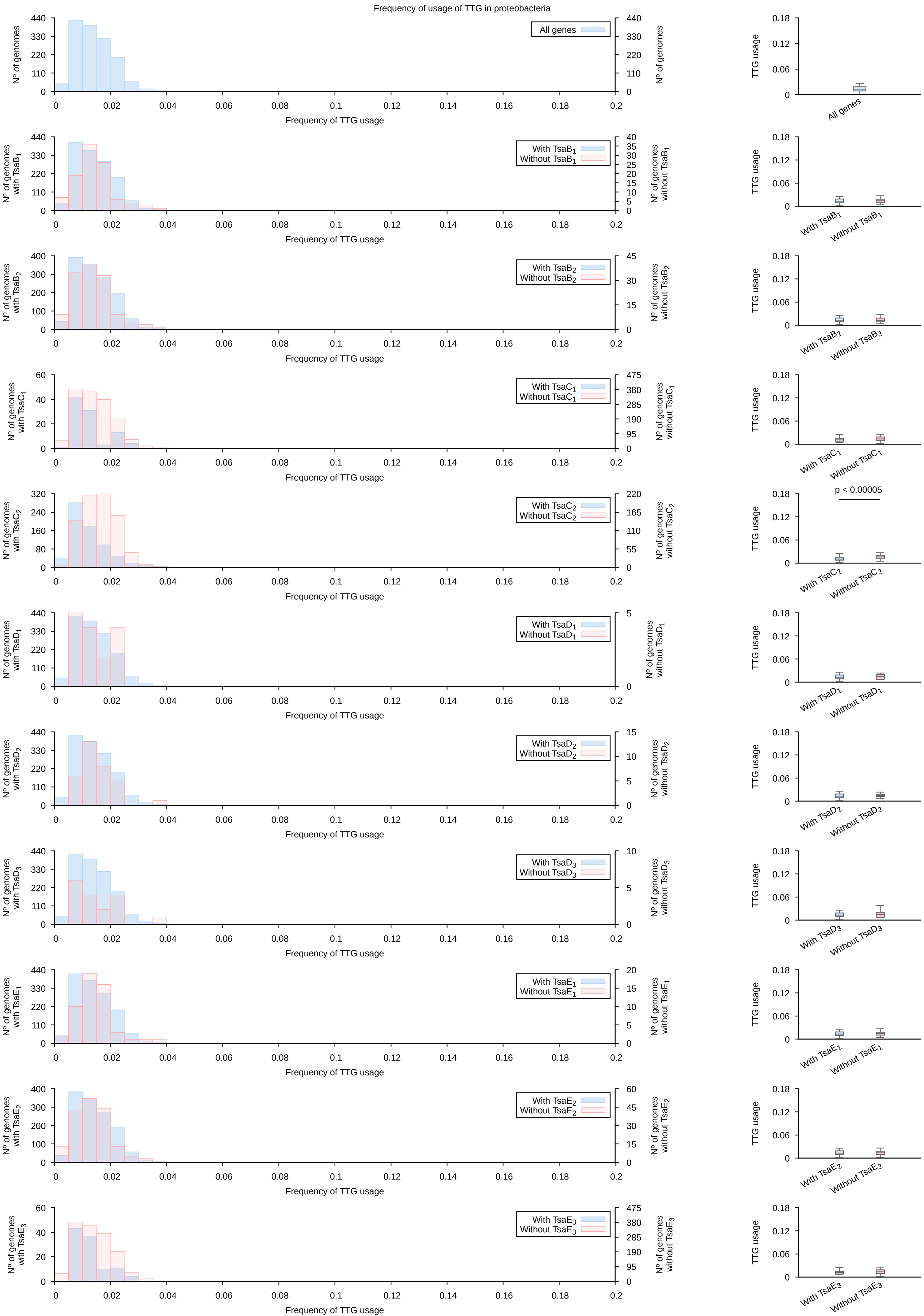

Frequency of usage of TTG in proteobacteria
All genes
Nº of genomes
Frequency of TTG usage
TTG usage
With TsaB$_1$
Without TsaB$_1$
Nº of genomes with TsaB$_1$
Nº of genomes without TsaB$_1$
With TsaB$_2$
Without TsaB$_2$
Nº of genomes with TsaB$_2$
Nº of genomes without TsaB$_2$
With TsaC$_1$
Without TsaC$_1$
Nº of genomes with TsaC$_1$
Nº of genomes without TsaC$_1$
With TsaC$_2$
Without TsaC$_2$
Nº of genomes with TsaC$_2$
Nº of genomes without TsaC$_2$
p < 0.00005
With TsaD$_1$
Without TsaD$_1$
Nº of genomes with TsaD$_1$
Nº of genomes without TsaD$_1$
With TsaD$_2$
Without TsaD$_2$
Nº of genomes with TsaD$_2$
Nº of genomes without TsaD$_2$
With TsaD$_3$
Without TsaD$_3$
Nº of genomes with TsaD$_3$
Nº of genomes without TsaD$_3$
With TsaE$_1$
Without TsaE$_1$
Nº of genomes with TsaE$_1$
Nº of genomes without TsaE$_1$
With TsaE$_2$
Without TsaE$_2$
Nº of genomes with TsaE$_2$
Nº of genomes without TsaE$_2$
With TsaE$_3$
Without TsaE$_3$
Nº of genomes with TsaE$_3$
Nº of genomes without TsaE$_3$

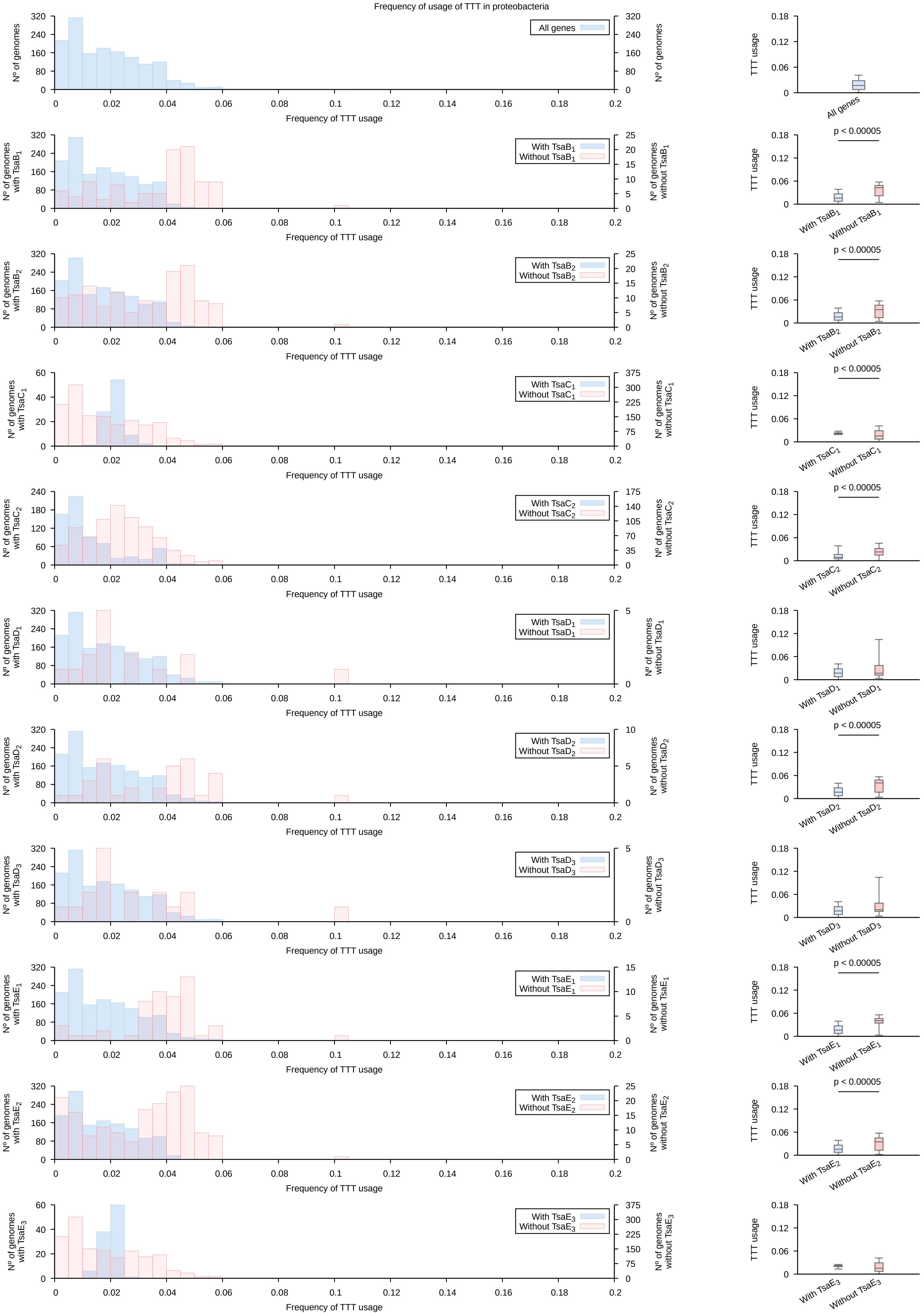

Frequency of usage of TTT in proteobacteria
All genes
Nº of genomes
Frequency of TTT usage
TTT usage
With TsaB1
Without TsaB1
Nº of genomes with TsaB1
Nº of genomes without TsaB1
p < 0.00005
With TsaB2
Without TsaB2
Nº of genomes with TsaB2
Nº of genomes without TsaB2
p < 0.00005
With TsaC1
Without TsaC1
Nº of genomes with TsaC1
Nº of genomes without TsaC1
p < 0.00005
With TsaC2
Without TsaC2
Nº of genomes with TsaC2
Nº of genomes without TsaC2
p < 0.00005
With TsaD1
Without TsaD1
Nº of genomes with TsaD1
Nº of genomes without TsaD1
With TsaD2
Without TsaD2
Nº of genomes with TsaD2
Nº of genomes without TsaD2
p < 0.00005
With TsaD3
Without TsaD3
Nº of genomes with TsaD3
Nº of genomes without TsaD3
With TsaE1
Without TsaE1
Nº of genomes with TsaE1
Nº of genomes without TsaE1
p < 0.00005
With TsaE2
Without TsaE2
Nº of genomes with TsaE2
Nº of genomes without TsaE2
p < 0.00005
With TsaE3
Without TsaE3
Nº of genomes with TsaE3
Nº of genomes without TsaE3
