# Supplementary material for: Impact of the chemical modification of tRNAs anticodon loop on the variability and evolution of codon usage in proteobacteria
Source: Front Microbiol. 2024 Aug 5;15:1412318. doi: 10.3389/fmicb.2024.1412318 (PMC11332805; doi:10.3389/fmicb.2024.1412318)
Supplement: Supplementary file 1 [file Data_Sheet_1.zip › Supp_figures/Fig_S12.pdf]

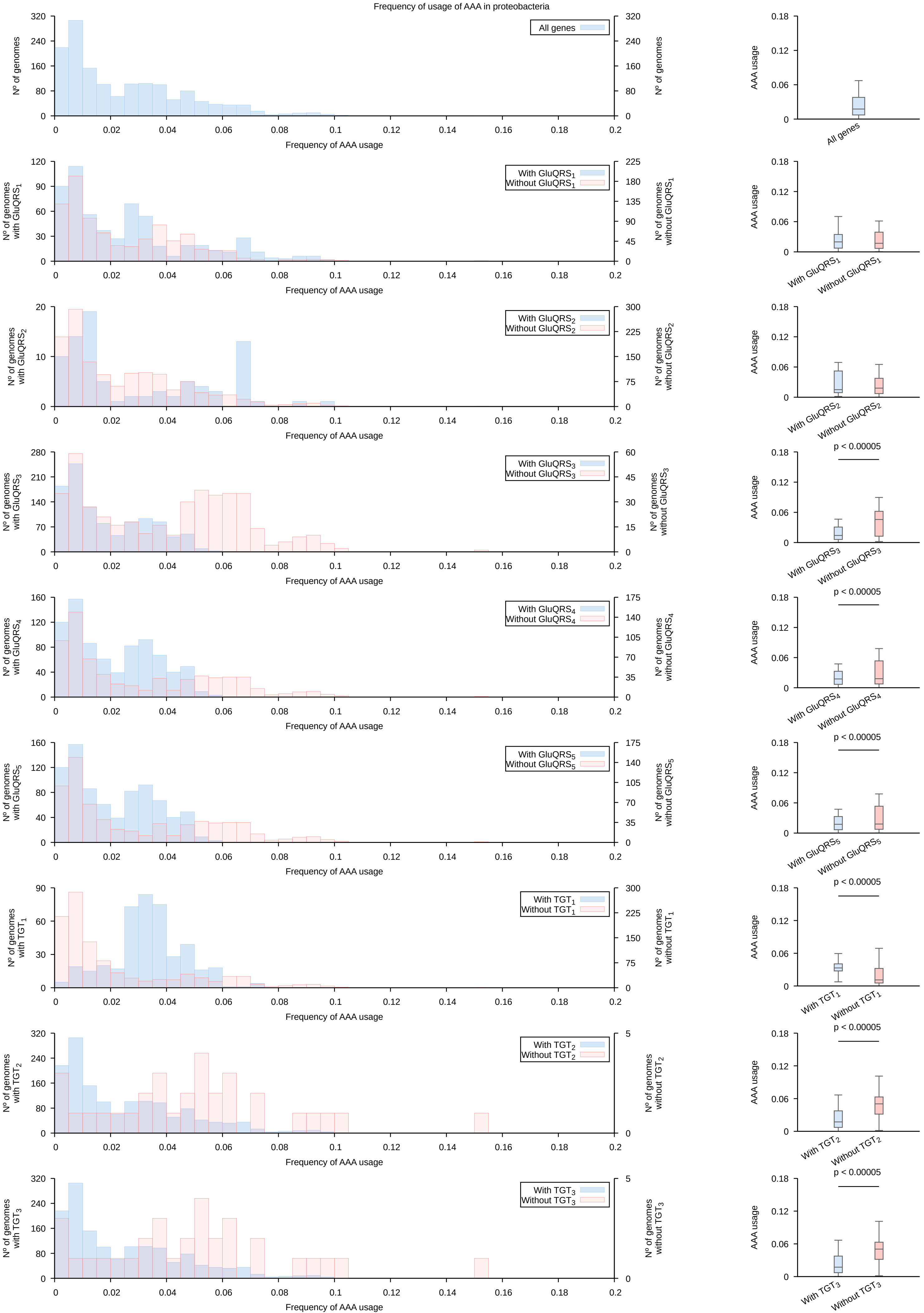
Frequency of usage of AAA in proteobacteria
All genes
Nº of genomes
Frequency of AAA usage
AAA usage
With GluQRS$_1$
Without GluQRS$_1$
Nº of genomes with GluQRS$_1$
Nº of genomes without GluQRS$_1$
With GluQRS$_2$
Without GluQRS$_2$
Nº of genomes with GluQRS$_2$
Nº of genomes without GluQRS$_2$
With GluQRS$_3$
Without GluQRS$_3$
Nº of genomes with GluQRS$_3$
Nº of genomes without GluQRS$_3$
With GluQRS$_4$
Without GluQRS$_4$
Nº of genomes with GluQRS$_4$
Nº of genomes without GluQRS$_4$
With GluQRS$_5$
Without GluQRS$_5$
Nº of genomes with GluQRS$_5$
Nº of genomes without GluQRS$_5$
With TGT$_1$
Without TGT$_1$
Nº of genomes with TGT$_1$
Nº of genomes without TGT$_1$
With TGT$_2$
Without TGT$_2$
Nº of genomes with TGT$_2$
Nº of genomes without TGT$_2$
With TGT$_3$
Without TGT$_3$
Nº of genomes with TGT$_3$
Nº of genomes without TGT$_3$
p < 0.00005

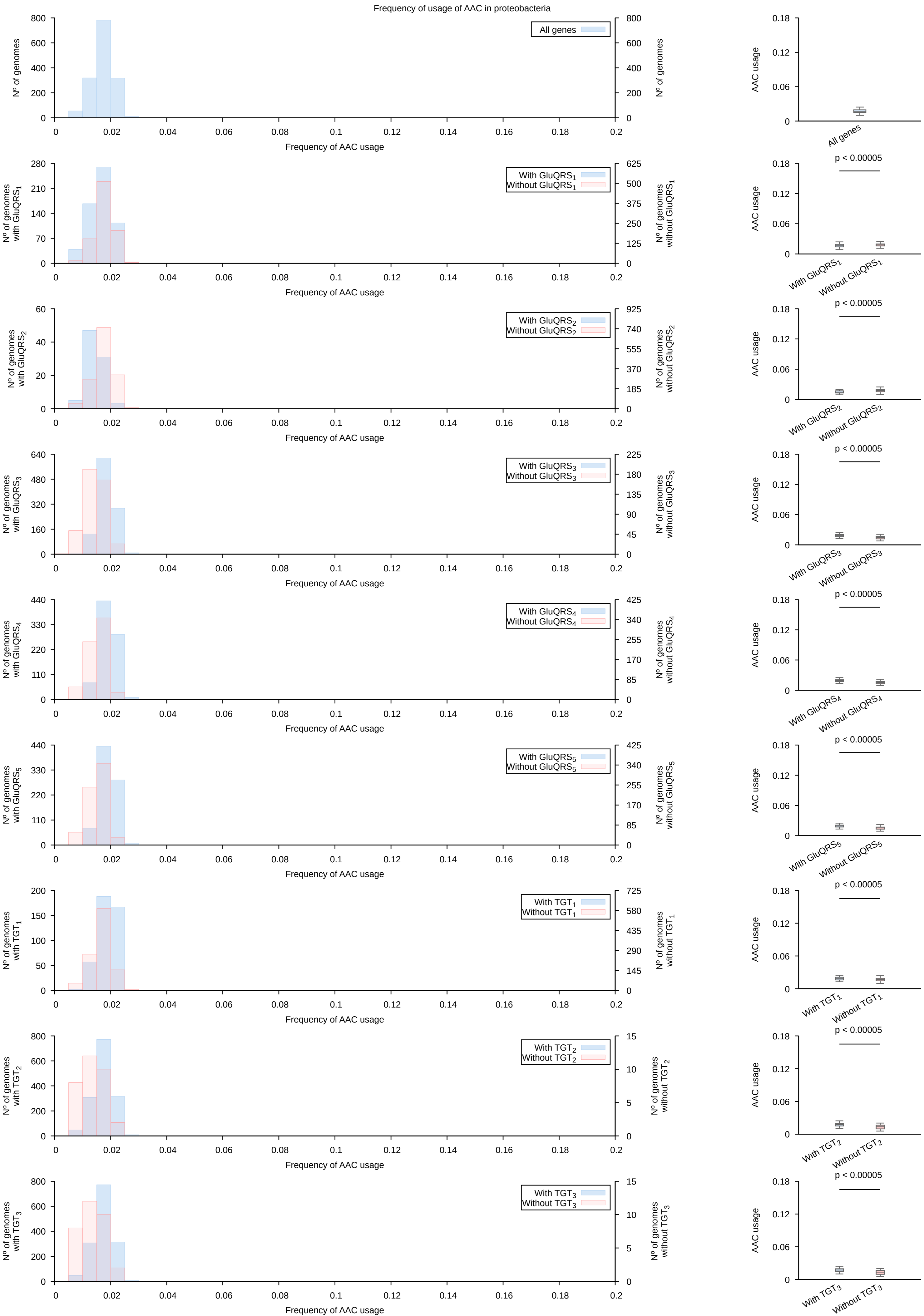

Frequency of usage of AAC in proteobacteria
Nº of genomes
All genes
Frequency of AAC usage
AAC usage
Nº of genomes with GluQRS1
Nº of genomes without GluQRS1
With GluQRS1
Without GluQRS1
p < 0.00005
Nº of genomes with GluQRS2
Nº of genomes without GluQRS2
With GluQRS2
Without GluQRS2
Nº of genomes with GluQRS3
Nº of genomes without GluQRS3
With GluQRS3
Without GluQRS3
Nº of genomes with GluQRS4
Nº of genomes without GluQRS4
With GluQRS4
Without GluQRS4
Nº of genomes with GluQRS5
Nº of genomes without GluQRS5
With GluQRS5
Without GluQRS5
Nº of genomes with TGT1
Nº of genomes without TGT1
With TGT1
Without TGT1
Nº of genomes with TGT2
Nº of genomes without TGT2
With TGT2
Without TGT2
Nº of genomes with TGT3
Nº of genomes without TGT3
With TGT3
Without TGT3

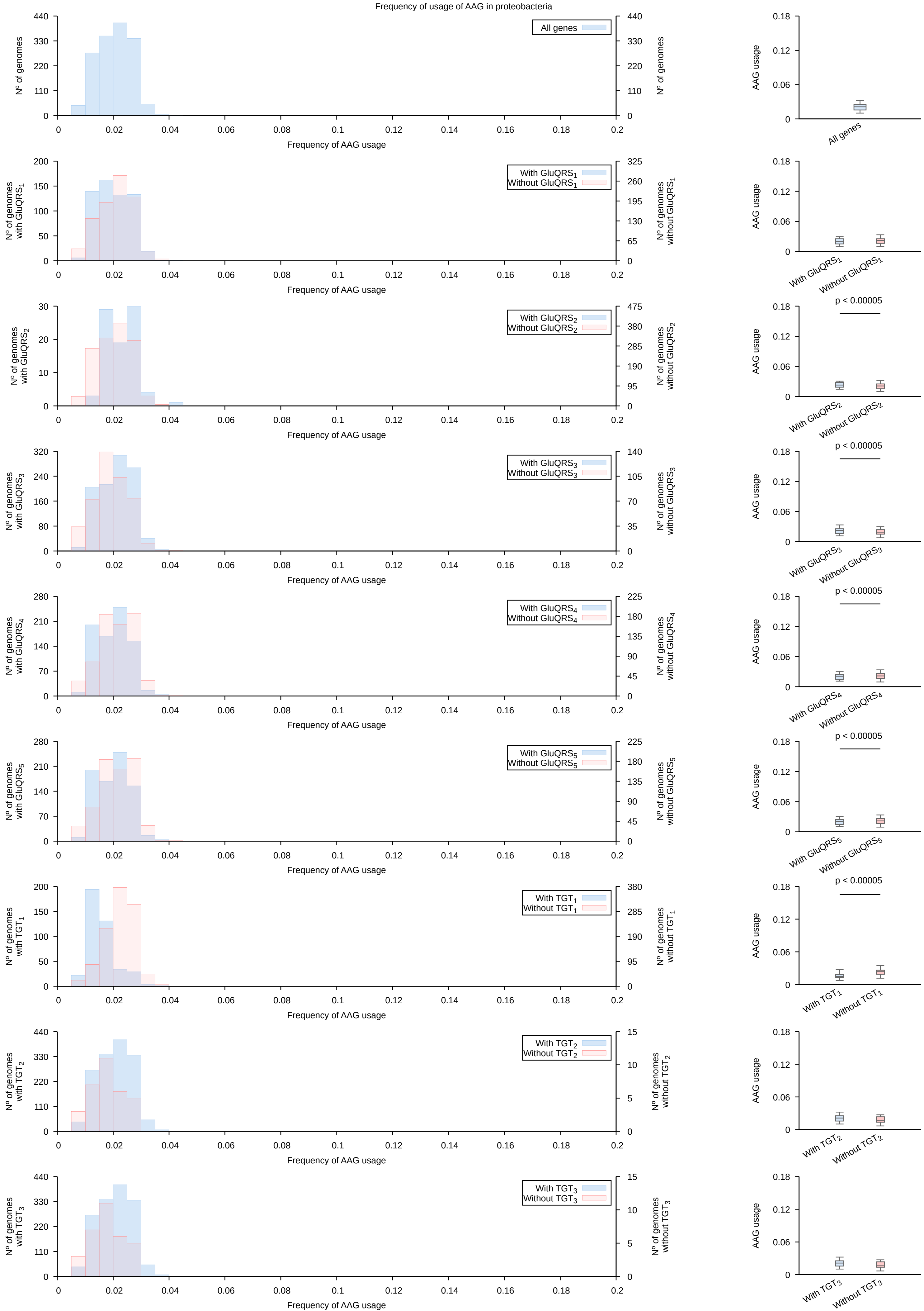
Frequency of usage of AAG in proteobacteria
All genes
With GluQRS$_1$
Without GluQRS$_1$
With GluQRS$_2$
Without GluQRS$_2$
With GluQRS$_3$
Without GluQRS$_3$
With GluQRS$_4$
Without GluQRS$_4$
With GluQRS$_5$
Without GluQRS$_5$
With TGT$_1$
Without TGT$_1$
With TGT$_2$
Without TGT$_2$
With TGT$_3$
Without TGT$_3$
Nº of genomes
Frequency of AAG usage
AAG usage
p < 0.00005

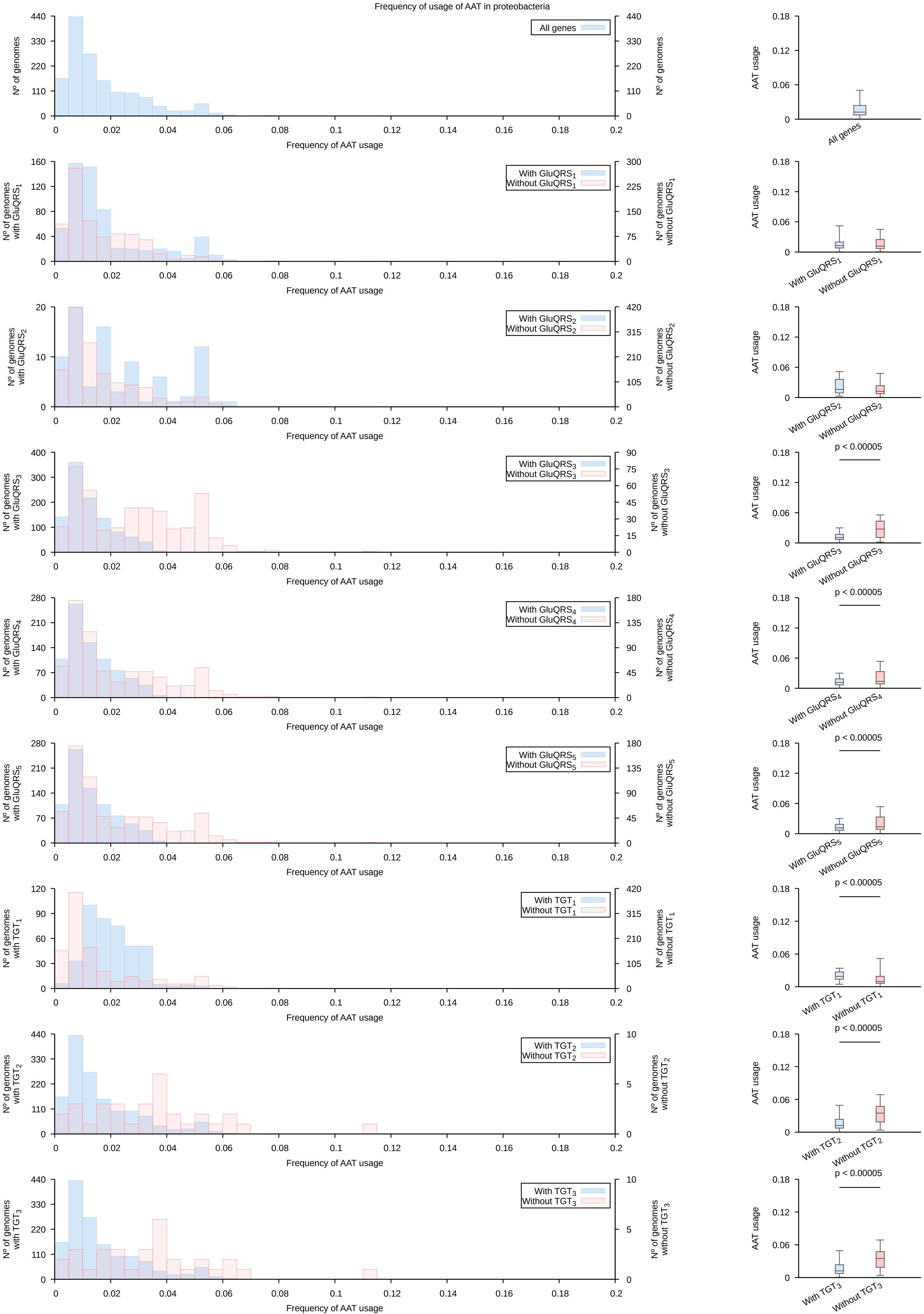
Frequency of usage of AAT in proteobacteria
Nº of genomes
All genes
AAT usage
Frequency of AAT usage
Nº of genomes with GluQRS1
Nº of genomes without GluQRS1
With GluQRS1
Without GluQRS1
Nº of genomes with GluQRS2
Nº of genomes without GluQRS2
With GluQRS2
Without GluQRS2
Nº of genomes with GluQRS3
Nº of genomes without GluQRS3
With GluQRS3
Without GluQRS3
p < 0.00005
Nº of genomes with GluQRS4
Nº of genomes without GluQRS4
With GluQRS4
Without GluQRS4
Nº of genomes with GluQRS5
Nº of genomes without GluQRS5
With GluQRS5
Without GluQRS5
Nº of genomes with TGT1
Nº of genomes without TGT1
With TGT1
Without TGT1
Nº of genomes with TGT2
Nº of genomes without TGT2
With TGT2
Without TGT2
Nº of genomes with TGT3
Nº of genomes without TGT3
With TGT3
Without TGT3

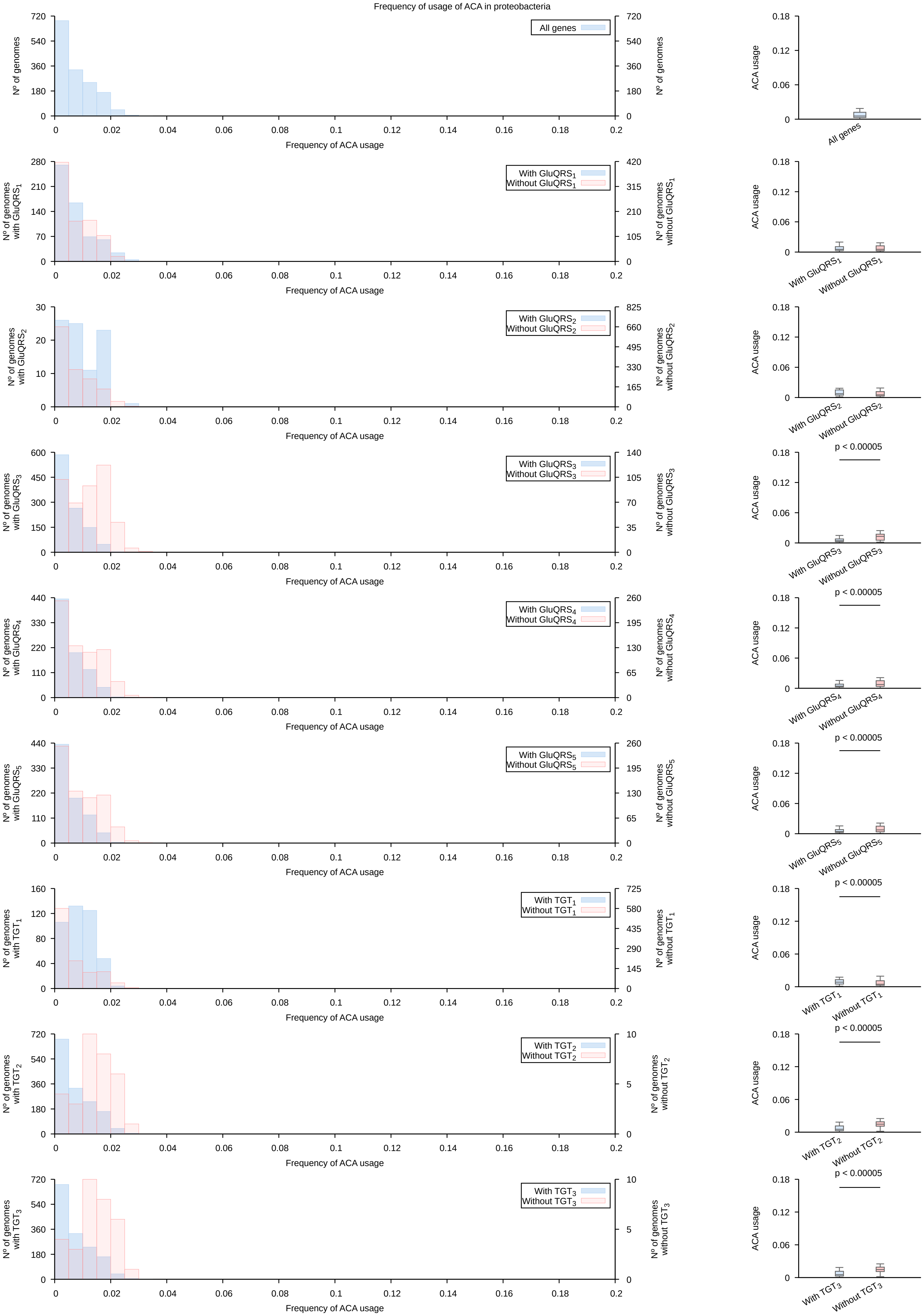

Frequency of usage of ACA in proteobacteria
All genes
Nº of genomes
Frequency of ACA usage
ACA usage
With GluQRS$_1$
Without GluQRS$_1$
Nº of genomes with GluQRS$_1$
Nº of genomes without GluQRS$_1$
With GluQRS$_2$
Without GluQRS$_2$
Nº of genomes with GluQRS$_2$
Nº of genomes without GluQRS$_2$
With GluQRS$_3$
Without GluQRS$_3$
Nº of genomes with GluQRS$_3$
Nº of genomes without GluQRS$_3$
p < 0.00005
With GluQRS$_4$
Without GluQRS$_4$
Nº of genomes with GluQRS$_4$
Nº of genomes without GluQRS$_4$
p < 0.00005
With GluQRS$_5$
Without GluQRS$_5$
Nº of genomes with GluQRS$_5$
Nº of genomes without GluQRS$_5$
p < 0.00005
With TGT$_1$
Without TGT$_1$
Nº of genomes with TGT$_1$
Nº of genomes without TGT$_1$
p < 0.00005
With TGT$_2$
Without TGT$_2$
Nº of genomes with TGT$_2$
Nº of genomes without TGT$_2$
p < 0.00005
With TGT$_3$
Without TGT$_3$
Nº of genomes with TGT$_3$
Nº of genomes without TGT$_3$
p < 0.00005

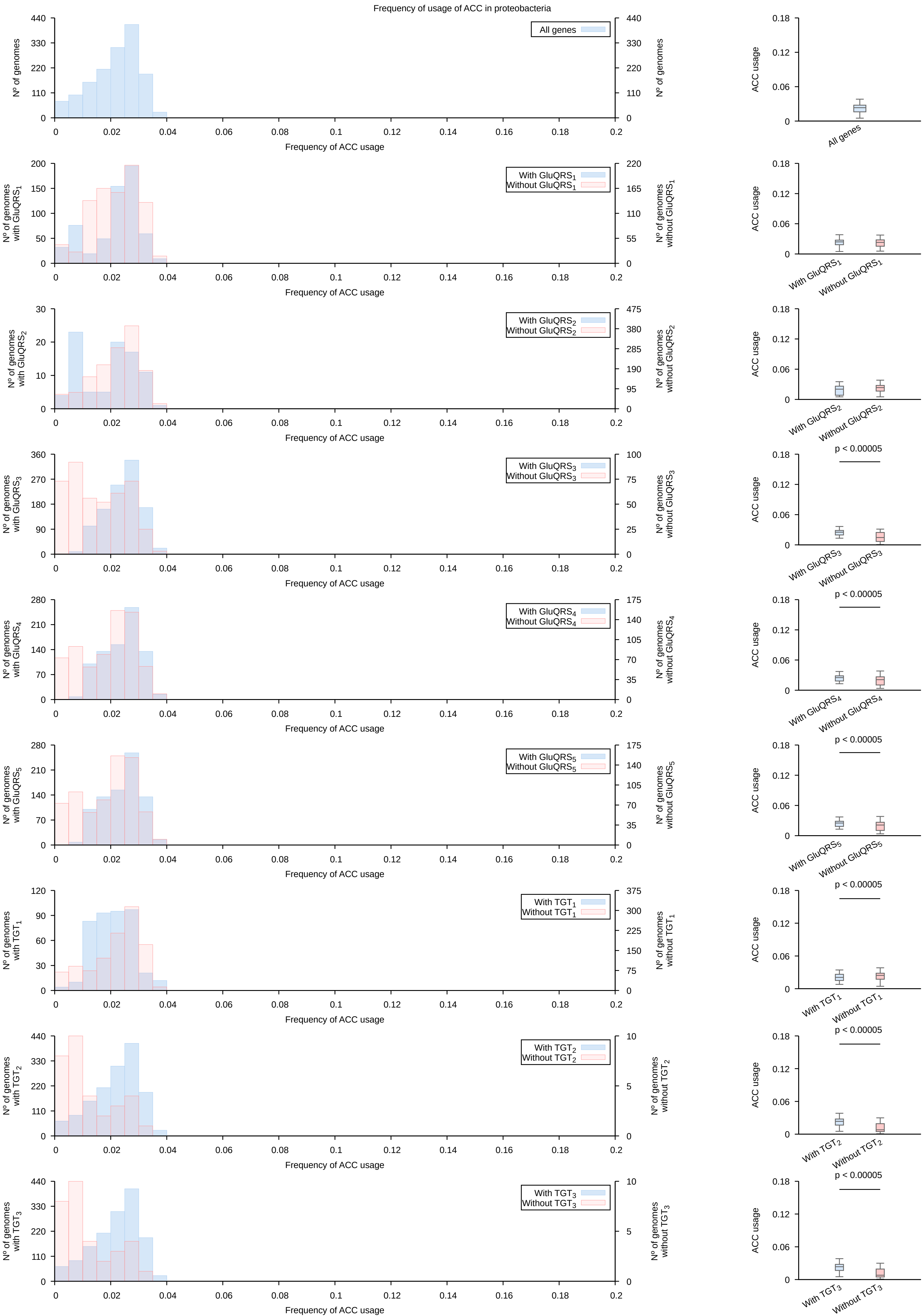

Frequency of usage of ACC in proteobacteria
Nº of genomes
Frequency of ACC usage
All genes
ACC usage
Nº of genomes with GluQRS$_1$
Nº of genomes without GluQRS$_1$
With GluQRS$_1$
Without GluQRS$_1$
Nº of genomes with GluQRS$_2$
Nº of genomes without GluQRS$_2$
With GluQRS$_2$
Without GluQRS$_2$
Nº of genomes with GluQRS$_3$
Nº of genomes without GluQRS$_3$
With GluQRS$_3$
Without GluQRS$_3$
p < 0.00005
Nº of genomes with GluQRS$_4$
Nº of genomes without GluQRS$_4$
With GluQRS$_4$
Without GluQRS$_4$
p < 0.00005
Nº of genomes with GluQRS$_5$
Nº of genomes without GluQRS$_5$
With GluQRS$_5$
Without GluQRS$_5$
p < 0.00005
Nº of genomes with TGT$_1$
Nº of genomes without TGT$_1$
With TGT$_1$
Without TGT$_1$
p < 0.00005
Nº of genomes with TGT$_2$
Nº of genomes without TGT$_2$
With TGT$_2$
Without TGT$_2$
p < 0.00005
Nº of genomes with TGT$_3$
Nº of genomes without TGT$_3$
With TGT$_3$
Without TGT$_3$
p < 0.00005

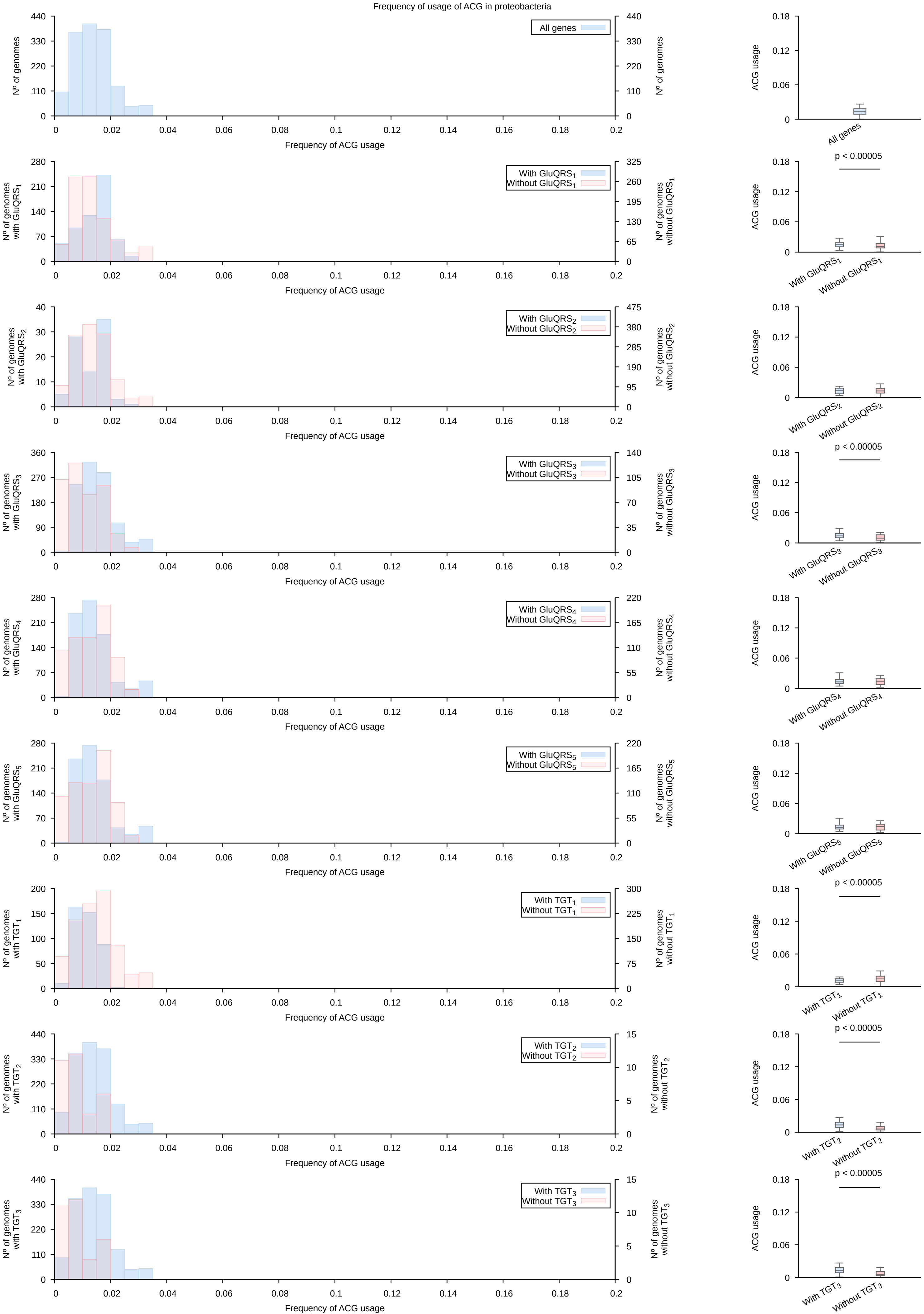
Frequency of usage of ACG in proteobacteria
All genes
With GluQRS1
Without GluQRS1
With GluQRS2
Without GluQRS2
With GluQRS3
Without GluQRS3
With GluQRS4
Without GluQRS4
With GluQRS5
Without GluQRS5
With TGT1
Without TGT1
With TGT2
Without TGT2
With TGT3
Without TGT3
Nº of genomes
Nº of genomes with GluQRS1
Nº of genomes without GluQRS1
Nº of genomes with GluQRS2
Nº of genomes without GluQRS2
Nº of genomes with GluQRS3
Nº of genomes without GluQRS3
Nº of genomes with GluQRS4
Nº of genomes without GluQRS4
Nº of genomes with GluQRS5
Nº of genomes without GluQRS5
Nº of genomes with TGT1
Nº of genomes without TGT1
Nº of genomes with TGT2
Nº of genomes without TGT2
Nº of genomes with TGT3
Nº of genomes without TGT3
Frequency of ACG usage
ACG usage
p < 0.00005

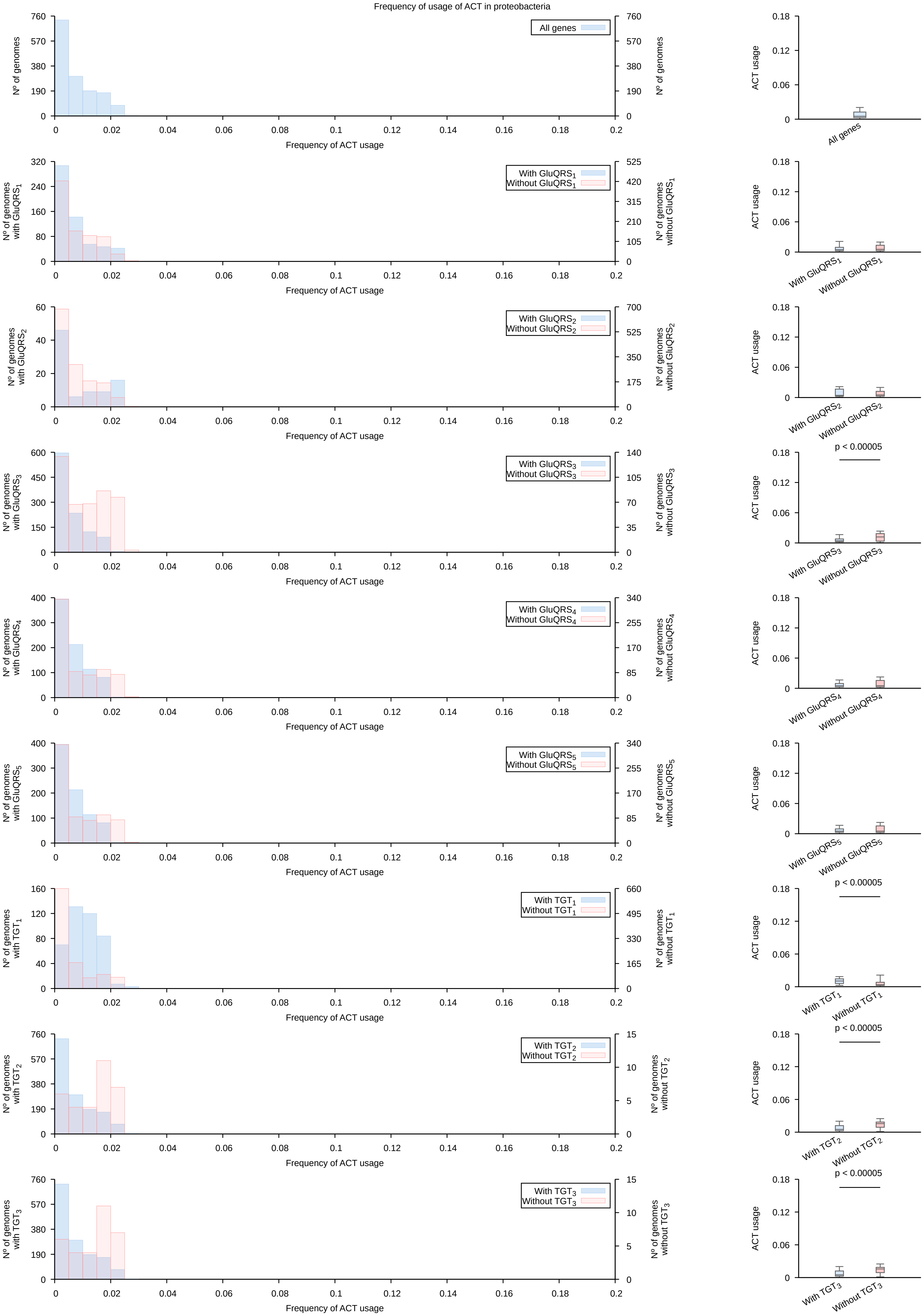
Frequency of usage of ACT in proteobacteria
Nº of genomes
Frequency of ACT usage
ACT usage
All genes
Nº of genomes with $GluQRS_1$
Nº of genomes without $GluQRS_1$
With $GluQRS_1$
Without $GluQRS_1$
Nº of genomes with $GluQRS_2$
Nº of genomes without $GluQRS_2$
With $GluQRS_2$
Without $GluQRS_2$
Nº of genomes with $GluQRS_3$
Nº of genomes without $GluQRS_3$
With $GluQRS_3$
Without $GluQRS_3$
$p < 0.00005$
Nº of genomes with $GluQRS_4$
Nº of genomes without $GluQRS_4$
With $GluQRS_4$
Without $GluQRS_4$
Nº of genomes with $GluQRS_5$
Nº of genomes without $GluQRS_5$
With $GluQRS_5$
Without $GluQRS_5$
Nº of genomes with TGT_1
Nº of genomes without TGT_1
With TGT_1
Without TGT_1
$p < 0.00005$
Nº of genomes with TGT_2
Nº of genomes without TGT_2
With TGT_2
Without TGT_2
$p < 0.00005$
Nº of genomes with TGT_3
Nº of genomes without TGT_3
With TGT_3
Without TGT_3
$p < 0.00005$

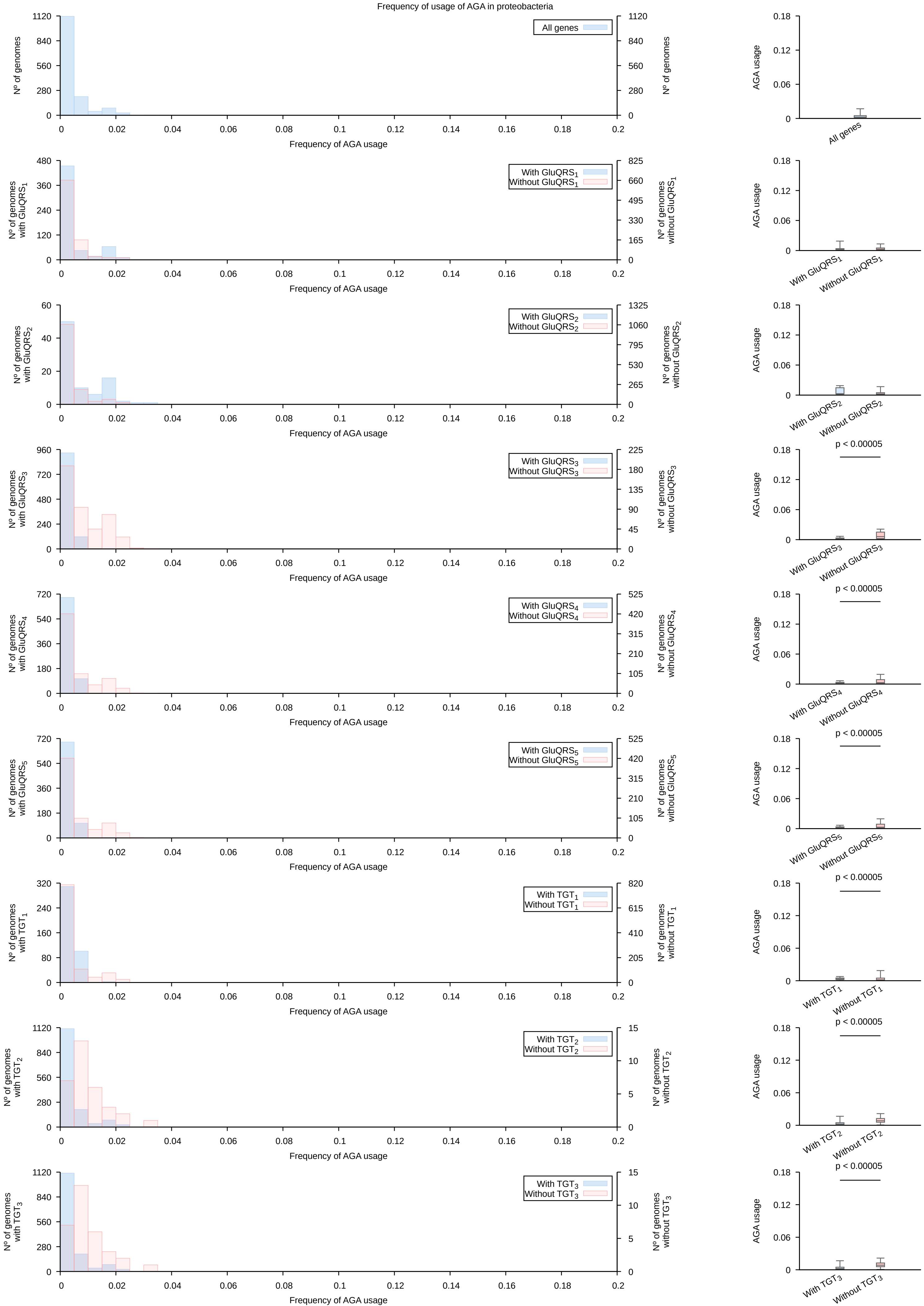

Frequency of usage of AGA in proteobacteria
All genes
Nº of genomes
Frequency of AGA usage
AGA usage
With GluQRS$_1$
Without GluQRS$_1$
Nº of genomes with GluQRS$_1$
Nº of genomes without GluQRS$_1$
With GluQRS$_2$
Without GluQRS$_2$
Nº of genomes with GluQRS$_2$
Nº of genomes without GluQRS$_2$
With GluQRS$_3$
Without GluQRS$_3$
Nº of genomes with GluQRS$_3$
Nº of genomes without GluQRS$_3$
p < 0.00005
With GluQRS$_4$
Without GluQRS$_4$
Nº of genomes with GluQRS$_4$
Nº of genomes without GluQRS$_4$
p < 0.00005
With GluQRS$_5$
Without GluQRS$_5$
Nº of genomes with GluQRS$_5$
Nº of genomes without GluQRS$_5$
p < 0.00005
With TGT$_1$
Without TGT$_1$
Nº of genomes with TGT$_1$
Nº of genomes without TGT$_1$
p < 0.00005
With TGT$_2$
Without TGT$_2$
Nº of genomes with TGT$_2$
Nº of genomes without TGT$_2$
p < 0.00005
With TGT$_3$
Without TGT$_3$
Nº of genomes with TGT$_3$
Nº of genomes without TGT$_3$

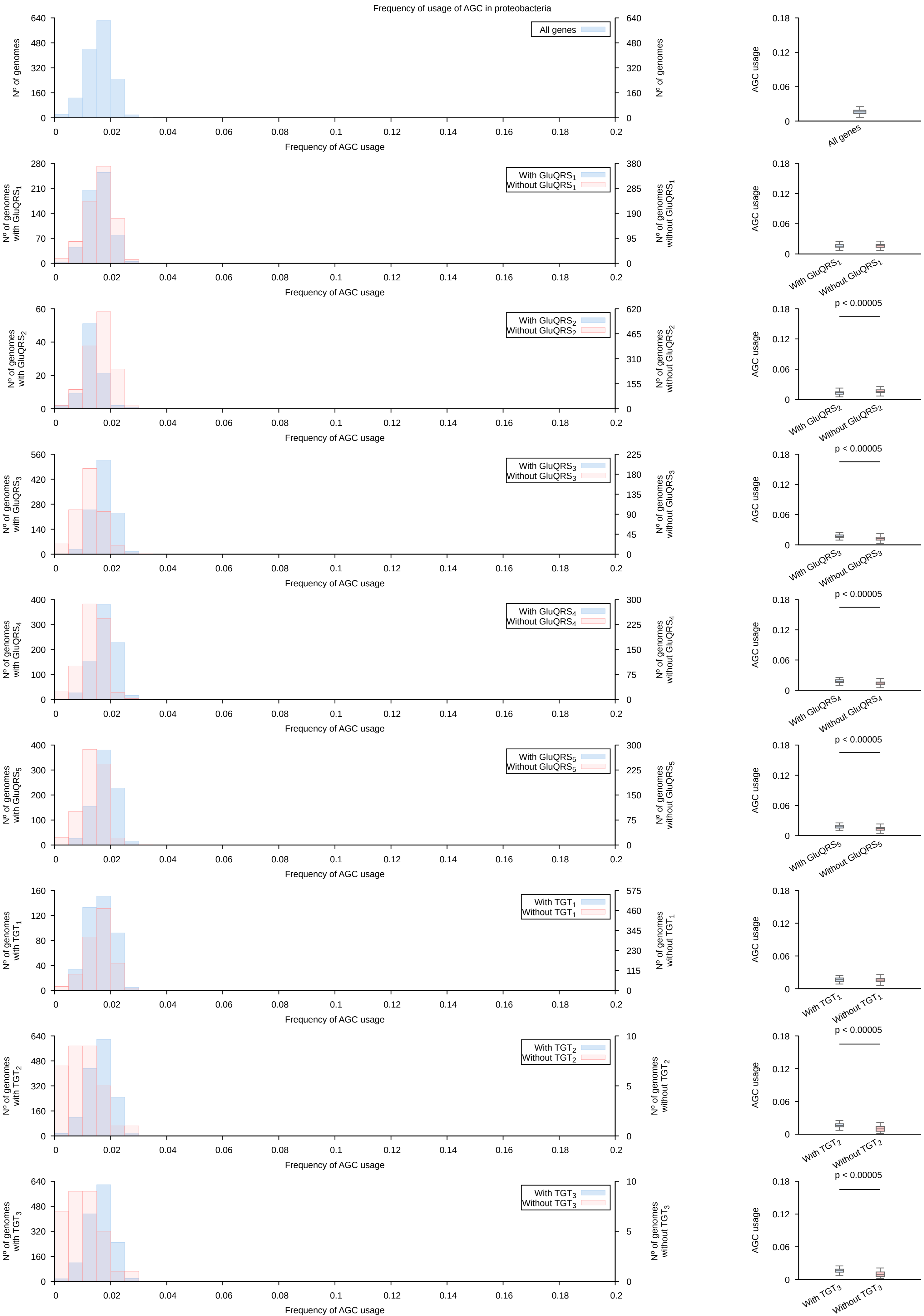

Frequency of usage of AGC in proteobacteria
All genes
With $GluQRS_1$
Without $GluQRS_1$
With $GluQRS_2$
Without $GluQRS_2$
With $GluQRS_3$
Without $GluQRS_3$
With $GluQRS_4$
Without $GluQRS_4$
With $GluQRS_5$
Without $GluQRS_5$
With TGT_1
Without TGT_1
With TGT_2
Without TGT_2
With TGT_3
Without TGT_3
Nº of genomes
Nº of genomes with $GluQRS_1$
Nº of genomes without $GluQRS_1$
Nº of genomes with $GluQRS_2$
Nº of genomes without $GluQRS_2$
Nº of genomes with $GluQRS_3$
Nº of genomes without $GluQRS_3$
Nº of genomes with $GluQRS_4$
Nº of genomes without $GluQRS_4$
Nº of genomes with $GluQRS_5$
Nº of genomes without $GluQRS_5$
Nº of genomes with TGT_1
Nº of genomes without TGT_1
Nº of genomes with TGT_2
Nº of genomes without TGT_2
Nº of genomes with TGT_3
Nº of genomes without TGT_3
Frequency of AGC usage
AGC usage
$p < 0.00005$

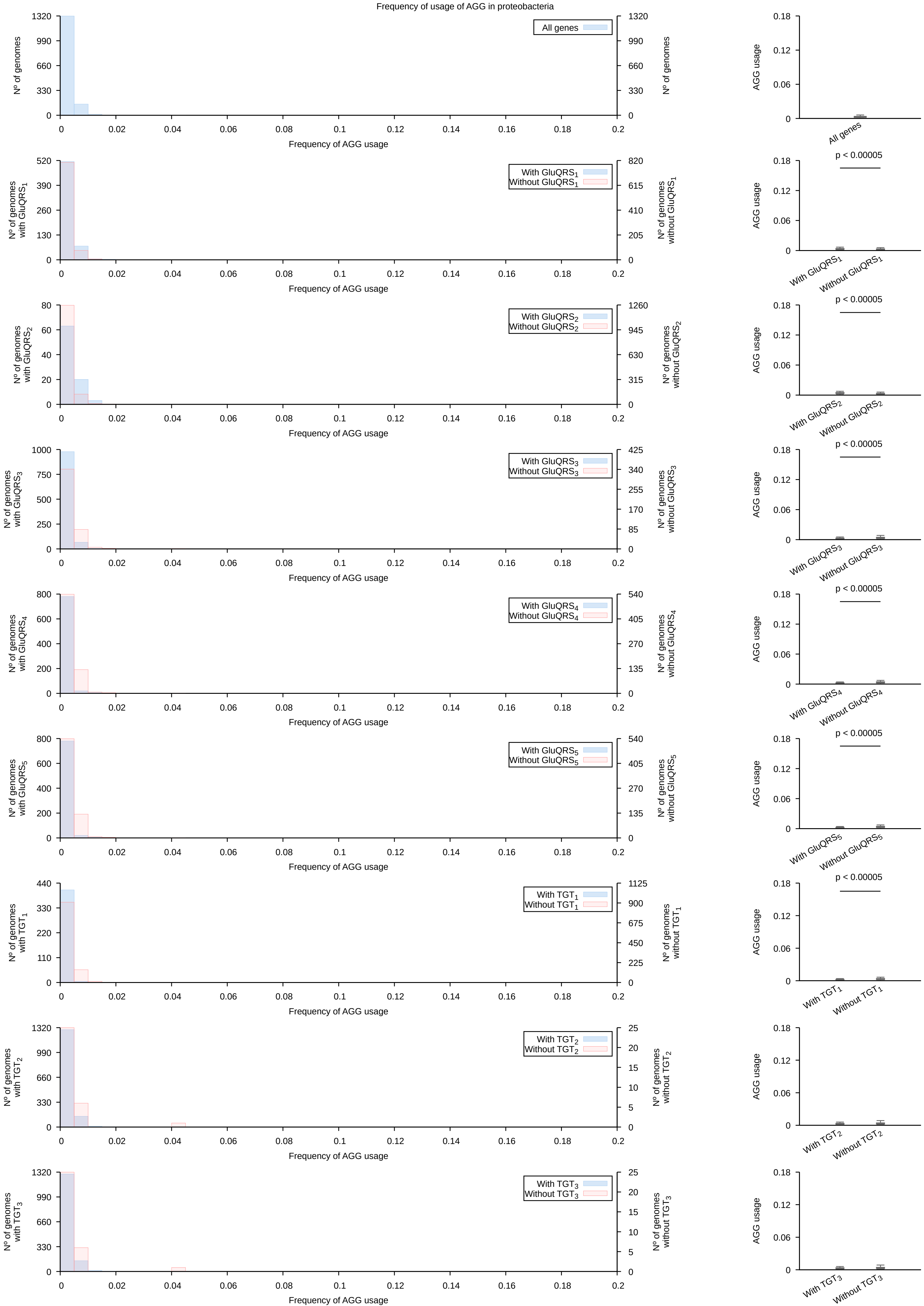
Frequency of usage of AGG in proteobacteria
All genes
With GluQRS1
Without GluQRS1
With GluQRS2
Without GluQRS2
With GluQRS3
Without GluQRS3
With GluQRS4
Without GluQRS4
With GluQRS5
Without GluQRS5
With TGT1
Without TGT1
With TGT2
Without TGT2
With TGT3
Without TGT3
Nº of genomes
Frequency of AGG usage
AGG usage
p < 0.00005

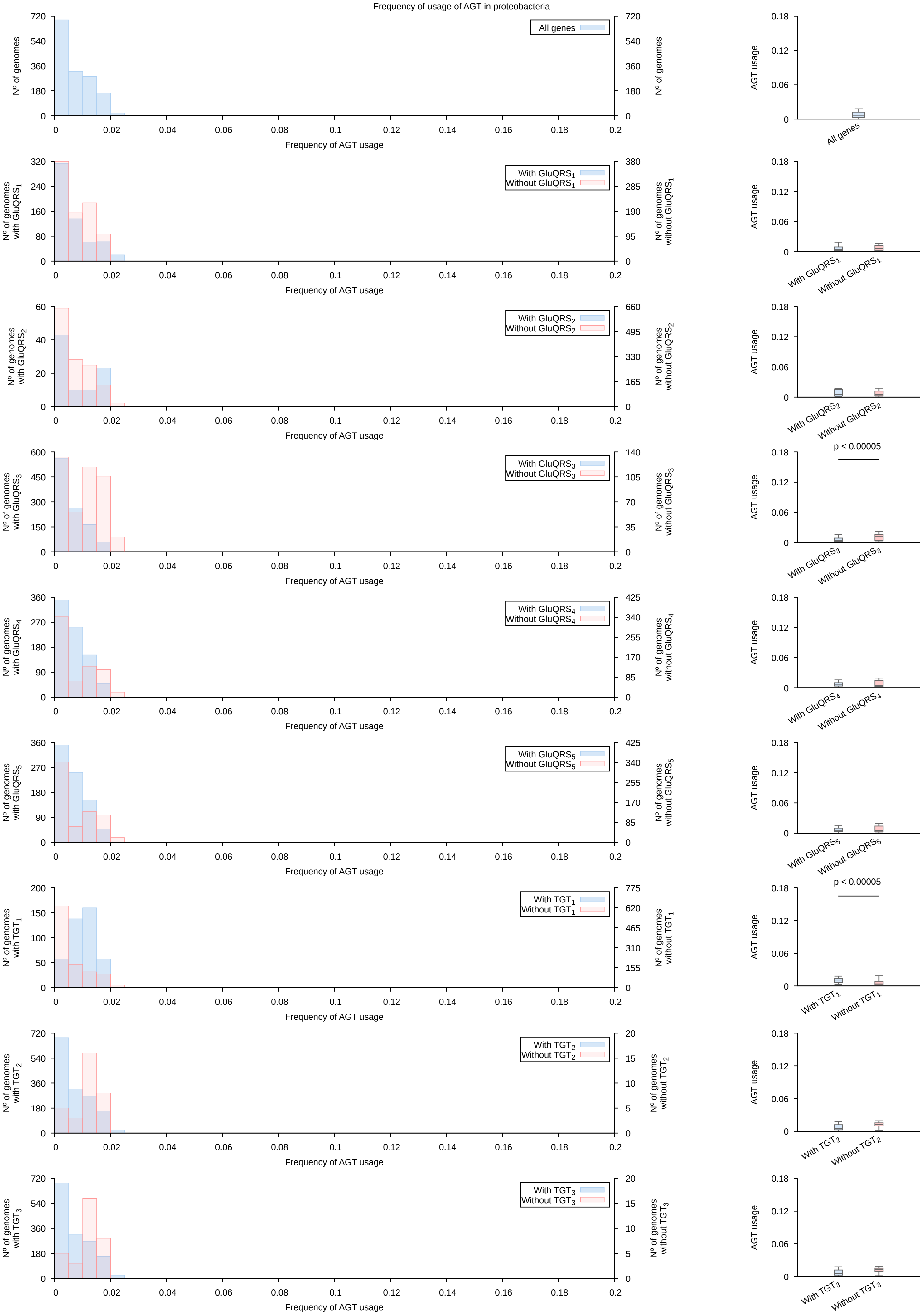

Frequency of usage of AGT in proteobacteria
All genes
Nº of genomes
Frequency of AGT usage
AGT usage
With GluQRS$_1$
Without GluQRS$_1$
Nº of genomes with GluQRS$_1$
Nº of genomes without GluQRS$_1$
With GluQRS$_2$
Without GluQRS$_2$
Nº of genomes with GluQRS$_2$
Nº of genomes without GluQRS$_2$
With GluQRS$_3$
Without GluQRS$_3$
Nº of genomes with GluQRS$_3$
Nº of genomes without GluQRS$_3$
p < 0.00005
With GluQRS$_4$
Without GluQRS$_4$
Nº of genomes with GluQRS$_4$
Nº of genomes without GluQRS$_4$
With GluQRS$_5$
Without GluQRS$_5$
Nº of genomes with GluQRS$_5$
Nº of genomes without GluQRS$_5$
With TGT$_1$
Without TGT$_1$
Nº of genomes with TGT$_1$
Nº of genomes without TGT$_1$
p < 0.00005
With TGT$_2$
Without TGT$_2$
Nº of genomes with TGT$_2$
Nº of genomes without TGT$_2$
With TGT$_3$
Without TGT$_3$
Nº of genomes with TGT$_3$
Nº of genomes without TGT$_3$

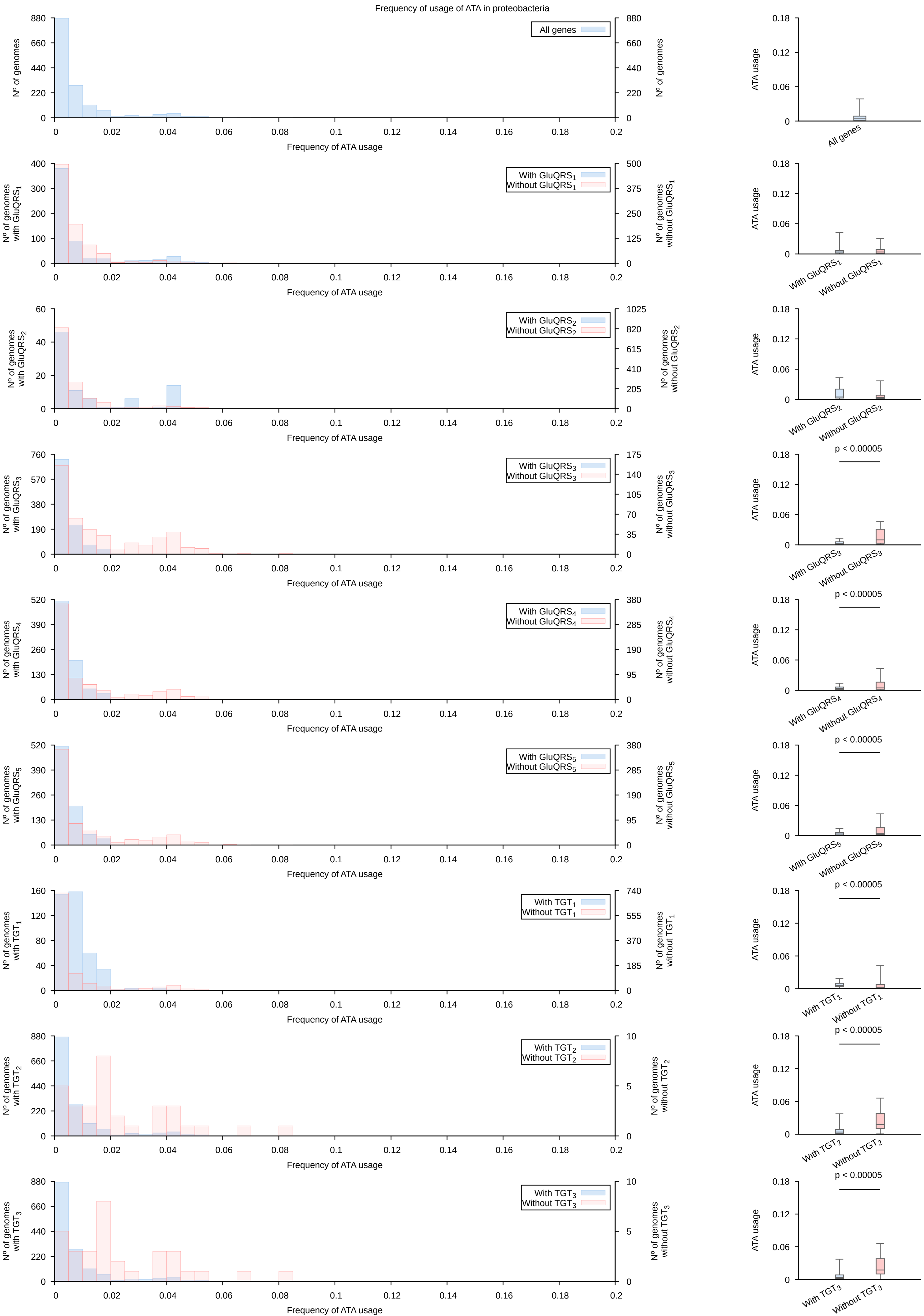

Frequency of usage of ATA in proteobacteria
Nº of genomes
Frequency of ATA usage
All genes
ATA usage
Nº of genomes with GluQRS$_1$
Nº of genomes without GluQRS$_1$
With GluQRS$_1$
Without GluQRS$_1$
Nº of genomes with GluQRS$_2$
Nº of genomes without GluQRS$_2$
With GluQRS$_2$
Without GluQRS$_2$
Nº of genomes with GluQRS$_3$
Nº of genomes without GluQRS$_3$
With GluQRS$_3$
Without GluQRS$_3$
p < 0.00005
Nº of genomes with GluQRS$_4$
Nº of genomes without GluQRS$_4$
With GluQRS$_4$
Without GluQRS$_4$
p < 0.00005
Nº of genomes with GluQRS$_5$
Nº of genomes without GluQRS$_5$
With GluQRS$_5$
Without GluQRS$_5$
p < 0.00005
Nº of genomes with TGT$_1$
Nº of genomes without TGT$_1$
With TGT$_1$
Without TGT$_1$
p < 0.00005
Nº of genomes with TGT$_2$
Nº of genomes without TGT$_2$
With TGT$_2$
Without TGT$_2$
p < 0.00005
Nº of genomes with TGT$_3$
Nº of genomes without TGT$_3$
With TGT$_3$
Without TGT$_3$
p < 0.00005

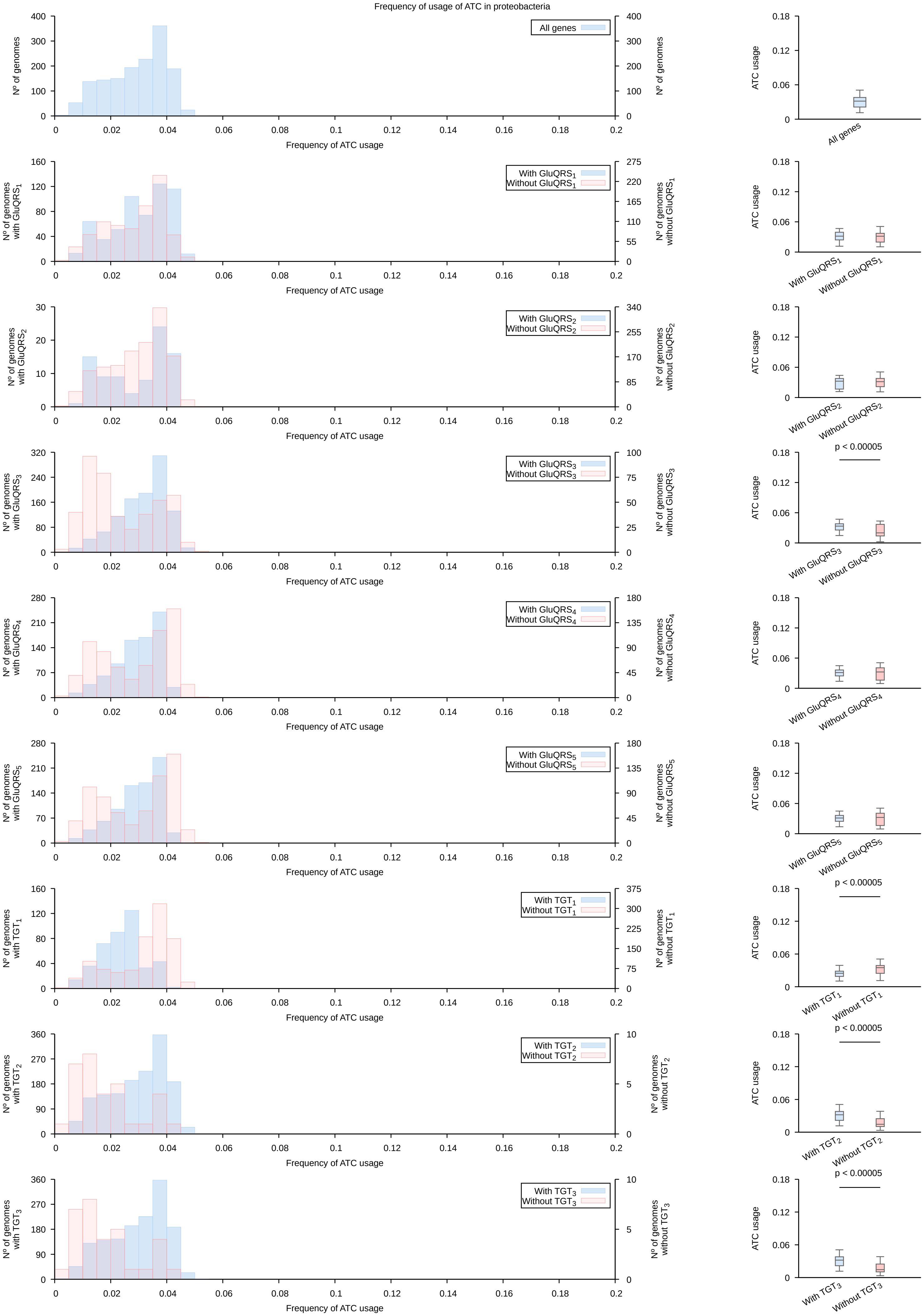

Frequency of usage of ATC in proteobacteria
Nº of genomes
Frequency of ATC usage
All genes
ATC usage
With GluQRS1
Without GluQRS1
With GluQRS2
Without GluQRS2
With GluQRS3
Without GluQRS3
With GluQRS4
Without GluQRS4
With GluQRS5
Without GluQRS5
With TGT1
Without TGT1
With TGT2
Without TGT2
With TGT3
Without TGT3
p < 0.00005

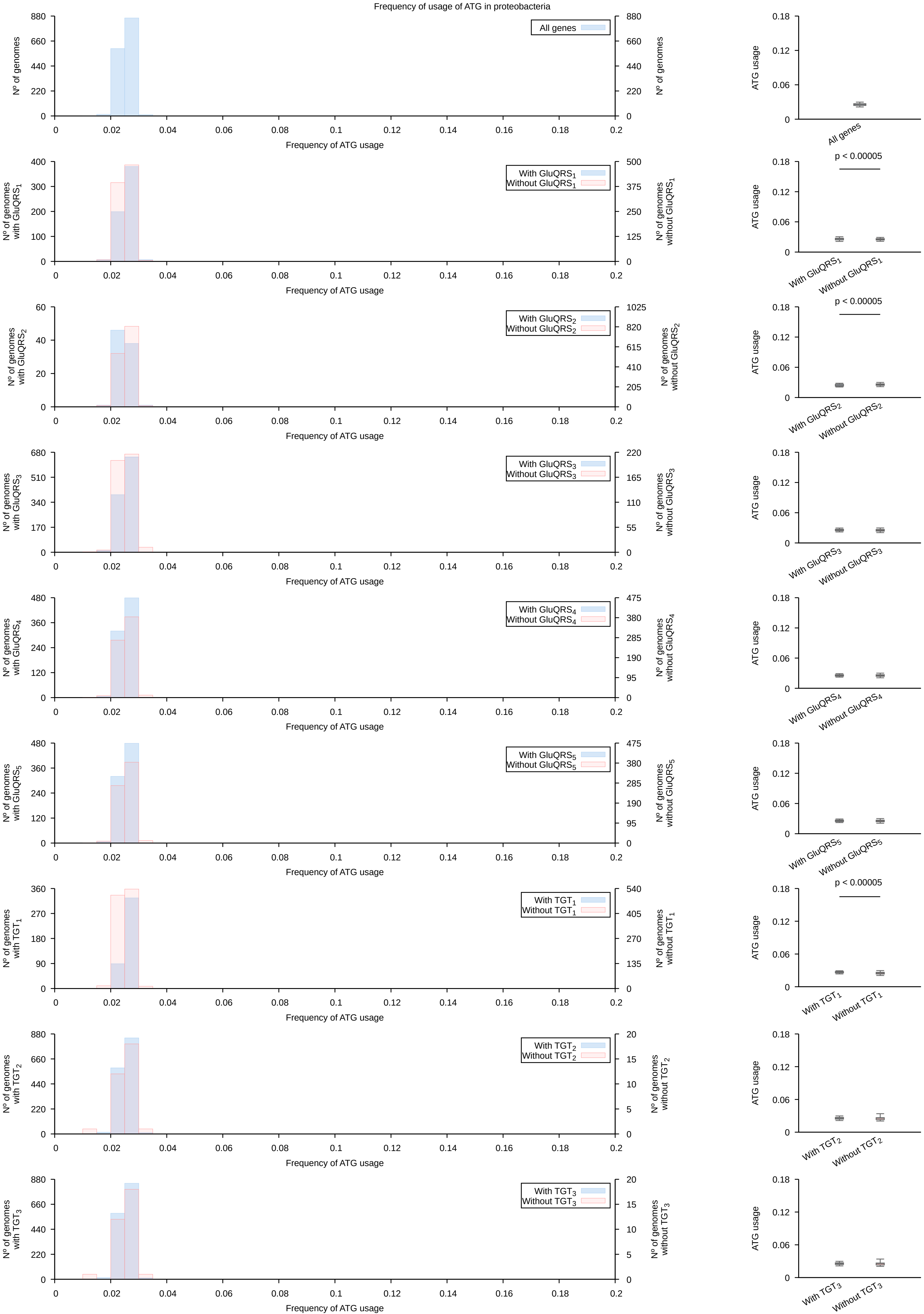

Frequency of usage of ATG in proteobacteria
All genes
Nº of genomes
Frequency of ATG usage
ATG usage
With GluQRS$_1$
Without GluQRS$_1$
Nº of genomes with GluQRS$_1$
Nº of genomes without GluQRS$_1$
p < 0.00005
With GluQRS$_2$
Without GluQRS$_2$
Nº of genomes with GluQRS$_2$
Nº of genomes without GluQRS$_2$
p < 0.00005
With GluQRS$_3$
Without GluQRS$_3$
Nº of genomes with GluQRS$_3$
Nº of genomes without GluQRS$_3$
With GluQRS$_4$
Without GluQRS$_4$
Nº of genomes with GluQRS$_4$
Nº of genomes without GluQRS$_4$
With GluQRS$_5$
Without GluQRS$_5$
Nº of genomes with GluQRS$_5$
Nº of genomes without GluQRS$_5$
With TGT$_1$
Without TGT$_1$
Nº of genomes with TGT$_1$
Nº of genomes without TGT$_1$
p < 0.00005
With TGT$_2$
Without TGT$_2$
Nº of genomes with TGT$_2$
Nº of genomes without TGT$_2$
With TGT$_3$
Without TGT$_3$
Nº of genomes with TGT$_3$
Nº of genomes without TGT$_3$

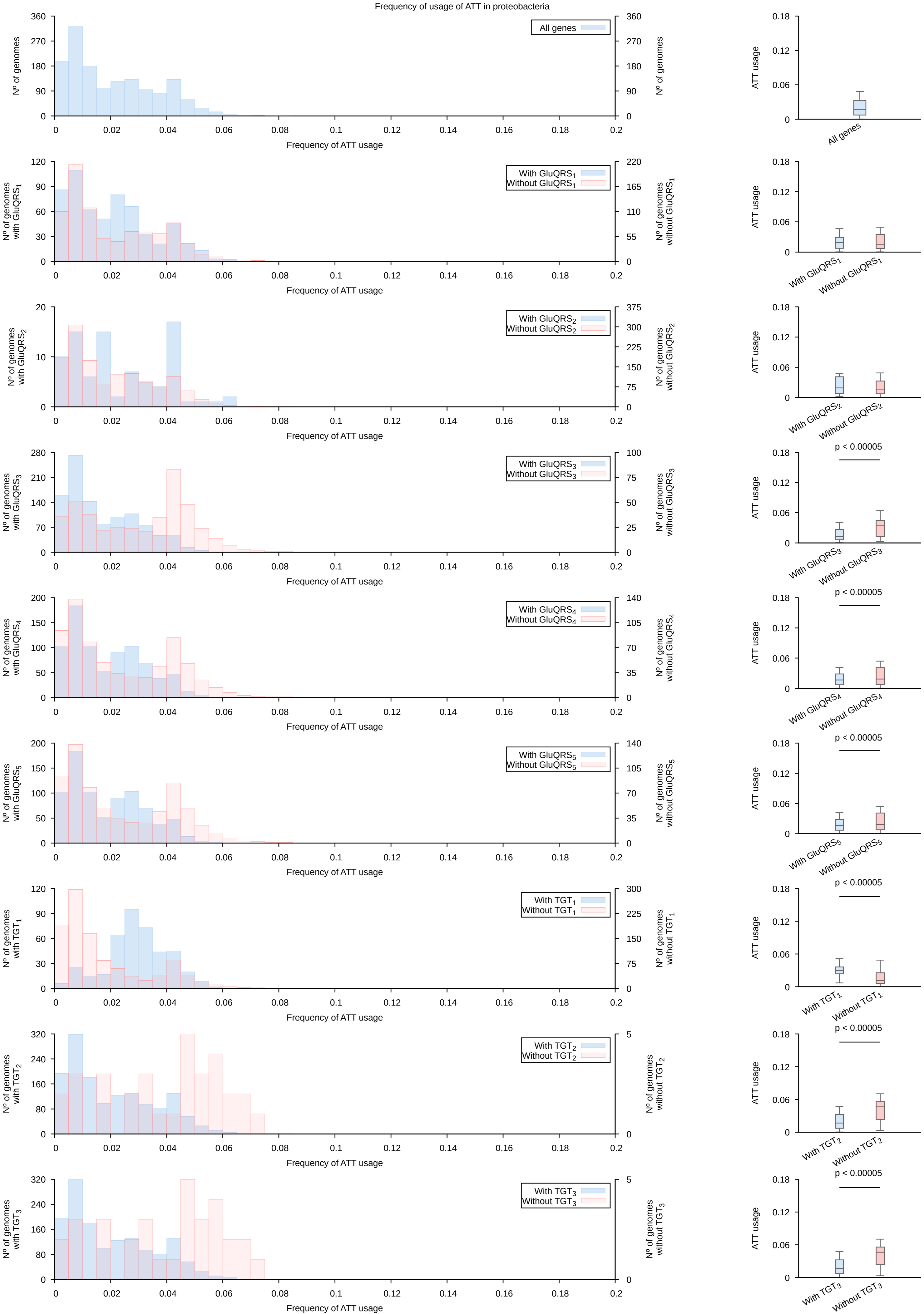
Frequency of usage of ATT in proteobacteria
All genes
With GluQRS$_1$
Without GluQRS$_1$
With GluQRS$_2$
Without GluQRS$_2$
With GluQRS$_3$
Without GluQRS$_3$
With GluQRS$_4$
Without GluQRS$_4$
With GluQRS$_5$
Without GluQRS$_5$
With TGT$_1$
Without TGT$_1$
With TGT$_2$
Without TGT$_2$
With TGT$_3$
Without TGT$_3$
Frequency of ATT usage
Nº of genomes
ATT usage
p < 0.00005

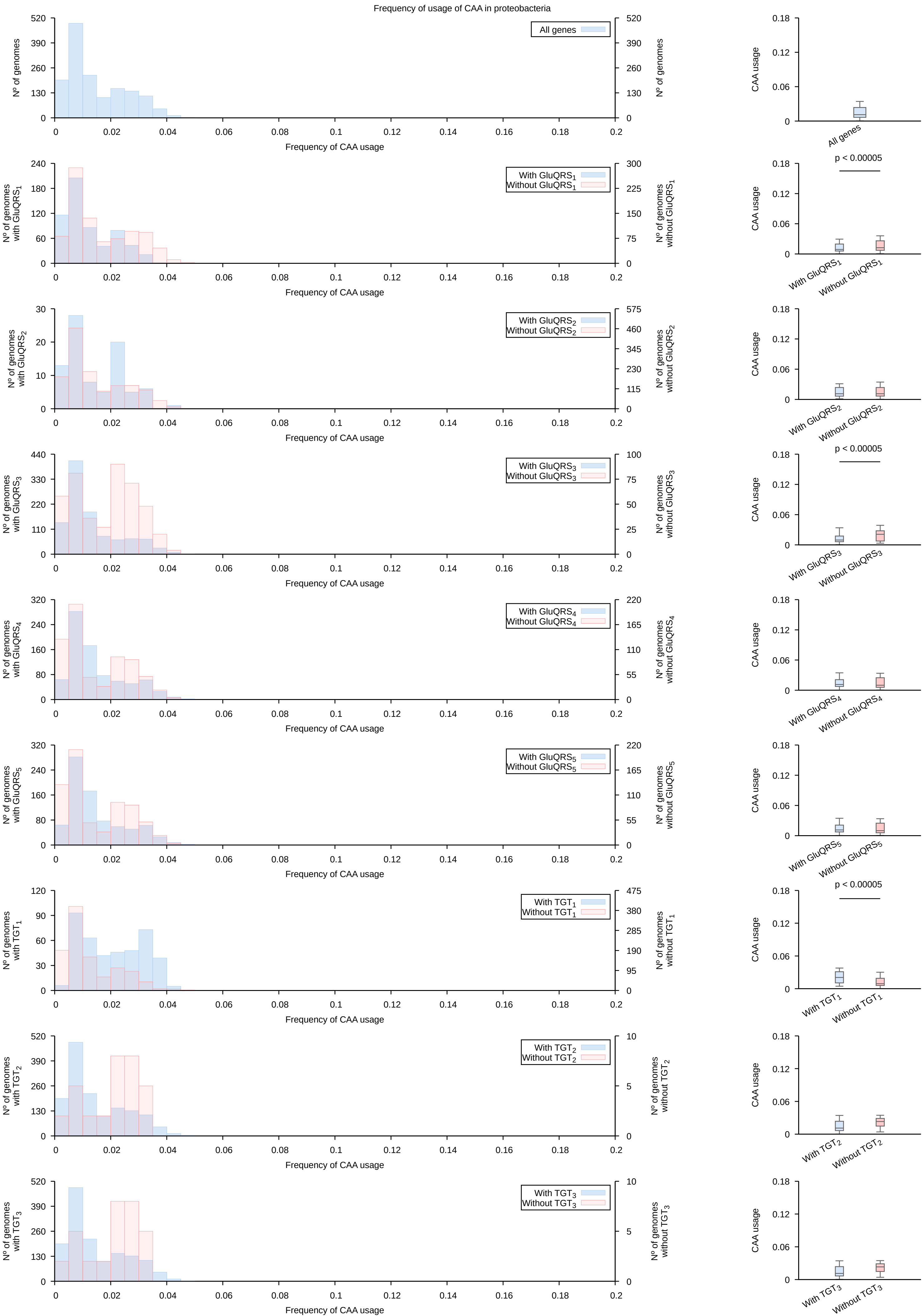
Frequency of usage of CAA in proteobacteria
All genes
With GluQRS1
Without GluQRS1
With GluQRS2
Without GluQRS2
With GluQRS3
Without GluQRS3
With GluQRS4
Without GluQRS4
With GluQRS5
Without GluQRS5
With TGT1
Without TGT1
With TGT2
Without TGT2
With TGT3
Without TGT3
Nº of genomes
Frequency of CAA usage
CAA usage
p < 0.00005

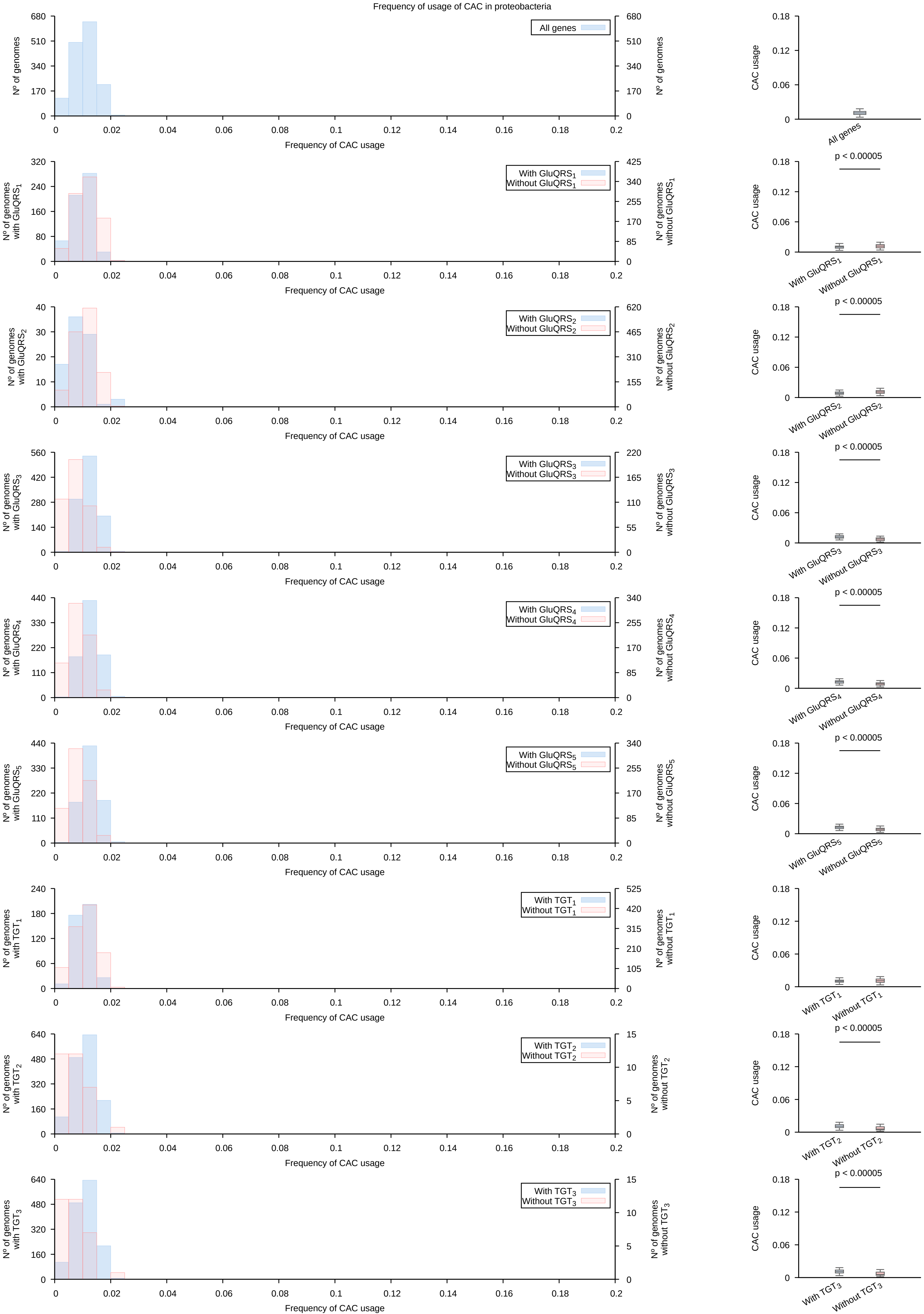

Frequency of usage of CAC in proteobacteria
Nº of genomes
All genes
Frequency of CAC usage
CAC usage
Nº of genomes with GluQRS$_1$
Nº of genomes without GluQRS$_1$
With GluQRS$_1$
Without GluQRS$_1$
p < 0.00005
Nº of genomes with GluQRS$_2$
Nº of genomes without GluQRS$_2$
With GluQRS$_2$
Without GluQRS$_2$
p < 0.00005
Nº of genomes with GluQRS$_3$
Nº of genomes without GluQRS$_3$
With GluQRS$_3$
Without GluQRS$_3$
p < 0.00005
Nº of genomes with GluQRS$_4$
Nº of genomes without GluQRS$_4$
With GluQRS$_4$
Without GluQRS$_4$
p < 0.00005
Nº of genomes with GluQRS$_5$
Nº of genomes without GluQRS$_5$
With GluQRS$_5$
Without GluQRS$_5$
p < 0.00005
Nº of genomes with TGT$_1$
Nº of genomes without TGT$_1$
With TGT$_1$
Without TGT$_1$
Nº of genomes with TGT$_2$
Nº of genomes without TGT$_2$
With TGT$_2$
Without TGT$_2$
p < 0.00005
Nº of genomes with TGT$_3$
Nº of genomes without TGT$_3$
With TGT$_3$
Without TGT$_3$
p < 0.00005

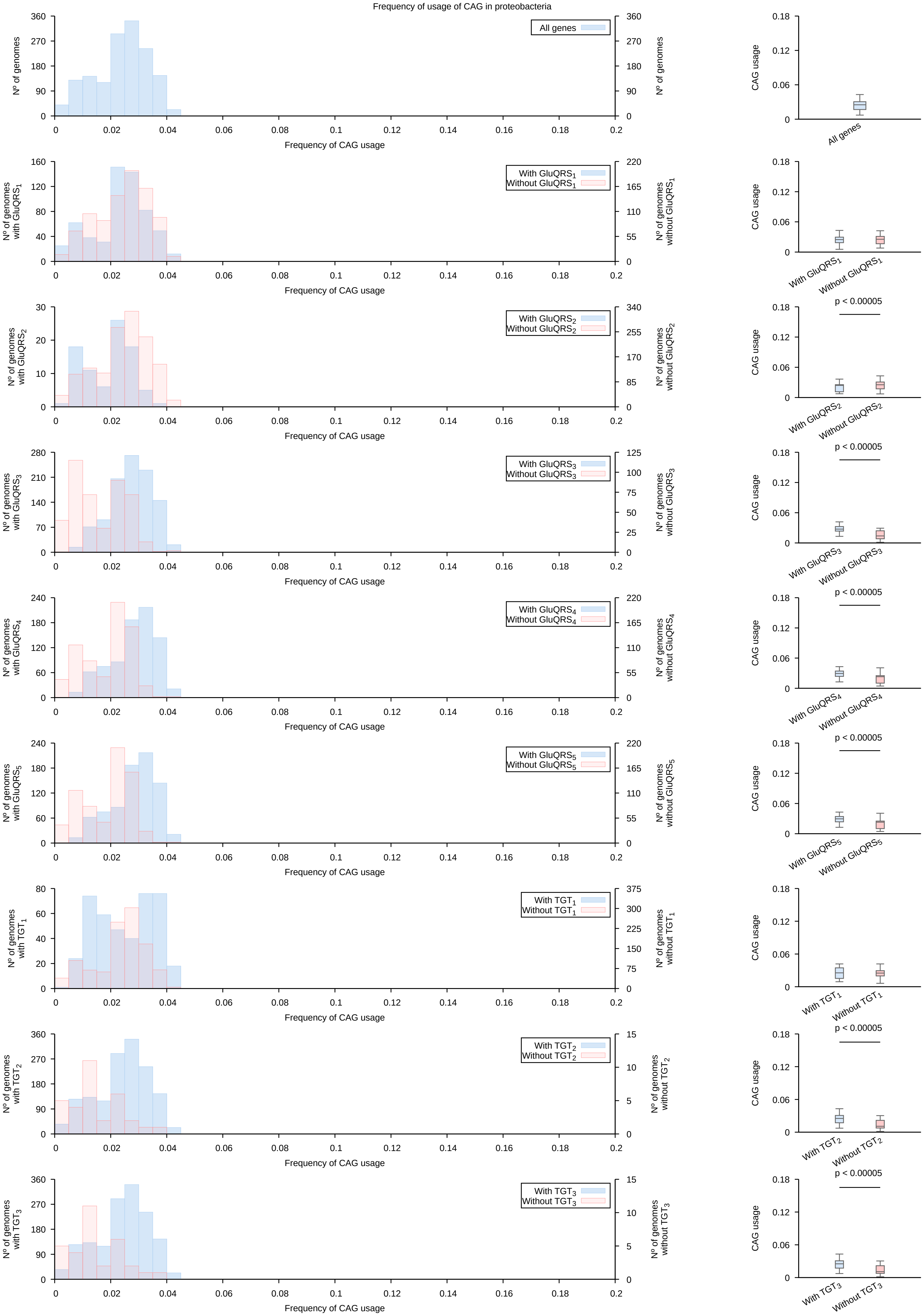
Frequency of usage of CAG in proteobacteria
Nº of genomes
Frequency of CAG usage
CAG usage
All genes
With GluQRS$_1$
Without GluQRS$_1$
Nº of genomes with GluQRS$_1$
Nº of genomes without GluQRS$_1$
With GluQRS$_2$
Without GluQRS$_2$
Nº of genomes with GluQRS$_2$
Nº of genomes without GluQRS$_2$
p < 0.00005
With GluQRS$_3$
Without GluQRS$_3$
Nº of genomes with GluQRS$_3$
Nº of genomes without GluQRS$_3$
p < 0.00005
With GluQRS$_4$
Without GluQRS$_4$
Nº of genomes with GluQRS$_4$
Nº of genomes without GluQRS$_4$
p < 0.00005
With GluQRS$_5$
Without GluQRS$_5$
Nº of genomes with GluQRS$_5$
Nº of genomes without GluQRS$_5$
p < 0.00005
With TGT$_1$
Without TGT$_1$
Nº of genomes with TGT$_1$
Nº of genomes without TGT$_1$
With TGT$_2$
Without TGT$_2$
Nº of genomes with TGT$_2$
Nº of genomes without TGT$_2$
p < 0.00005
With TGT$_3$
Without TGT$_3$
Nº of genomes with TGT$_3$
Nº of genomes without TGT$_3$
p < 0.00005

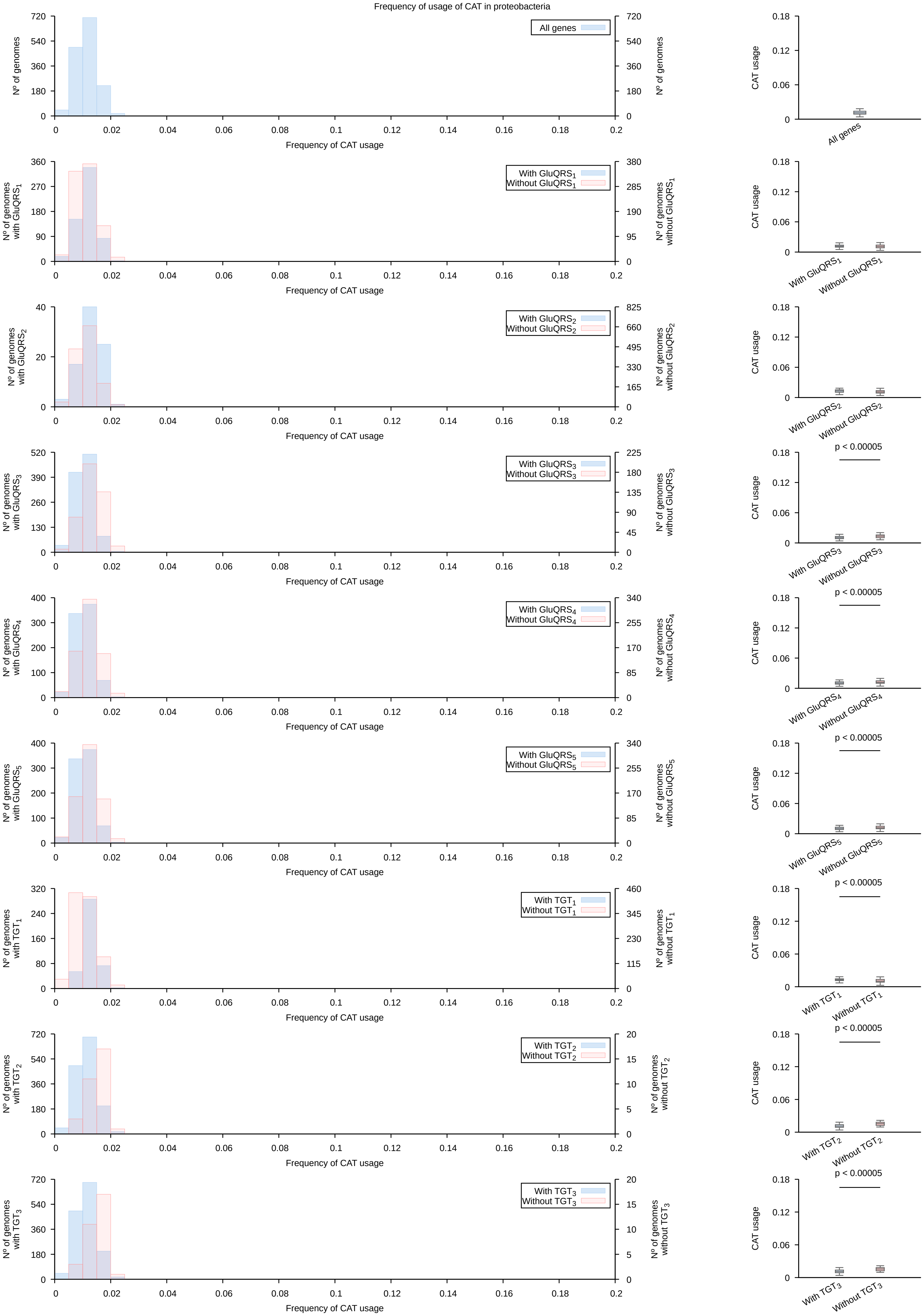

Frequency of usage of CAT in proteobacteria
Frequency of CAT usage
Nº of genomes
CAT usage
All genes
Nº of genomes with GluQRS$_1$
Nº of genomes without GluQRS$_1$
With GluQRS$_1$
Without GluQRS$_1$
Nº of genomes with GluQRS$_2$
Nº of genomes without GluQRS$_2$
With GluQRS$_2$
Without GluQRS$_2$
Nº of genomes with GluQRS$_3$
Nº of genomes without GluQRS$_3$
With GluQRS$_3$
Without GluQRS$_3$
Nº of genomes with GluQRS$_4$
Nº of genomes without GluQRS$_4$
With GluQRS$_4$
Without GluQRS$_4$
Nº of genomes with GluQRS$_5$
Nº of genomes without GluQRS$_5$
With GluQRS$_5$
Without GluQRS$_5$
Nº of genomes with TGT$_1$
Nº of genomes without TGT$_1$
With TGT$_1$
Without TGT$_1$
Nº of genomes with TGT$_2$
Nº of genomes without TGT$_2$
With TGT$_2$
Without TGT$_2$
Nº of genomes with TGT$_3$
Nº of genomes without TGT$_3$
With TGT$_3$
Without TGT$_3$
p < 0.00005

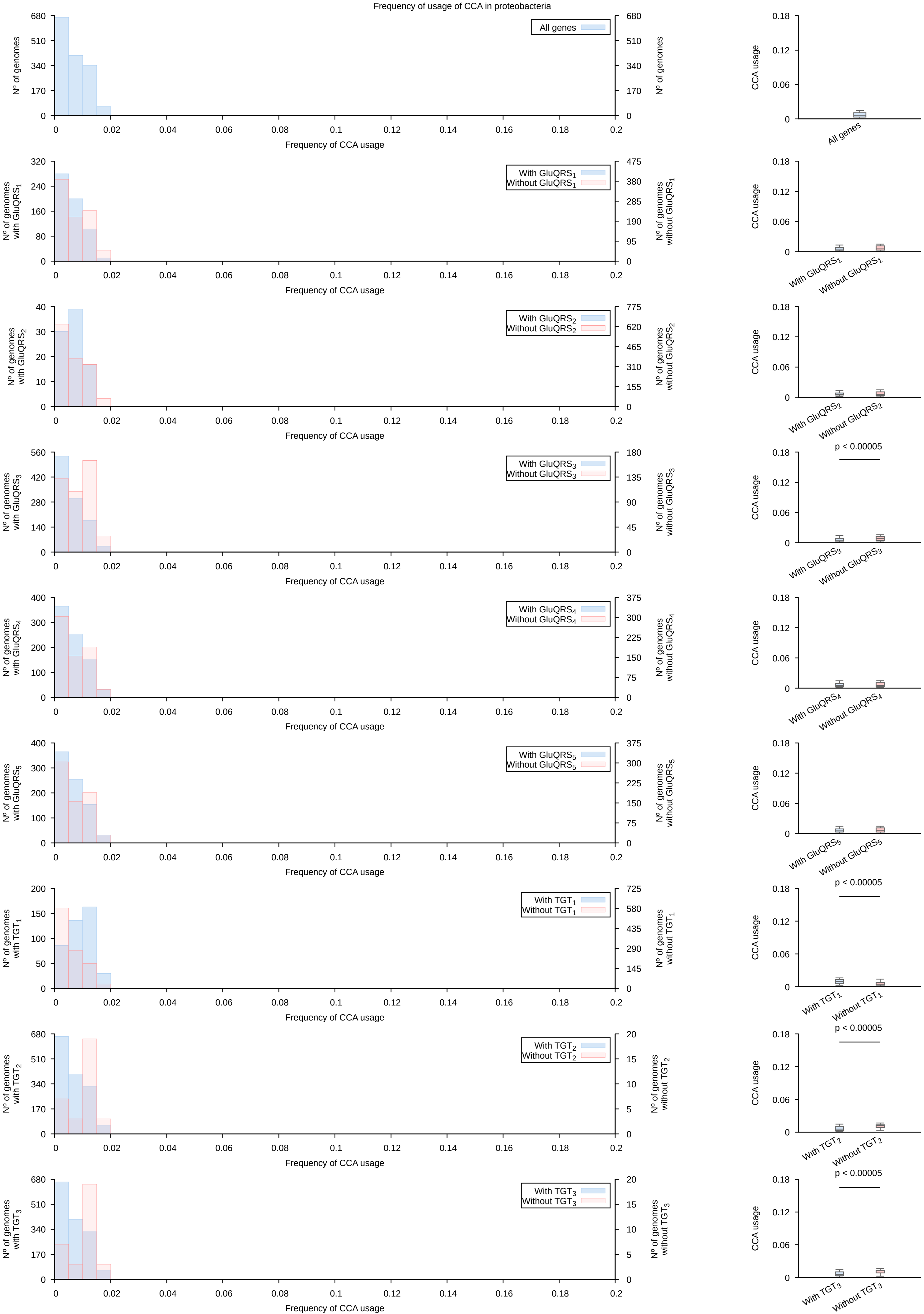
Frequency of usage of CCA in proteobacteria
All genes
Nº of genomes
Frequency of CCA usage
CCA usage
With GluQRS1
Without GluQRS1
Nº of genomes with GluQRS1
Nº of genomes without GluQRS1
With GluQRS2
Without GluQRS2
Nº of genomes with GluQRS2
Nº of genomes without GluQRS2
With GluQRS3
Without GluQRS3
Nº of genomes with GluQRS3
Nº of genomes without GluQRS3
p < 0.00005
With GluQRS4
Without GluQRS4
Nº of genomes with GluQRS4
Nº of genomes without GluQRS4
With GluQRS5
Without GluQRS5
Nº of genomes with GluQRS5
Nº of genomes without GluQRS5
With TGT1
Without TGT1
Nº of genomes with TGT1
Nº of genomes without TGT1
p < 0.00005
With TGT2
Without TGT2
Nº of genomes with TGT2
Nº of genomes without TGT2
p < 0.00005
With TGT3
Without TGT3
Nº of genomes with TGT3
Nº of genomes without TGT3
p < 0.00005

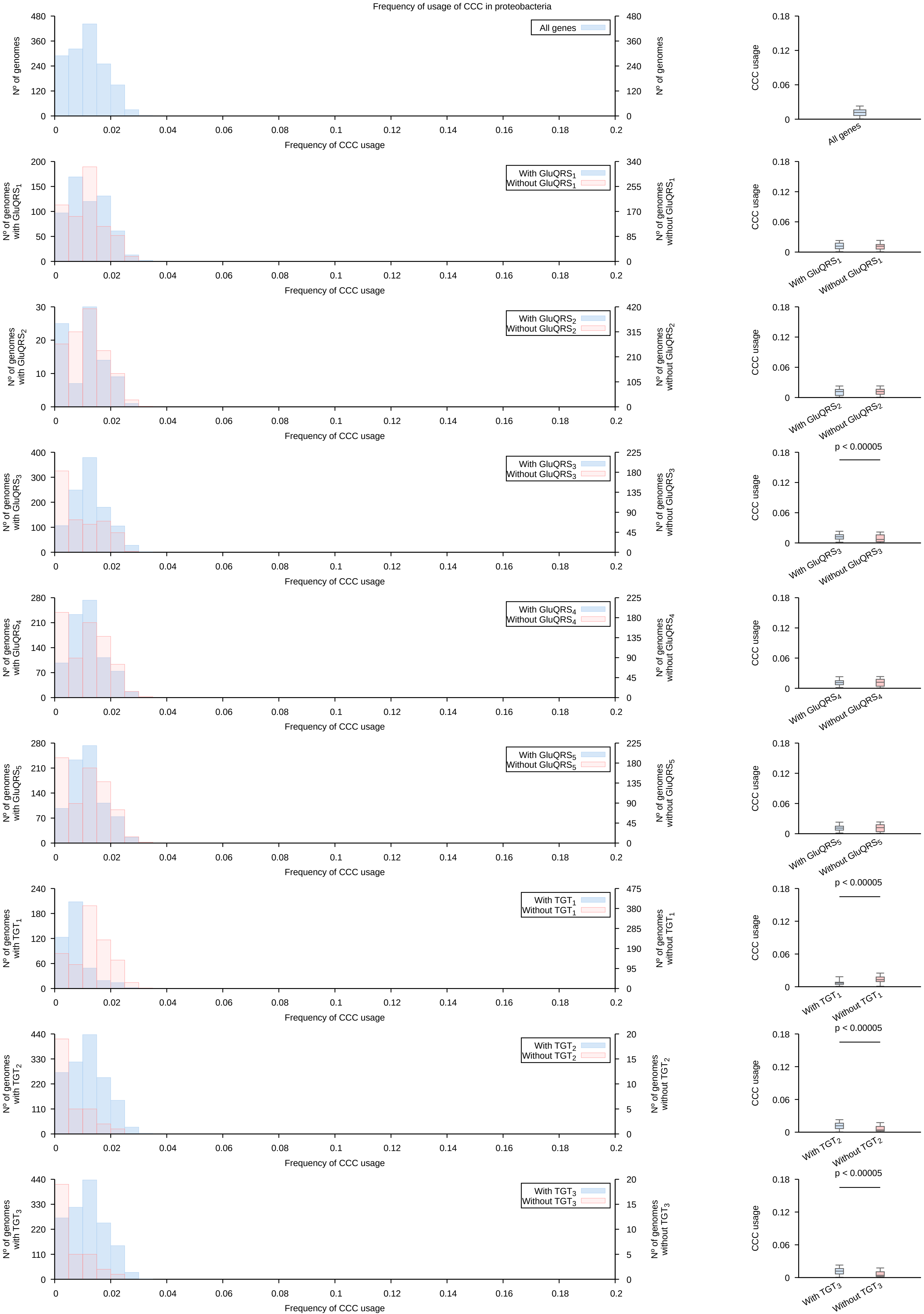

Frequency of usage of CCC in proteobacteria
All genes
With GluQRS$_1$
Without GluQRS$_1$
With GluQRS$_2$
Without GluQRS$_2$
With GluQRS$_3$
Without GluQRS$_3$
With GluQRS$_4$
Without GluQRS$_4$
With GluQRS$_5$
Without GluQRS$_5$
With TGT$_1$
Without TGT$_1$
With TGT$_2$
Without TGT$_2$
With TGT$_3$
Without TGT$_3$
Nº of genomes
Nº of genomes with GluQRS$_1$
Nº of genomes without GluQRS$_1$
Nº of genomes with GluQRS$_2$
Nº of genomes without GluQRS$_2$
Nº of genomes with GluQRS$_3$
Nº of genomes without GluQRS$_3$
Nº of genomes with GluQRS$_4$
Nº of genomes without GluQRS$_4$
Nº of genomes with GluQRS$_5$
Nº of genomes without GluQRS$_5$
Nº of genomes with TGT$_1$
Nº of genomes without TGT$_1$
Nº of genomes with TGT$_2$
Nº of genomes without TGT$_2$
Nº of genomes with TGT$_3$
Nº of genomes without TGT$_3$
Frequency of CCC usage
CCC usage
p < 0.00005

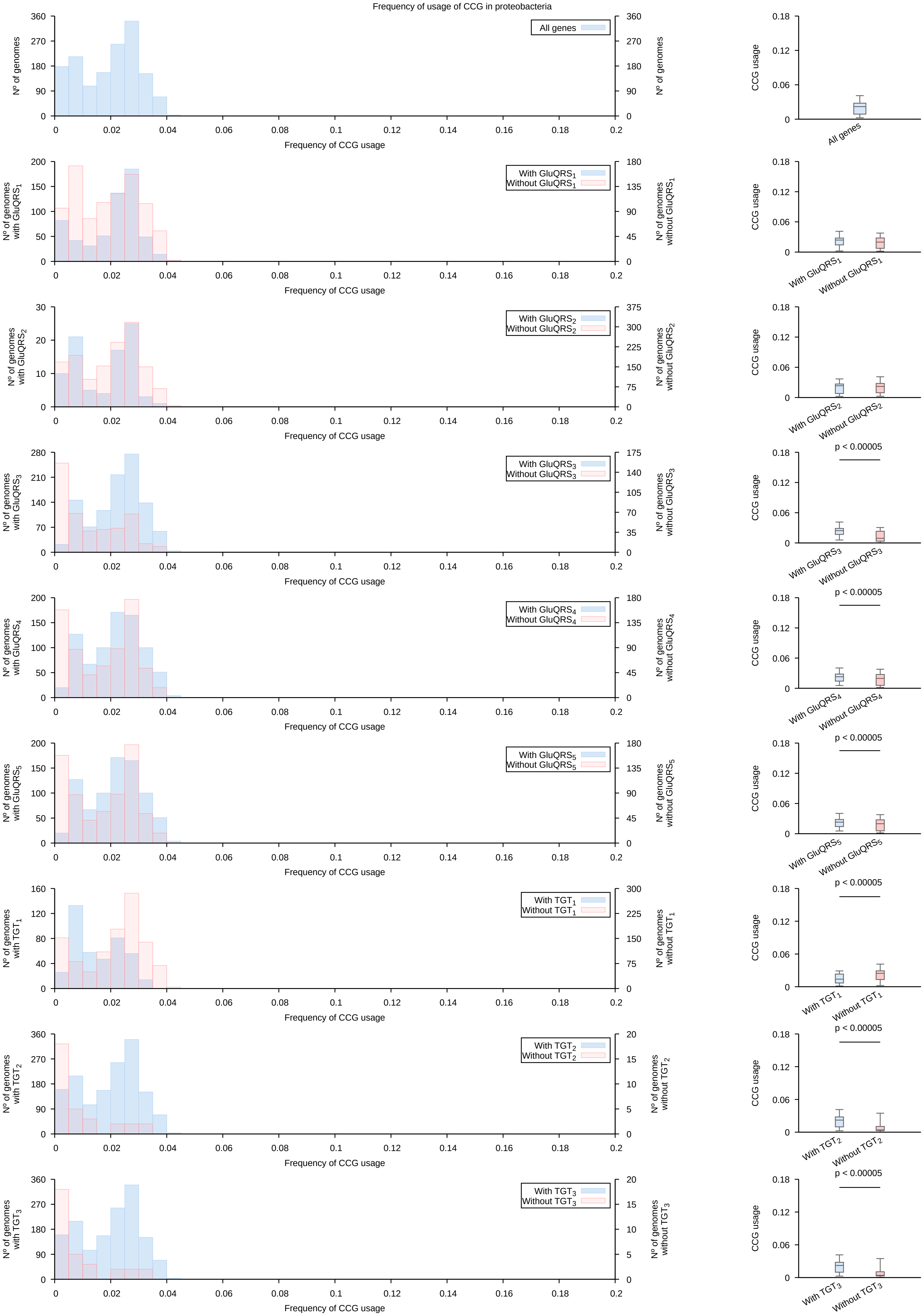

Frequency of usage of CCG in proteobacteria
All genes
With GluQRS$_1$
Without GluQRS$_1$
With GluQRS$_2$
Without GluQRS$_2$
With GluQRS$_3$
Without GluQRS$_3$
With GluQRS$_4$
Without GluQRS$_4$
With GluQRS$_5$
Without GluQRS$_5$
With TGT$_1$
Without TGT$_1$
With TGT$_2$
Without TGT$_2$
With TGT$_3$
Without TGT$_3$
Nº of genomes
Nº of genomes with GluQRS$_1$
Nº of genomes without GluQRS$_1$
Nº of genomes with GluQRS$_2$
Nº of genomes without GluQRS$_2$
Nº of genomes with GluQRS$_3$
Nº of genomes without GluQRS$_3$
Nº of genomes with GluQRS$_4$
Nº of genomes without GluQRS$_4$
Nº of genomes with GluQRS$_5$
Nº of genomes without GluQRS$_5$
Nº of genomes with TGT$_1$
Nº of genomes without TGT$_1$
Nº of genomes with TGT$_2$
Nº of genomes without TGT$_2$
Nº of genomes with TGT$_3$
Nº of genomes without TGT$_3$
Frequency of CCG usage
0 0.02 0.04 0.06 0.08 0.1 0.12 0.14 0.16 0.18 0.2
CCG usage
0 0.06 0.12 0.18
p < 0.00005

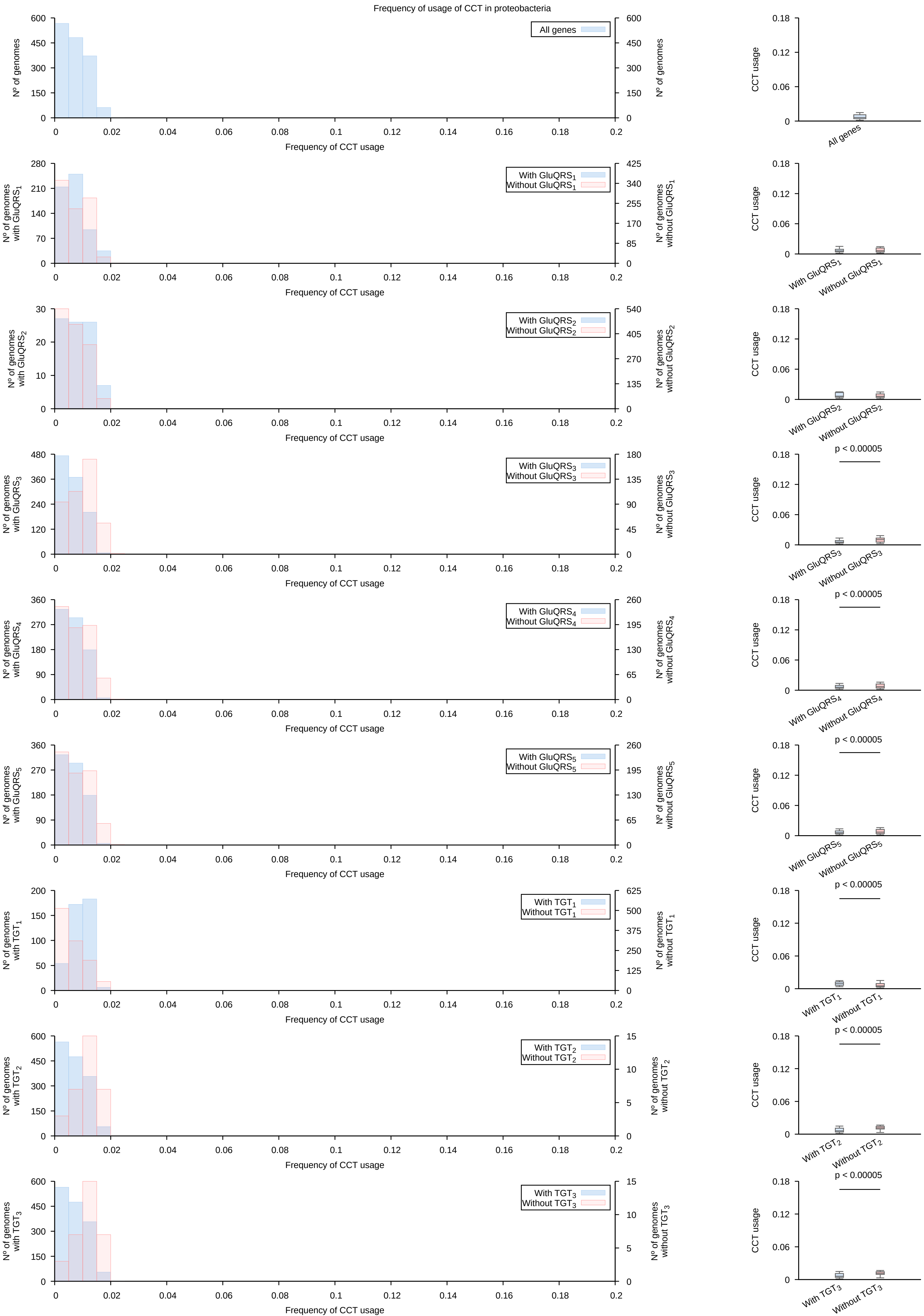

Frequency of usage of CCT in proteobacteria
All genes
Nº of genomes
Frequency of CCT usage
CCT usage
With GluQRS$_1$
Without GluQRS$_1$
Nº of genomes with GluQRS$_1$
Nº of genomes without GluQRS$_1$
With GluQRS$_2$
Without GluQRS$_2$
Nº of genomes with GluQRS$_2$
Nº of genomes without GluQRS$_2$
With GluQRS$_3$
Without GluQRS$_3$
Nº of genomes with GluQRS$_3$
Nº of genomes without GluQRS$_3$
$p < 0.00005$
With GluQRS$_4$
Without GluQRS$_4$
Nº of genomes with GluQRS$_4$
Nº of genomes without GluQRS$_4$
$p < 0.00005$
With GluQRS$_5$
Without GluQRS$_5$
Nº of genomes with GluQRS$_5$
Nº of genomes without GluQRS$_5$
$p < 0.00005$
With TGT$_1$
Without TGT$_1$
Nº of genomes with TGT$_1$
Nº of genomes without TGT$_1$
$p < 0.00005$
With TGT$_2$
Without TGT$_2$
Nº of genomes with TGT$_2$
Nº of genomes without TGT$_2$
$p < 0.00005$
With TGT$_3$
Without TGT$_3$
Nº of genomes with TGT$_3$
Nº of genomes without TGT$_3$
$p < 0.00005$

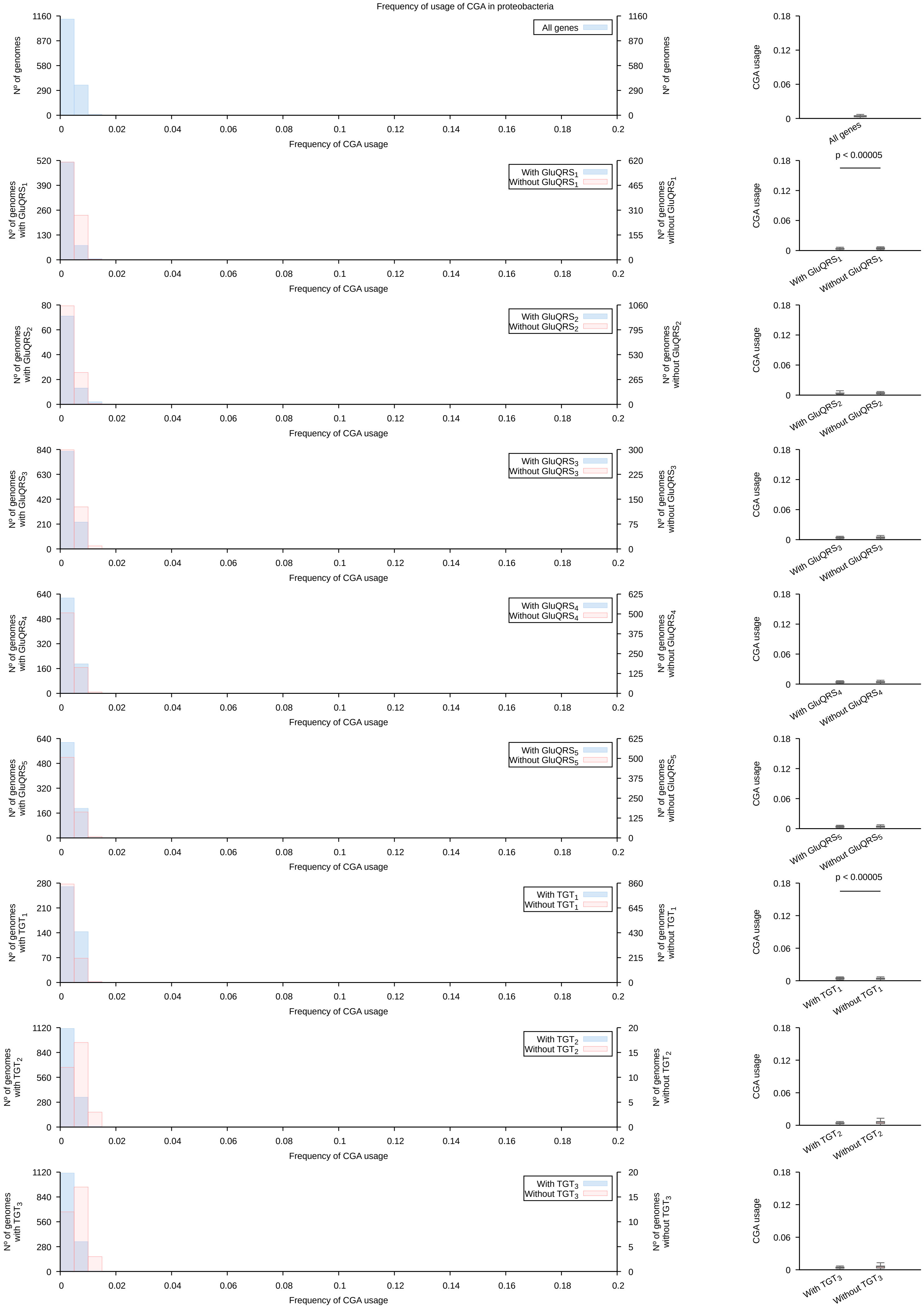

Frequency of usage of CGA in proteobacteria
All genes
Nº of genomes
Frequency of CGA usage
CGA usage
With GluQRS$_1$
Without GluQRS$_1$
Nº of genomes with GluQRS$_1$
Nº of genomes without GluQRS$_1$
p < 0.00005
With GluQRS$_2$
Without GluQRS$_2$
Nº of genomes with GluQRS$_2$
Nº of genomes without GluQRS$_2$
With GluQRS$_3$
Without GluQRS$_3$
Nº of genomes with GluQRS$_3$
Nº of genomes without GluQRS$_3$
With GluQRS$_4$
Without GluQRS$_4$
Nº of genomes with GluQRS$_4$
Nº of genomes without GluQRS$_4$
With GluQRS$_5$
Without GluQRS$_5$
Nº of genomes with GluQRS$_5$
Nº of genomes without GluQRS$_5$
With TGT$_1$
Without TGT$_1$
Nº of genomes with TGT$_1$
Nº of genomes without TGT$_1$
p < 0.00005
With TGT$_2$
Without TGT$_2$
Nº of genomes with TGT$_2$
Nº of genomes without TGT$_2$
With TGT$_3$
Without TGT$_3$
Nº of genomes with TGT$_3$
Nº of genomes without TGT$_3$

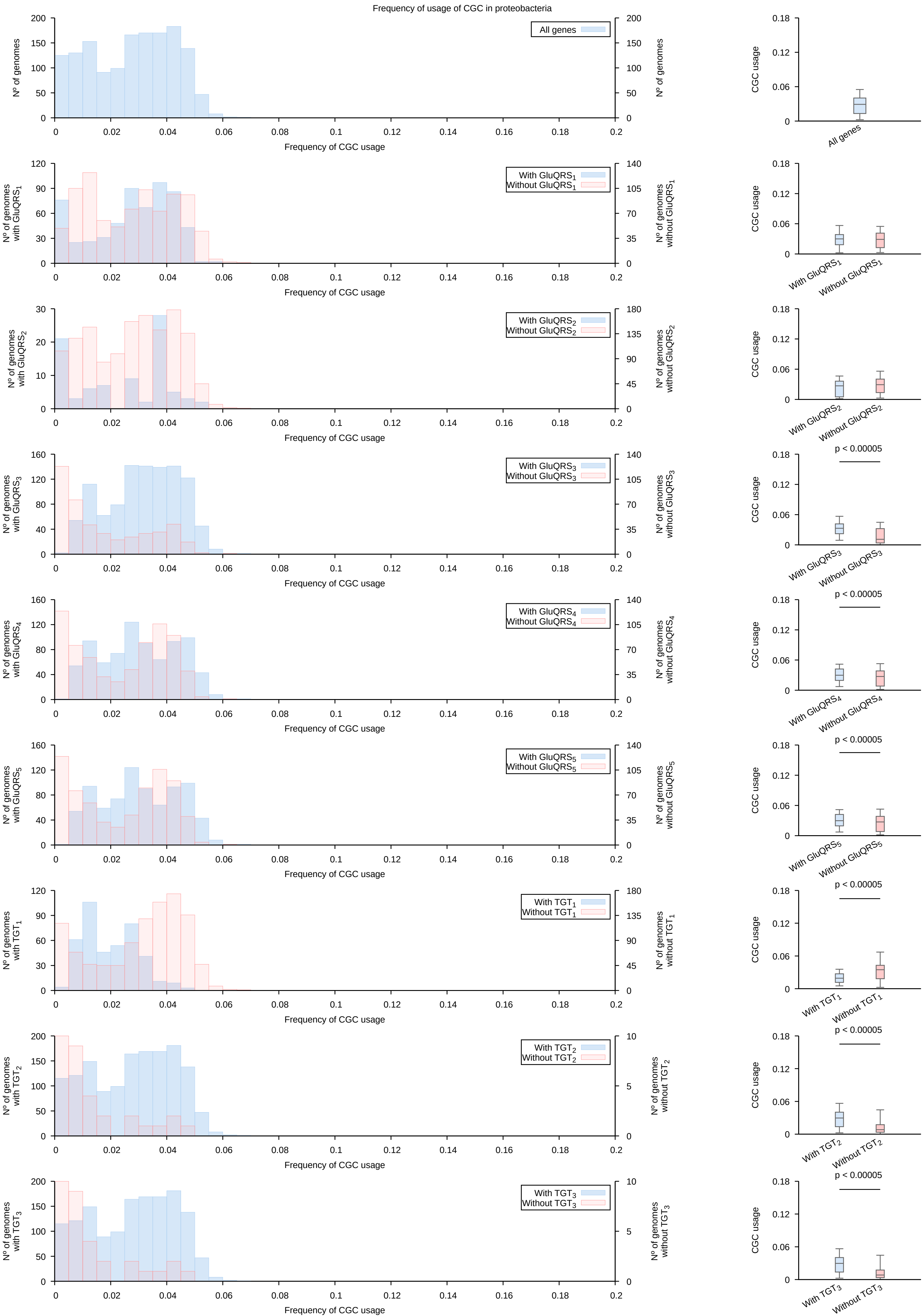

Frequency of usage of CGC in proteobacteria
All genes
With GluQRS$_1$
Without GluQRS$_1$
With GluQRS$_2$
Without GluQRS$_2$
With GluQRS$_3$
Without GluQRS$_3$
With GluQRS$_4$
Without GluQRS$_4$
With GluQRS$_5$
Without GluQRS$_5$
With TGT$_1$
Without TGT$_1$
With TGT$_2$
Without TGT$_2$
With TGT$_3$
Without TGT$_3$
Nº of genomes
Frequency of CGC usage
CGC usage
p < 0.00005

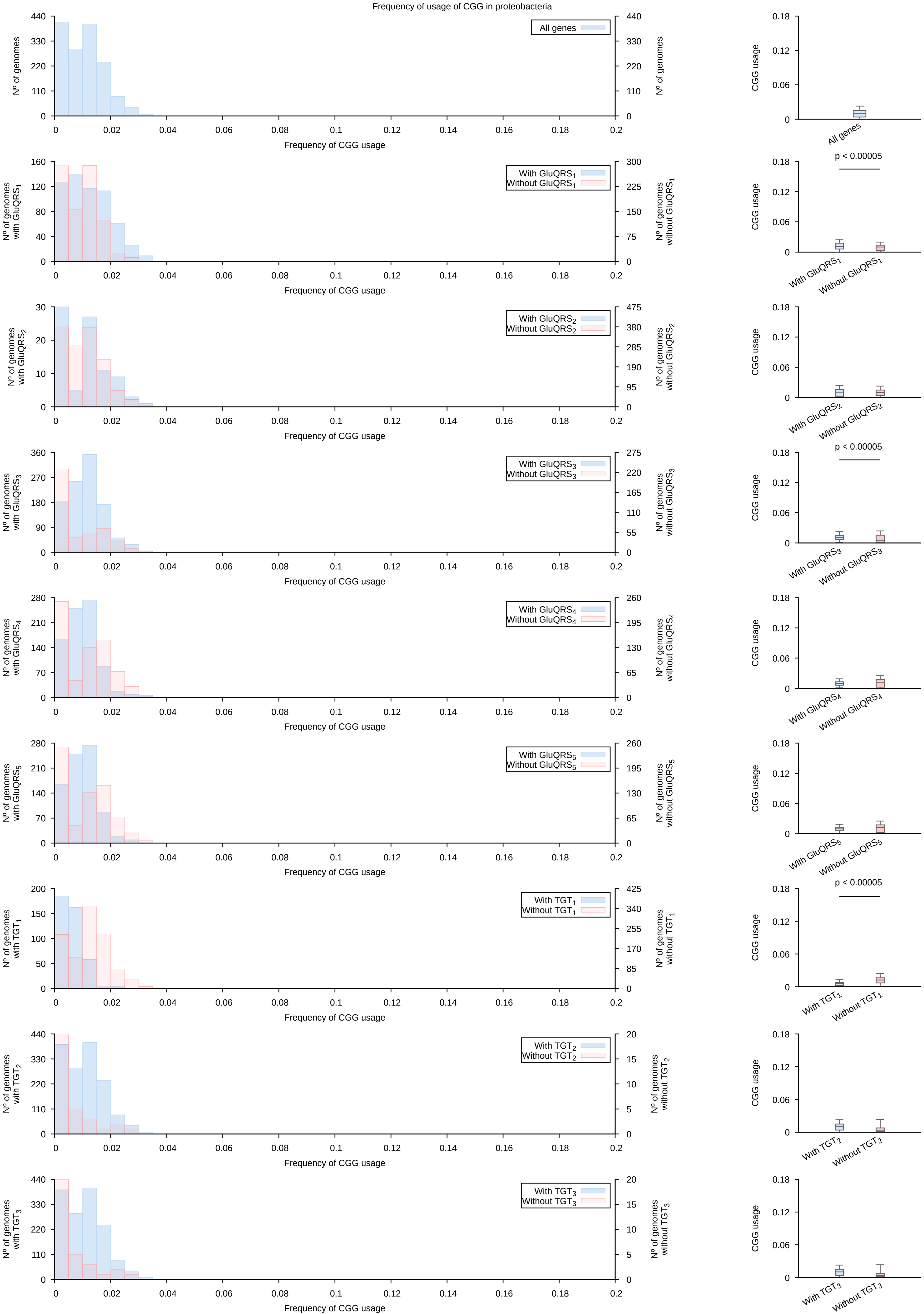

Frequency of usage of CGG in proteobacteria
All genes
With GluQRS1
Without GluQRS1
With GluQRS2
Without GluQRS2
With GluQRS3
Without GluQRS3
With GluQRS4
Without GluQRS4
With GluQRS5
Without GluQRS5
With TGT1
Without TGT1
With TGT2
Without TGT2
With TGT3
Without TGT3
Nº of genomes
Frequency of CGG usage
CGG usage
p < 0.00005

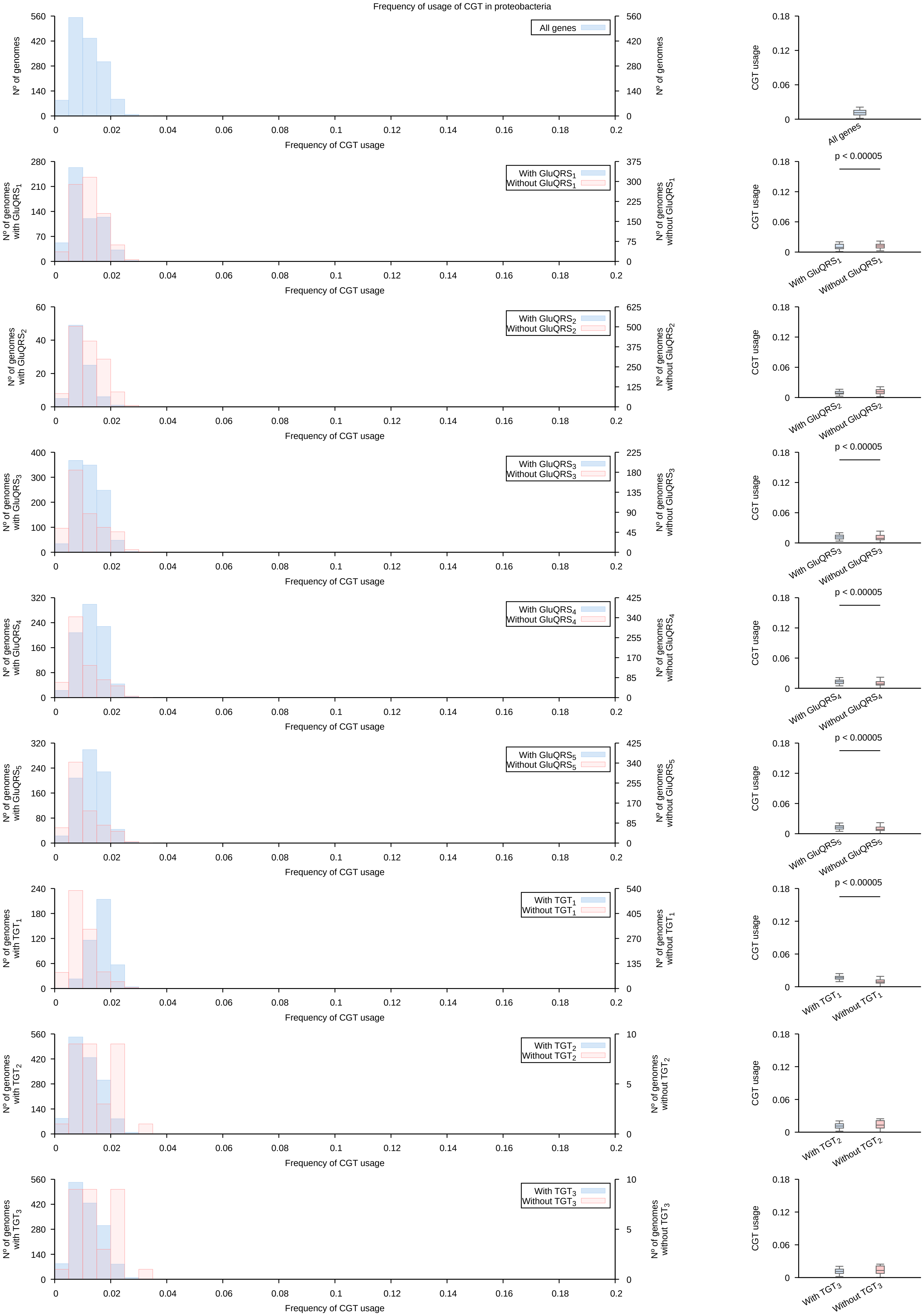
Frequency of usage of CGT in proteobacteria
Nº of genomes
Frequency of CGT usage
All genes
CGT usage
Nº of genomes with GluQRS$_1$
Nº of genomes without GluQRS$_1$
With GluQRS$_1$
Without GluQRS$_1$
p < 0.00005
Nº of genomes with GluQRS$_2$
Nº of genomes without GluQRS$_2$
With GluQRS$_2$
Without GluQRS$_2$
Nº of genomes with GluQRS$_3$
Nº of genomes without GluQRS$_3$
With GluQRS$_3$
Without GluQRS$_3$
p < 0.00005
Nº of genomes with GluQRS$_4$
Nº of genomes without GluQRS$_4$
With GluQRS$_4$
Without GluQRS$_4$
p < 0.00005
Nº of genomes with GluQRS$_5$
Nº of genomes without GluQRS$_5$
With GluQRS$_5$
Without GluQRS$_5$
p < 0.00005
Nº of genomes with TGT$_1$
Nº of genomes without TGT$_1$
With TGT$_1$
Without TGT$_1$
p < 0.00005
Nº of genomes with TGT$_2$
Nº of genomes without TGT$_2$
With TGT$_2$
Without TGT$_2$
Nº of genomes with TGT$_3$
Nº of genomes without TGT$_3$
With TGT$_3$
Without TGT$_3$

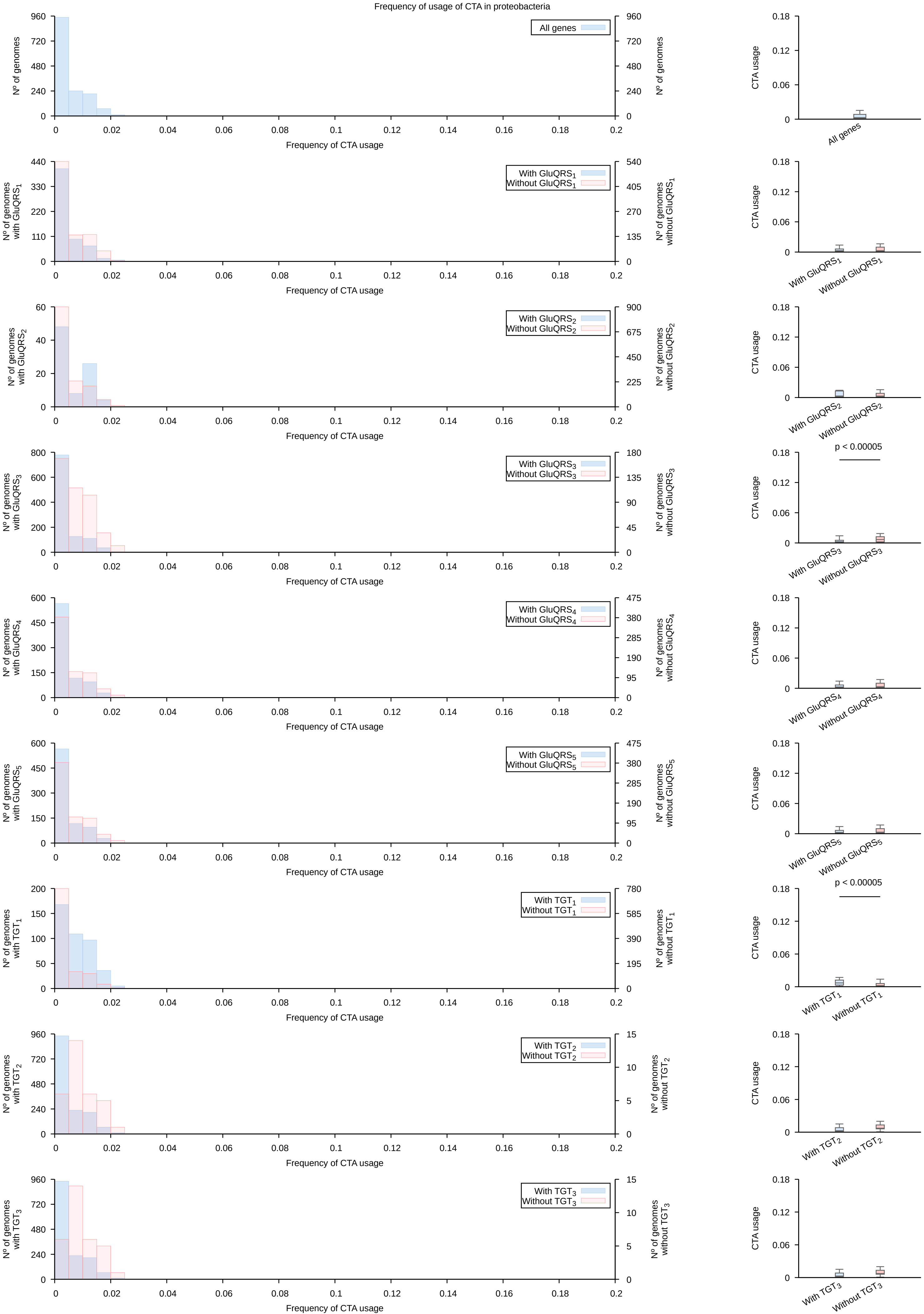
Frequency of usage of CTA in proteobacteria
All genes
With GluQRS1
Without GluQRS1
With GluQRS2
Without GluQRS2
With GluQRS3
Without GluQRS3
With GluQRS4
Without GluQRS4
With GluQRS5
Without GluQRS5
With TGT1
Without TGT1
With TGT2
Without TGT2
With TGT3
Without TGT3
Nº of genomes
Frequency of CTA usage
CTA usage
p < 0.00005
p < 0.00005

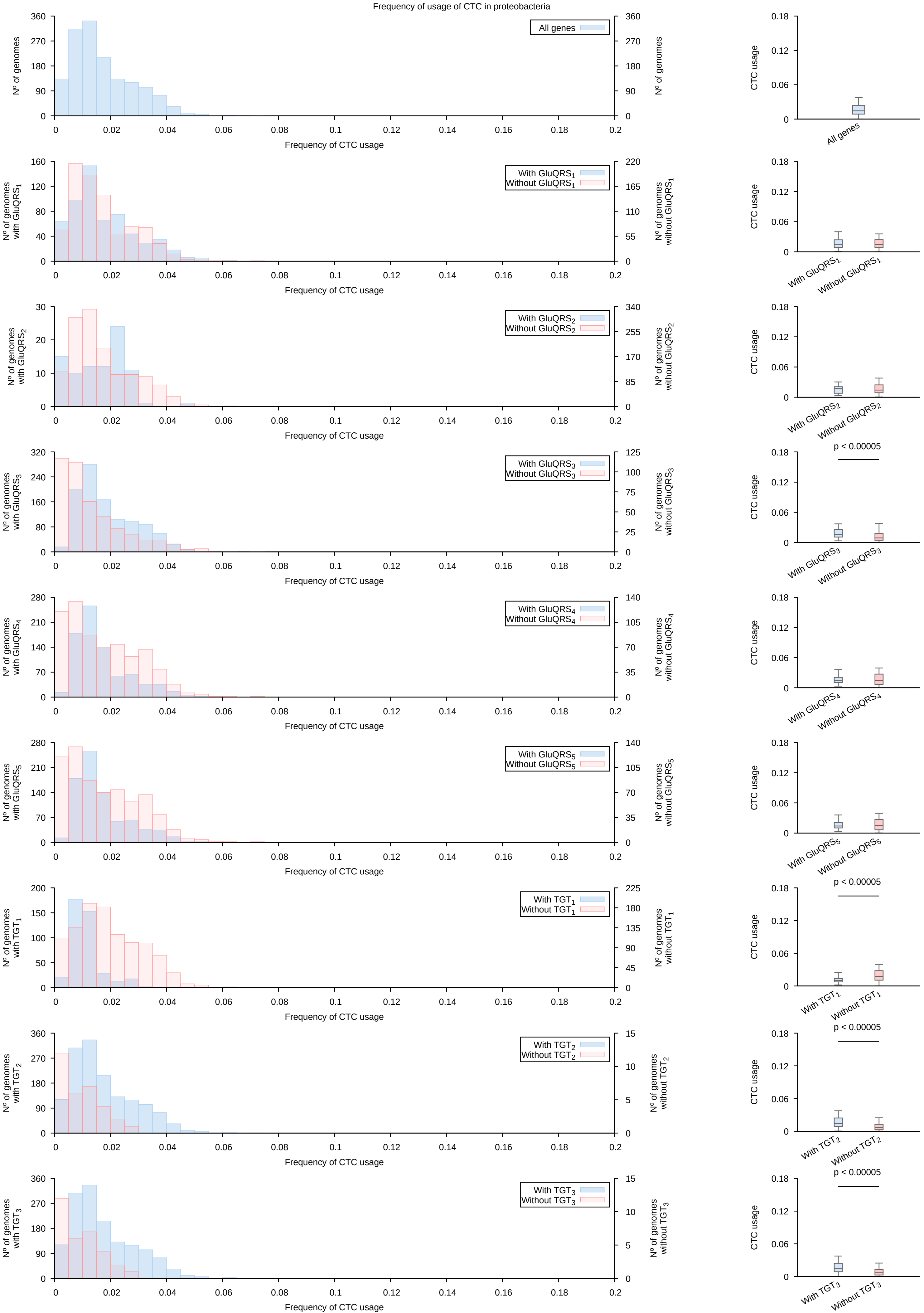Frequency of usage of CTC in proteobacteria
All genes
Nº of genomes
CTC usage
Frequency of CTC usage
With GluQRS$_1$
Without GluQRS$_1$
Nº of genomes with GluQRS$_1$
Nº of genomes without GluQRS$_1$
With GluQRS$_2$
Without GluQRS$_2$
Nº of genomes with GluQRS$_2$
Nº of genomes without GluQRS$_2$
With GluQRS$_3$
Without GluQRS$_3$
Nº of genomes with GluQRS$_3$
Nº of genomes without GluQRS$_3$
p < 0.00005
With GluQRS$_4$
Without GluQRS$_4$
Nº of genomes with GluQRS$_4$
Nº of genomes without GluQRS$_4$
With GluQRS$_5$
Without GluQRS$_5$
Nº of genomes with GluQRS$_5$
Nº of genomes without GluQRS$_5$
With TGT$_1$
Without TGT$_1$
Nº of genomes with TGT$_1$
Nº of genomes without TGT$_1$
p < 0.00005
With TGT$_2$
Without TGT$_2$
Nº of genomes with TGT$_2$
Nº of genomes without TGT$_2$
p < 0.00005
With TGT$_3$
Without TGT$_3$
Nº of genomes with TGT$_3$
Nº of genomes without TGT$_3$
p < 0.00005

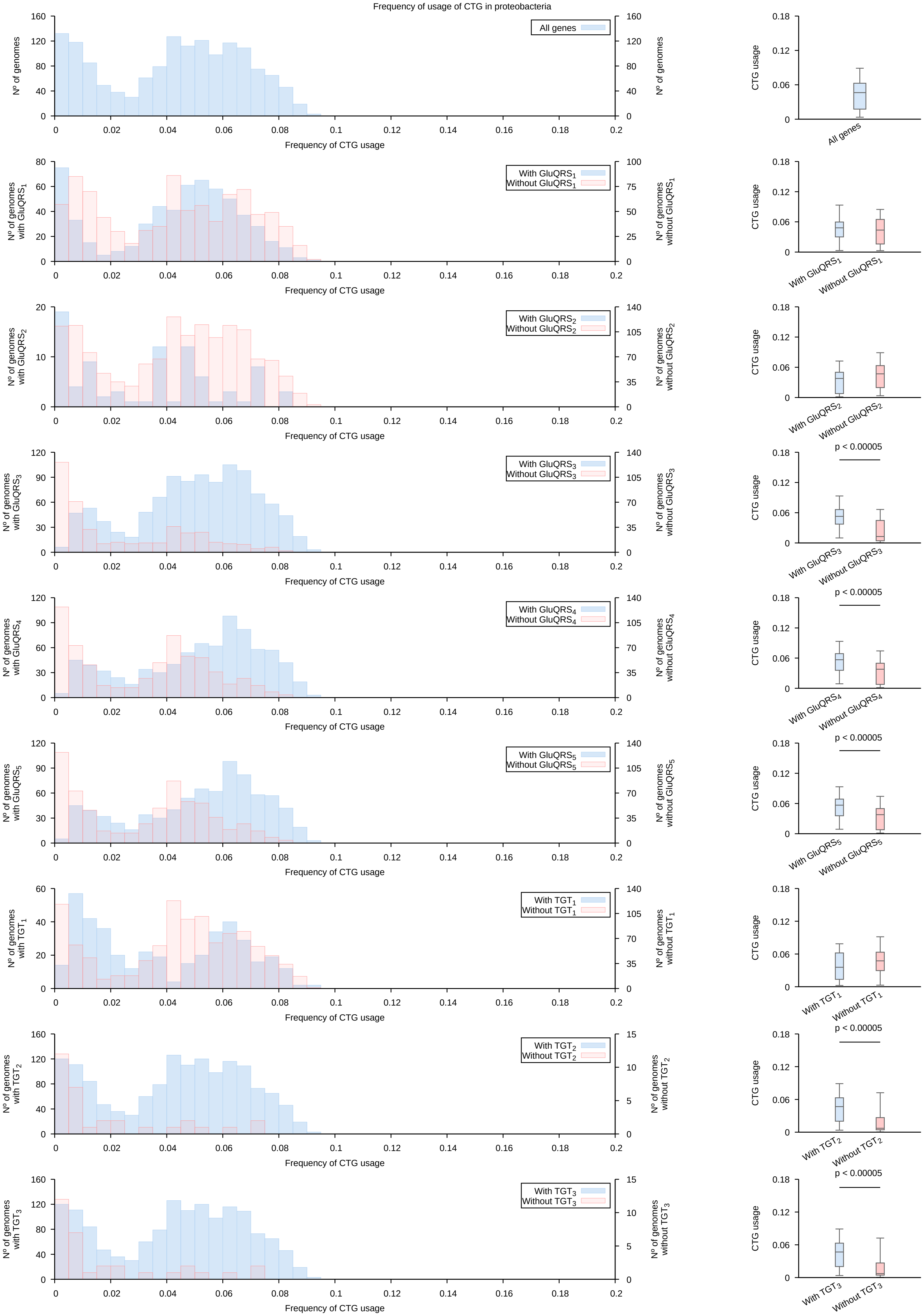
Frequency of usage of CTG in proteobacteria
All genes
Nº of genomes
Frequency of CTG usage
CTG usage
With GluQRS$_1$
Without GluQRS$_1$
Nº of genomes with GluQRS$_1$
Nº of genomes without GluQRS$_1$
With GluQRS$_2$
Without GluQRS$_2$
Nº of genomes with GluQRS$_2$
Nº of genomes without GluQRS$_2$
With GluQRS$_3$
Without GluQRS$_3$
Nº of genomes with GluQRS$_3$
Nº of genomes without GluQRS$_3$
p < 0.00005
With GluQRS$_4$
Without GluQRS$_4$
Nº of genomes with GluQRS$_4$
Nº of genomes without GluQRS$_4$
p < 0.00005
With GluQRS$_5$
Without GluQRS$_5$
Nº of genomes with GluQRS$_5$
Nº of genomes without GluQRS$_5$
p < 0.00005
With TGT$_1$
Without TGT$_1$
Nº of genomes with TGT$_1$
Nº of genomes without TGT$_1$
With TGT$_2$
Without TGT$_2$
Nº of genomes with TGT$_2$
Nº of genomes without TGT$_2$
p < 0.00005
With TGT$_3$
Without TGT$_3$
Nº of genomes with TGT$_3$
Nº of genomes without TGT$_3$
p < 0.00005

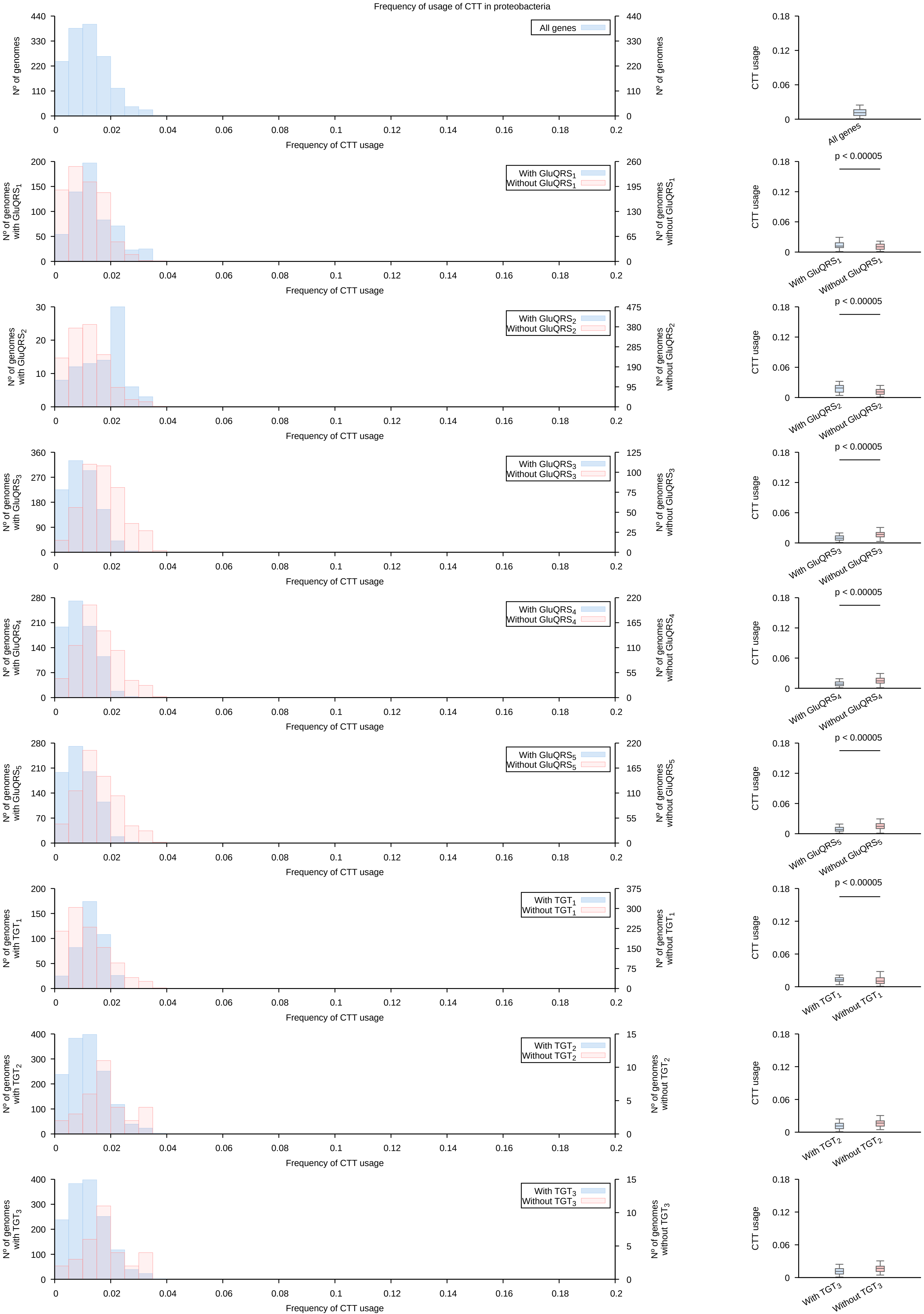
Frequency of usage of CTT in proteobacteria
All genes
Nº of genomes
Frequency of CTT usage
CTT usage
With GluQRS$_1$
Without GluQRS$_1$
Nº of genomes with GluQRS$_1$
Nº of genomes without GluQRS$_1$
p < 0.00005
With GluQRS$_2$
Without GluQRS$_2$
Nº of genomes with GluQRS$_2$
Nº of genomes without GluQRS$_2$
p < 0.00005
With GluQRS$_3$
Without GluQRS$_3$
Nº of genomes with GluQRS$_3$
Nº of genomes without GluQRS$_3$
p < 0.00005
With GluQRS$_4$
Without GluQRS$_4$
Nº of genomes with GluQRS$_4$
Nº of genomes without GluQRS$_4$
p < 0.00005
With GluQRS$_5$
Without GluQRS$_5$
Nº of genomes with GluQRS$_5$
Nº of genomes without GluQRS$_5$
p < 0.00005
With TGT$_1$
Without TGT$_1$
Nº of genomes with TGT$_1$
Nº of genomes without TGT$_1$
p < 0.00005
With TGT$_2$
Without TGT$_2$
Nº of genomes with TGT$_2$
Nº of genomes without TGT$_2$
With TGT$_3$
Without TGT$_3$
Nº of genomes with TGT$_3$
Nº of genomes without TGT$_3$

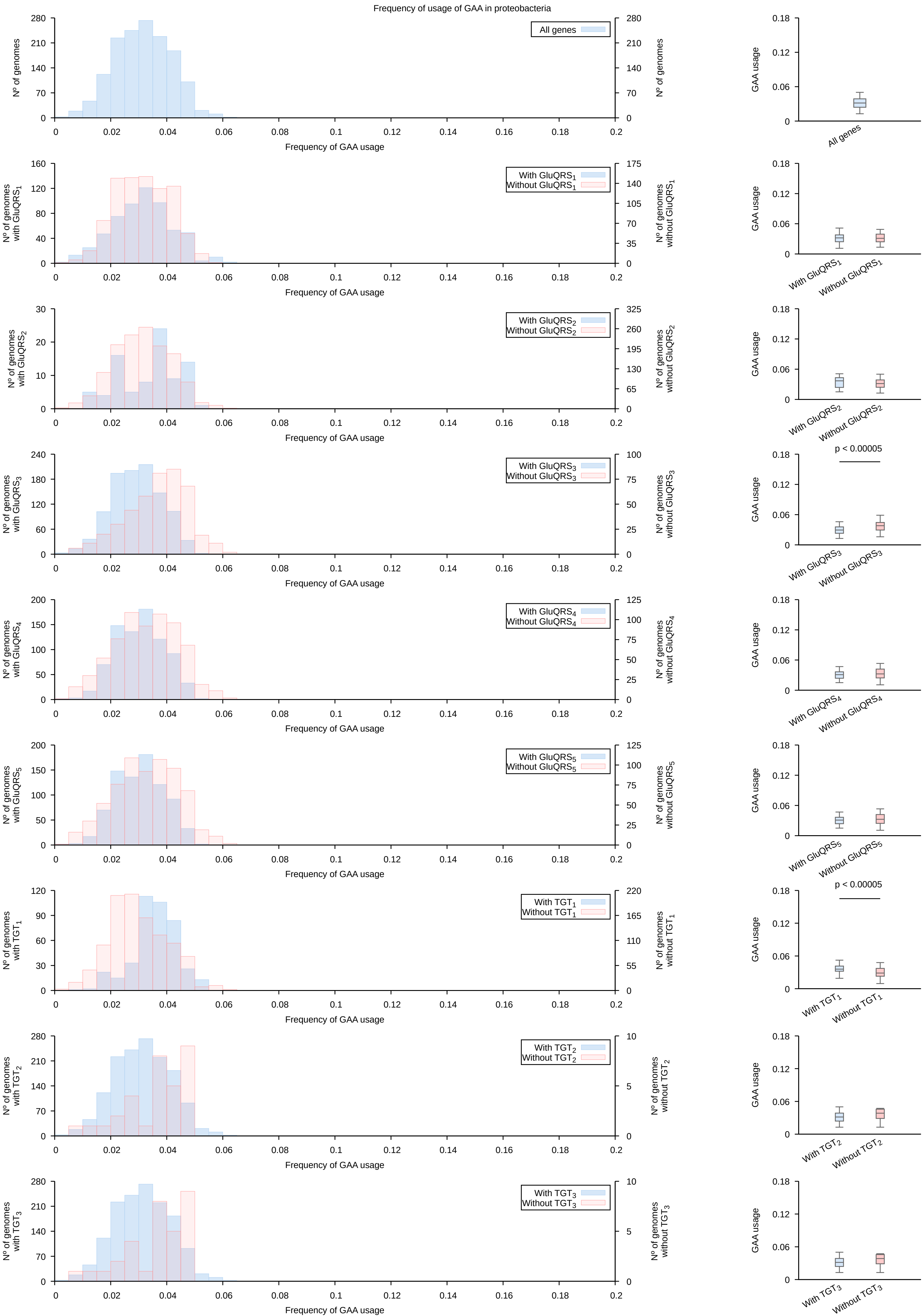

Frequency of usage of GAA in proteobacteria
All genes
Nº of genomes
Frequency of GAA usage
GAA usage
With GluQRS$_1$
Without GluQRS$_1$
Nº of genomes with GluQRS$_1$
Nº of genomes without GluQRS$_1$
With GluQRS$_2$
Without GluQRS$_2$
Nº of genomes with GluQRS$_2$
Nº of genomes without GluQRS$_2$
With GluQRS$_3$
Without GluQRS$_3$
Nº of genomes with GluQRS$_3$
Nº of genomes without GluQRS$_3$
p < 0.00005
With GluQRS$_4$
Without GluQRS$_4$
Nº of genomes with GluQRS$_4$
Nº of genomes without GluQRS$_4$
With GluQRS$_5$
Without GluQRS$_5$
Nº of genomes with GluQRS$_5$
Nº of genomes without GluQRS$_5$
With TGT$_1$
Without TGT$_1$
Nº of genomes with TGT$_1$
Nº of genomes without TGT$_1$
p < 0.00005
With TGT$_2$
Without TGT$_2$
Nº of genomes with TGT$_2$
Nº of genomes without TGT$_2$
With TGT$_3$
Without TGT$_3$
Nº of genomes with TGT$_3$
Nº of genomes without TGT$_3$

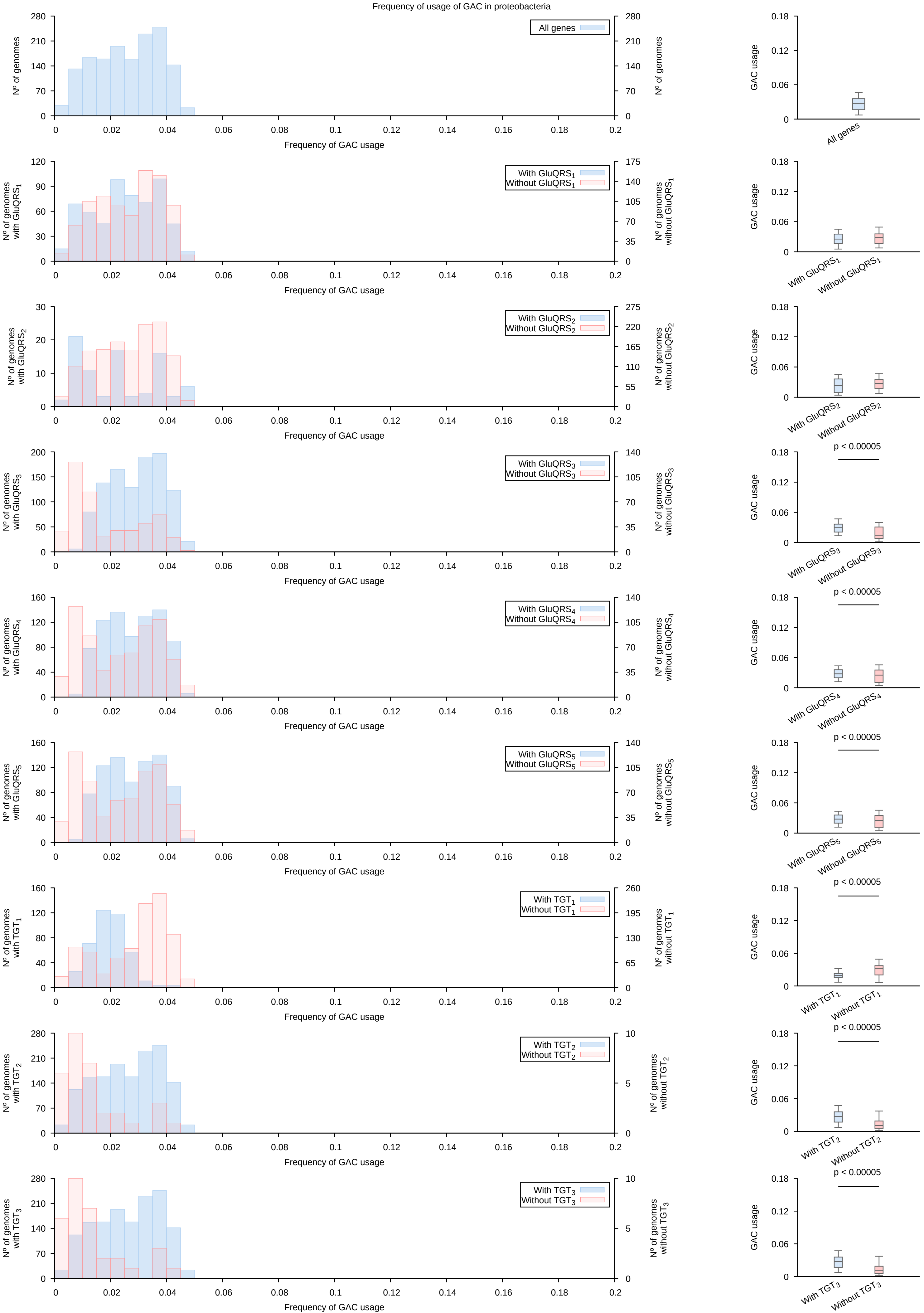
Frequency of usage of GAC in proteobacteria
All genes
Nº of genomes
GAC usage
Frequency of GAC usage
With GluQRS$_1$
Without GluQRS$_1$
Nº of genomes with GluQRS$_1$
Nº of genomes without GluQRS$_1$
With GluQRS$_2$
Without GluQRS$_2$
Nº of genomes with GluQRS$_2$
Nº of genomes without GluQRS$_2$
With GluQRS$_3$
Without GluQRS$_3$
Nº of genomes with GluQRS$_3$
Nº of genomes without GluQRS$_3$
p < 0.00005
With GluQRS$_4$
Without GluQRS$_4$
Nº of genomes with GluQRS$_4$
Nº of genomes without GluQRS$_4$
p < 0.00005
With GluQRS$_5$
Without GluQRS$_5$
Nº of genomes with GluQRS$_5$
Nº of genomes without GluQRS$_5$
p < 0.00005
With TGT$_1$
Without TGT$_1$
Nº of genomes with TGT$_1$
Nº of genomes without TGT$_1$
p < 0.00005
With TGT$_2$
Without TGT$_2$
Nº of genomes with TGT$_2$
Nº of genomes without TGT$_2$
p < 0.00005
With TGT$_3$
Without TGT$_3$
Nº of genomes with TGT$_3$
Nº of genomes without TGT$_3$
p < 0.00005

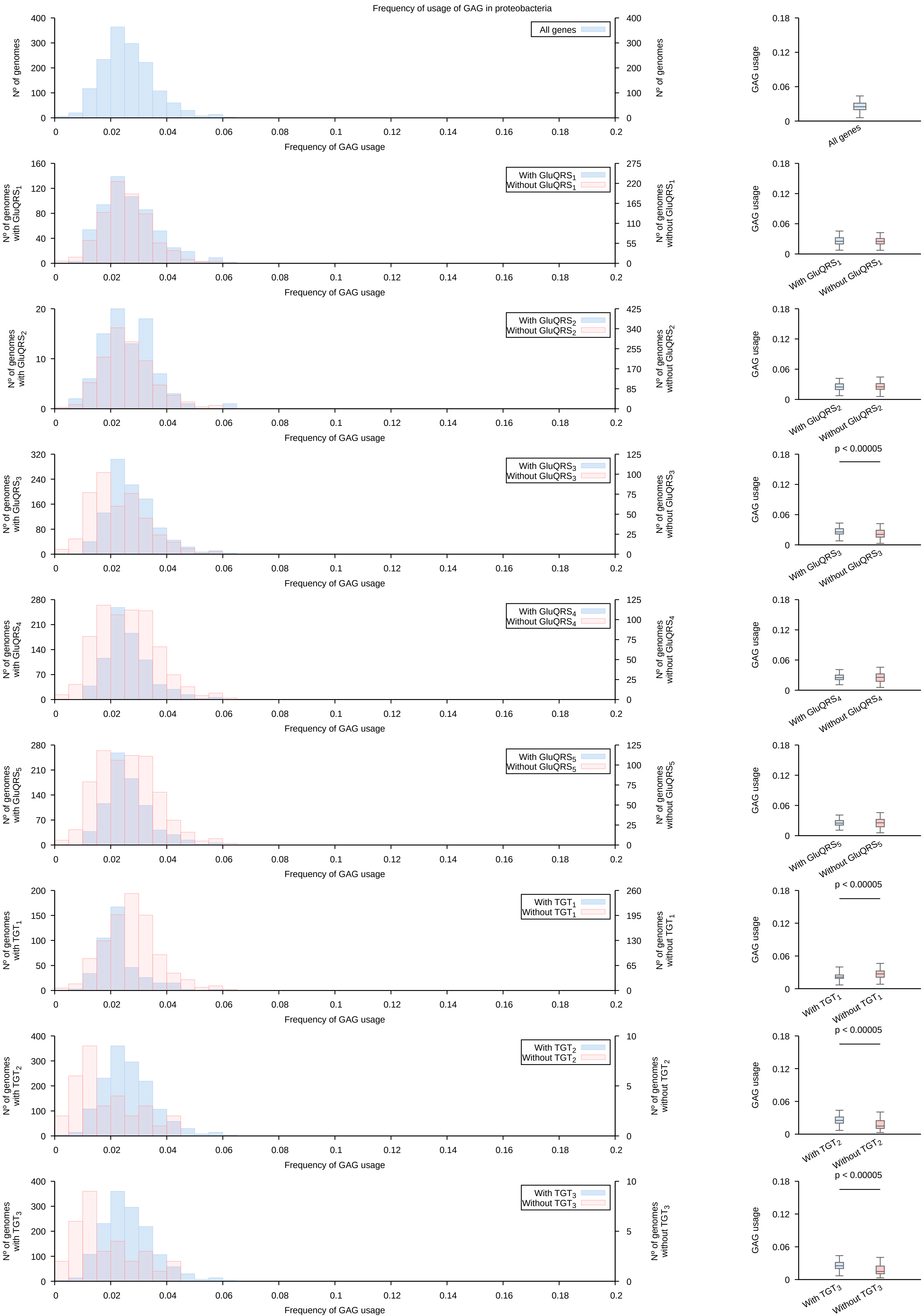
Frequency of usage of GAG in proteobacteria
All genes
Nº of genomes
Frequency of GAG usage
GAG usage
0
0.02
0.04
0.06
0.08
0.1
0.12
0.14
0.16
0.18
0.2
0.06
0.12
0.18
With GluQRS$_1$
Without GluQRS$_1$
Nº of genomes with GluQRS$_1$
Nº of genomes without GluQRS$_1$
With GluQRS$_2$
Without GluQRS$_2$
Nº of genomes with GluQRS$_2$
Nº of genomes without GluQRS$_2$
With GluQRS$_3$
Without GluQRS$_3$
Nº of genomes with GluQRS$_3$
Nº of genomes without GluQRS$_3$
p < 0.00005
With GluQRS$_4$
Without GluQRS$_4$
Nº of genomes with GluQRS$_4$
Nº of genomes without GluQRS$_4$
With GluQRS$_5$
Without GluQRS$_5$
Nº of genomes with GluQRS$_5$
Nº of genomes without GluQRS$_5$
With TGT$_1$
Without TGT$_1$
Nº of genomes with TGT$_1$
Nº of genomes without TGT$_1$
p < 0.00005
With TGT$_2$
Without TGT$_2$
Nº of genomes with TGT$_2$
Nº of genomes without TGT$_2$
p < 0.00005
With TGT$_3$
Without TGT$_3$
Nº of genomes with TGT$_3$
Nº of genomes without TGT$_3$
p < 0.00005

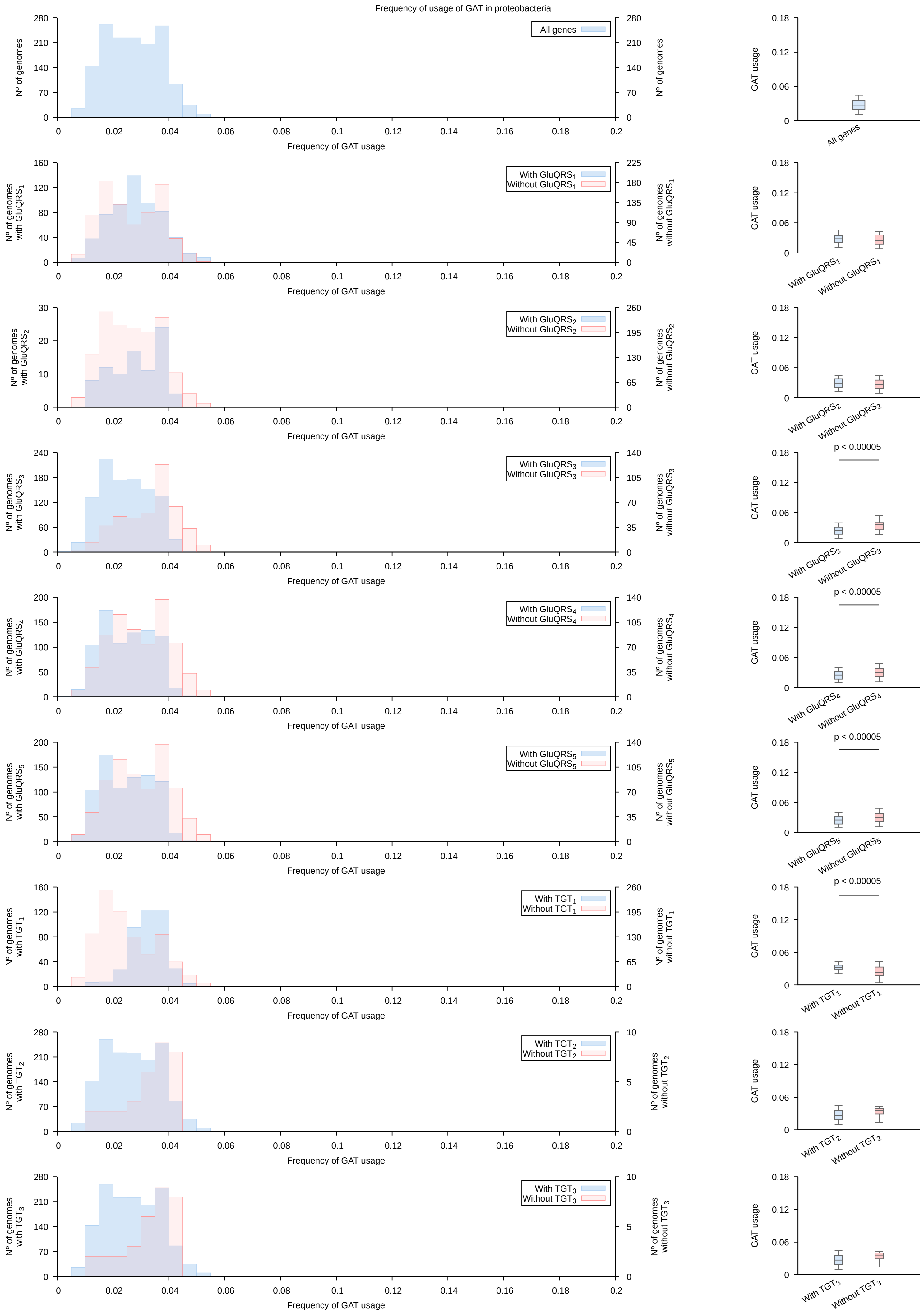

Frequency of usage of GAT in proteobacteria
All genes
With GluQRS1
Without GluQRS1
With GluQRS2
Without GluQRS2
With GluQRS3
Without GluQRS3
With GluQRS4
Without GluQRS4
With GluQRS5
Without GluQRS5
With TGT1
Without TGT1
With TGT2
Without TGT2
With TGT3
Without TGT3
Nº of genomes
Frequency of GAT usage
GAT usage
p < 0.00005

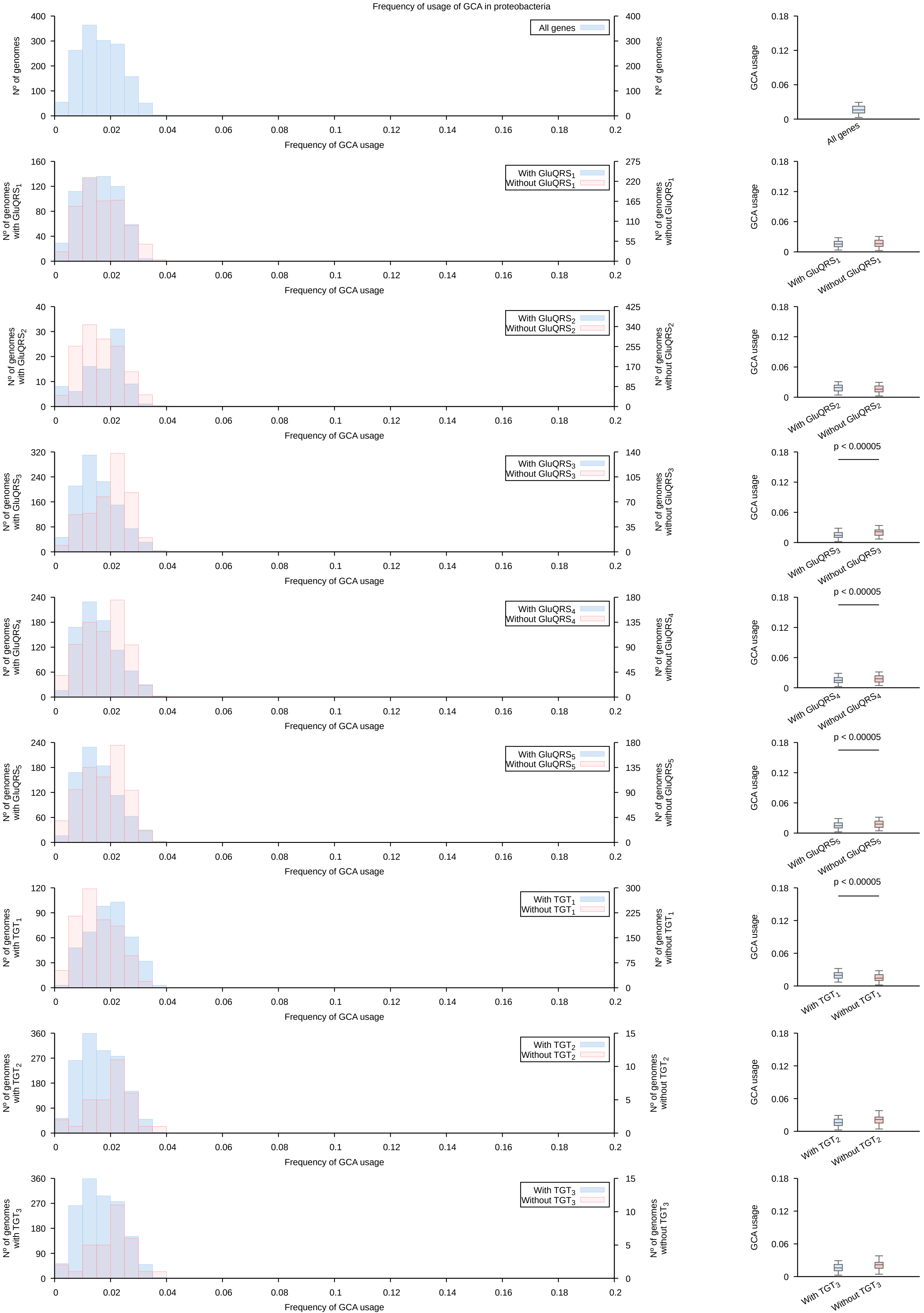

Frequency of usage of GCA in proteobacteria
All genes
With GluQRS$_1$
Without GluQRS$_1$
With GluQRS$_2$
Without GluQRS$_2$
With GluQRS$_3$
Without GluQRS$_3$
With GluQRS$_4$
Without GluQRS$_4$
With GluQRS$_5$
Without GluQRS$_5$
With TGT$_1$
Without TGT$_1$
With TGT$_2$
Without TGT$_2$
With TGT$_3$
Without TGT$_3$
Nº of genomes
Nº of genomes with GluQRS$_1$
Nº of genomes without GluQRS$_1$
Nº of genomes with GluQRS$_2$
Nº of genomes without GluQRS$_2$
Nº of genomes with GluQRS$_3$
Nº of genomes without GluQRS$_3$
Nº of genomes with GluQRS$_4$
Nº of genomes without GluQRS$_4$
Nº of genomes with GluQRS$_5$
Nº of genomes without GluQRS$_5$
Nº of genomes with TGT$_1$
Nº of genomes without TGT$_1$
Nº of genomes with TGT$_2$
Nº of genomes without TGT$_2$
Nº of genomes with TGT$_3$
Nº of genomes without TGT$_3$
Frequency of GCA usage
GCA usage
$p < 0.00005$

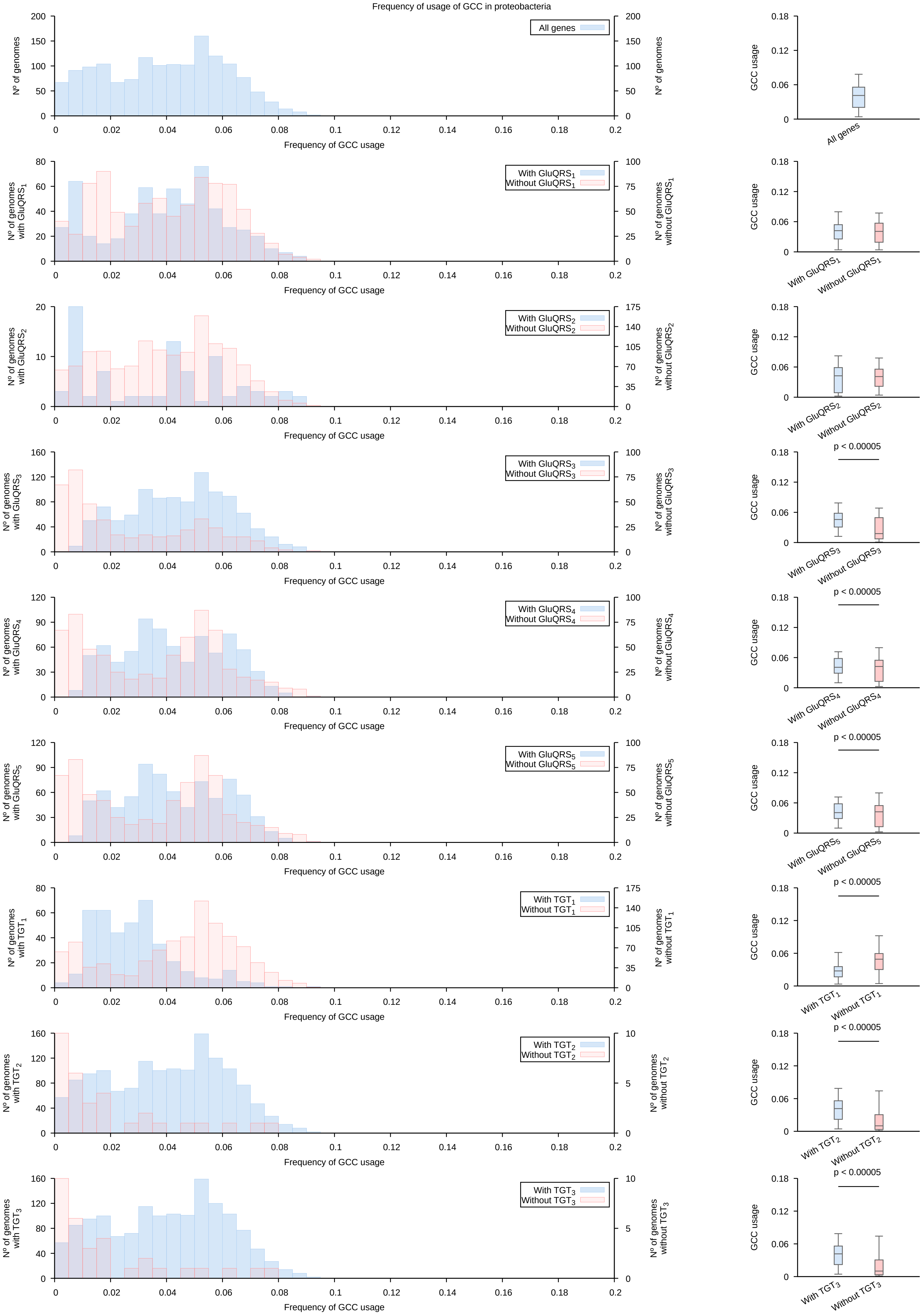
Frequency of usage of GCC in proteobacteria
All genes
Nº of genomes
Frequency of GCC usage
GCC usage
With GluQRS1
Without GluQRS1
Nº of genomes with GluQRS1
Nº of genomes without GluQRS1
With GluQRS2
Without GluQRS2
Nº of genomes with GluQRS2
Nº of genomes without GluQRS2
With GluQRS3
Without GluQRS3
Nº of genomes with GluQRS3
Nº of genomes without GluQRS3
With GluQRS4
Without GluQRS4
Nº of genomes with GluQRS4
Nº of genomes without GluQRS4
With GluQRS5
Without GluQRS5
Nº of genomes with GluQRS5
Nº of genomes without GluQRS5
With TGT1
Without TGT1
Nº of genomes with TGT1
Nº of genomes without TGT1
With TGT2
Without TGT2
Nº of genomes with TGT2
Nº of genomes without TGT2
With TGT3
Without TGT3
Nº of genomes with TGT3
Nº of genomes without TGT3
p < 0.00005

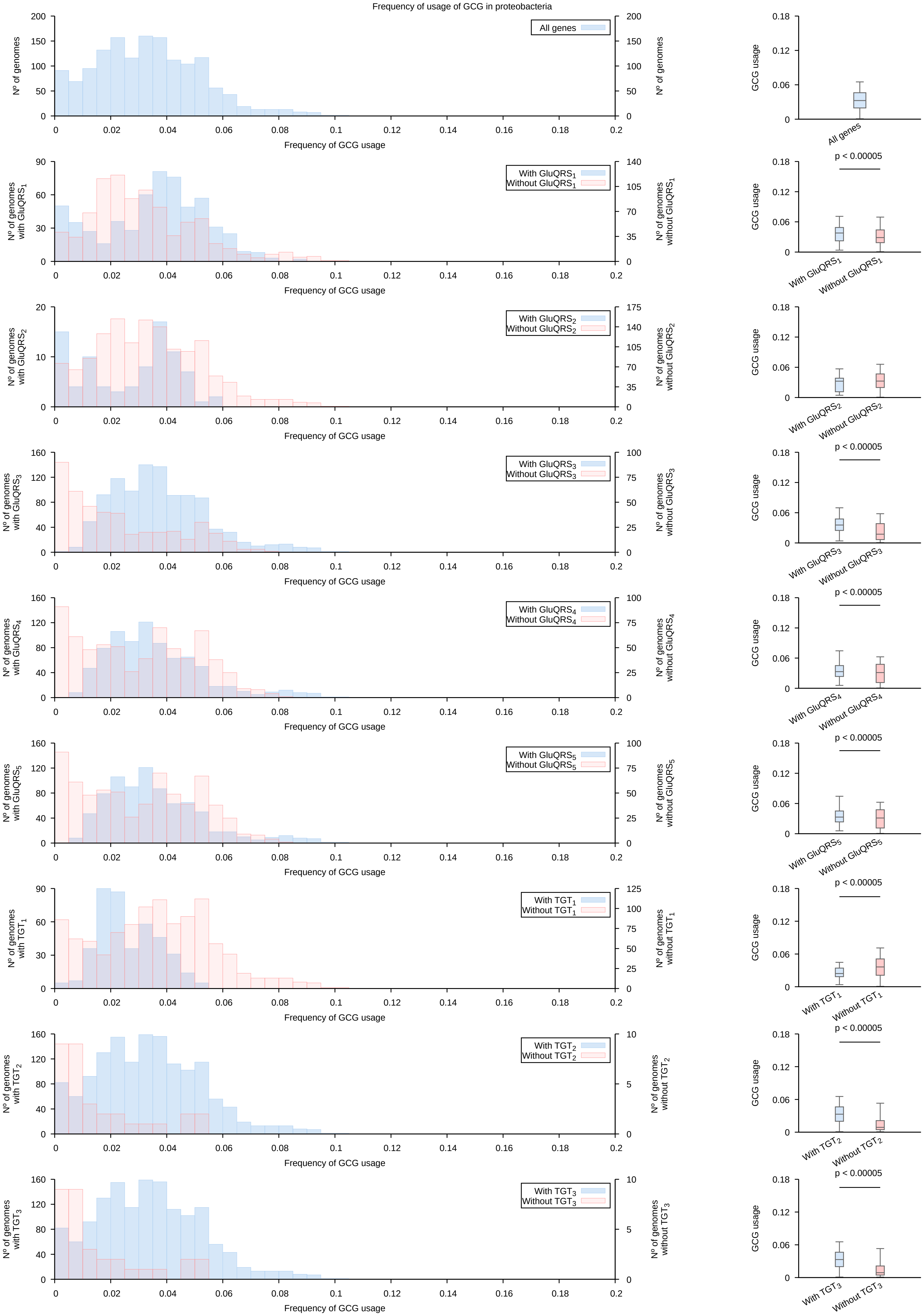
Frequency of usage of GCG in proteobacteria
Nº of genomes
All genes
Frequency of GCG usage
GCG usage
Nº of genomes with GluQRS$_1$
Nº of genomes without GluQRS$_1$
With GluQRS$_1$
Without GluQRS$_1$
p < 0.00005
Nº of genomes with GluQRS$_2$
Nº of genomes without GluQRS$_2$
With GluQRS$_2$
Without GluQRS$_2$
Nº of genomes with GluQRS$_3$
Nº of genomes without GluQRS$_3$
With GluQRS$_3$
Without GluQRS$_3$
Nº of genomes with GluQRS$_4$
Nº of genomes without GluQRS$_4$
With GluQRS$_4$
Without GluQRS$_4$
Nº of genomes with GluQRS$_5$
Nº of genomes without GluQRS$_5$
With GluQRS$_5$
Without GluQRS$_5$
Nº of genomes with TGT$_1$
Nº of genomes without TGT$_1$
With TGT$_1$
Without TGT$_1$
Nº of genomes with TGT$_2$
Nº of genomes without TGT$_2$
With TGT$_2$
Without TGT$_2$
Nº of genomes with TGT$_3$
Nº of genomes without TGT$_3$
With TGT$_3$
Without TGT$_3$

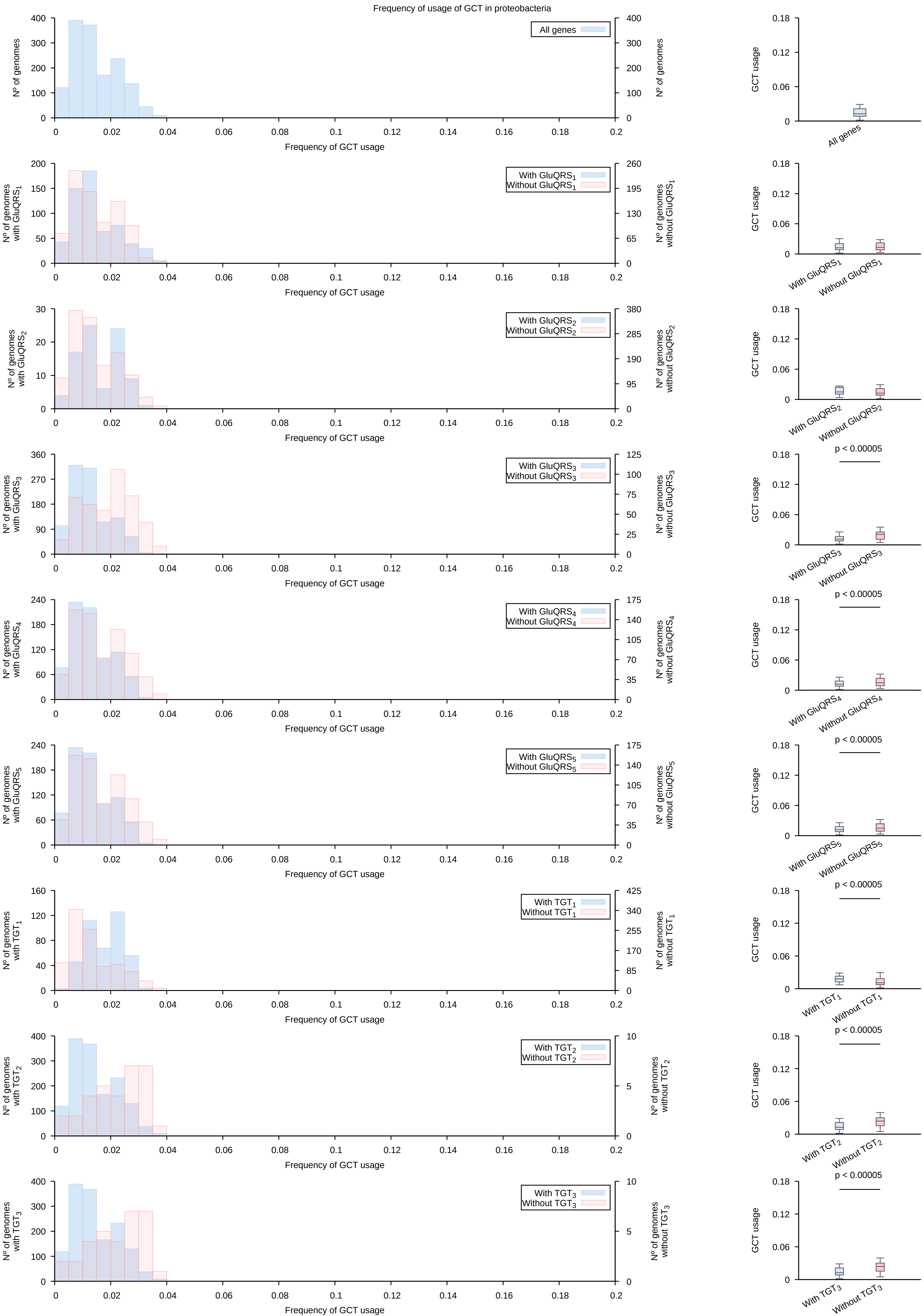

Frequency of usage of GCT in proteobacteria
Nº of genomes
Frequency of GCT usage
GCT usage
All genes
Nº of genomes with GluQRS$_1$
Nº of genomes without GluQRS$_1$
With GluQRS$_1$
Without GluQRS$_1$
Nº of genomes with GluQRS$_2$
Nº of genomes without GluQRS$_2$
With GluQRS$_2$
Without GluQRS$_2$
Nº of genomes with GluQRS$_3$
Nº of genomes without GluQRS$_3$
With GluQRS$_3$
Without GluQRS$_3$
p < 0.00005
Nº of genomes with GluQRS$_4$
Nº of genomes without GluQRS$_4$
With GluQRS$_4$
Without GluQRS$_4$
p < 0.00005
Nº of genomes with GluQRS$_5$
Nº of genomes without GluQRS$_5$
With GluQRS$_5$
Without GluQRS$_5$
p < 0.00005
Nº of genomes with TGT$_1$
Nº of genomes without TGT$_1$
With TGT$_1$
Without TGT$_1$
p < 0.00005
Nº of genomes with TGT$_2$
Nº of genomes without TGT$_2$
With TGT$_2$
Without TGT$_2$
p < 0.00005
Nº of genomes with TGT$_3$
Nº of genomes without TGT$_3$
With TGT$_3$
Without TGT$_3$
p < 0.00005

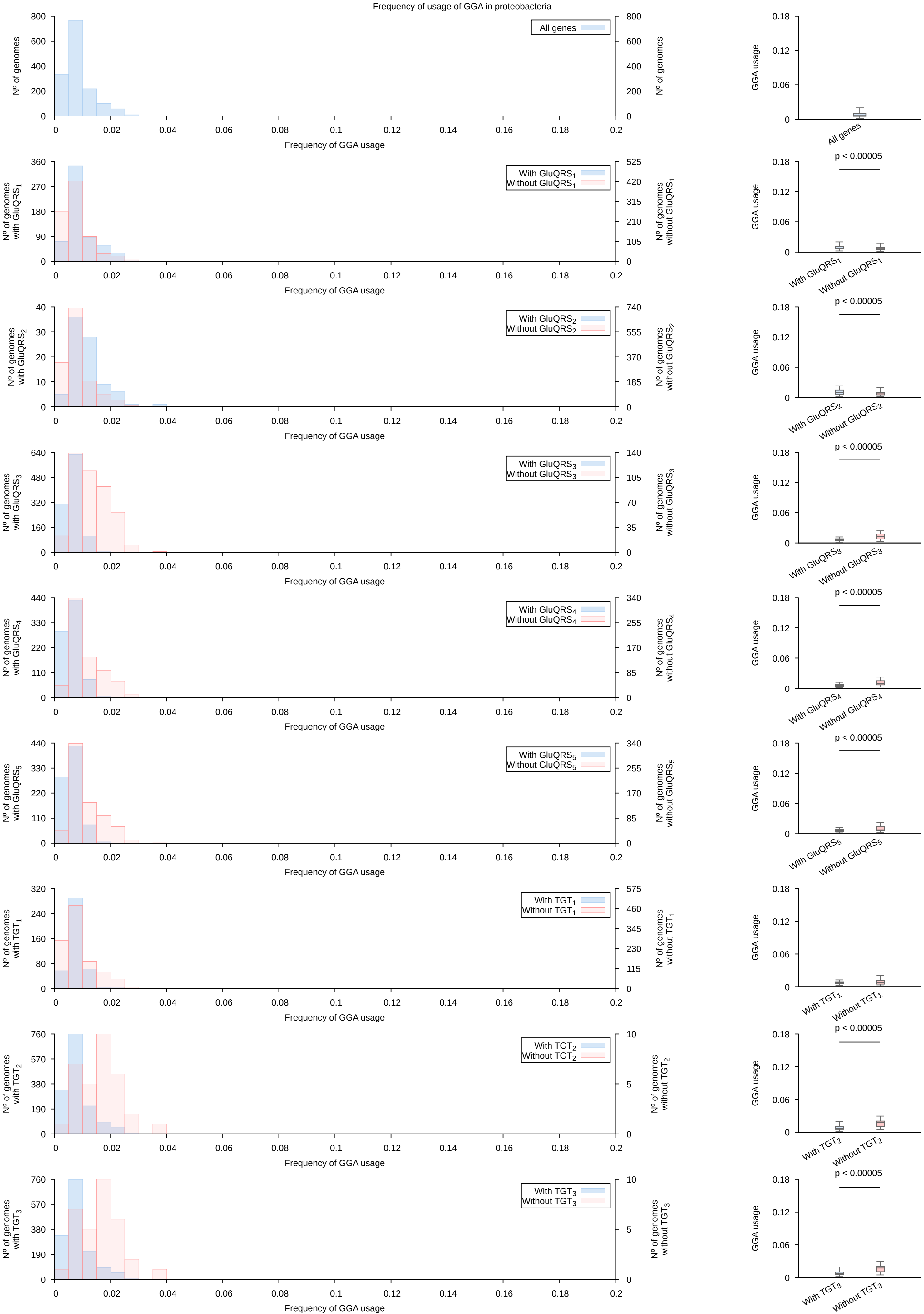

Frequency of usage of GGA in proteobacteria
Nº of genomes
All genes
Frequency of GGA usage
GGA usage
All genes
Nº of genomes with GluQRS$_1$
Nº of genomes without GluQRS$_1$
With GluQRS$_1$
Without GluQRS$_1$
p < 0.00005
Nº of genomes with GluQRS$_2$
Nº of genomes without GluQRS$_2$
With GluQRS$_2$
Without GluQRS$_2$
p < 0.00005
Nº of genomes with GluQRS$_3$
Nº of genomes without GluQRS$_3$
With GluQRS$_3$
Without GluQRS$_3$
p < 0.00005
Nº of genomes with GluQRS$_4$
Nº of genomes without GluQRS$_4$
With GluQRS$_4$
Without GluQRS$_4$
p < 0.00005
Nº of genomes with GluQRS$_5$
Nº of genomes without GluQRS$_5$
With GluQRS$_5$
Without GluQRS$_5$
p < 0.00005
Nº of genomes with TGT$_1$
Nº of genomes without TGT$_1$
With TGT$_1$
Without TGT$_1$
Nº of genomes with TGT$_2$
Nº of genomes without TGT$_2$
With TGT$_2$
Without TGT$_2$
p < 0.00005
Nº of genomes with TGT$_3$
Nº of genomes without TGT$_3$
With TGT$_3$
Without TGT$_3$
p < 0.00005

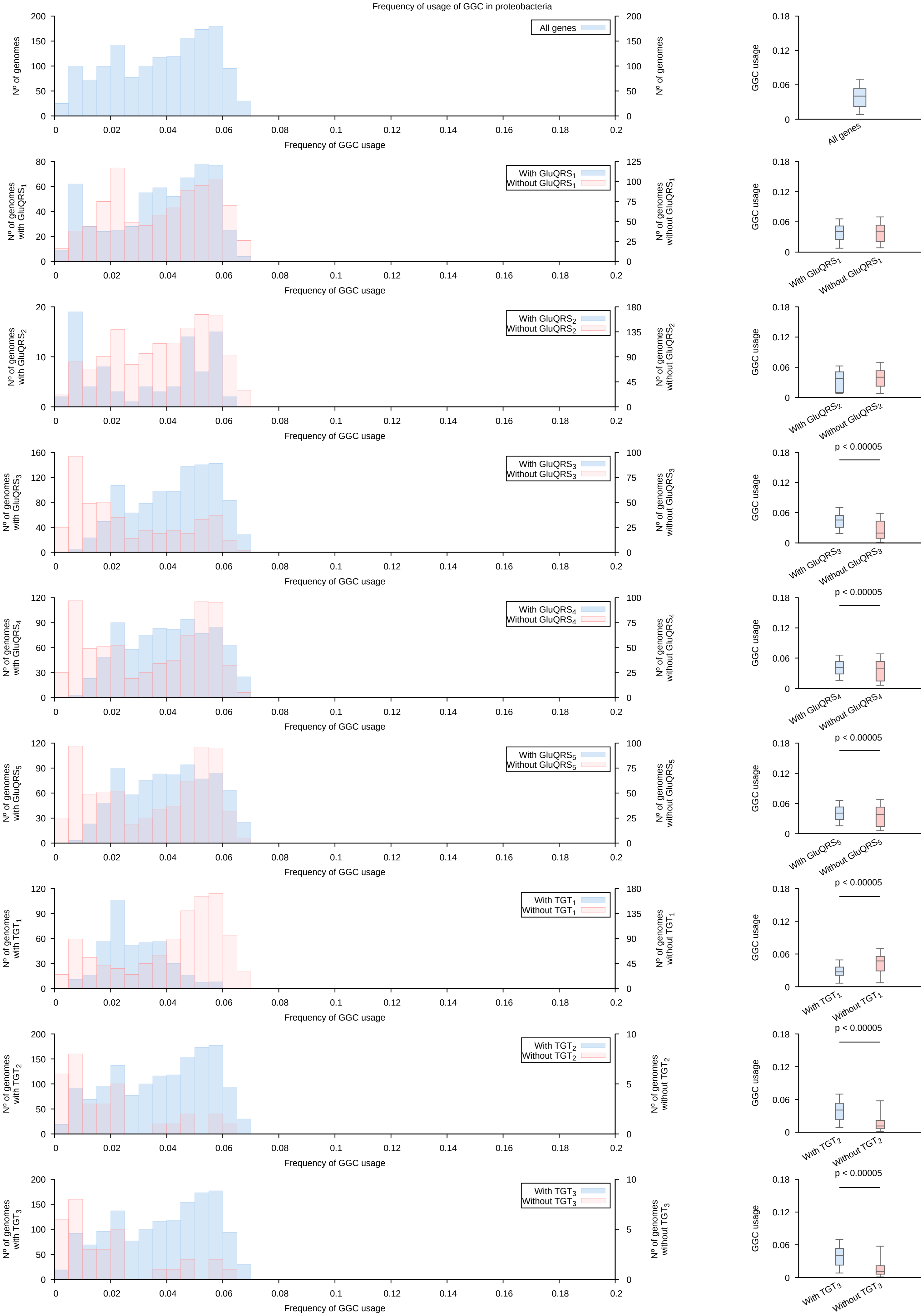
Frequency of usage of GGC in proteobacteria
All genes
Nº of genomes
Frequency of GGC usage
GGC usage
With GluQRS$_1$
Without GluQRS$_1$
Nº of genomes with GluQRS$_1$
Nº of genomes without GluQRS$_1$
With GluQRS$_2$
Without GluQRS$_2$
Nº of genomes with GluQRS$_2$
Nº of genomes without GluQRS$_2$
With GluQRS$_3$
Without GluQRS$_3$
Nº of genomes with GluQRS$_3$
Nº of genomes without GluQRS$_3$
p < 0.00005
With GluQRS$_4$
Without GluQRS$_4$
Nº of genomes with GluQRS$_4$
Nº of genomes without GluQRS$_4$
p < 0.00005
With GluQRS$_5$
Without GluQRS$_5$
Nº of genomes with GluQRS$_5$
Nº of genomes without GluQRS$_5$
p < 0.00005
With TGT$_1$
Without TGT$_1$
Nº of genomes with TGT$_1$
Nº of genomes without TGT$_1$
p < 0.00005
With TGT$_2$
Without TGT$_2$
Nº of genomes with TGT$_2$
Nº of genomes without TGT$_2$
p < 0.00005
With TGT$_3$
Without TGT$_3$
Nº of genomes with TGT$_3$
Nº of genomes without TGT$_3$
p < 0.00005

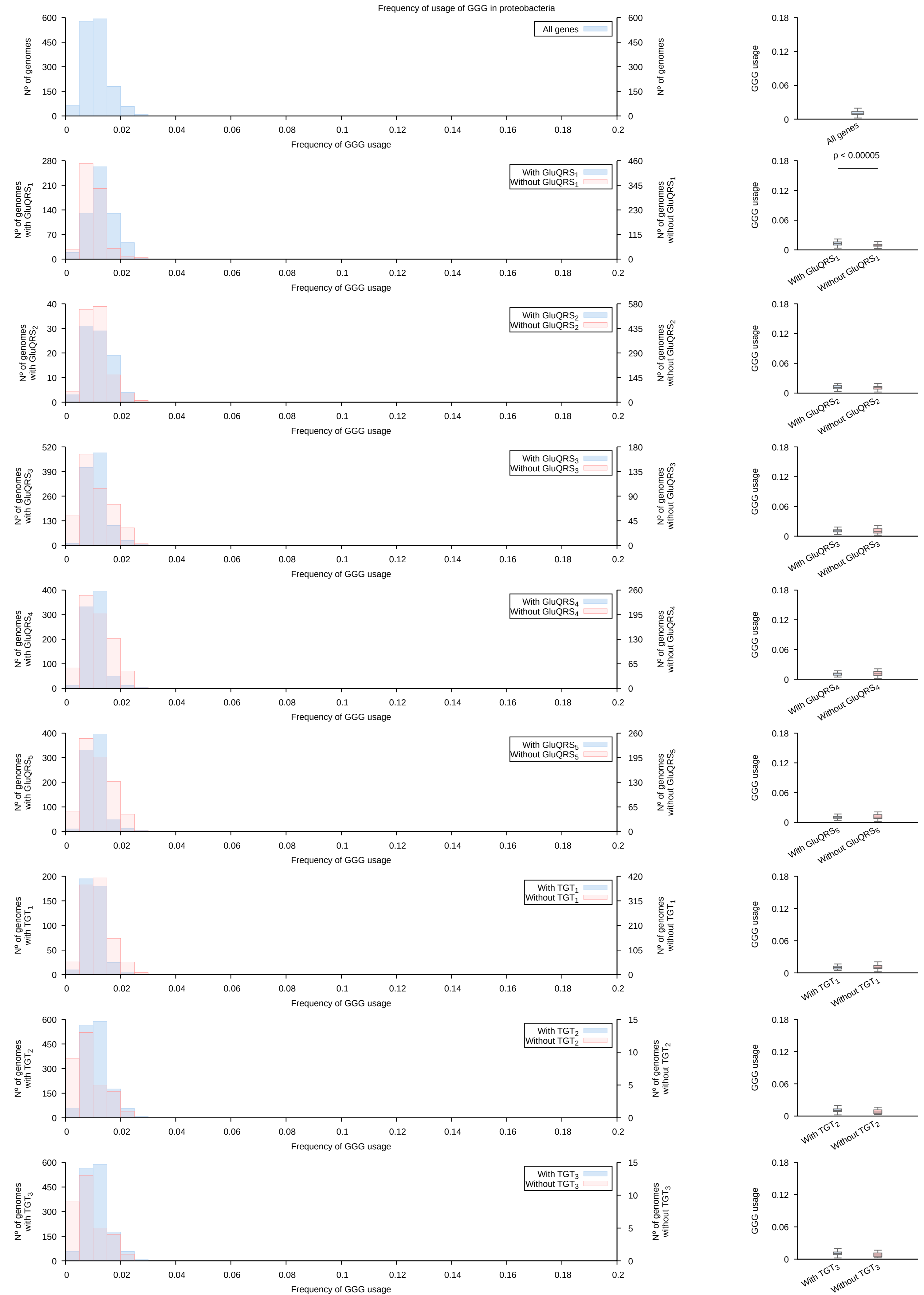
Frequency of usage of GGG in proteobacteria
All genes
With GluQRS$_1$
Without GluQRS$_1$
With GluQRS$_2$
Without GluQRS$_2$
With GluQRS$_3$
Without GluQRS$_3$
With GluQRS$_4$
Without GluQRS$_4$
With GluQRS$_5$
Without GluQRS$_5$
With TGT$_1$
Without TGT$_1$
With TGT$_2$
Without TGT$_2$
With TGT$_3$
Without TGT$_3$
Nº of genomes
Nº of genomes with GluQRS$_1$
Nº of genomes without GluQRS$_1$
Nº of genomes with GluQRS$_2$
Nº of genomes without GluQRS$_2$
Nº of genomes with GluQRS$_3$
Nº of genomes without GluQRS$_3$
Nº of genomes with GluQRS$_4$
Nº of genomes without GluQRS$_4$
Nº of genomes with GluQRS$_5$
Nº of genomes without GluQRS$_5$
Nº of genomes with TGT$_1$
Nº of genomes without TGT$_1$
Nº of genomes with TGT$_2$
Nº of genomes without TGT$_2$
Nº of genomes with TGT$_3$
Nº of genomes without TGT$_3$
Frequency of GGG usage
GGG usage
p < 0.00005

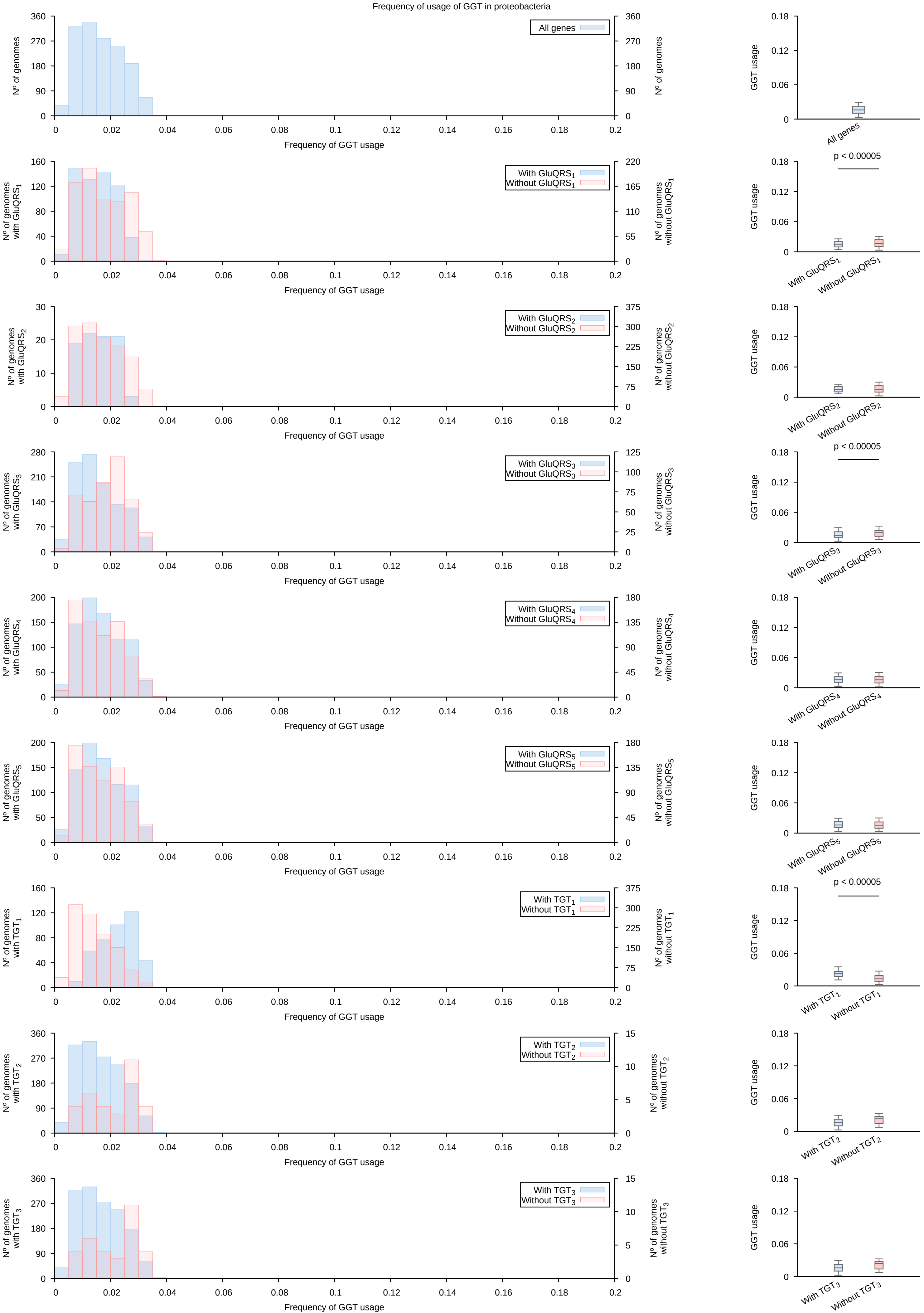

Frequency of usage of GGT in proteobacteria
Nº of genomes
All genes
Frequency of GGT usage
GGT usage
All genes
Nº of genomes with GluQRS$_1$
Nº of genomes without GluQRS$_1$
With GluQRS$_1$
Without GluQRS$_1$
p < 0.00005
Nº of genomes with GluQRS$_2$
Nº of genomes without GluQRS$_2$
With GluQRS$_2$
Without GluQRS$_2$
Nº of genomes with GluQRS$_3$
Nº of genomes without GluQRS$_3$
With GluQRS$_3$
Without GluQRS$_3$
p < 0.00005
Nº of genomes with GluQRS$_4$
Nº of genomes without GluQRS$_4$
With GluQRS$_4$
Without GluQRS$_4$
Nº of genomes with GluQRS$_5$
Nº of genomes without GluQRS$_5$
With GluQRS$_5$
Without GluQRS$_5$
Nº of genomes with TGT$_1$
Nº of genomes without TGT$_1$
With TGT$_1$
Without TGT$_1$
p < 0.00005
Nº of genomes with TGT$_2$
Nº of genomes without TGT$_2$
With TGT$_2$
Without TGT$_2$
Nº of genomes with TGT$_3$
Nº of genomes without TGT$_3$
With TGT$_3$
Without TGT$_3$

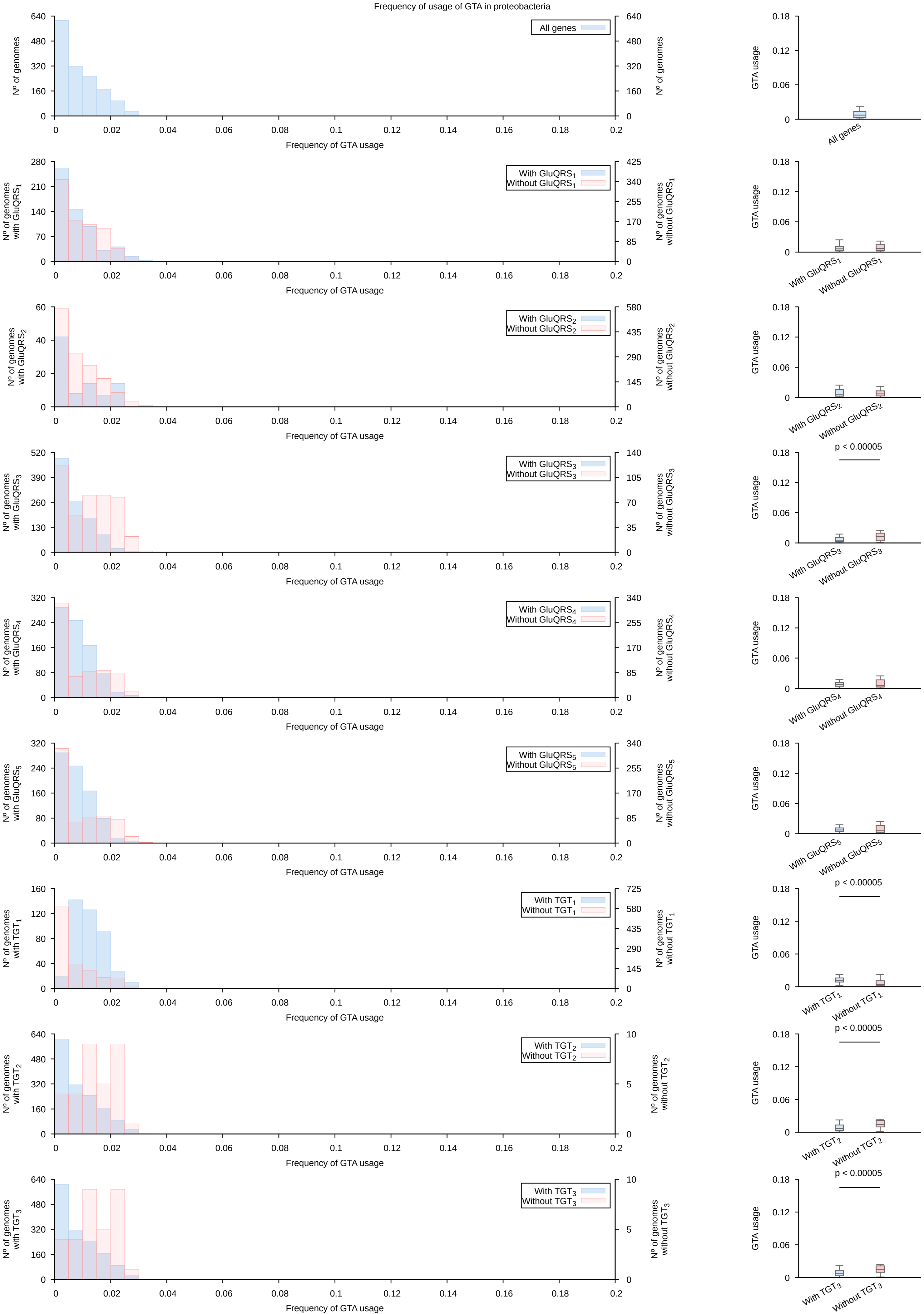
Frequency of usage of GTA in proteobacteria
All genes
With GluQRS$_1$
Without GluQRS$_1$
With GluQRS$_2$
Without GluQRS$_2$
With GluQRS$_3$
Without GluQRS$_3$
With GluQRS$_4$
Without GluQRS$_4$
With GluQRS$_5$
Without GluQRS$_5$
With TGT$_1$
Without TGT$_1$
With TGT$_2$
Without TGT$_2$
With TGT$_3$
Without TGT$_3$
Nº of genomes
Nº of genomes with GluQRS$_1$
Nº of genomes without GluQRS$_1$
Nº of genomes with GluQRS$_2$
Nº of genomes without GluQRS$_2$
Nº of genomes with GluQRS$_3$
Nº of genomes without GluQRS$_3$
Nº of genomes with GluQRS$_4$
Nº of genomes without GluQRS$_4$
Nº of genomes with GluQRS$_5$
Nº of genomes without GluQRS$_5$
Nº of genomes with TGT$_1$
Nº of genomes without TGT$_1$
Nº of genomes with TGT$_2$
Nº of genomes without TGT$_2$
Nº of genomes with TGT$_3$
Nº of genomes without TGT$_3$
Frequency of GTA usage
GTA usage
p < 0.00005

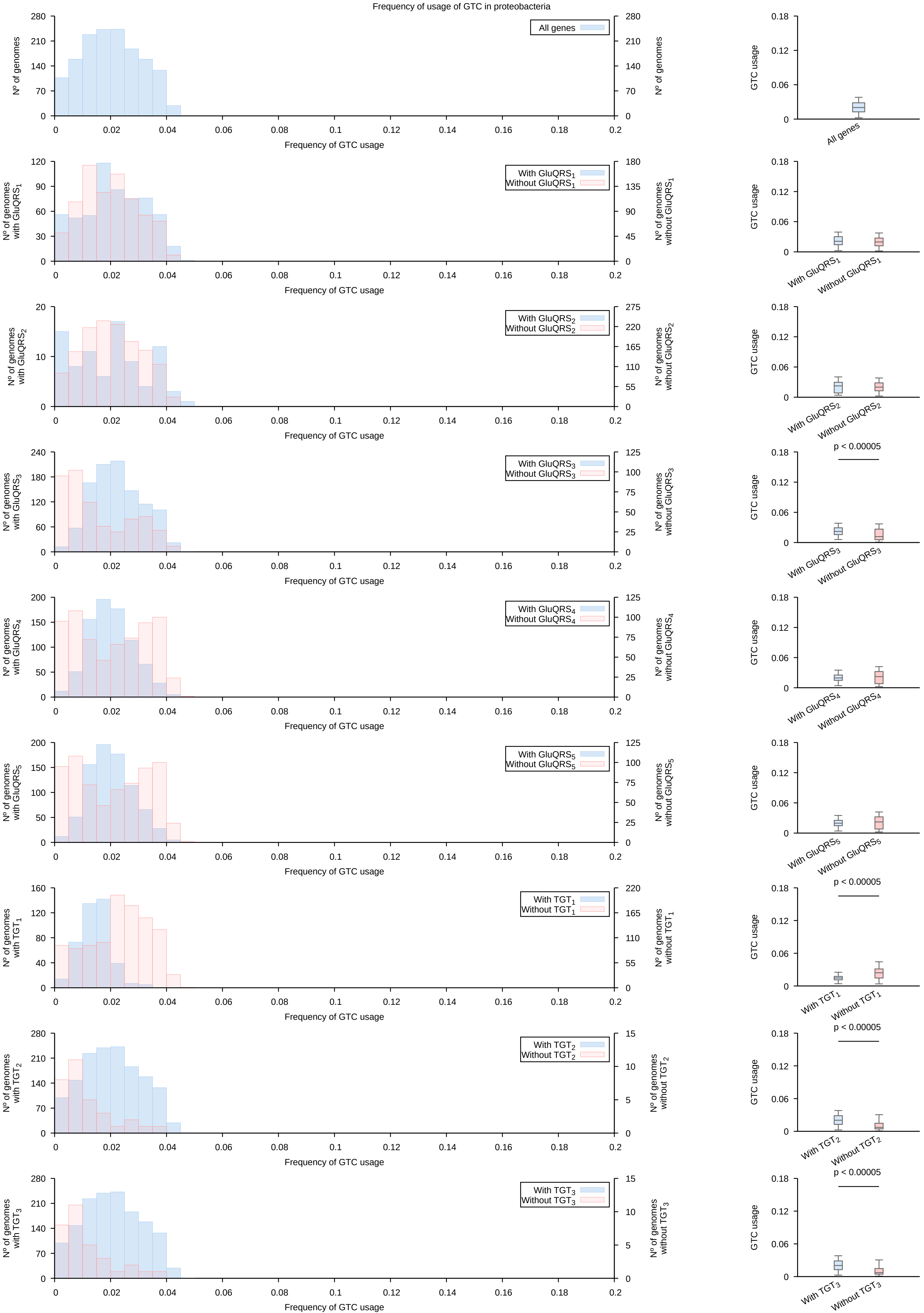
Frequency of usage of GTC in proteobacteria
All genes
Nº of genomes
Frequency of GTC usage
GTC usage
With GluQRS$_1$
Without GluQRS$_1$
Nº of genomes with GluQRS$_1$
Nº of genomes without GluQRS$_1$
With GluQRS$_2$
Without GluQRS$_2$
Nº of genomes with GluQRS$_2$
Nº of genomes without GluQRS$_2$
With GluQRS$_3$
Without GluQRS$_3$
Nº of genomes with GluQRS$_3$
Nº of genomes without GluQRS$_3$
p < 0.00005
With GluQRS$_4$
Without GluQRS$_4$
Nº of genomes with GluQRS$_4$
Nº of genomes without GluQRS$_4$
With GluQRS$_5$
Without GluQRS$_5$
Nº of genomes with GluQRS$_5$
Nº of genomes without GluQRS$_5$
With TGT$_1$
Without TGT$_1$
Nº of genomes with TGT$_1$
Nº of genomes without TGT$_1$
p < 0.00005
With TGT$_2$
Without TGT$_2$
Nº of genomes with TGT$_2$
Nº of genomes without TGT$_2$
p < 0.00005
With TGT$_3$
Without TGT$_3$
Nº of genomes with TGT$_3$
Nº of genomes without TGT$_3$
p < 0.00005

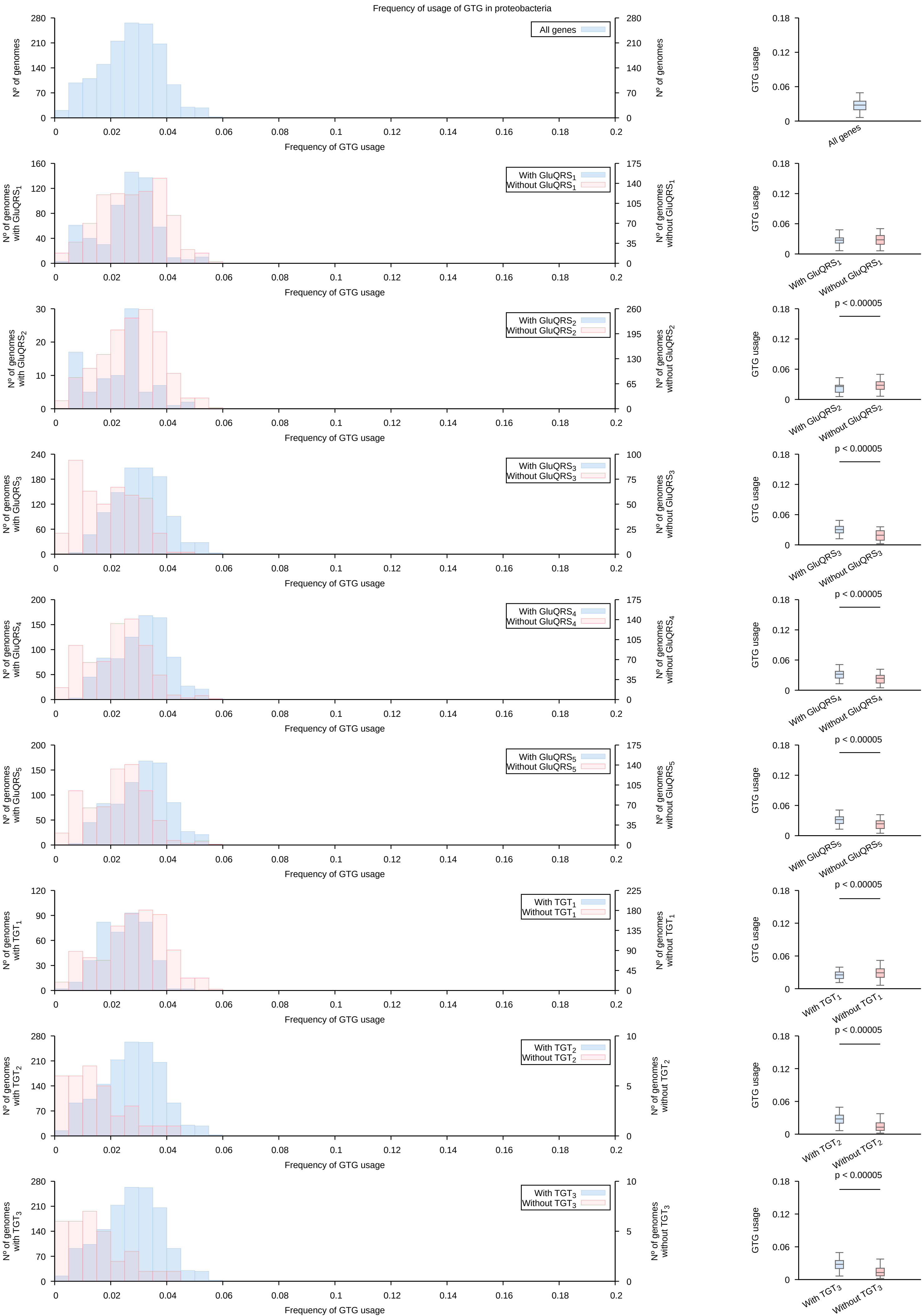

Frequency of usage of GTG in proteobacteria
All genes
Nº of genomes
Frequency of GTG usage
GTG usage
With GluQRS$_1$
Without GluQRS$_1$
Nº of genomes with GluQRS$_1$
Nº of genomes without GluQRS$_1$
With GluQRS$_2$
Without GluQRS$_2$
Nº of genomes with GluQRS$_2$
Nº of genomes without GluQRS$_2$
p < 0.00005
With GluQRS$_3$
Without GluQRS$_3$
Nº of genomes with GluQRS$_3$
Nº of genomes without GluQRS$_3$
p < 0.00005
With GluQRS$_4$
Without GluQRS$_4$
Nº of genomes with GluQRS$_4$
Nº of genomes without GluQRS$_4$
p < 0.00005
With GluQRS$_5$
Without GluQRS$_5$
Nº of genomes with GluQRS$_5$
Nº of genomes without GluQRS$_5$
p < 0.00005
With TGT$_1$
Without TGT$_1$
Nº of genomes with TGT$_1$
Nº of genomes without TGT$_1$
p < 0.00005
With TGT$_2$
Without TGT$_2$
Nº of genomes with TGT$_2$
Nº of genomes without TGT$_2$
p < 0.00005
With TGT$_3$
Without TGT$_3$
Nº of genomes with TGT$_3$
Nº of genomes without TGT$_3$
p < 0.00005
0 0.02 0.04 0.06 0.08 0.1 0.12 0.14 0.16 0.18 0.2
0 0.06 0.12 0.18

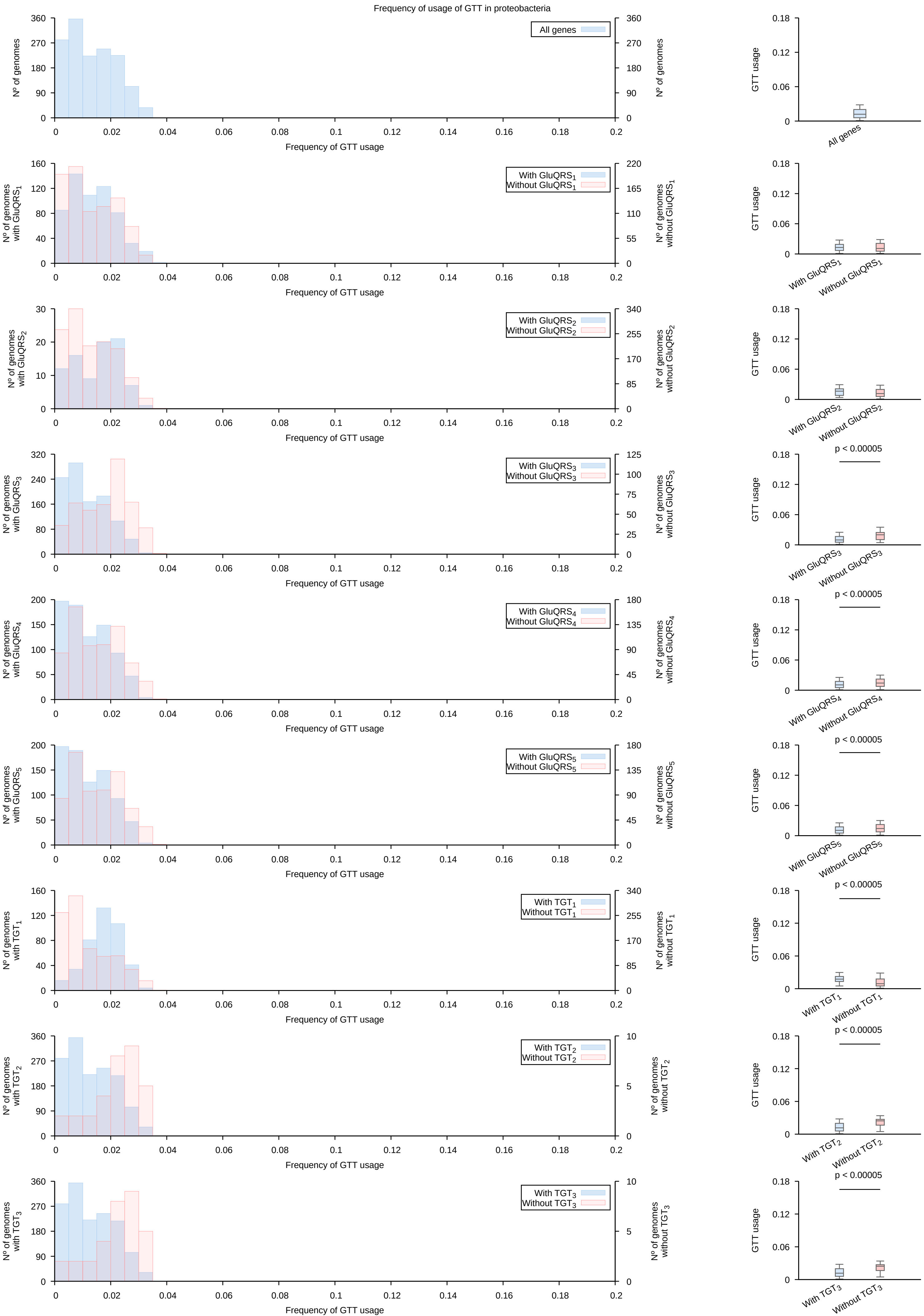

Frequency of usage of GTT in proteobacteria
All genes
With GluQRS1
Without GluQRS1
With GluQRS2
Without GluQRS2
With GluQRS3
Without GluQRS3
With GluQRS4
Without GluQRS4
With GluQRS5
Without GluQRS5
With TGT1
Without TGT1
With TGT2
Without TGT2
With TGT3
Without TGT3
Nº of genomes
Nº of genomes with GluQRS1
Nº of genomes without GluQRS1
Nº of genomes with GluQRS2
Nº of genomes without GluQRS2
Nº of genomes with GluQRS3
Nº of genomes without GluQRS3
Nº of genomes with GluQRS4
Nº of genomes without GluQRS4
Nº of genomes with GluQRS5
Nº of genomes without GluQRS5
Nº of genomes with TGT1
Nº of genomes without TGT1
Nº of genomes with TGT2
Nº of genomes without TGT2
Nº of genomes with TGT3
Nº of genomes without TGT3
Frequency of GTT usage
GTT usage
p < 0.00005

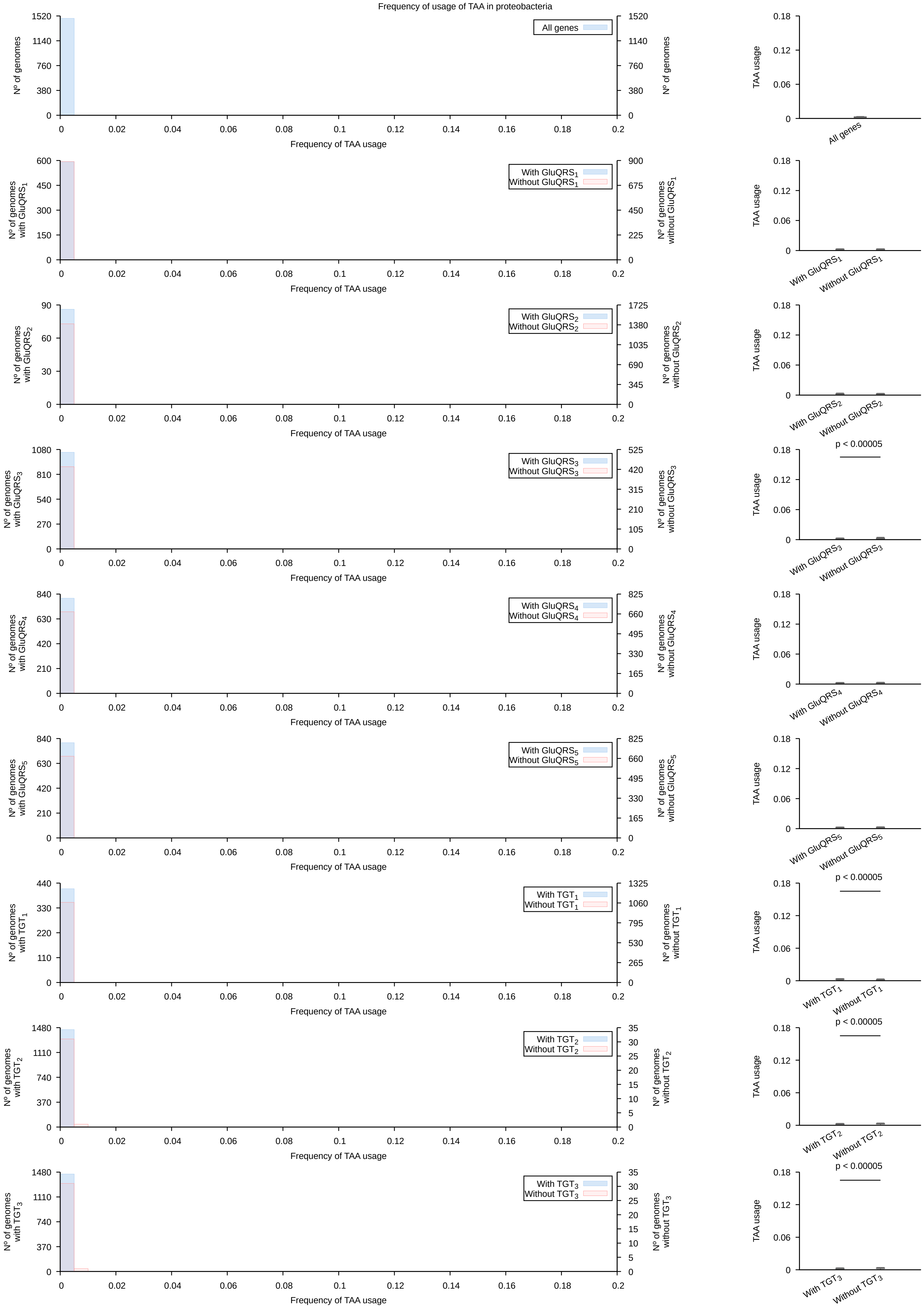
Frequency of usage of TAA in proteobacteria
Nº of genomes
All genes
Frequency of TAA usage
TAA usage
With GluQRS$_1$
Without GluQRS$_1$
Nº of genomes with GluQRS$_1$
Nº of genomes without GluQRS$_1$
With GluQRS$_2$
Without GluQRS$_2$
With GluQRS$_3$
Without GluQRS$_3$
p < 0.00005
With GluQRS$_4$
Without GluQRS$_4$
With GluQRS$_5$
Without GluQRS$_5$
With TGT$_1$
Without TGT$_1$
With TGT$_2$
Without TGT$_2$
With TGT$_3$
Without TGT$_3$

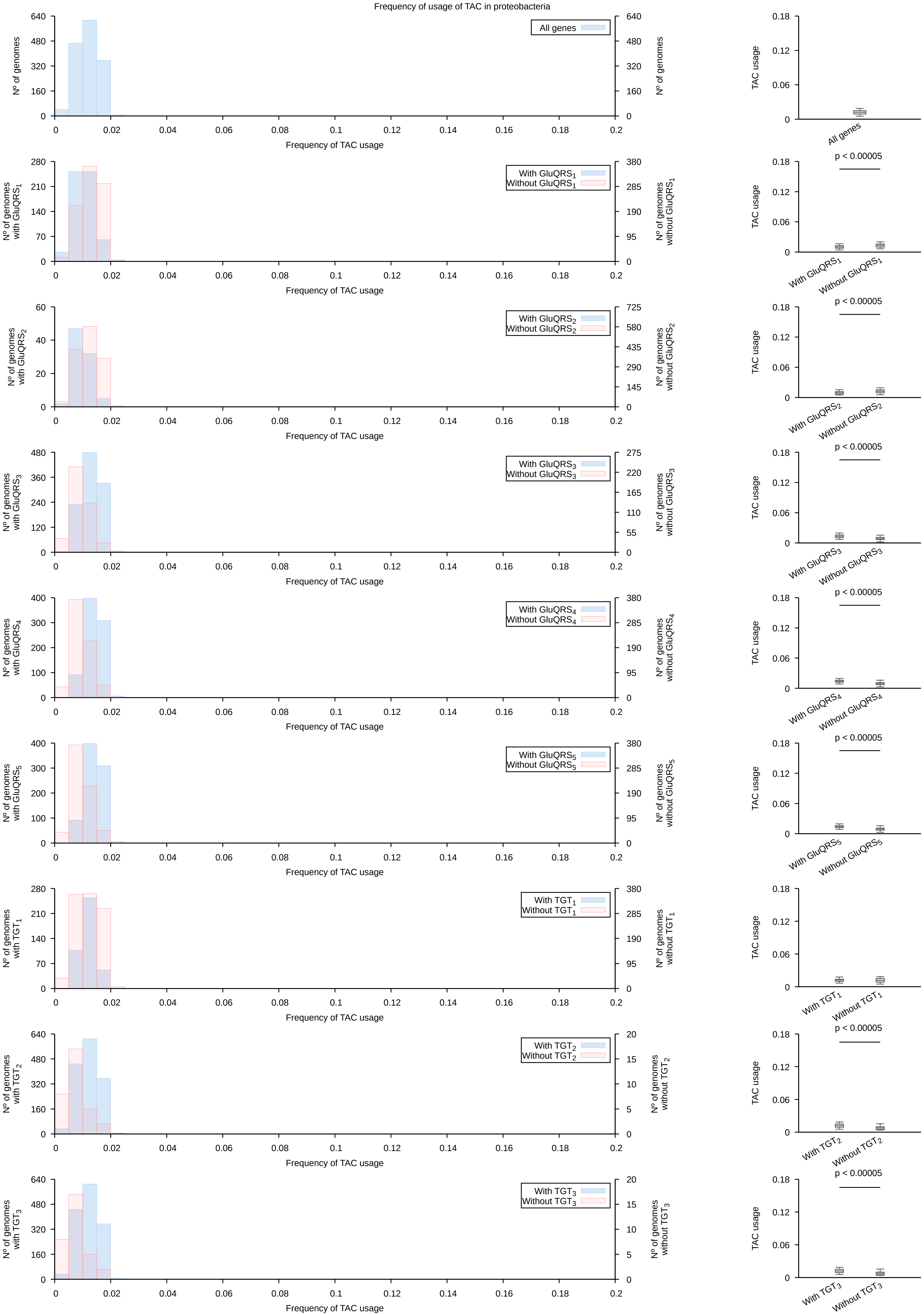

Frequency of usage of TAC in proteobacteria
All genes
With GluQRS$_1$
Without GluQRS$_1$
With GluQRS$_2$
Without GluQRS$_2$
With GluQRS$_3$
Without GluQRS$_3$
With GluQRS$_4$
Without GluQRS$_4$
With GluQRS$_5$
Without GluQRS$_5$
With TGT$_1$
Without TGT$_1$
With TGT$_2$
Without TGT$_2$
With TGT$_3$
Without TGT$_3$
Nº of genomes
Frequency of TAC usage
TAC usage
p < 0.00005

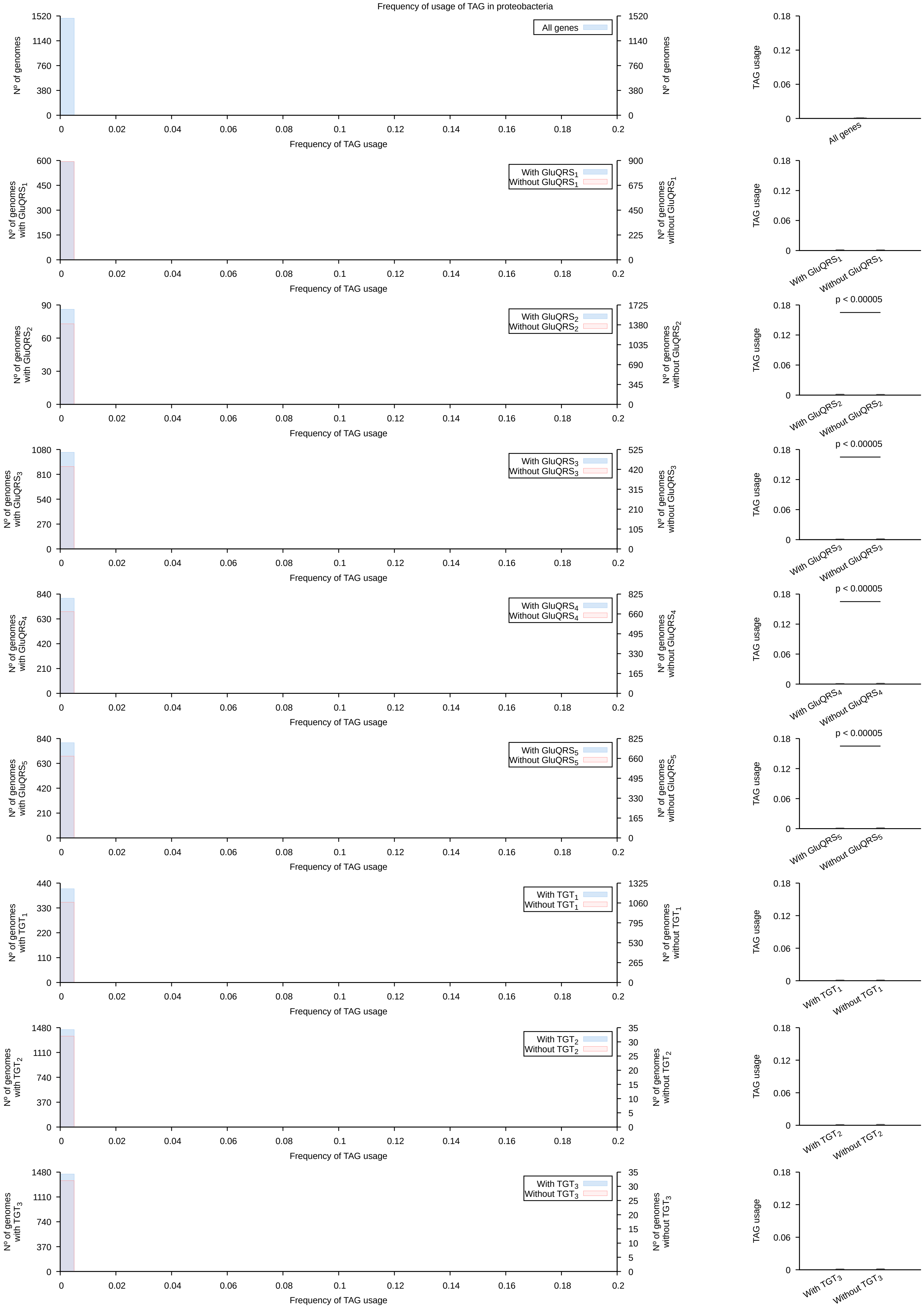
Frequency of usage of TAG in proteobacteria
All genes
With GluQRS1
Without GluQRS1
With GluQRS2
Without GluQRS2
With GluQRS3
Without GluQRS3
With GluQRS4
Without GluQRS4
With GluQRS5
Without GluQRS5
With TGT1
Without TGT1
With TGT2
Without TGT2
With TGT3
Without TGT3
Nº of genomes
Frequency of TAG usage
TAG usage
p < 0.00005

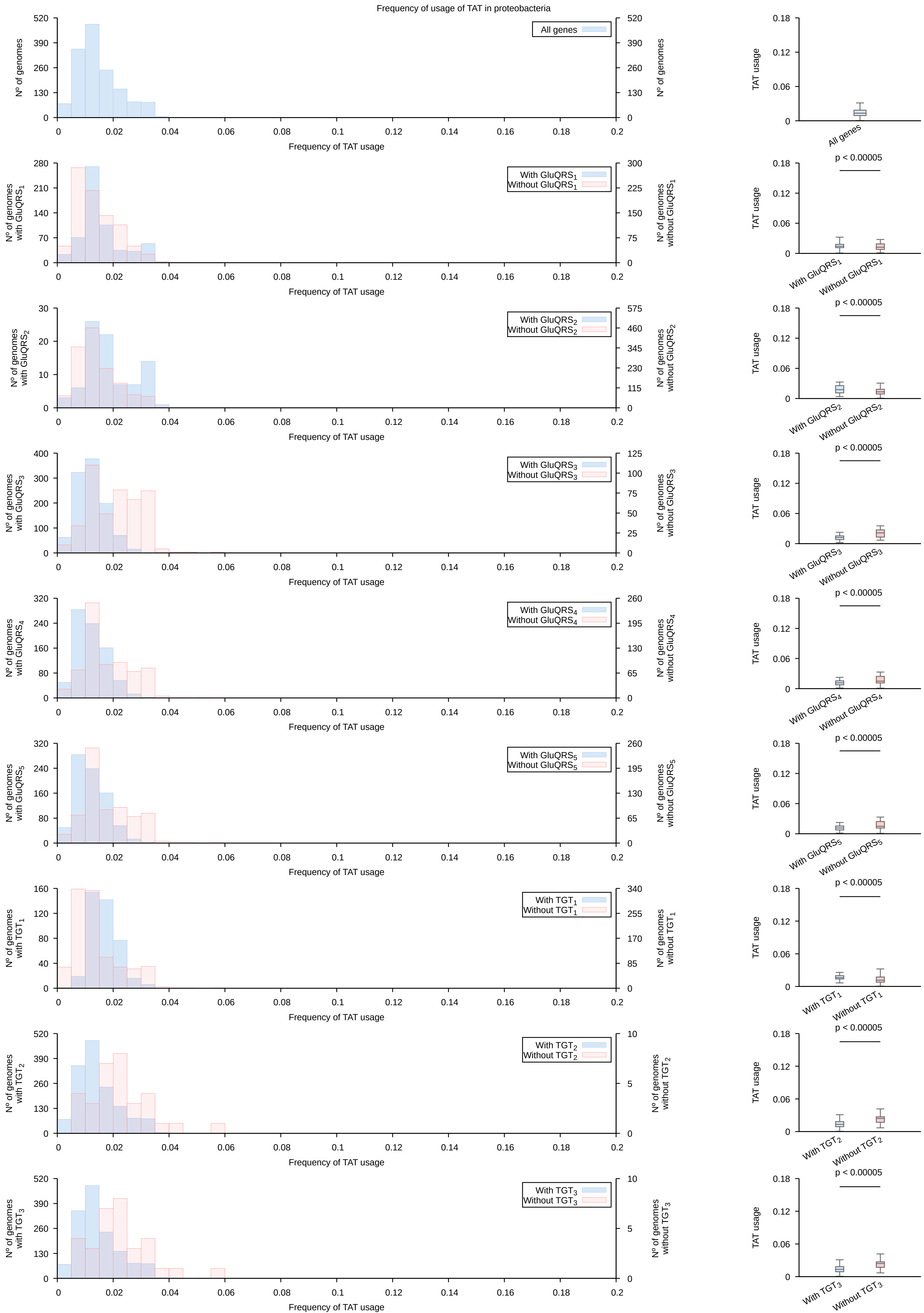

Frequency of usage of TAT in proteobacteria
All genes
With $GluQRS_1$
Without $GluQRS_1$
With $GluQRS_2$
Without $GluQRS_2$
With $GluQRS_3$
Without $GluQRS_3$
With $GluQRS_4$
Without $GluQRS_4$
With $GluQRS_5$
Without $GluQRS_5$
With TGT_1
Without TGT_1
With TGT_2
Without TGT_2
With TGT_3
Without TGT_3
Nº of genomes
Frequency of TAT usage
TAT usage
p < 0.00005

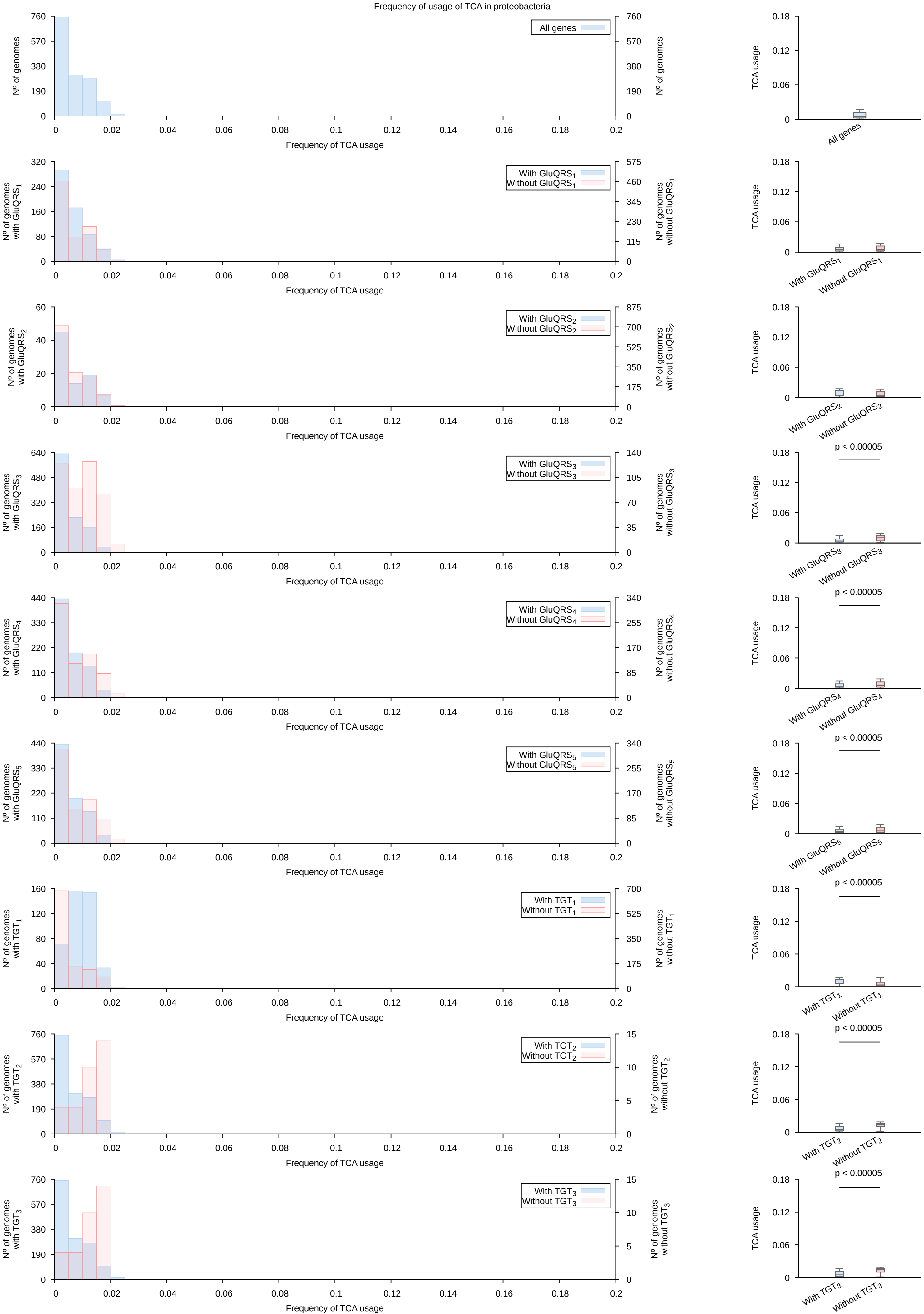
Frequency of usage of TCA in proteobacteria
All genes
With GluQRS1
Without GluQRS1
With GluQRS2
Without GluQRS2
With GluQRS3
Without GluQRS3
With GluQRS4
Without GluQRS4
With GluQRS5
Without GluQRS5
With TGT1
Without TGT1
With TGT2
Without TGT2
With TGT3
Without TGT3
Nº of genomes
Frequency of TCA usage
TCA usage
p < 0.00005

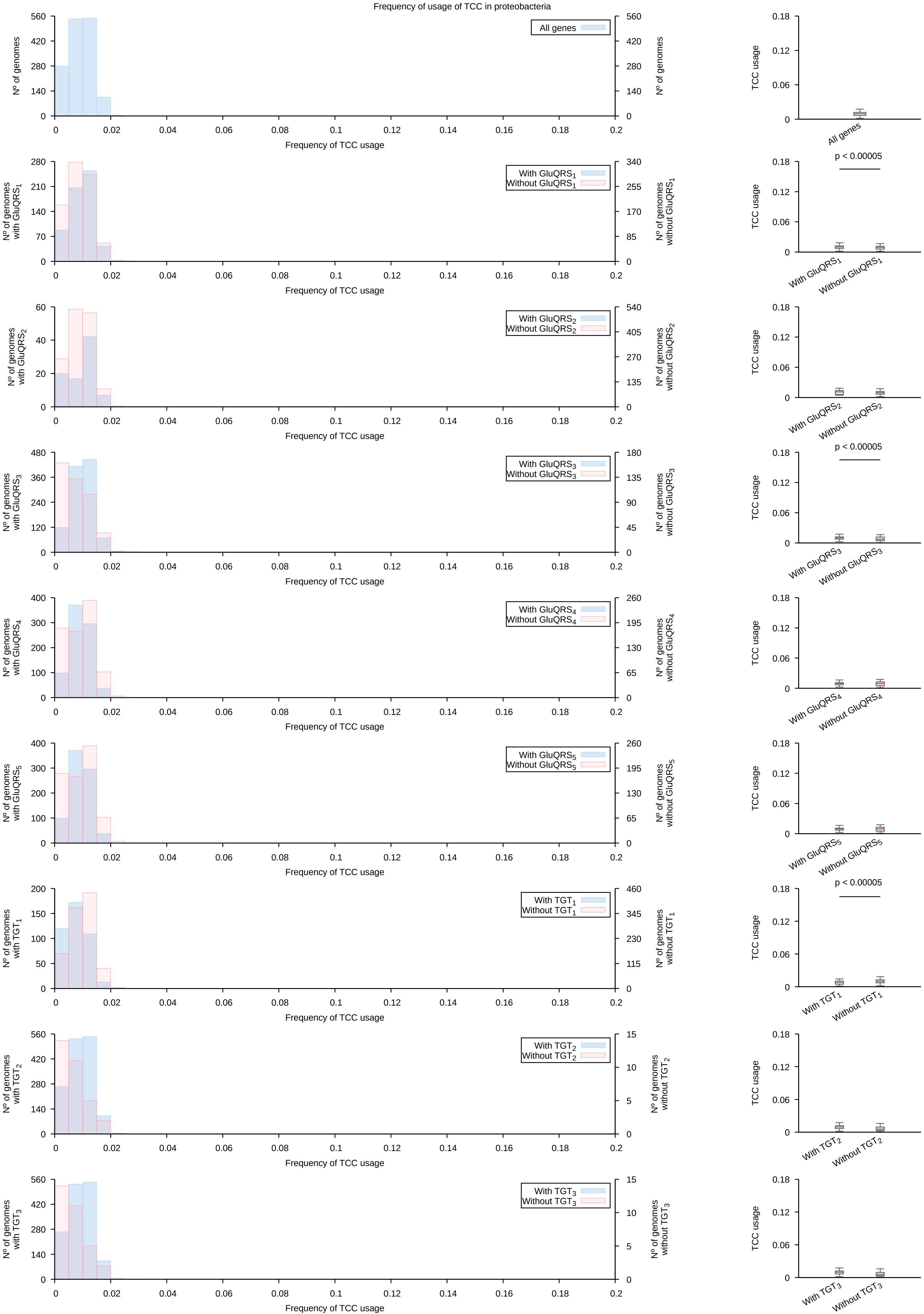
Frequency of usage of TCC in proteobacteria
All genes
Nº of genomes
Frequency of TCC usage
TCC usage
With GluQRS$_1$
Without GluQRS$_1$
Nº of genomes with GluQRS$_1$
Nº of genomes without GluQRS$_1$
p < 0.00005
With GluQRS$_2$
Without GluQRS$_2$
Nº of genomes with GluQRS$_2$
Nº of genomes without GluQRS$_2$
With GluQRS$_3$
Without GluQRS$_3$
Nº of genomes with GluQRS$_3$
Nº of genomes without GluQRS$_3$
p < 0.00005
With GluQRS$_4$
Without GluQRS$_4$
Nº of genomes with GluQRS$_4$
Nº of genomes without GluQRS$_4$
With GluQRS$_5$
Without GluQRS$_5$
Nº of genomes with GluQRS$_5$
Nº of genomes without GluQRS$_5$
With TGT$_1$
Without TGT$_1$
Nº of genomes with TGT$_1$
Nº of genomes without TGT$_1$
p < 0.00005
With TGT$_2$
Without TGT$_2$
Nº of genomes with TGT$_2$
Nº of genomes without TGT$_2$
With TGT$_3$
Without TGT$_3$
Nº of genomes with TGT$_3$
Nº of genomes without TGT$_3$
0 0.02 0.04 0.06 0.08 0.1 0.12 0.14 0.16 0.18 0.2
0 0.06 0.12 0.18

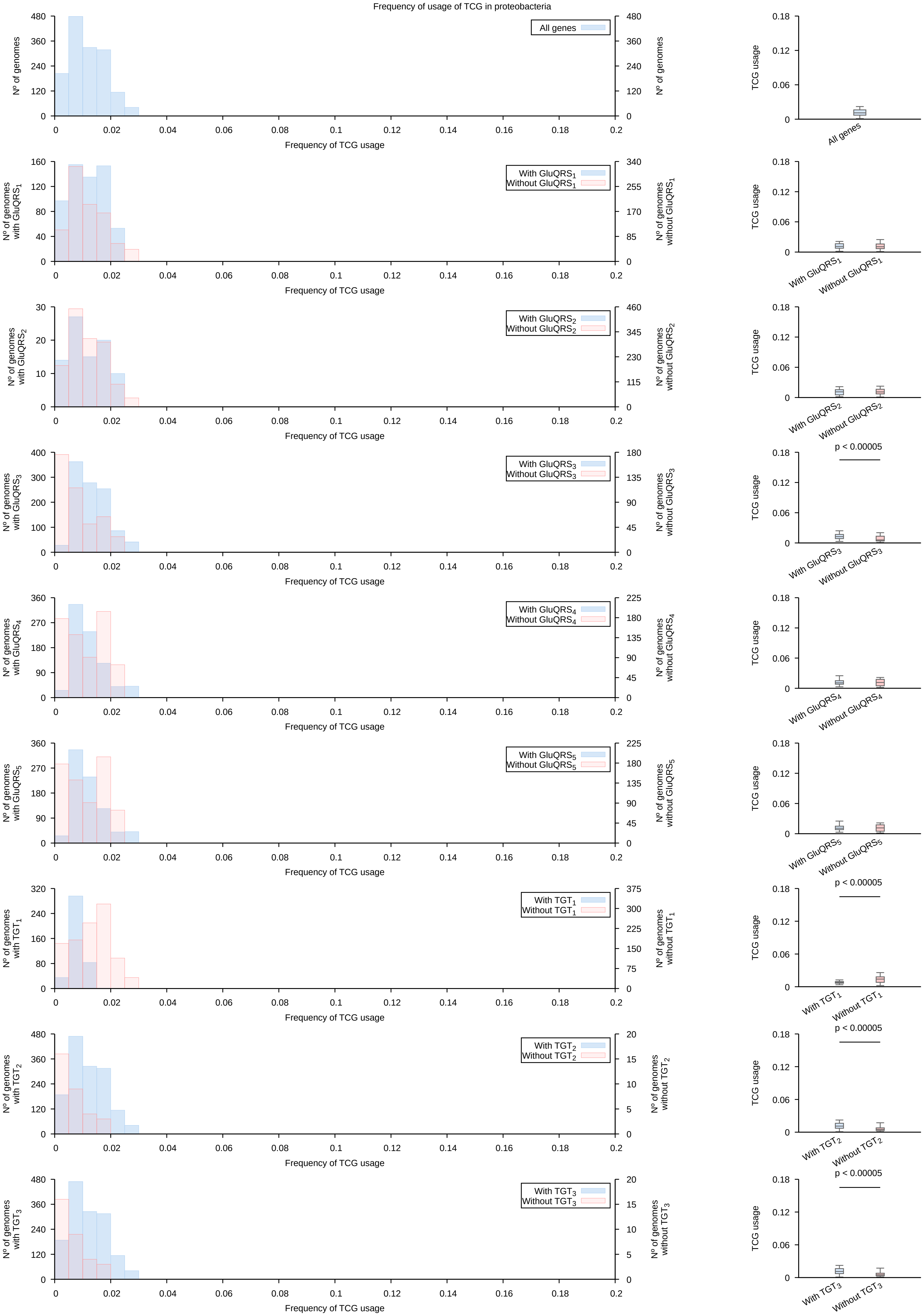
Frequency of usage of TCG in proteobacteria
Nº of genomes
Frequency of TCG usage
TCG usage
All genes
With GluQRS$_1$
Without GluQRS$_1$
Nº of genomes with GluQRS$_1$
Nº of genomes without GluQRS$_1$
With GluQRS$_2$
Without GluQRS$_2$
Nº of genomes with GluQRS$_2$
Nº of genomes without GluQRS$_2$
With GluQRS$_3$
Without GluQRS$_3$
Nº of genomes with GluQRS$_3$
Nº of genomes without GluQRS$_3$
p < 0.00005
With GluQRS$_4$
Without GluQRS$_4$
Nº of genomes with GluQRS$_4$
Nº of genomes without GluQRS$_4$
With GluQRS$_5$
Without GluQRS$_5$
Nº of genomes with GluQRS$_5$
Nº of genomes without GluQRS$_5$
With TGT$_1$
Without TGT$_1$
Nº of genomes with TGT$_1$
Nº of genomes without TGT$_1$
p < 0.00005
With TGT$_2$
Without TGT$_2$
Nº of genomes with TGT$_2$
Nº of genomes without TGT$_2$
p < 0.00005
With TGT$_3$
Without TGT$_3$
Nº of genomes with TGT$_3$
Nº of genomes without TGT$_3$
p < 0.00005

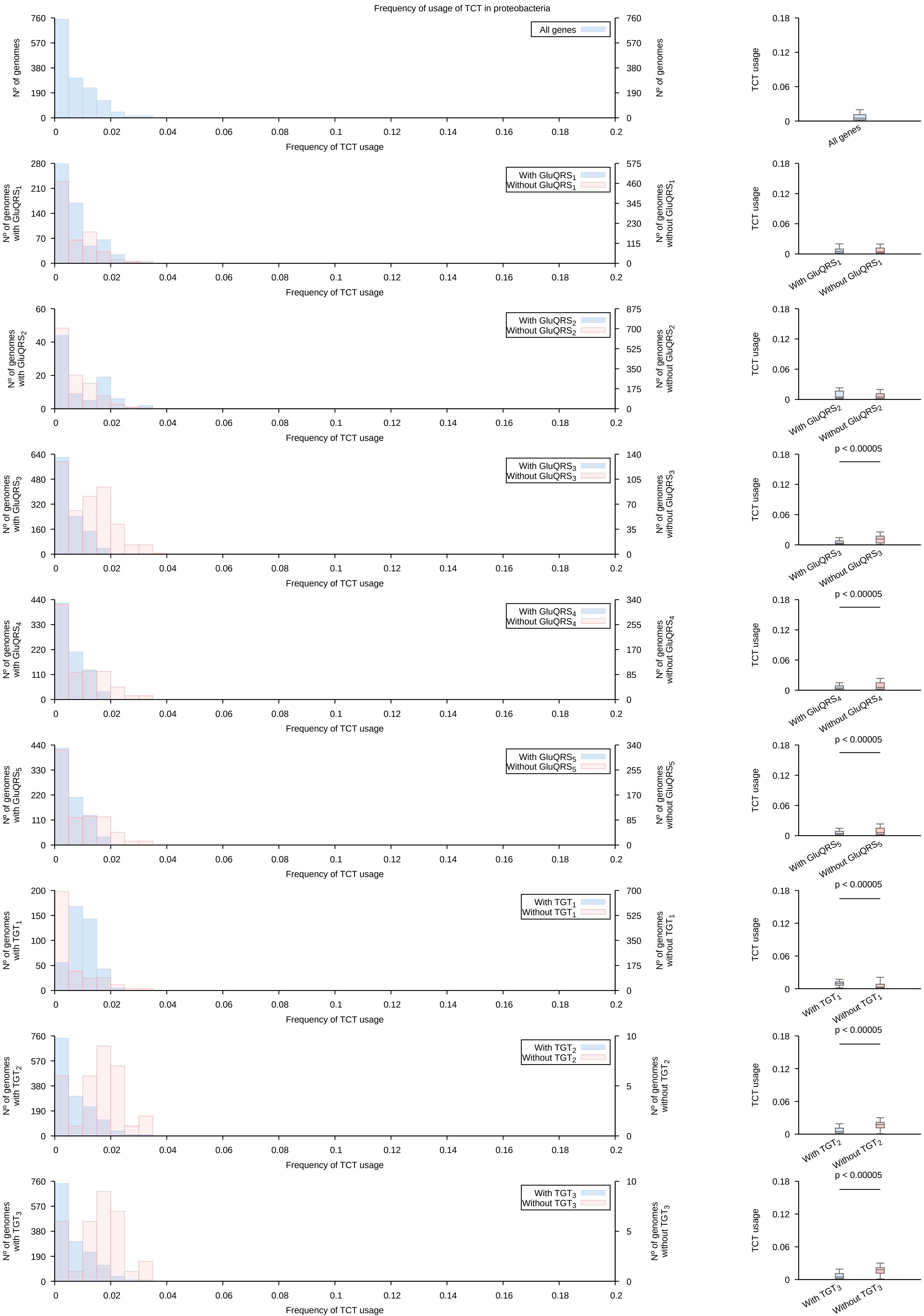
Frequency of usage of TCT in proteobacteria
All genes
Nº of genomes
Frequency of TCT usage
TCT usage
With GluQRS1
Without GluQRS1
Nº of genomes with GluQRS1
Nº of genomes without GluQRS1
With GluQRS2
Without GluQRS2
Nº of genomes with GluQRS2
Nº of genomes without GluQRS2
With GluQRS3
Without GluQRS3
Nº of genomes with GluQRS3
Nº of genomes without GluQRS3
With GluQRS4
Without GluQRS4
Nº of genomes with GluQRS4
Nº of genomes without GluQRS4
With GluQRS5
Without GluQRS5
Nº of genomes with GluQRS5
Nº of genomes without GluQRS5
With TGT1
Without TGT1
Nº of genomes with TGT1
Nº of genomes without TGT1
With TGT2
Without TGT2
Nº of genomes with TGT2
Nº of genomes without TGT2
With TGT3
Without TGT3
Nº of genomes with TGT3
Nº of genomes without TGT3
p < 0.00005

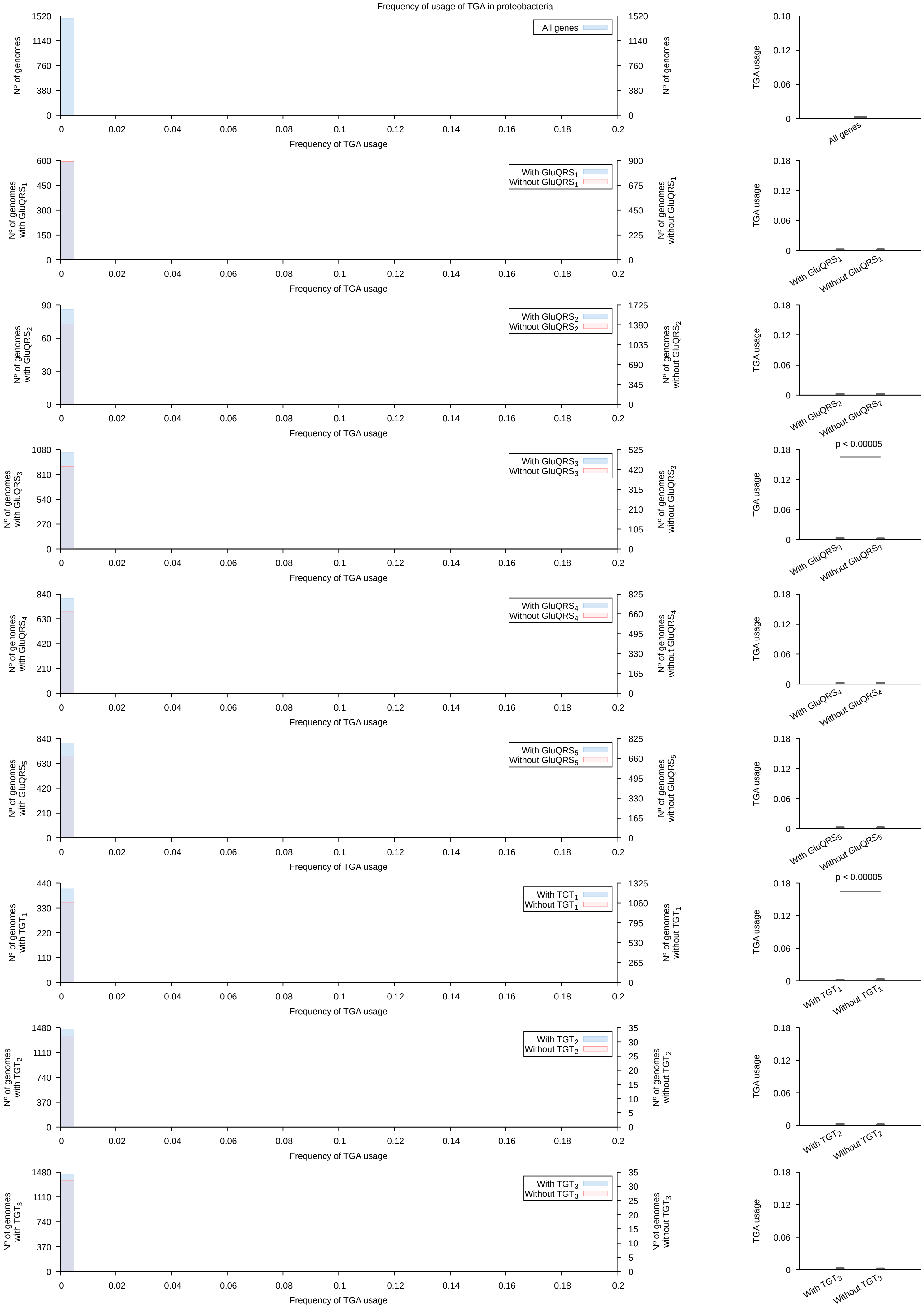

Frequency of usage of TGA in proteobacteria
All genes
With GluQRS$_1$
Without GluQRS$_1$
With GluQRS$_2$
Without GluQRS$_2$
With GluQRS$_3$
Without GluQRS$_3$
With GluQRS$_4$
Without GluQRS$_4$
With GluQRS$_5$
Without GluQRS$_5$
With TGT$_1$
Without TGT$_1$
With TGT$_2$
Without TGT$_2$
With TGT$_3$
Without TGT$_3$
Nº of genomes
Frequency of TGA usage
TGA usage
p < 0.00005
p < 0.00005

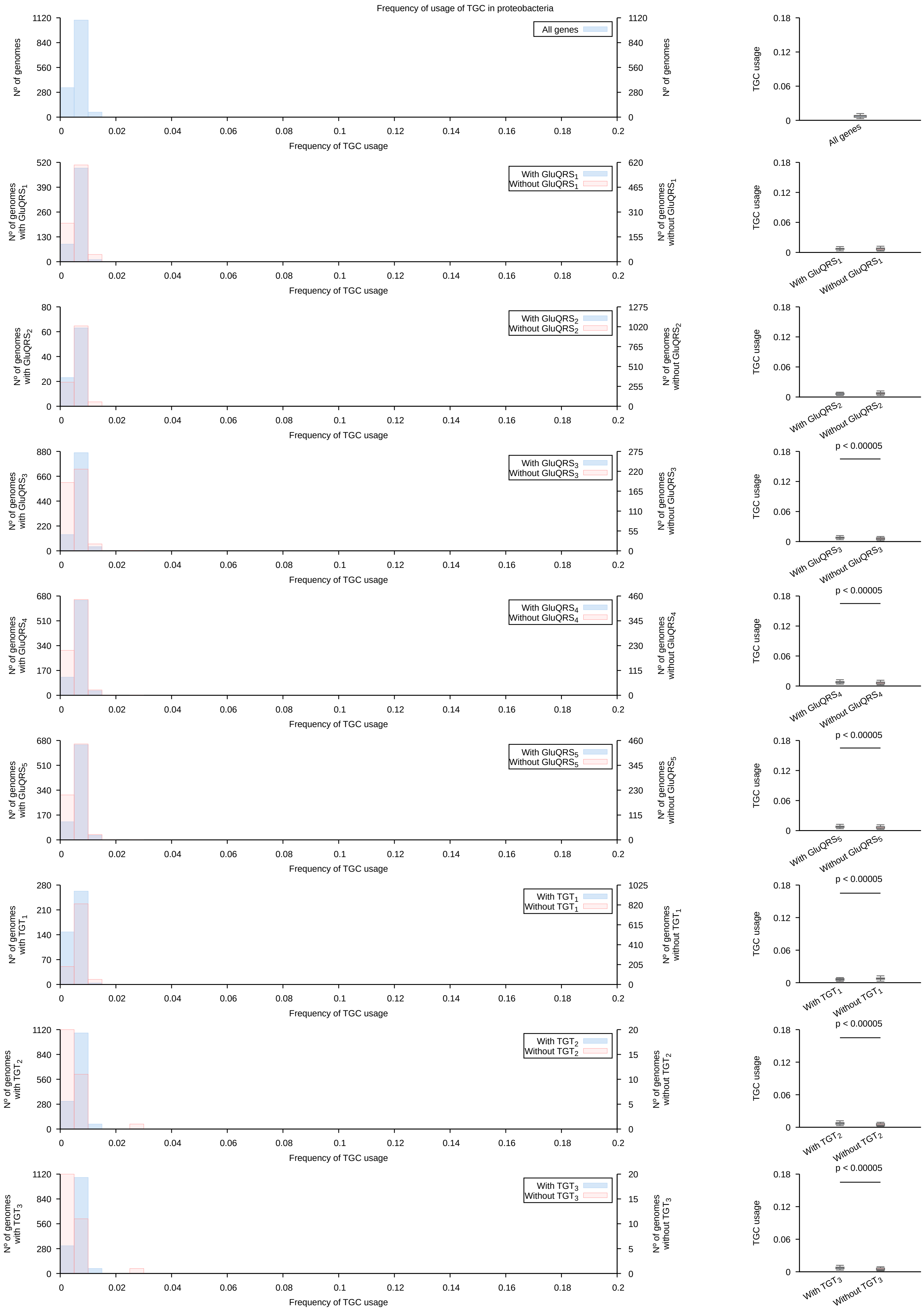
Frequency of usage of TGC in proteobacteria
Frequency of TGC usage
Nº of genomes
TGC usage
All genes
With GluQRS1
Without GluQRS1
With GluQRS2
Without GluQRS2
With GluQRS3
Without GluQRS3
With GluQRS4
Without GluQRS4
With GluQRS5
Without GluQRS5
With TGT1
Without TGT1
With TGT2
Without TGT2
With TGT3
Without TGT3
p < 0.00005

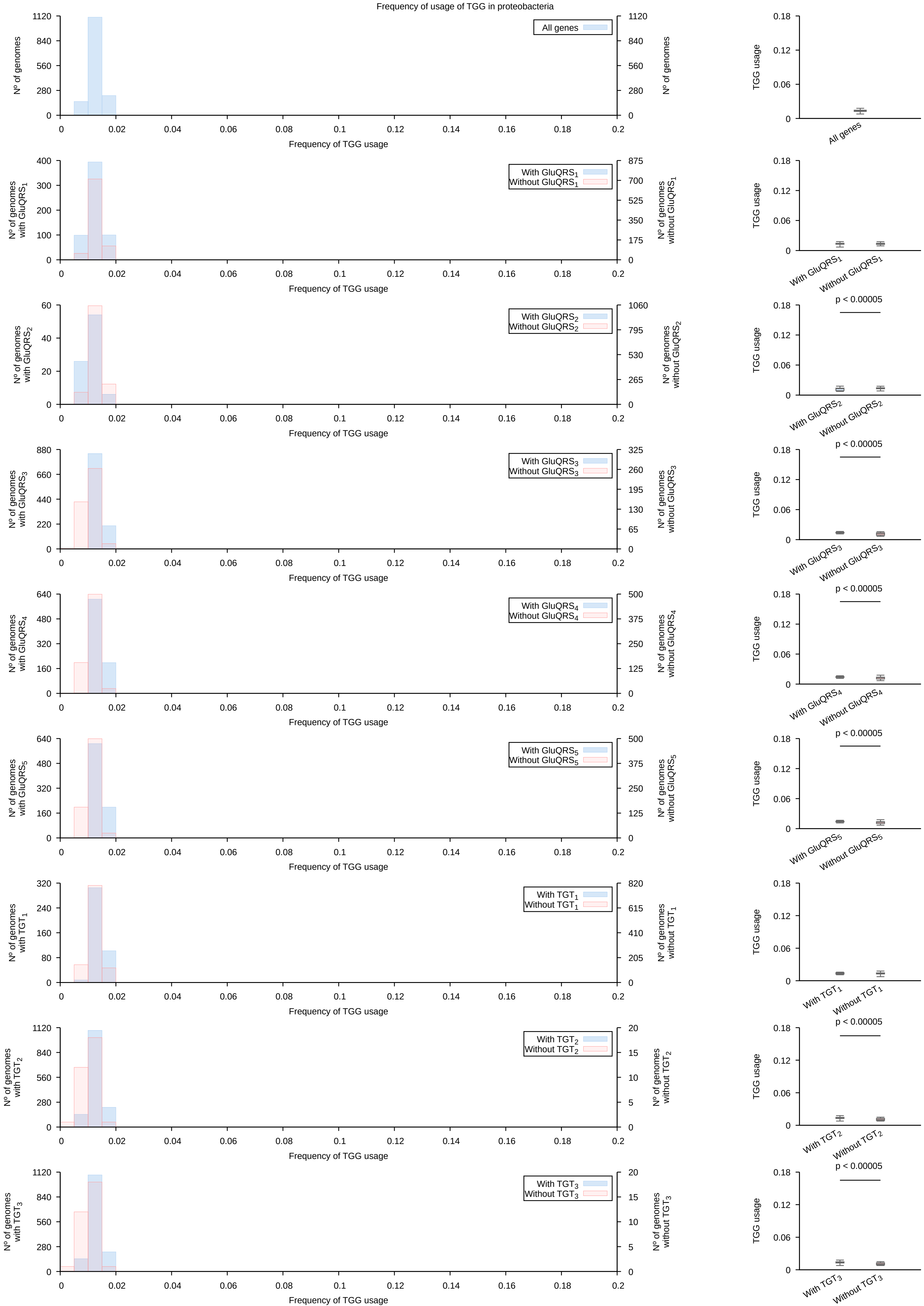
Frequency of usage of TGG in proteobacteria
All genes
With GluQRS1
Without GluQRS1
With GluQRS2
Without GluQRS2
With GluQRS3
Without GluQRS3
With GluQRS4
Without GluQRS4
With GluQRS5
Without GluQRS5
With TGT1
Without TGT1
With TGT2
Without TGT2
With TGT3
Without TGT3
Nº of genomes
Frequency of TGG usage
TGG usage
p < 0.00005

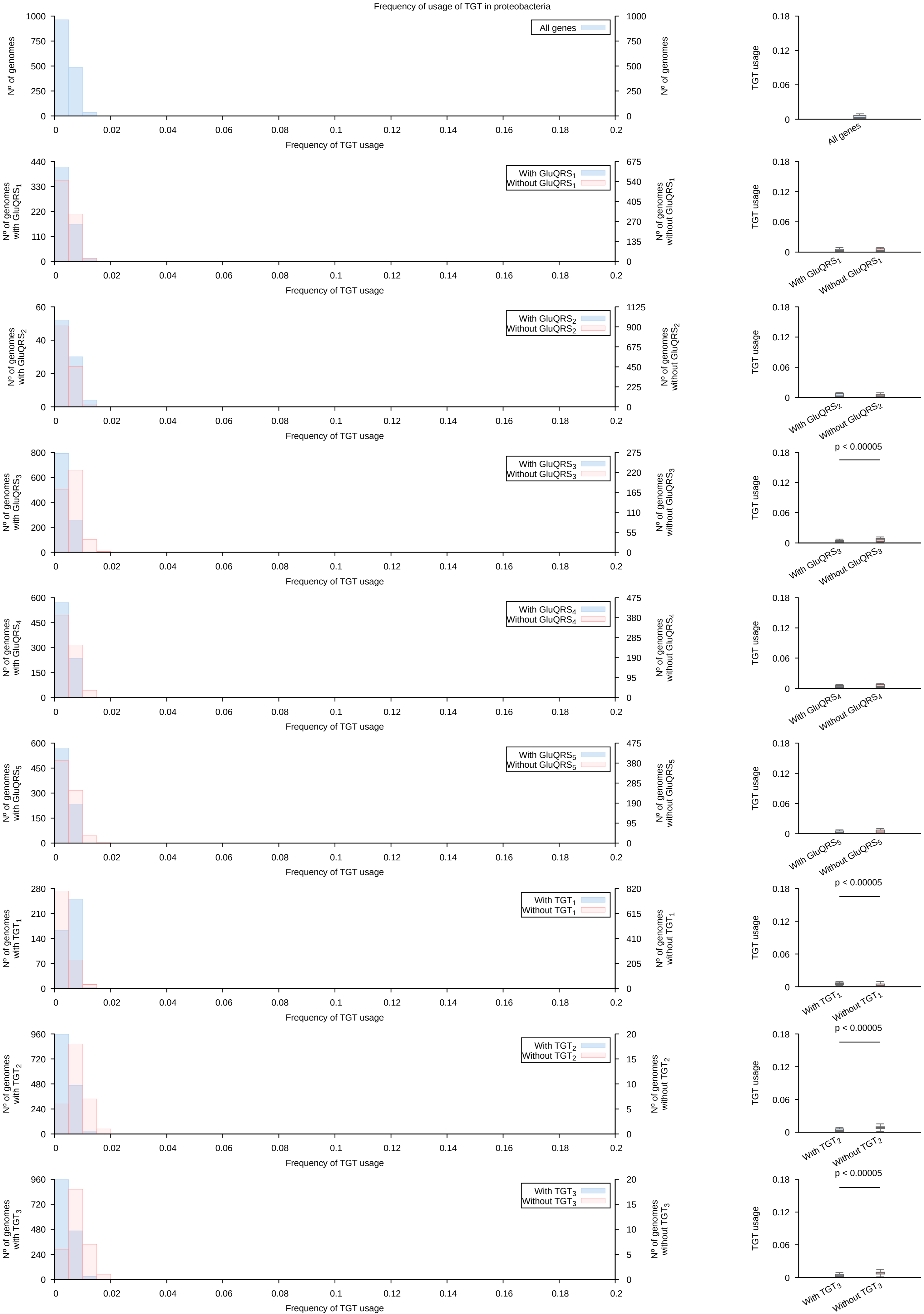
Frequency of usage of TGT in proteobacteria
Nº of genomes
Frequency of TGT usage
All genes
TGT usage
Nº of genomes with GluQRS$_1$
Nº of genomes without GluQRS$_1$
With GluQRS$_1$
Without GluQRS$_1$
Nº of genomes with GluQRS$_2$
Nº of genomes without GluQRS$_2$
With GluQRS$_2$
Without GluQRS$_2$
Nº of genomes with GluQRS$_3$
Nº of genomes without GluQRS$_3$
With GluQRS$_3$
Without GluQRS$_3$
p < 0.00005
Nº of genomes with GluQRS$_4$
Nº of genomes without GluQRS$_4$
With GluQRS$_4$
Without GluQRS$_4$
Nº of genomes with GluQRS$_5$
Nº of genomes without GluQRS$_5$
With GluQRS$_5$
Without GluQRS$_5$
Nº of genomes with TGT$_1$
Nº of genomes without TGT$_1$
With TGT$_1$
Without TGT$_1$
p < 0.00005
Nº of genomes with TGT$_2$
Nº of genomes without TGT$_2$
With TGT$_2$
Without TGT$_2$
p < 0.00005
Nº of genomes with TGT$_3$
Nº of genomes without TGT$_3$
With TGT$_3$
Without TGT$_3$
p < 0.00005

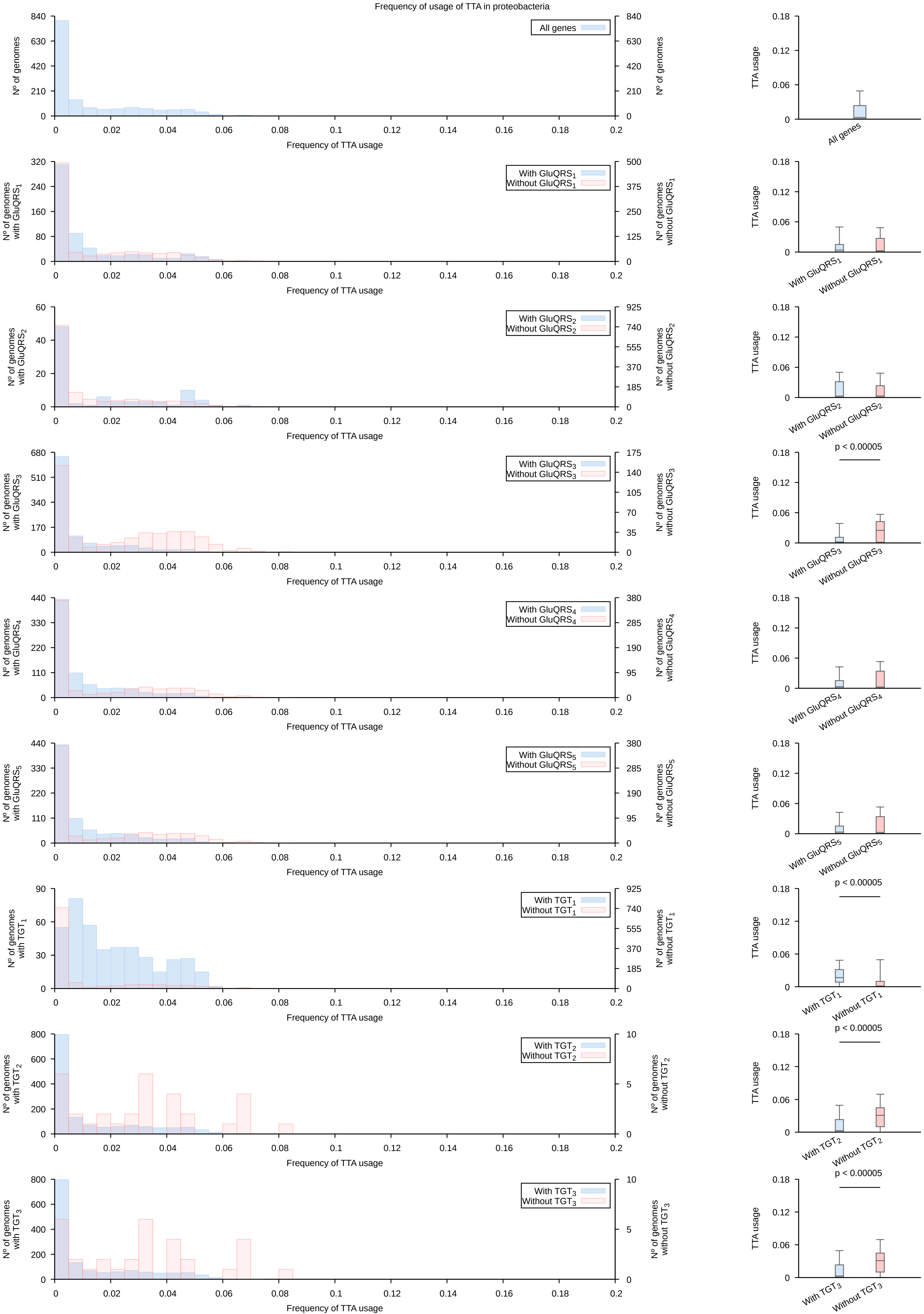
Frequency of usage of TTA in proteobacteria
All genes
With GluQRS$_1$
Without GluQRS$_1$
With GluQRS$_2$
Without GluQRS$_2$
With GluQRS$_3$
Without GluQRS$_3$
With GluQRS$_4$
Without GluQRS$_4$
With GluQRS$_5$
Without GluQRS$_5$
With TGT$_1$
Without TGT$_1$
With TGT$_2$
Without TGT$_2$
With TGT$_3$
Without TGT$_3$
Nº of genomes
Nº of genomes with GluQRS$_1$
Nº of genomes without GluQRS$_1$
Nº of genomes with GluQRS$_2$
Nº of genomes without GluQRS$_2$
Nº of genomes with GluQRS$_3$
Nº of genomes without GluQRS$_3$
Nº of genomes with GluQRS$_4$
Nº of genomes without GluQRS$_4$
Nº of genomes with GluQRS$_5$
Nº of genomes without GluQRS$_5$
Nº of genomes with TGT$_1$
Nº of genomes without TGT$_1$
Nº of genomes with TGT$_2$
Nº of genomes without TGT$_2$
Nº of genomes with TGT$_3$
Nº of genomes without TGT$_3$
Frequency of TTA usage
TTA usage
$p < 0.00005$
0
0.02
0.04
0.06
0.08
0.1
0.12
0.14
0.16
0.18
0.2

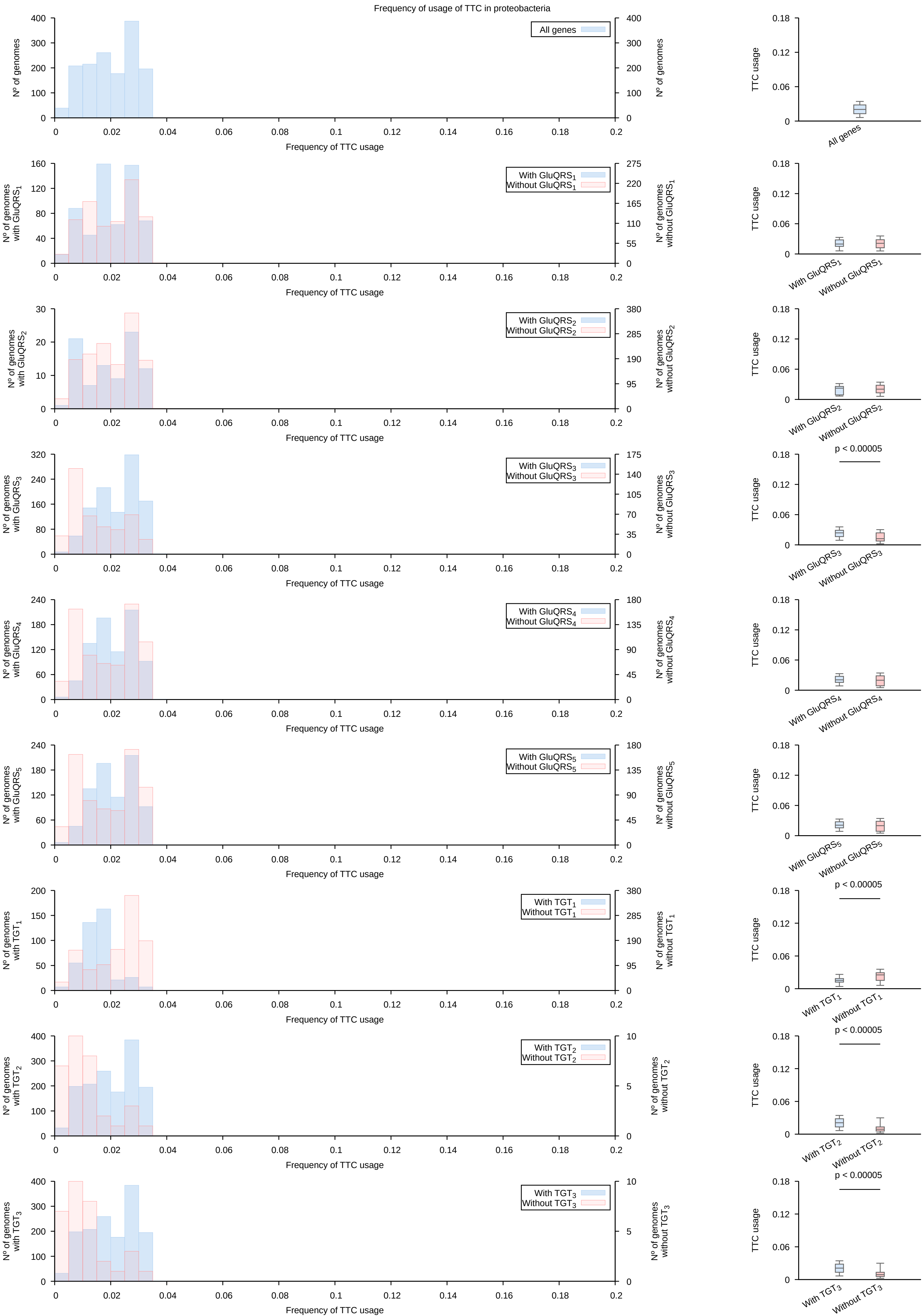
Frequency of usage of TTC in proteobacteria
All genes
With GluQRS$_1$
Without GluQRS$_1$
With GluQRS$_2$
Without GluQRS$_2$
With GluQRS$_3$
Without GluQRS$_3$
With GluQRS$_4$
Without GluQRS$_4$
With GluQRS$_5$
Without GluQRS$_5$
With TGT$_1$
Without TGT$_1$
With TGT$_2$
Without TGT$_2$
With TGT$_3$
Without TGT$_3$
Nº of genomes
Nº of genomes with GluQRS$_1$
Nº of genomes without GluQRS$_1$
Nº of genomes with GluQRS$_2$
Nº of genomes without GluQRS$_2$
Nº of genomes with GluQRS$_3$
Nº of genomes without GluQRS$_3$
Nº of genomes with GluQRS$_4$
Nº of genomes without GluQRS$_4$
Nº of genomes with GluQRS$_5$
Nº of genomes without GluQRS$_5$
Nº of genomes with TGT$_1$
Nº of genomes without TGT$_1$
Nº of genomes with TGT$_2$
Nº of genomes without TGT$_2$
Nº of genomes with TGT$_3$
Nº of genomes without TGT$_3$
Frequency of TTC usage
TTC usage
p < 0.00005
0
0.02
0.04
0.06
0.08
0.1
0.12
0.14
0.16
0.18
0.2

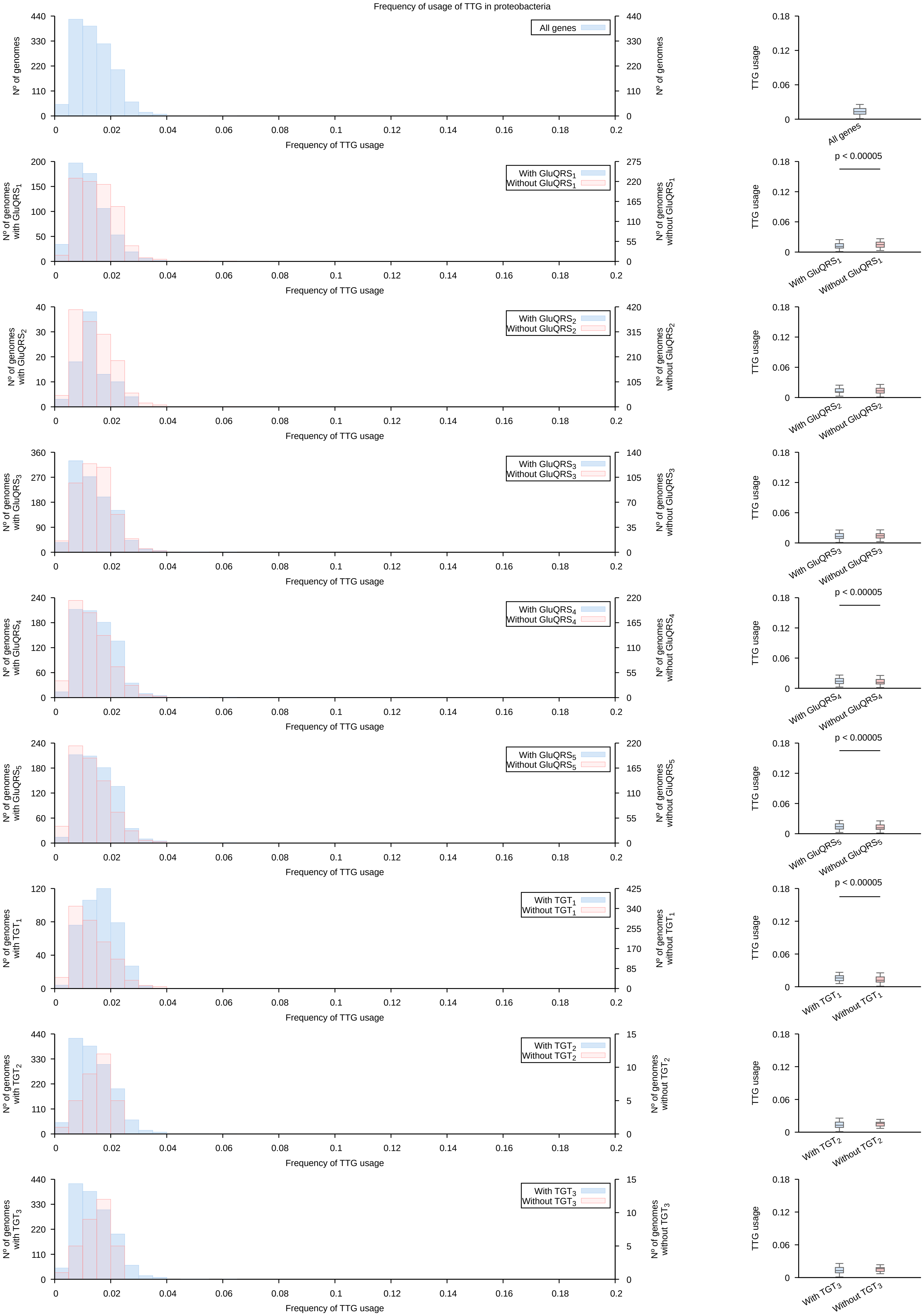

Frequency of usage of TTG in proteobacteria
Nº of genomes
Frequency of TTG usage
TTG usage
All genes
Nº of genomes with GluQRS$_1$
Nº of genomes without GluQRS$_1$
With GluQRS$_1$
Without GluQRS$_1$
p < 0.00005
Nº of genomes with GluQRS$_2$
Nº of genomes without GluQRS$_2$
With GluQRS$_2$
Without GluQRS$_2$
Nº of genomes with GluQRS$_3$
Nº of genomes without GluQRS$_3$
With GluQRS$_3$
Without GluQRS$_3$
Nº of genomes with GluQRS$_4$
Nº of genomes without GluQRS$_4$
With GluQRS$_4$
Without GluQRS$_4$
p < 0.00005
Nº of genomes with GluQRS$_5$
Nº of genomes without GluQRS$_5$
With GluQRS$_5$
Without GluQRS$_5$
p < 0.00005
Nº of genomes with TGT$_1$
Nº of genomes without TGT$_1$
With TGT$_1$
Without TGT$_1$
p < 0.00005
Nº of genomes with TGT$_2$
Nº of genomes without TGT$_2$
With TGT$_2$
Without TGT$_2$
Nº of genomes with TGT$_3$
Nº of genomes without TGT$_3$
With TGT$_3$
Without TGT$_3$

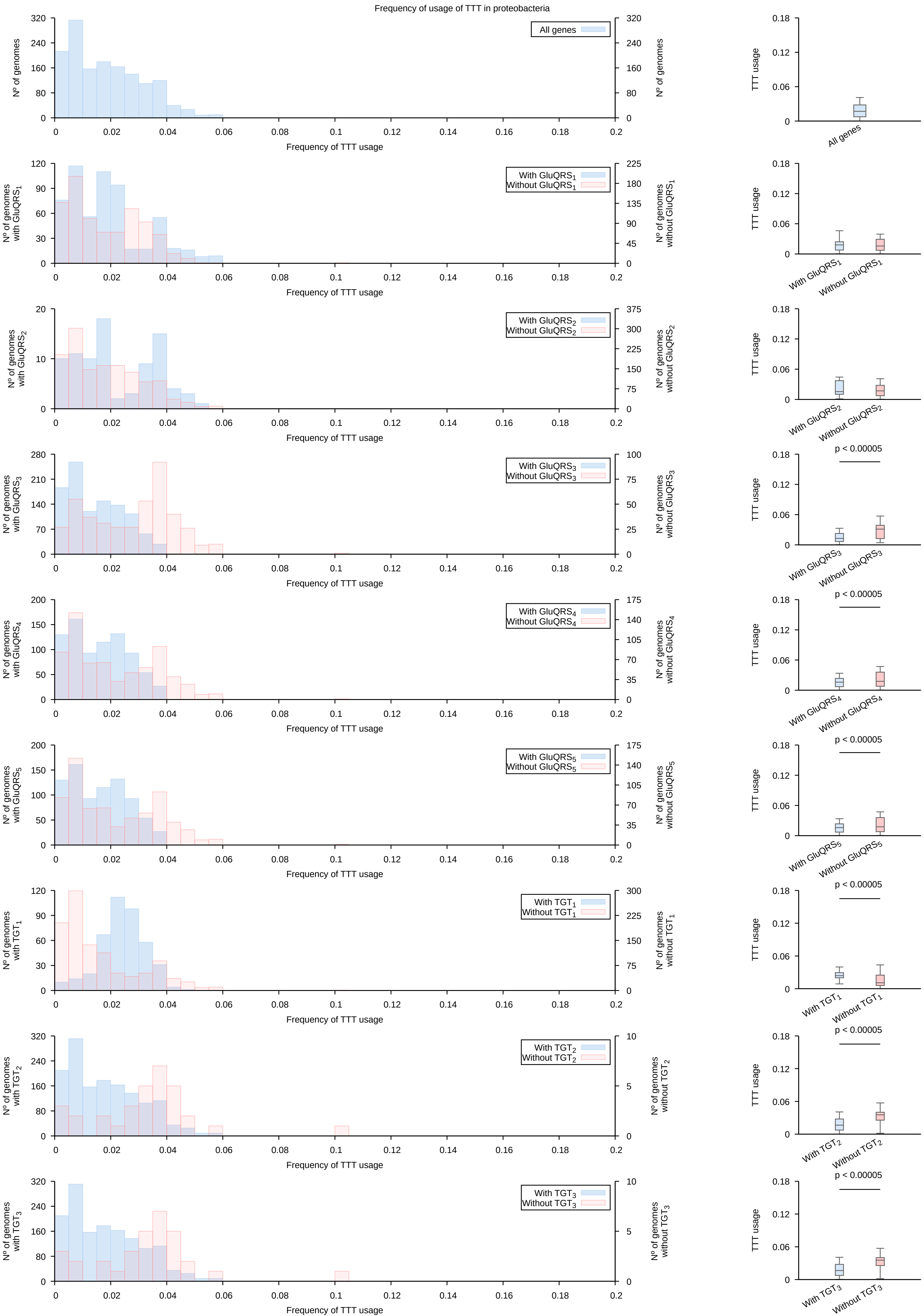
Frequency of usage of TTT in proteobacteria
Nº of genomes
All genes
TTT usage
Frequency of TTT usage
Nº of genomes with $GluQRS_1$
Nº of genomes without $GluQRS_1$
With $GluQRS_1$
Without $GluQRS_1$
Nº of genomes with $GluQRS_2$
Nº of genomes without $GluQRS_2$
With $GluQRS_2$
Without $GluQRS_2$
Nº of genomes with $GluQRS_3$
Nº of genomes without $GluQRS_3$
With $GluQRS_3$
Without $GluQRS_3$
$p < 0.00005$
Nº of genomes with $GluQRS_4$
Nº of genomes without $GluQRS_4$
With $GluQRS_4$
Without $GluQRS_4$
$p < 0.00005$
Nº of genomes with $GluQRS_5$
Nº of genomes without $GluQRS_5$
With $GluQRS_5$
Without $GluQRS_5$
$p < 0.00005$
Nº of genomes with TGT_1
Nº of genomes without TGT_1
With TGT_1
Without TGT_1
$p < 0.00005$
Nº of genomes with TGT_2
Nº of genomes without TGT_2
With TGT_2
Without TGT_2
$p < 0.00005$
Nº of genomes with TGT_3
Nº of genomes without TGT_3
With TGT_3
Without TGT_3
$p < 0.00005$
