# Supplementary material for: Impact of the chemical modification of tRNAs anticodon loop on the variability and evolution of codon usage in proteobacteria
Source: Front Microbiol. 2024 Aug 5;15:1412318. doi: 10.3389/fmicb.2024.1412318 (PMC11332805; doi:10.3389/fmicb.2024.1412318)
Supplement: Supplementary file 1 [file Data_Sheet_1.zip › Supp_figures/Fig_S17.pdf]

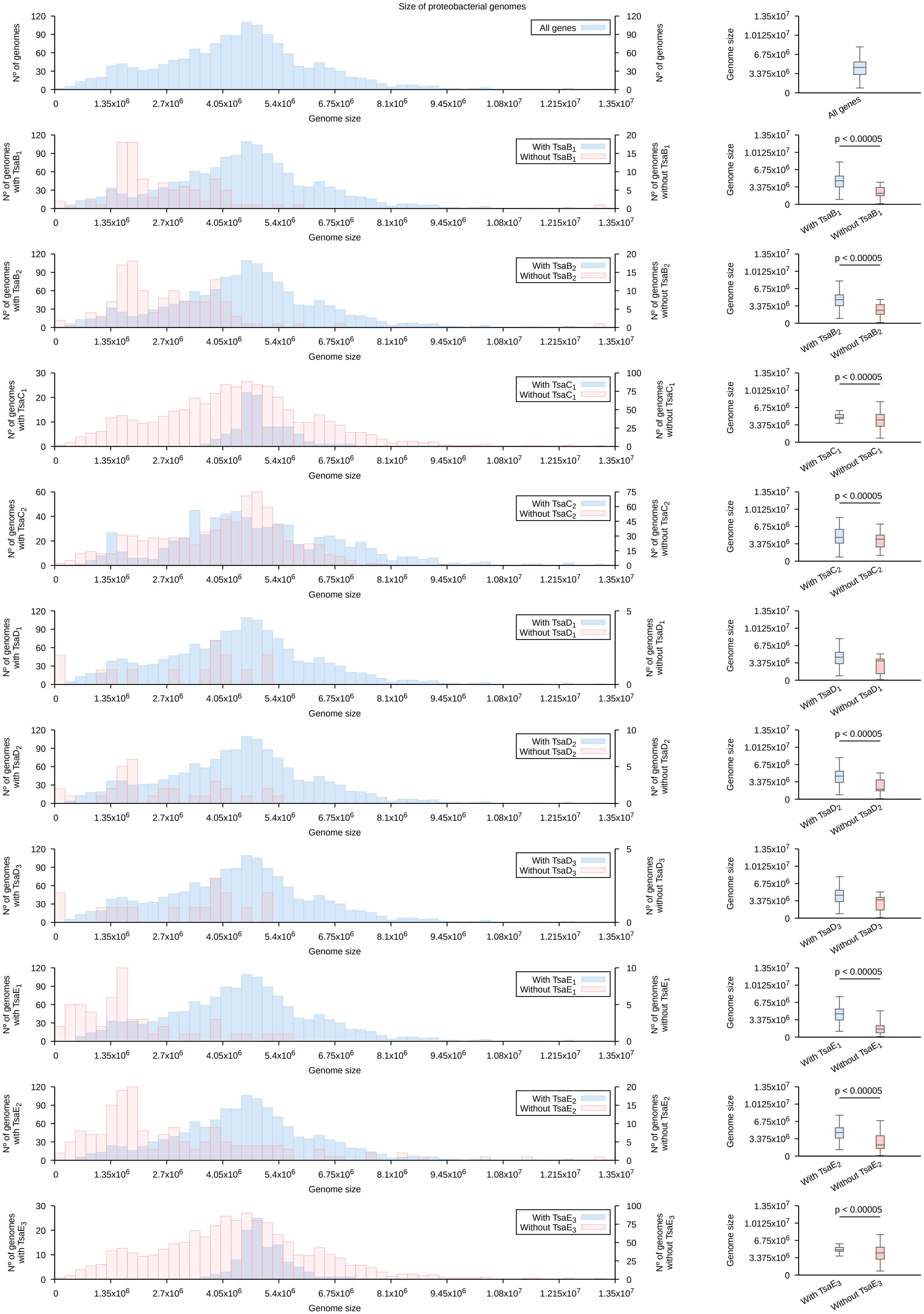

Size of proteobacterial genomes
Nº of genomes
All genes
Genome size
0
1.35x10^6
2.7x10^6
4.05x10^6
5.4x10^6
6.75x10^6
8.1x10^6
9.45x10^6
1.08x10^7
1.215x10^7
1.35x10^7
3.375x10^6
1.0125x10^7
Nº of genomes with TsaB$_1$
Nº of genomes without TsaB$_1$
With TsaB$_1$
Without TsaB$_1$
p < 0.00005
Nº of genomes with TsaB$_2$
Nº of genomes without TsaB$_2$
With TsaB$_2$
Without TsaB$_2$
p < 0.00005
Nº of genomes with TsaC$_1$
Nº of genomes without TsaC$_1$
With TsaC$_1$
Without TsaC$_1$
p < 0.00005
Nº of genomes with TsaC$_2$
Nº of genomes without TsaC$_2$
With TsaC$_2$
Without TsaC$_2$
p < 0.00005
Nº of genomes with TsaD$_1$
Nº of genomes without TsaD$_1$
With TsaD$_1$
Without TsaD$_1$
Nº of genomes with TsaD$_2$
Nº of genomes without TsaD$_2$
With TsaD$_2$
Without TsaD$_2$
p < 0.00005
Nº of genomes with TsaD$_3$
Nº of genomes without TsaD$_3$
With TsaD$_3$
Without TsaD$_3$
Nº of genomes with TsaE$_1$
Nº of genomes without TsaE$_1$
With TsaE$_1$
Without TsaE$_1$
p < 0.00005
Nº of genomes with TsaE$_2$
Nº of genomes without TsaE$_2$
With TsaE$_2$
Without TsaE$_2$
p < 0.00005
Nº of genomes with TsaE$_3$
Nº of genomes without TsaE$_3$
With TsaE$_3$
Without TsaE$_3$
p < 0.00005

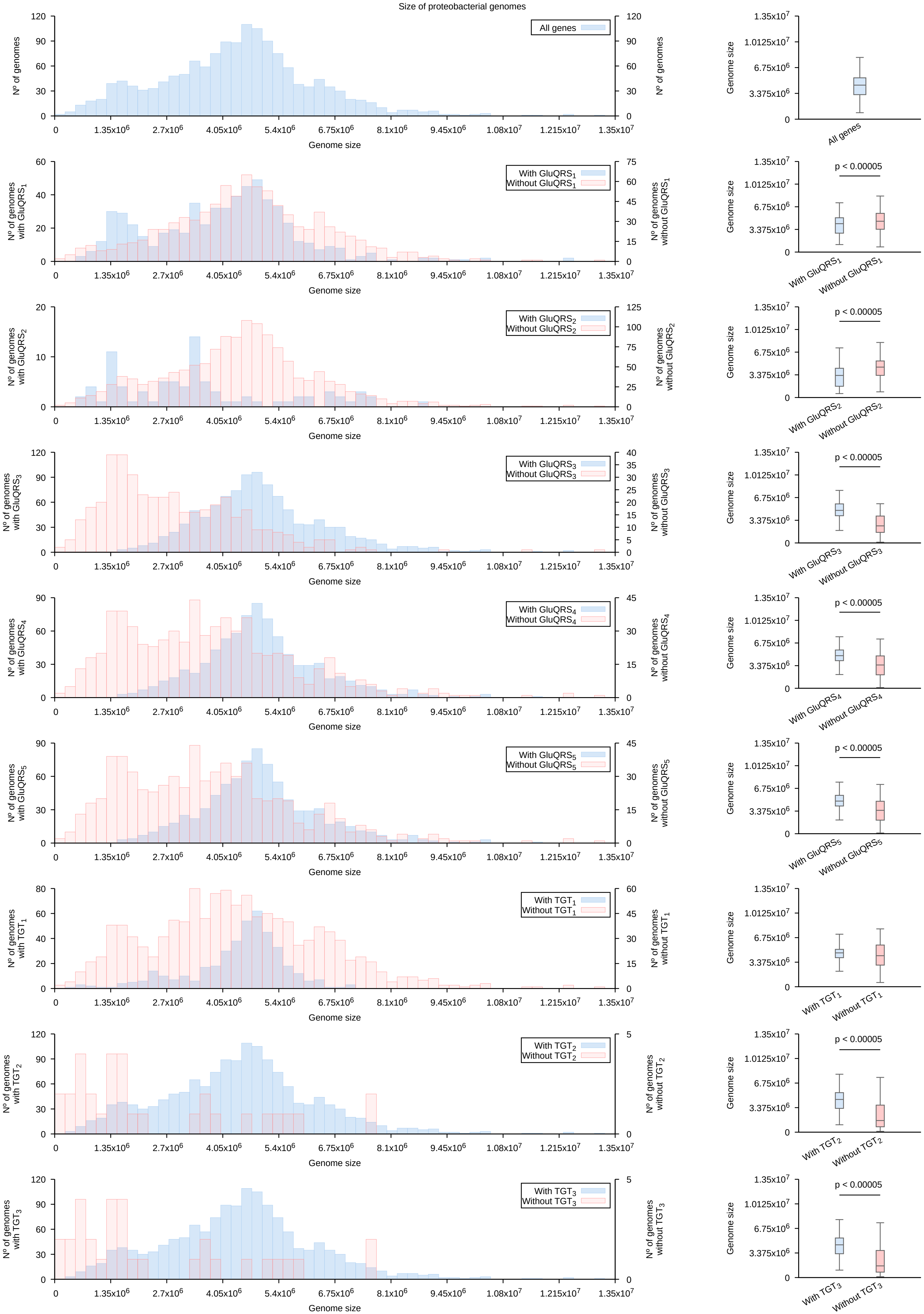

Size of proteobacterial genomes
All genes
Nº of genomes
Genome size
0
1.35x10^6
2.7x10^6
4.05x10^6
5.4x10^6
6.75x10^6
8.1x10^6
9.45x10^6
1.08x10^7
1.215x10^7
1.35x10^7
3.375x10^6
1.0125x10^7
With GluQRS$_1$
Without GluQRS$_1$
Nº of genomes with GluQRS$_1$
Nº of genomes without GluQRS$_1$
With GluQRS$_2$
Without GluQRS$_2$
Nº of genomes with GluQRS$_2$
Nº of genomes without GluQRS$_2$
With GluQRS$_3$
Without GluQRS$_3$
Nº of genomes with GluQRS$_3$
Nº of genomes without GluQRS$_3$
With GluQRS$_4$
Without GluQRS$_4$
Nº of genomes with GluQRS$_4$
Nº of genomes without GluQRS$_4$
With GluQRS$_5$
Without GluQRS$_5$
Nº of genomes with GluQRS$_5$
Nº of genomes without GluQRS$_5$
With TGT$_1$
Without TGT$_1$
Nº of genomes with TGT$_1$
Nº of genomes without TGT$_1$
With TGT$_2$
Without TGT$_2$
Nº of genomes with TGT$_2$
Nº of genomes without TGT$_2$
With TGT$_3$
Without TGT$_3$
Nº of genomes with TGT$_3$
Nº of genomes without TGT$_3$
p < 0.00005

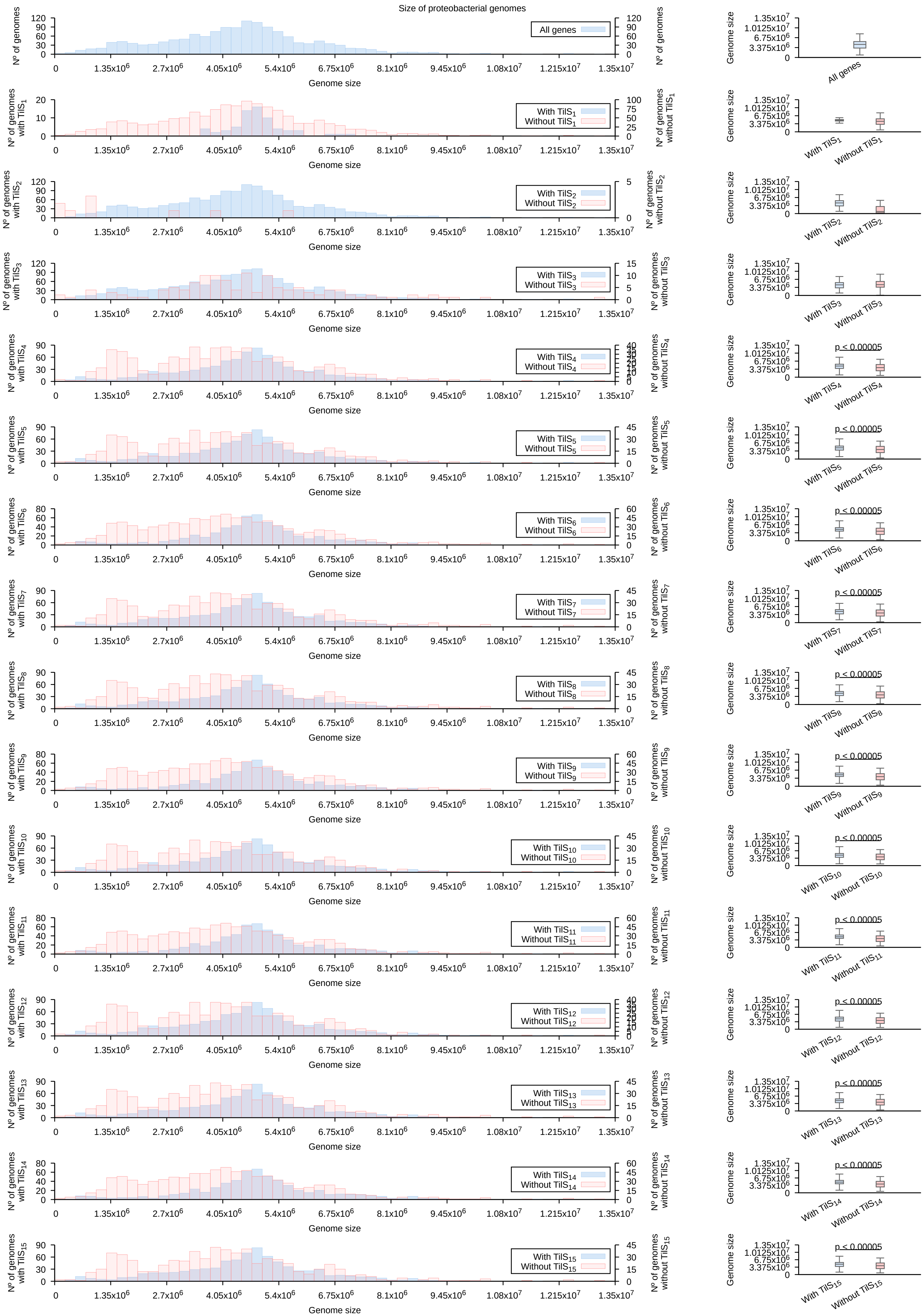
Size of proteobacterial genomes
Nº of genomes
Genome size
All genes
0
1.35x10^6
2.7x10^6
4.05x10^6
5.4x10^6
6.75x10^6
8.1x10^6
9.45x10^6
1.08x10^7
1.215x10^7
1.35x10^7
1.0125x10^7
3.375x10^6
Nº of genomes with TilS1
Nº of genomes without TilS1
With TilS1
Without TilS1
Nº of genomes with TilS2
Nº of genomes without TilS2
With TilS2
Without TilS2
Nº of genomes with TilS3
Nº of genomes without TilS3
With TilS3
Without TilS3
Nº of genomes with TilS4
Nº of genomes without TilS4
With TilS4
Without TilS4
Nº of genomes with TilS5
Nº of genomes without TilS5
With TilS5
Without TilS5
Nº of genomes with TilS6
Nº of genomes without TilS6
With TilS6
Without TilS6
Nº of genomes with TilS7
Nº of genomes without TilS7
With TilS7
Without TilS7
Nº of genomes with TilS8
Nº of genomes without TilS8
With TilS8
Without TilS8
Nº of genomes with TilS9
Nº of genomes without TilS9
With TilS9
Without TilS9
Nº of genomes with TilS10
Nº of genomes without TilS10
With TilS10
Without TilS10
Nº of genomes with TilS11
Nº of genomes without TilS11
With TilS11
Without TilS11
Nº of genomes with TilS12
Nº of genomes without TilS12
With TilS12
Without TilS12
Nº of genomes with TilS13
Nº of genomes without TilS13
With TilS13
Without TilS13
Nº of genomes with TilS14
Nº of genomes without TilS14
With TilS14
Without TilS14
Nº of genomes with TilS15
Nº of genomes without TilS15
With TilS15
Without TilS15
p < 0.00005

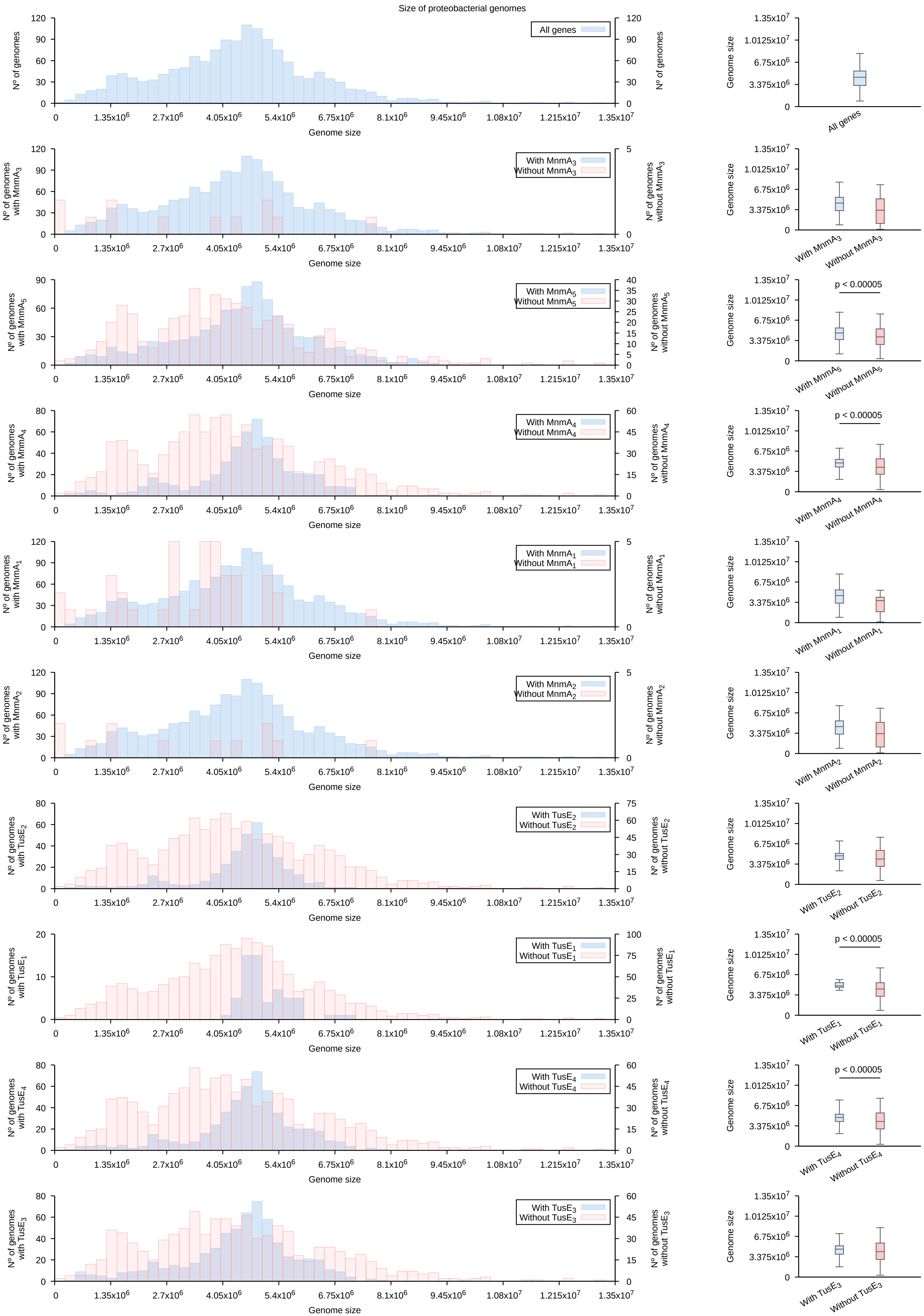
Size of proteobacterial genomes
All genes
Nº of genomes
Genome size
0
1.35x10^6
2.7x10^6
4.05x10^6
5.4x10^6
6.75x10^6
8.1x10^6
9.45x10^6
1.08x10^7
1.215x10^7
1.35x10^7
3.375x10^6
1.0125x10^7
With MnmA3
Without MnmA3
Nº of genomes with MnmA3
Nº of genomes without MnmA3
With MnmA5
Without MnmA5
Nº of genomes with MnmA5
Nº of genomes without MnmA5
p < 0.00005
With MnmA4
Without MnmA4
Nº of genomes with MnmA4
Nº of genomes without MnmA4
p < 0.00005
With MnmA1
Without MnmA1
Nº of genomes with MnmA1
Nº of genomes without MnmA1
With MnmA2
Without MnmA2
Nº of genomes with MnmA2
Nº of genomes without MnmA2
With TusE2
Without TusE2
Nº of genomes with TusE2
Nº of genomes without TusE2
With TusE1
Without TusE1
Nº of genomes with TusE1
Nº of genomes without TusE1
p < 0.00005
With TusE4
Without TusE4
Nº of genomes with TusE4
Nº of genomes without TusE4
p < 0.00005
With TusE3
Without TusE3
Nº of genomes with TusE3
Nº of genomes without TusE3
