# Supplementary material for: Impact of the chemical modification of tRNAs anticodon loop on the variability and evolution of codon usage in proteobacteria
Source: Front Microbiol. 2024 Aug 5;15:1412318. doi: 10.3389/fmicb.2024.1412318 (PMC11332805; doi:10.3389/fmicb.2024.1412318)
Supplement: Supplementary file 1 [file Data_Sheet_1.zip › Supp_figures/Fig_S18A.pdf]

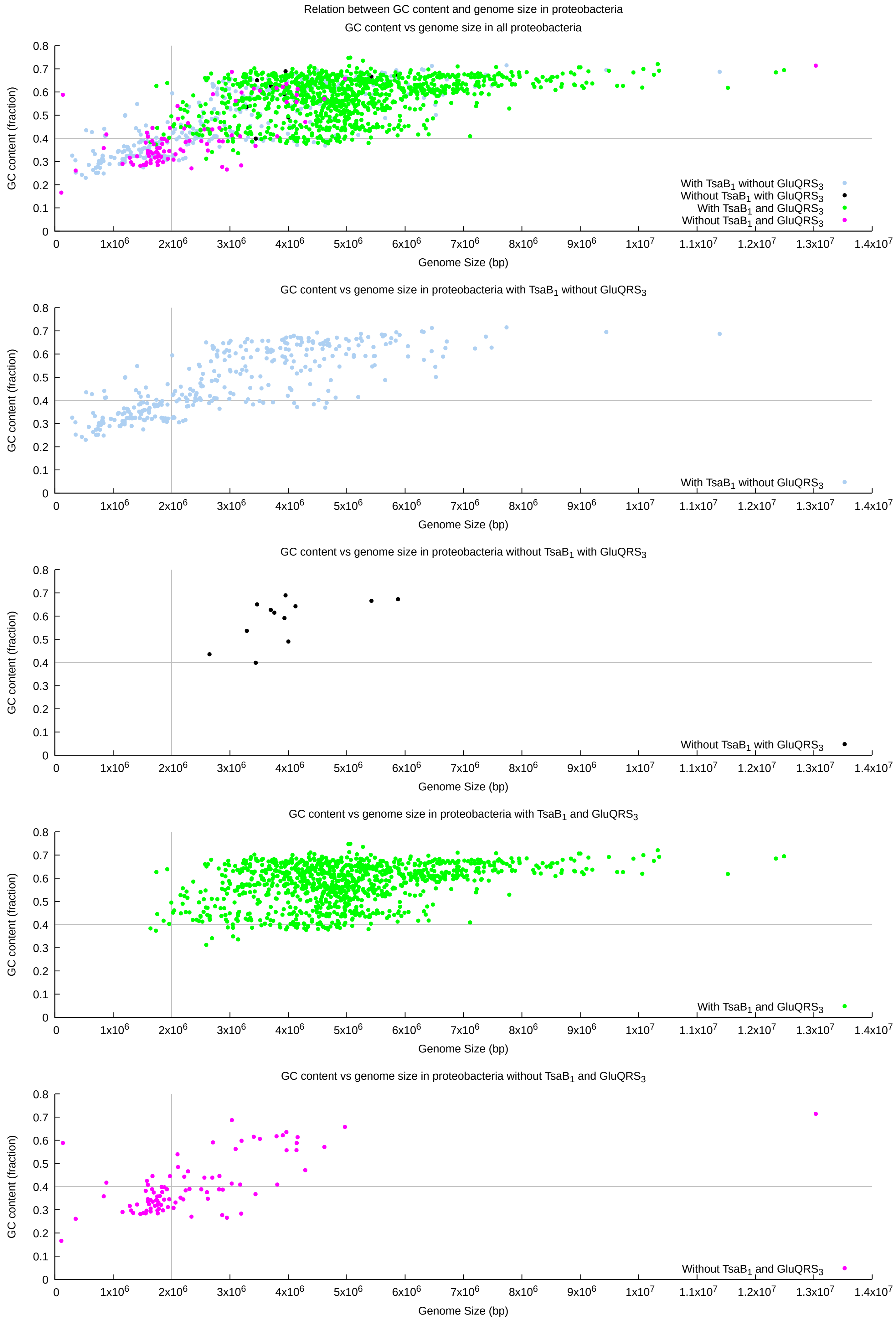

Relation between GC content and genome size in proteobacteria
GC content vs genome size in all proteobacteria
GC content (fraction)
Genome Size (bp)
With TsaB$_1$ without GluQRS$_3$
Without TsaB$_1$ with GluQRS$_3$
With TsaB$_1$ and GluQRS$_3$
Without TsaB$_1$ and GluQRS$_3$
GC content vs genome size in proteobacteria with TsaB$_1$ without GluQRS$_3$
GC content vs genome size in proteobacteria without TsaB$_1$ with GluQRS$_3$
GC content vs genome size in proteobacteria with TsaB$_1$ and GluQRS$_3$
GC content vs genome size in proteobacteria without TsaB$_1$ and GluQRS$_3$
0
0.1
0.2
0.3
0.4
0.5
0.6
0.7
0.8
$1x10^6$
$2x10^6$
$3x10^6$
$4x10^6$
$5x10^6$
$6x10^6$
$7x10^6$
$8x10^6$
$9x10^6$
$1x10^7$
$1.1x10^7$
$1.2x10^7$
$1.3x10^7$
$1.4x10^7$
