# Supplementary material for: Impact of the chemical modification of tRNAs anticodon loop on the variability and evolution of codon usage in proteobacteria
Source: Front Microbiol. 2024 Aug 5;15:1412318. doi: 10.3389/fmicb.2024.1412318 (PMC11332805; doi:10.3389/fmicb.2024.1412318)
Supplement: Supplementary file 1 [file Data_Sheet_1.zip › Supp_figures/Fig_S15.pdf]

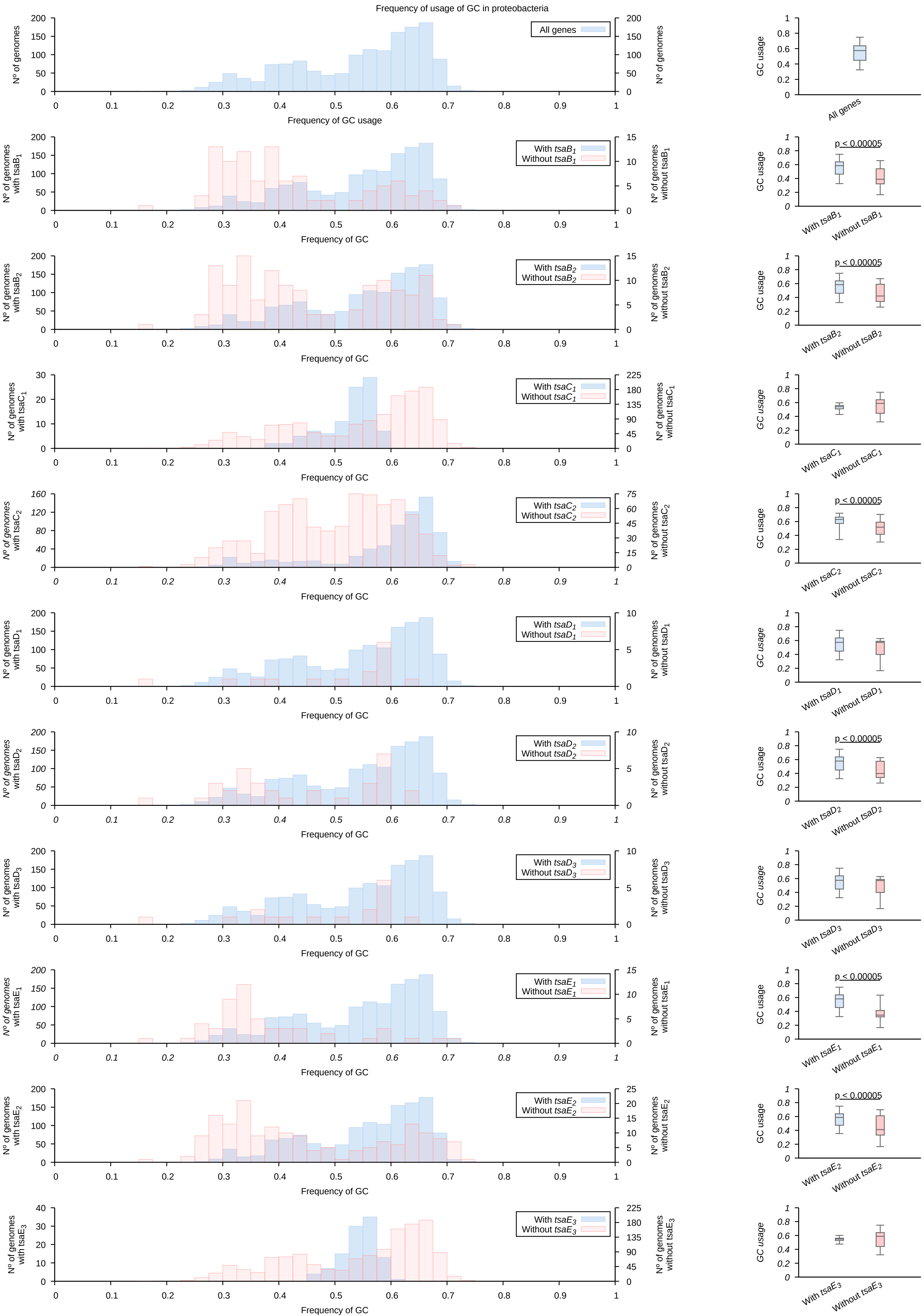

Frequency of usage of GC in proteobacteria
Nº of genomes
Frequency of GC usage
All genes
GC usage
Nº of genomes with tsaB1
Nº of genomes without tsaB1
With tsaB1
Without tsaB1
p < 0.00005
Nº of genomes with tsaB2
Nº of genomes without tsaB2
With tsaB2
Without tsaB2
p < 0.00005
Nº of genomes with tsaC1
Nº of genomes without tsaC1
With tsaC1
Without tsaC1
Nº of genomes with tsaC2
Nº of genomes without tsaC2
With tsaC2
Without tsaC2
p < 0.00005
Nº of genomes with tsaD1
Nº of genomes without tsaD1
With tsaD1
Without tsaD1
Nº of genomes with tsaD2
Nº of genomes without tsaD2
With tsaD2
Without tsaD2
p < 0.00005
Nº of genomes with tsaD3
Nº of genomes without tsaD3
With tsaD3
Without tsaD3
Nº of genomes with tsaE1
Nº of genomes without tsaE1
With tsaE1
Without tsaE1
p < 0.00005
Nº of genomes with tsaE2
Nº of genomes without tsaE2
With tsaE2
Without tsaE2
p < 0.00005
Nº of genomes with tsaE3
Nº of genomes without tsaE3
With tsaE3
Without tsaE3
Frequency of GC

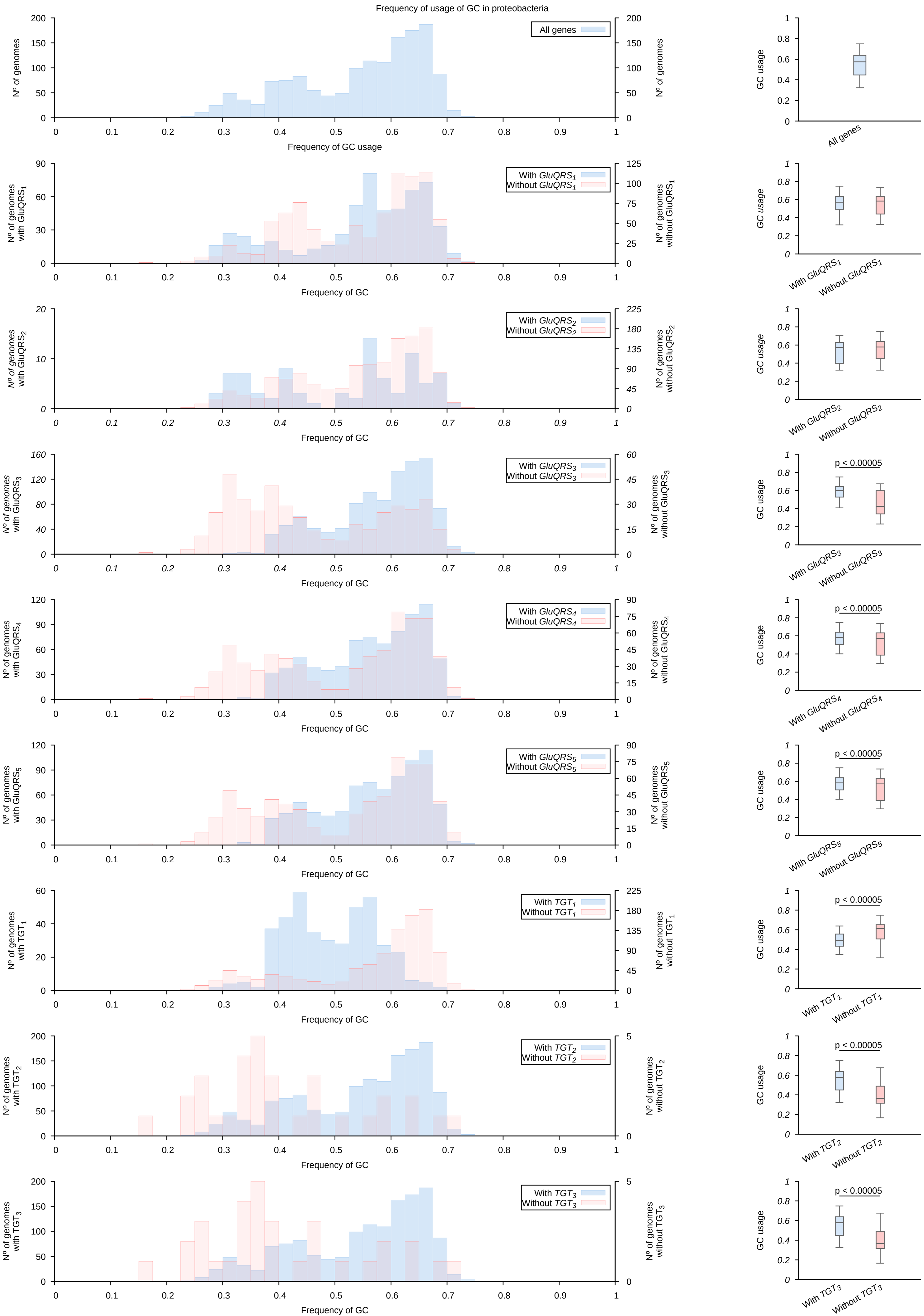

Frequency of usage of GC in proteobacteria
All genes
Nº of genomes
Frequency of GC usage
GC usage
With GluQRS1
Without GluQRS1
Nº of genomes with GluQRS1
Nº of genomes without GluQRS1
Frequency of GC
With GluQRS2
Without GluQRS2
Nº of genomes with GluQRS2
Nº of genomes without GluQRS2
With GluQRS3
Without GluQRS3
Nº of genomes with GluQRS3
Nº of genomes without GluQRS3
p < 0.00005
With GluQRS4
Without GluQRS4
Nº of genomes with GluQRS4
Nº of genomes without GluQRS4
p < 0.00005
With GluQRS5
Without GluQRS5
Nº of genomes with GluQRS5
Nº of genomes without GluQRS5
p < 0.00005
With TGT1
Without TGT1
Nº of genomes with TGT1
Nº of genomes without TGT1
p < 0.00005
With TGT2
Without TGT2
Nº of genomes with TGT2
Nº of genomes without TGT2
p < 0.00005
With TGT3
Without TGT3
Nº of genomes with TGT3
Nº of genomes without TGT3
p < 0.00005

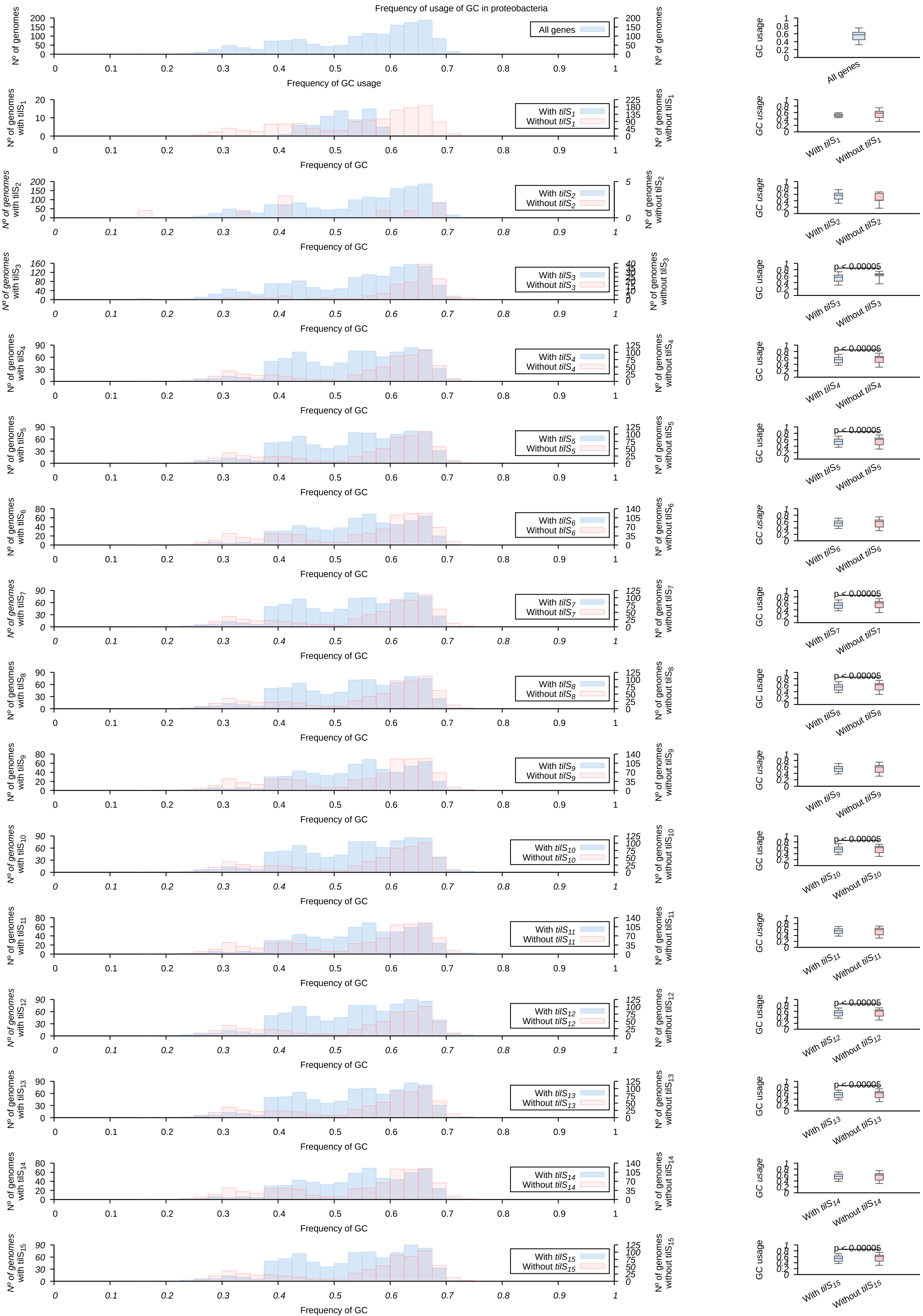

Frequency of usage of GC in proteobacteria
Nº of genomes
All genes
Frequency of GC usage
GC usage
Nº of genomes with $tilS_1$
With $tilS_1$
Without $tilS_1$
Nº of genomes without $tilS_1$
Frequency of GC
Nº of genomes with $tilS_2$
With $tilS_2$
Without $tilS_2$
Nº of genomes without $tilS_2$
Nº of genomes with $tilS_3$
With $tilS_3$
Without $tilS_3$
Nº of genomes without $tilS_3$
$p < 0.00005$
Nº of genomes with $tilS_4$
With $tilS_4$
Without $tilS_4$
Nº of genomes without $tilS_4$
Nº of genomes with $tilS_5$
With $tilS_5$
Without $tilS_5$
Nº of genomes without $tilS_5$
Nº of genomes with $tilS_6$
With $tilS_6$
Without $tilS_6$
Nº of genomes without $tilS_6$
Nº of genomes with $tilS_7$
With $tilS_7$
Without $tilS_7$
Nº of genomes without $tilS_7$
Nº of genomes with $tilS_8$
With $tilS_8$
Without $tilS_8$
Nº of genomes without $tilS_8$
Nº of genomes with $tilS_9$
With $tilS_9$
Without $tilS_9$
Nº of genomes without $tilS_9$
Nº of genomes with $tilS_{10}$
With $tilS_{10}$
Without $tilS_{10}$
Nº of genomes without $tilS_{10}$
Nº of genomes with $tilS_{11}$
With $tilS_{11}$
Without $tilS_{11}$
Nº of genomes without $tilS_{11}$
Nº of genomes with $tilS_{12}$
With $tilS_{12}$
Without $tilS_{12}$
Nº of genomes without $tilS_{12}$
Nº of genomes with $tilS_{13}$
With $tilS_{13}$
Without $tilS_{13}$
Nº of genomes without $tilS_{13}$
Nº of genomes with $tilS_{14}$
With $tilS_{14}$
Without $tilS_{14}$
Nº of genomes without $tilS_{14}$
Nº of genomes with $tilS_{15}$
With $tilS_{15}$
Without $tilS_{15}$
Nº of genomes without $tilS_{15}$

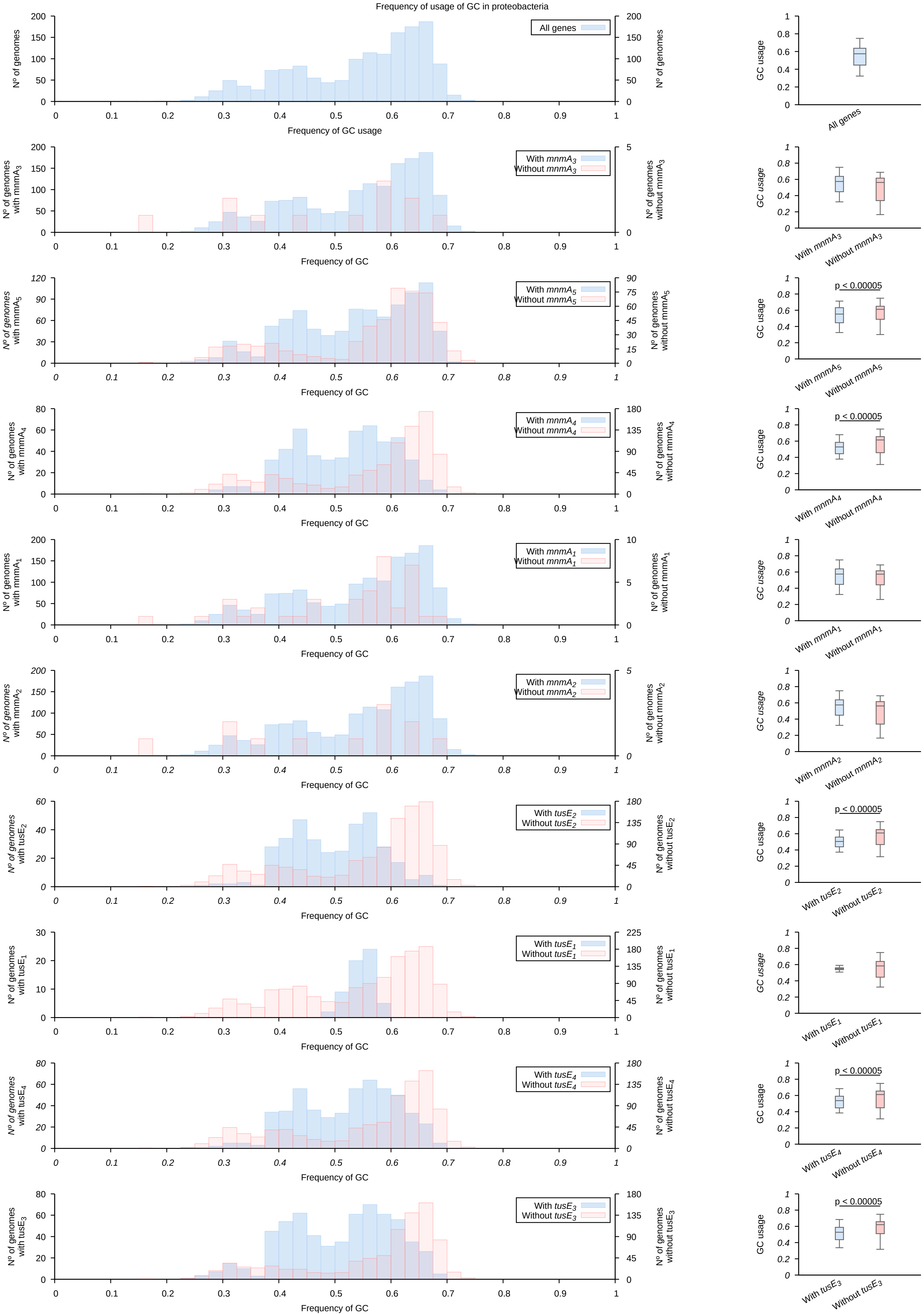

Frequency of usage of GC in proteobacteria
All genes
Nº of genomes
Frequency of GC usage
GC usage
With mnmA3
Without mnmA3
Nº of genomes with mnmA3
Nº of genomes without mnmA3
Frequency of GC
With mnmA5
Without mnmA5
Nº of genomes with mnmA5
Nº of genomes without mnmA5
p < 0.00005
With mnmA4
Without mnmA4
Nº of genomes with mnmA4
Nº of genomes without mnmA4
p < 0.00005
With mnmA1
Without mnmA1
Nº of genomes with mnmA1
Nº of genomes without mnmA1
With mnmA2
Without mnmA2
Nº of genomes with mnmA2
Nº of genomes without mnmA2
With tusE2
Without tusE2
Nº of genomes with tusE2
Nº of genomes without tusE2
p < 0.00005
With tusE1
Without tusE1
Nº of genomes with tusE1
Nº of genomes without tusE1
With tusE4
Without tusE4
Nº of genomes with tusE4
Nº of genomes without tusE4
p < 0.00005
With tusE3
Without tusE3
Nº of genomes with tusE3
Nº of genomes without tusE3
p < 0.00005
