# Supplementary material for: Impact of the chemical modification of tRNAs anticodon loop on the variability and evolution of codon usage in proteobacteria
Source: Front Microbiol. 2024 Aug 5;15:1412318. doi: 10.3389/fmicb.2024.1412318 (PMC11332805; doi:10.3389/fmicb.2024.1412318)
Supplement: Supplementary file 1 [file Data_Sheet_1.zip › Supp_figures/Fig_S2.pdf]

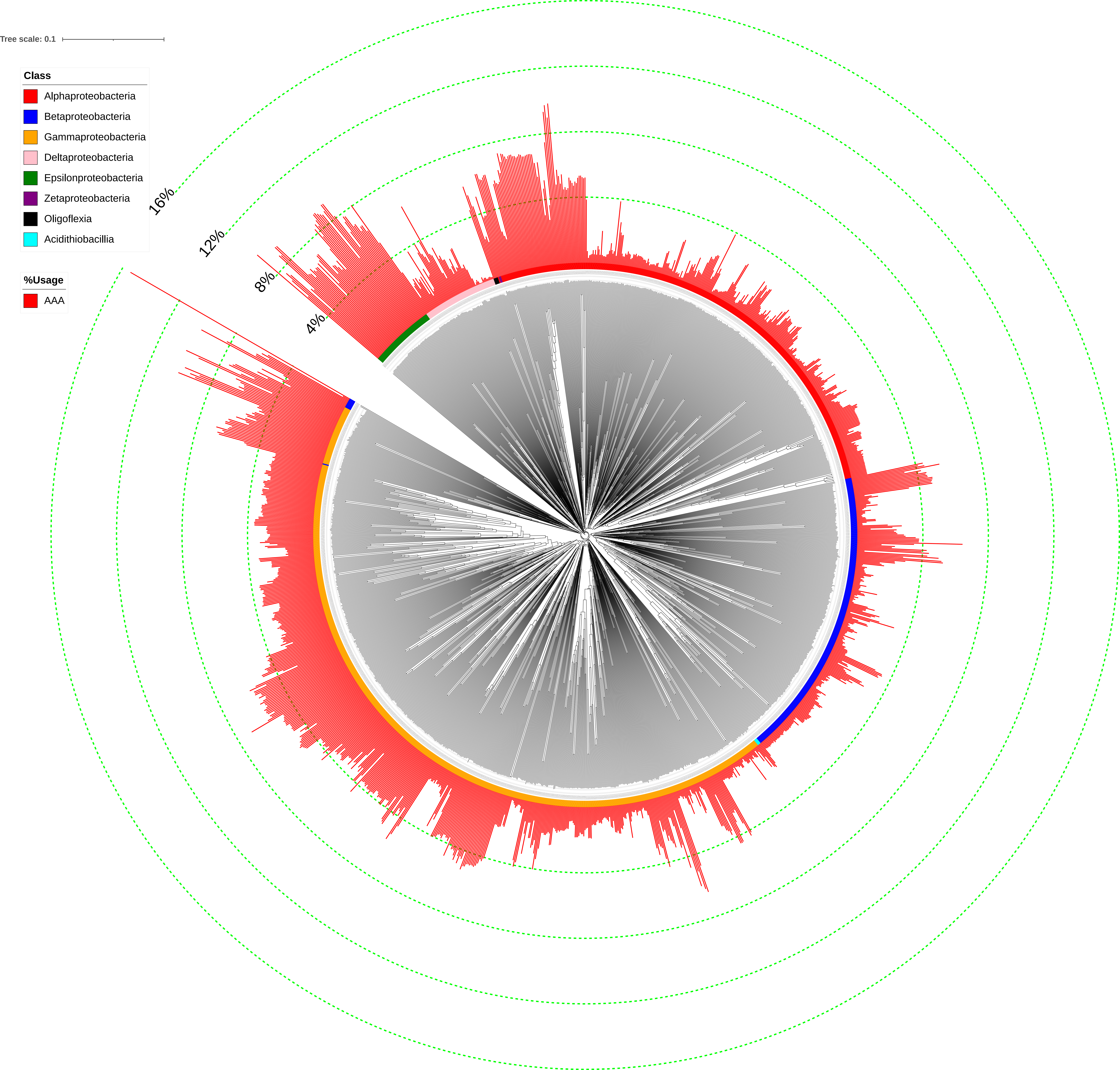
Tree scale: 0.1
Class
Alphaproteobacteria
Betaproteobacteria
Gammaproteobacteria
Deltaproteobacteria
Epsilonproteobacteria
Zetaproteobacteria
Oligoflexia
Acidithiobacillia
%Usage
AAA
4%
8%
12%
16%

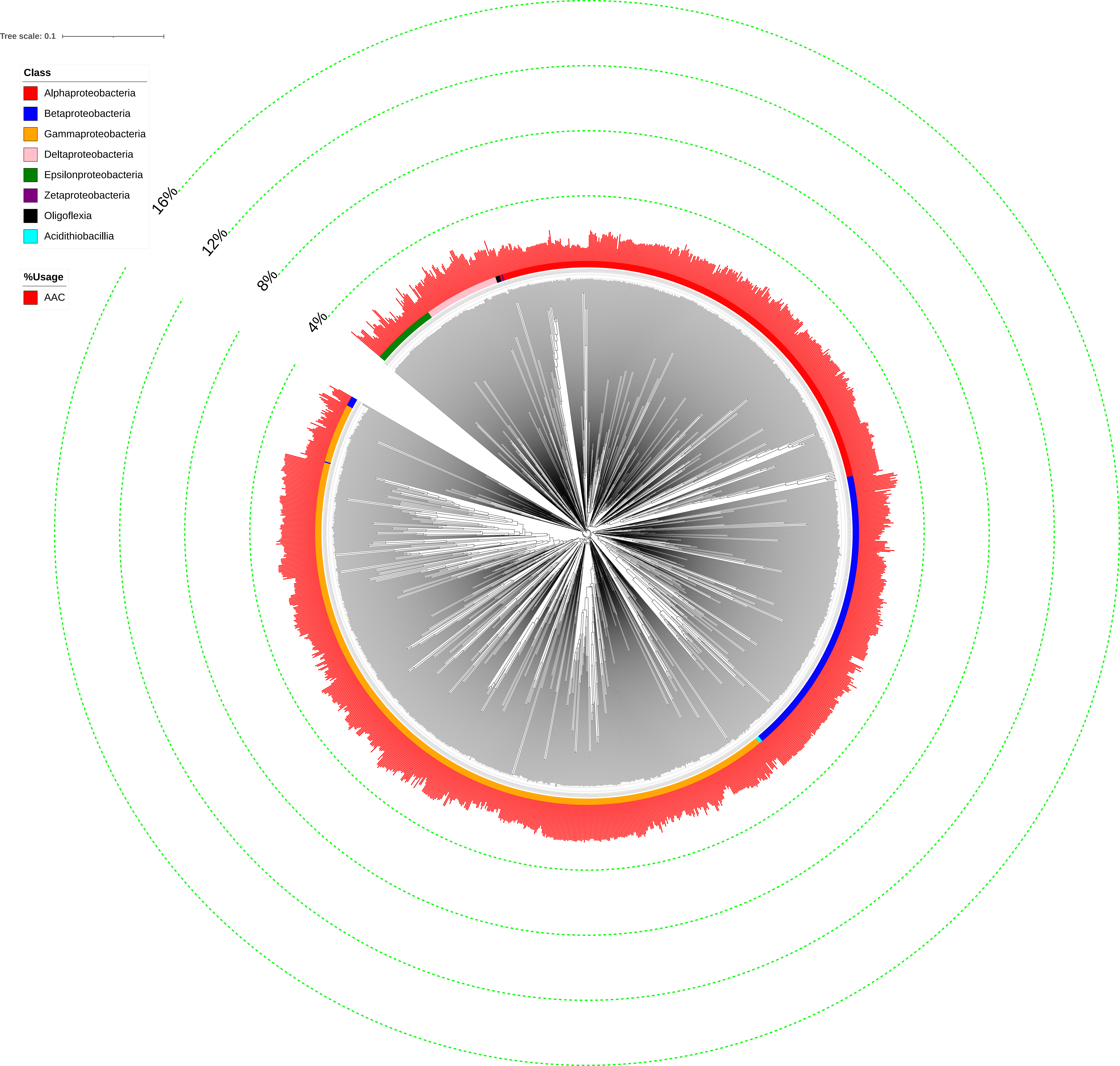

Tree scale: 0.1
Class
Alphaproteobacteria
Betaproteobacteria
Gammaproteobacteria
Deltaproteobacteria
Epsilonproteobacteria
Zetaproteobacteria
Oligoflexia
Acidithiobacillia
%Usage
AAC
4%
8%
12%
16%

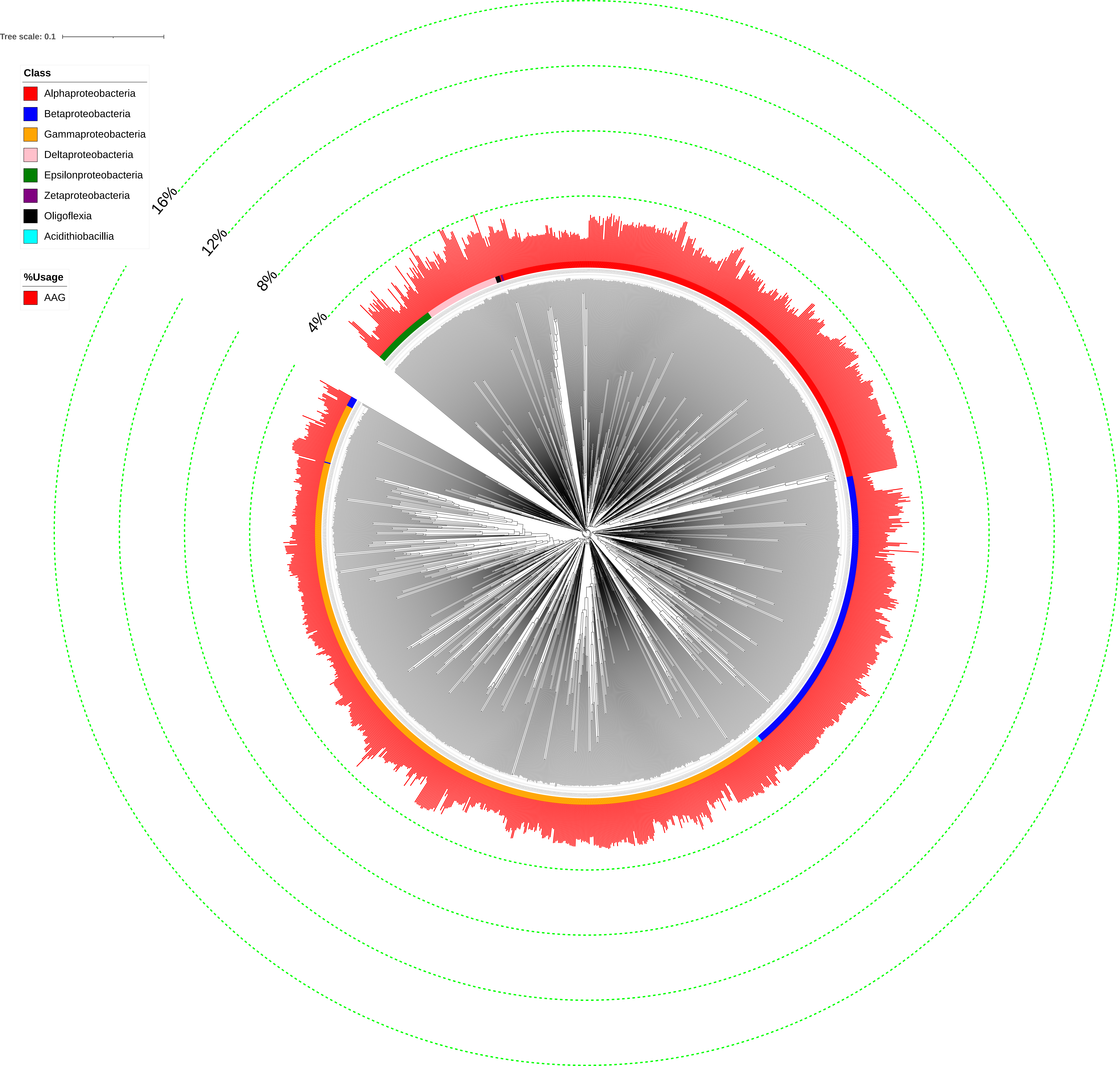

Tree scale: 0.1
Class
Alphaproteobacteria
Betaproteobacteria
Gammaproteobacteria
Deltaproteobacteria
Epsilonproteobacteria
Zetaproteobacteria
Oligoflexia
Acidithiobacillia
%Usage
AAG
4%
8%
12%
16%

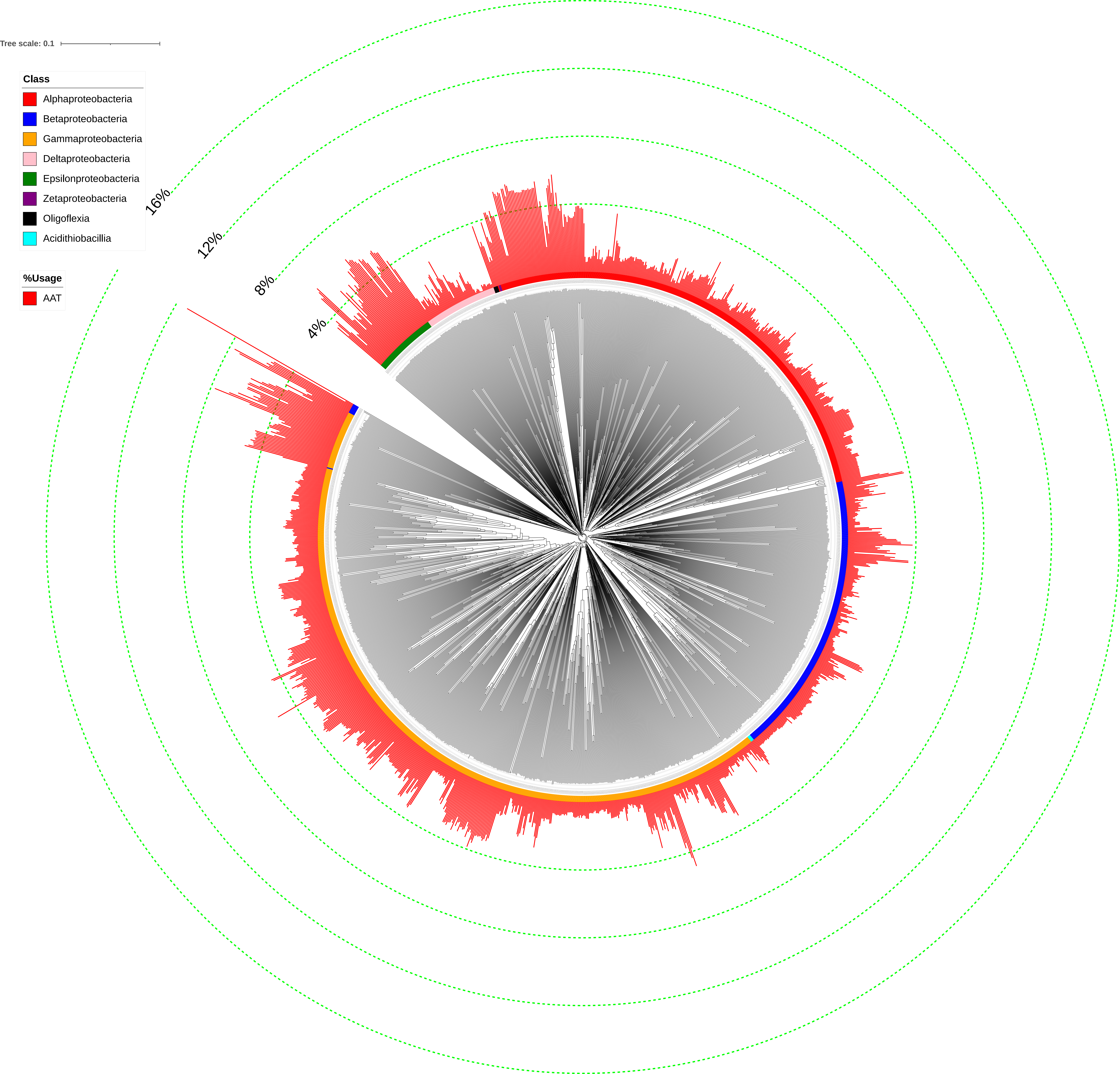

Tree scale: 0.1
Class
Alphaproteobacteria
Betaproteobacteria
Gammaproteobacteria
Deltaproteobacteria
Epsilonproteobacteria
Zetaproteobacteria
Oligoflexia
Acidithiobacillia
%Usage
AAT
4%
8%
12%
16%

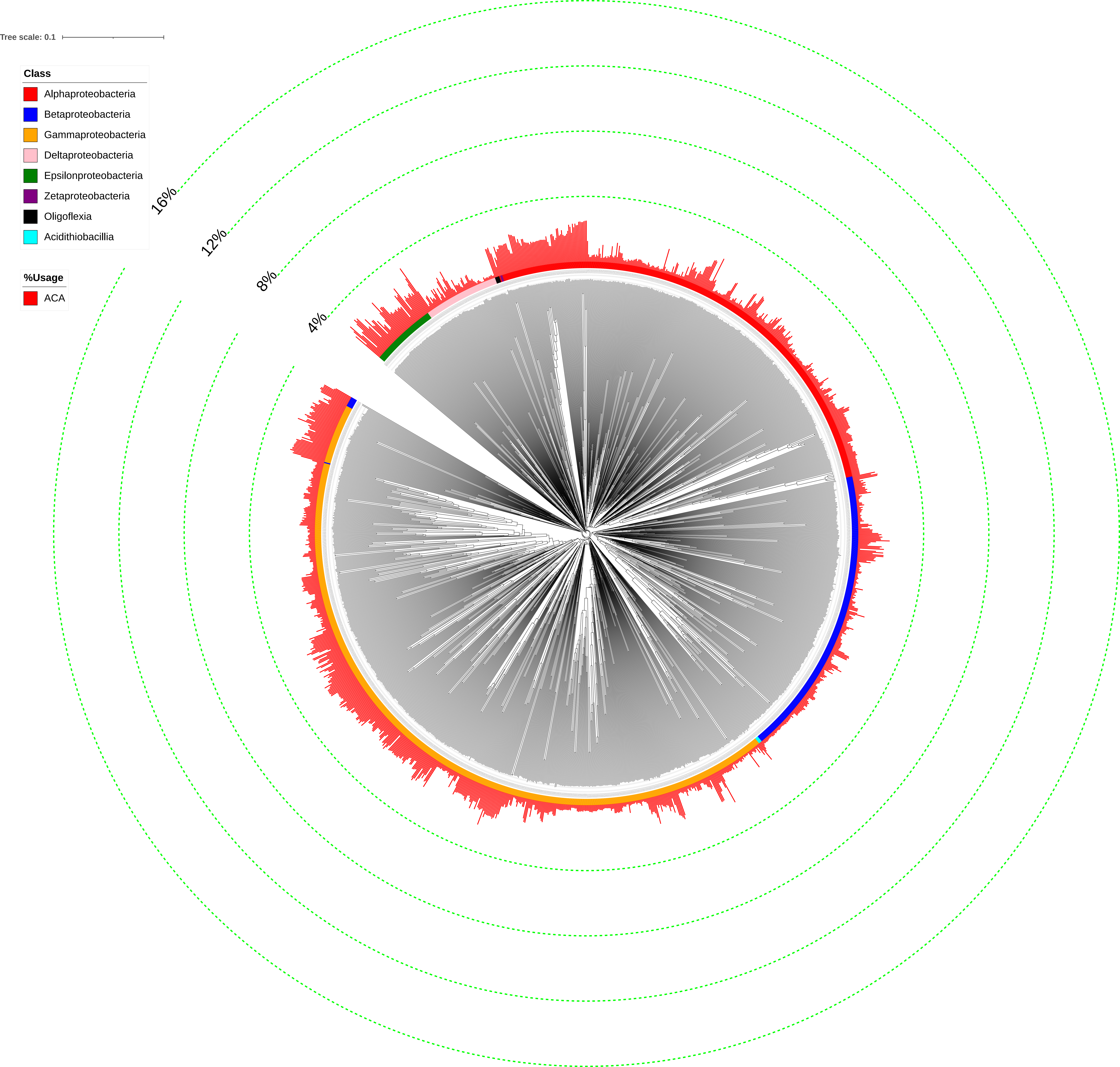

Tree scale: 0.1
Class
Alphaproteobacteria
Betaproteobacteria
Gammaproteobacteria
Deltaproteobacteria
Epsilonproteobacteria
Zetaproteobacteria
Oligoflexia
Acidithiobacillia
%Usage
ACA
4%
8%
12%
16%

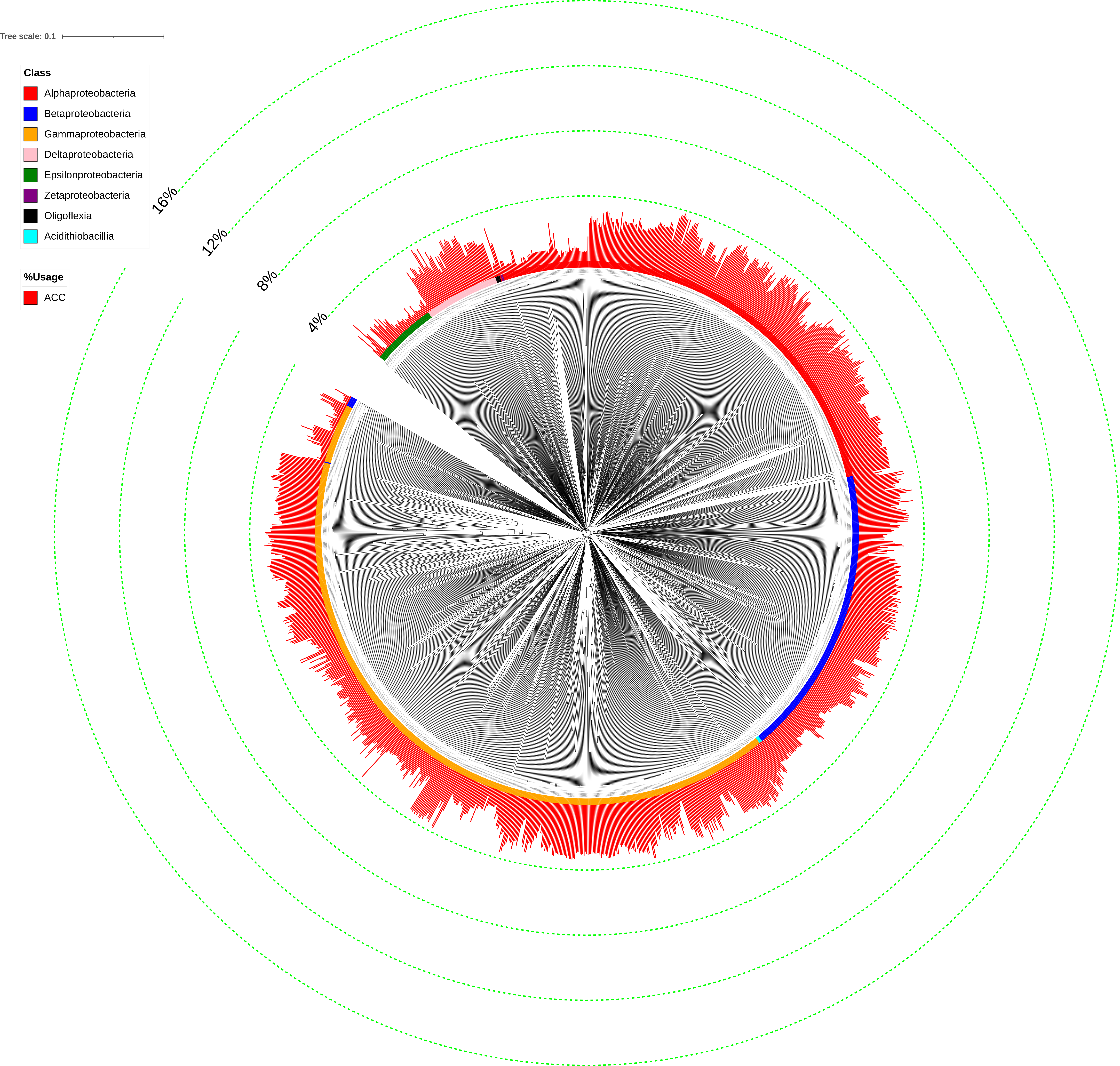

Tree scale: 0.1
Class
Alphaproteobacteria
Betaproteobacteria
Gammaproteobacteria
Deltaproteobacteria
Epsilonproteobacteria
Zetaproteobacteria
Oligoflexia
Acidithiobacillia
%Usage
ACC
16%
12%
8%
4%

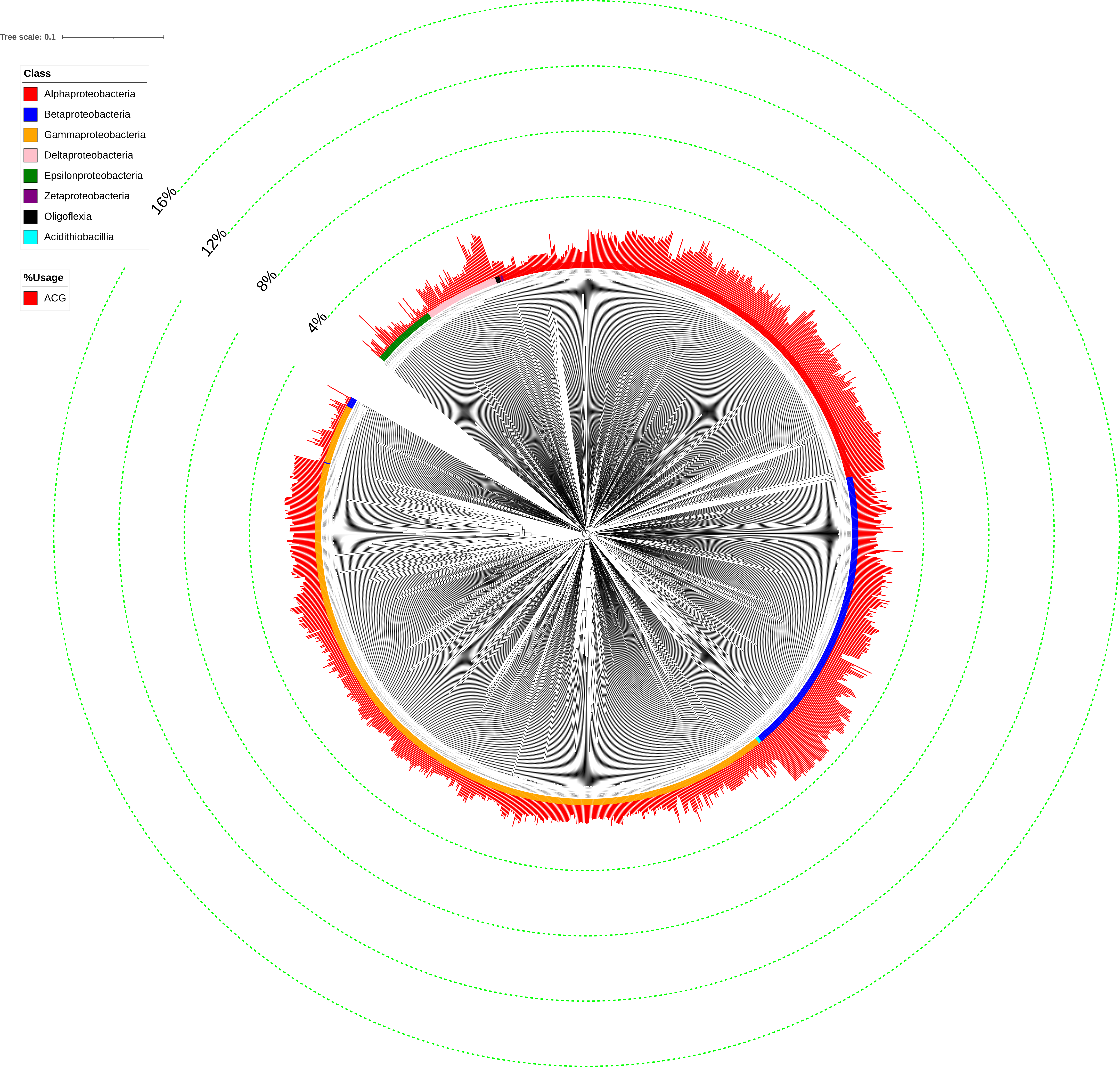

Tree scale: 0.1
Class
Alphaproteobacteria
Betaproteobacteria
Gammaproteobacteria
Deltaproteobacteria
Epsilonproteobacteria
Zetaproteobacteria
Oligoflexia
Acidithiobacillia
%Usage
ACG
4%
8%
12%
16%

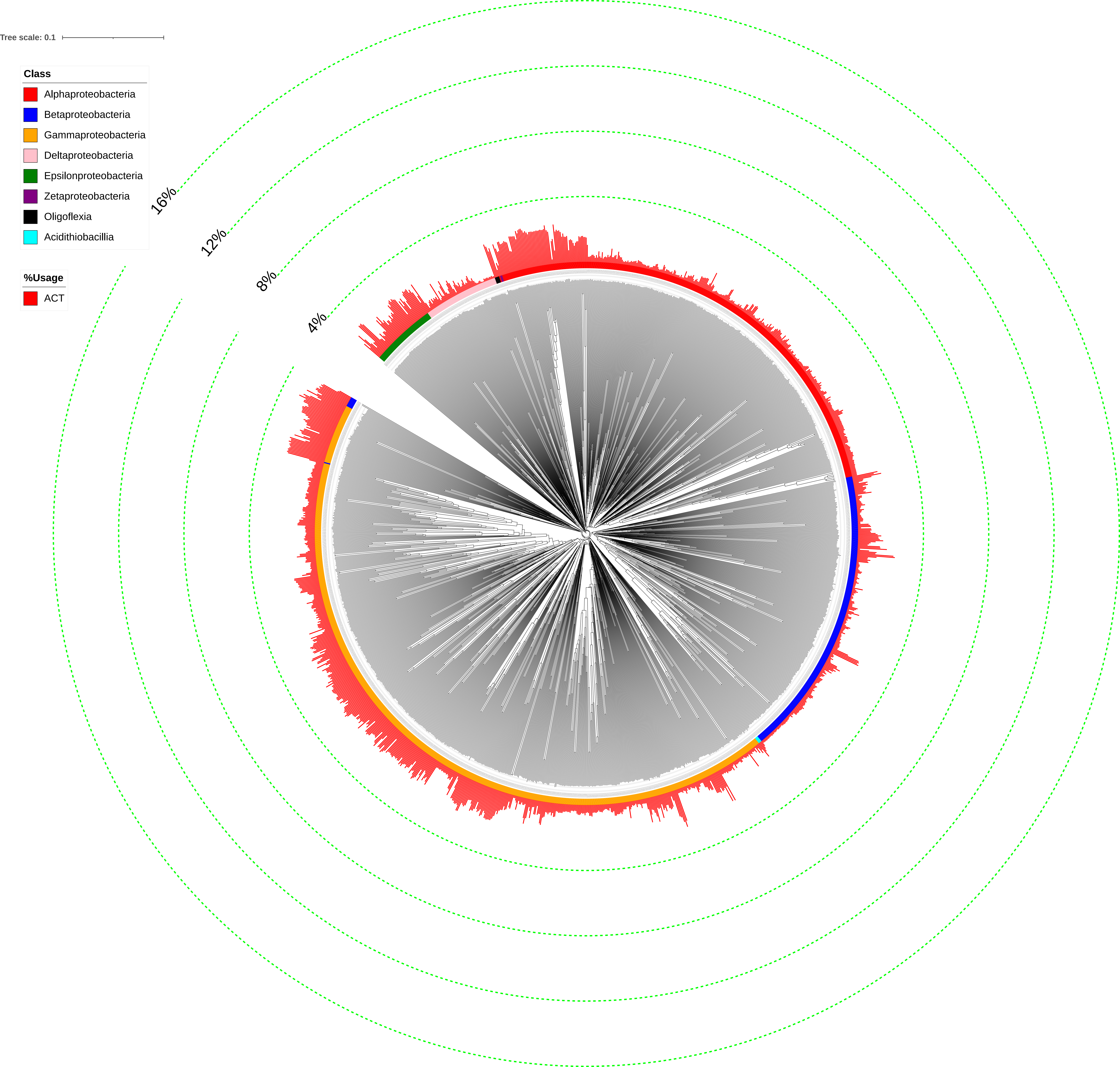

Tree scale: 0.1
Class
Alphaproteobacteria
Betaproteobacteria
Gammaproteobacteria
Deltaproteobacteria
Epsilonproteobacteria
Zetaproteobacteria
Oligoflexia
Acidithiobacillia
%Usage
ACT
16%
12%
8%
4%

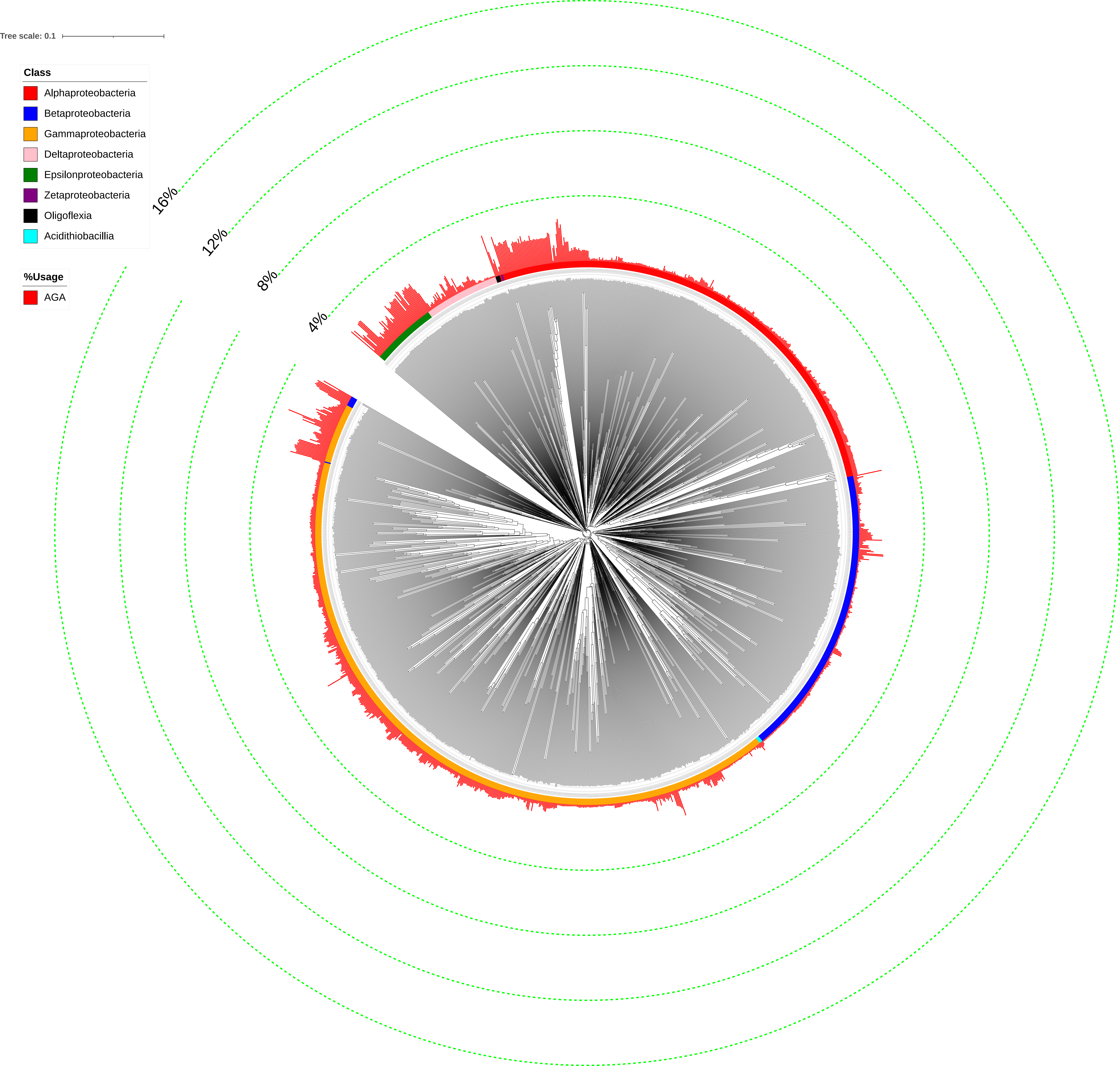

Tree scale: 0.1
Class
Alphaproteobacteria
Betaproteobacteria
Gammaproteobacteria
Deltaproteobacteria
Epsilonproteobacteria
Zetaproteobacteria
Oligoflexia
Acidithiobacillia
%Usage
AGA
16%
12%
8%
4%

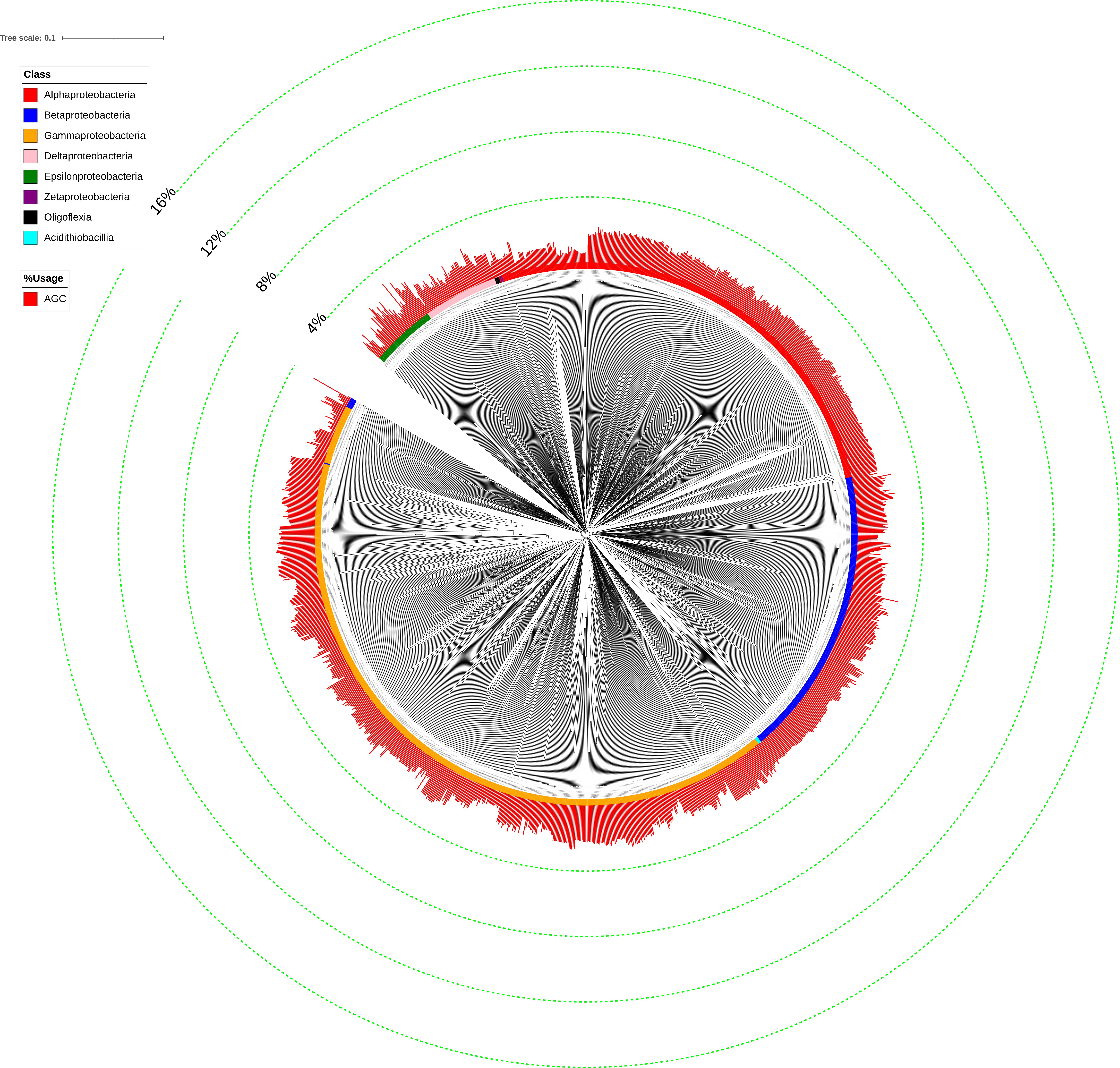

Tree scale: 0.1
Class
Alphaproteobacteria
Betaproteobacteria
Gammaproteobacteria
Deltaproteobacteria
Epsilonproteobacteria
Zetaproteobacteria
Oligoflexia
Acidithiobacillia
%Usage
AGC
16%
12%
8%
4%

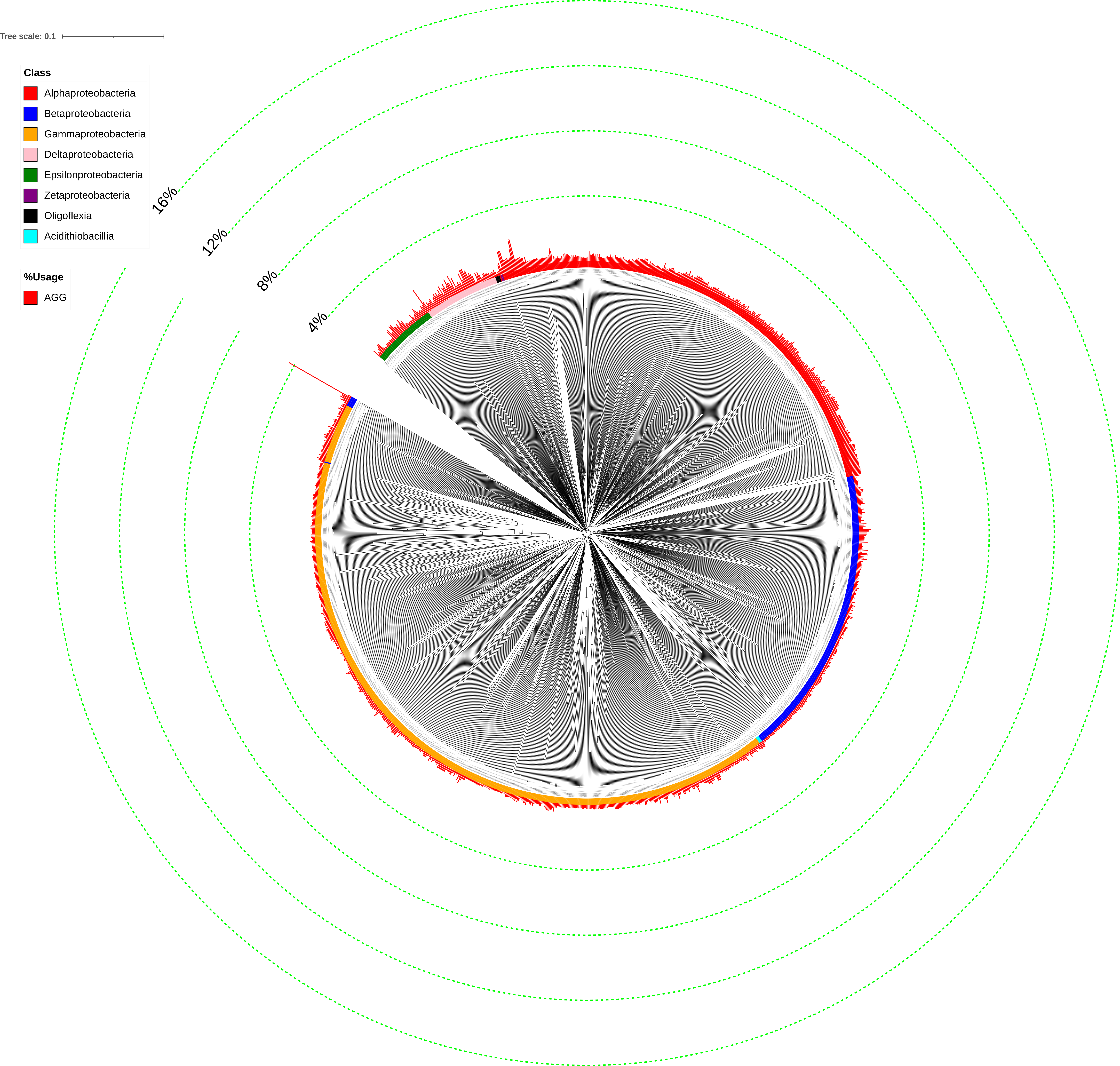

Tree scale: 0.1
Class
Alphaproteobacteria
Betaproteobacteria
Gammaproteobacteria
Deltaproteobacteria
Epsilonproteobacteria
Zetaproteobacteria
Oligoflexia
Acidithiobacillia
%Usage
AGG
16%
12%
8%
4%

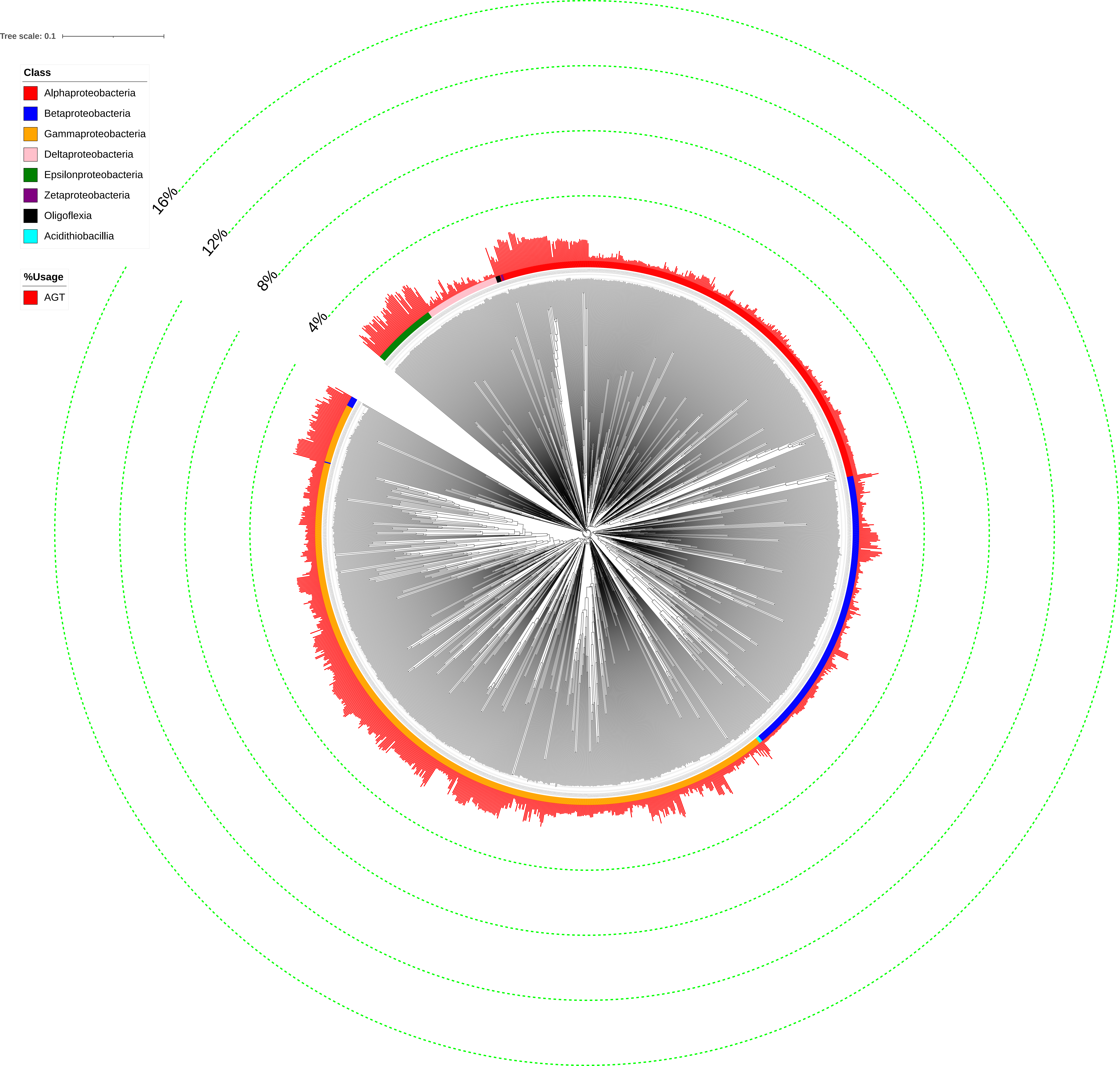

Tree scale: 0.1
Class
Alphaproteobacteria
Betaproteobacteria
Gammaproteobacteria
Deltaproteobacteria
Epsilonproteobacteria
Zetaproteobacteria
Oligoflexia
Acidithiobacillia
%Usage
AGT
4%
8%
12%
16%

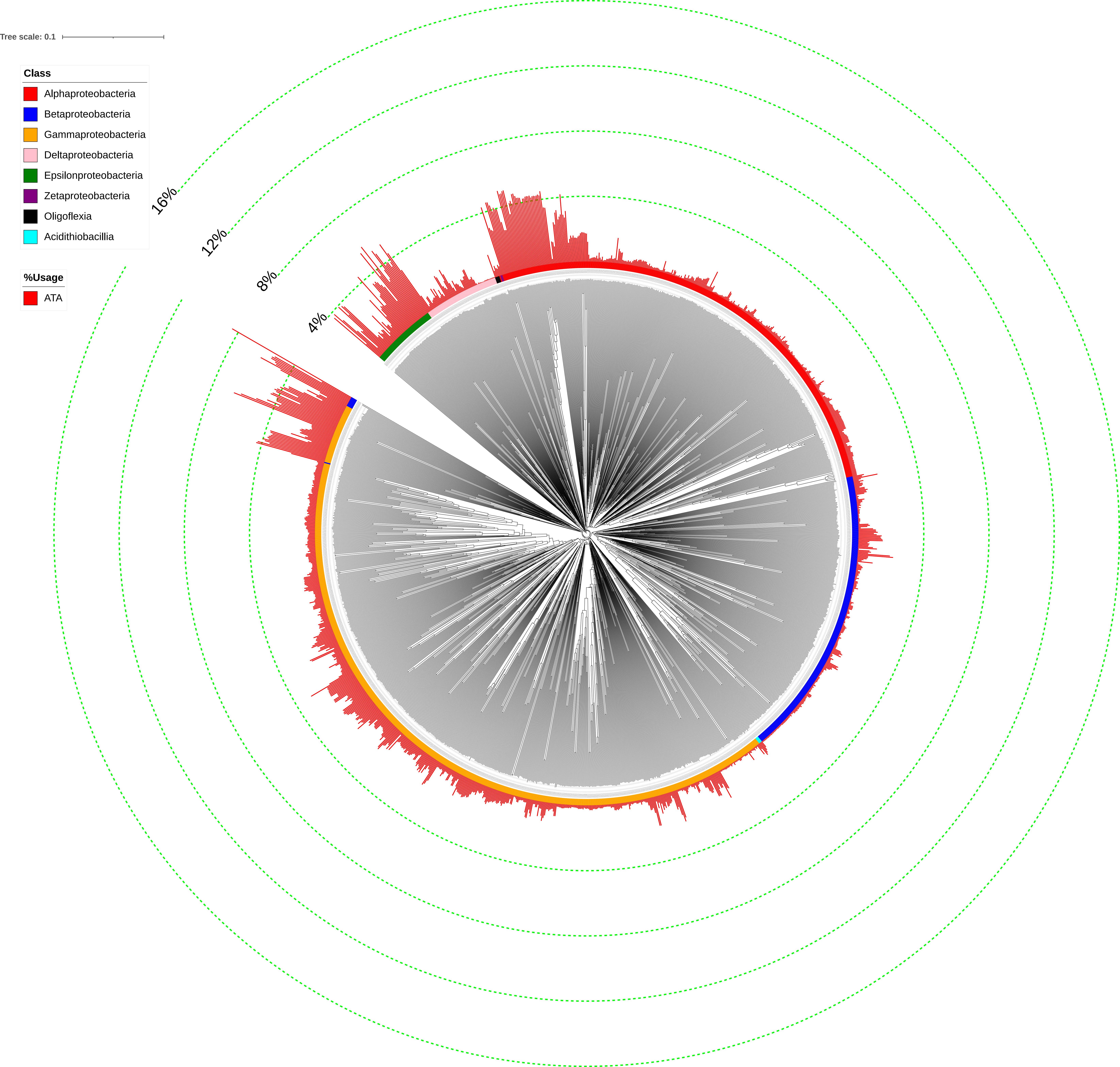

Tree scale: 0.1
Class
Alphaproteobacteria
Betaproteobacteria
Gammaproteobacteria
Deltaproteobacteria
Epsilonproteobacteria
Zetaproteobacteria
Oligoflexia
Acidithiobacillia
%Usage
ATA
4%
8%
12%
16%

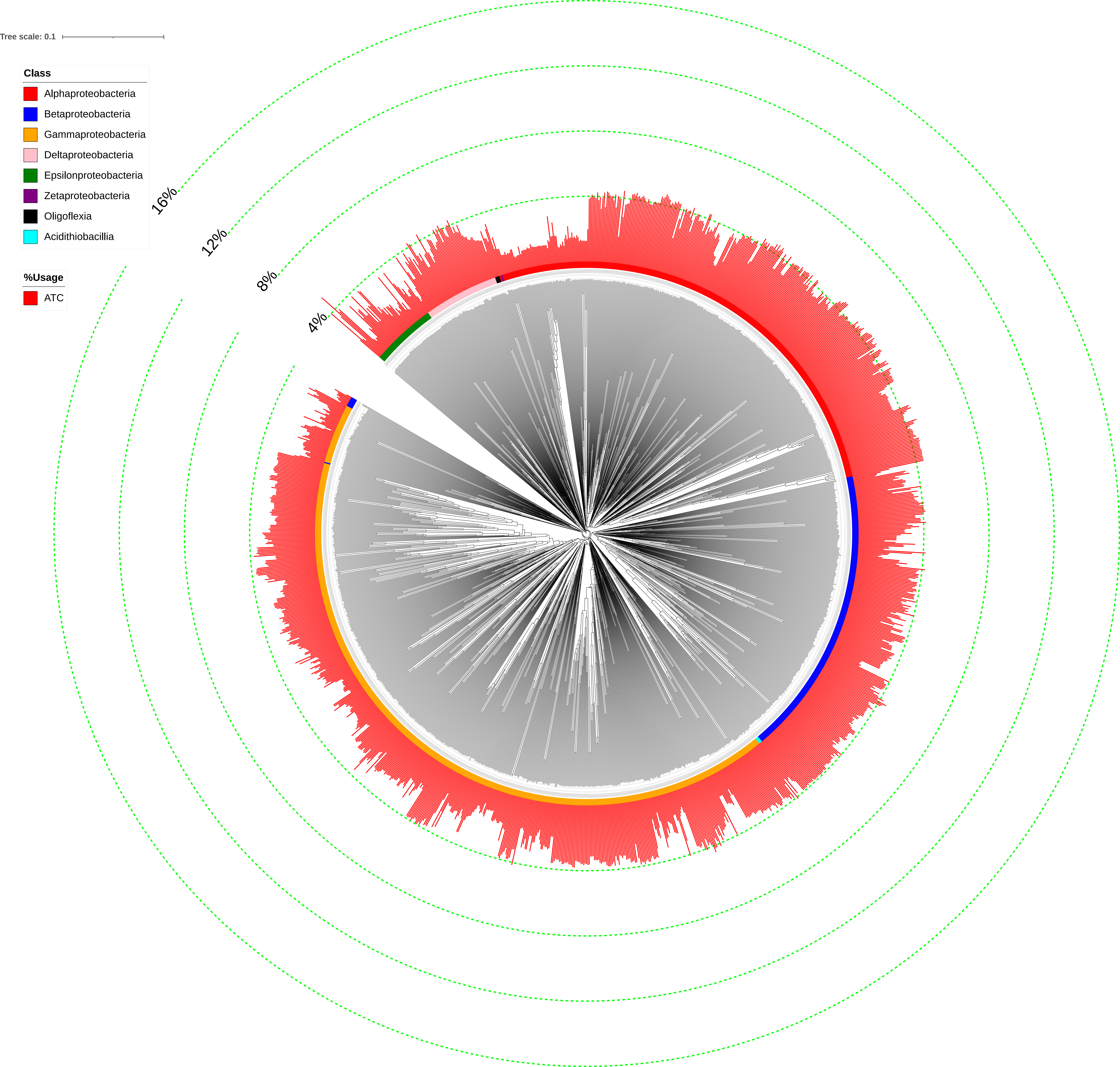

Tree scale: 0.1
Class
Alphaproteobacteria
Betaproteobacteria
Gammaproteobacteria
Deltaproteobacteria
Epsilonproteobacteria
Zetaproteobacteria
Oligoflexia
Acidithiobacillia
%Usage
ATC
16%
12%
8%
4%

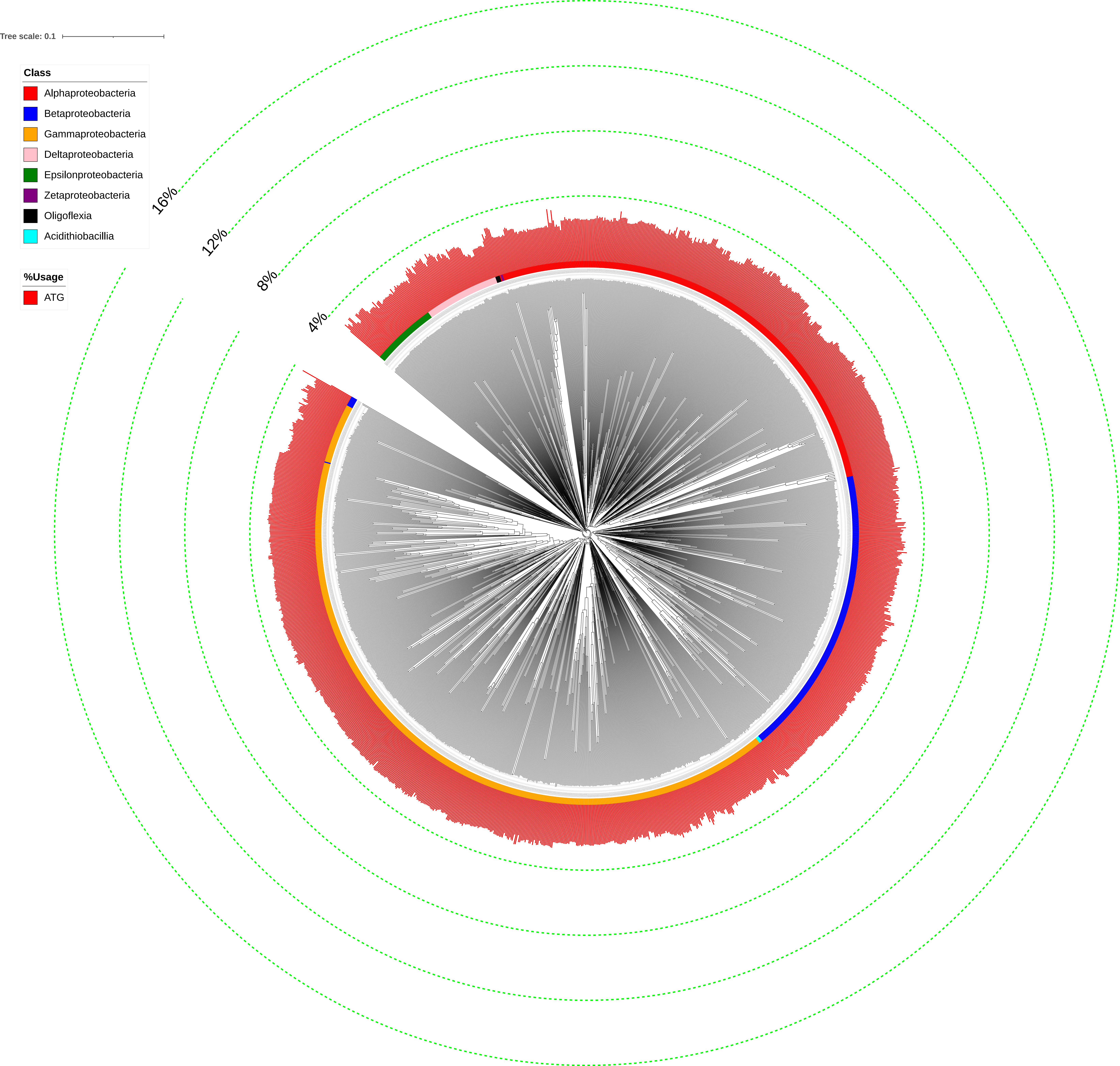
Tree scale: 0.1
Class
Alphaproteobacteria
Betaproteobacteria
Gammaproteobacteria
Deltaproteobacteria
Epsilonproteobacteria
Zetaproteobacteria
Oligoflexia
Acidithiobacillia
%Usage
ATG
16%
12%
8%
4%

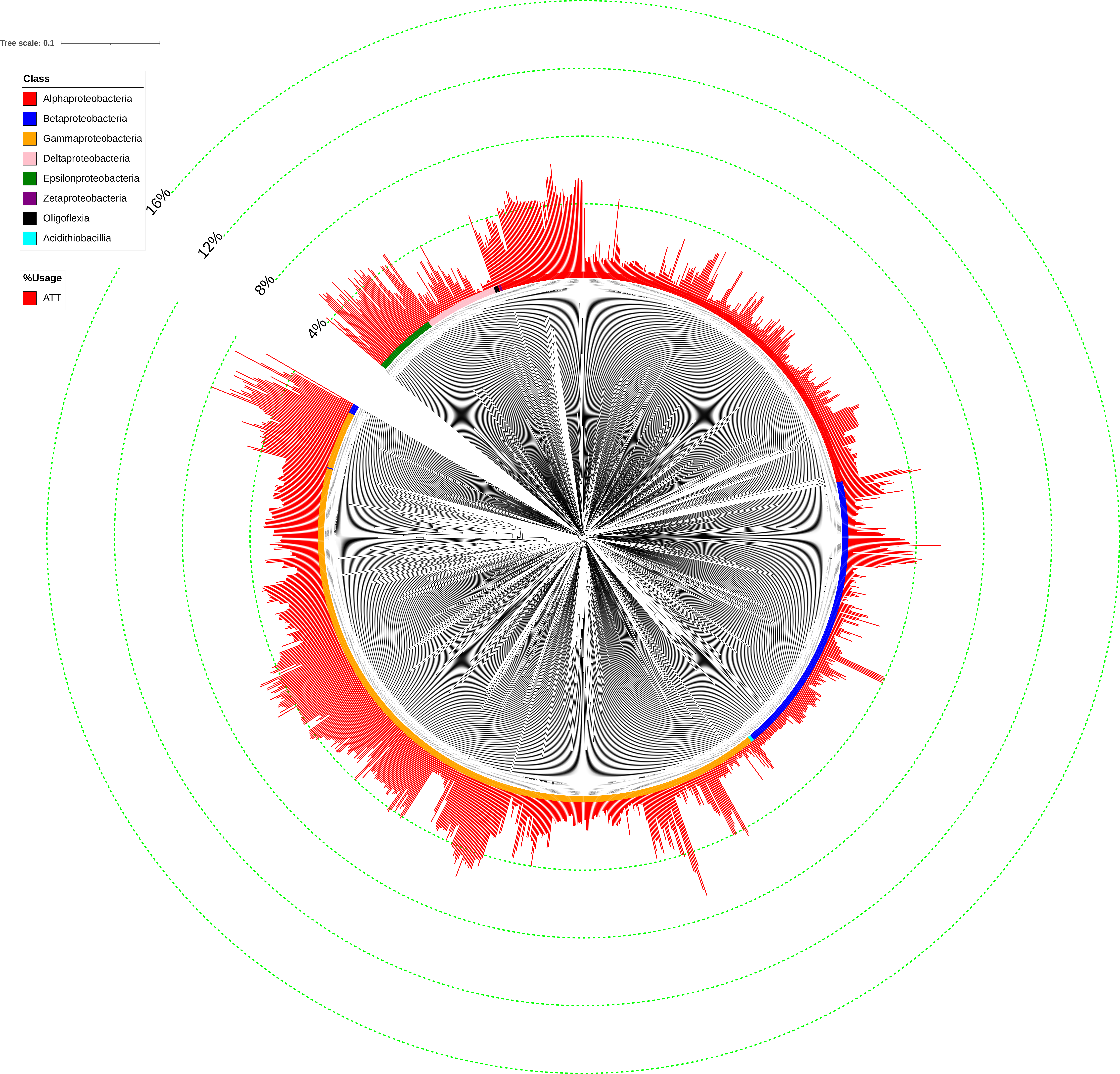

Tree scale: 0.1
Class
Alphaproteobacteria
Betaproteobacteria
Gammaproteobacteria
Deltaproteobacteria
Epsilonproteobacteria
Zetaproteobacteria
Oligoflexia
Acidithiobacillia
%Usage
ATT
4%
8%
12%
16%

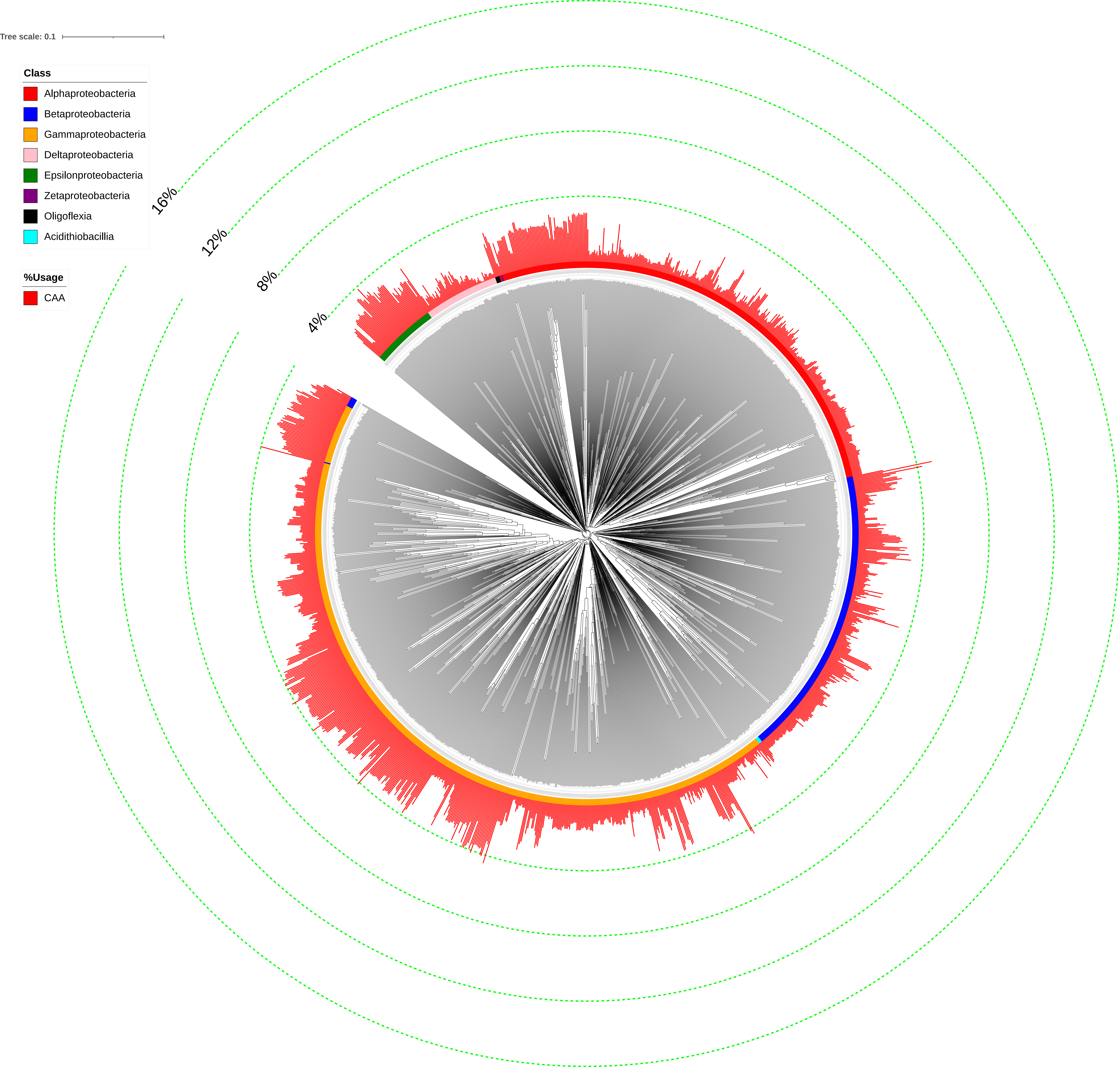

Tree scale: 0.1
Class
Alphaproteobacteria
Betaproteobacteria
Gammaproteobacteria
Deltaproteobacteria
Epsilonproteobacteria
Zetaproteobacteria
Oligoflexia
Acidithiobacillia
%Usage
CAA
16%
12%
8%
4%

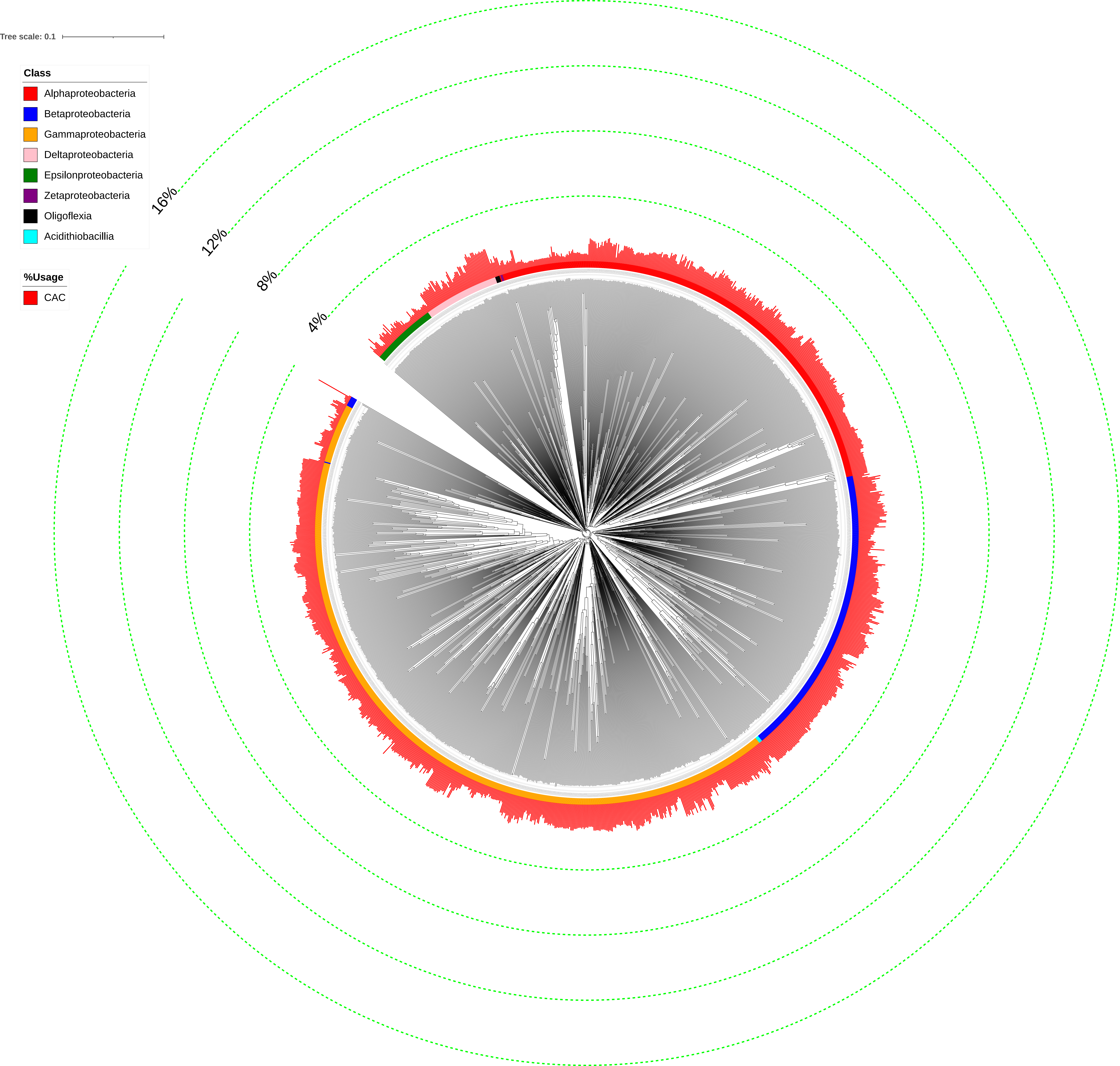

Tree scale: 0.1
Class
Alphaproteobacteria
Betaproteobacteria
Gammaproteobacteria
Deltaproteobacteria
Epsilonproteobacteria
Zetaproteobacteria
Oligoflexia
Acidithiobacillia
%Usage
CAC
4%
8%
12%
16%

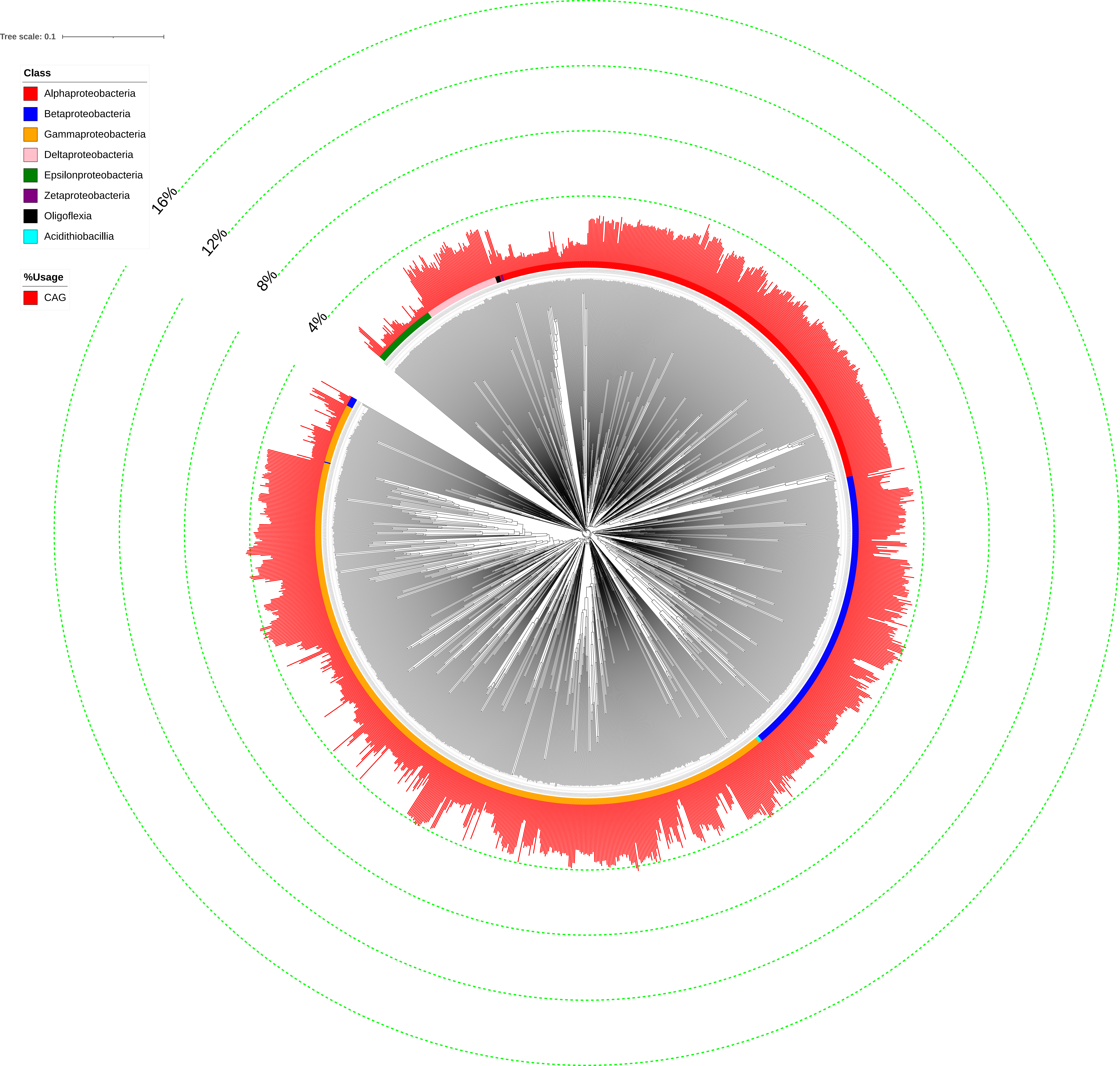

Tree scale: 0.1
Class
Alphaproteobacteria
Betaproteobacteria
Gammaproteobacteria
Deltaproteobacteria
Epsilonproteobacteria
Zetaproteobacteria
Oligoflexia
Acidithiobacillia
%Usage
CAG
16%
12%
8%
4%

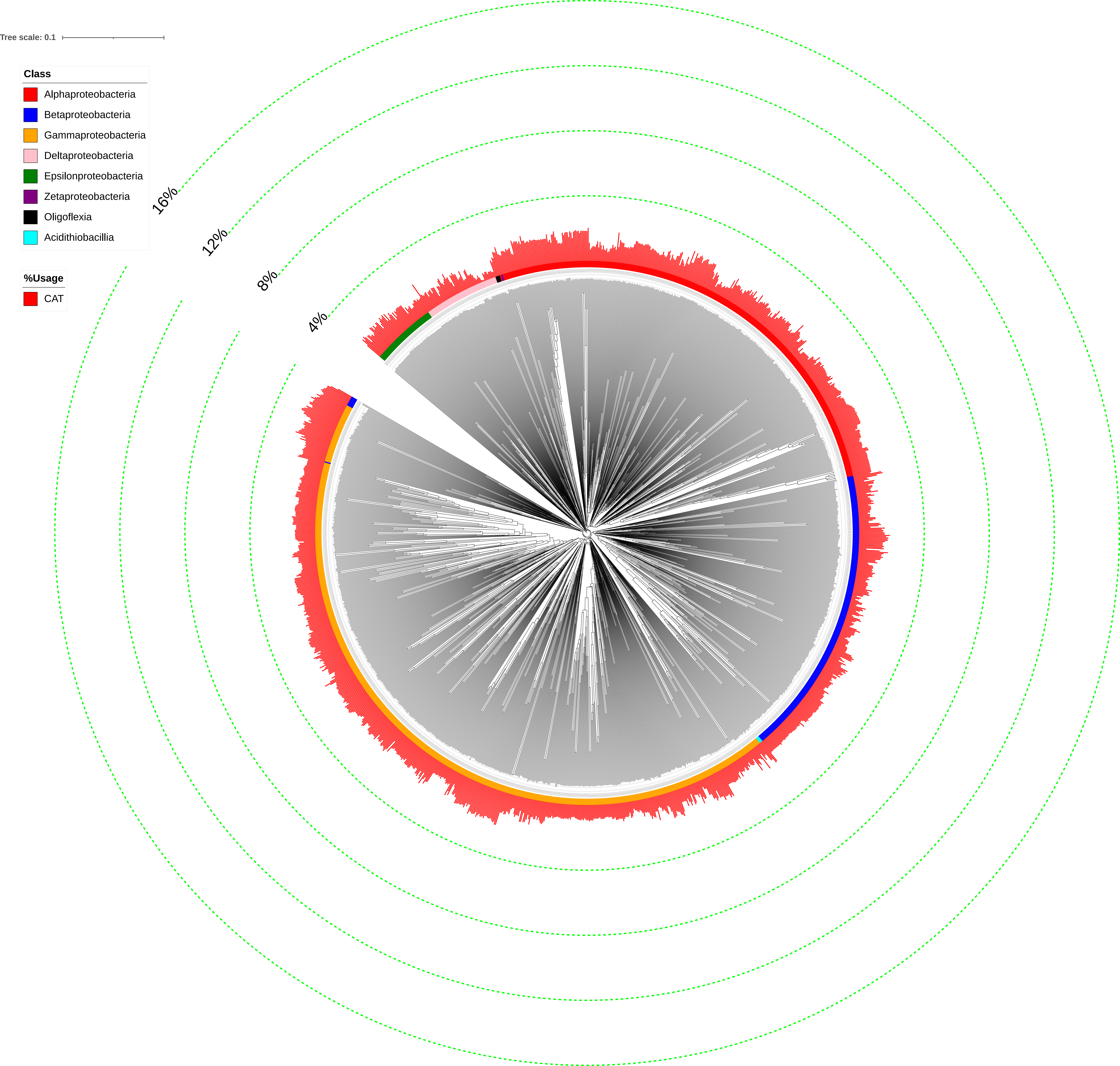

Tree scale: 0.1
Class
Alphaproteobacteria
Betaproteobacteria
Gammaproteobacteria
Deltaproteobacteria
Epsilonproteobacteria
Zetaproteobacteria
Oligoflexia
Acidithiobacillia
%Usage
CAT
16%
12%
8%
4%

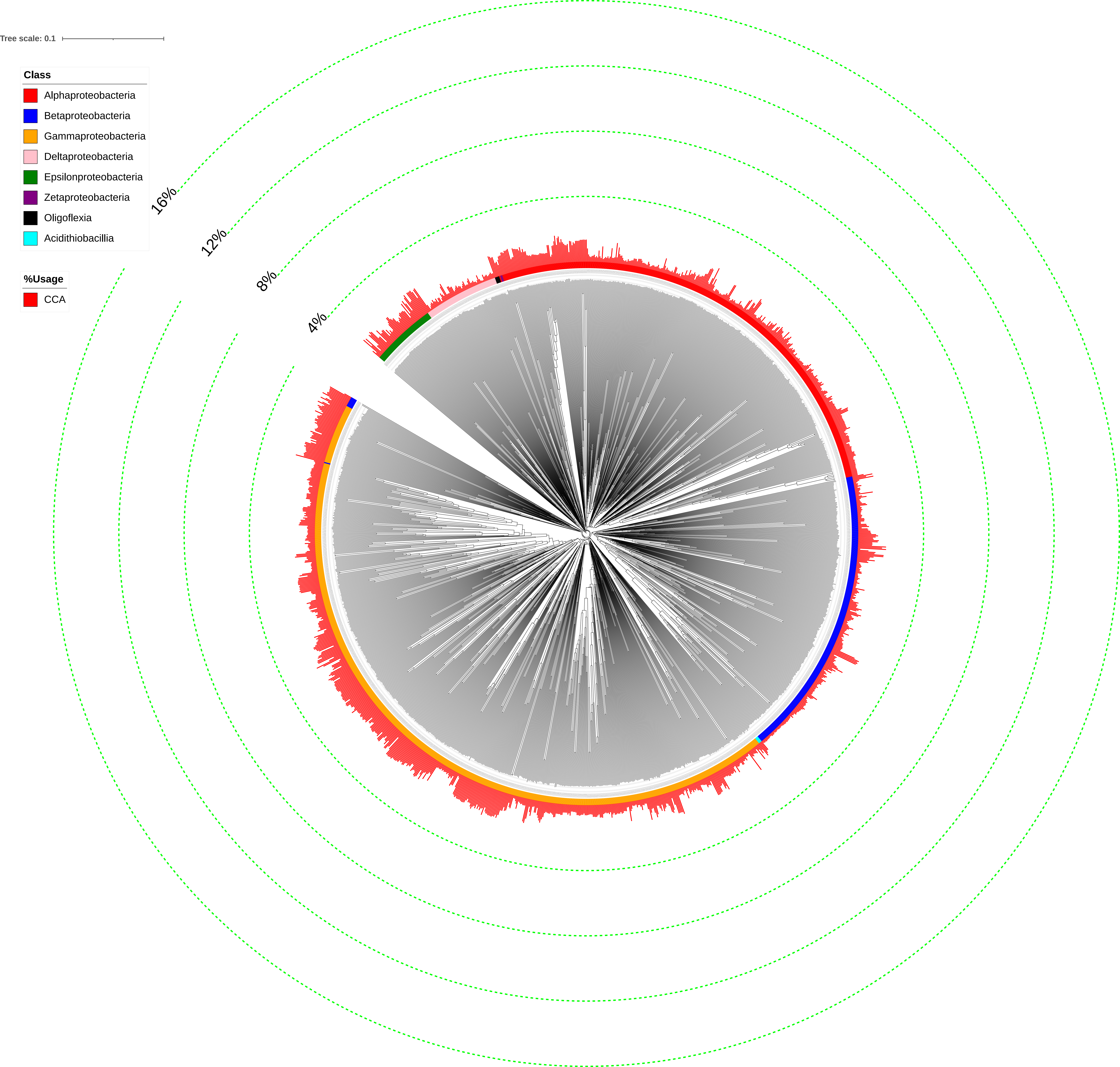
Tree scale: 0.1
Class
Alphaproteobacteria
Betaproteobacteria
Gammaproteobacteria
Deltaproteobacteria
Epsilonproteobacteria
Zetaproteobacteria
Oligoflexia
Acidithiobacillia
%Usage
CCA
16%
12%
8%
4%

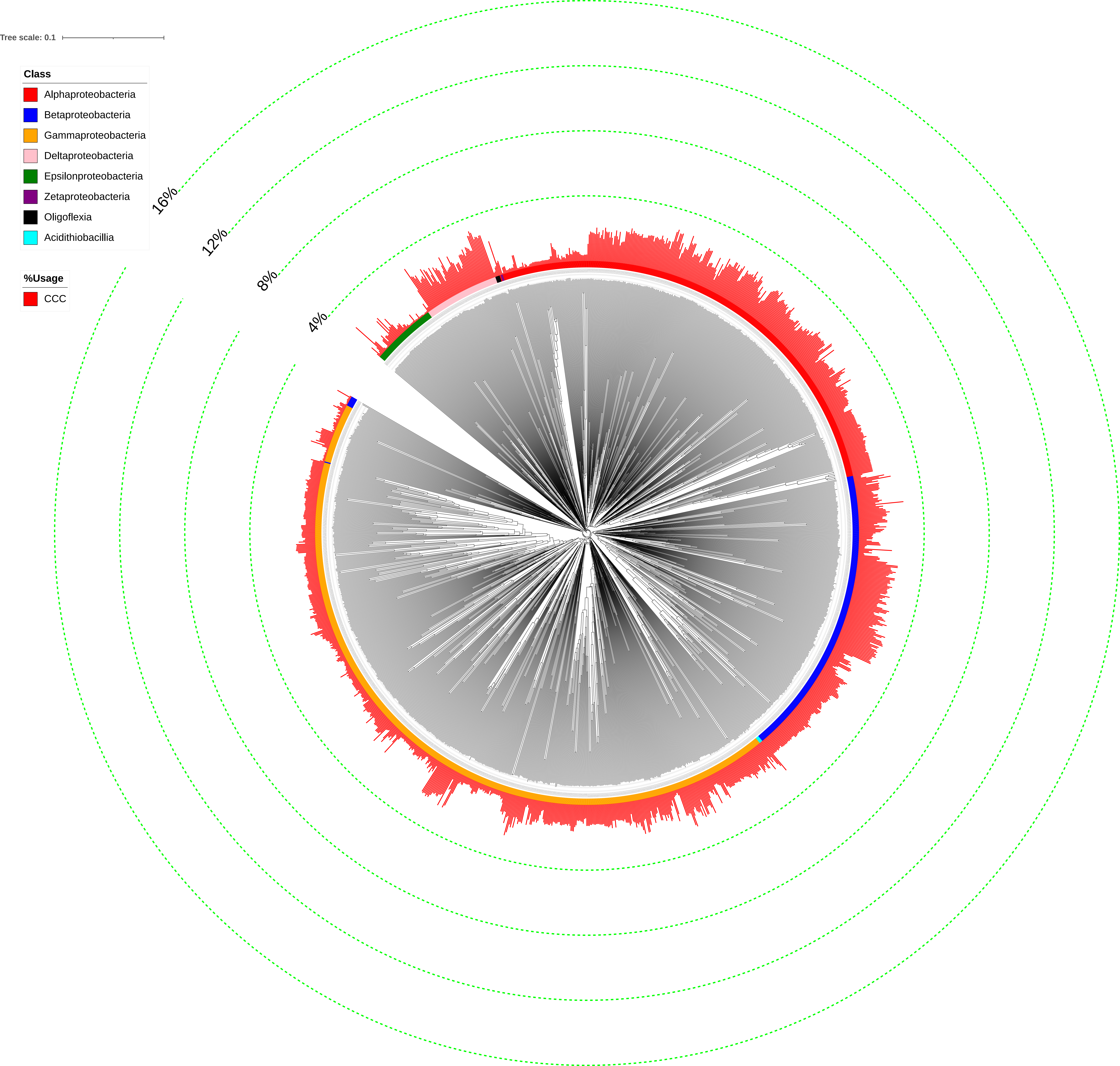

Tree scale: 0.1
Class
Alphaproteobacteria
Betaproteobacteria
Gammaproteobacteria
Deltaproteobacteria
Epsilonproteobacteria
Zetaproteobacteria
Oligoflexia
Acidithiobacillia
%Usage
CCC
4%
8%
12%
16%

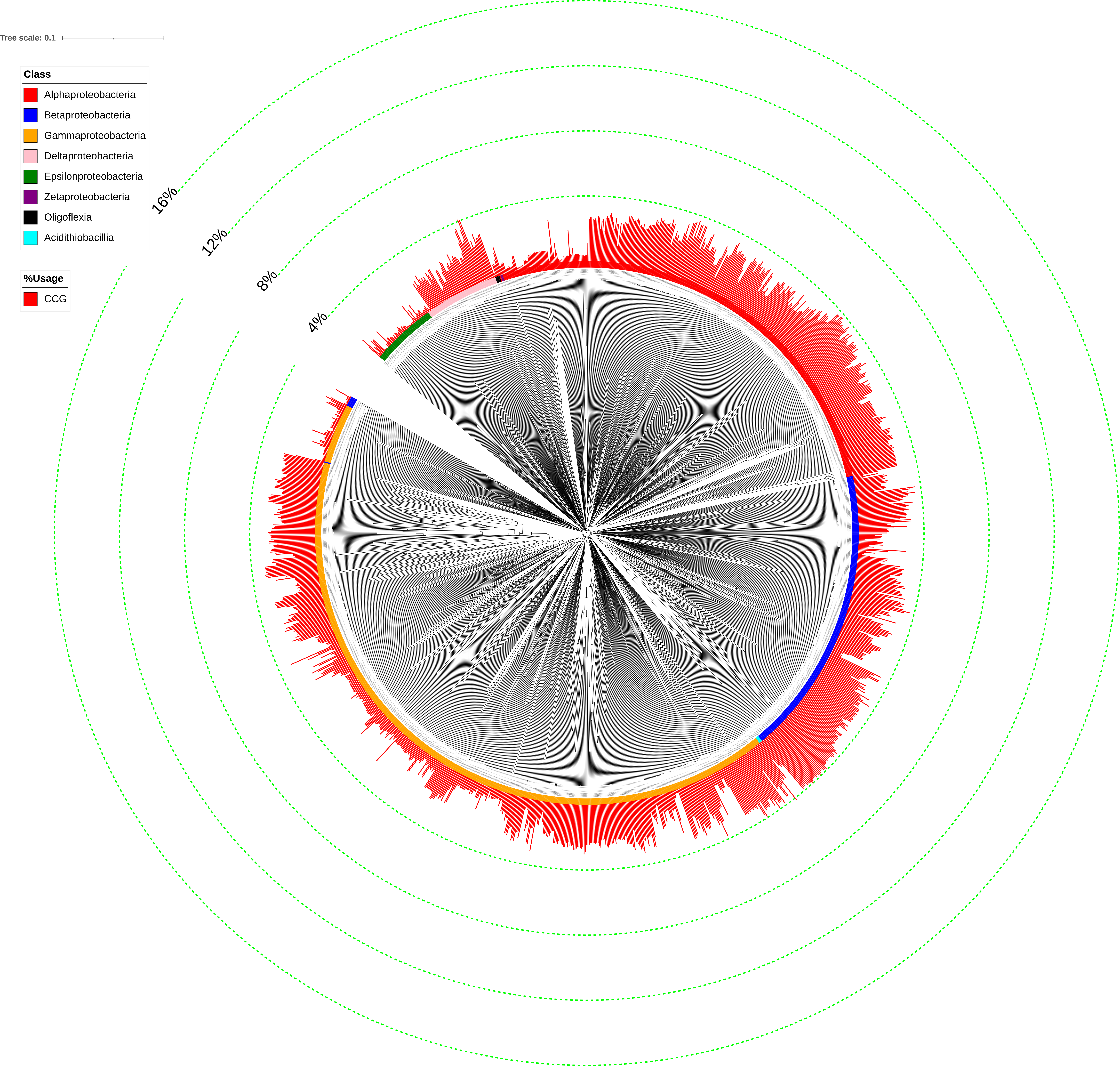

Tree scale: 0.1
Class
Alphaproteobacteria
Betaproteobacteria
Gammaproteobacteria
Deltaproteobacteria
Epsilonproteobacteria
Zetaproteobacteria
Oligoflexia
Acidithiobacillia
%Usage
CCG
16%
12%
8%
4%

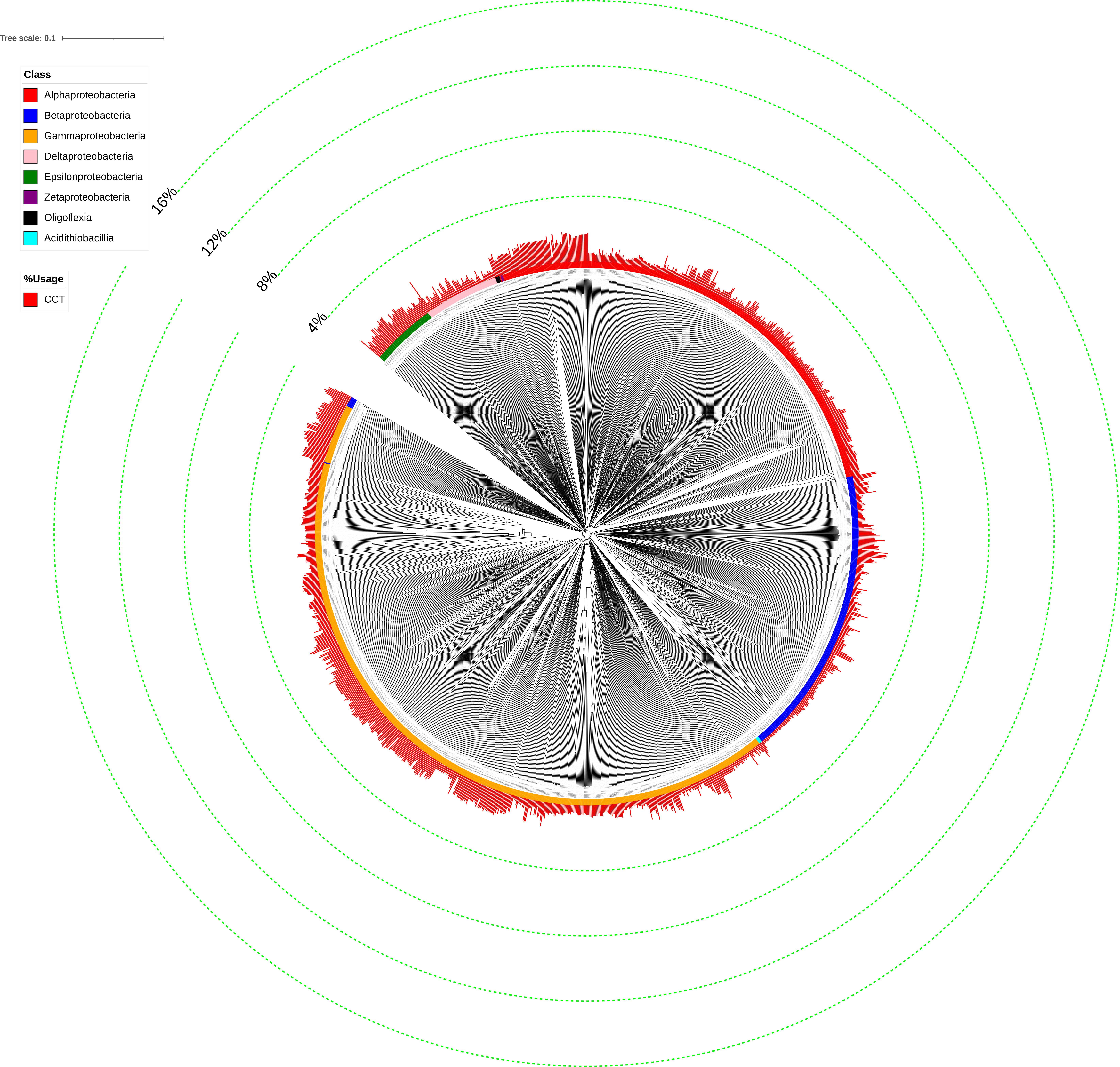

Tree scale: 0.1
Class
Alphaproteobacteria
Betaproteobacteria
Gammaproteobacteria
Deltaproteobacteria
Epsilonproteobacteria
Zetaproteobacteria
Oligoflexia
Acidithiobacillia
%Usage
CCT
4%
8%
12%
16%

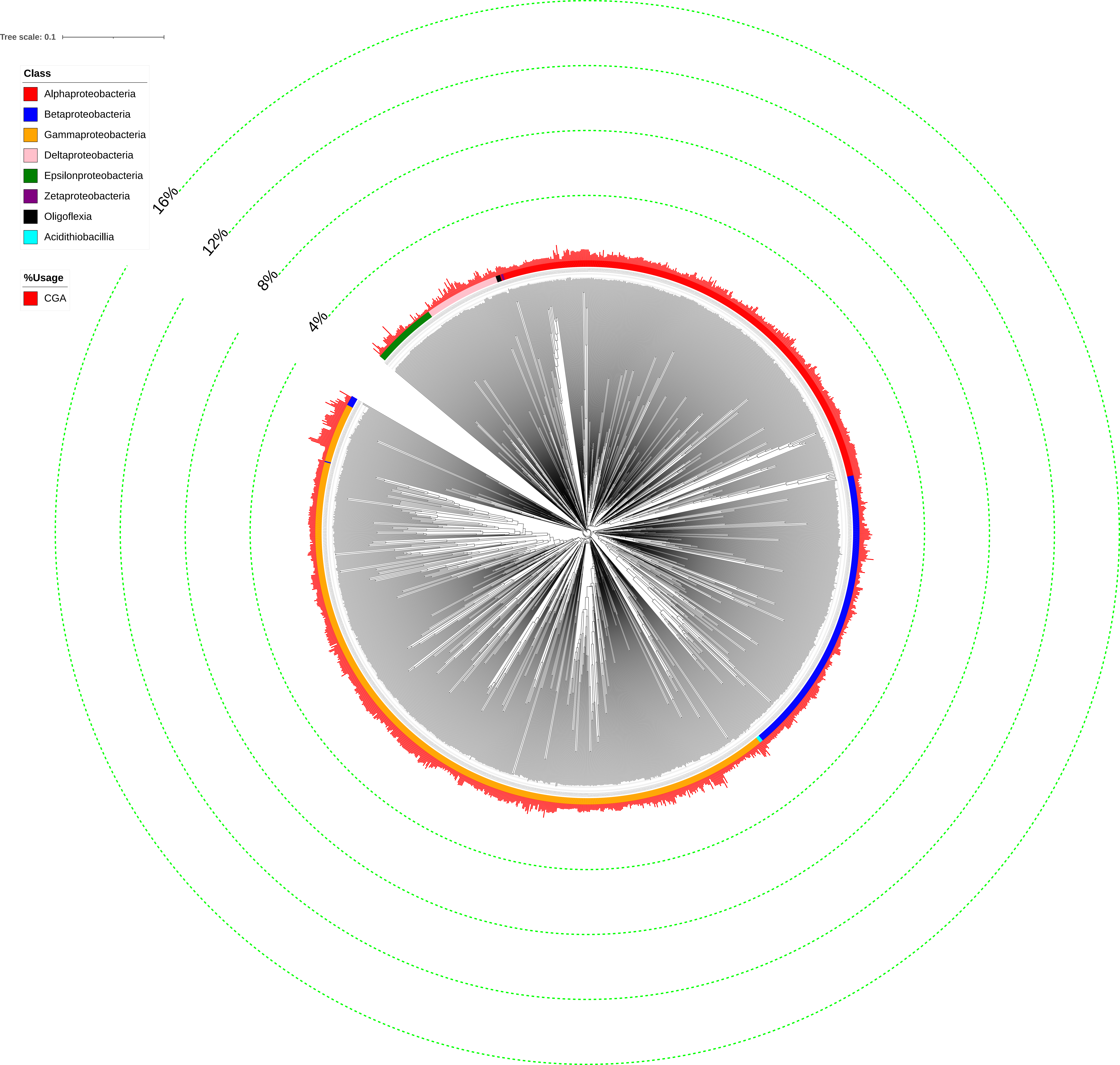

Tree scale: 0.1
Class
Alphaproteobacteria
Betaproteobacteria
Gammaproteobacteria
Deltaproteobacteria
Epsilonproteobacteria
Zetaproteobacteria
Oligoflexia
Acidithiobacillia
%Usage
CGA
16%
12%
8%
4%

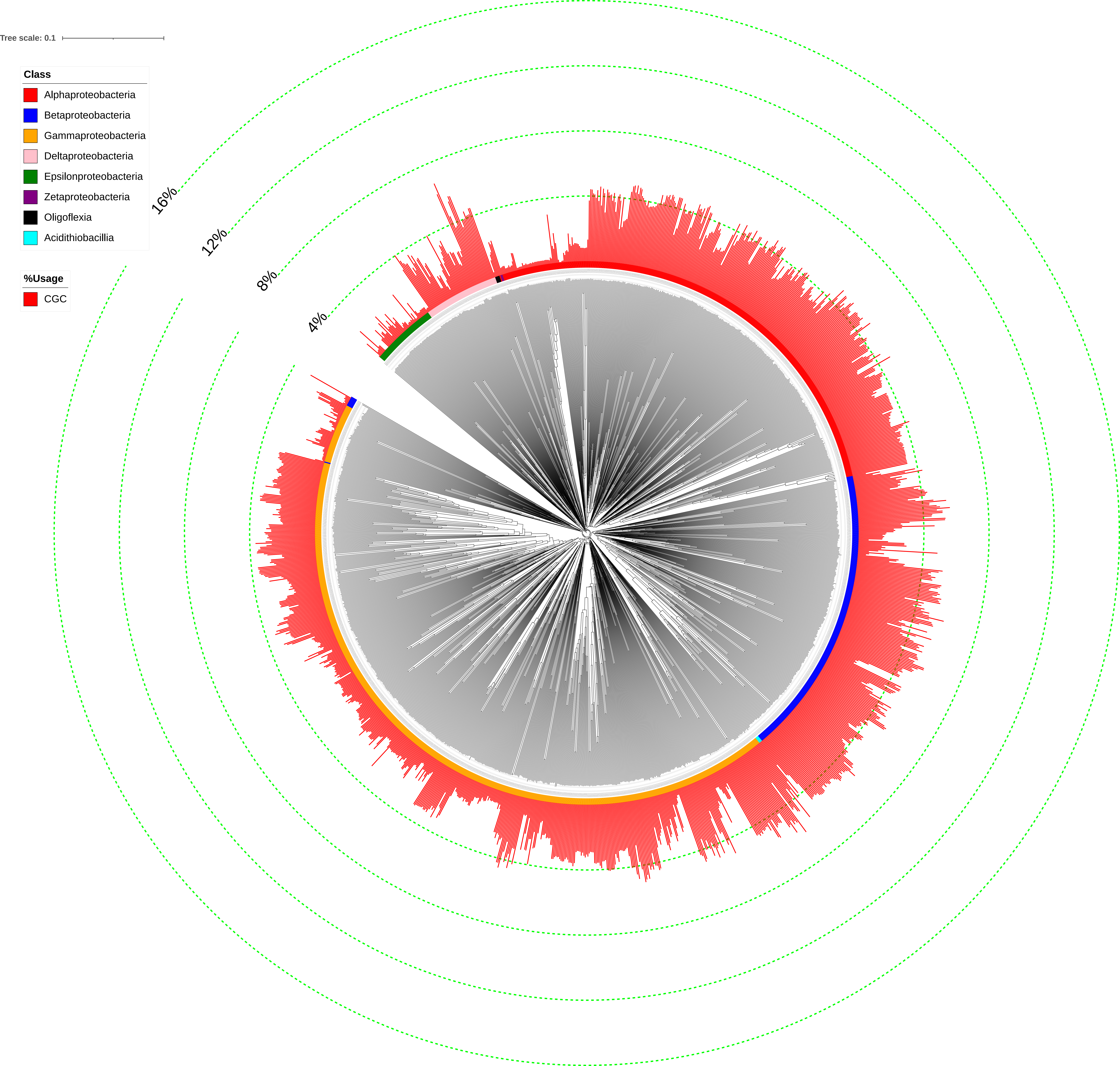
Tree scale: 0.1
Class
Alphaproteobacteria
Betaproteobacteria
Gammaproteobacteria
Deltaproteobacteria
Epsilonproteobacteria
Zetaproteobacteria
Oligoflexia
Acidithiobacillia
%Usage
CGC
16%
12%
8%
4%

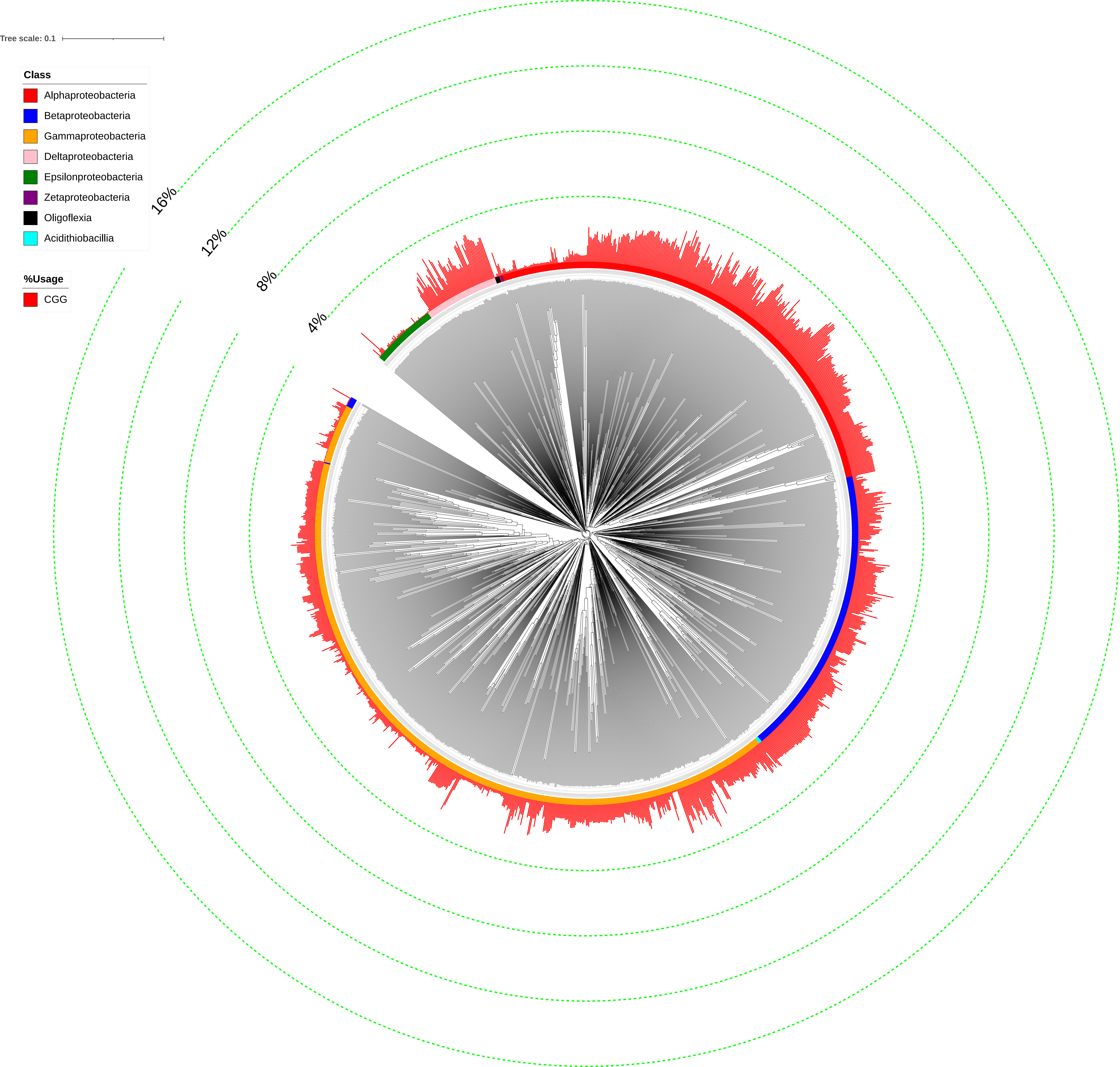

Tree scale: 0.1
Class
Alphaproteobacteria
Betaproteobacteria
Gammaproteobacteria
Deltaproteobacteria
Epsilonproteobacteria
Zetaproteobacteria
Oligoflexia
Acidithiobacillia
%Usage
CGG
16%
12%
8%
4%

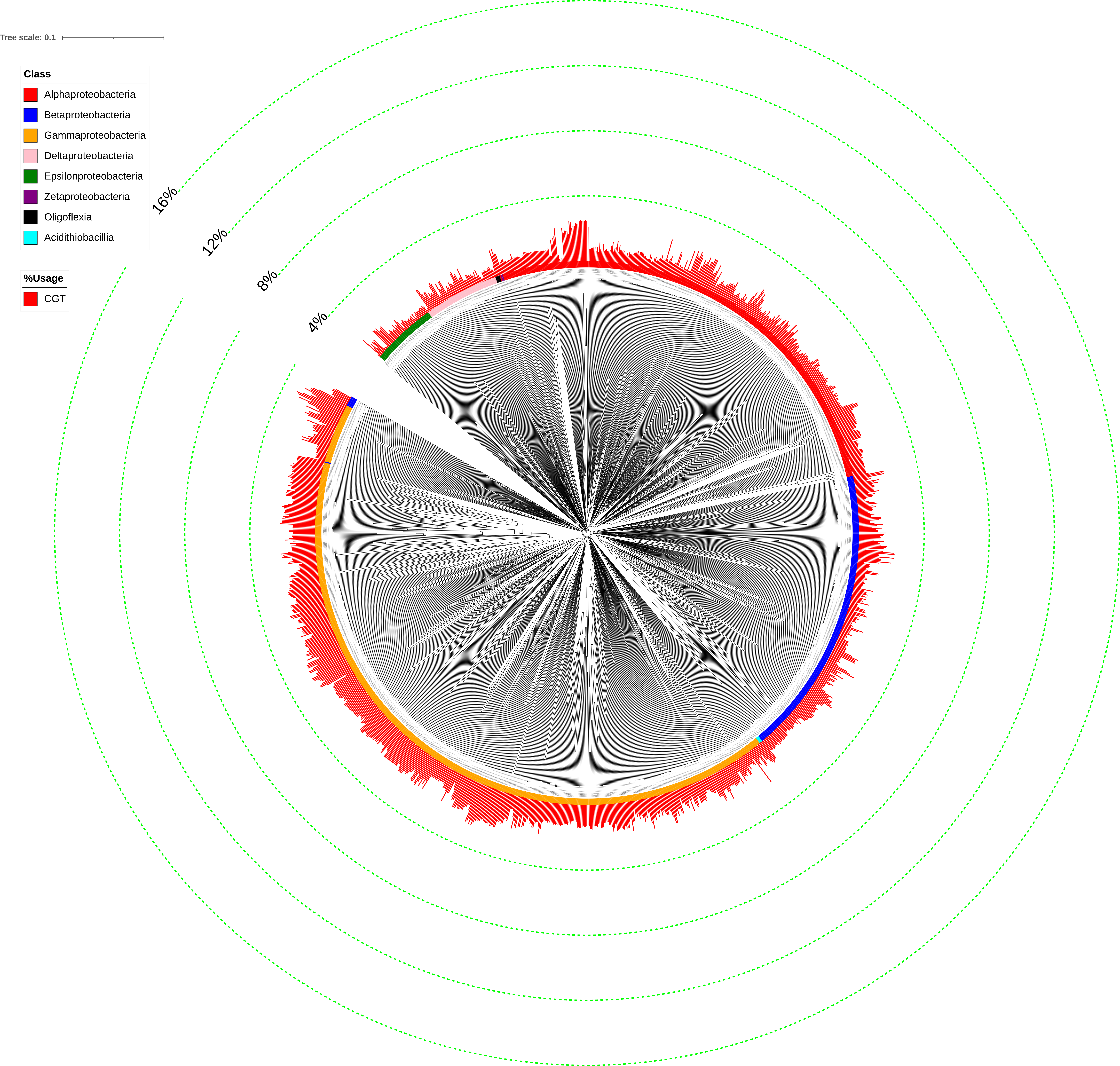
Tree scale: 0.1
Class
Alphaproteobacteria
Betaproteobacteria
Gammaproteobacteria
Deltaproteobacteria
Epsilonproteobacteria
Zetaproteobacteria
Oligoflexia
Acidithiobacillia
%Usage
CGT
16%
12%
8%
4%

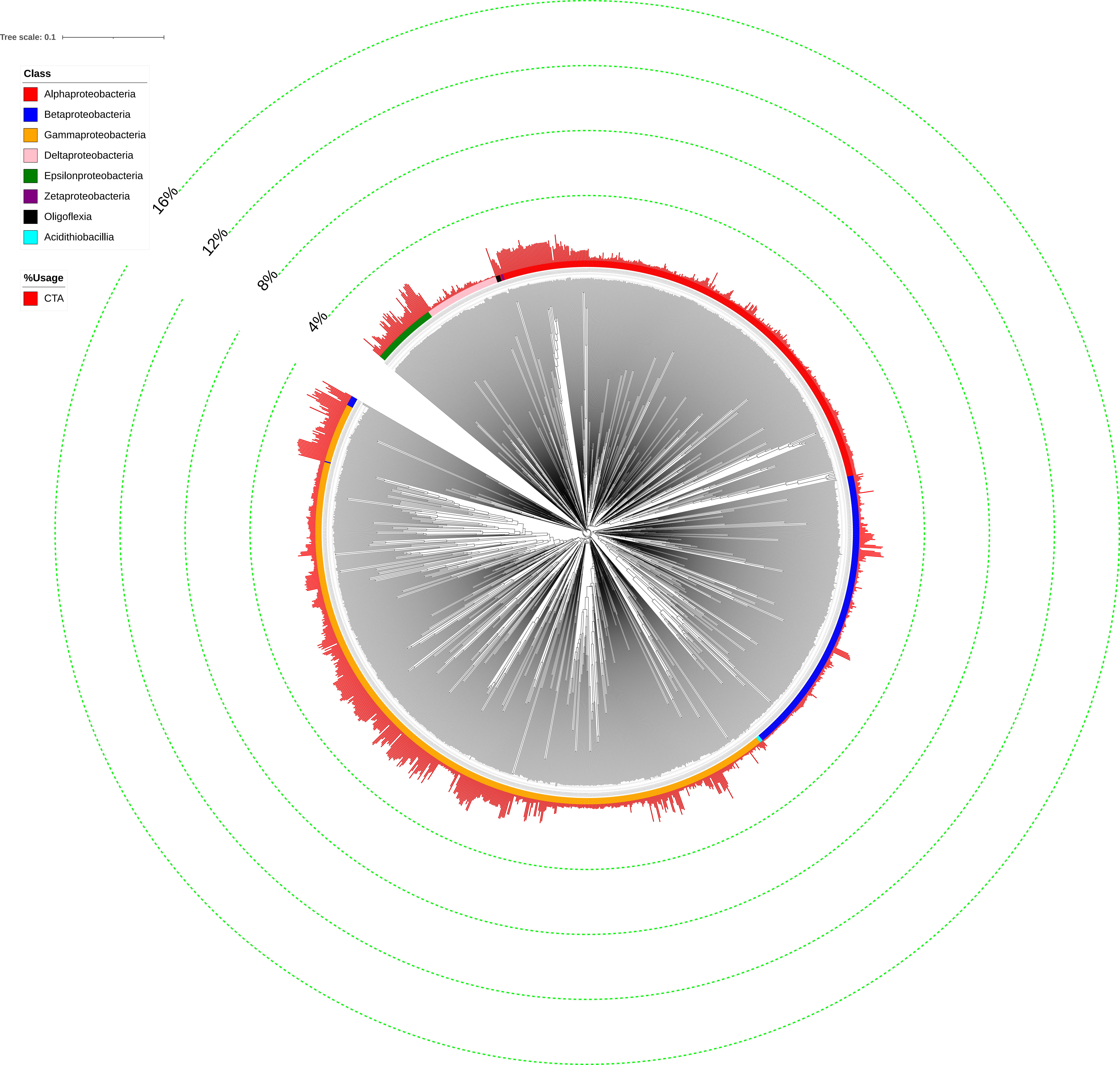
Tree scale: 0.1
Class
Alphaproteobacteria
Betaproteobacteria
Gammaproteobacteria
Deltaproteobacteria
Epsilonproteobacteria
Zetaproteobacteria
Oligoflexia
Acidithiobacillia
%Usage
CTA
16%
12%
8%
4%

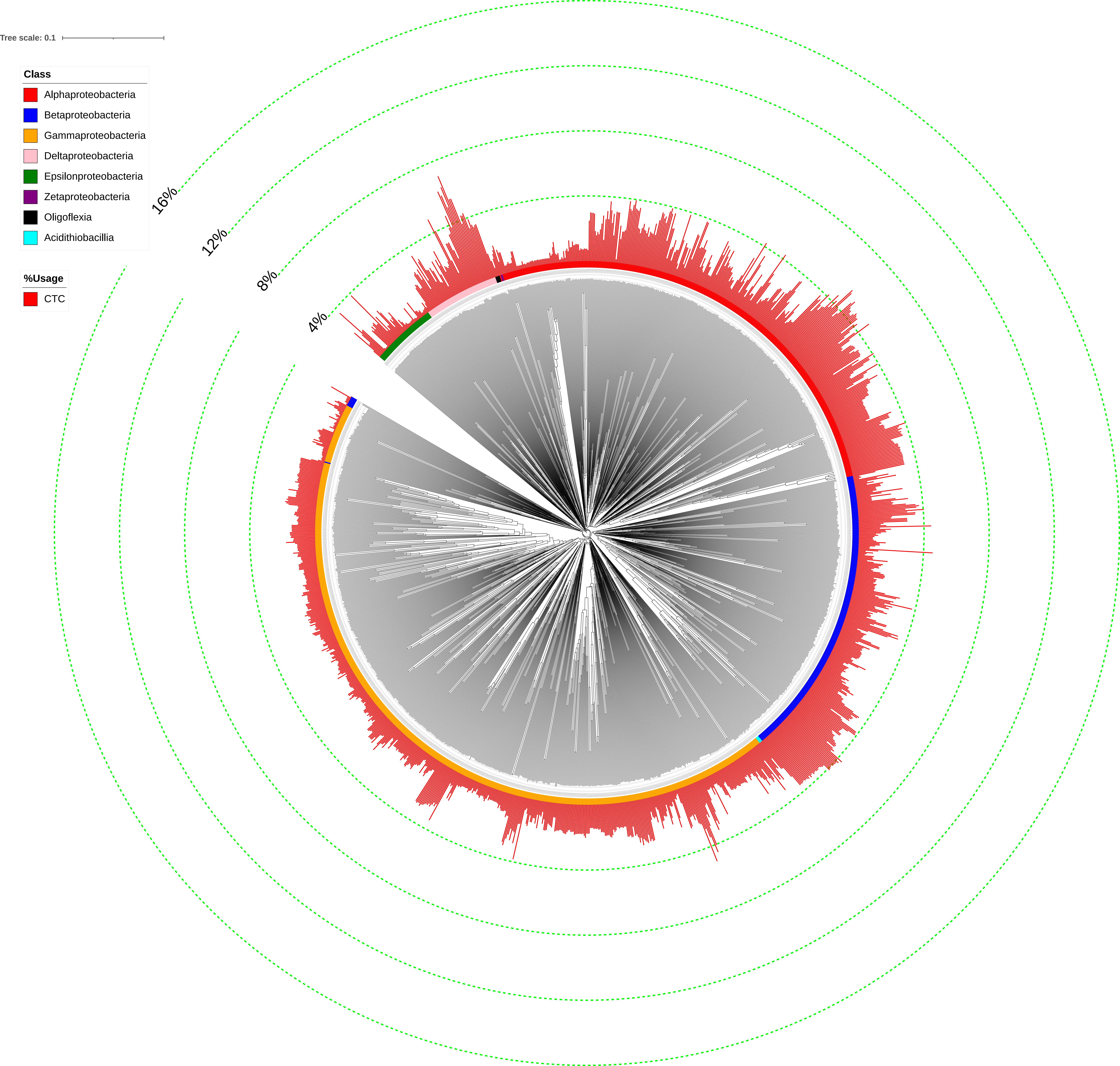
Tree scale: 0.1
Class
Alphaproteobacteria
Betaproteobacteria
Gammaproteobacteria
Deltaproteobacteria
Epsilonproteobacteria
Zetaproteobacteria
Oligoflexia
Acidithiobacillia
%Usage
CTC
16%
12%
8%
4%

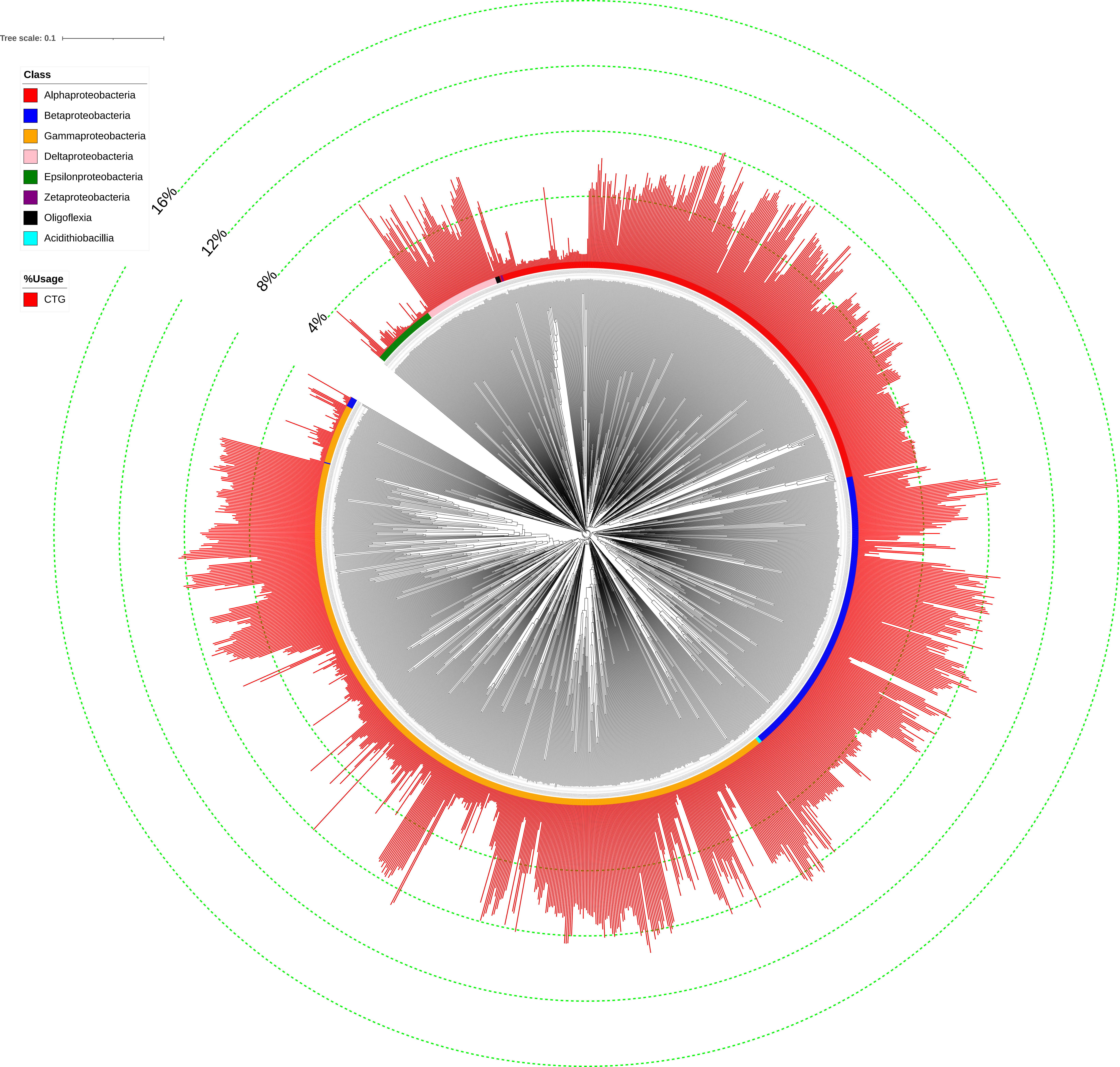

Tree scale: 0.1
Class
Alphaproteobacteria
Betaproteobacteria
Gammaproteobacteria
Deltaproteobacteria
Epsilonproteobacteria
Zetaproteobacteria
Oligoflexia
Acidithiobacillia
%Usage
CTG
16%
12%
8%
4%

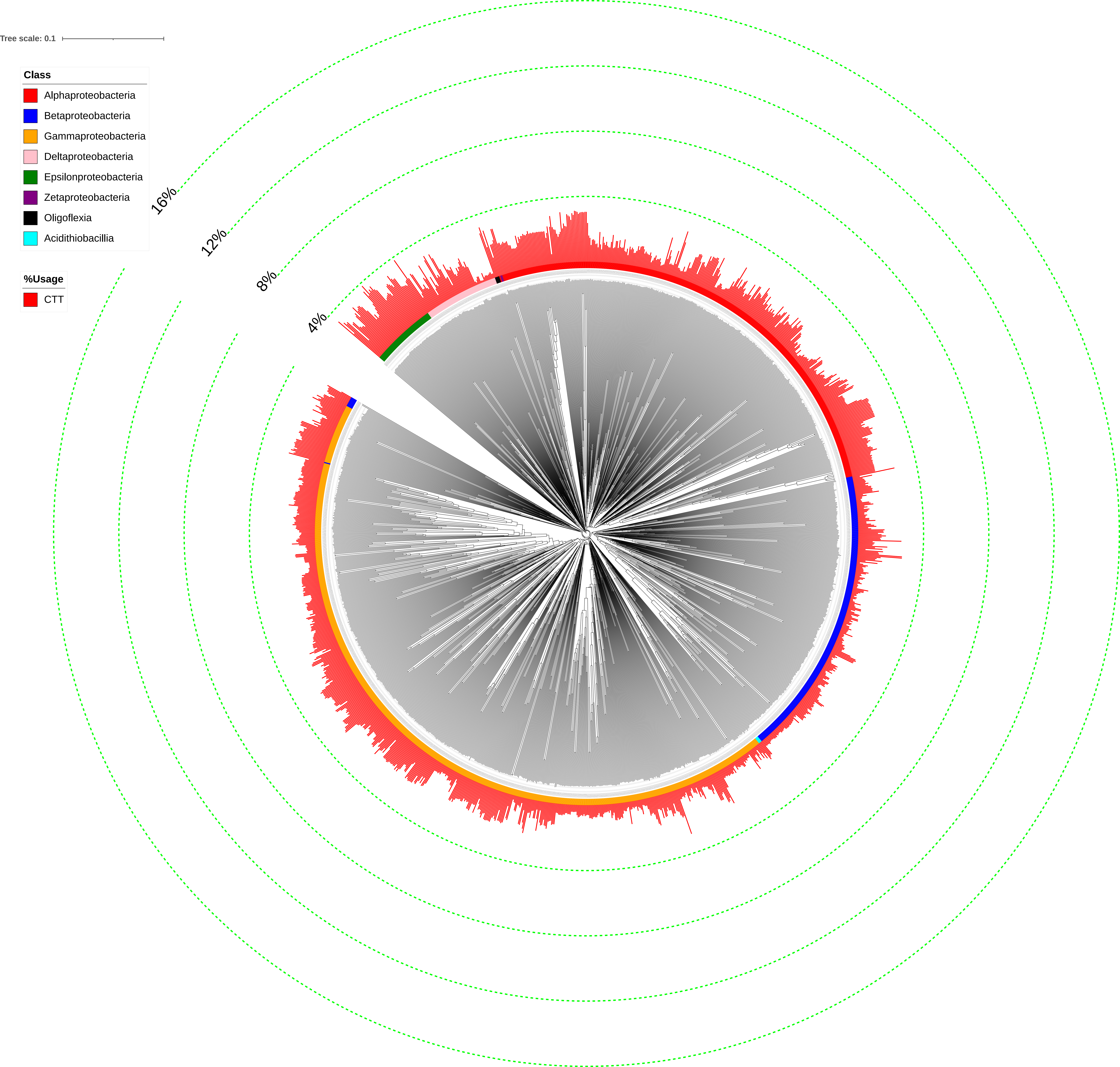

Tree scale: 0.1
Class
Alphaproteobacteria
Betaproteobacteria
Gammaproteobacteria
Deltaproteobacteria
Epsilonproteobacteria
Zetaproteobacteria
Oligoflexia
Acidithiobacillia
%Usage
CTT
4%
8%
12%
16%

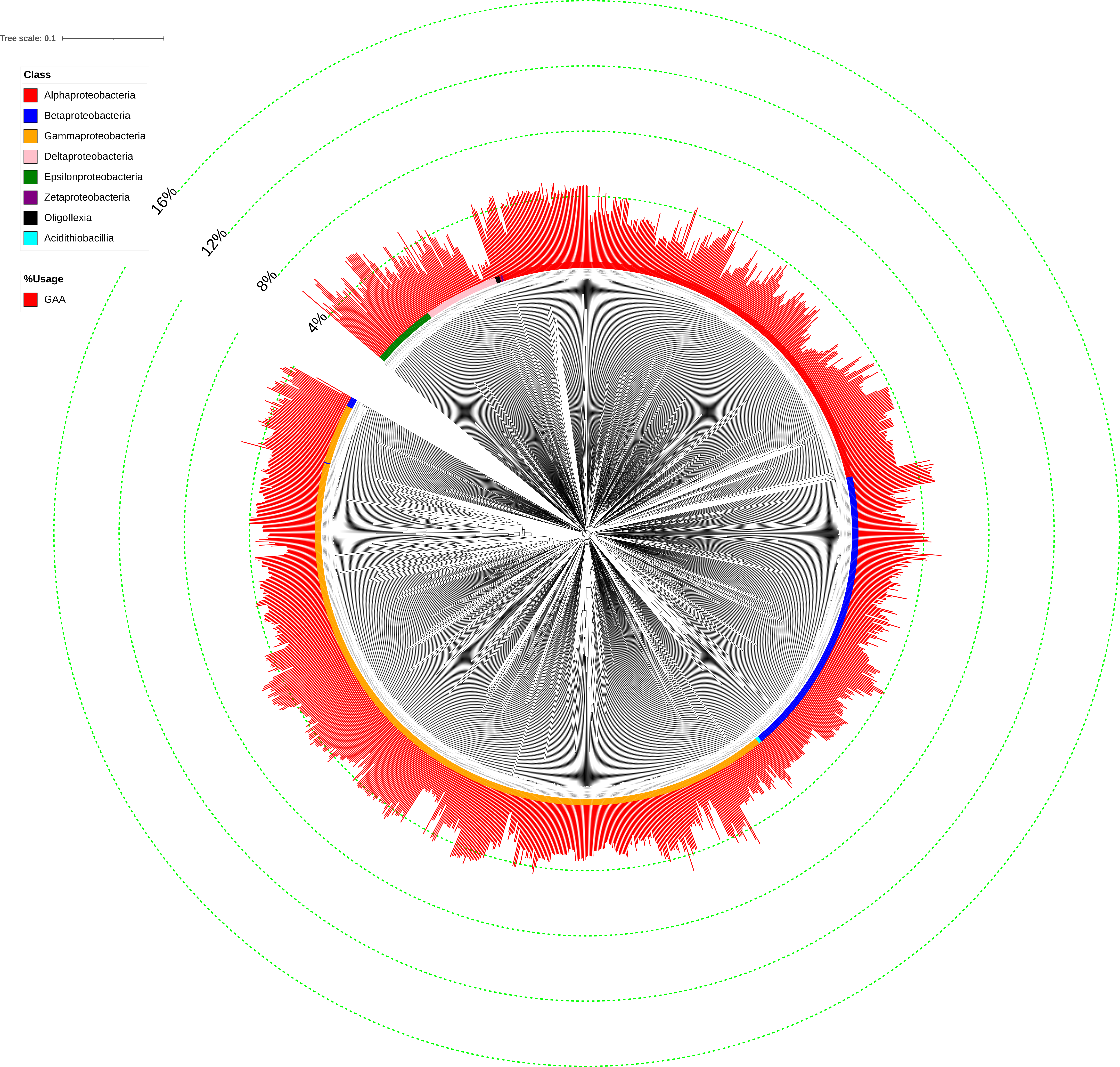

Tree scale: 0.1
Class
Alphaproteobacteria
Betaproteobacteria
Gammaproteobacteria
Deltaproteobacteria
Epsilonproteobacteria
Zetaproteobacteria
Oligoflexia
Acidithiobacillia
%Usage
GAA
4%
8%
12%
16%

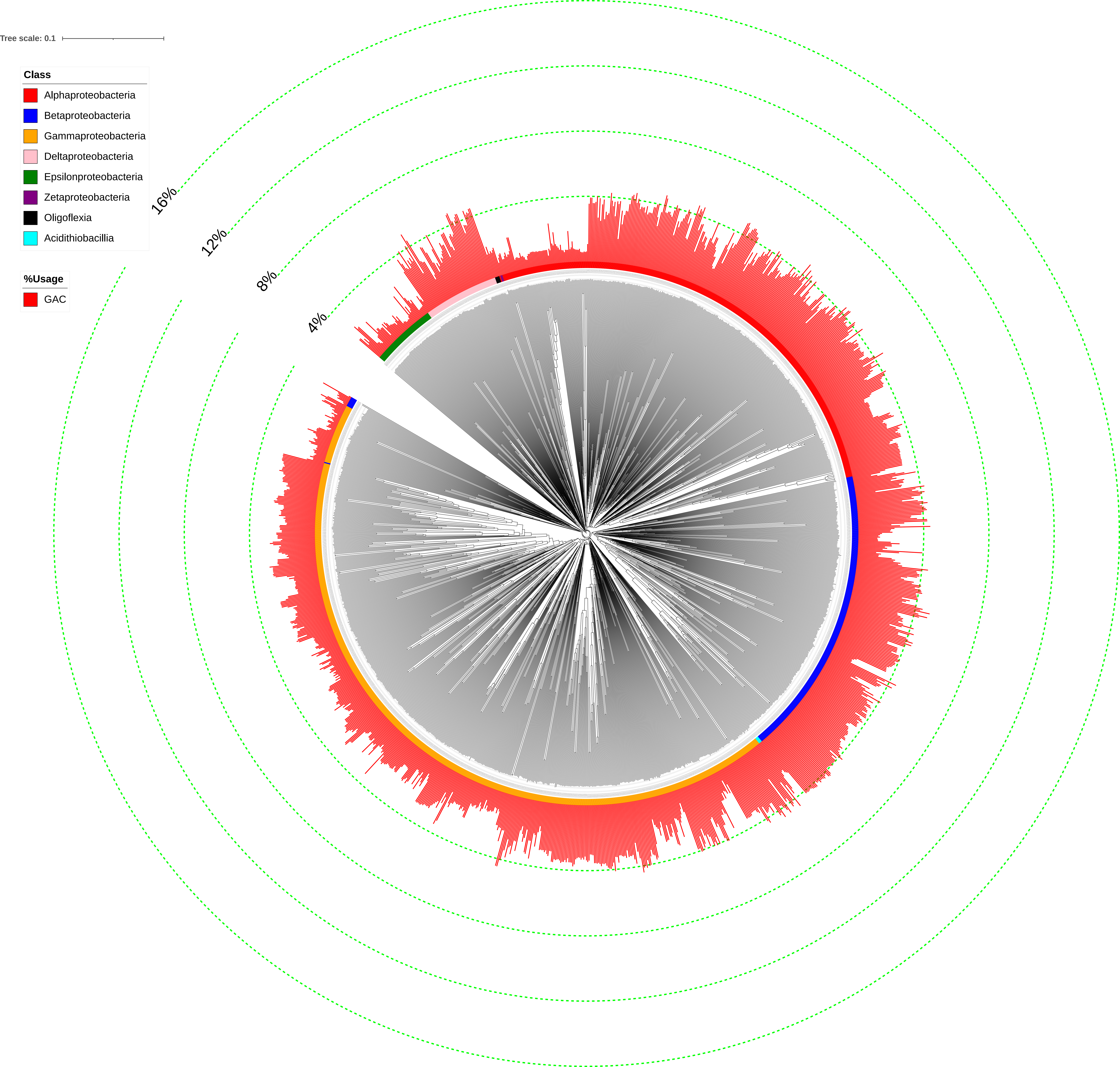

Tree scale: 0.1
Class
Alphaproteobacteria
Betaproteobacteria
Gammaproteobacteria
Deltaproteobacteria
Epsilonproteobacteria
Zetaproteobacteria
Oligoflexia
Acidithiobacillia
%Usage
GAC
16%
12%
8%
4%

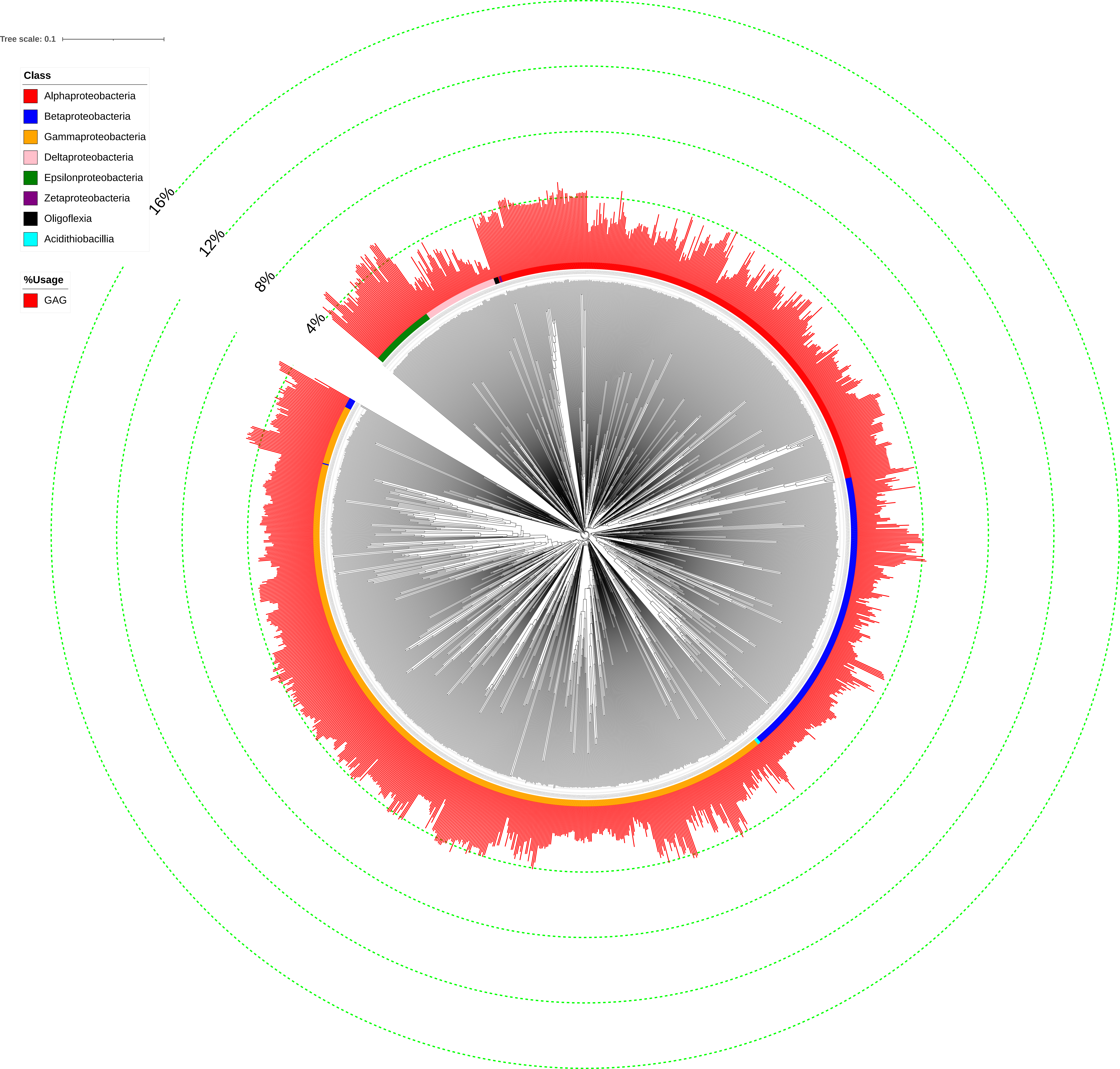

Tree scale: 0.1
Class
Alphaproteobacteria
Betaproteobacteria
Gammaproteobacteria
Deltaproteobacteria
Epsilonproteobacteria
Zetaproteobacteria
Oligoflexia
Acidithiobacillia
%Usage
GAG
4%
8%
12%
16%

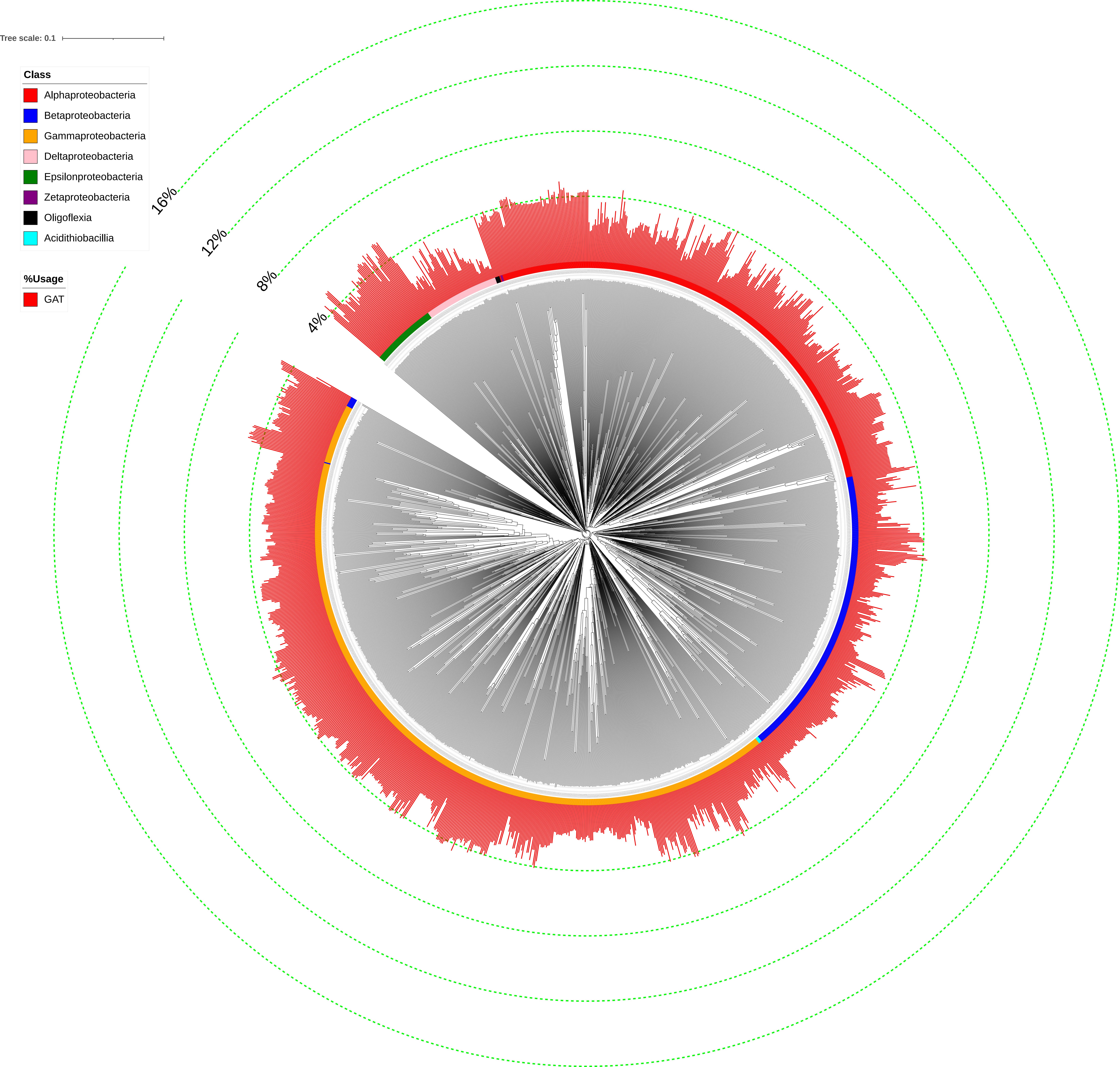

Tree scale: 0.1
Class
Alphaproteobacteria
Betaproteobacteria
Gammaproteobacteria
Deltaproteobacteria
Epsilonproteobacteria
Zetaproteobacteria
Oligoflexia
Acidithiobacillia
%Usage
GAT
16%
12%
8%
4%

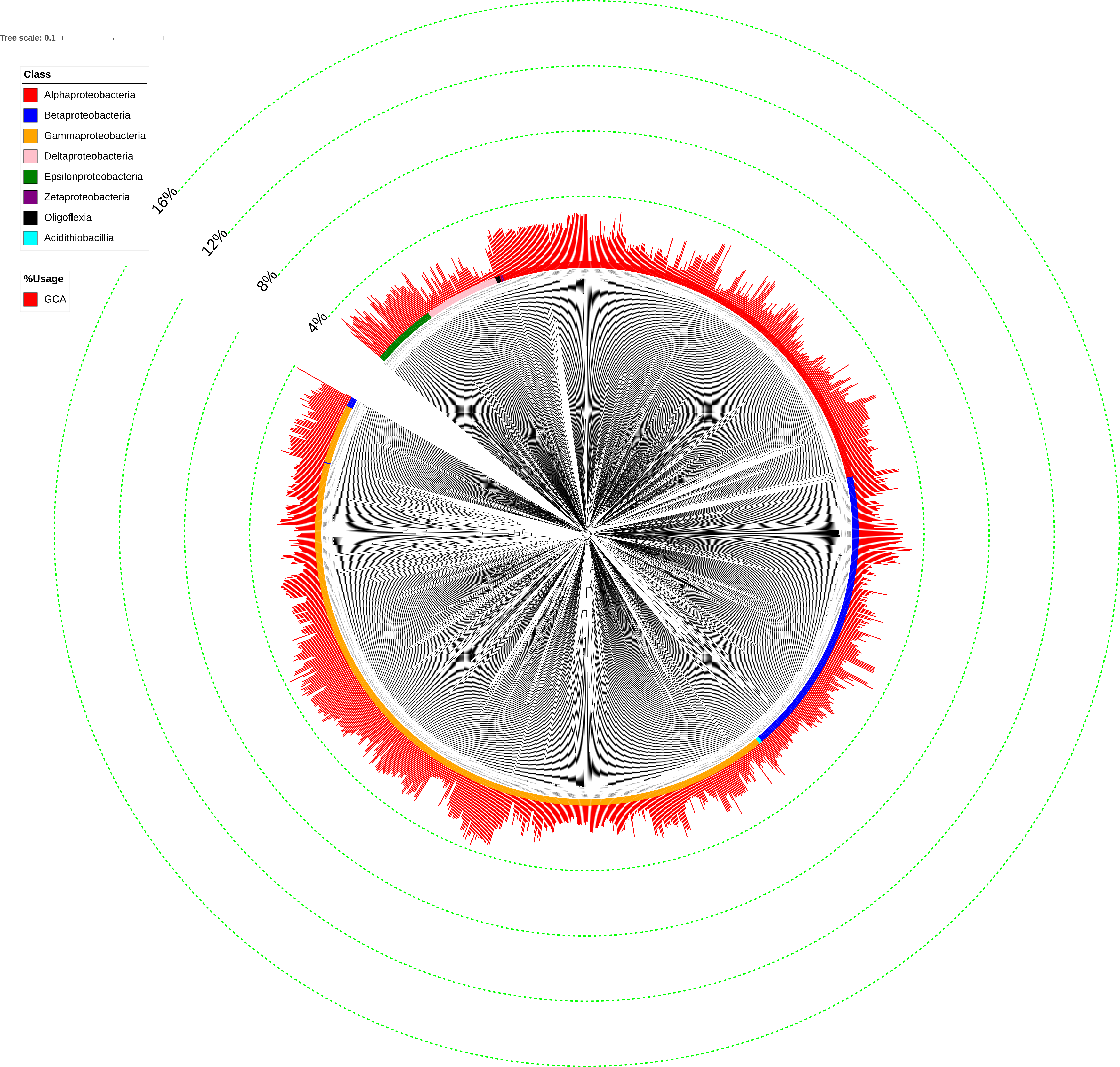

Tree scale: 0.1
Class
Alphaproteobacteria
Betaproteobacteria
Gammaproteobacteria
Deltaproteobacteria
Epsilonproteobacteria
Zetaproteobacteria
Oligoflexia
Acidithiobacillia
%Usage
GCA
16%
12%
8%
4%

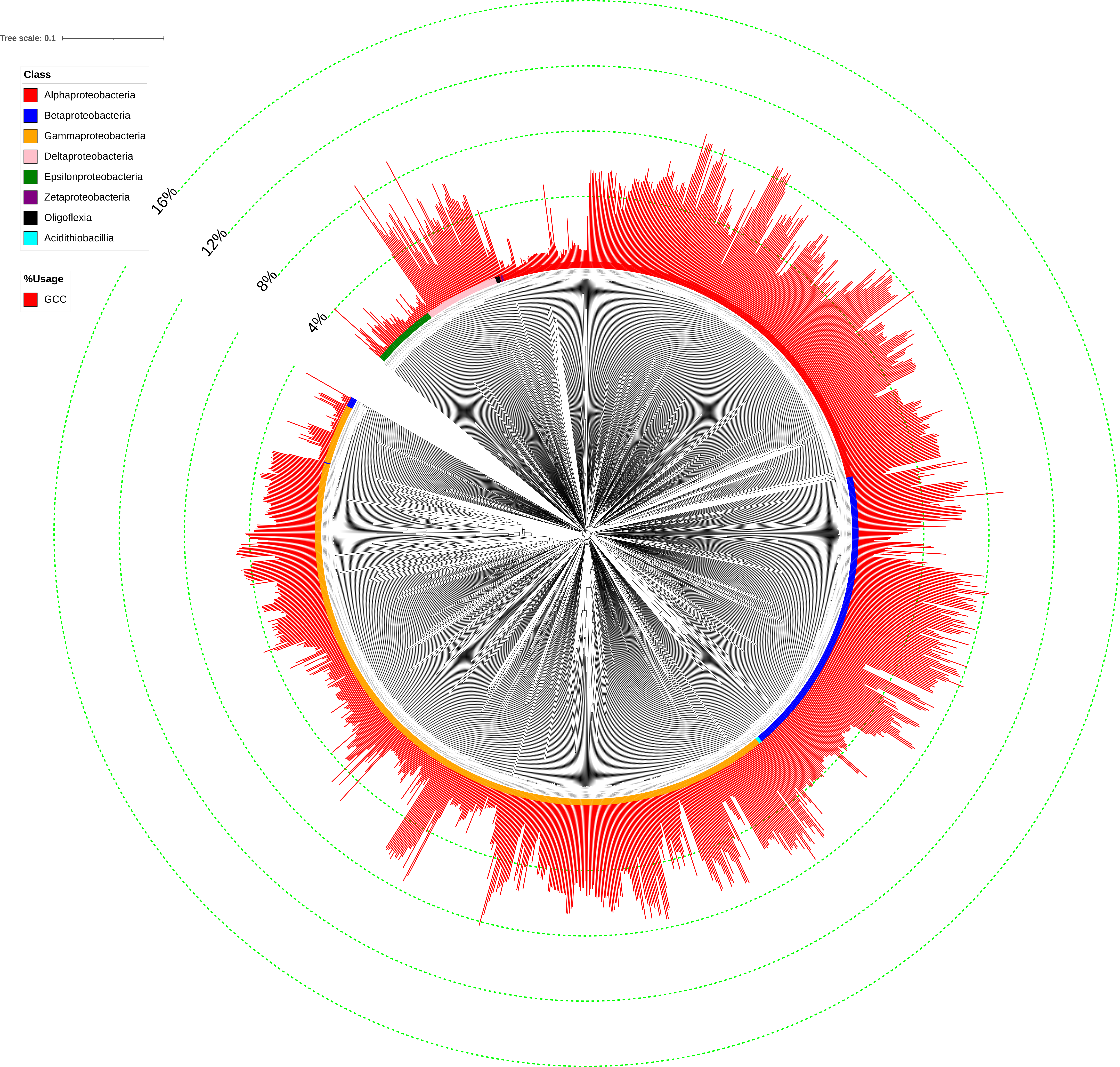

Tree scale: 0.1
Class
Alphaproteobacteria
Betaproteobacteria
Gammaproteobacteria
Deltaproteobacteria
Epsilonproteobacteria
Zetaproteobacteria
Oligoflexia
Acidithiobacillia
%Usage
GCC
4%
8%
12%
16%

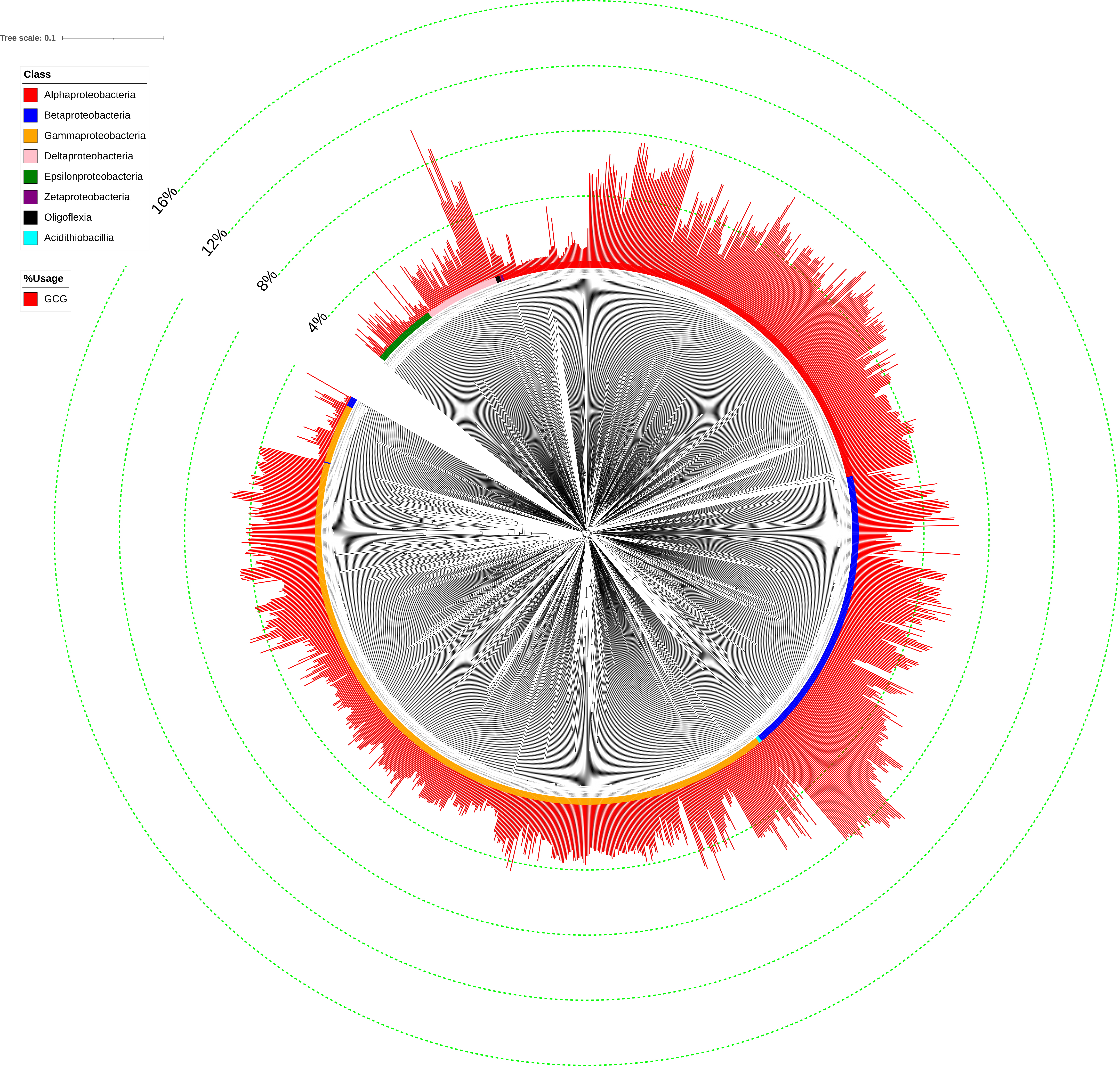

Tree scale: 0.1
Class
Alphaproteobacteria
Betaproteobacteria
Gammaproteobacteria
Deltaproteobacteria
Epsilonproteobacteria
Zetaproteobacteria
Oligoflexia
Acidithiobacillia
%Usage
GCG
4%
8%
12%
16%

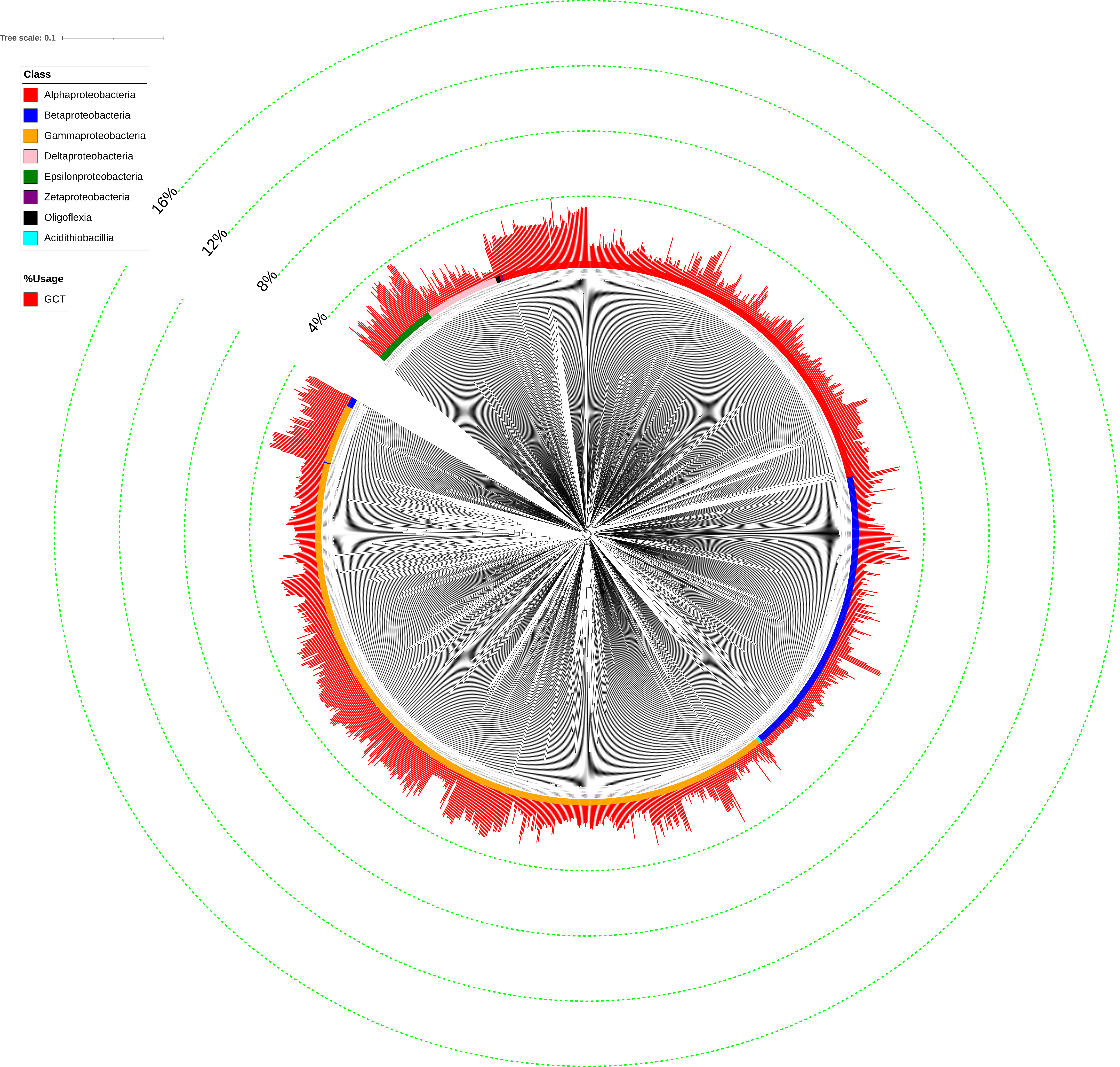

Tree scale: 0.1
Class
Alphaproteobacteria
Betaproteobacteria
Gammaproteobacteria
Deltaproteobacteria
Epsilonproteobacteria
Zetaproteobacteria
Oligoflexia
Acidithiobacillia
%Usage
GCT
16%
12%
8%
4%

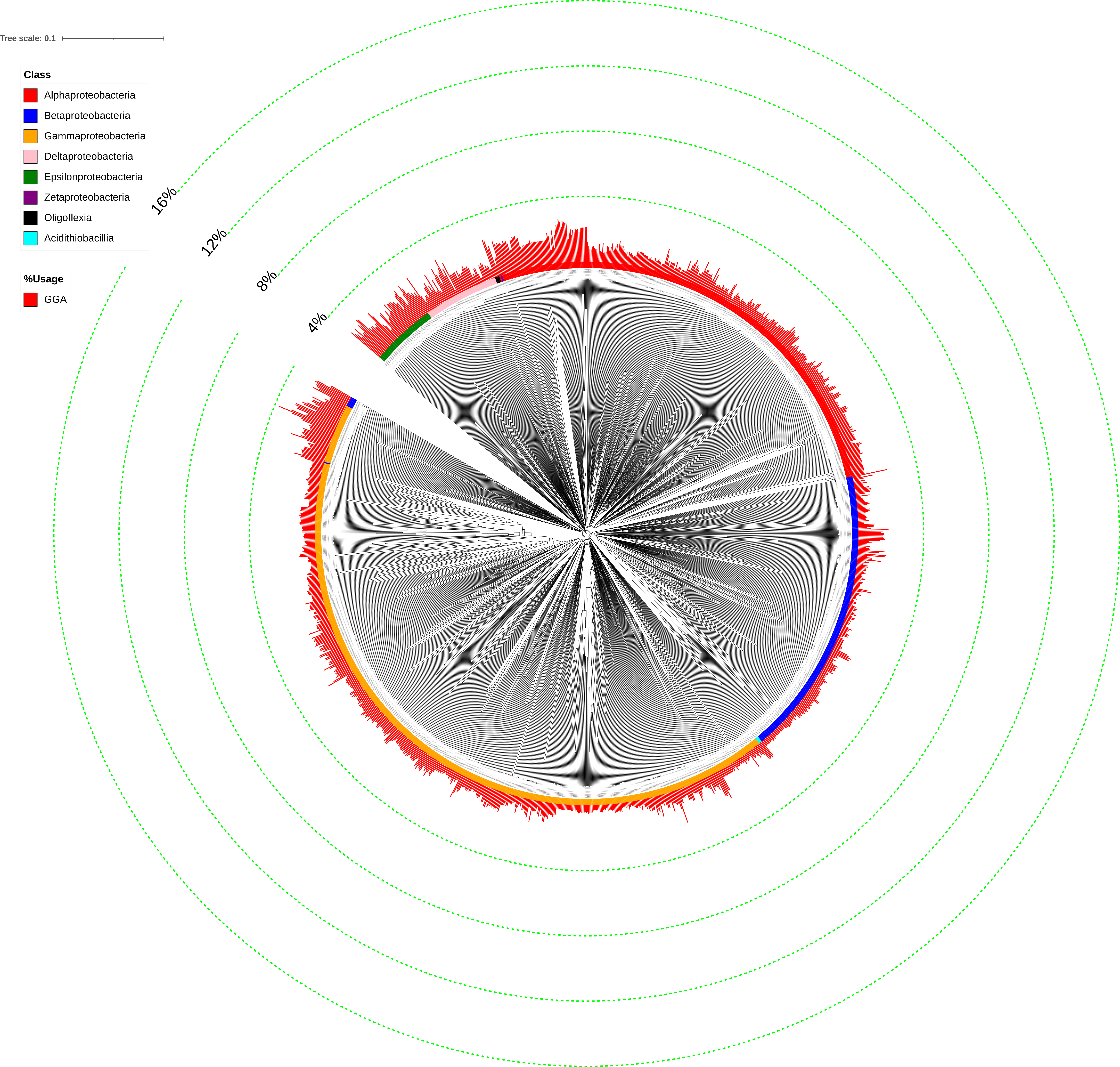

Tree scale: 0.1
Class
Alphaproteobacteria
Betaproteobacteria
Gammaproteobacteria
Deltaproteobacteria
Epsilonproteobacteria
Zetaproteobacteria
Oligoflexia
Acidithiobacillia
%Usage
GGA
16%
12%
8%
4%

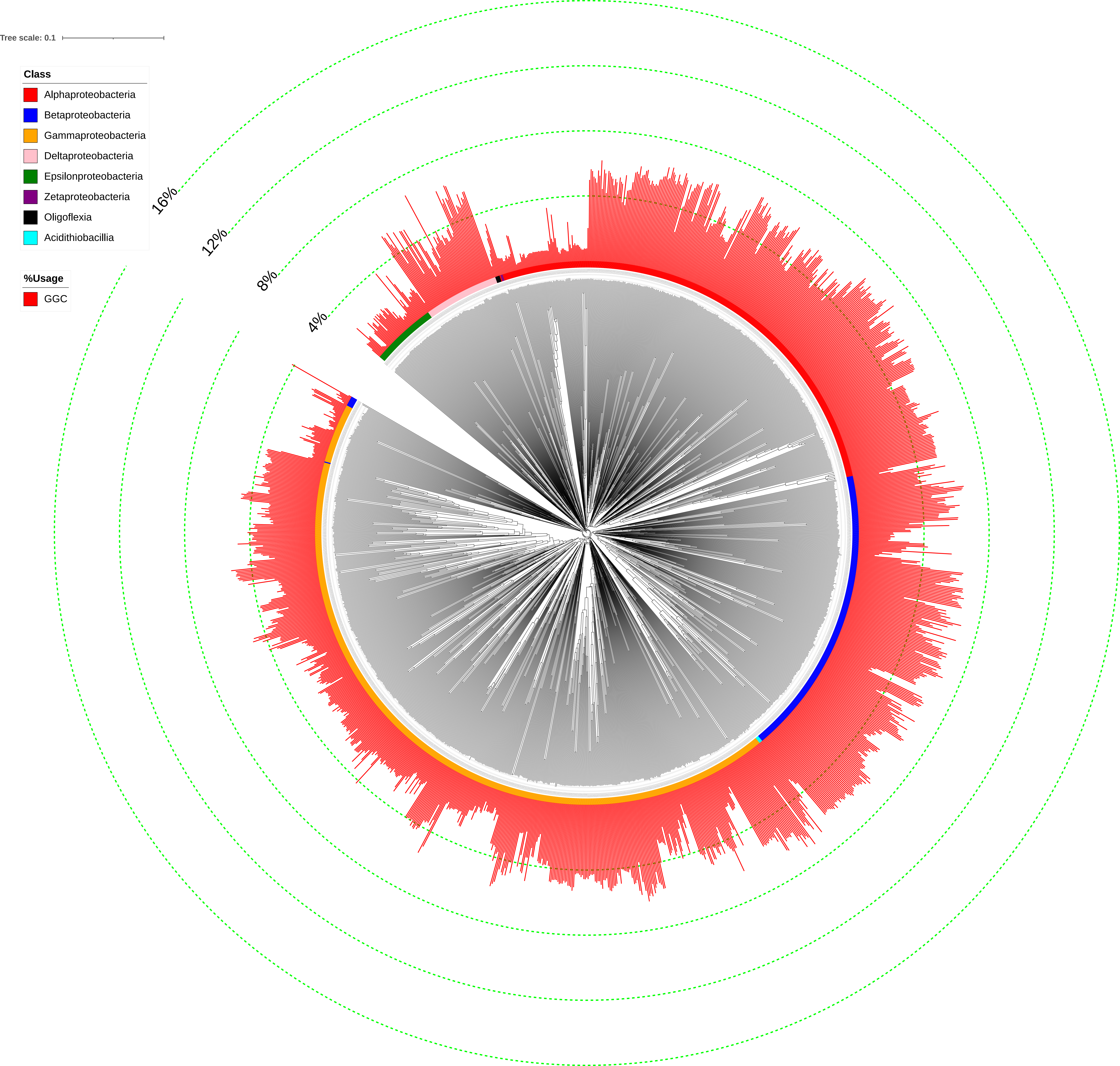

Tree scale: 0.1
Class
Alphaproteobacteria
Betaproteobacteria
Gammaproteobacteria
Deltaproteobacteria
Epsilonproteobacteria
Zetaproteobacteria
Oligoflexia
Acidithiobacillia
%Usage
GGC
4%
8%
12%
16%

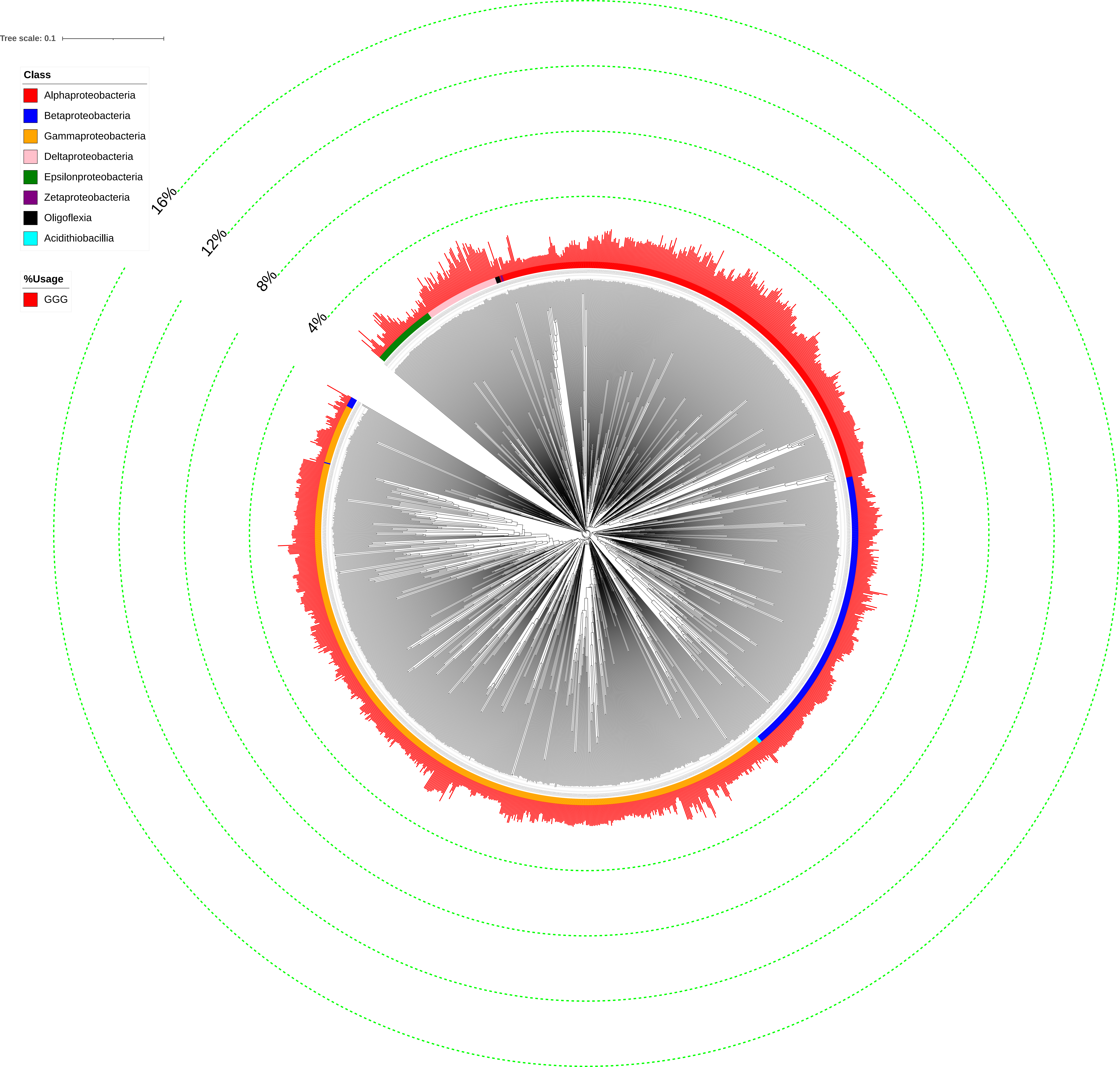

Tree scale: 0.1
Class
Alphaproteobacteria
Betaproteobacteria
Gammaproteobacteria
Deltaproteobacteria
Epsilonproteobacteria
Zetaproteobacteria
Oligoflexia
Acidithiobacillia
%Usage
GGG
16%
12%
8%
4%

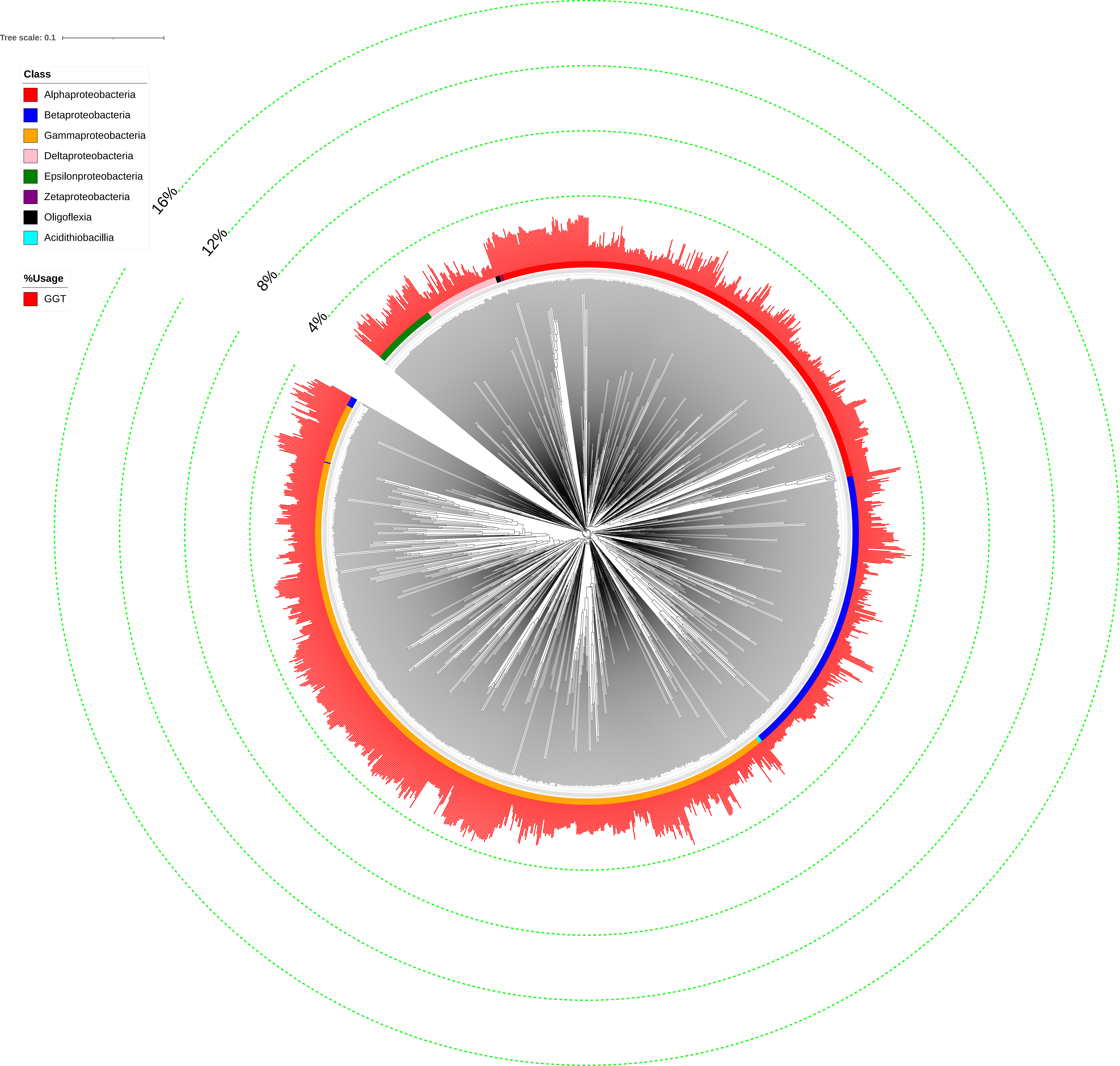

Tree scale: 0.1
Class
Alphaproteobacteria
Betaproteobacteria
Gammaproteobacteria
Deltaproteobacteria
Epsilonproteobacteria
Zetaproteobacteria
Oligoflexia
Acidithiobacillia
%Usage
GGT
16%
12%
8%
4%

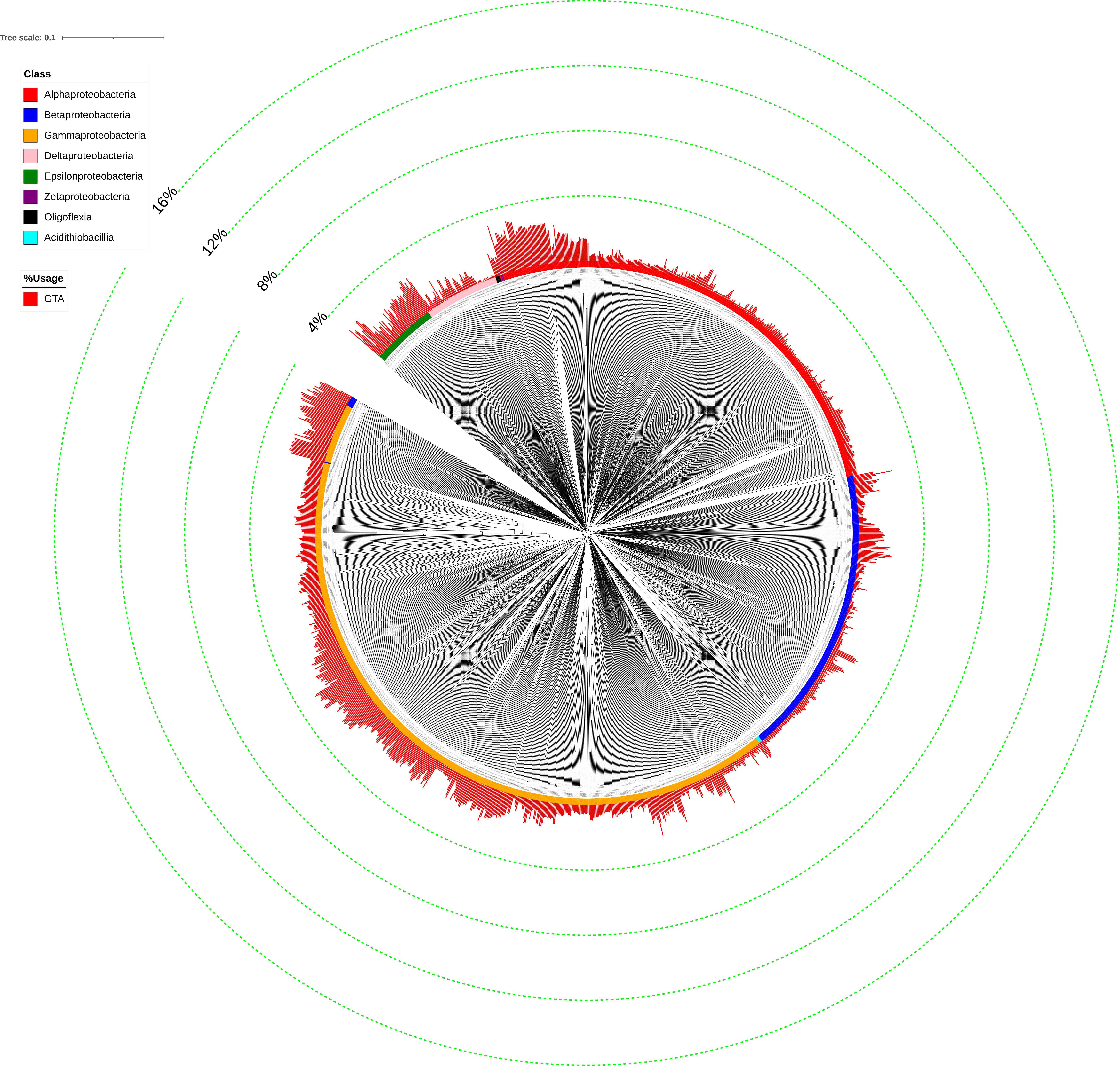

Tree scale: 0.1
Class
Alphaproteobacteria
Betaproteobacteria
Gammaproteobacteria
Deltaproteobacteria
Epsilonproteobacteria
Zetaproteobacteria
Oligoflexia
Acidithiobacillia
%Usage
GTA
16%
12%
8%
4%

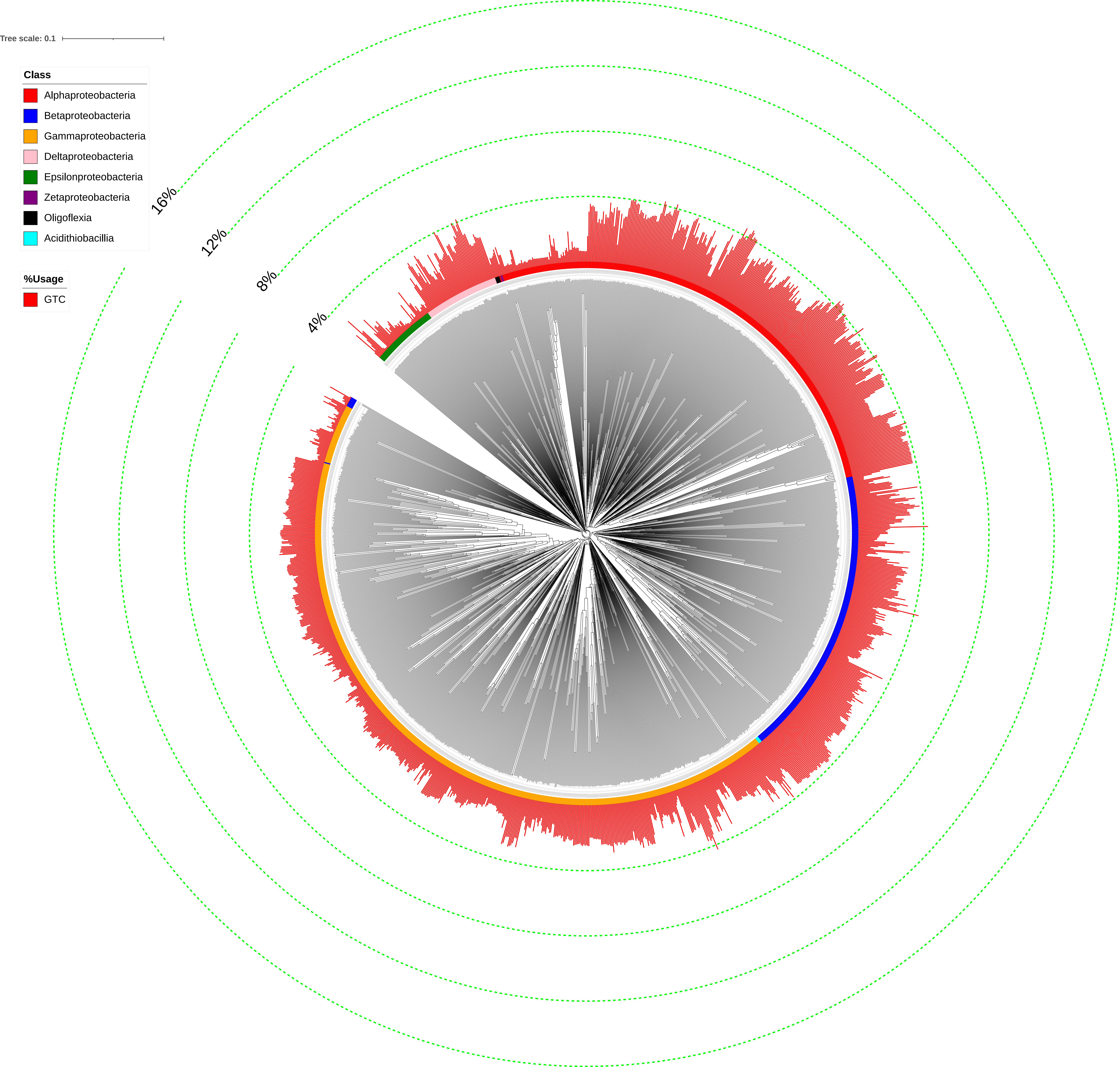

Tree scale: 0.1
Class
Alphaproteobacteria
Betaproteobacteria
Gammaproteobacteria
Deltaproteobacteria
Epsilonproteobacteria
Zetaproteobacteria
Oligoflexia
Acidithiobacillia
%Usage
GTC
16%
12%
8%
4%

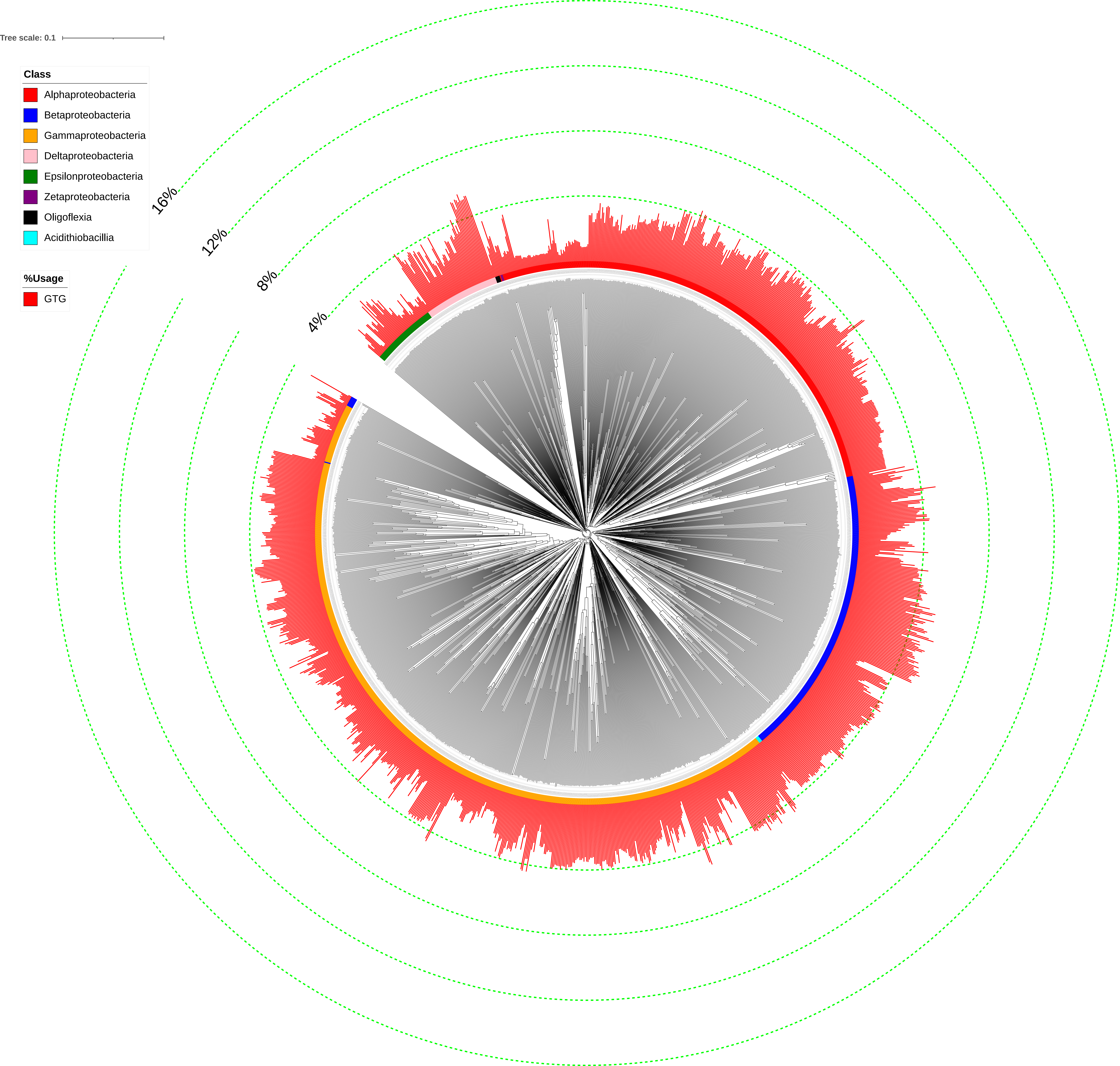

Tree scale: 0.1
Class
Alphaproteobacteria
Betaproteobacteria
Gammaproteobacteria
Deltaproteobacteria
Epsilonproteobacteria
Zetaproteobacteria
Oligoflexia
Acidithiobacillia
%Usage
GTG
16%
12%
8%
4%

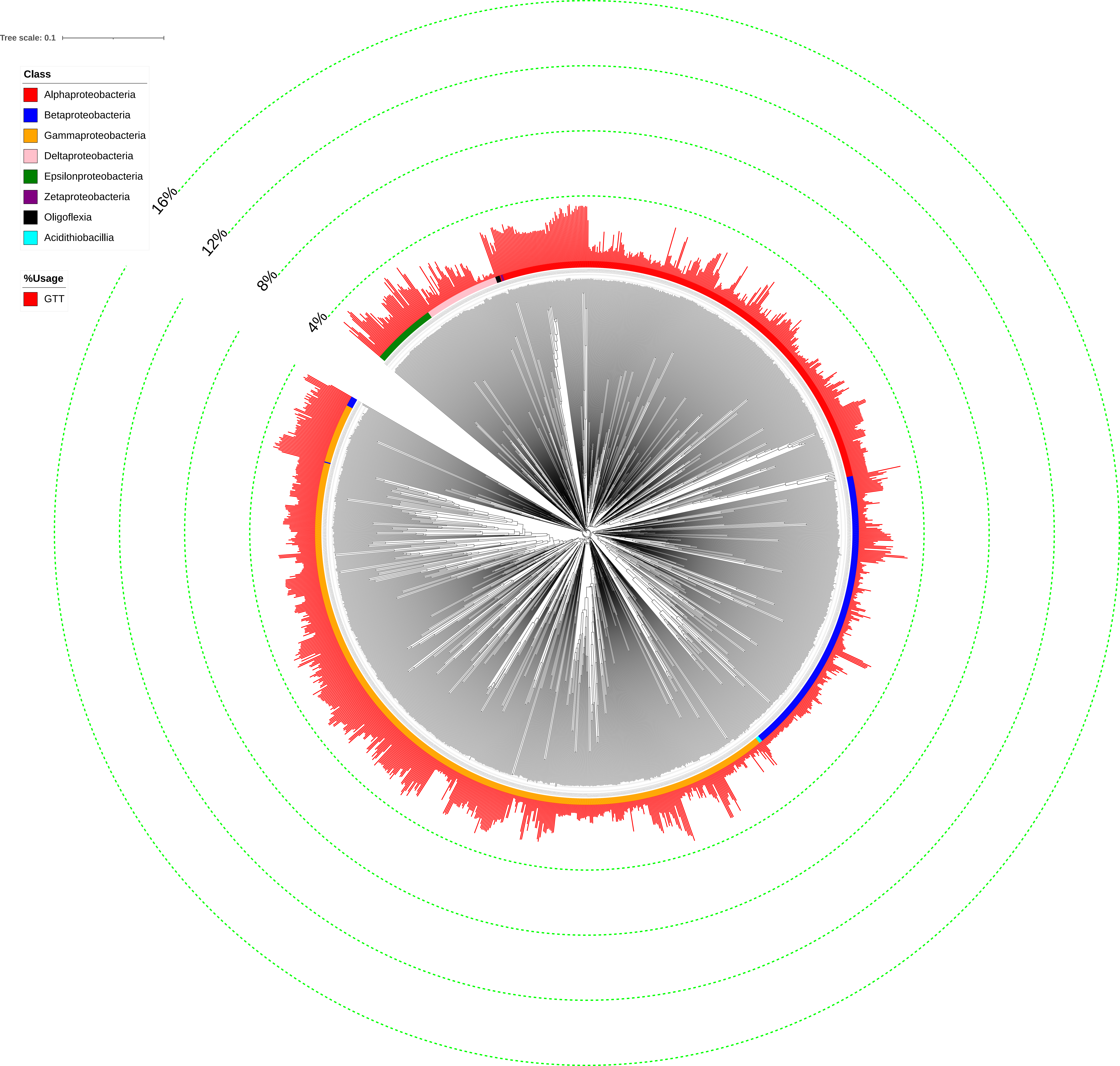

Tree scale: 0.1
Class
Alphaproteobacteria
Betaproteobacteria
Gammaproteobacteria
Deltaproteobacteria
Epsilonproteobacteria
Zetaproteobacteria
Oligoflexia
Acidithiobacillia
%Usage
GTT
4%
8%
12%
16%

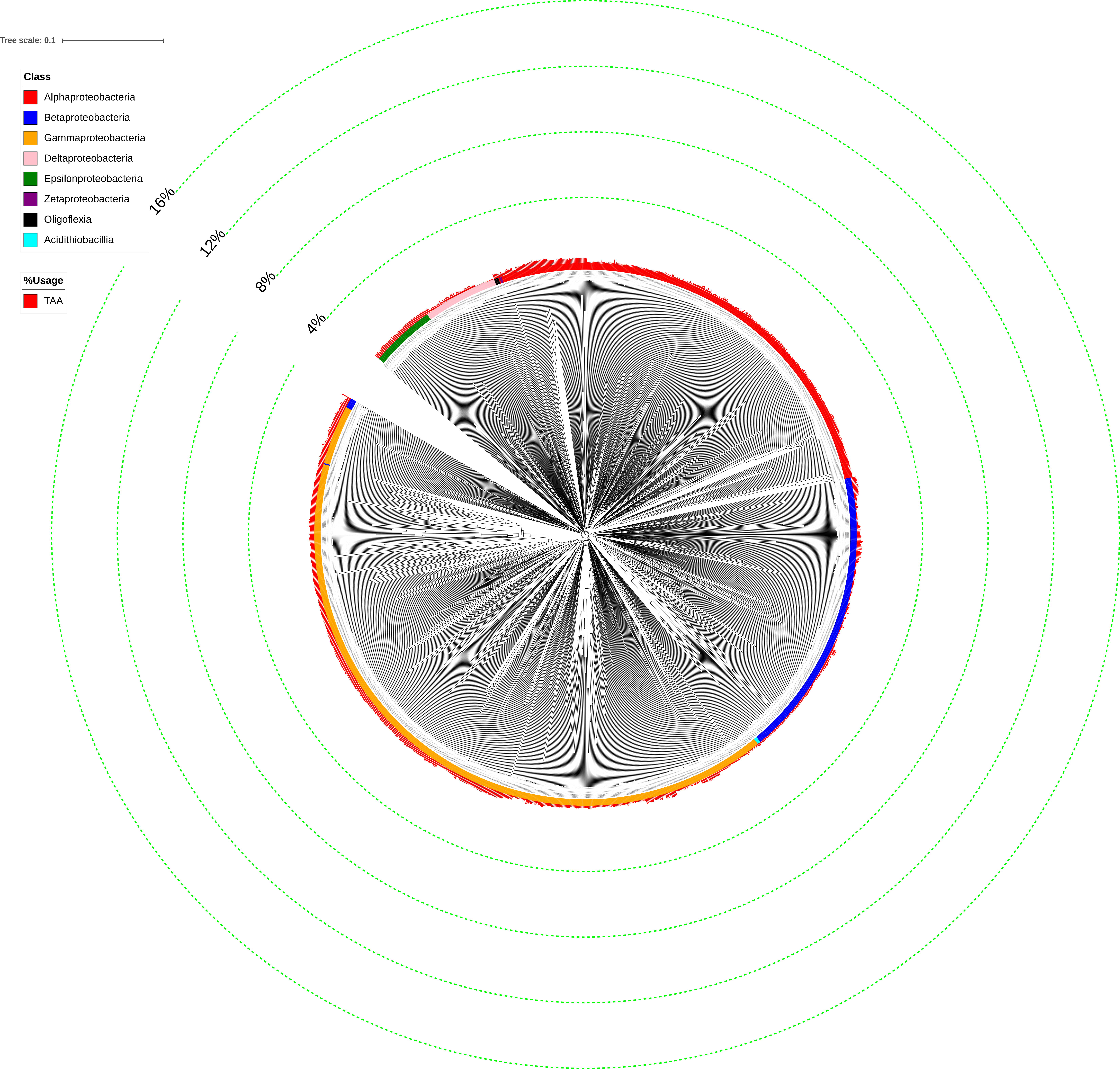
Tree scale: 0.1
Class
Alphaproteobacteria
Betaproteobacteria
Gammaproteobacteria
Deltaproteobacteria
Epsilonproteobacteria
Zetaproteobacteria
Oligoflexia
Acidithiobacillia
%Usage
TAA
16%
12%
8%
4%

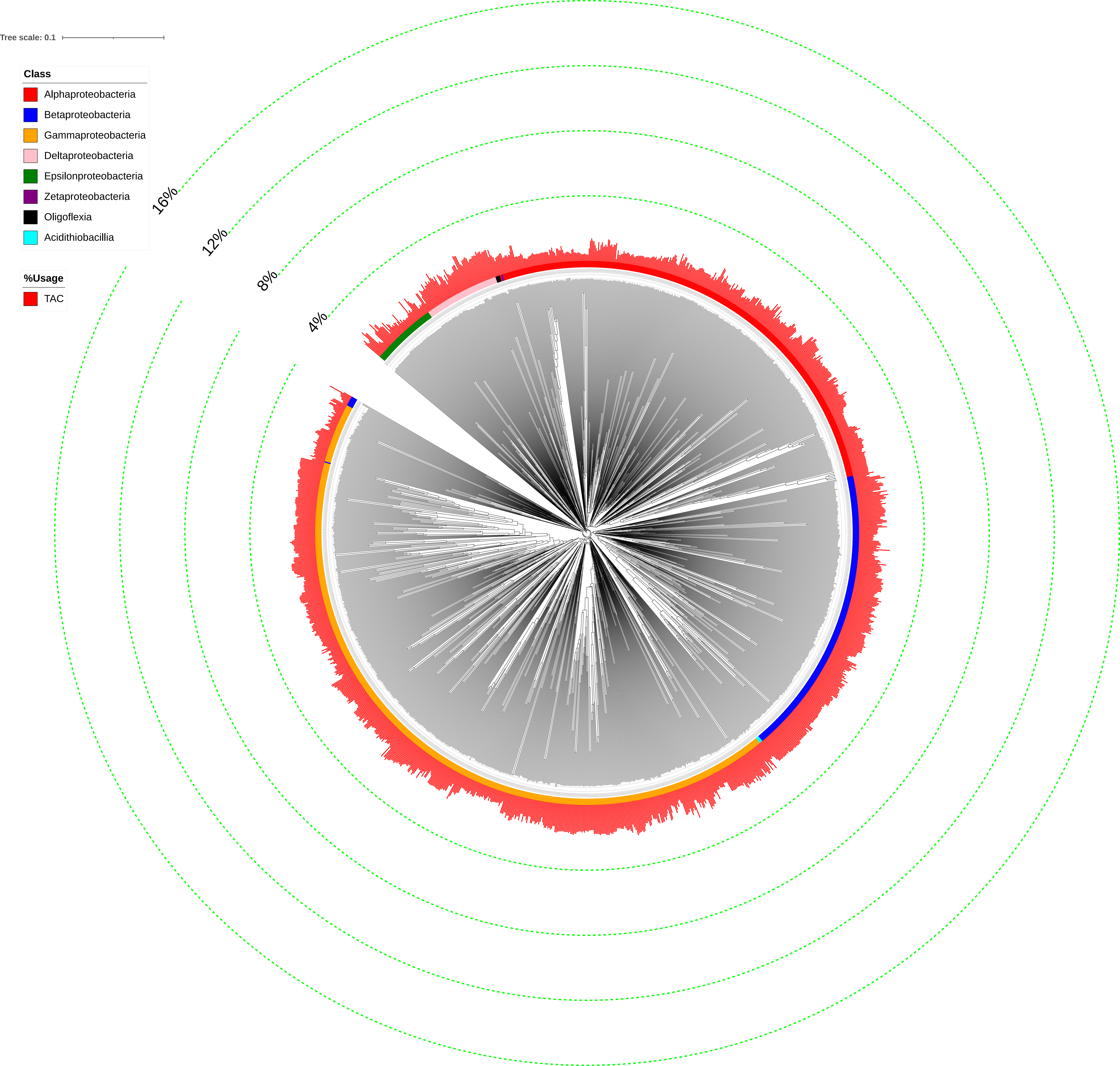

Tree scale: 0.1
Class
Alphaproteobacteria
Betaproteobacteria
Gammaproteobacteria
Deltaproteobacteria
Epsilonproteobacteria
Zetaproteobacteria
Oligoflexia
Acidithiobacillia
%Usage
TAC
16%
12%
8%
4%

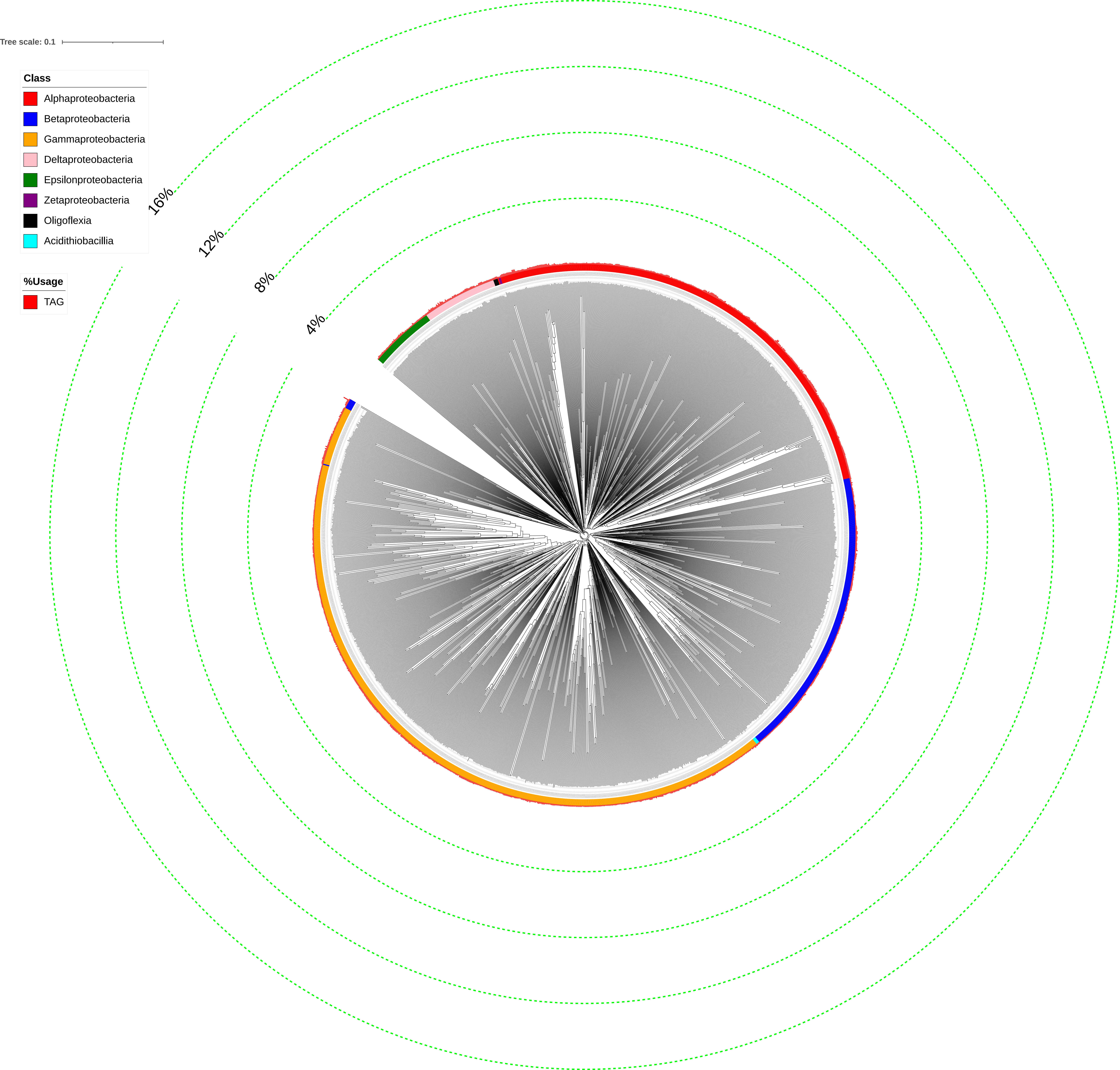

Tree scale: 0.1
Class
Alphaproteobacteria
Betaproteobacteria
Gammaproteobacteria
Deltaproteobacteria
Epsilonproteobacteria
Zetaproteobacteria
Oligoflexia
Acidithiobacillia
%Usage
TAG
4%
8%
12%
16%

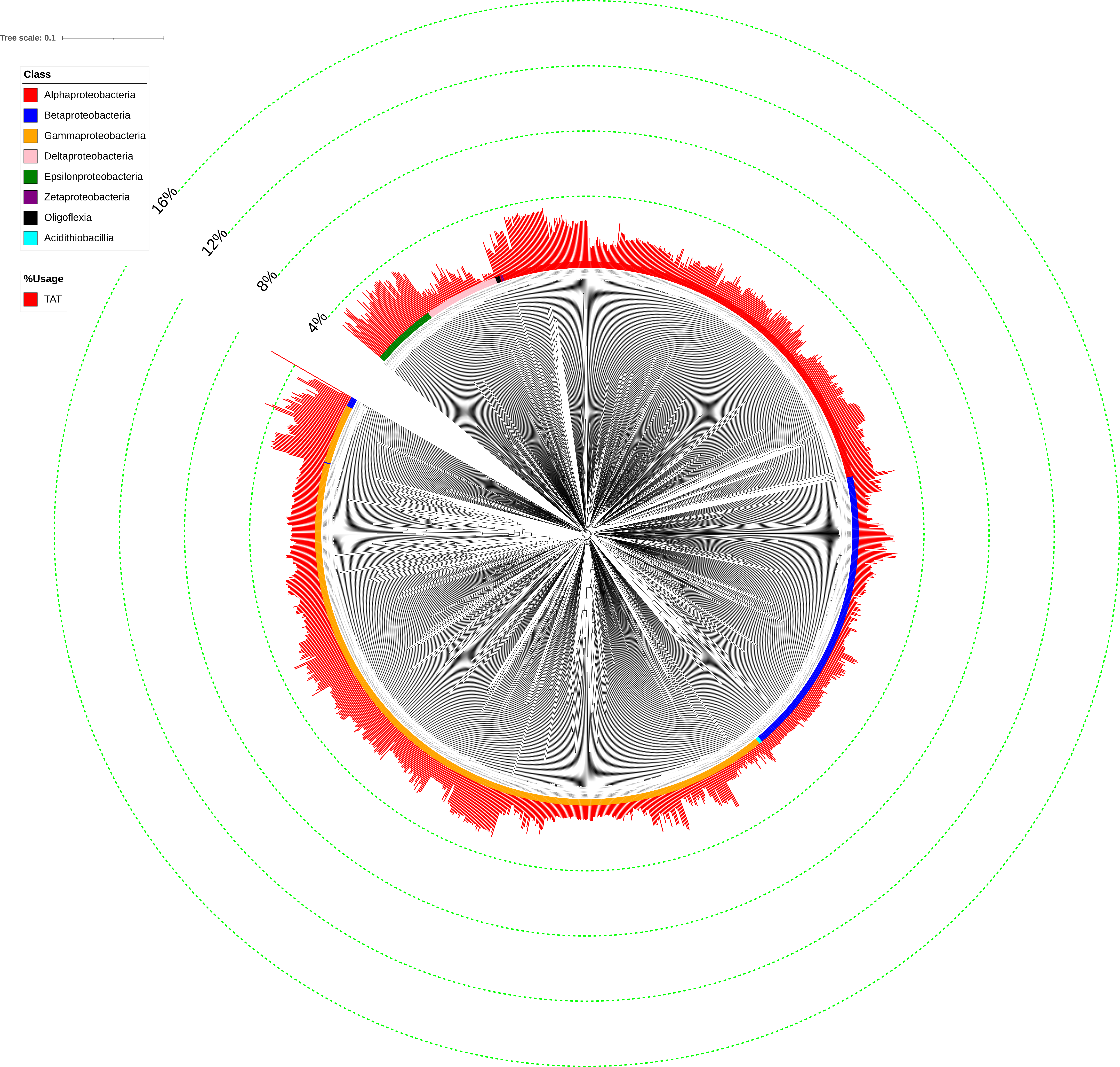

Tree scale: 0.1
Class
Alphaproteobacteria
Betaproteobacteria
Gammaproteobacteria
Deltaproteobacteria
Epsilonproteobacteria
Zetaproteobacteria
Oligoflexia
Acidithiobacillia
%Usage
TAT
16%
12%
8%
4%

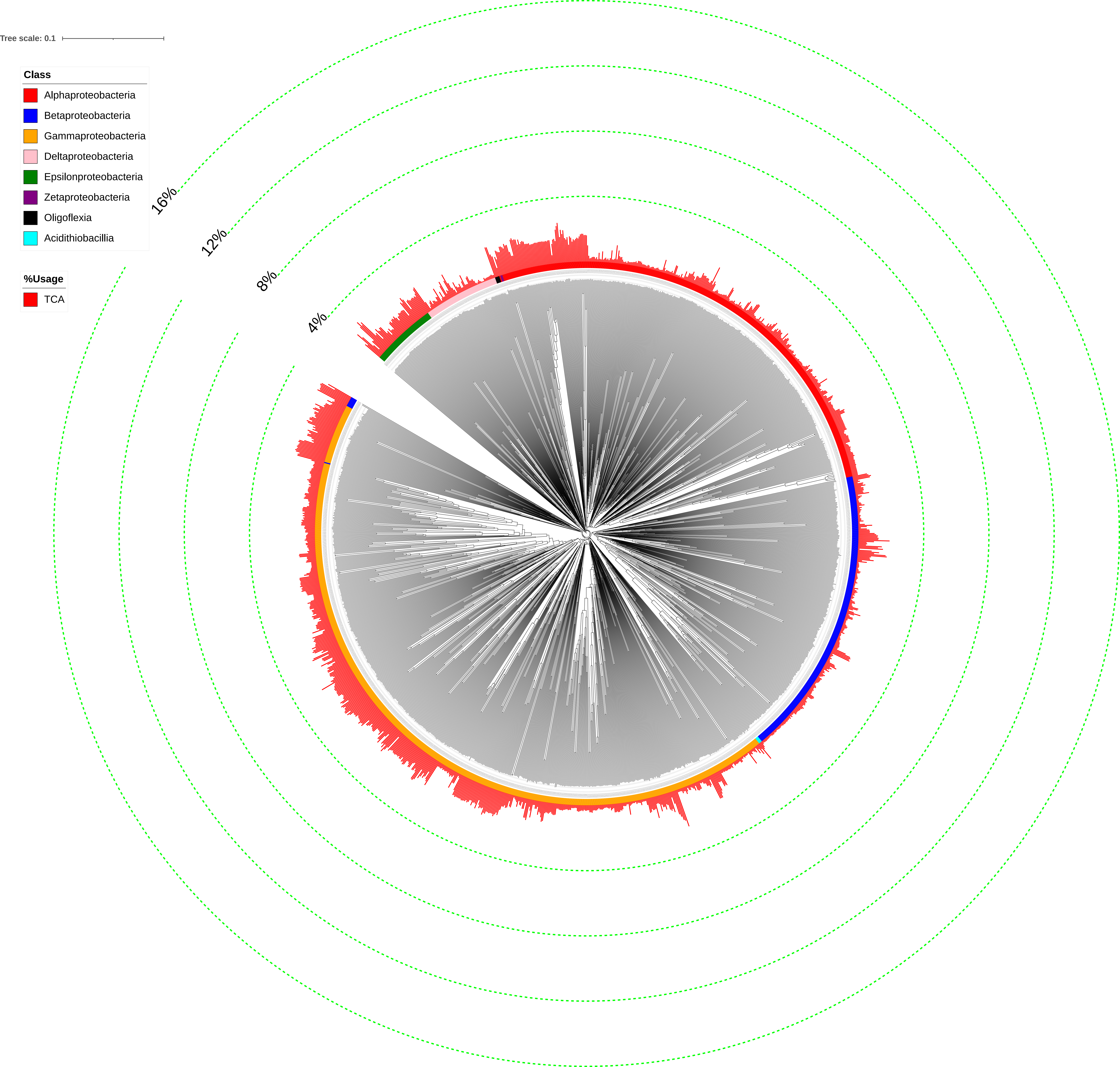

Tree scale: 0.1
Class
Alphaproteobacteria
Betaproteobacteria
Gammaproteobacteria
Deltaproteobacteria
Epsilonproteobacteria
Zetaproteobacteria
Oligoflexia
Acidithiobacillia
%Usage
TCA
16%
12%
8%
4%

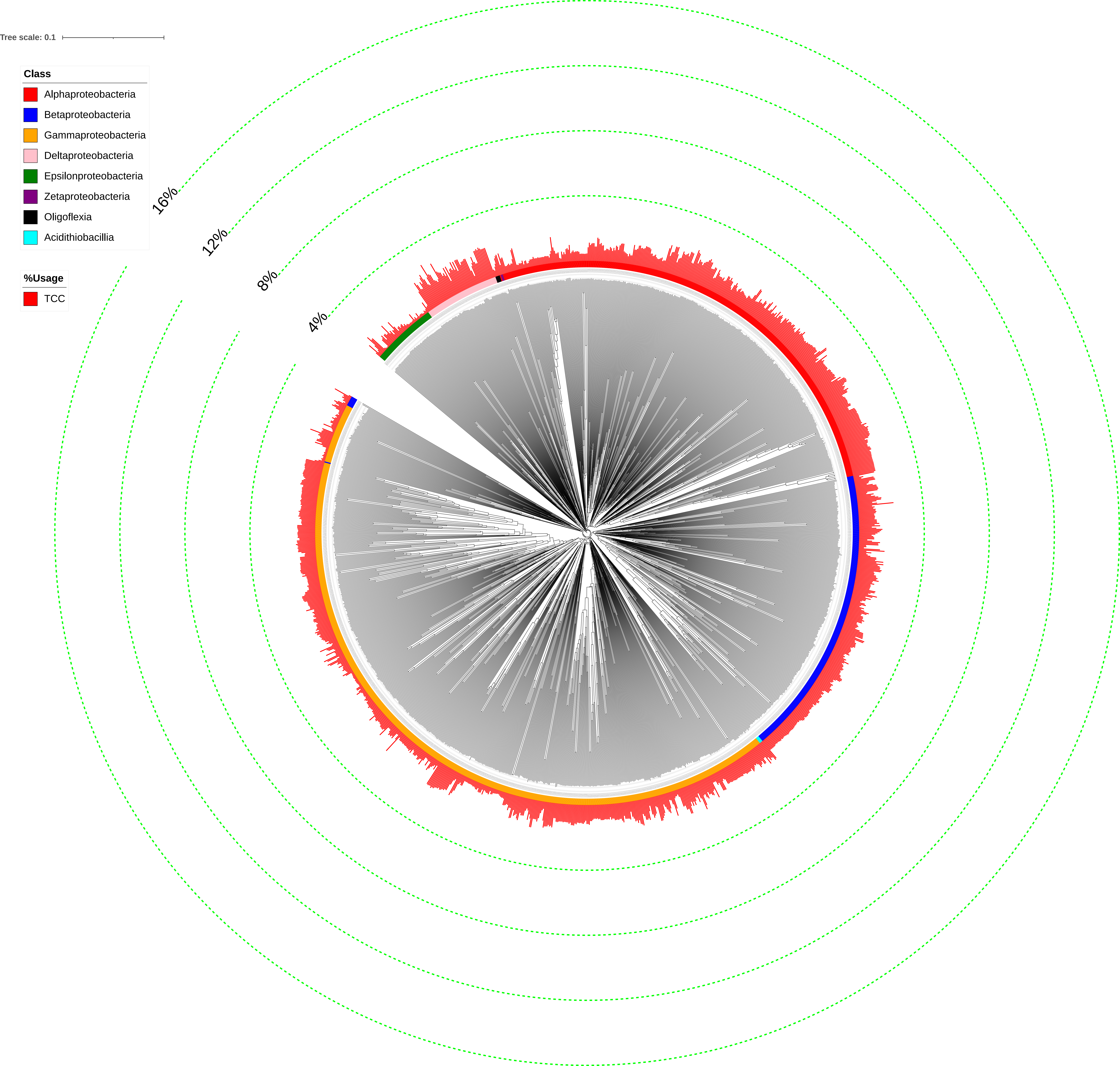

Tree scale: 0.1
Class
Alphaproteobacteria
Betaproteobacteria
Gammaproteobacteria
Deltaproteobacteria
Epsilonproteobacteria
Zetaproteobacteria
Oligoflexia
Acidithiobacillia
%Usage
TCC
16%
12%
8%
4%

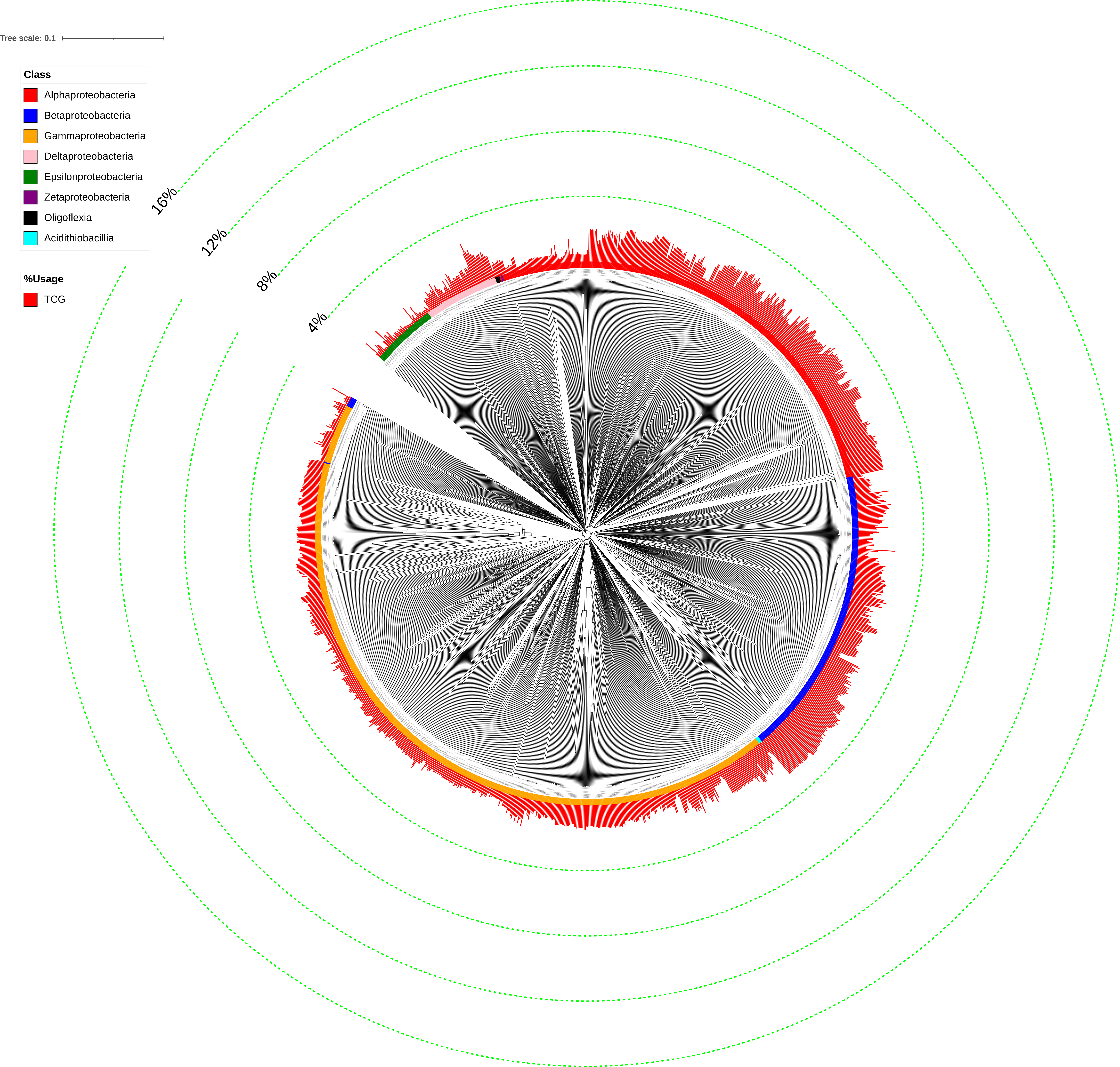

Tree scale: 0.1
Class
Alphaproteobacteria
Betaproteobacteria
Gammaproteobacteria
Deltaproteobacteria
Epsilonproteobacteria
Zetaproteobacteria
Oligoflexia
Acidithiobacillia
%Usage
TCG
16%
12%
8%
4%

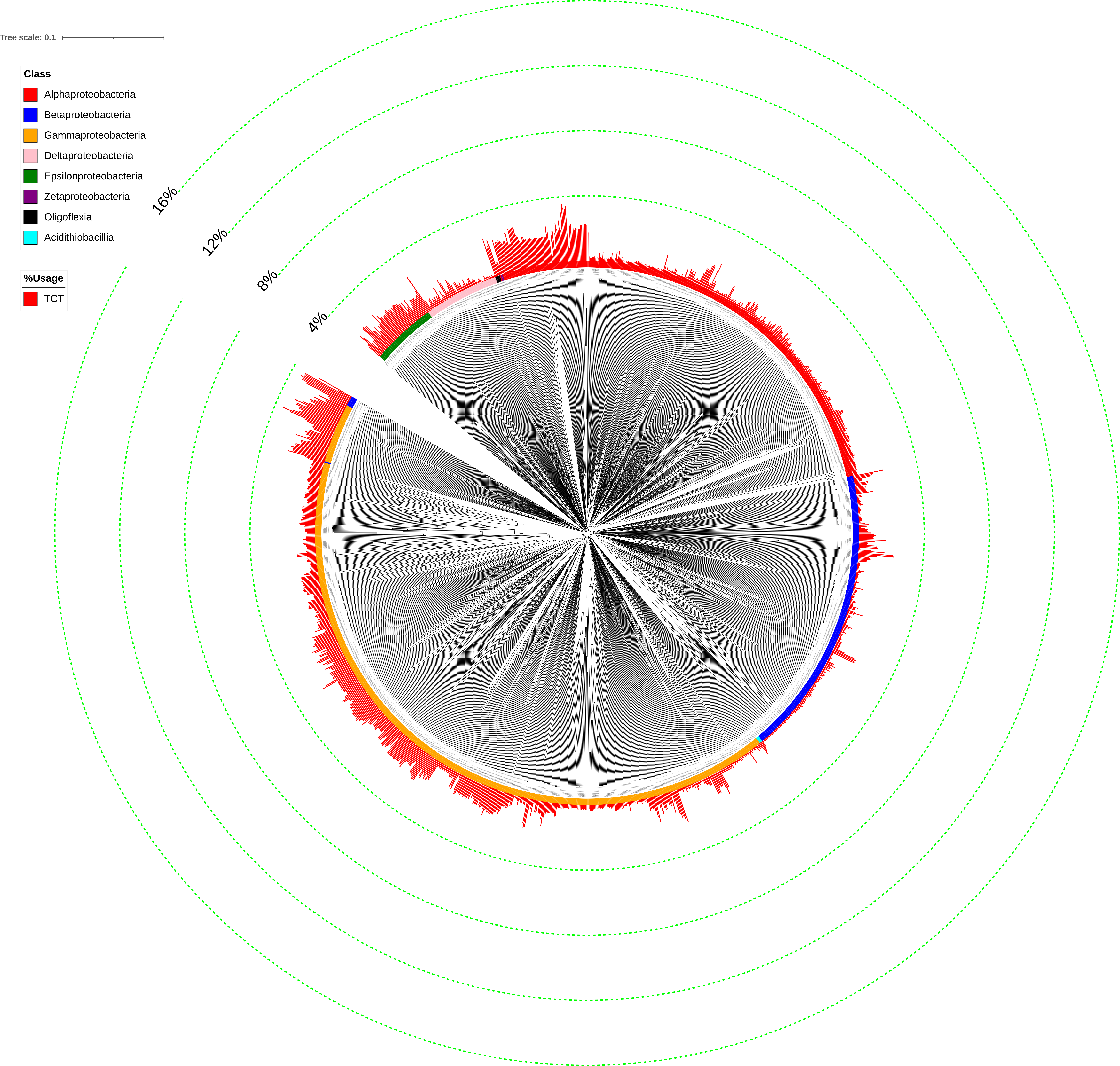

Tree scale: 0.1
Class
Alphaproteobacteria
Betaproteobacteria
Gammaproteobacteria
Deltaproteobacteria
Epsilonproteobacteria
Zetaproteobacteria
Oligoflexia
Acidithiobacillia
%Usage
TCT
16%
12%
8%
4%

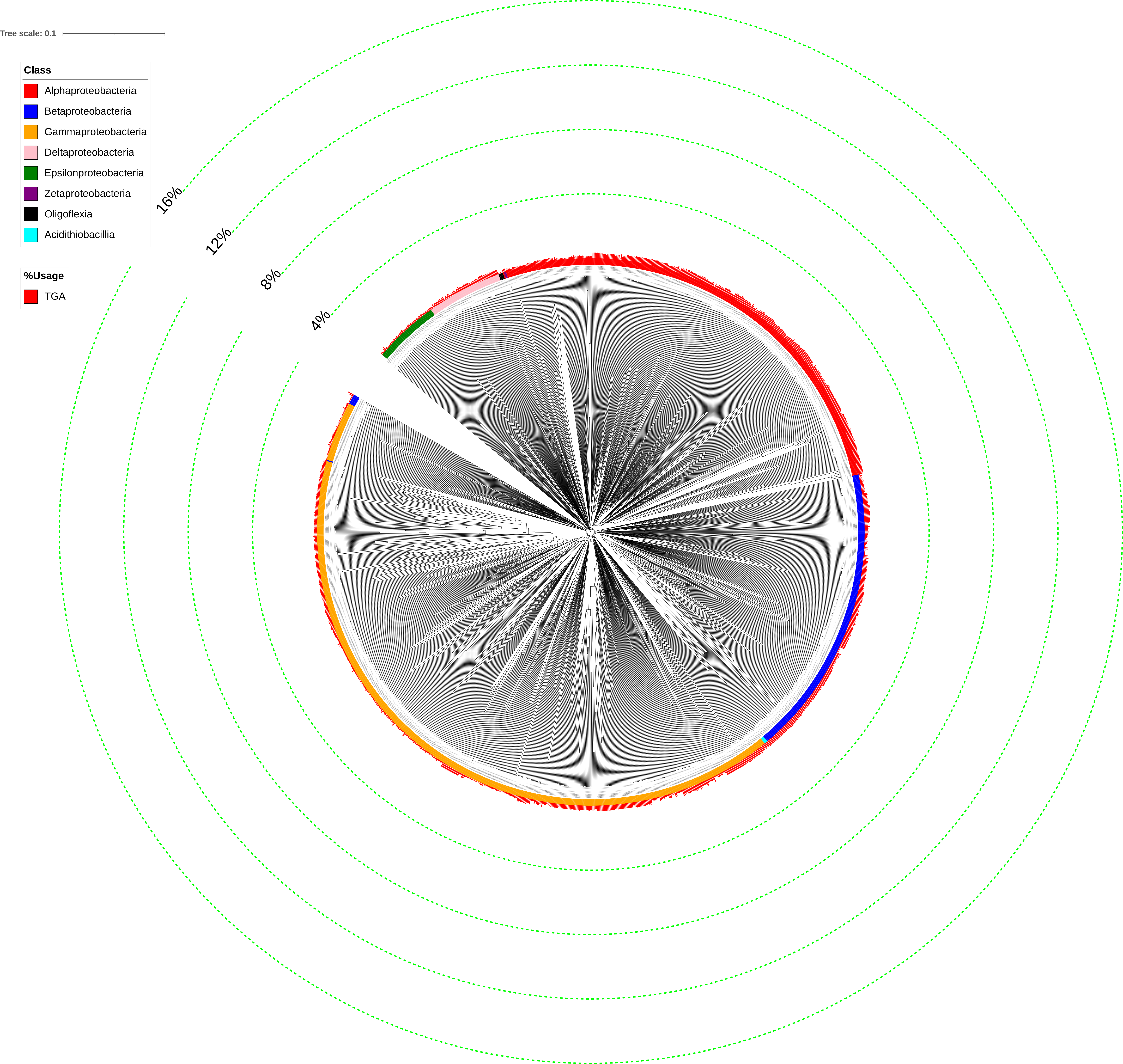

Tree scale: 0.1
Class
Alphaproteobacteria
Betaproteobacteria
Gammaproteobacteria
Deltaproteobacteria
Epsilonproteobacteria
Zetaproteobacteria
Oligoflexia
Acidithiobacillia
%Usage
TGA
4%
8%
12%
16%

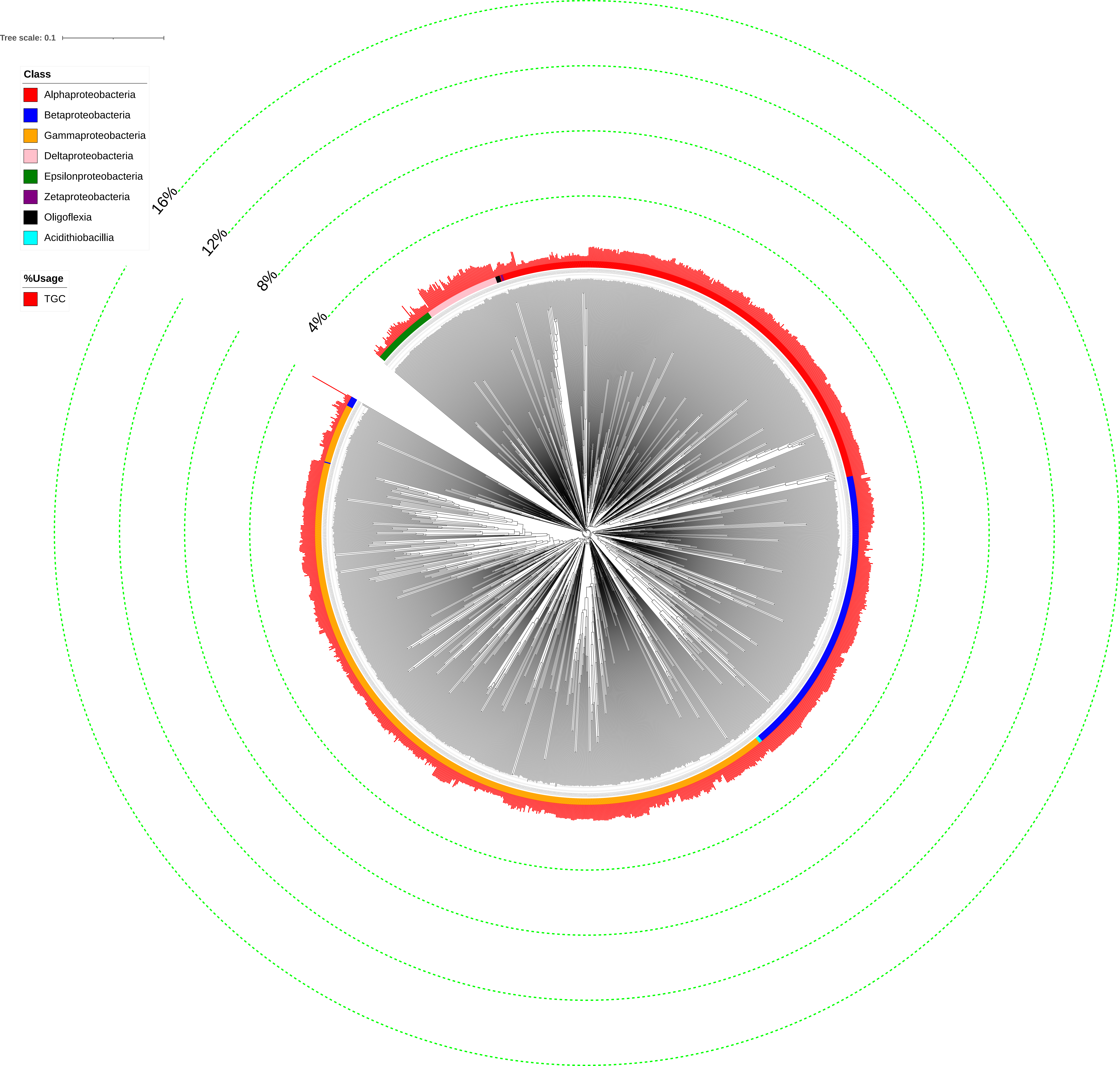

Tree scale: 0.1
Class
Alphaproteobacteria
Betaproteobacteria
Gammaproteobacteria
Deltaproteobacteria
Epsilonproteobacteria
Zetaproteobacteria
Oligoflexia
Acidithiobacillia
%Usage
TGC
4%
8%
12%
16%

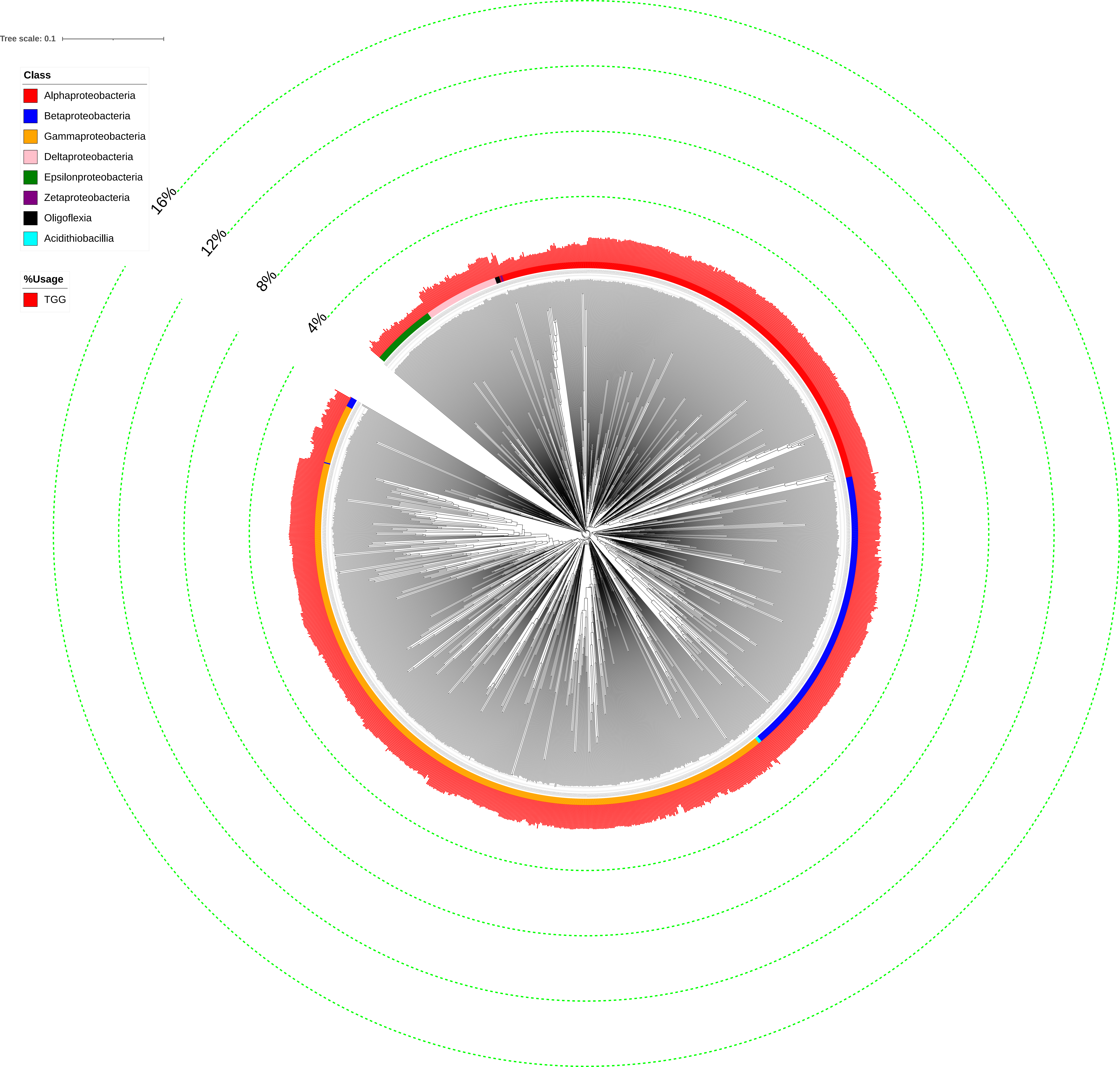
Tree scale: 0.1
Class
Alphaproteobacteria
Betaproteobacteria
Gammaproteobacteria
Deltaproteobacteria
Epsilonproteobacteria
Zetaproteobacteria
Oligoflexia
Acidithiobacillia
%Usage
TGG
16%
12%
8%
4%

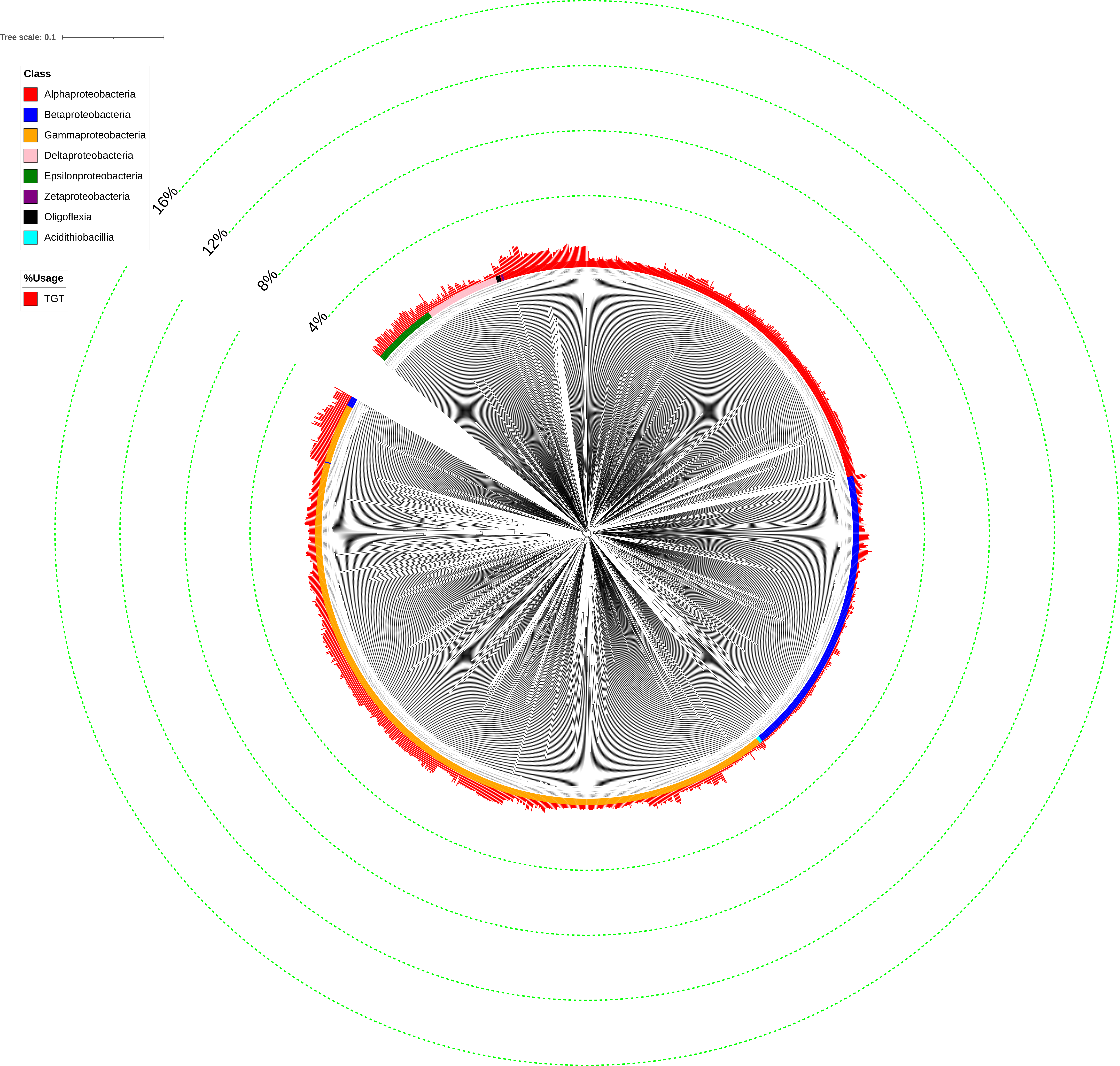
Tree scale: 0.1
Class
Alphaproteobacteria
Betaproteobacteria
Gammaproteobacteria
Deltaproteobacteria
Epsilonproteobacteria
Zetaproteobacteria
Oligoflexia
Acidithiobacillia
%Usage
TGT
16%
12%
8%
4%

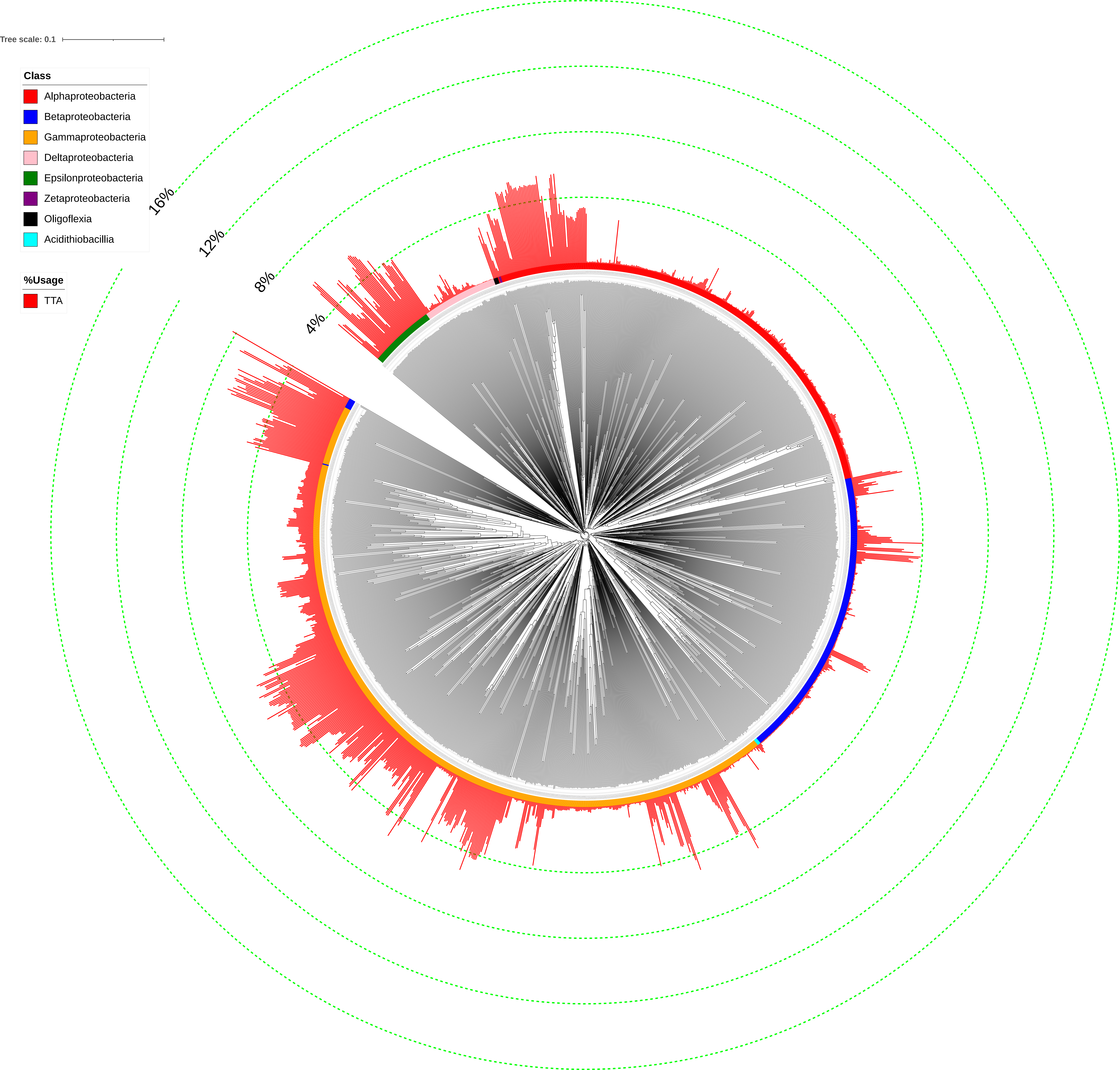
Tree scale: 0.1
Class
Alphaproteobacteria
Betaproteobacteria
Gammaproteobacteria
Deltaproteobacteria
Epsilonproteobacteria
Zetaproteobacteria
Oligoflexia
Acidithiobacillia
%Usage
TTA
16%
12%
8%
4%

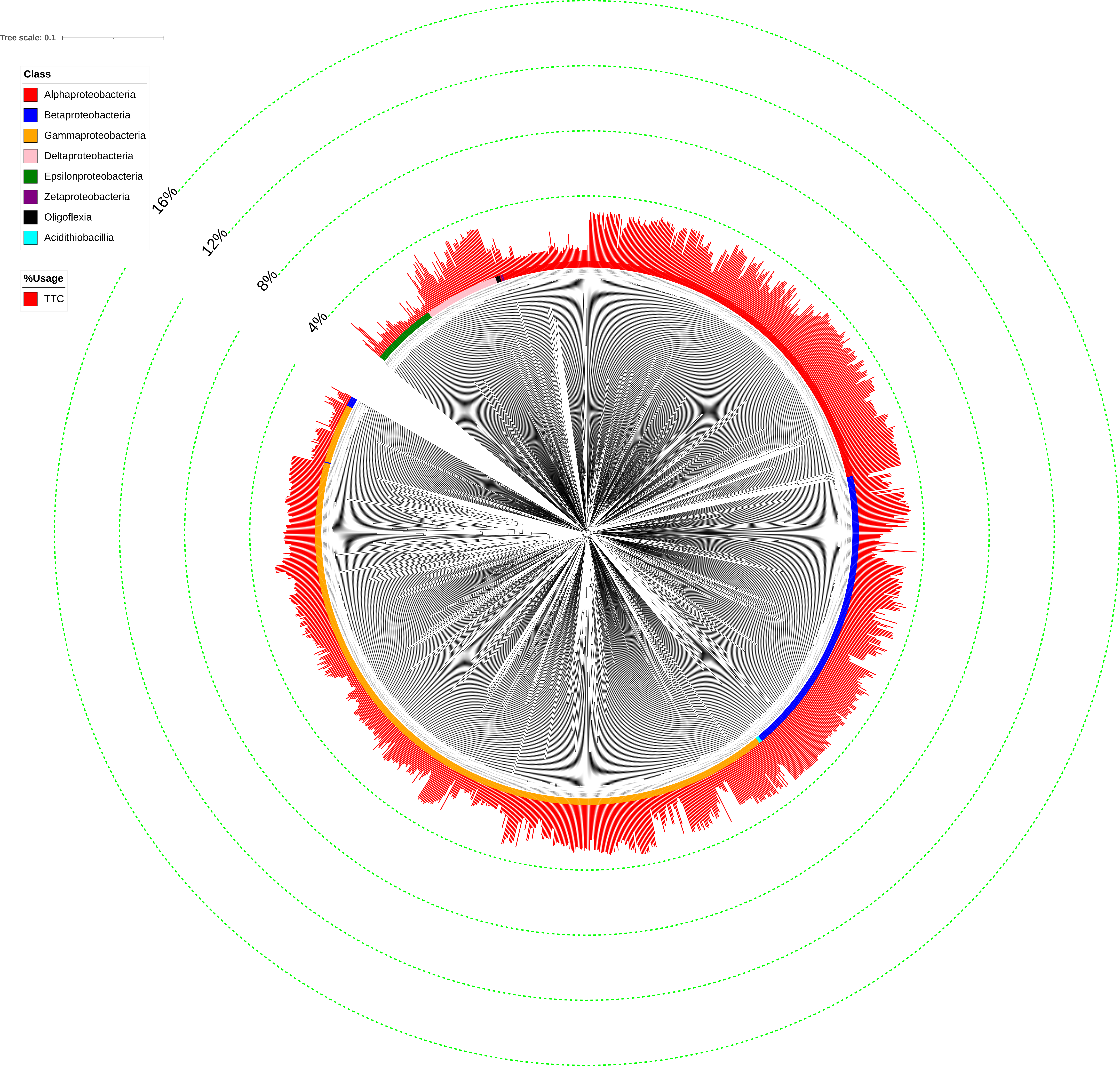

Tree scale: 0.1
Class
Alphaproteobacteria
Betaproteobacteria
Gammaproteobacteria
Deltaproteobacteria
Epsilonproteobacteria
Zetaproteobacteria
Oligoflexia
Acidithiobacillia
%Usage
TTC
16%
12%
8%
4%

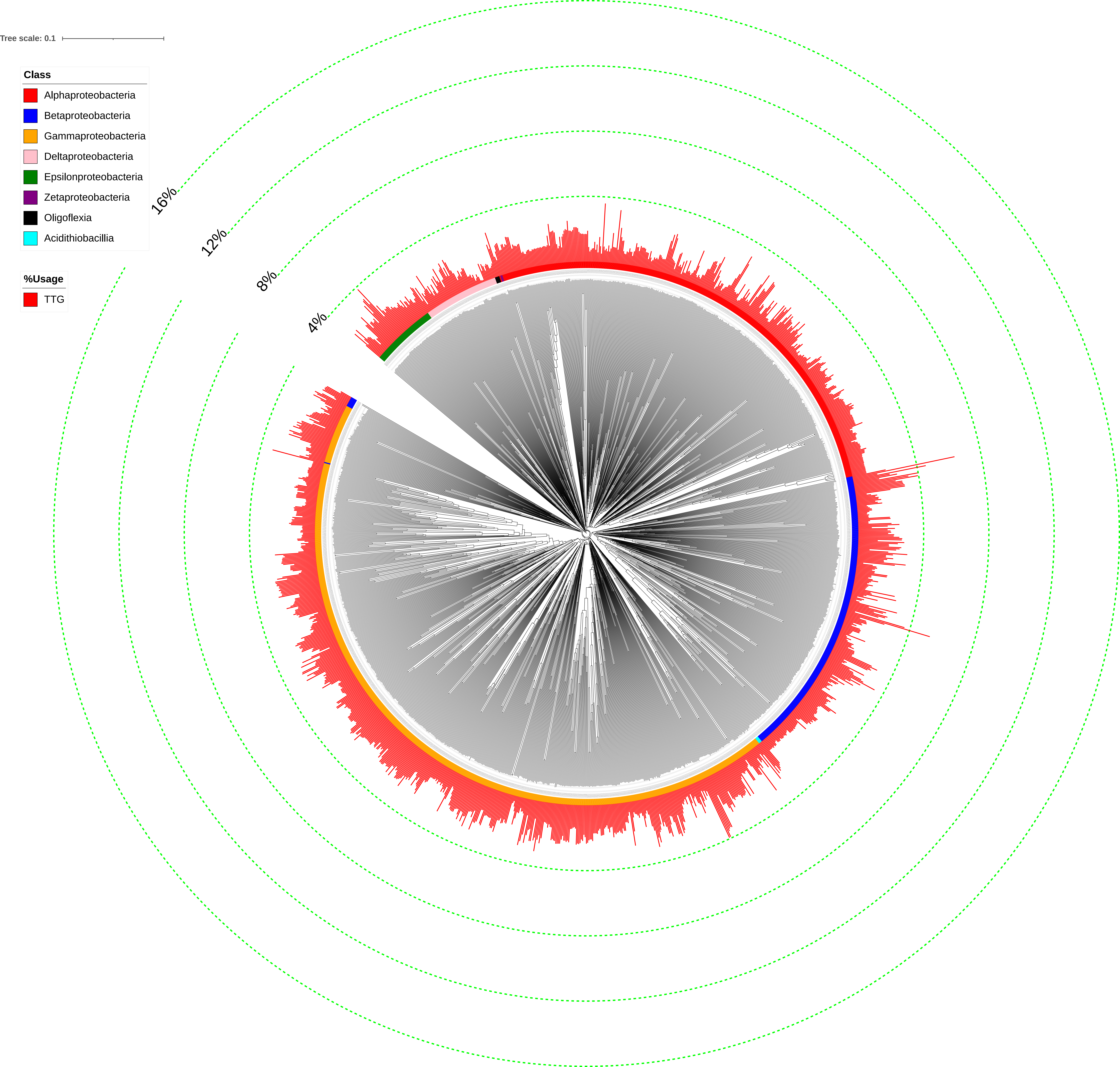

Tree scale: 0.1
Class
Alphaproteobacteria
Betaproteobacteria
Gammaproteobacteria
Deltaproteobacteria
Epsilonproteobacteria
Zetaproteobacteria
Oligoflexia
Acidithiobacillia
%Usage
TTG
4%
8%
12%
16%

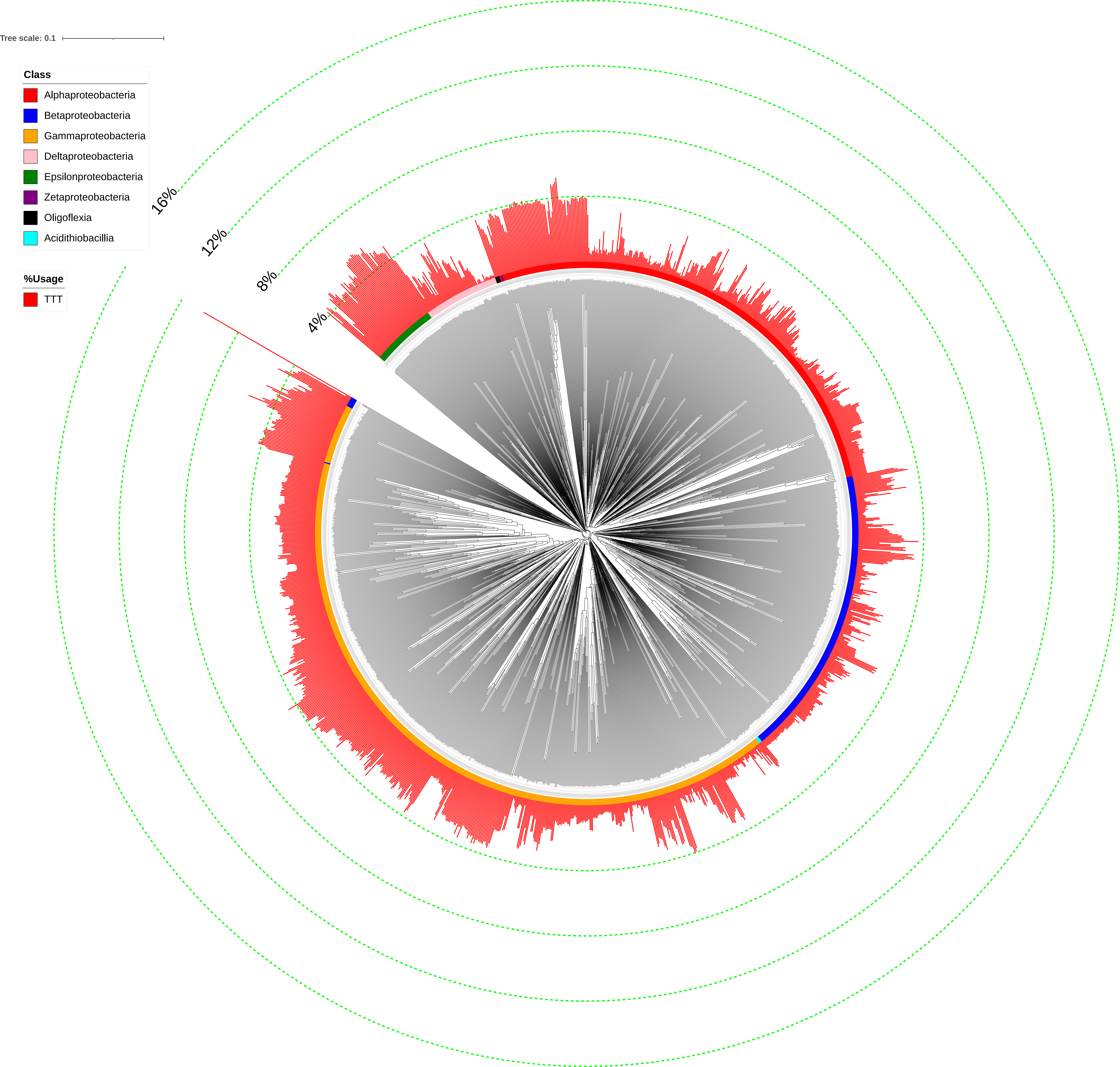

Tree scale: 0.1
Class
Alphaproteobacteria
Betaproteobacteria
Gammaproteobacteria
Deltaproteobacteria
Epsilonproteobacteria
Zetaproteobacteria
Oligoflexia
Acidithiobacillia
%Usage
TTT
16%
12%
8%
4%
