# Supplementary figures and images for: Impact of the chemical modification of tRNAs anticodon loop on the variability and evolution of codon usage in proteobacteria
Source: Front Microbiol. 2024 Aug 5;15:1412318. doi: 10.3389/fmicb.2024.1412318 (PMC11332805; doi:10.3389/fmicb.2024.1412318)

Histogram of GC%

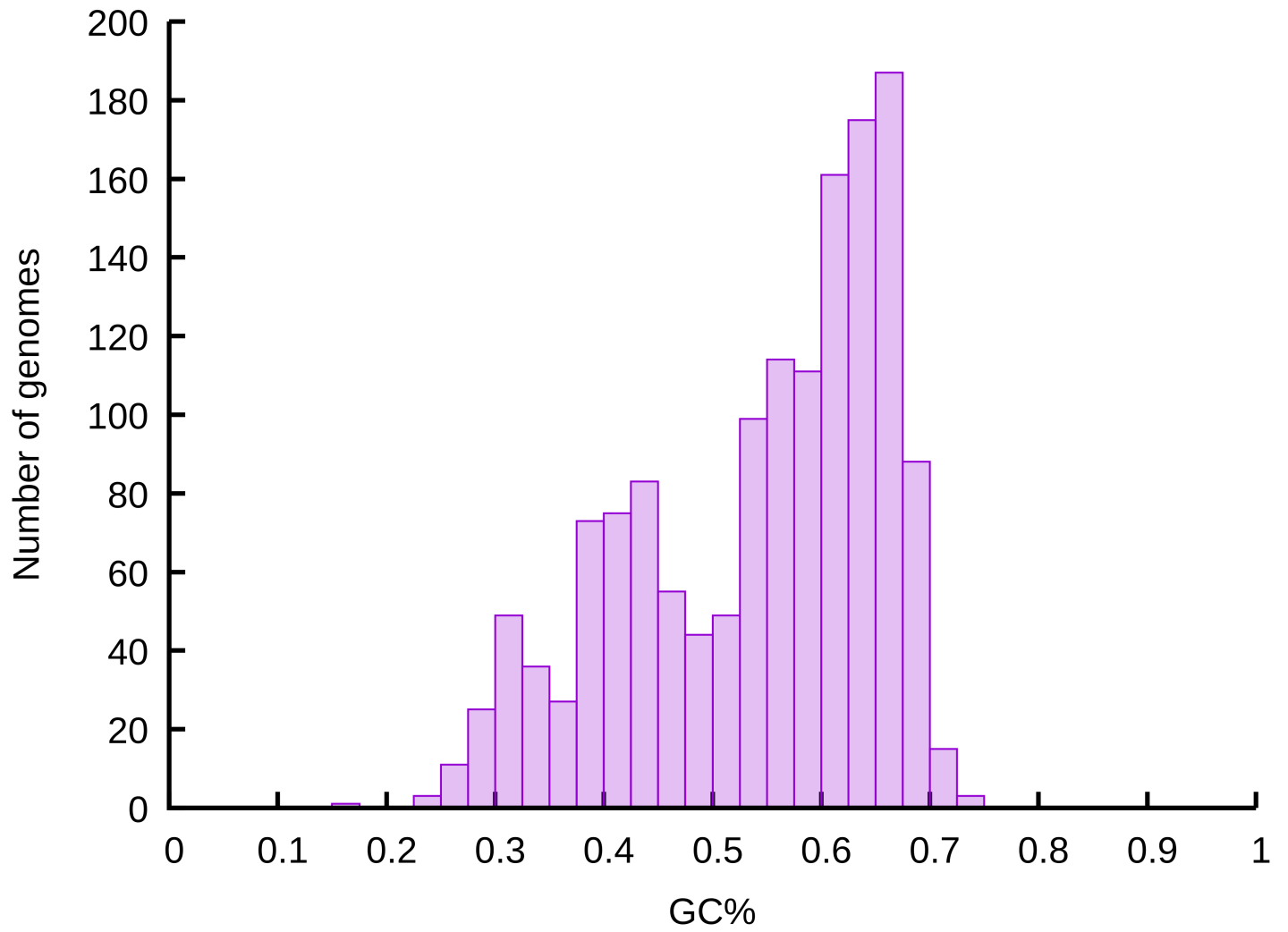

Supplement: Supplementary file 1 [file Data_Sheet_1.zip › Supp_figures/Fig_S6.pdf]

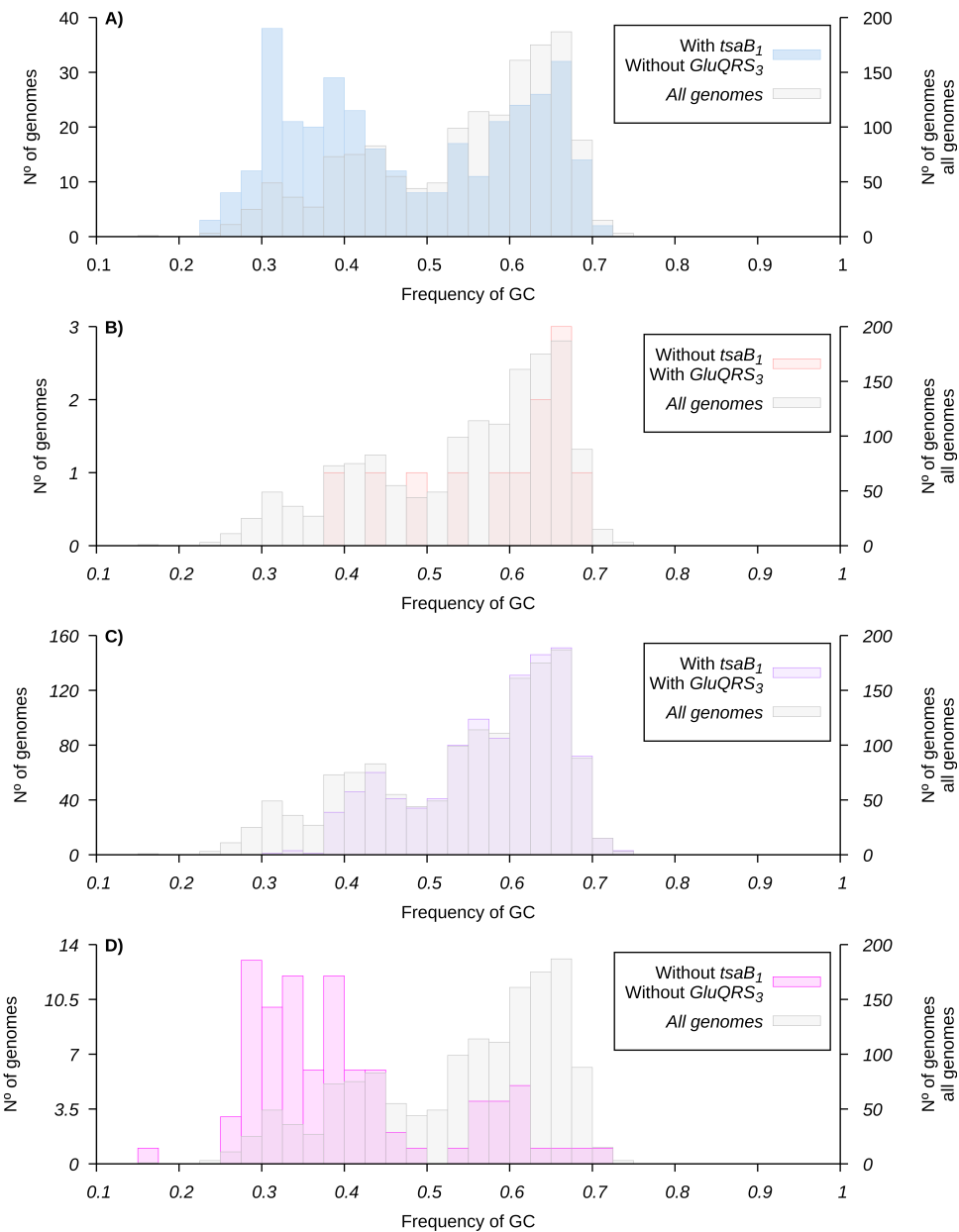

Supplement: Supplementary file 1 [file Data_Sheet_1.zip › Supp_figures/Fig_S16.pdf]
