# Supplementary material for: Impact of the chemical modification of tRNAs anticodon loop on the variability and evolution of codon usage in proteobacteria
Source: Front Microbiol. 2024 Aug 5;15:1412318. doi: 10.3389/fmicb.2024.1412318 (PMC11332805; doi:10.3389/fmicb.2024.1412318)
Supplement: Supplementary file 2 [file Data_Sheet_2.zip › Supp_tables/Table_S4.pdf]

Supplementary table S4. Criteria used to screen for genes encoding tRNA modification enzymes using rpsBlast

Gene	Modification	Index	CDD patterns expected to be found in gene*	CDD patterns expected to be absent in gene*
tsaB	t6A	1	TIGR03725	-
tsaB	t6A	2	COG1214	-
tsaC	t6A	1	PRK10634	-
tsaC	t6A	2	COG0009, pfam03481	-
tsaD	t6A	1	COG0533	-
tsaD	t6A	2	TIGR03723	-
tsaD	t6A	3	PRK09604	-
tsaE	t6A	1	pfam02367	-
tsaE	t6A	2	COG0802	-
tsaE	t6A	3	PRK10646	-
gluQRS	GluQ	1	COG0008	TIGR00464<br>PRK05347<br>TIGR00440<br>pfam19269
gluQRS	GluQ	2	cd00808	TIGR00464<br>PRK05347<br>TIGR00440<br>pfam19269
gluQRS	GluQ	3	PRK05710	-
gluQRS	GluQ	4	TIGR03838	-
tgt	GluQ	1	TIGR00430	-
tgt	GluQ	2	pfam01702	-
tgt	GluQ	3	COG0343	-
tusE	2sU	1	PRK11508	-
tusE	2sU	2	COG2920	-
tusE	2sU	3	pfam04358	-
tusE	2sU	4	TIGR03342	-
mnmA	2sU	1	PRK00143	-
mnmA	2sU	2	cd01998	-
mnmA	2sU	3	COG0482	-
mnmA	2sU	4	TIGR00420	-
mnmA	2sU	5	pfam03054	-
tilS	K2C	1	PRK10660	-
tilS	K2C	2	COG0037	-
tilS	K2C	3	TIGR02432	-
tilS	K2C	4	TIGR02432<br>smart00977	-

tilS	K2C	5	TIGR02432<br>TIGR02433	-
tilS	K2C	6	TIGR02432<br>pfam11734	-
tilS	K2C	7	TIGR02432<br>smart00977<br>pfam09179	-
tilS	K2C	8	TIGR02432<br>TIGR02433<br>pfam09179	-
tilS	K2C	9	TIGR02432<br>pfam11734<br>pfam09179	-
tilS	K2C	10	COG0037<br>TIGR02433	-
tilS	K2C	11	COG0037<br>pfam11734	-
tilS	K2C	12	COG0037<br>smart00977	-
tilS	K2C	13	COG0037<br>TIGR02433<br>pfam09179	-
tilS	K2C	14	COG0037<br>pfam11734<br>pfam09179	-
tilS	K2C	15	COG0037<br>smart00977<br>pfam09179	-

*CDD uses patterns obtained from diverse sources. Here patterns are named using the code presented in CDD database, conserving upper and lower cases as used in the database.
