# Supplementary material for: Impact of the chemical modification of tRNAs anticodon loop on the variability and evolution of codon usage in proteobacteria
Source: Front Microbiol. 2024 Aug 5;15:1412318. doi: 10.3389/fmicb.2024.1412318 (PMC11332805; doi:10.3389/fmicb.2024.1412318)
Supplement: Supplementary file 3 [file Data_Sheet_3.zip › Supp_scripts/README.pdf]

Introduction

The set of attached scripts correspond to those used in the manuscript "Impact of the chemical modification of tRNAs anticodon loop on the variability and evolution of codon usage in proteobacteria" by Delgado et al. (2024). They were written to analyze codon usage in multiple genomes and its relation to other parameters such as GC%, number of tRNA genes or the presence/absence of a given gene. These are all Perl scripts tested to run in Debian Linux, although they should run in any Linux distribution. Some scripts require the installation of additional programs or Perl modules.

Required programs:

Before using the scripts make sure the following programs are installed and in the path

tRNAscan-SE (tested with version 2.0)

Gnuplot (tested with version 5.2)

rpsblast (tested with version 2.8.1+, included in Debian package ncbi-blast+)

Required Perl modules:

Before using the scripts make sure the following Perl modules have been installed

Statistics::Descriptive

Statistics::Basic

Statistics::LineFit

Statistics::Ttest

Statistics::Test::WilcoxonRankSum

Installation

To use any of the scripts simply copy the file to the desired directory and call them from the command line with the required command line arguments.

Scripts usage

In the following lines we describe the usage of all scripts. If you call any of them from command line it will print to the prompt a small message indicating the required arguments and sometimes a brief description of its intended usage. Many of the scripts are very verbose which allows to look for intermediate results and helps to find potential errors, but is usually unnecessary. I recommend to forward the results to a log file that can be erased if not required.

===

Codon_counter-V1.pl

The script will count the number of codons in every gene in each fasta file of the folder and produce a result file for each genome containing the fraction of codons of each gene that correspond to each codon. Genes annotated as pseudogenes or presenting a number of nucleotides not dividable by three are not considered. The program is designed to use the “_cds_from_genomic.fna” files from GeneBank or RefSeq that have fasta headers in genebank format. This allows the script to recognize genes annotated with the “pseudo” flag as “true”. In order to work, the script requires the text that will be added to each result file and a file with the list of codons that it should look for. We have added a sample file named codons.txt that contains all possible codons, but users may want to erase stop codons from the list.

Usage: perl Codon_counter-V1.pl <word_to_add_to_result_files_name> <codons_list_file e.g codons.txt>

===

Codon_statistics-V2.pl

Script produce files with average, mean, mode and standard deviation of codon usages in each genome. It uses ratios of codon usages indicated in each files in the running folder that presents the text “-1-norm.tab” that can be added by Codon_counter-V1.pl script at the end of each genomes codon count file. This text may be changed by modifying the value of the “$pat” variable in the script. Script requires that the Perl module “Statistics::Basic” is installed.

The script produces 5 result files (average.txt; average-b.txt; std_desv.txt; median.txt; mode.txt). Only “average-b.txt” was used for further analyses in this work.

Usage: perl Codon_statistics-V2.pl

===

AGCT_count_V2.pl

Script counts the number of G, C, A and T present in all fasta entries of each genome fasta file present in the running folder. Additionally, the program calculates the fraction of nucleotides that correspond to each nucleotide type and the fraction of nucleotides that correspond to either G or C. The script is designed to select all fasta files with the text “_genomic.fna” in order to select full genomes and not annotated features such as RNAs or CDS. If you prefer to determine these parameters considering only CDS, please comment variables $pat, $notpat and $notpat2 in lines 15 to 17 of the script and uncomment the corresponding variables in lines 20 to 22 or change variables according to the text in the file names you are are analyzing.

Usage: perl AGCT_count_V2.pl <Results_file_name>

===

statistics-all-columns-V2.pl

Script calculates a series of statistical values for each column of the indicated file. In this work it was used to estimate variability of codon usage between proteobacterial genomes based on standard deviations and the distance between the values of percentiles 90 and 10. The program additionally calculate other simple statistical values of columns such as mean, median, skewness, kurtosis and the value of selected percentiles. The script requires that the Perl module “Statistics::Descriptive” is installed.

The file with data to be analyzed should be organized in tab separated columns. First column will be skipped (considering it contains the genome names or accession codes). First line in the file has to be commented and should contain the column titles. Other commented lines will be ignored.

Usage: perl statistics-all-columns-V2.pl <tab delimited file with data to analyze> <result file base name> <OPTATIVE: results folder>

===

trnascan_all_v2.pl

Script execute programs tRNAscan-SE with arguments -B -I on all fasta files in the directory containing the text “genomic.fna” in their file names and not containing the text “cds_from_genomic” or “rna_from_genomic”. For each analyzed file it will generate a result file with “.tscan” appended to the original file name. This script requires tRNAscan-SE installed and in the path. It has been tested with version 2.0 of tRNAscan-SE.

Usage: perl trnascan_all_v2.pl

===

count_trnascan.pl

Script will read any file in the current directory that was produced by script trnascan_all_v2.pl. Files are recognized if ending with “.fna.tscan”. It will construct a results file indicating the number of genes coding for each tRNA in each of the analyzed genomes.

Usage: perl count_trnascan.pl <Name of results file>

===

Histogram-all-columns-compare-all_vs_high-bars.pl

Program will take data on two tab delimited files that contain the frequencies of codon usage in all genes or in highly expressed genes from each of the analyzed genomes (<tab delimited file all genes> and <tab delimited file high expression genes>). Data files must be tab delimited. Lines with "#" will be skipped. Both files should have the same amount of columns and use the same column names in similar order. First line in the file has to be commented and have the column titles.

Using these files it will construct three results files for each column. These results files will contain the number of genomes that present a codon usage that falls in each of the defined ranges. Each range is constructed based on the given values. Ranges start at <beginning> value and make <steps> steps of <range> size

Based on these results files, the script will produce a series of histogram plots comparing the distributions on each given file using GnuPlot. This script requires GnuPlot installed and in the path. It has been tested with version 5.2 of GnuPlot.
Plots will be saved in the "./histogram_All_vs_High" directory, unless another folder is specified a the end of the command line

Usage: perl Histogram-all-columns-compare-all_vs_high-bars.pl <tab delimited file all genes> <tab delimited file high expression genes> <range> <beginning> <steps> <result file base name> <Histograms folder (optional)>

===

plot-codonusage-vs-GC-V3-High_expression-vs-all-genes.pl

Program will take data on files <data file all genes> and <data file high expression genes> and make plots comparing codon usage with frequency of nucleotides in genomes. First line in each file has to be commented and indicate the column titles. This line must use the same titles as given by previous scripts so that the program can recognize them. Also, both files must use the same names for fields. Each file should contain the columns presenting the usage of each codon as well as columns presenting the content of each nucleotide. In linux, you can use the paste command to construct this file pasting the average codon file and the GC content files produced by the following scripts:

    Codon_statistics-V2.pl
    AGCT_count_V2.pl

For example:

    paste average-b.txt genomic_AGCT.tab > usage-genomic_AGCT.tab

Results files will use the <result file base name> to construct result files in <results directory>
This program calculate linear regression using perl “Statistics::LineFit” module and uses GnuPlot to construct plots. Both must be installed.
After finishing, the program will unite all pdf files of the folder in <summary file> and add to that file name a ".pdf" extension.
Additionally it will make a text file with the same name, but finishing in ".tab", that contains the slope, intercept and R^2 for each codon

Usage: perl plot-codonusage-vs-GC-V3-High_expression-vs-all-genes.pl <data file all genes> <data file high expression genes> <result file base name> <results directory> <summary file>

===

plot-codonusage-vs-tRNAgenes-V3.pl

Program will take data on file <data> and make plots comparing codon usage with the number of tRNA genes with complementary anticodons. First line in the data file has to be commented and have the column titles. This line must use the same titles as given by previous scripts so that this program recognize them. Each file should contain the columns presenting the usage of each codon as well as columns presenting the number of tRNA genes. In Linux, you can use the paste command to construct this file pasting the average codon file and the tRNA genes content files produced by the following scripts:

    Codon_statistics-V2.pl
    count_trnascan.pl

For example:

    paste average-b.txt tRNA_genes.tab > usage-tRNAs.tab

Results files will use the <result file base name> to construct result files in <results directory>
This program calculate linear regression using perl “Statistics::LineFit” module and produces plots using GnuPlot. Both should be installed. After finishing, the program will unite all pdf files of the folder in <summary file> and add to that file name a ".pdf" extension
Additionally it will make a text file with the same name, but finishing in ".tab", that contains the slope, intercept and R^2 for each codon. Some additional plots are made based on this file

Usage: perl plot-codonusage-vs-tRNAgenes-V3.pl <data file> <result file base name> <results directory> <summary file>

==

rpsblast-summary-V4.pl

The aim of this script is to screen for a set of sequence patterns in all genomes present in the current directory. Screens are performed using rpsblast (tested with version 2.8.1+, included in Debian package ncbi-blast+) and patterns can be obtained from the CDD database at NCBI webpage. Nevertheless, if using the complete database the program will take too long to run for a data base with thousands of genomes. In such cases, it is recommended to construct a specific database that only includes the patterns that should be found in the genes of interest, as well as those expected to be absent from those genes. Full instructions for constructing your databases can be found at the CDD database webpage at NCBI. Briefly, you should run the makeprofiledb program. For example, supposing you have already downloaded the patterns of interest:

makeprofiledb -in patterns_of_interest.txt -out database_name

where "patterns_of_interest.txt" is the file containing the names of the patterns of interest and "database_name" is the name your database will have.

Program will look for the indicated pattern(s) in all the files of the directory that follow the indicated rules. Information such as the patterns file and gene selection rules must be indicated in a <Criteria File>. Please look at RPSB-criteria-example.txt for an example of <Criteria File>. Additionally, it is necessary to indicate the base for the name of the results directory and for the results files. <Results directory base name> must end with "/".

Each gene in the analyzed genomes will be selected if it has any of the required patterns in the inclusion criteria

Each gene in the analyzed genomes will be rejected if it has any of the patterns in the rejection criteria

Usage: perl rpsblast-summary-V4.pl <Criteria file> <Results directory base name> <Results base name>

==

batch-separate_file_by_gene_presence_and_plot_histograms.pl

Program checks for all files in directory <directory> that start with <pattern> and end with <extension>. The aim is to select results files from rpsblast-summary-V4.pl. Then it runs separate_file_by_gene_presence-v2.pl for each of this files using the program indicated in variable $separate_prog and the codon usage file indicated in <usage file>. This separates the codon usage of each genomes based on the presence or absence of the gen of interest. Then it uses the results files to run program indicated in variable $plot_prog (codon_usage_by_gene_presence-ALL-V3.1.pl) to construct superposed histograms and box plots comparing the distribution of codon usages in genomes that present or lack each gene. It additionally calculates statistical significance of differences between populations based on T-test (suppose normal distribution, if populations are not normally distributed, use batch-separate_file_by_gene_presence_and_plot_histograms-Wilcoxon.pl).
All files in the indicated directory will be used to make plots in the same page of each pdf.
Please correct the path in variables $separate_prog and $plot_prog if the corresponding scripts are not in the path or current directory

Usage: perl batch-separate_file_by_gene_presence_and_plot_histograms.pl <directory> <pattern> <extension> <usage file> <results directory>

==

separate_file_by_gene_presence-v2.pl

The aim of this script is to separate a file presenting the codon usage of a set of genomes in two files, one having the data of genomes that present a gene and another the data of genomes that lack a gene.
Program will check in each line of <data_file>

if it starts with a # symbol or the word genome it will be skipped
each line must have a genome name followed by a tab and a number. It might have items after that if separated by a tab
if a genome is followed by a 0 then it is put in the no haves list
if it has any other number, then it is put in the haves list
Program will check each line in <in_file>
if it starts with a # symbol it will copy it in the to results files
if it starts with a genome name from the haves list, it will copy it to the haves result file
if it starts with a genome name from the don't haves list, it will copy it to the don't haves result file
Results file will be constructed based on the <in_file> name and the <results_base_name>. You additionally must indicate the results directory at <results_dir>

Usage: perl separate_file_by_gene_presence-v2.pl <data_file> <in_file> <results_base_name> <results_dir>

===
codon_usage_by_gene_presence-ALL-V3.1.pl

Program will make histograms and boxplots for the distribution of values of each column of several <data-files>
The list of <data-files> must be in the file <data-file>
its format must be:
lines beginning with # are not considered
first line not starting with # must have a single file containing the codon usages in all genomes format like in file average-b.txt
following lines (not starting with #) must have three fields separated by tabs:
First field : name of the gene
Second field: first file, has data for genomes with the gene
Third field : second file, has data for genomes withOUT the gen

All data files need to be in the same format. Data divided be tab separated columns
First line starts with "#" and indicates the name of each field

<gene_name> corresponds to the gene that has been analyzed. Will be used in plots names
Results files will be constructed based on the <results_base_name> and the names of each column and <gene_name>
A file with all plots will be saved to a file that additionally contains <summary_file_base_name>
Results will be saved in <Results_directory>. It must end with "/"

Additionally, the program adds an histogram. For that, the <Range> (size of bins), as well as the minimum value to count (<Beginning>) and the number of bins to include (<Steps>) must also be added to the command line. If <Beginning> is set to zero, please use 0.0 instead of 0.

This script requires the following modules
Statistics::Descriptive
Statistics::Ttest
Please consider that this script uses T-test to define statistical confidence of differences. This suppose a normal distribution of data. If your data is not normally distributed, please use codon_usage_by_gene_presence-ALL-Wilcoxon-V3.1.pl instead. P value is set to 0.00005. If a different value is required, please change the variable $pvalue accordingly.

Usage: perl codon_usage_by_gene_presence-ALL-V3.1.pl <data-file> <results_base_name> <summary_file_base_name> <Results_directory> <Range> <Beginning> <Steps>

===
batch-separate_file_by_gene_presence_and_plot_histograms-Wilcoxon.pl

Program checks for all files in directory <directory> that start with <pattern> and end with <extension>. The aim is to select results files from rpsblast-summary-V4.pl. Then it runs separate_file_by_gene_presence-v2.pl for each of this files using the program indicated in variable $separate_prog and the codon usage file indicated in <usage file>. This separates the codon usage of each genomes based on the presence or absence of the gen of interest. Then it uses the results files to run program indicated in variable $plot_prog (codon_usage_by_gene_presence-ALL-Wilcoxon-V3.1.pl) to construct superposed histograms and box plots comparing the distribution of codon usages in genomes that present or lack each gene. It additionally calculates statistical significance of differences between populations based on Mann-Whitney test also called Wilcoxon-Mann-Whitney test or Wilcoxon Rank Sum (non parametric, can be used to test differences between non-normal distributions).
All files in the indicated directory will be used to make plots in the same page of each pdf.
Please correct the path in variables $separate_prog and $plot_prog if the corresponding scripts are not in the path or current directory

Usage: perl batch-separate_file_by_gene_presence_and_plot_histograms-Wilcoxon.pl <directory> <pattern> <extension> <usage file> <results directory>

==

codon_usage_by_gene_presence-ALL-Wilcoxon-V3.1.pl

Program will make histograms and boxplots for the distribution of values of each column of several <data-files>
   The list of <data-files> must be in the file <data-file>
   its format must be:
   lines beginning with # are not considered
   first line not starting with # must have a single file containing the codon usages in all genomes format like in file average-b.txt
   following lines (not starting with #) must have three fields separated by tabs:
      First field : name of the gene
      Second field: first file, has data for genomes with the gene
      Third field : second file, has data for genomes withOUT the gen

   All data files need to be in the same format. Data divided be tab separated columns
   First line starts with "#" and indicates the name of each field

<gene_name> corresponds to the gene that has been analyzed. Will be used in plots names
Results files will be constructed based on the <results_base_name> and the names of each column and <gene_name>
   A file with all plots will be saved to a file that additionally contains <summary_file_base_name>
Results will be saved in <Results_directory>. It must end with "/"

Additionally, the program adds an histogram. For that, the <Range> (size of bins), as well as the minimum value to count (<Beginning>) and the number of bins to include (<Steps>) must also be added to the command line. If <Beginning> is set to zero, please use 0.0 instead of 0.

This script requires the following modules
   Statistics::Descriptive
   Statistics::Test::WilcoxonRankSum
This script do not suppose normal distribution as it uses Wilcoxon Rank Sum test. P value is set to 0.00005. If a different value is required, please change the variable $pvalue accordingly.

Usage: perl codon_usage_by_gene_presence-ALL-Wilcoxon-V3.1.pl <data-file> <results_base_name> <summary_file_base_name> <Results_directory> <Range> <Beggining> <Steps>

==
