# Supplementary material for: Impact of the chemical modification of tRNAs anticodon loop on the variability and evolution of codon usage in proteobacteria
Source: Front Microbiol. 2024 Aug 5;15:1412318. doi: 10.3389/fmicb.2024.1412318 (PMC11332805; doi:10.3389/fmicb.2024.1412318)
Supplement: Supplementary file 1 [file Compiled_supplementary_figures.pdf › Supp_figures/Compiled_supplementary_figures.pdf]

Compiled supplementary figures

This is a compiled figure containing all individual supplementary figures with their legends. Given that some figures are slow to open, a copy of each individual supplementary figure is included in additional files. Similarly, tables can be found in individual files or as a single compiled file.

All supplementary files can be accessed at https://doi.org/10.34691/UCHILE/AYDRZL

Figure S1. Histograms of codon usage in proteobacteria. The average usage of each codon was calculated for 1484 species of proteobacteria. The histograms show the number of genomes that present each range of codon usage considering either all genes of each genome (purple) or a subset of genes expected to be highly expressed (green). Codon usages are expressed as the ratio between the number of codon of each identity and the total number of codons. Each page of the pdf file presents the histogram of the indicated codons.

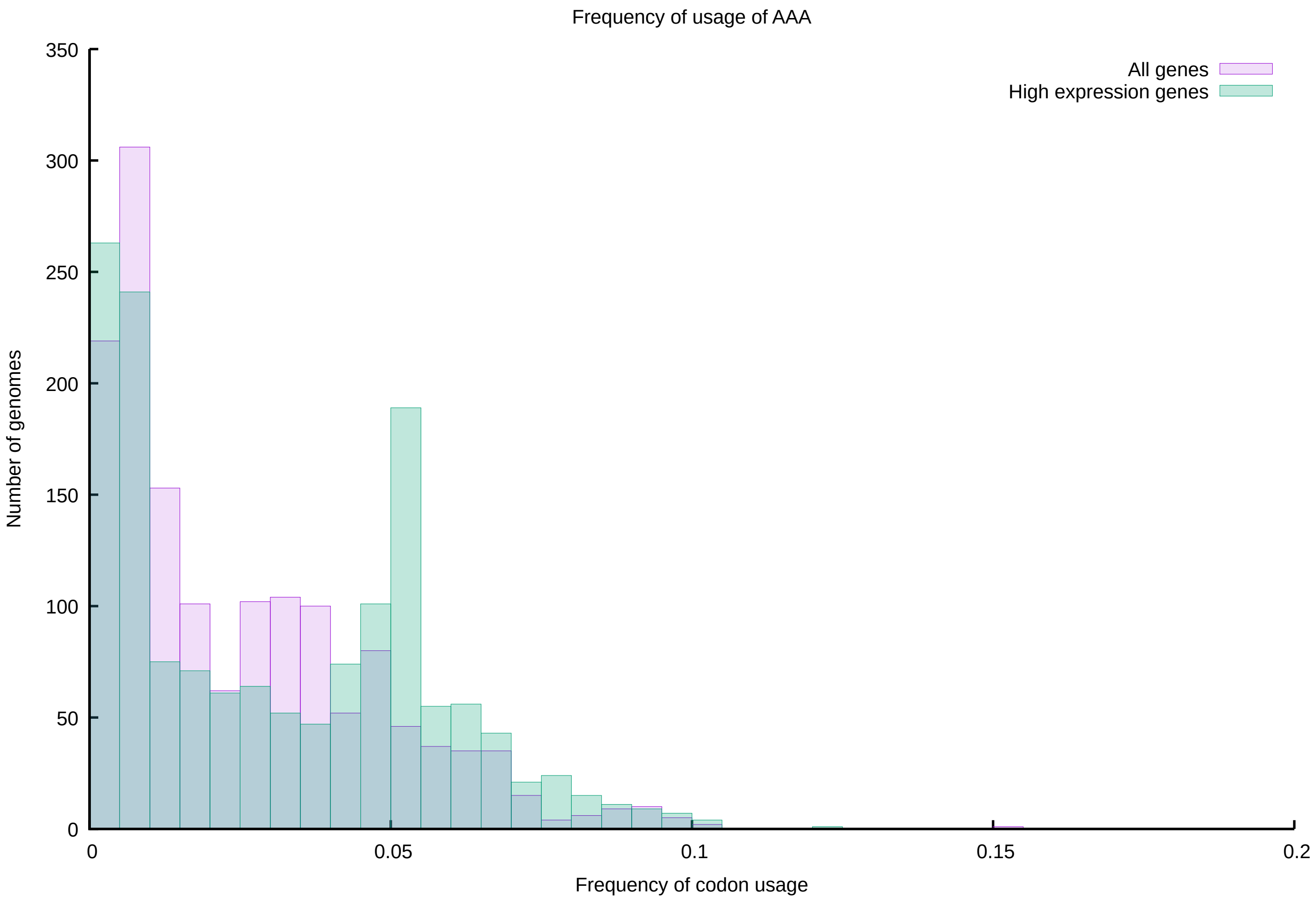

Frequency of usage of AAA
All genes
High expression genes
Number of genomes
350
300
250
200
150
100
50
0
0
0.05
0.1
0.15
0.2
Frequency of codon usage

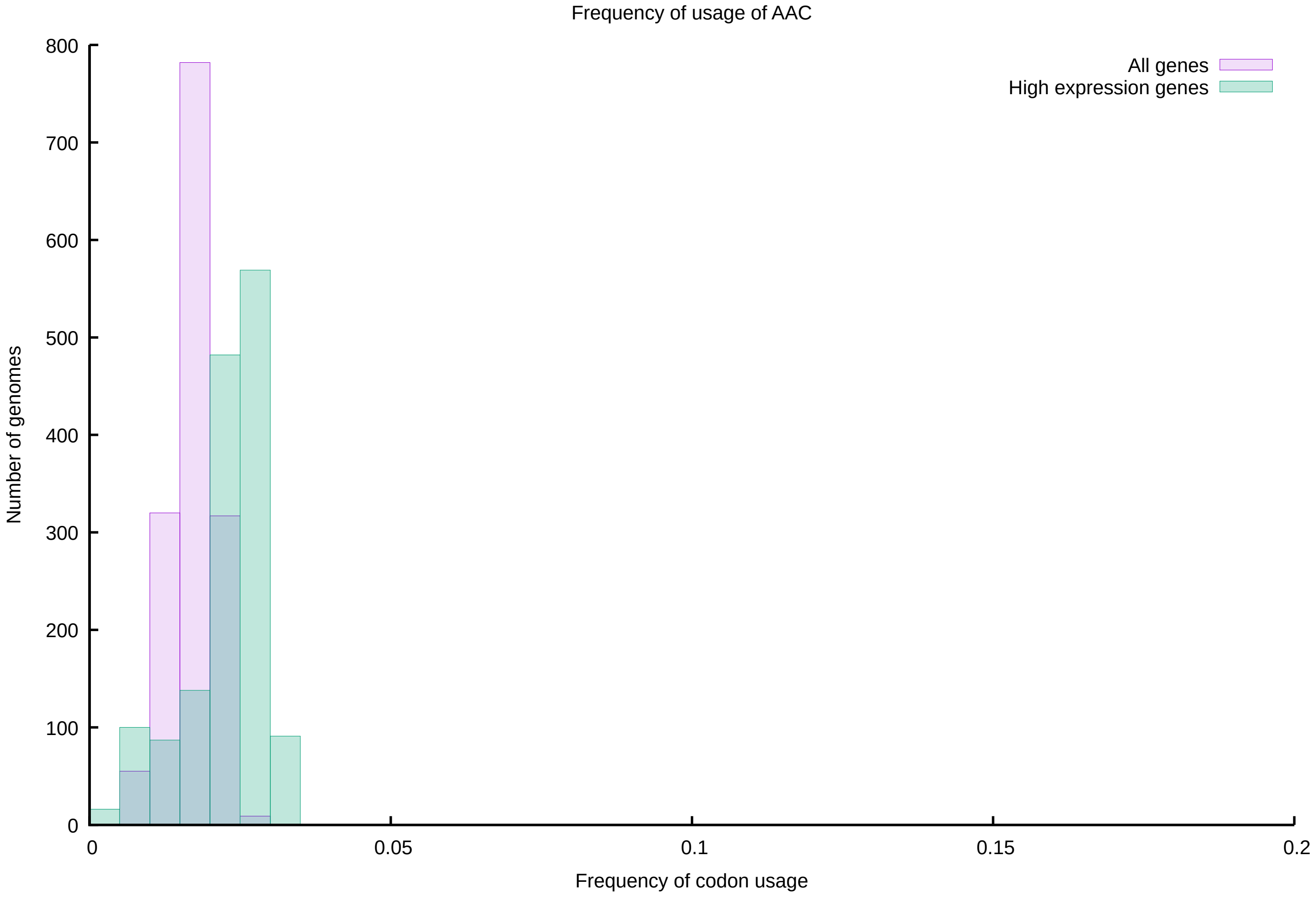

Frequency of usage of AAC
All genes
High expression genes
Number of genomes
0
100
200
300
400
500
600
700
800
0
0.05
0.1
0.15
0.2
Frequency of codon usage

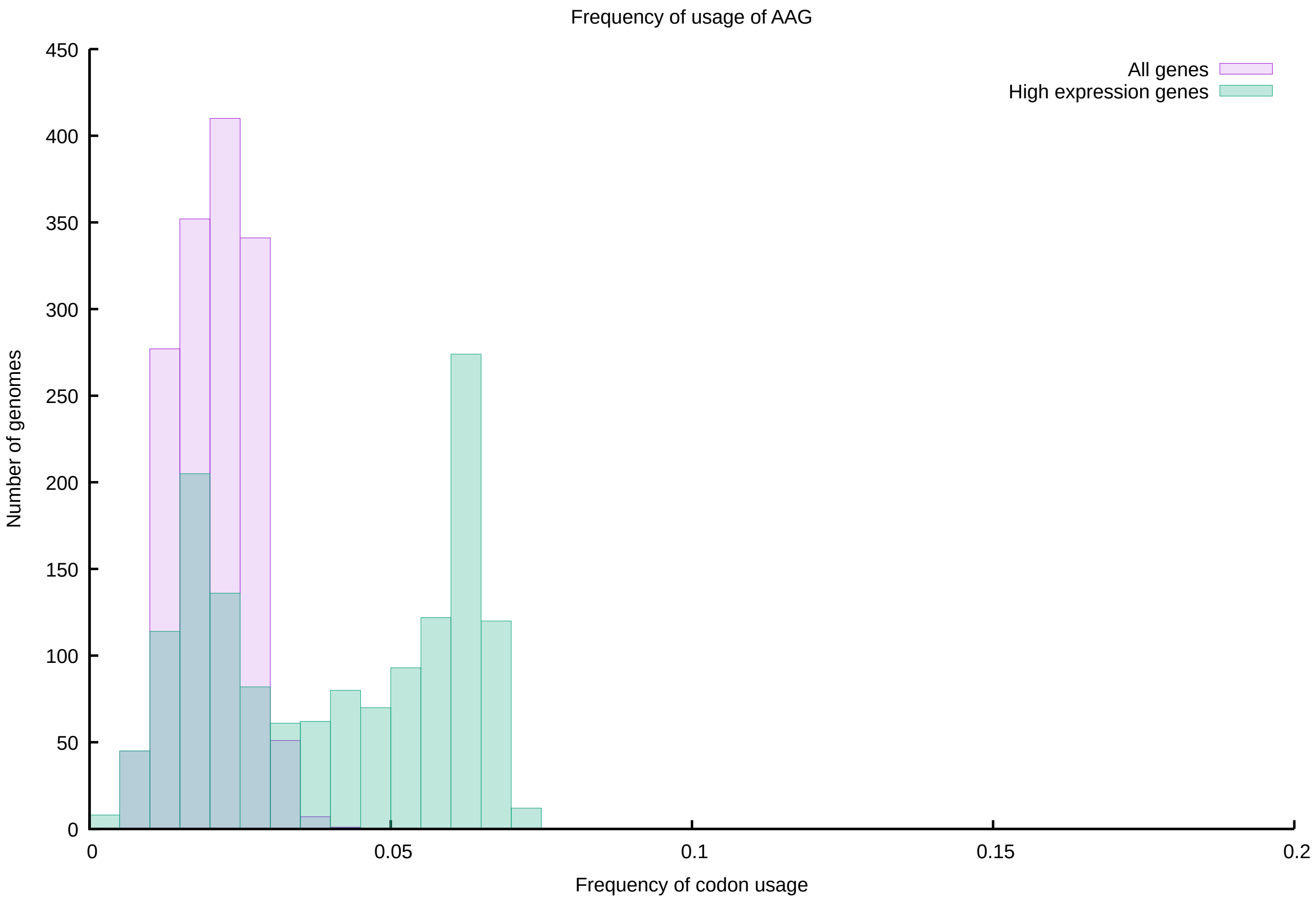

Frequency of usage of AAG
All genes
High expression genes
Number of genomes
0
50
100
150
200
250
300
350
400
450
0
0.05
0.1
0.15
0.2
Frequency of codon usage

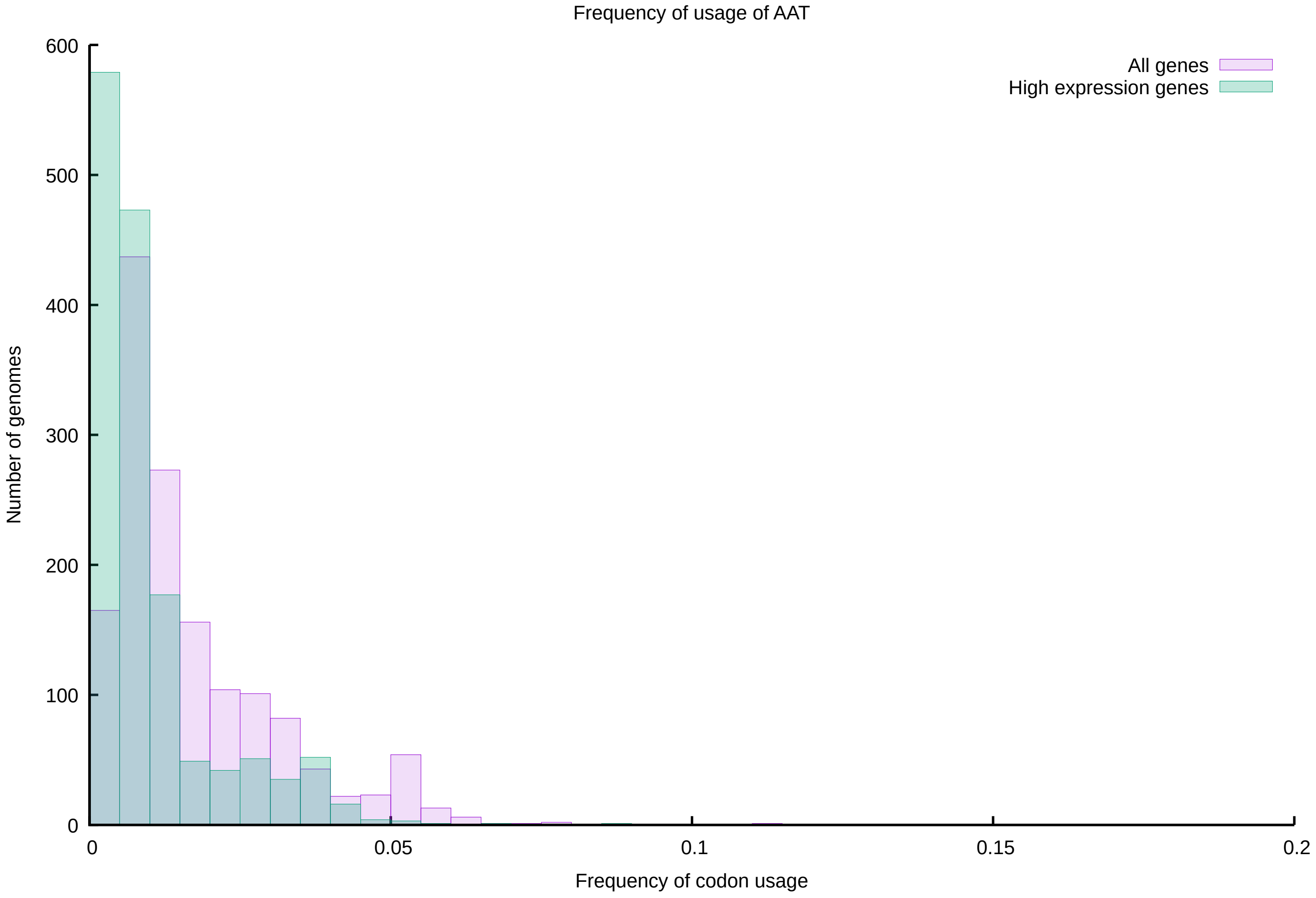
Frequency of usage of AAT
All genes
High expression genes
Number of genomes
0
100
200
300
400
500
600
0
0.05
0.1
0.15
0.2
Frequency of codon usage

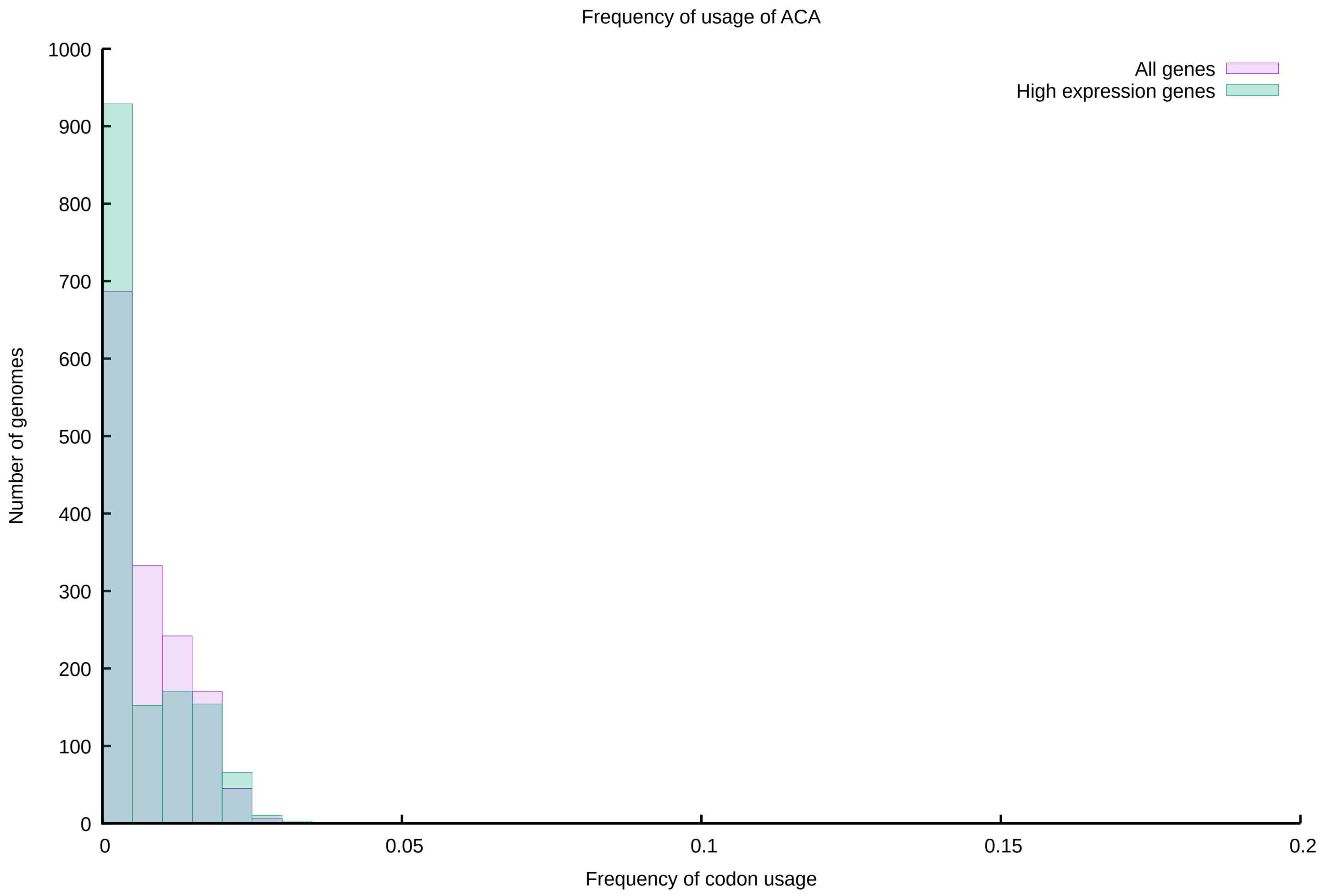

Frequency of usage of ACA
All genes
High expression genes
Number of genomes
1000
900
800
700
600
500
400
300
200
100
0
0
0.05
0.1
0.15
0.2
Frequency of codon usage

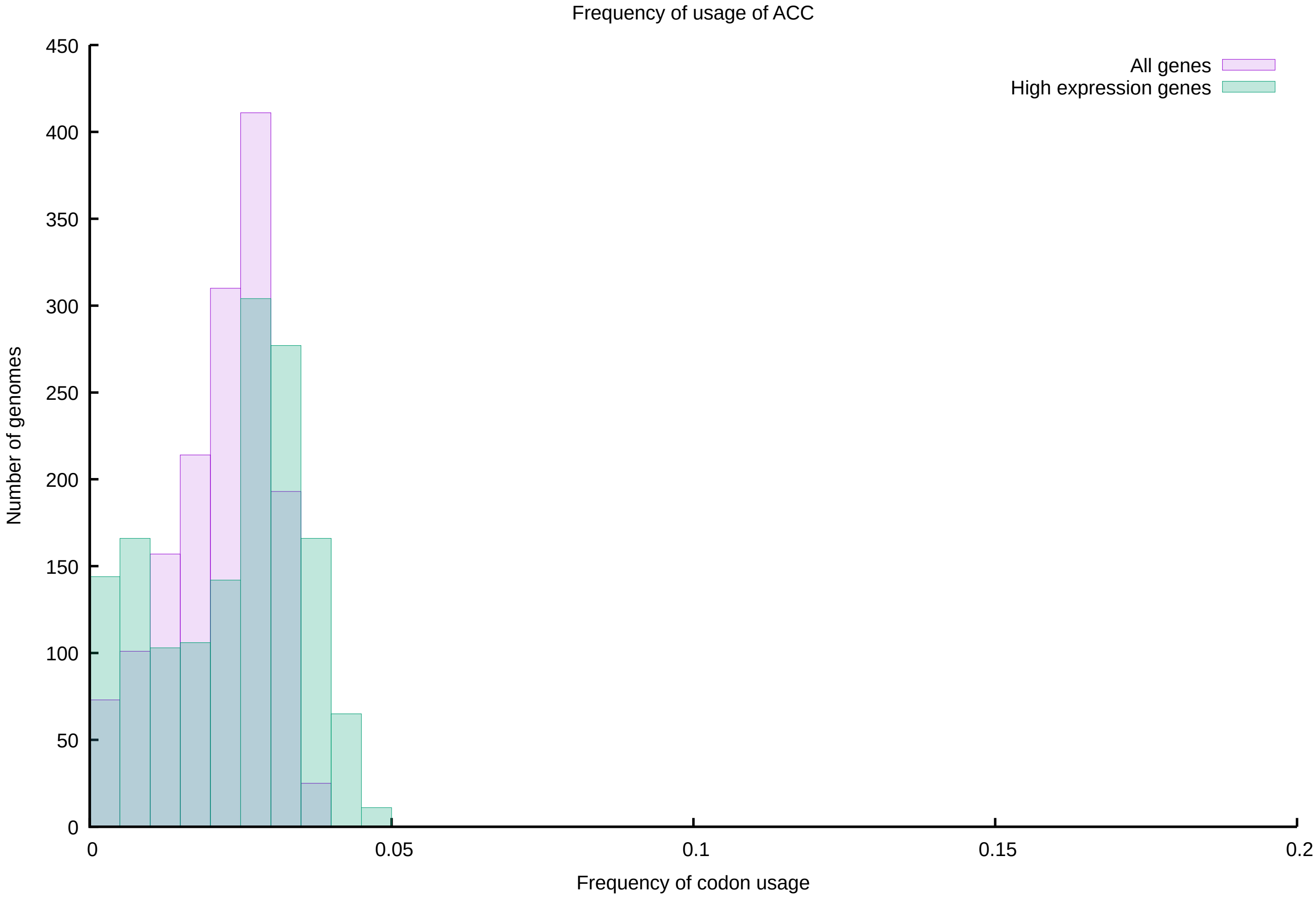

Frequency of usage of ACC
All genes
High expression genes
Number of genomes
450
400
350
300
250
200
150
100
50
0
0
0.05
0.1
0.15
0.2
Frequency of codon usage

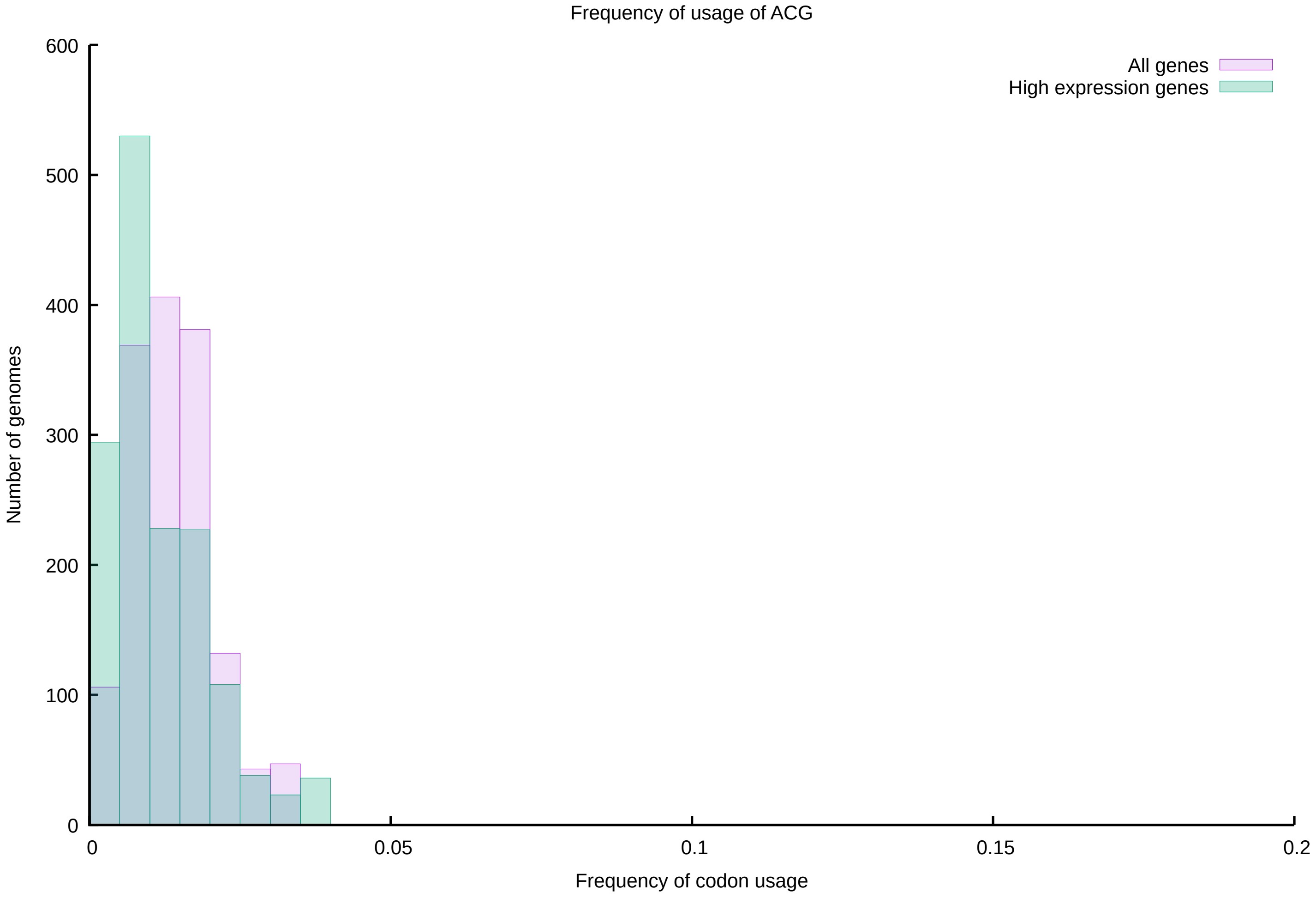
Frequency of usage of ACG
All genes
High expression genes
Number of genomes
0
100
200
300
400
500
600
0
0.05
0.1
0.15
0.2
Frequency of codon usage

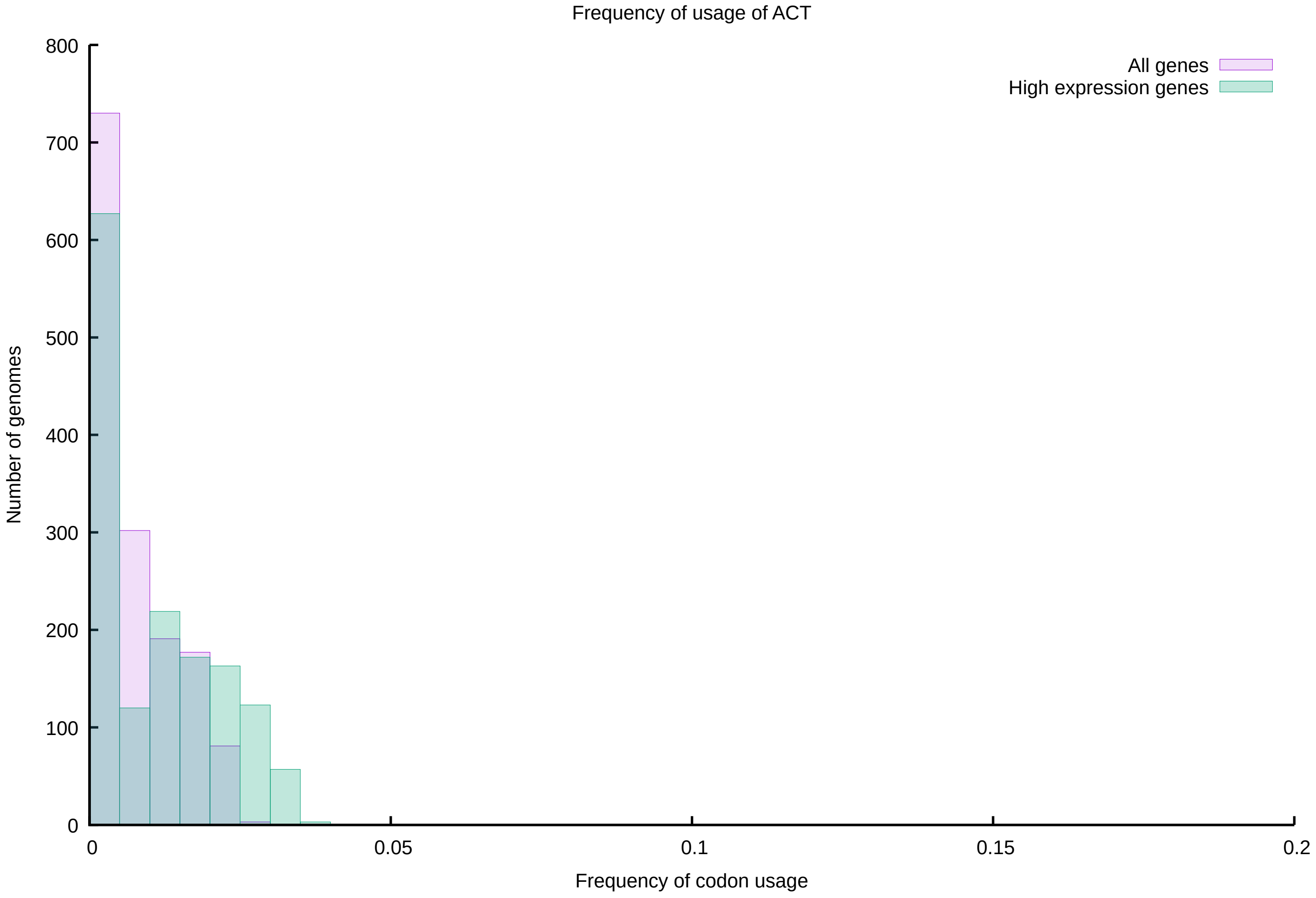
Frequency of usage of ACT
All genes
High expression genes
Number of genomes
Frequency of codon usage
800
700
600
500
400
300
200
100
0
0
0.05
0.1
0.15
0.2

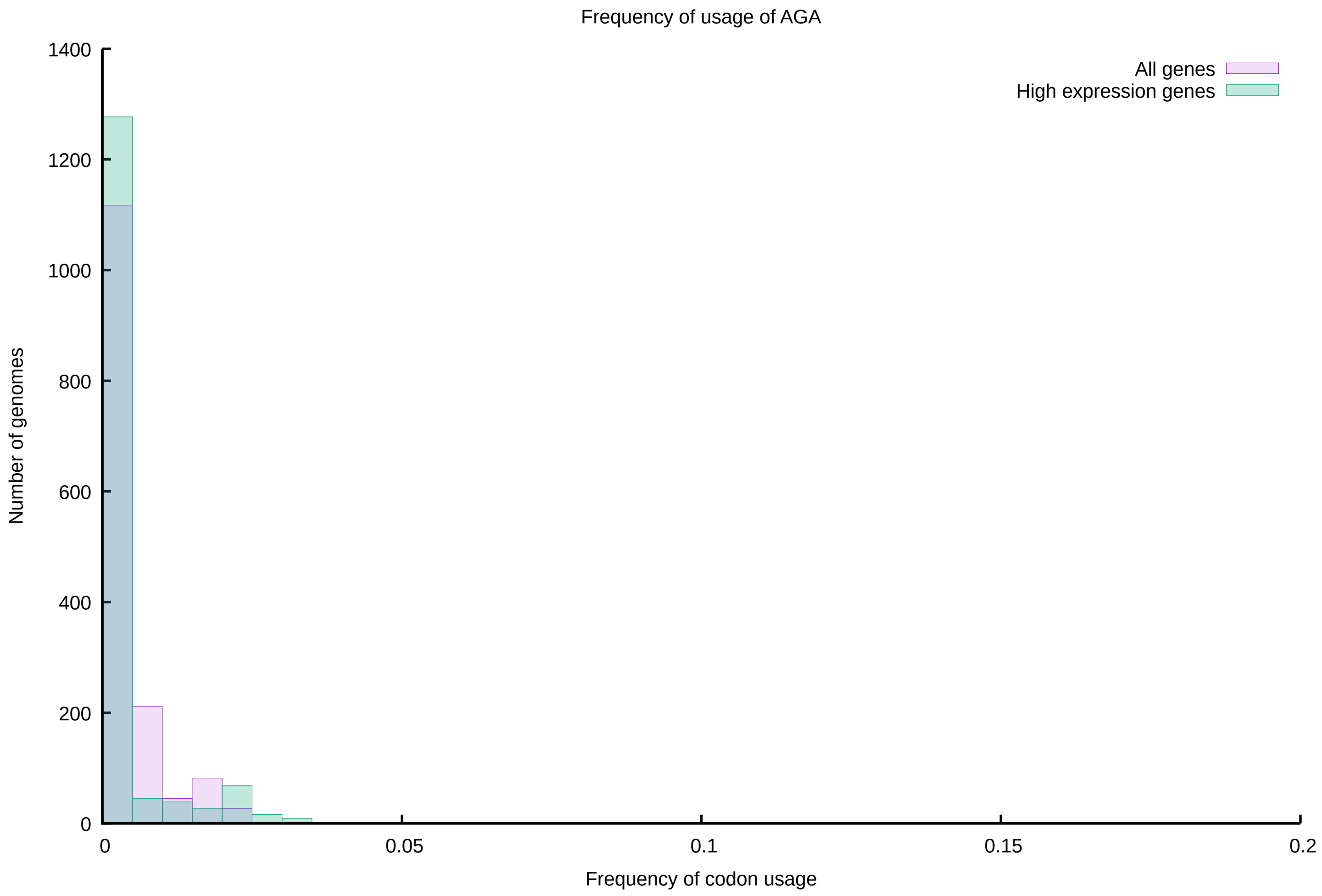

Frequency of usage of AGA
All genes
High expression genes
Number of genomes
1400
1200
1000
800
600
400
200
0
0
0.05
0.1
0.15
0.2
Frequency of codon usage

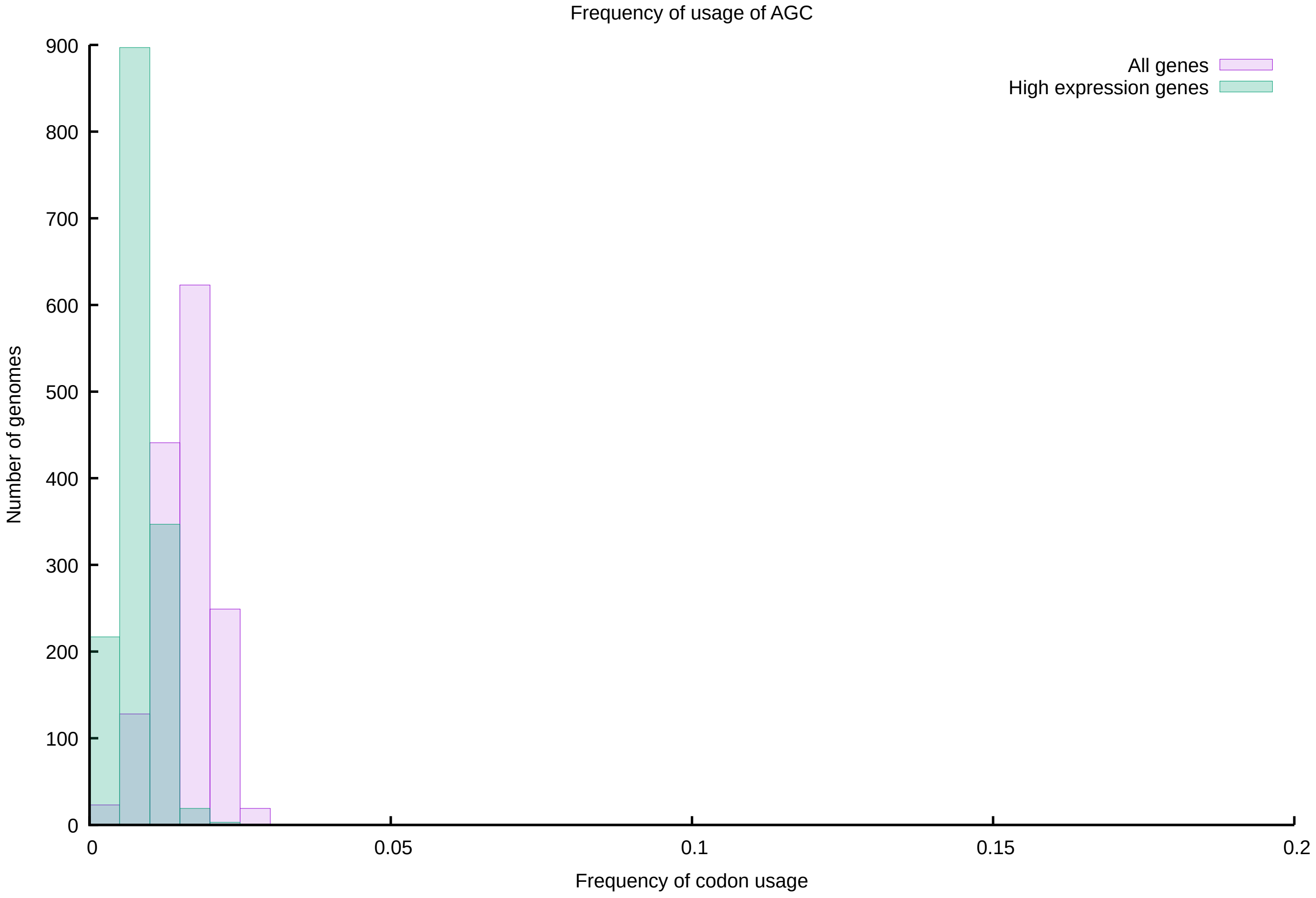
Frequency of usage of AGC
All genes
High expression genes
Number of genomes
900
800
700
600
500
400
300
200
100
0
0
0.05
0.1
0.15
0.2
Frequency of codon usage

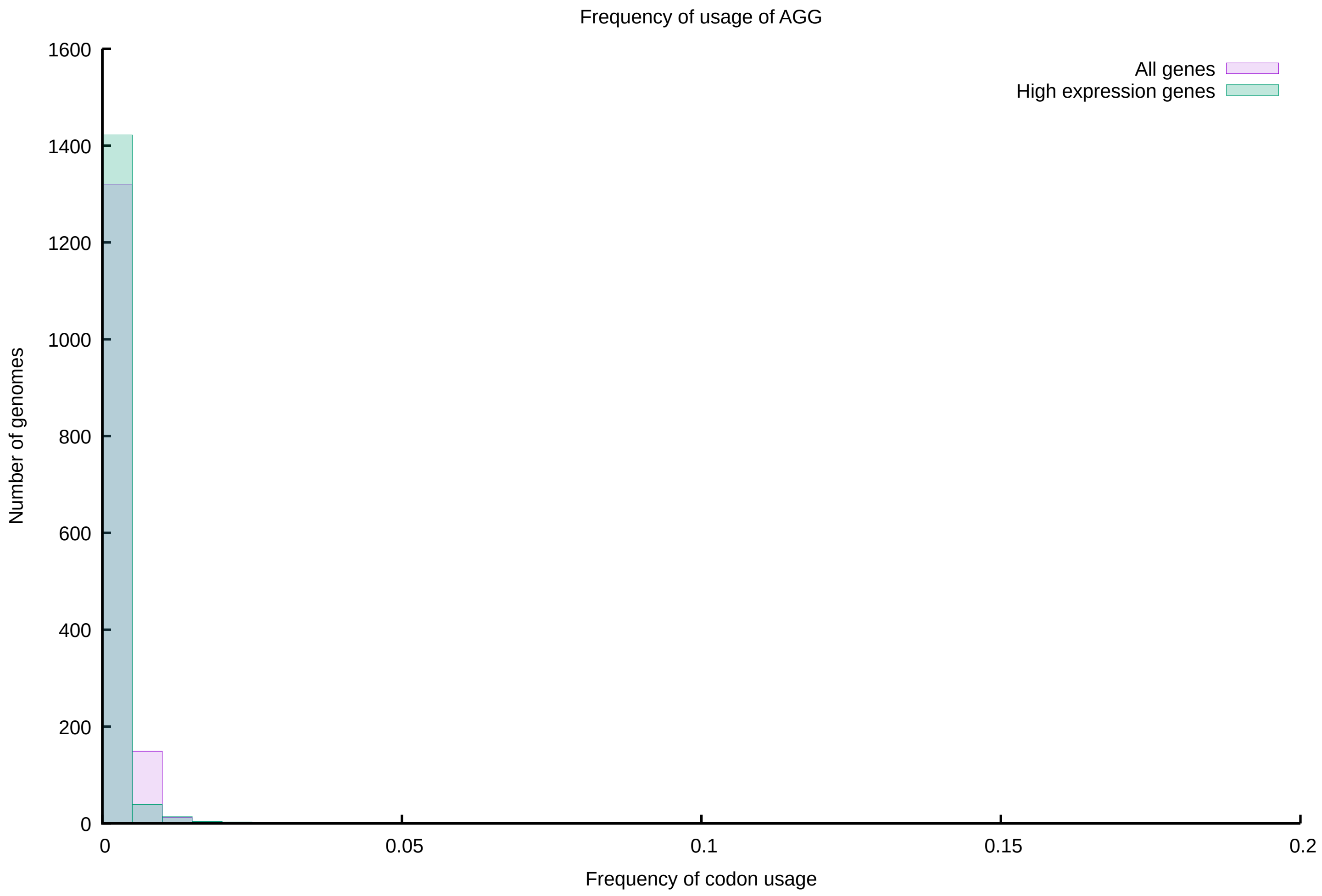

Frequency of usage of AGG
All genes
High expression genes
Number of genomes
1600
1400
1200
1000
800
600
400
200
0
0
0.05
0.1
0.15
0.2
Frequency of codon usage

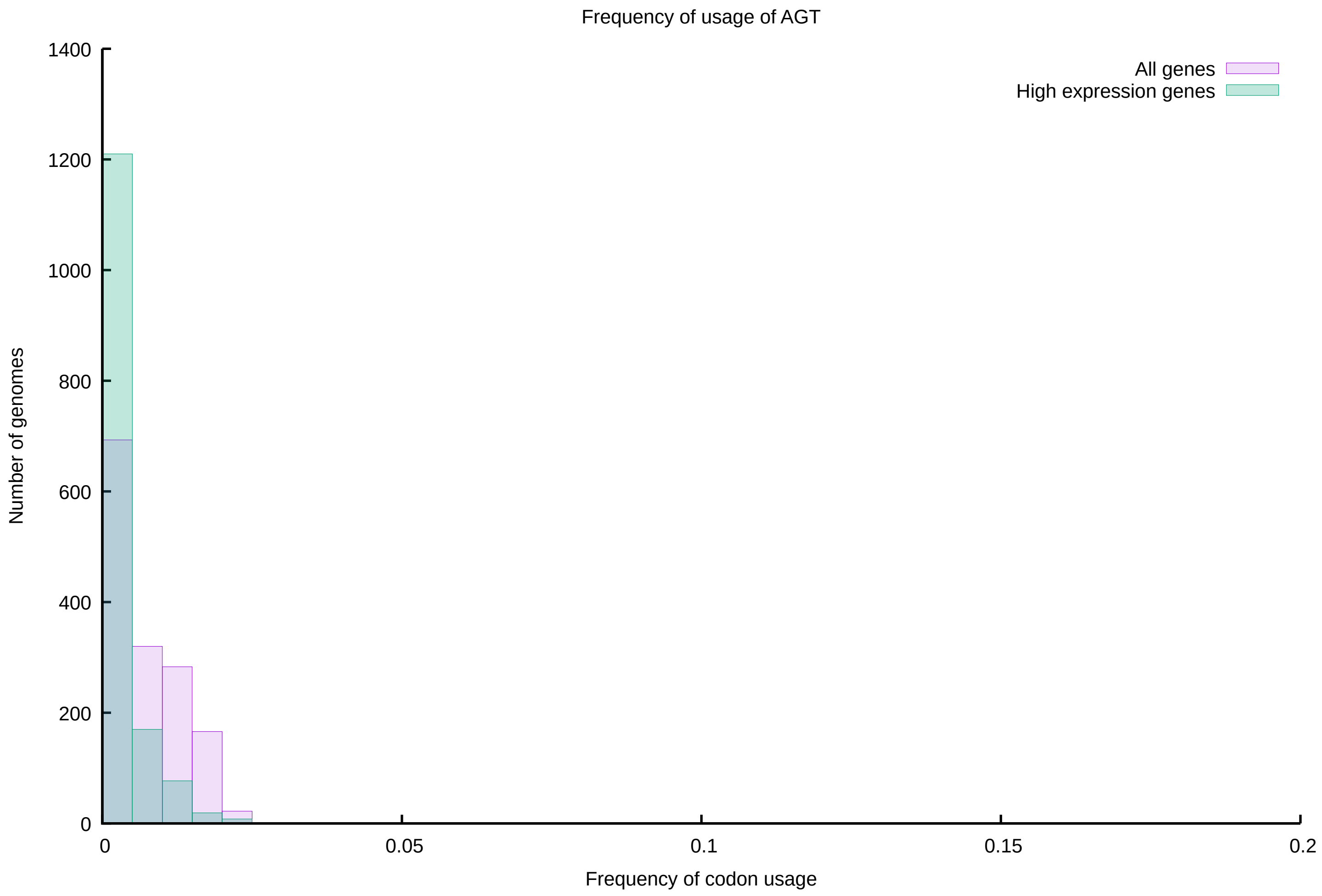

Frequency of usage of AGT
All genes
High expression genes
Number of genomes
Frequency of codon usage
0
200
400
600
800
1000
1200
1400
0.05
0.1
0.15
0.2

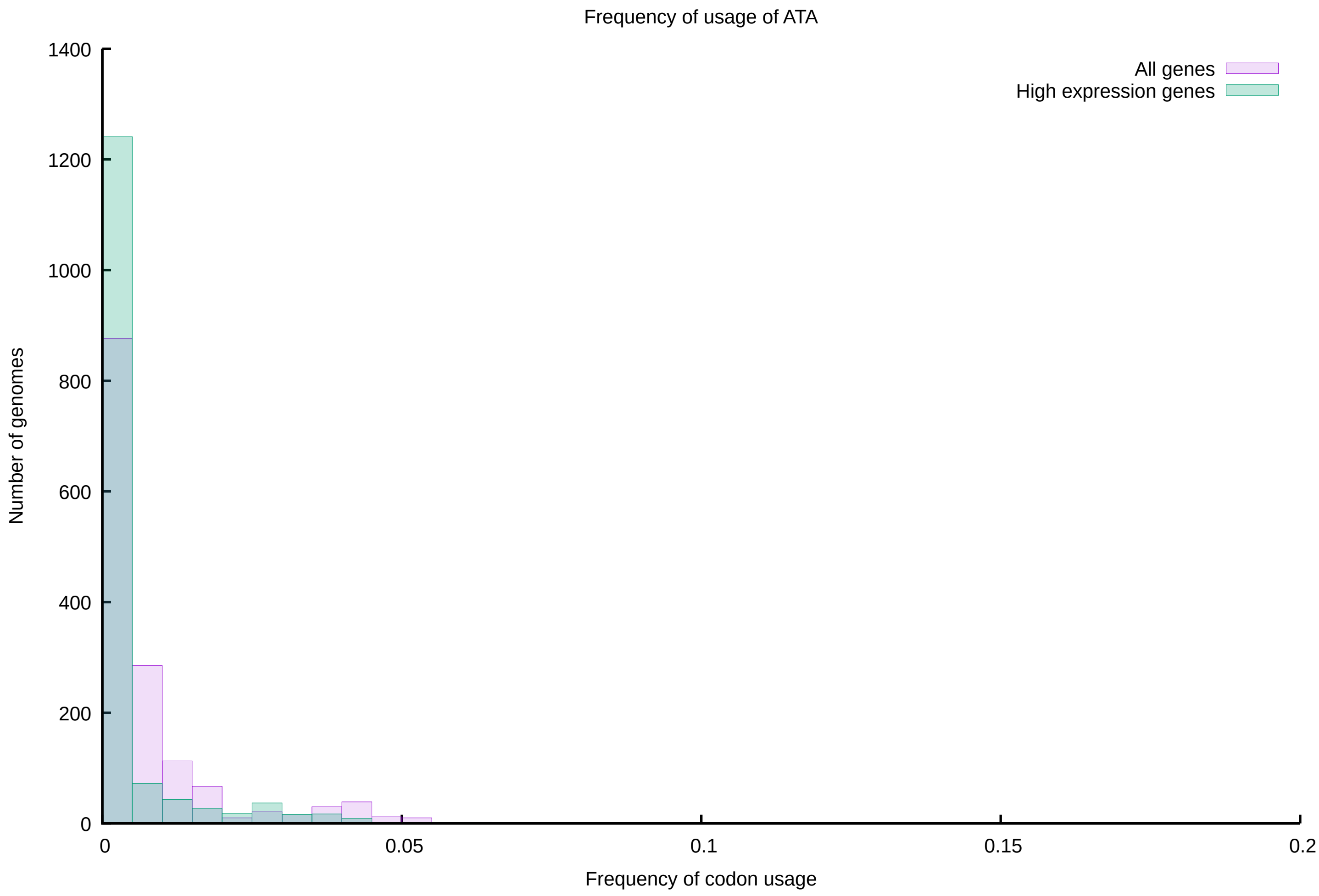
Frequency of usage of ATA
All genes
High expression genes
Number of genomes
0
200
400
600
800
1000
1200
1400
0
0.05
0.1
0.15
0.2
Frequency of codon usage

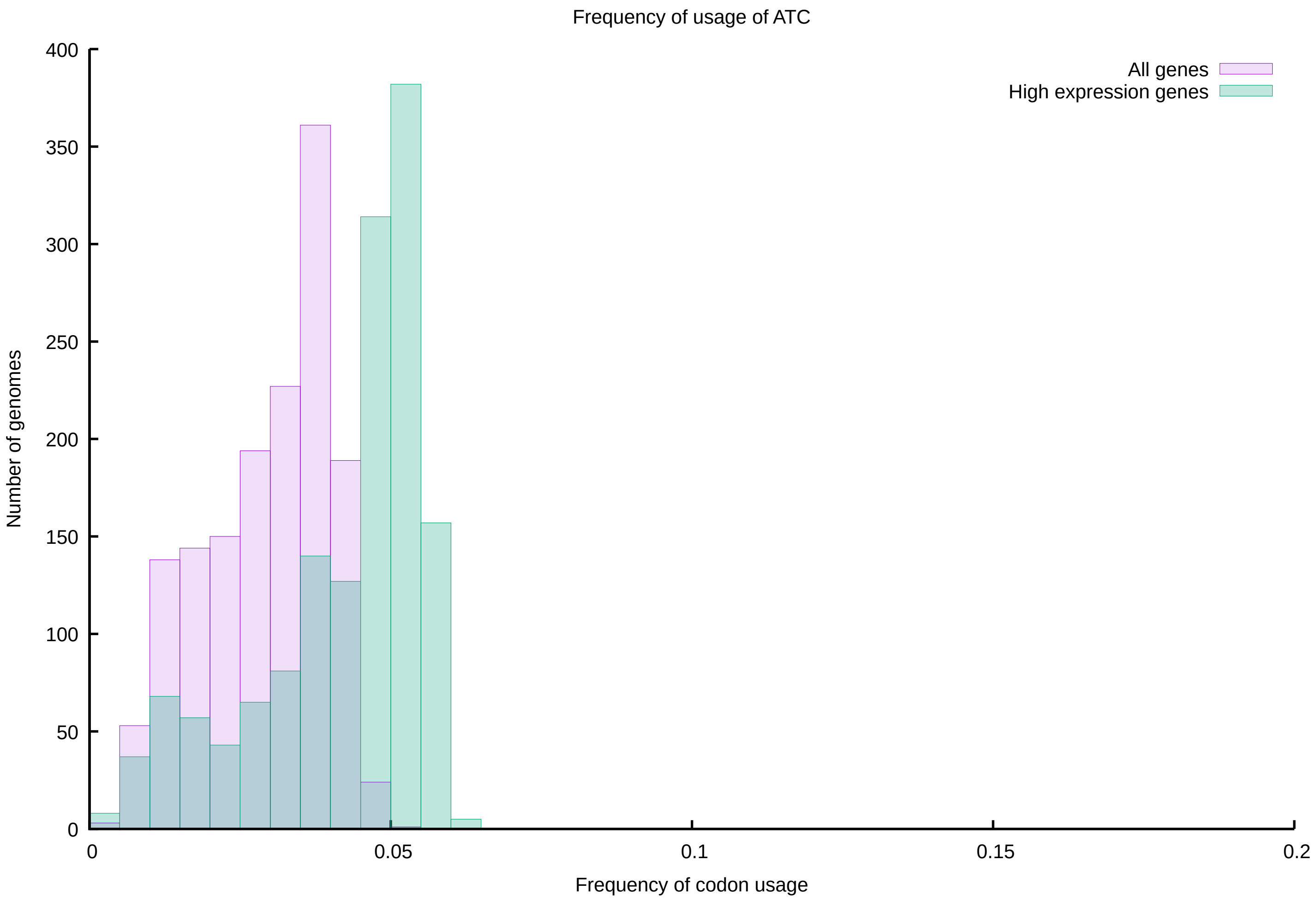
Frequency of usage of ATC
All genes
High expression genes
Number of genomes
Frequency of codon usage
0
50
100
150
200
250
300
350
400
0
0.05
0.1
0.15
0.2

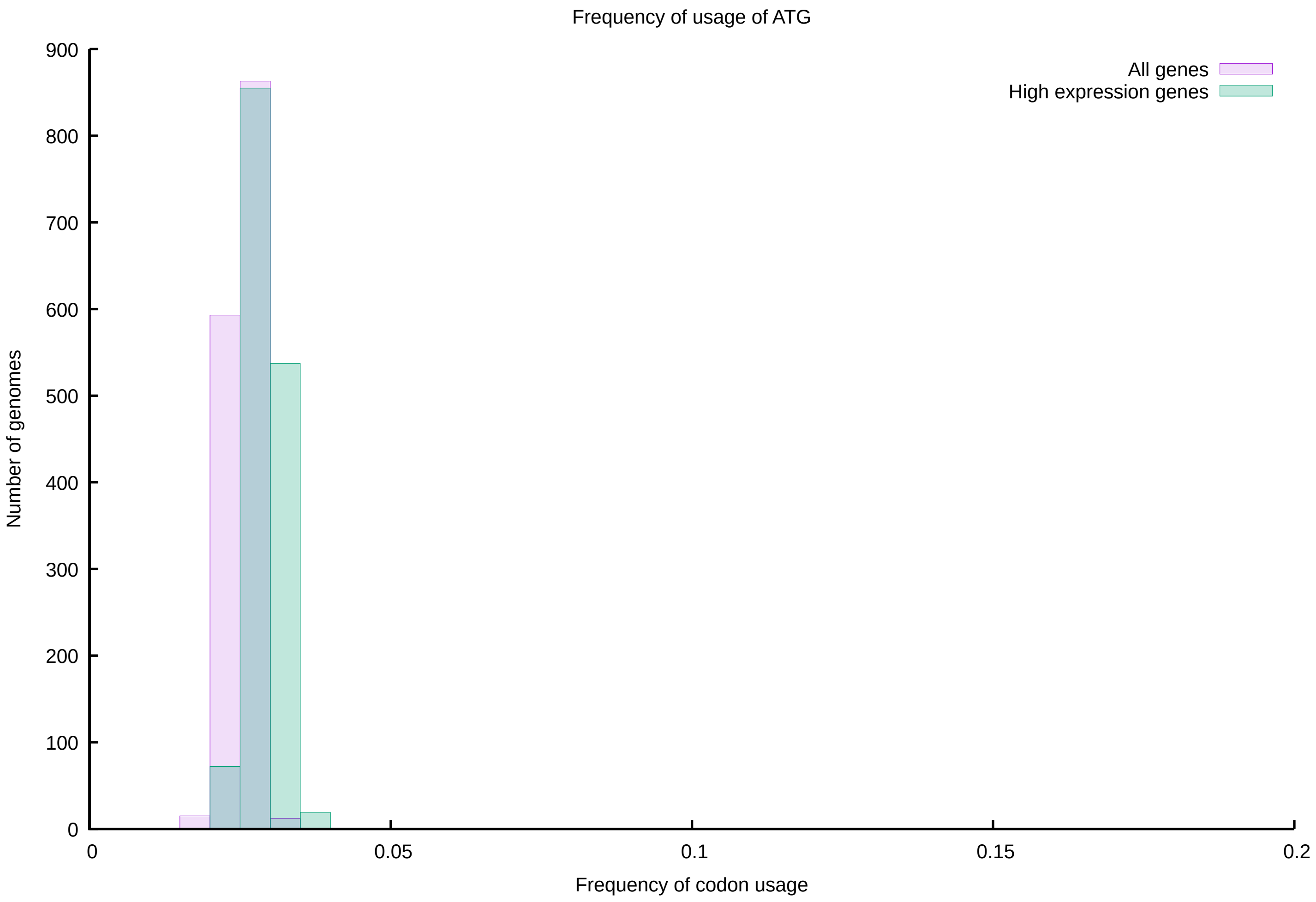

Frequency of usage of ATG
All genes
High expression genes
Number of genomes
900
800
700
600
500
400
300
200
100
0
0
0.05
0.1
0.15
0.2
Frequency of codon usage

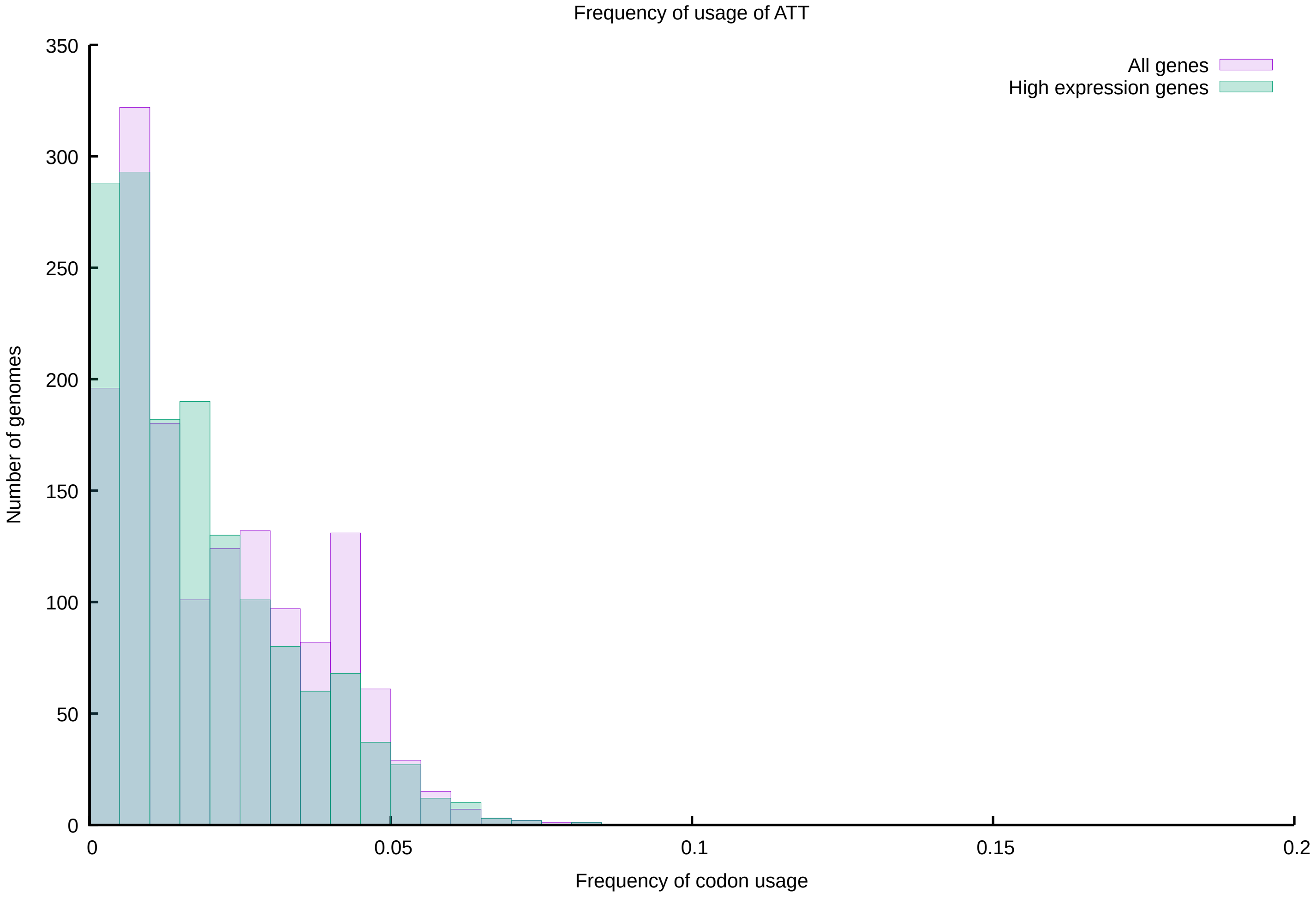

Frequency of usage of ATT
All genes
High expression genes
Number of genomes
350
300
250
200
150
100
50
0
0
0.05
0.1
0.15
0.2
Frequency of codon usage

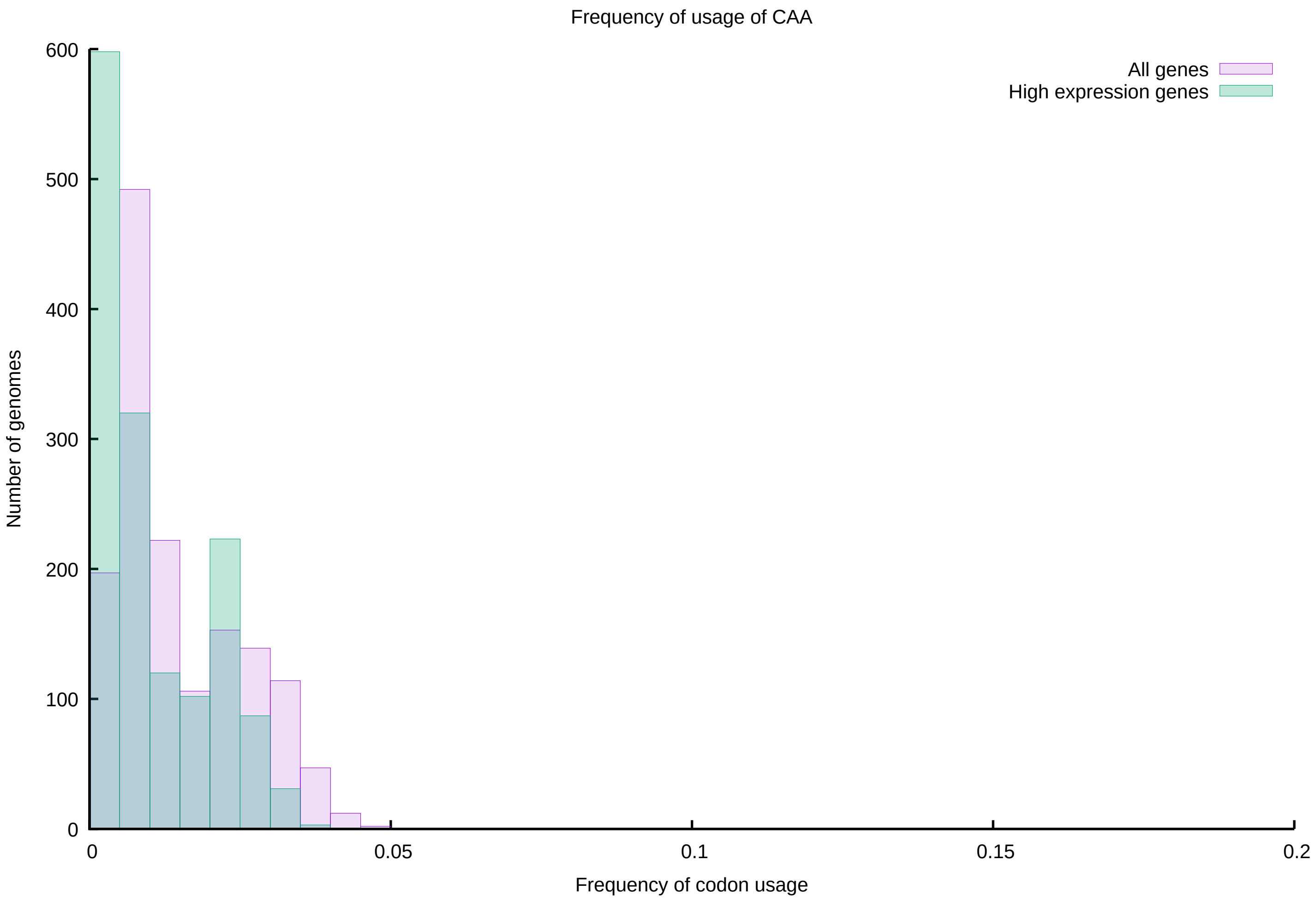

Frequency of usage of CAA
All genes
High expression genes
Number of genomes
0
100
200
300
400
500
600
0
0.05
0.1
0.15
0.2
Frequency of codon usage

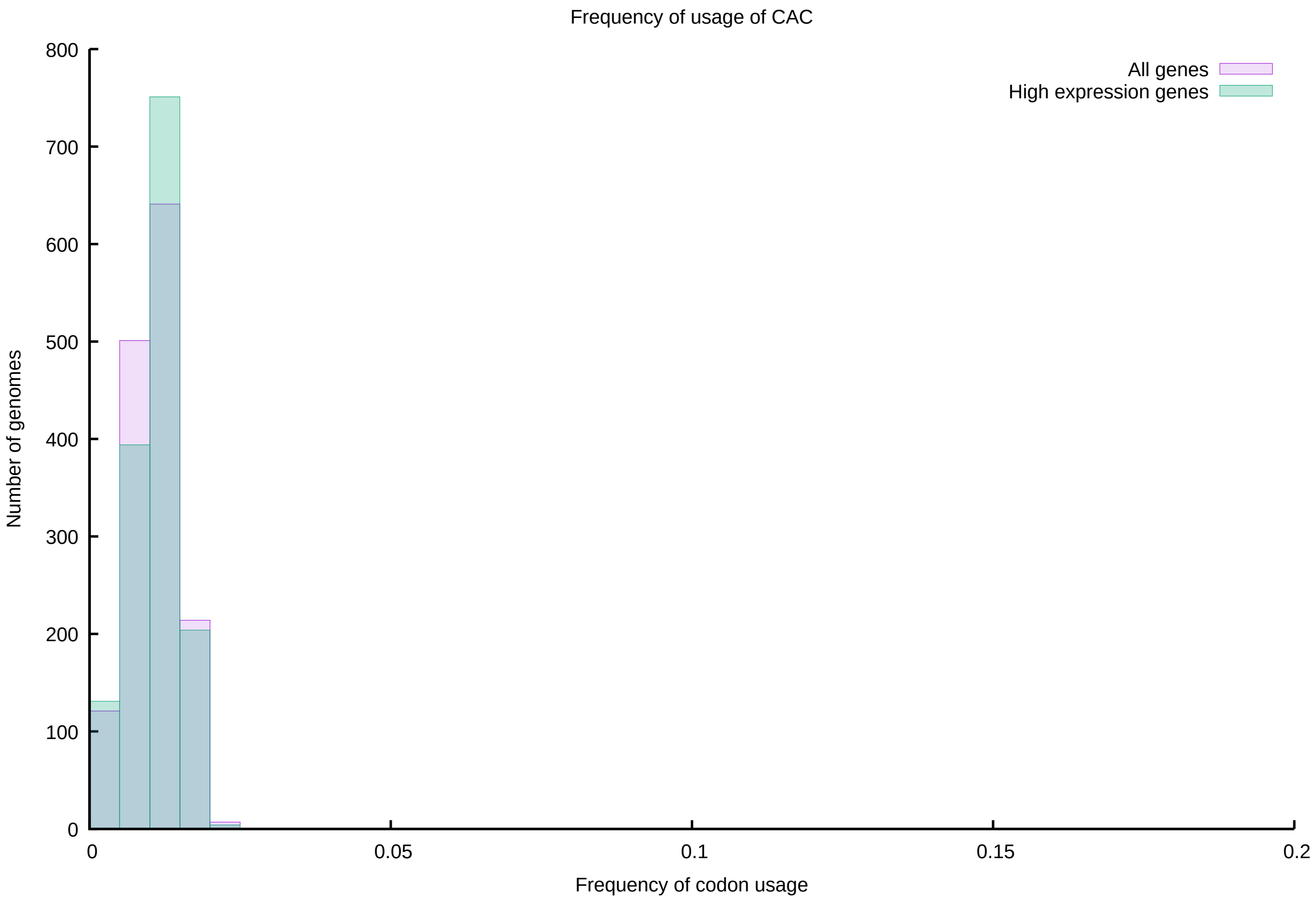

Frequency of usage of CAC
All genes
High expression genes
Number of genomes
Frequency of codon usage
0
100
200
300
400
500
600
700
800
0.05
0.1
0.15
0.2

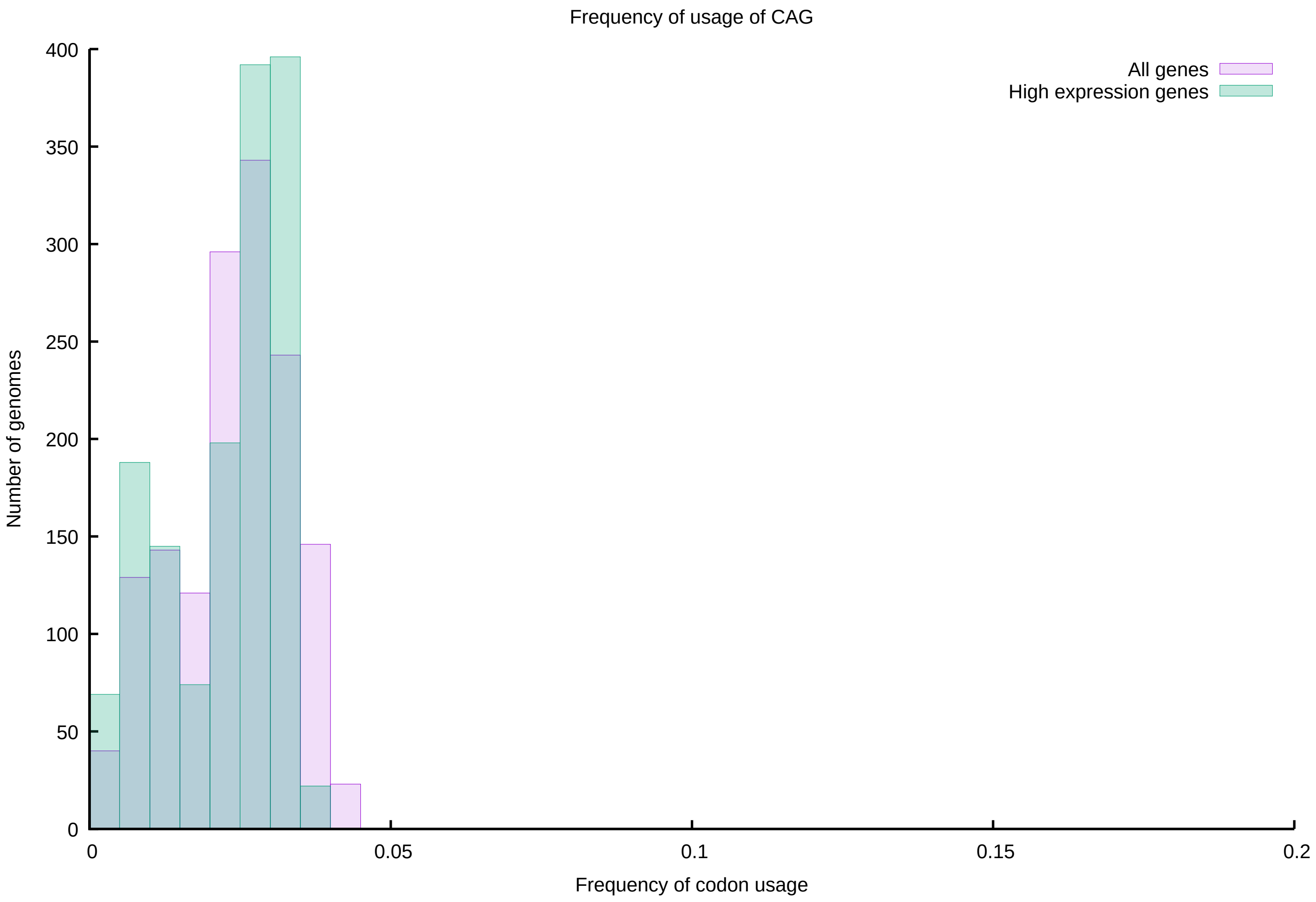

Frequency of usage of CAG
All genes
High expression genes
Number of genomes
Frequency of codon usage
0
50
100
150
200
250
300
350
400
0
0.05
0.1
0.15
0.2

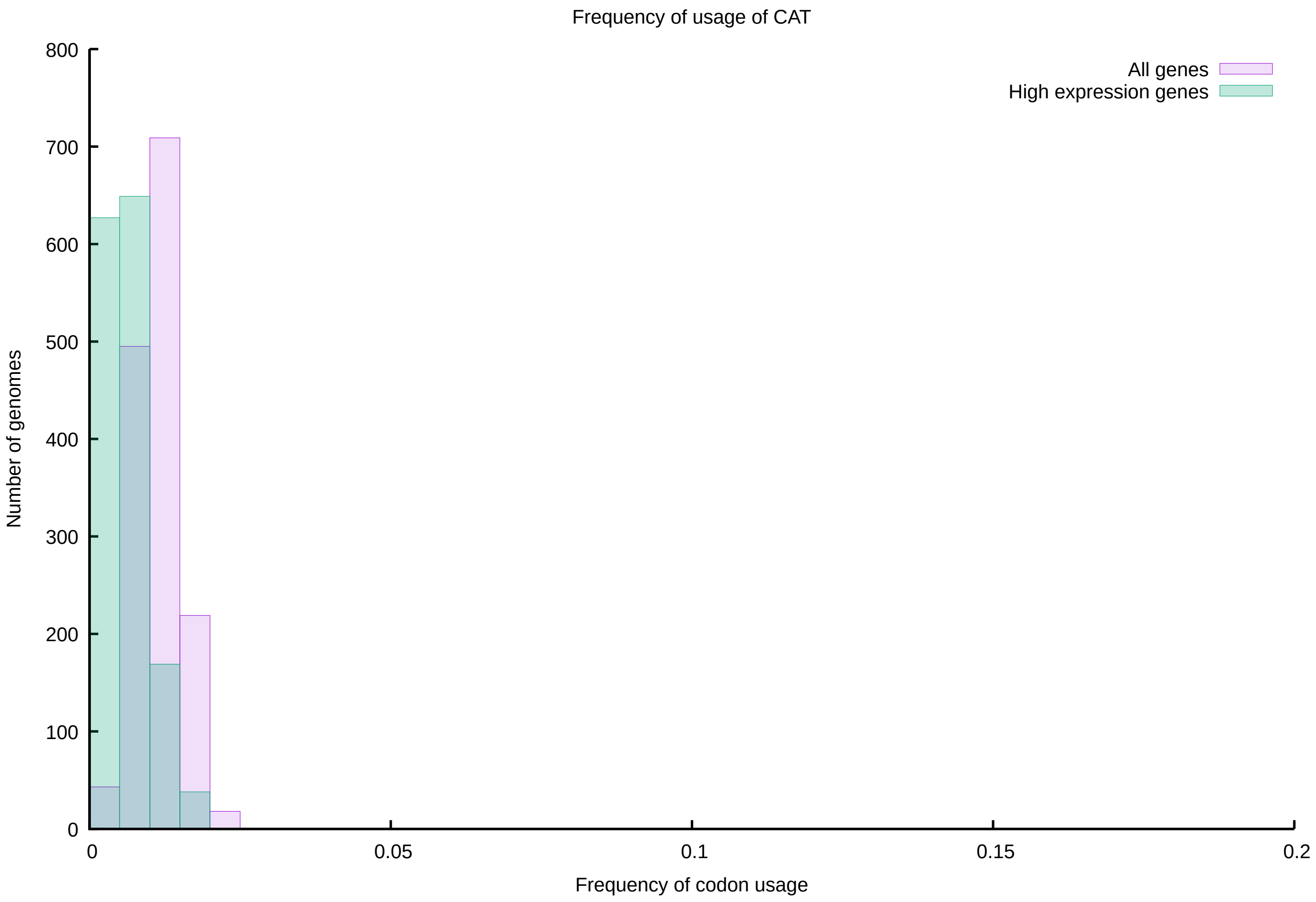
Frequency of usage of CAT
All genes
High expression genes
Number of genomes
0
100
200
300
400
500
600
700
800
0
0.05
0.1
0.15
0.2
Frequency of codon usage

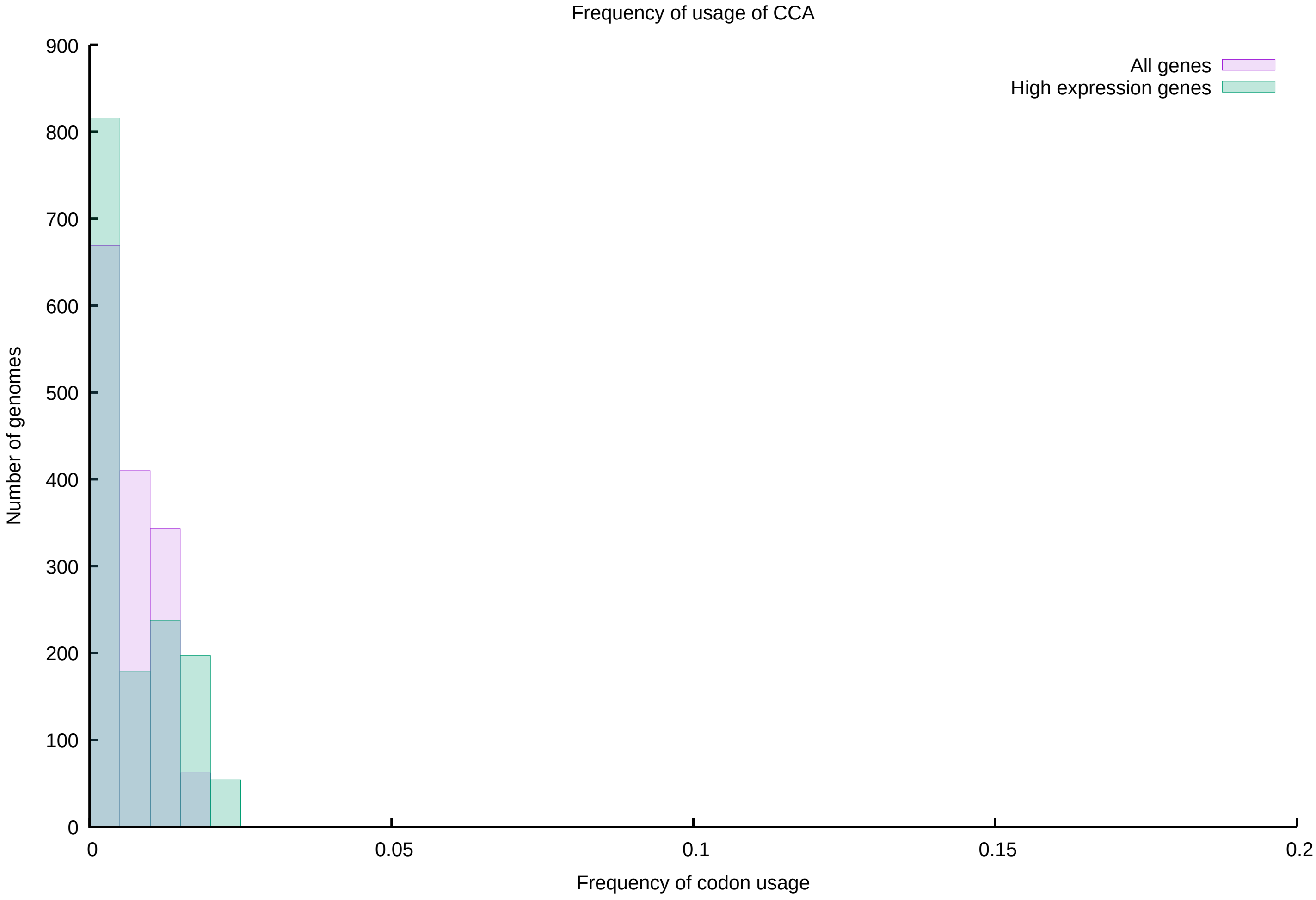
Frequency of usage of CCA
All genes
High expression genes
Number of genomes
Frequency of codon usage
0
100
200
300
400
500
600
700
800
900
0
0.05
0.1
0.15
0.2

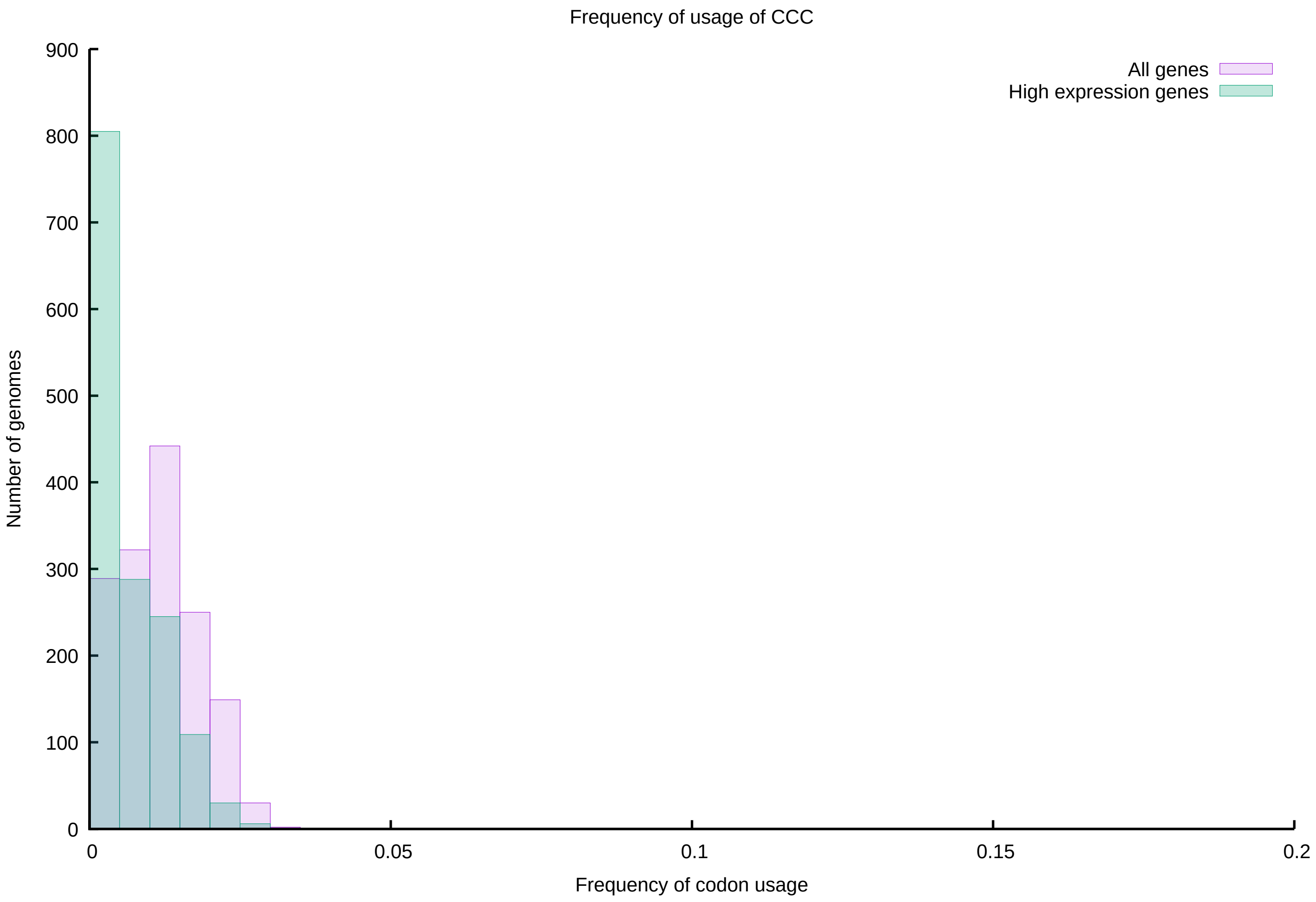
Frequency of usage of CCC
All genes
High expression genes
Number of genomes
0
100
200
300
400
500
600
700
800
900
0
0.05
0.1
0.15
0.2
Frequency of codon usage

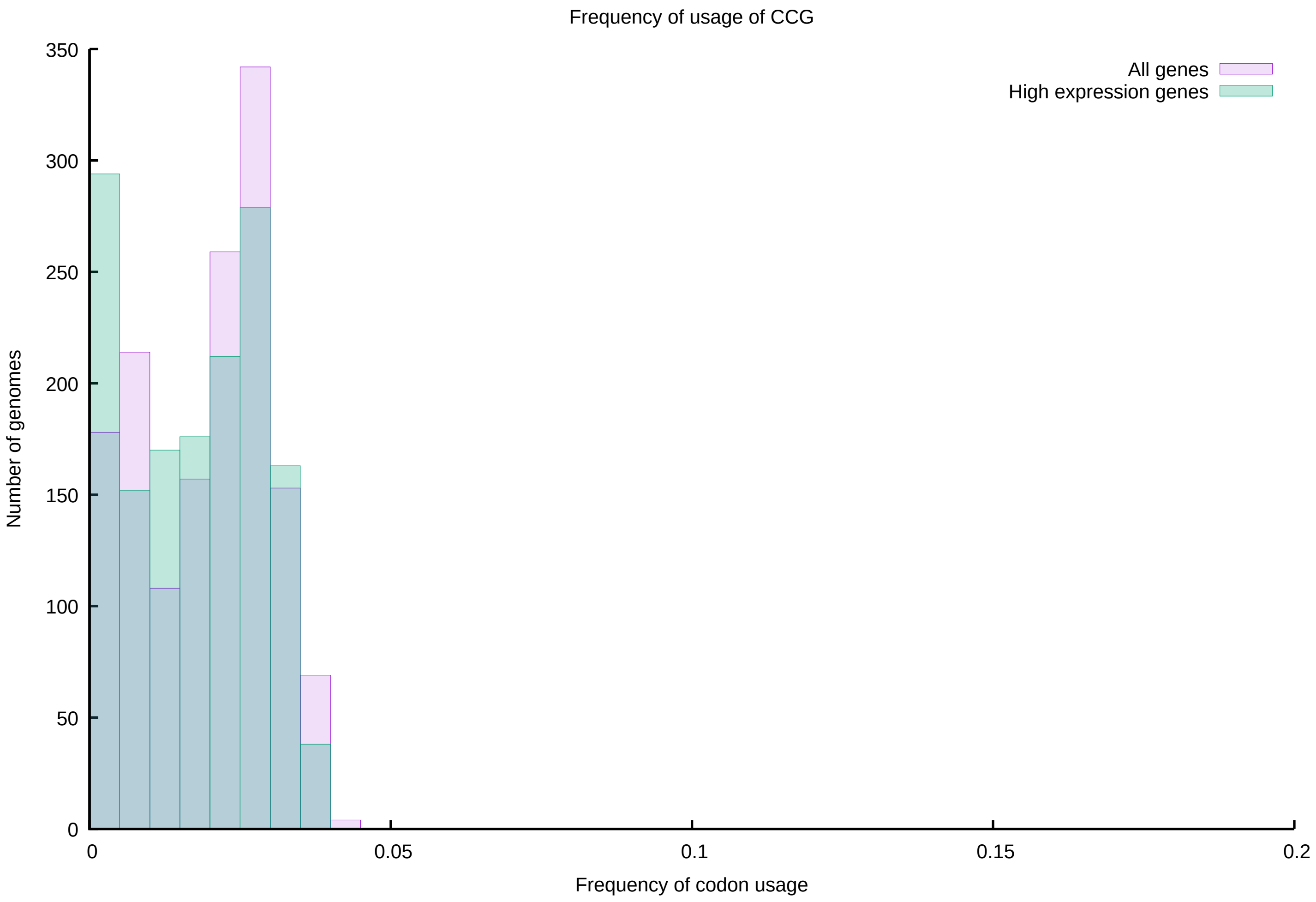
Frequency of usage of CCG
All genes
High expression genes
Number of genomes
350
300
250
200
150
100
50
0
0
0.05
0.1
0.15
0.2
Frequency of codon usage

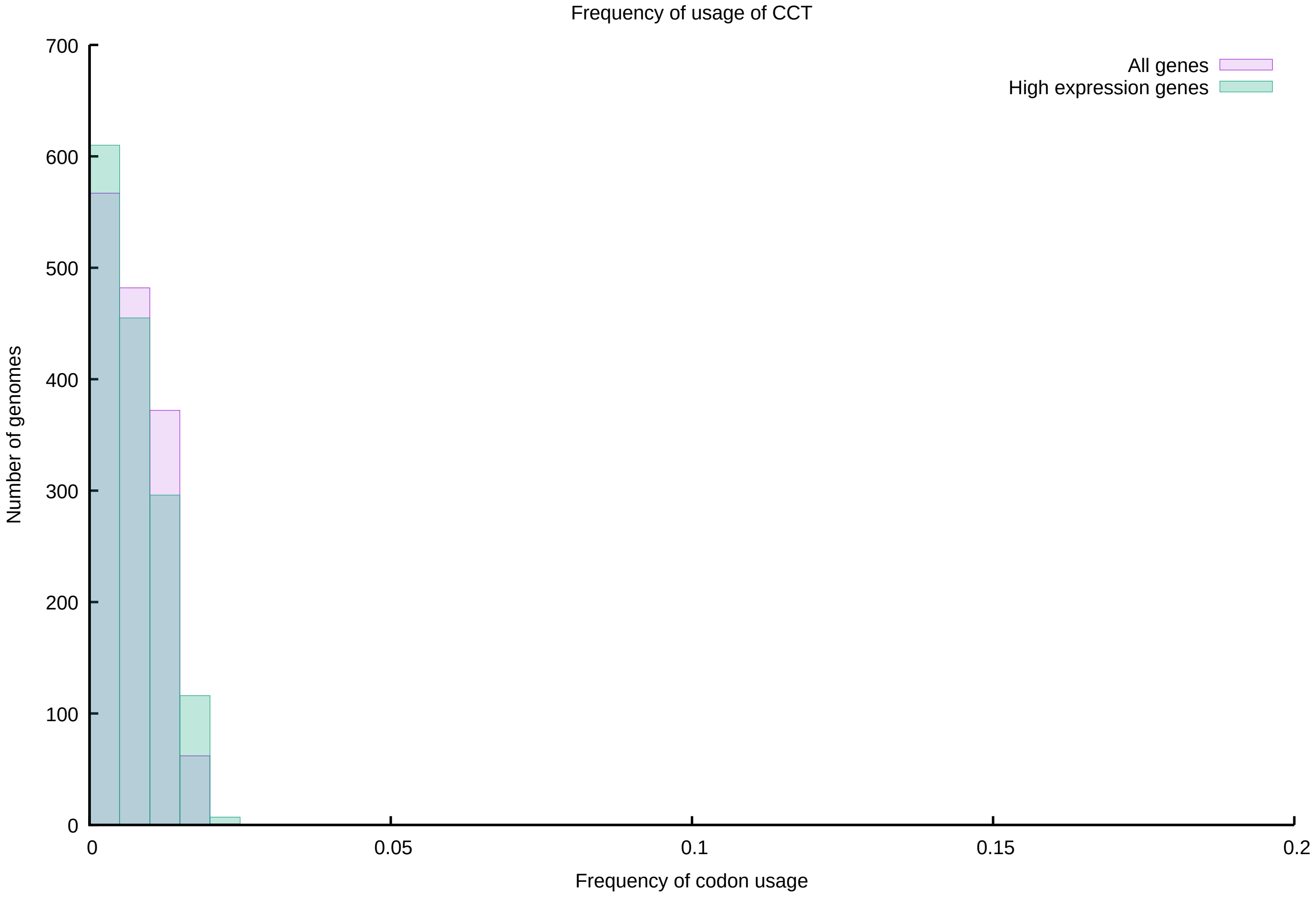

Frequency of usage of CCT
All genes
High expression genes
Number of genomes
Frequency of codon usage
0
100
200
300
400
500
600
700
0
0.05
0.1
0.15
0.2

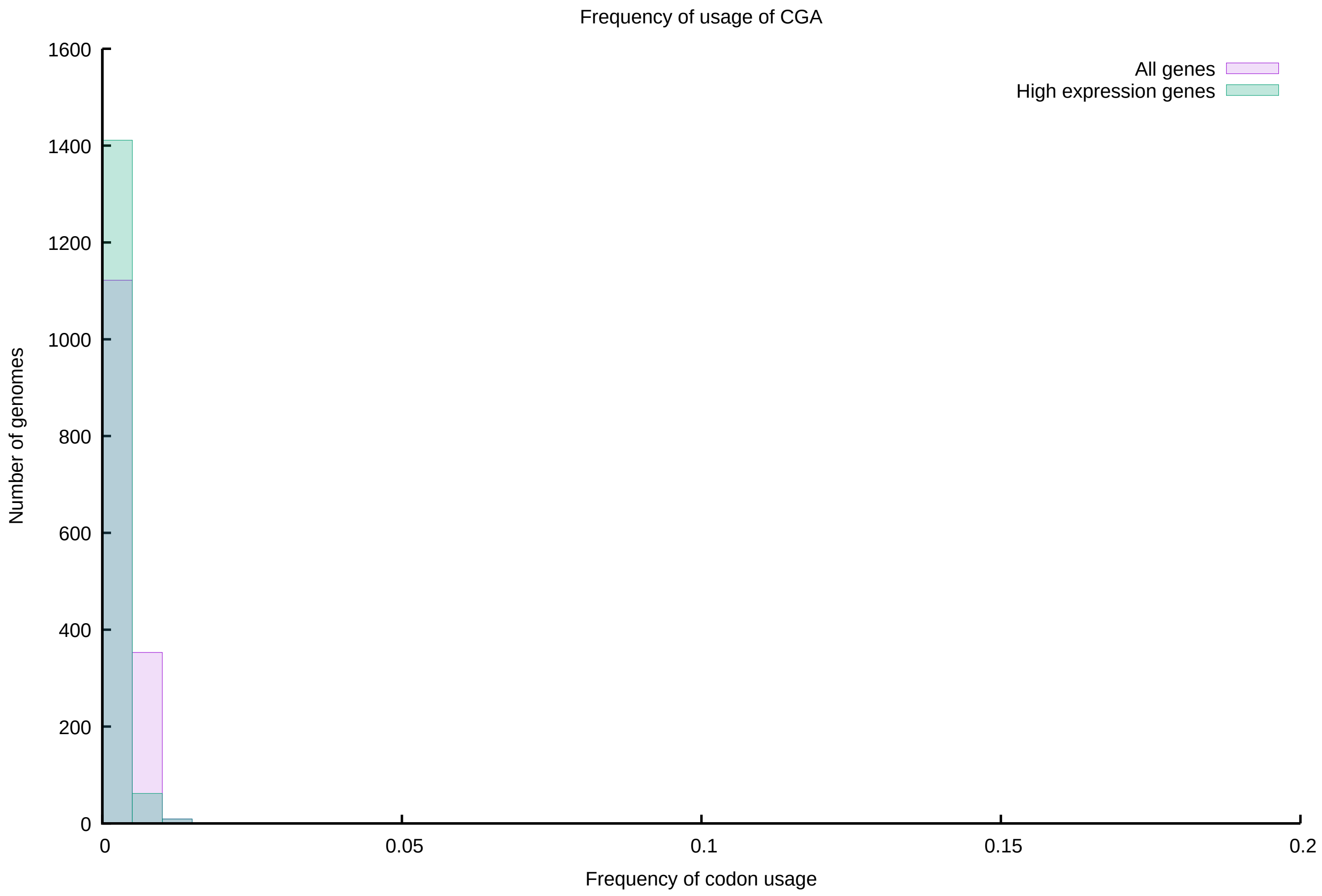
Frequency of usage of CGA
All genes
High expression genes
Number of genomes
1600
1400
1200
1000
800
600
400
200
0
0
0.05
0.1
0.15
0.2
Frequency of codon usage

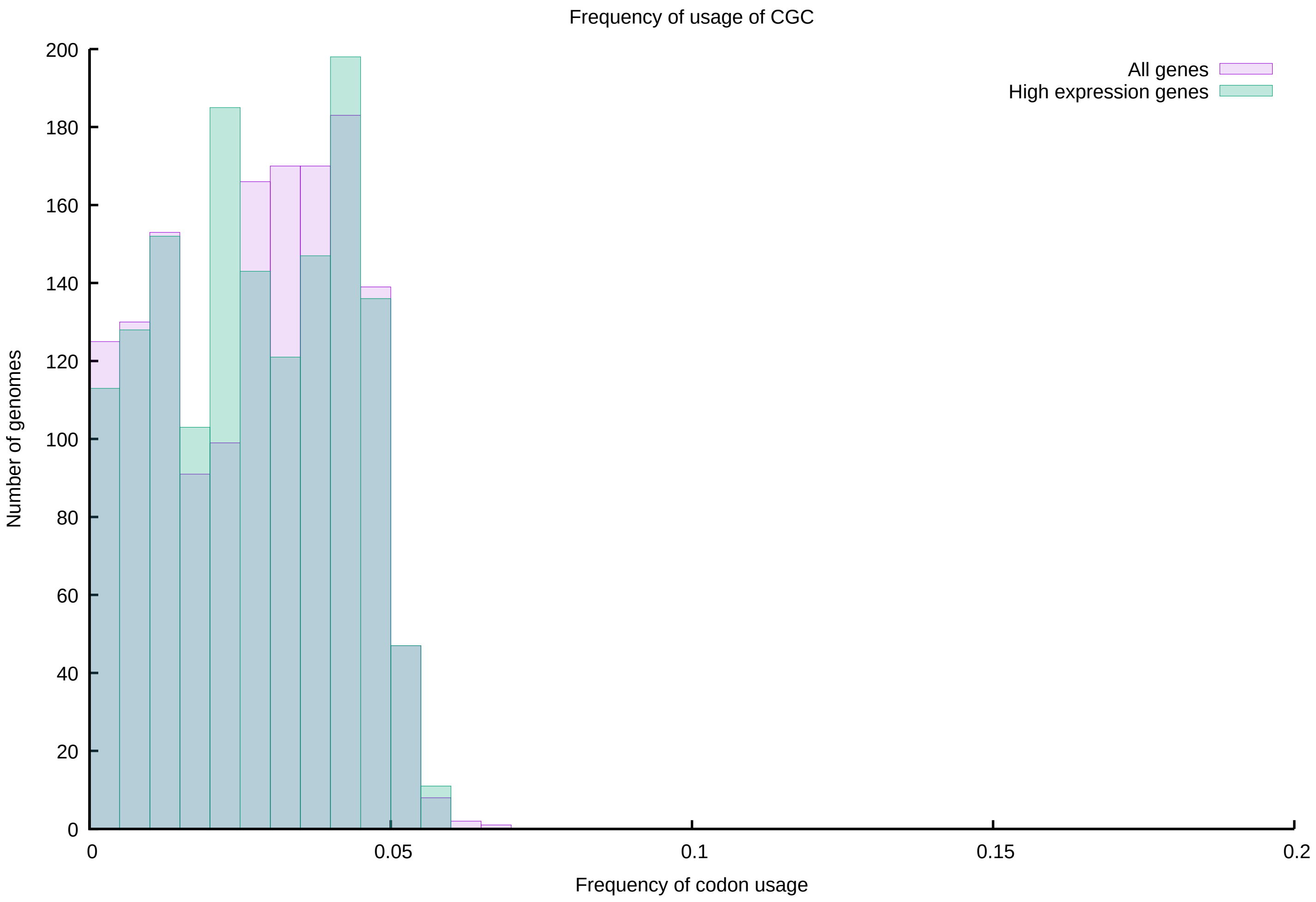

Frequency of usage of CGC
All genes
High expression genes
Number of genomes
Frequency of codon usage
0
20
40
60
80
100
120
140
160
180
200
0
0.05
0.1
0.15
0.2

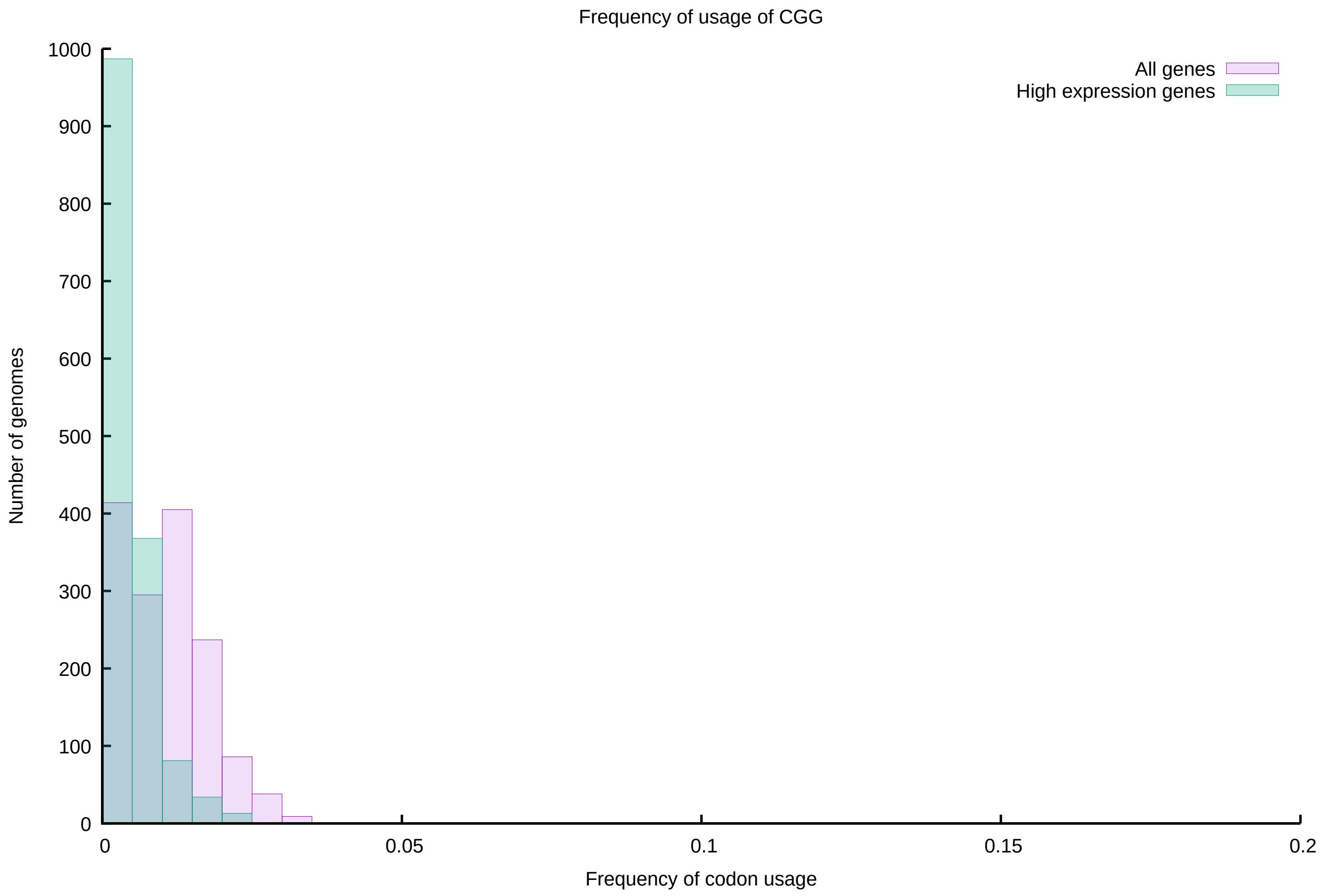

Frequency of usage of CGG
All genes
High expression genes
Number of genomes
Frequency of codon usage
0
100
200
300
400
500
600
700
800
900
1000
0
0.05
0.1
0.15
0.2

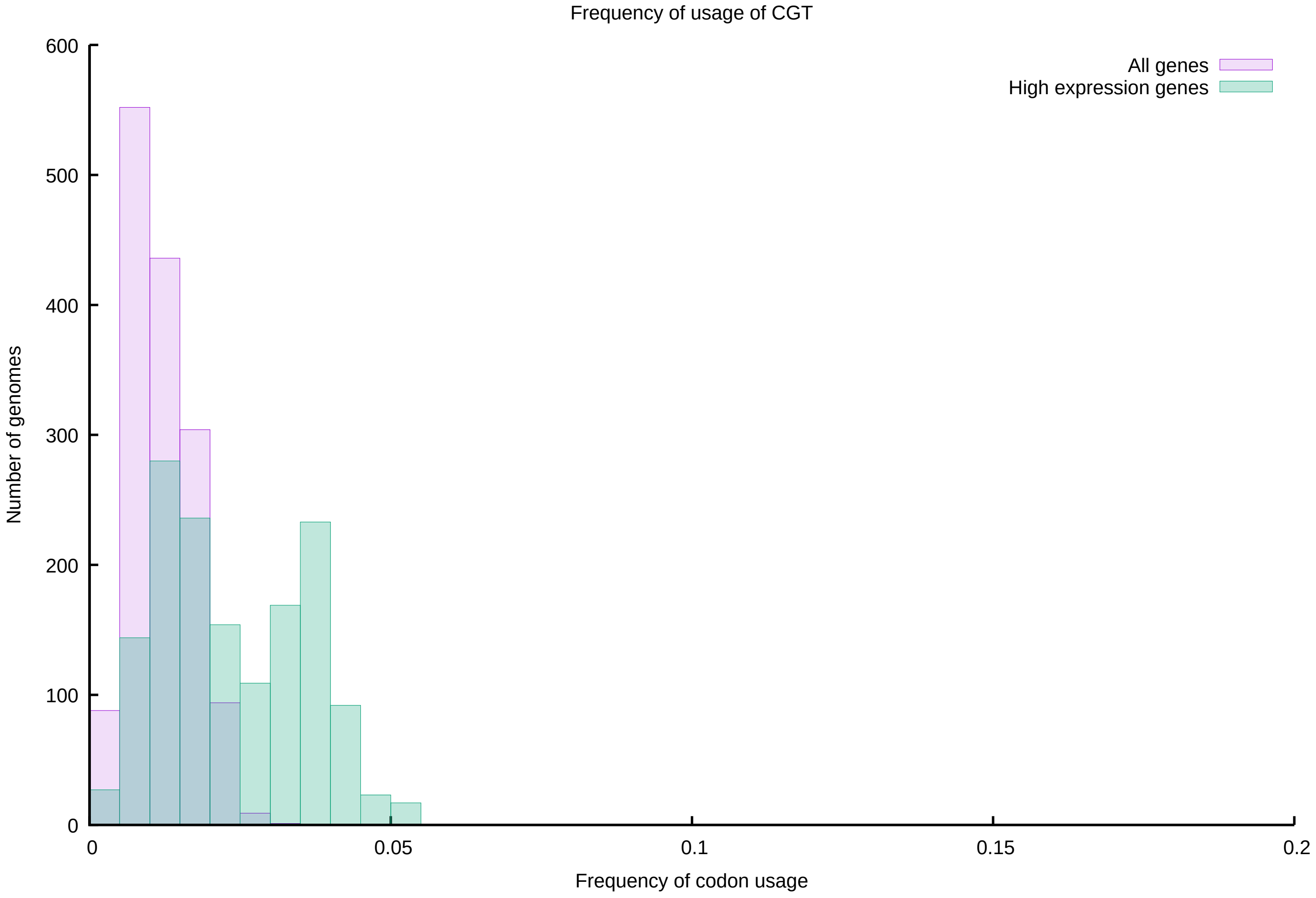
Frequency of usage of CGT
All genes
High expression genes
Number of genomes
Frequency of codon usage
0
100
200
300
400
500
600
0
0.05
0.1
0.15
0.2

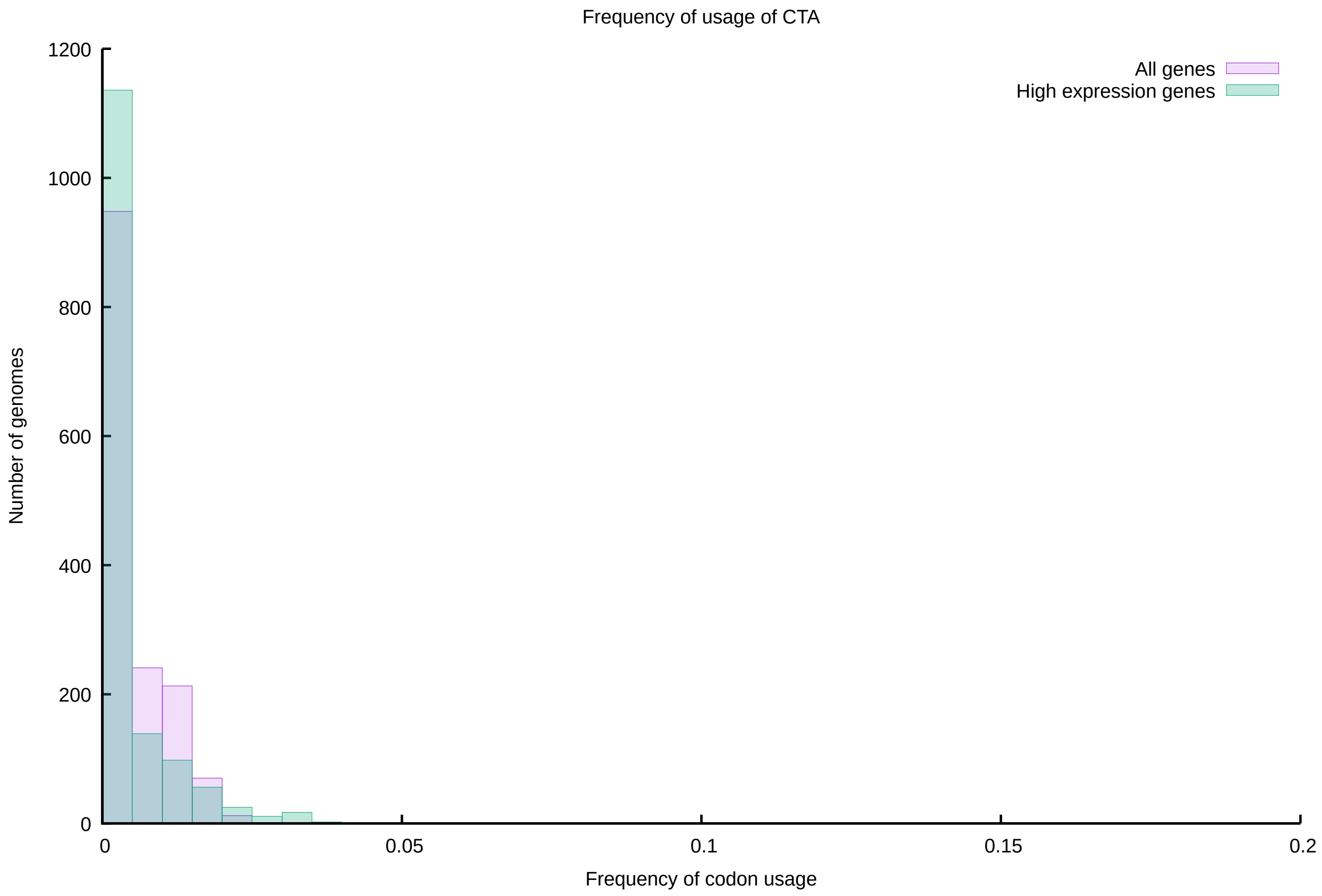

Frequency of usage of CTA
All genes
High expression genes
Number of genomes
1200
1000
800
600
400
200
0
0
0.05
0.1
0.15
0.2
Frequency of codon usage

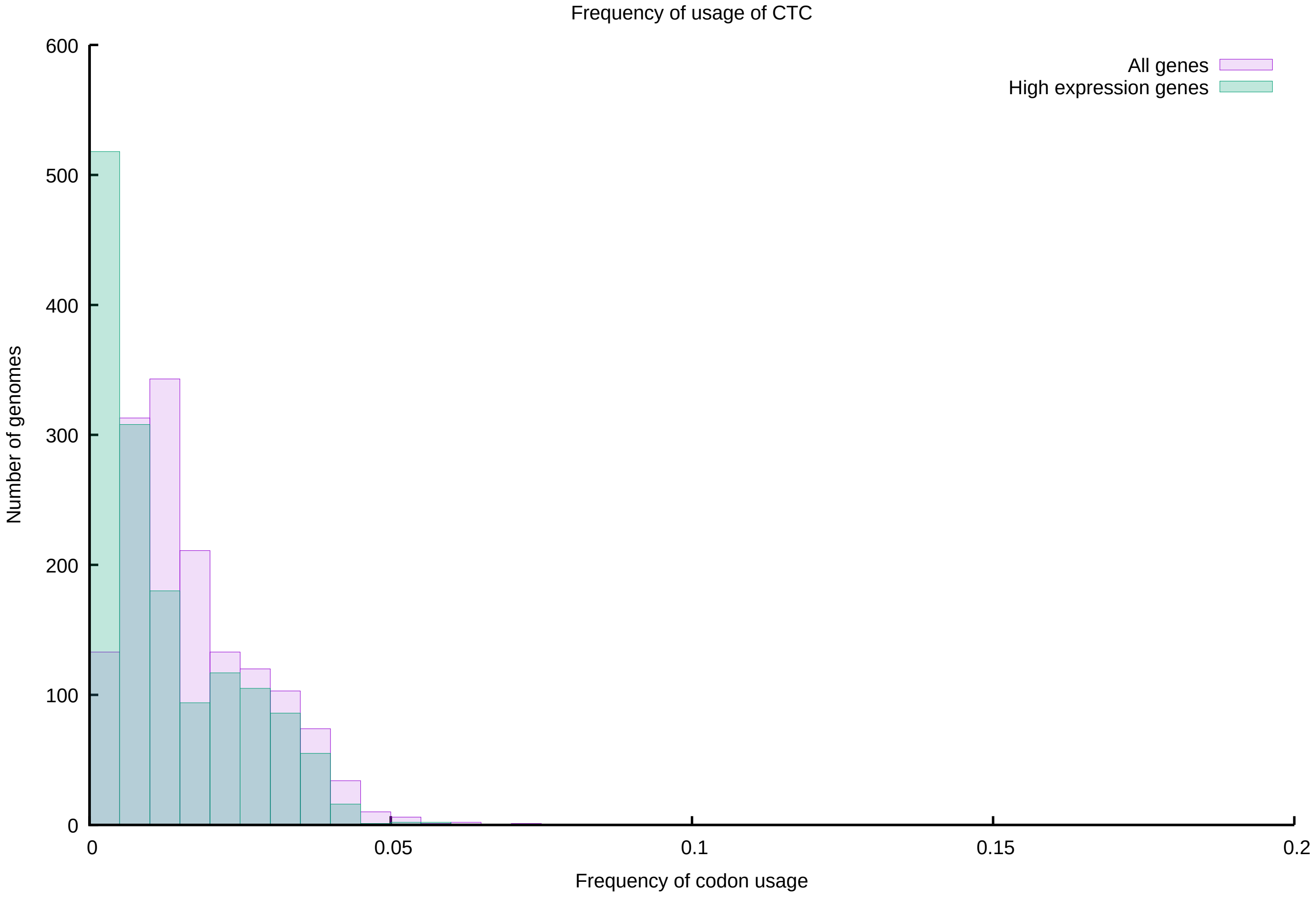
Frequency of usage of CTC
All genes
High expression genes
Number of genomes
Frequency of codon usage
0
100
200
300
400
500
600
0
0.05
0.1
0.15
0.2

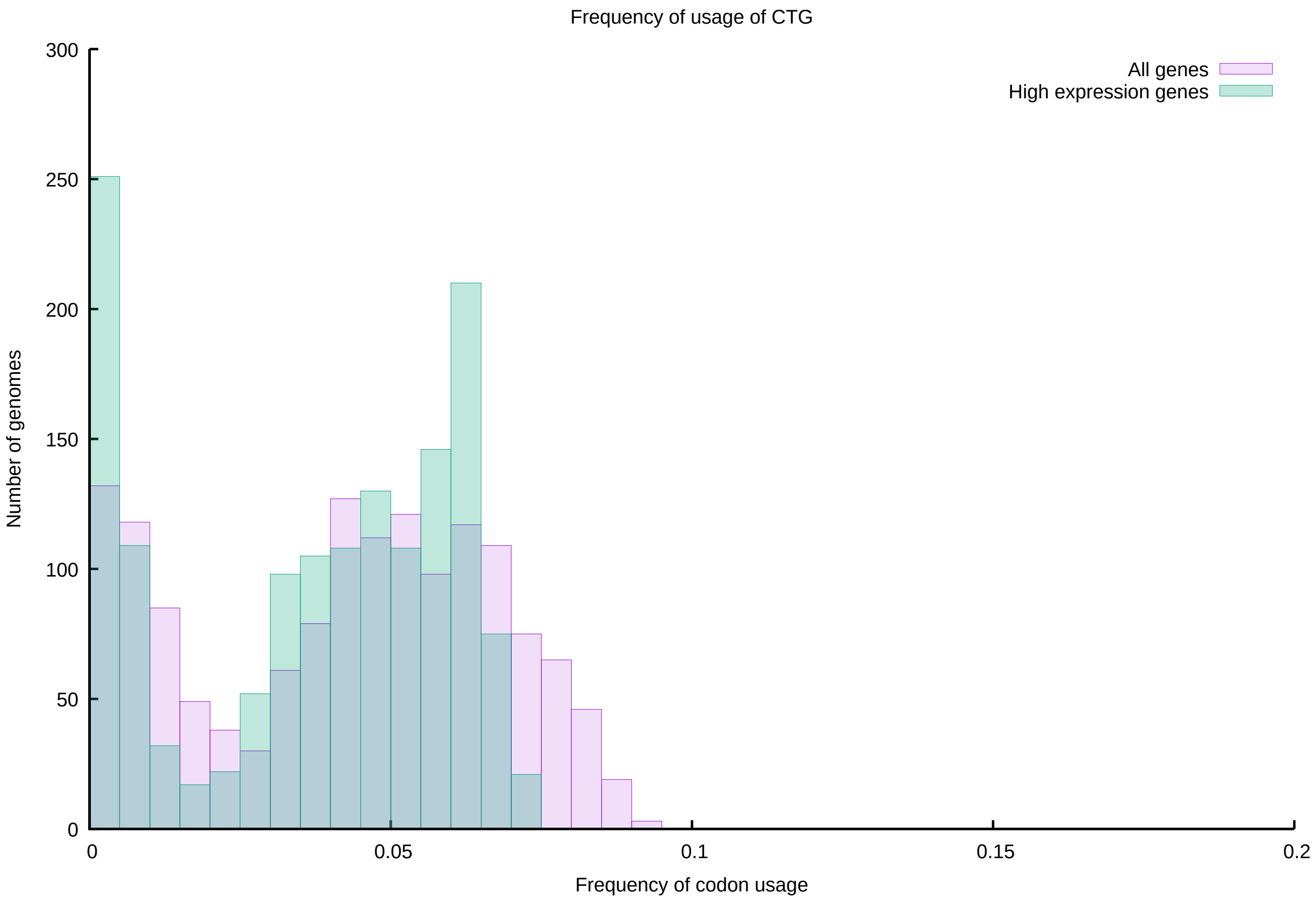

Frequency of usage of CTG
All genes
High expression genes
Number of genomes
300
250
200
150
100
50
0
0
0.05
0.1
0.15
0.2
Frequency of codon usage

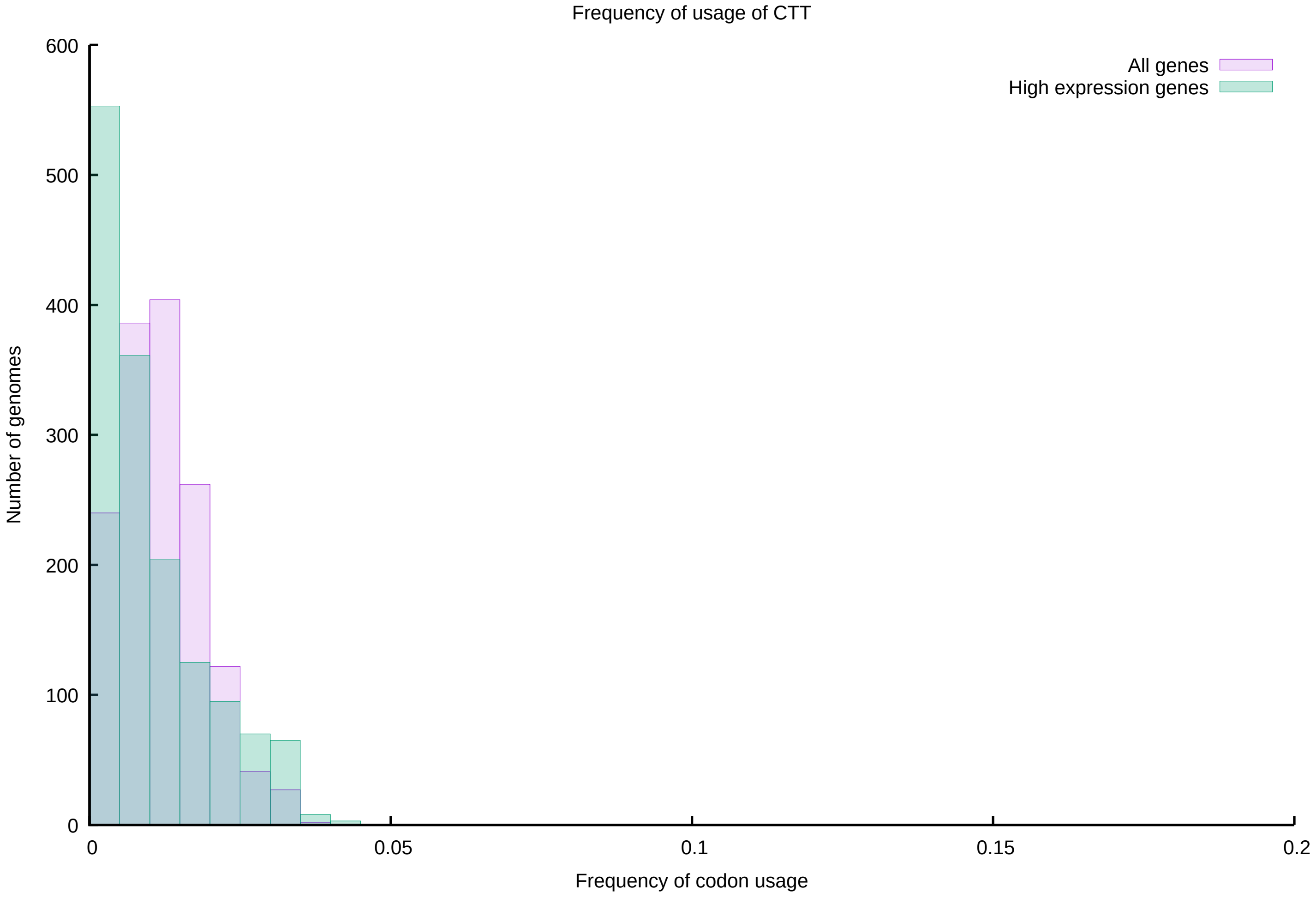

Frequency of usage of CTT
All genes
High expression genes
Number of genomes
600
500
400
300
200
100
0
0
0.05
0.1
0.15
0.2
Frequency of codon usage

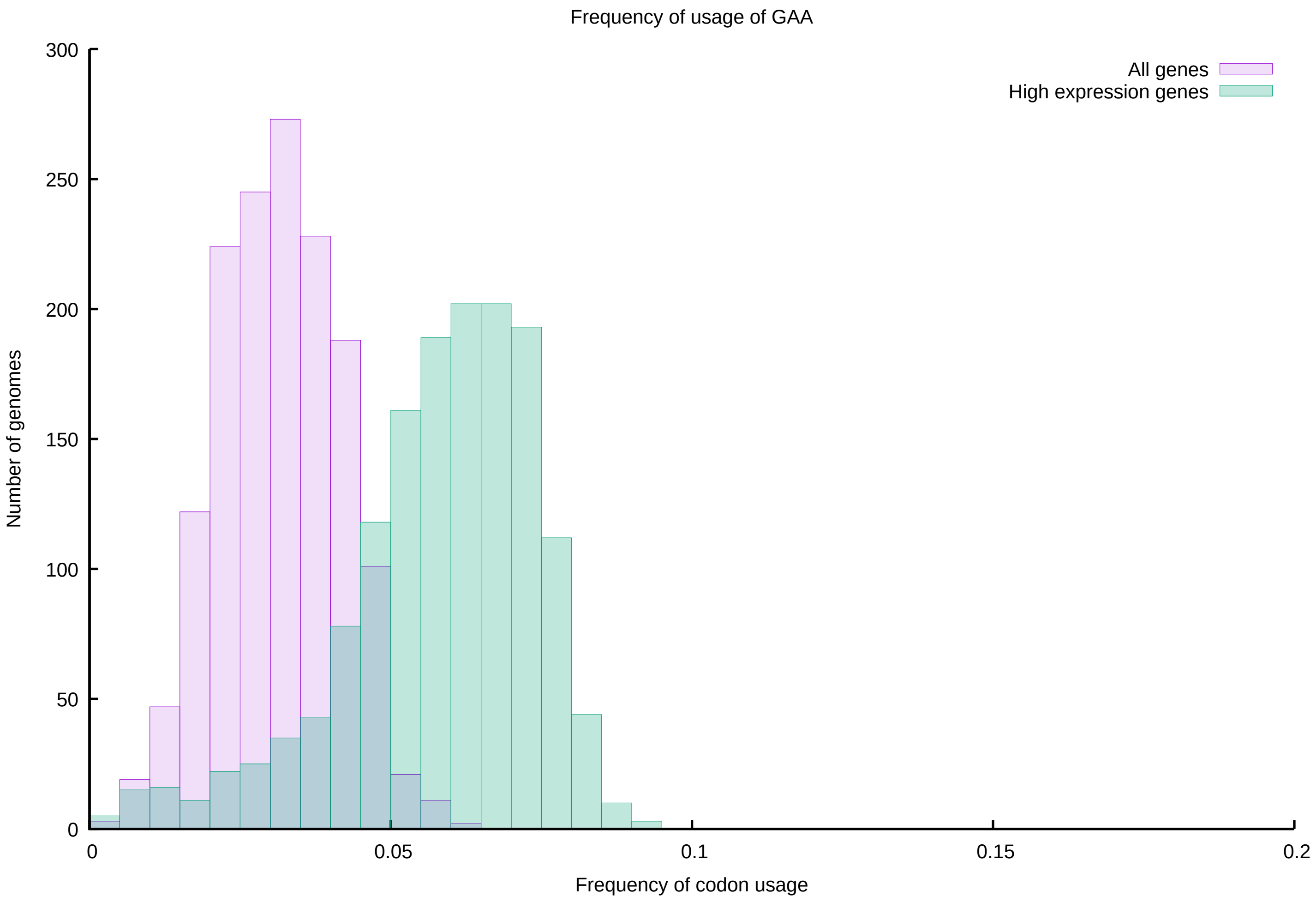
Frequency of usage of GAA
All genes
High expression genes
Number of genomes
0
50
100
150
200
250
300
0
0.05
0.1
0.15
0.2
Frequency of codon usage

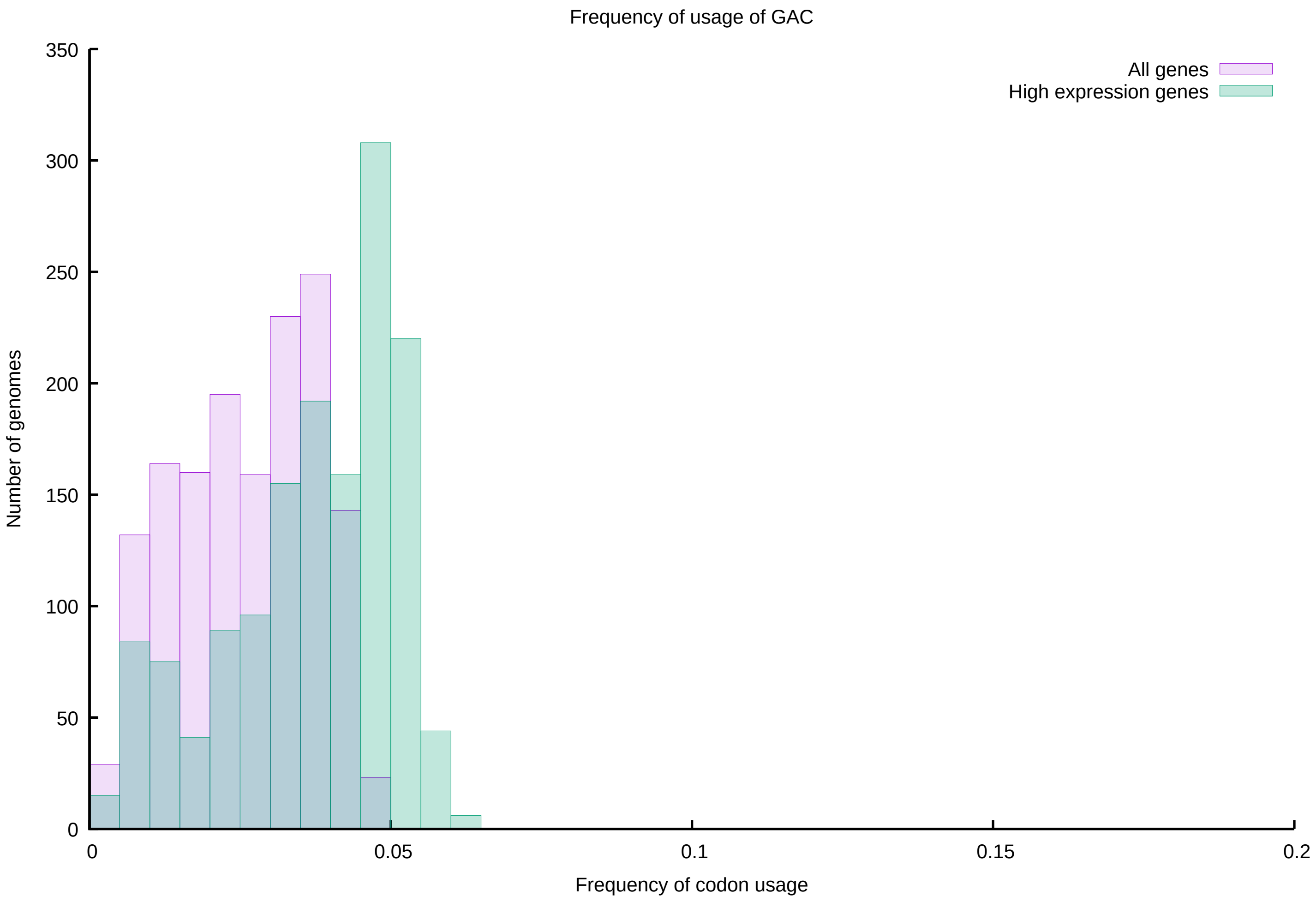
Frequency of usage of GAC
All genes
High expression genes
Number of genomes
Frequency of codon usage
0
50
100
150
200
250
300
350
0
0.05
0.1
0.15
0.2

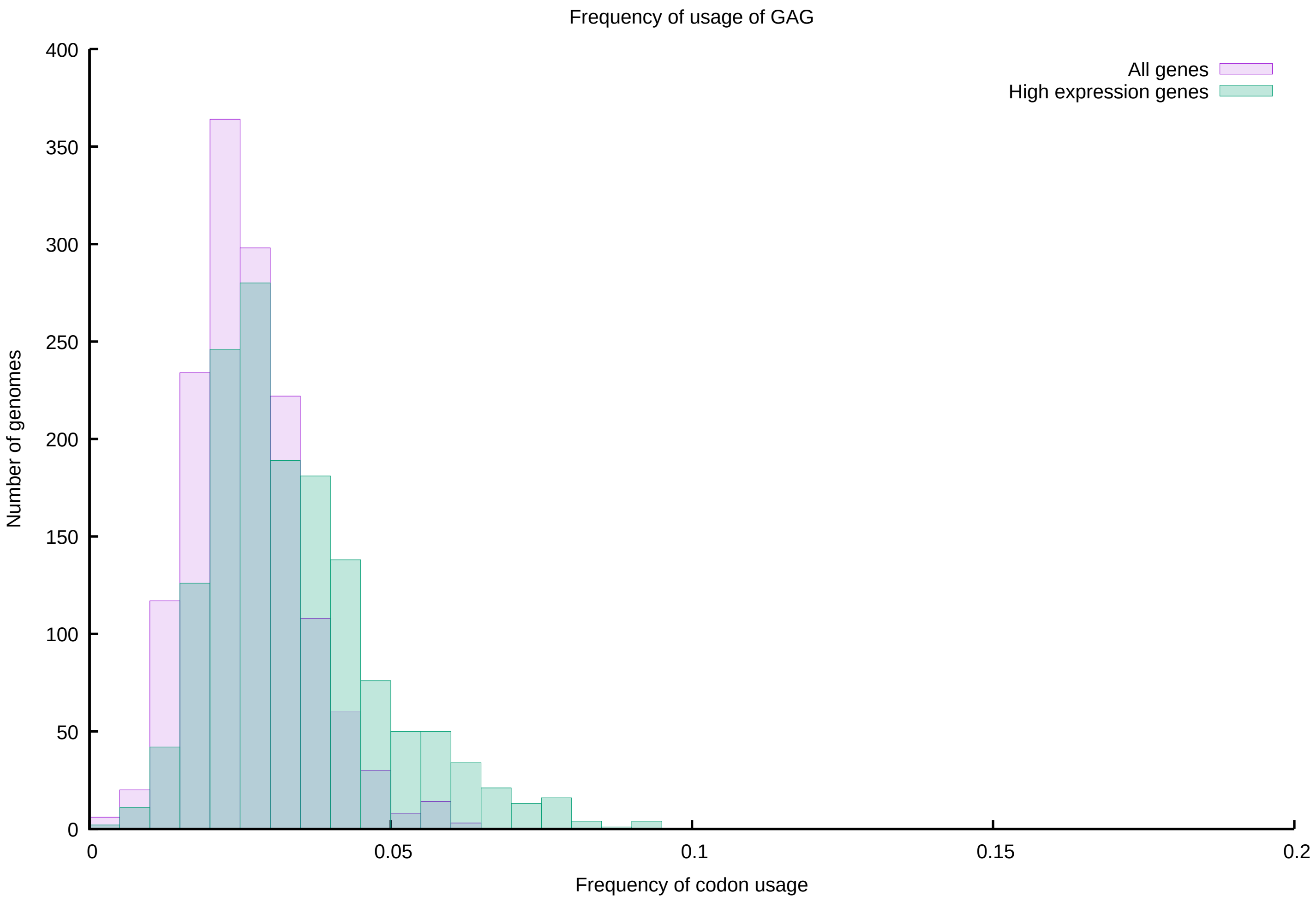

Frequency of usage of GAG
All genes
High expression genes
Number of genomes
400
350
300
250
200
150
100
50
0
0
0.05
0.1
0.15
0.2
Frequency of codon usage

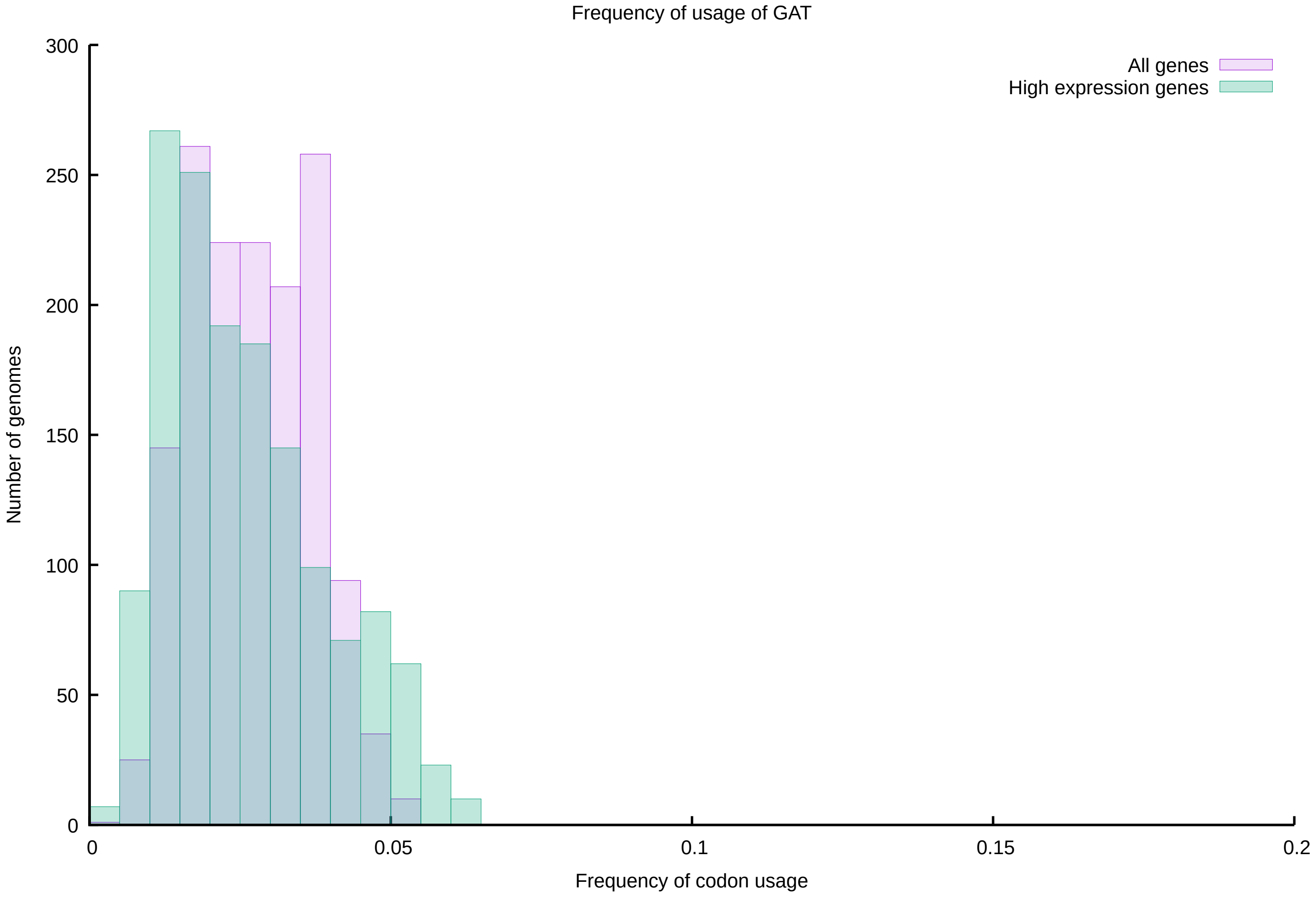

Frequency of usage of GAT
All genes
High expression genes
Number of genomes
Frequency of codon usage
0
50
100
150
200
250
300
0
0.05
0.1
0.15
0.2

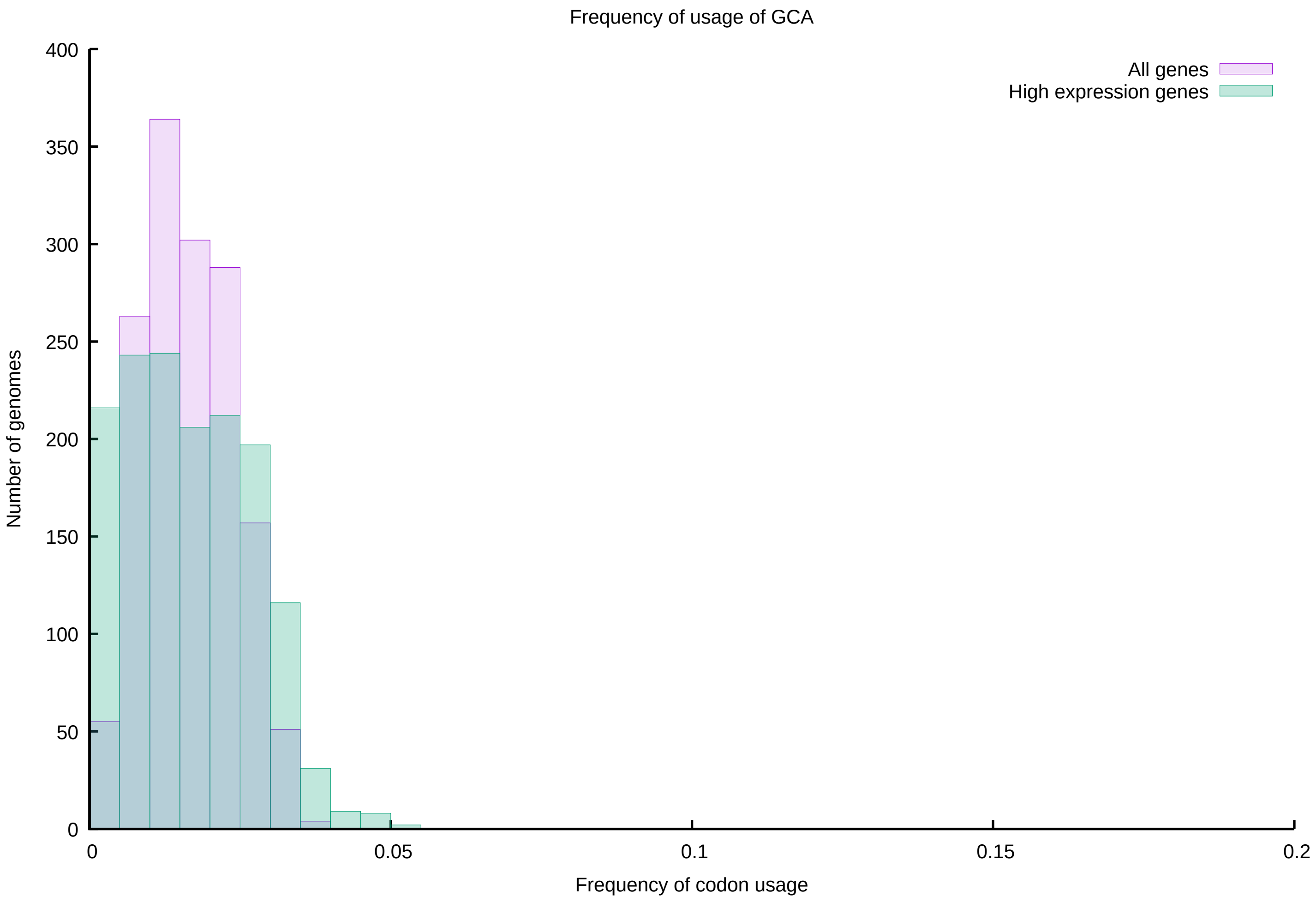
Frequency of usage of GCA
All genes
High expression genes
Number of genomes
400
350
300
250
200
150
100
50
0
0
0.05
0.1
0.15
0.2
Frequency of codon usage

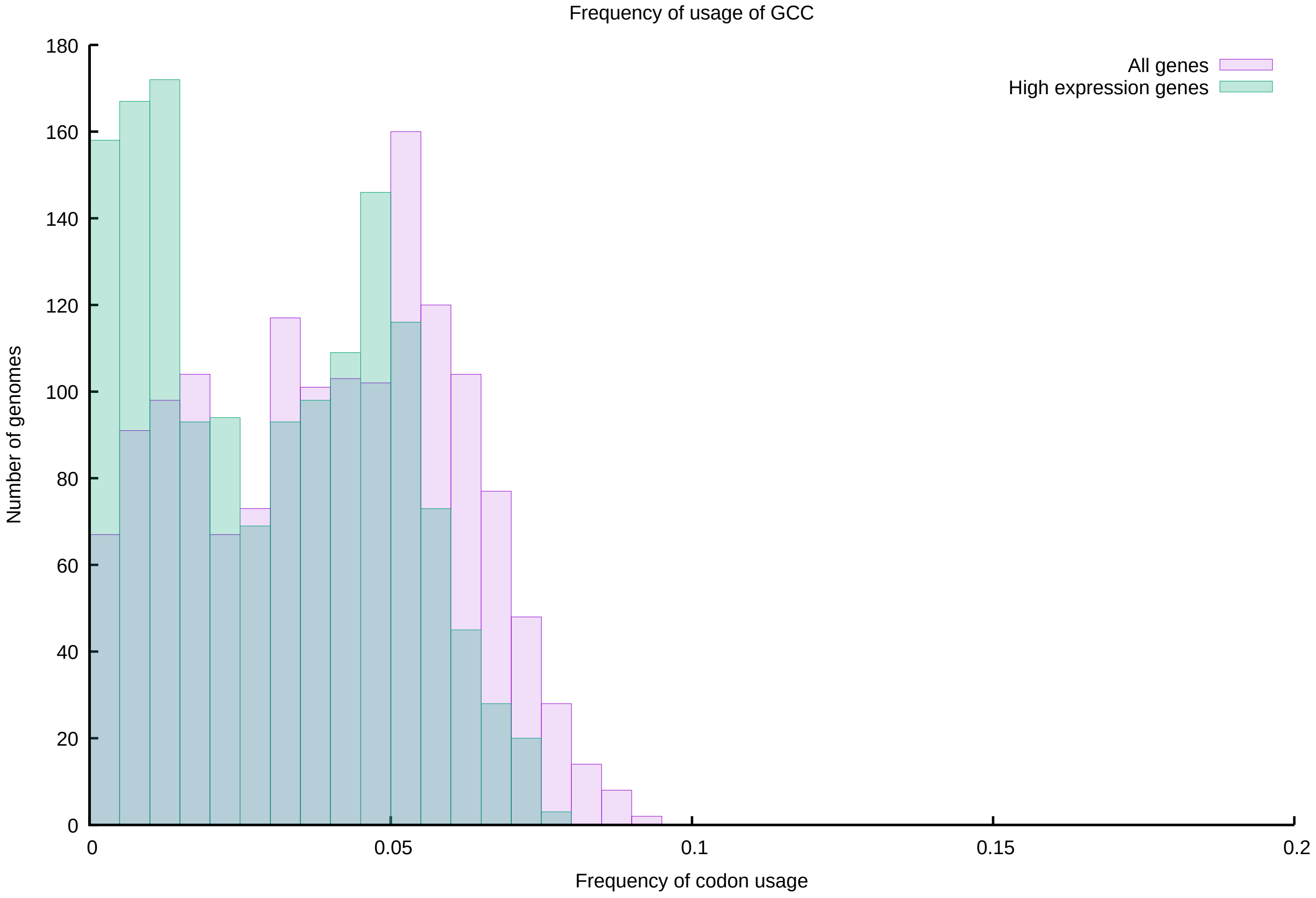
Frequency of usage of GCC
All genes
High expression genes
Number of genomes
180
160
140
120
100
80
60
40
20
0
0
0.05
0.1
0.15
0.2
Frequency of codon usage

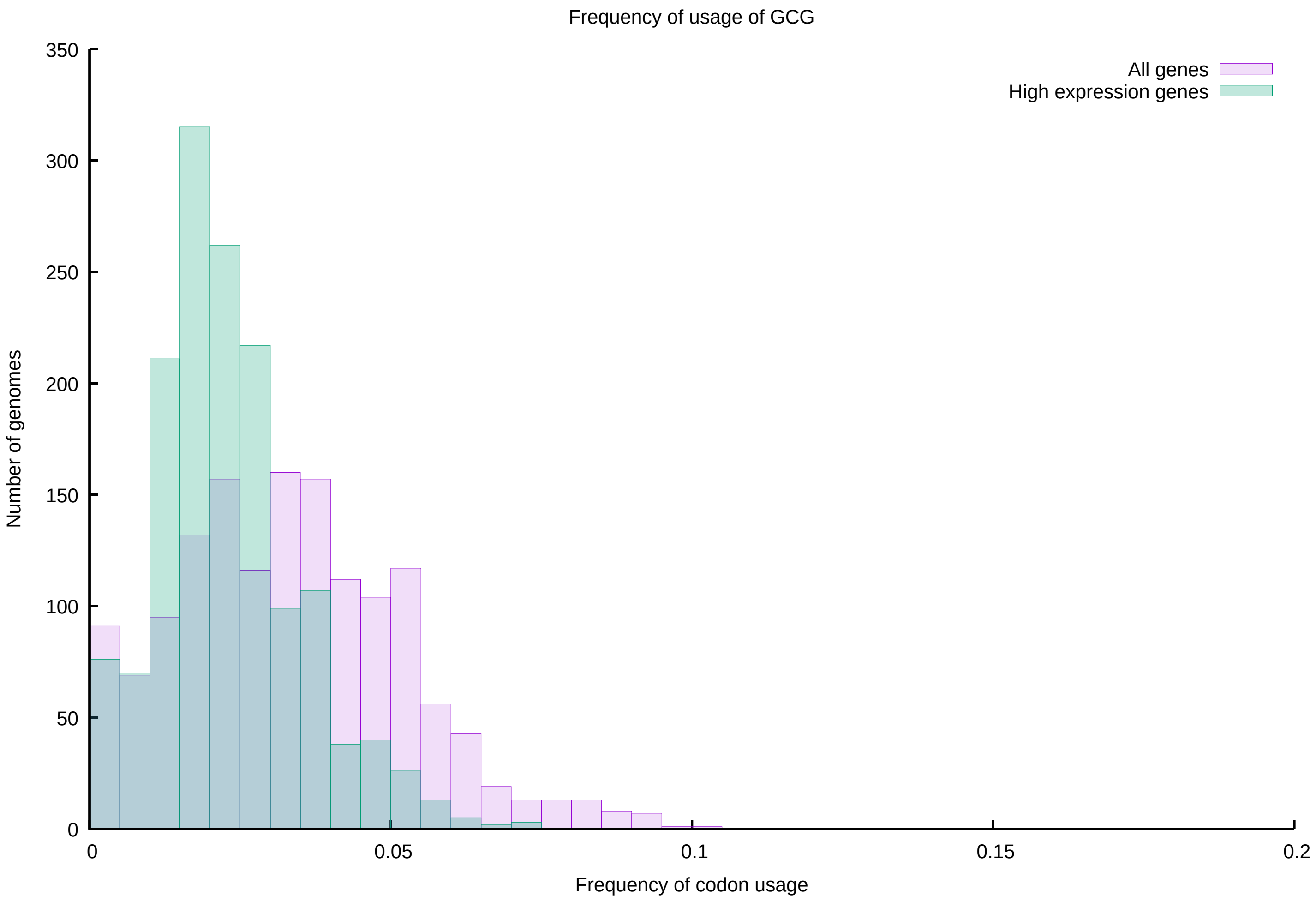

Frequency of usage of GCG
All genes
High expression genes
Number of genomes
350
300
250
200
150
100
50
0
0
0.05
0.1
0.15
0.2
Frequency of codon usage

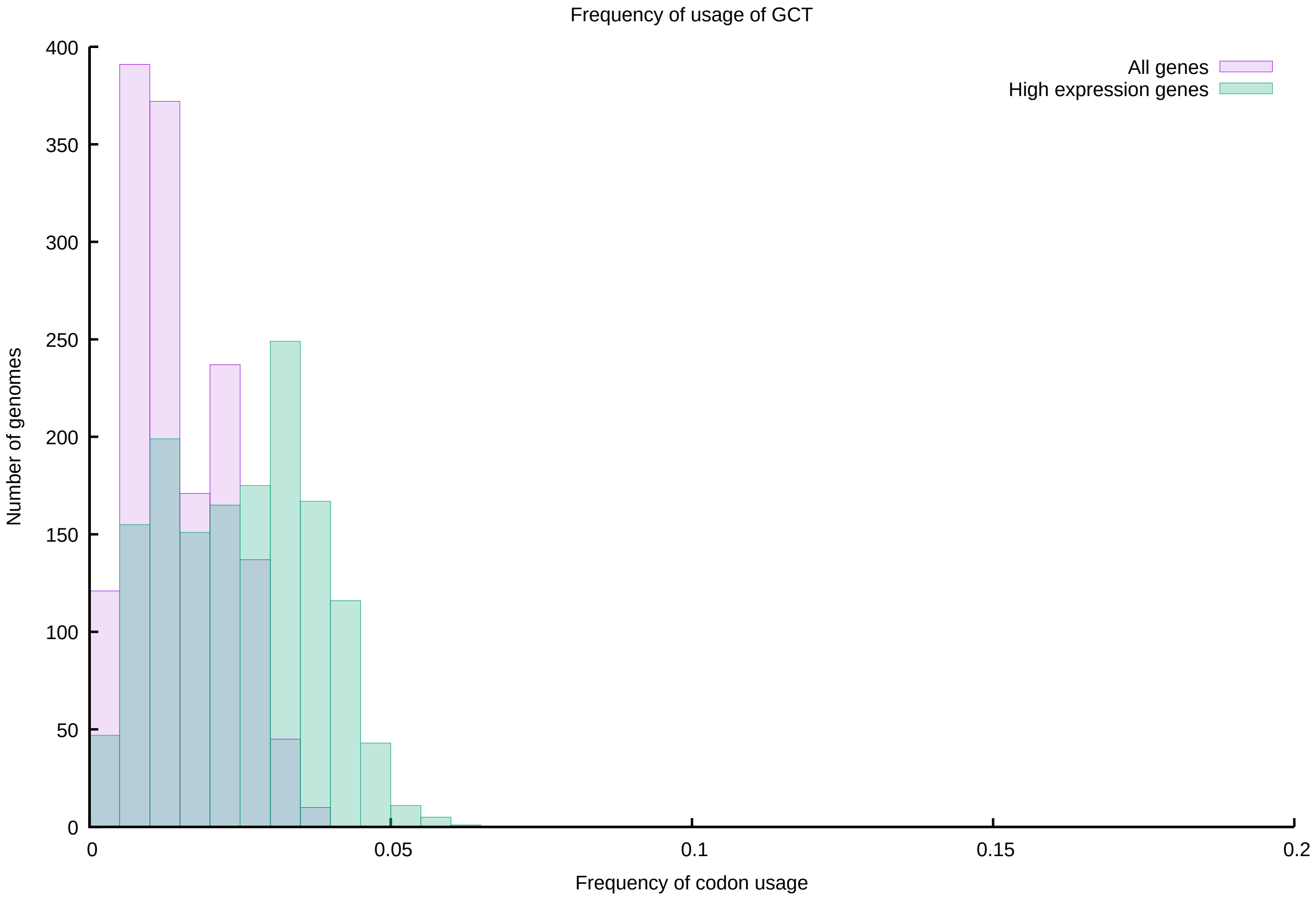
Frequency of usage of GCT
All genes
High expression genes
Number of genomes
Frequency of codon usage
0
50
100
150
200
250
300
350
400
0
0.05
0.1
0.15
0.2

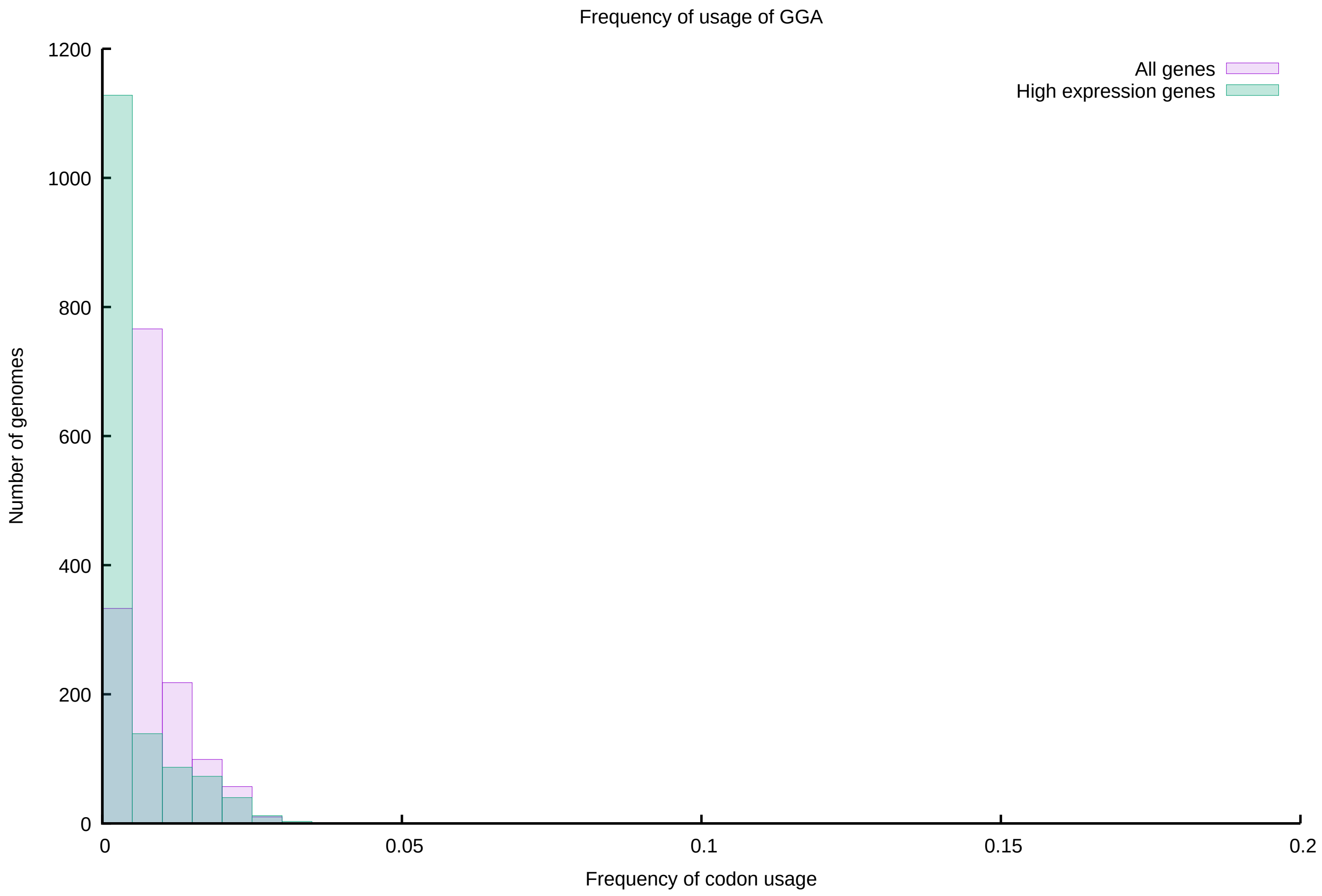

Frequency of usage of GGA
All genes
High expression genes
Number of genomes
0
200
400
600
800
1000
1200
0
0.05
0.1
0.15
0.2
Frequency of codon usage

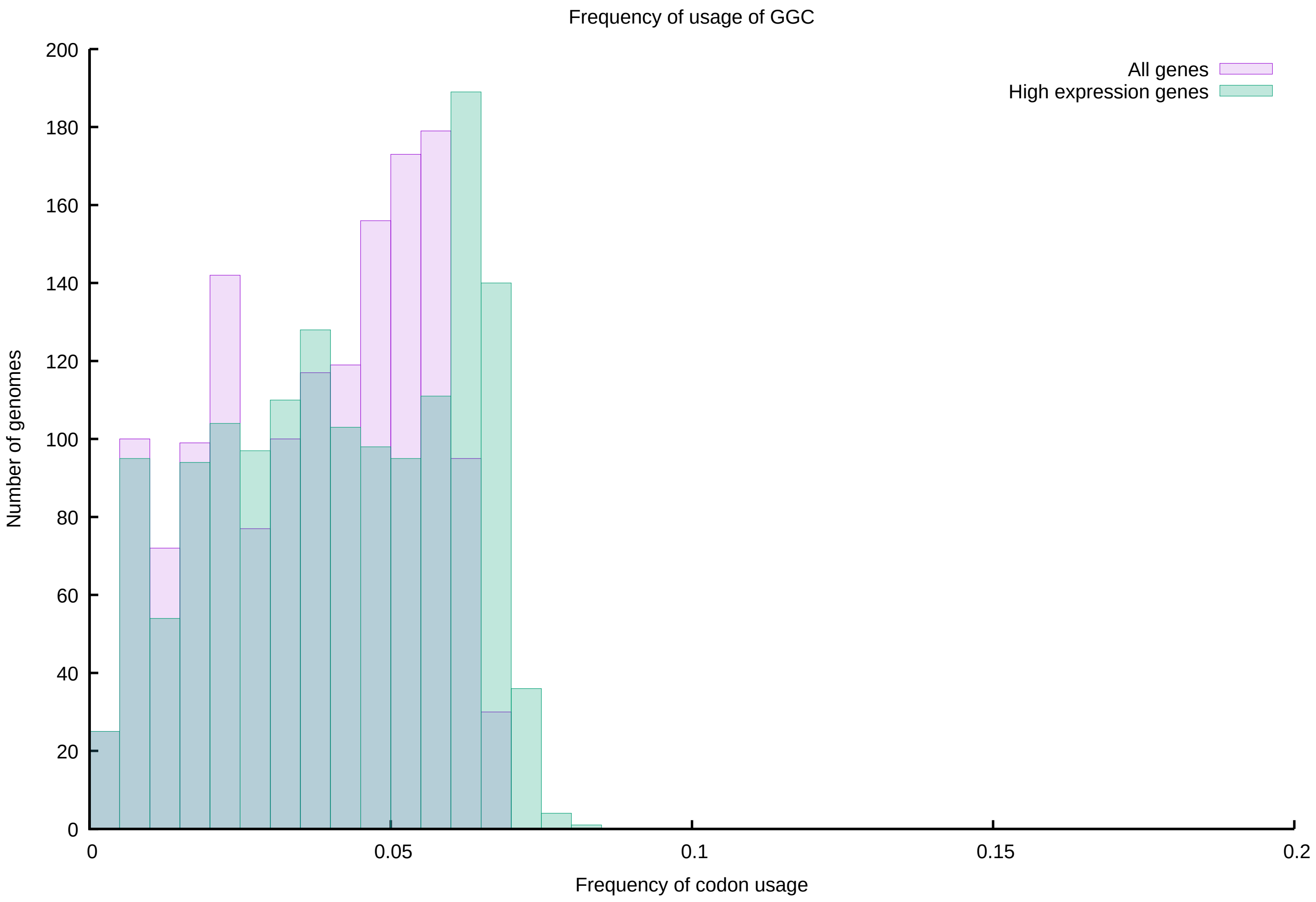
Frequency of usage of GGC
All genes
High expression genes
Number of genomes
Frequency of codon usage
0
20
40
60
80
100
120
140
160
180
200
0
0.05
0.1
0.15
0.2

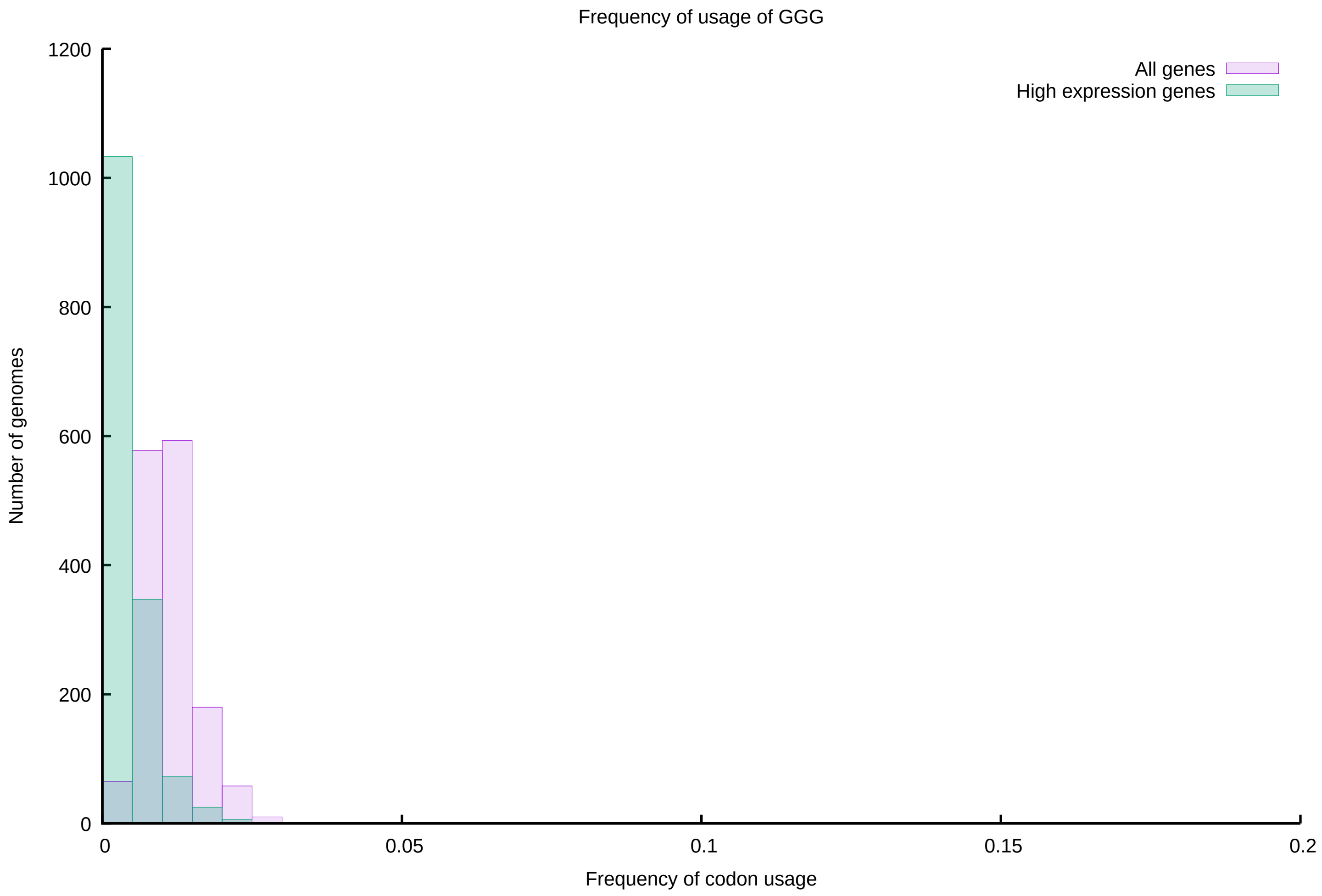
Frequency of usage of GGG
All genes
High expression genes
Number of genomes
0
200
400
600
800
1000
1200
0
0.05
0.1
0.15
0.2
Frequency of codon usage

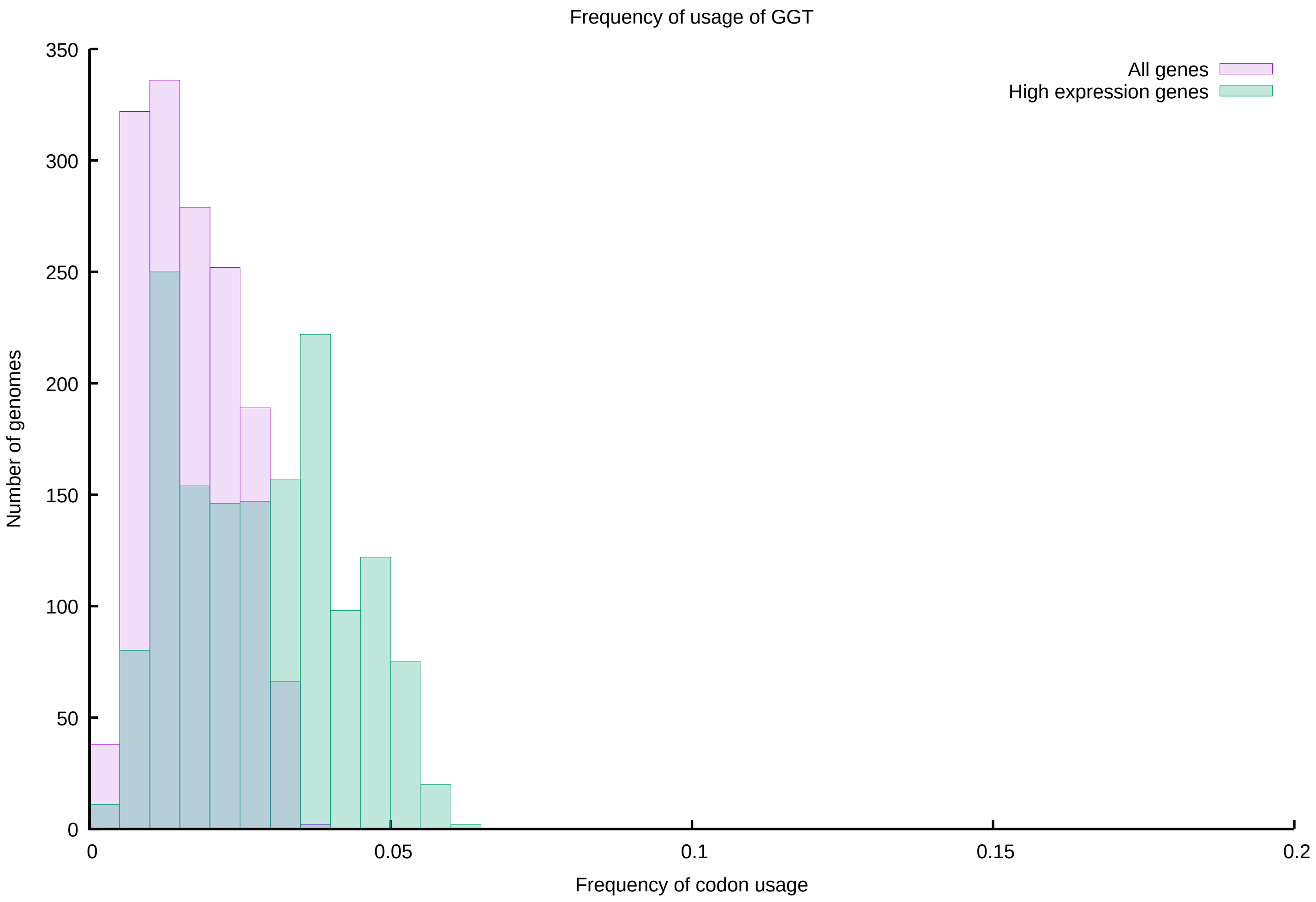
Frequency of usage of GGT
All genes
High expression genes
Number of genomes
Frequency of codon usage
0
50
100
150
200
250
300
350
0
0.05
0.1
0.15
0.2

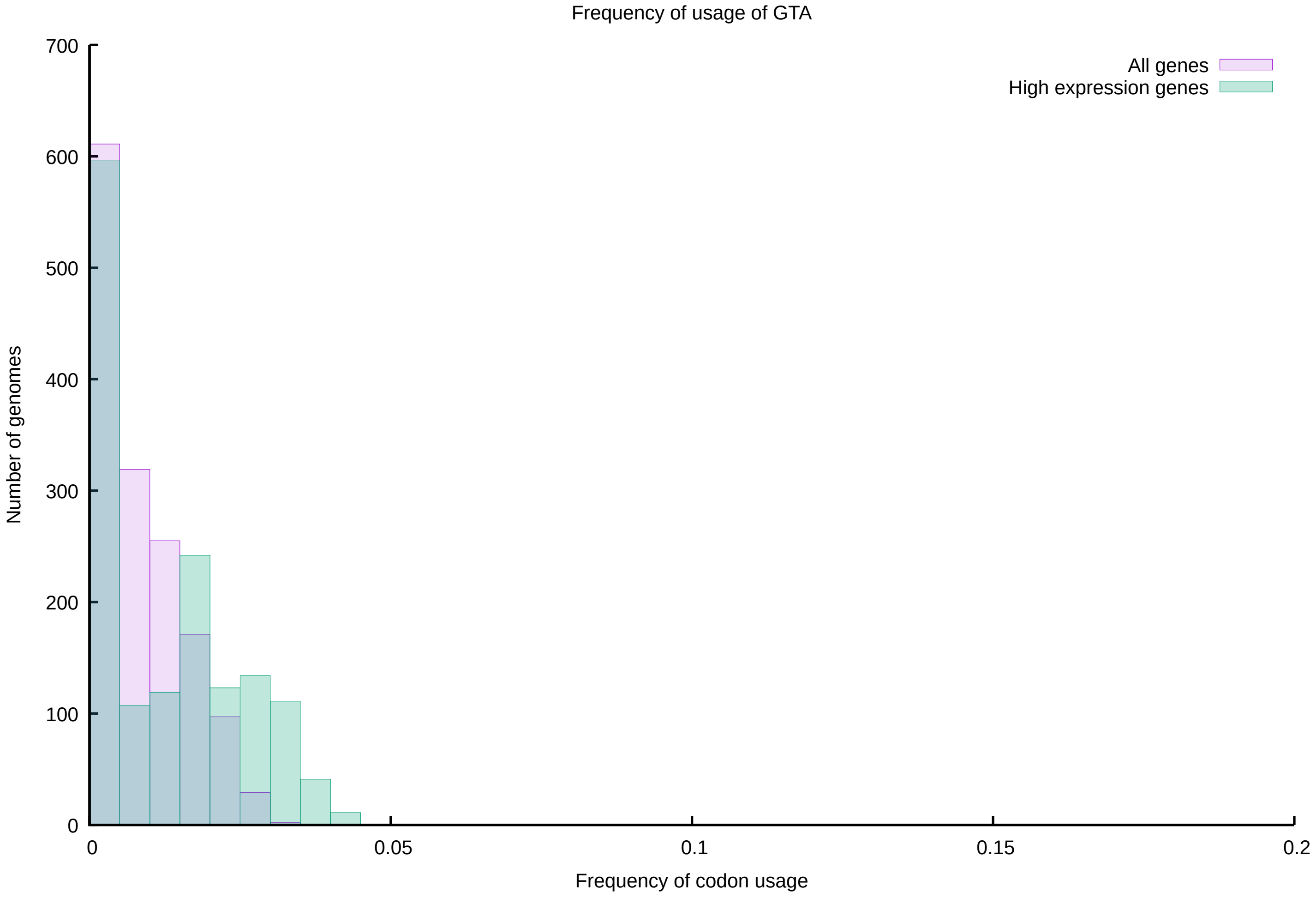

Frequency of usage of GTA
All genes
High expression genes
Number of genomes
700
600
500
400
300
200
100
0
0
0.05
0.1
0.15
0.2
Frequency of codon usage

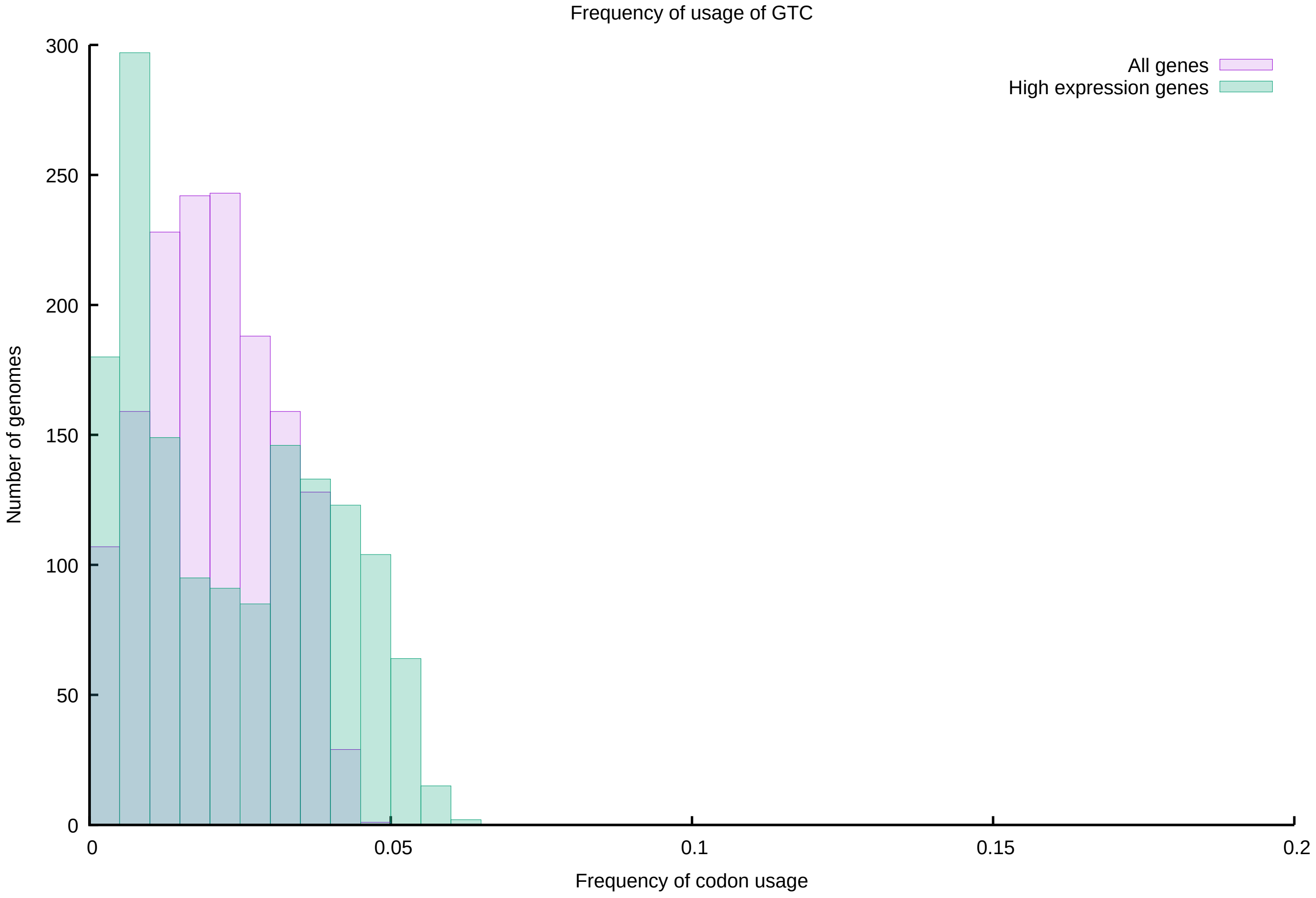
Frequency of usage of GTC
All genes
High expression genes
Number of genomes
Frequency of codon usage
0
50
100
150
200
250
300
0
0.05
0.1
0.15
0.2

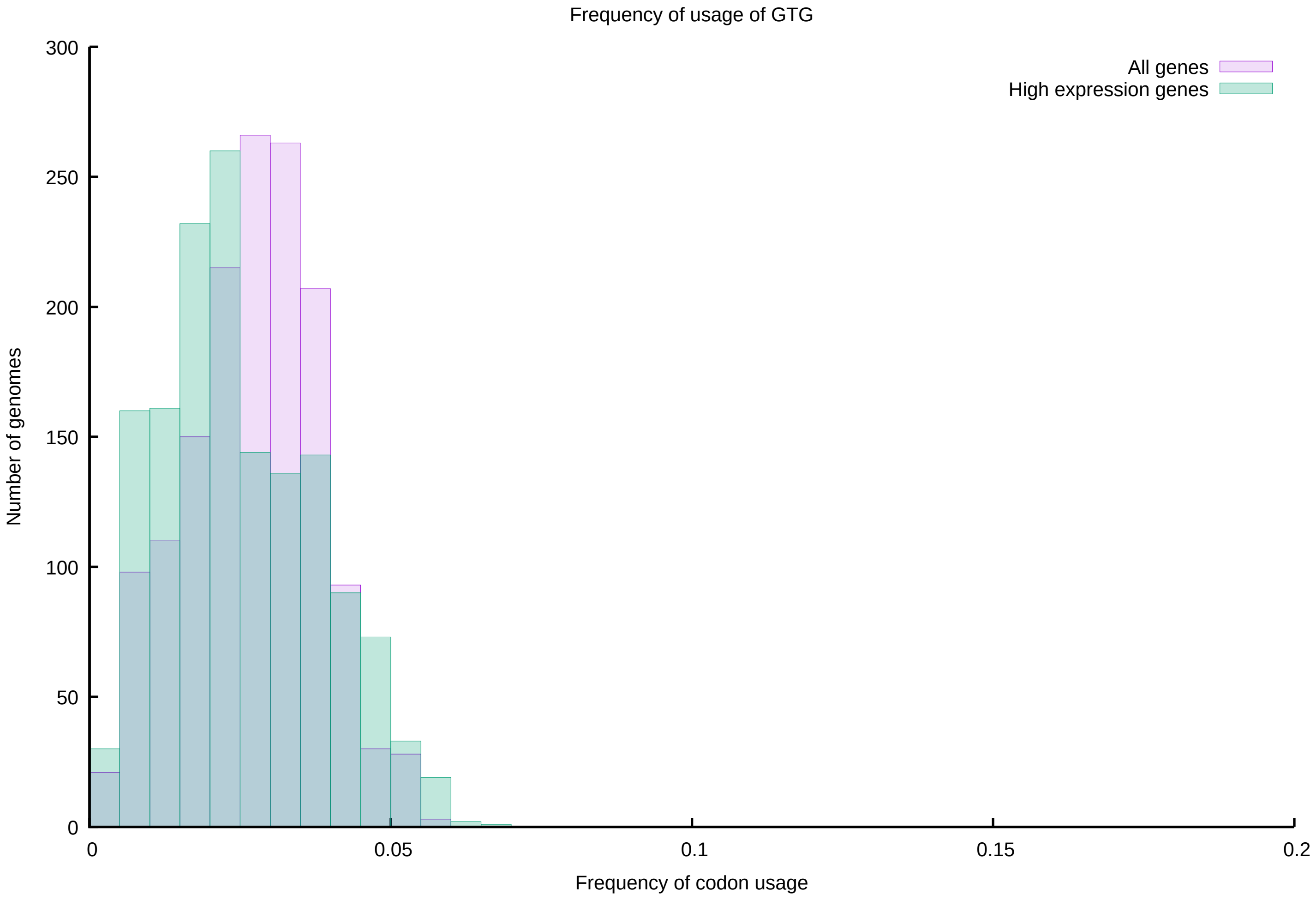

Frequency of usage of GTG
All genes
High expression genes
Number of genomes
300
250
200
150
100
50
0
0
0.05
0.1
0.15
0.2
Frequency of codon usage

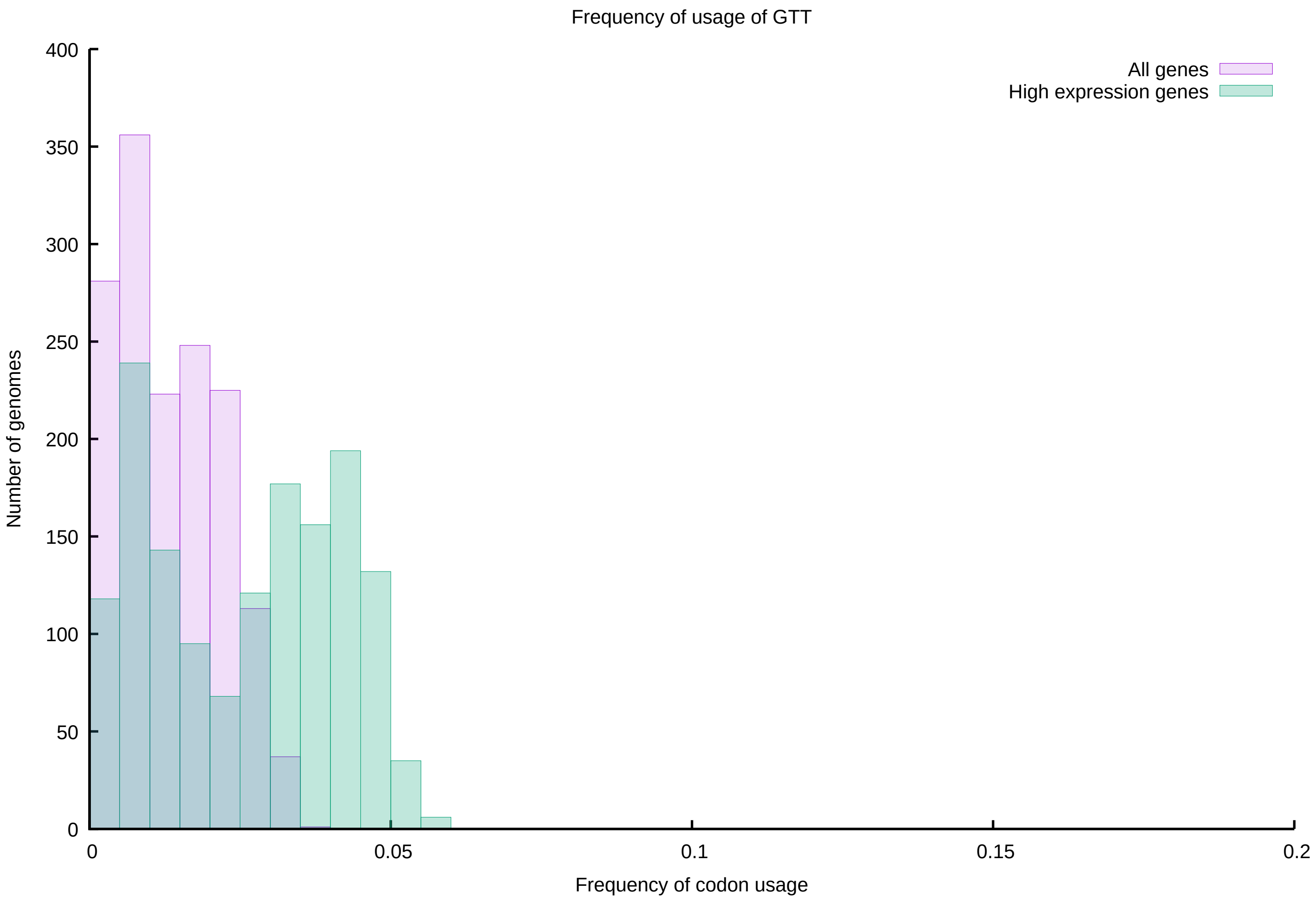
Frequency of usage of GTT
All genes
High expression genes
Number of genomes
Frequency of codon usage
0
50
100
150
200
250
300
350
400
0
0.05
0.1
0.15
0.2

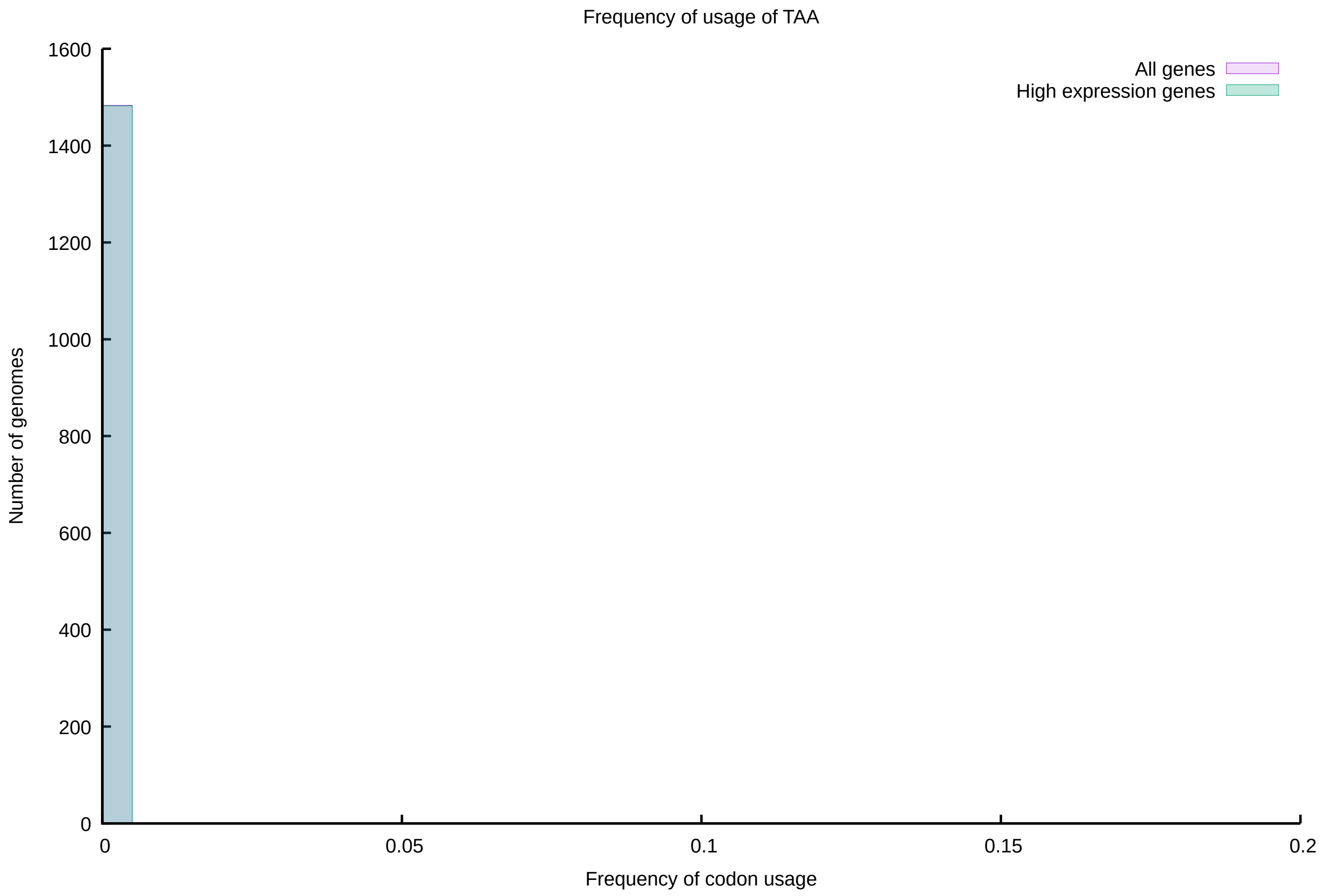

Frequency of usage of TAA
All genes
High expression genes
Number of genomes
1600
1400
1200
1000
800
600
400
200
0
0
0.05
0.1
0.15
0.2
Frequency of codon usage

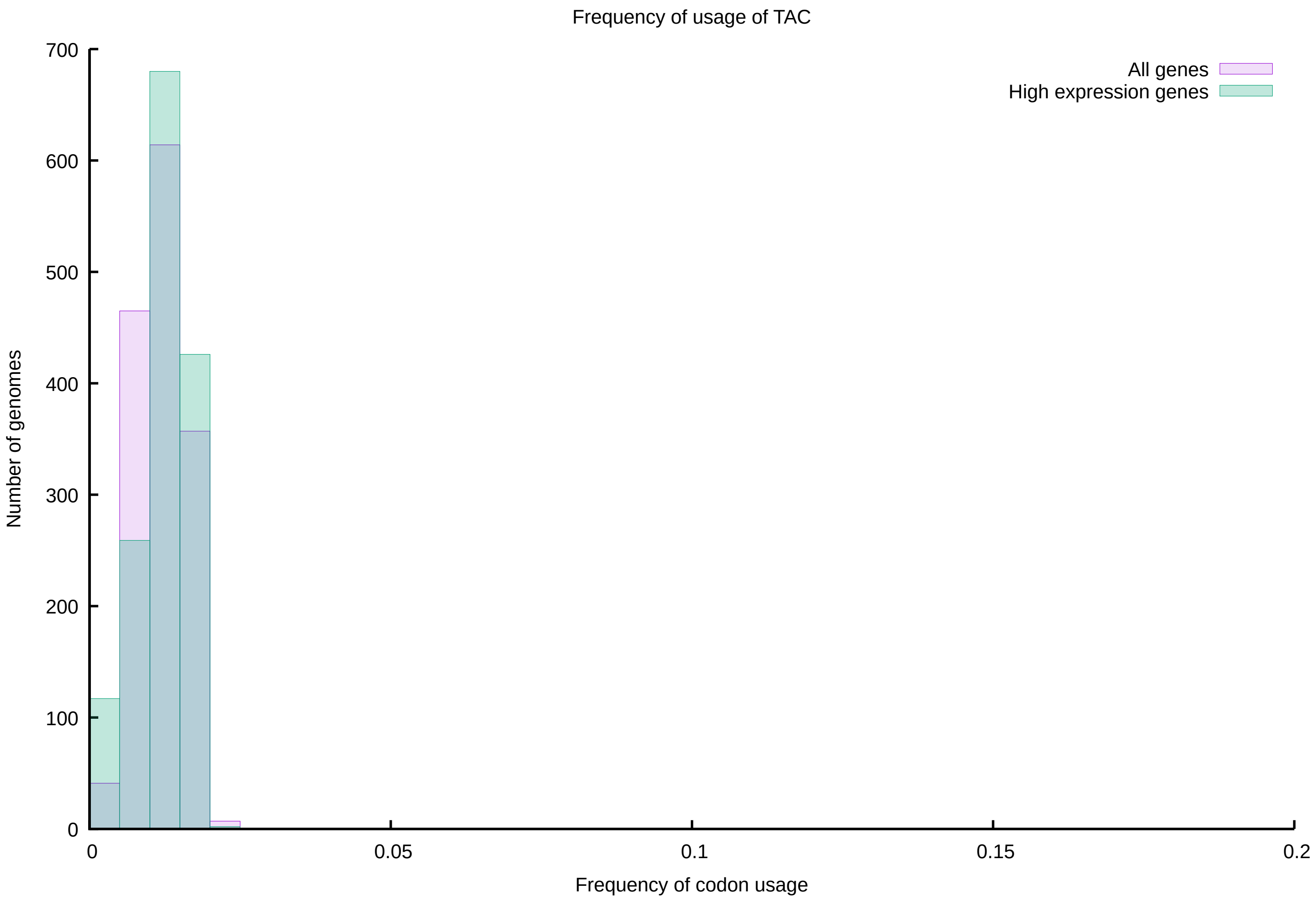
Frequency of usage of TAC
All genes
High expression genes
Number of genomes
0
100
200
300
400
500
600
700
0
0.05
0.1
0.15
0.2
Frequency of codon usage

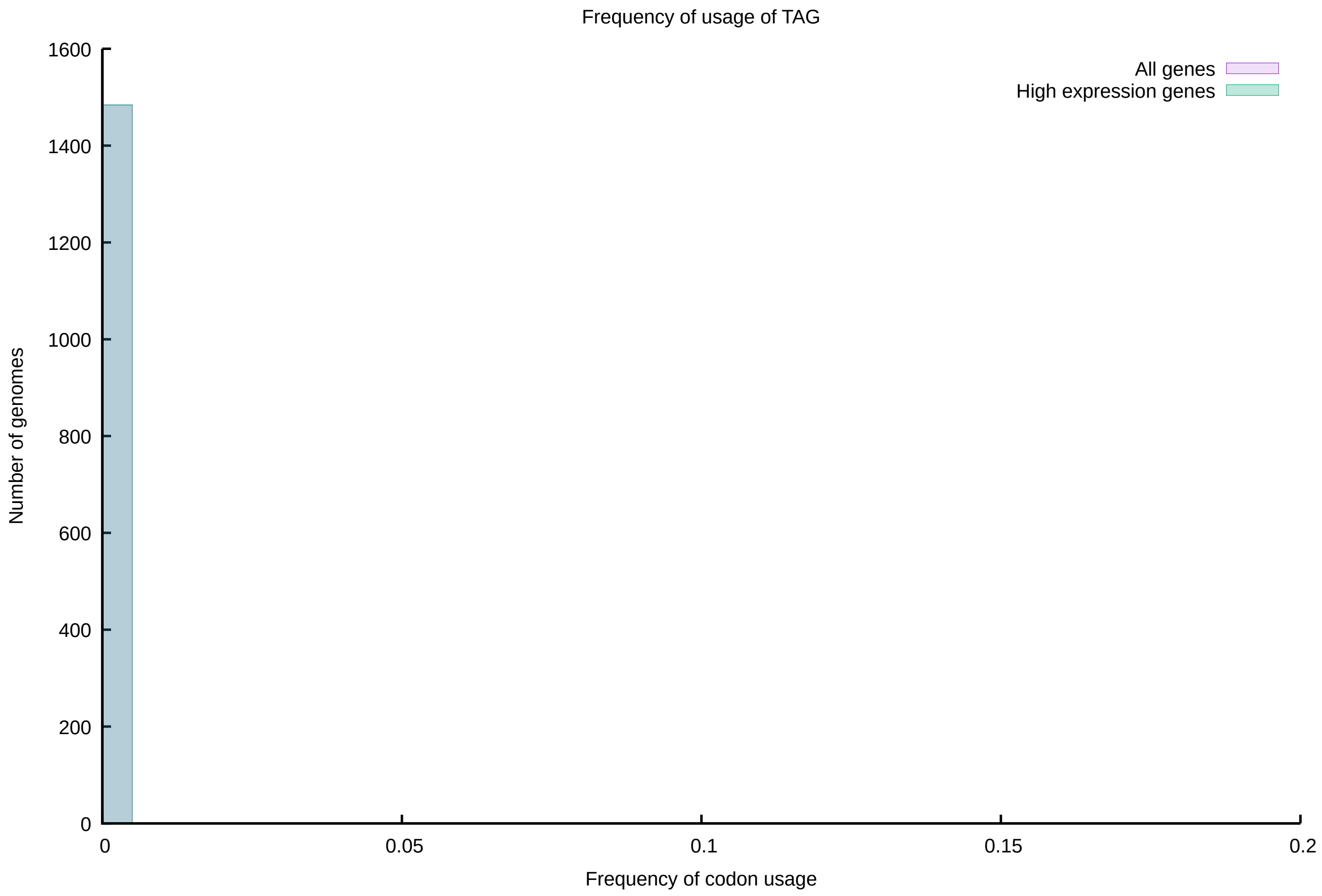
Frequency of usage of TAG
All genes
High expression genes
Number of genomes
1600
1400
1200
1000
800
600
400
200
0
0
0.05
0.1
0.15
0.2
Frequency of codon usage

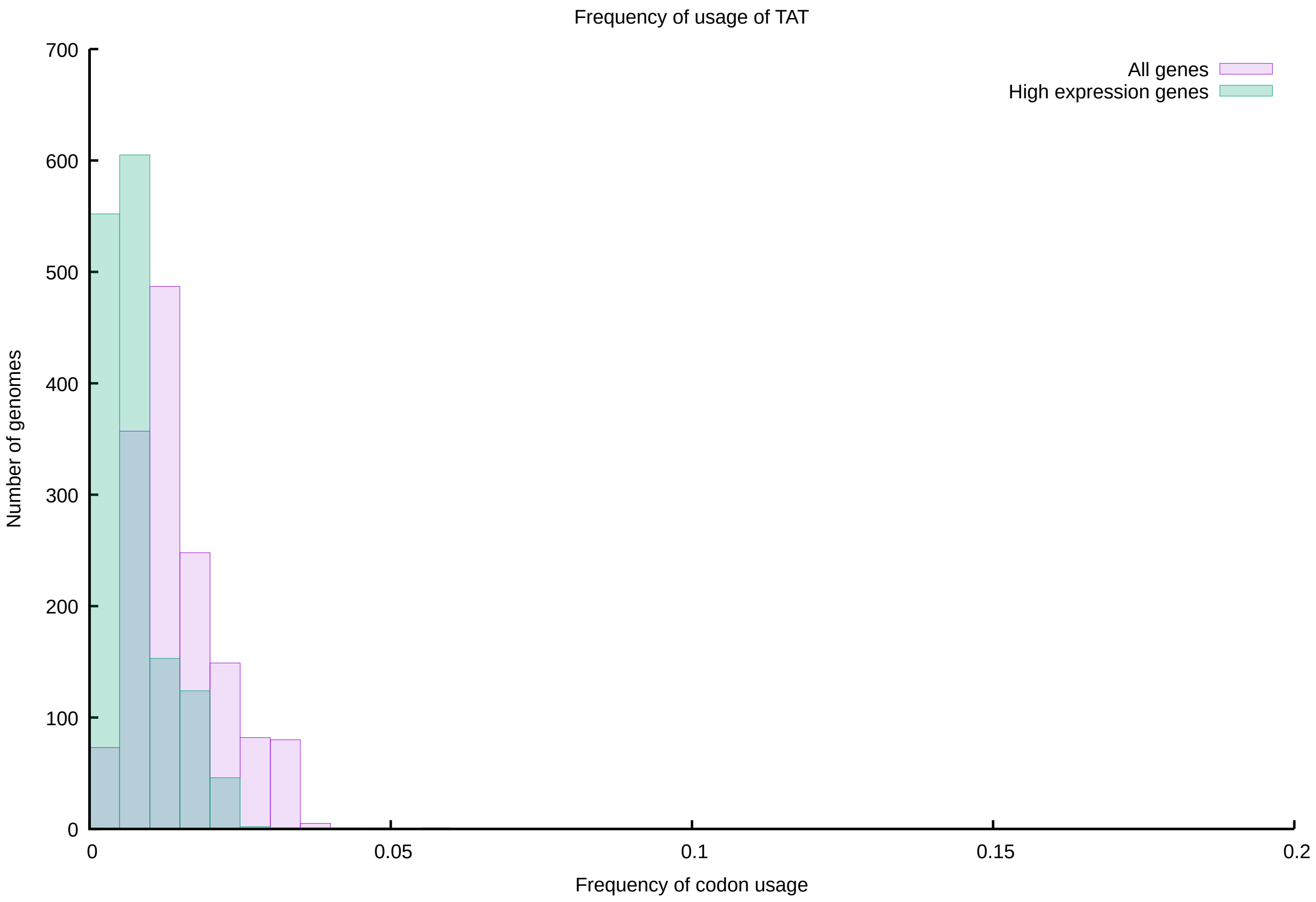

Frequency of usage of TAT
All genes
High expression genes
Number of genomes
700
600
500
400
300
200
100
0
0
0.05
0.1
0.15
0.2
Frequency of codon usage

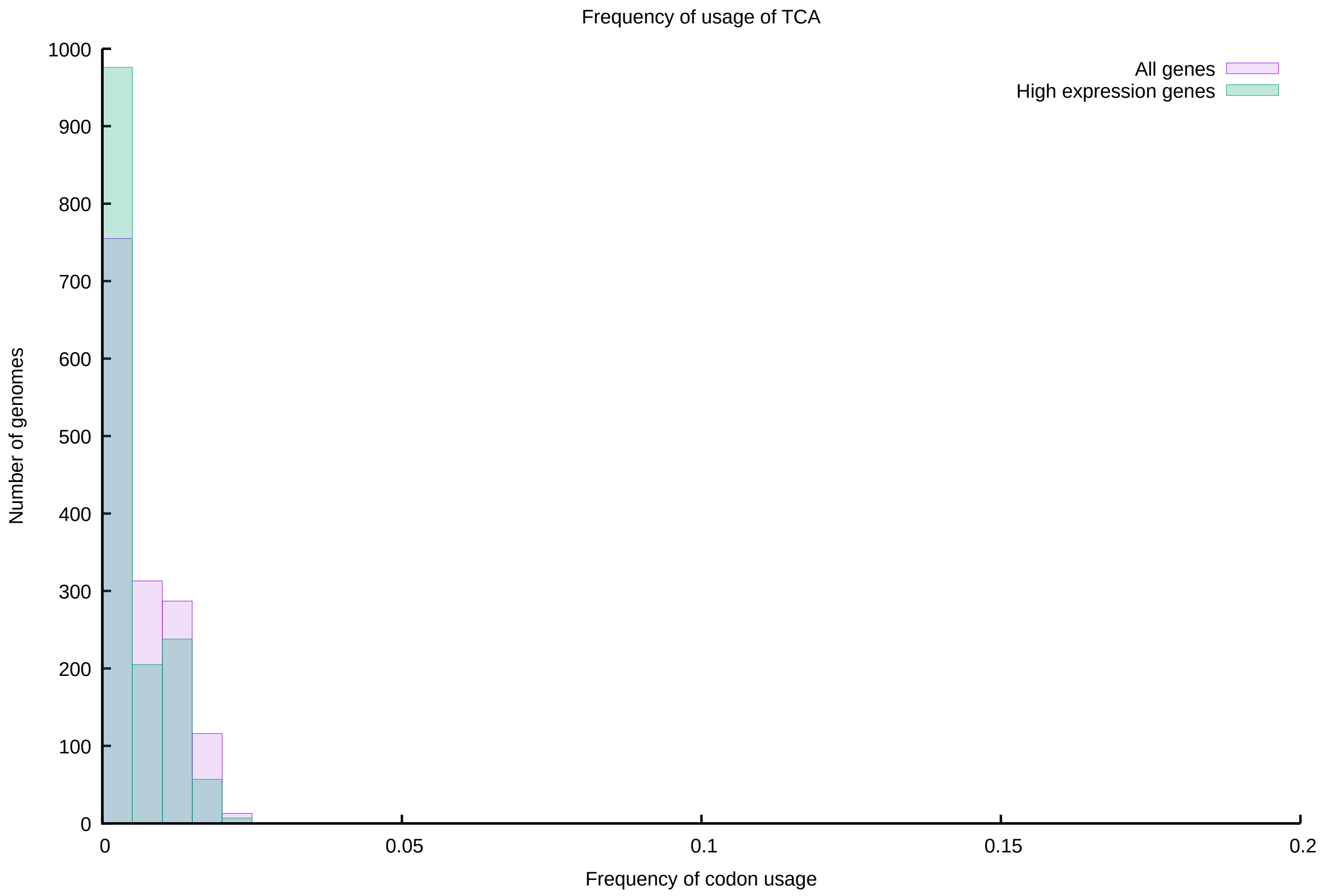

Frequency of usage of TCA
All genes
High expression genes
Number of genomes
0
100
200
300
400
500
600
700
800
900
1000
0
0.05
0.1
0.15
0.2
Frequency of codon usage

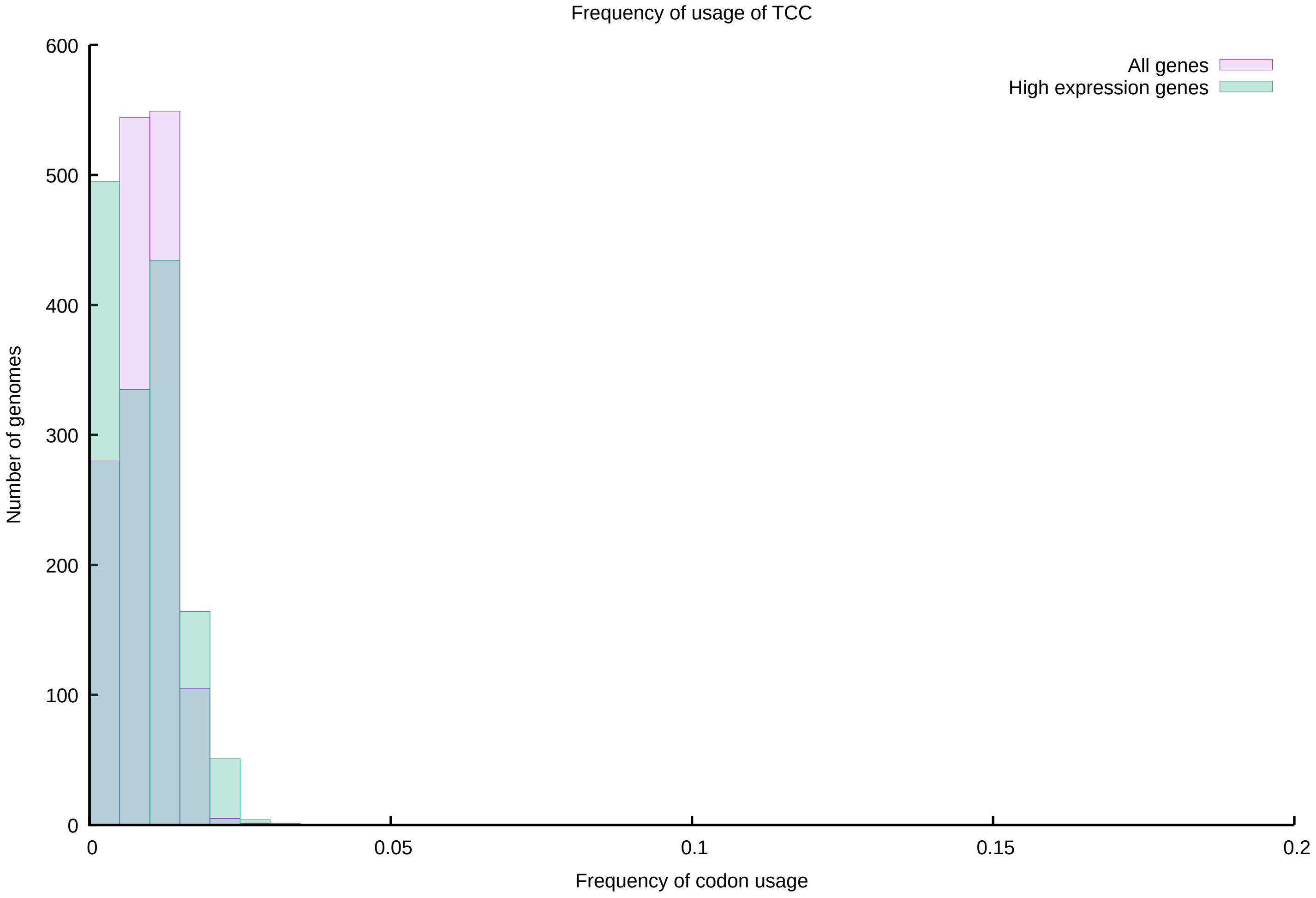

Frequency of usage of TCC
All genes
High expression genes
Number of genomes
0
100
200
300
400
500
600
0
0.05
0.1
0.15
0.2
Frequency of codon usage

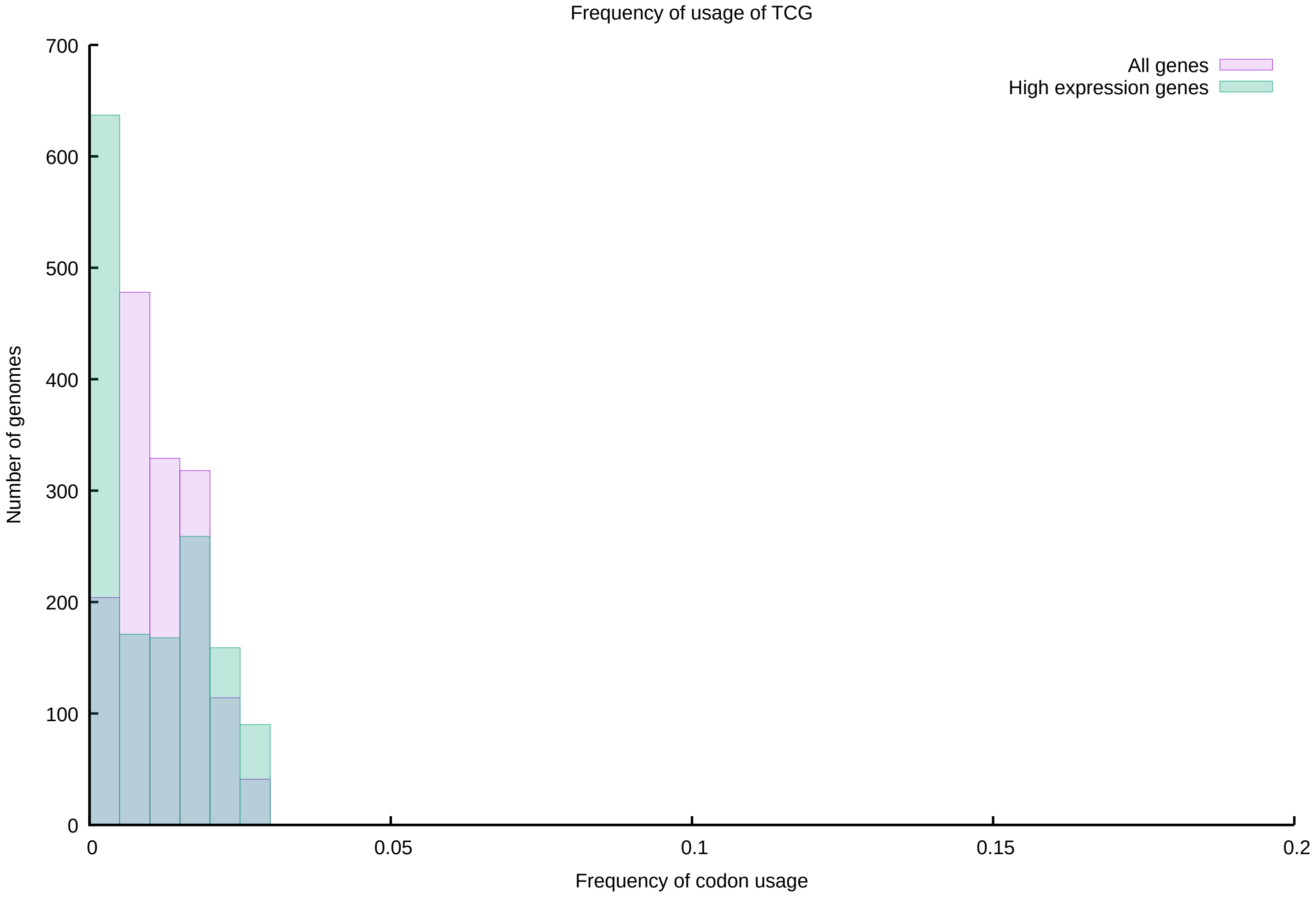
Frequency of usage of TCG
All genes
High expression genes
Number of genomes
0
100
200
300
400
500
600
700
0
0.05
0.1
0.15
0.2
Frequency of codon usage

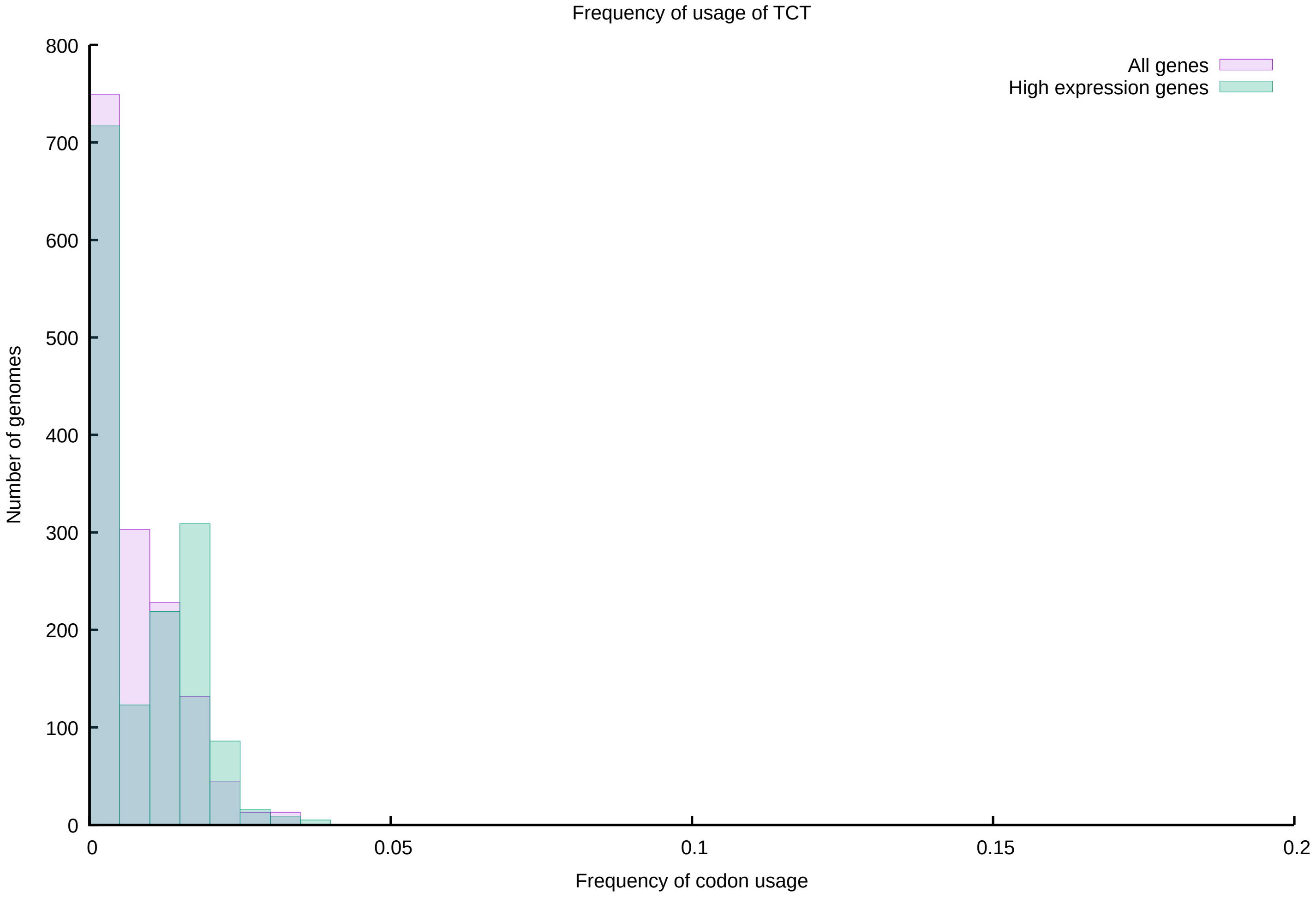

Frequency of usage of TCT
All genes
High expression genes
Number of genomes
800
700
600
500
400
300
200
100
0
0
0.05
0.1
0.15
0.2
Frequency of codon usage

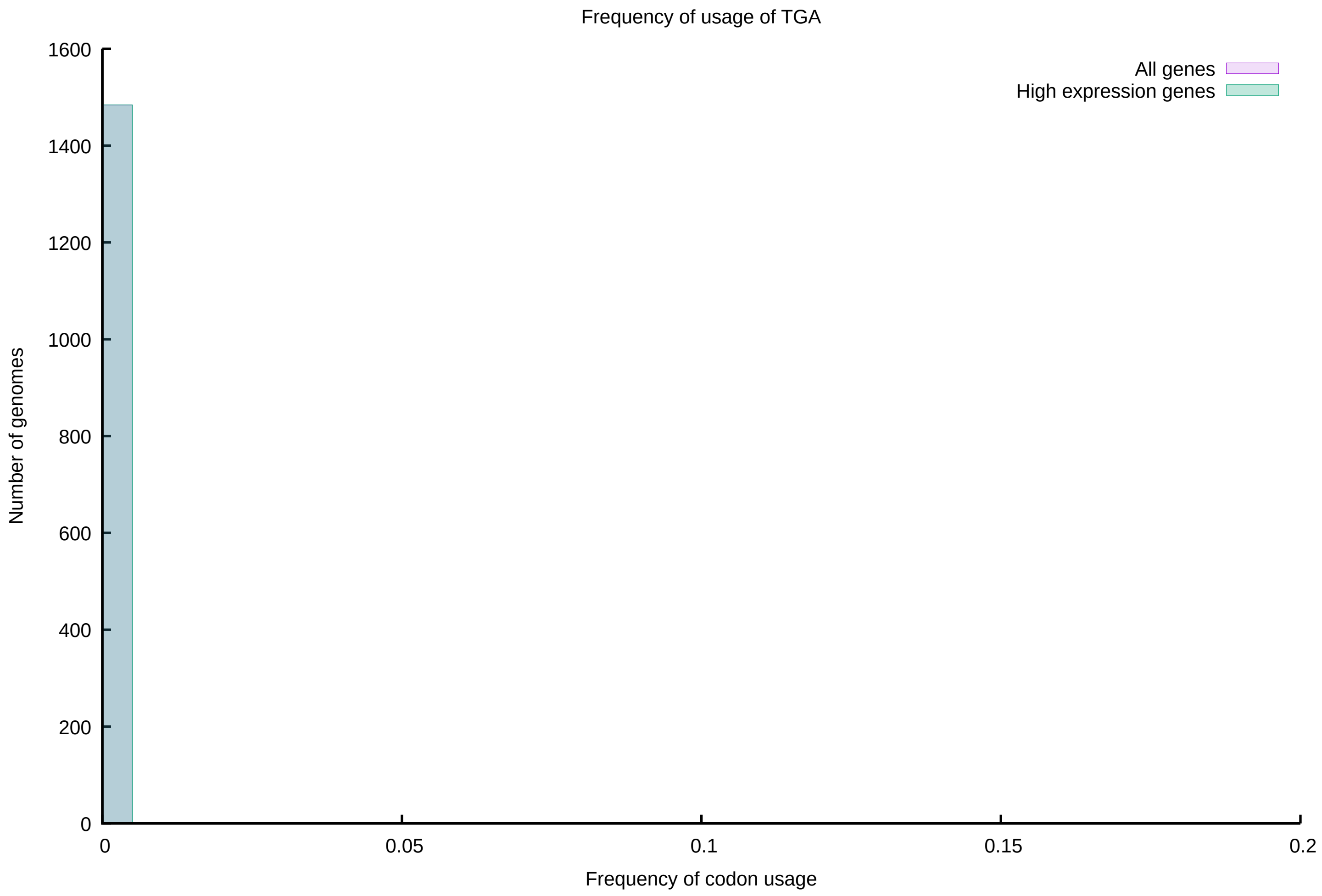

Frequency of usage of TGA
All genes
High expression genes
Number of genomes
1600
1400
1200
1000
800
600
400
200
0
0
0.05
0.1
0.15
0.2
Frequency of codon usage

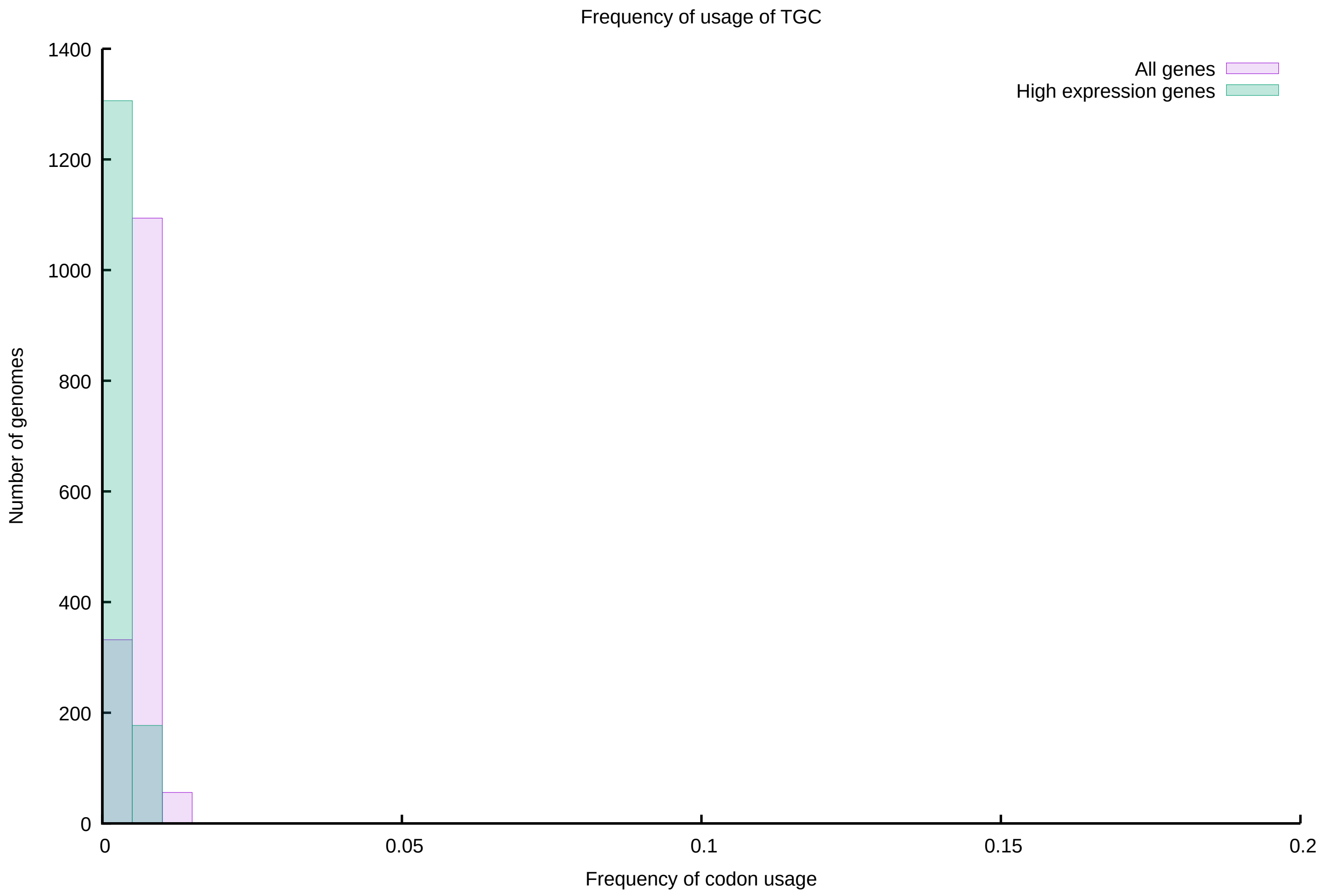

Frequency of usage of TGC
All genes
High expression genes
Number of genomes
0
200
400
600
800
1000
1200
1400
0
0.05
0.1
0.15
0.2
Frequency of codon usage

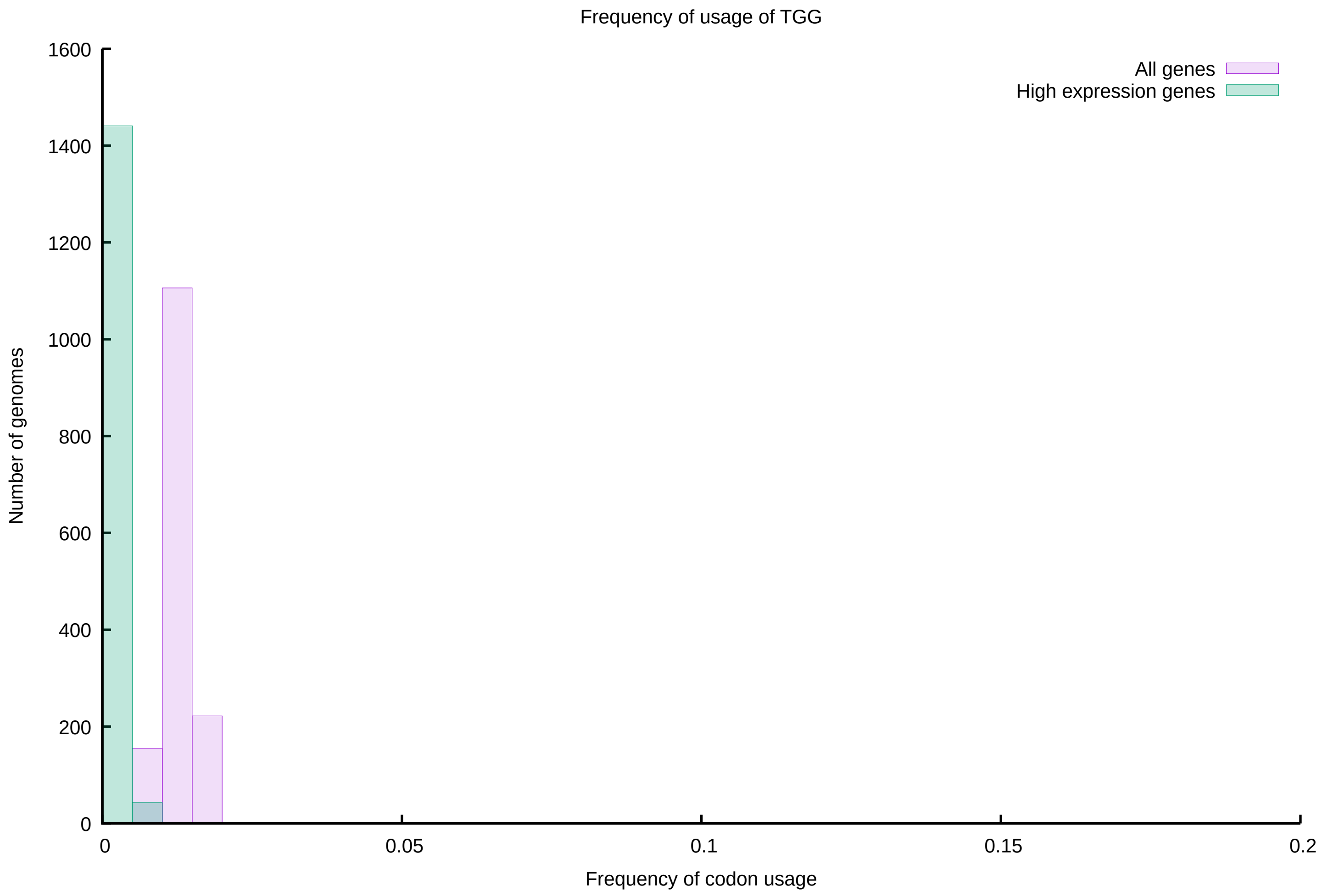

Frequency of usage of TGG
All genes
High expression genes
Number of genomes
0
200
400
600
800
1000
1200
1400
1600
0
0.05
0.1
0.15
0.2
Frequency of codon usage

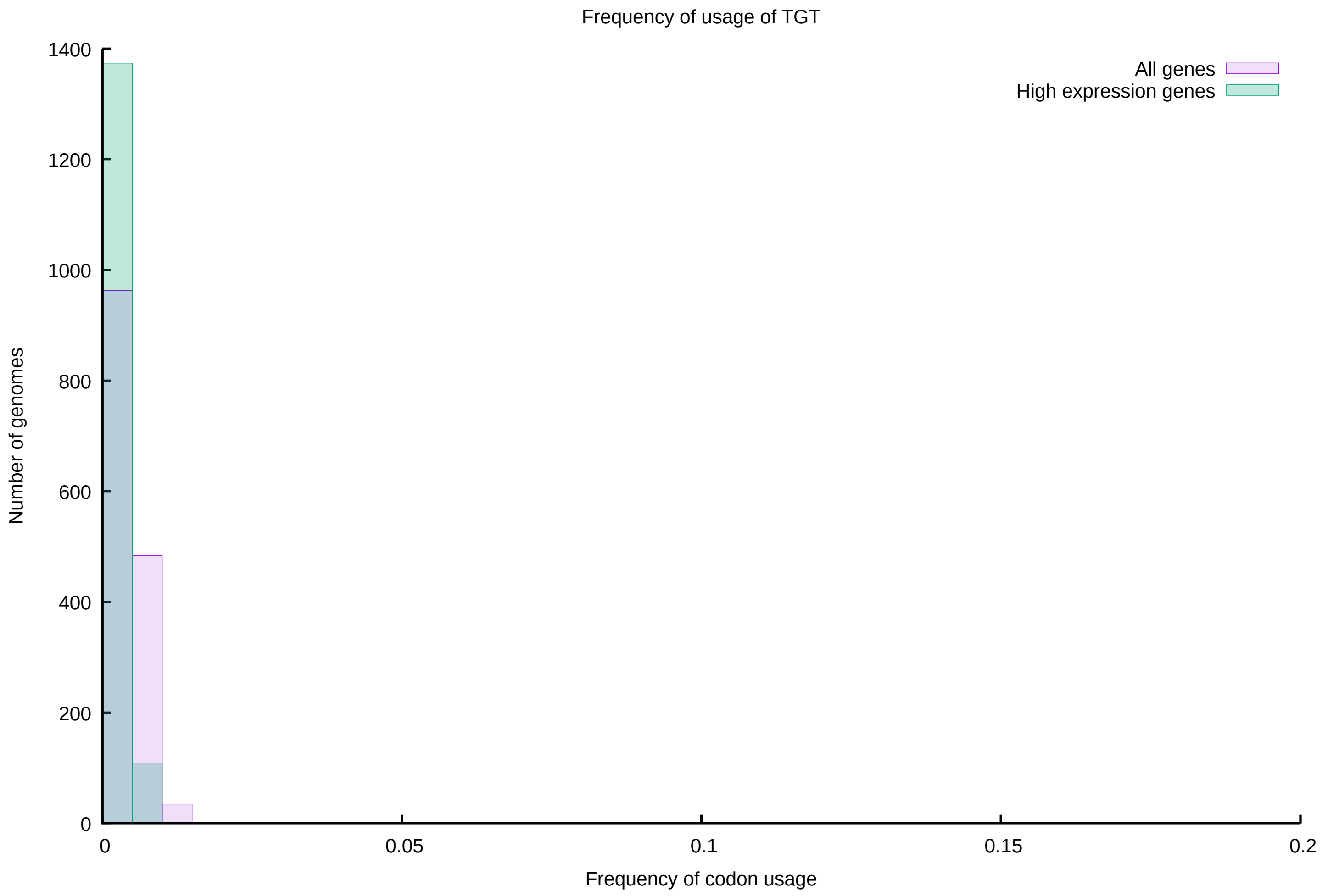
Frequency of usage of TGT
All genes
High expression genes
Number of genomes
1400
1200
1000
800
600
400
200
0
0
0.05
0.1
0.15
0.2
Frequency of codon usage

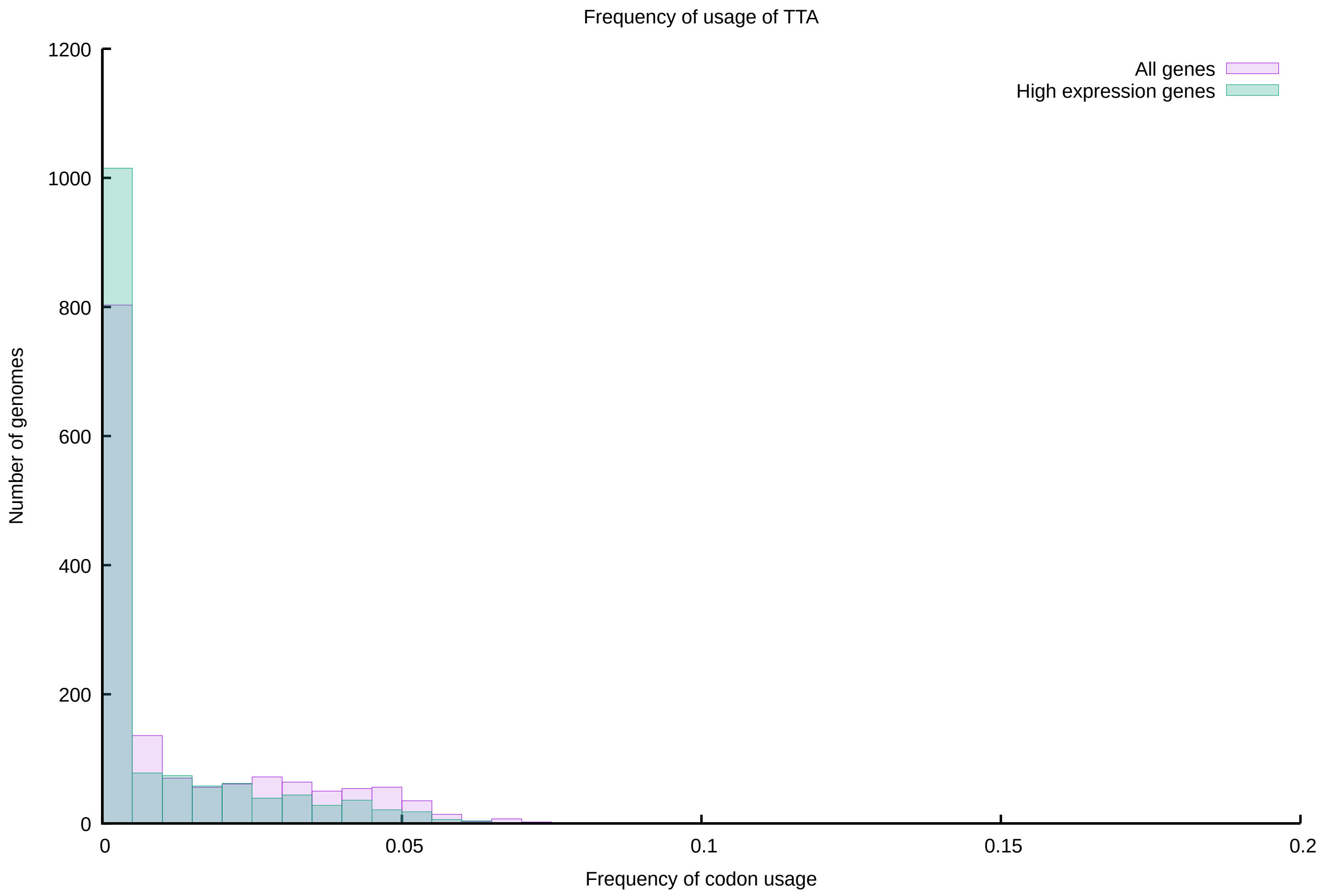
Frequency of usage of TTA
All genes
High expression genes
Number of genomes
0
200
400
600
800
1000
1200
0
0.05
0.1
0.15
0.2
Frequency of codon usage

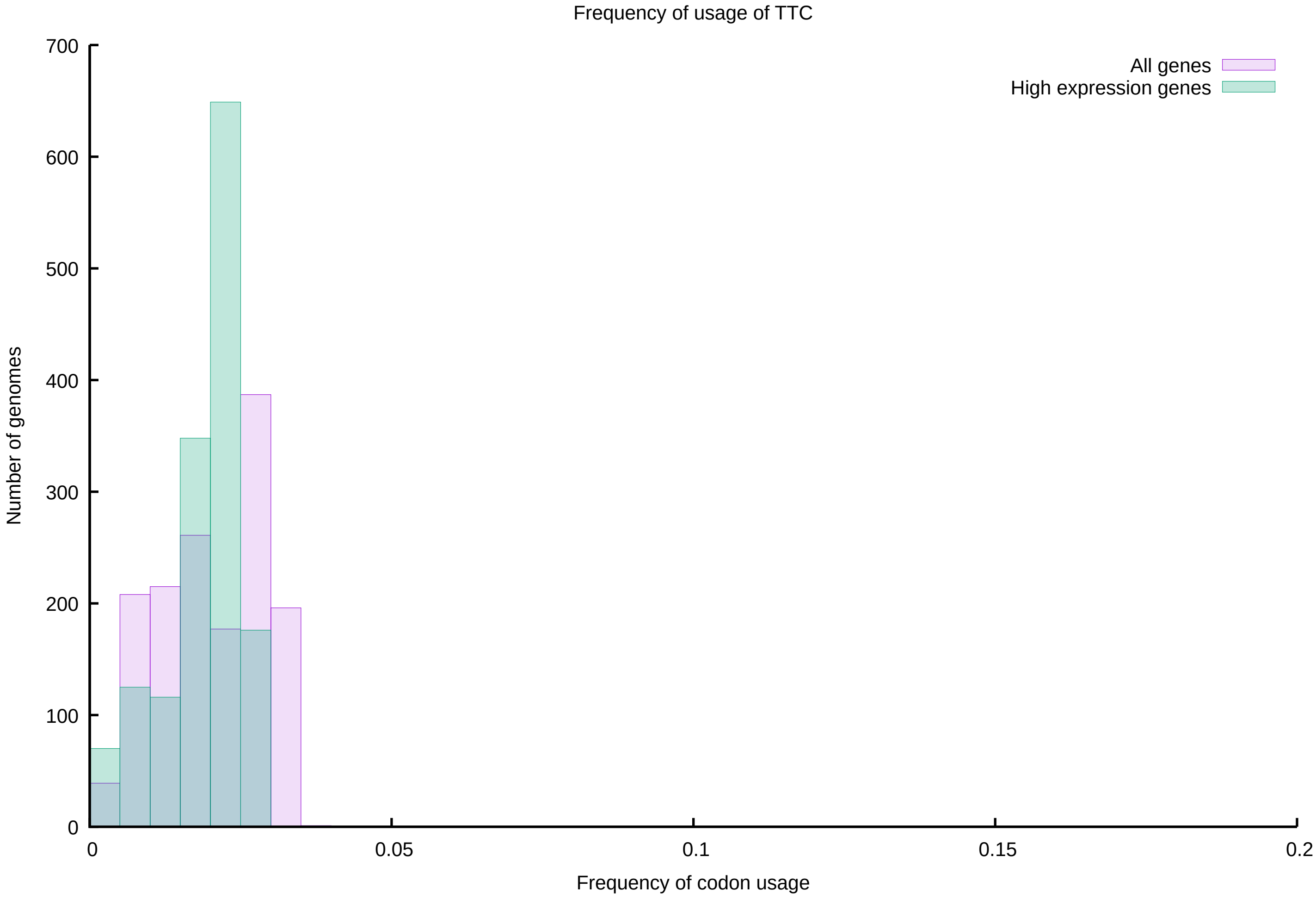

Frequency of usage of TTC
All genes
High expression genes
Number of genomes
0
100
200
300
400
500
600
700
0
0.05
0.1
0.15
0.2
Frequency of codon usage

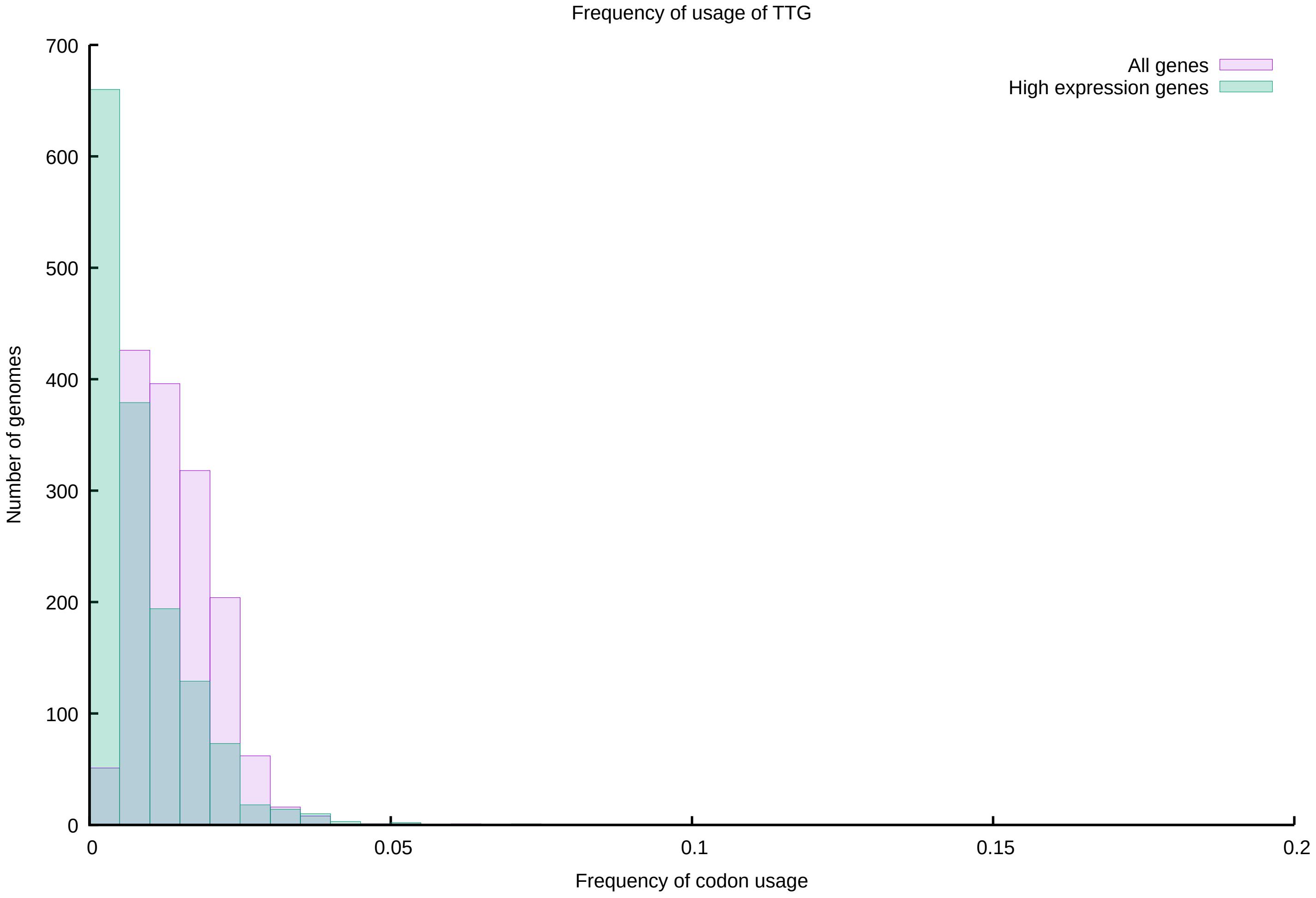
Frequency of usage of TTG
All genes
High expression genes
Number of genomes
0
100
200
300
400
500
600
700
0
0.05
0.1
0.15
0.2
Frequency of codon usage

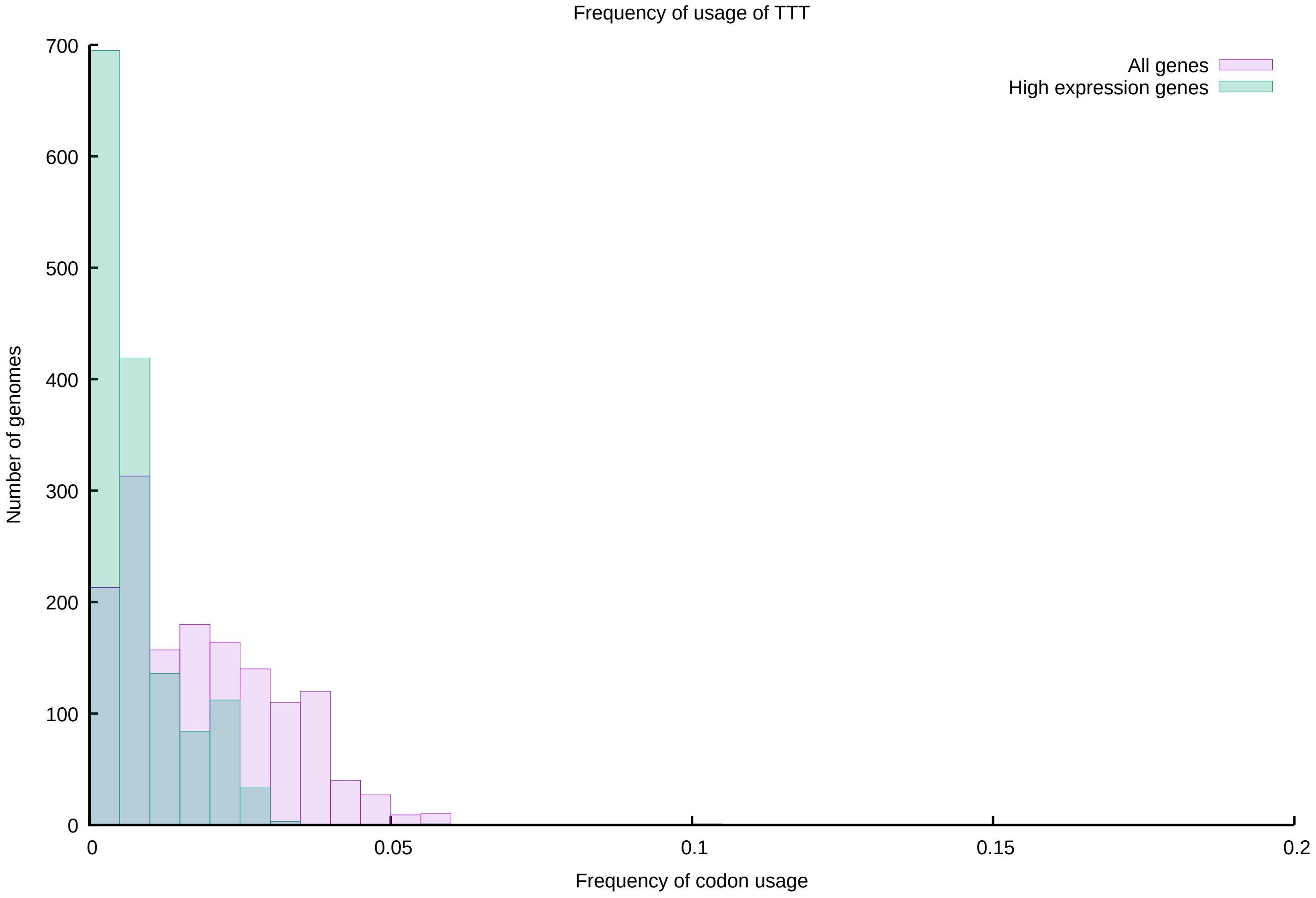
Frequency of usage of TTT
All genes
High expression genes
Number of genomes
0
100
200
300
400
500
600
700
0
0.05
0.1
0.15
0.2
Frequency of codon usage

Figure S2. Phylogenetic distribution of the frequency of usage of each codon in proteobacteria. A phylogenetic tree was constructed and the average frequency of usage for each codon in all genes of each genome is indicated using external red bars. Additionally, the class of each specie is indicated in a color strip below the bars (Red: alfa-protebacteria; Blue: beta-proteobacteria; Orange: gamma-proteobacteria; Pink: Delta-proteobacteria; Green: epsilon-proteobacteria; Purple: zeta-proteobacteria; Black: oligoflexia; Lightblue: acidithiobacillia). *Clostridium botulinum* and *Bacillus subtilis* were used as out group for tree construction. Codon usage and class are not presented for these genomes. An interactive version of this figure can be accessed at https://itol.embl.de/tree/200896812311881706546712.

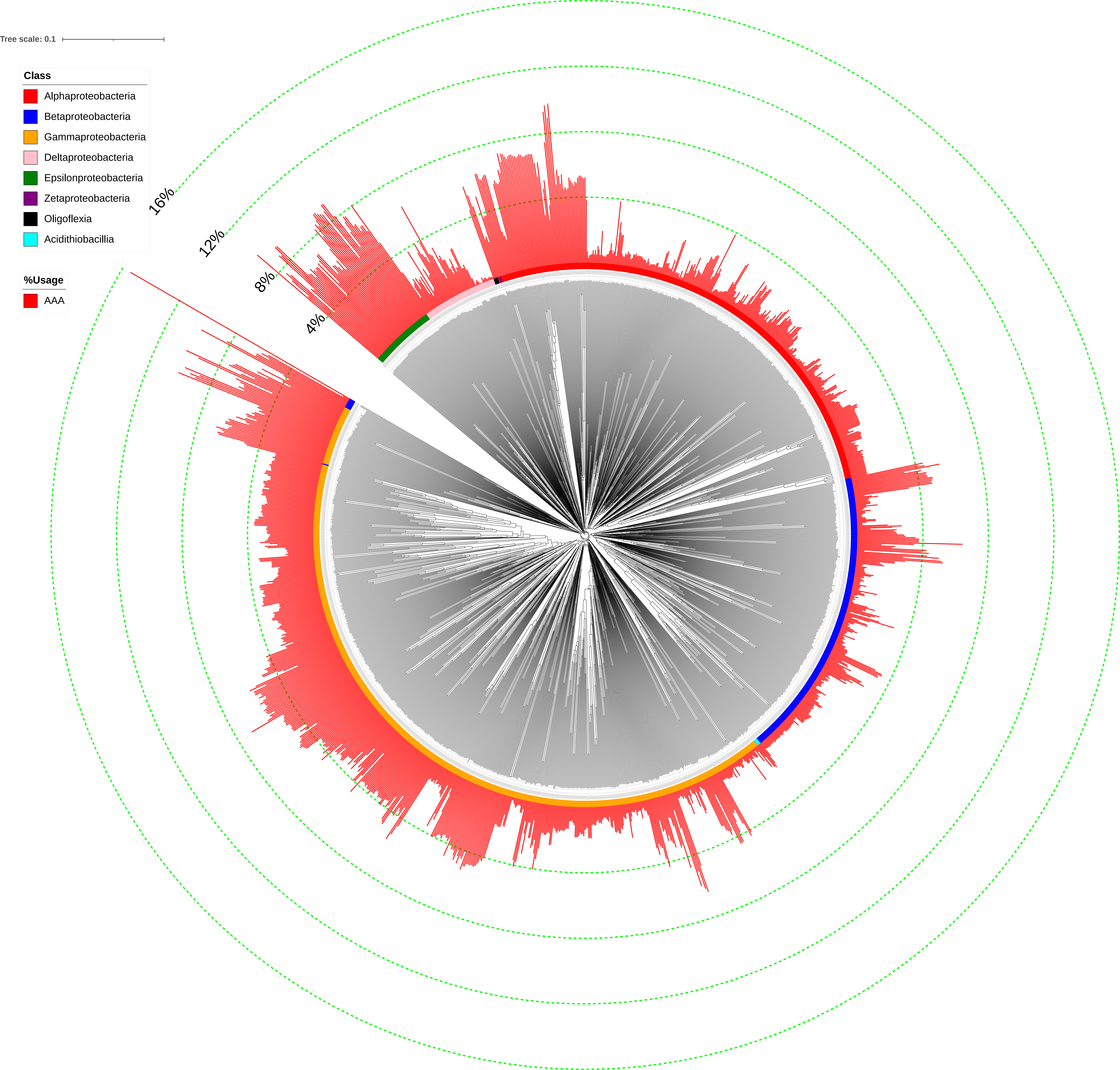

Tree scale: 0.1
Class
Alphaproteobacteria
Betaproteobacteria
Gammaproteobacteria
Deltaproteobacteria
Epsilonproteobacteria
Zetaproteobacteria
Oligoflexia
Acidithiobacillia
%Usage
AAA
16%
12%
8%
4%

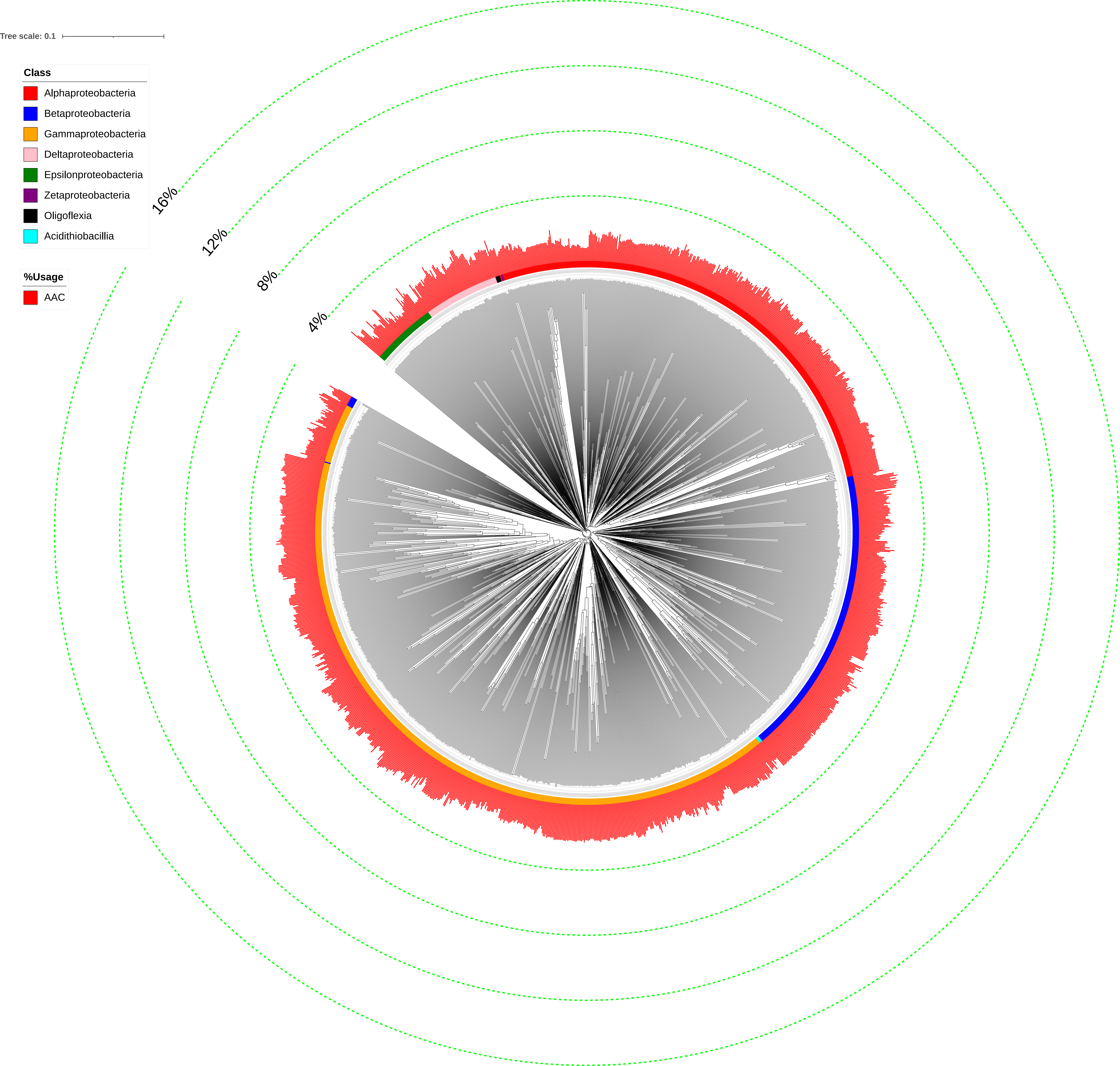
Tree scale: 0.1
Class
Alphaproteobacteria
Betaproteobacteria
Gammaproteobacteria
Deltaproteobacteria
Epsilonproteobacteria
Zetaproteobacteria
Oligoflexia
Acidithiobacillia
%Usage
AAC
4%
8%
12%
16%

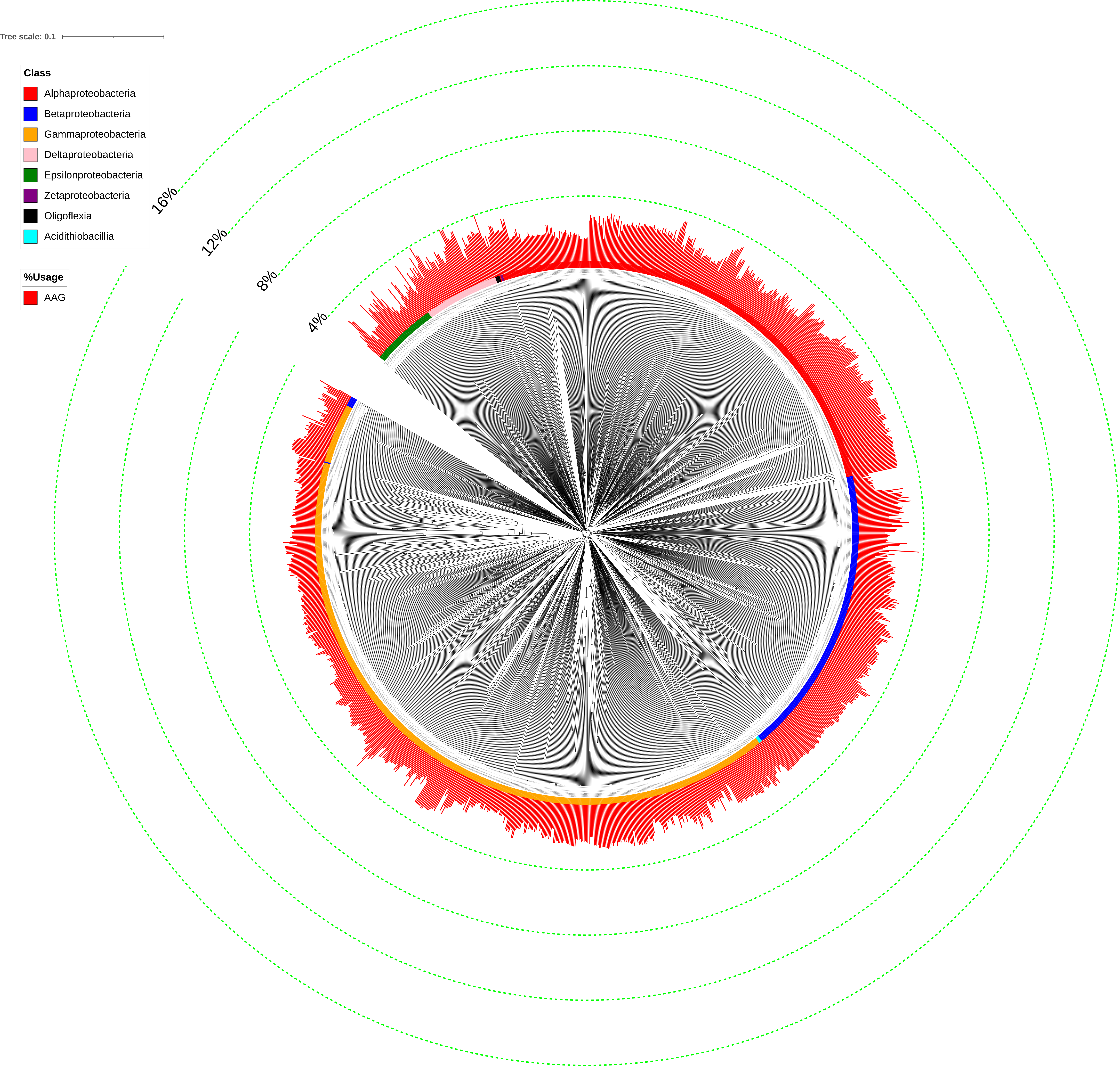
Tree scale: 0.1
Class
Alphaproteobacteria
Betaproteobacteria
Gammaproteobacteria
Deltaproteobacteria
Epsilonproteobacteria
Zetaproteobacteria
Oligoflexia
Acidithiobacillia
%Usage
AAG
4%
8%
12%
16%

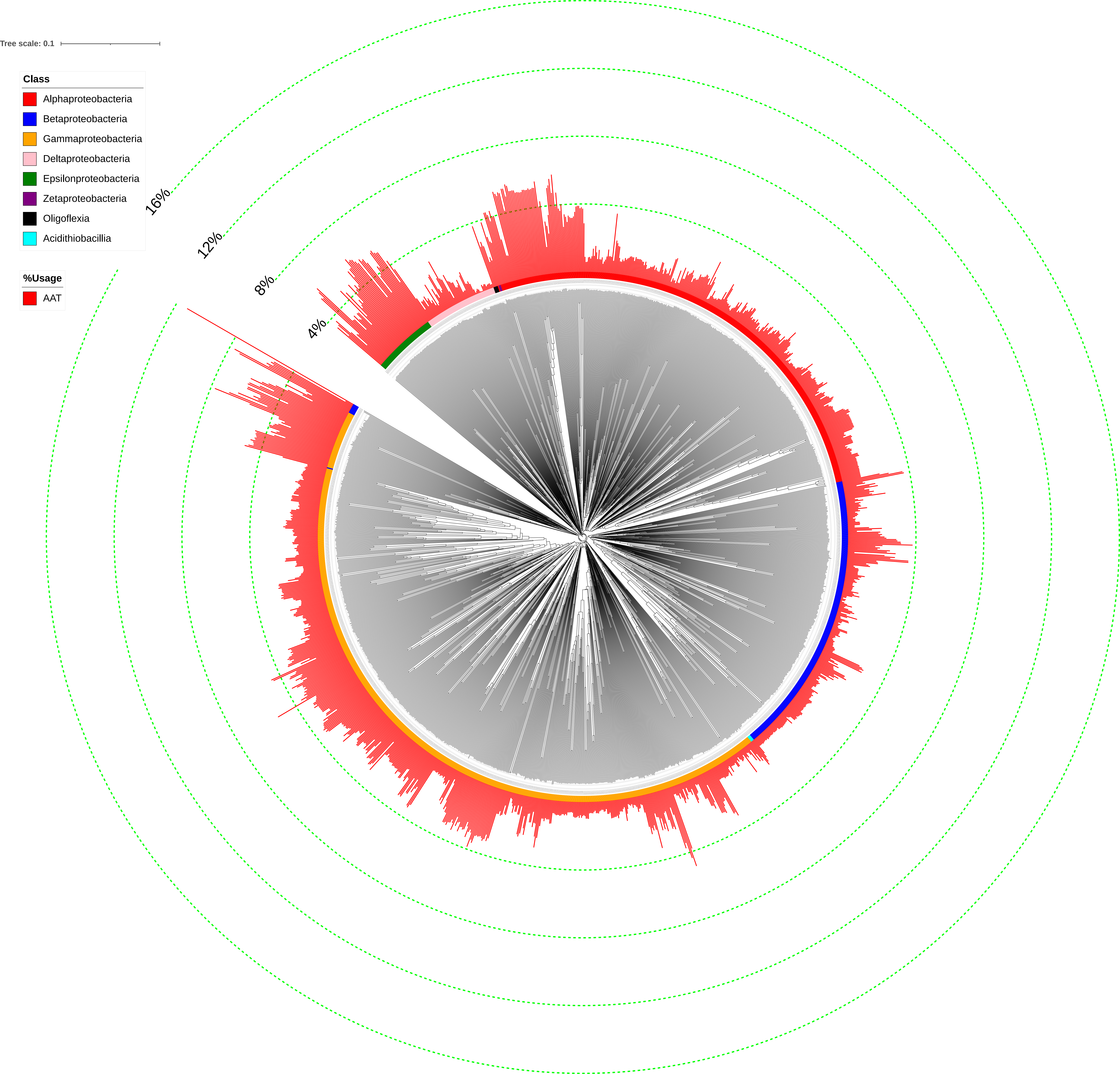
Tree scale: 0.1
Class
Alphaproteobacteria
Betaproteobacteria
Gammaproteobacteria
Deltaproteobacteria
Epsilonproteobacteria
Zetaproteobacteria
Oligoflexia
Acidithiobacillia
%Usage
AAT
16%
12%
8%
4%

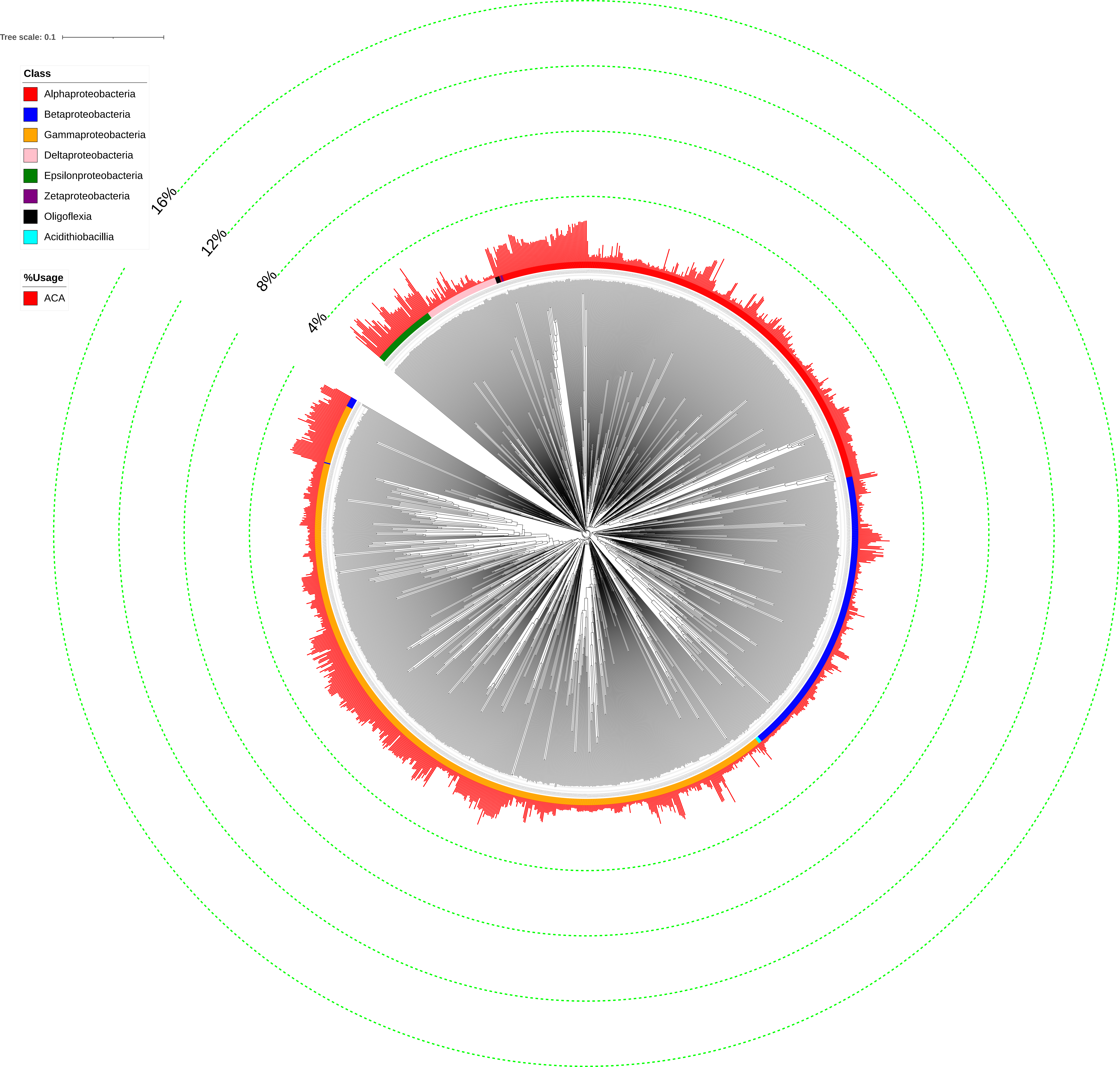

Tree scale: 0.1
Class
Alphaproteobacteria
Betaproteobacteria
Gammaproteobacteria
Deltaproteobacteria
Epsilonproteobacteria
Zetaproteobacteria
Oligoflexia
Acidithiobacillia
%Usage
ACA
16%
12%
8%
4%

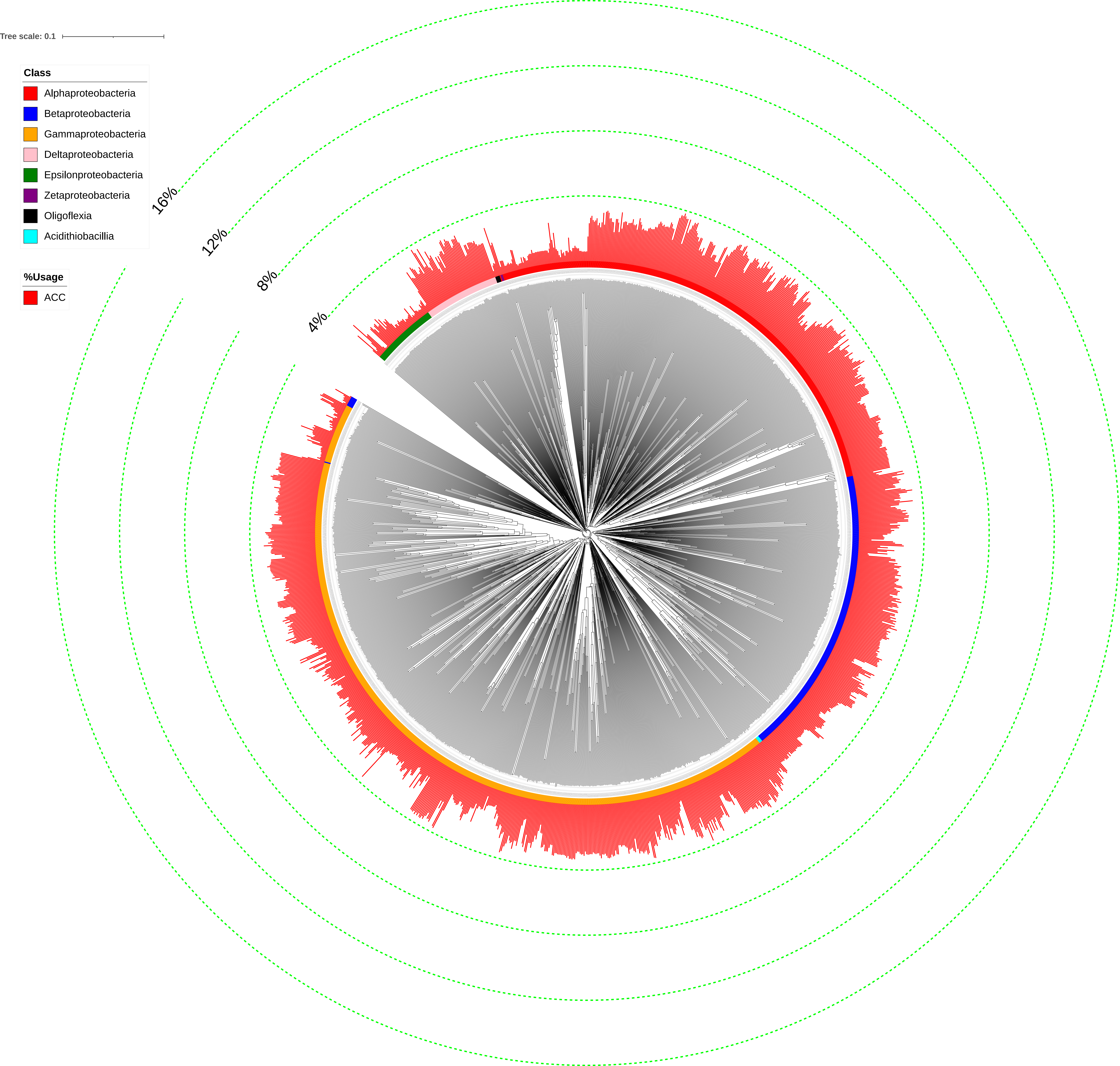

Tree scale: 0.1
Class
Alphaproteobacteria
Betaproteobacteria
Gammaproteobacteria
Deltaproteobacteria
Epsilonproteobacteria
Zetaproteobacteria
Oligoflexia
Acidithiobacillia
%Usage
ACC
16%
12%
8%
4%

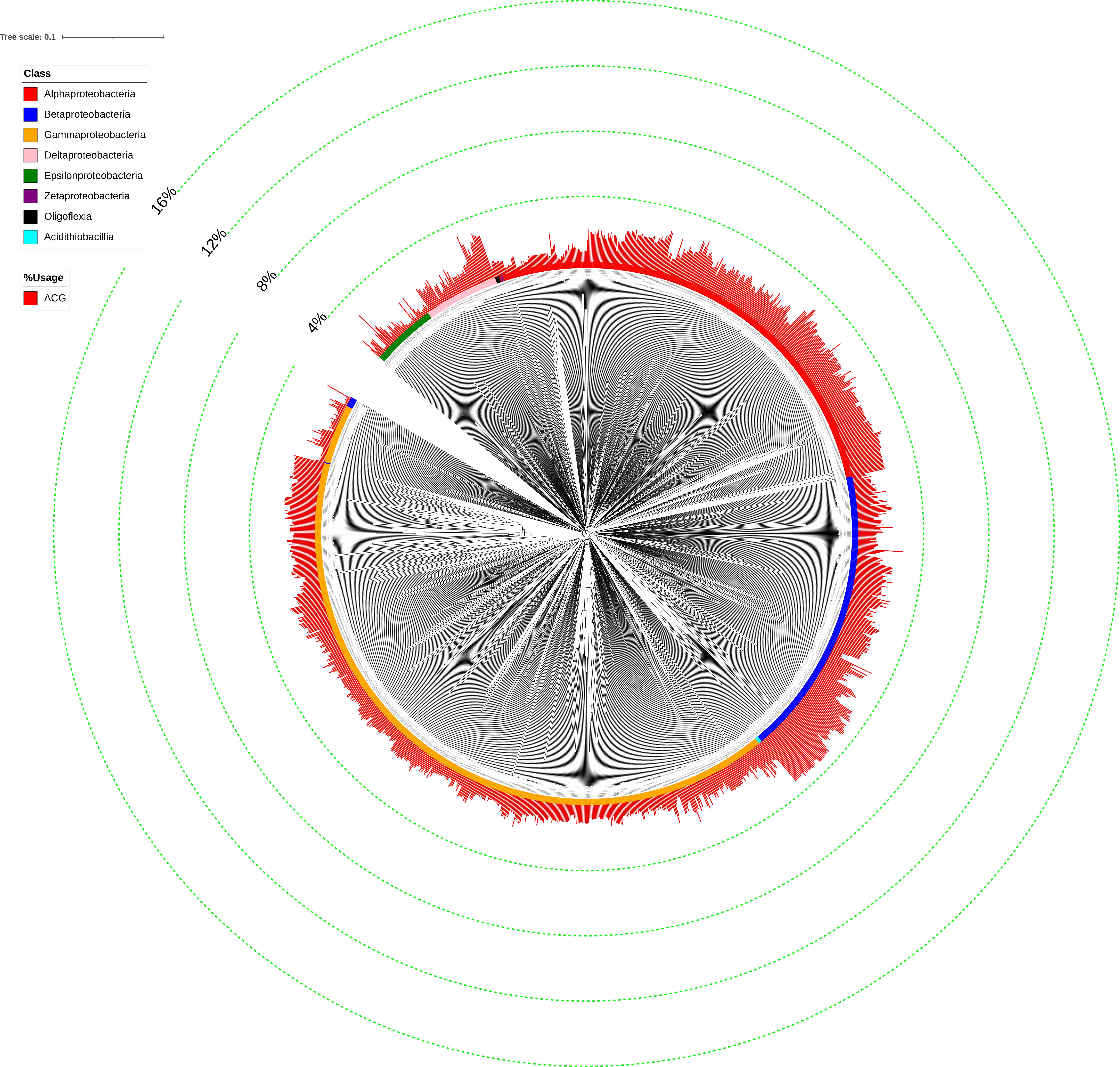

Tree scale: 0.1
Class
Alphaproteobacteria
Betaproteobacteria
Gammaproteobacteria
Deltaproteobacteria
Epsilonproteobacteria
Zetaproteobacteria
Oligoflexia
Acidithiobacillia
%Usage
ACG
16%
12%
8%
4%

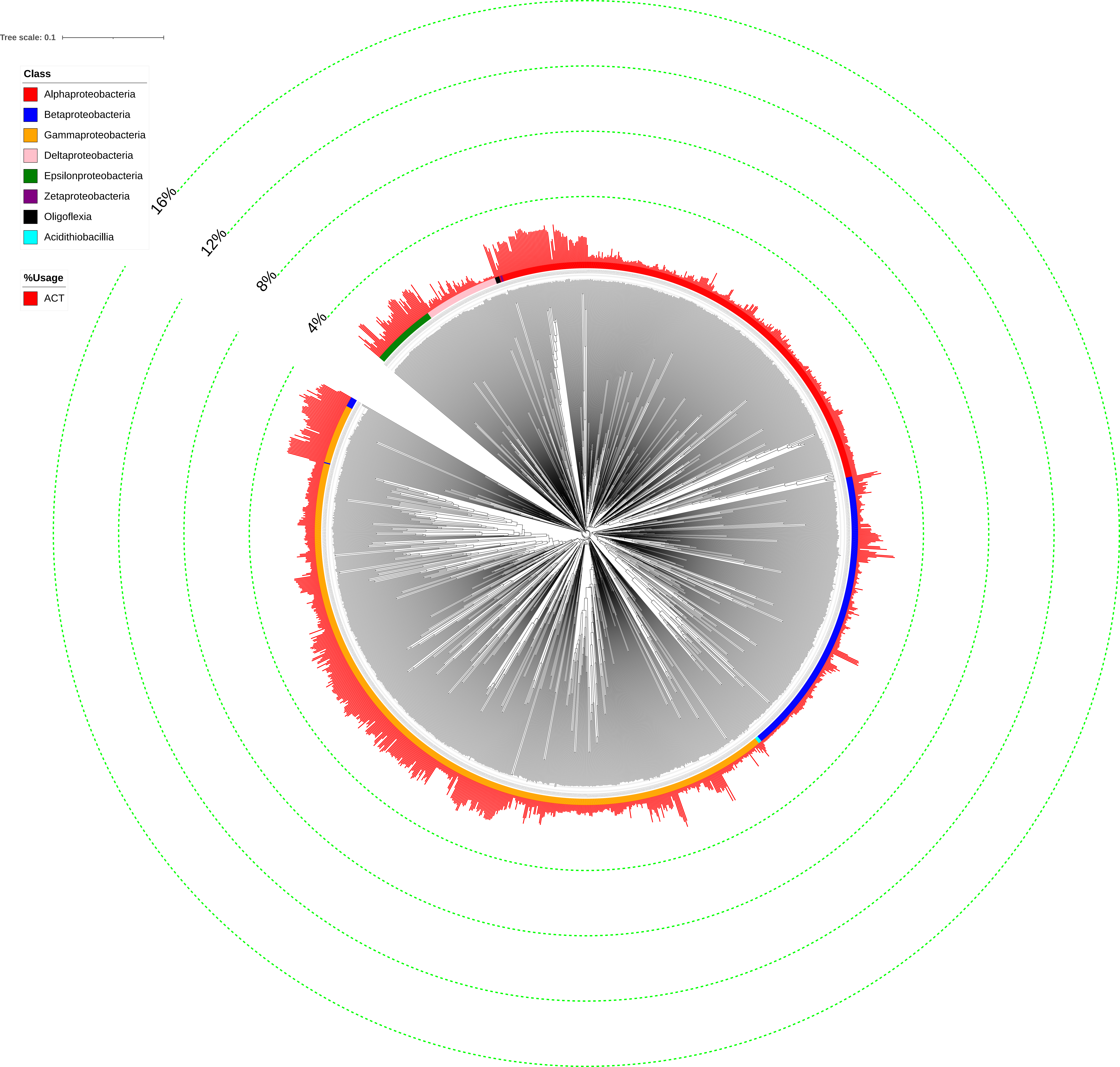

Tree scale: 0.1
Class
Alphaproteobacteria
Betaproteobacteria
Gammaproteobacteria
Deltaproteobacteria
Epsilonproteobacteria
Zetaproteobacteria
Oligoflexia
Acidithiobacillia
%Usage
ACT
4%
8%
12%
16%

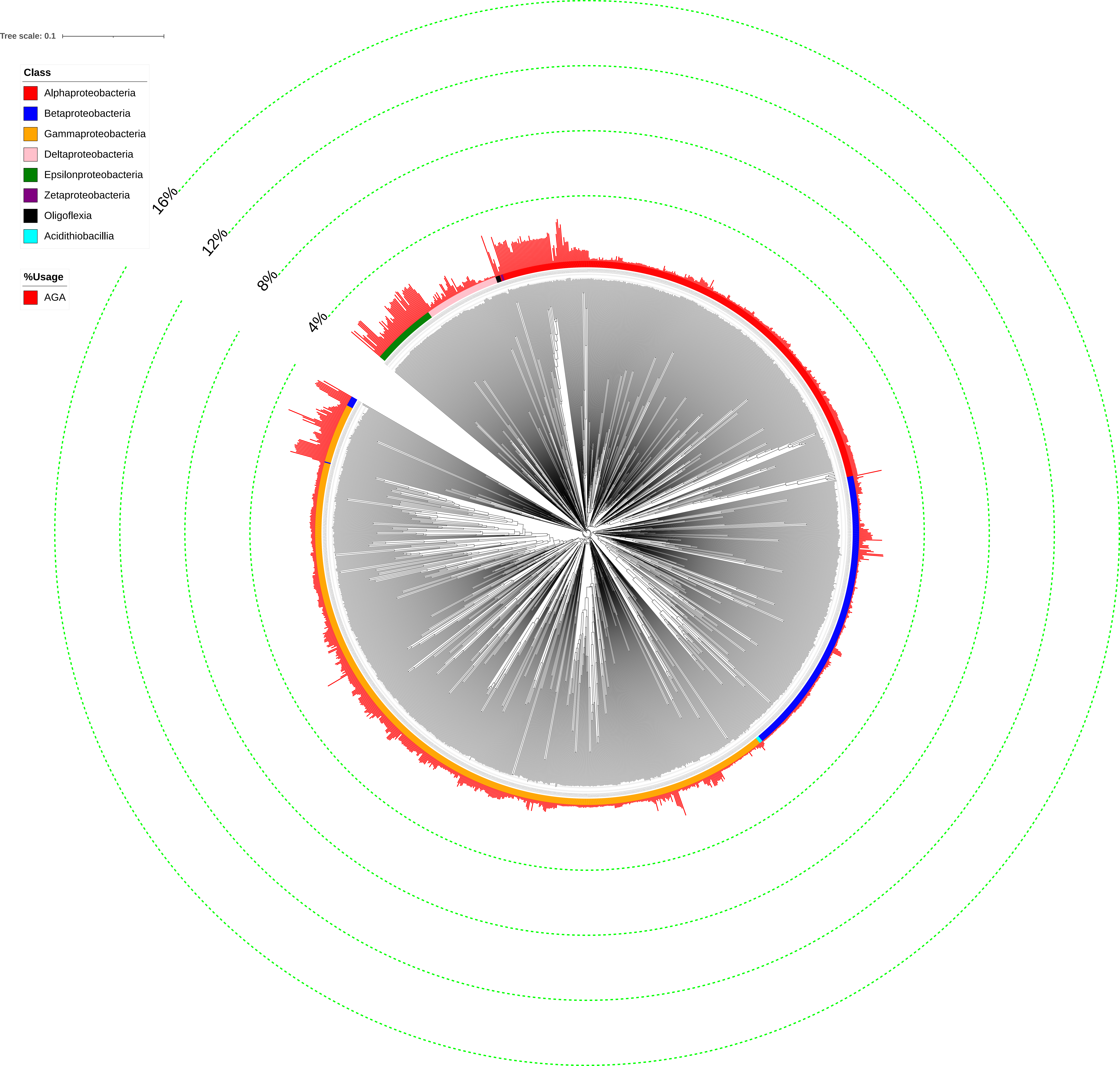

Tree scale: 0.1
Class
Alphaproteobacteria
Betaproteobacteria
Gammaproteobacteria
Deltaproteobacteria
Epsilonproteobacteria
Zetaproteobacteria
Oligoflexia
Acidithiobacillia
%Usage
AGA
16%
12%
8%
4%

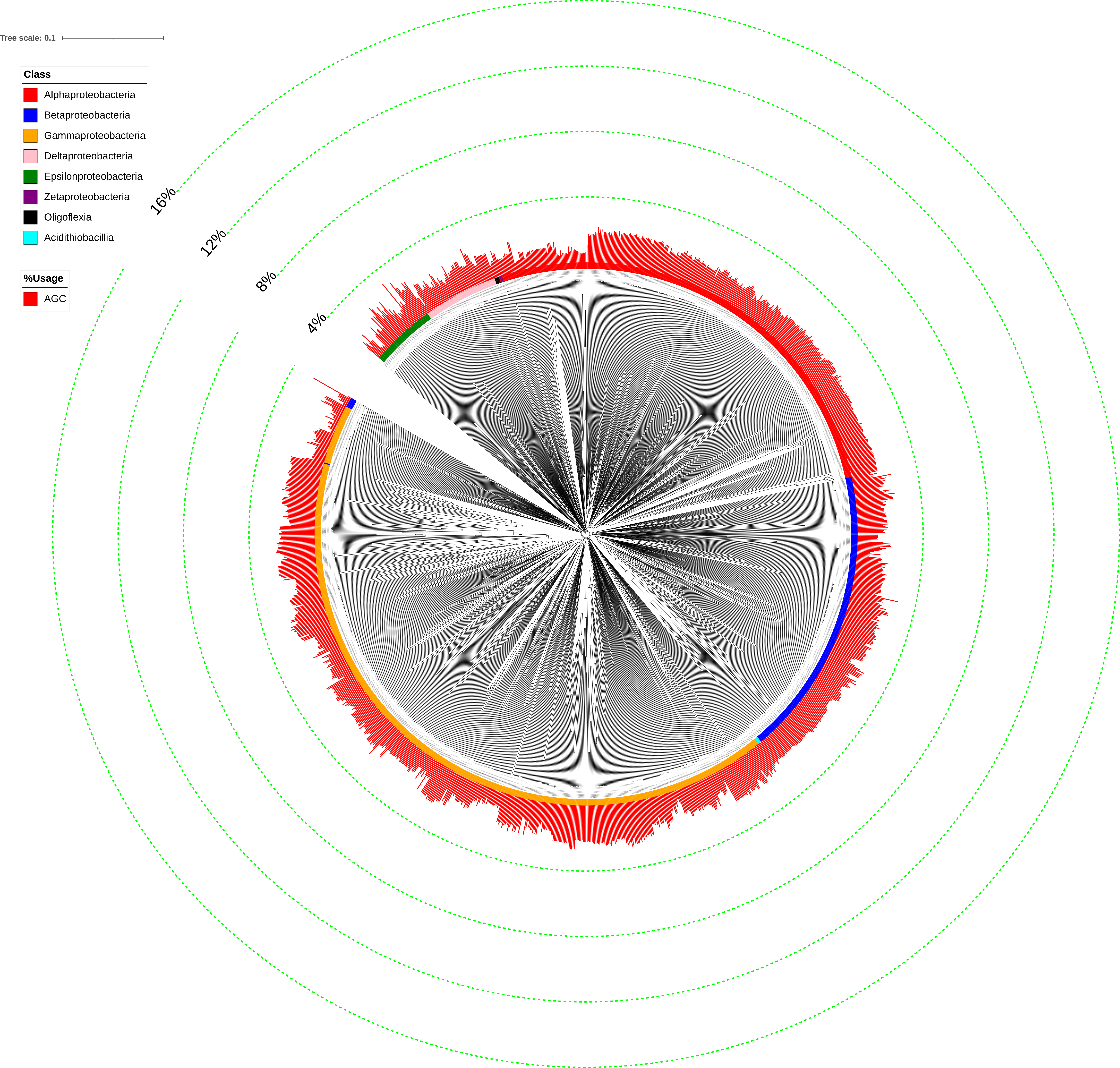

Tree scale: 0.1
Class
Alphaproteobacteria
Betaproteobacteria
Gammaproteobacteria
Deltaproteobacteria
Epsilonproteobacteria
Zetaproteobacteria
Oligoflexia
Acidithiobacillia
%Usage
AGC
16%
12%
8%
4%

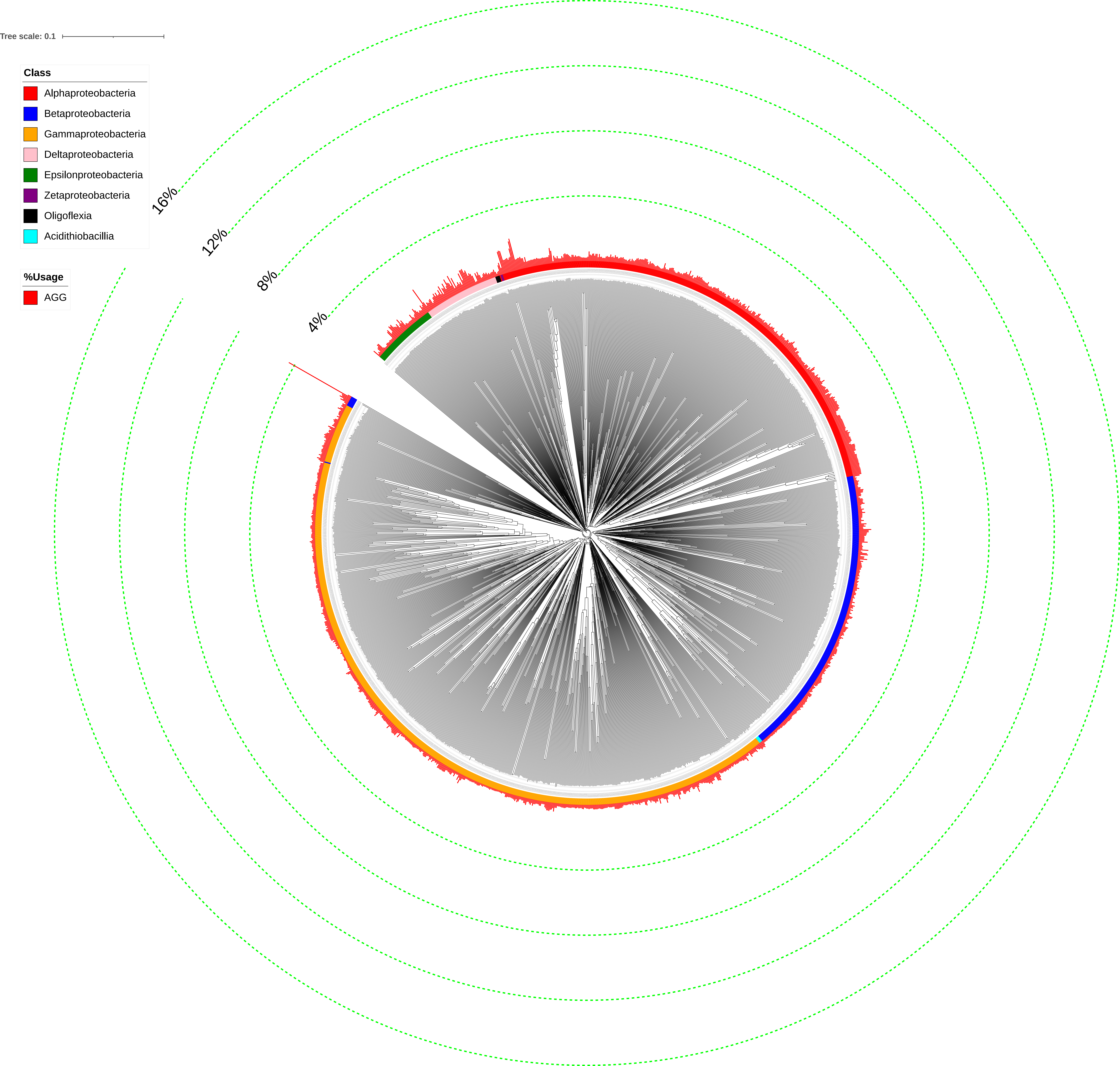

Tree scale: 0.1
Class
Alphaproteobacteria
Betaproteobacteria
Gammaproteobacteria
Deltaproteobacteria
Epsilonproteobacteria
Zetaproteobacteria
Oligoflexia
Acidithiobacillia
%Usage
AGG
16%
12%
8%
4%

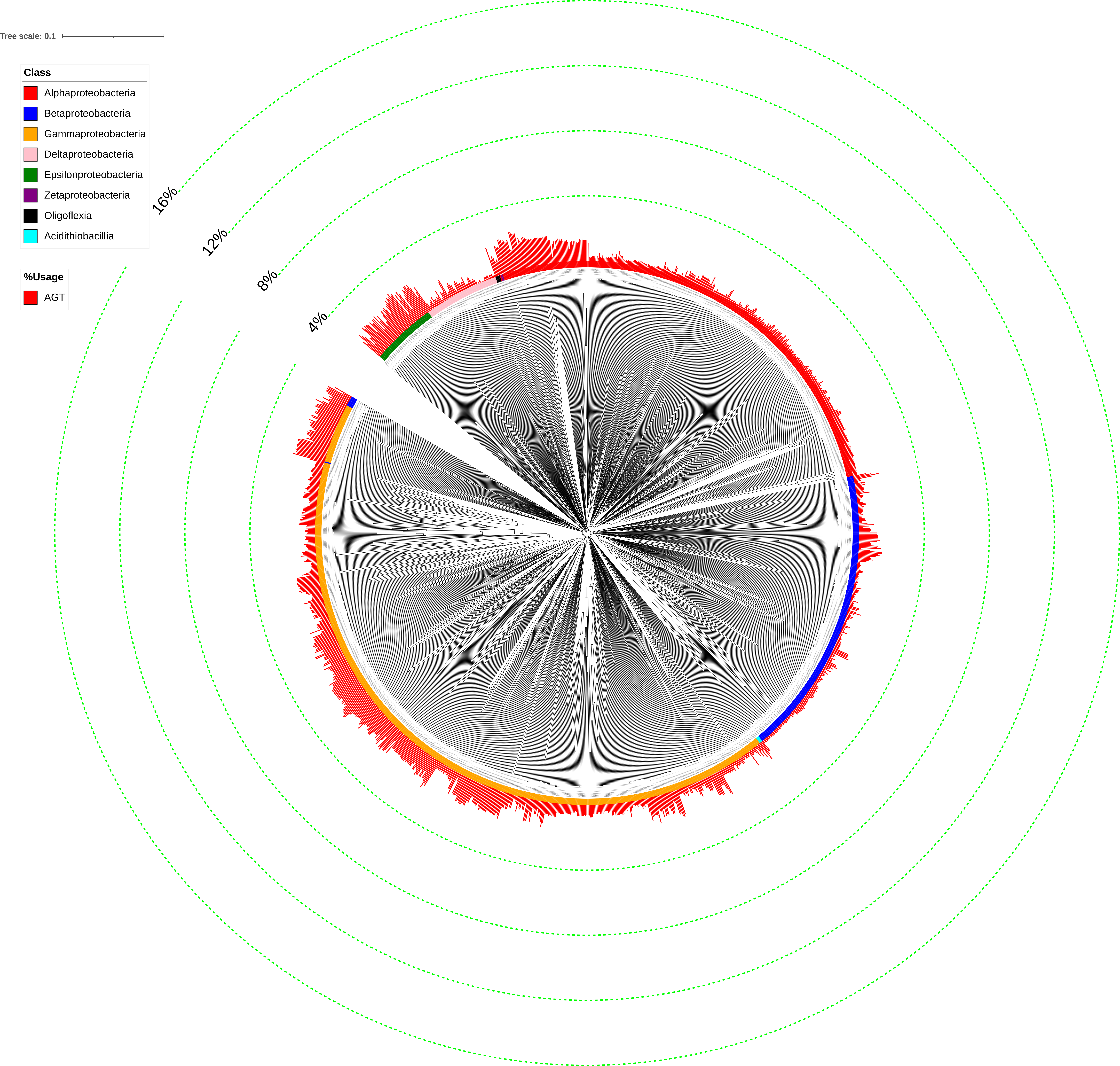
Tree scale: 0.1
Class
Alphaproteobacteria
Betaproteobacteria
Gammaproteobacteria
Deltaproteobacteria
Epsilonproteobacteria
Zetaproteobacteria
Oligoflexia
Acidithiobacillia
%Usage
AGT
16%
12%
8%
4%

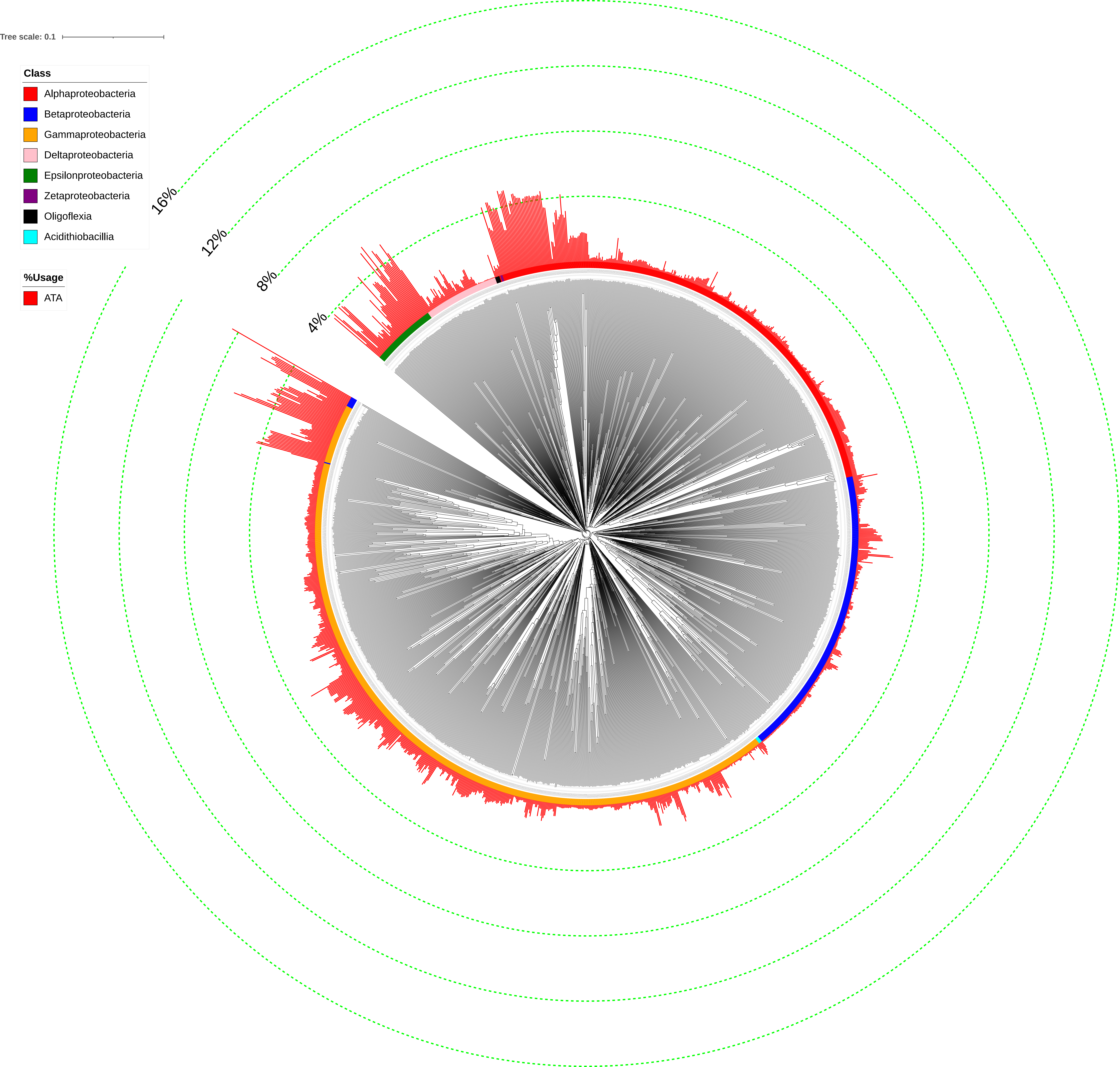

Tree scale: 0.1
Class
Alphaproteobacteria
Betaproteobacteria
Gammaproteobacteria
Deltaproteobacteria
Epsilonproteobacteria
Zetaproteobacteria
Oligoflexia
Acidithiobacillia
%Usage
ATA
4%
8%
12%
16%

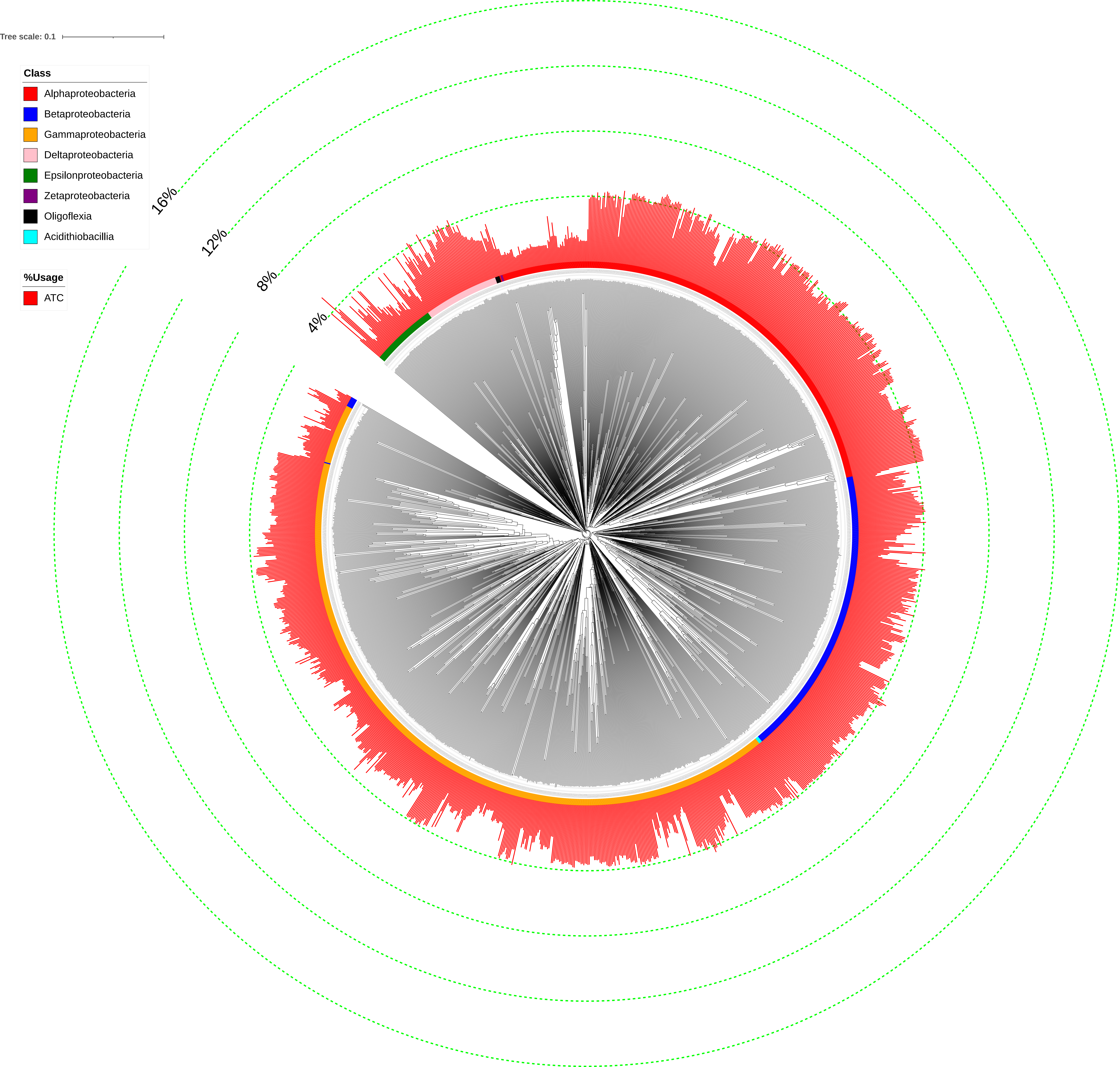

Tree scale: 0.1
Class
Alphaproteobacteria
Betaproteobacteria
Gammaproteobacteria
Deltaproteobacteria
Epsilonproteobacteria
Zetaproteobacteria
Oligoflexia
Acidithiobacillia
%Usage
ATC
4%
8%
12%
16%

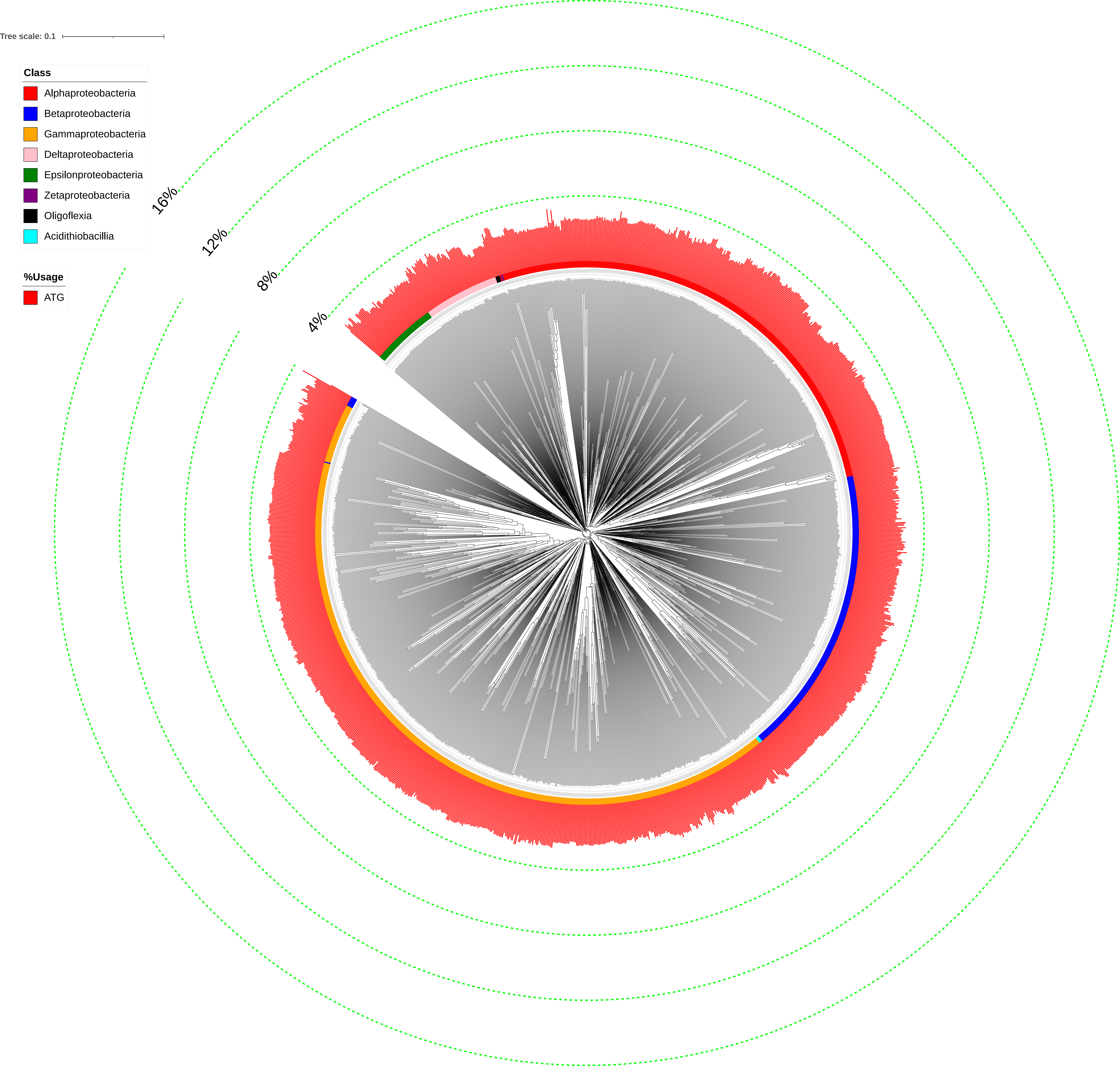

Tree scale: 0.1
Class
Alphaproteobacteria
Betaproteobacteria
Gammaproteobacteria
Deltaproteobacteria
Epsilonproteobacteria
Zetaproteobacteria
Oligoflexia
Acidithiobacillia
%Usage
ATG
4%
8%
12%
16%

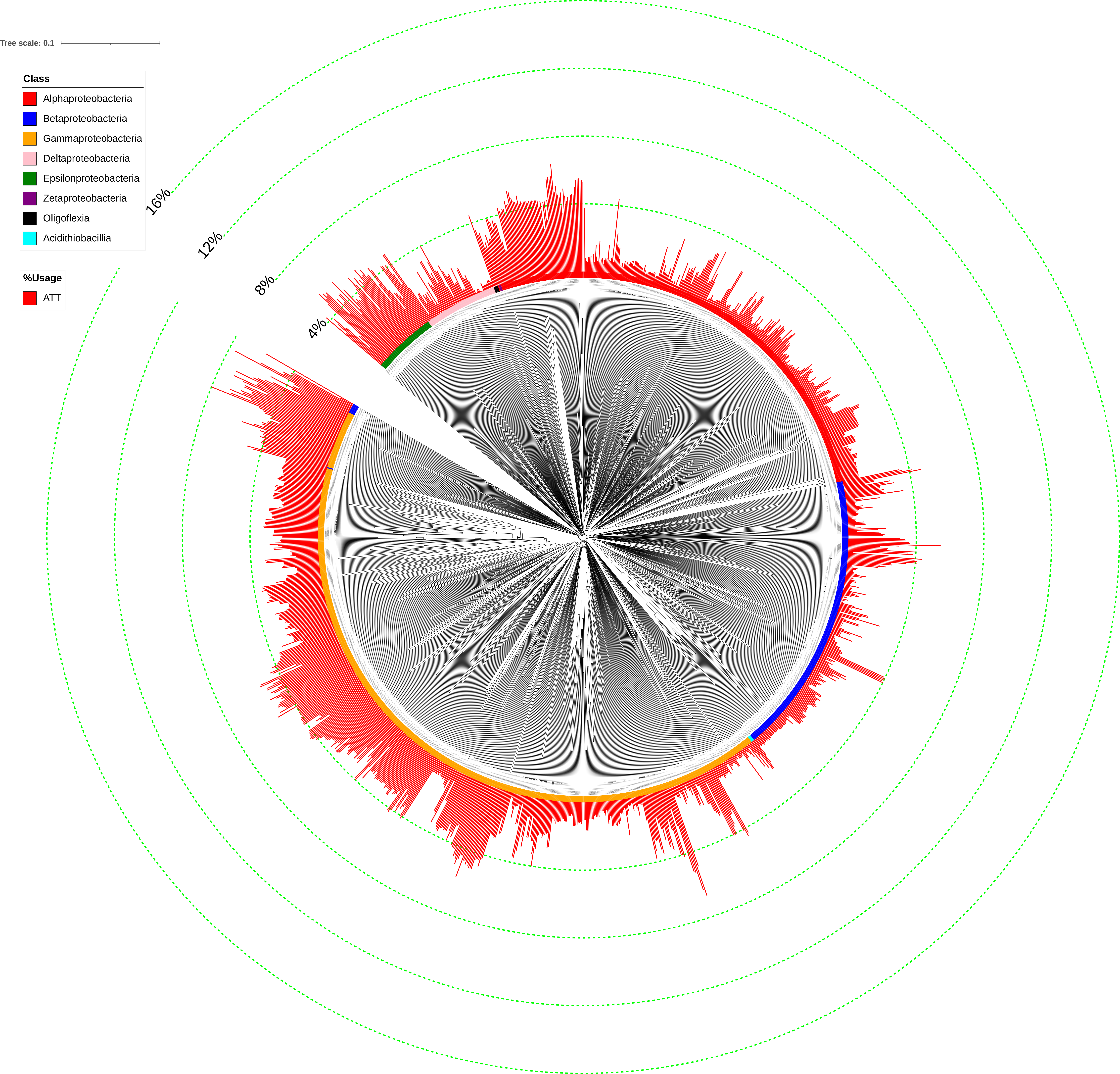

Tree scale: 0.1
Class
Alphaproteobacteria
Betaproteobacteria
Gammaproteobacteria
Deltaproteobacteria
Epsilonproteobacteria
Zetaproteobacteria
Oligoflexia
Acidithiobacillia
%Usage
ATT
16%
12%
8%
4%

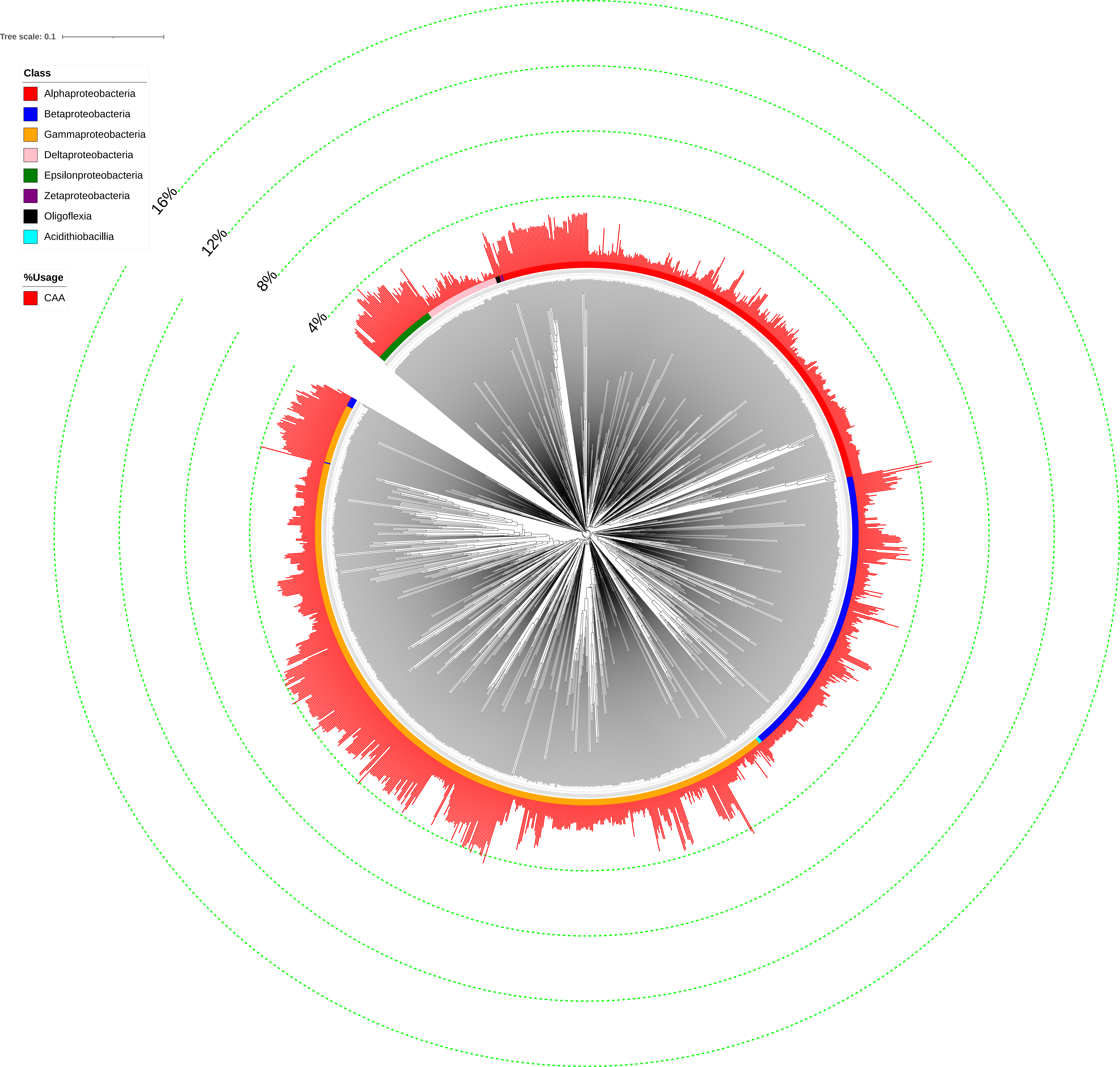

Tree scale: 0.1
Class
Alphaproteobacteria
Betaproteobacteria
Gammaproteobacteria
Deltaproteobacteria
Epsilonproteobacteria
Zetaproteobacteria
Oligoflexia
Acidithiobacillia
%Usage
CAA
4%
8%
12%
16%

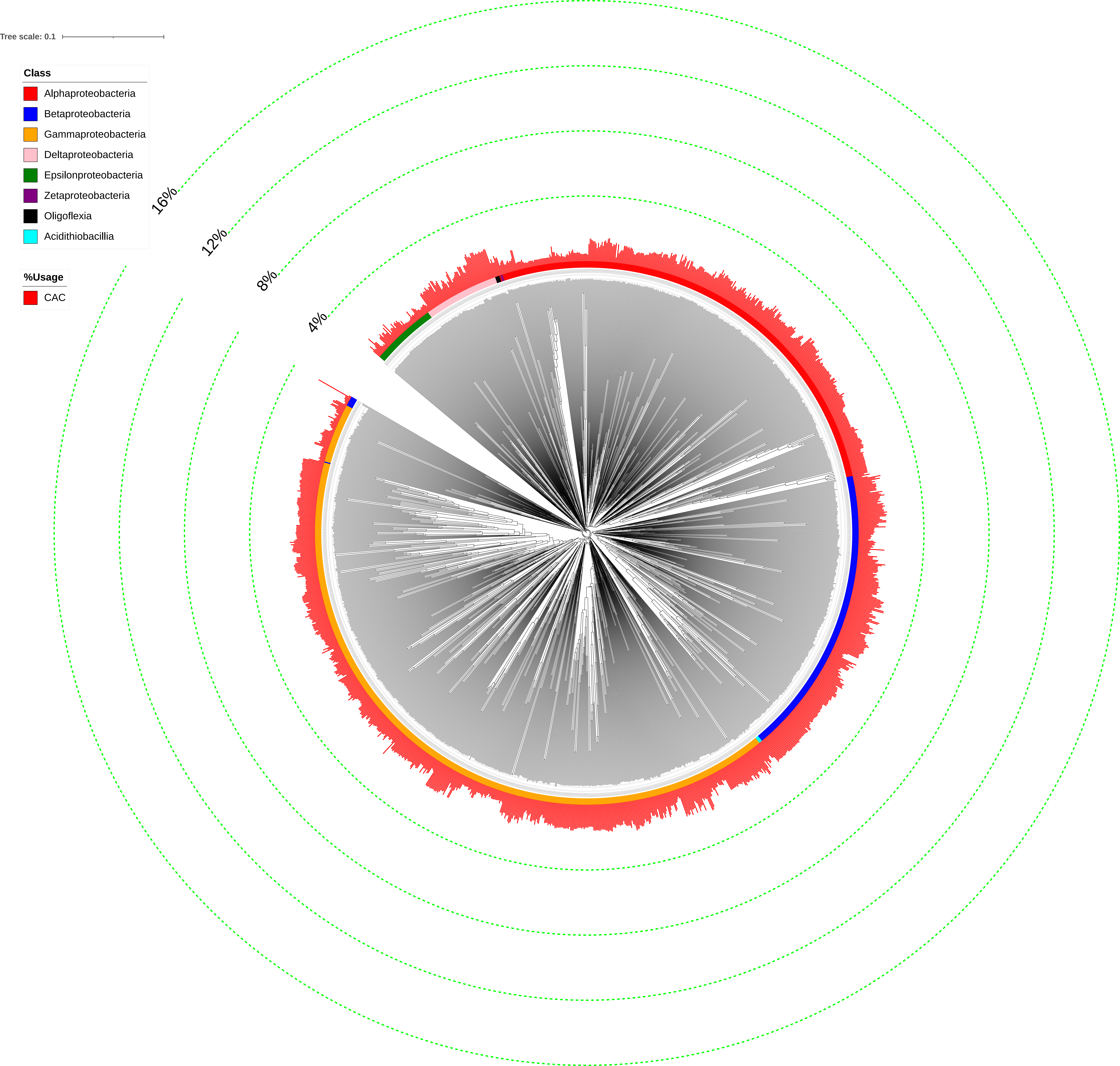

Tree scale: 0.1
Class
Alphaproteobacteria
Betaproteobacteria
Gammaproteobacteria
Deltaproteobacteria
Epsilonproteobacteria
Zetaproteobacteria
Oligoflexia
Acidithiobacillia
%Usage
CAC
4%
8%
12%
16%

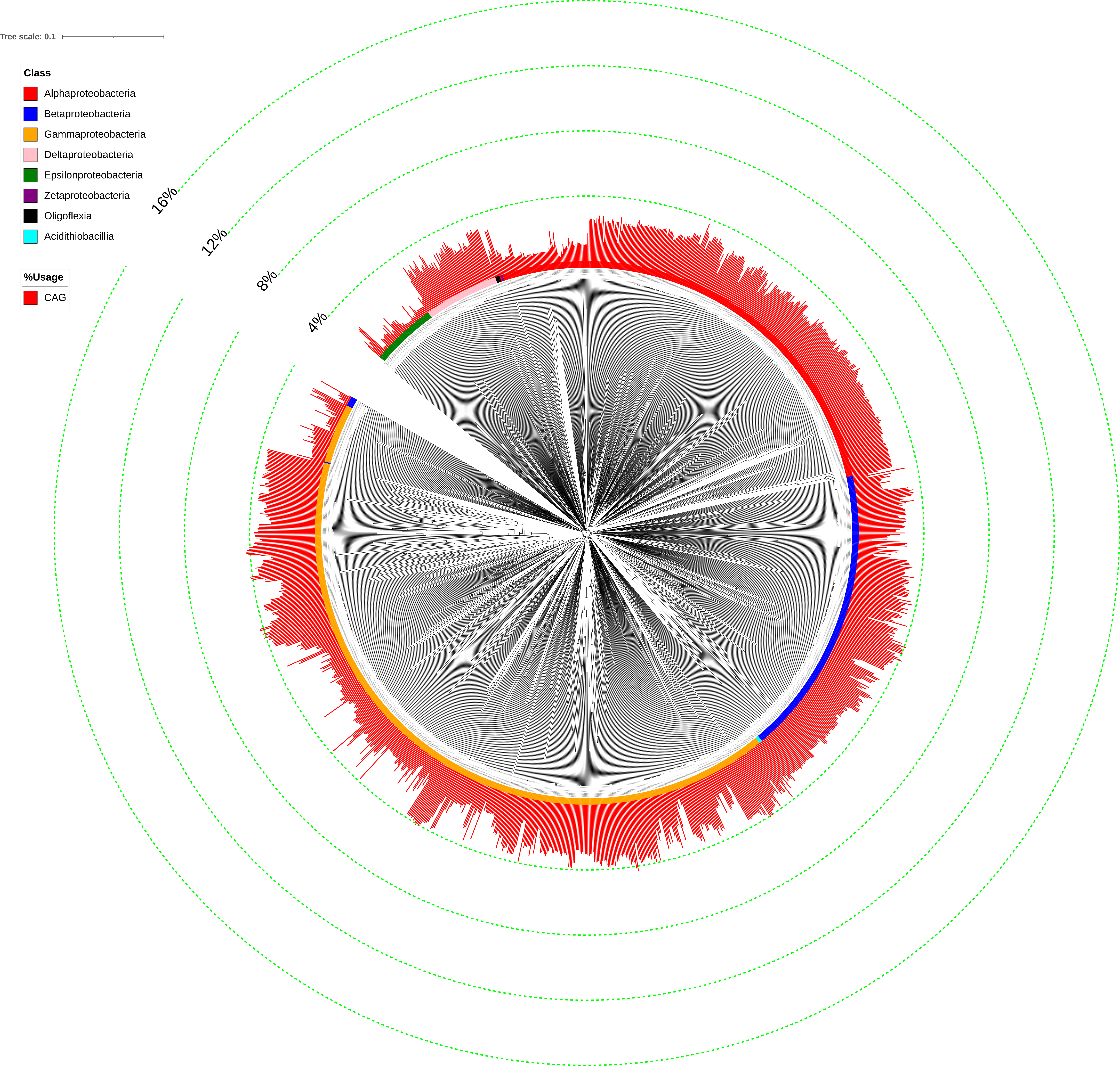
Tree scale: 0.1
Class
Alphaproteobacteria
Betaproteobacteria
Gammaproteobacteria
Deltaproteobacteria
Epsilonproteobacteria
Zetaproteobacteria
Oligoflexia
Acidithiobacillia
%Usage
CAG
4%
8%
12%
16%

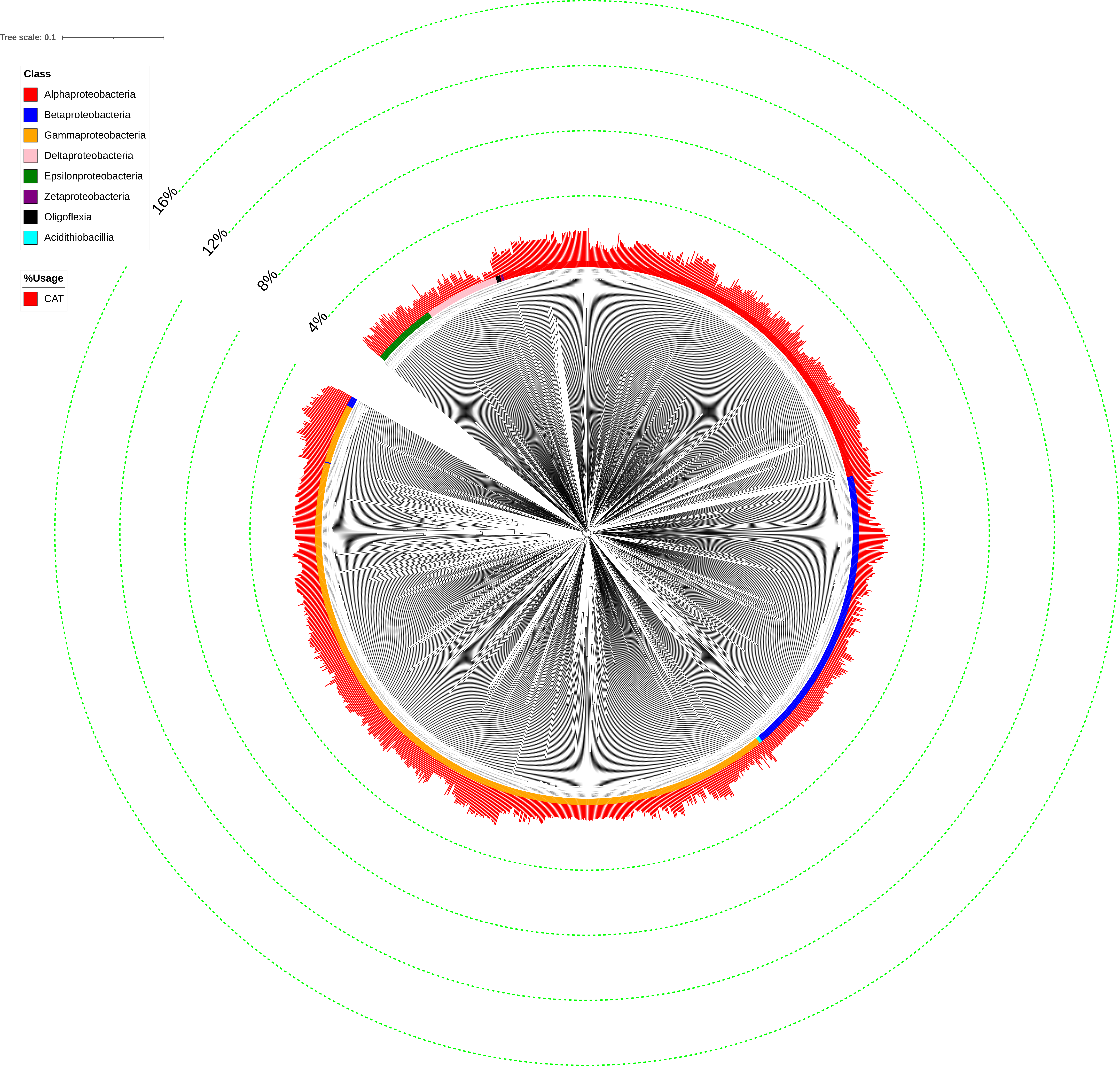
Tree scale: 0.1
Class
Alphaproteobacteria
Betaproteobacteria
Gammaproteobacteria
Deltaproteobacteria
Epsilonproteobacteria
Zetaproteobacteria
Oligoflexia
Acidithiobacillia
%Usage
CAT
16%
12%
8%
4%

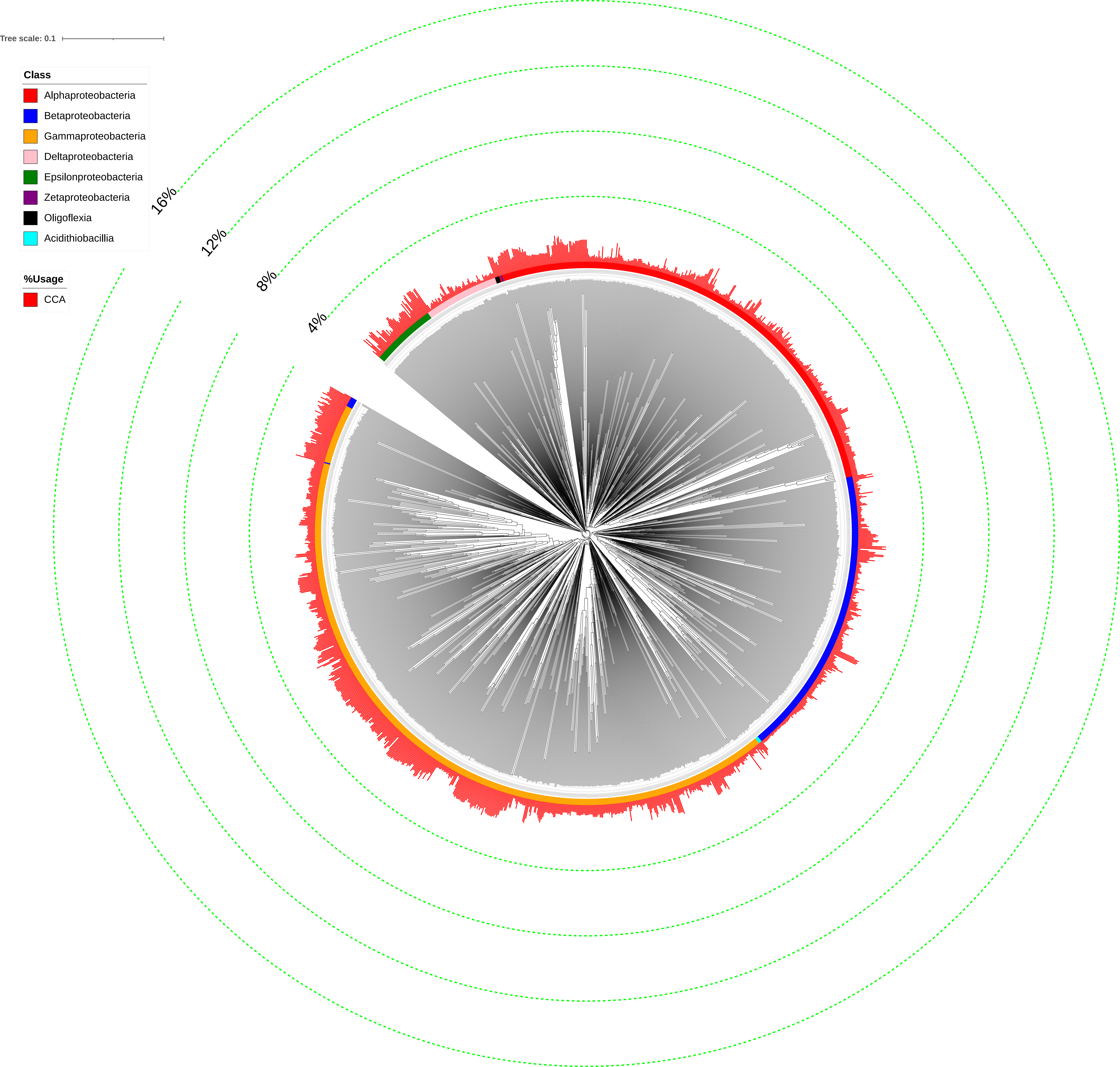

Tree scale: 0.1
Class
Alphaproteobacteria
Betaproteobacteria
Gammaproteobacteria
Deltaproteobacteria
Epsilonproteobacteria
Zetaproteobacteria
Oligoflexia
Acidithiobacillia
%Usage
CCA
4%
8%
12%
16%

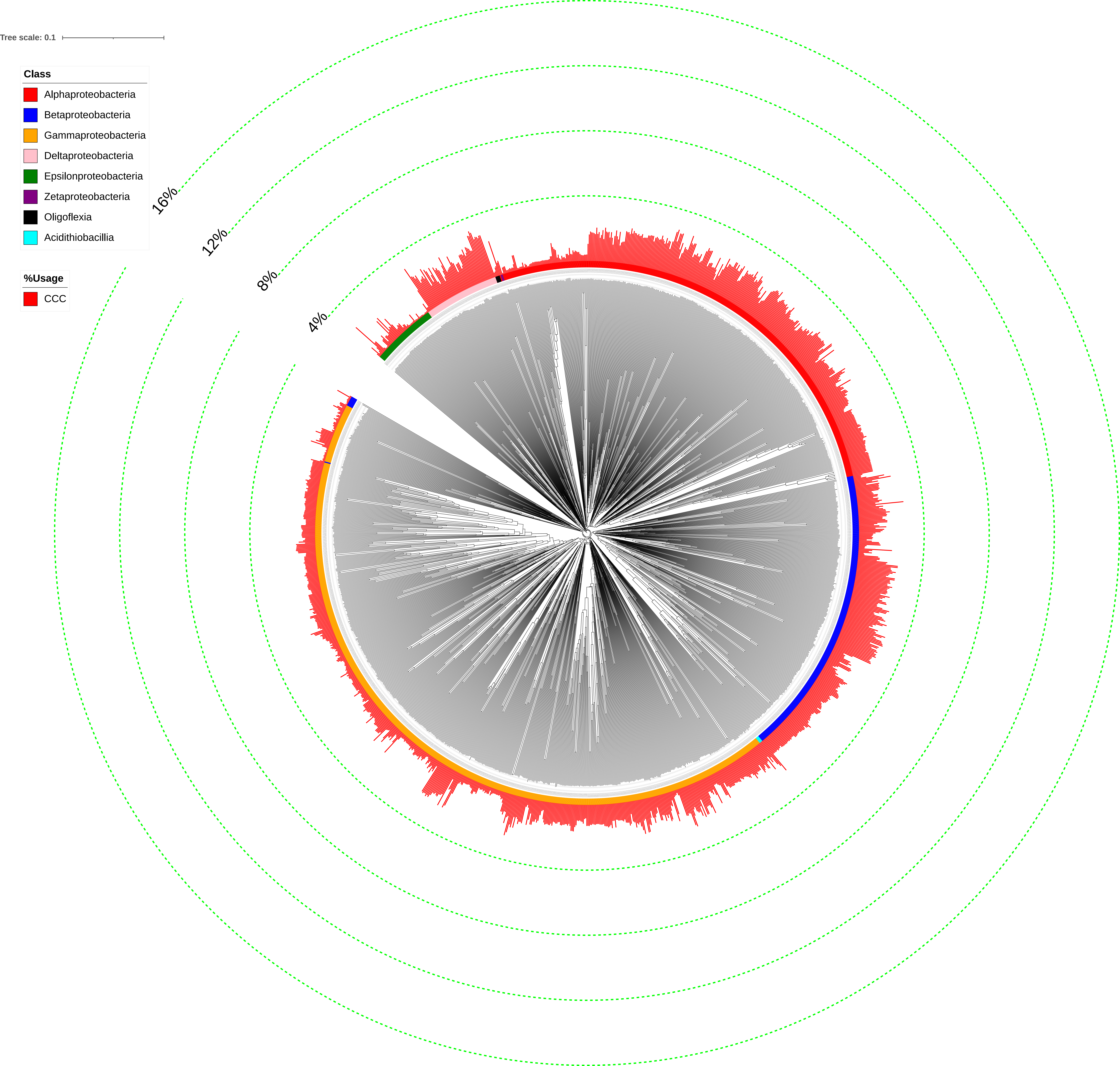

Tree scale: 0.1
Class
Alphaproteobacteria
Betaproteobacteria
Gammaproteobacteria
Deltaproteobacteria
Epsilonproteobacteria
Zetaproteobacteria
Oligoflexia
Acidithiobacillia
%Usage
CCC
4%
8%
12%
16%

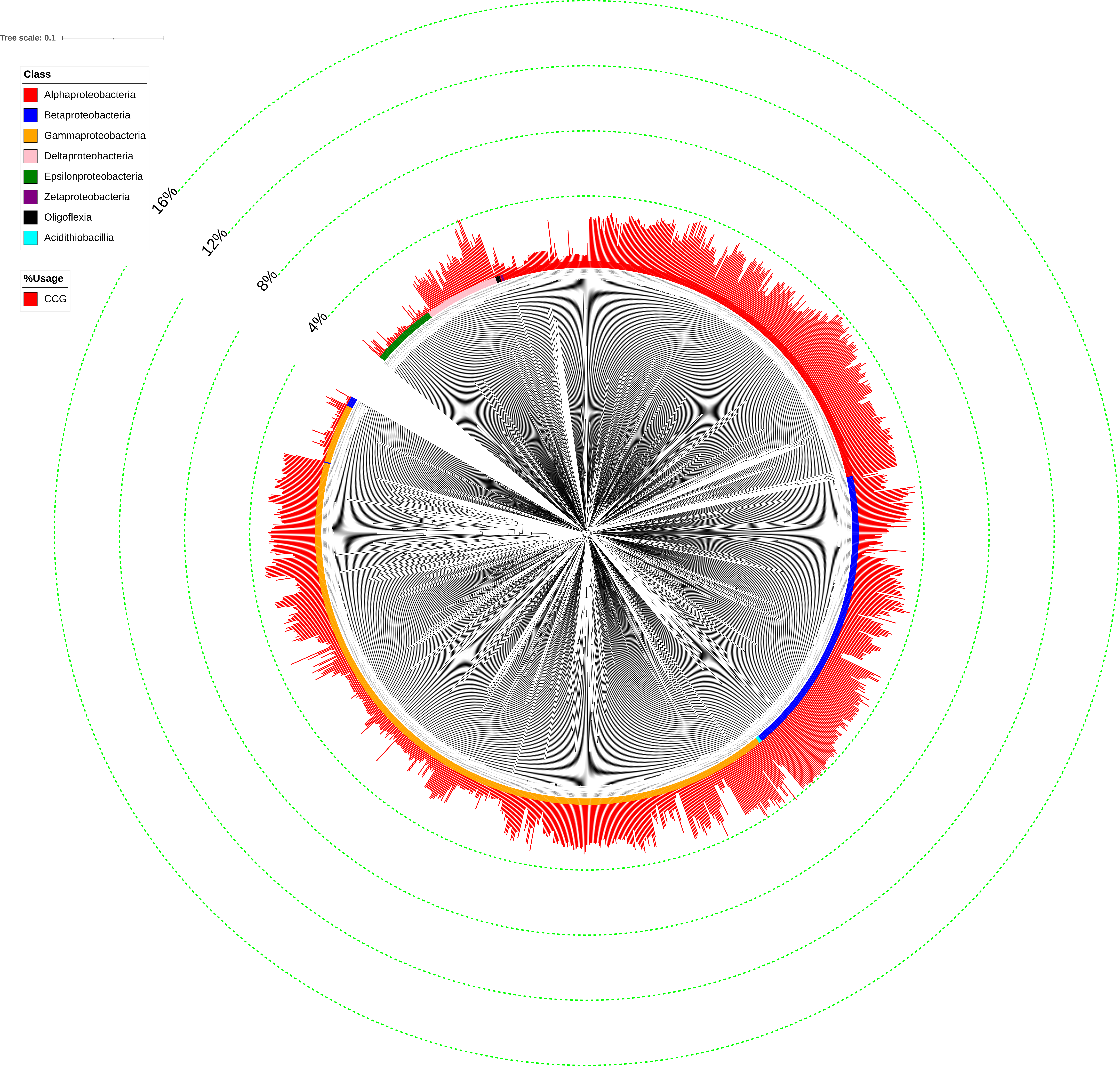
Tree scale: 0.1
Class
Alphaproteobacteria
Betaproteobacteria
Gammaproteobacteria
Deltaproteobacteria
Epsilonproteobacteria
Zetaproteobacteria
Oligoflexia
Acidithiobacillia
%Usage
CCG
16%
12%
8%
4%

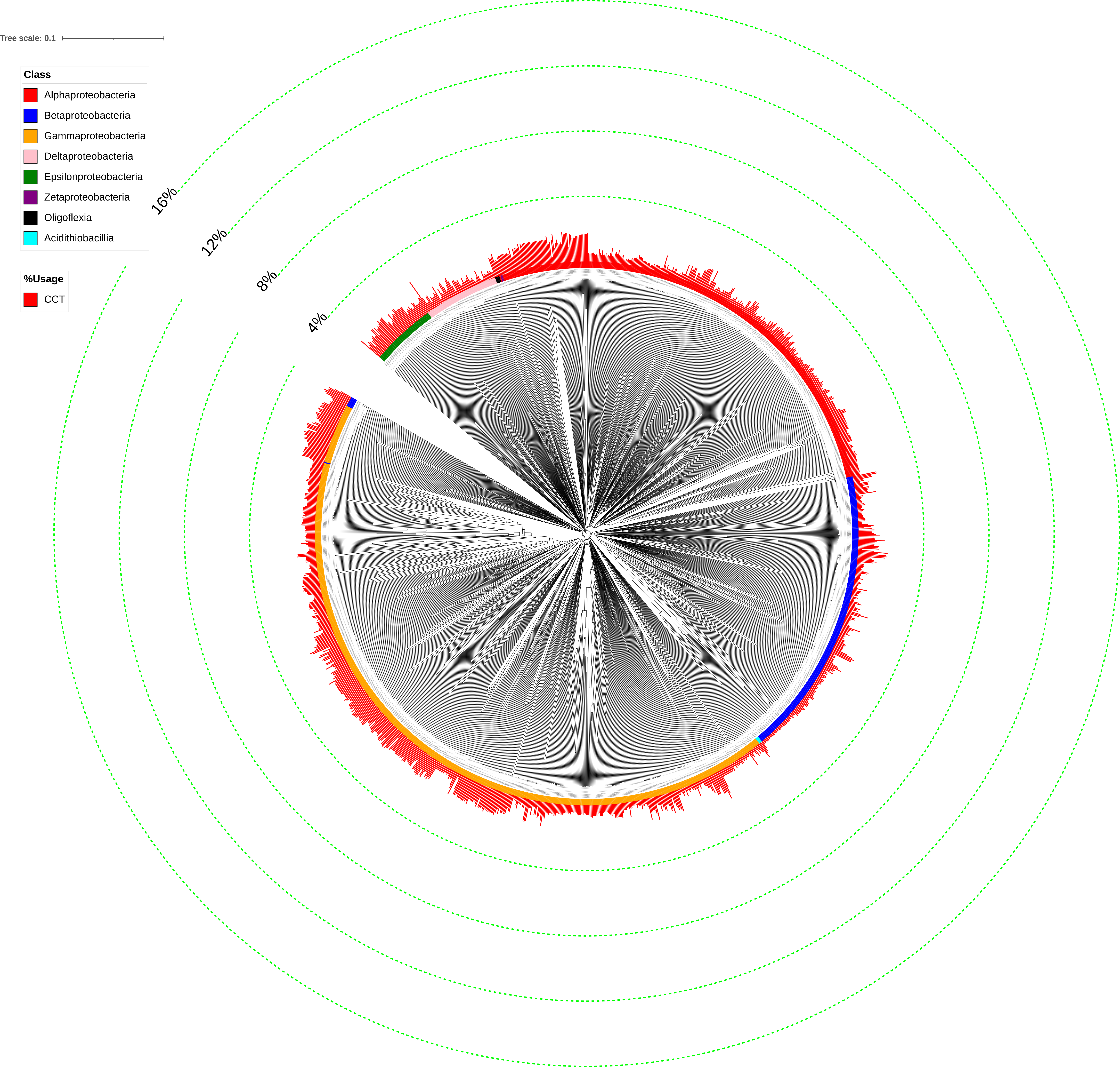

Tree scale: 0.1
Class
Alphaproteobacteria
Betaproteobacteria
Gammaproteobacteria
Deltaproteobacteria
Epsilonproteobacteria
Zetaproteobacteria
Oligoflexia
Acidithiobacillia
%Usage
CCT
16%
12%
8%
4%

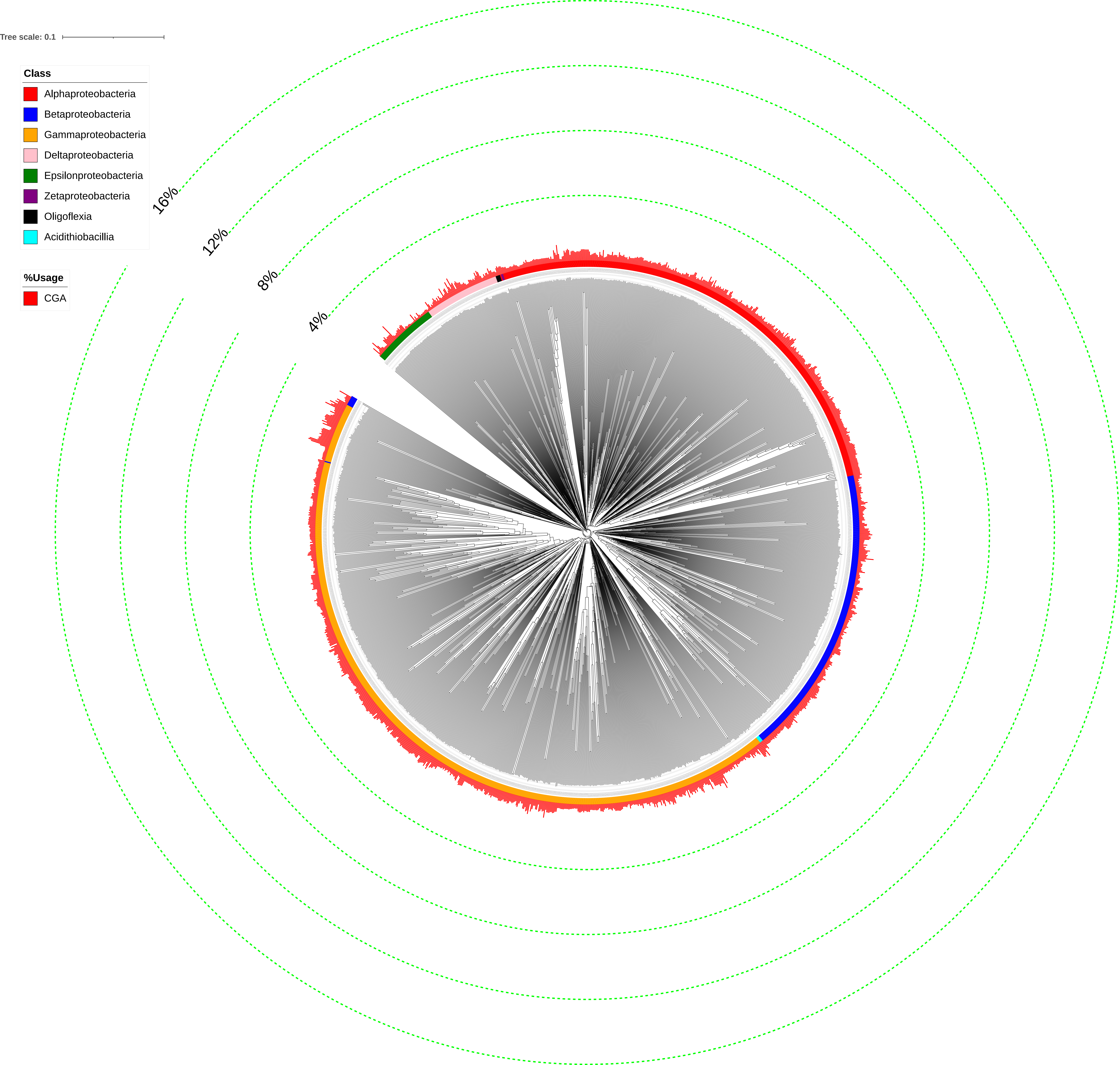

Tree scale: 0.1
Class
Alphaproteobacteria
Betaproteobacteria
Gammaproteobacteria
Deltaproteobacteria
Epsilonproteobacteria
Zetaproteobacteria
Oligoflexia
Acidithiobacillia
%Usage
CGA
16%
12%
8%
4%

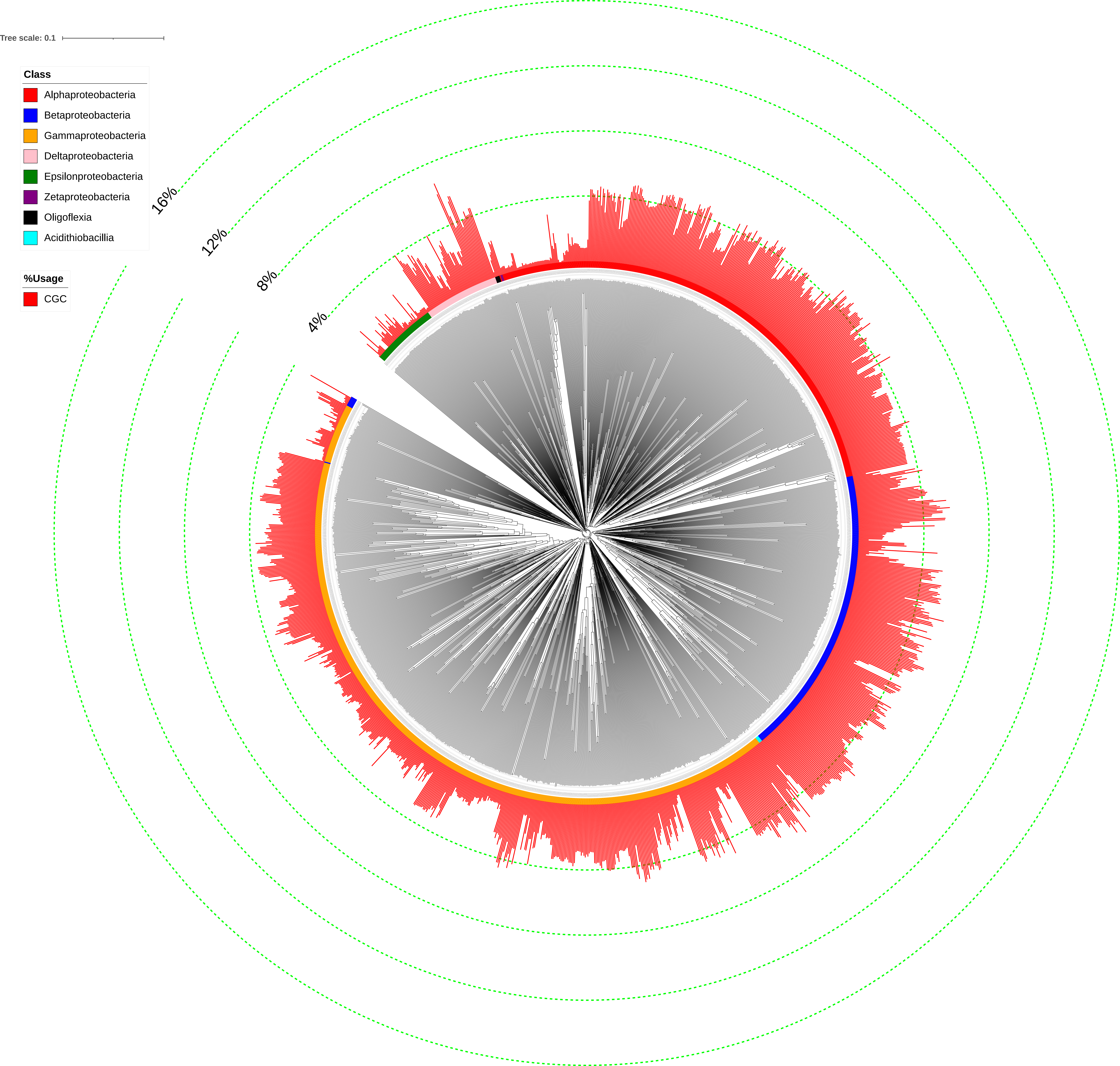
Tree scale: 0.1
Class
Alphaproteobacteria
Betaproteobacteria
Gammaproteobacteria
Deltaproteobacteria
Epsilonproteobacteria
Zetaproteobacteria
Oligoflexia
Acidithiobacillia
%Usage
CGC
16%
12%
8%
4%

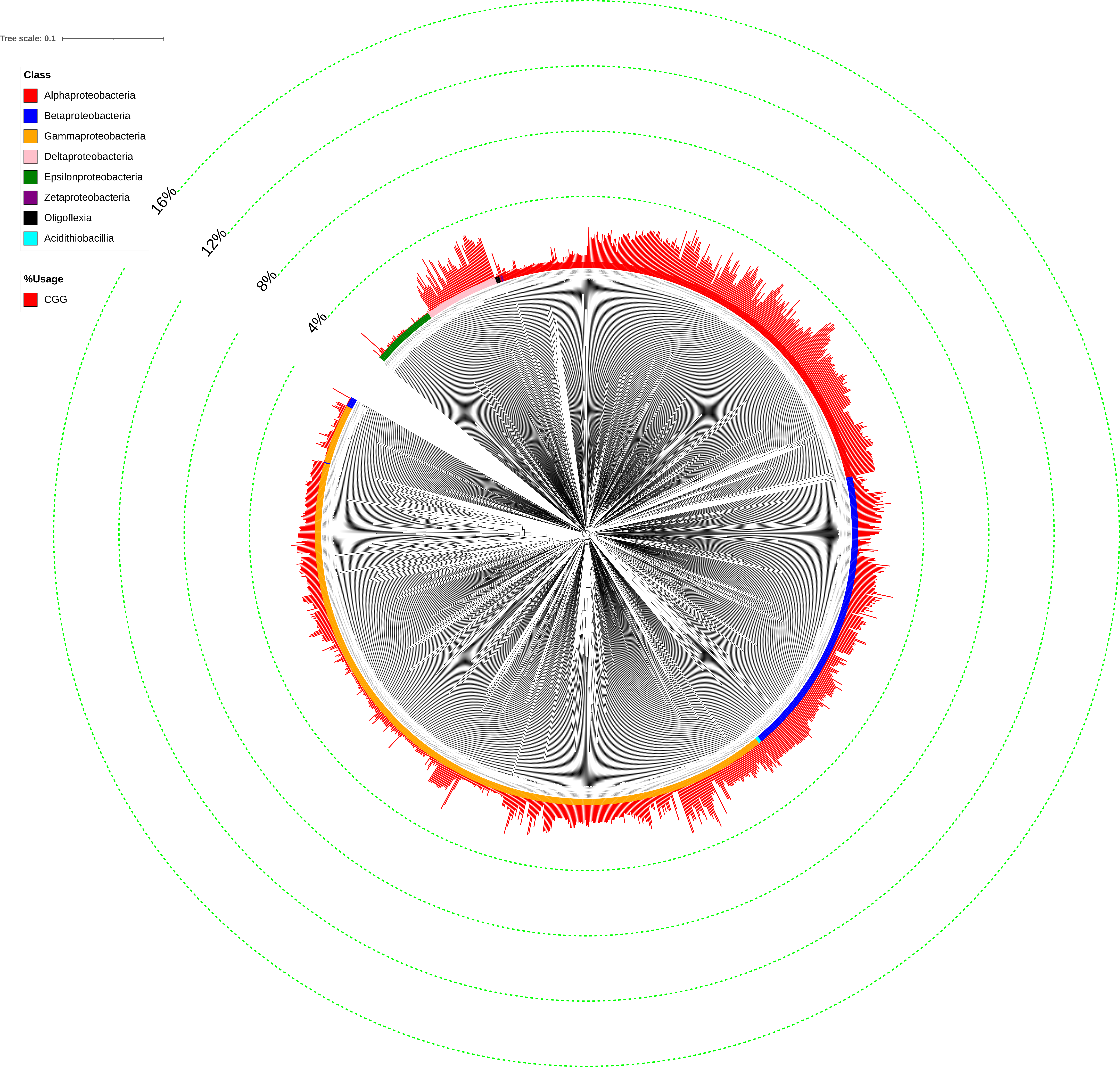

Tree scale: 0.1
Class
Alphaproteobacteria
Betaproteobacteria
Gammaproteobacteria
Deltaproteobacteria
Epsilonproteobacteria
Zetaproteobacteria
Oligoflexia
Acidithiobacillia
%Usage
CGG
16%
12%
8%
4%

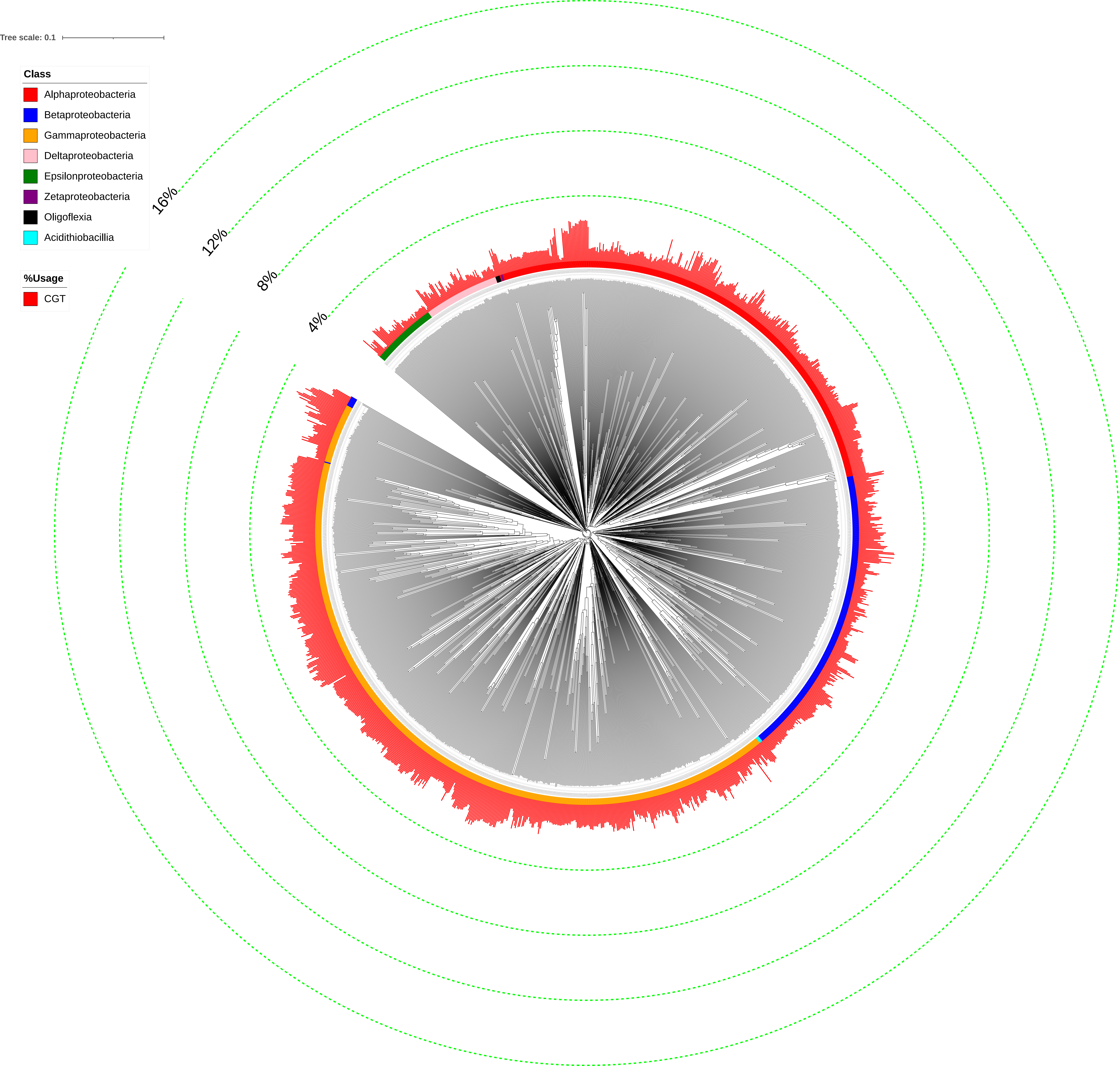
Tree scale: 0.1
Class
Alphaproteobacteria
Betaproteobacteria
Gammaproteobacteria
Deltaproteobacteria
Epsilonproteobacteria
Zetaproteobacteria
Oligoflexia
Acidithiobacillia
%Usage
CGT
4%
8%
12%
16%

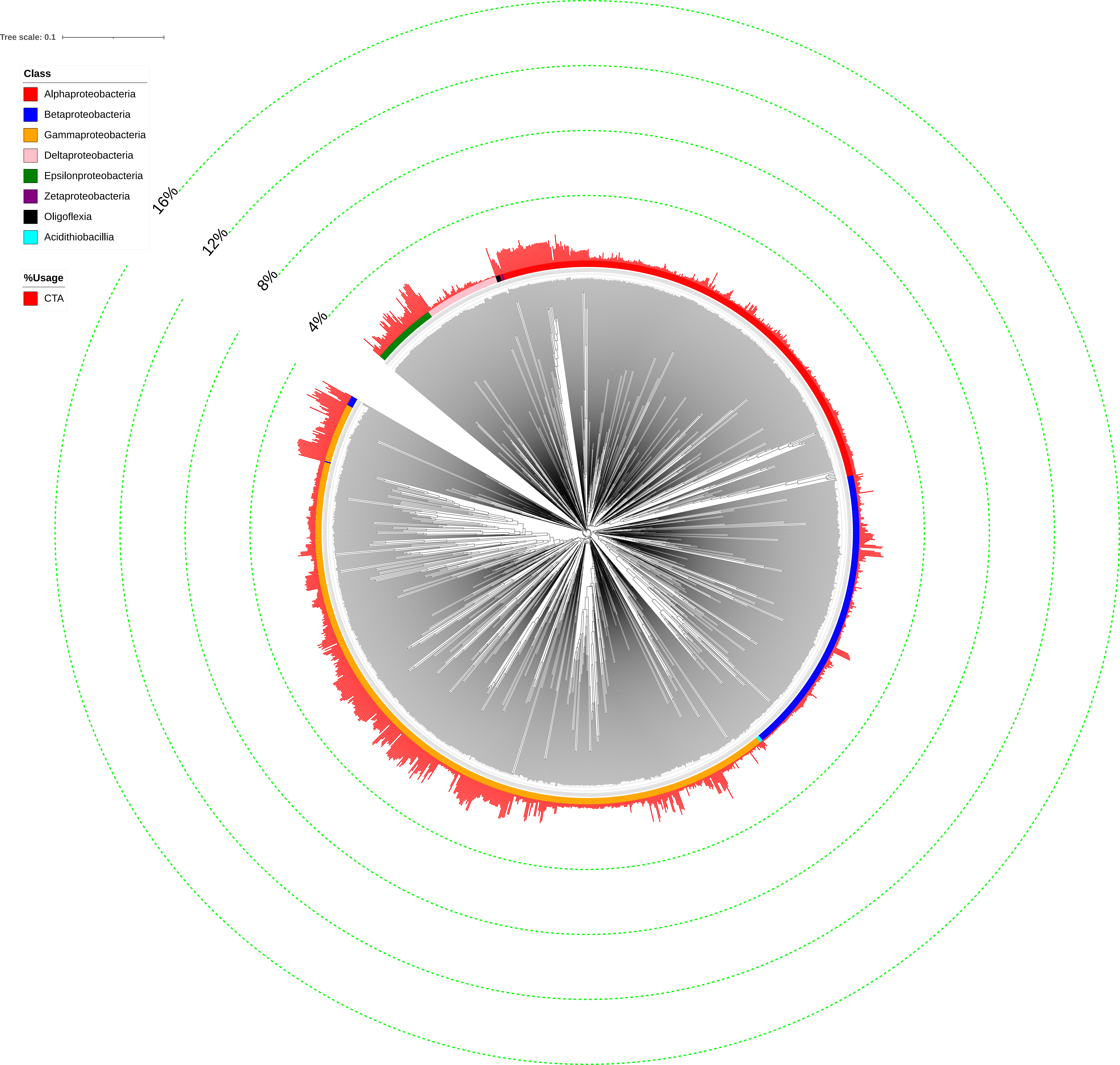
Tree scale: 0.1
Class
Alphaproteobacteria
Betaproteobacteria
Gammaproteobacteria
Deltaproteobacteria
Epsilonproteobacteria
Zetaproteobacteria
Oligoflexia
Acidithiobacillia
%Usage
CTA
4%
8%
12%
16%

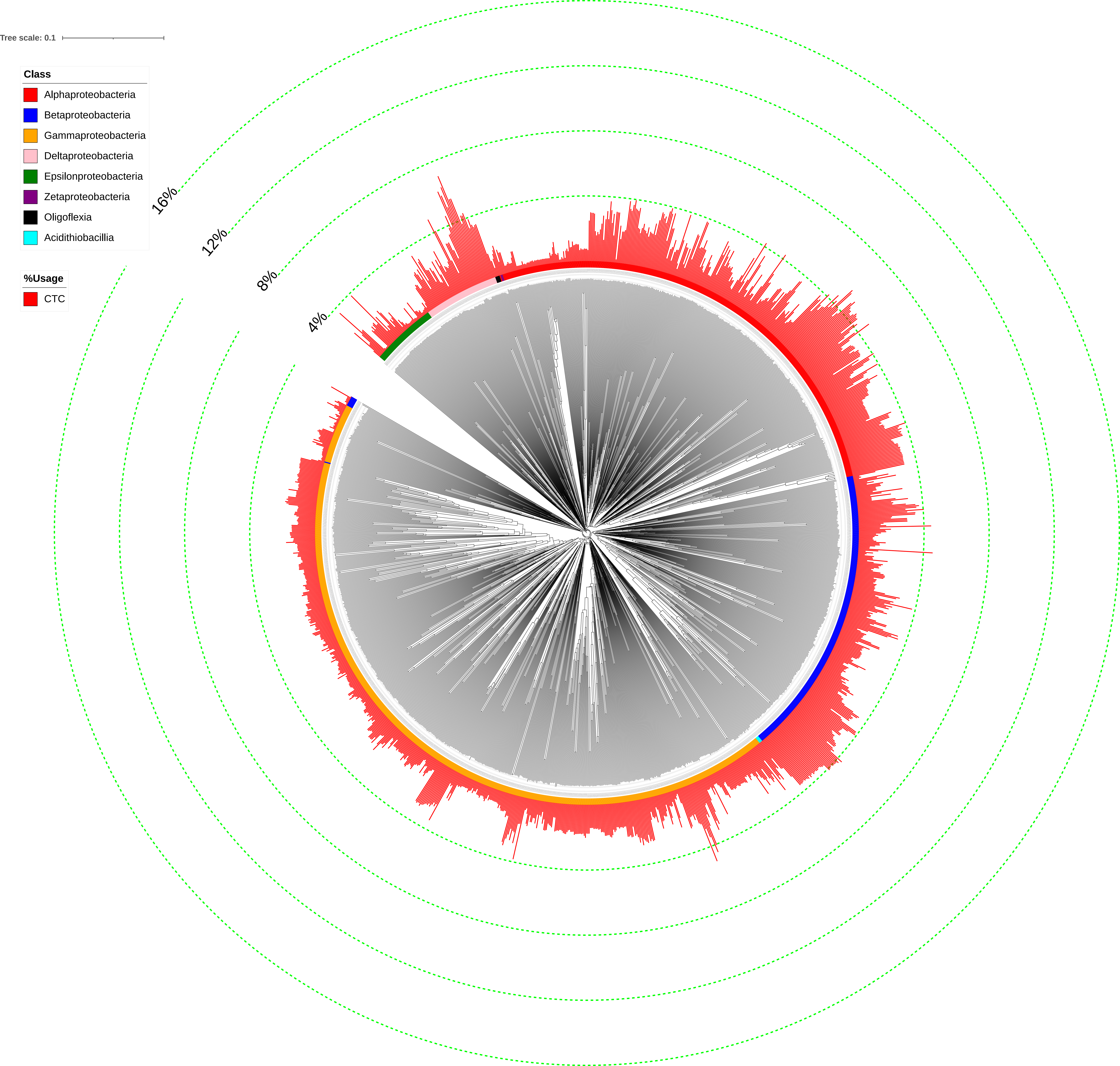
Tree scale: 0.1
Class
Alphaproteobacteria
Betaproteobacteria
Gammaproteobacteria
Deltaproteobacteria
Epsilonproteobacteria
Zetaproteobacteria
Oligoflexia
Acidithiobacillia
%Usage
CTC
4%
8%
12%
16%

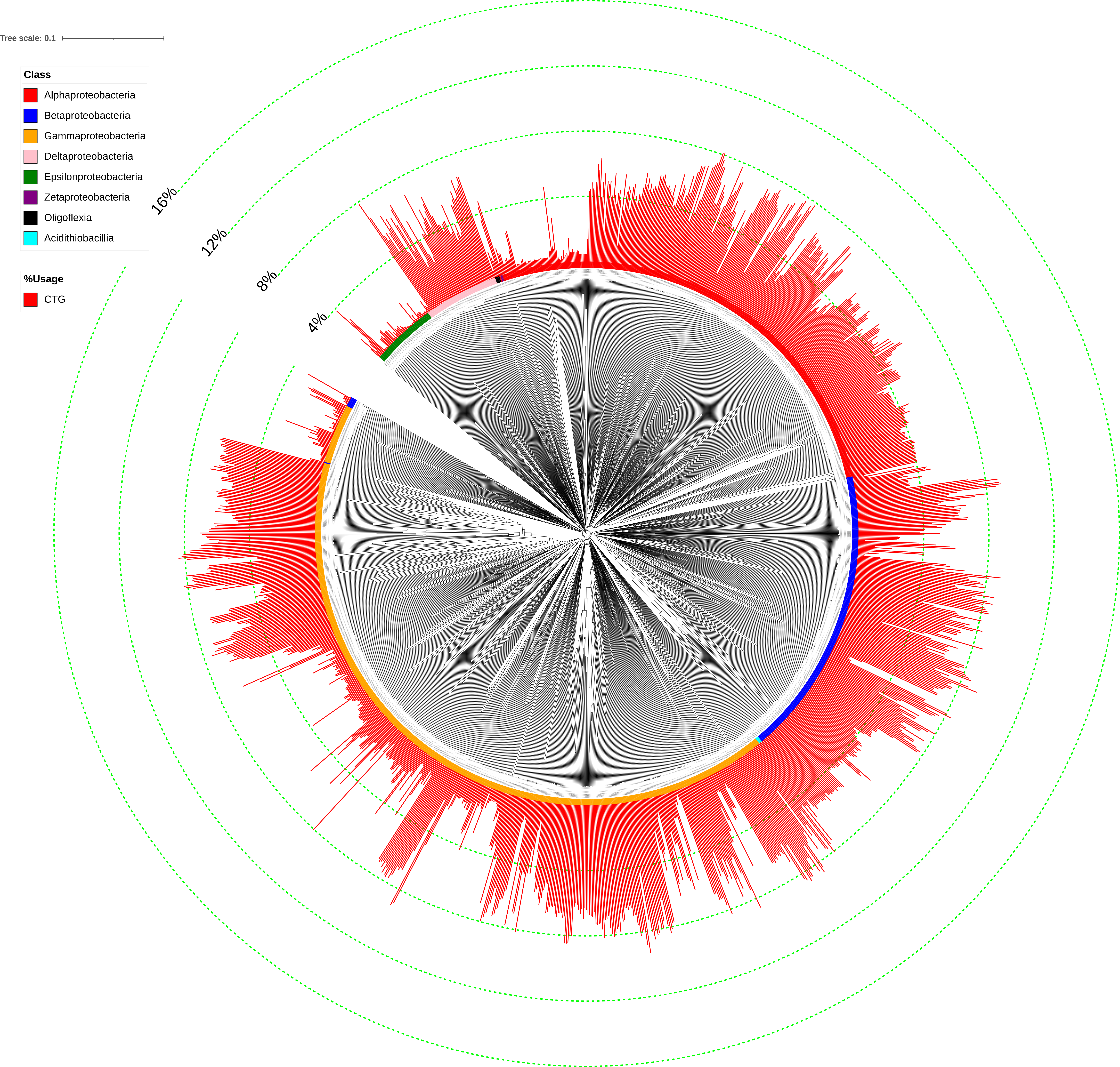

Tree scale: 0.1
Class
Alphaproteobacteria
Betaproteobacteria
Gammaproteobacteria
Deltaproteobacteria
Epsilonproteobacteria
Zetaproteobacteria
Oligoflexia
Acidithiobacillia
%Usage
CTG
4%
8%
12%
16%

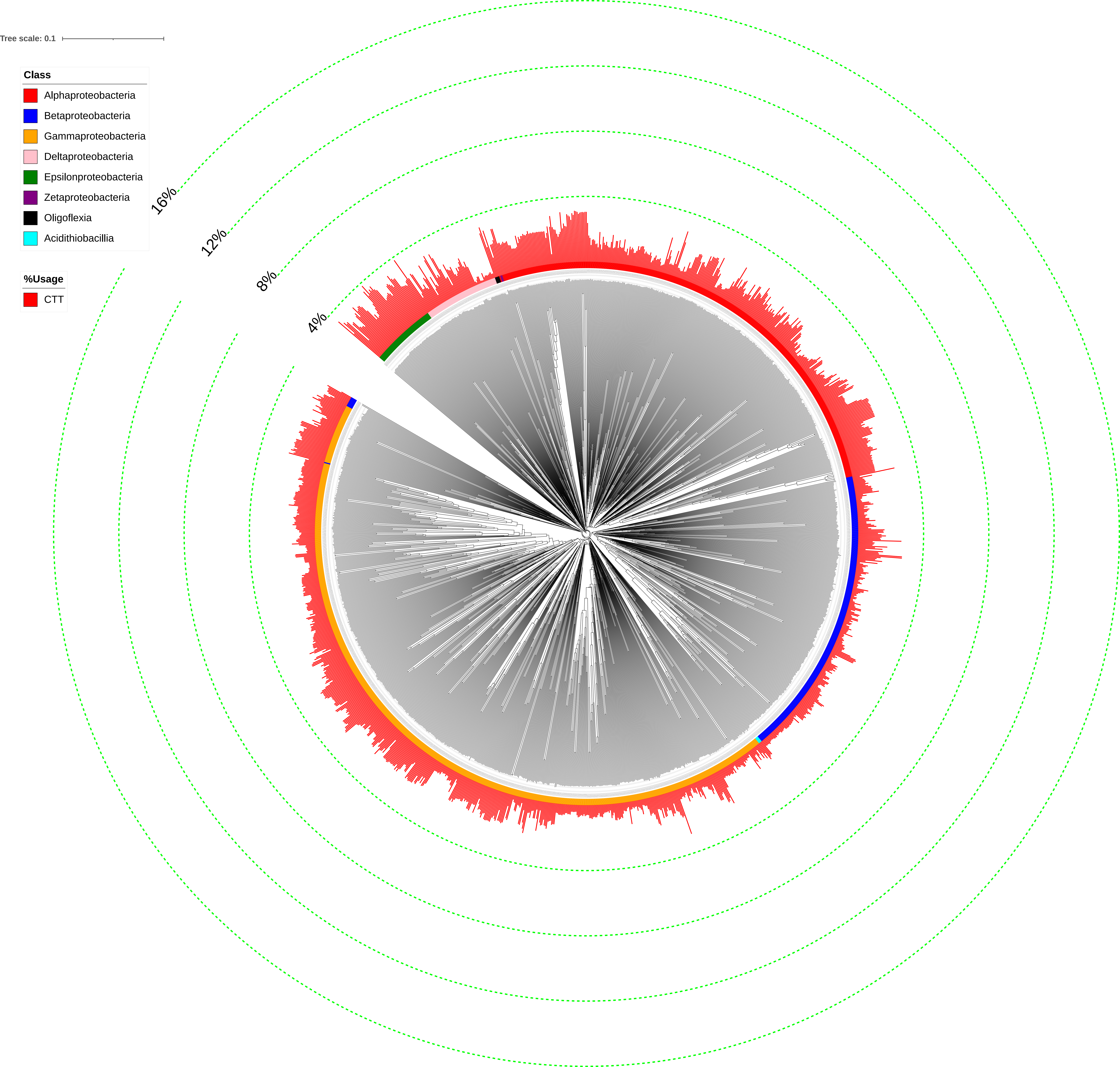

Tree scale: 0.1
Class
Alphaproteobacteria
Betaproteobacteria
Gammaproteobacteria
Deltaproteobacteria
Epsilonproteobacteria
Zetaproteobacteria
Oligoflexia
Acidithiobacillia
%Usage
CTT
16%
12%
8%
4%

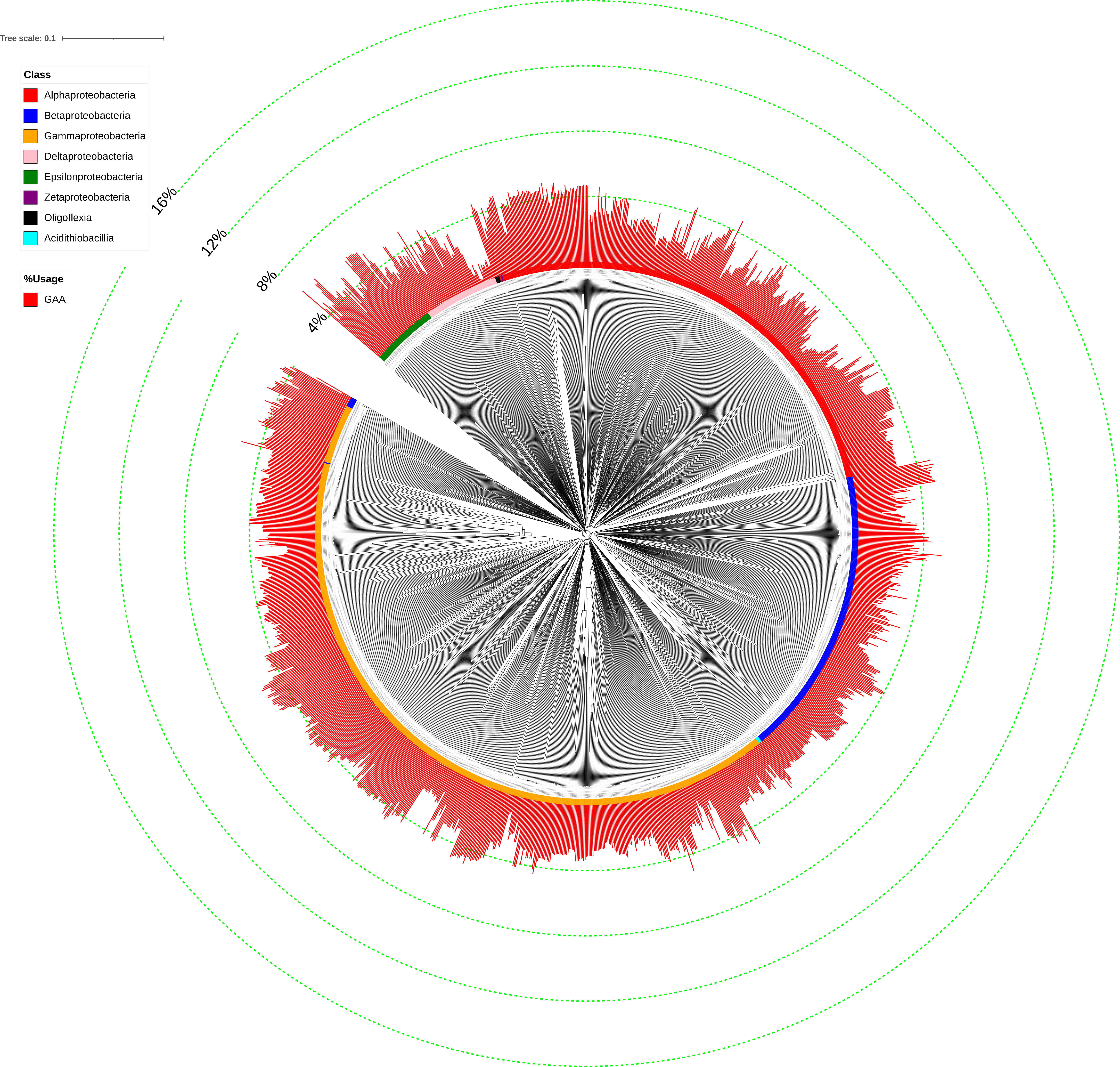

Tree scale: 0.1
Class
Alphaproteobacteria
Betaproteobacteria
Gammaproteobacteria
Deltaproteobacteria
Epsilonproteobacteria
Zetaproteobacteria
Oligoflexia
Acidithiobacillia
%Usage
GAA
4%
8%
12%
16%

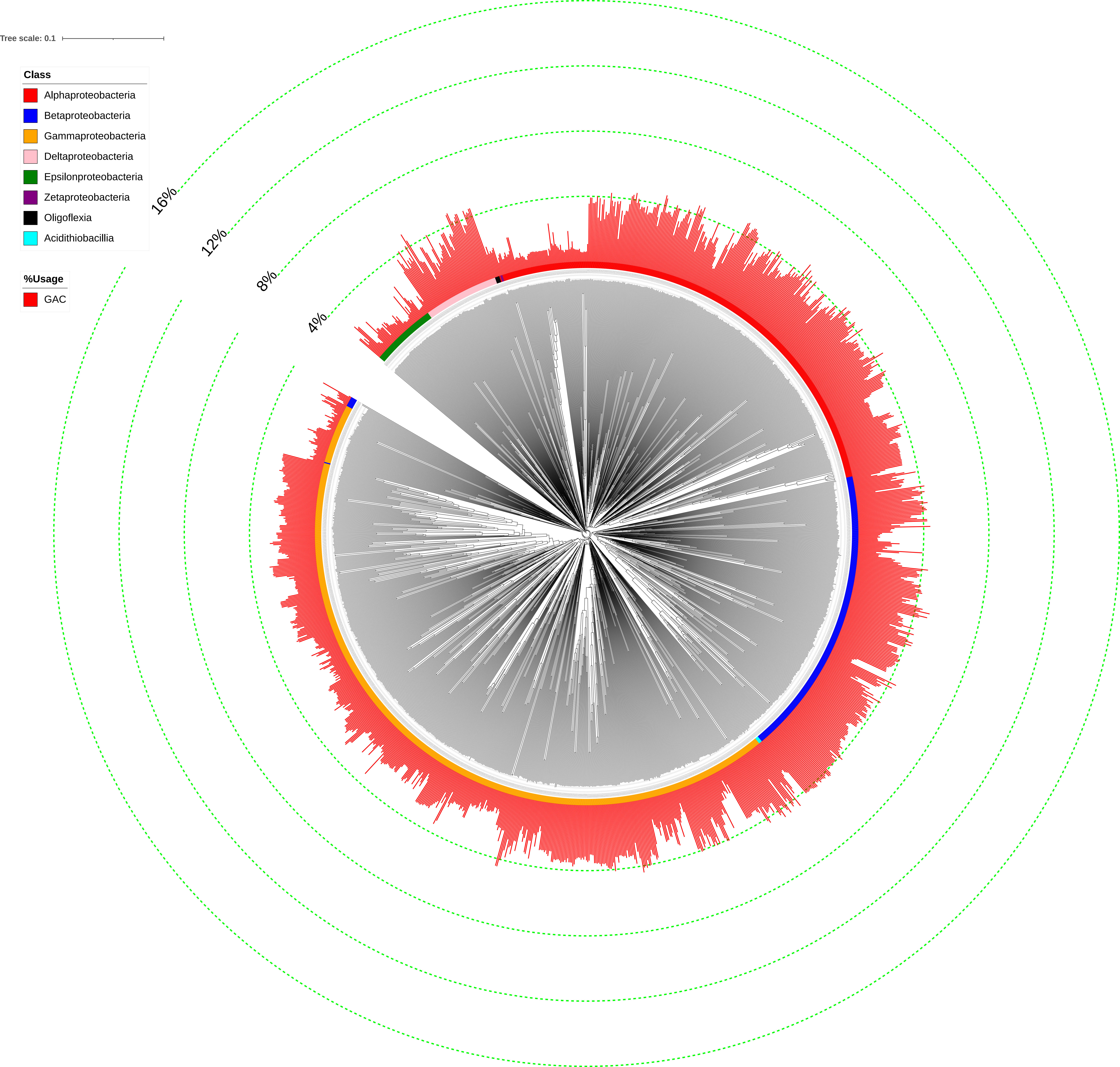
Tree scale: 0.1
Class
Alphaproteobacteria
Betaproteobacteria
Gammaproteobacteria
Deltaproteobacteria
Epsilonproteobacteria
Zetaproteobacteria
Oligoflexia
Acidithiobacillia
%Usage
GAC
16%
12%
8%
4%

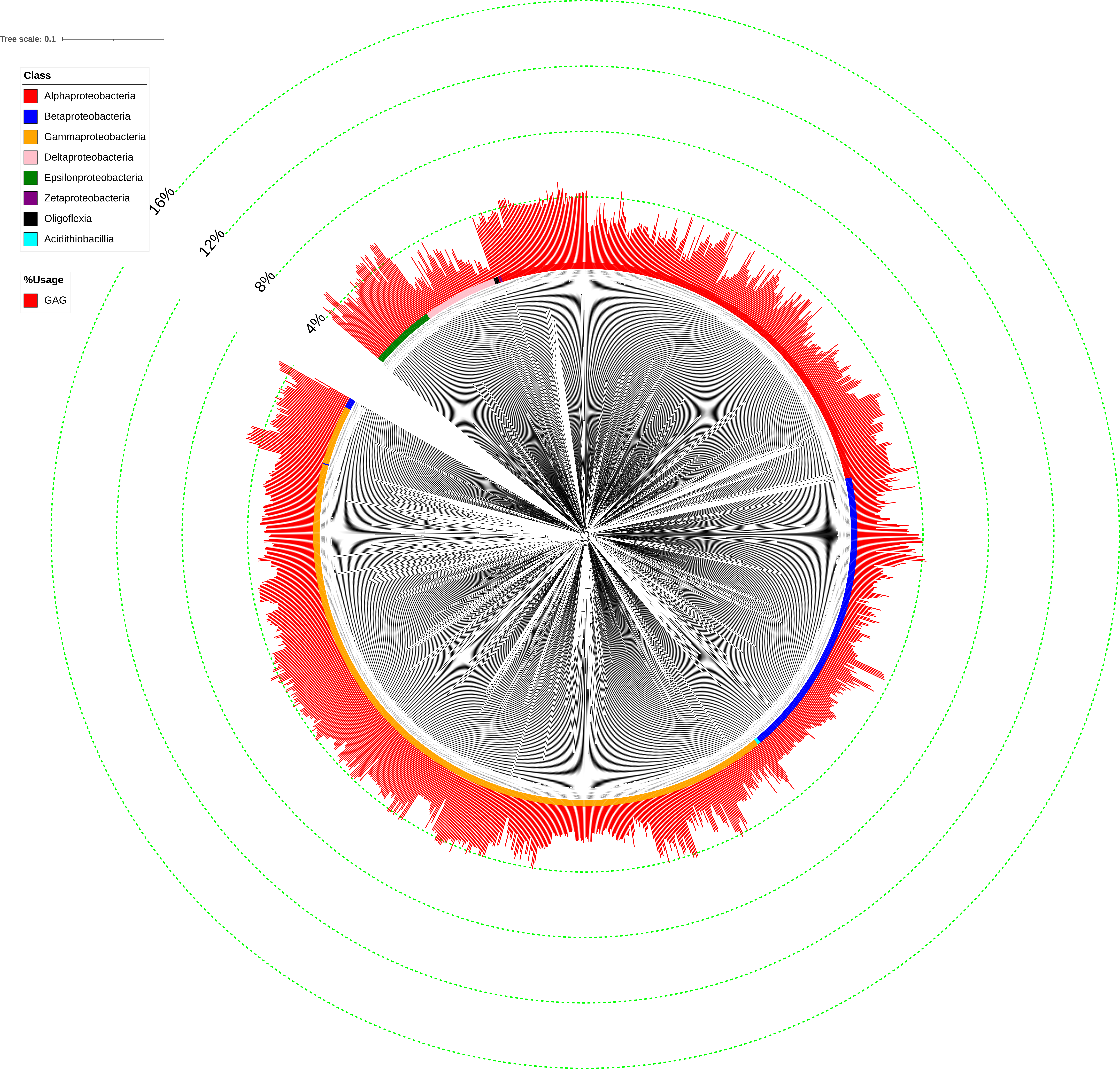

Tree scale: 0.1
Class
Alphaproteobacteria
Betaproteobacteria
Gammaproteobacteria
Deltaproteobacteria
Epsilonproteobacteria
Zetaproteobacteria
Oligoflexia
Acidithiobacillia
%Usage
GAG
4%
8%
12%
16%

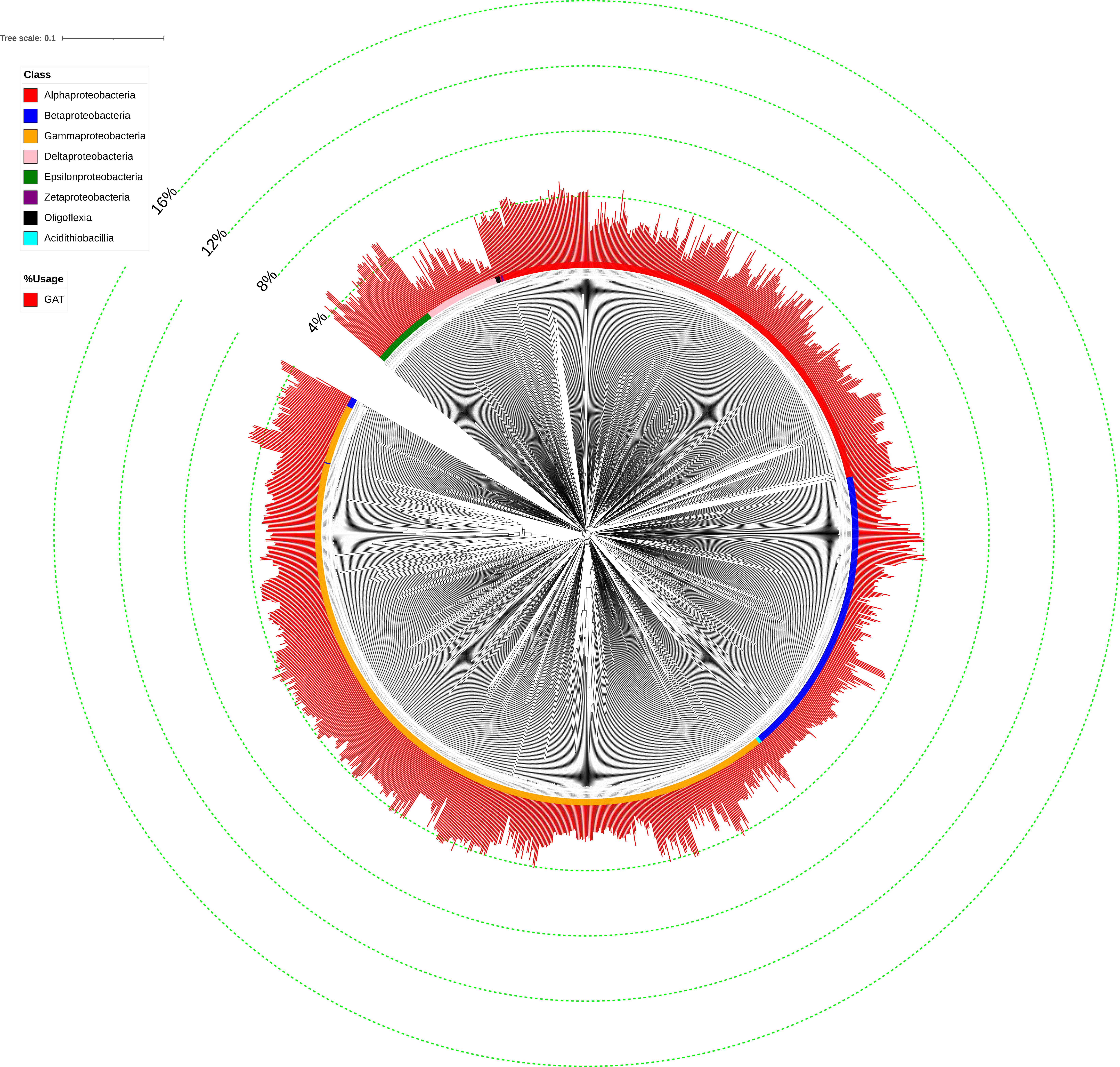
Tree scale: 0.1
Class
Alphaproteobacteria
Betaproteobacteria
Gammaproteobacteria
Deltaproteobacteria
Epsilonproteobacteria
Zetaproteobacteria
Oligoflexia
Acidithiobacillia
%Usage
GAT
16%
12%
8%
4%

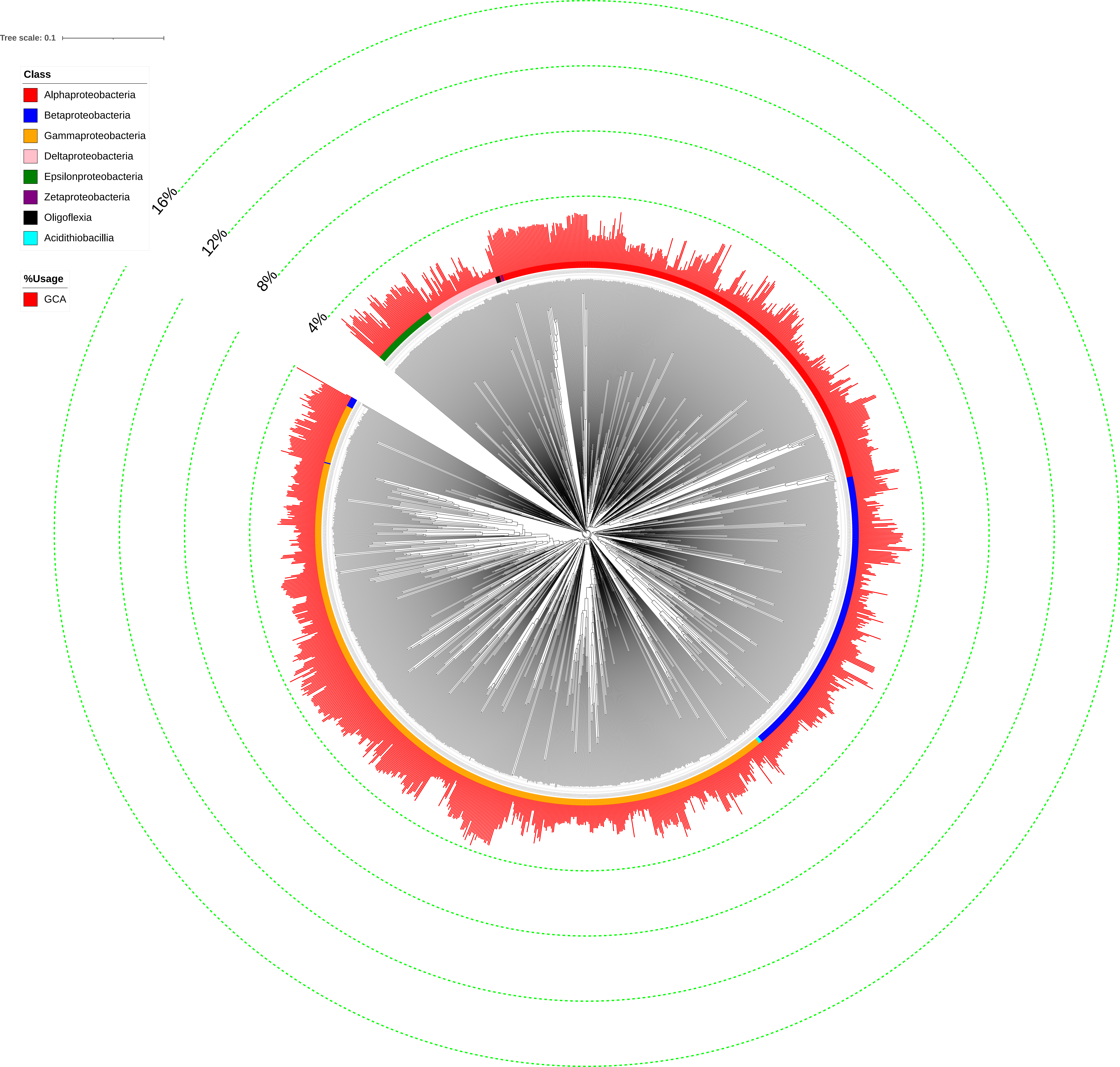

Tree scale: 0.1
Class
Alphaproteobacteria
Betaproteobacteria
Gammaproteobacteria
Deltaproteobacteria
Epsilonproteobacteria
Zetaproteobacteria
Oligoflexia
Acidithiobacillia
%Usage
GCA
16%
12%
8%
4%

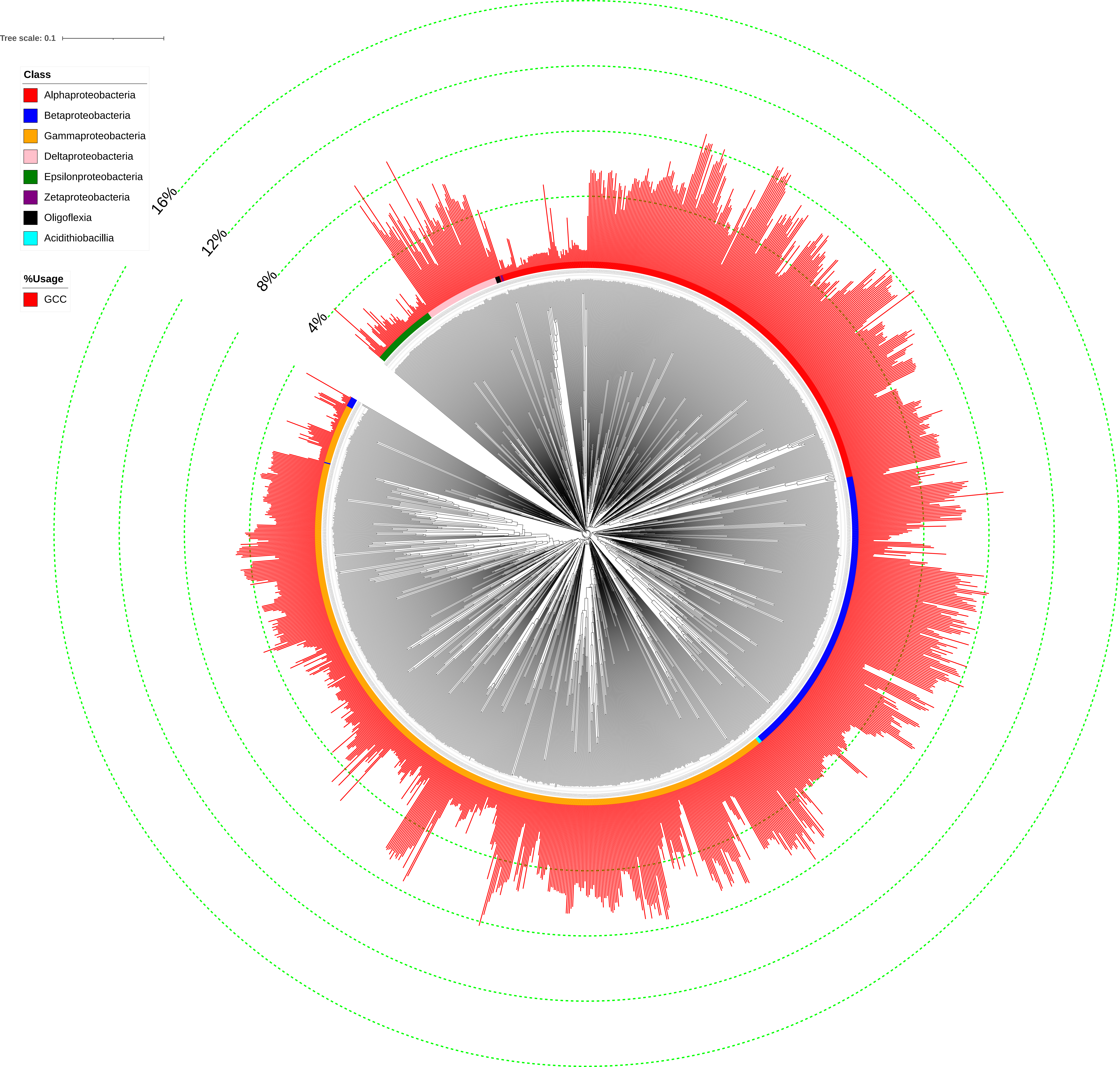

Tree scale: 0.1
Class
Alphaproteobacteria
Betaproteobacteria
Gammaproteobacteria
Deltaproteobacteria
Epsilonproteobacteria
Zetaproteobacteria
Oligoflexia
Acidithiobacillia
%Usage
GCC
16%
12%
8%
4%

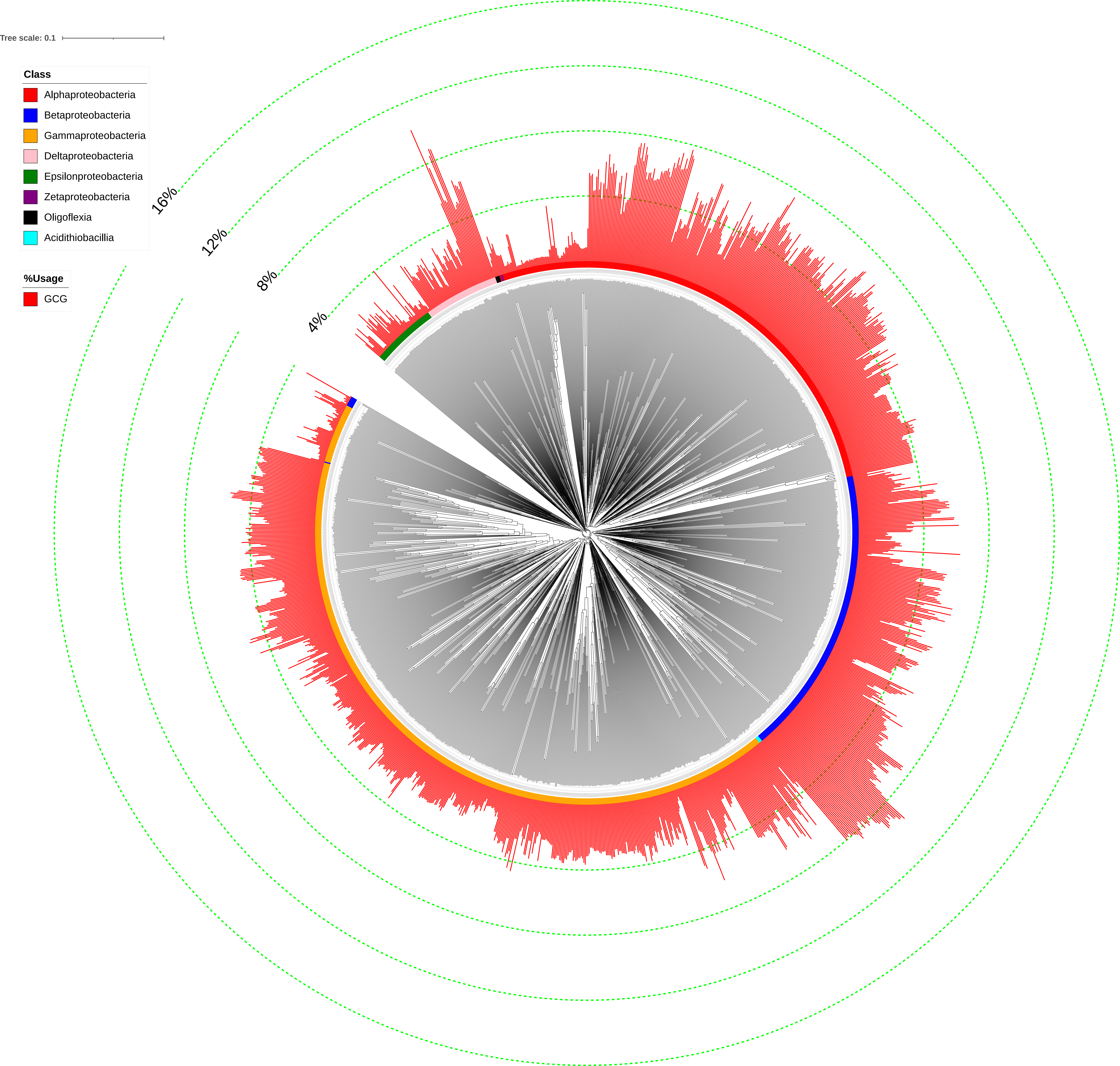
Tree scale: 0.1
Class
Alphaproteobacteria
Betaproteobacteria
Gammaproteobacteria
Deltaproteobacteria
Epsilonproteobacteria
Zetaproteobacteria
Oligoflexia
Acidithiobacillia
%Usage
GCG
16%
12%
8%
4%

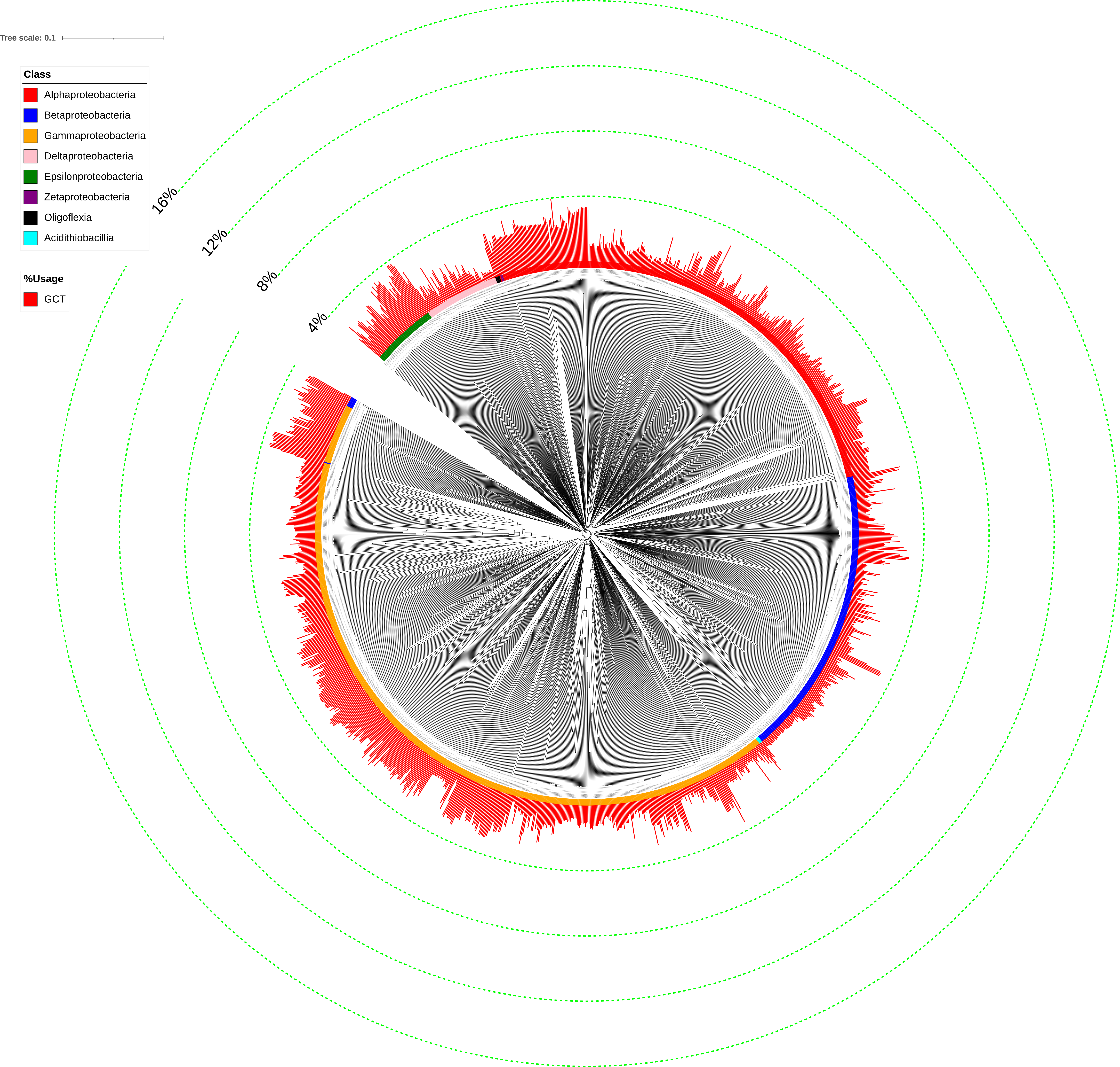
Tree scale: 0.1
Class
Alphaproteobacteria
Betaproteobacteria
Gammaproteobacteria
Deltaproteobacteria
Epsilonproteobacteria
Zetaproteobacteria
Oligoflexia
Acidithiobacillia
%Usage
GCT
4%
8%
12%
16%

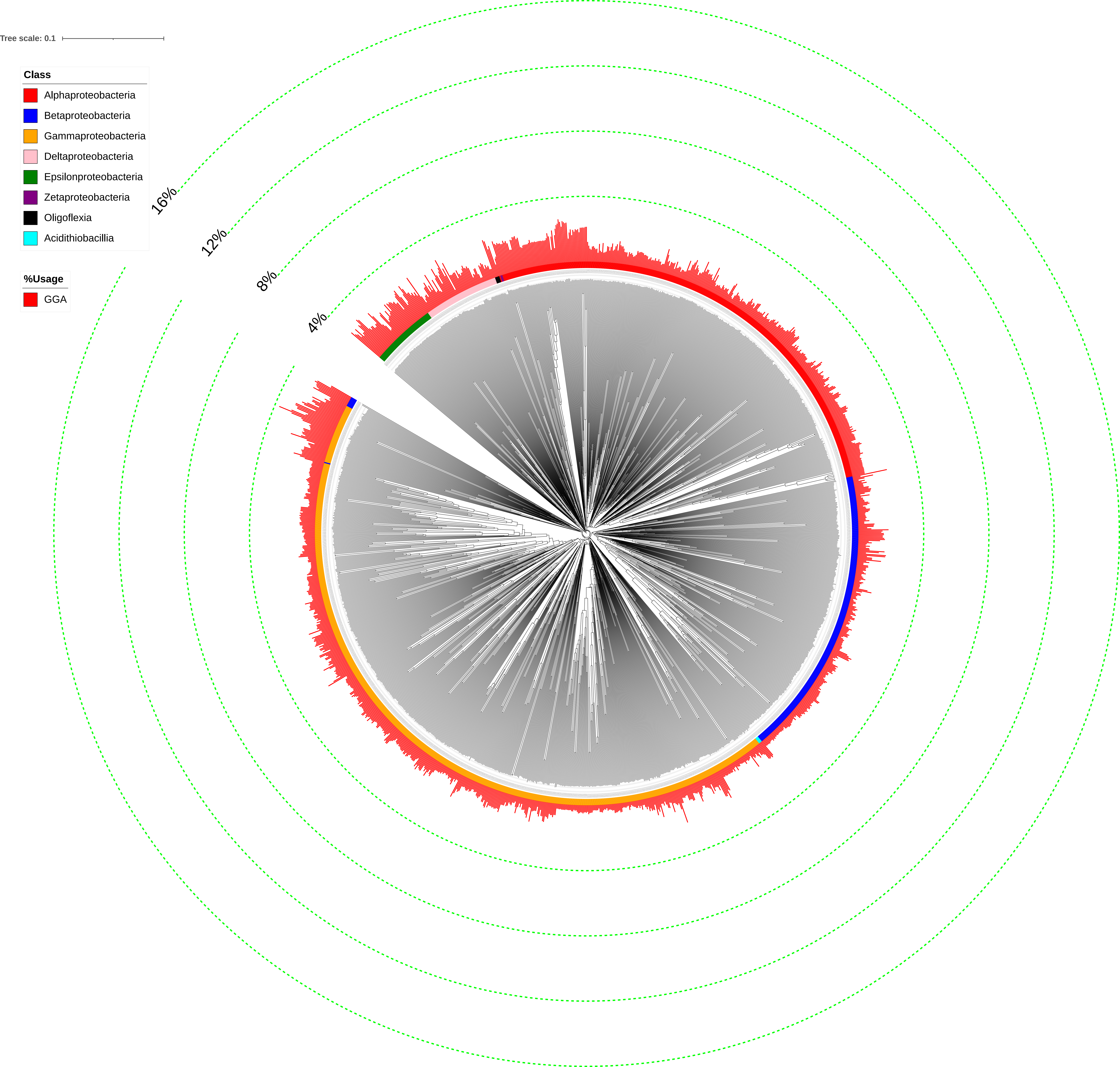

Tree scale: 0.1
Class
Alphaproteobacteria
Betaproteobacteria
Gammaproteobacteria
Deltaproteobacteria
Epsilonproteobacteria
Zetaproteobacteria
Oligoflexia
Acidithiobacillia
%Usage
GGA
16%
12%
8%
4%

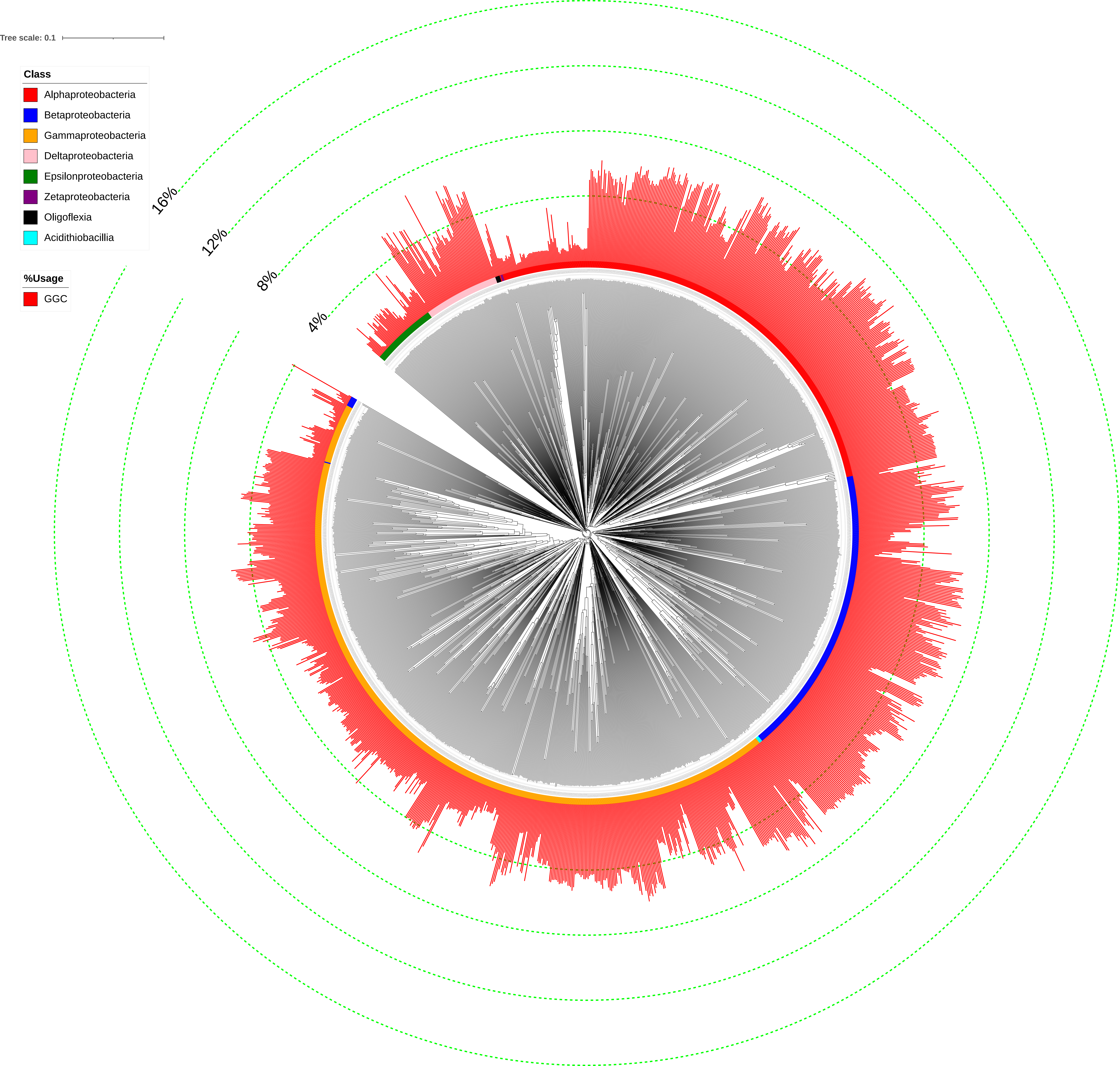

Tree scale: 0.1
Class
Alphaproteobacteria
Betaproteobacteria
Gammaproteobacteria
Deltaproteobacteria
Epsilonproteobacteria
Zetaproteobacteria
Oligoflexia
Acidithiobacillia
%Usage
GGC
16%
12%
8%
4%

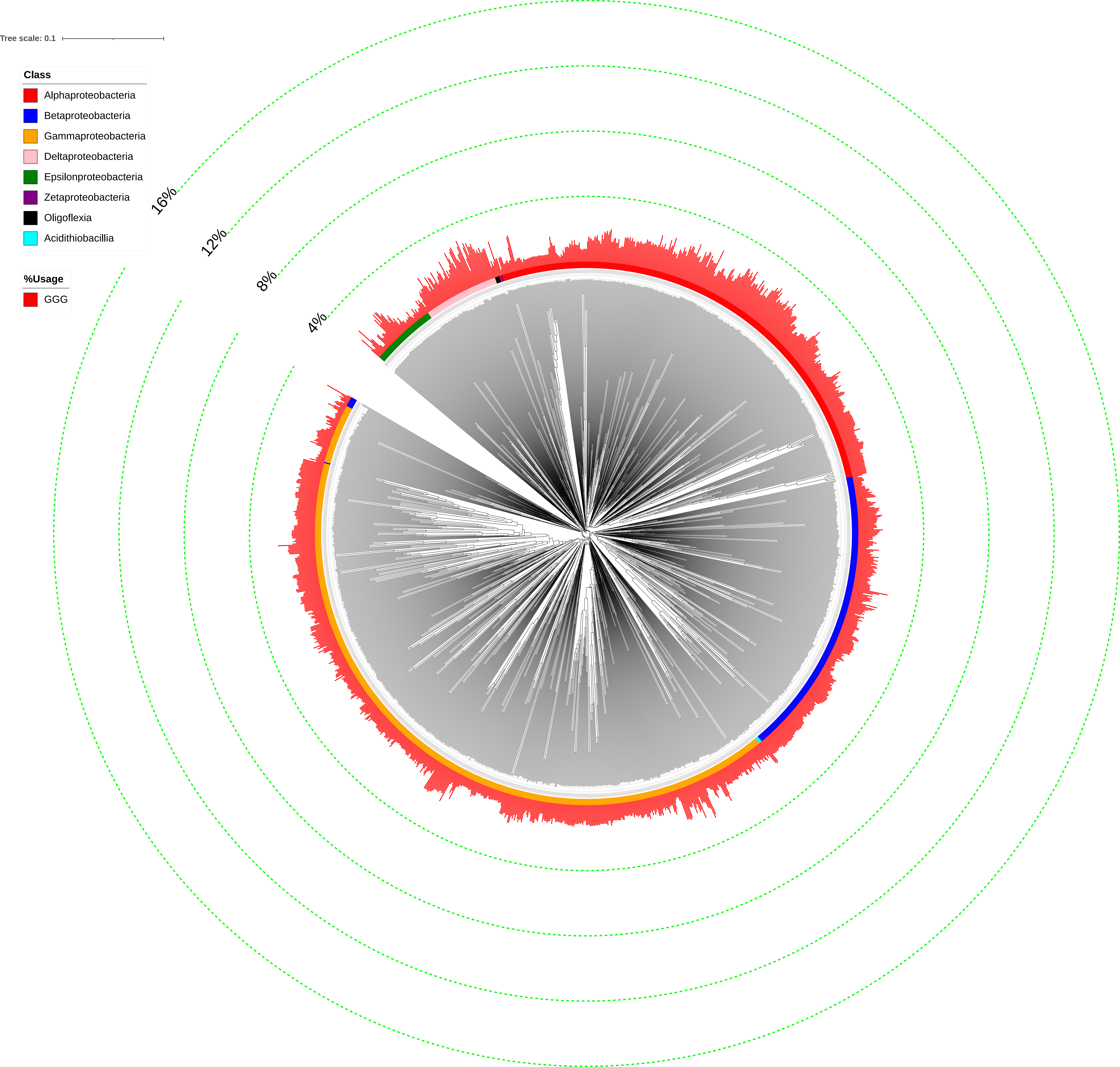
Tree scale: 0.1
Class
Alphaproteobacteria
Betaproteobacteria
Gammaproteobacteria
Deltaproteobacteria
Epsilonproteobacteria
Zetaproteobacteria
Oligoflexia
Acidithiobacillia
%Usage
GGG
4%
8%
12%
16%

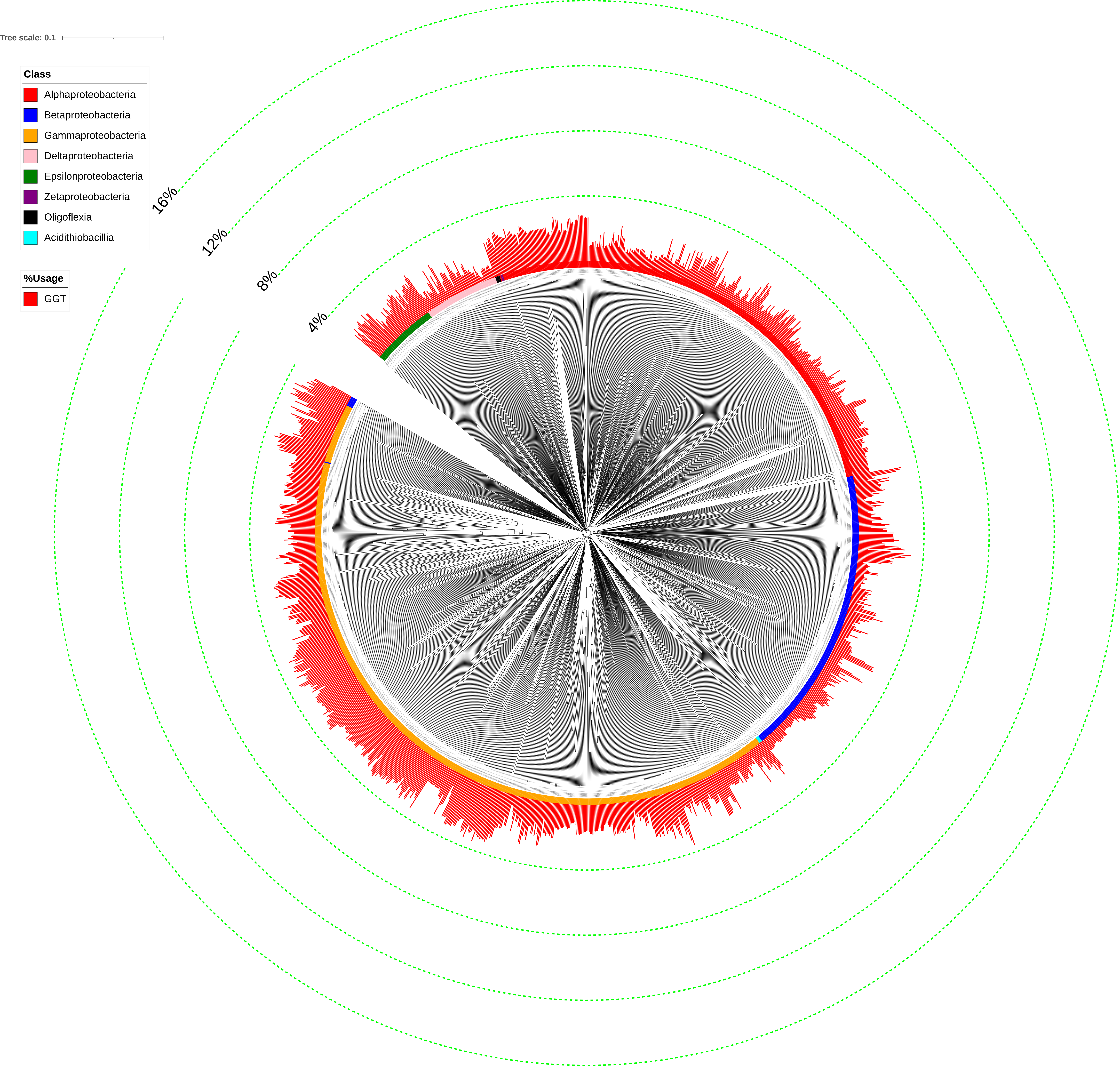
Tree scale: 0.1
Class
Alphaproteobacteria
Betaproteobacteria
Gammaproteobacteria
Deltaproteobacteria
Epsilonproteobacteria
Zetaproteobacteria
Oligoflexia
Acidithiobacillia
%Usage
GGT
16%
12%
8%
4%

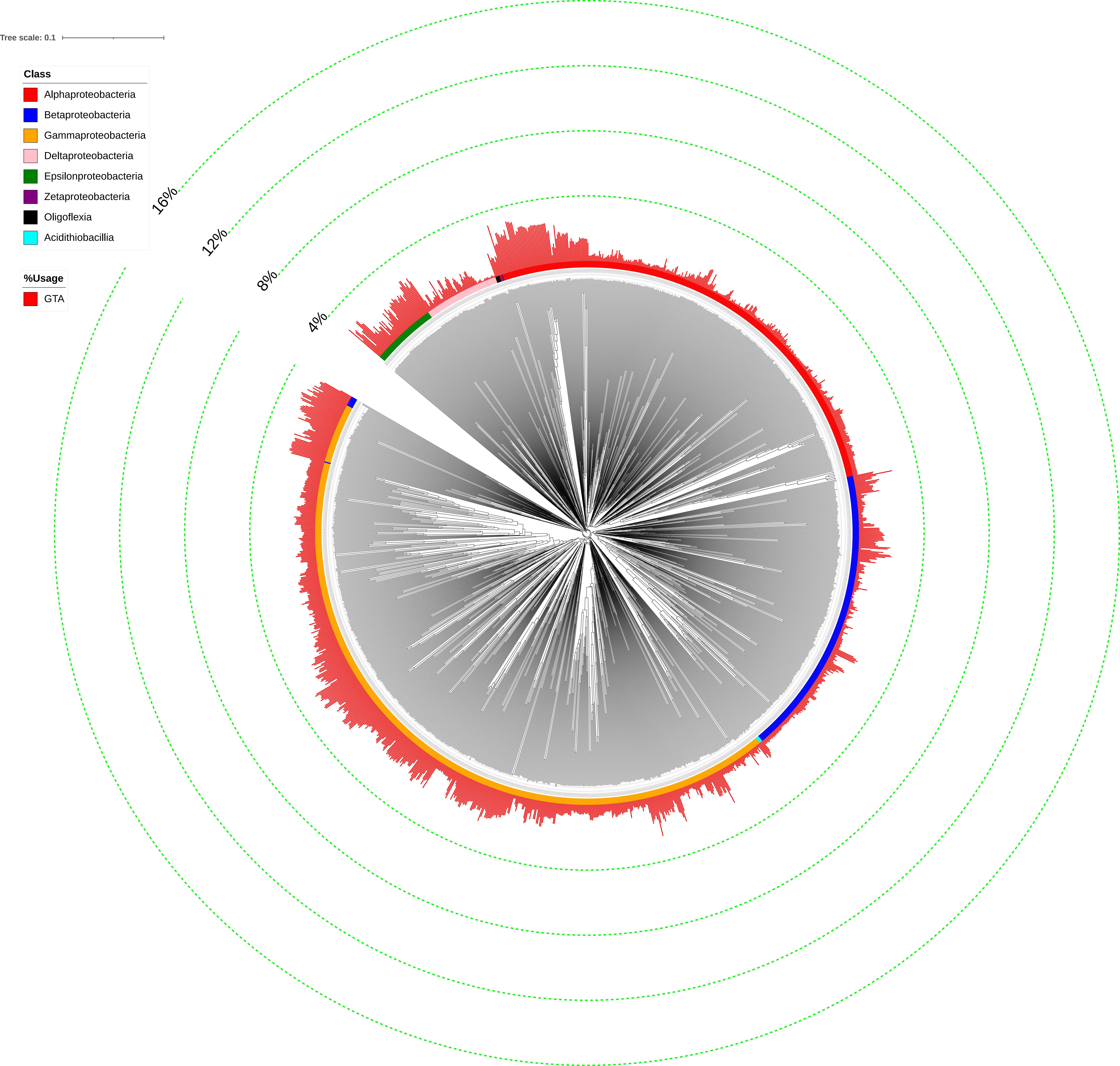

Tree scale: 0.1
Class
Alphaproteobacteria
Betaproteobacteria
Gammaproteobacteria
Deltaproteobacteria
Epsilonproteobacteria
Zetaproteobacteria
Oligoflexia
Acidithiobacillia
%Usage
GTA
4%
8%
12%
16%

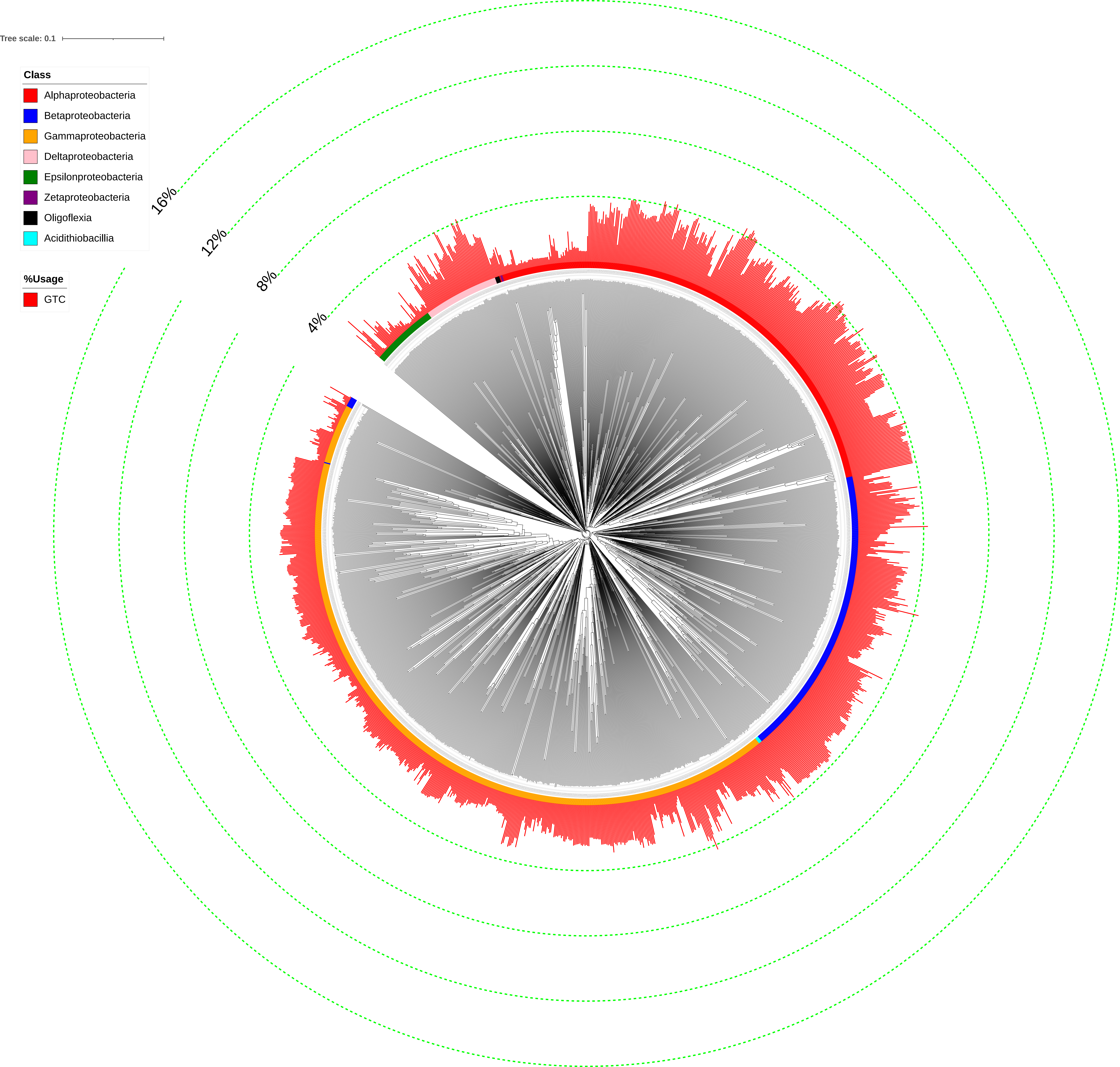

Tree scale: 0.1
Class
Alphaproteobacteria
Betaproteobacteria
Gammaproteobacteria
Deltaproteobacteria
Epsilonproteobacteria
Zetaproteobacteria
Oligoflexia
Acidithiobacillia
%Usage
GTC
16%
12%
8%
4%

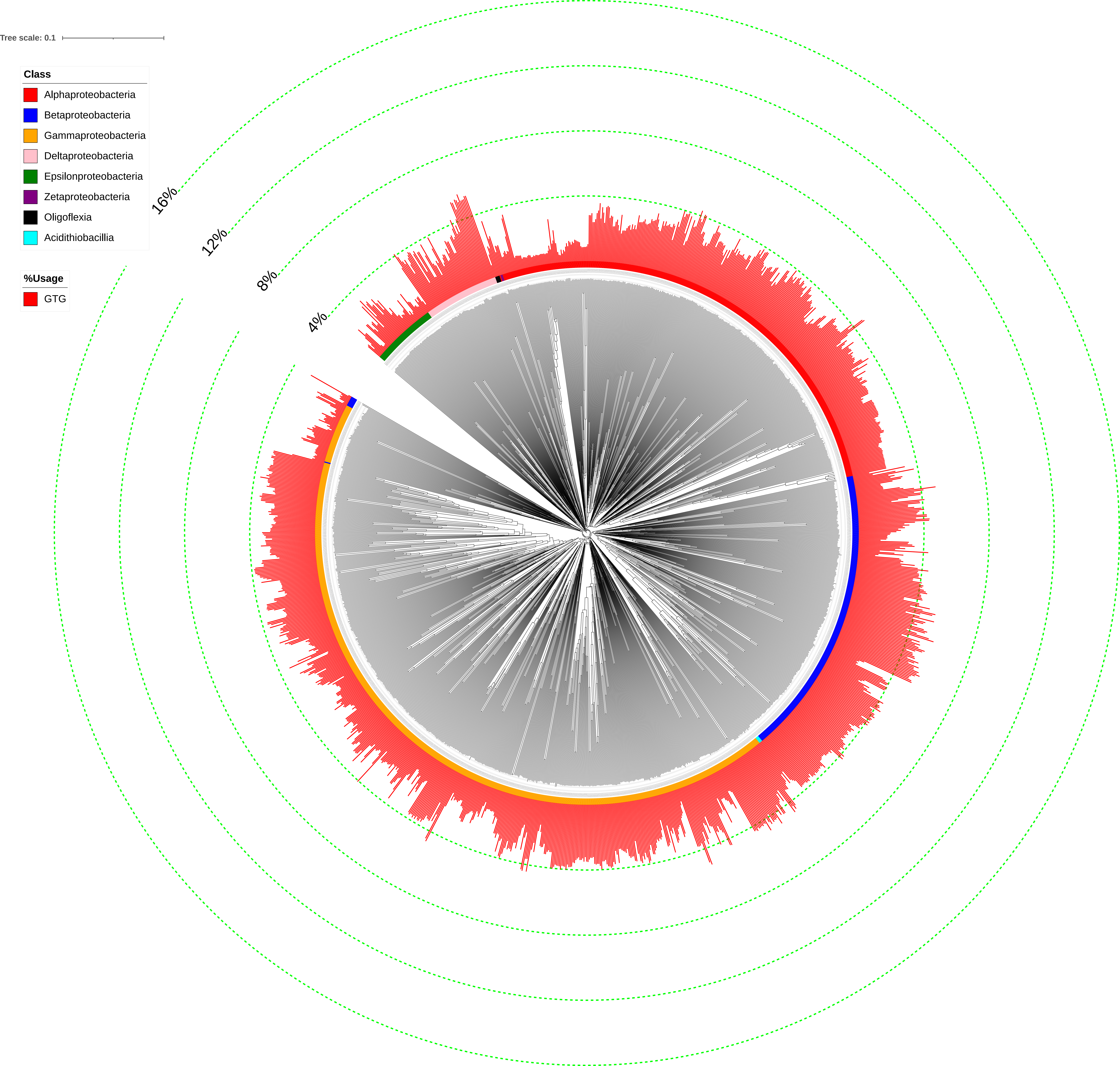
Tree scale: 0.1
Class
Alphaproteobacteria
Betaproteobacteria
Gammaproteobacteria
Deltaproteobacteria
Epsilonproteobacteria
Zetaproteobacteria
Oligoflexia
Acidithiobacillia
%Usage
GTG
16%
12%
8%
4%

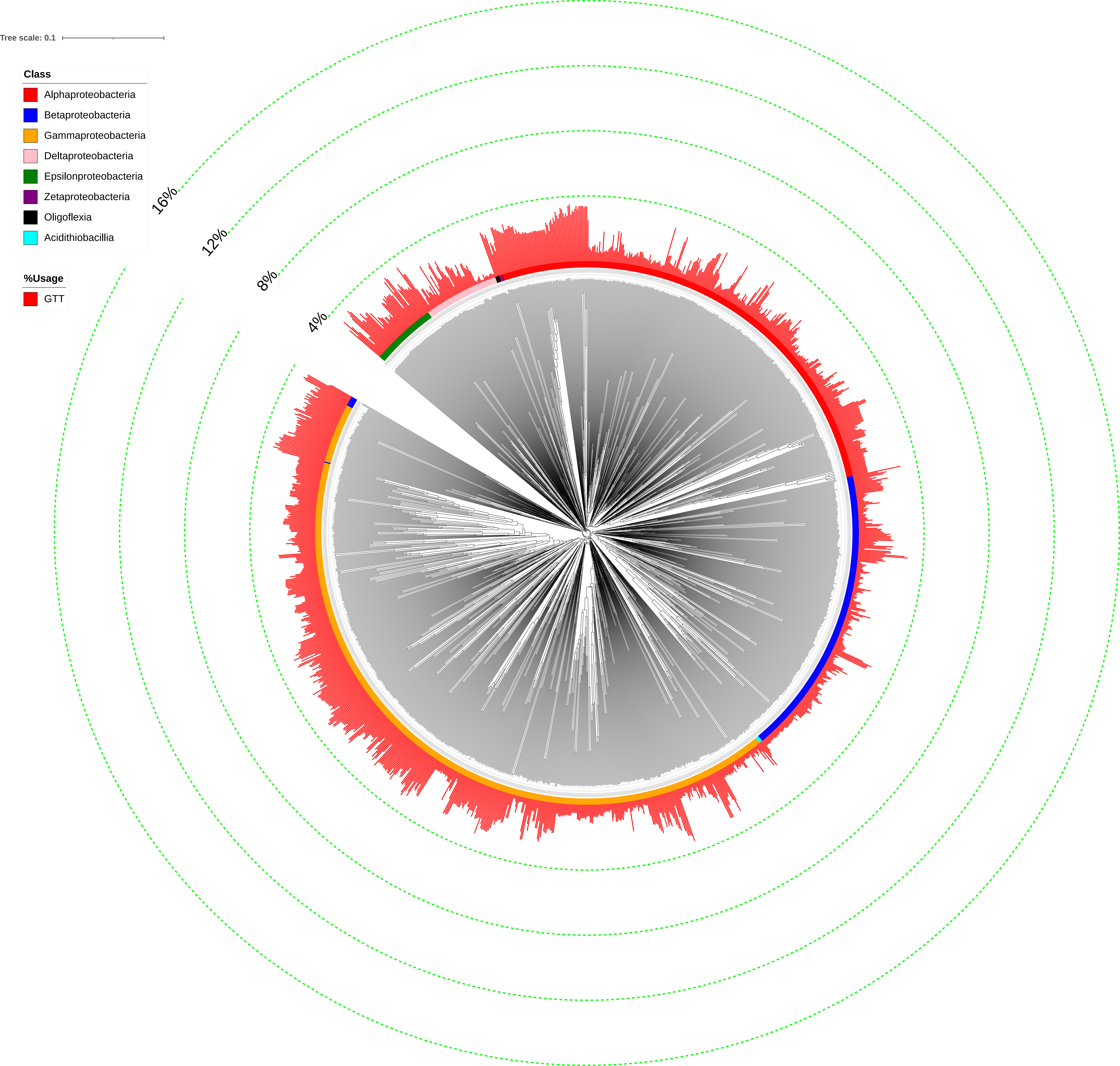

Tree scale: 0.1
Class
Alphaproteobacteria
Betaproteobacteria
Gammaproteobacteria
Deltaproteobacteria
Epsilonproteobacteria
Zetaproteobacteria
Oligoflexia
Acidithiobacillia
%Usage
GTT
4%
8%
12%
16%

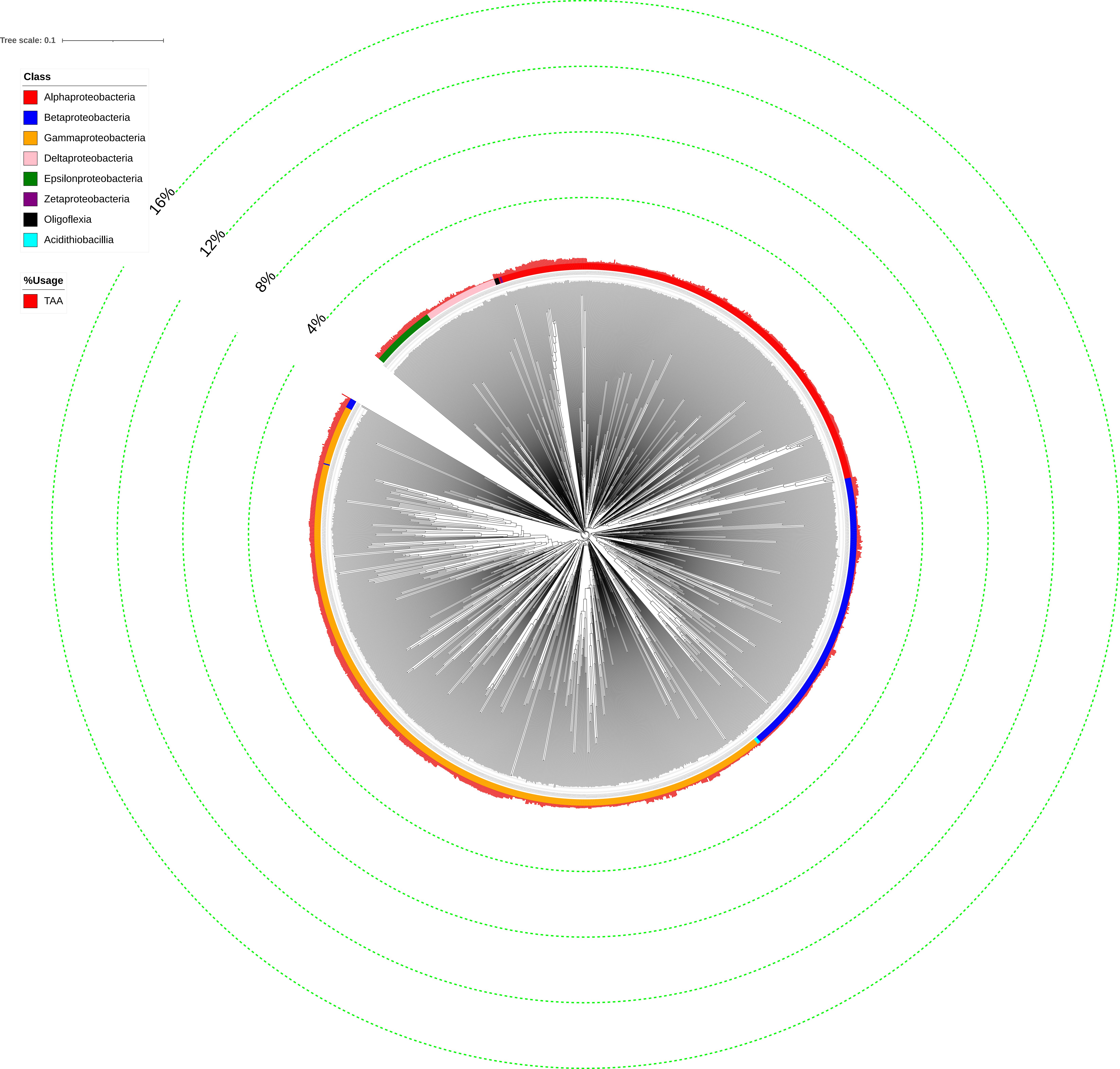
Tree scale: 0.1
Class
Alphaproteobacteria
Betaproteobacteria
Gammaproteobacteria
Deltaproteobacteria
Epsilonproteobacteria
Zetaproteobacteria
Oligoflexia
Acidithiobacillia
%Usage
TAA
16%
12%
8%
4%

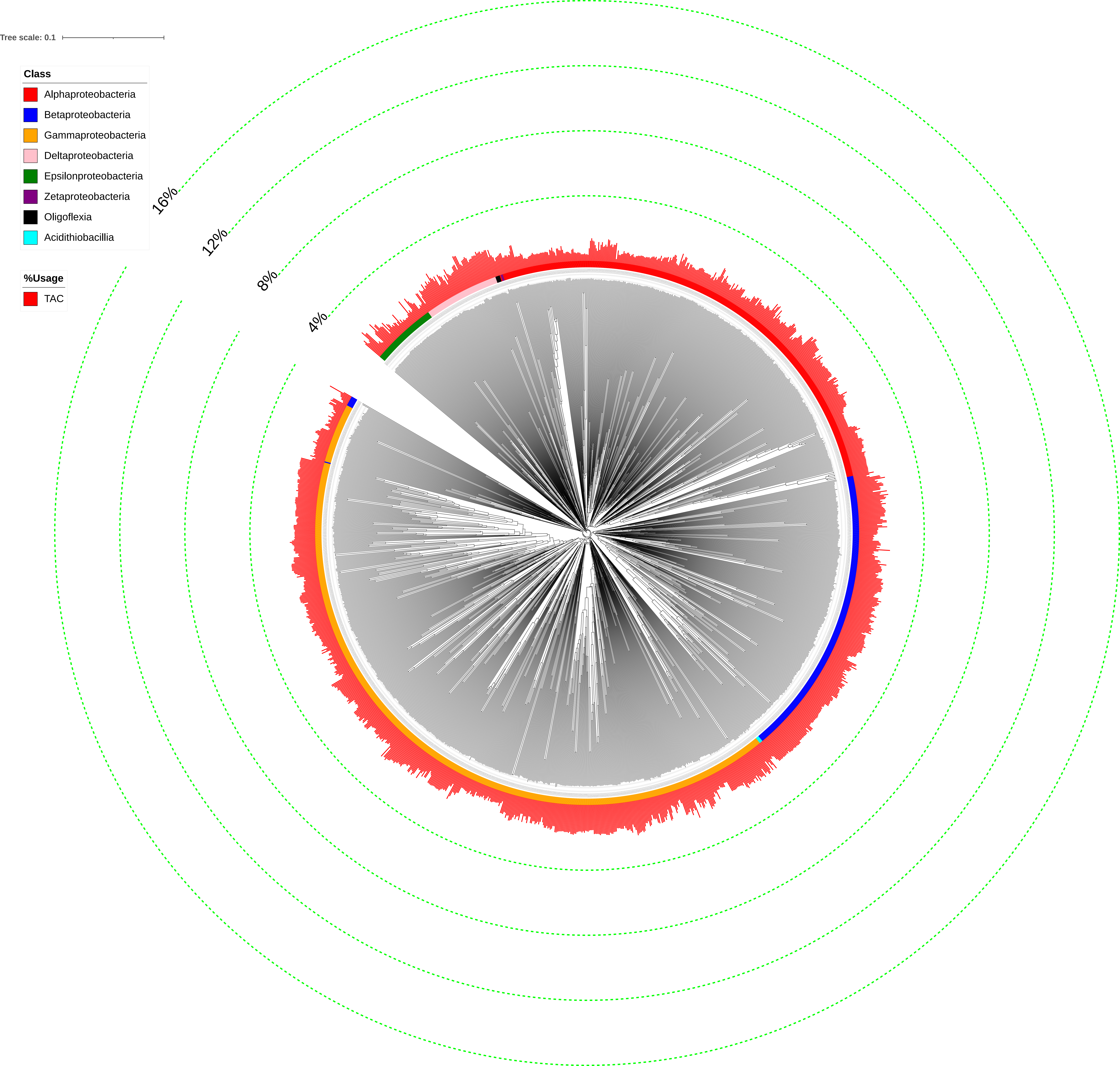

Tree scale: 0.1
Class
Alphaproteobacteria
Betaproteobacteria
Gammaproteobacteria
Deltaproteobacteria
Epsilonproteobacteria
Zetaproteobacteria
Oligoflexia
Acidithiobacillia
%Usage
TAC
4%
8%
12%
16%

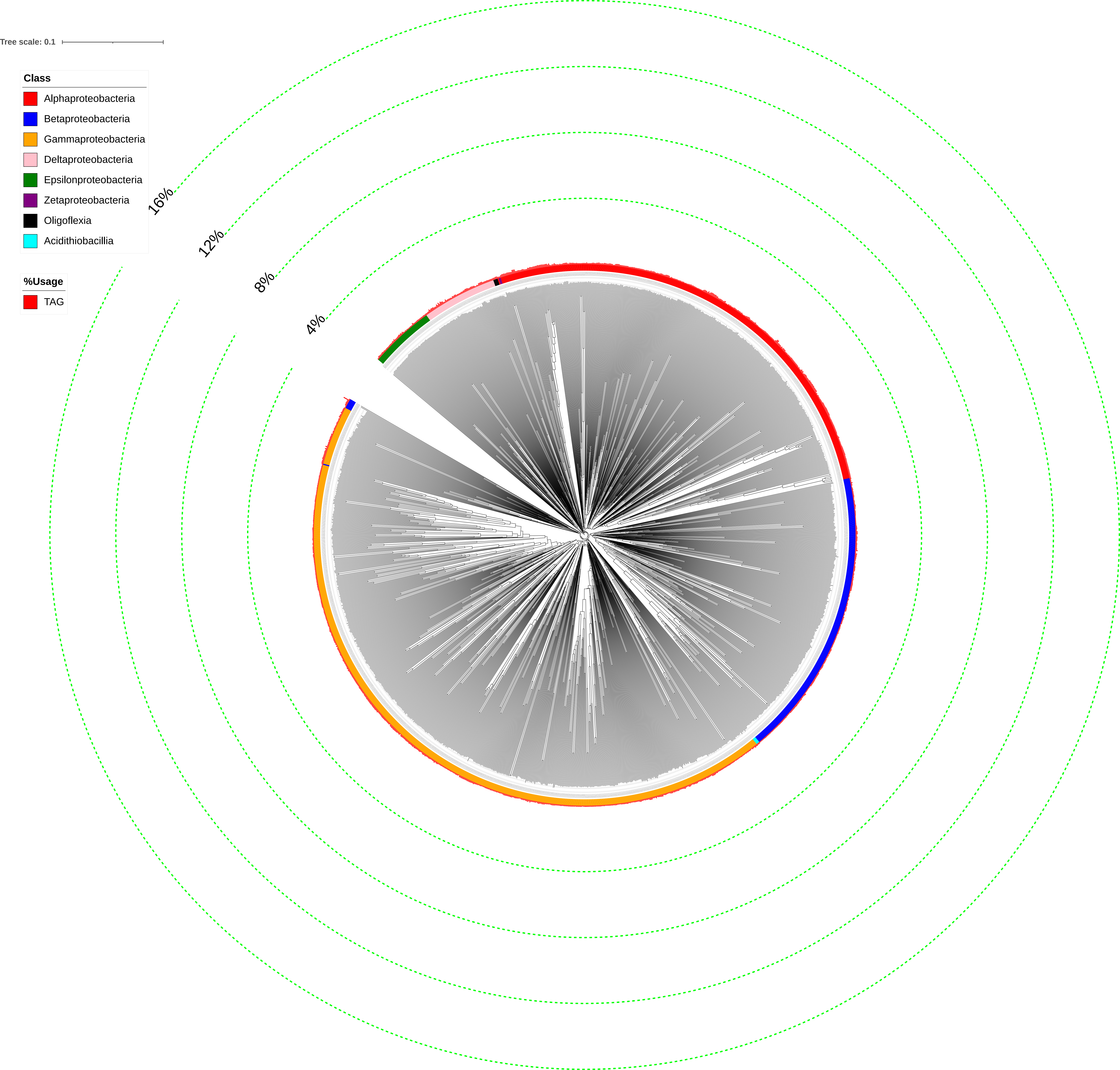

Tree scale: 0.1
Class
Alphaproteobacteria
Betaproteobacteria
Gammaproteobacteria
Deltaproteobacteria
Epsilonproteobacteria
Zetaproteobacteria
Oligoflexia
Acidithiobacillia
%Usage
TAG
16%
12%
8%
4%

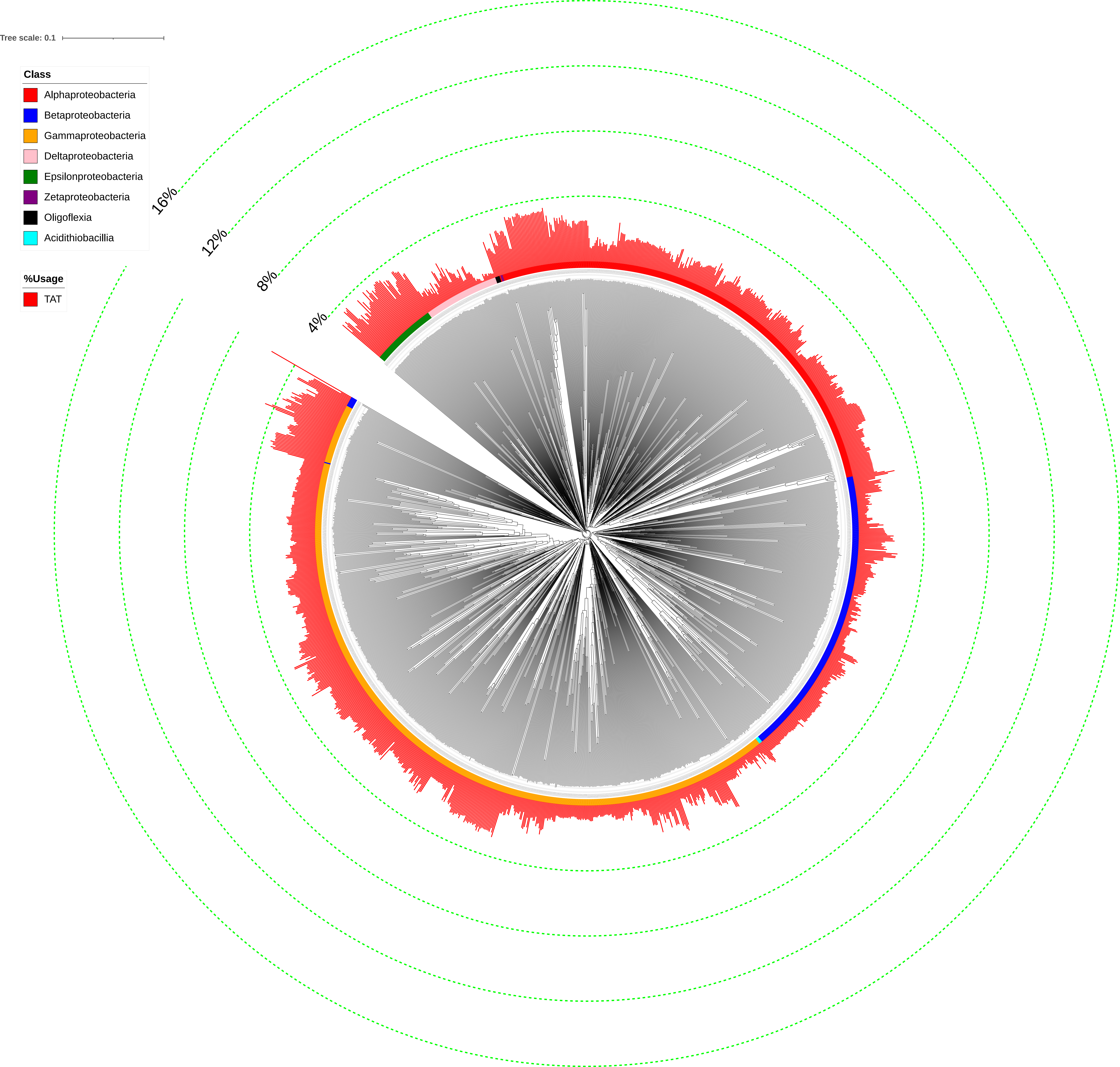

Tree scale: 0.1
Class
Alphaproteobacteria
Betaproteobacteria
Gammaproteobacteria
Deltaproteobacteria
Epsilonproteobacteria
Zetaproteobacteria
Oligoflexia
Acidithiobacillia
%Usage
TAT
16%
12%
8%
4%

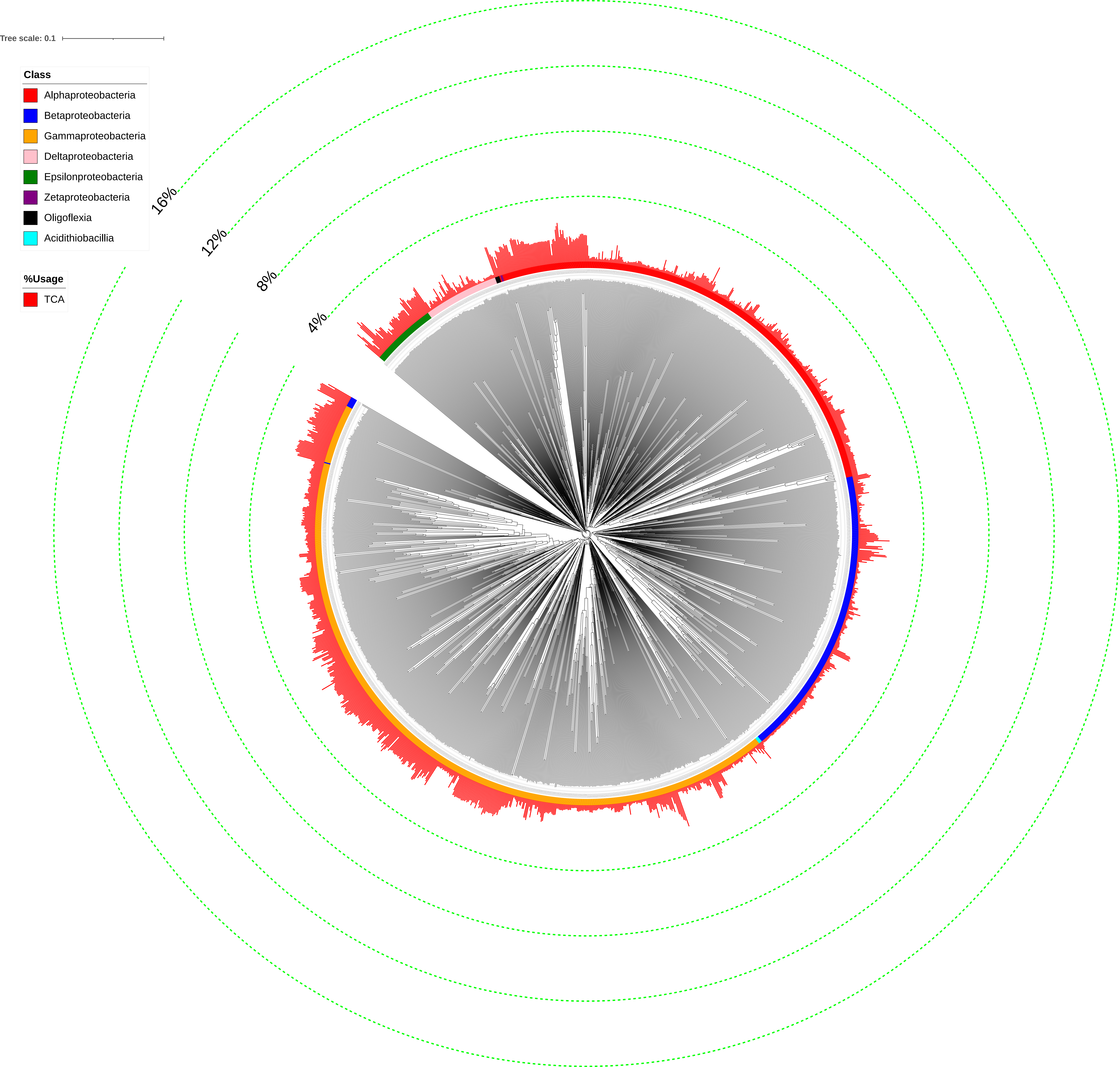
Tree scale: 0.1
Class
Alphaproteobacteria
Betaproteobacteria
Gammaproteobacteria
Deltaproteobacteria
Epsilonproteobacteria
Zetaproteobacteria
Oligoflexia
Acidithiobacillia
%Usage
TCA
16%
12%
8%
4%

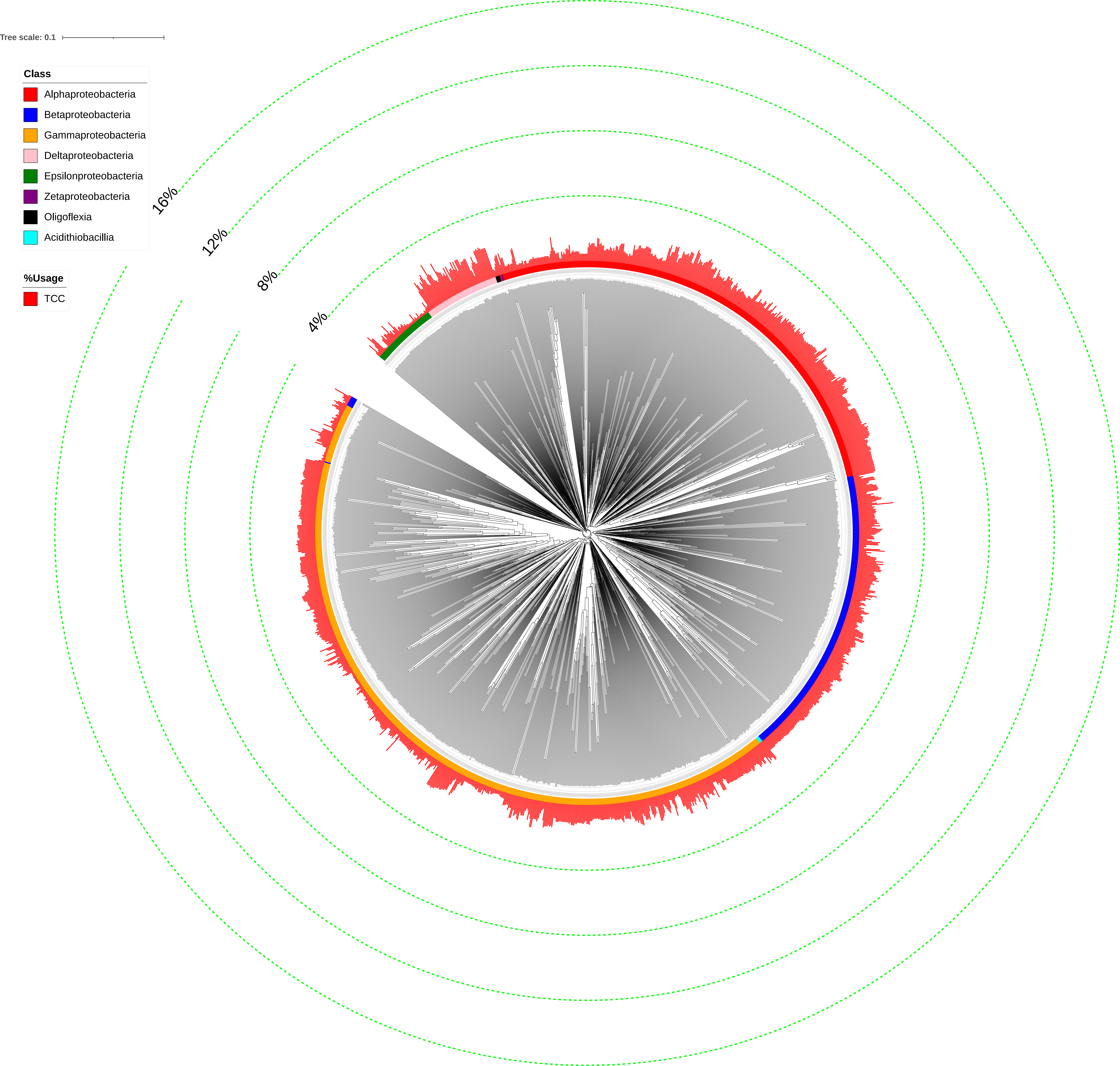
Tree scale: 0.1
Class
Alphaproteobacteria
Betaproteobacteria
Gammaproteobacteria
Deltaproteobacteria
Epsilonproteobacteria
Zetaproteobacteria
Oligoflexia
Acidithiobacillia
%Usage
TCC
16%
12%
8%
4%

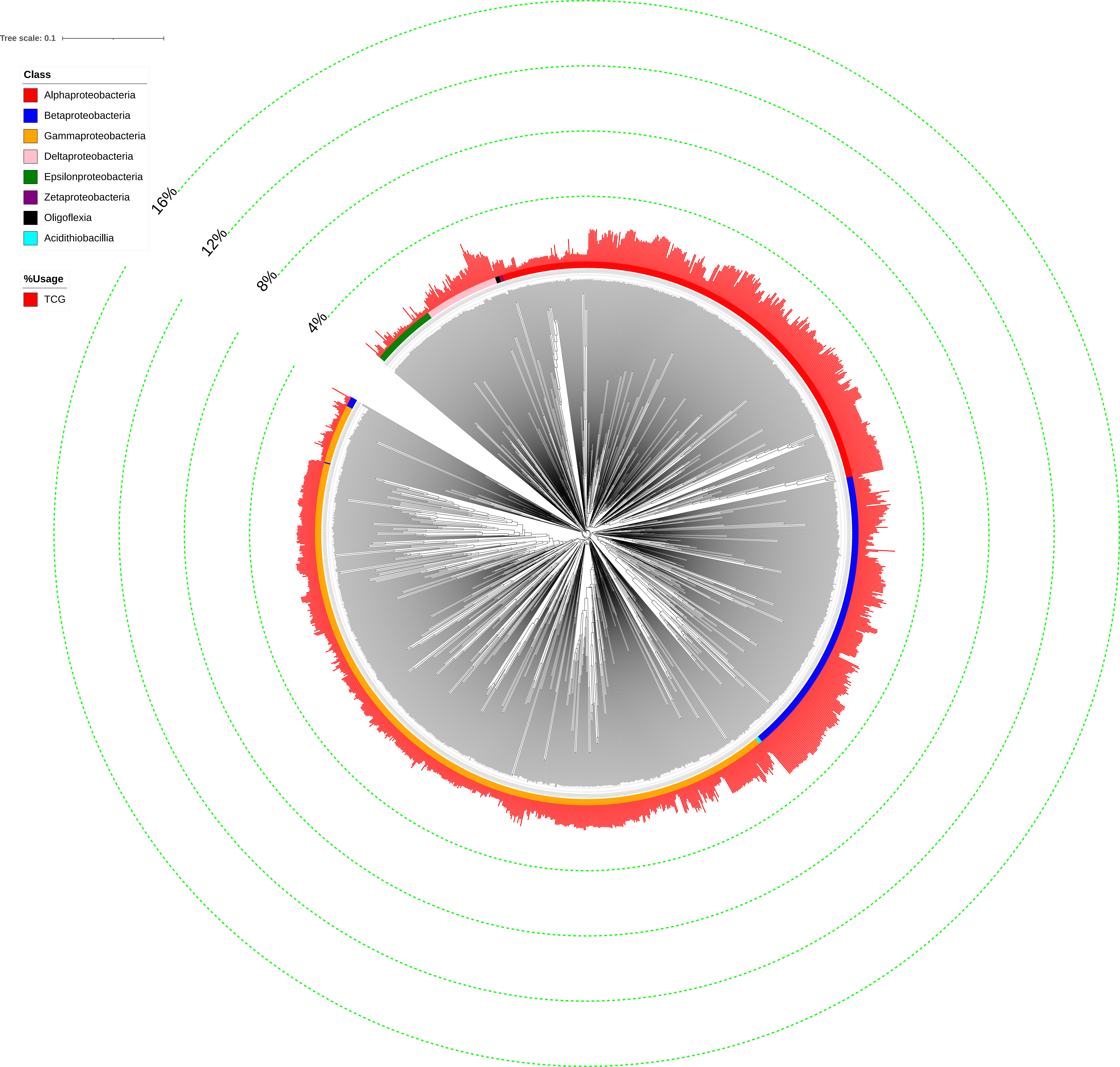
Tree scale: 0.1
Class
Alphaproteobacteria
Betaproteobacteria
Gammaproteobacteria
Deltaproteobacteria
Epsilonproteobacteria
Zetaproteobacteria
Oligoflexia
Acidithiobacillia
%Usage
TCG
16%
12%
8%
4%

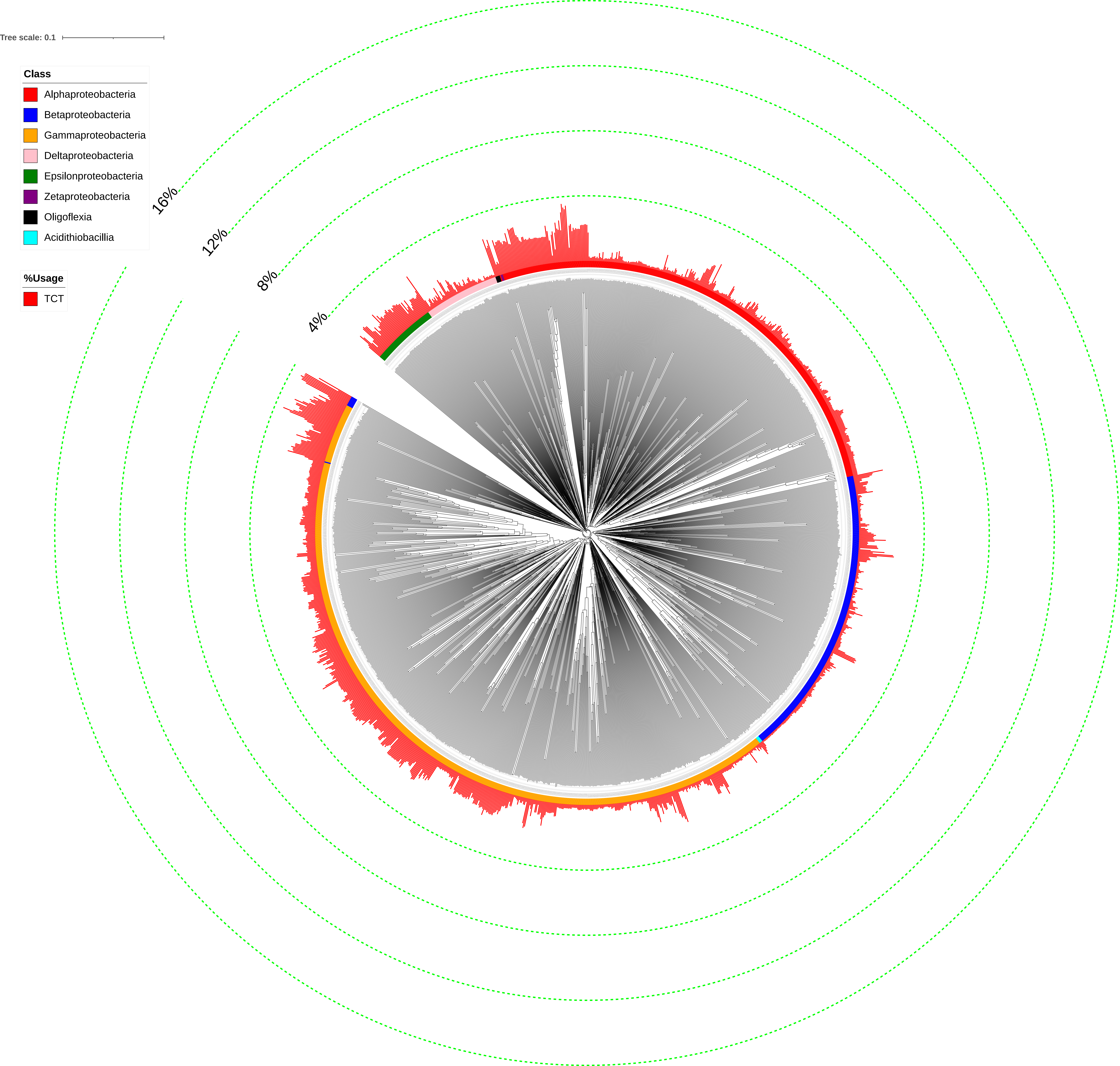

Tree scale: 0.1
Class
Alphaproteobacteria
Betaproteobacteria
Gammaproteobacteria
Deltaproteobacteria
Epsilonproteobacteria
Zetaproteobacteria
Oligoflexia
Acidithiobacillia
%Usage
TCT
4%
8%
12%
16%

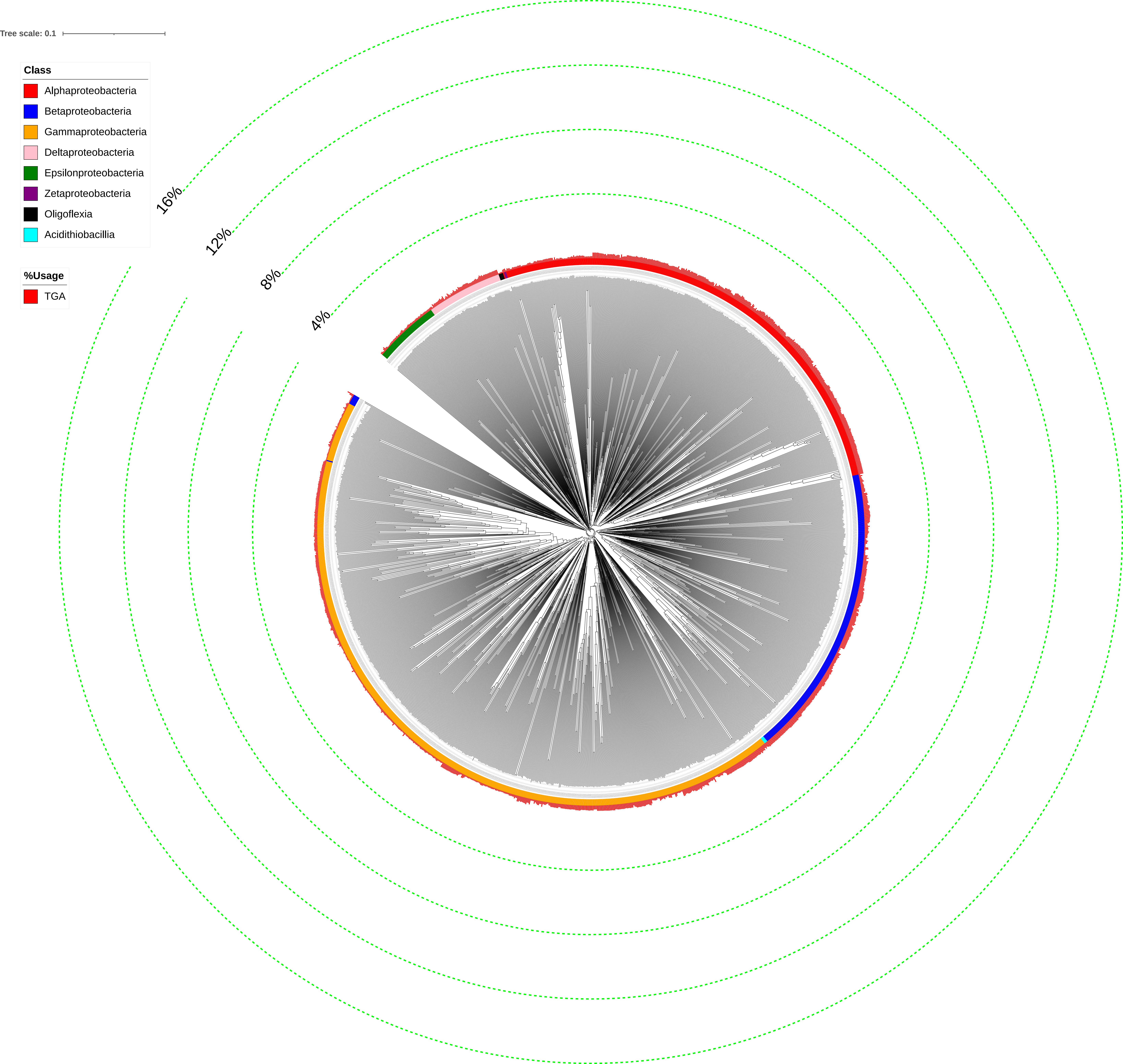

Tree scale: 0.1
Class
Alphaproteobacteria
Betaproteobacteria
Gammaproteobacteria
Deltaproteobacteria
Epsilonproteobacteria
Zetaproteobacteria
Oligoflexia
Acidithiobacillia
%Usage
TGA
4%
8%
12%
16%

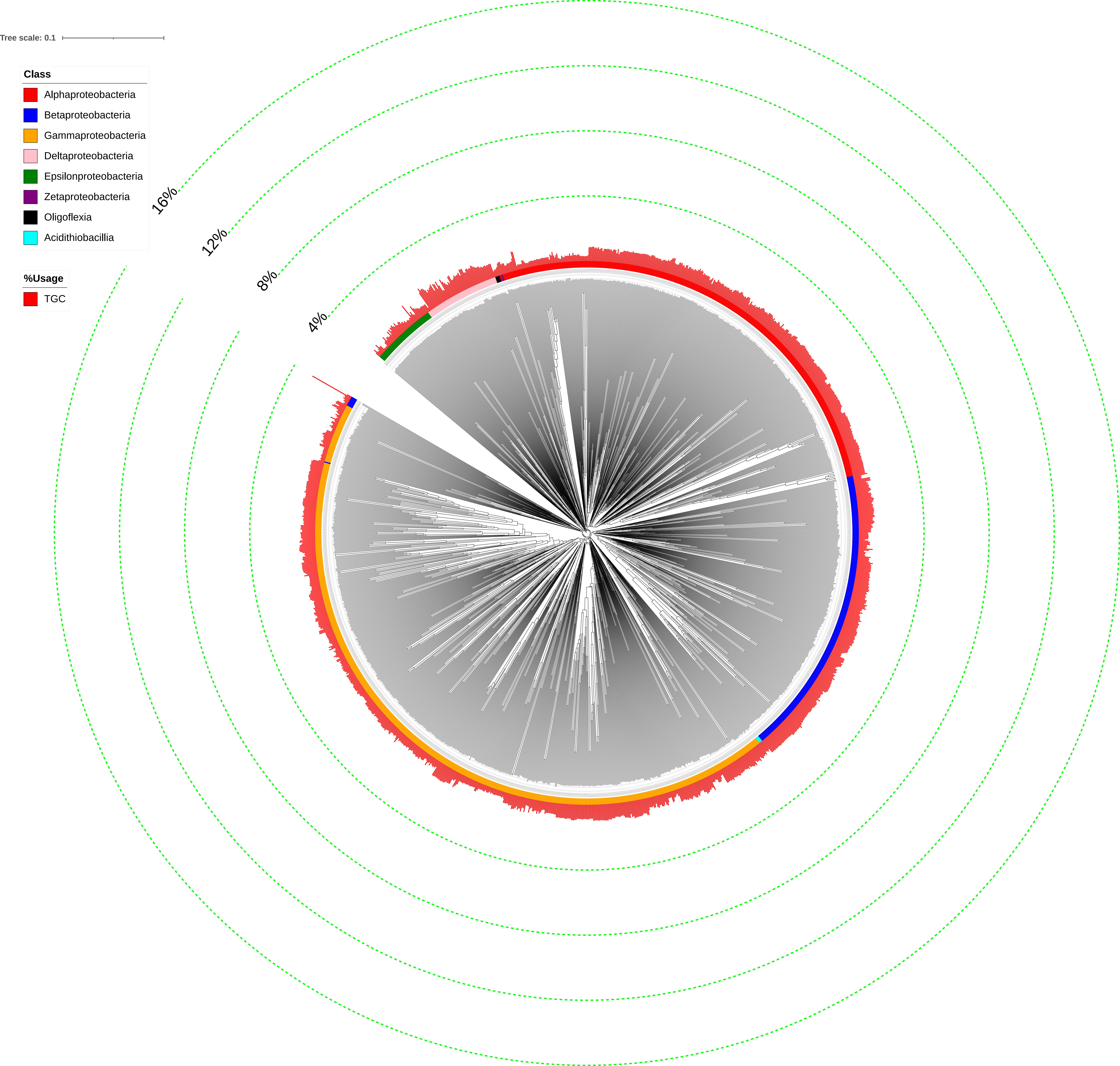
Tree scale: 0.1
Class
Alphaproteobacteria
Betaproteobacteria
Gammaproteobacteria
Deltaproteobacteria
Epsilonproteobacteria
Zetaproteobacteria
Oligoflexia
Acidithiobacillia
%Usage
TGC
16%
12%
8%
4%

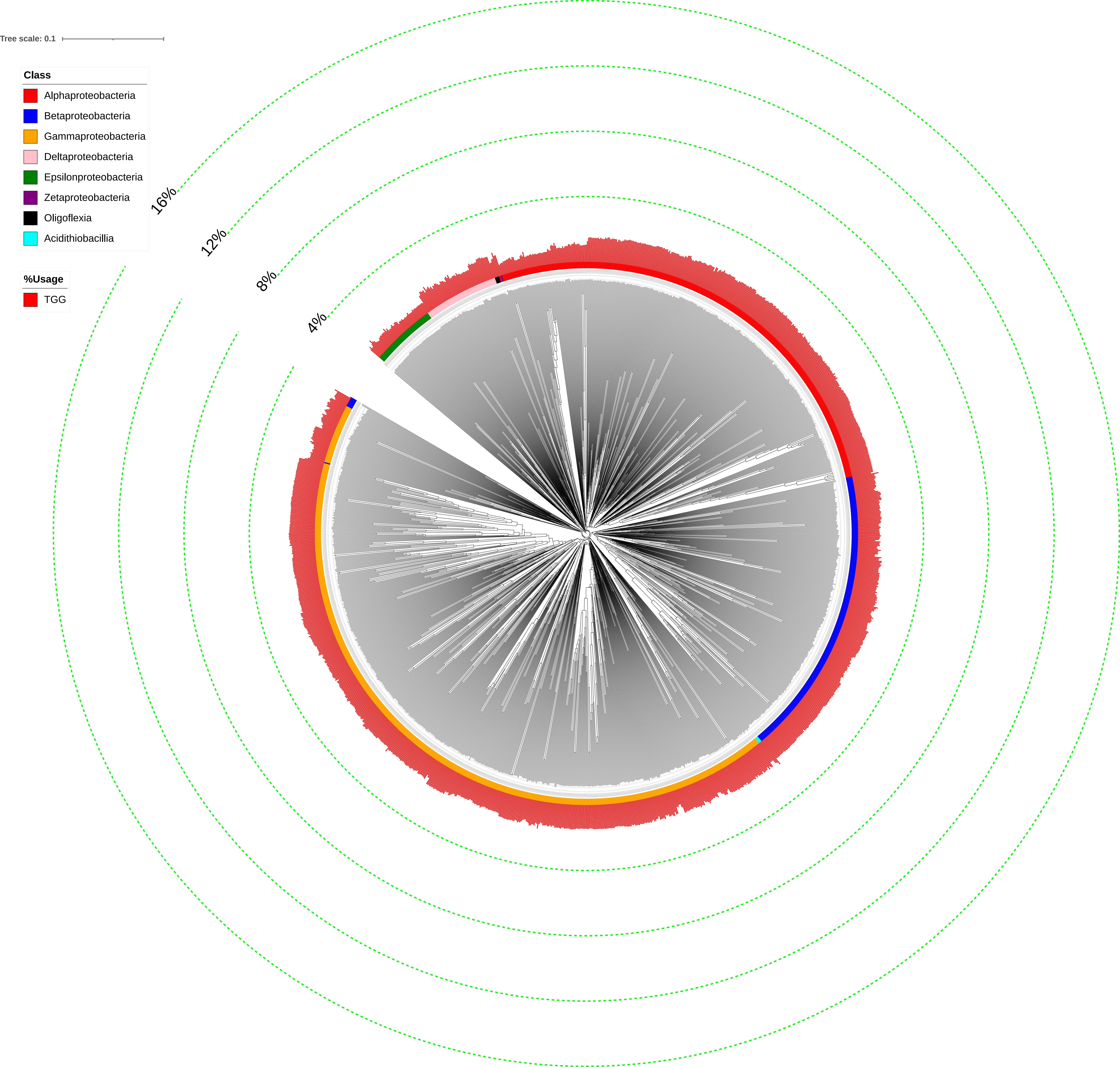
Tree scale: 0.1
Class
Alphaproteobacteria
Betaproteobacteria
Gammaproteobacteria
Deltaproteobacteria
Epsilonproteobacteria
Zetaproteobacteria
Oligoflexia
Acidithiobacillia
%Usage
TGG
16%
12%
8%
4%

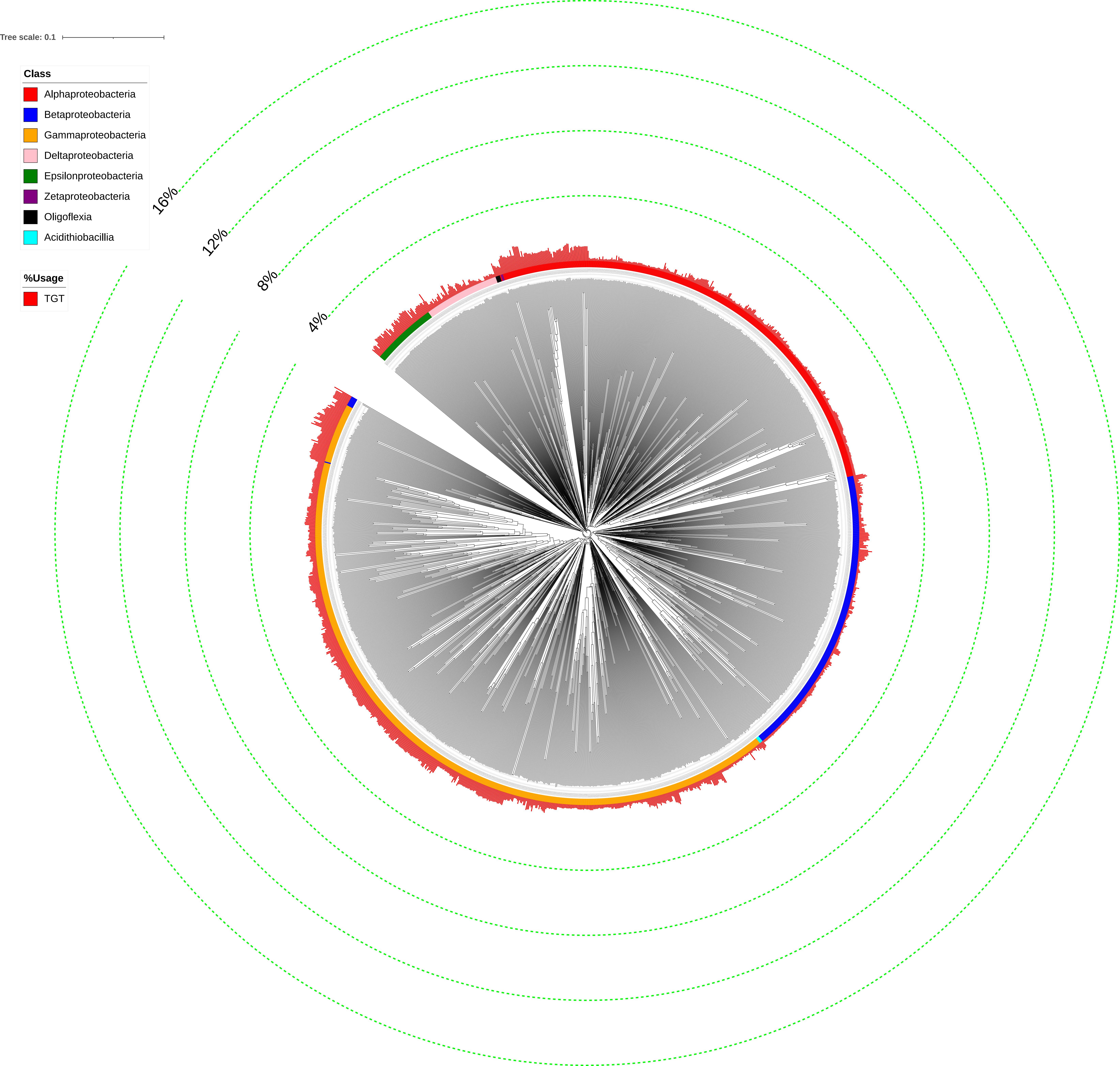
Tree scale: 0.1
Class
Alphaproteobacteria
Betaproteobacteria
Gammaproteobacteria
Deltaproteobacteria
Epsilonproteobacteria
Zetaproteobacteria
Oligoflexia
Acidithiobacillia
%Usage
TGT
16%
12%
8%
4%

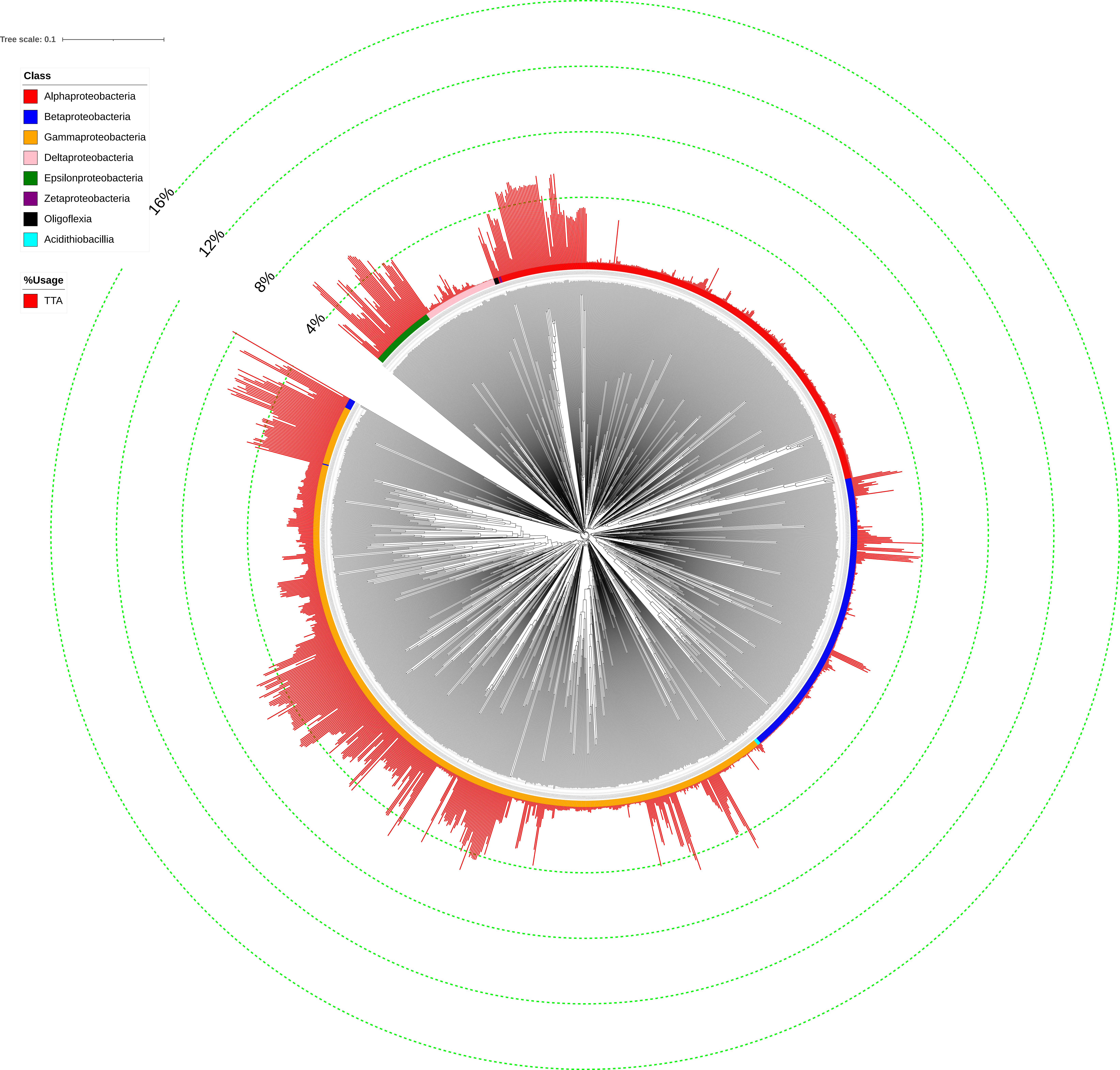

Tree scale: 0.1
Class
Alphaproteobacteria
Betaproteobacteria
Gammaproteobacteria
Deltaproteobacteria
Epsilonproteobacteria
Zetaproteobacteria
Oligoflexia
Acidithiobacillia
%Usage
TTA
16%
12%
8%
4%

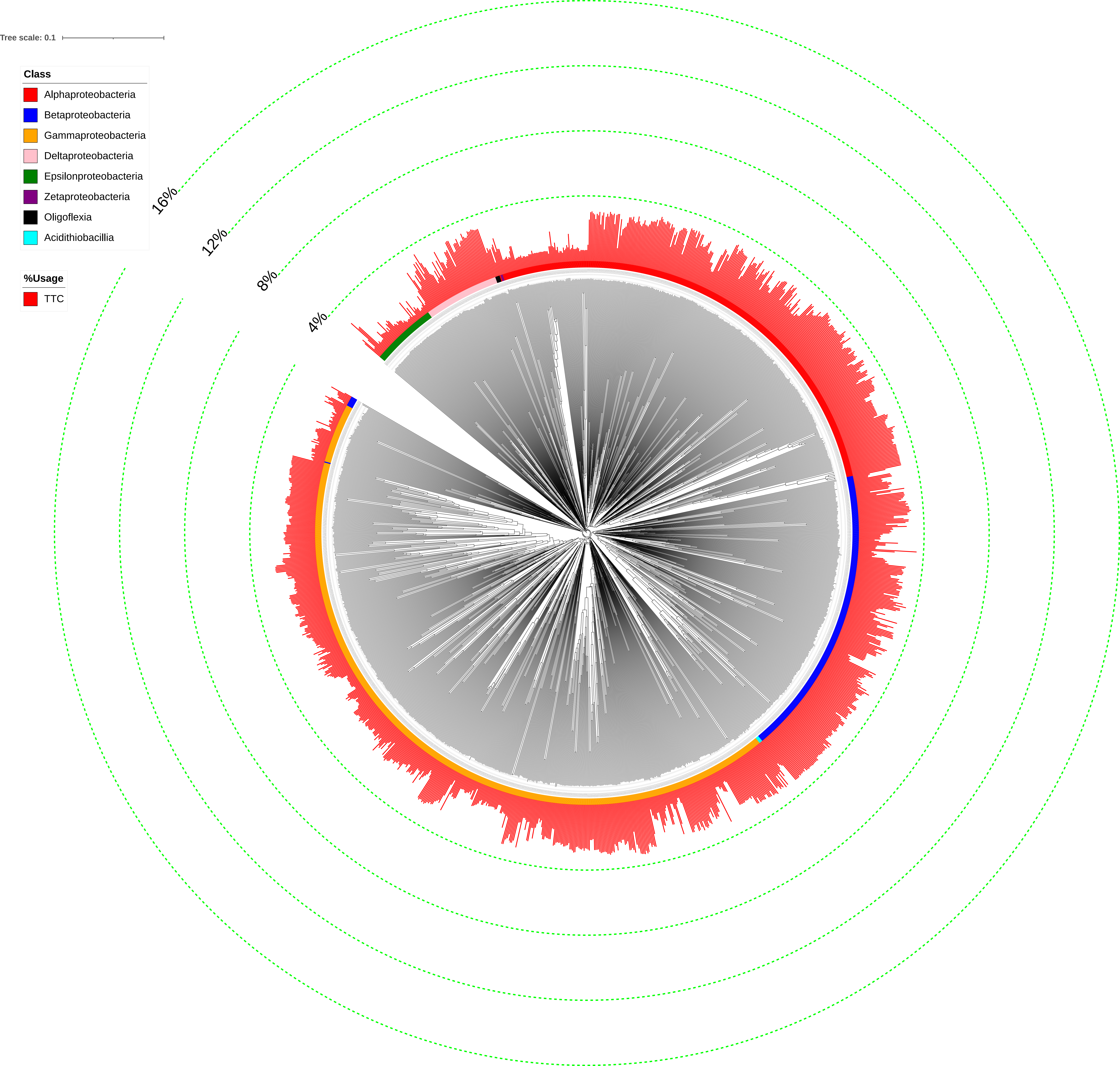

Tree scale: 0.1
Class
Alphaproteobacteria
Betaproteobacteria
Gammaproteobacteria
Deltaproteobacteria
Epsilonproteobacteria
Zetaproteobacteria
Oligoflexia
Acidithiobacillia
%Usage
TTC
16%
12%
8%
4%

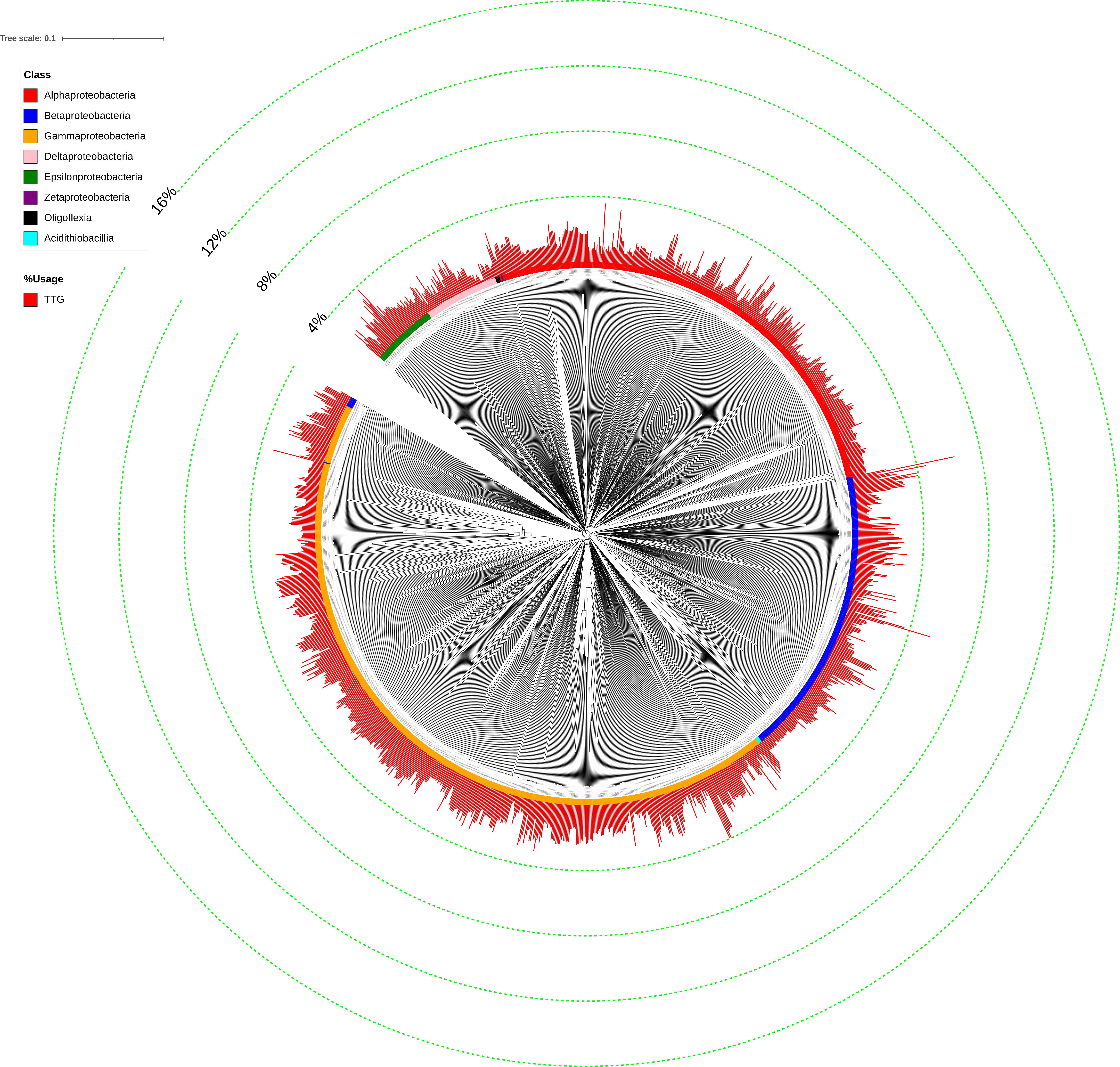

Tree scale: 0.1
Class
Alphaproteobacteria
Betaproteobacteria
Gammaproteobacteria
Deltaproteobacteria
Epsilonproteobacteria
Zetaproteobacteria
Oligoflexia
Acidithiobacillia
%Usage
TTG
16%
12%
8%
4%

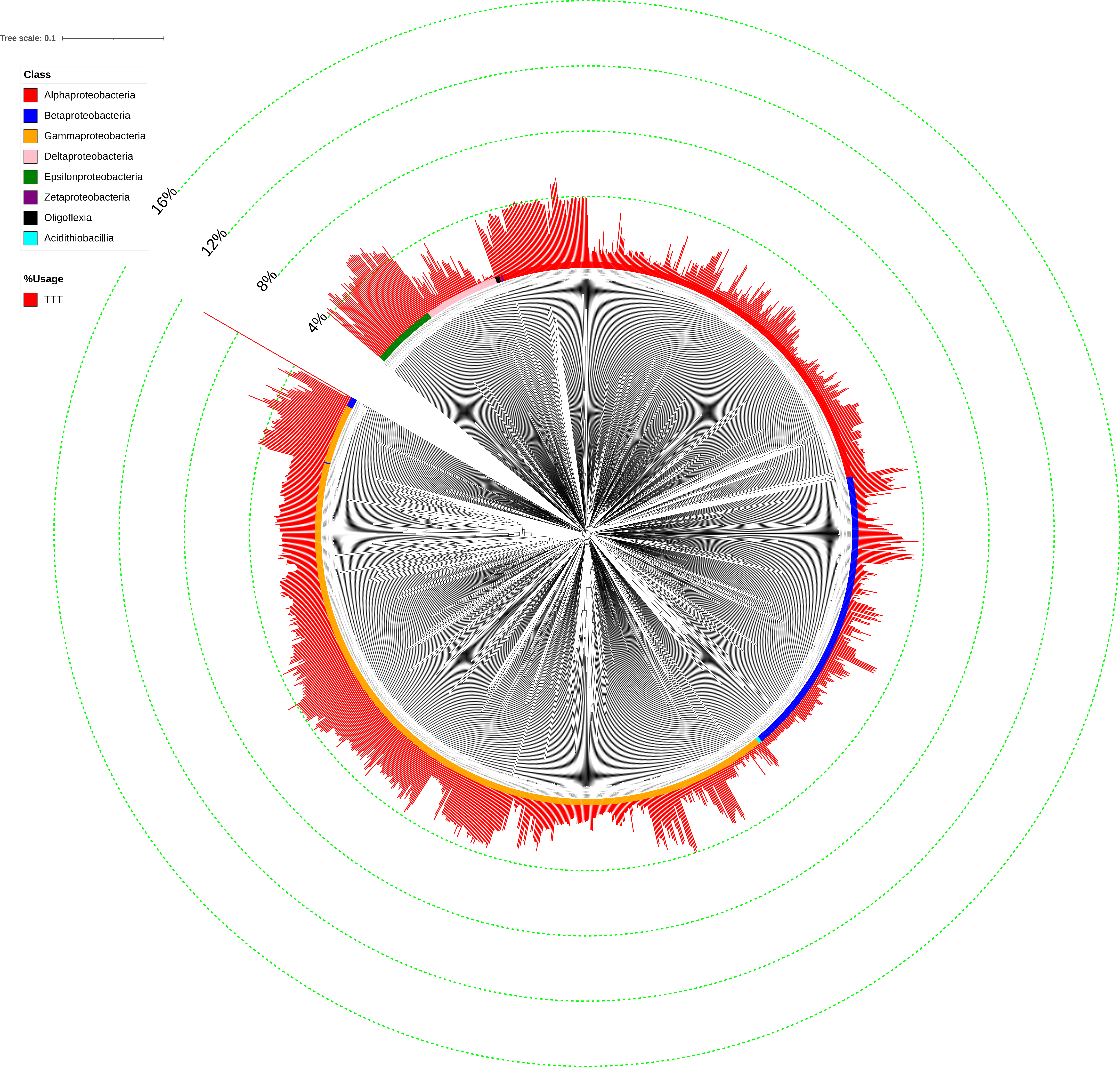

Tree scale: 0.1
Class
Alphaproteobacteria
Betaproteobacteria
Gammaproteobacteria
Deltaproteobacteria
Epsilonproteobacteria
Zetaproteobacteria
Oligoflexia
Acidithiobacillia
%Usage
TTT
4%
8%
12%
16%

Figure S3. Phylogenetic distribution of the frequency of usage of each codon in highly expressed genes of proteobacteria. A phylogenetic tree was constructed and the average frequency of usage for each codon in genes expected to be highly expressed of each genome is indicated using external red bars. Additionally, the class of each specie is indicated in a color strip below the bars (Red: alfa-protebacteria; Blue: beta-proteobacteria; Orange: gamma-proteobacteria; Pink: Delta-proteobacteria; Green: epsilon-proteobacteria; Purple: zeta-proteobacteria; Black: oligoflexia; Lightblue: acidithiobacillia). *Clostridium botulinum* and *Bacillus subtilis* were used as out group for tree construction. Codon usage and class are not presented for these genomes. Interactive version of this figure can be accessed at https://itol.embl.de/tree/200896812320501706546749.

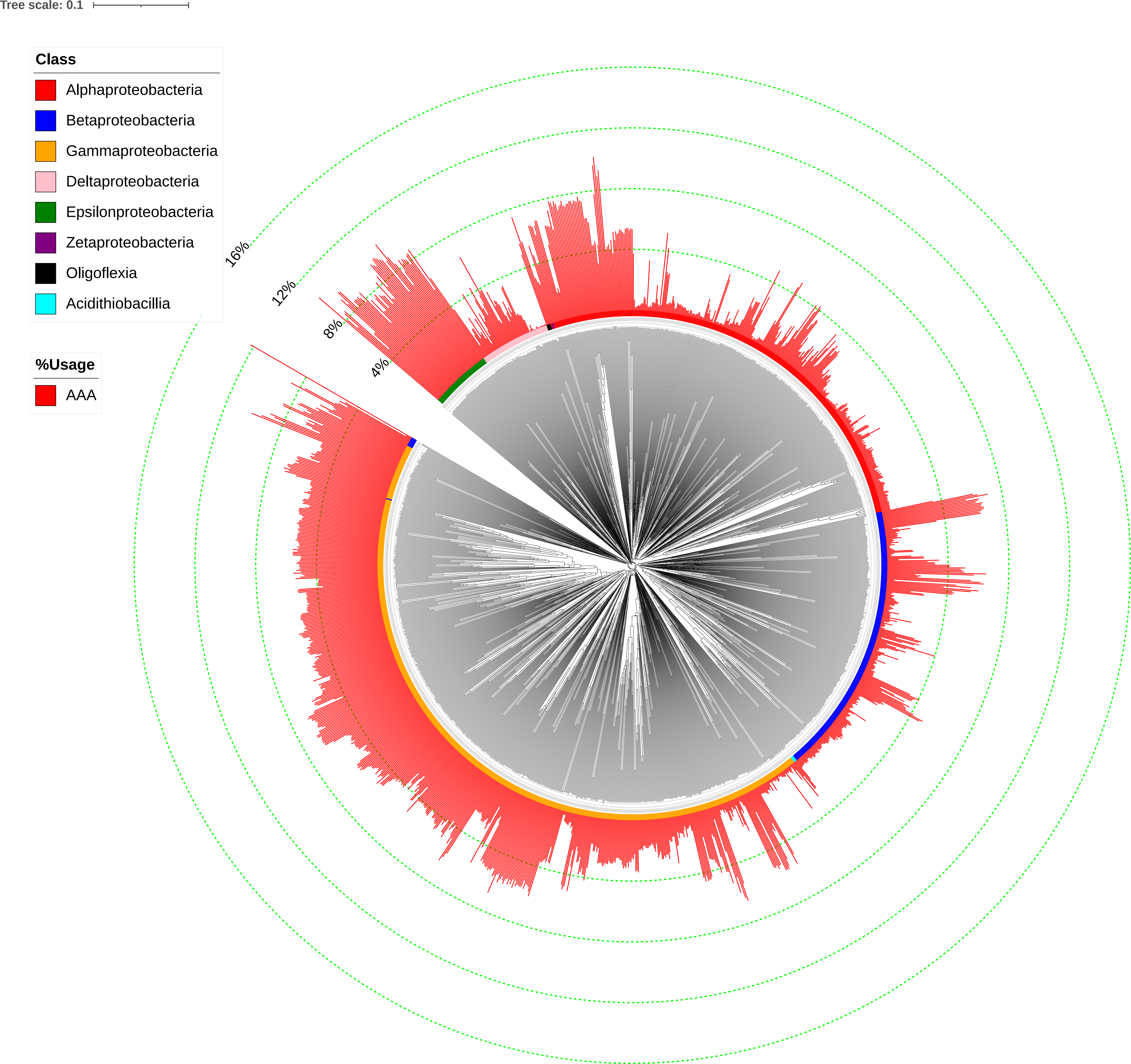

Tree scale: 0.1
Class
Alphaproteobacteria
Betaproteobacteria
Gammaproteobacteria
Deltaproteobacteria
Epsilonproteobacteria
Zetaproteobacteria
Oligoflexia
Acidithiobacillia
%Usage
AAA
16%
12%
8%
4%

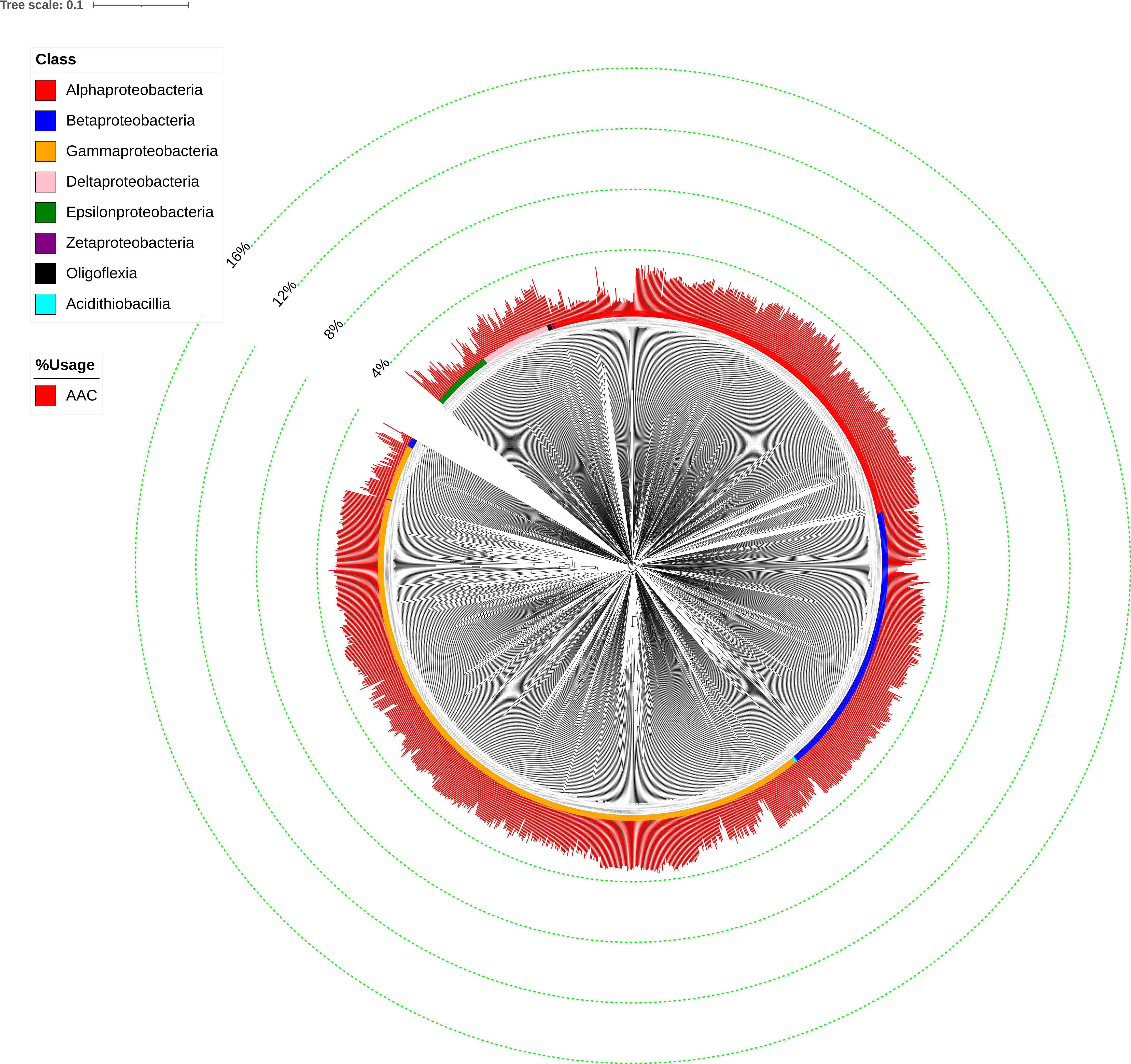
Tree scale: 0.1
Class
Alphaproteobacteria
Betaproteobacteria
Gammaproteobacteria
Deltaproteobacteria
Epsilonproteobacteria
Zetaproteobacteria
Oligoflexia
Acidithiobacillia
%Usage
AAC
16%
12%
8%
4%

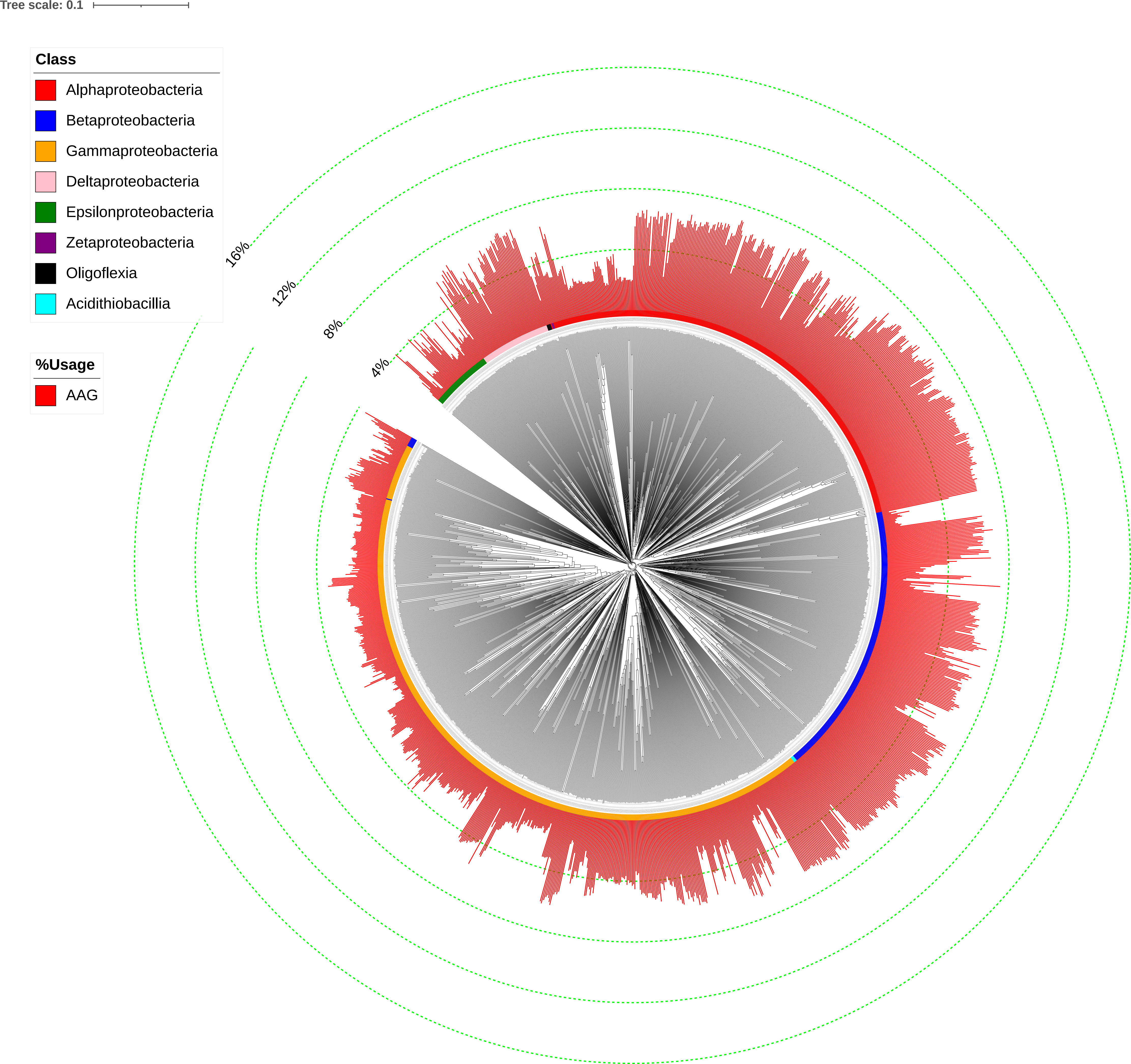

Tree scale: 0.1
Class
Alphaproteobacteria
Betaproteobacteria
Gammaproteobacteria
Deltaproteobacteria
Epsilonproteobacteria
Zetaproteobacteria
Oligoflexia
Acidithiobacillia
%Usage
AAG
4%
8%
12%
16%

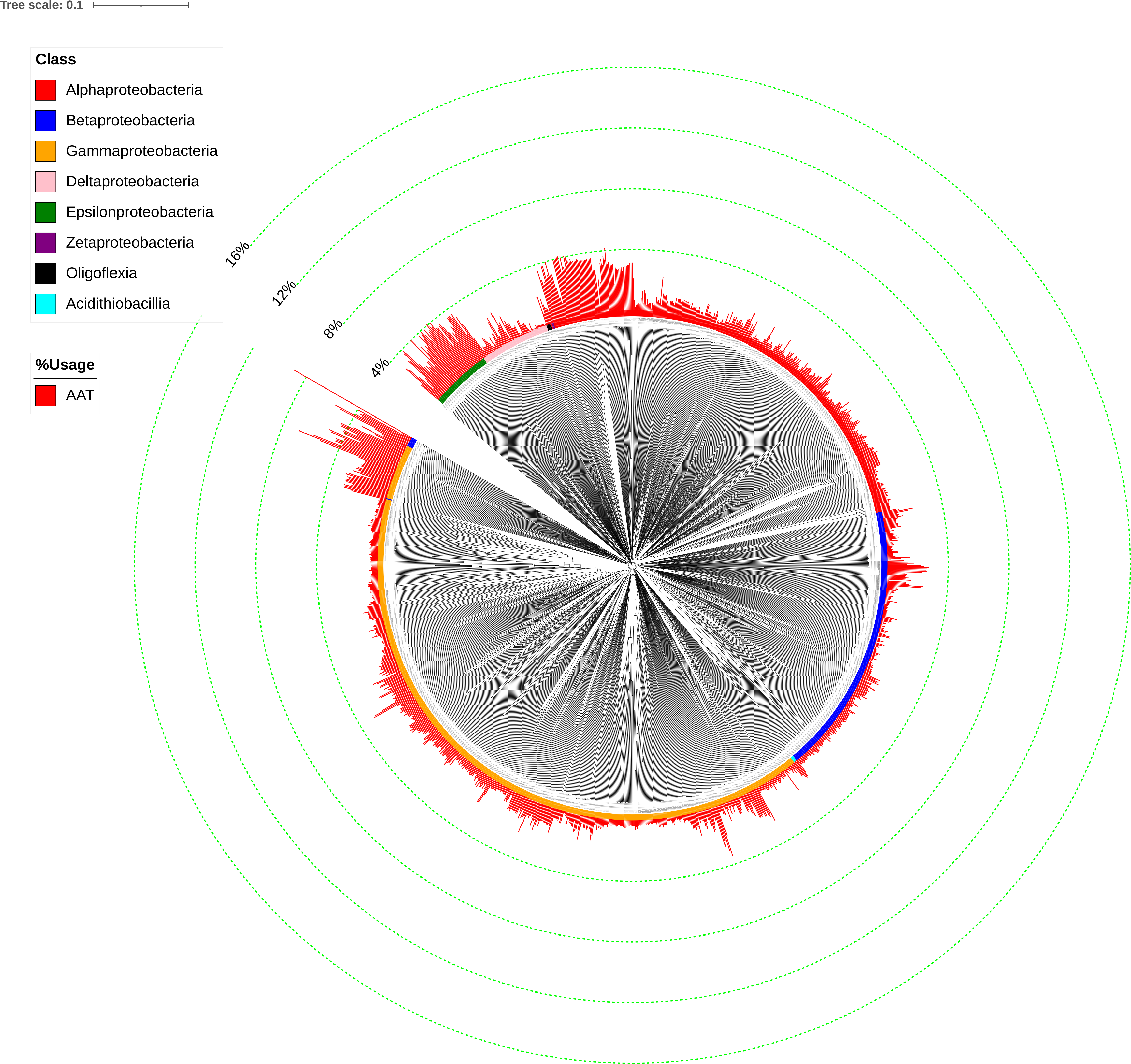

Tree scale: 0.1
Class
Alphaproteobacteria
Betaproteobacteria
Gammaproteobacteria
Deltaproteobacteria
Epsilonproteobacteria
Zetaproteobacteria
Oligoflexia
Acidithiobacillia
%Usage
AAT
4%
8%
12%
16%

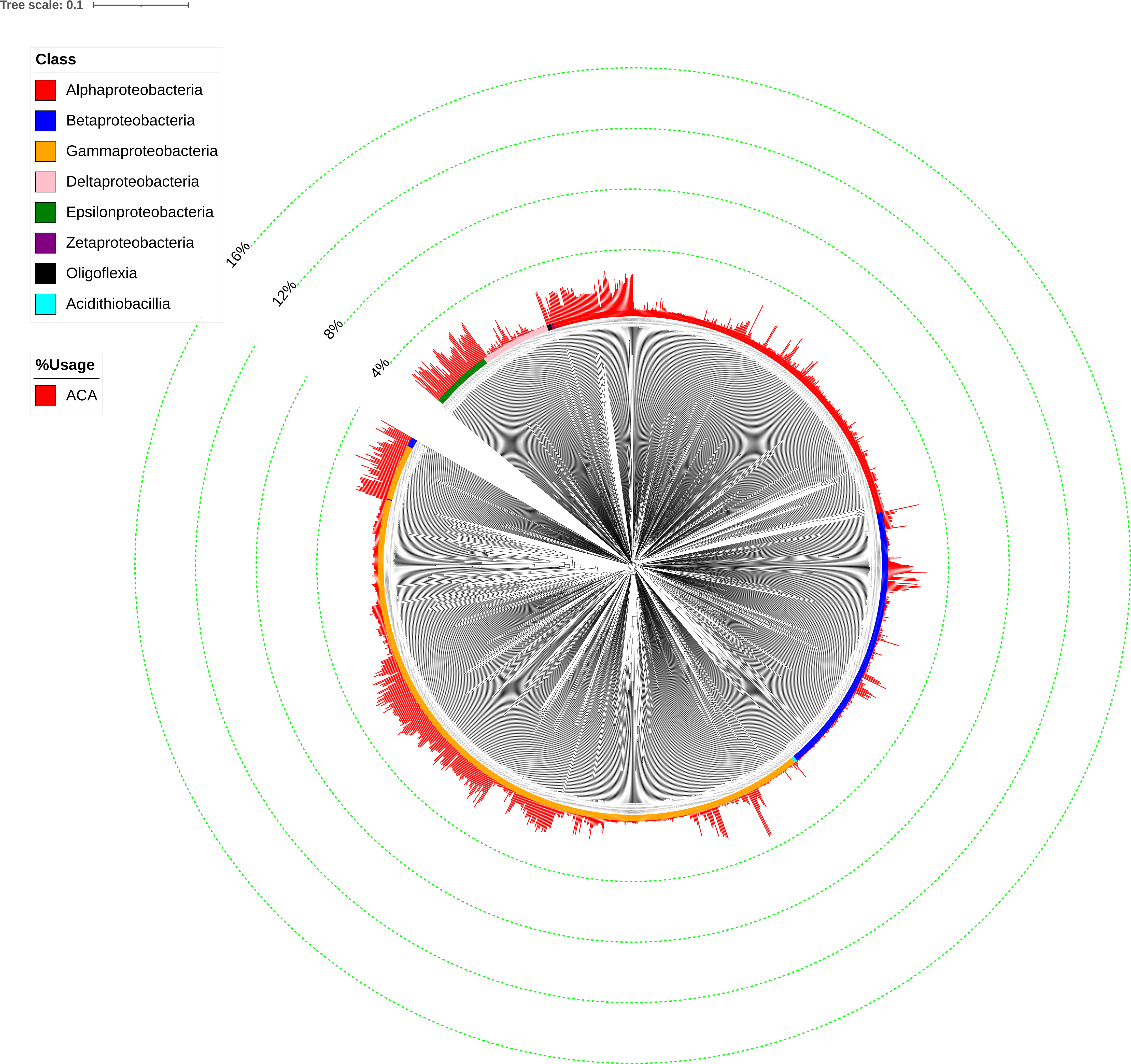
Tree scale: 0.1
Class
Alphaproteobacteria
Betaproteobacteria
Gammaproteobacteria
Deltaproteobacteria
Epsilonproteobacteria
Zetaproteobacteria
Oligoflexia
Acidithiobacillia
%Usage
ACA
16%
12%
8%
4%

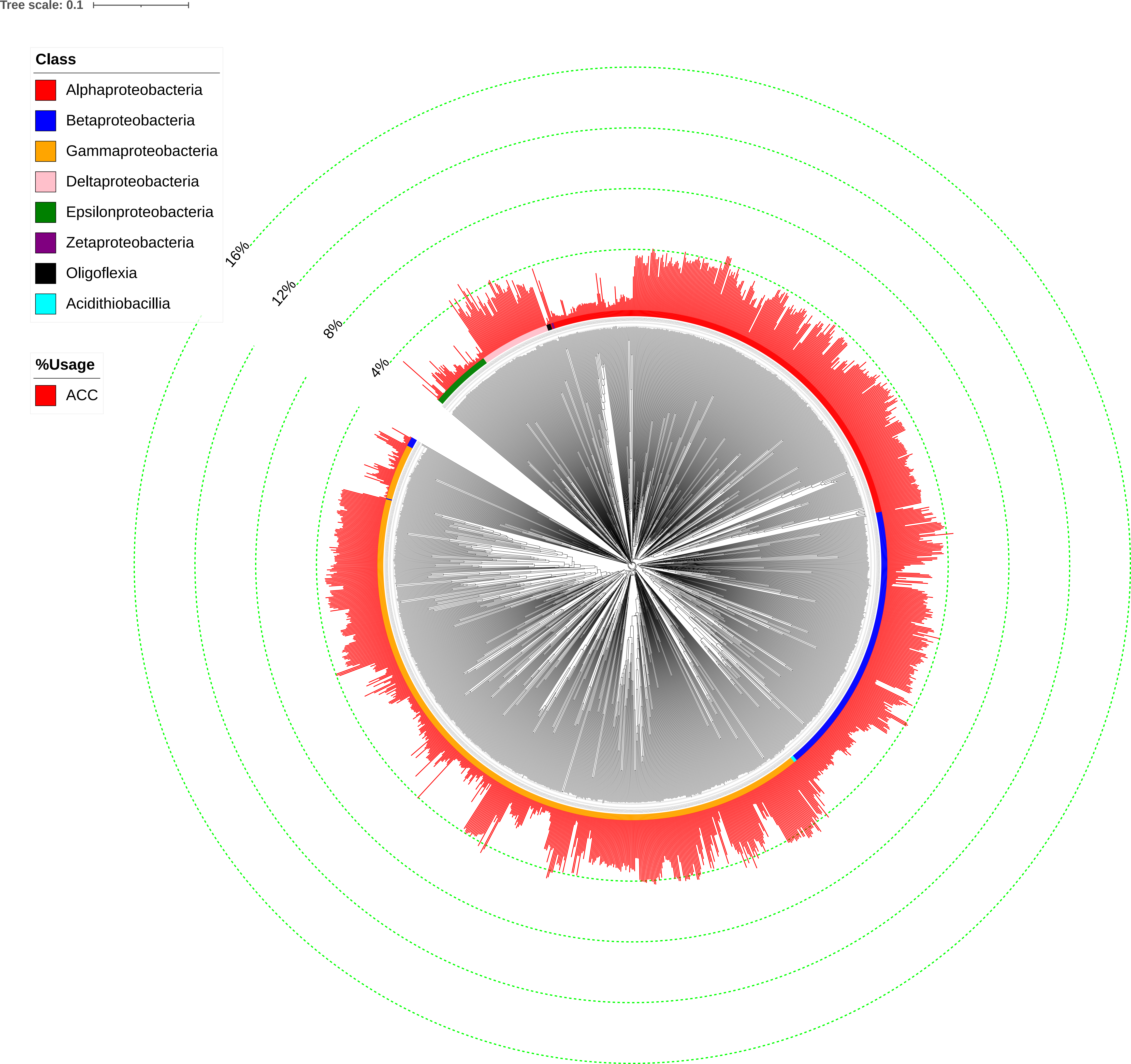
Tree scale: 0.1
Class
Alphaproteobacteria
Betaproteobacteria
Gammaproteobacteria
Deltaproteobacteria
Epsilonproteobacteria
Zetaproteobacteria
Oligoflexia
Acidithiobacillia
%Usage
ACC
16%
12%
8%
4%

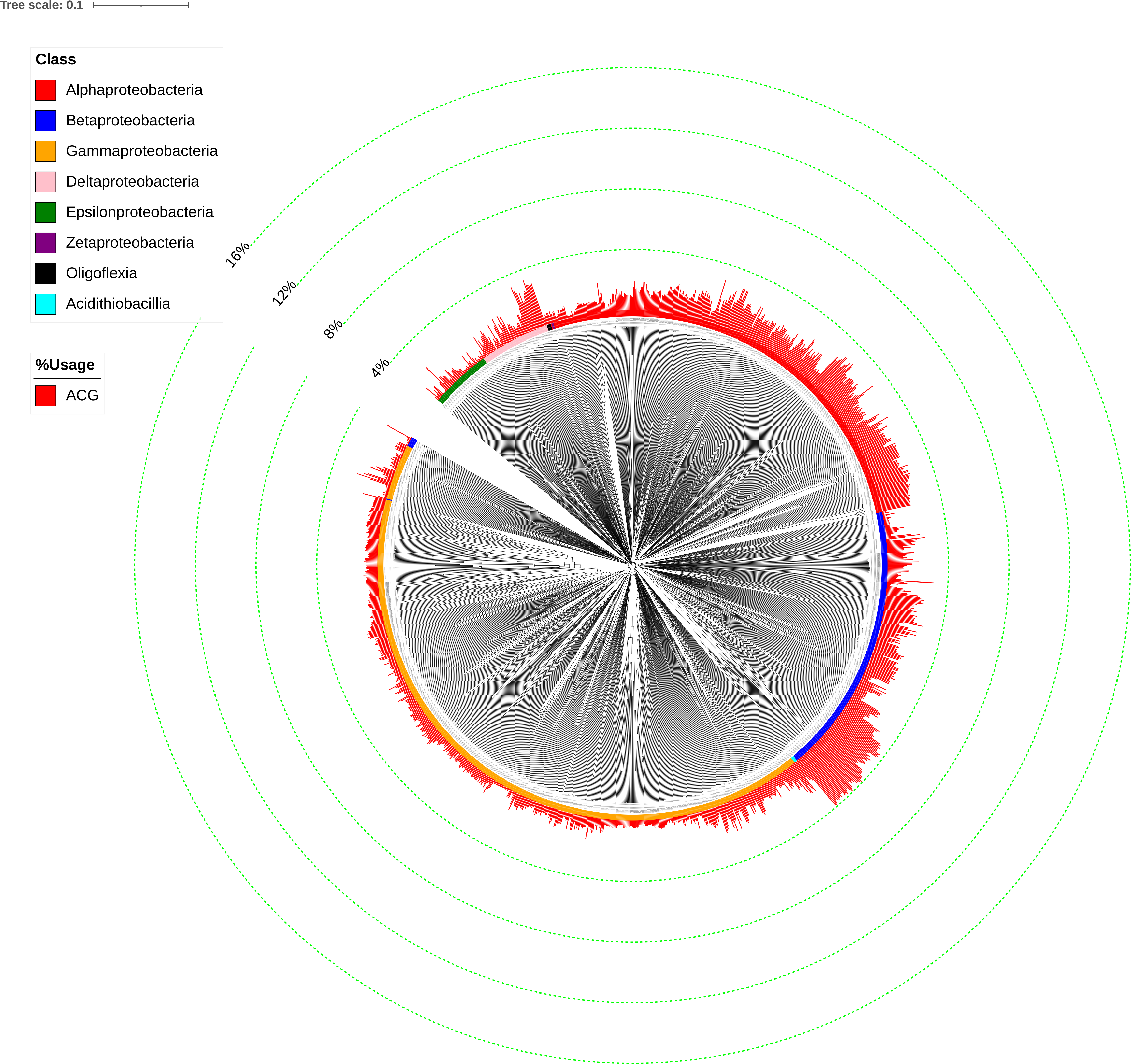
Tree scale: 0.1
Class
Alphaproteobacteria
Betaproteobacteria
Gammaproteobacteria
Deltaproteobacteria
Epsilonproteobacteria
Zetaproteobacteria
Oligoflexia
Acidithiobacillia
%Usage
ACG
16%
12%
8%
4%

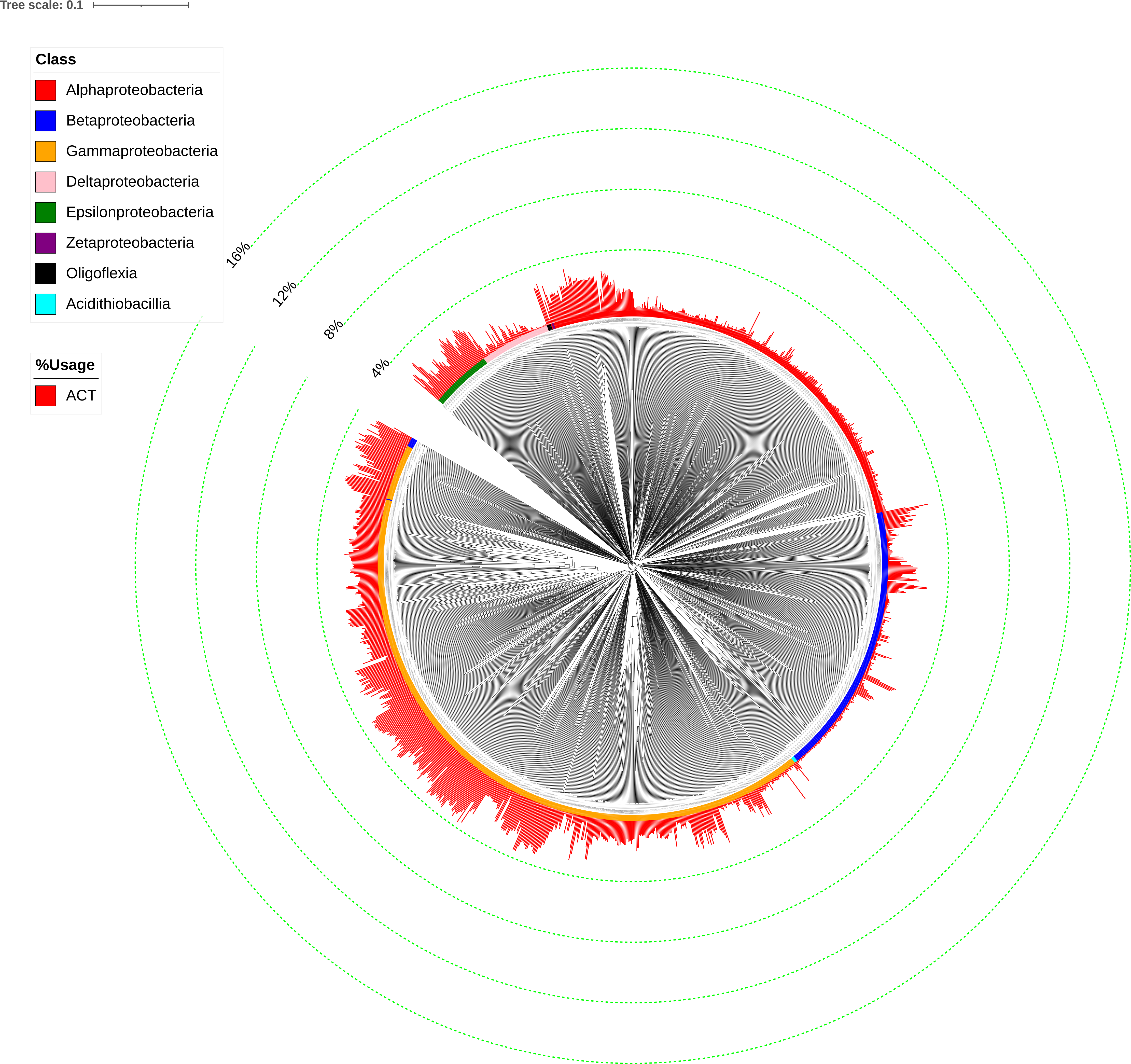
Tree scale: 0.1
Class
Alphaproteobacteria
Betaproteobacteria
Gammaproteobacteria
Deltaproteobacteria
Epsilonproteobacteria
Zetaproteobacteria
Oligoflexia
Acidithiobacillia
%Usage
ACT
16%
12%
8%
4%

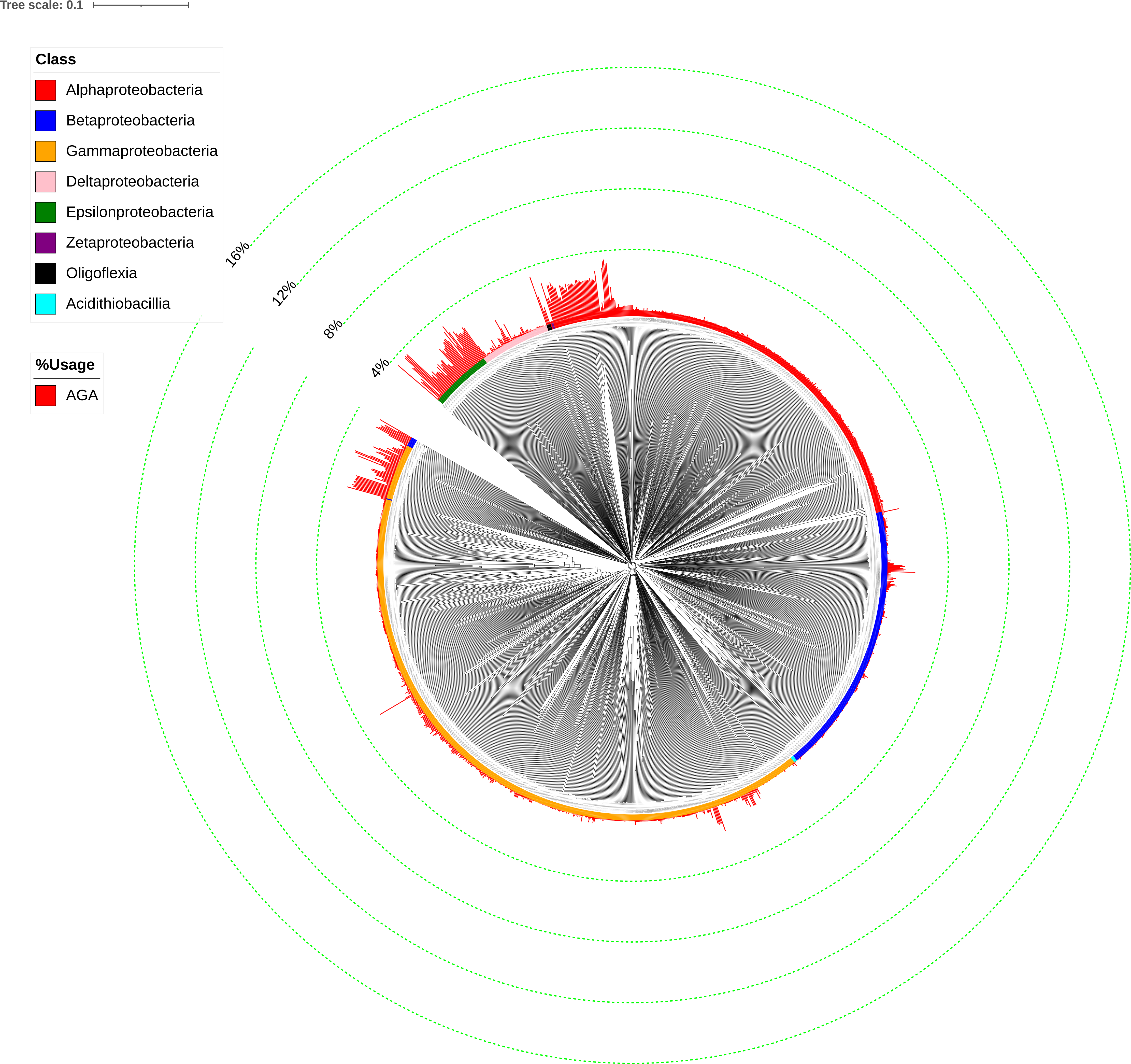

Tree scale: 0.1
Class
Alphaproteobacteria
Betaproteobacteria
Gammaproteobacteria
Deltaproteobacteria
Epsilonproteobacteria
Zetaproteobacteria
Oligoflexia
Acidithiobacillia
%Usage
AGA
16%
12%
8%
4%

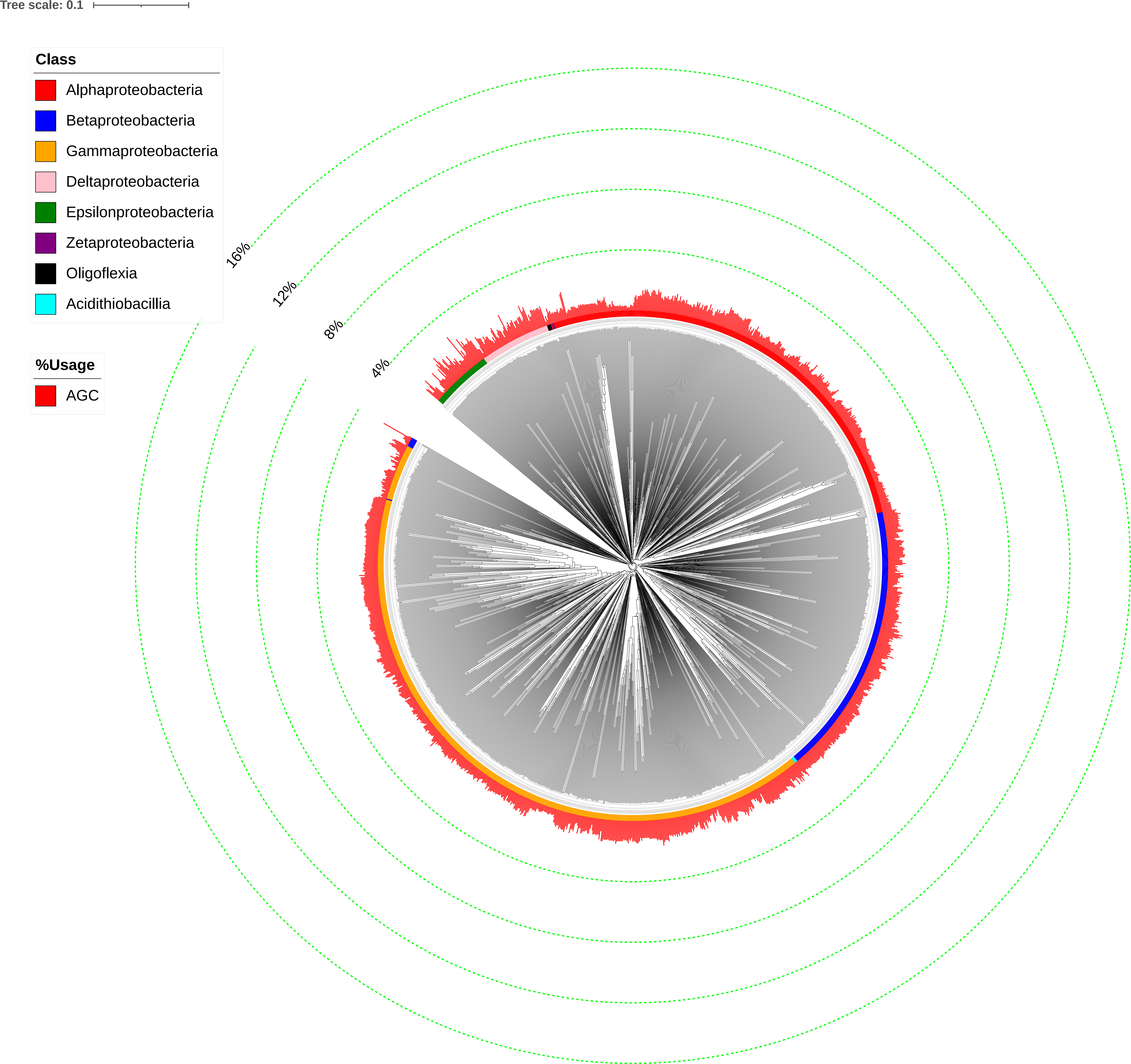

Tree scale: 0.1
Class
Alphaproteobacteria
Betaproteobacteria
Gammaproteobacteria
Deltaproteobacteria
Epsilonproteobacteria
Zetaproteobacteria
Oligoflexia
Acidithiobacillia
%Usage
AGC
4%
8%
12%
16%

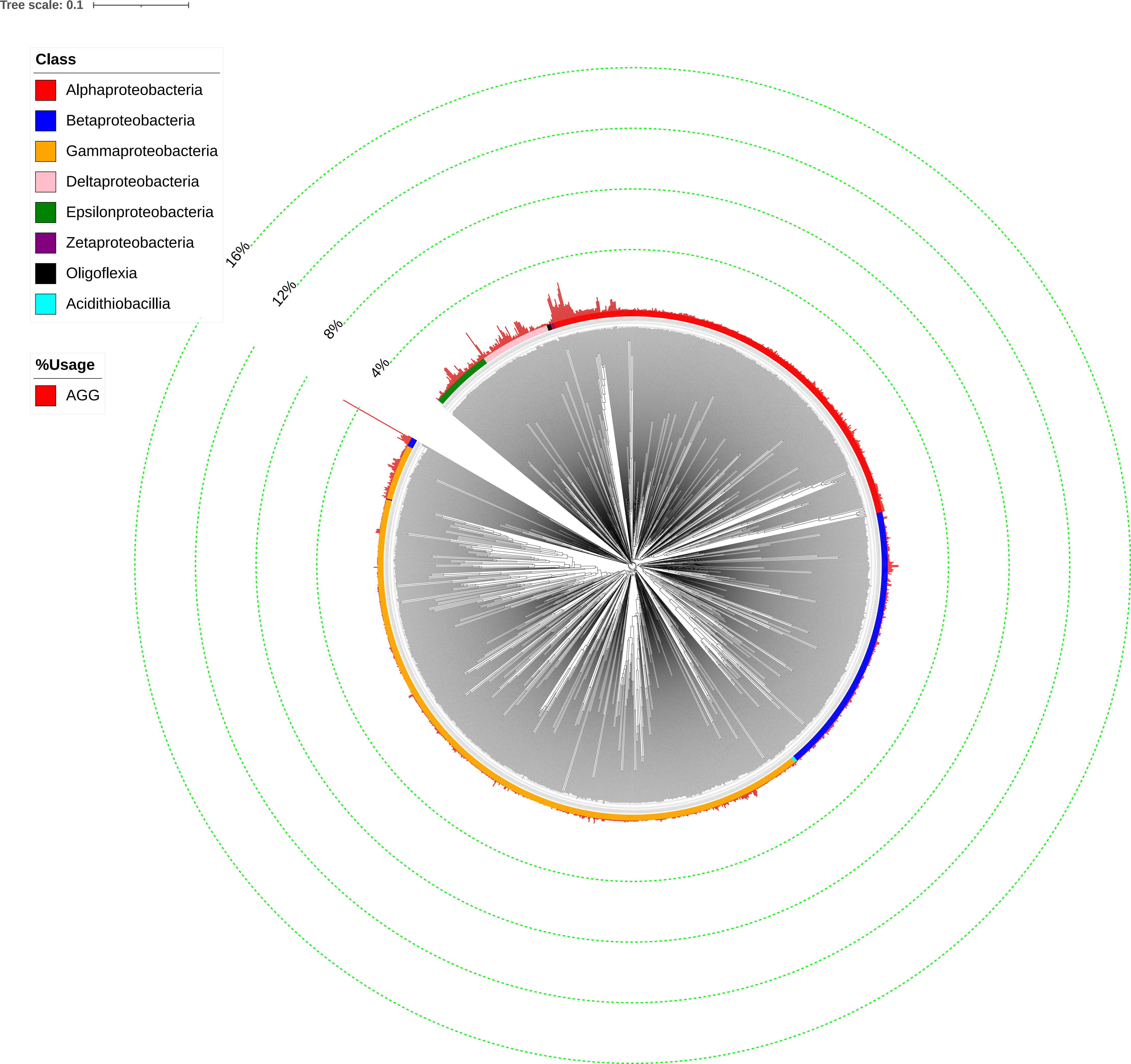

Tree scale: 0.1
Class
Alphaproteobacteria
Betaproteobacteria
Gammaproteobacteria
Deltaproteobacteria
Epsilonproteobacteria
Zetaproteobacteria
Oligoflexia
Acidithiobacillia
%Usage
AGG
4%
8%
12%
16%

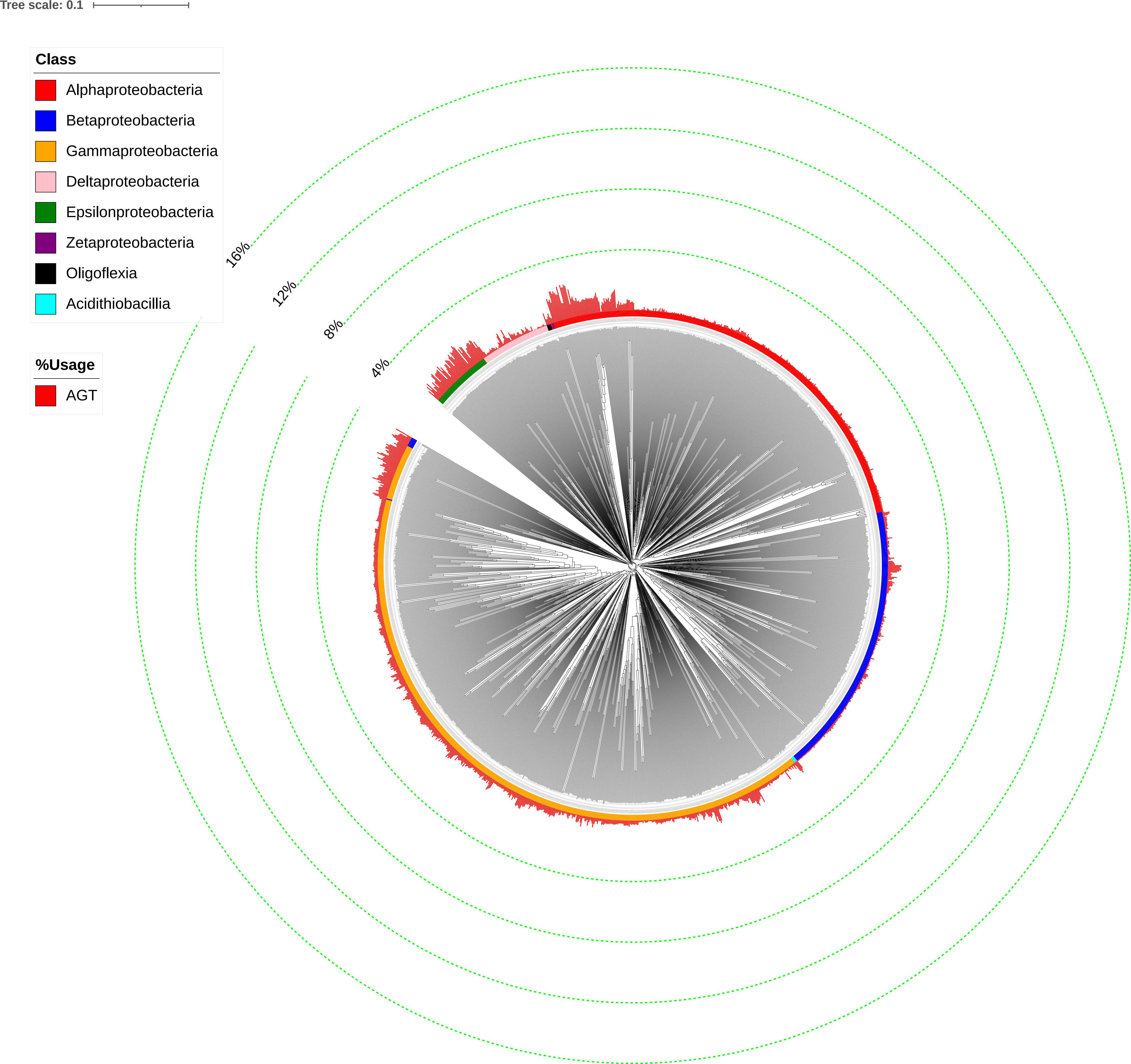

Tree scale: 0.1
Class
Alphaproteobacteria
Betaproteobacteria
Gammaproteobacteria
Deltaproteobacteria
Epsilonproteobacteria
Zetaproteobacteria
Oligoflexia
Acidithiobacillia
%Usage
AGT
16%
12%
8%
4%

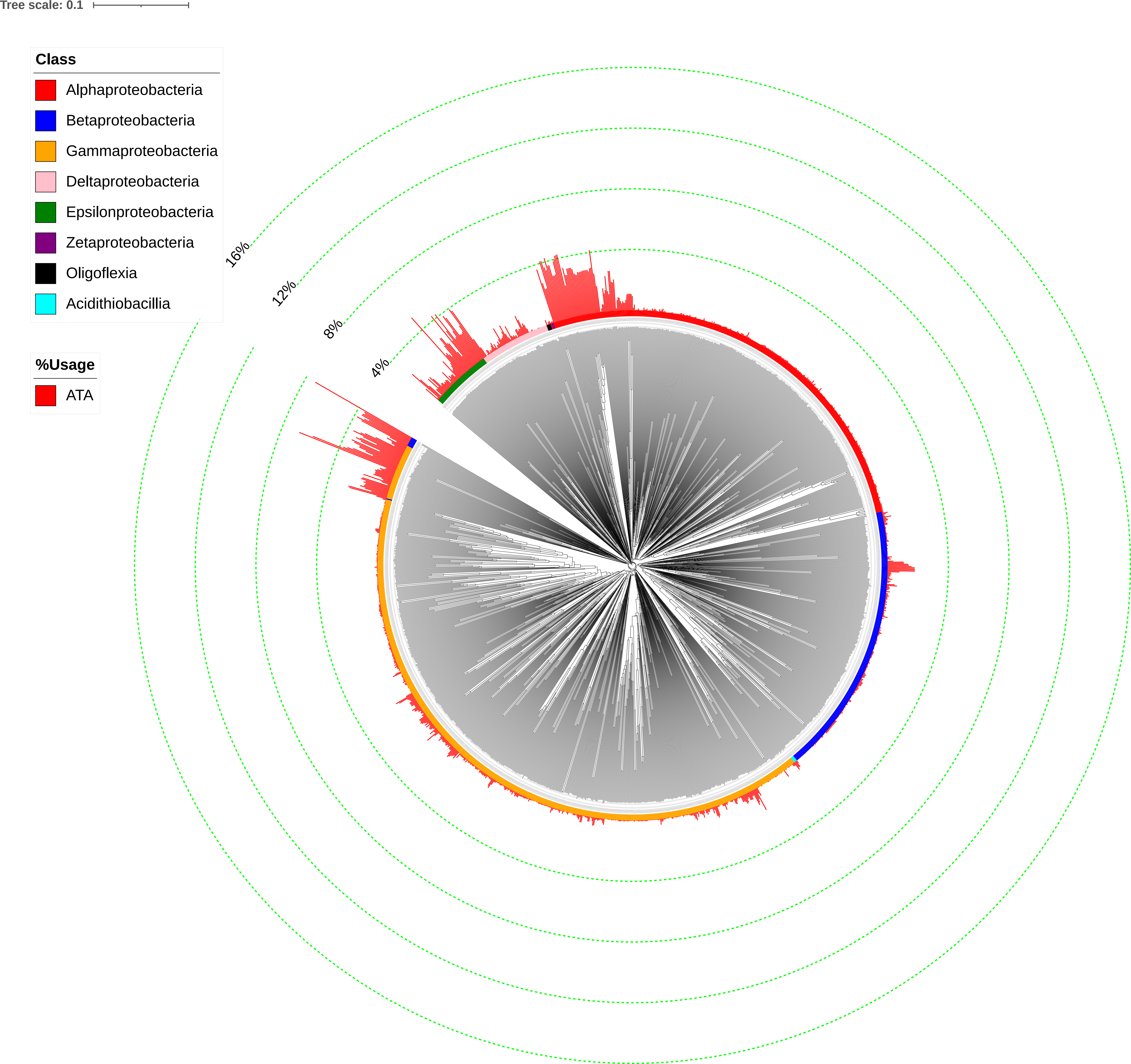
Tree scale: 0.1
Class
Alphaproteobacteria
Betaproteobacteria
Gammaproteobacteria
Deltaproteobacteria
Epsilonproteobacteria
Zetaproteobacteria
Oligoflexia
Acidithiobacillia
%Usage
ATA
4%
8%
12%
16%

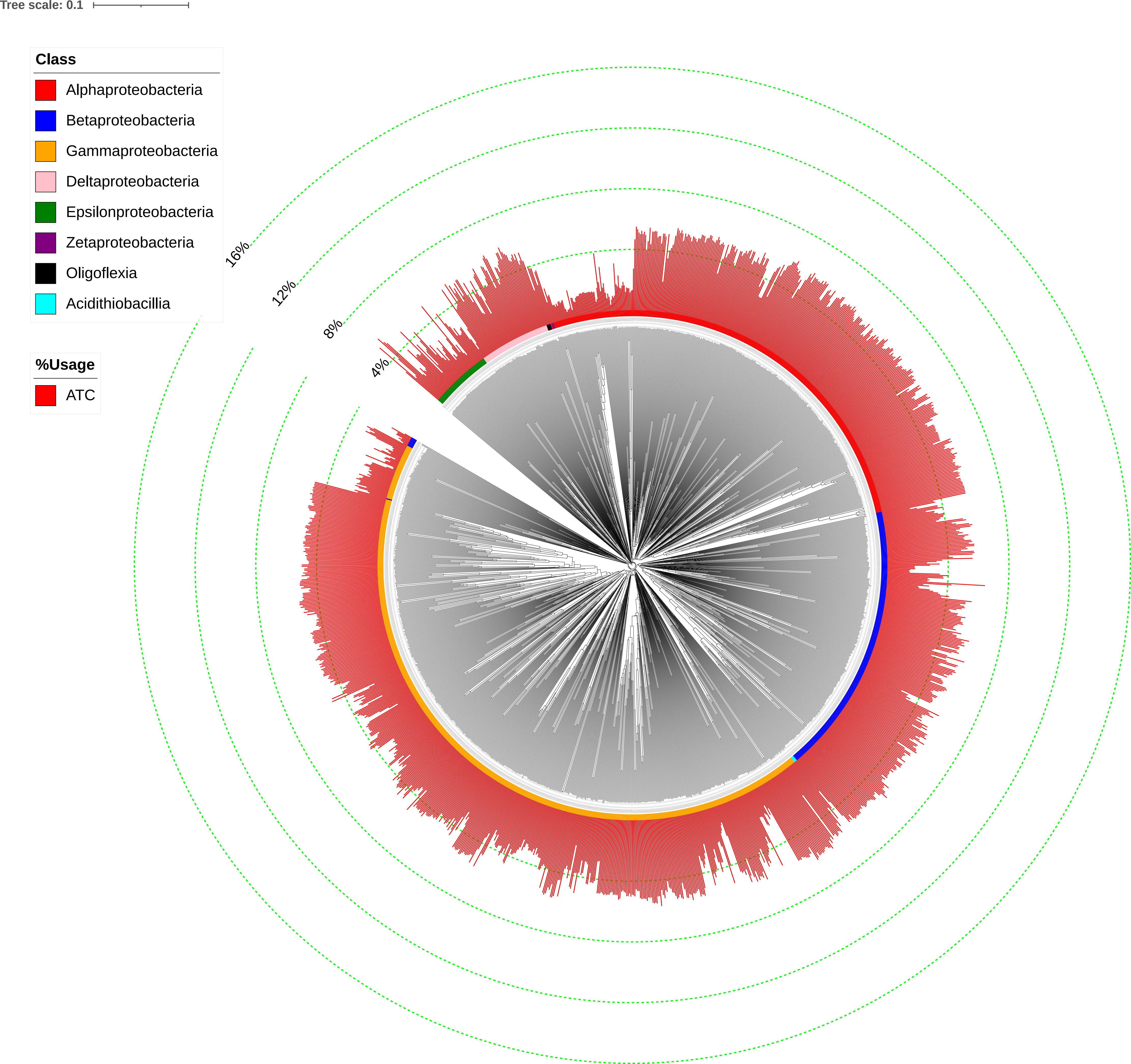
Tree scale: 0.1
Class
Alphaproteobacteria
Betaproteobacteria
Gammaproteobacteria
Deltaproteobacteria
Epsilonproteobacteria
Zetaproteobacteria
Oligoflexia
Acidithiobacillia
%Usage
ATC
16%
12%
8%
4%

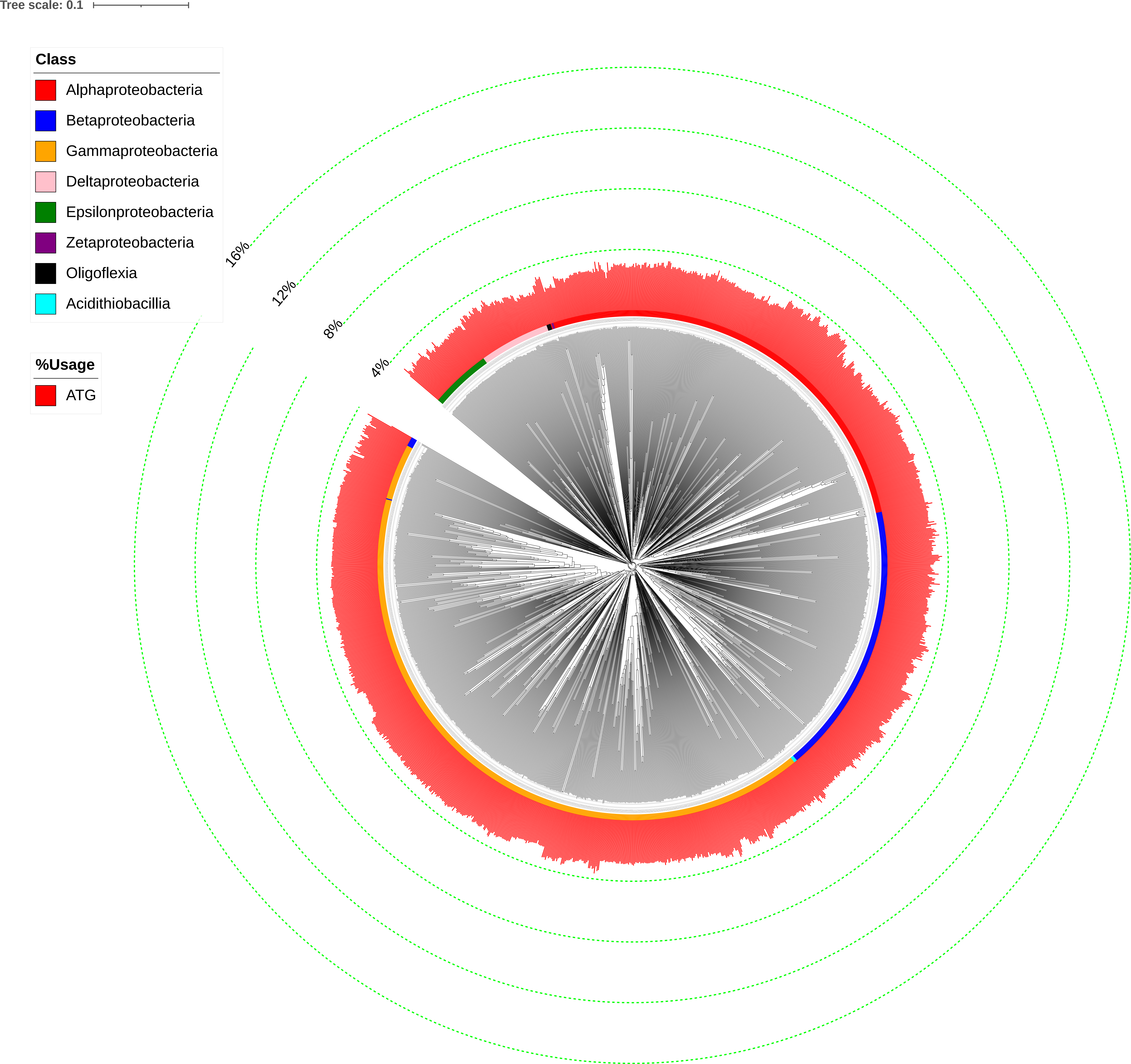
Tree scale: 0.1
Class
Alphaproteobacteria
Betaproteobacteria
Gammaproteobacteria
Deltaproteobacteria
Epsilonproteobacteria
Zetaproteobacteria
Oligoflexia
Acidithiobacillia
%Usage
ATG
16%
12%
8%
4%

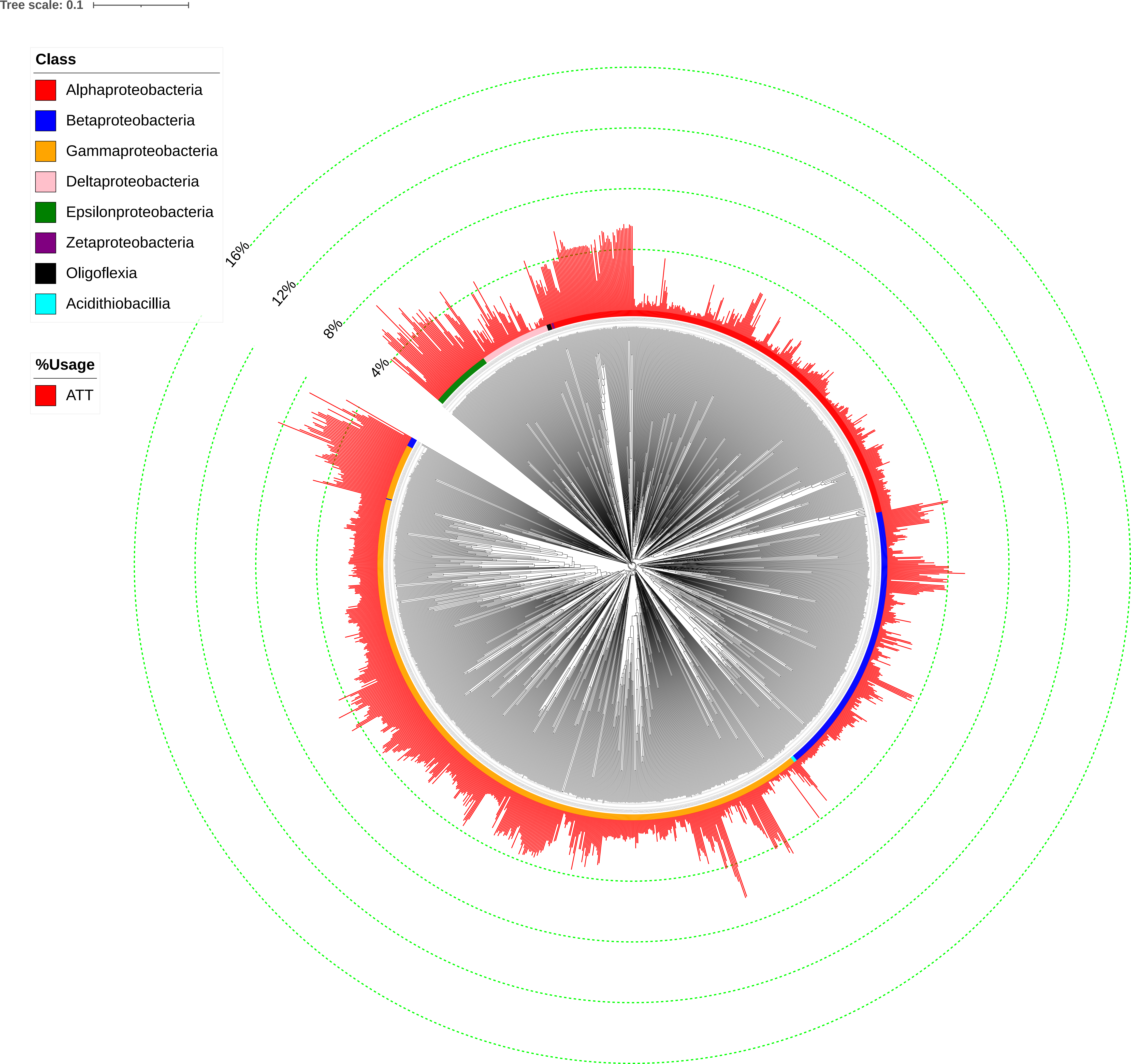

Tree scale: 0.1
Class
Alphaproteobacteria
Betaproteobacteria
Gammaproteobacteria
Deltaproteobacteria
Epsilonproteobacteria
Zetaproteobacteria
Oligoflexia
Acidithiobacillia
%Usage
ATT
4%
8%
12%
16%

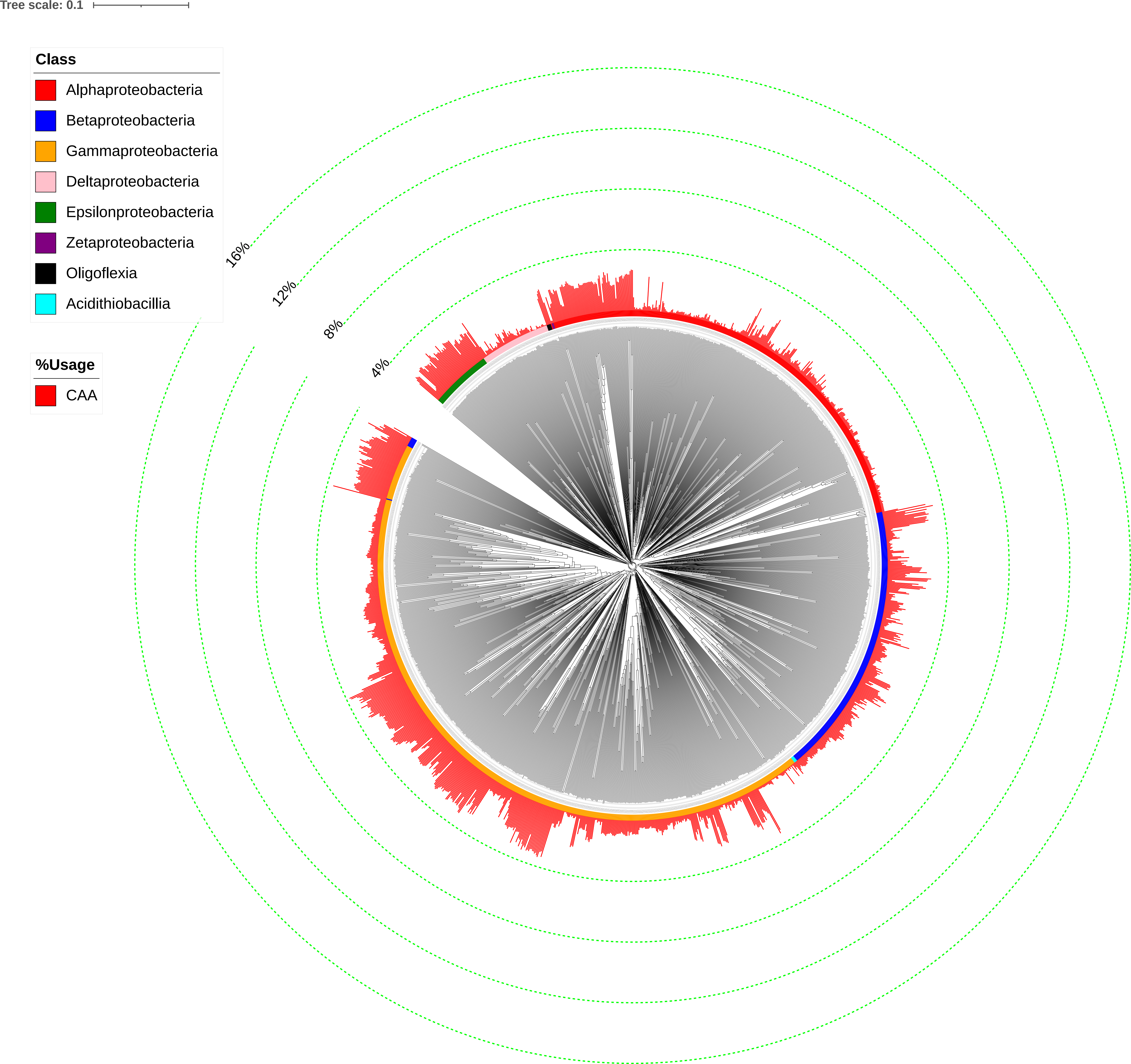
Tree scale: 0.1
Class
Alphaproteobacteria
Betaproteobacteria
Gammaproteobacteria
Deltaproteobacteria
Epsilonproteobacteria
Zetaproteobacteria
Oligoflexia
Acidithiobacillia
%Usage
CAA
4%
8%
12%
16%

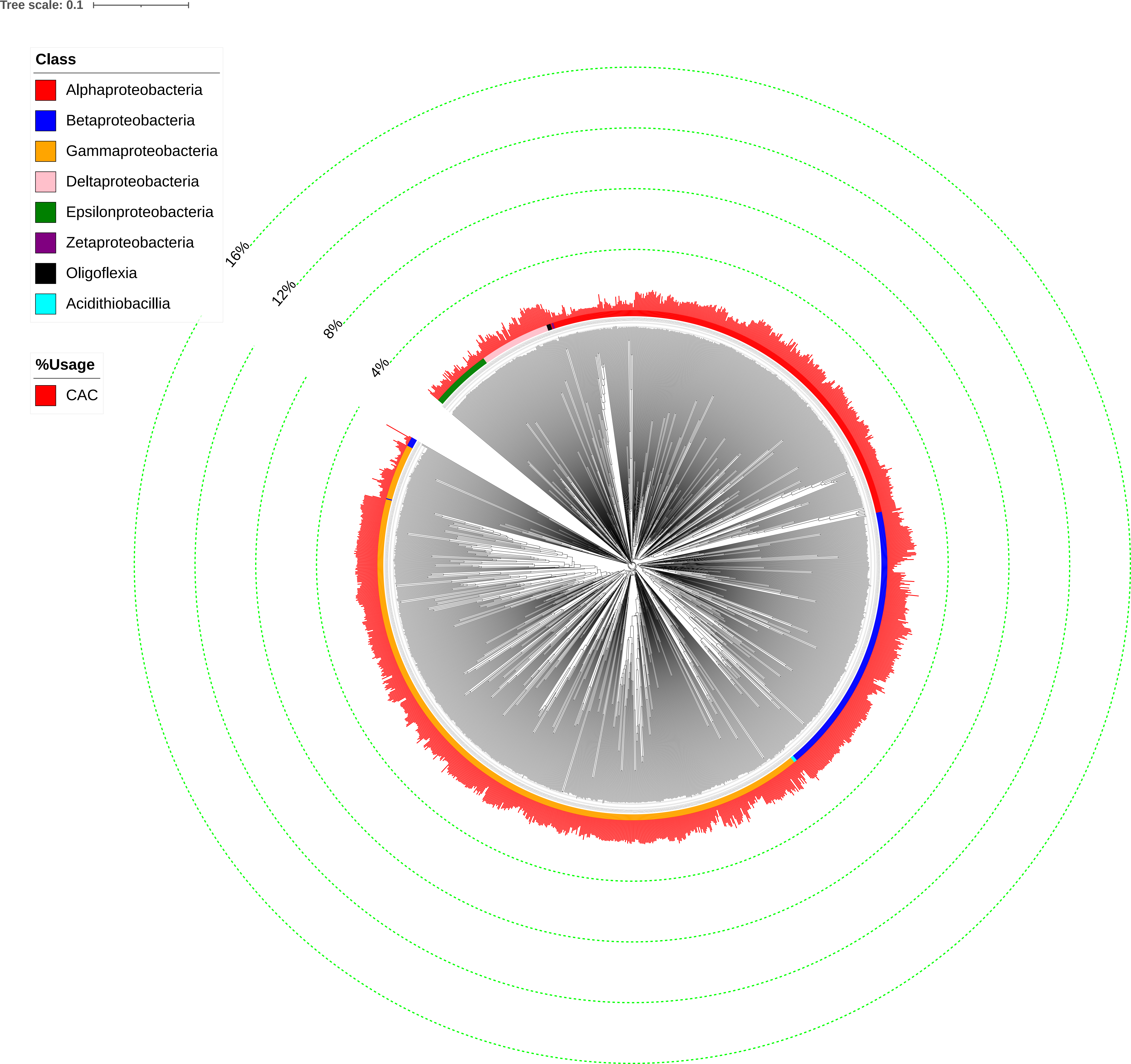
Tree scale: 0.1
Class
Alphaproteobacteria
Betaproteobacteria
Gammaproteobacteria
Deltaproteobacteria
Epsilonproteobacteria
Zetaproteobacteria
Oligoflexia
Acidithiobacillia
%Usage
CAC
16%
12%
8%
4%

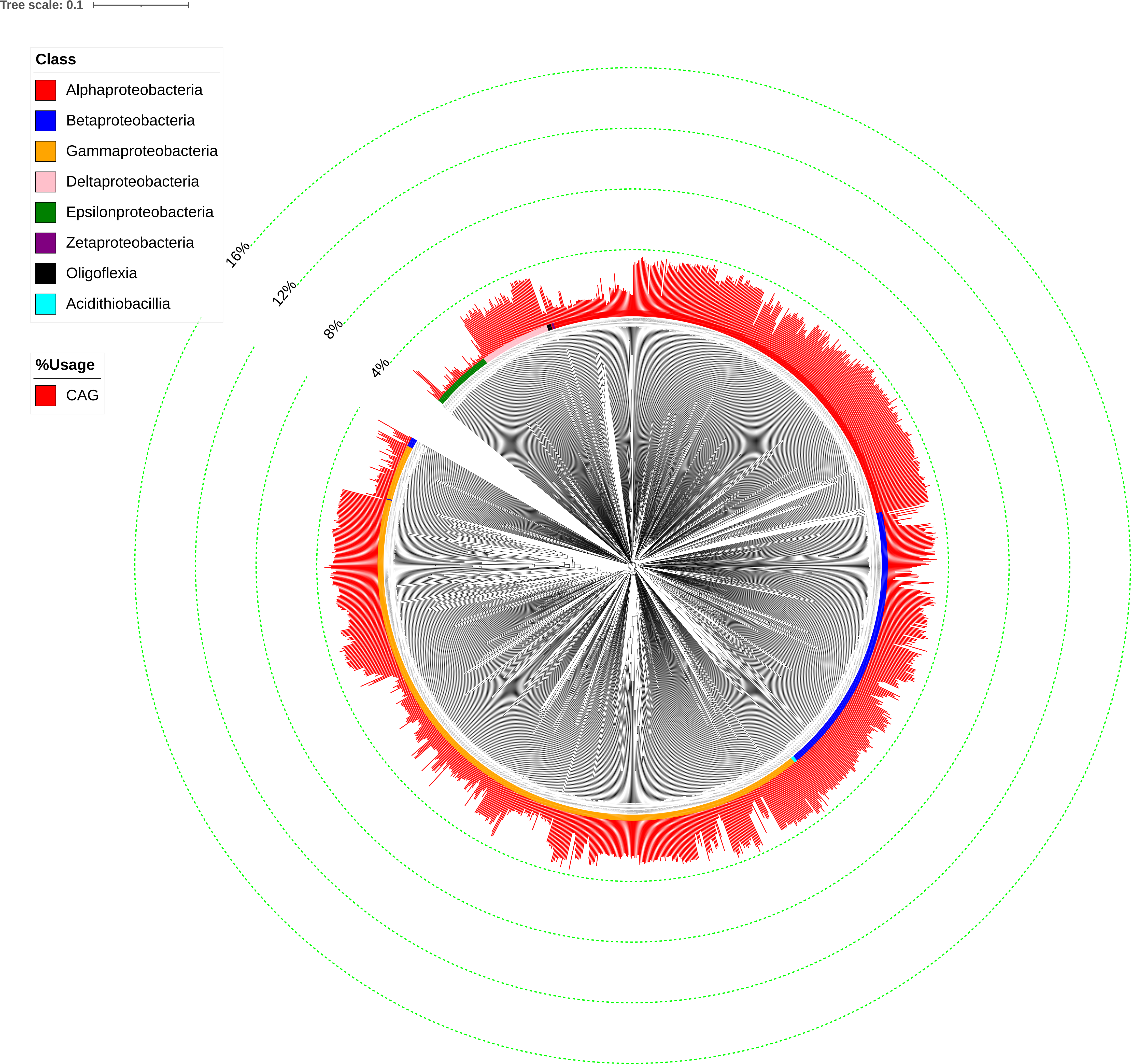

Tree scale: 0.1
Class
Alphaproteobacteria
Betaproteobacteria
Gammaproteobacteria
Deltaproteobacteria
Epsilonproteobacteria
Zetaproteobacteria
Oligoflexia
Acidithiobacillia
%Usage
CAG
16%
12%
8%
4%

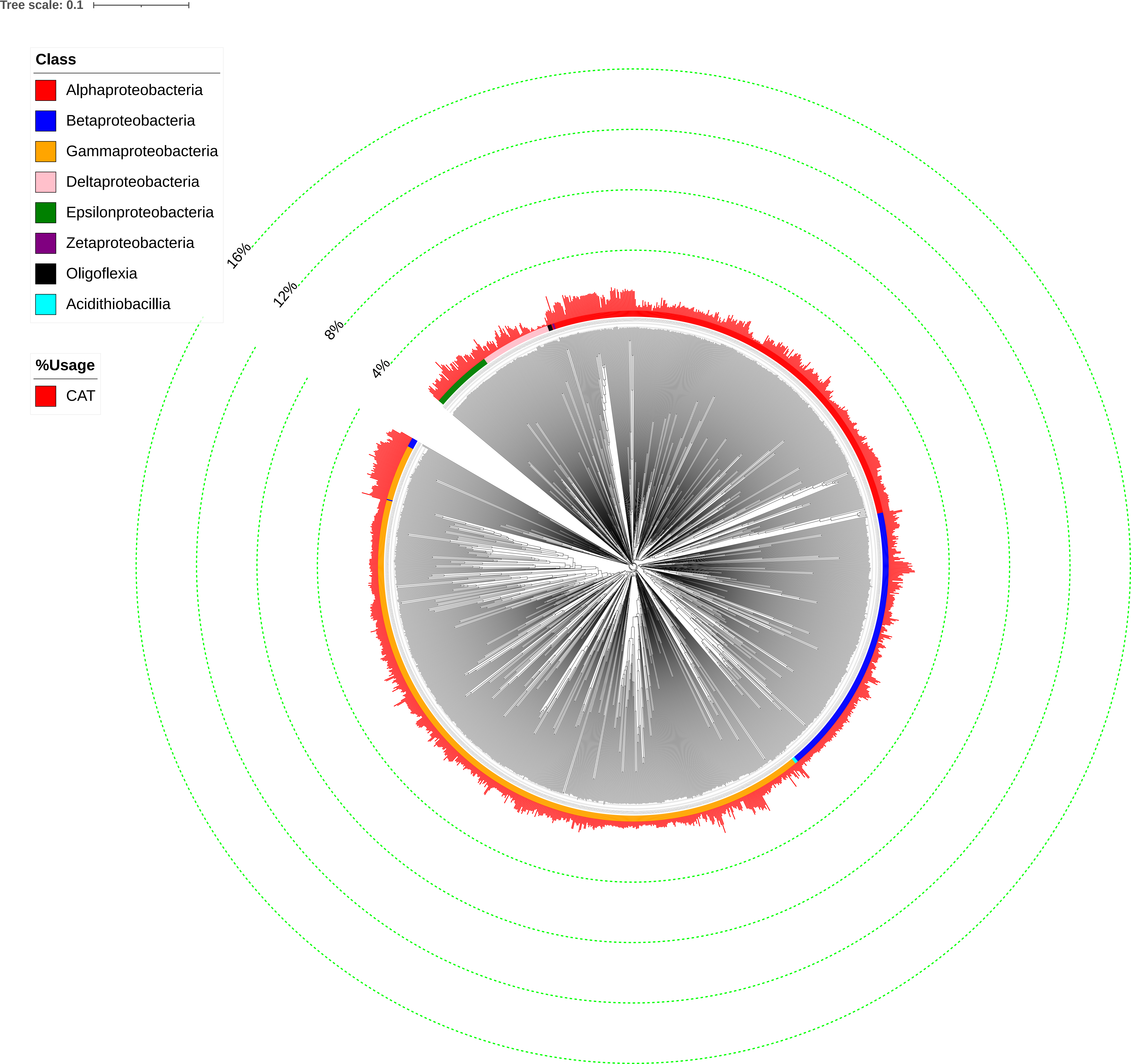

Tree scale: 0.1
Class
Alphaproteobacteria
Betaproteobacteria
Gammaproteobacteria
Deltaproteobacteria
Epsilonproteobacteria
Zetaproteobacteria
Oligoflexia
Acidithiobacillia
%Usage
CAT
16%
12%
8%
4%

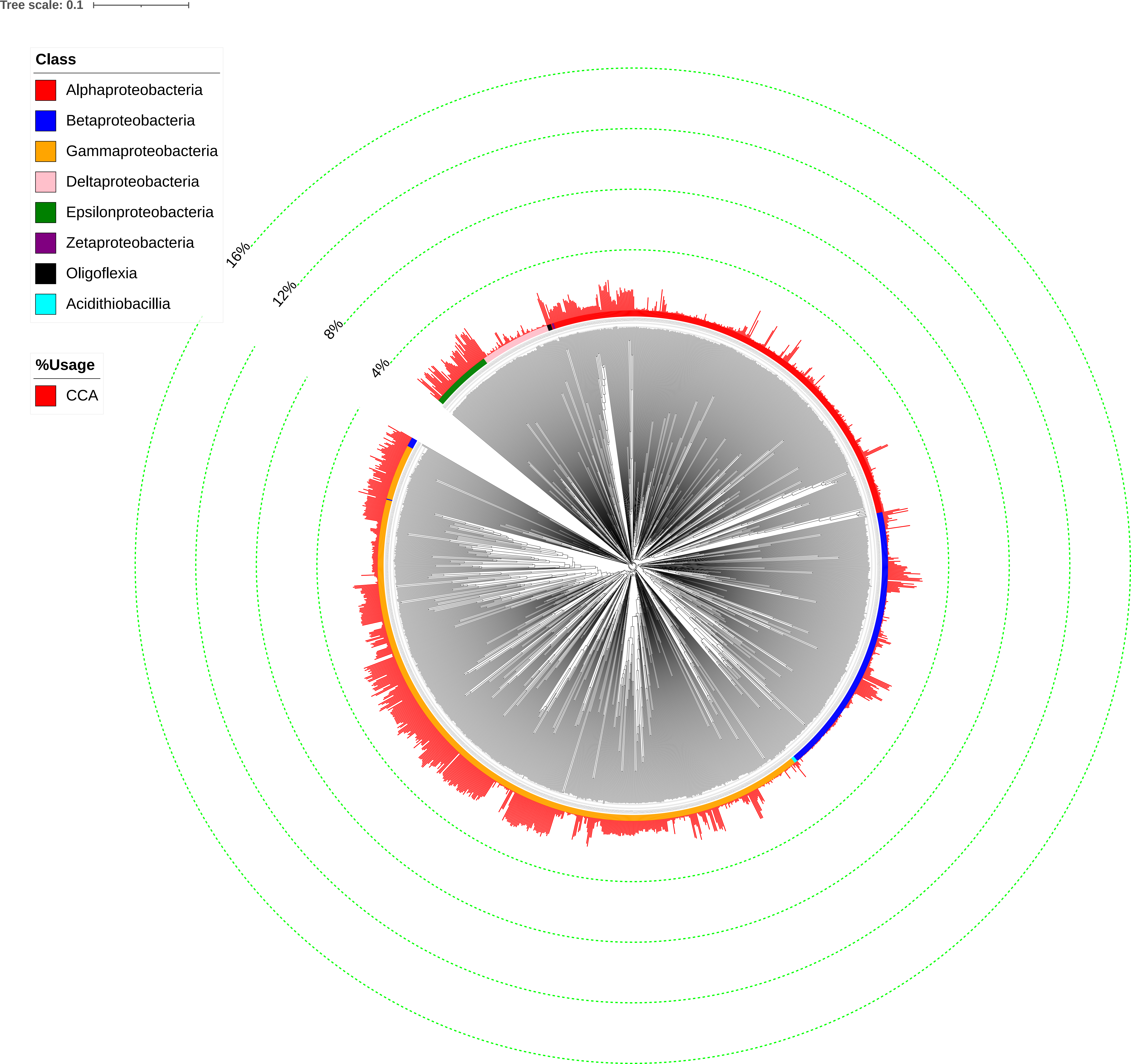
Tree scale: 0.1
Class
Alphaproteobacteria
Betaproteobacteria
Gammaproteobacteria
Deltaproteobacteria
Epsilonproteobacteria
Zetaproteobacteria
Oligoflexia
Acidithiobacillia
%Usage
CCA
16%
12%
8%
4%

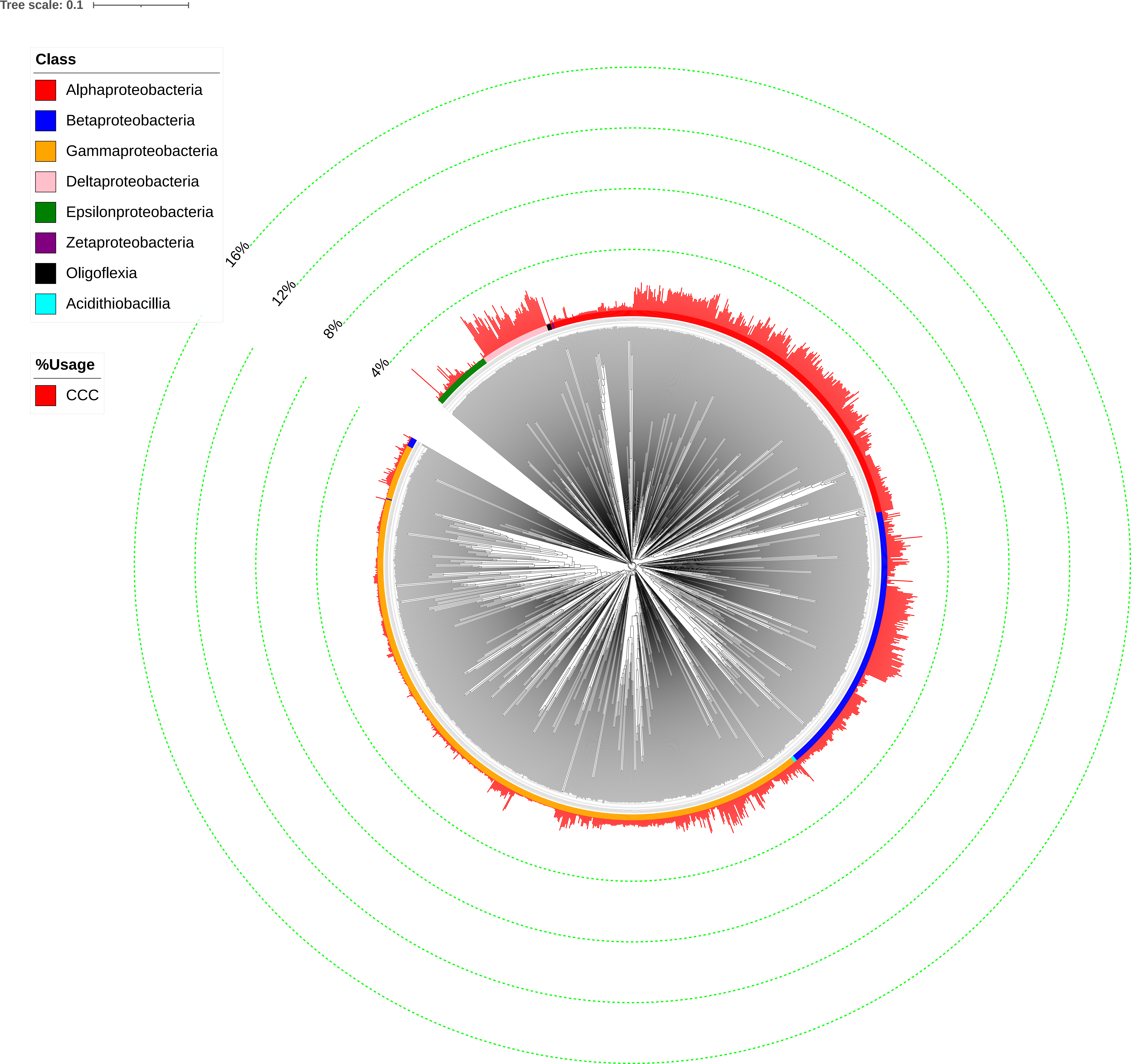
Tree scale: 0.1
Class
Alphaproteobacteria
Betaproteobacteria
Gammaproteobacteria
Deltaproteobacteria
Epsilonproteobacteria
Zetaproteobacteria
Oligoflexia
Acidithiobacillia
%Usage
CCC
16%
12%
8%
4%

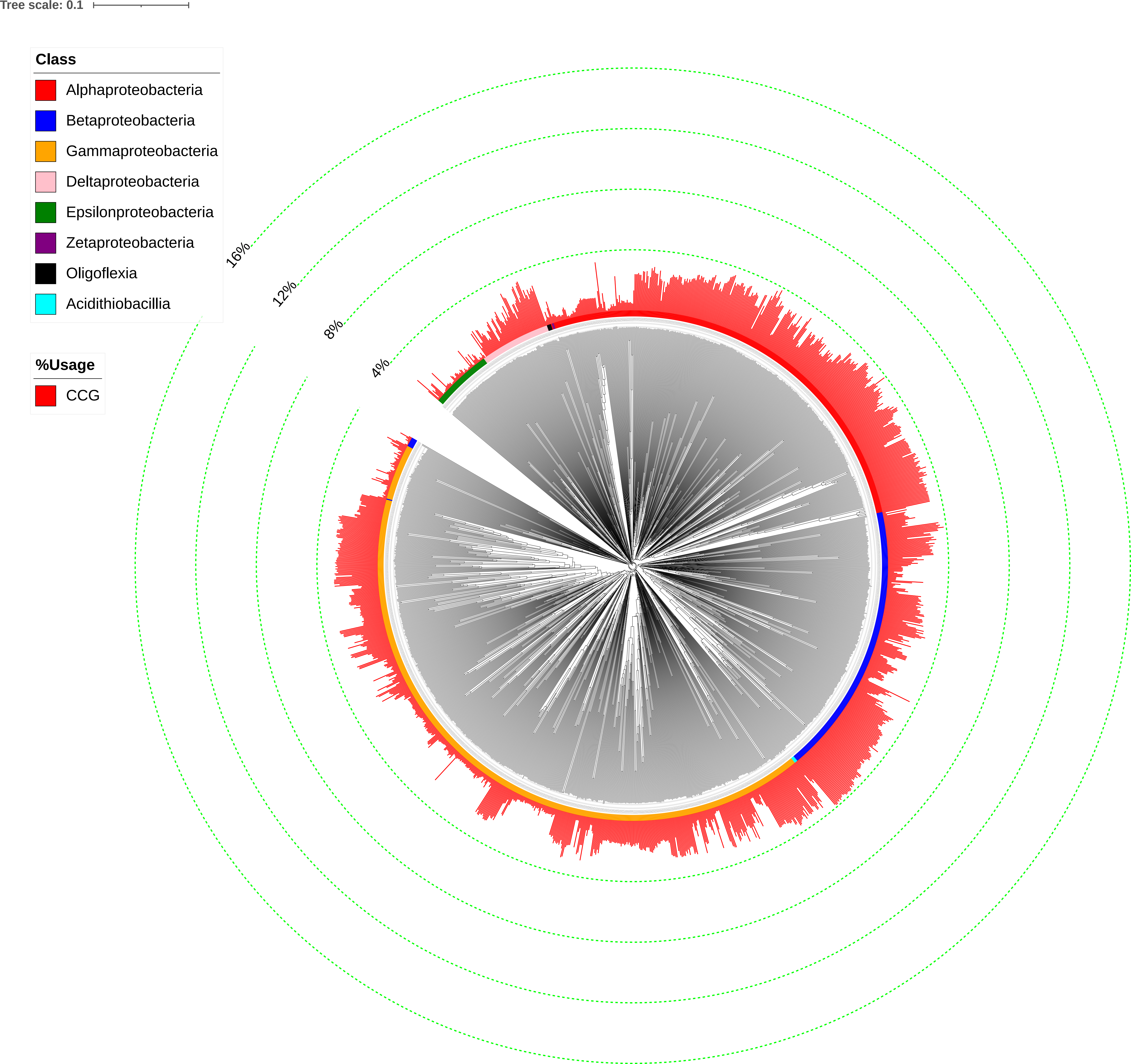

Tree scale: 0.1
Class
Alphaproteobacteria
Betaproteobacteria
Gammaproteobacteria
Deltaproteobacteria
Epsilonproteobacteria
Zetaproteobacteria
Oligoflexia
Acidithiobacillia
%Usage
CCG
4%
8%
12%
16%

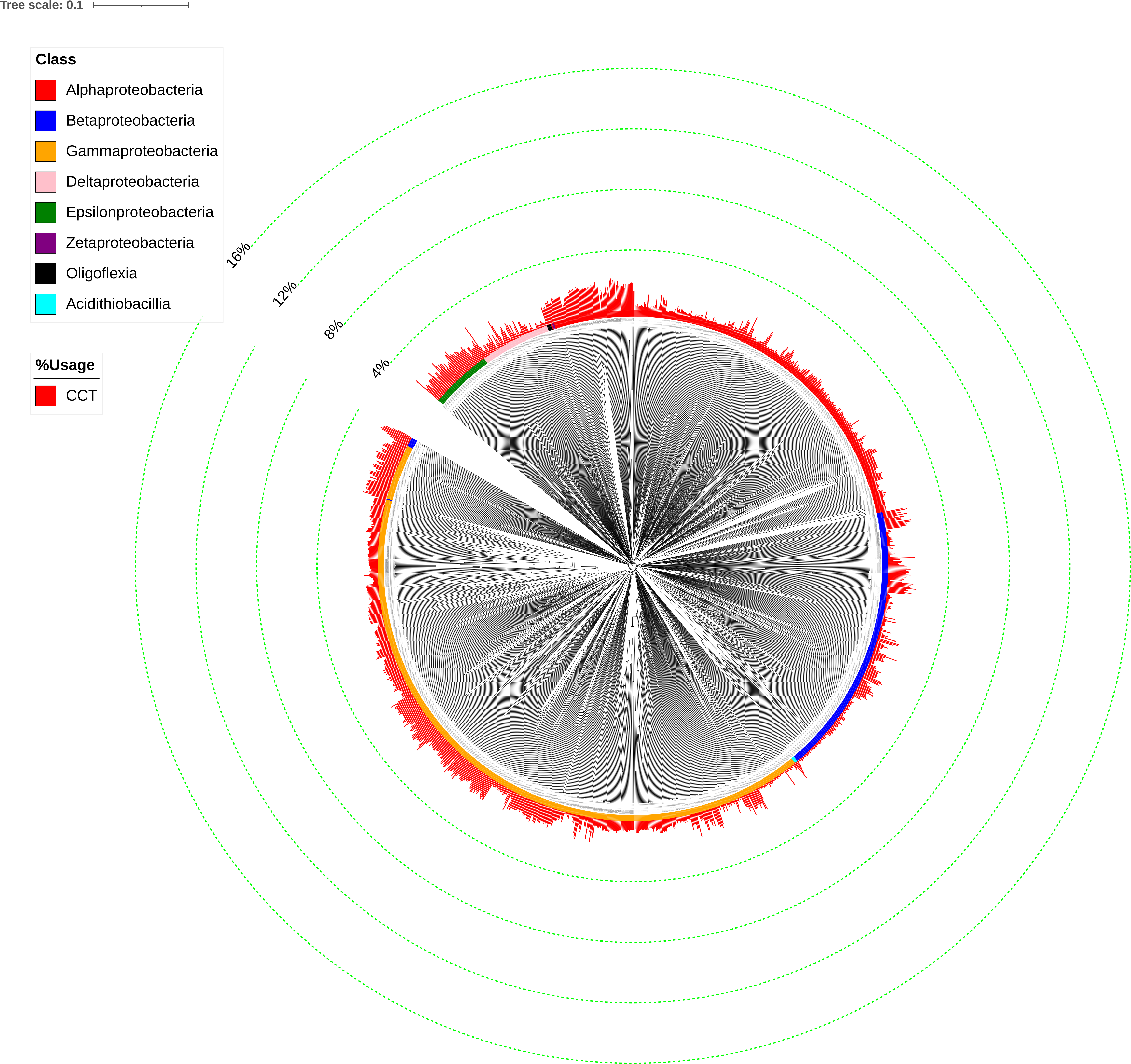
Tree scale: 0.1
Class
Alphaproteobacteria
Betaproteobacteria
Gammaproteobacteria
Deltaproteobacteria
Epsilonproteobacteria
Zetaproteobacteria
Oligoflexia
Acidithiobacillia
%Usage
CCT
4%
8%
12%
16%

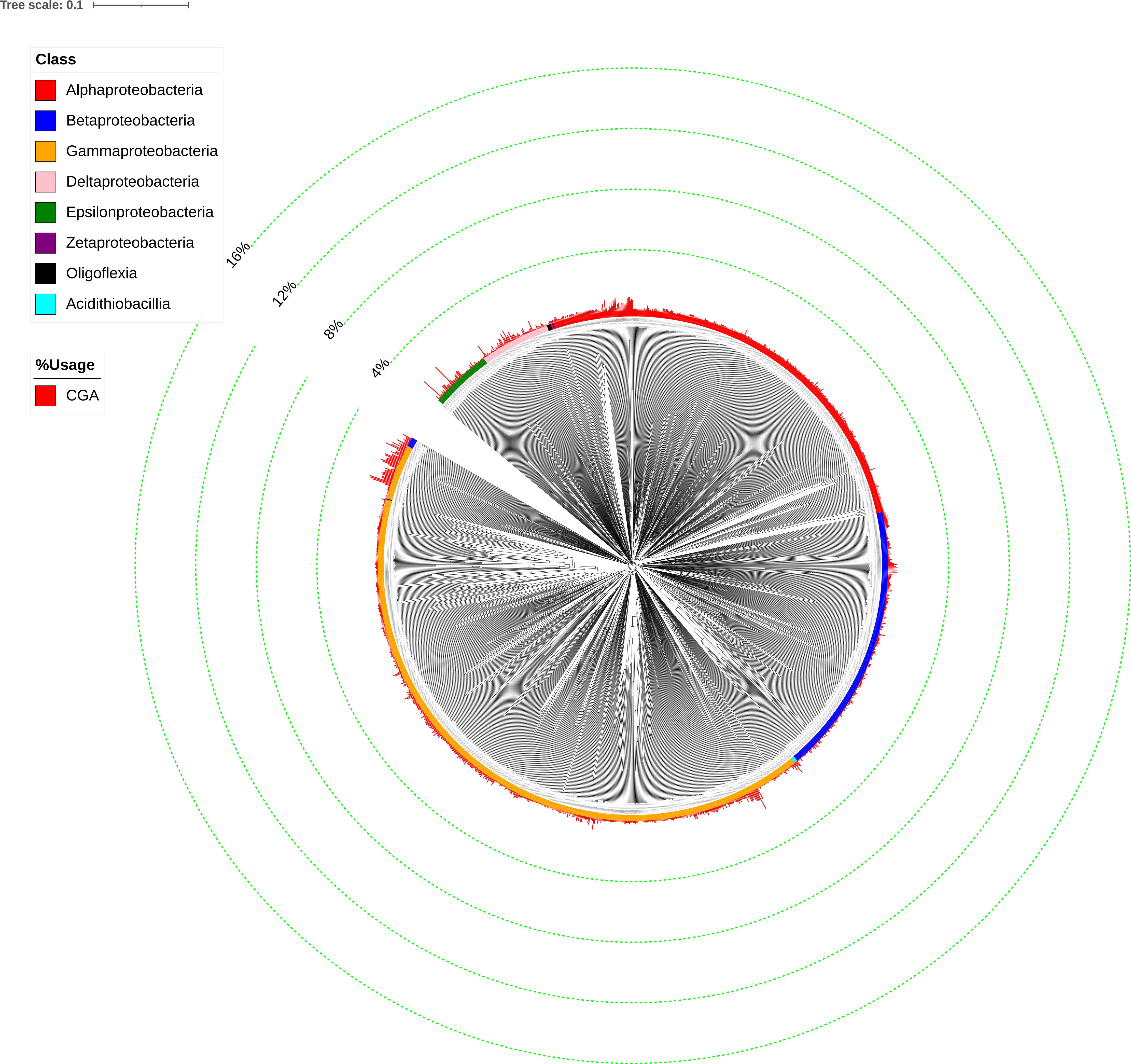

Tree scale: 0.1
Class
Alphaproteobacteria
Betaproteobacteria
Gammaproteobacteria
Deltaproteobacteria
Epsilonproteobacteria
Zetaproteobacteria
Oligoflexia
Acidithiobacillia
%Usage
CGA
16%
12%
8%
4%

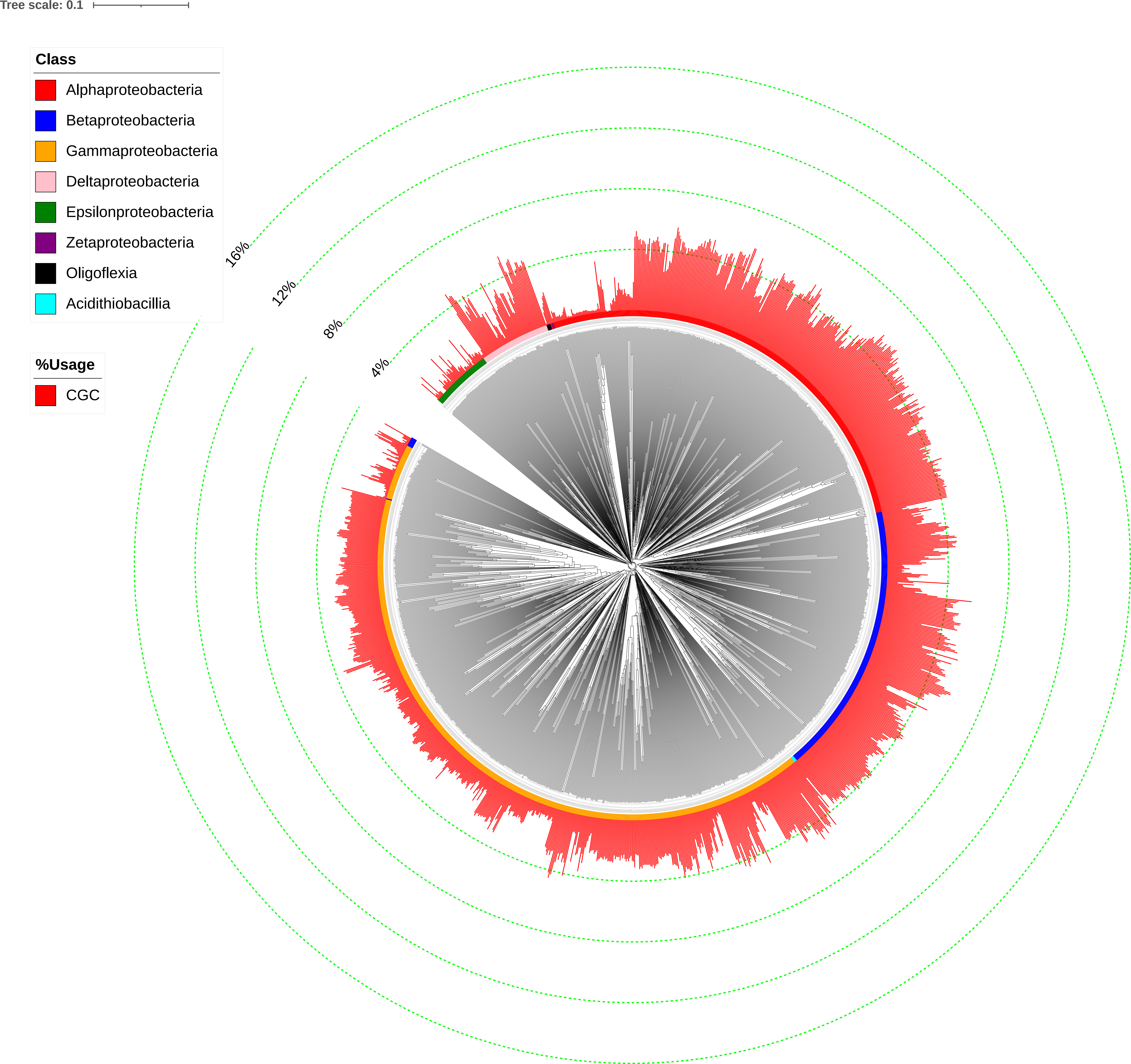

Tree scale: 0.1
Class
Alphaproteobacteria
Betaproteobacteria
Gammaproteobacteria
Deltaproteobacteria
Epsilonproteobacteria
Zetaproteobacteria
Oligoflexia
Acidithiobacillia
%Usage
CGC
16%
12%
8%
4%

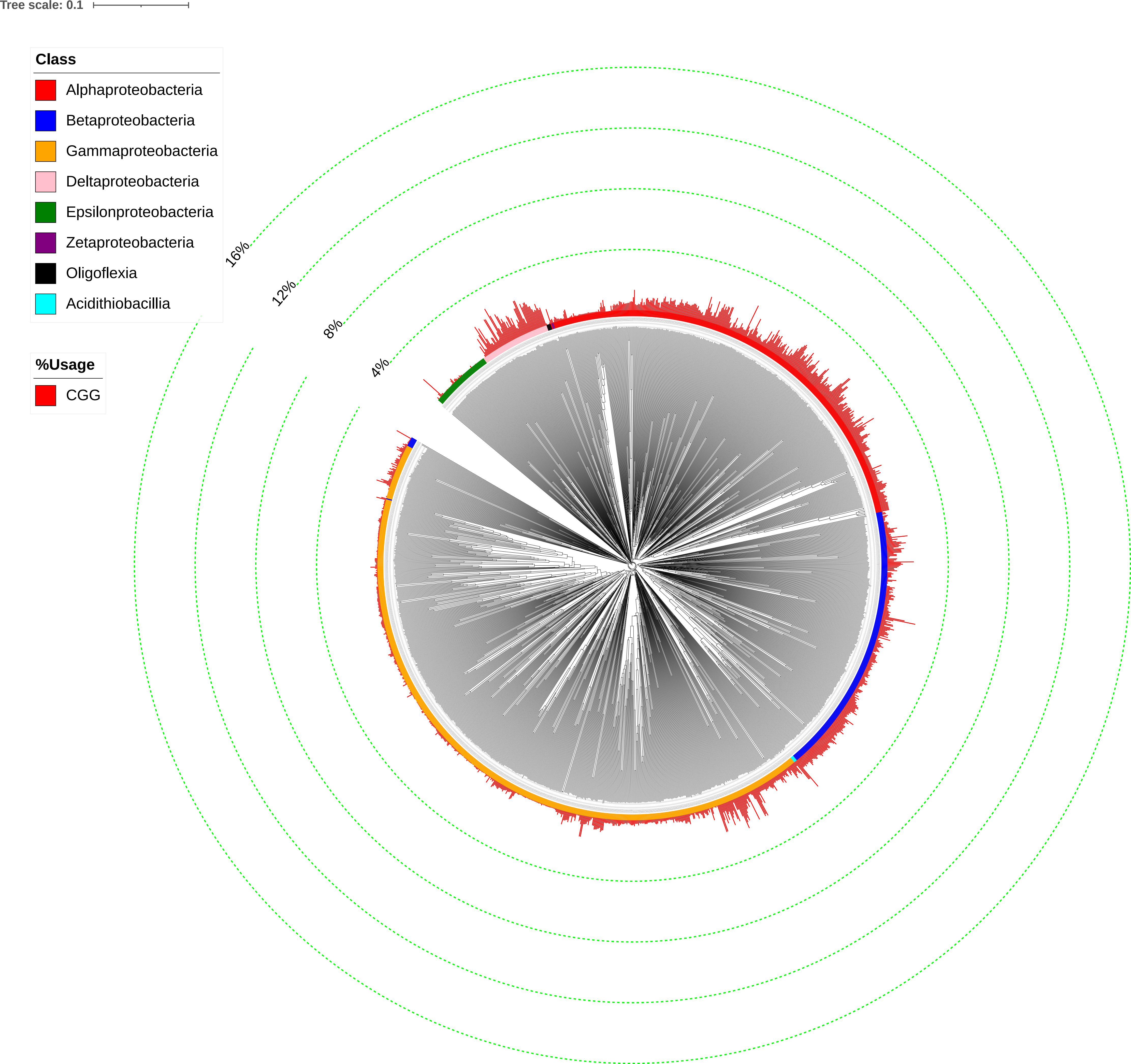

Tree scale: 0.1
Class
Alphaproteobacteria
Betaproteobacteria
Gammaproteobacteria
Deltaproteobacteria
Epsilonproteobacteria
Zetaproteobacteria
Oligoflexia
Acidithiobacillia
%Usage
CGG
16%
12%
8%
4%

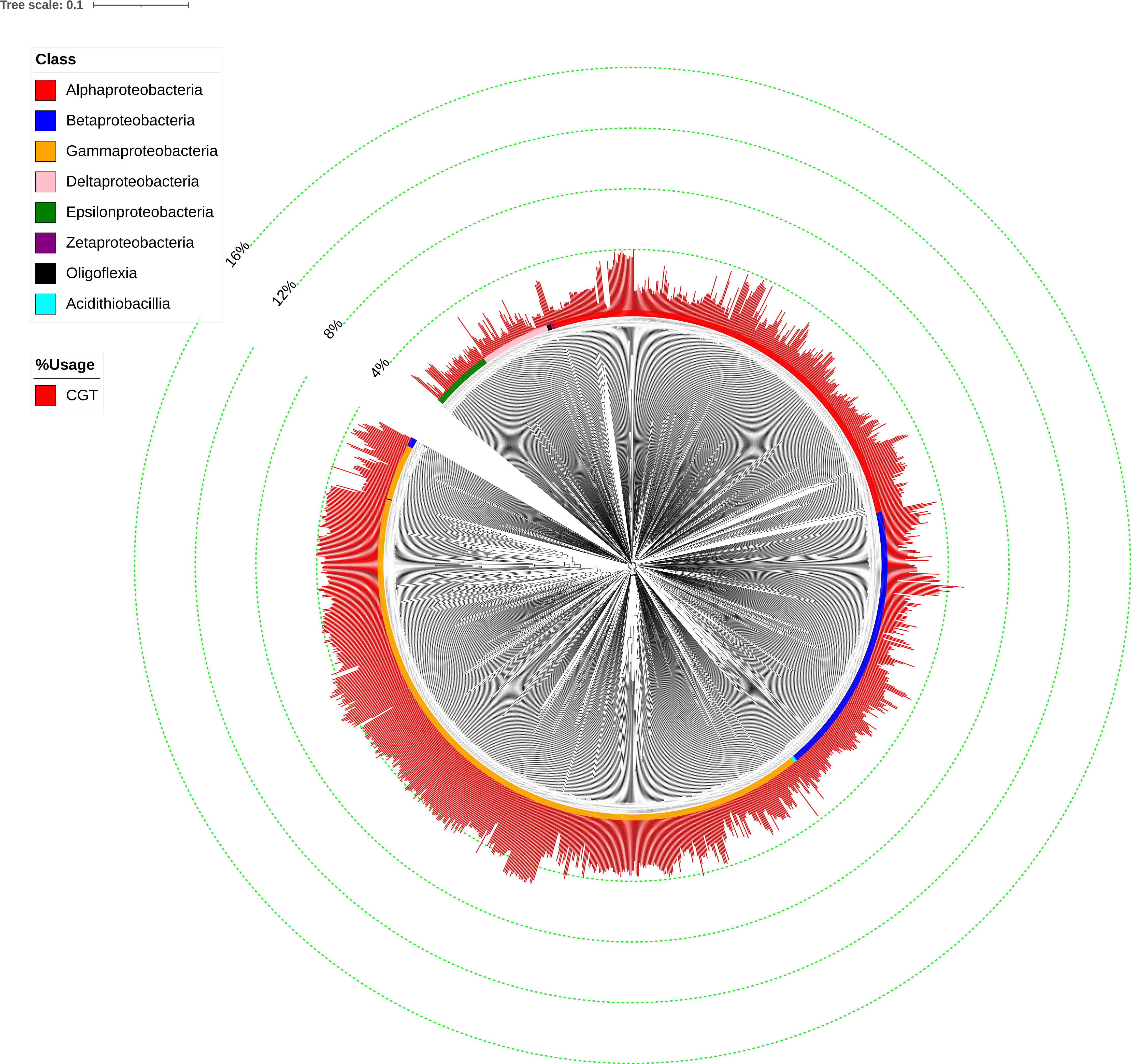

Tree scale: 0.1
Class
Alphaproteobacteria
Betaproteobacteria
Gammaproteobacteria
Deltaproteobacteria
Epsilonproteobacteria
Zetaproteobacteria
Oligoflexia
Acidithiobacillia
%Usage
CGT
16%
12%
8%
4%

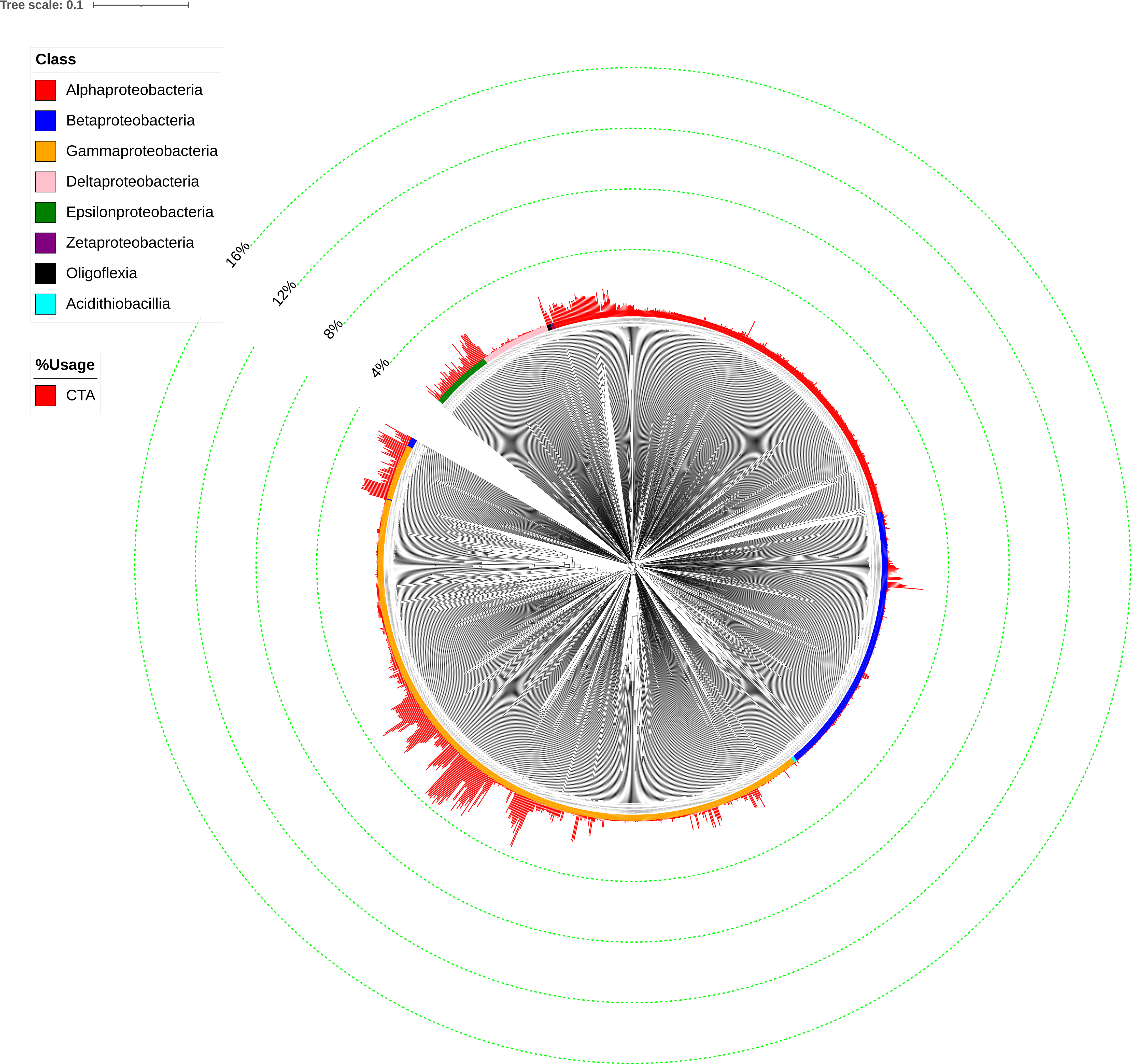
Tree scale: 0.1
Class
Alphaproteobacteria
Betaproteobacteria
Gammaproteobacteria
Deltaproteobacteria
Epsilonproteobacteria
Zetaproteobacteria
Oligoflexia
Acidithiobacillia
%Usage
CTA
16%
12%
8%
4%

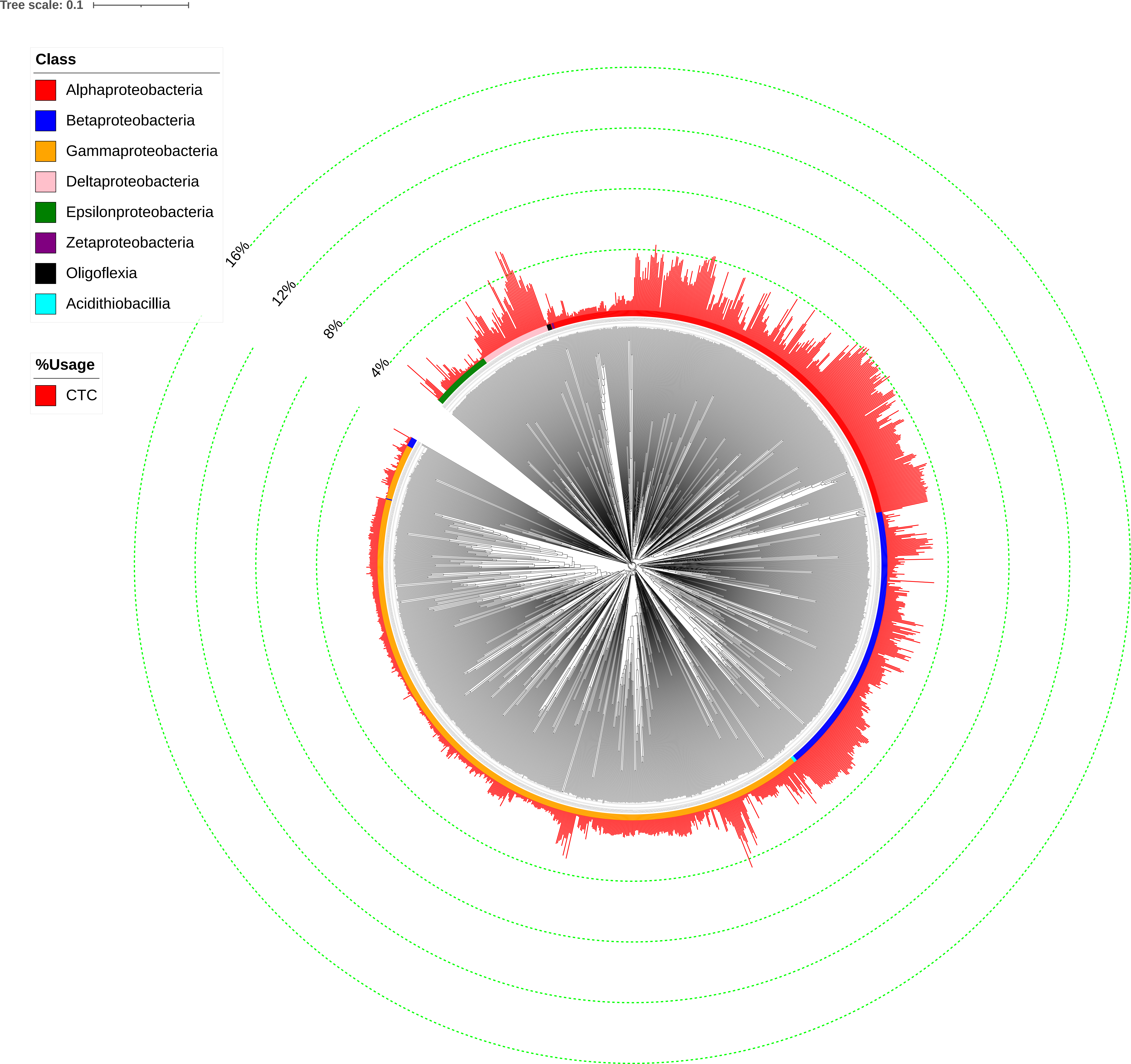
Tree scale: 0.1
Class
Alphaproteobacteria
Betaproteobacteria
Gammaproteobacteria
Deltaproteobacteria
Epsilonproteobacteria
Zetaproteobacteria
Oligoflexia
Acidithiobacillia
%Usage
CTC
16%
12%
8%
4%

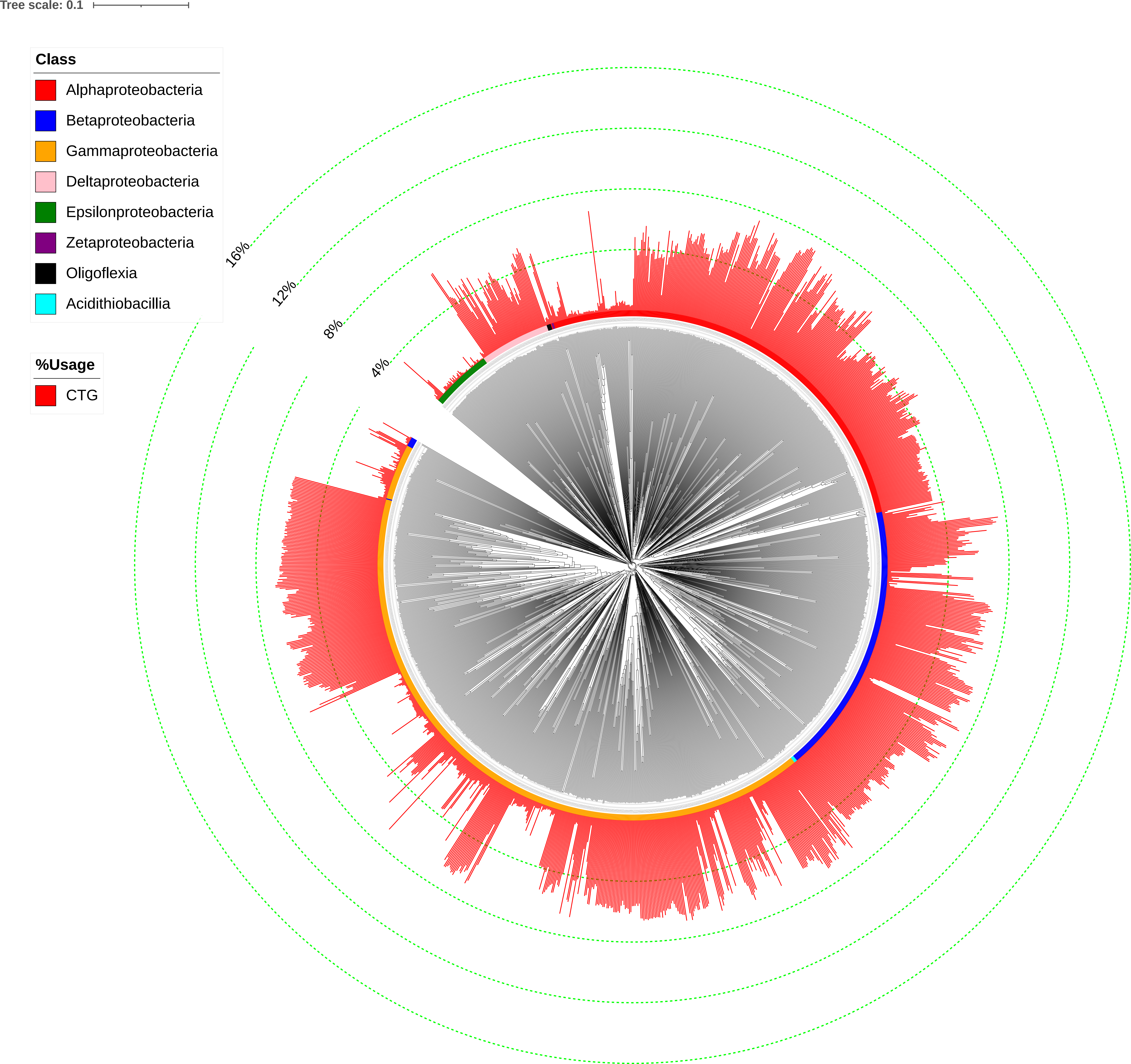
Tree scale: 0.1
Class
Alphaproteobacteria
Betaproteobacteria
Gammaproteobacteria
Deltaproteobacteria
Epsilonproteobacteria
Zetaproteobacteria
Oligoflexia
Acidithiobacillia
%Usage
CTG
16%
12%
8%
4%

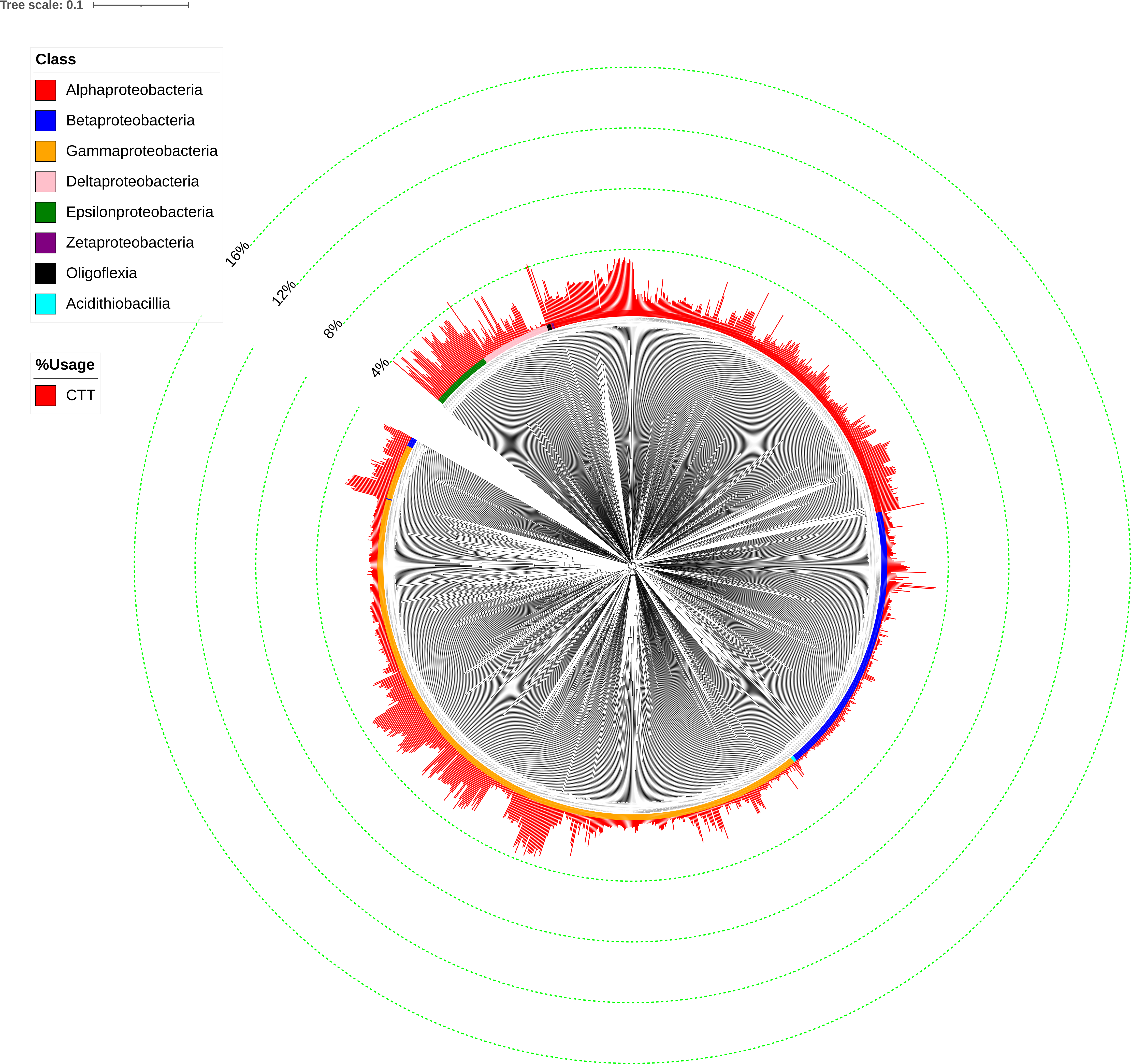
Tree scale: 0.1
Class
Alphaproteobacteria
Betaproteobacteria
Gammaproteobacteria
Deltaproteobacteria
Epsilonproteobacteria
Zetaproteobacteria
Oligoflexia
Acidithiobacillia
%Usage
CTT
16%
12%
8%
4%

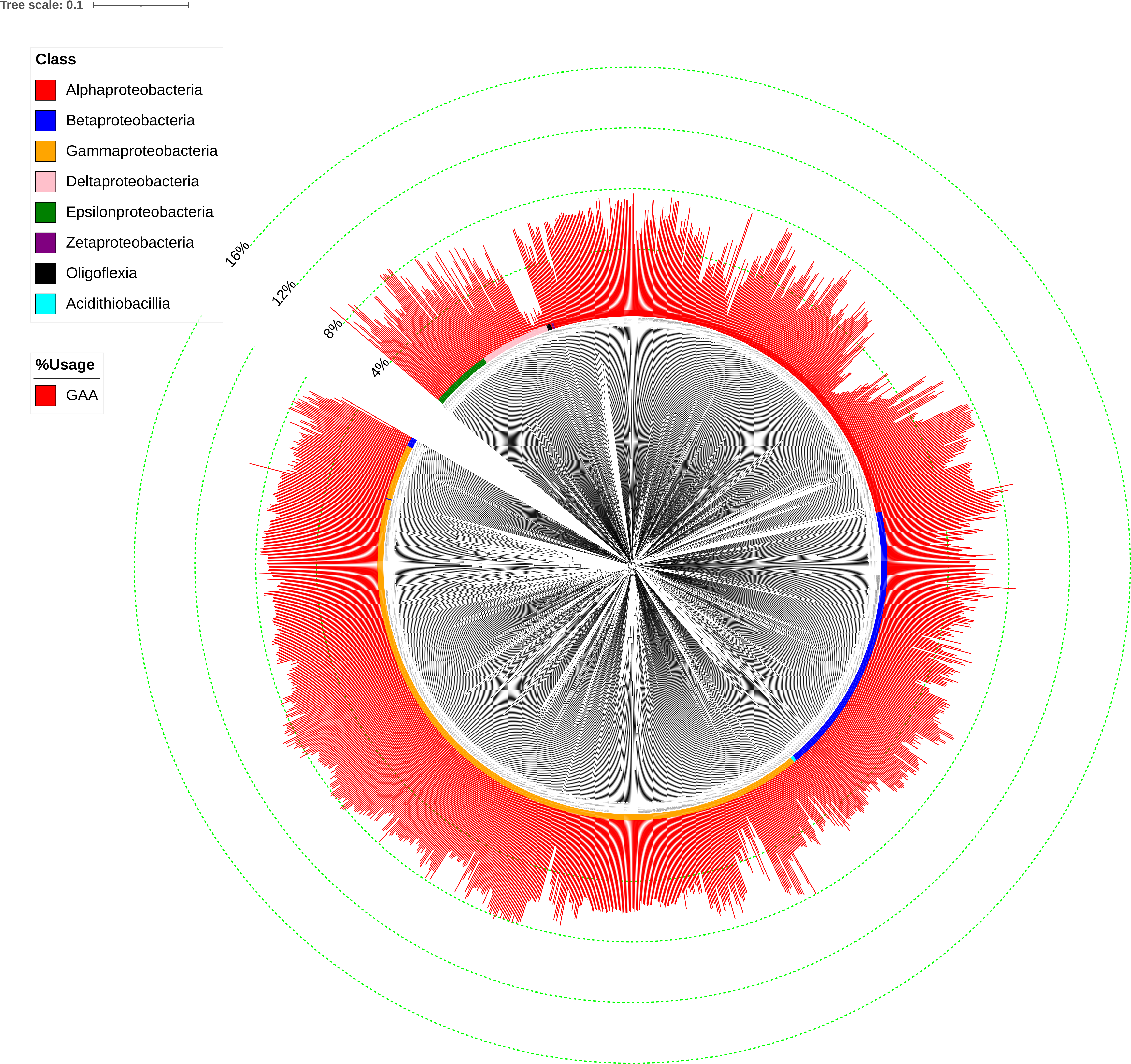
Tree scale: 0.1
Class
Alphaproteobacteria
Betaproteobacteria
Gammaproteobacteria
Deltaproteobacteria
Epsilonproteobacteria
Zetaproteobacteria
Oligoflexia
Acidithiobacillia
%Usage
GAA
4%
8%
12%
16%

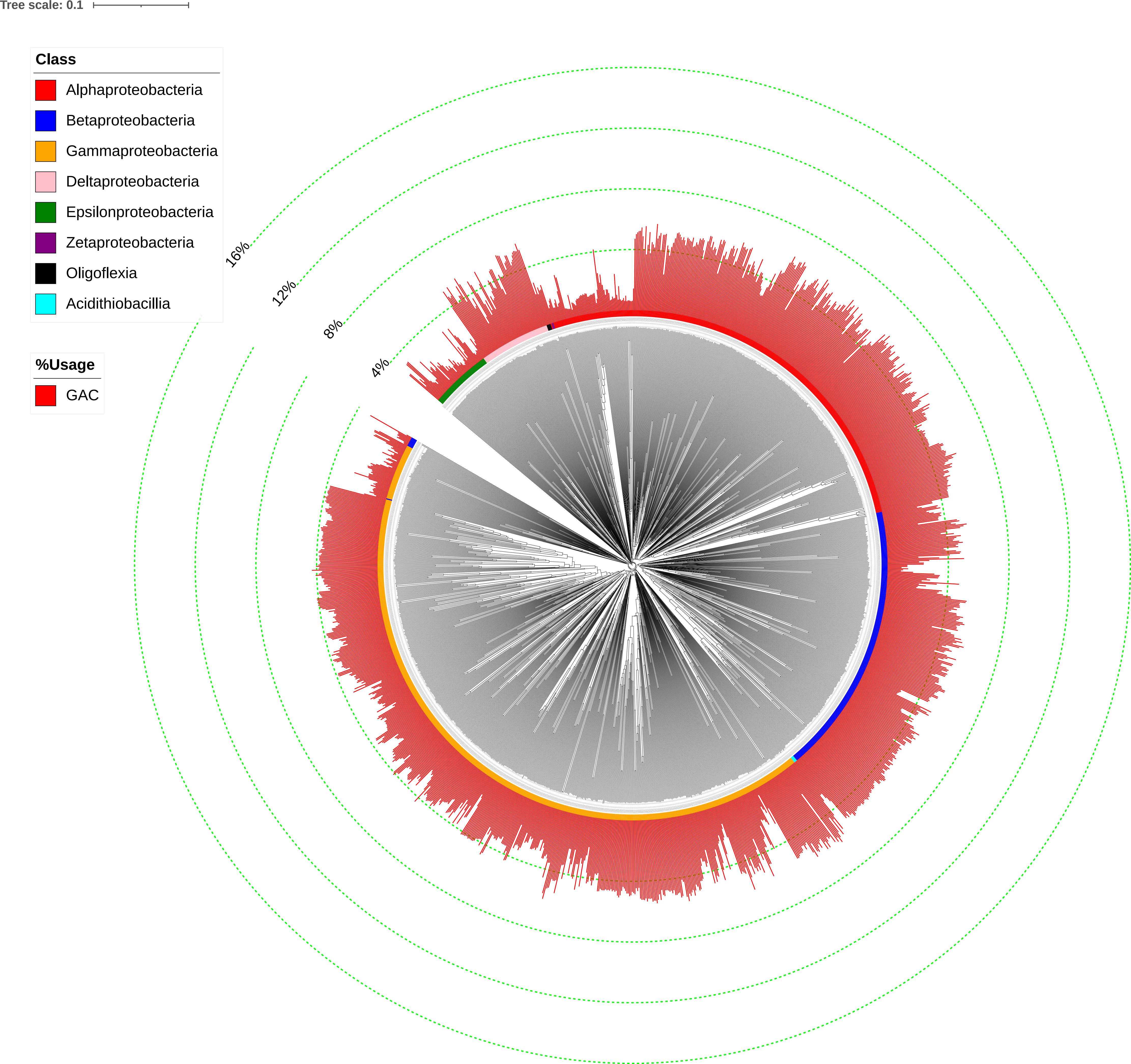
Tree scale: 0.1
Class
Alphaproteobacteria
Betaproteobacteria
Gammaproteobacteria
Deltaproteobacteria
Epsilonproteobacteria
Zetaproteobacteria
Oligoflexia
Acidithiobacillia
%Usage
GAC
4%
8%
12%
16%

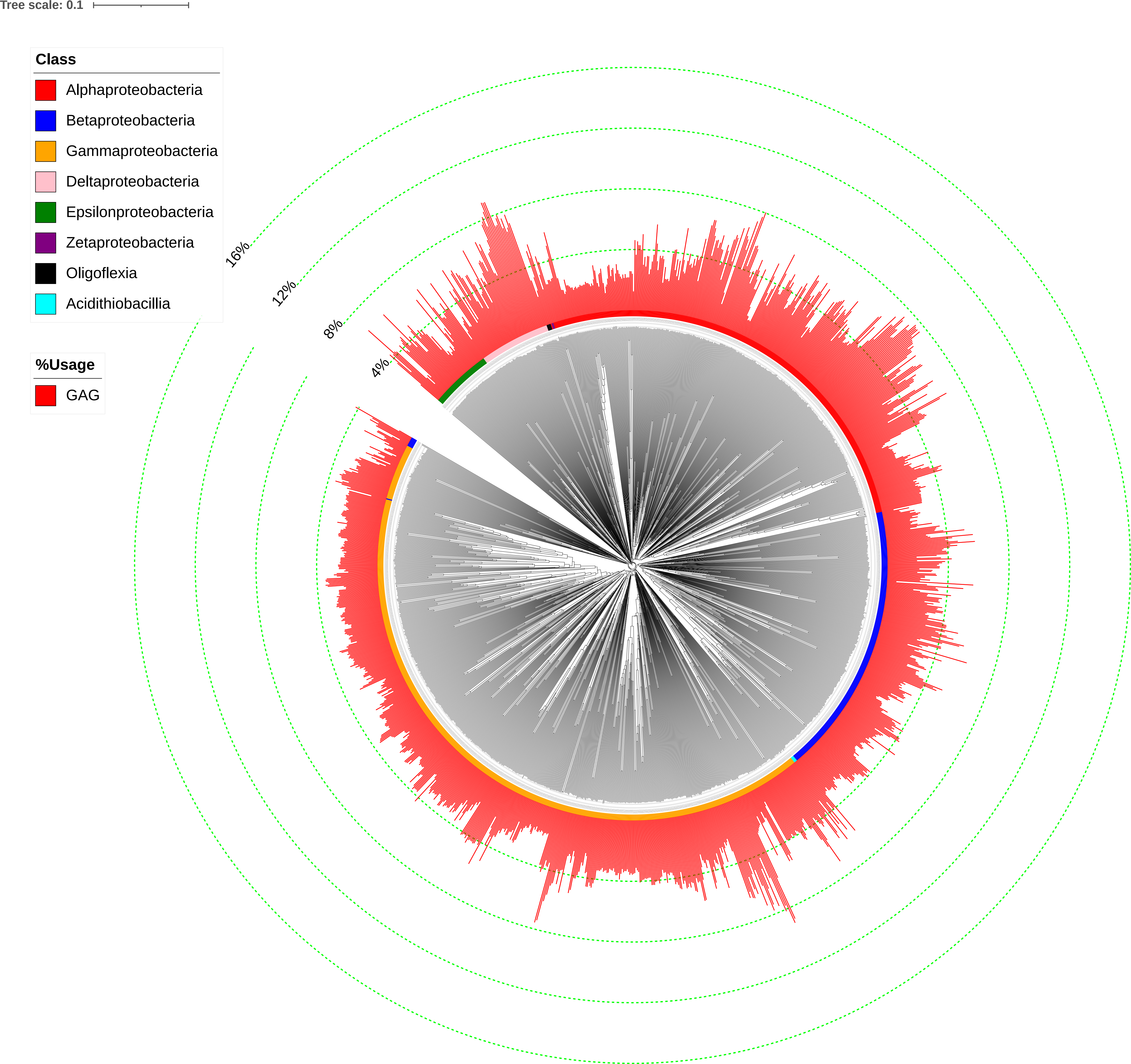

Tree scale: 0.1
Class
Alphaproteobacteria
Betaproteobacteria
Gammaproteobacteria
Deltaproteobacteria
Epsilonproteobacteria
Zetaproteobacteria
Oligoflexia
Acidithiobacillia
%Usage
GAG
16%
12%
8%
4%

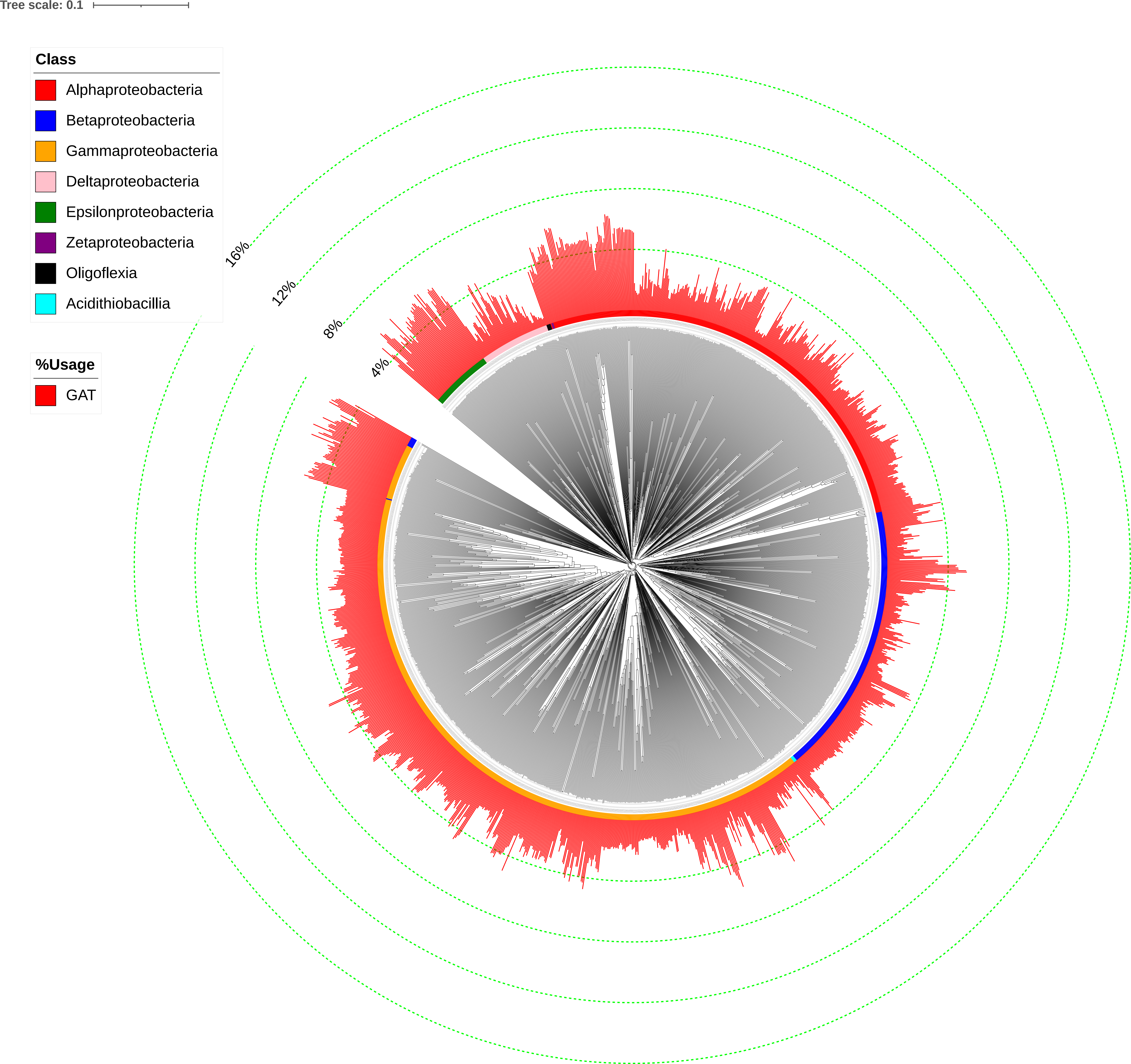

Tree scale: 0.1
Class
Alphaproteobacteria
Betaproteobacteria
Gammaproteobacteria
Deltaproteobacteria
Epsilonproteobacteria
Zetaproteobacteria
Oligoflexia
Acidithiobacillia
%Usage
GAT
16%
12%
8%
4%

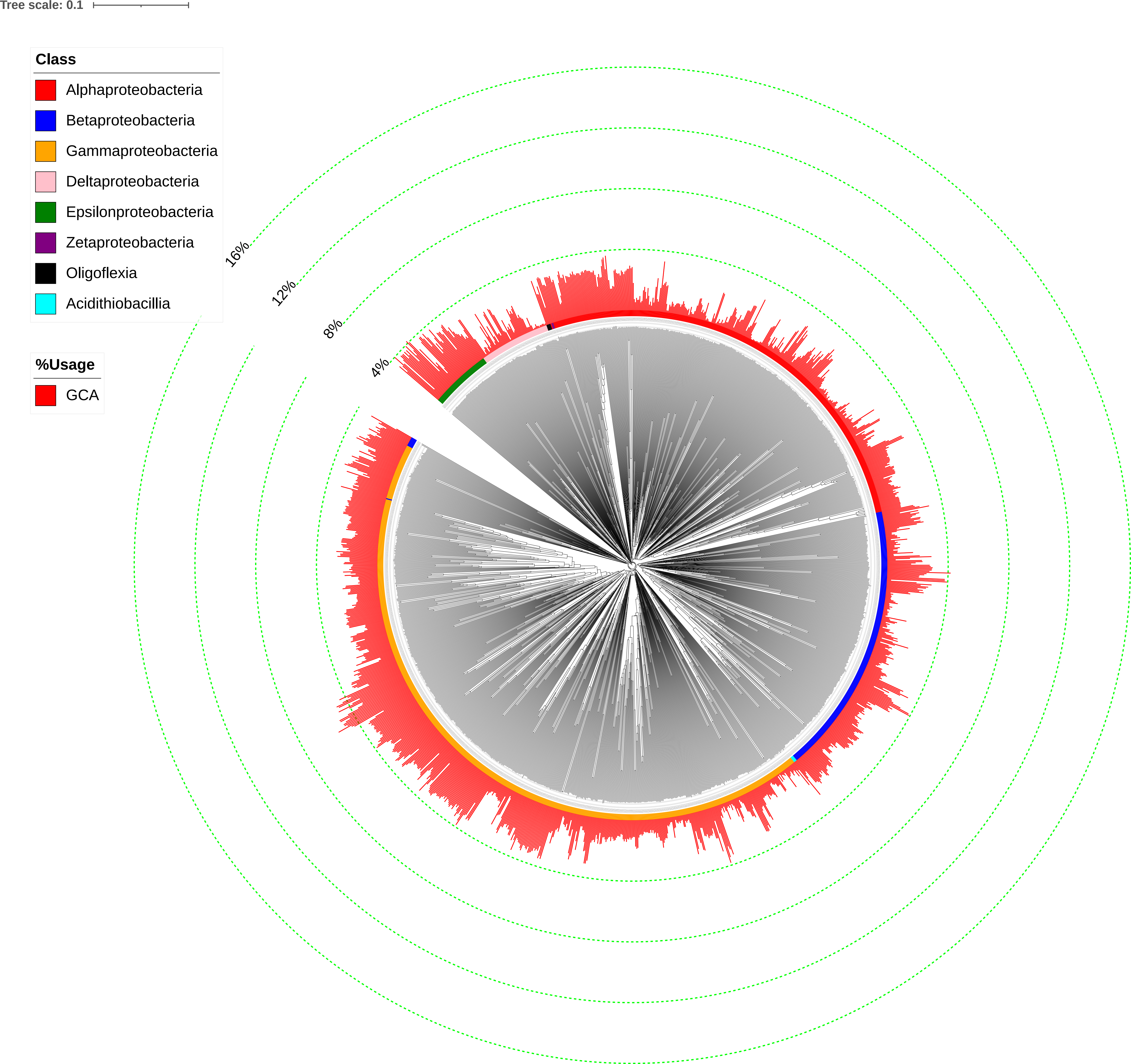

Tree scale: 0.1
Class
Alphaproteobacteria
Betaproteobacteria
Gammaproteobacteria
Deltaproteobacteria
Epsilonproteobacteria
Zetaproteobacteria
Oligoflexia
Acidithiobacillia
%Usage
GCA
16%
12%
8%
4%

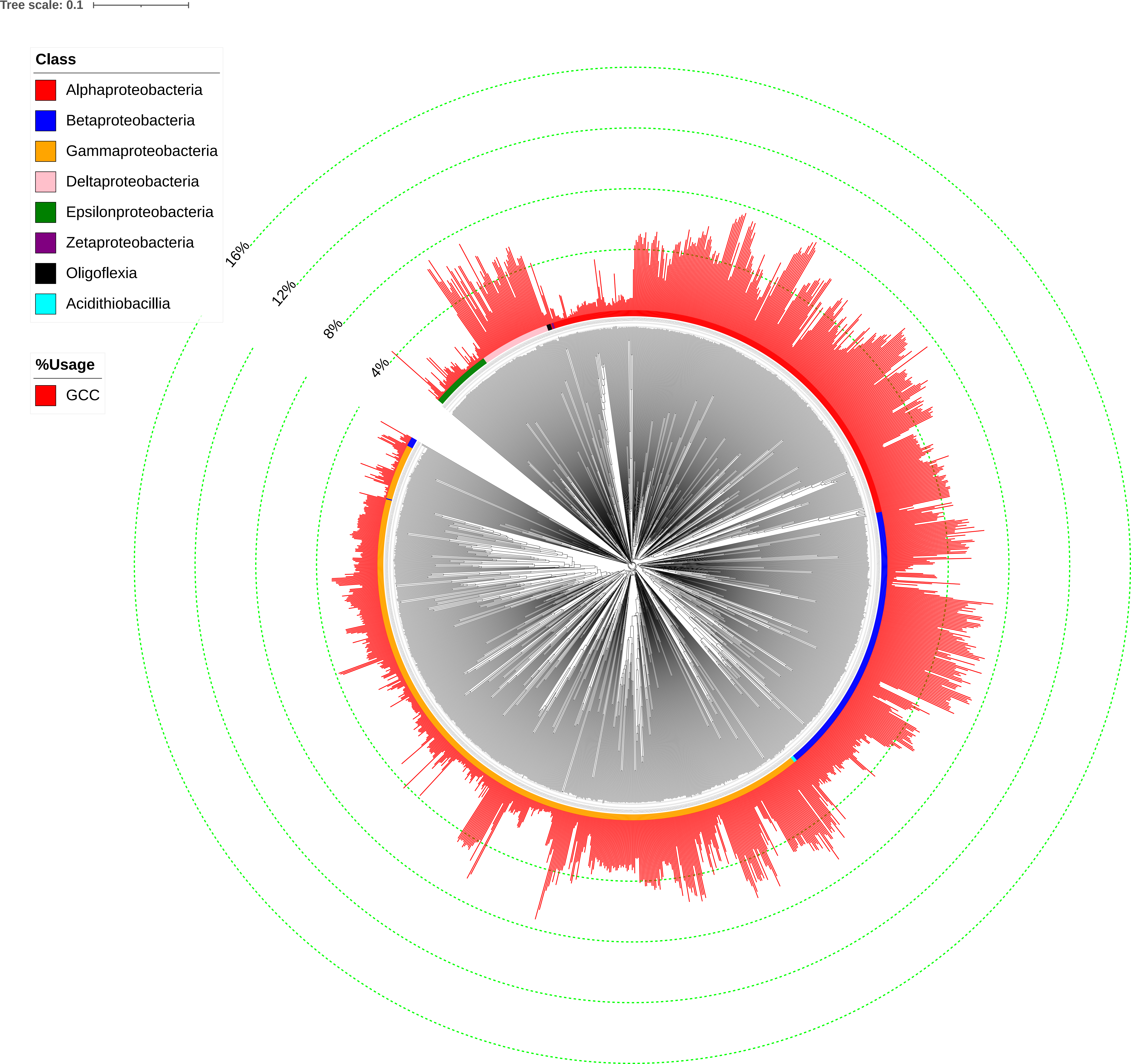

Tree scale: 0.1
Class
Alphaproteobacteria
Betaproteobacteria
Gammaproteobacteria
Deltaproteobacteria
Epsilonproteobacteria
Zetaproteobacteria
Oligoflexia
Acidithiobacillia
%Usage
GCC
16%
12%
8%
4%

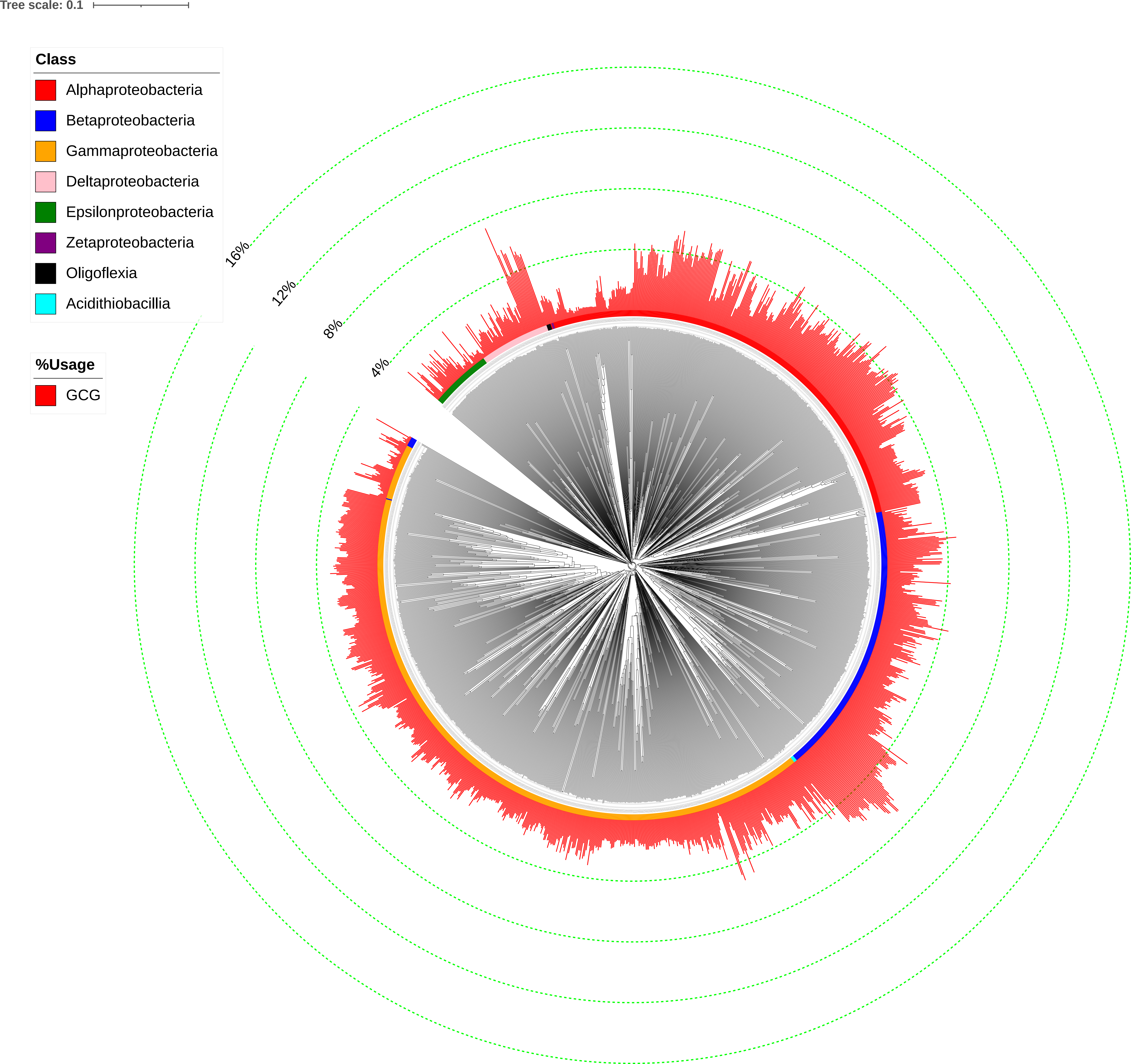
Tree scale: 0.1
Class
Alphaproteobacteria
Betaproteobacteria
Gammaproteobacteria
Deltaproteobacteria
Epsilonproteobacteria
Zetaproteobacteria
Oligoflexia
Acidithiobacillia
%Usage
GCG
4%
8%
12%
16%

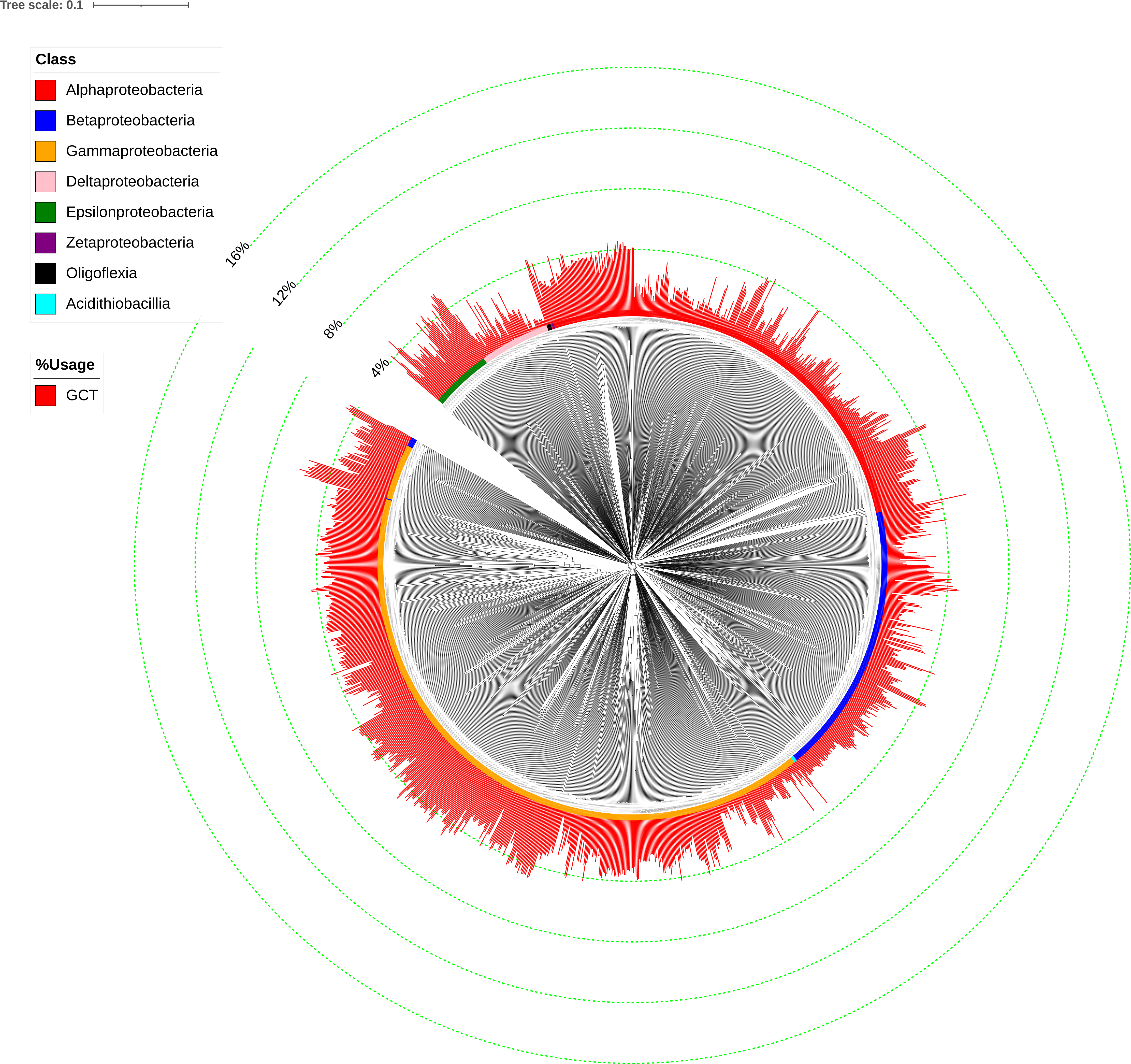

Tree scale: 0.1
Class
Alphaproteobacteria
Betaproteobacteria
Gammaproteobacteria
Deltaproteobacteria
Epsilonproteobacteria
Zetaproteobacteria
Oligoflexia
Acidithiobacillia
%Usage
GCT
16%
12%
8%
4%

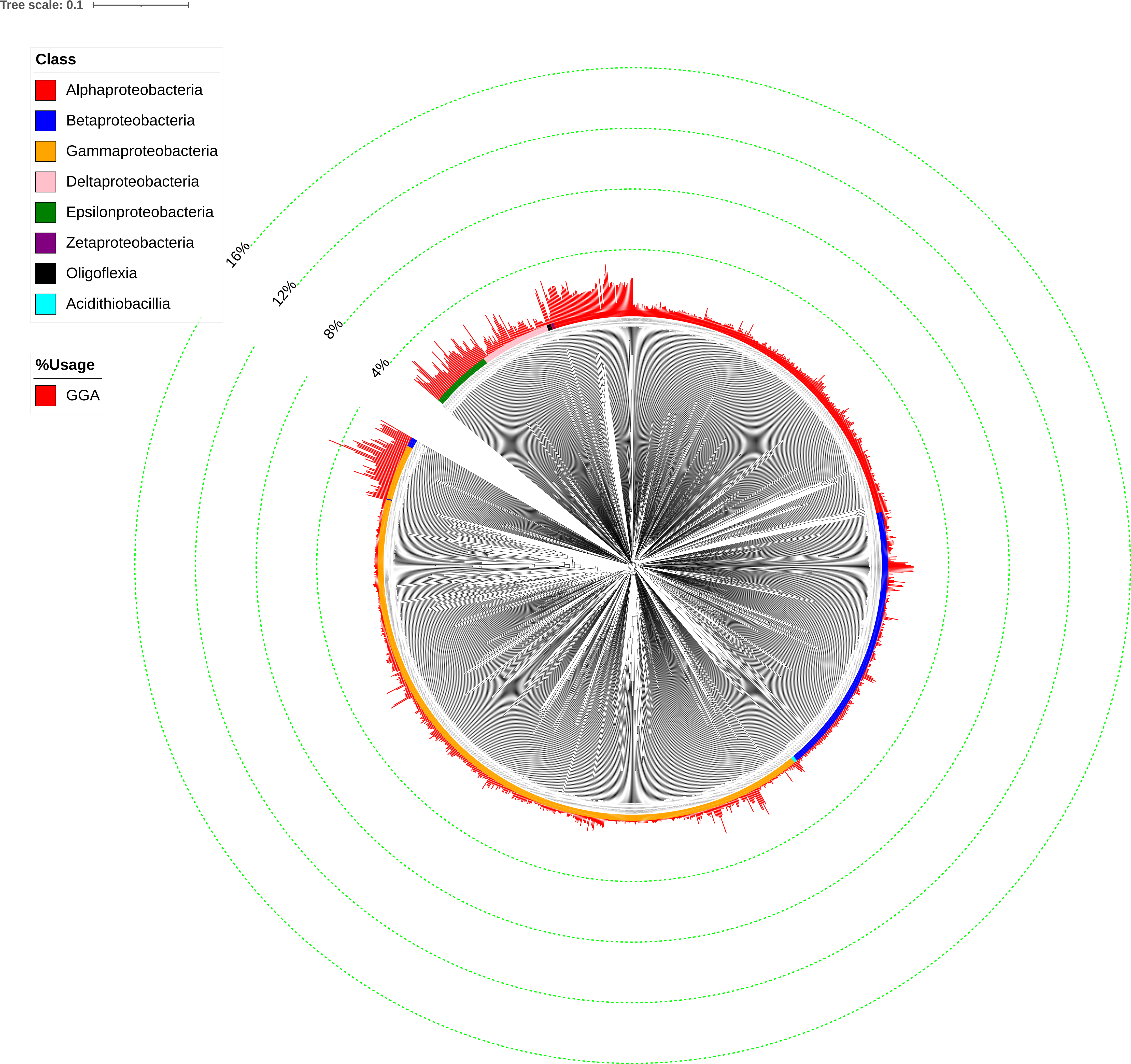

Tree scale: 0.1
Class
Alphaproteobacteria
Betaproteobacteria
Gammaproteobacteria
Deltaproteobacteria
Epsilonproteobacteria
Zetaproteobacteria
Oligoflexia
Acidithiobacillia
%Usage
GGA
16%
12%
8%
4%

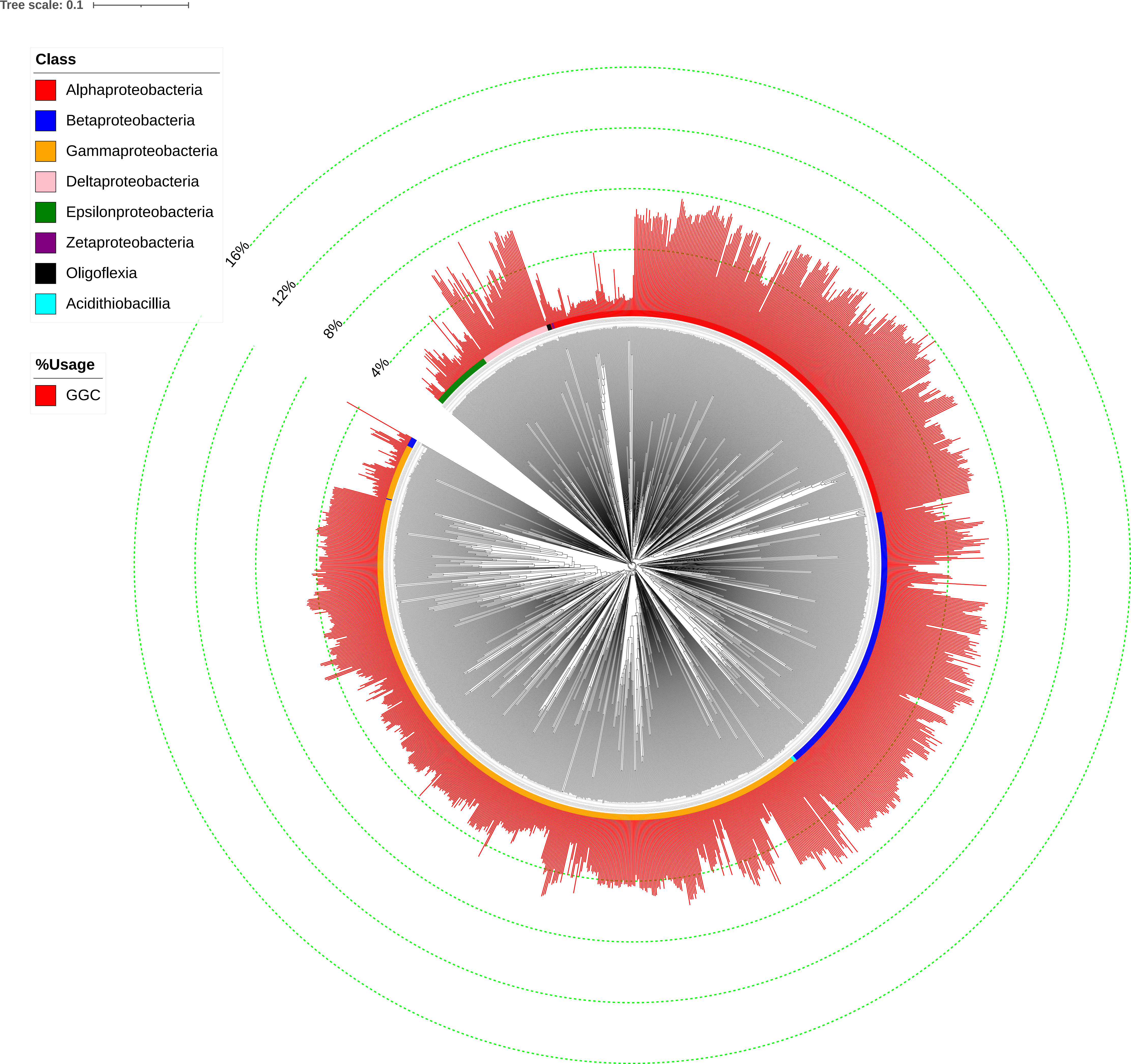
Tree scale: 0.1
Class
Alphaproteobacteria
Betaproteobacteria
Gammaproteobacteria
Deltaproteobacteria
Epsilonproteobacteria
Zetaproteobacteria
Oligoflexia
Acidithiobacillia
%Usage
GGC
16%
12%
8%
4%

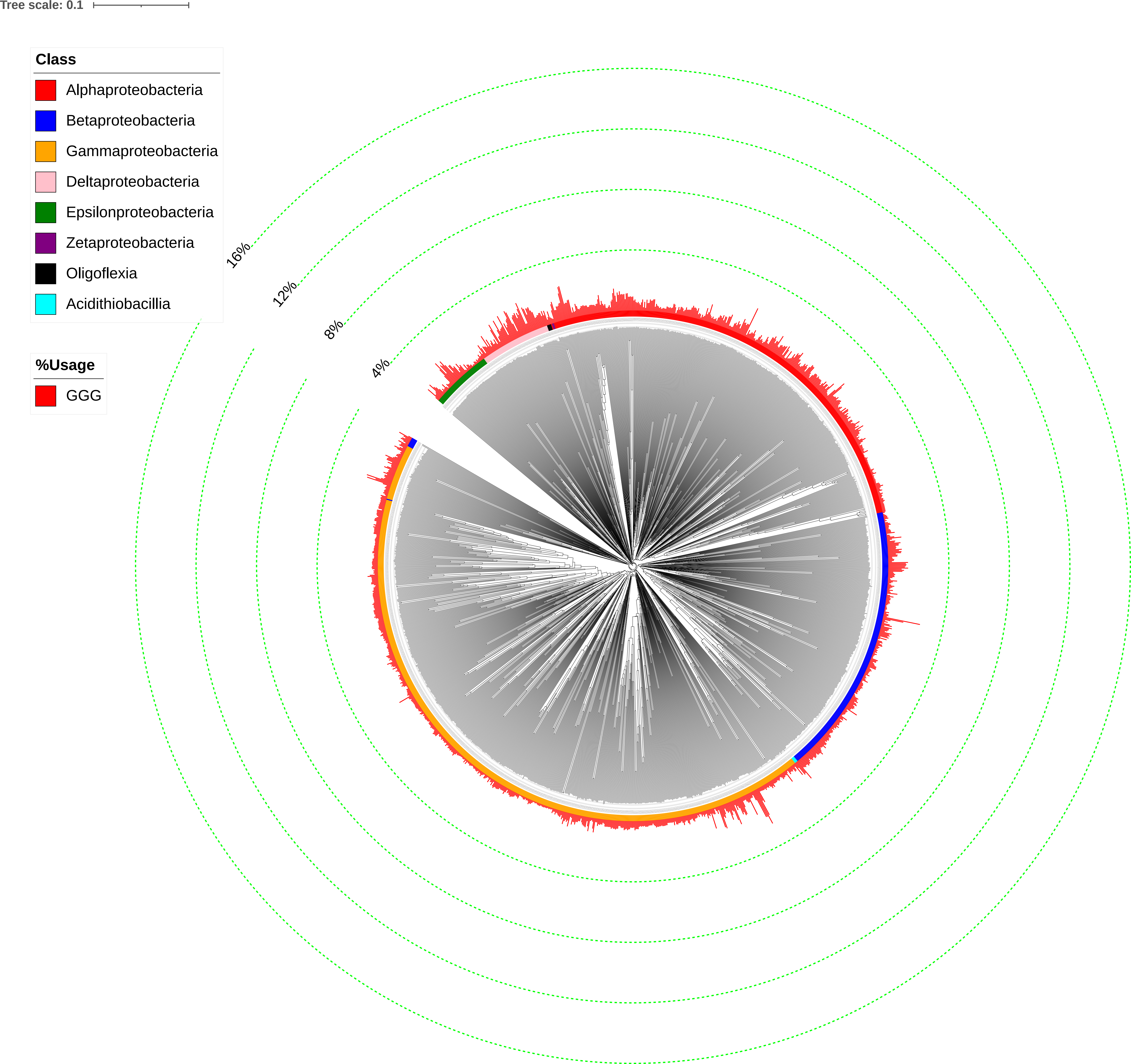
Tree scale: 0.1
Class
Alphaproteobacteria
Betaproteobacteria
Gammaproteobacteria
Deltaproteobacteria
Epsilonproteobacteria
Zetaproteobacteria
Oligoflexia
Acidithiobacillia
%Usage
GGG
16%
12%
8%
4%

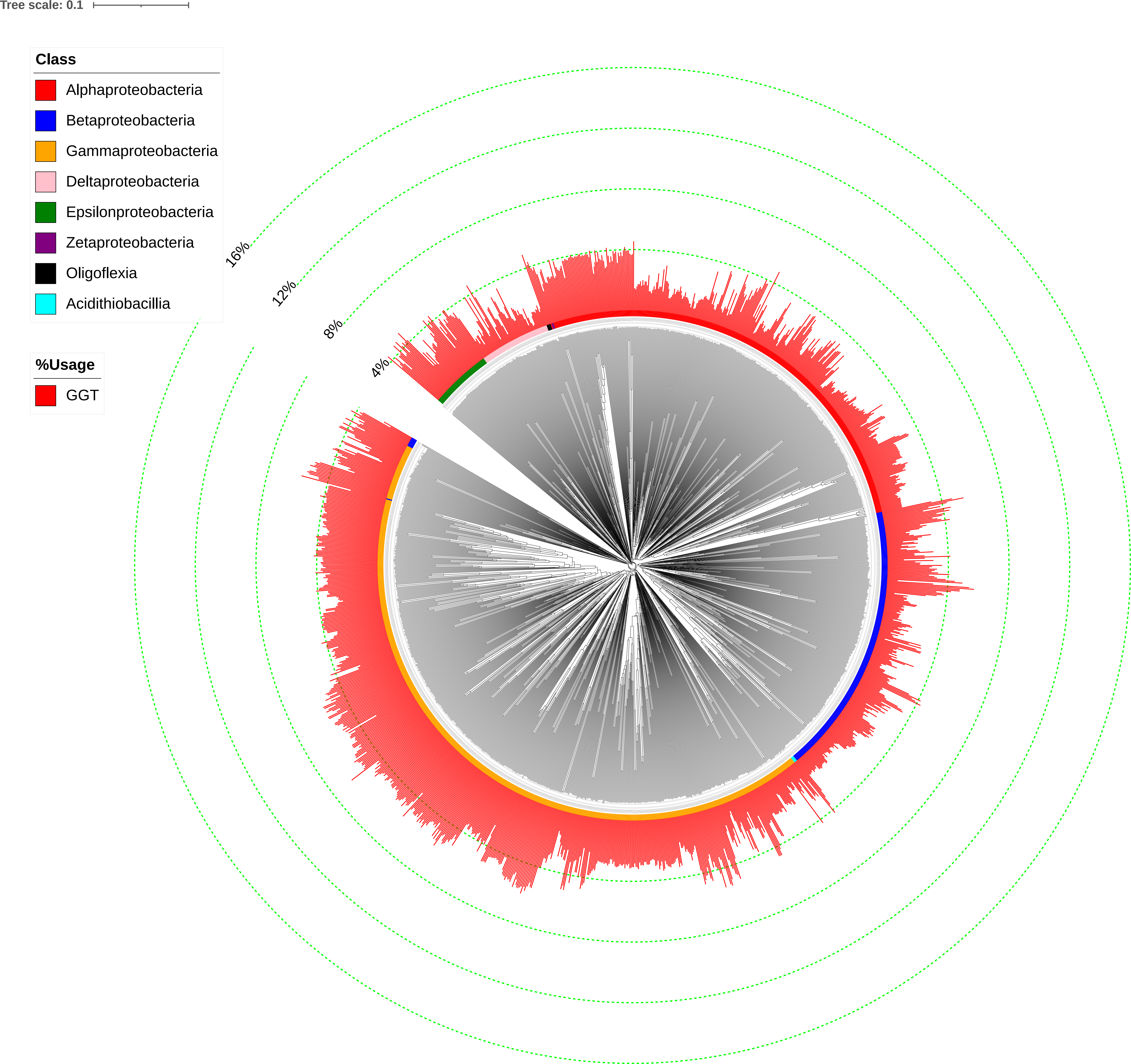
Tree scale: 0.1
Class
Alphaproteobacteria
Betaproteobacteria
Gammaproteobacteria
Deltaproteobacteria
Epsilonproteobacteria
Zetaproteobacteria
Oligoflexia
Acidithiobacillia
%Usage
GGT
16%
12%
8%
4%

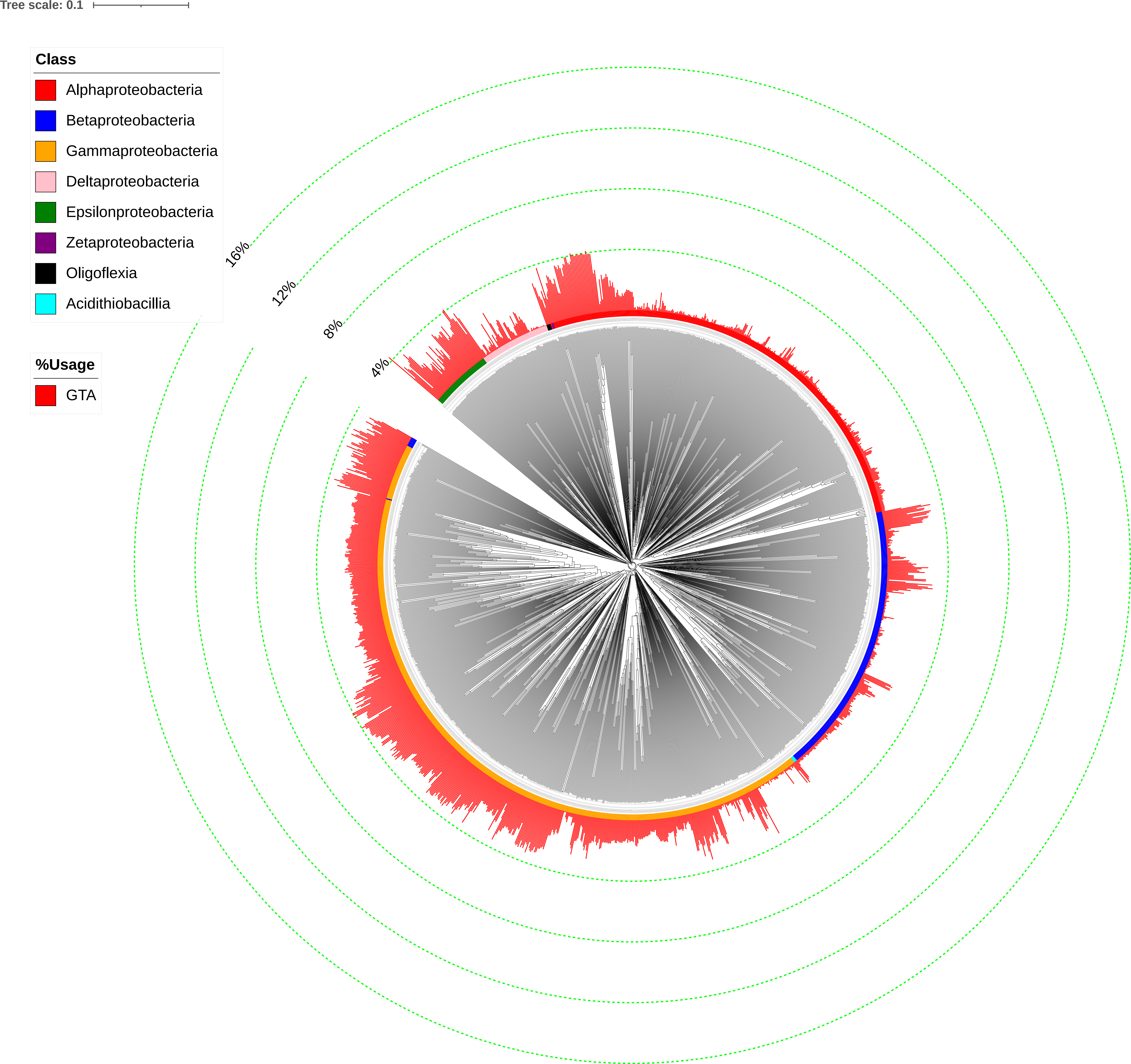
Tree scale: 0.1
Class
Alphaproteobacteria
Betaproteobacteria
Gammaproteobacteria
Deltaproteobacteria
Epsilonproteobacteria
Zetaproteobacteria
Oligoflexia
Acidithiobacillia
%Usage
GTA
4%
8%
12%
16%

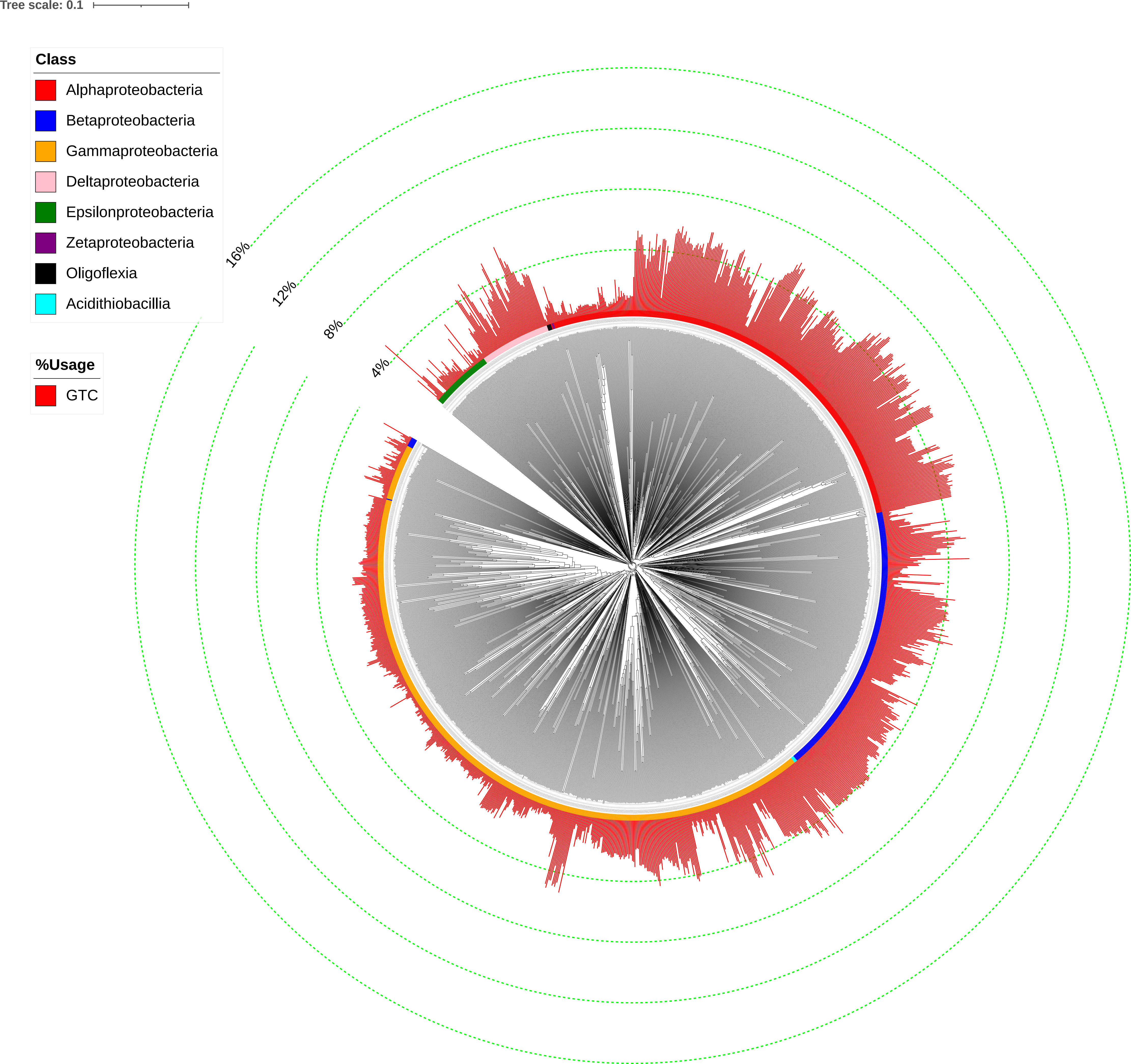

Tree scale: 0.1
Class
Alphaproteobacteria
Betaproteobacteria
Gammaproteobacteria
Deltaproteobacteria
Epsilonproteobacteria
Zetaproteobacteria
Oligoflexia
Acidithiobacillia
%Usage
GTC
16%
12%
8%
4%

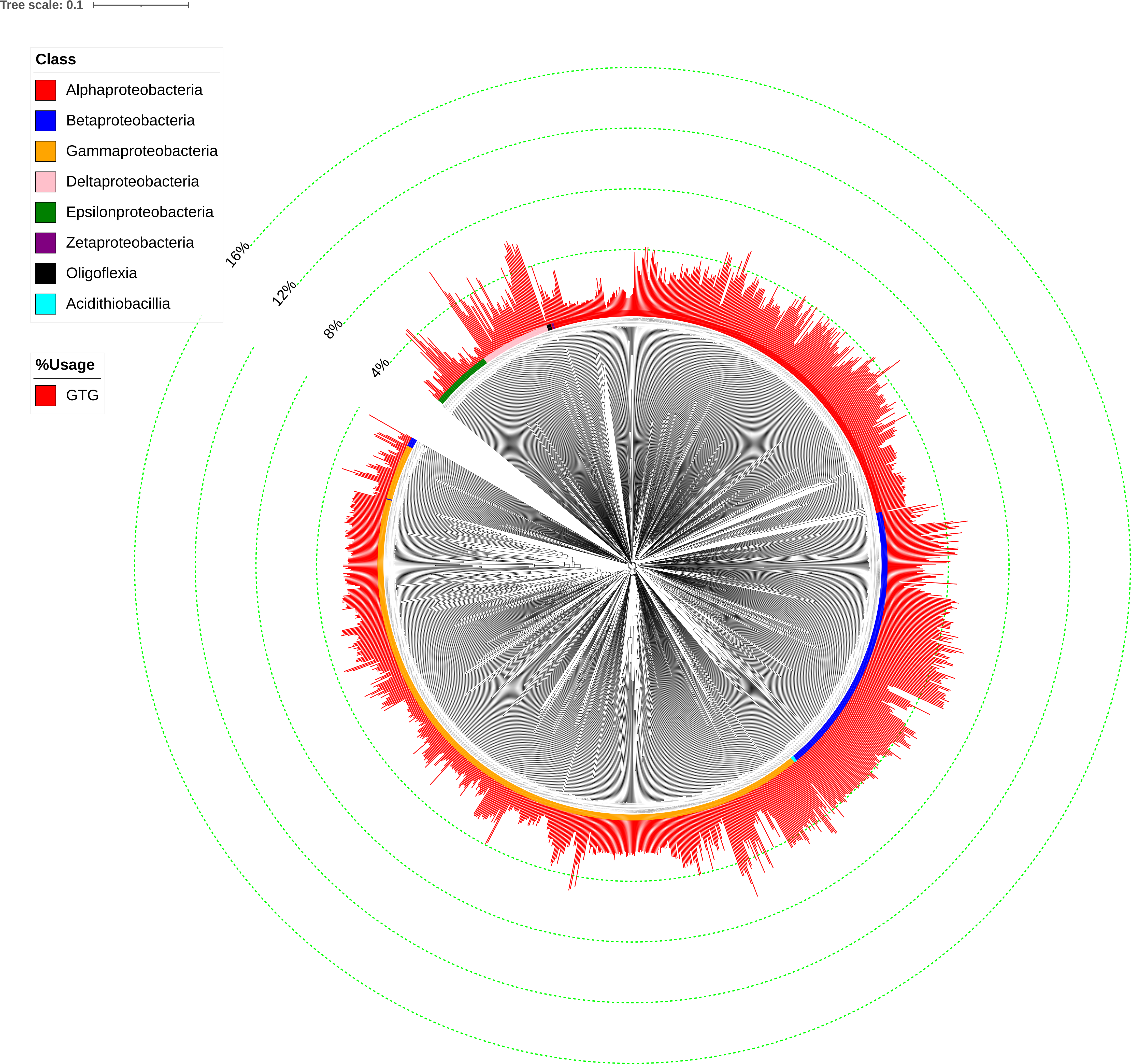

Tree scale: 0.1
Class
Alphaproteobacteria
Betaproteobacteria
Gammaproteobacteria
Deltaproteobacteria
Epsilonproteobacteria
Zetaproteobacteria
Oligoflexia
Acidithiobacillia
%Usage
GTG
16%
12%
8%
4%

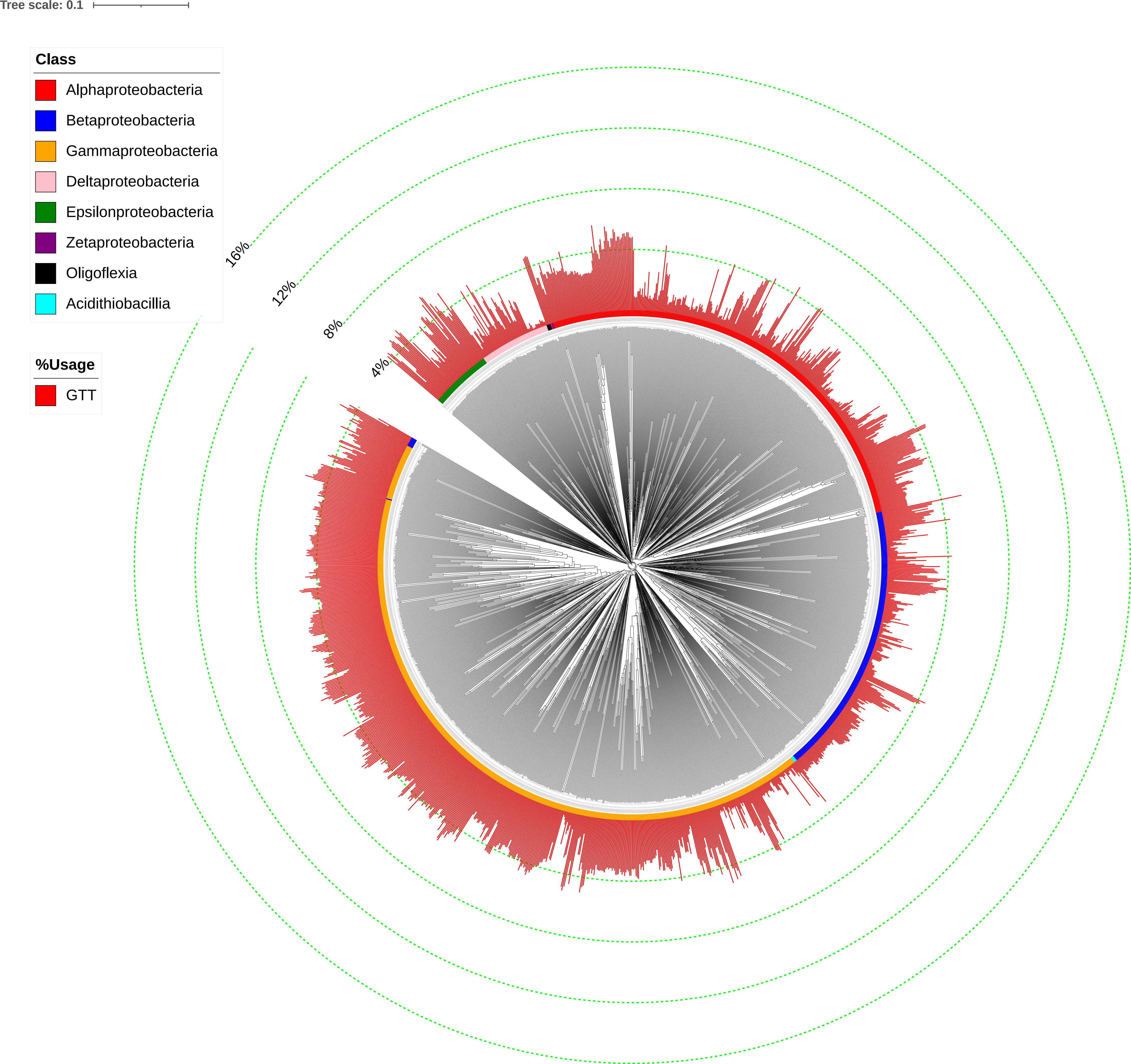

Tree scale: 0.1
Class
Alphaproteobacteria
Betaproteobacteria
Gammaproteobacteria
Deltaproteobacteria
Epsilonproteobacteria
Zetaproteobacteria
Oligoflexia
Acidithiobacillia
%Usage
GTT
16%
12%
8%
4%

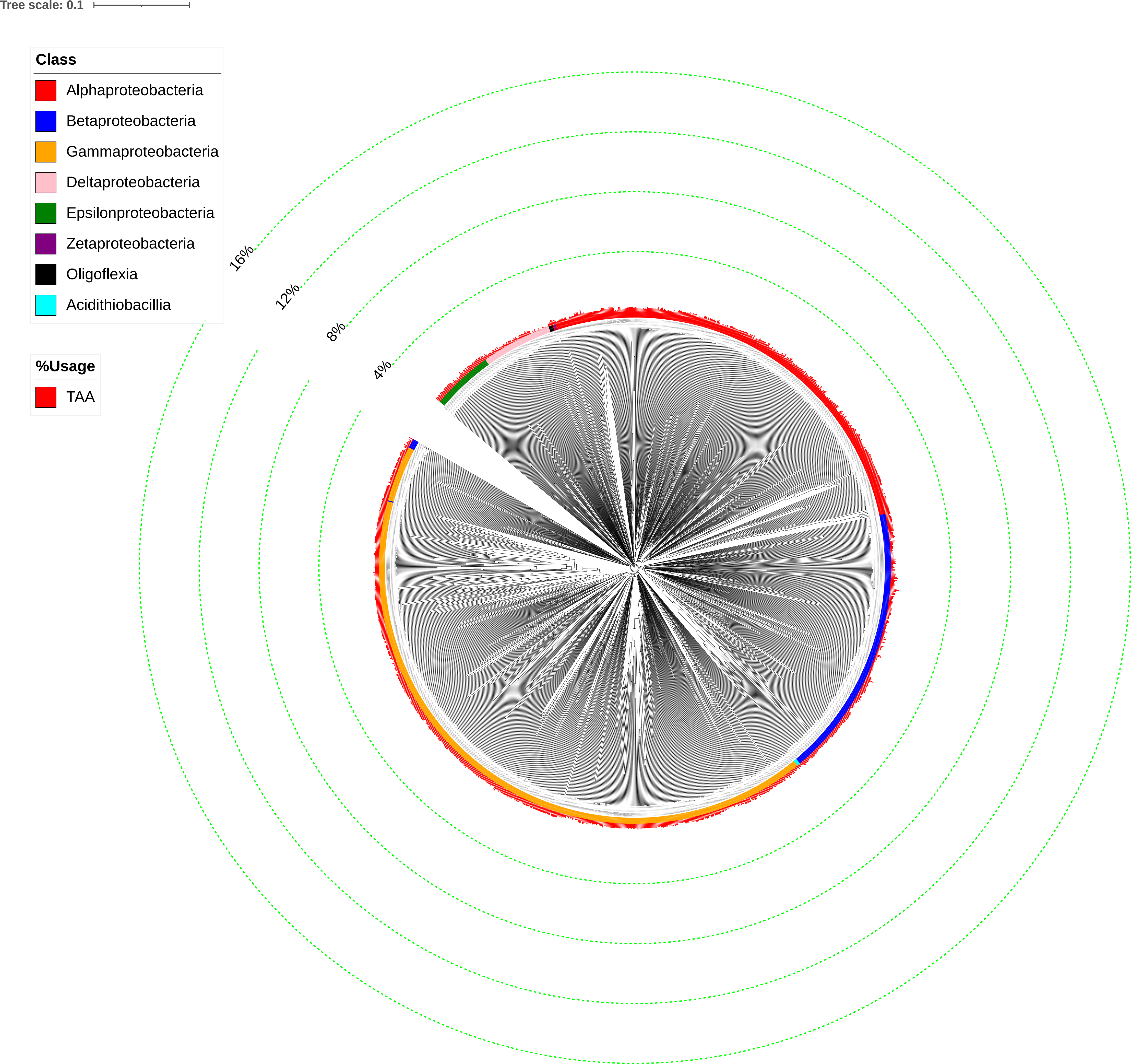

Tree scale: 0.1
Class
Alphaproteobacteria
Betaproteobacteria
Gammaproteobacteria
Deltaproteobacteria
Epsilonproteobacteria
Zetaproteobacteria
Oligoflexia
Acidithiobacillia
%Usage
TAA
16%
12%
8%
4%

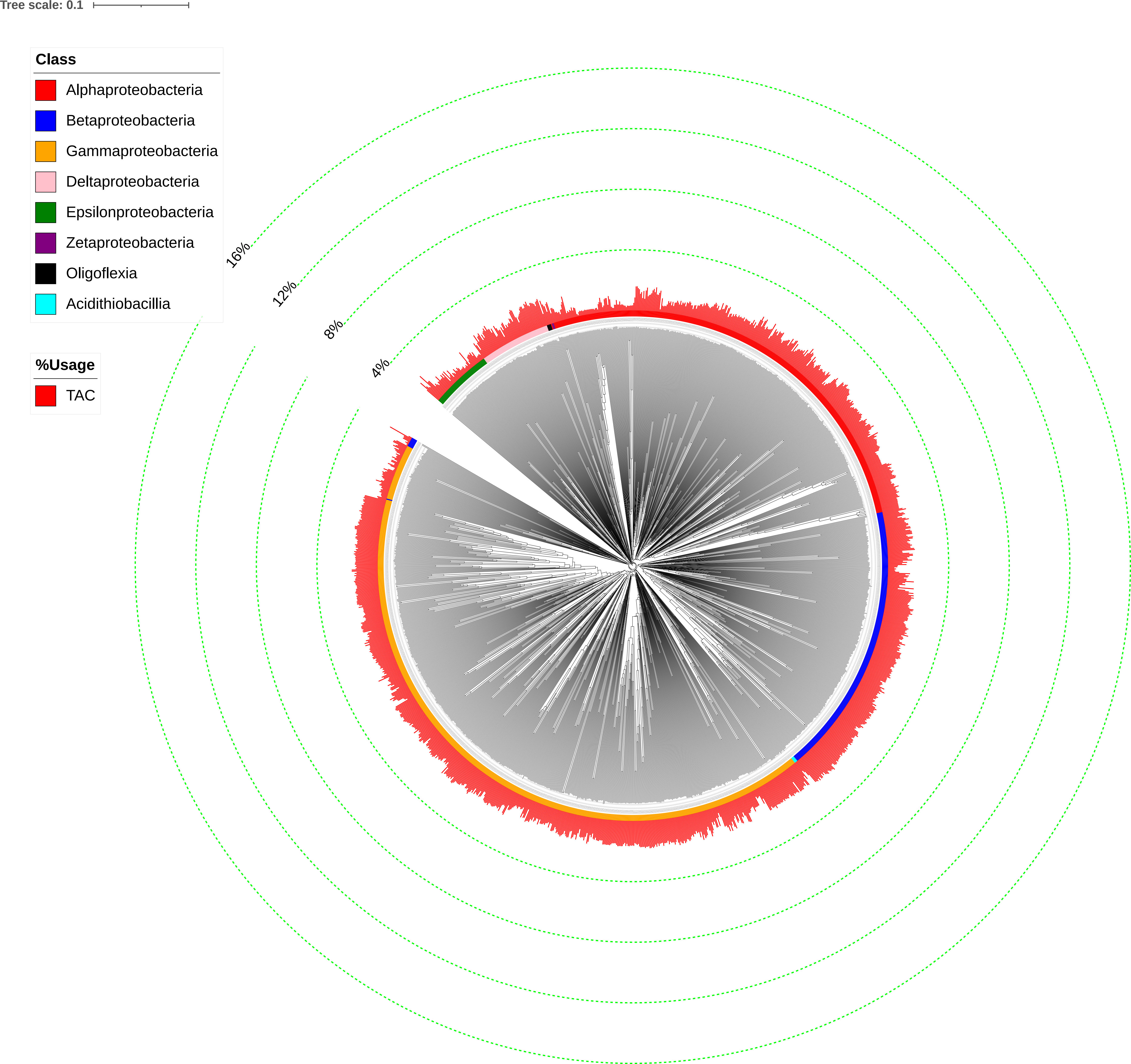
Tree scale: 0.1
Class
Alphaproteobacteria
Betaproteobacteria
Gammaproteobacteria
Deltaproteobacteria
Epsilonproteobacteria
Zetaproteobacteria
Oligoflexia
Acidithiobacillia
%Usage
TAC
16%
12%
8%
4%

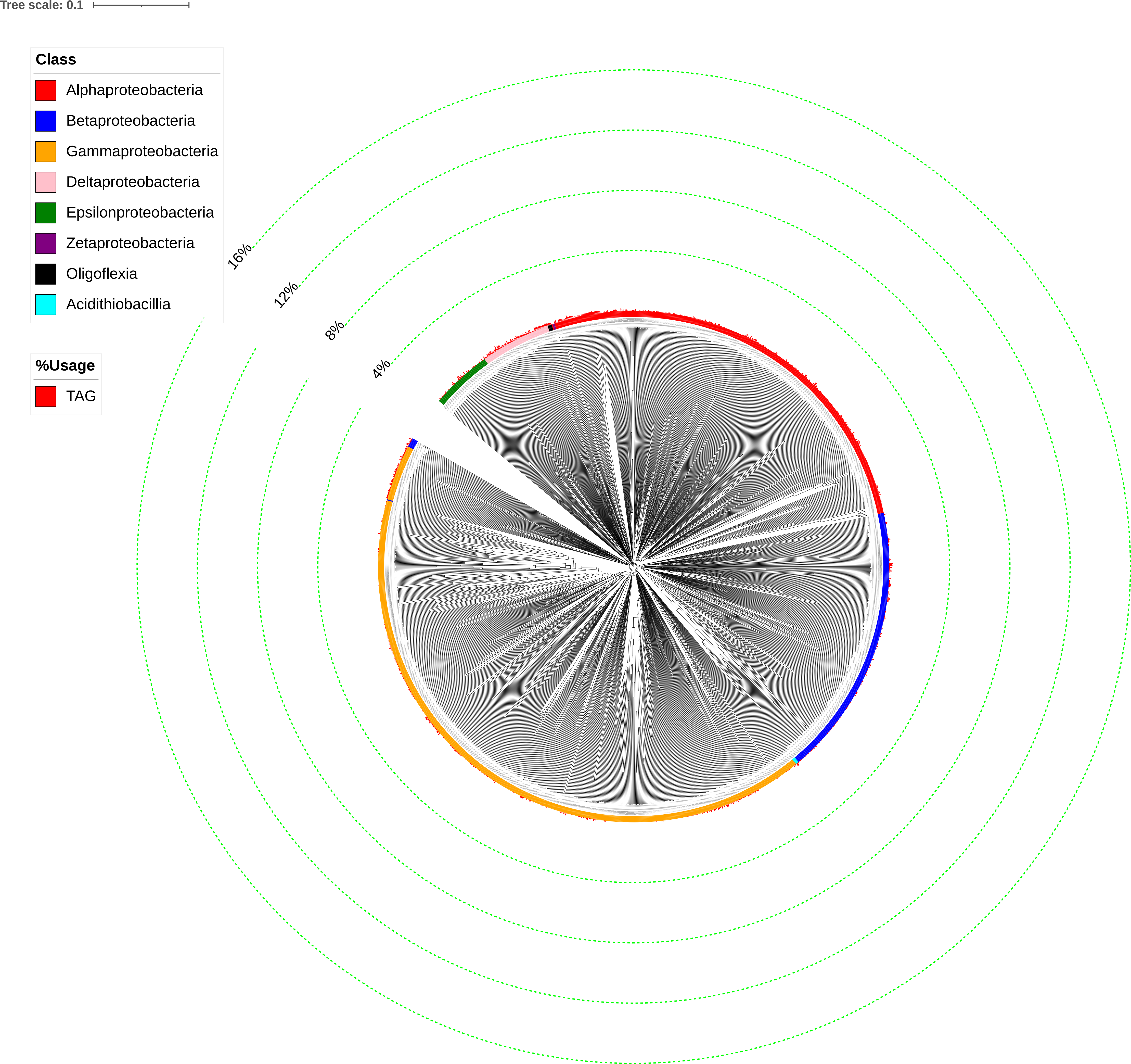
Tree scale: 0.1
Class
Alphaproteobacteria
Betaproteobacteria
Gammaproteobacteria
Deltaproteobacteria
Epsilonproteobacteria
Zetaproteobacteria
Oligoflexia
Acidithiobacillia
%Usage
TAG
16%
12%
8%
4%

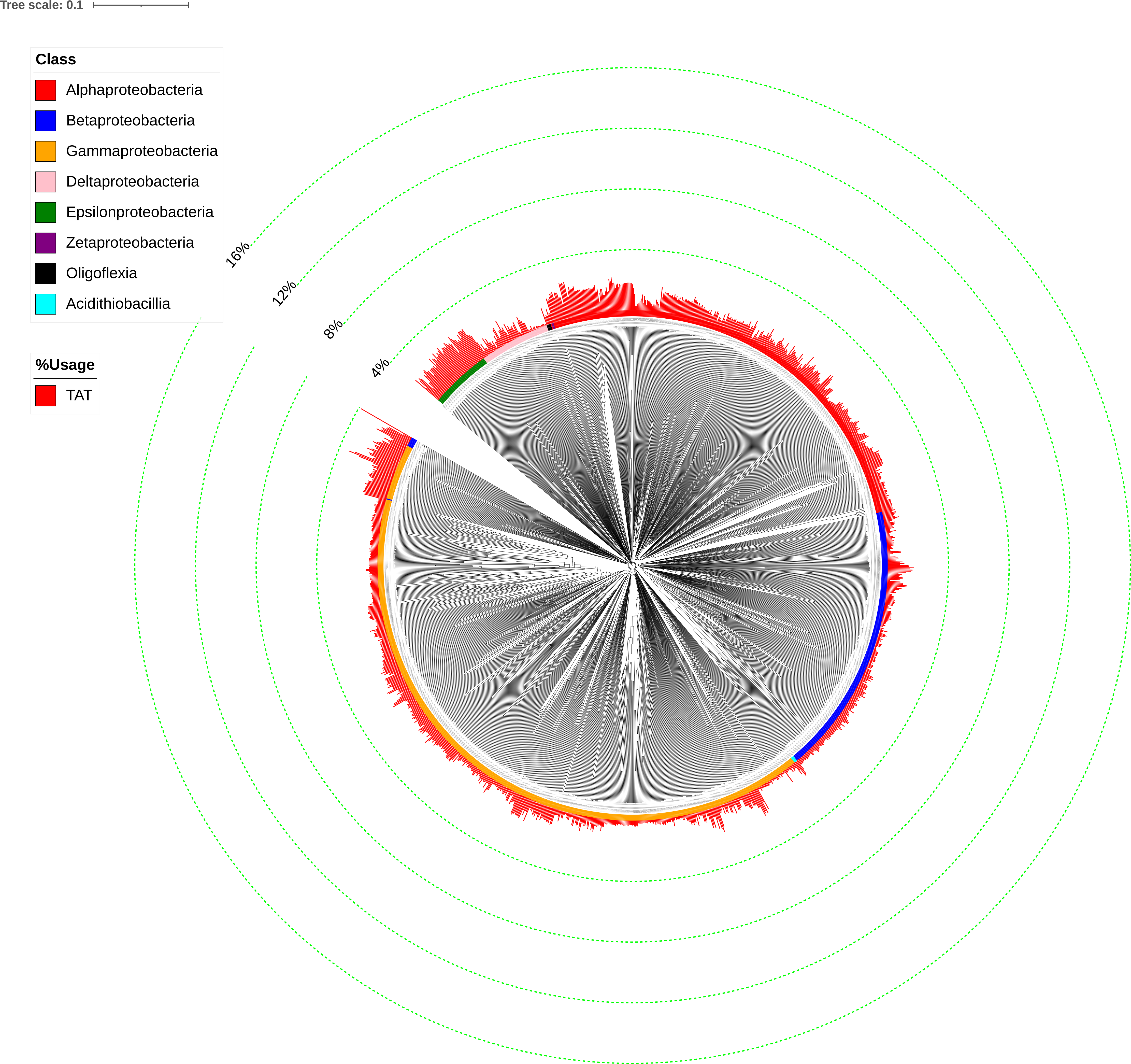

Tree scale: 0.1
Class
Alphaproteobacteria
Betaproteobacteria
Gammaproteobacteria
Deltaproteobacteria
Epsilonproteobacteria
Zetaproteobacteria
Oligoflexia
Acidithiobacillia
%Usage
TAT
16%
12%
8%
4%

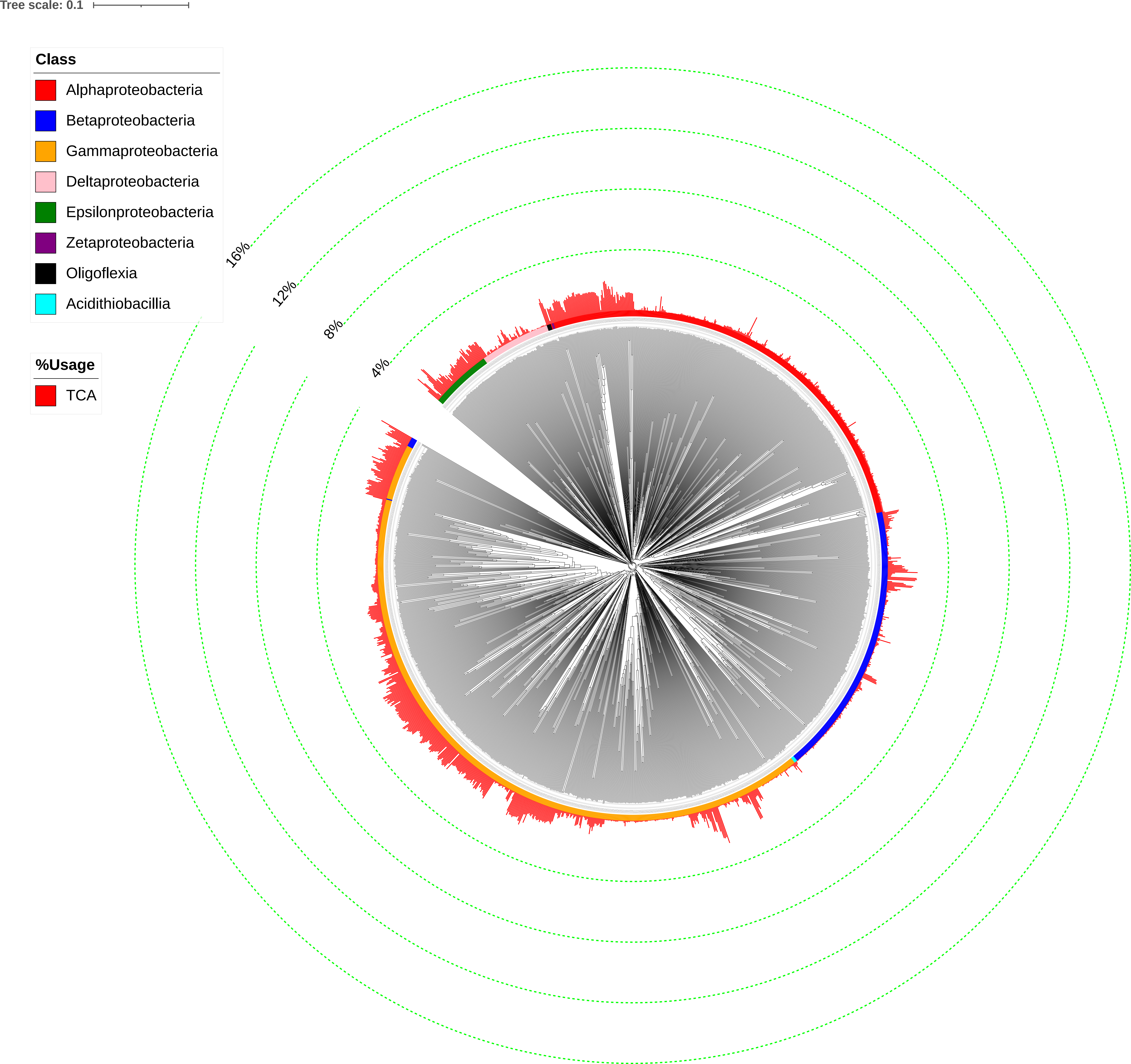
Tree scale: 0.1
Class
Alphaproteobacteria
Betaproteobacteria
Gammaproteobacteria
Deltaproteobacteria
Epsilonproteobacteria
Zetaproteobacteria
Oligoflexia
Acidithiobacillia
%Usage
TCA
16%
12%
8%
4%

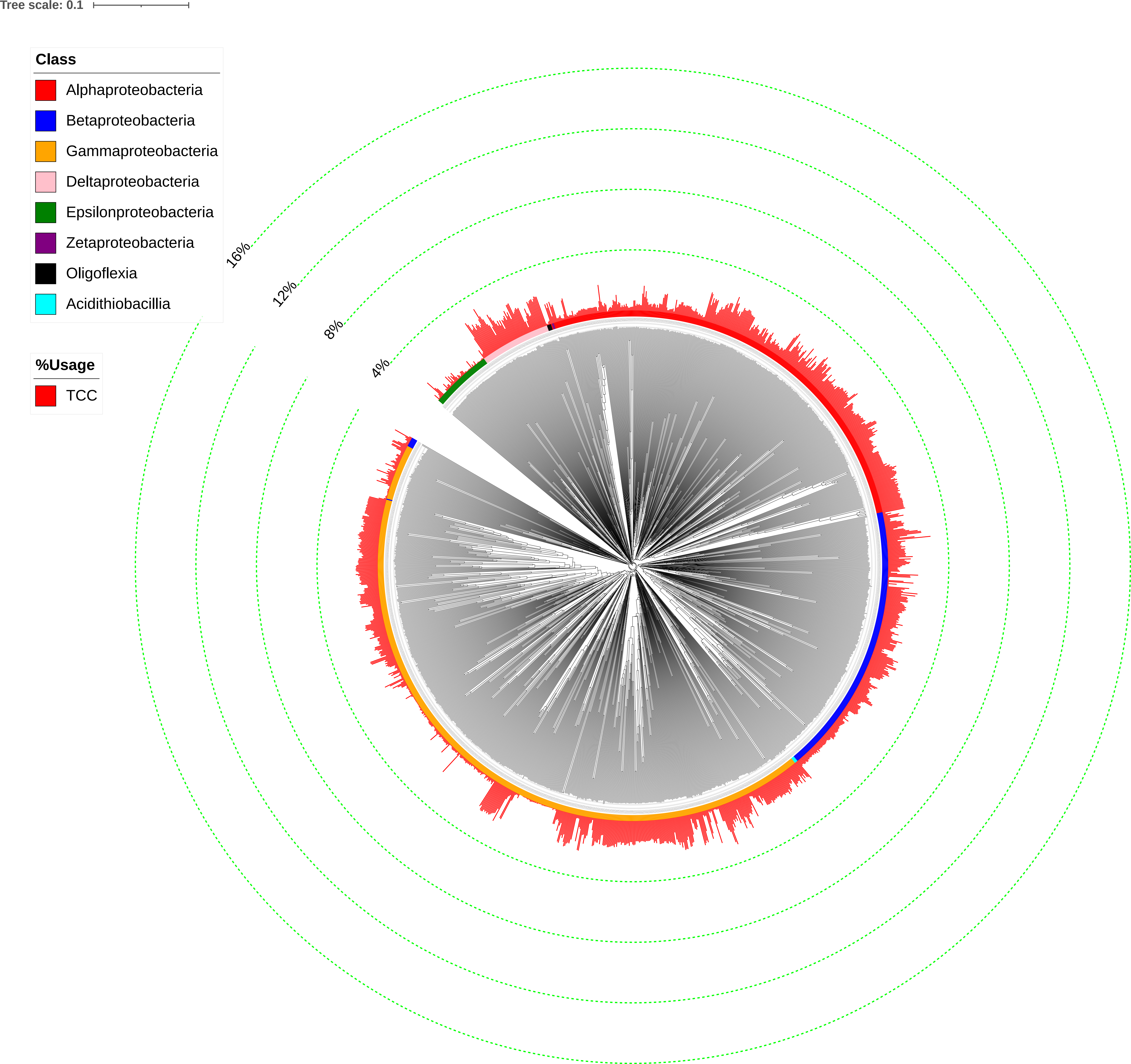

Tree scale: 0.1
Class
Alphaproteobacteria
Betaproteobacteria
Gammaproteobacteria
Deltaproteobacteria
Epsilonproteobacteria
Zetaproteobacteria
Oligoflexia
Acidithiobacillia
%Usage
TCC
4%
8%
12%
16%

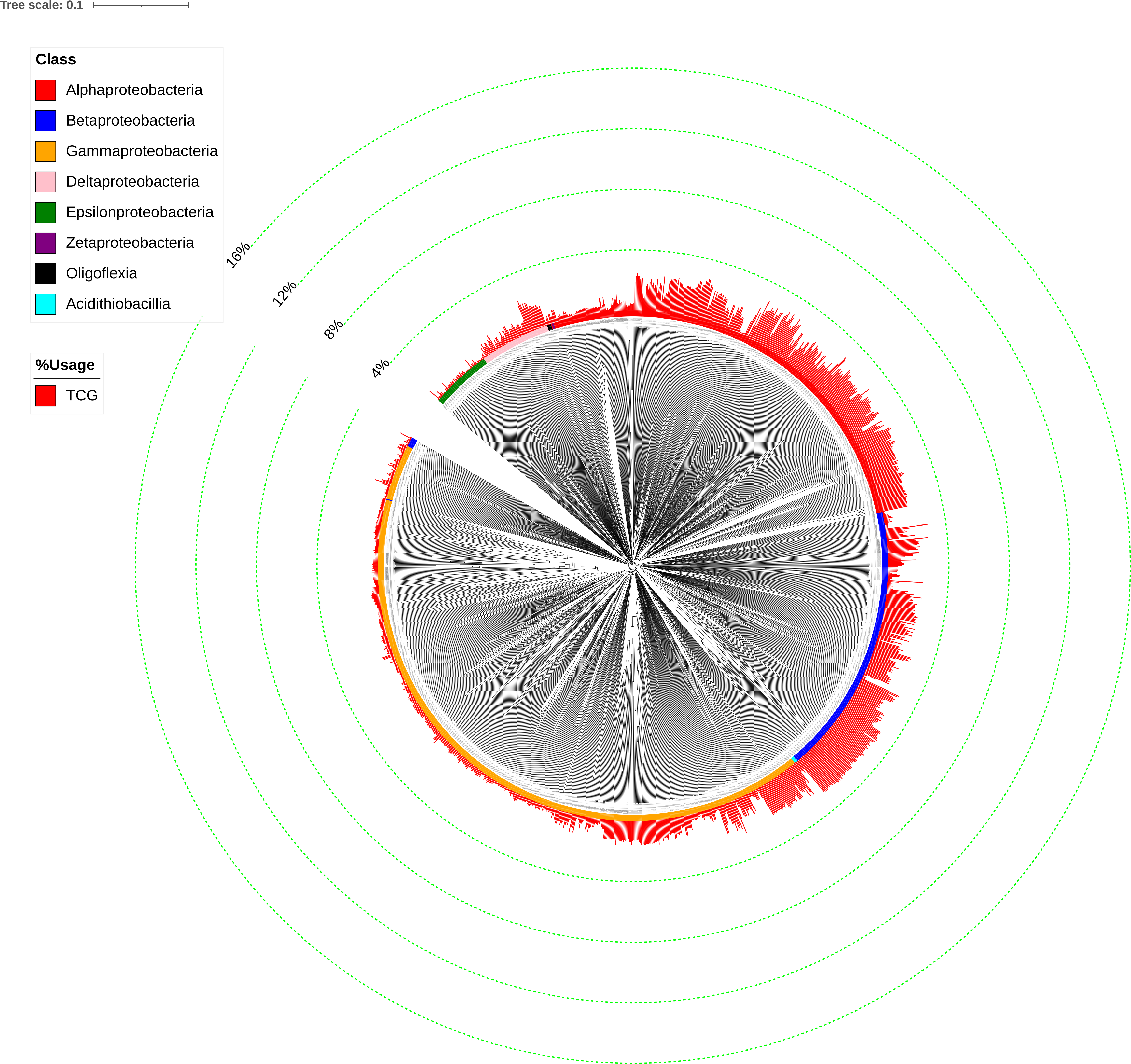
Tree scale: 0.1
Class
Alphaproteobacteria
Betaproteobacteria
Gammaproteobacteria
Deltaproteobacteria
Epsilonproteobacteria
Zetaproteobacteria
Oligoflexia
Acidithiobacillia
%Usage
TCG
4%
8%
12%
16%

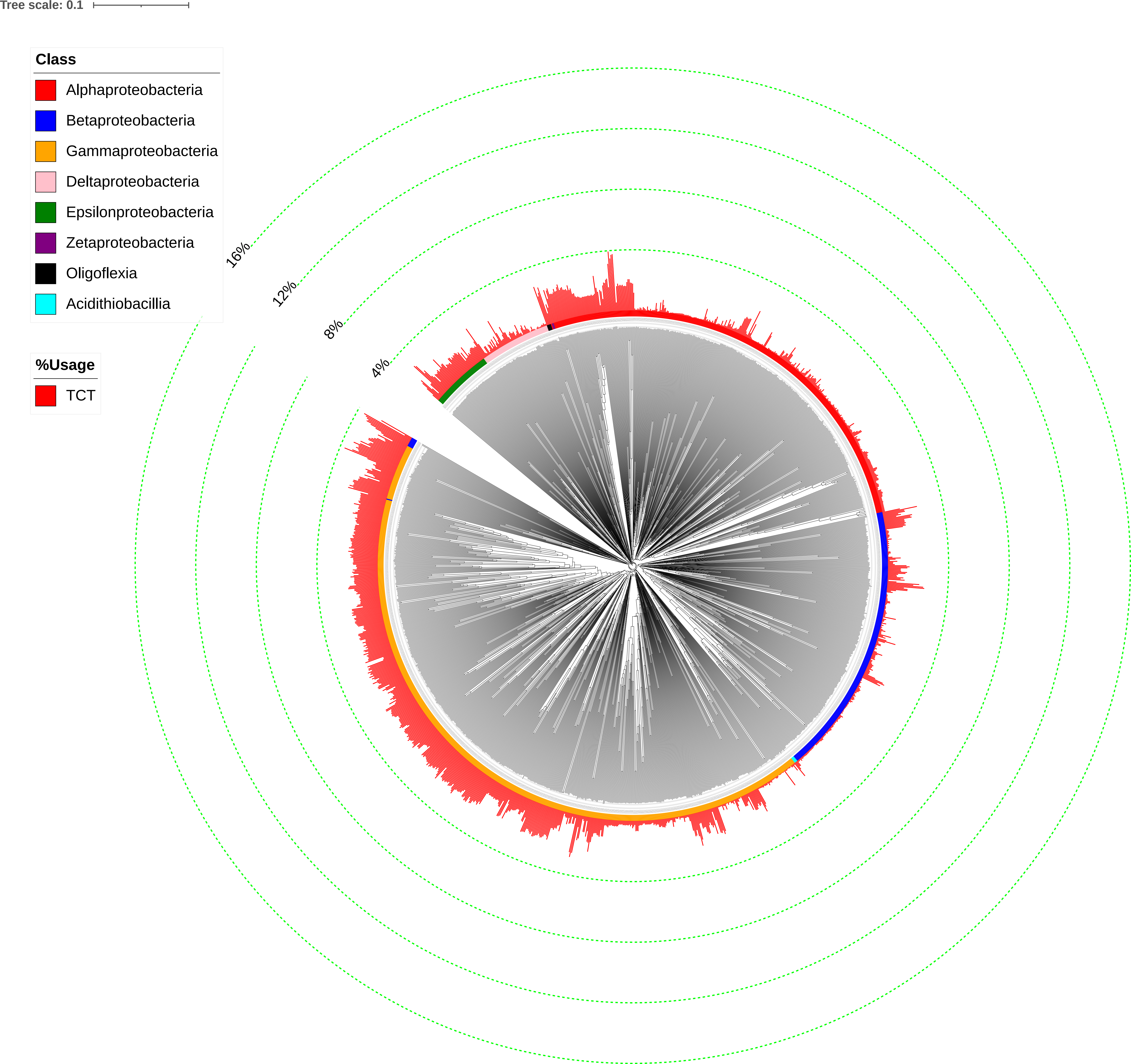

Tree scale: 0.1
Class
Alphaproteobacteria
Betaproteobacteria
Gammaproteobacteria
Deltaproteobacteria
Epsilonproteobacteria
Zetaproteobacteria
Oligoflexia
Acidithiobacillia
%Usage
TCT
16%
12%
8%
4%

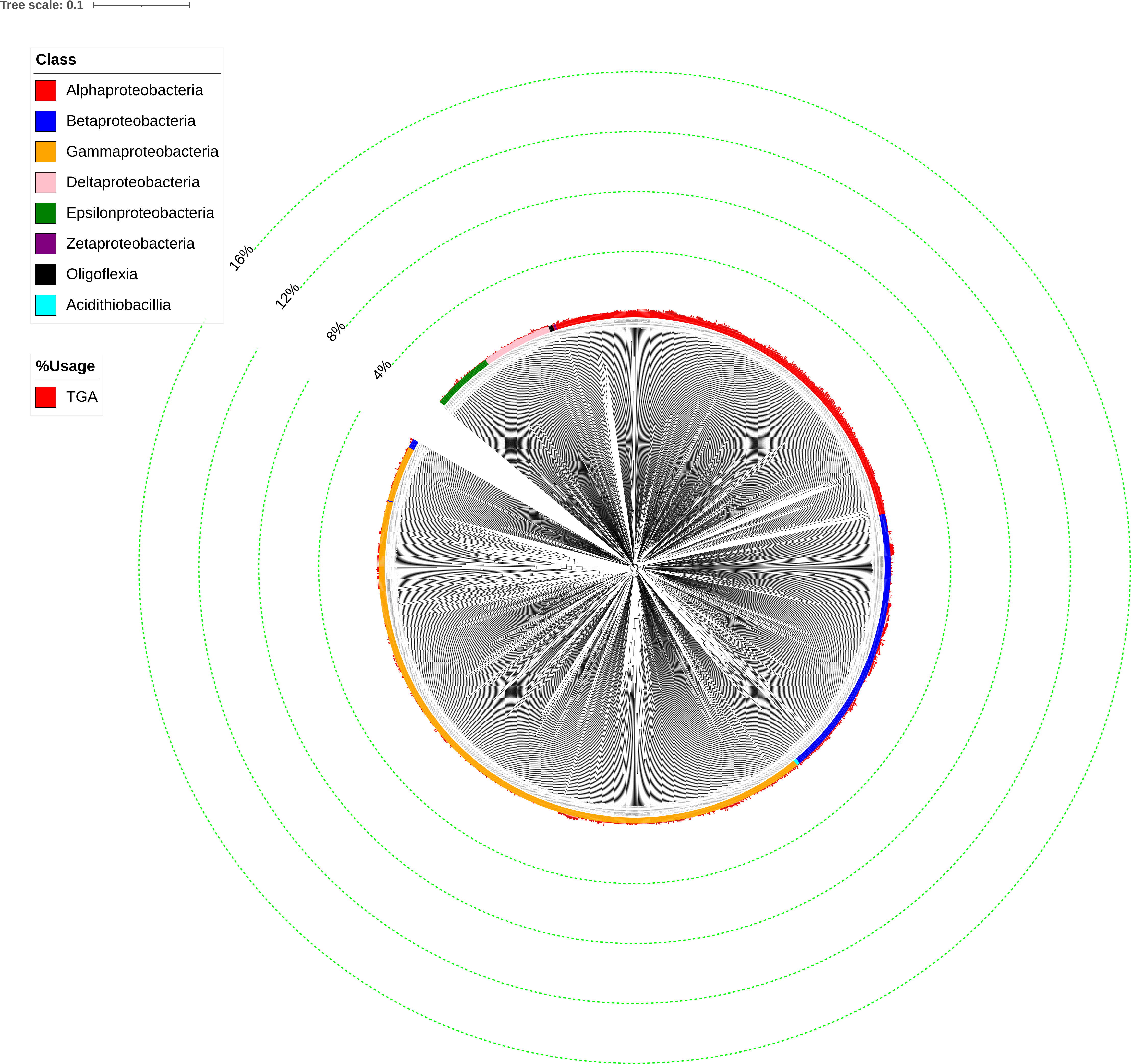

Tree scale: 0.1
Class
Alphaproteobacteria
Betaproteobacteria
Gammaproteobacteria
Deltaproteobacteria
Epsilonproteobacteria
Zetaproteobacteria
Oligoflexia
Acidithiobacillia
%Usage
TGA
16%
12%
8%
4%

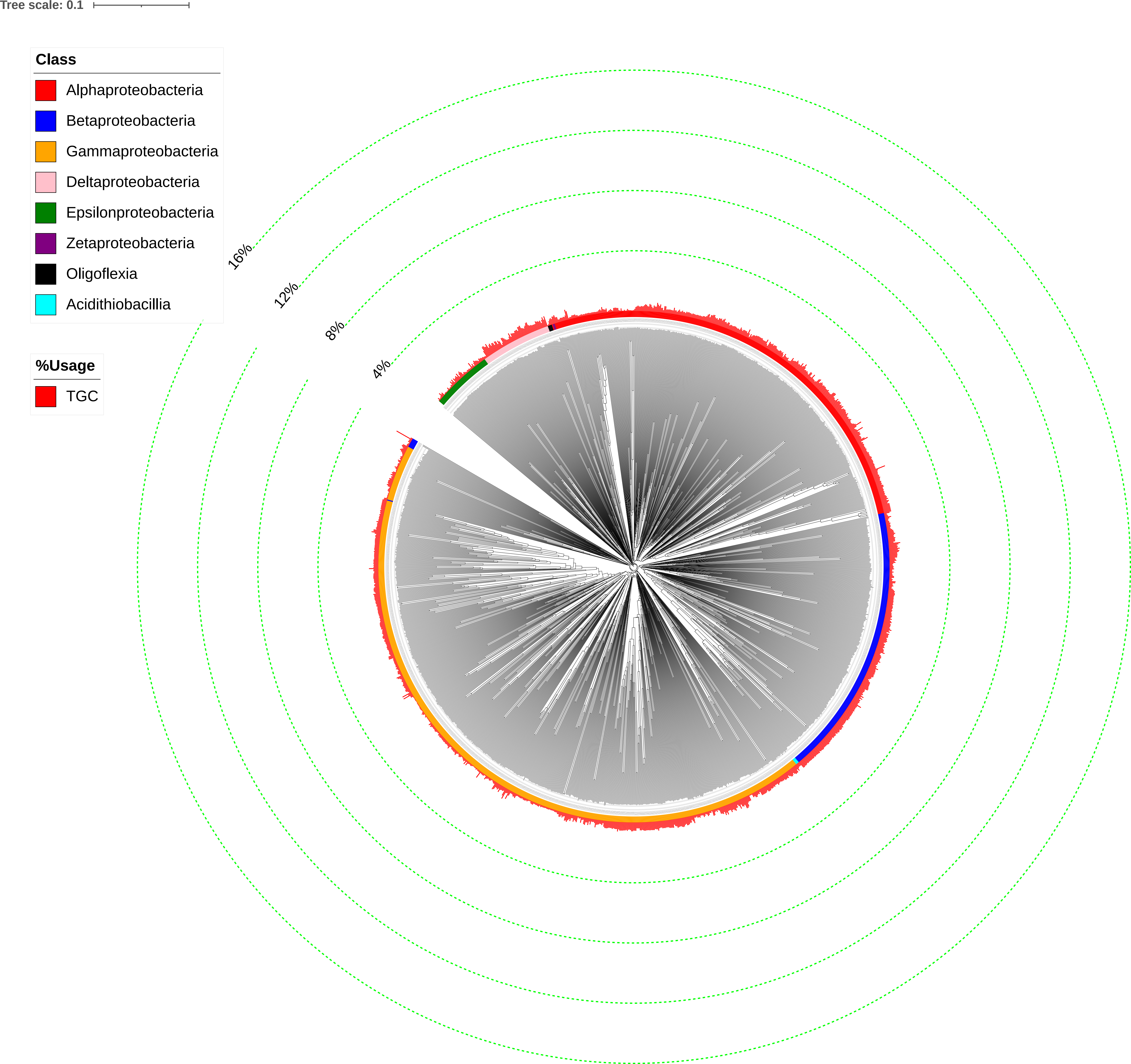
Tree scale: 0.1
Class
Alphaproteobacteria
Betaproteobacteria
Gammaproteobacteria
Deltaproteobacteria
Epsilonproteobacteria
Zetaproteobacteria
Oligoflexia
Acidithiobacillia
%Usage
TGC
16%
12%
8%
4%

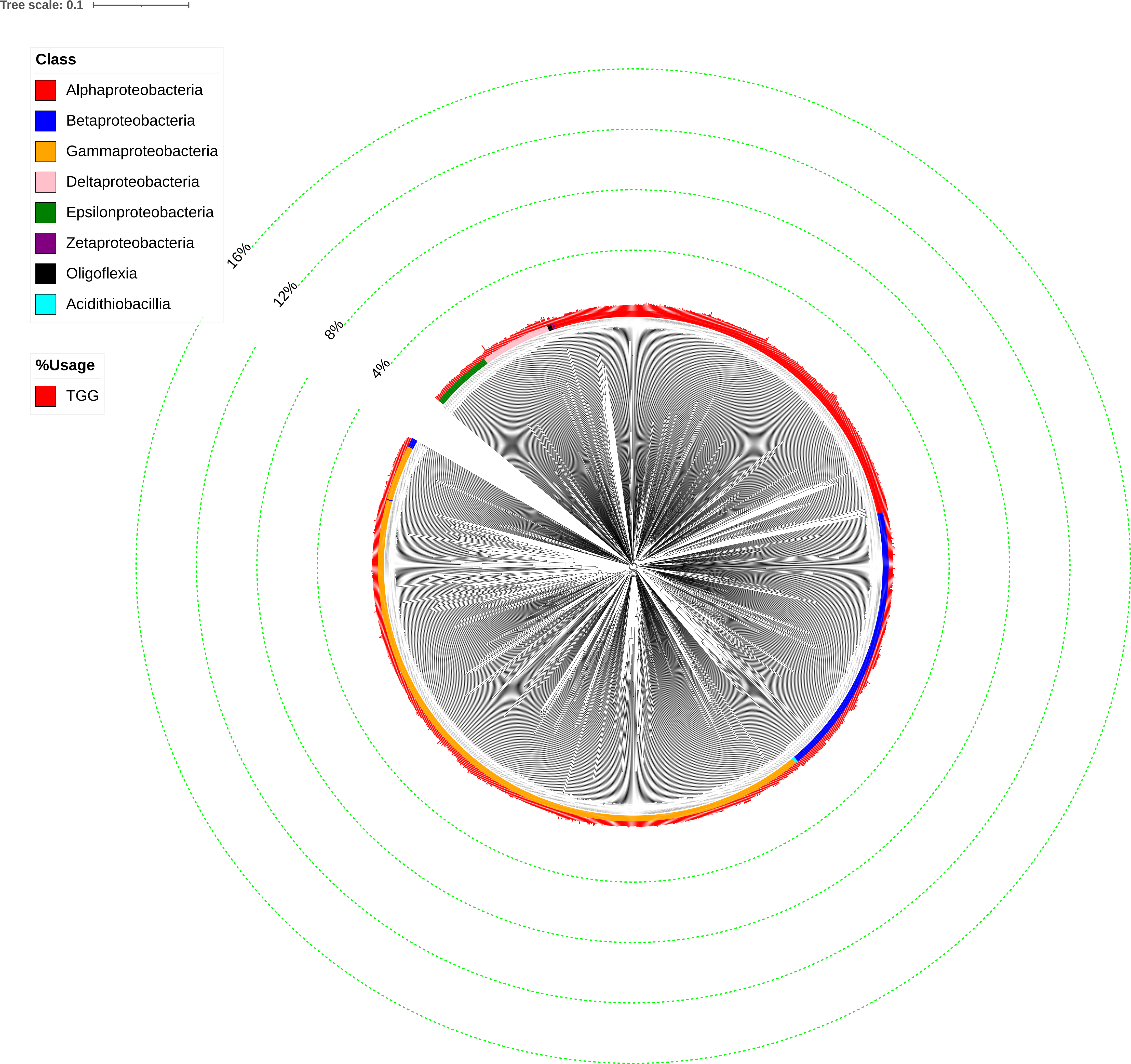

Tree scale: 0.1
Class
Alphaproteobacteria
Betaproteobacteria
Gammaproteobacteria
Deltaproteobacteria
Epsilonproteobacteria
Zetaproteobacteria
Oligoflexia
Acidithiobacillia
%Usage
TGG
16%
12%
8%
4%

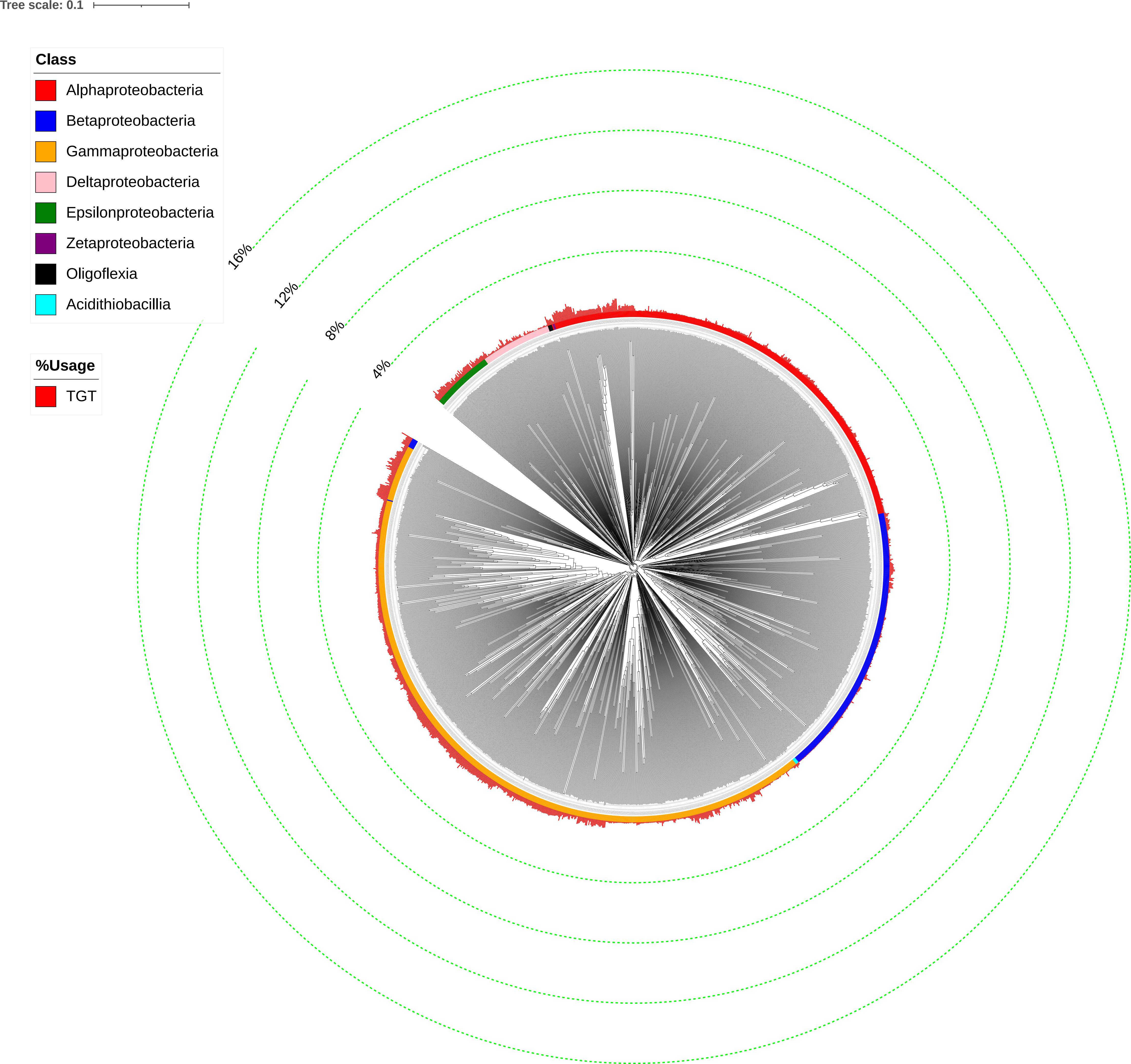

Tree scale: 0.1
Class
Alphaproteobacteria
Betaproteobacteria
Gammaproteobacteria
Deltaproteobacteria
Epsilonproteobacteria
Zetaproteobacteria
Oligoflexia
Acidithiobacillia
%Usage
TGT
4%
8%
12%
16%

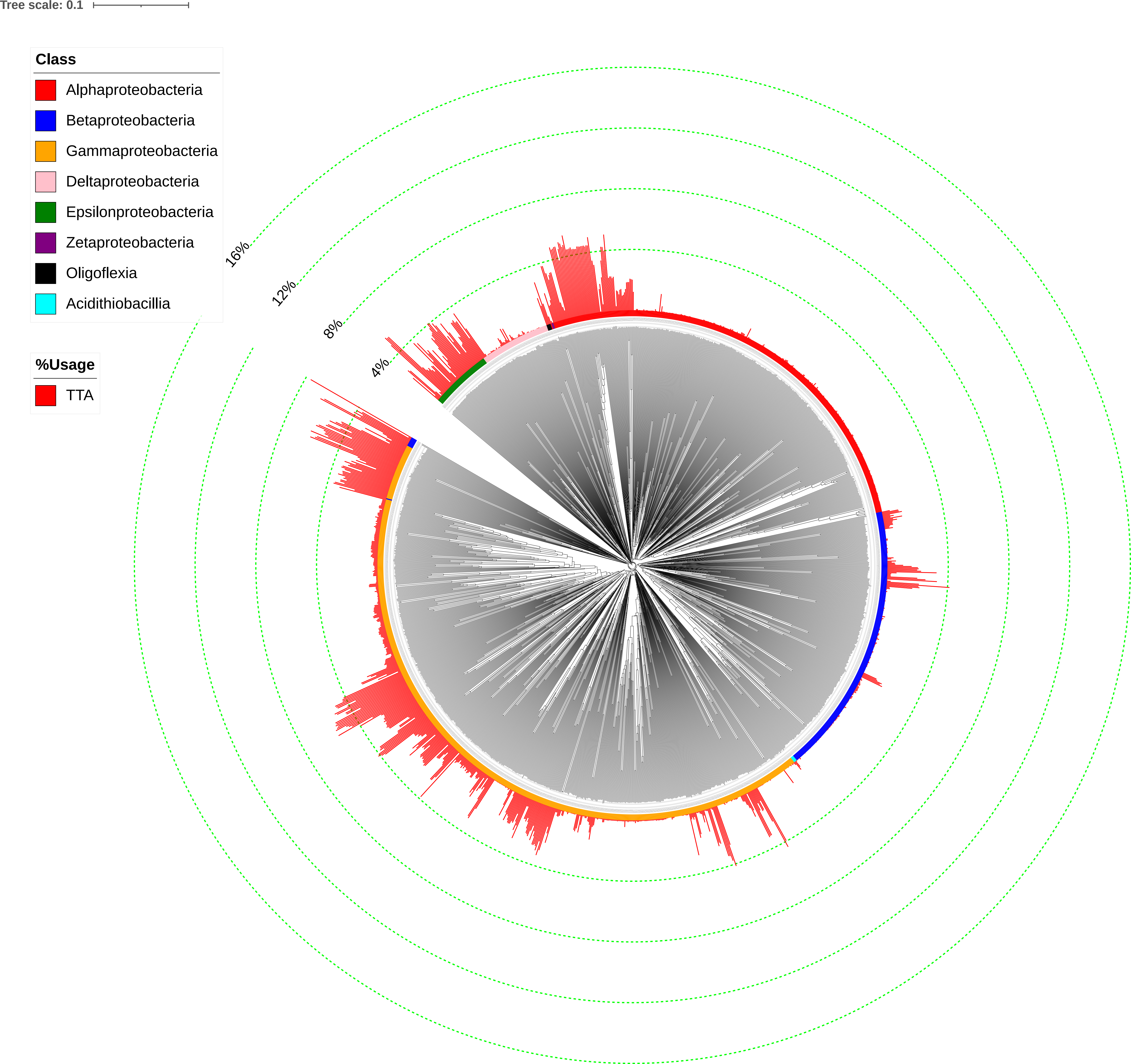

Tree scale: 0.1
Class
Alphaproteobacteria
Betaproteobacteria
Gammaproteobacteria
Deltaproteobacteria
Epsilonproteobacteria
Zetaproteobacteria
Oligoflexia
Acidithiobacillia
%Usage
TTA
16%
12%
8%
4%

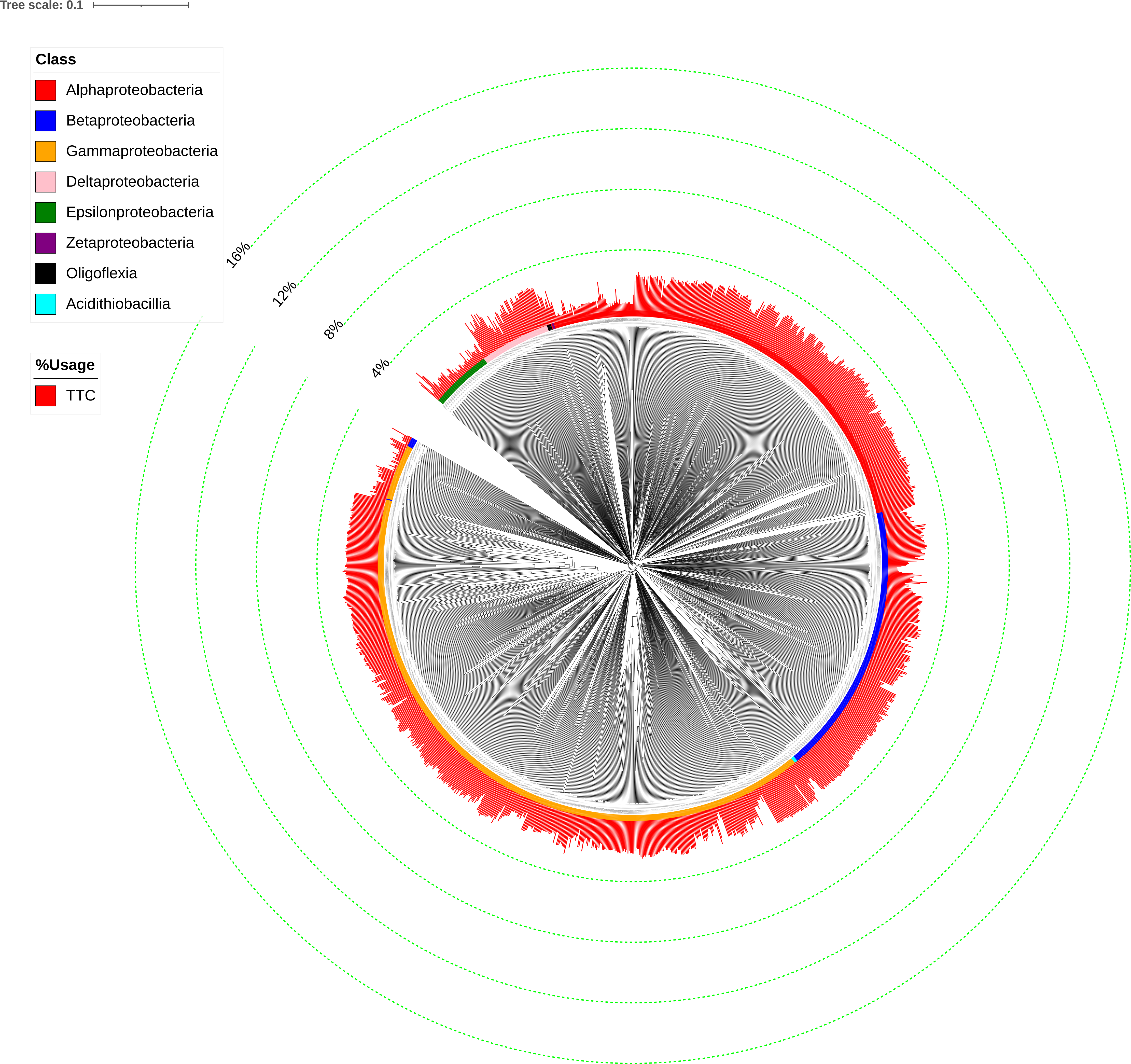

Tree scale: 0.1
Class
Alphaproteobacteria
Betaproteobacteria
Gammaproteobacteria
Deltaproteobacteria
Epsilonproteobacteria
Zetaproteobacteria
Oligoflexia
Acidithiobacillia
%Usage
TTC
16%
12%
8%
4%

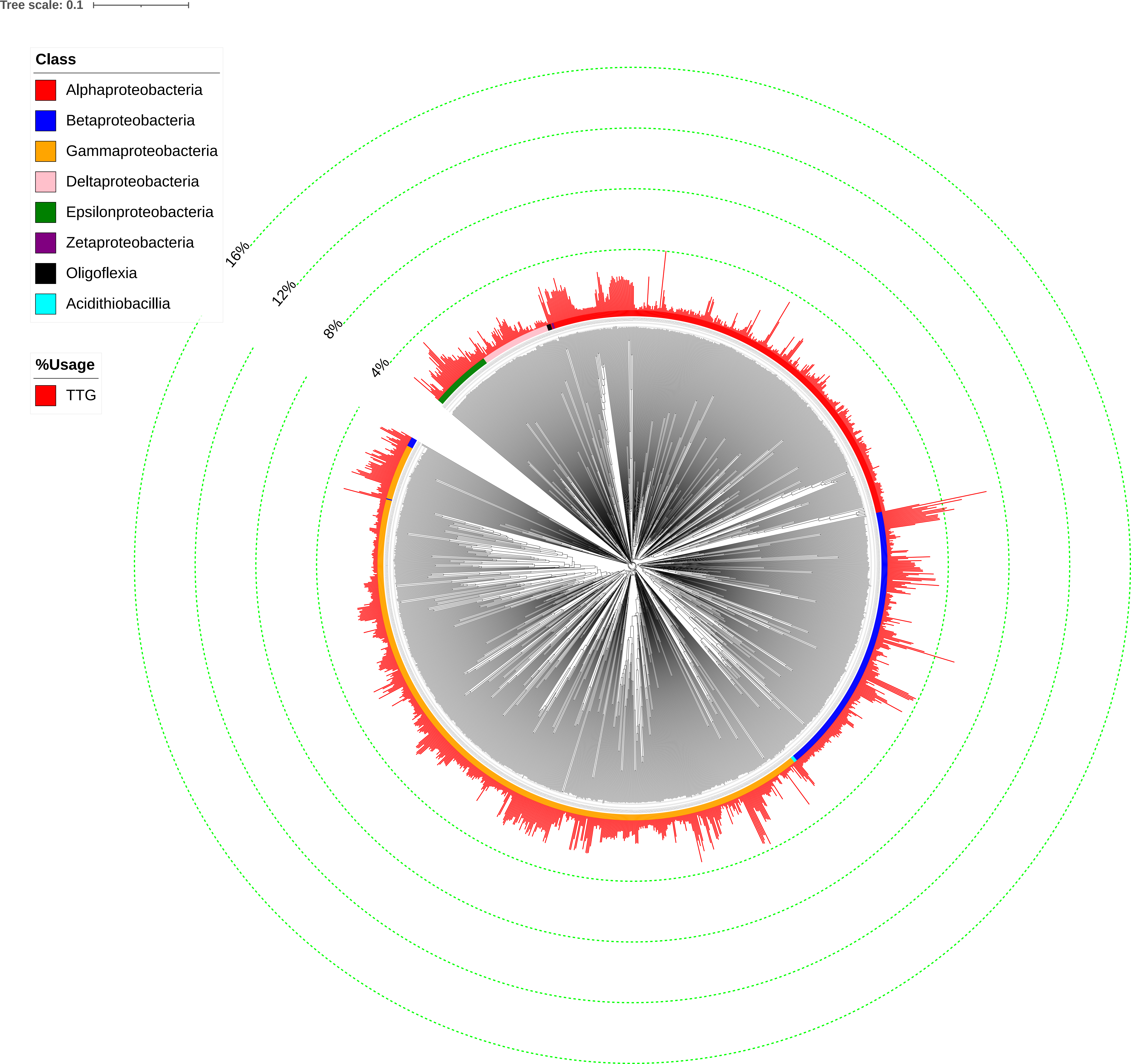

Tree scale: 0.1
Class
Alphaproteobacteria
Betaproteobacteria
Gammaproteobacteria
Deltaproteobacteria
Epsilonproteobacteria
Zetaproteobacteria
Oligoflexia
Acidithiobacillia
%Usage
TTG
4%
8%
12%
16%

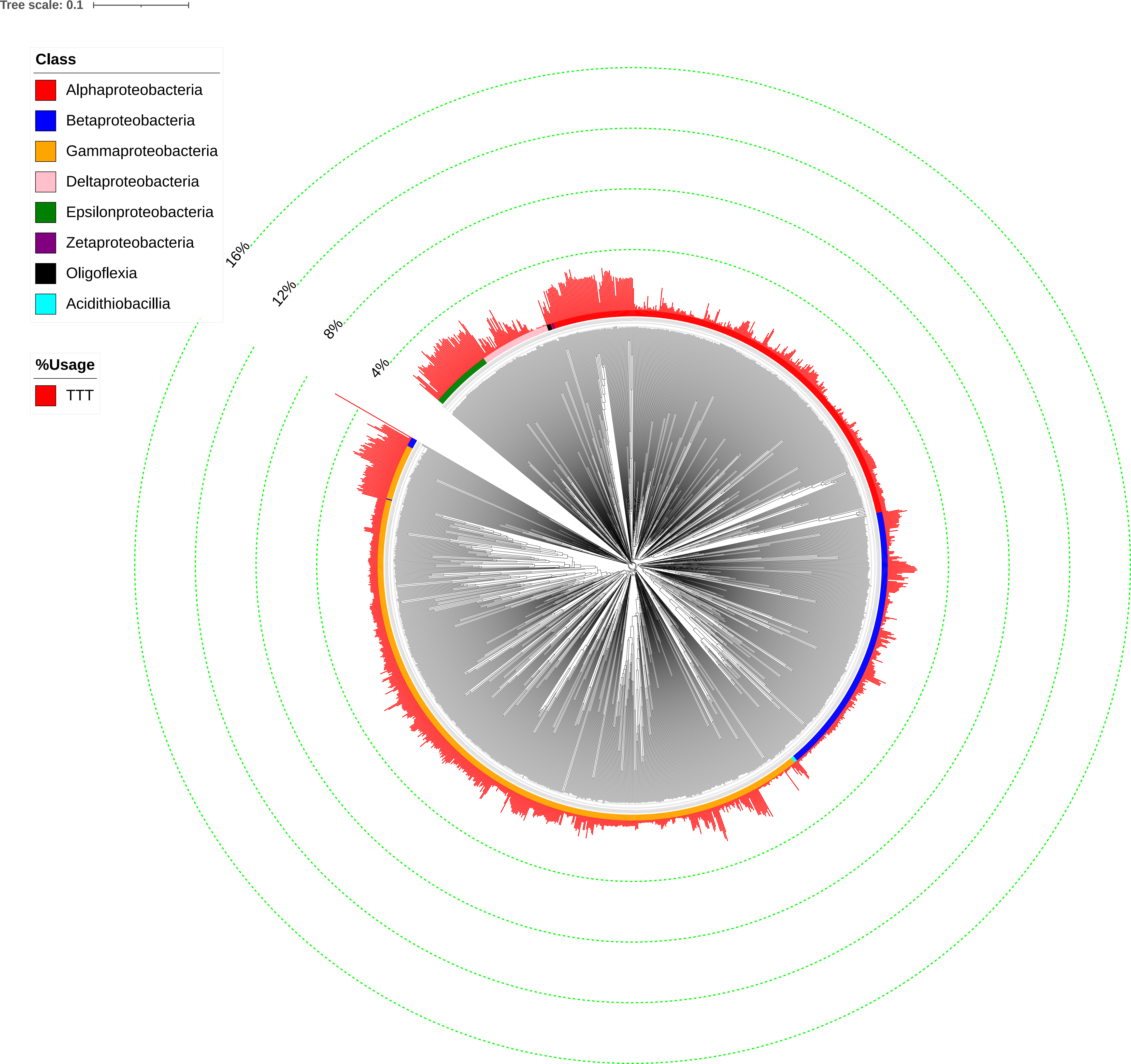

Tree scale: 0.1
Class
Alphaproteobacteria
Betaproteobacteria
Gammaproteobacteria
Deltaproteobacteria
Epsilonproteobacteria
Zetaproteobacteria
Oligoflexia
Acidithiobacillia
%Usage
TTT
16%
12%
8%
4%

Figure S4. Variability of codon usage in proteobacteria. As an estimation of the variability in frequencies of codon usage in proteobacteria the difference between codon usages at percentiles 90 and 10 of the complete population was calculated. Data is presented in the context of the genetic code and values are colored with respect to variability, where the most variable codons are highlighted with blue colors and the least variable codons in red colors. **A)** Delta 90-10 of codon usages considering all genes of genomes. **B)** Delta 90-10 of codon usages considering a set of genes expected to be highly expressed.

A)

1st base	2nd base T			2nd base C			2nd base A			2nd base G			3rd base
T	TTT	0.0339	Phe	TCT	0.0152	Ser	TAT	0.0190	Tyr	TGT	0.0070	Cys	T
	TTC	0.0225		TCC	0.0108		TAC	0.0100		TGC	0.0056		C
	TTA	0.0423	Leu	TCA	0.0133		TAA	0.0027	Stop	TGA	0.0027	Stop	A
	TTG	0.0165		TCG	0.0156		TAG	0.0007		TGG	0.0053	Trp	G
C	CTT	0.0167	Leu	CCT	0.0110	Pro	CAT	0.0091	His	CGT	0.0135	Arg	T
	CTC	0.0287		CCC	0.0177		CAC	0.0103		CGC	0.0401		C
	CTA	0.0125		CCA	0.0114		CAA	0.0266	Gln	CGA	0.0043		A
	CTG	0.0677		CCG	0.0273		CAG	0.0259		CGG	0.0179		G
A	ATT	0.0395	Ile	ACT	0.0161	Thr	AAT	0.0320	Asn	AGT	0.0142	Ser	T
	ATC	0.0280		ACC	0.0229		AAC	0.0094		AGC	0.0116		C
	ATA	0.0187		ACA	0.0148		AAA	0.0519	Lys	AGA	0.0098	Arg	A
	ATG	0.0055	Met	ACG	0.0153		AAG	0.0156		AGG	0.0037		G
G	GTT	0.0212	Val	GCT	0.0204	Ala	GAT	0.0253	Asp	GGT	0.0202	Gly	T
	GTC	0.0287		GCC	0.0570		GAC	0.0310		GGC	0.0474		C
	GTA	0.0172		GCA	0.0188		GAA	0.0257	Glu	GGA	0.0119		A
	GTG	0.0286		GCG	0.0475		GAG	0.0231		GGG	0.0112		G

B)

1st base	2nd base T			2nd base C			2nd base A			2nd base G			3rd base
T	TTT	0.0188	Phe	TCT	0.0188	Ser	TAT	0.0135	Tyr	TGT	0.0046	Cys	T
	TTC	0.0174		TCC	0.0154		TAC	0.0110		TGC	0.0041		C
	TTA	0.0313	Leu	TCA	0.0132		TAA	0.0022	Stop	TGA	0.0018	Stop	A
	TTG	0.0170		TCG	0.0205		TAG	0.0011		TGG	0.0015	Trp	G
C	CTT	0.0228	Leu	CCT	0.0131	Pro	CAT	0.0083	His	CGT	0.0301	Arg	T
	CTC	0.0286		CCC	0.0146		CAC	0.0098		CGC	0.0399		C
	CTA	0.0121		CCA	0.0178		CAA	0.0229	Gln	CGA	0.0030		A
	CTG	0.0608		CCG	0.0278		CAG	0.0246		CGG	0.0092		G
A	ATT	0.0375	Ile	ACT	0.0252	Thr	AAT	0.0243	Asn	AGT	0.0080	Ser	T
	ATC	0.0367		ACC	0.0322		AAC	0.0170		AGC	0.0077		C
	ATA	0.0120		ACA	0.0171		AAA	0.0607	Lys	AGA	0.0113	Arg	A
	ATG	0.0060	Met	ACG	0.0185		AAG	0.0502		AGG	0.0027		G
G	GTT	0.0398	Val	GCT	0.0328	Ala	GAT	0.0351	Asp	GGT	0.0364	Gly	T
	GTC	0.0423		GCC	0.0512		GAC	0.0387		GGC	0.0535		C
	GTA	0.0300		GCA	0.0269		GAA	0.0381	Glu	GGA	0.0135		A
	GTG	0.0346		GCG	0.0289		GAG	0.0346		GGG	0.0072		G

Figure S5. The range of codon usage variability is similar when analyzing codon usages in complete genomes or a set of highly expressed genes. The variability of codon usage of each codon measured based on either complete genomes or genes expected to be highly expressed is compared in scatter plots. Variabilties were estimated in standard deviations and the distance between values at percentiles 10 and 90. To allow a better analysis, a comparison of mean values is also included.

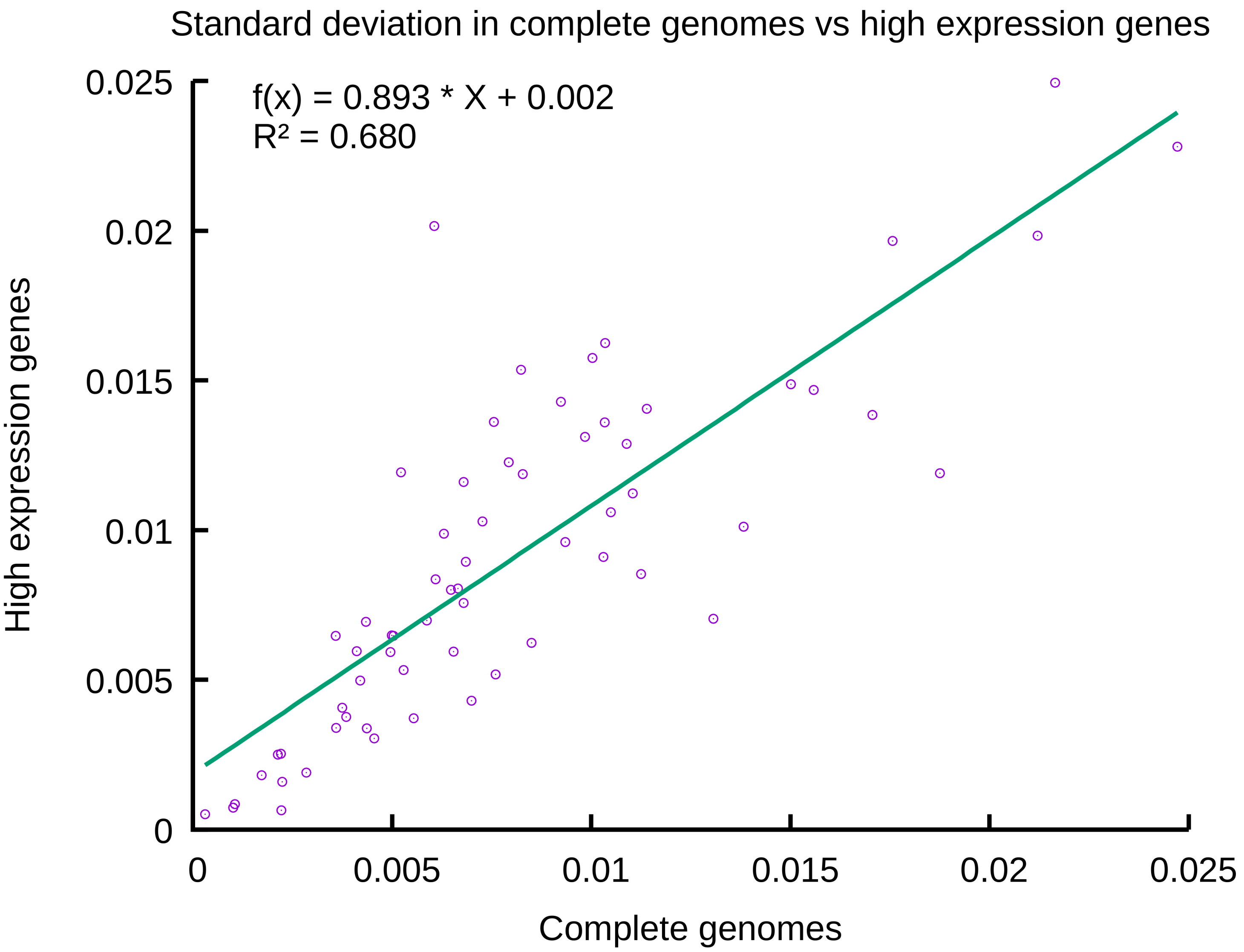
Standard deviation in complete genomes vs high expression genes
f(x) = 0.893 * X + 0.002
R² = 0.680
High expression genes
0.025
0.02
0.015
0.01
0.005
0
0
0.005
0.01
0.015
0.02
0.025
Complete genomes

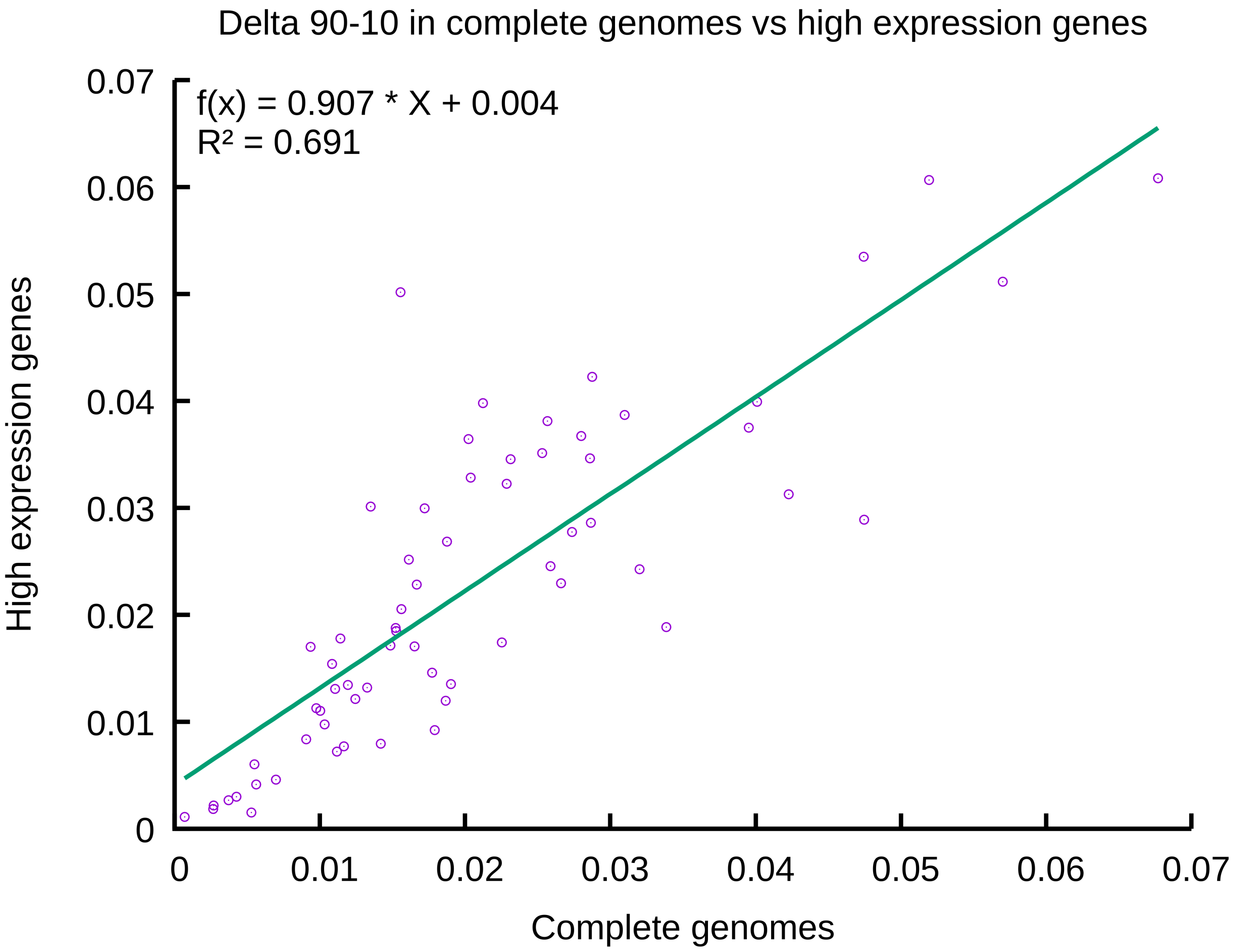
Delta 90-10 in complete genomes vs high expression genes
f(x) = 0.907 * X + 0.004
R² = 0.691
High expression genes
Complete genomes
0
0.01
0.02
0.03
0.04
0.05
0.06
0.07

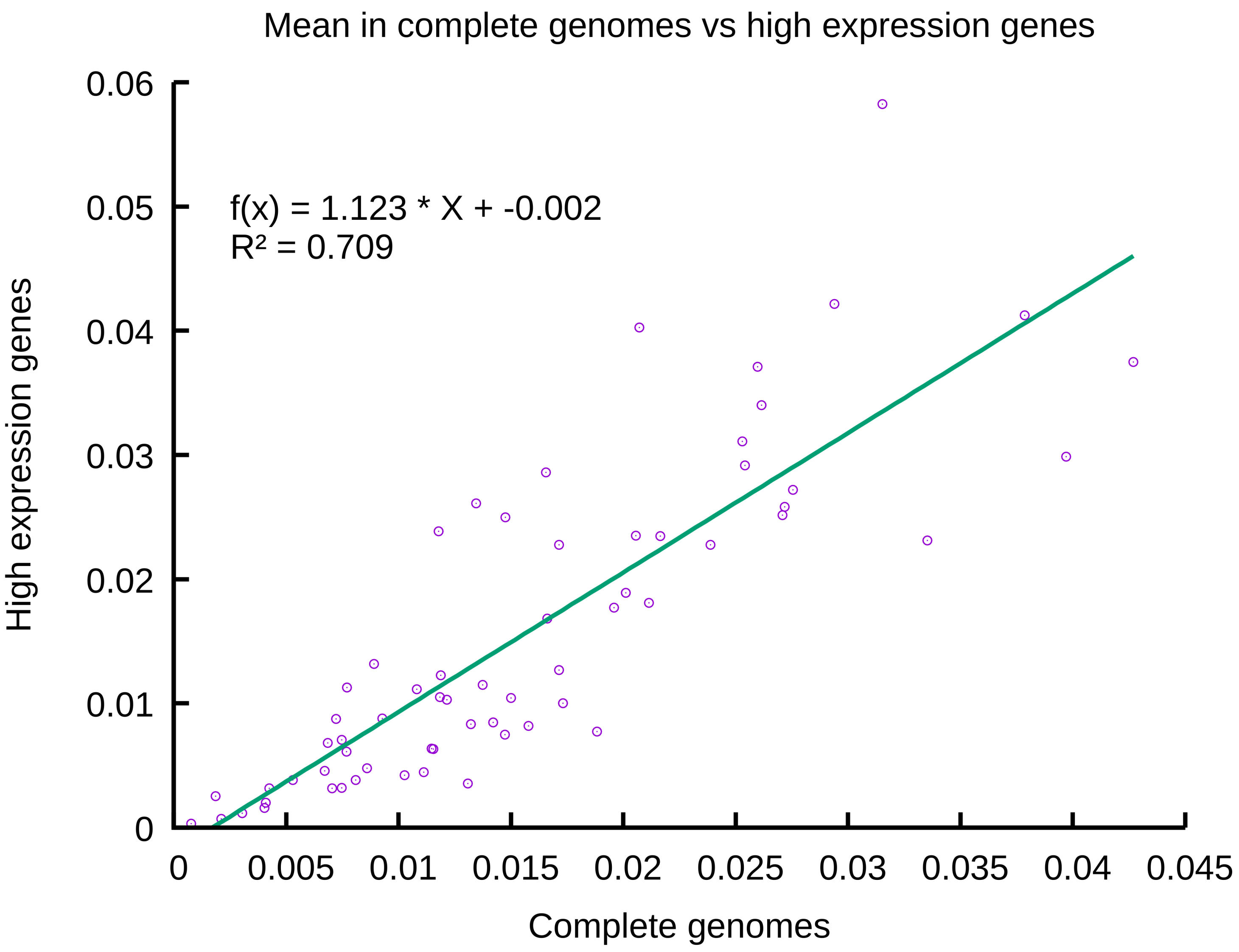
Mean in complete genomes vs high expression genes
f(x) = 1.123 * X + -0.002
R² = 0.709
High expression genes
0
0.01
0.02
0.03
0.04
0.05
0.06
0
0.005
0.01
0.015
0.02
0.025
0.03
0.035
0.04
0.045
Complete genomes

Figure S6. Histogram of the GC% in genomes of proteobacteria. The distribution of GC content on each of the analyzed proteobacterial genomes is represented in a histogram. GC content is represented as a fraction of the total nucleotides in genomes.

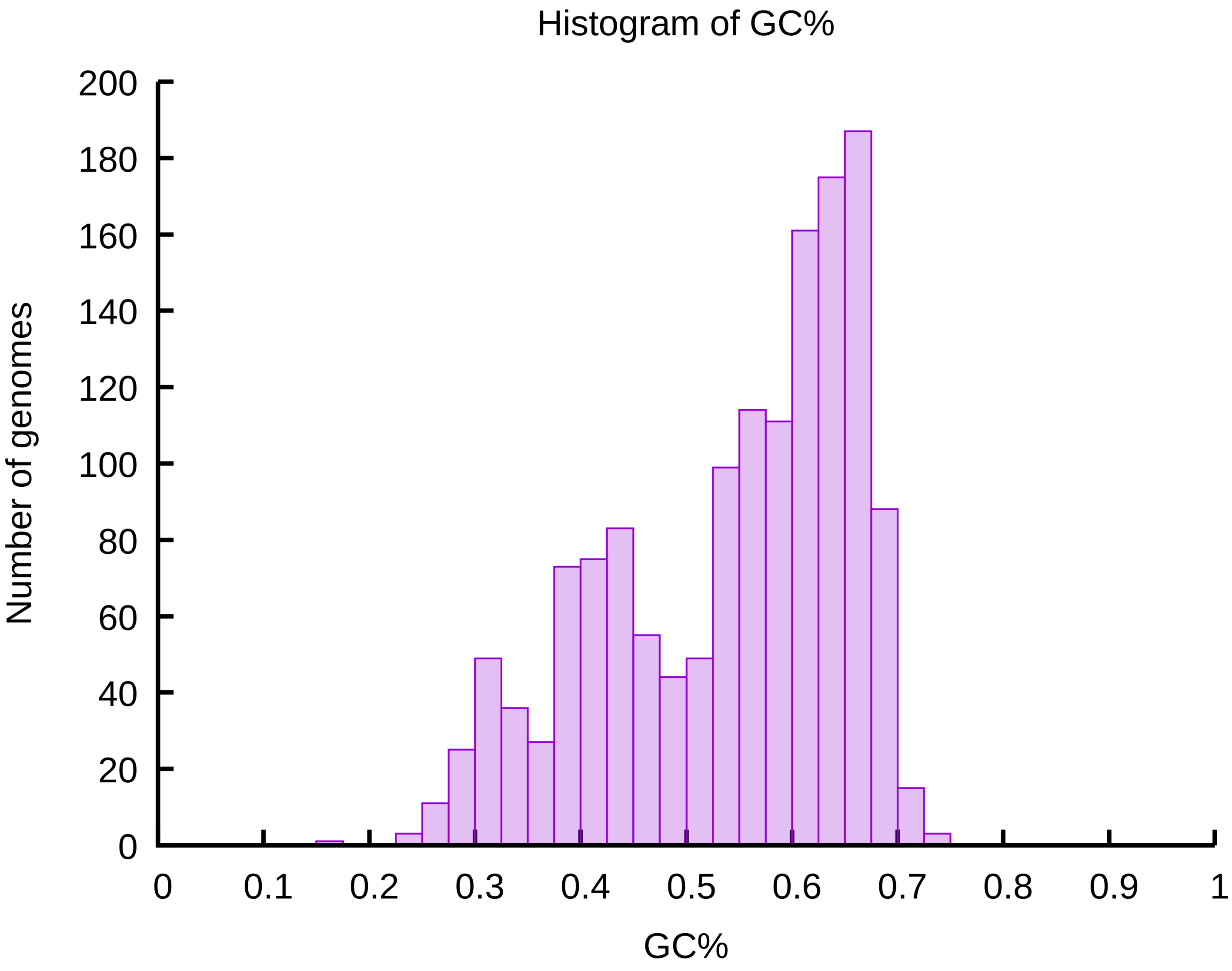
Histogram of GC%
Number of genomes
0
20
40
60
80
100
120
140
160
180
200
0
0.1
0.2
0.3
0.4
0.5
0.6
0.7
0.8
0.9
1
GC%

Figure S7. Histogram of codon usage in high expression genes of genomes that present or lack genes involved in t^6A formation. Top left graph: histogram of the usage of the indicated codon in highly expressed genes of proteobacterial genomes. **Top right graph:** box plot indicating the distribution of codon usage in the same population. **Lower graphs:** The collection of 1484 proteobacterial genomes were separated in two groups, based on the presence (light blue) or absence (orange) of the indicated pattern in any gene of the analyzed genomes. Patterns are indicated using subscript numbers. See table S4 for a detailed description of the used patterns. **Left:** Data is represented in superposed histograms. **Right:** Data is represented in a boxplot. An horizontal bar was added when the two populations are different based on Wilcoxon-Mann-Whitney test and $p < 0.00005$. Whiskers include 95% of data.

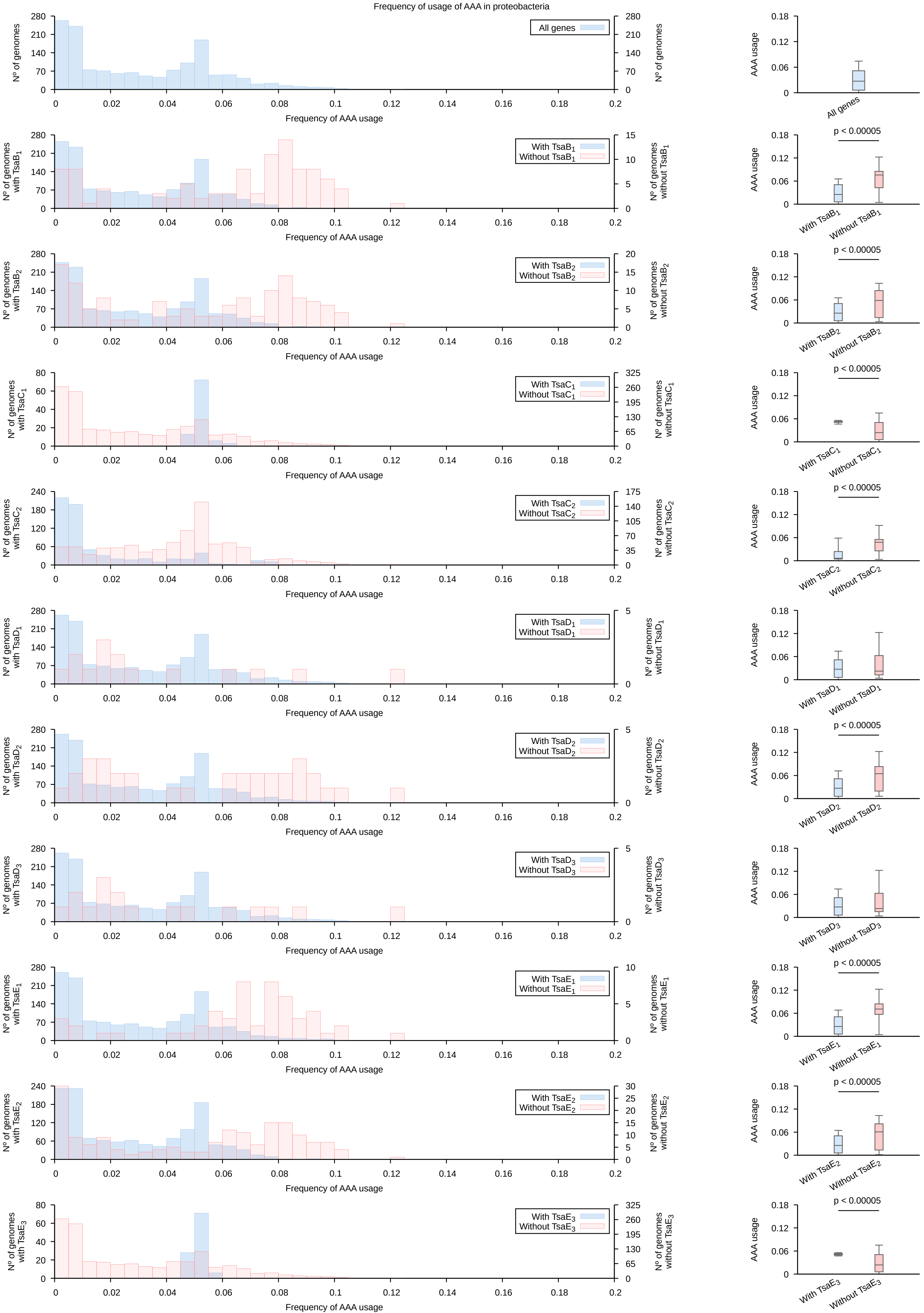

Frequency of usage of AAA in proteobacteria
All genes
Nº of genomes
Frequency of AAA usage
AAA usage
With TsaB1
Without TsaB1
Nº of genomes with TsaB1
Nº of genomes without TsaB1
With TsaB2
Without TsaB2
Nº of genomes with TsaB2
Nº of genomes without TsaB2
With TsaC1
Without TsaC1
Nº of genomes with TsaC1
Nº of genomes without TsaC1
With TsaC2
Without TsaC2
Nº of genomes with TsaC2
Nº of genomes without TsaC2
With TsaD1
Without TsaD1
Nº of genomes with TsaD1
Nº of genomes without TsaD1
With TsaD2
Without TsaD2
Nº of genomes with TsaD2
Nº of genomes without TsaD2
With TsaD3
Without TsaD3
Nº of genomes with TsaD3
Nº of genomes without TsaD3
With TsaE1
Without TsaE1
Nº of genomes with TsaE1
Nº of genomes without TsaE1
With TsaE2
Without TsaE2
Nº of genomes with TsaE2
Nº of genomes without TsaE2
With TsaE3
Without TsaE3
Nº of genomes with TsaE3
Nº of genomes without TsaE3
p < 0.00005
0 0.02 0.04 0.06 0.08 0.1 0.12 0.14 0.16 0.18 0.2
0 0.06 0.12 0.18

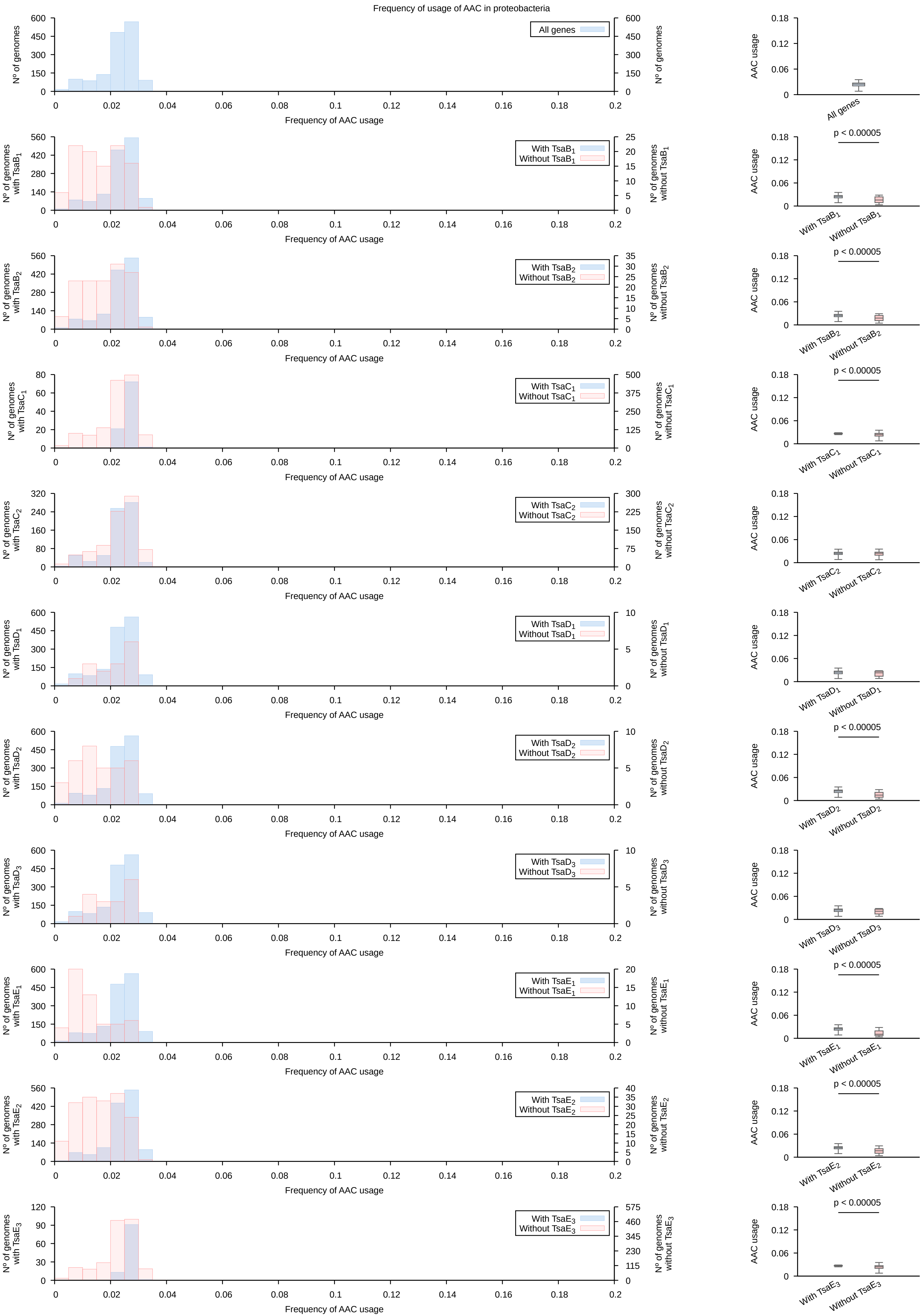

Frequency of usage of AAC in proteobacteria
All genes
Nº of genomes
Frequency of AAC usage
AAC usage
With TsaB1
Without TsaB1
Nº of genomes with TsaB1
Nº of genomes without TsaB1
p < 0.00005
With TsaB2
Without TsaB2
Nº of genomes with TsaB2
Nº of genomes without TsaB2
p < 0.00005
With TsaC1
Without TsaC1
Nº of genomes with TsaC1
Nº of genomes without TsaC1
p < 0.00005
With TsaC2
Without TsaC2
Nº of genomes with TsaC2
Nº of genomes without TsaC2
With TsaD1
Without TsaD1
Nº of genomes with TsaD1
Nº of genomes without TsaD1
With TsaD2
Without TsaD2
Nº of genomes with TsaD2
Nº of genomes without TsaD2
p < 0.00005
With TsaD3
Without TsaD3
Nº of genomes with TsaD3
Nº of genomes without TsaD3
With TsaE1
Without TsaE1
Nº of genomes with TsaE1
Nº of genomes without TsaE1
p < 0.00005
With TsaE2
Without TsaE2
Nº of genomes with TsaE2
Nº of genomes without TsaE2
p < 0.00005
With TsaE3
Without TsaE3
Nº of genomes with TsaE3
Nº of genomes without TsaE3
p < 0.00005

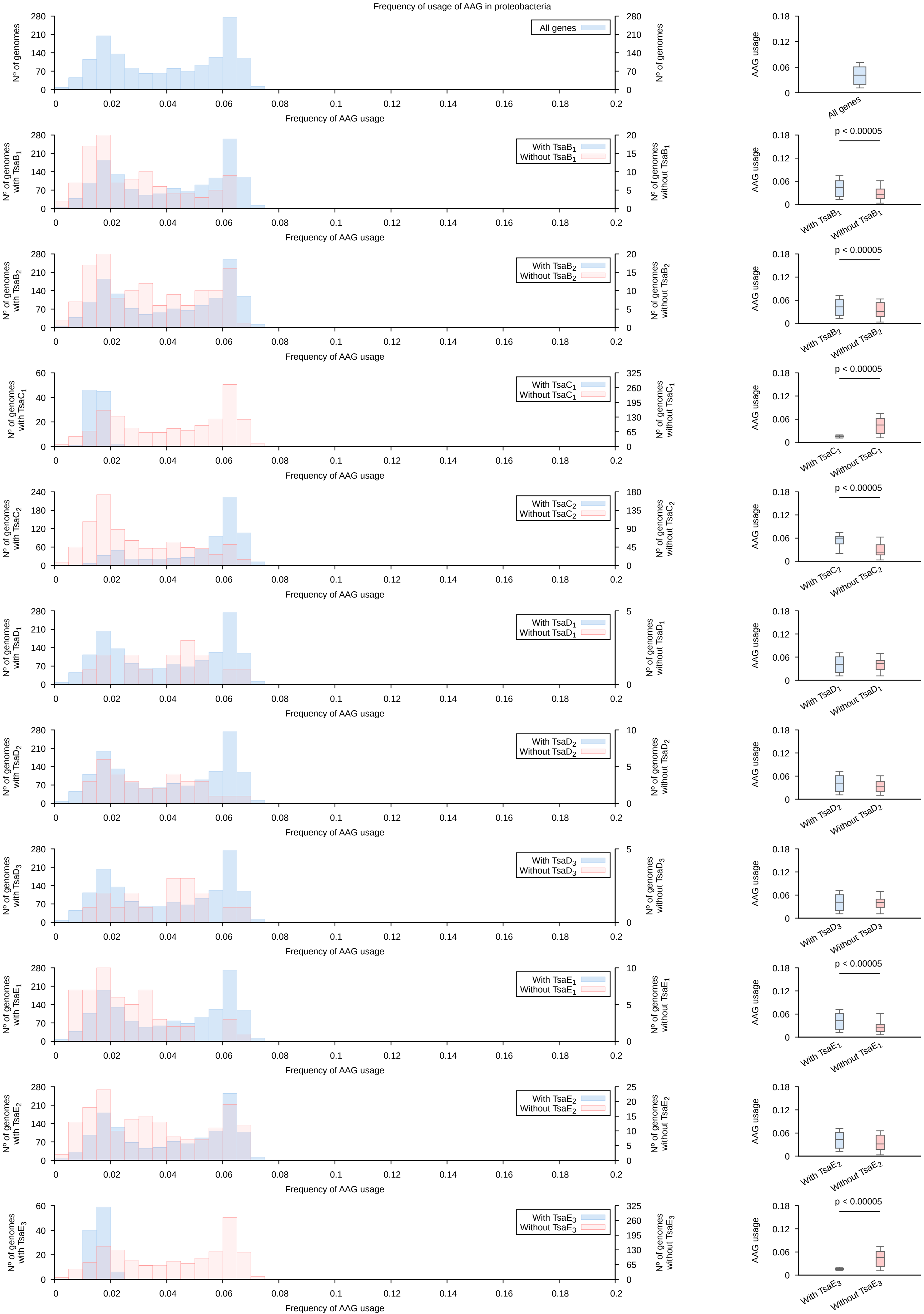
Frequency of usage of AAG in proteobacteria
All genes
Nº of genomes
AAG usage
Frequency of AAG usage
With TsaB$_1$
Without TsaB$_1$
Nº of genomes with TsaB$_1$
Nº of genomes without TsaB$_1$
p < 0.00005
With TsaB$_2$
Without TsaB$_2$
Nº of genomes with TsaB$_2$
Nº of genomes without TsaB$_2$
p < 0.00005
With TsaC$_1$
Without TsaC$_1$
Nº of genomes with TsaC$_1$
Nº of genomes without TsaC$_1$
p < 0.00005
With TsaC$_2$
Without TsaC$_2$
Nº of genomes with TsaC$_2$
Nº of genomes without TsaC$_2$
p < 0.00005
With TsaD$_1$
Without TsaD$_1$
Nº of genomes with TsaD$_1$
Nº of genomes without TsaD$_1$
With TsaD$_2$
Without TsaD$_2$
Nº of genomes with TsaD$_2$
Nº of genomes without TsaD$_2$
With TsaD$_3$
Without TsaD$_3$
Nº of genomes with TsaD$_3$
Nº of genomes without TsaD$_3$
With TsaE$_1$
Without TsaE$_1$
Nº of genomes with TsaE$_1$
Nº of genomes without TsaE$_1$
p < 0.00005
With TsaE$_2$
Without TsaE$_2$
Nº of genomes with TsaE$_2$
Nº of genomes without TsaE$_2$
With TsaE$_3$
Without TsaE$_3$
Nº of genomes with TsaE$_3$
Nº of genomes without TsaE$_3$
p < 0.00005

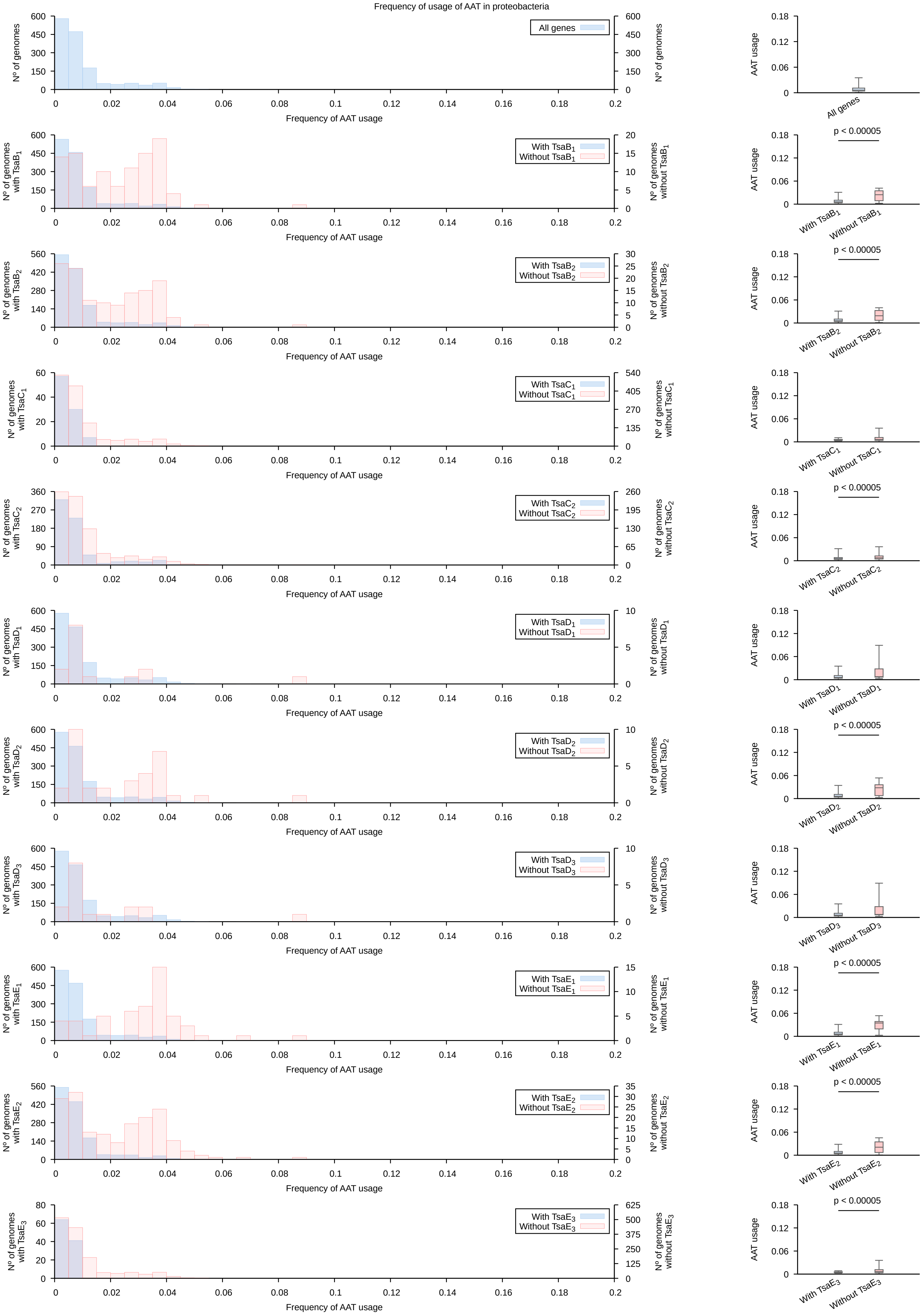

Frequency of usage of AAT in proteobacteria
Nº of genomes
All genes
Frequency of AAT usage
AAT usage
Nº of genomes with TsaB$_1$
Nº of genomes without TsaB$_1$
With TsaB$_1$
Without TsaB$_1$
p < 0.00005
Nº of genomes with TsaB$_2$
Nº of genomes without TsaB$_2$
With TsaB$_2$
Without TsaB$_2$
p < 0.00005
Nº of genomes with TsaC$_1$
Nº of genomes without TsaC$_1$
With TsaC$_1$
Without TsaC$_1$
Nº of genomes with TsaC$_2$
Nº of genomes without TsaC$_2$
With TsaC$_2$
Without TsaC$_2$
p < 0.00005
Nº of genomes with TsaD$_1$
Nº of genomes without TsaD$_1$
With TsaD$_1$
Without TsaD$_1$
Nº of genomes with TsaD$_2$
Nº of genomes without TsaD$_2$
With TsaD$_2$
Without TsaD$_2$
p < 0.00005
Nº of genomes with TsaD$_3$
Nº of genomes without TsaD$_3$
With TsaD$_3$
Without TsaD$_3$
Nº of genomes with TsaE$_1$
Nº of genomes without TsaE$_1$
With TsaE$_1$
Without TsaE$_1$
p < 0.00005
Nº of genomes with TsaE$_2$
Nº of genomes without TsaE$_2$
With TsaE$_2$
Without TsaE$_2$
p < 0.00005
Nº of genomes with TsaE$_3$
Nº of genomes without TsaE$_3$
With TsaE$_3$
Without TsaE$_3$
p < 0.00005

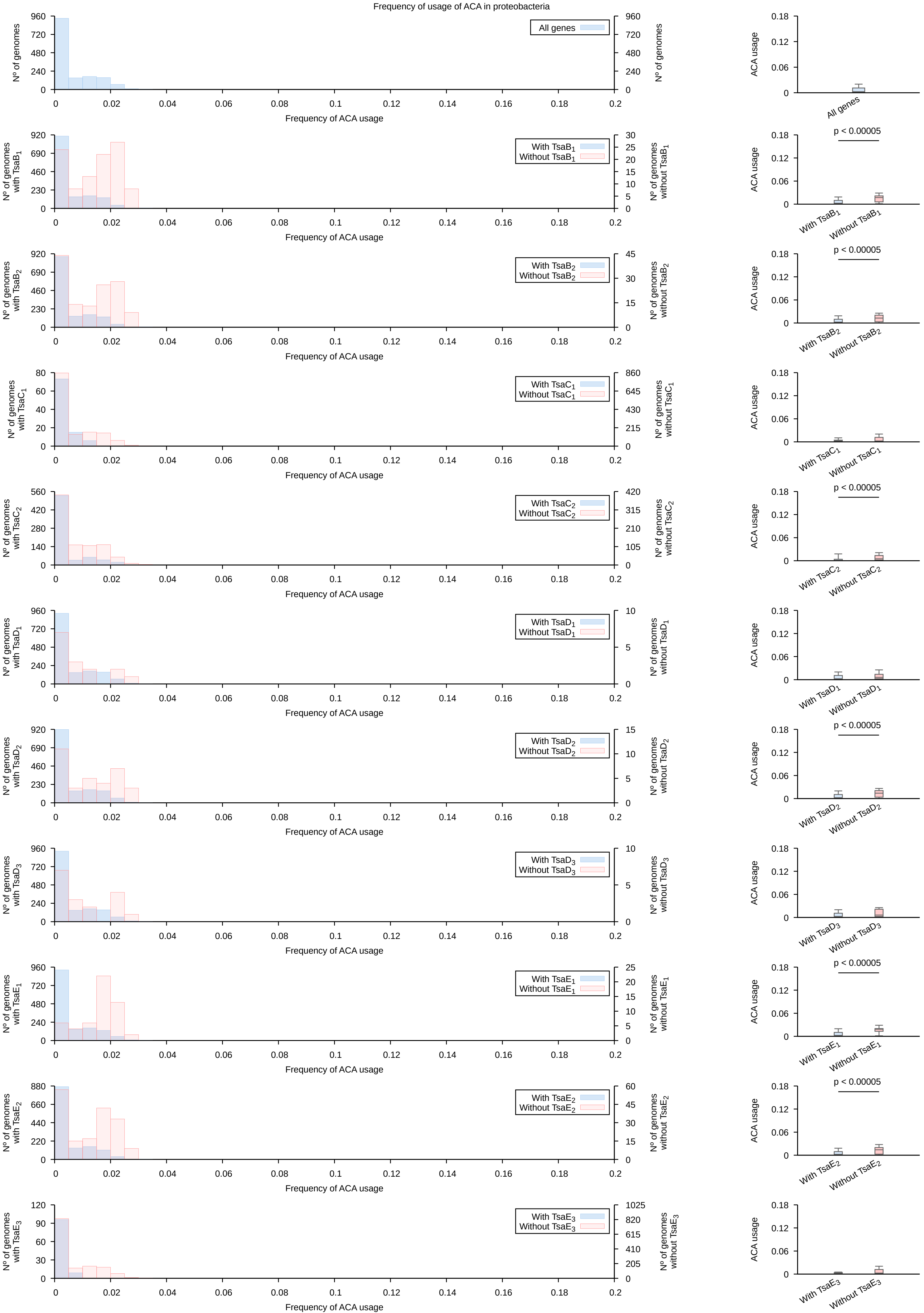

Frequency of usage of ACA in proteobacteria
Nº of genomes
All genes
Frequency of ACA usage
ACA usage
Nº of genomes with TsaB1
Nº of genomes without TsaB1
With TsaB1
Without TsaB1
p < 0.00005
Nº of genomes with TsaB2
Nº of genomes without TsaB2
With TsaB2
Without TsaB2
p < 0.00005
Nº of genomes with TsaC1
Nº of genomes without TsaC1
With TsaC1
Without TsaC1
Nº of genomes with TsaC2
Nº of genomes without TsaC2
With TsaC2
Without TsaC2
p < 0.00005
Nº of genomes with TsaD1
Nº of genomes without TsaD1
With TsaD1
Without TsaD1
Nº of genomes with TsaD2
Nº of genomes without TsaD2
With TsaD2
Without TsaD2
p < 0.00005
Nº of genomes with TsaD3
Nº of genomes without TsaD3
With TsaD3
Without TsaD3
Nº of genomes with TsaE1
Nº of genomes without TsaE1
With TsaE1
Without TsaE1
p < 0.00005
Nº of genomes with TsaE2
Nº of genomes without TsaE2
With TsaE2
Without TsaE2
p < 0.00005
Nº of genomes with TsaE3
Nº of genomes without TsaE3
With TsaE3
Without TsaE3

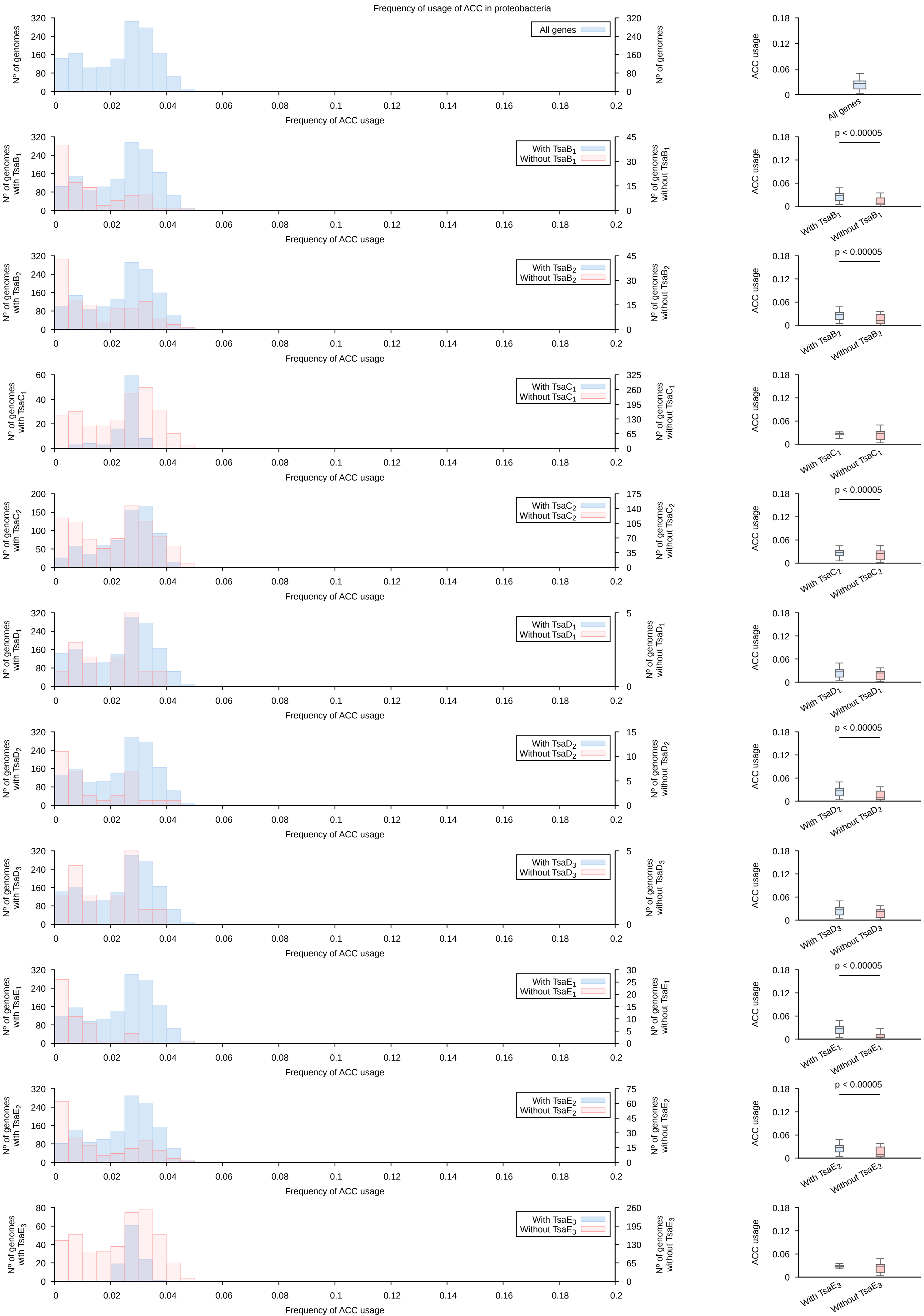
Frequency of usage of ACC in proteobacteria
Nº of genomes
Frequency of ACC usage
ACC usage
All genes
With TsaB1
Without TsaB1
Nº of genomes with TsaB1
Nº of genomes without TsaB1
With TsaB2
Without TsaB2
Nº of genomes with TsaB2
Nº of genomes without TsaB2
With TsaC1
Without TsaC1
Nº of genomes with TsaC1
Nº of genomes without TsaC1
With TsaC2
Without TsaC2
Nº of genomes with TsaC2
Nº of genomes without TsaC2
With TsaD1
Without TsaD1
Nº of genomes with TsaD1
Nº of genomes without TsaD1
With TsaD2
Without TsaD2
Nº of genomes with TsaD2
Nº of genomes without TsaD2
With TsaD3
Without TsaD3
Nº of genomes with TsaD3
Nº of genomes without TsaD3
With TsaE1
Without TsaE1
Nº of genomes with TsaE1
Nº of genomes without TsaE1
With TsaE2
Without TsaE2
Nº of genomes with TsaE2
Nº of genomes without TsaE2
With TsaE3
Without TsaE3
Nº of genomes with TsaE3
Nº of genomes without TsaE3
p < 0.00005

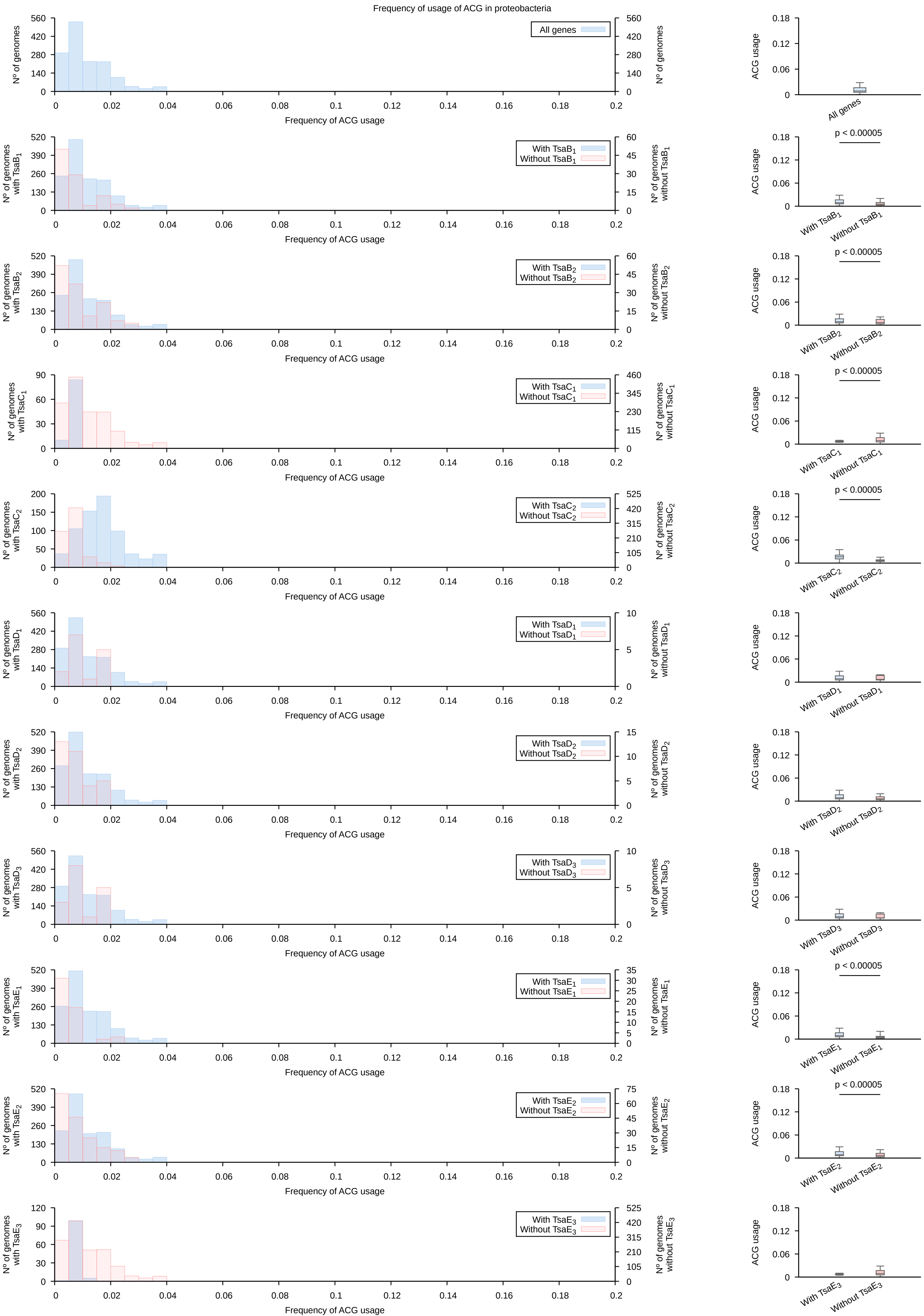
Frequency of usage of ACG in proteobacteria
Nº of genomes
Frequency of ACG usage
All genes
ACG usage
Nº of genomes with TsaB$_1$
Nº of genomes without TsaB$_1$
With TsaB$_1$
Without TsaB$_1$
p < 0.00005
Nº of genomes with TsaB$_2$
Nº of genomes without TsaB$_2$
With TsaB$_2$
Without TsaB$_2$
p < 0.00005
Nº of genomes with TsaC$_1$
Nº of genomes without TsaC$_1$
With TsaC$_1$
Without TsaC$_1$
p < 0.00005
Nº of genomes with TsaC$_2$
Nº of genomes without TsaC$_2$
With TsaC$_2$
Without TsaC$_2$
p < 0.00005
Nº of genomes with TsaD$_1$
Nº of genomes without TsaD$_1$
With TsaD$_1$
Without TsaD$_1$
Nº of genomes with TsaD$_2$
Nº of genomes without TsaD$_2$
With TsaD$_2$
Without TsaD$_2$
Nº of genomes with TsaD$_3$
Nº of genomes without TsaD$_3$
With TsaD$_3$
Without TsaD$_3$
Nº of genomes with TsaE$_1$
Nº of genomes without TsaE$_1$
With TsaE$_1$
Without TsaE$_1$
p < 0.00005
Nº of genomes with TsaE$_2$
Nº of genomes without TsaE$_2$
With TsaE$_2$
Without TsaE$_2$
p < 0.00005
Nº of genomes with TsaE$_3$
Nº of genomes without TsaE$_3$
With TsaE$_3$
Without TsaE$_3$

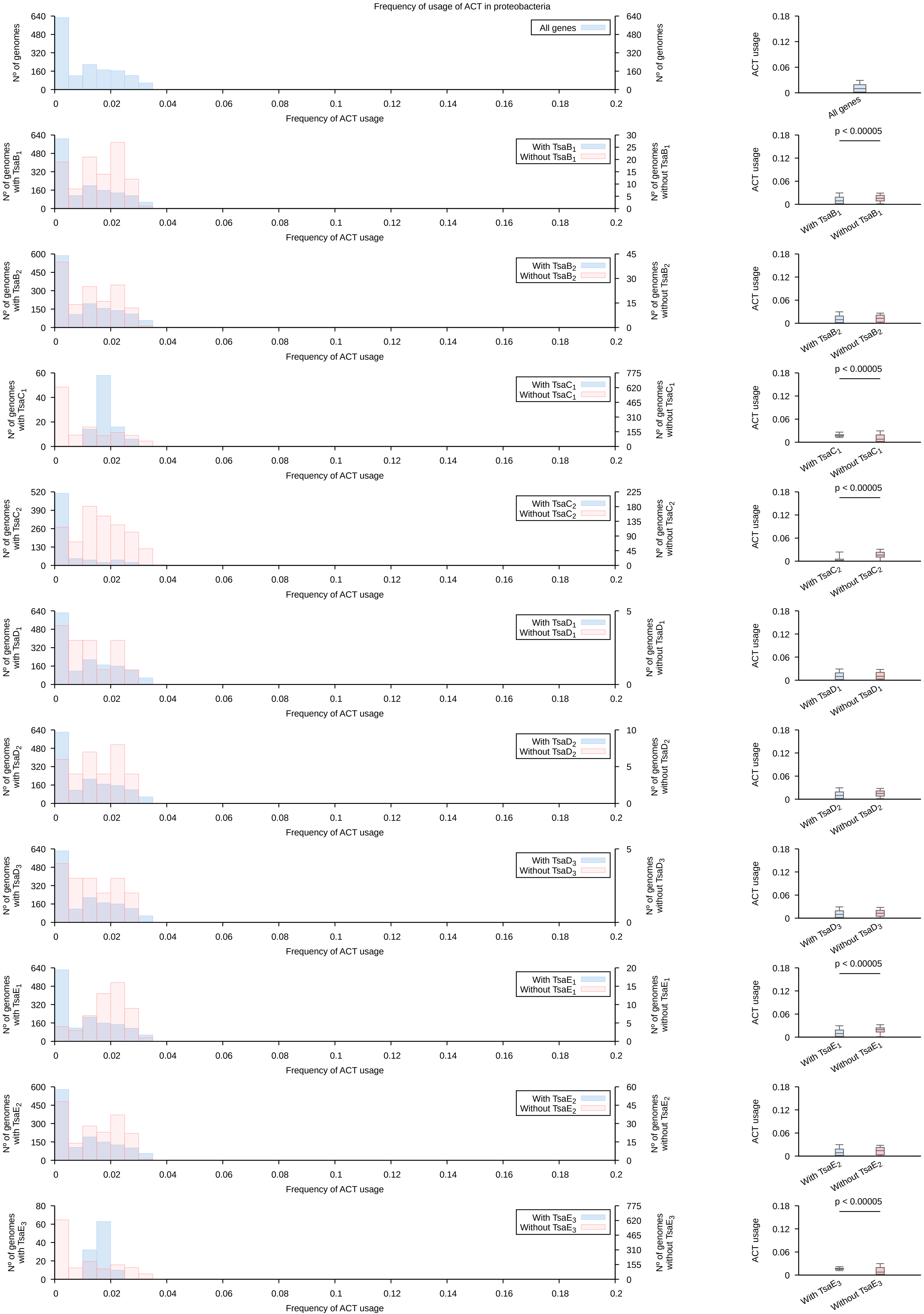
Frequency of usage of ACT in proteobacteria
All genes
With TsaB$_1$
Without TsaB$_1$
With TsaB$_2$
Without TsaB$_2$
With TsaC$_1$
Without TsaC$_1$
With TsaC$_2$
Without TsaC$_2$
With TsaD$_1$
Without TsaD$_1$
With TsaD$_2$
Without TsaD$_2$
With TsaD$_3$
Without TsaD$_3$
With TsaE$_1$
Without TsaE$_1$
With TsaE$_2$
Without TsaE$_2$
With TsaE$_3$
Without TsaE$_3$
Nº of genomes
Frequency of ACT usage
ACT usage
p < 0.00005

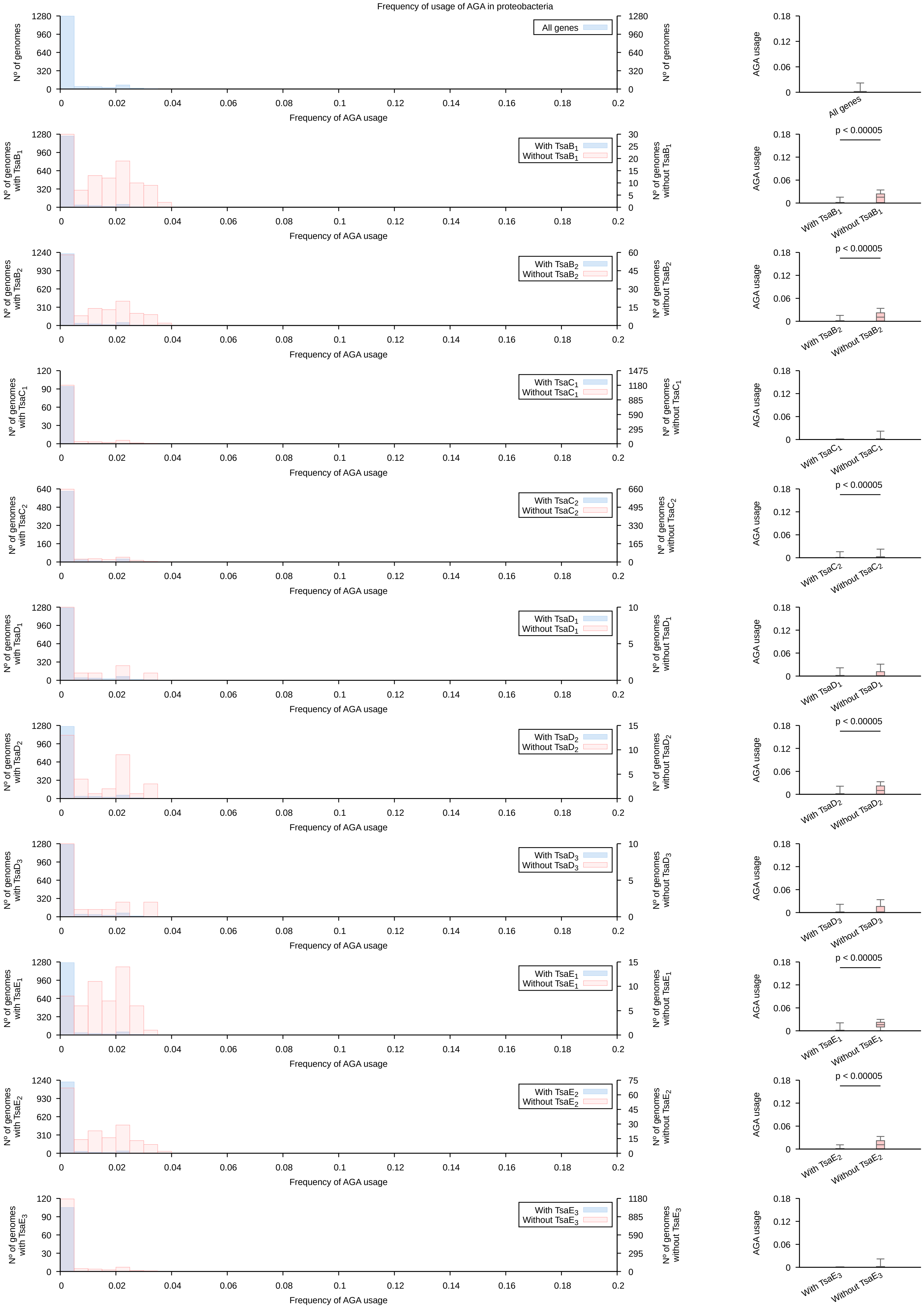

Frequency of usage of AGA in proteobacteria
All genes
Nº of genomes
Frequency of AGA usage
AGA usage
With TsaB$_1$
Without TsaB$_1$
Nº of genomes with TsaB$_1$
Nº of genomes without TsaB$_1$
p < 0.00005
With TsaB$_2$
Without TsaB$_2$
Nº of genomes with TsaB$_2$
Nº of genomes without TsaB$_2$
p < 0.00005
With TsaC$_1$
Without TsaC$_1$
Nº of genomes with TsaC$_1$
Nº of genomes without TsaC$_1$
With TsaC$_2$
Without TsaC$_2$
Nº of genomes with TsaC$_2$
Nº of genomes without TsaC$_2$
p < 0.00005
With TsaD$_1$
Without TsaD$_1$
Nº of genomes with TsaD$_1$
Nº of genomes without TsaD$_1$
With TsaD$_2$
Without TsaD$_2$
Nº of genomes with TsaD$_2$
Nº of genomes without TsaD$_2$
p < 0.00005
With TsaD$_3$
Without TsaD$_3$
Nº of genomes with TsaD$_3$
Nº of genomes without TsaD$_3$
With TsaE$_1$
Without TsaE$_1$
Nº of genomes with TsaE$_1$
Nº of genomes without TsaE$_1$
p < 0.00005
With TsaE$_2$
Without TsaE$_2$
Nº of genomes with TsaE$_2$
Nº of genomes without TsaE$_2$
p < 0.00005
With TsaE$_3$
Without TsaE$_3$
Nº of genomes with TsaE$_3$
Nº of genomes without TsaE$_3$

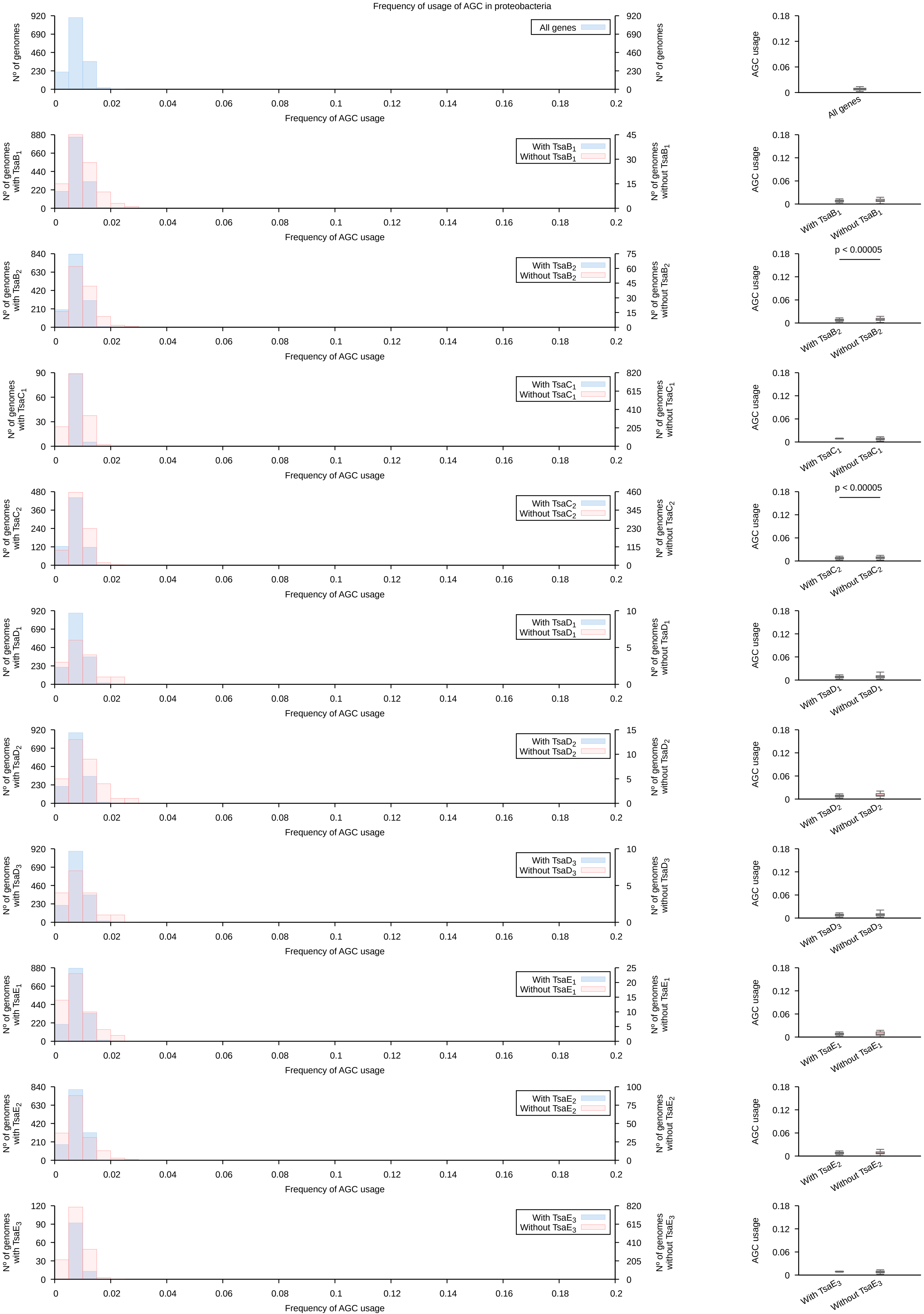

Frequency of usage of AGC in proteobacteria
Nº of genomes
Frequency of AGC usage
All genes
AGC usage
Nº of genomes with TsaB$_1$
Nº of genomes without TsaB$_1$
With TsaB$_1$
Without TsaB$_1$
Nº of genomes with TsaB$_2$
Nº of genomes without TsaB$_2$
With TsaB$_2$
Without TsaB$_2$
p < 0.00005
Nº of genomes with TsaC$_1$
Nº of genomes without TsaC$_1$
With TsaC$_1$
Without TsaC$_1$
Nº of genomes with TsaC$_2$
Nº of genomes without TsaC$_2$
With TsaC$_2$
Without TsaC$_2$
p < 0.00005
Nº of genomes with TsaD$_1$
Nº of genomes without TsaD$_1$
With TsaD$_1$
Without TsaD$_1$
Nº of genomes with TsaD$_2$
Nº of genomes without TsaD$_2$
With TsaD$_2$
Without TsaD$_2$
Nº of genomes with TsaD$_3$
Nº of genomes without TsaD$_3$
With TsaD$_3$
Without TsaD$_3$
Nº of genomes with TsaE$_1$
Nº of genomes without TsaE$_1$
With TsaE$_1$
Without TsaE$_1$
Nº of genomes with TsaE$_2$
Nº of genomes without TsaE$_2$
With TsaE$_2$
Without TsaE$_2$
Nº of genomes with TsaE$_3$
Nº of genomes without TsaE$_3$
With TsaE$_3$
Without TsaE$_3$

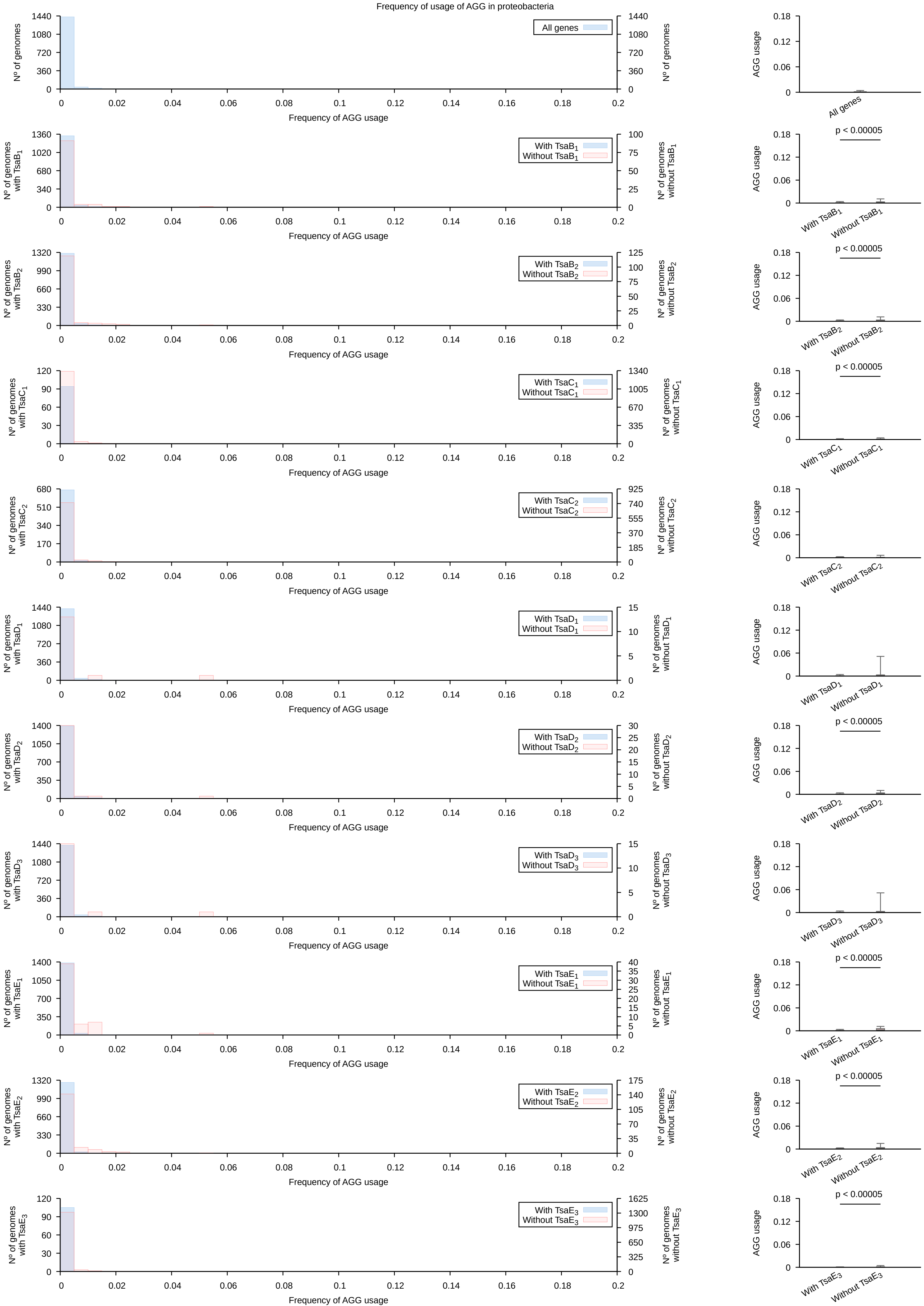
Frequency of usage of AGG in proteobacteria
All genes
Nº of genomes
Frequency of AGG usage
AGG usage
With TsaB$_1$
Without TsaB$_1$
Nº of genomes with TsaB$_1$
Nº of genomes without TsaB$_1$
p < 0.00005
With TsaB$_2$
Without TsaB$_2$
Nº of genomes with TsaB$_2$
Nº of genomes without TsaB$_2$
p < 0.00005
With TsaC$_1$
Without TsaC$_1$
Nº of genomes with TsaC$_1$
Nº of genomes without TsaC$_1$
p < 0.00005
With TsaC$_2$
Without TsaC$_2$
Nº of genomes with TsaC$_2$
Nº of genomes without TsaC$_2$
With TsaD$_1$
Without TsaD$_1$
Nº of genomes with TsaD$_1$
Nº of genomes without TsaD$_1$
With TsaD$_2$
Without TsaD$_2$
Nº of genomes with TsaD$_2$
Nº of genomes without TsaD$_2$
p < 0.00005
With TsaD$_3$
Without TsaD$_3$
Nº of genomes with TsaD$_3$
Nº of genomes without TsaD$_3$
With TsaE$_1$
Without TsaE$_1$
Nº of genomes with TsaE$_1$
Nº of genomes without TsaE$_1$
p < 0.00005
With TsaE$_2$
Without TsaE$_2$
Nº of genomes with TsaE$_2$
Nº of genomes without TsaE$_2$
p < 0.00005
With TsaE$_3$
Without TsaE$_3$
Nº of genomes with TsaE$_3$
Nº of genomes without TsaE$_3$
p < 0.00005
0 0.02 0.04 0.06 0.08 0.1 0.12 0.14 0.16 0.18 0.2
0 0.06 0.12 0.18

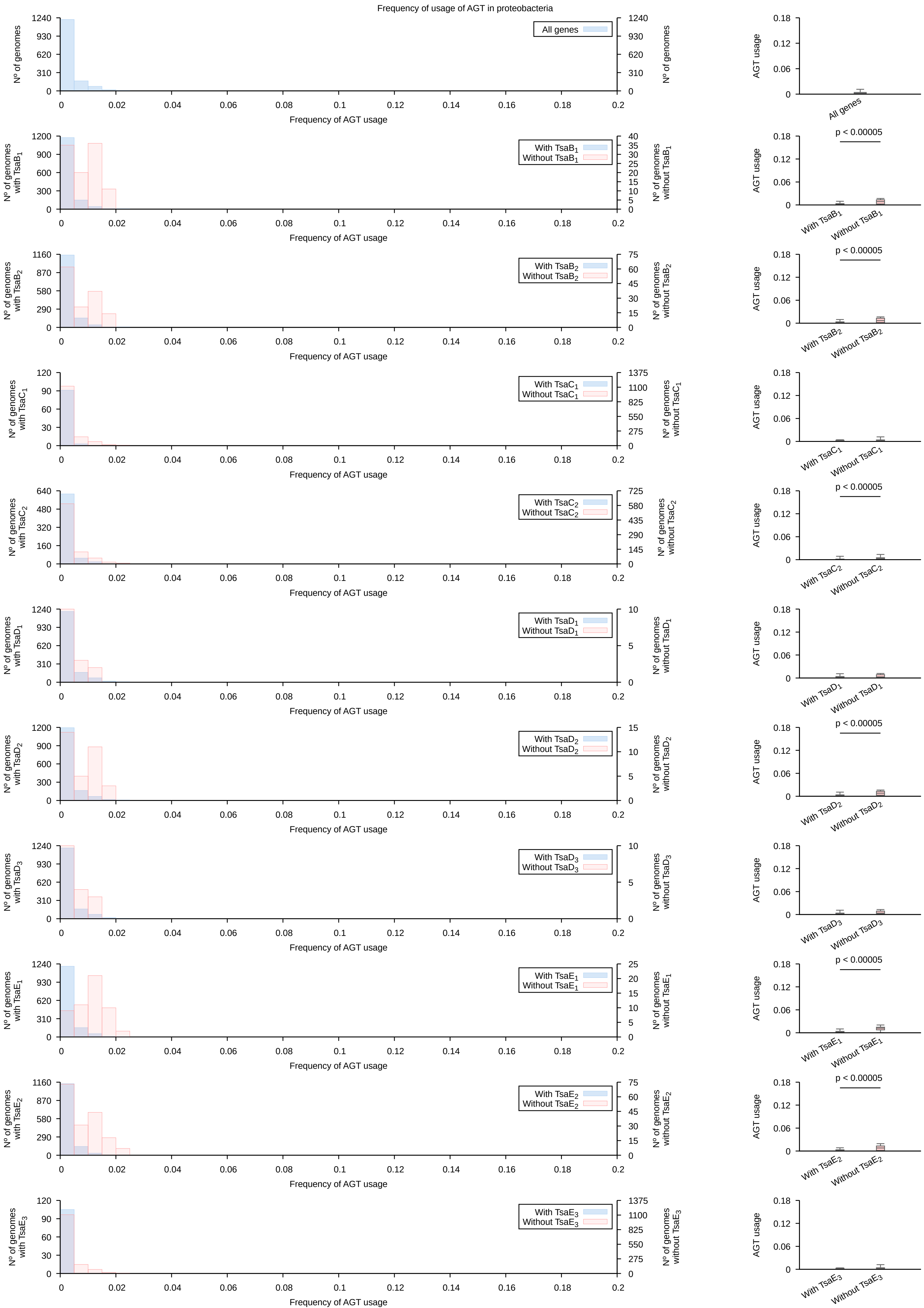
Frequency of usage of AGT in proteobacteria
Nº of genomes
All genes
Frequency of AGT usage
AGT usage
Nº of genomes with TsaB$_1$
Nº of genomes without TsaB$_1$
With TsaB$_1$
Without TsaB$_1$
p < 0.00005
Nº of genomes with TsaB$_2$
Nº of genomes without TsaB$_2$
With TsaB$_2$
Without TsaB$_2$
p < 0.00005
Nº of genomes with TsaC$_1$
Nº of genomes without TsaC$_1$
With TsaC$_1$
Without TsaC$_1$
Nº of genomes with TsaC$_2$
Nº of genomes without TsaC$_2$
With TsaC$_2$
Without TsaC$_2$
p < 0.00005
Nº of genomes with TsaD$_1$
Nº of genomes without TsaD$_1$
With TsaD$_1$
Without TsaD$_1$
Nº of genomes with TsaD$_2$
Nº of genomes without TsaD$_2$
With TsaD$_2$
Without TsaD$_2$
p < 0.00005
Nº of genomes with TsaD$_3$
Nº of genomes without TsaD$_3$
With TsaD$_3$
Without TsaD$_3$
Nº of genomes with TsaE$_1$
Nº of genomes without TsaE$_1$
With TsaE$_1$
Without TsaE$_1$
p < 0.00005
Nº of genomes with TsaE$_2$
Nº of genomes without TsaE$_2$
With TsaE$_2$
Without TsaE$_2$
p < 0.00005
Nº of genomes with TsaE$_3$
Nº of genomes without TsaE$_3$
With TsaE$_3$
Without TsaE$_3$

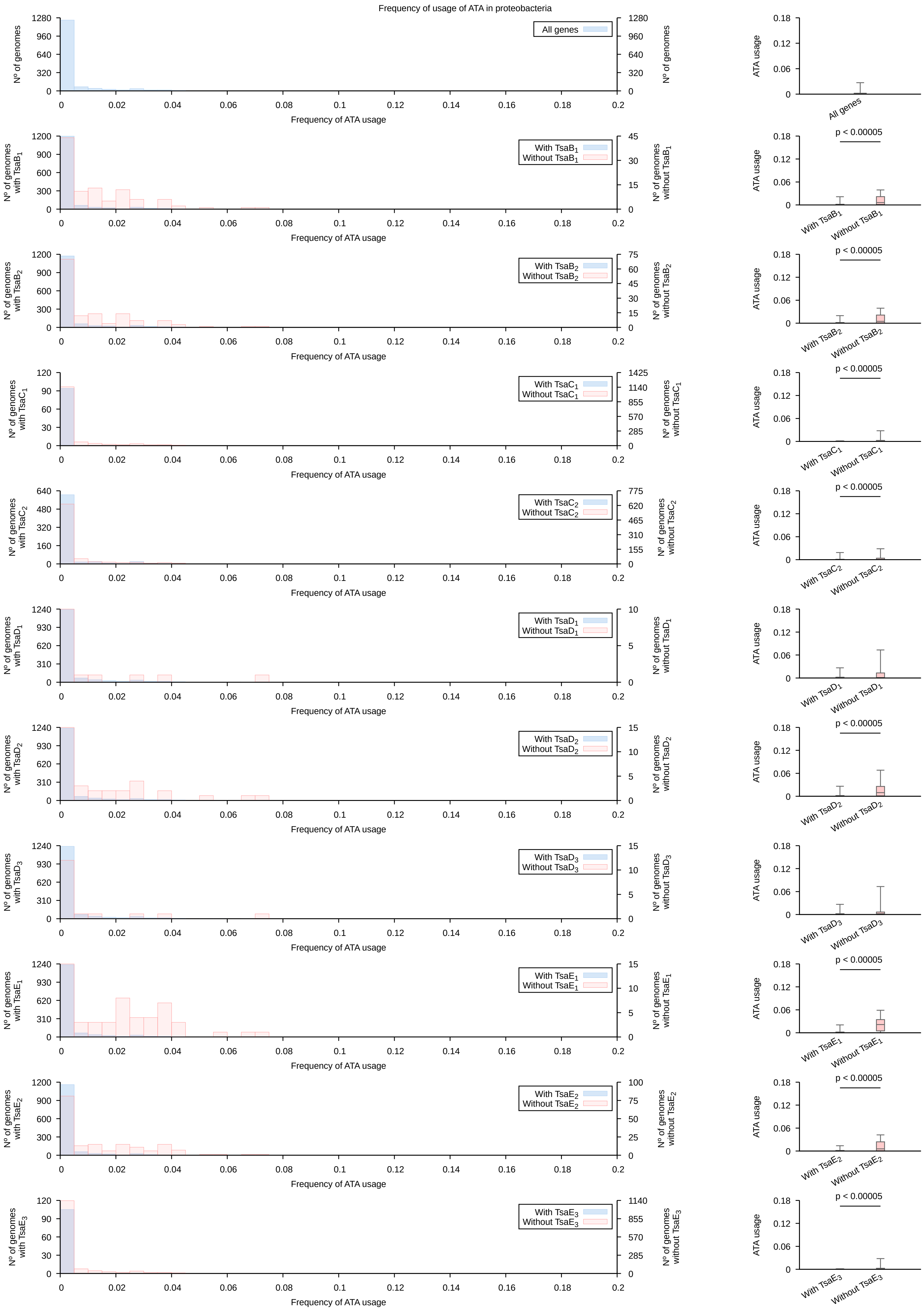
Frequency of usage of ATA in proteobacteria
Nº of genomes
Frequency of ATA usage
ATA usage
All genes
With TsaB1
Without TsaB1
Nº of genomes with TsaB1
Nº of genomes without TsaB1
With TsaB2
Without TsaB2
Nº of genomes with TsaB2
Nº of genomes without TsaB2
With TsaC1
Without TsaC1
Nº of genomes with TsaC1
Nº of genomes without TsaC1
With TsaC2
Without TsaC2
Nº of genomes with TsaC2
Nº of genomes without TsaC2
With TsaD1
Without TsaD1
Nº of genomes with TsaD1
Nº of genomes without TsaD1
With TsaD2
Without TsaD2
Nº of genomes with TsaD2
Nº of genomes without TsaD2
With TsaD3
Without TsaD3
Nº of genomes with TsaD3
Nº of genomes without TsaD3
With TsaE1
Without TsaE1
Nº of genomes with TsaE1
Nº of genomes without TsaE1
With TsaE2
Without TsaE2
Nº of genomes with TsaE2
Nº of genomes without TsaE2
With TsaE3
Without TsaE3
Nº of genomes with TsaE3
Nº of genomes without TsaE3
p < 0.00005
0 0.02 0.04 0.06 0.08 0.1 0.12 0.14 0.16 0.18 0.2
0 0.06 0.12 0.18

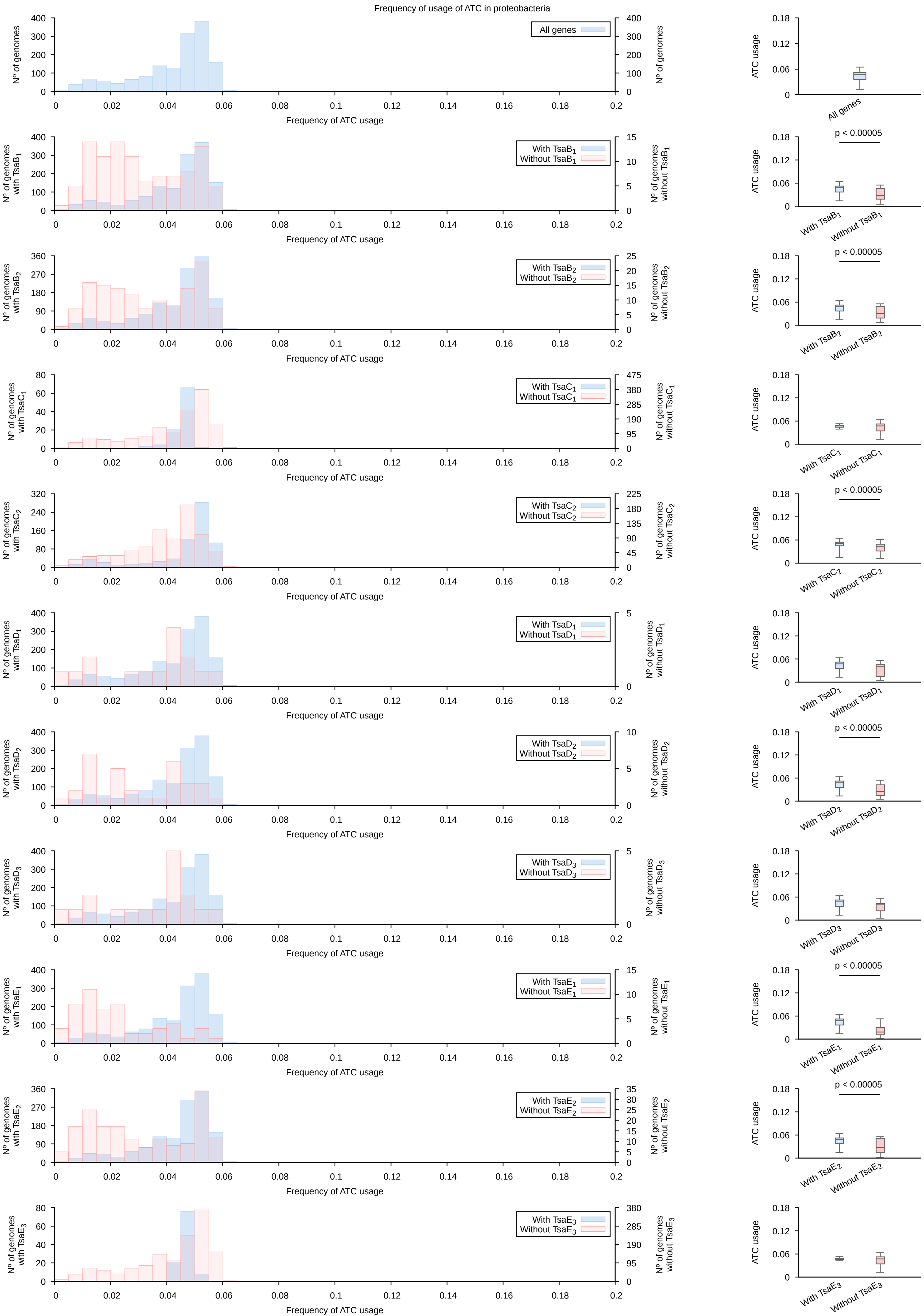

Frequency of usage of ATC in proteobacteria
Nº of genomes
All genes
Frequency of ATC usage
ATC usage
All genes
Nº of genomes with TsaB$_1$
Nº of genomes without TsaB$_1$
With TsaB$_1$
Without TsaB$_1$
p < 0.00005
Nº of genomes with TsaB$_2$
Nº of genomes without TsaB$_2$
With TsaB$_2$
Without TsaB$_2$
p < 0.00005
Nº of genomes with TsaC$_1$
Nº of genomes without TsaC$_1$
With TsaC$_1$
Without TsaC$_1$
Nº of genomes with TsaC$_2$
Nº of genomes without TsaC$_2$
With TsaC$_2$
Without TsaC$_2$
p < 0.00005
Nº of genomes with TsaD$_1$
Nº of genomes without TsaD$_1$
With TsaD$_1$
Without TsaD$_1$
Nº of genomes with TsaD$_2$
Nº of genomes without TsaD$_2$
With TsaD$_2$
Without TsaD$_2$
p < 0.00005
Nº of genomes with TsaD$_3$
Nº of genomes without TsaD$_3$
With TsaD$_3$
Without TsaD$_3$
Nº of genomes with TsaE$_1$
Nº of genomes without TsaE$_1$
With TsaE$_1$
Without TsaE$_1$
p < 0.00005
Nº of genomes with TsaE$_2$
Nº of genomes without TsaE$_2$
With TsaE$_2$
Without TsaE$_2$
p < 0.00005
Nº of genomes with TsaE$_3$
Nº of genomes without TsaE$_3$
With TsaE$_3$
Without TsaE$_3$

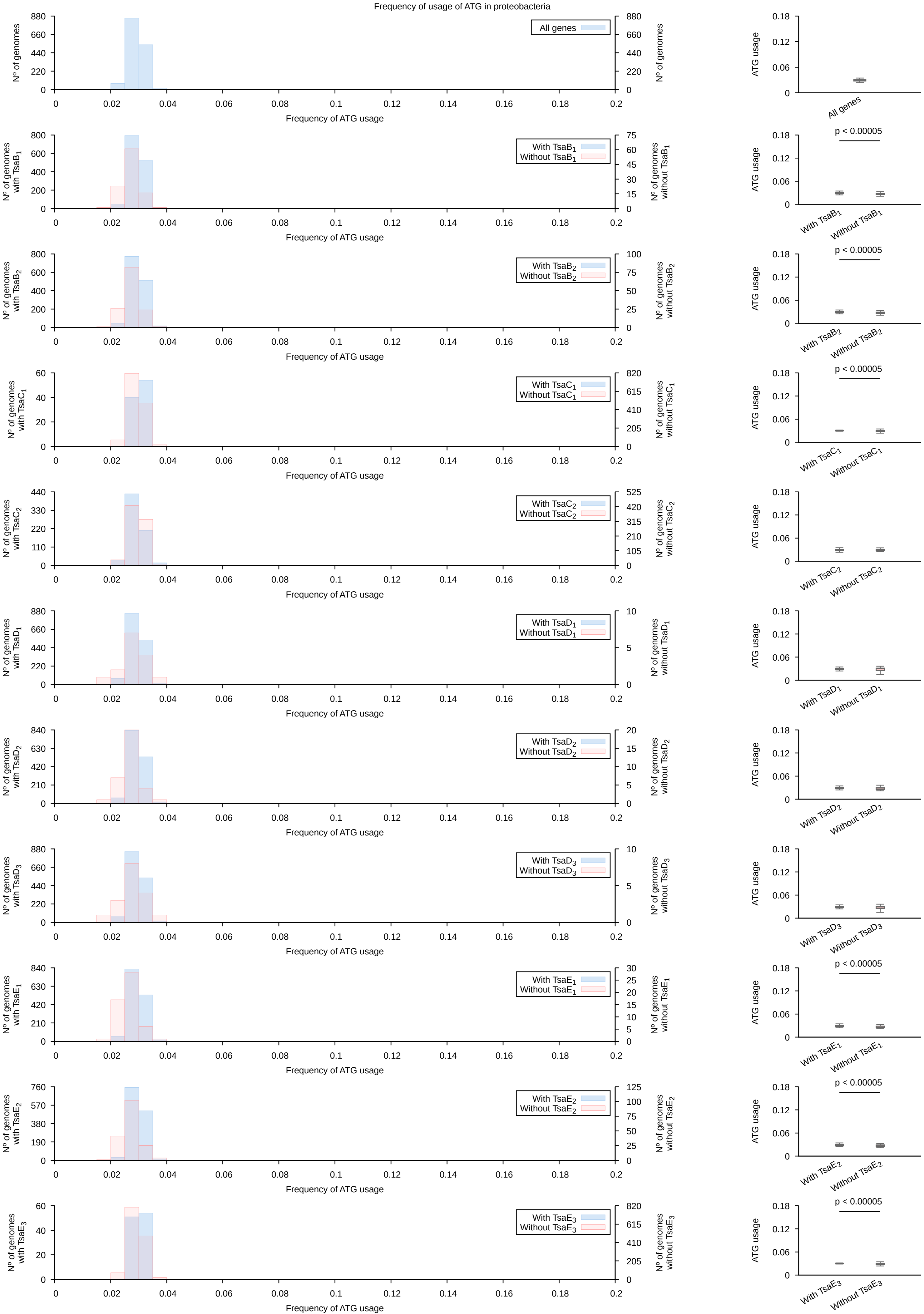
Frequency of usage of ATG in proteobacteria
All genes
Nº of genomes
Frequency of ATG usage
ATG usage
With TsaB$_1$
Without TsaB$_1$
Nº of genomes with TsaB$_1$
Nº of genomes without TsaB$_1$
p < 0.00005
With TsaB$_2$
Without TsaB$_2$
Nº of genomes with TsaB$_2$
Nº of genomes without TsaB$_2$
p < 0.00005
With TsaC$_1$
Without TsaC$_1$
Nº of genomes with TsaC$_1$
Nº of genomes without TsaC$_1$
p < 0.00005
With TsaC$_2$
Without TsaC$_2$
Nº of genomes with TsaC$_2$
Nº of genomes without TsaC$_2$
With TsaD$_1$
Without TsaD$_1$
Nº of genomes with TsaD$_1$
Nº of genomes without TsaD$_1$
With TsaD$_2$
Without TsaD$_2$
Nº of genomes with TsaD$_2$
Nº of genomes without TsaD$_2$
With TsaD$_3$
Without TsaD$_3$
Nº of genomes with TsaD$_3$
Nº of genomes without TsaD$_3$
With TsaE$_1$
Without TsaE$_1$
Nº of genomes with TsaE$_1$
Nº of genomes without TsaE$_1$
p < 0.00005
With TsaE$_2$
Without TsaE$_2$
Nº of genomes with TsaE$_2$
Nº of genomes without TsaE$_2$
p < 0.00005
With TsaE$_3$
Without TsaE$_3$
Nº of genomes with TsaE$_3$
Nº of genomes without TsaE$_3$
p < 0.00005

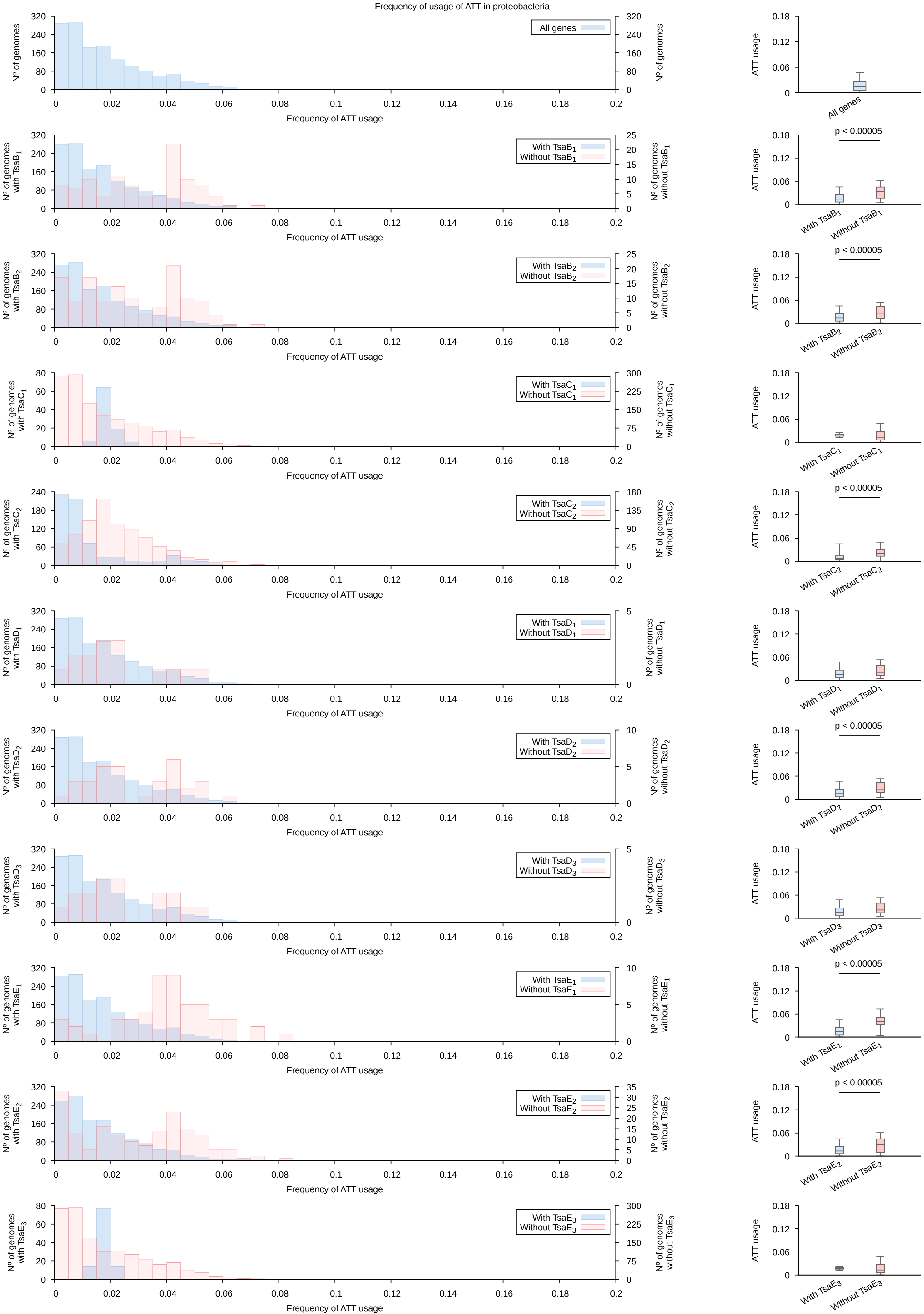
Frequency of usage of ATT in proteobacteria
Nº of genomes
All genes
Frequency of ATT usage
ATT usage
All genes
Nº of genomes with TsaB1
With TsaB1
Without TsaB1
Nº of genomes without TsaB1
p < 0.00005
Nº of genomes with TsaB2
With TsaB2
Without TsaB2
Nº of genomes without TsaB2
p < 0.00005
Nº of genomes with TsaC1
With TsaC1
Without TsaC1
Nº of genomes without TsaC1
Nº of genomes with TsaC2
With TsaC2
Without TsaC2
Nº of genomes without TsaC2
p < 0.00005
Nº of genomes with TsaD1
With TsaD1
Without TsaD1
Nº of genomes without TsaD1
Nº of genomes with TsaD2
With TsaD2
Without TsaD2
Nº of genomes without TsaD2
p < 0.00005
Nº of genomes with TsaD3
With TsaD3
Without TsaD3
Nº of genomes without TsaD3
Nº of genomes with TsaE1
With TsaE1
Without TsaE1
Nº of genomes without TsaE1
p < 0.00005
Nº of genomes with TsaE2
With TsaE2
Without TsaE2
Nº of genomes without TsaE2
p < 0.00005
Nº of genomes with TsaE3
With TsaE3
Without TsaE3
Nº of genomes without TsaE3

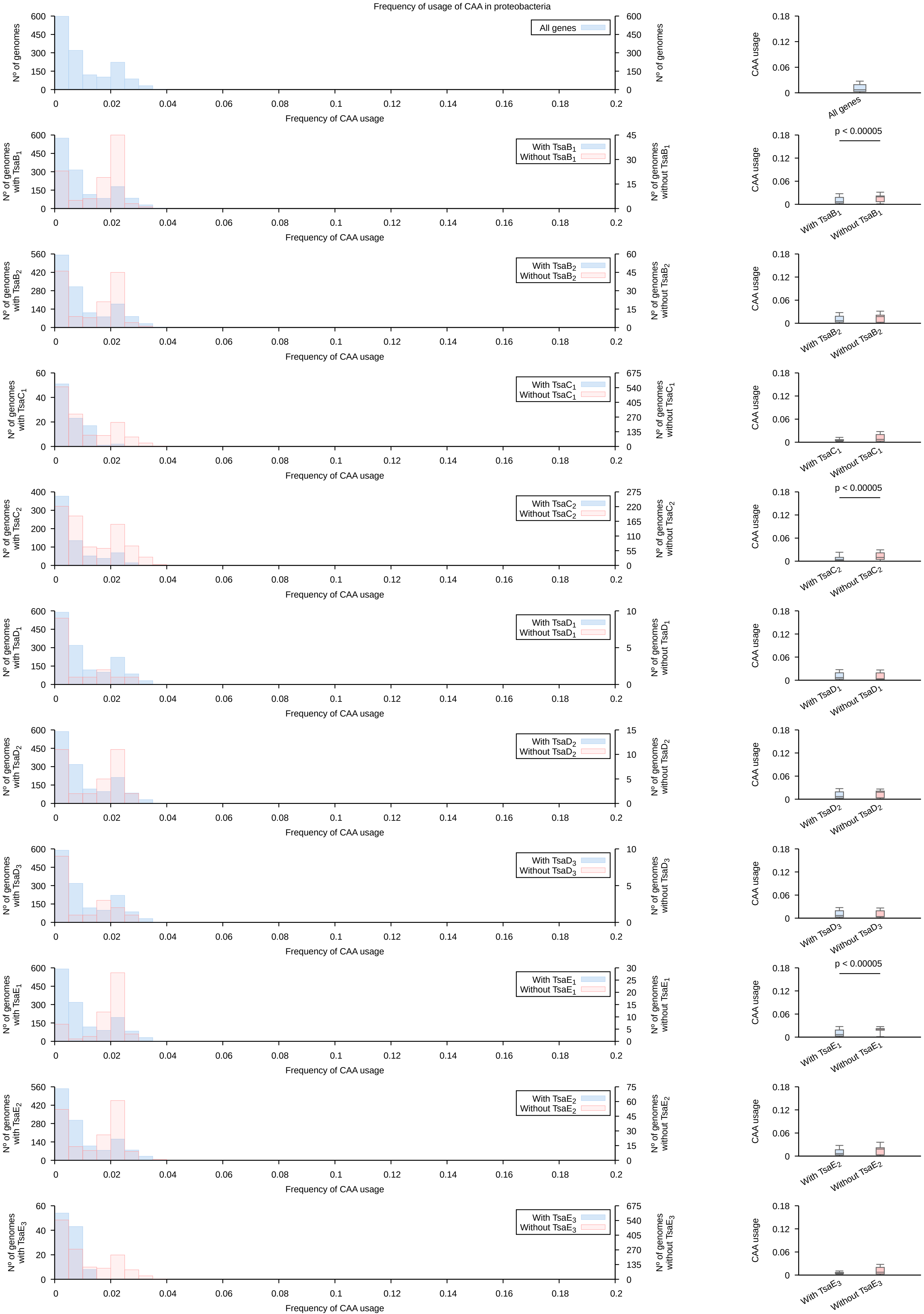

Frequency of usage of CAA in proteobacteria
Nº of genomes
Frequency of CAA usage
All genes
CAA usage
With TsaB$_1$
Without TsaB$_1$
Nº of genomes with TsaB$_1$
Nº of genomes without TsaB$_1$
p < 0.00005
With TsaB$_2$
Without TsaB$_2$
Nº of genomes with TsaB$_2$
Nº of genomes without TsaB$_2$
With TsaC$_1$
Without TsaC$_1$
Nº of genomes with TsaC$_1$
Nº of genomes without TsaC$_1$
With TsaC$_2$
Without TsaC$_2$
Nº of genomes with TsaC$_2$
Nº of genomes without TsaC$_2$
p < 0.00005
With TsaD$_1$
Without TsaD$_1$
Nº of genomes with TsaD$_1$
Nº of genomes without TsaD$_1$
With TsaD$_2$
Without TsaD$_2$
Nº of genomes with TsaD$_2$
Nº of genomes without TsaD$_2$
With TsaD$_3$
Without TsaD$_3$
Nº of genomes with TsaD$_3$
Nº of genomes without TsaD$_3$
With TsaE$_1$
Without TsaE$_1$
Nº of genomes with TsaE$_1$
Nº of genomes without TsaE$_1$
p < 0.00005
With TsaE$_2$
Without TsaE$_2$
Nº of genomes with TsaE$_2$
Nº of genomes without TsaE$_2$
With TsaE$_3$
Without TsaE$_3$
Nº of genomes with TsaE$_3$
Nº of genomes without TsaE$_3$

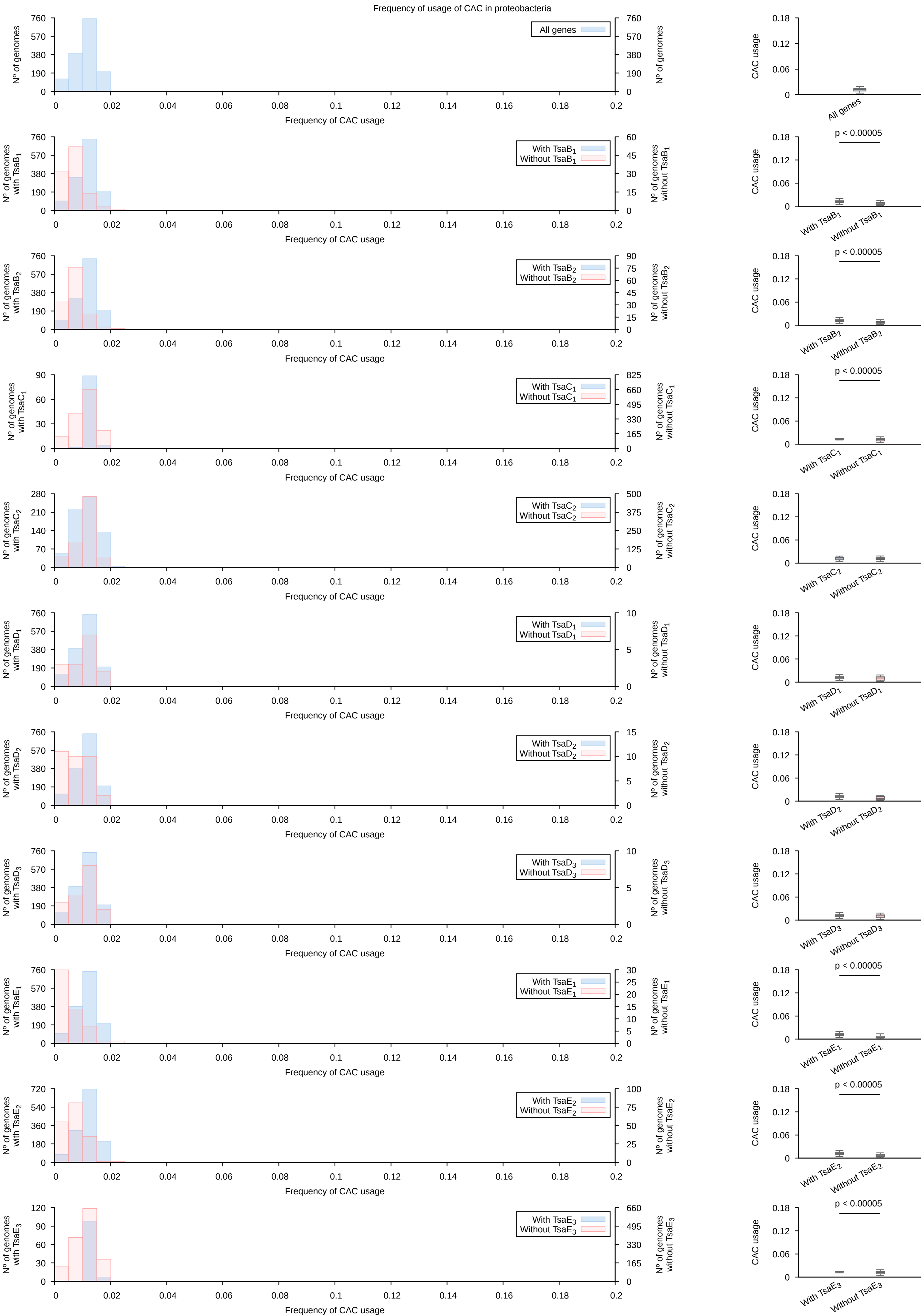
Frequency of usage of CAC in proteobacteria
Nº of genomes
All genes
Frequency of CAC usage
CAC usage
With TsaB$_1$
Without TsaB$_1$
Nº of genomes with TsaB$_1$
Nº of genomes without TsaB$_1$
With TsaB$_2$
Without TsaB$_2$
Nº of genomes with TsaB$_2$
Nº of genomes without TsaB$_2$
With TsaC$_1$
Without TsaC$_1$
Nº of genomes with TsaC$_1$
Nº of genomes without TsaC$_1$
With TsaC$_2$
Without TsaC$_2$
Nº of genomes with TsaC$_2$
Nº of genomes without TsaC$_2$
With TsaD$_1$
Without TsaD$_1$
Nº of genomes with TsaD$_1$
Nº of genomes without TsaD$_1$
With TsaD$_2$
Without TsaD$_2$
Nº of genomes with TsaD$_2$
Nº of genomes without TsaD$_2$
With TsaD$_3$
Without TsaD$_3$
Nº of genomes with TsaD$_3$
Nº of genomes without TsaD$_3$
With TsaE$_1$
Without TsaE$_1$
Nº of genomes with TsaE$_1$
Nº of genomes without TsaE$_1$
With TsaE$_2$
Without TsaE$_2$
Nº of genomes with TsaE$_2$
Nº of genomes without TsaE$_2$
With TsaE$_3$
Without TsaE$_3$
Nº of genomes with TsaE$_3$
Nº of genomes without TsaE$_3$
p < 0.00005

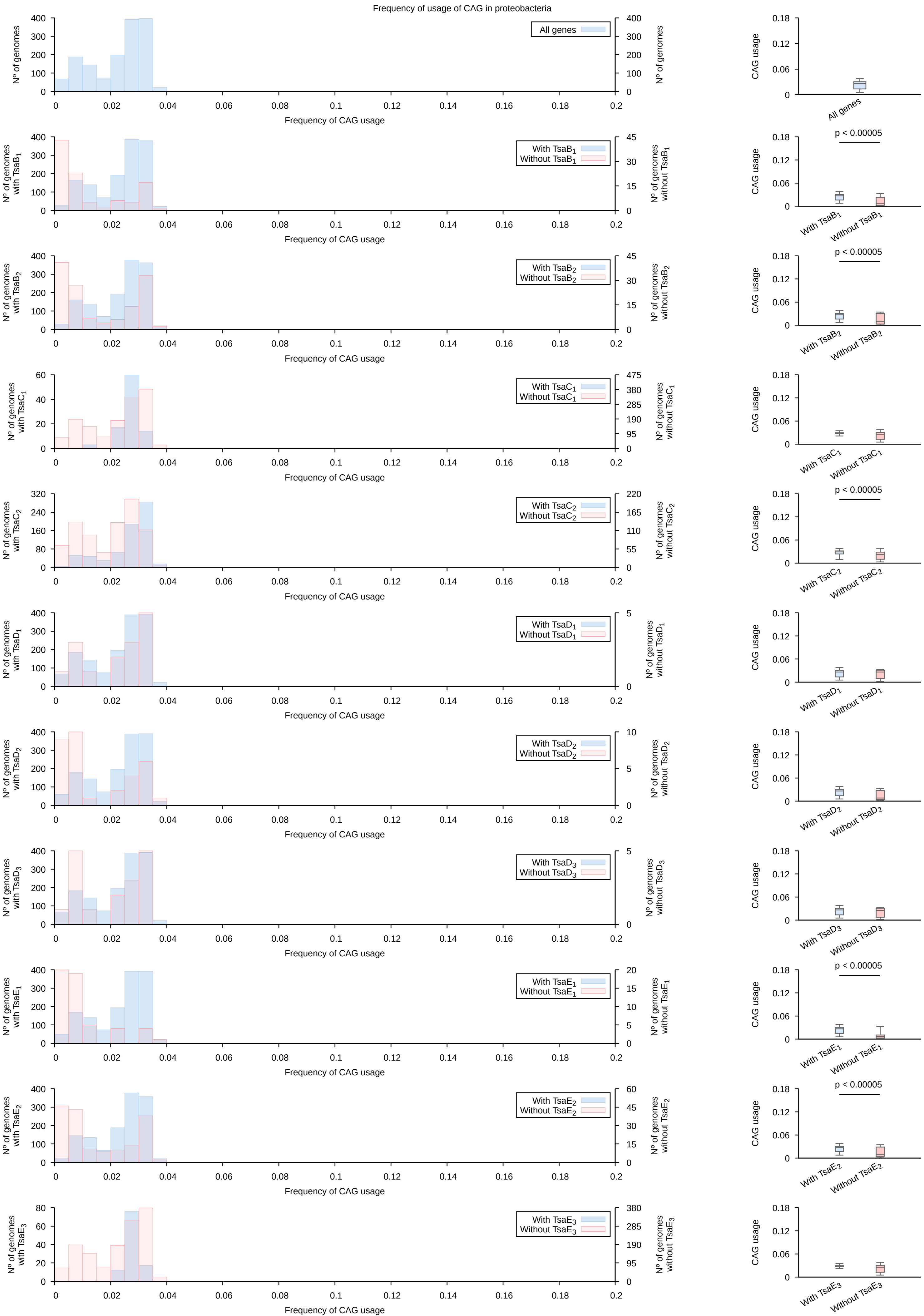

Frequency of usage of CAG in proteobacteria
All genes
Nº of genomes
Frequency of CAG usage
CAG usage
All genes
With TsaB1
Without TsaB1
Nº of genomes with TsaB1
Nº of genomes without TsaB1
p < 0.00005
With TsaB2
Without TsaB2
Nº of genomes with TsaB2
Nº of genomes without TsaB2
p < 0.00005
With TsaC1
Without TsaC1
Nº of genomes with TsaC1
Nº of genomes without TsaC1
With TsaC2
Without TsaC2
Nº of genomes with TsaC2
Nº of genomes without TsaC2
p < 0.00005
With TsaD1
Without TsaD1
Nº of genomes with TsaD1
Nº of genomes without TsaD1
With TsaD2
Without TsaD2
Nº of genomes with TsaD2
Nº of genomes without TsaD2
With TsaD3
Without TsaD3
Nº of genomes with TsaD3
Nº of genomes without TsaD3
With TsaE1
Without TsaE1
Nº of genomes with TsaE1
Nº of genomes without TsaE1
p < 0.00005
With TsaE2
Without TsaE2
Nº of genomes with TsaE2
Nº of genomes without TsaE2
p < 0.00005
With TsaE3
Without TsaE3
Nº of genomes with TsaE3
Nº of genomes without TsaE3
0 0.02 0.04 0.06 0.08 0.1 0.12 0.14 0.16 0.18 0.2
0 0.06 0.12 0.18

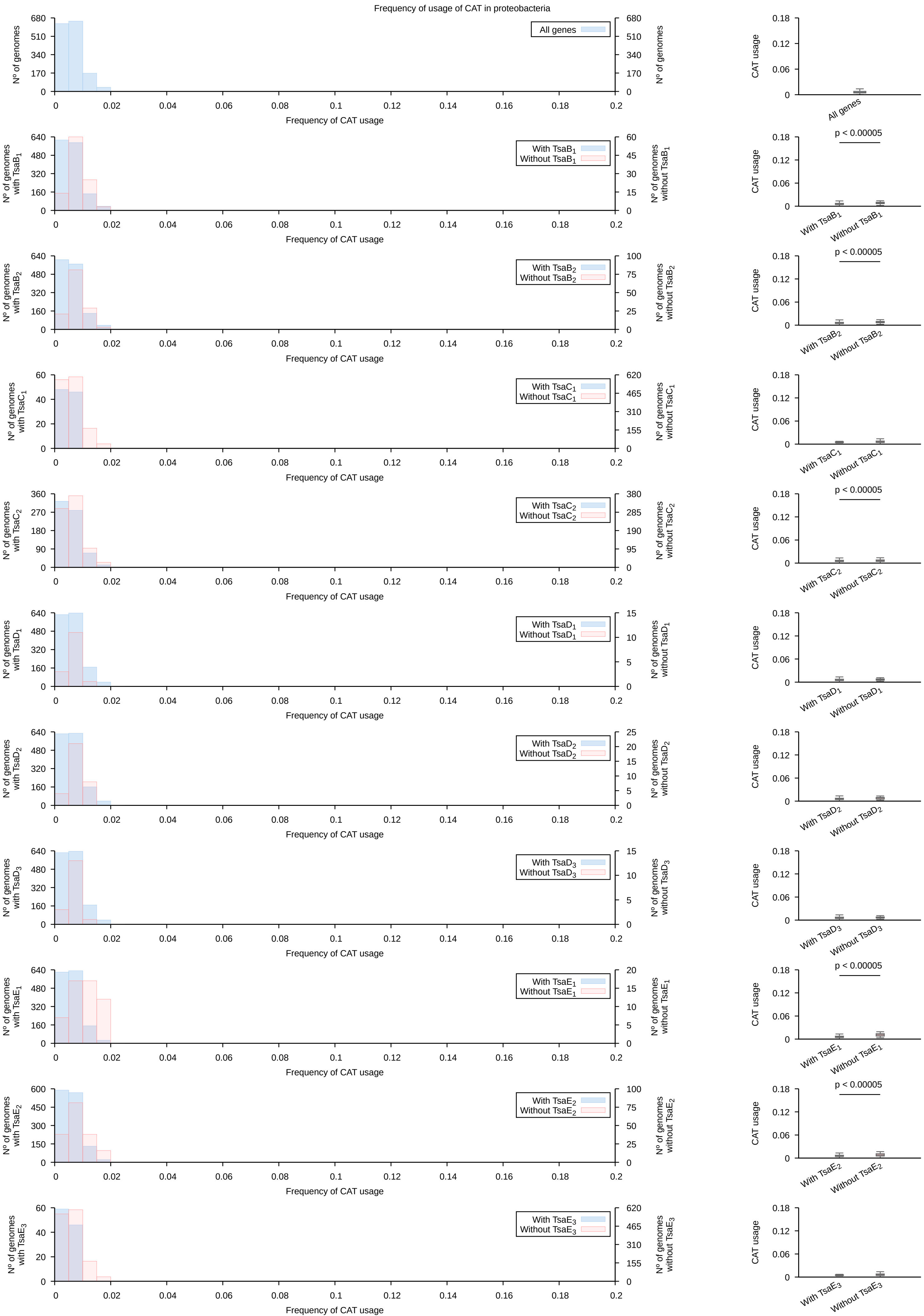

Frequency of usage of CAT in proteobacteria
All genes
Nº of genomes
Frequency of CAT usage
CAT usage
With TsaB$_1$
Without TsaB$_1$
Nº of genomes with TsaB$_1$
Nº of genomes without TsaB$_1$
p < 0.00005
With TsaB$_2$
Without TsaB$_2$
Nº of genomes with TsaB$_2$
Nº of genomes without TsaB$_2$
p < 0.00005
With TsaC$_1$
Without TsaC$_1$
Nº of genomes with TsaC$_1$
Nº of genomes without TsaC$_1$
With TsaC$_2$
Without TsaC$_2$
Nº of genomes with TsaC$_2$
Nº of genomes without TsaC$_2$
p < 0.00005
With TsaD$_1$
Without TsaD$_1$
Nº of genomes with TsaD$_1$
Nº of genomes without TsaD$_1$
With TsaD$_2$
Without TsaD$_2$
Nº of genomes with TsaD$_2$
Nº of genomes without TsaD$_2$
With TsaD$_3$
Without TsaD$_3$
Nº of genomes with TsaD$_3$
Nº of genomes without TsaD$_3$
With TsaE$_1$
Without TsaE$_1$
Nº of genomes with TsaE$_1$
Nº of genomes without TsaE$_1$
p < 0.00005
With TsaE$_2$
Without TsaE$_2$
Nº of genomes with TsaE$_2$
Nº of genomes without TsaE$_2$
p < 0.00005
With TsaE$_3$
Without TsaE$_3$
Nº of genomes with TsaE$_3$
Nº of genomes without TsaE$_3$

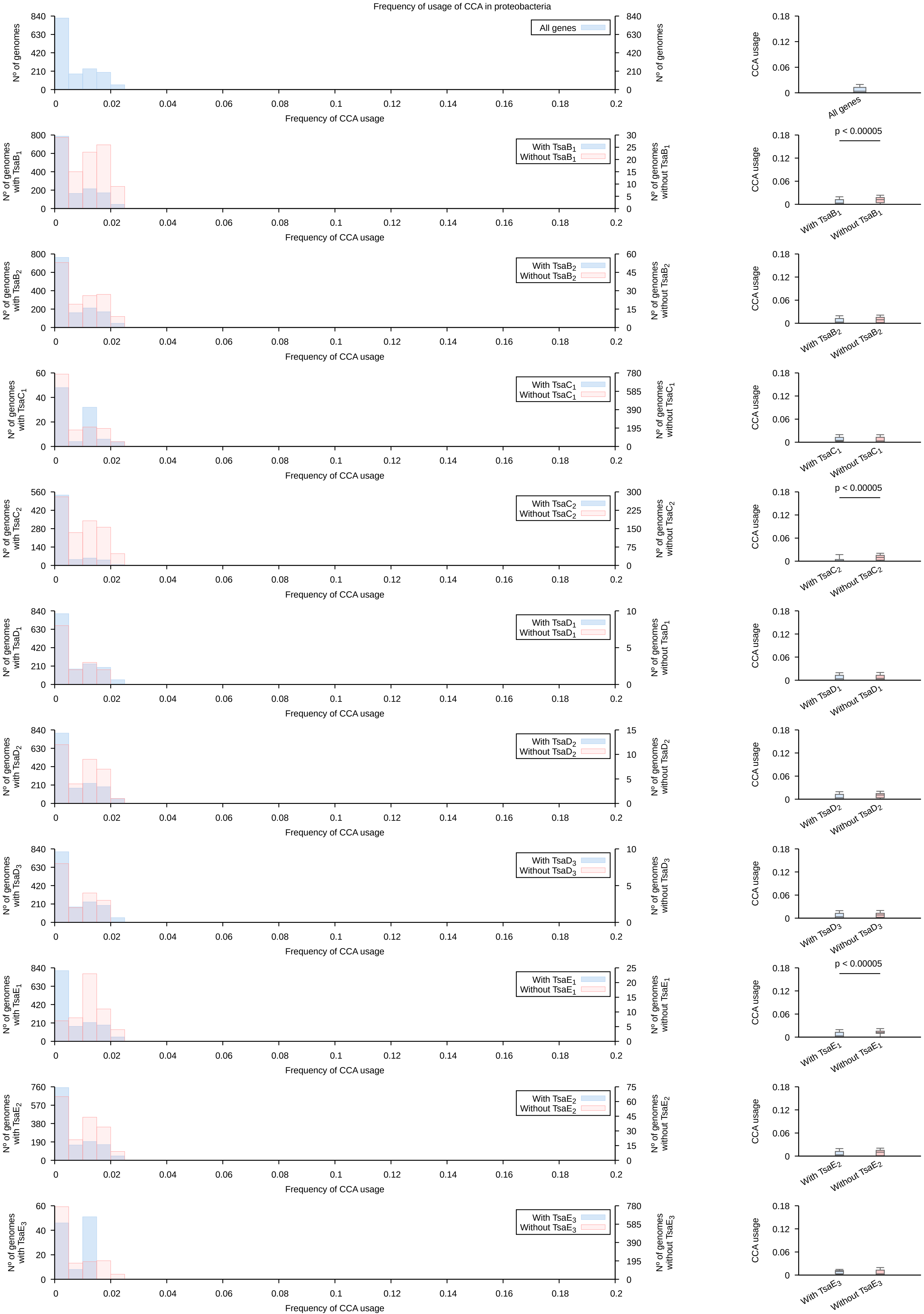
Frequency of usage of CCA in proteobacteria
Nº of genomes
All genes
Frequency of CCA usage
CCA usage
Nº of genomes with TsaB$_1$
Nº of genomes without TsaB$_1$
With TsaB$_1$
Without TsaB$_1$
p < 0.00005
Nº of genomes with TsaB$_2$
Nº of genomes without TsaB$_2$
With TsaB$_2$
Without TsaB$_2$
Nº of genomes with TsaC$_1$
Nº of genomes without TsaC$_1$
With TsaC$_1$
Without TsaC$_1$
Nº of genomes with TsaC$_2$
Nº of genomes without TsaC$_2$
With TsaC$_2$
Without TsaC$_2$
p < 0.00005
Nº of genomes with TsaD$_1$
Nº of genomes without TsaD$_1$
With TsaD$_1$
Without TsaD$_1$
Nº of genomes with TsaD$_2$
Nº of genomes without TsaD$_2$
With TsaD$_2$
Without TsaD$_2$
Nº of genomes with TsaD$_3$
Nº of genomes without TsaD$_3$
With TsaD$_3$
Without TsaD$_3$
Nº of genomes with TsaE$_1$
Nº of genomes without TsaE$_1$
With TsaE$_1$
Without TsaE$_1$
p < 0.00005
Nº of genomes with TsaE$_2$
Nº of genomes without TsaE$_2$
With TsaE$_2$
Without TsaE$_2$
Nº of genomes with TsaE$_3$
Nº of genomes without TsaE$_3$
With TsaE$_3$
Without TsaE$_3$

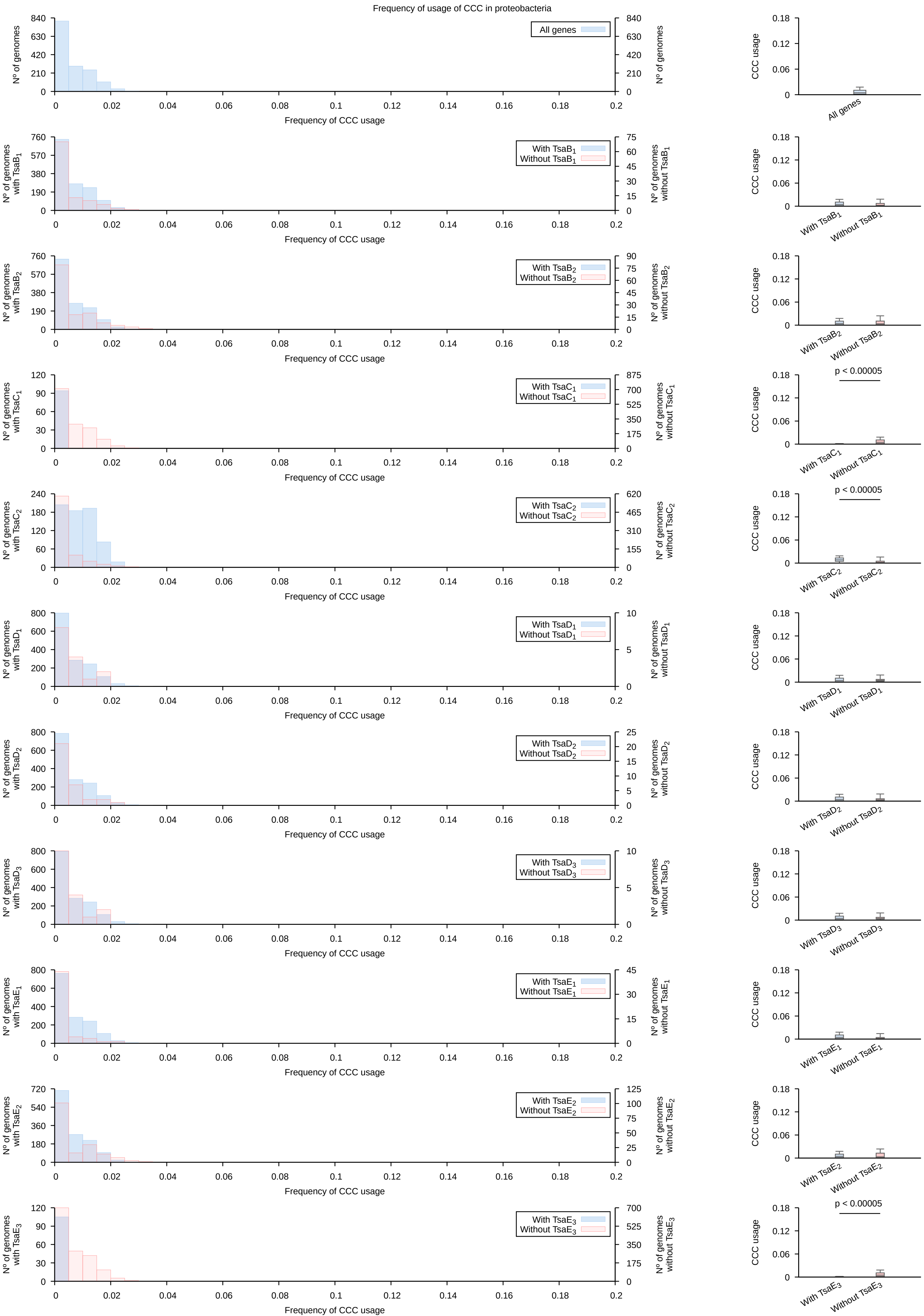

Frequency of usage of CCC in proteobacteria
Nº of genomes
Frequency of CCC usage
CCC usage
All genes
Nº of genomes with TsaB$_1$
Nº of genomes without TsaB$_1$
With TsaB$_1$
Without TsaB$_1$
Nº of genomes with TsaB$_2$
Nº of genomes without TsaB$_2$
With TsaB$_2$
Without TsaB$_2$
Nº of genomes with TsaC$_1$
Nº of genomes without TsaC$_1$
With TsaC$_1$
Without TsaC$_1$
p < 0.00005
Nº of genomes with TsaC$_2$
Nº of genomes without TsaC$_2$
With TsaC$_2$
Without TsaC$_2$
p < 0.00005
Nº of genomes with TsaD$_1$
Nº of genomes without TsaD$_1$
With TsaD$_1$
Without TsaD$_1$
Nº of genomes with TsaD$_2$
Nº of genomes without TsaD$_2$
With TsaD$_2$
Without TsaD$_2$
Nº of genomes with TsaD$_3$
Nº of genomes without TsaD$_3$
With TsaD$_3$
Without TsaD$_3$
Nº of genomes with TsaE$_1$
Nº of genomes without TsaE$_1$
With TsaE$_1$
Without TsaE$_1$
Nº of genomes with TsaE$_2$
Nº of genomes without TsaE$_2$
With TsaE$_2$
Without TsaE$_2$
Nº of genomes with TsaE$_3$
Nº of genomes without TsaE$_3$
With TsaE$_3$
Without TsaE$_3$
p < 0.00005

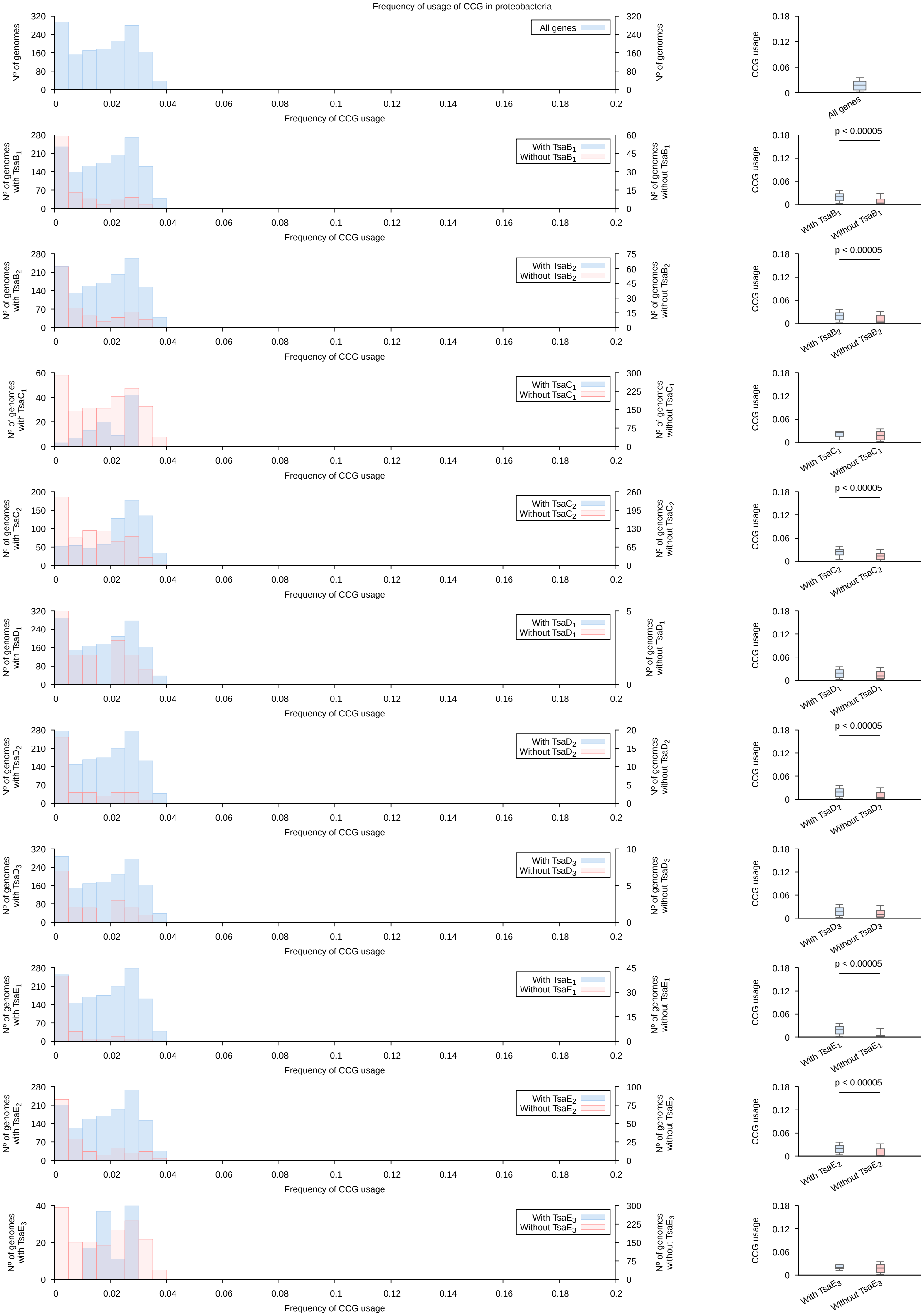

Frequency of usage of CCG in proteobacteria
All genes
Nº of genomes
CCG usage
Frequency of CCG usage
With TsaB1
Without TsaB1
Nº of genomes with TsaB1
Nº of genomes without TsaB1
p < 0.00005
With TsaB2
Without TsaB2
Nº of genomes with TsaB2
Nº of genomes without TsaB2
p < 0.00005
With TsaC1
Without TsaC1
Nº of genomes with TsaC1
Nº of genomes without TsaC1
With TsaC2
Without TsaC2
Nº of genomes with TsaC2
Nº of genomes without TsaC2
p < 0.00005
With TsaD1
Without TsaD1
Nº of genomes with TsaD1
Nº of genomes without TsaD1
With TsaD2
Without TsaD2
Nº of genomes with TsaD2
Nº of genomes without TsaD2
p < 0.00005
With TsaD3
Without TsaD3
Nº of genomes with TsaD3
Nº of genomes without TsaD3
With TsaE1
Without TsaE1
Nº of genomes with TsaE1
Nº of genomes without TsaE1
p < 0.00005
With TsaE2
Without TsaE2
Nº of genomes with TsaE2
Nº of genomes without TsaE2
p < 0.00005
With TsaE3
Without TsaE3
Nº of genomes with TsaE3
Nº of genomes without TsaE3

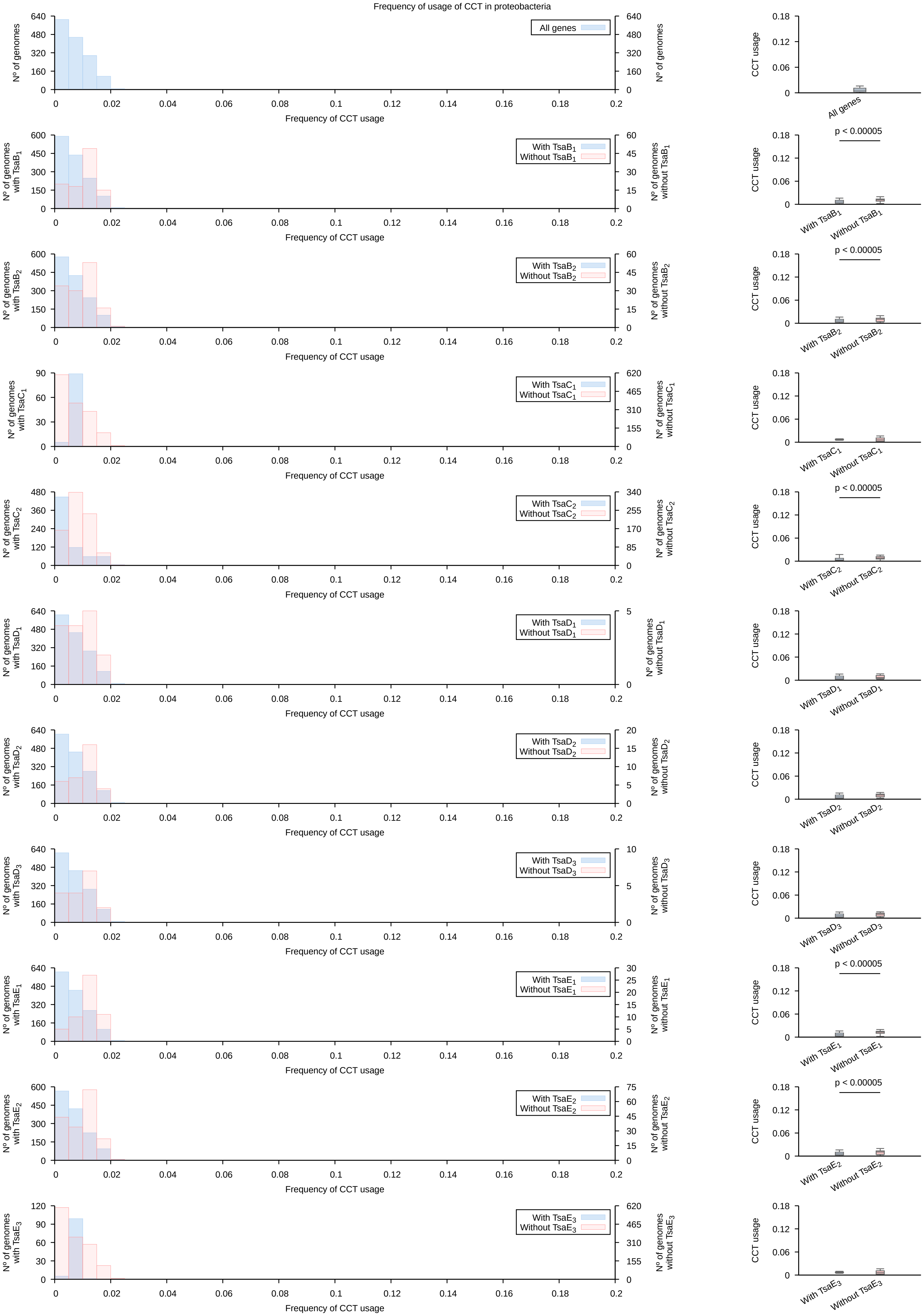
Frequency of usage of CCT in proteobacteria
All genes
Nº of genomes
Frequency of CCT usage
CCT usage
With TsaB$_1$
Without TsaB$_1$
Nº of genomes with TsaB$_1$
Nº of genomes without TsaB$_1$
p < 0.00005
With TsaB$_2$
Without TsaB$_2$
Nº of genomes with TsaB$_2$
Nº of genomes without TsaB$_2$
p < 0.00005
With TsaC$_1$
Without TsaC$_1$
Nº of genomes with TsaC$_1$
Nº of genomes without TsaC$_1$
With TsaC$_2$
Without TsaC$_2$
Nº of genomes with TsaC$_2$
Nº of genomes without TsaC$_2$
p < 0.00005
With TsaD$_1$
Without TsaD$_1$
Nº of genomes with TsaD$_1$
Nº of genomes without TsaD$_1$
With TsaD$_2$
Without TsaD$_2$
Nº of genomes with TsaD$_2$
Nº of genomes without TsaD$_2$
With TsaD$_3$
Without TsaD$_3$
Nº of genomes with TsaD$_3$
Nº of genomes without TsaD$_3$
With TsaE$_1$
Without TsaE$_1$
Nº of genomes with TsaE$_1$
Nº of genomes without TsaE$_1$
p < 0.00005
With TsaE$_2$
Without TsaE$_2$
Nº of genomes with TsaE$_2$
Nº of genomes without TsaE$_2$
p < 0.00005
With TsaE$_3$
Without TsaE$_3$
Nº of genomes with TsaE$_3$
Nº of genomes without TsaE$_3$

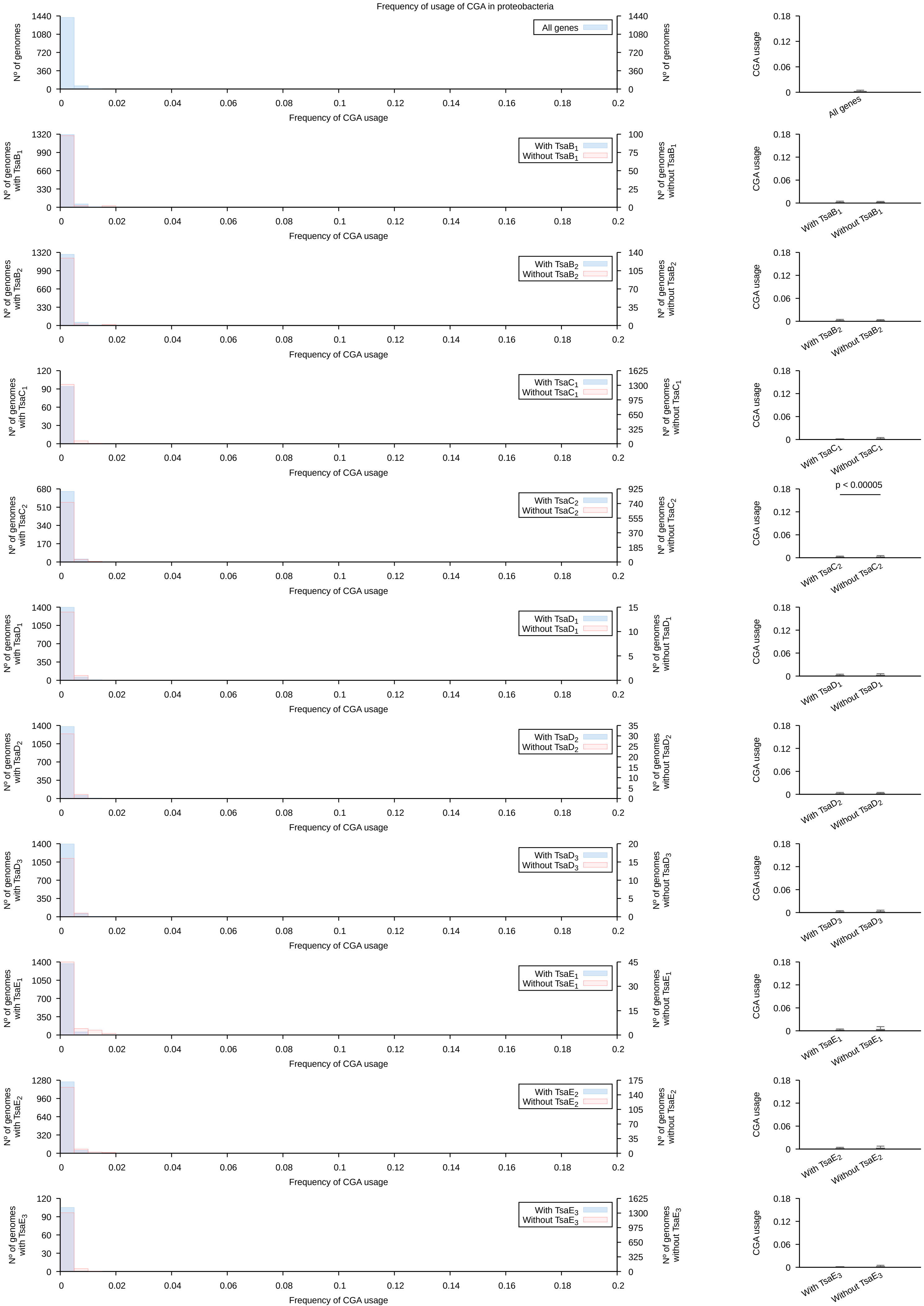

Frequency of usage of CGA in proteobacteria
All genes
Nº of genomes
Frequency of CGA usage
CGA usage
With TsaB1
Without TsaB1
Nº of genomes with TsaB1
Nº of genomes without TsaB1
With TsaB2
Without TsaB2
Nº of genomes with TsaB2
Nº of genomes without TsaB2
With TsaC1
Without TsaC1
Nº of genomes with TsaC1
Nº of genomes without TsaC1
With TsaC2
Without TsaC2
Nº of genomes with TsaC2
Nº of genomes without TsaC2
p < 0.00005
With TsaD1
Without TsaD1
Nº of genomes with TsaD1
Nº of genomes without TsaD1
With TsaD2
Without TsaD2
Nº of genomes with TsaD2
Nº of genomes without TsaD2
With TsaD3
Without TsaD3
Nº of genomes with TsaD3
Nº of genomes without TsaD3
With TsaE1
Without TsaE1
Nº of genomes with TsaE1
Nº of genomes without TsaE1
With TsaE2
Without TsaE2
Nº of genomes with TsaE2
Nº of genomes without TsaE2
With TsaE3
Without TsaE3
Nº of genomes with TsaE3
Nº of genomes without TsaE3

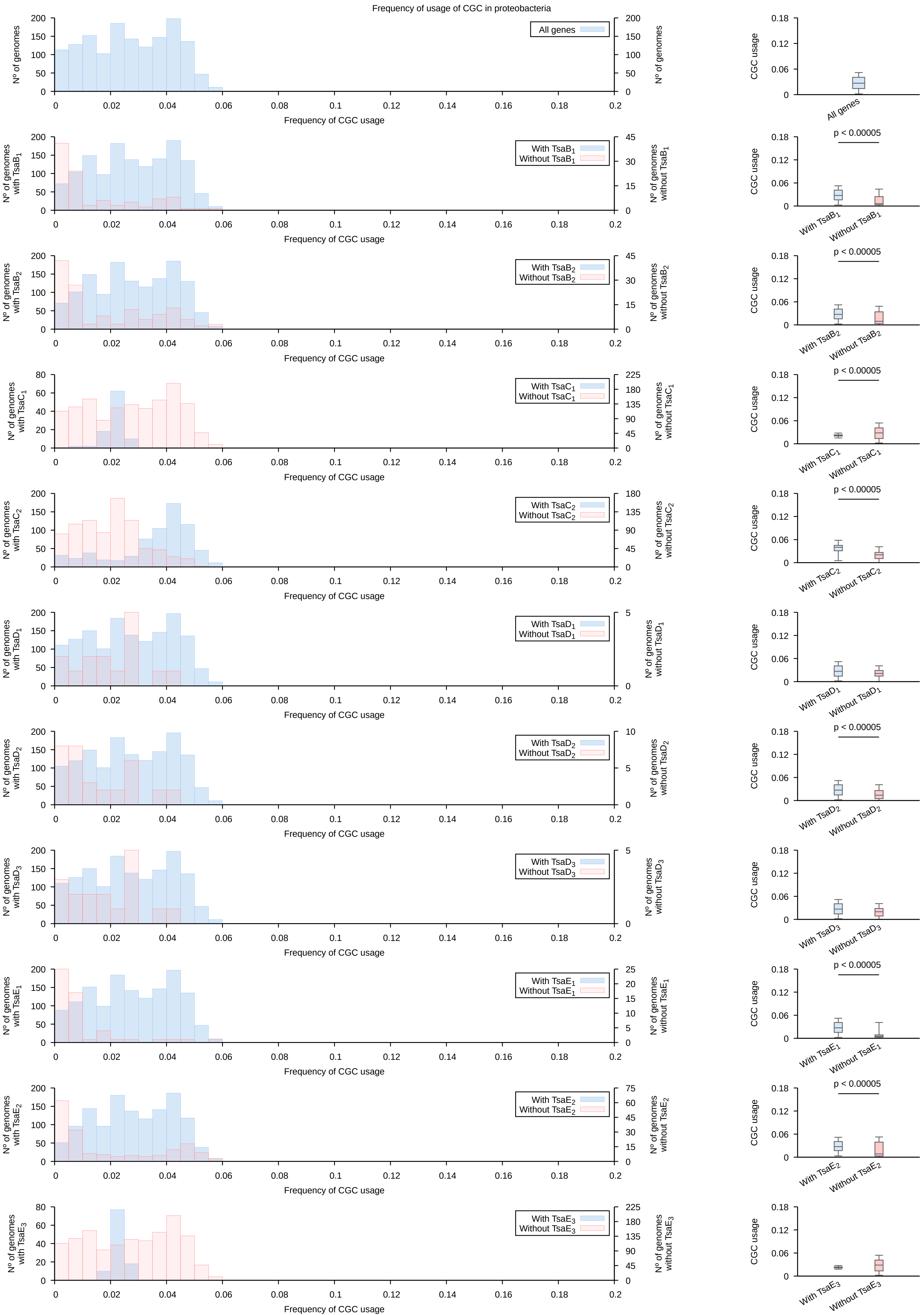
Frequency of usage of CGC in proteobacteria
All genes
Nº of genomes
Frequency of CGC usage
CGC usage
With TsaB$_1$
Without TsaB$_1$
With TsaB$_2$
Without TsaB$_2$
With TsaC$_1$
Without TsaC$_1$
With TsaC$_2$
Without TsaC$_2$
With TsaD$_1$
Without TsaD$_1$
With TsaD$_2$
Without TsaD$_2$
With TsaD$_3$
Without TsaD$_3$
With TsaE$_1$
Without TsaE$_1$
With TsaE$_2$
Without TsaE$_2$
With TsaE$_3$
Without TsaE$_3$
p < 0.00005
0
0.02
0.04
0.06
0.08
0.1
0.12
0.14
0.16
0.18
0.2

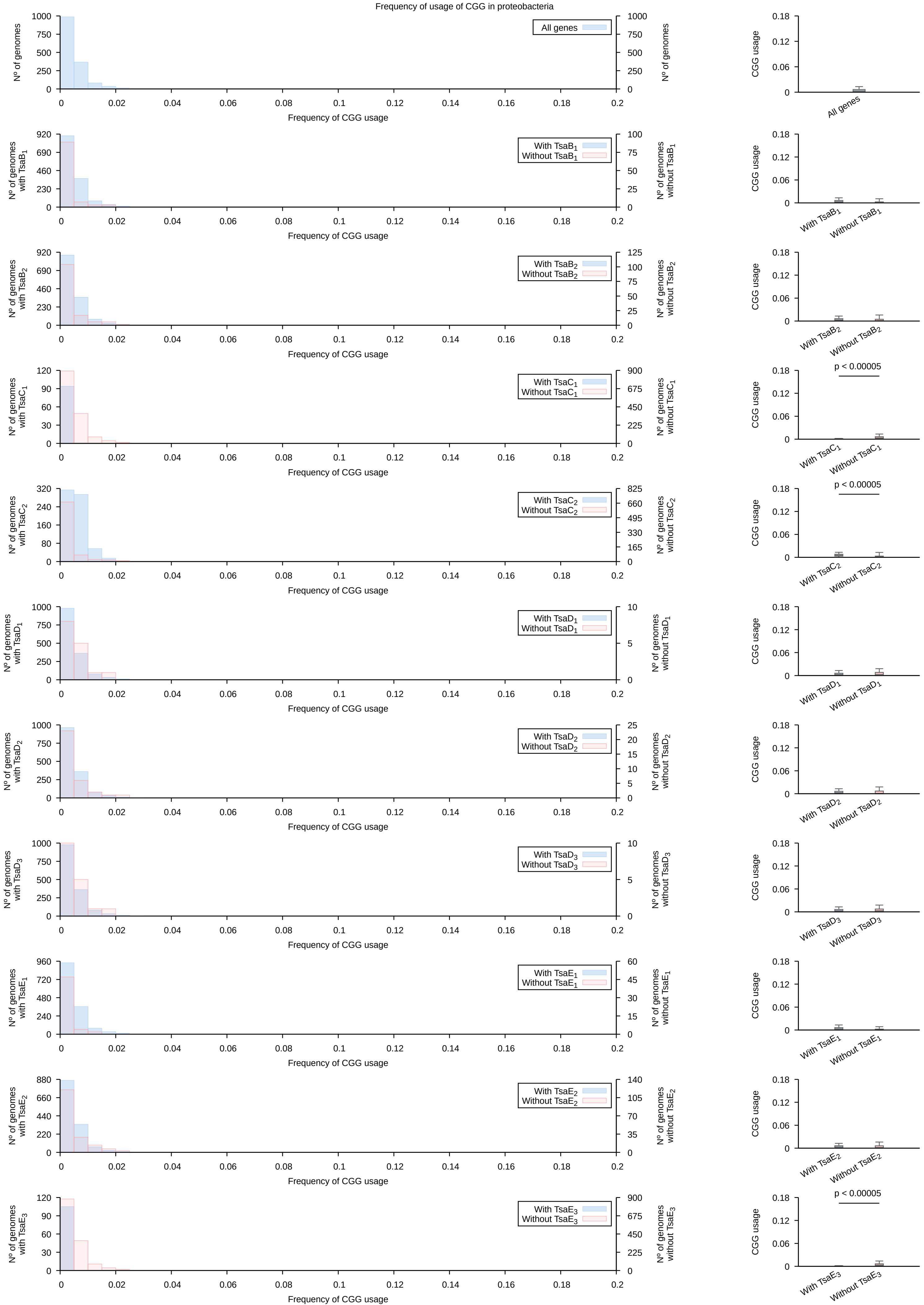

Frequency of usage of CGG in proteobacteria
Nº of genomes
All genes
Frequency of CGG usage
CGG usage
Nº of genomes with TsaB$_1$
Nº of genomes without TsaB$_1$
With TsaB$_1$
Without TsaB$_1$
Nº of genomes with TsaB$_2$
Nº of genomes without TsaB$_2$
With TsaB$_2$
Without TsaB$_2$
Nº of genomes with TsaC$_1$
Nº of genomes without TsaC$_1$
With TsaC$_1$
Without TsaC$_1$
p < 0.00005
Nº of genomes with TsaC$_2$
Nº of genomes without TsaC$_2$
With TsaC$_2$
Without TsaC$_2$
p < 0.00005
Nº of genomes with TsaD$_1$
Nº of genomes without TsaD$_1$
With TsaD$_1$
Without TsaD$_1$
Nº of genomes with TsaD$_2$
Nº of genomes without TsaD$_2$
With TsaD$_2$
Without TsaD$_2$
Nº of genomes with TsaD$_3$
Nº of genomes without TsaD$_3$
With TsaD$_3$
Without TsaD$_3$
Nº of genomes with TsaE$_1$
Nº of genomes without TsaE$_1$
With TsaE$_1$
Without TsaE$_1$
Nº of genomes with TsaE$_2$
Nº of genomes without TsaE$_2$
With TsaE$_2$
Without TsaE$_2$
Nº of genomes with TsaE$_3$
Nº of genomes without TsaE$_3$
With TsaE$_3$
Without TsaE$_3$
p < 0.00005

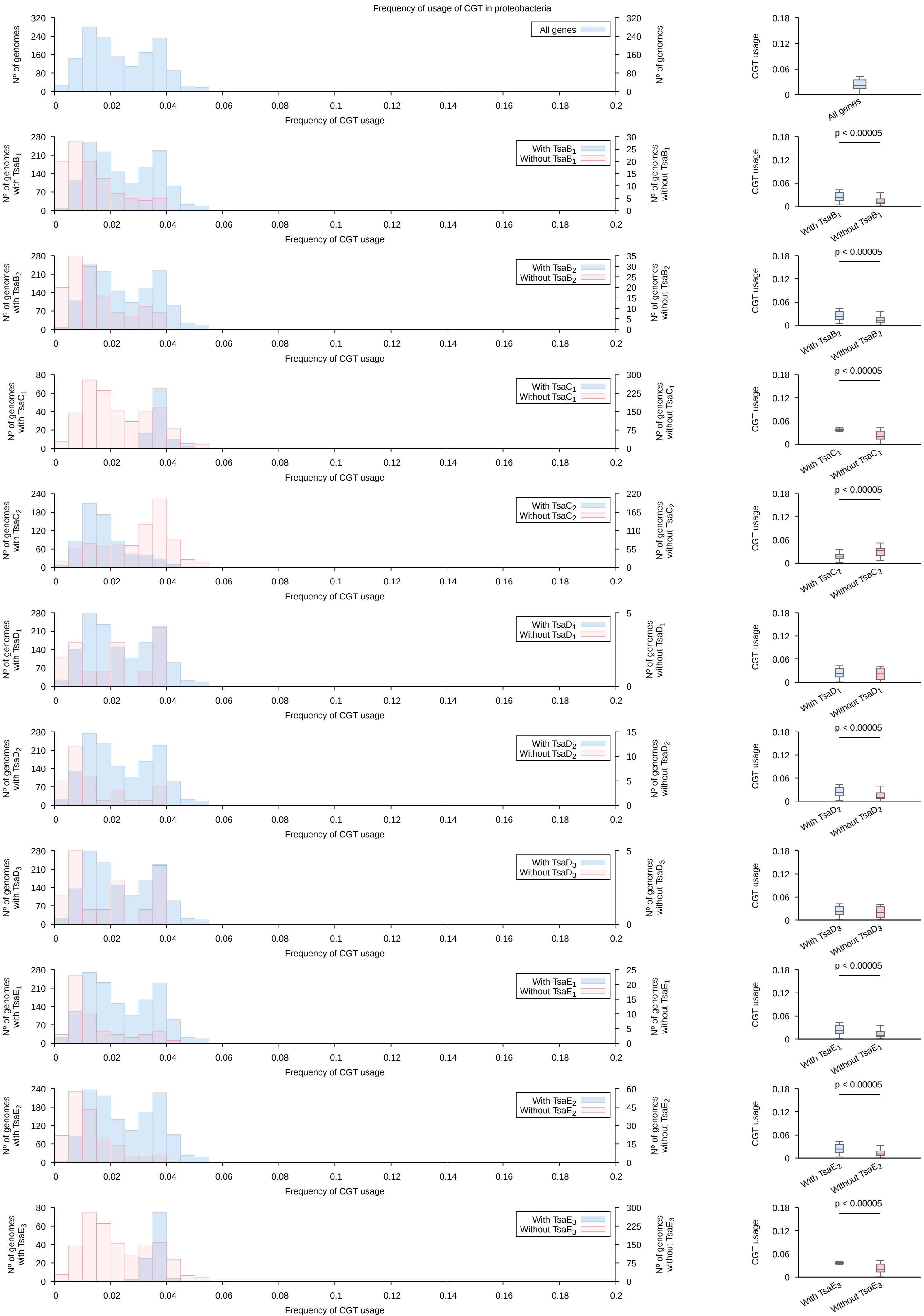
Frequency of usage of CGT in proteobacteria
All genes
With TsaB1
Without TsaB1
With TsaB2
Without TsaB2
With TsaC1
Without TsaC1
With TsaC2
Without TsaC2
With TsaD1
Without TsaD1
With TsaD2
Without TsaD2
With TsaD3
Without TsaD3
With TsaE1
Without TsaE1
With TsaE2
Without TsaE2
With TsaE3
Without TsaE3
Nº of genomes
Frequency of CGT usage
CGT usage
p < 0.00005

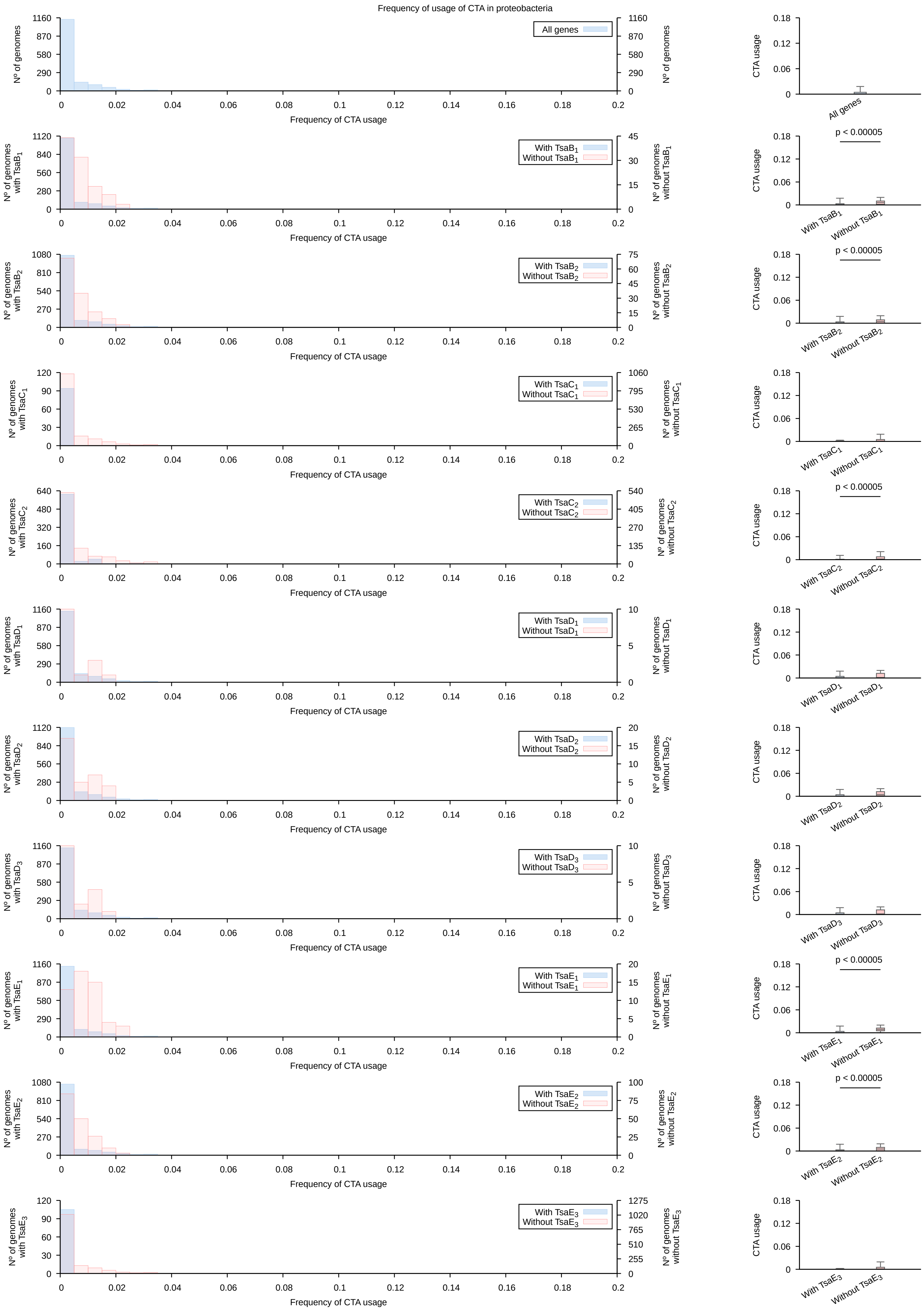
Frequency of usage of CTA in proteobacteria
All genes
With TsaB1
Without TsaB1
With TsaB2
Without TsaB2
With TsaC1
Without TsaC1
With TsaC2
Without TsaC2
With TsaD1
Without TsaD1
With TsaD2
Without TsaD2
With TsaD3
Without TsaD3
With TsaE1
Without TsaE1
With TsaE2
Without TsaE2
With TsaE3
Without TsaE3
Nº of genomes
Frequency of CTA usage
CTA usage
p < 0.00005
0
0.02
0.04
0.06
0.08
0.1
0.12
0.14
0.16
0.18
0.2

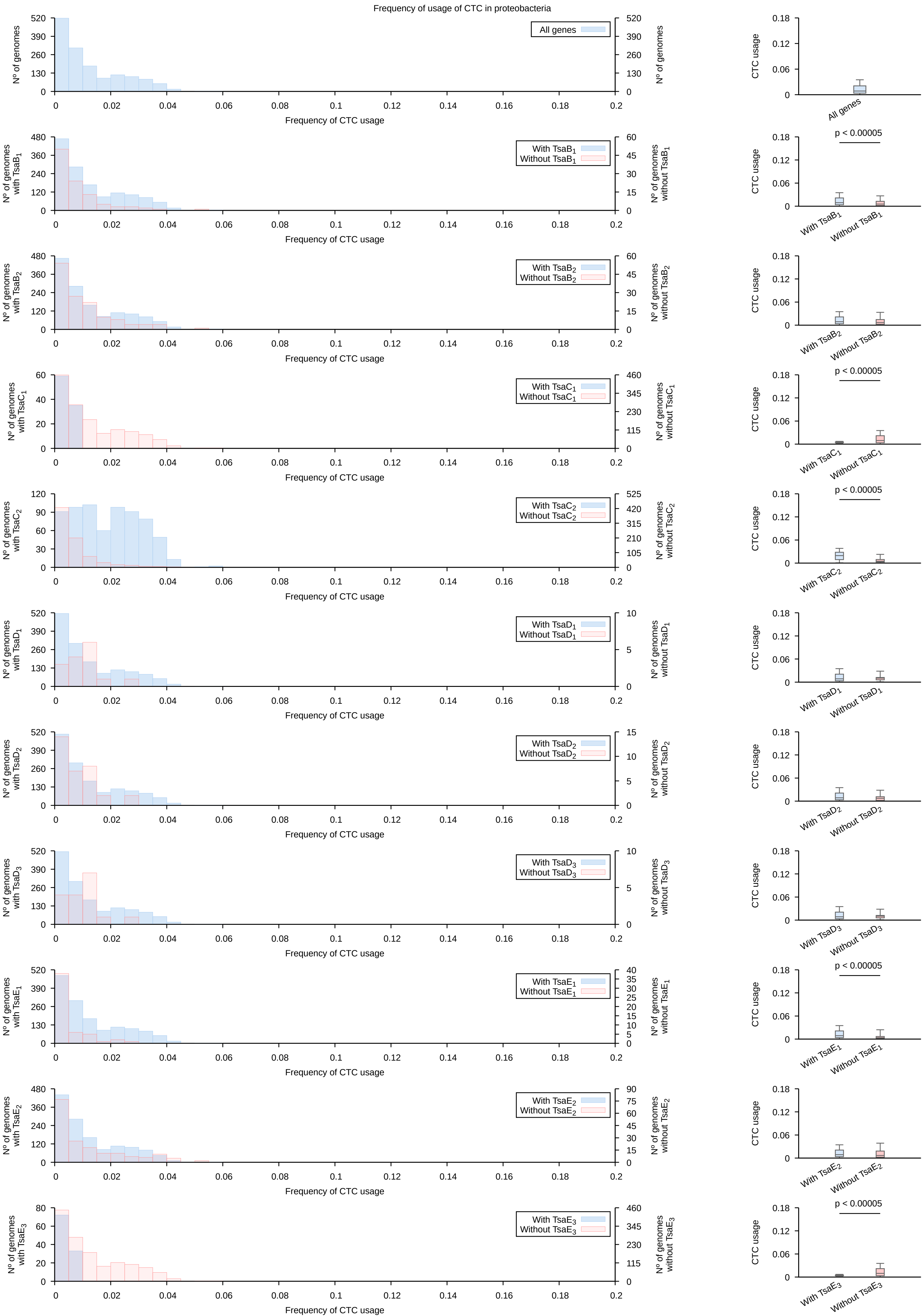
Frequency of usage of CTC in proteobacteria
Nº of genomes
Frequency of CTC usage
All genes
CTC usage
Nº of genomes with TsaB$_1$
Nº of genomes without TsaB$_1$
With TsaB$_1$
Without TsaB$_1$
p < 0.00005
Nº of genomes with TsaB$_2$
Nº of genomes without TsaB$_2$
With TsaB$_2$
Without TsaB$_2$
Nº of genomes with TsaC$_1$
Nº of genomes without TsaC$_1$
With TsaC$_1$
Without TsaC$_1$
p < 0.00005
Nº of genomes with TsaC$_2$
Nº of genomes without TsaC$_2$
With TsaC$_2$
Without TsaC$_2$
p < 0.00005
Nº of genomes with TsaD$_1$
Nº of genomes without TsaD$_1$
With TsaD$_1$
Without TsaD$_1$
Nº of genomes with TsaD$_2$
Nº of genomes without TsaD$_2$
With TsaD$_2$
Without TsaD$_2$
Nº of genomes with TsaD$_3$
Nº of genomes without TsaD$_3$
With TsaD$_3$
Without TsaD$_3$
Nº of genomes with TsaE$_1$
Nº of genomes without TsaE$_1$
With TsaE$_1$
Without TsaE$_1$
p < 0.00005
Nº of genomes with TsaE$_2$
Nº of genomes without TsaE$_2$
With TsaE$_2$
Without TsaE$_2$
Nº of genomes with TsaE$_3$
Nº of genomes without TsaE$_3$
With TsaE$_3$
Without TsaE$_3$
p < 0.00005

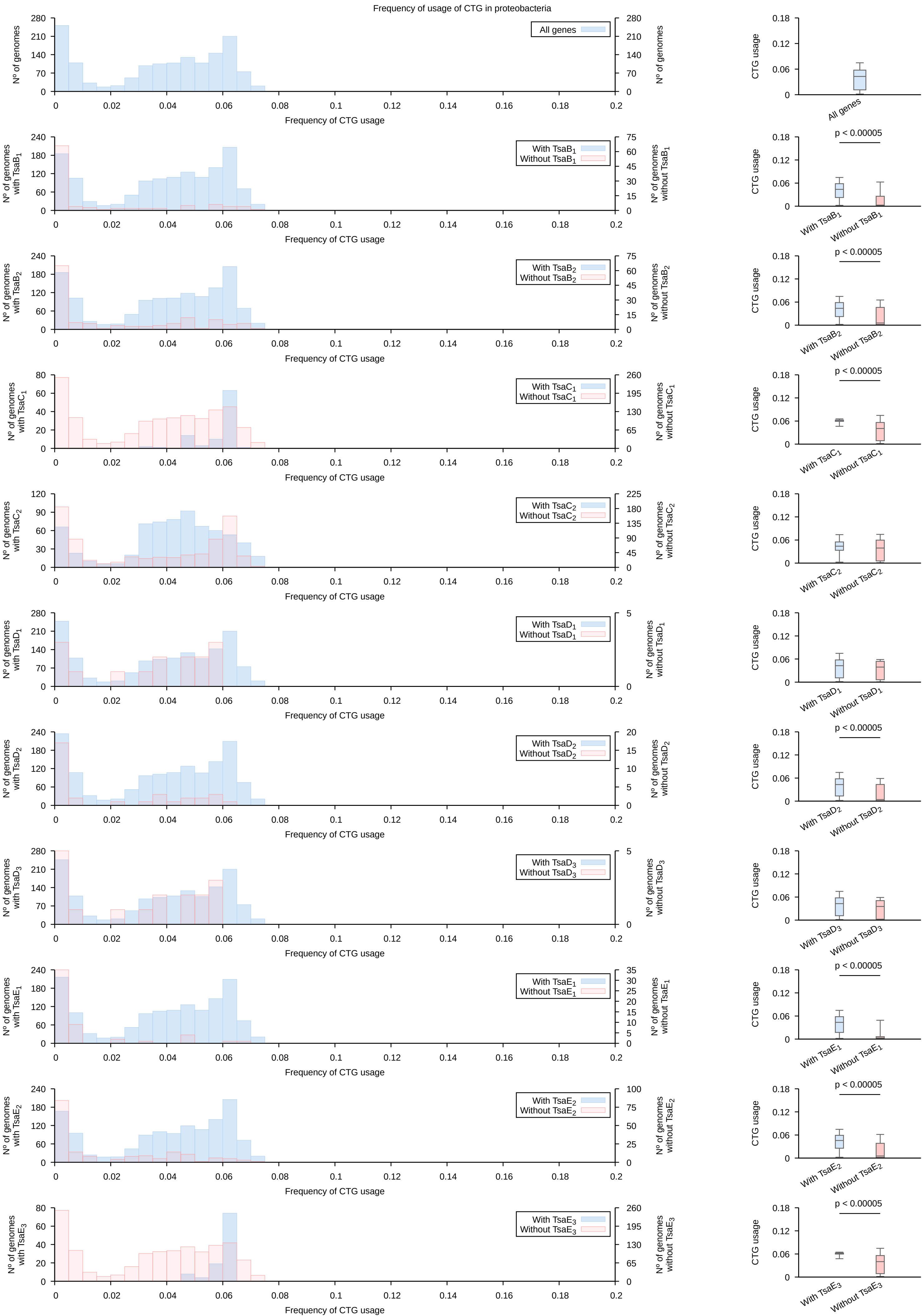
Frequency of usage of CTG in proteobacteria
All genes
Nº of genomes
Frequency of CTG usage
CTG usage
All genes
With TsaB$_1$
Without TsaB$_1$
Nº of genomes with TsaB$_1$
Nº of genomes without TsaB$_1$
p < 0.00005
With TsaB$_2$
Without TsaB$_2$
Nº of genomes with TsaB$_2$
Nº of genomes without TsaB$_2$
p < 0.00005
With TsaC$_1$
Without TsaC$_1$
Nº of genomes with TsaC$_1$
Nº of genomes without TsaC$_1$
p < 0.00005
With TsaC$_2$
Without TsaC$_2$
Nº of genomes with TsaC$_2$
Nº of genomes without TsaC$_2$
With TsaD$_1$
Without TsaD$_1$
Nº of genomes with TsaD$_1$
Nº of genomes without TsaD$_1$
With TsaD$_2$
Without TsaD$_2$
Nº of genomes with TsaD$_2$
Nº of genomes without TsaD$_2$
p < 0.00005
With TsaD$_3$
Without TsaD$_3$
Nº of genomes with TsaD$_3$
Nº of genomes without TsaD$_3$
With TsaE$_1$
Without TsaE$_1$
Nº of genomes with TsaE$_1$
Nº of genomes without TsaE$_1$
p < 0.00005
With TsaE$_2$
Without TsaE$_2$
Nº of genomes with TsaE$_2$
Nº of genomes without TsaE$_2$
p < 0.00005
With TsaE$_3$
Without TsaE$_3$
Nº of genomes with TsaE$_3$
Nº of genomes without TsaE$_3$
p < 0.00005

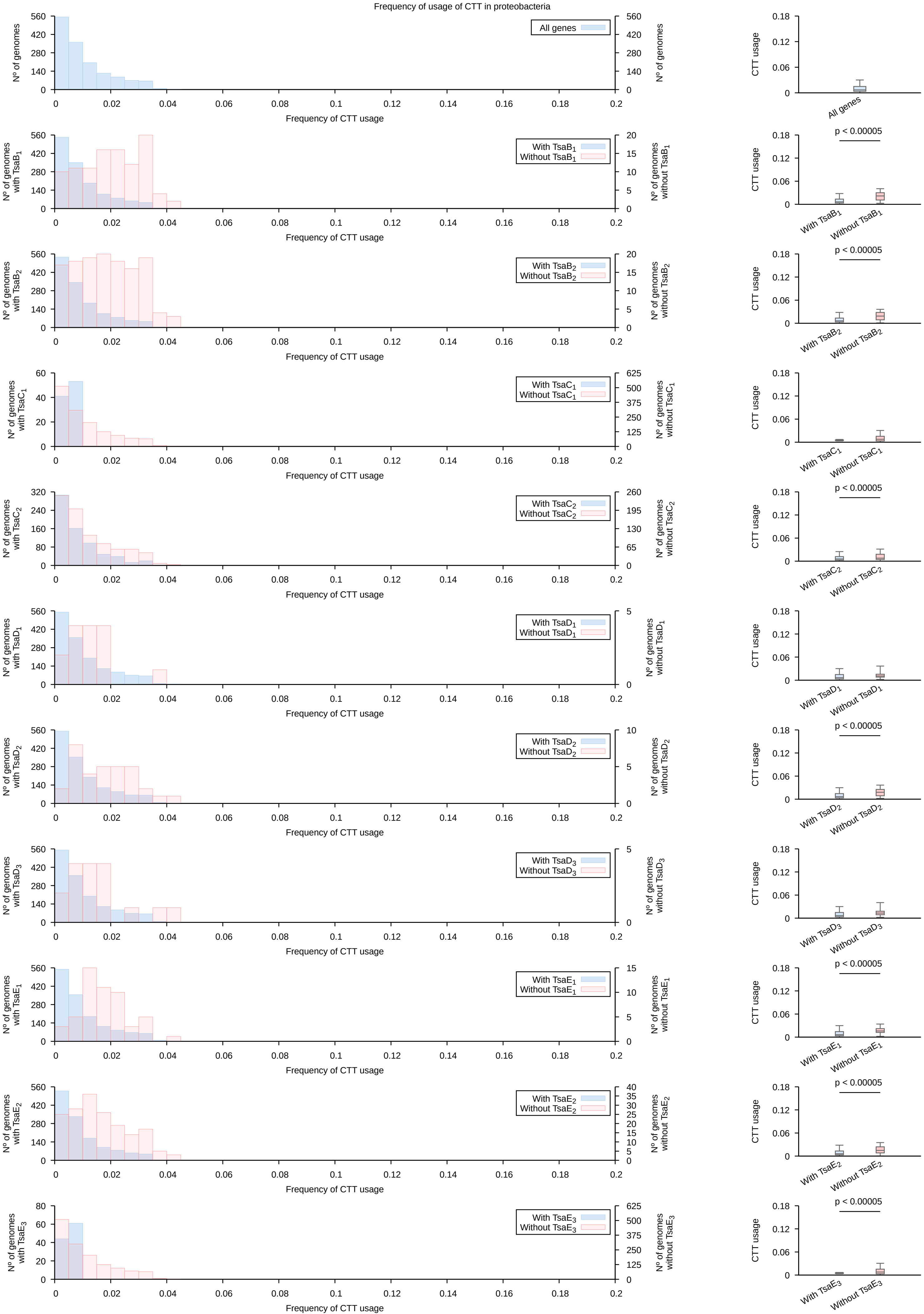

Frequency of usage of CTT in proteobacteria
All genes
Nº of genomes
Frequency of CTT usage
CTT usage
With TsaB1
Without TsaB1
Nº of genomes with TsaB1
Nº of genomes without TsaB1
p < 0.00005
With TsaB2
Without TsaB2
Nº of genomes with TsaB2
Nº of genomes without TsaB2
p < 0.00005
With TsaC1
Without TsaC1
Nº of genomes with TsaC1
Nº of genomes without TsaC1
With TsaC2
Without TsaC2
Nº of genomes with TsaC2
Nº of genomes without TsaC2
p < 0.00005
With TsaD1
Without TsaD1
Nº of genomes with TsaD1
Nº of genomes without TsaD1
With TsaD2
Without TsaD2
Nº of genomes with TsaD2
Nº of genomes without TsaD2
p < 0.00005
With TsaD3
Without TsaD3
Nº of genomes with TsaD3
Nº of genomes without TsaD3
With TsaE1
Without TsaE1
Nº of genomes with TsaE1
Nº of genomes without TsaE1
p < 0.00005
With TsaE2
Without TsaE2
Nº of genomes with TsaE2
Nº of genomes without TsaE2
p < 0.00005
With TsaE3
Without TsaE3
Nº of genomes with TsaE3
Nº of genomes without TsaE3
p < 0.00005

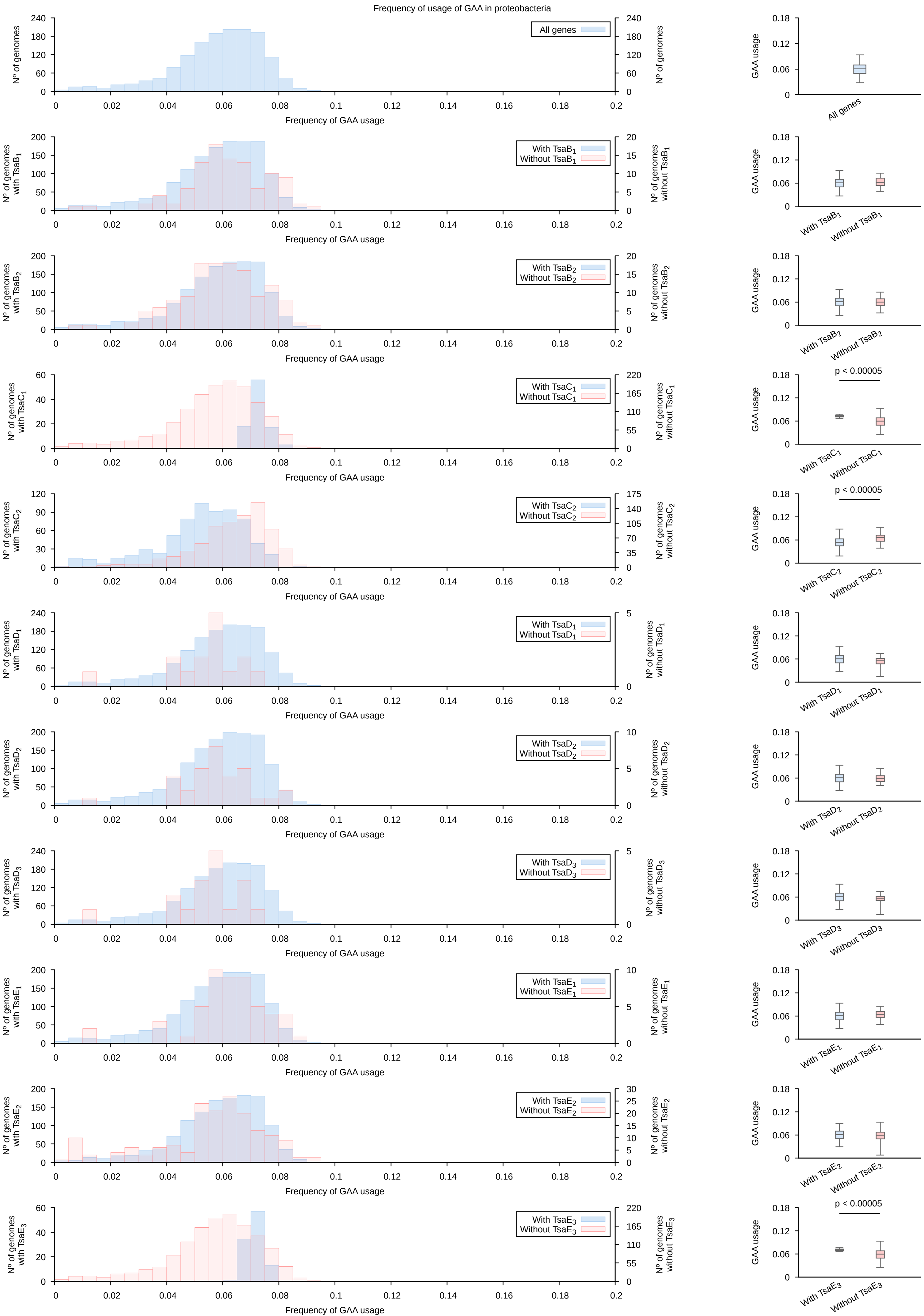

Frequency of usage of GAA in proteobacteria
Nº of genomes
All genes
Frequency of GAA usage
GAA usage
All genes
Nº of genomes with TsaB1
Nº of genomes without TsaB1
With TsaB1
Without TsaB1
Frequency of GAA usage
GAA usage
Nº of genomes with TsaB2
Nº of genomes without TsaB2
With TsaB2
Without TsaB2
Frequency of GAA usage
GAA usage
Nº of genomes with TsaC1
Nº of genomes without TsaC1
With TsaC1
Without TsaC1
Frequency of GAA usage
GAA usage
p < 0.00005
Nº of genomes with TsaC2
Nº of genomes without TsaC2
With TsaC2
Without TsaC2
Frequency of GAA usage
GAA usage
p < 0.00005
Nº of genomes with TsaD1
Nº of genomes without TsaD1
With TsaD1
Without TsaD1
Frequency of GAA usage
GAA usage
Nº of genomes with TsaD2
Nº of genomes without TsaD2
With TsaD2
Without TsaD2
Frequency of GAA usage
GAA usage
Nº of genomes with TsaD3
Nº of genomes without TsaD3
With TsaD3
Without TsaD3
Frequency of GAA usage
GAA usage
Nº of genomes with TsaE1
Nº of genomes without TsaE1
With TsaE1
Without TsaE1
Frequency of GAA usage
GAA usage
Nº of genomes with TsaE2
Nº of genomes without TsaE2
With TsaE2
Without TsaE2
Frequency of GAA usage
GAA usage
Nº of genomes with TsaE3
Nº of genomes without TsaE3
With TsaE3
Without TsaE3
Frequency of GAA usage
GAA usage
p < 0.00005

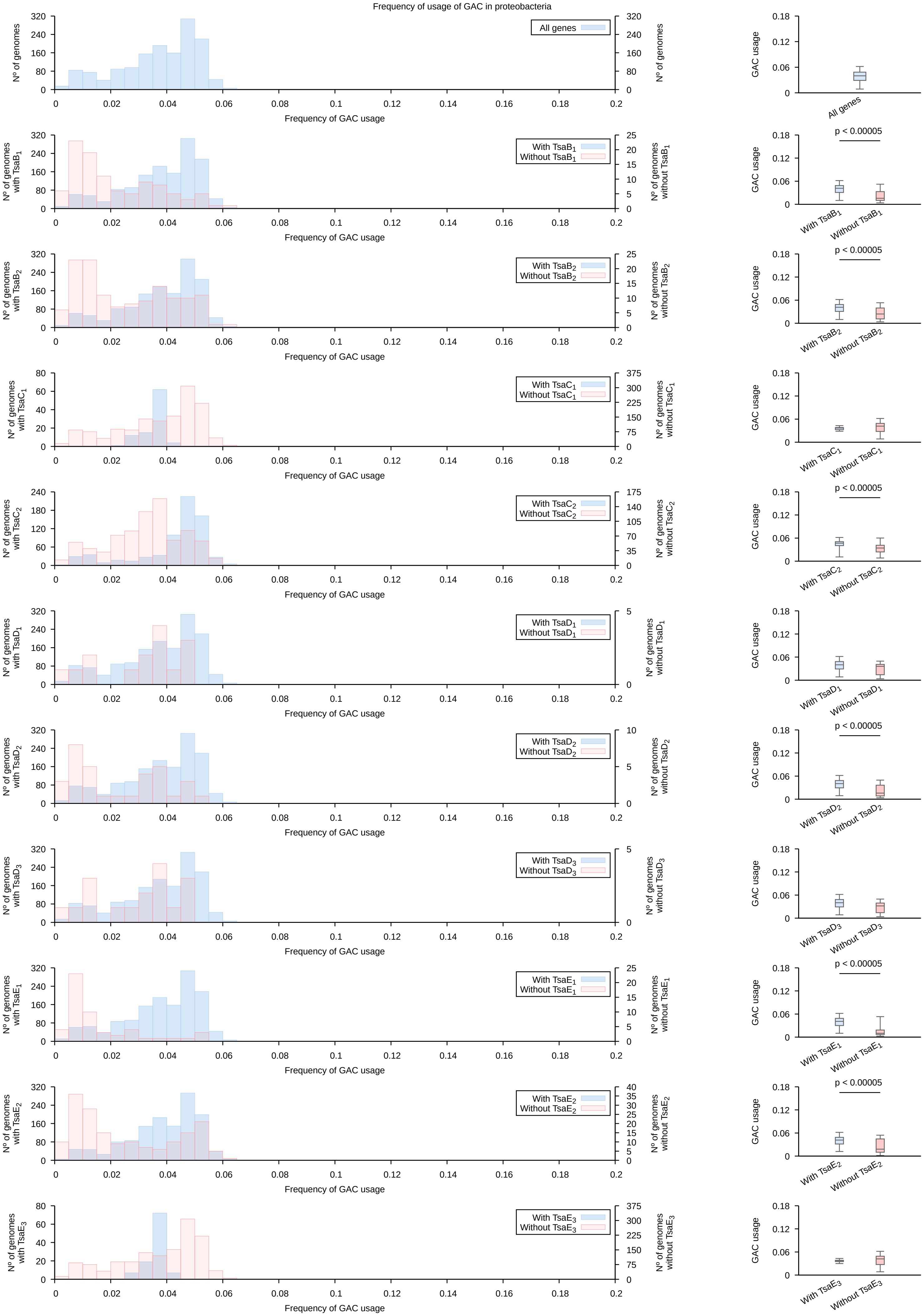

Frequency of usage of GAC in proteobacteria
All genes
Nº of genomes
Frequency of GAC usage
GAC usage
With TsaB1
Without TsaB1
Nº of genomes with TsaB1
Nº of genomes without TsaB1
p < 0.00005
With TsaB2
Without TsaB2
Nº of genomes with TsaB2
Nº of genomes without TsaB2
p < 0.00005
With TsaC1
Without TsaC1
Nº of genomes with TsaC1
Nº of genomes without TsaC1
With TsaC2
Without TsaC2
Nº of genomes with TsaC2
Nº of genomes without TsaC2
p < 0.00005
With TsaD1
Without TsaD1
Nº of genomes with TsaD1
Nº of genomes without TsaD1
With TsaD2
Without TsaD2
Nº of genomes with TsaD2
Nº of genomes without TsaD2
p < 0.00005
With TsaD3
Without TsaD3
Nº of genomes with TsaD3
Nº of genomes without TsaD3
With TsaE1
Without TsaE1
Nº of genomes with TsaE1
Nº of genomes without TsaE1
p < 0.00005
With TsaE2
Without TsaE2
Nº of genomes with TsaE2
Nº of genomes without TsaE2
p < 0.00005
With TsaE3
Without TsaE3
Nº of genomes with TsaE3
Nº of genomes without TsaE3

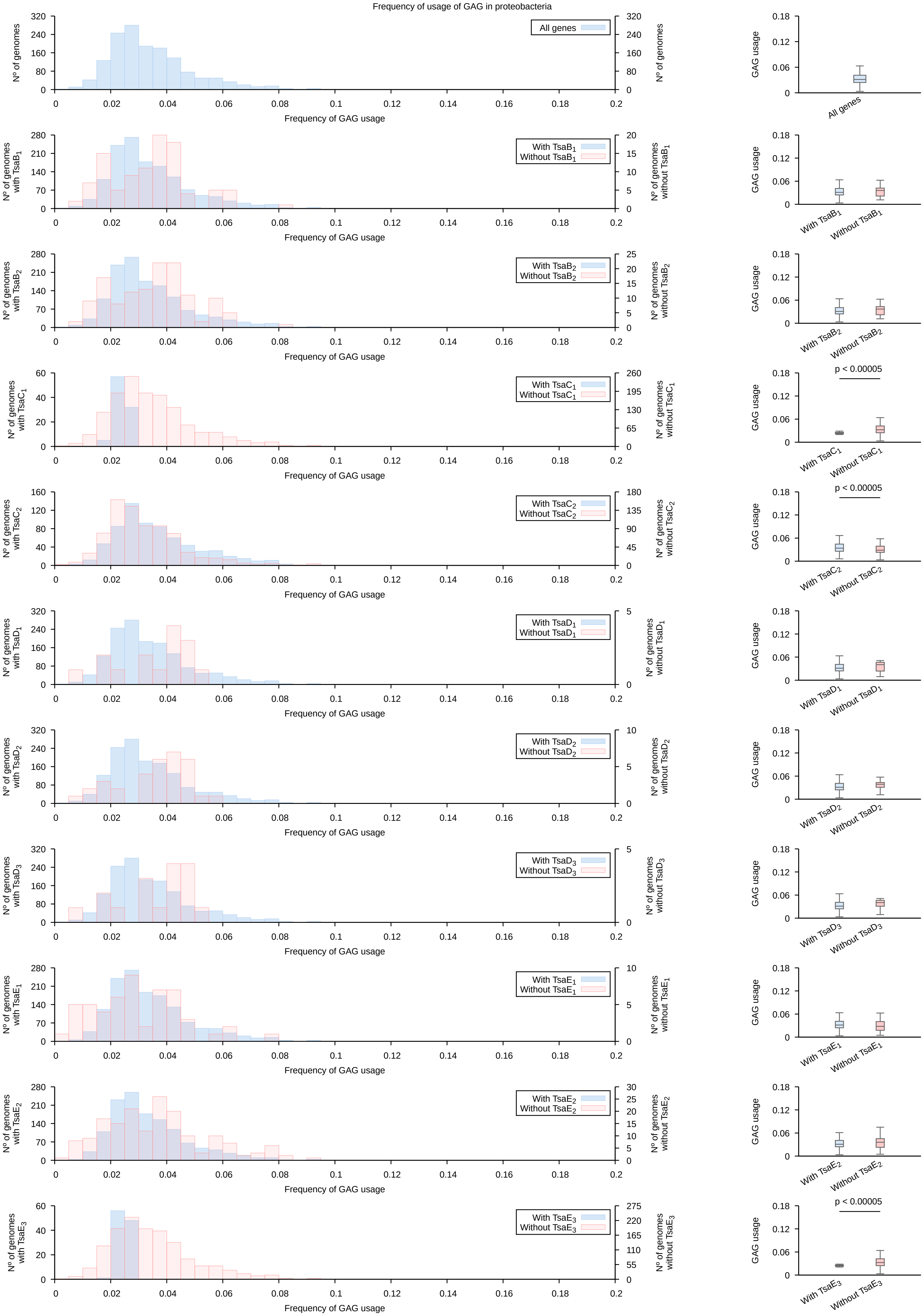

Frequency of usage of GAG in proteobacteria
Nº of genomes
All genes
Frequency of GAG usage
GAG usage
All genes
Nº of genomes with TsaB1
Nº of genomes without TsaB1
With TsaB1
Without TsaB1
Nº of genomes with TsaB2
Nº of genomes without TsaB2
With TsaB2
Without TsaB2
Nº of genomes with TsaC1
Nº of genomes without TsaC1
With TsaC1
Without TsaC1
p < 0.00005
Nº of genomes with TsaC2
Nº of genomes without TsaC2
With TsaC2
Without TsaC2
p < 0.00005
Nº of genomes with TsaD1
Nº of genomes without TsaD1
With TsaD1
Without TsaD1
Nº of genomes with TsaD2
Nº of genomes without TsaD2
With TsaD2
Without TsaD2
Nº of genomes with TsaD3
Nº of genomes without TsaD3
With TsaD3
Without TsaD3
Nº of genomes with TsaE1
Nº of genomes without TsaE1
With TsaE1
Without TsaE1
Nº of genomes with TsaE2
Nº of genomes without TsaE2
With TsaE2
Without TsaE2
Nº of genomes with TsaE3
Nº of genomes without TsaE3
With TsaE3
Without TsaE3
p < 0.00005

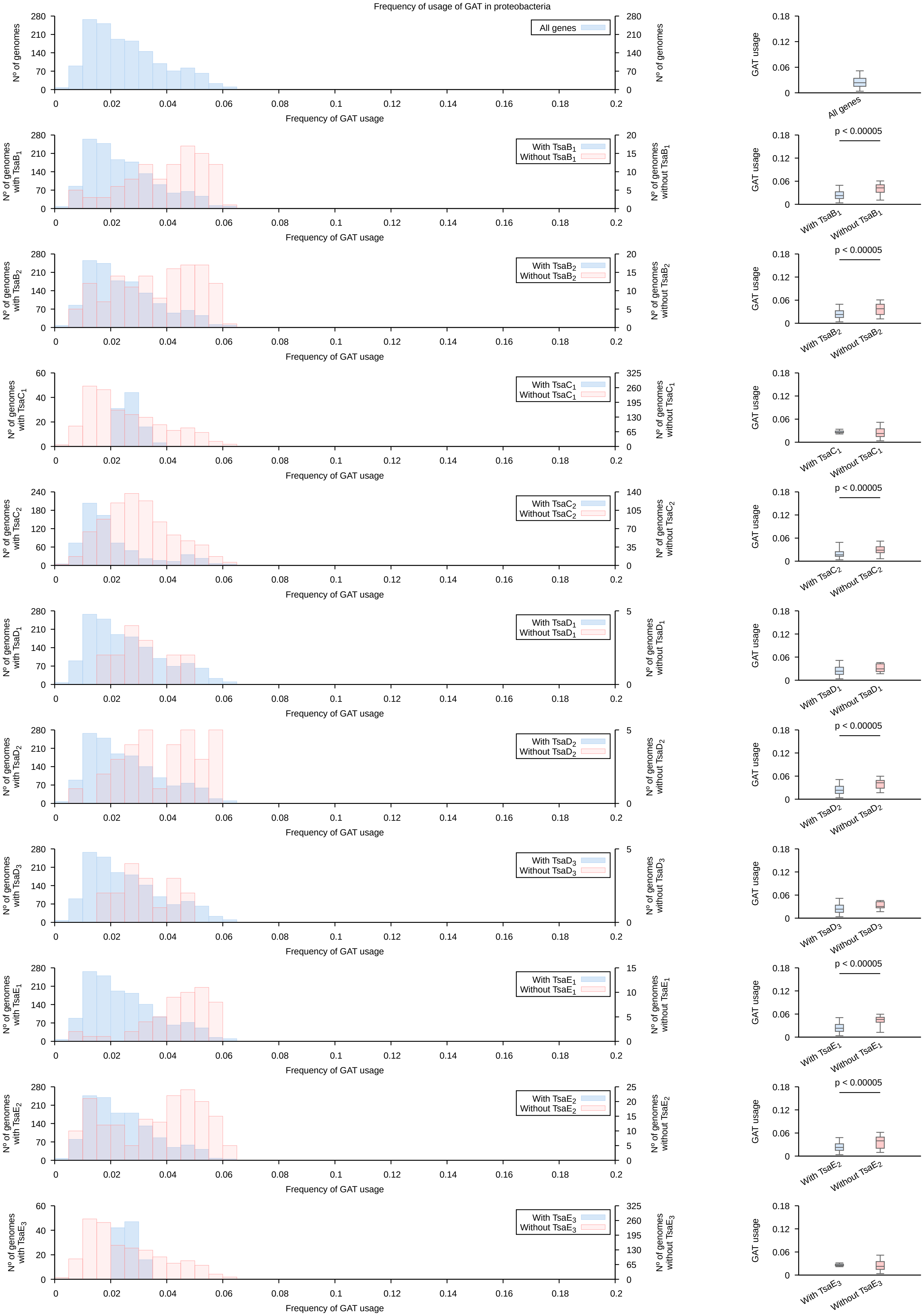

Frequency of usage of GAT in proteobacteria
Nº of genomes
All genes
Frequency of GAT usage
GAT usage
Nº of genomes with TsaB$_1$
Nº of genomes without TsaB$_1$
With TsaB$_1$
Without TsaB$_1$
p < 0.00005
Nº of genomes with TsaB$_2$
Nº of genomes without TsaB$_2$
With TsaB$_2$
Without TsaB$_2$
p < 0.00005
Nº of genomes with TsaC$_1$
Nº of genomes without TsaC$_1$
With TsaC$_1$
Without TsaC$_1$
Nº of genomes with TsaC$_2$
Nº of genomes without TsaC$_2$
With TsaC$_2$
Without TsaC$_2$
p < 0.00005
Nº of genomes with TsaD$_1$
Nº of genomes without TsaD$_1$
With TsaD$_1$
Without TsaD$_1$
Nº of genomes with TsaD$_2$
Nº of genomes without TsaD$_2$
With TsaD$_2$
Without TsaD$_2$
p < 0.00005
Nº of genomes with TsaD$_3$
Nº of genomes without TsaD$_3$
With TsaD$_3$
Without TsaD$_3$
Nº of genomes with TsaE$_1$
Nº of genomes without TsaE$_1$
With TsaE$_1$
Without TsaE$_1$
p < 0.00005
Nº of genomes with TsaE$_2$
Nº of genomes without TsaE$_2$
With TsaE$_2$
Without TsaE$_2$
p < 0.00005
Nº of genomes with TsaE$_3$
Nº of genomes without TsaE$_3$
With TsaE$_3$
Without TsaE$_3$

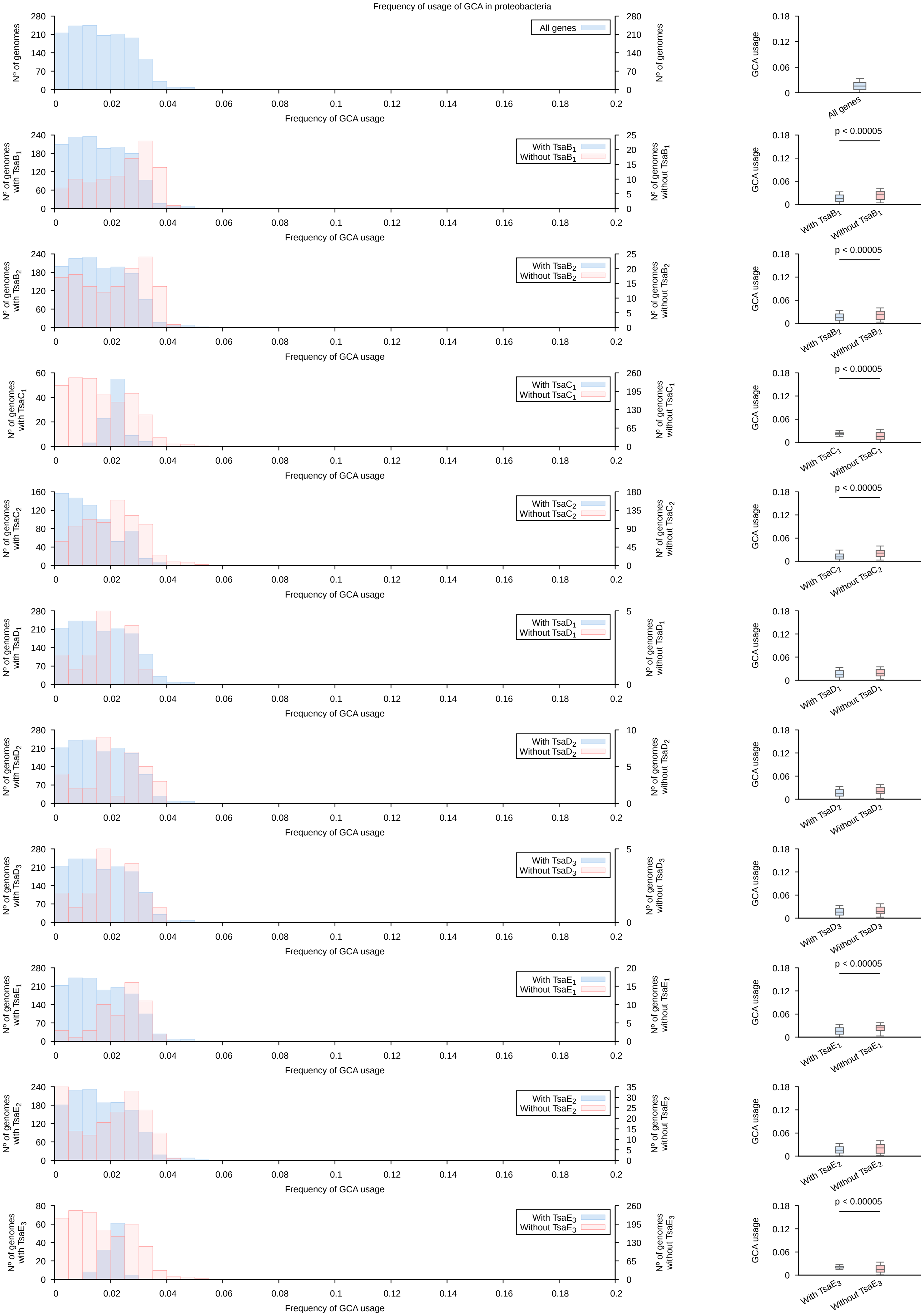

Frequency of usage of GCA in proteobacteria
Nº of genomes
All genes
Frequency of GCA usage
GCA usage
Nº of genomes with TsaB1
Nº of genomes without TsaB1
With TsaB1
Without TsaB1
p < 0.00005
Nº of genomes with TsaB2
Nº of genomes without TsaB2
With TsaB2
Without TsaB2
p < 0.00005
Nº of genomes with TsaC1
Nº of genomes without TsaC1
With TsaC1
Without TsaC1
p < 0.00005
Nº of genomes with TsaC2
Nº of genomes without TsaC2
With TsaC2
Without TsaC2
p < 0.00005
Nº of genomes with TsaD1
Nº of genomes without TsaD1
With TsaD1
Without TsaD1
Nº of genomes with TsaD2
Nº of genomes without TsaD2
With TsaD2
Without TsaD2
Nº of genomes with TsaD3
Nº of genomes without TsaD3
With TsaD3
Without TsaD3
Nº of genomes with TsaE1
Nº of genomes without TsaE1
With TsaE1
Without TsaE1
p < 0.00005
Nº of genomes with TsaE2
Nº of genomes without TsaE2
With TsaE2
Without TsaE2
Nº of genomes with TsaE3
Nº of genomes without TsaE3
With TsaE3
Without TsaE3
p < 0.00005

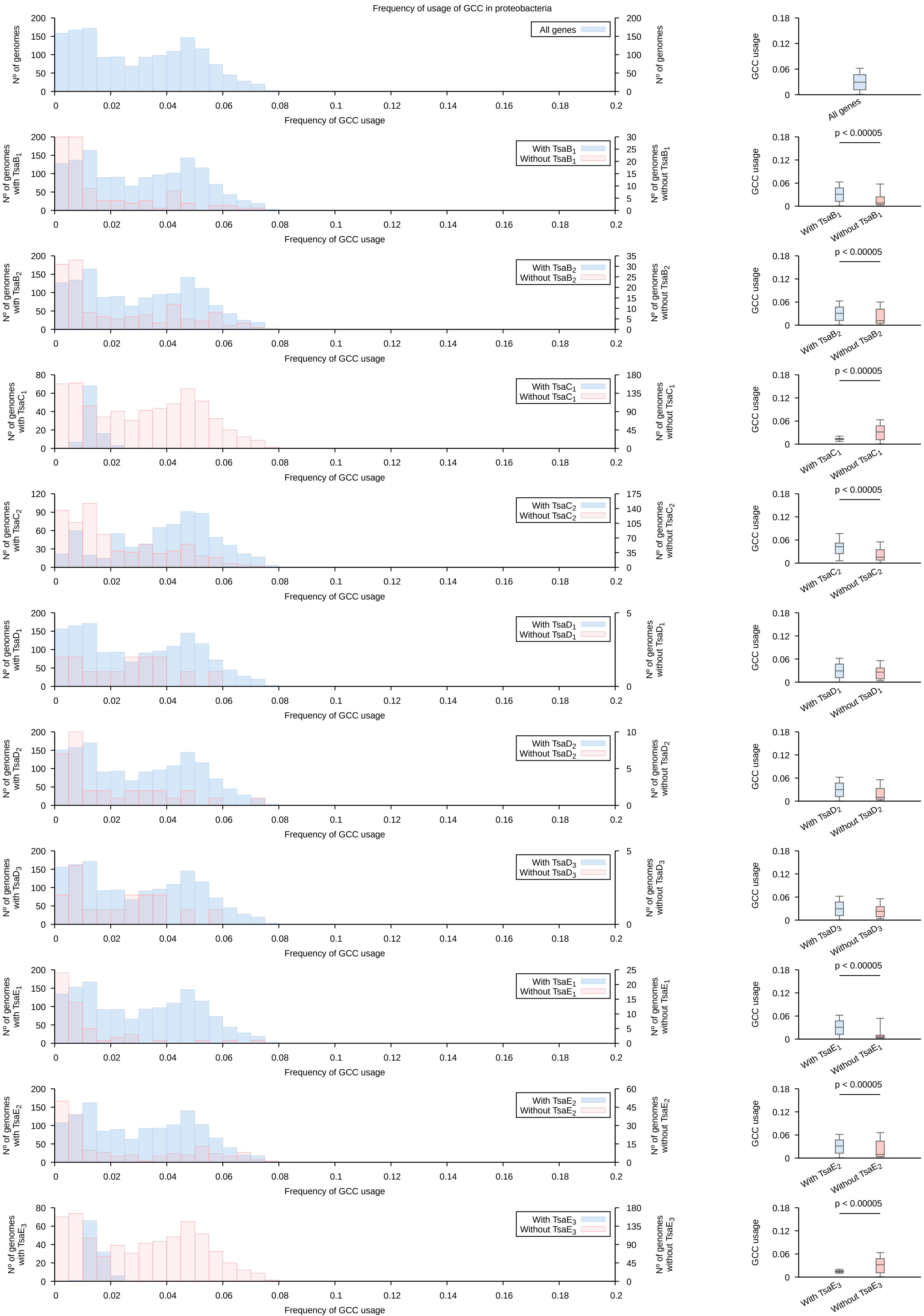
Frequency of usage of GCC in proteobacteria
Nº of genomes
All genes
GCC usage
Frequency of GCC usage
Nº of genomes with TsaB$_1$
Nº of genomes without TsaB$_1$
With TsaB$_1$
Without TsaB$_1$
p < 0.00005
Nº of genomes with TsaB$_2$
Nº of genomes without TsaB$_2$
With TsaB$_2$
Without TsaB$_2$
p < 0.00005
Nº of genomes with TsaC$_1$
Nº of genomes without TsaC$_1$
With TsaC$_1$
Without TsaC$_1$
p < 0.00005
Nº of genomes with TsaC$_2$
Nº of genomes without TsaC$_2$
With TsaC$_2$
Without TsaC$_2$
p < 0.00005
Nº of genomes with TsaD$_1$
Nº of genomes without TsaD$_1$
With TsaD$_1$
Without TsaD$_1$
Nº of genomes with TsaD$_2$
Nº of genomes without TsaD$_2$
With TsaD$_2$
Without TsaD$_2$
Nº of genomes with TsaD$_3$
Nº of genomes without TsaD$_3$
With TsaD$_3$
Without TsaD$_3$
Nº of genomes with TsaE$_1$
Nº of genomes without TsaE$_1$
With TsaE$_1$
Without TsaE$_1$
p < 0.00005
Nº of genomes with TsaE$_2$
Nº of genomes without TsaE$_2$
With TsaE$_2$
Without TsaE$_2$
p < 0.00005
Nº of genomes with TsaE$_3$
Nº of genomes without TsaE$_3$
With TsaE$_3$
Without TsaE$_3$
p < 0.00005

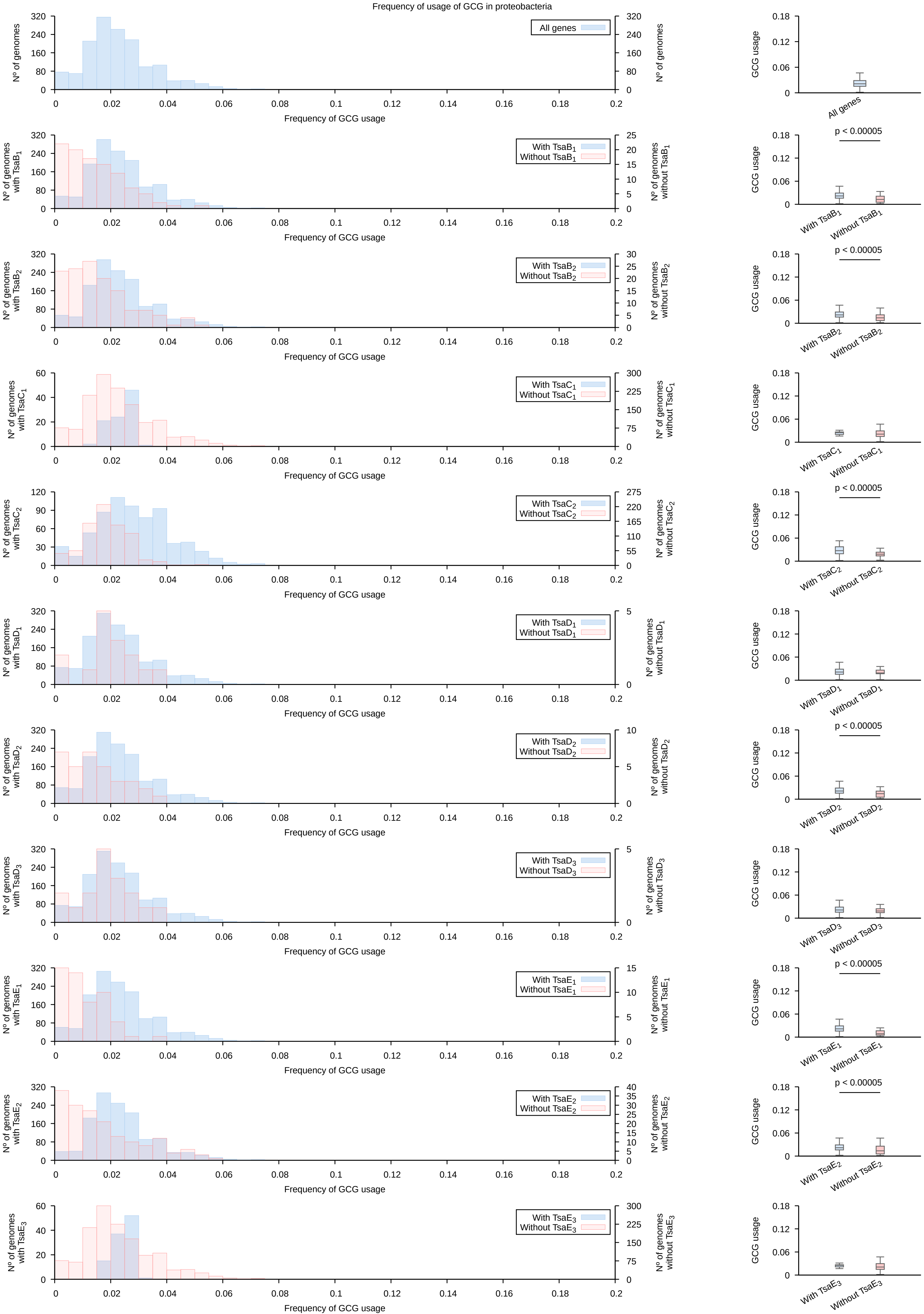

Frequency of usage of GCG in proteobacteria
All genes
Nº of genomes
Frequency of GCG usage
GCG usage
With TsaB1
Without TsaB1
Nº of genomes with TsaB1
Nº of genomes without TsaB1
p < 0.00005
With TsaB2
Without TsaB2
Nº of genomes with TsaB2
Nº of genomes without TsaB2
p < 0.00005
With TsaC1
Without TsaC1
Nº of genomes with TsaC1
Nº of genomes without TsaC1
With TsaC2
Without TsaC2
Nº of genomes with TsaC2
Nº of genomes without TsaC2
p < 0.00005
With TsaD1
Without TsaD1
Nº of genomes with TsaD1
Nº of genomes without TsaD1
With TsaD2
Without TsaD2
Nº of genomes with TsaD2
Nº of genomes without TsaD2
p < 0.00005
With TsaD3
Without TsaD3
Nº of genomes with TsaD3
Nº of genomes without TsaD3
With TsaE1
Without TsaE1
Nº of genomes with TsaE1
Nº of genomes without TsaE1
p < 0.00005
With TsaE2
Without TsaE2
Nº of genomes with TsaE2
Nº of genomes without TsaE2
p < 0.00005
With TsaE3
Without TsaE3
Nº of genomes with TsaE3
Nº of genomes without TsaE3

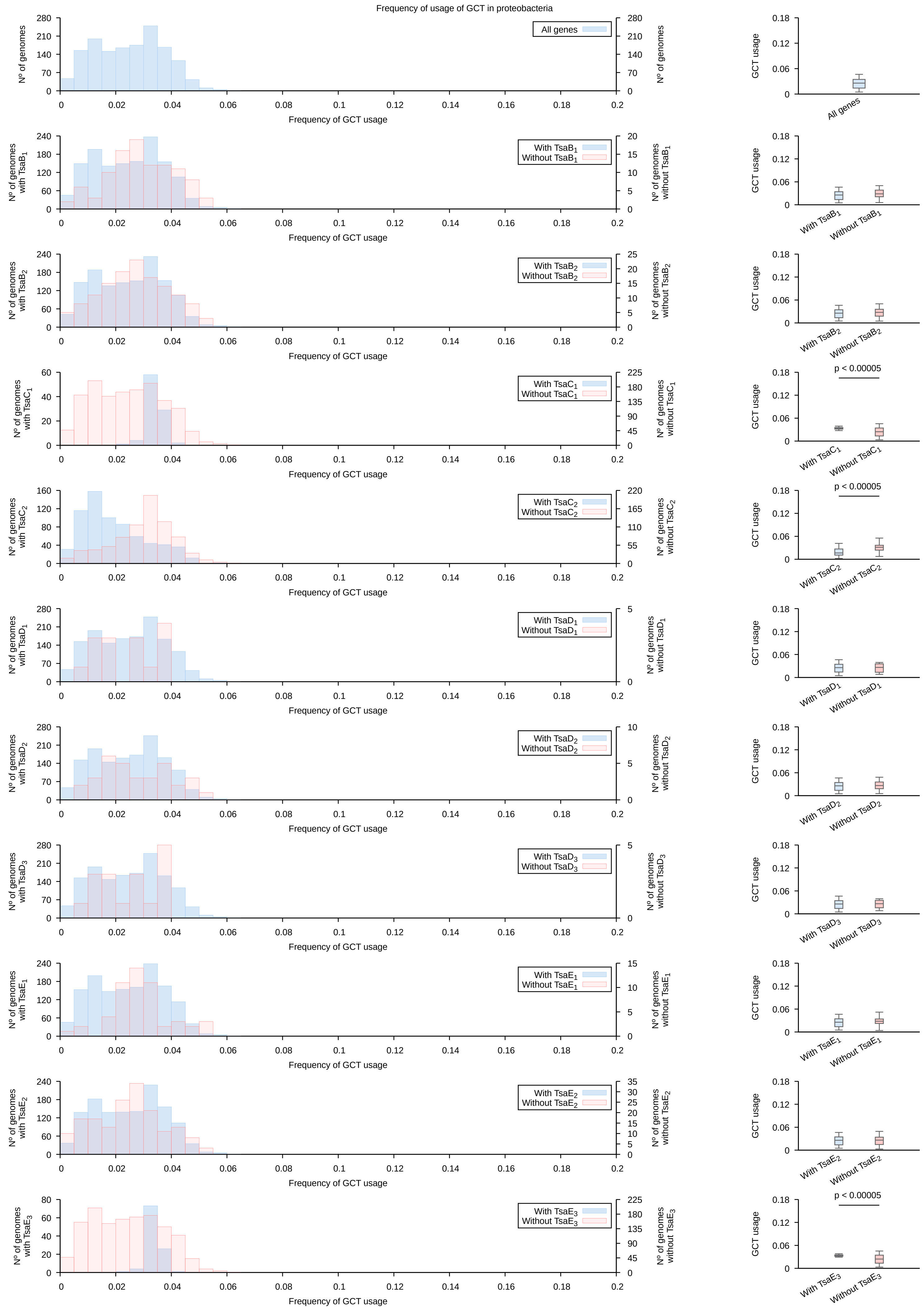

Frequency of usage of GCT in proteobacteria
All genes
Nº of genomes
Frequency of GCT usage
GCT usage
With $TsaB_1$
Without $TsaB_1$
Nº of genomes with $TsaB_1$
Nº of genomes without $TsaB_1$
With $TsaB_2$
Without $TsaB_2$
Nº of genomes with $TsaB_2$
Nº of genomes without $TsaB_2$
With $TsaC_1$
Without $TsaC_1$
Nº of genomes with $TsaC_1$
Nº of genomes without $TsaC_1$
p < 0.00005
With $TsaC_2$
Without $TsaC_2$
Nº of genomes with $TsaC_2$
Nº of genomes without $TsaC_2$
p < 0.00005
With $TsaD_1$
Without $TsaD_1$
Nº of genomes with $TsaD_1$
Nº of genomes without $TsaD_1$
With $TsaD_2$
Without $TsaD_2$
Nº of genomes with $TsaD_2$
Nº of genomes without $TsaD_2$
With $TsaD_3$
Without $TsaD_3$
Nº of genomes with $TsaD_3$
Nº of genomes without $TsaD_3$
With $TsaE_1$
Without $TsaE_1$
Nº of genomes with $TsaE_1$
Nº of genomes without $TsaE_1$
With $TsaE_2$
Without $TsaE_2$
Nº of genomes with $TsaE_2$
Nº of genomes without $TsaE_2$
With $TsaE_3$
Without $TsaE_3$
Nº of genomes with $TsaE_3$
Nº of genomes without $TsaE_3$
p < 0.00005

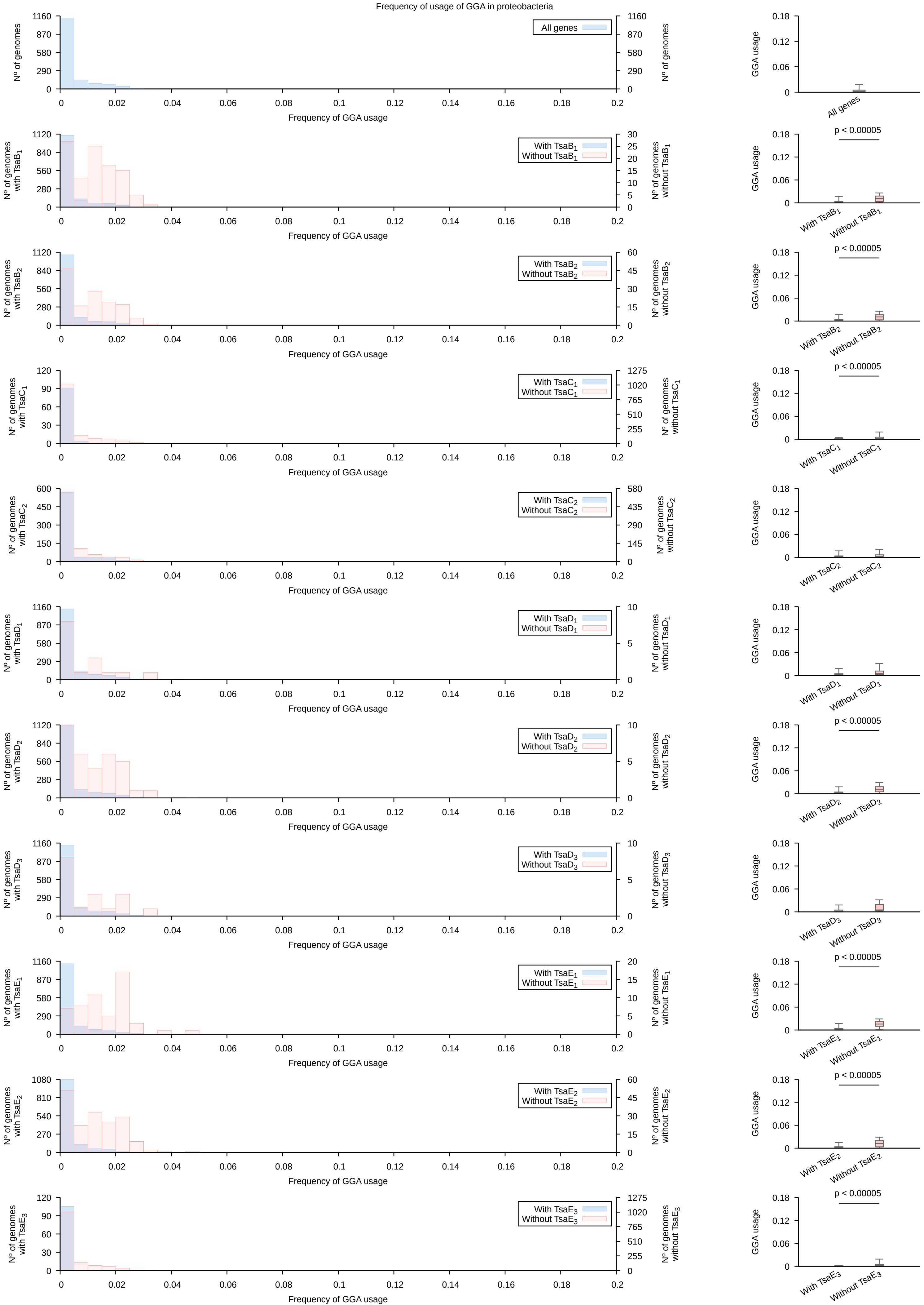

Frequency of usage of GGA in proteobacteria
All genes
Nº of genomes
Frequency of GGA usage
GGA usage
With TsaB$_1$
Without TsaB$_1$
Nº of genomes with TsaB$_1$
Nº of genomes without TsaB$_1$
p < 0.00005
With TsaB$_2$
Without TsaB$_2$
Nº of genomes with TsaB$_2$
Nº of genomes without TsaB$_2$
p < 0.00005
With TsaC$_1$
Without TsaC$_1$
Nº of genomes with TsaC$_1$
Nº of genomes without TsaC$_1$
p < 0.00005
With TsaC$_2$
Without TsaC$_2$
Nº of genomes with TsaC$_2$
Nº of genomes without TsaC$_2$
With TsaD$_1$
Without TsaD$_1$
Nº of genomes with TsaD$_1$
Nº of genomes without TsaD$_1$
With TsaD$_2$
Without TsaD$_2$
Nº of genomes with TsaD$_2$
Nº of genomes without TsaD$_2$
p < 0.00005
With TsaD$_3$
Without TsaD$_3$
Nº of genomes with TsaD$_3$
Nº of genomes without TsaD$_3$
With TsaE$_1$
Without TsaE$_1$
Nº of genomes with TsaE$_1$
Nº of genomes without TsaE$_1$
p < 0.00005
With TsaE$_2$
Without TsaE$_2$
Nº of genomes with TsaE$_2$
Nº of genomes without TsaE$_2$
p < 0.00005
With TsaE$_3$
Without TsaE$_3$
Nº of genomes with TsaE$_3$
Nº of genomes without TsaE$_3$
p < 0.00005

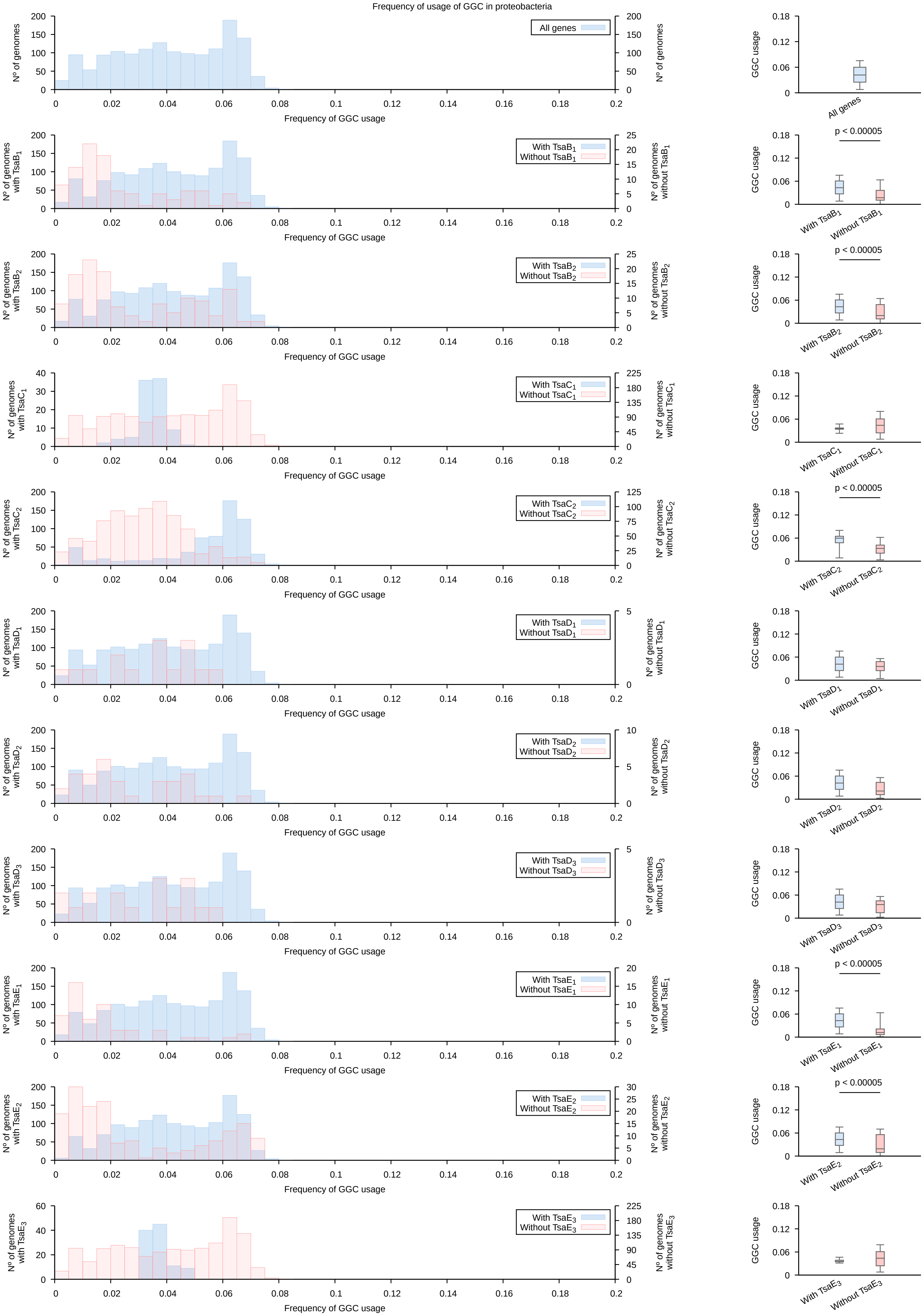
Frequency of usage of GGC in proteobacteria
All genes
Nº of genomes
Frequency of GGC usage
GGC usage
With TsaB1
Without TsaB1
Nº of genomes with TsaB1
Nº of genomes without TsaB1
p < 0.00005
With TsaB2
Without TsaB2
Nº of genomes with TsaB2
Nº of genomes without TsaB2
p < 0.00005
With TsaC1
Without TsaC1
Nº of genomes with TsaC1
Nº of genomes without TsaC1
With TsaC2
Without TsaC2
Nº of genomes with TsaC2
Nº of genomes without TsaC2
p < 0.00005
With TsaD1
Without TsaD1
Nº of genomes with TsaD1
Nº of genomes without TsaD1
With TsaD2
Without TsaD2
Nº of genomes with TsaD2
Nº of genomes without TsaD2
With TsaD3
Without TsaD3
Nº of genomes with TsaD3
Nº of genomes without TsaD3
With TsaE1
Without TsaE1
Nº of genomes with TsaE1
Nº of genomes without TsaE1
p < 0.00005
With TsaE2
Without TsaE2
Nº of genomes with TsaE2
Nº of genomes without TsaE2
p < 0.00005
With TsaE3
Without TsaE3
Nº of genomes with TsaE3
Nº of genomes without TsaE3

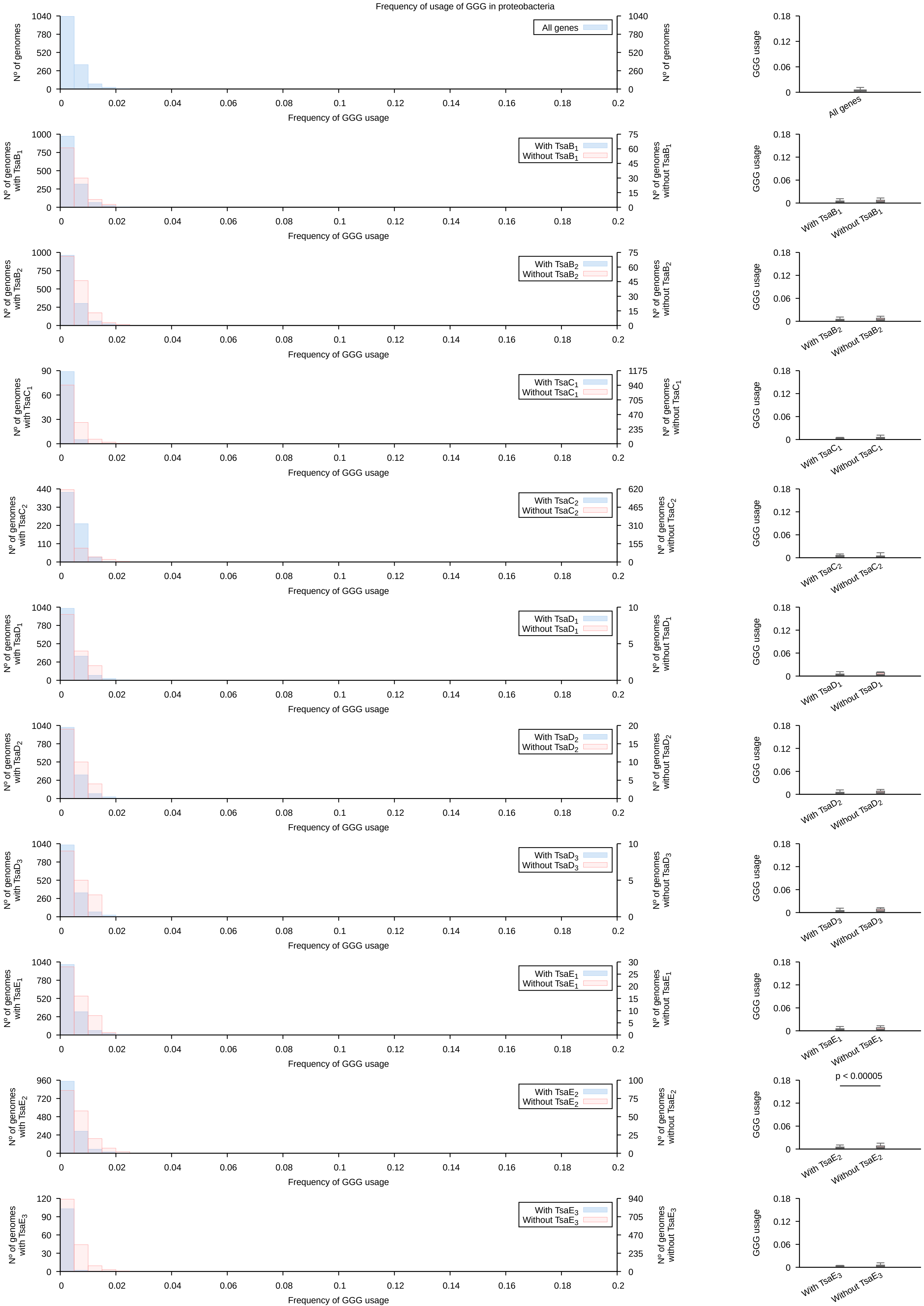
Frequency of usage of GGG in proteobacteria
All genes
Nº of genomes
Frequency of GGG usage
GGG usage
With TsaB$_1$
Without TsaB$_1$
Nº of genomes with TsaB$_1$
Nº of genomes without TsaB$_1$
With TsaB$_2$
Without TsaB$_2$
Nº of genomes with TsaB$_2$
Nº of genomes without TsaB$_2$
With TsaC$_1$
Without TsaC$_1$
Nº of genomes with TsaC$_1$
Nº of genomes without TsaC$_1$
With TsaC$_2$
Without TsaC$_2$
Nº of genomes with TsaC$_2$
Nº of genomes without TsaC$_2$
With TsaD$_1$
Without TsaD$_1$
Nº of genomes with TsaD$_1$
Nº of genomes without TsaD$_1$
With TsaD$_2$
Without TsaD$_2$
Nº of genomes with TsaD$_2$
Nº of genomes without TsaD$_2$
With TsaD$_3$
Without TsaD$_3$
Nº of genomes with TsaD$_3$
Nº of genomes without TsaD$_3$
With TsaE$_1$
Without TsaE$_1$
Nº of genomes with TsaE$_1$
Nº of genomes without TsaE$_1$
With TsaE$_2$
Without TsaE$_2$
Nº of genomes with TsaE$_2$
Nº of genomes without TsaE$_2$
p < 0.00005
With TsaE$_3$
Without TsaE$_3$
Nº of genomes with TsaE$_3$
Nº of genomes without TsaE$_3$

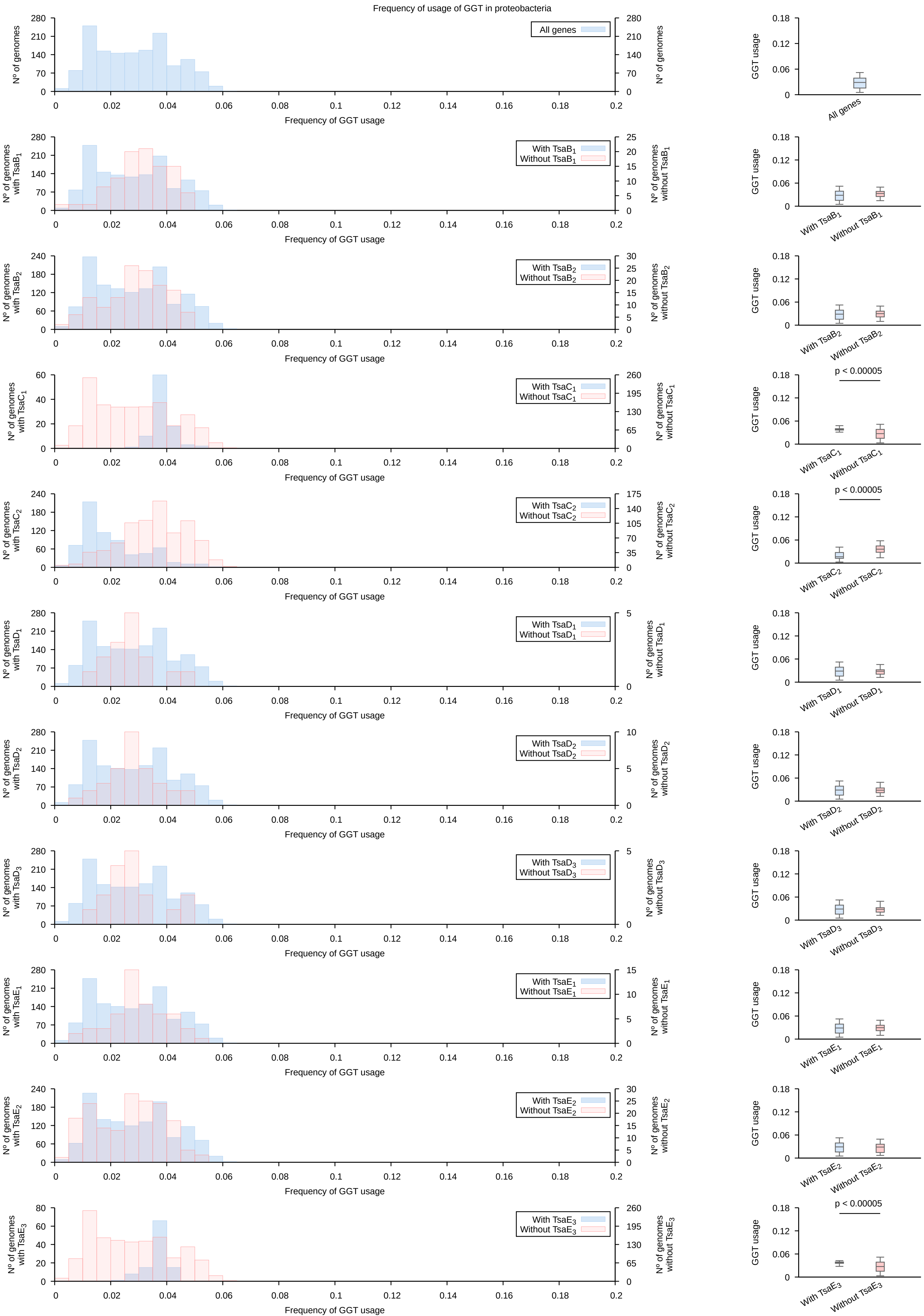
Frequency of usage of GGT in proteobacteria
All genes
With TsaB1
Without TsaB1
With TsaB2
Without TsaB2
With TsaC1
Without TsaC1
With TsaC2
Without TsaC2
With TsaD1
Without TsaD1
With TsaD2
Without TsaD2
With TsaD3
Without TsaD3
With TsaE1
Without TsaE1
With TsaE2
Without TsaE2
With TsaE3
Without TsaE3
Nº of genomes
Frequency of GGT usage
GGT usage
p < 0.00005

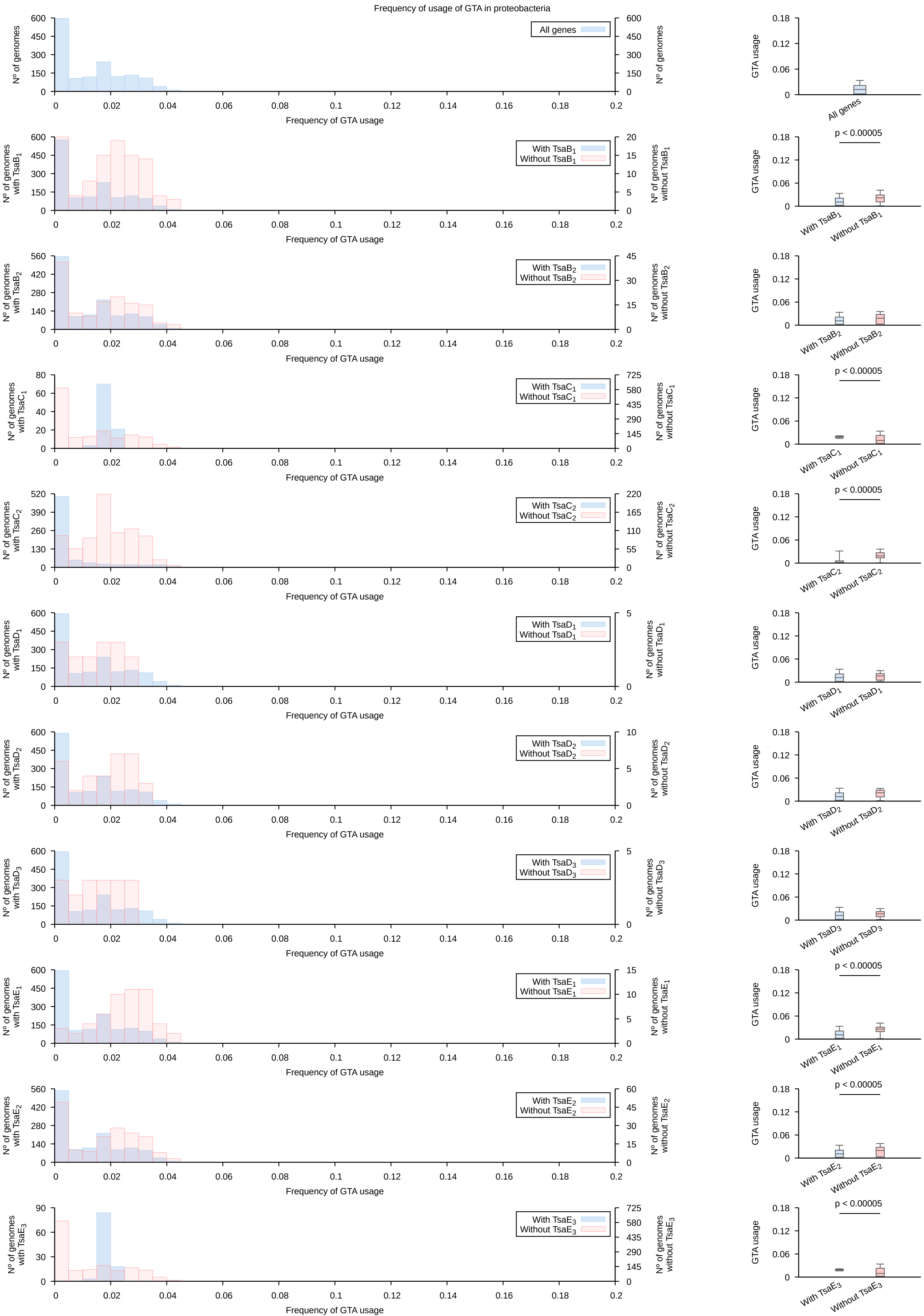

Frequency of usage of GTA in proteobacteria
All genes
Nº of genomes
Frequency of GTA usage
GTA usage
With TsaB$_1$
Without TsaB$_1$
Nº of genomes with TsaB$_1$
Nº of genomes without TsaB$_1$
p < 0.00005
With TsaB$_2$
Without TsaB$_2$
Nº of genomes with TsaB$_2$
Nº of genomes without TsaB$_2$
With TsaC$_1$
Without TsaC$_1$
Nº of genomes with TsaC$_1$
Nº of genomes without TsaC$_1$
p < 0.00005
With TsaC$_2$
Without TsaC$_2$
Nº of genomes with TsaC$_2$
Nº of genomes without TsaC$_2$
p < 0.00005
With TsaD$_1$
Without TsaD$_1$
Nº of genomes with TsaD$_1$
Nº of genomes without TsaD$_1$
With TsaD$_2$
Without TsaD$_2$
Nº of genomes with TsaD$_2$
Nº of genomes without TsaD$_2$
With TsaD$_3$
Without TsaD$_3$
Nº of genomes with TsaD$_3$
Nº of genomes without TsaD$_3$
With TsaE$_1$
Without TsaE$_1$
Nº of genomes with TsaE$_1$
Nº of genomes without TsaE$_1$
p < 0.00005
With TsaE$_2$
Without TsaE$_2$
Nº of genomes with TsaE$_2$
Nº of genomes without TsaE$_2$
p < 0.00005
With TsaE$_3$
Without TsaE$_3$
Nº of genomes with TsaE$_3$
Nº of genomes without TsaE$_3$
p < 0.00005

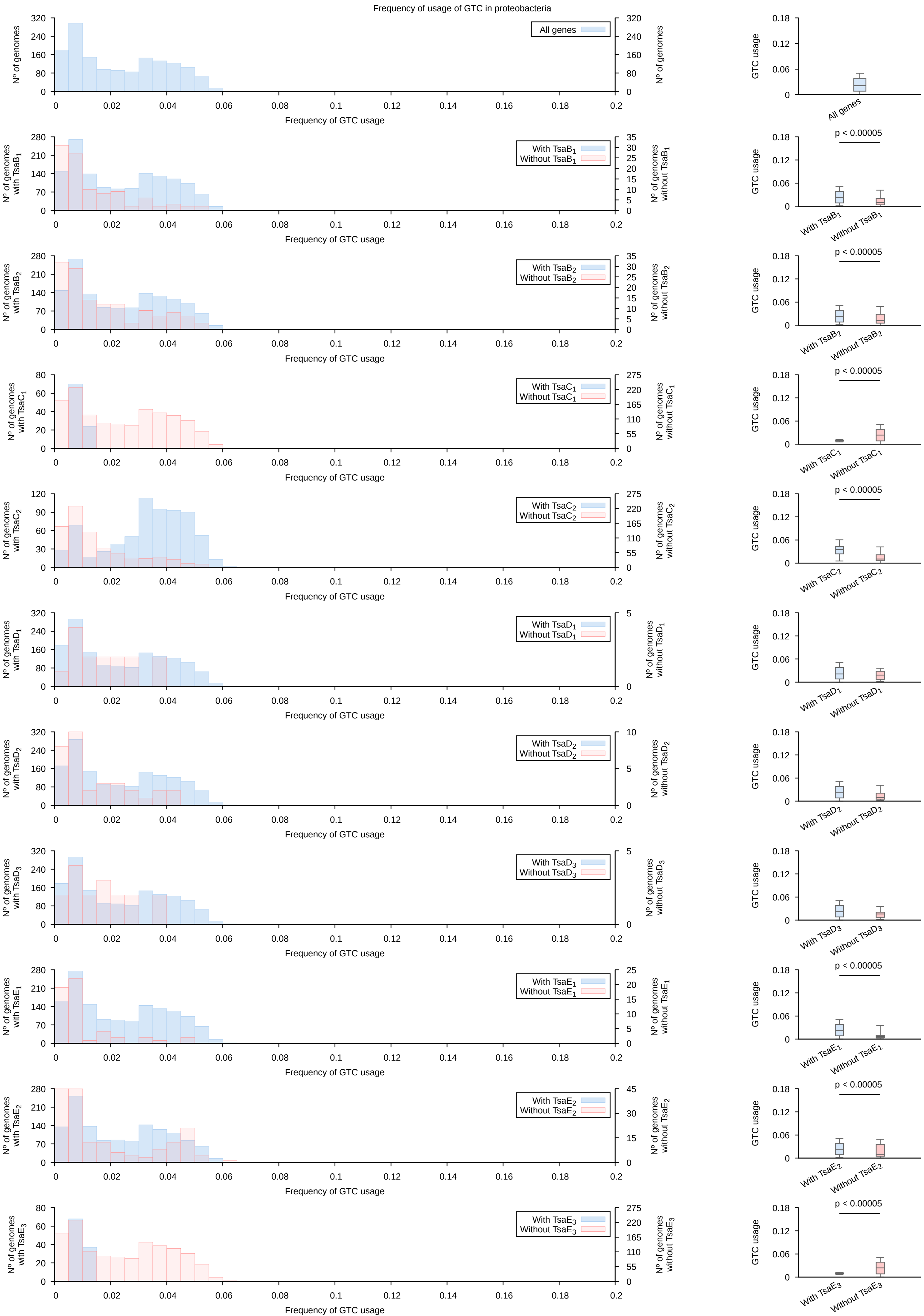
Frequency of usage of GTC in proteobacteria
All genes
Nº of genomes
Frequency of GTC usage
GTC usage
With TsaB$_1$
Without TsaB$_1$
Nº of genomes with TsaB$_1$
Nº of genomes without TsaB$_1$
p < 0.00005
With TsaB$_2$
Without TsaB$_2$
Nº of genomes with TsaB$_2$
Nº of genomes without TsaB$_2$
p < 0.00005
With TsaC$_1$
Without TsaC$_1$
Nº of genomes with TsaC$_1$
Nº of genomes without TsaC$_1$
p < 0.00005
With TsaC$_2$
Without TsaC$_2$
Nº of genomes with TsaC$_2$
Nº of genomes without TsaC$_2$
p < 0.00005
With TsaD$_1$
Without TsaD$_1$
Nº of genomes with TsaD$_1$
Nº of genomes without TsaD$_1$
With TsaD$_2$
Without TsaD$_2$
Nº of genomes with TsaD$_2$
Nº of genomes without TsaD$_2$
With TsaD$_3$
Without TsaD$_3$
Nº of genomes with TsaD$_3$
Nº of genomes without TsaD$_3$
With TsaE$_1$
Without TsaE$_1$
Nº of genomes with TsaE$_1$
Nº of genomes without TsaE$_1$
p < 0.00005
With TsaE$_2$
Without TsaE$_2$
Nº of genomes with TsaE$_2$
Nº of genomes without TsaE$_2$
p < 0.00005
With TsaE$_3$
Without TsaE$_3$
Nº of genomes with TsaE$_3$
Nº of genomes without TsaE$_3$
p < 0.00005

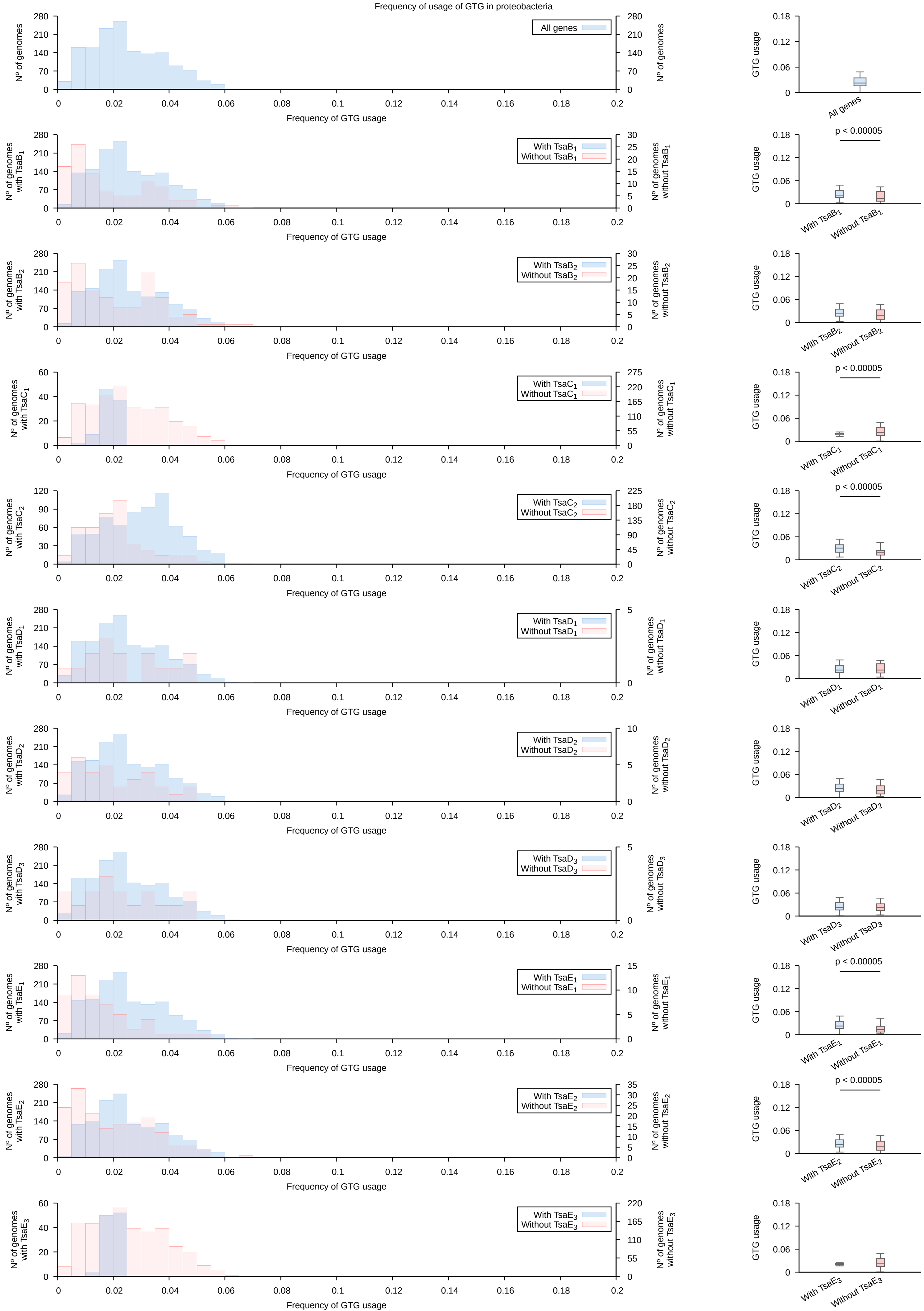
Frequency of usage of GTG in proteobacteria
All genes
With TsaB$_1$
Without TsaB$_1$
With TsaB$_2$
Without TsaB$_2$
With TsaC$_1$
Without TsaC$_1$
With TsaC$_2$
Without TsaC$_2$
With TsaD$_1$
Without TsaD$_1$
With TsaD$_2$
Without TsaD$_2$
With TsaD$_3$
Without TsaD$_3$
With TsaE$_1$
Without TsaE$_1$
With TsaE$_2$
Without TsaE$_2$
With TsaE$_3$
Without TsaE$_3$
Nº of genomes
Frequency of GTG usage
GTG usage
p < 0.00005

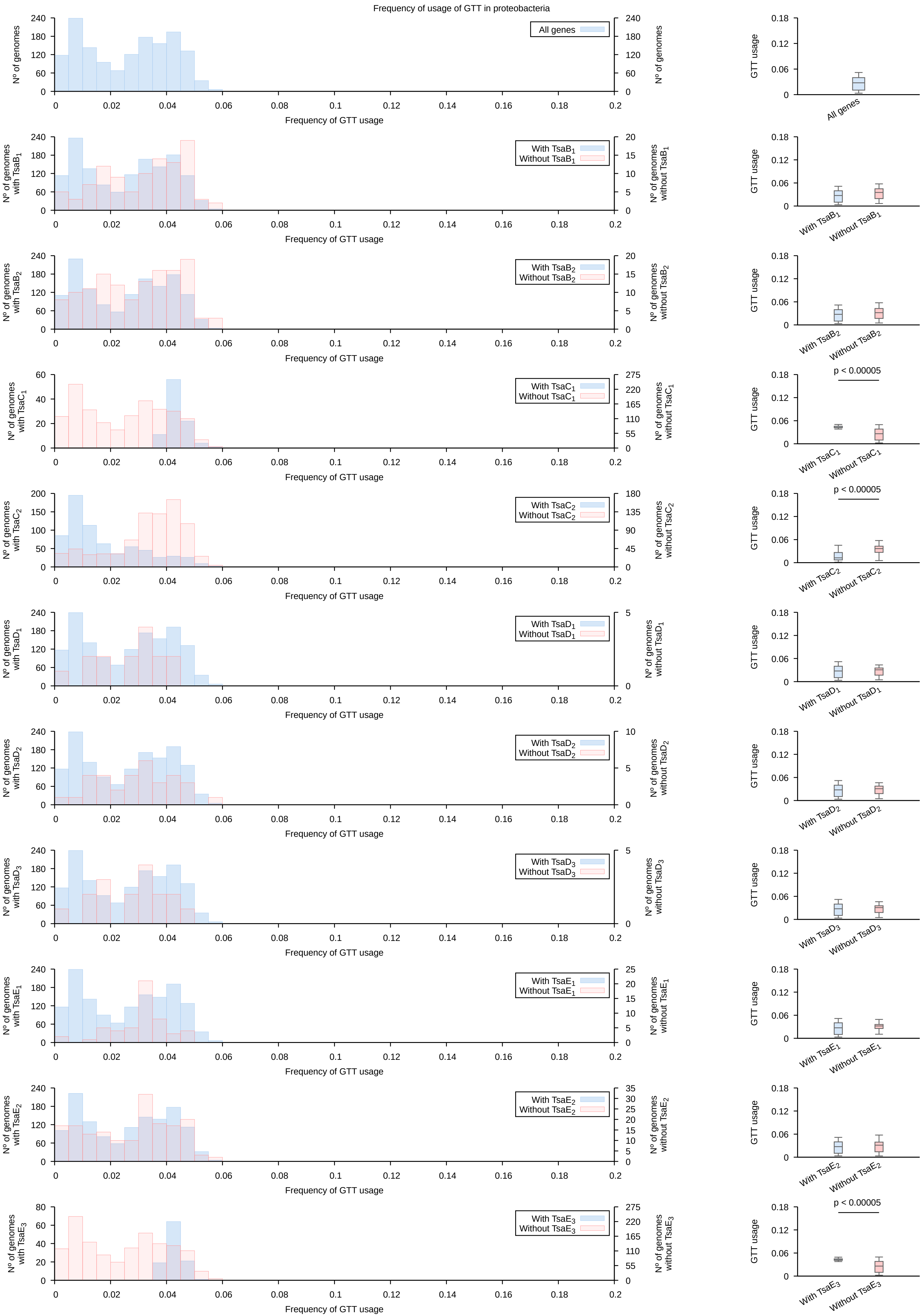
Frequency of usage of GTT in proteobacteria
Nº of genomes
Frequency of GTT usage
All genes
GTT usage
p < 0.00005
With TsaB1
Without TsaB1
With TsaB2
Without TsaB2
With TsaC1
Without TsaC1
With TsaC2
Without TsaC2
With TsaD1
Without TsaD1
With TsaD2
Without TsaD2
With TsaD3
Without TsaD3
With TsaE1
Without TsaE1
With TsaE2
Without TsaE2
With TsaE3
Without TsaE3

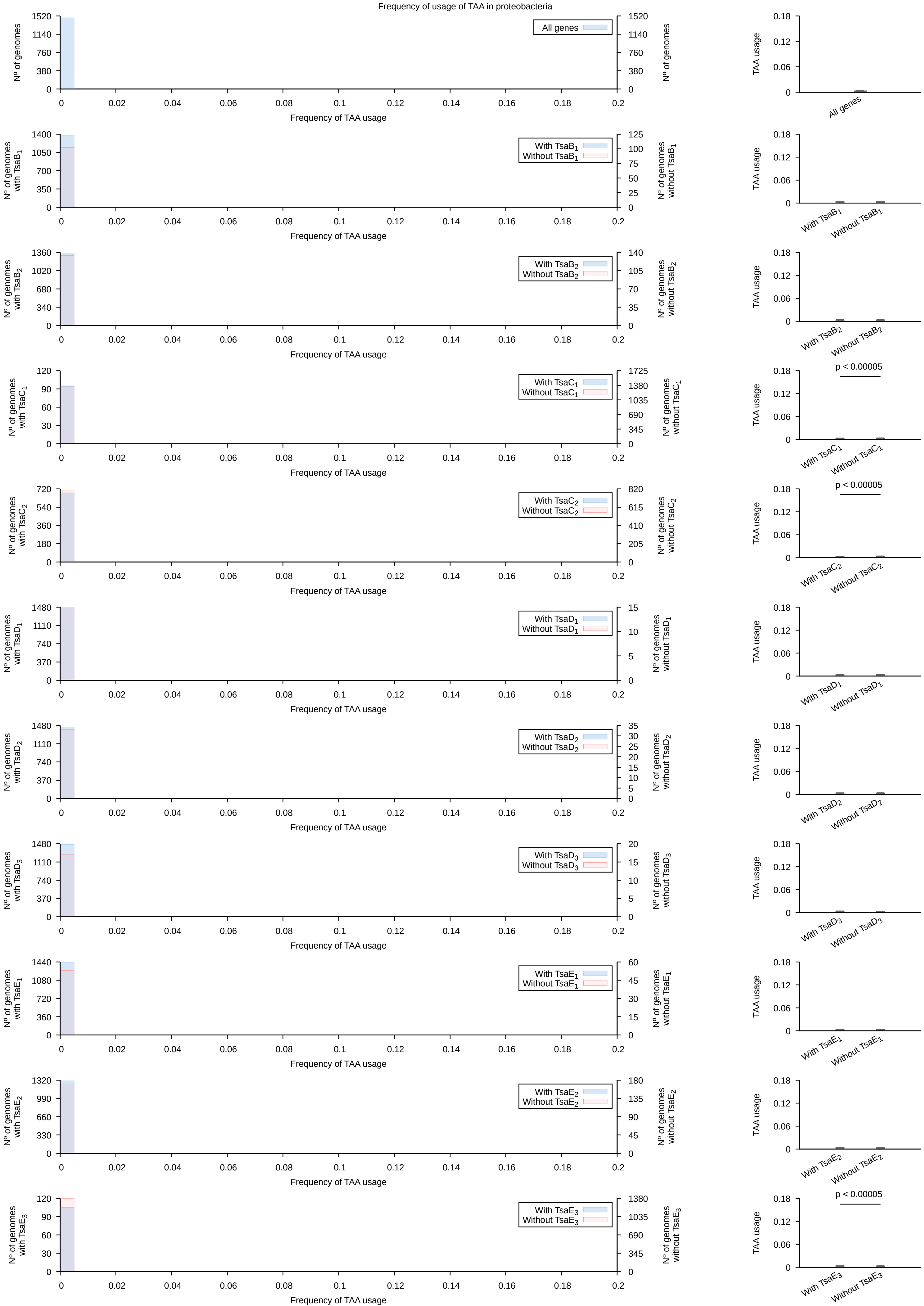
Frequency of usage of TAA in proteobacteria
Nº of genomes
All genes
Frequency of TAA usage
TAA usage
All genes
Nº of genomes with TsaB1
Nº of genomes without TsaB1
With TsaB1
Without TsaB1
Nº of genomes with TsaB2
Nº of genomes without TsaB2
With TsaB2
Without TsaB2
Nº of genomes with TsaC1
Nº of genomes without TsaC1
With TsaC1
Without TsaC1
p < 0.00005
Nº of genomes with TsaC2
Nº of genomes without TsaC2
With TsaC2
Without TsaC2
p < 0.00005
Nº of genomes with TsaD1
Nº of genomes without TsaD1
With TsaD1
Without TsaD1
Nº of genomes with TsaD2
Nº of genomes without TsaD2
With TsaD2
Without TsaD2
Nº of genomes with TsaD3
Nº of genomes without TsaD3
With TsaD3
Without TsaD3
Nº of genomes with TsaE1
Nº of genomes without TsaE1
With TsaE1
Without TsaE1
Nº of genomes with TsaE2
Nº of genomes without TsaE2
With TsaE2
Without TsaE2
Nº of genomes with TsaE3
Nº of genomes without TsaE3
With TsaE3
Without TsaE3
p < 0.00005

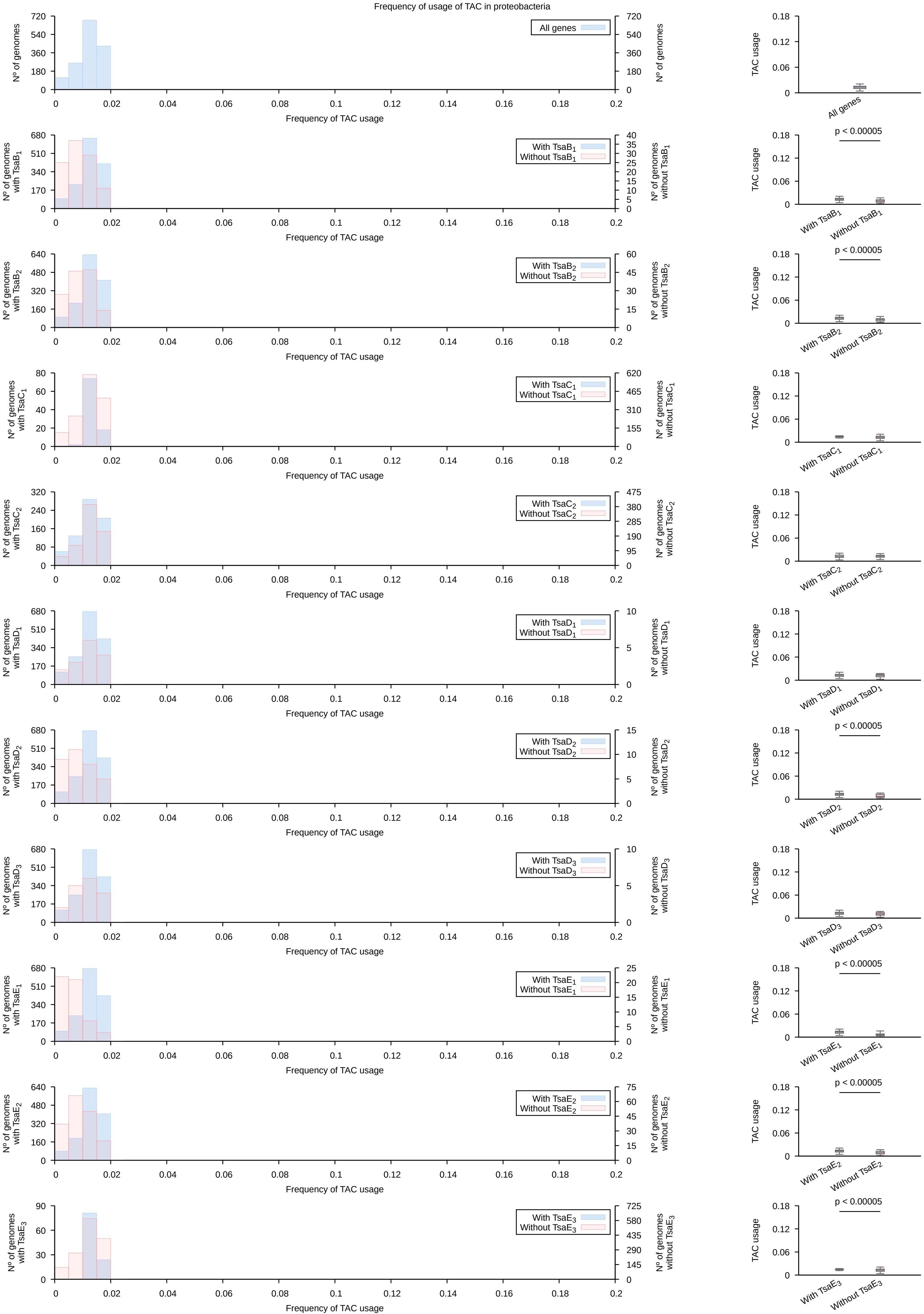
Frequency of usage of TAC in proteobacteria
All genes
Nº of genomes
Frequency of TAC usage
TAC usage
With TsaB$_1$
Without TsaB$_1$
Nº of genomes with TsaB$_1$
Nº of genomes without TsaB$_1$
p < 0.00005
With TsaB$_2$
Without TsaB$_2$
Nº of genomes with TsaB$_2$
Nº of genomes without TsaB$_2$
p < 0.00005
With TsaC$_1$
Without TsaC$_1$
Nº of genomes with TsaC$_1$
Nº of genomes without TsaC$_1$
With TsaC$_2$
Without TsaC$_2$
Nº of genomes with TsaC$_2$
Nº of genomes without TsaC$_2$
With TsaD$_1$
Without TsaD$_1$
Nº of genomes with TsaD$_1$
Nº of genomes without TsaD$_1$
With TsaD$_2$
Without TsaD$_2$
Nº of genomes with TsaD$_2$
Nº of genomes without TsaD$_2$
p < 0.00005
With TsaD$_3$
Without TsaD$_3$
Nº of genomes with TsaD$_3$
Nº of genomes without TsaD$_3$
With TsaE$_1$
Without TsaE$_1$
Nº of genomes with TsaE$_1$
Nº of genomes without TsaE$_1$
p < 0.00005
With TsaE$_2$
Without TsaE$_2$
Nº of genomes with TsaE$_2$
Nº of genomes without TsaE$_2$
p < 0.00005
With TsaE$_3$
Without TsaE$_3$
Nº of genomes with TsaE$_3$
Nº of genomes without TsaE$_3$
p < 0.00005

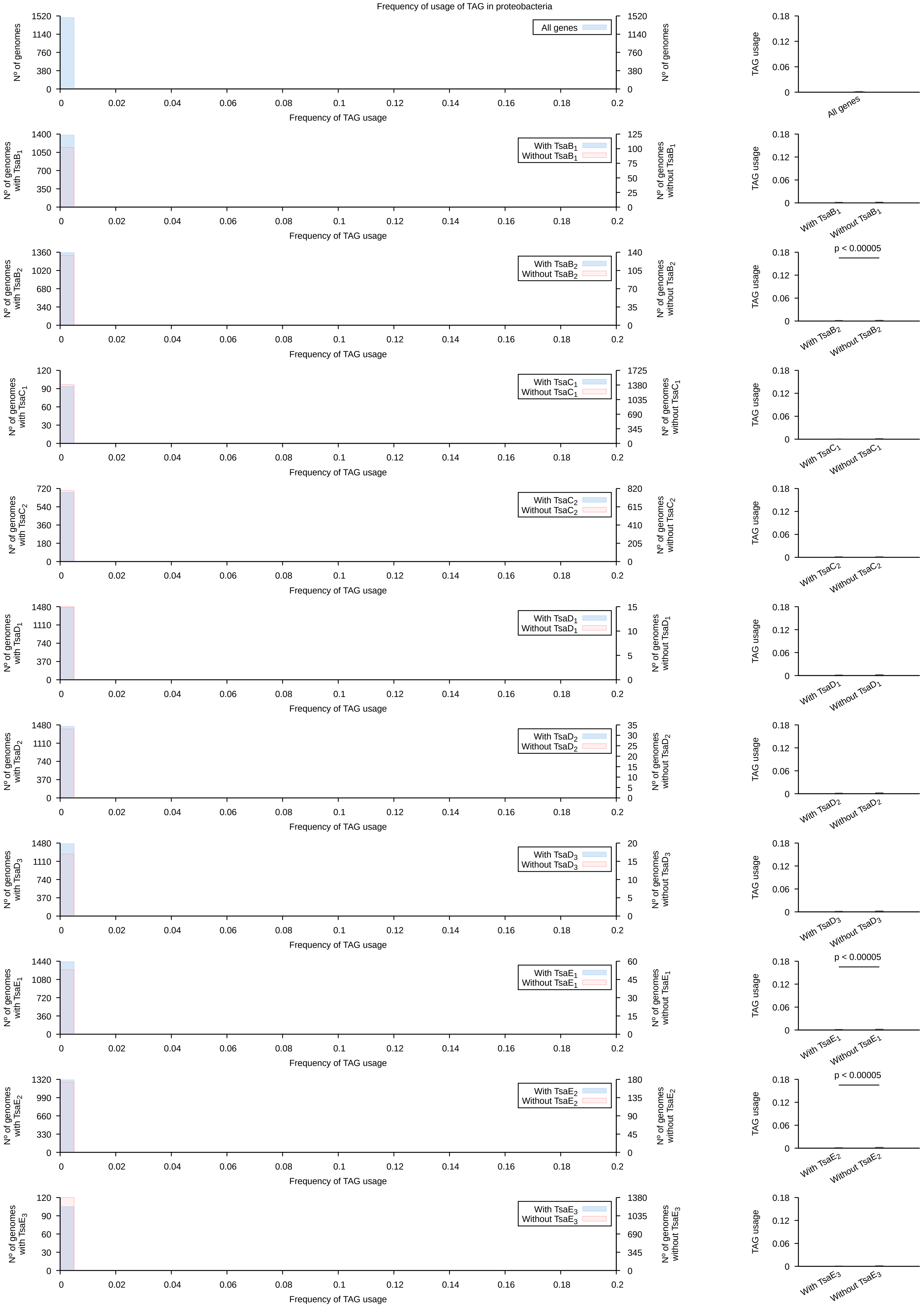

Frequency of usage of TAG in proteobacteria
Nº of genomes
Frequency of TAG usage
TAG usage
All genes
p < 0.00005
With TsaB$_1$
Without TsaB$_1$
Nº of genomes with TsaB$_1$
Nº of genomes without TsaB$_1$
With TsaB$_2$
Without TsaB$_2$
Nº of genomes with TsaB$_2$
Nº of genomes without TsaB$_2$
With TsaC$_1$
Without TsaC$_1$
Nº of genomes with TsaC$_1$
Nº of genomes without TsaC$_1$
With TsaC$_2$
Without TsaC$_2$
Nº of genomes with TsaC$_2$
Nº of genomes without TsaC$_2$
With TsaD$_1$
Without TsaD$_1$
Nº of genomes with TsaD$_1$
Nº of genomes without TsaD$_1$
With TsaD$_2$
Without TsaD$_2$
Nº of genomes with TsaD$_2$
Nº of genomes without TsaD$_2$
With TsaD$_3$
Without TsaD$_3$
Nº of genomes with TsaD$_3$
Nº of genomes without TsaD$_3$
With TsaE$_1$
Without TsaE$_1$
Nº of genomes with TsaE$_1$
Nº of genomes without TsaE$_1$
With TsaE$_2$
Without TsaE$_2$
Nº of genomes with TsaE$_2$
Nº of genomes without TsaE$_2$
With TsaE$_3$
Without TsaE$_3$
Nº of genomes with TsaE$_3$
Nº of genomes without TsaE$_3$

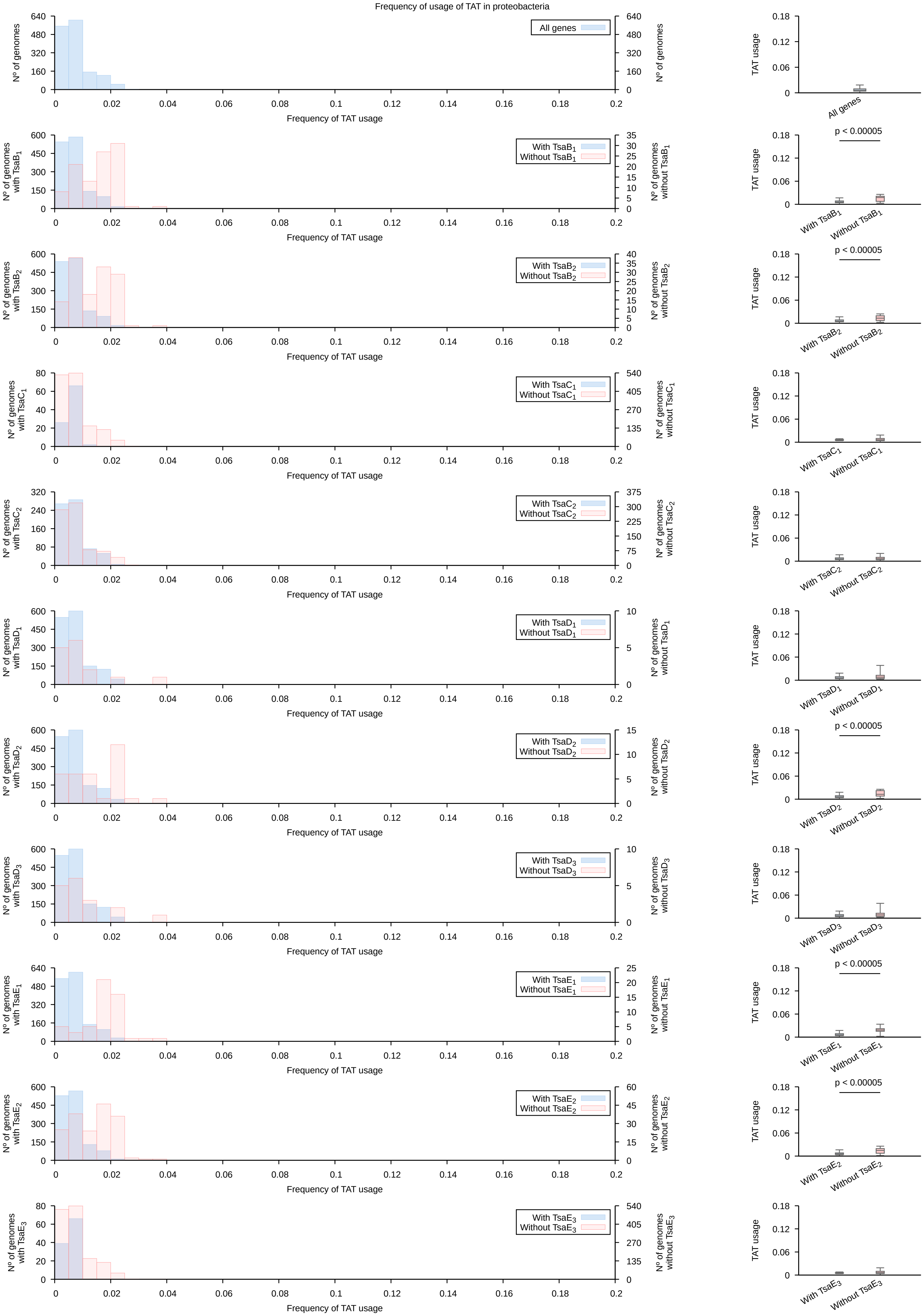

Frequency of usage of TAT in proteobacteria
Nº of genomes
Frequency of TAT usage
All genes
TAT usage
Nº of genomes with $TsaB_1$
Nº of genomes without $TsaB_1$
With $TsaB_1$
Without $TsaB_1$
p < 0.00005
Nº of genomes with $TsaB_2$
Nº of genomes without $TsaB_2$
With $TsaB_2$
Without $TsaB_2$
p < 0.00005
Nº of genomes with $TsaC_1$
Nº of genomes without $TsaC_1$
With $TsaC_1$
Without $TsaC_1$
Nº of genomes with $TsaC_2$
Nº of genomes without $TsaC_2$
With $TsaC_2$
Without $TsaC_2$
Nº of genomes with $TsaD_1$
Nº of genomes without $TsaD_1$
With $TsaD_1$
Without $TsaD_1$
Nº of genomes with $TsaD_2$
Nº of genomes without $TsaD_2$
With $TsaD_2$
Without $TsaD_2$
p < 0.00005
Nº of genomes with $TsaD_3$
Nº of genomes without $TsaD_3$
With $TsaD_3$
Without $TsaD_3$
Nº of genomes with $TsaE_1$
Nº of genomes without $TsaE_1$
With $TsaE_1$
Without $TsaE_1$
p < 0.00005
Nº of genomes with $TsaE_2$
Nº of genomes without $TsaE_2$
With $TsaE_2$
Without $TsaE_2$
p < 0.00005
Nº of genomes with $TsaE_3$
Nº of genomes without $TsaE_3$
With $TsaE_3$
Without $TsaE_3$

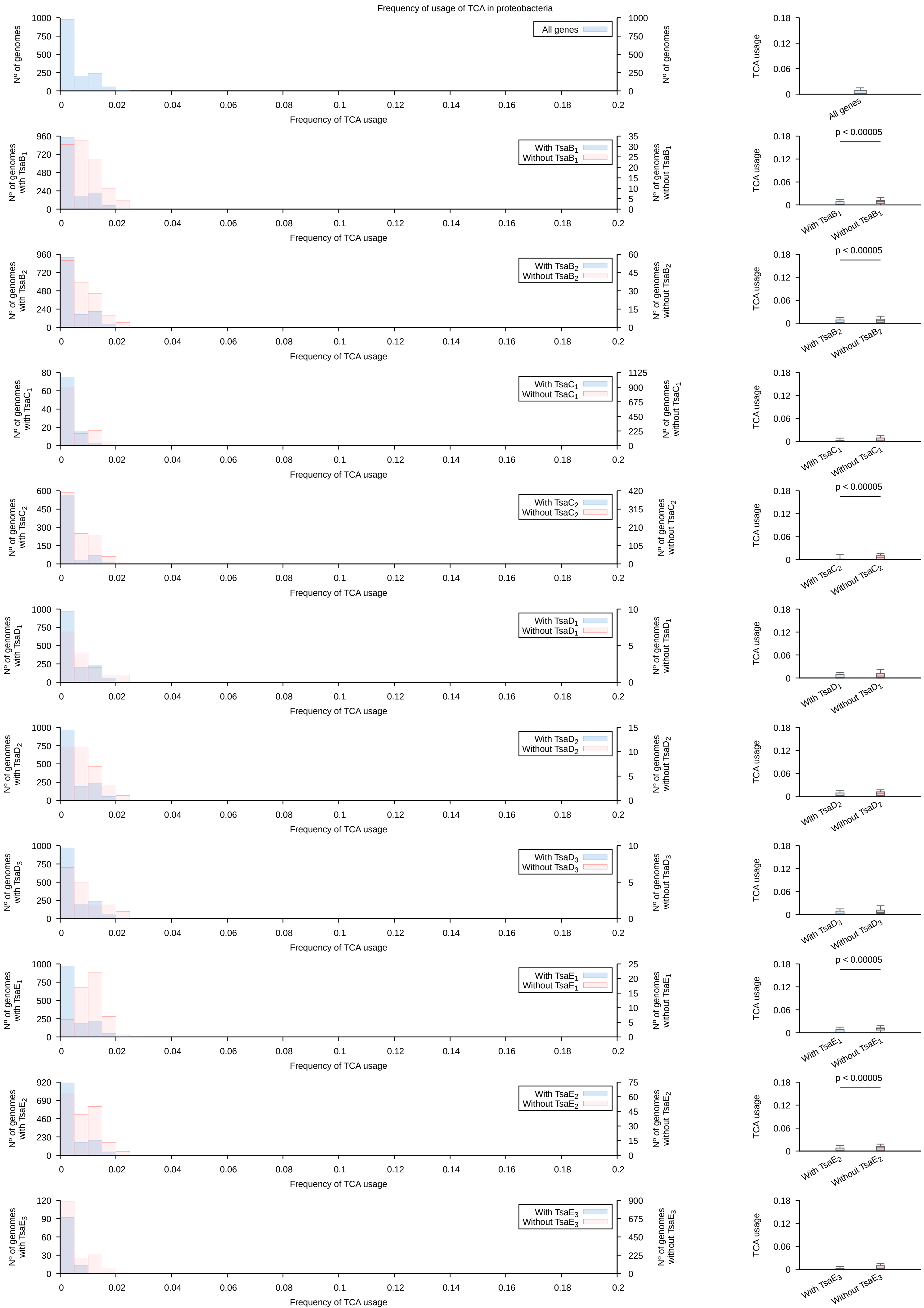

Frequency of usage of TCA in proteobacteria
All genes
Nº of genomes
Frequency of TCA usage
TCA usage
With TsaB$_1$
Without TsaB$_1$
Nº of genomes with TsaB$_1$
Nº of genomes without TsaB$_1$
p < 0.00005
With TsaB$_2$
Without TsaB$_2$
Nº of genomes with TsaB$_2$
Nº of genomes without TsaB$_2$
p < 0.00005
With TsaC$_1$
Without TsaC$_1$
Nº of genomes with TsaC$_1$
Nº of genomes without TsaC$_1$
With TsaC$_2$
Without TsaC$_2$
Nº of genomes with TsaC$_2$
Nº of genomes without TsaC$_2$
p < 0.00005
With TsaD$_1$
Without TsaD$_1$
Nº of genomes with TsaD$_1$
Nº of genomes without TsaD$_1$
With TsaD$_2$
Without TsaD$_2$
Nº of genomes with TsaD$_2$
Nº of genomes without TsaD$_2$
With TsaD$_3$
Without TsaD$_3$
Nº of genomes with TsaD$_3$
Nº of genomes without TsaD$_3$
With TsaE$_1$
Without TsaE$_1$
Nº of genomes with TsaE$_1$
Nº of genomes without TsaE$_1$
p < 0.00005
With TsaE$_2$
Without TsaE$_2$
Nº of genomes with TsaE$_2$
Nº of genomes without TsaE$_2$
p < 0.00005
With TsaE$_3$
Without TsaE$_3$
Nº of genomes with TsaE$_3$
Nº of genomes without TsaE$_3$

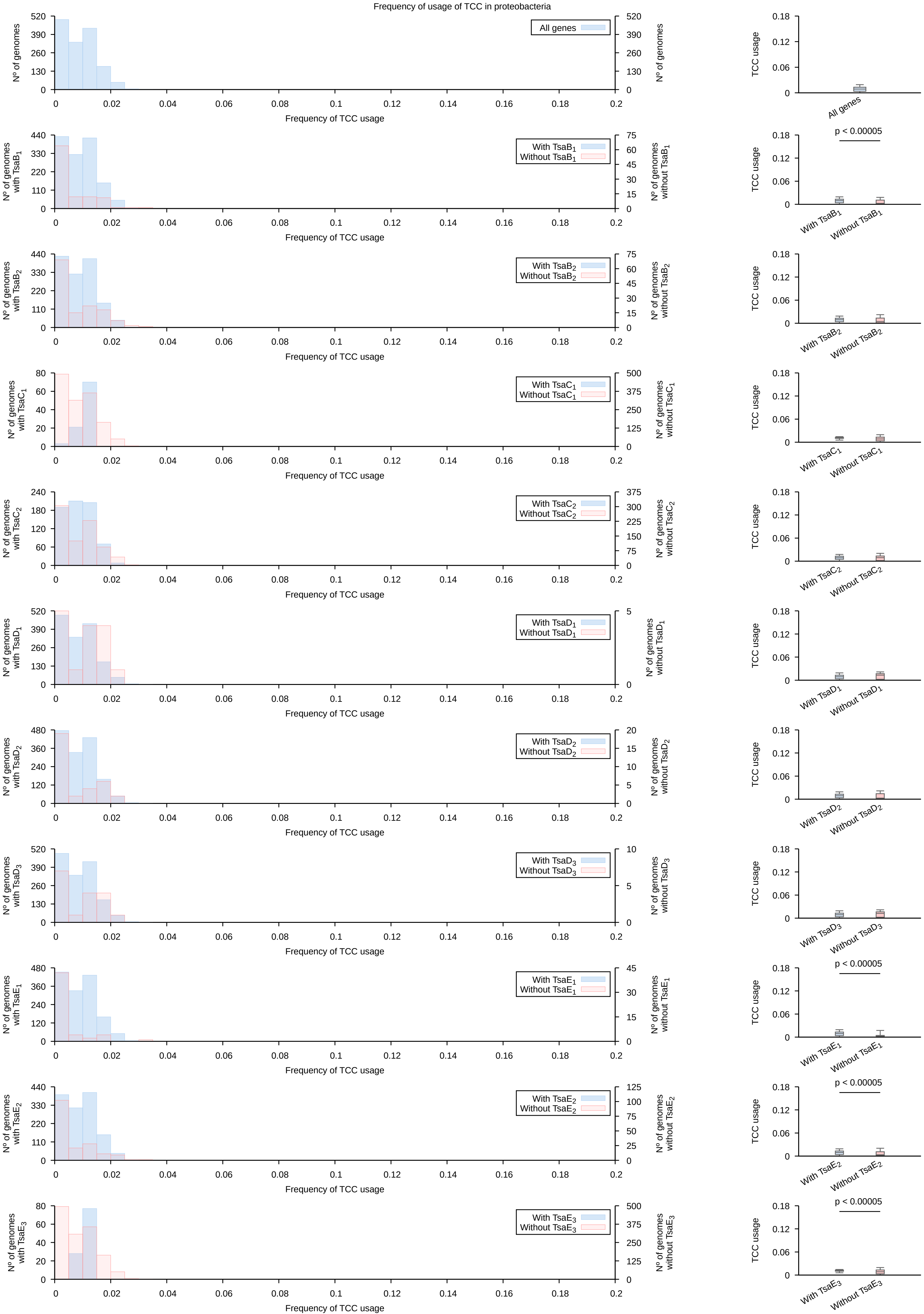

Frequency of usage of TCC in proteobacteria
All genes
Nº of genomes
Frequency of TCC usage
TCC usage
With TsaB1
Without TsaB1
Nº of genomes with TsaB1
Nº of genomes without TsaB1
p < 0.00005
With TsaB2
Without TsaB2
Nº of genomes with TsaB2
Nº of genomes without TsaB2
With TsaC1
Without TsaC1
Nº of genomes with TsaC1
Nº of genomes without TsaC1
With TsaC2
Without TsaC2
Nº of genomes with TsaC2
Nº of genomes without TsaC2
With TsaD1
Without TsaD1
Nº of genomes with TsaD1
Nº of genomes without TsaD1
With TsaD2
Without TsaD2
Nº of genomes with TsaD2
Nº of genomes without TsaD2
With TsaD3
Without TsaD3
Nº of genomes with TsaD3
Nº of genomes without TsaD3
With TsaE1
Without TsaE1
Nº of genomes with TsaE1
Nº of genomes without TsaE1
p < 0.00005
With TsaE2
Without TsaE2
Nº of genomes with TsaE2
Nº of genomes without TsaE2
p < 0.00005
With TsaE3
Without TsaE3
Nº of genomes with TsaE3
Nº of genomes without TsaE3
p < 0.00005

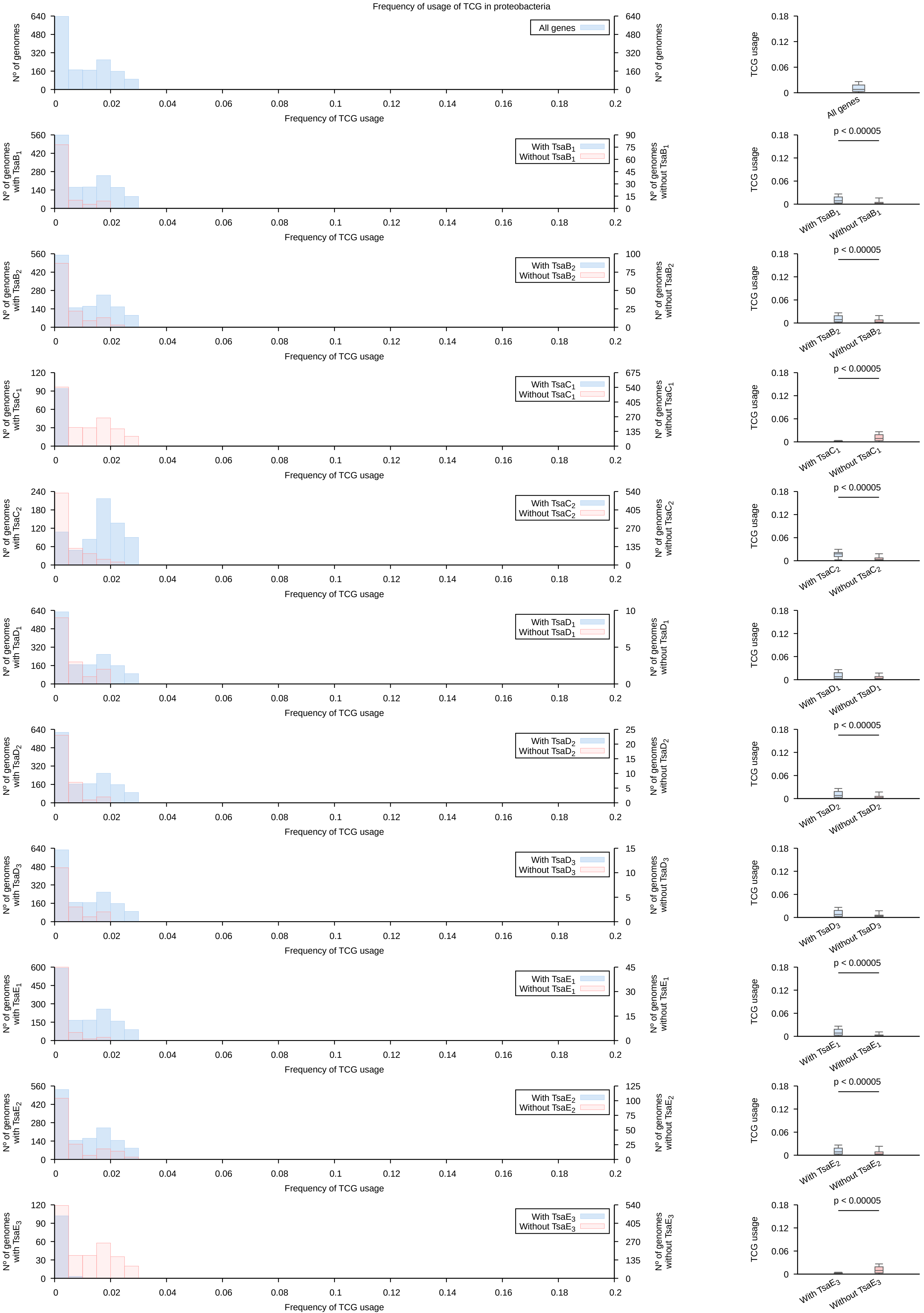

Frequency of usage of TCG in proteobacteria
All genes
Nº of genomes
Frequency of TCG usage
TCG usage
0
0.02
0.04
0.06
0.08
0.1
0.12
0.14
0.16
0.18
0.2
0.06
0.12
0.18
With TsaB$_1$
Without TsaB$_1$
Nº of genomes with TsaB$_1$
Nº of genomes without TsaB$_1$
p < 0.00005
With TsaB$_2$
Without TsaB$_2$
Nº of genomes with TsaB$_2$
Nº of genomes without TsaB$_2$
p < 0.00005
With TsaC$_1$
Without TsaC$_1$
Nº of genomes with TsaC$_1$
Nº of genomes without TsaC$_1$
p < 0.00005
With TsaC$_2$
Without TsaC$_2$
Nº of genomes with TsaC$_2$
Nº of genomes without TsaC$_2$
p < 0.00005
With TsaD$_1$
Without TsaD$_1$
Nº of genomes with TsaD$_1$
Nº of genomes without TsaD$_1$
With TsaD$_2$
Without TsaD$_2$
Nº of genomes with TsaD$_2$
Nº of genomes without TsaD$_2$
p < 0.00005
With TsaD$_3$
Without TsaD$_3$
Nº of genomes with TsaD$_3$
Nº of genomes without TsaD$_3$
With TsaE$_1$
Without TsaE$_1$
Nº of genomes with TsaE$_1$
Nº of genomes without TsaE$_1$
p < 0.00005
With TsaE$_2$
Without TsaE$_2$
Nº of genomes with TsaE$_2$
Nº of genomes without TsaE$_2$
p < 0.00005
With TsaE$_3$
Without TsaE$_3$
Nº of genomes with TsaE$_3$
Nº of genomes without TsaE$_3$
p < 0.00005

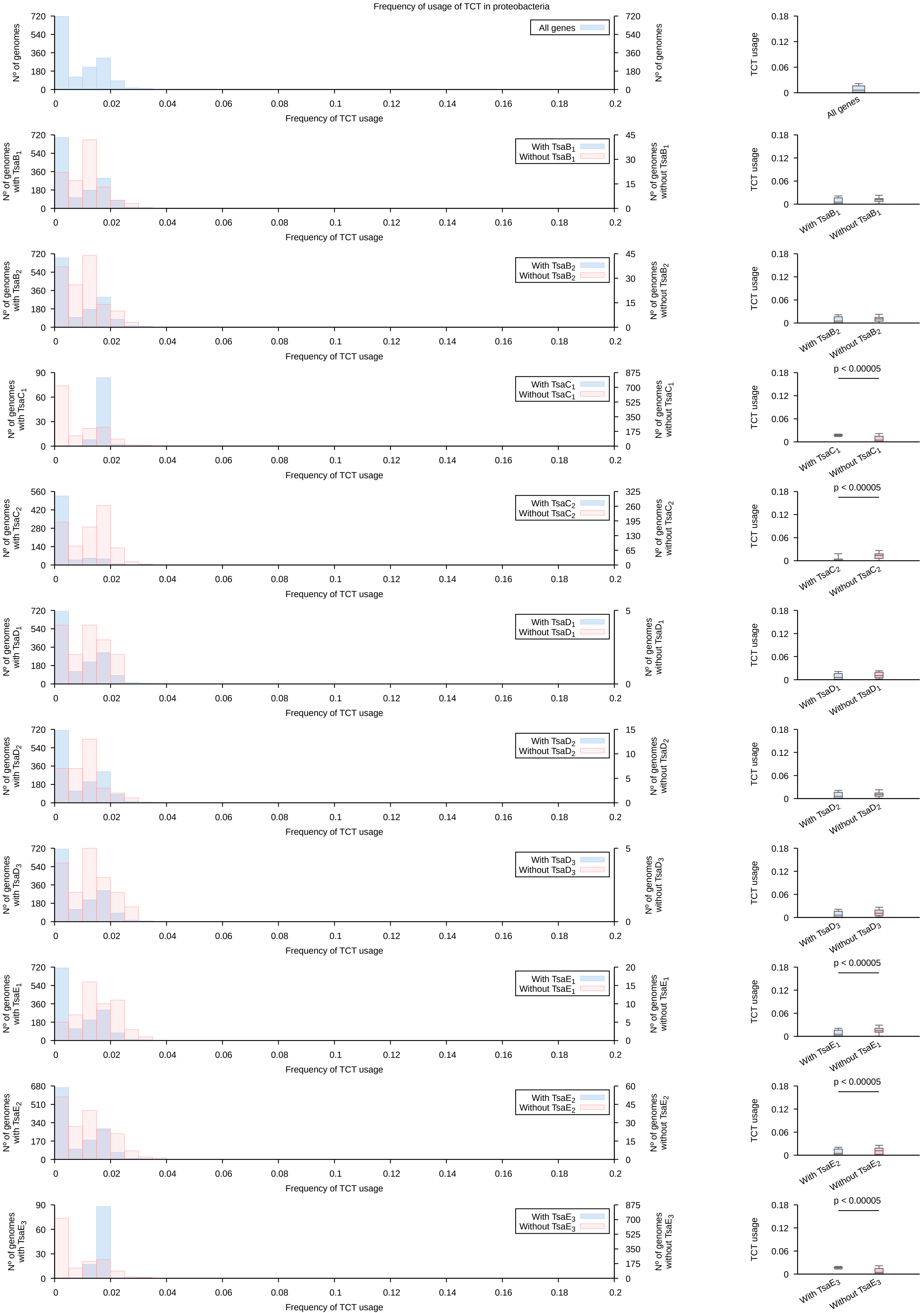

Frequency of usage of TCT in proteobacteria
All genes
Nº of genomes
TCT usage
Frequency of TCT usage
With TsaB$_1$
Without TsaB$_1$
Nº of genomes with TsaB$_1$
Nº of genomes without TsaB$_1$
With TsaB$_2$
Without TsaB$_2$
Nº of genomes with TsaB$_2$
Nº of genomes without TsaB$_2$
With TsaC$_1$
Without TsaC$_1$
Nº of genomes with TsaC$_1$
Nº of genomes without TsaC$_1$
With TsaC$_2$
Without TsaC$_2$
Nº of genomes with TsaC$_2$
Nº of genomes without TsaC$_2$
With TsaD$_1$
Without TsaD$_1$
Nº of genomes with TsaD$_1$
Nº of genomes without TsaD$_1$
With TsaD$_2$
Without TsaD$_2$
Nº of genomes with TsaD$_2$
Nº of genomes without TsaD$_2$
With TsaD$_3$
Without TsaD$_3$
Nº of genomes with TsaD$_3$
Nº of genomes without TsaD$_3$
With TsaE$_1$
Without TsaE$_1$
Nº of genomes with TsaE$_1$
Nº of genomes without TsaE$_1$
With TsaE$_2$
Without TsaE$_2$
Nº of genomes with TsaE$_2$
Nº of genomes without TsaE$_2$
With TsaE$_3$
Without TsaE$_3$
Nº of genomes with TsaE$_3$
Nº of genomes without TsaE$_3$
p < 0.00005

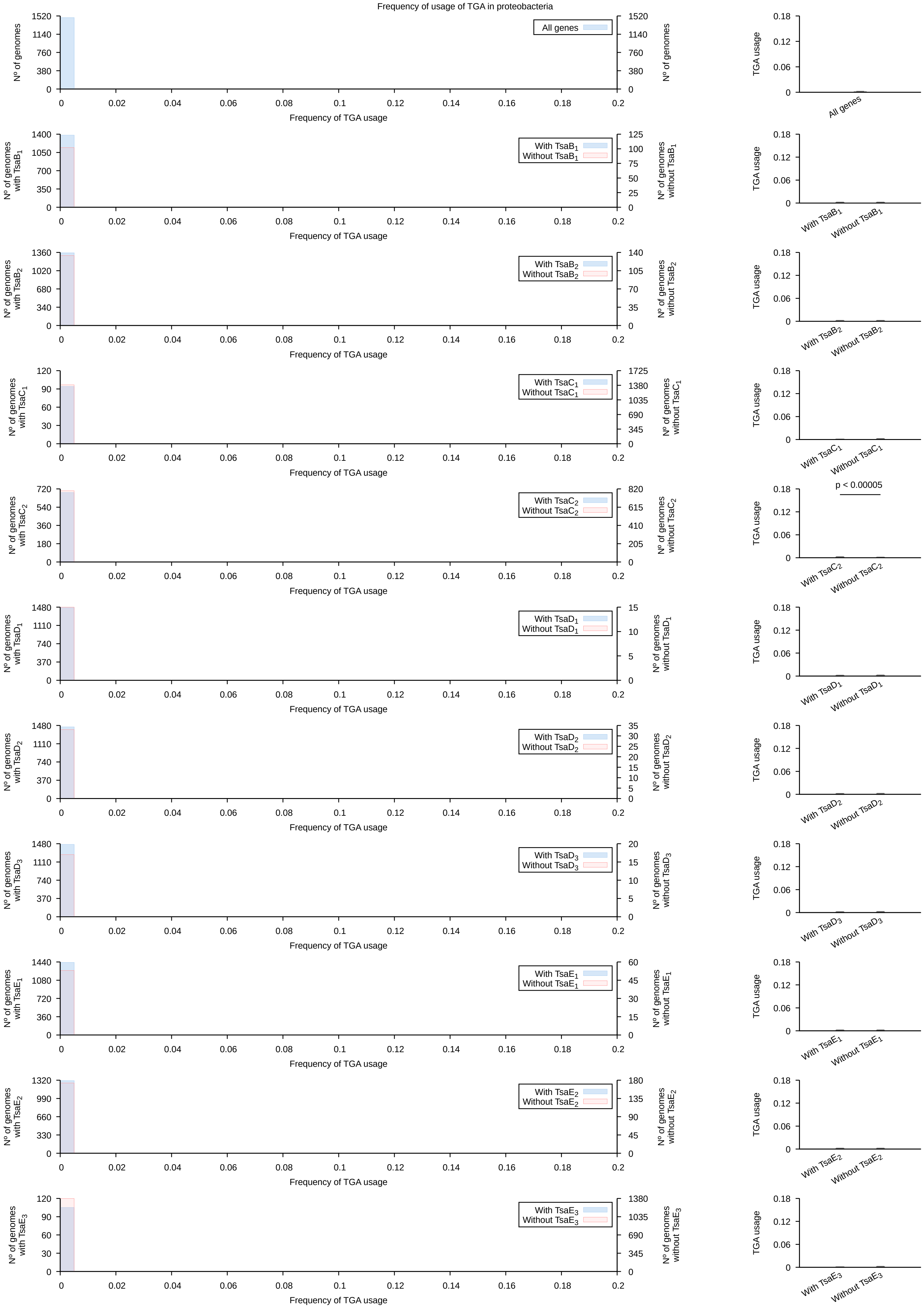
Frequency of usage of TGA in proteobacteria
All genes
Nº of genomes
Frequency of TGA usage
TGA usage
With TsaB$_1$
Without TsaB$_1$
Nº of genomes with TsaB$_1$
Nº of genomes without TsaB$_1$
With TsaB$_2$
Without TsaB$_2$
Nº of genomes with TsaB$_2$
Nº of genomes without TsaB$_2$
With TsaC$_1$
Without TsaC$_1$
Nº of genomes with TsaC$_1$
Nº of genomes without TsaC$_1$
With TsaC$_2$
Without TsaC$_2$
Nº of genomes with TsaC$_2$
Nº of genomes without TsaC$_2$
p < 0.00005
With TsaD$_1$
Without TsaD$_1$
Nº of genomes with TsaD$_1$
Nº of genomes without TsaD$_1$
With TsaD$_2$
Without TsaD$_2$
Nº of genomes with TsaD$_2$
Nº of genomes without TsaD$_2$
With TsaD$_3$
Without TsaD$_3$
Nº of genomes with TsaD$_3$
Nº of genomes without TsaD$_3$
With TsaE$_1$
Without TsaE$_1$
Nº of genomes with TsaE$_1$
Nº of genomes without TsaE$_1$
With TsaE$_2$
Without TsaE$_2$
Nº of genomes with TsaE$_2$
Nº of genomes without TsaE$_2$
With TsaE$_3$
Without TsaE$_3$
Nº of genomes with TsaE$_3$
Nº of genomes without TsaE$_3$

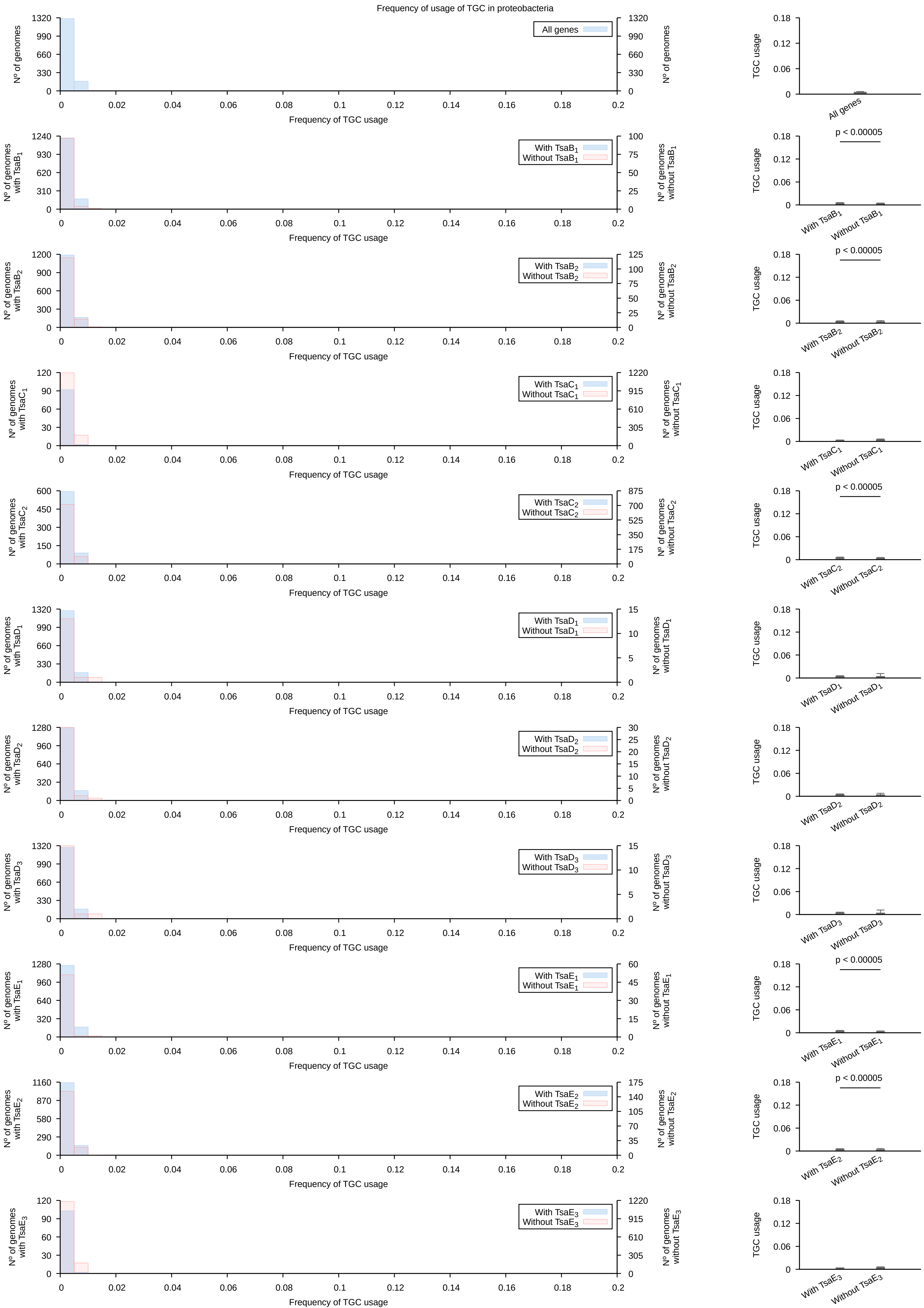
Frequency of usage of TGC in proteobacteria
Nº of genomes
Frequency of TGC usage
TGC usage
All genes
With TsaB1
Without TsaB1
With TsaB2
Without TsaB2
With TsaC1
Without TsaC1
With TsaC2
Without TsaC2
With TsaD1
Without TsaD1
With TsaD2
Without TsaD2
With TsaD3
Without TsaD3
With TsaE1
Without TsaE1
With TsaE2
Without TsaE2
With TsaE3
Without TsaE3
p < 0.00005

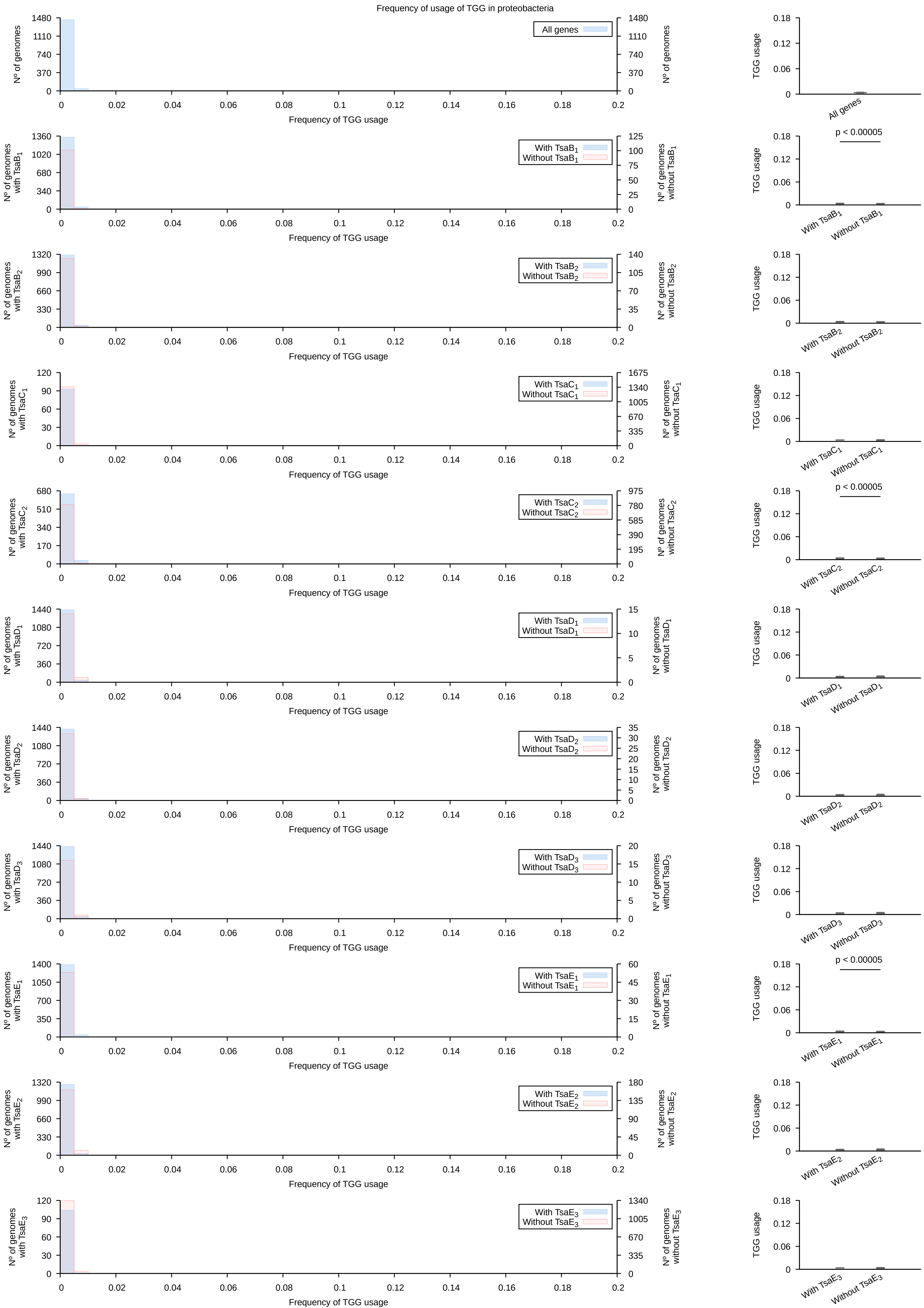

Frequency of usage of TGG in proteobacteria
All genes
Nº of genomes
Frequency of TGG usage
TGG usage
With TsaB$_1$
Without TsaB$_1$
Nº of genomes with TsaB$_1$
Nº of genomes without TsaB$_1$
p < 0.00005
With TsaB$_2$
Without TsaB$_2$
Nº of genomes with TsaB$_2$
Nº of genomes without TsaB$_2$
With TsaC$_1$
Without TsaC$_1$
Nº of genomes with TsaC$_1$
Nº of genomes without TsaC$_1$
With TsaC$_2$
Without TsaC$_2$
Nº of genomes with TsaC$_2$
Nº of genomes without TsaC$_2$
p < 0.00005
With TsaD$_1$
Without TsaD$_1$
Nº of genomes with TsaD$_1$
Nº of genomes without TsaD$_1$
With TsaD$_2$
Without TsaD$_2$
Nº of genomes with TsaD$_2$
Nº of genomes without TsaD$_2$
With TsaD$_3$
Without TsaD$_3$
Nº of genomes with TsaD$_3$
Nº of genomes without TsaD$_3$
With TsaE$_1$
Without TsaE$_1$
Nº of genomes with TsaE$_1$
Nº of genomes without TsaE$_1$
p < 0.00005
With TsaE$_2$
Without TsaE$_2$
Nº of genomes with TsaE$_2$
Nº of genomes without TsaE$_2$
With TsaE$_3$
Without TsaE$_3$
Nº of genomes with TsaE$_3$
Nº of genomes without TsaE$_3$

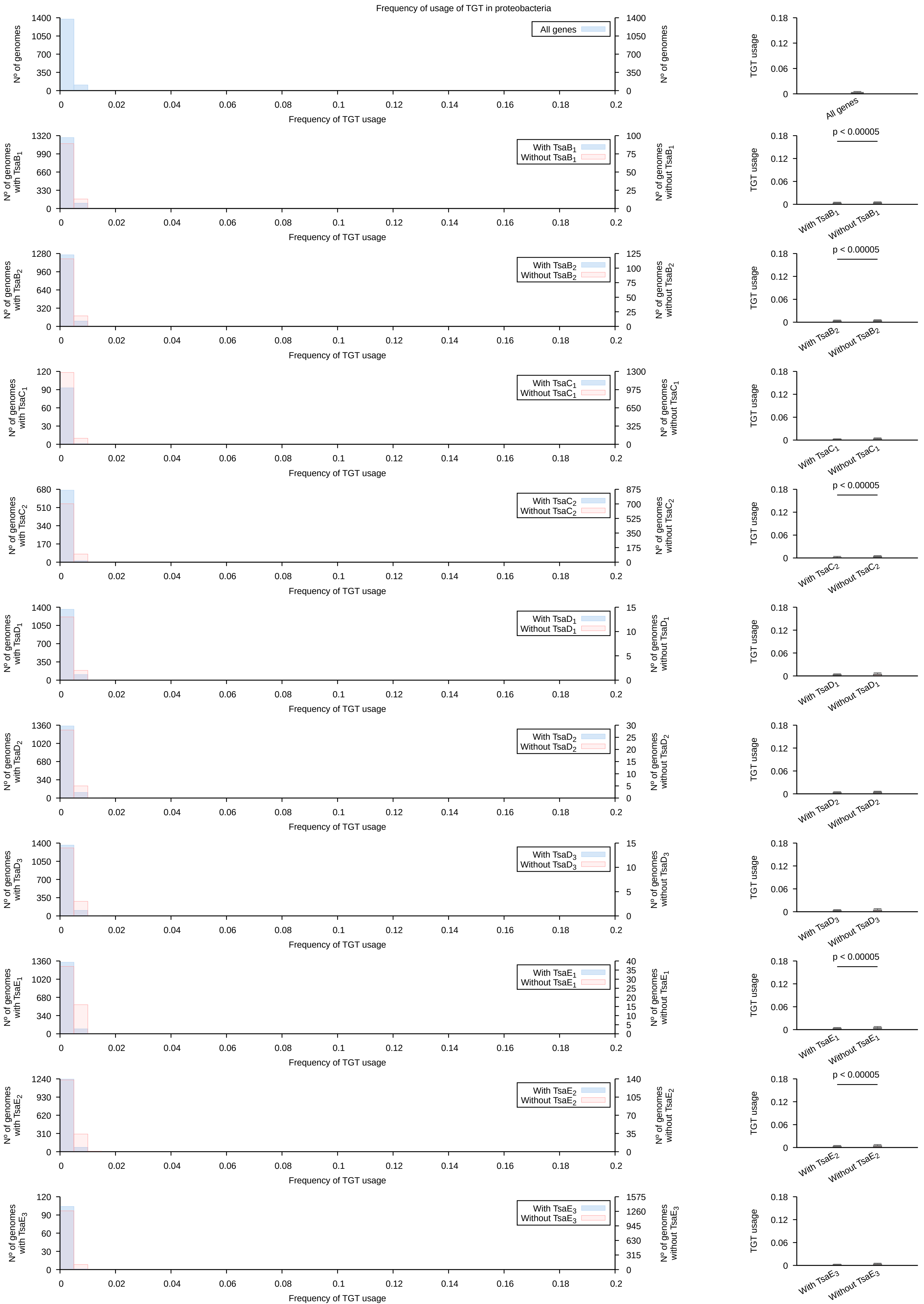

Frequency of usage of TGT in proteobacteria
Nº of genomes
All genes
Frequency of TGT usage
TGT usage
With TsaB1
Without TsaB1
With TsaB2
Without TsaB2
With TsaC1
Without TsaC1
With TsaC2
Without TsaC2
With TsaD1
Without TsaD1
With TsaD2
Without TsaD2
With TsaD3
Without TsaD3
With TsaE1
Without TsaE1
With TsaE2
Without TsaE2
With TsaE3
Without TsaE3
p < 0.00005

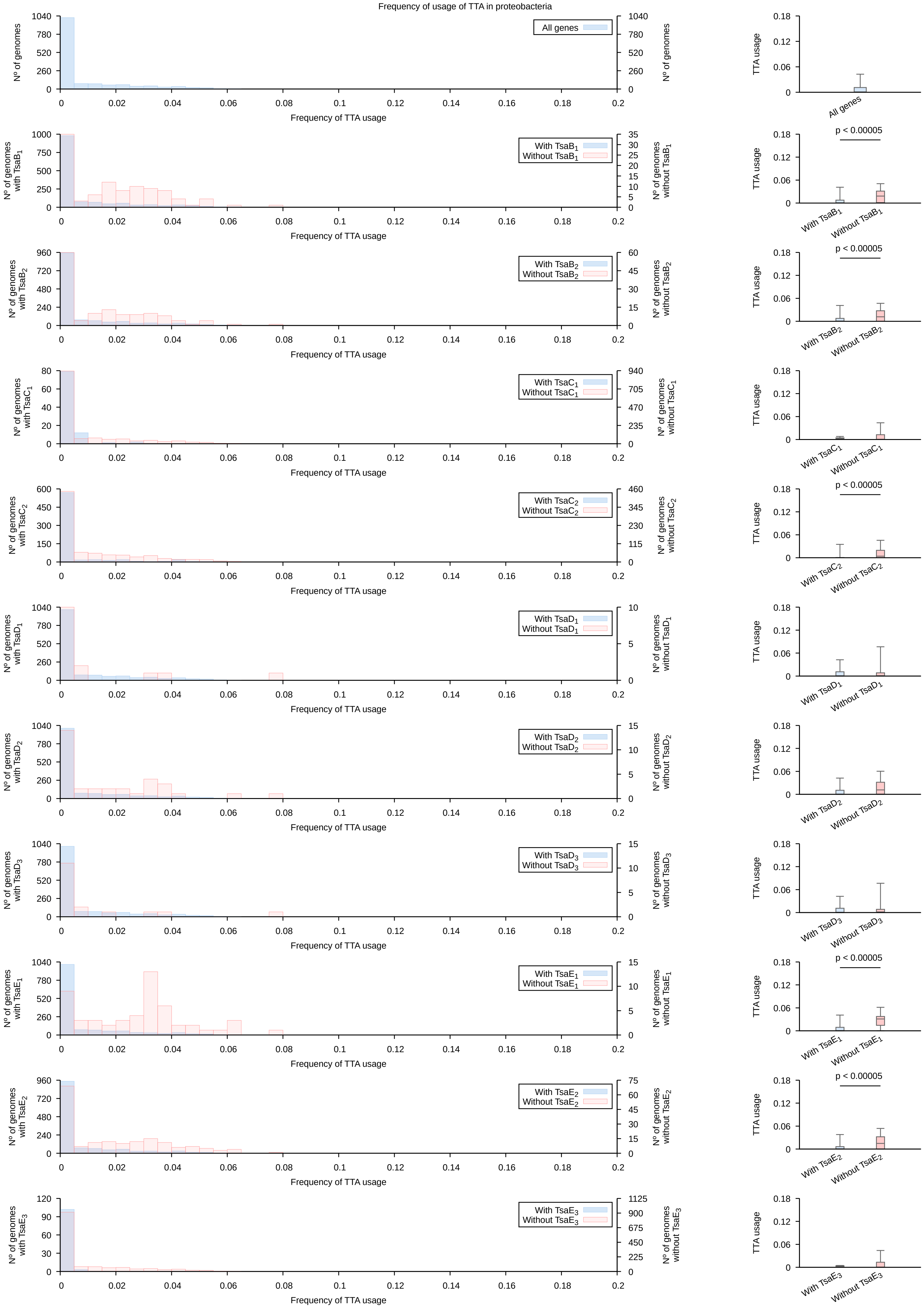
Frequency of usage of TTA in proteobacteria
All genes
Nº of genomes
Frequency of TTA usage
TTA usage
With TsaB1
Without TsaB1
Nº of genomes with TsaB1
Nº of genomes without TsaB1
p < 0.00005
With TsaB2
Without TsaB2
Nº of genomes with TsaB2
Nº of genomes without TsaB2
p < 0.00005
With TsaC1
Without TsaC1
Nº of genomes with TsaC1
Nº of genomes without TsaC1
With TsaC2
Without TsaC2
Nº of genomes with TsaC2
Nº of genomes without TsaC2
p < 0.00005
With TsaD1
Without TsaD1
Nº of genomes with TsaD1
Nº of genomes without TsaD1
With TsaD2
Without TsaD2
Nº of genomes with TsaD2
Nº of genomes without TsaD2
With TsaD3
Without TsaD3
Nº of genomes with TsaD3
Nº of genomes without TsaD3
With TsaE1
Without TsaE1
Nº of genomes with TsaE1
Nº of genomes without TsaE1
p < 0.00005
With TsaE2
Without TsaE2
Nº of genomes with TsaE2
Nº of genomes without TsaE2
p < 0.00005
With TsaE3
Without TsaE3
Nº of genomes with TsaE3
Nº of genomes without TsaE3

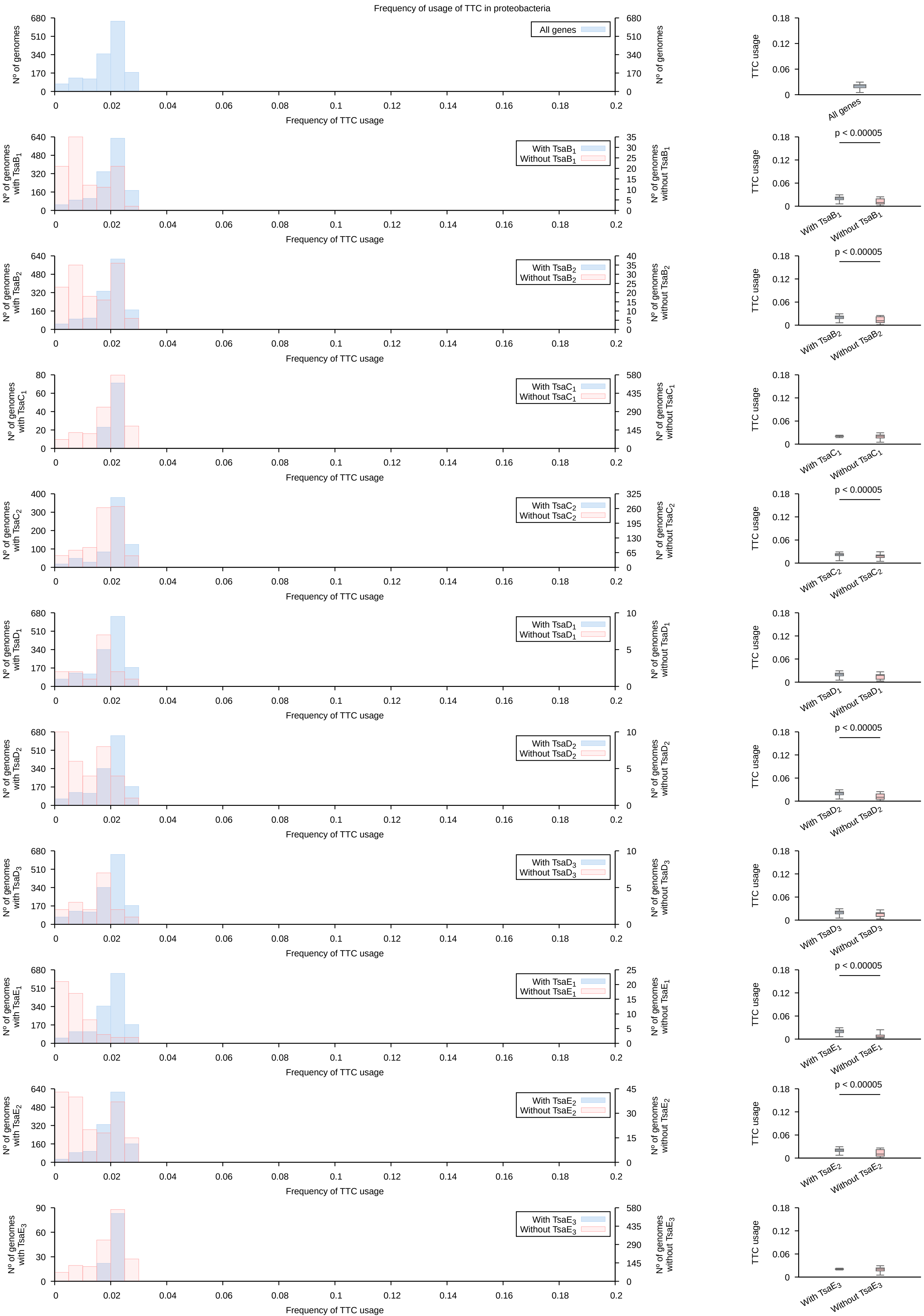
Frequency of usage of TTC in proteobacteria
Nº of genomes
All genes
Frequency of TTC usage
TTC usage
p < 0.00005
Nº of genomes with TsaB$_1$
Nº of genomes without TsaB$_1$
With TsaB$_1$
Without TsaB$_1$
Nº of genomes with TsaB$_2$
Nº of genomes without TsaB$_2$
With TsaB$_2$
Without TsaB$_2$
Nº of genomes with TsaC$_1$
Nº of genomes without TsaC$_1$
With TsaC$_1$
Without TsaC$_1$
Nº of genomes with TsaC$_2$
Nº of genomes without TsaC$_2$
With TsaC$_2$
Without TsaC$_2$
Nº of genomes with TsaD$_1$
Nº of genomes without TsaD$_1$
With TsaD$_1$
Without TsaD$_1$
Nº of genomes with TsaD$_2$
Nº of genomes without TsaD$_2$
With TsaD$_2$
Without TsaD$_2$
Nº of genomes with TsaD$_3$
Nº of genomes without TsaD$_3$
With TsaD$_3$
Without TsaD$_3$
Nº of genomes with TsaE$_1$
Nº of genomes without TsaE$_1$
With TsaE$_1$
Without TsaE$_1$
Nº of genomes with TsaE$_2$
Nº of genomes without TsaE$_2$
With TsaE$_2$
Without TsaE$_2$
Nº of genomes with TsaE$_3$
Nº of genomes without TsaE$_3$
With TsaE$_3$
Without TsaE$_3$

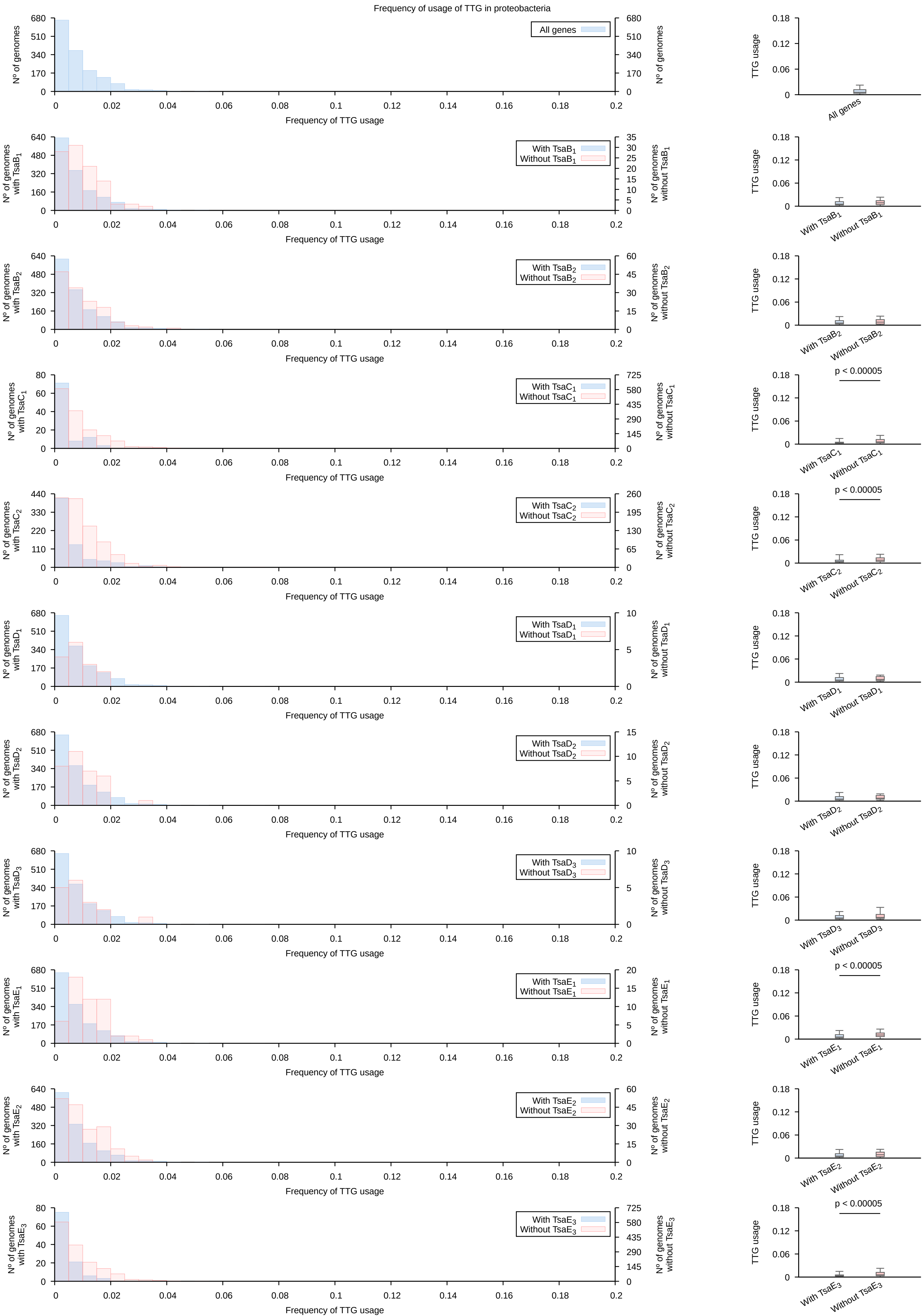
Frequency of usage of TTG in proteobacteria
All genes
Nº of genomes
TTG usage
Frequency of TTG usage
With TsaB1
Without TsaB1
Nº of genomes with TsaB1
Nº of genomes without TsaB1
With TsaB2
Without TsaB2
Nº of genomes with TsaB2
Nº of genomes without TsaB2
With TsaC1
Without TsaC1
Nº of genomes with TsaC1
Nº of genomes without TsaC1
p < 0.00005
With TsaC2
Without TsaC2
Nº of genomes with TsaC2
Nº of genomes without TsaC2
p < 0.00005
With TsaD1
Without TsaD1
Nº of genomes with TsaD1
Nº of genomes without TsaD1
With TsaD2
Without TsaD2
Nº of genomes with TsaD2
Nº of genomes without TsaD2
With TsaD3
Without TsaD3
Nº of genomes with TsaD3
Nº of genomes without TsaD3
With TsaE1
Without TsaE1
Nº of genomes with TsaE1
Nº of genomes without TsaE1
p < 0.00005
With TsaE2
Without TsaE2
Nº of genomes with TsaE2
Nº of genomes without TsaE2
With TsaE3
Without TsaE3
Nº of genomes with TsaE3
Nº of genomes without TsaE3
p < 0.00005

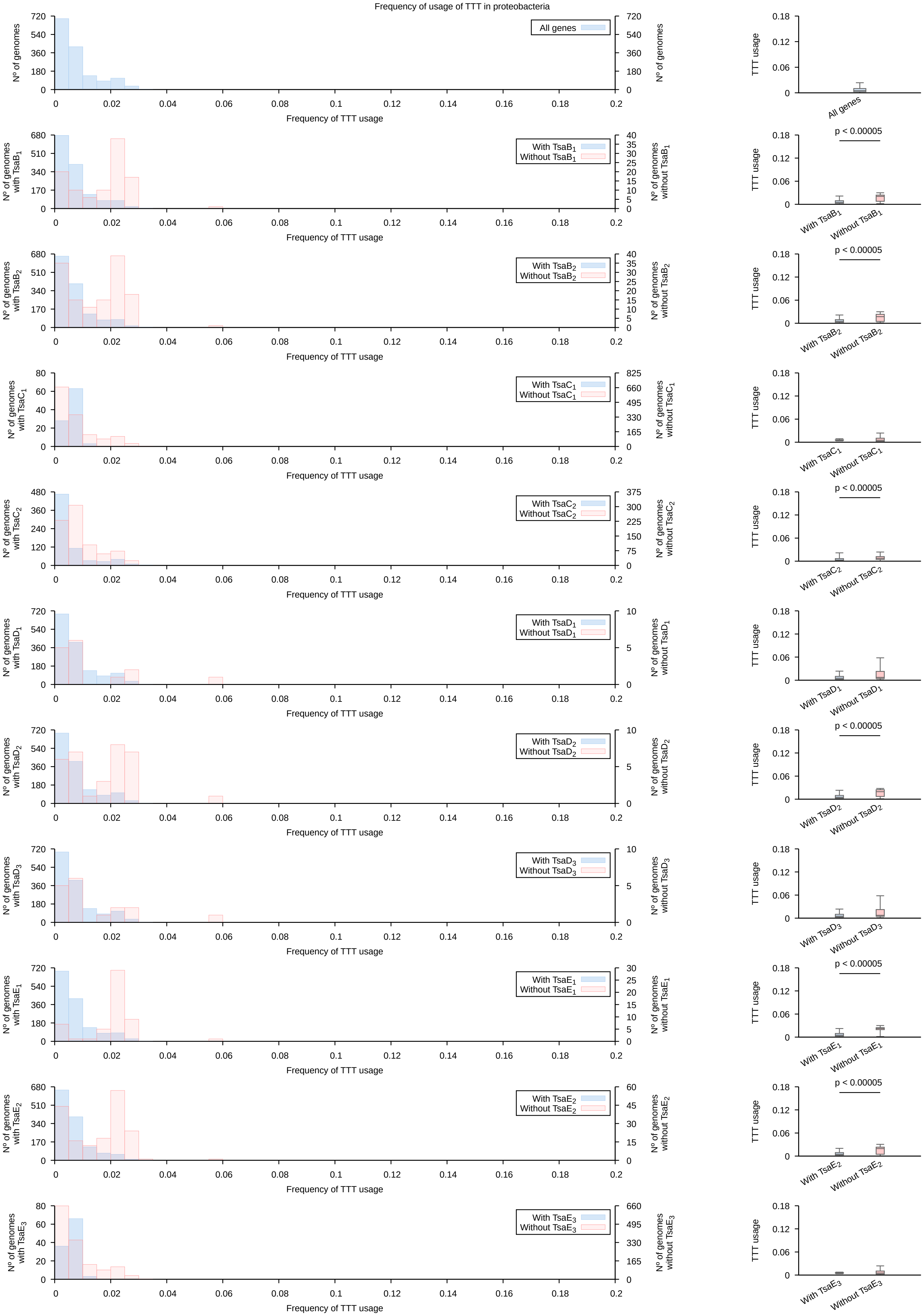

Frequency of usage of TTT in proteobacteria
Nº of genomes
All genes
Frequency of TTT usage
TTT usage
Nº of genomes with TsaB$_1$
Nº of genomes without TsaB$_1$
With TsaB$_1$
Without TsaB$_1$
p < 0.00005
Nº of genomes with TsaB$_2$
Nº of genomes without TsaB$_2$
With TsaB$_2$
Without TsaB$_2$
p < 0.00005
Nº of genomes with TsaC$_1$
Nº of genomes without TsaC$_1$
With TsaC$_1$
Without TsaC$_1$
Nº of genomes with TsaC$_2$
Nº of genomes without TsaC$_2$
With TsaC$_2$
Without TsaC$_2$
p < 0.00005
Nº of genomes with TsaD$_1$
Nº of genomes without TsaD$_1$
With TsaD$_1$
Without TsaD$_1$
Nº of genomes with TsaD$_2$
Nº of genomes without TsaD$_2$
With TsaD$_2$
Without TsaD$_2$
p < 0.00005
Nº of genomes with TsaD$_3$
Nº of genomes without TsaD$_3$
With TsaD$_3$
Without TsaD$_3$
Nº of genomes with TsaE$_1$
Nº of genomes without TsaE$_1$
With TsaE$_1$
Without TsaE$_1$
p < 0.00005
Nº of genomes with TsaE$_2$
Nº of genomes without TsaE$_2$
With TsaE$_2$
Without TsaE$_2$
p < 0.00005
Nº of genomes with TsaE$_3$
Nº of genomes without TsaE$_3$
With TsaE$_3$
Without TsaE$_3$

Figure S8. Histogram of codon usage in high expression genes of genomes that present or lack genes involved in GluQ formation. Top left graph: histogram of the usage of the indicated codon in highly expressed genes of proteobacterial genomes. **Top right graph:** box plot indicating the distribution of codon usage in the same population. **Lower graphs:** The collection of 1484 proteobacterial genomes were separated in two groups, based on the presence (light blue) or absence (orange) of the indicated pattern in any gene of the analyzed genomes. Patterns are indicated using subscript numbers. See table S4 for a detailed description of the used patterns. **Left:** Data is represented in superposed histograms. **Right:** Data is represented in a boxplot. An horizontal bar was added when the two populations are different based on Wilcoxon-Mann-Whitney test and $p < 0.00005$. Whiskers include 95% of data.

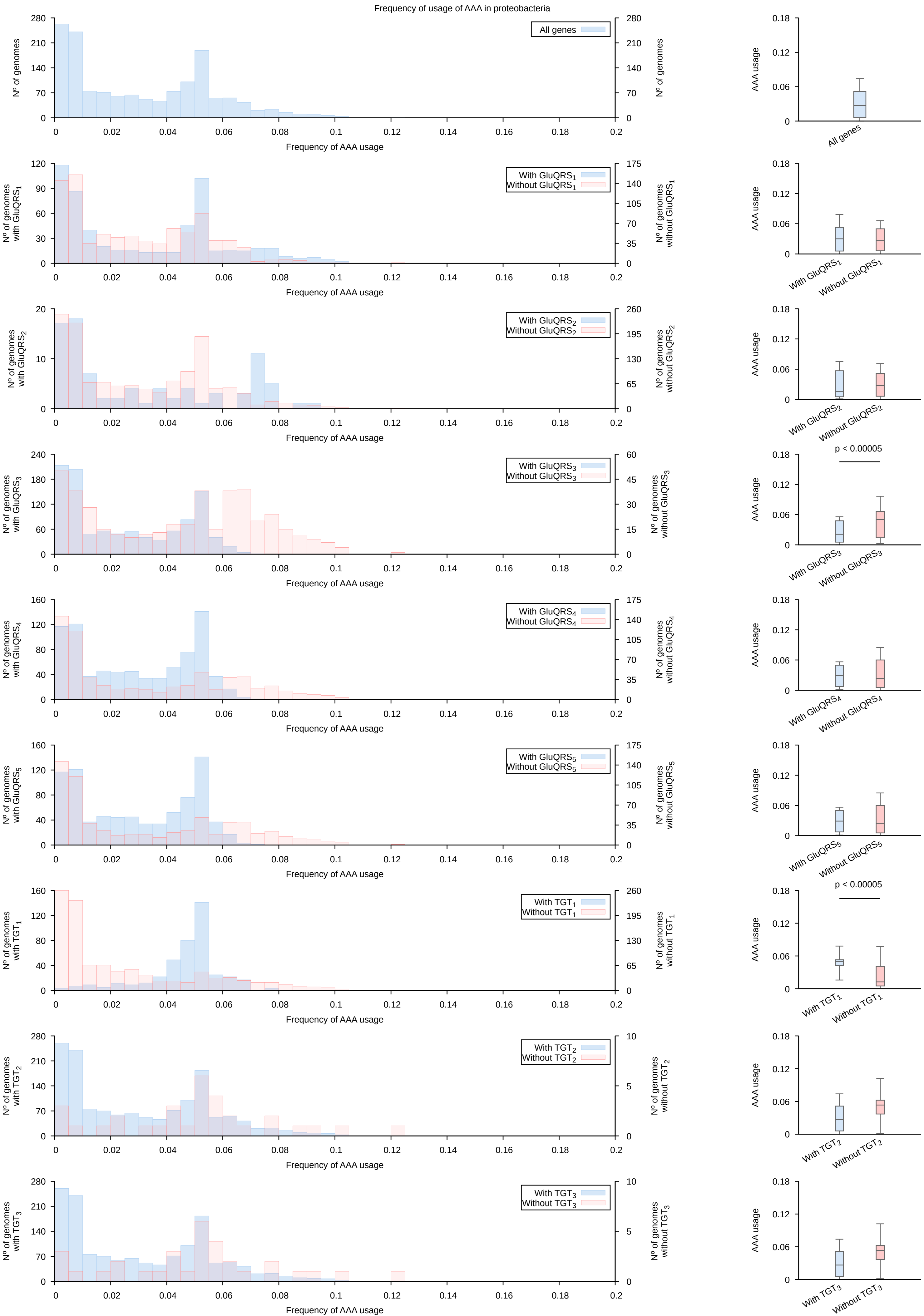
Frequency of usage of AAA in proteobacteria
All genes
Nº of genomes
Frequency of AAA usage
AAA usage
With GluQRS1
Without GluQRS1
Nº of genomes with GluQRS1
Nº of genomes without GluQRS1
With GluQRS2
Without GluQRS2
Nº of genomes with GluQRS2
Nº of genomes without GluQRS2
With GluQRS3
Without GluQRS3
Nº of genomes with GluQRS3
Nº of genomes without GluQRS3
p < 0.00005
With GluQRS4
Without GluQRS4
Nº of genomes with GluQRS4
Nº of genomes without GluQRS4
With GluQRS5
Without GluQRS5
Nº of genomes with GluQRS5
Nº of genomes without GluQRS5
With TGT1
Without TGT1
Nº of genomes with TGT1
Nº of genomes without TGT1
p < 0.00005
With TGT2
Without TGT2
Nº of genomes with TGT2
Nº of genomes without TGT2
With TGT3
Without TGT3
Nº of genomes with TGT3
Nº of genomes without TGT3

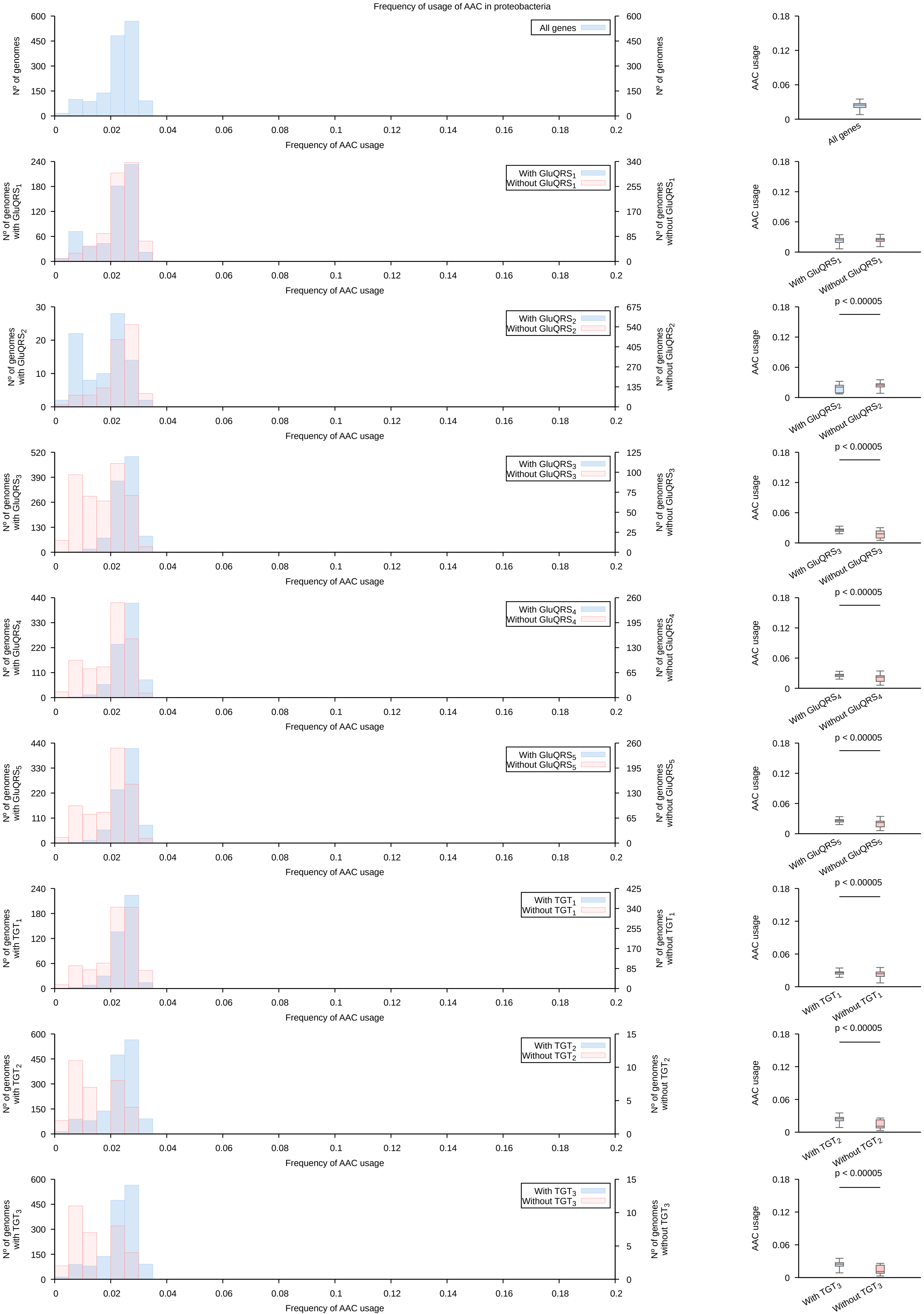
Frequency of usage of AAC in proteobacteria
All genes
With GluQRS1
Without GluQRS1
With GluQRS2
Without GluQRS2
With GluQRS3
Without GluQRS3
With GluQRS4
Without GluQRS4
With GluQRS5
Without GluQRS5
With TGT1
Without TGT1
With TGT2
Without TGT2
With TGT3
Without TGT3
Nº of genomes
Nº of genomes with GluQRS1
Nº of genomes without GluQRS1
Nº of genomes with GluQRS2
Nº of genomes without GluQRS2
Nº of genomes with GluQRS3
Nº of genomes without GluQRS3
Nº of genomes with GluQRS4
Nº of genomes without GluQRS4
Nº of genomes with GluQRS5
Nº of genomes without GluQRS5
Nº of genomes with TGT1
Nº of genomes without TGT1
Nº of genomes with TGT2
Nº of genomes without TGT2
Nº of genomes with TGT3
Nº of genomes without TGT3
Frequency of AAC usage
AAC usage
p < 0.00005

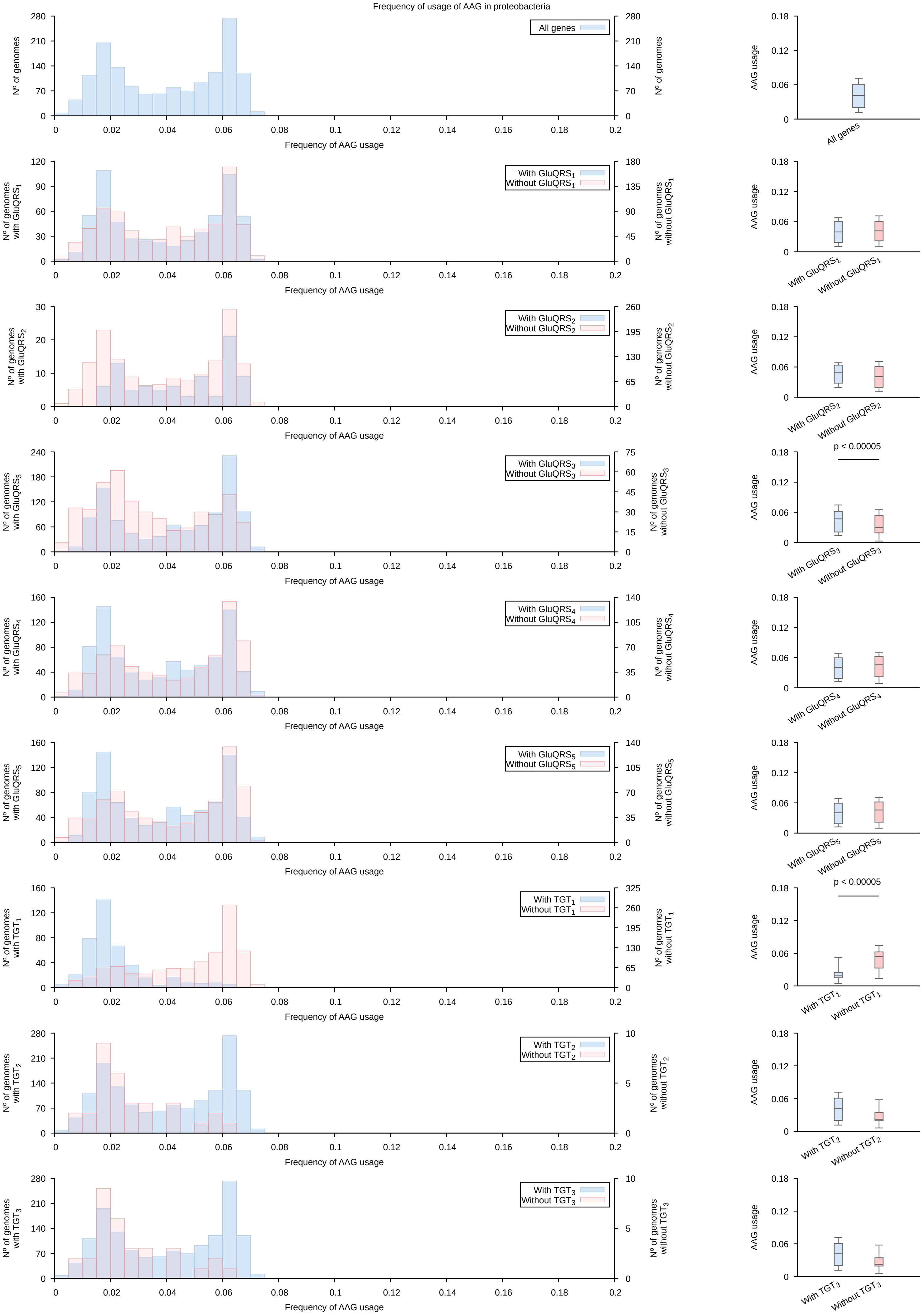

Frequency of usage of AAG in proteobacteria
All genes
Nº of genomes
Frequency of AAG usage
AAG usage
With GluQRS$_1$
Without GluQRS$_1$
Nº of genomes with GluQRS$_1$
Nº of genomes without GluQRS$_1$
With GluQRS$_2$
Without GluQRS$_2$
Nº of genomes with GluQRS$_2$
Nº of genomes without GluQRS$_2$
With GluQRS$_3$
Without GluQRS$_3$
Nº of genomes with GluQRS$_3$
Nº of genomes without GluQRS$_3$
$p < 0.00005$
With GluQRS$_4$
Without GluQRS$_4$
Nº of genomes with GluQRS$_4$
Nº of genomes without GluQRS$_4$
With GluQRS$_5$
Without GluQRS$_5$
Nº of genomes with GluQRS$_5$
Nº of genomes without GluQRS$_5$
With TGT$_1$
Without TGT$_1$
Nº of genomes with TGT$_1$
Nº of genomes without TGT$_1$
$p < 0.00005$
With TGT$_2$
Without TGT$_2$
Nº of genomes with TGT$_2$
Nº of genomes without TGT$_2$
With TGT$_3$
Without TGT$_3$
Nº of genomes with TGT$_3$
Nº of genomes without TGT$_3$

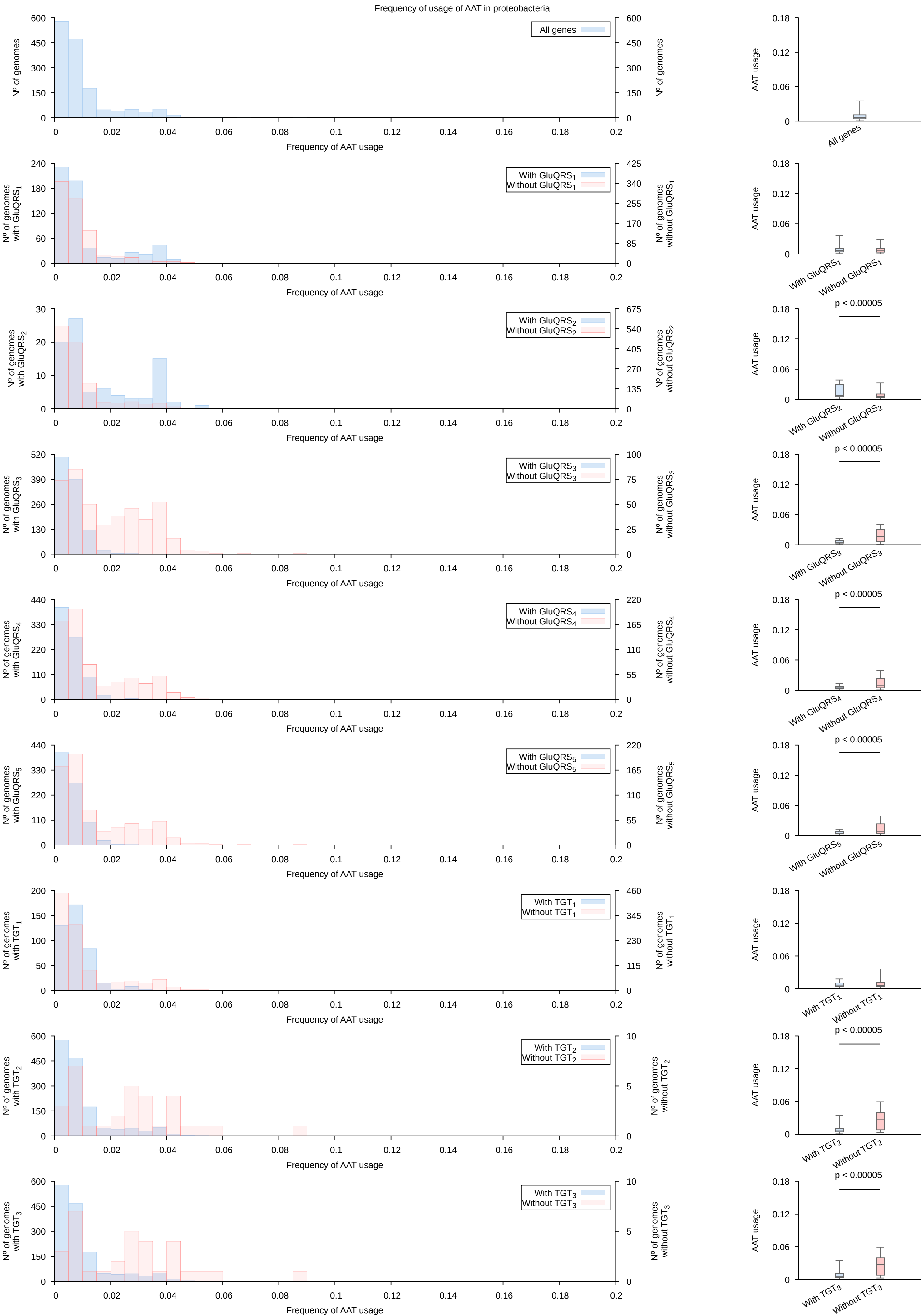
Frequency of usage of AAT in proteobacteria
Nº of genomes
Frequency of AAT usage
All genes
AAT usage
Nº of genomes with GluQRS$_1$
Nº of genomes without GluQRS$_1$
With GluQRS$_1$
Without GluQRS$_1$
Nº of genomes with GluQRS$_2$
Nº of genomes without GluQRS$_2$
With GluQRS$_2$
Without GluQRS$_2$
p < 0.00005
Nº of genomes with GluQRS$_3$
Nº of genomes without GluQRS$_3$
With GluQRS$_3$
Without GluQRS$_3$
p < 0.00005
Nº of genomes with GluQRS$_4$
Nº of genomes without GluQRS$_4$
With GluQRS$_4$
Without GluQRS$_4$
p < 0.00005
Nº of genomes with GluQRS$_5$
Nº of genomes without GluQRS$_5$
With GluQRS$_5$
Without GluQRS$_5$
p < 0.00005
Nº of genomes with TGT$_1$
Nº of genomes without TGT$_1$
With TGT$_1$
Without TGT$_1$
Nº of genomes with TGT$_2$
Nº of genomes without TGT$_2$
With TGT$_2$
Without TGT$_2$
p < 0.00005
Nº of genomes with TGT$_3$
Nº of genomes without TGT$_3$
With TGT$_3$
Without TGT$_3$
p < 0.00005

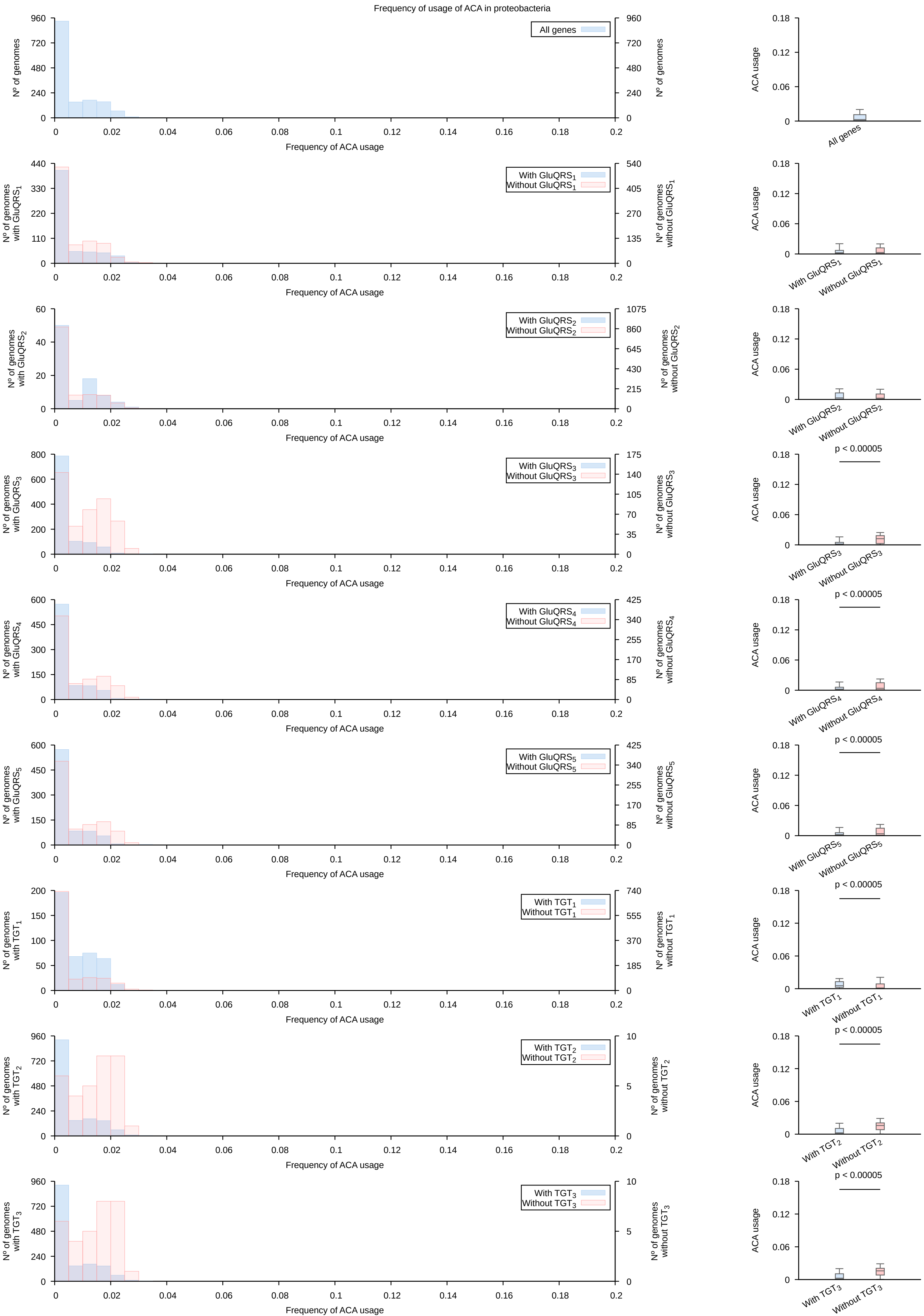

Frequency of usage of ACA in proteobacteria
All genes
With GluQRS1
Without GluQRS1
With GluQRS2
Without GluQRS2
With GluQRS3
Without GluQRS3
With GluQRS4
Without GluQRS4
With GluQRS5
Without GluQRS5
With TGT1
Without TGT1
With TGT2
Without TGT2
With TGT3
Without TGT3
Nº of genomes
Nº of genomes with GluQRS1
Nº of genomes without GluQRS1
Nº of genomes with GluQRS2
Nº of genomes without GluQRS2
Nº of genomes with GluQRS3
Nº of genomes without GluQRS3
Nº of genomes with GluQRS4
Nº of genomes without GluQRS4
Nº of genomes with GluQRS5
Nº of genomes without GluQRS5
Nº of genomes with TGT1
Nº of genomes without TGT1
Nº of genomes with TGT2
Nº of genomes without TGT2
Nº of genomes with TGT3
Nº of genomes without TGT3
Frequency of ACA usage
ACA usage
p < 0.00005

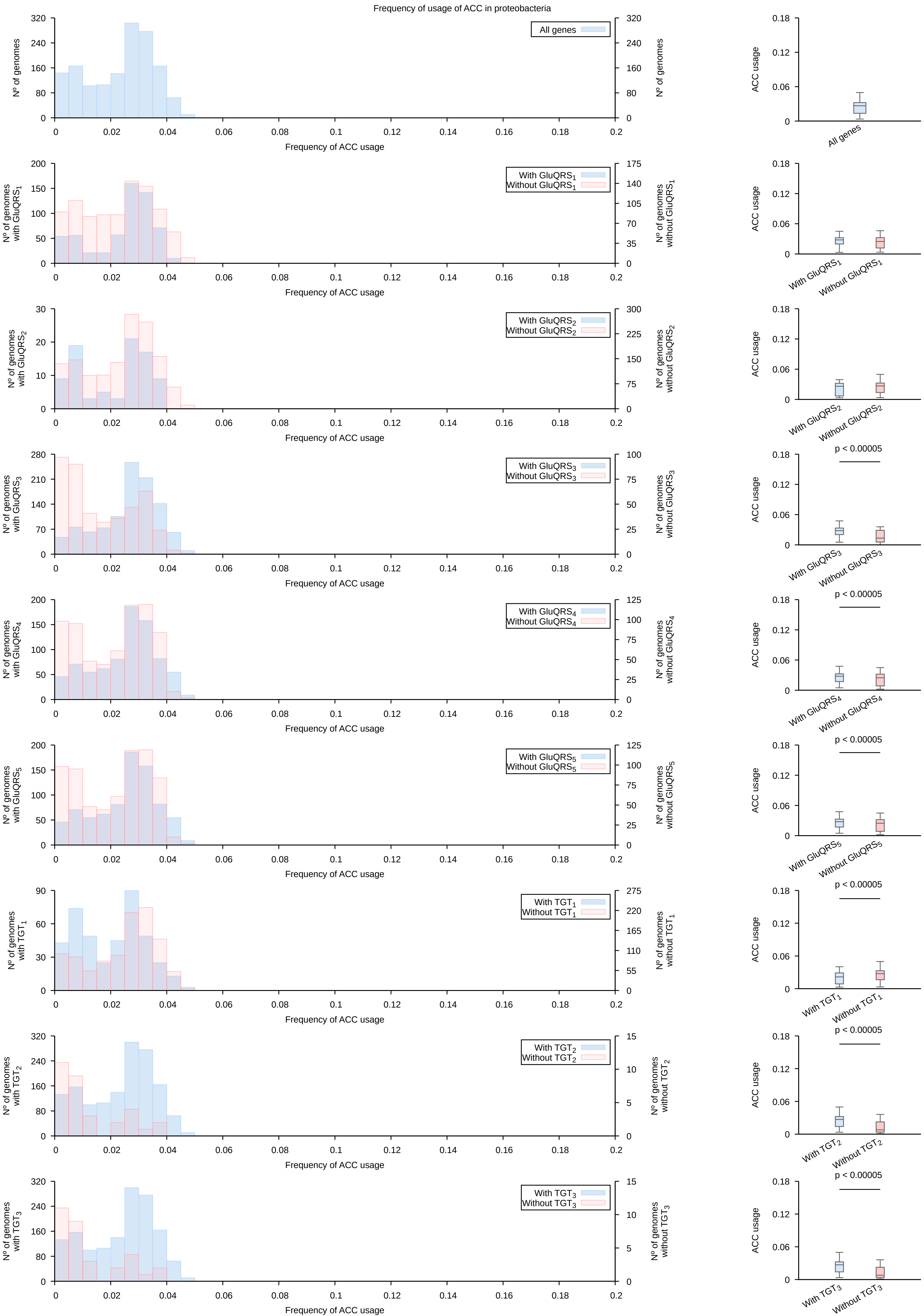

Frequency of usage of ACC in proteobacteria
Nº of genomes
All genes
Frequency of ACC usage
ACC usage
0
0.02
0.04
0.06
0.08
0.1
0.12
0.14
0.16
0.18
0.2
Nº of genomes with GluQRS$_1$
Nº of genomes without GluQRS$_1$
With GluQRS$_1$
Without GluQRS$_1$
Nº of genomes with GluQRS$_2$
Nº of genomes without GluQRS$_2$
With GluQRS$_2$
Without GluQRS$_2$
Nº of genomes with GluQRS$_3$
Nº of genomes without GluQRS$_3$
With GluQRS$_3$
Without GluQRS$_3$
p < 0.00005
Nº of genomes with GluQRS$_4$
Nº of genomes without GluQRS$_4$
With GluQRS$_4$
Without GluQRS$_4$
p < 0.00005
Nº of genomes with GluQRS$_5$
Nº of genomes without GluQRS$_5$
With GluQRS$_5$
Without GluQRS$_5$
p < 0.00005
Nº of genomes with TGT$_1$
Nº of genomes without TGT$_1$
With TGT$_1$
Without TGT$_1$
p < 0.00005
Nº of genomes with TGT$_2$
Nº of genomes without TGT$_2$
With TGT$_2$
Without TGT$_2$
p < 0.00005
Nº of genomes with TGT$_3$
Nº of genomes without TGT$_3$
With TGT$_3$
Without TGT$_3$
p < 0.00005

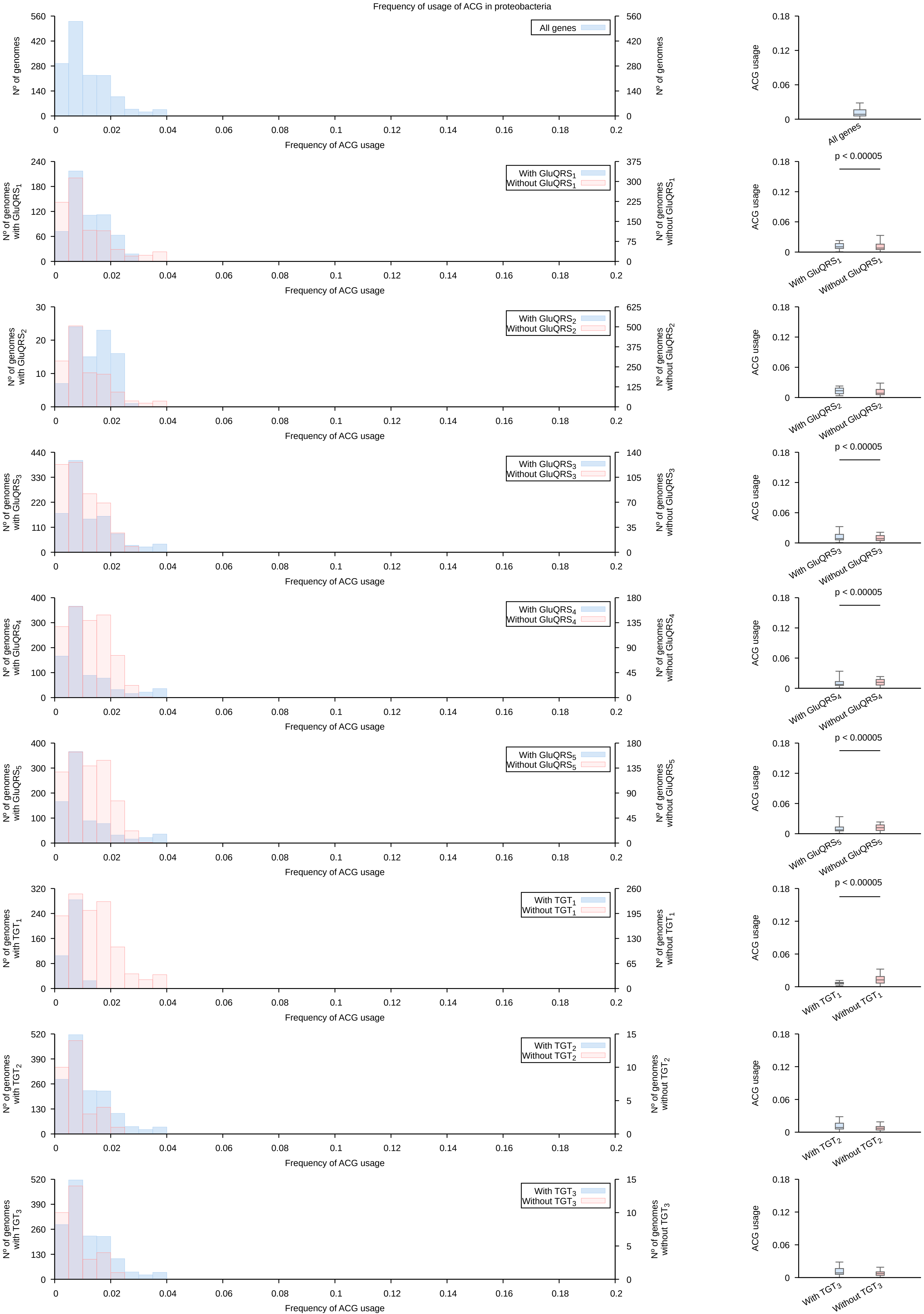
Frequency of usage of ACG in proteobacteria
Nº of genomes
Frequency of ACG usage
All genes
ACG usage
Nº of genomes with GluQRS$_1$
Nº of genomes without GluQRS$_1$
With GluQRS$_1$
Without GluQRS$_1$
p < 0.00005
Nº of genomes with GluQRS$_2$
Nº of genomes without GluQRS$_2$
With GluQRS$_2$
Without GluQRS$_2$
Nº of genomes with GluQRS$_3$
Nº of genomes without GluQRS$_3$
With GluQRS$_3$
Without GluQRS$_3$
p < 0.00005
Nº of genomes with GluQRS$_4$
Nº of genomes without GluQRS$_4$
With GluQRS$_4$
Without GluQRS$_4$
p < 0.00005
Nº of genomes with GluQRS$_5$
Nº of genomes without GluQRS$_5$
With GluQRS$_5$
Without GluQRS$_5$
p < 0.00005
Nº of genomes with TGT$_1$
Nº of genomes without TGT$_1$
With TGT$_1$
Without TGT$_1$
p < 0.00005
Nº of genomes with TGT$_2$
Nº of genomes without TGT$_2$
With TGT$_2$
Without TGT$_2$
Nº of genomes with TGT$_3$
Nº of genomes without TGT$_3$
With TGT$_3$
Without TGT$_3$

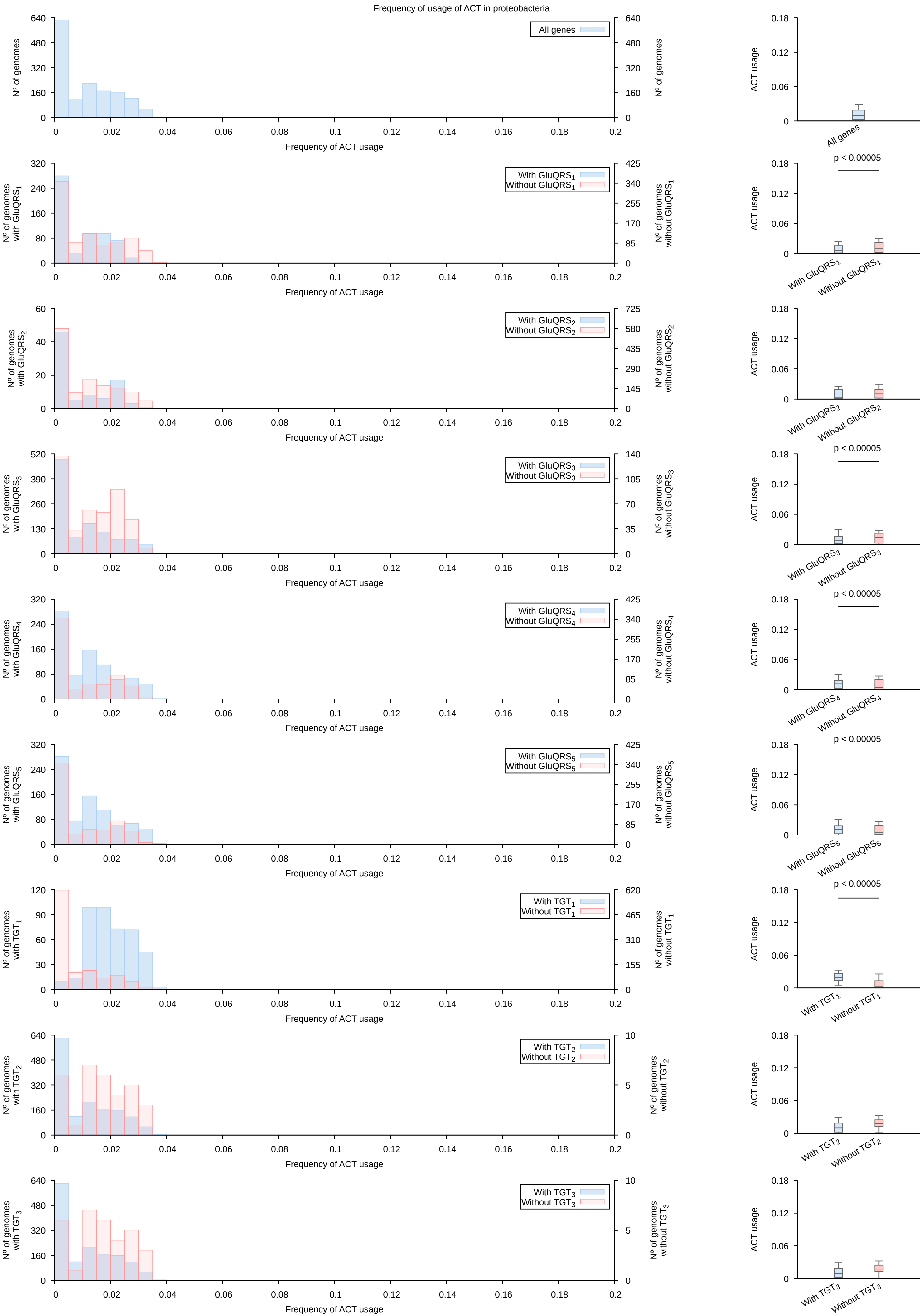

Frequency of usage of ACT in proteobacteria
All genes
Nº of genomes
Frequency of ACT usage
ACT usage
With GluQRS$_1$
Without GluQRS$_1$
Nº of genomes with GluQRS$_1$
Nº of genomes without GluQRS$_1$
p < 0.00005
With GluQRS$_2$
Without GluQRS$_2$
Nº of genomes with GluQRS$_2$
Nº of genomes without GluQRS$_2$
With GluQRS$_3$
Without GluQRS$_3$
Nº of genomes with GluQRS$_3$
Nº of genomes without GluQRS$_3$
p < 0.00005
With GluQRS$_4$
Without GluQRS$_4$
Nº of genomes with GluQRS$_4$
Nº of genomes without GluQRS$_4$
p < 0.00005
With GluQRS$_5$
Without GluQRS$_5$
Nº of genomes with GluQRS$_5$
Nº of genomes without GluQRS$_5$
p < 0.00005
With TGT$_1$
Without TGT$_1$
Nº of genomes with TGT$_1$
Nº of genomes without TGT$_1$
p < 0.00005
With TGT$_2$
Without TGT$_2$
Nº of genomes with TGT$_2$
Nº of genomes without TGT$_2$
With TGT$_3$
Without TGT$_3$
Nº of genomes with TGT$_3$
Nº of genomes without TGT$_3$

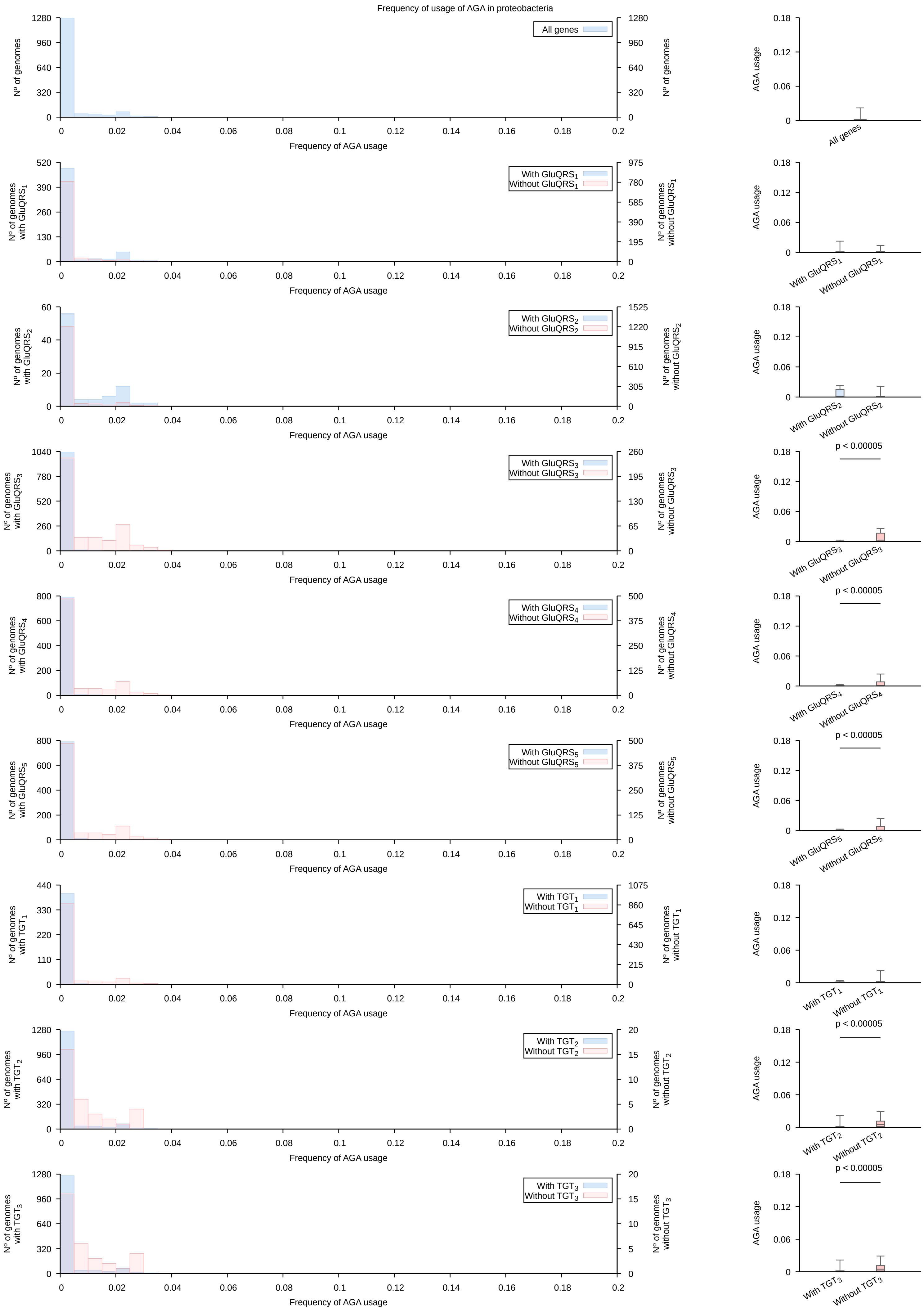

Frequency of usage of AGA in proteobacteria
Nº of genomes
All genes
Frequency of AGA usage
AGA usage
All genes
Nº of genomes with GluQRS$_1$
Nº of genomes without GluQRS$_1$
With GluQRS$_1$
Without GluQRS$_1$
Nº of genomes with GluQRS$_2$
Nº of genomes without GluQRS$_2$
With GluQRS$_2$
Without GluQRS$_2$
Nº of genomes with GluQRS$_3$
Nº of genomes without GluQRS$_3$
With GluQRS$_3$
Without GluQRS$_3$
p < 0.00005
Nº of genomes with GluQRS$_4$
Nº of genomes without GluQRS$_4$
With GluQRS$_4$
Without GluQRS$_4$
p < 0.00005
Nº of genomes with GluQRS$_5$
Nº of genomes without GluQRS$_5$
With GluQRS$_5$
Without GluQRS$_5$
p < 0.00005
Nº of genomes with TGT$_1$
Nº of genomes without TGT$_1$
With TGT$_1$
Without TGT$_1$
Nº of genomes with TGT$_2$
Nº of genomes without TGT$_2$
With TGT$_2$
Without TGT$_2$
p < 0.00005
Nº of genomes with TGT$_3$
Nº of genomes without TGT$_3$
With TGT$_3$
Without TGT$_3$
p < 0.00005

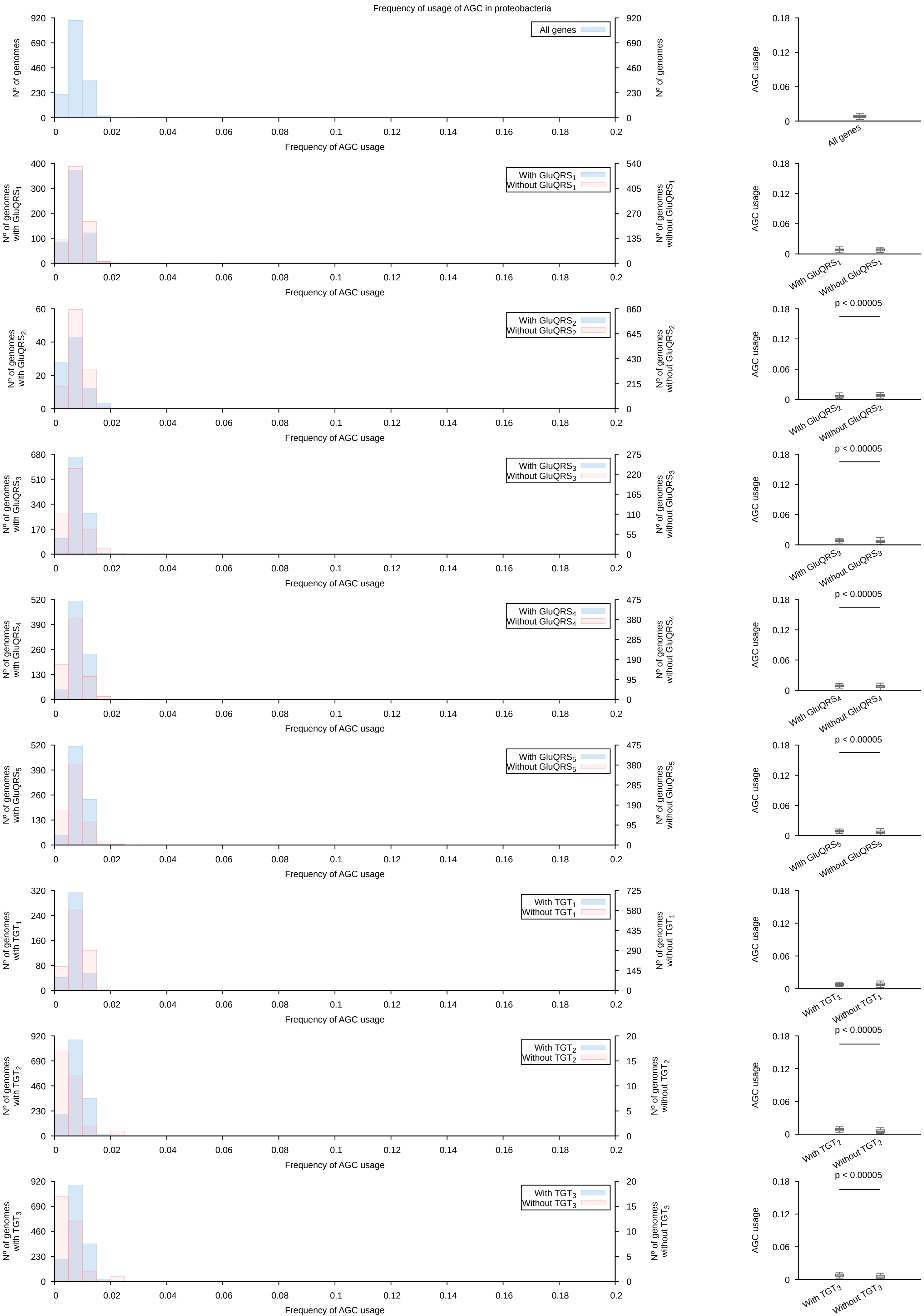
Frequency of usage of AGC in proteobacteria
All genes
Nº of genomes
Frequency of AGC usage
AGC usage
With GluQRS$_1$
Without GluQRS$_1$
Nº of genomes with GluQRS$_1$
Nº of genomes without GluQRS$_1$
With GluQRS$_2$
Without GluQRS$_2$
Nº of genomes with GluQRS$_2$
Nº of genomes without GluQRS$_2$
p < 0.00005
With GluQRS$_3$
Without GluQRS$_3$
Nº of genomes with GluQRS$_3$
Nº of genomes without GluQRS$_3$
p < 0.00005
With GluQRS$_4$
Without GluQRS$_4$
Nº of genomes with GluQRS$_4$
Nº of genomes without GluQRS$_4$
p < 0.00005
With GluQRS$_5$
Without GluQRS$_5$
Nº of genomes with GluQRS$_5$
Nº of genomes without GluQRS$_5$
p < 0.00005
With TGT$_1$
Without TGT$_1$
Nº of genomes with TGT$_1$
Nº of genomes without TGT$_1$
With TGT$_2$
Without TGT$_2$
Nº of genomes with TGT$_2$
Nº of genomes without TGT$_2$
p < 0.00005
With TGT$_3$
Without TGT$_3$
Nº of genomes with TGT$_3$
Nº of genomes without TGT$_3$
p < 0.00005

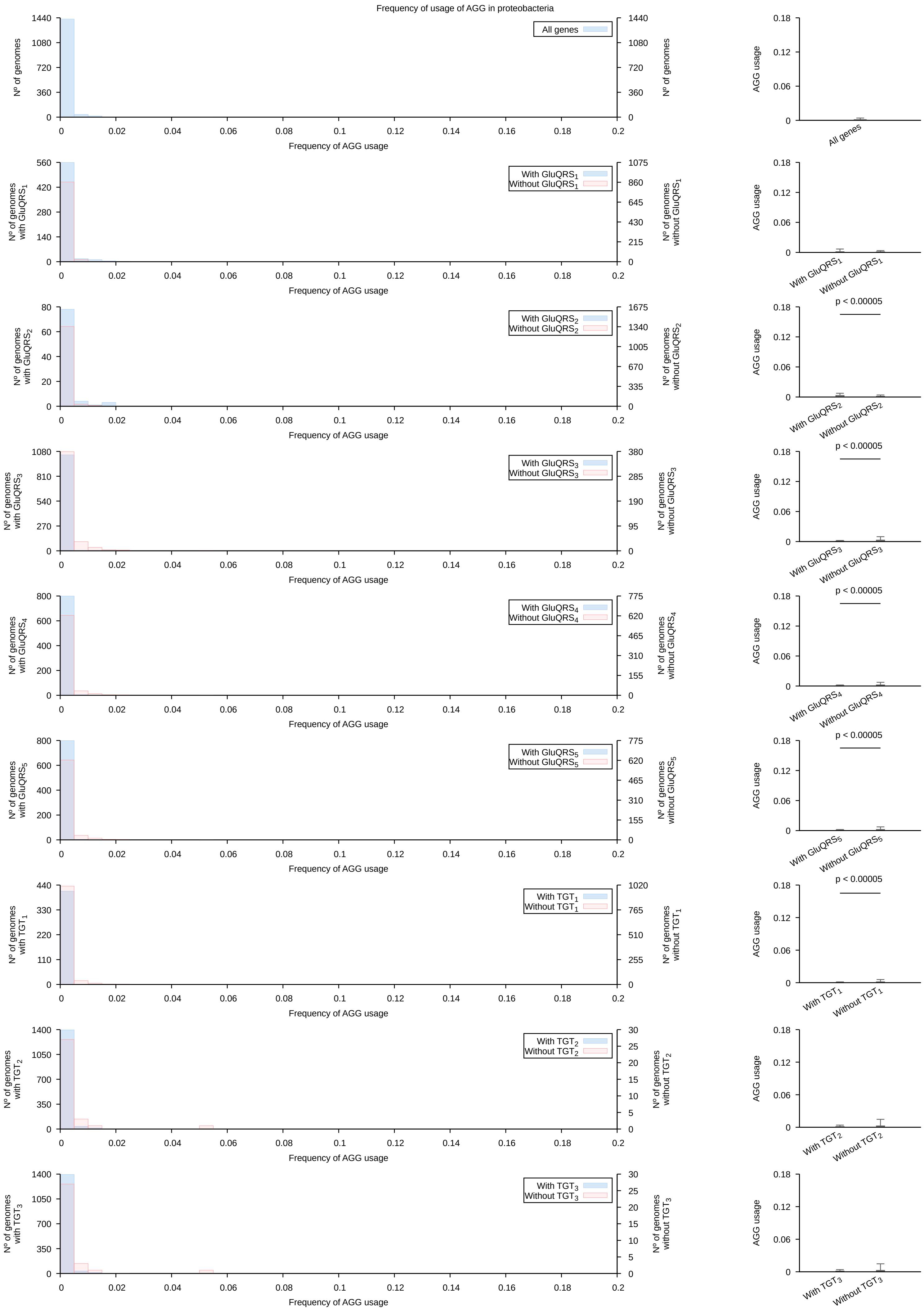

Frequency of usage of AGG in proteobacteria
All genes
With GluQRS$_1$
Without GluQRS$_1$
With GluQRS$_2$
Without GluQRS$_2$
With GluQRS$_3$
Without GluQRS$_3$
With GluQRS$_4$
Without GluQRS$_4$
With GluQRS$_5$
Without GluQRS$_5$
With TGT$_1$
Without TGT$_1$
With TGT$_2$
Without TGT$_2$
With TGT$_3$
Without TGT$_3$
Nº of genomes
Nº of genomes with GluQRS$_1$
Nº of genomes without GluQRS$_1$
Nº of genomes with GluQRS$_2$
Nº of genomes without GluQRS$_2$
Nº of genomes with GluQRS$_3$
Nº of genomes without GluQRS$_3$
Nº of genomes with GluQRS$_4$
Nº of genomes without GluQRS$_4$
Nº of genomes with GluQRS$_5$
Nº of genomes without GluQRS$_5$
Nº of genomes with TGT$_1$
Nº of genomes without TGT$_1$
Nº of genomes with TGT$_2$
Nº of genomes without TGT$_2$
Nº of genomes with TGT$_3$
Nº of genomes without TGT$_3$
Frequency of AGG usage
0 0.02 0.04 0.06 0.08 0.1 0.12 0.14 0.16 0.18 0.2
AGG usage
0 0.06 0.12 0.18
p < 0.00005

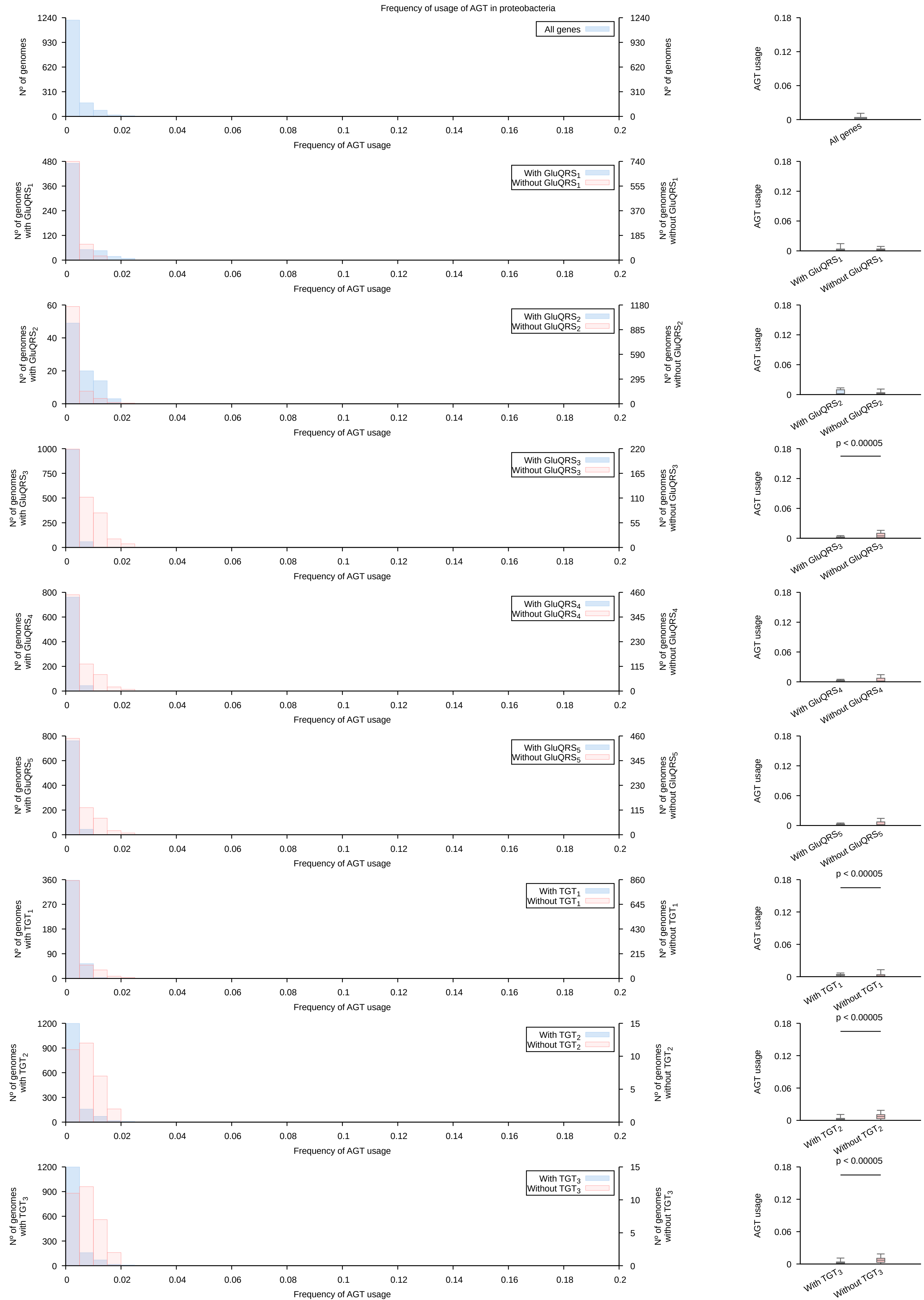
Frequency of usage of AGT in proteobacteria
All genes
Nº of genomes
AGT usage
Frequency of AGT usage
With GluQRS1
Without GluQRS1
Nº of genomes with GluQRS1
Nº of genomes without GluQRS1
With GluQRS2
Without GluQRS2
Nº of genomes with GluQRS2
Nº of genomes without GluQRS2
With GluQRS3
Without GluQRS3
Nº of genomes with GluQRS3
Nº of genomes without GluQRS3
p < 0.00005
With GluQRS4
Without GluQRS4
Nº of genomes with GluQRS4
Nº of genomes without GluQRS4
With GluQRS5
Without GluQRS5
Nº of genomes with GluQRS5
Nº of genomes without GluQRS5
With TGT1
Without TGT1
Nº of genomes with TGT1
Nº of genomes without TGT1
p < 0.00005
With TGT2
Without TGT2
Nº of genomes with TGT2
Nº of genomes without TGT2
p < 0.00005
With TGT3
Without TGT3
Nº of genomes with TGT3
Nº of genomes without TGT3
p < 0.00005

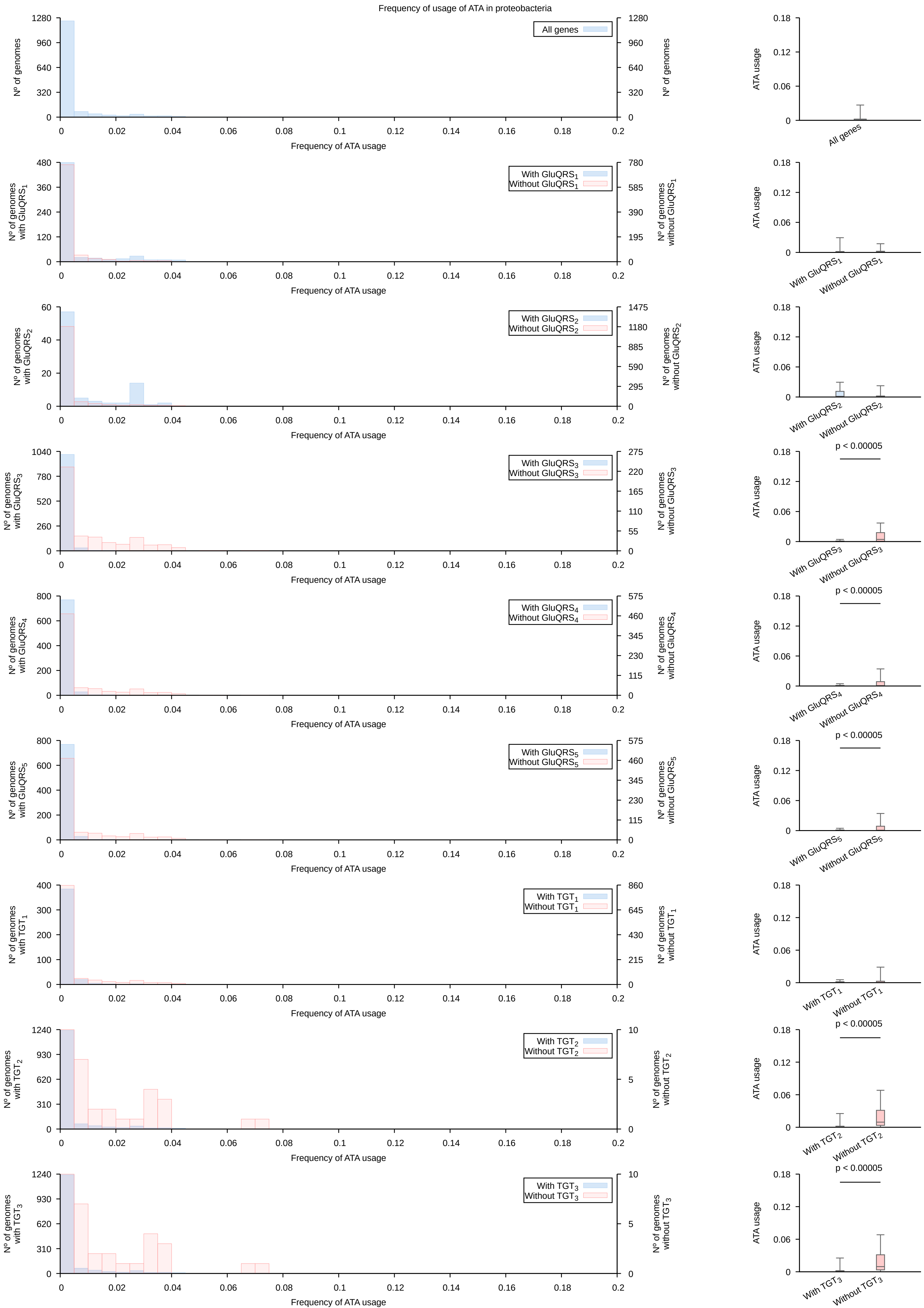
Frequency of usage of ATA in proteobacteria
All genes
Nº of genomes
Frequency of ATA usage
ATA usage
With GluQRS1
Without GluQRS1
Nº of genomes with GluQRS1
Nº of genomes without GluQRS1
With GluQRS2
Without GluQRS2
Nº of genomes with GluQRS2
Nº of genomes without GluQRS2
With GluQRS3
Without GluQRS3
Nº of genomes with GluQRS3
Nº of genomes without GluQRS3
p < 0.00005
With GluQRS4
Without GluQRS4
Nº of genomes with GluQRS4
Nº of genomes without GluQRS4
p < 0.00005
With GluQRS5
Without GluQRS5
Nº of genomes with GluQRS5
Nº of genomes without GluQRS5
p < 0.00005
With TGT1
Without TGT1
Nº of genomes with TGT1
Nº of genomes without TGT1
With TGT2
Without TGT2
Nº of genomes with TGT2
Nº of genomes without TGT2
p < 0.00005
With TGT3
Without TGT3
Nº of genomes with TGT3
Nº of genomes without TGT3
p < 0.00005

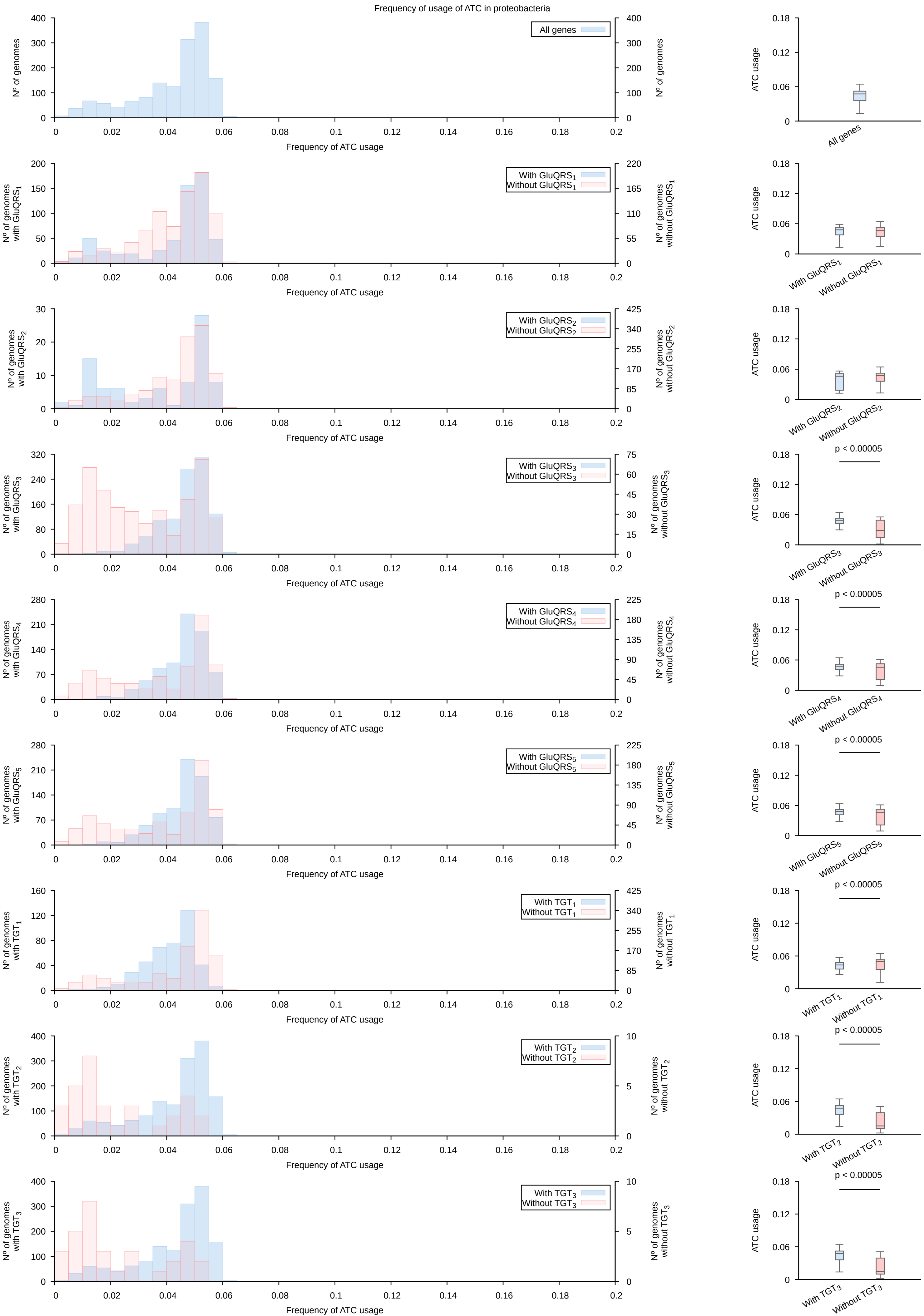
Frequency of usage of ATC in proteobacteria
All genes
With GluQRS$_1$
Without GluQRS$_1$
With GluQRS$_2$
Without GluQRS$_2$
With GluQRS$_3$
Without GluQRS$_3$
With GluQRS$_4$
Without GluQRS$_4$
With GluQRS$_5$
Without GluQRS$_5$
With TGT$_1$
Without TGT$_1$
With TGT$_2$
Without TGT$_2$
With TGT$_3$
Without TGT$_3$
Nº of genomes
Nº of genomes with GluQRS$_1$
Nº of genomes without GluQRS$_1$
Nº of genomes with GluQRS$_2$
Nº of genomes without GluQRS$_2$
Nº of genomes with GluQRS$_3$
Nº of genomes without GluQRS$_3$
Nº of genomes with GluQRS$_4$
Nº of genomes without GluQRS$_4$
Nº of genomes with GluQRS$_5$
Nº of genomes without GluQRS$_5$
Nº of genomes with TGT$_1$
Nº of genomes without TGT$_1$
Nº of genomes with TGT$_2$
Nº of genomes without TGT$_2$
Nº of genomes with TGT$_3$
Nº of genomes without TGT$_3$
Frequency of ATC usage
0 0.02 0.04 0.06 0.08 0.1 0.12 0.14 0.16 0.18 0.2
ATC usage
0 0.06 0.12 0.18
p < 0.00005

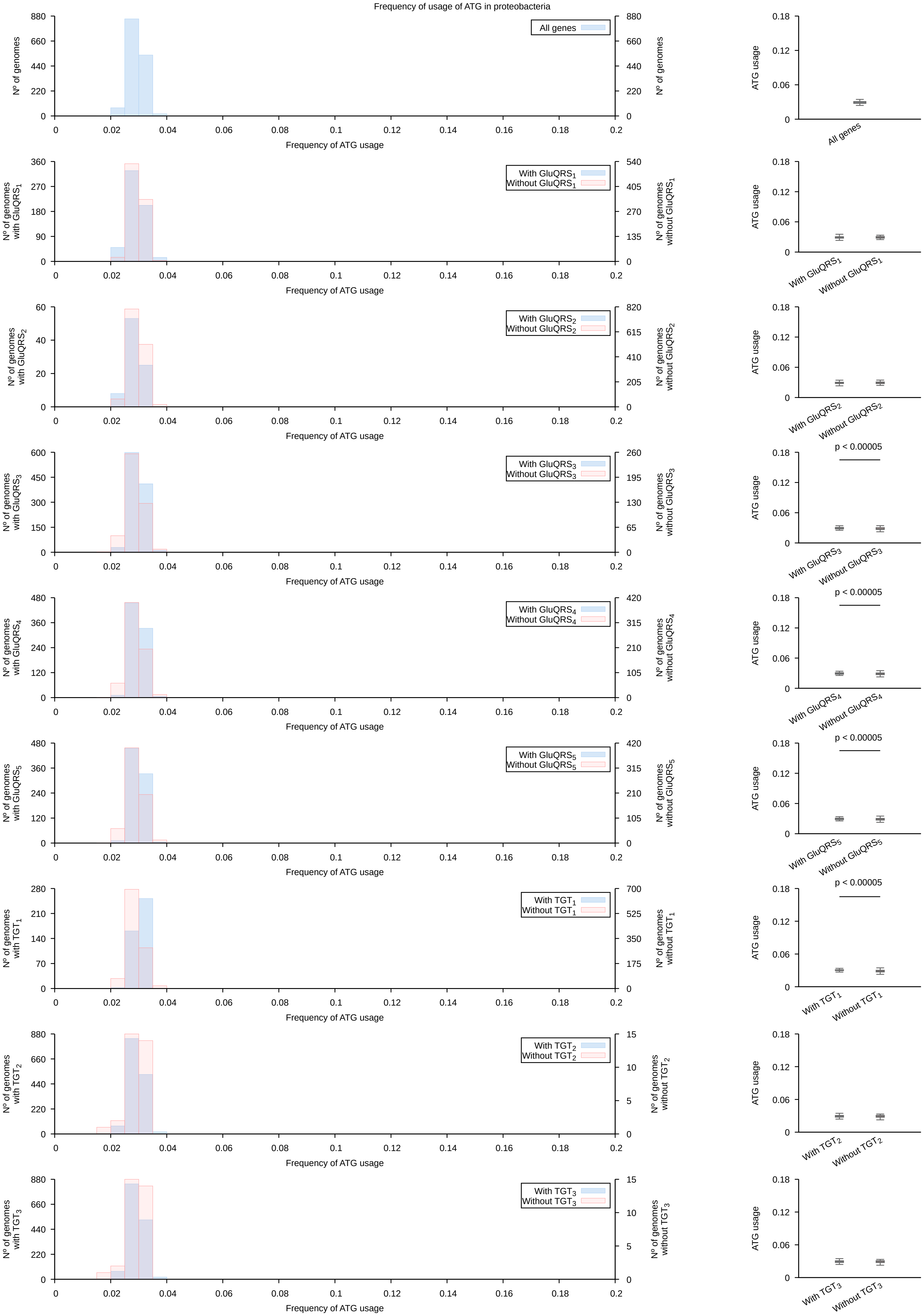
Frequency of usage of ATG in proteobacteria
All genes
With GluQRS1
Without GluQRS1
With GluQRS2
Without GluQRS2
With GluQRS3
Without GluQRS3
With GluQRS4
Without GluQRS4
With GluQRS5
Without GluQRS5
With TGT1
Without TGT1
With TGT2
Without TGT2
With TGT3
Without TGT3
Frequency of ATG usage
Nº of genomes
ATG usage
p < 0.00005

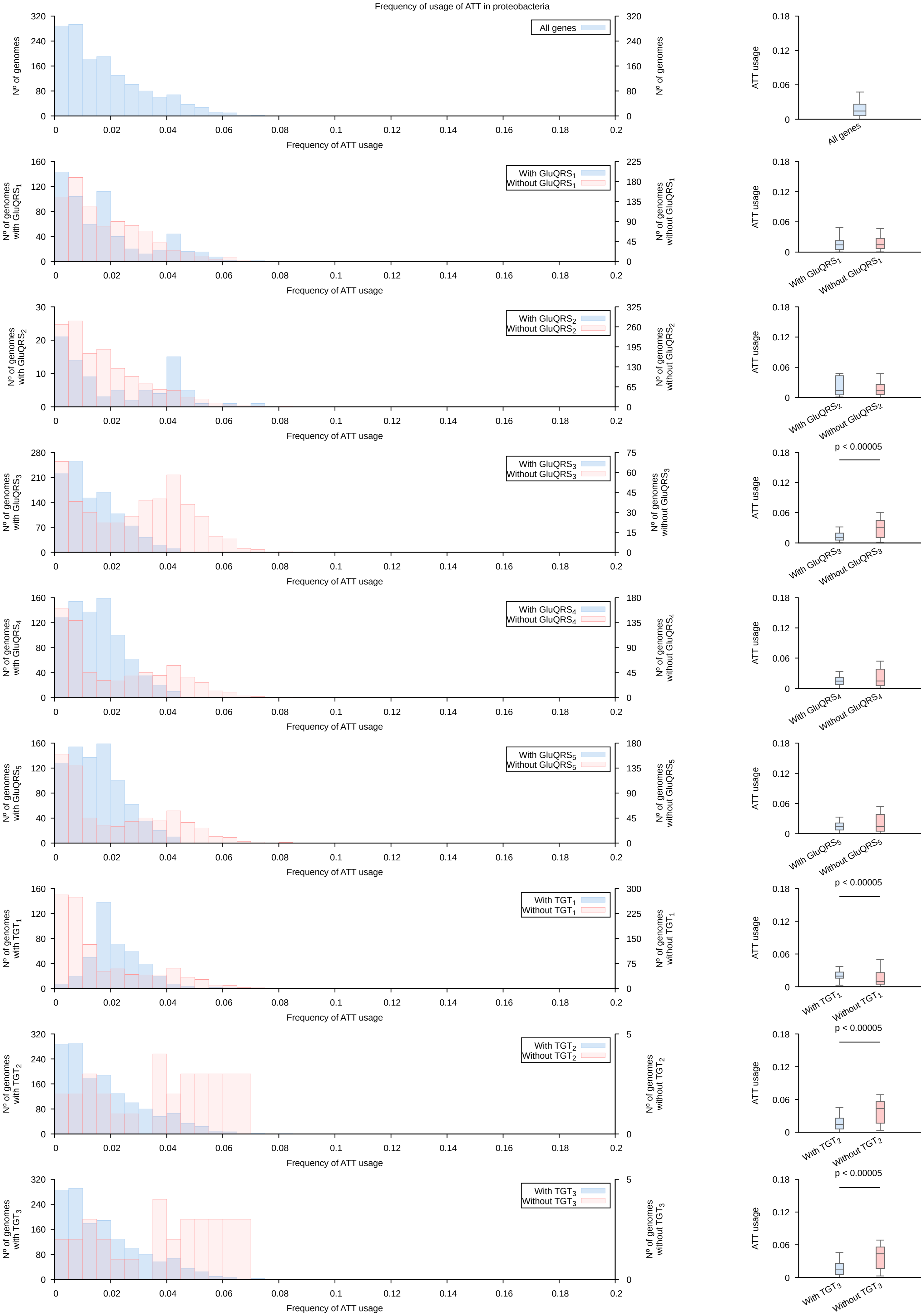

Frequency of usage of ATT in proteobacteria
All genes
Nº of genomes
Frequency of ATT usage
ATT usage
With GluQRS1
Without GluQRS1
Nº of genomes with GluQRS1
Nº of genomes without GluQRS1
With GluQRS2
Without GluQRS2
Nº of genomes with GluQRS2
Nº of genomes without GluQRS2
With GluQRS3
Without GluQRS3
Nº of genomes with GluQRS3
Nº of genomes without GluQRS3
p < 0.00005
With GluQRS4
Without GluQRS4
Nº of genomes with GluQRS4
Nº of genomes without GluQRS4
With GluQRS5
Without GluQRS5
Nº of genomes with GluQRS5
Nº of genomes without GluQRS5
With TGT1
Without TGT1
Nº of genomes with TGT1
Nº of genomes without TGT1
p < 0.00005
With TGT2
Without TGT2
Nº of genomes with TGT2
Nº of genomes without TGT2
p < 0.00005
With TGT3
Without TGT3
Nº of genomes with TGT3
Nº of genomes without TGT3
p < 0.00005

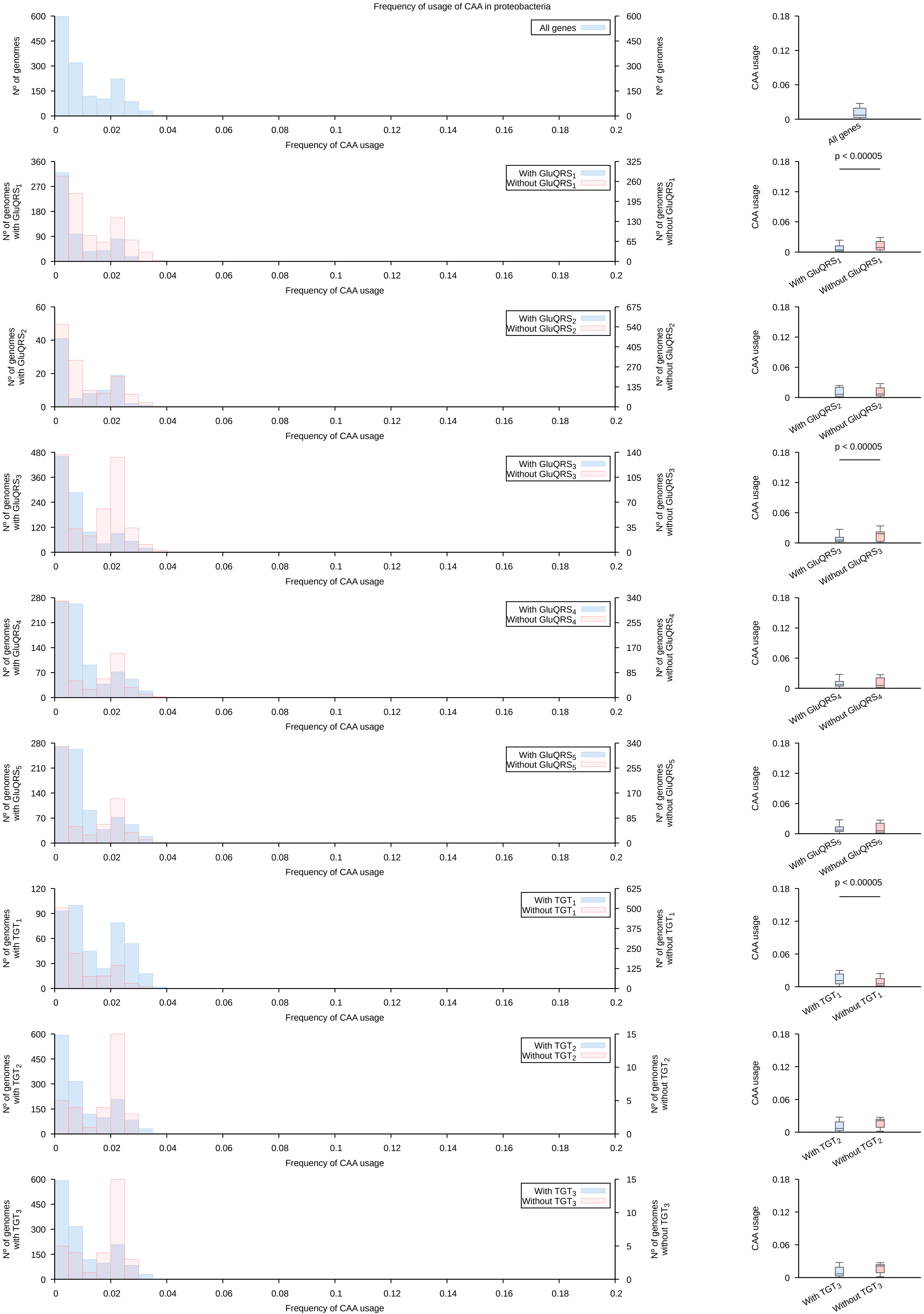
Frequency of usage of CAA in proteobacteria
All genes
Nº of genomes
Frequency of CAA usage
CAA usage
With GluQRS$_1$
Without GluQRS$_1$
Nº of genomes with GluQRS$_1$
Nº of genomes without GluQRS$_1$
p < 0.00005
With GluQRS$_2$
Without GluQRS$_2$
Nº of genomes with GluQRS$_2$
Nº of genomes without GluQRS$_2$
With GluQRS$_3$
Without GluQRS$_3$
Nº of genomes with GluQRS$_3$
Nº of genomes without GluQRS$_3$
p < 0.00005
With GluQRS$_4$
Without GluQRS$_4$
Nº of genomes with GluQRS$_4$
Nº of genomes without GluQRS$_4$
With GluQRS$_5$
Without GluQRS$_5$
Nº of genomes with GluQRS$_5$
Nº of genomes without GluQRS$_5$
With TGT$_1$
Without TGT$_1$
Nº of genomes with TGT$_1$
Nº of genomes without TGT$_1$
p < 0.00005
With TGT$_2$
Without TGT$_2$
Nº of genomes with TGT$_2$
Nº of genomes without TGT$_2$
With TGT$_3$
Without TGT$_3$
Nº of genomes with TGT$_3$
Nº of genomes without TGT$_3$

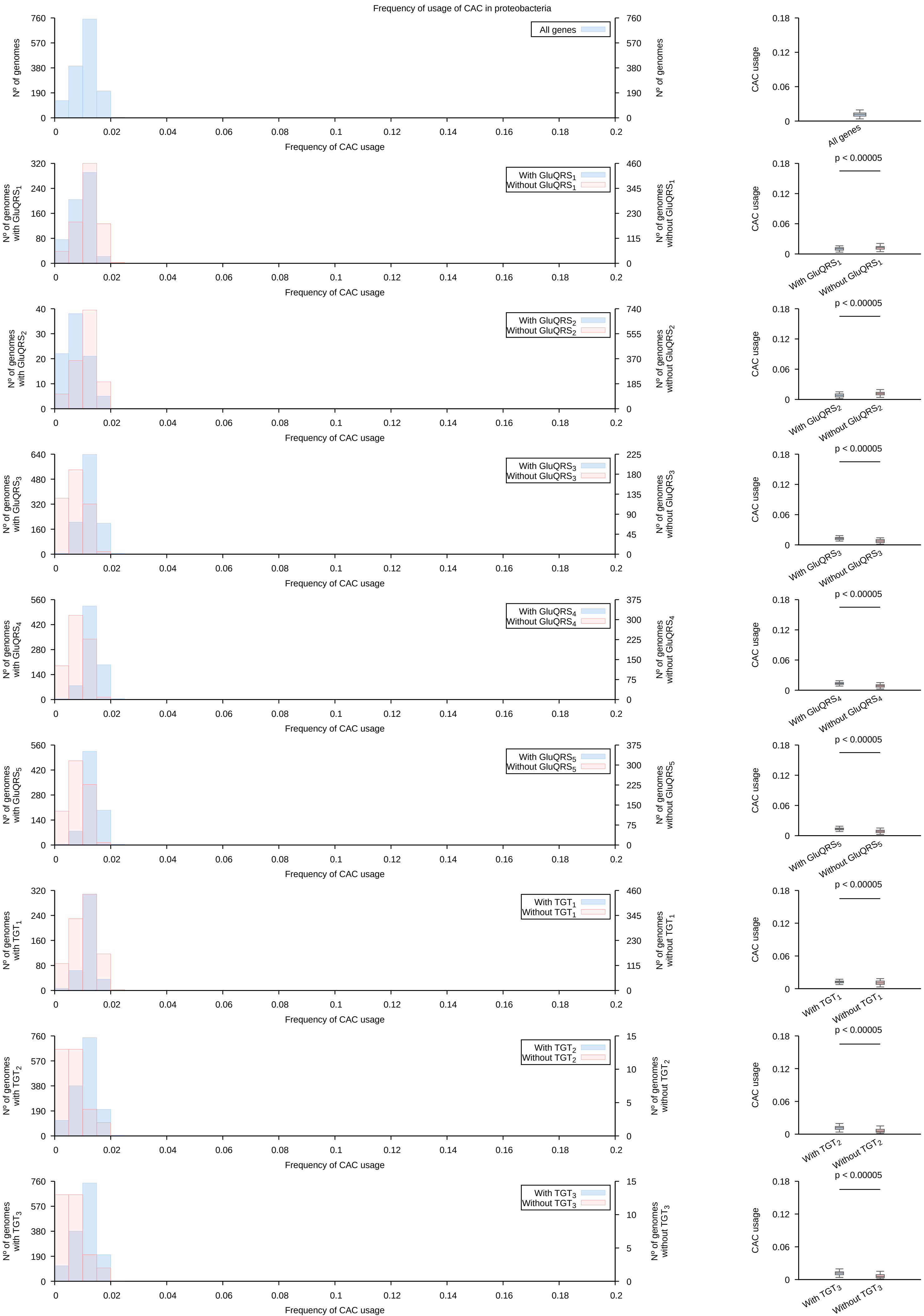
Frequency of usage of CAC in proteobacteria
Nº of genomes
760
570
380
190
0
All genes
0
0.02
0.04
0.06
0.08
0.1
0.12
0.14
0.16
0.18
0.2
Frequency of CAC usage
CAC usage
0.18
0.12
0.06
All genes
Nº of genomes with GluQRS1
Nº of genomes without GluQRS1
320
240
160
80
460
345
230
115
With GluQRS1
Without GluQRS1
p < 0.00005
Nº of genomes with GluQRS2
Nº of genomes without GluQRS2
40
30
20
10
740
555
370
185
With GluQRS2
Without GluQRS2
Nº of genomes with GluQRS3
Nº of genomes without GluQRS3
640
480
320
160
225
180
135
90
45
With GluQRS3
Without GluQRS3
Nº of genomes with GluQRS4
Nº of genomes without GluQRS4
560
420
280
140
375
300
225
150
75
With GluQRS4
Without GluQRS4
Nº of genomes with GluQRS5
Nº of genomes without GluQRS5
With GluQRS5
Without GluQRS5
Nº of genomes with TGT1
Nº of genomes without TGT1
With TGT1
Without TGT1
Nº of genomes with TGT2
Nº of genomes without TGT2
15
10
5
With TGT2
Without TGT2
Nº of genomes with TGT3
Nº of genomes without TGT3
With TGT3
Without TGT3

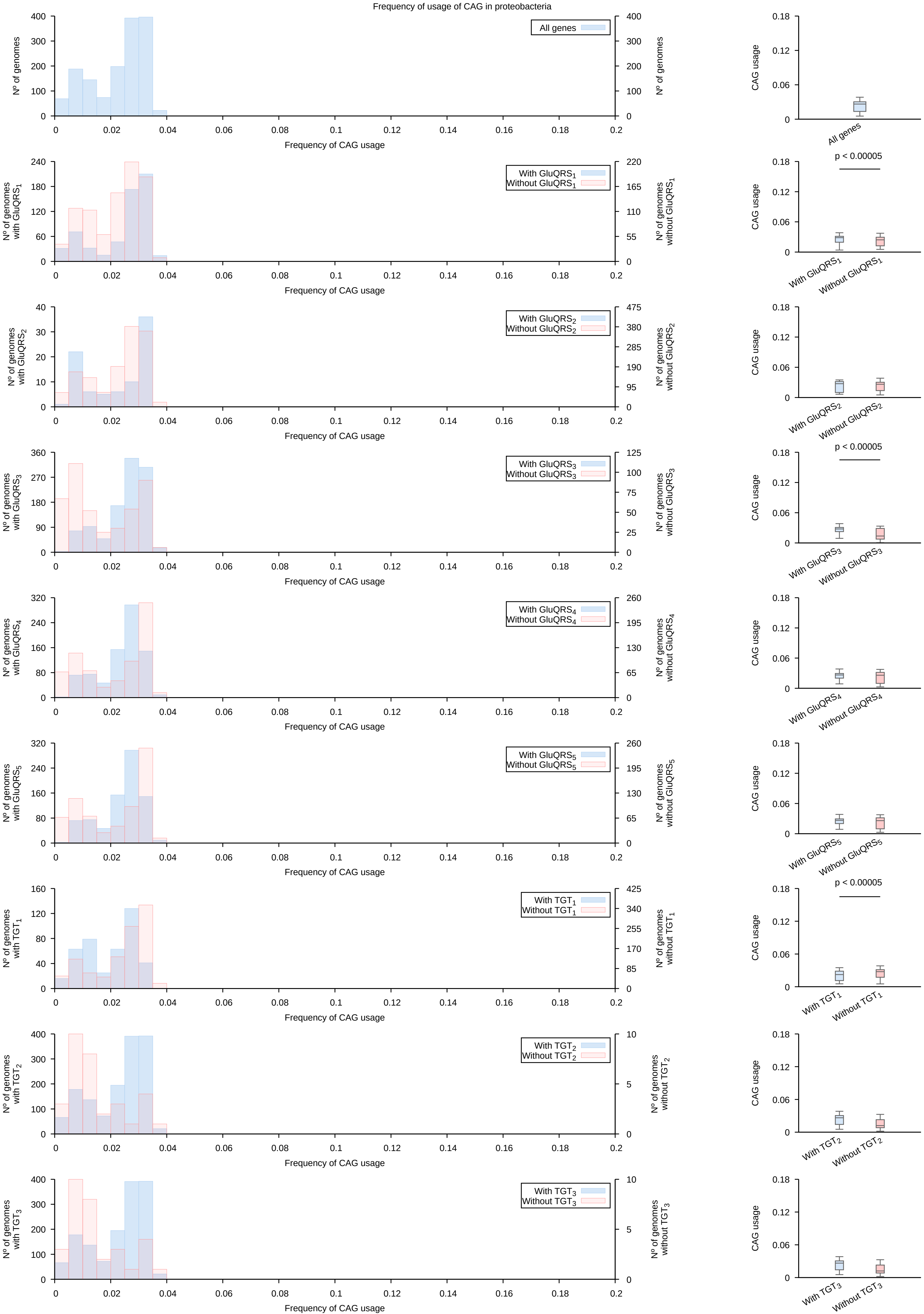
Frequency of usage of CAG in proteobacteria
All genes
With GluQRS1
Without GluQRS1
With GluQRS2
Without GluQRS2
With GluQRS3
Without GluQRS3
With GluQRS4
Without GluQRS4
With GluQRS5
Without GluQRS5
With TGT1
Without TGT1
With TGT2
Without TGT2
With TGT3
Without TGT3
Nº of genomes
Frequency of CAG usage
CAG usage
p < 0.00005

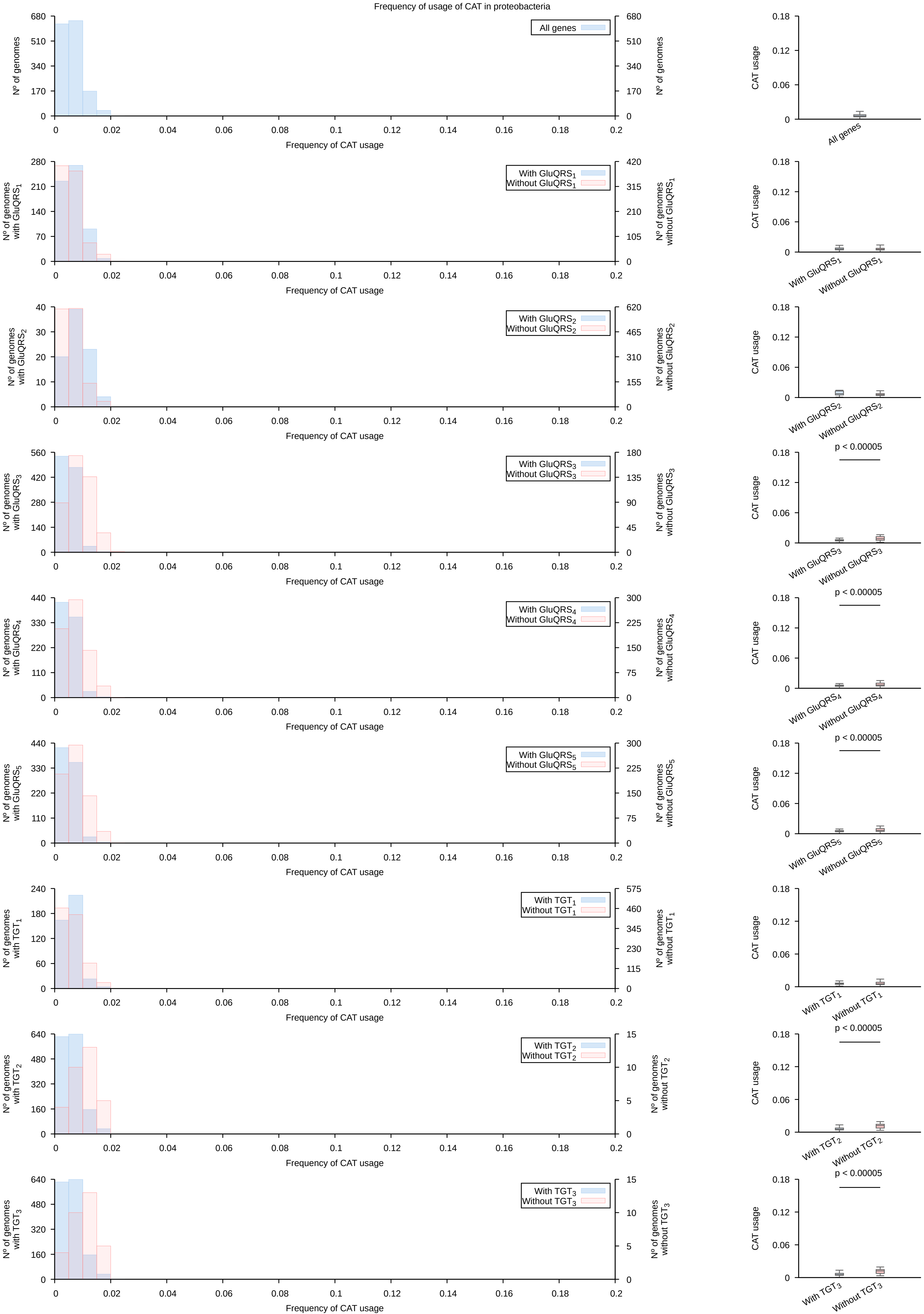
Frequency of usage of CAT in proteobacteria
Nº of genomes
Frequency of CAT usage
All genes
CAT usage
Nº of genomes with GluQRS$_1$
Nº of genomes without GluQRS$_1$
With GluQRS$_1$
Without GluQRS$_1$
Nº of genomes with GluQRS$_2$
Nº of genomes without GluQRS$_2$
With GluQRS$_2$
Without GluQRS$_2$
Nº of genomes with GluQRS$_3$
Nº of genomes without GluQRS$_3$
With GluQRS$_3$
Without GluQRS$_3$
p < 0.00005
Nº of genomes with GluQRS$_4$
Nº of genomes without GluQRS$_4$
With GluQRS$_4$
Without GluQRS$_4$
p < 0.00005
Nº of genomes with GluQRS$_5$
Nº of genomes without GluQRS$_5$
With GluQRS$_5$
Without GluQRS$_5$
p < 0.00005
Nº of genomes with TGT$_1$
Nº of genomes without TGT$_1$
With TGT$_1$
Without TGT$_1$
Nº of genomes with TGT$_2$
Nº of genomes without TGT$_2$
With TGT$_2$
Without TGT$_2$
p < 0.00005
Nº of genomes with TGT$_3$
Nº of genomes without TGT$_3$
With TGT$_3$
Without TGT$_3$
p < 0.00005

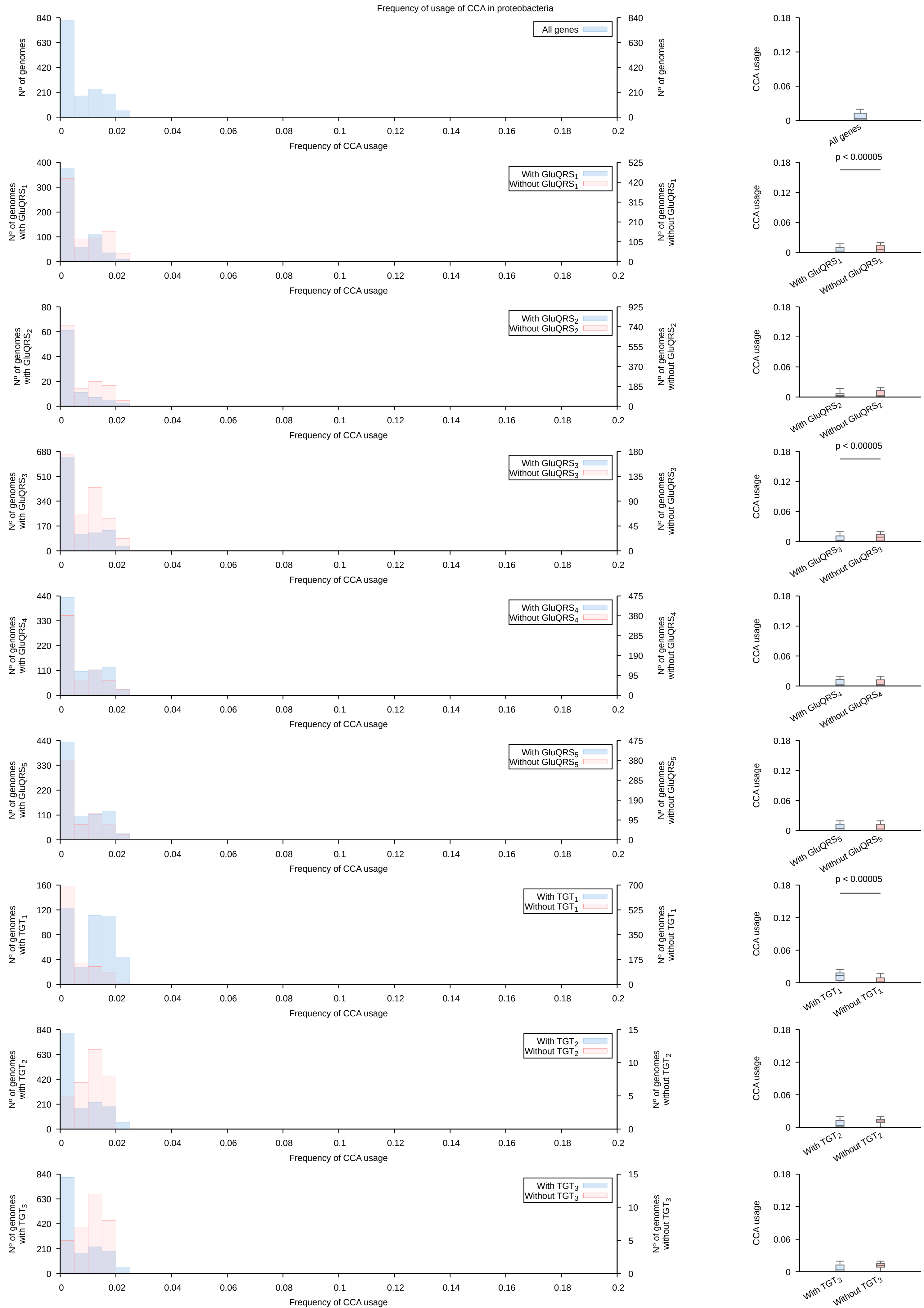
Frequency of usage of CCA in proteobacteria
Nº of genomes
All genes
Frequency of CCA usage
CCA usage
All genes
Nº of genomes with GluQRS$_1$
Nº of genomes without GluQRS$_1$
With GluQRS$_1$
Without GluQRS$_1$
p < 0.00005
Nº of genomes with GluQRS$_2$
Nº of genomes without GluQRS$_2$
With GluQRS$_2$
Without GluQRS$_2$
Nº of genomes with GluQRS$_3$
Nº of genomes without GluQRS$_3$
With GluQRS$_3$
Without GluQRS$_3$
p < 0.00005
Nº of genomes with GluQRS$_4$
Nº of genomes without GluQRS$_4$
With GluQRS$_4$
Without GluQRS$_4$
Nº of genomes with GluQRS$_5$
Nº of genomes without GluQRS$_5$
With GluQRS$_5$
Without GluQRS$_5$
Nº of genomes with TGT$_1$
Nº of genomes without TGT$_1$
With TGT$_1$
Without TGT$_1$
p < 0.00005
Nº of genomes with TGT$_2$
Nº of genomes without TGT$_2$
With TGT$_2$
Without TGT$_2$
Nº of genomes with TGT$_3$
Nº of genomes without TGT$_3$
With TGT$_3$
Without TGT$_3$

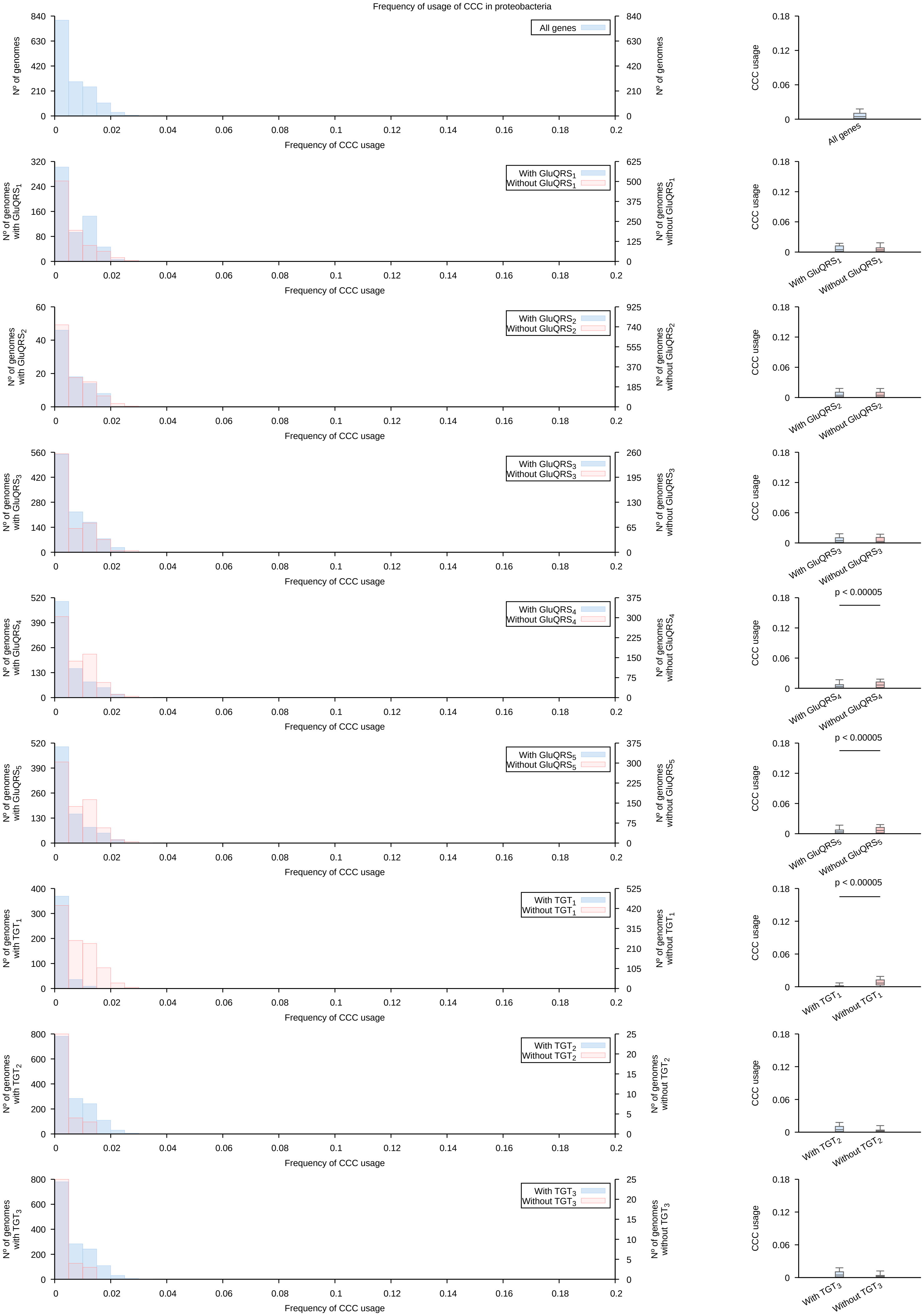

Frequency of usage of CCC in proteobacteria
All genes
Nº of genomes
Frequency of CCC usage
CCC usage
With GluQRS$_1$
Without GluQRS$_1$
Nº of genomes with GluQRS$_1$
Nº of genomes without GluQRS$_1$
With GluQRS$_2$
Without GluQRS$_2$
Nº of genomes with GluQRS$_2$
Nº of genomes without GluQRS$_2$
With GluQRS$_3$
Without GluQRS$_3$
Nº of genomes with GluQRS$_3$
Nº of genomes without GluQRS$_3$
With GluQRS$_4$
Without GluQRS$_4$
Nº of genomes with GluQRS$_4$
Nº of genomes without GluQRS$_4$
p < 0.00005
With GluQRS$_5$
Without GluQRS$_5$
Nº of genomes with GluQRS$_5$
Nº of genomes without GluQRS$_5$
p < 0.00005
With TGT$_1$
Without TGT$_1$
Nº of genomes with TGT$_1$
Nº of genomes without TGT$_1$
p < 0.00005
With TGT$_2$
Without TGT$_2$
Nº of genomes with TGT$_2$
Nº of genomes without TGT$_2$
With TGT$_3$
Without TGT$_3$
Nº of genomes with TGT$_3$
Nº of genomes without TGT$_3$

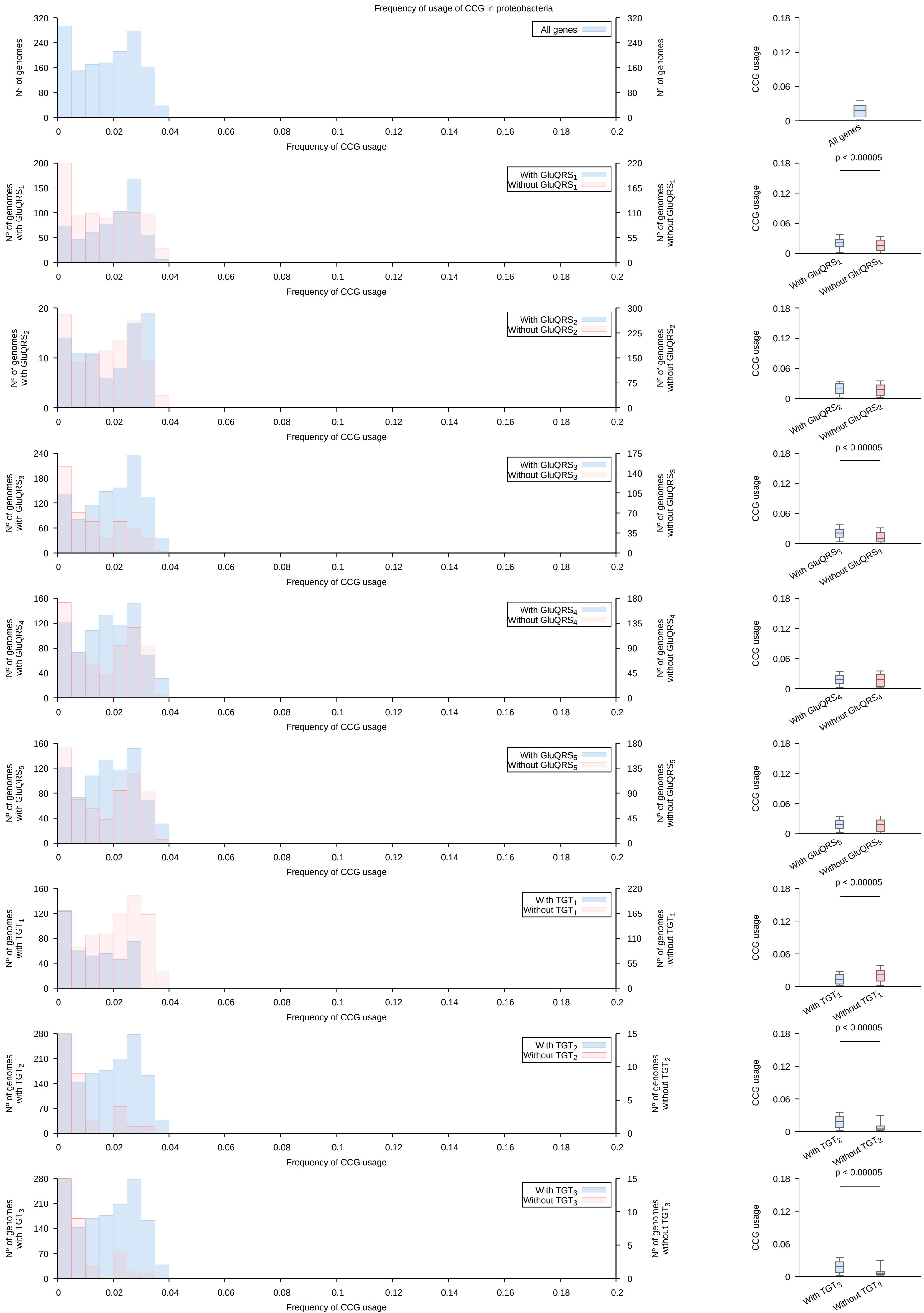

Frequency of usage of CCG in proteobacteria
Nº of genomes
Frequency of CCG usage
CCG usage
All genes
With GluQRS$_1$
Without GluQRS$_1$
Nº of genomes with GluQRS$_1$
Nº of genomes without GluQRS$_1$
p < 0.00005
With GluQRS$_2$
Without GluQRS$_2$
Nº of genomes with GluQRS$_2$
Nº of genomes without GluQRS$_2$
With GluQRS$_3$
Without GluQRS$_3$
Nº of genomes with GluQRS$_3$
Nº of genomes without GluQRS$_3$
p < 0.00005
With GluQRS$_4$
Without GluQRS$_4$
Nº of genomes with GluQRS$_4$
Nº of genomes without GluQRS$_4$
With GluQRS$_5$
Without GluQRS$_5$
Nº of genomes with GluQRS$_5$
Nº of genomes without GluQRS$_5$
With TGT$_1$
Without TGT$_1$
Nº of genomes with TGT$_1$
Nº of genomes without TGT$_1$
p < 0.00005
With TGT$_2$
Without TGT$_2$
Nº of genomes with TGT$_2$
Nº of genomes without TGT$_2$
p < 0.00005
With TGT$_3$
Without TGT$_3$
Nº of genomes with TGT$_3$
Nº of genomes without TGT$_3$
p < 0.00005

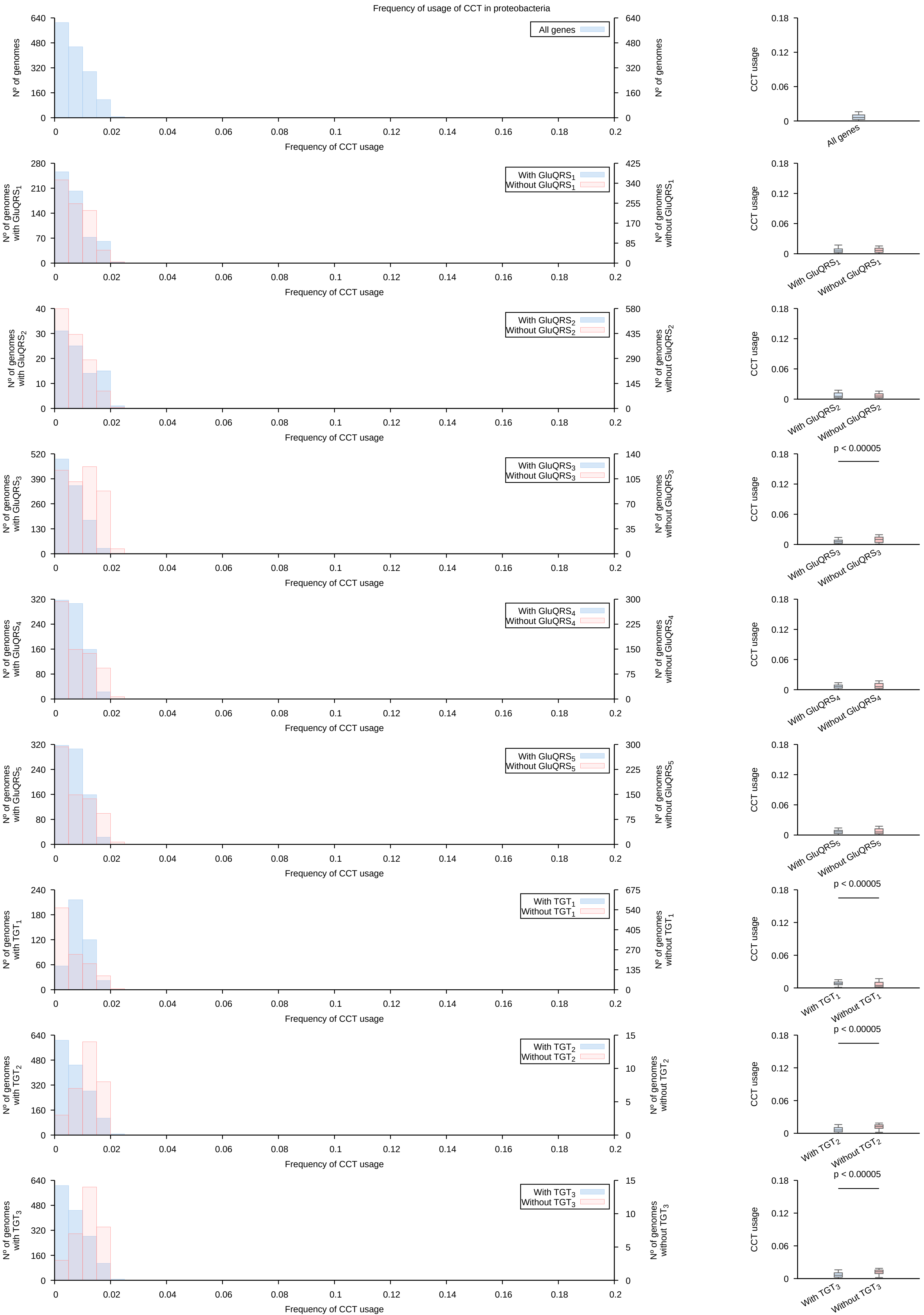

Frequency of usage of CCT in proteobacteria
All genes
Nº of genomes
Frequency of CCT usage
CCT usage
With GluQRS1
Without GluQRS1
Nº of genomes with GluQRS1
Nº of genomes without GluQRS1
With GluQRS2
Without GluQRS2
Nº of genomes with GluQRS2
Nº of genomes without GluQRS2
With GluQRS3
Without GluQRS3
Nº of genomes with GluQRS3
Nº of genomes without GluQRS3
p < 0.00005
With GluQRS4
Without GluQRS4
Nº of genomes with GluQRS4
Nº of genomes without GluQRS4
With GluQRS5
Without GluQRS5
Nº of genomes with GluQRS5
Nº of genomes without GluQRS5
With TGT1
Without TGT1
Nº of genomes with TGT1
Nº of genomes without TGT1
p < 0.00005
With TGT2
Without TGT2
Nº of genomes with TGT2
Nº of genomes without TGT2
p < 0.00005
With TGT3
Without TGT3
Nº of genomes with TGT3
Nº of genomes without TGT3
p < 0.00005

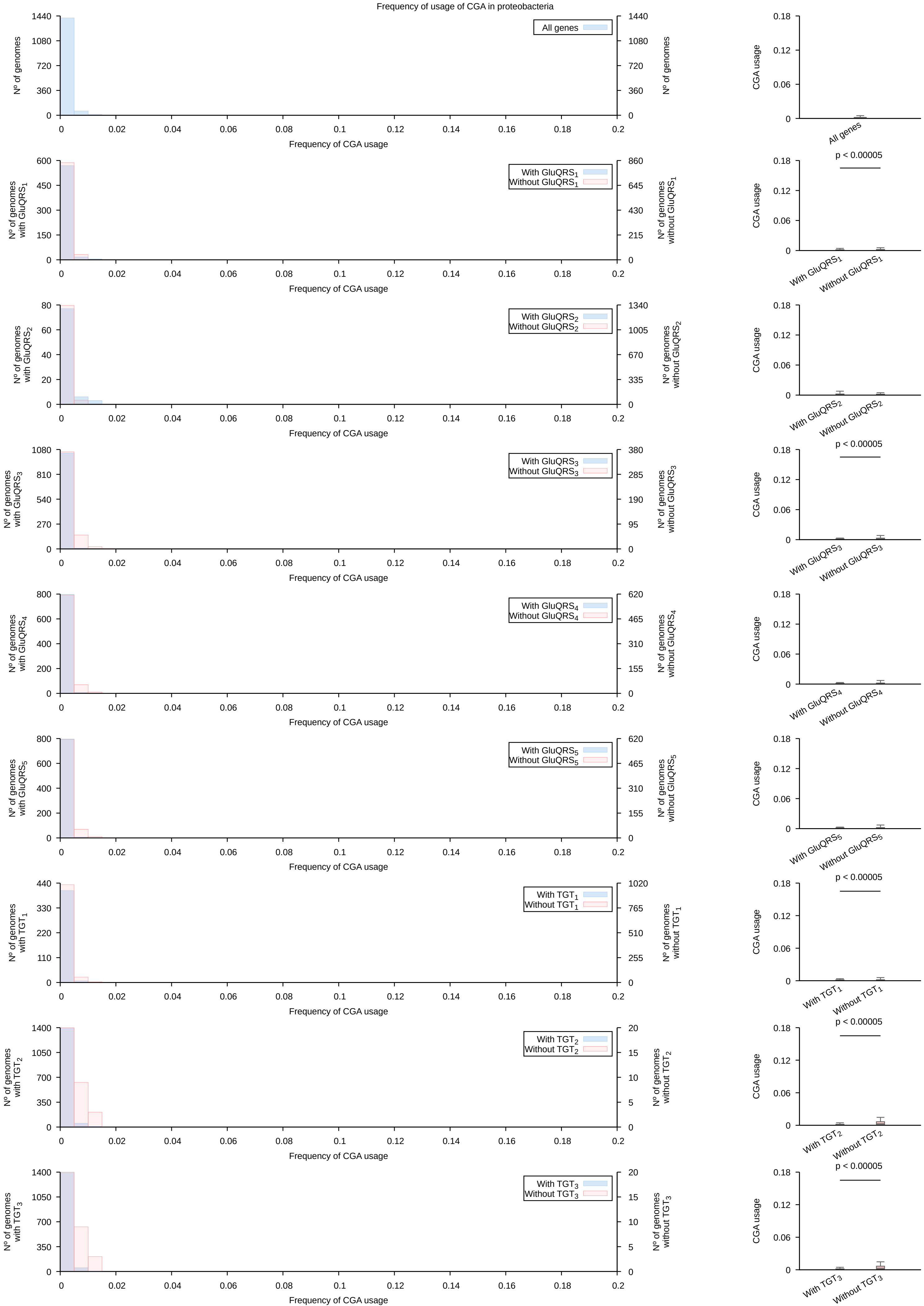
Frequency of usage of CGA in proteobacteria
All genes
With GluQRS$_1$
Without GluQRS$_1$
With GluQRS$_2$
Without GluQRS$_2$
With GluQRS$_3$
Without GluQRS$_3$
With GluQRS$_4$
Without GluQRS$_4$
With GluQRS$_5$
Without GluQRS$_5$
With TGT$_1$
Without TGT$_1$
With TGT$_2$
Without TGT$_2$
With TGT$_3$
Without TGT$_3$
Nº of genomes
Nº of genomes with GluQRS$_1$
Nº of genomes without GluQRS$_1$
Nº of genomes with GluQRS$_2$
Nº of genomes without GluQRS$_2$
Nº of genomes with GluQRS$_3$
Nº of genomes without GluQRS$_3$
Nº of genomes with GluQRS$_4$
Nº of genomes without GluQRS$_4$
Nº of genomes with GluQRS$_5$
Nº of genomes without GluQRS$_5$
Nº of genomes with TGT$_1$
Nº of genomes without TGT$_1$
Nº of genomes with TGT$_2$
Nº of genomes without TGT$_2$
Nº of genomes with TGT$_3$
Nº of genomes without TGT$_3$
Frequency of CGA usage
CGA usage
p < 0.00005
0
0.02
0.04
0.06
0.08
0.1
0.12
0.14
0.16
0.18
0.2

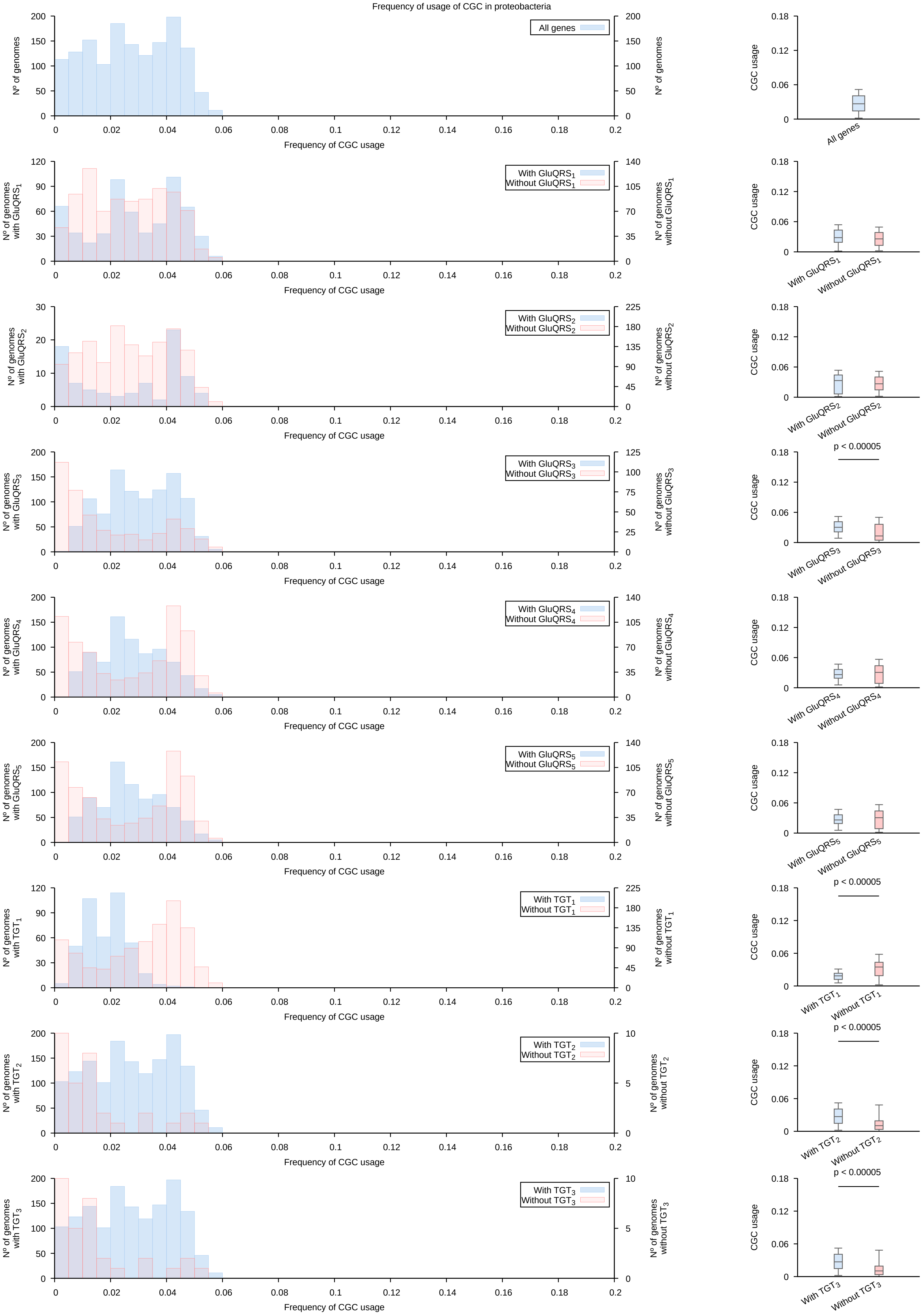

Frequency of usage of CGC in proteobacteria
All genes
Nº of genomes
Frequency of CGC usage
CGC usage
With GluQRS$_1$
Without GluQRS$_1$
Nº of genomes with GluQRS$_1$
Nº of genomes without GluQRS$_1$
With GluQRS$_2$
Without GluQRS$_2$
Nº of genomes with GluQRS$_2$
Nº of genomes without GluQRS$_2$
With GluQRS$_3$
Without GluQRS$_3$
Nº of genomes with GluQRS$_3$
Nº of genomes without GluQRS$_3$
p < 0.00005
With GluQRS$_4$
Without GluQRS$_4$
Nº of genomes with GluQRS$_4$
Nº of genomes without GluQRS$_4$
With GluQRS$_5$
Without GluQRS$_5$
Nº of genomes with GluQRS$_5$
Nº of genomes without GluQRS$_5$
With TGT$_1$
Without TGT$_1$
Nº of genomes with TGT$_1$
Nº of genomes without TGT$_1$
p < 0.00005
With TGT$_2$
Without TGT$_2$
Nº of genomes with TGT$_2$
Nº of genomes without TGT$_2$
p < 0.00005
With TGT$_3$
Without TGT$_3$
Nº of genomes with TGT$_3$
Nº of genomes without TGT$_3$
p < 0.00005

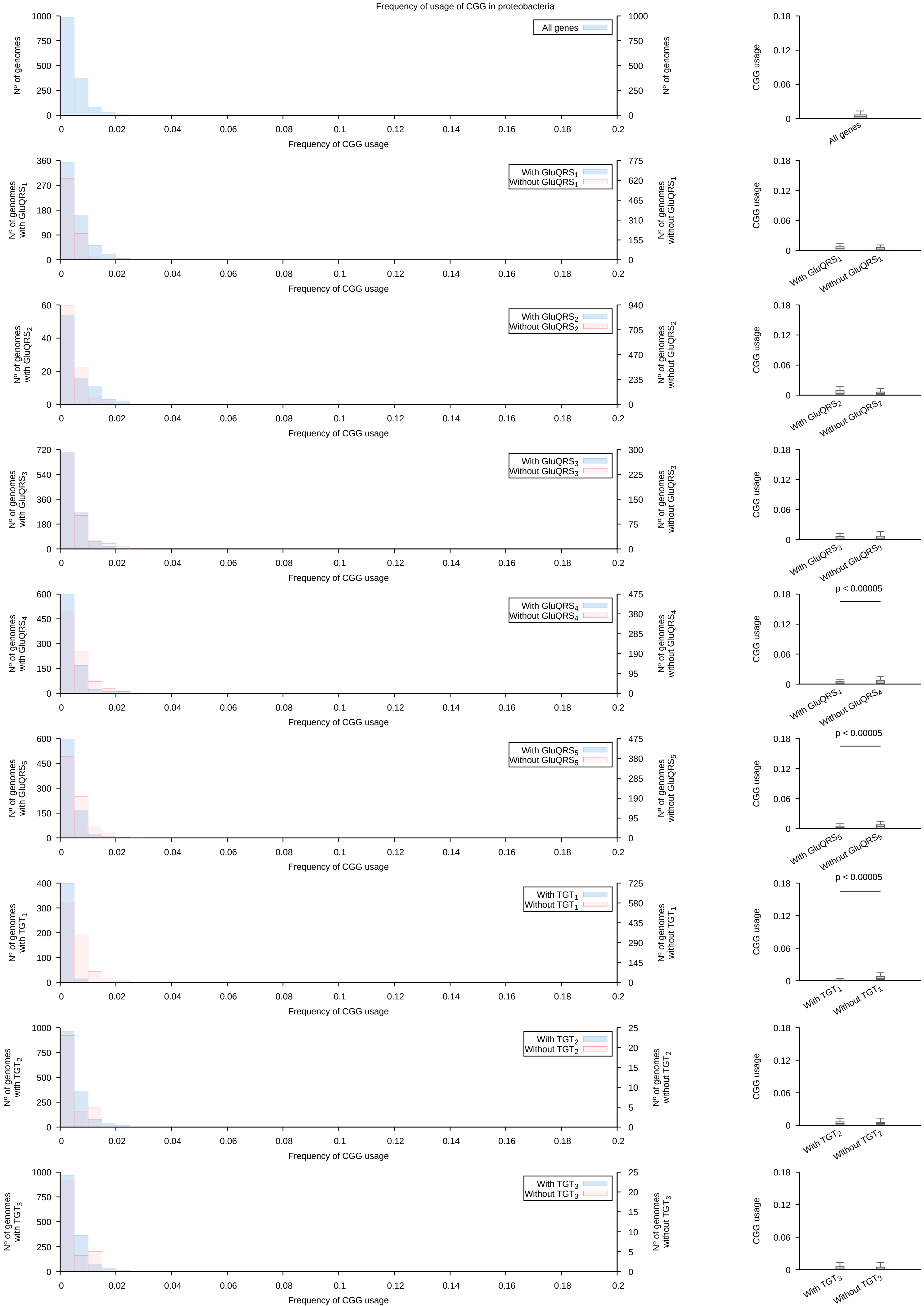
Frequency of usage of CGG in proteobacteria
Frequency of CGG usage
Nº of genomes
CGG usage
All genes
With GluQRS$_1$
Without GluQRS$_1$
With GluQRS$_2$
Without GluQRS$_2$
With GluQRS$_3$
Without GluQRS$_3$
With GluQRS$_4$
Without GluQRS$_4$
With GluQRS$_5$
Without GluQRS$_5$
With TGT$_1$
Without TGT$_1$
With TGT$_2$
Without TGT$_2$
With TGT$_3$
Without TGT$_3$
p < 0.00005

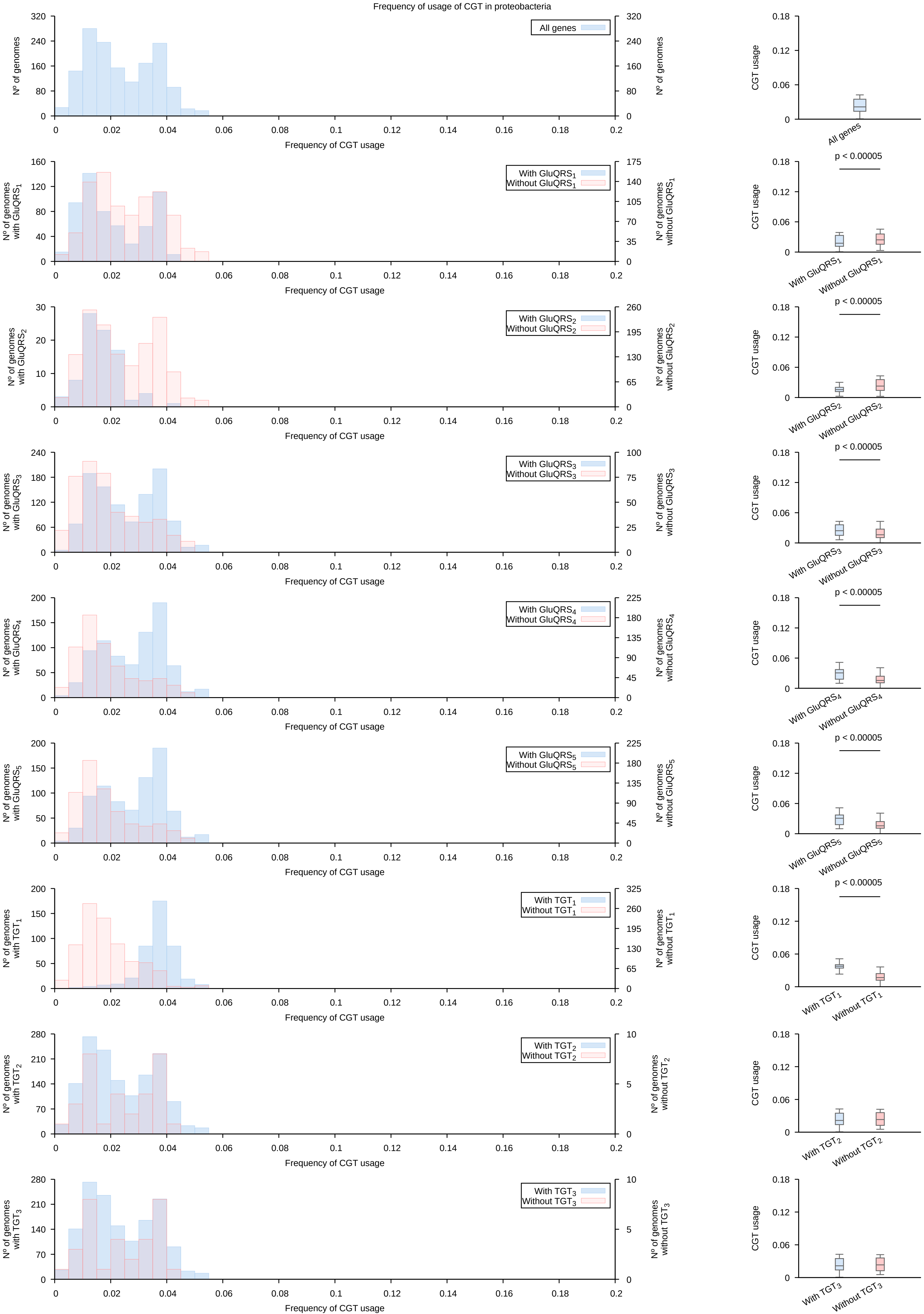
Frequency of usage of CGT in proteobacteria
Nº of genomes
All genes
Frequency of CGT usage
CGT usage
Nº of genomes with GluQRS$_1$
Nº of genomes without GluQRS$_1$
With GluQRS$_1$
Without GluQRS$_1$
p < 0.00005
Nº of genomes with GluQRS$_2$
Nº of genomes without GluQRS$_2$
With GluQRS$_2$
Without GluQRS$_2$
p < 0.00005
Nº of genomes with GluQRS$_3$
Nº of genomes without GluQRS$_3$
With GluQRS$_3$
Without GluQRS$_3$
p < 0.00005
Nº of genomes with GluQRS$_4$
Nº of genomes without GluQRS$_4$
With GluQRS$_4$
Without GluQRS$_4$
p < 0.00005
Nº of genomes with GluQRS$_5$
Nº of genomes without GluQRS$_5$
With GluQRS$_5$
Without GluQRS$_5$
p < 0.00005
Nº of genomes with TGT$_1$
Nº of genomes without TGT$_1$
With TGT$_1$
Without TGT$_1$
p < 0.00005
Nº of genomes with TGT$_2$
Nº of genomes without TGT$_2$
With TGT$_2$
Without TGT$_2$
Nº of genomes with TGT$_3$
Nº of genomes without TGT$_3$
With TGT$_3$
Without TGT$_3$

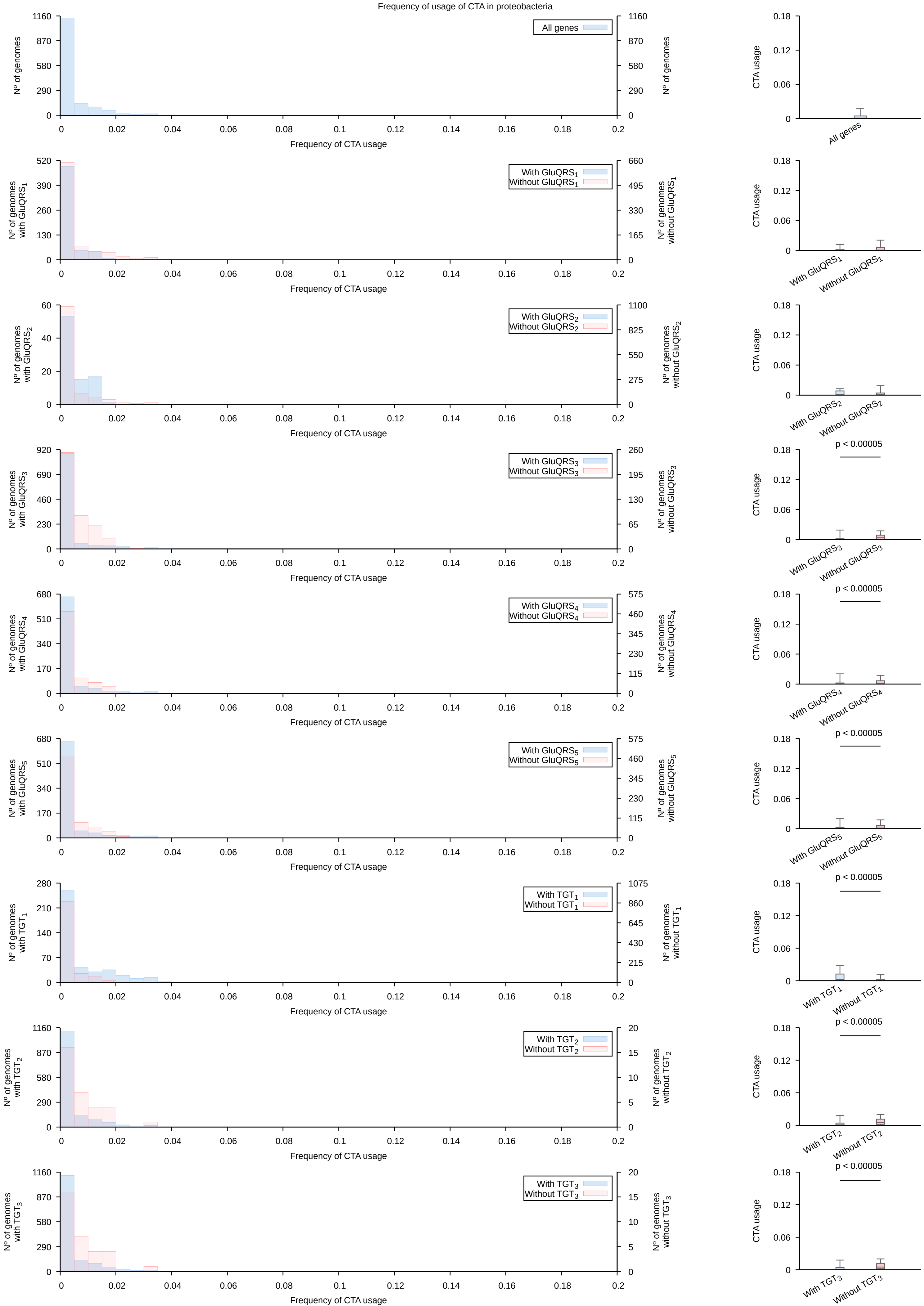
Frequency of usage of CTA in proteobacteria
All genes
Nº of genomes
Frequency of CTA usage
CTA usage
With GluQRS$_1$
Without GluQRS$_1$
Nº of genomes with GluQRS$_1$
Nº of genomes without GluQRS$_1$
With GluQRS$_2$
Without GluQRS$_2$
Nº of genomes with GluQRS$_2$
Nº of genomes without GluQRS$_2$
With GluQRS$_3$
Without GluQRS$_3$
Nº of genomes with GluQRS$_3$
Nº of genomes without GluQRS$_3$
p < 0.00005
With GluQRS$_4$
Without GluQRS$_4$
Nº of genomes with GluQRS$_4$
Nº of genomes without GluQRS$_4$
p < 0.00005
With GluQRS$_5$
Without GluQRS$_5$
Nº of genomes with GluQRS$_5$
Nº of genomes without GluQRS$_5$
p < 0.00005
With TGT$_1$
Without TGT$_1$
Nº of genomes with TGT$_1$
Nº of genomes without TGT$_1$
p < 0.00005
With TGT$_2$
Without TGT$_2$
Nº of genomes with TGT$_2$
Nº of genomes without TGT$_2$
p < 0.00005
With TGT$_3$
Without TGT$_3$
Nº of genomes with TGT$_3$
Nº of genomes without TGT$_3$
p < 0.00005

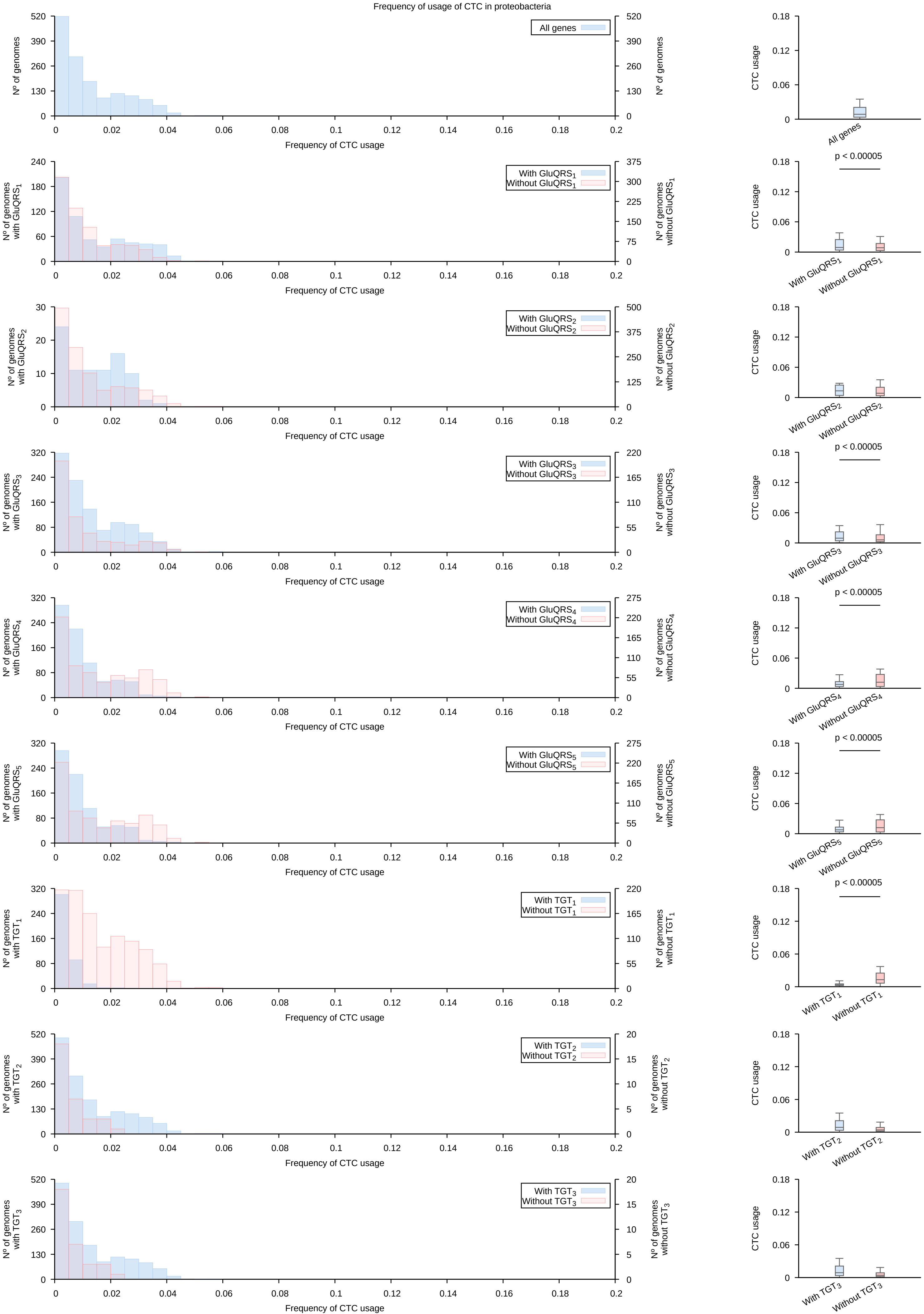

Frequency of usage of CTC in proteobacteria
All genes
Nº of genomes
Frequency of CTC usage
CTC usage
With GluQRS$_1$
Without GluQRS$_1$
Nº of genomes with GluQRS$_1$
Nº of genomes without GluQRS$_1$
p < 0.00005
With GluQRS$_2$
Without GluQRS$_2$
Nº of genomes with GluQRS$_2$
Nº of genomes without GluQRS$_2$
With GluQRS$_3$
Without GluQRS$_3$
Nº of genomes with GluQRS$_3$
Nº of genomes without GluQRS$_3$
p < 0.00005
With GluQRS$_4$
Without GluQRS$_4$
Nº of genomes with GluQRS$_4$
Nº of genomes without GluQRS$_4$
p < 0.00005
With GluQRS$_5$
Without GluQRS$_5$
Nº of genomes with GluQRS$_5$
Nº of genomes without GluQRS$_5$
p < 0.00005
With TGT$_1$
Without TGT$_1$
Nº of genomes with TGT$_1$
Nº of genomes without TGT$_1$
p < 0.00005
With TGT$_2$
Without TGT$_2$
Nº of genomes with TGT$_2$
Nº of genomes without TGT$_2$
With TGT$_3$
Without TGT$_3$
Nº of genomes with TGT$_3$
Nº of genomes without TGT$_3$

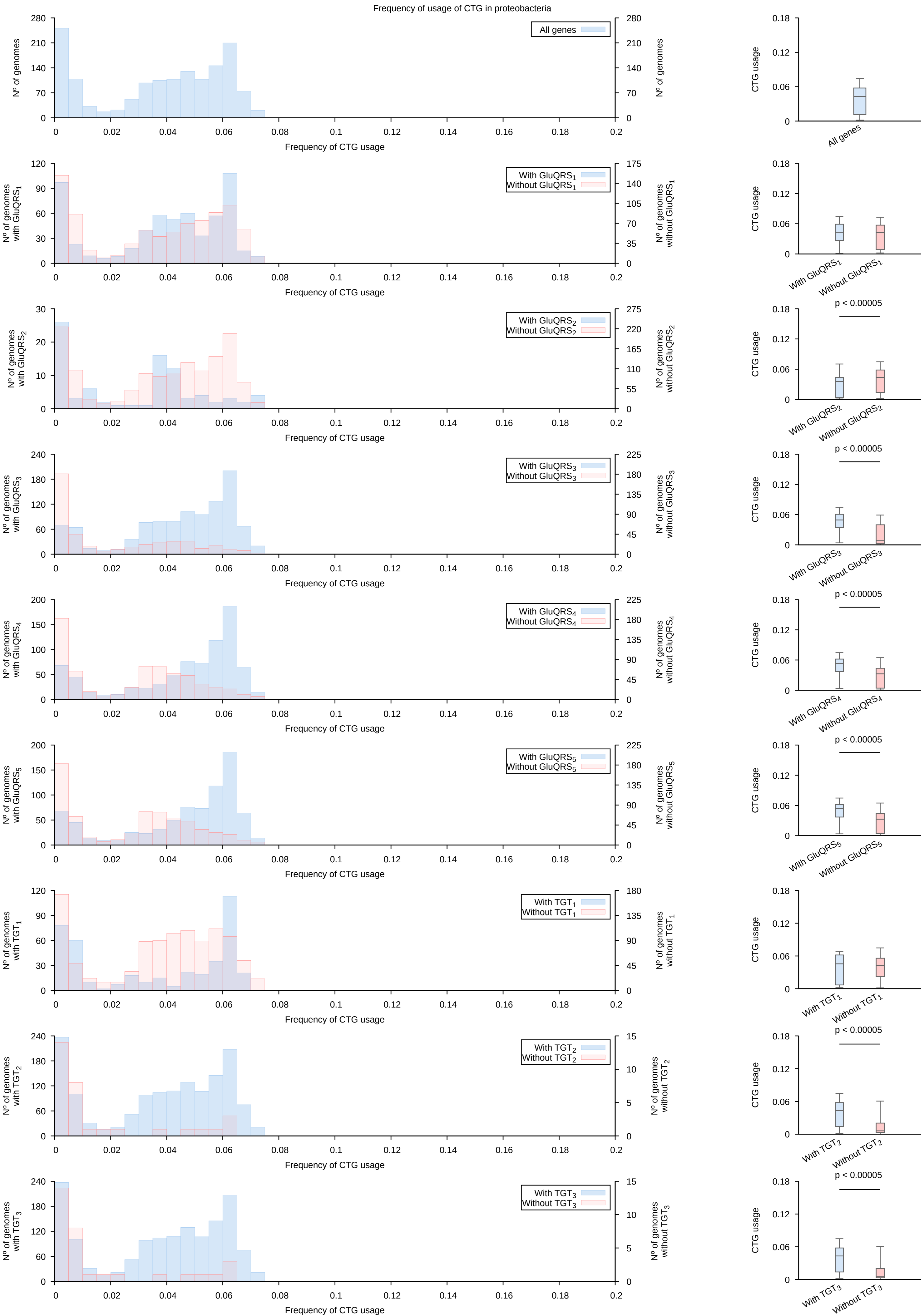

Frequency of usage of CTG in proteobacteria
All genes
Nº of genomes
Frequency of CTG usage
CTG usage
With GluQRS$_1$
Without GluQRS$_1$
Nº of genomes with GluQRS$_1$
Nº of genomes without GluQRS$_1$
With GluQRS$_2$
Without GluQRS$_2$
Nº of genomes with GluQRS$_2$
Nº of genomes without GluQRS$_2$
p < 0.00005
With GluQRS$_3$
Without GluQRS$_3$
Nº of genomes with GluQRS$_3$
Nº of genomes without GluQRS$_3$
p < 0.00005
With GluQRS$_4$
Without GluQRS$_4$
Nº of genomes with GluQRS$_4$
Nº of genomes without GluQRS$_4$
p < 0.00005
With GluQRS$_5$
Without GluQRS$_5$
Nº of genomes with GluQRS$_5$
Nº of genomes without GluQRS$_5$
p < 0.00005
With TGT$_1$
Without TGT$_1$
Nº of genomes with TGT$_1$
Nº of genomes without TGT$_1$
With TGT$_2$
Without TGT$_2$
Nº of genomes with TGT$_2$
Nº of genomes without TGT$_2$
p < 0.00005
With TGT$_3$
Without TGT$_3$
Nº of genomes with TGT$_3$
Nº of genomes without TGT$_3$
p < 0.00005

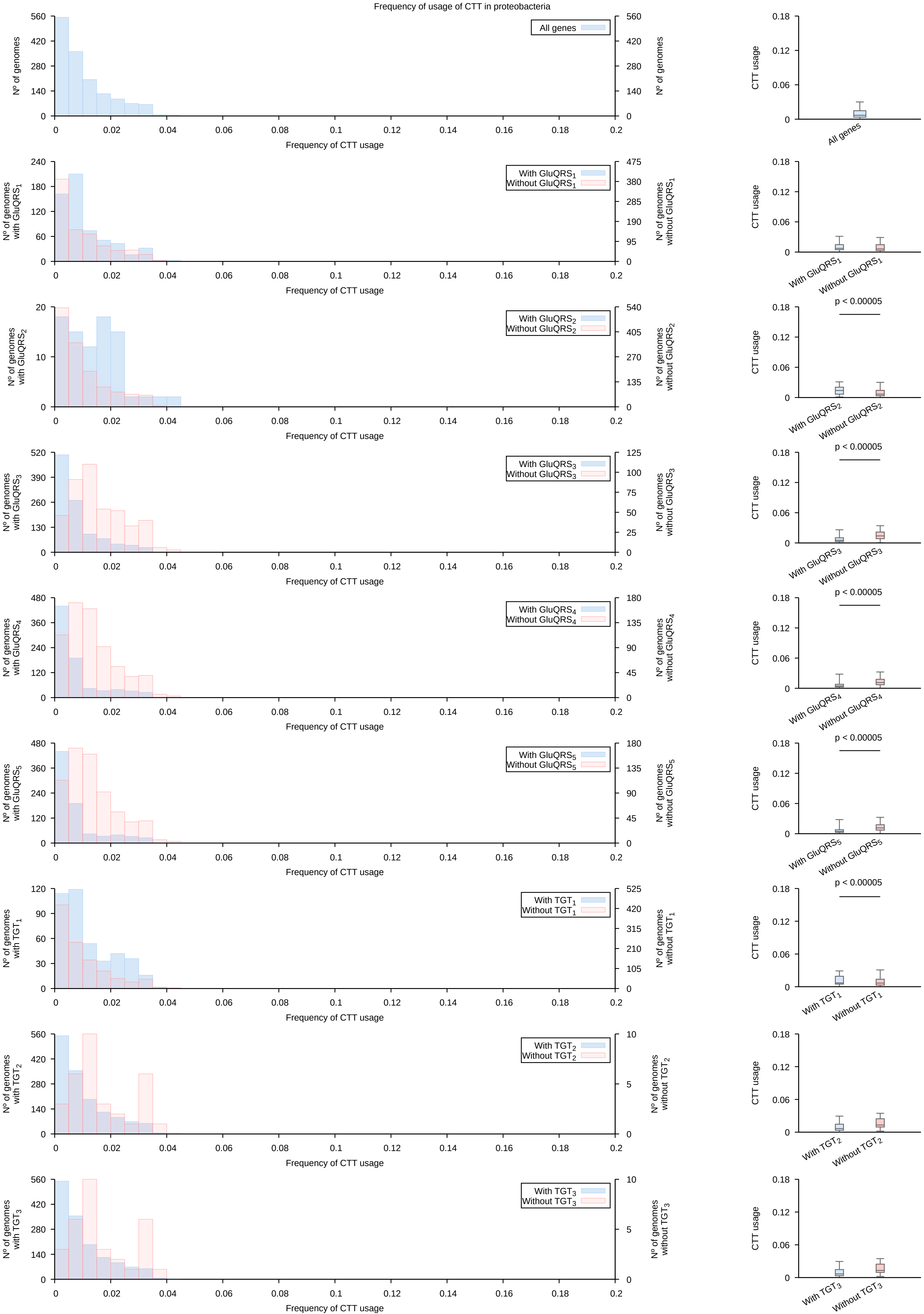
Frequency of usage of CTT in proteobacteria
Nº of genomes
Frequency of CTT usage
CTT usage
All genes
With GluQRS1
Without GluQRS1
Nº of genomes with GluQRS1
Nº of genomes without GluQRS1
With GluQRS2
Without GluQRS2
p < 0.00005
With GluQRS3
Without GluQRS3
With GluQRS4
Without GluQRS4
With GluQRS5
Without GluQRS5
With TGT1
Without TGT1
With TGT2
Without TGT2
With TGT3
Without TGT3

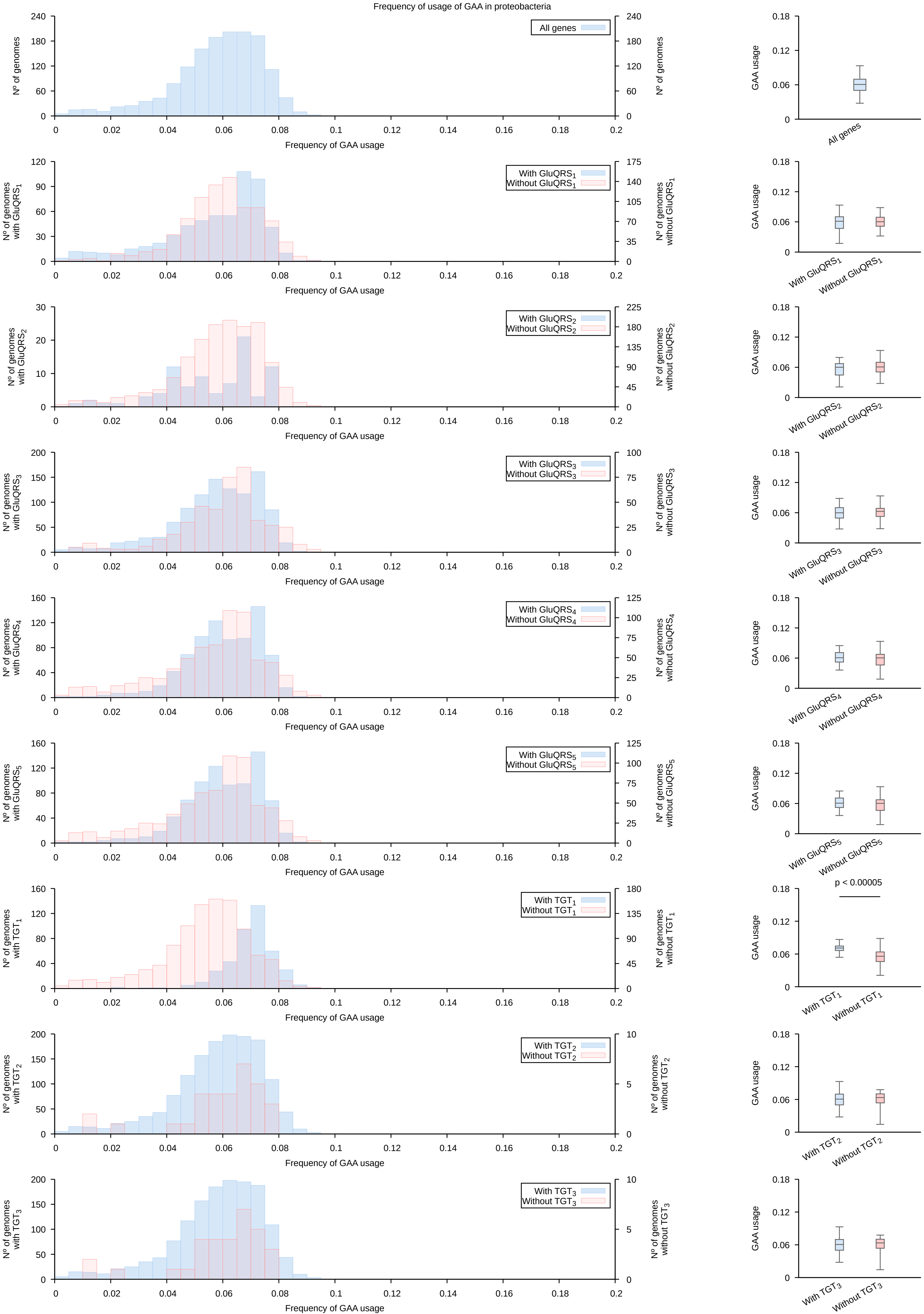
Frequency of usage of GAA in proteobacteria
Nº of genomes
All genes
Frequency of GAA usage
GAA usage
All genes
Nº of genomes with GluQRS$_1$
Nº of genomes without GluQRS$_1$
With GluQRS$_1$
Without GluQRS$_1$
Nº of genomes with GluQRS$_2$
Nº of genomes without GluQRS$_2$
With GluQRS$_2$
Without GluQRS$_2$
Nº of genomes with GluQRS$_3$
Nº of genomes without GluQRS$_3$
With GluQRS$_3$
Without GluQRS$_3$
Nº of genomes with GluQRS$_4$
Nº of genomes without GluQRS$_4$
With GluQRS$_4$
Without GluQRS$_4$
Nº of genomes with GluQRS$_5$
Nº of genomes without GluQRS$_5$
With GluQRS$_5$
Without GluQRS$_5$
Nº of genomes with TGT$_1$
Nº of genomes without TGT$_1$
With TGT$_1$
Without TGT$_1$
p < 0.00005
Nº of genomes with TGT$_2$
Nº of genomes without TGT$_2$
With TGT$_2$
Without TGT$_2$
Nº of genomes with TGT$_3$
Nº of genomes without TGT$_3$
With TGT$_3$
Without TGT$_3$

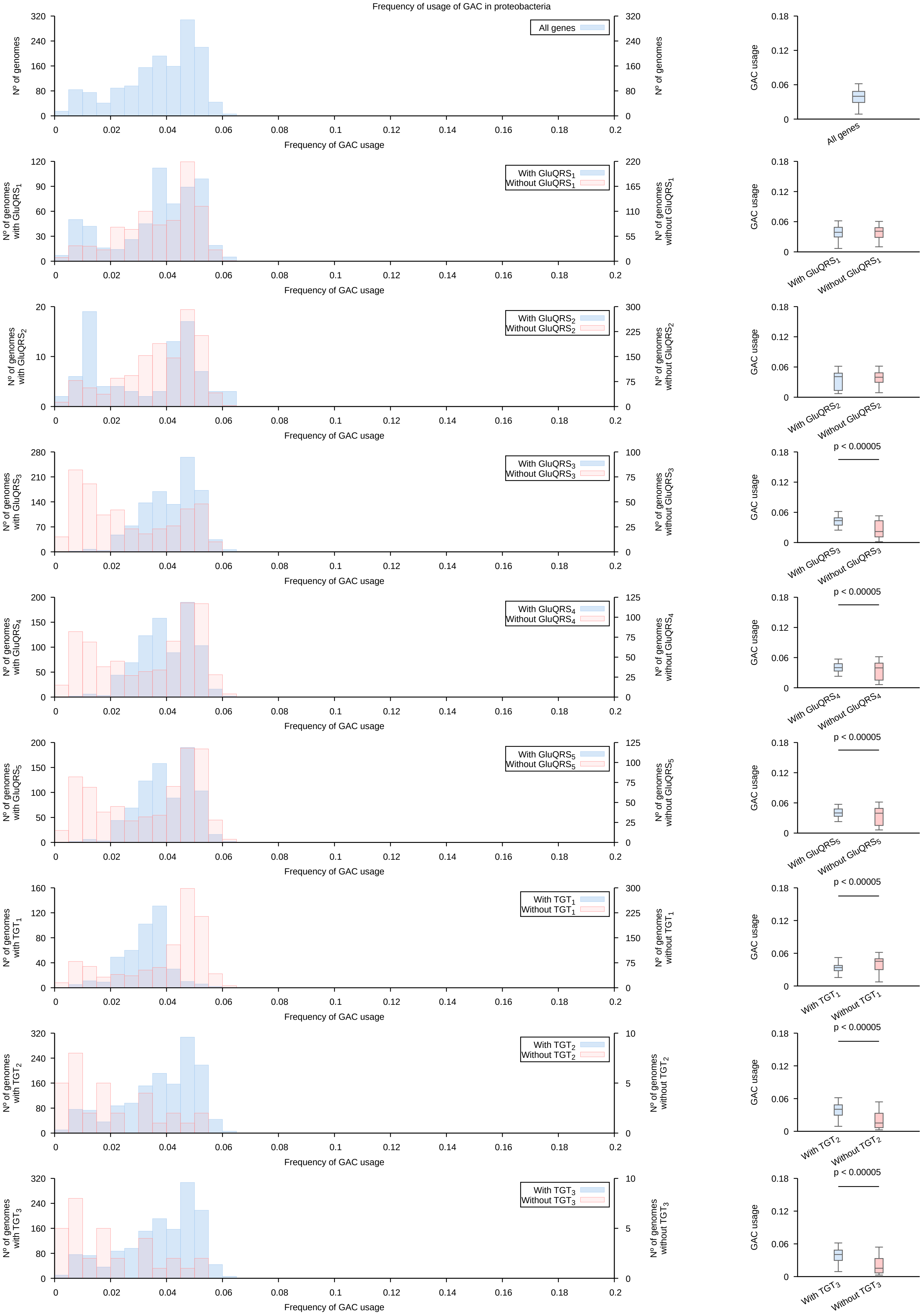
Frequency of usage of GAC in proteobacteria
All genes
With GluQRS$_1$
Without GluQRS$_1$
With GluQRS$_2$
Without GluQRS$_2$
With GluQRS$_3$
Without GluQRS$_3$
With GluQRS$_4$
Without GluQRS$_4$
With GluQRS$_5$
Without GluQRS$_5$
With TGT$_1$
Without TGT$_1$
With TGT$_2$
Without TGT$_2$
With TGT$_3$
Without TGT$_3$
Nº of genomes
Nº of genomes with GluQRS$_1$
Nº of genomes without GluQRS$_1$
Nº of genomes with GluQRS$_2$
Nº of genomes without GluQRS$_2$
Nº of genomes with GluQRS$_3$
Nº of genomes without GluQRS$_3$
Nº of genomes with GluQRS$_4$
Nº of genomes without GluQRS$_4$
Nº of genomes with GluQRS$_5$
Nº of genomes without GluQRS$_5$
Nº of genomes with TGT$_1$
Nº of genomes without TGT$_1$
Nº of genomes with TGT$_2$
Nº of genomes without TGT$_2$
Nº of genomes with TGT$_3$
Nº of genomes without TGT$_3$
Frequency of GAC usage
GAC usage
p < 0.00005

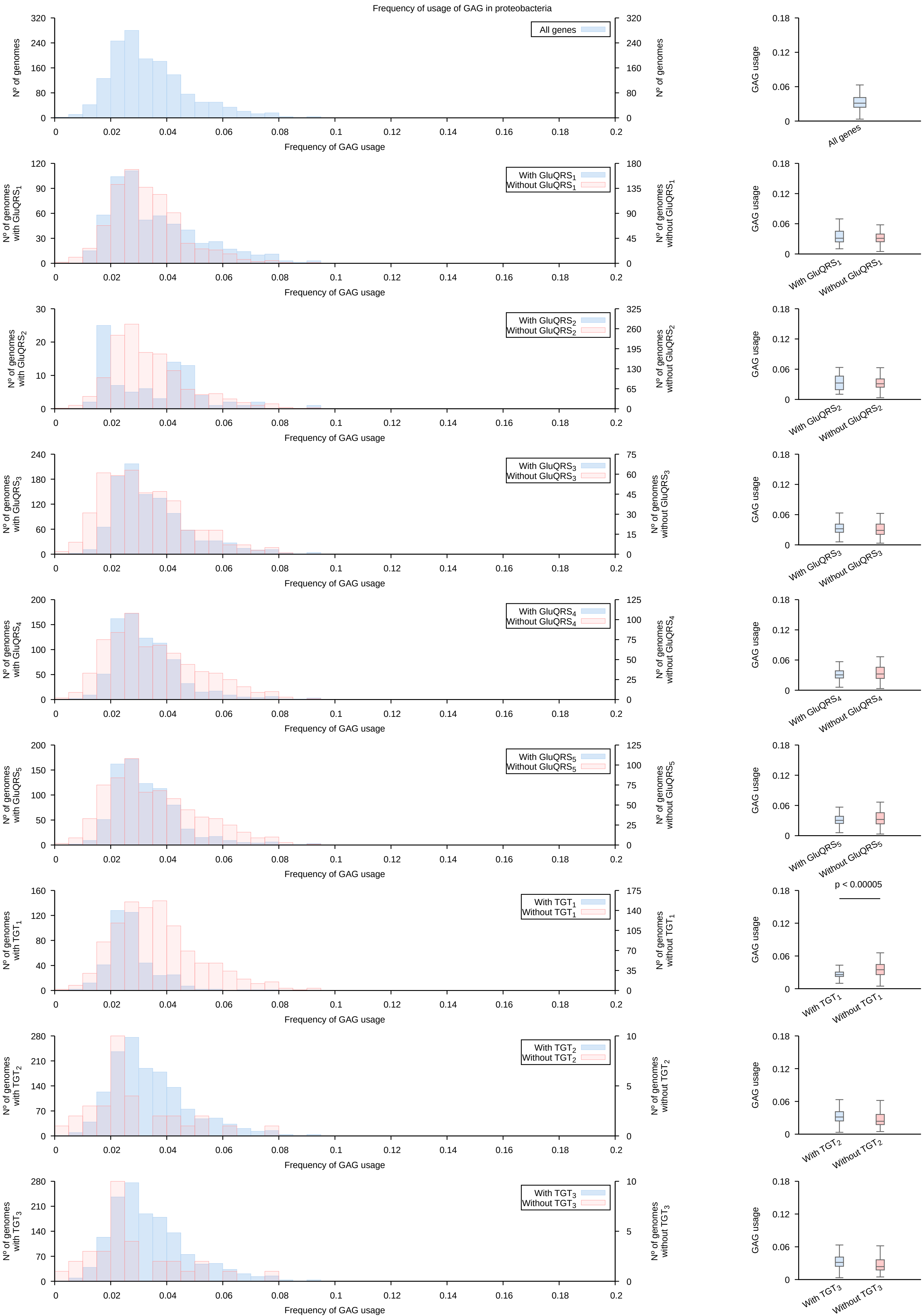
Frequency of usage of GAG in proteobacteria
All genes
Nº of genomes
Frequency of GAG usage
GAG usage
With GluQRS$_1$
Without GluQRS$_1$
Nº of genomes with GluQRS$_1$
Nº of genomes without GluQRS$_1$
With GluQRS$_2$
Without GluQRS$_2$
Nº of genomes with GluQRS$_2$
Nº of genomes without GluQRS$_2$
With GluQRS$_3$
Without GluQRS$_3$
Nº of genomes with GluQRS$_3$
Nº of genomes without GluQRS$_3$
With GluQRS$_4$
Without GluQRS$_4$
Nº of genomes with GluQRS$_4$
Nº of genomes without GluQRS$_4$
With GluQRS$_5$
Without GluQRS$_5$
Nº of genomes with GluQRS$_5$
Nº of genomes without GluQRS$_5$
With TGT$_1$
Without TGT$_1$
Nº of genomes with TGT$_1$
Nº of genomes without TGT$_1$
p < 0.00005
With TGT$_2$
Without TGT$_2$
Nº of genomes with TGT$_2$
Nº of genomes without TGT$_2$
With TGT$_3$
Without TGT$_3$
Nº of genomes with TGT$_3$
Nº of genomes without TGT$_3$

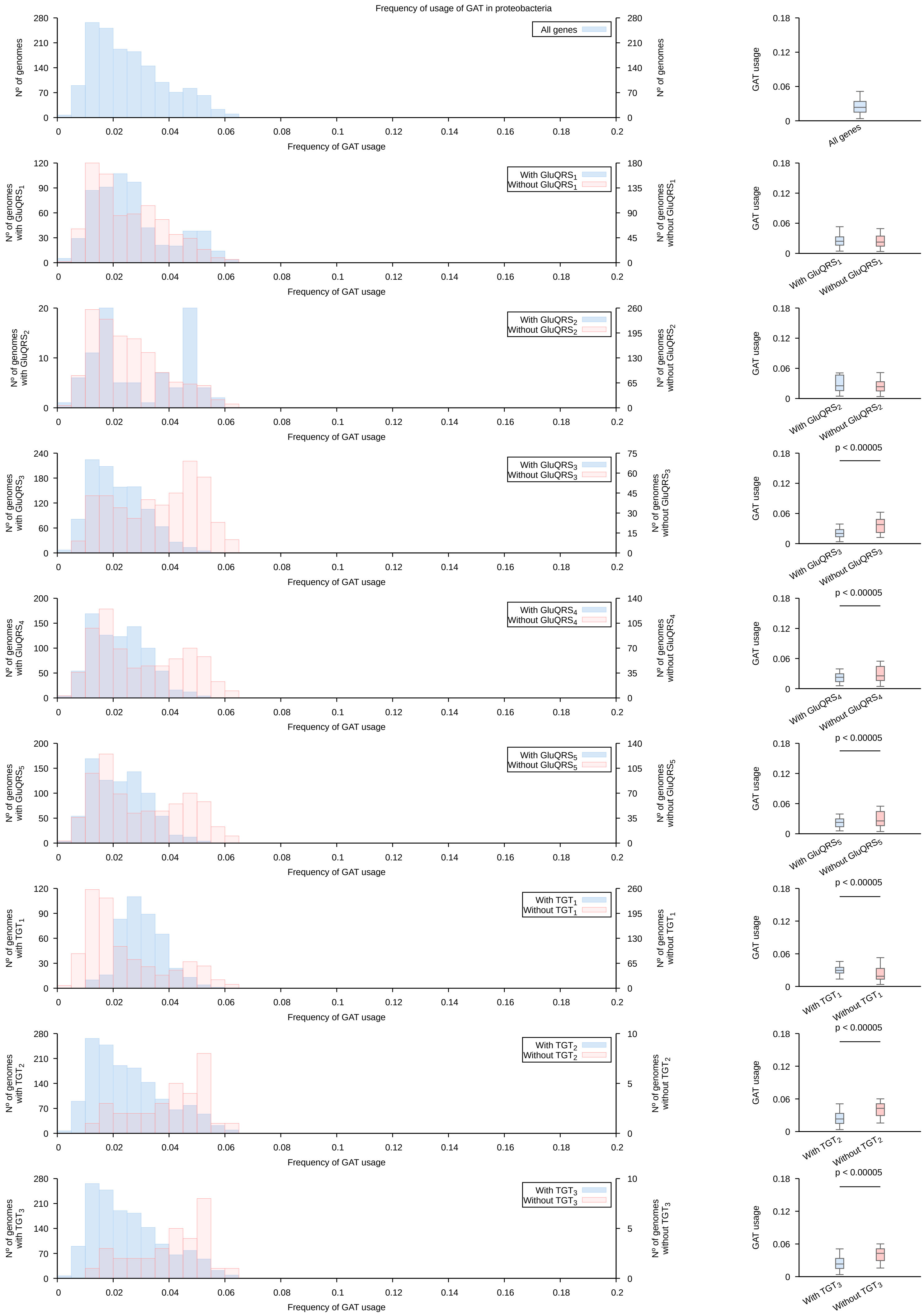

Frequency of usage of GAT in proteobacteria
Nº of genomes
Frequency of GAT usage
GAT usage
All genes
Nº of genomes with GluQRS$_1$
Nº of genomes without GluQRS$_1$
With GluQRS$_1$
Without GluQRS$_1$
Nº of genomes with GluQRS$_2$
Nº of genomes without GluQRS$_2$
With GluQRS$_2$
Without GluQRS$_2$
Nº of genomes with GluQRS$_3$
Nº of genomes without GluQRS$_3$
With GluQRS$_3$
Without GluQRS$_3$
Nº of genomes with GluQRS$_4$
Nº of genomes without GluQRS$_4$
With GluQRS$_4$
Without GluQRS$_4$
Nº of genomes with GluQRS$_5$
Nº of genomes without GluQRS$_5$
With GluQRS$_5$
Without GluQRS$_5$
Nº of genomes with TGT$_1$
Nº of genomes without TGT$_1$
With TGT$_1$
Without TGT$_1$
Nº of genomes with TGT$_2$
Nº of genomes without TGT$_2$
With TGT$_2$
Without TGT$_2$
Nº of genomes with TGT$_3$
Nº of genomes without TGT$_3$
With TGT$_3$
Without TGT$_3$
$p < 0.00005$

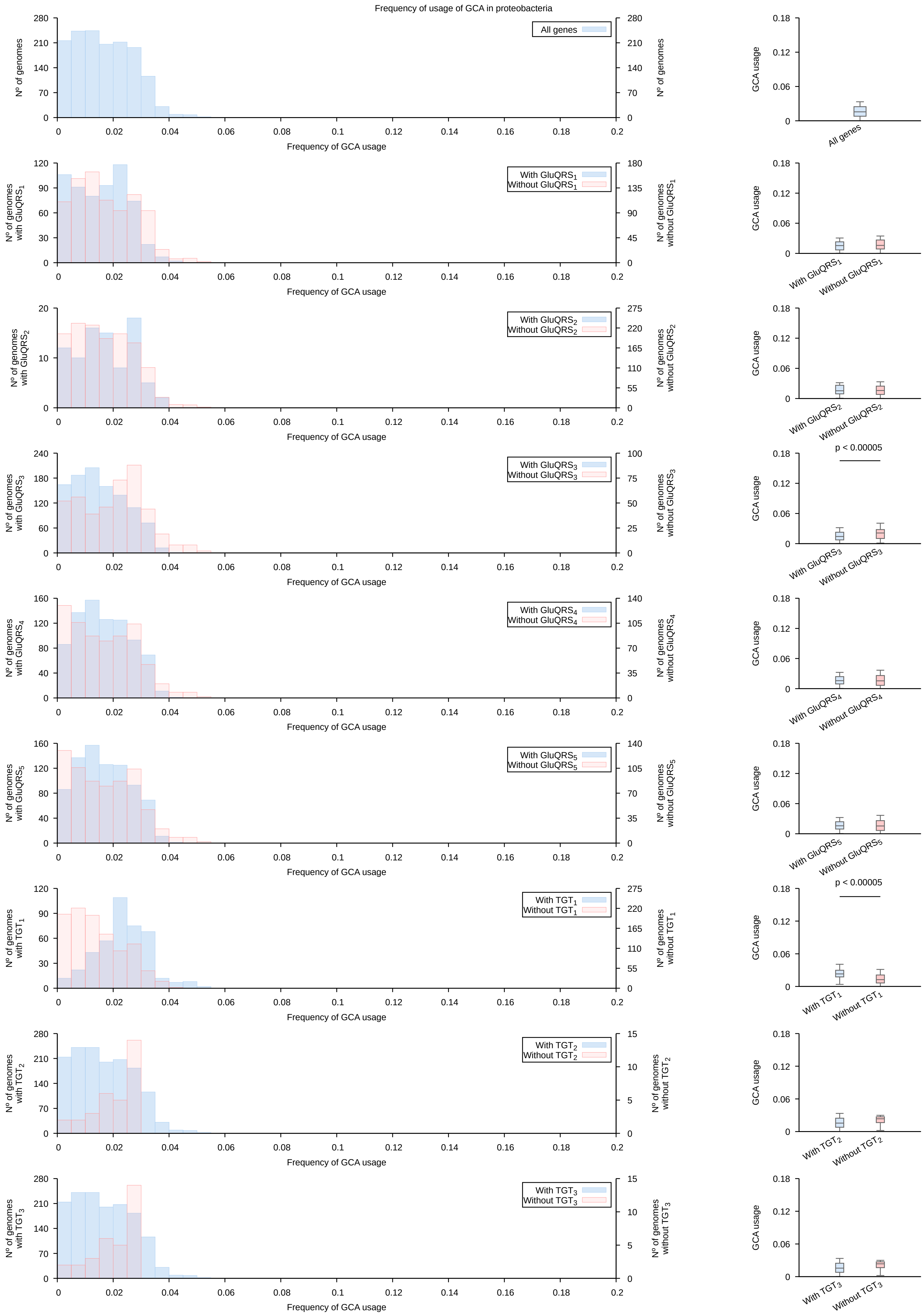
Frequency of usage of GCA in proteobacteria
Nº of genomes
Frequency of GCA usage
GCA usage
All genes
Nº of genomes with GluQRS$_1$
Nº of genomes without GluQRS$_1$
With GluQRS$_1$
Without GluQRS$_1$
Nº of genomes with GluQRS$_2$
Nº of genomes without GluQRS$_2$
With GluQRS$_2$
Without GluQRS$_2$
Nº of genomes with GluQRS$_3$
Nº of genomes without GluQRS$_3$
With GluQRS$_3$
Without GluQRS$_3$
p < 0.00005
Nº of genomes with GluQRS$_4$
Nº of genomes without GluQRS$_4$
With GluQRS$_4$
Without GluQRS$_4$
Nº of genomes with GluQRS$_5$
Nº of genomes without GluQRS$_5$
With GluQRS$_5$
Without GluQRS$_5$
Nº of genomes with TGT$_1$
Nº of genomes without TGT$_1$
With TGT$_1$
Without TGT$_1$
p < 0.00005
Nº of genomes with TGT$_2$
Nº of genomes without TGT$_2$
With TGT$_2$
Without TGT$_2$
Nº of genomes with TGT$_3$
Nº of genomes without TGT$_3$
With TGT$_3$
Without TGT$_3$

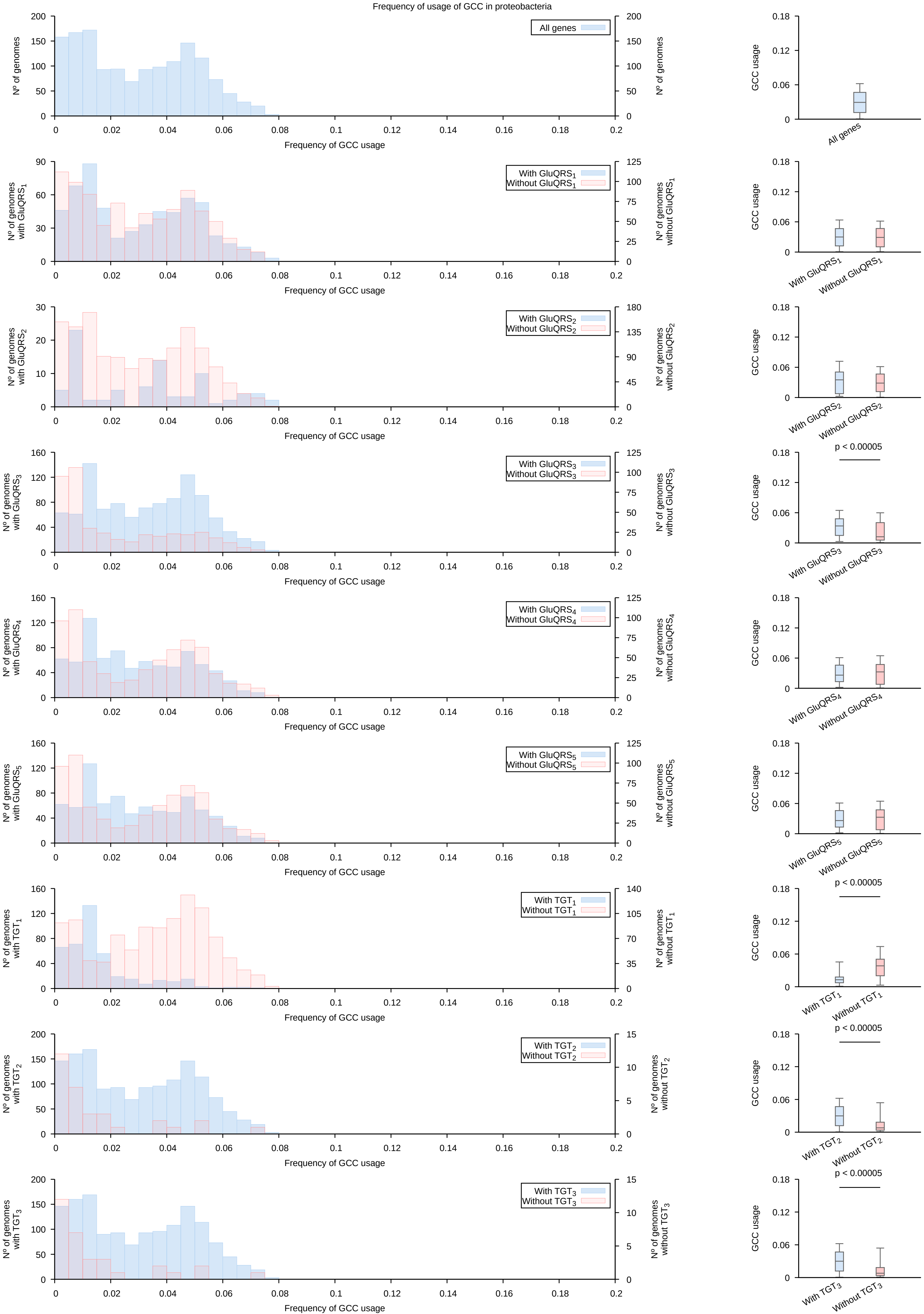

Frequency of usage of GCC in proteobacteria
Frequency of GCC usage
Nº of genomes
All genes
GCC usage
Nº of genomes with GluQRS$_1$
Nº of genomes without GluQRS$_1$
With GluQRS$_1$
Without GluQRS$_1$
Nº of genomes with GluQRS$_2$
Nº of genomes without GluQRS$_2$
With GluQRS$_2$
Without GluQRS$_2$
Nº of genomes with GluQRS$_3$
Nº of genomes without GluQRS$_3$
With GluQRS$_3$
Without GluQRS$_3$
p < 0.00005
Nº of genomes with GluQRS$_4$
Nº of genomes without GluQRS$_4$
With GluQRS$_4$
Without GluQRS$_4$
Nº of genomes with GluQRS$_5$
Nº of genomes without GluQRS$_5$
With GluQRS$_5$
Without GluQRS$_5$
Nº of genomes with TGT$_1$
Nº of genomes without TGT$_1$
With TGT$_1$
Without TGT$_1$
p < 0.00005
Nº of genomes with TGT$_2$
Nº of genomes without TGT$_2$
With TGT$_2$
Without TGT$_2$
p < 0.00005
Nº of genomes with TGT$_3$
Nº of genomes without TGT$_3$
With TGT$_3$
Without TGT$_3$
p < 0.00005

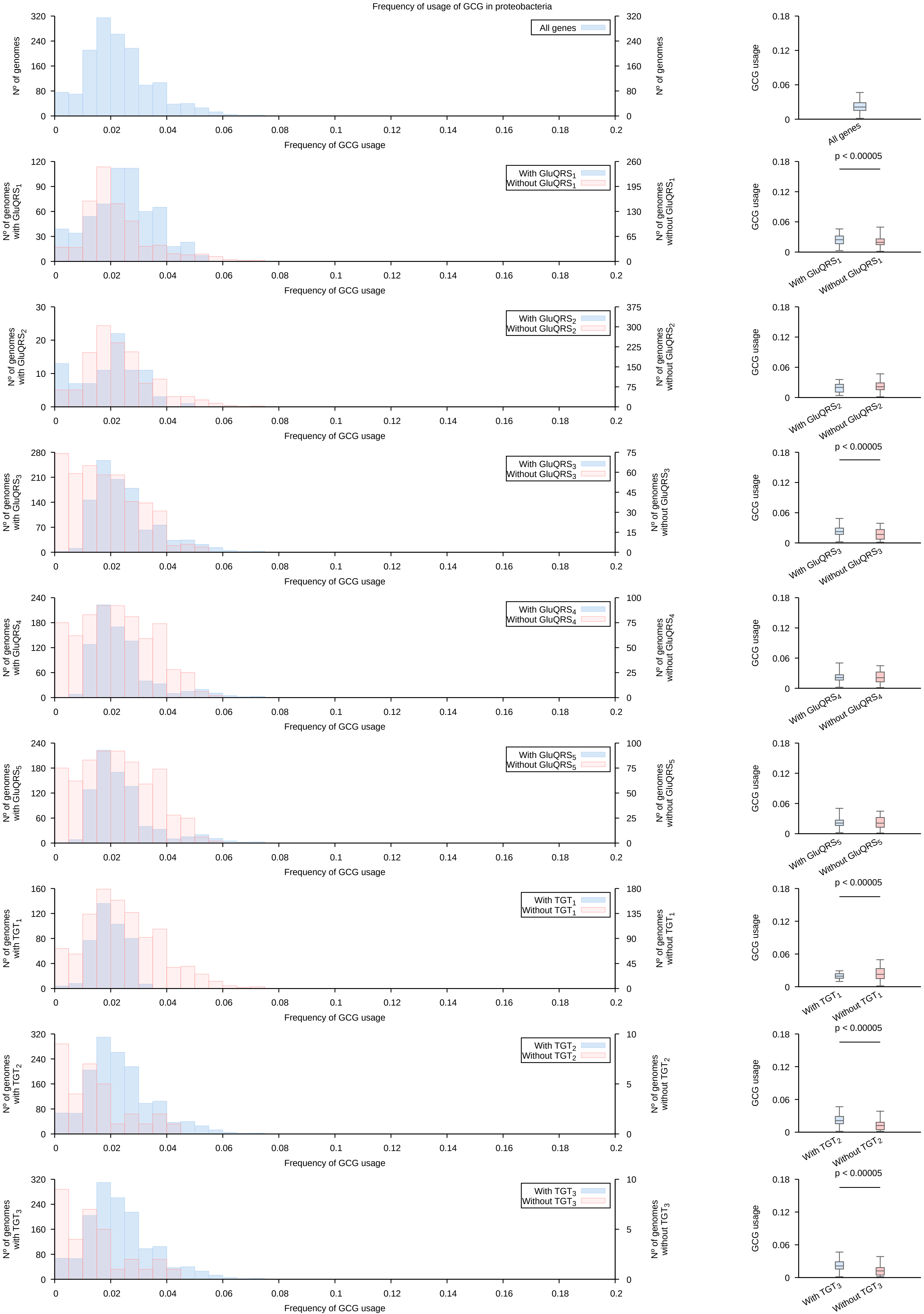
Frequency of usage of GCG in proteobacteria
All genes
With GluQRS1
Without GluQRS1
With GluQRS2
Without GluQRS2
With GluQRS3
Without GluQRS3
With GluQRS4
Without GluQRS4
With GluQRS5
Without GluQRS5
With TGT1
Without TGT1
With TGT2
Without TGT2
With TGT3
Without TGT3
Nº of genomes
Nº of genomes with GluQRS1
Nº of genomes without GluQRS1
Nº of genomes with GluQRS2
Nº of genomes without GluQRS2
Nº of genomes with GluQRS3
Nº of genomes without GluQRS3
Nº of genomes with GluQRS4
Nº of genomes without GluQRS4
Nº of genomes with GluQRS5
Nº of genomes without GluQRS5
Nº of genomes with TGT1
Nº of genomes without TGT1
Nº of genomes with TGT2
Nº of genomes without TGT2
Nº of genomes with TGT3
Nº of genomes without TGT3
Frequency of GCG usage
GCG usage
p < 0.00005

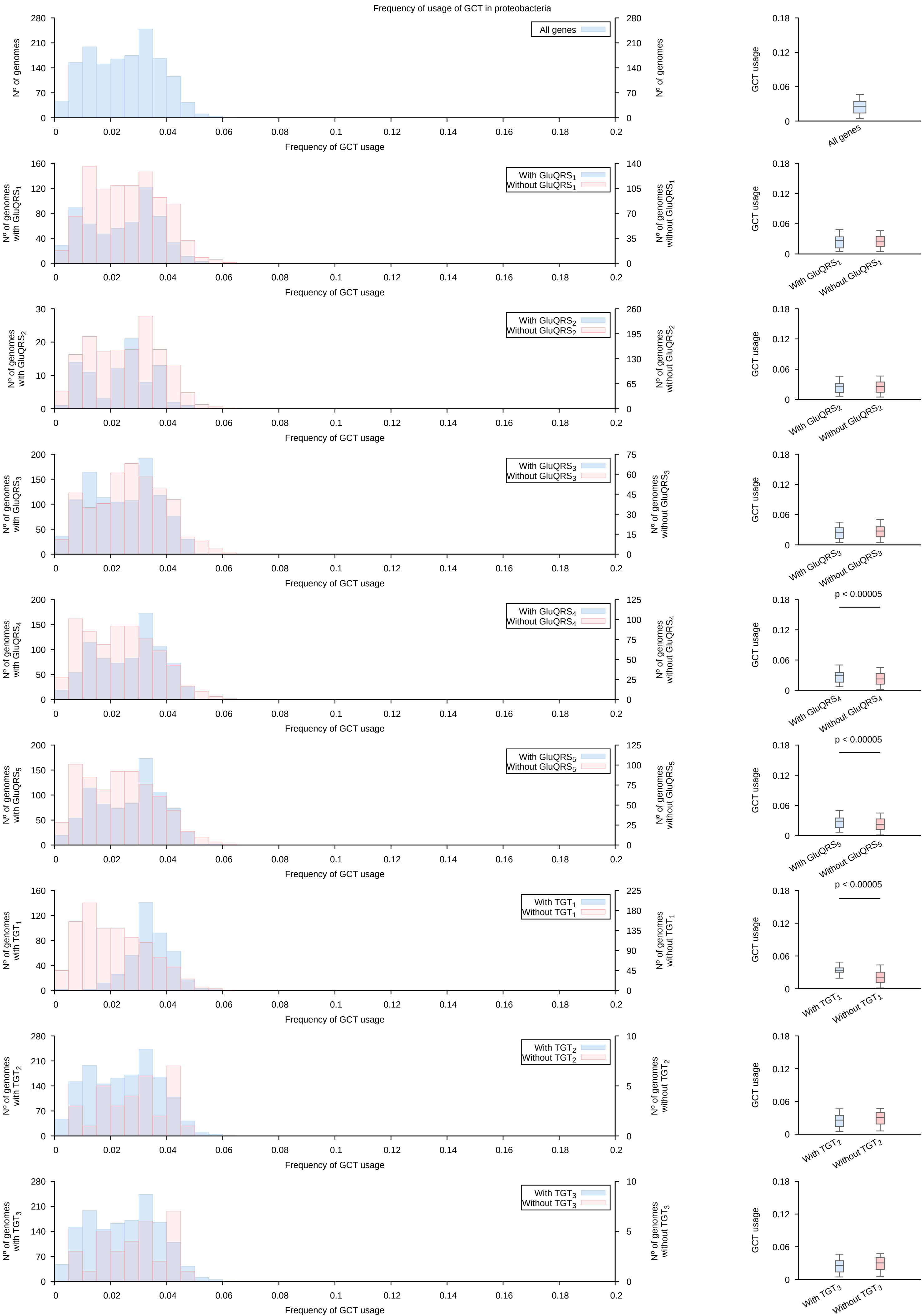
Frequency of usage of GCT in proteobacteria
All genes
Nº of genomes
Frequency of GCT usage
GCT usage
With GluQRS$_1$
Without GluQRS$_1$
Nº of genomes with GluQRS$_1$
Nº of genomes without GluQRS$_1$
With GluQRS$_2$
Without GluQRS$_2$
Nº of genomes with GluQRS$_2$
Nº of genomes without GluQRS$_2$
With GluQRS$_3$
Without GluQRS$_3$
Nº of genomes with GluQRS$_3$
Nº of genomes without GluQRS$_3$
With GluQRS$_4$
Without GluQRS$_4$
Nº of genomes with GluQRS$_4$
Nº of genomes without GluQRS$_4$
p < 0.00005
With GluQRS$_5$
Without GluQRS$_5$
Nº of genomes with GluQRS$_5$
Nº of genomes without GluQRS$_5$
p < 0.00005
With TGT$_1$
Without TGT$_1$
Nº of genomes with TGT$_1$
Nº of genomes without TGT$_1$
p < 0.00005
With TGT$_2$
Without TGT$_2$
Nº of genomes with TGT$_2$
Nº of genomes without TGT$_2$
With TGT$_3$
Without TGT$_3$
Nº of genomes with TGT$_3$
Nº of genomes without TGT$_3$

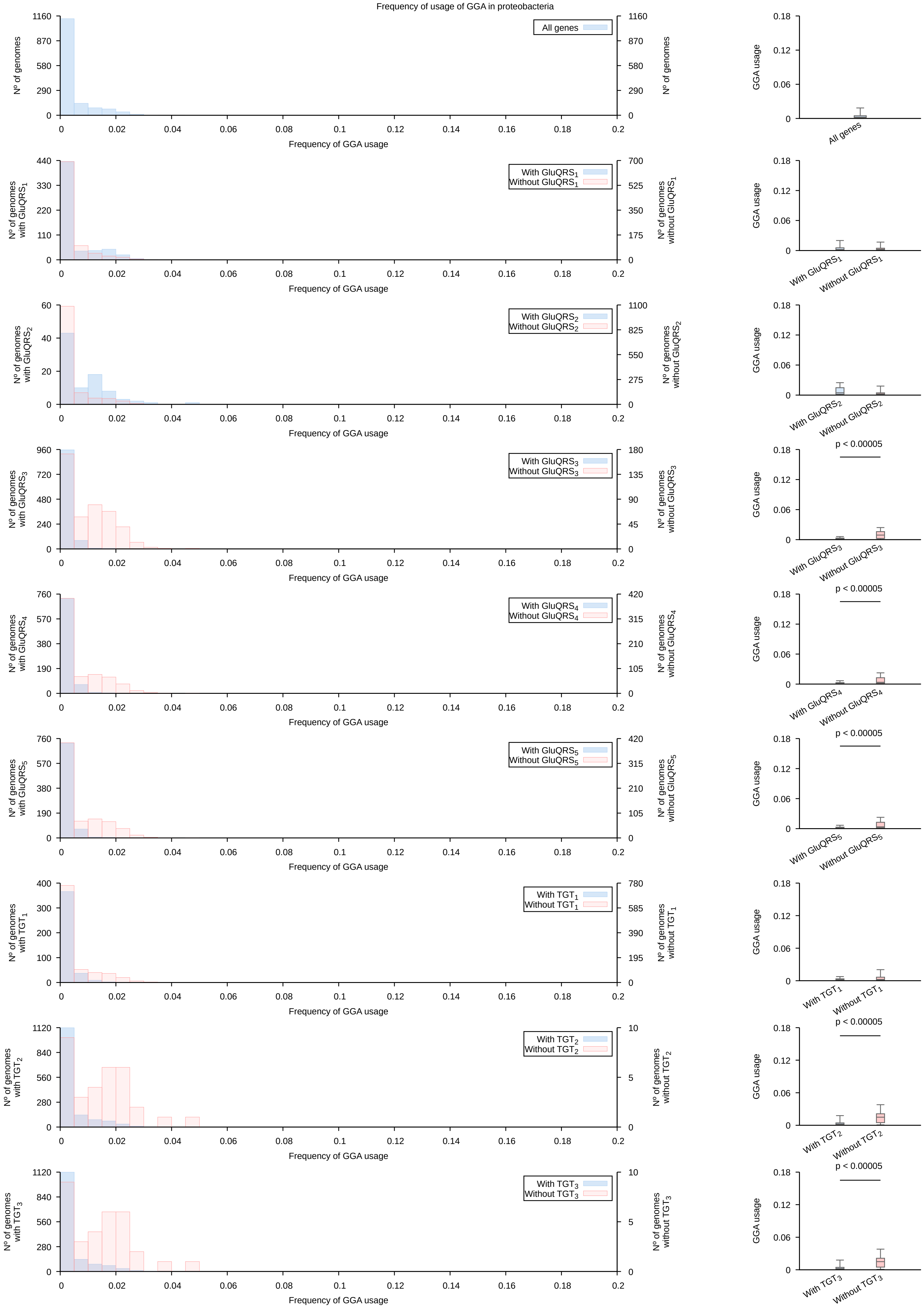

Frequency of usage of GGA in proteobacteria
All genes
With GluQRS$_1$
Without GluQRS$_1$
With GluQRS$_2$
Without GluQRS$_2$
With GluQRS$_3$
Without GluQRS$_3$
With GluQRS$_4$
Without GluQRS$_4$
With GluQRS$_5$
Without GluQRS$_5$
With TGT$_1$
Without TGT$_1$
With TGT$_2$
Without TGT$_2$
With TGT$_3$
Without TGT$_3$
Nº of genomes
Nº of genomes with GluQRS$_1$
Nº of genomes without GluQRS$_1$
Nº of genomes with GluQRS$_2$
Nº of genomes without GluQRS$_2$
Nº of genomes with GluQRS$_3$
Nº of genomes without GluQRS$_3$
Nº of genomes with GluQRS$_4$
Nº of genomes without GluQRS$_4$
Nº of genomes with GluQRS$_5$
Nº of genomes without GluQRS$_5$
Nº of genomes with TGT$_1$
Nº of genomes without TGT$_1$
Nº of genomes with TGT$_2$
Nº of genomes without TGT$_2$
Nº of genomes with TGT$_3$
Nº of genomes without TGT$_3$
Frequency of GGA usage
GGA usage
p < 0.00005

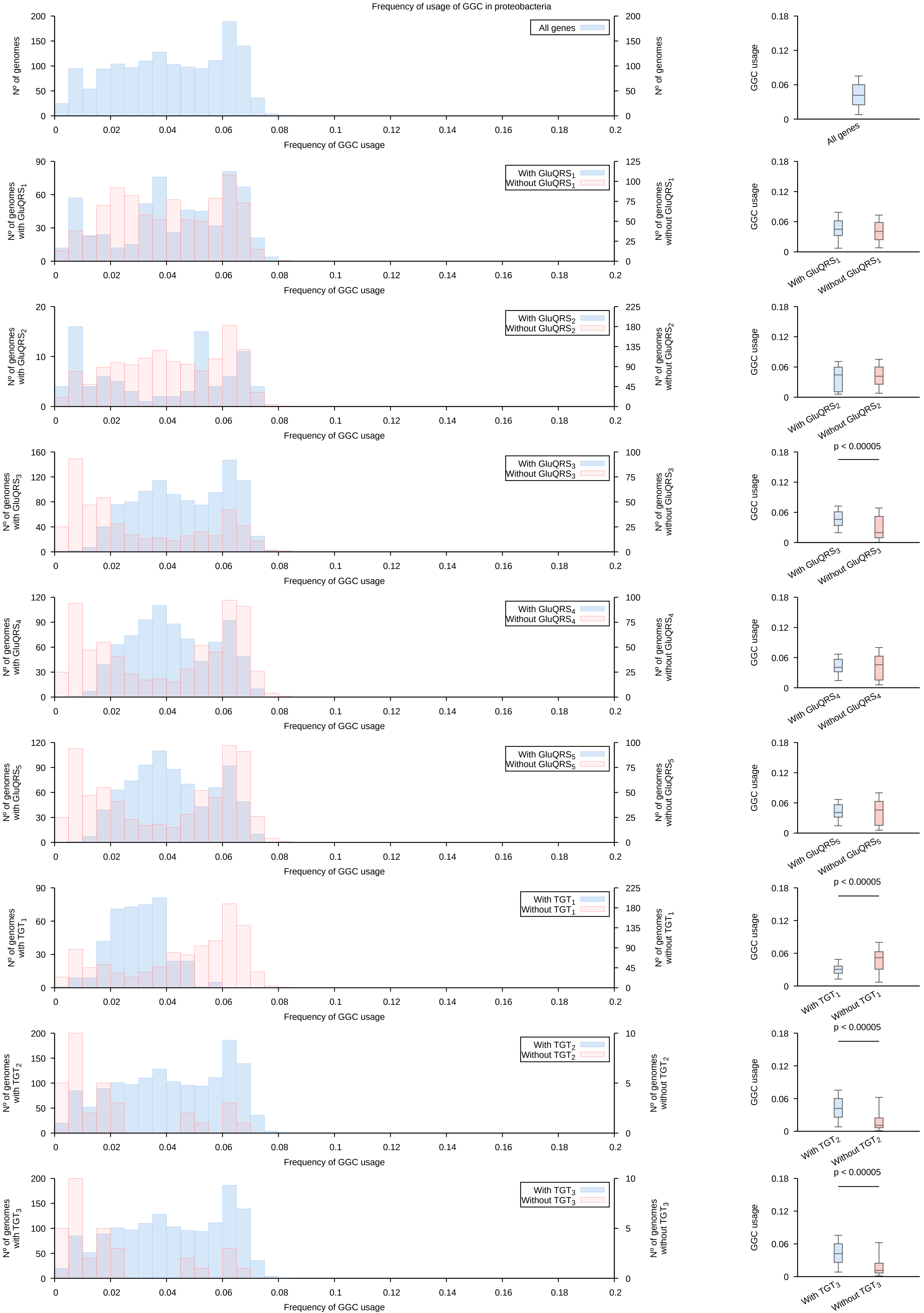

Frequency of usage of GGC in proteobacteria
All genes
Nº of genomes
Frequency of GGC usage
GGC usage
Nº of genomes with GluQRS$_1$
Nº of genomes without GluQRS$_1$
With GluQRS$_1$
Without GluQRS$_1$
Nº of genomes with GluQRS$_2$
Nº of genomes without GluQRS$_2$
With GluQRS$_2$
Without GluQRS$_2$
Nº of genomes with GluQRS$_3$
Nº of genomes without GluQRS$_3$
With GluQRS$_3$
Without GluQRS$_3$
p < 0.00005
Nº of genomes with GluQRS$_4$
Nº of genomes without GluQRS$_4$
With GluQRS$_4$
Without GluQRS$_4$
Nº of genomes with GluQRS$_5$
Nº of genomes without GluQRS$_5$
With GluQRS$_5$
Without GluQRS$_5$
Nº of genomes with TGT$_1$
Nº of genomes without TGT$_1$
With TGT$_1$
Without TGT$_1$
p < 0.00005
Nº of genomes with TGT$_2$
Nº of genomes without TGT$_2$
With TGT$_2$
Without TGT$_2$
p < 0.00005
Nº of genomes with TGT$_3$
Nº of genomes without TGT$_3$
With TGT$_3$
Without TGT$_3$
p < 0.00005

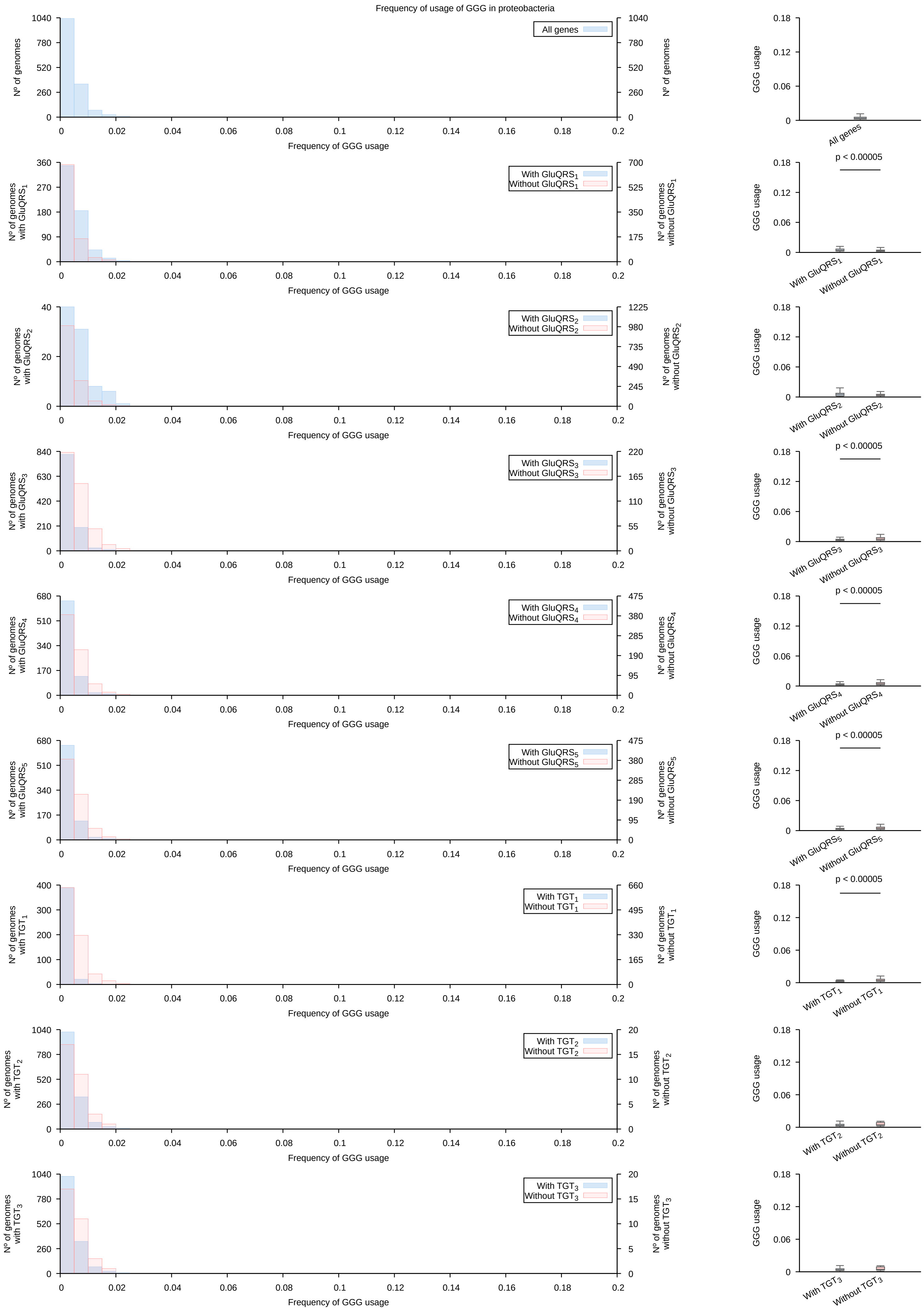

Frequency of usage of GGG in proteobacteria
All genes
Nº of genomes
Frequency of GGG usage
GGG usage
With GluQRS$_1$
Without GluQRS$_1$
Nº of genomes with GluQRS$_1$
Nº of genomes without GluQRS$_1$
p < 0.00005
With GluQRS$_2$
Without GluQRS$_2$
Nº of genomes with GluQRS$_2$
Nº of genomes without GluQRS$_2$
With GluQRS$_3$
Without GluQRS$_3$
Nº of genomes with GluQRS$_3$
Nº of genomes without GluQRS$_3$
p < 0.00005
With GluQRS$_4$
Without GluQRS$_4$
Nº of genomes with GluQRS$_4$
Nº of genomes without GluQRS$_4$
p < 0.00005
With GluQRS$_5$
Without GluQRS$_5$
Nº of genomes with GluQRS$_5$
Nº of genomes without GluQRS$_5$
p < 0.00005
With TGT$_1$
Without TGT$_1$
Nº of genomes with TGT$_1$
Nº of genomes without TGT$_1$
p < 0.00005
With TGT$_2$
Without TGT$_2$
Nº of genomes with TGT$_2$
Nº of genomes without TGT$_2$
With TGT$_3$
Without TGT$_3$
Nº of genomes with TGT$_3$
Nº of genomes without TGT$_3$

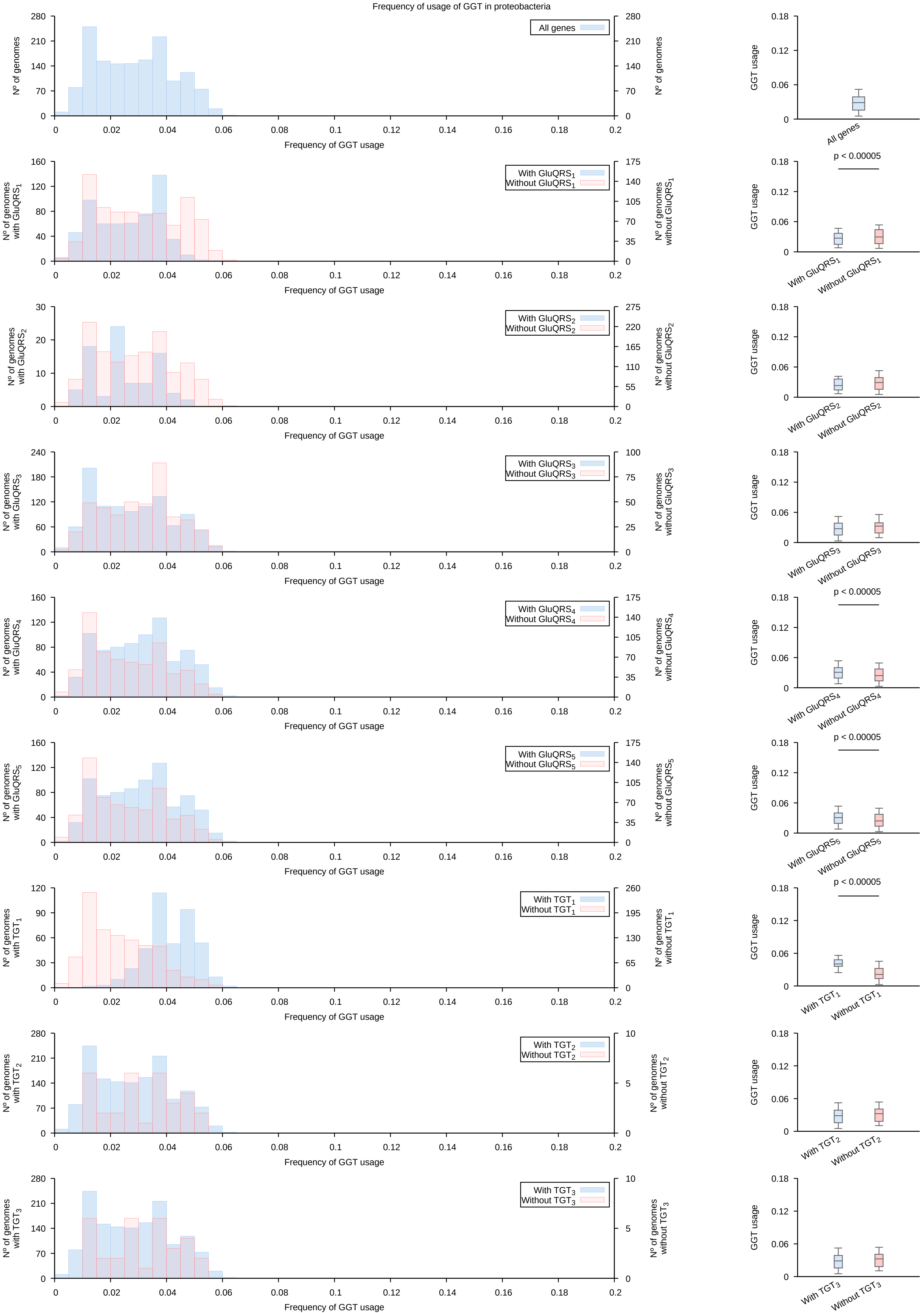

Frequency of usage of GGT in proteobacteria
All genes
Nº of genomes
Frequency of GGT usage
GGT usage
With GluQRS$_1$
Without GluQRS$_1$
Nº of genomes with GluQRS$_1$
Nº of genomes without GluQRS$_1$
p < 0.00005
With GluQRS$_2$
Without GluQRS$_2$
Nº of genomes with GluQRS$_2$
Nº of genomes without GluQRS$_2$
With GluQRS$_3$
Without GluQRS$_3$
Nº of genomes with GluQRS$_3$
Nº of genomes without GluQRS$_3$
With GluQRS$_4$
Without GluQRS$_4$
Nº of genomes with GluQRS$_4$
Nº of genomes without GluQRS$_4$
p < 0.00005
With GluQRS$_5$
Without GluQRS$_5$
Nº of genomes with GluQRS$_5$
Nº of genomes without GluQRS$_5$
p < 0.00005
With TGT$_1$
Without TGT$_1$
Nº of genomes with TGT$_1$
Nº of genomes without TGT$_1$
p < 0.00005
With TGT$_2$
Without TGT$_2$
Nº of genomes with TGT$_2$
Nº of genomes without TGT$_2$
With TGT$_3$
Without TGT$_3$
Nº of genomes with TGT$_3$
Nº of genomes without TGT$_3$

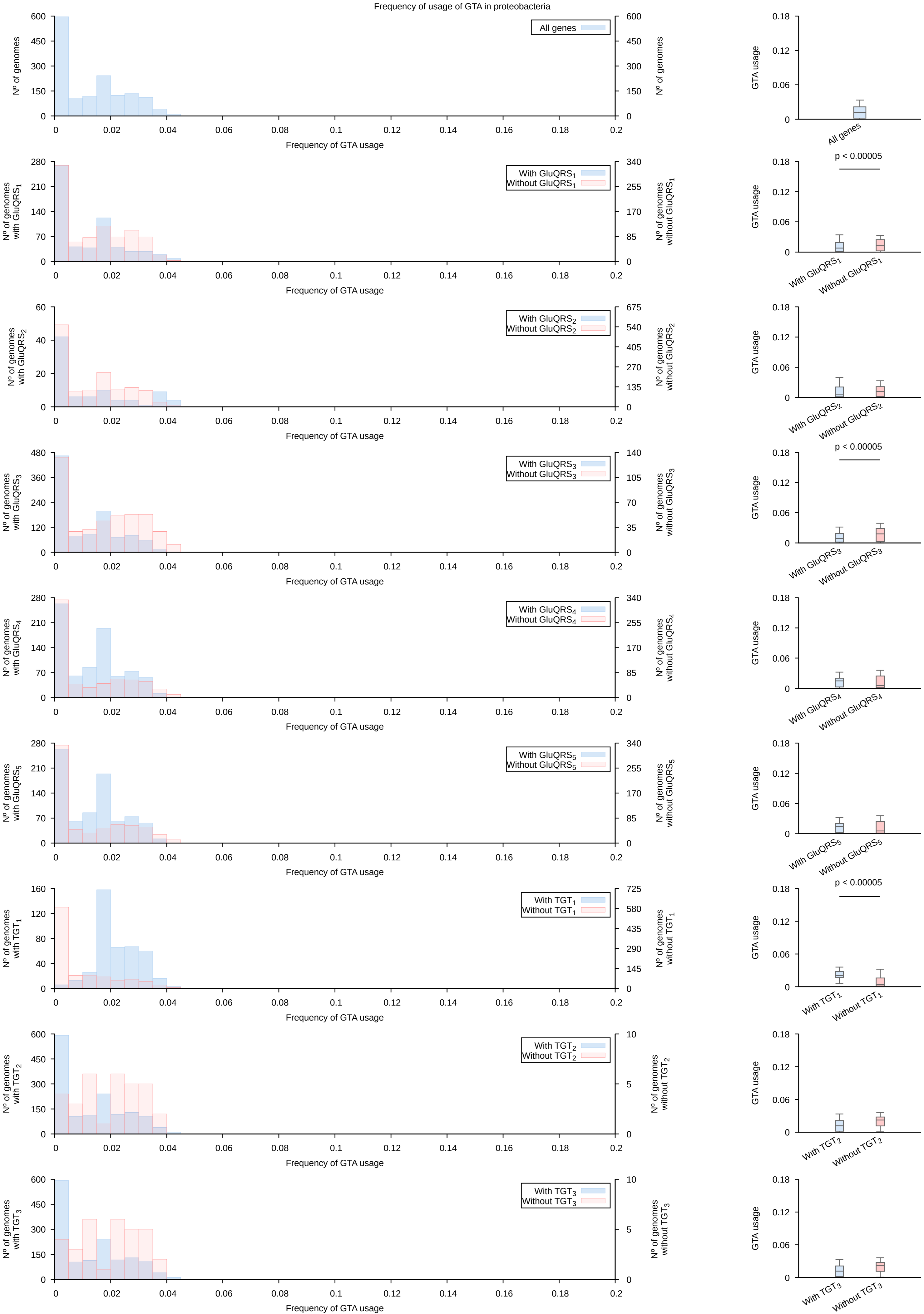
Frequency of usage of GTA in proteobacteria
All genes
Nº of genomes
Frequency of GTA usage
GTA usage
With GluQRS$_1$
Without GluQRS$_1$
Nº of genomes with GluQRS$_1$
Nº of genomes without GluQRS$_1$
p < 0.00005
With GluQRS$_2$
Without GluQRS$_2$
Nº of genomes with GluQRS$_2$
Nº of genomes without GluQRS$_2$
With GluQRS$_3$
Without GluQRS$_3$
Nº of genomes with GluQRS$_3$
Nº of genomes without GluQRS$_3$
p < 0.00005
With GluQRS$_4$
Without GluQRS$_4$
Nº of genomes with GluQRS$_4$
Nº of genomes without GluQRS$_4$
With GluQRS$_5$
Without GluQRS$_5$
Nº of genomes with GluQRS$_5$
Nº of genomes without GluQRS$_5$
With TGT$_1$
Without TGT$_1$
Nº of genomes with TGT$_1$
Nº of genomes without TGT$_1$
p < 0.00005
With TGT$_2$
Without TGT$_2$
Nº of genomes with TGT$_2$
Nº of genomes without TGT$_2$
With TGT$_3$
Without TGT$_3$
Nº of genomes with TGT$_3$
Nº of genomes without TGT$_3$

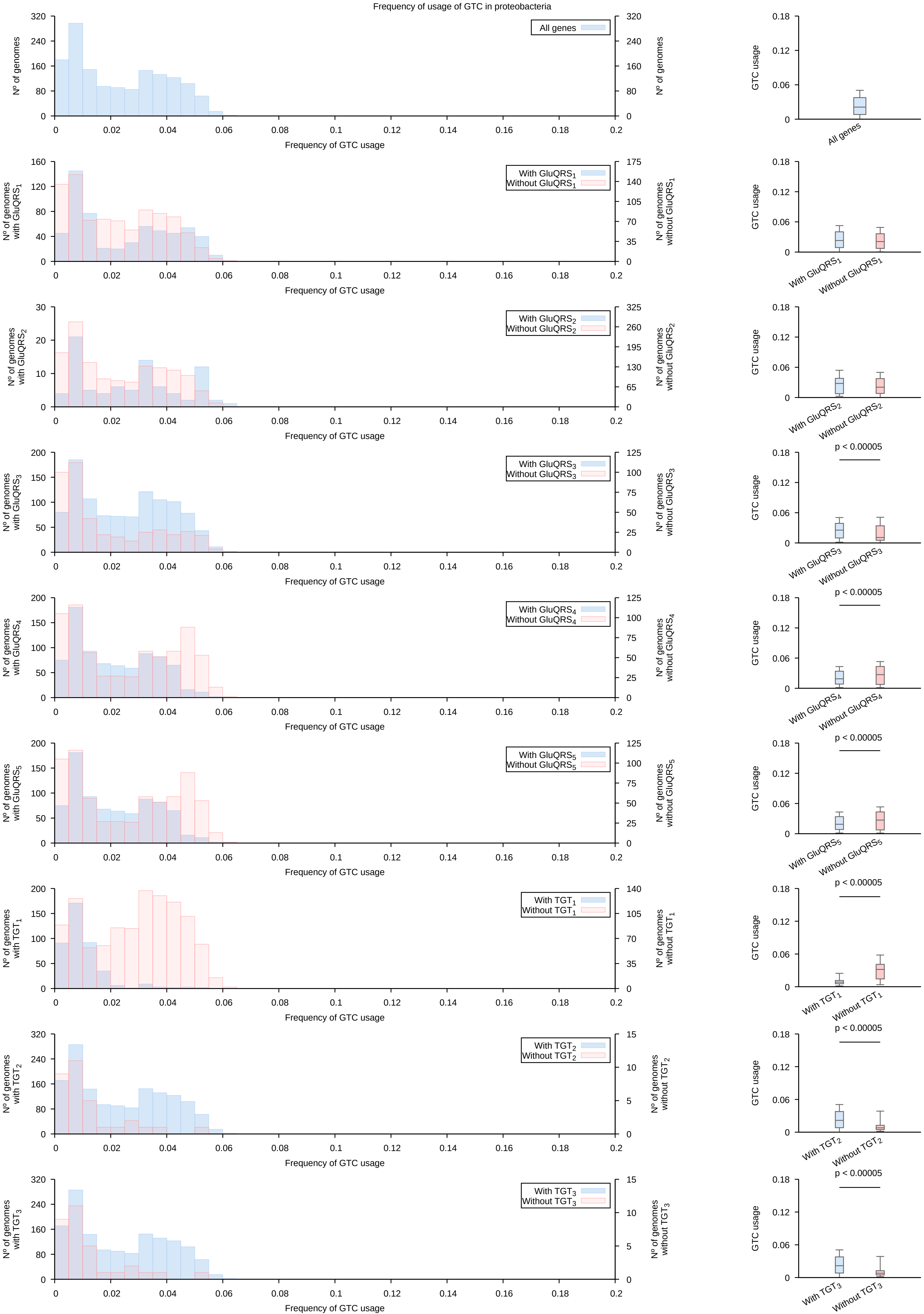

Frequency of usage of GTC in proteobacteria
All genes
Nº of genomes
Frequency of GTC usage
GTC usage
With GluQRS$_1$
Without GluQRS$_1$
Nº of genomes with GluQRS$_1$
Nº of genomes without GluQRS$_1$
With GluQRS$_2$
Without GluQRS$_2$
Nº of genomes with GluQRS$_2$
Nº of genomes without GluQRS$_2$
With GluQRS$_3$
Without GluQRS$_3$
Nº of genomes with GluQRS$_3$
Nº of genomes without GluQRS$_3$
p < 0.00005
With GluQRS$_4$
Without GluQRS$_4$
Nº of genomes with GluQRS$_4$
Nº of genomes without GluQRS$_4$
p < 0.00005
With GluQRS$_5$
Without GluQRS$_5$
Nº of genomes with GluQRS$_5$
Nº of genomes without GluQRS$_5$
p < 0.00005
With TGT$_1$
Without TGT$_1$
Nº of genomes with TGT$_1$
Nº of genomes without TGT$_1$
p < 0.00005
With TGT$_2$
Without TGT$_2$
Nº of genomes with TGT$_2$
Nº of genomes without TGT$_2$
p < 0.00005
With TGT$_3$
Without TGT$_3$
Nº of genomes with TGT$_3$
Nº of genomes without TGT$_3$
p < 0.00005

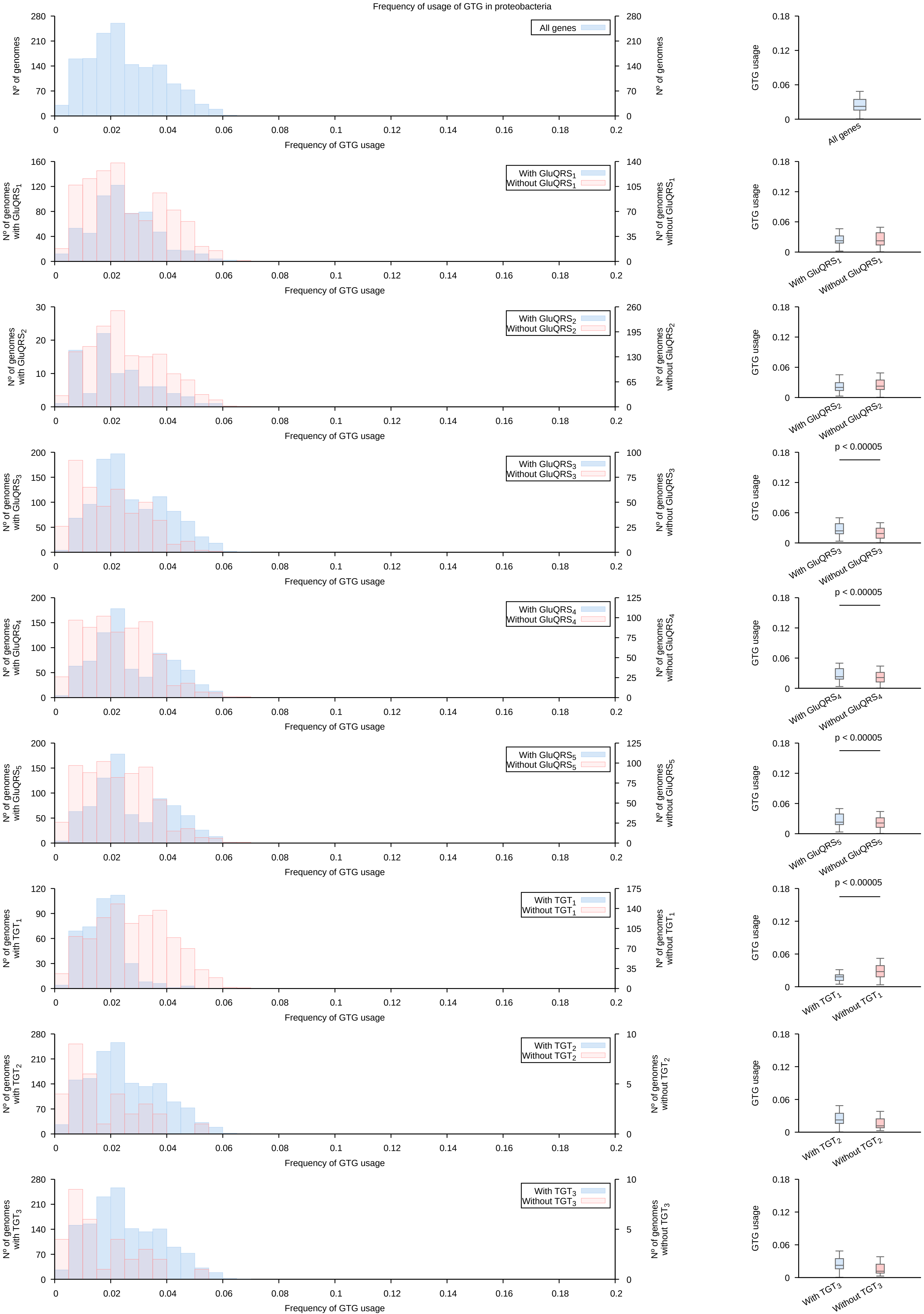

Frequency of usage of GTG in proteobacteria
All genes
Nº of genomes
Frequency of GTG usage
GTG usage
With GluQRS$_1$
Without GluQRS$_1$
Nº of genomes with GluQRS$_1$
Nº of genomes without GluQRS$_1$
With GluQRS$_2$
Without GluQRS$_2$
Nº of genomes with GluQRS$_2$
Nº of genomes without GluQRS$_2$
With GluQRS$_3$
Without GluQRS$_3$
Nº of genomes with GluQRS$_3$
Nº of genomes without GluQRS$_3$
p < 0.00005
With GluQRS$_4$
Without GluQRS$_4$
Nº of genomes with GluQRS$_4$
Nº of genomes without GluQRS$_4$
p < 0.00005
With GluQRS$_5$
Without GluQRS$_5$
Nº of genomes with GluQRS$_5$
Nº of genomes without GluQRS$_5$
p < 0.00005
With TGT$_1$
Without TGT$_1$
Nº of genomes with TGT$_1$
Nº of genomes without TGT$_1$
p < 0.00005
With TGT$_2$
Without TGT$_2$
Nº of genomes with TGT$_2$
Nº of genomes without TGT$_2$
With TGT$_3$
Without TGT$_3$
Nº of genomes with TGT$_3$
Nº of genomes without TGT$_3$
0 0.02 0.04 0.06 0.08 0.1 0.12 0.14 0.16 0.18 0.2
0 0.06 0.12 0.18

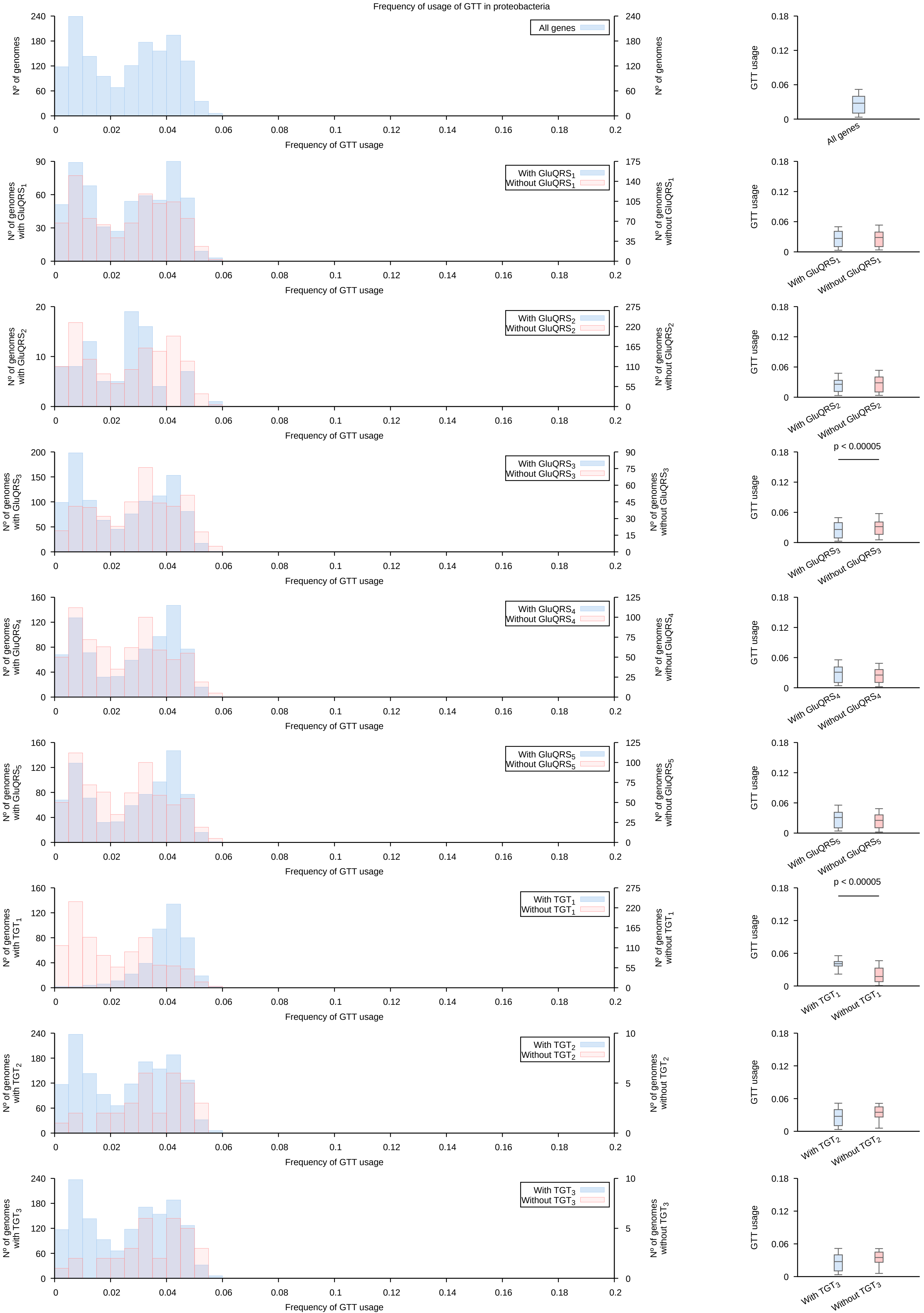
Frequency of usage of GTT in proteobacteria
All genes
Nº of genomes
Frequency of GTT usage
GTT usage
With GluQRS$_1$
Without GluQRS$_1$
Nº of genomes with GluQRS$_1$
Nº of genomes without GluQRS$_1$
With GluQRS$_2$
Without GluQRS$_2$
Nº of genomes with GluQRS$_2$
Nº of genomes without GluQRS$_2$
With GluQRS$_3$
Without GluQRS$_3$
Nº of genomes with GluQRS$_3$
Nº of genomes without GluQRS$_3$
p < 0.00005
With GluQRS$_4$
Without GluQRS$_4$
Nº of genomes with GluQRS$_4$
Nº of genomes without GluQRS$_4$
With GluQRS$_5$
Without GluQRS$_5$
Nº of genomes with GluQRS$_5$
Nº of genomes without GluQRS$_5$
With TGT$_1$
Without TGT$_1$
Nº of genomes with TGT$_1$
Nº of genomes without TGT$_1$
p < 0.00005
With TGT$_2$
Without TGT$_2$
Nº of genomes with TGT$_2$
Nº of genomes without TGT$_2$
With TGT$_3$
Without TGT$_3$
Nº of genomes with TGT$_3$
Nº of genomes without TGT$_3$

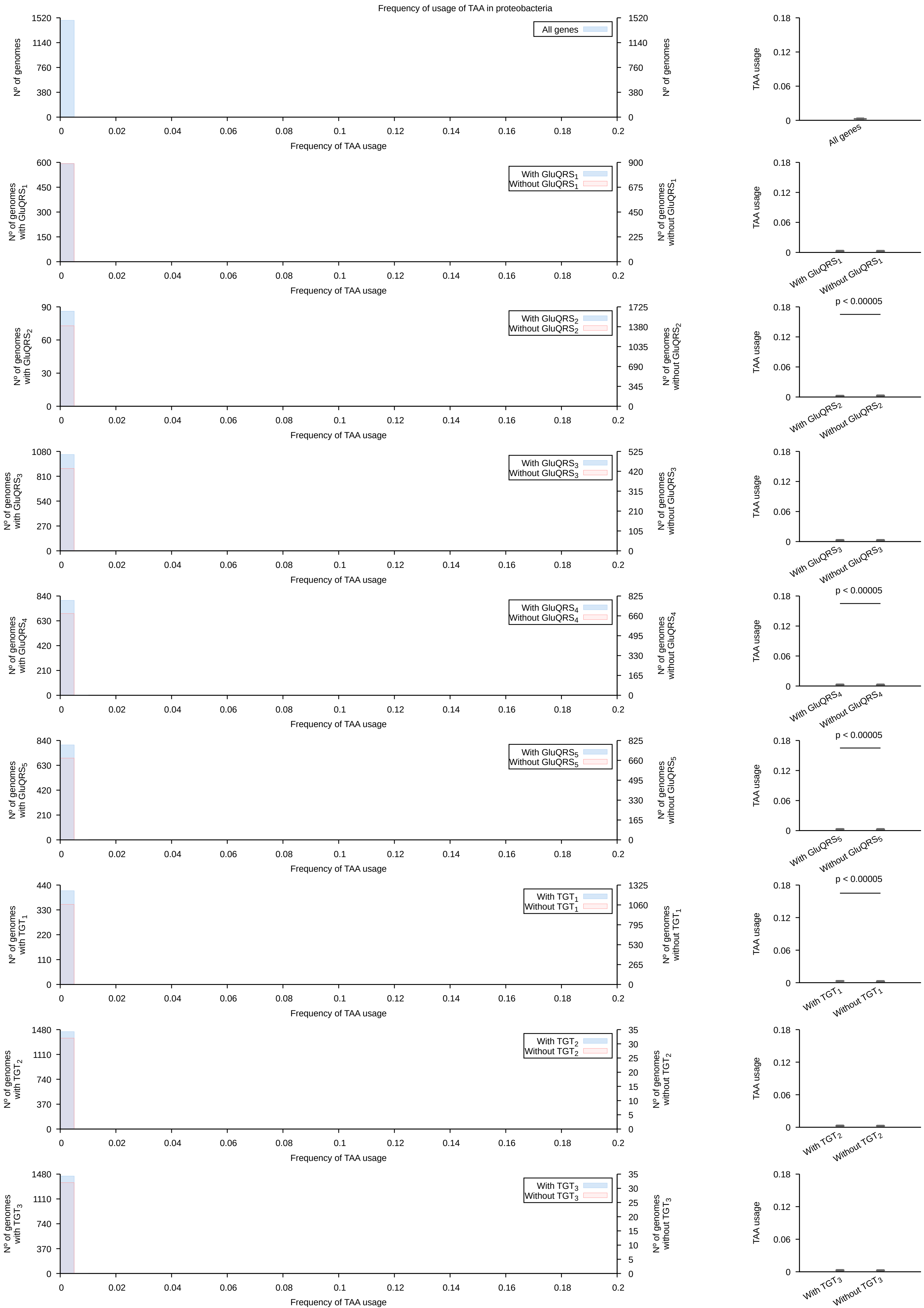

Frequency of usage of TAA in proteobacteria
All genes
Nº of genomes
Frequency of TAA usage
TAA usage
With GluQRS$_1$
Without GluQRS$_1$
Nº of genomes with GluQRS$_1$
Nº of genomes without GluQRS$_1$
With GluQRS$_2$
Without GluQRS$_2$
Nº of genomes with GluQRS$_2$
Nº of genomes without GluQRS$_2$
p < 0.00005
With GluQRS$_3$
Without GluQRS$_3$
Nº of genomes with GluQRS$_3$
Nº of genomes without GluQRS$_3$
With GluQRS$_4$
Without GluQRS$_4$
Nº of genomes with GluQRS$_4$
Nº of genomes without GluQRS$_4$
p < 0.00005
With GluQRS$_5$
Without GluQRS$_5$
Nº of genomes with GluQRS$_5$
Nº of genomes without GluQRS$_5$
p < 0.00005
With TGT$_1$
Without TGT$_1$
Nº of genomes with TGT$_1$
Nº of genomes without TGT$_1$
p < 0.00005
With TGT$_2$
Without TGT$_2$
Nº of genomes with TGT$_2$
Nº of genomes without TGT$_2$
With TGT$_3$
Without TGT$_3$
Nº of genomes with TGT$_3$
Nº of genomes without TGT$_3$

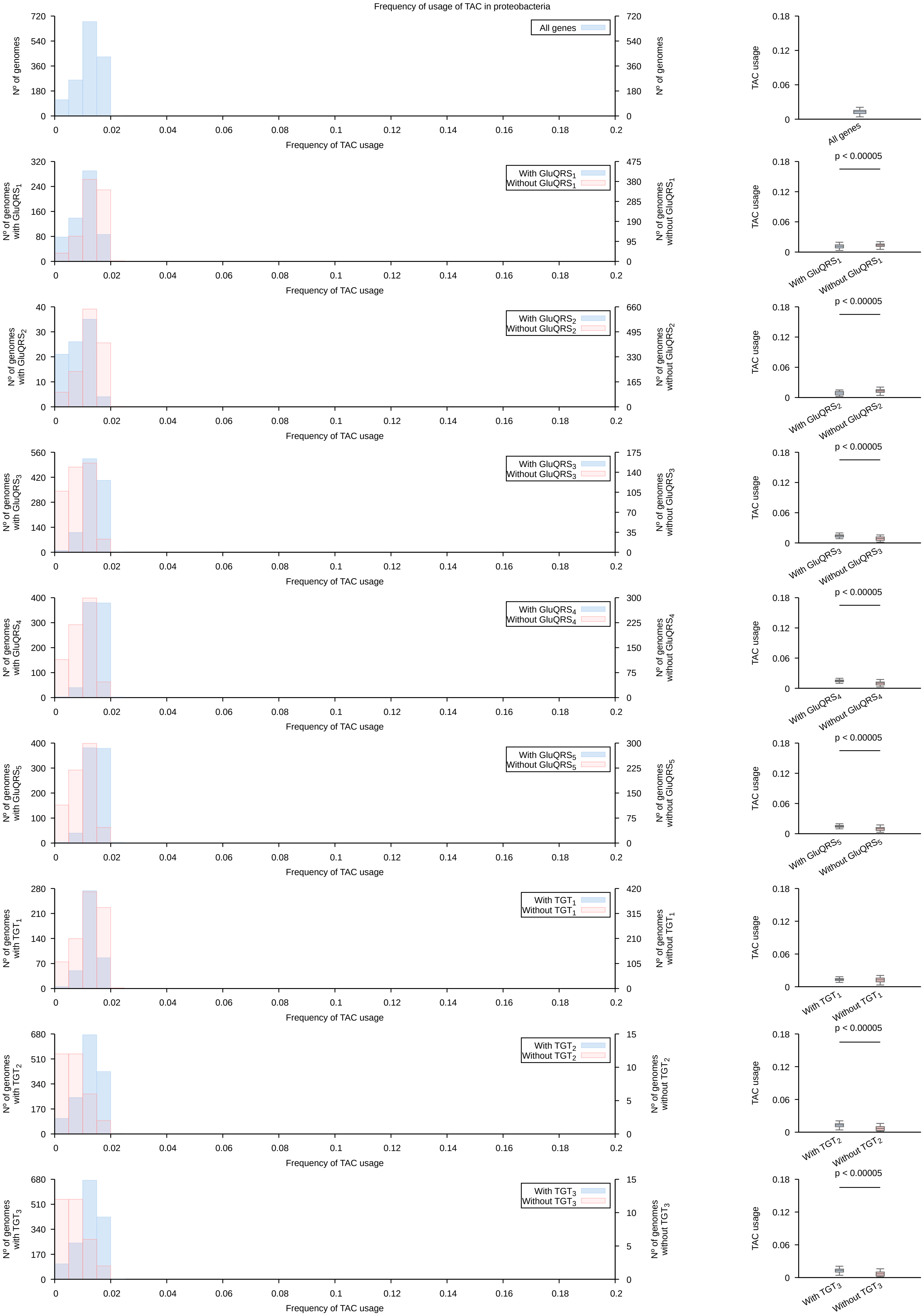

Frequency of usage of TAC in proteobacteria
Nº of genomes
Frequency of TAC usage
All genes
TAC usage
With GluQRS$_1$
Without GluQRS$_1$
Nº of genomes with GluQRS$_1$
Nº of genomes without GluQRS$_1$
p < 0.00005
With GluQRS$_2$
Without GluQRS$_2$
Nº of genomes with GluQRS$_2$
Nº of genomes without GluQRS$_2$
p < 0.00005
With GluQRS$_3$
Without GluQRS$_3$
Nº of genomes with GluQRS$_3$
Nº of genomes without GluQRS$_3$
p < 0.00005
With GluQRS$_4$
Without GluQRS$_4$
Nº of genomes with GluQRS$_4$
Nº of genomes without GluQRS$_4$
p < 0.00005
With GluQRS$_5$
Without GluQRS$_5$
Nº of genomes with GluQRS$_5$
Nº of genomes without GluQRS$_5$
p < 0.00005
With TGT$_1$
Without TGT$_1$
Nº of genomes with TGT$_1$
Nº of genomes without TGT$_1$
With TGT$_2$
Without TGT$_2$
Nº of genomes with TGT$_2$
Nº of genomes without TGT$_2$
p < 0.00005
With TGT$_3$
Without TGT$_3$
Nº of genomes with TGT$_3$
Nº of genomes without TGT$_3$
p < 0.00005

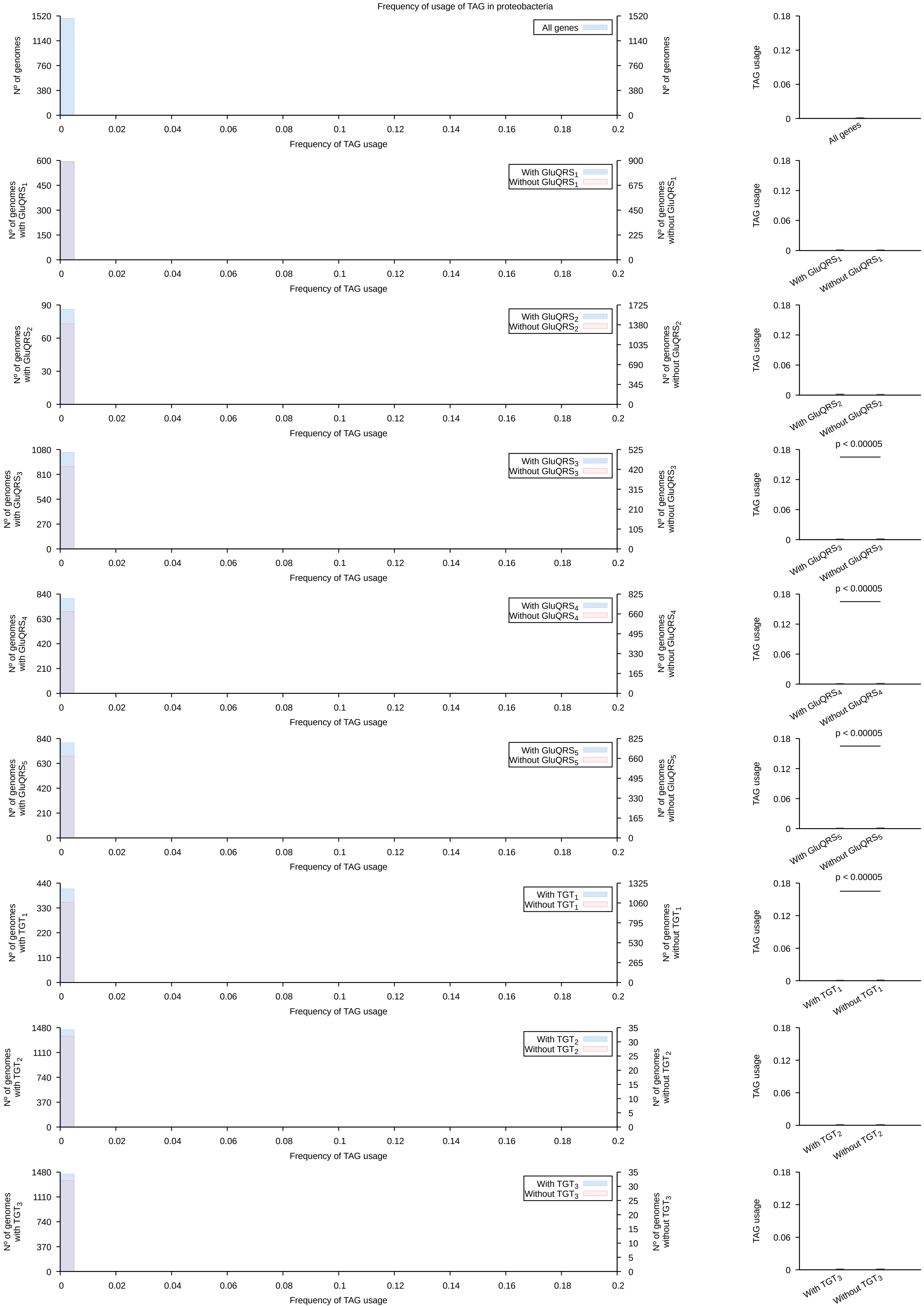

Frequency of usage of TAG in proteobacteria
All genes
Nº of genomes
Frequency of TAG usage
TAG usage
With GluQRS1
Without GluQRS1
Nº of genomes with GluQRS1
Nº of genomes without GluQRS1
With GluQRS2
Without GluQRS2
Nº of genomes with GluQRS2
Nº of genomes without GluQRS2
With GluQRS3
Without GluQRS3
Nº of genomes with GluQRS3
Nº of genomes without GluQRS3
p < 0.00005
With GluQRS4
Without GluQRS4
Nº of genomes with GluQRS4
Nº of genomes without GluQRS4
p < 0.00005
With GluQRS5
Without GluQRS5
Nº of genomes with GluQRS5
Nº of genomes without GluQRS5
p < 0.00005
With TGT1
Without TGT1
Nº of genomes with TGT1
Nº of genomes without TGT1
p < 0.00005
With TGT2
Without TGT2
Nº of genomes with TGT2
Nº of genomes without TGT2
With TGT3
Without TGT3
Nº of genomes with TGT3
Nº of genomes without TGT3

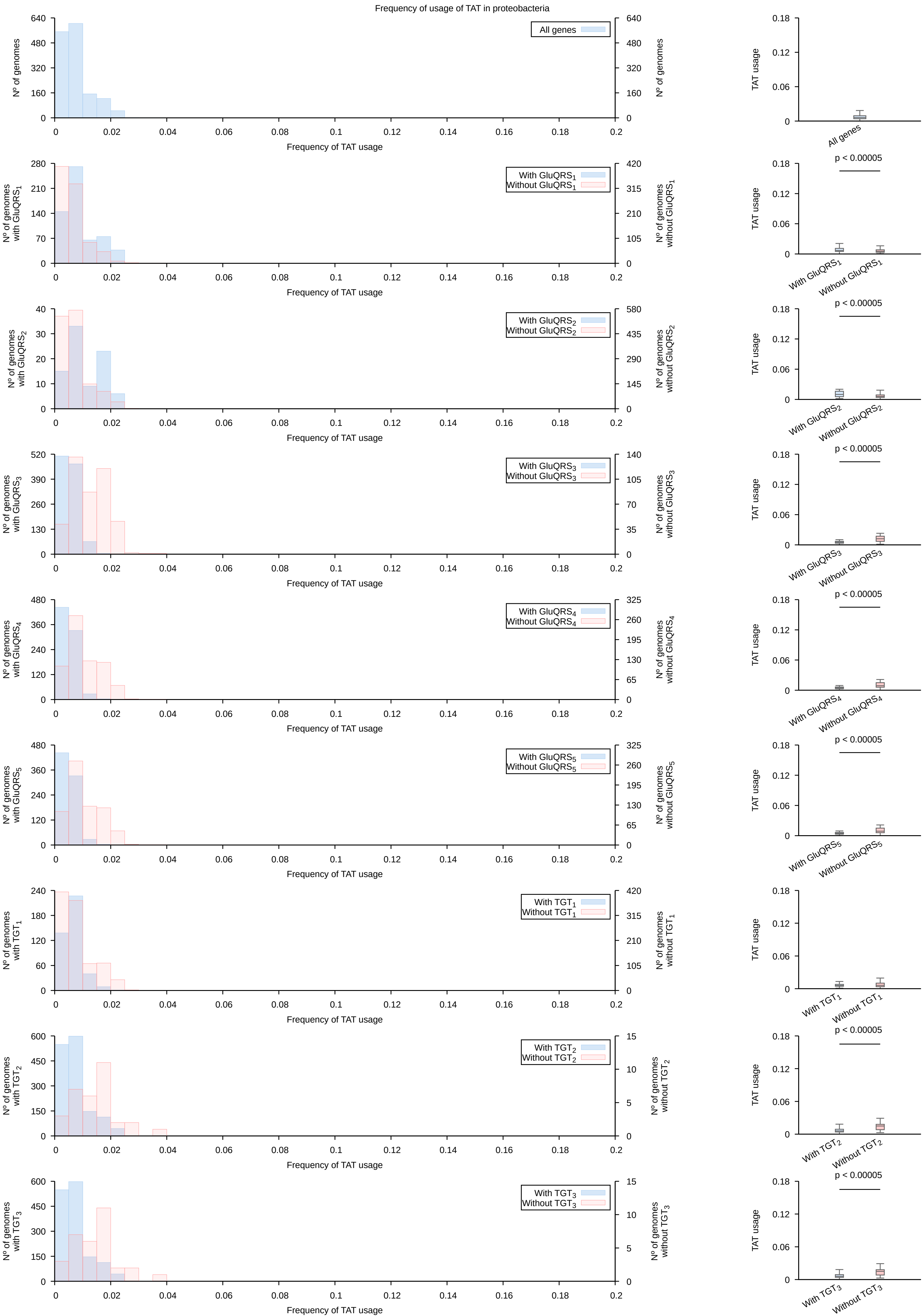
Frequency of usage of TAT in proteobacteria
All genes
Nº of genomes
Frequency of TAT usage
TAT usage
With GluQRS$_1$
Without GluQRS$_1$
Nº of genomes with GluQRS$_1$
Nº of genomes without GluQRS$_1$
p < 0.00005
With GluQRS$_2$
Without GluQRS$_2$
Nº of genomes with GluQRS$_2$
Nº of genomes without GluQRS$_2$
p < 0.00005
With GluQRS$_3$
Without GluQRS$_3$
Nº of genomes with GluQRS$_3$
Nº of genomes without GluQRS$_3$
p < 0.00005
With GluQRS$_4$
Without GluQRS$_4$
Nº of genomes with GluQRS$_4$
Nº of genomes without GluQRS$_4$
p < 0.00005
With GluQRS$_5$
Without GluQRS$_5$
Nº of genomes with GluQRS$_5$
Nº of genomes without GluQRS$_5$
p < 0.00005
With TGT$_1$
Without TGT$_1$
Nº of genomes with TGT$_1$
Nº of genomes without TGT$_1$
With TGT$_2$
Without TGT$_2$
Nº of genomes with TGT$_2$
Nº of genomes without TGT$_2$
p < 0.00005
With TGT$_3$
Without TGT$_3$
Nº of genomes with TGT$_3$
Nº of genomes without TGT$_3$
p < 0.00005

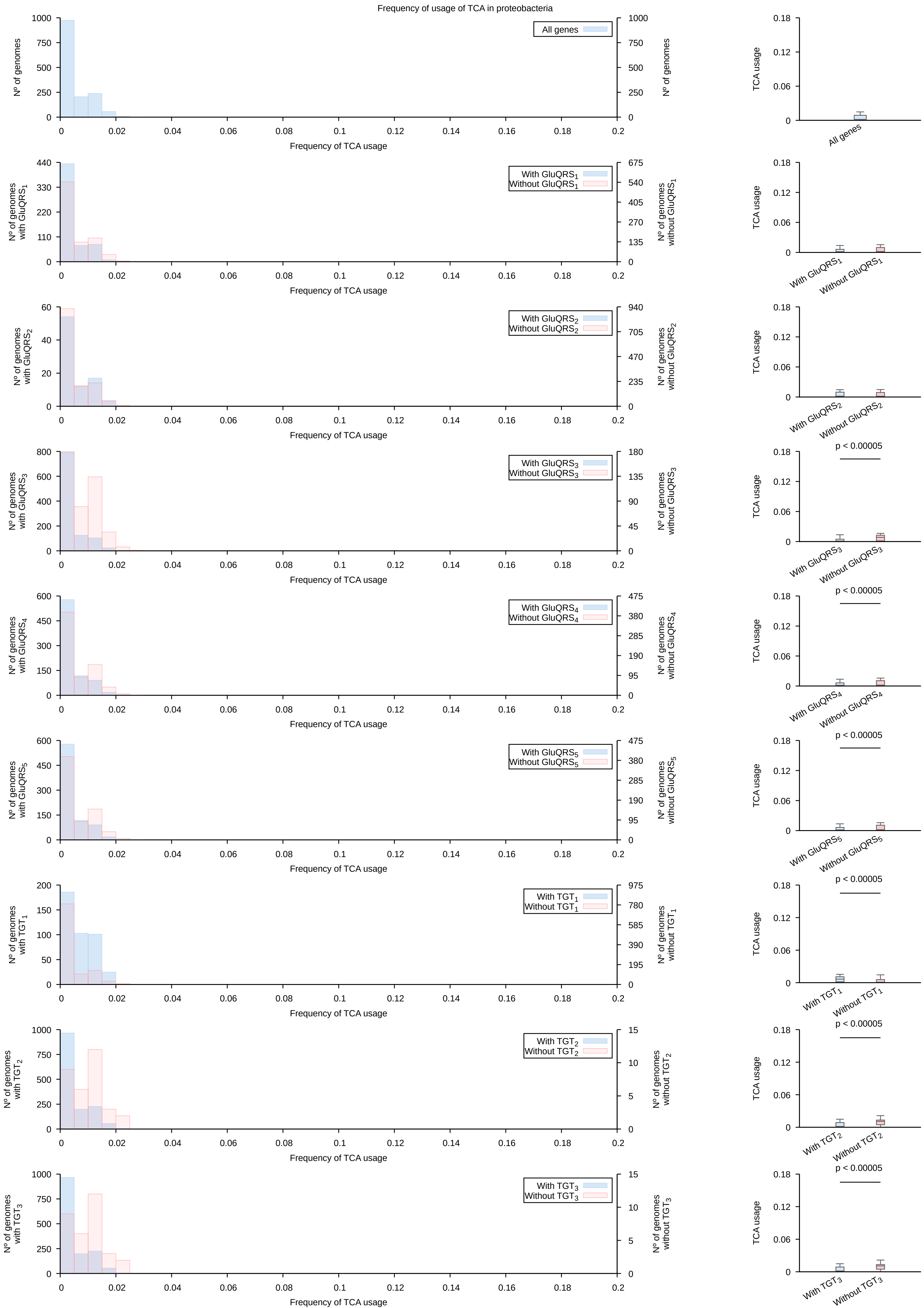

Frequency of usage of TCA in proteobacteria
Nº of genomes
Frequency of TCA usage
All genes
TCA usage
With GluQRS1
Without GluQRS1
With GluQRS2
Without GluQRS2
With GluQRS3
Without GluQRS3
With GluQRS4
Without GluQRS4
With GluQRS5
Without GluQRS5
With TGT1
Without TGT1
With TGT2
Without TGT2
With TGT3
Without TGT3
p < 0.00005

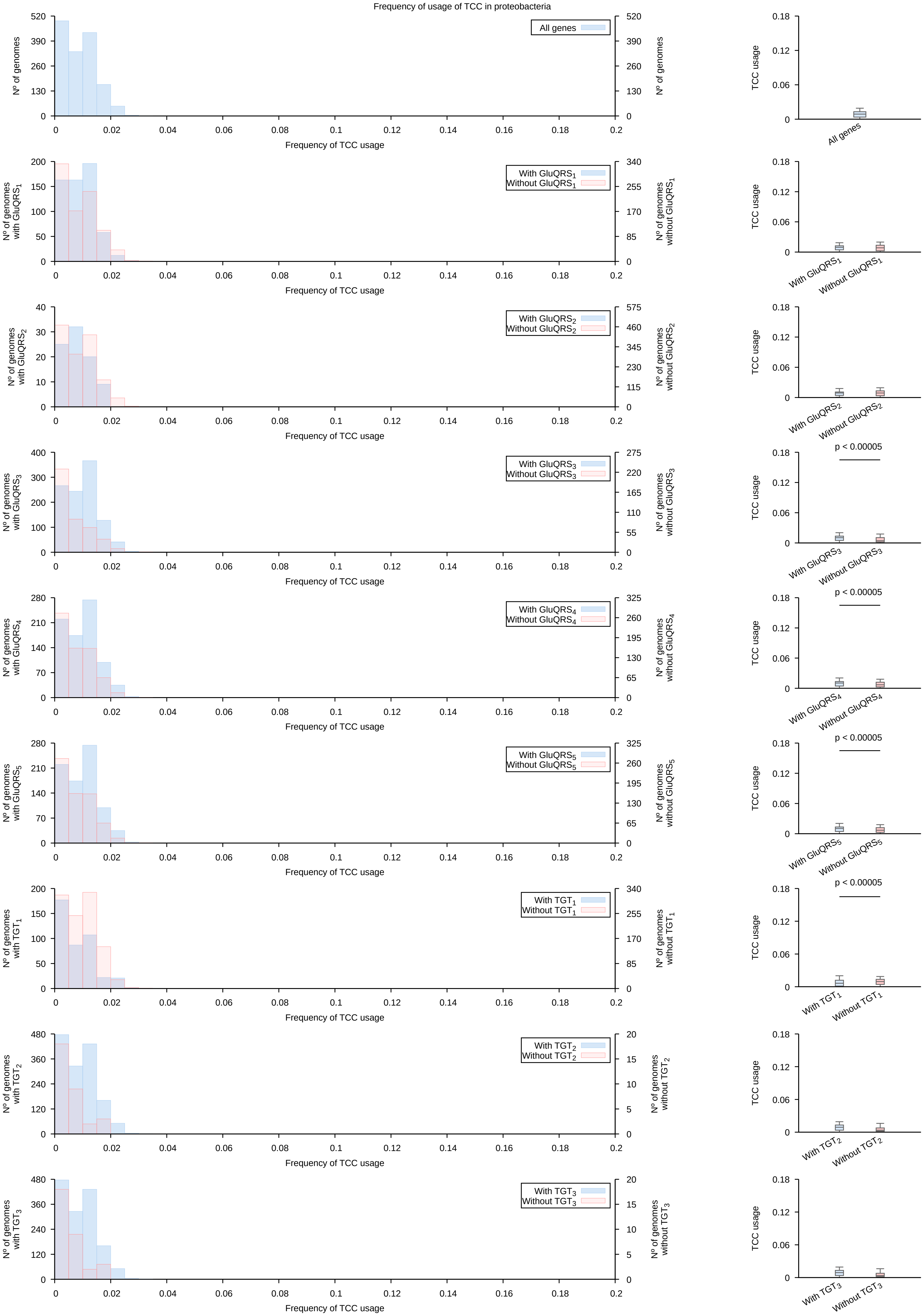
Frequency of usage of TCC in proteobacteria
All genes
Nº of genomes
Frequency of TCC usage
TCC usage
With $GluQRS_1$
Without $GluQRS_1$
Nº of genomes with $GluQRS_1$
Nº of genomes without $GluQRS_1$
With $GluQRS_2$
Without $GluQRS_2$
Nº of genomes with $GluQRS_2$
Nº of genomes without $GluQRS_2$
With $GluQRS_3$
Without $GluQRS_3$
Nº of genomes with $GluQRS_3$
Nº of genomes without $GluQRS_3$
$p < 0.00005$
With $GluQRS_4$
Without $GluQRS_4$
Nº of genomes with $GluQRS_4$
Nº of genomes without $GluQRS_4$
$p < 0.00005$
With $GluQRS_5$
Without $GluQRS_5$
Nº of genomes with $GluQRS_5$
Nº of genomes without $GluQRS_5$
$p < 0.00005$
With TGT_1
Without TGT_1
Nº of genomes with TGT_1
Nº of genomes without TGT_1
$p < 0.00005$
With TGT_2
Without TGT_2
Nº of genomes with TGT_2
Nº of genomes without TGT_2
With TGT_3
Without TGT_3
Nº of genomes with TGT_3
Nº of genomes without TGT_3

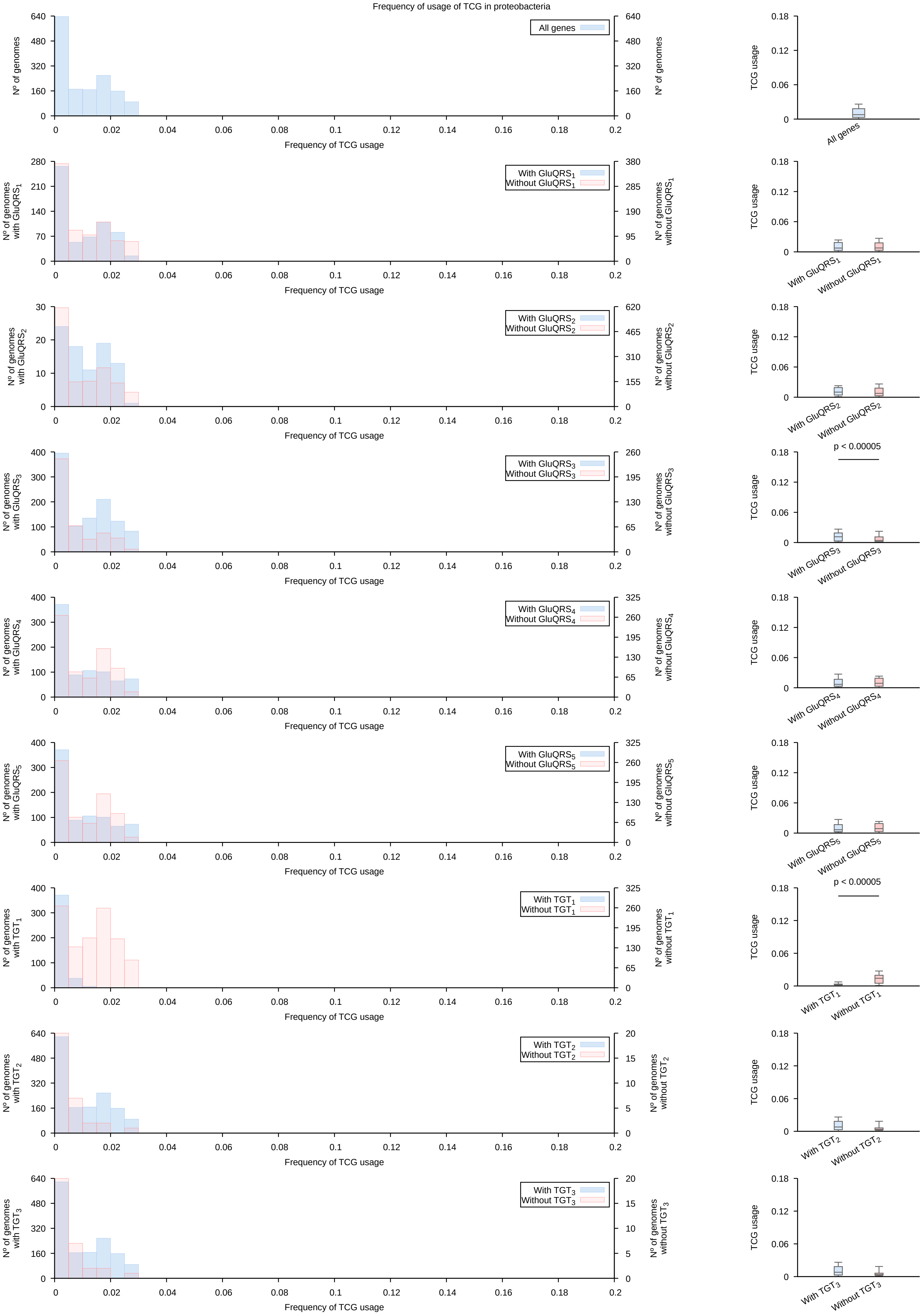

Frequency of usage of TCG in proteobacteria
Nº of genomes
All genes
Frequency of TCG usage
TCG usage
Nº of genomes with GluQRS$_1$
Nº of genomes without GluQRS$_1$
With GluQRS$_1$
Without GluQRS$_1$
Nº of genomes with GluQRS$_2$
Nº of genomes without GluQRS$_2$
With GluQRS$_2$
Without GluQRS$_2$
Nº of genomes with GluQRS$_3$
Nº of genomes without GluQRS$_3$
With GluQRS$_3$
Without GluQRS$_3$
$p < 0.00005$
Nº of genomes with GluQRS$_4$
Nº of genomes without GluQRS$_4$
With GluQRS$_4$
Without GluQRS$_4$
Nº of genomes with GluQRS$_5$
Nº of genomes without GluQRS$_5$
With GluQRS$_5$
Without GluQRS$_5$
Nº of genomes with TGT$_1$
Nº of genomes without TGT$_1$
With TGT$_1$
Without TGT$_1$
$p < 0.00005$
Nº of genomes with TGT$_2$
Nº of genomes without TGT$_2$
With TGT$_2$
Without TGT$_2$
Nº of genomes with TGT$_3$
Nº of genomes without TGT$_3$
With TGT$_3$
Without TGT$_3$

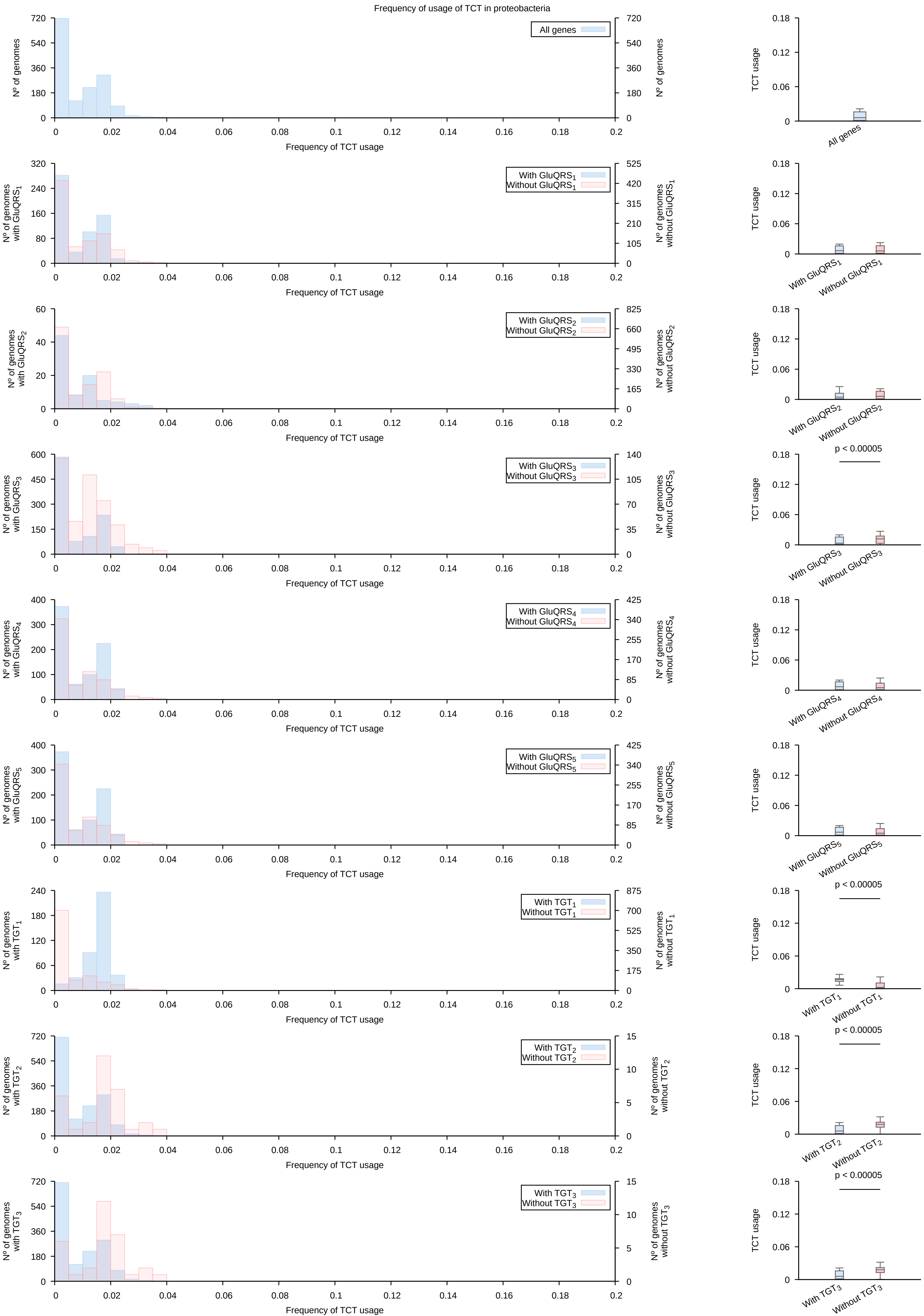
Frequency of usage of TCT in proteobacteria
All genes
Nº of genomes
Frequency of TCT usage
TCT usage
With GluQRS$_1$
Without GluQRS$_1$
Nº of genomes with GluQRS$_1$
Nº of genomes without GluQRS$_1$
With GluQRS$_2$
Without GluQRS$_2$
Nº of genomes with GluQRS$_2$
Nº of genomes without GluQRS$_2$
With GluQRS$_3$
Without GluQRS$_3$
Nº of genomes with GluQRS$_3$
Nº of genomes without GluQRS$_3$
p < 0.00005
With GluQRS$_4$
Without GluQRS$_4$
Nº of genomes with GluQRS$_4$
Nº of genomes without GluQRS$_4$
With GluQRS$_5$
Without GluQRS$_5$
Nº of genomes with GluQRS$_5$
Nº of genomes without GluQRS$_5$
With TGT$_1$
Without TGT$_1$
Nº of genomes with TGT$_1$
Nº of genomes without TGT$_1$
p < 0.00005
With TGT$_2$
Without TGT$_2$
Nº of genomes with TGT$_2$
Nº of genomes without TGT$_2$
p < 0.00005
With TGT$_3$
Without TGT$_3$
Nº of genomes with TGT$_3$
Nº of genomes without TGT$_3$
p < 0.00005

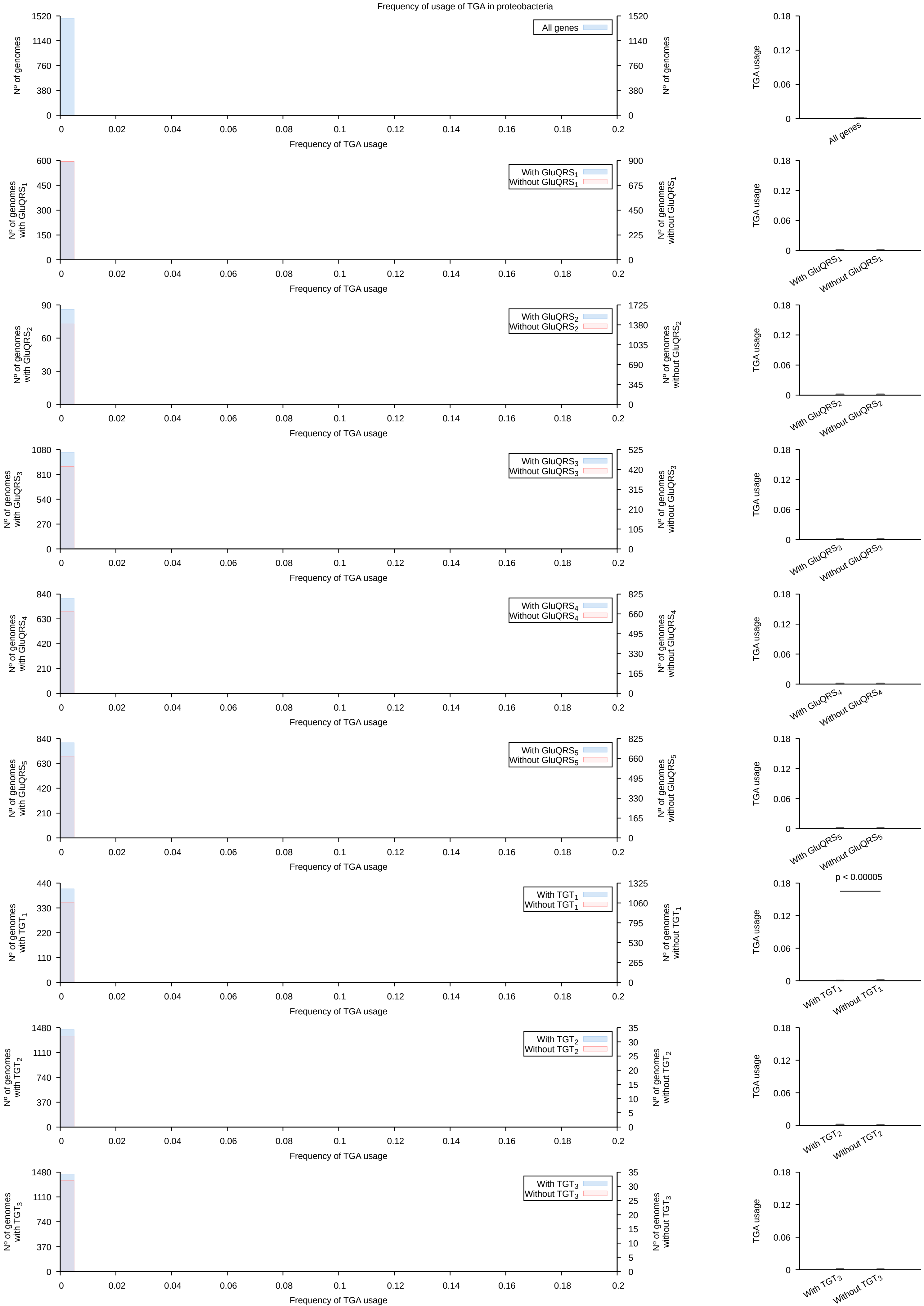
Frequency of usage of TGA in proteobacteria
All genes
With GluQRS$_1$
Without GluQRS$_1$
With GluQRS$_2$
Without GluQRS$_2$
With GluQRS$_3$
Without GluQRS$_3$
With GluQRS$_4$
Without GluQRS$_4$
With GluQRS$_5$
Without GluQRS$_5$
With TGT$_1$
Without TGT$_1$
With TGT$_2$
Without TGT$_2$
With TGT$_3$
Without TGT$_3$
Nº of genomes
Nº of genomes with GluQRS$_1$
Nº of genomes without GluQRS$_1$
Nº of genomes with GluQRS$_2$
Nº of genomes without GluQRS$_2$
Nº of genomes with GluQRS$_3$
Nº of genomes without GluQRS$_3$
Nº of genomes with GluQRS$_4$
Nº of genomes without GluQRS$_4$
Nº of genomes with GluQRS$_5$
Nº of genomes without GluQRS$_5$
Nº of genomes with TGT$_1$
Nº of genomes without TGT$_1$
Nº of genomes with TGT$_2$
Nº of genomes without TGT$_2$
Nº of genomes with TGT$_3$
Nº of genomes without TGT$_3$
Frequency of TGA usage
TGA usage
p < 0.00005

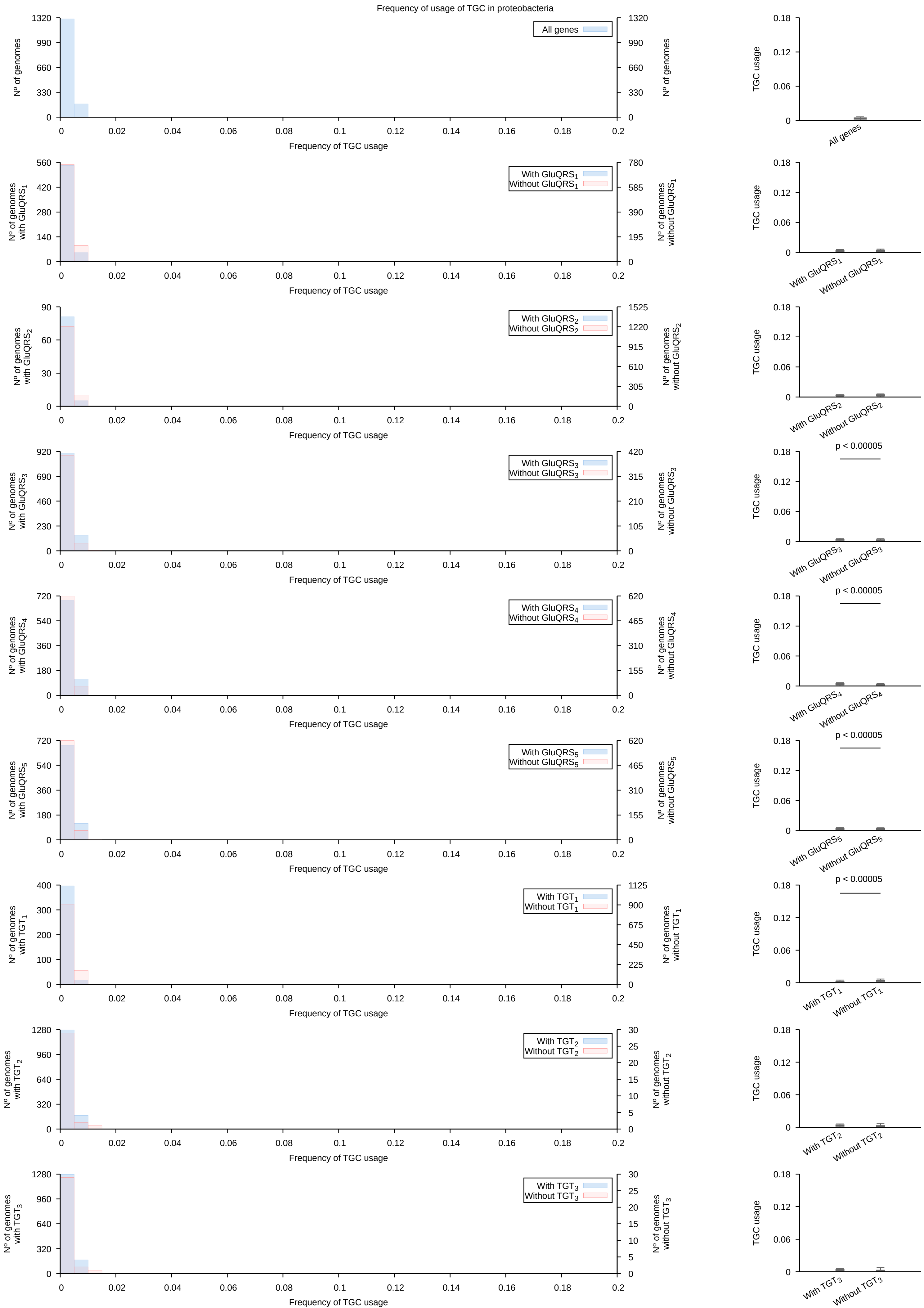
Frequency of usage of TGC in proteobacteria
Nº of genomes
Frequency of TGC usage
All genes
TGC usage
Nº of genomes with GluQRS$_1$
Nº of genomes without GluQRS$_1$
With GluQRS$_1$
Without GluQRS$_1$
Nº of genomes with GluQRS$_2$
Nº of genomes without GluQRS$_2$
With GluQRS$_2$
Without GluQRS$_2$
Nº of genomes with GluQRS$_3$
Nº of genomes without GluQRS$_3$
With GluQRS$_3$
Without GluQRS$_3$
p < 0.00005
Nº of genomes with GluQRS$_4$
Nº of genomes without GluQRS$_4$
With GluQRS$_4$
Without GluQRS$_4$
p < 0.00005
Nº of genomes with GluQRS$_5$
Nº of genomes without GluQRS$_5$
With GluQRS$_5$
Without GluQRS$_5$
p < 0.00005
Nº of genomes with TGT$_1$
Nº of genomes without TGT$_1$
With TGT$_1$
Without TGT$_1$
p < 0.00005
Nº of genomes with TGT$_2$
Nº of genomes without TGT$_2$
With TGT$_2$
Without TGT$_2$
Nº of genomes with TGT$_3$
Nº of genomes without TGT$_3$
With TGT$_3$
Without TGT$_3$

Frequency of usage of TGG in proteobacteria

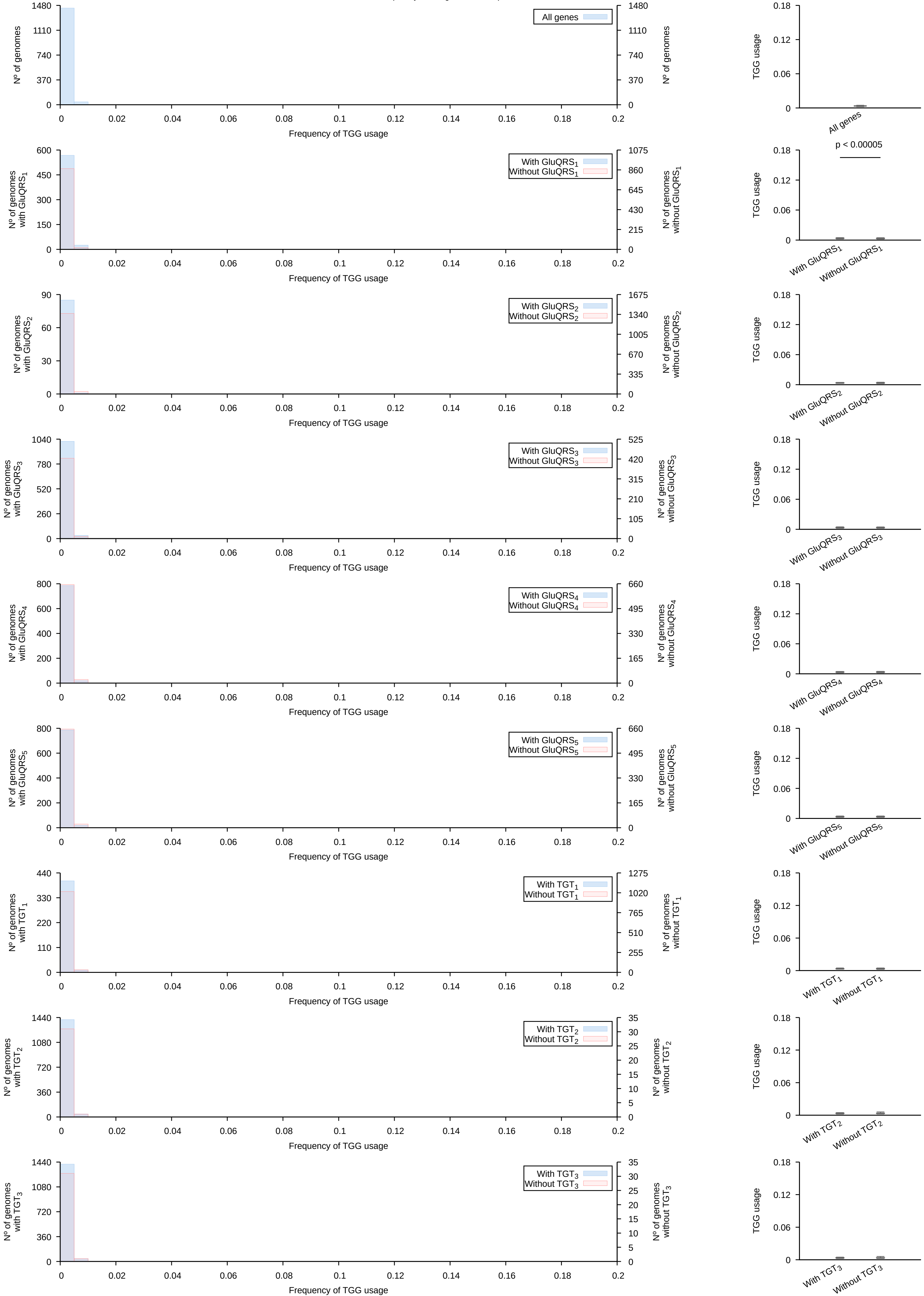

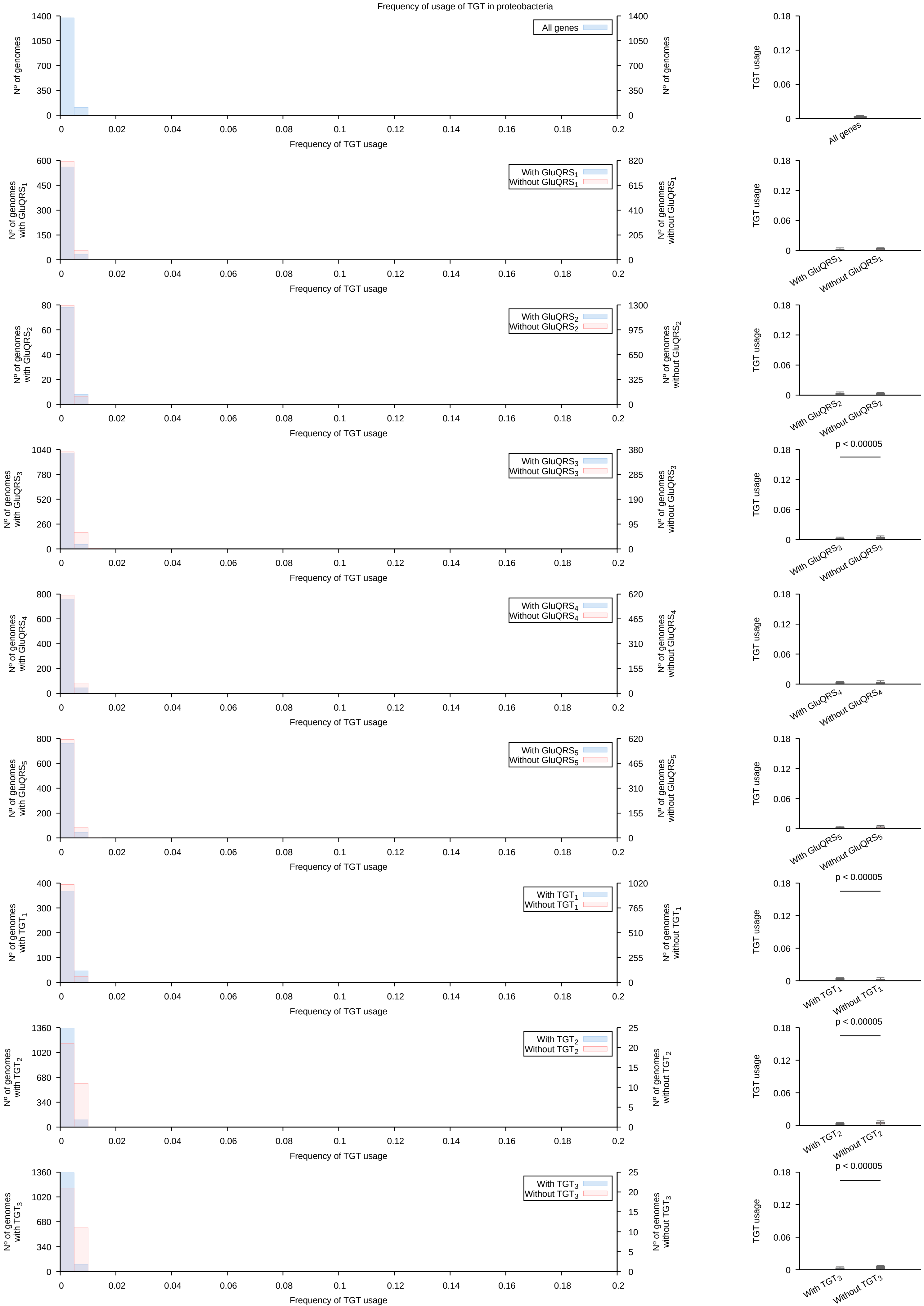

Frequency of usage of TGT in proteobacteria
All genes
Nº of genomes
Frequency of TGT usage
TGT usage
With GluQRS$_1$
Without GluQRS$_1$
Nº of genomes with GluQRS$_1$
Nº of genomes without GluQRS$_1$
With GluQRS$_2$
Without GluQRS$_2$
Nº of genomes with GluQRS$_2$
Nº of genomes without GluQRS$_2$
With GluQRS$_3$
Without GluQRS$_3$
Nº of genomes with GluQRS$_3$
Nº of genomes without GluQRS$_3$
p < 0.00005
With GluQRS$_4$
Without GluQRS$_4$
Nº of genomes with GluQRS$_4$
Nº of genomes without GluQRS$_4$
With GluQRS$_5$
Without GluQRS$_5$
Nº of genomes with GluQRS$_5$
Nº of genomes without GluQRS$_5$
With TGT$_1$
Without TGT$_1$
Nº of genomes with TGT$_1$
Nº of genomes without TGT$_1$
p < 0.00005
With TGT$_2$
Without TGT$_2$
Nº of genomes with TGT$_2$
Nº of genomes without TGT$_2$
p < 0.00005
With TGT$_3$
Without TGT$_3$
Nº of genomes with TGT$_3$
Nº of genomes without TGT$_3$
p < 0.00005

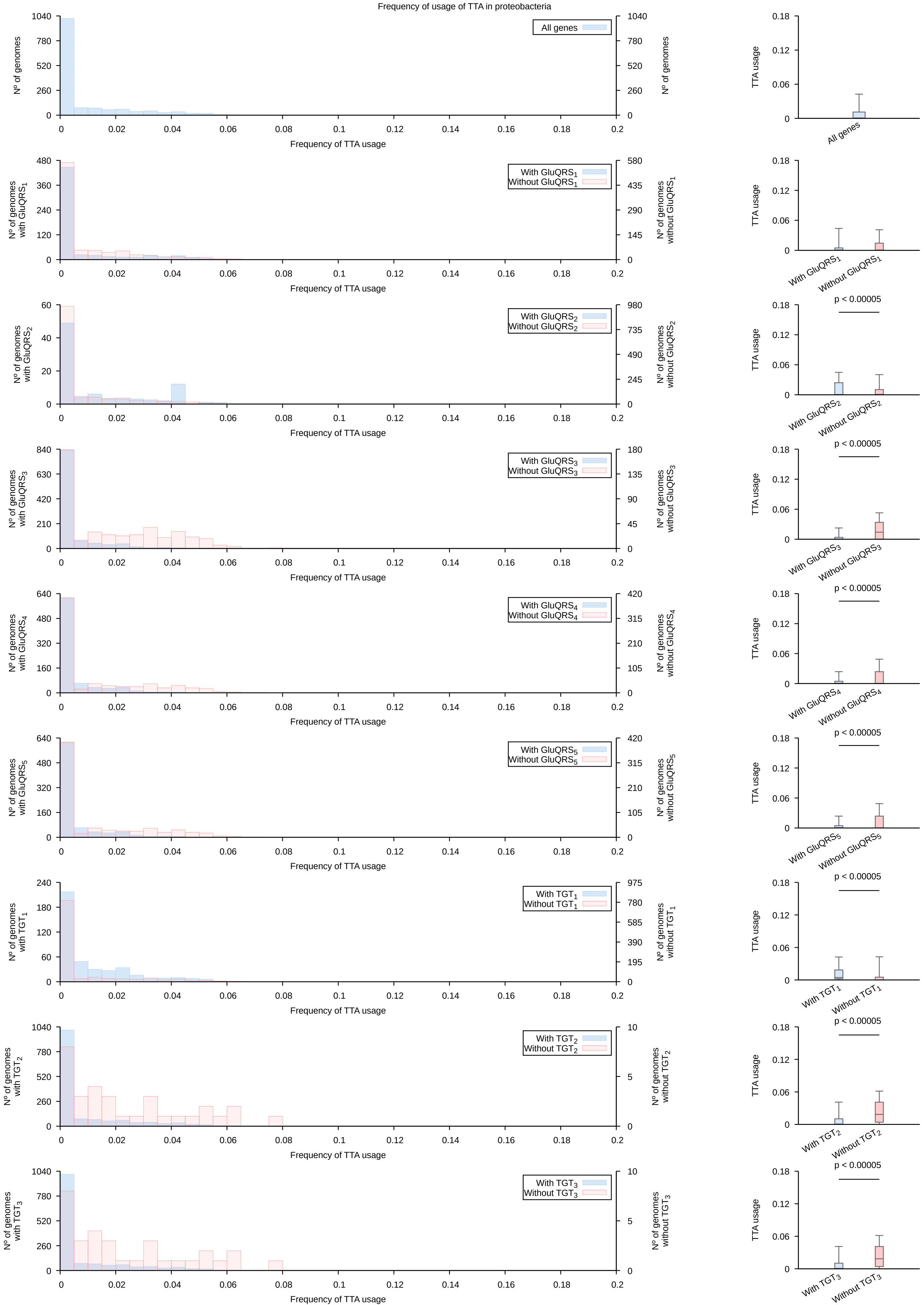
Frequency of usage of TTA in proteobacteria
Nº of genomes
Frequency of TTA usage
All genes
TTA usage
Nº of genomes with GluQRS$_1$
Nº of genomes without GluQRS$_1$
With GluQRS$_1$
Without GluQRS$_1$
Nº of genomes with GluQRS$_2$
Nº of genomes without GluQRS$_2$
With GluQRS$_2$
Without GluQRS$_2$
p < 0.00005
Nº of genomes with GluQRS$_3$
Nº of genomes without GluQRS$_3$
With GluQRS$_3$
Without GluQRS$_3$
p < 0.00005
Nº of genomes with GluQRS$_4$
Nº of genomes without GluQRS$_4$
With GluQRS$_4$
Without GluQRS$_4$
p < 0.00005
Nº of genomes with GluQRS$_5$
Nº of genomes without GluQRS$_5$
With GluQRS$_5$
Without GluQRS$_5$
p < 0.00005
Nº of genomes with TGT$_1$
Nº of genomes without TGT$_1$
With TGT$_1$
Without TGT$_1$
p < 0.00005
Nº of genomes with TGT$_2$
Nº of genomes without TGT$_2$
With TGT$_2$
Without TGT$_2$
p < 0.00005
Nº of genomes with TGT$_3$
Nº of genomes without TGT$_3$
With TGT$_3$
Without TGT$_3$
p < 0.00005

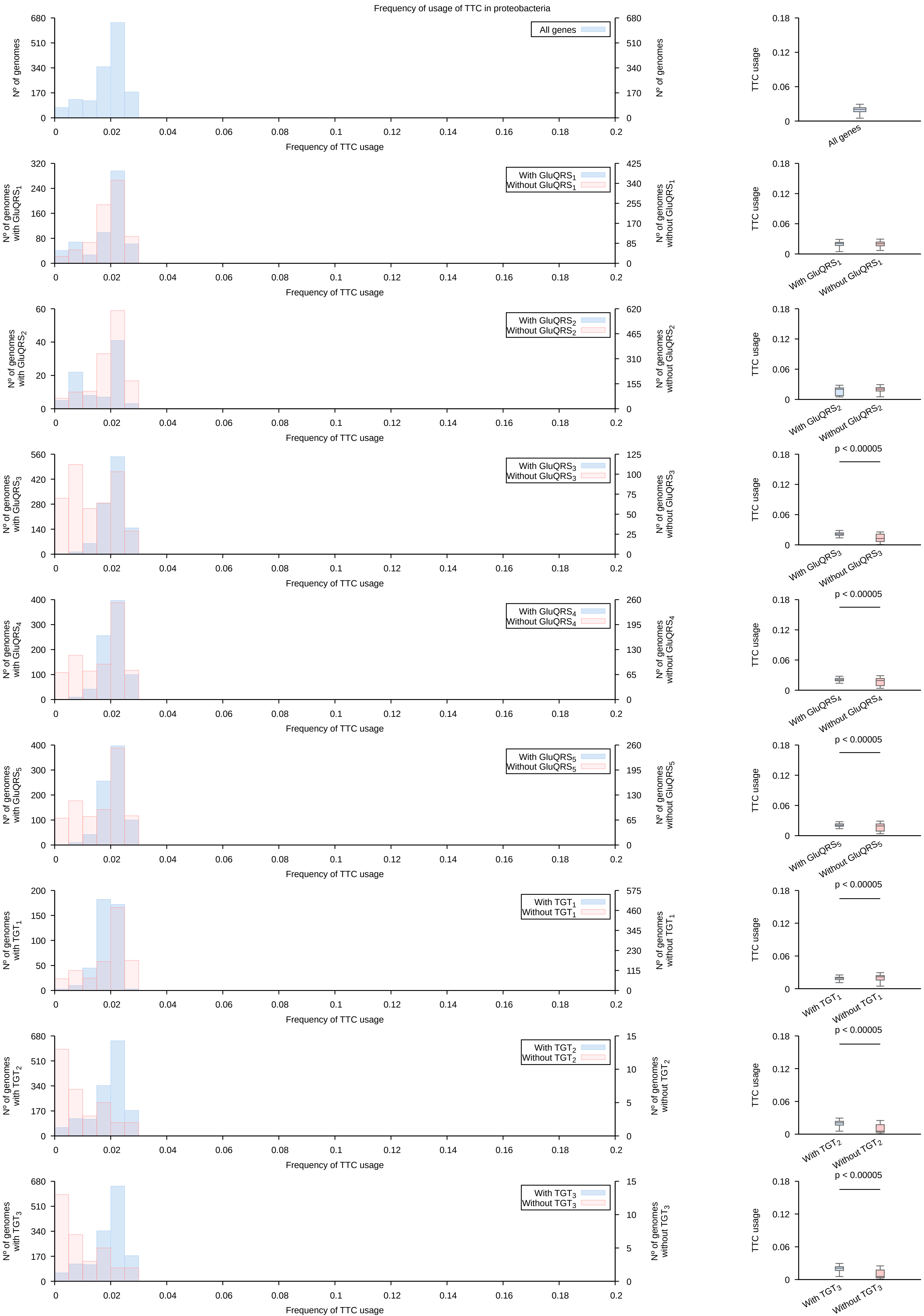

Frequency of usage of TTC in proteobacteria
Nº of genomes
Frequency of TTC usage
All genes
TTC usage
With GluQRS$_1$
Without GluQRS$_1$
Nº of genomes with GluQRS$_1$
Nº of genomes without GluQRS$_1$
With GluQRS$_2$
Without GluQRS$_2$
Nº of genomes with GluQRS$_2$
Nº of genomes without GluQRS$_2$
With GluQRS$_3$
Without GluQRS$_3$
Nº of genomes with GluQRS$_3$
Nº of genomes without GluQRS$_3$
$p < 0.00005$
With GluQRS$_4$
Without GluQRS$_4$
Nº of genomes with GluQRS$_4$
Nº of genomes without GluQRS$_4$
$p < 0.00005$
With GluQRS$_5$
Without GluQRS$_5$
Nº of genomes with GluQRS$_5$
Nº of genomes without GluQRS$_5$
$p < 0.00005$
With TGT$_1$
Without TGT$_1$
Nº of genomes with TGT$_1$
Nº of genomes without TGT$_1$
$p < 0.00005$
With TGT$_2$
Without TGT$_2$
Nº of genomes with TGT$_2$
Nº of genomes without TGT$_2$
$p < 0.00005$
With TGT$_3$
Without TGT$_3$
Nº of genomes with TGT$_3$
Nº of genomes without TGT$_3$
$p < 0.00005$

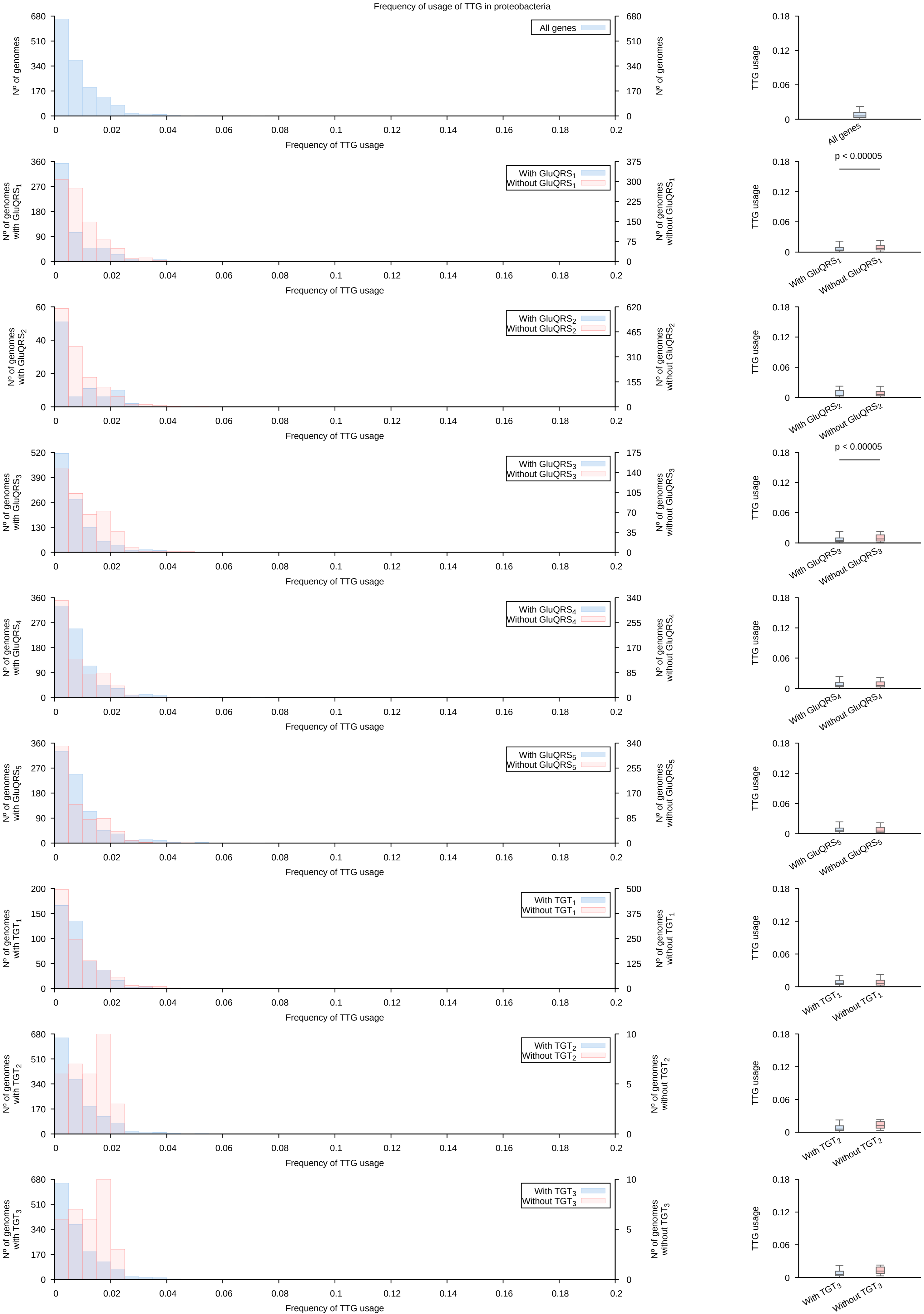
Frequency of usage of TTG in proteobacteria
Nº of genomes
All genes
Frequency of TTG usage
TTG usage
Nº of genomes with GluQRS$_1$
Nº of genomes without GluQRS$_1$
With GluQRS$_1$
Without GluQRS$_1$
p < 0.00005
Nº of genomes with GluQRS$_2$
Nº of genomes without GluQRS$_2$
With GluQRS$_2$
Without GluQRS$_2$
Nº of genomes with GluQRS$_3$
Nº of genomes without GluQRS$_3$
With GluQRS$_3$
Without GluQRS$_3$
p < 0.00005
Nº of genomes with GluQRS$_4$
Nº of genomes without GluQRS$_4$
With GluQRS$_4$
Without GluQRS$_4$
Nº of genomes with GluQRS$_5$
Nº of genomes without GluQRS$_5$
With GluQRS$_5$
Without GluQRS$_5$
Nº of genomes with TGT$_1$
Nº of genomes without TGT$_1$
With TGT$_1$
Without TGT$_1$
Nº of genomes with TGT$_2$
Nº of genomes without TGT$_2$
With TGT$_2$
Without TGT$_2$
Nº of genomes with TGT$_3$
Nº of genomes without TGT$_3$
With TGT$_3$
Without TGT$_3$

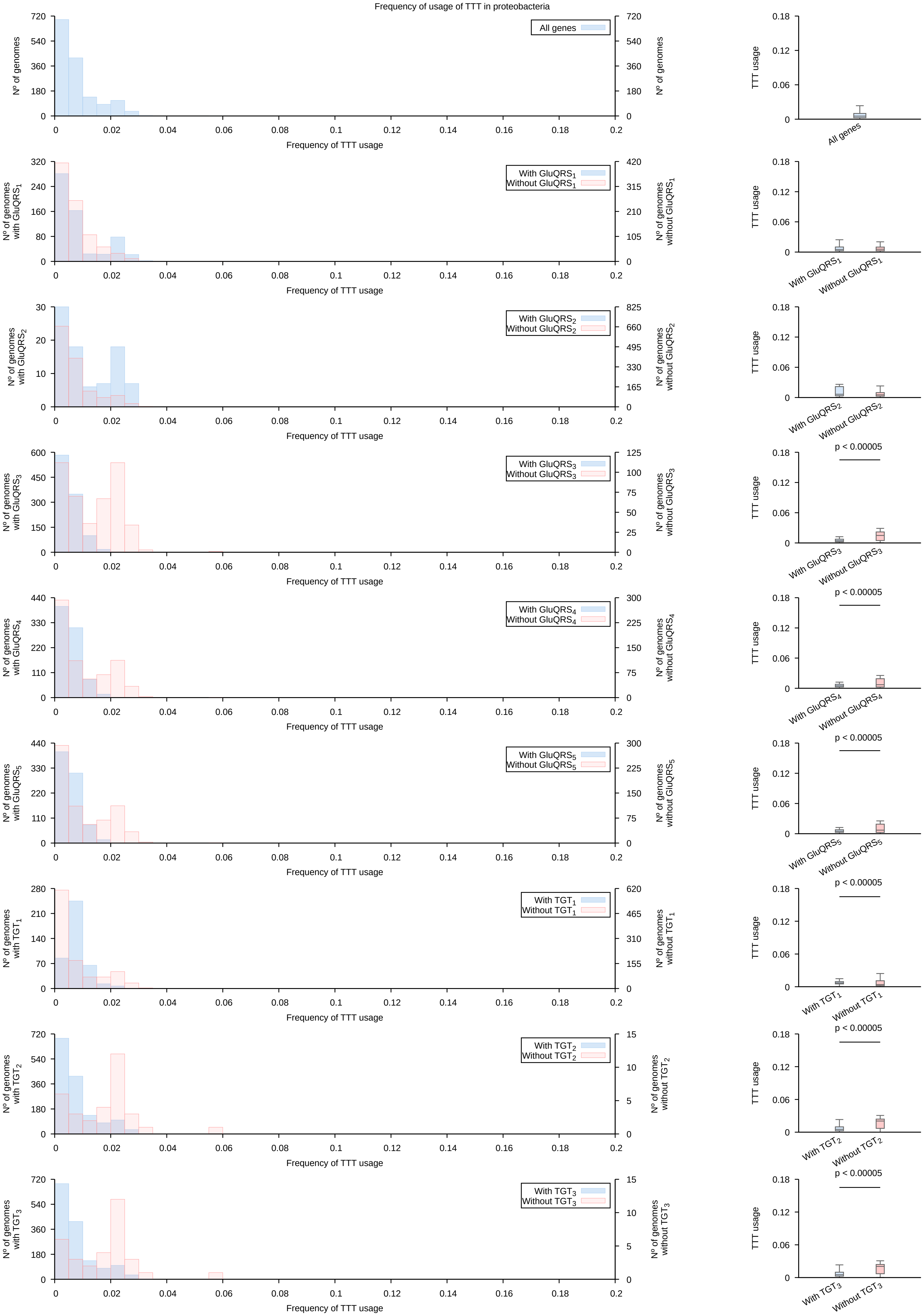

Frequency of usage of TTT in proteobacteria
All genes
Nº of genomes
Frequency of TTT usage
TTT usage
With GluQRS1
Without GluQRS1
Nº of genomes with GluQRS1
Nº of genomes without GluQRS1
With GluQRS2
Without GluQRS2
Nº of genomes with GluQRS2
Nº of genomes without GluQRS2
With GluQRS3
Without GluQRS3
Nº of genomes with GluQRS3
Nº of genomes without GluQRS3
With GluQRS4
Without GluQRS4
Nº of genomes with GluQRS4
Nº of genomes without GluQRS4
With GluQRS5
Without GluQRS5
Nº of genomes with GluQRS5
Nº of genomes without GluQRS5
With TGT1
Without TGT1
Nº of genomes with TGT1
Nº of genomes without TGT1
With TGT2
Without TGT2
Nº of genomes with TGT2
Nº of genomes without TGT2
With TGT3
Without TGT3
Nº of genomes with TGT3
Nº of genomes without TGT3
p < 0.00005

Figure S9. Histogram of codon usage in high expression genes of genomes that present or lack genes involved in k^2C formation. Top left graph: histogram of the usage of the indicated codon in highly expressed genes of proteobacterial genomes. **Top right graph:** box plot indicating the distribution of codon usage in the same population. **Lower graphs:** The collection of 1484 proteobacterial genomes were separated in two groups, based on the presence (light blue) or absence (orange) of the indicated pattern in any gene of the analyzed genomes. Patterns are indicated using subscript numbers. See table S4 for a detailed description of the used patterns. **Left:** Data is represented in superposed histograms. **Right:** Data is represented in a boxplot. An horizontal bar was added when the two populations are different based on Wilcoxon-Mann-Whitney test and $p < 0.00005$. Whiskers include 95% of data.

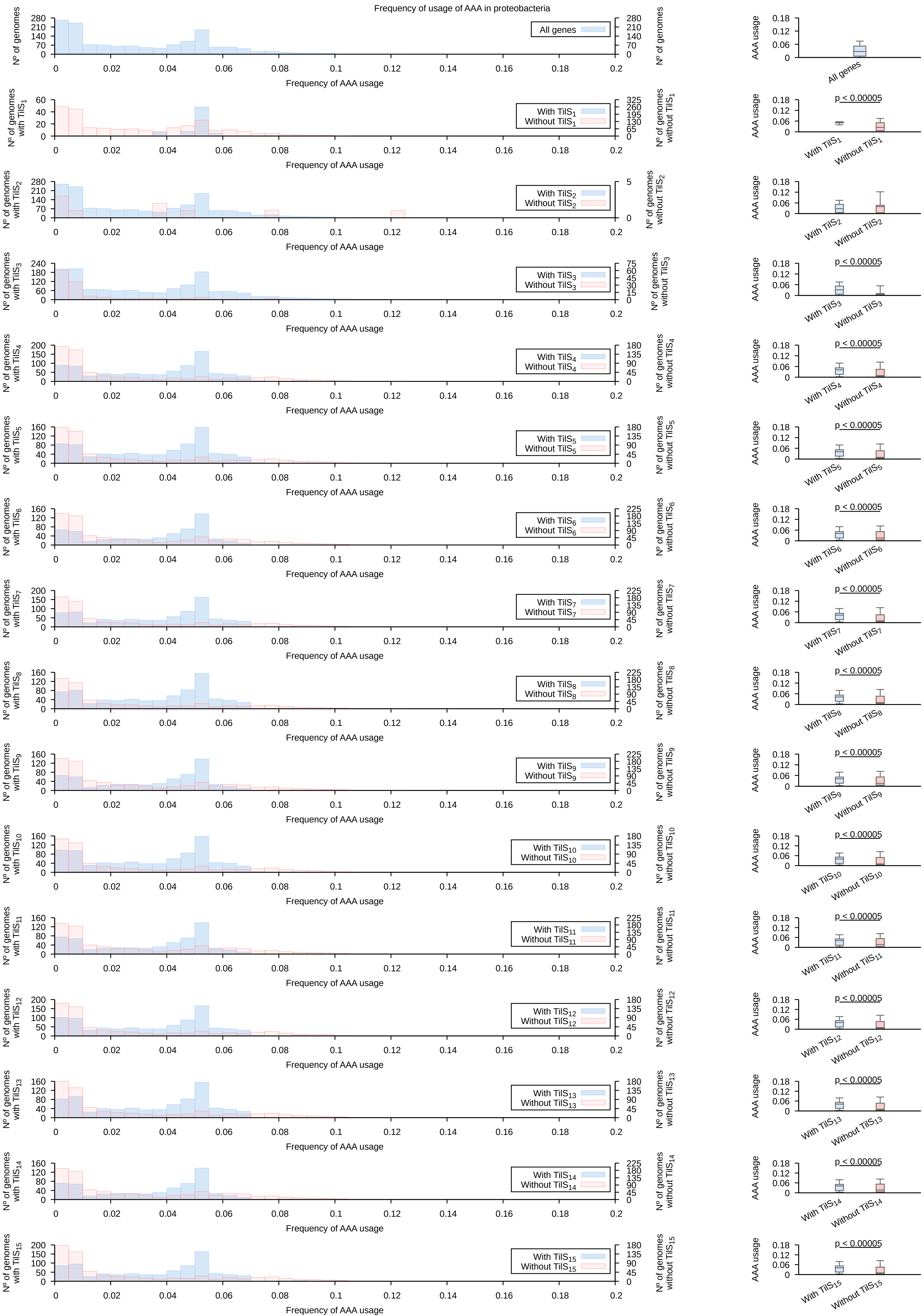
Frequency of usage of AAA in proteobacteria
Nº of genomes
All genes
Frequency of AAA usage
AAA usage
Nº of genomes with $TilS_1$
Nº of genomes without $TilS_1$
With $TilS_1$
Without $TilS_1$
p < 0.00005
Nº of genomes with $TilS_2$
Nº of genomes without $TilS_2$
With $TilS_2$
Without $TilS_2$
Nº of genomes with $TilS_3$
Nº of genomes without $TilS_3$
With $TilS_3$
Without $TilS_3$
Nº of genomes with $TilS_4$
Nº of genomes without $TilS_4$
With $TilS_4$
Without $TilS_4$
Nº of genomes with $TilS_5$
Nº of genomes without $TilS_5$
With $TilS_5$
Without $TilS_5$
Nº of genomes with $TilS_6$
Nº of genomes without $TilS_6$
With $TilS_6$
Without $TilS_6$
Nº of genomes with $TilS_7$
Nº of genomes without $TilS_7$
With $TilS_7$
Without $TilS_7$
Nº of genomes with $TilS_8$
Nº of genomes without $TilS_8$
With $TilS_8$
Without $TilS_8$
Nº of genomes with $TilS_9$
Nº of genomes without $TilS_9$
With $TilS_9$
Without $TilS_9$
Nº of genomes with $TilS_{10}$
Nº of genomes without $TilS_{10}$
With $TilS_{10}$
Without $TilS_{10}$
Nº of genomes with $TilS_{11}$
Nº of genomes without $TilS_{11}$
With $TilS_{11}$
Without $TilS_{11}$
Nº of genomes with $TilS_{12}$
Nº of genomes without $TilS_{12}$
With $TilS_{12}$
Without $TilS_{12}$
Nº of genomes with $TilS_{13}$
Nº of genomes without $TilS_{13}$
With $TilS_{13}$
Without $TilS_{13}$
Nº of genomes with $TilS_{14}$
Nº of genomes without $TilS_{14}$
With $TilS_{14}$
Without $TilS_{14}$
Nº of genomes with $TilS_{15}$
Nº of genomes without $TilS_{15}$
With $TilS_{15}$
Without $TilS_{15}$

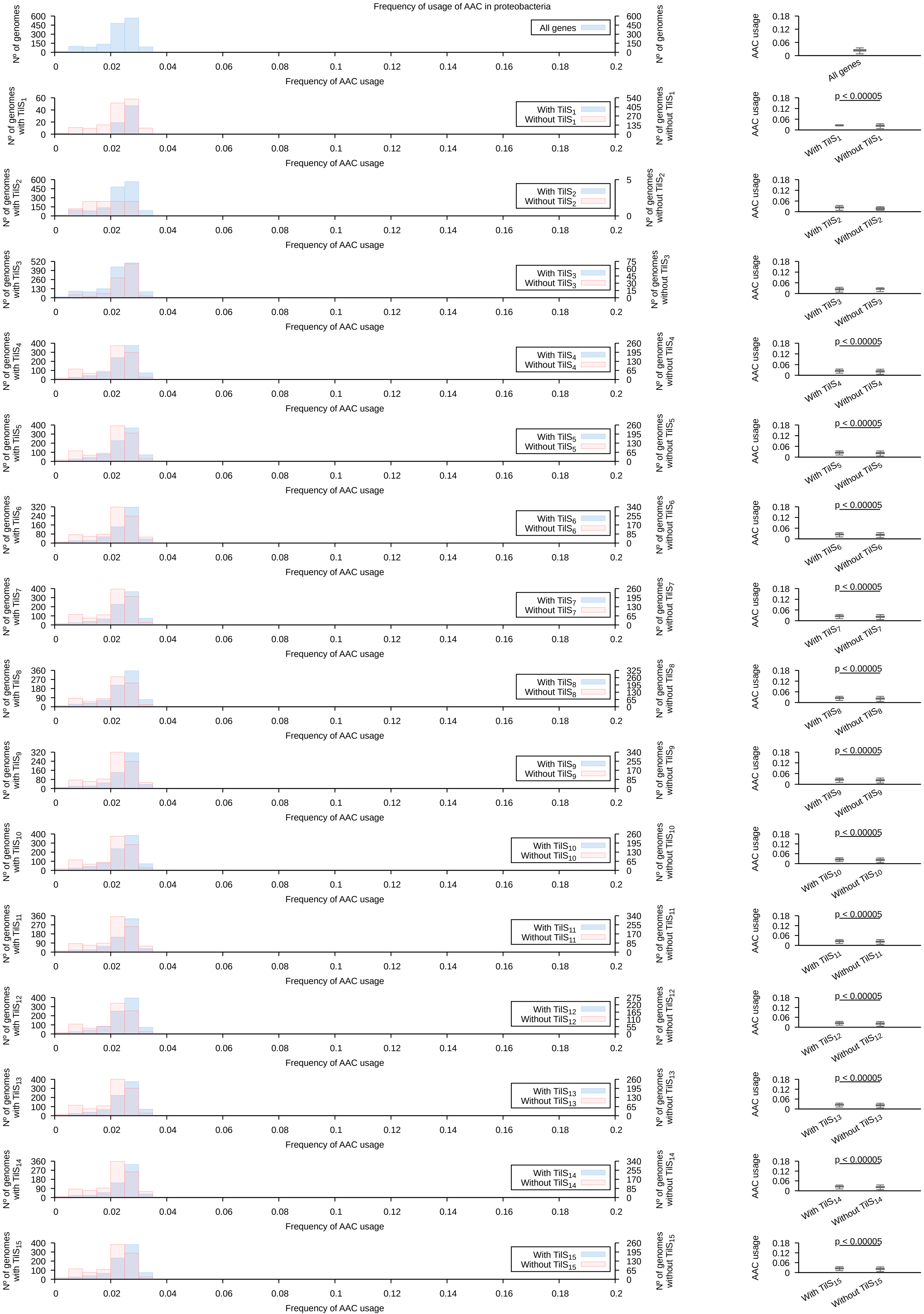

Frequency of usage of AAC in proteobacteria
Nº of genomes
All genes
Frequency of AAC usage
AAC usage
Nº of genomes with TilS$_1$
Nº of genomes without TilS$_1$
With TilS$_1$
Without TilS$_1$
p < 0.00005
Nº of genomes with TilS$_2$
Nº of genomes without TilS$_2$
With TilS$_2$
Without TilS$_2$
Nº of genomes with TilS$_3$
Nº of genomes without TilS$_3$
With TilS$_3$
Without TilS$_3$
Nº of genomes with TilS$_4$
Nº of genomes without TilS$_4$
With TilS$_4$
Without TilS$_4$
p < 0.00005
Nº of genomes with TilS$_5$
Nº of genomes without TilS$_5$
With TilS$_5$
Without TilS$_5$
p < 0.00005
Nº of genomes with TilS$_6$
Nº of genomes without TilS$_6$
With TilS$_6$
Without TilS$_6$
p < 0.00005
Nº of genomes with TilS$_7$
Nº of genomes without TilS$_7$
With TilS$_7$
Without TilS$_7$
p < 0.00005
Nº of genomes with TilS$_8$
Nº of genomes without TilS$_8$
With TilS$_8$
Without TilS$_8$
p < 0.00005
Nº of genomes with TilS$_9$
Nº of genomes without TilS$_9$
With TilS$_9$
Without TilS$_9$
p < 0.00005
Nº of genomes with TilS$_{10}$
Nº of genomes without TilS$_{10}$
With TilS$_{10}$
Without TilS$_{10}$
p < 0.00005
Nº of genomes with TilS$_{11}$
Nº of genomes without TilS$_{11}$
With TilS$_{11}$
Without TilS$_{11}$
p < 0.00005
Nº of genomes with TilS$_{12}$
Nº of genomes without TilS$_{12}$
With TilS$_{12}$
Without TilS$_{12}$
p < 0.00005
Nº of genomes with TilS$_{13}$
Nº of genomes without TilS$_{13}$
With TilS$_{13}$
Without TilS$_{13}$
p < 0.00005
Nº of genomes with TilS$_{14}$
Nº of genomes without TilS$_{14}$
With TilS$_{14}$
Without TilS$_{14}$
p < 0.00005
Nº of genomes with TilS$_{15}$
Nº of genomes without TilS$_{15}$
With TilS$_{15}$
Without TilS$_{15}$
p < 0.00005

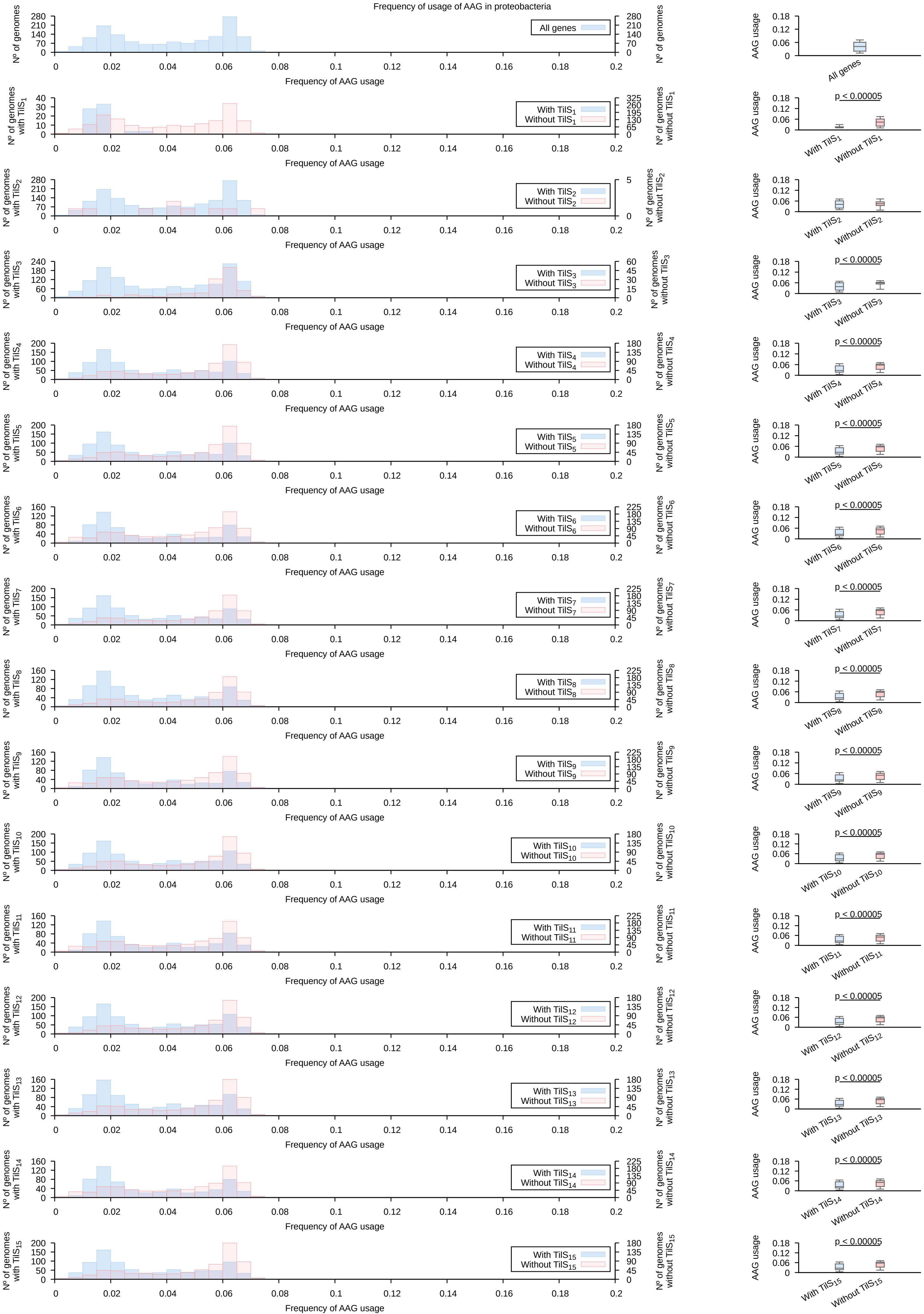

Frequency of usage of AAG in proteobacteria
Nº of genomes
All genes
Frequency of AAG usage
AAG usage
p < 0.00005
Nº of genomes with $TilS_1$
Nº of genomes without $TilS_1$
With $TilS_1$
Without $TilS_1$
With $TilS_2$
Without $TilS_2$
With $TilS_3$
Without $TilS_3$
With $TilS_4$
Without $TilS_4$
With $TilS_5$
Without $TilS_5$
With $TilS_6$
Without $TilS_6$
With $TilS_7$
Without $TilS_7$
With $TilS_8$
Without $TilS_8$
With $TilS_9$
Without $TilS_9$
With $TilS_{10}$
Without $TilS_{10}$
With $TilS_{11}$
Without $TilS_{11}$
With $TilS_{12}$
Without $TilS_{12}$
With $TilS_{13}$
Without $TilS_{13}$
With $TilS_{14}$
Without $TilS_{14}$
With $TilS_{15}$
Without $TilS_{15}$

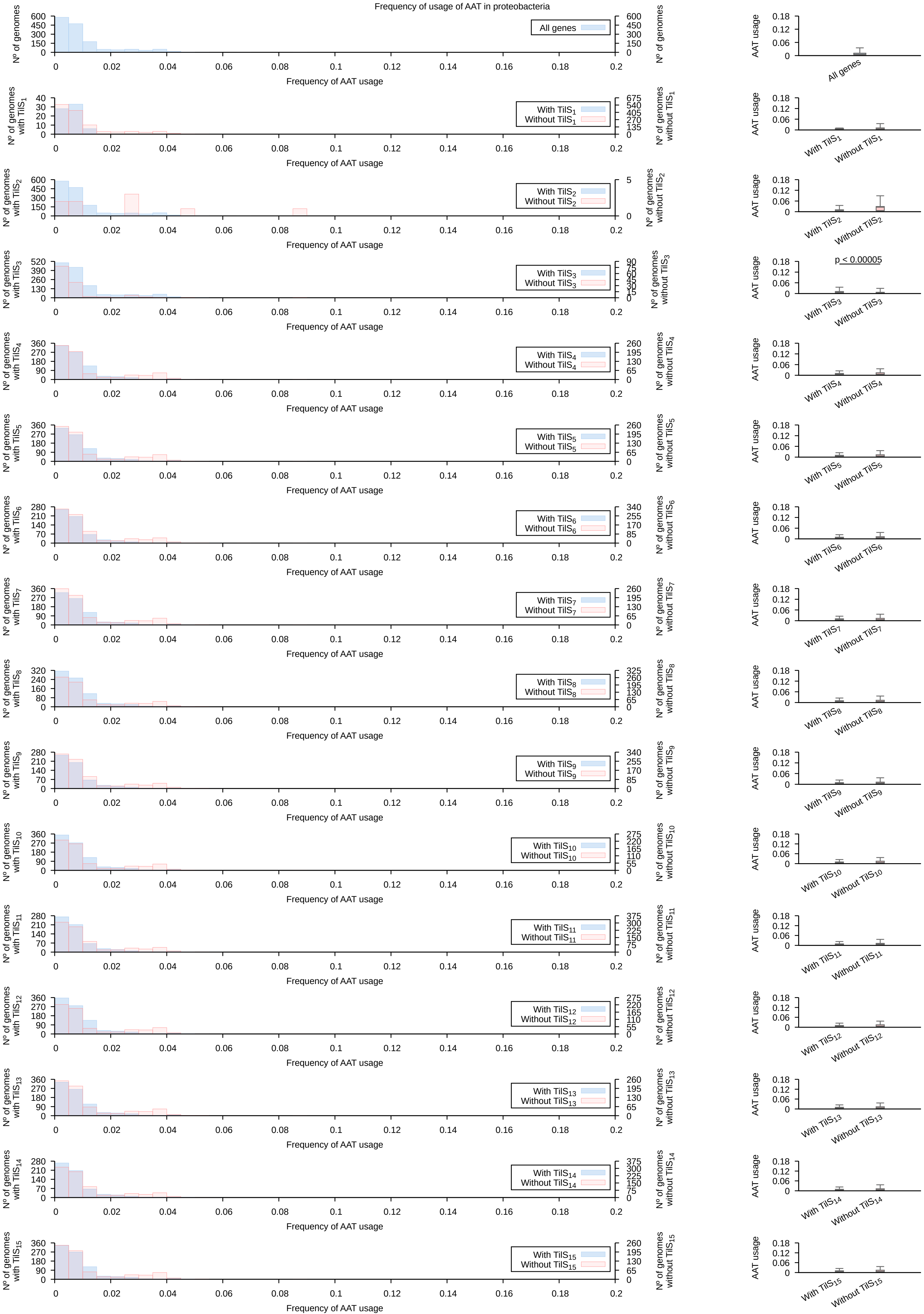
Frequency of usage of AAT in proteobacteria
Nº of genomes
All genes
Frequency of AAT usage
AAT usage
All genes
Nº of genomes with TilS$_1$
Nº of genomes without TilS$_1$
With TilS$_1$
Without TilS$_1$
Nº of genomes with TilS$_2$
Nº of genomes without TilS$_2$
With TilS$_2$
Without TilS$_2$
Nº of genomes with TilS$_3$
Nº of genomes without TilS$_3$
With TilS$_3$
Without TilS$_3$
p < 0.00005
Nº of genomes with TilS$_4$
Nº of genomes without TilS$_4$
With TilS$_4$
Without TilS$_4$
Nº of genomes with TilS$_5$
Nº of genomes without TilS$_5$
With TilS$_5$
Without TilS$_5$
Nº of genomes with TilS$_6$
Nº of genomes without TilS$_6$
With TilS$_6$
Without TilS$_6$
Nº of genomes with TilS$_7$
Nº of genomes without TilS$_7$
With TilS$_7$
Without TilS$_7$
Nº of genomes with TilS$_8$
Nº of genomes without TilS$_8$
With TilS$_8$
Without TilS$_8$
Nº of genomes with TilS$_9$
Nº of genomes without TilS$_9$
With TilS$_9$
Without TilS$_9$
Nº of genomes with TilS$_{10}$
Nº of genomes without TilS$_{10}$
With TilS$_{10}$
Without TilS$_{10}$
Nº of genomes with TilS$_{11}$
Nº of genomes without TilS$_{11}$
With TilS$_{11}$
Without TilS$_{11}$
Nº of genomes with TilS$_{12}$
Nº of genomes without TilS$_{12}$
With TilS$_{12}$
Without TilS$_{12}$
Nº of genomes with TilS$_{13}$
Nº of genomes without TilS$_{13}$
With TilS$_{13}$
Without TilS$_{13}$
Nº of genomes with TilS$_{14}$
Nº of genomes without TilS$_{14}$
With TilS$_{14}$
Without TilS$_{14}$
Nº of genomes with TilS$_{15}$
Nº of genomes without TilS$_{15}$
With TilS$_{15}$
Without TilS$_{15}$

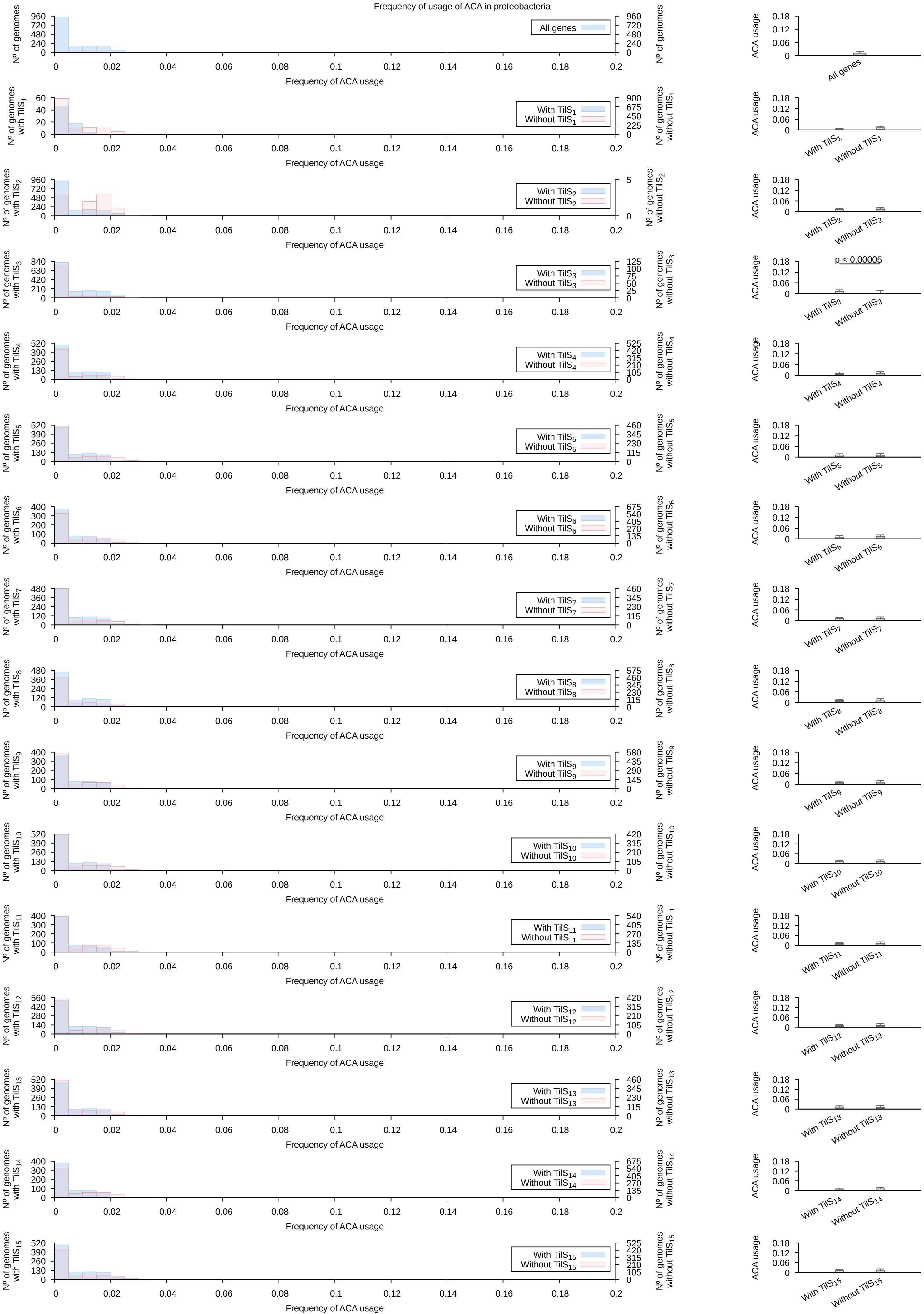
Frequency of usage of ACA in proteobacteria
Nº of genomes
Frequency of ACA usage
ACA usage
All genes
With TilS$_1$
Without TilS$_1$
With TilS$_2$
Without TilS$_2$
With TilS$_3$
Without TilS$_3$
p < 0.00005
With TilS$_4$
Without TilS$_4$
With TilS$_5$
Without TilS$_5$
With TilS$_6$
Without TilS$_6$
With TilS$_7$
Without TilS$_7$
With TilS$_8$
Without TilS$_8$
With TilS$_9$
Without TilS$_9$
With TilS$_{10}$
Without TilS$_{10}$
With TilS$_{11}$
Without TilS$_{11}$
With TilS$_{12}$
Without TilS$_{12}$
With TilS$_{13}$
Without TilS$_{13}$
With TilS$_{14}$
Without TilS$_{14}$
With TilS$_{15}$
Without TilS$_{15}$
Nº of genomes with TilS
Nº of genomes without TilS

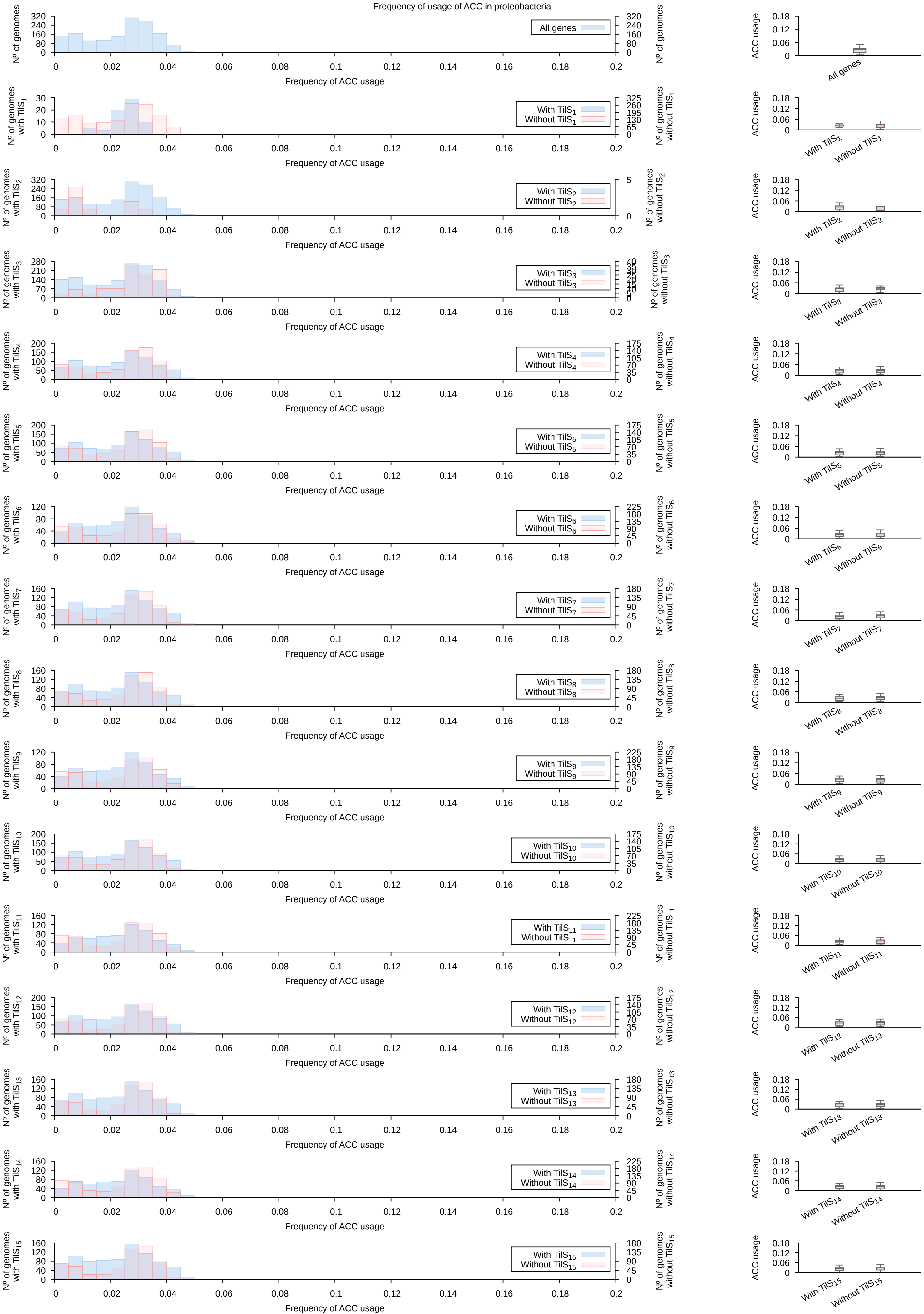

Frequency of usage of ACC in proteobacteria
Nº of genomes
All genes
Frequency of ACC usage
ACC usage
Nº of genomes with TilS$_1$
Nº of genomes without TilS$_1$
With TilS$_1$
Without TilS$_1$
Nº of genomes with TilS$_2$
Nº of genomes without TilS$_2$
With TilS$_2$
Without TilS$_2$
Nº of genomes with TilS$_3$
Nº of genomes without TilS$_3$
With TilS$_3$
Without TilS$_3$
Nº of genomes with TilS$_4$
Nº of genomes without TilS$_4$
With TilS$_4$
Without TilS$_4$
Nº of genomes with TilS$_5$
Nº of genomes without TilS$_5$
With TilS$_5$
Without TilS$_5$
Nº of genomes with TilS$_6$
Nº of genomes without TilS$_6$
With TilS$_6$
Without TilS$_6$
Nº of genomes with TilS$_7$
Nº of genomes without TilS$_7$
With TilS$_7$
Without TilS$_7$
Nº of genomes with TilS$_8$
Nº of genomes without TilS$_8$
With TilS$_8$
Without TilS$_8$
Nº of genomes with TilS$_9$
Nº of genomes without TilS$_9$
With TilS$_9$
Without TilS$_9$
Nº of genomes with TilS$_{10}$
Nº of genomes without TilS$_{10}$
With TilS$_{10}$
Without TilS$_{10}$
Nº of genomes with TilS$_{11}$
Nº of genomes without TilS$_{11}$
With TilS$_{11}$
Without TilS$_{11}$
Nº of genomes with TilS$_{12}$
Nº of genomes without TilS$_{12}$
With TilS$_{12}$
Without TilS$_{12}$
Nº of genomes with TilS$_{13}$
Nº of genomes without TilS$_{13}$
With TilS$_{13}$
Without TilS$_{13}$
Nº of genomes with TilS$_{14}$
Nº of genomes without TilS$_{14}$
With TilS$_{14}$
Without TilS$_{14}$
Nº of genomes with TilS$_{15}$
Nº of genomes without TilS$_{15}$
With TilS$_{15}$
Without TilS$_{15}$

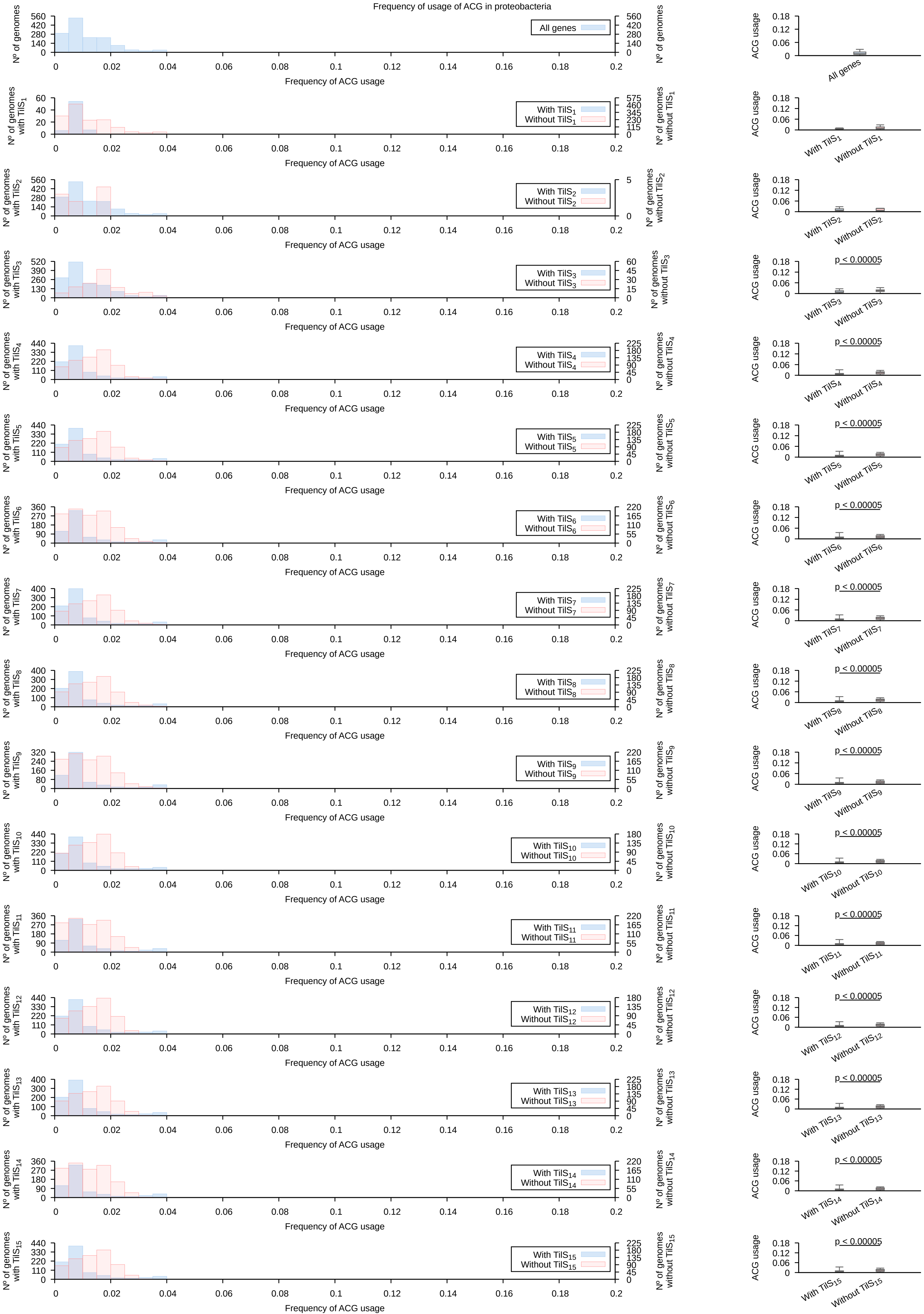

Frequency of usage of ACG in proteobacteria
Nº of genomes
All genes
Frequency of ACG usage
ACG usage
Nº of genomes with TilS$_1$
Nº of genomes without TilS$_1$
With TilS$_1$
Without TilS$_1$
With TilS$_2$
Without TilS$_2$
With TilS$_3$
Without TilS$_3$
With TilS$_4$
Without TilS$_4$
With TilS$_5$
Without TilS$_5$
With TilS$_6$
Without TilS$_6$
With TilS$_7$
Without TilS$_7$
With TilS$_8$
Without TilS$_8$
With TilS$_9$
Without TilS$_9$
With TilS$_{10}$
Without TilS$_{10}$
With TilS$_{11}$
Without TilS$_{11}$
With TilS$_{12}$
Without TilS$_{12}$
With TilS$_{13}$
Without TilS$_{13}$
With TilS$_{14}$
Without TilS$_{14}$
With TilS$_{15}$
Without TilS$_{15}$
p < 0.00005

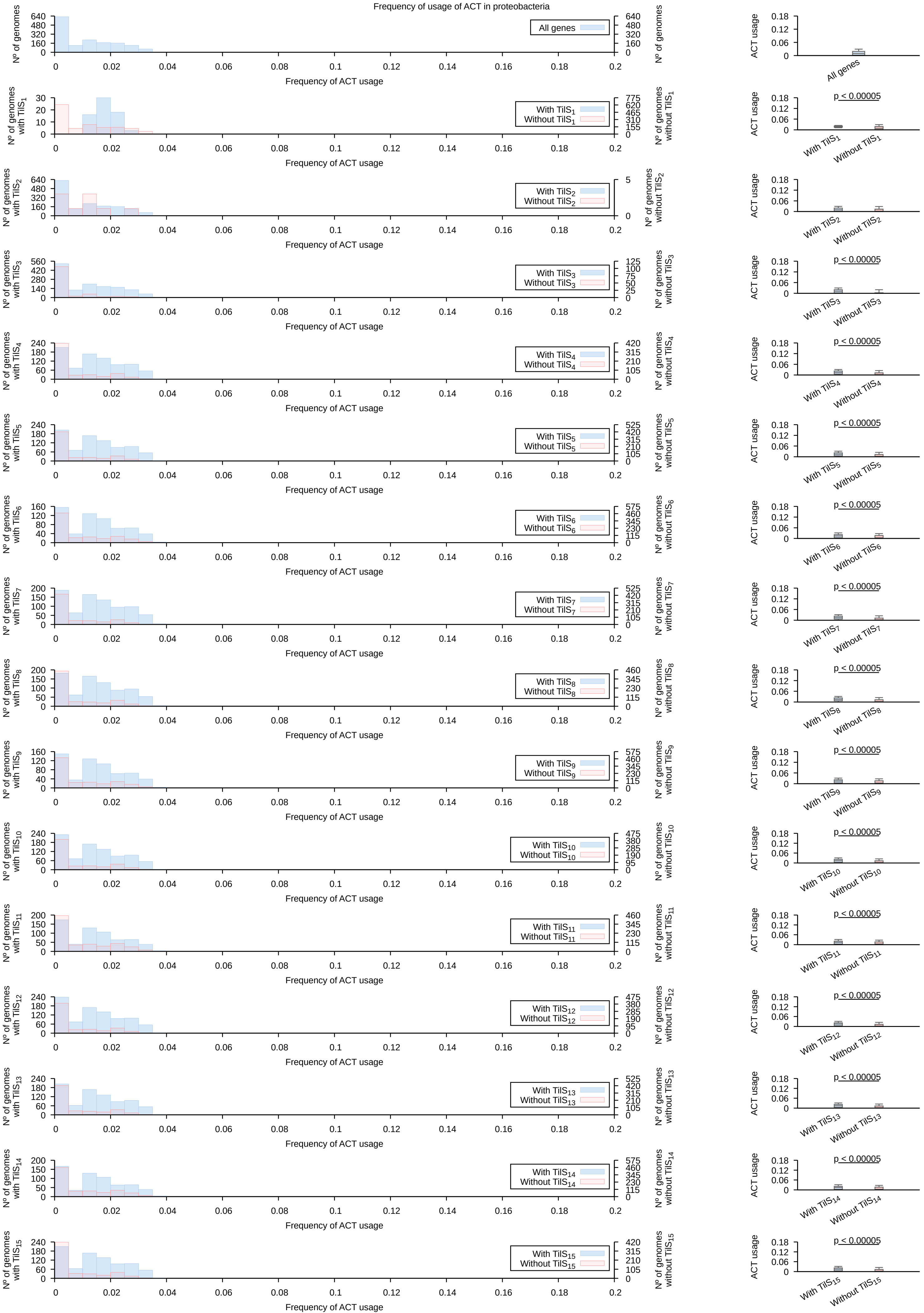

Frequency of usage of ACT in proteobacteria
Nº of genomes
All genes
Frequency of ACT usage
ACT usage
Nº of genomes with $TilS_1$
Nº of genomes without $TilS_1$
With $TilS_1$
Without $TilS_1$
p < 0.00005
Nº of genomes with $TilS_2$
Nº of genomes without $TilS_2$
With $TilS_2$
Without $TilS_2$
Nº of genomes with $TilS_3$
Nº of genomes without $TilS_3$
With $TilS_3$
Without $TilS_3$
p < 0.00005
Nº of genomes with $TilS_4$
Nº of genomes without $TilS_4$
With $TilS_4$
Without $TilS_4$
p < 0.00005
Nº of genomes with $TilS_5$
Nº of genomes without $TilS_5$
With $TilS_5$
Without $TilS_5$
p < 0.00005
Nº of genomes with $TilS_6$
Nº of genomes without $TilS_6$
With $TilS_6$
Without $TilS_6$
p < 0.00005
Nº of genomes with $TilS_7$
Nº of genomes without $TilS_7$
With $TilS_7$
Without $TilS_7$
p < 0.00005
Nº of genomes with $TilS_8$
Nº of genomes without $TilS_8$
With $TilS_8$
Without $TilS_8$
p < 0.00005
Nº of genomes with $TilS_9$
Nº of genomes without $TilS_9$
With $TilS_9$
Without $TilS_9$
p < 0.00005
Nº of genomes with $TilS_{10}$
Nº of genomes without $TilS_{10}$
With $TilS_{10}$
Without $TilS_{10}$
p < 0.00005
Nº of genomes with $TilS_{11}$
Nº of genomes without $TilS_{11}$
With $TilS_{11}$
Without $TilS_{11}$
p < 0.00005
Nº of genomes with $TilS_{12}$
Nº of genomes without $TilS_{12}$
With $TilS_{12}$
Without $TilS_{12}$
p < 0.00005
Nº of genomes with $TilS_{13}$
Nº of genomes without $TilS_{13}$
With $TilS_{13}$
Without $TilS_{13}$
p < 0.00005
Nº of genomes with $TilS_{14}$
Nº of genomes without $TilS_{14}$
With $TilS_{14}$
Without $TilS_{14}$
p < 0.00005
Nº of genomes with $TilS_{15}$
Nº of genomes without $TilS_{15}$
With $TilS_{15}$
Without $TilS_{15}$
p < 0.00005

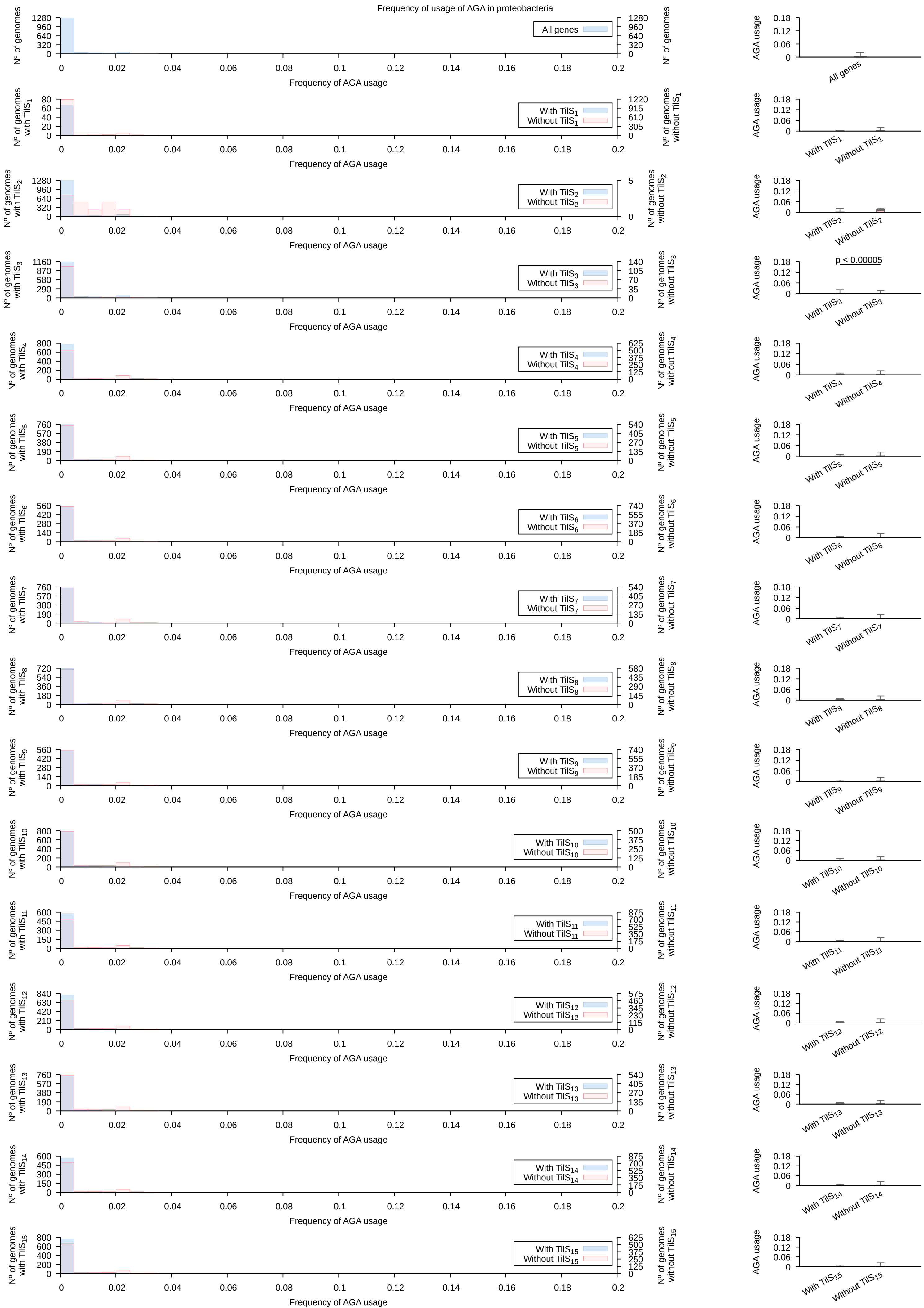

Frequency of usage of AGA in proteobacteria
Nº of genomes
All genes
Frequency of AGA usage
AGA usage
Nº of genomes with TilS$_1$
Nº of genomes without TilS$_1$
With TilS$_1$
Without TilS$_1$
Nº of genomes with TilS$_2$
Nº of genomes without TilS$_2$
With TilS$_2$
Without TilS$_2$
Nº of genomes with TilS$_3$
Nº of genomes without TilS$_3$
With TilS$_3$
Without TilS$_3$
p < 0.00005
Nº of genomes with TilS$_4$
Nº of genomes without TilS$_4$
With TilS$_4$
Without TilS$_4$
Nº of genomes with TilS$_5$
Nº of genomes without TilS$_5$
With TilS$_5$
Without TilS$_5$
Nº of genomes with TilS$_6$
Nº of genomes without TilS$_6$
With TilS$_6$
Without TilS$_6$
Nº of genomes with TilS$_7$
Nº of genomes without TilS$_7$
With TilS$_7$
Without TilS$_7$
Nº of genomes with TilS$_8$
Nº of genomes without TilS$_8$
With TilS$_8$
Without TilS$_8$
Nº of genomes with TilS$_9$
Nº of genomes without TilS$_9$
With TilS$_9$
Without TilS$_9$
Nº of genomes with TilS$_{10}$
Nº of genomes without TilS$_{10}$
With TilS$_{10}$
Without TilS$_{10}$
Nº of genomes with TilS$_{11}$
Nº of genomes without TilS$_{11}$
With TilS$_{11}$
Without TilS$_{11}$
Nº of genomes with TilS$_{12}$
Nº of genomes without TilS$_{12}$
With TilS$_{12}$
Without TilS$_{12}$
Nº of genomes with TilS$_{13}$
Nº of genomes without TilS$_{13}$
With TilS$_{13}$
Without TilS$_{13}$
Nº of genomes with TilS$_{14}$
Nº of genomes without TilS$_{14}$
With TilS$_{14}$
Without TilS$_{14}$
Nº of genomes with TilS$_{15}$
Nº of genomes without TilS$_{15}$
With TilS$_{15}$
Without TilS$_{15}$

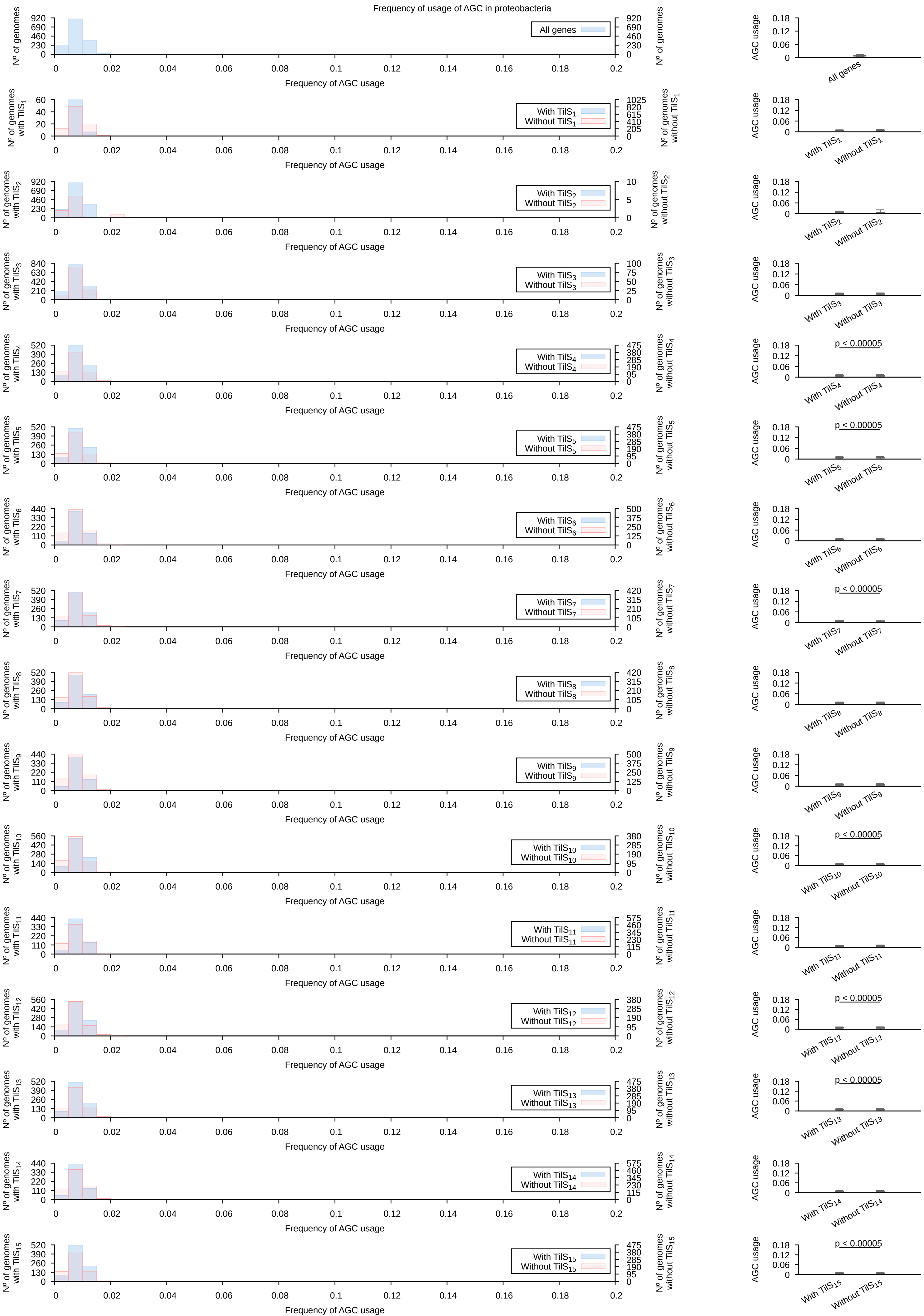

Frequency of usage of AGC in proteobacteria
Nº of genomes
All genes
Frequency of AGC usage
AGC usage
Nº of genomes with TilS1
Nº of genomes without TilS1
With TilS1
Without TilS1
Nº of genomes with TilS2
Nº of genomes without TilS2
With TilS2
Without TilS2
Nº of genomes with TilS3
Nº of genomes without TilS3
With TilS3
Without TilS3
Nº of genomes with TilS4
Nº of genomes without TilS4
With TilS4
Without TilS4
p < 0.00005
Nº of genomes with TilS5
Nº of genomes without TilS5
With TilS5
Without TilS5
p < 0.00005
Nº of genomes with TilS6
Nº of genomes without TilS6
With TilS6
Without TilS6
Nº of genomes with TilS7
Nº of genomes without TilS7
With TilS7
Without TilS7
p < 0.00005
Nº of genomes with TilS8
Nº of genomes without TilS8
With TilS8
Without TilS8
Nº of genomes with TilS9
Nº of genomes without TilS9
With TilS9
Without TilS9
Nº of genomes with TilS10
Nº of genomes without TilS10
With TilS10
Without TilS10
p < 0.00005
Nº of genomes with TilS11
Nº of genomes without TilS11
With TilS11
Without TilS11
Nº of genomes with TilS12
Nº of genomes without TilS12
With TilS12
Without TilS12
p < 0.00005
Nº of genomes with TilS13
Nº of genomes without TilS13
With TilS13
Without TilS13
p < 0.00005
Nº of genomes with TilS14
Nº of genomes without TilS14
With TilS14
Without TilS14
Nº of genomes with TilS15
Nº of genomes without TilS15
With TilS15
Without TilS15
p < 0.00005

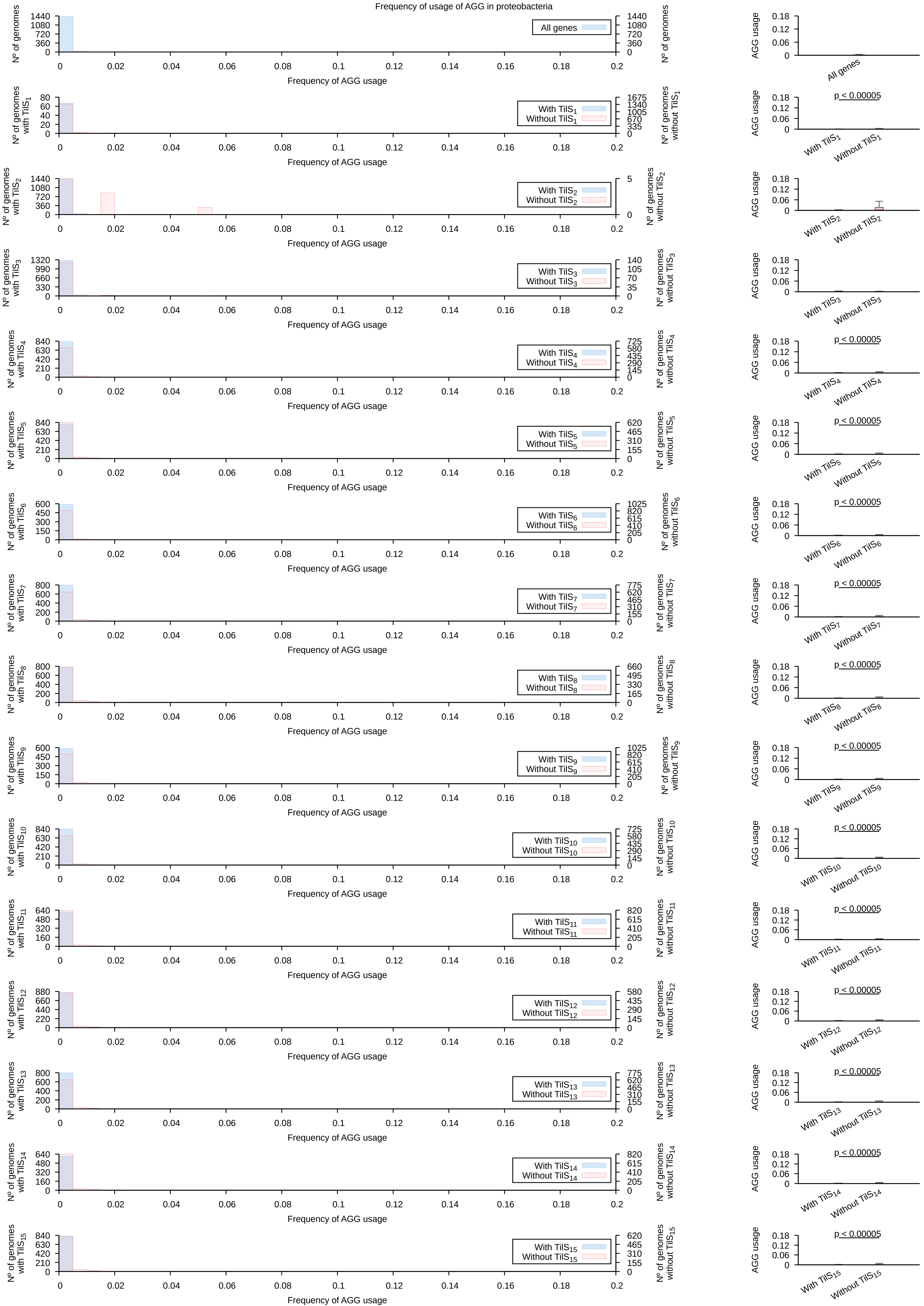
Frequency of usage of AGG in proteobacteria
Nº of genomes
All genes
Frequency of AGG usage
AGG usage
Nº of genomes with TilS1
Nº of genomes without TilS1
With TilS1
Without TilS1
p < 0.00005
Nº of genomes with TilS2
Nº of genomes without TilS2
With TilS2
Without TilS2
Nº of genomes with TilS3
Nº of genomes without TilS3
With TilS3
Without TilS3
Nº of genomes with TilS4
Nº of genomes without TilS4
With TilS4
Without TilS4
p < 0.00005
Nº of genomes with TilS5
Nº of genomes without TilS5
With TilS5
Without TilS5
p < 0.00005
Nº of genomes with TilS6
Nº of genomes without TilS6
With TilS6
Without TilS6
p < 0.00005
Nº of genomes with TilS7
Nº of genomes without TilS7
With TilS7
Without TilS7
p < 0.00005
Nº of genomes with TilS8
Nº of genomes without TilS8
With TilS8
Without TilS8
p < 0.00005
Nº of genomes with TilS9
Nº of genomes without TilS9
With TilS9
Without TilS9
p < 0.00005
Nº of genomes with TilS10
Nº of genomes without TilS10
With TilS10
Without TilS10
p < 0.00005
Nº of genomes with TilS11
Nº of genomes without TilS11
With TilS11
Without TilS11
p < 0.00005
Nº of genomes with TilS12
Nº of genomes without TilS12
With TilS12
Without TilS12
p < 0.00005
Nº of genomes with TilS13
Nº of genomes without TilS13
With TilS13
Without TilS13
p < 0.00005
Nº of genomes with TilS14
Nº of genomes without TilS14
With TilS14
Without TilS14
p < 0.00005
Nº of genomes with TilS15
Nº of genomes without TilS15
With TilS15
Without TilS15
p < 0.00005

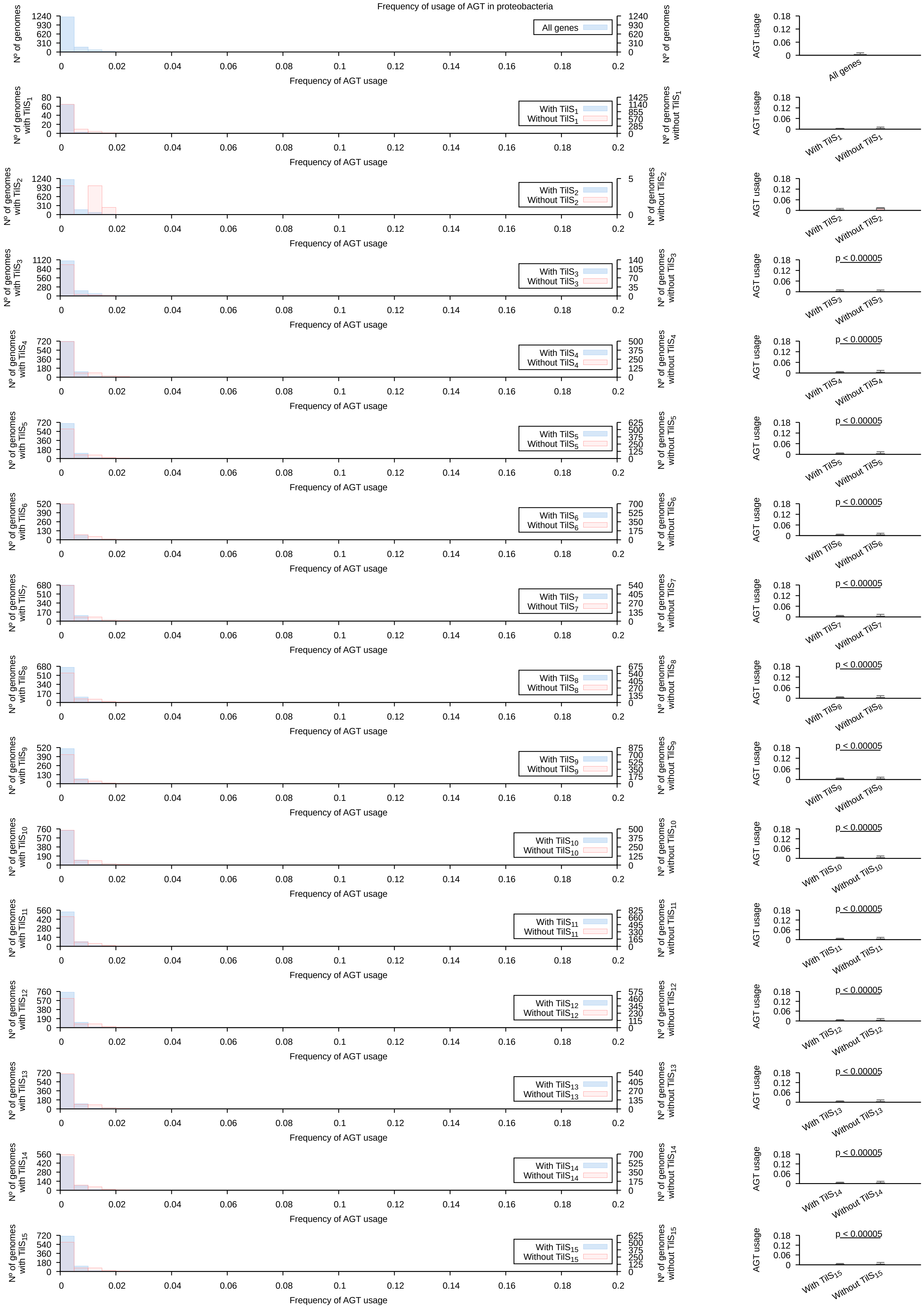
Frequency of usage of AGT in proteobacteria
Nº of genomes
All genes
Frequency of AGT usage
AGT usage
Nº of genomes with TilS1
Nº of genomes without TilS1
With TilS1
Without TilS1
Nº of genomes with TilS2
Nº of genomes without TilS2
With TilS2
Without TilS2
Nº of genomes with TilS3
Nº of genomes without TilS3
With TilS3
Without TilS3
p < 0.00005
Nº of genomes with TilS4
Nº of genomes without TilS4
With TilS4
Without TilS4
Nº of genomes with TilS5
Nº of genomes without TilS5
With TilS5
Without TilS5
Nº of genomes with TilS6
Nº of genomes without TilS6
With TilS6
Without TilS6
Nº of genomes with TilS7
Nº of genomes without TilS7
With TilS7
Without TilS7
Nº of genomes with TilS8
Nº of genomes without TilS8
With TilS8
Without TilS8
Nº of genomes with TilS9
Nº of genomes without TilS9
With TilS9
Without TilS9
Nº of genomes with TilS10
Nº of genomes without TilS10
With TilS10
Without TilS10
Nº of genomes with TilS11
Nº of genomes without TilS11
With TilS11
Without TilS11
Nº of genomes with TilS12
Nº of genomes without TilS12
With TilS12
Without TilS12
Nº of genomes with TilS13
Nº of genomes without TilS13
With TilS13
Without TilS13
Nº of genomes with TilS14
Nº of genomes without TilS14
With TilS14
Without TilS14
Nº of genomes with TilS15
Nº of genomes without TilS15
With TilS15
Without TilS15

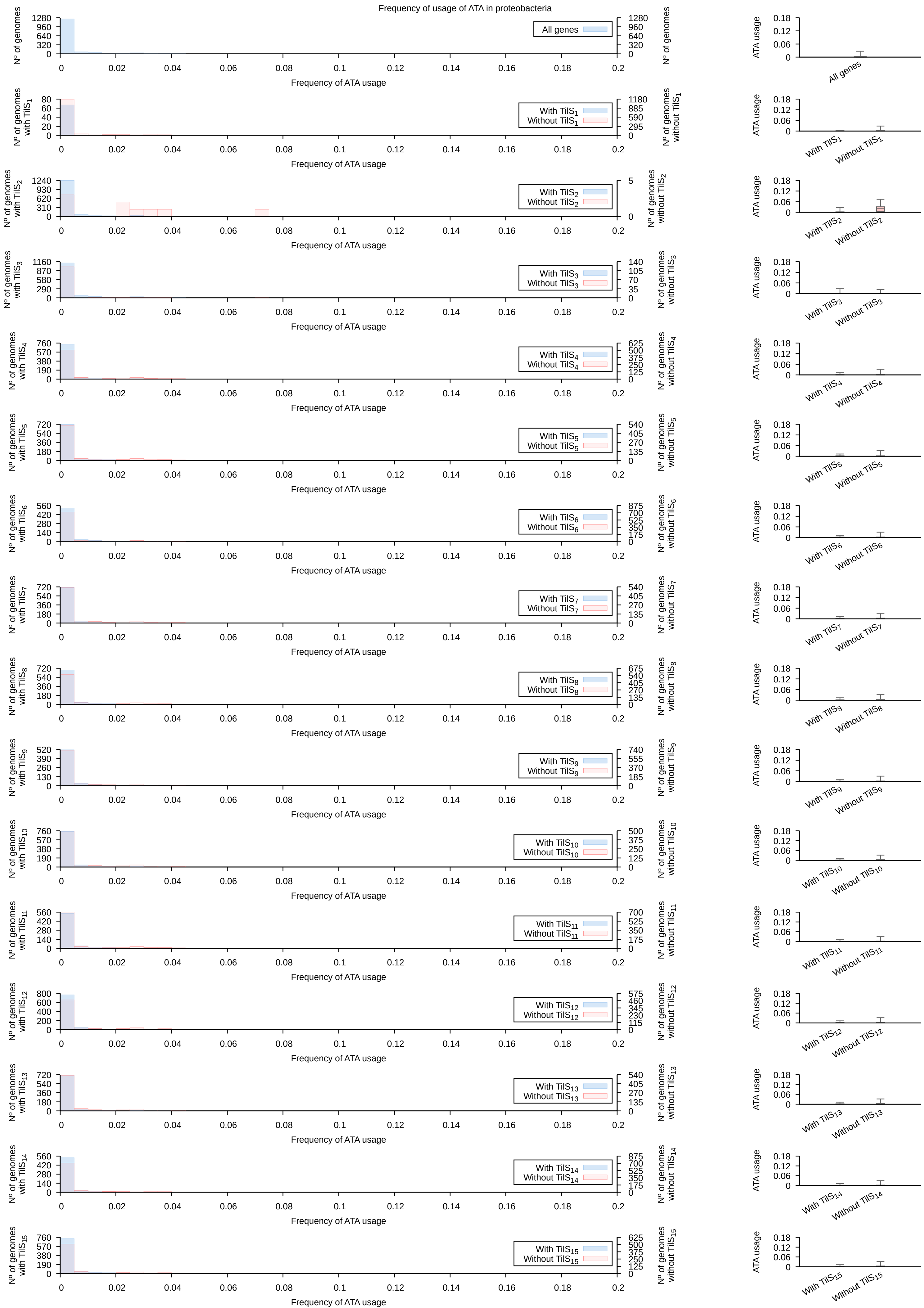

Frequency of usage of ATA in proteobacteria
Nº of genomes
All genes
Frequency of ATA usage
ATA usage
Nº of genomes with TilS$_1$
Nº of genomes without TilS$_1$
With TilS$_1$
Without TilS$_1$
With TilS$_2$
Without TilS$_2$
With TilS$_3$
Without TilS$_3$
With TilS$_4$
Without TilS$_4$
With TilS$_5$
Without TilS$_5$
With TilS$_6$
Without TilS$_6$
With TilS$_7$
Without TilS$_7$
With TilS$_8$
Without TilS$_8$
With TilS$_9$
Without TilS$_9$
With TilS$_{10}$
Without TilS$_{10}$
With TilS$_{11}$
Without TilS$_{11}$
With TilS$_{12}$
Without TilS$_{12}$
With TilS$_{13}$
Without TilS$_{13}$
With TilS$_{14}$
Without TilS$_{14}$
With TilS$_{15}$
Without TilS$_{15}$

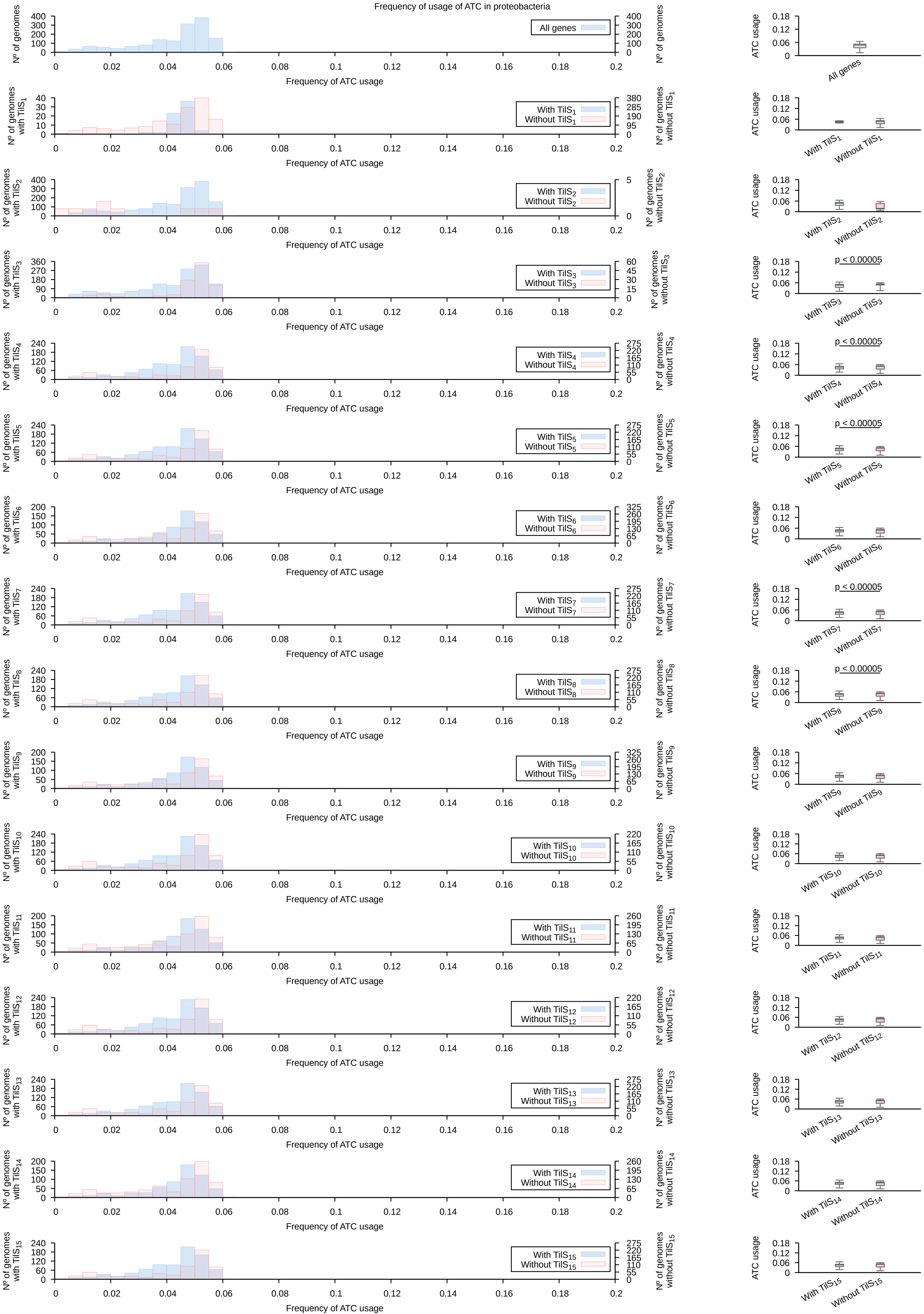

Frequency of usage of ATC in proteobacteria
Nº of genomes
All genes
Frequency of ATC usage
ATC usage
With TilS$_1$
Without TilS$_1$
With TilS$_2$
Without TilS$_2$
With TilS$_3$
Without TilS$_3$
p < 0.00005
With TilS$_4$
Without TilS$_4$
With TilS$_5$
Without TilS$_5$
With TilS$_6$
Without TilS$_6$
With TilS$_7$
Without TilS$_7$
With TilS$_8$
Without TilS$_8$
With TilS$_9$
Without TilS$_9$
With TilS$_{10}$
Without TilS$_{10}$
With TilS$_{11}$
Without TilS$_{11}$
With TilS$_{12}$
Without TilS$_{12}$
With TilS$_{13}$
Without TilS$_{13}$
With TilS$_{14}$
Without TilS$_{14}$
With TilS$_{15}$
Without TilS$_{15}$

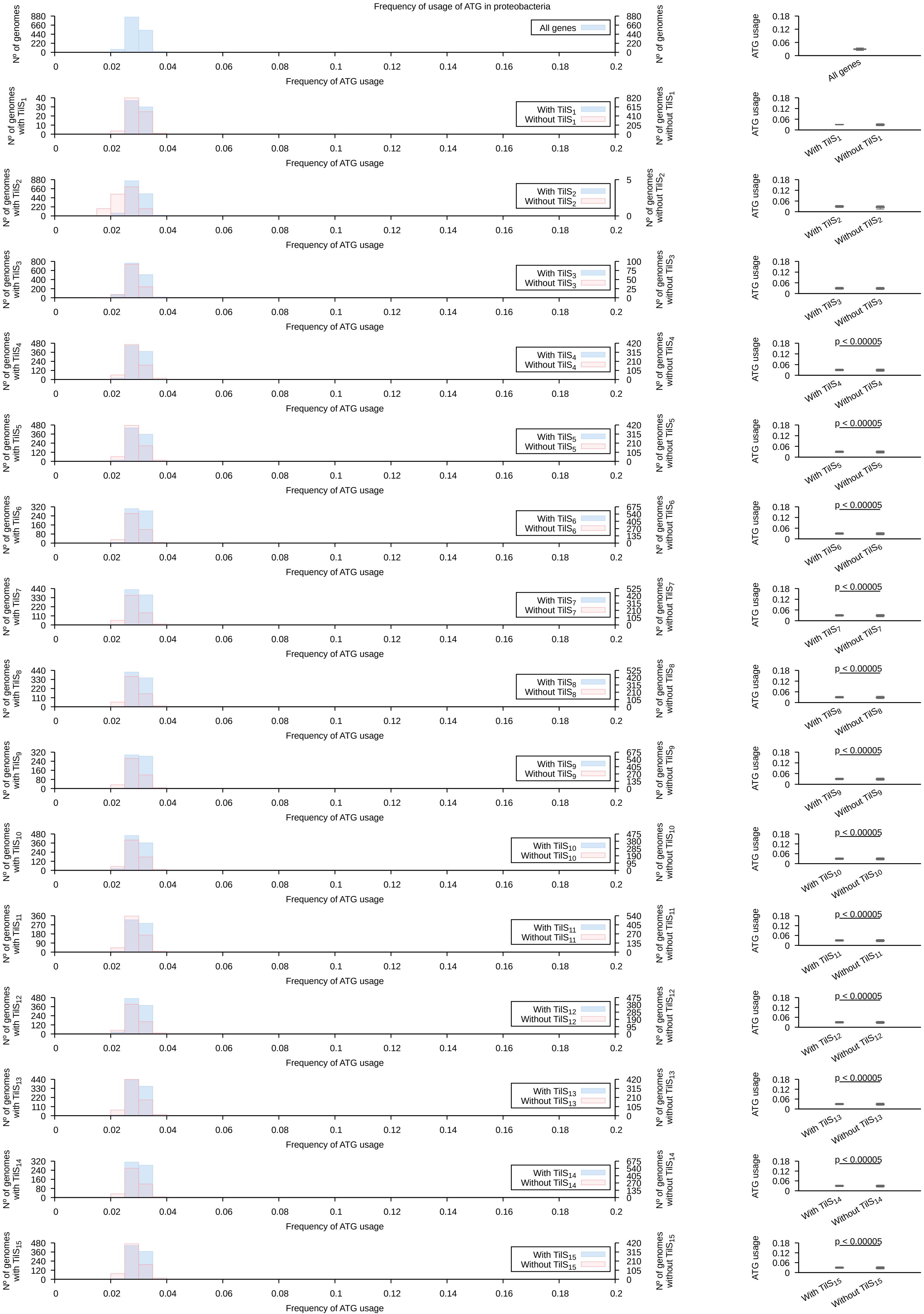

Frequency of usage of ATG in proteobacteria
Nº of genomes
All genes
Frequency of ATG usage
ATG usage
Nº of genomes with TilS1
Nº of genomes without TilS1
With TilS1
Without TilS1
Nº of genomes with TilS2
Nº of genomes without TilS2
With TilS2
Without TilS2
Nº of genomes with TilS3
Nº of genomes without TilS3
With TilS3
Without TilS3
Nº of genomes with TilS4
Nº of genomes without TilS4
With TilS4
Without TilS4
p < 0.00005
Nº of genomes with TilS5
Nº of genomes without TilS5
With TilS5
Without TilS5
p < 0.00005
Nº of genomes with TilS6
Nº of genomes without TilS6
With TilS6
Without TilS6
p < 0.00005
Nº of genomes with TilS7
Nº of genomes without TilS7
With TilS7
Without TilS7
p < 0.00005
Nº of genomes with TilS8
Nº of genomes without TilS8
With TilS8
Without TilS8
p < 0.00005
Nº of genomes with TilS9
Nº of genomes without TilS9
With TilS9
Without TilS9
p < 0.00005
Nº of genomes with TilS10
Nº of genomes without TilS10
With TilS10
Without TilS10
p < 0.00005
Nº of genomes with TilS11
Nº of genomes without TilS11
With TilS11
Without TilS11
p < 0.00005
Nº of genomes with TilS12
Nº of genomes without TilS12
With TilS12
Without TilS12
p < 0.00005
Nº of genomes with TilS13
Nº of genomes without TilS13
With TilS13
Without TilS13
p < 0.00005
Nº of genomes with TilS14
Nº of genomes without TilS14
With TilS14
Without TilS14
p < 0.00005
Nº of genomes with TilS15
Nº of genomes without TilS15
With TilS15
Without TilS15
p < 0.00005

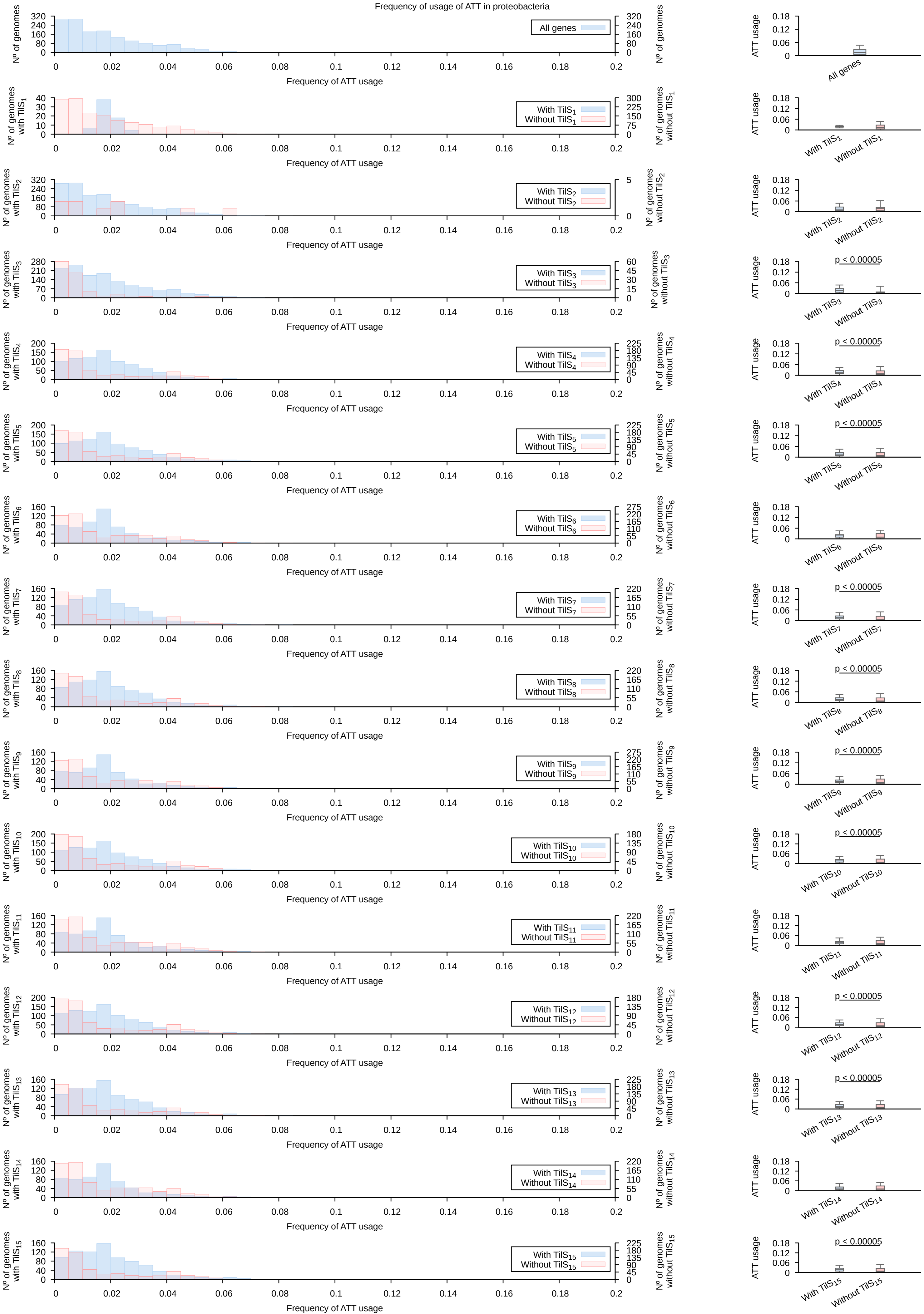

Frequency of usage of ATT in proteobacteria
Nº of genomes
All genes
Frequency of ATT usage
ATT usage
Nº of genomes with TilS1
With TilS1
Without TilS1
Nº of genomes without TilS1
Nº of genomes with TilS2
With TilS2
Without TilS2
Nº of genomes without TilS2
Nº of genomes with TilS3
With TilS3
Without TilS3
Nº of genomes without TilS3
p < 0.00005
Nº of genomes with TilS4
With TilS4
Without TilS4
Nº of genomes without TilS4
p < 0.00005
Nº of genomes with TilS5
With TilS5
Without TilS5
Nº of genomes without TilS5
p < 0.00005
Nº of genomes with TilS6
With TilS6
Without TilS6
Nº of genomes without TilS6
Nº of genomes with TilS7
With TilS7
Without TilS7
Nº of genomes without TilS7
p < 0.00005
Nº of genomes with TilS8
With TilS8
Without TilS8
Nº of genomes without TilS8
p < 0.00005
Nº of genomes with TilS9
With TilS9
Without TilS9
Nº of genomes without TilS9
p < 0.00005
Nº of genomes with TilS10
With TilS10
Without TilS10
Nº of genomes without TilS10
p < 0.00005
Nº of genomes with TilS11
With TilS11
Without TilS11
Nº of genomes without TilS11
Nº of genomes with TilS12
With TilS12
Without TilS12
Nº of genomes without TilS12
p < 0.00005
Nº of genomes with TilS13
With TilS13
Without TilS13
Nº of genomes without TilS13
p < 0.00005
Nº of genomes with TilS14
With TilS14
Without TilS14
Nº of genomes without TilS14
Nº of genomes with TilS15
With TilS15
Without TilS15
Nº of genomes without TilS15
p < 0.00005

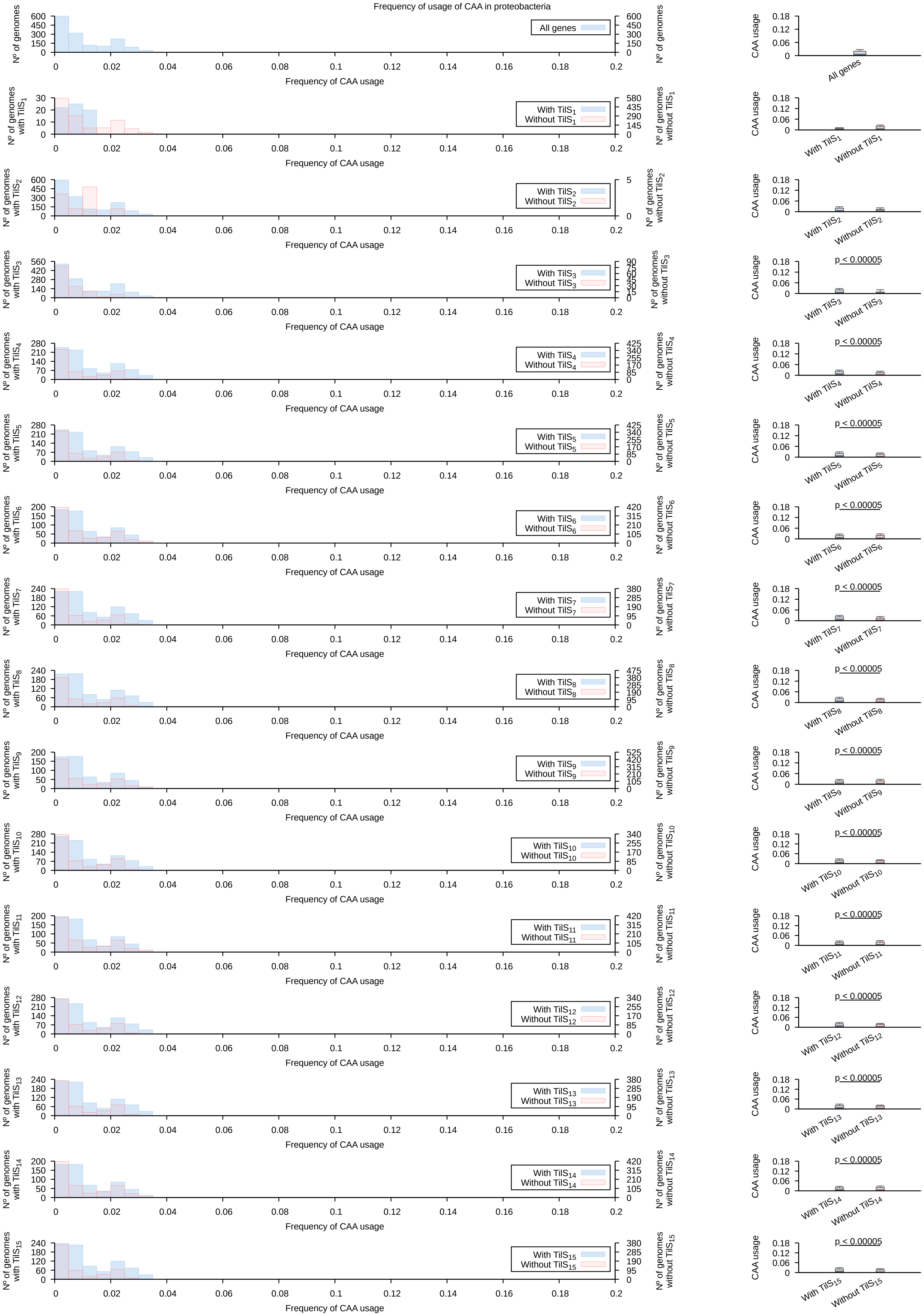

Frequency of usage of CAA in proteobacteria
Nº of genomes
All genes
Frequency of CAA usage
CAA usage
With TilS1
Without TilS1
With TilS2
Without TilS2
With TilS3
Without TilS3
With TilS4
Without TilS4
With TilS5
Without TilS5
With TilS6
Without TilS6
With TilS7
Without TilS7
With TilS8
Without TilS8
With TilS9
Without TilS9
With TilS10
Without TilS10
With TilS11
Without TilS11
With TilS12
Without TilS12
With TilS13
Without TilS13
With TilS14
Without TilS14
With TilS15
Without TilS15
p < 0.00005

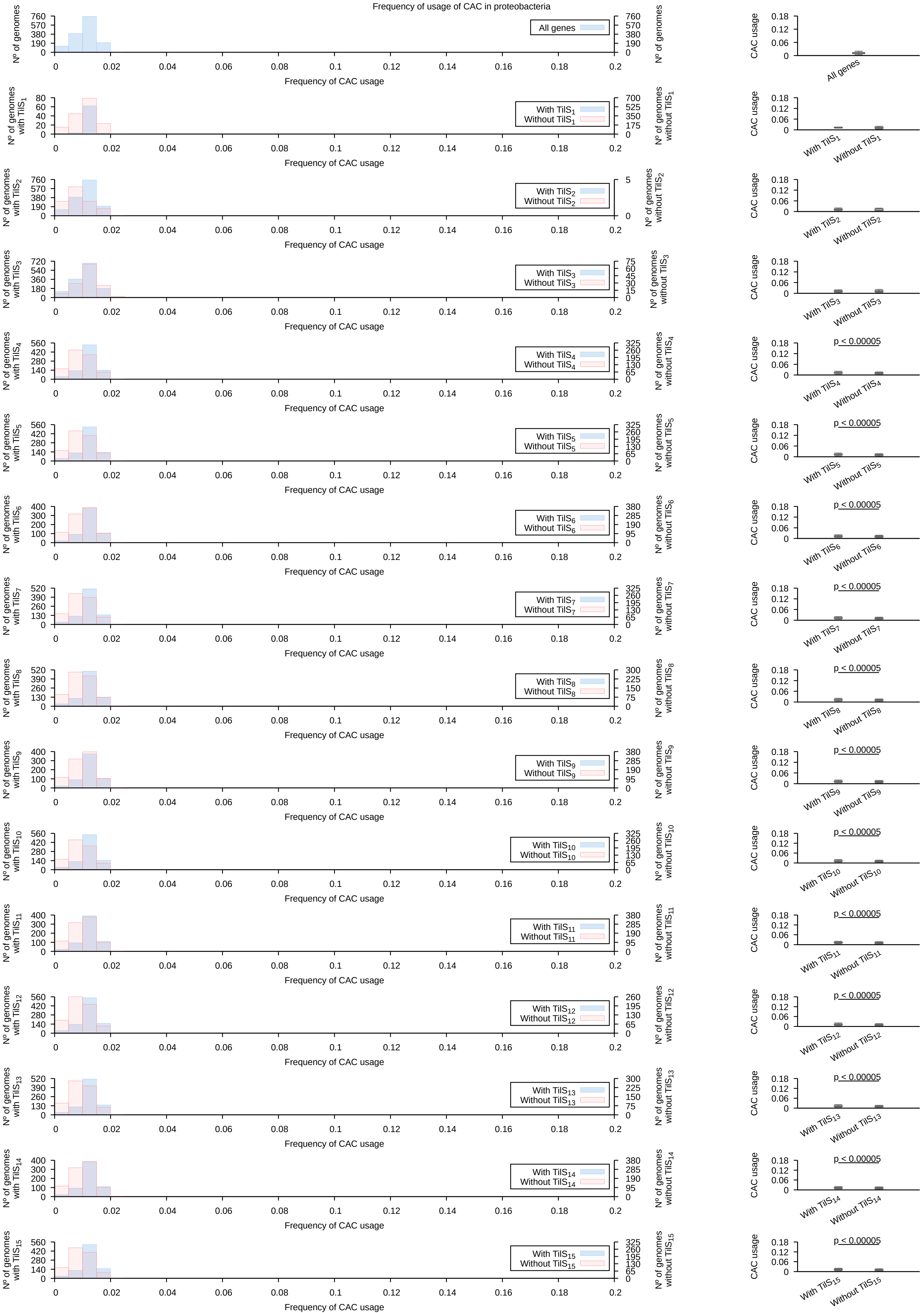

Frequency of usage of CAC in proteobacteria
Nº of genomes
All genes
Frequency of CAC usage
CAC usage
0 0.02 0.04 0.06 0.08 0.1 0.12 0.14 0.16 0.18 0.2
0 0.06 0.12 0.18
Nº of genomes with TilS$_1$
Nº of genomes without TilS$_1$
With TilS$_1$
Without TilS$_1$
Nº of genomes with TilS$_2$
Nº of genomes without TilS$_2$
With TilS$_2$
Without TilS$_2$
Nº of genomes with TilS$_3$
Nº of genomes without TilS$_3$
With TilS$_3$
Without TilS$_3$
Nº of genomes with TilS$_4$
Nº of genomes without TilS$_4$
With TilS$_4$
Without TilS$_4$
p < 0.00005
Nº of genomes with TilS$_5$
Nº of genomes without TilS$_5$
With TilS$_5$
Without TilS$_5$
p < 0.00005
Nº of genomes with TilS$_6$
Nº of genomes without TilS$_6$
With TilS$_6$
Without TilS$_6$
p < 0.00005
Nº of genomes with TilS$_7$
Nº of genomes without TilS$_7$
With TilS$_7$
Without TilS$_7$
p < 0.00005
Nº of genomes with TilS$_8$
Nº of genomes without TilS$_8$
With TilS$_8$
Without TilS$_8$
p < 0.00005
Nº of genomes with TilS$_9$
Nº of genomes without TilS$_9$
With TilS$_9$
Without TilS$_9$
p < 0.00005
Nº of genomes with TilS$_{10}$
Nº of genomes without TilS$_{10}$
With TilS$_{10}$
Without TilS$_{10}$
p < 0.00005
Nº of genomes with TilS$_{11}$
Nº of genomes without TilS$_{11}$
With TilS$_{11}$
Without TilS$_{11}$
p < 0.00005
Nº of genomes with TilS$_{12}$
Nº of genomes without TilS$_{12}$
With TilS$_{12}$
Without TilS$_{12}$
p < 0.00005
Nº of genomes with TilS$_{13}$
Nº of genomes without TilS$_{13}$
With TilS$_{13}$
Without TilS$_{13}$
p < 0.00005
Nº of genomes with TilS$_{14}$
Nº of genomes without TilS$_{14}$
With TilS$_{14}$
Without TilS$_{14}$
p < 0.00005
Nº of genomes with TilS$_{15}$
Nº of genomes without TilS$_{15}$
With TilS$_{15}$
Without TilS$_{15}$
p < 0.00005

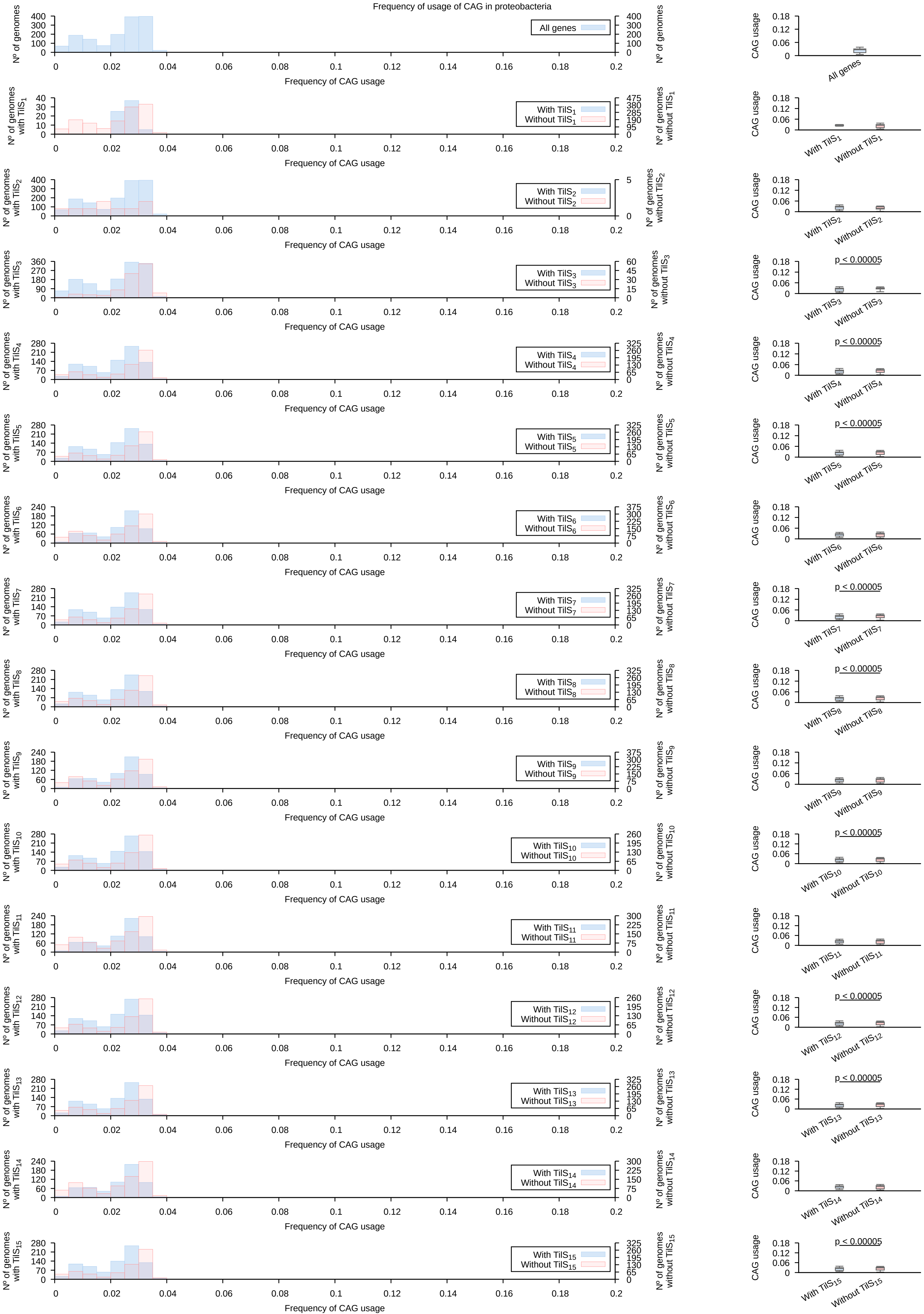
Frequency of usage of CAG in proteobacteria
Nº of genomes
All genes
Frequency of CAG usage
CAG usage
p < 0.00005
Nº of genomes with TilS$_1$
Nº of genomes without TilS$_1$
With TilS$_1$
Without TilS$_1$
With TilS$_2$
Without TilS$_2$
With TilS$_3$
Without TilS$_3$
With TilS$_4$
Without TilS$_4$
With TilS$_5$
Without TilS$_5$
With TilS$_6$
Without TilS$_6$
With TilS$_7$
Without TilS$_7$
With TilS$_8$
Without TilS$_8$
With TilS$_9$
Without TilS$_9$
With TilS$_{10}$
Without TilS$_{10}$
With TilS$_{11}$
Without TilS$_{11}$
With TilS$_{12}$
Without TilS$_{12}$
With TilS$_{13}$
Without TilS$_{13}$
With TilS$_{14}$
Without TilS$_{14}$
With TilS$_{15}$
Without TilS$_{15}$

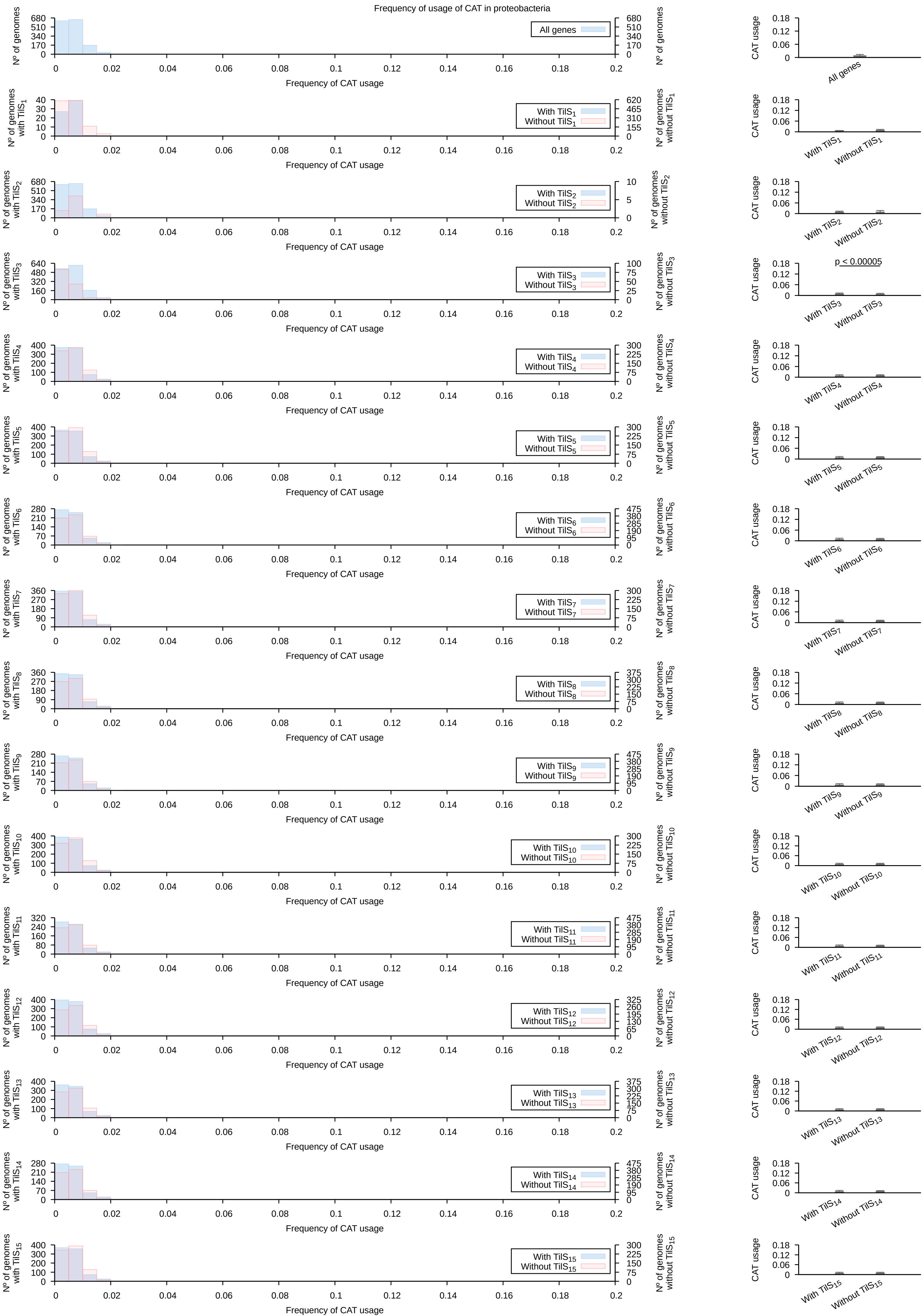

Frequency of usage of CAT in proteobacteria
Nº of genomes
All genes
Frequency of CAT usage
CAT usage
0
0.02
0.04
0.06
0.08
0.1
0.12
0.14
0.16
0.18
0.2
Nº of genomes with TilS1
Nº of genomes without TilS1
With TilS1
Without TilS1
Nº of genomes with TilS2
Nº of genomes without TilS2
With TilS2
Without TilS2
Nº of genomes with TilS3
Nº of genomes without TilS3
With TilS3
Without TilS3
p < 0.00005
Nº of genomes with TilS4
Nº of genomes without TilS4
With TilS4
Without TilS4
Nº of genomes with TilS5
Nº of genomes without TilS5
With TilS5
Without TilS5
Nº of genomes with TilS6
Nº of genomes without TilS6
With TilS6
Without TilS6
Nº of genomes with TilS7
Nº of genomes without TilS7
With TilS7
Without TilS7
Nº of genomes with TilS8
Nº of genomes without TilS8
With TilS8
Without TilS8
Nº of genomes with TilS9
Nº of genomes without TilS9
With TilS9
Without TilS9
Nº of genomes with TilS10
Nº of genomes without TilS10
With TilS10
Without TilS10
Nº of genomes with TilS11
Nº of genomes without TilS11
With TilS11
Without TilS11
Nº of genomes with TilS12
Nº of genomes without TilS12
With TilS12
Without TilS12
Nº of genomes with TilS13
Nº of genomes without TilS13
With TilS13
Without TilS13
Nº of genomes with TilS14
Nº of genomes without TilS14
With TilS14
Without TilS14
Nº of genomes with TilS15
Nº of genomes without TilS15
With TilS15
Without TilS15

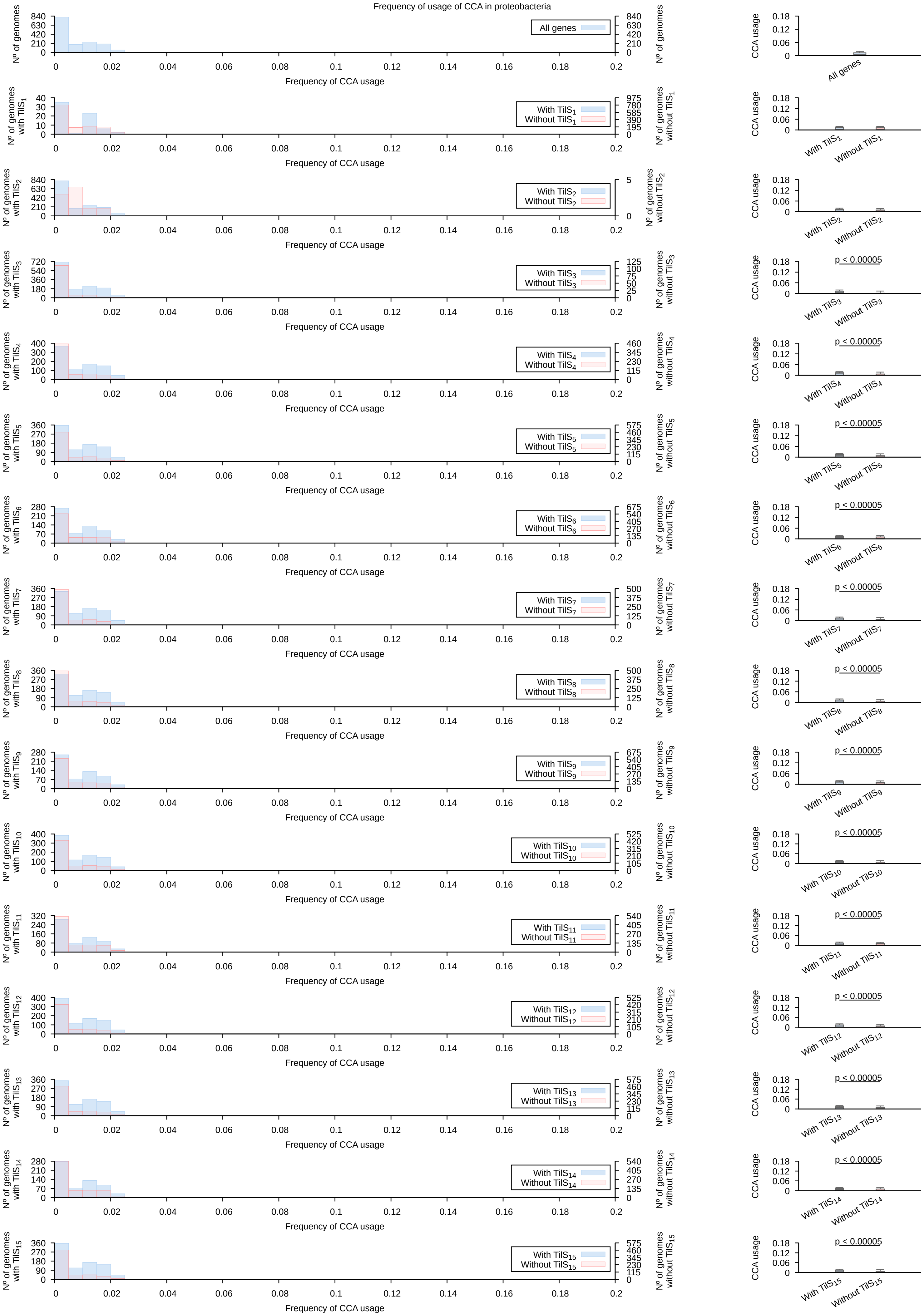

Frequency of usage of CCA in proteobacteria
Nº of genomes
Frequency of CCA usage
CCA usage
All genes
With TilS1
Without TilS1
With TilS2
Without TilS2
With TilS3
Without TilS3
With TilS4
Without TilS4
With TilS5
Without TilS5
With TilS6
Without TilS6
With TilS7
Without TilS7
With TilS8
Without TilS8
With TilS9
Without TilS9
With TilS10
Without TilS10
With TilS11
Without TilS11
With TilS12
Without TilS12
With TilS13
Without TilS13
With TilS14
Without TilS14
With TilS15
Without TilS15
p < 0.00005

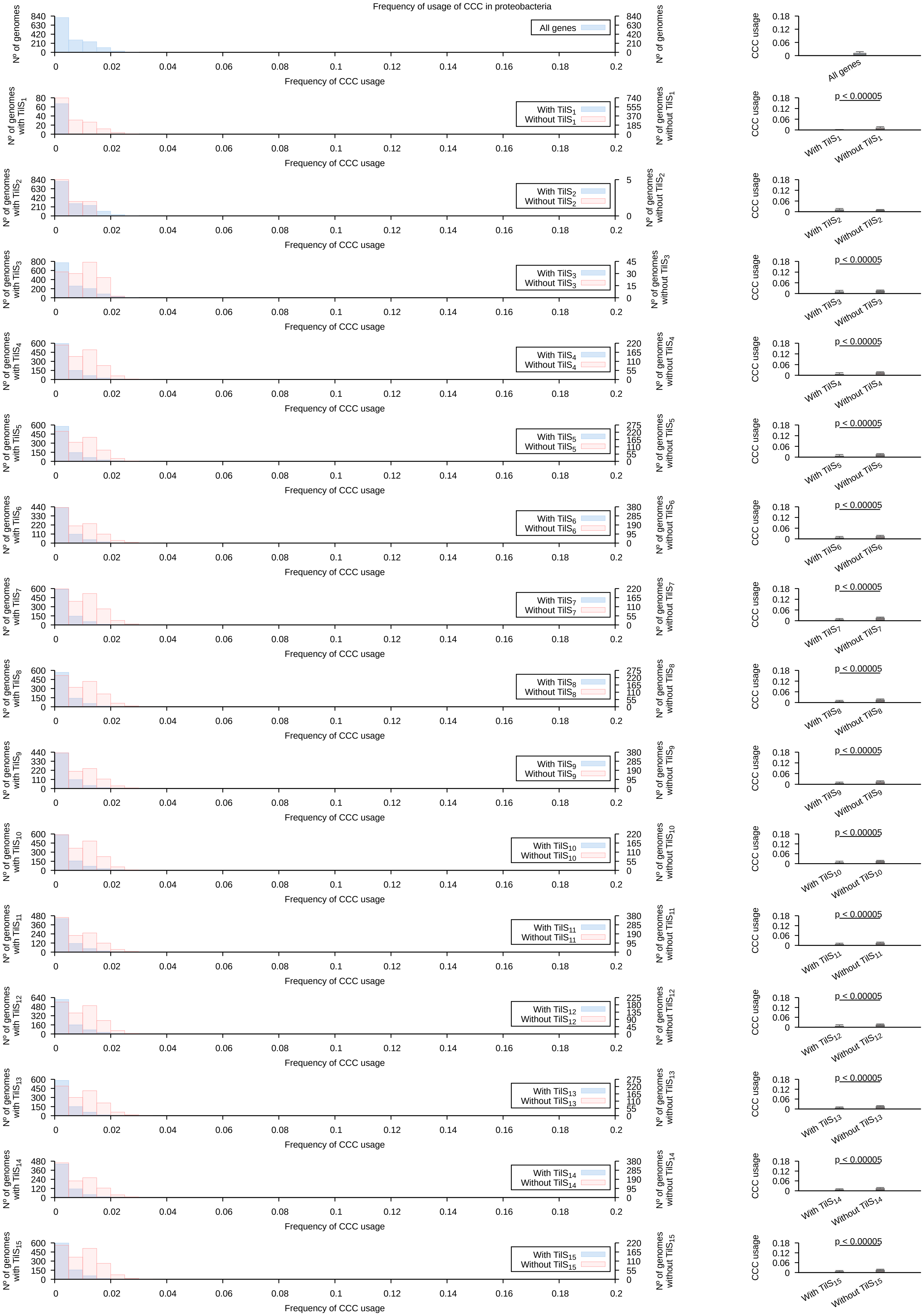

Frequency of usage of CCC in proteobacteria
Nº of genomes
All genes
Frequency of CCC usage
CCC usage
All genes
Nº of genomes with TilS$_1$
With TilS$_1$
Without TilS$_1$
Nº of genomes without TilS$_1$
p < 0.00005
Nº of genomes with TilS$_2$
With TilS$_2$
Without TilS$_2$
Nº of genomes without TilS$_2$
Nº of genomes with TilS$_3$
With TilS$_3$
Without TilS$_3$
Nº of genomes without TilS$_3$
Nº of genomes with TilS$_4$
With TilS$_4$
Without TilS$_4$
Nº of genomes without TilS$_4$
Nº of genomes with TilS$_5$
With TilS$_5$
Without TilS$_5$
Nº of genomes without TilS$_5$
Nº of genomes with TilS$_6$
With TilS$_6$
Without TilS$_6$
Nº of genomes without TilS$_6$
Nº of genomes with TilS$_7$
With TilS$_7$
Without TilS$_7$
Nº of genomes without TilS$_7$
Nº of genomes with TilS$_8$
With TilS$_8$
Without TilS$_8$
Nº of genomes without TilS$_8$
Nº of genomes with TilS$_9$
With TilS$_9$
Without TilS$_9$
Nº of genomes without TilS$_9$
Nº of genomes with TilS$_{10}$
With TilS$_{10}$
Without TilS$_{10}$
Nº of genomes without TilS$_{10}$
Nº of genomes with TilS$_{11}$
With TilS$_{11}$
Without TilS$_{11}$
Nº of genomes without TilS$_{11}$
Nº of genomes with TilS$_{12}$
With TilS$_{12}$
Without TilS$_{12}$
Nº of genomes without TilS$_{12}$
Nº of genomes with TilS$_{13}$
With TilS$_{13}$
Without TilS$_{13}$
Nº of genomes without TilS$_{13}$
Nº of genomes with TilS$_{14}$
With TilS$_{14}$
Without TilS$_{14}$
Nº of genomes without TilS$_{14}$
Nº of genomes with TilS$_{15}$
With TilS$_{15}$
Without TilS$_{15}$
Nº of genomes without TilS$_{15}$

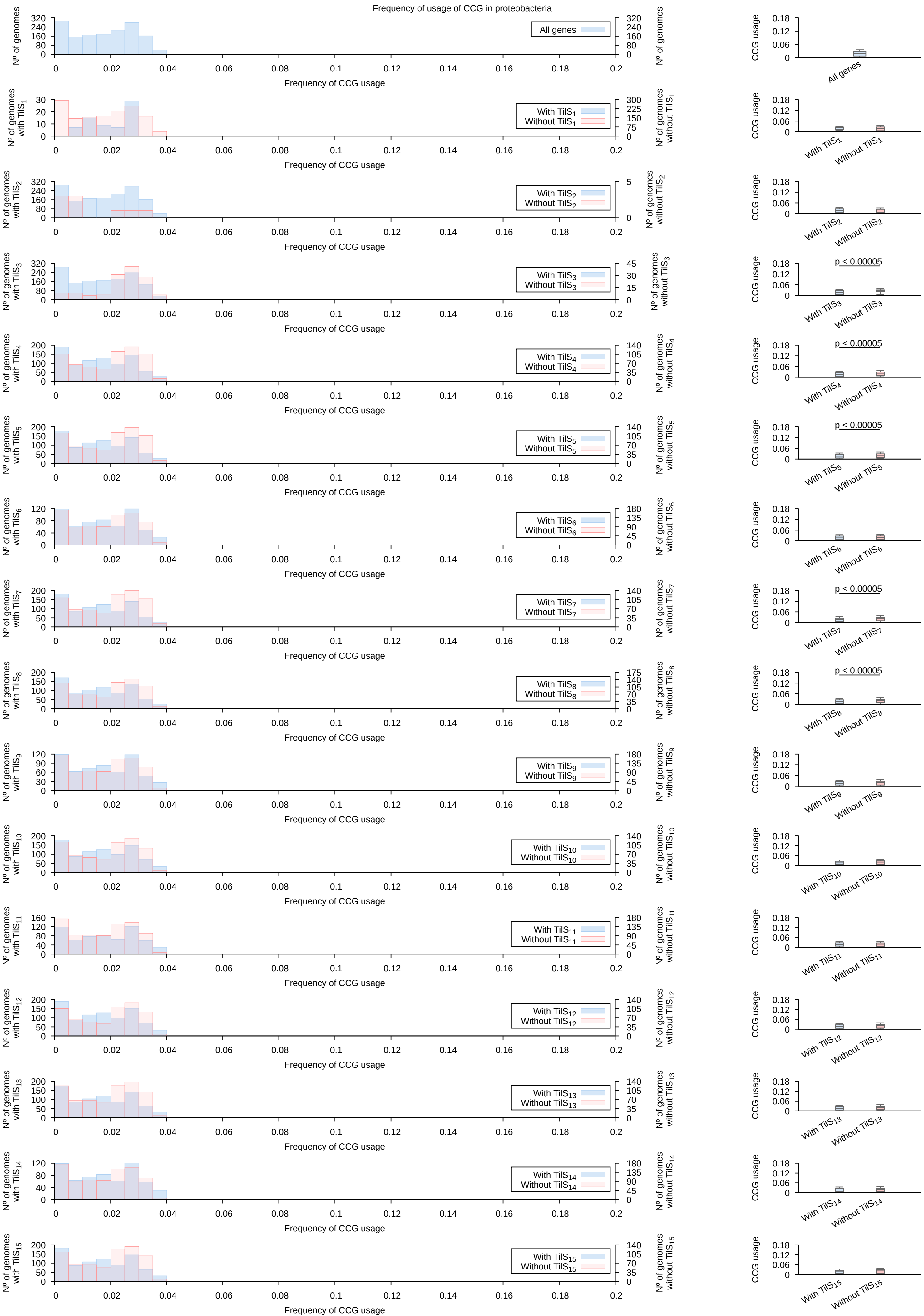

Frequency of usage of CCG in proteobacteria
Nº of genomes
All genes
Frequency of CCG usage
CCG usage
Nº of genomes with TilS1
Nº of genomes without TilS1
With TilS1
Without TilS1
Nº of genomes with TilS2
Nº of genomes without TilS2
With TilS2
Without TilS2
Nº of genomes with TilS3
Nº of genomes without TilS3
With TilS3
Without TilS3
p < 0.00005
Nº of genomes with TilS4
Nº of genomes without TilS4
With TilS4
Without TilS4
p < 0.00005
Nº of genomes with TilS5
Nº of genomes without TilS5
With TilS5
Without TilS5
p < 0.00005
Nº of genomes with TilS6
Nº of genomes without TilS6
With TilS6
Without TilS6
Nº of genomes with TilS7
Nº of genomes without TilS7
With TilS7
Without TilS7
p < 0.00005
Nº of genomes with TilS8
Nº of genomes without TilS8
With TilS8
Without TilS8
p < 0.00005
Nº of genomes with TilS9
Nº of genomes without TilS9
With TilS9
Without TilS9
Nº of genomes with TilS10
Nº of genomes without TilS10
With TilS10
Without TilS10
Nº of genomes with TilS11
Nº of genomes without TilS11
With TilS11
Without TilS11
Nº of genomes with TilS12
Nº of genomes without TilS12
With TilS12
Without TilS12
Nº of genomes with TilS13
Nº of genomes without TilS13
With TilS13
Without TilS13
Nº of genomes with TilS14
Nº of genomes without TilS14
With TilS14
Without TilS14
Nº of genomes with TilS15
Nº of genomes without TilS15
With TilS15
Without TilS15

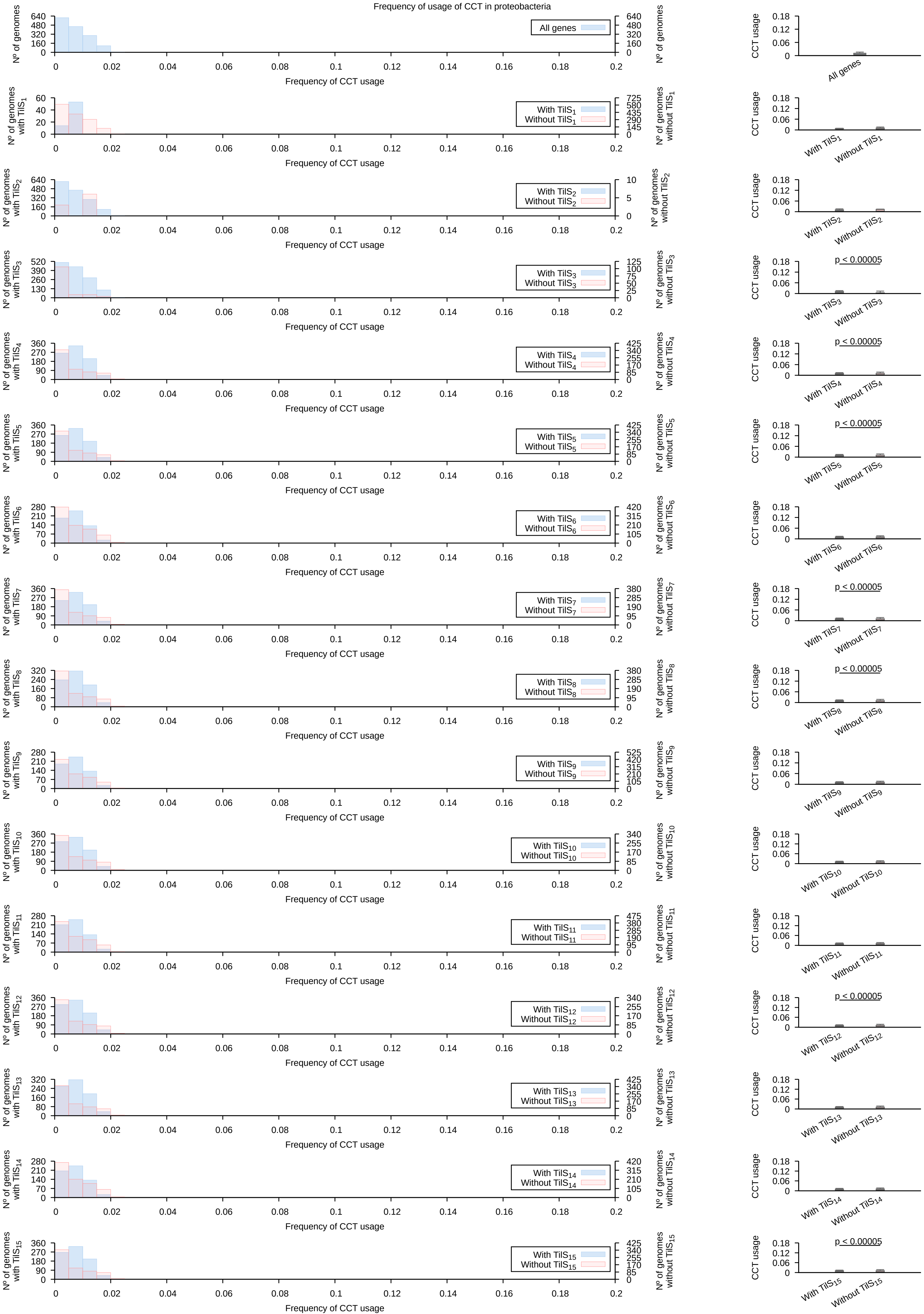

Frequency of usage of CCT in proteobacteria
Nº of genomes
All genes
Frequency of CCT usage
CCT usage
All genes
Nº of genomes with TilS$_1$
Nº of genomes without TilS$_1$
With TilS$_1$
Without TilS$_1$
With TilS$_2$
Without TilS$_2$
With TilS$_3$
Without TilS$_3$
p < 0.00005
With TilS$_4$
Without TilS$_4$
p < 0.00005
With TilS$_5$
Without TilS$_5$
p < 0.00005
With TilS$_6$
Without TilS$_6$
With TilS$_7$
Without TilS$_7$
p < 0.00005
With TilS$_8$
Without TilS$_8$
p < 0.00005
With TilS$_9$
Without TilS$_9$
With TilS$_{10}$
Without TilS$_{10}$
With TilS$_{11}$
Without TilS$_{11}$
With TilS$_{12}$
Without TilS$_{12}$
p < 0.00005
With TilS$_{13}$
Without TilS$_{13}$
With TilS$_{14}$
Without TilS$_{14}$
With TilS$_{15}$
Without TilS$_{15}$
p < 0.00005

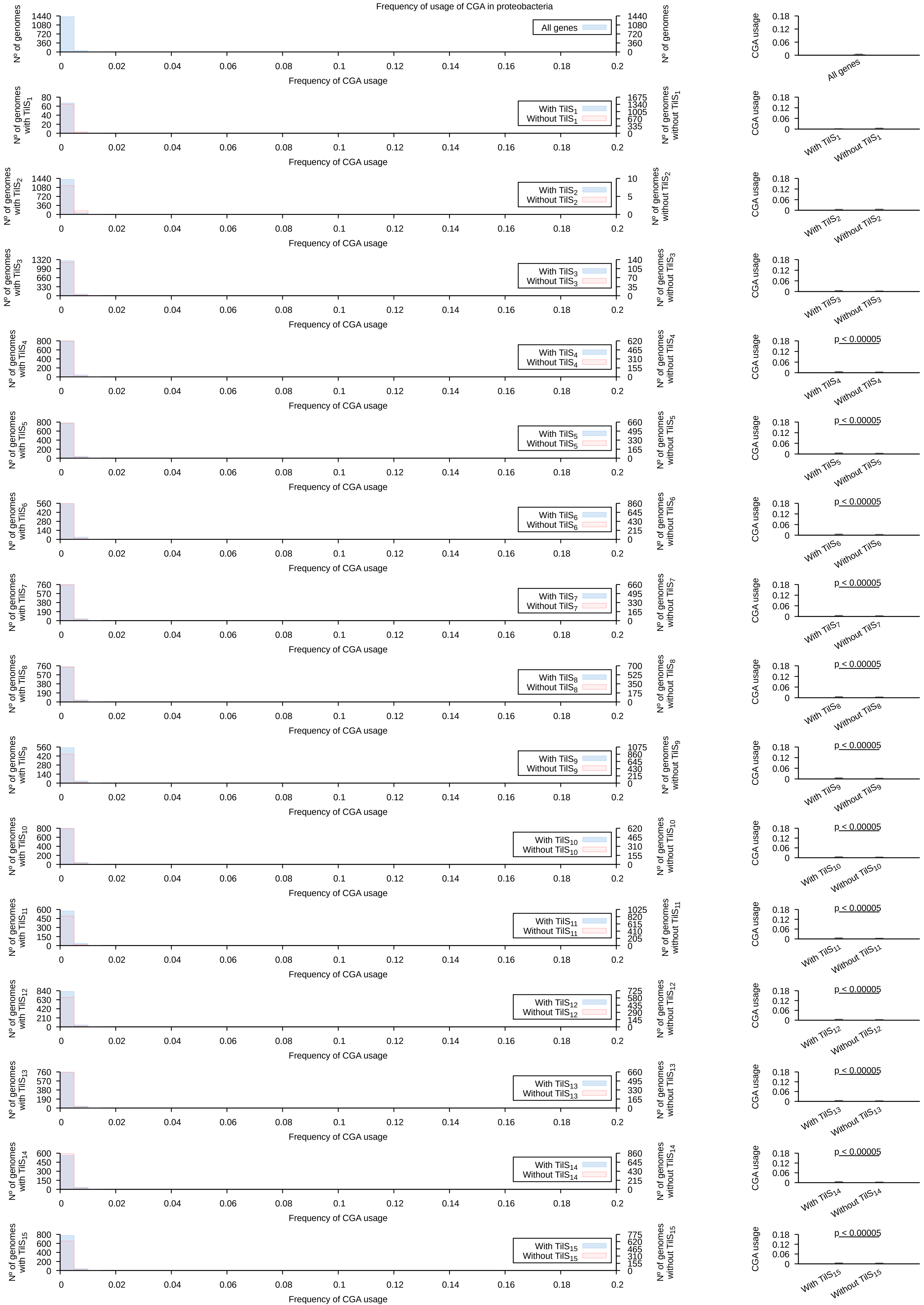

Frequency of usage of CGA in proteobacteria
Nº of genomes
All genes
Frequency of CGA usage
CGA usage
Nº of genomes with TilS$_1$
Nº of genomes without TilS$_1$
With TilS$_1$
Without TilS$_1$
Nº of genomes with TilS$_2$
Nº of genomes without TilS$_2$
With TilS$_2$
Without TilS$_2$
Nº of genomes with TilS$_3$
Nº of genomes without TilS$_3$
With TilS$_3$
Without TilS$_3$
Nº of genomes with TilS$_4$
Nº of genomes without TilS$_4$
With TilS$_4$
Without TilS$_4$
p < 0.00005
Nº of genomes with TilS$_5$
Nº of genomes without TilS$_5$
With TilS$_5$
Without TilS$_5$
p < 0.00005
Nº of genomes with TilS$_6$
Nº of genomes without TilS$_6$
With TilS$_6$
Without TilS$_6$
p < 0.00005
Nº of genomes with TilS$_7$
Nº of genomes without TilS$_7$
With TilS$_7$
Without TilS$_7$
p < 0.00005
Nº of genomes with TilS$_8$
Nº of genomes without TilS$_8$
With TilS$_8$
Without TilS$_8$
p < 0.00005
Nº of genomes with TilS$_9$
Nº of genomes without TilS$_9$
With TilS$_9$
Without TilS$_9$
p < 0.00005
Nº of genomes with TilS$_{10}$
Nº of genomes without TilS$_{10}$
With TilS$_{10}$
Without TilS$_{10}$
p < 0.00005
Nº of genomes with TilS$_{11}$
Nº of genomes without TilS$_{11}$
With TilS$_{11}$
Without TilS$_{11}$
p < 0.00005
Nº of genomes with TilS$_{12}$
Nº of genomes without TilS$_{12}$
With TilS$_{12}$
Without TilS$_{12}$
p < 0.00005
Nº of genomes with TilS$_{13}$
Nº of genomes without TilS$_{13}$
With TilS$_{13}$
Without TilS$_{13}$
p < 0.00005
Nº of genomes with TilS$_{14}$
Nº of genomes without TilS$_{14}$
With TilS$_{14}$
Without TilS$_{14}$
p < 0.00005
Nº of genomes with TilS$_{15}$
Nº of genomes without TilS$_{15}$
With TilS$_{15}$
Without TilS$_{15}$
p < 0.00005

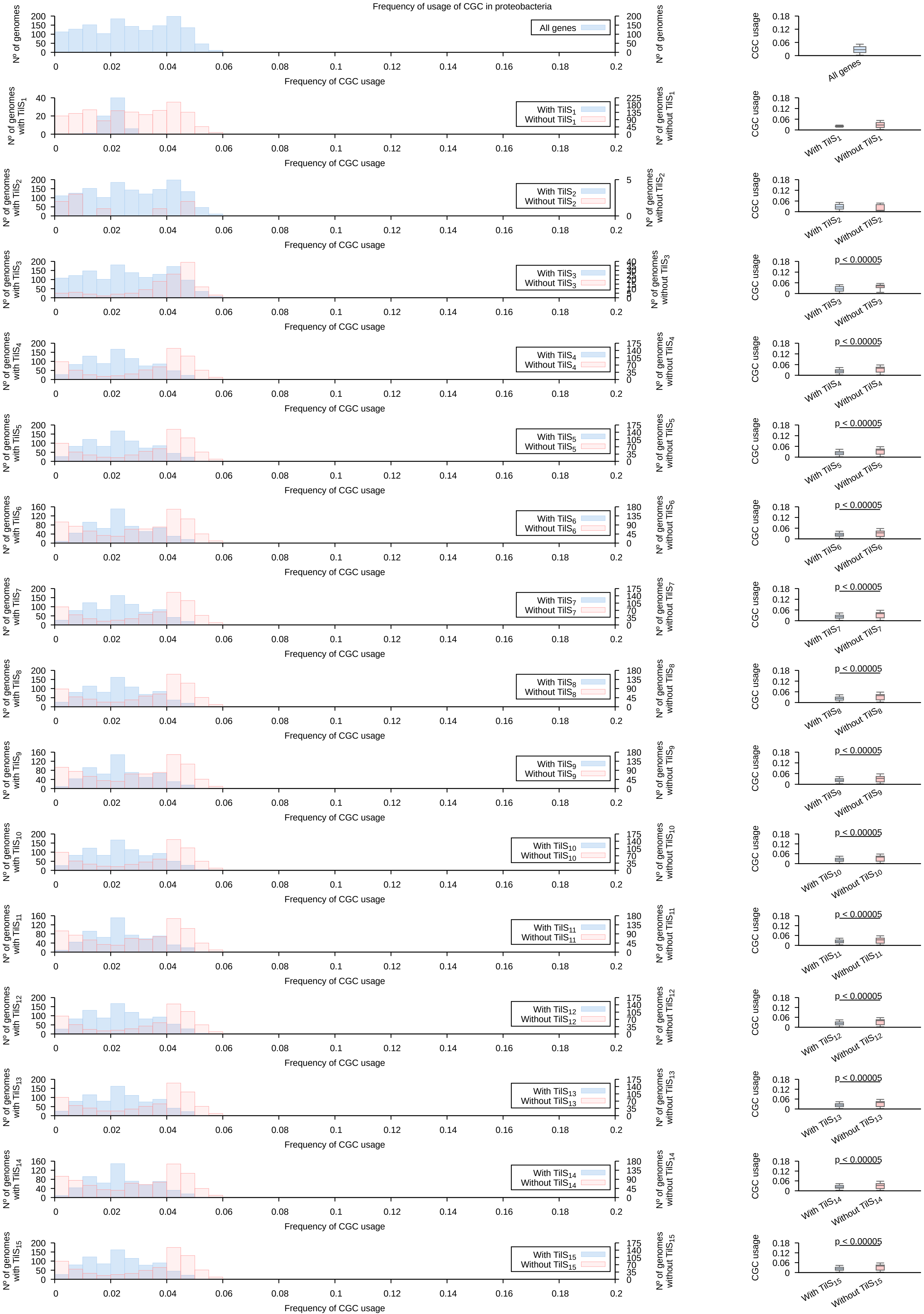
Frequency of usage of CGC in proteobacteria
Nº of genomes
All genes
CGC usage
Frequency of CGC usage
Nº of genomes with TilS$_1$
Nº of genomes without TilS$_1$
With TilS$_1$
Without TilS$_1$
Nº of genomes with TilS$_2$
Nº of genomes without TilS$_2$
With TilS$_2$
Without TilS$_2$
Nº of genomes with TilS$_3$
Nº of genomes without TilS$_3$
With TilS$_3$
Without TilS$_3$
p < 0.00005
Nº of genomes with TilS$_4$
Nº of genomes without TilS$_4$
With TilS$_4$
Without TilS$_4$
p < 0.00005
Nº of genomes with TilS$_5$
Nº of genomes without TilS$_5$
With TilS$_5$
Without TilS$_5$
p < 0.00005
Nº of genomes with TilS$_6$
Nº of genomes without TilS$_6$
With TilS$_6$
Without TilS$_6$
p < 0.00005
Nº of genomes with TilS$_7$
Nº of genomes without TilS$_7$
With TilS$_7$
Without TilS$_7$
p < 0.00005
Nº of genomes with TilS$_8$
Nº of genomes without TilS$_8$
With TilS$_8$
Without TilS$_8$
p < 0.00005
Nº of genomes with TilS$_9$
Nº of genomes without TilS$_9$
With TilS$_9$
Without TilS$_9$
p < 0.00005
Nº of genomes with TilS$_{10}$
Nº of genomes without TilS$_{10}$
With TilS$_{10}$
Without TilS$_{10}$
p < 0.00005
Nº of genomes with TilS$_{11}$
Nº of genomes without TilS$_{11}$
With TilS$_{11}$
Without TilS$_{11}$
p < 0.00005
Nº of genomes with TilS$_{12}$
Nº of genomes without TilS$_{12}$
With TilS$_{12}$
Without TilS$_{12}$
p < 0.00005
Nº of genomes with TilS$_{13}$
Nº of genomes without TilS$_{13}$
With TilS$_{13}$
Without TilS$_{13}$
p < 0.00005
Nº of genomes with TilS$_{14}$
Nº of genomes without TilS$_{14}$
With TilS$_{14}$
Without TilS$_{14}$
p < 0.00005
Nº of genomes with TilS$_{15}$
Nº of genomes without TilS$_{15}$
With TilS$_{15}$
Without TilS$_{15}$
p < 0.00005

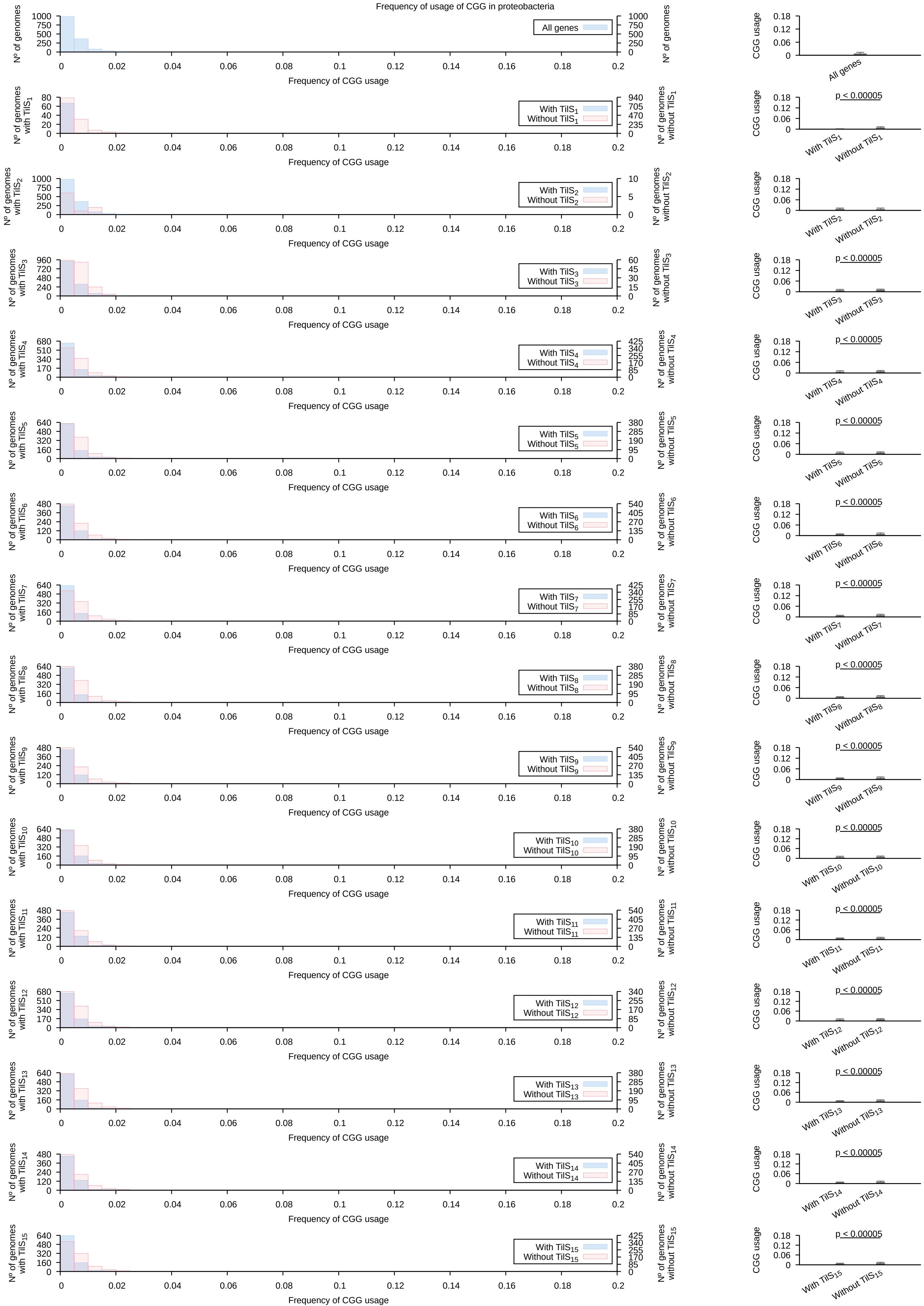
Frequency of usage of CGG in proteobacteria
Nº of genomes
1000 750 500 250 0
All genes
Frequency of CGG usage
0 0.02 0.04 0.06 0.08 0.1 0.12 0.14 0.16 0.18 0.2
CGG usage
0.18 0.12 0.06 0
All genes
Nº of genomes with TilS$_1$
80 60 40 20 0
With TilS$_1$
Without TilS$_1$
940 705 470 235 0
Nº of genomes without TilS$_1$
p < 0.00005
Nº of genomes with TilS$_2$
1000 750 500 250 0
With TilS$_2$
Without TilS$_2$
10 5 0
Nº of genomes without TilS$_2$
Nº of genomes with TilS$_3$
960 720 480 240 0
With TilS$_3$
Without TilS$_3$
60 45 30 15 0
Nº of genomes without TilS$_3$
p < 0.00005
Nº of genomes with TilS$_4$
680 510 340 170 0
With TilS$_4$
Without TilS$_4$
425 340 255 170 85 0
Nº of genomes without TilS$_4$
p < 0.00005
Nº of genomes with TilS$_5$
640 480 320 160 0
With TilS$_5$
Without TilS$_5$
380 285 190 95 0
Nº of genomes without TilS$_5$
p < 0.00005
Nº of genomes with TilS$_6$
480 360 240 120 0
With TilS$_6$
Without TilS$_6$
540 405 270 135 0
Nº of genomes without TilS$_6$
p < 0.00005
Nº of genomes with TilS$_7$
640 480 320 160 0
With TilS$_7$
Without TilS$_7$
425 340 255 170 85 0
Nº of genomes without TilS$_7$
p < 0.00005
Nº of genomes with TilS$_8$
640 480 320 160 0
With TilS$_8$
Without TilS$_8$
380 285 190 95 0
Nº of genomes without TilS$_8$
p < 0.00005
Nº of genomes with TilS$_9$
480 360 240 120 0
With TilS$_9$
Without TilS$_9$
540 405 270 135 0
Nº of genomes without TilS$_9$
p < 0.00005
Nº of genomes with TilS$_{10}$
640 480 320 160 0
With TilS$_{10}$
Without TilS$_{10}$
380 285 190 95 0
Nº of genomes without TilS$_{10}$
p < 0.00005
Nº of genomes with TilS$_{11}$
480 360 240 120 0
With TilS$_{11}$
Without TilS$_{11}$
540 405 270 135 0
Nº of genomes without TilS$_{11}$
p < 0.00005
Nº of genomes with TilS$_{12}$
680 510 340 170 0
With TilS$_{12}$
Without TilS$_{12}$
340 255 170 85 0
Nº of genomes without TilS$_{12}$
p < 0.00005
Nº of genomes with TilS$_{13}$
640 480 320 160 0
With TilS$_{13}$
Without TilS$_{13}$
380 285 190 95 0
Nº of genomes without TilS$_{13}$
p < 0.00005
Nº of genomes with TilS$_{14}$
480 360 240 120 0
With TilS$_{14}$
Without TilS$_{14}$
540 405 270 135 0
Nº of genomes without TilS$_{14}$
p < 0.00005
Nº of genomes with TilS$_{15}$
640 480 320 160 0
With TilS$_{15}$
Without TilS$_{15}$
425 340 255 170 85 0
Nº of genomes without TilS$_{15}$
p < 0.00005

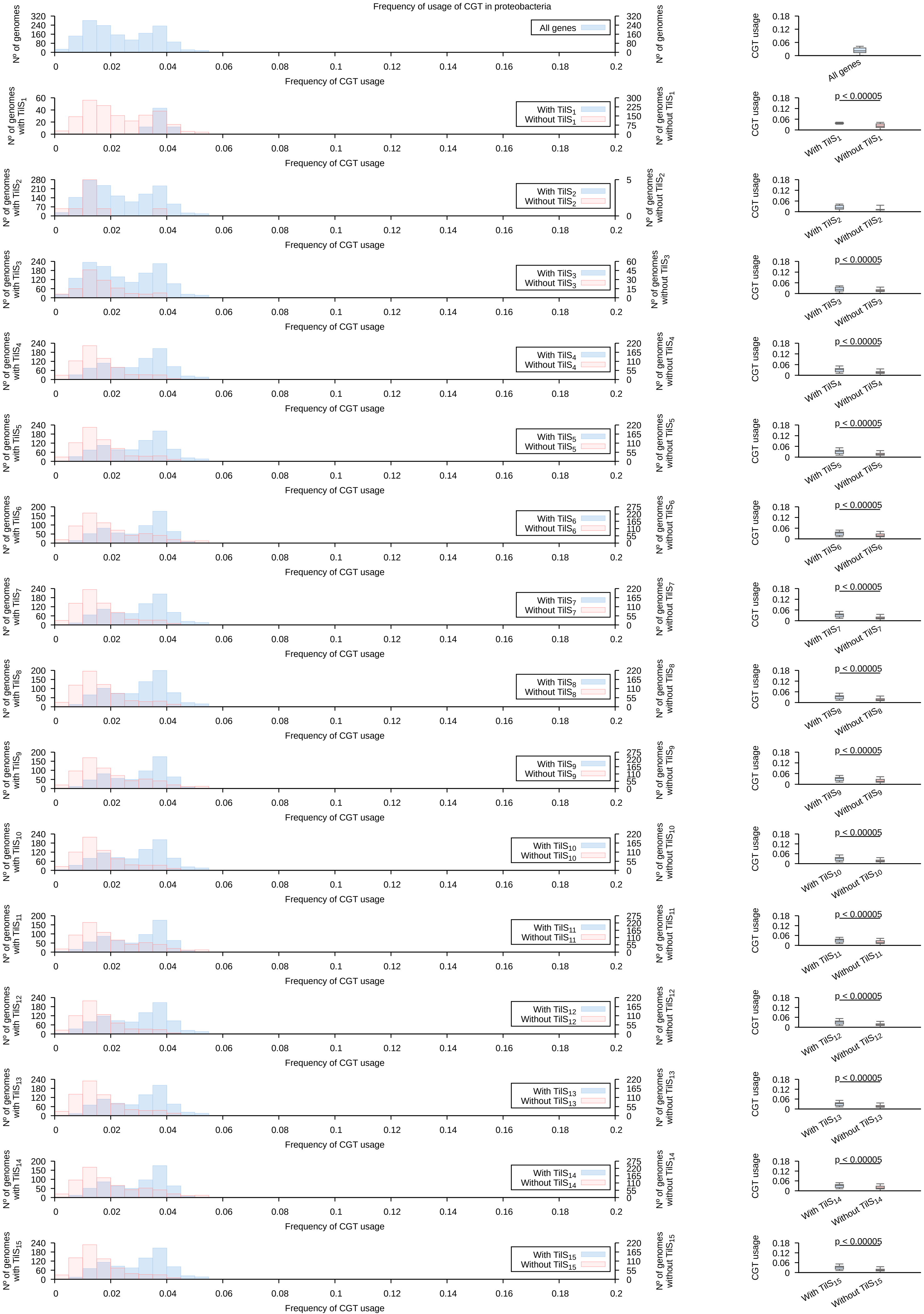

Frequency of usage of CGT in proteobacteria
Nº of genomes
Frequency of CGT usage
All genes
CGT usage
With TilS$_1$
Without TilS$_1$
Nº of genomes with TilS$_1$
Nº of genomes without TilS$_1$
p < 0.00005
With TilS$_2$
Without TilS$_2$
Nº of genomes with TilS$_2$
Nº of genomes without TilS$_2$
With TilS$_3$
Without TilS$_3$
Nº of genomes with TilS$_3$
Nº of genomes without TilS$_3$
p < 0.00005
With TilS$_4$
Without TilS$_4$
Nº of genomes with TilS$_4$
Nº of genomes without TilS$_4$
p < 0.00005
With TilS$_5$
Without TilS$_5$
Nº of genomes with TilS$_5$
Nº of genomes without TilS$_5$
p < 0.00005
With TilS$_6$
Without TilS$_6$
Nº of genomes with TilS$_6$
Nº of genomes without TilS$_6$
p < 0.00005
With TilS$_7$
Without TilS$_7$
Nº of genomes with TilS$_7$
Nº of genomes without TilS$_7$
p < 0.00005
With TilS$_8$
Without TilS$_8$
Nº of genomes with TilS$_8$
Nº of genomes without TilS$_8$
p < 0.00005
With TilS$_9$
Without TilS$_9$
Nº of genomes with TilS$_9$
Nº of genomes without TilS$_9$
p < 0.00005
With TilS$_{10}$
Without TilS$_{10}$
Nº of genomes with TilS$_{10}$
Nº of genomes without TilS$_{10}$
p < 0.00005
With TilS$_{11}$
Without TilS$_{11}$
Nº of genomes with TilS$_{11}$
Nº of genomes without TilS$_{11}$
p < 0.00005
With TilS$_{12}$
Without TilS$_{12}$
Nº of genomes with TilS$_{12}$
Nº of genomes without TilS$_{12}$
p < 0.00005
With TilS$_{13}$
Without TilS$_{13}$
Nº of genomes with TilS$_{13}$
Nº of genomes without TilS$_{13}$
p < 0.00005
With TilS$_{14}$
Without TilS$_{14}$
Nº of genomes with TilS$_{14}$
Nº of genomes without TilS$_{14}$
p < 0.00005
With TilS$_{15}$
Without TilS$_{15}$
Nº of genomes with TilS$_{15}$
Nº of genomes without TilS$_{15}$
p < 0.00005
0 0.02 0.04 0.06 0.08 0.1 0.12 0.14 0.16 0.18 0.2
0 0.06 0.12 0.18

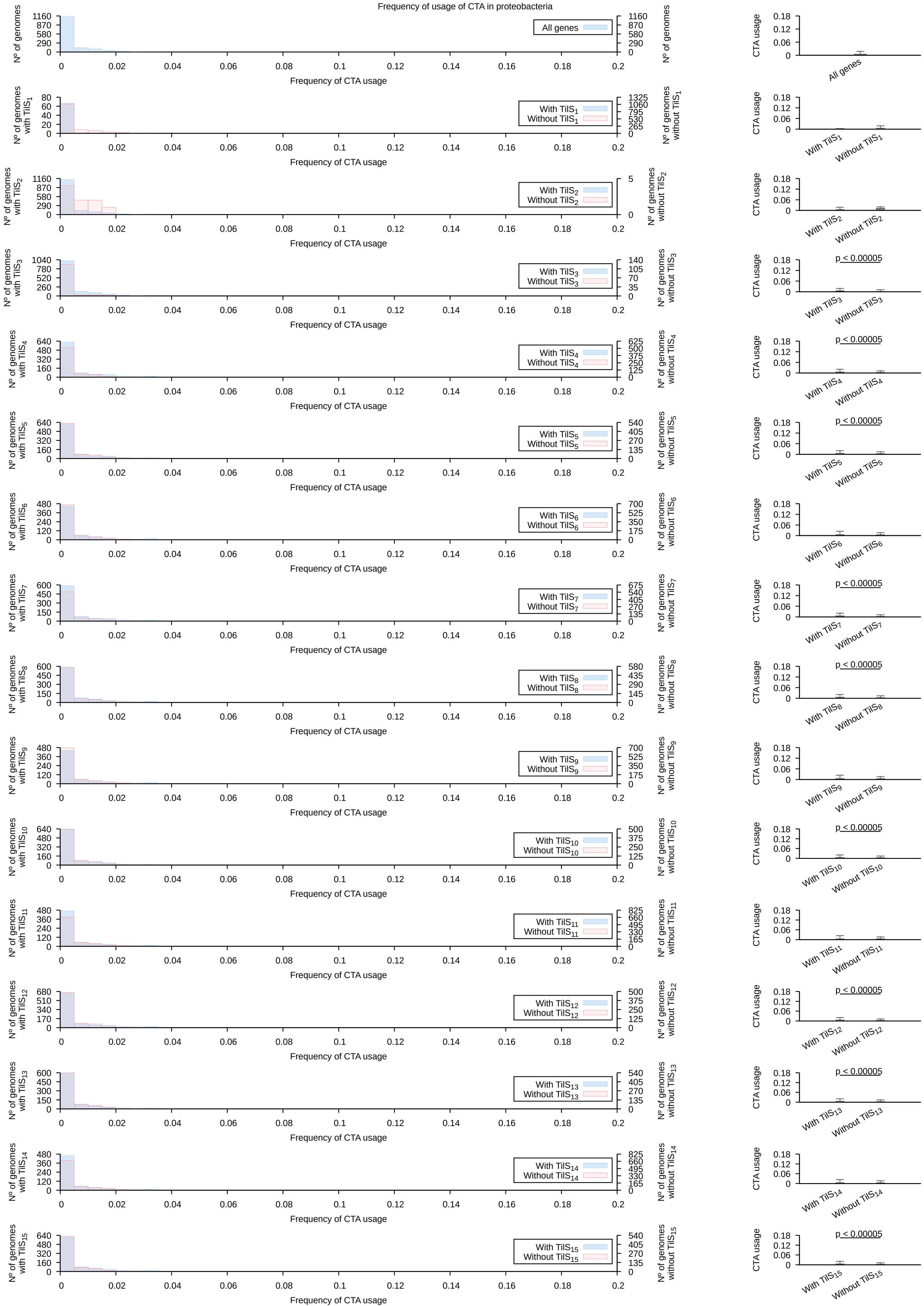
Frequency of usage of CTA in proteobacteria
Nº of genomes
Frequency of CTA usage
CTA usage
All genes
With TilS$_1$
Without TilS$_1$
With TilS$_2$
Without TilS$_2$
With TilS$_3$
Without TilS$_3$
With TilS$_4$
Without TilS$_4$
With TilS$_5$
Without TilS$_5$
With TilS$_6$
Without TilS$_6$
With TilS$_7$
Without TilS$_7$
With TilS$_8$
Without TilS$_8$
With TilS$_9$
Without TilS$_9$
With TilS$_{10}$
Without TilS$_{10}$
With TilS$_{11}$
Without TilS$_{11}$
With TilS$_{12}$
Without TilS$_{12}$
With TilS$_{13}$
Without TilS$_{13}$
With TilS$_{14}$
Without TilS$_{14}$
With TilS$_{15}$
Without TilS$_{15}$
p < 0.00005

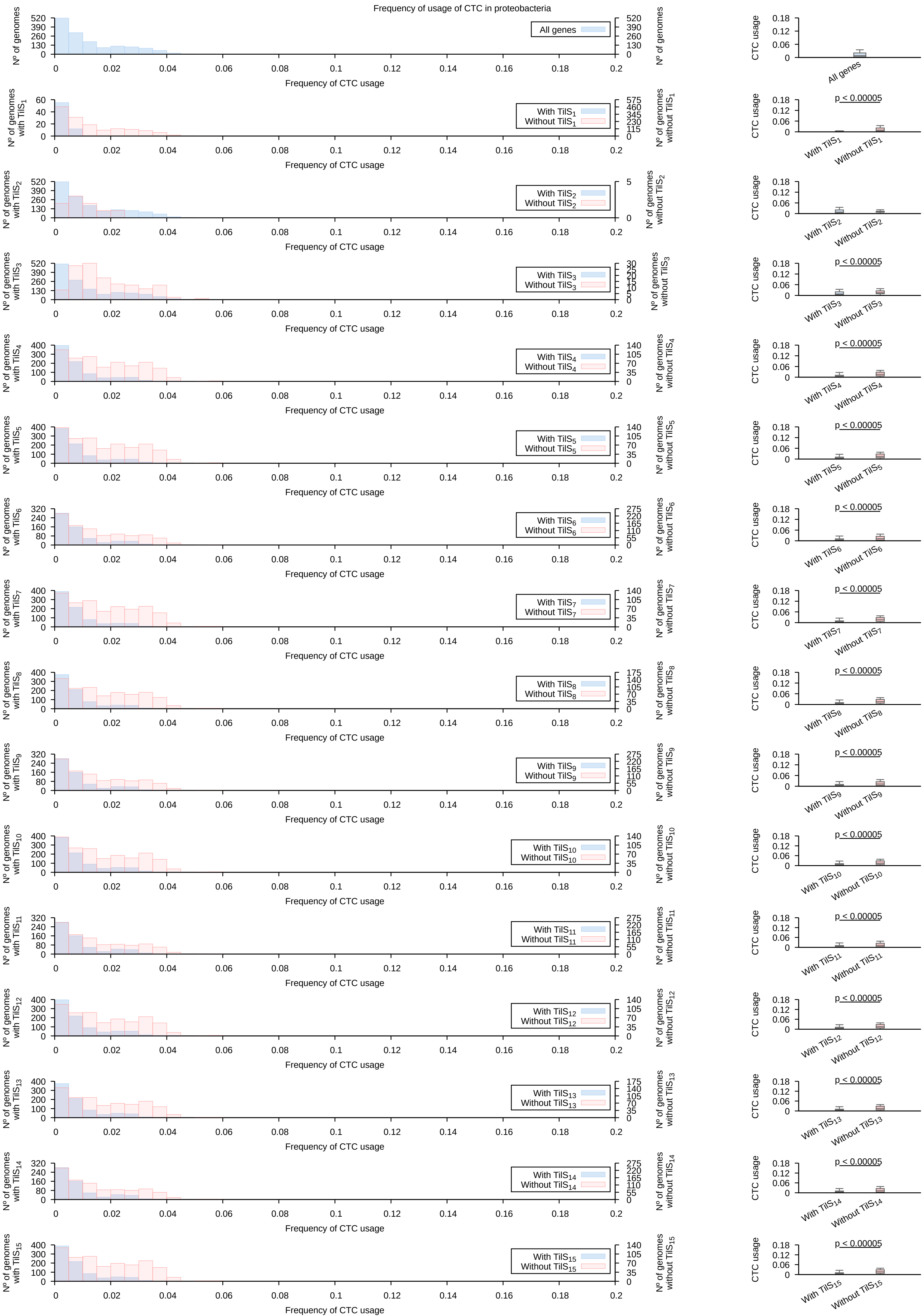
Frequency of usage of CTC in proteobacteria
Nº of genomes
520 390 260 130 0
All genes
520 390 260 130 0
Nº of genomes
0 0.02 0.04 0.06 0.08 0.1 0.12 0.14 0.16 0.18 0.2
Frequency of CTC usage
CTC usage
0.18 0.12 0.06 0
All genes
Nº of genomes with TilS$_1$
60 40 20 0
With TilS$_1$
Without TilS$_1$
575 460 345 230 115 0
Nº of genomes without TilS$_1$
0 0.02 0.04 0.06 0.08 0.1 0.12 0.14 0.16 0.18 0.2
Frequency of CTC usage
CTC usage
$p < 0.00005$
0.18 0.12 0.06 0
With TilS$_1$
Without TilS$_1$
Nº of genomes with TilS$_2$
520 390 260 130 0
With TilS$_2$
Without TilS$_2$
5 0
Nº of genomes without TilS$_2$
0 0.02 0.04 0.06 0.08 0.1 0.12 0.14 0.16 0.18 0.2
Frequency of CTC usage
CTC usage
0.18 0.12 0.06 0
With TilS$_2$
Without TilS$_2$
Nº of genomes with TilS$_3$
520 390 260 130 0
With TilS$_3$
Without TilS$_3$
30 25 20 15 10 5 0
Nº of genomes without TilS$_3$
0 0.02 0.04 0.06 0.08 0.1 0.12 0.14 0.16 0.18 0.2
Frequency of CTC usage
CTC usage
$p < 0.00005$
0.18 0.12 0.06 0
With TilS$_3$
Without TilS$_3$
Nº of genomes with TilS$_4$
400 300 200 100 0
With TilS$_4$
Without TilS$_4$
140 105 70 35 0
Nº of genomes without TilS$_4$
0 0.02 0.04 0.06 0.08 0.1 0.12 0.14 0.16 0.18 0.2
Frequency of CTC usage
CTC usage
$p < 0.00005$
0.18 0.12 0.06 0
With TilS$_4$
Without TilS$_4$
Nº of genomes with TilS$_5$
400 300 200 100 0
With TilS$_5$
Without TilS$_5$
140 105 70 35 0
Nº of genomes without TilS$_5$
0 0.02 0.04 0.06 0.08 0.1 0.12 0.14 0.16 0.18 0.2
Frequency of CTC usage
CTC usage
$p < 0.00005$
0.18 0.12 0.06 0
With TilS$_5$
Without TilS$_5$
Nº of genomes with TilS$_6$
320 240 160 80 0
With TilS$_6$
Without TilS$_6$
275 220 165 110 55 0
Nº of genomes without TilS$_6$
0 0.02 0.04 0.06 0.08 0.1 0.12 0.14 0.16 0.18 0.2
Frequency of CTC usage
CTC usage
$p < 0.00005$
0.18 0.12 0.06 0
With TilS$_6$
Without TilS$_6$
Nº of genomes with TilS$_7$
400 300 200 100 0
With TilS$_7$
Without TilS$_7$
140 105 70 35 0
Nº of genomes without TilS$_7$
0 0.02 0.04 0.06 0.08 0.1 0.12 0.14 0.16 0.18 0.2
Frequency of CTC usage
CTC usage
$p < 0.00005$
0.18 0.12 0.06 0
With TilS$_7$
Without TilS$_7$
Nº of genomes with TilS$_8$
400 300 200 100 0
With TilS$_8$
Without TilS$_8$
175 140 105 70 35 0
Nº of genomes without TilS$_8$
0 0.02 0.04 0.06 0.08 0.1 0.12 0.14 0.16 0.18 0.2
Frequency of CTC usage
CTC usage
$p < 0.00005$
0.18 0.12 0.06 0
With TilS$_8$
Without TilS$_8$
Nº of genomes with TilS$_9$
320 240 160 80 0
With TilS$_9$
Without TilS$_9$
275 220 165 110 55 0
Nº of genomes without TilS$_9$
0 0.02 0.04 0.06 0.08 0.1 0.12 0.14 0.16 0.18 0.2
Frequency of CTC usage
CTC usage
$p < 0.00005$
0.18 0.12 0.06 0
With TilS$_9$
Without TilS$_9$
Nº of genomes with TilS$_{10}$
400 300 200 100 0
With TilS$_{10}$
Without TilS$_{10}$
140 105 70 35 0
Nº of genomes without TilS$_{10}$
0 0.02 0.04 0.06 0.08 0.1 0.12 0.14 0.16 0.18 0.2
Frequency of CTC usage
CTC usage
$p < 0.00005$
0.18 0.12 0.06 0
With TilS$_{10}$
Without TilS$_{10}$
Nº of genomes with TilS$_{11}$
320 240 160 80 0
With TilS$_{11}$
Without TilS$_{11}$
275 220 165 110 55 0
Nº of genomes without TilS$_{11}$
0 0.02 0.04 0.06 0.08 0.1 0.12 0.14 0.16 0.18 0.2
Frequency of CTC usage
CTC usage
$p < 0.00005$
0.18 0.12 0.06 0
With TilS$_{11}$
Without TilS$_{11}$
Nº of genomes with TilS$_{12}$
400 300 200 100 0
With TilS$_{12}$
Without TilS$_{12}$
140 105 70 35 0
Nº of genomes without TilS$_{12}$
0 0.02 0.04 0.06 0.08 0.1 0.12 0.14 0.16 0.18 0.2
Frequency of CTC usage
CTC usage
$p < 0.00005$
0.18 0.12 0.06 0
With TilS$_{12}$
Without TilS$_{12}$
Nº of genomes with TilS$_{13}$
400 300 200 100 0
With TilS$_{13}$
Without TilS$_{13}$
175 140 105 70 35 0
Nº of genomes without TilS$_{13}$
0 0.02 0.04 0.06 0.08 0.1 0.12 0.14 0.16 0.18 0.2
Frequency of CTC usage
CTC usage
$p < 0.00005$
0.18 0.12 0.06 0
With TilS$_{13}$
Without TilS$_{13}$
Nº of genomes with TilS$_{14}$
320 240 160 80 0
With TilS$_{14}$
Without TilS$_{14}$
275 220 165 110 55 0
Nº of genomes without TilS$_{14}$
0 0.02 0.04 0.06 0.08 0.1 0.12 0.14 0.16 0.18 0.2
Frequency of CTC usage
CTC usage
$p < 0.00005$
0.18 0.12 0.06 0
With TilS$_{14}$
Without TilS$_{14}$
Nº of genomes with TilS$_{15}$
400 300 200 100 0
With TilS$_{15}$
Without TilS$_{15}$
140 105 70 35 0
Nº of genomes without TilS$_{15}$
0 0.02 0.04 0.06 0.08 0.1 0.12 0.14 0.16 0.18 0.2
Frequency of CTC usage
CTC usage
$p < 0.00005$
0.18 0.12 0.06 0
With TilS$_{15}$
Without TilS$_{15}$

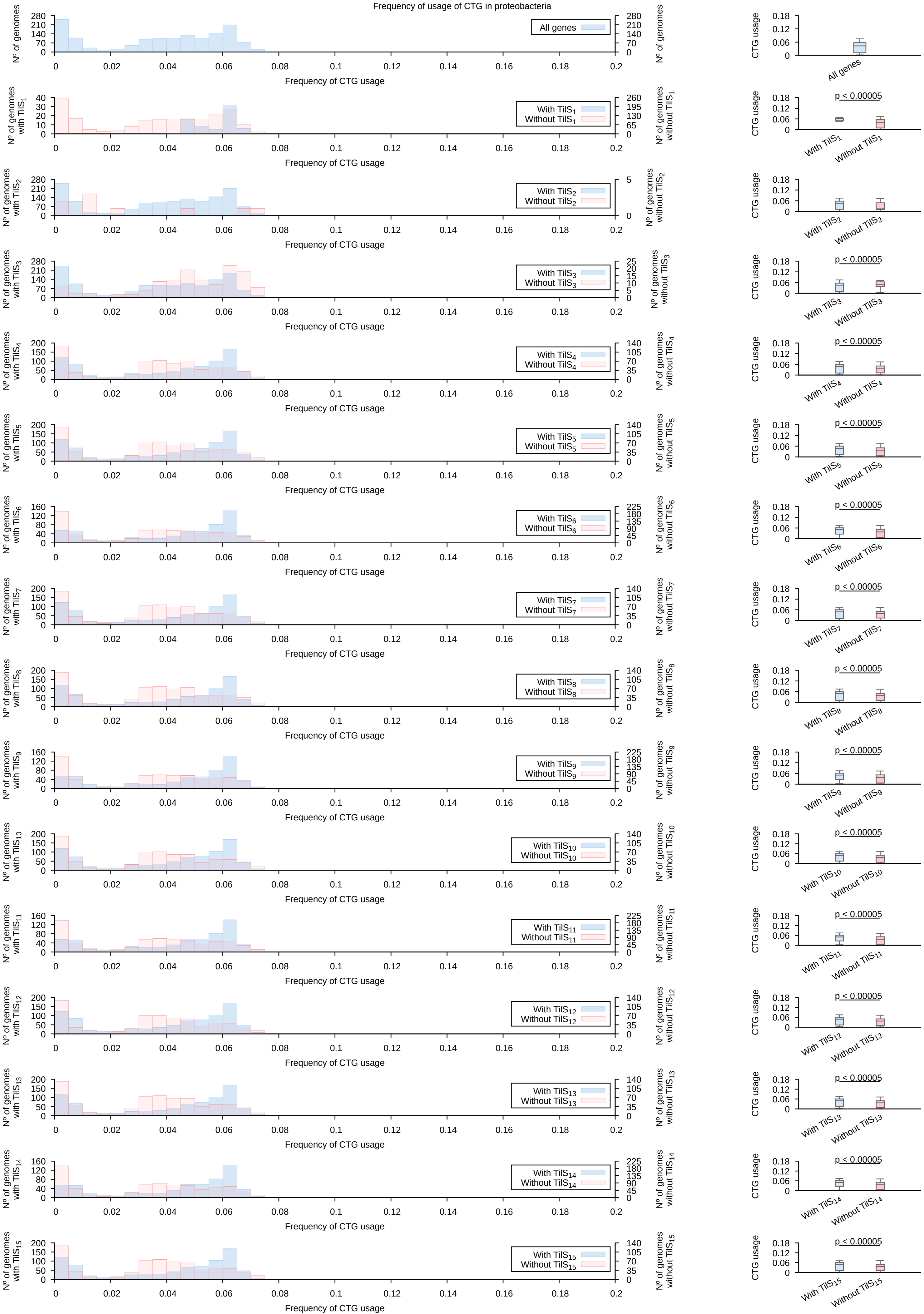
Frequency of usage of CTG in proteobacteria
Nº of genomes
All genes
Frequency of CTG usage
CTG usage
All genes
Nº of genomes with TilS1
Nº of genomes without TilS1
With TilS1
Without TilS1
p < 0.00005
Nº of genomes with TilS2
Nº of genomes without TilS2
With TilS2
Without TilS2
Nº of genomes with TilS3
Nº of genomes without TilS3
With TilS3
Without TilS3
p < 0.00005
Nº of genomes with TilS4
Nº of genomes without TilS4
With TilS4
Without TilS4
p < 0.00005
Nº of genomes with TilS5
Nº of genomes without TilS5
With TilS5
Without TilS5
p < 0.00005
Nº of genomes with TilS6
Nº of genomes without TilS6
With TilS6
Without TilS6
p < 0.00005
Nº of genomes with TilS7
Nº of genomes without TilS7
With TilS7
Without TilS7
p < 0.00005
Nº of genomes with TilS8
Nº of genomes without TilS8
With TilS8
Without TilS8
p < 0.00005
Nº of genomes with TilS9
Nº of genomes without TilS9
With TilS9
Without TilS9
p < 0.00005
Nº of genomes with TilS10
Nº of genomes without TilS10
With TilS10
Without TilS10
p < 0.00005
Nº of genomes with TilS11
Nº of genomes without TilS11
With TilS11
Without TilS11
p < 0.00005
Nº of genomes with TilS12
Nº of genomes without TilS12
With TilS12
Without TilS12
p < 0.00005
Nº of genomes with TilS13
Nº of genomes without TilS13
With TilS13
Without TilS13
p < 0.00005
Nº of genomes with TilS14
Nº of genomes without TilS14
With TilS14
Without TilS14
p < 0.00005
Nº of genomes with TilS15
Nº of genomes without TilS15
With TilS15
Without TilS15
p < 0.00005

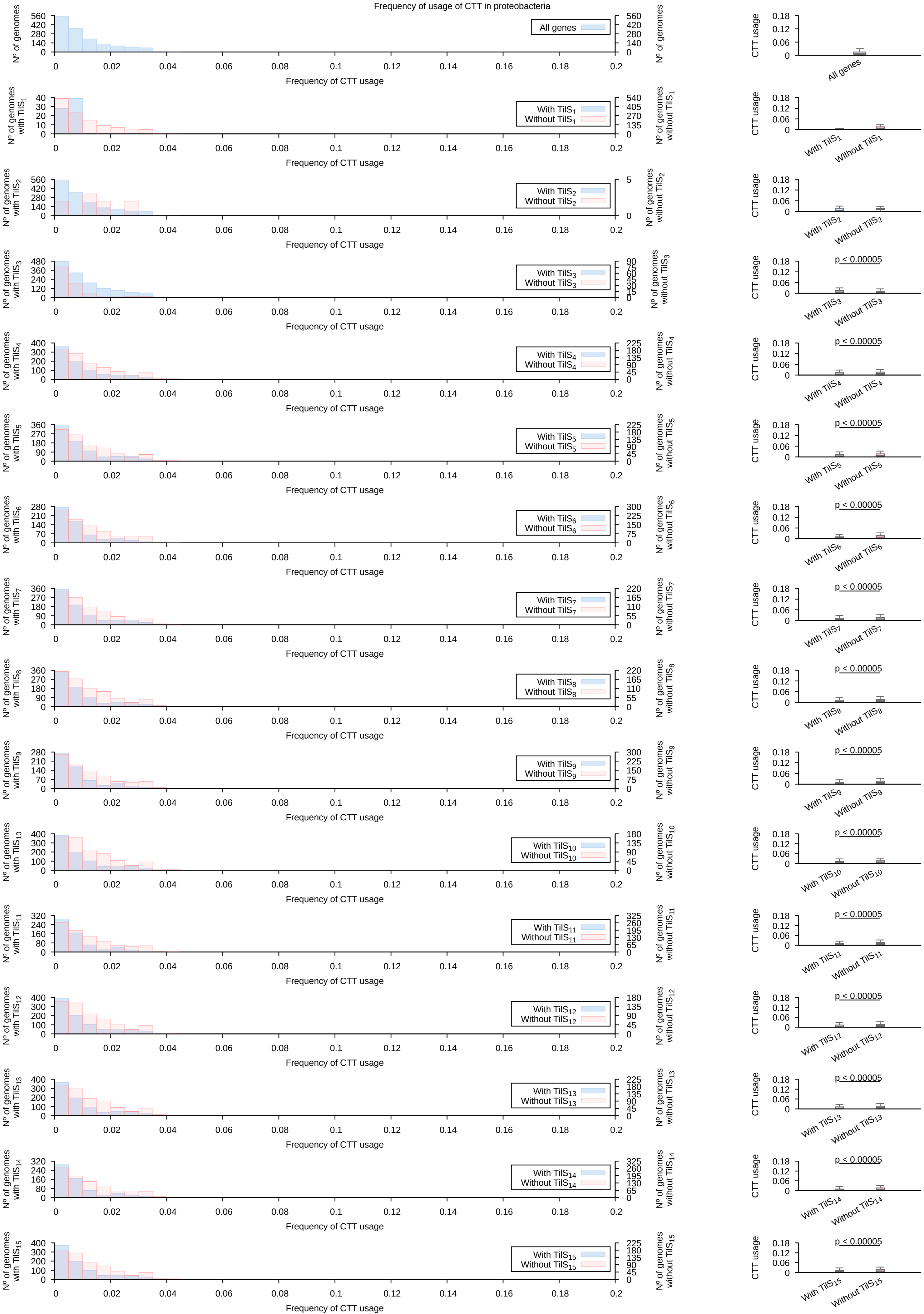
Frequency of usage of CTT in proteobacteria
Nº of genomes
Frequency of CTT usage
CTT usage
All genes
Nº of genomes with TilS1
Nº of genomes without TilS1
With TilS1
Without TilS1
Nº of genomes with TilS2
Nº of genomes without TilS2
With TilS2
Without TilS2
Nº of genomes with TilS3
Nº of genomes without TilS3
With TilS3
Without TilS3
p < 0.00005
Nº of genomes with TilS4
Nº of genomes without TilS4
With TilS4
Without TilS4
p < 0.00005
Nº of genomes with TilS5
Nº of genomes without TilS5
With TilS5
Without TilS5
p < 0.00005
Nº of genomes with TilS6
Nº of genomes without TilS6
With TilS6
Without TilS6
p < 0.00005
Nº of genomes with TilS7
Nº of genomes without TilS7
With TilS7
Without TilS7
p < 0.00005
Nº of genomes with TilS8
Nº of genomes without TilS8
With TilS8
Without TilS8
p < 0.00005
Nº of genomes with TilS9
Nº of genomes without TilS9
With TilS9
Without TilS9
p < 0.00005
Nº of genomes with TilS10
Nº of genomes without TilS10
With TilS10
Without TilS10
p < 0.00005
Nº of genomes with TilS11
Nº of genomes without TilS11
With TilS11
Without TilS11
p < 0.00005
Nº of genomes with TilS12
Nº of genomes without TilS12
With TilS12
Without TilS12
p < 0.00005
Nº of genomes with TilS13
Nº of genomes without TilS13
With TilS13
Without TilS13
p < 0.00005
Nº of genomes with TilS14
Nº of genomes without TilS14
With TilS14
Without TilS14
p < 0.00005
Nº of genomes with TilS15
Nº of genomes without TilS15
With TilS15
Without TilS15
p < 0.00005

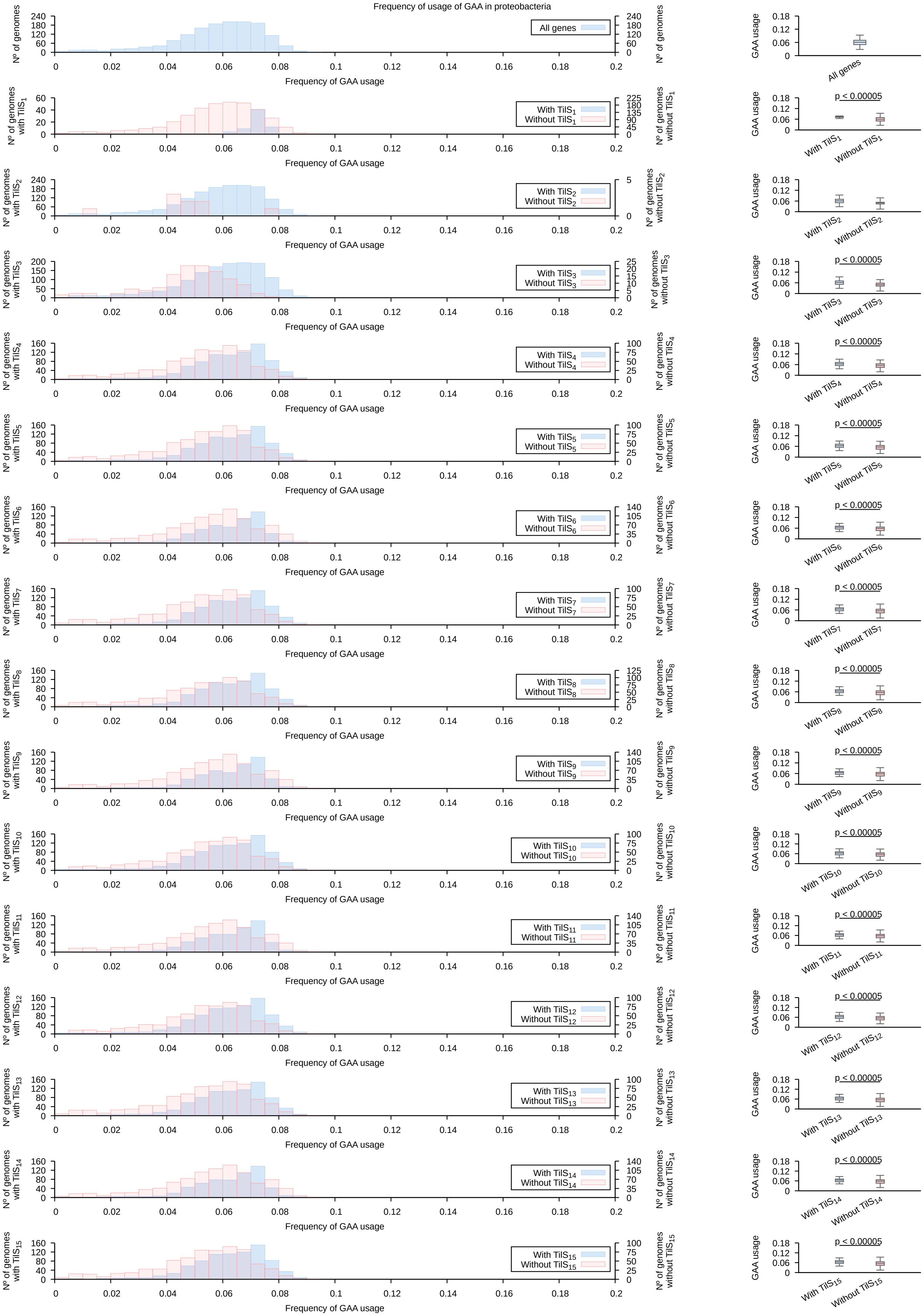
Frequency of usage of GAA in proteobacteria
Nº of genomes
All genes
Frequency of GAA usage
GAA usage
Nº of genomes with TilS$_1$
Nº of genomes without TilS$_1$
With TilS$_1$
Without TilS$_1$
p < 0.00005
Nº of genomes with TilS$_2$
Nº of genomes without TilS$_2$
With TilS$_2$
Without TilS$_2$
Nº of genomes with TilS$_3$
Nº of genomes without TilS$_3$
With TilS$_3$
Without TilS$_3$
Nº of genomes with TilS$_4$
Nº of genomes without TilS$_4$
With TilS$_4$
Without TilS$_4$
Nº of genomes with TilS$_5$
Nº of genomes without TilS$_5$
With TilS$_5$
Without TilS$_5$
Nº of genomes with TilS$_6$
Nº of genomes without TilS$_6$
With TilS$_6$
Without TilS$_6$
Nº of genomes with TilS$_7$
Nº of genomes without TilS$_7$
With TilS$_7$
Without TilS$_7$
Nº of genomes with TilS$_8$
Nº of genomes without TilS$_8$
With TilS$_8$
Without TilS$_8$
Nº of genomes with TilS$_9$
Nº of genomes without TilS$_9$
With TilS$_9$
Without TilS$_9$
Nº of genomes with TilS$_{10}$
Nº of genomes without TilS$_{10}$
With TilS$_{10}$
Without TilS$_{10}$
Nº of genomes with TilS$_{11}$
Nº of genomes without TilS$_{11}$
With TilS$_{11}$
Without TilS$_{11}$
Nº of genomes with TilS$_{12}$
Nº of genomes without TilS$_{12}$
With TilS$_{12}$
Without TilS$_{12}$
Nº of genomes with TilS$_{13}$
Nº of genomes without TilS$_{13}$
With TilS$_{13}$
Without TilS$_{13}$
Nº of genomes with TilS$_{14}$
Nº of genomes without TilS$_{14}$
With TilS$_{14}$
Without TilS$_{14}$
Nº of genomes with TilS$_{15}$
Nº of genomes without TilS$_{15}$
With TilS$_{15}$
Without TilS$_{15}$

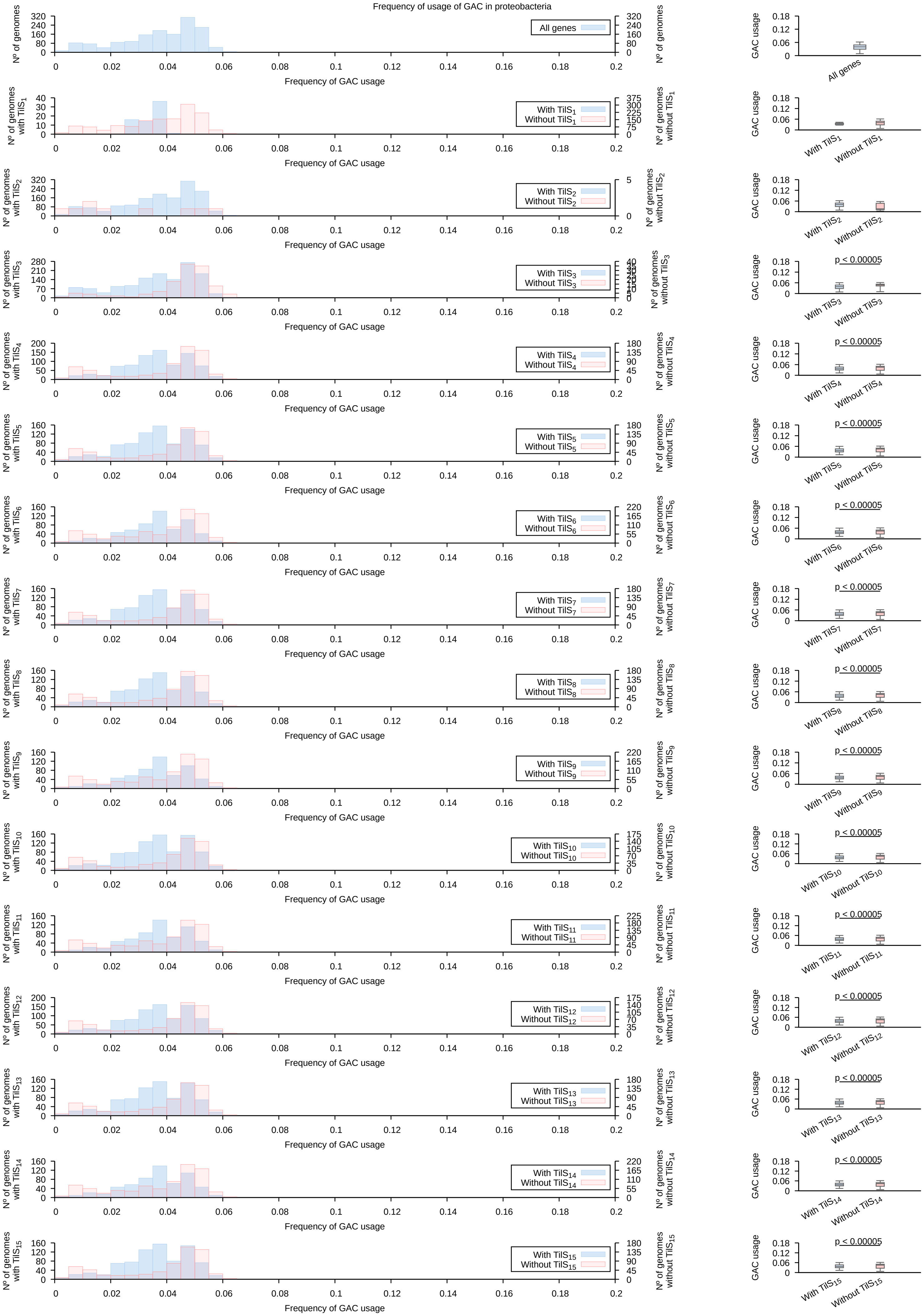

Frequency of usage of GAC in proteobacteria
Nº of genomes
All genes
Frequency of GAC usage
GAC usage
0
0.02
0.04
0.06
0.08
0.1
0.12
0.14
0.16
0.18
0.2
Nº of genomes with TilS$_1$
Nº of genomes without TilS$_1$
With TilS$_1$
Without TilS$_1$
Nº of genomes with TilS$_2$
Nº of genomes without TilS$_2$
With TilS$_2$
Without TilS$_2$
Nº of genomes with TilS$_3$
Nº of genomes without TilS$_3$
With TilS$_3$
Without TilS$_3$
p < 0.00005
Nº of genomes with TilS$_4$
Nº of genomes without TilS$_4$
With TilS$_4$
Without TilS$_4$
p < 0.00005
Nº of genomes with TilS$_5$
Nº of genomes without TilS$_5$
With TilS$_5$
Without TilS$_5$
p < 0.00005
Nº of genomes with TilS$_6$
Nº of genomes without TilS$_6$
With TilS$_6$
Without TilS$_6$
p < 0.00005
Nº of genomes with TilS$_7$
Nº of genomes without TilS$_7$
With TilS$_7$
Without TilS$_7$
p < 0.00005
Nº of genomes with TilS$_8$
Nº of genomes without TilS$_8$
With TilS$_8$
Without TilS$_8$
p < 0.00005
Nº of genomes with TilS$_9$
Nº of genomes without TilS$_9$
With TilS$_9$
Without TilS$_9$
p < 0.00005
Nº of genomes with TilS$_{10}$
Nº of genomes without TilS$_{10}$
With TilS$_{10}$
Without TilS$_{10}$
p < 0.00005
Nº of genomes with TilS$_{11}$
Nº of genomes without TilS$_{11}$
With TilS$_{11}$
Without TilS$_{11}$
p < 0.00005
Nº of genomes with TilS$_{12}$
Nº of genomes without TilS$_{12}$
With TilS$_{12}$
Without TilS$_{12}$
p < 0.00005
Nº of genomes with TilS$_{13}$
Nº of genomes without TilS$_{13}$
With TilS$_{13}$
Without TilS$_{13}$
p < 0.00005
Nº of genomes with TilS$_{14}$
Nº of genomes without TilS$_{14}$
With TilS$_{14}$
Without TilS$_{14}$
p < 0.00005
Nº of genomes with TilS$_{15}$
Nº of genomes without TilS$_{15}$
With TilS$_{15}$
Without TilS$_{15}$
p < 0.00005

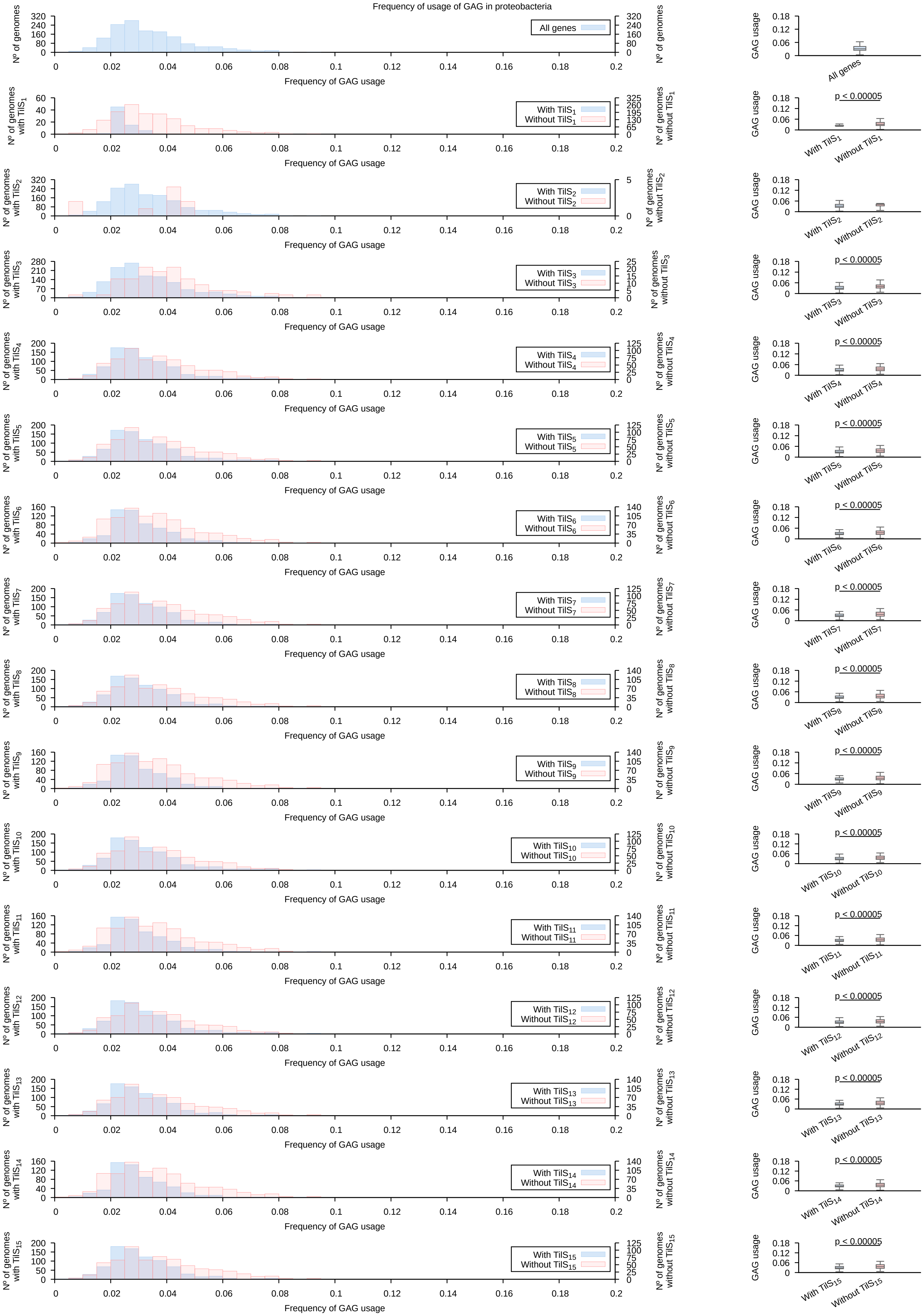
Frequency of usage of GAG in proteobacteria
Nº of genomes
All genes
Frequency of GAG usage
GAG usage
Nº of genomes with TilS$_1$
Nº of genomes without TilS$_1$
With TilS$_1$
Without TilS$_1$
p < 0.00005
With TilS$_2$
Without TilS$_2$
With TilS$_3$
Without TilS$_3$
With TilS$_4$
Without TilS$_4$
With TilS$_5$
Without TilS$_5$
With TilS$_6$
Without TilS$_6$
With TilS$_7$
Without TilS$_7$
With TilS$_8$
Without TilS$_8$
With TilS$_9$
Without TilS$_9$
With TilS$_{10}$
Without TilS$_{10}$
With TilS$_{11}$
Without TilS$_{11}$
With TilS$_{12}$
Without TilS$_{12}$
With TilS$_{13}$
Without TilS$_{13}$
With TilS$_{14}$
Without TilS$_{14}$
With TilS$_{15}$
Without TilS$_{15}$

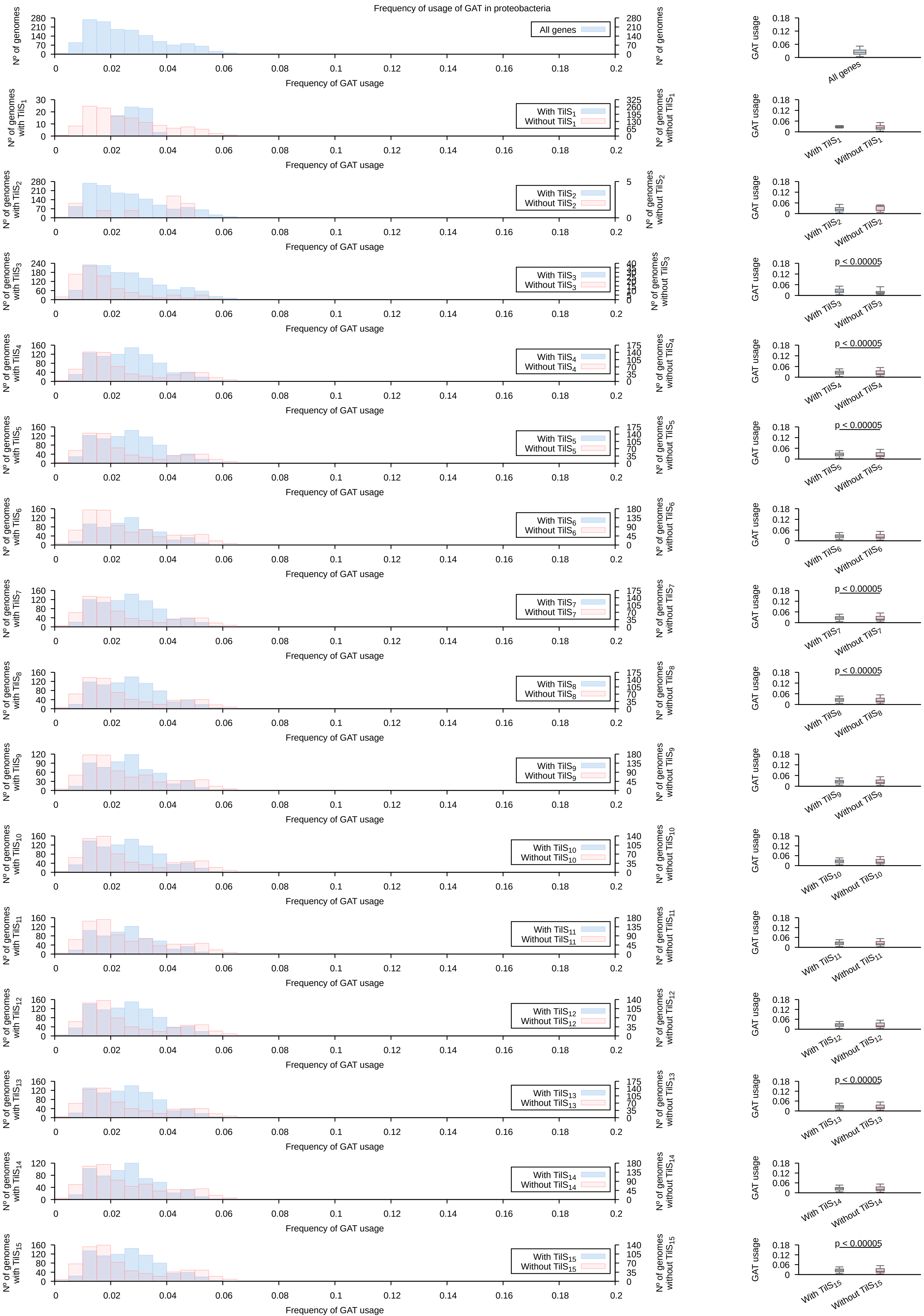

Frequency of usage of GAT in proteobacteria
Nº of genomes
All genes
GAT usage
Frequency of GAT usage
Nº of genomes with TilS$_1$
Nº of genomes without TilS$_1$
With TilS$_1$
Without TilS$_1$
Nº of genomes with TilS$_2$
Nº of genomes without TilS$_2$
With TilS$_2$
Without TilS$_2$
Nº of genomes with TilS$_3$
Nº of genomes without TilS$_3$
With TilS$_3$
Without TilS$_3$
p < 0.00005
Nº of genomes with TilS$_4$
Nº of genomes without TilS$_4$
With TilS$_4$
Without TilS$_4$
p < 0.00005
Nº of genomes with TilS$_5$
Nº of genomes without TilS$_5$
With TilS$_5$
Without TilS$_5$
p < 0.00005
Nº of genomes with TilS$_6$
Nº of genomes without TilS$_6$
With TilS$_6$
Without TilS$_6$
Nº of genomes with TilS$_7$
Nº of genomes without TilS$_7$
With TilS$_7$
Without TilS$_7$
p < 0.00005
Nº of genomes with TilS$_8$
Nº of genomes without TilS$_8$
With TilS$_8$
Without TilS$_8$
p < 0.00005
Nº of genomes with TilS$_9$
Nº of genomes without TilS$_9$
With TilS$_9$
Without TilS$_9$
Nº of genomes with TilS$_{10}$
Nº of genomes without TilS$_{10}$
With TilS$_{10}$
Without TilS$_{10}$
Nº of genomes with TilS$_{11}$
Nº of genomes without TilS$_{11}$
With TilS$_{11}$
Without TilS$_{11}$
Nº of genomes with TilS$_{12}$
Nº of genomes without TilS$_{12}$
With TilS$_{12}$
Without TilS$_{12}$
Nº of genomes with TilS$_{13}$
Nº of genomes without TilS$_{13}$
With TilS$_{13}$
Without TilS$_{13}$
p < 0.00005
Nº of genomes with TilS$_{14}$
Nº of genomes without TilS$_{14}$
With TilS$_{14}$
Without TilS$_{14}$
Nº of genomes with TilS$_{15}$
Nº of genomes without TilS$_{15}$
With TilS$_{15}$
Without TilS$_{15}$
p < 0.00005

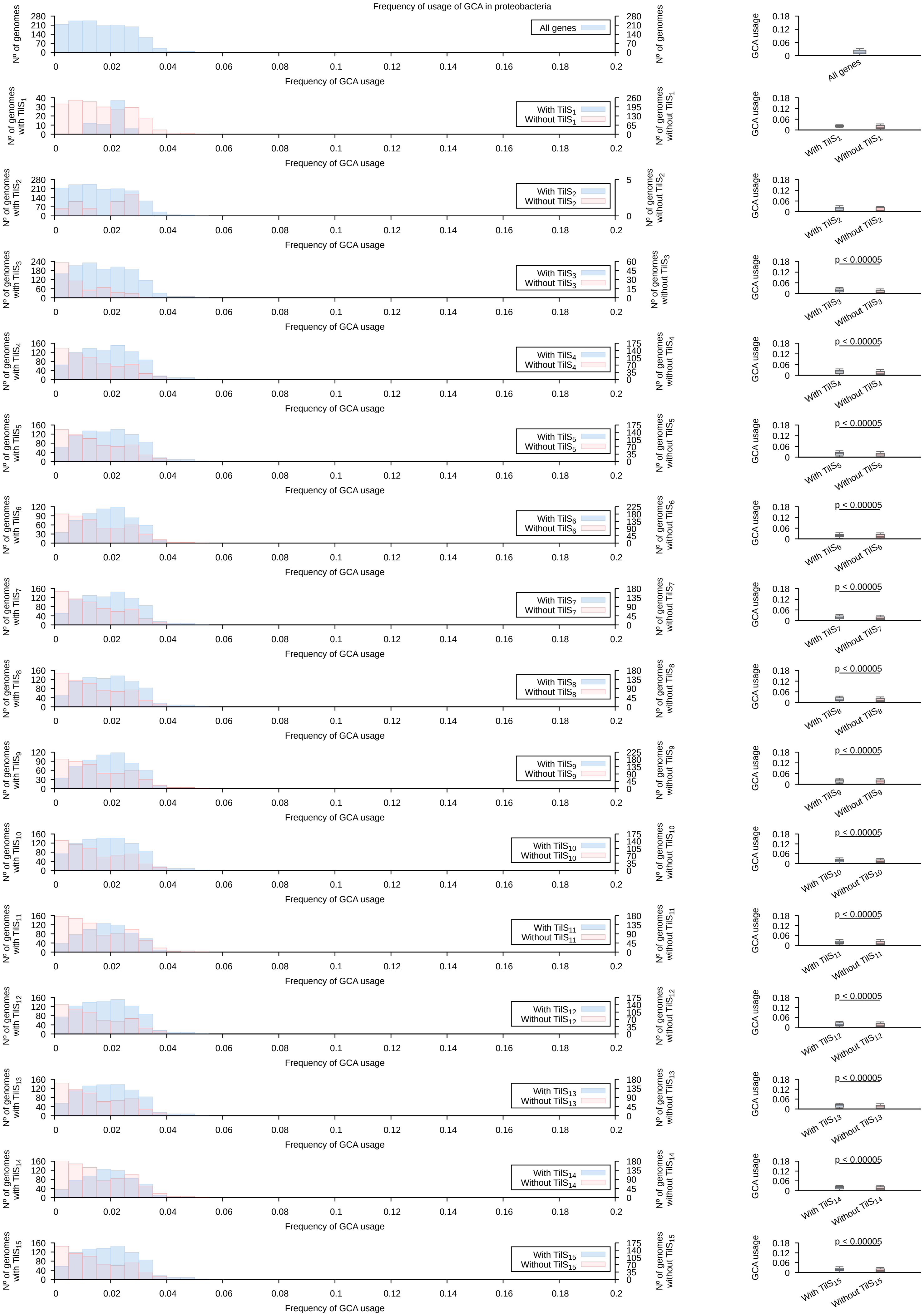
Frequency of usage of GCA in proteobacteria
Nº of genomes
All genes
Frequency of GCA usage
GCA usage
Nº of genomes with TilS$_1$
Nº of genomes without TilS$_1$
With TilS$_1$
Without TilS$_1$
Nº of genomes with TilS$_2$
Nº of genomes without TilS$_2$
With TilS$_2$
Without TilS$_2$
Nº of genomes with TilS$_3$
Nº of genomes without TilS$_3$
With TilS$_3$
Without TilS$_3$
p < 0.00005
Nº of genomes with TilS$_4$
Nº of genomes without TilS$_4$
With TilS$_4$
Without TilS$_4$
p < 0.00005
Nº of genomes with TilS$_5$
Nº of genomes without TilS$_5$
With TilS$_5$
Without TilS$_5$
p < 0.00005
Nº of genomes with TilS$_6$
Nº of genomes without TilS$_6$
With TilS$_6$
Without TilS$_6$
p < 0.00005
Nº of genomes with TilS$_7$
Nº of genomes without TilS$_7$
With TilS$_7$
Without TilS$_7$
p < 0.00005
Nº of genomes with TilS$_8$
Nº of genomes without TilS$_8$
With TilS$_8$
Without TilS$_8$
p < 0.00005
Nº of genomes with TilS$_9$
Nº of genomes without TilS$_9$
With TilS$_9$
Without TilS$_9$
p < 0.00005
Nº of genomes with TilS$_{10}$
Nº of genomes without TilS$_{10}$
With TilS$_{10}$
Without TilS$_{10}$
p < 0.00005
Nº of genomes with TilS$_{11}$
Nº of genomes without TilS$_{11}$
With TilS$_{11}$
Without TilS$_{11}$
p < 0.00005
Nº of genomes with TilS$_{12}$
Nº of genomes without TilS$_{12}$
With TilS$_{12}$
Without TilS$_{12}$
p < 0.00005
Nº of genomes with TilS$_{13}$
Nº of genomes without TilS$_{13}$
With TilS$_{13}$
Without TilS$_{13}$
p < 0.00005
Nº of genomes with TilS$_{14}$
Nº of genomes without TilS$_{14}$
With TilS$_{14}$
Without TilS$_{14}$
p < 0.00005
Nº of genomes with TilS$_{15}$
Nº of genomes without TilS$_{15}$
With TilS$_{15}$
Without TilS$_{15}$
p < 0.00005

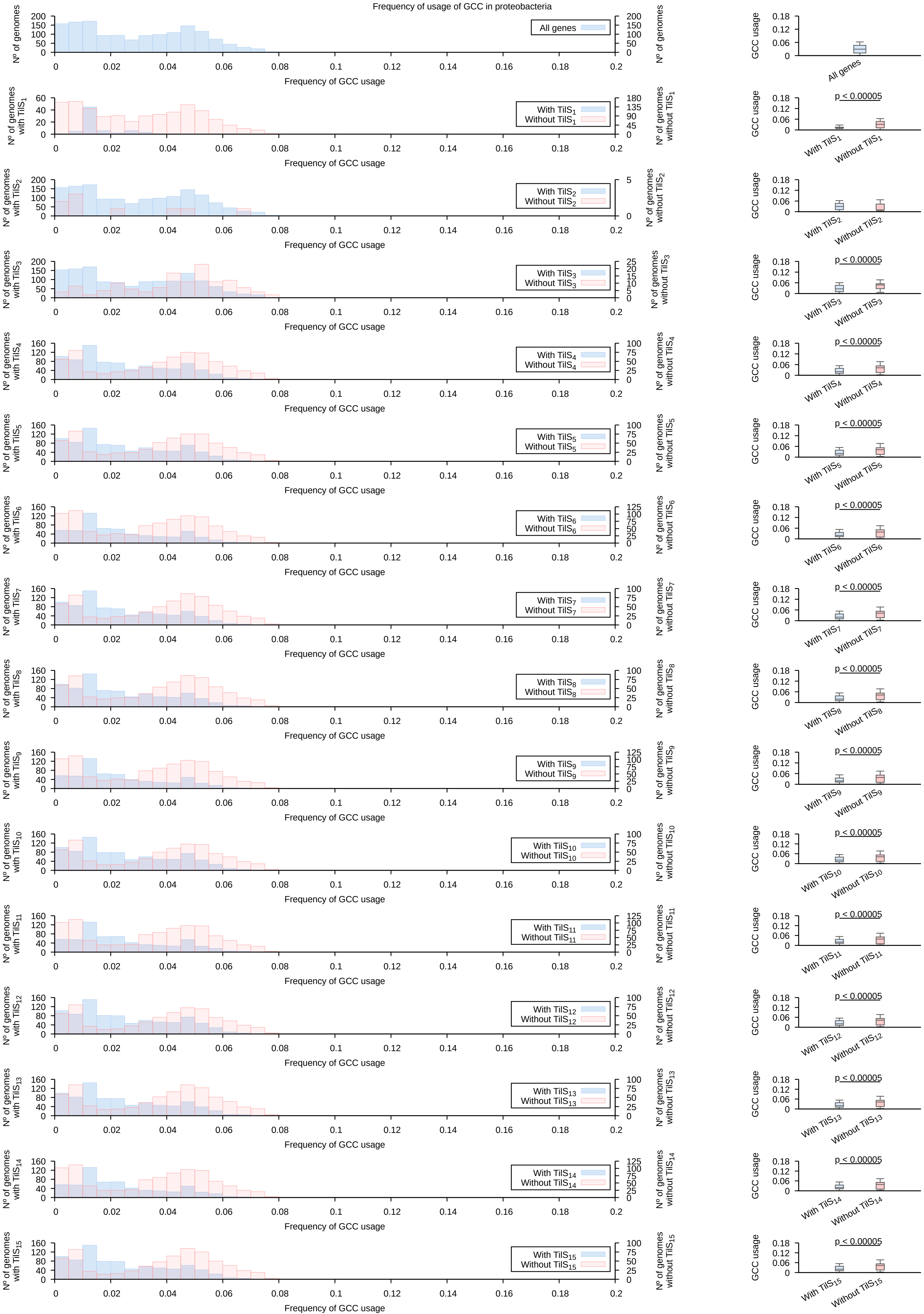

Frequency of usage of GCC in proteobacteria
Nº of genomes
All genes
Frequency of GCC usage
GCC usage
Nº of genomes with TilS$_1$
Nº of genomes without TilS$_1$
With TilS$_1$
Without TilS$_1$
p < 0.00005
Nº of genomes with TilS$_2$
Nº of genomes without TilS$_2$
With TilS$_2$
Without TilS$_2$
Nº of genomes with TilS$_3$
Nº of genomes without TilS$_3$
With TilS$_3$
Without TilS$_3$
p < 0.00005
Nº of genomes with TilS$_4$
Nº of genomes without TilS$_4$
With TilS$_4$
Without TilS$_4$
p < 0.00005
Nº of genomes with TilS$_5$
Nº of genomes without TilS$_5$
With TilS$_5$
Without TilS$_5$
p < 0.00005
Nº of genomes with TilS$_6$
Nº of genomes without TilS$_6$
With TilS$_6$
Without TilS$_6$
p < 0.00005
Nº of genomes with TilS$_7$
Nº of genomes without TilS$_7$
With TilS$_7$
Without TilS$_7$
p < 0.00005
Nº of genomes with TilS$_8$
Nº of genomes without TilS$_8$
With TilS$_8$
Without TilS$_8$
p < 0.00005
Nº of genomes with TilS$_9$
Nº of genomes without TilS$_9$
With TilS$_9$
Without TilS$_9$
p < 0.00005
Nº of genomes with TilS$_{10}$
Nº of genomes without TilS$_{10}$
With TilS$_{10}$
Without TilS$_{10}$
p < 0.00005
Nº of genomes with TilS$_{11}$
Nº of genomes without TilS$_{11}$
With TilS$_{11}$
Without TilS$_{11}$
p < 0.00005
Nº of genomes with TilS$_{12}$
Nº of genomes without TilS$_{12}$
With TilS$_{12}$
Without TilS$_{12}$
p < 0.00005
Nº of genomes with TilS$_{13}$
Nº of genomes without TilS$_{13}$
With TilS$_{13}$
Without TilS$_{13}$
p < 0.00005
Nº of genomes with TilS$_{14}$
Nº of genomes without TilS$_{14}$
With TilS$_{14}$
Without TilS$_{14}$
p < 0.00005
Nº of genomes with TilS$_{15}$
Nº of genomes without TilS$_{15}$
With TilS$_{15}$
Without TilS$_{15}$
p < 0.00005

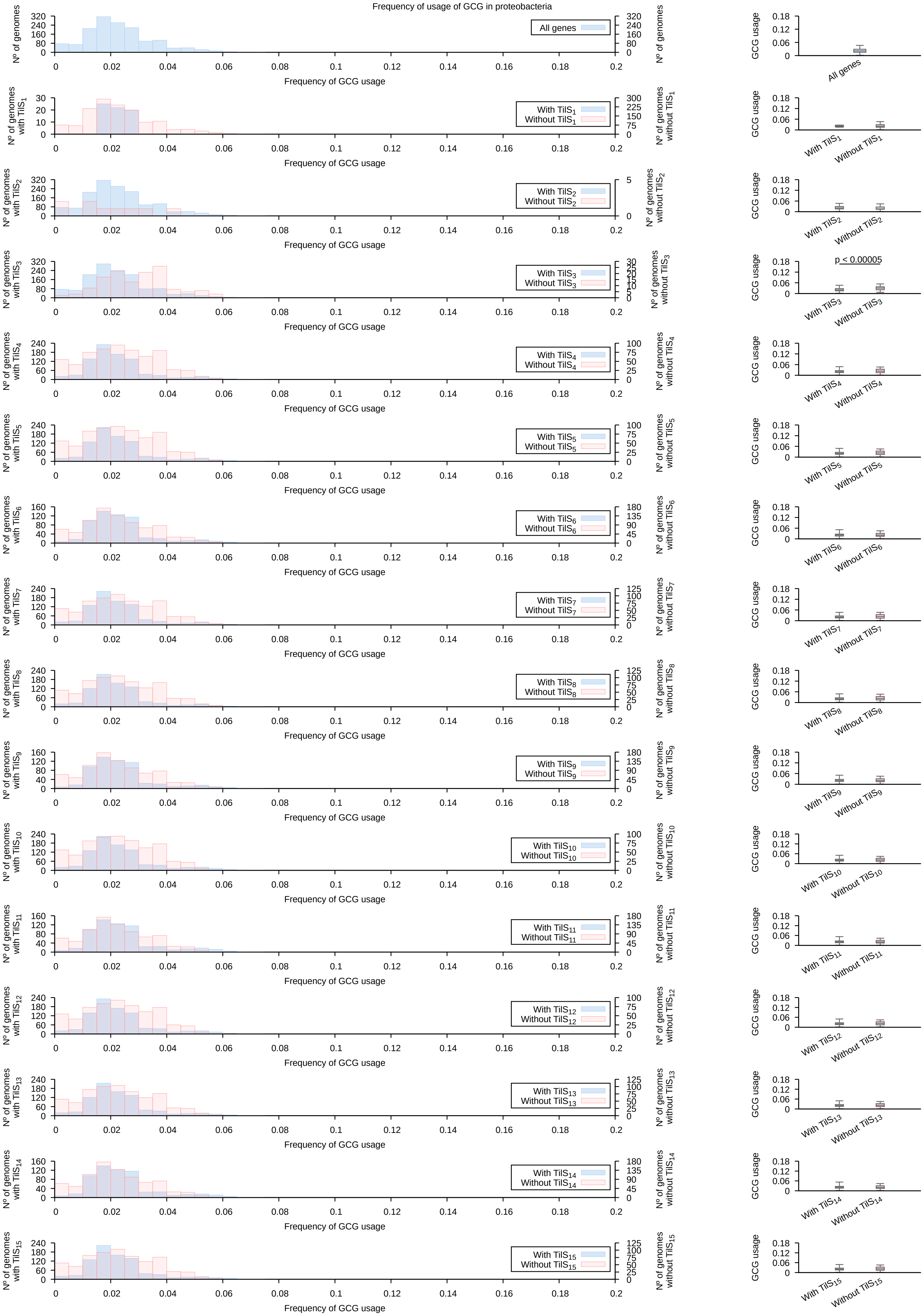
Frequency of usage of GCG in proteobacteria
Nº of genomes
All genes
Frequency of GCG usage
0 0.02 0.04 0.06 0.08 0.1 0.12 0.14 0.16 0.18 0.2
GCG usage
0 0.06 0.12 0.18
All genes
Nº of genomes with $TilS_1$
With $TilS_1$
Without $TilS_1$
Nº of genomes without $TilS_1$
Nº of genomes with $TilS_2$
With $TilS_2$
Without $TilS_2$
Nº of genomes without $TilS_2$
Nº of genomes with $TilS_3$
With $TilS_3$
Without $TilS_3$
Nº of genomes without $TilS_3$
p < 0.00005
Nº of genomes with $TilS_4$
With $TilS_4$
Without $TilS_4$
Nº of genomes without $TilS_4$
Nº of genomes with $TilS_5$
With $TilS_5$
Without $TilS_5$
Nº of genomes without $TilS_5$
Nº of genomes with $TilS_6$
With $TilS_6$
Without $TilS_6$
Nº of genomes without $TilS_6$
Nº of genomes with $TilS_7$
With $TilS_7$
Without $TilS_7$
Nº of genomes without $TilS_7$
Nº of genomes with $TilS_8$
With $TilS_8$
Without $TilS_8$
Nº of genomes without $TilS_8$
Nº of genomes with $TilS_9$
With $TilS_9$
Without $TilS_9$
Nº of genomes without $TilS_9$
Nº of genomes with $TilS_{10}$
With $TilS_{10}$
Without $TilS_{10}$
Nº of genomes without $TilS_{10}$
Nº of genomes with $TilS_{11}$
With $TilS_{11}$
Without $TilS_{11}$
Nº of genomes without $TilS_{11}$
Nº of genomes with $TilS_{12}$
With $TilS_{12}$
Without $TilS_{12}$
Nº of genomes without $TilS_{12}$
Nº of genomes with $TilS_{13}$
With $TilS_{13}$
Without $TilS_{13}$
Nº of genomes without $TilS_{13}$
Nº of genomes with $TilS_{14}$
With $TilS_{14}$
Without $TilS_{14}$
Nº of genomes without $TilS_{14}$
Nº of genomes with $TilS_{15}$
With $TilS_{15}$
Without $TilS_{15}$
Nº of genomes without $TilS_{15}$

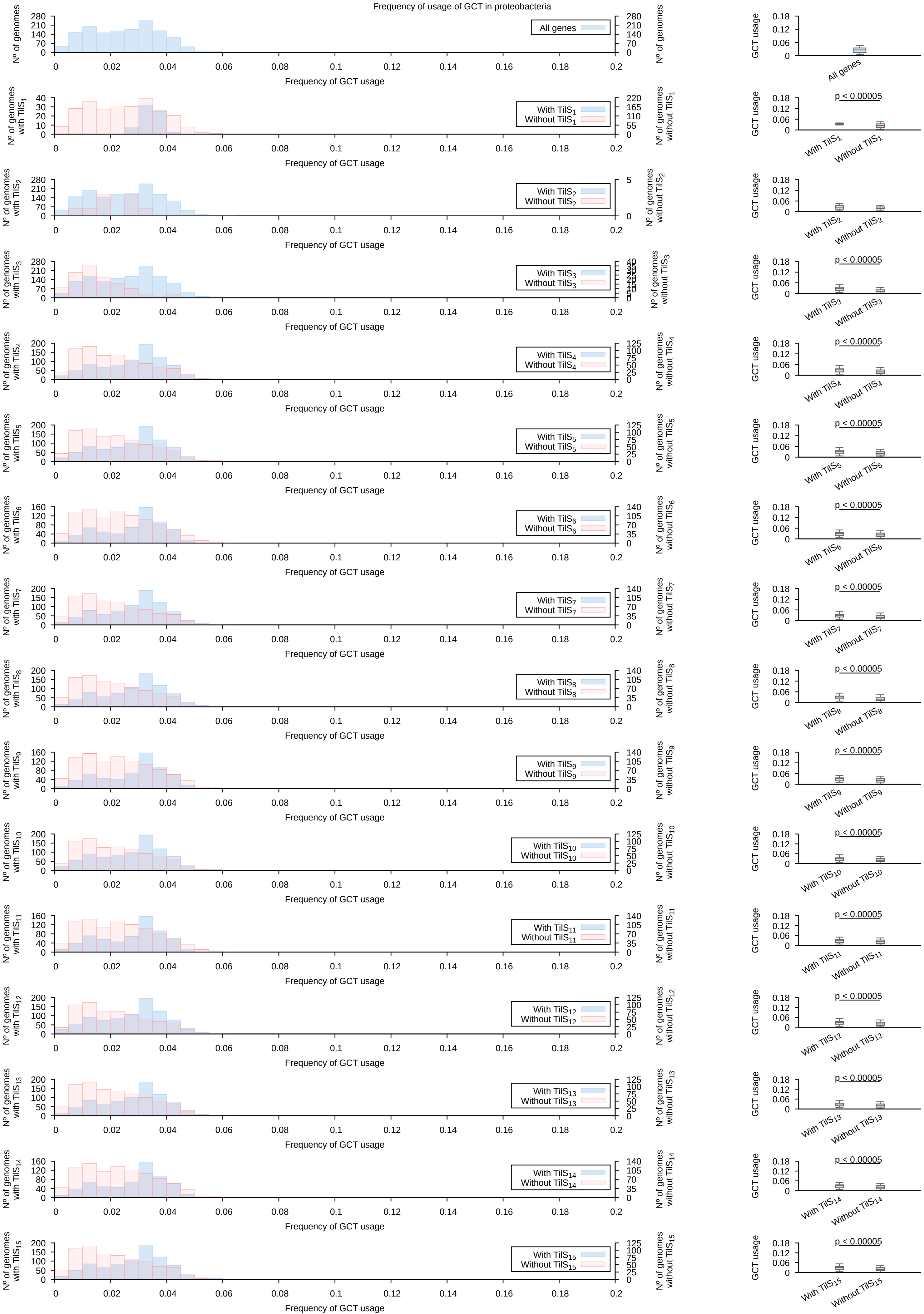

Frequency of usage of GCT in proteobacteria
Nº of genomes
All genes
Frequency of GCT usage
GCT usage
Nº of genomes with TilS$_1$
Nº of genomes without TilS$_1$
With TilS$_1$
Without TilS$_1$
p < 0.00005
Nº of genomes with TilS$_2$
Nº of genomes without TilS$_2$
With TilS$_2$
Without TilS$_2$
Nº of genomes with TilS$_3$
Nº of genomes without TilS$_3$
With TilS$_3$
Without TilS$_3$
Nº of genomes with TilS$_4$
Nº of genomes without TilS$_4$
With TilS$_4$
Without TilS$_4$
Nº of genomes with TilS$_5$
Nº of genomes without TilS$_5$
With TilS$_5$
Without TilS$_5$
Nº of genomes with TilS$_6$
Nº of genomes without TilS$_6$
With TilS$_6$
Without TilS$_6$
Nº of genomes with TilS$_7$
Nº of genomes without TilS$_7$
With TilS$_7$
Without TilS$_7$
Nº of genomes with TilS$_8$
Nº of genomes without TilS$_8$
With TilS$_8$
Without TilS$_8$
Nº of genomes with TilS$_9$
Nº of genomes without TilS$_9$
With TilS$_9$
Without TilS$_9$
Nº of genomes with TilS$_{10}$
Nº of genomes without TilS$_{10}$
With TilS$_{10}$
Without TilS$_{10}$
Nº of genomes with TilS$_{11}$
Nº of genomes without TilS$_{11}$
With TilS$_{11}$
Without TilS$_{11}$
Nº of genomes with TilS$_{12}$
Nº of genomes without TilS$_{12}$
With TilS$_{12}$
Without TilS$_{12}$
Nº of genomes with TilS$_{13}$
Nº of genomes without TilS$_{13}$
With TilS$_{13}$
Without TilS$_{13}$
Nº of genomes with TilS$_{14}$
Nº of genomes without TilS$_{14}$
With TilS$_{14}$
Without TilS$_{14}$
Nº of genomes with TilS$_{15}$
Nº of genomes without TilS$_{15}$
With TilS$_{15}$
Without TilS$_{15}$

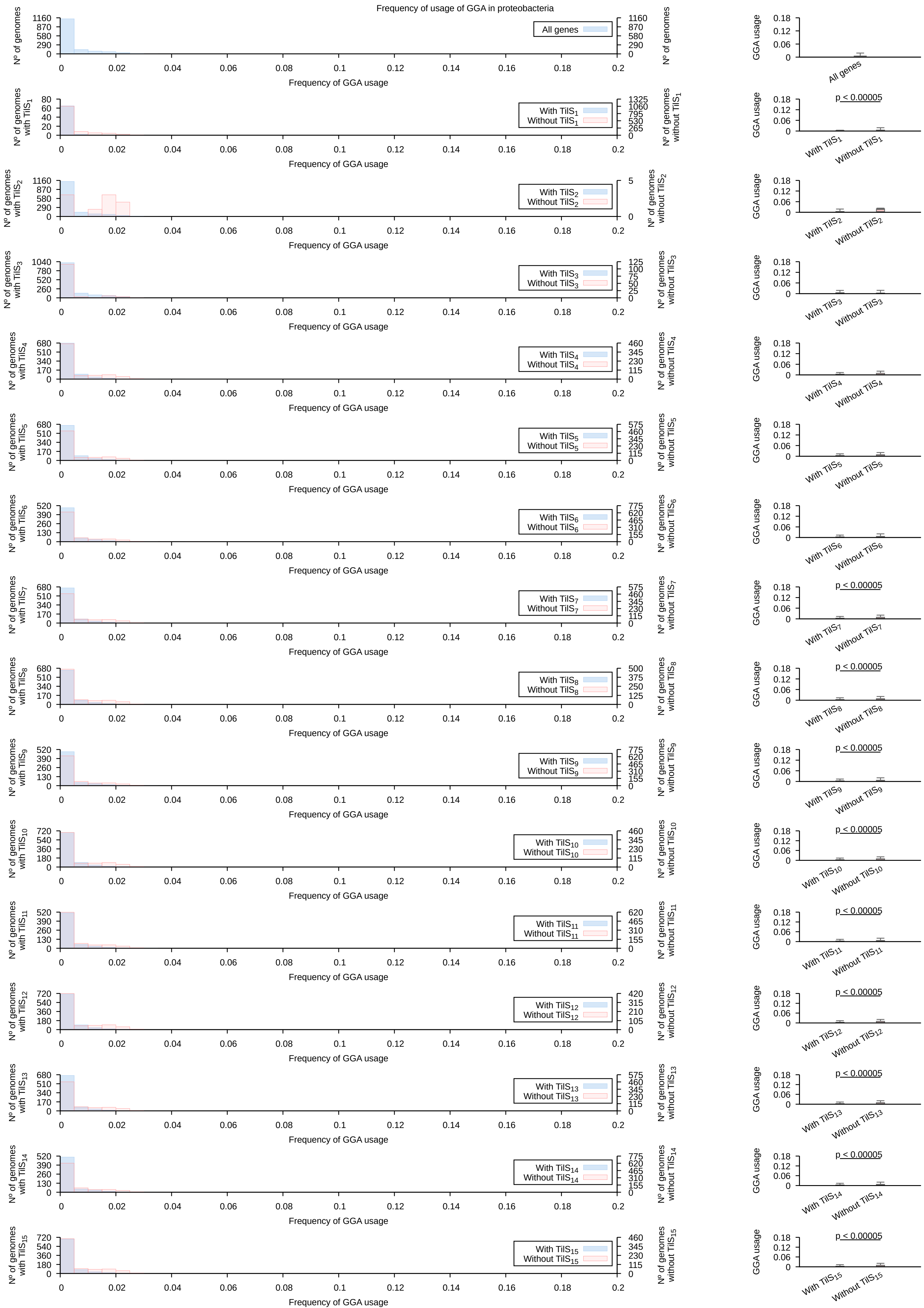

Frequency of usage of GGA in proteobacteria
Nº of genomes
All genes
Frequency of GGA usage
GGA usage
Nº of genomes with TilS$_1$
Nº of genomes without TilS$_1$
With TilS$_1$
Without TilS$_1$
p < 0.00005
Nº of genomes with TilS$_2$
Nº of genomes without TilS$_2$
With TilS$_2$
Without TilS$_2$
Nº of genomes with TilS$_3$
Nº of genomes without TilS$_3$
With TilS$_3$
Without TilS$_3$
Nº of genomes with TilS$_4$
Nº of genomes without TilS$_4$
With TilS$_4$
Without TilS$_4$
Nº of genomes with TilS$_5$
Nº of genomes without TilS$_5$
With TilS$_5$
Without TilS$_5$
Nº of genomes with TilS$_6$
Nº of genomes without TilS$_6$
With TilS$_6$
Without TilS$_6$
Nº of genomes with TilS$_7$
Nº of genomes without TilS$_7$
With TilS$_7$
Without TilS$_7$
p < 0.00005
Nº of genomes with TilS$_8$
Nº of genomes without TilS$_8$
With TilS$_8$
Without TilS$_8$
p < 0.00005
Nº of genomes with TilS$_9$
Nº of genomes without TilS$_9$
With TilS$_9$
Without TilS$_9$
p < 0.00005
Nº of genomes with TilS$_{10}$
Nº of genomes without TilS$_{10}$
With TilS$_{10}$
Without TilS$_{10}$
p < 0.00005
Nº of genomes with TilS$_{11}$
Nº of genomes without TilS$_{11}$
With TilS$_{11}$
Without TilS$_{11}$
p < 0.00005
Nº of genomes with TilS$_{12}$
Nº of genomes without TilS$_{12}$
With TilS$_{12}$
Without TilS$_{12}$
p < 0.00005
Nº of genomes with TilS$_{13}$
Nº of genomes without TilS$_{13}$
With TilS$_{13}$
Without TilS$_{13}$
p < 0.00005
Nº of genomes with TilS$_{14}$
Nº of genomes without TilS$_{14}$
With TilS$_{14}$
Without TilS$_{14}$
p < 0.00005
Nº of genomes with TilS$_{15}$
Nº of genomes without TilS$_{15}$
With TilS$_{15}$
Without TilS$_{15}$
p < 0.00005

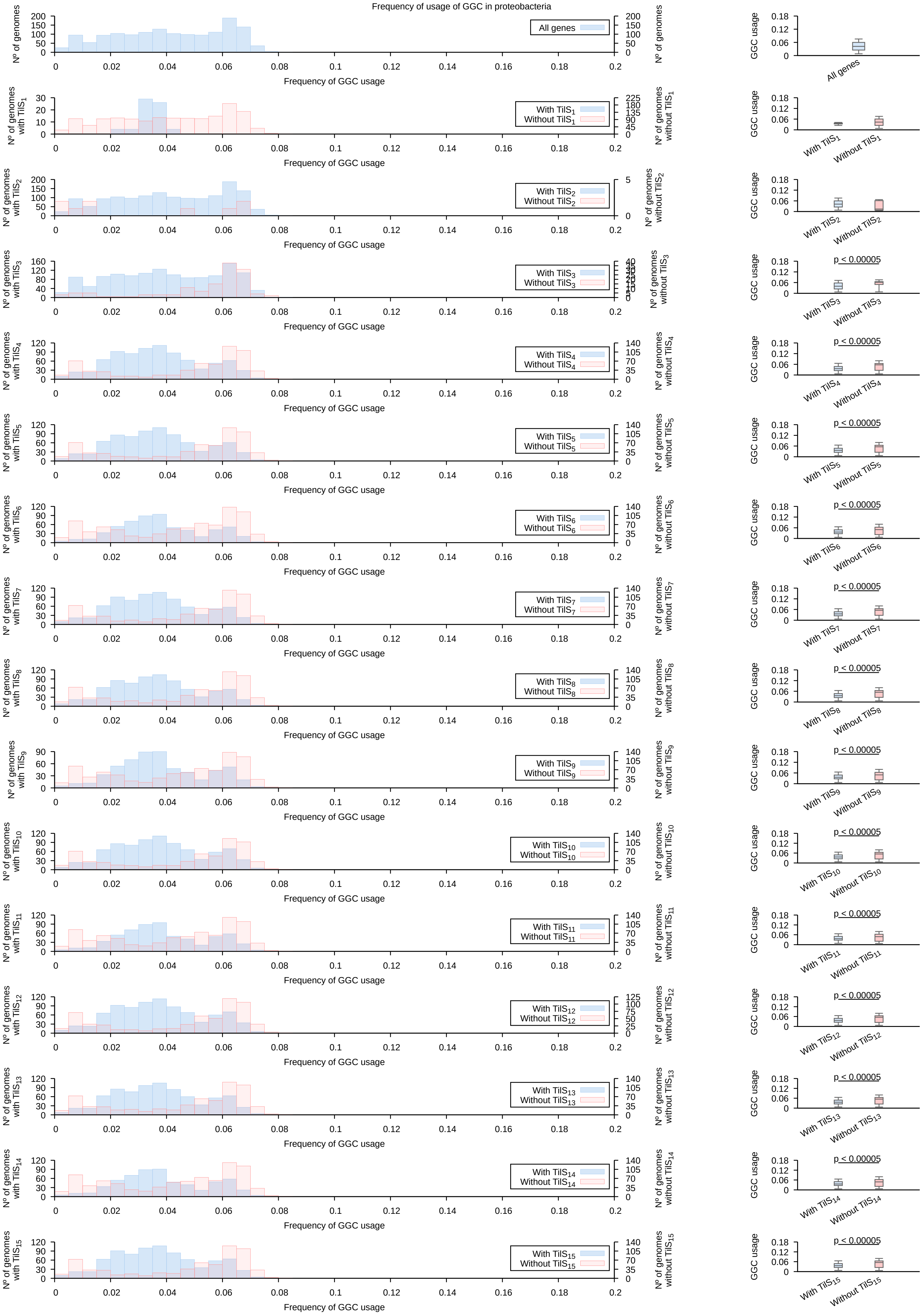

Frequency of usage of GGC in proteobacteria
Nº of genomes
All genes
GGC usage
Frequency of GGC usage
Nº of genomes with TilS$_1$
Nº of genomes without TilS$_1$
With TilS$_1$
Without TilS$_1$
Nº of genomes with TilS$_2$
Nº of genomes without TilS$_2$
With TilS$_2$
Without TilS$_2$
Nº of genomes with TilS$_3$
Nº of genomes without TilS$_3$
With TilS$_3$
Without TilS$_3$
p < 0.00005
Nº of genomes with TilS$_4$
Nº of genomes without TilS$_4$
With TilS$_4$
Without TilS$_4$
p < 0.00005
Nº of genomes with TilS$_5$
Nº of genomes without TilS$_5$
With TilS$_5$
Without TilS$_5$
p < 0.00005
Nº of genomes with TilS$_6$
Nº of genomes without TilS$_6$
With TilS$_6$
Without TilS$_6$
p < 0.00005
Nº of genomes with TilS$_7$
Nº of genomes without TilS$_7$
With TilS$_7$
Without TilS$_7$
p < 0.00005
Nº of genomes with TilS$_8$
Nº of genomes without TilS$_8$
With TilS$_8$
Without TilS$_8$
p < 0.00005
Nº of genomes with TilS$_9$
Nº of genomes without TilS$_9$
With TilS$_9$
Without TilS$_9$
p < 0.00005
Nº of genomes with TilS$_{10}$
Nº of genomes without TilS$_{10}$
With TilS$_{10}$
Without TilS$_{10}$
p < 0.00005
Nº of genomes with TilS$_{11}$
Nº of genomes without TilS$_{11}$
With TilS$_{11}$
Without TilS$_{11}$
p < 0.00005
Nº of genomes with TilS$_{12}$
Nº of genomes without TilS$_{12}$
With TilS$_{12}$
Without TilS$_{12}$
p < 0.00005
Nº of genomes with TilS$_{13}$
Nº of genomes without TilS$_{13}$
With TilS$_{13}$
Without TilS$_{13}$
p < 0.00005
Nº of genomes with TilS$_{14}$
Nº of genomes without TilS$_{14}$
With TilS$_{14}$
Without TilS$_{14}$
p < 0.00005
Nº of genomes with TilS$_{15}$
Nº of genomes without TilS$_{15}$
With TilS$_{15}$
Without TilS$_{15}$
p < 0.00005

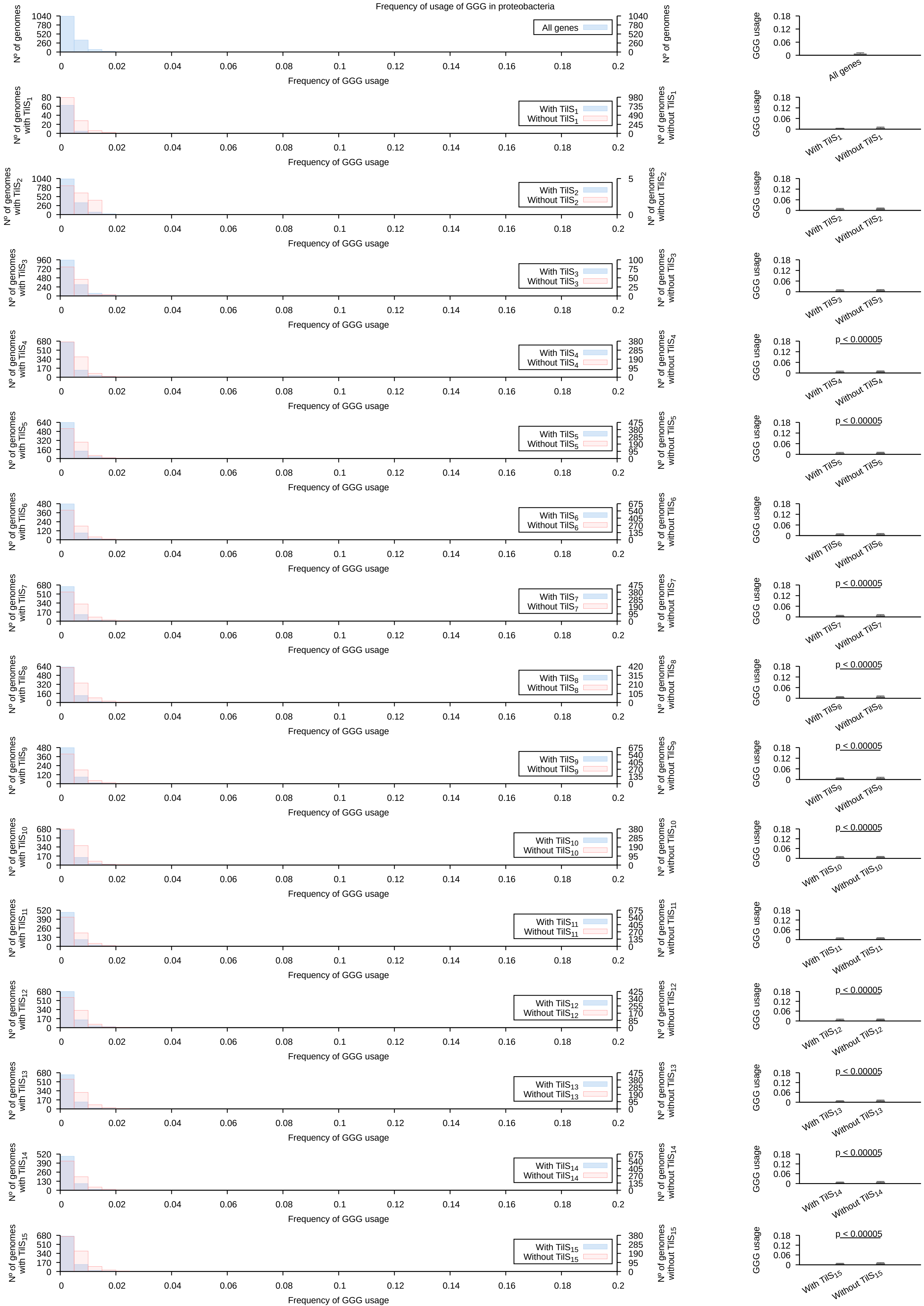
Frequency of usage of GGG in proteobacteria
Nº of genomes
All genes
Frequency of GGG usage
GGG usage
Nº of genomes with TilS$_1$
Nº of genomes without TilS$_1$
With TilS$_1$
Without TilS$_1$
Nº of genomes with TilS$_2$
Nº of genomes without TilS$_2$
With TilS$_2$
Without TilS$_2$
Nº of genomes with TilS$_3$
Nº of genomes without TilS$_3$
With TilS$_3$
Without TilS$_3$
Nº of genomes with TilS$_4$
Nº of genomes without TilS$_4$
With TilS$_4$
Without TilS$_4$
p < 0.00005
Nº of genomes with TilS$_5$
Nº of genomes without TilS$_5$
With TilS$_5$
Without TilS$_5$
p < 0.00005
Nº of genomes with TilS$_6$
Nº of genomes without TilS$_6$
With TilS$_6$
Without TilS$_6$
Nº of genomes with TilS$_7$
Nº of genomes without TilS$_7$
With TilS$_7$
Without TilS$_7$
p < 0.00005
Nº of genomes with TilS$_8$
Nº of genomes without TilS$_8$
With TilS$_8$
Without TilS$_8$
p < 0.00005
Nº of genomes with TilS$_9$
Nº of genomes without TilS$_9$
With TilS$_9$
Without TilS$_9$
p < 0.00005
Nº of genomes with TilS$_{10}$
Nº of genomes without TilS$_{10}$
With TilS$_{10}$
Without TilS$_{10}$
p < 0.00005
Nº of genomes with TilS$_{11}$
Nº of genomes without TilS$_{11}$
With TilS$_{11}$
Without TilS$_{11}$
Nº of genomes with TilS$_{12}$
Nº of genomes without TilS$_{12}$
With TilS$_{12}$
Without TilS$_{12}$
p < 0.00005
Nº of genomes with TilS$_{13}$
Nº of genomes without TilS$_{13}$
With TilS$_{13}$
Without TilS$_{13}$
p < 0.00005
Nº of genomes with TilS$_{14}$
Nº of genomes without TilS$_{14}$
With TilS$_{14}$
Without TilS$_{14}$
p < 0.00005
Nº of genomes with TilS$_{15}$
Nº of genomes without TilS$_{15}$
With TilS$_{15}$
Without TilS$_{15}$
p < 0.00005

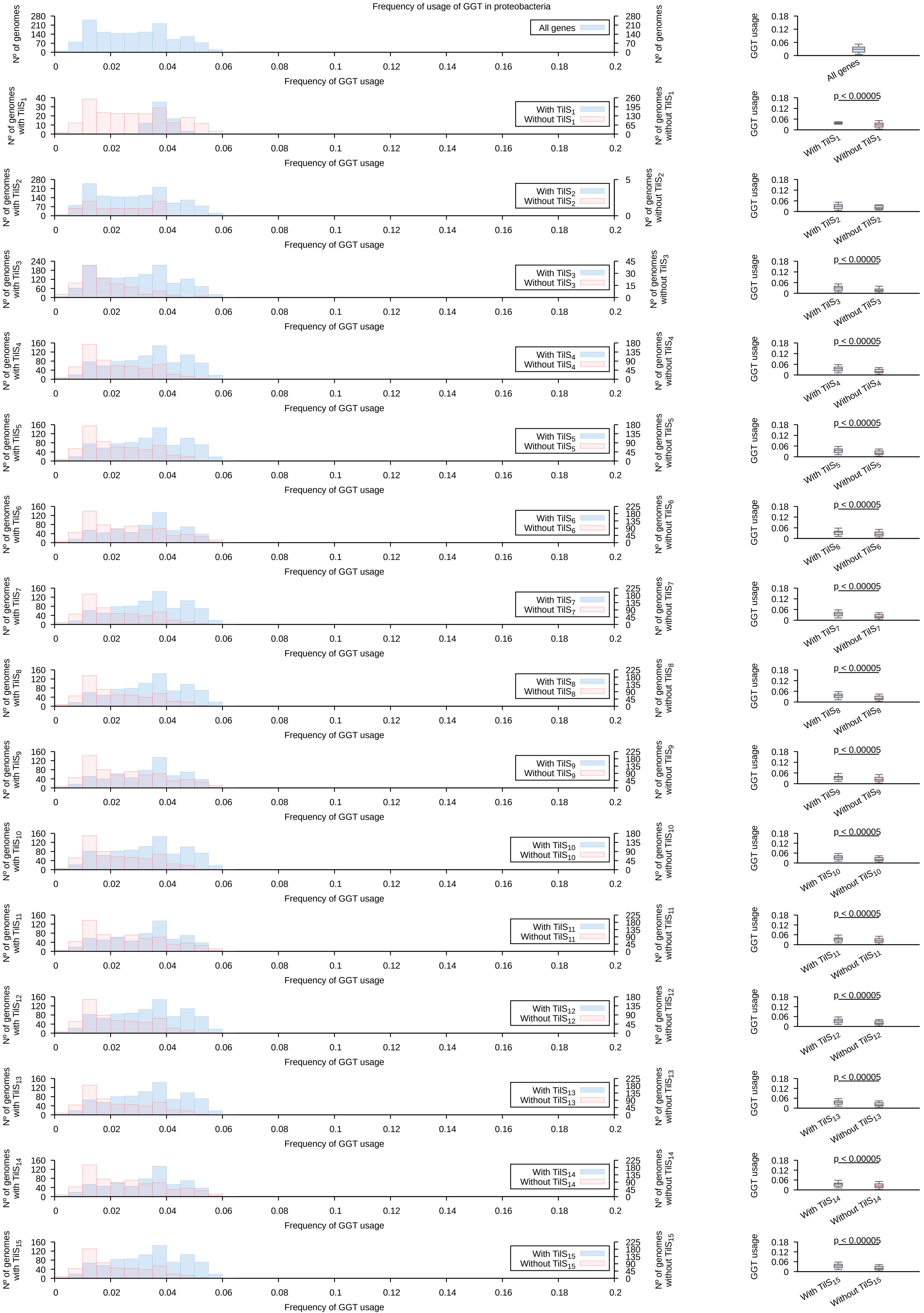

Frequency of usage of GGT in proteobacteria
Nº of genomes
All genes
Frequency of GGT usage
GGT usage
0.18 0.12 0.06 0
0 0.02 0.04 0.06 0.08 0.1 0.12 0.14 0.16 0.18 0.2
Nº of genomes with TilS$_1$
Nº of genomes without TilS$_1$
With TilS$_1$
Without TilS$_1$
p < 0.00005
Nº of genomes with TilS$_2$
Nº of genomes without TilS$_2$
With TilS$_2$
Without TilS$_2$
Nº of genomes with TilS$_3$
Nº of genomes without TilS$_3$
With TilS$_3$
Without TilS$_3$
p < 0.00005
Nº of genomes with TilS$_4$
Nº of genomes without TilS$_4$
With TilS$_4$
Without TilS$_4$
p < 0.00005
Nº of genomes with TilS$_5$
Nº of genomes without TilS$_5$
With TilS$_5$
Without TilS$_5$
p < 0.00005
Nº of genomes with TilS$_6$
Nº of genomes without TilS$_6$
With TilS$_6$
Without TilS$_6$
p < 0.00005
Nº of genomes with TilS$_7$
Nº of genomes without TilS$_7$
With TilS$_7$
Without TilS$_7$
p < 0.00005
Nº of genomes with TilS$_8$
Nº of genomes without TilS$_8$
With TilS$_8$
Without TilS$_8$
p < 0.00005
Nº of genomes with TilS$_9$
Nº of genomes without TilS$_9$
With TilS$_9$
Without TilS$_9$
p < 0.00005
Nº of genomes with TilS$_{10}$
Nº of genomes without TilS$_{10}$
With TilS$_{10}$
Without TilS$_{10}$
p < 0.00005
Nº of genomes with TilS$_{11}$
Nº of genomes without TilS$_{11}$
With TilS$_{11}$
Without TilS$_{11}$
p < 0.00005
Nº of genomes with TilS$_{12}$
Nº of genomes without TilS$_{12}$
With TilS$_{12}$
Without TilS$_{12}$
p < 0.00005
Nº of genomes with TilS$_{13}$
Nº of genomes without TilS$_{13}$
With TilS$_{13}$
Without TilS$_{13}$
p < 0.00005
Nº of genomes with TilS$_{14}$
Nº of genomes without TilS$_{14}$
With TilS$_{14}$
Without TilS$_{14}$
p < 0.00005
Nº of genomes with TilS$_{15}$
Nº of genomes without TilS$_{15}$
With TilS$_{15}$
Without TilS$_{15}$
p < 0.00005

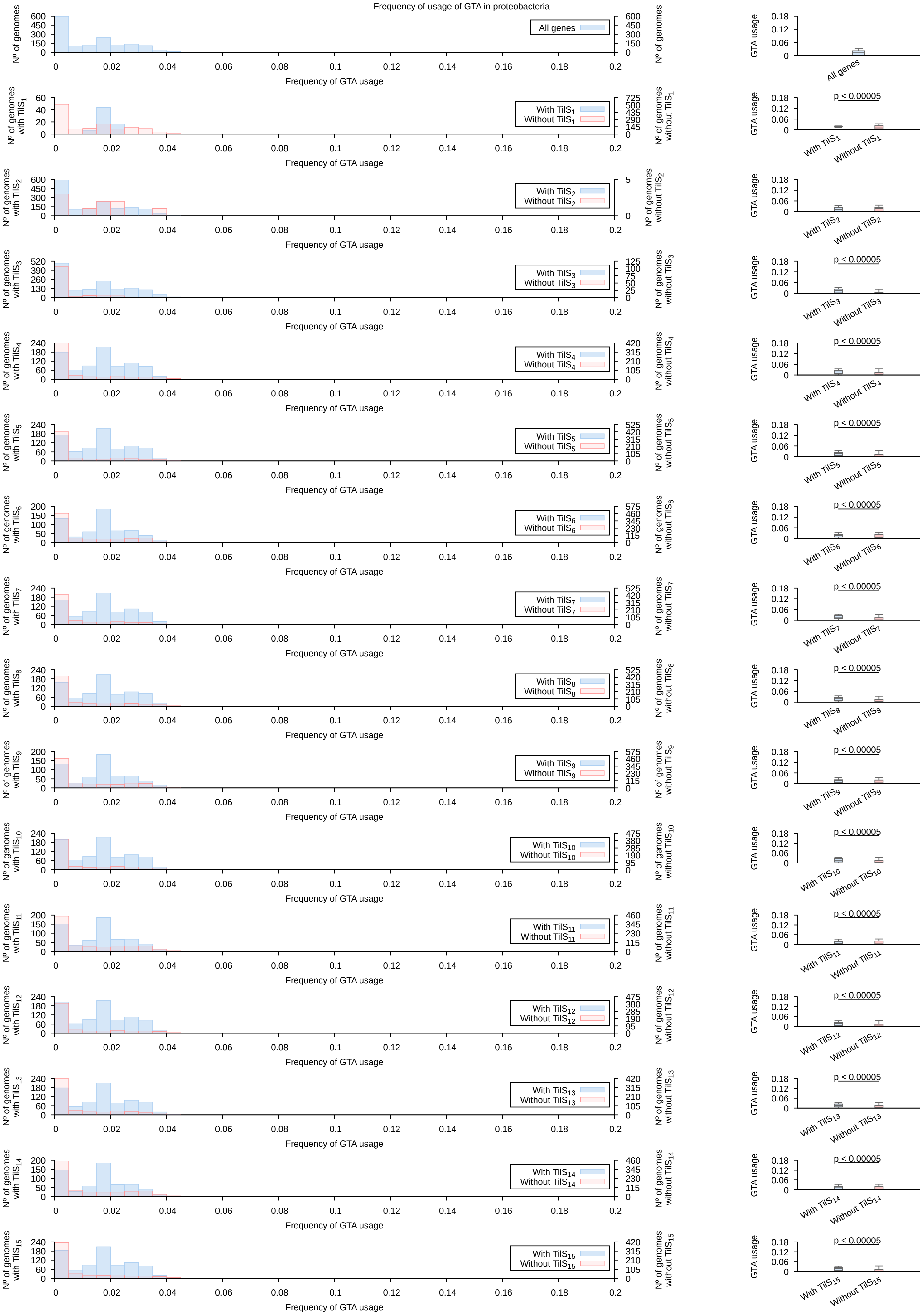

Frequency of usage of GTA in proteobacteria
Nº of genomes
Frequency of GTA usage
GTA usage
All genes
With $TilS_1$
Without $TilS_1$
Nº of genomes with $TilS_1$
Nº of genomes without $TilS_1$
$p < 0.00005$
With $TilS_2$
Without $TilS_2$
Nº of genomes with $TilS_2$
Nº of genomes without $TilS_2$
With $TilS_3$
Without $TilS_3$
Nº of genomes with $TilS_3$
Nº of genomes without $TilS_3$
$p < 0.00005$
With $TilS_4$
Without $TilS_4$
Nº of genomes with $TilS_4$
Nº of genomes without $TilS_4$
$p < 0.00005$
With $TilS_5$
Without $TilS_5$
Nº of genomes with $TilS_5$
Nº of genomes without $TilS_5$
$p < 0.00005$
With $TilS_6$
Without $TilS_6$
Nº of genomes with $TilS_6$
Nº of genomes without $TilS_6$
$p < 0.00005$
With $TilS_7$
Without $TilS_7$
Nº of genomes with $TilS_7$
Nº of genomes without $TilS_7$
$p < 0.00005$
With $TilS_8$
Without $TilS_8$
Nº of genomes with $TilS_8$
Nº of genomes without $TilS_8$
$p < 0.00005$
With $TilS_9$
Without $TilS_9$
Nº of genomes with $TilS_9$
Nº of genomes without $TilS_9$
$p < 0.00005$
With $TilS_{10}$
Without $TilS_{10}$
Nº of genomes with $TilS_{10}$
Nº of genomes without $TilS_{10}$
$p < 0.00005$
With $TilS_{11}$
Without $TilS_{11}$
Nº of genomes with $TilS_{11}$
Nº of genomes without $TilS_{11}$
$p < 0.00005$
With $TilS_{12}$
Without $TilS_{12}$
Nº of genomes with $TilS_{12}$
Nº of genomes without $TilS_{12}$
$p < 0.00005$
With $TilS_{13}$
Without $TilS_{13}$
Nº of genomes with $TilS_{13}$
Nº of genomes without $TilS_{13}$
$p < 0.00005$
With $TilS_{14}$
Without $TilS_{14}$
Nº of genomes with $TilS_{14}$
Nº of genomes without $TilS_{14}$
$p < 0.00005$
With $TilS_{15}$
Without $TilS_{15}$
Nº of genomes with $TilS_{15}$
Nº of genomes without $TilS_{15}$
$p < 0.00005$

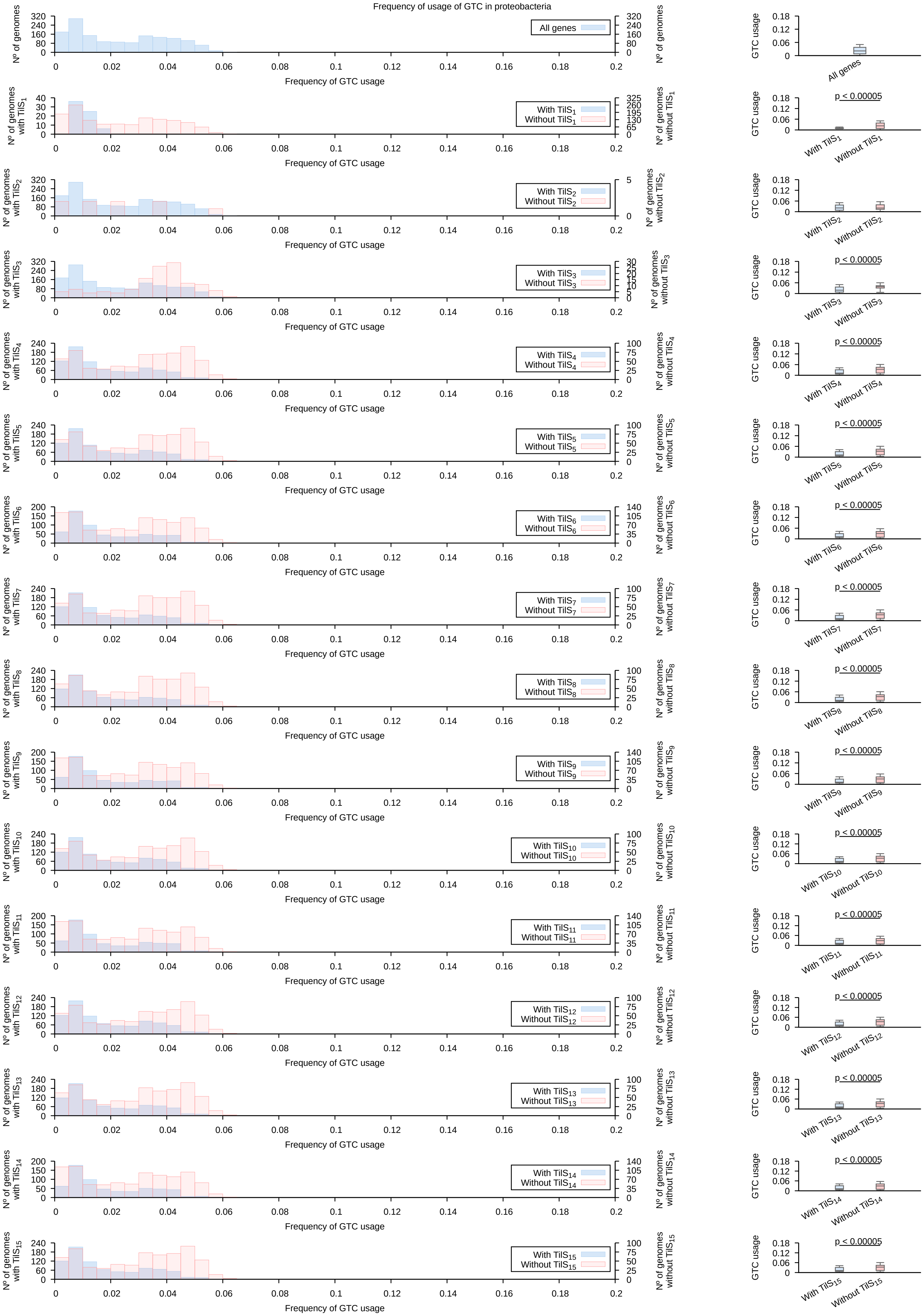
Frequency of usage of GTC in proteobacteria
Nº of genomes
All genes
Frequency of GTC usage
GTC usage
All genes
Nº of genomes with TilS1
Nº of genomes without TilS1
With TilS1
Without TilS1
p < 0.00005
Nº of genomes with TilS2
Nº of genomes without TilS2
With TilS2
Without TilS2
Nº of genomes with TilS3
Nº of genomes without TilS3
With TilS3
Without TilS3
p < 0.00005
Nº of genomes with TilS4
Nº of genomes without TilS4
With TilS4
Without TilS4
p < 0.00005
Nº of genomes with TilS5
Nº of genomes without TilS5
With TilS5
Without TilS5
p < 0.00005
Nº of genomes with TilS6
Nº of genomes without TilS6
With TilS6
Without TilS6
p < 0.00005
Nº of genomes with TilS7
Nº of genomes without TilS7
With TilS7
Without TilS7
p < 0.00005
Nº of genomes with TilS8
Nº of genomes without TilS8
With TilS8
Without TilS8
p < 0.00005
Nº of genomes with TilS9
Nº of genomes without TilS9
With TilS9
Without TilS9
p < 0.00005
Nº of genomes with TilS10
Nº of genomes without TilS10
With TilS10
Without TilS10
p < 0.00005
Nº of genomes with TilS11
Nº of genomes without TilS11
With TilS11
Without TilS11
p < 0.00005
Nº of genomes with TilS12
Nº of genomes without TilS12
With TilS12
Without TilS12
p < 0.00005
Nº of genomes with TilS13
Nº of genomes without TilS13
With TilS13
Without TilS13
p < 0.00005
Nº of genomes with TilS14
Nº of genomes without TilS14
With TilS14
Without TilS14
p < 0.00005
Nº of genomes with TilS15
Nº of genomes without TilS15
With TilS15
Without TilS15
p < 0.00005

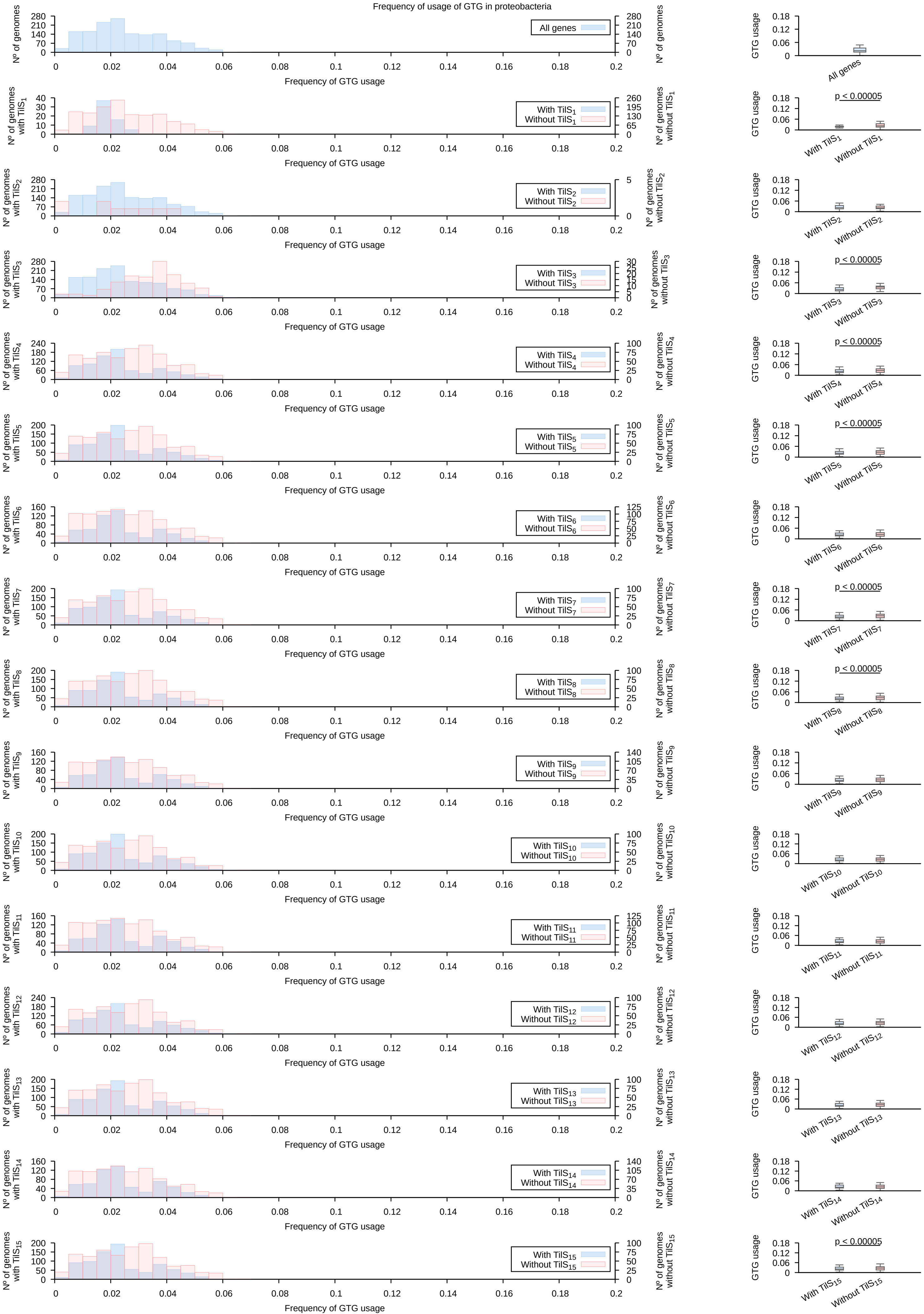

Frequency of usage of GTG in proteobacteria
Nº of genomes
All genes
Frequency of GTG usage
GTG usage
Nº of genomes with TilS$_1$
Nº of genomes without TilS$_1$
With TilS$_1$
Without TilS$_1$
p < 0.00005
Nº of genomes with TilS$_2$
Nº of genomes without TilS$_2$
With TilS$_2$
Without TilS$_2$
Nº of genomes with TilS$_3$
Nº of genomes without TilS$_3$
With TilS$_3$
Without TilS$_3$
p < 0.00005
Nº of genomes with TilS$_4$
Nº of genomes without TilS$_4$
With TilS$_4$
Without TilS$_4$
p < 0.00005
Nº of genomes with TilS$_5$
Nº of genomes without TilS$_5$
With TilS$_5$
Without TilS$_5$
p < 0.00005
Nº of genomes with TilS$_6$
Nº of genomes without TilS$_6$
With TilS$_6$
Without TilS$_6$
Nº of genomes with TilS$_7$
Nº of genomes without TilS$_7$
With TilS$_7$
Without TilS$_7$
p < 0.00005
Nº of genomes with TilS$_8$
Nº of genomes without TilS$_8$
With TilS$_8$
Without TilS$_8$
p < 0.00005
Nº of genomes with TilS$_9$
Nº of genomes without TilS$_9$
With TilS$_9$
Without TilS$_9$
Nº of genomes with TilS$_{10}$
Nº of genomes without TilS$_{10}$
With TilS$_{10}$
Without TilS$_{10}$
Nº of genomes with TilS$_{11}$
Nº of genomes without TilS$_{11}$
With TilS$_{11}$
Without TilS$_{11}$
Nº of genomes with TilS$_{12}$
Nº of genomes without TilS$_{12}$
With TilS$_{12}$
Without TilS$_{12}$
Nº of genomes with TilS$_{13}$
Nº of genomes without TilS$_{13}$
With TilS$_{13}$
Without TilS$_{13}$
Nº of genomes with TilS$_{14}$
Nº of genomes without TilS$_{14}$
With TilS$_{14}$
Without TilS$_{14}$
Nº of genomes with TilS$_{15}$
Nº of genomes without TilS$_{15}$
With TilS$_{15}$
Without TilS$_{15}$
p < 0.00005

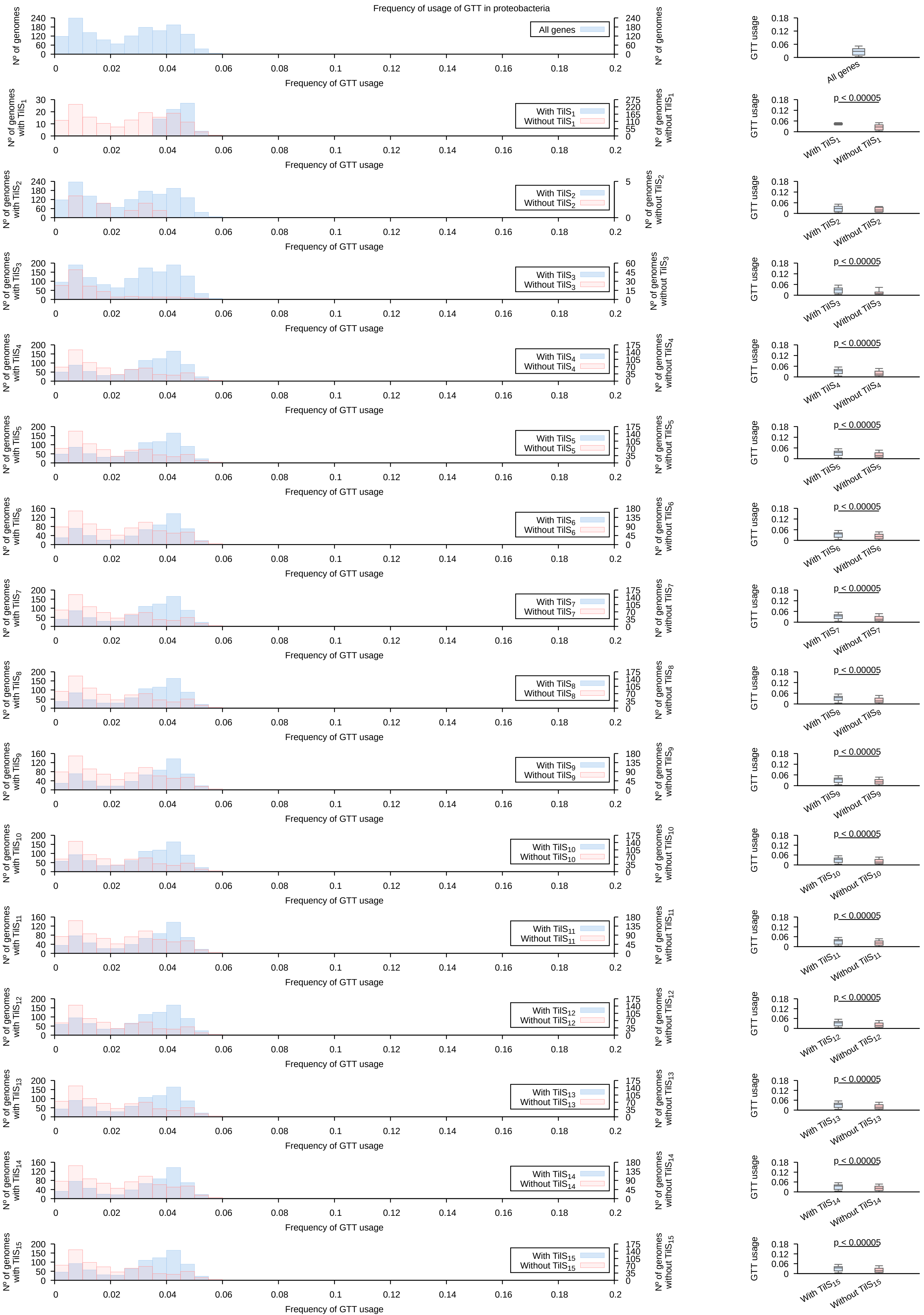

Frequency of usage of GTT in proteobacteria
Nº of genomes
All genes
Frequency of GTT usage
GTT usage
All genes
Nº of genomes with TilS$_1$
Nº of genomes without TilS$_1$
With TilS$_1$
Without TilS$_1$
p < 0.00005
Nº of genomes with TilS$_2$
Nº of genomes without TilS$_2$
With TilS$_2$
Without TilS$_2$
Nº of genomes with TilS$_3$
Nº of genomes without TilS$_3$
With TilS$_3$
Without TilS$_3$
p < 0.00005
Nº of genomes with TilS$_4$
Nº of genomes without TilS$_4$
With TilS$_4$
Without TilS$_4$
p < 0.00005
Nº of genomes with TilS$_5$
Nº of genomes without TilS$_5$
With TilS$_5$
Without TilS$_5$
p < 0.00005
Nº of genomes with TilS$_6$
Nº of genomes without TilS$_6$
With TilS$_6$
Without TilS$_6$
p < 0.00005
Nº of genomes with TilS$_7$
Nº of genomes without TilS$_7$
With TilS$_7$
Without TilS$_7$
p < 0.00005
Nº of genomes with TilS$_8$
Nº of genomes without TilS$_8$
With TilS$_8$
Without TilS$_8$
p < 0.00005
Nº of genomes with TilS$_9$
Nº of genomes without TilS$_9$
With TilS$_9$
Without TilS$_9$
p < 0.00005
Nº of genomes with TilS$_{10}$
Nº of genomes without TilS$_{10}$
With TilS$_{10}$
Without TilS$_{10}$
p < 0.00005
Nº of genomes with TilS$_{11}$
Nº of genomes without TilS$_{11}$
With TilS$_{11}$
Without TilS$_{11}$
p < 0.00005
Nº of genomes with TilS$_{12}$
Nº of genomes without TilS$_{12}$
With TilS$_{12}$
Without TilS$_{12}$
p < 0.00005
Nº of genomes with TilS$_{13}$
Nº of genomes without TilS$_{13}$
With TilS$_{13}$
Without TilS$_{13}$
p < 0.00005
Nº of genomes with TilS$_{14}$
Nº of genomes without TilS$_{14}$
With TilS$_{14}$
Without TilS$_{14}$
p < 0.00005
Nº of genomes with TilS$_{15}$
Nº of genomes without TilS$_{15}$
With TilS$_{15}$
Without TilS$_{15}$
p < 0.00005

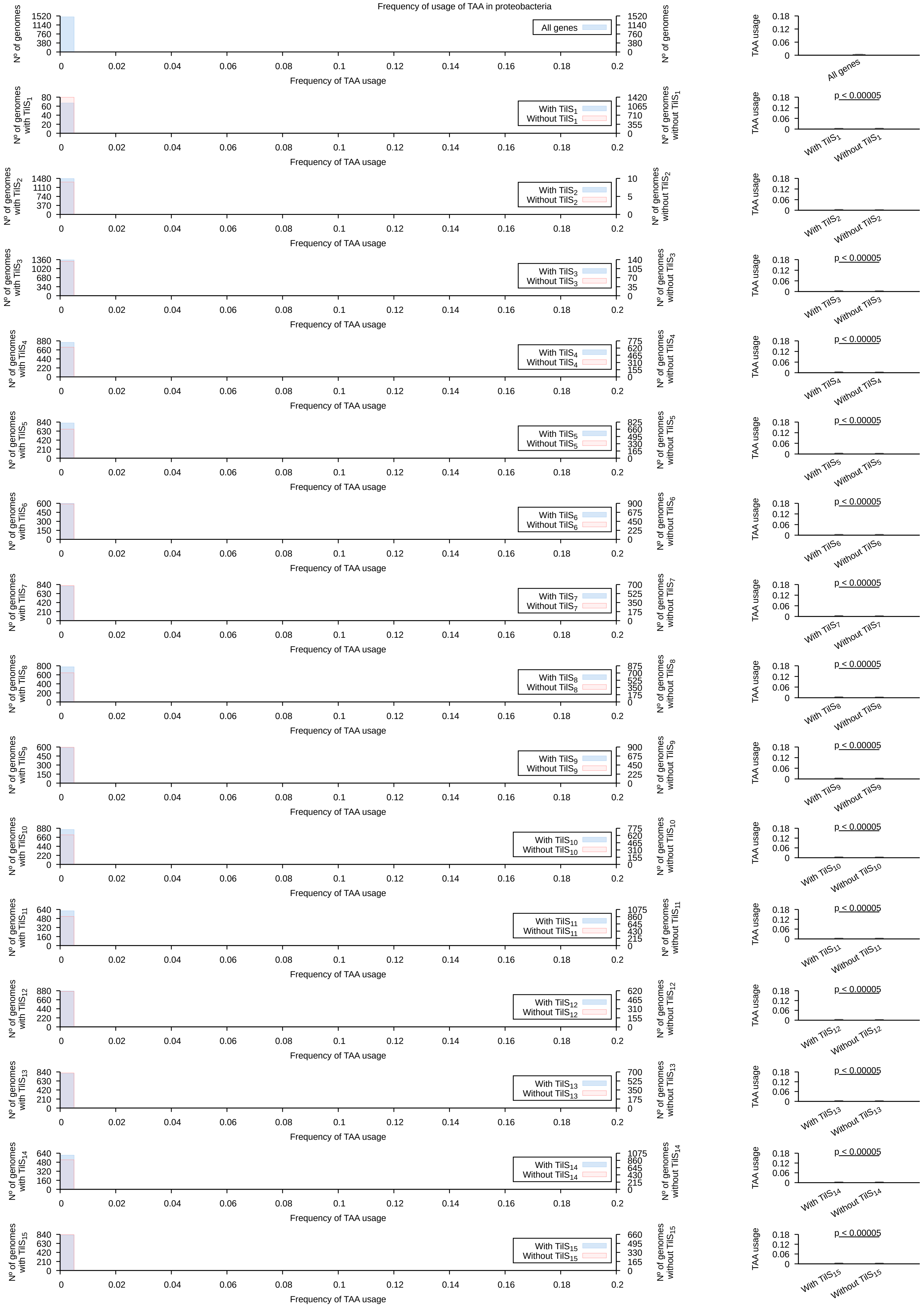

Frequency of usage of TAA in proteobacteria
Nº of genomes
All genes
Frequency of TAA usage
TAA usage
With TilS$_1$
Without TilS$_1$
With TilS$_2$
Without TilS$_2$
With TilS$_3$
Without TilS$_3$
With TilS$_4$
Without TilS$_4$
With TilS$_5$
Without TilS$_5$
With TilS$_6$
Without TilS$_6$
With TilS$_7$
Without TilS$_7$
With TilS$_8$
Without TilS$_8$
With TilS$_9$
Without TilS$_9$
With TilS$_{10}$
Without TilS$_{10}$
With TilS$_{11}$
Without TilS$_{11}$
With TilS$_{12}$
Without TilS$_{12}$
With TilS$_{13}$
Without TilS$_{13}$
With TilS$_{14}$
Without TilS$_{14}$
With TilS$_{15}$
Without TilS$_{15}$
$p < 0.00005$

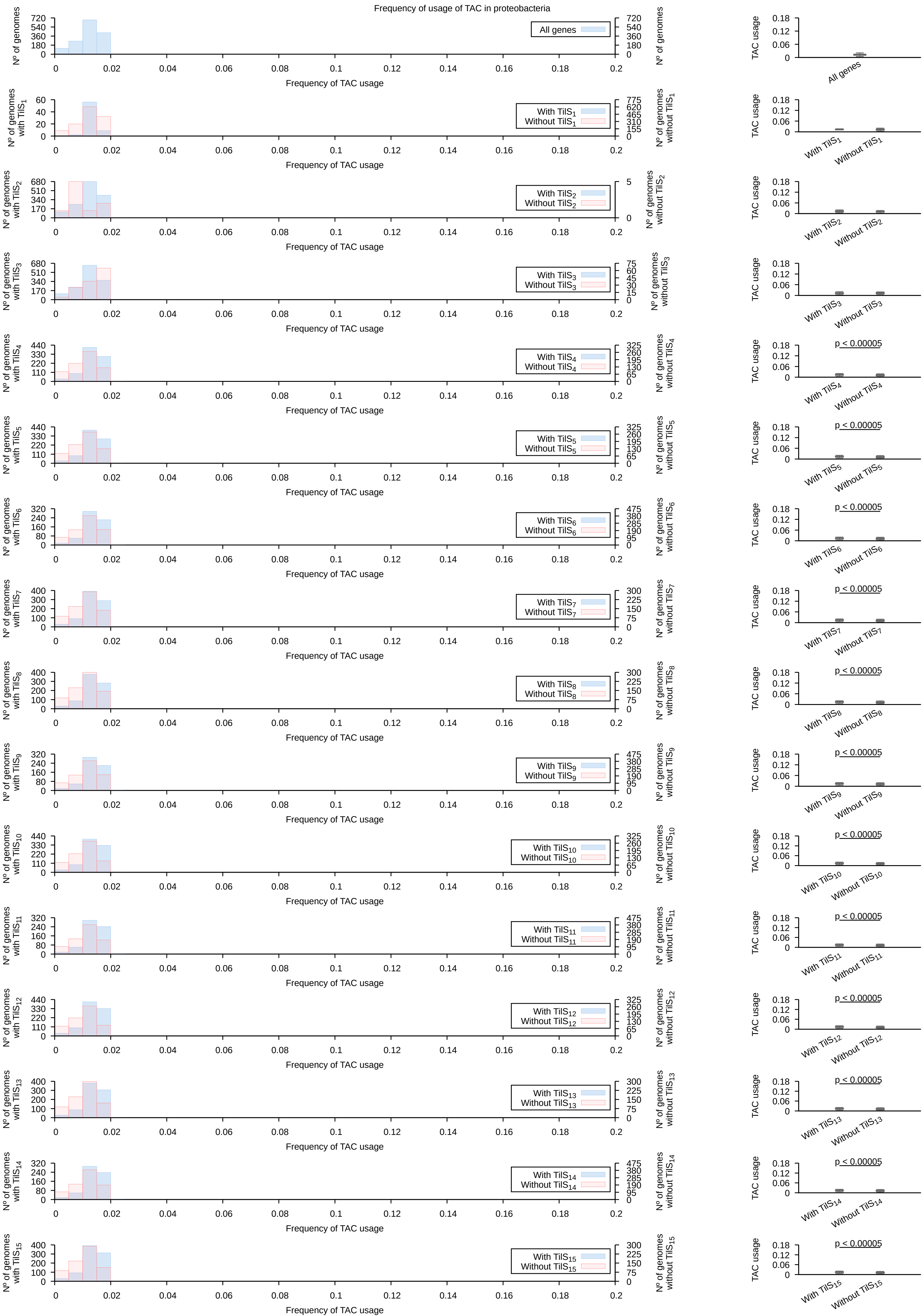

Frequency of usage of TAC in proteobacteria
Nº of genomes
All genes
Frequency of TAC usage
TAC usage
With TilS
Without TilS
Nº of genomes with TilS
Nº of genomes without TilS
p < 0.00005

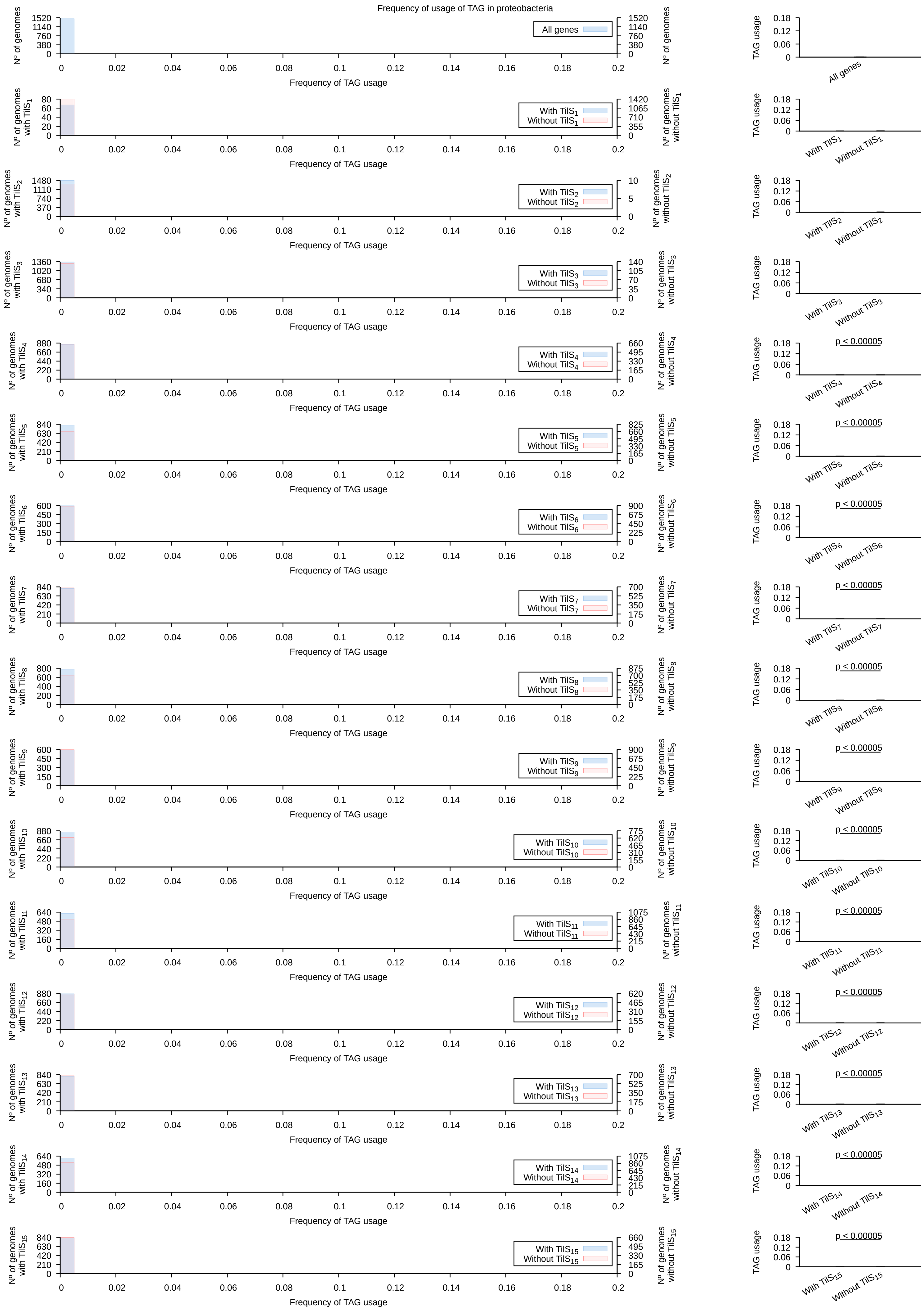

Frequency of usage of TAG in proteobacteria
Nº of genomes
Frequency of TAG usage
TAG usage
All genes
With TilS1
Without TilS1
With TilS2
Without TilS2
With TilS3
Without TilS3
With TilS4
Without TilS4
With TilS5
Without TilS5
With TilS6
Without TilS6
With TilS7
Without TilS7
With TilS8
Without TilS8
With TilS9
Without TilS9
With TilS10
Without TilS10
With TilS11
Without TilS11
With TilS12
Without TilS12
With TilS13
Without TilS13
With TilS14
Without TilS14
With TilS15
Without TilS15
p < 0.00005

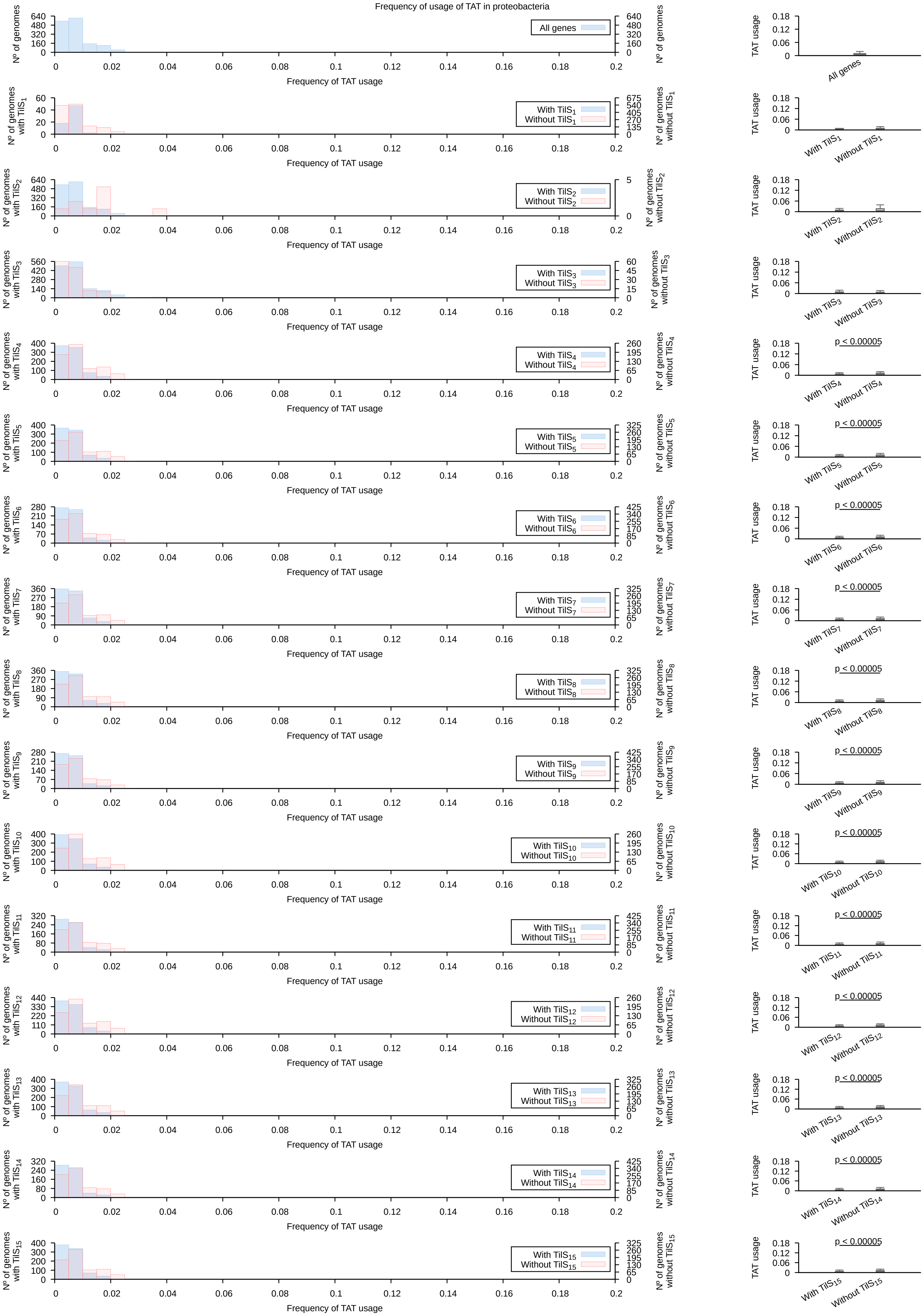
Frequency of usage of TAT in proteobacteria
Nº of genomes
Frequency of TAT usage
TAT usage
All genes
With TilS$_1$
Without TilS$_1$
With TilS$_2$
Without TilS$_2$
With TilS$_3$
Without TilS$_3$
With TilS$_4$
Without TilS$_4$
With TilS$_5$
Without TilS$_5$
With TilS$_6$
Without TilS$_6$
With TilS$_7$
Without TilS$_7$
With TilS$_8$
Without TilS$_8$
With TilS$_9$
Without TilS$_9$
With TilS$_{10}$
Without TilS$_{10}$
With TilS$_{11}$
Without TilS$_{11}$
With TilS$_{12}$
Without TilS$_{12}$
With TilS$_{13}$
Without TilS$_{13}$
With TilS$_{14}$
Without TilS$_{14}$
With TilS$_{15}$
Without TilS$_{15}$
p < 0.00005

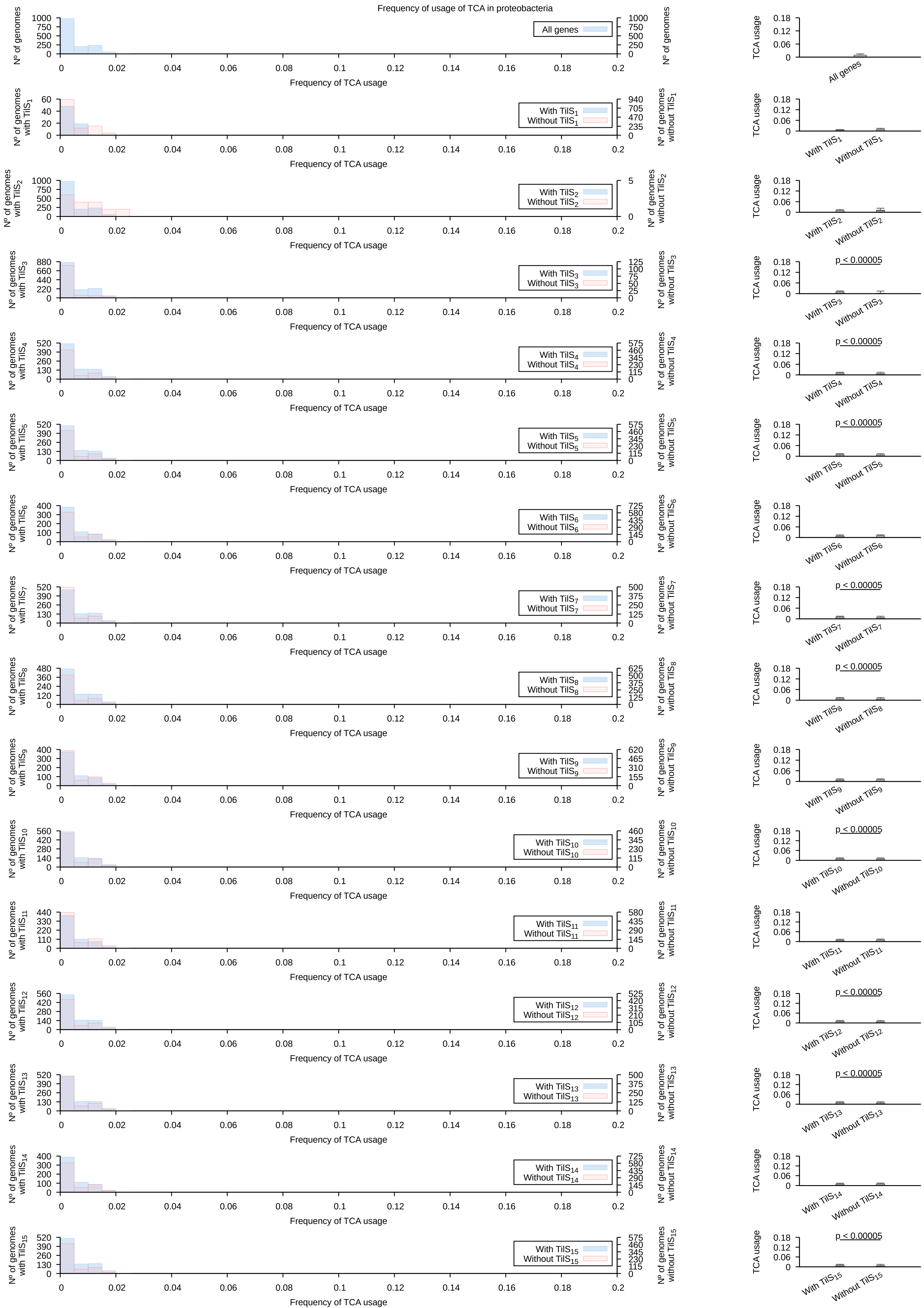
Frequency of usage of TCA in proteobacteria
Nº of genomes
All genes
Frequency of TCA usage
TCA usage
0 0.02 0.04 0.06 0.08 0.1 0.12 0.14 0.16 0.18 0.2
0 0.06 0.12 0.18
Nº of genomes with TilS$_1$
Nº of genomes without TilS$_1$
With TilS$_1$
Without TilS$_1$
Nº of genomes with TilS$_2$
Nº of genomes without TilS$_2$
With TilS$_2$
Without TilS$_2$
Nº of genomes with TilS$_3$
Nº of genomes without TilS$_3$
With TilS$_3$
Without TilS$_3$
p < 0.00005
Nº of genomes with TilS$_4$
Nº of genomes without TilS$_4$
With TilS$_4$
Without TilS$_4$
p < 0.00005
Nº of genomes with TilS$_5$
Nº of genomes without TilS$_5$
With TilS$_5$
Without TilS$_5$
p < 0.00005
Nº of genomes with TilS$_6$
Nº of genomes without TilS$_6$
With TilS$_6$
Without TilS$_6$
Nº of genomes with TilS$_7$
Nº of genomes without TilS$_7$
With TilS$_7$
Without TilS$_7$
p < 0.00005
Nº of genomes with TilS$_8$
Nº of genomes without TilS$_8$
With TilS$_8$
Without TilS$_8$
p < 0.00005
Nº of genomes with TilS$_9$
Nº of genomes without TilS$_9$
With TilS$_9$
Without TilS$_9$
Nº of genomes with TilS$_{10}$
Nº of genomes without TilS$_{10}$
With TilS$_{10}$
Without TilS$_{10}$
p < 0.00005
Nº of genomes with TilS$_{11}$
Nº of genomes without TilS$_{11}$
With TilS$_{11}$
Without TilS$_{11}$
Nº of genomes with TilS$_{12}$
Nº of genomes without TilS$_{12}$
With TilS$_{12}$
Without TilS$_{12}$
p < 0.00005
Nº of genomes with TilS$_{13}$
Nº of genomes without TilS$_{13}$
With TilS$_{13}$
Without TilS$_{13}$
p < 0.00005
Nº of genomes with TilS$_{14}$
Nº of genomes without TilS$_{14}$
With TilS$_{14}$
Without TilS$_{14}$
Nº of genomes with TilS$_{15}$
Nº of genomes without TilS$_{15}$
With TilS$_{15}$
Without TilS$_{15}$
p < 0.00005

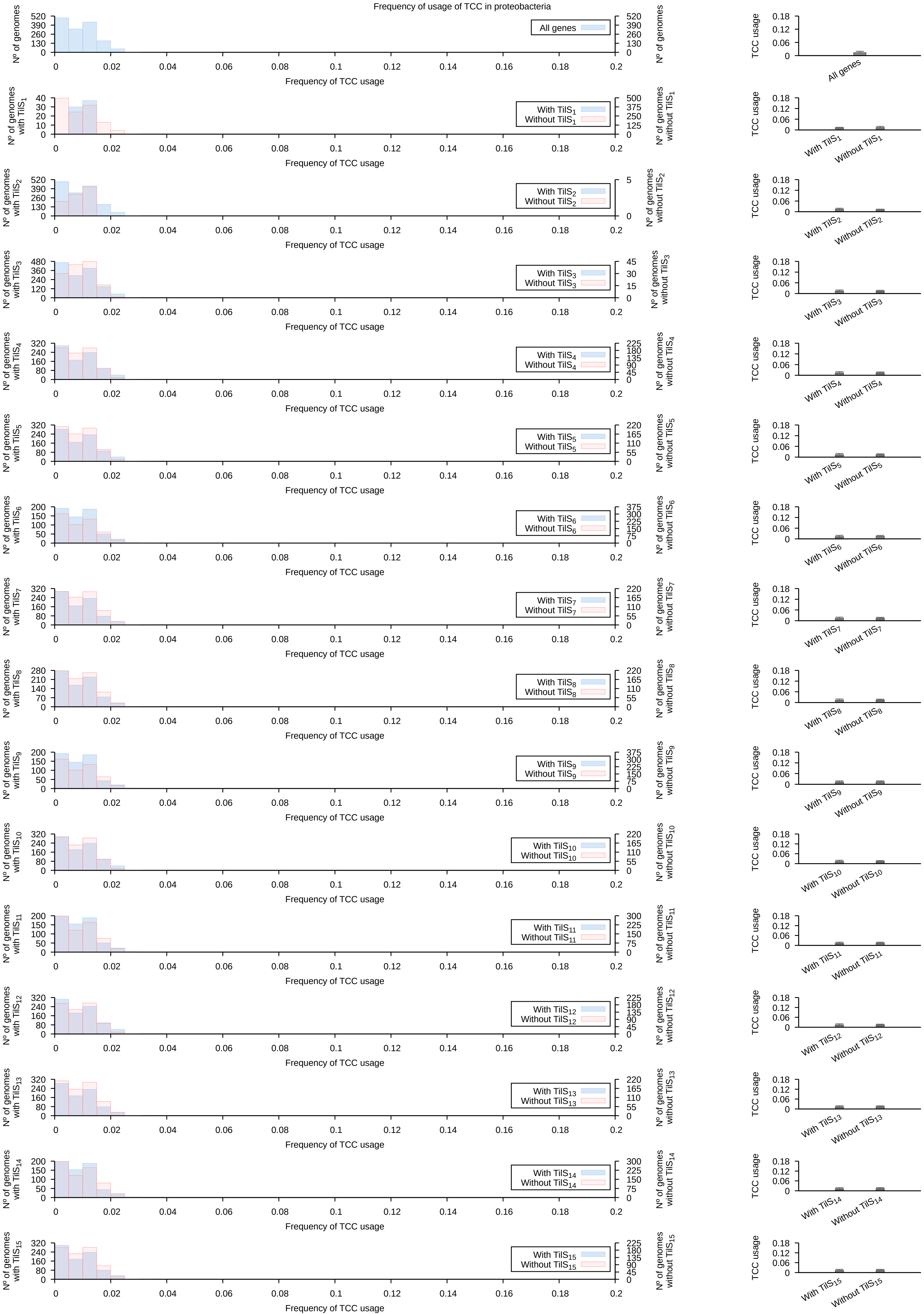
Frequency of usage of TCC in proteobacteria
Nº of genomes
All genes
Frequency of TCC usage
TCC usage
Nº of genomes with TilS$_1$
Nº of genomes without TilS$_1$
With TilS$_1$
Without TilS$_1$
Nº of genomes with TilS$_2$
Nº of genomes without TilS$_2$
With TilS$_2$
Without TilS$_2$
Nº of genomes with TilS$_3$
Nº of genomes without TilS$_3$
With TilS$_3$
Without TilS$_3$
Nº of genomes with TilS$_4$
Nº of genomes without TilS$_4$
With TilS$_4$
Without TilS$_4$
Nº of genomes with TilS$_5$
Nº of genomes without TilS$_5$
With TilS$_5$
Without TilS$_5$
Nº of genomes with TilS$_6$
Nº of genomes without TilS$_6$
With TilS$_6$
Without TilS$_6$
Nº of genomes with TilS$_7$
Nº of genomes without TilS$_7$
With TilS$_7$
Without TilS$_7$
Nº of genomes with TilS$_8$
Nº of genomes without TilS$_8$
With TilS$_8$
Without TilS$_8$
Nº of genomes with TilS$_9$
Nº of genomes without TilS$_9$
With TilS$_9$
Without TilS$_9$
Nº of genomes with TilS$_{10}$
Nº of genomes without TilS$_{10}$
With TilS$_{10}$
Without TilS$_{10}$
Nº of genomes with TilS$_{11}$
Nº of genomes without TilS$_{11}$
With TilS$_{11}$
Without TilS$_{11}$
Nº of genomes with TilS$_{12}$
Nº of genomes without TilS$_{12}$
With TilS$_{12}$
Without TilS$_{12}$
Nº of genomes with TilS$_{13}$
Nº of genomes without TilS$_{13}$
With TilS$_{13}$
Without TilS$_{13}$
Nº of genomes with TilS$_{14}$
Nº of genomes without TilS$_{14}$
With TilS$_{14}$
Without TilS$_{14}$
Nº of genomes with TilS$_{15}$
Nº of genomes without TilS$_{15}$
With TilS$_{15}$
Without TilS$_{15}$

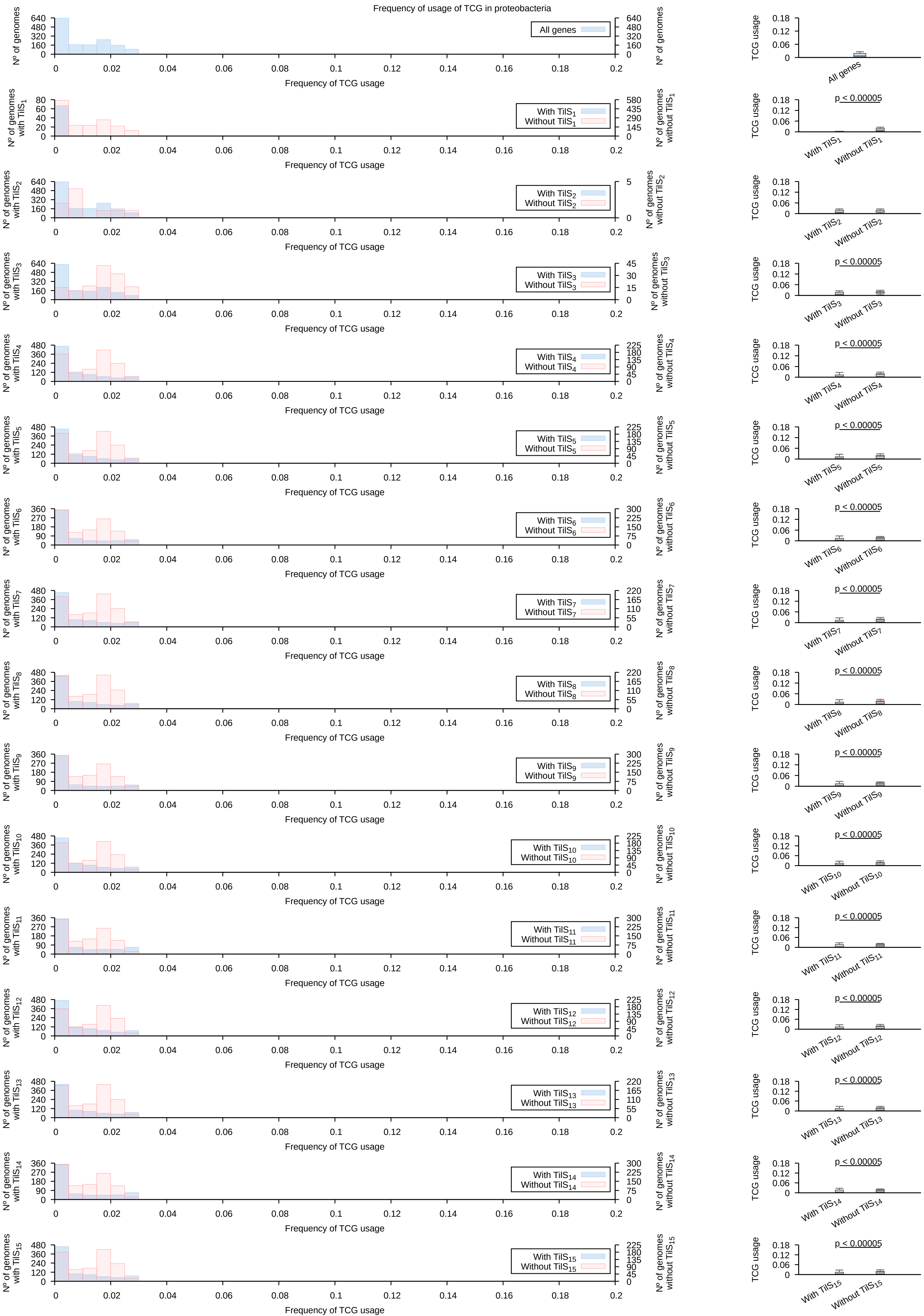
Frequency of usage of TCG in proteobacteria
Nº of genomes
All genes
Frequency of TCG usage
TCG usage
0 0.02 0.04 0.06 0.08 0.1 0.12 0.14 0.16 0.18 0.2
0 0.06 0.12 0.18
Nº of genomes with TilS$_1$
Nº of genomes without TilS$_1$
With TilS$_1$
Without TilS$_1$
p < 0.00005
Nº of genomes with TilS$_2$
Nº of genomes without TilS$_2$
With TilS$_2$
Without TilS$_2$
Nº of genomes with TilS$_3$
Nº of genomes without TilS$_3$
With TilS$_3$
Without TilS$_3$
p < 0.00005
Nº of genomes with TilS$_4$
Nº of genomes without TilS$_4$
With TilS$_4$
Without TilS$_4$
p < 0.00005
Nº of genomes with TilS$_5$
Nº of genomes without TilS$_5$
With TilS$_5$
Without TilS$_5$
p < 0.00005
Nº of genomes with TilS$_6$
Nº of genomes without TilS$_6$
With TilS$_6$
Without TilS$_6$
p < 0.00005
Nº of genomes with TilS$_7$
Nº of genomes without TilS$_7$
With TilS$_7$
Without TilS$_7$
p < 0.00005
Nº of genomes with TilS$_8$
Nº of genomes without TilS$_8$
With TilS$_8$
Without TilS$_8$
p < 0.00005
Nº of genomes with TilS$_9$
Nº of genomes without TilS$_9$
With TilS$_9$
Without TilS$_9$
p < 0.00005
Nº of genomes with TilS$_{10}$
Nº of genomes without TilS$_{10}$
With TilS$_{10}$
Without TilS$_{10}$
p < 0.00005
Nº of genomes with TilS$_{11}$
Nº of genomes without TilS$_{11}$
With TilS$_{11}$
Without TilS$_{11}$
p < 0.00005
Nº of genomes with TilS$_{12}$
Nº of genomes without TilS$_{12}$
With TilS$_{12}$
Without TilS$_{12}$
p < 0.00005
Nº of genomes with TilS$_{13}$
Nº of genomes without TilS$_{13}$
With TilS$_{13}$
Without TilS$_{13}$
p < 0.00005
Nº of genomes with TilS$_{14}$
Nº of genomes without TilS$_{14}$
With TilS$_{14}$
Without TilS$_{14}$
p < 0.00005
Nº of genomes with TilS$_{15}$
Nº of genomes without TilS$_{15}$
With TilS$_{15}$
Without TilS$_{15}$
p < 0.00005

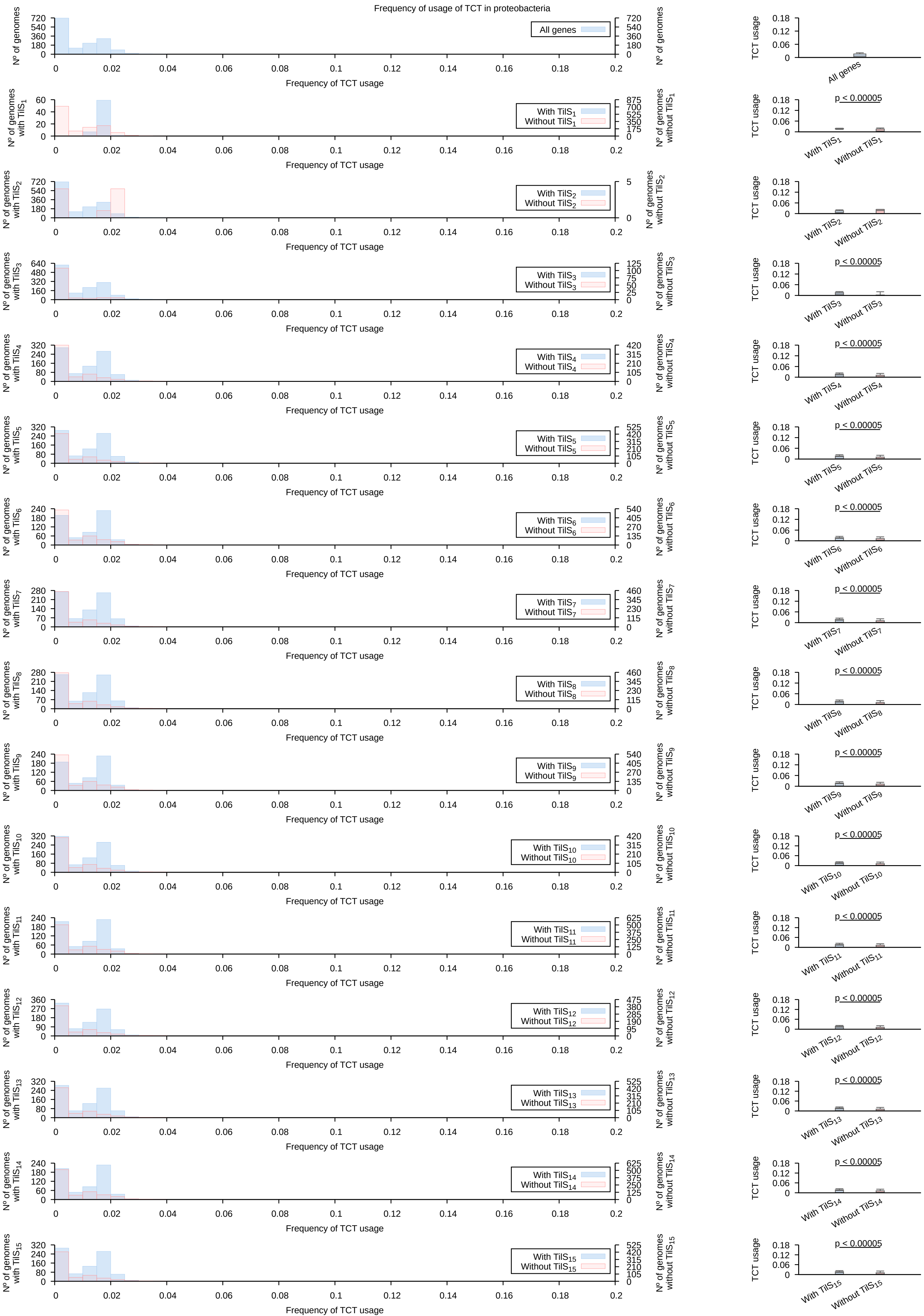

Frequency of usage of TCT in proteobacteria
Nº of genomes
Frequency of TCT usage
TCT usage
All genes
With TilS$_1$
Without TilS$_1$
With TilS$_2$
Without TilS$_2$
With TilS$_3$
Without TilS$_3$
With TilS$_4$
Without TilS$_4$
With TilS$_5$
Without TilS$_5$
With TilS$_6$
Without TilS$_6$
With TilS$_7$
Without TilS$_7$
With TilS$_8$
Without TilS$_8$
With TilS$_9$
Without TilS$_9$
With TilS$_{10}$
Without TilS$_{10}$
With TilS$_{11}$
Without TilS$_{11}$
With TilS$_{12}$
Without TilS$_{12}$
With TilS$_{13}$
Without TilS$_{13}$
With TilS$_{14}$
Without TilS$_{14}$
With TilS$_{15}$
Without TilS$_{15}$
$p < 0.00005$

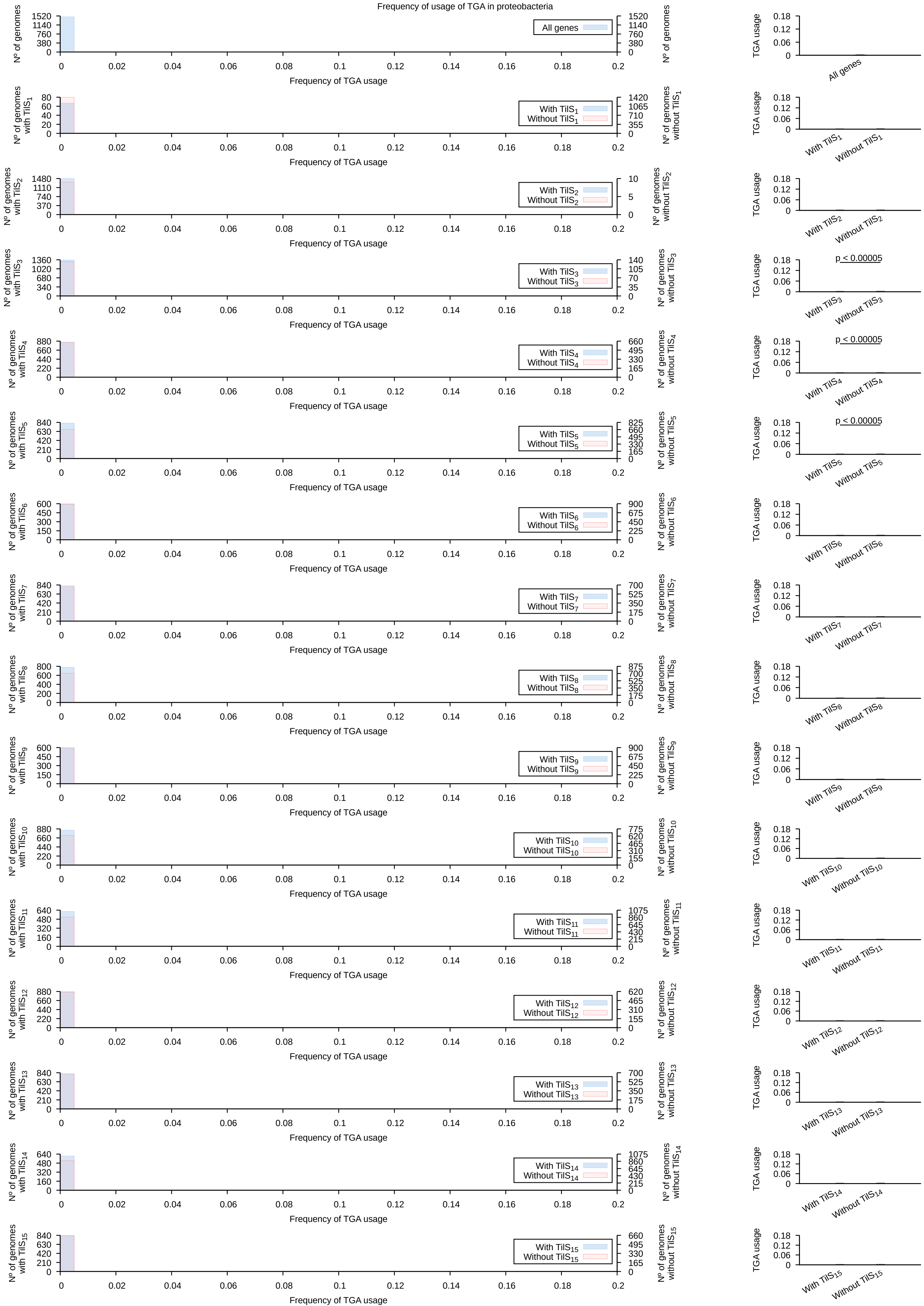

Frequency of usage of TGA in proteobacteria
Nº of genomes
All genes
Frequency of TGA usage
TGA usage
Nº of genomes with TilS$_1$
Nº of genomes without TilS$_1$
With TilS$_1$
Without TilS$_1$
p < 0.00005

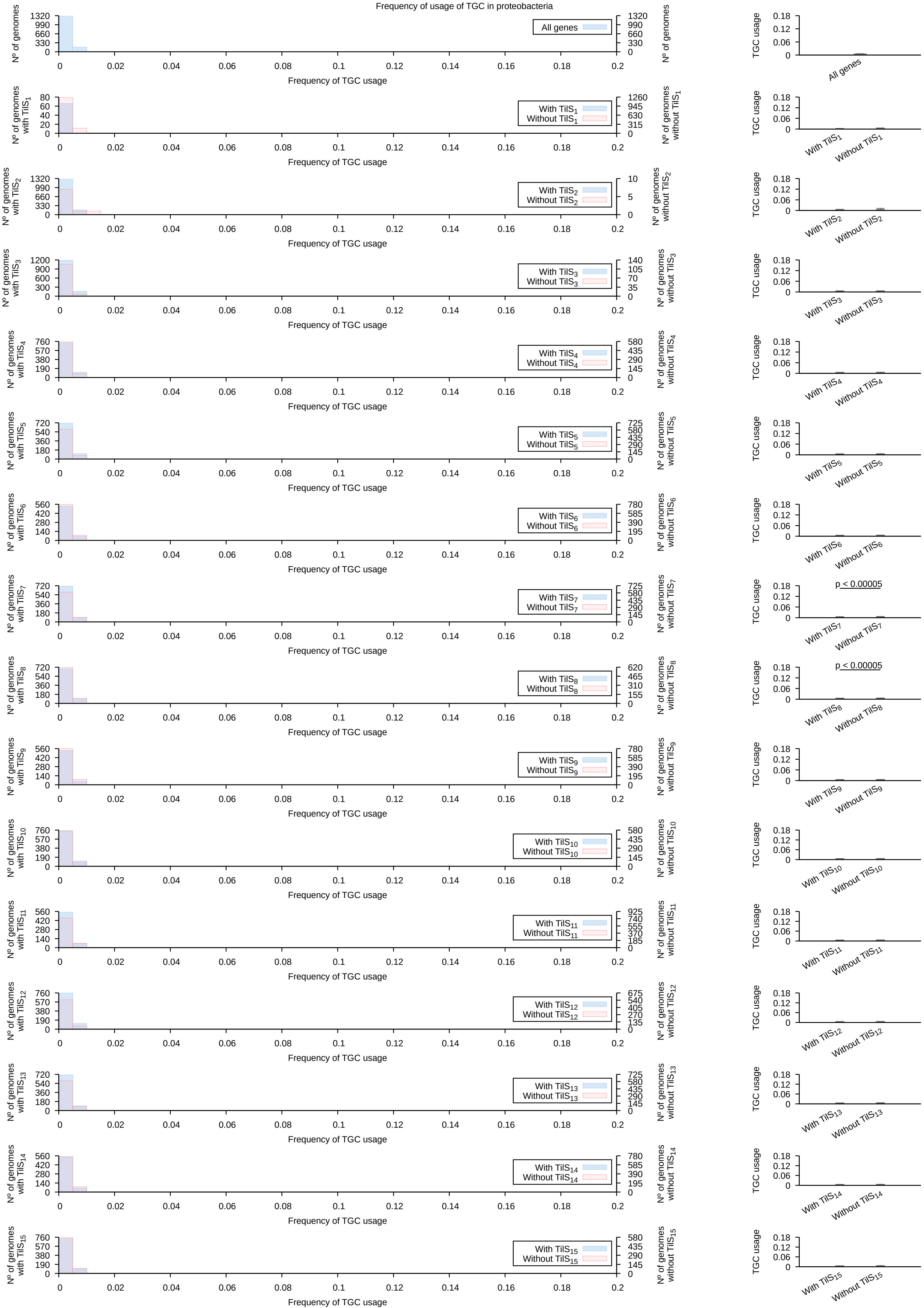
Frequency of usage of TGC in proteobacteria
Nº of genomes
All genes
Frequency of TGC usage
TGC usage
Nº of genomes with TilS$_1$
With TilS$_1$
Without TilS$_1$
Nº of genomes without TilS$_1$
Nº of genomes with TilS$_2$
With TilS$_2$
Without TilS$_2$
Nº of genomes without TilS$_2$
Nº of genomes with TilS$_3$
With TilS$_3$
Without TilS$_3$
Nº of genomes without TilS$_3$
Nº of genomes with TilS$_4$
With TilS$_4$
Without TilS$_4$
Nº of genomes without TilS$_4$
Nº of genomes with TilS$_5$
With TilS$_5$
Without TilS$_5$
Nº of genomes without TilS$_5$
Nº of genomes with TilS$_6$
With TilS$_6$
Without TilS$_6$
Nº of genomes without TilS$_6$
Nº of genomes with TilS$_7$
With TilS$_7$
Without TilS$_7$
Nº of genomes without TilS$_7$
$p < 0.00005$
Nº of genomes with TilS$_8$
With TilS$_8$
Without TilS$_8$
Nº of genomes without TilS$_8$
$p < 0.00005$
Nº of genomes with TilS$_9$
With TilS$_9$
Without TilS$_9$
Nº of genomes without TilS$_9$
Nº of genomes with TilS$_{10}$
With TilS$_{10}$
Without TilS$_{10}$
Nº of genomes without TilS$_{10}$
Nº of genomes with TilS$_{11}$
With TilS$_{11}$
Without TilS$_{11}$
Nº of genomes without TilS$_{11}$
Nº of genomes with TilS$_{12}$
With TilS$_{12}$
Without TilS$_{12}$
Nº of genomes without TilS$_{12}$
Nº of genomes with TilS$_{13}$
With TilS$_{13}$
Without TilS$_{13}$
Nº of genomes without TilS$_{13}$
Nº of genomes with TilS$_{14}$
With TilS$_{14}$
Without TilS$_{14}$
Nº of genomes without TilS$_{14}$
Nº of genomes with TilS$_{15}$
With TilS$_{15}$
Without TilS$_{15}$
Nº of genomes without TilS$_{15}$

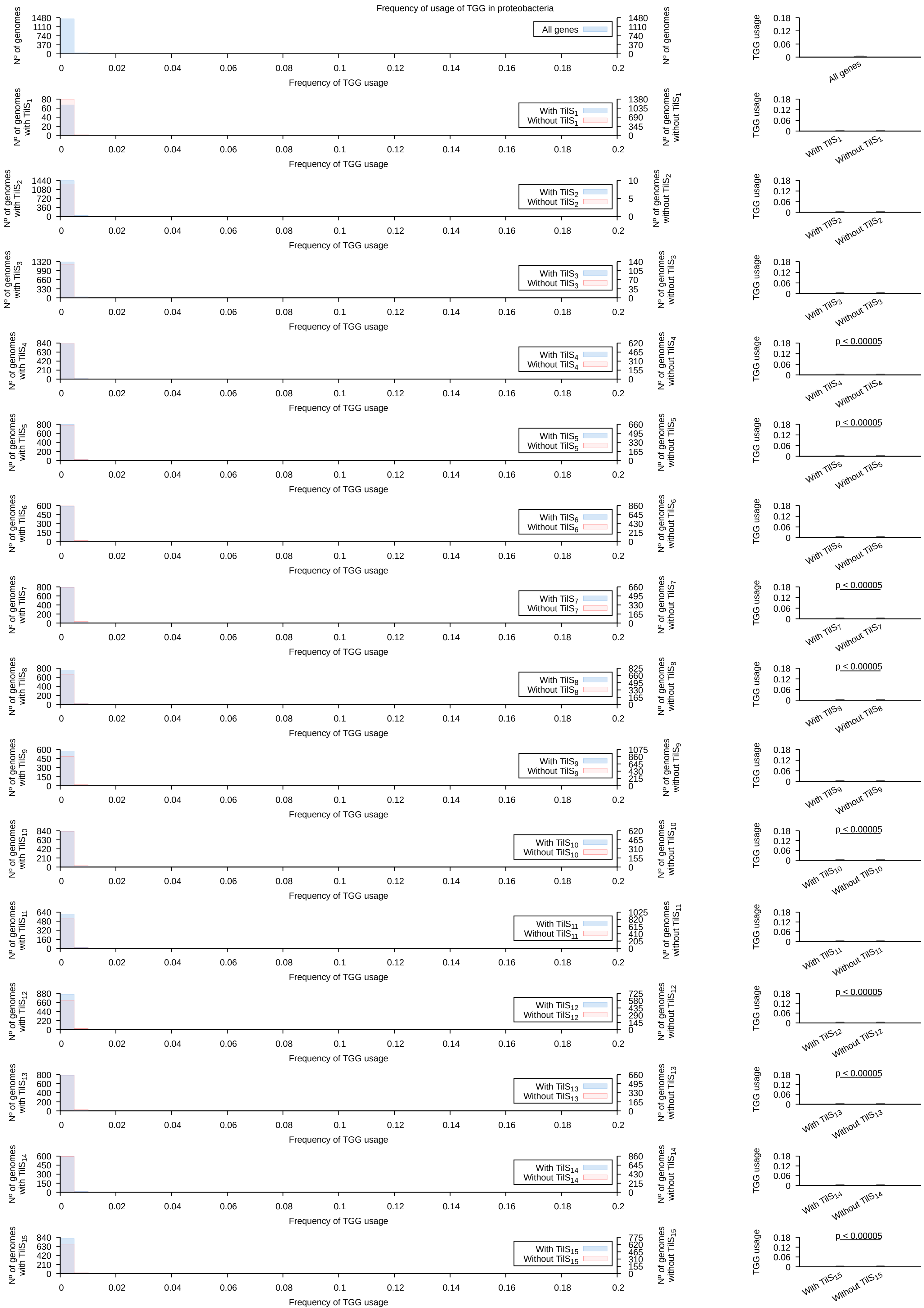

Frequency of usage of TGG in proteobacteria
Nº of genomes
All genes
Frequency of TGG usage
TGG usage
Nº of genomes with TilS$_1$
Nº of genomes without TilS$_1$
With TilS$_1$
Without TilS$_1$
Nº of genomes with TilS$_2$
Nº of genomes without TilS$_2$
With TilS$_2$
Without TilS$_2$
Nº of genomes with TilS$_3$
Nº of genomes without TilS$_3$
With TilS$_3$
Without TilS$_3$
Nº of genomes with TilS$_4$
Nº of genomes without TilS$_4$
With TilS$_4$
Without TilS$_4$
p < 0.00005
Nº of genomes with TilS$_5$
Nº of genomes without TilS$_5$
With TilS$_5$
Without TilS$_5$
p < 0.00005
Nº of genomes with TilS$_6$
Nº of genomes without TilS$_6$
With TilS$_6$
Without TilS$_6$
Nº of genomes with TilS$_7$
Nº of genomes without TilS$_7$
With TilS$_7$
Without TilS$_7$
p < 0.00005
Nº of genomes with TilS$_8$
Nº of genomes without TilS$_8$
With TilS$_8$
Without TilS$_8$
p < 0.00005
Nº of genomes with TilS$_9$
Nº of genomes without TilS$_9$
With TilS$_9$
Without TilS$_9$
Nº of genomes with TilS$_{10}$
Nº of genomes without TilS$_{10}$
With TilS$_{10}$
Without TilS$_{10}$
p < 0.00005
Nº of genomes with TilS$_{11}$
Nº of genomes without TilS$_{11}$
With TilS$_{11}$
Without TilS$_{11}$
Nº of genomes with TilS$_{12}$
Nº of genomes without TilS$_{12}$
With TilS$_{12}$
Without TilS$_{12}$
p < 0.00005
Nº of genomes with TilS$_{13}$
Nº of genomes without TilS$_{13}$
With TilS$_{13}$
Without TilS$_{13}$
p < 0.00005
Nº of genomes with TilS$_{14}$
Nº of genomes without TilS$_{14}$
With TilS$_{14}$
Without TilS$_{14}$
Nº of genomes with TilS$_{15}$
Nº of genomes without TilS$_{15}$
With TilS$_{15}$
Without TilS$_{15}$
p < 0.00005

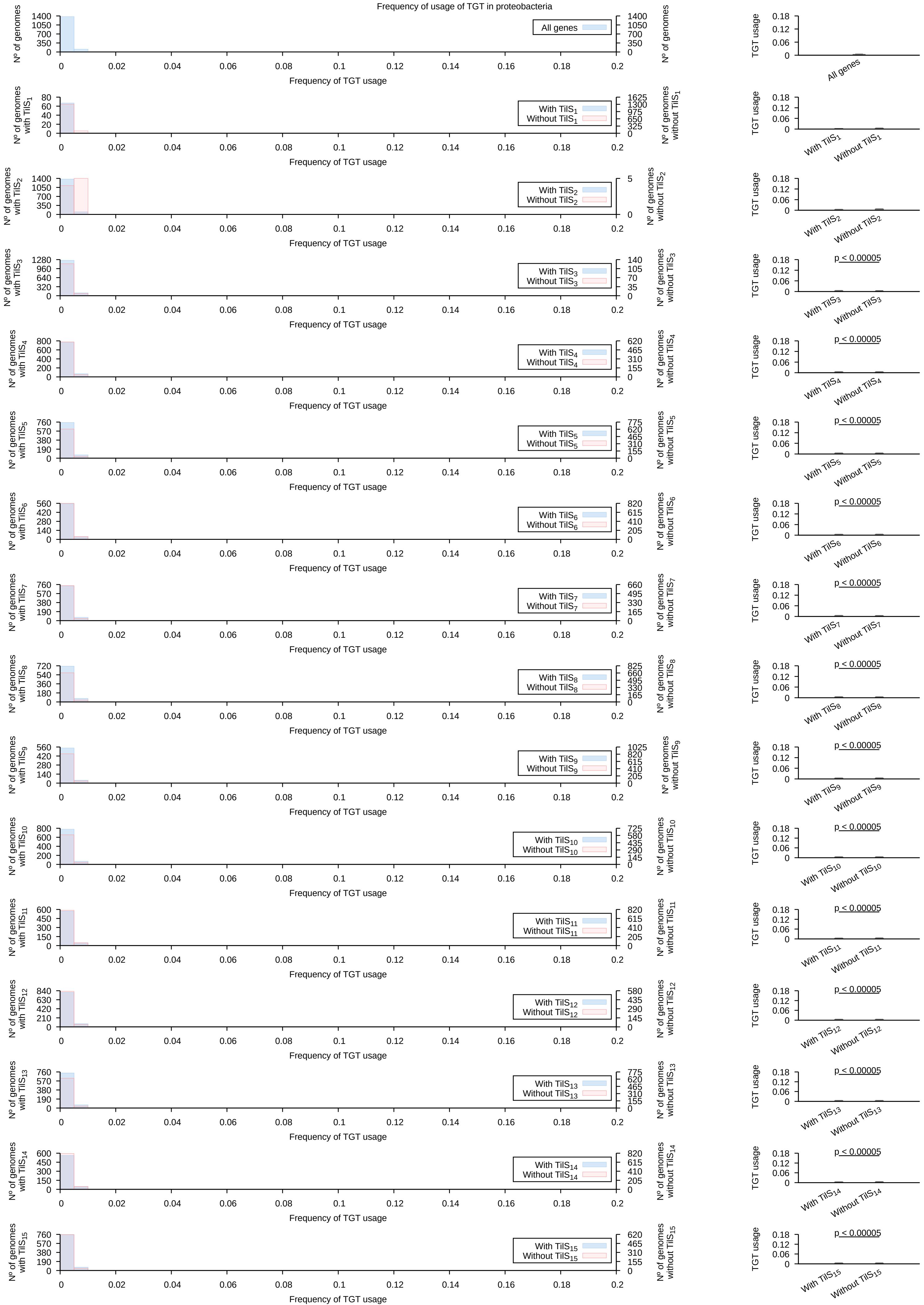

Frequency of usage of TGT in proteobacteria
Nº of genomes
All genes
Frequency of TGT usage
TGT usage
All genes
Nº of genomes with TilS1
With TilS1
Without TilS1
Nº of genomes without TilS1
Nº of genomes with TilS2
With TilS2
Without TilS2
Nº of genomes without TilS2
Nº of genomes with TilS3
With TilS3
Without TilS3
Nº of genomes without TilS3
p < 0.00005
Nº of genomes with TilS4
With TilS4
Without TilS4
Nº of genomes without TilS4
Nº of genomes with TilS5
With TilS5
Without TilS5
Nº of genomes without TilS5
Nº of genomes with TilS6
With TilS6
Without TilS6
Nº of genomes without TilS6
Nº of genomes with TilS7
With TilS7
Without TilS7
Nº of genomes without TilS7
Nº of genomes with TilS8
With TilS8
Without TilS8
Nº of genomes without TilS8
Nº of genomes with TilS9
With TilS9
Without TilS9
Nº of genomes without TilS9
Nº of genomes with TilS10
With TilS10
Without TilS10
Nº of genomes without TilS10
Nº of genomes with TilS11
With TilS11
Without TilS11
Nº of genomes without TilS11
Nº of genomes with TilS12
With TilS12
Without TilS12
Nº of genomes without TilS12
Nº of genomes with TilS13
With TilS13
Without TilS13
Nº of genomes without TilS13
Nº of genomes with TilS14
With TilS14
Without TilS14
Nº of genomes without TilS14
Nº of genomes with TilS15
With TilS15
Without TilS15
Nº of genomes without TilS15

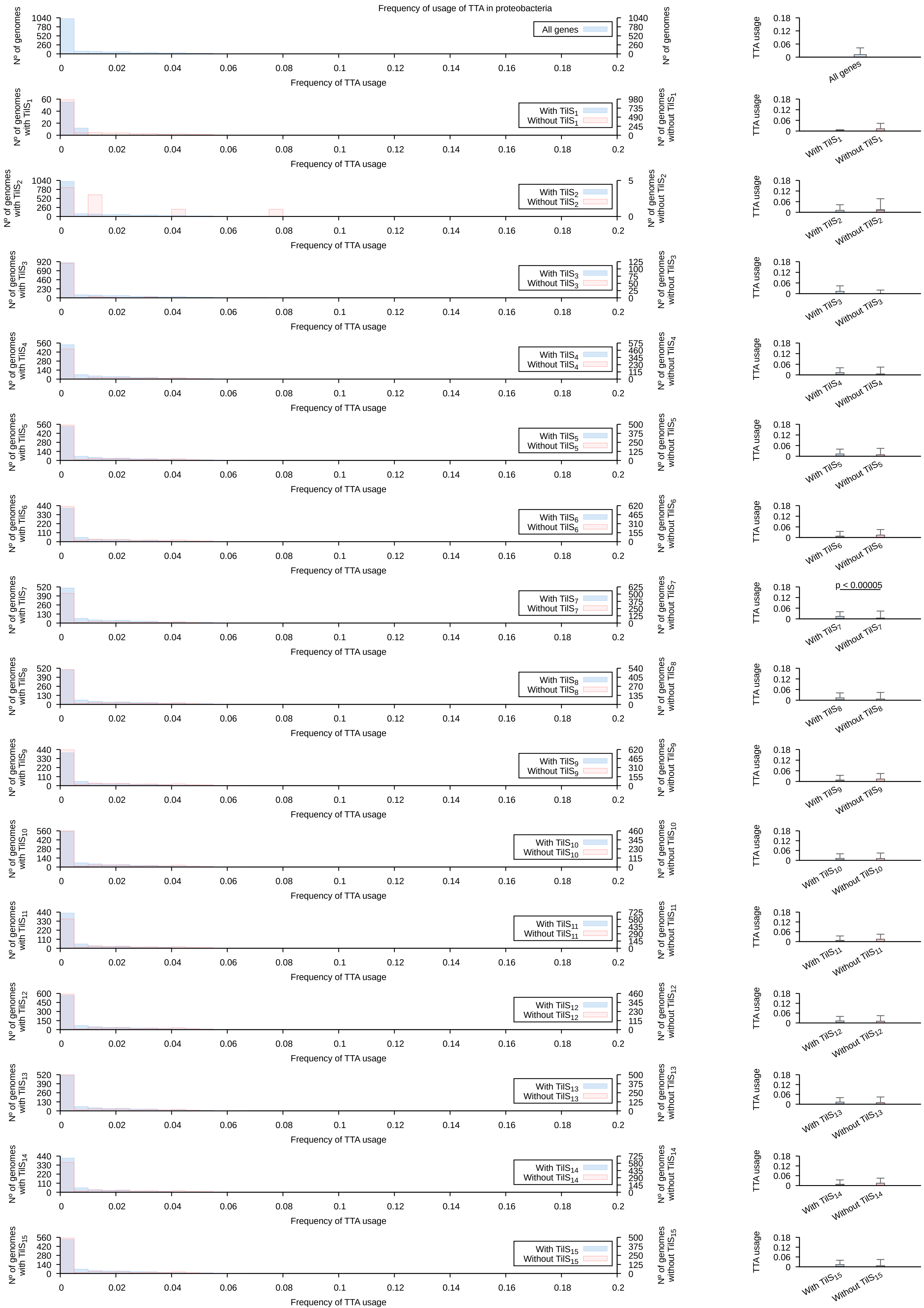
Frequency of usage of TTA in proteobacteria
Nº of genomes
All genes
Frequency of TTA usage
TTA usage
Nº of genomes with TilS1
Nº of genomes without TilS1
With TilS1
Without TilS1
Nº of genomes with TilS2
Nº of genomes without TilS2
With TilS2
Without TilS2
Nº of genomes with TilS3
Nº of genomes without TilS3
With TilS3
Without TilS3
Nº of genomes with TilS4
Nº of genomes without TilS4
With TilS4
Without TilS4
Nº of genomes with TilS5
Nº of genomes without TilS5
With TilS5
Without TilS5
Nº of genomes with TilS6
Nº of genomes without TilS6
With TilS6
Without TilS6
Nº of genomes with TilS7
Nº of genomes without TilS7
With TilS7
Without TilS7
p < 0.00005
Nº of genomes with TilS8
Nº of genomes without TilS8
With TilS8
Without TilS8
Nº of genomes with TilS9
Nº of genomes without TilS9
With TilS9
Without TilS9
Nº of genomes with TilS10
Nº of genomes without TilS10
With TilS10
Without TilS10
Nº of genomes with TilS11
Nº of genomes without TilS11
With TilS11
Without TilS11
Nº of genomes with TilS12
Nº of genomes without TilS12
With TilS12
Without TilS12
Nº of genomes with TilS13
Nº of genomes without TilS13
With TilS13
Without TilS13
Nº of genomes with TilS14
Nº of genomes without TilS14
With TilS14
Without TilS14
Nº of genomes with TilS15
Nº of genomes without TilS15
With TilS15
Without TilS15

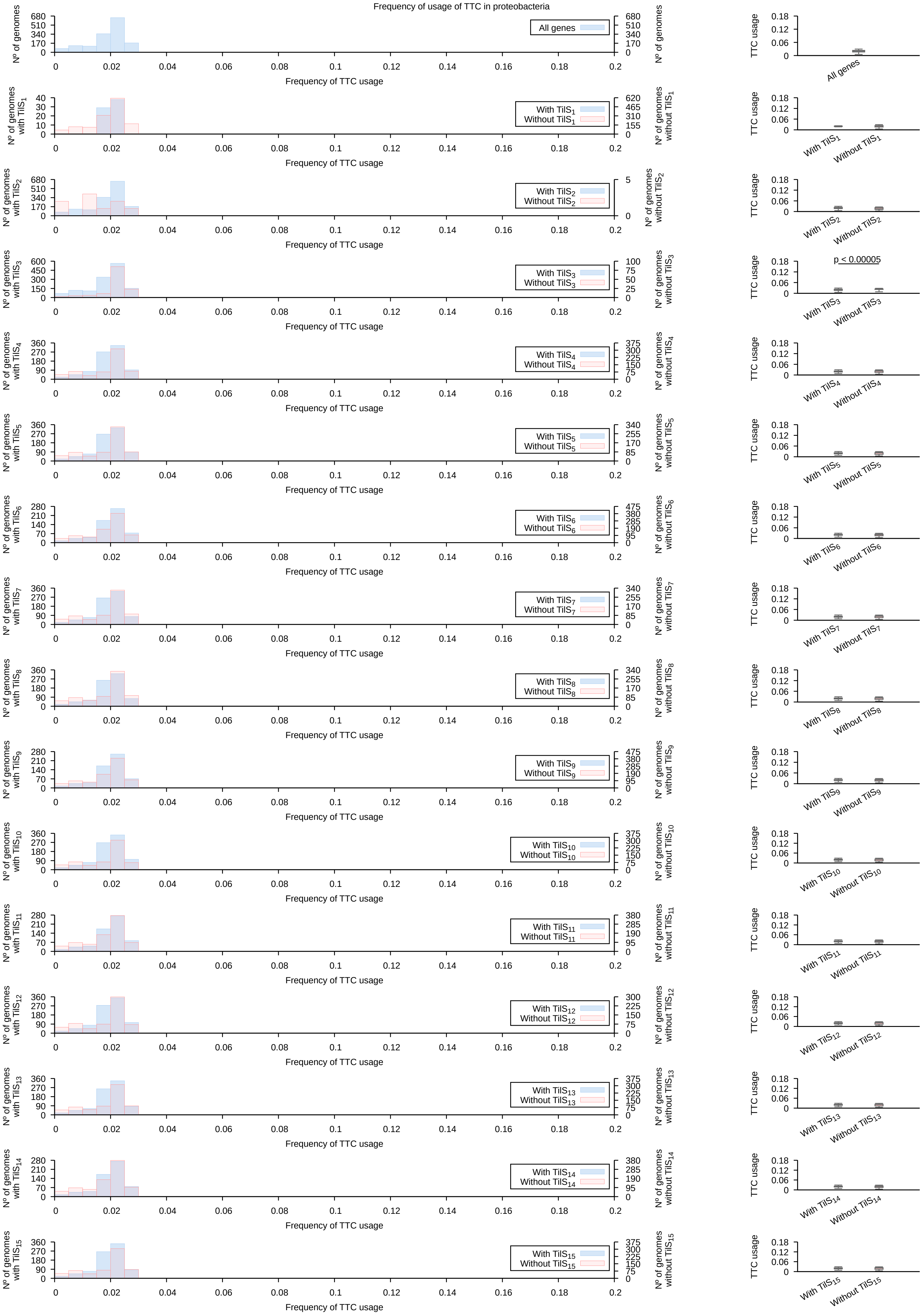
Frequency of usage of TTC in proteobacteria
Nº of genomes
All genes
Frequency of TTC usage
TTC usage
With TilS1
Without TilS1
With TilS2
Without TilS2
With TilS3
Without TilS3
p < 0.00005
With TilS4
Without TilS4
With TilS5
Without TilS5
With TilS6
Without TilS6
With TilS7
Without TilS7
With TilS8
Without TilS8
With TilS9
Without TilS9
With TilS10
Without TilS10
With TilS11
Without TilS11
With TilS12
Without TilS12
With TilS13
Without TilS13
With TilS14
Without TilS14
With TilS15
Without TilS15

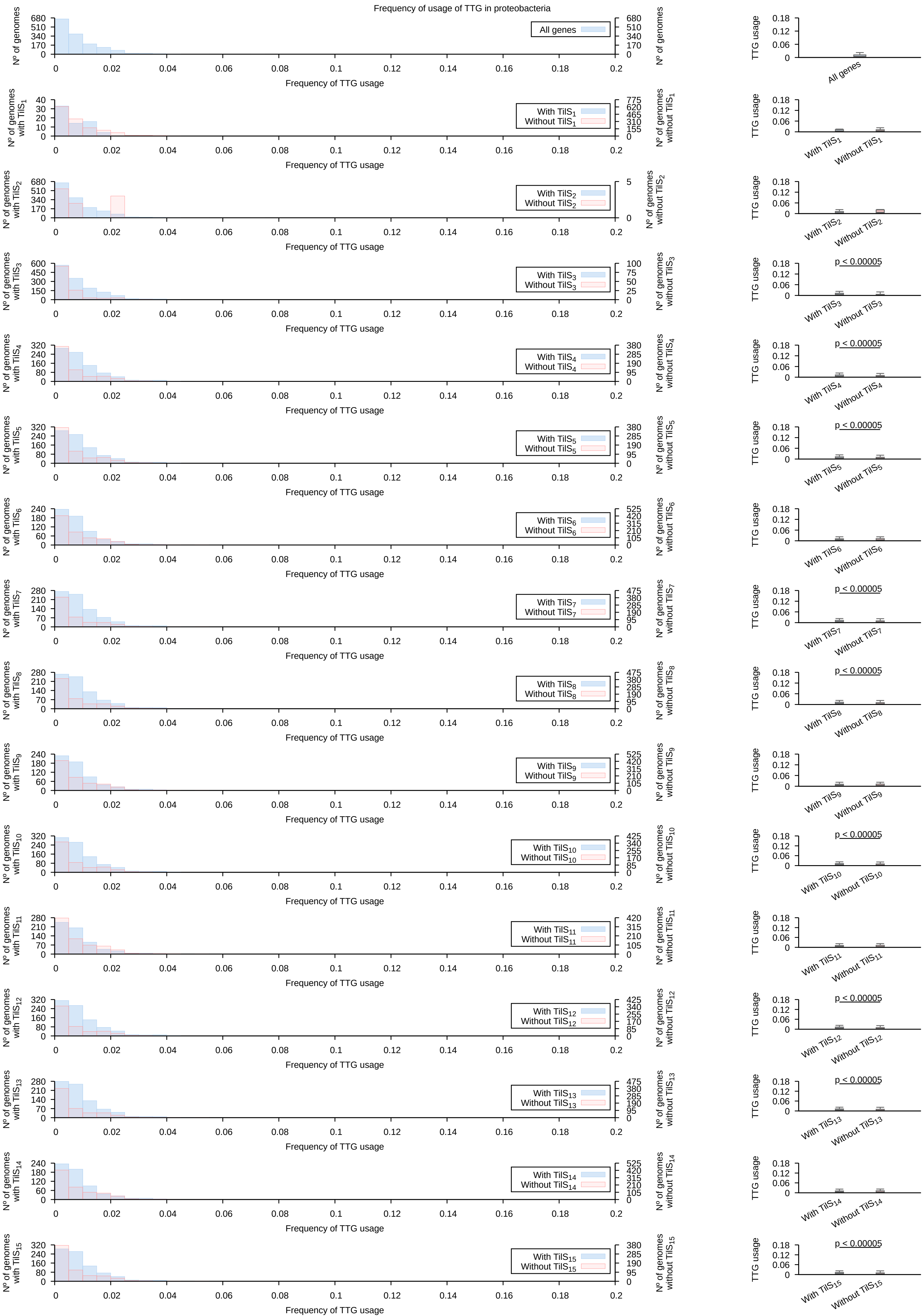
Frequency of usage of TTG in proteobacteria
Nº of genomes
All genes
Frequency of TTG usage
TTG usage
Nº of genomes with TilS$_1$
Nº of genomes without TilS$_1$
With TilS$_1$
Without TilS$_1$
Nº of genomes with TilS$_2$
Nº of genomes without TilS$_2$
With TilS$_2$
Without TilS$_2$
Nº of genomes with TilS$_3$
Nº of genomes without TilS$_3$
With TilS$_3$
Without TilS$_3$
p < 0.00005
Nº of genomes with TilS$_4$
Nº of genomes without TilS$_4$
With TilS$_4$
Without TilS$_4$
p < 0.00005
Nº of genomes with TilS$_5$
Nº of genomes without TilS$_5$
With TilS$_5$
Without TilS$_5$
p < 0.00005
Nº of genomes with TilS$_6$
Nº of genomes without TilS$_6$
With TilS$_6$
Without TilS$_6$
Nº of genomes with TilS$_7$
Nº of genomes without TilS$_7$
With TilS$_7$
Without TilS$_7$
p < 0.00005
Nº of genomes with TilS$_8$
Nº of genomes without TilS$_8$
With TilS$_8$
Without TilS$_8$
p < 0.00005
Nº of genomes with TilS$_9$
Nº of genomes without TilS$_9$
With TilS$_9$
Without TilS$_9$
Nº of genomes with TilS$_{10}$
Nº of genomes without TilS$_{10}$
With TilS$_{10}$
Without TilS$_{10}$
p < 0.00005
Nº of genomes with TilS$_{11}$
Nº of genomes without TilS$_{11}$
With TilS$_{11}$
Without TilS$_{11}$
Nº of genomes with TilS$_{12}$
Nº of genomes without TilS$_{12}$
With TilS$_{12}$
Without TilS$_{12}$
p < 0.00005
Nº of genomes with TilS$_{13}$
Nº of genomes without TilS$_{13}$
With TilS$_{13}$
Without TilS$_{13}$
p < 0.00005
Nº of genomes with TilS$_{14}$
Nº of genomes without TilS$_{14}$
With TilS$_{14}$
Without TilS$_{14}$
Nº of genomes with TilS$_{15}$
Nº of genomes without TilS$_{15}$
With TilS$_{15}$
Without TilS$_{15}$
p < 0.00005

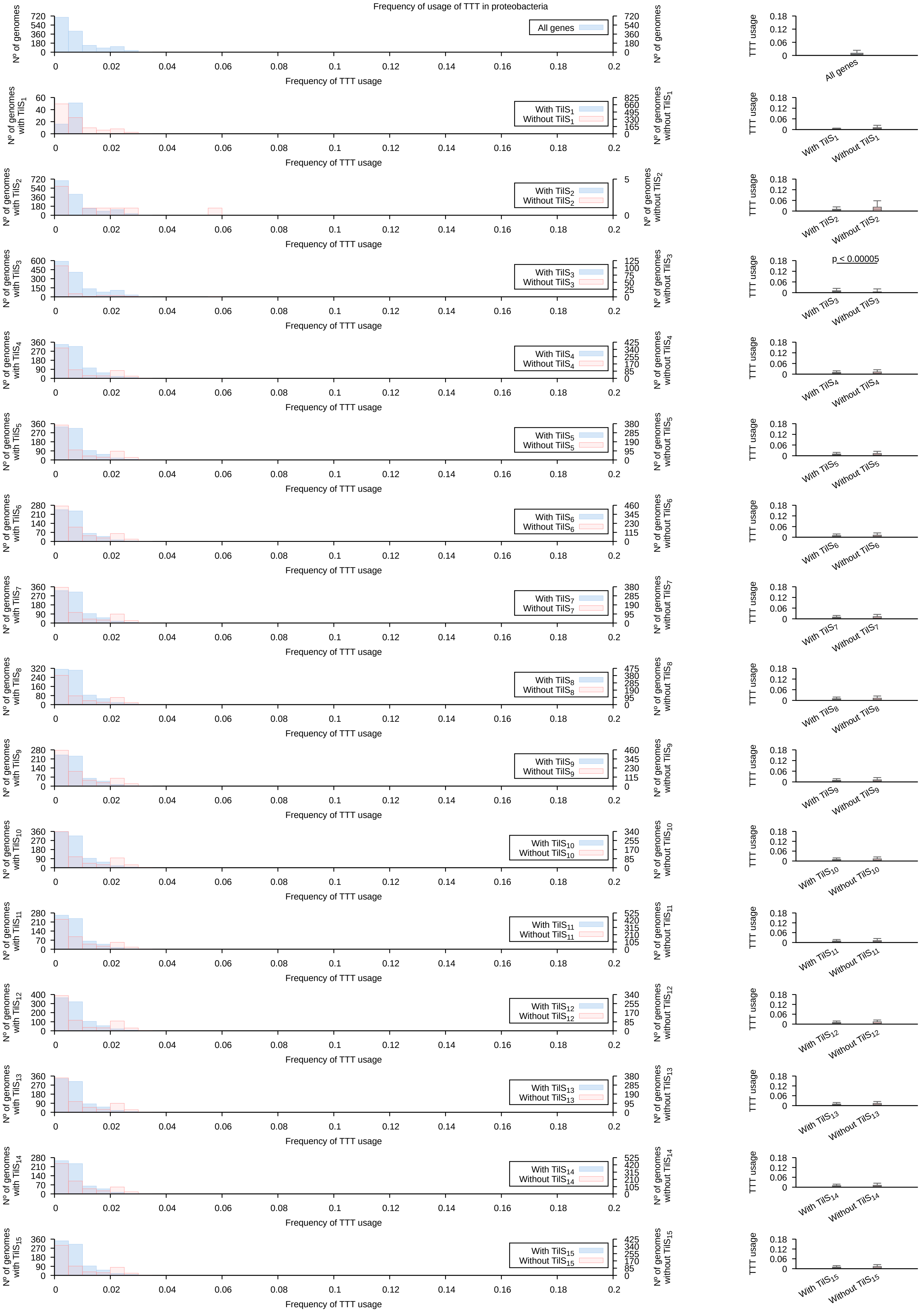

Frequency of usage of TTT in proteobacteria
Nº of genomes
All genes
Frequency of TTT usage
TTT usage
With TilS1
Without TilS1
Nº of genomes with TilS1
Nº of genomes without TilS1
With TilS2
Without TilS2
With TilS3
Without TilS3
p < 0.00005
With TilS4
Without TilS4
With TilS5
Without TilS5
With TilS6
Without TilS6
With TilS7
Without TilS7
With TilS8
Without TilS8
With TilS9
Without TilS9
With TilS10
Without TilS10
With TilS11
Without TilS11
With TilS12
Without TilS12
With TilS13
Without TilS13
With TilS14
Without TilS14
With TilS15
Without TilS15

Figure S10. Histogram of codon usage in high expression genes of genomes that present or lack genes involved in s^2U formation. Top left graph: histogram of the usage of the indicated codon in highly expressed genes of proteobacterial genomes. **Top right graph:** box plot indicating the distribution of codon usage in the same population. **Lower graphs:** The collection of 1484 proteobacterial genomes were separated in two groups, based on the presence (light blue) or absence (orange) of the indicated pattern in any gene of the analyzed genomes. Patterns are indicated using subscript numbers. See table S4 for a detailed description of the used patterns. **Left:** Data is represented in superposed histograms. **Right:** Data is represented in a boxplot. An horizontal bar was added when the two populations are different based on Wilcoxon-Mann-Whitney test and $p < 0.00005$. Whiskers include 95% of data.

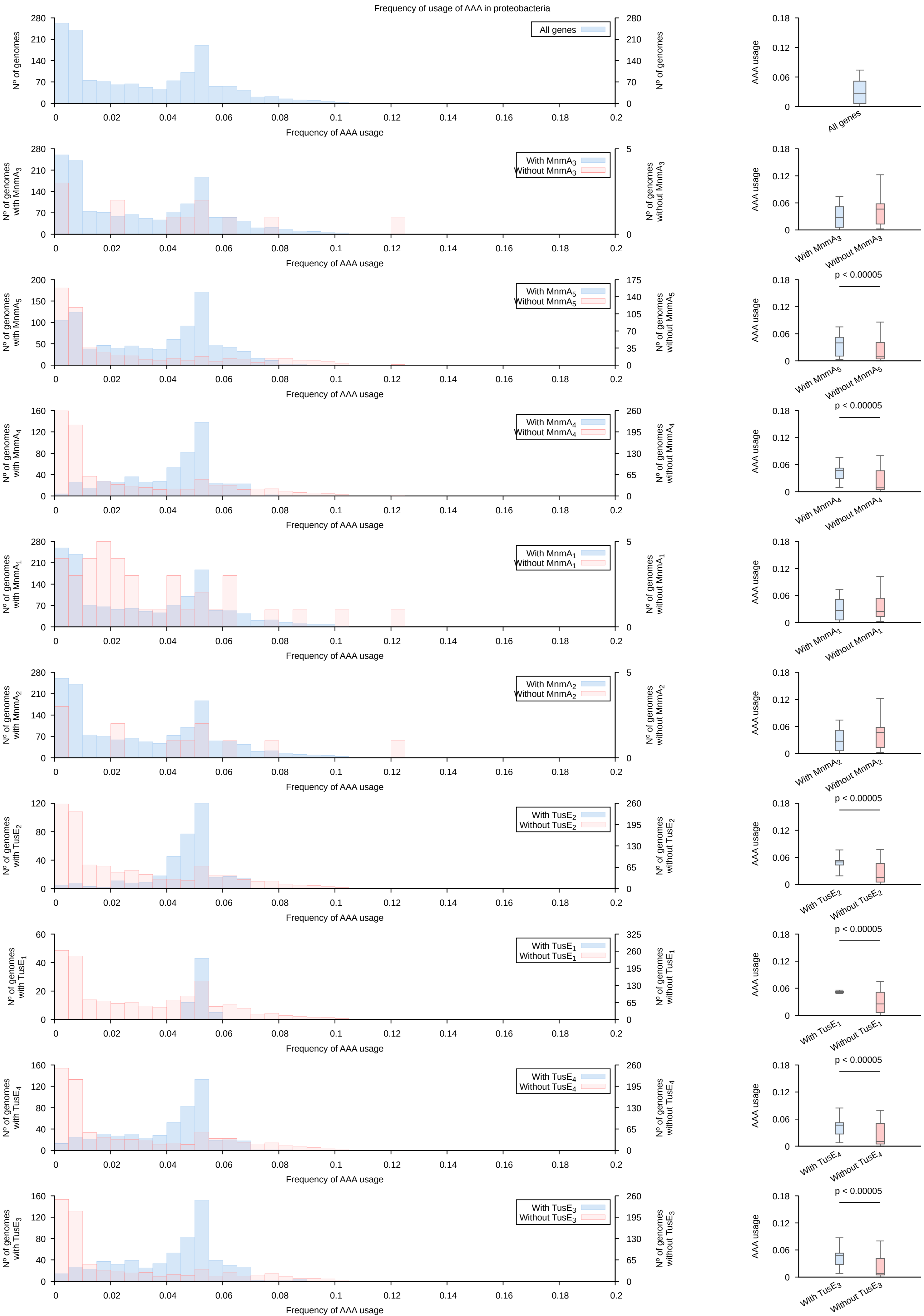

Frequency of usage of AAA in proteobacteria
All genes
Nº of genomes
Frequency of AAA usage
AAA usage
With MnmA$_3$
Without MnmA$_3$
Nº of genomes with MnmA$_3$
Nº of genomes without MnmA$_3$
With MnmA$_5$
Without MnmA$_5$
Nº of genomes with MnmA$_5$
Nº of genomes without MnmA$_5$
p < 0.00005
With MnmA$_4$
Without MnmA$_4$
Nº of genomes with MnmA$_4$
Nº of genomes without MnmA$_4$
p < 0.00005
With MnmA$_1$
Without MnmA$_1$
Nº of genomes with MnmA$_1$
Nº of genomes without MnmA$_1$
With MnmA$_2$
Without MnmA$_2$
Nº of genomes with MnmA$_2$
Nº of genomes without MnmA$_2$
With TusE$_2$
Without TusE$_2$
Nº of genomes with TusE$_2$
Nº of genomes without TusE$_2$
p < 0.00005
With TusE$_1$
Without TusE$_1$
Nº of genomes with TusE$_1$
Nº of genomes without TusE$_1$
p < 0.00005
With TusE$_4$
Without TusE$_4$
Nº of genomes with TusE$_4$
Nº of genomes without TusE$_4$
p < 0.00005
With TusE$_3$
Without TusE$_3$
Nº of genomes with TusE$_3$
Nº of genomes without TusE$_3$
p < 0.00005

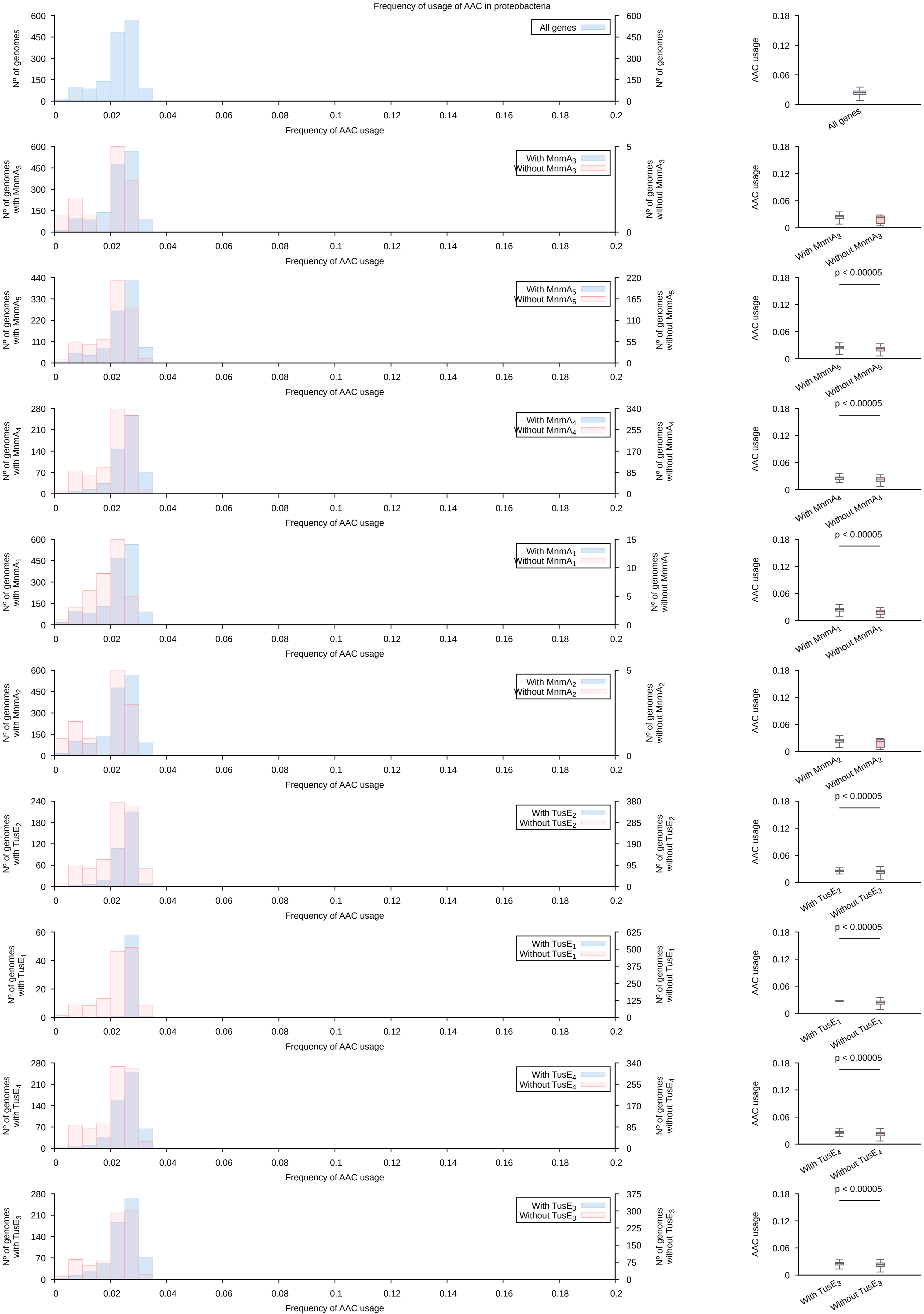

Frequency of usage of AAC in proteobacteria
All genes
Nº of genomes
Frequency of AAC usage
AAC usage
All genes
With MnmA3
Without MnmA3
Nº of genomes with MnmA3
Nº of genomes without MnmA3
With MnmA5
Without MnmA5
Nº of genomes with MnmA5
Nº of genomes without MnmA5
p < 0.00005
With MnmA4
Without MnmA4
Nº of genomes with MnmA4
Nº of genomes without MnmA4
p < 0.00005
With MnmA1
Without MnmA1
Nº of genomes with MnmA1
Nº of genomes without MnmA1
p < 0.00005
With MnmA2
Without MnmA2
Nº of genomes with MnmA2
Nº of genomes without MnmA2
With TusE2
Without TusE2
Nº of genomes with TusE2
Nº of genomes without TusE2
p < 0.00005
With TusE1
Without TusE1
Nº of genomes with TusE1
Nº of genomes without TusE1
p < 0.00005
With TusE4
Without TusE4
Nº of genomes with TusE4
Nº of genomes without TusE4
p < 0.00005
With TusE3
Without TusE3
Nº of genomes with TusE3
Nº of genomes without TusE3
p < 0.00005

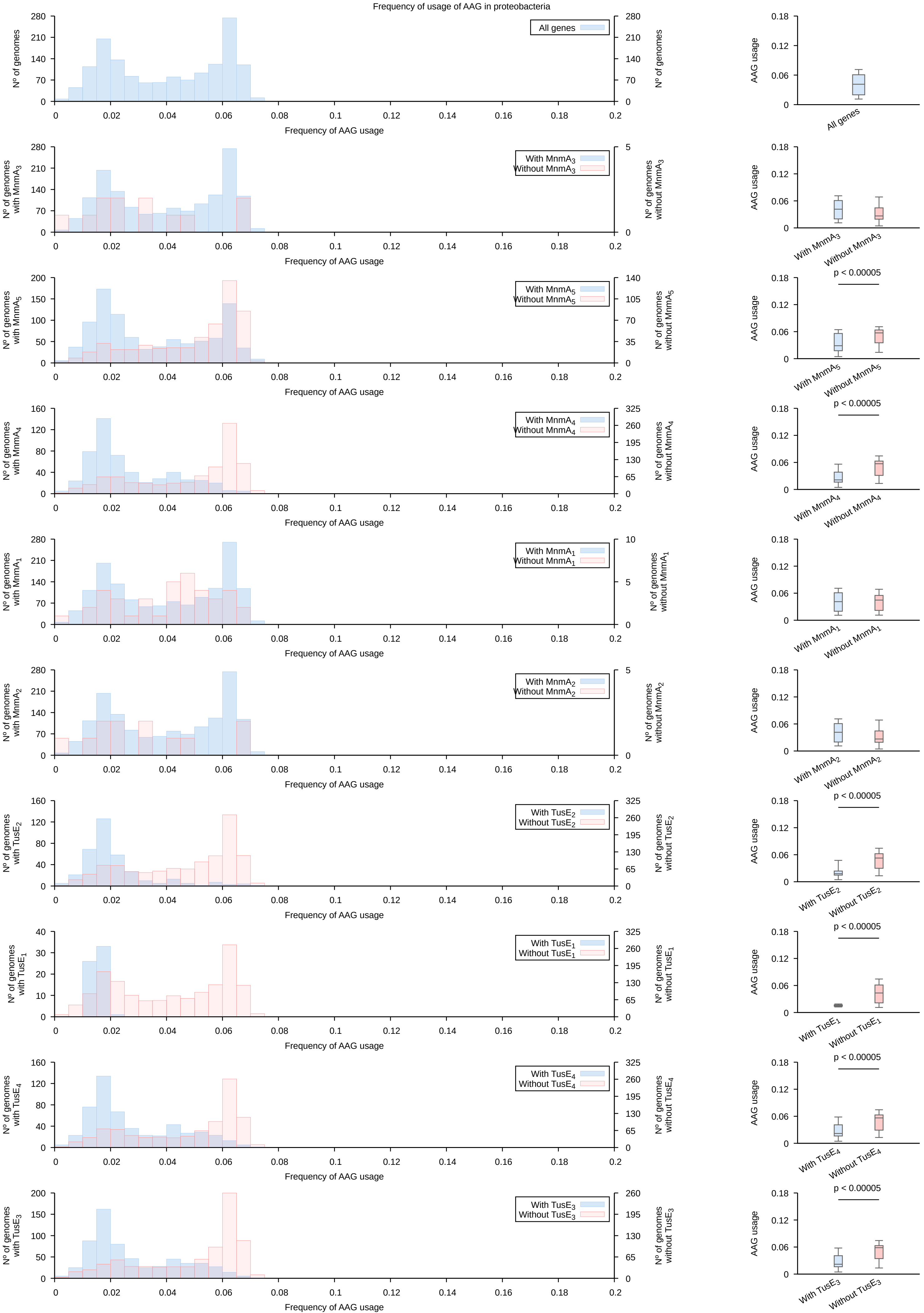

Frequency of usage of AAG in proteobacteria
All genes
With $MnmA_3$
Without $MnmA_3$
With $MnmA_5$
Without $MnmA_5$
With $MnmA_4$
Without $MnmA_4$
With $MnmA_1$
Without $MnmA_1$
With $MnmA_2$
Without $MnmA_2$
With $TusE_2$
Without $TusE_2$
With $TusE_1$
Without $TusE_1$
With $TusE_4$
Without $TusE_4$
With $TusE_3$
Without $TusE_3$
Nº of genomes
Nº of genomes with $MnmA_3$
Nº of genomes without $MnmA_3$
Nº of genomes with $MnmA_5$
Nº of genomes without $MnmA_5$
Nº of genomes with $MnmA_4$
Nº of genomes without $MnmA_4$
Nº of genomes with $MnmA_1$
Nº of genomes without $MnmA_1$
Nº of genomes with $MnmA_2$
Nº of genomes without $MnmA_2$
Nº of genomes with $TusE_2$
Nº of genomes without $TusE_2$
Nº of genomes with $TusE_1$
Nº of genomes without $TusE_1$
Nº of genomes with $TusE_4$
Nº of genomes without $TusE_4$
Nº of genomes with $TusE_3$
Nº of genomes without $TusE_3$
Frequency of AAG usage
AAG usage
p < 0.00005

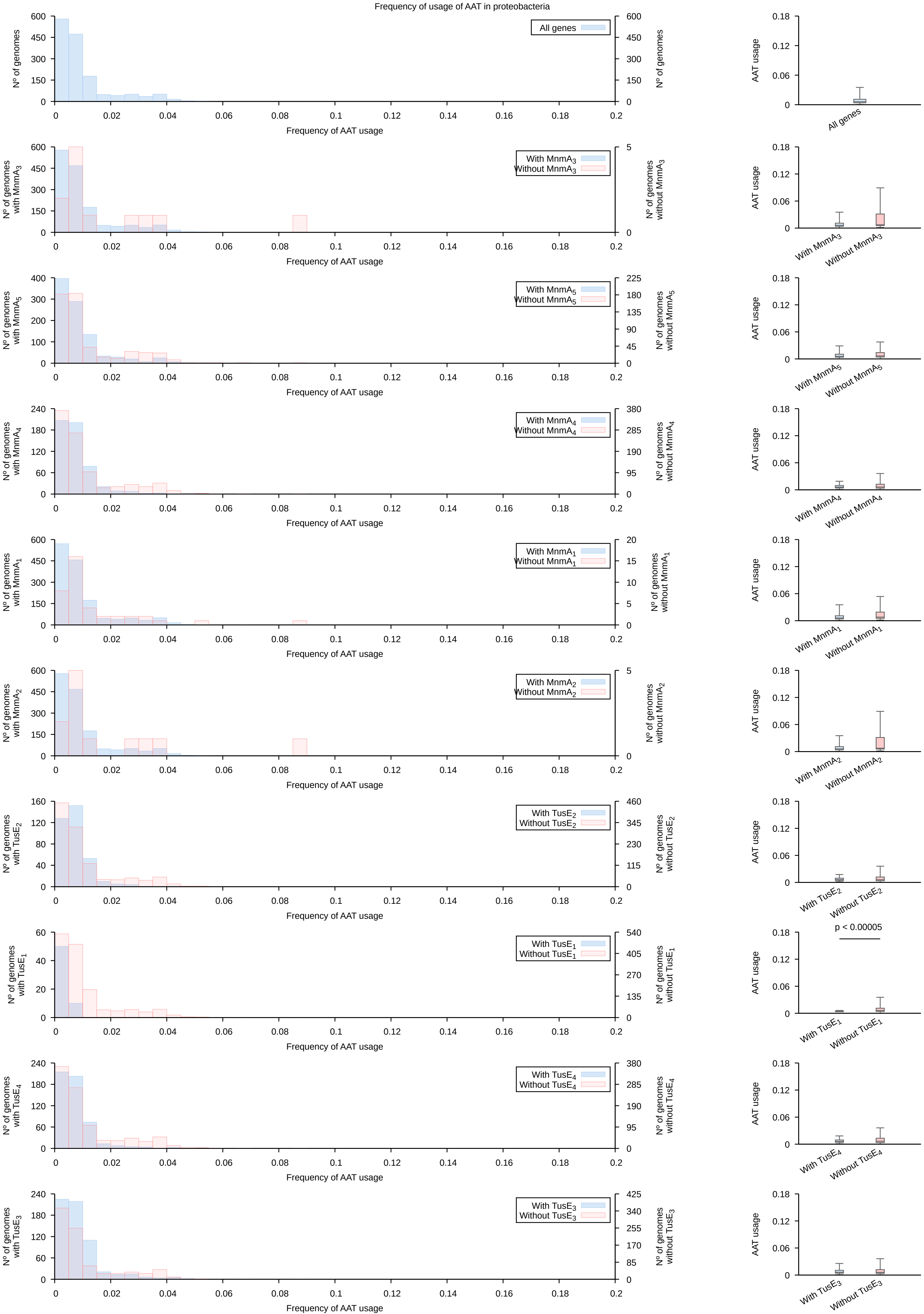
Frequency of usage of AAT in proteobacteria
Nº of genomes
All genes
Frequency of AAT usage
AAT usage
With MnmA$_3$
Without MnmA$_3$
Nº of genomes with MnmA$_3$
Nº of genomes without MnmA$_3$
With MnmA$_5$
Without MnmA$_5$
Nº of genomes with MnmA$_5$
Nº of genomes without MnmA$_5$
With MnmA$_4$
Without MnmA$_4$
Nº of genomes with MnmA$_4$
Nº of genomes without MnmA$_4$
With MnmA$_1$
Without MnmA$_1$
Nº of genomes with MnmA$_1$
Nº of genomes without MnmA$_1$
With MnmA$_2$
Without MnmA$_2$
Nº of genomes with MnmA$_2$
Nº of genomes without MnmA$_2$
With TusE$_2$
Without TusE$_2$
Nº of genomes with TusE$_2$
Nº of genomes without TusE$_2$
With TusE$_1$
Without TusE$_1$
Nº of genomes with TusE$_1$
Nº of genomes without TusE$_1$
p < 0.00005
With TusE$_4$
Without TusE$_4$
Nº of genomes with TusE$_4$
Nº of genomes without TusE$_4$
With TusE$_3$
Without TusE$_3$
Nº of genomes with TusE$_3$
Nº of genomes without TusE$_3$
0 0.02 0.04 0.06 0.08 0.1 0.12 0.14 0.16 0.18 0.2
0 0.06 0.12 0.18

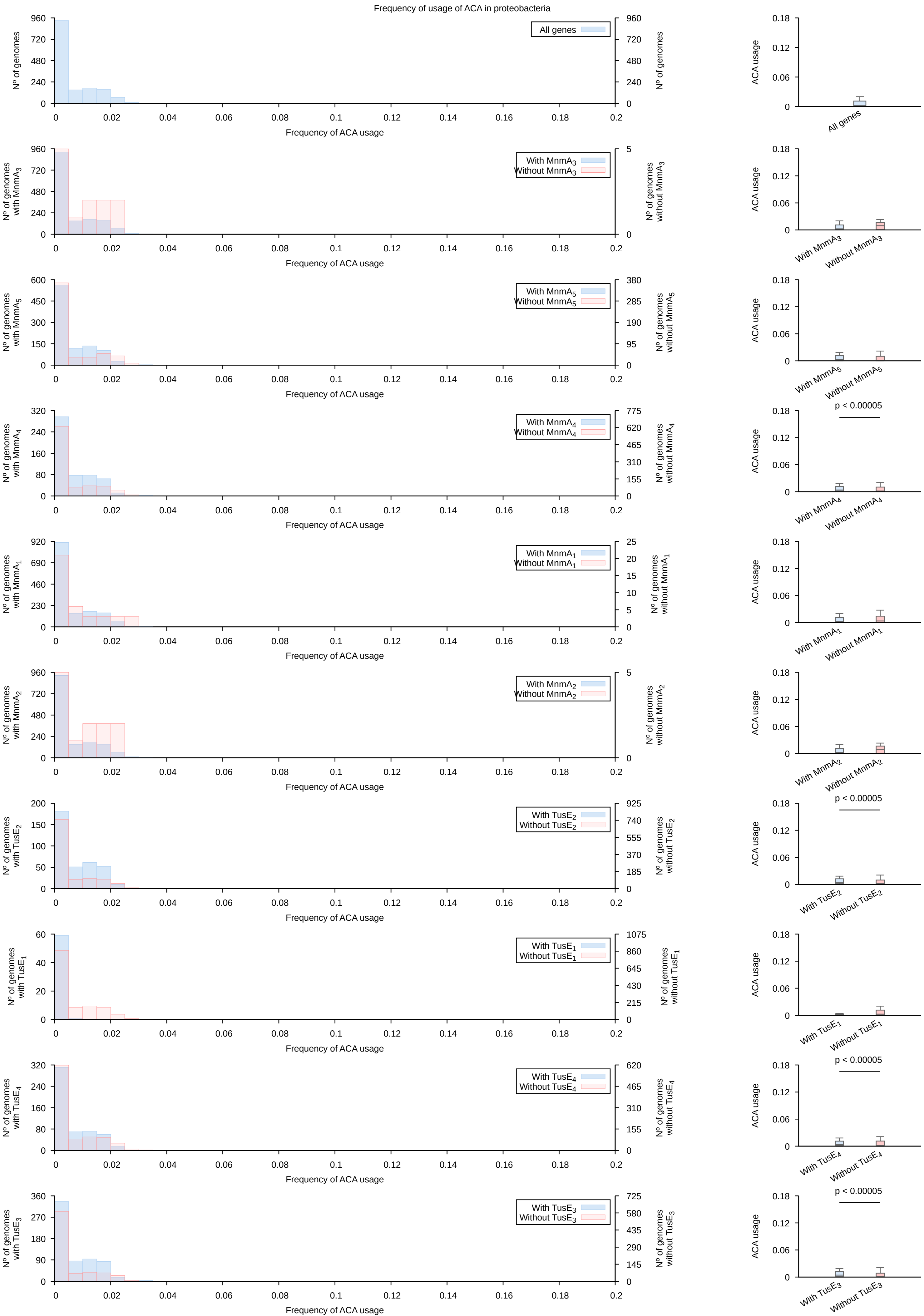
Frequency of usage of ACA in proteobacteria
Nº of genomes
Frequency of ACA usage
ACA usage
All genes
Nº of genomes with MnmA$_3$
Nº of genomes without MnmA$_3$
With MnmA$_3$
Without MnmA$_3$
Nº of genomes with MnmA$_5$
Nº of genomes without MnmA$_5$
With MnmA$_5$
Without MnmA$_5$
Nº of genomes with MnmA$_4$
Nº of genomes without MnmA$_4$
With MnmA$_4$
Without MnmA$_4$
p < 0.00005
Nº of genomes with MnmA$_1$
Nº of genomes without MnmA$_1$
With MnmA$_1$
Without MnmA$_1$
Nº of genomes with MnmA$_2$
Nº of genomes without MnmA$_2$
With MnmA$_2$
Without MnmA$_2$
Nº of genomes with TusE$_2$
Nº of genomes without TusE$_2$
With TusE$_2$
Without TusE$_2$
p < 0.00005
Nº of genomes with TusE$_1$
Nº of genomes without TusE$_1$
With TusE$_1$
Without TusE$_1$
Nº of genomes with TusE$_4$
Nº of genomes without TusE$_4$
With TusE$_4$
Without TusE$_4$
p < 0.00005
Nº of genomes with TusE$_3$
Nº of genomes without TusE$_3$
With TusE$_3$
Without TusE$_3$
p < 0.00005

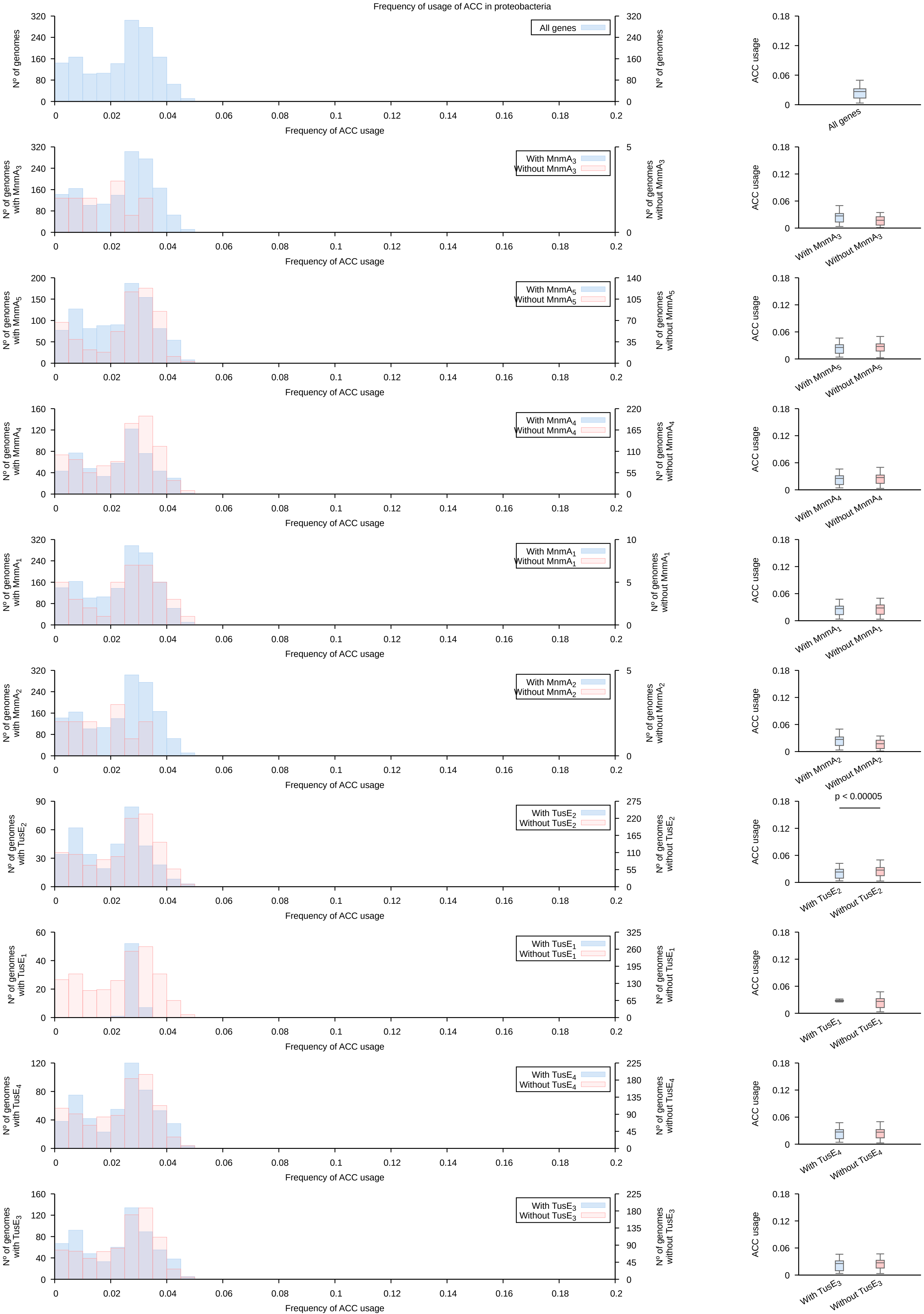

Frequency of usage of ACC in proteobacteria
All genes
With MnmA3
Without MnmA3
With MnmA5
Without MnmA5
With MnmA4
Without MnmA4
With MnmA1
Without MnmA1
With MnmA2
Without MnmA2
With TusE2
Without TusE2
p < 0.00005
With TusE1
Without TusE1
With TusE4
Without TusE4
With TusE3
Without TusE3
Nº of genomes
ACC usage
Frequency of ACC usage

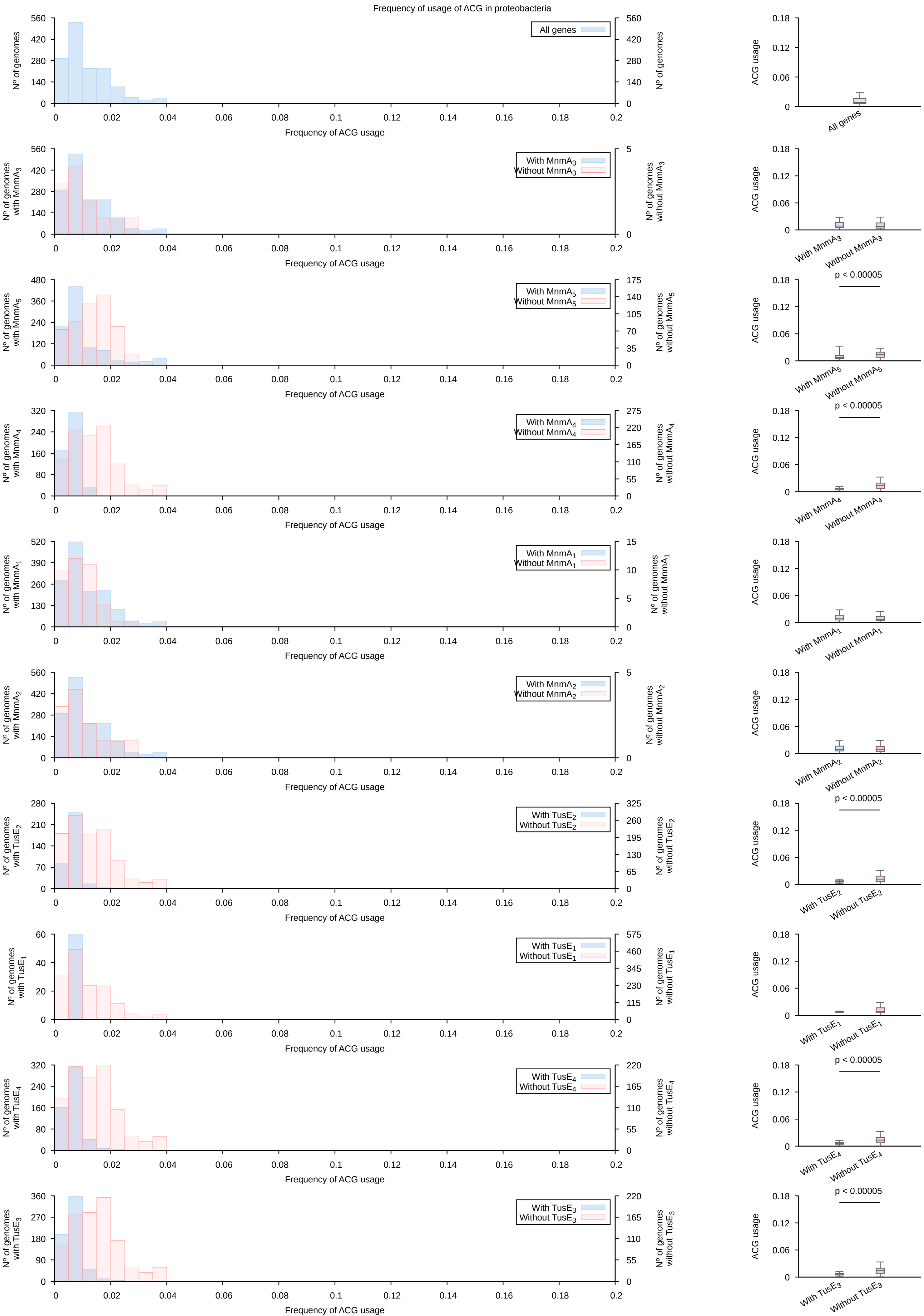
Frequency of usage of ACG in proteobacteria
Nº of genomes
Frequency of ACG usage
ACG usage
All genes
Nº of genomes with MnmA$_3$
Nº of genomes without MnmA$_3$
With MnmA$_3$
Without MnmA$_3$
Nº of genomes with MnmA$_5$
Nº of genomes without MnmA$_5$
With MnmA$_5$
Without MnmA$_5$
p < 0.00005
Nº of genomes with MnmA$_4$
Nº of genomes without MnmA$_4$
With MnmA$_4$
Without MnmA$_4$
p < 0.00005
Nº of genomes with MnmA$_1$
Nº of genomes without MnmA$_1$
With MnmA$_1$
Without MnmA$_1$
Nº of genomes with MnmA$_2$
Nº of genomes without MnmA$_2$
With MnmA$_2$
Without MnmA$_2$
Nº of genomes with TusE$_2$
Nº of genomes without TusE$_2$
With TusE$_2$
Without TusE$_2$
p < 0.00005
Nº of genomes with TusE$_1$
Nº of genomes without TusE$_1$
With TusE$_1$
Without TusE$_1$
Nº of genomes with TusE$_4$
Nº of genomes without TusE$_4$
With TusE$_4$
Without TusE$_4$
p < 0.00005
Nº of genomes with TusE$_3$
Nº of genomes without TusE$_3$
With TusE$_3$
Without TusE$_3$
p < 0.00005

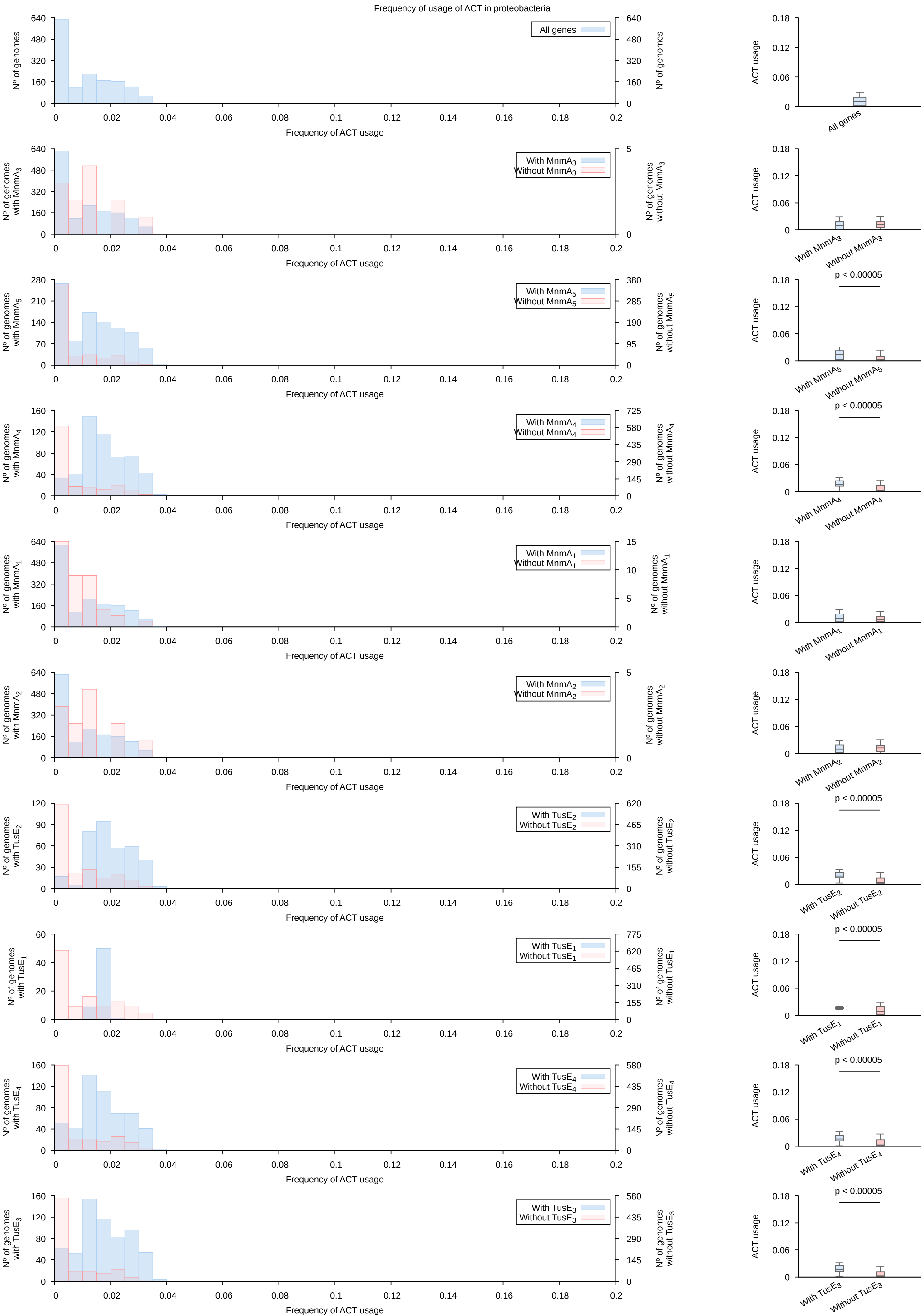
Frequency of usage of ACT in proteobacteria
All genes
Nº of genomes
Frequency of ACT usage
ACT usage
With MnmA3
Without MnmA3
Nº of genomes with MnmA3
Nº of genomes without MnmA3
With MnmA5
Without MnmA5
Nº of genomes with MnmA5
Nº of genomes without MnmA5
With MnmA4
Without MnmA4
Nº of genomes with MnmA4
Nº of genomes without MnmA4
With MnmA1
Without MnmA1
Nº of genomes with MnmA1
Nº of genomes without MnmA1
With MnmA2
Without MnmA2
Nº of genomes with MnmA2
Nº of genomes without MnmA2
With TusE2
Without TusE2
Nº of genomes with TusE2
Nº of genomes without TusE2
With TusE1
Without TusE1
Nº of genomes with TusE1
Nº of genomes without TusE1
With TusE4
Without TusE4
Nº of genomes with TusE4
Nº of genomes without TusE4
With TusE3
Without TusE3
Nº of genomes with TusE3
Nº of genomes without TusE3
p < 0.00005

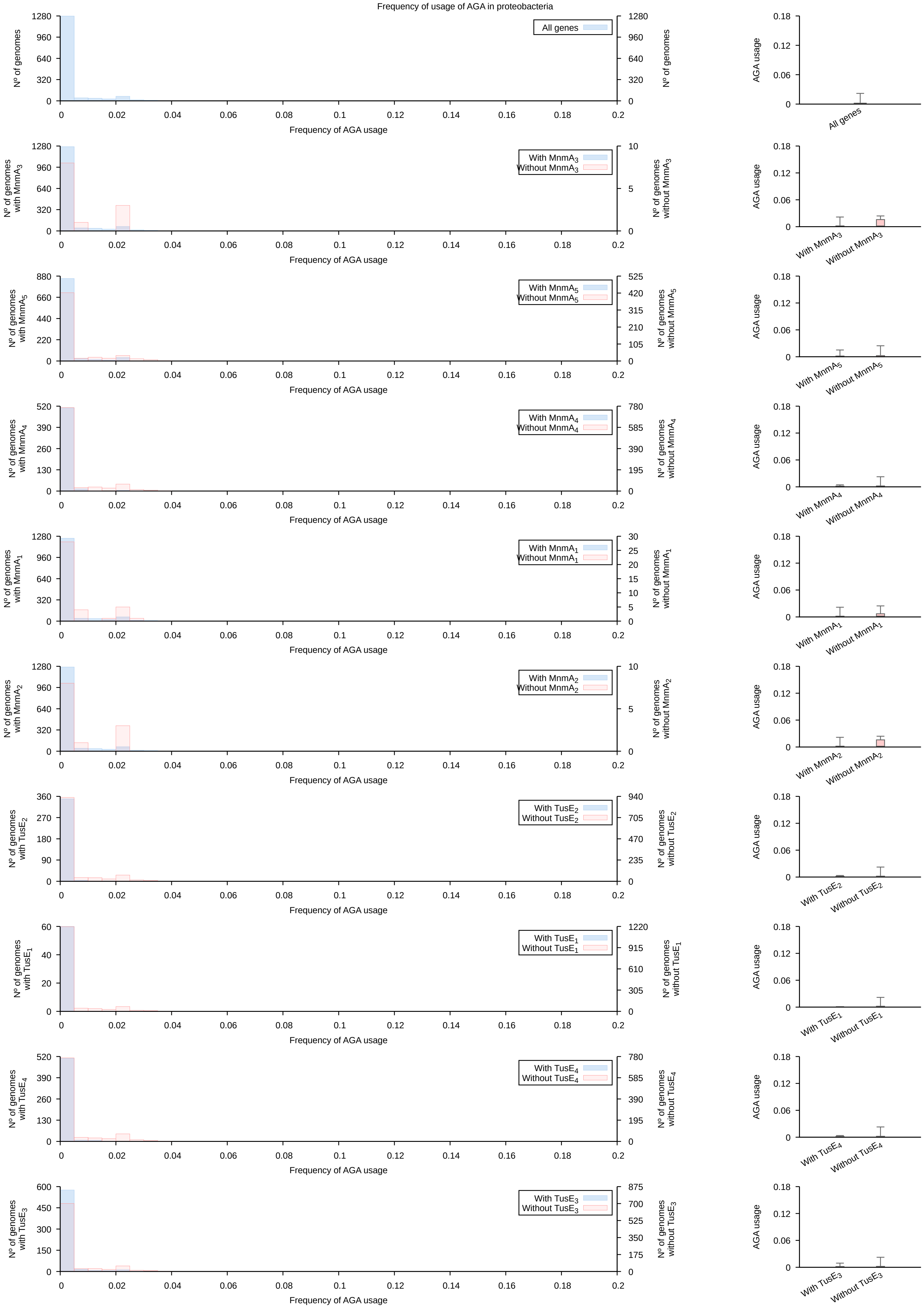

Frequency of usage of AGA in proteobacteria
Nº of genomes
Frequency of AGA usage
All genes
AGA usage
Nº of genomes with MnmA$_3$
Nº of genomes without MnmA$_3$
With MnmA$_3$
Without MnmA$_3$
Nº of genomes with MnmA$_5$
Nº of genomes without MnmA$_5$
With MnmA$_5$
Without MnmA$_5$
Nº of genomes with MnmA$_4$
Nº of genomes without MnmA$_4$
With MnmA$_4$
Without MnmA$_4$
Nº of genomes with MnmA$_1$
Nº of genomes without MnmA$_1$
With MnmA$_1$
Without MnmA$_1$
Nº of genomes with MnmA$_2$
Nº of genomes without MnmA$_2$
With MnmA$_2$
Without MnmA$_2$
Nº of genomes with TusE$_2$
Nº of genomes without TusE$_2$
With TusE$_2$
Without TusE$_2$
Nº of genomes with TusE$_1$
Nº of genomes without TusE$_1$
With TusE$_1$
Without TusE$_1$
Nº of genomes with TusE$_4$
Nº of genomes without TusE$_4$
With TusE$_4$
Without TusE$_4$
Nº of genomes with TusE$_3$
Nº of genomes without TusE$_3$
With TusE$_3$
Without TusE$_3$

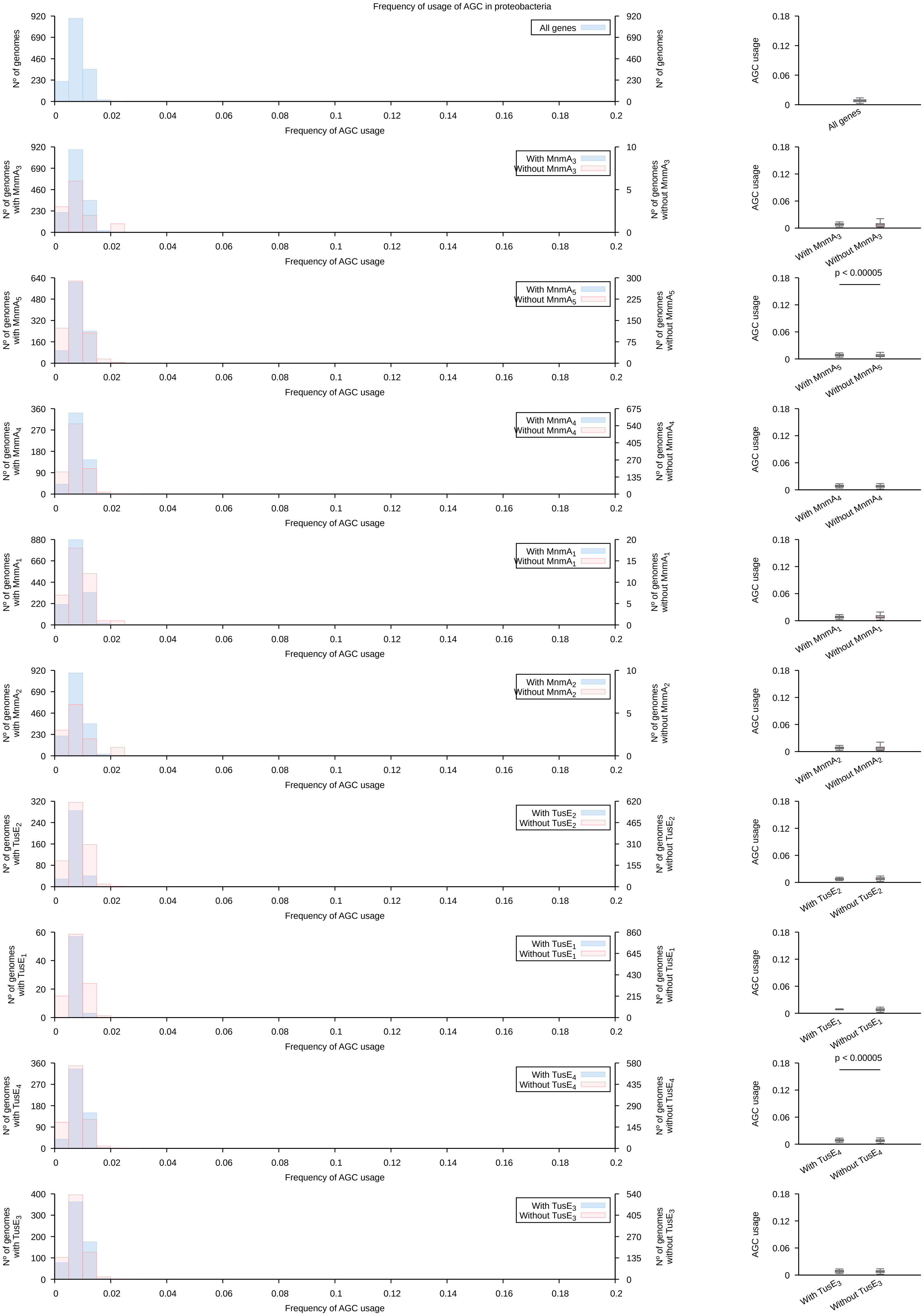
Frequency of usage of AGC in proteobacteria
Nº of genomes
All genes
Frequency of AGC usage
AGC usage
Nº of genomes with MnmA$_3$
Nº of genomes without MnmA$_3$
With MnmA$_3$
Without MnmA$_3$
Nº of genomes with MnmA$_5$
Nº of genomes without MnmA$_5$
With MnmA$_5$
Without MnmA$_5$
p < 0.00005
Nº of genomes with MnmA$_4$
Nº of genomes without MnmA$_4$
With MnmA$_4$
Without MnmA$_4$
Nº of genomes with MnmA$_1$
Nº of genomes without MnmA$_1$
With MnmA$_1$
Without MnmA$_1$
Nº of genomes with MnmA$_2$
Nº of genomes without MnmA$_2$
With MnmA$_2$
Without MnmA$_2$
Nº of genomes with TusE$_2$
Nº of genomes without TusE$_2$
With TusE$_2$
Without TusE$_2$
Nº of genomes with TusE$_1$
Nº of genomes without TusE$_1$
With TusE$_1$
Without TusE$_1$
Nº of genomes with TusE$_4$
Nº of genomes without TusE$_4$
With TusE$_4$
Without TusE$_4$
p < 0.00005
Nº of genomes with TusE$_3$
Nº of genomes without TusE$_3$
With TusE$_3$
Without TusE$_3$

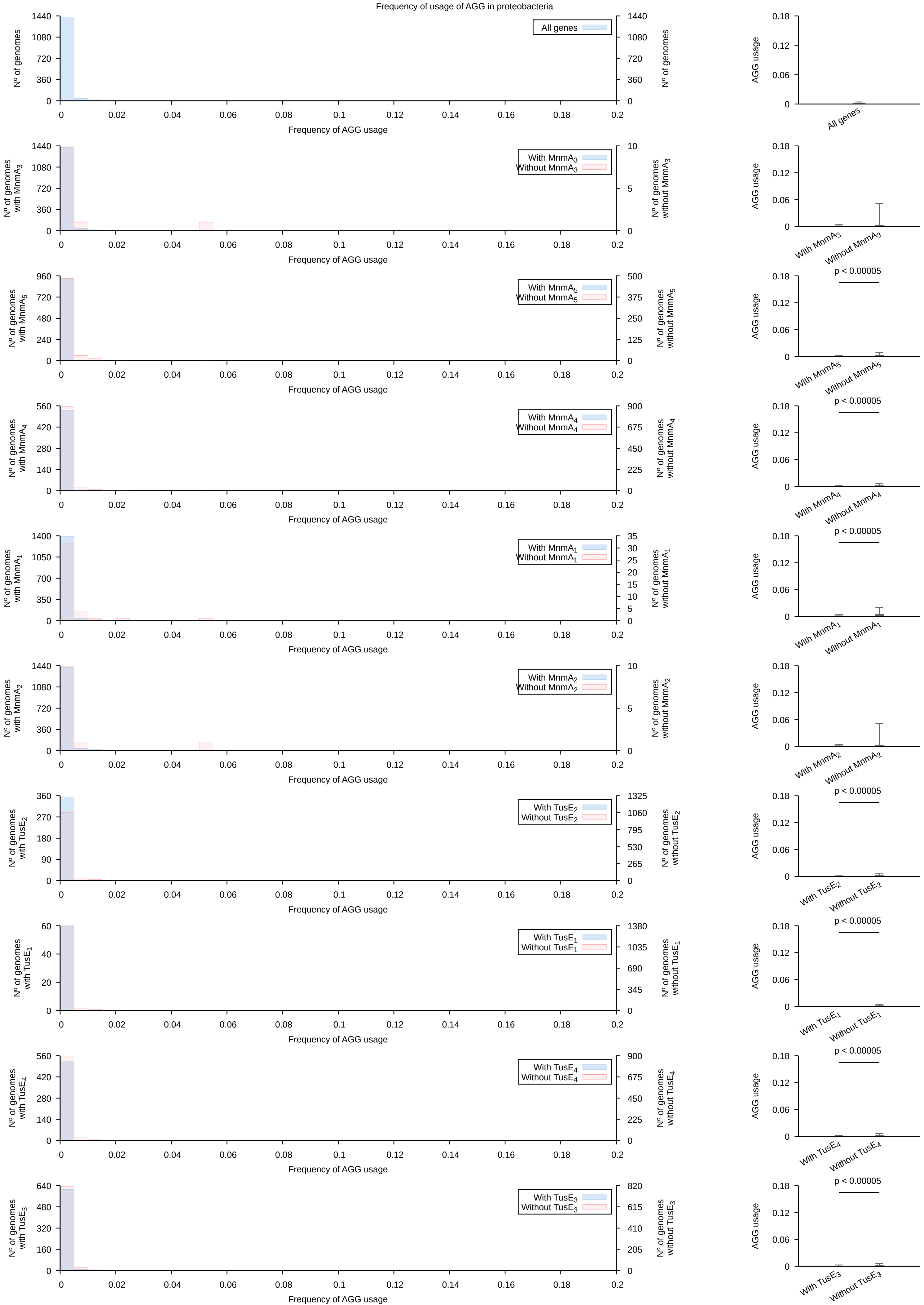
Frequency of usage of AGG in proteobacteria
All genes
Nº of genomes
Frequency of AGG usage
AGG usage
With MnmA$_3$
Without MnmA$_3$
Nº of genomes with MnmA$_3$
Nº of genomes without MnmA$_3$
With MnmA$_5$
Without MnmA$_5$
Nº of genomes with MnmA$_5$
Nº of genomes without MnmA$_5$
p < 0.00005
With MnmA$_4$
Without MnmA$_4$
Nº of genomes with MnmA$_4$
Nº of genomes without MnmA$_4$
p < 0.00005
With MnmA$_1$
Without MnmA$_1$
Nº of genomes with MnmA$_1$
Nº of genomes without MnmA$_1$
p < 0.00005
With MnmA$_2$
Without MnmA$_2$
Nº of genomes with MnmA$_2$
Nº of genomes without MnmA$_2$
With TusE$_2$
Without TusE$_2$
Nº of genomes with TusE$_2$
Nº of genomes without TusE$_2$
p < 0.00005
With TusE$_1$
Without TusE$_1$
Nº of genomes with TusE$_1$
Nº of genomes without TusE$_1$
p < 0.00005
With TusE$_4$
Without TusE$_4$
Nº of genomes with TusE$_4$
Nº of genomes without TusE$_4$
p < 0.00005
With TusE$_3$
Without TusE$_3$
Nº of genomes with TusE$_3$
Nº of genomes without TusE$_3$
p < 0.00005

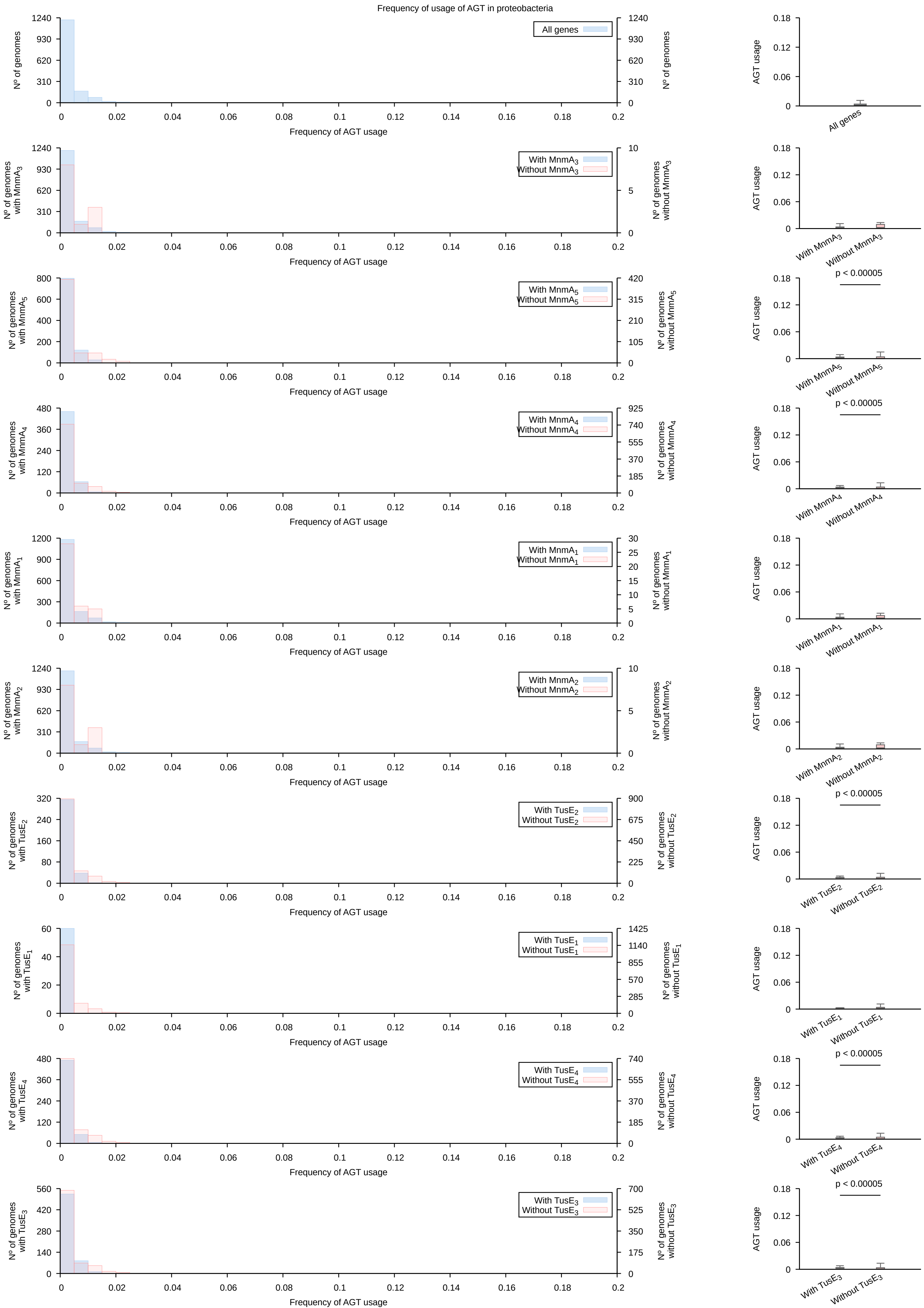
Frequency of usage of AGT in proteobacteria
All genes
Nº of genomes
Frequency of AGT usage
AGT usage
With $MnmA_3$
Without $MnmA_3$
Nº of genomes with $MnmA_3$
Nº of genomes without $MnmA_3$
With $MnmA_5$
Without $MnmA_5$
Nº of genomes with $MnmA_5$
Nº of genomes without $MnmA_5$
p < 0.00005
With $MnmA_4$
Without $MnmA_4$
Nº of genomes with $MnmA_4$
Nº of genomes without $MnmA_4$
p < 0.00005
With $MnmA_1$
Without $MnmA_1$
Nº of genomes with $MnmA_1$
Nº of genomes without $MnmA_1$
With $MnmA_2$
Without $MnmA_2$
Nº of genomes with $MnmA_2$
Nº of genomes without $MnmA_2$
With $TusE_2$
Without $TusE_2$
Nº of genomes with $TusE_2$
Nº of genomes without $TusE_2$
p < 0.00005
With $TusE_1$
Without $TusE_1$
Nº of genomes with $TusE_1$
Nº of genomes without $TusE_1$
With $TusE_4$
Without $TusE_4$
Nº of genomes with $TusE_4$
Nº of genomes without $TusE_4$
p < 0.00005
With $TusE_3$
Without $TusE_3$
Nº of genomes with $TusE_3$
Nº of genomes without $TusE_3$
p < 0.00005

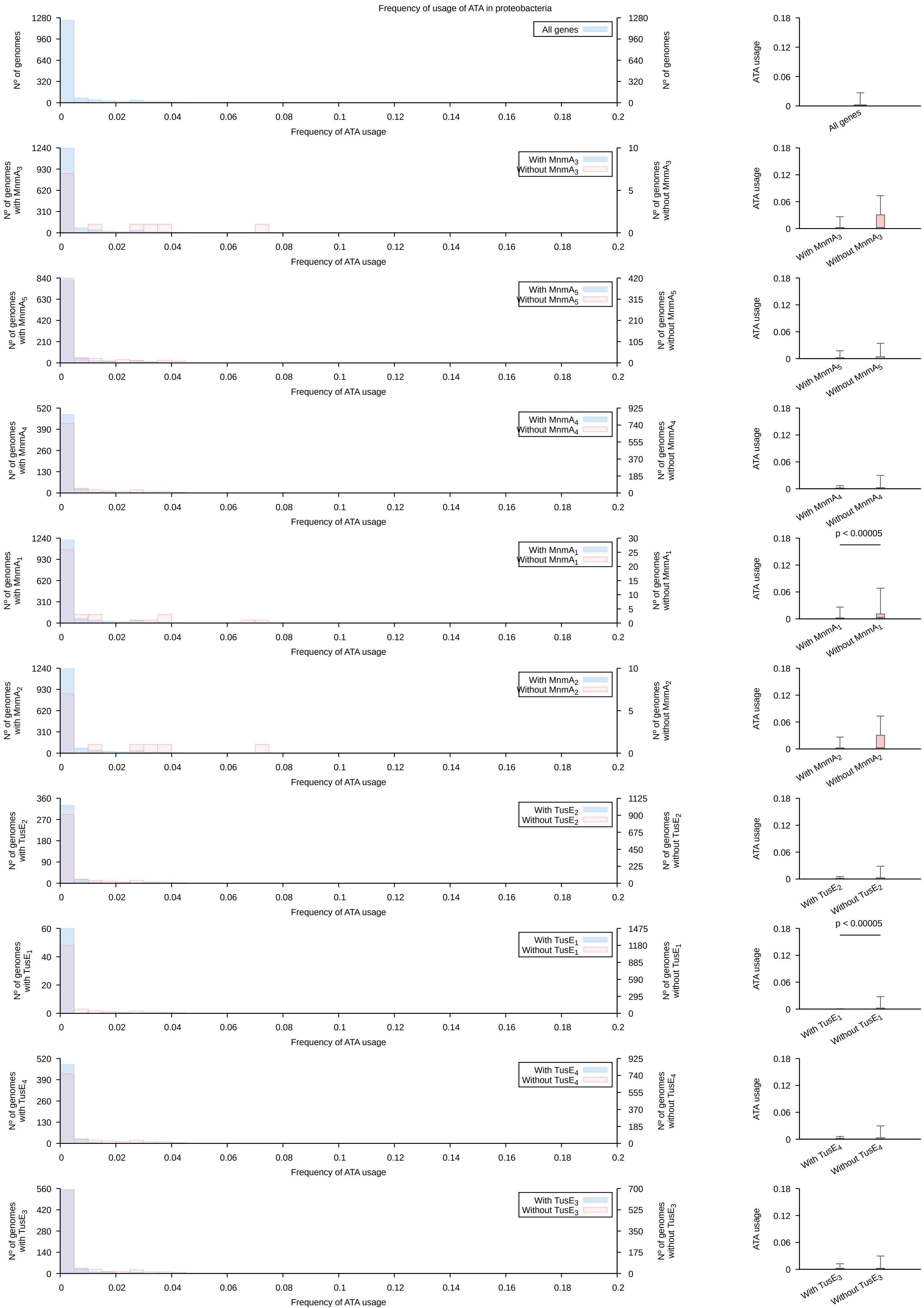
Frequency of usage of ATA in proteobacteria
Nº of genomes
Frequency of ATA usage
ATA usage
All genes
With MnmA3
Without MnmA3
Nº of genomes with MnmA3
Nº of genomes without MnmA3
With MnmA5
Without MnmA5
Nº of genomes with MnmA5
Nº of genomes without MnmA5
With MnmA4
Without MnmA4
Nº of genomes with MnmA4
Nº of genomes without MnmA4
With MnmA1
Without MnmA1
Nº of genomes with MnmA1
Nº of genomes without MnmA1
p < 0.00005
With MnmA2
Without MnmA2
Nº of genomes with MnmA2
Nº of genomes without MnmA2
With TusE2
Without TusE2
Nº of genomes with TusE2
Nº of genomes without TusE2
With TusE1
Without TusE1
Nº of genomes with TusE1
Nº of genomes without TusE1
p < 0.00005
With TusE4
Without TusE4
Nº of genomes with TusE4
Nº of genomes without TusE4
With TusE3
Without TusE3
Nº of genomes with TusE3
Nº of genomes without TusE3

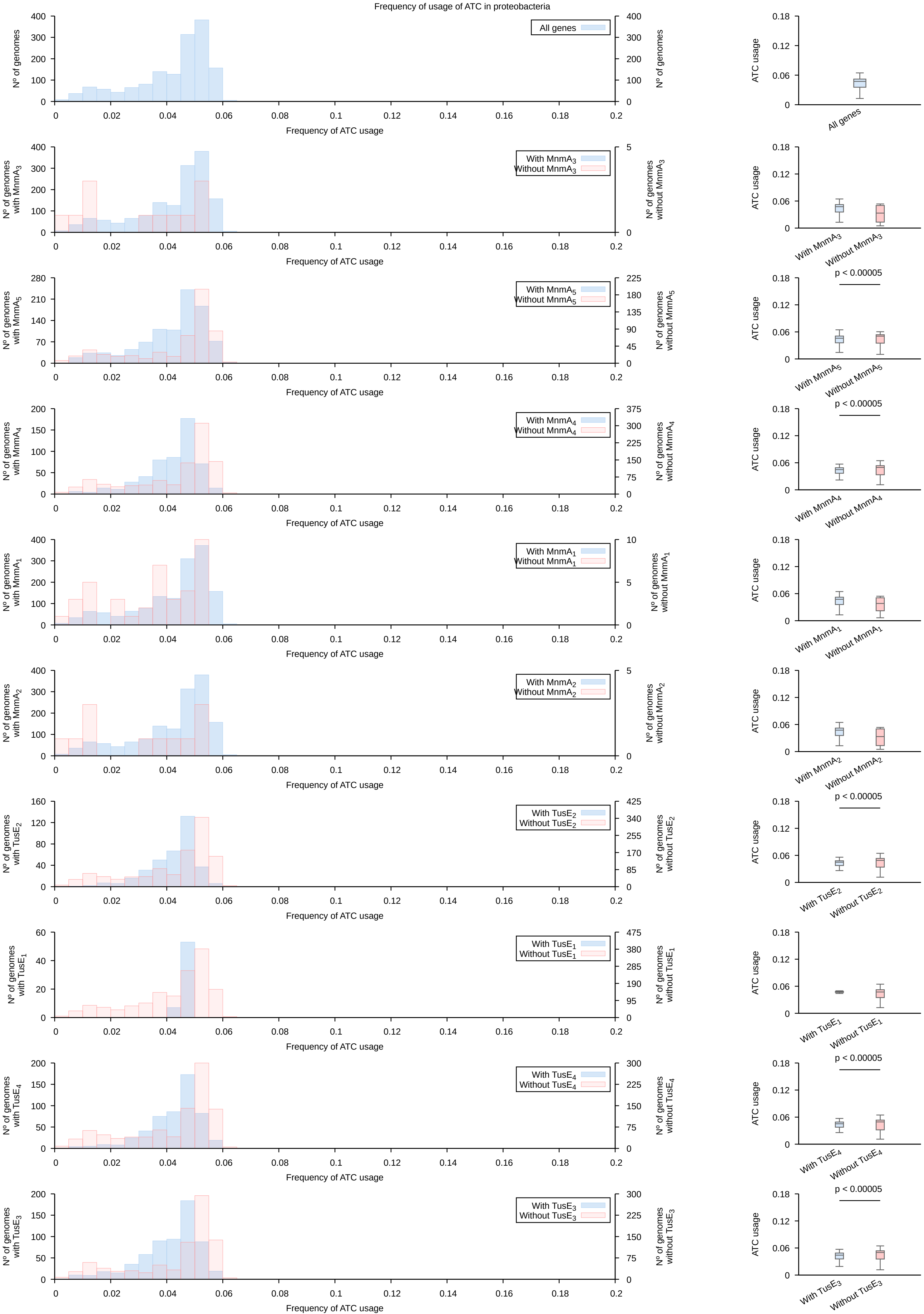

Frequency of usage of ATC in proteobacteria
Nº of genomes
All genes
Frequency of ATC usage
ATC usage
Nº of genomes with $MnmA_3$
Nº of genomes without $MnmA_3$
With $MnmA_3$
Without $MnmA_3$
Nº of genomes with $MnmA_5$
Nº of genomes without $MnmA_5$
With $MnmA_5$
Without $MnmA_5$
p < 0.00005
Nº of genomes with $MnmA_4$
Nº of genomes without $MnmA_4$
With $MnmA_4$
Without $MnmA_4$
p < 0.00005
Nº of genomes with $MnmA_1$
Nº of genomes without $MnmA_1$
With $MnmA_1$
Without $MnmA_1$
Nº of genomes with $MnmA_2$
Nº of genomes without $MnmA_2$
With $MnmA_2$
Without $MnmA_2$
Nº of genomes with $TusE_2$
Nº of genomes without $TusE_2$
With $TusE_2$
Without $TusE_2$
p < 0.00005
Nº of genomes with $TusE_1$
Nº of genomes without $TusE_1$
With $TusE_1$
Without $TusE_1$
Nº of genomes with $TusE_4$
Nº of genomes without $TusE_4$
With $TusE_4$
Without $TusE_4$
p < 0.00005
Nº of genomes with $TusE_3$
Nº of genomes without $TusE_3$
With $TusE_3$
Without $TusE_3$
p < 0.00005

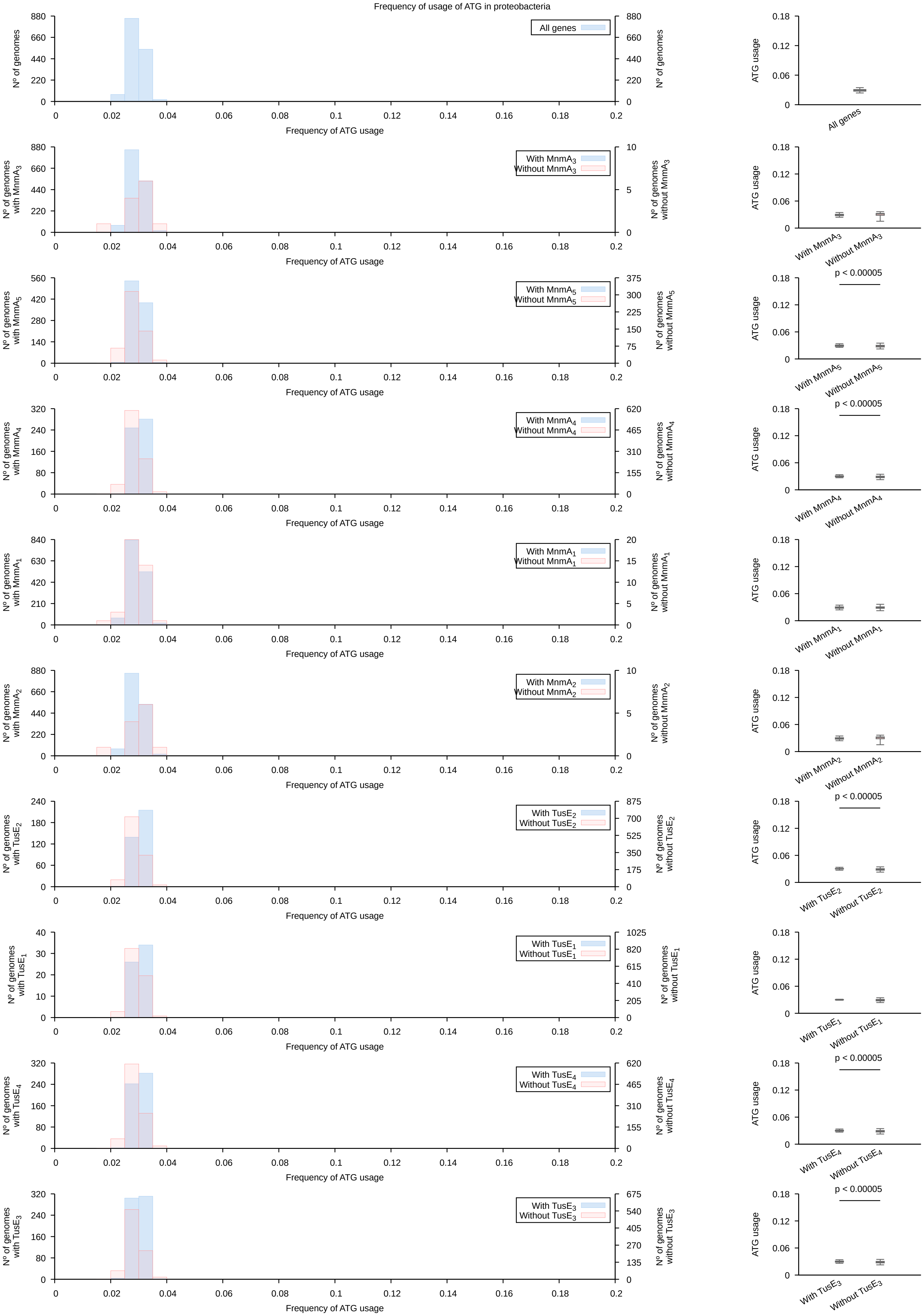
Frequency of usage of ATG in proteobacteria
All genes
Nº of genomes
Frequency of ATG usage
ATG usage
With MnmA$_3$
Without MnmA$_3$
Nº of genomes with MnmA$_3$
Nº of genomes without MnmA$_3$
With MnmA$_5$
Without MnmA$_5$
Nº of genomes with MnmA$_5$
Nº of genomes without MnmA$_5$
p < 0.00005
With MnmA$_4$
Without MnmA$_4$
Nº of genomes with MnmA$_4$
Nº of genomes without MnmA$_4$
p < 0.00005
With MnmA$_1$
Without MnmA$_1$
Nº of genomes with MnmA$_1$
Nº of genomes without MnmA$_1$
With MnmA$_2$
Without MnmA$_2$
Nº of genomes with MnmA$_2$
Nº of genomes without MnmA$_2$
With TusE$_2$
Without TusE$_2$
Nº of genomes with TusE$_2$
Nº of genomes without TusE$_2$
p < 0.00005
With TusE$_1$
Without TusE$_1$
Nº of genomes with TusE$_1$
Nº of genomes without TusE$_1$
With TusE$_4$
Without TusE$_4$
Nº of genomes with TusE$_4$
Nº of genomes without TusE$_4$
p < 0.00005
With TusE$_3$
Without TusE$_3$
Nº of genomes with TusE$_3$
Nº of genomes without TusE$_3$
p < 0.00005

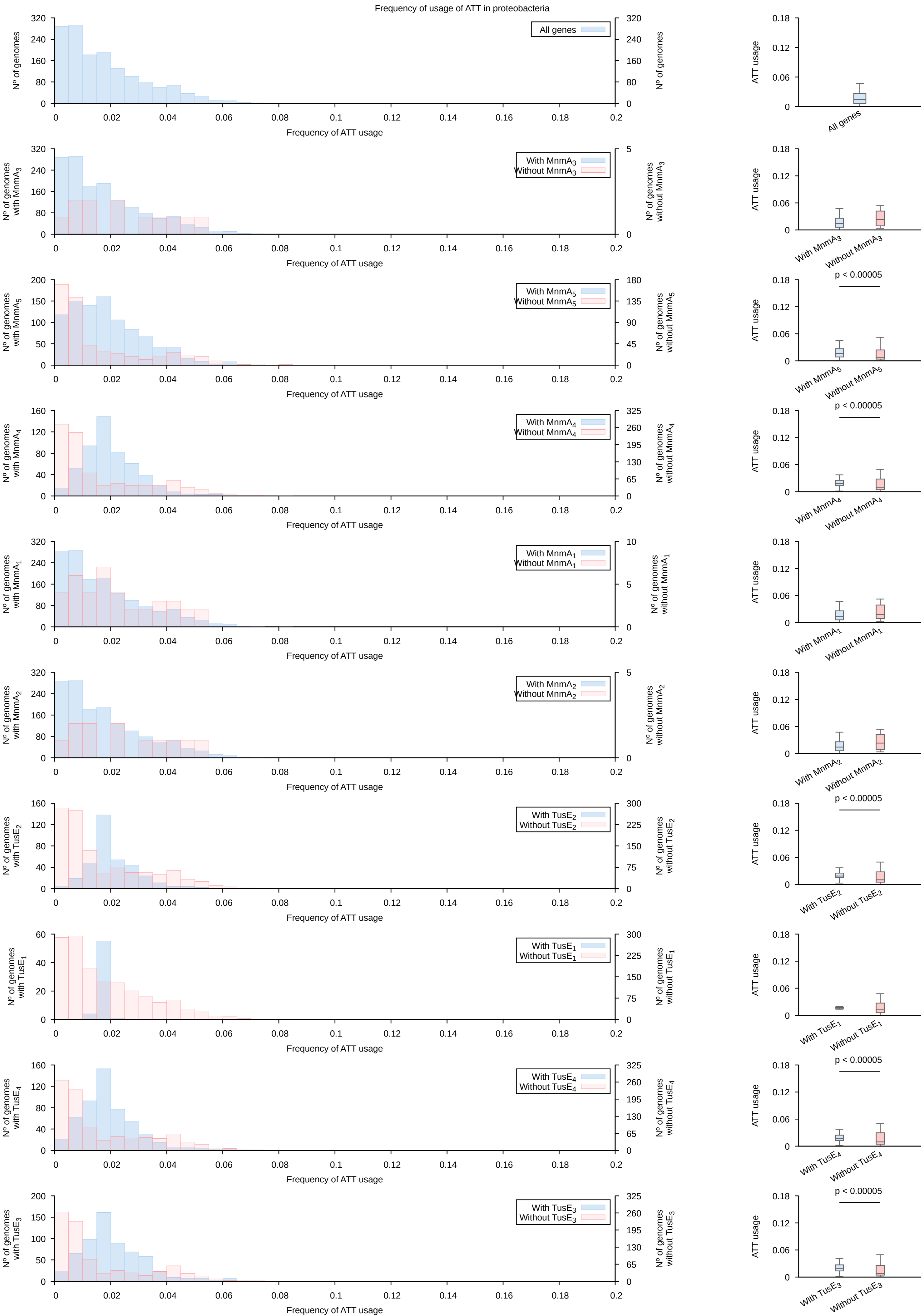

Frequency of usage of ATT in proteobacteria
Nº of genomes
All genes
Frequency of ATT usage
ATT usage
Nº of genomes with MnmA$_3$
Nº of genomes without MnmA$_3$
With MnmA$_3$
Without MnmA$_3$
Nº of genomes with MnmA$_5$
Nº of genomes without MnmA$_5$
With MnmA$_5$
Without MnmA$_5$
p < 0.00005
Nº of genomes with MnmA$_4$
Nº of genomes without MnmA$_4$
With MnmA$_4$
Without MnmA$_4$
p < 0.00005
Nº of genomes with MnmA$_1$
Nº of genomes without MnmA$_1$
With MnmA$_1$
Without MnmA$_1$
Nº of genomes with MnmA$_2$
Nº of genomes without MnmA$_2$
With MnmA$_2$
Without MnmA$_2$
Nº of genomes with TusE$_2$
Nº of genomes without TusE$_2$
With TusE$_2$
Without TusE$_2$
p < 0.00005
Nº of genomes with TusE$_1$
Nº of genomes without TusE$_1$
With TusE$_1$
Without TusE$_1$
Nº of genomes with TusE$_4$
Nº of genomes without TusE$_4$
With TusE$_4$
Without TusE$_4$
p < 0.00005
Nº of genomes with TusE$_3$
Nº of genomes without TusE$_3$
With TusE$_3$
Without TusE$_3$
p < 0.00005

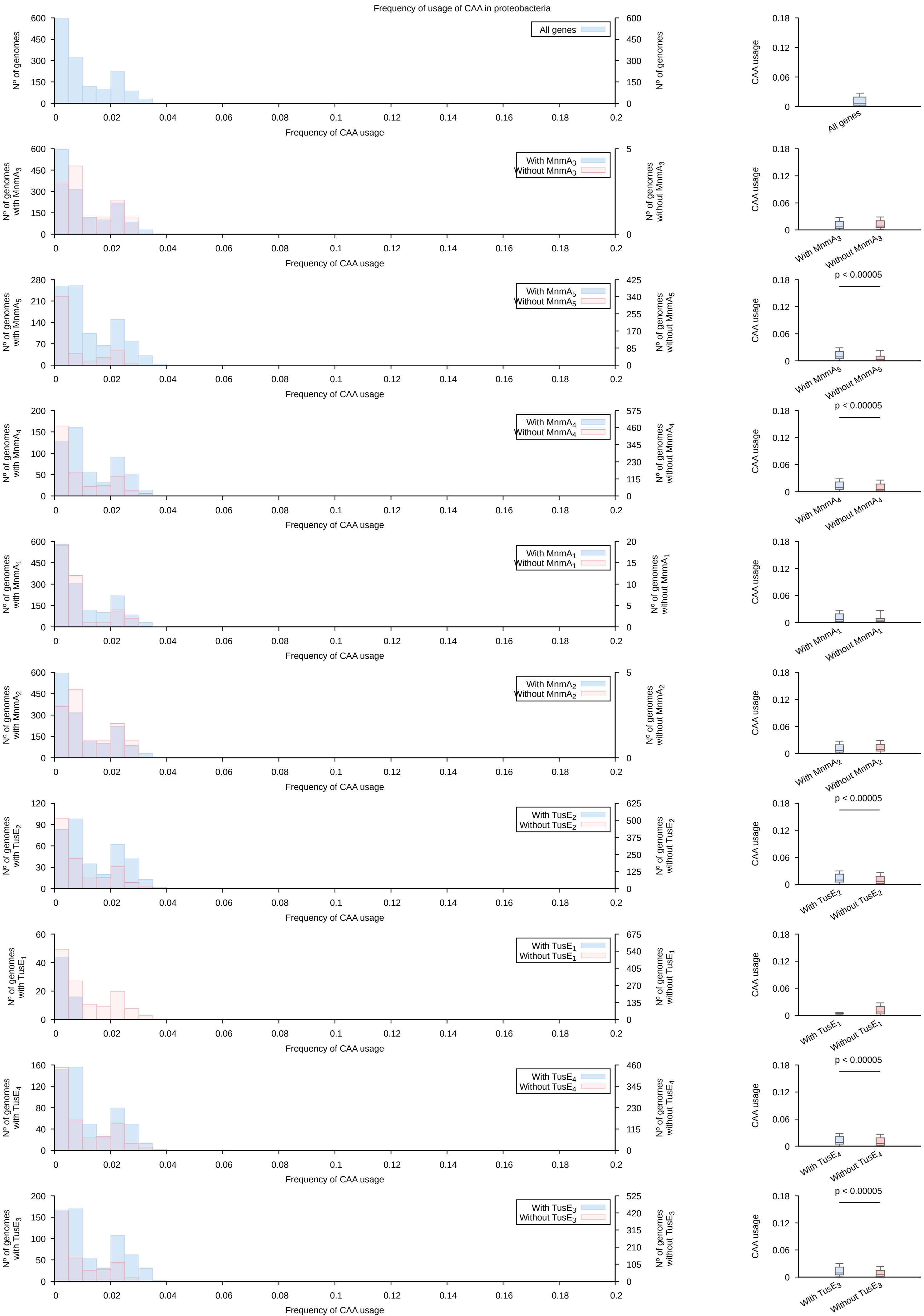

Frequency of usage of CAA in proteobacteria
Nº of genomes
All genes
Frequency of CAA usage
CAA usage
Nº of genomes with MnmA$_3$
Nº of genomes without MnmA$_3$
With MnmA$_3$
Without MnmA$_3$
Nº of genomes with MnmA$_5$
Nº of genomes without MnmA$_5$
With MnmA$_5$
Without MnmA$_5$
p < 0.00005
Nº of genomes with MnmA$_4$
Nº of genomes without MnmA$_4$
With MnmA$_4$
Without MnmA$_4$
p < 0.00005
Nº of genomes with MnmA$_1$
Nº of genomes without MnmA$_1$
With MnmA$_1$
Without MnmA$_1$
Nº of genomes with MnmA$_2$
Nº of genomes without MnmA$_2$
With MnmA$_2$
Without MnmA$_2$
Nº of genomes with TusE$_2$
Nº of genomes without TusE$_2$
With TusE$_2$
Without TusE$_2$
p < 0.00005
Nº of genomes with TusE$_1$
Nº of genomes without TusE$_1$
With TusE$_1$
Without TusE$_1$
Nº of genomes with TusE$_4$
Nº of genomes without TusE$_4$
With TusE$_4$
Without TusE$_4$
p < 0.00005
Nº of genomes with TusE$_3$
Nº of genomes without TusE$_3$
With TusE$_3$
Without TusE$_3$
p < 0.00005

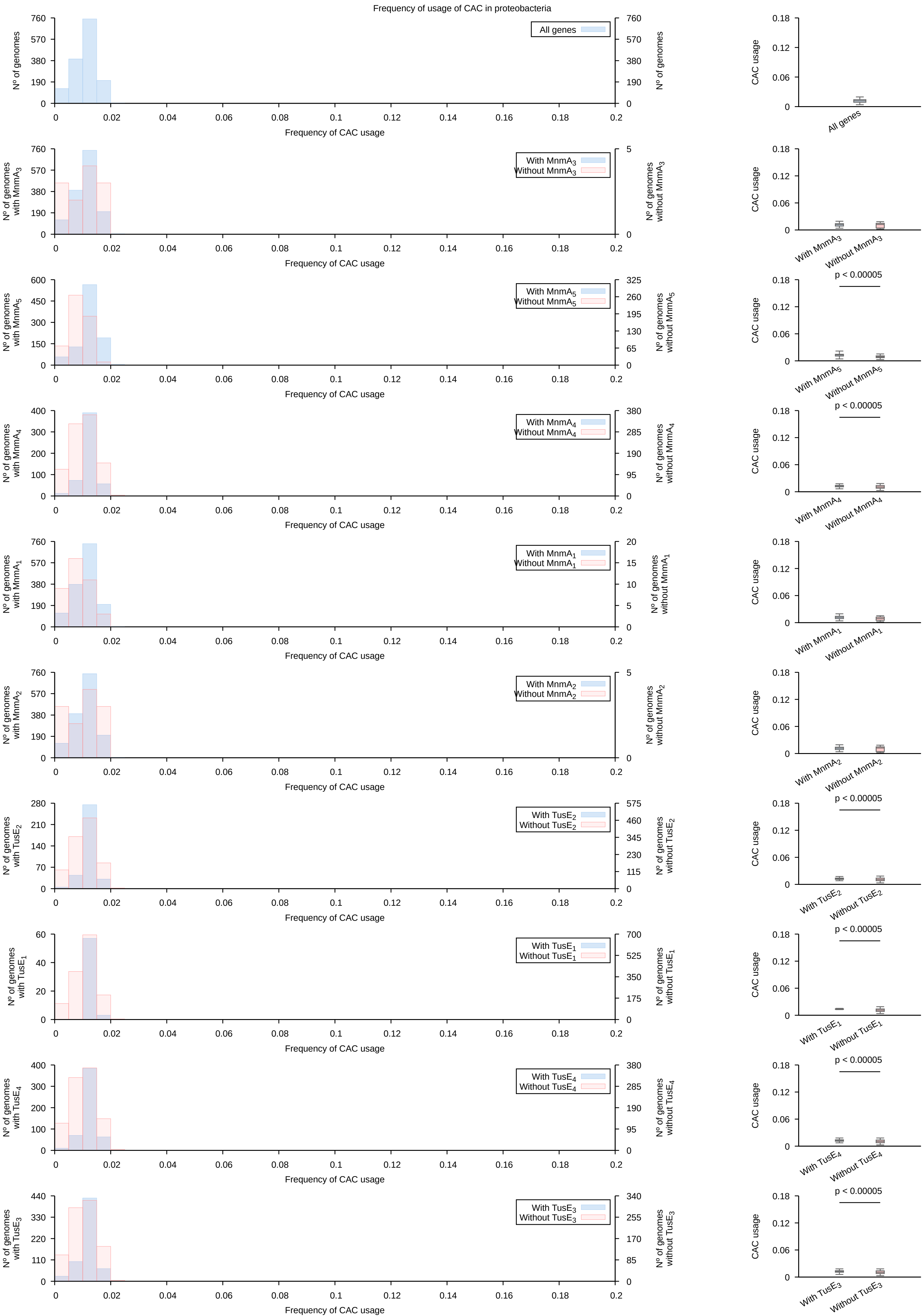

Frequency of usage of CAC in proteobacteria
All genes
Nº of genomes
Frequency of CAC usage
CAC usage
With MnmA$_3$
Without MnmA$_3$
Nº of genomes with MnmA$_3$
Nº of genomes without MnmA$_3$
With MnmA$_5$
Without MnmA$_5$
Nº of genomes with MnmA$_5$
Nº of genomes without MnmA$_5$
p < 0.00005
With MnmA$_4$
Without MnmA$_4$
Nº of genomes with MnmA$_4$
Nº of genomes without MnmA$_4$
p < 0.00005
With MnmA$_1$
Without MnmA$_1$
Nº of genomes with MnmA$_1$
Nº of genomes without MnmA$_1$
With MnmA$_2$
Without MnmA$_2$
Nº of genomes with MnmA$_2$
Nº of genomes without MnmA$_2$
With TusE$_2$
Without TusE$_2$
Nº of genomes with TusE$_2$
Nº of genomes without TusE$_2$
p < 0.00005
With TusE$_1$
Without TusE$_1$
Nº of genomes with TusE$_1$
Nº of genomes without TusE$_1$
p < 0.00005
With TusE$_4$
Without TusE$_4$
Nº of genomes with TusE$_4$
Nº of genomes without TusE$_4$
p < 0.00005
With TusE$_3$
Without TusE$_3$
Nº of genomes with TusE$_3$
Nº of genomes without TusE$_3$
p < 0.00005

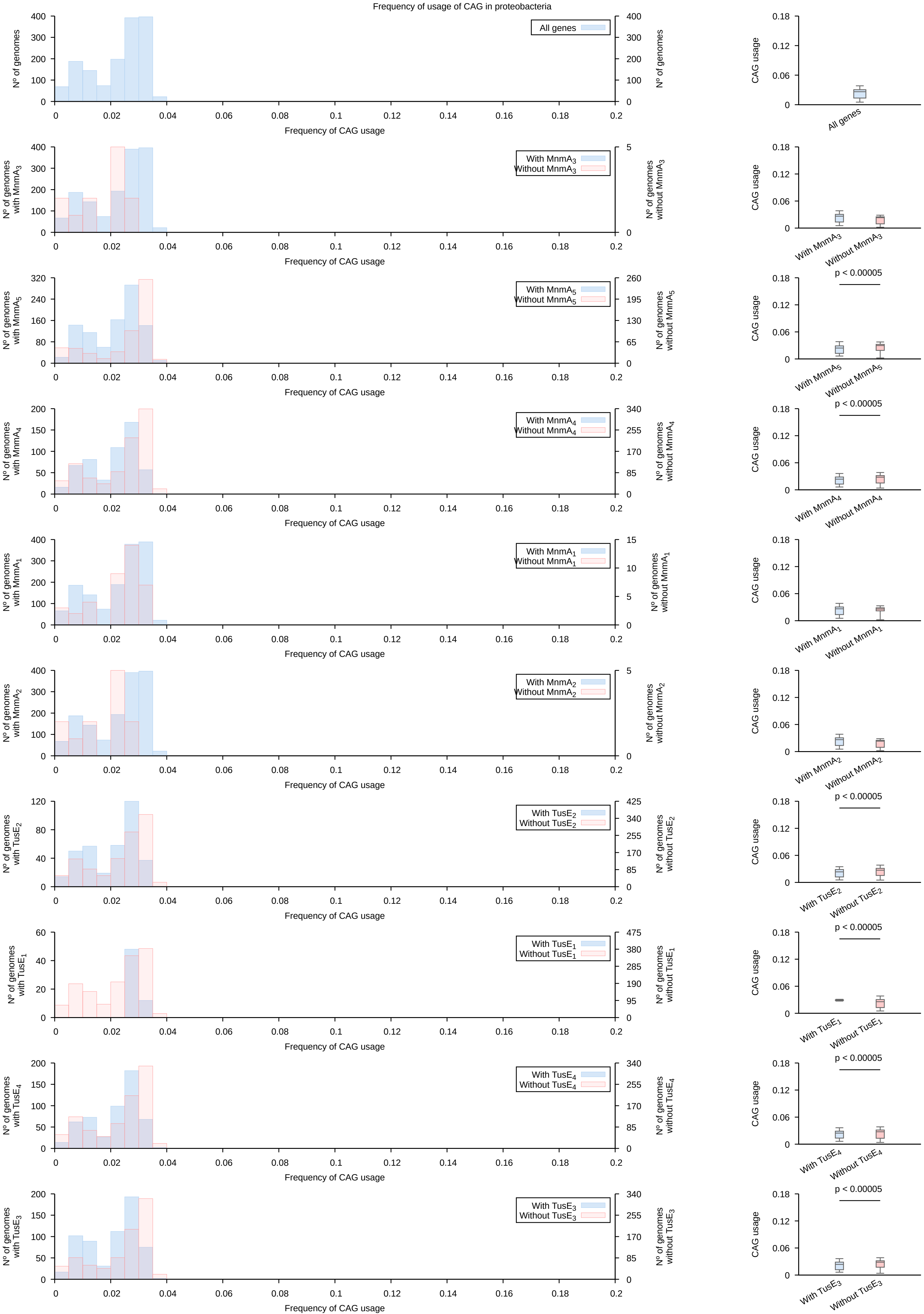
Frequency of usage of CAG in proteobacteria
Nº of genomes
All genes
Frequency of CAG usage
CAG usage
All genes
Nº of genomes with MnmA3
With MnmA3
Without MnmA3
Nº of genomes without MnmA3
Nº of genomes with MnmA5
With MnmA5
Without MnmA5
Nº of genomes without MnmA5
p < 0.00005
Nº of genomes with MnmA4
With MnmA4
Without MnmA4
Nº of genomes without MnmA4
p < 0.00005
Nº of genomes with MnmA1
With MnmA1
Without MnmA1
Nº of genomes without MnmA1
Nº of genomes with MnmA2
With MnmA2
Without MnmA2
Nº of genomes without MnmA2
Nº of genomes with TusE2
With TusE2
Without TusE2
Nº of genomes without TusE2
p < 0.00005
Nº of genomes with TusE1
With TusE1
Without TusE1
Nº of genomes without TusE1
p < 0.00005
Nº of genomes with TusE4
With TusE4
Without TusE4
Nº of genomes without TusE4
p < 0.00005
Nº of genomes with TusE3
With TusE3
Without TusE3
Nº of genomes without TusE3
p < 0.00005

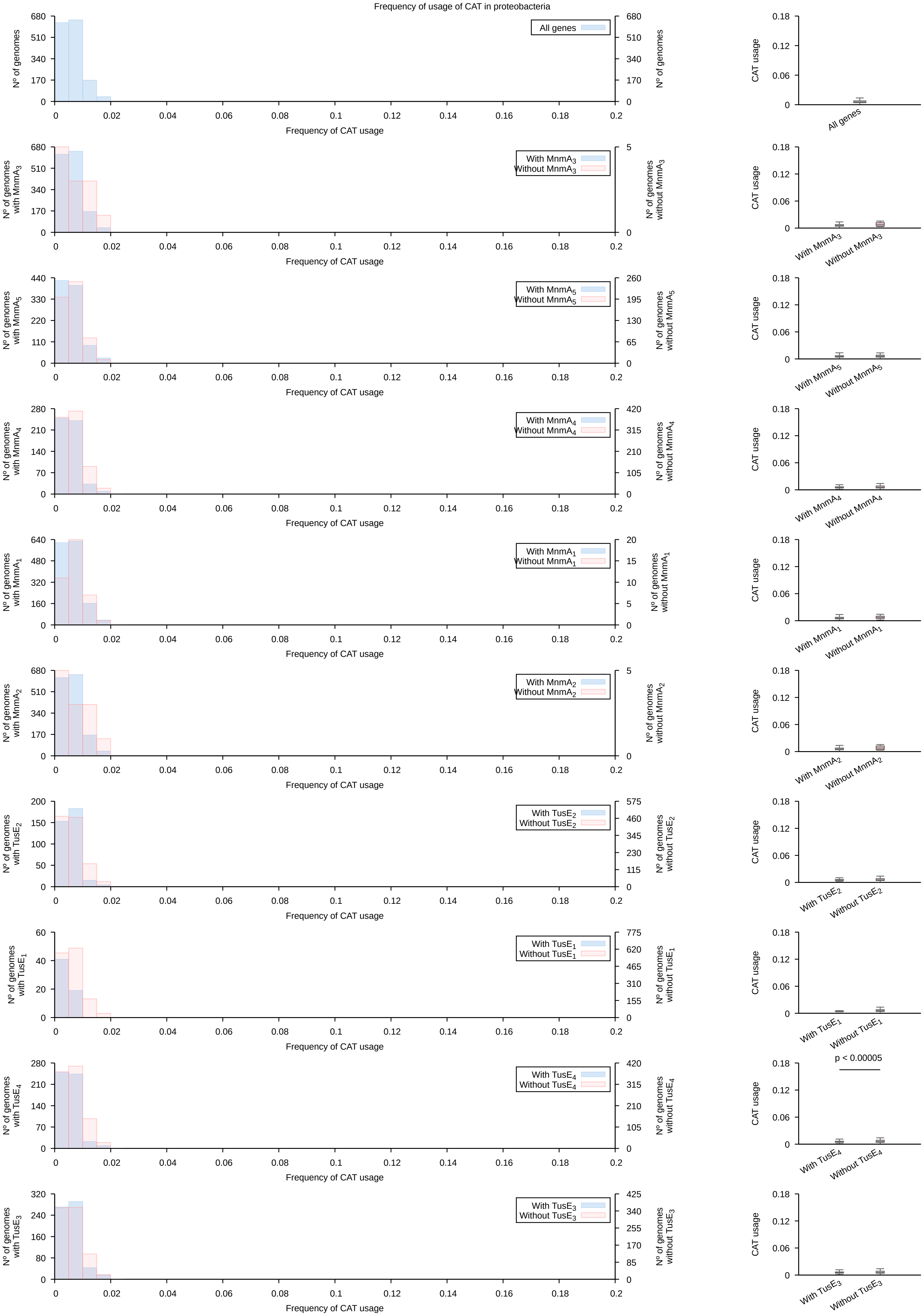
Frequency of usage of CAT in proteobacteria
All genes
With MnmA3
Without MnmA3
With MnmA5
Without MnmA5
With MnmA4
Without MnmA4
With MnmA1
Without MnmA1
With MnmA2
Without MnmA2
With TusE2
Without TusE2
With TusE1
Without TusE1
With TusE4
Without TusE4
p < 0.00005
With TusE3
Without TusE3
Nº of genomes
Frequency of CAT usage
CAT usage

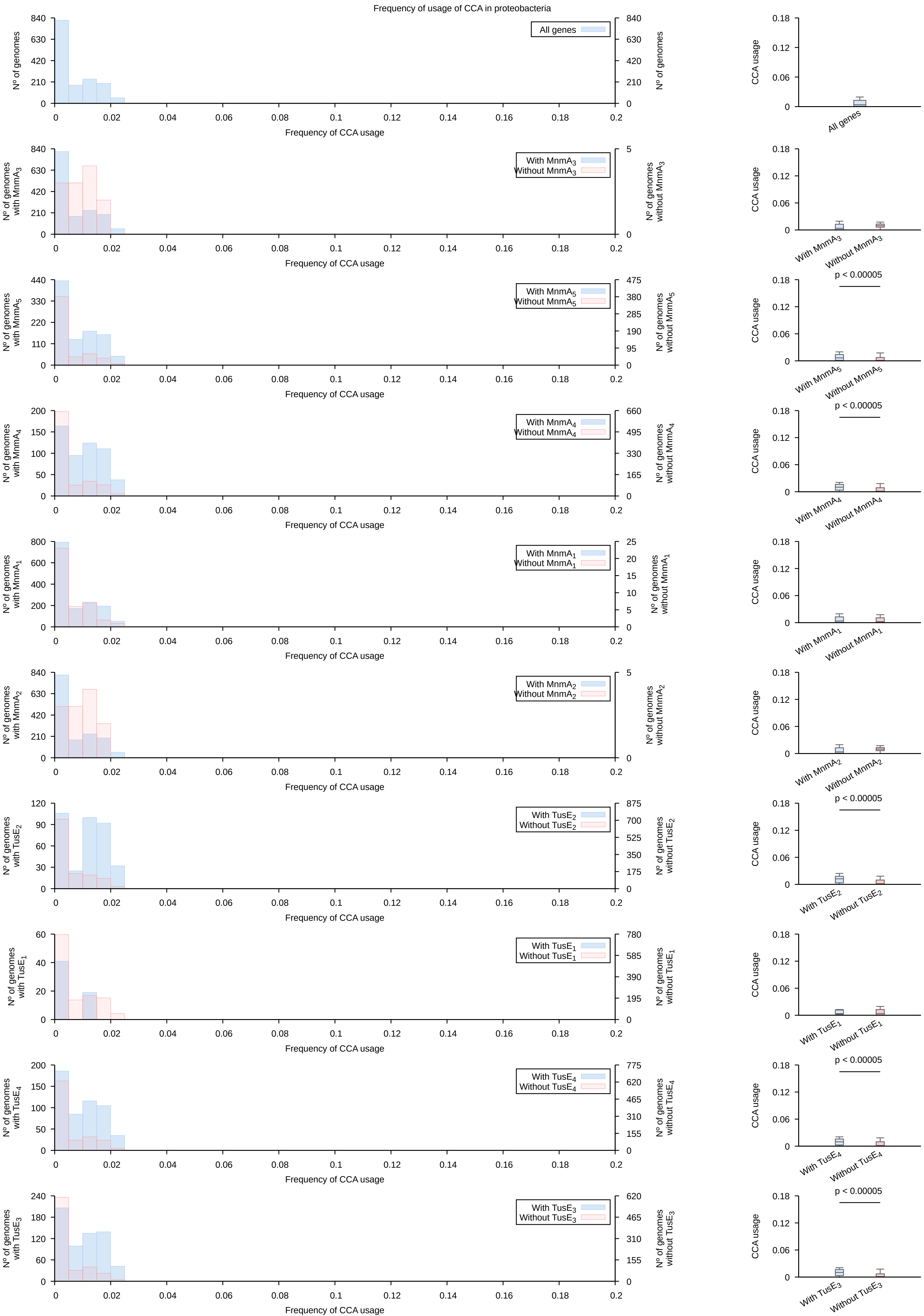

Frequency of usage of CCA in proteobacteria
All genes
Nº of genomes
Frequency of CCA usage
CCA usage
With MnmA$_3$
Without MnmA$_3$
Nº of genomes with MnmA$_3$
Nº of genomes without MnmA$_3$
With MnmA$_5$
Without MnmA$_5$
Nº of genomes with MnmA$_5$
Nº of genomes without MnmA$_5$
p < 0.00005
With MnmA$_4$
Without MnmA$_4$
Nº of genomes with MnmA$_4$
Nº of genomes without MnmA$_4$
p < 0.00005
With MnmA$_1$
Without MnmA$_1$
Nº of genomes with MnmA$_1$
Nº of genomes without MnmA$_1$
With MnmA$_2$
Without MnmA$_2$
Nº of genomes with MnmA$_2$
Nº of genomes without MnmA$_2$
With TusE$_2$
Without TusE$_2$
Nº of genomes with TusE$_2$
Nº of genomes without TusE$_2$
p < 0.00005
With TusE$_1$
Without TusE$_1$
Nº of genomes with TusE$_1$
Nº of genomes without TusE$_1$
With TusE$_4$
Without TusE$_4$
Nº of genomes with TusE$_4$
Nº of genomes without TusE$_4$
p < 0.00005
With TusE$_3$
Without TusE$_3$
Nº of genomes with TusE$_3$
Nº of genomes without TusE$_3$
p < 0.00005

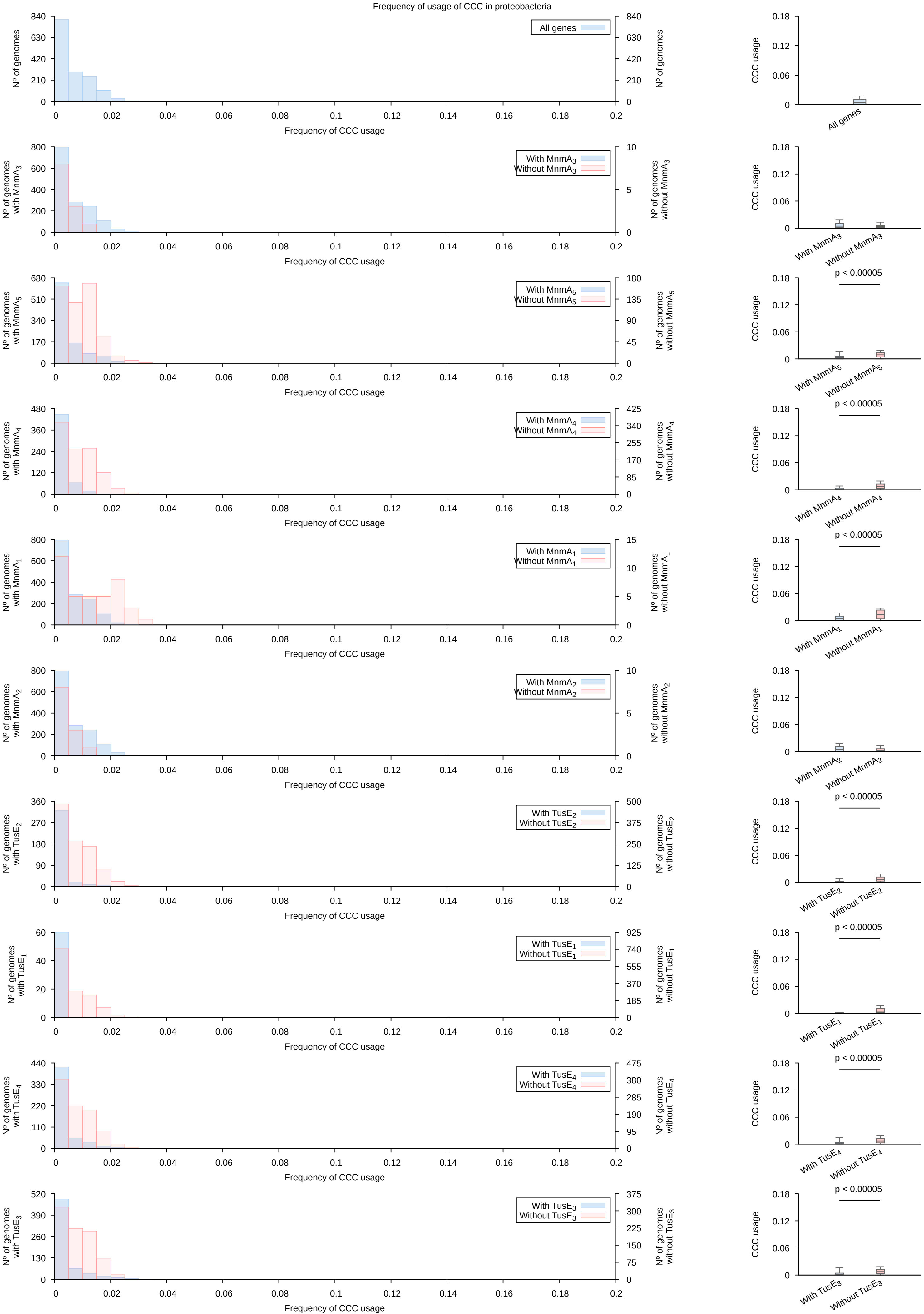

Frequency of usage of CCC in proteobacteria
All genes
Nº of genomes
Frequency of CCC usage
CCC usage
With $MnmA_3$
Without $MnmA_3$
Nº of genomes with $MnmA_3$
Nº of genomes without $MnmA_3$
With $MnmA_5$
Without $MnmA_5$
Nº of genomes with $MnmA_5$
Nº of genomes without $MnmA_5$
p < 0.00005
With $MnmA_4$
Without $MnmA_4$
Nº of genomes with $MnmA_4$
Nº of genomes without $MnmA_4$
p < 0.00005
With $MnmA_1$
Without $MnmA_1$
Nº of genomes with $MnmA_1$
Nº of genomes without $MnmA_1$
p < 0.00005
With $MnmA_2$
Without $MnmA_2$
Nº of genomes with $MnmA_2$
Nº of genomes without $MnmA_2$
With $TusE_2$
Without $TusE_2$
Nº of genomes with $TusE_2$
Nº of genomes without $TusE_2$
p < 0.00005
With $TusE_1$
Without $TusE_1$
Nº of genomes with $TusE_1$
Nº of genomes without $TusE_1$
p < 0.00005
With $TusE_4$
Without $TusE_4$
Nº of genomes with $TusE_4$
Nº of genomes without $TusE_4$
p < 0.00005
With $TusE_3$
Without $TusE_3$
Nº of genomes with $TusE_3$
Nº of genomes without $TusE_3$
p < 0.00005

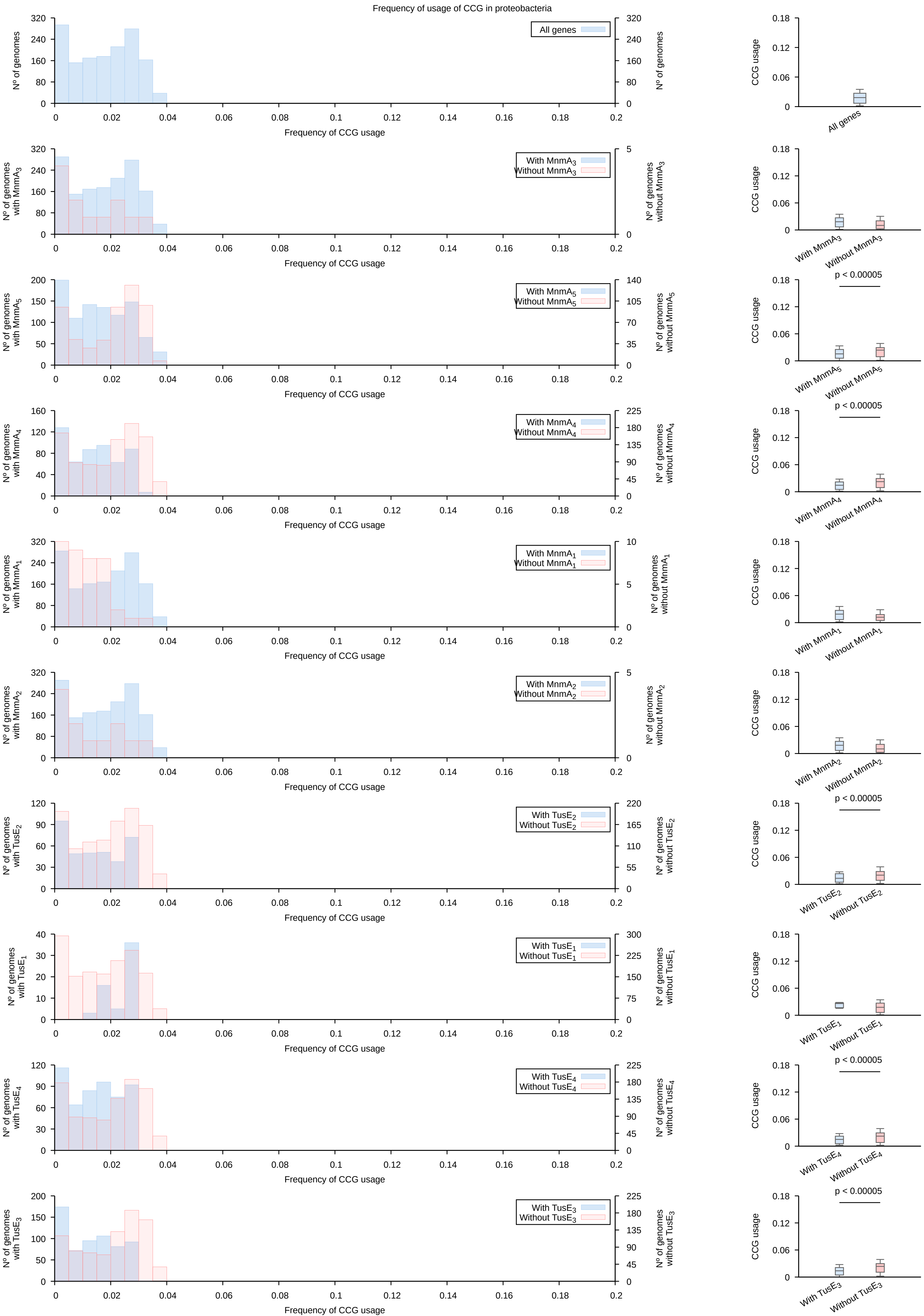
Frequency of usage of CCG in proteobacteria
Nº of genomes
All genes
Frequency of CCG usage
CCG usage
Nº of genomes with $MnmA_3$
Nº of genomes without $MnmA_3$
With $MnmA_3$
Without $MnmA_3$
Nº of genomes with $MnmA_5$
Nº of genomes without $MnmA_5$
With $MnmA_5$
Without $MnmA_5$
p < 0.00005
Nº of genomes with $MnmA_4$
Nº of genomes without $MnmA_4$
With $MnmA_4$
Without $MnmA_4$
p < 0.00005
Nº of genomes with $MnmA_1$
Nº of genomes without $MnmA_1$
With $MnmA_1$
Without $MnmA_1$
Nº of genomes with $MnmA_2$
Nº of genomes without $MnmA_2$
With $MnmA_2$
Without $MnmA_2$
Nº of genomes with $TusE_2$
Nº of genomes without $TusE_2$
With $TusE_2$
Without $TusE_2$
p < 0.00005
Nº of genomes with $TusE_1$
Nº of genomes without $TusE_1$
With $TusE_1$
Without $TusE_1$
Nº of genomes with $TusE_4$
Nº of genomes without $TusE_4$
With $TusE_4$
Without $TusE_4$
p < 0.00005
Nº of genomes with $TusE_3$
Nº of genomes without $TusE_3$
With $TusE_3$
Without $TusE_3$
p < 0.00005

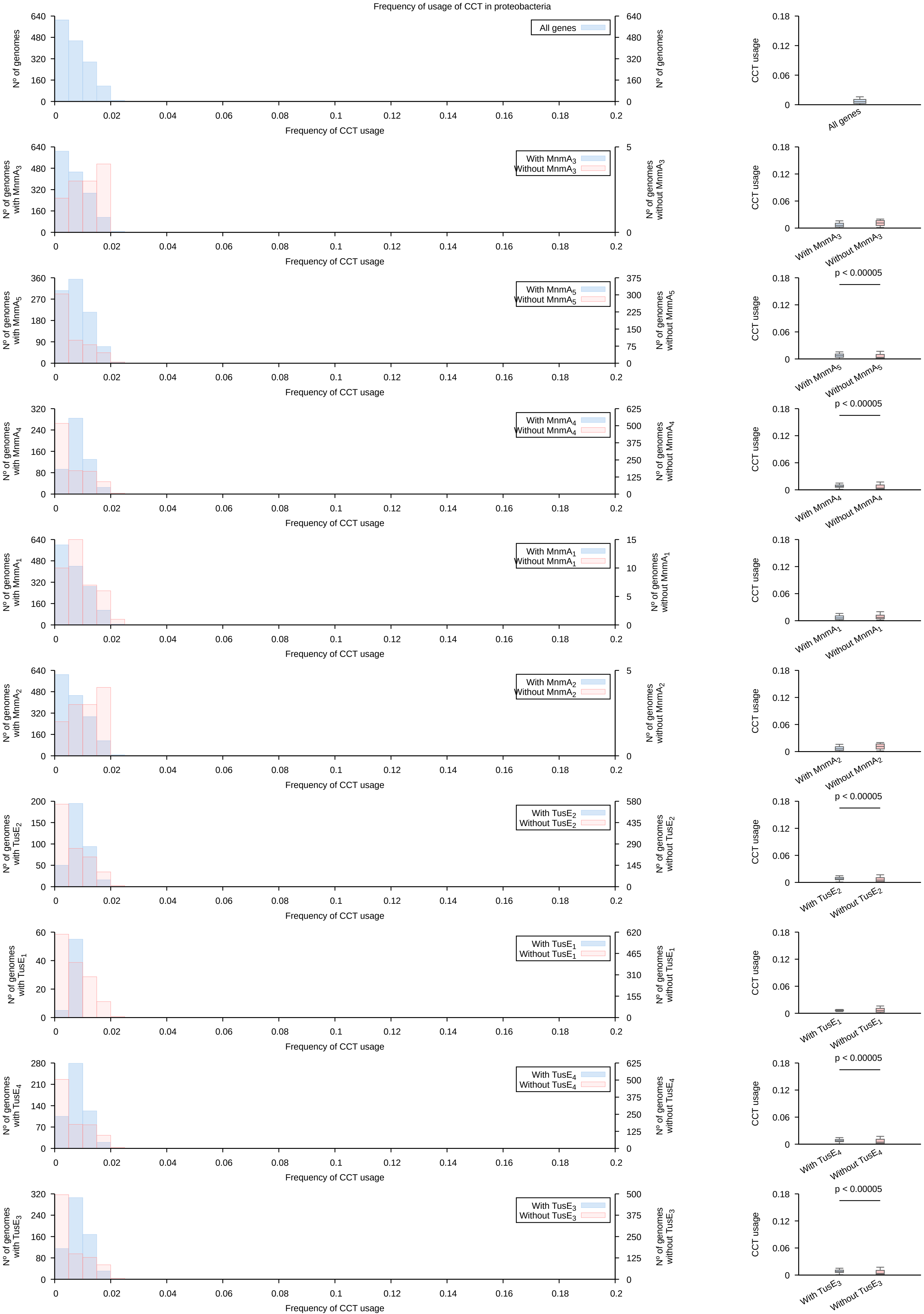

Frequency of usage of CCT in proteobacteria
Nº of genomes
All genes
Frequency of CCT usage
CCT usage
Nº of genomes with MnmA$_3$
Nº of genomes without MnmA$_3$
With MnmA$_3$
Without MnmA$_3$
Nº of genomes with MnmA$_5$
Nº of genomes without MnmA$_5$
With MnmA$_5$
Without MnmA$_5$
p < 0.00005
Nº of genomes with MnmA$_4$
Nº of genomes without MnmA$_4$
With MnmA$_4$
Without MnmA$_4$
p < 0.00005
Nº of genomes with MnmA$_1$
Nº of genomes without MnmA$_1$
With MnmA$_1$
Without MnmA$_1$
Nº of genomes with MnmA$_2$
Nº of genomes without MnmA$_2$
With MnmA$_2$
Without MnmA$_2$
Nº of genomes with TusE$_2$
Nº of genomes without TusE$_2$
With TusE$_2$
Without TusE$_2$
p < 0.00005
Nº of genomes with TusE$_1$
Nº of genomes without TusE$_1$
With TusE$_1$
Without TusE$_1$
Nº of genomes with TusE$_4$
Nº of genomes without TusE$_4$
With TusE$_4$
Without TusE$_4$
p < 0.00005
Nº of genomes with TusE$_3$
Nº of genomes without TusE$_3$
With TusE$_3$
Without TusE$_3$
p < 0.00005

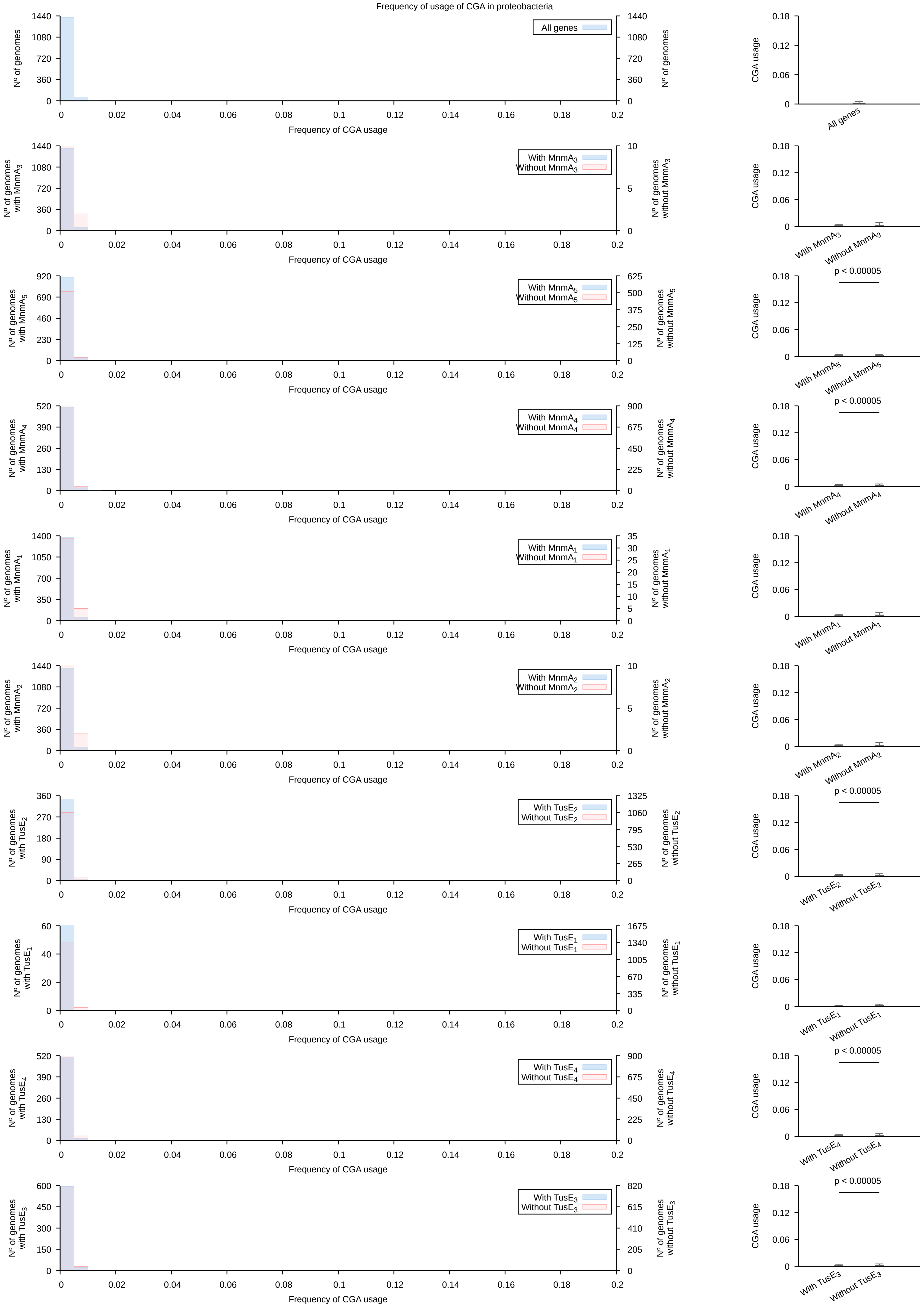
Frequency of usage of CGA in proteobacteria
All genes
With $MnmA_3$
Without $MnmA_3$
With $MnmA_5$
Without $MnmA_5$
With $MnmA_4$
Without $MnmA_4$
With $MnmA_1$
Without $MnmA_1$
With $MnmA_2$
Without $MnmA_2$
With $TusE_2$
Without $TusE_2$
With $TusE_1$
Without $TusE_1$
With $TusE_4$
Without $TusE_4$
With $TusE_3$
Without $TusE_3$
Nº of genomes
Frequency of CGA usage
CGA usage
p < 0.00005

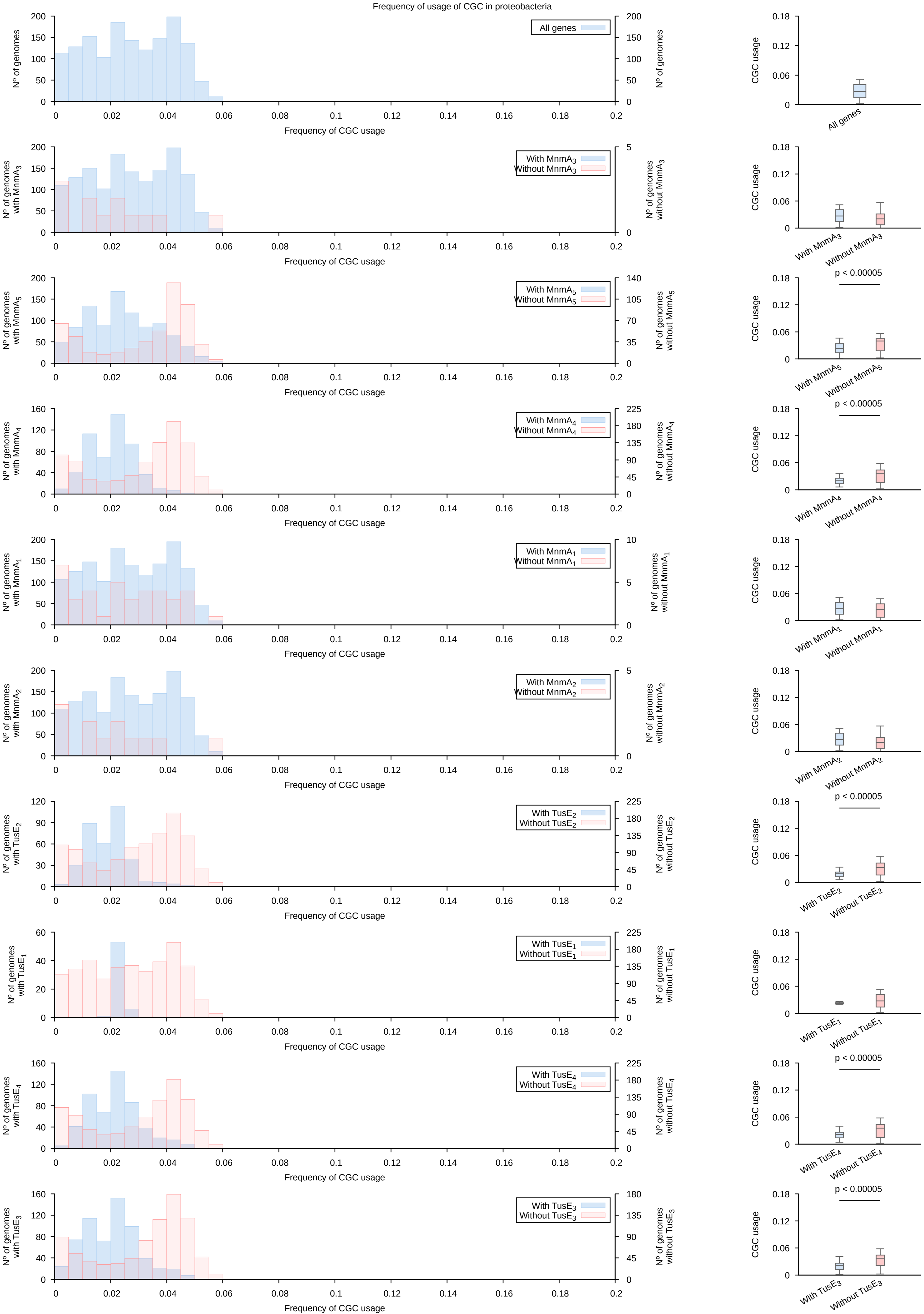

Frequency of usage of CGC in proteobacteria
Nº of genomes
All genes
Frequency of CGC usage
CGC usage
All genes
Nº of genomes with MnmA$_3$
With MnmA$_3$
Without MnmA$_3$
Nº of genomes without MnmA$_3$
Nº of genomes with MnmA$_5$
With MnmA$_5$
Without MnmA$_5$
Nº of genomes without MnmA$_5$
p < 0.00005
Nº of genomes with MnmA$_4$
With MnmA$_4$
Without MnmA$_4$
Nº of genomes without MnmA$_4$
p < 0.00005
Nº of genomes with MnmA$_1$
With MnmA$_1$
Without MnmA$_1$
Nº of genomes without MnmA$_1$
Nº of genomes with MnmA$_2$
With MnmA$_2$
Without MnmA$_2$
Nº of genomes without MnmA$_2$
Nº of genomes with TusE$_2$
With TusE$_2$
Without TusE$_2$
Nº of genomes without TusE$_2$
p < 0.00005
Nº of genomes with TusE$_1$
With TusE$_1$
Without TusE$_1$
Nº of genomes without TusE$_1$
Nº of genomes with TusE$_4$
With TusE$_4$
Without TusE$_4$
Nº of genomes without TusE$_4$
p < 0.00005
Nº of genomes with TusE$_3$
With TusE$_3$
Without TusE$_3$
Nº of genomes without TusE$_3$
p < 0.00005

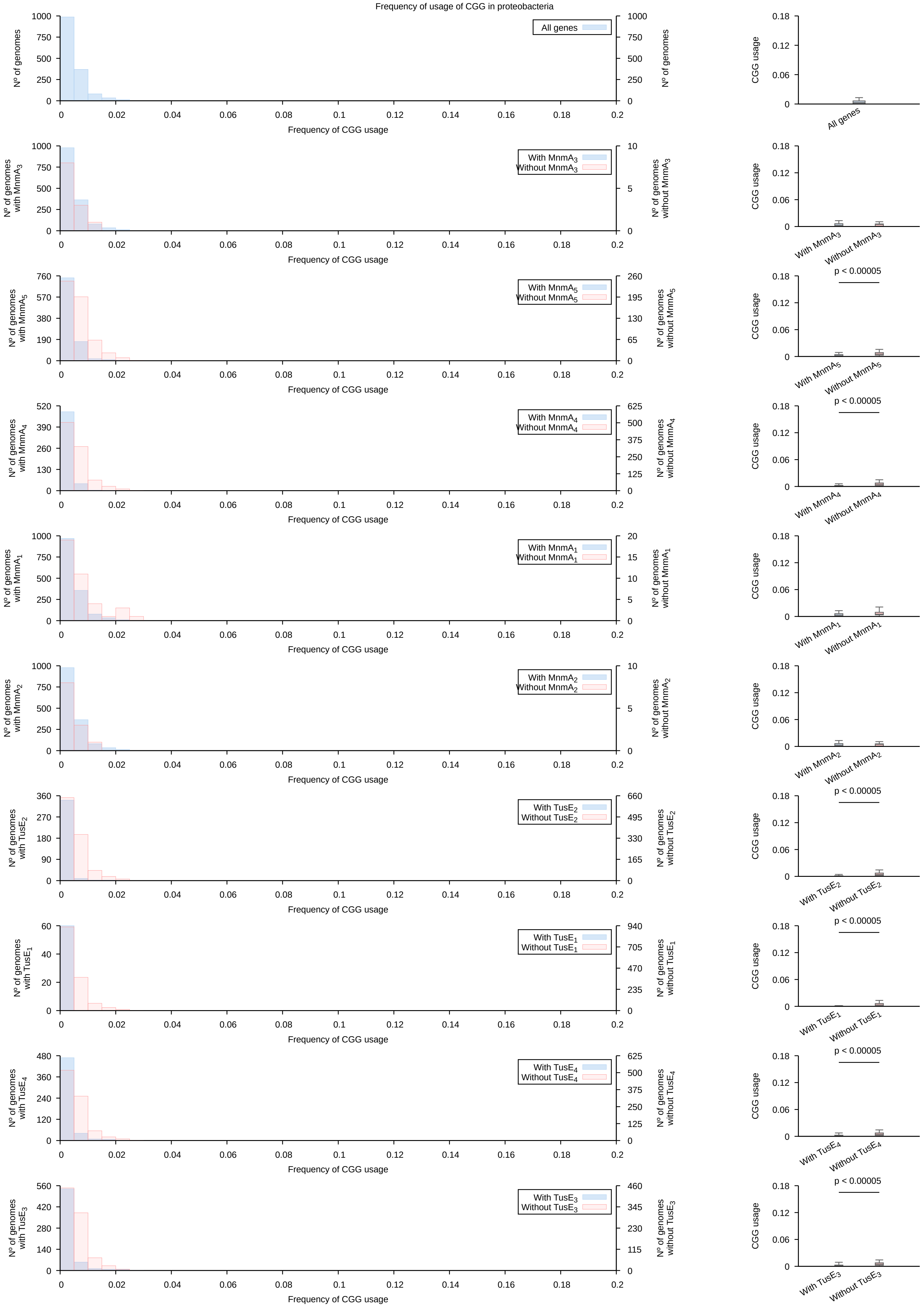
Frequency of usage of CGG in proteobacteria
All genes
Nº of genomes
Frequency of CGG usage
CGG usage
With MnmA$_3$
Without MnmA$_3$
Nº of genomes with MnmA$_3$
Nº of genomes without MnmA$_3$
With MnmA$_5$
Without MnmA$_5$
Nº of genomes with MnmA$_5$
Nº of genomes without MnmA$_5$
p < 0.00005
With MnmA$_4$
Without MnmA$_4$
Nº of genomes with MnmA$_4$
Nº of genomes without MnmA$_4$
p < 0.00005
With MnmA$_1$
Without MnmA$_1$
Nº of genomes with MnmA$_1$
Nº of genomes without MnmA$_1$
With MnmA$_2$
Without MnmA$_2$
Nº of genomes with MnmA$_2$
Nº of genomes without MnmA$_2$
With TusE$_2$
Without TusE$_2$
Nº of genomes with TusE$_2$
Nº of genomes without TusE$_2$
p < 0.00005
With TusE$_1$
Without TusE$_1$
Nº of genomes with TusE$_1$
Nº of genomes without TusE$_1$
p < 0.00005
With TusE$_4$
Without TusE$_4$
Nº of genomes with TusE$_4$
Nº of genomes without TusE$_4$
p < 0.00005
With TusE$_3$
Without TusE$_3$
Nº of genomes with TusE$_3$
Nº of genomes without TusE$_3$
p < 0.00005

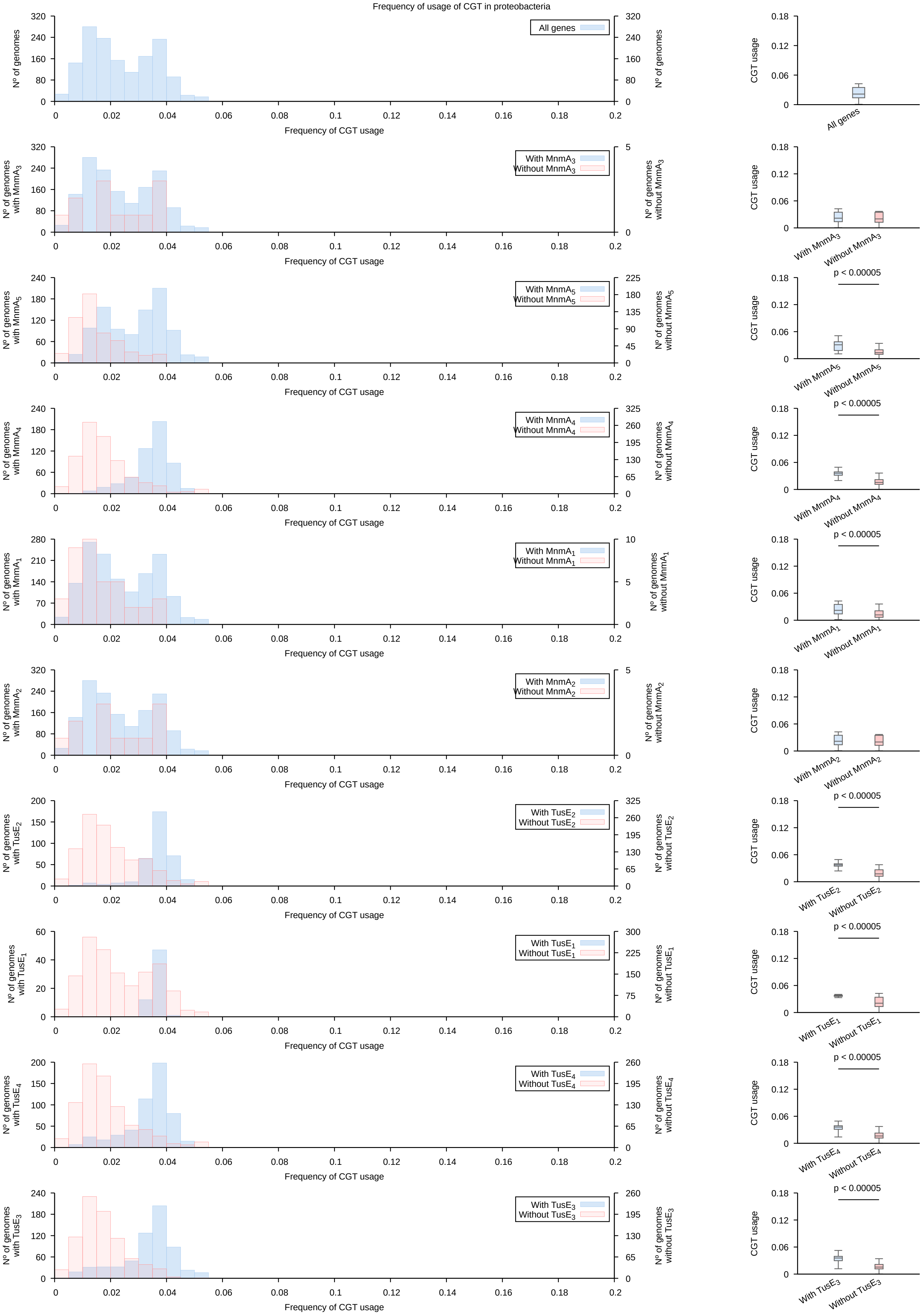
Frequency of usage of CGT in proteobacteria
Nº of genomes
All genes
Frequency of CGT usage
CGT usage
Nº of genomes with MnmA$_3$
Nº of genomes without MnmA$_3$
With MnmA$_3$
Without MnmA$_3$
Nº of genomes with MnmA$_5$
Nº of genomes without MnmA$_5$
With MnmA$_5$
Without MnmA$_5$
p < 0.00005
Nº of genomes with MnmA$_4$
Nº of genomes without MnmA$_4$
With MnmA$_4$
Without MnmA$_4$
p < 0.00005
Nº of genomes with MnmA$_1$
Nº of genomes without MnmA$_1$
With MnmA$_1$
Without MnmA$_1$
p < 0.00005
Nº of genomes with MnmA$_2$
Nº of genomes without MnmA$_2$
With MnmA$_2$
Without MnmA$_2$
Nº of genomes with TusE$_2$
Nº of genomes without TusE$_2$
With TusE$_2$
Without TusE$_2$
p < 0.00005
Nº of genomes with TusE$_1$
Nº of genomes without TusE$_1$
With TusE$_1$
Without TusE$_1$
p < 0.00005
Nº of genomes with TusE$_4$
Nº of genomes without TusE$_4$
With TusE$_4$
Without TusE$_4$
p < 0.00005
Nº of genomes with TusE$_3$
Nº of genomes without TusE$_3$
With TusE$_3$
Without TusE$_3$
p < 0.00005

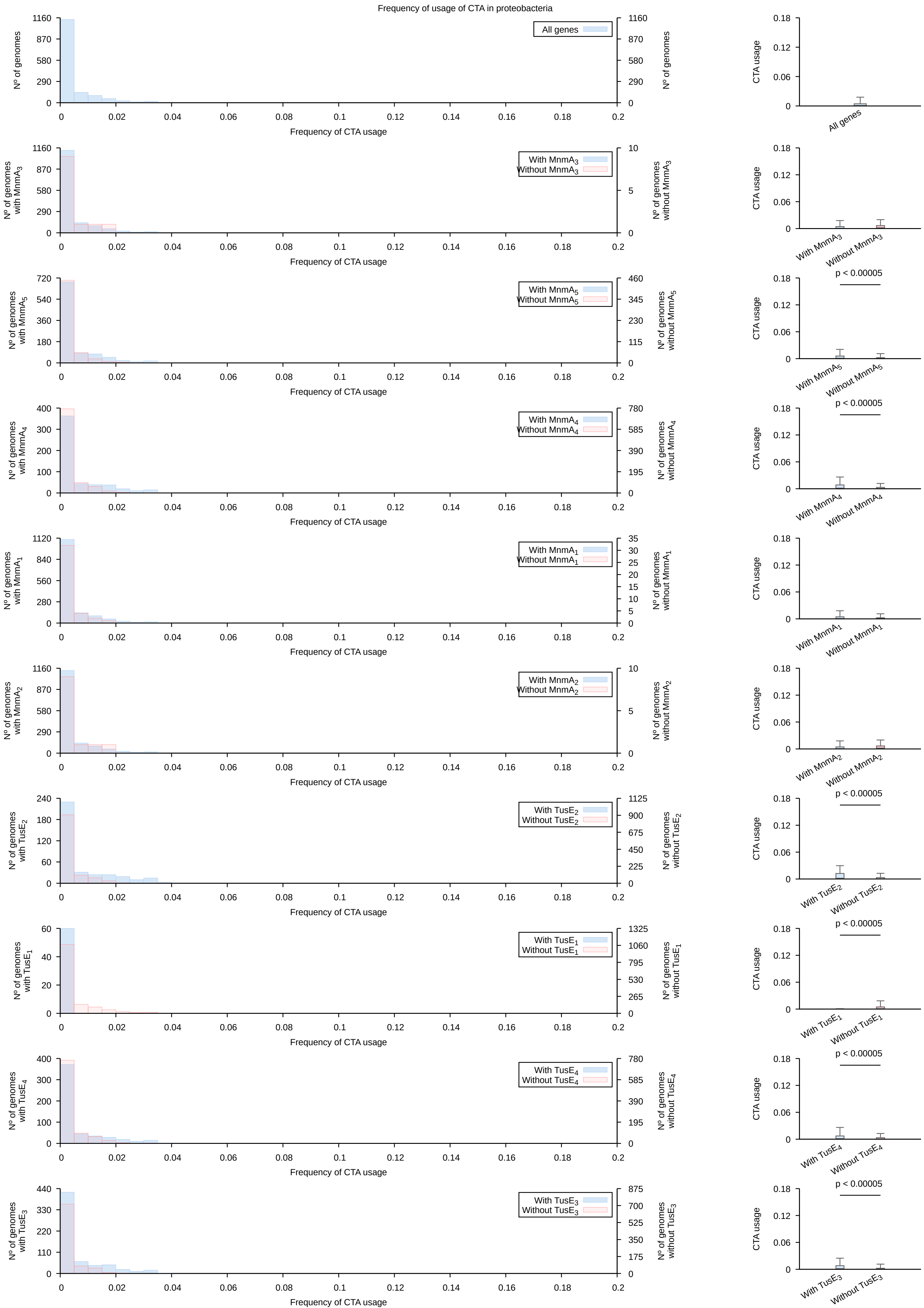
Frequency of usage of CTA in proteobacteria
Nº of genomes
All genes
Frequency of CTA usage
CTA usage
Nº of genomes with MnmA$_3$
Nº of genomes without MnmA$_3$
With MnmA$_3$
Without MnmA$_3$
Nº of genomes with MnmA$_5$
Nº of genomes without MnmA$_5$
With MnmA$_5$
Without MnmA$_5$
p < 0.00005
Nº of genomes with MnmA$_4$
Nº of genomes without MnmA$_4$
With MnmA$_4$
Without MnmA$_4$
p < 0.00005
Nº of genomes with MnmA$_1$
Nº of genomes without MnmA$_1$
With MnmA$_1$
Without MnmA$_1$
Nº of genomes with MnmA$_2$
Nº of genomes without MnmA$_2$
With MnmA$_2$
Without MnmA$_2$
Nº of genomes with TusE$_2$
Nº of genomes without TusE$_2$
With TusE$_2$
Without TusE$_2$
p < 0.00005
Nº of genomes with TusE$_1$
Nº of genomes without TusE$_1$
With TusE$_1$
Without TusE$_1$
p < 0.00005
Nº of genomes with TusE$_4$
Nº of genomes without TusE$_4$
With TusE$_4$
Without TusE$_4$
p < 0.00005
Nº of genomes with TusE$_3$
Nº of genomes without TusE$_3$
With TusE$_3$
Without TusE$_3$
p < 0.00005

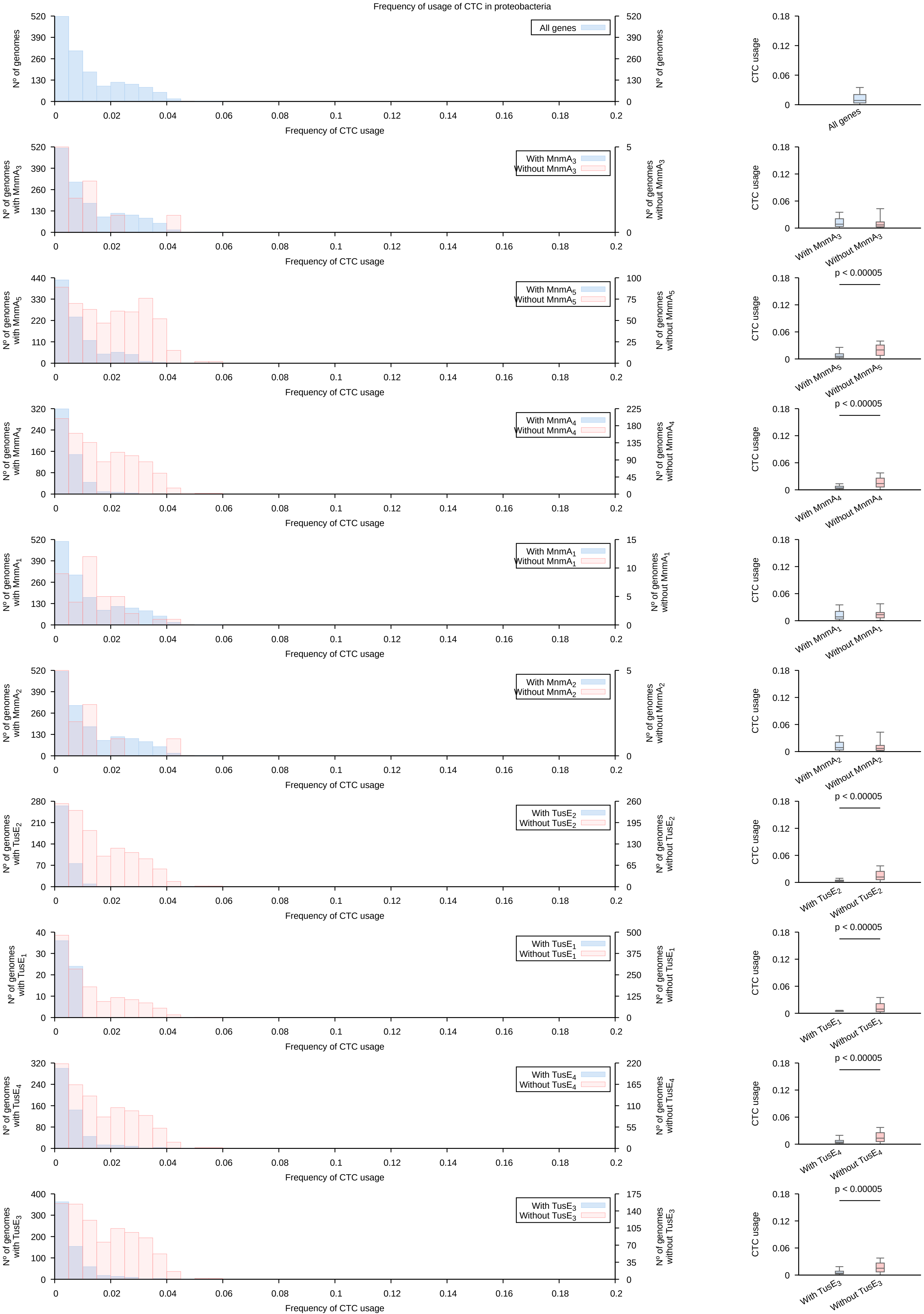

Frequency of usage of CTC in proteobacteria
Nº of genomes
Frequency of CTC usage
CTC usage
All genes
With MnmA3
Without MnmA3
With MnmA5
Without MnmA5
With MnmA4
Without MnmA4
With MnmA1
Without MnmA1
With MnmA2
Without MnmA2
With TusE2
Without TusE2
With TusE1
Without TusE1
With TusE4
Without TusE4
With TusE3
Without TusE3
p < 0.00005

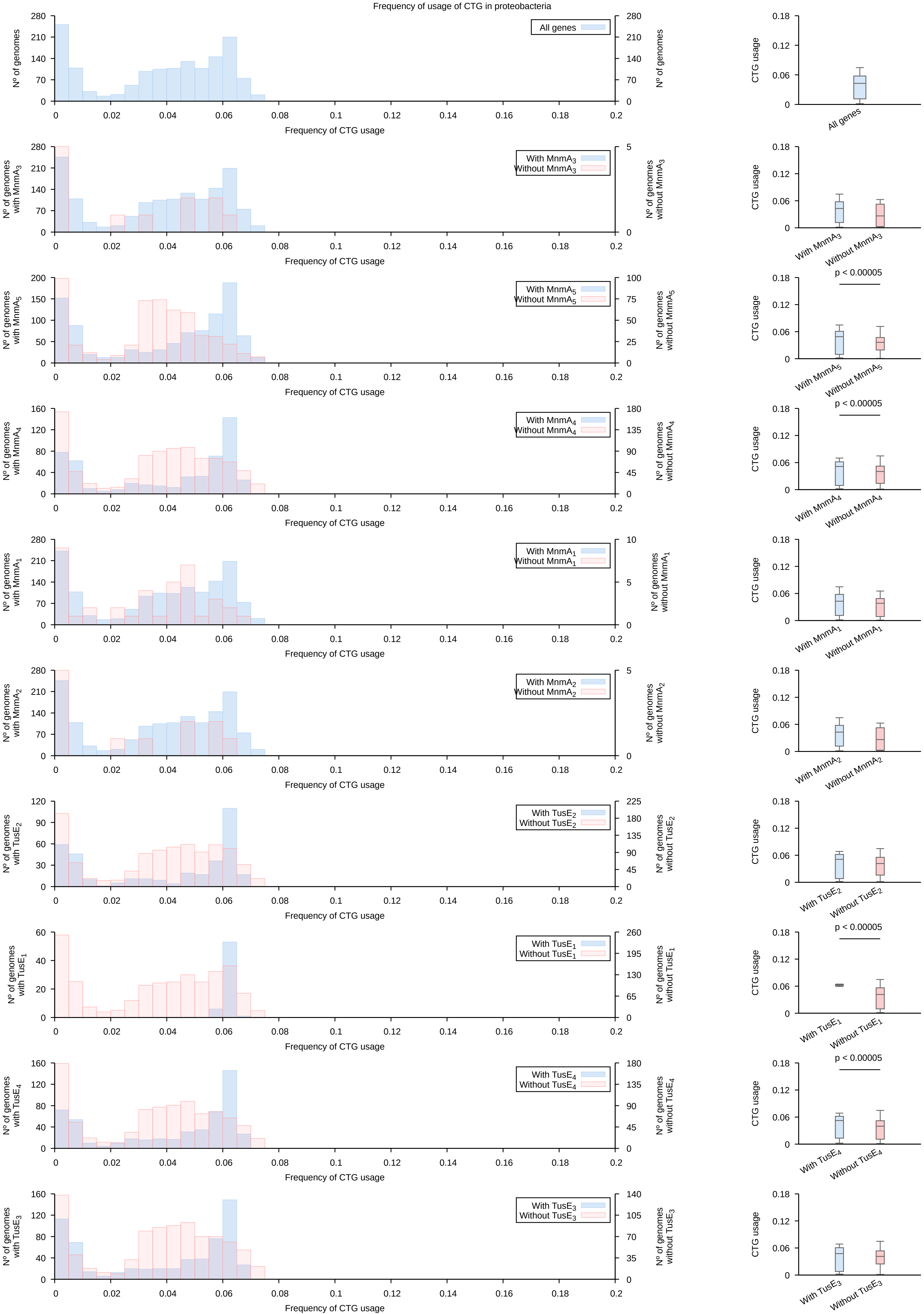
Frequency of usage of CTG in proteobacteria
Nº of genomes
All genes
Frequency of CTG usage
CTG usage
Nº of genomes with MnmA3
Nº of genomes without MnmA3
With MnmA3
Without MnmA3
Nº of genomes with MnmA5
Nº of genomes without MnmA5
With MnmA5
Without MnmA5
p < 0.00005
Nº of genomes with MnmA4
Nº of genomes without MnmA4
With MnmA4
Without MnmA4
p < 0.00005
Nº of genomes with MnmA1
Nº of genomes without MnmA1
With MnmA1
Without MnmA1
Nº of genomes with MnmA2
Nº of genomes without MnmA2
With MnmA2
Without MnmA2
Nº of genomes with TusE2
Nº of genomes without TusE2
With TusE2
Without TusE2
Nº of genomes with TusE1
Nº of genomes without TusE1
With TusE1
Without TusE1
p < 0.00005
Nº of genomes with TusE4
Nº of genomes without TusE4
With TusE4
Without TusE4
p < 0.00005
Nº of genomes with TusE3
Nº of genomes without TusE3
With TusE3
Without TusE3

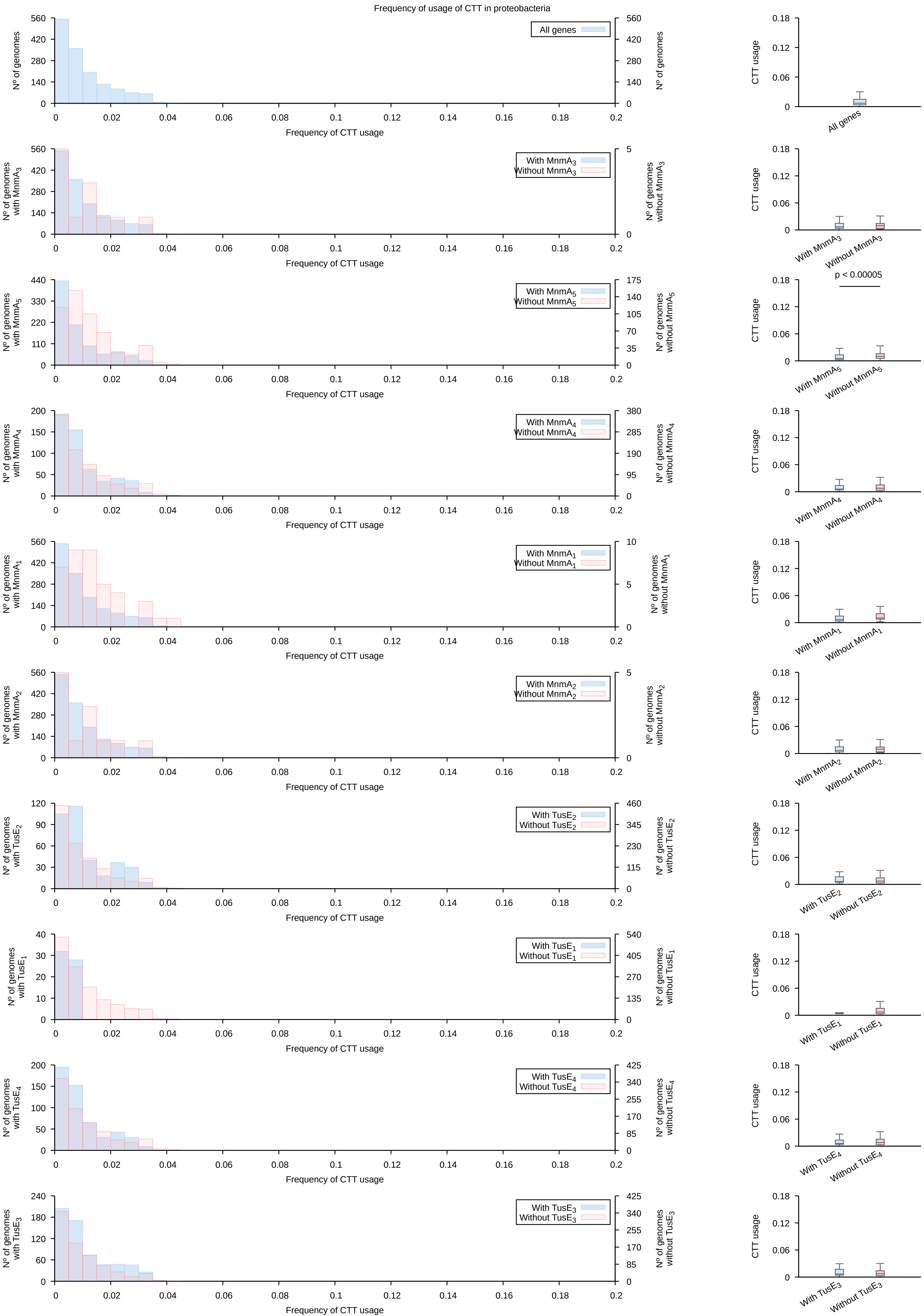

Frequency of usage of CTT in proteobacteria
Nº of genomes
All genes
Frequency of CTT usage
CTT usage
Nº of genomes with MnmA3
Nº of genomes without MnmA3
With MnmA3
Without MnmA3
Nº of genomes with MnmA5
Nº of genomes without MnmA5
With MnmA5
Without MnmA5
p < 0.00005
Nº of genomes with MnmA4
Nº of genomes without MnmA4
With MnmA4
Without MnmA4
Nº of genomes with MnmA1
Nº of genomes without MnmA1
With MnmA1
Without MnmA1
Nº of genomes with MnmA2
Nº of genomes without MnmA2
With MnmA2
Without MnmA2
Nº of genomes with TusE2
Nº of genomes without TusE2
With TusE2
Without TusE2
Nº of genomes with TusE1
Nº of genomes without TusE1
With TusE1
Without TusE1
Nº of genomes with TusE4
Nº of genomes without TusE4
With TusE4
Without TusE4
Nº of genomes with TusE3
Nº of genomes without TusE3
With TusE3
Without TusE3

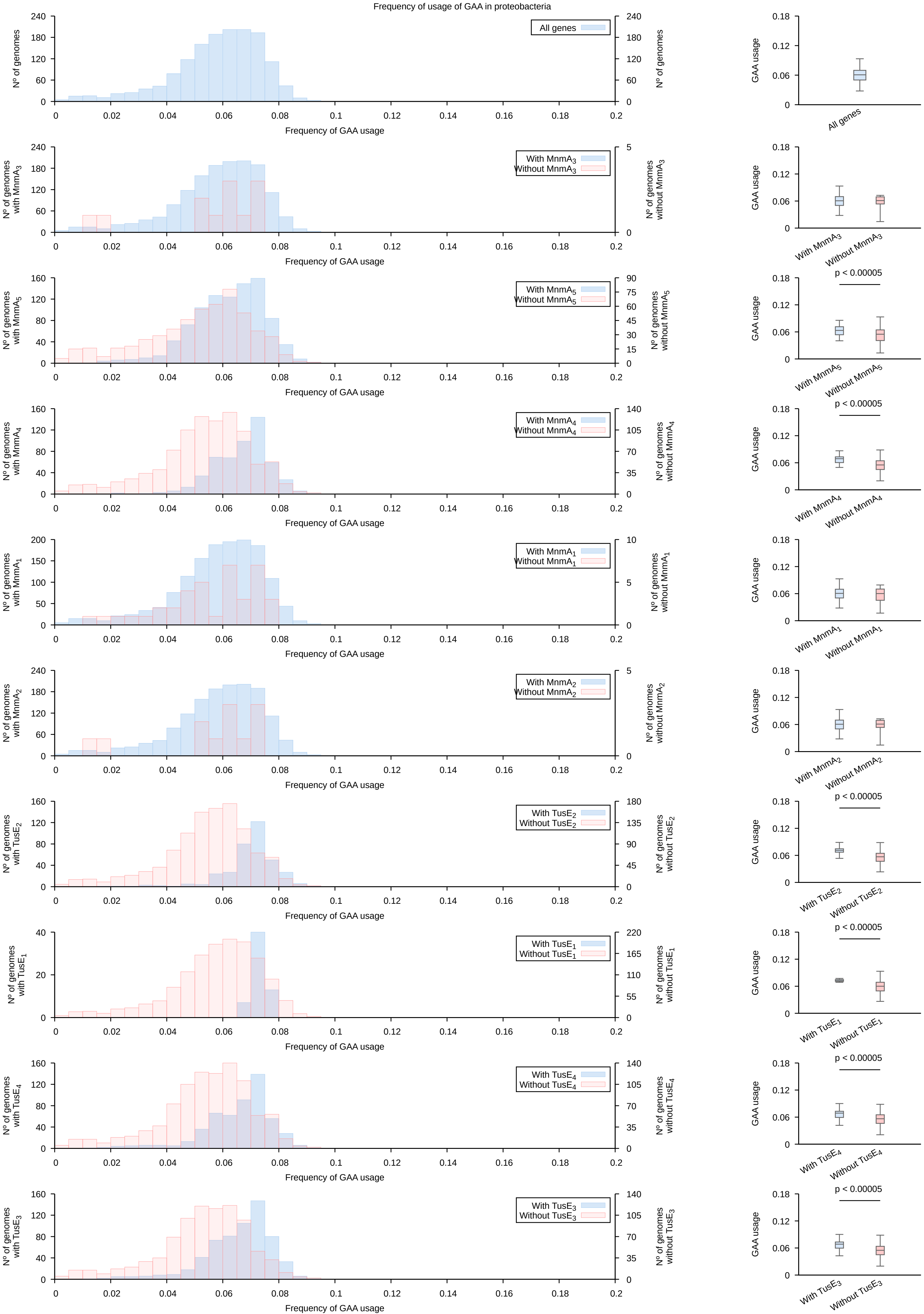
Frequency of usage of GAA in proteobacteria
All genes
Nº of genomes
Frequency of GAA usage
GAA usage
With MnmA$_3$
Without MnmA$_3$
Nº of genomes with MnmA$_3$
Nº of genomes without MnmA$_3$
With MnmA$_5$
Without MnmA$_5$
Nº of genomes with MnmA$_5$
Nº of genomes without MnmA$_5$
p < 0.00005
With MnmA$_4$
Without MnmA$_4$
Nº of genomes with MnmA$_4$
Nº of genomes without MnmA$_4$
p < 0.00005
With MnmA$_1$
Without MnmA$_1$
Nº of genomes with MnmA$_1$
Nº of genomes without MnmA$_1$
With MnmA$_2$
Without MnmA$_2$
Nº of genomes with MnmA$_2$
Nº of genomes without MnmA$_2$
With TusE$_2$
Without TusE$_2$
Nº of genomes with TusE$_2$
Nº of genomes without TusE$_2$
p < 0.00005
With TusE$_1$
Without TusE$_1$
Nº of genomes with TusE$_1$
Nº of genomes without TusE$_1$
p < 0.00005
With TusE$_4$
Without TusE$_4$
Nº of genomes with TusE$_4$
Nº of genomes without TusE$_4$
p < 0.00005
With TusE$_3$
Without TusE$_3$
Nº of genomes with TusE$_3$
Nº of genomes without TusE$_3$
p < 0.00005

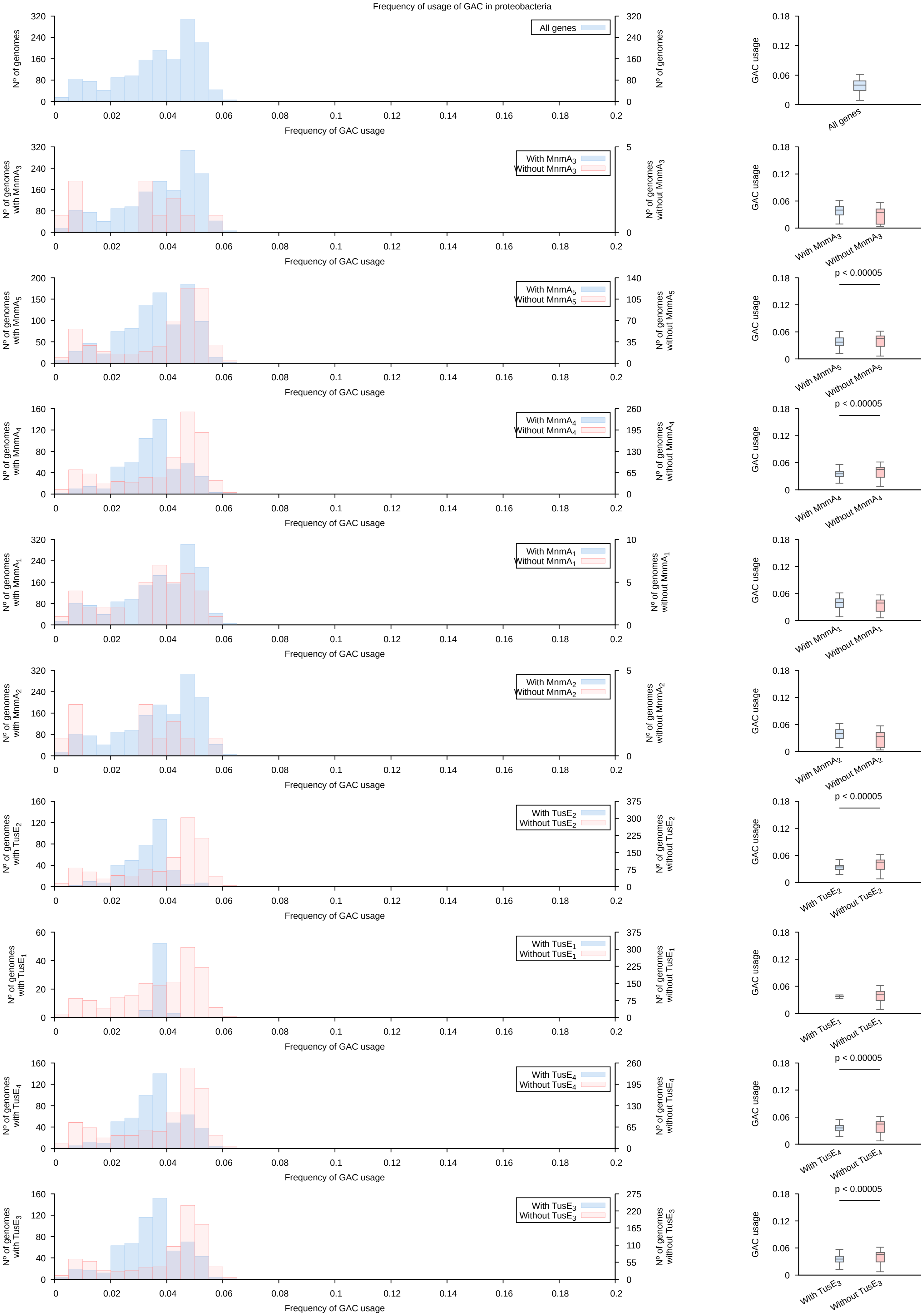

Frequency of usage of GAC in proteobacteria
Nº of genomes
All genes
Frequency of GAC usage
GAC usage
Nº of genomes with MnmA3
Nº of genomes without MnmA3
With MnmA3
Without MnmA3
Nº of genomes with MnmA5
Nº of genomes without MnmA5
With MnmA5
Without MnmA5
p < 0.00005
Nº of genomes with MnmA4
Nº of genomes without MnmA4
With MnmA4
Without MnmA4
p < 0.00005
Nº of genomes with MnmA1
Nº of genomes without MnmA1
With MnmA1
Without MnmA1
Nº of genomes with MnmA2
Nº of genomes without MnmA2
With MnmA2
Without MnmA2
Nº of genomes with TusE2
Nº of genomes without TusE2
With TusE2
Without TusE2
p < 0.00005
Nº of genomes with TusE1
Nº of genomes without TusE1
With TusE1
Without TusE1
Nº of genomes with TusE4
Nº of genomes without TusE4
With TusE4
Without TusE4
p < 0.00005
Nº of genomes with TusE3
Nº of genomes without TusE3
With TusE3
Without TusE3
p < 0.00005

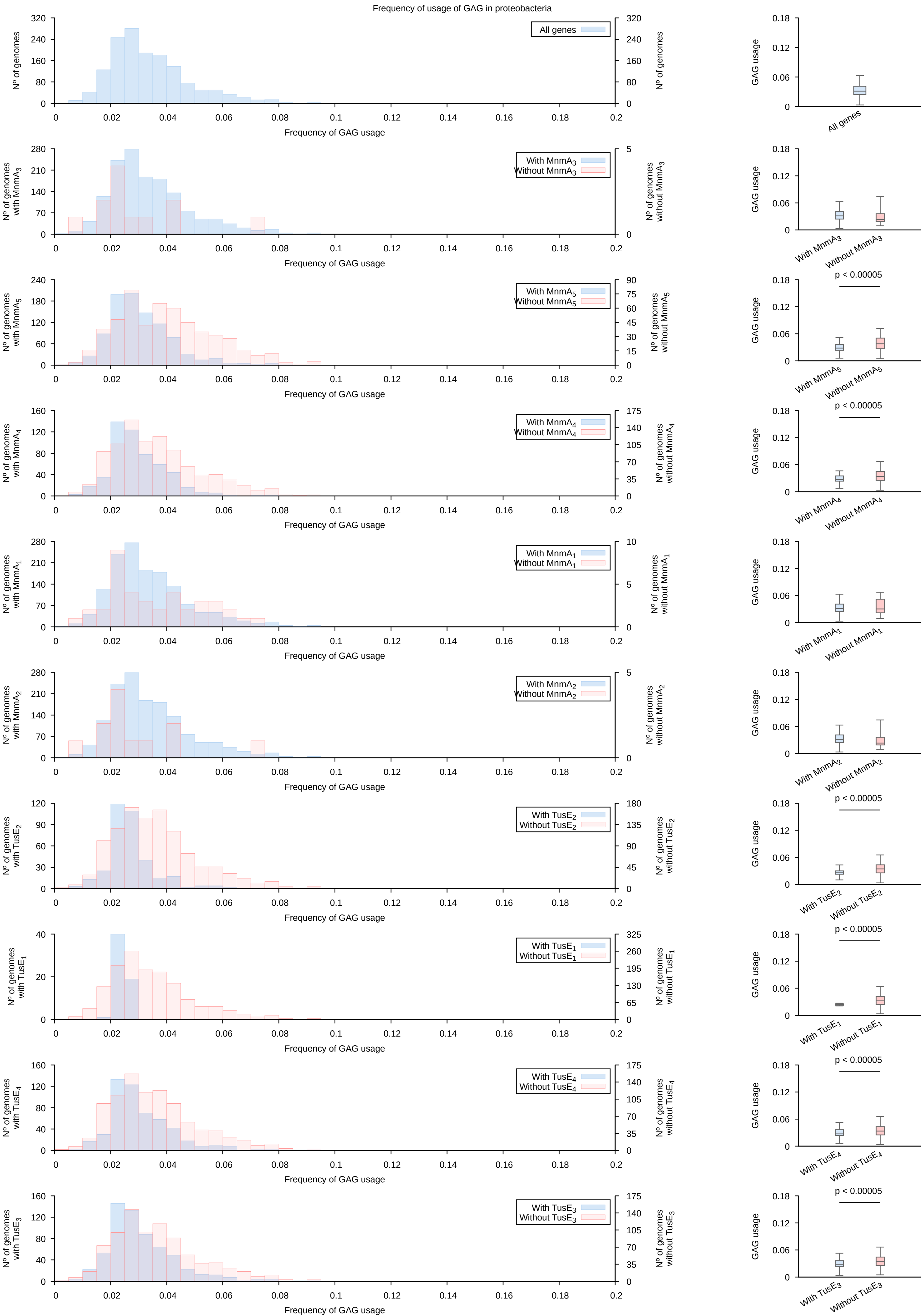
Frequency of usage of GAG in proteobacteria
Nº of genomes
All genes
Frequency of GAG usage
GAG usage
Nº of genomes with MnmA3
Nº of genomes without MnmA3
With MnmA3
Without MnmA3
Nº of genomes with MnmA5
Nº of genomes without MnmA5
With MnmA5
Without MnmA5
p < 0.00005
Nº of genomes with MnmA4
Nº of genomes without MnmA4
With MnmA4
Without MnmA4
p < 0.00005
Nº of genomes with MnmA1
Nº of genomes without MnmA1
With MnmA1
Without MnmA1
Nº of genomes with MnmA2
Nº of genomes without MnmA2
With MnmA2
Without MnmA2
Nº of genomes with TusE2
Nº of genomes without TusE2
With TusE2
Without TusE2
p < 0.00005
Nº of genomes with TusE1
Nº of genomes without TusE1
With TusE1
Without TusE1
p < 0.00005
Nº of genomes with TusE4
Nº of genomes without TusE4
With TusE4
Without TusE4
p < 0.00005
Nº of genomes with TusE3
Nº of genomes without TusE3
With TusE3
Without TusE3
p < 0.00005

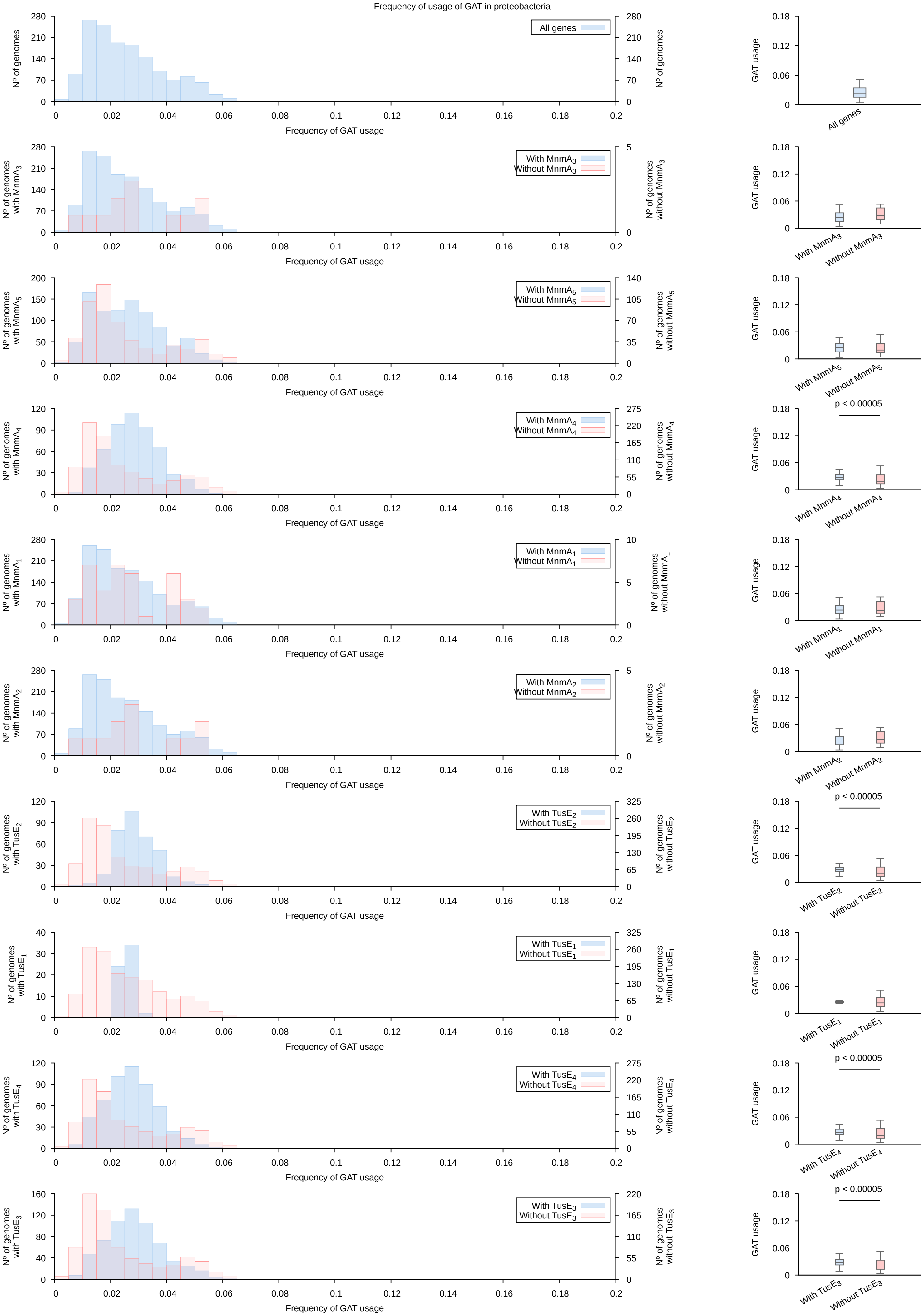

Frequency of usage of GAT in proteobacteria
All genes
With MnmA3
Without MnmA3
With MnmA5
Without MnmA5
With MnmA4
Without MnmA4
With MnmA1
Without MnmA1
With MnmA2
Without MnmA2
With TusE2
Without TusE2
With TusE1
Without TusE1
With TusE4
Without TusE4
With TusE3
Without TusE3
Nº of genomes
Frequency of GAT usage
GAT usage
p < 0.00005

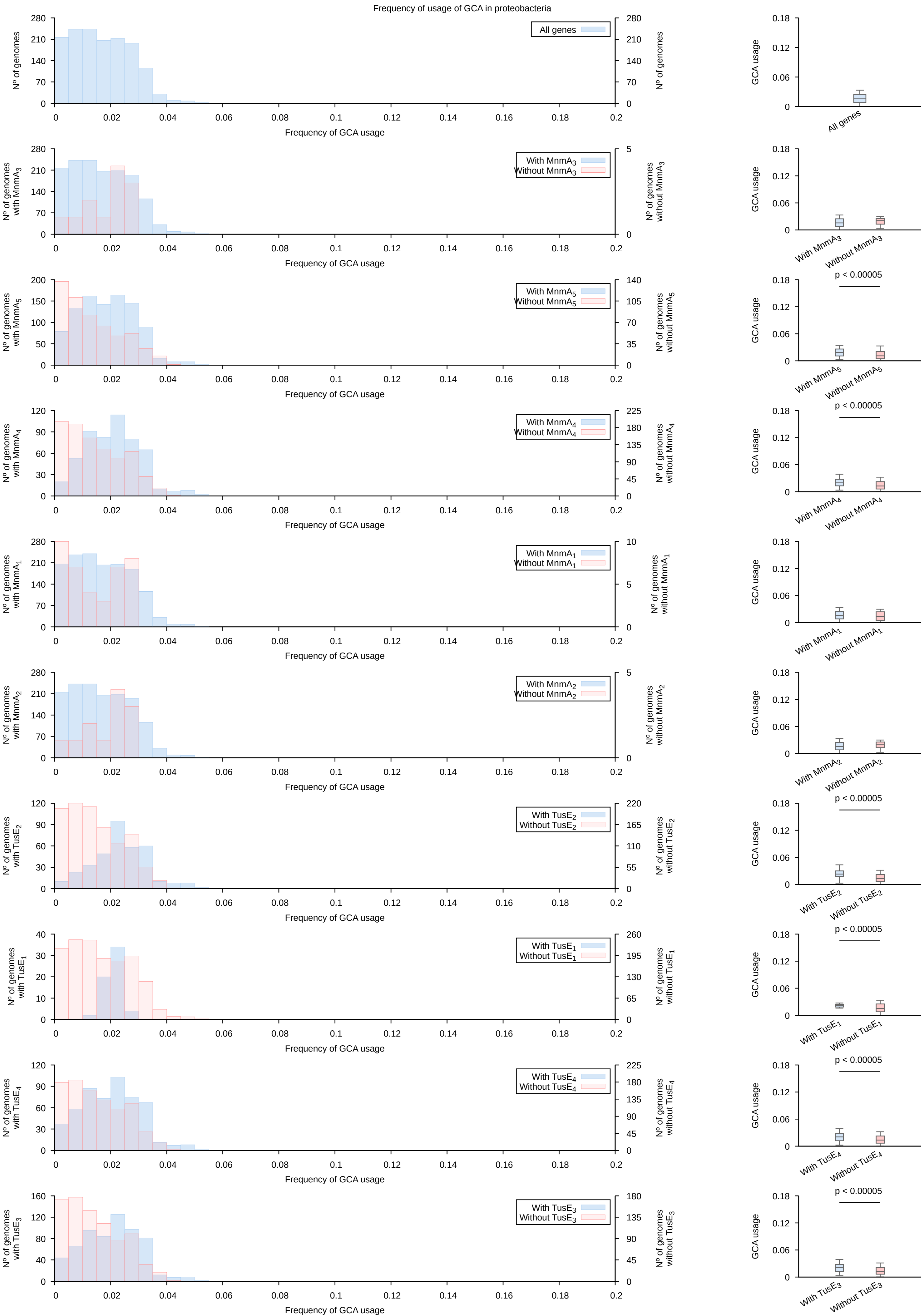
Frequency of usage of GCA in proteobacteria
All genes
Nº of genomes
Frequency of GCA usage
GCA usage
With MnmA$_3$
Without MnmA$_3$
Nº of genomes with MnmA$_3$
Nº of genomes without MnmA$_3$
With MnmA$_5$
Without MnmA$_5$
Nº of genomes with MnmA$_5$
Nº of genomes without MnmA$_5$
p < 0.00005
With MnmA$_4$
Without MnmA$_4$
Nº of genomes with MnmA$_4$
Nº of genomes without MnmA$_4$
p < 0.00005
With MnmA$_1$
Without MnmA$_1$
Nº of genomes with MnmA$_1$
Nº of genomes without MnmA$_1$
With MnmA$_2$
Without MnmA$_2$
Nº of genomes with MnmA$_2$
Nº of genomes without MnmA$_2$
With TusE$_2$
Without TusE$_2$
Nº of genomes with TusE$_2$
Nº of genomes without TusE$_2$
p < 0.00005
With TusE$_1$
Without TusE$_1$
Nº of genomes with TusE$_1$
Nº of genomes without TusE$_1$
p < 0.00005
With TusE$_4$
Without TusE$_4$
Nº of genomes with TusE$_4$
Nº of genomes without TusE$_4$
p < 0.00005
With TusE$_3$
Without TusE$_3$
Nº of genomes with TusE$_3$
Nº of genomes without TusE$_3$
p < 0.00005

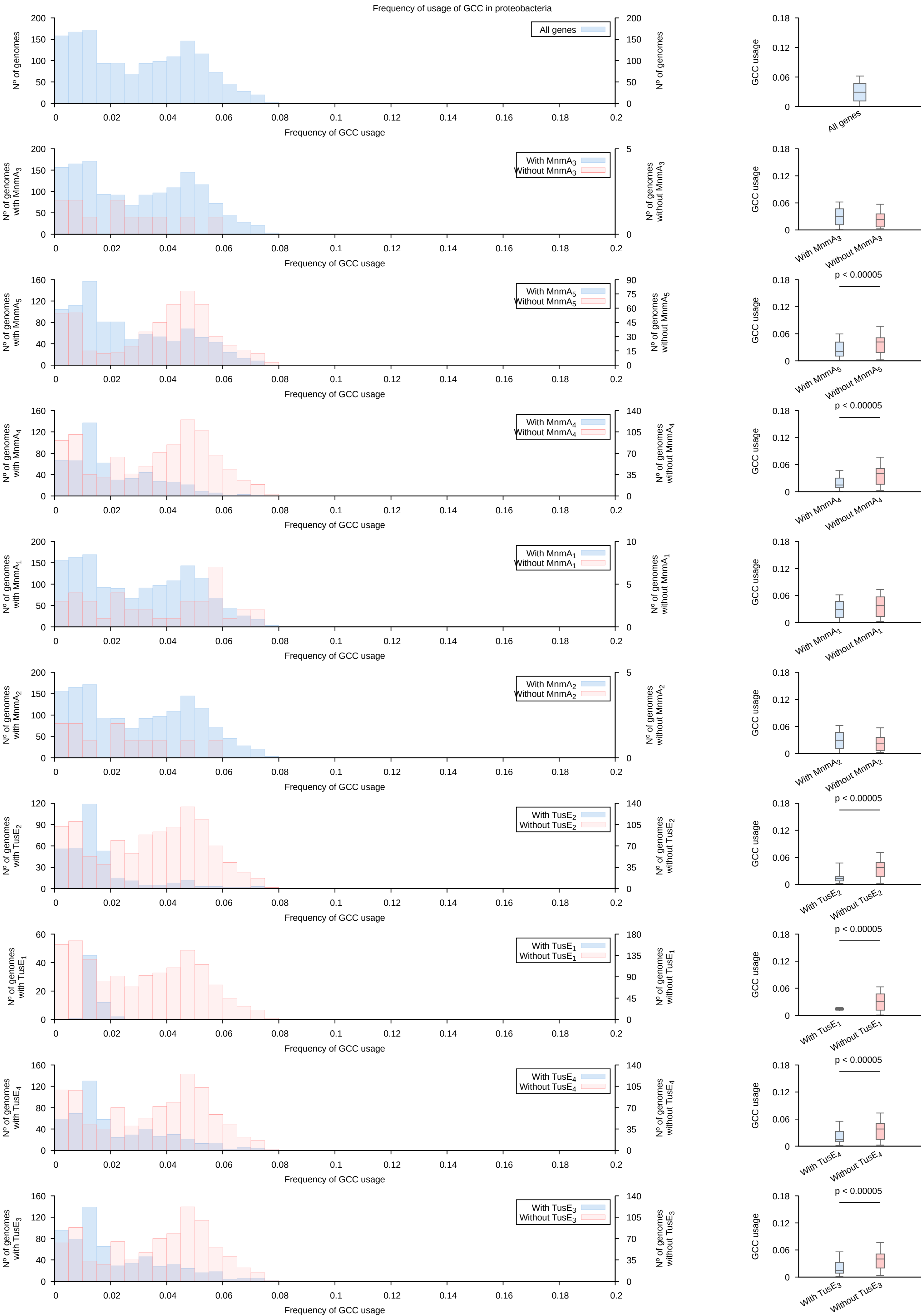

Frequency of usage of GCC in proteobacteria
Nº of genomes
All genes
Frequency of GCC usage
GCC usage
All genes
Nº of genomes with MnmA$_3$
Nº of genomes without MnmA$_3$
With MnmA$_3$
Without MnmA$_3$
Nº of genomes with MnmA$_5$
Nº of genomes without MnmA$_5$
With MnmA$_5$
Without MnmA$_5$
p < 0.00005
Nº of genomes with MnmA$_4$
Nº of genomes without MnmA$_4$
With MnmA$_4$
Without MnmA$_4$
p < 0.00005
Nº of genomes with MnmA$_1$
Nº of genomes without MnmA$_1$
With MnmA$_1$
Without MnmA$_1$
Nº of genomes with MnmA$_2$
Nº of genomes without MnmA$_2$
With MnmA$_2$
Without MnmA$_2$
Nº of genomes with TusE$_2$
Nº of genomes without TusE$_2$
With TusE$_2$
Without TusE$_2$
p < 0.00005
Nº of genomes with TusE$_1$
Nº of genomes without TusE$_1$
With TusE$_1$
Without TusE$_1$
p < 0.00005
Nº of genomes with TusE$_4$
Nº of genomes without TusE$_4$
With TusE$_4$
Without TusE$_4$
p < 0.00005
Nº of genomes with TusE$_3$
Nº of genomes without TusE$_3$
With TusE$_3$
Without TusE$_3$
p < 0.00005

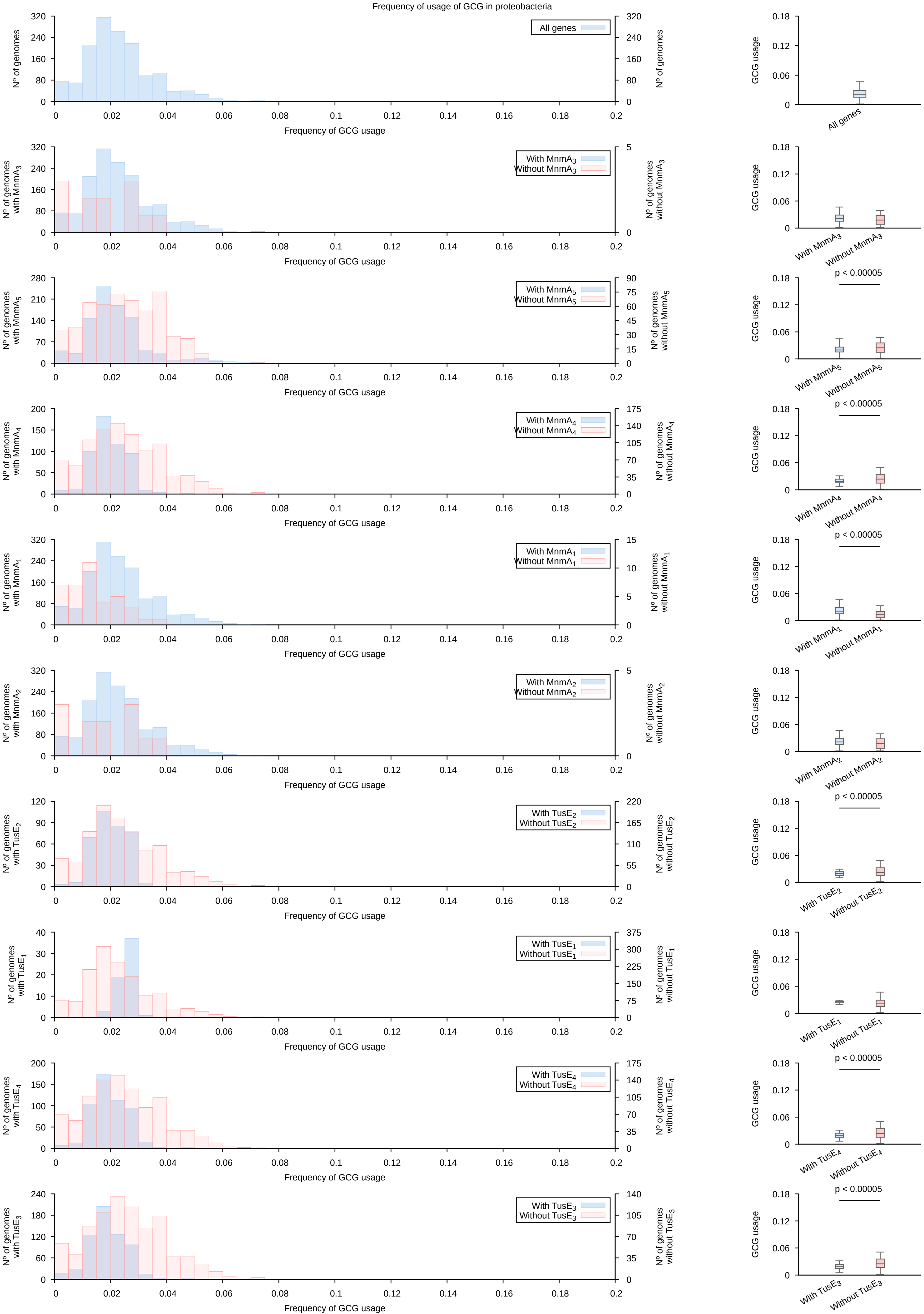
Frequency of usage of GCG in proteobacteria
Nº of genomes
Frequency of GCG usage
GCG usage
All genes
Nº of genomes with MnmA3
Nº of genomes without MnmA3
With MnmA3
Without MnmA3
Nº of genomes with MnmA5
Nº of genomes without MnmA5
With MnmA5
Without MnmA5
p < 0.00005
Nº of genomes with MnmA4
Nº of genomes without MnmA4
With MnmA4
Without MnmA4
p < 0.00005
Nº of genomes with MnmA1
Nº of genomes without MnmA1
With MnmA1
Without MnmA1
p < 0.00005
Nº of genomes with MnmA2
Nº of genomes without MnmA2
With MnmA2
Without MnmA2
Nº of genomes with TusE2
Nº of genomes without TusE2
With TusE2
Without TusE2
p < 0.00005
Nº of genomes with TusE1
Nº of genomes without TusE1
With TusE1
Without TusE1
Nº of genomes with TusE4
Nº of genomes without TusE4
With TusE4
Without TusE4
p < 0.00005
Nº of genomes with TusE3
Nº of genomes without TusE3
With TusE3
Without TusE3
p < 0.00005

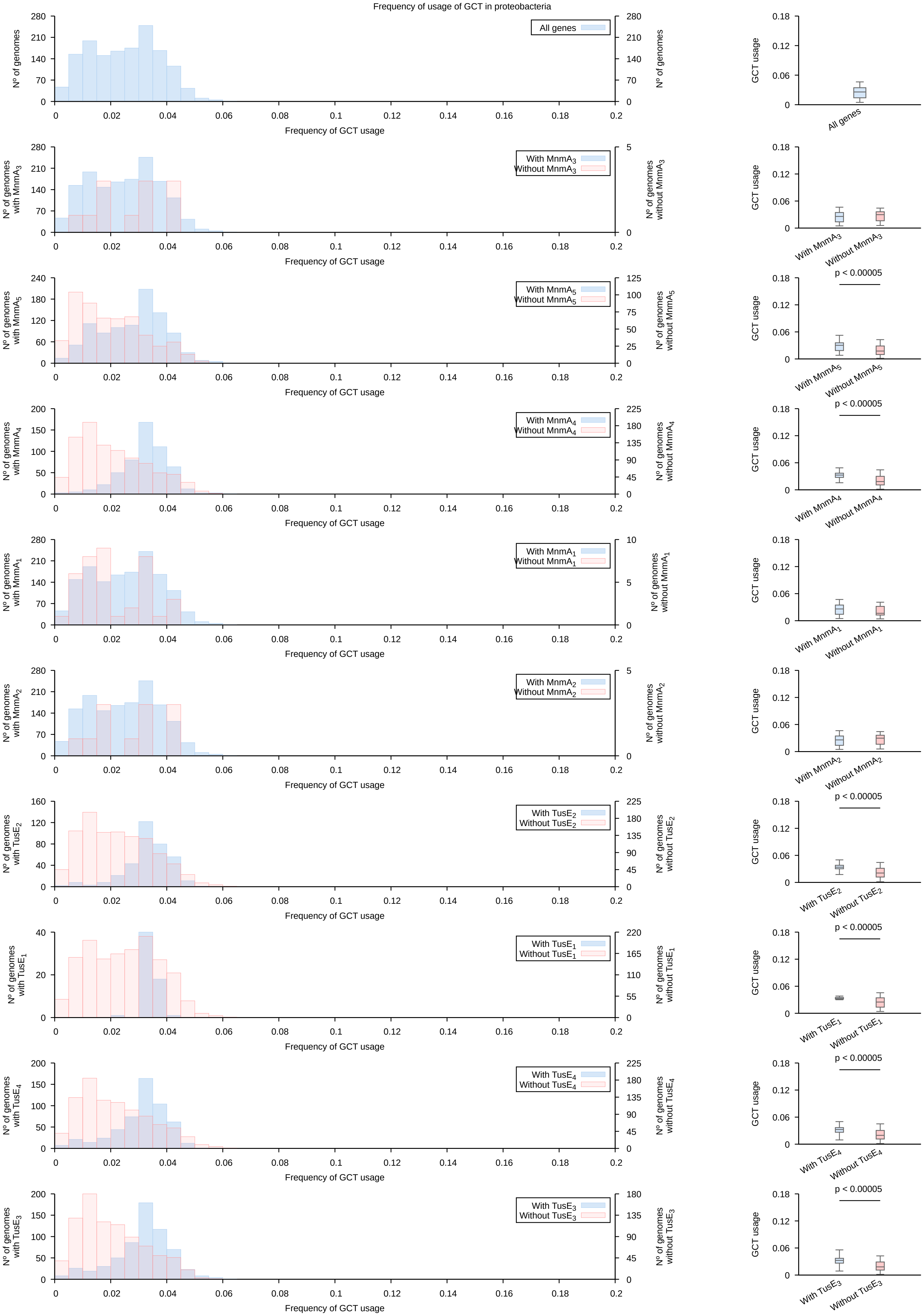
Frequency of usage of GCT in proteobacteria
Nº of genomes
All genes
Frequency of GCT usage
GCT usage
All genes
Nº of genomes with MnmA$_3$
Nº of genomes without MnmA$_3$
With MnmA$_3$
Without MnmA$_3$
Nº of genomes with MnmA$_5$
Nº of genomes without MnmA$_5$
With MnmA$_5$
Without MnmA$_5$
p < 0.00005
Nº of genomes with MnmA$_4$
Nº of genomes without MnmA$_4$
With MnmA$_4$
Without MnmA$_4$
p < 0.00005
Nº of genomes with MnmA$_1$
Nº of genomes without MnmA$_1$
With MnmA$_1$
Without MnmA$_1$
Nº of genomes with MnmA$_2$
Nº of genomes without MnmA$_2$
With MnmA$_2$
Without MnmA$_2$
Nº of genomes with TusE$_2$
Nº of genomes without TusE$_2$
With TusE$_2$
Without TusE$_2$
p < 0.00005
Nº of genomes with TusE$_1$
Nº of genomes without TusE$_1$
With TusE$_1$
Without TusE$_1$
p < 0.00005
Nº of genomes with TusE$_4$
Nº of genomes without TusE$_4$
With TusE$_4$
Without TusE$_4$
p < 0.00005
Nº of genomes with TusE$_3$
Nº of genomes without TusE$_3$
With TusE$_3$
Without TusE$_3$
p < 0.00005

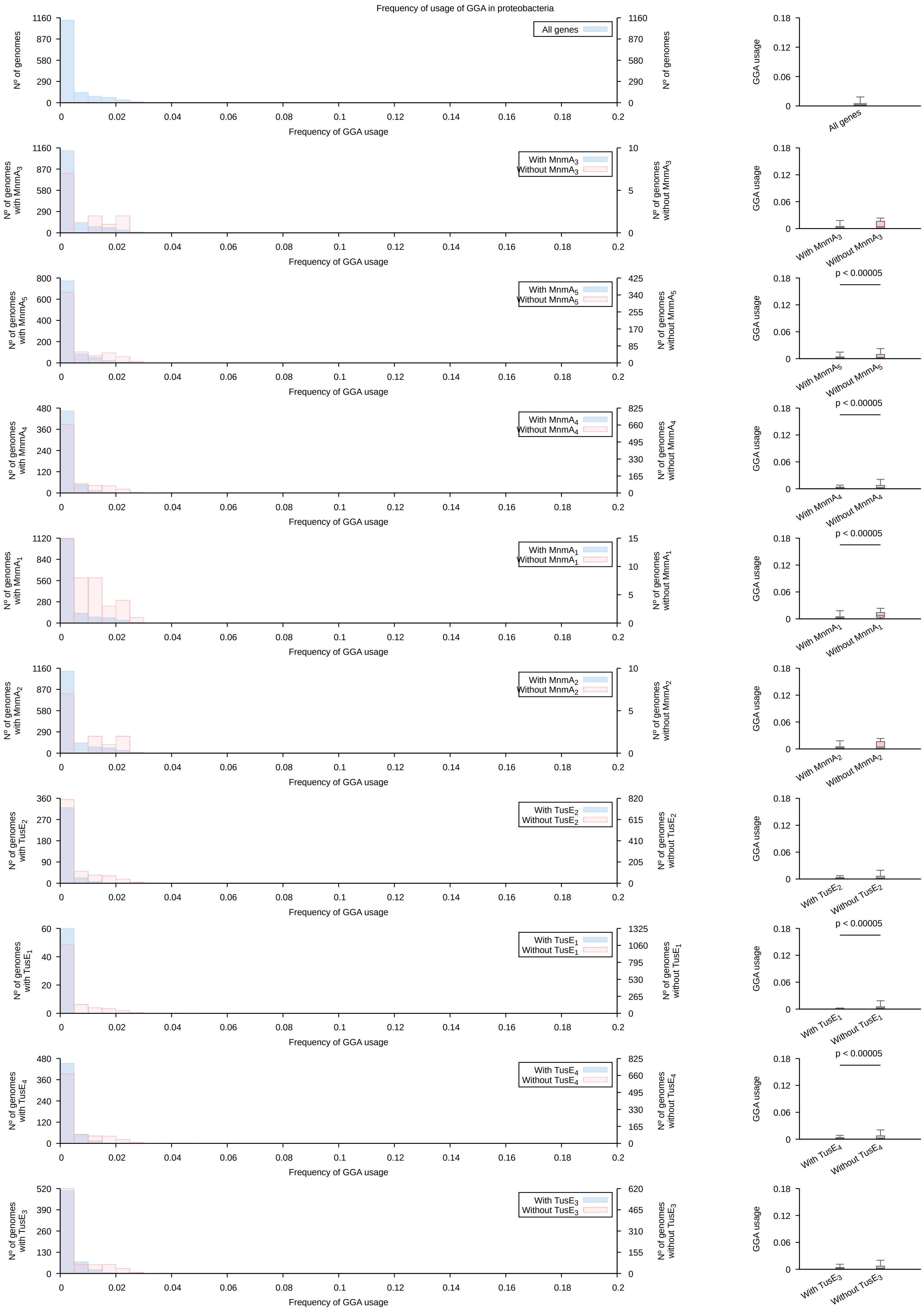
Frequency of usage of GGA in proteobacteria
Nº of genomes
Frequency of GGA usage
All genes
GGA usage
Nº of genomes with MnmA3
Nº of genomes without MnmA3
With MnmA3
Without MnmA3
Nº of genomes with MnmA5
Nº of genomes without MnmA5
With MnmA5
Without MnmA5
p < 0.00005
Nº of genomes with MnmA4
Nº of genomes without MnmA4
With MnmA4
Without MnmA4
p < 0.00005
Nº of genomes with MnmA1
Nº of genomes without MnmA1
With MnmA1
Without MnmA1
p < 0.00005
Nº of genomes with MnmA2
Nº of genomes without MnmA2
With MnmA2
Without MnmA2
Nº of genomes with TusE2
Nº of genomes without TusE2
With TusE2
Without TusE2
Nº of genomes with TusE1
Nº of genomes without TusE1
With TusE1
Without TusE1
p < 0.00005
Nº of genomes with TusE4
Nº of genomes without TusE4
With TusE4
Without TusE4
p < 0.00005
Nº of genomes with TusE3
Nº of genomes without TusE3
With TusE3
Without TusE3

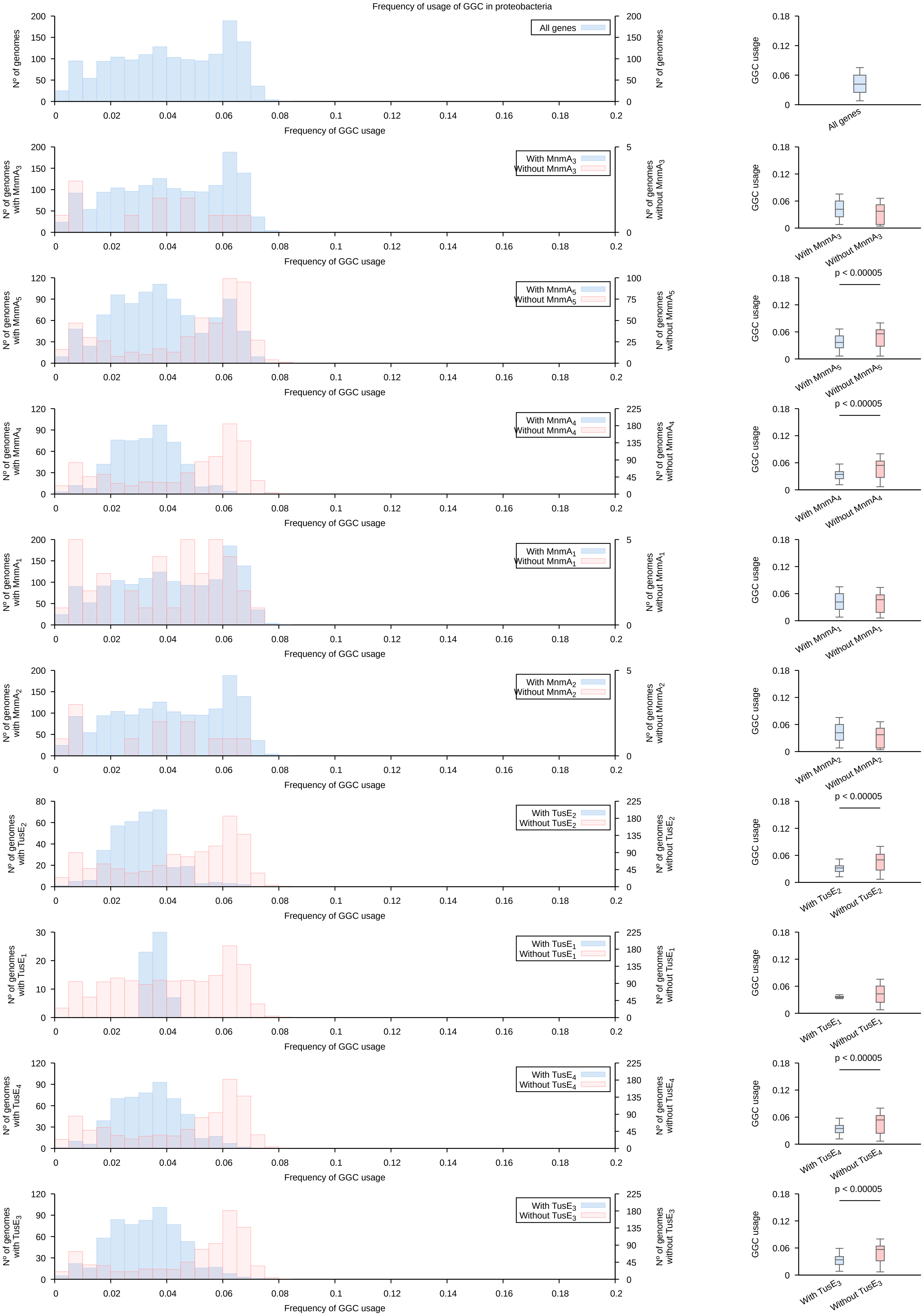
Frequency of usage of GGC in proteobacteria
All genes
Nº of genomes
Frequency of GGC usage
GGC usage
With MnmA$_3$
Without MnmA$_3$
Nº of genomes with MnmA$_3$
Nº of genomes without MnmA$_3$
With MnmA$_5$
Without MnmA$_5$
Nº of genomes with MnmA$_5$
Nº of genomes without MnmA$_5$
p < 0.00005
With MnmA$_4$
Without MnmA$_4$
Nº of genomes with MnmA$_4$
Nº of genomes without MnmA$_4$
p < 0.00005
With MnmA$_1$
Without MnmA$_1$
Nº of genomes with MnmA$_1$
Nº of genomes without MnmA$_1$
With MnmA$_2$
Without MnmA$_2$
Nº of genomes with MnmA$_2$
Nº of genomes without MnmA$_2$
With TusE$_2$
Without TusE$_2$
Nº of genomes with TusE$_2$
Nº of genomes without TusE$_2$
p < 0.00005
With TusE$_1$
Without TusE$_1$
Nº of genomes with TusE$_1$
Nº of genomes without TusE$_1$
With TusE$_4$
Without TusE$_4$
Nº of genomes with TusE$_4$
Nº of genomes without TusE$_4$
p < 0.00005
With TusE$_3$
Without TusE$_3$
Nº of genomes with TusE$_3$
Nº of genomes without TusE$_3$
p < 0.00005

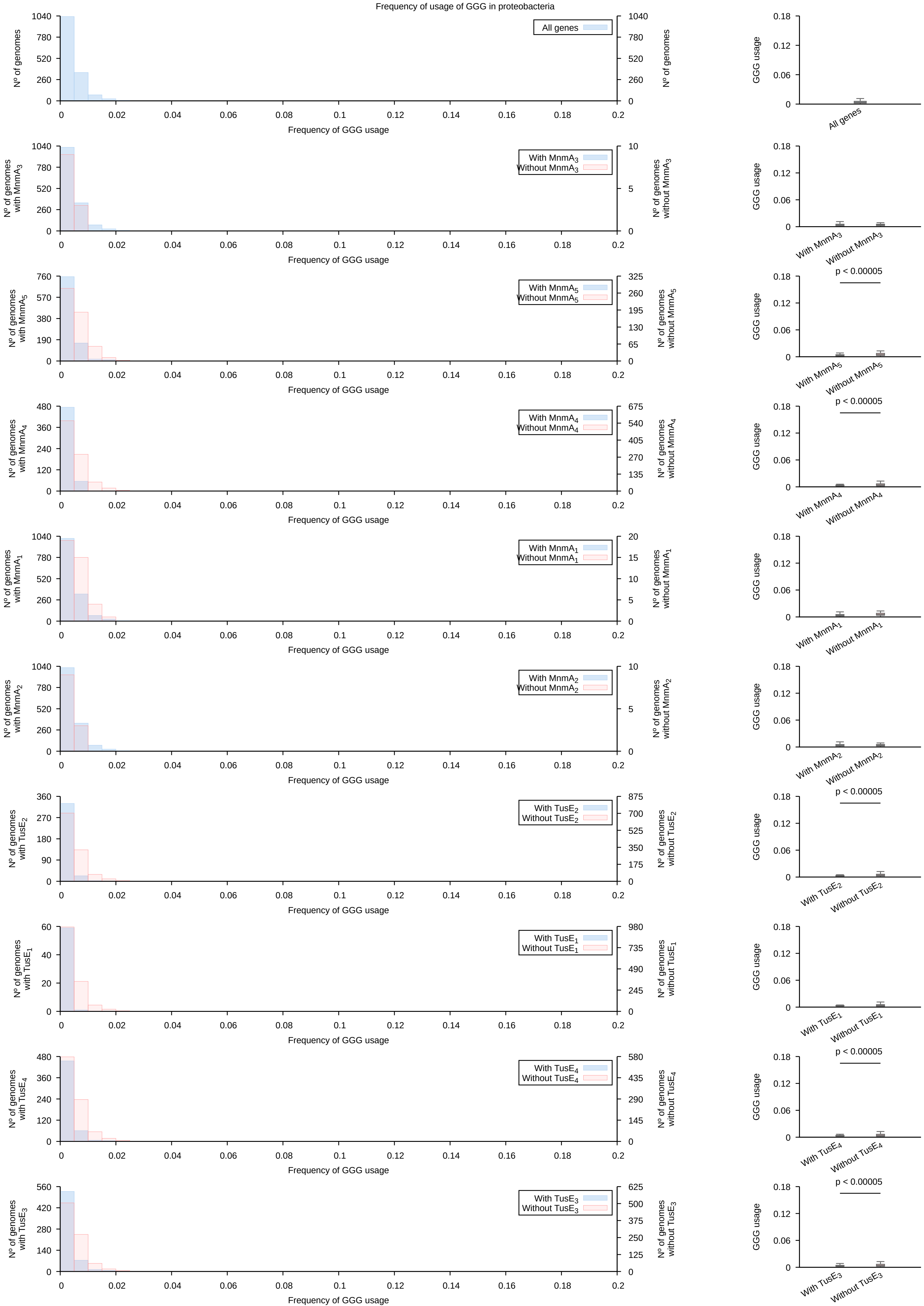
Frequency of usage of GGG in proteobacteria
All genes
Nº of genomes
Frequency of GGG usage
GGG usage
With $MnmA_3$
Without $MnmA_3$
Nº of genomes with $MnmA_3$
Nº of genomes without $MnmA_3$
With $MnmA_5$
Without $MnmA_5$
Nº of genomes with $MnmA_5$
Nº of genomes without $MnmA_5$
p < 0.00005
With $MnmA_4$
Without $MnmA_4$
Nº of genomes with $MnmA_4$
Nº of genomes without $MnmA_4$
p < 0.00005
With $MnmA_1$
Without $MnmA_1$
Nº of genomes with $MnmA_1$
Nº of genomes without $MnmA_1$
With $MnmA_2$
Without $MnmA_2$
Nº of genomes with $MnmA_2$
Nº of genomes without $MnmA_2$
With $TusE_2$
Without $TusE_2$
Nº of genomes with $TusE_2$
Nº of genomes without $TusE_2$
p < 0.00005
With $TusE_1$
Without $TusE_1$
Nº of genomes with $TusE_1$
Nº of genomes without $TusE_1$
With $TusE_4$
Without $TusE_4$
Nº of genomes with $TusE_4$
Nº of genomes without $TusE_4$
p < 0.00005
With $TusE_3$
Without $TusE_3$
Nº of genomes with $TusE_3$
Nº of genomes without $TusE_3$
p < 0.00005

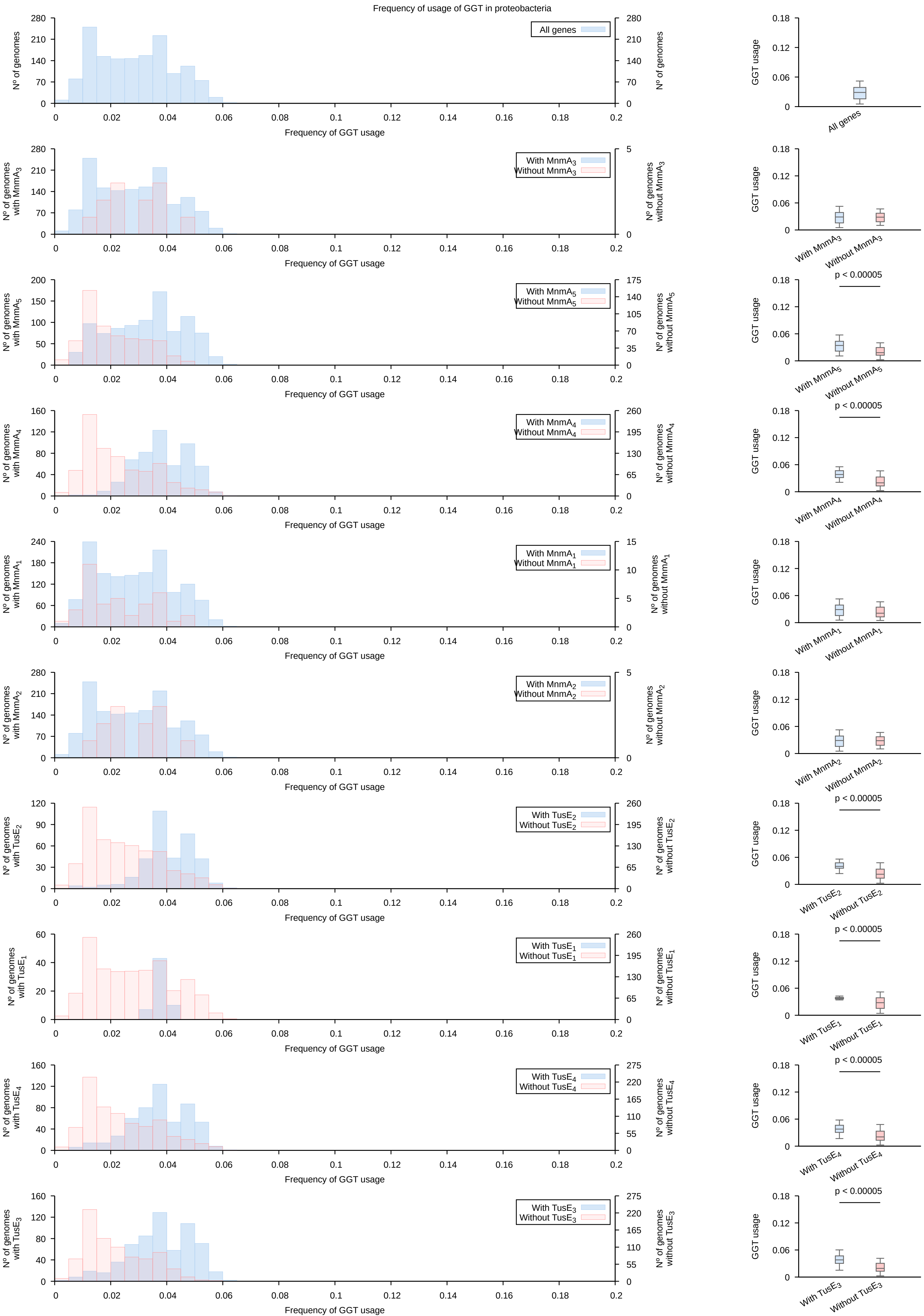
Frequency of usage of GGT in proteobacteria
Nº of genomes
All genes
Frequency of GGT usage
GGT usage
All genes
Nº of genomes with $MnmA_3$
Nº of genomes without $MnmA_3$
With $MnmA_3$
Without $MnmA_3$
Nº of genomes with $MnmA_5$
Nº of genomes without $MnmA_5$
With $MnmA_5$
Without $MnmA_5$
p < 0.00005
Nº of genomes with $MnmA_4$
Nº of genomes without $MnmA_4$
With $MnmA_4$
Without $MnmA_4$
p < 0.00005
Nº of genomes with $MnmA_1$
Nº of genomes without $MnmA_1$
With $MnmA_1$
Without $MnmA_1$
Nº of genomes with $MnmA_2$
Nº of genomes without $MnmA_2$
With $MnmA_2$
Without $MnmA_2$
Nº of genomes with $TusE_2$
Nº of genomes without $TusE_2$
With $TusE_2$
Without $TusE_2$
p < 0.00005
Nº of genomes with $TusE_1$
Nº of genomes without $TusE_1$
With $TusE_1$
Without $TusE_1$
p < 0.00005
Nº of genomes with $TusE_4$
Nº of genomes without $TusE_4$
With $TusE_4$
Without $TusE_4$
p < 0.00005
Nº of genomes with $TusE_3$
Nº of genomes without $TusE_3$
With $TusE_3$
Without $TusE_3$
p < 0.00005

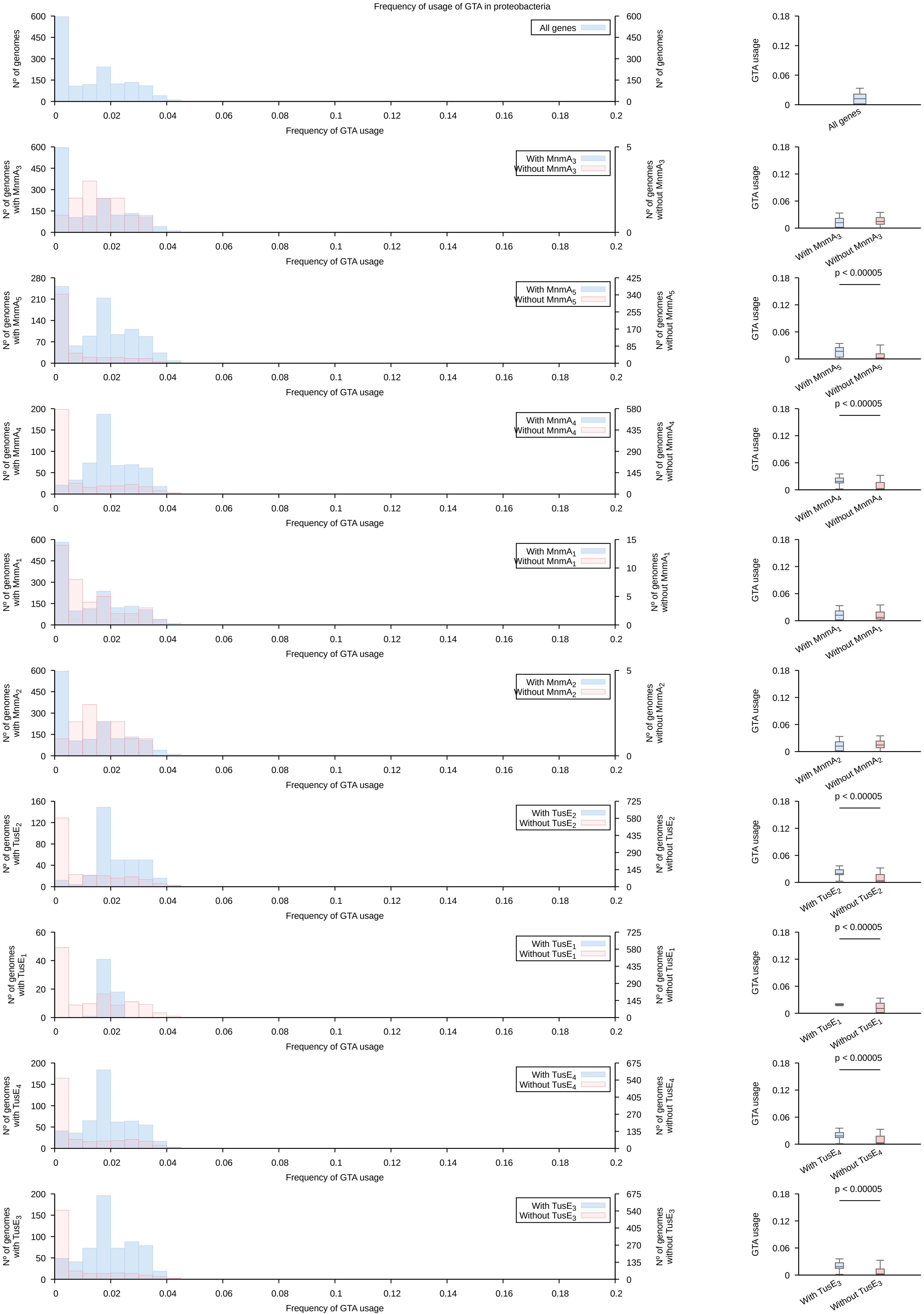
Frequency of usage of GTA in proteobacteria
All genes
Nº of genomes
Frequency of GTA usage
GTA usage
With $MnmA_3$
Without $MnmA_3$
Nº of genomes with $MnmA_3$
Nº of genomes without $MnmA_3$
With $MnmA_5$
Without $MnmA_5$
Nº of genomes with $MnmA_5$
Nº of genomes without $MnmA_5$
p < 0.00005
With $MnmA_4$
Without $MnmA_4$
Nº of genomes with $MnmA_4$
Nº of genomes without $MnmA_4$
p < 0.00005
With $MnmA_1$
Without $MnmA_1$
Nº of genomes with $MnmA_1$
Nº of genomes without $MnmA_1$
With $MnmA_2$
Without $MnmA_2$
Nº of genomes with $MnmA_2$
Nº of genomes without $MnmA_2$
With $TusE_2$
Without $TusE_2$
Nº of genomes with $TusE_2$
Nº of genomes without $TusE_2$
p < 0.00005
With $TusE_1$
Without $TusE_1$
Nº of genomes with $TusE_1$
Nº of genomes without $TusE_1$
p < 0.00005
With $TusE_4$
Without $TusE_4$
Nº of genomes with $TusE_4$
Nº of genomes without $TusE_4$
p < 0.00005
With $TusE_3$
Without $TusE_3$
Nº of genomes with $TusE_3$
Nº of genomes without $TusE_3$
p < 0.00005

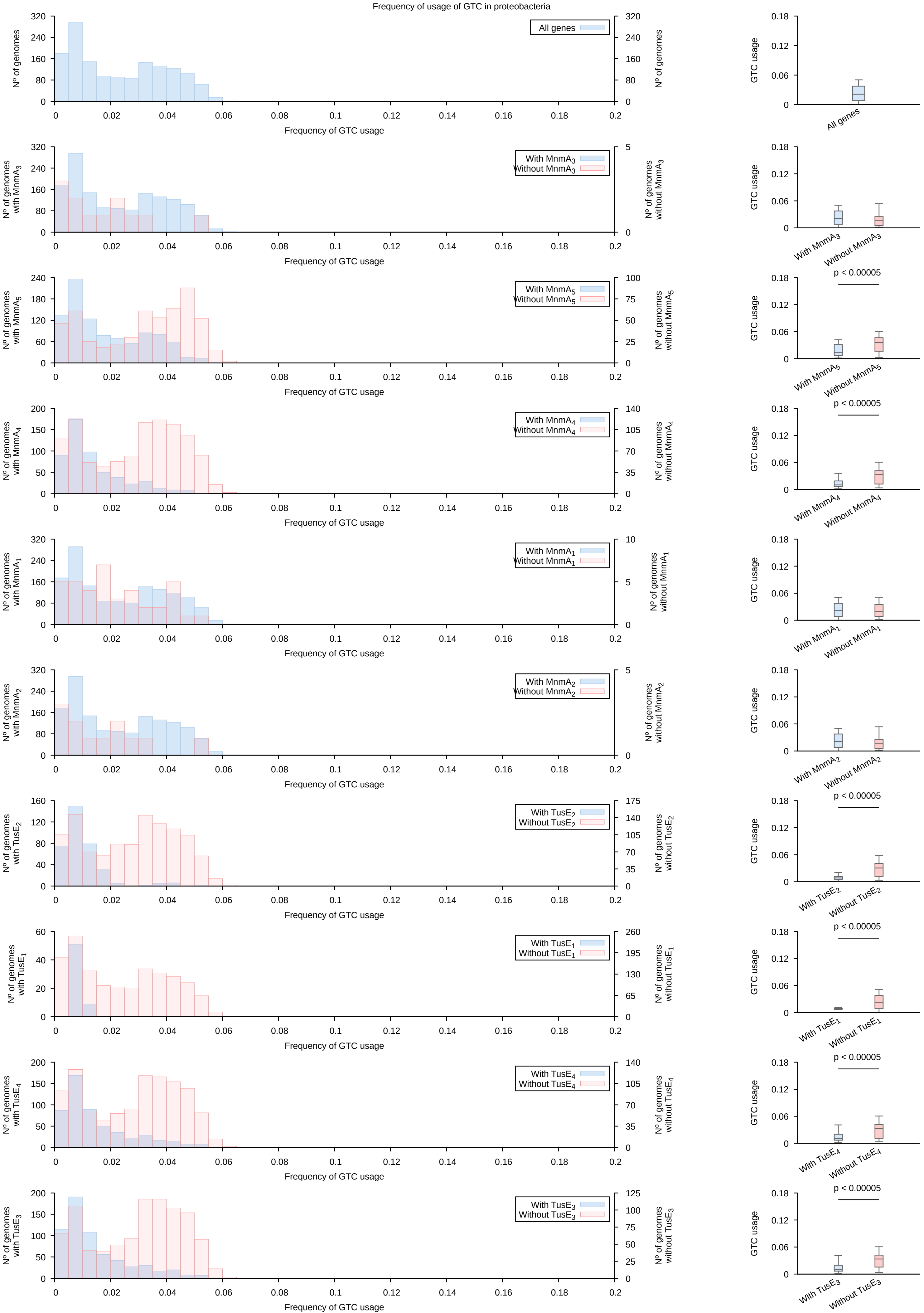

Frequency of usage of GTC in proteobacteria
All genes
Nº of genomes
GTC usage
Frequency of GTC usage
With MnmA3
Without MnmA3
Nº of genomes with MnmA3
Nº of genomes without MnmA3
With MnmA5
Without MnmA5
Nº of genomes with MnmA5
Nº of genomes without MnmA5
p < 0.00005
With MnmA4
Without MnmA4
Nº of genomes with MnmA4
Nº of genomes without MnmA4
With MnmA1
Without MnmA1
Nº of genomes with MnmA1
Nº of genomes without MnmA1
With MnmA2
Without MnmA2
Nº of genomes with MnmA2
Nº of genomes without MnmA2
With TusE2
Without TusE2
Nº of genomes with TusE2
Nº of genomes without TusE2
With TusE1
Without TusE1
Nº of genomes with TusE1
Nº of genomes without TusE1
With TusE4
Without TusE4
Nº of genomes with TusE4
Nº of genomes without TusE4
With TusE3
Without TusE3
Nº of genomes with TusE3
Nº of genomes without TusE3

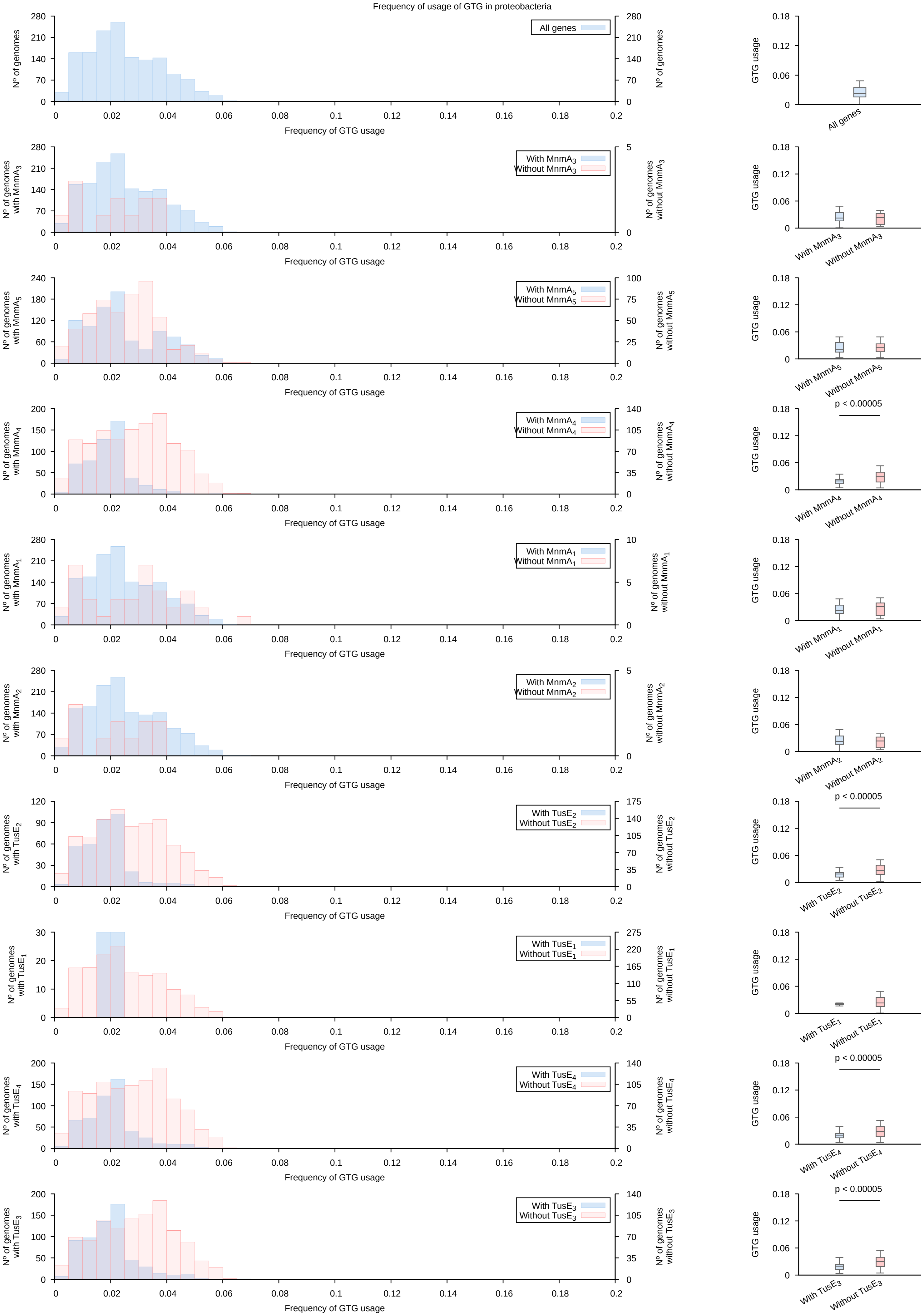

Frequency of usage of GTG in proteobacteria
Nº of genomes
All genes
Frequency of GTG usage
GTG usage
Nº of genomes with MnmA3
Nº of genomes without MnmA3
With MnmA3
Without MnmA3
Nº of genomes with MnmA5
Nº of genomes without MnmA5
With MnmA5
Without MnmA5
Nº of genomes with MnmA4
Nº of genomes without MnmA4
With MnmA4
Without MnmA4
p < 0.00005
Nº of genomes with MnmA1
Nº of genomes without MnmA1
With MnmA1
Without MnmA1
Nº of genomes with MnmA2
Nº of genomes without MnmA2
With MnmA2
Without MnmA2
Nº of genomes with TusE2
Nº of genomes without TusE2
With TusE2
Without TusE2
p < 0.00005
Nº of genomes with TusE1
Nº of genomes without TusE1
With TusE1
Without TusE1
Nº of genomes with TusE4
Nº of genomes without TusE4
With TusE4
Without TusE4
p < 0.00005
Nº of genomes with TusE3
Nº of genomes without TusE3
With TusE3
Without TusE3
p < 0.00005

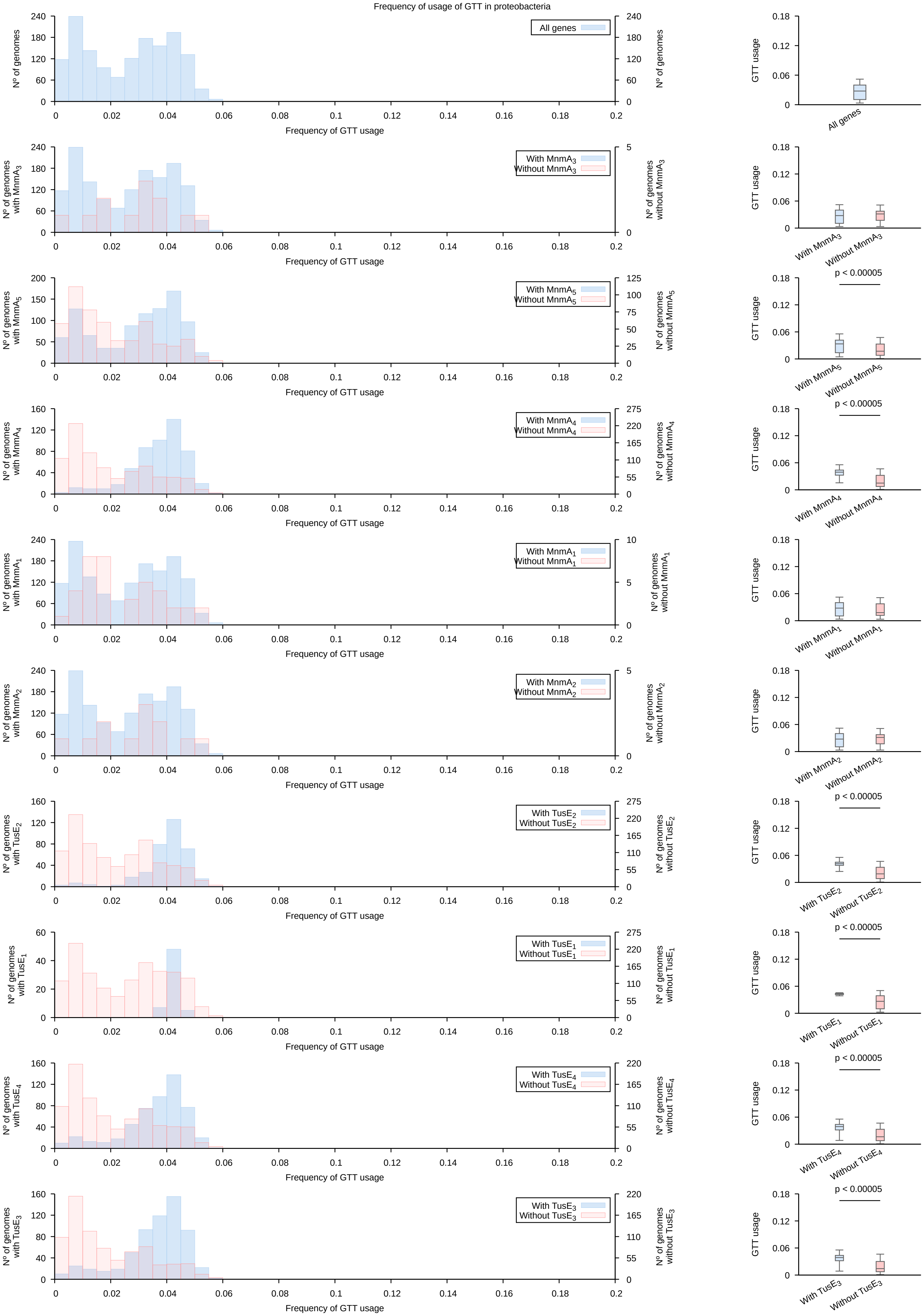
Frequency of usage of GTT in proteobacteria
Nº of genomes
All genes
Frequency of GTT usage
GTT usage
Nº of genomes with MnmA$_3$
Nº of genomes without MnmA$_3$
With MnmA$_3$
Without MnmA$_3$
Nº of genomes with MnmA$_5$
Nº of genomes without MnmA$_5$
With MnmA$_5$
Without MnmA$_5$
p < 0.00005
Nº of genomes with MnmA$_4$
Nº of genomes without MnmA$_4$
With MnmA$_4$
Without MnmA$_4$
p < 0.00005
Nº of genomes with MnmA$_1$
Nº of genomes without MnmA$_1$
With MnmA$_1$
Without MnmA$_1$
Nº of genomes with MnmA$_2$
Nº of genomes without MnmA$_2$
With MnmA$_2$
Without MnmA$_2$
Nº of genomes with TusE$_2$
Nº of genomes without TusE$_2$
With TusE$_2$
Without TusE$_2$
p < 0.00005
Nº of genomes with TusE$_1$
Nº of genomes without TusE$_1$
With TusE$_1$
Without TusE$_1$
p < 0.00005
Nº of genomes with TusE$_4$
Nº of genomes without TusE$_4$
With TusE$_4$
Without TusE$_4$
p < 0.00005
Nº of genomes with TusE$_3$
Nº of genomes without TusE$_3$
With TusE$_3$
Without TusE$_3$
p < 0.00005

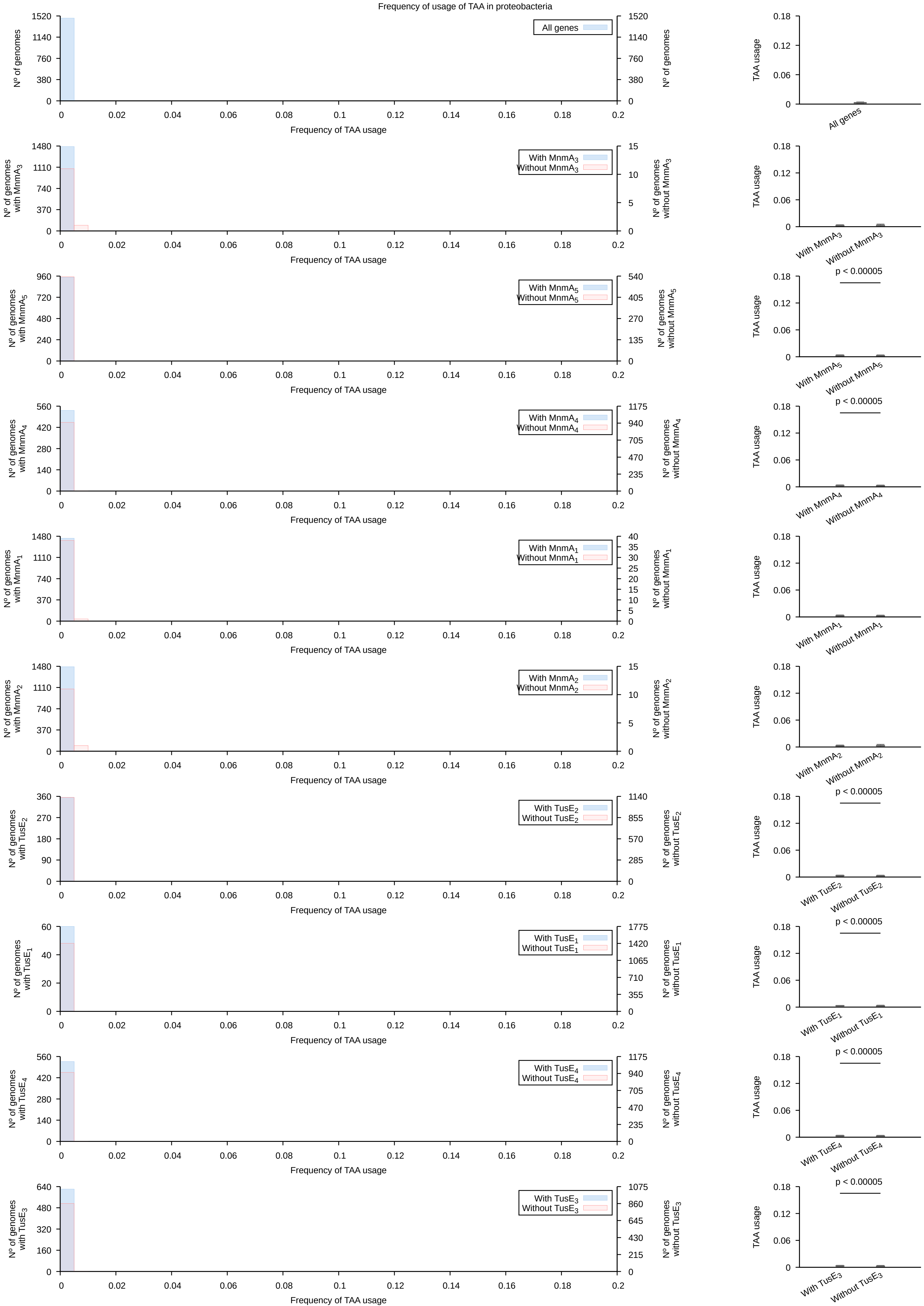
Frequency of usage of TAA in proteobacteria
All genes
Nº of genomes
Frequency of TAA usage
TAA usage
With MnmA3
Without MnmA3
Nº of genomes with MnmA3
Nº of genomes without MnmA3
With MnmA5
Without MnmA5
Nº of genomes with MnmA5
Nº of genomes without MnmA5
p < 0.00005
With MnmA4
Without MnmA4
Nº of genomes with MnmA4
Nº of genomes without MnmA4
p < 0.00005
With MnmA1
Without MnmA1
Nº of genomes with MnmA1
Nº of genomes without MnmA1
With MnmA2
Without MnmA2
Nº of genomes with MnmA2
Nº of genomes without MnmA2
With TusE2
Without TusE2
Nº of genomes with TusE2
Nº of genomes without TusE2
p < 0.00005
With TusE1
Without TusE1
Nº of genomes with TusE1
Nº of genomes without TusE1
p < 0.00005
With TusE4
Without TusE4
Nº of genomes with TusE4
Nº of genomes without TusE4
p < 0.00005
With TusE3
Without TusE3
Nº of genomes with TusE3
Nº of genomes without TusE3
p < 0.00005

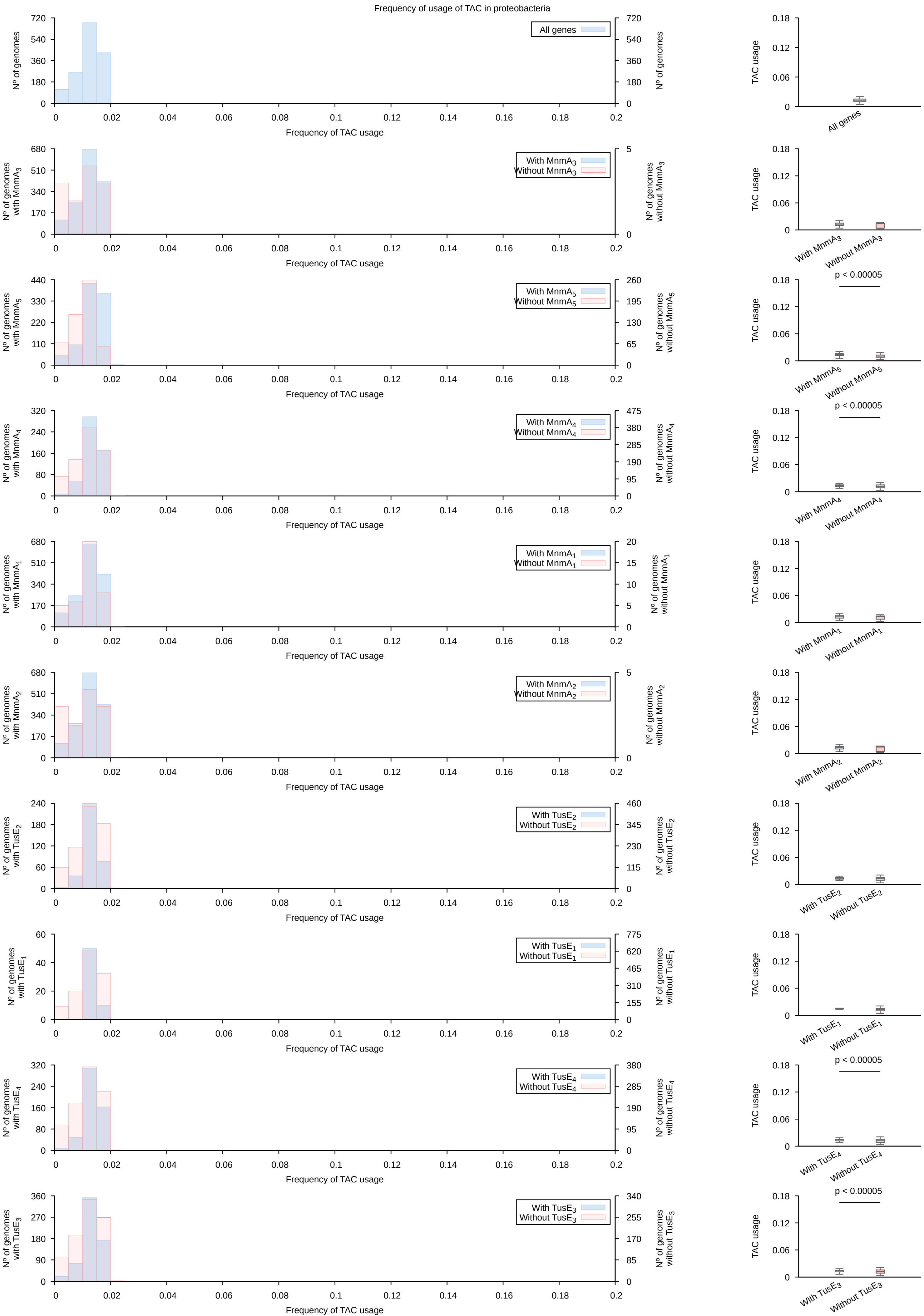
Frequency of usage of TAC in proteobacteria
All genes
Nº of genomes
Frequency of TAC usage
TAC usage
With $MnmA_3$
Without $MnmA_3$
Nº of genomes with $MnmA_3$
Nº of genomes without $MnmA_3$
With $MnmA_5$
Without $MnmA_5$
Nº of genomes with $MnmA_5$
Nº of genomes without $MnmA_5$
p < 0.00005
With $MnmA_4$
Without $MnmA_4$
Nº of genomes with $MnmA_4$
Nº of genomes without $MnmA_4$
p < 0.00005
With $MnmA_1$
Without $MnmA_1$
Nº of genomes with $MnmA_1$
Nº of genomes without $MnmA_1$
With $MnmA_2$
Without $MnmA_2$
Nº of genomes with $MnmA_2$
Nº of genomes without $MnmA_2$
With $TusE_2$
Without $TusE_2$
Nº of genomes with $TusE_2$
Nº of genomes without $TusE_2$
With $TusE_1$
Without $TusE_1$
Nº of genomes with $TusE_1$
Nº of genomes without $TusE_1$
With $TusE_4$
Without $TusE_4$
Nº of genomes with $TusE_4$
Nº of genomes without $TusE_4$
p < 0.00005
With $TusE_3$
Without $TusE_3$
Nº of genomes with $TusE_3$
Nº of genomes without $TusE_3$
p < 0.00005

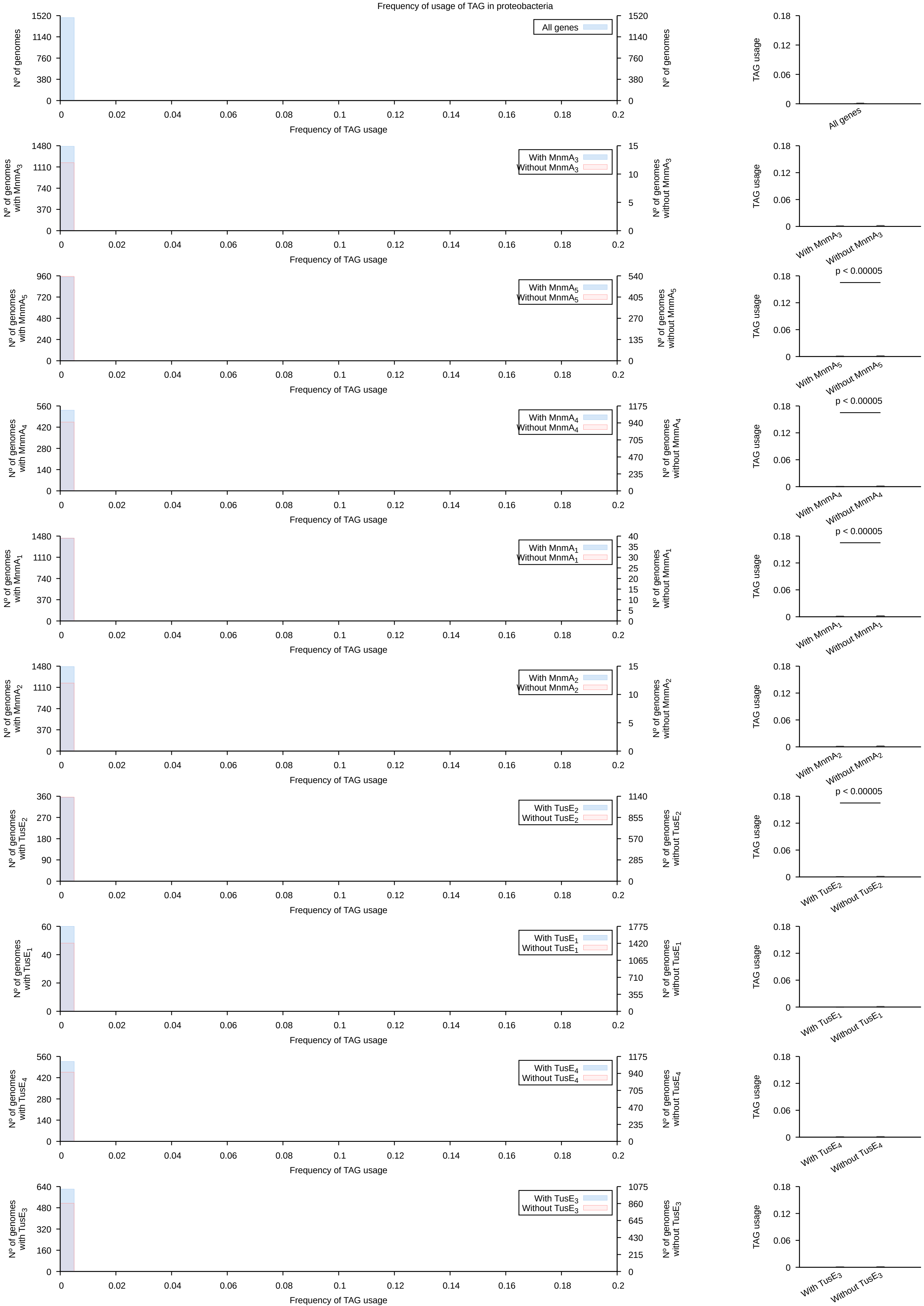

Frequency of usage of TAG in proteobacteria
Nº of genomes
Frequency of TAG usage
TAG usage
All genes
With MnmA$_3$
Without MnmA$_3$
With MnmA$_5$
Without MnmA$_5$
With MnmA$_4$
Without MnmA$_4$
With MnmA$_1$
Without MnmA$_1$
With MnmA$_2$
Without MnmA$_2$
With TusE$_2$
Without TusE$_2$
With TusE$_1$
Without TusE$_1$
With TusE$_4$
Without TusE$_4$
With TusE$_3$
Without TusE$_3$
p < 0.00005

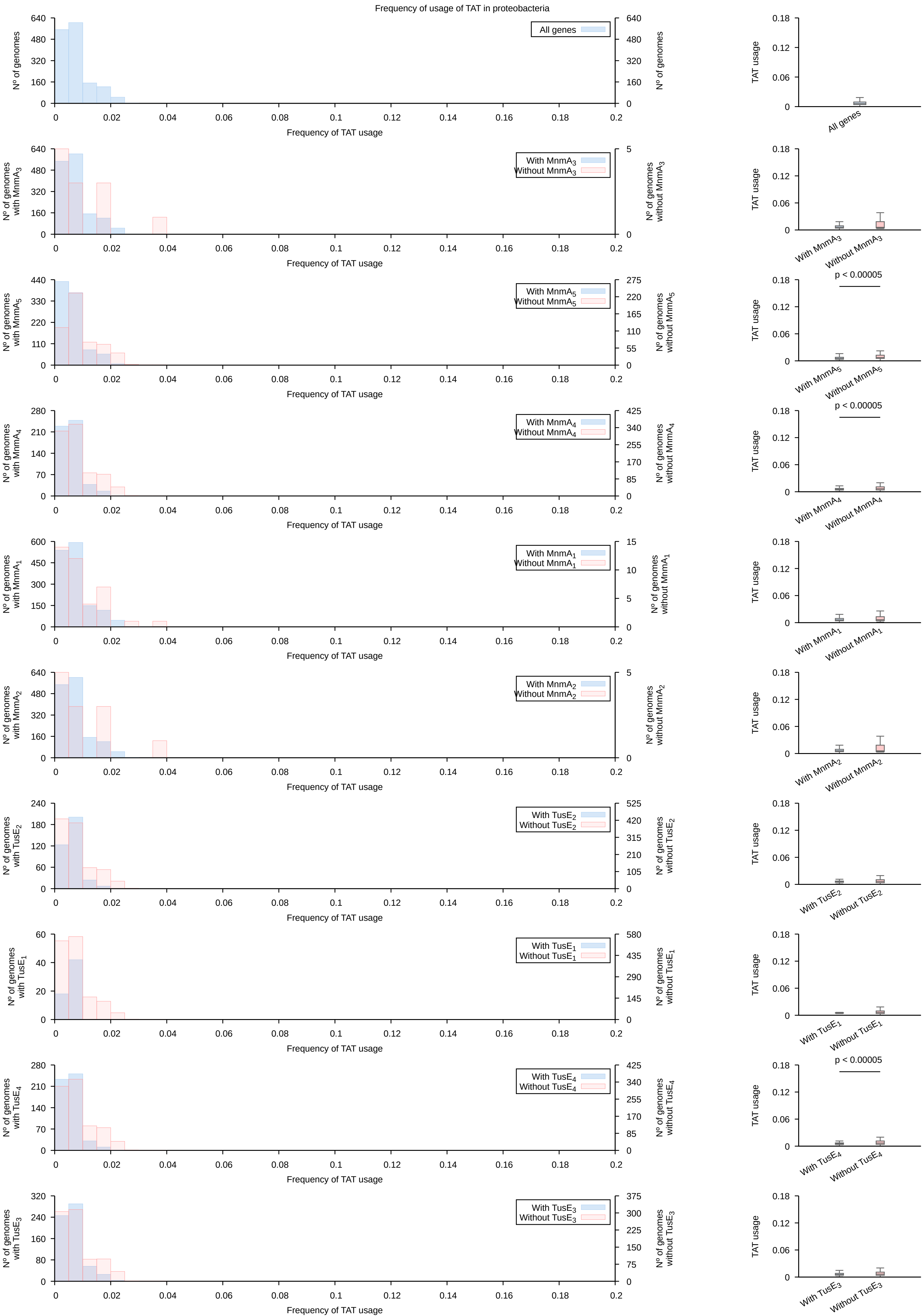
Frequency of usage of TAT in proteobacteria
All genes
Nº of genomes
Frequency of TAT usage
TAT usage
With MnmA3
Without MnmA3
Nº of genomes with MnmA3
Nº of genomes without MnmA3
With MnmA5
Without MnmA5
Nº of genomes with MnmA5
Nº of genomes without MnmA5
p < 0.00005
With MnmA4
Without MnmA4
Nº of genomes with MnmA4
Nº of genomes without MnmA4
p < 0.00005
With MnmA1
Without MnmA1
Nº of genomes with MnmA1
Nº of genomes without MnmA1
With MnmA2
Without MnmA2
Nº of genomes with MnmA2
Nº of genomes without MnmA2
With TusE2
Without TusE2
Nº of genomes with TusE2
Nº of genomes without TusE2
With TusE1
Without TusE1
Nº of genomes with TusE1
Nº of genomes without TusE1
With TusE4
Without TusE4
Nº of genomes with TusE4
Nº of genomes without TusE4
p < 0.00005
With TusE3
Without TusE3
Nº of genomes with TusE3
Nº of genomes without TusE3

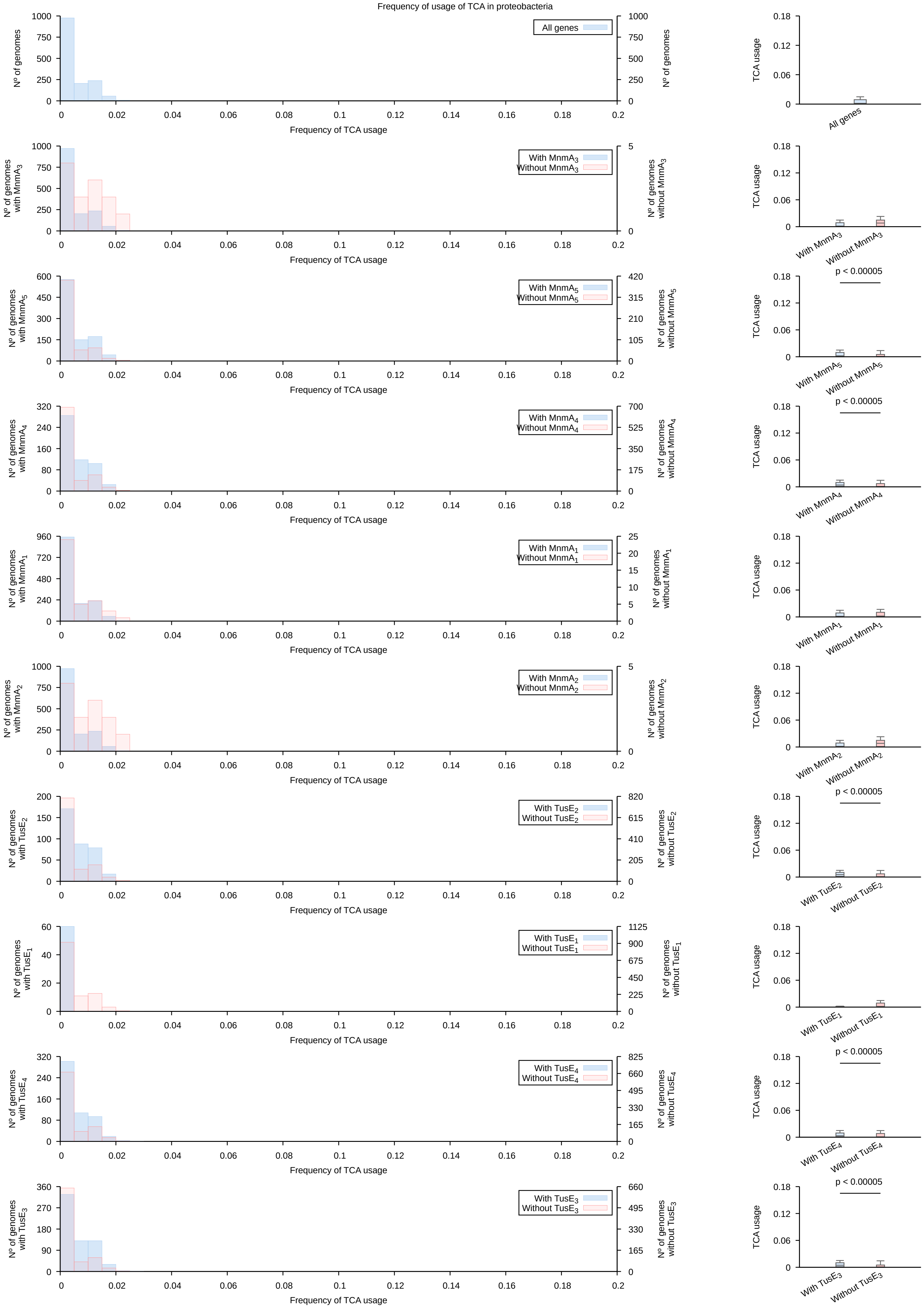
Frequency of usage of TCA in proteobacteria
All genes
With MnmA3
Without MnmA3
With MnmA5
Without MnmA5
With MnmA4
Without MnmA4
With MnmA1
Without MnmA1
With MnmA2
Without MnmA2
With TusE2
Without TusE2
With TusE1
Without TusE1
With TusE4
Without TusE4
With TusE3
Without TusE3
Nº of genomes
Frequency of TCA usage
TCA usage
p < 0.00005

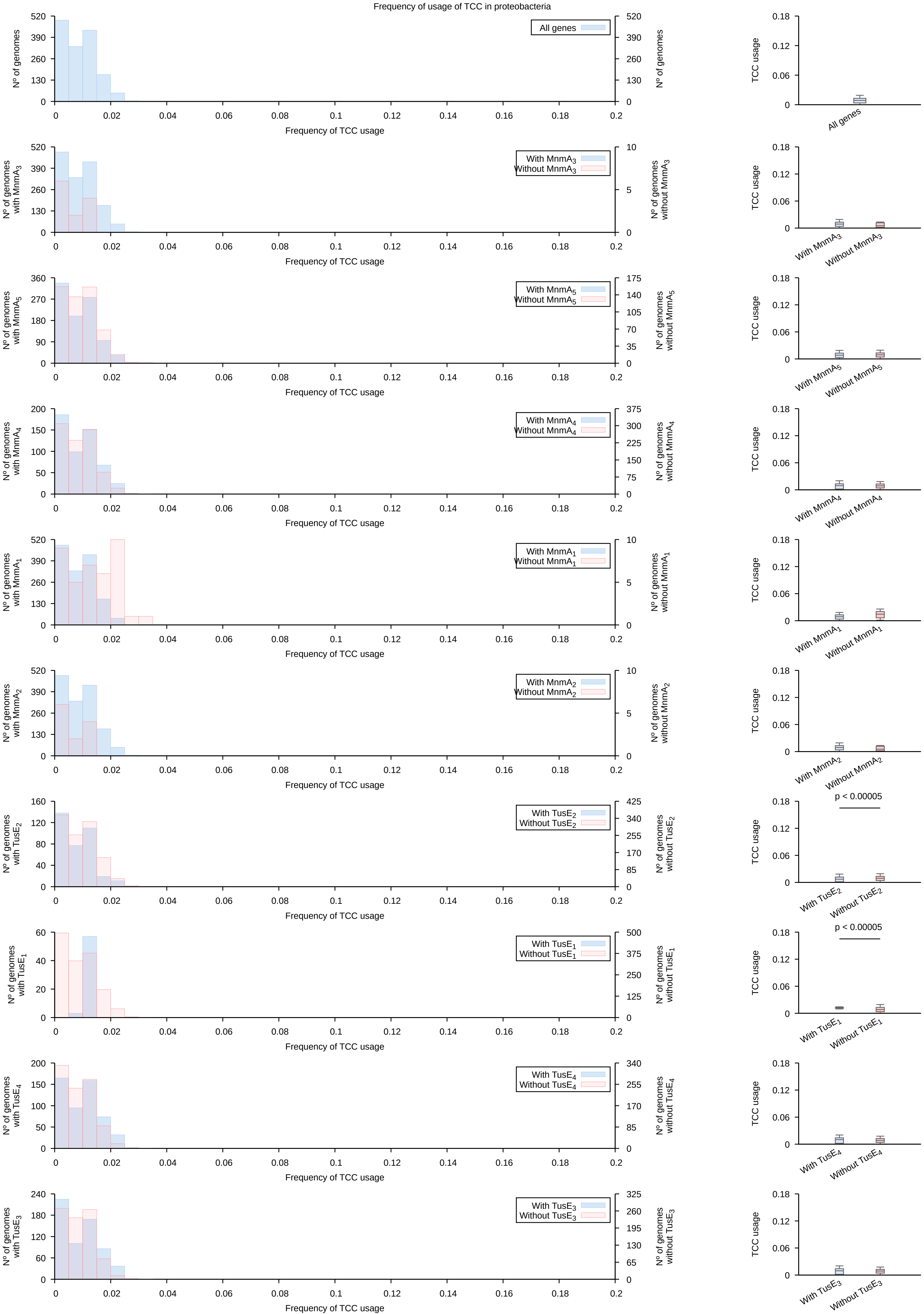

Frequency of usage of TCC in proteobacteria
All genes
Nº of genomes
Frequency of TCC usage
TCC usage
With $MnmA_3$
Without $MnmA_3$
Nº of genomes with $MnmA_3$
Nº of genomes without $MnmA_3$
With $MnmA_5$
Without $MnmA_5$
Nº of genomes with $MnmA_5$
Nº of genomes without $MnmA_5$
With $MnmA_4$
Without $MnmA_4$
Nº of genomes with $MnmA_4$
Nº of genomes without $MnmA_4$
With $MnmA_1$
Without $MnmA_1$
Nº of genomes with $MnmA_1$
Nº of genomes without $MnmA_1$
With $MnmA_2$
Without $MnmA_2$
Nº of genomes with $MnmA_2$
Nº of genomes without $MnmA_2$
With $TusE_2$
Without $TusE_2$
Nº of genomes with $TusE_2$
Nº of genomes without $TusE_2$
p < 0.00005
With $TusE_1$
Without $TusE_1$
Nº of genomes with $TusE_1$
Nº of genomes without $TusE_1$
p < 0.00005
With $TusE_4$
Without $TusE_4$
Nº of genomes with $TusE_4$
Nº of genomes without $TusE_4$
With $TusE_3$
Without $TusE_3$
Nº of genomes with $TusE_3$
Nº of genomes without $TusE_3$

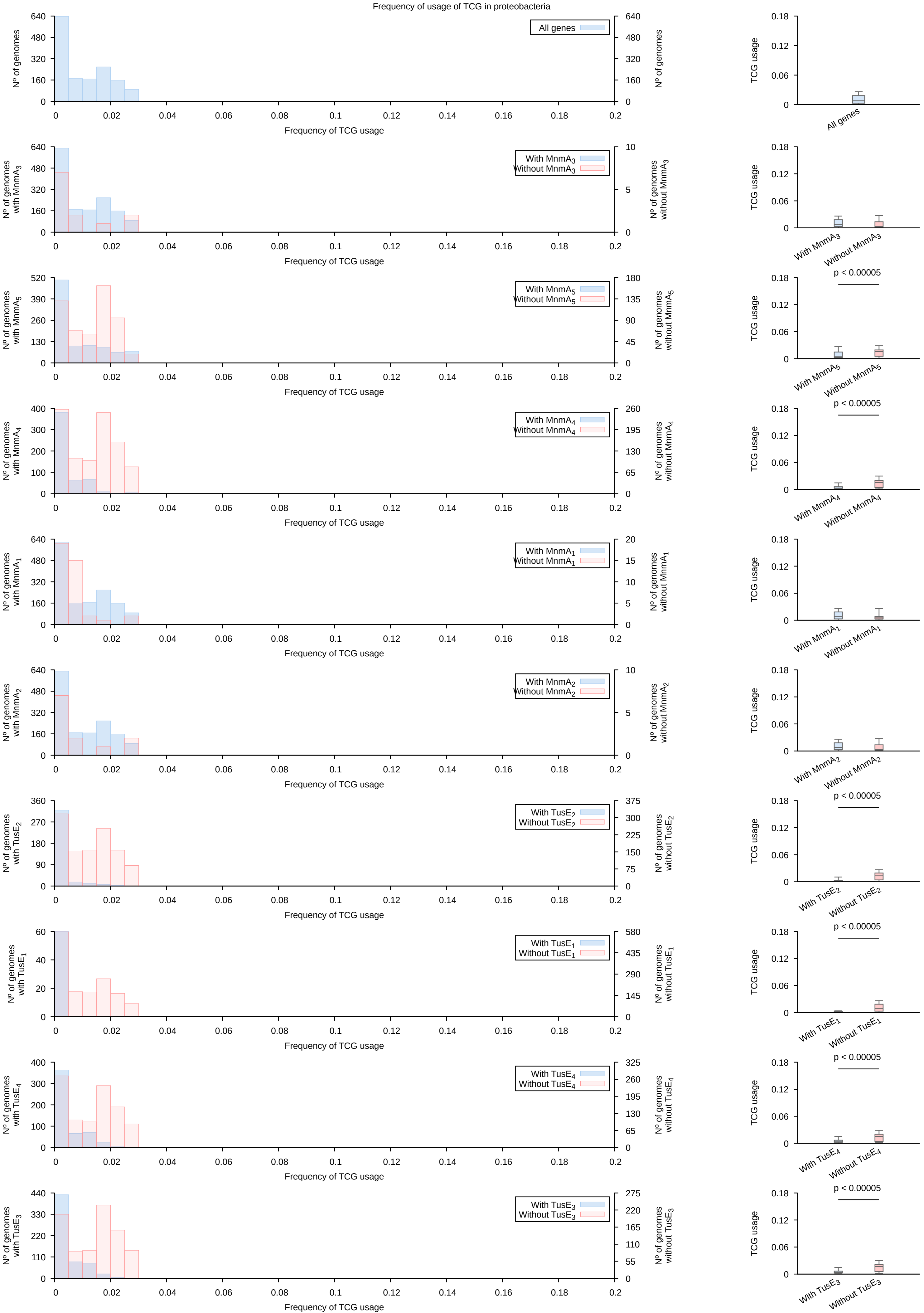

Frequency of usage of TCG in proteobacteria
Nº of genomes
All genes
Frequency of TCG usage
TCG usage
Nº of genomes with MnmA3
Nº of genomes without MnmA3
With MnmA3
Without MnmA3
Nº of genomes with MnmA5
Nº of genomes without MnmA5
With MnmA5
Without MnmA5
p < 0.00005
Nº of genomes with MnmA4
Nº of genomes without MnmA4
With MnmA4
Without MnmA4
p < 0.00005
Nº of genomes with MnmA1
Nº of genomes without MnmA1
With MnmA1
Without MnmA1
Nº of genomes with MnmA2
Nº of genomes without MnmA2
With MnmA2
Without MnmA2
Nº of genomes with TusE2
Nº of genomes without TusE2
With TusE2
Without TusE2
p < 0.00005
Nº of genomes with TusE1
Nº of genomes without TusE1
With TusE1
Without TusE1
p < 0.00005
Nº of genomes with TusE4
Nº of genomes without TusE4
With TusE4
Without TusE4
p < 0.00005
Nº of genomes with TusE3
Nº of genomes without TusE3
With TusE3
Without TusE3
p < 0.00005

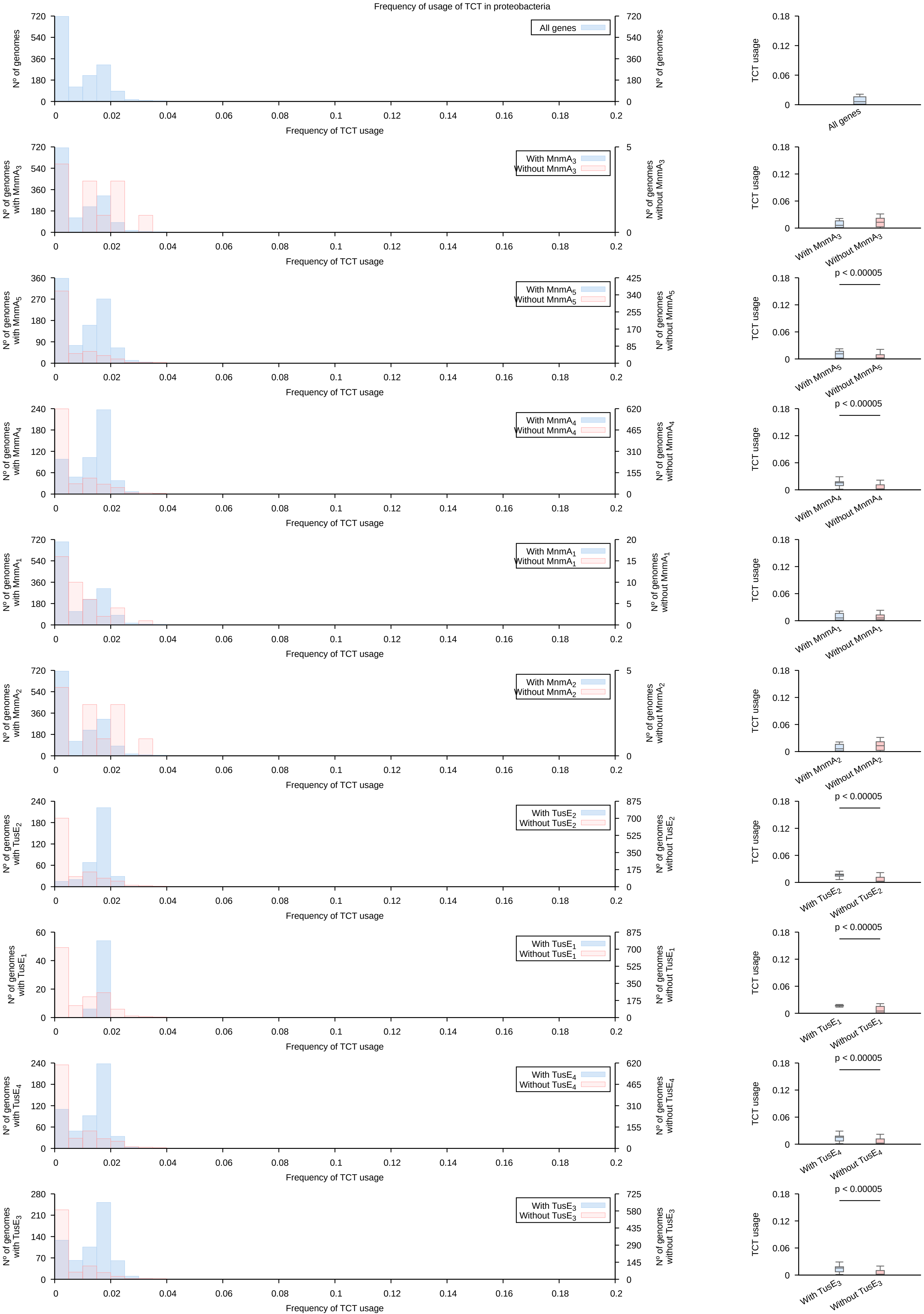
Frequency of usage of TCT in proteobacteria
All genes
Nº of genomes
Frequency of TCT usage
TCT usage
With MnmA3
Without MnmA3
Nº of genomes with MnmA3
Nº of genomes without MnmA3
With MnmA5
Without MnmA5
Nº of genomes with MnmA5
Nº of genomes without MnmA5
p < 0.00005
With MnmA4
Without MnmA4
Nº of genomes with MnmA4
Nº of genomes without MnmA4
p < 0.00005
With MnmA1
Without MnmA1
Nº of genomes with MnmA1
Nº of genomes without MnmA1
With MnmA2
Without MnmA2
Nº of genomes with MnmA2
Nº of genomes without MnmA2
With TusE2
Without TusE2
Nº of genomes with TusE2
Nº of genomes without TusE2
p < 0.00005
With TusE1
Without TusE1
Nº of genomes with TusE1
Nº of genomes without TusE1
p < 0.00005
With TusE4
Without TusE4
Nº of genomes with TusE4
Nº of genomes without TusE4
p < 0.00005
With TusE3
Without TusE3
Nº of genomes with TusE3
Nº of genomes without TusE3
p < 0.00005

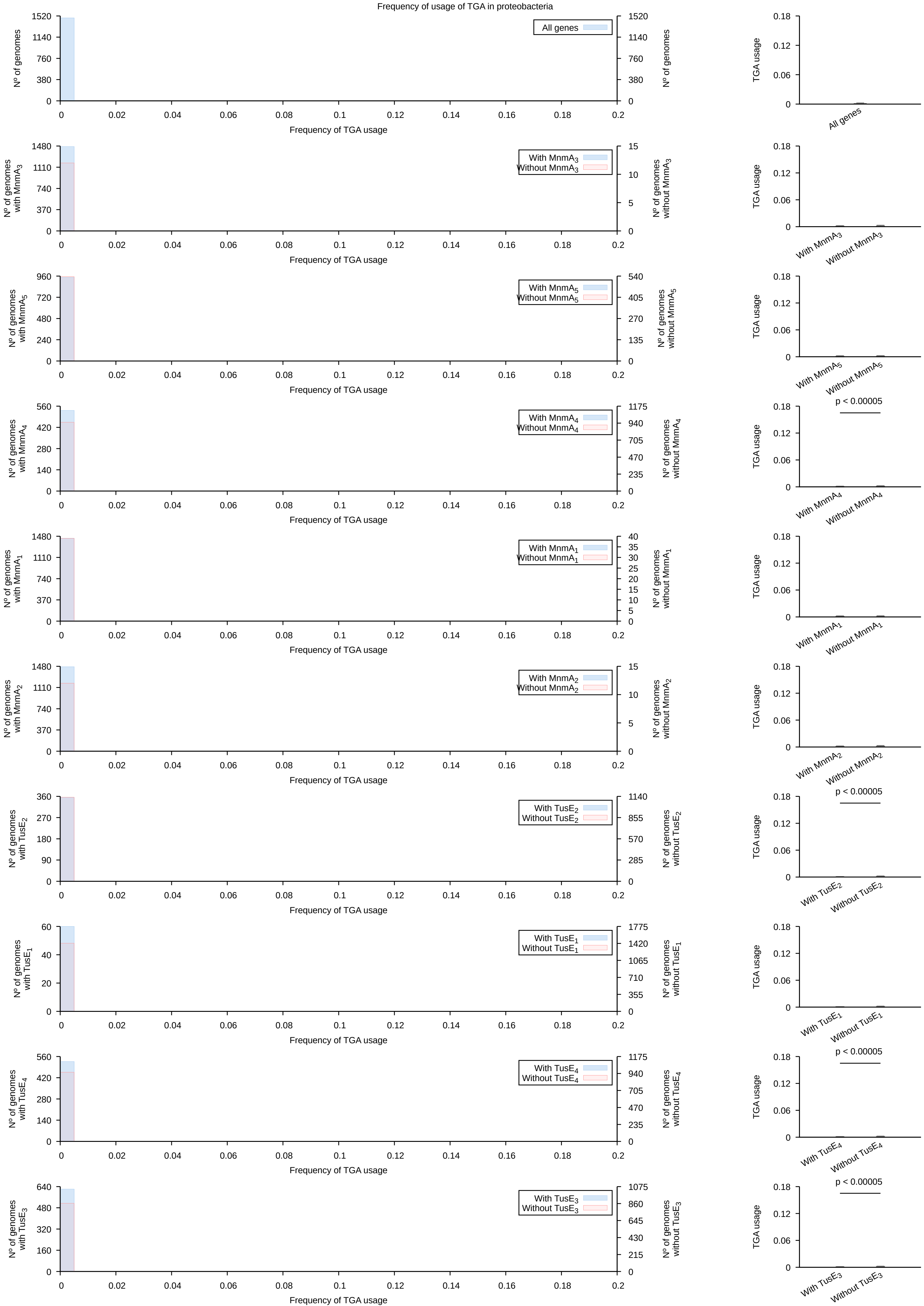
Frequency of usage of TGA in proteobacteria
All genes
Nº of genomes
Frequency of TGA usage
TGA usage
0
0.02
0.04
0.06
0.08
0.1
0.12
0.14
0.16
0.18
0.2
1520
1140
760
380
With MnmA$_3$
Without MnmA$_3$
Nº of genomes with MnmA$_3$
Nº of genomes without MnmA$_3$
With MnmA$_5$
Without MnmA$_5$
Nº of genomes with MnmA$_5$
Nº of genomes without MnmA$_5$
With MnmA$_4$
Without MnmA$_4$
Nº of genomes with MnmA$_4$
Nº of genomes without MnmA$_4$
p < 0.00005
With MnmA$_1$
Without MnmA$_1$
Nº of genomes with MnmA$_1$
Nº of genomes without MnmA$_1$
With MnmA$_2$
Without MnmA$_2$
Nº of genomes with MnmA$_2$
Nº of genomes without MnmA$_2$
With TusE$_2$
Without TusE$_2$
Nº of genomes with TusE$_2$
Nº of genomes without TusE$_2$
p < 0.00005
With TusE$_1$
Without TusE$_1$
Nº of genomes with TusE$_1$
Nº of genomes without TusE$_1$
With TusE$_4$
Without TusE$_4$
Nº of genomes with TusE$_4$
Nº of genomes without TusE$_4$
p < 0.00005
With TusE$_3$
Without TusE$_3$
Nº of genomes with TusE$_3$
Nº of genomes without TusE$_3$
p < 0.00005

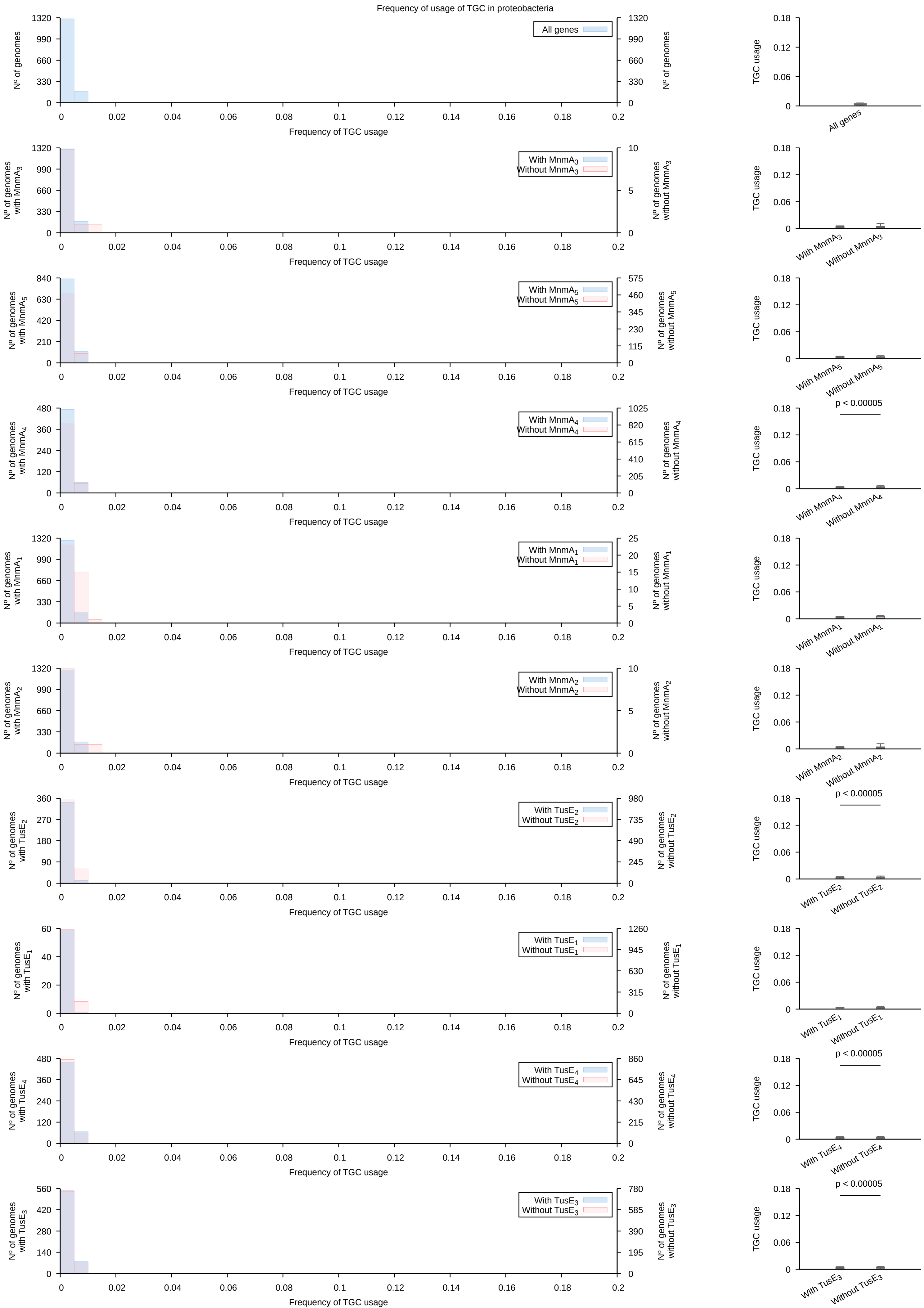
Frequency of usage of TGC in proteobacteria
All genes
Nº of genomes
Frequency of TGC usage
TGC usage
With $MnmA_3$
Without $MnmA_3$
Nº of genomes with $MnmA_3$
Nº of genomes without $MnmA_3$
With $MnmA_5$
Without $MnmA_5$
Nº of genomes with $MnmA_5$
Nº of genomes without $MnmA_5$
With $MnmA_4$
Without $MnmA_4$
Nº of genomes with $MnmA_4$
Nº of genomes without $MnmA_4$
$p < 0.00005$
With $MnmA_1$
Without $MnmA_1$
Nº of genomes with $MnmA_1$
Nº of genomes without $MnmA_1$
With $MnmA_2$
Without $MnmA_2$
Nº of genomes with $MnmA_2$
Nº of genomes without $MnmA_2$
With $TusE_2$
Without $TusE_2$
Nº of genomes with $TusE_2$
Nº of genomes without $TusE_2$
$p < 0.00005$
With $TusE_1$
Without $TusE_1$
Nº of genomes with $TusE_1$
Nº of genomes without $TusE_1$
With $TusE_4$
Without $TusE_4$
Nº of genomes with $TusE_4$
Nº of genomes without $TusE_4$
$p < 0.00005$
With $TusE_3$
Without $TusE_3$
Nº of genomes with $TusE_3$
Nº of genomes without $TusE_3$
$p < 0.00005$

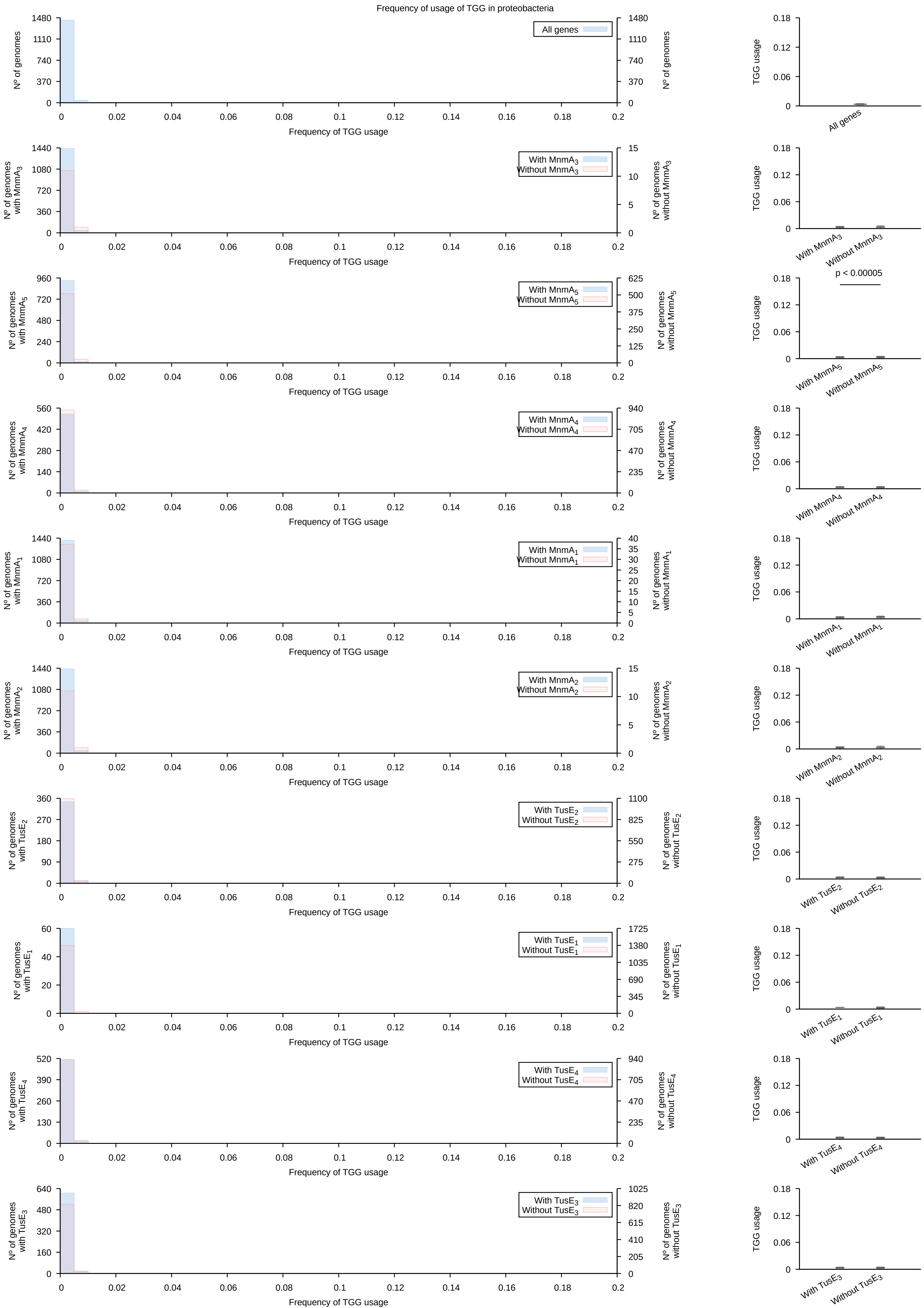
Frequency of usage of TGG in proteobacteria
All genes
With MnmA$_3$
Without MnmA$_3$
With MnmA$_5$
Without MnmA$_5$
p < 0.00005
With MnmA$_4$
Without MnmA$_4$
With MnmA$_1$
Without MnmA$_1$
With MnmA$_2$
Without MnmA$_2$
With TusE$_2$
Without TusE$_2$
With TusE$_1$
Without TusE$_1$
With TusE$_4$
Without TusE$_4$
With TusE$_3$
Without TusE$_3$
Nº of genomes
Frequency of TGG usage
TGG usage

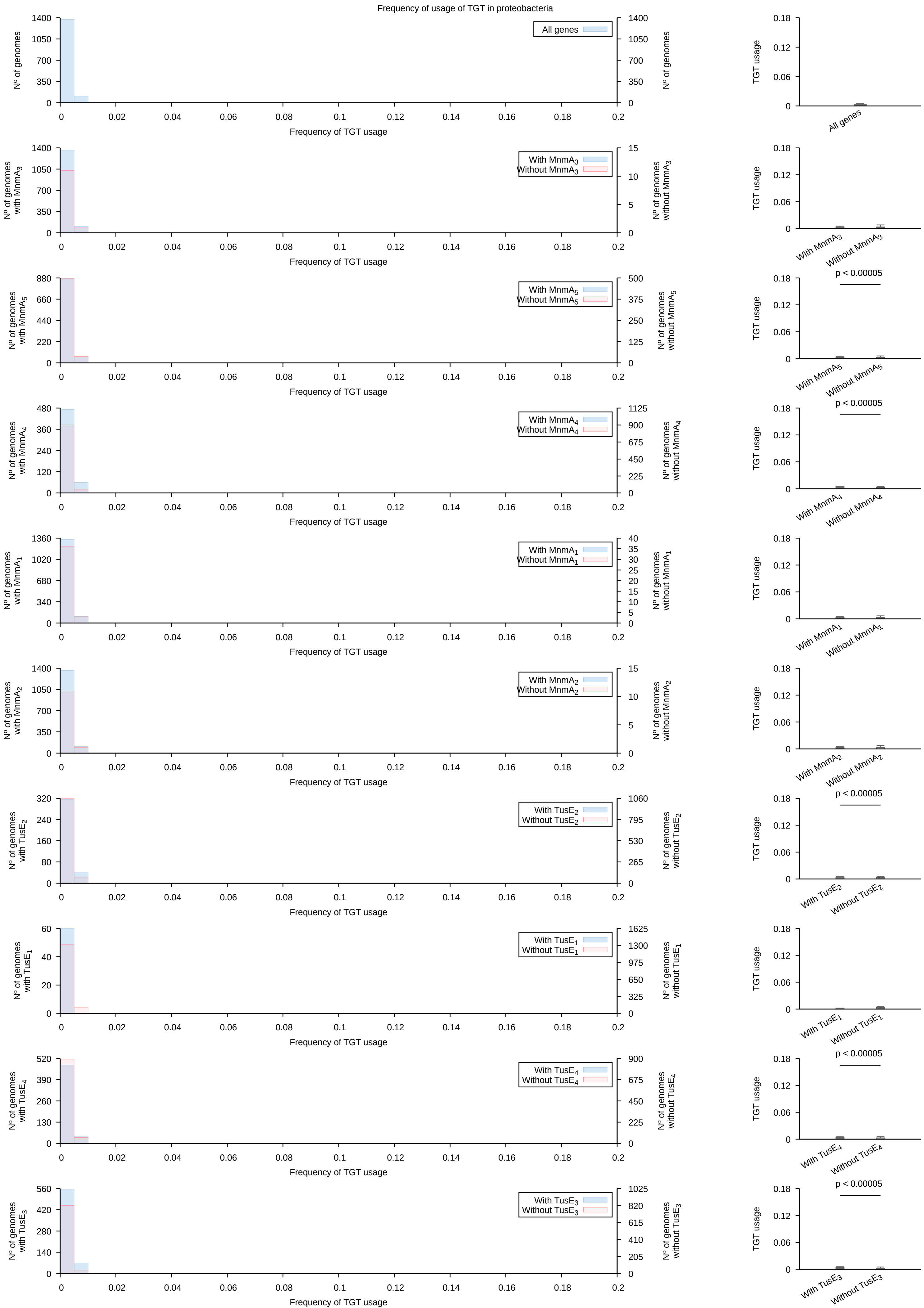

Frequency of usage of TGT in proteobacteria
All genes
Nº of genomes
Frequency of TGT usage
TGT usage
With MnmA3
Without MnmA3
Nº of genomes with MnmA3
Nº of genomes without MnmA3
With MnmA5
Without MnmA5
Nº of genomes with MnmA5
Nº of genomes without MnmA5
p < 0.00005
With MnmA4
Without MnmA4
Nº of genomes with MnmA4
Nº of genomes without MnmA4
p < 0.00005
With MnmA1
Without MnmA1
Nº of genomes with MnmA1
Nº of genomes without MnmA1
With MnmA2
Without MnmA2
Nº of genomes with MnmA2
Nº of genomes without MnmA2
With TusE2
Without TusE2
Nº of genomes with TusE2
Nº of genomes without TusE2
p < 0.00005
With TusE1
Without TusE1
Nº of genomes with TusE1
Nº of genomes without TusE1
With TusE4
Without TusE4
Nº of genomes with TusE4
Nº of genomes without TusE4
p < 0.00005
With TusE3
Without TusE3
Nº of genomes with TusE3
Nº of genomes without TusE3
p < 0.00005

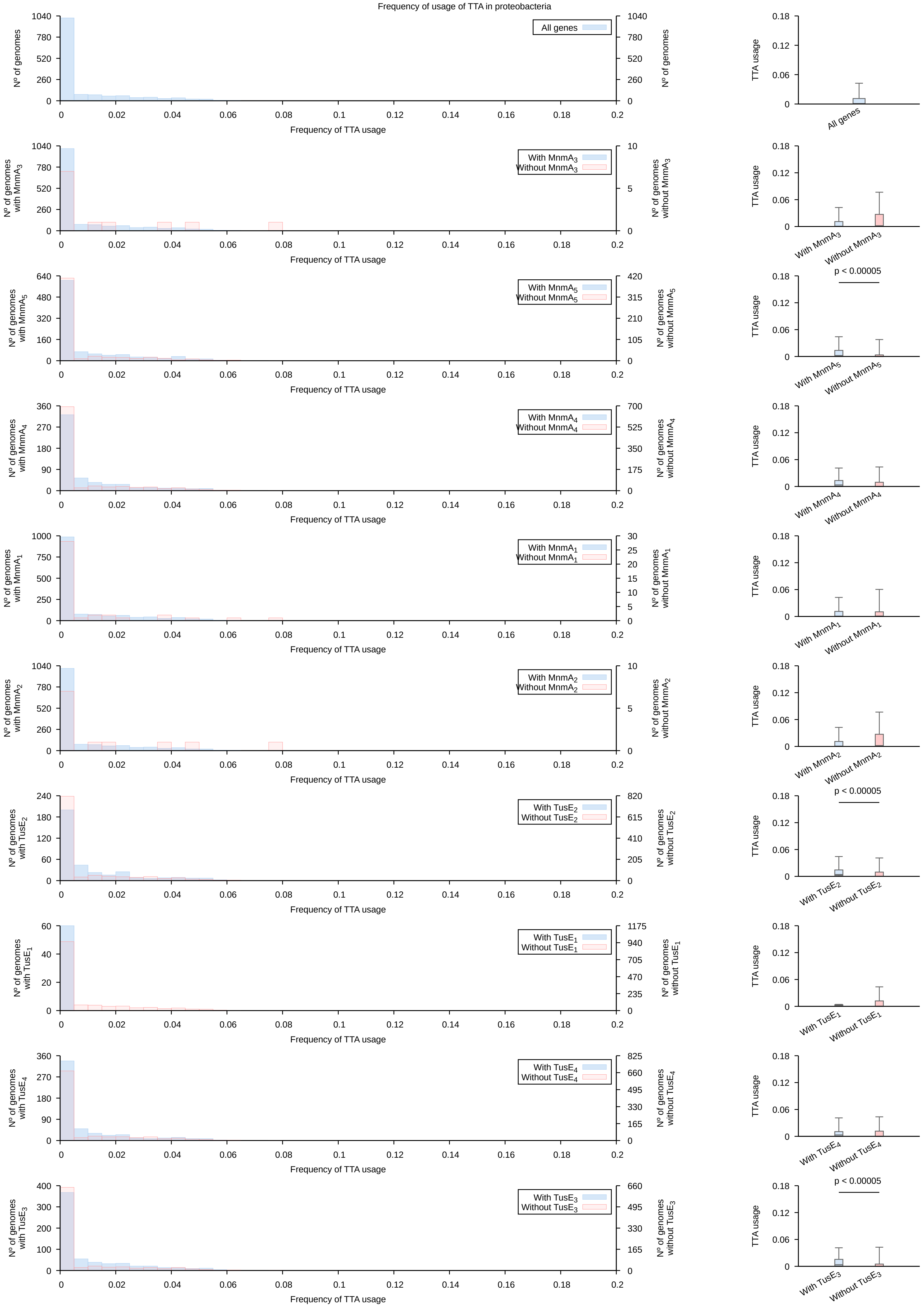

Frequency of usage of TTA in proteobacteria
Nº of genomes
Frequency of TTA usage
TTA usage
All genes
With MnmA3
Without MnmA3
Nº of genomes with MnmA3
Nº of genomes without MnmA3
With MnmA5
Without MnmA5
Nº of genomes with MnmA5
Nº of genomes without MnmA5
p < 0.00005
With MnmA4
Without MnmA4
Nº of genomes with MnmA4
Nº of genomes without MnmA4
With MnmA1
Without MnmA1
Nº of genomes with MnmA1
Nº of genomes without MnmA1
With MnmA2
Without MnmA2
Nº of genomes with MnmA2
Nº of genomes without MnmA2
With TusE2
Without TusE2
Nº of genomes with TusE2
Nº of genomes without TusE2
p < 0.00005
With TusE1
Without TusE1
Nº of genomes with TusE1
Nº of genomes without TusE1
With TusE4
Without TusE4
Nº of genomes with TusE4
Nº of genomes without TusE4
With TusE3
Without TusE3
Nº of genomes with TusE3
Nº of genomes without TusE3
p < 0.00005

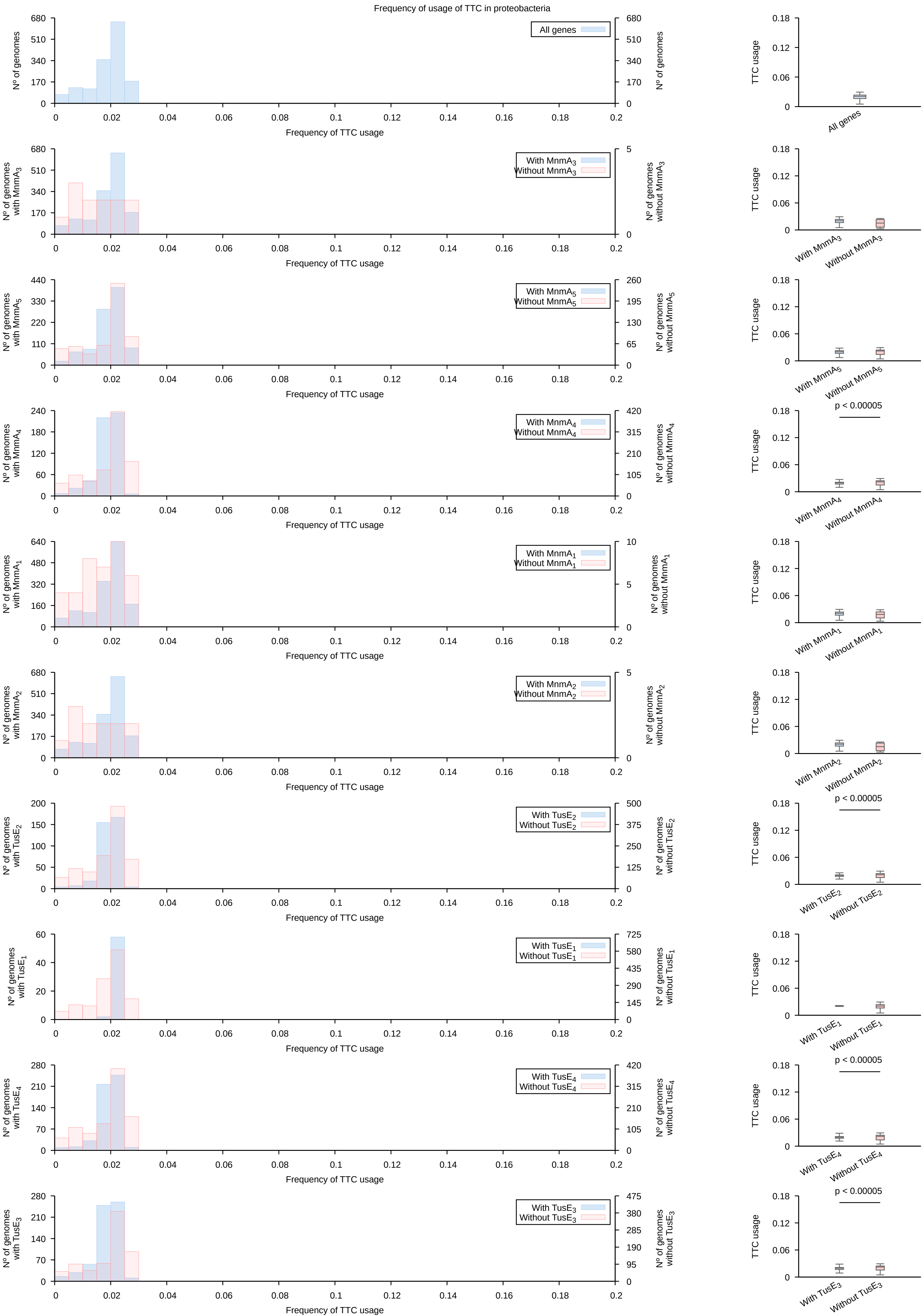
Frequency of usage of TTC in proteobacteria
All genes
Nº of genomes
Frequency of TTC usage
TTC usage
With MnmA$_3$
Without MnmA$_3$
Nº of genomes with MnmA$_3$
Nº of genomes without MnmA$_3$
With MnmA$_5$
Without MnmA$_5$
Nº of genomes with MnmA$_5$
Nº of genomes without MnmA$_5$
With MnmA$_4$
Without MnmA$_4$
Nº of genomes with MnmA$_4$
Nº of genomes without MnmA$_4$
p < 0.00005
With MnmA$_1$
Without MnmA$_1$
Nº of genomes with MnmA$_1$
Nº of genomes without MnmA$_1$
With MnmA$_2$
Without MnmA$_2$
Nº of genomes with MnmA$_2$
Nº of genomes without MnmA$_2$
With TusE$_2$
Without TusE$_2$
Nº of genomes with TusE$_2$
Nº of genomes without TusE$_2$
p < 0.00005
With TusE$_1$
Without TusE$_1$
Nº of genomes with TusE$_1$
Nº of genomes without TusE$_1$
With TusE$_4$
Without TusE$_4$
Nº of genomes with TusE$_4$
Nº of genomes without TusE$_4$
p < 0.00005
With TusE$_3$
Without TusE$_3$
Nº of genomes with TusE$_3$
Nº of genomes without TusE$_3$
p < 0.00005

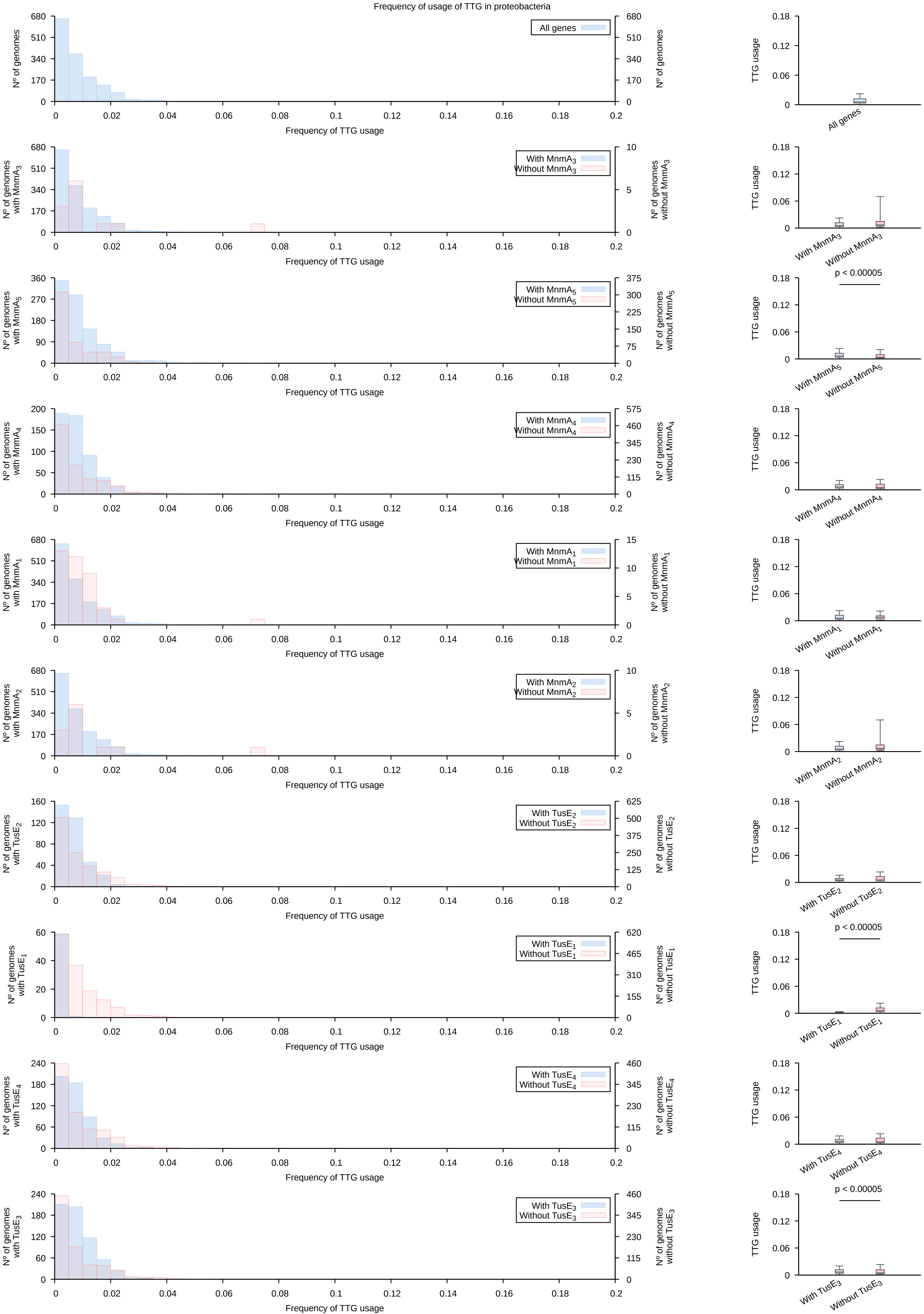
Frequency of usage of TTG in proteobacteria
All genes
Nº of genomes
Frequency of TTG usage
TTG usage
With MnmA$_3$
Without MnmA$_3$
Nº of genomes with MnmA$_3$
Nº of genomes without MnmA$_3$
With MnmA$_5$
Without MnmA$_5$
Nº of genomes with MnmA$_5$
Nº of genomes without MnmA$_5$
p < 0.00005
With MnmA$_4$
Without MnmA$_4$
Nº of genomes with MnmA$_4$
Nº of genomes without MnmA$_4$
With MnmA$_1$
Without MnmA$_1$
Nº of genomes with MnmA$_1$
Nº of genomes without MnmA$_1$
With MnmA$_2$
Without MnmA$_2$
Nº of genomes with MnmA$_2$
Nº of genomes without MnmA$_2$
With TusE$_2$
Without TusE$_2$
Nº of genomes with TusE$_2$
Nº of genomes without TusE$_2$
With TusE$_1$
Without TusE$_1$
Nº of genomes with TusE$_1$
Nº of genomes without TusE$_1$
p < 0.00005
With TusE$_4$
Without TusE$_4$
Nº of genomes with TusE$_4$
Nº of genomes without TusE$_4$
With TusE$_3$
Without TusE$_3$
Nº of genomes with TusE$_3$
Nº of genomes without TusE$_3$
p < 0.00005

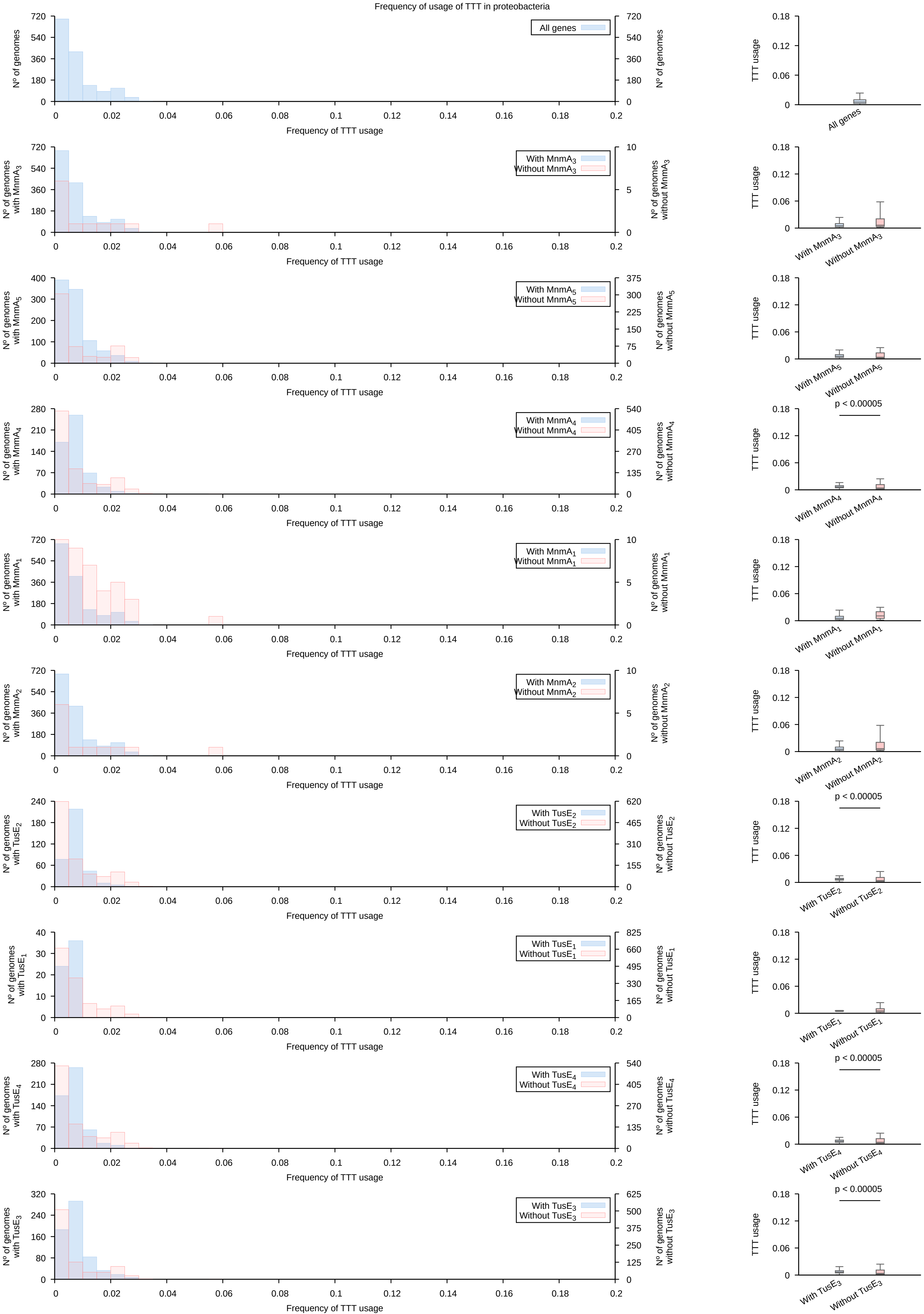

Frequency of usage of TTT in proteobacteria
All genes
Nº of genomes
Frequency of TTT usage
TTT usage
With MnmA$_3$
Without MnmA$_3$
Nº of genomes with MnmA$_3$
Nº of genomes without MnmA$_3$
With MnmA$_5$
Without MnmA$_5$
Nº of genomes with MnmA$_5$
Nº of genomes without MnmA$_5$
With MnmA$_4$
Without MnmA$_4$
Nº of genomes with MnmA$_4$
Nº of genomes without MnmA$_4$
p < 0.00005
With MnmA$_1$
Without MnmA$_1$
Nº of genomes with MnmA$_1$
Nº of genomes without MnmA$_1$
With MnmA$_2$
Without MnmA$_2$
Nº of genomes with MnmA$_2$
Nº of genomes without MnmA$_2$
With TusE$_2$
Without TusE$_2$
Nº of genomes with TusE$_2$
Nº of genomes without TusE$_2$
p < 0.00005
With TusE$_1$
Without TusE$_1$
Nº of genomes with TusE$_1$
Nº of genomes without TusE$_1$
With TusE$_4$
Without TusE$_4$
Nº of genomes with TusE$_4$
Nº of genomes without TusE$_4$
p < 0.00005
With TusE$_3$
Without TusE$_3$
Nº of genomes with TusE$_3$
Nº of genomes without TusE$_3$
p < 0.00005

Figure S11. Histogram of codon usage in all genes of genomes that present or lack genes involved in t^6A formation. Top left graph: histogram of the usage of the indicated codon in highly expressed genes of proteobacterial genomes. **Top right graph:** box plot indicating the distribution of codon usage in the same population. **Lower graphs:** The collection of 1484 proteobacterial genomes were separated in two groups, based on the presence (light blue) or absence (orange) of the indicated pattern in any gene of the analyzed genomes. Patterns are indicated using subscript numbers. See table S4 for a detailed description of the used patterns. **Left:** Data is represented in superposed histograms. **Right:** Data is represented in a boxplot. An horizontal bar was added when the two populations are different based on Wilcoxon-Mann-Whitney test and $p < 0.00005$. Whiskers include 95% of data.

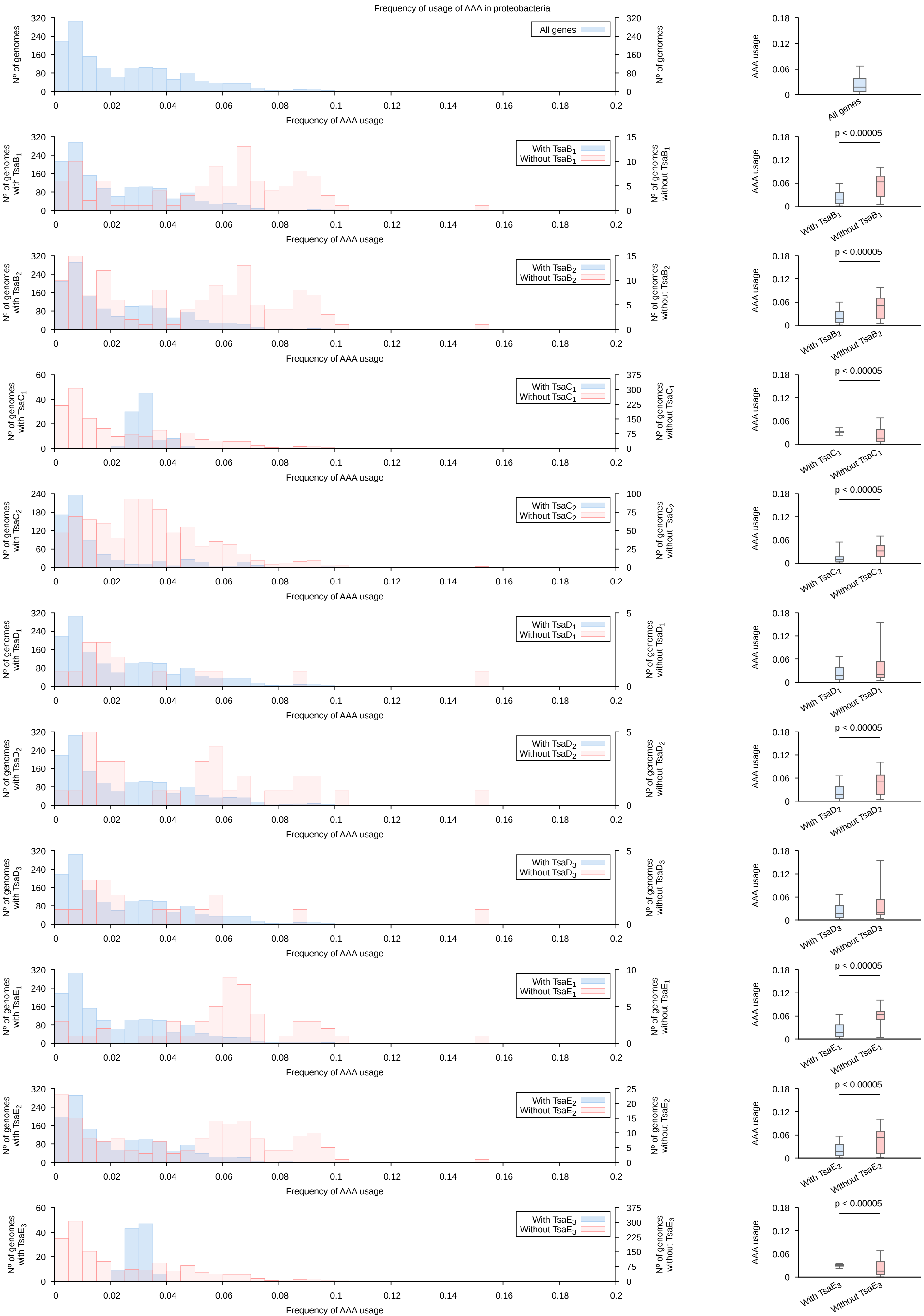
Frequency of usage of AAA in proteobacteria
All genes
Nº of genomes
Frequency of AAA usage
AAA usage
With TsaB$_1$
Without TsaB$_1$
With TsaB$_2$
Without TsaB$_2$
With TsaC$_1$
Without TsaC$_1$
With TsaC$_2$
Without TsaC$_2$
With TsaD$_1$
Without TsaD$_1$
With TsaD$_2$
Without TsaD$_2$
With TsaD$_3$
Without TsaD$_3$
With TsaE$_1$
Without TsaE$_1$
With TsaE$_2$
Without TsaE$_2$
With TsaE$_3$
Without TsaE$_3$
Nº of genomes with TsaB$_1$
Nº of genomes without TsaB$_1$
Nº of genomes with TsaB$_2$
Nº of genomes without TsaB$_2$
Nº of genomes with TsaC$_1$
Nº of genomes without TsaC$_1$
Nº of genomes with TsaC$_2$
Nº of genomes without TsaC$_2$
Nº of genomes with TsaD$_1$
Nº of genomes without TsaD$_1$
Nº of genomes with TsaD$_2$
Nº of genomes without TsaD$_2$
Nº of genomes with TsaD$_3$
Nº of genomes without TsaD$_3$
Nº of genomes with TsaE$_1$
Nº of genomes without TsaE$_1$
Nº of genomes with TsaE$_2$
Nº of genomes without TsaE$_2$
Nº of genomes with TsaE$_3$
Nº of genomes without TsaE$_3$
p < 0.00005

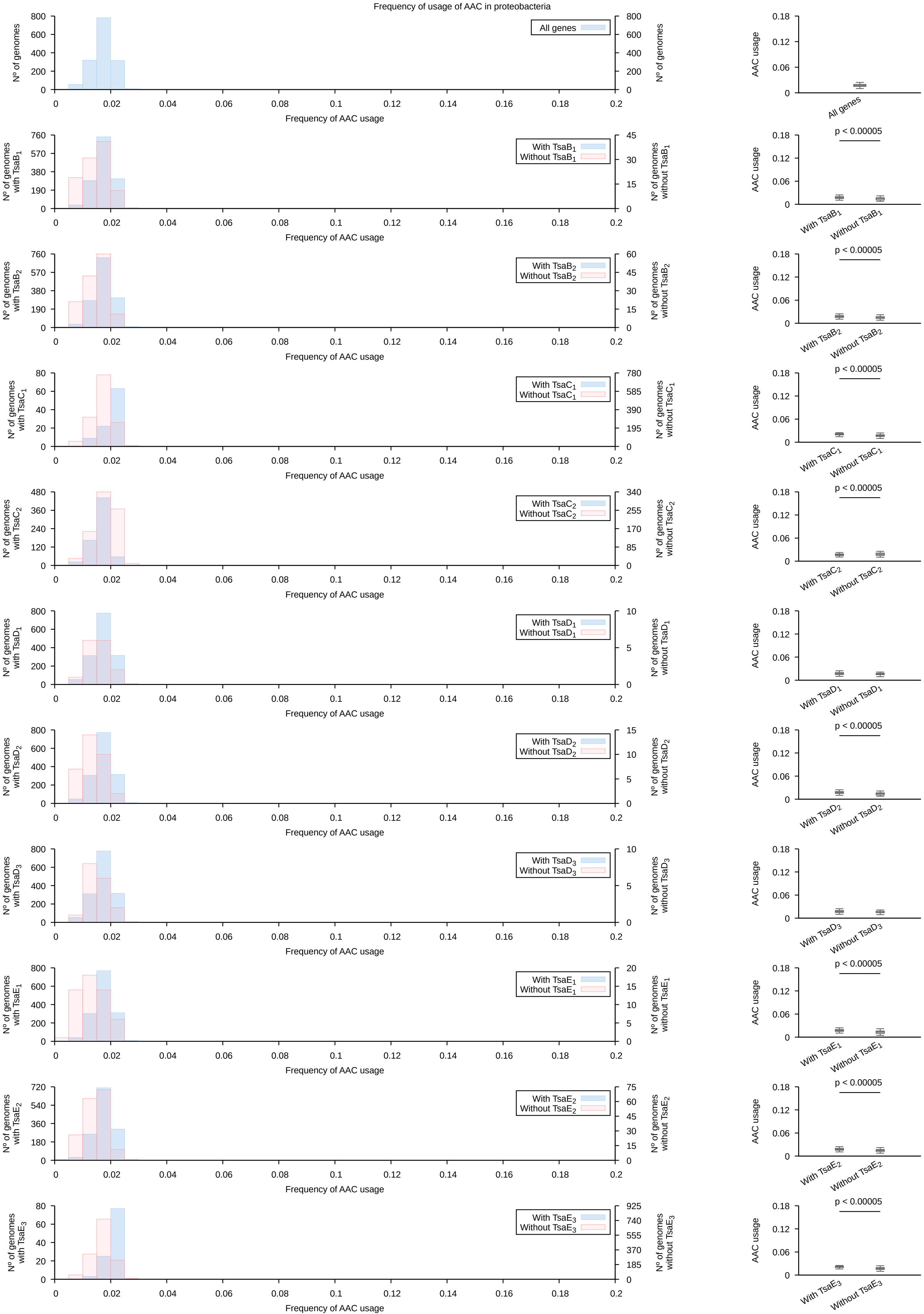
Frequency of usage of AAC in proteobacteria
All genes
Nº of genomes
Frequency of AAC usage
AAC usage
With TsaB1
Without TsaB1
Nº of genomes with TsaB1
Nº of genomes without TsaB1
p < 0.00005
With TsaB2
Without TsaB2
Nº of genomes with TsaB2
Nº of genomes without TsaB2
p < 0.00005
With TsaC1
Without TsaC1
Nº of genomes with TsaC1
Nº of genomes without TsaC1
p < 0.00005
With TsaC2
Without TsaC2
Nº of genomes with TsaC2
Nº of genomes without TsaC2
p < 0.00005
With TsaD1
Without TsaD1
Nº of genomes with TsaD1
Nº of genomes without TsaD1
With TsaD2
Without TsaD2
Nº of genomes with TsaD2
Nº of genomes without TsaD2
p < 0.00005
With TsaD3
Without TsaD3
Nº of genomes with TsaD3
Nº of genomes without TsaD3
With TsaE1
Without TsaE1
Nº of genomes with TsaE1
Nº of genomes without TsaE1
p < 0.00005
With TsaE2
Without TsaE2
Nº of genomes with TsaE2
Nº of genomes without TsaE2
p < 0.00005
With TsaE3
Without TsaE3
Nº of genomes with TsaE3
Nº of genomes without TsaE3
p < 0.00005

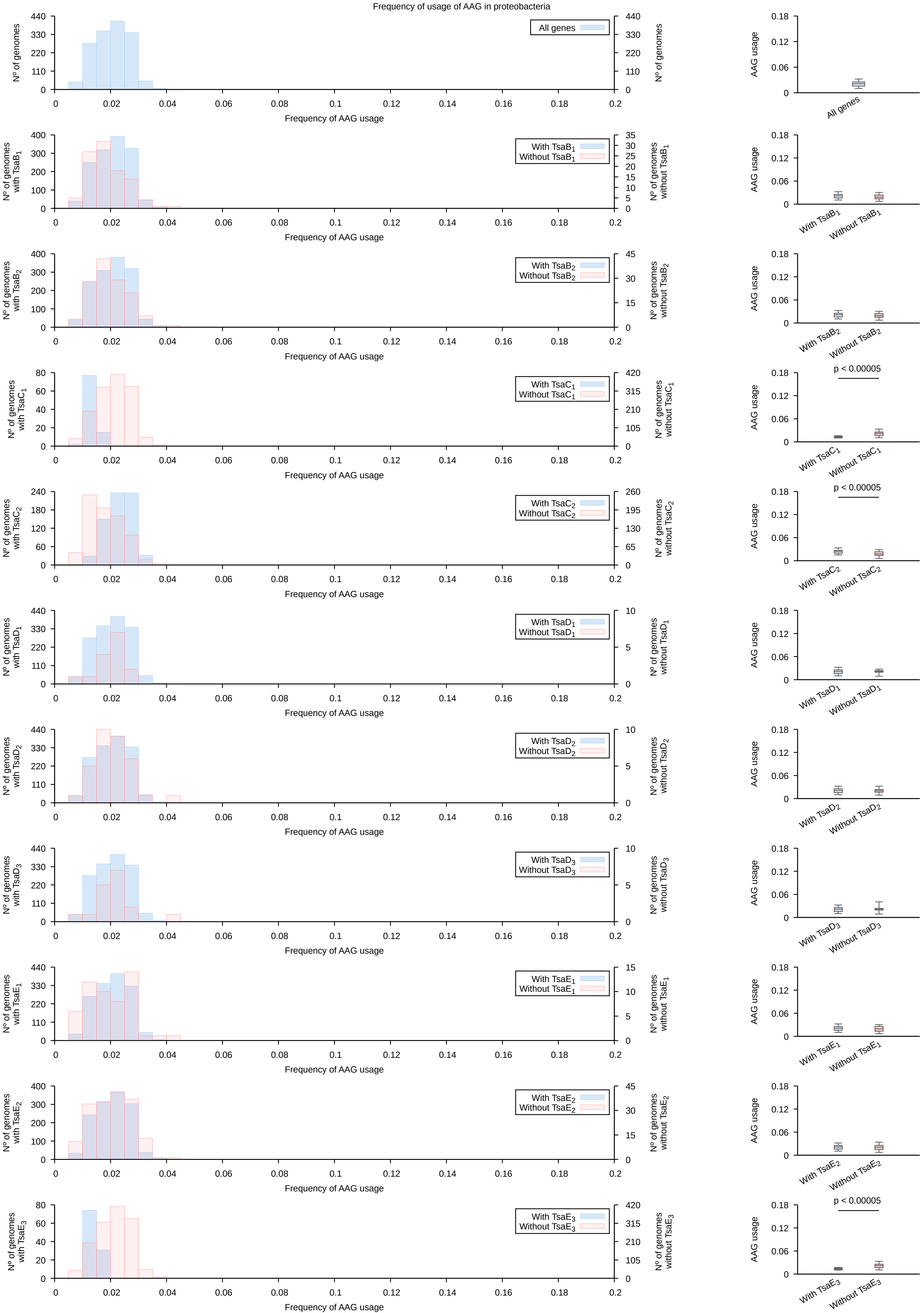
Frequency of usage of AAG in proteobacteria
Nº of genomes
All genes
Frequency of AAG usage
AAG usage
Nº of genomes with TsaB1
Nº of genomes without TsaB1
With TsaB1
Without TsaB1
Nº of genomes with TsaB2
Nº of genomes without TsaB2
With TsaB2
Without TsaB2
Nº of genomes with TsaC1
Nº of genomes without TsaC1
With TsaC1
Without TsaC1
p < 0.00005
Nº of genomes with TsaC2
Nº of genomes without TsaC2
With TsaC2
Without TsaC2
p < 0.00005
Nº of genomes with TsaD1
Nº of genomes without TsaD1
With TsaD1
Without TsaD1
Nº of genomes with TsaD2
Nº of genomes without TsaD2
With TsaD2
Without TsaD2
Nº of genomes with TsaD3
Nº of genomes without TsaD3
With TsaD3
Without TsaD3
Nº of genomes with TsaE1
Nº of genomes without TsaE1
With TsaE1
Without TsaE1
Nº of genomes with TsaE2
Nº of genomes without TsaE2
With TsaE2
Without TsaE2
Nº of genomes with TsaE3
Nº of genomes without TsaE3
With TsaE3
Without TsaE3
p < 0.00005

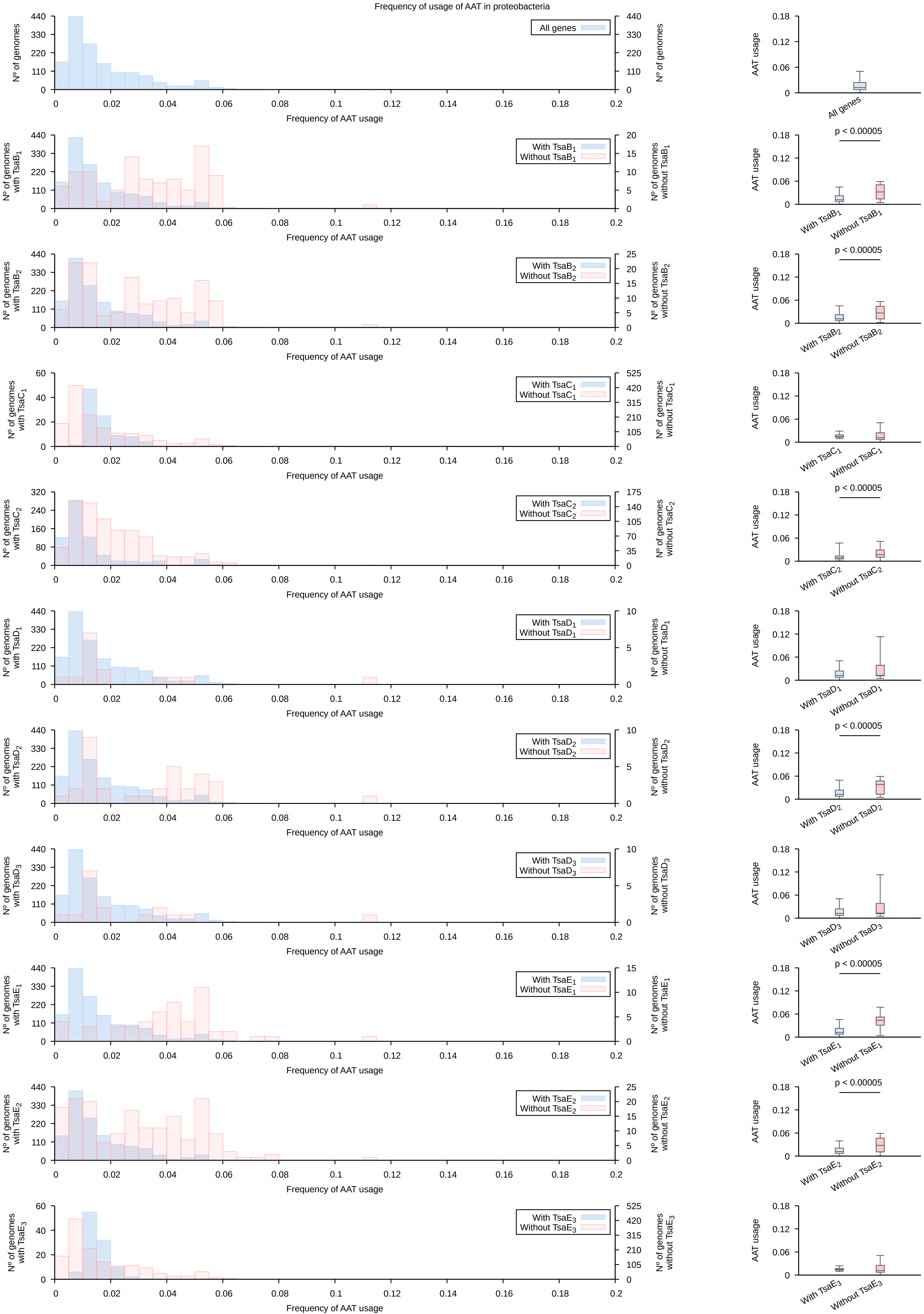
Frequency of usage of AAT in proteobacteria
All genes
Nº of genomes
Frequency of AAT usage
AAT usage
With TsaB$_1$
Without TsaB$_1$
Nº of genomes with TsaB$_1$
Nº of genomes without TsaB$_1$
p < 0.00005
With TsaB$_2$
Without TsaB$_2$
Nº of genomes with TsaB$_2$
Nº of genomes without TsaB$_2$
p < 0.00005
With TsaC$_1$
Without TsaC$_1$
Nº of genomes with TsaC$_1$
Nº of genomes without TsaC$_1$
With TsaC$_2$
Without TsaC$_2$
Nº of genomes with TsaC$_2$
Nº of genomes without TsaC$_2$
p < 0.00005
With TsaD$_1$
Without TsaD$_1$
Nº of genomes with TsaD$_1$
Nº of genomes without TsaD$_1$
With TsaD$_2$
Without TsaD$_2$
Nº of genomes with TsaD$_2$
Nº of genomes without TsaD$_2$
p < 0.00005
With TsaD$_3$
Without TsaD$_3$
Nº of genomes with TsaD$_3$
Nº of genomes without TsaD$_3$
With TsaE$_1$
Without TsaE$_1$
Nº of genomes with TsaE$_1$
Nº of genomes without TsaE$_1$
p < 0.00005
With TsaE$_2$
Without TsaE$_2$
Nº of genomes with TsaE$_2$
Nº of genomes without TsaE$_2$
p < 0.00005
With TsaE$_3$
Without TsaE$_3$
Nº of genomes with TsaE$_3$
Nº of genomes without TsaE$_3$

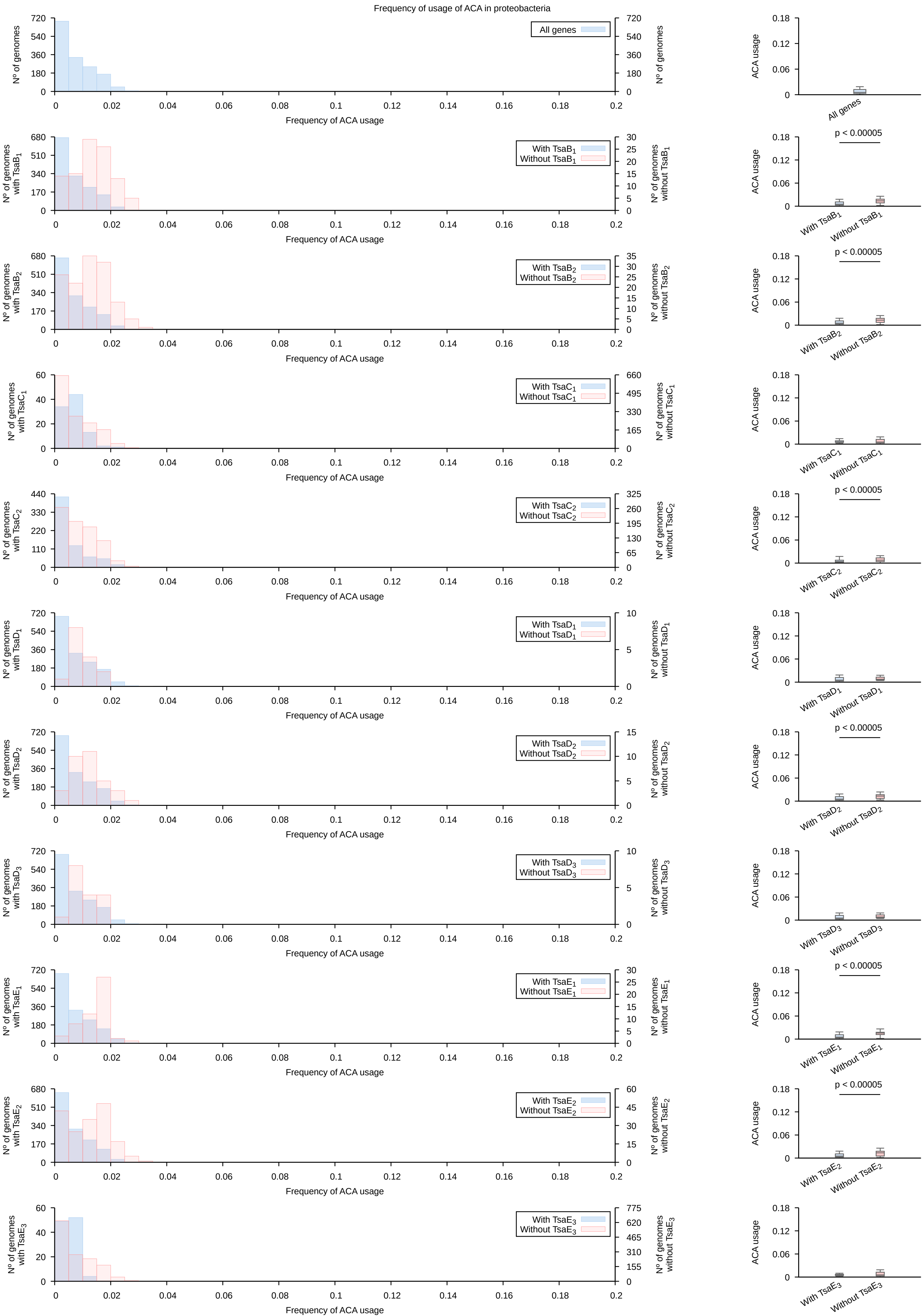
Frequency of usage of ACA in proteobacteria
Nº of genomes
All genes
Frequency of ACA usage
ACA usage
Nº of genomes with TsaB$_1$
Nº of genomes without TsaB$_1$
With TsaB$_1$
Without TsaB$_1$
p < 0.00005
Nº of genomes with TsaB$_2$
Nº of genomes without TsaB$_2$
With TsaB$_2$
Without TsaB$_2$
p < 0.00005
Nº of genomes with TsaC$_1$
Nº of genomes without TsaC$_1$
With TsaC$_1$
Without TsaC$_1$
Nº of genomes with TsaC$_2$
Nº of genomes without TsaC$_2$
With TsaC$_2$
Without TsaC$_2$
p < 0.00005
Nº of genomes with TsaD$_1$
Nº of genomes without TsaD$_1$
With TsaD$_1$
Without TsaD$_1$
Nº of genomes with TsaD$_2$
Nº of genomes without TsaD$_2$
With TsaD$_2$
Without TsaD$_2$
p < 0.00005
Nº of genomes with TsaD$_3$
Nº of genomes without TsaD$_3$
With TsaD$_3$
Without TsaD$_3$
Nº of genomes with TsaE$_1$
Nº of genomes without TsaE$_1$
With TsaE$_1$
Without TsaE$_1$
p < 0.00005
Nº of genomes with TsaE$_2$
Nº of genomes without TsaE$_2$
With TsaE$_2$
Without TsaE$_2$
p < 0.00005
Nº of genomes with TsaE$_3$
Nº of genomes without TsaE$_3$
With TsaE$_3$
Without TsaE$_3$

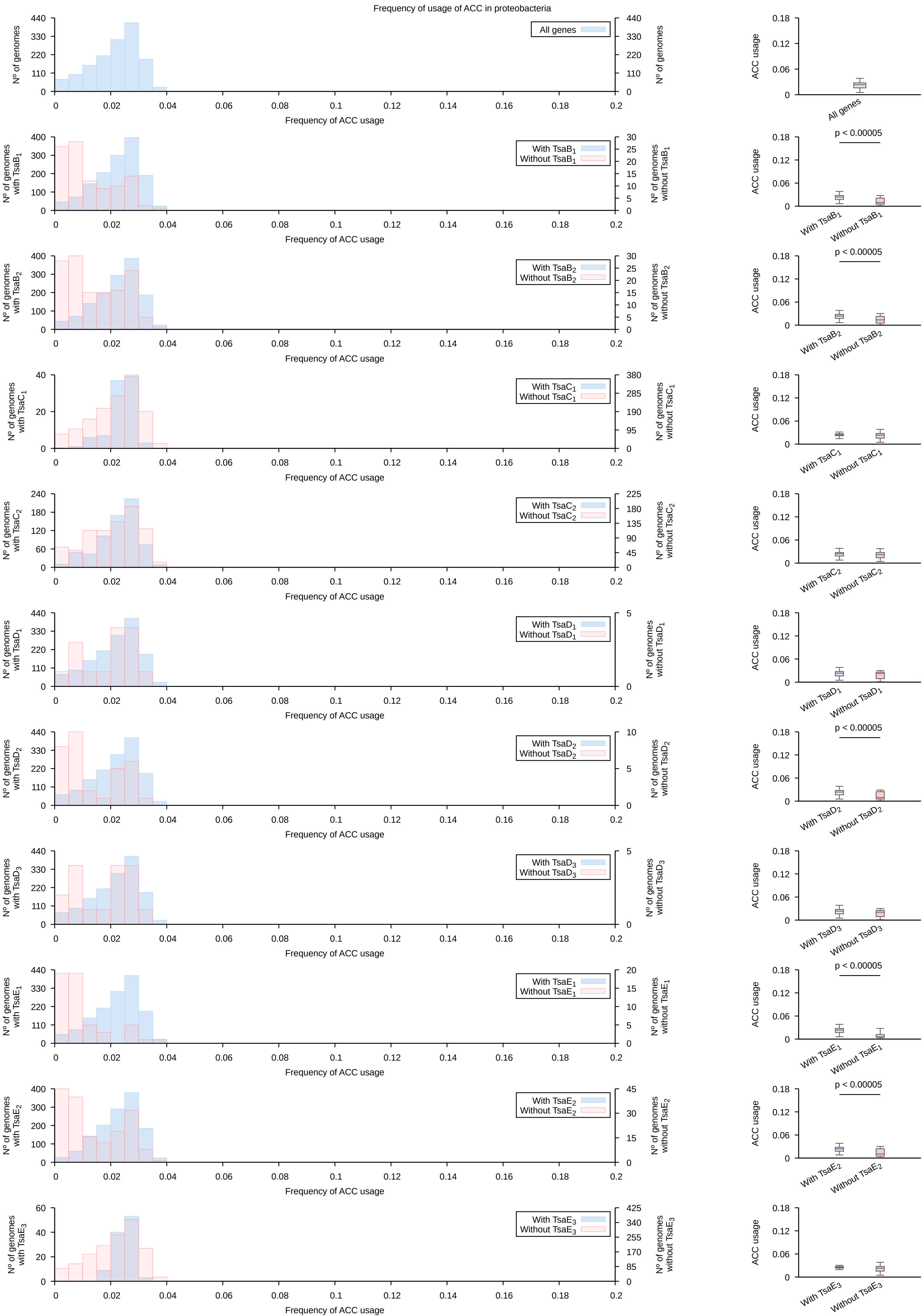

Frequency of usage of ACC in proteobacteria
All genes
Nº of genomes
Frequency of ACC usage
ACC usage
With TsaB$_1$
Without TsaB$_1$
Nº of genomes with TsaB$_1$
Nº of genomes without TsaB$_1$
p < 0.00005
With TsaB$_2$
Without TsaB$_2$
Nº of genomes with TsaB$_2$
Nº of genomes without TsaB$_2$
p < 0.00005
With TsaC$_1$
Without TsaC$_1$
Nº of genomes with TsaC$_1$
Nº of genomes without TsaC$_1$
With TsaC$_2$
Without TsaC$_2$
Nº of genomes with TsaC$_2$
Nº of genomes without TsaC$_2$
With TsaD$_1$
Without TsaD$_1$
Nº of genomes with TsaD$_1$
Nº of genomes without TsaD$_1$
With TsaD$_2$
Without TsaD$_2$
Nº of genomes with TsaD$_2$
Nº of genomes without TsaD$_2$
p < 0.00005
With TsaD$_3$
Without TsaD$_3$
Nº of genomes with TsaD$_3$
Nº of genomes without TsaD$_3$
With TsaE$_1$
Without TsaE$_1$
Nº of genomes with TsaE$_1$
Nº of genomes without TsaE$_1$
p < 0.00005
With TsaE$_2$
Without TsaE$_2$
Nº of genomes with TsaE$_2$
Nº of genomes without TsaE$_2$
p < 0.00005
With TsaE$_3$
Without TsaE$_3$
Nº of genomes with TsaE$_3$
Nº of genomes without TsaE$_3$

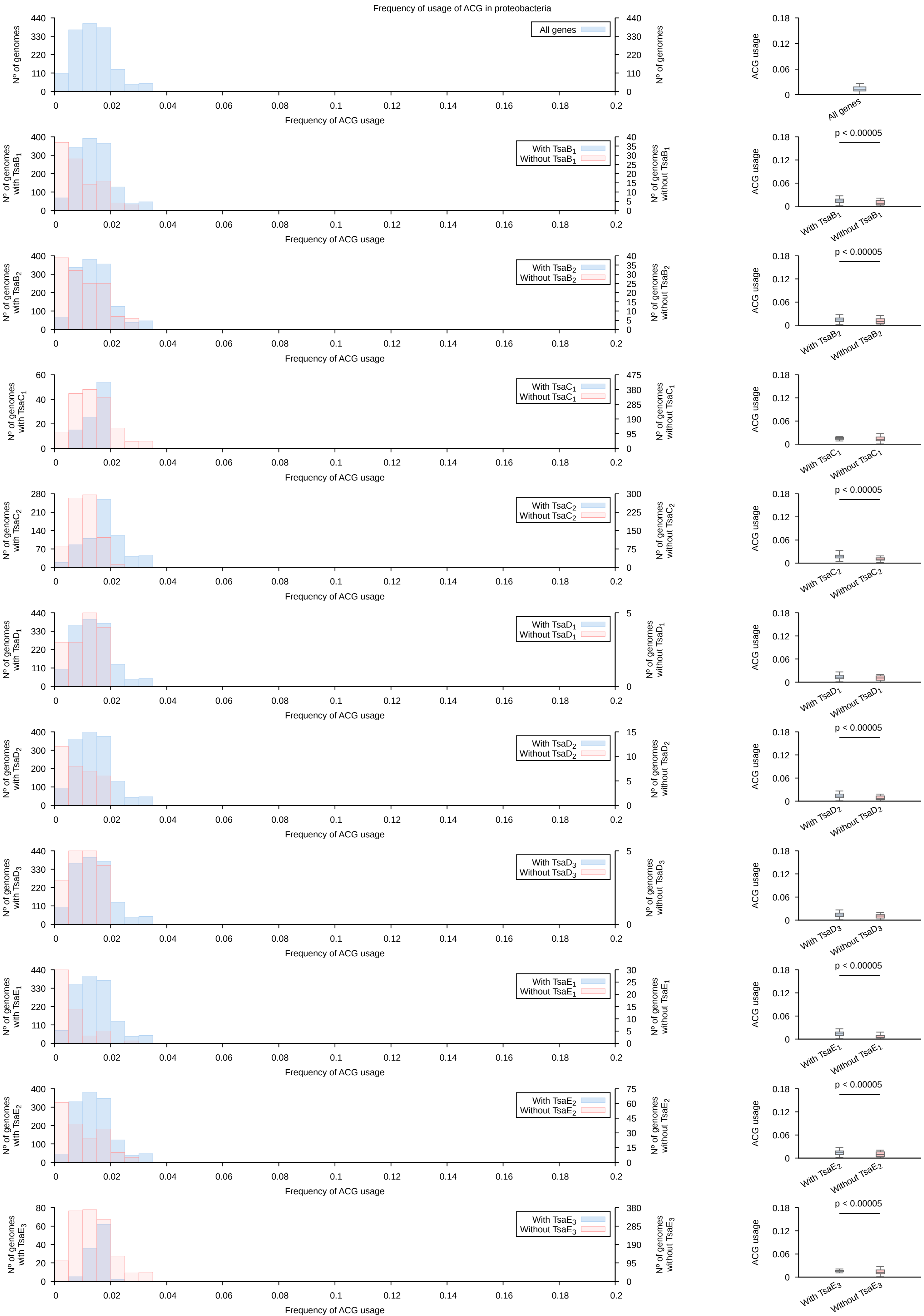
Frequency of usage of ACG in proteobacteria
Nº of genomes
Frequency of ACG usage
ACG usage
All genes
With TsaB1
Without TsaB1
Nº of genomes with TsaB1
Nº of genomes without TsaB1
p < 0.00005
With TsaB2
Without TsaB2
Nº of genomes with TsaB2
Nº of genomes without TsaB2
p < 0.00005
With TsaC1
Without TsaC1
Nº of genomes with TsaC1
Nº of genomes without TsaC1
With TsaC2
Without TsaC2
Nº of genomes with TsaC2
Nº of genomes without TsaC2
p < 0.00005
With TsaD1
Without TsaD1
Nº of genomes with TsaD1
Nº of genomes without TsaD1
With TsaD2
Without TsaD2
Nº of genomes with TsaD2
Nº of genomes without TsaD2
p < 0.00005
With TsaD3
Without TsaD3
Nº of genomes with TsaD3
Nº of genomes without TsaD3
With TsaE1
Without TsaE1
Nº of genomes with TsaE1
Nº of genomes without TsaE1
p < 0.00005
With TsaE2
Without TsaE2
Nº of genomes with TsaE2
Nº of genomes without TsaE2
p < 0.00005
With TsaE3
Without TsaE3
Nº of genomes with TsaE3
Nº of genomes without TsaE3
p < 0.00005

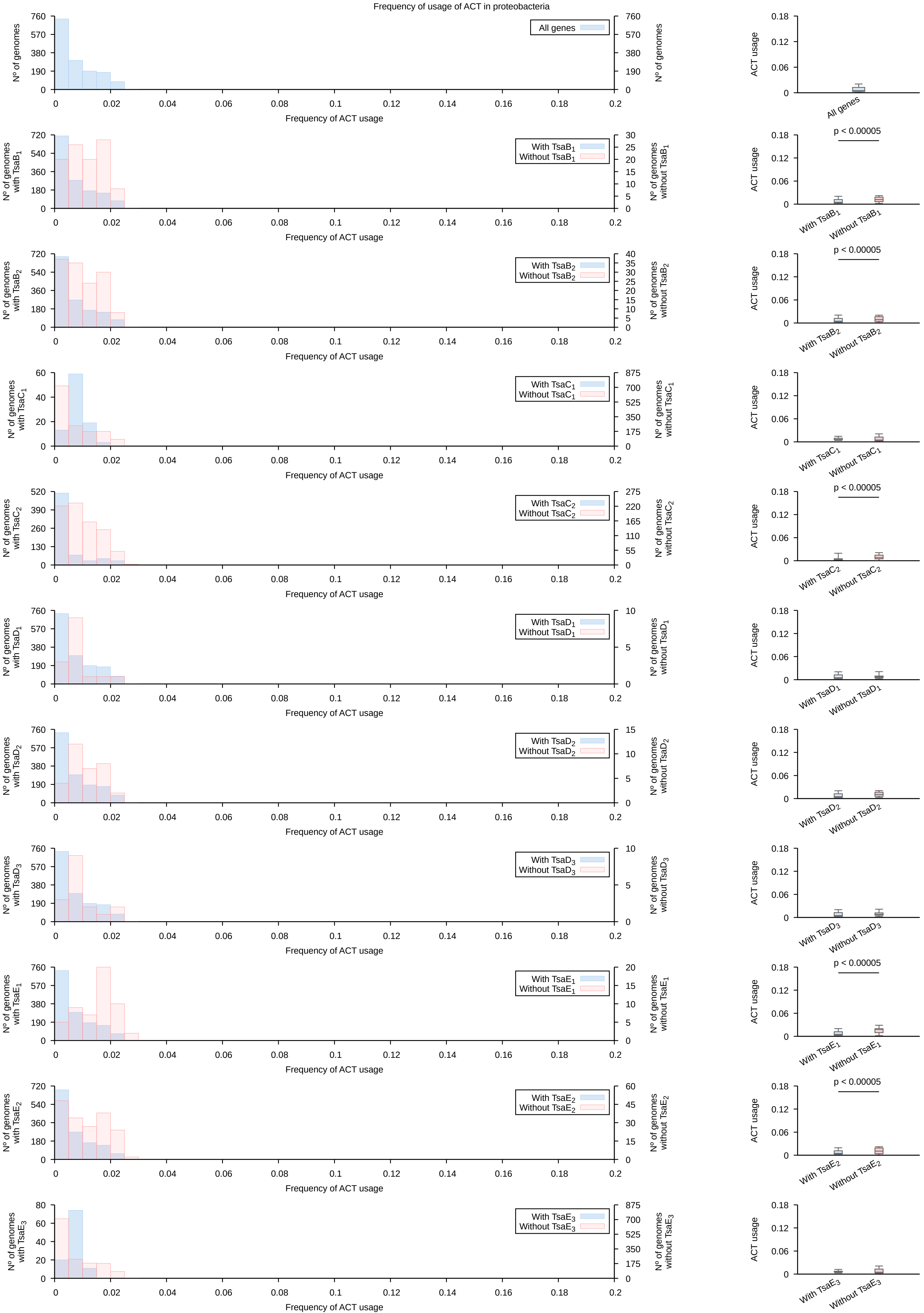

Frequency of usage of ACT in proteobacteria
Nº of genomes
All genes
Frequency of ACT usage
ACT usage
Nº of genomes with TsaB1
Nº of genomes without TsaB1
With TsaB1
Without TsaB1
p < 0.00005
Nº of genomes with TsaB2
Nº of genomes without TsaB2
With TsaB2
Without TsaB2
p < 0.00005
Nº of genomes with TsaC1
Nº of genomes without TsaC1
With TsaC1
Without TsaC1
Nº of genomes with TsaC2
Nº of genomes without TsaC2
With TsaC2
Without TsaC2
p < 0.00005
Nº of genomes with TsaD1
Nº of genomes without TsaD1
With TsaD1
Without TsaD1
Nº of genomes with TsaD2
Nº of genomes without TsaD2
With TsaD2
Without TsaD2
Nº of genomes with TsaD3
Nº of genomes without TsaD3
With TsaD3
Without TsaD3
Nº of genomes with TsaE1
Nº of genomes without TsaE1
With TsaE1
Without TsaE1
p < 0.00005
Nº of genomes with TsaE2
Nº of genomes without TsaE2
With TsaE2
Without TsaE2
p < 0.00005
Nº of genomes with TsaE3
Nº of genomes without TsaE3
With TsaE3
Without TsaE3

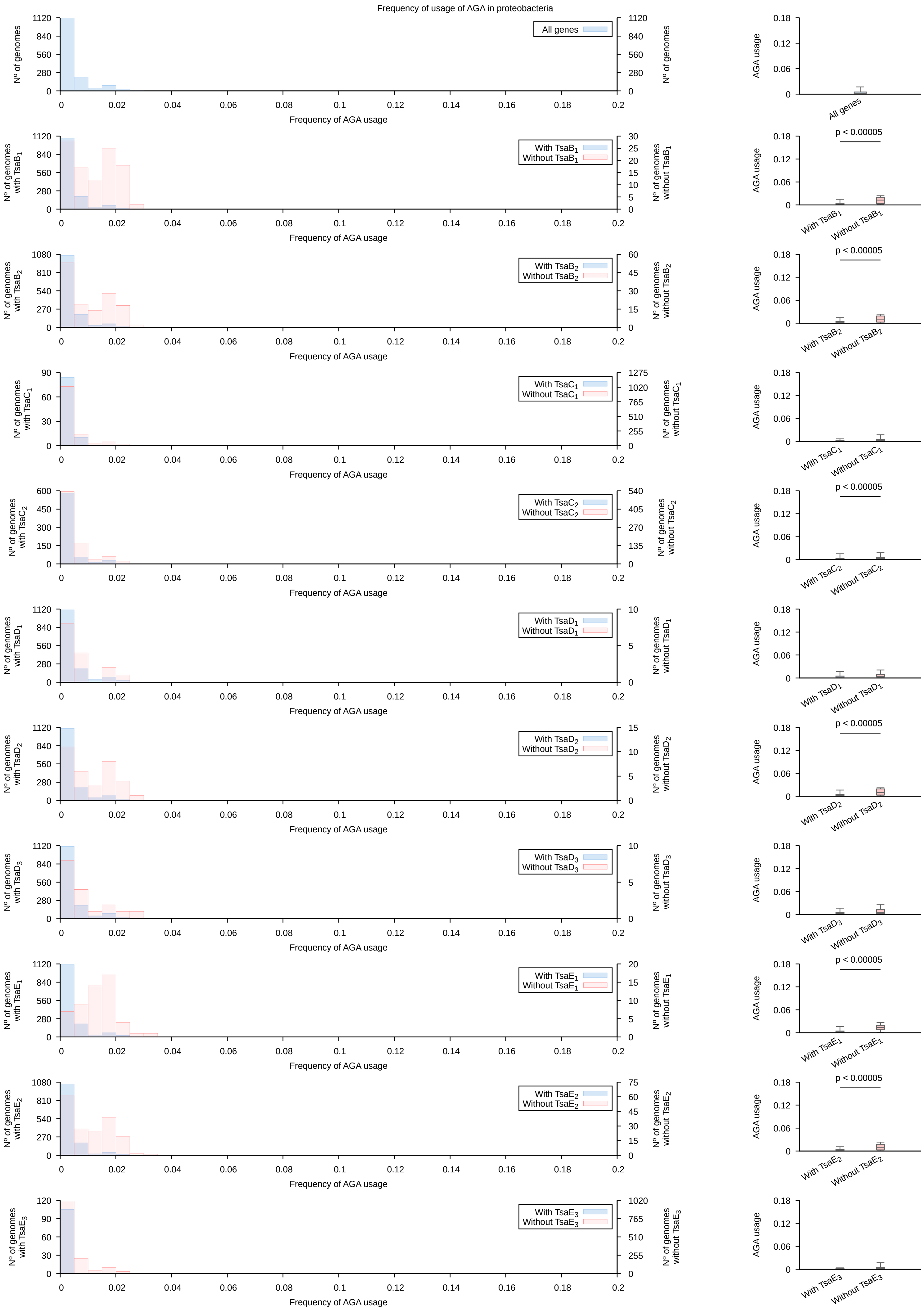
Frequency of usage of AGA in proteobacteria
All genes
With $TsaB_1$
Without $TsaB_1$
With $TsaB_2$
Without $TsaB_2$
With $TsaC_1$
Without $TsaC_1$
With $TsaC_2$
Without $TsaC_2$
With $TsaD_1$
Without $TsaD_1$
With $TsaD_2$
Without $TsaD_2$
With $TsaD_3$
Without $TsaD_3$
With $TsaE_1$
Without $TsaE_1$
With $TsaE_2$
Without $TsaE_2$
With $TsaE_3$
Without $TsaE_3$
Frequency of AGA usage
Nº of genomes
Nº of genomes with $TsaB_1$
Nº of genomes without $TsaB_1$
Nº of genomes with $TsaB_2$
Nº of genomes without $TsaB_2$
Nº of genomes with $TsaC_1$
Nº of genomes without $TsaC_1$
Nº of genomes with $TsaC_2$
Nº of genomes without $TsaC_2$
Nº of genomes with $TsaD_1$
Nº of genomes without $TsaD_1$
Nº of genomes with $TsaD_2$
Nº of genomes without $TsaD_2$
Nº of genomes with $TsaD_3$
Nº of genomes without $TsaD_3$
Nº of genomes with $TsaE_1$
Nº of genomes without $TsaE_1$
Nº of genomes with $TsaE_2$
Nº of genomes without $TsaE_2$
Nº of genomes with $TsaE_3$
Nº of genomes without $TsaE_3$
AGA usage
$p < 0.00005$

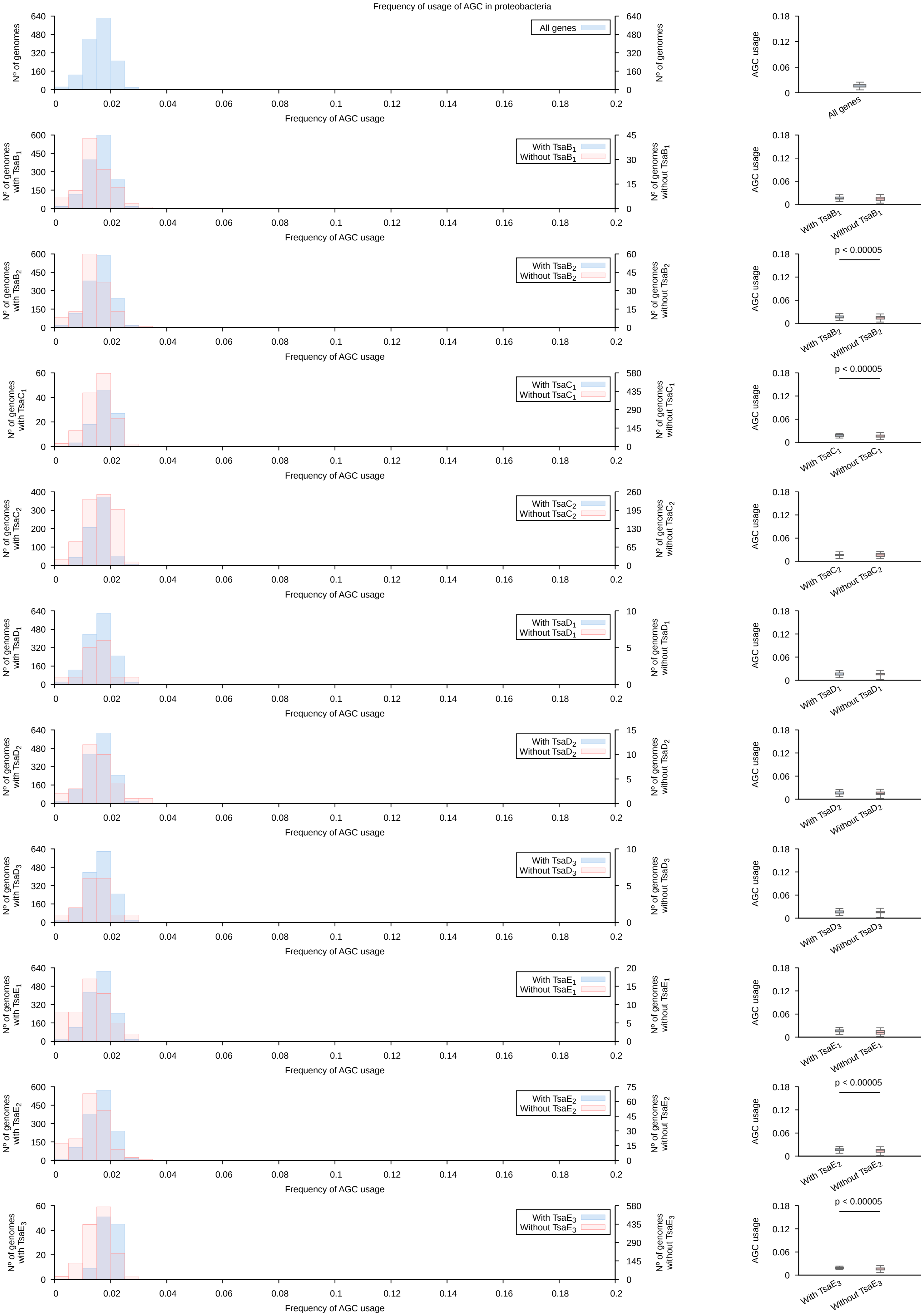

Frequency of usage of AGC in proteobacteria
Nº of genomes
All genes
Frequency of AGC usage
AGC usage
Nº of genomes with TsaB1
Nº of genomes without TsaB1
With TsaB1
Without TsaB1
Nº of genomes with TsaB2
Nº of genomes without TsaB2
With TsaB2
Without TsaB2
p < 0.00005
Nº of genomes with TsaC1
Nº of genomes without TsaC1
With TsaC1
Without TsaC1
p < 0.00005
Nº of genomes with TsaC2
Nº of genomes without TsaC2
With TsaC2
Without TsaC2
Nº of genomes with TsaD1
Nº of genomes without TsaD1
With TsaD1
Without TsaD1
Nº of genomes with TsaD2
Nº of genomes without TsaD2
With TsaD2
Without TsaD2
Nº of genomes with TsaD3
Nº of genomes without TsaD3
With TsaD3
Without TsaD3
Nº of genomes with TsaE1
Nº of genomes without TsaE1
With TsaE1
Without TsaE1
Nº of genomes with TsaE2
Nº of genomes without TsaE2
With TsaE2
Without TsaE2
p < 0.00005
Nº of genomes with TsaE3
Nº of genomes without TsaE3
With TsaE3
Without TsaE3
p < 0.00005

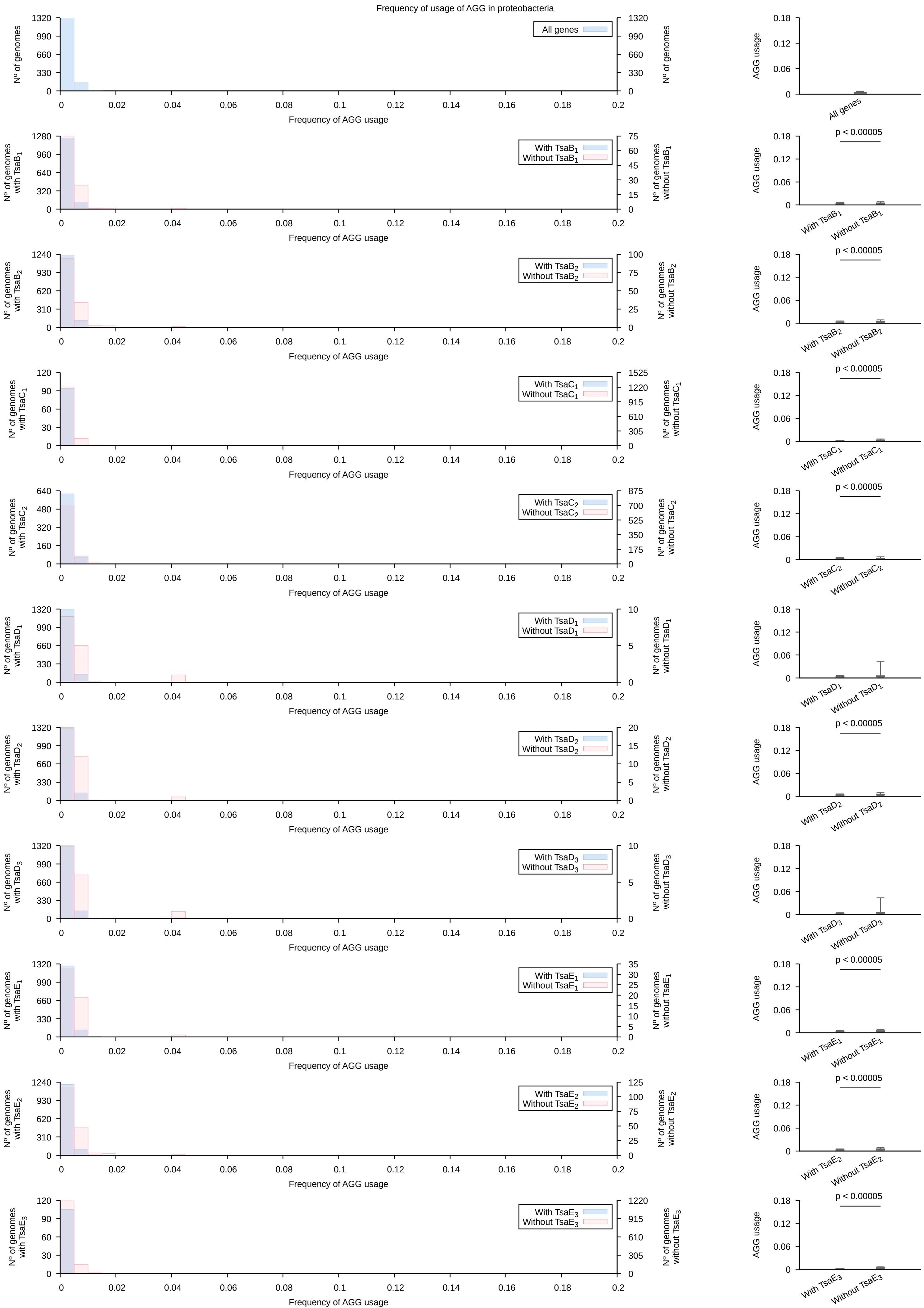

Frequency of usage of AGG in proteobacteria
Nº of genomes
Frequency of AGG usage
AGG usage
All genes
Nº of genomes with $TsaB_1$
Nº of genomes without $TsaB_1$
With $TsaB_1$
Without $TsaB_1$
p < 0.00005
Nº of genomes with $TsaB_2$
Nº of genomes without $TsaB_2$
With $TsaB_2$
Without $TsaB_2$
p < 0.00005
Nº of genomes with $TsaC_1$
Nº of genomes without $TsaC_1$
With $TsaC_1$
Without $TsaC_1$
p < 0.00005
Nº of genomes with $TsaC_2$
Nº of genomes without $TsaC_2$
With $TsaC_2$
Without $TsaC_2$
p < 0.00005
Nº of genomes with $TsaD_1$
Nº of genomes without $TsaD_1$
With $TsaD_1$
Without $TsaD_1$
Nº of genomes with $TsaD_2$
Nº of genomes without $TsaD_2$
With $TsaD_2$
Without $TsaD_2$
p < 0.00005
Nº of genomes with $TsaD_3$
Nº of genomes without $TsaD_3$
With $TsaD_3$
Without $TsaD_3$
Nº of genomes with $TsaE_1$
Nº of genomes without $TsaE_1$
With $TsaE_1$
Without $TsaE_1$
p < 0.00005
Nº of genomes with $TsaE_2$
Nº of genomes without $TsaE_2$
With $TsaE_2$
Without $TsaE_2$
p < 0.00005
Nº of genomes with $TsaE_3$
Nº of genomes without $TsaE_3$
With $TsaE_3$
Without $TsaE_3$
p < 0.00005

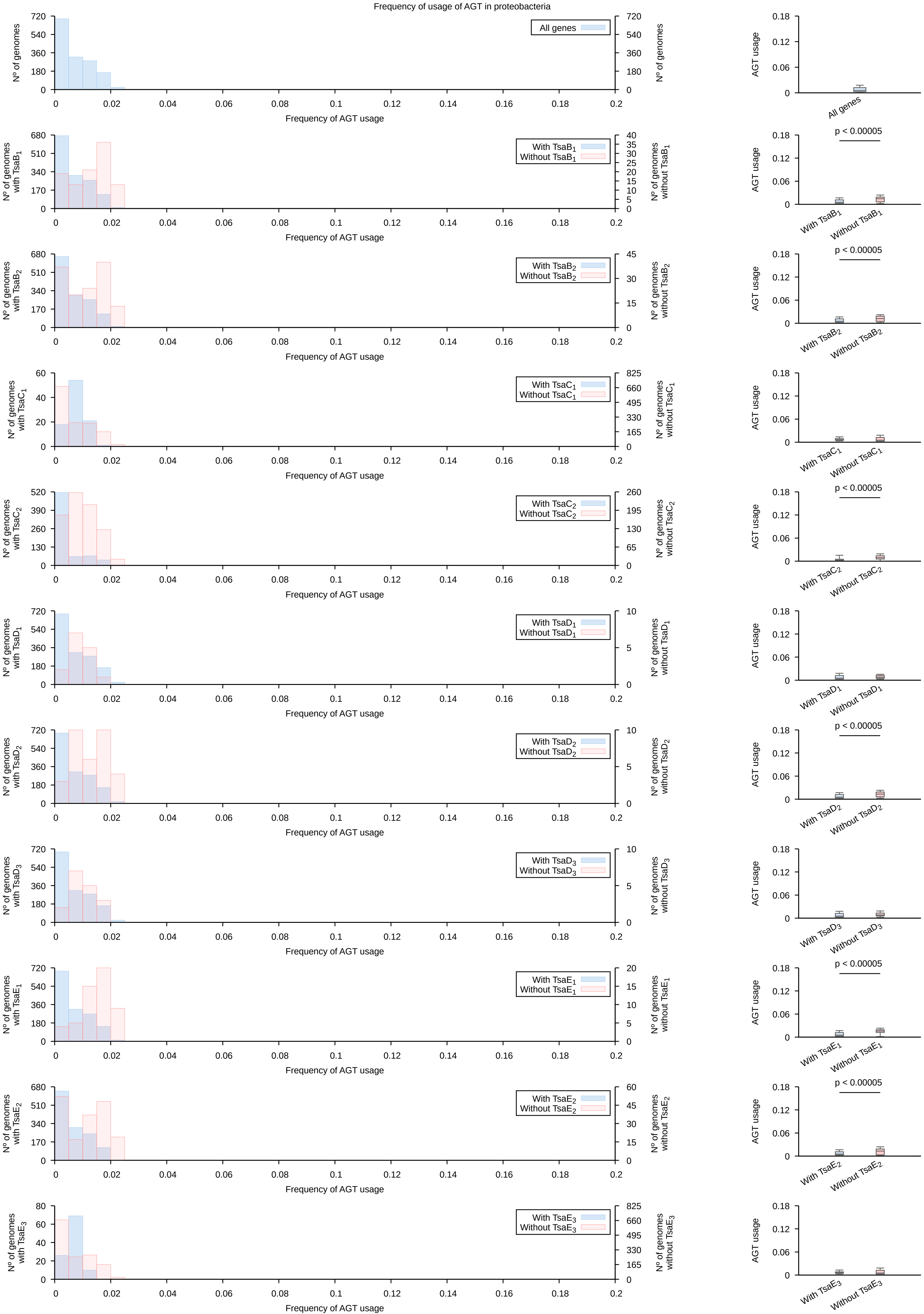
Frequency of usage of AGT in proteobacteria
All genes
Nº of genomes
Frequency of AGT usage
AGT usage
With TsaB$_1$
Without TsaB$_1$
Nº of genomes with TsaB$_1$
Nº of genomes without TsaB$_1$
p < 0.00005
With TsaB$_2$
Without TsaB$_2$
Nº of genomes with TsaB$_2$
Nº of genomes without TsaB$_2$
p < 0.00005
With TsaC$_1$
Without TsaC$_1$
Nº of genomes with TsaC$_1$
Nº of genomes without TsaC$_1$
With TsaC$_2$
Without TsaC$_2$
Nº of genomes with TsaC$_2$
Nº of genomes without TsaC$_2$
p < 0.00005
With TsaD$_1$
Without TsaD$_1$
Nº of genomes with TsaD$_1$
Nº of genomes without TsaD$_1$
With TsaD$_2$
Without TsaD$_2$
Nº of genomes with TsaD$_2$
Nº of genomes without TsaD$_2$
p < 0.00005
With TsaD$_3$
Without TsaD$_3$
Nº of genomes with TsaD$_3$
Nº of genomes without TsaD$_3$
With TsaE$_1$
Without TsaE$_1$
Nº of genomes with TsaE$_1$
Nº of genomes without TsaE$_1$
p < 0.00005
With TsaE$_2$
Without TsaE$_2$
Nº of genomes with TsaE$_2$
Nº of genomes without TsaE$_2$
p < 0.00005
With TsaE$_3$
Without TsaE$_3$
Nº of genomes with TsaE$_3$
Nº of genomes without TsaE$_3$
0 0.02 0.04 0.06 0.08 0.1 0.12 0.14 0.16 0.18 0.2
0 0.06 0.12 0.18

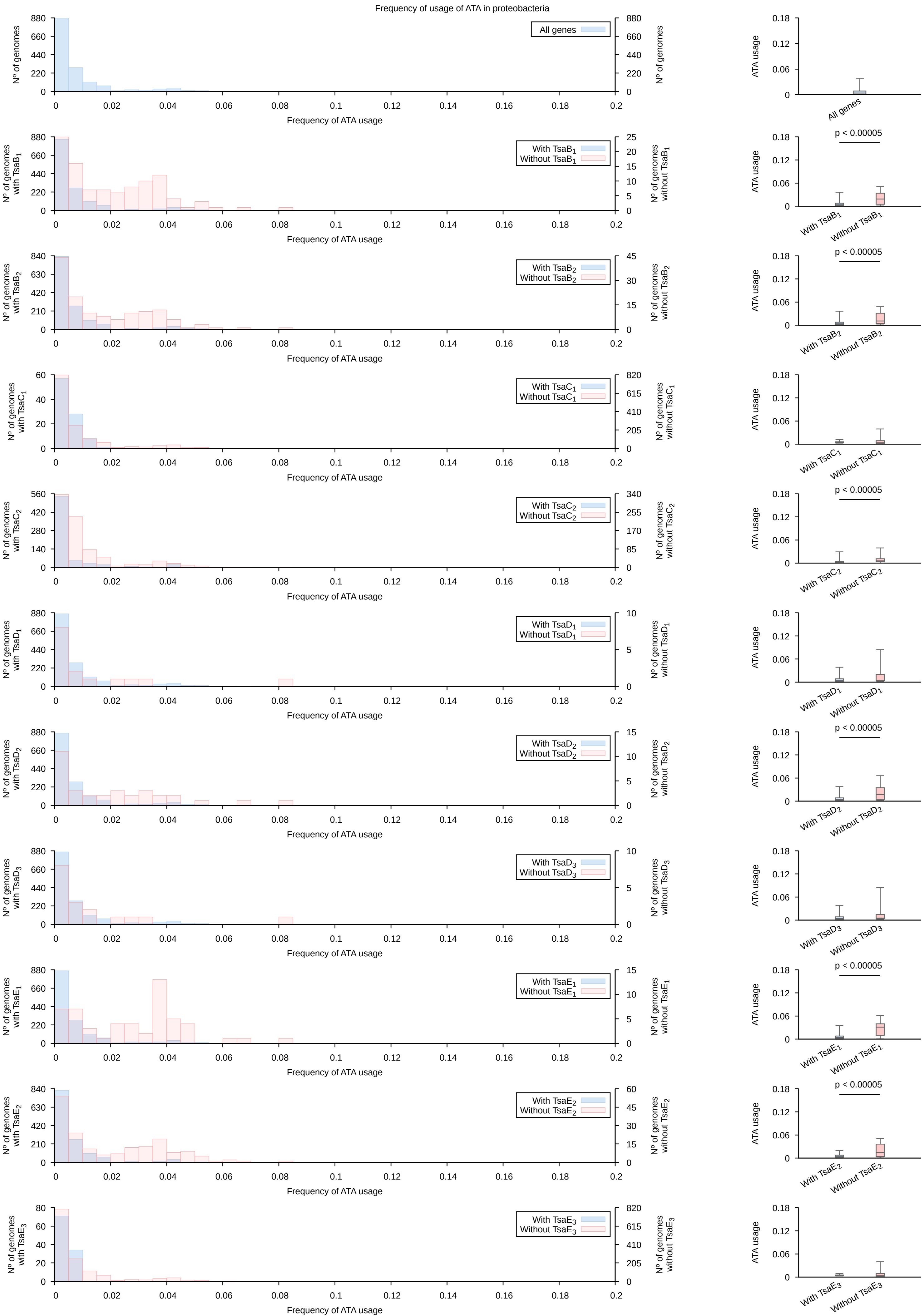

Frequency of usage of ATA in proteobacteria
Nº of genomes
All genes
Frequency of ATA usage
ATA usage
Nº of genomes with TsaB$_1$
Nº of genomes without TsaB$_1$
With TsaB$_1$
Without TsaB$_1$
p < 0.00005
Nº of genomes with TsaB$_2$
Nº of genomes without TsaB$_2$
With TsaB$_2$
Without TsaB$_2$
p < 0.00005
Nº of genomes with TsaC$_1$
Nº of genomes without TsaC$_1$
With TsaC$_1$
Without TsaC$_1$
Nº of genomes with TsaC$_2$
Nº of genomes without TsaC$_2$
With TsaC$_2$
Without TsaC$_2$
p < 0.00005
Nº of genomes with TsaD$_1$
Nº of genomes without TsaD$_1$
With TsaD$_1$
Without TsaD$_1$
Nº of genomes with TsaD$_2$
Nº of genomes without TsaD$_2$
With TsaD$_2$
Without TsaD$_2$
p < 0.00005
Nº of genomes with TsaD$_3$
Nº of genomes without TsaD$_3$
With TsaD$_3$
Without TsaD$_3$
Nº of genomes with TsaE$_1$
Nº of genomes without TsaE$_1$
With TsaE$_1$
Without TsaE$_1$
p < 0.00005
Nº of genomes with TsaE$_2$
Nº of genomes without TsaE$_2$
With TsaE$_2$
Without TsaE$_2$
p < 0.00005
Nº of genomes with TsaE$_3$
Nº of genomes without TsaE$_3$
With TsaE$_3$
Without TsaE$_3$

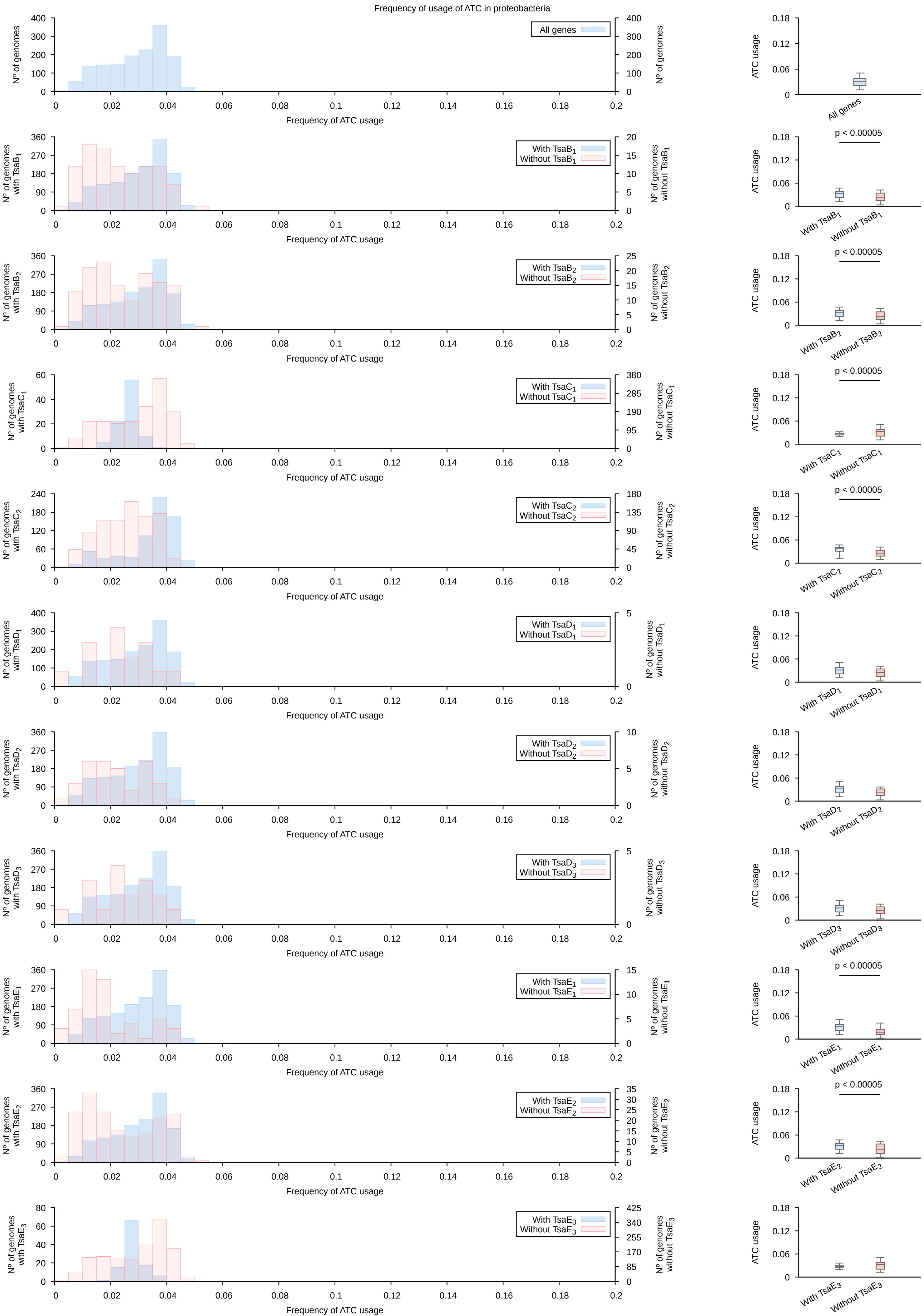
Frequency of usage of ATC in proteobacteria
All genes
Nº of genomes
Frequency of ATC usage
ATC usage
With TsaB1
Without TsaB1
Nº of genomes with TsaB1
Nº of genomes without TsaB1
p < 0.00005
With TsaB2
Without TsaB2
Nº of genomes with TsaB2
Nº of genomes without TsaB2
p < 0.00005
With TsaC1
Without TsaC1
Nº of genomes with TsaC1
Nº of genomes without TsaC1
p < 0.00005
With TsaC2
Without TsaC2
Nº of genomes with TsaC2
Nº of genomes without TsaC2
p < 0.00005
With TsaD1
Without TsaD1
Nº of genomes with TsaD1
Nº of genomes without TsaD1
With TsaD2
Without TsaD2
Nº of genomes with TsaD2
Nº of genomes without TsaD2
With TsaD3
Without TsaD3
Nº of genomes with TsaD3
Nº of genomes without TsaD3
With TsaE1
Without TsaE1
Nº of genomes with TsaE1
Nº of genomes without TsaE1
p < 0.00005
With TsaE2
Without TsaE2
Nº of genomes with TsaE2
Nº of genomes without TsaE2
p < 0.00005
With TsaE3
Without TsaE3
Nº of genomes with TsaE3
Nº of genomes without TsaE3

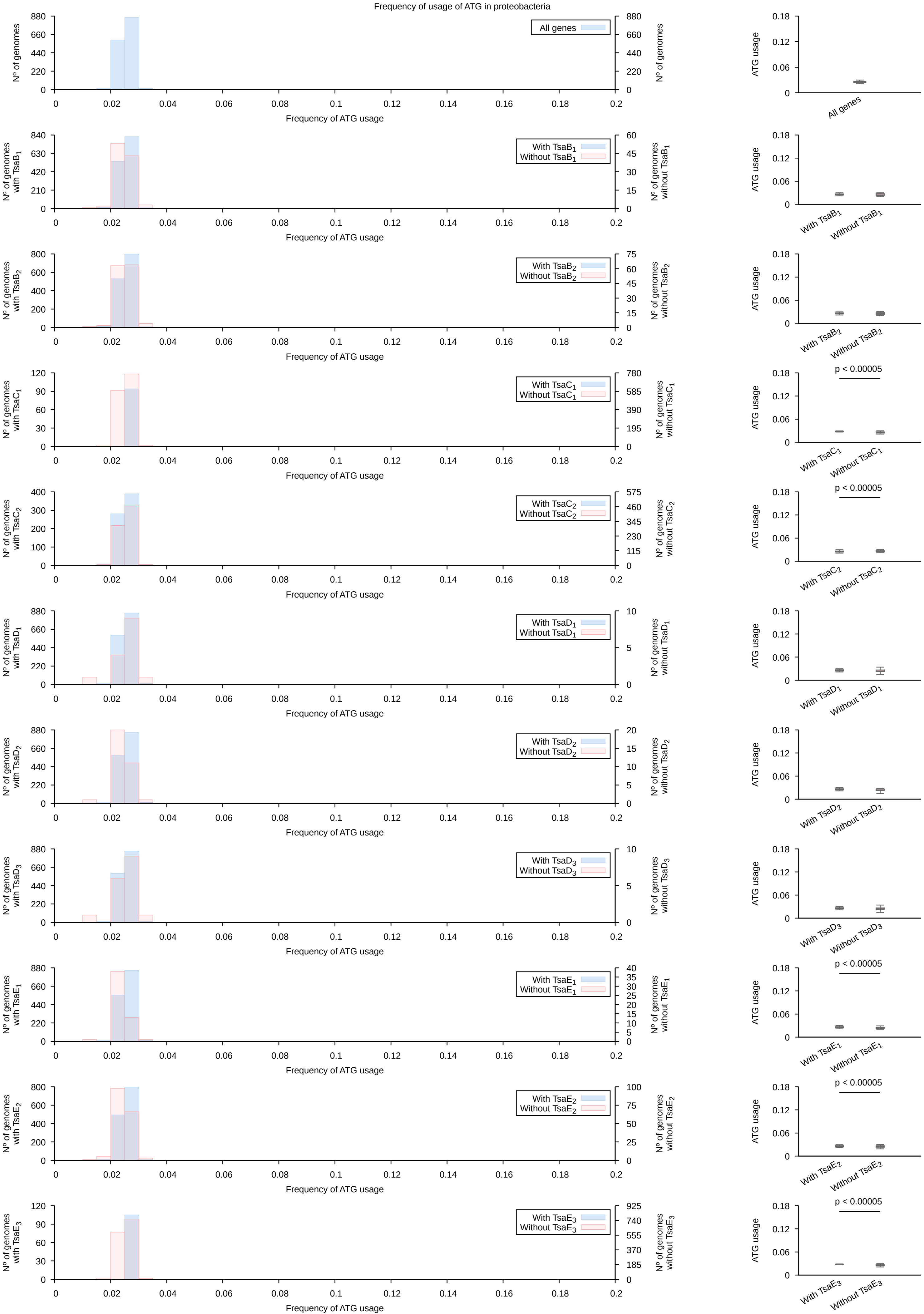

Frequency of usage of ATG in proteobacteria
Nº of genomes
All genes
Frequency of ATG usage
ATG usage
Nº of genomes with TsaB$_1$
Nº of genomes without TsaB$_1$
With TsaB$_1$
Without TsaB$_1$
Nº of genomes with TsaB$_2$
Nº of genomes without TsaB$_2$
With TsaB$_2$
Without TsaB$_2$
Nº of genomes with TsaC$_1$
Nº of genomes without TsaC$_1$
With TsaC$_1$
Without TsaC$_1$
p < 0.00005
Nº of genomes with TsaC$_2$
Nº of genomes without TsaC$_2$
With TsaC$_2$
Without TsaC$_2$
p < 0.00005
Nº of genomes with TsaD$_1$
Nº of genomes without TsaD$_1$
With TsaD$_1$
Without TsaD$_1$
Nº of genomes with TsaD$_2$
Nº of genomes without TsaD$_2$
With TsaD$_2$
Without TsaD$_2$
Nº of genomes with TsaD$_3$
Nº of genomes without TsaD$_3$
With TsaD$_3$
Without TsaD$_3$
Nº of genomes with TsaE$_1$
Nº of genomes without TsaE$_1$
With TsaE$_1$
Without TsaE$_1$
p < 0.00005
Nº of genomes with TsaE$_2$
Nº of genomes without TsaE$_2$
With TsaE$_2$
Without TsaE$_2$
p < 0.00005
Nº of genomes with TsaE$_3$
Nº of genomes without TsaE$_3$
With TsaE$_3$
Without TsaE$_3$
p < 0.00005

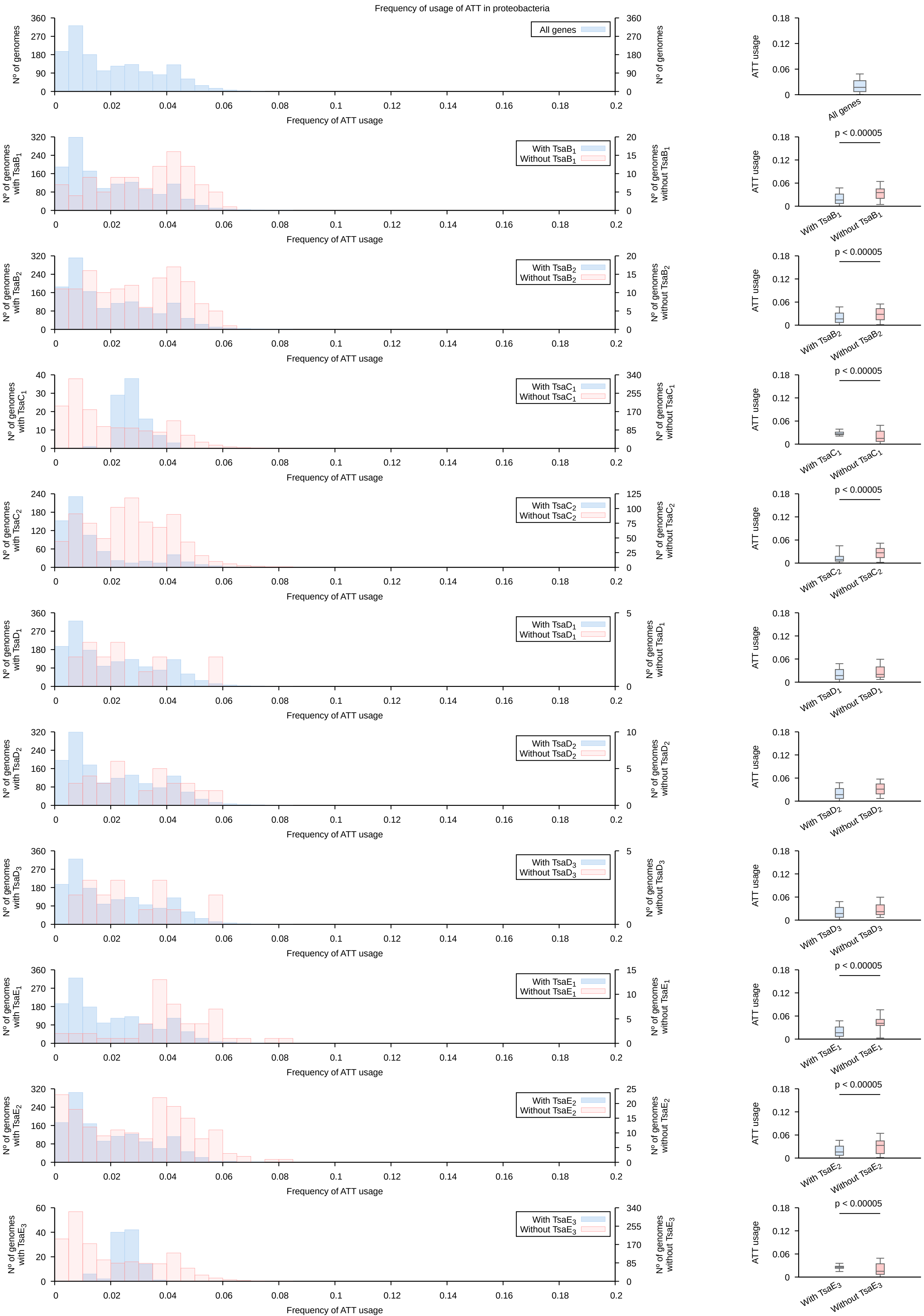

Frequency of usage of ATT in proteobacteria
All genes
Nº of genomes
Frequency of ATT usage
ATT usage
With TsaB$_1$
Without TsaB$_1$
Nº of genomes with TsaB$_1$
Nº of genomes without TsaB$_1$
p < 0.00005
With TsaB$_2$
Without TsaB$_2$
Nº of genomes with TsaB$_2$
Nº of genomes without TsaB$_2$
p < 0.00005
With TsaC$_1$
Without TsaC$_1$
Nº of genomes with TsaC$_1$
Nº of genomes without TsaC$_1$
p < 0.00005
With TsaC$_2$
Without TsaC$_2$
Nº of genomes with TsaC$_2$
Nº of genomes without TsaC$_2$
p < 0.00005
With TsaD$_1$
Without TsaD$_1$
Nº of genomes with TsaD$_1$
Nº of genomes without TsaD$_1$
With TsaD$_2$
Without TsaD$_2$
Nº of genomes with TsaD$_2$
Nº of genomes without TsaD$_2$
With TsaD$_3$
Without TsaD$_3$
Nº of genomes with TsaD$_3$
Nº of genomes without TsaD$_3$
With TsaE$_1$
Without TsaE$_1$
Nº of genomes with TsaE$_1$
Nº of genomes without TsaE$_1$
p < 0.00005
With TsaE$_2$
Without TsaE$_2$
Nº of genomes with TsaE$_2$
Nº of genomes without TsaE$_2$
p < 0.00005
With TsaE$_3$
Without TsaE$_3$
Nº of genomes with TsaE$_3$
Nº of genomes without TsaE$_3$
p < 0.00005

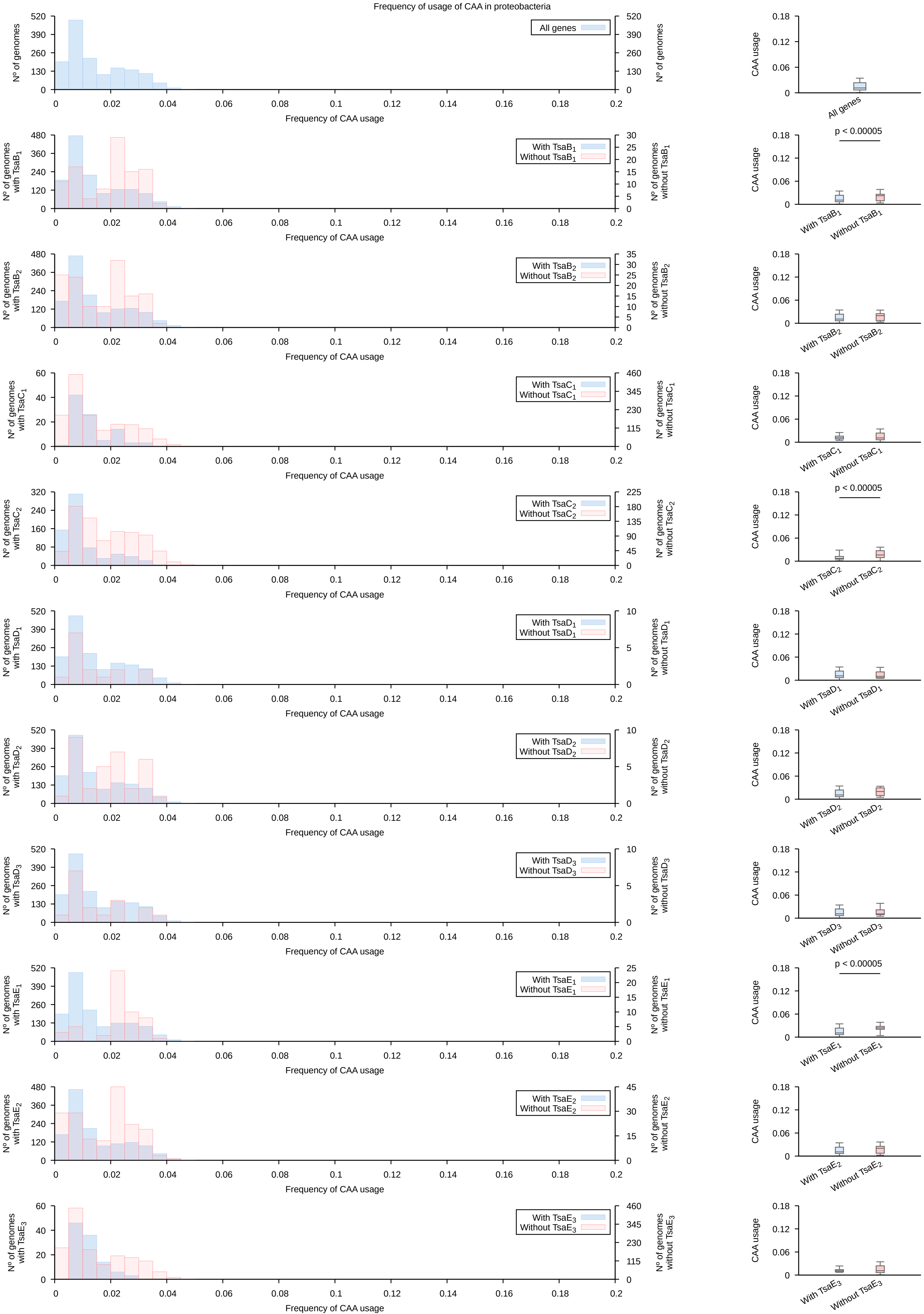

Frequency of usage of CAA in proteobacteria
All genes
Nº of genomes
Frequency of CAA usage
CAA usage
With $TsaB_1$
Without $TsaB_1$
Nº of genomes with $TsaB_1$
Nº of genomes without $TsaB_1$
p < 0.00005
With $TsaB_2$
Without $TsaB_2$
Nº of genomes with $TsaB_2$
Nº of genomes without $TsaB_2$
With $TsaC_1$
Without $TsaC_1$
Nº of genomes with $TsaC_1$
Nº of genomes without $TsaC_1$
With $TsaC_2$
Without $TsaC_2$
Nº of genomes with $TsaC_2$
Nº of genomes without $TsaC_2$
p < 0.00005
With $TsaD_1$
Without $TsaD_1$
Nº of genomes with $TsaD_1$
Nº of genomes without $TsaD_1$
With $TsaD_2$
Without $TsaD_2$
Nº of genomes with $TsaD_2$
Nº of genomes without $TsaD_2$
With $TsaD_3$
Without $TsaD_3$
Nº of genomes with $TsaD_3$
Nº of genomes without $TsaD_3$
With $TsaE_1$
Without $TsaE_1$
Nº of genomes with $TsaE_1$
Nº of genomes without $TsaE_1$
p < 0.00005
With $TsaE_2$
Without $TsaE_2$
Nº of genomes with $TsaE_2$
Nº of genomes without $TsaE_2$
With $TsaE_3$
Without $TsaE_3$
Nº of genomes with $TsaE_3$
Nº of genomes without $TsaE_3$

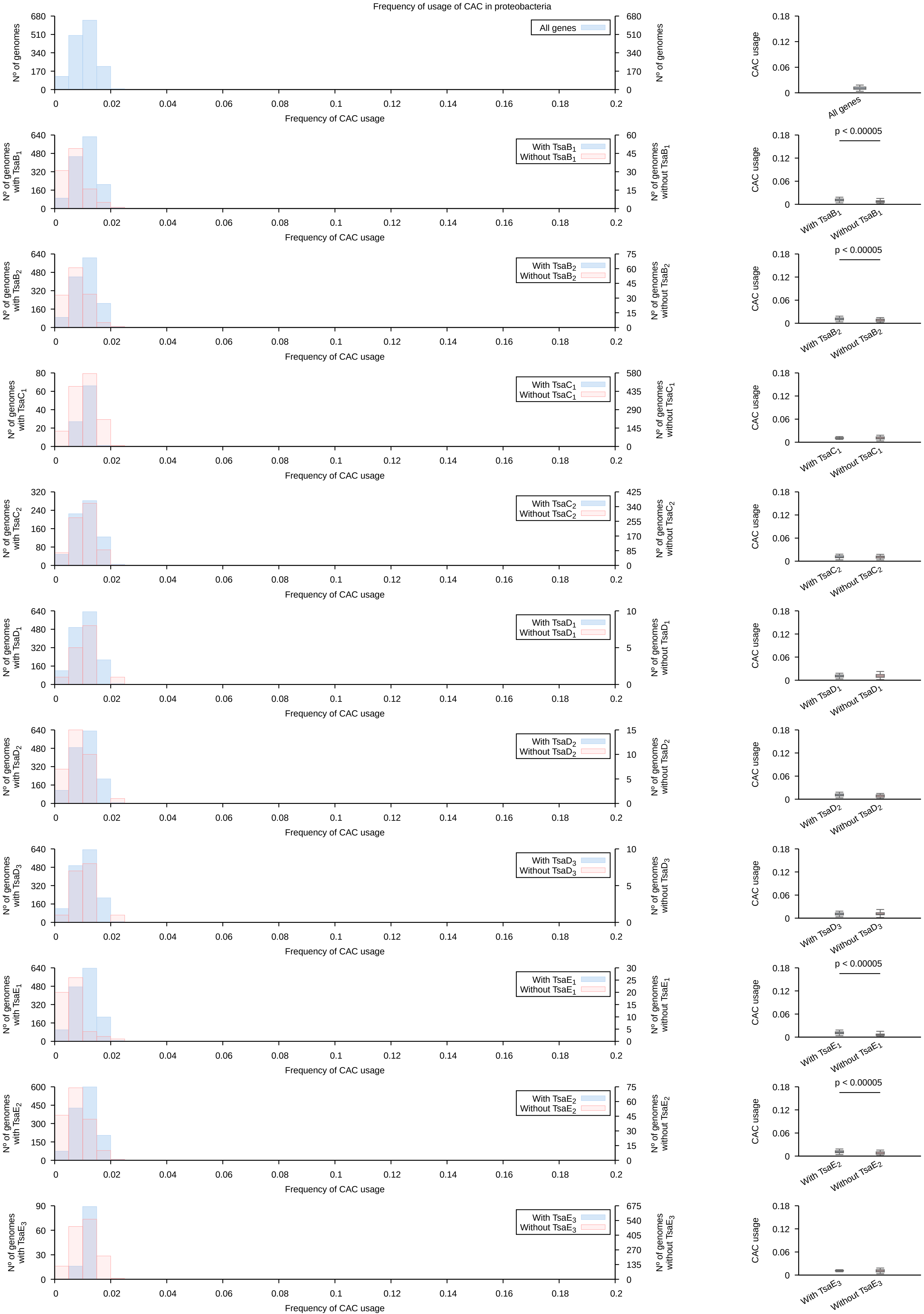
Frequency of usage of CAC in proteobacteria
All genes
With TsaB$_1$
Without TsaB$_1$
With TsaB$_2$
Without TsaB$_2$
With TsaC$_1$
Without TsaC$_1$
With TsaC$_2$
Without TsaC$_2$
With TsaD$_1$
Without TsaD$_1$
With TsaD$_2$
Without TsaD$_2$
With TsaD$_3$
Without TsaD$_3$
With TsaE$_1$
Without TsaE$_1$
With TsaE$_2$
Without TsaE$_2$
With TsaE$_3$
Without TsaE$_3$
Nº of genomes
Frequency of CAC usage
CAC usage
p < 0.00005

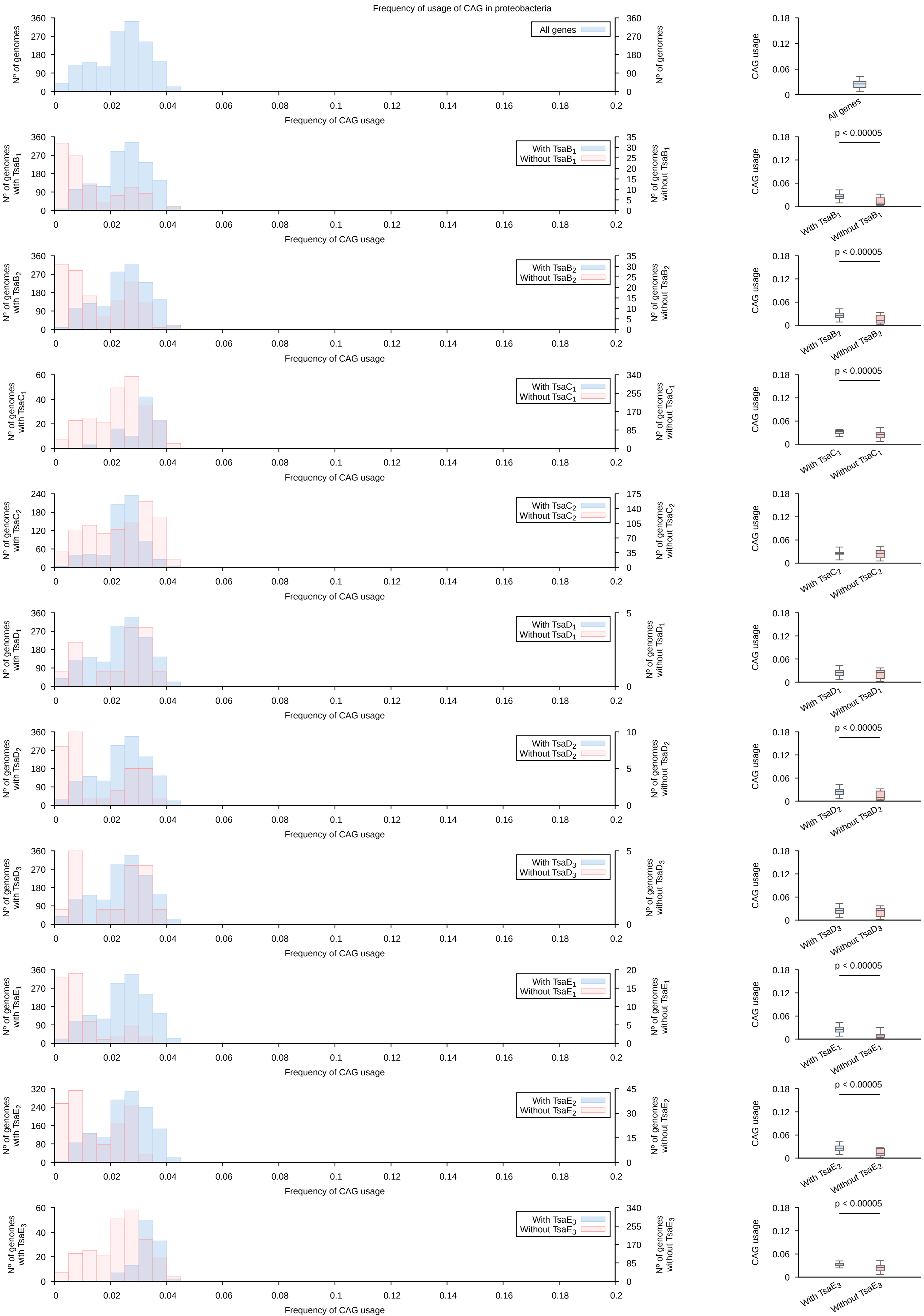

Frequency of usage of CAG in proteobacteria
All genes
Nº of genomes
Frequency of CAG usage
CAG usage
With TsaB$_1$
Without TsaB$_1$
Nº of genomes with TsaB$_1$
Nº of genomes without TsaB$_1$
p < 0.00005
With TsaB$_2$
Without TsaB$_2$
Nº of genomes with TsaB$_2$
Nº of genomes without TsaB$_2$
p < 0.00005
With TsaC$_1$
Without TsaC$_1$
Nº of genomes with TsaC$_1$
Nº of genomes without TsaC$_1$
p < 0.00005
With TsaC$_2$
Without TsaC$_2$
Nº of genomes with TsaC$_2$
Nº of genomes without TsaC$_2$
With TsaD$_1$
Without TsaD$_1$
Nº of genomes with TsaD$_1$
Nº of genomes without TsaD$_1$
With TsaD$_2$
Without TsaD$_2$
Nº of genomes with TsaD$_2$
Nº of genomes without TsaD$_2$
p < 0.00005
With TsaD$_3$
Without TsaD$_3$
Nº of genomes with TsaD$_3$
Nº of genomes without TsaD$_3$
With TsaE$_1$
Without TsaE$_1$
Nº of genomes with TsaE$_1$
Nº of genomes without TsaE$_1$
p < 0.00005
With TsaE$_2$
Without TsaE$_2$
Nº of genomes with TsaE$_2$
Nº of genomes without TsaE$_2$
p < 0.00005
With TsaE$_3$
Without TsaE$_3$
Nº of genomes with TsaE$_3$
Nº of genomes without TsaE$_3$
p < 0.00005

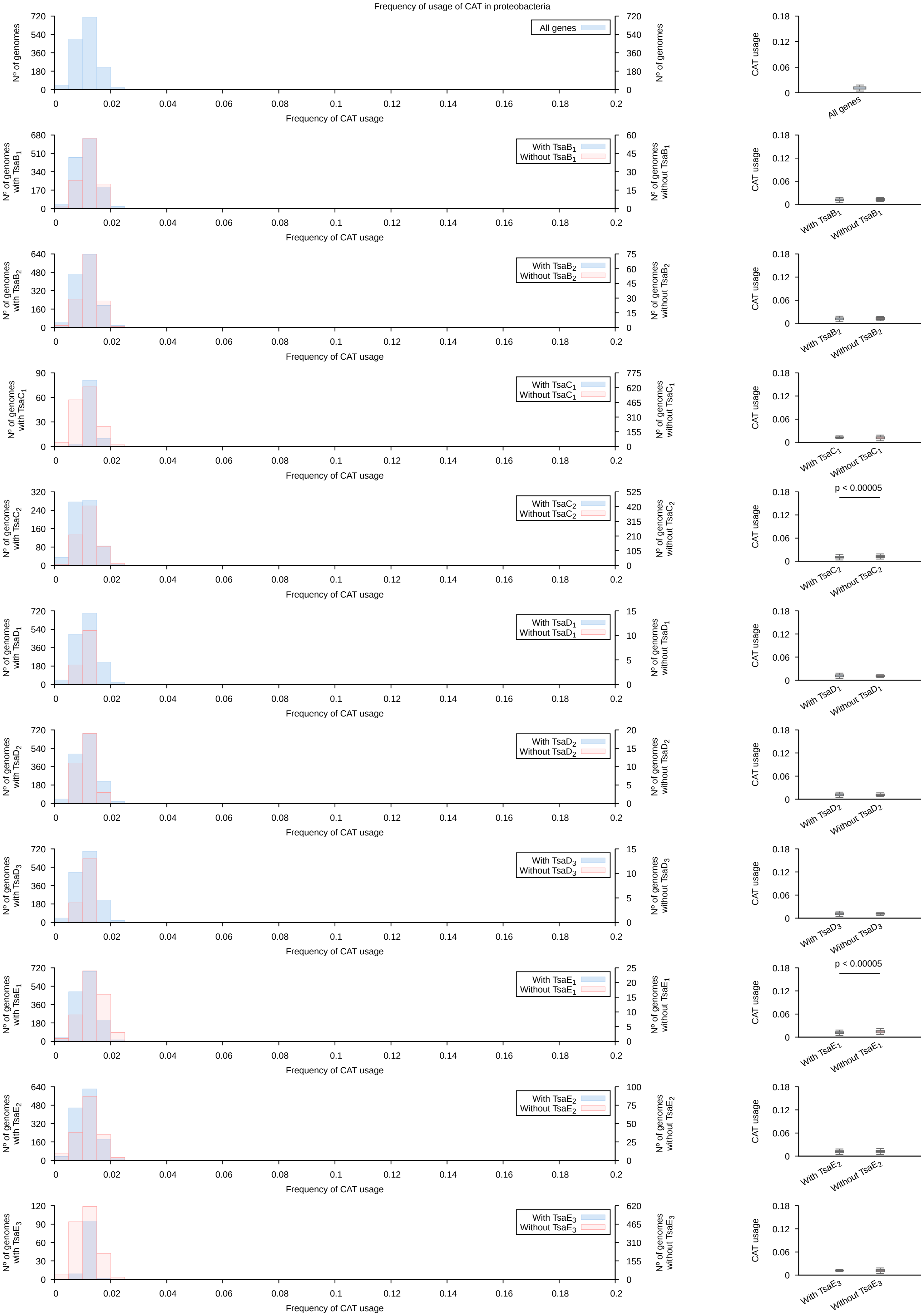

Frequency of usage of CAT in proteobacteria
All genes
With TsaB1
Without TsaB1
With TsaB2
Without TsaB2
With TsaC1
Without TsaC1
With TsaC2
Without TsaC2
With TsaD1
Without TsaD1
With TsaD2
Without TsaD2
With TsaD3
Without TsaD3
With TsaE1
Without TsaE1
With TsaE2
Without TsaE2
With TsaE3
Without TsaE3
Nº of genomes
Frequency of CAT usage
CAT usage
p < 0.00005

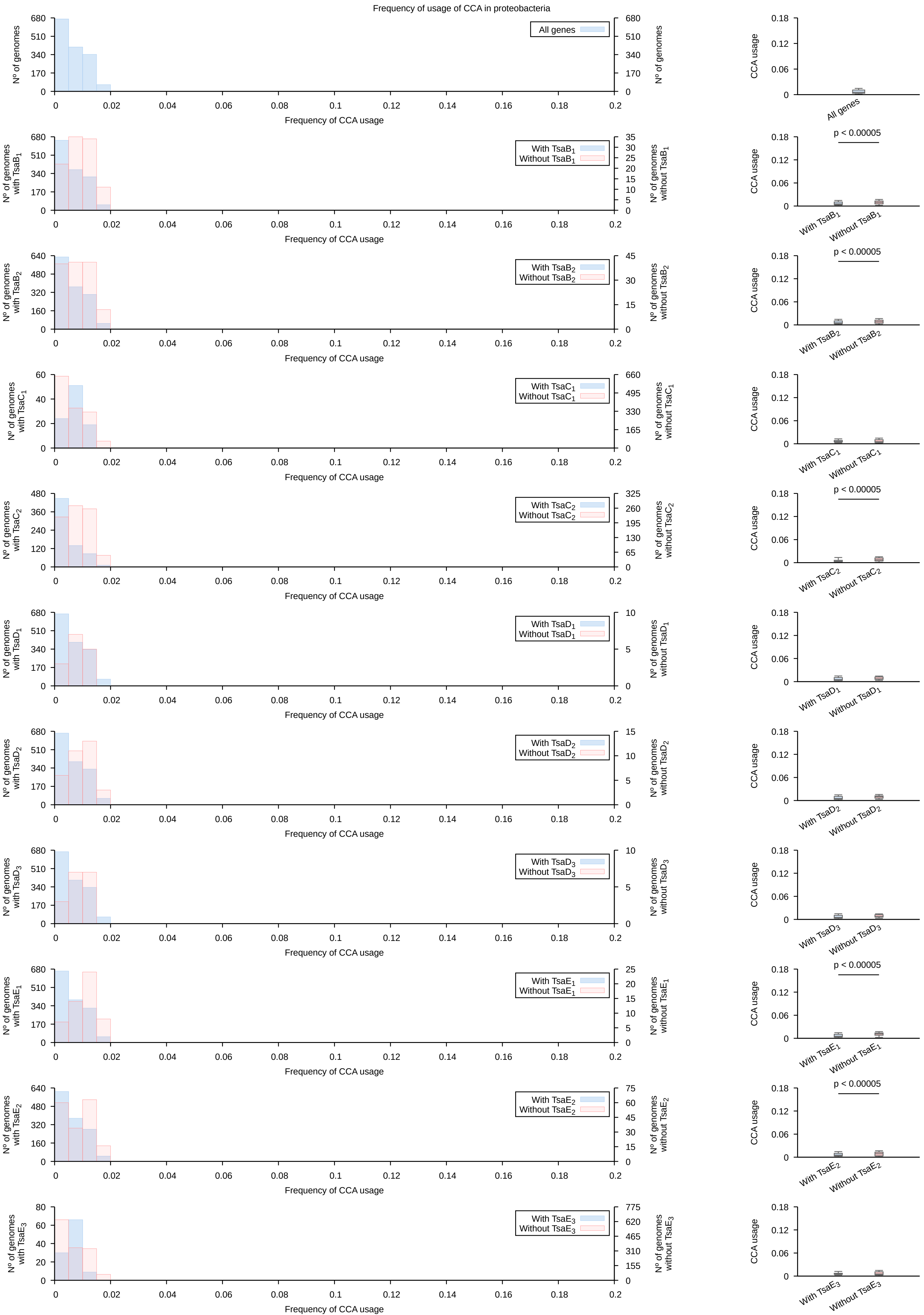
Frequency of usage of CCA in proteobacteria
All genes
Nº of genomes
Frequency of CCA usage
CCA usage
0 0.02 0.04 0.06 0.08 0.1 0.12 0.14 0.16 0.18 0.2
0 0.06 0.12 0.18
With TsaB1
Without TsaB1
Nº of genomes with TsaB1
Nº of genomes without TsaB1
p < 0.00005
With TsaB2
Without TsaB2
Nº of genomes with TsaB2
Nº of genomes without TsaB2
p < 0.00005
With TsaC1
Without TsaC1
Nº of genomes with TsaC1
Nº of genomes without TsaC1
With TsaC2
Without TsaC2
Nº of genomes with TsaC2
Nº of genomes without TsaC2
p < 0.00005
With TsaD1
Without TsaD1
Nº of genomes with TsaD1
Nº of genomes without TsaD1
With TsaD2
Without TsaD2
Nº of genomes with TsaD2
Nº of genomes without TsaD2
With TsaD3
Without TsaD3
Nº of genomes with TsaD3
Nº of genomes without TsaD3
With TsaE1
Without TsaE1
Nº of genomes with TsaE1
Nº of genomes without TsaE1
p < 0.00005
With TsaE2
Without TsaE2
Nº of genomes with TsaE2
Nº of genomes without TsaE2
p < 0.00005
With TsaE3
Without TsaE3
Nº of genomes with TsaE3
Nº of genomes without TsaE3

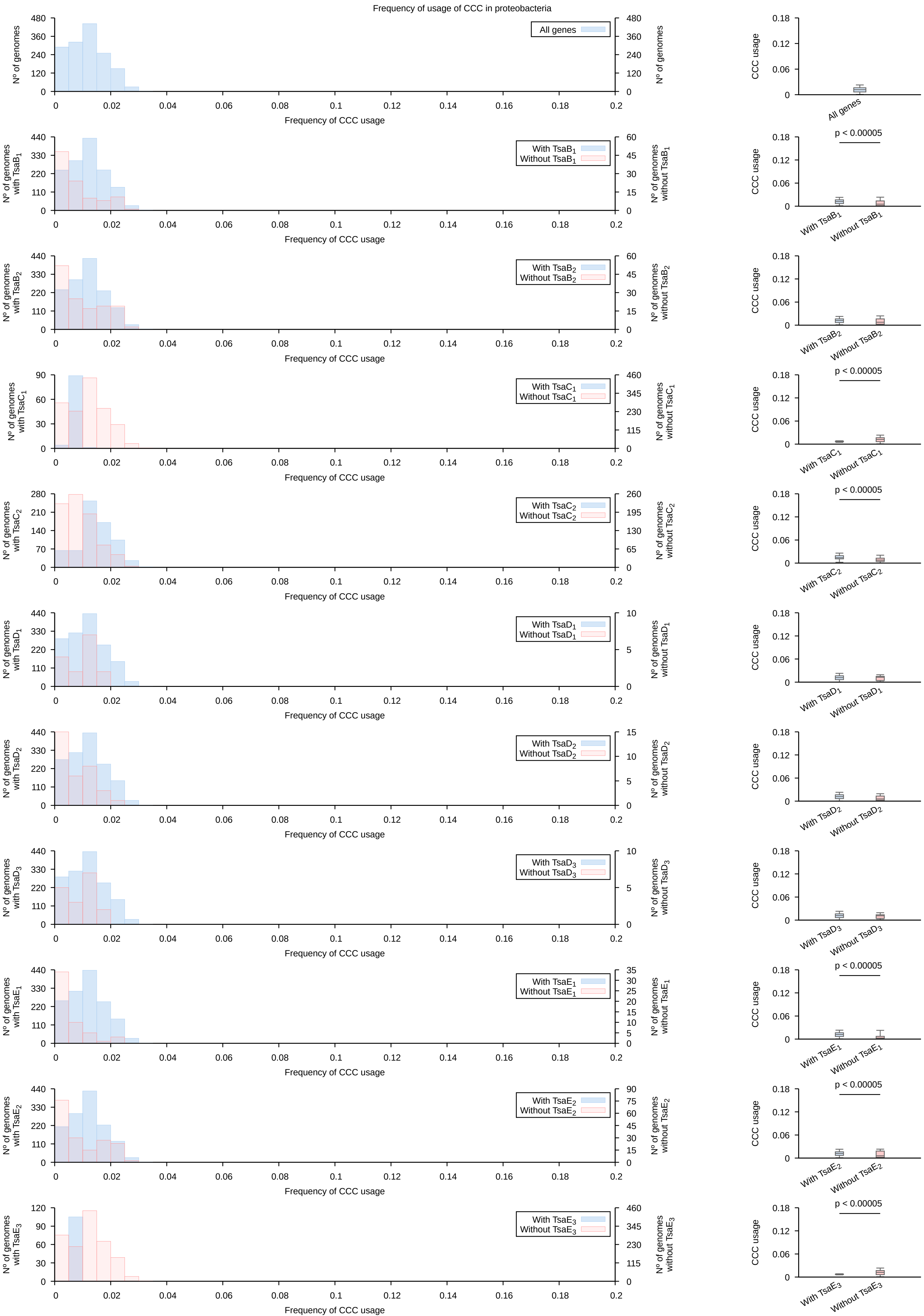
Frequency of usage of CCC in proteobacteria
Nº of genomes
All genes
Frequency of CCC usage
CCC usage
Nº of genomes with TsaB1
Nº of genomes without TsaB1
With TsaB1
Without TsaB1
p < 0.00005
Nº of genomes with TsaB2
Nº of genomes without TsaB2
With TsaB2
Without TsaB2
Nº of genomes with TsaC1
Nº of genomes without TsaC1
With TsaC1
Without TsaC1
p < 0.00005
Nº of genomes with TsaC2
Nº of genomes without TsaC2
With TsaC2
Without TsaC2
p < 0.00005
Nº of genomes with TsaD1
Nº of genomes without TsaD1
With TsaD1
Without TsaD1
Nº of genomes with TsaD2
Nº of genomes without TsaD2
With TsaD2
Without TsaD2
Nº of genomes with TsaD3
Nº of genomes without TsaD3
With TsaD3
Without TsaD3
Nº of genomes with TsaE1
Nº of genomes without TsaE1
With TsaE1
Without TsaE1
p < 0.00005
Nº of genomes with TsaE2
Nº of genomes without TsaE2
With TsaE2
Without TsaE2
p < 0.00005
Nº of genomes with TsaE3
Nº of genomes without TsaE3
With TsaE3
Without TsaE3
p < 0.00005
0 0.02 0.04 0.06 0.08 0.1 0.12 0.14 0.16 0.18 0.2
0 0.06 0.12 0.18

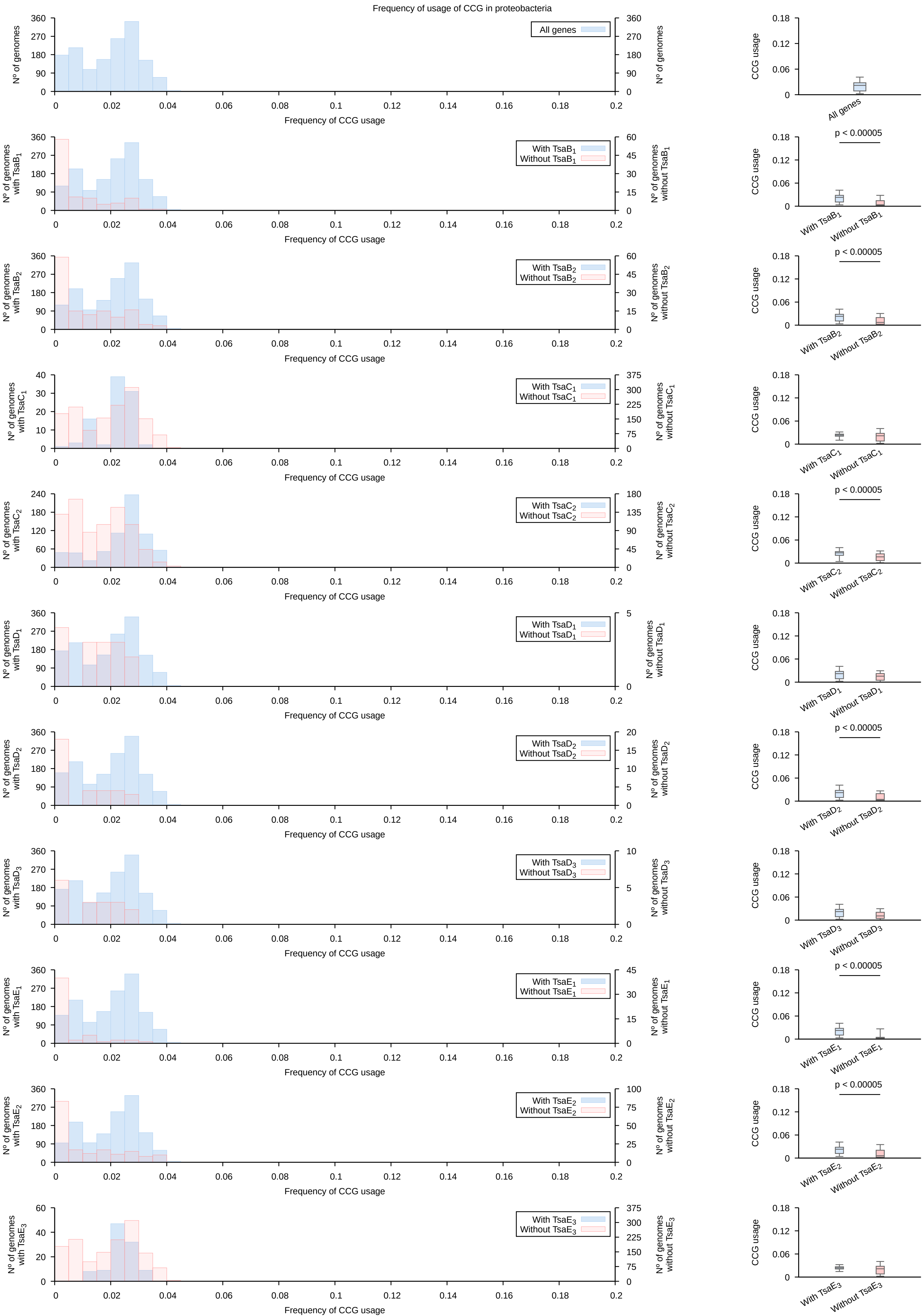

Frequency of usage of CCG in proteobacteria
Nº of genomes
All genes
Frequency of CCG usage
CCG usage
Nº of genomes with TsaB$_1$
Nº of genomes without TsaB$_1$
With TsaB$_1$
Without TsaB$_1$
p < 0.00005
Nº of genomes with TsaB$_2$
Nº of genomes without TsaB$_2$
With TsaB$_2$
Without TsaB$_2$
p < 0.00005
Nº of genomes with TsaC$_1$
Nº of genomes without TsaC$_1$
With TsaC$_1$
Without TsaC$_1$
Nº of genomes with TsaC$_2$
Nº of genomes without TsaC$_2$
With TsaC$_2$
Without TsaC$_2$
p < 0.00005
Nº of genomes with TsaD$_1$
Nº of genomes without TsaD$_1$
With TsaD$_1$
Without TsaD$_1$
Nº of genomes with TsaD$_2$
Nº of genomes without TsaD$_2$
With TsaD$_2$
Without TsaD$_2$
p < 0.00005
Nº of genomes with TsaD$_3$
Nº of genomes without TsaD$_3$
With TsaD$_3$
Without TsaD$_3$
Nº of genomes with TsaE$_1$
Nº of genomes without TsaE$_1$
With TsaE$_1$
Without TsaE$_1$
p < 0.00005
Nº of genomes with TsaE$_2$
Nº of genomes without TsaE$_2$
With TsaE$_2$
Without TsaE$_2$
p < 0.00005
Nº of genomes with TsaE$_3$
Nº of genomes without TsaE$_3$
With TsaE$_3$
Without TsaE$_3$

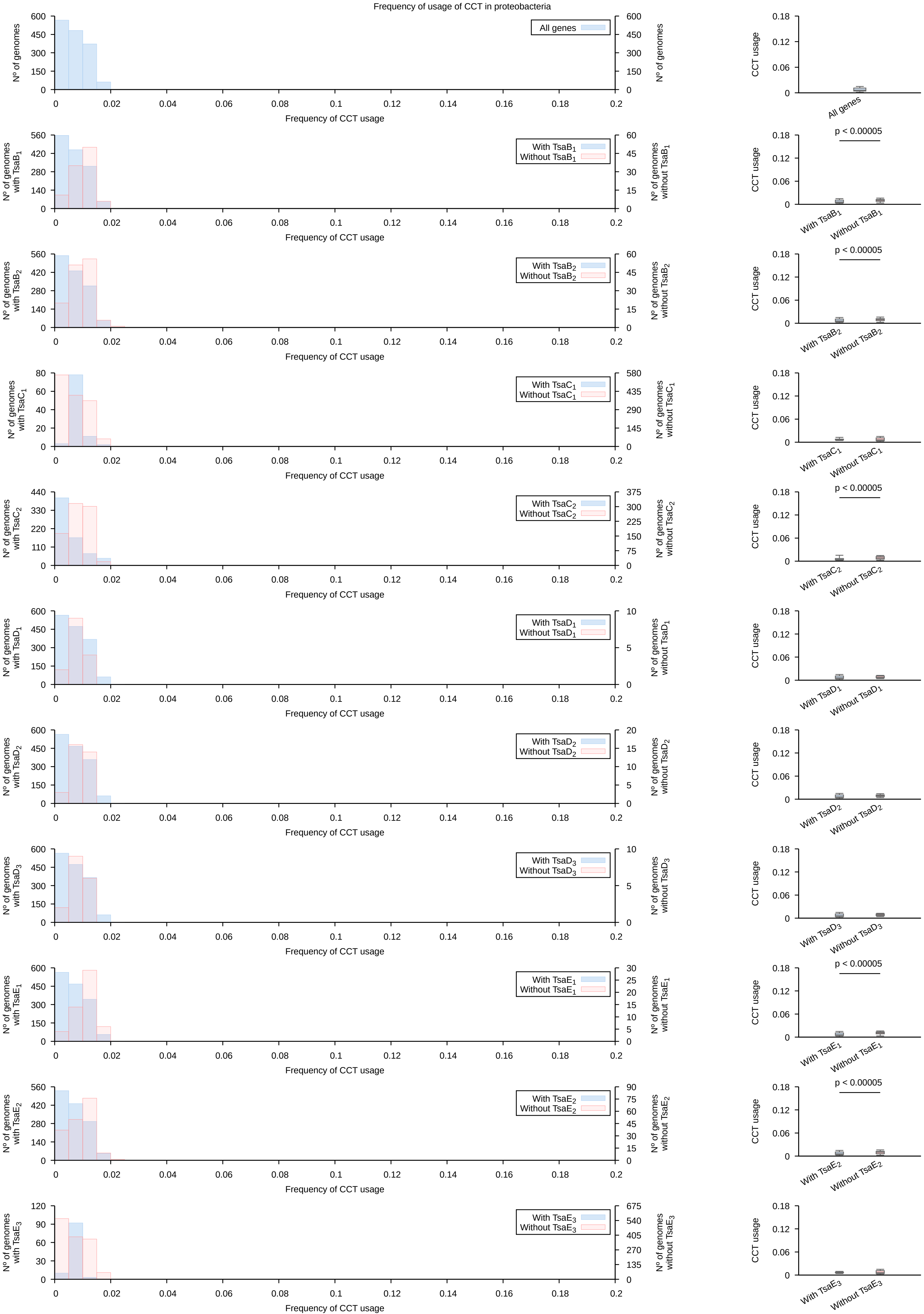
Frequency of usage of CCT in proteobacteria
All genes
Nº of genomes
Frequency of CCT usage
CCT usage
With TsaB$_1$
Without TsaB$_1$
Nº of genomes with TsaB$_1$
Nº of genomes without TsaB$_1$
p < 0.00005
With TsaB$_2$
Without TsaB$_2$
Nº of genomes with TsaB$_2$
Nº of genomes without TsaB$_2$
p < 0.00005
With TsaC$_1$
Without TsaC$_1$
Nº of genomes with TsaC$_1$
Nº of genomes without TsaC$_1$
With TsaC$_2$
Without TsaC$_2$
Nº of genomes with TsaC$_2$
Nº of genomes without TsaC$_2$
p < 0.00005
With TsaD$_1$
Without TsaD$_1$
Nº of genomes with TsaD$_1$
Nº of genomes without TsaD$_1$
With TsaD$_2$
Without TsaD$_2$
Nº of genomes with TsaD$_2$
Nº of genomes without TsaD$_2$
With TsaD$_3$
Without TsaD$_3$
Nº of genomes with TsaD$_3$
Nº of genomes without TsaD$_3$
With TsaE$_1$
Without TsaE$_1$
Nº of genomes with TsaE$_1$
Nº of genomes without TsaE$_1$
p < 0.00005
With TsaE$_2$
Without TsaE$_2$
Nº of genomes with TsaE$_2$
Nº of genomes without TsaE$_2$
p < 0.00005
With TsaE$_3$
Without TsaE$_3$
Nº of genomes with TsaE$_3$
Nº of genomes without TsaE$_3$

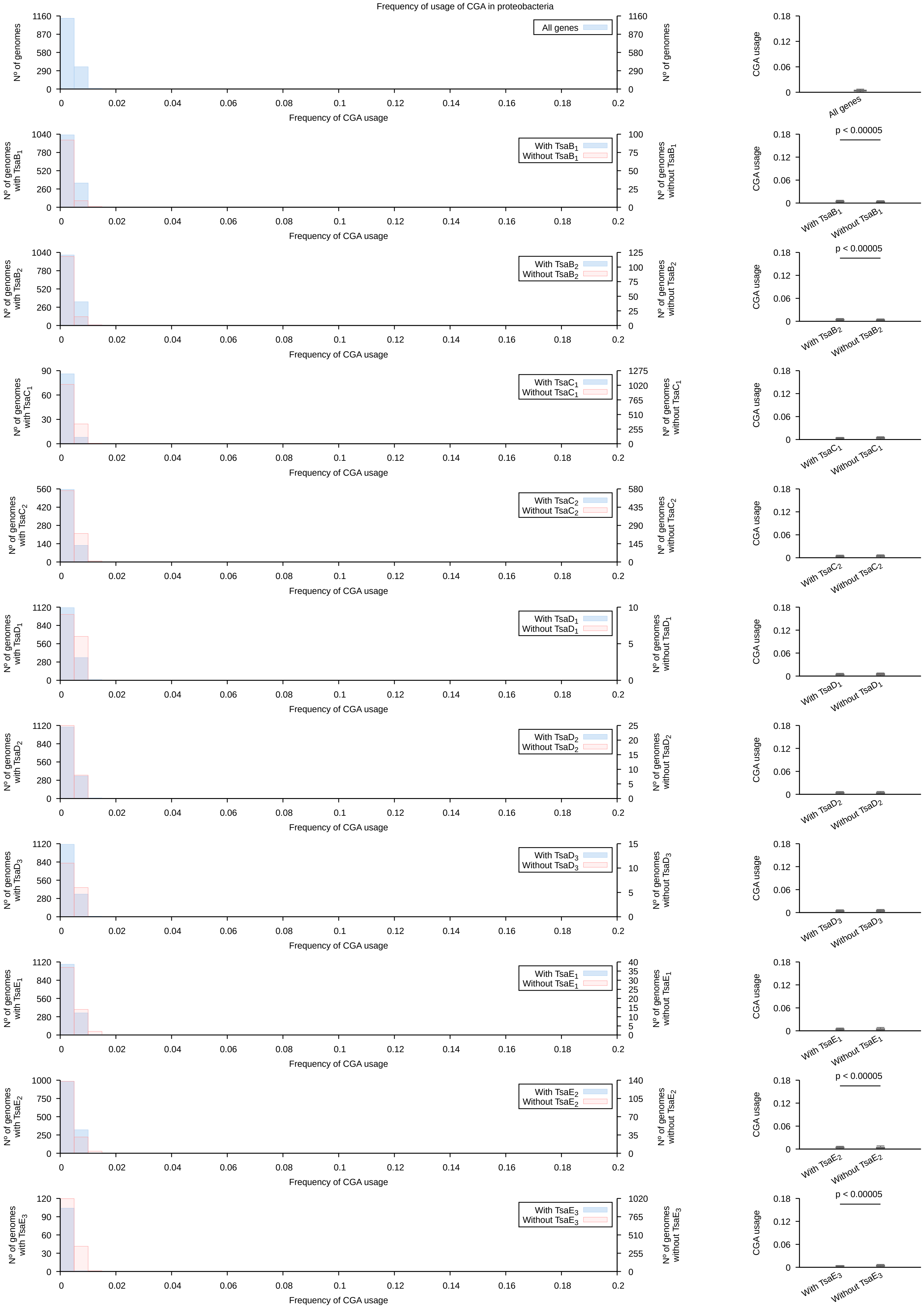
Frequency of usage of CGA in proteobacteria
Nº of genomes
All genes
Frequency of CGA usage
CGA usage
Nº of genomes with TsaB$_1$
Nº of genomes without TsaB$_1$
With TsaB$_1$
Without TsaB$_1$
p < 0.00005
Nº of genomes with TsaB$_2$
Nº of genomes without TsaB$_2$
With TsaB$_2$
Without TsaB$_2$
p < 0.00005
Nº of genomes with TsaC$_1$
Nº of genomes without TsaC$_1$
With TsaC$_1$
Without TsaC$_1$
Nº of genomes with TsaC$_2$
Nº of genomes without TsaC$_2$
With TsaC$_2$
Without TsaC$_2$
Nº of genomes with TsaD$_1$
Nº of genomes without TsaD$_1$
With TsaD$_1$
Without TsaD$_1$
Nº of genomes with TsaD$_2$
Nº of genomes without TsaD$_2$
With TsaD$_2$
Without TsaD$_2$
Nº of genomes with TsaD$_3$
Nº of genomes without TsaD$_3$
With TsaD$_3$
Without TsaD$_3$
Nº of genomes with TsaE$_1$
Nº of genomes without TsaE$_1$
With TsaE$_1$
Without TsaE$_1$
Nº of genomes with TsaE$_2$
Nº of genomes without TsaE$_2$
With TsaE$_2$
Without TsaE$_2$
p < 0.00005
Nº of genomes with TsaE$_3$
Nº of genomes without TsaE$_3$
With TsaE$_3$
Without TsaE$_3$
p < 0.00005

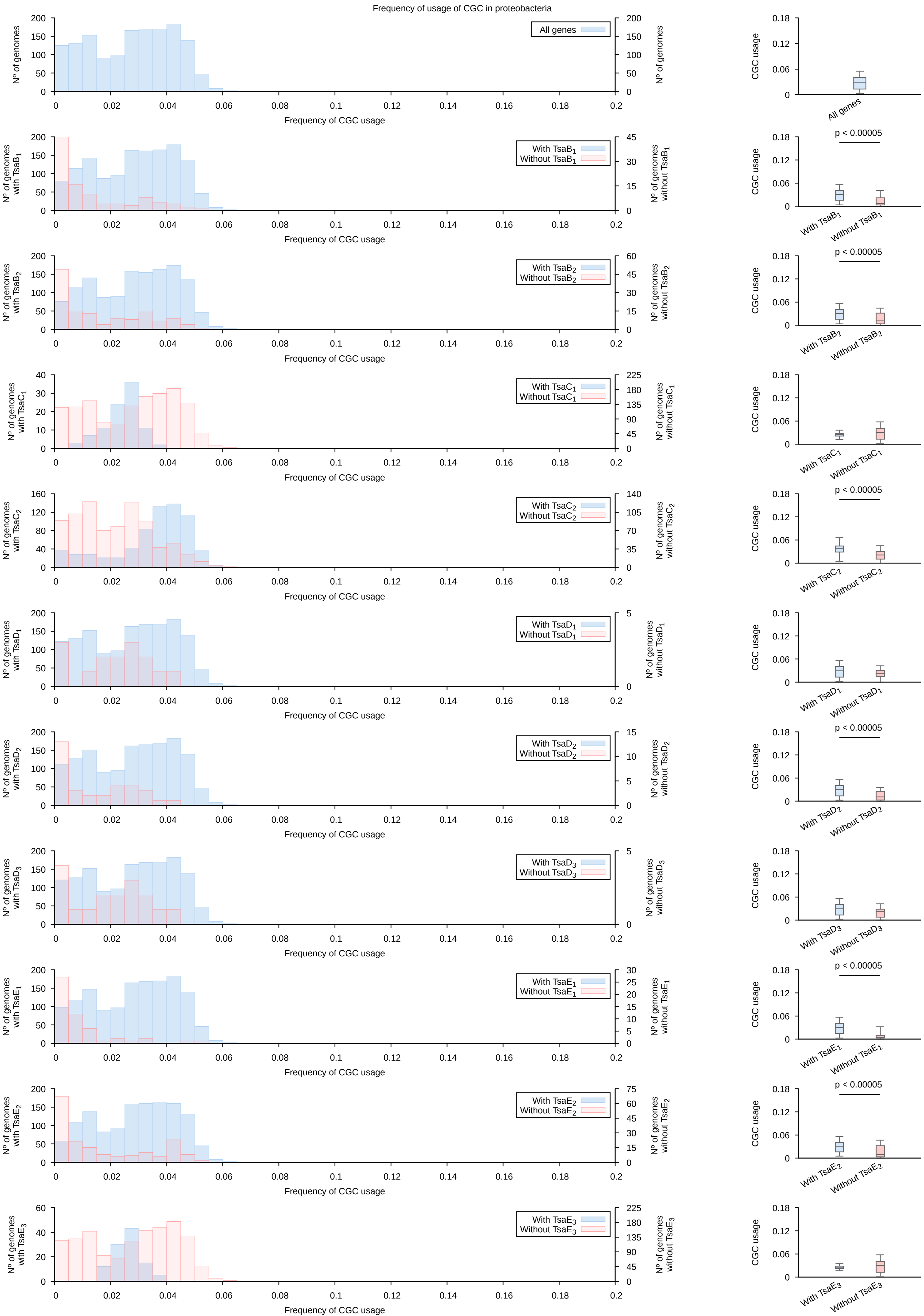
Frequency of usage of CGC in proteobacteria
All genes
Nº of genomes
Frequency of CGC usage
CGC usage
With TsaB$_1$
Without TsaB$_1$
Nº of genomes with TsaB$_1$
Nº of genomes without TsaB$_1$
p < 0.00005
With TsaB$_2$
Without TsaB$_2$
Nº of genomes with TsaB$_2$
Nº of genomes without TsaB$_2$
p < 0.00005
With TsaC$_1$
Without TsaC$_1$
Nº of genomes with TsaC$_1$
Nº of genomes without TsaC$_1$
With TsaC$_2$
Without TsaC$_2$
Nº of genomes with TsaC$_2$
Nº of genomes without TsaC$_2$
p < 0.00005
With TsaD$_1$
Without TsaD$_1$
Nº of genomes with TsaD$_1$
Nº of genomes without TsaD$_1$
With TsaD$_2$
Without TsaD$_2$
Nº of genomes with TsaD$_2$
Nº of genomes without TsaD$_2$
p < 0.00005
With TsaD$_3$
Without TsaD$_3$
Nº of genomes with TsaD$_3$
Nº of genomes without TsaD$_3$
With TsaE$_1$
Without TsaE$_1$
Nº of genomes with TsaE$_1$
Nº of genomes without TsaE$_1$
p < 0.00005
With TsaE$_2$
Without TsaE$_2$
Nº of genomes with TsaE$_2$
Nº of genomes without TsaE$_2$
p < 0.00005
With TsaE$_3$
Without TsaE$_3$
Nº of genomes with TsaE$_3$
Nº of genomes without TsaE$_3$

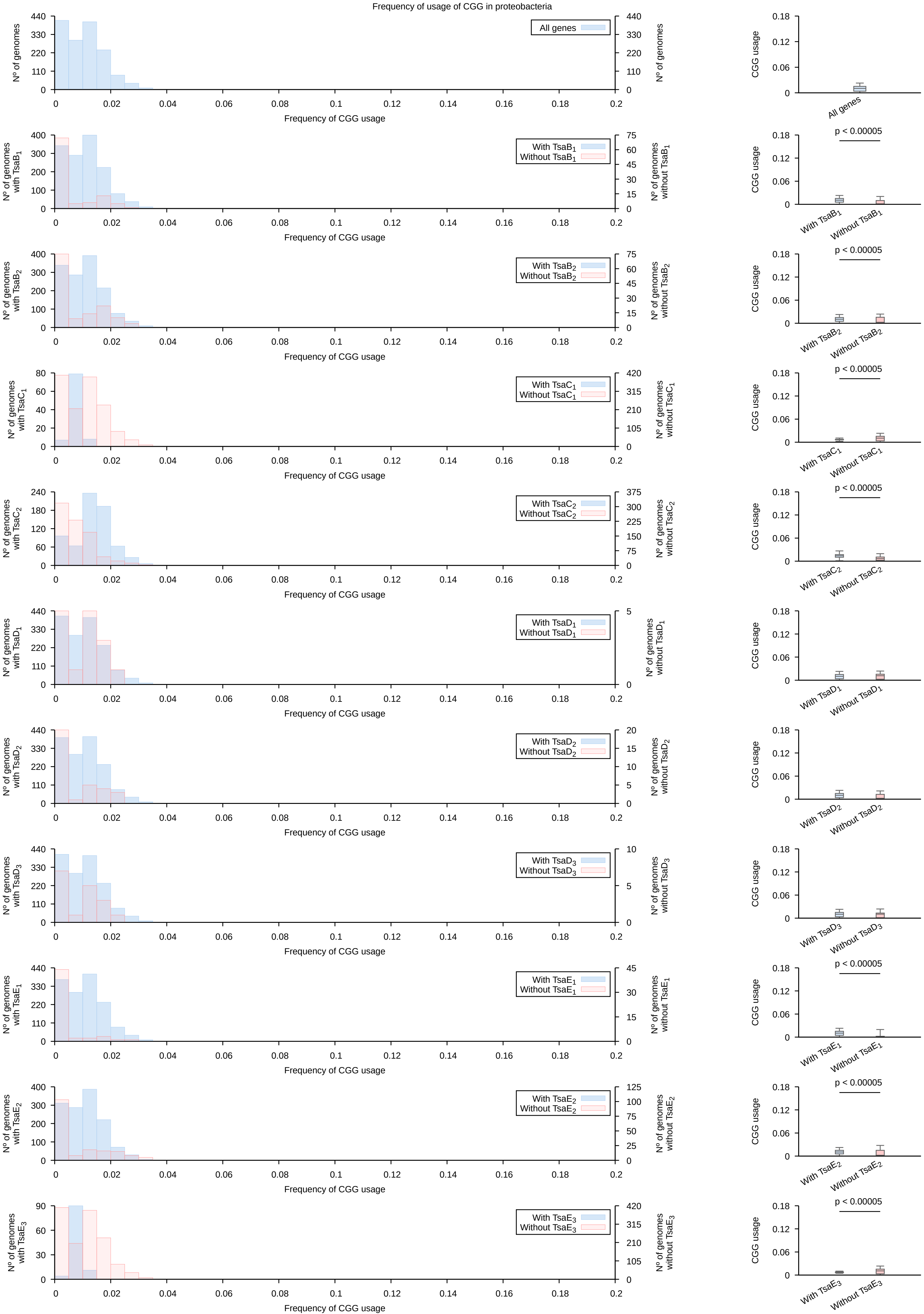

Frequency of usage of CGG in proteobacteria
All genes
Nº of genomes
Frequency of CGG usage
CGG usage
0
0.02
0.04
0.06
0.08
0.1
0.12
0.14
0.16
0.18
0.2
With TsaB$_1$
Without TsaB$_1$
Nº of genomes with TsaB$_1$
Nº of genomes without TsaB$_1$
p < 0.00005
With TsaB$_2$
Without TsaB$_2$
Nº of genomes with TsaB$_2$
Nº of genomes without TsaB$_2$
p < 0.00005
With TsaC$_1$
Without TsaC$_1$
Nº of genomes with TsaC$_1$
Nº of genomes without TsaC$_1$
p < 0.00005
With TsaC$_2$
Without TsaC$_2$
Nº of genomes with TsaC$_2$
Nº of genomes without TsaC$_2$
p < 0.00005
With TsaD$_1$
Without TsaD$_1$
Nº of genomes with TsaD$_1$
Nº of genomes without TsaD$_1$
With TsaD$_2$
Without TsaD$_2$
Nº of genomes with TsaD$_2$
Nº of genomes without TsaD$_2$
With TsaD$_3$
Without TsaD$_3$
Nº of genomes with TsaD$_3$
Nº of genomes without TsaD$_3$
With TsaE$_1$
Without TsaE$_1$
Nº of genomes with TsaE$_1$
Nº of genomes without TsaE$_1$
p < 0.00005
With TsaE$_2$
Without TsaE$_2$
Nº of genomes with TsaE$_2$
Nº of genomes without TsaE$_2$
p < 0.00005
With TsaE$_3$
Without TsaE$_3$
Nº of genomes with TsaE$_3$
Nº of genomes without TsaE$_3$
p < 0.00005

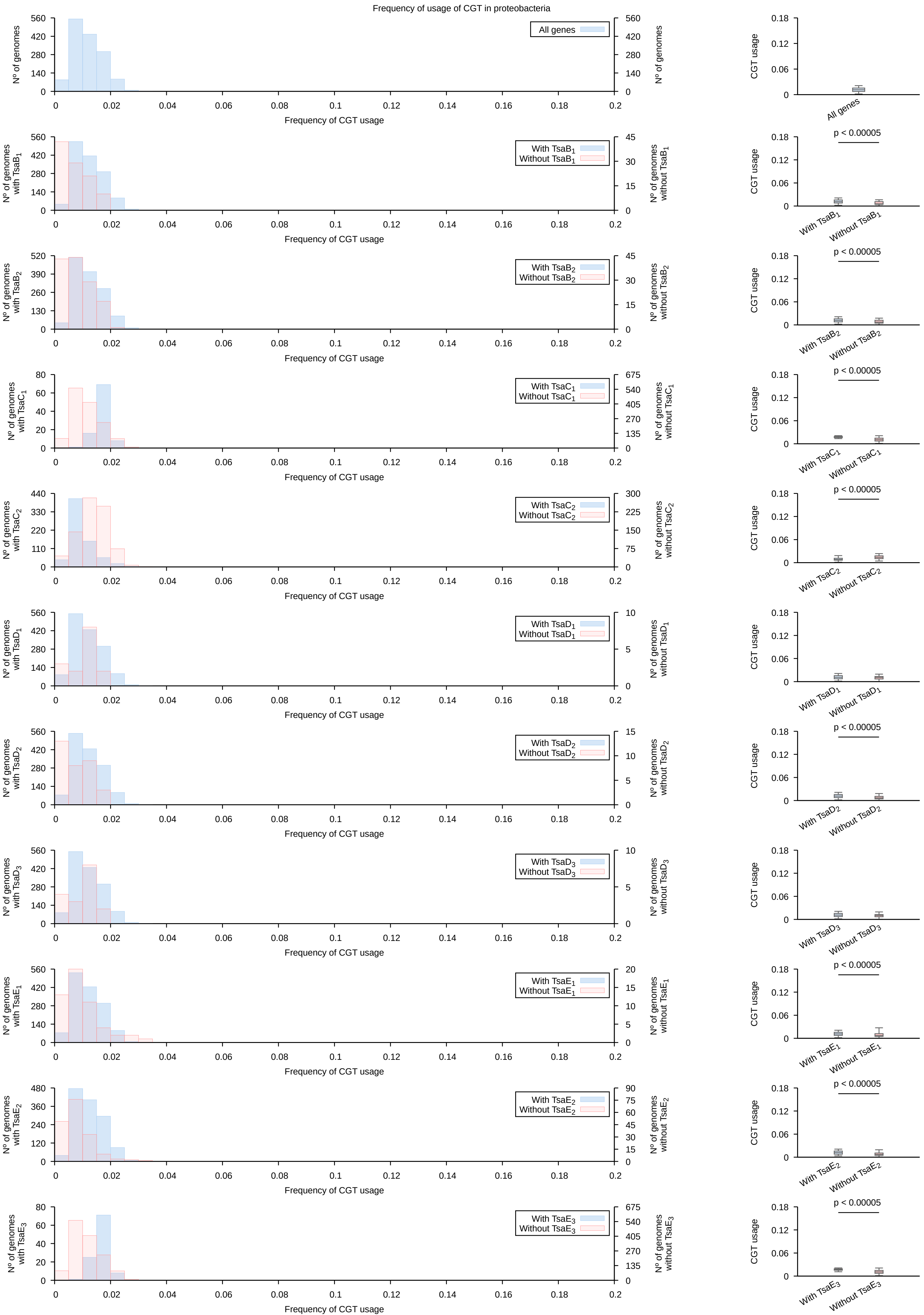
Frequency of usage of CGT in proteobacteria
Nº of genomes
All genes
Frequency of CGT usage
CGT usage
Nº of genomes with TsaB$_1$
Nº of genomes without TsaB$_1$
With TsaB$_1$
Without TsaB$_1$
p < 0.00005
Nº of genomes with TsaB$_2$
Nº of genomes without TsaB$_2$
With TsaB$_2$
Without TsaB$_2$
p < 0.00005
Nº of genomes with TsaC$_1$
Nº of genomes without TsaC$_1$
With TsaC$_1$
Without TsaC$_1$
p < 0.00005
Nº of genomes with TsaC$_2$
Nº of genomes without TsaC$_2$
With TsaC$_2$
Without TsaC$_2$
p < 0.00005
Nº of genomes with TsaD$_1$
Nº of genomes without TsaD$_1$
With TsaD$_1$
Without TsaD$_1$
Nº of genomes with TsaD$_2$
Nº of genomes without TsaD$_2$
With TsaD$_2$
Without TsaD$_2$
p < 0.00005
Nº of genomes with TsaD$_3$
Nº of genomes without TsaD$_3$
With TsaD$_3$
Without TsaD$_3$
Nº of genomes with TsaE$_1$
Nº of genomes without TsaE$_1$
With TsaE$_1$
Without TsaE$_1$
p < 0.00005
Nº of genomes with TsaE$_2$
Nº of genomes without TsaE$_2$
With TsaE$_2$
Without TsaE$_2$
p < 0.00005
Nº of genomes with TsaE$_3$
Nº of genomes without TsaE$_3$
With TsaE$_3$
Without TsaE$_3$
p < 0.00005

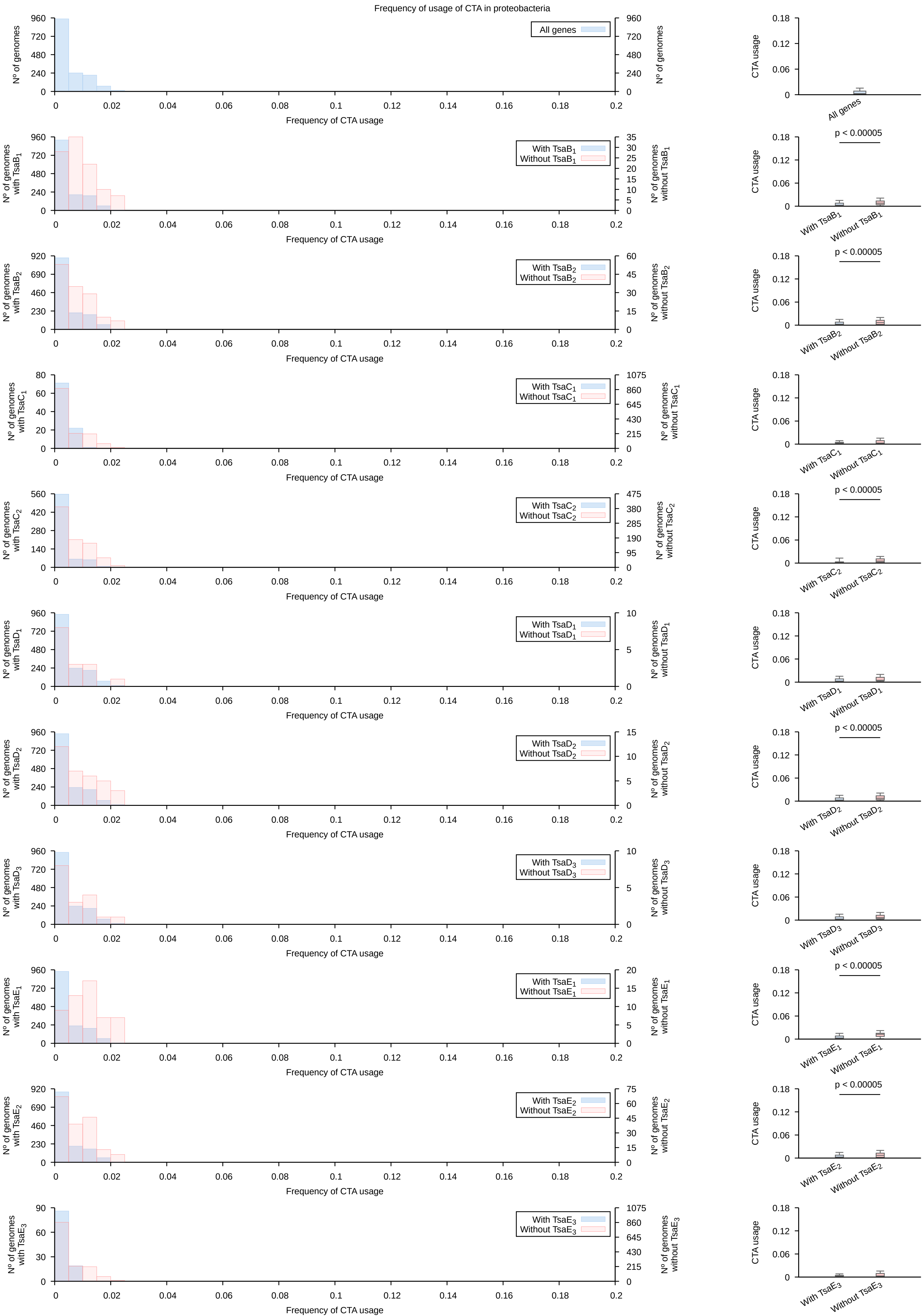
Frequency of usage of CTA in proteobacteria
All genes
Nº of genomes
Frequency of CTA usage
CTA usage
With TsaB1
Without TsaB1
Nº of genomes with TsaB1
Nº of genomes without TsaB1
p < 0.00005
With TsaB2
Without TsaB2
Nº of genomes with TsaB2
Nº of genomes without TsaB2
p < 0.00005
With TsaC1
Without TsaC1
Nº of genomes with TsaC1
Nº of genomes without TsaC1
With TsaC2
Without TsaC2
Nº of genomes with TsaC2
Nº of genomes without TsaC2
p < 0.00005
With TsaD1
Without TsaD1
Nº of genomes with TsaD1
Nº of genomes without TsaD1
With TsaD2
Without TsaD2
Nº of genomes with TsaD2
Nº of genomes without TsaD2
p < 0.00005
With TsaD3
Without TsaD3
Nº of genomes with TsaD3
Nº of genomes without TsaD3
With TsaE1
Without TsaE1
Nº of genomes with TsaE1
Nº of genomes without TsaE1
p < 0.00005
With TsaE2
Without TsaE2
Nº of genomes with TsaE2
Nº of genomes without TsaE2
p < 0.00005
With TsaE3
Without TsaE3
Nº of genomes with TsaE3
Nº of genomes without TsaE3

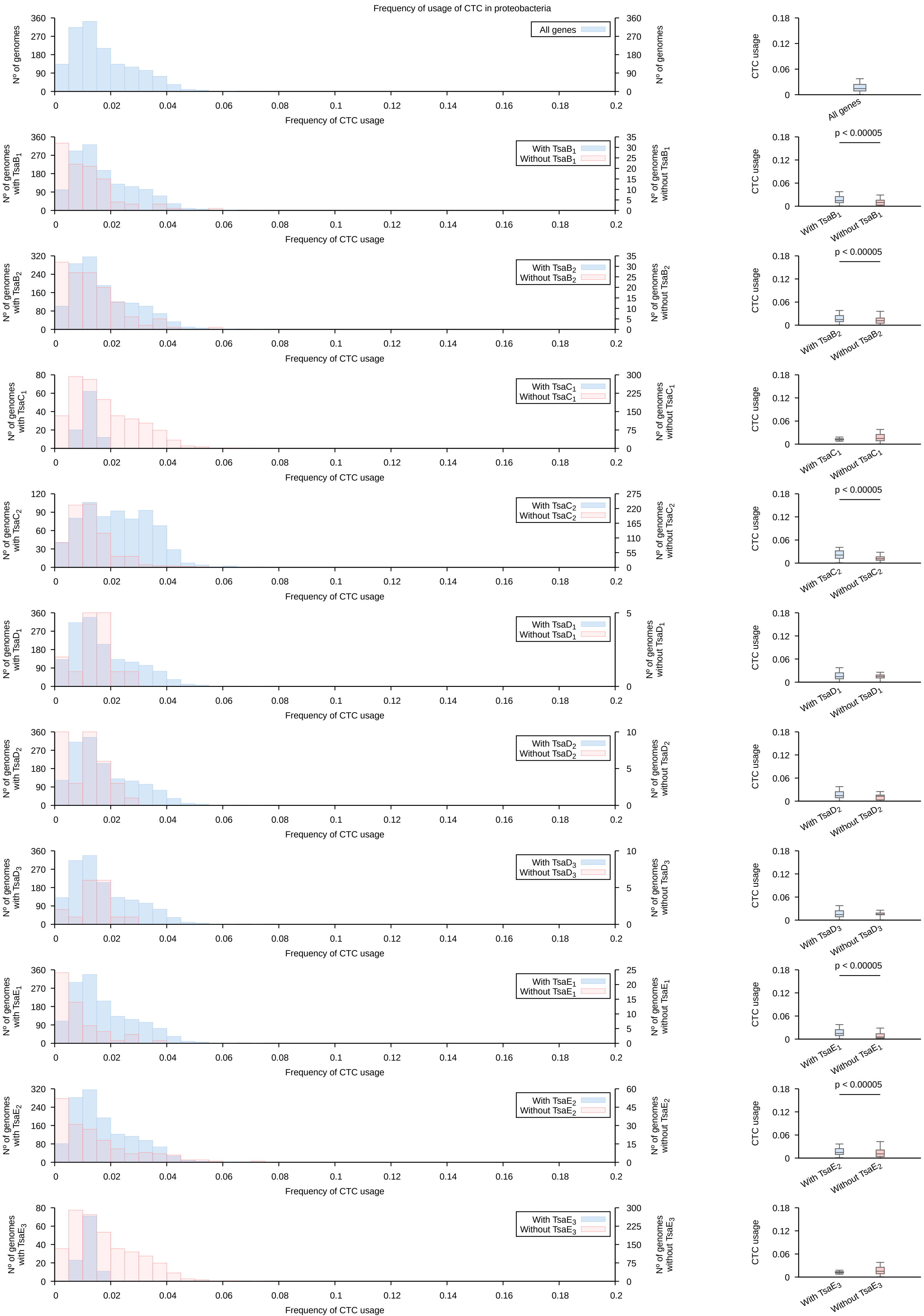
Frequency of usage of CTC in proteobacteria
Nº of genomes
Frequency of CTC usage
All genes
CTC usage
Nº of genomes with TsaB1
Nº of genomes without TsaB1
With TsaB1
Without TsaB1
p < 0.00005
Nº of genomes with TsaB2
Nº of genomes without TsaB2
With TsaB2
Without TsaB2
p < 0.00005
Nº of genomes with TsaC1
Nº of genomes without TsaC1
With TsaC1
Without TsaC1
Nº of genomes with TsaC2
Nº of genomes without TsaC2
With TsaC2
Without TsaC2
p < 0.00005
Nº of genomes with TsaD1
Nº of genomes without TsaD1
With TsaD1
Without TsaD1
Nº of genomes with TsaD2
Nº of genomes without TsaD2
With TsaD2
Without TsaD2
Nº of genomes with TsaD3
Nº of genomes without TsaD3
With TsaD3
Without TsaD3
Nº of genomes with TsaE1
Nº of genomes without TsaE1
With TsaE1
Without TsaE1
p < 0.00005
Nº of genomes with TsaE2
Nº of genomes without TsaE2
With TsaE2
Without TsaE2
p < 0.00005
Nº of genomes with TsaE3
Nº of genomes without TsaE3
With TsaE3
Without TsaE3

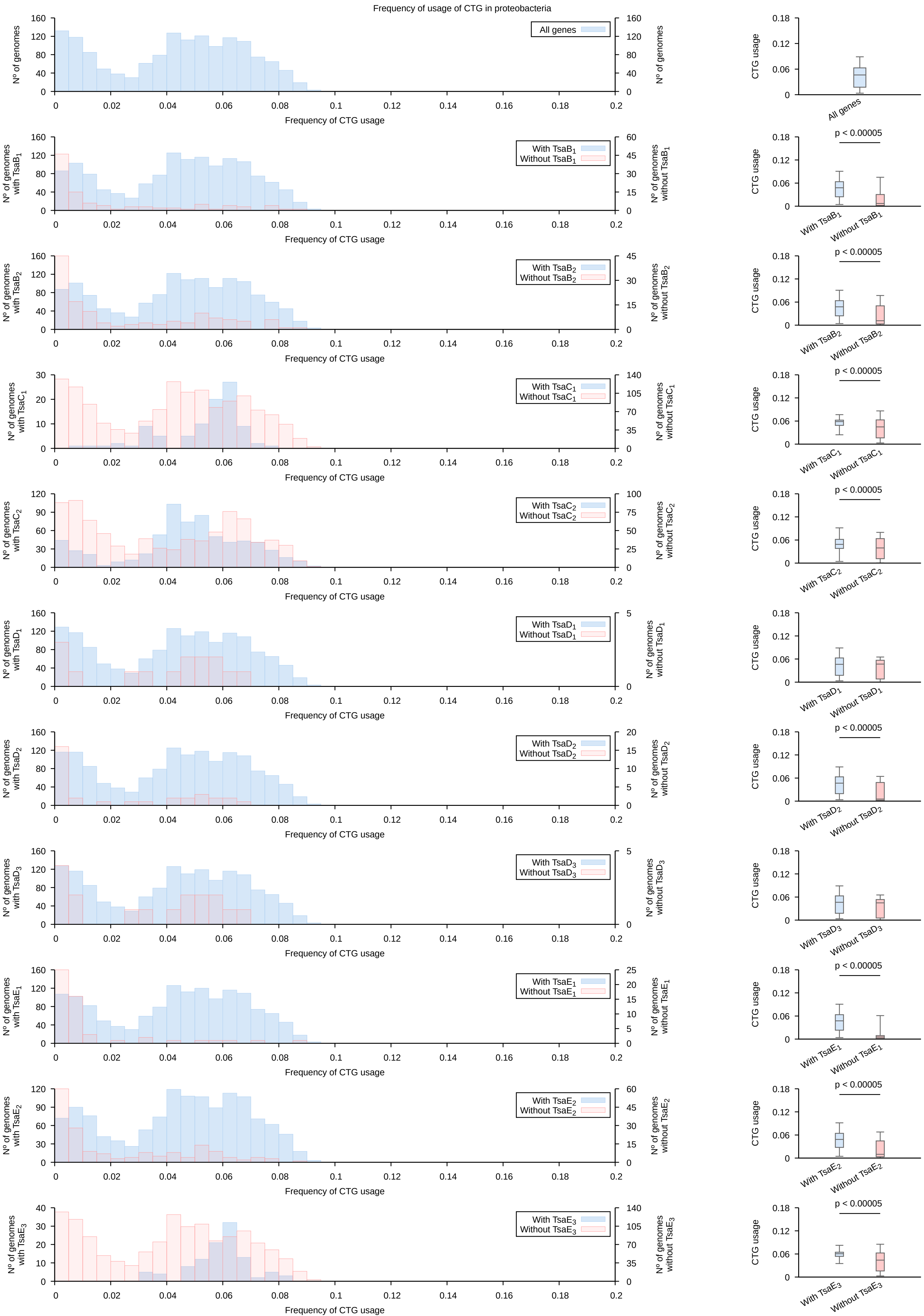

Frequency of usage of CTG in proteobacteria
Nº of genomes
All genes
Frequency of CTG usage
CTG usage
Nº of genomes with $TsaB_1$
Nº of genomes without $TsaB_1$
With $TsaB_1$
Without $TsaB_1$
p < 0.00005
Nº of genomes with $TsaB_2$
Nº of genomes without $TsaB_2$
With $TsaB_2$
Without $TsaB_2$
p < 0.00005
Nº of genomes with $TsaC_1$
Nº of genomes without $TsaC_1$
With $TsaC_1$
Without $TsaC_1$
p < 0.00005
Nº of genomes with $TsaC_2$
Nº of genomes without $TsaC_2$
With $TsaC_2$
Without $TsaC_2$
p < 0.00005
Nº of genomes with $TsaD_1$
Nº of genomes without $TsaD_1$
With $TsaD_1$
Without $TsaD_1$
Nº of genomes with $TsaD_2$
Nº of genomes without $TsaD_2$
With $TsaD_2$
Without $TsaD_2$
p < 0.00005
Nº of genomes with $TsaD_3$
Nº of genomes without $TsaD_3$
With $TsaD_3$
Without $TsaD_3$
Nº of genomes with $TsaE_1$
Nº of genomes without $TsaE_1$
With $TsaE_1$
Without $TsaE_1$
p < 0.00005
Nº of genomes with $TsaE_2$
Nº of genomes without $TsaE_2$
With $TsaE_2$
Without $TsaE_2$
p < 0.00005
Nº of genomes with $TsaE_3$
Nº of genomes without $TsaE_3$
With $TsaE_3$
Without $TsaE_3$
p < 0.00005

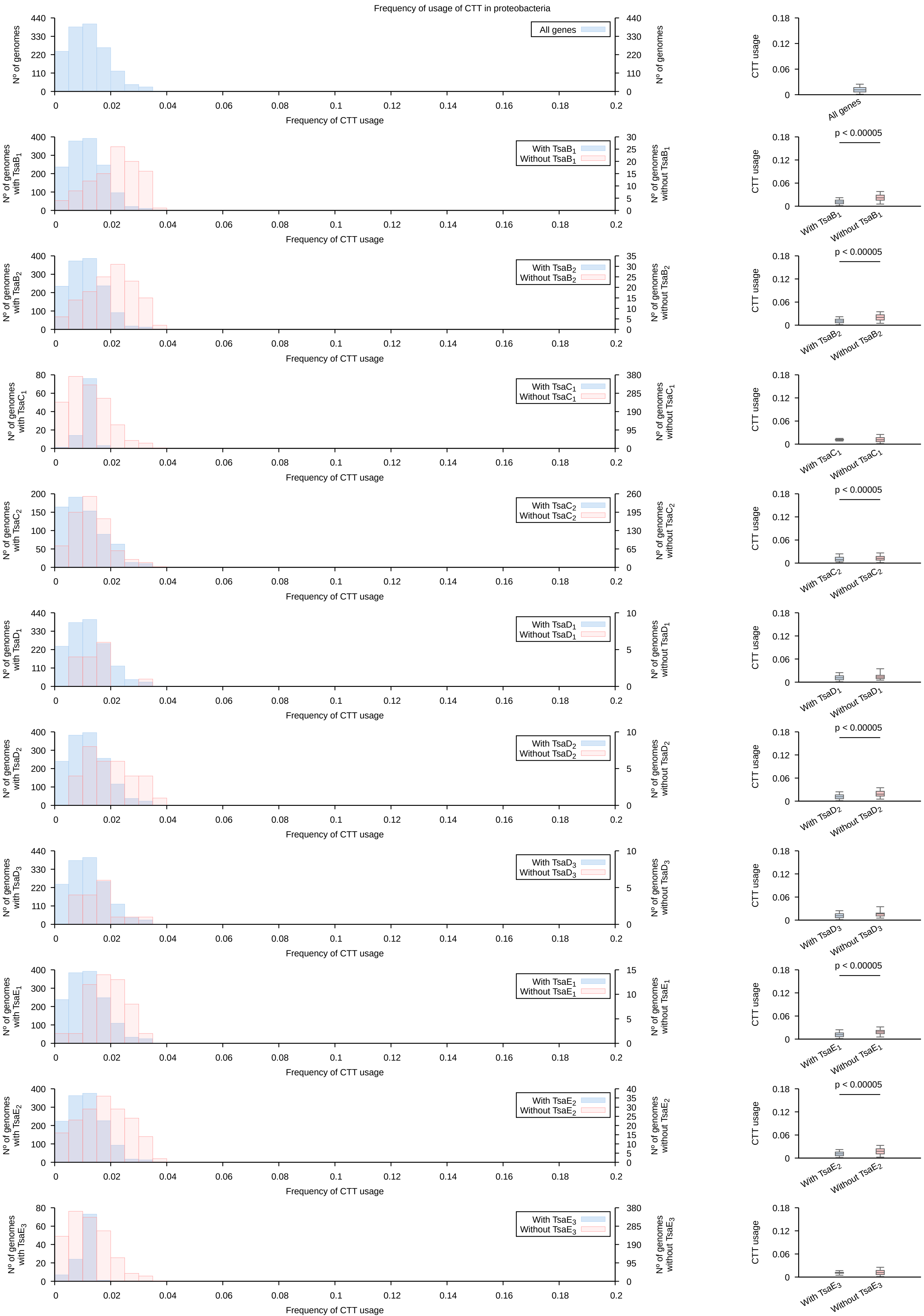
Frequency of usage of CTT in proteobacteria
Nº of genomes
Frequency of CTT usage
CTT usage
All genes
Nº of genomes with TsaB1
Nº of genomes without TsaB1
With TsaB1
Without TsaB1
p < 0.00005
Nº of genomes with TsaB2
Nº of genomes without TsaB2
With TsaB2
Without TsaB2
p < 0.00005
Nº of genomes with TsaC1
Nº of genomes without TsaC1
With TsaC1
Without TsaC1
Nº of genomes with TsaC2
Nº of genomes without TsaC2
With TsaC2
Without TsaC2
p < 0.00005
Nº of genomes with TsaD1
Nº of genomes without TsaD1
With TsaD1
Without TsaD1
Nº of genomes with TsaD2
Nº of genomes without TsaD2
With TsaD2
Without TsaD2
p < 0.00005
Nº of genomes with TsaD3
Nº of genomes without TsaD3
With TsaD3
Without TsaD3
Nº of genomes with TsaE1
Nº of genomes without TsaE1
With TsaE1
Without TsaE1
p < 0.00005
Nº of genomes with TsaE2
Nº of genomes without TsaE2
With TsaE2
Without TsaE2
p < 0.00005
Nº of genomes with TsaE3
Nº of genomes without TsaE3
With TsaE3
Without TsaE3
0
0.02
0.04
0.06
0.08
0.1
0.12
0.14
0.16
0.18
0.2

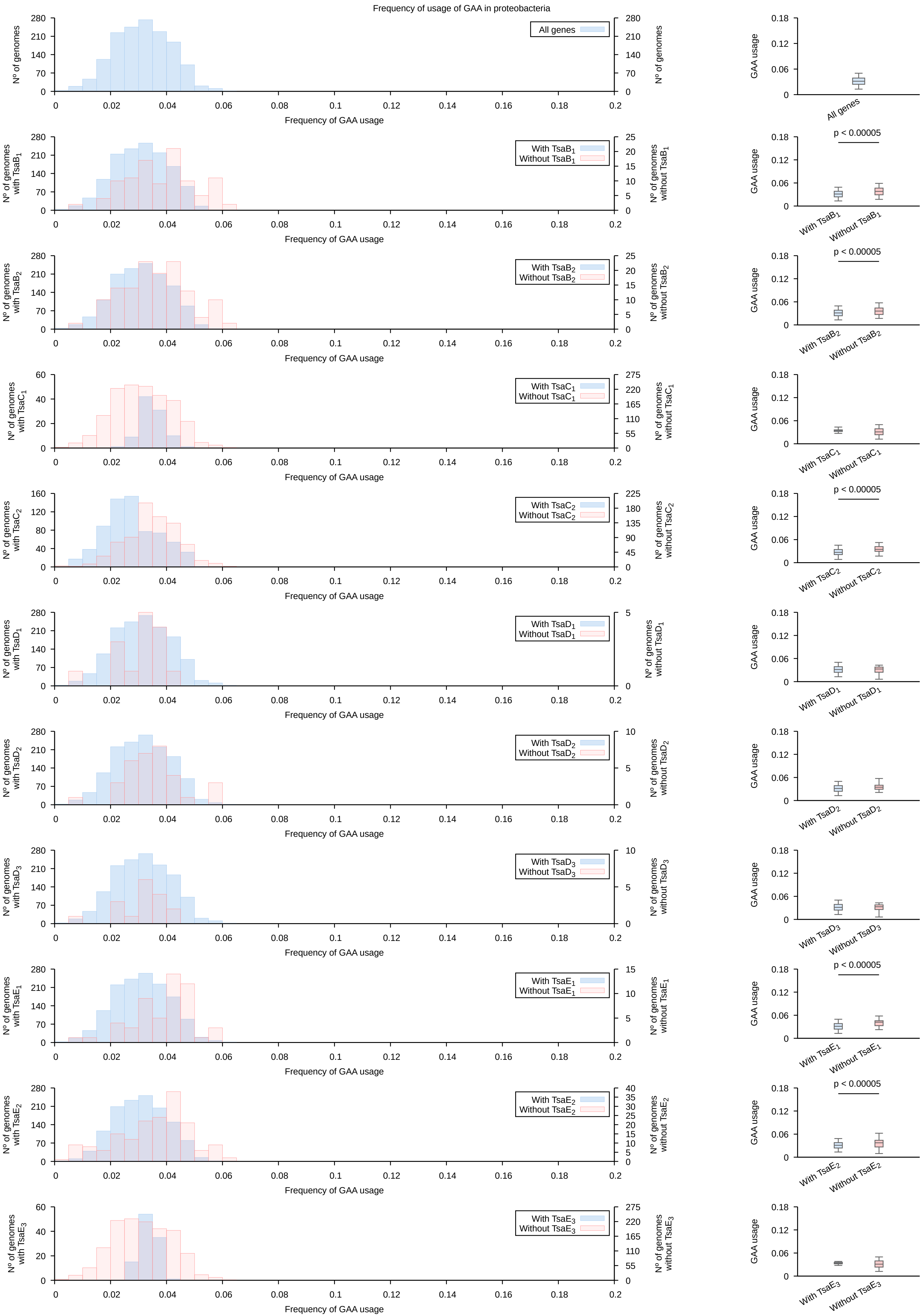
Frequency of usage of GAA in proteobacteria
All genes
With $TsaB_1$
Without $TsaB_1$
With $TsaB_2$
Without $TsaB_2$
With $TsaC_1$
Without $TsaC_1$
With $TsaC_2$
Without $TsaC_2$
With $TsaD_1$
Without $TsaD_1$
With $TsaD_2$
Without $TsaD_2$
With $TsaD_3$
Without $TsaD_3$
With $TsaE_1$
Without $TsaE_1$
With $TsaE_2$
Without $TsaE_2$
With $TsaE_3$
Without $TsaE_3$
Frequency of GAA usage
Nº of genomes
GAA usage
p < 0.00005

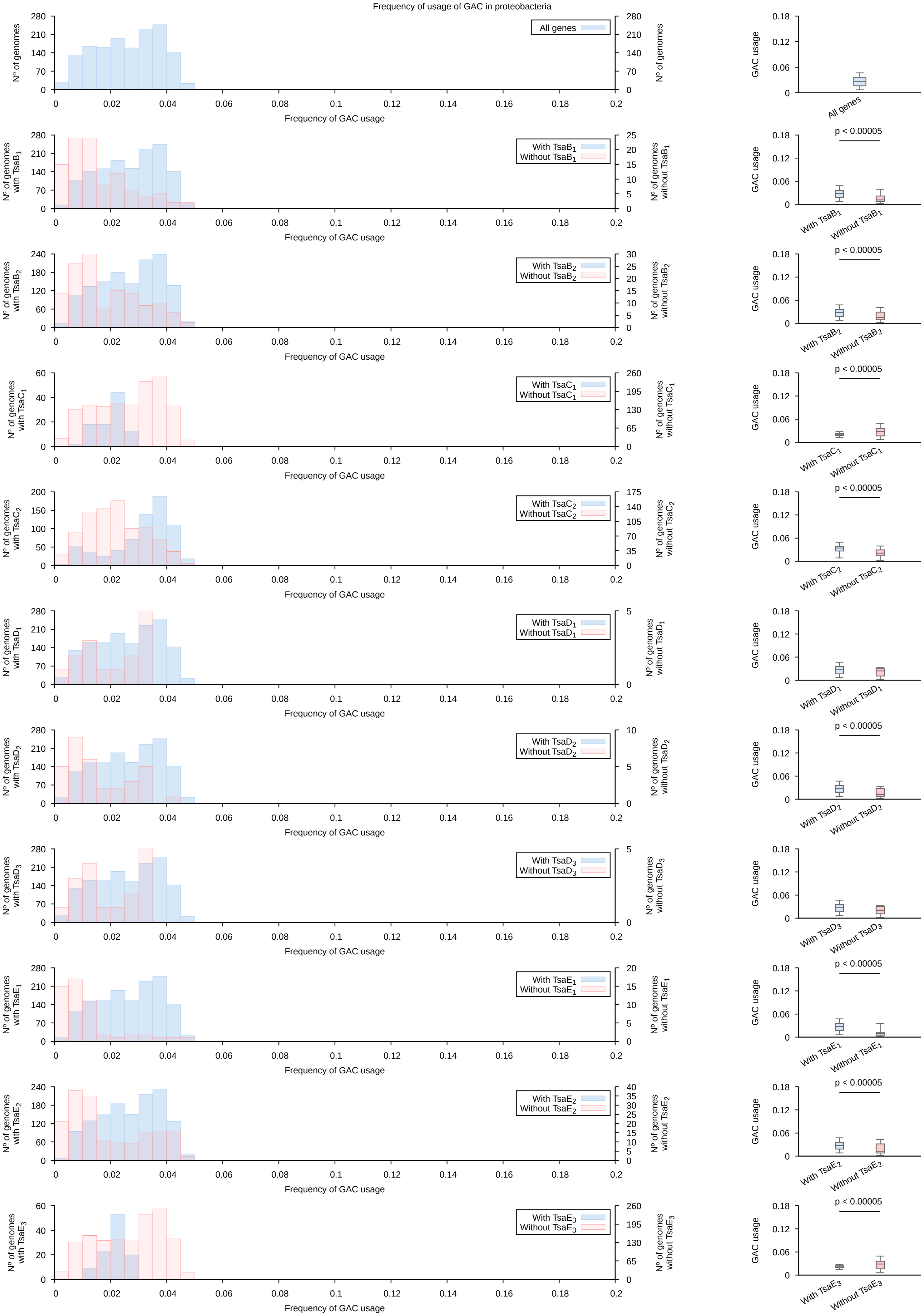
Frequency of usage of GAC in proteobacteria
All genes
With TsaB$_1$
Without TsaB$_1$
With TsaB$_2$
Without TsaB$_2$
With TsaC$_1$
Without TsaC$_1$
With TsaC$_2$
Without TsaC$_2$
With TsaD$_1$
Without TsaD$_1$
With TsaD$_2$
Without TsaD$_2$
With TsaD$_3$
Without TsaD$_3$
With TsaE$_1$
Without TsaE$_1$
With TsaE$_2$
Without TsaE$_2$
With TsaE$_3$
Without TsaE$_3$
Nº of genomes
Frequency of GAC usage
GAC usage
p < 0.00005

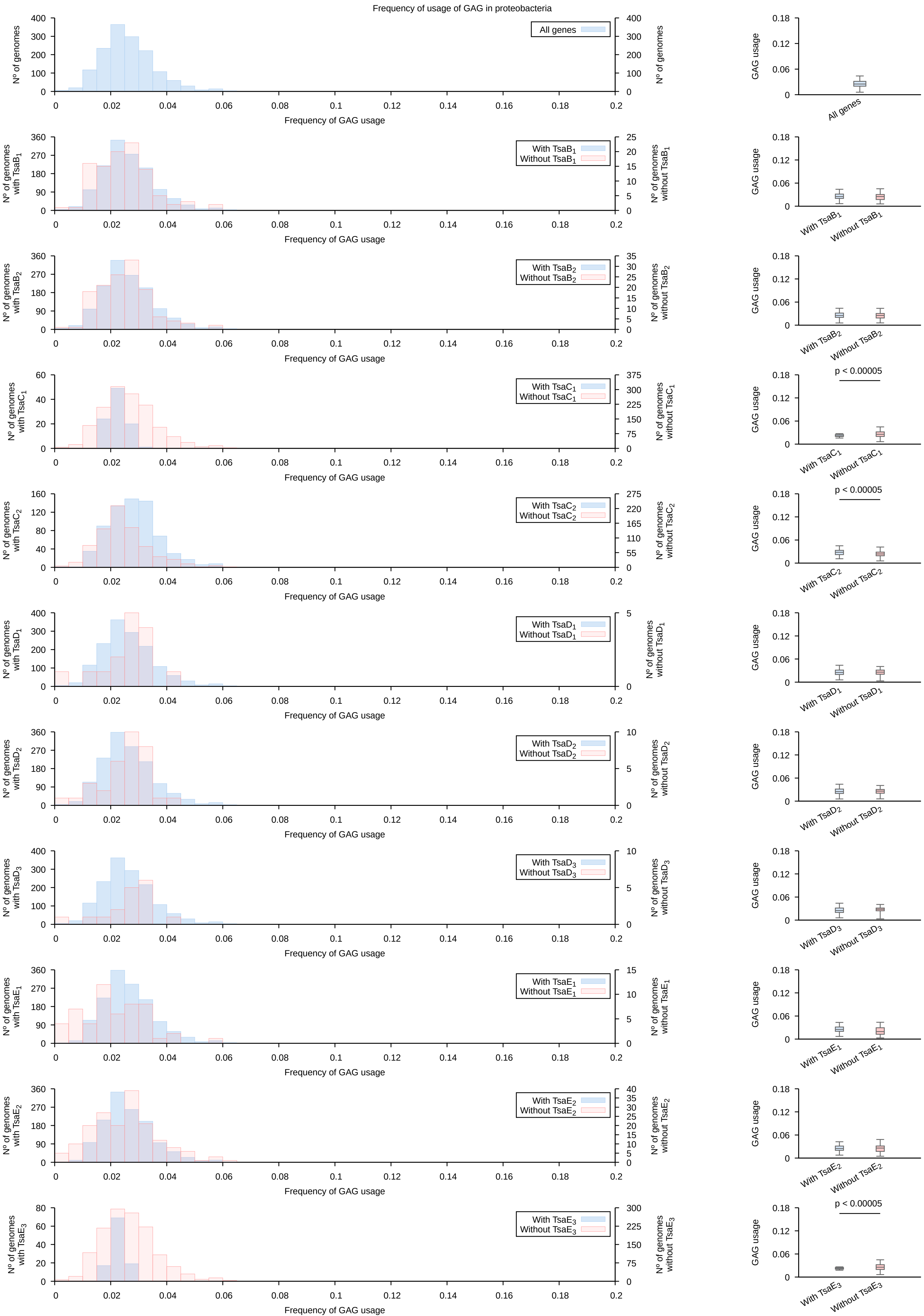

Frequency of usage of GAG in proteobacteria
Nº of genomes
All genes
Frequency of GAG usage
GAG usage
Nº of genomes with TsaB$_1$
Nº of genomes without TsaB$_1$
With TsaB$_1$
Without TsaB$_1$
Nº of genomes with TsaB$_2$
Nº of genomes without TsaB$_2$
With TsaB$_2$
Without TsaB$_2$
Nº of genomes with TsaC$_1$
Nº of genomes without TsaC$_1$
With TsaC$_1$
Without TsaC$_1$
p < 0.00005
Nº of genomes with TsaC$_2$
Nº of genomes without TsaC$_2$
With TsaC$_2$
Without TsaC$_2$
p < 0.00005
Nº of genomes with TsaD$_1$
Nº of genomes without TsaD$_1$
With TsaD$_1$
Without TsaD$_1$
Nº of genomes with TsaD$_2$
Nº of genomes without TsaD$_2$
With TsaD$_2$
Without TsaD$_2$
Nº of genomes with TsaD$_3$
Nº of genomes without TsaD$_3$
With TsaD$_3$
Without TsaD$_3$
Nº of genomes with TsaE$_1$
Nº of genomes without TsaE$_1$
With TsaE$_1$
Without TsaE$_1$
Nº of genomes with TsaE$_2$
Nº of genomes without TsaE$_2$
With TsaE$_2$
Without TsaE$_2$
Nº of genomes with TsaE$_3$
Nº of genomes without TsaE$_3$
With TsaE$_3$
Without TsaE$_3$
p < 0.00005

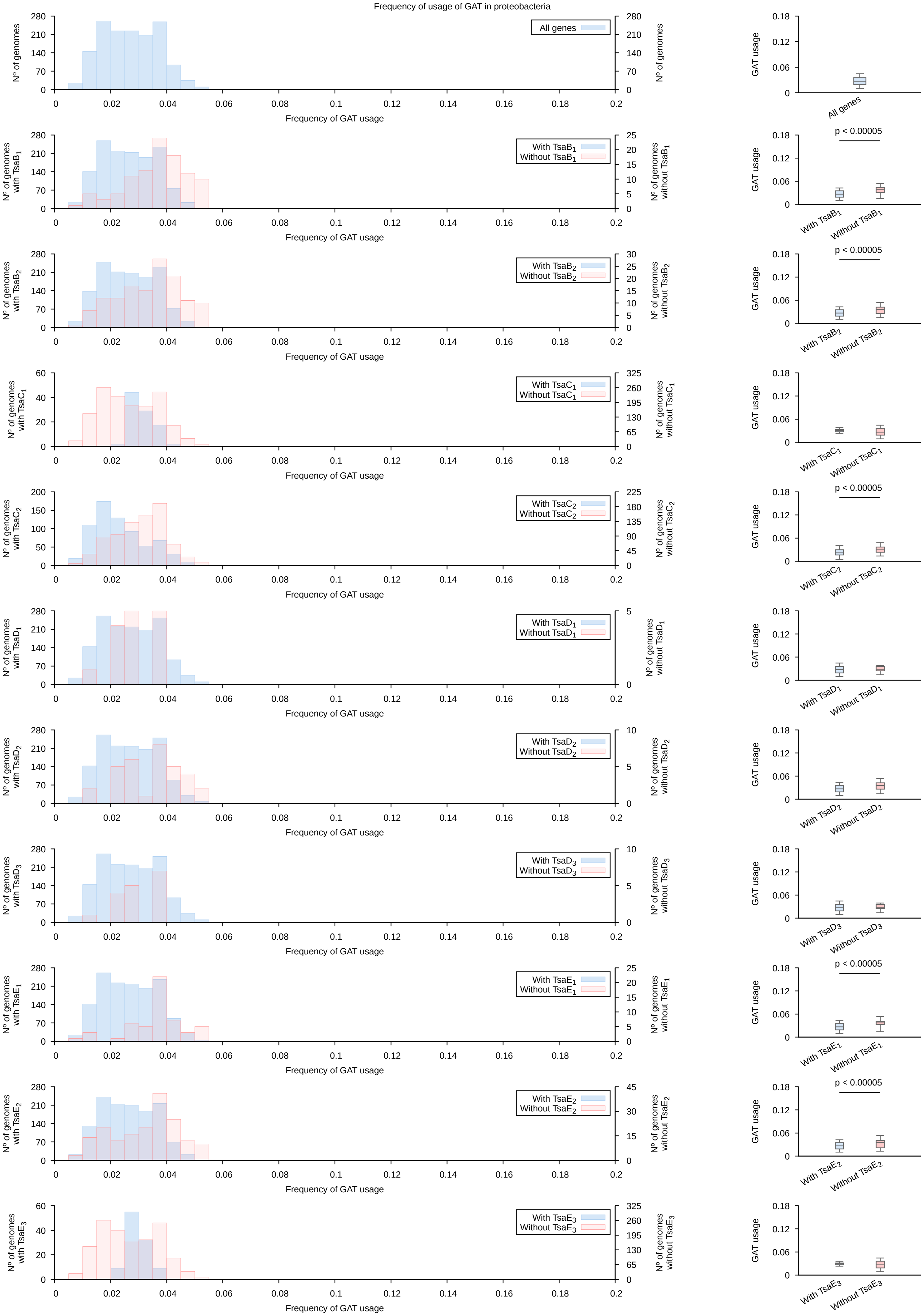
Frequency of usage of GAT in proteobacteria
Nº of genomes
All genes
Frequency of GAT usage
GAT usage
0
0.02
0.04
0.06
0.08
0.1
0.12
0.14
0.16
0.18
0.2
Nº of genomes with TsaB1
Nº of genomes without TsaB1
With TsaB1
Without TsaB1
p < 0.00005
Nº of genomes with TsaB2
Nº of genomes without TsaB2
With TsaB2
Without TsaB2
p < 0.00005
Nº of genomes with TsaC1
Nº of genomes without TsaC1
With TsaC1
Without TsaC1
Nº of genomes with TsaC2
Nº of genomes without TsaC2
With TsaC2
Without TsaC2
p < 0.00005
Nº of genomes with TsaD1
Nº of genomes without TsaD1
With TsaD1
Without TsaD1
Nº of genomes with TsaD2
Nº of genomes without TsaD2
With TsaD2
Without TsaD2
Nº of genomes with TsaD3
Nº of genomes without TsaD3
With TsaD3
Without TsaD3
Nº of genomes with TsaE1
Nº of genomes without TsaE1
With TsaE1
Without TsaE1
p < 0.00005
Nº of genomes with TsaE2
Nº of genomes without TsaE2
With TsaE2
Without TsaE2
p < 0.00005
Nº of genomes with TsaE3
Nº of genomes without TsaE3
With TsaE3
Without TsaE3

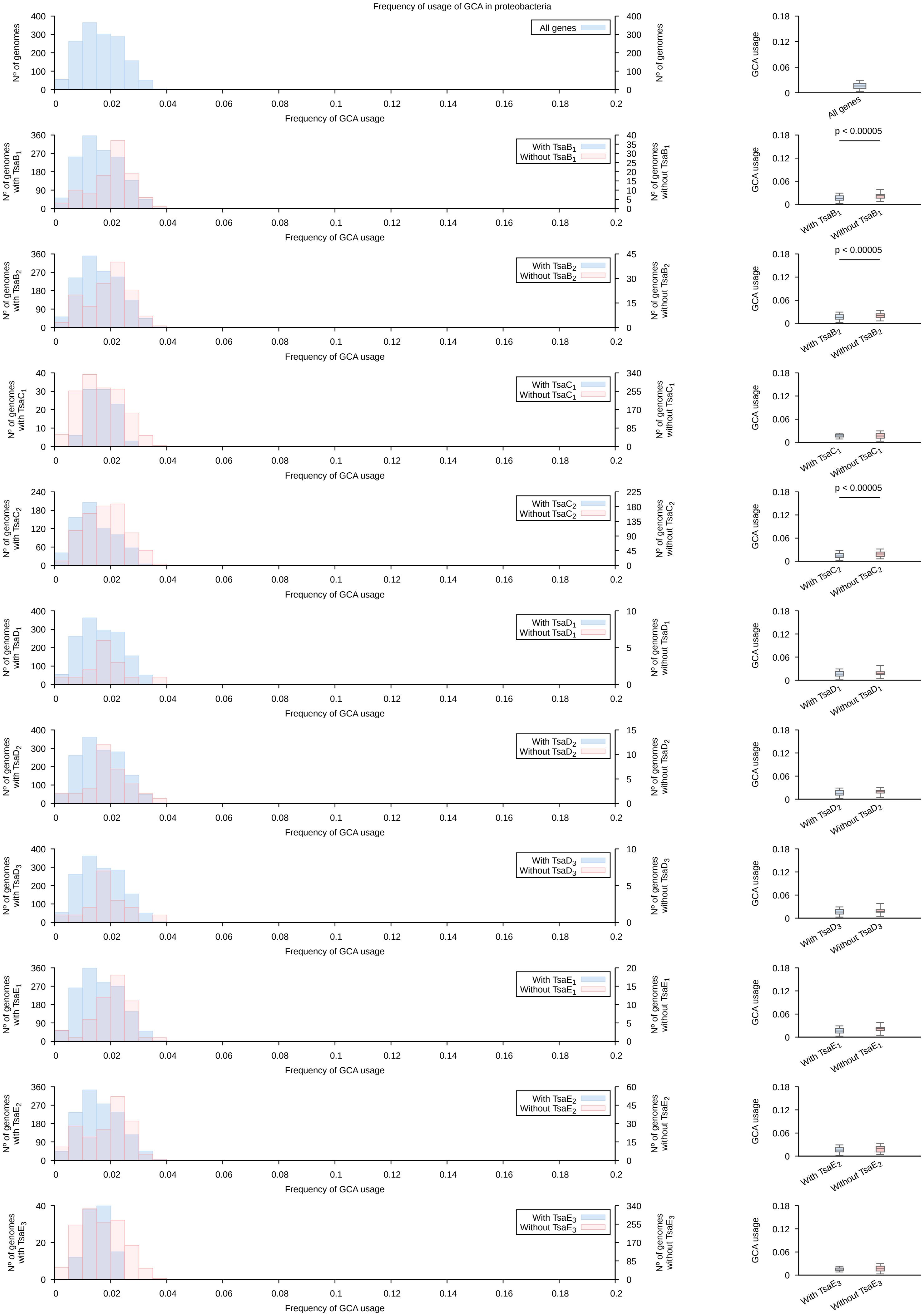
Frequency of usage of GCA in proteobacteria
All genes
Nº of genomes
Frequency of GCA usage
GCA usage
With TsaB1
Without TsaB1
Nº of genomes with TsaB1
Nº of genomes without TsaB1
p < 0.00005
With TsaB2
Without TsaB2
Nº of genomes with TsaB2
Nº of genomes without TsaB2
p < 0.00005
With TsaC1
Without TsaC1
Nº of genomes with TsaC1
Nº of genomes without TsaC1
With TsaC2
Without TsaC2
Nº of genomes with TsaC2
Nº of genomes without TsaC2
p < 0.00005
With TsaD1
Without TsaD1
Nº of genomes with TsaD1
Nº of genomes without TsaD1
With TsaD2
Without TsaD2
Nº of genomes with TsaD2
Nº of genomes without TsaD2
With TsaD3
Without TsaD3
Nº of genomes with TsaD3
Nº of genomes without TsaD3
With TsaE1
Without TsaE1
Nº of genomes with TsaE1
Nº of genomes without TsaE1
With TsaE2
Without TsaE2
Nº of genomes with TsaE2
Nº of genomes without TsaE2
With TsaE3
Without TsaE3
Nº of genomes with TsaE3
Nº of genomes without TsaE3

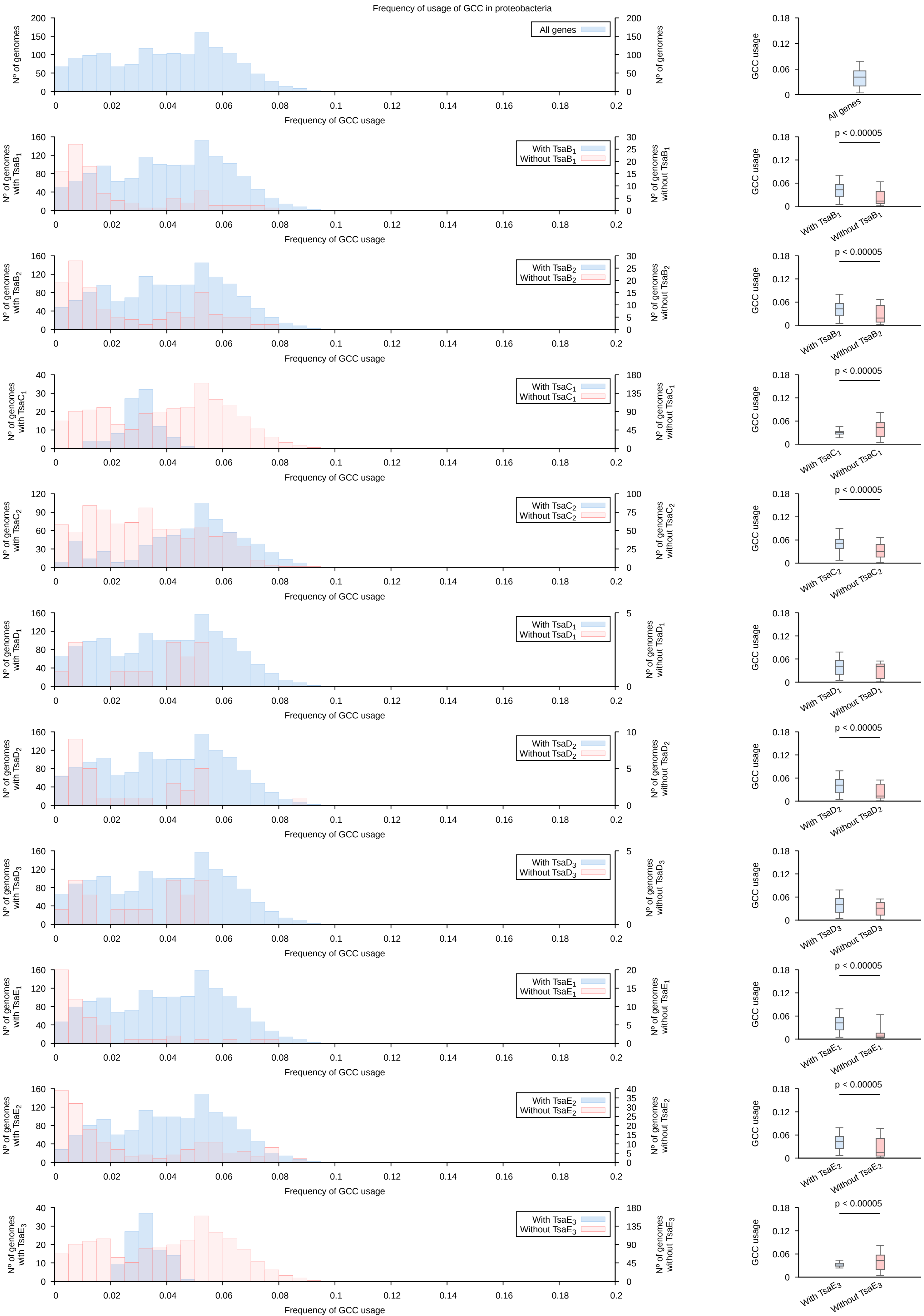
Frequency of usage of GCC in proteobacteria
All genes
Nº of genomes
Frequency of GCC usage
GCC usage
With TsaB$_1$
Without TsaB$_1$
Nº of genomes with TsaB$_1$
Nº of genomes without TsaB$_1$
p < 0.00005
With TsaB$_2$
Without TsaB$_2$
Nº of genomes with TsaB$_2$
Nº of genomes without TsaB$_2$
p < 0.00005
With TsaC$_1$
Without TsaC$_1$
Nº of genomes with TsaC$_1$
Nº of genomes without TsaC$_1$
p < 0.00005
With TsaC$_2$
Without TsaC$_2$
Nº of genomes with TsaC$_2$
Nº of genomes without TsaC$_2$
p < 0.00005
With TsaD$_1$
Without TsaD$_1$
Nº of genomes with TsaD$_1$
Nº of genomes without TsaD$_1$
With TsaD$_2$
Without TsaD$_2$
Nº of genomes with TsaD$_2$
Nº of genomes without TsaD$_2$
p < 0.00005
With TsaD$_3$
Without TsaD$_3$
Nº of genomes with TsaD$_3$
Nº of genomes without TsaD$_3$
With TsaE$_1$
Without TsaE$_1$
Nº of genomes with TsaE$_1$
Nº of genomes without TsaE$_1$
p < 0.00005
With TsaE$_2$
Without TsaE$_2$
Nº of genomes with TsaE$_2$
Nº of genomes without TsaE$_2$
p < 0.00005
With TsaE$_3$
Without TsaE$_3$
Nº of genomes with TsaE$_3$
Nº of genomes without TsaE$_3$
p < 0.00005

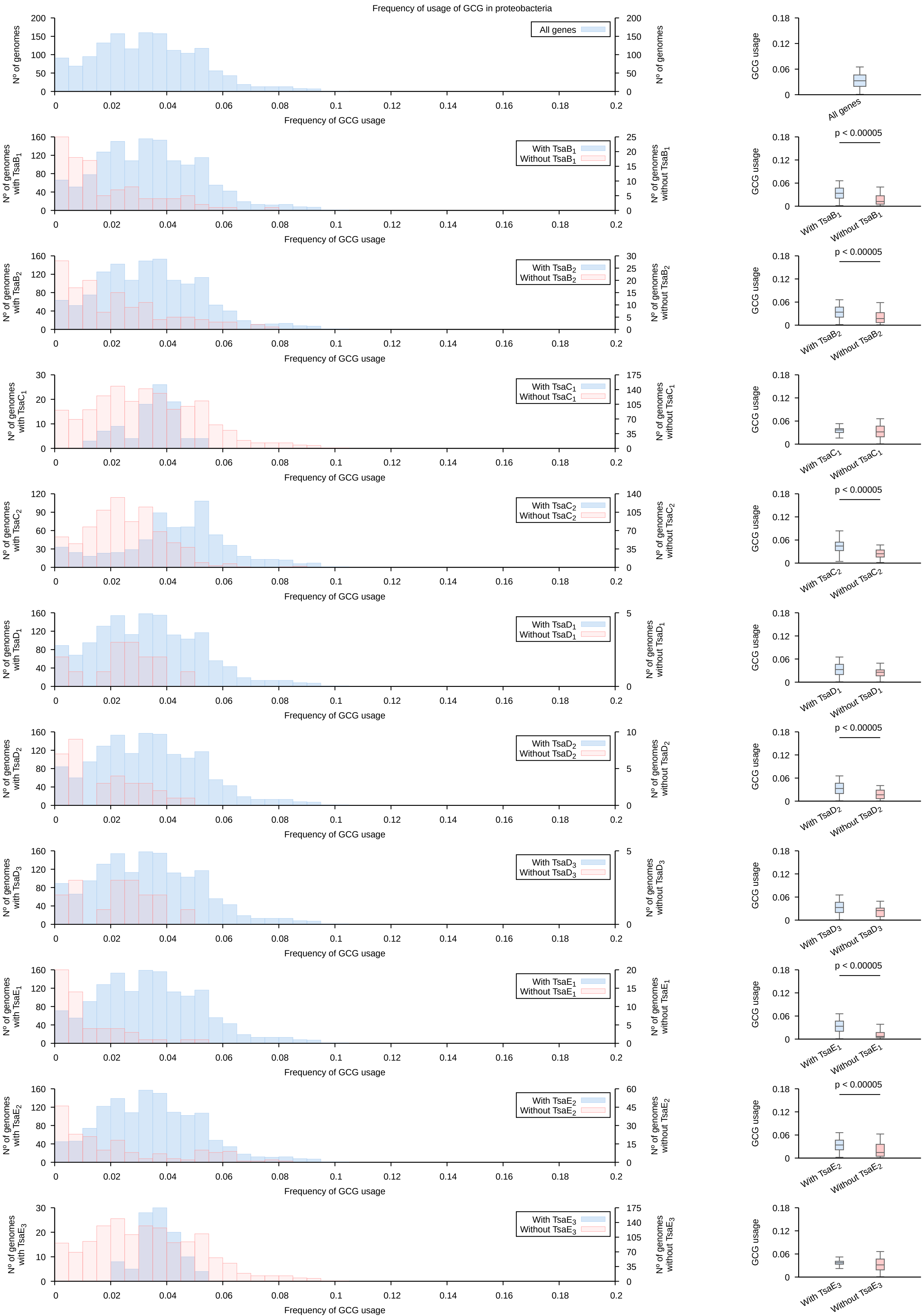
Frequency of usage of GCG in proteobacteria
Nº of genomes
All genes
Frequency of GCG usage
GCG usage
Nº of genomes with TsaB$_1$
Nº of genomes without TsaB$_1$
With TsaB$_1$
Without TsaB$_1$
p < 0.00005
Nº of genomes with TsaB$_2$
Nº of genomes without TsaB$_2$
With TsaB$_2$
Without TsaB$_2$
p < 0.00005
Nº of genomes with TsaC$_1$
Nº of genomes without TsaC$_1$
With TsaC$_1$
Without TsaC$_1$
Nº of genomes with TsaC$_2$
Nº of genomes without TsaC$_2$
With TsaC$_2$
Without TsaC$_2$
p < 0.00005
Nº of genomes with TsaD$_1$
Nº of genomes without TsaD$_1$
With TsaD$_1$
Without TsaD$_1$
Nº of genomes with TsaD$_2$
Nº of genomes without TsaD$_2$
With TsaD$_2$
Without TsaD$_2$
p < 0.00005
Nº of genomes with TsaD$_3$
Nº of genomes without TsaD$_3$
With TsaD$_3$
Without TsaD$_3$
Nº of genomes with TsaE$_1$
Nº of genomes without TsaE$_1$
With TsaE$_1$
Without TsaE$_1$
p < 0.00005
Nº of genomes with TsaE$_2$
Nº of genomes without TsaE$_2$
With TsaE$_2$
Without TsaE$_2$
p < 0.00005
Nº of genomes with TsaE$_3$
Nº of genomes without TsaE$_3$
With TsaE$_3$
Without TsaE$_3$

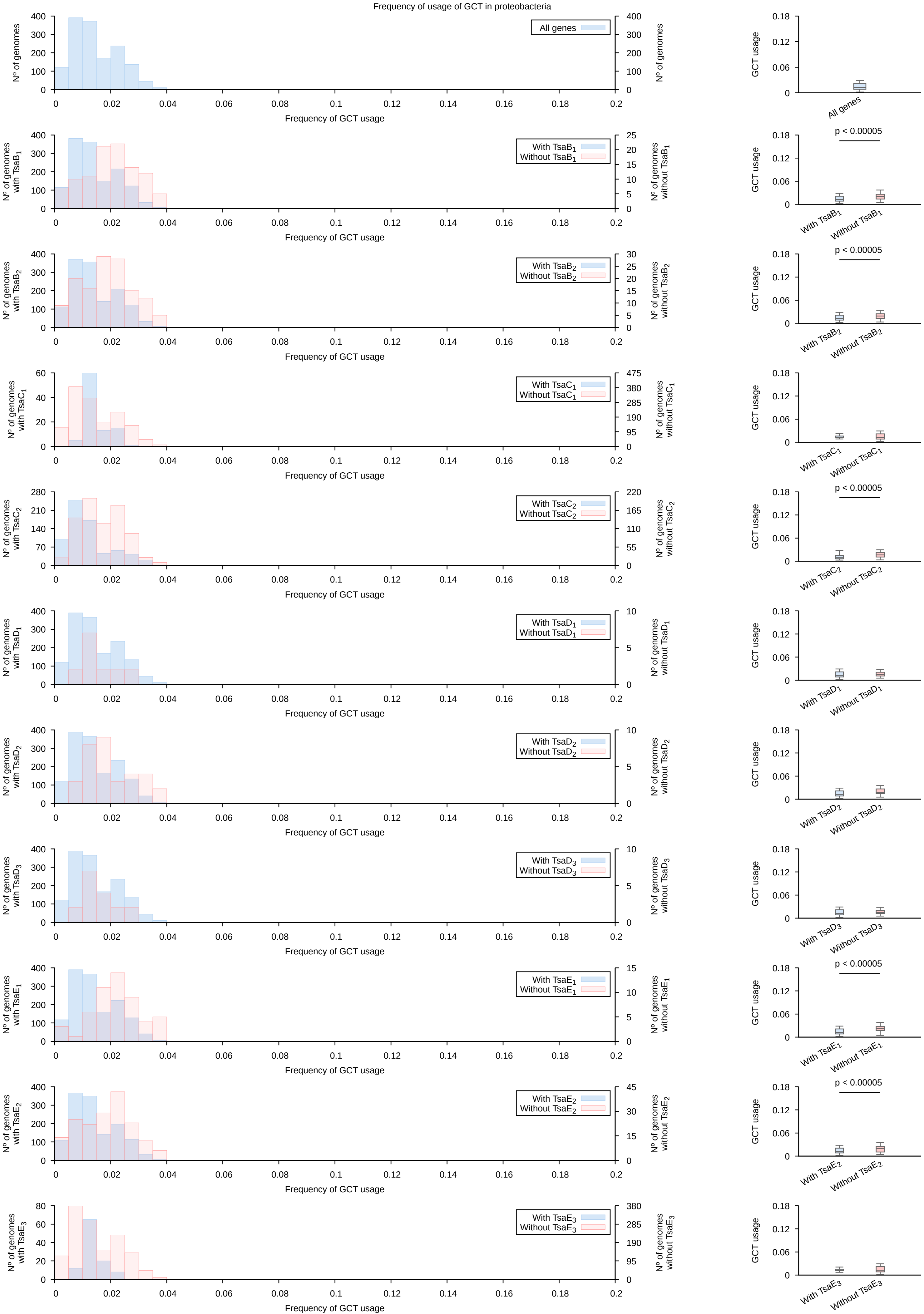
Frequency of usage of GCT in proteobacteria
All genes
Nº of genomes
Frequency of GCT usage
GCT usage
With TsaB$_1$
Without TsaB$_1$
Nº of genomes with TsaB$_1$
Nº of genomes without TsaB$_1$
p < 0.00005
With TsaB$_2$
Without TsaB$_2$
Nº of genomes with TsaB$_2$
Nº of genomes without TsaB$_2$
p < 0.00005
With TsaC$_1$
Without TsaC$_1$
Nº of genomes with TsaC$_1$
Nº of genomes without TsaC$_1$
With TsaC$_2$
Without TsaC$_2$
Nº of genomes with TsaC$_2$
Nº of genomes without TsaC$_2$
p < 0.00005
With TsaD$_1$
Without TsaD$_1$
Nº of genomes with TsaD$_1$
Nº of genomes without TsaD$_1$
With TsaD$_2$
Without TsaD$_2$
Nº of genomes with TsaD$_2$
Nº of genomes without TsaD$_2$
With TsaD$_3$
Without TsaD$_3$
Nº of genomes with TsaD$_3$
Nº of genomes without TsaD$_3$
With TsaE$_1$
Without TsaE$_1$
Nº of genomes with TsaE$_1$
Nº of genomes without TsaE$_1$
p < 0.00005
With TsaE$_2$
Without TsaE$_2$
Nº of genomes with TsaE$_2$
Nº of genomes without TsaE$_2$
p < 0.00005
With TsaE$_3$
Without TsaE$_3$
Nº of genomes with TsaE$_3$
Nº of genomes without TsaE$_3$

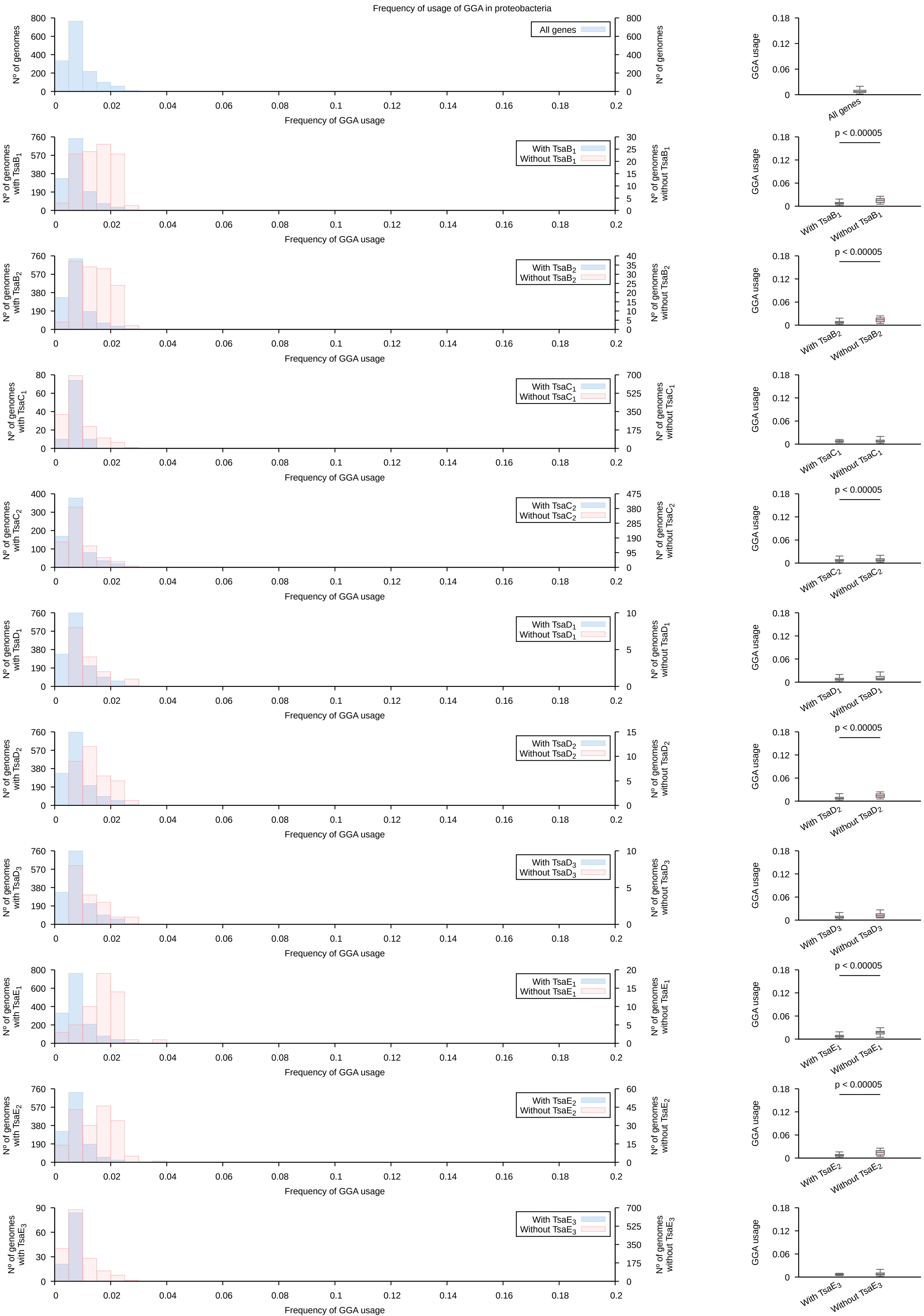
Frequency of usage of GGA in proteobacteria
Frequency of GGA usage
Nº of genomes
GGA usage
All genes
With $TsaB_1$
Without $TsaB_1$
With $TsaB_2$
Without $TsaB_2$
With $TsaC_1$
Without $TsaC_1$
With $TsaC_2$
Without $TsaC_2$
With $TsaD_1$
Without $TsaD_1$
With $TsaD_2$
Without $TsaD_2$
With $TsaD_3$
Without $TsaD_3$
With $TsaE_1$
Without $TsaE_1$
With $TsaE_2$
Without $TsaE_2$
With $TsaE_3$
Without $TsaE_3$
$p < 0.00005$

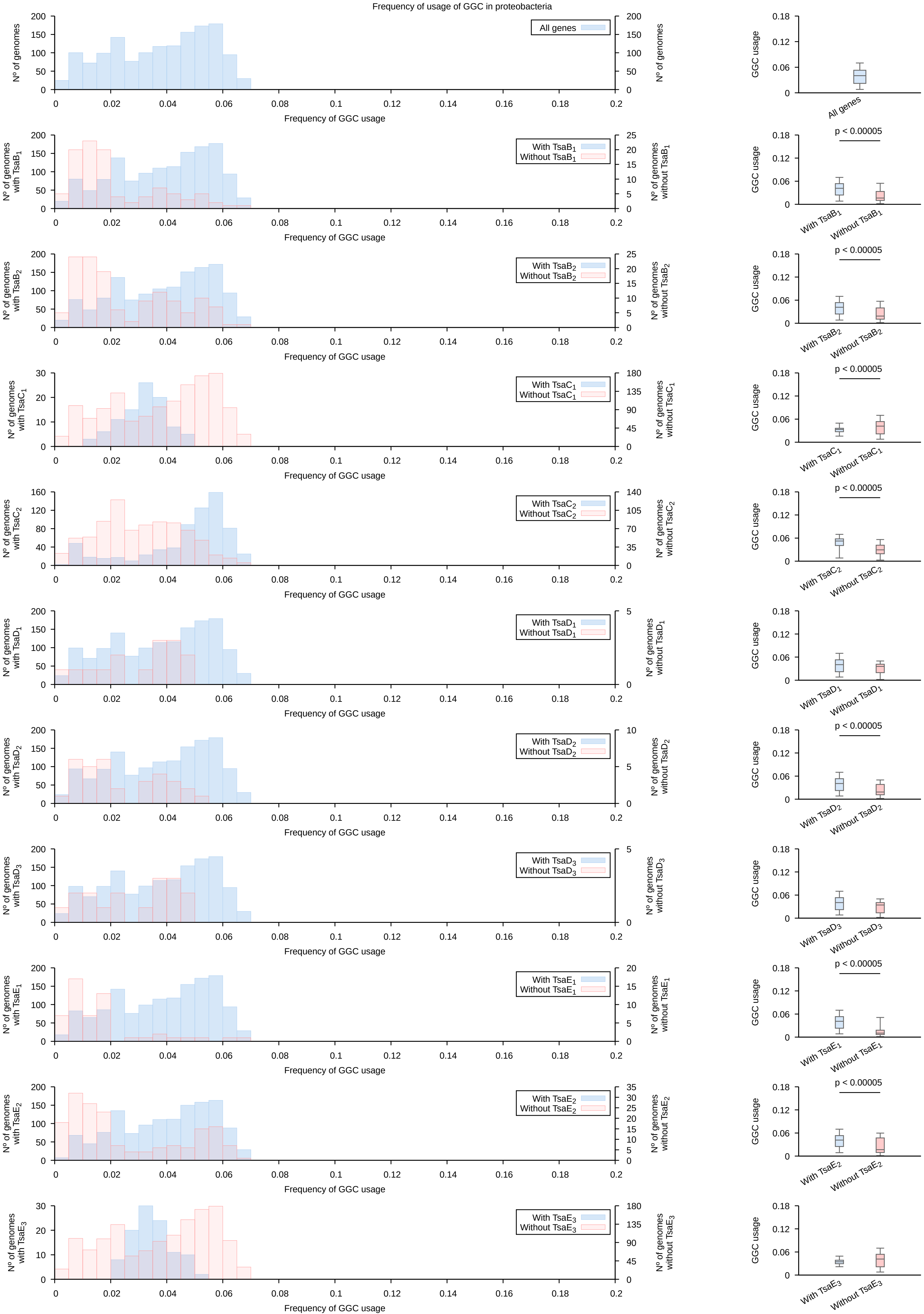
Frequency of usage of GGC in proteobacteria
All genes
With TsaB1
Without TsaB1
With TsaB2
Without TsaB2
With TsaC1
Without TsaC1
With TsaC2
Without TsaC2
With TsaD1
Without TsaD1
With TsaD2
Without TsaD2
With TsaD3
Without TsaD3
With TsaE1
Without TsaE1
With TsaE2
Without TsaE2
With TsaE3
Without TsaE3
Nº of genomes
Frequency of GGC usage
GGC usage
p < 0.00005

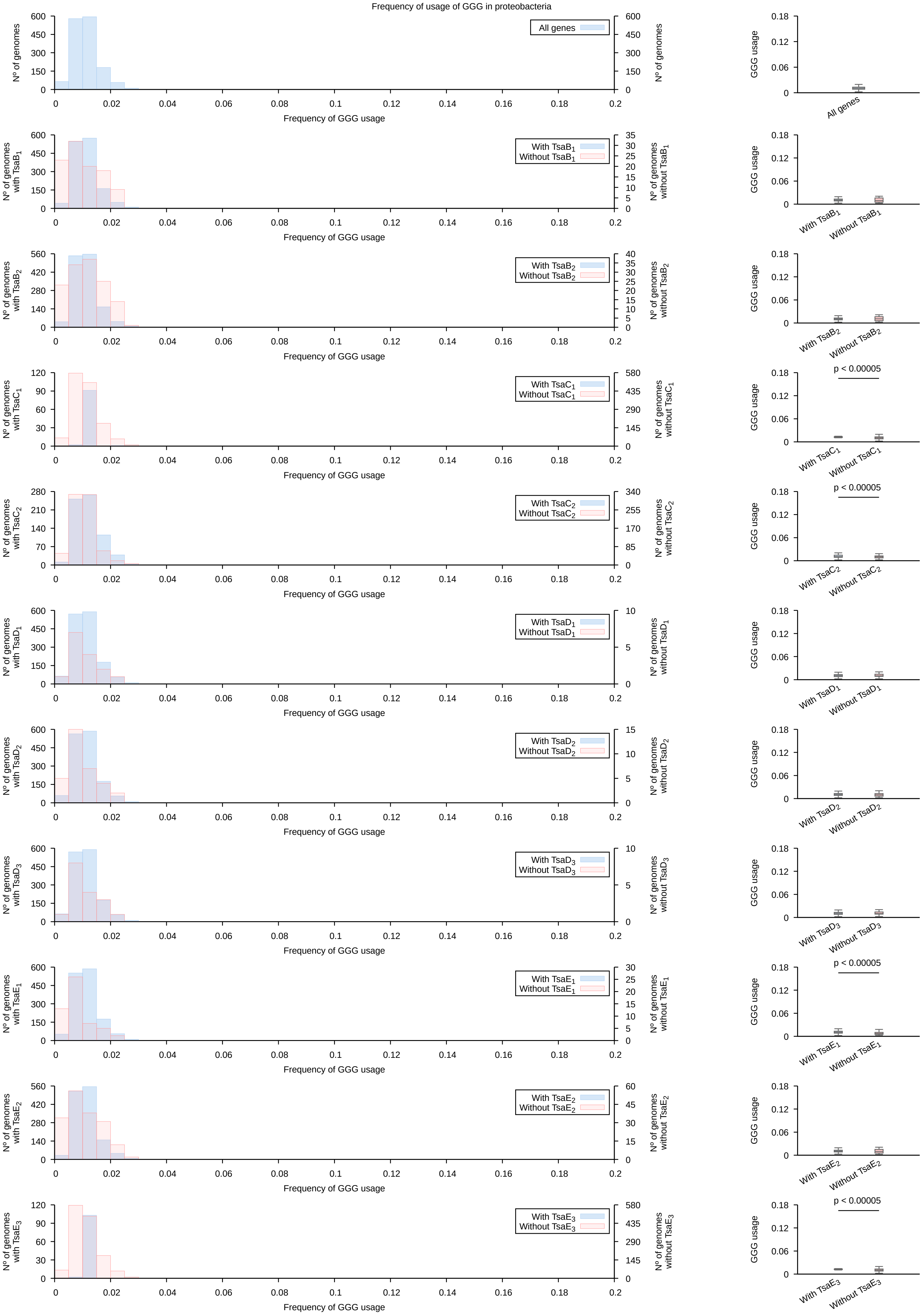
Frequency of usage of GGG in proteobacteria
Nº of genomes
Frequency of GGG usage
GGG usage
All genes
With TsaB1
Without TsaB1
With TsaB2
Without TsaB2
With TsaC1
Without TsaC1
With TsaC2
Without TsaC2
With TsaD1
Without TsaD1
With TsaD2
Without TsaD2
With TsaD3
Without TsaD3
With TsaE1
Without TsaE1
With TsaE2
Without TsaE2
With TsaE3
Without TsaE3
p < 0.00005

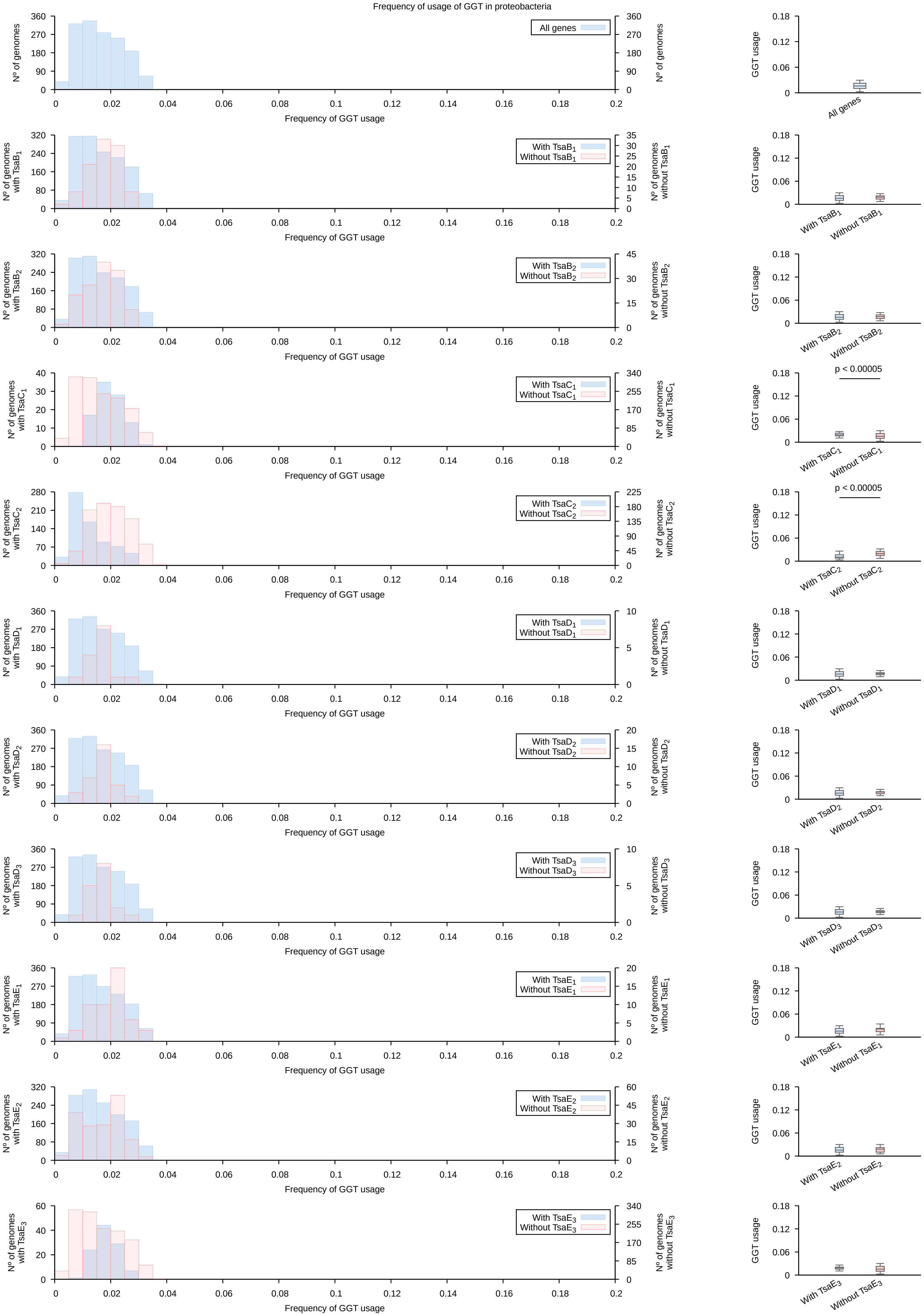
Frequency of usage of GGT in proteobacteria
Nº of genomes
Frequency of GGT usage
GGT usage
All genes
With TsaB$_1$
Without TsaB$_1$
Nº of genomes with TsaB$_1$
Nº of genomes without TsaB$_1$
With TsaB$_2$
Without TsaB$_2$
Nº of genomes with TsaB$_2$
Nº of genomes without TsaB$_2$
With TsaC$_1$
Without TsaC$_1$
Nº of genomes with TsaC$_1$
Nº of genomes without TsaC$_1$
p < 0.00005
With TsaC$_2$
Without TsaC$_2$
Nº of genomes with TsaC$_2$
Nº of genomes without TsaC$_2$
p < 0.00005
With TsaD$_1$
Without TsaD$_1$
Nº of genomes with TsaD$_1$
Nº of genomes without TsaD$_1$
With TsaD$_2$
Without TsaD$_2$
Nº of genomes with TsaD$_2$
Nº of genomes without TsaD$_2$
With TsaD$_3$
Without TsaD$_3$
Nº of genomes with TsaD$_3$
Nº of genomes without TsaD$_3$
With TsaE$_1$
Without TsaE$_1$
Nº of genomes with TsaE$_1$
Nº of genomes without TsaE$_1$
With TsaE$_2$
Without TsaE$_2$
Nº of genomes with TsaE$_2$
Nº of genomes without TsaE$_2$
With TsaE$_3$
Without TsaE$_3$
Nº of genomes with TsaE$_3$
Nº of genomes without TsaE$_3$

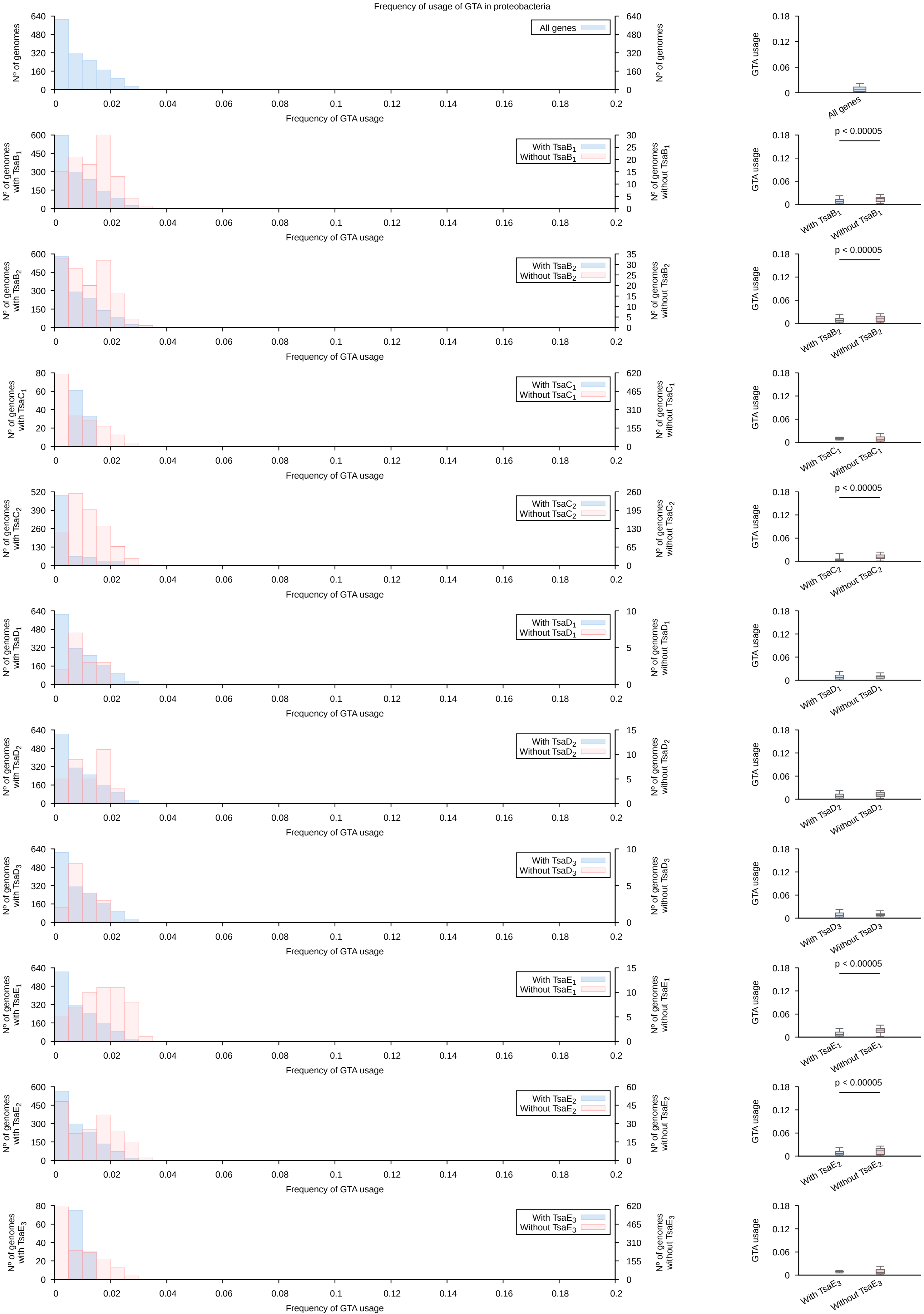
Frequency of usage of GTA in proteobacteria
All genes
Nº of genomes
Frequency of GTA usage
GTA usage
With $TsaB_1$
Without $TsaB_1$
Nº of genomes with $TsaB_1$
Nº of genomes without $TsaB_1$
p < 0.00005
With $TsaB_2$
Without $TsaB_2$
Nº of genomes with $TsaB_2$
Nº of genomes without $TsaB_2$
p < 0.00005
With $TsaC_1$
Without $TsaC_1$
Nº of genomes with $TsaC_1$
Nº of genomes without $TsaC_1$
With $TsaC_2$
Without $TsaC_2$
Nº of genomes with $TsaC_2$
Nº of genomes without $TsaC_2$
p < 0.00005
With $TsaD_1$
Without $TsaD_1$
Nº of genomes with $TsaD_1$
Nº of genomes without $TsaD_1$
With $TsaD_2$
Without $TsaD_2$
Nº of genomes with $TsaD_2$
Nº of genomes without $TsaD_2$
With $TsaD_3$
Without $TsaD_3$
Nº of genomes with $TsaD_3$
Nº of genomes without $TsaD_3$
With $TsaE_1$
Without $TsaE_1$
Nº of genomes with $TsaE_1$
Nº of genomes without $TsaE_1$
p < 0.00005
With $TsaE_2$
Without $TsaE_2$
Nº of genomes with $TsaE_2$
Nº of genomes without $TsaE_2$
p < 0.00005
With $TsaE_3$
Without $TsaE_3$
Nº of genomes with $TsaE_3$
Nº of genomes without $TsaE_3$

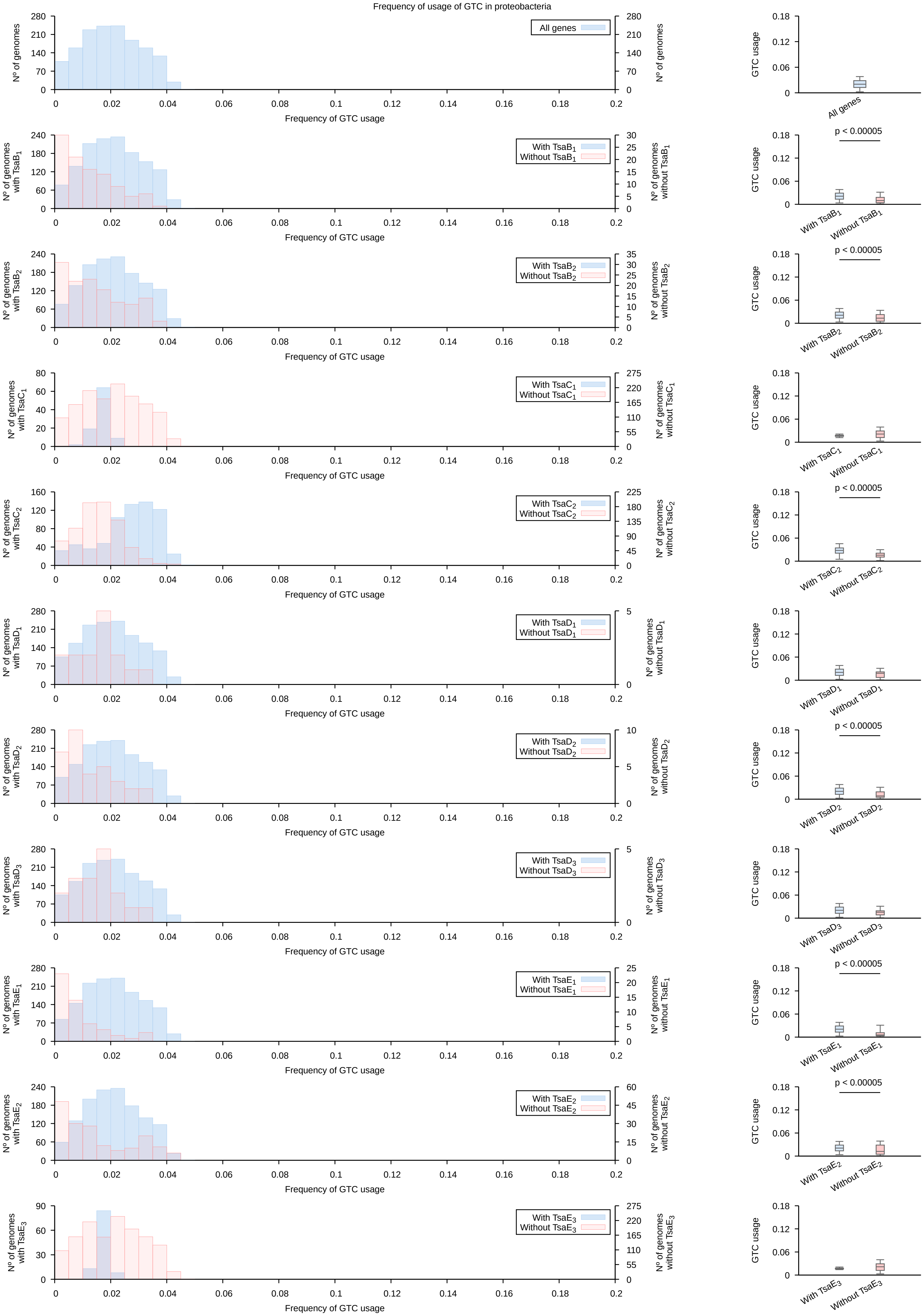
Frequency of usage of GTC in proteobacteria
All genes
With TsaB1
Without TsaB1
With TsaB2
Without TsaB2
With TsaC1
Without TsaC1
With TsaC2
Without TsaC2
With TsaD1
Without TsaD1
With TsaD2
Without TsaD2
With TsaD3
Without TsaD3
With TsaE1
Without TsaE1
With TsaE2
Without TsaE2
With TsaE3
Without TsaE3
Frequency of GTC usage
Nº of genomes
GTC usage
p < 0.00005

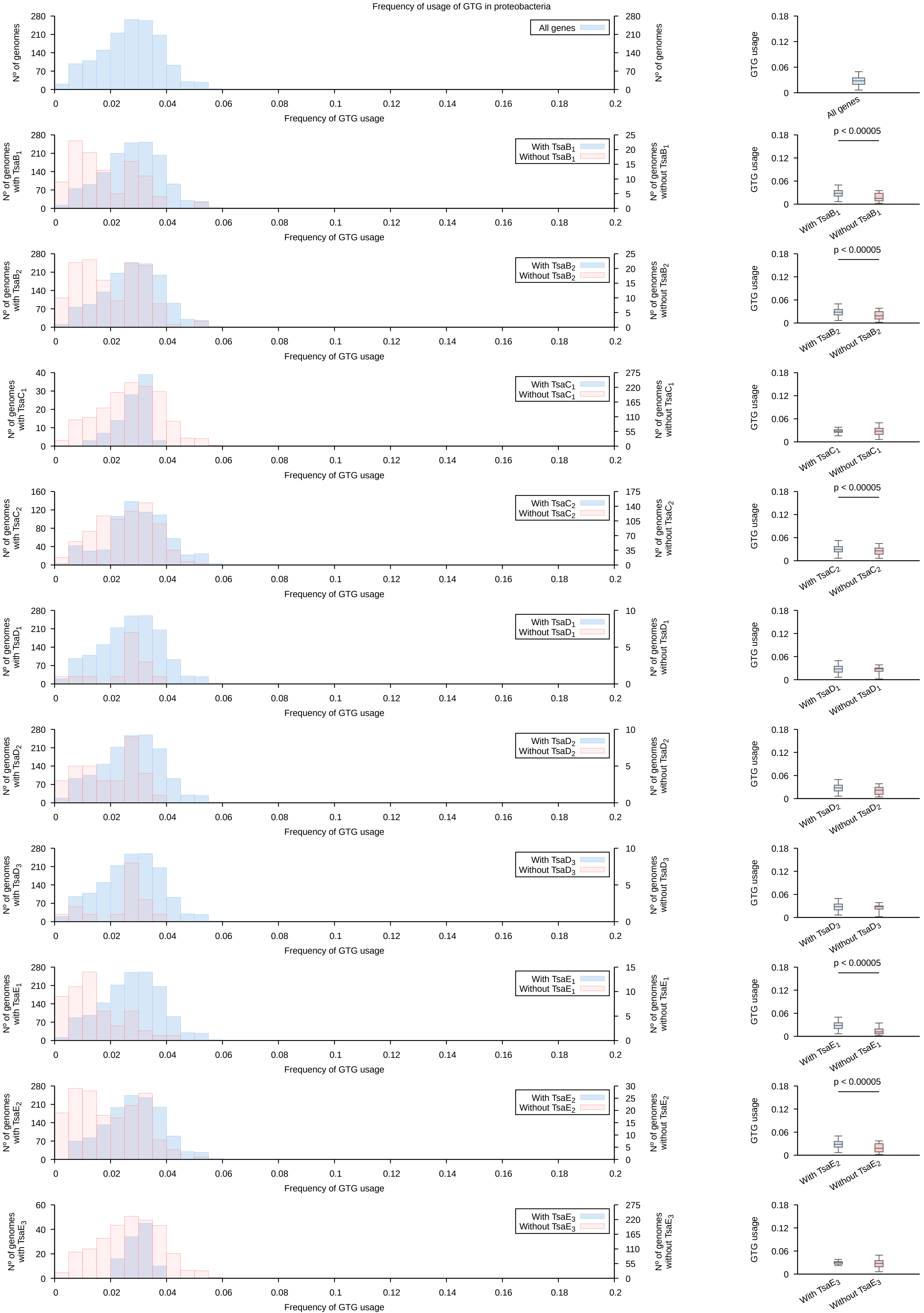
Frequency of usage of GTG in proteobacteria
All genes
Nº of genomes
GTG usage
Frequency of GTG usage
With TsaB1
Without TsaB1
Nº of genomes with TsaB1
Nº of genomes without TsaB1
p < 0.00005
With TsaB2
Without TsaB2
Nº of genomes with TsaB2
Nº of genomes without TsaB2
p < 0.00005
With TsaC1
Without TsaC1
Nº of genomes with TsaC1
Nº of genomes without TsaC1
With TsaC2
Without TsaC2
Nº of genomes with TsaC2
Nº of genomes without TsaC2
p < 0.00005
With TsaD1
Without TsaD1
Nº of genomes with TsaD1
Nº of genomes without TsaD1
With TsaD2
Without TsaD2
Nº of genomes with TsaD2
Nº of genomes without TsaD2
With TsaD3
Without TsaD3
Nº of genomes with TsaD3
Nº of genomes without TsaD3
With TsaE1
Without TsaE1
Nº of genomes with TsaE1
Nº of genomes without TsaE1
p < 0.00005
With TsaE2
Without TsaE2
Nº of genomes with TsaE2
Nº of genomes without TsaE2
p < 0.00005
With TsaE3
Without TsaE3
Nº of genomes with TsaE3
Nº of genomes without TsaE3

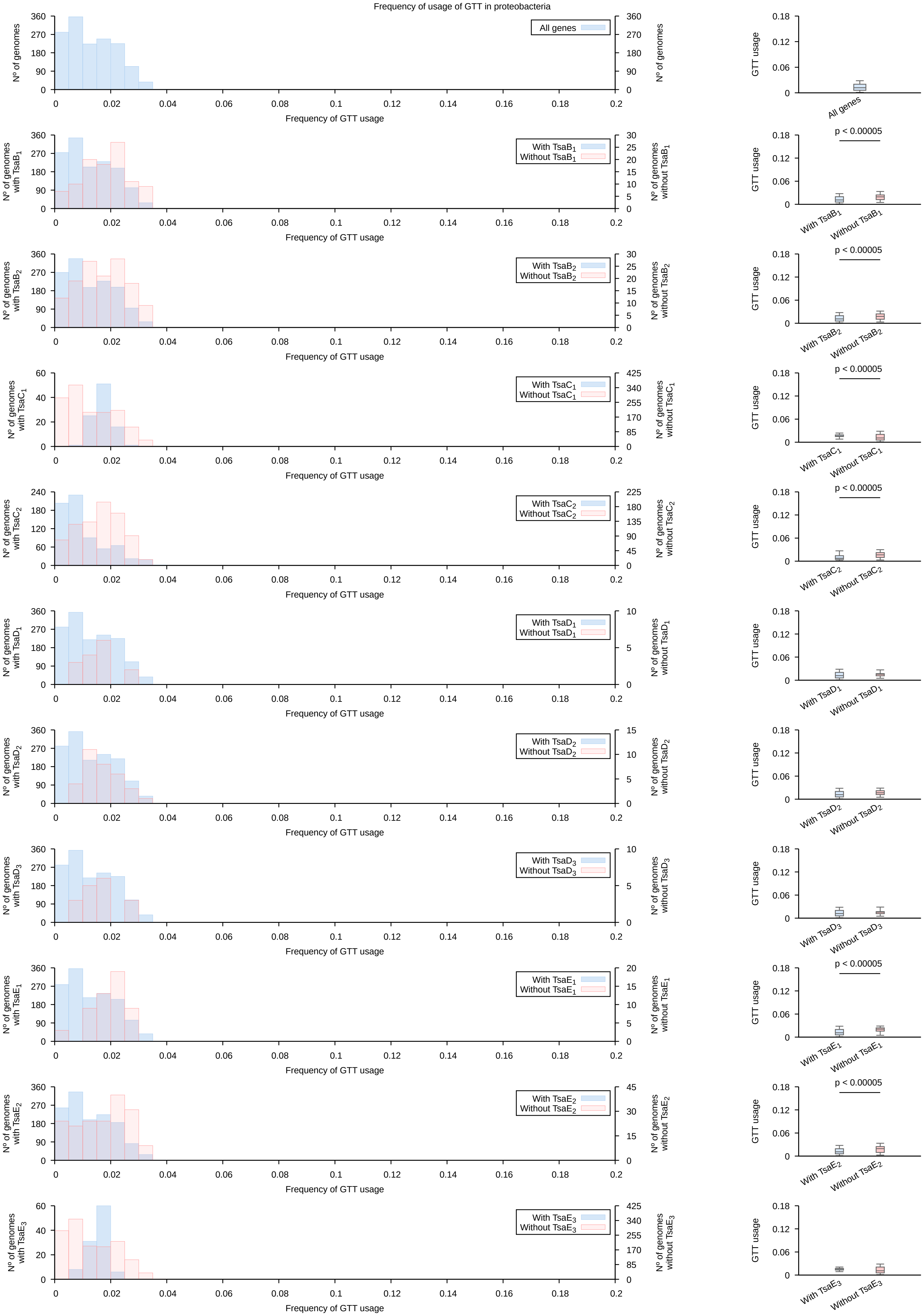
Frequency of usage of GTT in proteobacteria
Nº of genomes
All genes
Frequency of GTT usage
GTT usage
All genes
Nº of genomes with TsaB1
Nº of genomes without TsaB1
With TsaB1
Without TsaB1
p < 0.00005
Nº of genomes with TsaB2
Nº of genomes without TsaB2
With TsaB2
Without TsaB2
p < 0.00005
Nº of genomes with TsaC1
Nº of genomes without TsaC1
With TsaC1
Without TsaC1
p < 0.00005
Nº of genomes with TsaC2
Nº of genomes without TsaC2
With TsaC2
Without TsaC2
p < 0.00005
Nº of genomes with TsaD1
Nº of genomes without TsaD1
With TsaD1
Without TsaD1
Nº of genomes with TsaD2
Nº of genomes without TsaD2
With TsaD2
Without TsaD2
Nº of genomes with TsaD3
Nº of genomes without TsaD3
With TsaD3
Without TsaD3
Nº of genomes with TsaE1
Nº of genomes without TsaE1
With TsaE1
Without TsaE1
p < 0.00005
Nº of genomes with TsaE2
Nº of genomes without TsaE2
With TsaE2
Without TsaE2
p < 0.00005
Nº of genomes with TsaE3
Nº of genomes without TsaE3
With TsaE3
Without TsaE3

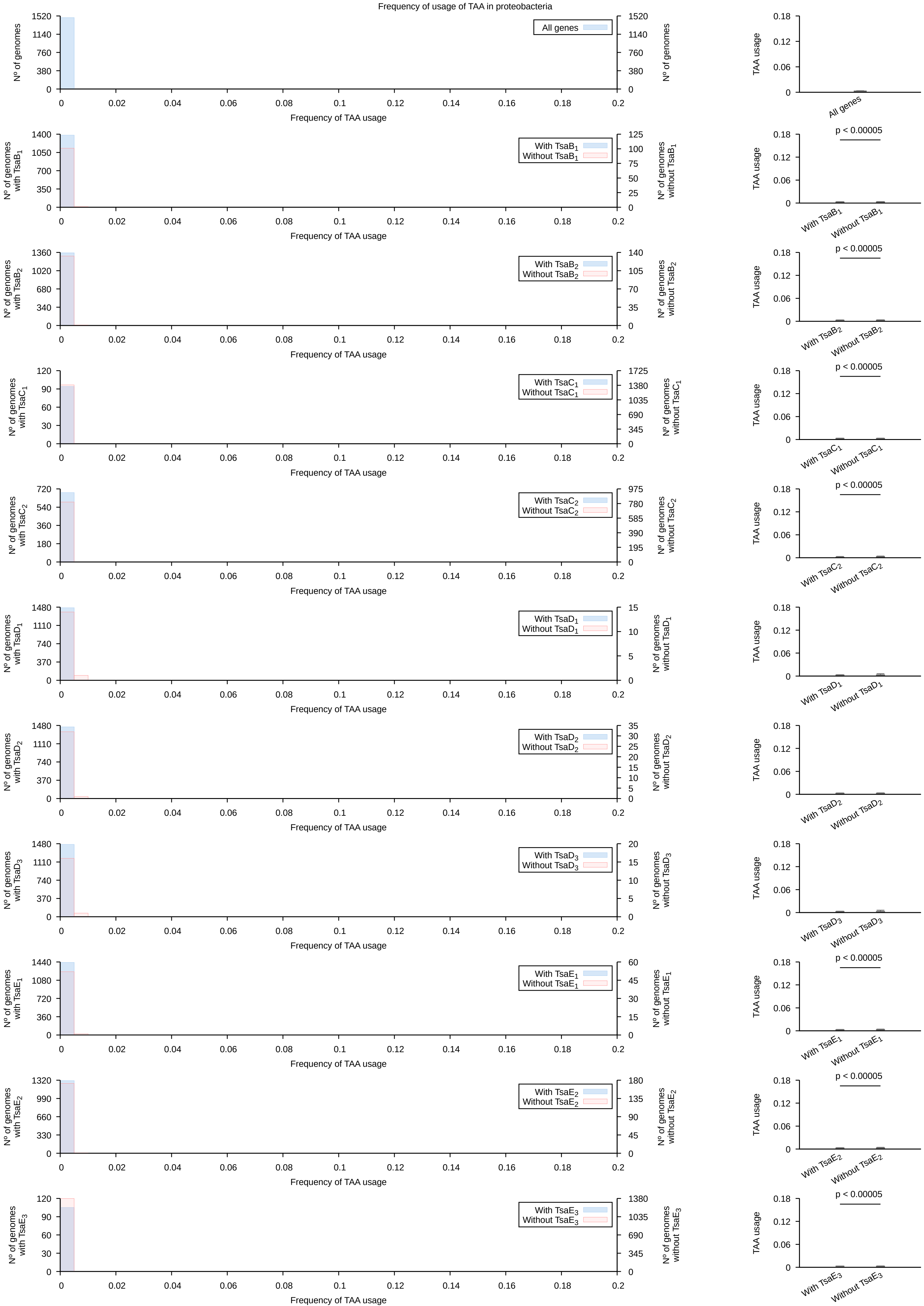
Frequency of usage of TAA in proteobacteria
Nº of genomes
Frequency of TAA usage
TAA usage
All genes
With TsaB1
Without TsaB1
With TsaB2
Without TsaB2
With TsaC1
Without TsaC1
With TsaC2
Without TsaC2
With TsaD1
Without TsaD1
With TsaD2
Without TsaD2
With TsaD3
Without TsaD3
With TsaE1
Without TsaE1
With TsaE2
Without TsaE2
With TsaE3
Without TsaE3
p < 0.00005

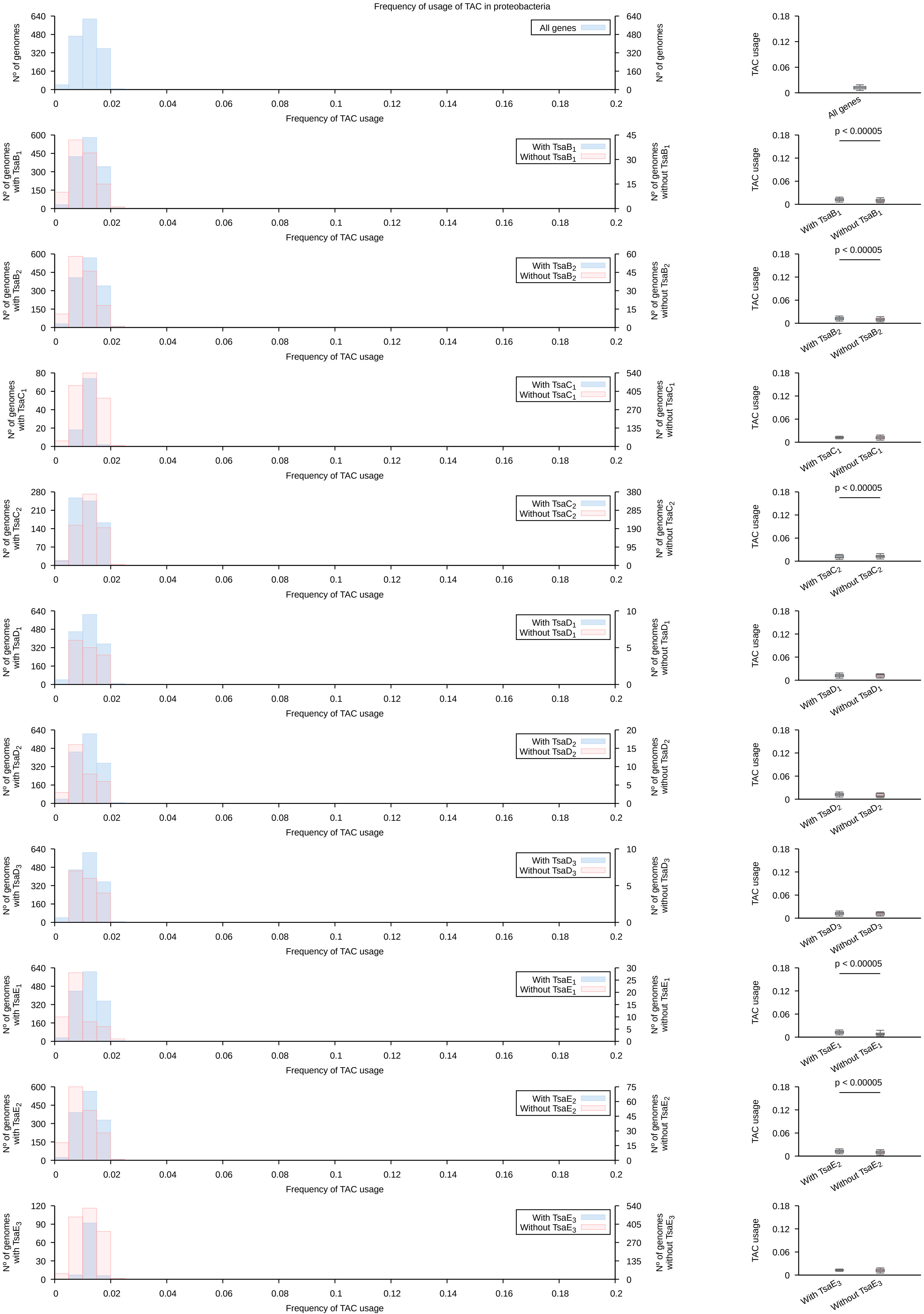
Frequency of usage of TAC in proteobacteria
Nº of genomes
All genes
Frequency of TAC usage
TAC usage
Nº of genomes with TsaB$_1$
Nº of genomes without TsaB$_1$
With TsaB$_1$
Without TsaB$_1$
p < 0.00005
Nº of genomes with TsaB$_2$
Nº of genomes without TsaB$_2$
With TsaB$_2$
Without TsaB$_2$
p < 0.00005
Nº of genomes with TsaC$_1$
Nº of genomes without TsaC$_1$
With TsaC$_1$
Without TsaC$_1$
Nº of genomes with TsaC$_2$
Nº of genomes without TsaC$_2$
With TsaC$_2$
Without TsaC$_2$
p < 0.00005
Nº of genomes with TsaD$_1$
Nº of genomes without TsaD$_1$
With TsaD$_1$
Without TsaD$_1$
Nº of genomes with TsaD$_2$
Nº of genomes without TsaD$_2$
With TsaD$_2$
Without TsaD$_2$
Nº of genomes with TsaD$_3$
Nº of genomes without TsaD$_3$
With TsaD$_3$
Without TsaD$_3$
Nº of genomes with TsaE$_1$
Nº of genomes without TsaE$_1$
With TsaE$_1$
Without TsaE$_1$
p < 0.00005
Nº of genomes with TsaE$_2$
Nº of genomes without TsaE$_2$
With TsaE$_2$
Without TsaE$_2$
p < 0.00005
Nº of genomes with TsaE$_3$
Nº of genomes without TsaE$_3$
With TsaE$_3$
Without TsaE$_3$

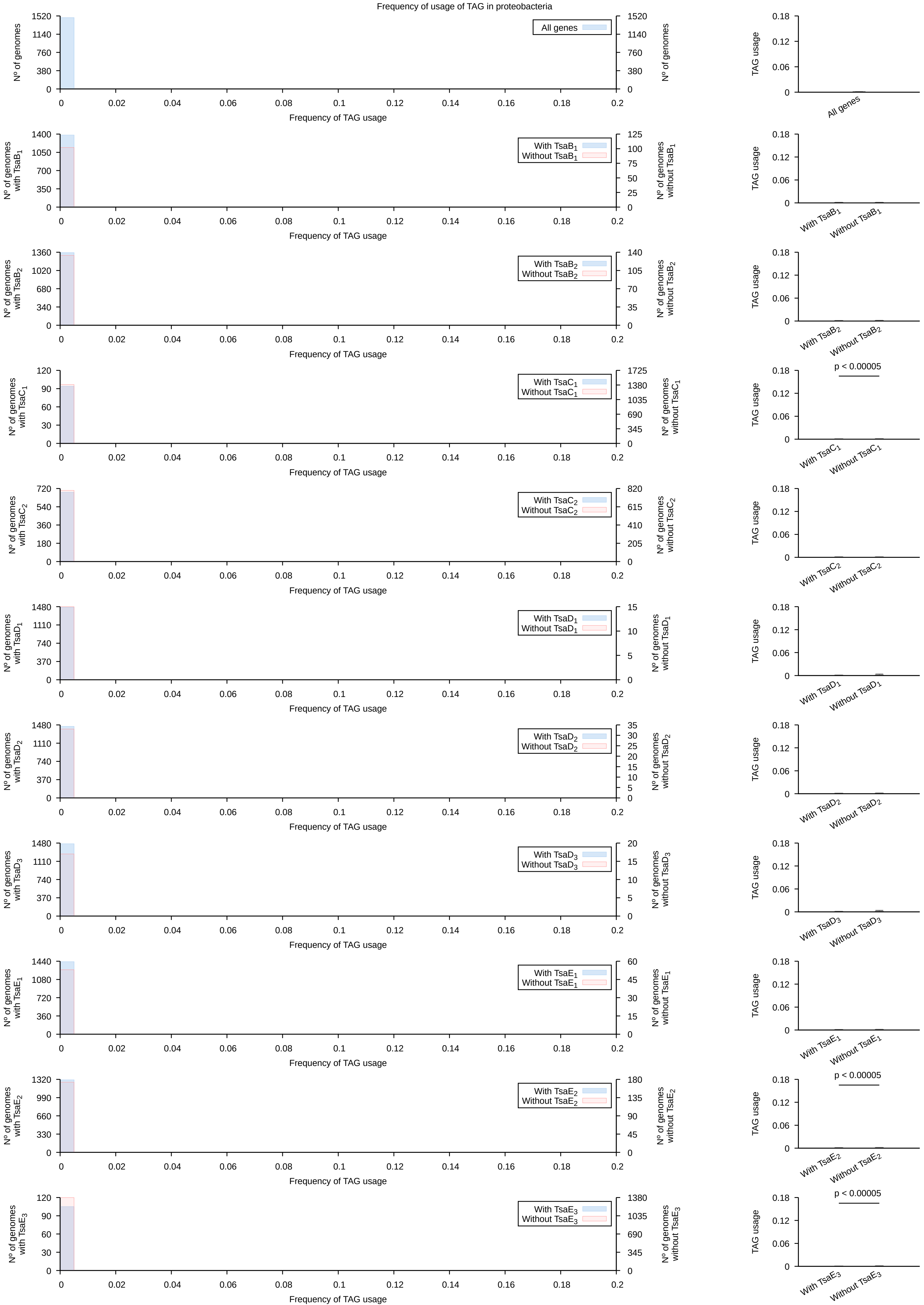

Frequency of usage of TAG in proteobacteria
Nº of genomes
All genes
Frequency of TAG usage
TAG usage
With $TsaB_1$
Without $TsaB_1$
Nº of genomes with $TsaB_1$
Nº of genomes without $TsaB_1$
With $TsaB_2$
Without $TsaB_2$
Nº of genomes with $TsaB_2$
Nº of genomes without $TsaB_2$
With $TsaC_1$
Without $TsaC_1$
Nº of genomes with $TsaC_1$
Nº of genomes without $TsaC_1$
p < 0.00005
With $TsaC_2$
Without $TsaC_2$
Nº of genomes with $TsaC_2$
Nº of genomes without $TsaC_2$
With $TsaD_1$
Without $TsaD_1$
Nº of genomes with $TsaD_1$
Nº of genomes without $TsaD_1$
With $TsaD_2$
Without $TsaD_2$
Nº of genomes with $TsaD_2$
Nº of genomes without $TsaD_2$
With $TsaD_3$
Without $TsaD_3$
Nº of genomes with $TsaD_3$
Nº of genomes without $TsaD_3$
With $TsaE_1$
Without $TsaE_1$
Nº of genomes with $TsaE_1$
Nº of genomes without $TsaE_1$
With $TsaE_2$
Without $TsaE_2$
Nº of genomes with $TsaE_2$
Nº of genomes without $TsaE_2$
p < 0.00005
With $TsaE_3$
Without $TsaE_3$
Nº of genomes with $TsaE_3$
Nº of genomes without $TsaE_3$
p < 0.00005

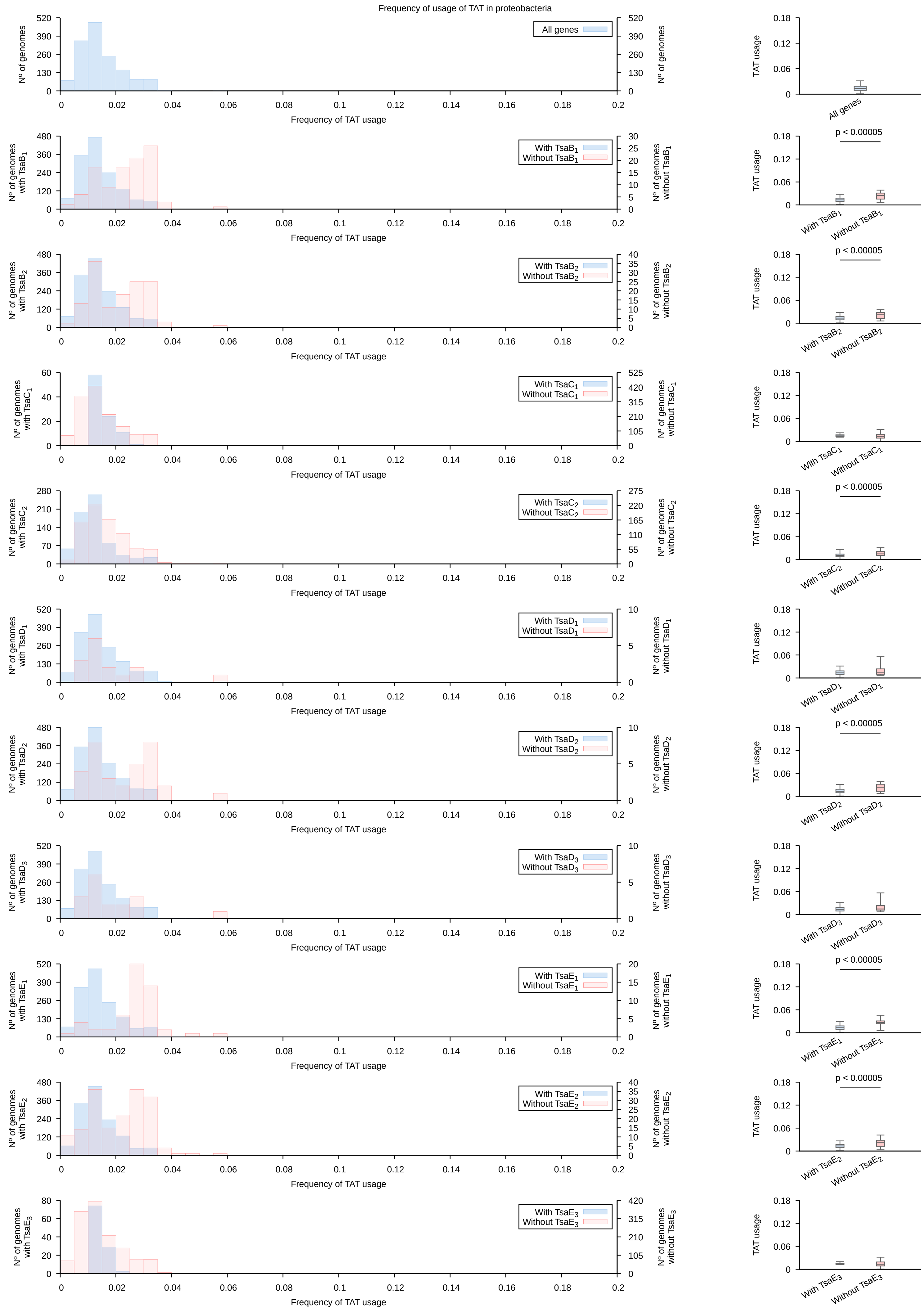

Frequency of usage of TAT in proteobacteria
Nº of genomes
Frequency of TAT usage
TAT usage
All genes
Nº of genomes with $TsaB_1$
Nº of genomes without $TsaB_1$
With $TsaB_1$
Without $TsaB_1$
$p < 0.00005$
Nº of genomes with $TsaB_2$
Nº of genomes without $TsaB_2$
With $TsaB_2$
Without $TsaB_2$
$p < 0.00005$
Nº of genomes with $TsaC_1$
Nº of genomes without $TsaC_1$
With $TsaC_1$
Without $TsaC_1$
Nº of genomes with $TsaC_2$
Nº of genomes without $TsaC_2$
With $TsaC_2$
Without $TsaC_2$
$p < 0.00005$
Nº of genomes with $TsaD_1$
Nº of genomes without $TsaD_1$
With $TsaD_1$
Without $TsaD_1$
Nº of genomes with $TsaD_2$
Nº of genomes without $TsaD_2$
With $TsaD_2$
Without $TsaD_2$
$p < 0.00005$
Nº of genomes with $TsaD_3$
Nº of genomes without $TsaD_3$
With $TsaD_3$
Without $TsaD_3$
Nº of genomes with $TsaE_1$
Nº of genomes without $TsaE_1$
With $TsaE_1$
Without $TsaE_1$
$p < 0.00005$
Nº of genomes with $TsaE_2$
Nº of genomes without $TsaE_2$
With $TsaE_2$
Without $TsaE_2$
$p < 0.00005$
Nº of genomes with $TsaE_3$
Nº of genomes without $TsaE_3$
With $TsaE_3$
Without $TsaE_3$

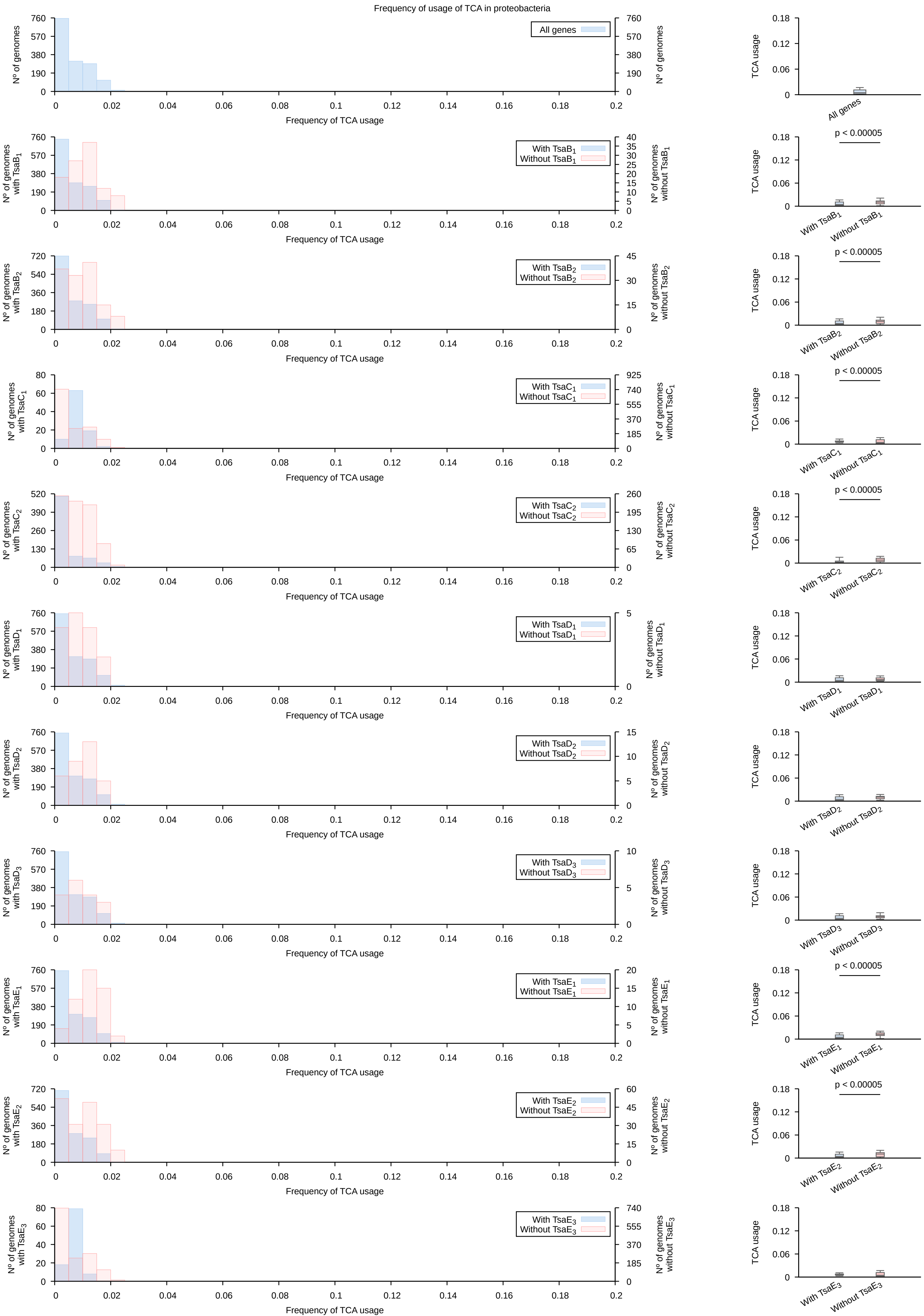
Frequency of usage of TCA in proteobacteria
All genes
Nº of genomes
Frequency of TCA usage
TCA usage
p < 0.00005
With TsaB$_1$
Without TsaB$_1$
Nº of genomes with TsaB$_1$
Nº of genomes without TsaB$_1$
With TsaB$_2$
Without TsaB$_2$
Nº of genomes with TsaB$_2$
Nº of genomes without TsaB$_2$
With TsaC$_1$
Without TsaC$_1$
Nº of genomes with TsaC$_1$
Nº of genomes without TsaC$_1$
With TsaC$_2$
Without TsaC$_2$
Nº of genomes with TsaC$_2$
Nº of genomes without TsaC$_2$
With TsaD$_1$
Without TsaD$_1$
Nº of genomes with TsaD$_1$
Nº of genomes without TsaD$_1$
With TsaD$_2$
Without TsaD$_2$
Nº of genomes with TsaD$_2$
Nº of genomes without TsaD$_2$
With TsaD$_3$
Without TsaD$_3$
Nº of genomes with TsaD$_3$
Nº of genomes without TsaD$_3$
With TsaE$_1$
Without TsaE$_1$
Nº of genomes with TsaE$_1$
Nº of genomes without TsaE$_1$
With TsaE$_2$
Without TsaE$_2$
Nº of genomes with TsaE$_2$
Nº of genomes without TsaE$_2$
With TsaE$_3$
Without TsaE$_3$
Nº of genomes with TsaE$_3$
Nº of genomes without TsaE$_3$

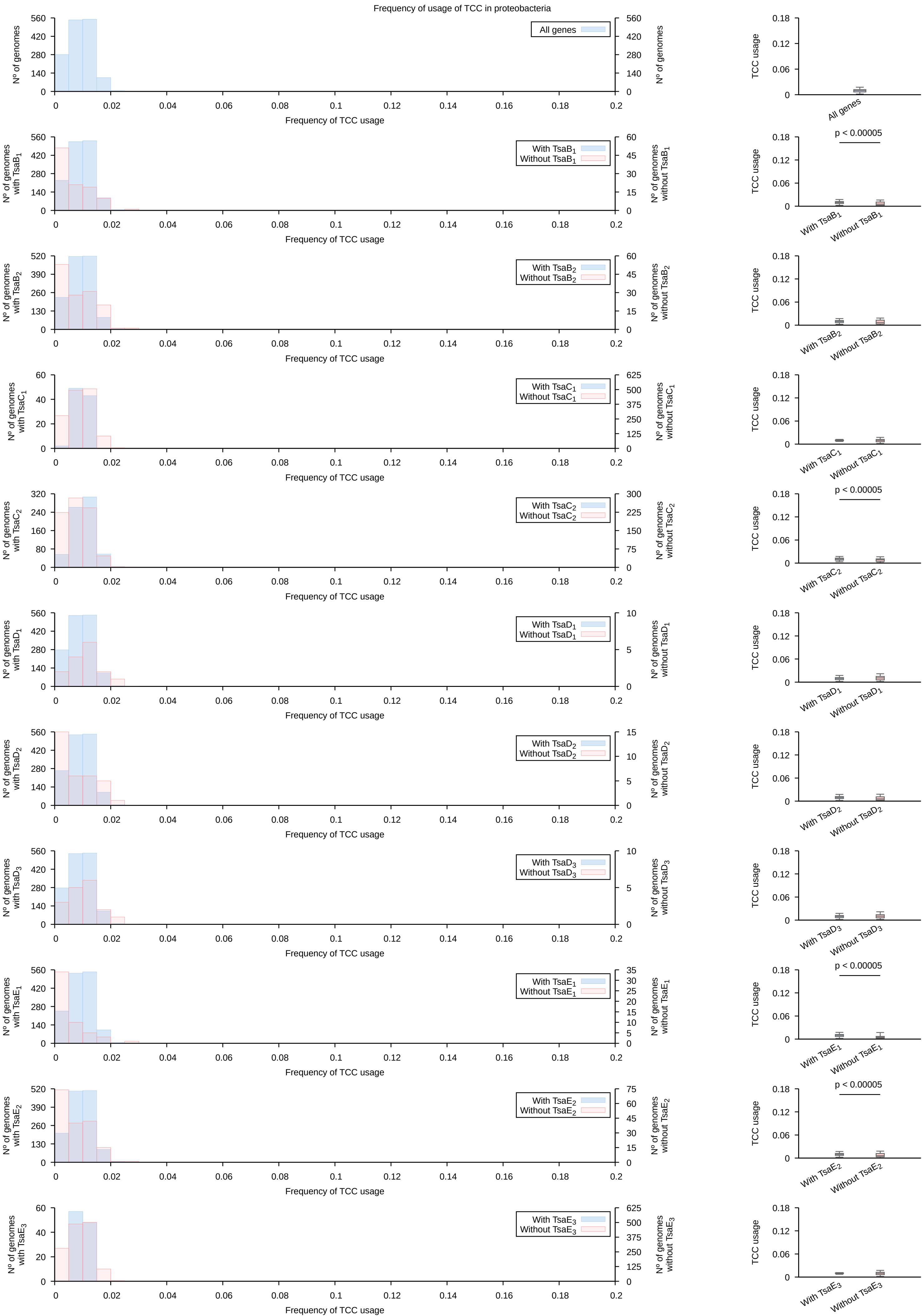
Frequency of usage of TCC in proteobacteria
All genes
Nº of genomes
Frequency of TCC usage
TCC usage
With TsaB$_1$
Without TsaB$_1$
Nº of genomes with TsaB$_1$
Nº of genomes without TsaB$_1$
p < 0.00005
With TsaB$_2$
Without TsaB$_2$
Nº of genomes with TsaB$_2$
Nº of genomes without TsaB$_2$
With TsaC$_1$
Without TsaC$_1$
Nº of genomes with TsaC$_1$
Nº of genomes without TsaC$_1$
With TsaC$_2$
Without TsaC$_2$
Nº of genomes with TsaC$_2$
Nº of genomes without TsaC$_2$
p < 0.00005
With TsaD$_1$
Without TsaD$_1$
Nº of genomes with TsaD$_1$
Nº of genomes without TsaD$_1$
With TsaD$_2$
Without TsaD$_2$
Nº of genomes with TsaD$_2$
Nº of genomes without TsaD$_2$
With TsaD$_3$
Without TsaD$_3$
Nº of genomes with TsaD$_3$
Nº of genomes without TsaD$_3$
With TsaE$_1$
Without TsaE$_1$
Nº of genomes with TsaE$_1$
Nº of genomes without TsaE$_1$
p < 0.00005
With TsaE$_2$
Without TsaE$_2$
Nº of genomes with TsaE$_2$
Nº of genomes without TsaE$_2$
p < 0.00005
With TsaE$_3$
Without TsaE$_3$
Nº of genomes with TsaE$_3$
Nº of genomes without TsaE$_3$

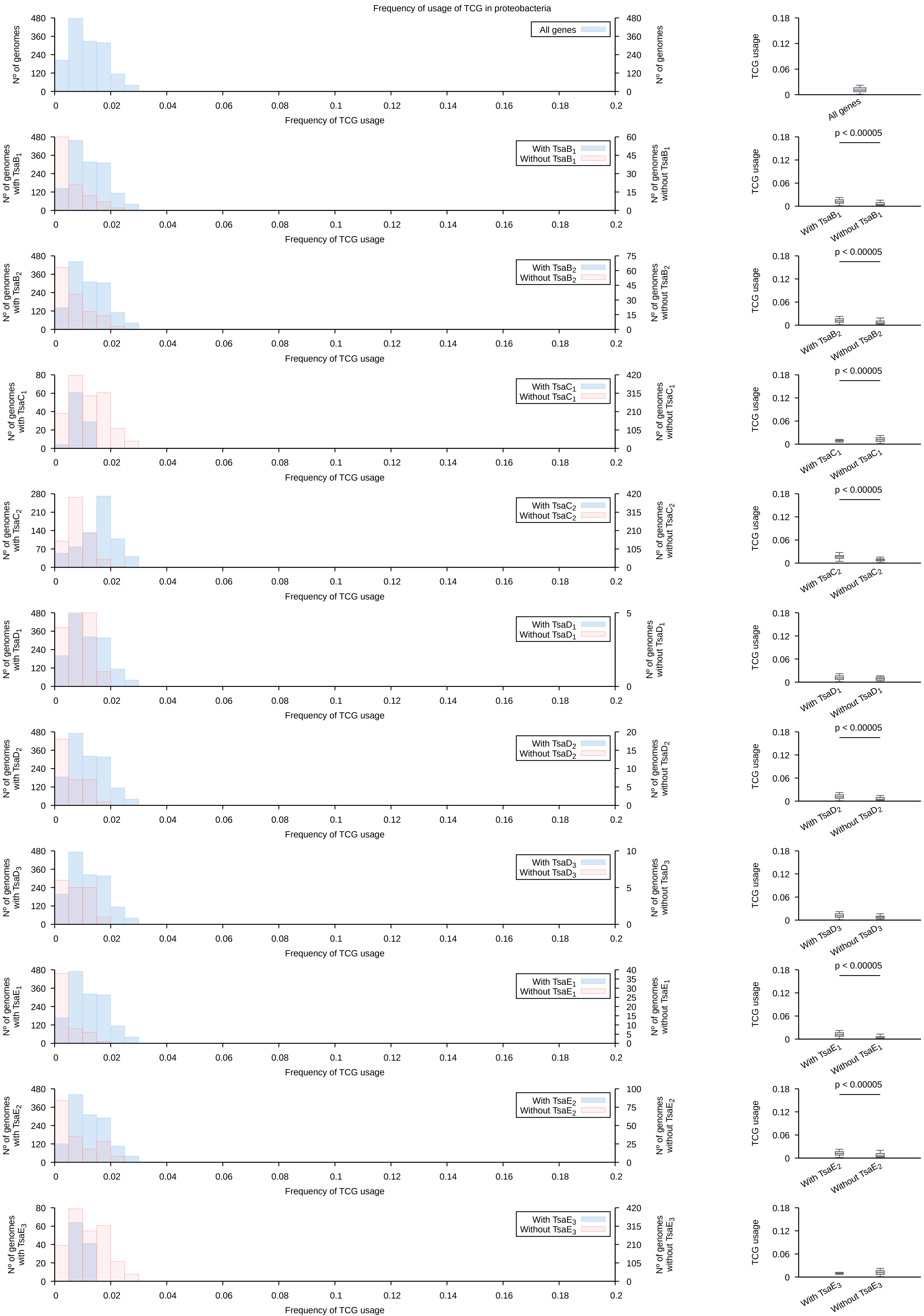

Frequency of usage of TCG in proteobacteria
All genes
Nº of genomes
Frequency of TCG usage
TCG usage
With TsaB$_1$
Without TsaB$_1$
Nº of genomes with TsaB$_1$
Nº of genomes without TsaB$_1$
p < 0.00005
With TsaB$_2$
Without TsaB$_2$
Nº of genomes with TsaB$_2$
Nº of genomes without TsaB$_2$
p < 0.00005
With TsaC$_1$
Without TsaC$_1$
Nº of genomes with TsaC$_1$
Nº of genomes without TsaC$_1$
p < 0.00005
With TsaC$_2$
Without TsaC$_2$
Nº of genomes with TsaC$_2$
Nº of genomes without TsaC$_2$
p < 0.00005
With TsaD$_1$
Without TsaD$_1$
Nº of genomes with TsaD$_1$
Nº of genomes without TsaD$_1$
With TsaD$_2$
Without TsaD$_2$
Nº of genomes with TsaD$_2$
Nº of genomes without TsaD$_2$
p < 0.00005
With TsaD$_3$
Without TsaD$_3$
Nº of genomes with TsaD$_3$
Nº of genomes without TsaD$_3$
With TsaE$_1$
Without TsaE$_1$
Nº of genomes with TsaE$_1$
Nº of genomes without TsaE$_1$
p < 0.00005
With TsaE$_2$
Without TsaE$_2$
Nº of genomes with TsaE$_2$
Nº of genomes without TsaE$_2$
p < 0.00005
With TsaE$_3$
Without TsaE$_3$
Nº of genomes with TsaE$_3$
Nº of genomes without TsaE$_3$

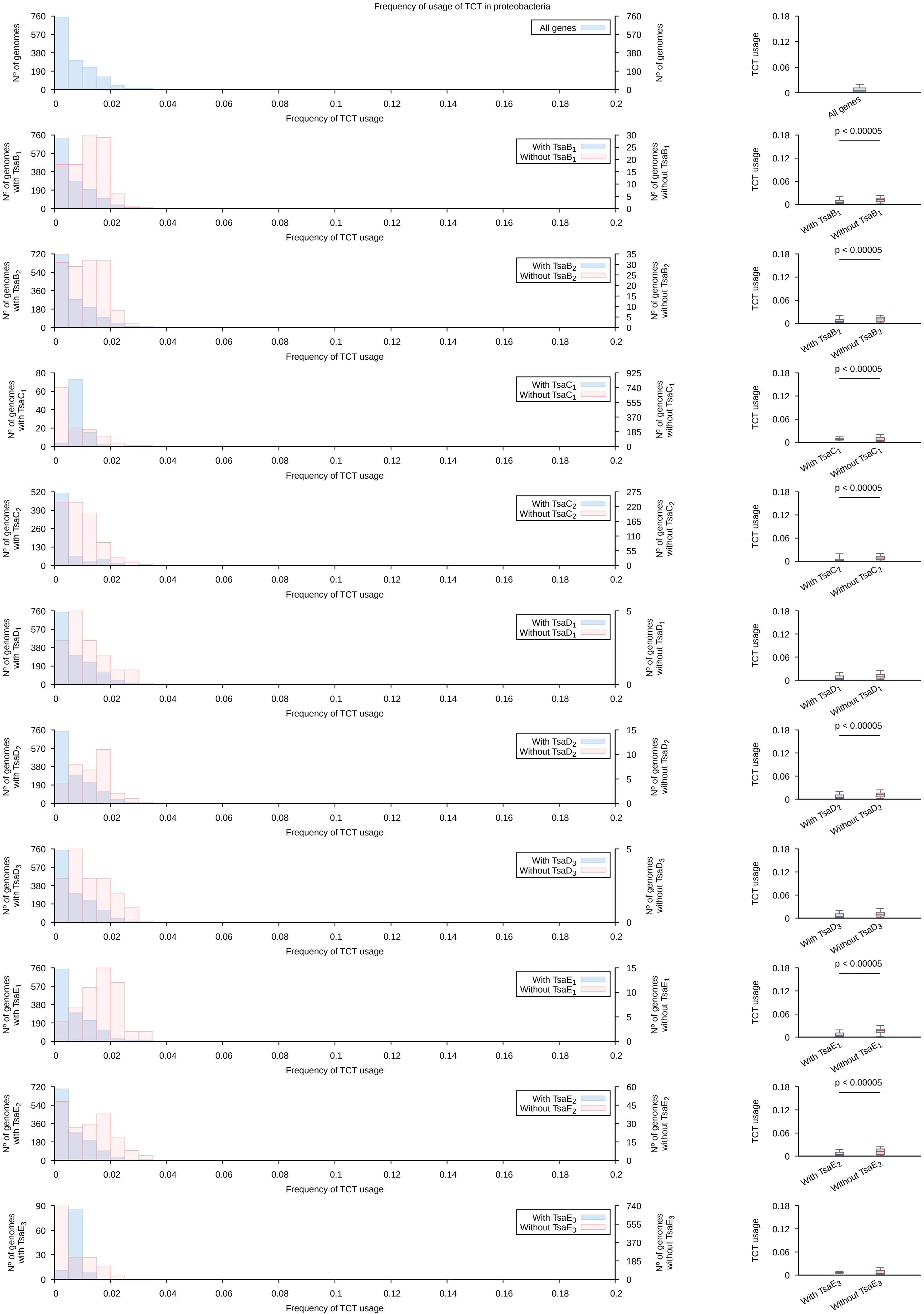

Frequency of usage of TCT in proteobacteria
All genes
Nº of genomes
Frequency of TCT usage
TCT usage
With TsaB1
Without TsaB1
Nº of genomes with TsaB1
Nº of genomes without TsaB1
p < 0.00005
With TsaB2
Without TsaB2
Nº of genomes with TsaB2
Nº of genomes without TsaB2
p < 0.00005
With TsaC1
Without TsaC1
Nº of genomes with TsaC1
Nº of genomes without TsaC1
p < 0.00005
With TsaC2
Without TsaC2
Nº of genomes with TsaC2
Nº of genomes without TsaC2
p < 0.00005
With TsaD1
Without TsaD1
Nº of genomes with TsaD1
Nº of genomes without TsaD1
With TsaD2
Without TsaD2
Nº of genomes with TsaD2
Nº of genomes without TsaD2
p < 0.00005
With TsaD3
Without TsaD3
Nº of genomes with TsaD3
Nº of genomes without TsaD3
With TsaE1
Without TsaE1
Nº of genomes with TsaE1
Nº of genomes without TsaE1
p < 0.00005
With TsaE2
Without TsaE2
Nº of genomes with TsaE2
Nº of genomes without TsaE2
p < 0.00005
With TsaE3
Without TsaE3
Nº of genomes with TsaE3
Nº of genomes without TsaE3

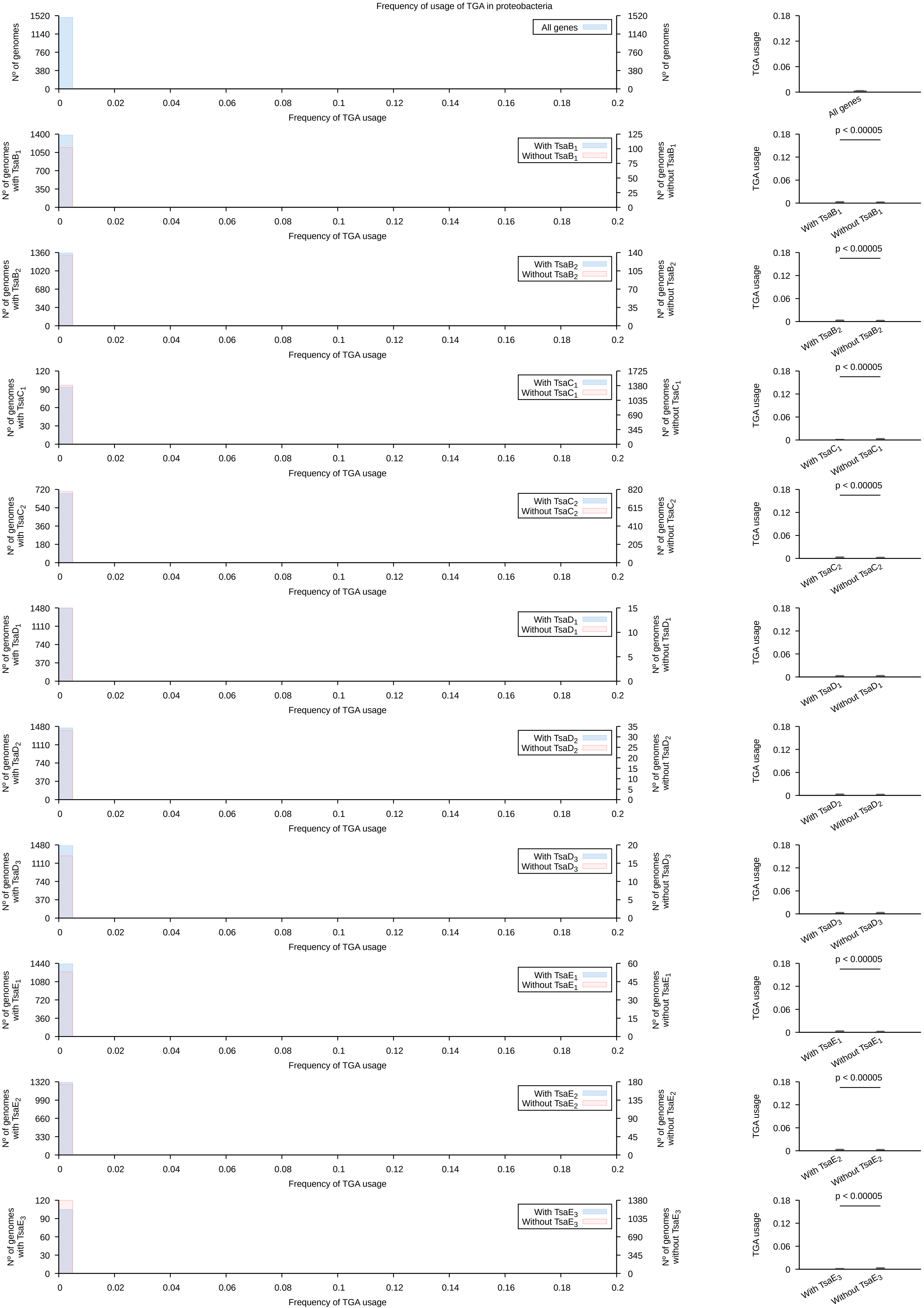

Frequency of usage of TGA in proteobacteria
All genes
Nº of genomes
Frequency of TGA usage
TGA usage
With TsaB$_1$
Without TsaB$_1$
Nº of genomes with TsaB$_1$
Nº of genomes without TsaB$_1$
p < 0.00005
With TsaB$_2$
Without TsaB$_2$
Nº of genomes with TsaB$_2$
Nº of genomes without TsaB$_2$
p < 0.00005
With TsaC$_1$
Without TsaC$_1$
Nº of genomes with TsaC$_1$
Nº of genomes without TsaC$_1$
p < 0.00005
With TsaC$_2$
Without TsaC$_2$
Nº of genomes with TsaC$_2$
Nº of genomes without TsaC$_2$
p < 0.00005
With TsaD$_1$
Without TsaD$_1$
Nº of genomes with TsaD$_1$
Nº of genomes without TsaD$_1$
With TsaD$_2$
Without TsaD$_2$
Nº of genomes with TsaD$_2$
Nº of genomes without TsaD$_2$
With TsaD$_3$
Without TsaD$_3$
Nº of genomes with TsaD$_3$
Nº of genomes without TsaD$_3$
With TsaE$_1$
Without TsaE$_1$
Nº of genomes with TsaE$_1$
Nº of genomes without TsaE$_1$
p < 0.00005
With TsaE$_2$
Without TsaE$_2$
Nº of genomes with TsaE$_2$
Nº of genomes without TsaE$_2$
p < 0.00005
With TsaE$_3$
Without TsaE$_3$
Nº of genomes with TsaE$_3$
Nº of genomes without TsaE$_3$
p < 0.00005

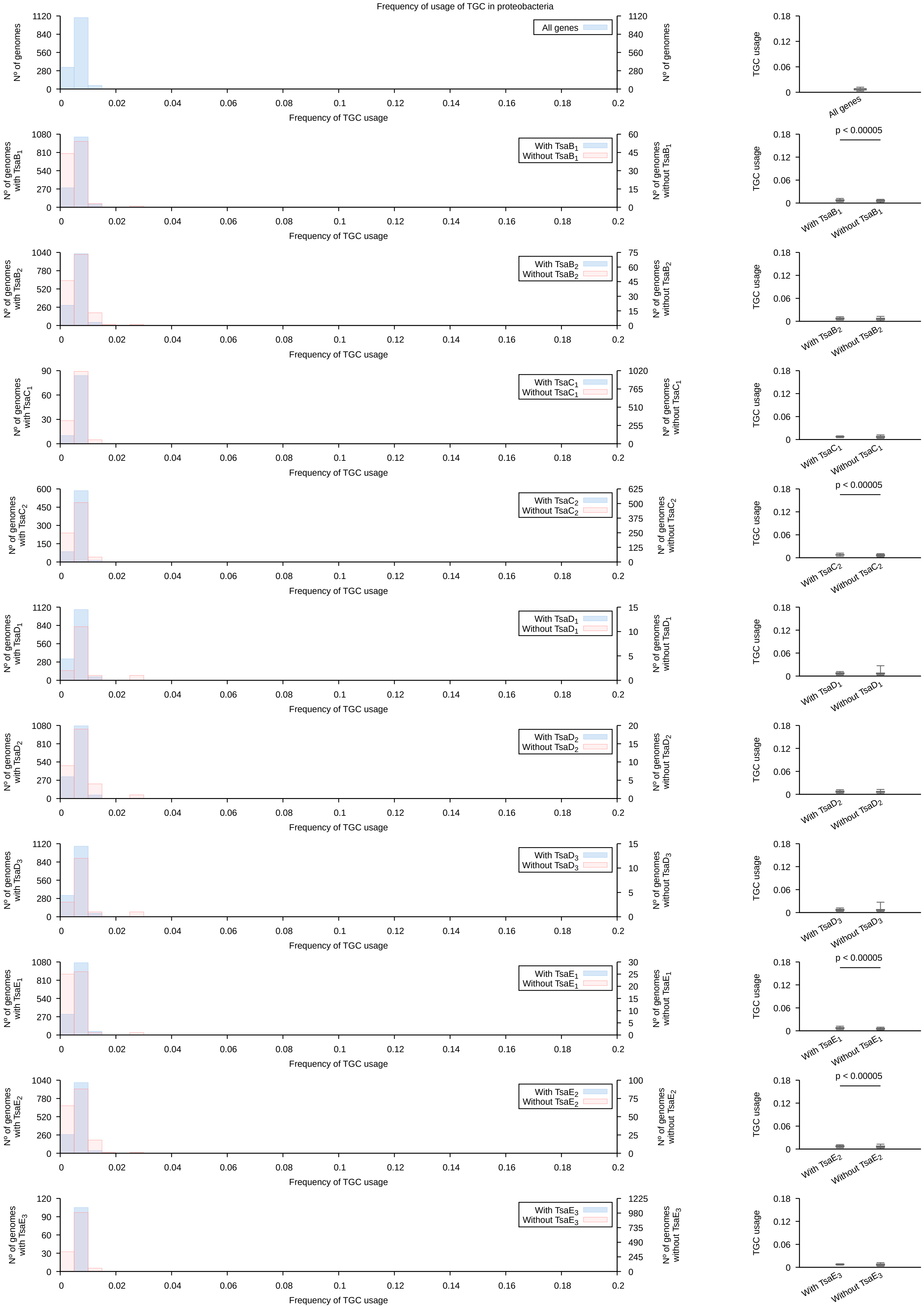
Frequency of usage of TGC in proteobacteria
Nº of genomes
Frequency of TGC usage
TGC usage
All genes
With $TsaB_1$
Without $TsaB_1$
Nº of genomes with $TsaB_1$
Nº of genomes without $TsaB_1$
p < 0.00005
With $TsaB_2$
Without $TsaB_2$
Nº of genomes with $TsaB_2$
Nº of genomes without $TsaB_2$
With $TsaC_1$
Without $TsaC_1$
Nº of genomes with $TsaC_1$
Nº of genomes without $TsaC_1$
With $TsaC_2$
Without $TsaC_2$
Nº of genomes with $TsaC_2$
Nº of genomes without $TsaC_2$
p < 0.00005
With $TsaD_1$
Without $TsaD_1$
Nº of genomes with $TsaD_1$
Nº of genomes without $TsaD_1$
With $TsaD_2$
Without $TsaD_2$
Nº of genomes with $TsaD_2$
Nº of genomes without $TsaD_2$
With $TsaD_3$
Without $TsaD_3$
Nº of genomes with $TsaD_3$
Nº of genomes without $TsaD_3$
With $TsaE_1$
Without $TsaE_1$
Nº of genomes with $TsaE_1$
Nº of genomes without $TsaE_1$
p < 0.00005
With $TsaE_2$
Without $TsaE_2$
Nº of genomes with $TsaE_2$
Nº of genomes without $TsaE_2$
p < 0.00005
With $TsaE_3$
Without $TsaE_3$
Nº of genomes with $TsaE_3$
Nº of genomes without $TsaE_3$

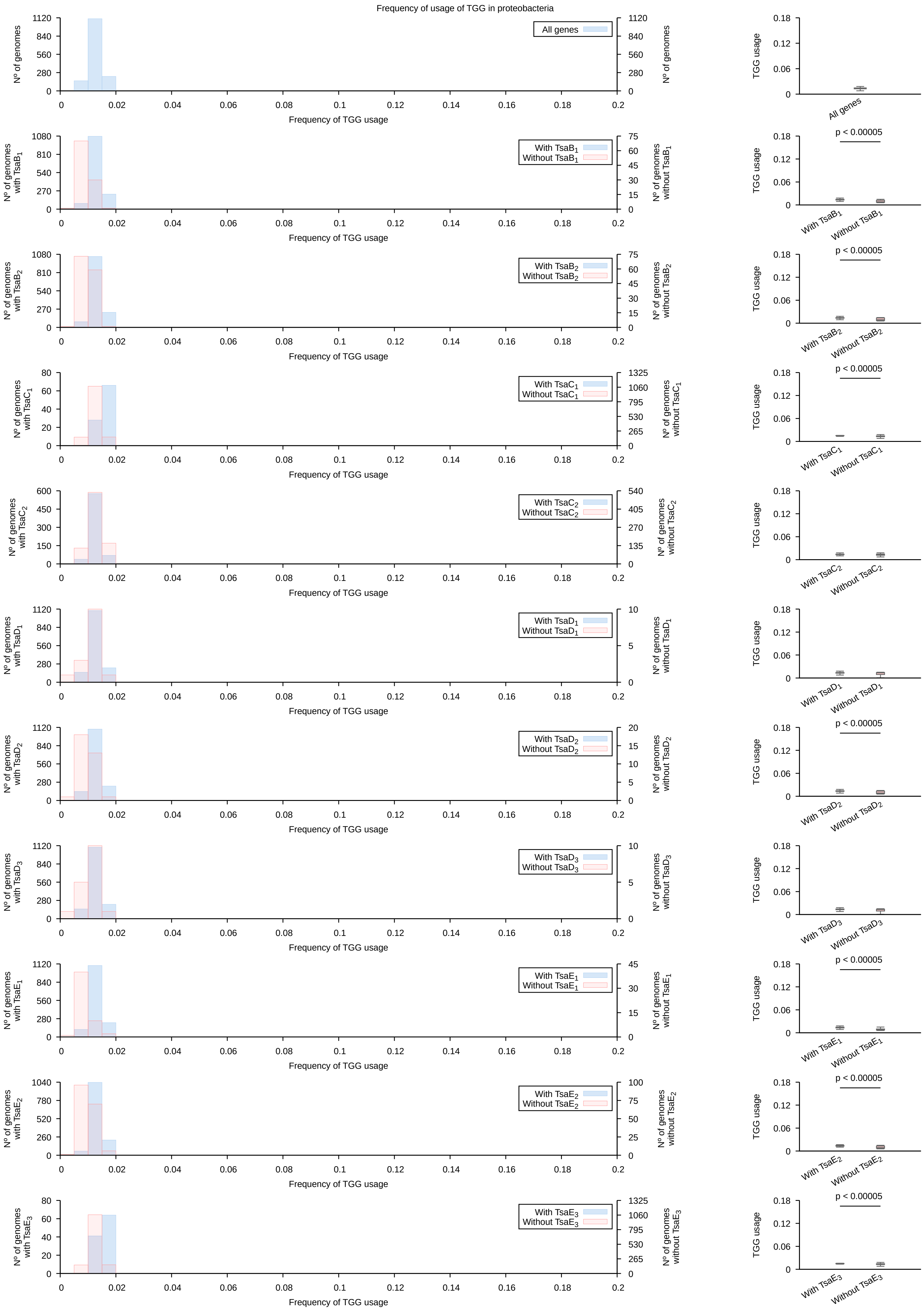

Frequency of usage of TGG in proteobacteria
Nº of genomes
All genes
Frequency of TGG usage
TGG usage
All genes
Nº of genomes with TsaB1
Nº of genomes without TsaB1
With TsaB1
Without TsaB1
p < 0.00005
Nº of genomes with TsaB2
Nº of genomes without TsaB2
With TsaB2
Without TsaB2
p < 0.00005
Nº of genomes with TsaC1
Nº of genomes without TsaC1
With TsaC1
Without TsaC1
p < 0.00005
Nº of genomes with TsaC2
Nº of genomes without TsaC2
With TsaC2
Without TsaC2
Nº of genomes with TsaD1
Nº of genomes without TsaD1
With TsaD1
Without TsaD1
Nº of genomes with TsaD2
Nº of genomes without TsaD2
With TsaD2
Without TsaD2
p < 0.00005
Nº of genomes with TsaD3
Nº of genomes without TsaD3
With TsaD3
Without TsaD3
Nº of genomes with TsaE1
Nº of genomes without TsaE1
With TsaE1
Without TsaE1
p < 0.00005
Nº of genomes with TsaE2
Nº of genomes without TsaE2
With TsaE2
Without TsaE2
p < 0.00005
Nº of genomes with TsaE3
Nº of genomes without TsaE3
With TsaE3
Without TsaE3
p < 0.00005

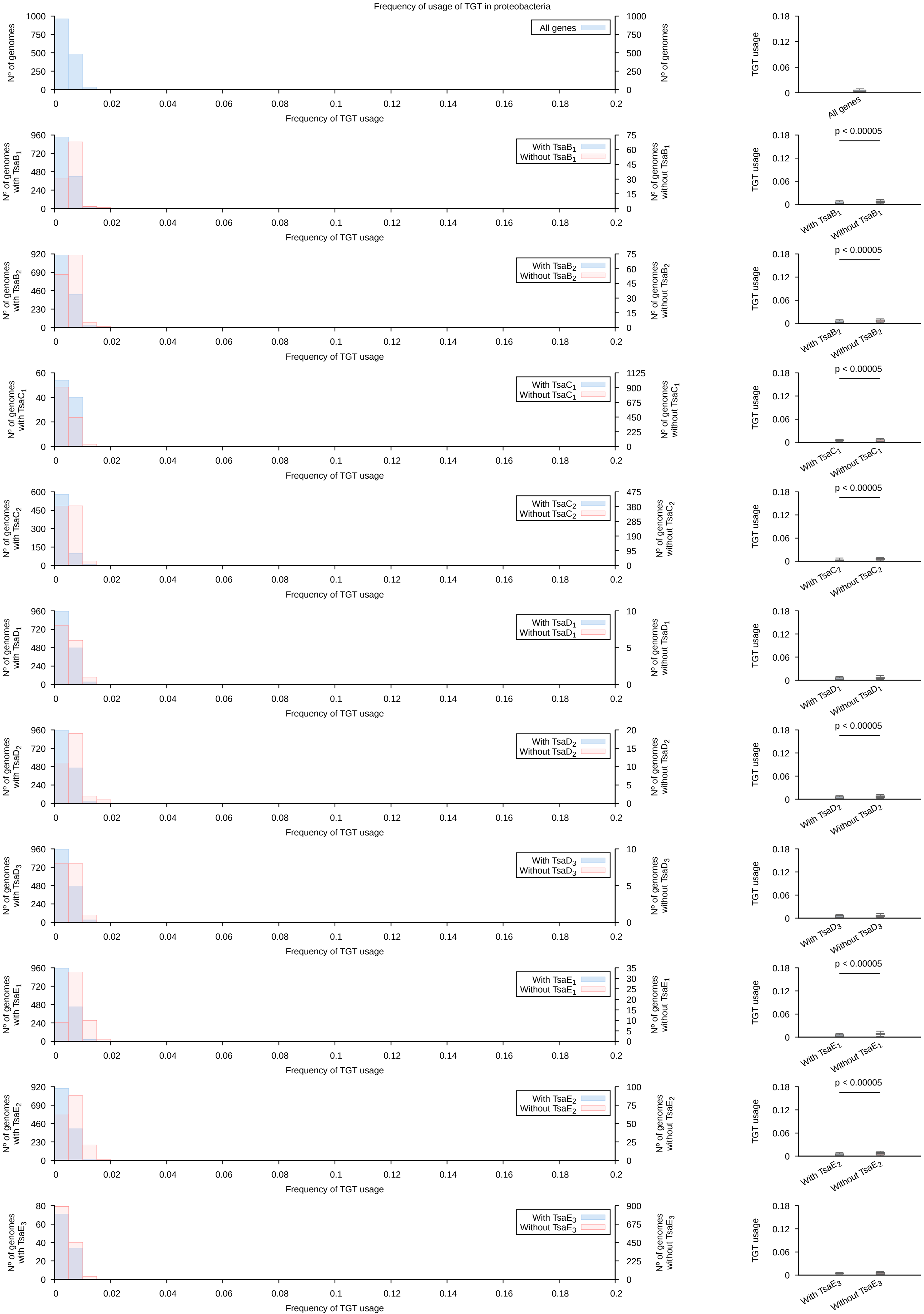

Frequency of usage of TGT in proteobacteria
Nº of genomes
All genes
Frequency of TGT usage
TGT usage
0 0.02 0.04 0.06 0.08 0.1 0.12 0.14 0.16 0.18 0.2
0 0.06 0.12 0.18
Nº of genomes with TsaB$_1$
Nº of genomes without TsaB$_1$
With TsaB$_1$
Without TsaB$_1$
p < 0.00005
Nº of genomes with TsaB$_2$
Nº of genomes without TsaB$_2$
With TsaB$_2$
Without TsaB$_2$
p < 0.00005
Nº of genomes with TsaC$_1$
Nº of genomes without TsaC$_1$
With TsaC$_1$
Without TsaC$_1$
p < 0.00005
Nº of genomes with TsaC$_2$
Nº of genomes without TsaC$_2$
With TsaC$_2$
Without TsaC$_2$
p < 0.00005
Nº of genomes with TsaD$_1$
Nº of genomes without TsaD$_1$
With TsaD$_1$
Without TsaD$_1$
Nº of genomes with TsaD$_2$
Nº of genomes without TsaD$_2$
With TsaD$_2$
Without TsaD$_2$
p < 0.00005
Nº of genomes with TsaD$_3$
Nº of genomes without TsaD$_3$
With TsaD$_3$
Without TsaD$_3$
Nº of genomes with TsaE$_1$
Nº of genomes without TsaE$_1$
With TsaE$_1$
Without TsaE$_1$
p < 0.00005
Nº of genomes with TsaE$_2$
Nº of genomes without TsaE$_2$
With TsaE$_2$
Without TsaE$_2$
p < 0.00005
Nº of genomes with TsaE$_3$
Nº of genomes without TsaE$_3$
With TsaE$_3$
Without TsaE$_3$

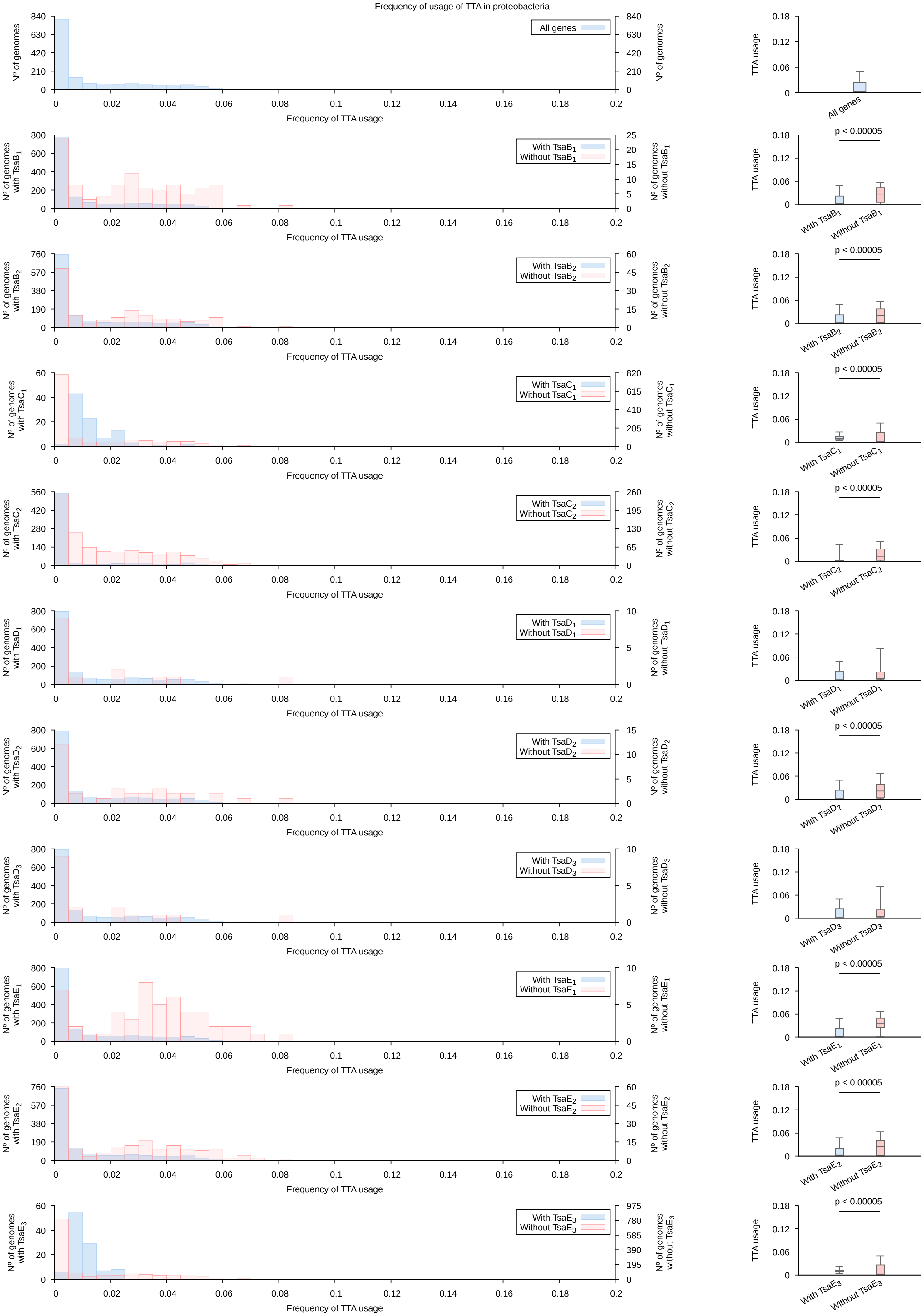
Frequency of usage of TTA in proteobacteria
Nº of genomes
Frequency of TTA usage
TTA usage
All genes
With TsaB$_1$
Without TsaB$_1$
Nº of genomes with TsaB$_1$
Nº of genomes without TsaB$_1$
With TsaB$_2$
Without TsaB$_2$
Nº of genomes with TsaB$_2$
Nº of genomes without TsaB$_2$
With TsaC$_1$
Without TsaC$_1$
Nº of genomes with TsaC$_1$
Nº of genomes without TsaC$_1$
With TsaC$_2$
Without TsaC$_2$
Nº of genomes with TsaC$_2$
Nº of genomes without TsaC$_2$
With TsaD$_1$
Without TsaD$_1$
Nº of genomes with TsaD$_1$
Nº of genomes without TsaD$_1$
With TsaD$_2$
Without TsaD$_2$
Nº of genomes with TsaD$_2$
Nº of genomes without TsaD$_2$
With TsaD$_3$
Without TsaD$_3$
Nº of genomes with TsaD$_3$
Nº of genomes without TsaD$_3$
With TsaE$_1$
Without TsaE$_1$
Nº of genomes with TsaE$_1$
Nº of genomes without TsaE$_1$
With TsaE$_2$
Without TsaE$_2$
Nº of genomes with TsaE$_2$
Nº of genomes without TsaE$_2$
With TsaE$_3$
Without TsaE$_3$
Nº of genomes with TsaE$_3$
Nº of genomes without TsaE$_3$
p < 0.00005

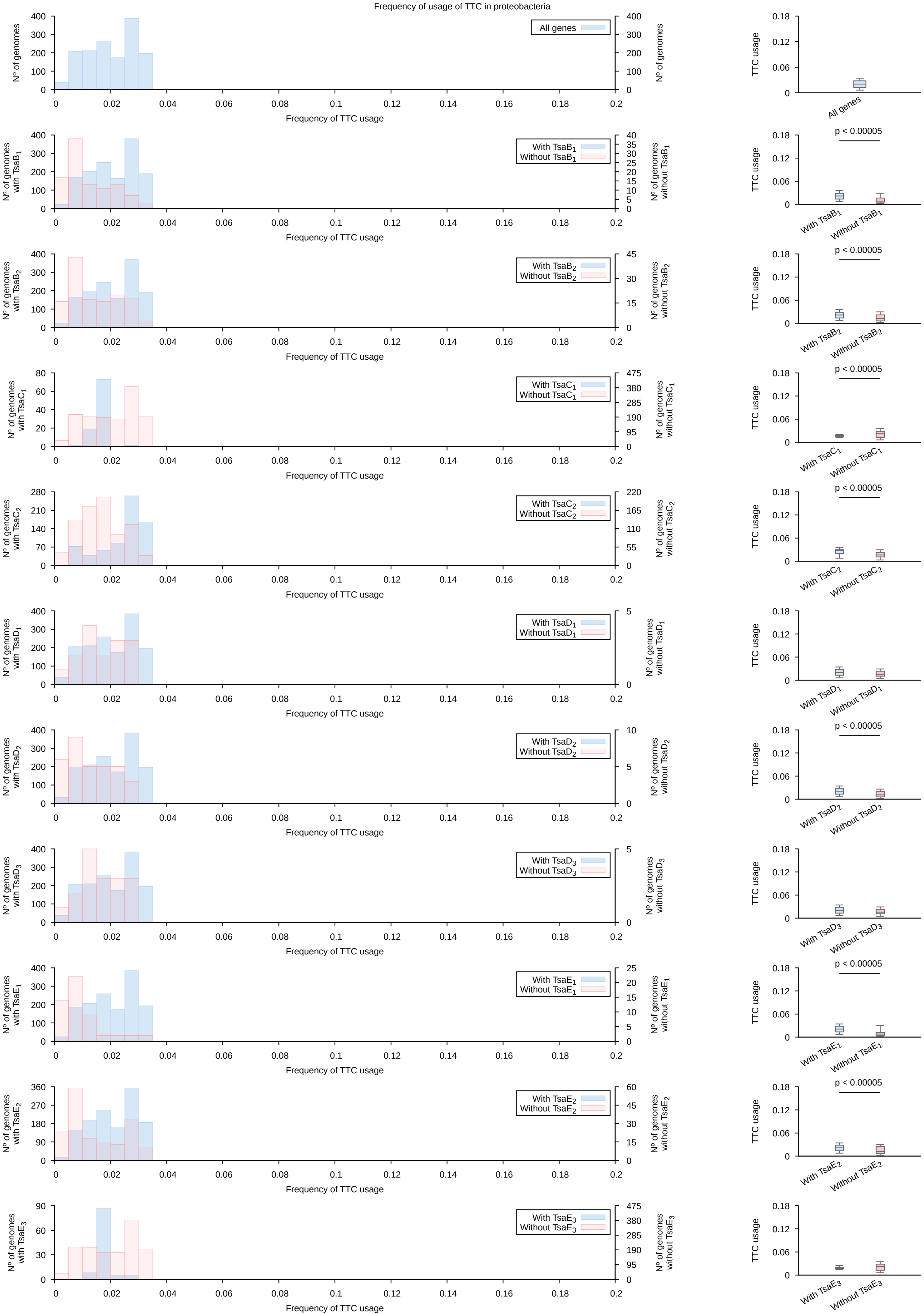

Frequency of usage of TTC in proteobacteria
All genes
Nº of genomes
Frequency of TTC usage
TTC usage
With TsaB1
Without TsaB1
Nº of genomes with TsaB1
Nº of genomes without TsaB1
p < 0.00005
With TsaB2
Without TsaB2
Nº of genomes with TsaB2
Nº of genomes without TsaB2
p < 0.00005
With TsaC1
Without TsaC1
Nº of genomes with TsaC1
Nº of genomes without TsaC1
p < 0.00005
With TsaC2
Without TsaC2
Nº of genomes with TsaC2
Nº of genomes without TsaC2
p < 0.00005
With TsaD1
Without TsaD1
Nº of genomes with TsaD1
Nº of genomes without TsaD1
With TsaD2
Without TsaD2
Nº of genomes with TsaD2
Nº of genomes without TsaD2
p < 0.00005
With TsaD3
Without TsaD3
Nº of genomes with TsaD3
Nº of genomes without TsaD3
With TsaE1
Without TsaE1
Nº of genomes with TsaE1
Nº of genomes without TsaE1
p < 0.00005
With TsaE2
Without TsaE2
Nº of genomes with TsaE2
Nº of genomes without TsaE2
p < 0.00005
With TsaE3
Without TsaE3
Nº of genomes with TsaE3
Nº of genomes without TsaE3

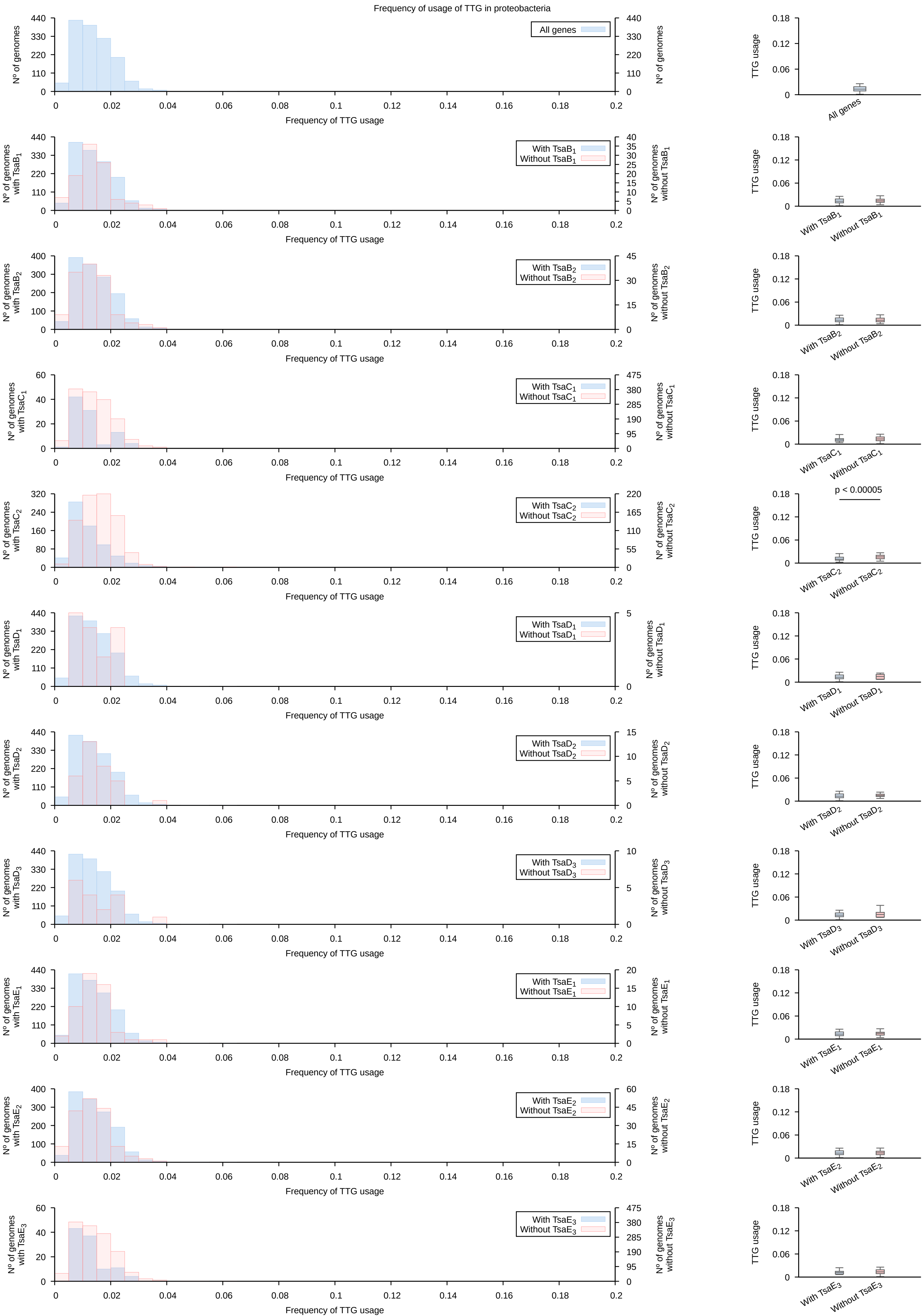
Frequency of usage of TTG in proteobacteria
All genes
Nº of genomes
Frequency of TTG usage
TTG usage
With TsaB$_1$
Without TsaB$_1$
Nº of genomes with TsaB$_1$
Nº of genomes without TsaB$_1$
With TsaB$_2$
Without TsaB$_2$
Nº of genomes with TsaB$_2$
Nº of genomes without TsaB$_2$
With TsaC$_1$
Without TsaC$_1$
Nº of genomes with TsaC$_1$
Nº of genomes without TsaC$_1$
With TsaC$_2$
Without TsaC$_2$
Nº of genomes with TsaC$_2$
Nº of genomes without TsaC$_2$
$p < 0.00005$
With TsaD$_1$
Without TsaD$_1$
Nº of genomes with TsaD$_1$
Nº of genomes without TsaD$_1$
With TsaD$_2$
Without TsaD$_2$
Nº of genomes with TsaD$_2$
Nº of genomes without TsaD$_2$
With TsaD$_3$
Without TsaD$_3$
Nº of genomes with TsaD$_3$
Nº of genomes without TsaD$_3$
With TsaE$_1$
Without TsaE$_1$
Nº of genomes with TsaE$_1$
Nº of genomes without TsaE$_1$
With TsaE$_2$
Without TsaE$_2$
Nº of genomes with TsaE$_2$
Nº of genomes without TsaE$_2$
With TsaE$_3$
Without TsaE$_3$
Nº of genomes with TsaE$_3$
Nº of genomes without TsaE$_3$

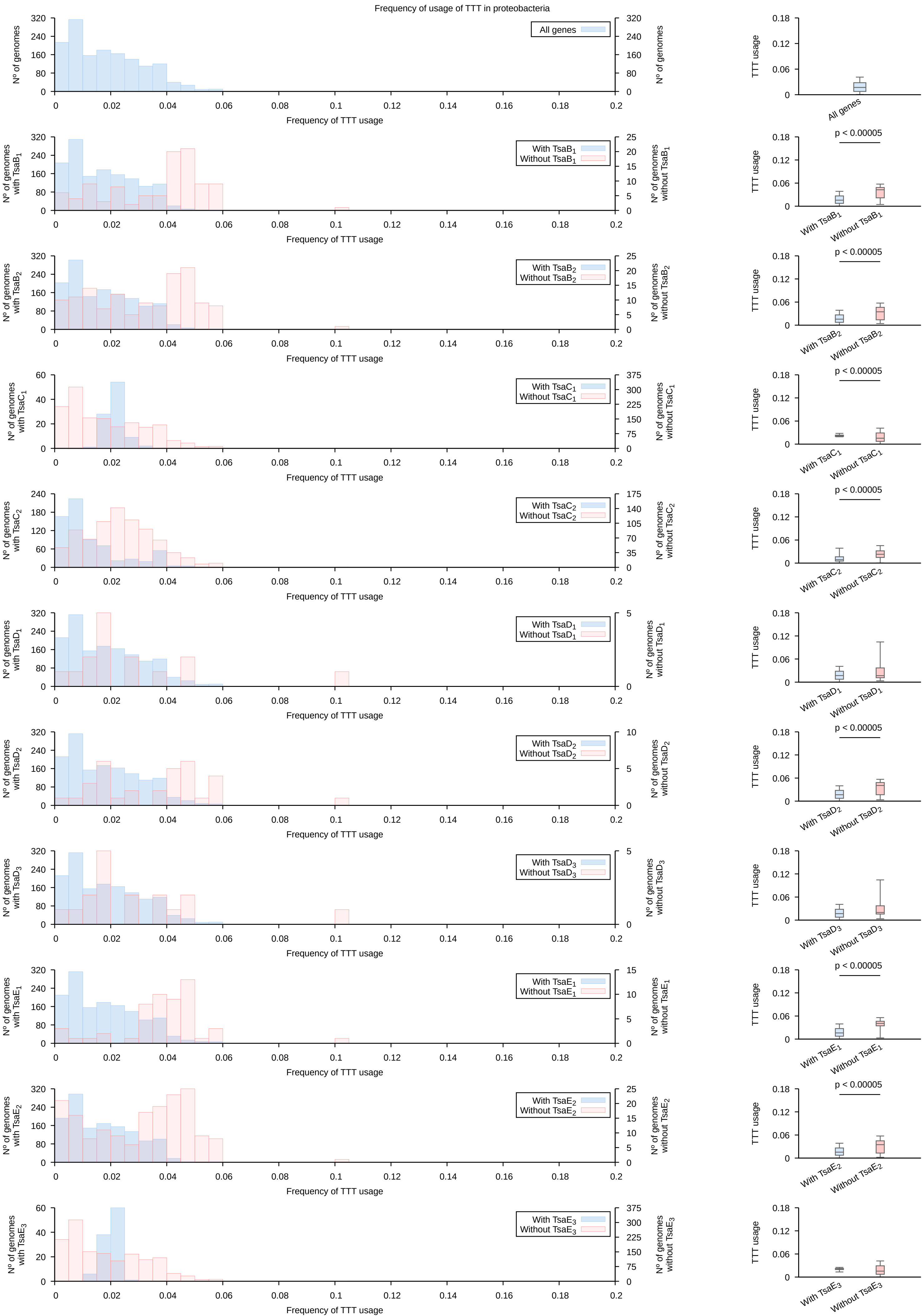
Frequency of usage of TTT in proteobacteria
Nº of genomes
All genes
Frequency of TTT usage
TTT usage
Nº of genomes with TsaB1
Nº of genomes without TsaB1
With TsaB1
Without TsaB1
p < 0.00005
Nº of genomes with TsaB2
Nº of genomes without TsaB2
With TsaB2
Without TsaB2
p < 0.00005
Nº of genomes with TsaC1
Nº of genomes without TsaC1
With TsaC1
Without TsaC1
p < 0.00005
Nº of genomes with TsaC2
Nº of genomes without TsaC2
With TsaC2
Without TsaC2
p < 0.00005
Nº of genomes with TsaD1
Nº of genomes without TsaD1
With TsaD1
Without TsaD1
Nº of genomes with TsaD2
Nº of genomes without TsaD2
With TsaD2
Without TsaD2
p < 0.00005
Nº of genomes with TsaD3
Nº of genomes without TsaD3
With TsaD3
Without TsaD3
Nº of genomes with TsaE1
Nº of genomes without TsaE1
With TsaE1
Without TsaE1
p < 0.00005
Nº of genomes with TsaE2
Nº of genomes without TsaE2
With TsaE2
Without TsaE2
p < 0.00005
Nº of genomes with TsaE3
Nº of genomes without TsaE3
With TsaE3
Without TsaE3

Figure S12. Histogram of codon usage in all genes of genomes that present or lack genes involved in GluQ formation. Top left graph: histogram of the usage of the indicated codon in highly expressed genes of proteobacterial genomes. **Top right graph:** box plot indicating the distribution of codon usage in the same population. **Lower graphs:** The collection of 1484 proteobacterial genomes were separated in two groups, based on the presence (light blue) or absence (orange) of the indicated pattern in any gene of the analyzed genomes. Patterns are indicated using subscript numbers. See table S4 for a detailed description of the used patterns. **Left:** Data is represented in superposed histograms. **Right:** Data is represented in a boxplot. An horizontal bar was added when the two populations are different based on Wilcoxon-Mann-Whitney test and $p < 0.00005$. Whiskers include 95% of data.

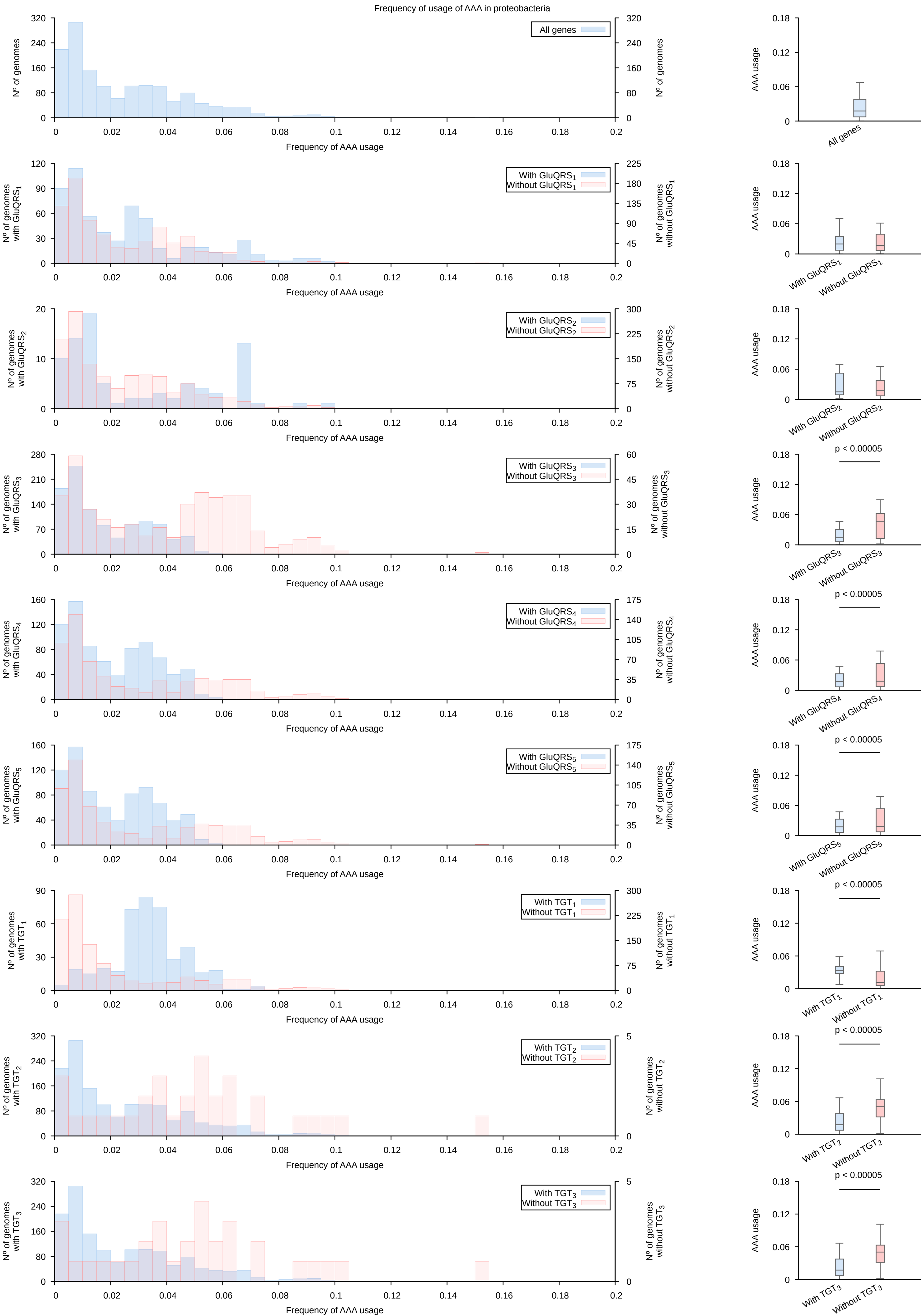
Frequency of usage of AAA in proteobacteria
Nº of genomes
All genes
Frequency of AAA usage
AAA usage
Nº of genomes with GluQRS$_1$
Nº of genomes without GluQRS$_1$
With GluQRS$_1$
Without GluQRS$_1$
Nº of genomes with GluQRS$_2$
Nº of genomes without GluQRS$_2$
With GluQRS$_2$
Without GluQRS$_2$
Nº of genomes with GluQRS$_3$
Nº of genomes without GluQRS$_3$
With GluQRS$_3$
Without GluQRS$_3$
p < 0.00005
Nº of genomes with GluQRS$_4$
Nº of genomes without GluQRS$_4$
With GluQRS$_4$
Without GluQRS$_4$
p < 0.00005
Nº of genomes with GluQRS$_5$
Nº of genomes without GluQRS$_5$
With GluQRS$_5$
Without GluQRS$_5$
p < 0.00005
Nº of genomes with TGT$_1$
Nº of genomes without TGT$_1$
With TGT$_1$
Without TGT$_1$
p < 0.00005
Nº of genomes with TGT$_2$
Nº of genomes without TGT$_2$
With TGT$_2$
Without TGT$_2$
p < 0.00005
Nº of genomes with TGT$_3$
Nº of genomes without TGT$_3$
With TGT$_3$
Without TGT$_3$
p < 0.00005

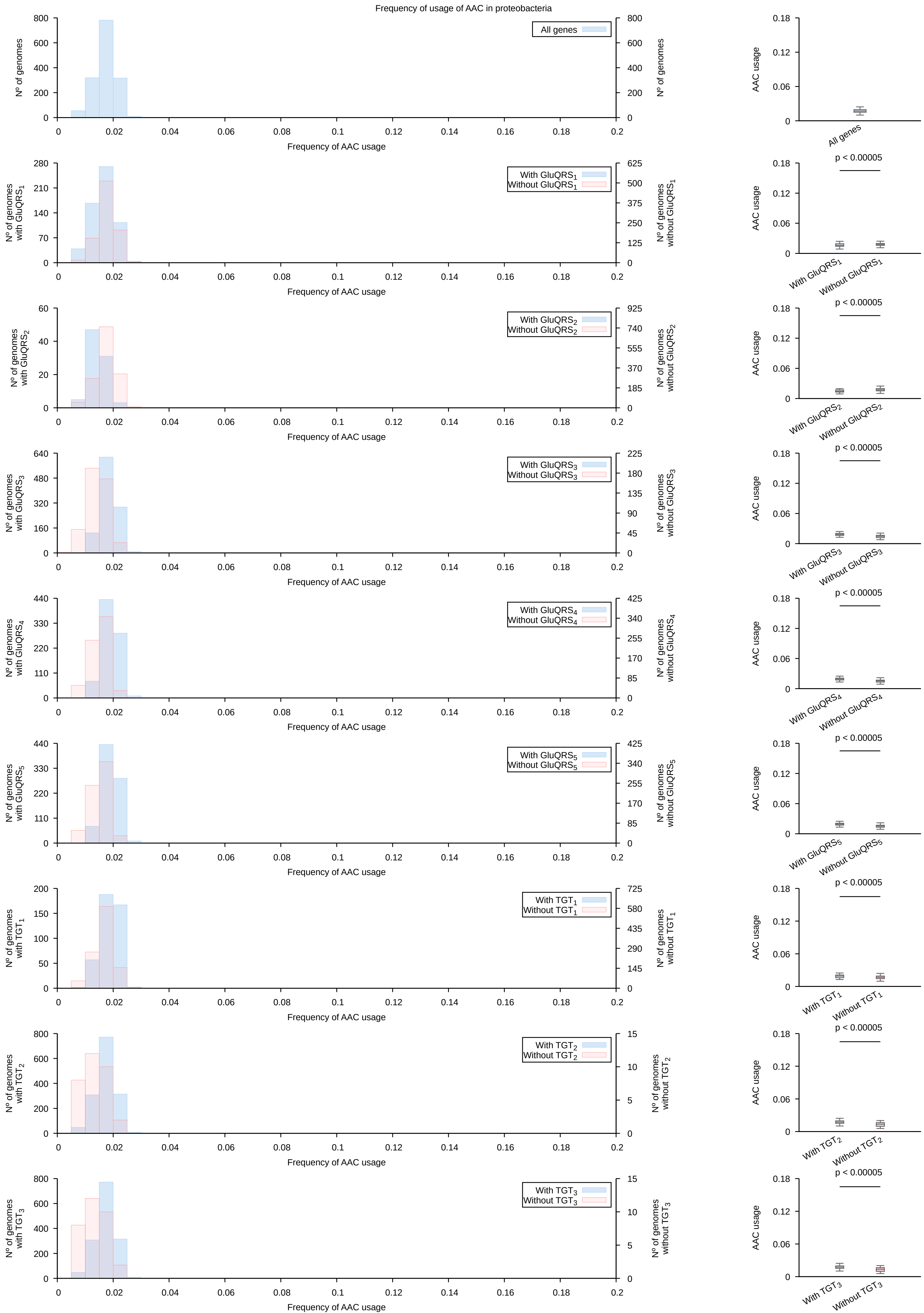
Frequency of usage of AAC in proteobacteria
Nº of genomes
All genes
Frequency of AAC usage
AAC usage
Nº of genomes with GluQRS$_1$
Nº of genomes without GluQRS$_1$
With GluQRS$_1$
Without GluQRS$_1$
p < 0.00005
Nº of genomes with GluQRS$_2$
Nº of genomes without GluQRS$_2$
With GluQRS$_2$
Without GluQRS$_2$
Nº of genomes with GluQRS$_3$
Nº of genomes without GluQRS$_3$
With GluQRS$_3$
Without GluQRS$_3$
Nº of genomes with GluQRS$_4$
Nº of genomes without GluQRS$_4$
With GluQRS$_4$
Without GluQRS$_4$
Nº of genomes with GluQRS$_5$
Nº of genomes without GluQRS$_5$
With GluQRS$_5$
Without GluQRS$_5$
Nº of genomes with TGT$_1$
Nº of genomes without TGT$_1$
With TGT$_1$
Without TGT$_1$
Nº of genomes with TGT$_2$
Nº of genomes without TGT$_2$
With TGT$_2$
Without TGT$_2$
Nº of genomes with TGT$_3$
Nº of genomes without TGT$_3$
With TGT$_3$
Without TGT$_3$

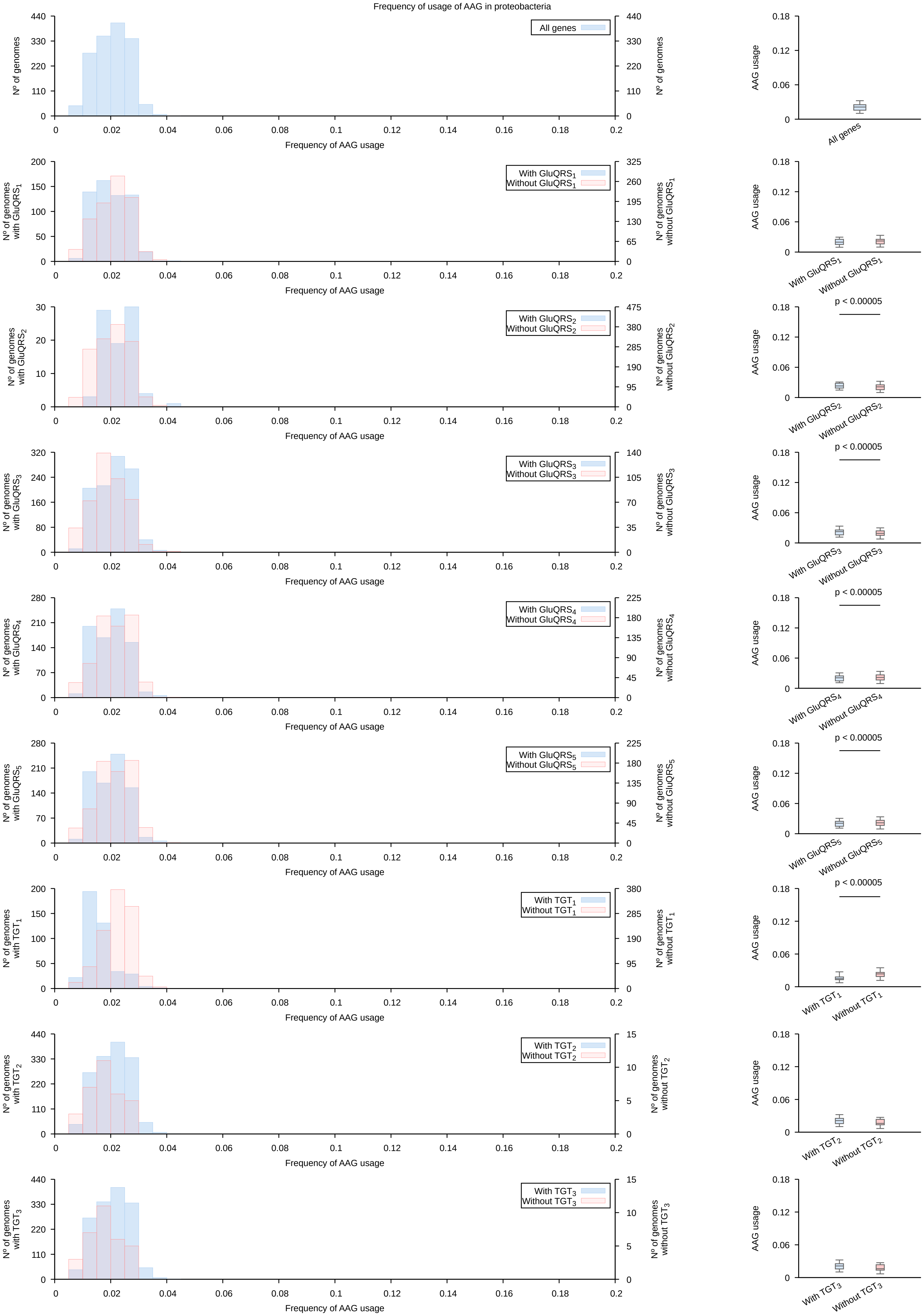

Frequency of usage of AAG in proteobacteria
All genes
With GluQRS1
Without GluQRS1
With GluQRS2
Without GluQRS2
With GluQRS3
Without GluQRS3
With GluQRS4
Without GluQRS4
With GluQRS5
Without GluQRS5
With TGT1
Without TGT1
With TGT2
Without TGT2
With TGT3
Without TGT3
Nº of genomes
Frequency of AAG usage
AAG usage
p < 0.00005

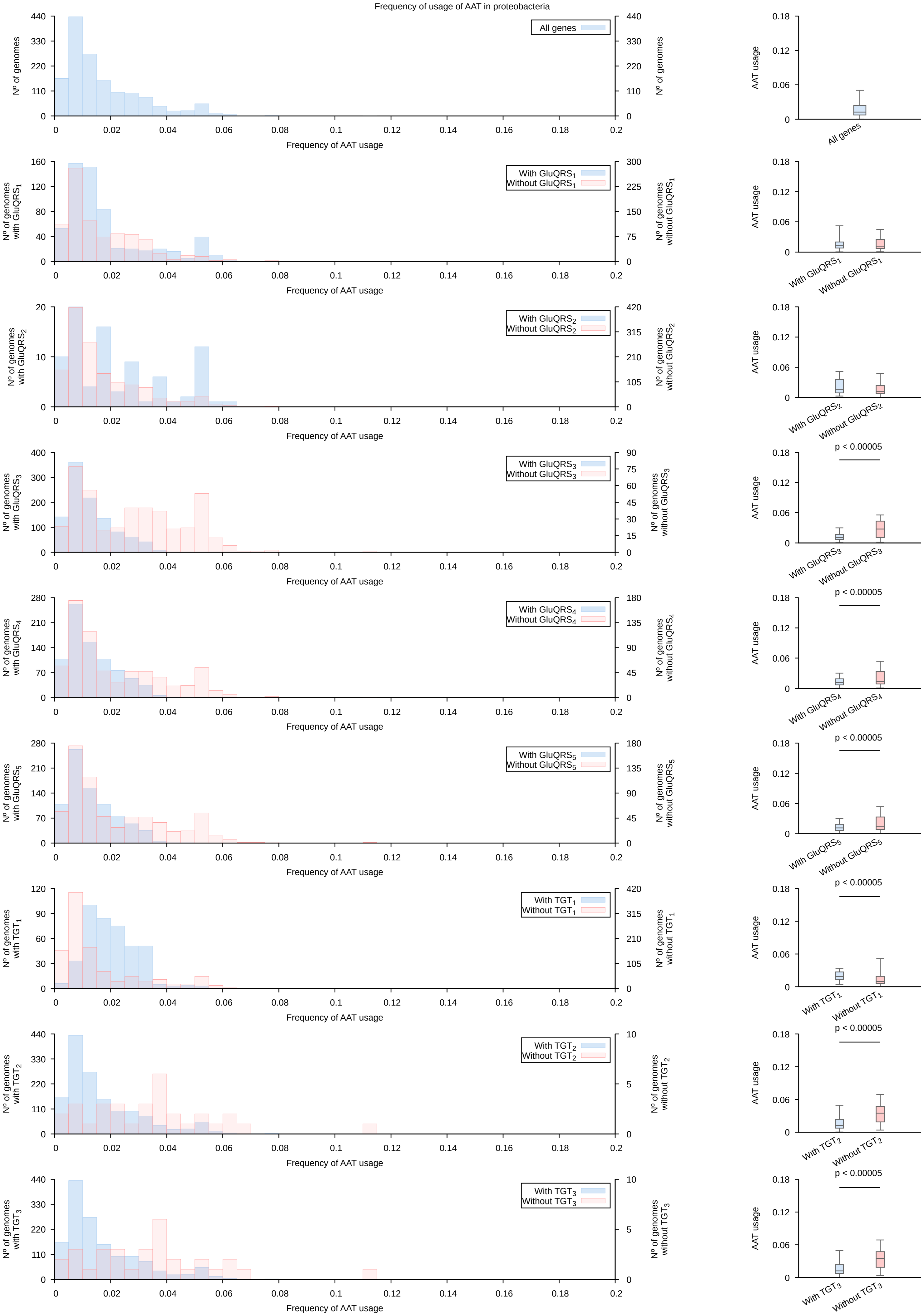

Frequency of usage of AAT in proteobacteria
Nº of genomes
Frequency of AAT usage
AAT usage
All genes
With GluQRS1
Without GluQRS1
Nº of genomes with GluQRS1
Nº of genomes without GluQRS1
With GluQRS2
Without GluQRS2
Nº of genomes with GluQRS2
Nº of genomes without GluQRS2
With GluQRS3
Without GluQRS3
Nº of genomes with GluQRS3
Nº of genomes without GluQRS3
With GluQRS4
Without GluQRS4
Nº of genomes with GluQRS4
Nº of genomes without GluQRS4
With GluQRS5
Without GluQRS5
Nº of genomes with GluQRS5
Nº of genomes without GluQRS5
With TGT1
Without TGT1
Nº of genomes with TGT1
Nº of genomes without TGT1
With TGT2
Without TGT2
Nº of genomes with TGT2
Nº of genomes without TGT2
With TGT3
Without TGT3
Nº of genomes with TGT3
Nº of genomes without TGT3
p < 0.00005

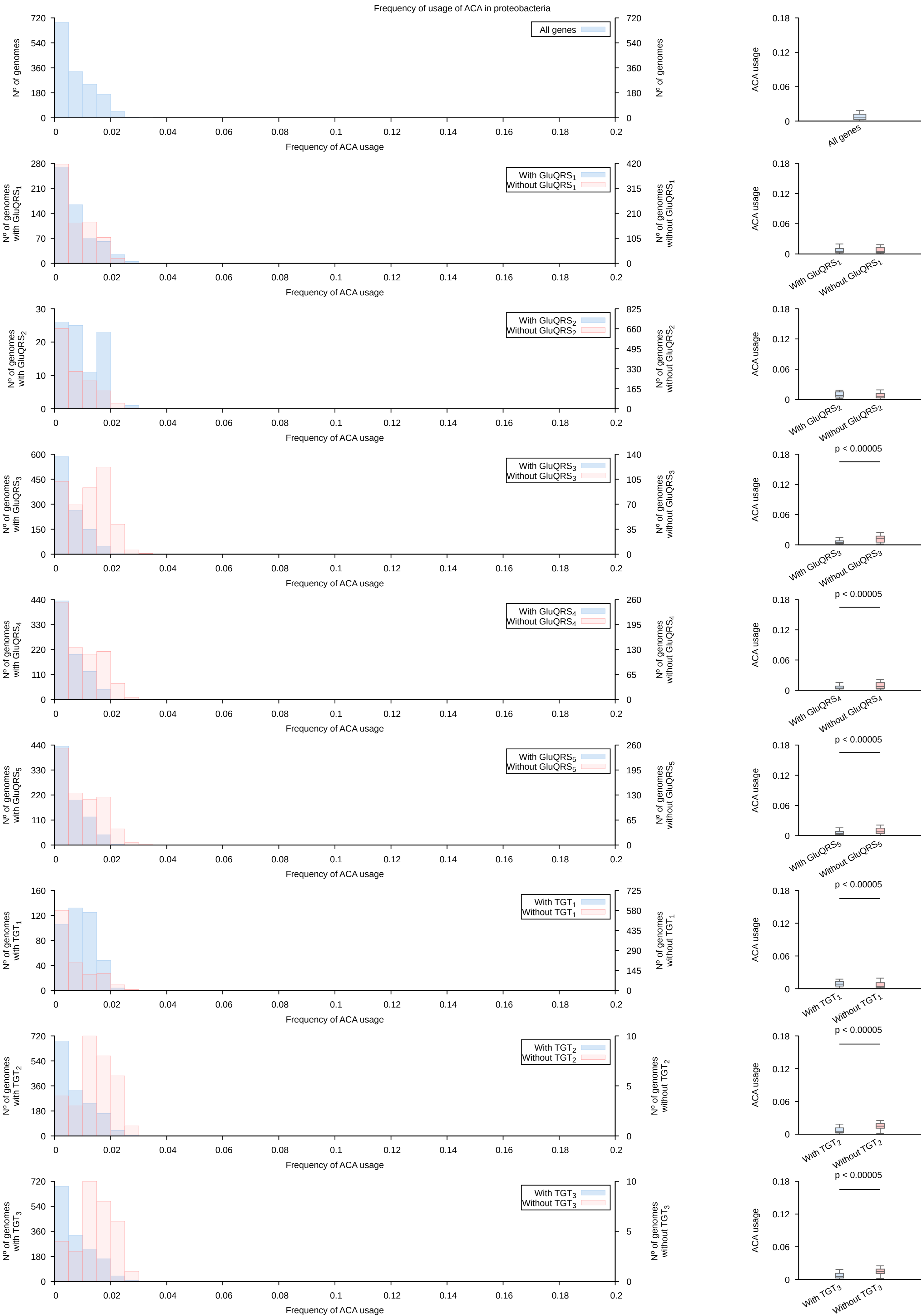
Frequency of usage of ACA in proteobacteria
Nº of genomes
Frequency of ACA usage
ACA usage
All genes
With GluQRS1
Without GluQRS1
Nº of genomes with GluQRS1
Nº of genomes without GluQRS1
With GluQRS2
Without GluQRS2
Nº of genomes with GluQRS2
Nº of genomes without GluQRS2
With GluQRS3
Without GluQRS3
Nº of genomes with GluQRS3
Nº of genomes without GluQRS3
With GluQRS4
Without GluQRS4
Nº of genomes with GluQRS4
Nº of genomes without GluQRS4
With GluQRS5
Without GluQRS5
Nº of genomes with GluQRS5
Nº of genomes without GluQRS5
With TGT1
Without TGT1
Nº of genomes with TGT1
Nº of genomes without TGT1
With TGT2
Without TGT2
Nº of genomes with TGT2
Nº of genomes without TGT2
With TGT3
Without TGT3
Nº of genomes with TGT3
Nº of genomes without TGT3
p < 0.00005

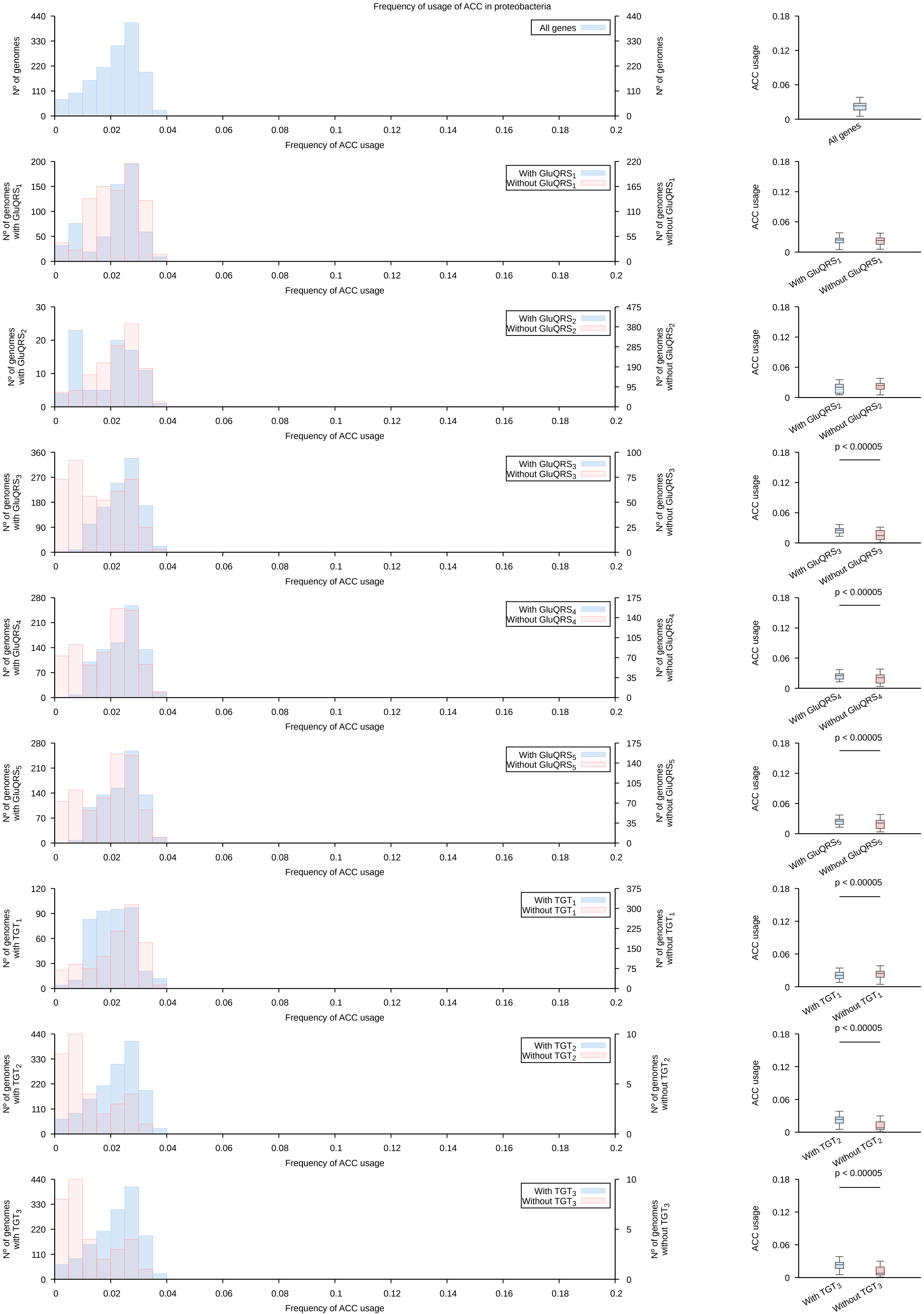

Frequency of usage of ACC in proteobacteria
All genes
Nº of genomes
Frequency of ACC usage
ACC usage
With GluQRS$_1$
Without GluQRS$_1$
Nº of genomes with GluQRS$_1$
Nº of genomes without GluQRS$_1$
With GluQRS$_2$
Without GluQRS$_2$
Nº of genomes with GluQRS$_2$
Nº of genomes without GluQRS$_2$
With GluQRS$_3$
Without GluQRS$_3$
Nº of genomes with GluQRS$_3$
Nº of genomes without GluQRS$_3$
p < 0.00005
With GluQRS$_4$
Without GluQRS$_4$
Nº of genomes with GluQRS$_4$
Nº of genomes without GluQRS$_4$
With GluQRS$_5$
Without GluQRS$_5$
Nº of genomes with GluQRS$_5$
Nº of genomes without GluQRS$_5$
With TGT$_1$
Without TGT$_1$
Nº of genomes with TGT$_1$
Nº of genomes without TGT$_1$
With TGT$_2$
Without TGT$_2$
Nº of genomes with TGT$_2$
Nº of genomes without TGT$_2$
With TGT$_3$
Without TGT$_3$
Nº of genomes with TGT$_3$
Nº of genomes without TGT$_3$
0 0.02 0.04 0.06 0.08 0.1 0.12 0.14 0.16 0.18 0.2
0 0.06 0.12 0.18

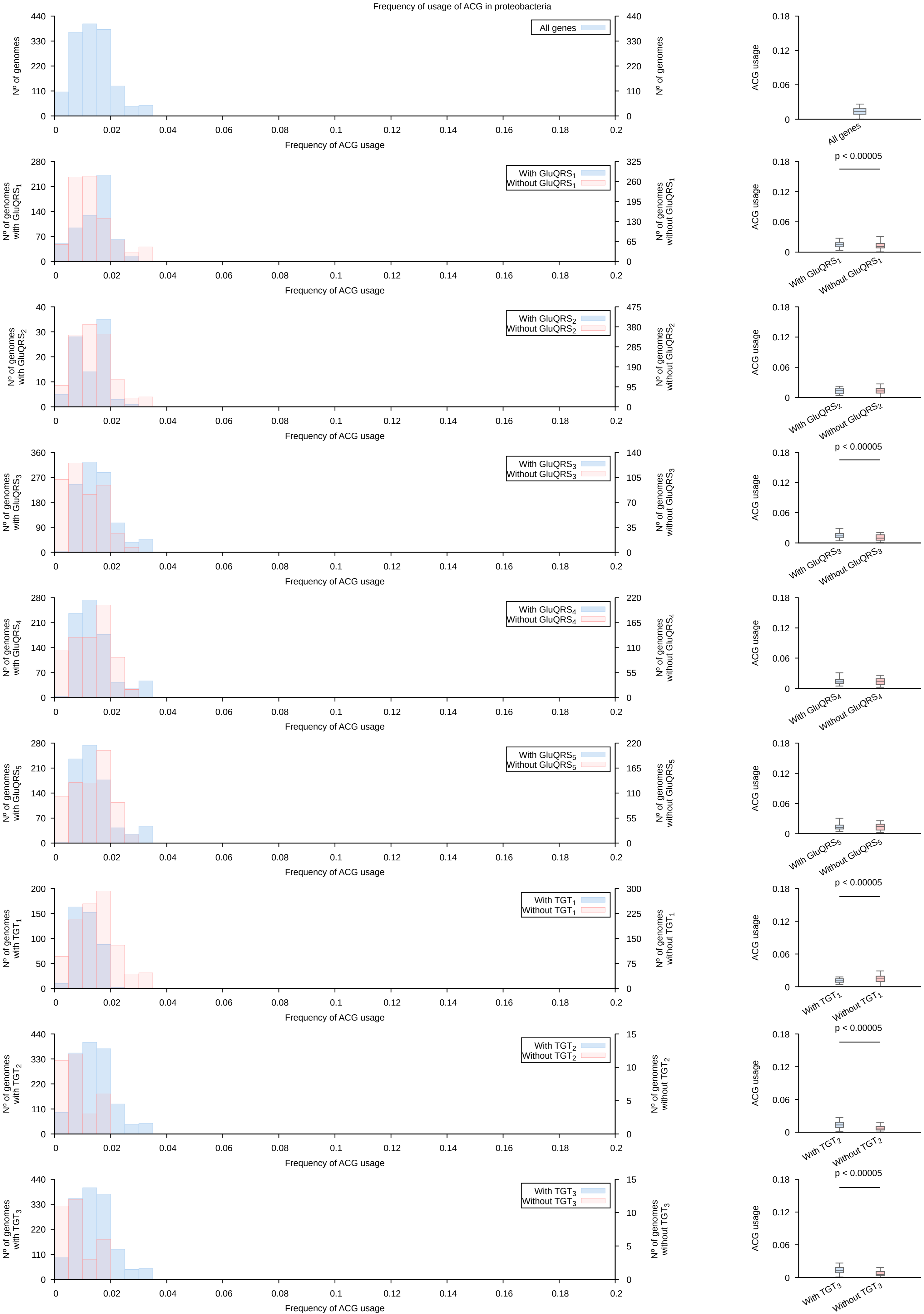

Frequency of usage of ACG in proteobacteria
All genes
Nº of genomes
Frequency of ACG usage
ACG usage
With GluQRS$_1$
Without GluQRS$_1$
Nº of genomes with GluQRS$_1$
Nº of genomes without GluQRS$_1$
p < 0.00005
With GluQRS$_2$
Without GluQRS$_2$
Nº of genomes with GluQRS$_2$
Nº of genomes without GluQRS$_2$
With GluQRS$_3$
Without GluQRS$_3$
Nº of genomes with GluQRS$_3$
Nº of genomes without GluQRS$_3$
p < 0.00005
With GluQRS$_4$
Without GluQRS$_4$
Nº of genomes with GluQRS$_4$
Nº of genomes without GluQRS$_4$
With GluQRS$_5$
Without GluQRS$_5$
Nº of genomes with GluQRS$_5$
Nº of genomes without GluQRS$_5$
With TGT$_1$
Without TGT$_1$
Nº of genomes with TGT$_1$
Nº of genomes without TGT$_1$
p < 0.00005
With TGT$_2$
Without TGT$_2$
Nº of genomes with TGT$_2$
Nº of genomes without TGT$_2$
p < 0.00005
With TGT$_3$
Without TGT$_3$
Nº of genomes with TGT$_3$
Nº of genomes without TGT$_3$
p < 0.00005

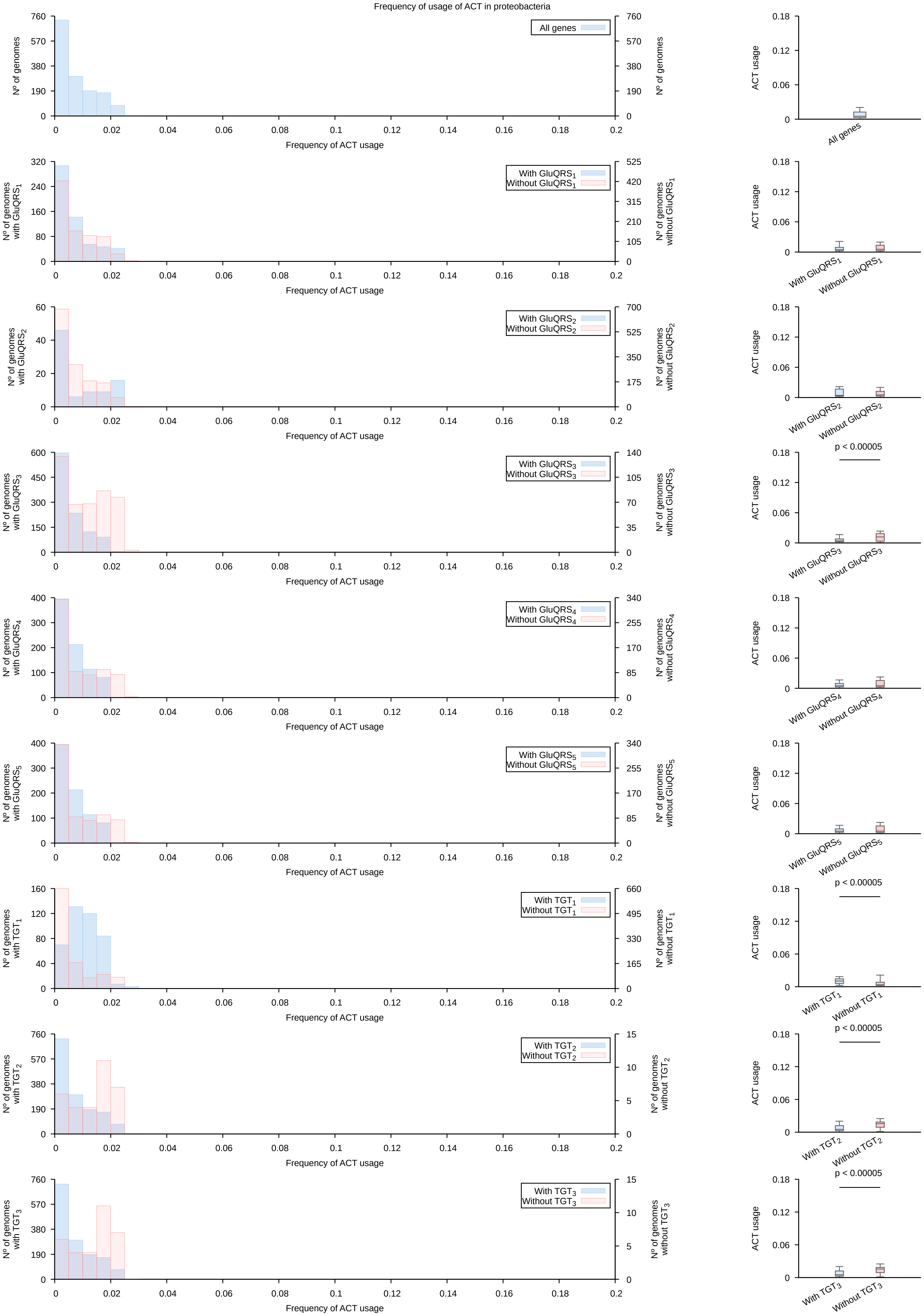
Frequency of usage of ACT in proteobacteria
All genes
Nº of genomes
Frequency of ACT usage
ACT usage
With GluQRS$_1$
Without GluQRS$_1$
Nº of genomes with GluQRS$_1$
Nº of genomes without GluQRS$_1$
With GluQRS$_2$
Without GluQRS$_2$
Nº of genomes with GluQRS$_2$
Nº of genomes without GluQRS$_2$
With GluQRS$_3$
Without GluQRS$_3$
Nº of genomes with GluQRS$_3$
Nº of genomes without GluQRS$_3$
p < 0.00005
With GluQRS$_4$
Without GluQRS$_4$
Nº of genomes with GluQRS$_4$
Nº of genomes without GluQRS$_4$
With GluQRS$_5$
Without GluQRS$_5$
Nº of genomes with GluQRS$_5$
Nº of genomes without GluQRS$_5$
With TGT$_1$
Without TGT$_1$
Nº of genomes with TGT$_1$
Nº of genomes without TGT$_1$
p < 0.00005
With TGT$_2$
Without TGT$_2$
Nº of genomes with TGT$_2$
Nº of genomes without TGT$_2$
p < 0.00005
With TGT$_3$
Without TGT$_3$
Nº of genomes with TGT$_3$
Nº of genomes without TGT$_3$
p < 0.00005

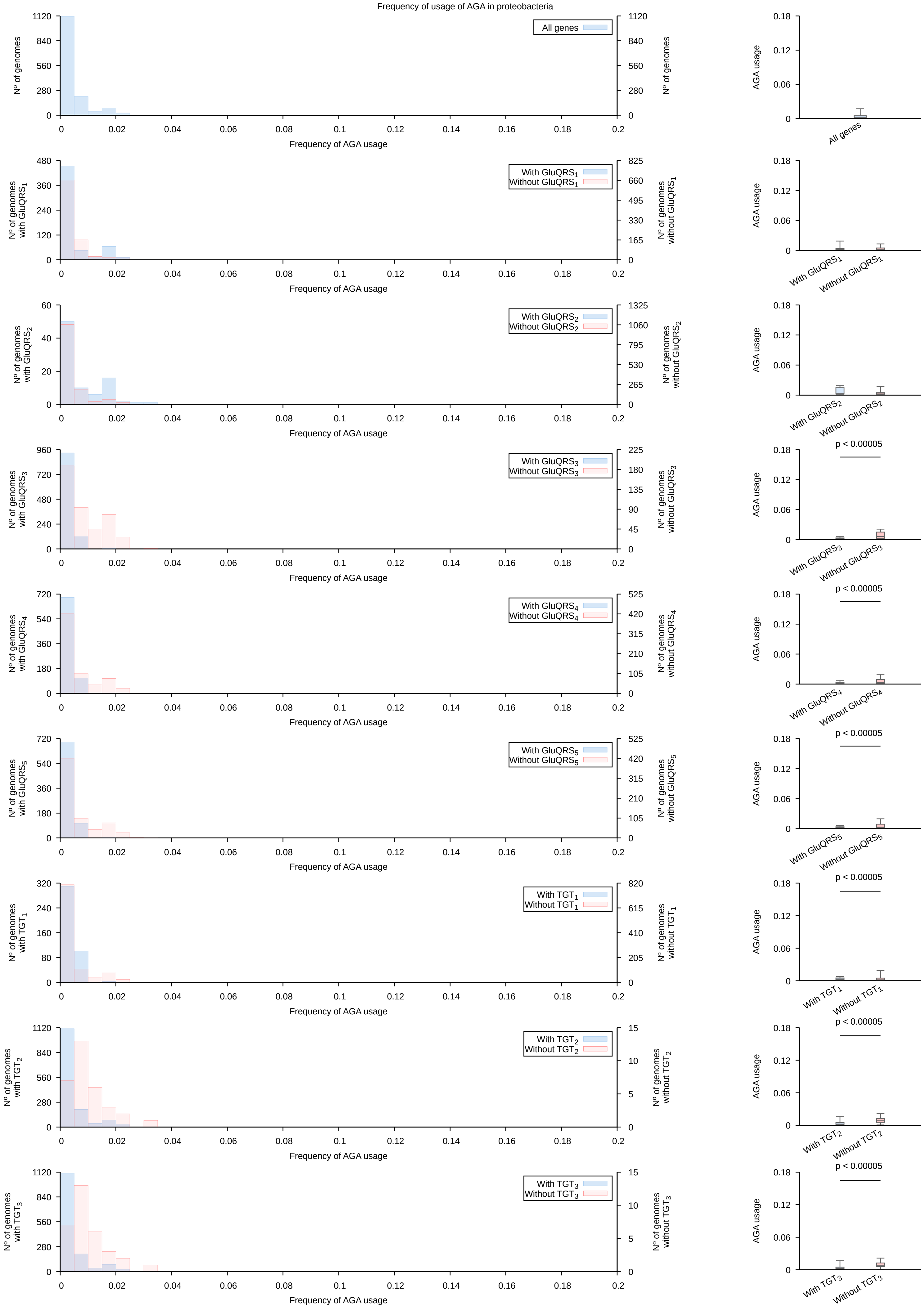
Frequency of usage of AGA in proteobacteria
All genes
With GluQRS1
Without GluQRS1
With GluQRS2
Without GluQRS2
With GluQRS3
Without GluQRS3
With GluQRS4
Without GluQRS4
With GluQRS5
Without GluQRS5
With TGT1
Without TGT1
With TGT2
Without TGT2
With TGT3
Without TGT3
Nº of genomes
Frequency of AGA usage
AGA usage
p < 0.00005

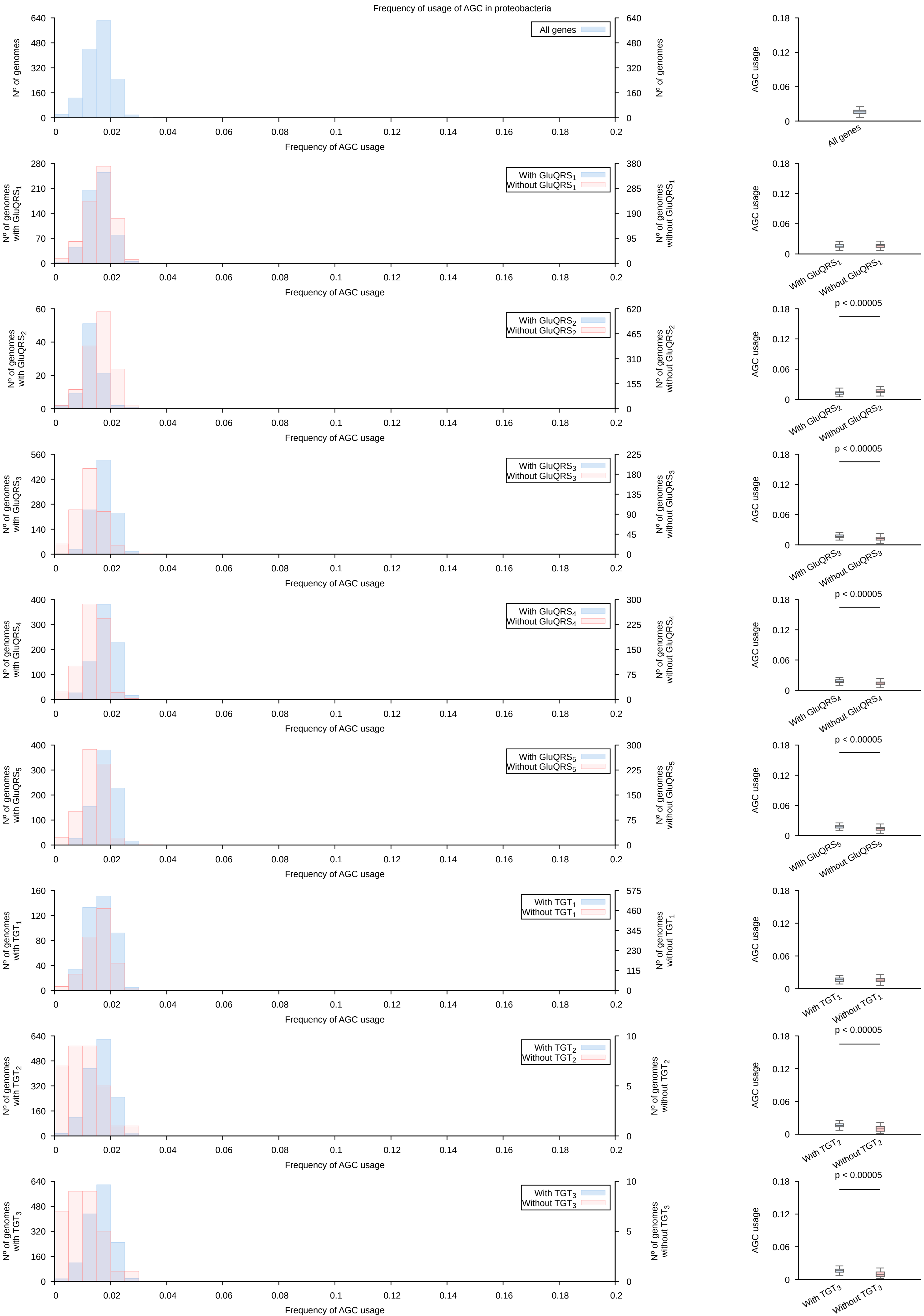
Frequency of usage of AGC in proteobacteria
All genes
Nº of genomes
Frequency of AGC usage
AGC usage
With GluQRS$_1$
Without GluQRS$_1$
Nº of genomes with GluQRS$_1$
Nº of genomes without GluQRS$_1$
With GluQRS$_2$
Without GluQRS$_2$
Nº of genomes with GluQRS$_2$
Nº of genomes without GluQRS$_2$
p < 0.00005
With GluQRS$_3$
Without GluQRS$_3$
Nº of genomes with GluQRS$_3$
Nº of genomes without GluQRS$_3$
p < 0.00005
With GluQRS$_4$
Without GluQRS$_4$
Nº of genomes with GluQRS$_4$
Nº of genomes without GluQRS$_4$
p < 0.00005
With GluQRS$_5$
Without GluQRS$_5$
Nº of genomes with GluQRS$_5$
Nº of genomes without GluQRS$_5$
p < 0.00005
With TGT$_1$
Without TGT$_1$
Nº of genomes with TGT$_1$
Nº of genomes without TGT$_1$
With TGT$_2$
Without TGT$_2$
Nº of genomes with TGT$_2$
Nº of genomes without TGT$_2$
p < 0.00005
With TGT$_3$
Without TGT$_3$
Nº of genomes with TGT$_3$
Nº of genomes without TGT$_3$
p < 0.00005

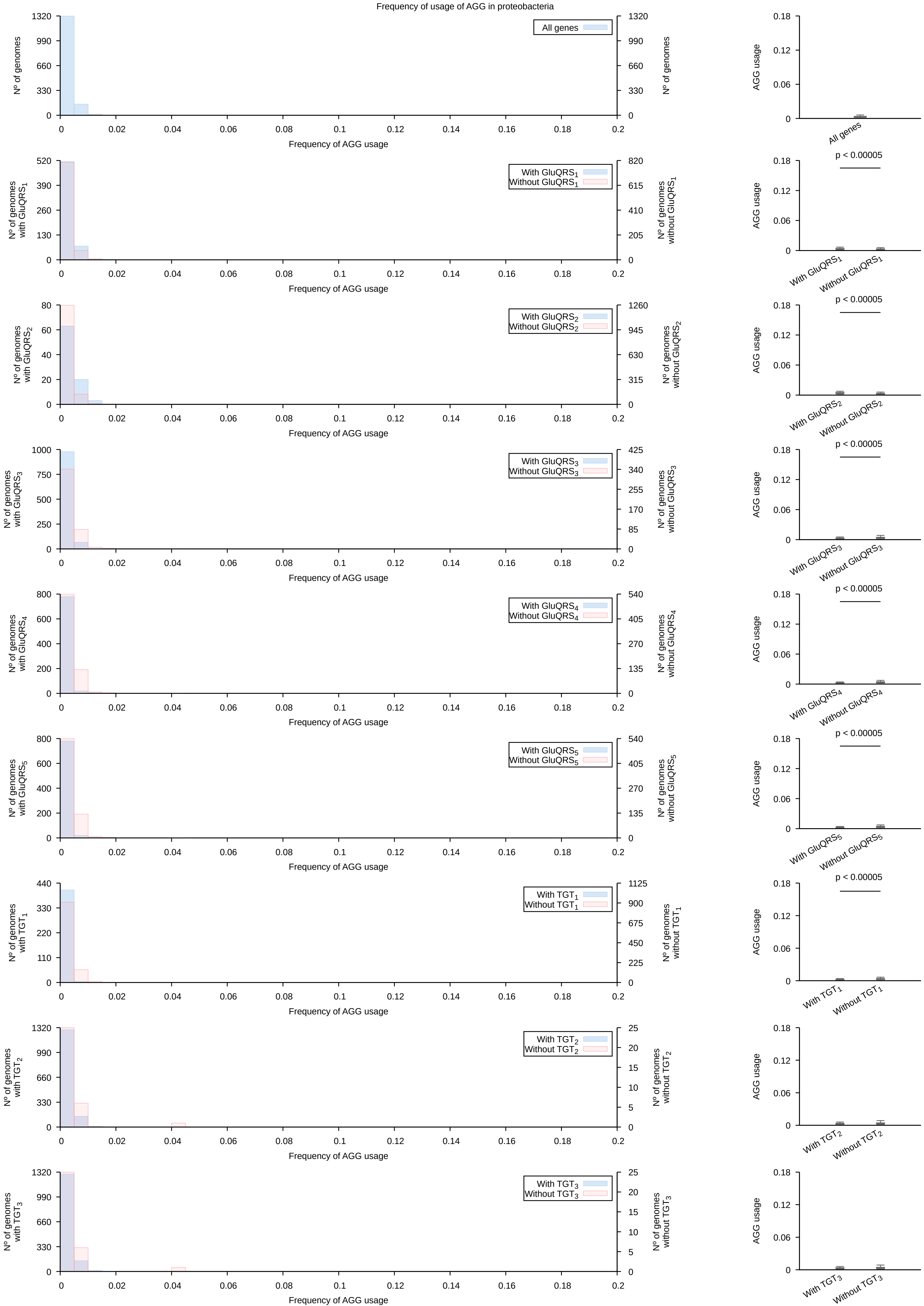

Frequency of usage of AGG in proteobacteria
All genes
Nº of genomes
Frequency of AGG usage
AGG usage
With GluQRS$_1$
Without GluQRS$_1$
Nº of genomes with GluQRS$_1$
Nº of genomes without GluQRS$_1$
p < 0.00005
With GluQRS$_2$
Without GluQRS$_2$
Nº of genomes with GluQRS$_2$
Nº of genomes without GluQRS$_2$
p < 0.00005
With GluQRS$_3$
Without GluQRS$_3$
Nº of genomes with GluQRS$_3$
Nº of genomes without GluQRS$_3$
p < 0.00005
With GluQRS$_4$
Without GluQRS$_4$
Nº of genomes with GluQRS$_4$
Nº of genomes without GluQRS$_4$
p < 0.00005
With GluQRS$_5$
Without GluQRS$_5$
Nº of genomes with GluQRS$_5$
Nº of genomes without GluQRS$_5$
p < 0.00005
With TGT$_1$
Without TGT$_1$
Nº of genomes with TGT$_1$
Nº of genomes without TGT$_1$
p < 0.00005
With TGT$_2$
Without TGT$_2$
Nº of genomes with TGT$_2$
Nº of genomes without TGT$_2$
With TGT$_3$
Without TGT$_3$
Nº of genomes with TGT$_3$
Nº of genomes without TGT$_3$

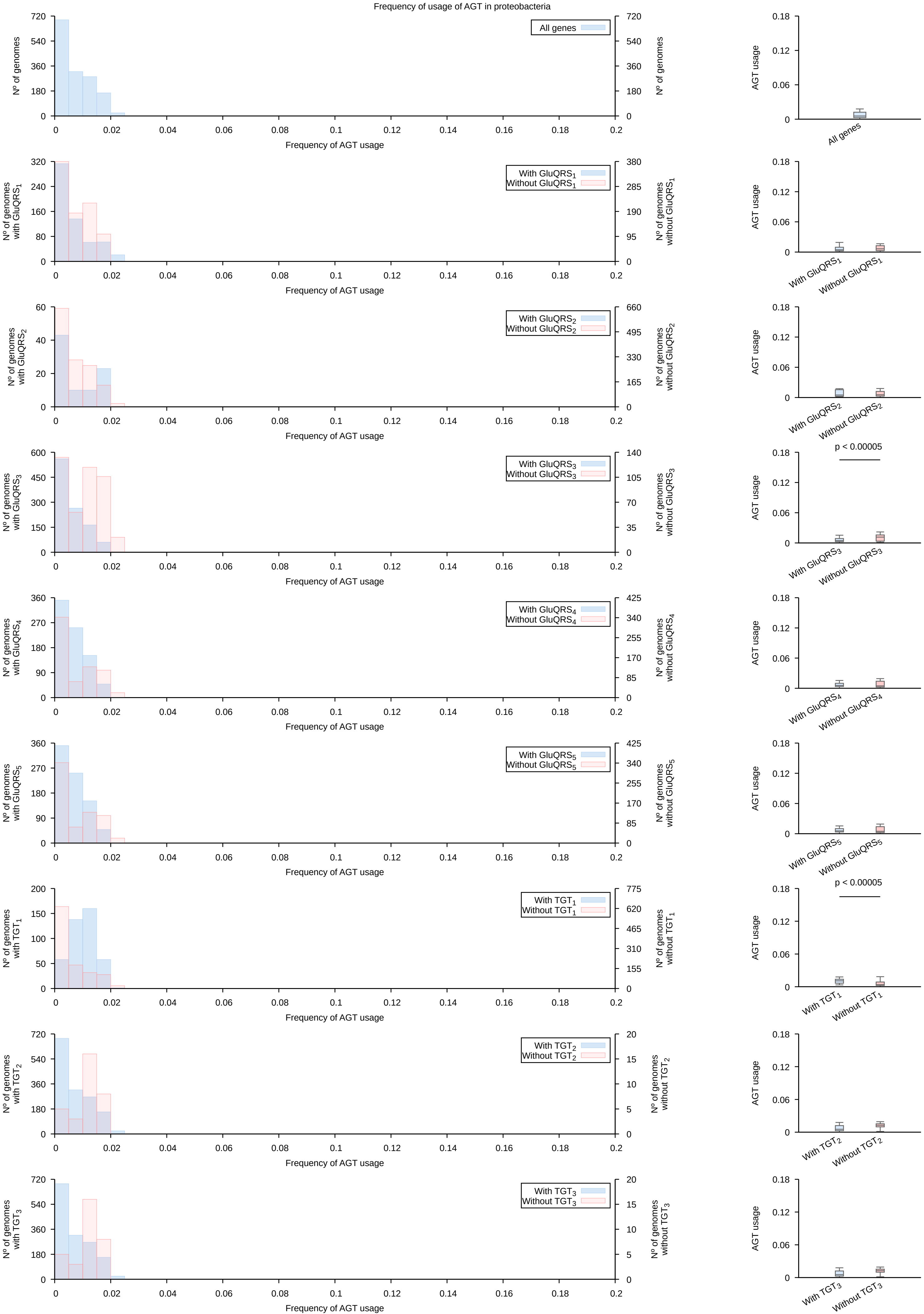
Frequency of usage of AGT in proteobacteria
All genes
Nº of genomes
Frequency of AGT usage
AGT usage
With GluQRS$_1$
Without GluQRS$_1$
Nº of genomes with GluQRS$_1$
Nº of genomes without GluQRS$_1$
With GluQRS$_2$
Without GluQRS$_2$
Nº of genomes with GluQRS$_2$
Nº of genomes without GluQRS$_2$
With GluQRS$_3$
Without GluQRS$_3$
Nº of genomes with GluQRS$_3$
Nº of genomes without GluQRS$_3$
$p < 0.00005$
With GluQRS$_4$
Without GluQRS$_4$
Nº of genomes with GluQRS$_4$
Nº of genomes without GluQRS$_4$
With GluQRS$_5$
Without GluQRS$_5$
Nº of genomes with GluQRS$_5$
Nº of genomes without GluQRS$_5$
With TGT$_1$
Without TGT$_1$
Nº of genomes with TGT$_1$
Nº of genomes without TGT$_1$
$p < 0.00005$
With TGT$_2$
Without TGT$_2$
Nº of genomes with TGT$_2$
Nº of genomes without TGT$_2$
With TGT$_3$
Without TGT$_3$
Nº of genomes with TGT$_3$
Nº of genomes without TGT$_3$

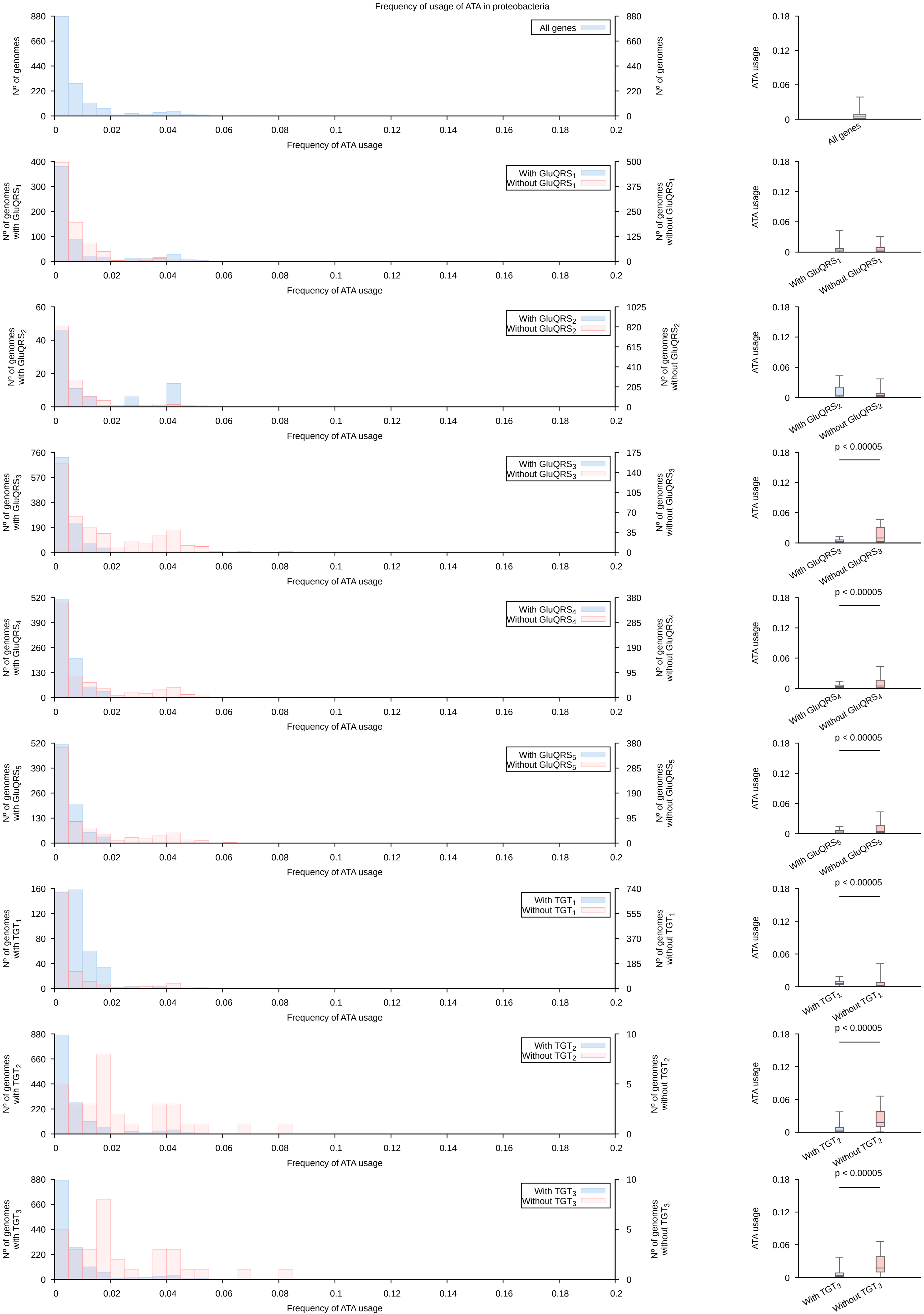
Frequency of usage of ATA in proteobacteria
Nº of genomes
Frequency of ATA usage
All genes
ATA usage
With $GluQRS_1$
Without $GluQRS_1$
Nº of genomes with $GluQRS_1$
Nº of genomes without $GluQRS_1$
With $GluQRS_2$
Without $GluQRS_2$
Nº of genomes with $GluQRS_2$
Nº of genomes without $GluQRS_2$
With $GluQRS_3$
Without $GluQRS_3$
Nº of genomes with $GluQRS_3$
Nº of genomes without $GluQRS_3$
With $GluQRS_4$
Without $GluQRS_4$
Nº of genomes with $GluQRS_4$
Nº of genomes without $GluQRS_4$
With $GluQRS_5$
Without $GluQRS_5$
Nº of genomes with $GluQRS_5$
Nº of genomes without $GluQRS_5$
With TGT_1
Without TGT_1
Nº of genomes with TGT_1
Nº of genomes without TGT_1
With TGT_2
Without TGT_2
Nº of genomes with TGT_2
Nº of genomes without TGT_2
With TGT_3
Without TGT_3
Nº of genomes with TGT_3
Nº of genomes without TGT_3
p < 0.00005

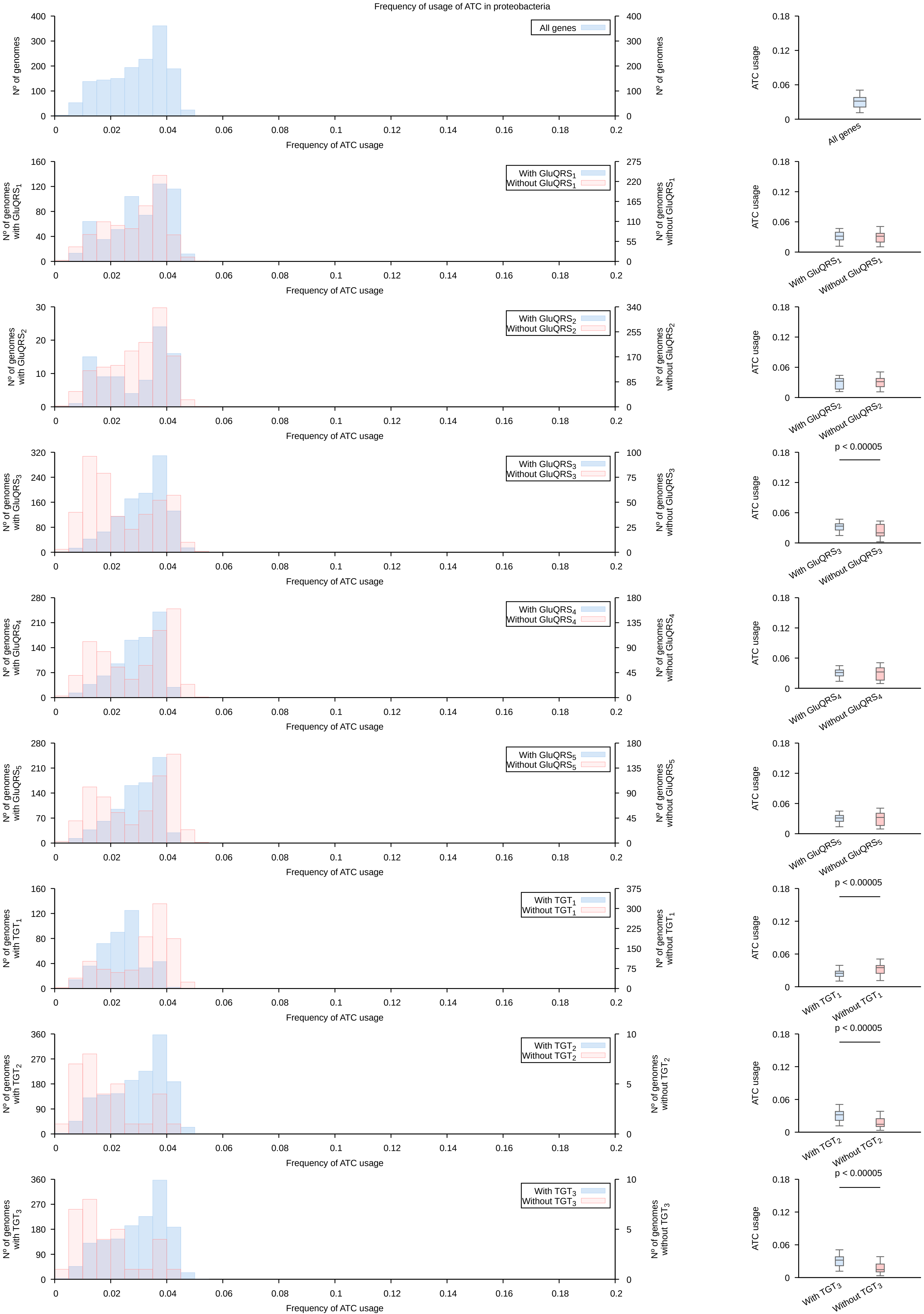
Frequency of usage of ATC in proteobacteria
Nº of genomes
Frequency of ATC usage
All genes
ATC usage
Nº of genomes with GluQRS$_1$
Nº of genomes without GluQRS$_1$
With GluQRS$_1$
Without GluQRS$_1$
Nº of genomes with GluQRS$_2$
Nº of genomes without GluQRS$_2$
With GluQRS$_2$
Without GluQRS$_2$
Nº of genomes with GluQRS$_3$
Nº of genomes without GluQRS$_3$
With GluQRS$_3$
Without GluQRS$_3$
p < 0.00005
Nº of genomes with GluQRS$_4$
Nº of genomes without GluQRS$_4$
With GluQRS$_4$
Without GluQRS$_4$
Nº of genomes with GluQRS$_5$
Nº of genomes without GluQRS$_5$
With GluQRS$_5$
Without GluQRS$_5$
Nº of genomes with TGT$_1$
Nº of genomes without TGT$_1$
With TGT$_1$
Without TGT$_1$
p < 0.00005
Nº of genomes with TGT$_2$
Nº of genomes without TGT$_2$
With TGT$_2$
Without TGT$_2$
p < 0.00005
Nº of genomes with TGT$_3$
Nº of genomes without TGT$_3$
With TGT$_3$
Without TGT$_3$
p < 0.00005

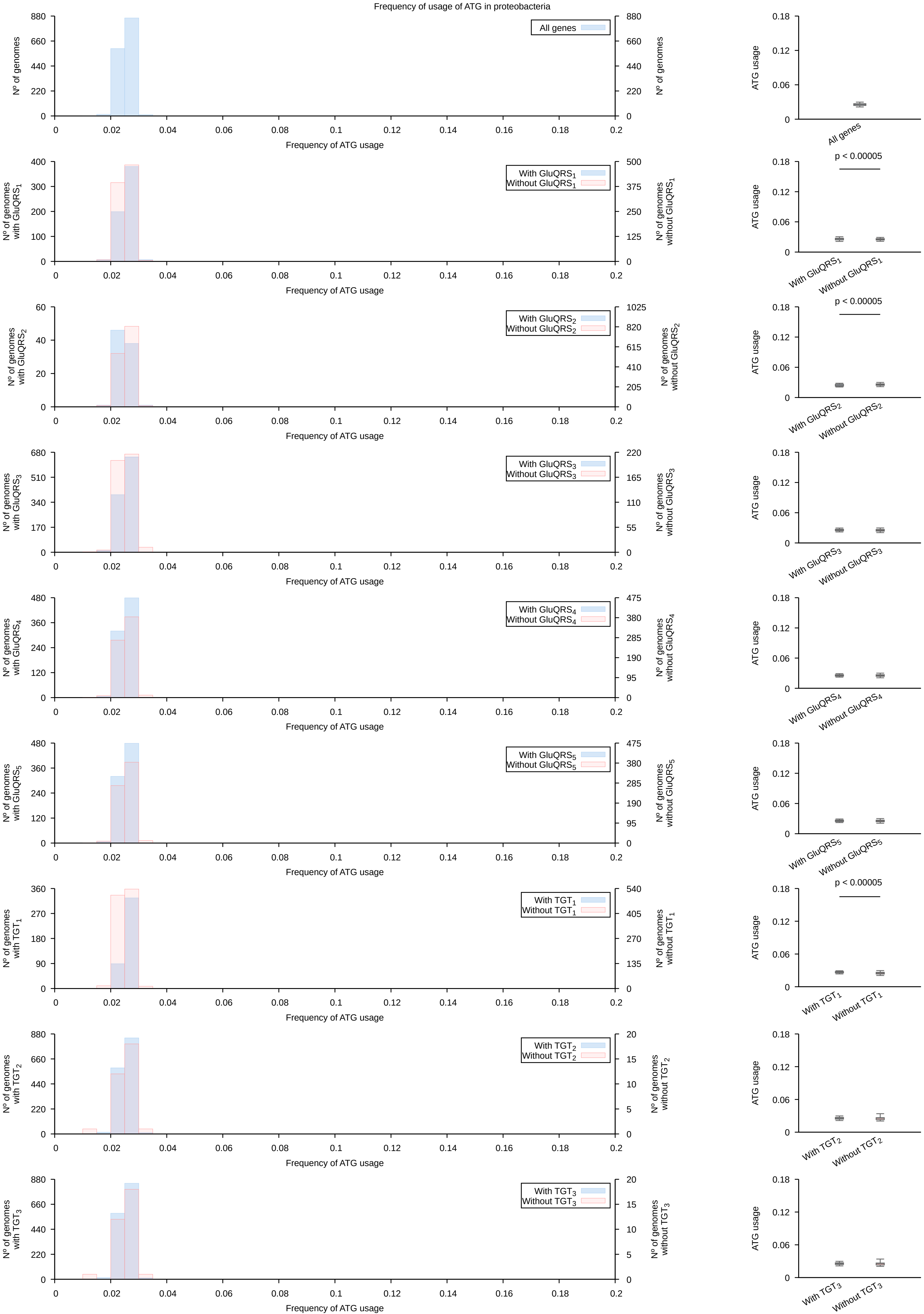
Frequency of usage of ATG in proteobacteria
Frequency of ATG usage
Nº of genomes
All genes
ATG usage
Nº of genomes with GluQRS$_1$
Nº of genomes without GluQRS$_1$
With GluQRS$_1$
Without GluQRS$_1$
p < 0.00005
Nº of genomes with GluQRS$_2$
Nº of genomes without GluQRS$_2$
With GluQRS$_2$
Without GluQRS$_2$
p < 0.00005
Nº of genomes with GluQRS$_3$
Nº of genomes without GluQRS$_3$
With GluQRS$_3$
Without GluQRS$_3$
Nº of genomes with GluQRS$_4$
Nº of genomes without GluQRS$_4$
With GluQRS$_4$
Without GluQRS$_4$
Nº of genomes with GluQRS$_5$
Nº of genomes without GluQRS$_5$
With GluQRS$_5$
Without GluQRS$_5$
Nº of genomes with TGT$_1$
Nº of genomes without TGT$_1$
With TGT$_1$
Without TGT$_1$
p < 0.00005
Nº of genomes with TGT$_2$
Nº of genomes without TGT$_2$
With TGT$_2$
Without TGT$_2$
Nº of genomes with TGT$_3$
Nº of genomes without TGT$_3$
With TGT$_3$
Without TGT$_3$

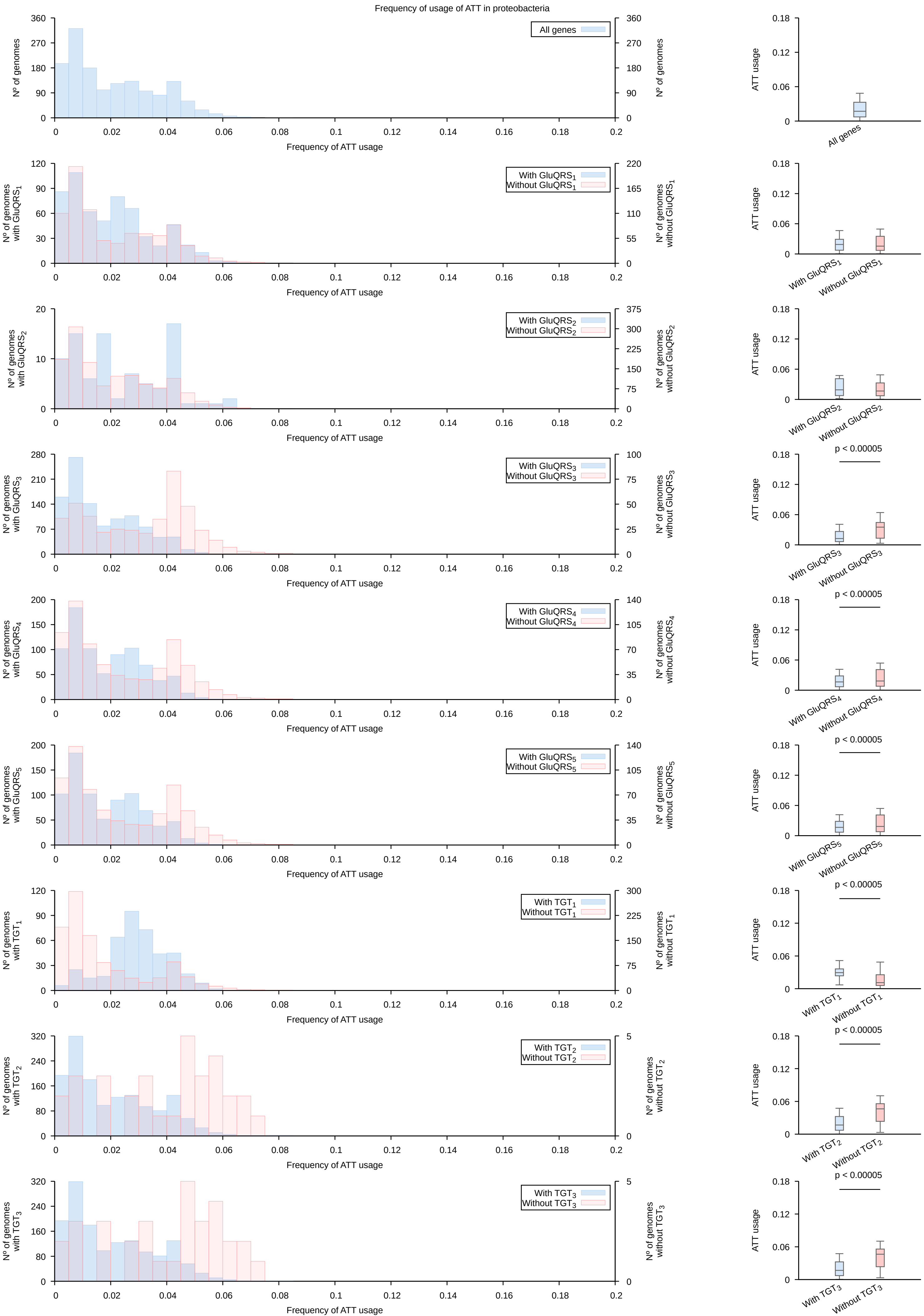

Frequency of usage of ATT in proteobacteria
All genes
Nº of genomes
Frequency of ATT usage
ATT usage
With GluQRS1
Without GluQRS1
Nº of genomes with GluQRS1
Nº of genomes without GluQRS1
With GluQRS2
Without GluQRS2
Nº of genomes with GluQRS2
Nº of genomes without GluQRS2
With GluQRS3
Without GluQRS3
Nº of genomes with GluQRS3
Nº of genomes without GluQRS3
p < 0.00005
With GluQRS4
Without GluQRS4
Nº of genomes with GluQRS4
Nº of genomes without GluQRS4
p < 0.00005
With GluQRS5
Without GluQRS5
Nº of genomes with GluQRS5
Nº of genomes without GluQRS5
p < 0.00005
With TGT1
Without TGT1
Nº of genomes with TGT1
Nº of genomes without TGT1
p < 0.00005
With TGT2
Without TGT2
Nº of genomes with TGT2
Nº of genomes without TGT2
p < 0.00005
With TGT3
Without TGT3
Nº of genomes with TGT3
Nº of genomes without TGT3
p < 0.00005

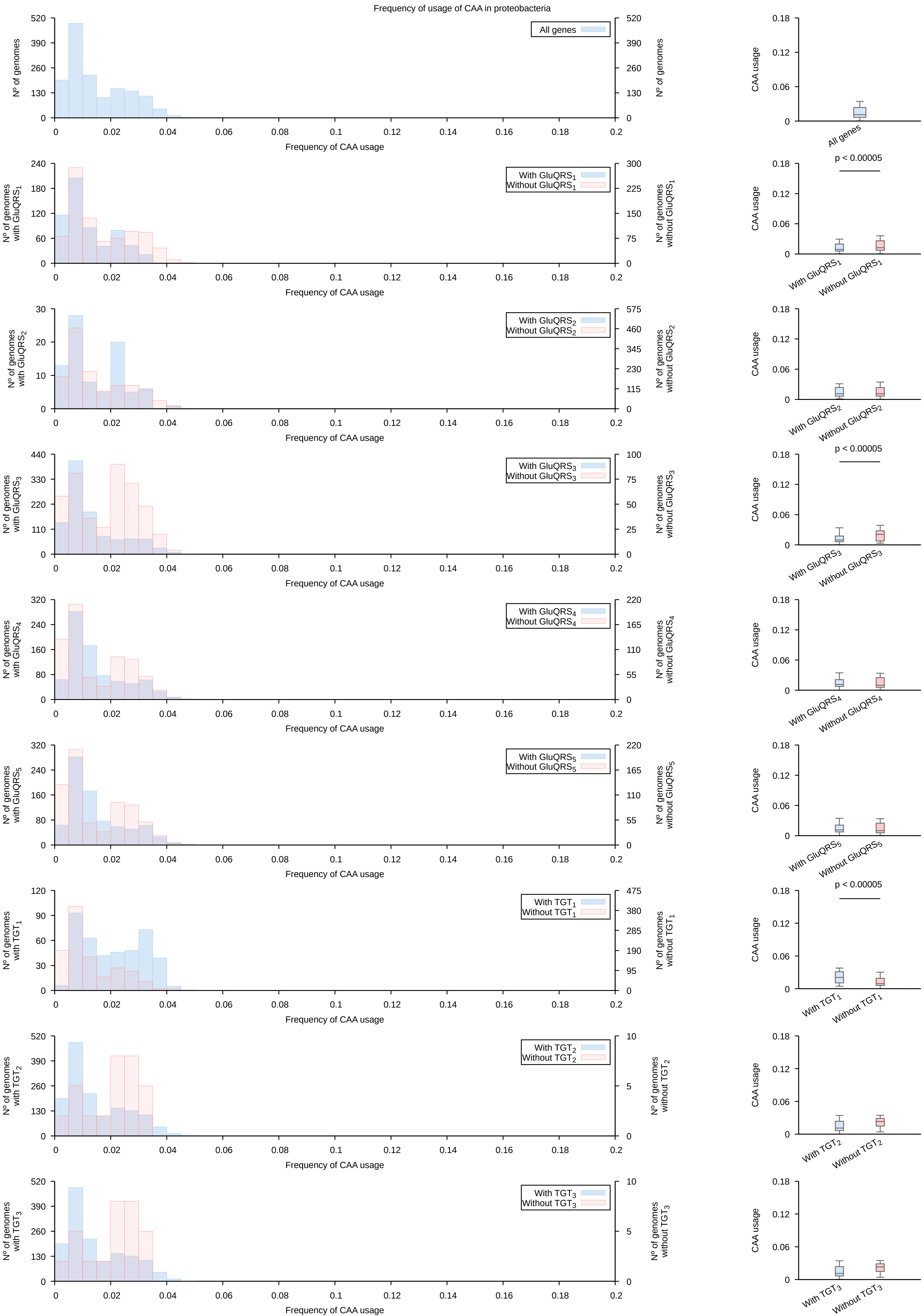
Frequency of usage of CAA in proteobacteria
All genes
Nº of genomes
Frequency of CAA usage
CAA usage
With GluQRS1
Without GluQRS1
Nº of genomes with GluQRS1
Nº of genomes without GluQRS1
p < 0.00005
With GluQRS2
Without GluQRS2
Nº of genomes with GluQRS2
Nº of genomes without GluQRS2
With GluQRS3
Without GluQRS3
Nº of genomes with GluQRS3
Nº of genomes without GluQRS3
p < 0.00005
With GluQRS4
Without GluQRS4
Nº of genomes with GluQRS4
Nº of genomes without GluQRS4
With GluQRS5
Without GluQRS5
Nº of genomes with GluQRS5
Nº of genomes without GluQRS5
With TGT1
Without TGT1
Nº of genomes with TGT1
Nº of genomes without TGT1
p < 0.00005
With TGT2
Without TGT2
Nº of genomes with TGT2
Nº of genomes without TGT2
With TGT3
Without TGT3
Nº of genomes with TGT3
Nº of genomes without TGT3

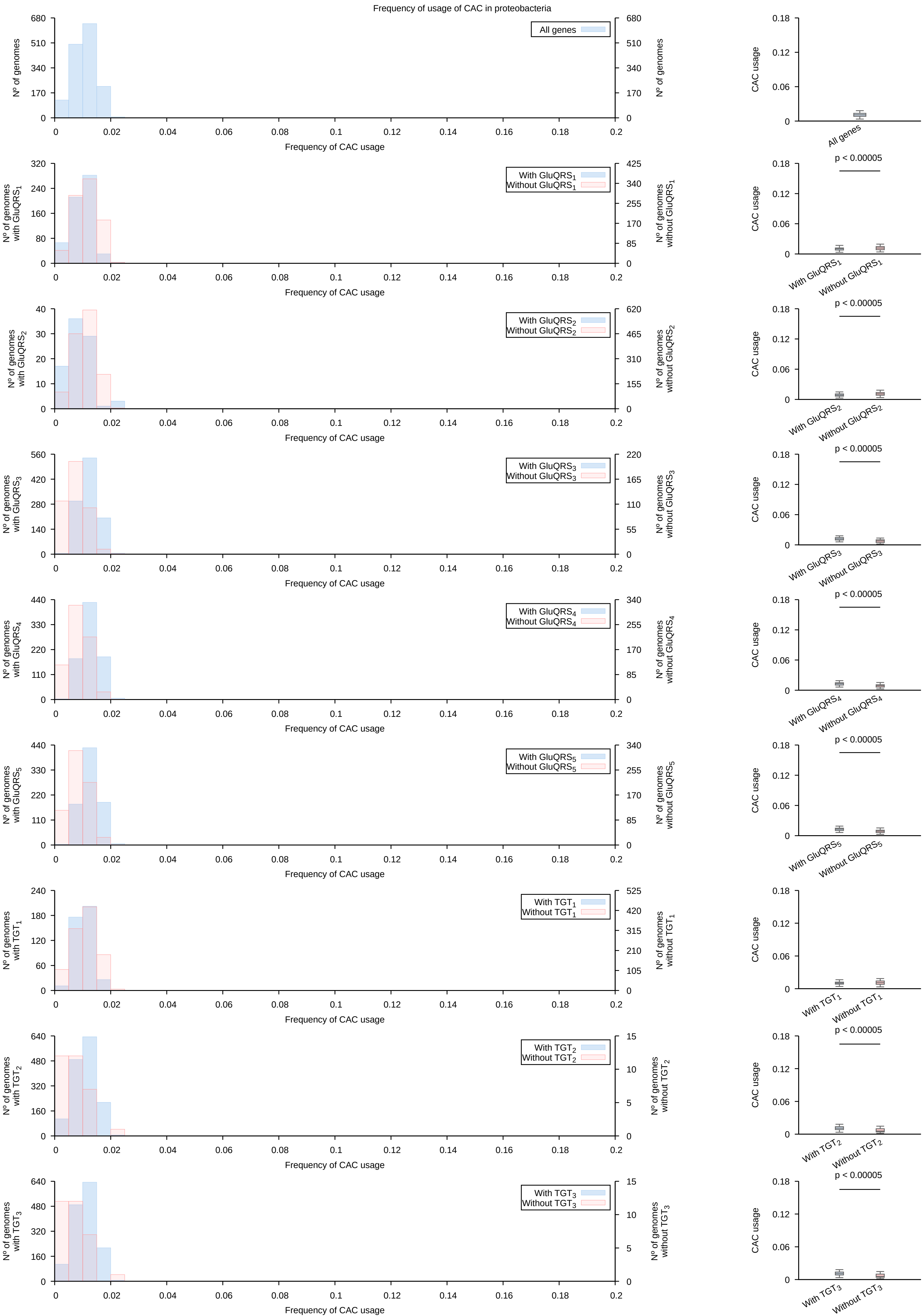

Frequency of usage of CAC in proteobacteria
Nº of genomes
All genes
Frequency of CAC usage
CAC usage
Nº of genomes with GluQRS1
Nº of genomes without GluQRS1
With GluQRS1
Without GluQRS1
p < 0.00005
Nº of genomes with GluQRS2
Nº of genomes without GluQRS2
With GluQRS2
Without GluQRS2
Nº of genomes with GluQRS3
Nº of genomes without GluQRS3
With GluQRS3
Without GluQRS3
Nº of genomes with GluQRS4
Nº of genomes without GluQRS4
With GluQRS4
Without GluQRS4
Nº of genomes with GluQRS5
Nº of genomes without GluQRS5
With GluQRS5
Without GluQRS5
Nº of genomes with TGT1
Nº of genomes without TGT1
With TGT1
Without TGT1
Nº of genomes with TGT2
Nº of genomes without TGT2
With TGT2
Without TGT2
Nº of genomes with TGT3
Nº of genomes without TGT3
With TGT3
Without TGT3

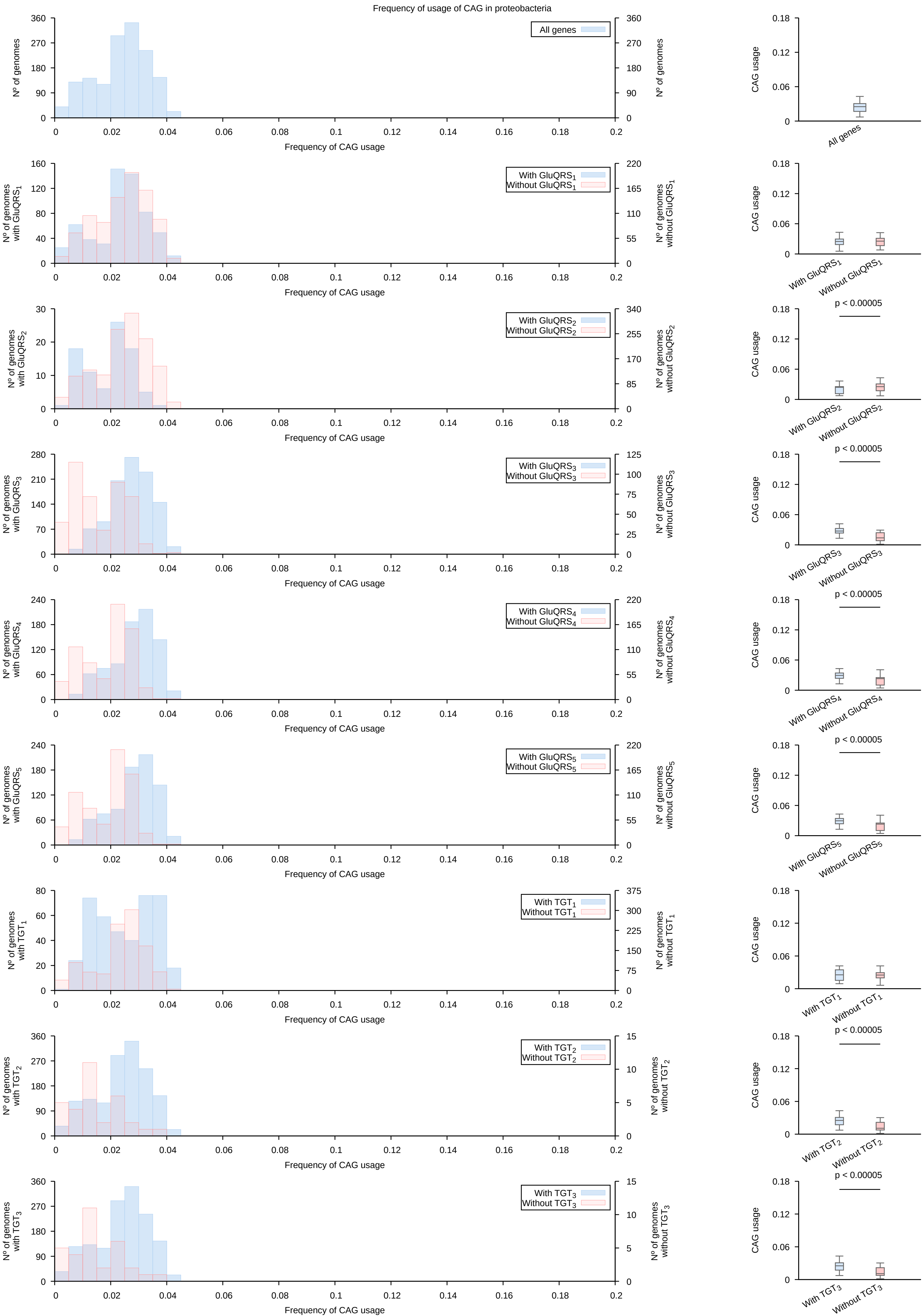

Frequency of usage of CAG in proteobacteria
All genes
With GluQRS1
Without GluQRS1
With GluQRS2
Without GluQRS2
With GluQRS3
Without GluQRS3
With GluQRS4
Without GluQRS4
With GluQRS5
Without GluQRS5
With TGT1
Without TGT1
With TGT2
Without TGT2
With TGT3
Without TGT3
Nº of genomes
Frequency of CAG usage
CAG usage
p < 0.00005

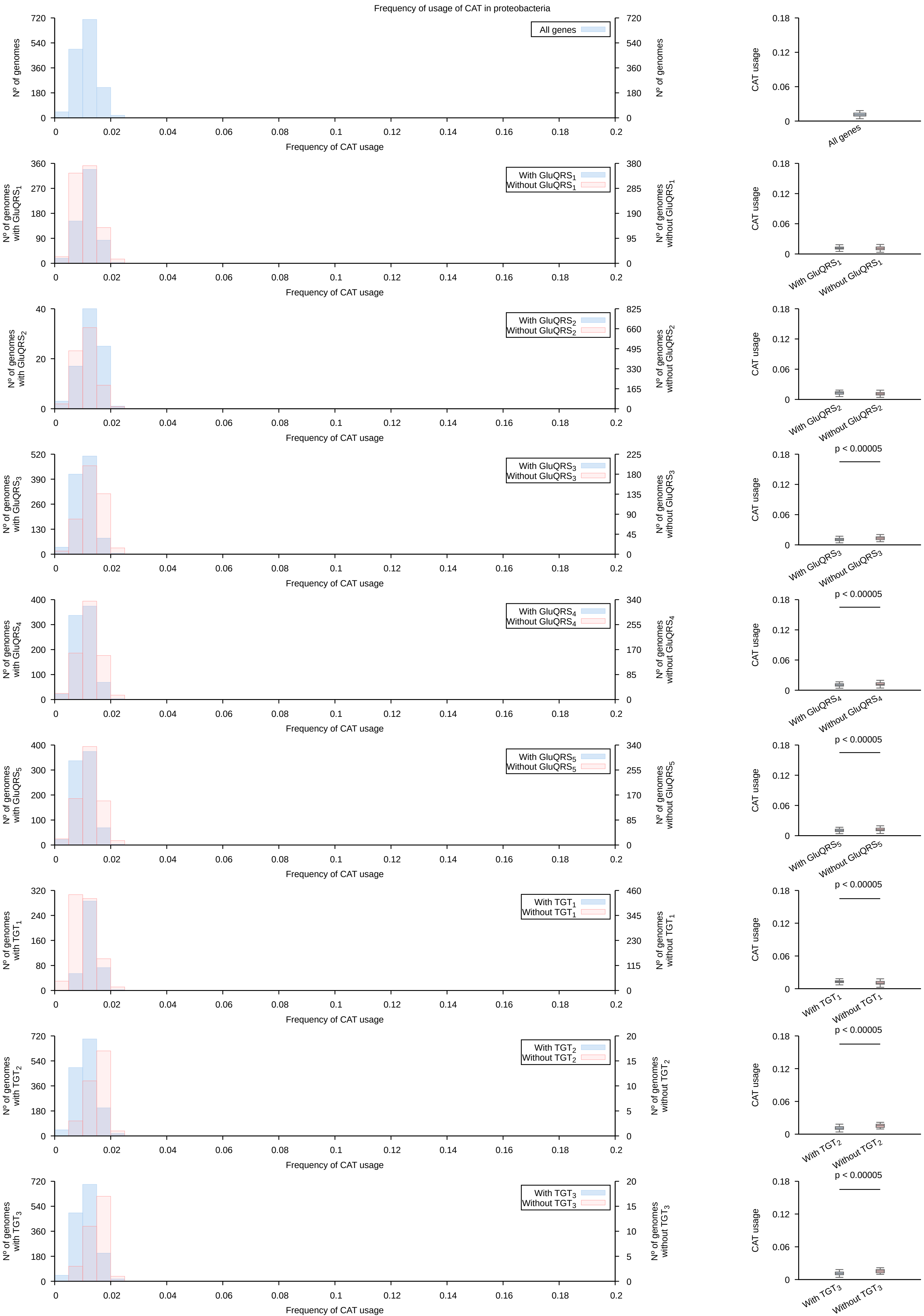

Frequency of usage of CAT in proteobacteria
All genes
With GluQRS$_1$
Without GluQRS$_1$
With GluQRS$_2$
Without GluQRS$_2$
With GluQRS$_3$
Without GluQRS$_3$
With GluQRS$_4$
Without GluQRS$_4$
With GluQRS$_5$
Without GluQRS$_5$
With TGT$_1$
Without TGT$_1$
With TGT$_2$
Without TGT$_2$
With TGT$_3$
Without TGT$_3$
Frequency of CAT usage
Nº of genomes
Nº of genomes with GluQRS$_1$
Nº of genomes without GluQRS$_1$
Nº of genomes with GluQRS$_2$
Nº of genomes without GluQRS$_2$
Nº of genomes with GluQRS$_3$
Nº of genomes without GluQRS$_3$
Nº of genomes with GluQRS$_4$
Nº of genomes without GluQRS$_4$
Nº of genomes with GluQRS$_5$
Nº of genomes without GluQRS$_5$
Nº of genomes with TGT$_1$
Nº of genomes without TGT$_1$
Nº of genomes with TGT$_2$
Nº of genomes without TGT$_2$
Nº of genomes with TGT$_3$
Nº of genomes without TGT$_3$
CAT usage
$p < 0.00005$

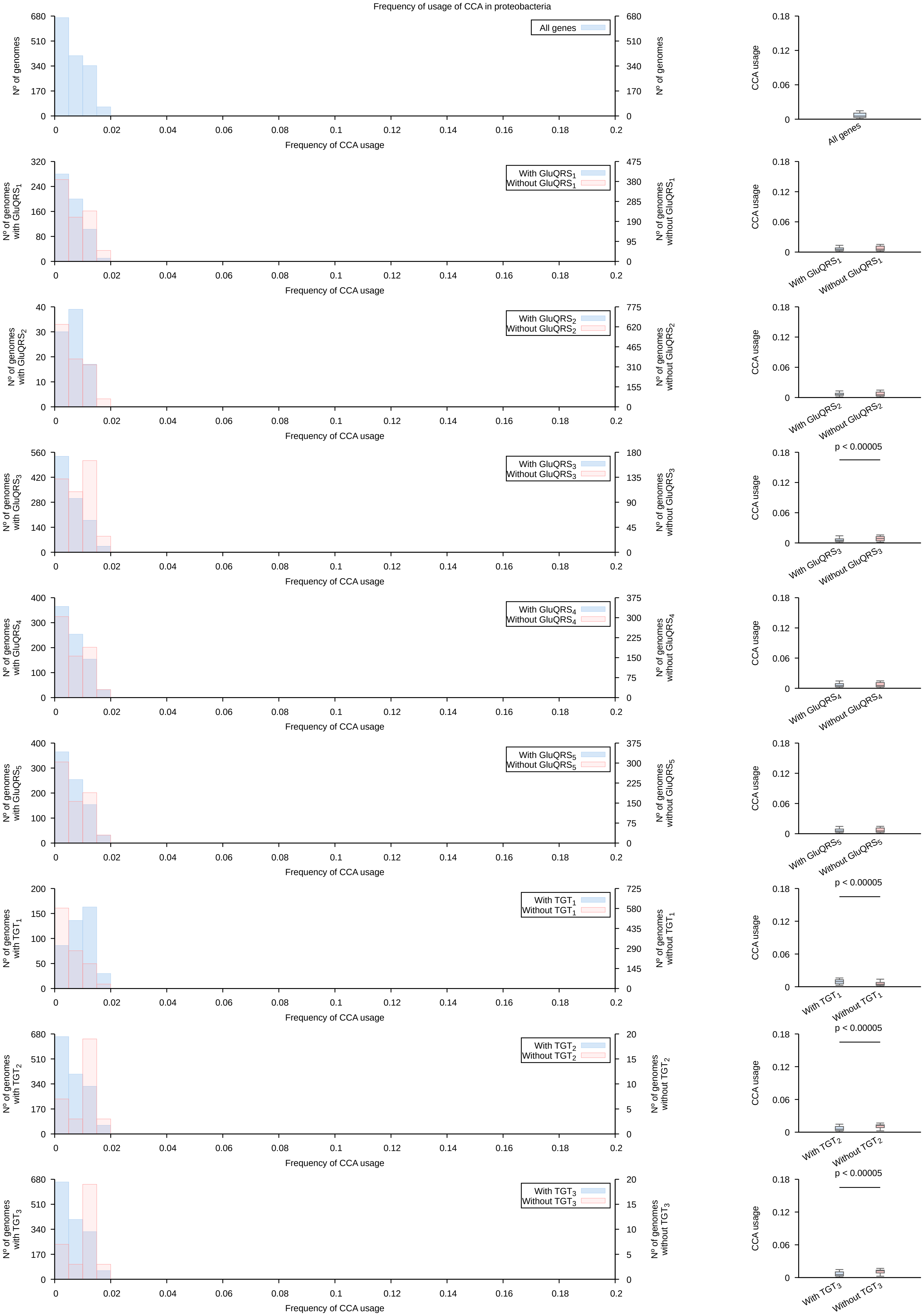

Frequency of usage of CCA in proteobacteria
All genes
Nº of genomes
Frequency of CCA usage
CCA usage
With GluQRS1
Without GluQRS1
Nº of genomes with GluQRS1
Nº of genomes without GluQRS1
With GluQRS2
Without GluQRS2
Nº of genomes with GluQRS2
Nº of genomes without GluQRS2
With GluQRS3
Without GluQRS3
Nº of genomes with GluQRS3
Nº of genomes without GluQRS3
p < 0.00005
With GluQRS4
Without GluQRS4
Nº of genomes with GluQRS4
Nº of genomes without GluQRS4
With GluQRS5
Without GluQRS5
Nº of genomes with GluQRS5
Nº of genomes without GluQRS5
With TGT1
Without TGT1
Nº of genomes with TGT1
Nº of genomes without TGT1
p < 0.00005
With TGT2
Without TGT2
Nº of genomes with TGT2
Nº of genomes without TGT2
p < 0.00005
With TGT3
Without TGT3
Nº of genomes with TGT3
Nº of genomes without TGT3
p < 0.00005

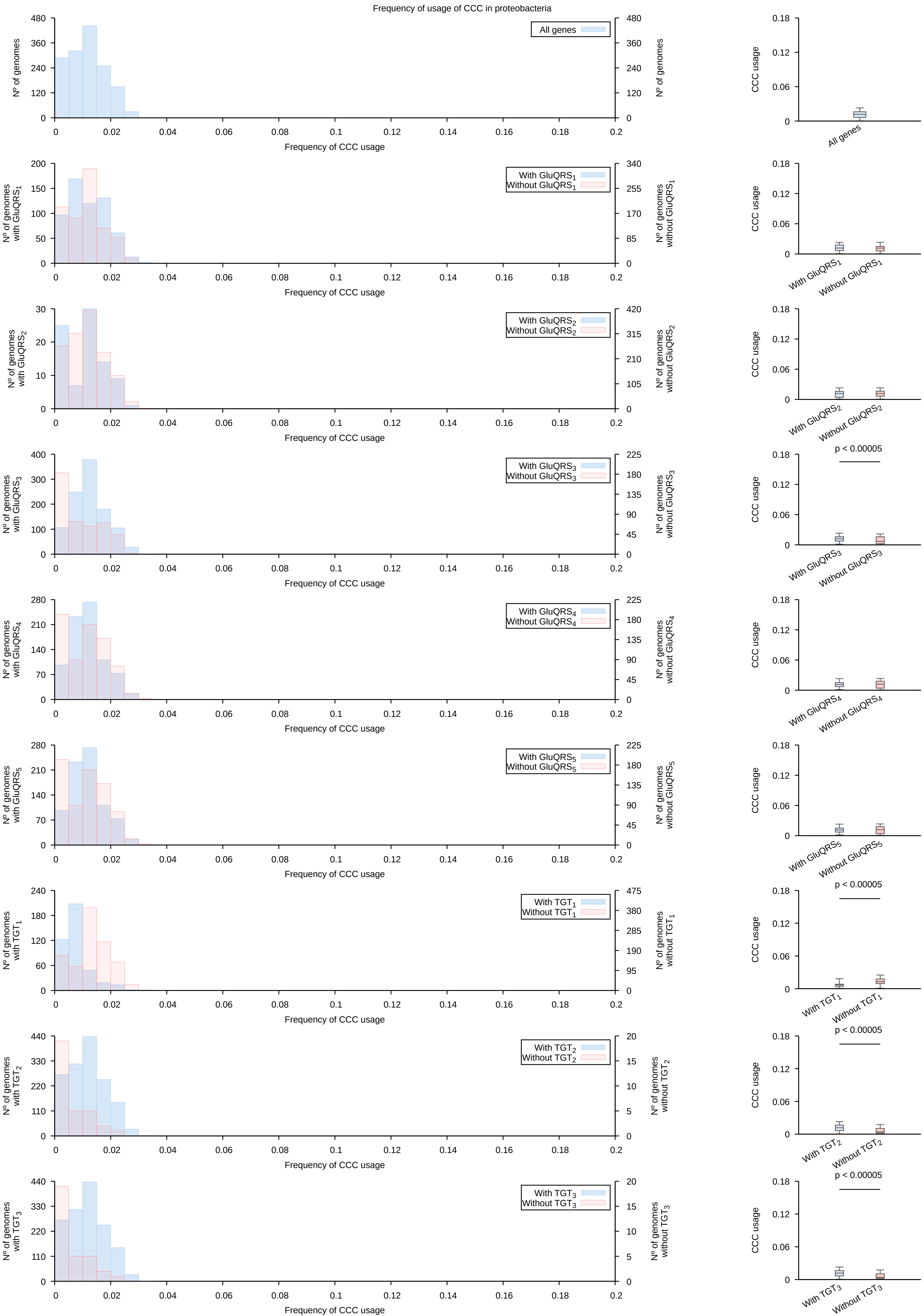

Frequency of usage of CCC in proteobacteria
Nº of genomes
All genes
Frequency of CCC usage
CCC usage
Nº of genomes with GluQRS1
Nº of genomes without GluQRS1
With GluQRS1
Without GluQRS1
Nº of genomes with GluQRS2
Nº of genomes without GluQRS2
With GluQRS2
Without GluQRS2
Nº of genomes with GluQRS3
Nº of genomes without GluQRS3
With GluQRS3
Without GluQRS3
p < 0.00005
Nº of genomes with GluQRS4
Nº of genomes without GluQRS4
With GluQRS4
Without GluQRS4
Nº of genomes with GluQRS5
Nº of genomes without GluQRS5
With GluQRS5
Without GluQRS5
Nº of genomes with TGT1
Nº of genomes without TGT1
With TGT1
Without TGT1
p < 0.00005
Nº of genomes with TGT2
Nº of genomes without TGT2
With TGT2
Without TGT2
p < 0.00005
Nº of genomes with TGT3
Nº of genomes without TGT3
With TGT3
Without TGT3
p < 0.00005

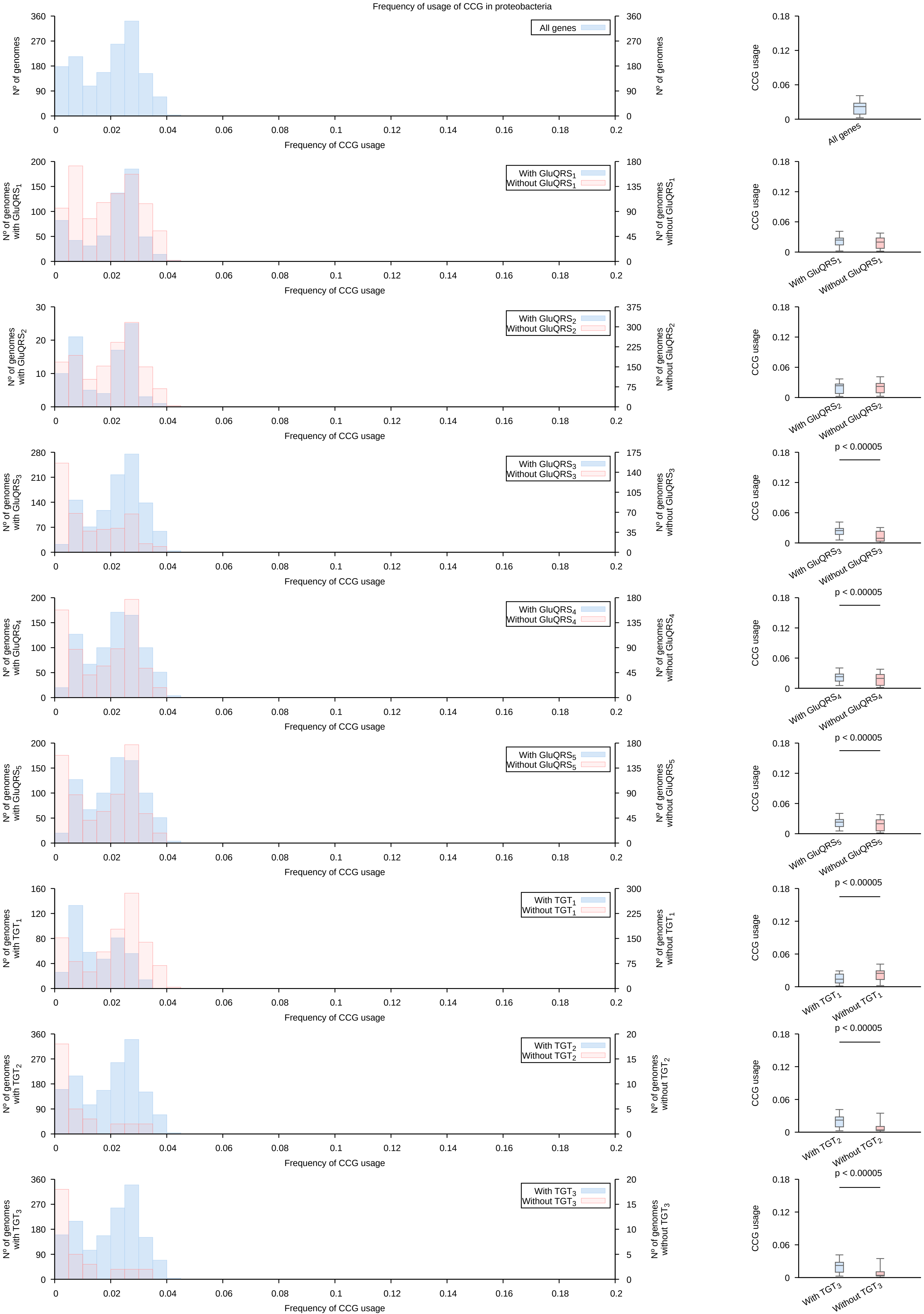

Frequency of usage of CCG in proteobacteria
All genes
Nº of genomes
Frequency of CCG usage
CCG usage
With GluQRS$_1$
Without GluQRS$_1$
Nº of genomes with GluQRS$_1$
Nº of genomes without GluQRS$_1$
With GluQRS$_2$
Without GluQRS$_2$
Nº of genomes with GluQRS$_2$
Nº of genomes without GluQRS$_2$
p < 0.00005
With GluQRS$_3$
Without GluQRS$_3$
Nº of genomes with GluQRS$_3$
Nº of genomes without GluQRS$_3$
With GluQRS$_4$
Without GluQRS$_4$
Nº of genomes with GluQRS$_4$
Nº of genomes without GluQRS$_4$
With GluQRS$_5$
Without GluQRS$_5$
Nº of genomes with GluQRS$_5$
Nº of genomes without GluQRS$_5$
With TGT$_1$
Without TGT$_1$
Nº of genomes with TGT$_1$
Nº of genomes without TGT$_1$
With TGT$_2$
Without TGT$_2$
Nº of genomes with TGT$_2$
Nº of genomes without TGT$_2$
With TGT$_3$
Without TGT$_3$
Nº of genomes with TGT$_3$
Nº of genomes without TGT$_3$

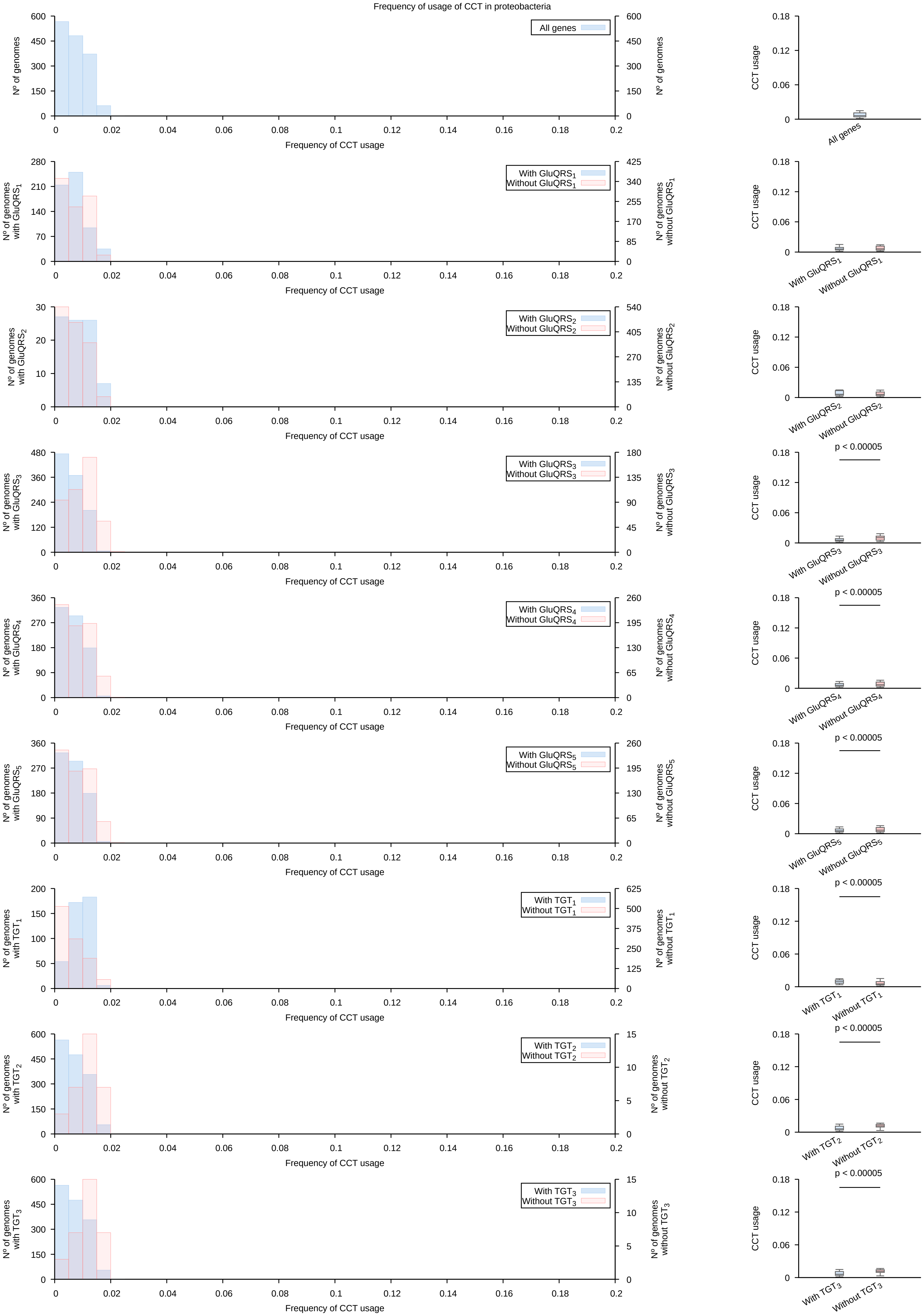
Frequency of usage of CCT in proteobacteria
Nº of genomes
Frequency of CCT usage
All genes
CCT usage
Nº of genomes with GluQRS1
Nº of genomes without GluQRS1
With GluQRS1
Without GluQRS1
Nº of genomes with GluQRS2
Nº of genomes without GluQRS2
With GluQRS2
Without GluQRS2
Nº of genomes with GluQRS3
Nº of genomes without GluQRS3
With GluQRS3
Without GluQRS3
p < 0.00005
Nº of genomes with GluQRS4
Nº of genomes without GluQRS4
With GluQRS4
Without GluQRS4
p < 0.00005
Nº of genomes with GluQRS5
Nº of genomes without GluQRS5
With GluQRS5
Without GluQRS5
p < 0.00005
Nº of genomes with TGT1
Nº of genomes without TGT1
With TGT1
Without TGT1
p < 0.00005
Nº of genomes with TGT2
Nº of genomes without TGT2
With TGT2
Without TGT2
p < 0.00005
Nº of genomes with TGT3
Nº of genomes without TGT3
With TGT3
Without TGT3
p < 0.00005

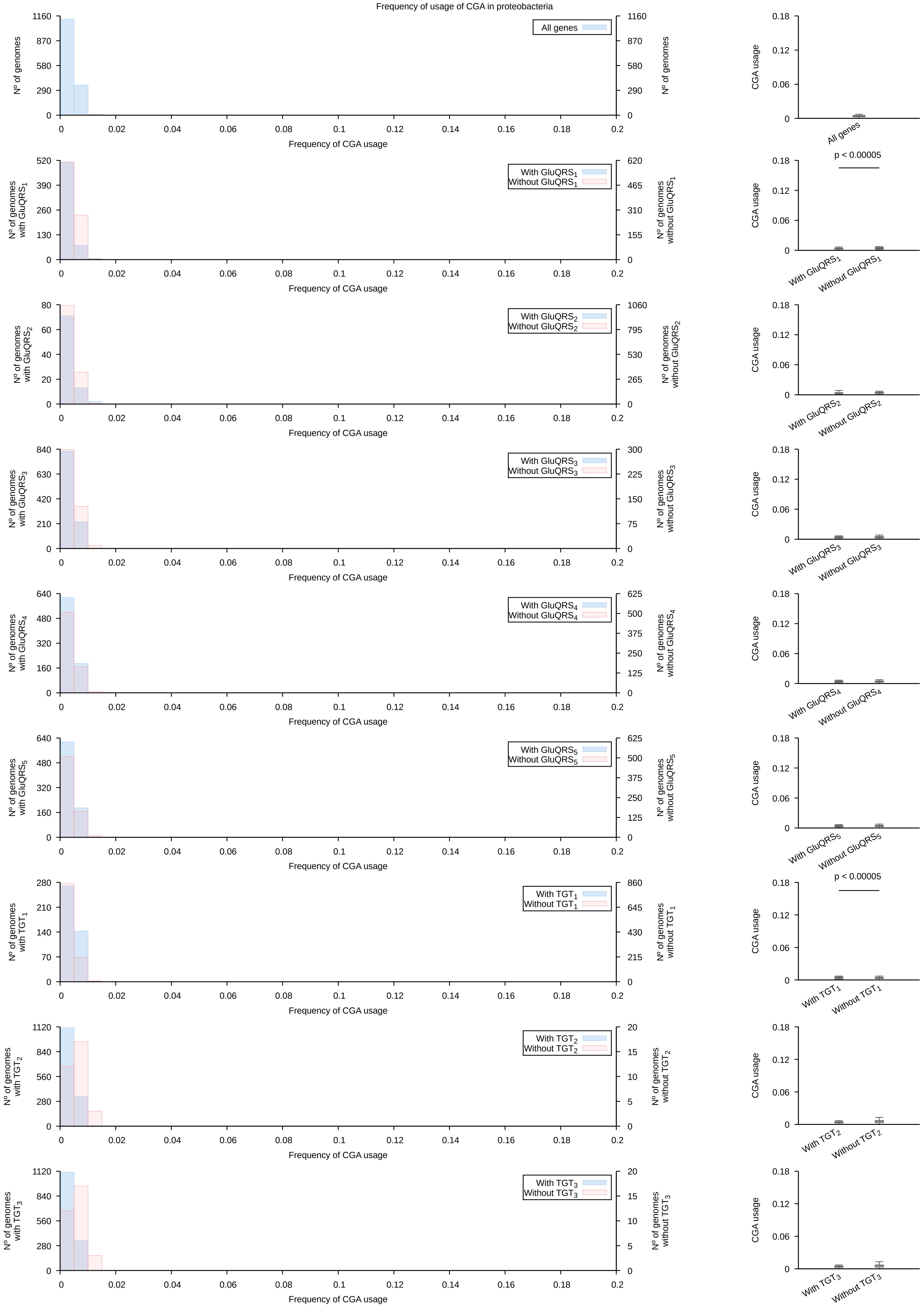
Frequency of usage of CGA in proteobacteria
Nº of genomes
All genes
Frequency of CGA usage
CGA usage
All genes
Nº of genomes with GluQRS$_1$
Nº of genomes without GluQRS$_1$
With GluQRS$_1$
Without GluQRS$_1$
p < 0.00005
Nº of genomes with GluQRS$_2$
Nº of genomes without GluQRS$_2$
With GluQRS$_2$
Without GluQRS$_2$
Nº of genomes with GluQRS$_3$
Nº of genomes without GluQRS$_3$
With GluQRS$_3$
Without GluQRS$_3$
Nº of genomes with GluQRS$_4$
Nº of genomes without GluQRS$_4$
With GluQRS$_4$
Without GluQRS$_4$
Nº of genomes with GluQRS$_5$
Nº of genomes without GluQRS$_5$
With GluQRS$_5$
Without GluQRS$_5$
Nº of genomes with TGT$_1$
Nº of genomes without TGT$_1$
With TGT$_1$
Without TGT$_1$
p < 0.00005
Nº of genomes with TGT$_2$
Nº of genomes without TGT$_2$
With TGT$_2$
Without TGT$_2$
Nº of genomes with TGT$_3$
Nº of genomes without TGT$_3$
With TGT$_3$
Without TGT$_3$
0 0.02 0.04 0.06 0.08 0.1 0.12 0.14 0.16 0.18 0.2
0 0.06 0.12 0.18

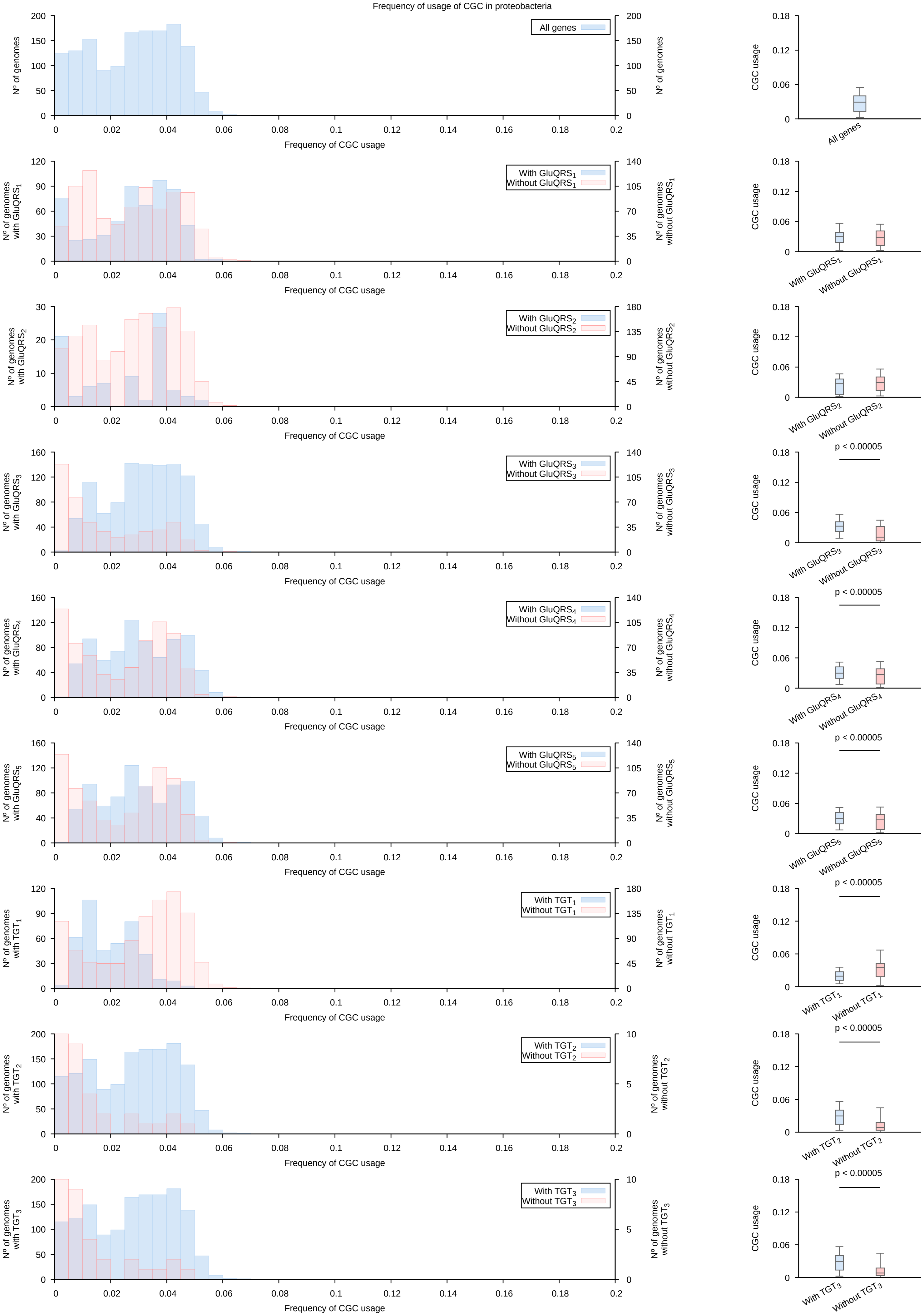
Frequency of usage of CGC in proteobacteria
All genes
Nº of genomes
Frequency of CGC usage
CGC usage
With GluQRS$_1$
Without GluQRS$_1$
Nº of genomes with GluQRS$_1$
Nº of genomes without GluQRS$_1$
With GluQRS$_2$
Without GluQRS$_2$
Nº of genomes with GluQRS$_2$
Nº of genomes without GluQRS$_2$
With GluQRS$_3$
Without GluQRS$_3$
Nº of genomes with GluQRS$_3$
Nº of genomes without GluQRS$_3$
p < 0.00005
With GluQRS$_4$
Without GluQRS$_4$
Nº of genomes with GluQRS$_4$
Nº of genomes without GluQRS$_4$
p < 0.00005
With GluQRS$_5$
Without GluQRS$_5$
Nº of genomes with GluQRS$_5$
Nº of genomes without GluQRS$_5$
p < 0.00005
With TGT$_1$
Without TGT$_1$
Nº of genomes with TGT$_1$
Nº of genomes without TGT$_1$
p < 0.00005
With TGT$_2$
Without TGT$_2$
Nº of genomes with TGT$_2$
Nº of genomes without TGT$_2$
p < 0.00005
With TGT$_3$
Without TGT$_3$
Nº of genomes with TGT$_3$
Nº of genomes without TGT$_3$
p < 0.00005

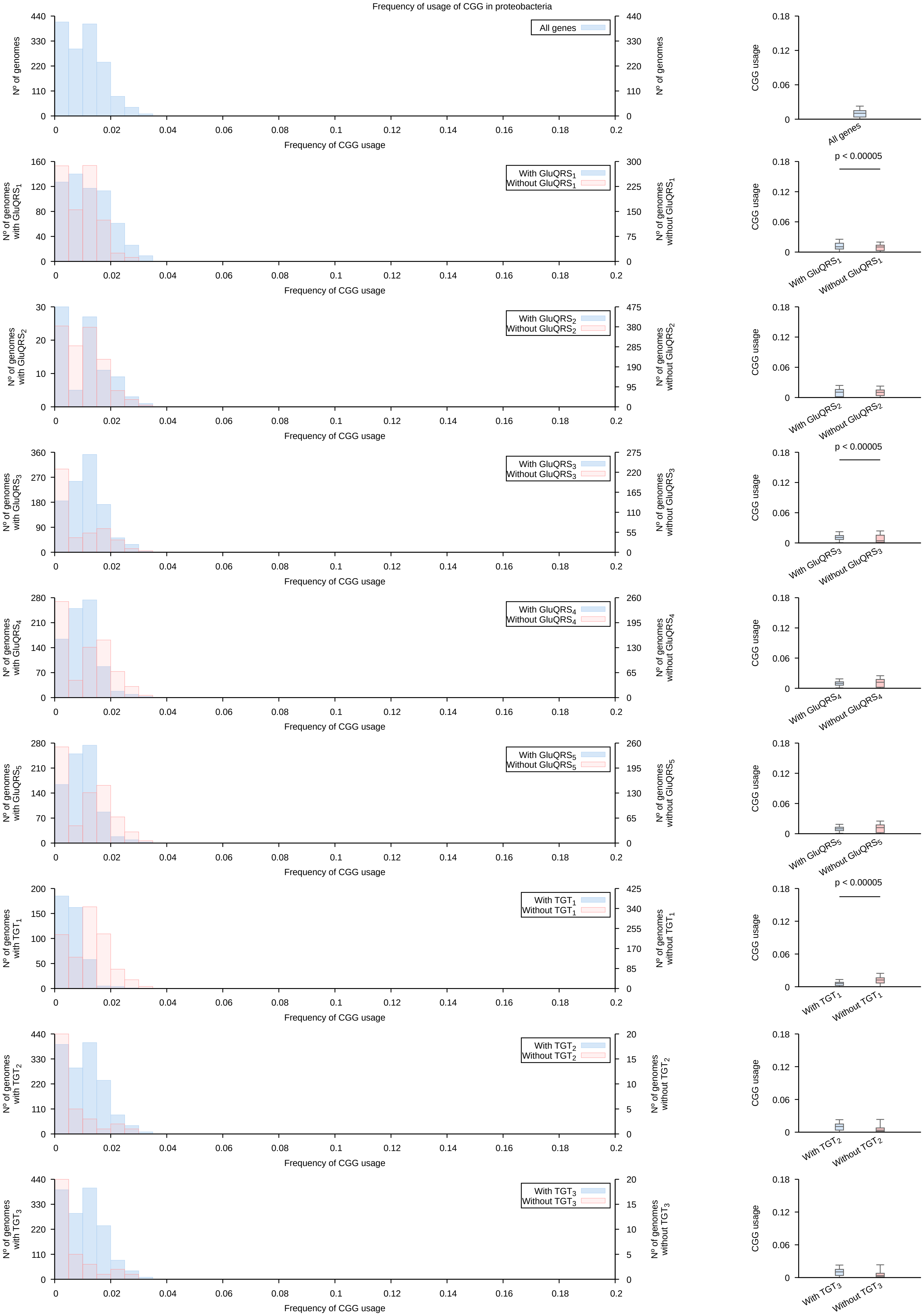
Frequency of usage of CGG in proteobacteria
All genes
Nº of genomes
Frequency of CGG usage
CGG usage
With GluQRS$_1$
Without GluQRS$_1$
Nº of genomes with GluQRS$_1$
Nº of genomes without GluQRS$_1$
p < 0.00005
With GluQRS$_2$
Without GluQRS$_2$
Nº of genomes with GluQRS$_2$
Nº of genomes without GluQRS$_2$
With GluQRS$_3$
Without GluQRS$_3$
Nº of genomes with GluQRS$_3$
Nº of genomes without GluQRS$_3$
p < 0.00005
With GluQRS$_4$
Without GluQRS$_4$
Nº of genomes with GluQRS$_4$
Nº of genomes without GluQRS$_4$
With GluQRS$_5$
Without GluQRS$_5$
Nº of genomes with GluQRS$_5$
Nº of genomes without GluQRS$_5$
With TGT$_1$
Without TGT$_1$
Nº of genomes with TGT$_1$
Nº of genomes without TGT$_1$
p < 0.00005
With TGT$_2$
Without TGT$_2$
Nº of genomes with TGT$_2$
Nº of genomes without TGT$_2$
With TGT$_3$
Without TGT$_3$
Nº of genomes with TGT$_3$
Nº of genomes without TGT$_3$

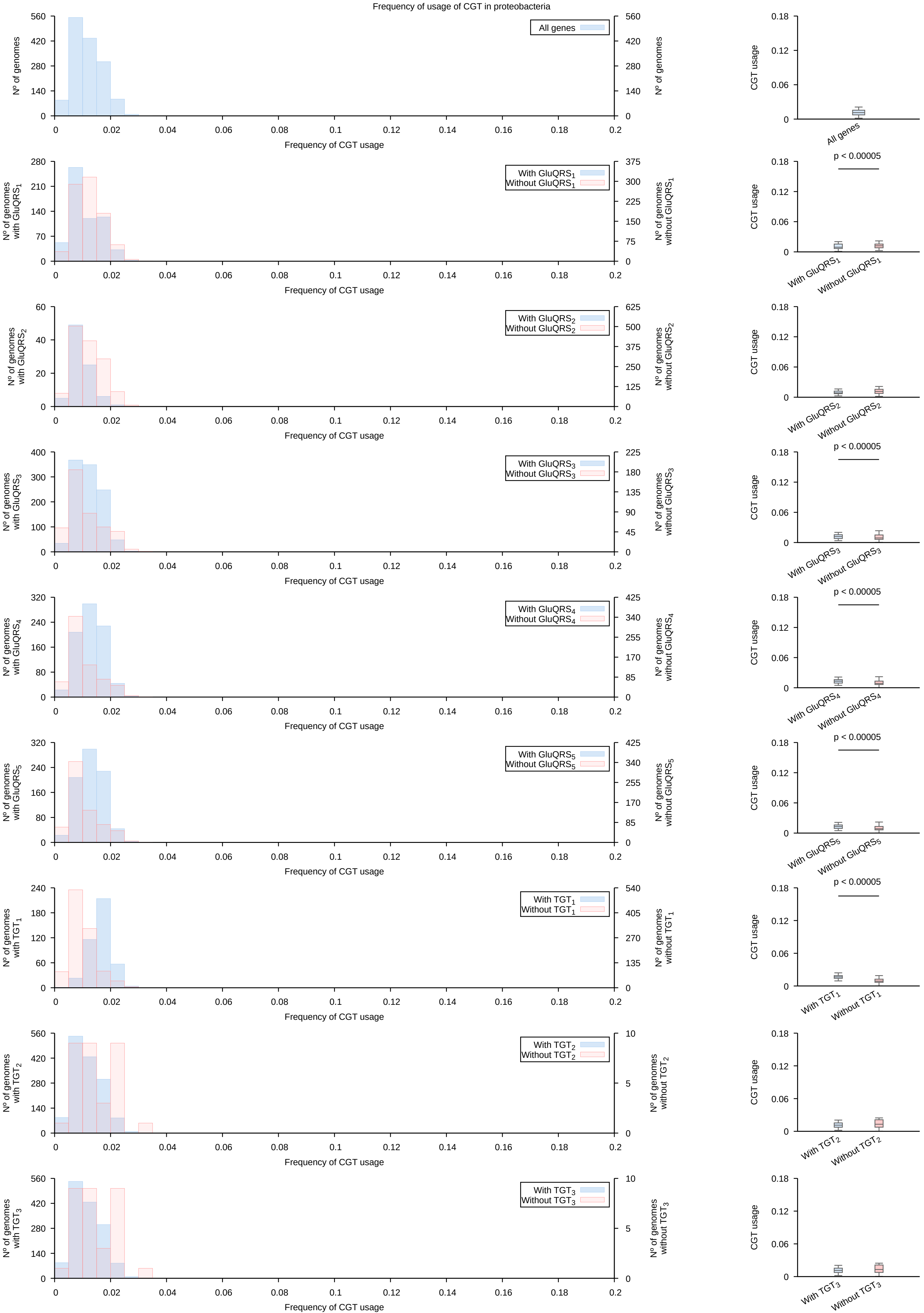
Frequency of usage of CGT in proteobacteria
All genes
Nº of genomes
Frequency of CGT usage
CGT usage
With GluQRS$_1$
Without GluQRS$_1$
Nº of genomes with GluQRS$_1$
Nº of genomes without GluQRS$_1$
p < 0.00005
With GluQRS$_2$
Without GluQRS$_2$
Nº of genomes with GluQRS$_2$
Nº of genomes without GluQRS$_2$
With GluQRS$_3$
Without GluQRS$_3$
Nº of genomes with GluQRS$_3$
Nº of genomes without GluQRS$_3$
p < 0.00005
With GluQRS$_4$
Without GluQRS$_4$
Nº of genomes with GluQRS$_4$
Nº of genomes without GluQRS$_4$
p < 0.00005
With GluQRS$_5$
Without GluQRS$_5$
Nº of genomes with GluQRS$_5$
Nº of genomes without GluQRS$_5$
p < 0.00005
With TGT$_1$
Without TGT$_1$
Nº of genomes with TGT$_1$
Nº of genomes without TGT$_1$
p < 0.00005
With TGT$_2$
Without TGT$_2$
Nº of genomes with TGT$_2$
Nº of genomes without TGT$_2$
With TGT$_3$
Without TGT$_3$
Nº of genomes with TGT$_3$
Nº of genomes without TGT$_3$

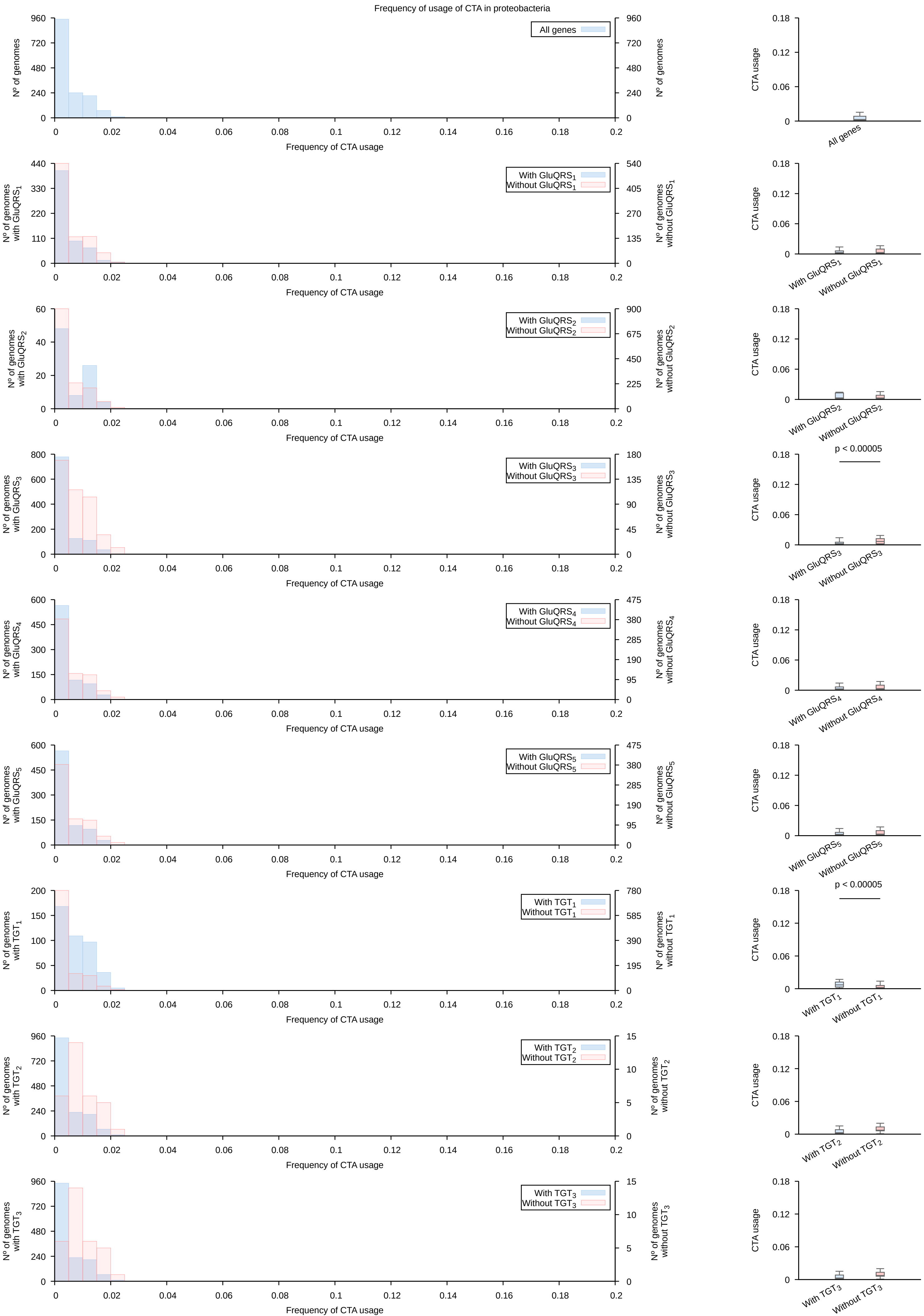
Frequency of usage of CTA in proteobacteria
All genes
With GluQRS$_1$
Without GluQRS$_1$
With GluQRS$_2$
Without GluQRS$_2$
With GluQRS$_3$
Without GluQRS$_3$
With GluQRS$_4$
Without GluQRS$_4$
With GluQRS$_5$
Without GluQRS$_5$
With TGT$_1$
Without TGT$_1$
With TGT$_2$
Without TGT$_2$
With TGT$_3$
Without TGT$_3$
Nº of genomes
Nº of genomes with GluQRS$_1$
Nº of genomes without GluQRS$_1$
Nº of genomes with GluQRS$_2$
Nº of genomes without GluQRS$_2$
Nº of genomes with GluQRS$_3$
Nº of genomes without GluQRS$_3$
Nº of genomes with GluQRS$_4$
Nº of genomes without GluQRS$_4$
Nº of genomes with GluQRS$_5$
Nº of genomes without GluQRS$_5$
Nº of genomes with TGT$_1$
Nº of genomes without TGT$_1$
Nº of genomes with TGT$_2$
Nº of genomes without TGT$_2$
Nº of genomes with TGT$_3$
Nº of genomes without TGT$_3$
Frequency of CTA usage
CTA usage
p < 0.00005
p < 0.00005

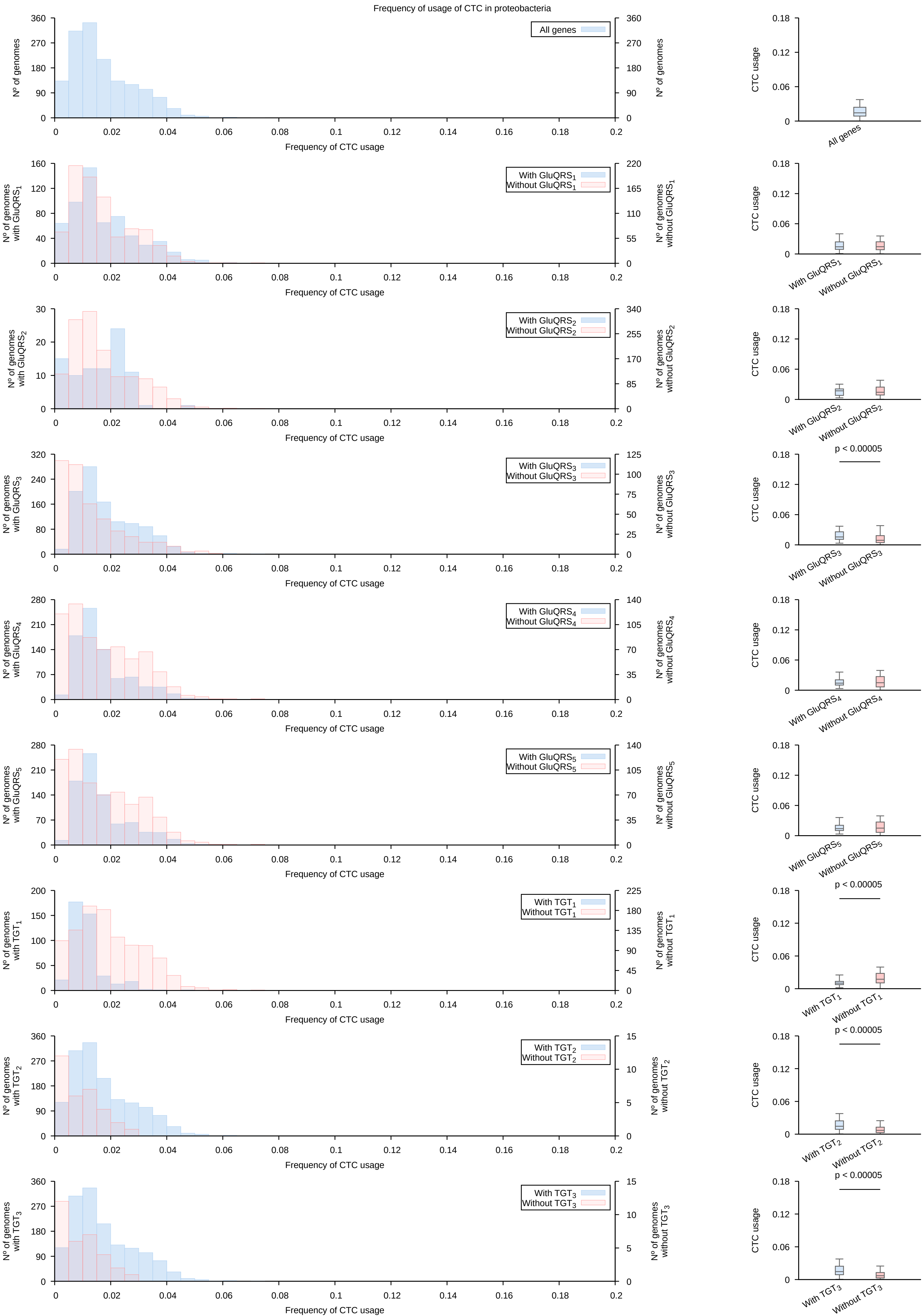
Frequency of usage of CTC in proteobacteria
Nº of genomes
Frequency of CTC usage
CTC usage
All genes
With GluQRS$_1$
Without GluQRS$_1$
With GluQRS$_2$
Without GluQRS$_2$
With GluQRS$_3$
Without GluQRS$_3$
p < 0.00005
With GluQRS$_4$
Without GluQRS$_4$
With GluQRS$_5$
Without GluQRS$_5$
With TGT$_1$
Without TGT$_1$
p < 0.00005
With TGT$_2$
Without TGT$_2$
p < 0.00005
With TGT$_3$
Without TGT$_3$
p < 0.00005

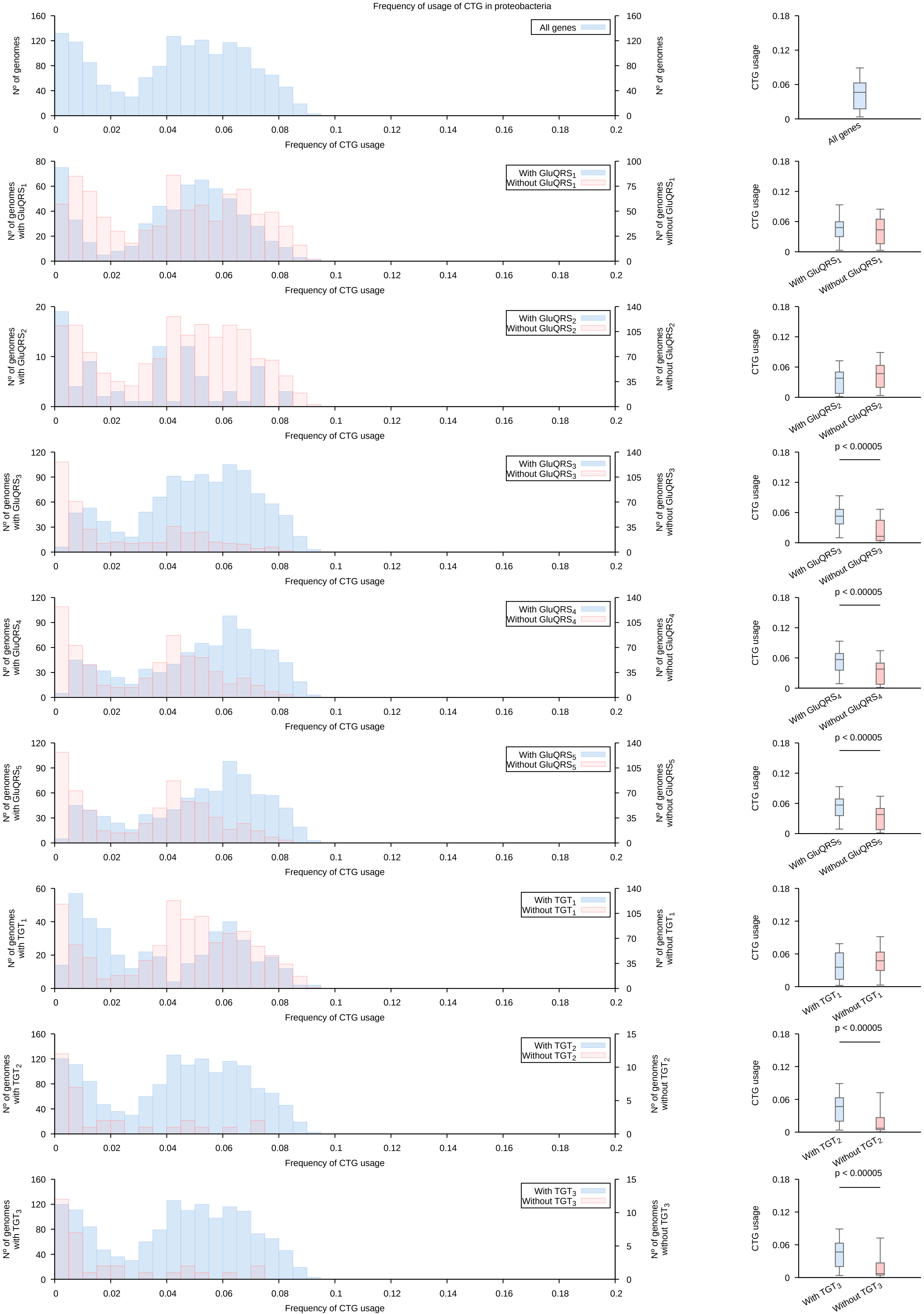
Frequency of usage of CTG in proteobacteria
All genes
Nº of genomes
Frequency of CTG usage
CTG usage
With GluQRS$_1$
Without GluQRS$_1$
Nº of genomes with GluQRS$_1$
Nº of genomes without GluQRS$_1$
With GluQRS$_2$
Without GluQRS$_2$
Nº of genomes with GluQRS$_2$
Nº of genomes without GluQRS$_2$
With GluQRS$_3$
Without GluQRS$_3$
Nº of genomes with GluQRS$_3$
Nº of genomes without GluQRS$_3$
p < 0.00005
With GluQRS$_4$
Without GluQRS$_4$
Nº of genomes with GluQRS$_4$
Nº of genomes without GluQRS$_4$
p < 0.00005
With GluQRS$_5$
Without GluQRS$_5$
Nº of genomes with GluQRS$_5$
Nº of genomes without GluQRS$_5$
p < 0.00005
With TGT$_1$
Without TGT$_1$
Nº of genomes with TGT$_1$
Nº of genomes without TGT$_1$
With TGT$_2$
Without TGT$_2$
Nº of genomes with TGT$_2$
Nº of genomes without TGT$_2$
p < 0.00005
With TGT$_3$
Without TGT$_3$
Nº of genomes with TGT$_3$
Nº of genomes without TGT$_3$
p < 0.00005

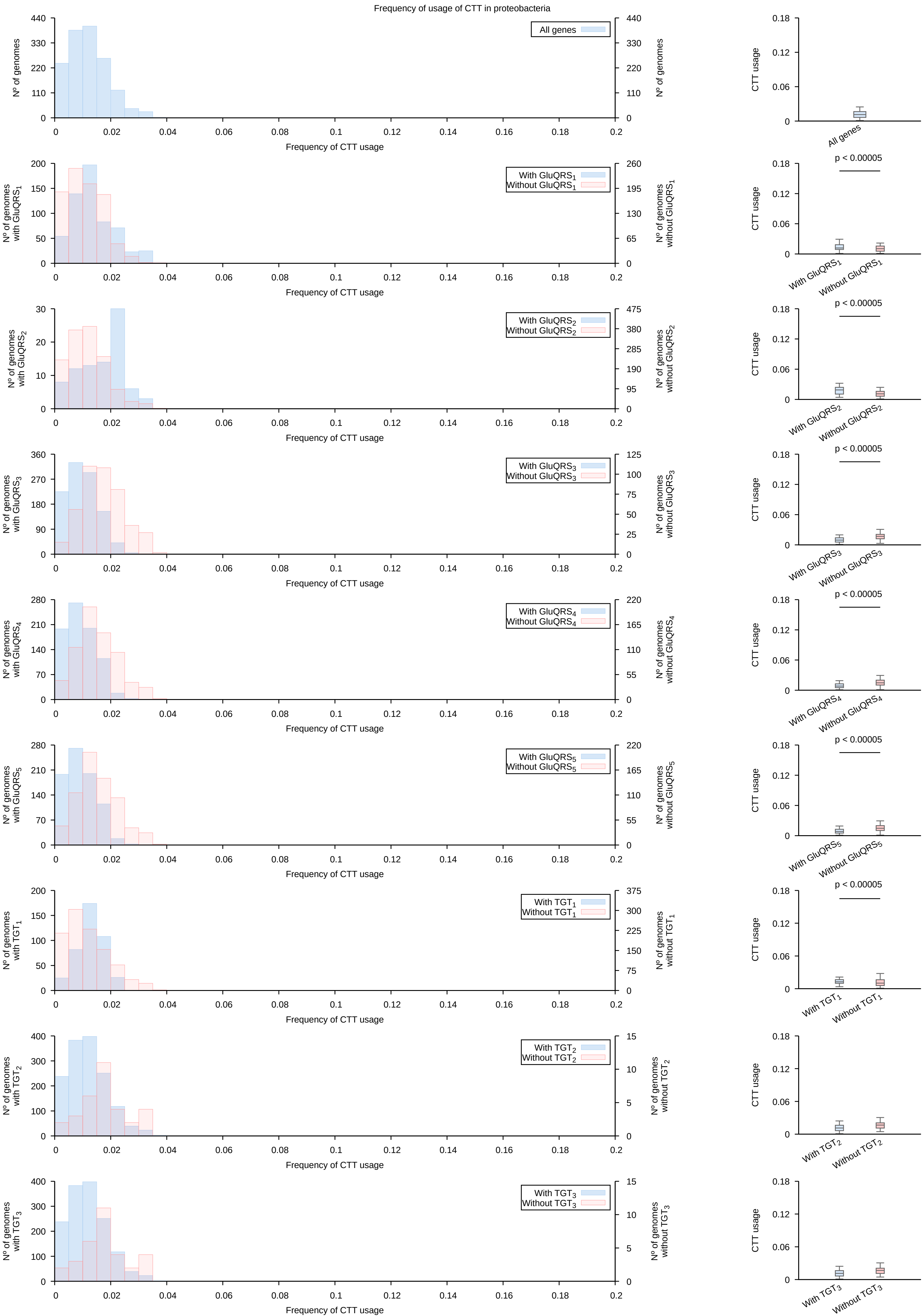
Frequency of usage of CTT in proteobacteria
Nº of genomes
All genes
Frequency of CTT usage
CTT usage
Nº of genomes with GluQRS1
Nº of genomes without GluQRS1
With GluQRS1
Without GluQRS1
p < 0.00005
Nº of genomes with GluQRS2
Nº of genomes without GluQRS2
With GluQRS2
Without GluQRS2
p < 0.00005
Nº of genomes with GluQRS3
Nº of genomes without GluQRS3
With GluQRS3
Without GluQRS3
p < 0.00005
Nº of genomes with GluQRS4
Nº of genomes without GluQRS4
With GluQRS4
Without GluQRS4
p < 0.00005
Nº of genomes with GluQRS5
Nº of genomes without GluQRS5
With GluQRS5
Without GluQRS5
p < 0.00005
Nº of genomes with TGT1
Nº of genomes without TGT1
With TGT1
Without TGT1
p < 0.00005
Nº of genomes with TGT2
Nº of genomes without TGT2
With TGT2
Without TGT2
Nº of genomes with TGT3
Nº of genomes without TGT3
With TGT3
Without TGT3
0
0.02
0.04
0.06
0.08
0.1
0.12
0.14
0.16
0.18
0.2

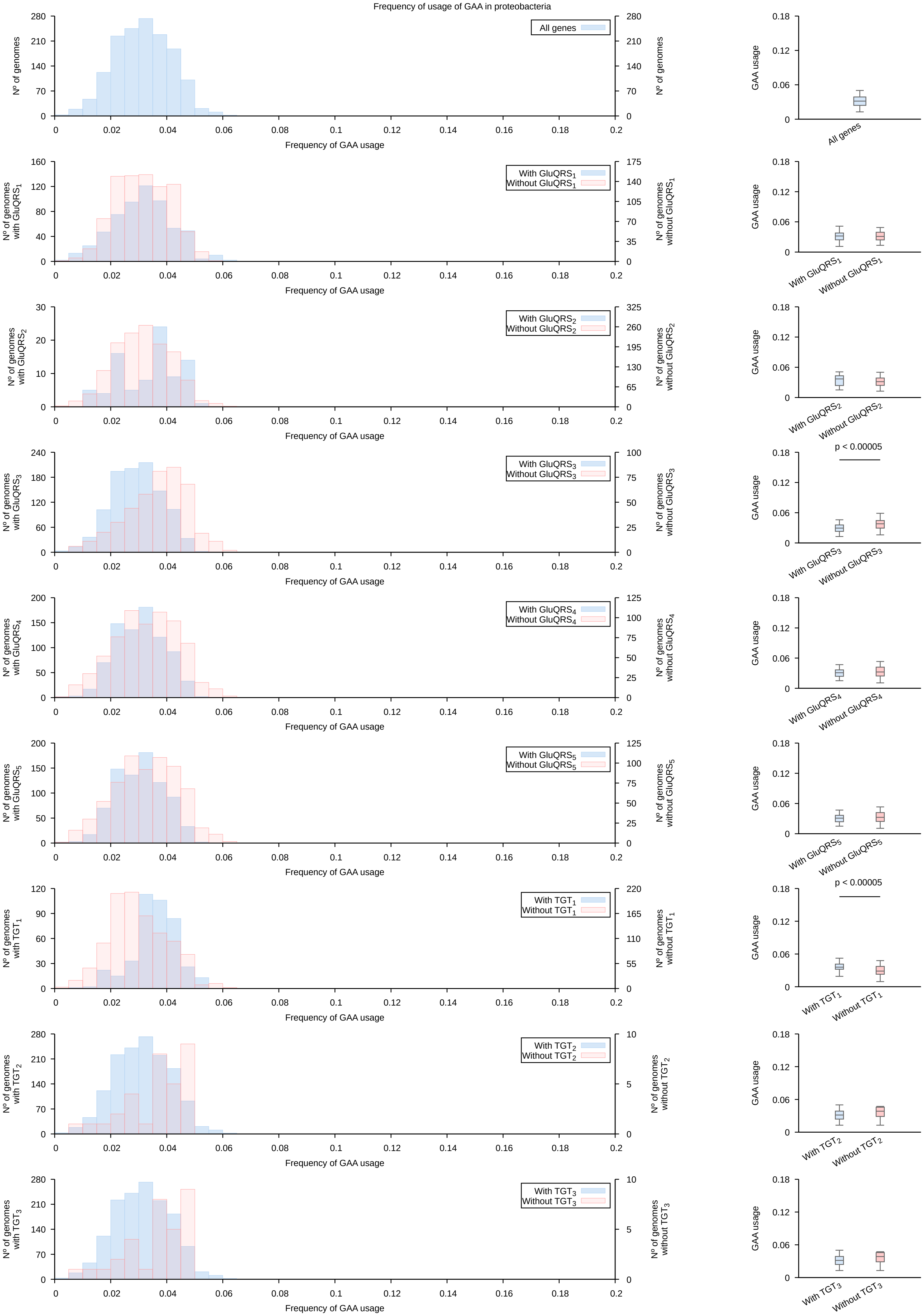
Frequency of usage of GAA in proteobacteria
All genes
Nº of genomes
Frequency of GAA usage
GAA usage
With GluQRS$_1$
Without GluQRS$_1$
Nº of genomes with GluQRS$_1$
Nº of genomes without GluQRS$_1$
With GluQRS$_2$
Without GluQRS$_2$
Nº of genomes with GluQRS$_2$
Nº of genomes without GluQRS$_2$
With GluQRS$_3$
Without GluQRS$_3$
Nº of genomes with GluQRS$_3$
Nº of genomes without GluQRS$_3$
p < 0.00005
With GluQRS$_4$
Without GluQRS$_4$
Nº of genomes with GluQRS$_4$
Nº of genomes without GluQRS$_4$
With GluQRS$_5$
Without GluQRS$_5$
Nº of genomes with GluQRS$_5$
Nº of genomes without GluQRS$_5$
With TGT$_1$
Without TGT$_1$
Nº of genomes with TGT$_1$
Nº of genomes without TGT$_1$
p < 0.00005
With TGT$_2$
Without TGT$_2$
Nº of genomes with TGT$_2$
Nº of genomes without TGT$_2$
With TGT$_3$
Without TGT$_3$
Nº of genomes with TGT$_3$
Nº of genomes without TGT$_3$

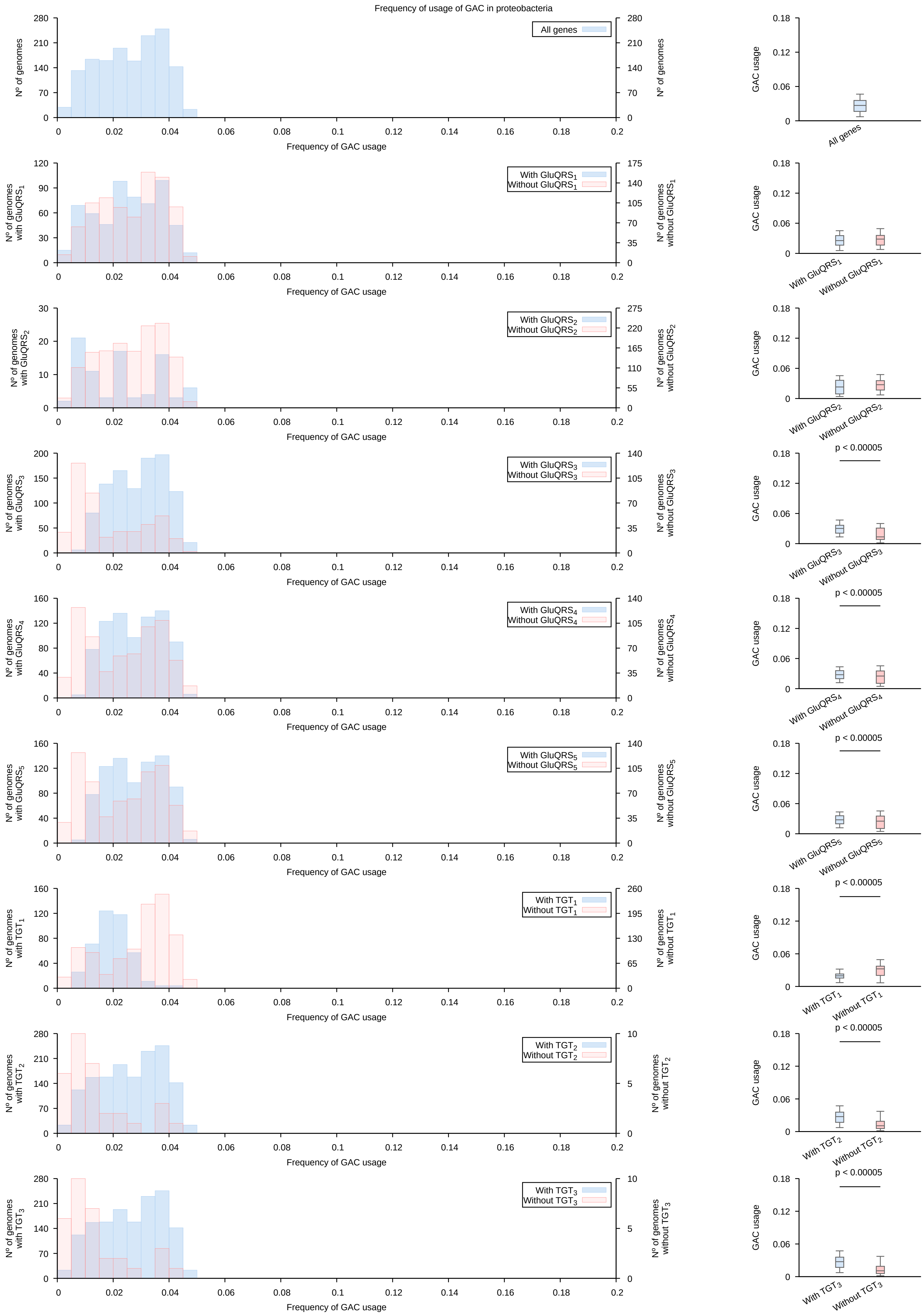

Frequency of usage of GAC in proteobacteria
All genes
Nº of genomes
GAC usage
Frequency of GAC usage
With GluQRS$_1$
Without GluQRS$_1$
Nº of genomes with GluQRS$_1$
Nº of genomes without GluQRS$_1$
With GluQRS$_2$
Without GluQRS$_2$
Nº of genomes with GluQRS$_2$
Nº of genomes without GluQRS$_2$
With GluQRS$_3$
Without GluQRS$_3$
Nº of genomes with GluQRS$_3$
Nº of genomes without GluQRS$_3$
p < 0.00005
With GluQRS$_4$
Without GluQRS$_4$
Nº of genomes with GluQRS$_4$
Nº of genomes without GluQRS$_4$
p < 0.00005
With GluQRS$_5$
Without GluQRS$_5$
Nº of genomes with GluQRS$_5$
Nº of genomes without GluQRS$_5$
p < 0.00005
With TGT$_1$
Without TGT$_1$
Nº of genomes with TGT$_1$
Nº of genomes without TGT$_1$
p < 0.00005
With TGT$_2$
Without TGT$_2$
Nº of genomes with TGT$_2$
Nº of genomes without TGT$_2$
p < 0.00005
With TGT$_3$
Without TGT$_3$
Nº of genomes with TGT$_3$
Nº of genomes without TGT$_3$
p < 0.00005

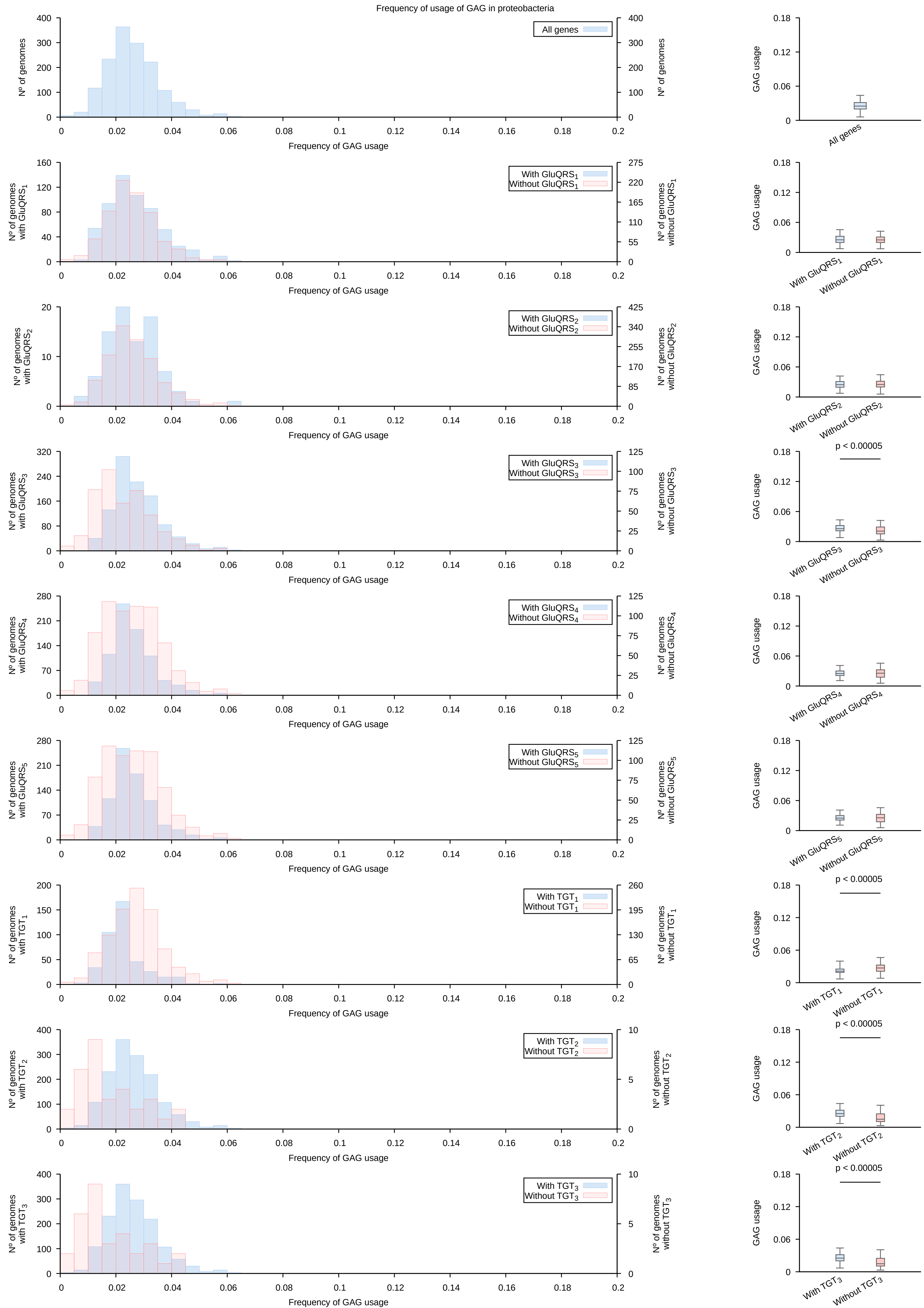
Frequency of usage of GAG in proteobacteria
All genes
Nº of genomes
Frequency of GAG usage
GAG usage
With GluQRS$_1$
Without GluQRS$_1$
Nº of genomes with GluQRS$_1$
Nº of genomes without GluQRS$_1$
With GluQRS$_2$
Without GluQRS$_2$
Nº of genomes with GluQRS$_2$
Nº of genomes without GluQRS$_2$
With GluQRS$_3$
Without GluQRS$_3$
Nº of genomes with GluQRS$_3$
Nº of genomes without GluQRS$_3$
p < 0.00005
With GluQRS$_4$
Without GluQRS$_4$
Nº of genomes with GluQRS$_4$
Nº of genomes without GluQRS$_4$
With GluQRS$_5$
Without GluQRS$_5$
Nº of genomes with GluQRS$_5$
Nº of genomes without GluQRS$_5$
With TGT$_1$
Without TGT$_1$
Nº of genomes with TGT$_1$
Nº of genomes without TGT$_1$
p < 0.00005
With TGT$_2$
Without TGT$_2$
Nº of genomes with TGT$_2$
Nº of genomes without TGT$_2$
p < 0.00005
With TGT$_3$
Without TGT$_3$
Nº of genomes with TGT$_3$
Nº of genomes without TGT$_3$
p < 0.00005

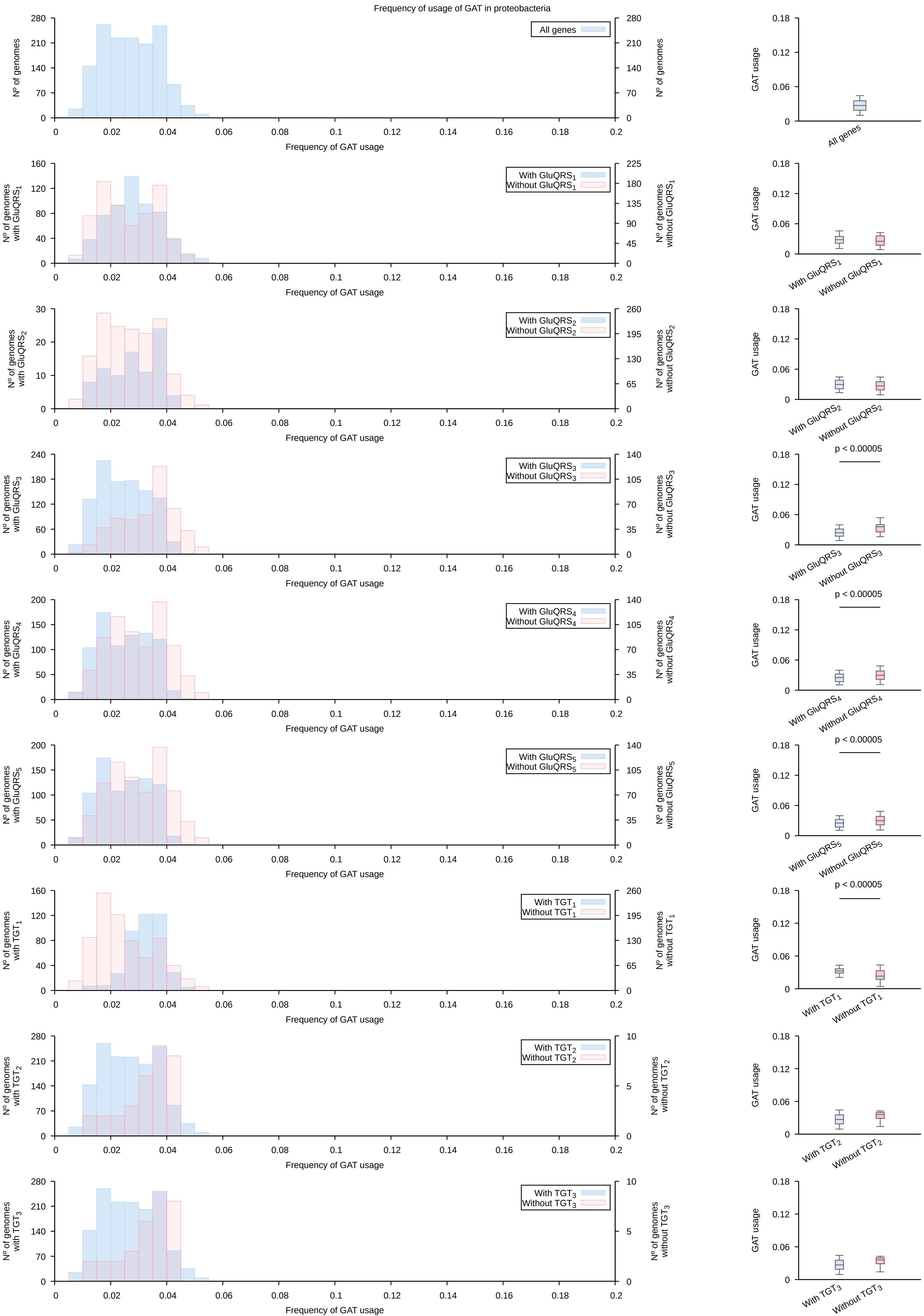
Frequency of usage of GAT in proteobacteria
All genes
Nº of genomes
Frequency of GAT usage
GAT usage
With GluQRS1
Without GluQRS1
Nº of genomes with GluQRS1
Nº of genomes without GluQRS1
With GluQRS2
Without GluQRS2
Nº of genomes with GluQRS2
Nº of genomes without GluQRS2
With GluQRS3
Without GluQRS3
Nº of genomes with GluQRS3
Nº of genomes without GluQRS3
p < 0.00005
With GluQRS4
Without GluQRS4
Nº of genomes with GluQRS4
Nº of genomes without GluQRS4
p < 0.00005
With GluQRS5
Without GluQRS5
Nº of genomes with GluQRS5
Nº of genomes without GluQRS5
p < 0.00005
With TGT1
Without TGT1
Nº of genomes with TGT1
Nº of genomes without TGT1
p < 0.00005
With TGT2
Without TGT2
Nº of genomes with TGT2
Nº of genomes without TGT2
With TGT3
Without TGT3
Nº of genomes with TGT3
Nº of genomes without TGT3

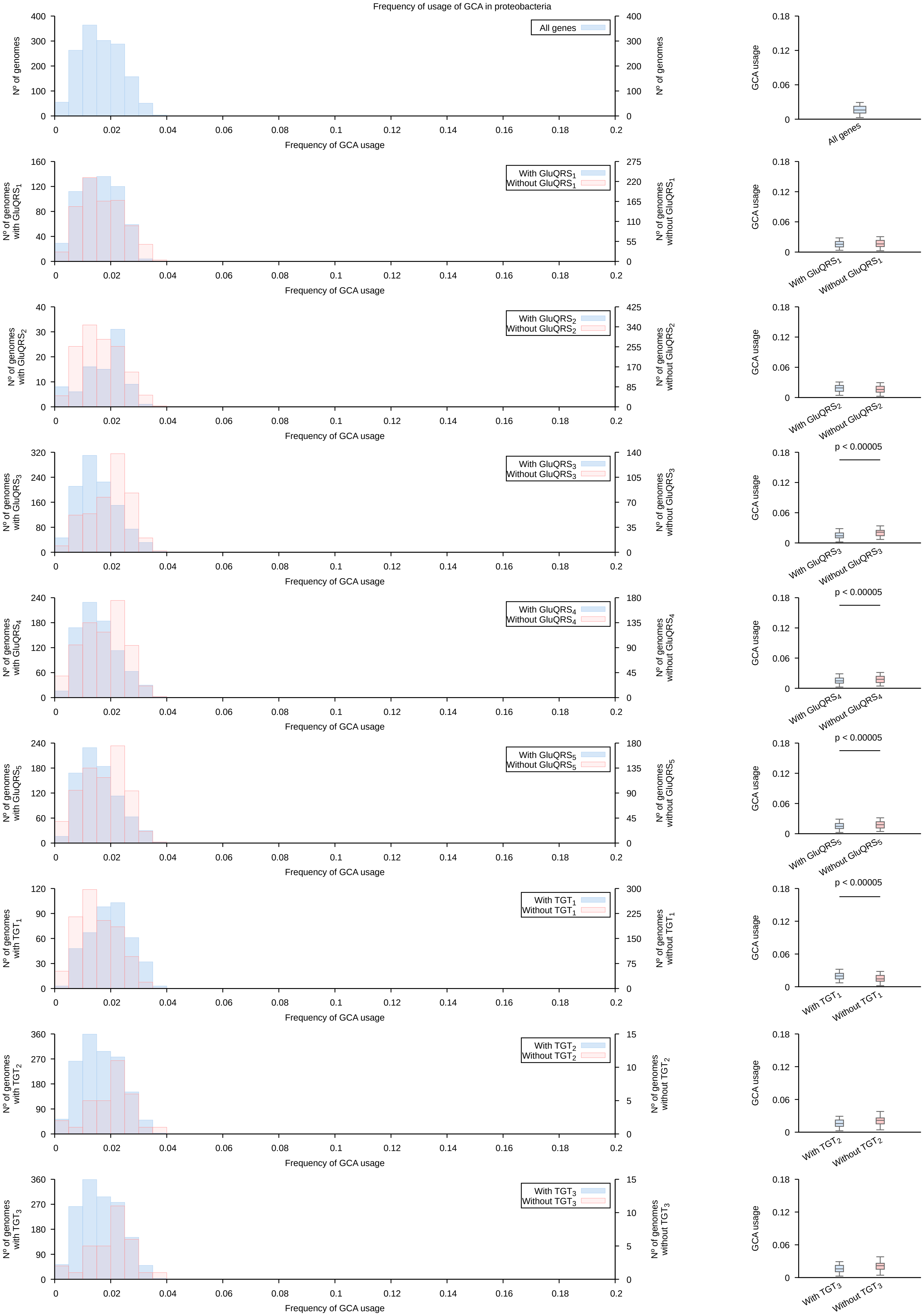
Frequency of usage of GCA in proteobacteria
All genes
With GluQRS1
Without GluQRS1
With GluQRS2
Without GluQRS2
With GluQRS3
Without GluQRS3
With GluQRS4
Without GluQRS4
With GluQRS5
Without GluQRS5
With TGT1
Without TGT1
With TGT2
Without TGT2
With TGT3
Without TGT3
Nº of genomes
Nº of genomes with GluQRS1
Nº of genomes without GluQRS1
Nº of genomes with GluQRS2
Nº of genomes without GluQRS2
Nº of genomes with GluQRS3
Nº of genomes without GluQRS3
Nº of genomes with GluQRS4
Nº of genomes without GluQRS4
Nº of genomes with GluQRS5
Nº of genomes without GluQRS5
Nº of genomes with TGT1
Nº of genomes without TGT1
Nº of genomes with TGT2
Nº of genomes without TGT2
Nº of genomes with TGT3
Nº of genomes without TGT3
Frequency of GCA usage
GCA usage
p < 0.00005

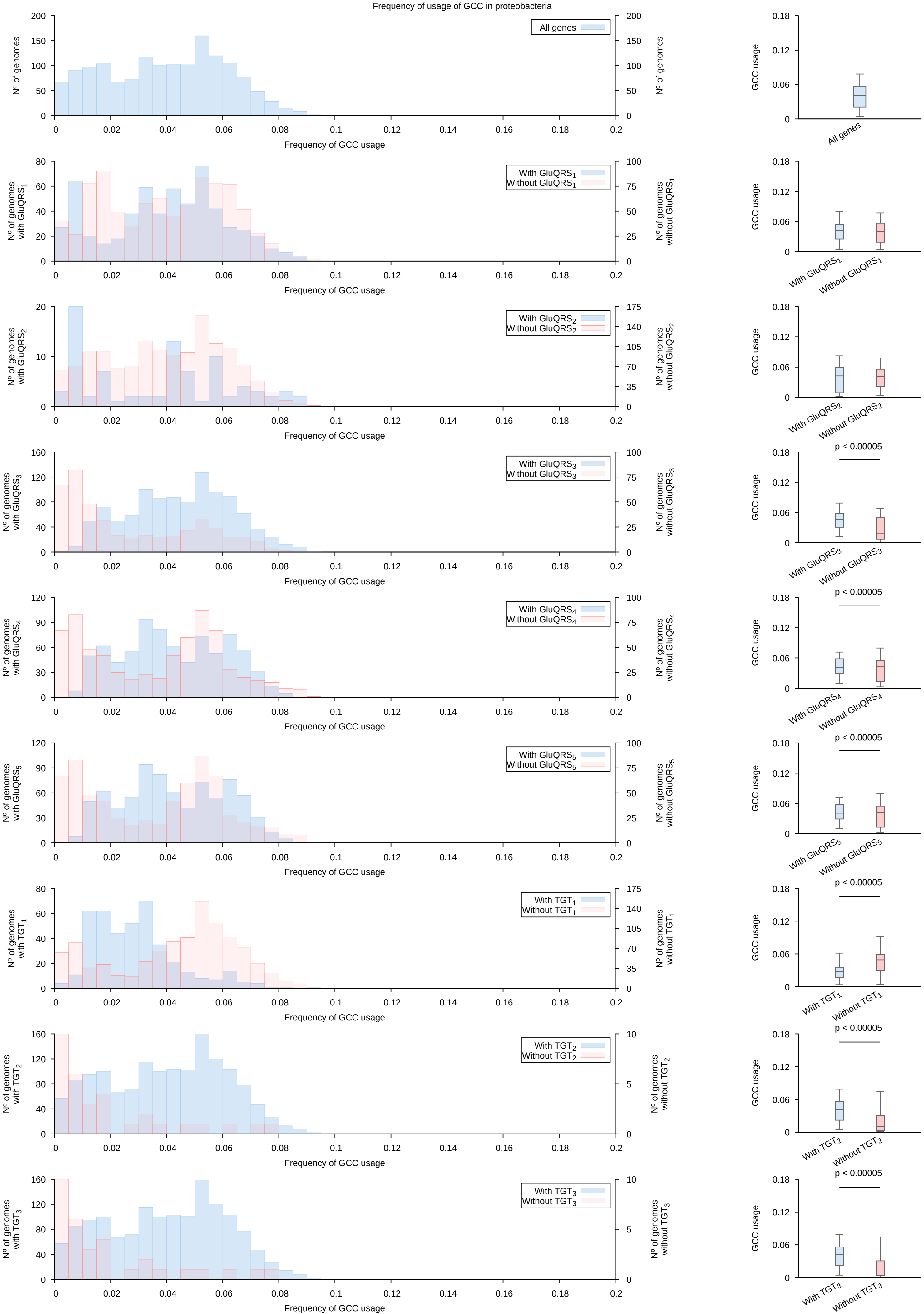
Frequency of usage of GCC in proteobacteria
Nº of genomes
All genes
Frequency of GCC usage
GCC usage
Nº of genomes with GluQRS$_1$
Nº of genomes without GluQRS$_1$
With GluQRS$_1$
Without GluQRS$_1$
Nº of genomes with GluQRS$_2$
Nº of genomes without GluQRS$_2$
With GluQRS$_2$
Without GluQRS$_2$
Nº of genomes with GluQRS$_3$
Nº of genomes without GluQRS$_3$
With GluQRS$_3$
Without GluQRS$_3$
p < 0.00005
Nº of genomes with GluQRS$_4$
Nº of genomes without GluQRS$_4$
With GluQRS$_4$
Without GluQRS$_4$
p < 0.00005
Nº of genomes with GluQRS$_5$
Nº of genomes without GluQRS$_5$
With GluQRS$_5$
Without GluQRS$_5$
p < 0.00005
Nº of genomes with TGT$_1$
Nº of genomes without TGT$_1$
With TGT$_1$
Without TGT$_1$
p < 0.00005
Nº of genomes with TGT$_2$
Nº of genomes without TGT$_2$
With TGT$_2$
Without TGT$_2$
p < 0.00005
Nº of genomes with TGT$_3$
Nº of genomes without TGT$_3$
With TGT$_3$
Without TGT$_3$
p < 0.00005

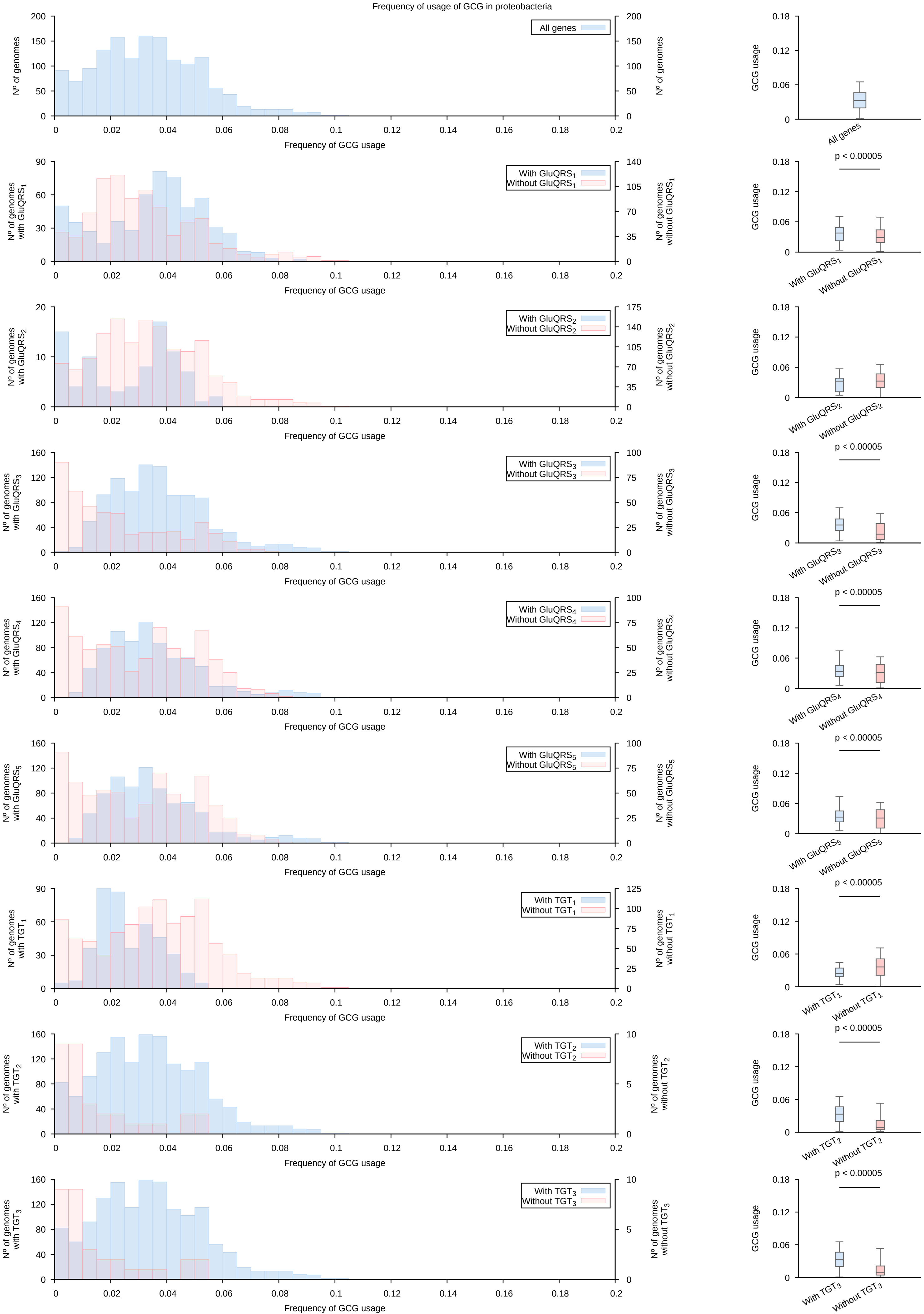

Frequency of usage of GCG in proteobacteria
All genes
Nº of genomes
Frequency of GCG usage
GCG usage
With GluQRS$_1$
Without GluQRS$_1$
Nº of genomes with GluQRS$_1$
Nº of genomes without GluQRS$_1$
With GluQRS$_2$
Without GluQRS$_2$
Nº of genomes with GluQRS$_2$
Nº of genomes without GluQRS$_2$
With GluQRS$_3$
Without GluQRS$_3$
Nº of genomes with GluQRS$_3$
Nº of genomes without GluQRS$_3$
With GluQRS$_4$
Without GluQRS$_4$
Nº of genomes with GluQRS$_4$
Nº of genomes without GluQRS$_4$
With GluQRS$_5$
Without GluQRS$_5$
Nº of genomes with GluQRS$_5$
Nº of genomes without GluQRS$_5$
With TGT$_1$
Without TGT$_1$
Nº of genomes with TGT$_1$
Nº of genomes without TGT$_1$
With TGT$_2$
Without TGT$_2$
Nº of genomes with TGT$_2$
Nº of genomes without TGT$_2$
With TGT$_3$
Without TGT$_3$
Nº of genomes with TGT$_3$
Nº of genomes without TGT$_3$
p < 0.00005

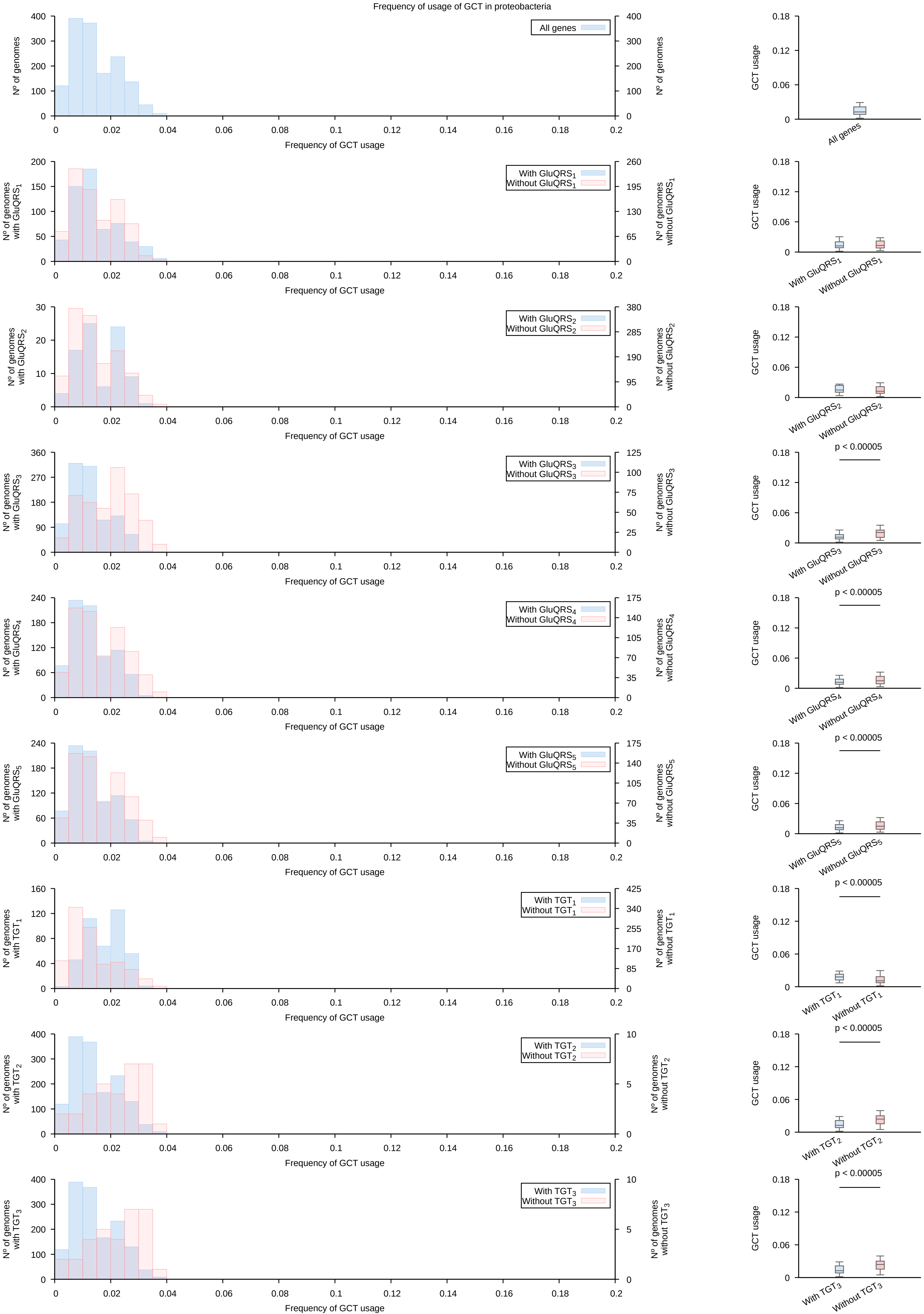

Frequency of usage of GCT in proteobacteria
All genes
With GluQRS1
Without GluQRS1
With GluQRS2
Without GluQRS2
With GluQRS3
Without GluQRS3
With GluQRS4
Without GluQRS4
With GluQRS5
Without GluQRS5
With TGT1
Without TGT1
With TGT2
Without TGT2
With TGT3
Without TGT3
Nº of genomes
Frequency of GCT usage
GCT usage
p < 0.00005

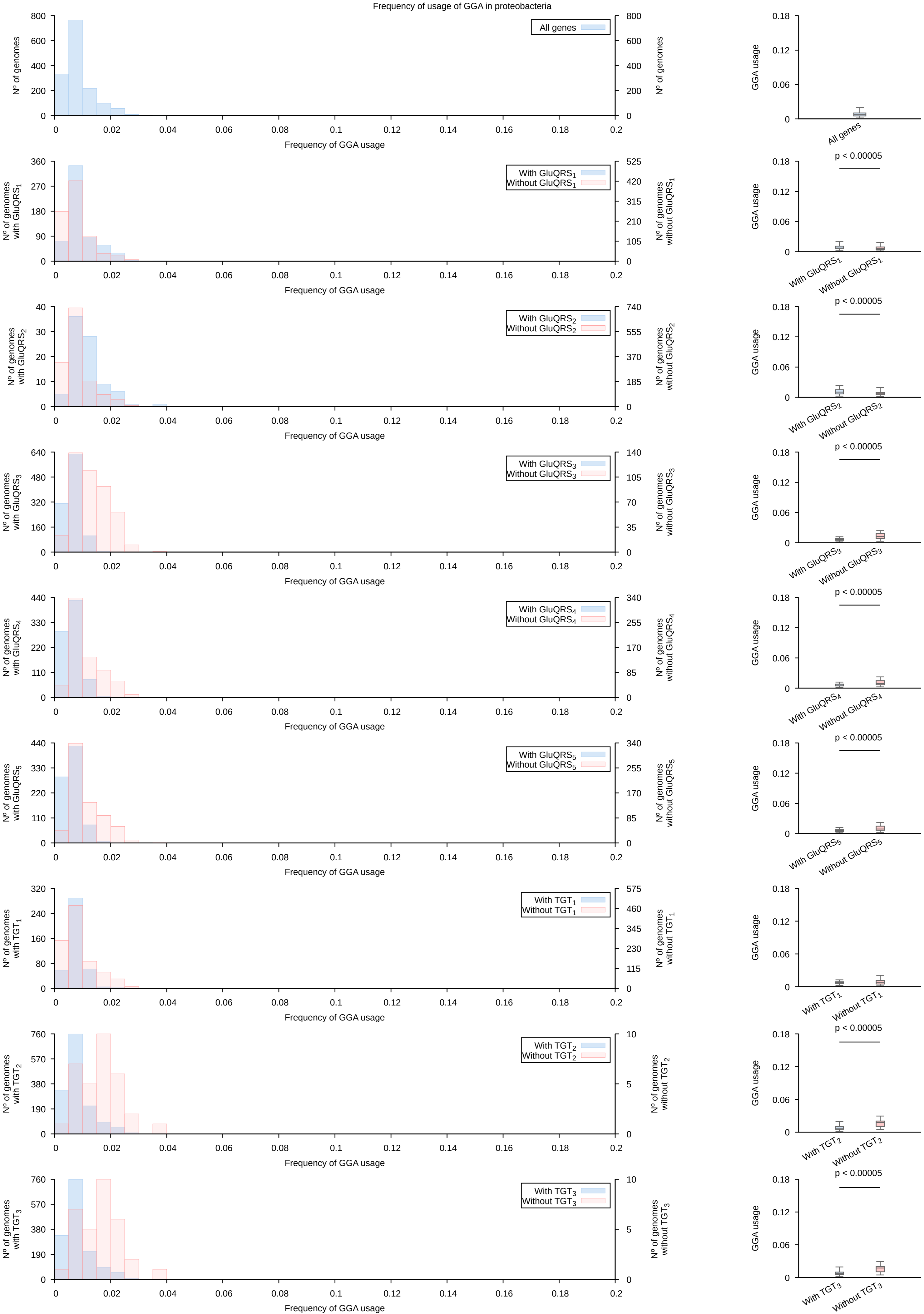

Frequency of usage of GGA in proteobacteria
Nº of genomes
All genes
Frequency of GGA usage
GGA usage
Nº of genomes with GluQRS1
Nº of genomes without GluQRS1
With GluQRS1
Without GluQRS1
p < 0.00005
Nº of genomes with GluQRS2
Nº of genomes without GluQRS2
With GluQRS2
Without GluQRS2
Nº of genomes with GluQRS3
Nº of genomes without GluQRS3
With GluQRS3
Without GluQRS3
Nº of genomes with GluQRS4
Nº of genomes without GluQRS4
With GluQRS4
Without GluQRS4
Nº of genomes with GluQRS5
Nº of genomes without GluQRS5
With GluQRS5
Without GluQRS5
Nº of genomes with TGT1
Nº of genomes without TGT1
With TGT1
Without TGT1
Nº of genomes with TGT2
Nº of genomes without TGT2
With TGT2
Without TGT2
Nº of genomes with TGT3
Nº of genomes without TGT3
With TGT3
Without TGT3

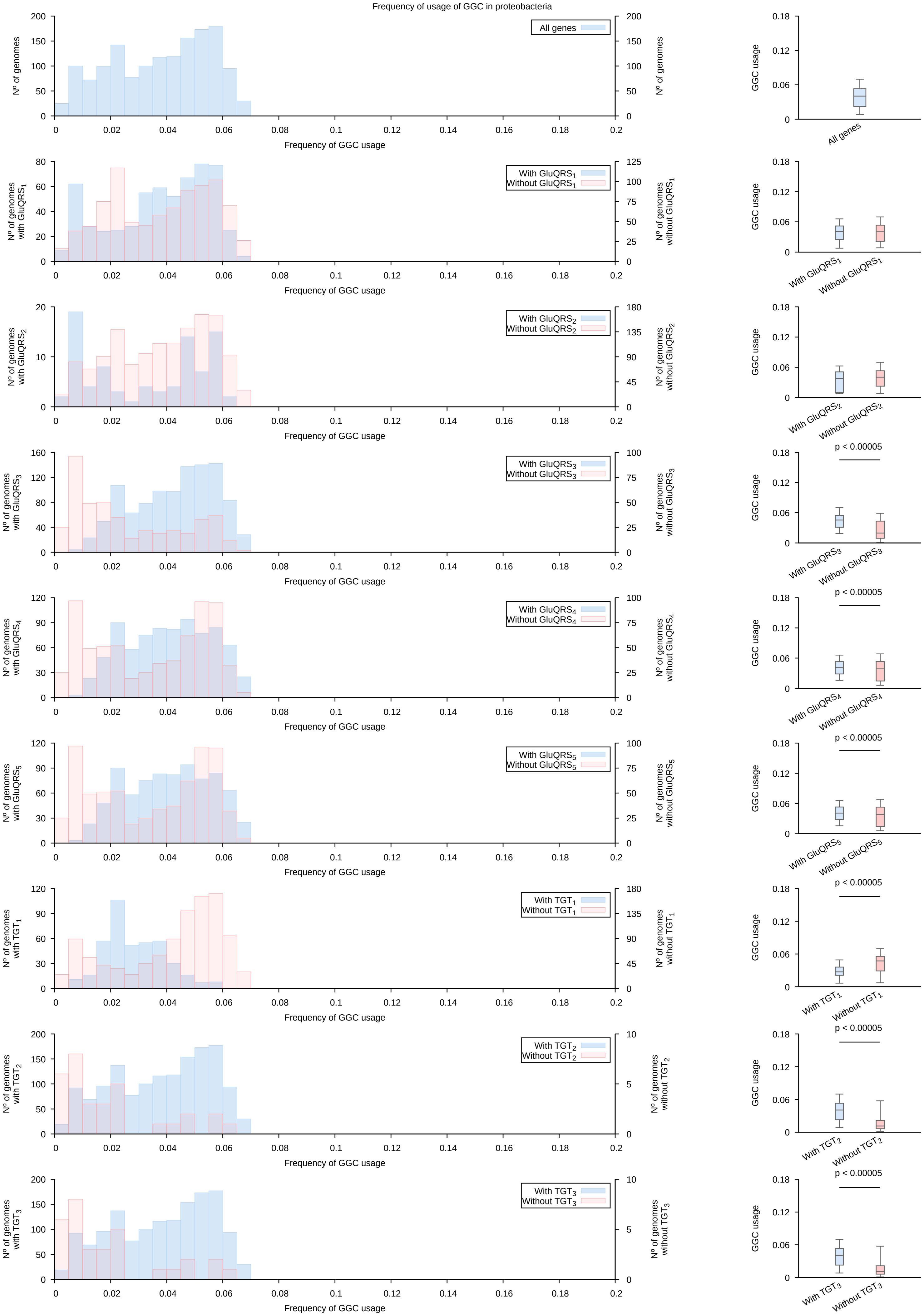
Frequency of usage of GGC in proteobacteria
All genes
Nº of genomes
Frequency of GGC usage
GGC usage
With GluQRS$_1$
Without GluQRS$_1$
Nº of genomes with GluQRS$_1$
Nº of genomes without GluQRS$_1$
With GluQRS$_2$
Without GluQRS$_2$
Nº of genomes with GluQRS$_2$
Nº of genomes without GluQRS$_2$
With GluQRS$_3$
Without GluQRS$_3$
Nº of genomes with GluQRS$_3$
Nº of genomes without GluQRS$_3$
p < 0.00005
With GluQRS$_4$
Without GluQRS$_4$
Nº of genomes with GluQRS$_4$
Nº of genomes without GluQRS$_4$
p < 0.00005
With GluQRS$_5$
Without GluQRS$_5$
Nº of genomes with GluQRS$_5$
Nº of genomes without GluQRS$_5$
p < 0.00005
With TGT$_1$
Without TGT$_1$
Nº of genomes with TGT$_1$
Nº of genomes without TGT$_1$
p < 0.00005
With TGT$_2$
Without TGT$_2$
Nº of genomes with TGT$_2$
Nº of genomes without TGT$_2$
p < 0.00005
With TGT$_3$
Without TGT$_3$
Nº of genomes with TGT$_3$
Nº of genomes without TGT$_3$
p < 0.00005

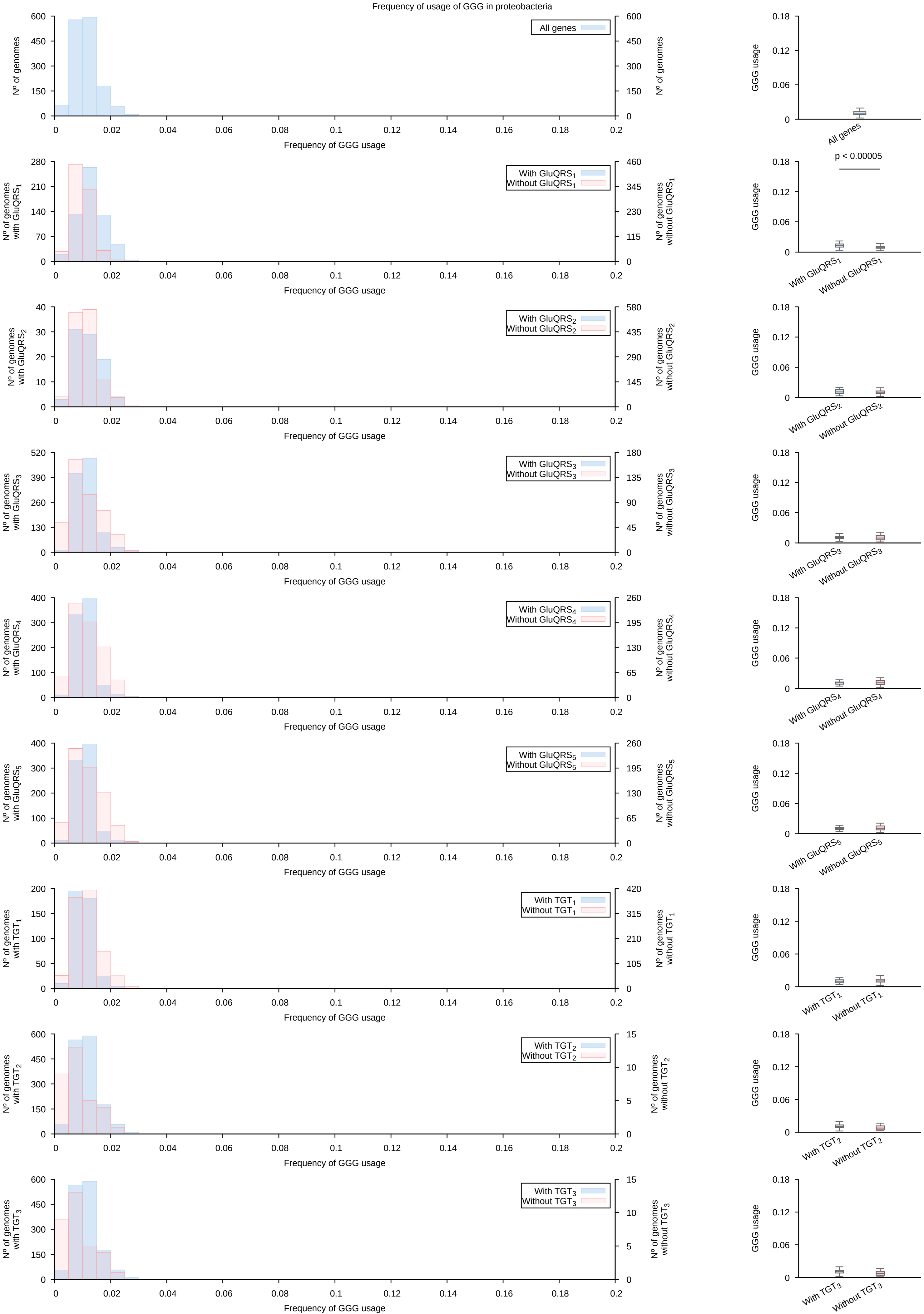

Frequency of usage of GGG in proteobacteria
All genes
With GluQRS$_1$
Without GluQRS$_1$
With GluQRS$_2$
Without GluQRS$_2$
With GluQRS$_3$
Without GluQRS$_3$
With GluQRS$_4$
Without GluQRS$_4$
With GluQRS$_5$
Without GluQRS$_5$
With TGT$_1$
Without TGT$_1$
With TGT$_2$
Without TGT$_2$
With TGT$_3$
Without TGT$_3$
Nº of genomes
Frequency of GGG usage
GGG usage
p < 0.00005

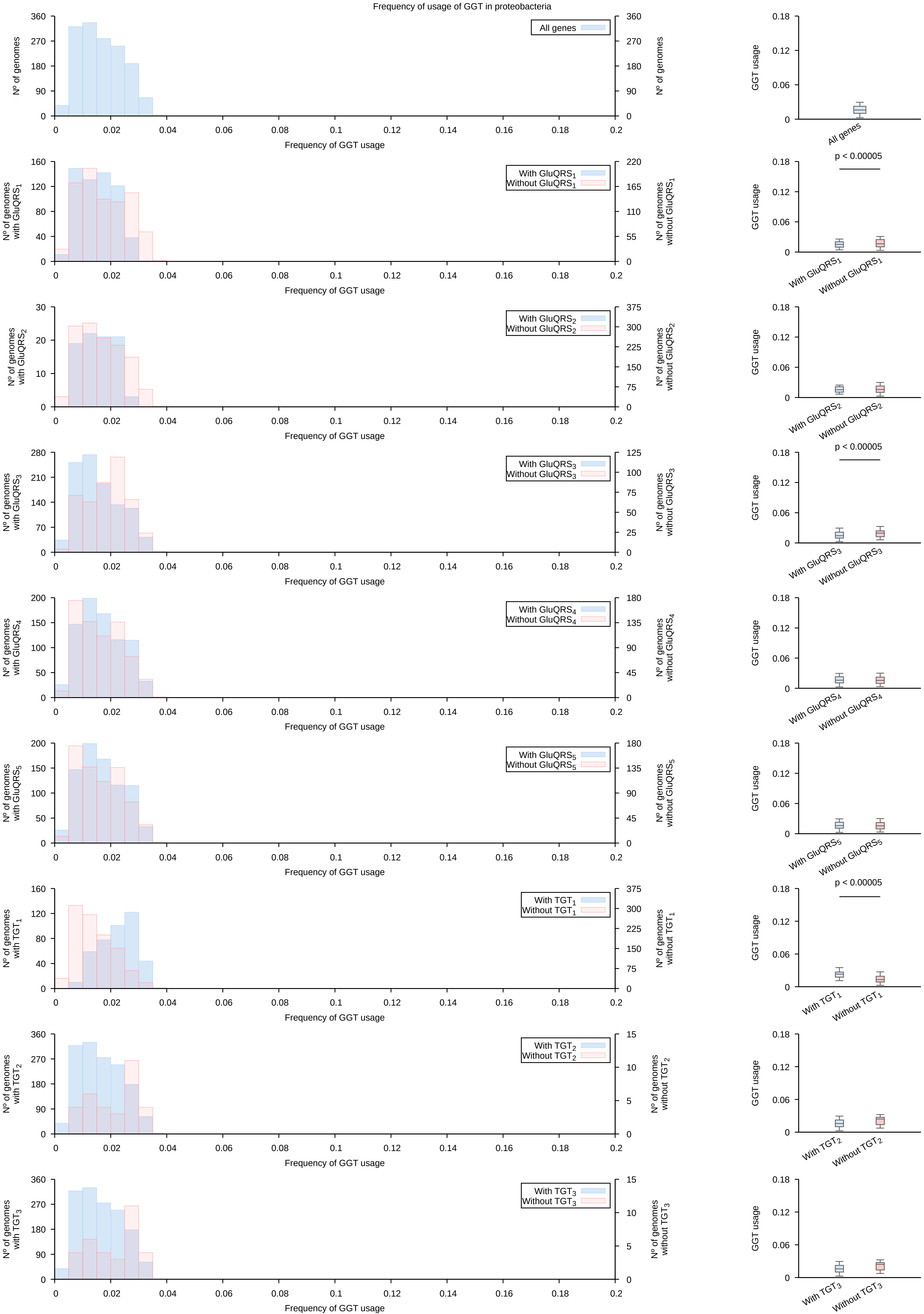

Frequency of usage of GGT in proteobacteria
All genes
Nº of genomes
Frequency of GGT usage
GGT usage
With GluQRS$_1$
Without GluQRS$_1$
Nº of genomes with GluQRS$_1$
Nº of genomes without GluQRS$_1$
p < 0.00005
With GluQRS$_2$
Without GluQRS$_2$
Nº of genomes with GluQRS$_2$
Nº of genomes without GluQRS$_2$
With GluQRS$_3$
Without GluQRS$_3$
Nº of genomes with GluQRS$_3$
Nº of genomes without GluQRS$_3$
p < 0.00005
With GluQRS$_4$
Without GluQRS$_4$
Nº of genomes with GluQRS$_4$
Nº of genomes without GluQRS$_4$
With GluQRS$_5$
Without GluQRS$_5$
Nº of genomes with GluQRS$_5$
Nº of genomes without GluQRS$_5$
With TGT$_1$
Without TGT$_1$
Nº of genomes with TGT$_1$
Nº of genomes without TGT$_1$
p < 0.00005
With TGT$_2$
Without TGT$_2$
Nº of genomes with TGT$_2$
Nº of genomes without TGT$_2$
With TGT$_3$
Without TGT$_3$
Nº of genomes with TGT$_3$
Nº of genomes without TGT$_3$

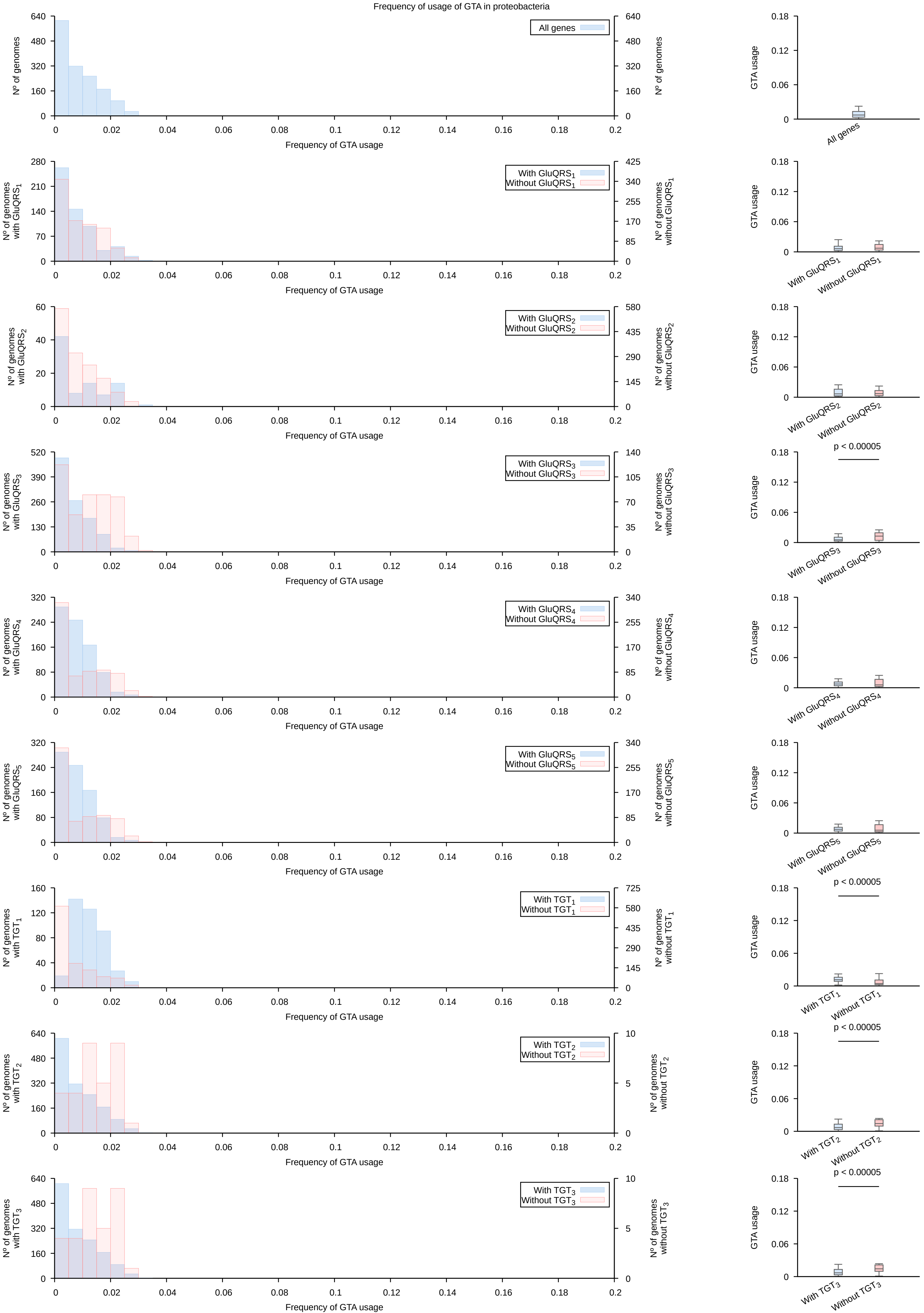

Frequency of usage of GTA in proteobacteria
All genes
Nº of genomes
Frequency of GTA usage
GTA usage
With GluQRS1
Without GluQRS1
Nº of genomes with GluQRS1
Nº of genomes without GluQRS1
With GluQRS2
Without GluQRS2
Nº of genomes with GluQRS2
Nº of genomes without GluQRS2
With GluQRS3
Without GluQRS3
Nº of genomes with GluQRS3
Nº of genomes without GluQRS3
p < 0.00005
With GluQRS4
Without GluQRS4
Nº of genomes with GluQRS4
Nº of genomes without GluQRS4
With GluQRS5
Without GluQRS5
Nº of genomes with GluQRS5
Nº of genomes without GluQRS5
With TGT1
Without TGT1
Nº of genomes with TGT1
Nº of genomes without TGT1
With TGT2
Without TGT2
Nº of genomes with TGT2
Nº of genomes without TGT2
With TGT3
Without TGT3
Nº of genomes with TGT3
Nº of genomes without TGT3

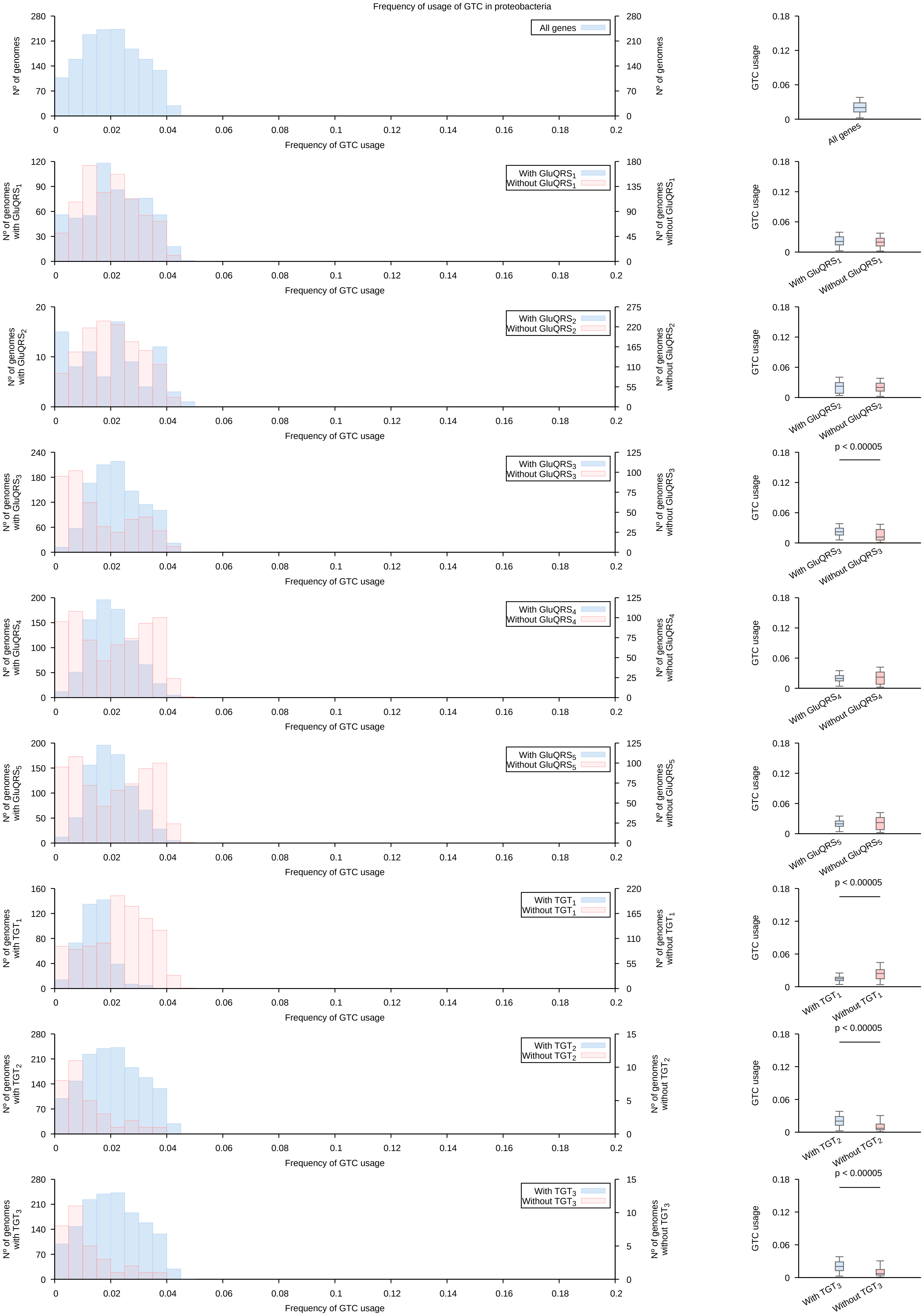

Frequency of usage of GTC in proteobacteria
All genes
Nº of genomes
Frequency of GTC usage
GTC usage
With GluQRS$_1$
Without GluQRS$_1$
Nº of genomes with GluQRS$_1$
Nº of genomes without GluQRS$_1$
With GluQRS$_2$
Without GluQRS$_2$
Nº of genomes with GluQRS$_2$
Nº of genomes without GluQRS$_2$
With GluQRS$_3$
Without GluQRS$_3$
Nº of genomes with GluQRS$_3$
Nº of genomes without GluQRS$_3$
p < 0.00005
With GluQRS$_4$
Without GluQRS$_4$
Nº of genomes with GluQRS$_4$
Nº of genomes without GluQRS$_4$
With GluQRS$_5$
Without GluQRS$_5$
Nº of genomes with GluQRS$_5$
Nº of genomes without GluQRS$_5$
With TGT$_1$
Without TGT$_1$
Nº of genomes with TGT$_1$
Nº of genomes without TGT$_1$
p < 0.00005
With TGT$_2$
Without TGT$_2$
Nº of genomes with TGT$_2$
Nº of genomes without TGT$_2$
p < 0.00005
With TGT$_3$
Without TGT$_3$
Nº of genomes with TGT$_3$
Nº of genomes without TGT$_3$
p < 0.00005

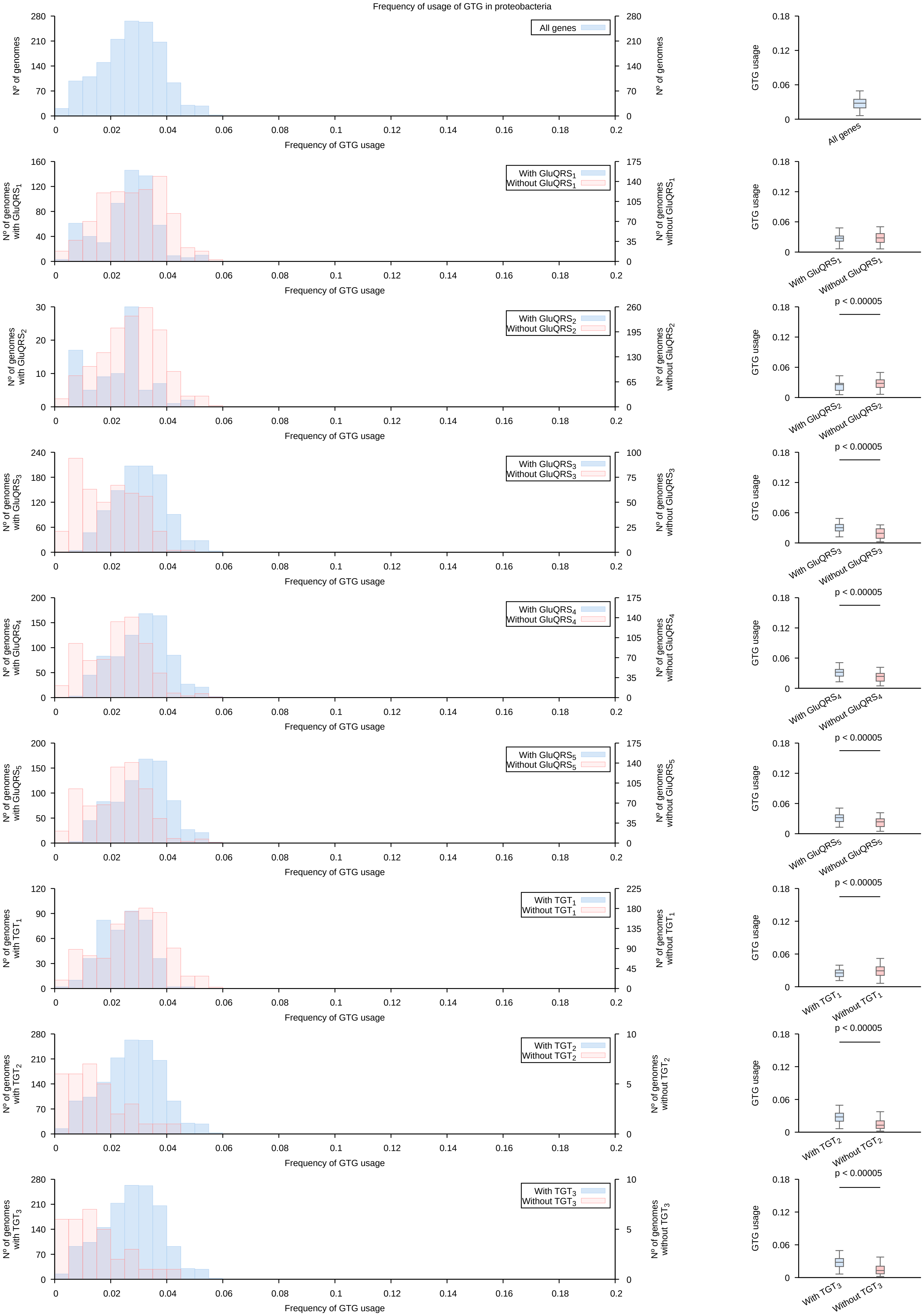

Frequency of usage of GTG in proteobacteria
All genes
Nº of genomes
GTG usage
Frequency of GTG usage
With GluQRS1
Without GluQRS1
Nº of genomes with GluQRS1
Nº of genomes without GluQRS1
With GluQRS2
Without GluQRS2
Nº of genomes with GluQRS2
Nº of genomes without GluQRS2
With GluQRS3
Without GluQRS3
Nº of genomes with GluQRS3
Nº of genomes without GluQRS3
With GluQRS4
Without GluQRS4
Nº of genomes with GluQRS4
Nº of genomes without GluQRS4
With GluQRS5
Without GluQRS5
Nº of genomes with GluQRS5
Nº of genomes without GluQRS5
With TGT1
Without TGT1
Nº of genomes with TGT1
Nº of genomes without TGT1
With TGT2
Without TGT2
Nº of genomes with TGT2
Nº of genomes without TGT2
With TGT3
Without TGT3
Nº of genomes with TGT3
Nº of genomes without TGT3
p < 0.00005

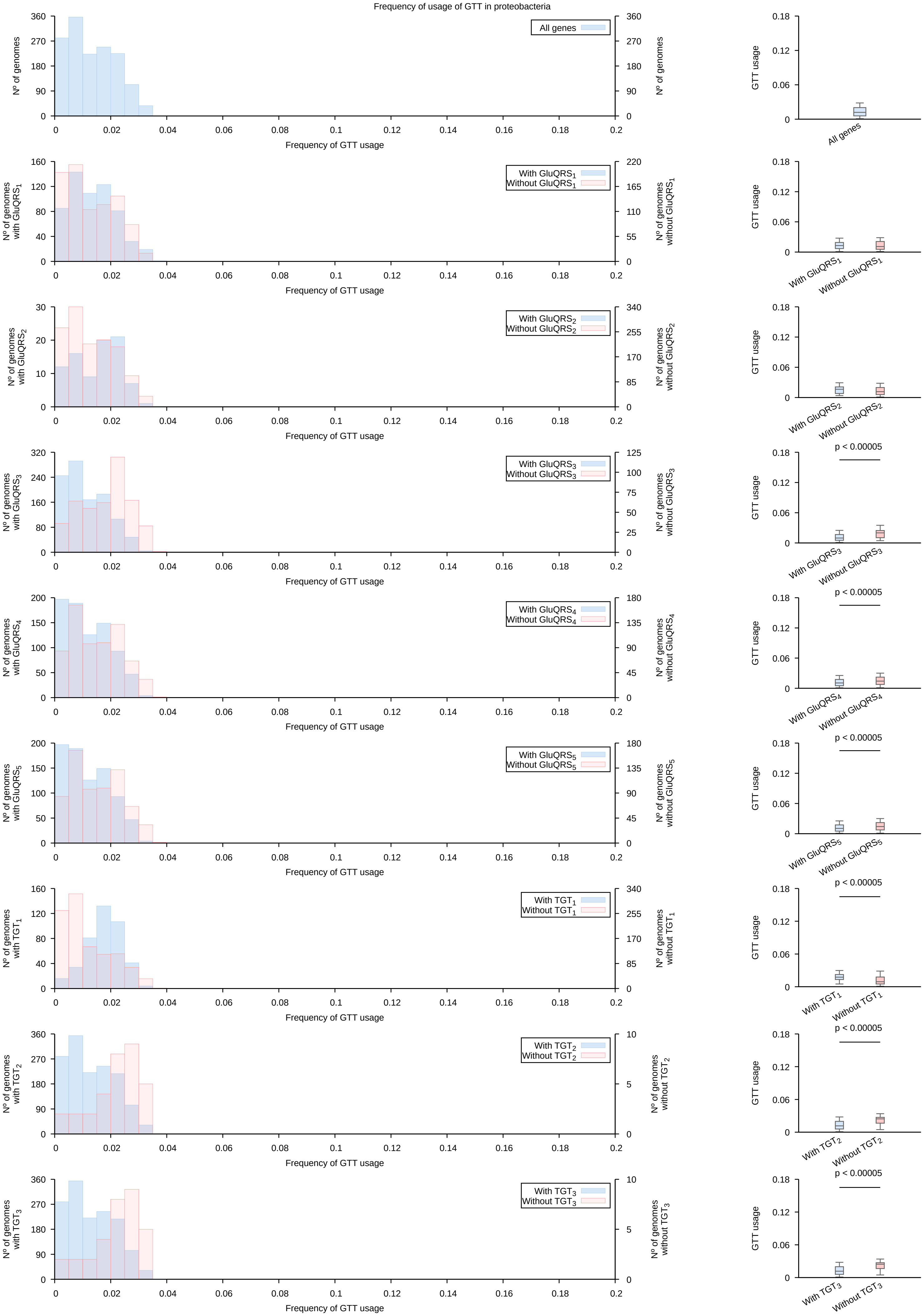
Frequency of usage of GTT in proteobacteria
Nº of genomes
All genes
Frequency of GTT usage
GTT usage
All genes
Nº of genomes with GluQRS$_1$
Nº of genomes without GluQRS$_1$
With GluQRS$_1$
Without GluQRS$_1$
Nº of genomes with GluQRS$_2$
Nº of genomes without GluQRS$_2$
With GluQRS$_2$
Without GluQRS$_2$
Nº of genomes with GluQRS$_3$
Nº of genomes without GluQRS$_3$
With GluQRS$_3$
Without GluQRS$_3$
p < 0.00005
Nº of genomes with GluQRS$_4$
Nº of genomes without GluQRS$_4$
With GluQRS$_4$
Without GluQRS$_4$
p < 0.00005
Nº of genomes with GluQRS$_5$
Nº of genomes without GluQRS$_5$
With GluQRS$_5$
Without GluQRS$_5$
p < 0.00005
Nº of genomes with TGT$_1$
Nº of genomes without TGT$_1$
With TGT$_1$
Without TGT$_1$
p < 0.00005
Nº of genomes with TGT$_2$
Nº of genomes without TGT$_2$
With TGT$_2$
Without TGT$_2$
p < 0.00005
Nº of genomes with TGT$_3$
Nº of genomes without TGT$_3$
With TGT$_3$
Without TGT$_3$
p < 0.00005

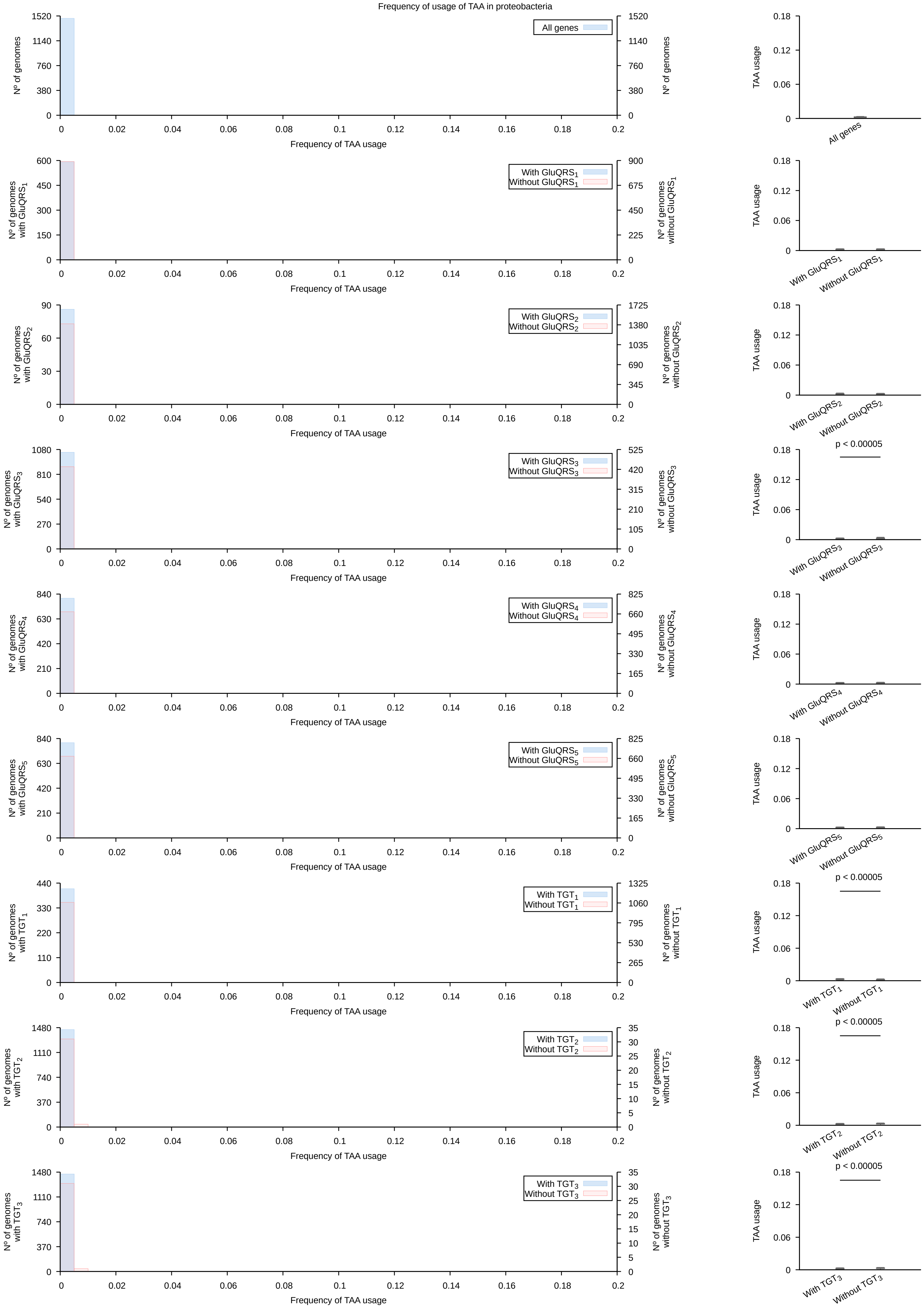

Frequency of usage of TAA in proteobacteria
All genes
With GluQRS$_1$
Without GluQRS$_1$
With GluQRS$_2$
Without GluQRS$_2$
With GluQRS$_3$
Without GluQRS$_3$
With GluQRS$_4$
Without GluQRS$_4$
With GluQRS$_5$
Without GluQRS$_5$
With TGT$_1$
Without TGT$_1$
With TGT$_2$
Without TGT$_2$
With TGT$_3$
Without TGT$_3$
Nº of genomes
Frequency of TAA usage
TAA usage
p < 0.00005

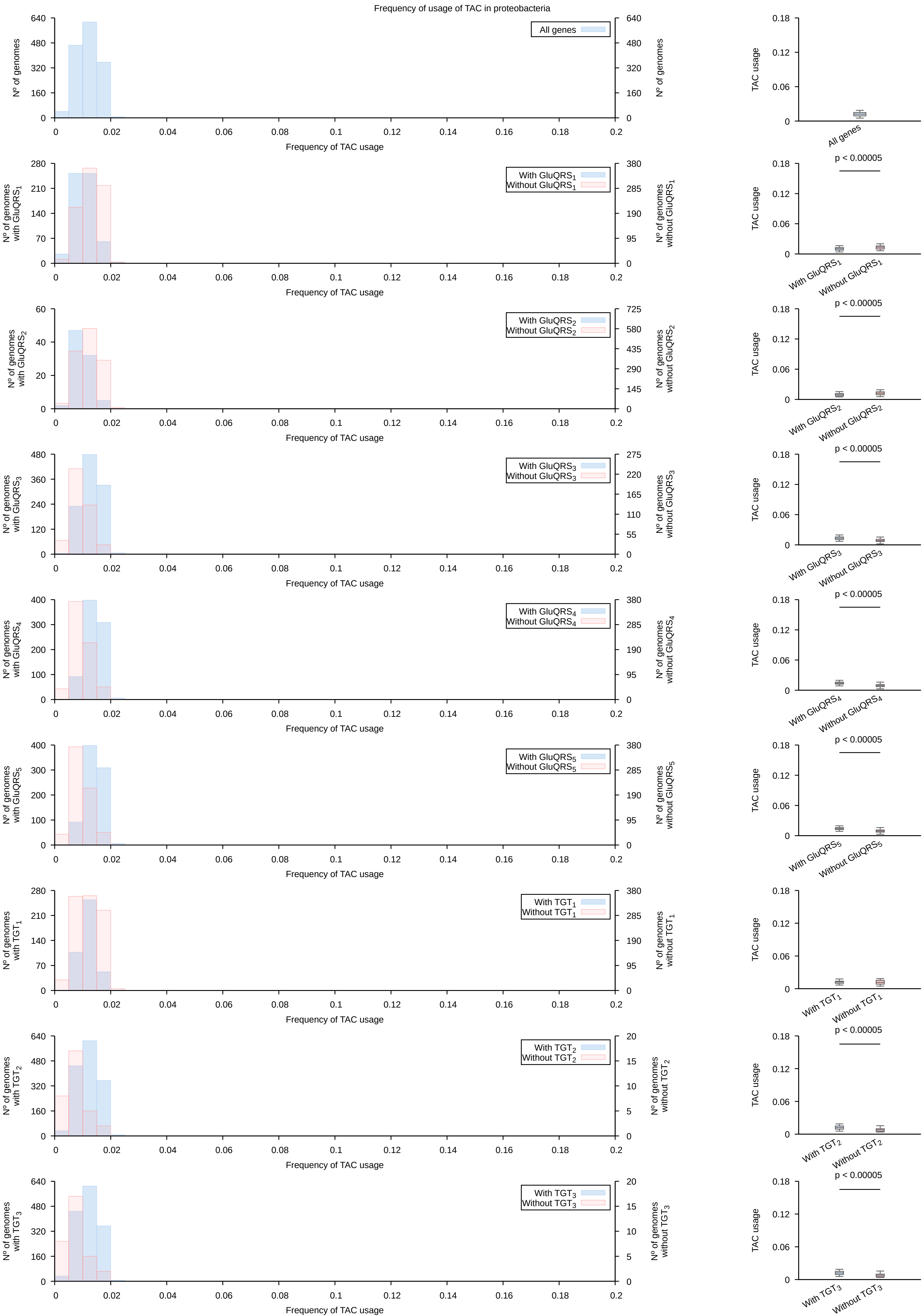
Frequency of usage of TAC in proteobacteria
All genes
Nº of genomes
Frequency of TAC usage
TAC usage
With GluQRS1
Without GluQRS1
Nº of genomes with GluQRS1
Nº of genomes without GluQRS1
p < 0.00005
With GluQRS2
Without GluQRS2
Nº of genomes with GluQRS2
Nº of genomes without GluQRS2
With GluQRS3
Without GluQRS3
Nº of genomes with GluQRS3
Nº of genomes without GluQRS3
With GluQRS4
Without GluQRS4
Nº of genomes with GluQRS4
Nº of genomes without GluQRS4
With GluQRS5
Without GluQRS5
Nº of genomes with GluQRS5
Nº of genomes without GluQRS5
With TGT1
Without TGT1
Nº of genomes with TGT1
Nº of genomes without TGT1
With TGT2
Without TGT2
Nº of genomes with TGT2
Nº of genomes without TGT2
With TGT3
Without TGT3
Nº of genomes with TGT3
Nº of genomes without TGT3
0
0.02
0.04
0.06
0.08
0.1
0.12
0.14
0.16
0.18
0.2

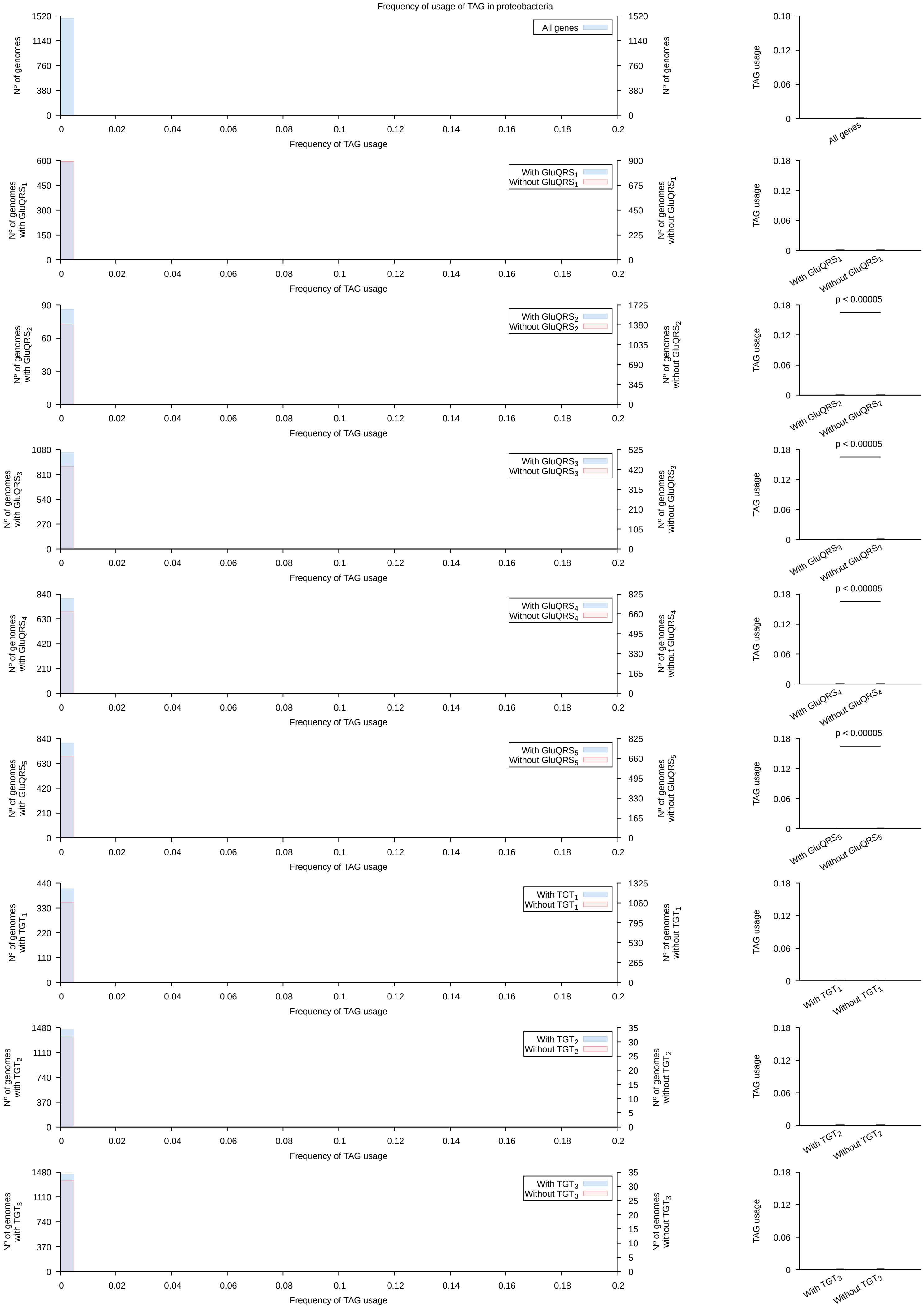

Frequency of usage of TAG in proteobacteria
Nº of genomes
Frequency of TAG usage
All genes
TAG usage
Nº of genomes with GluQRS$_1$
Nº of genomes without GluQRS$_1$
With GluQRS$_1$
Without GluQRS$_1$
Nº of genomes with GluQRS$_2$
Nº of genomes without GluQRS$_2$
With GluQRS$_2$
Without GluQRS$_2$
p < 0.00005
Nº of genomes with GluQRS$_3$
Nº of genomes without GluQRS$_3$
With GluQRS$_3$
Without GluQRS$_3$
p < 0.00005
Nº of genomes with GluQRS$_4$
Nº of genomes without GluQRS$_4$
With GluQRS$_4$
Without GluQRS$_4$
p < 0.00005
Nº of genomes with GluQRS$_5$
Nº of genomes without GluQRS$_5$
With GluQRS$_5$
Without GluQRS$_5$
p < 0.00005
Nº of genomes with TGT$_1$
Nº of genomes without TGT$_1$
With TGT$_1$
Without TGT$_1$
Nº of genomes with TGT$_2$
Nº of genomes without TGT$_2$
With TGT$_2$
Without TGT$_2$
Nº of genomes with TGT$_3$
Nº of genomes without TGT$_3$
With TGT$_3$
Without TGT$_3$

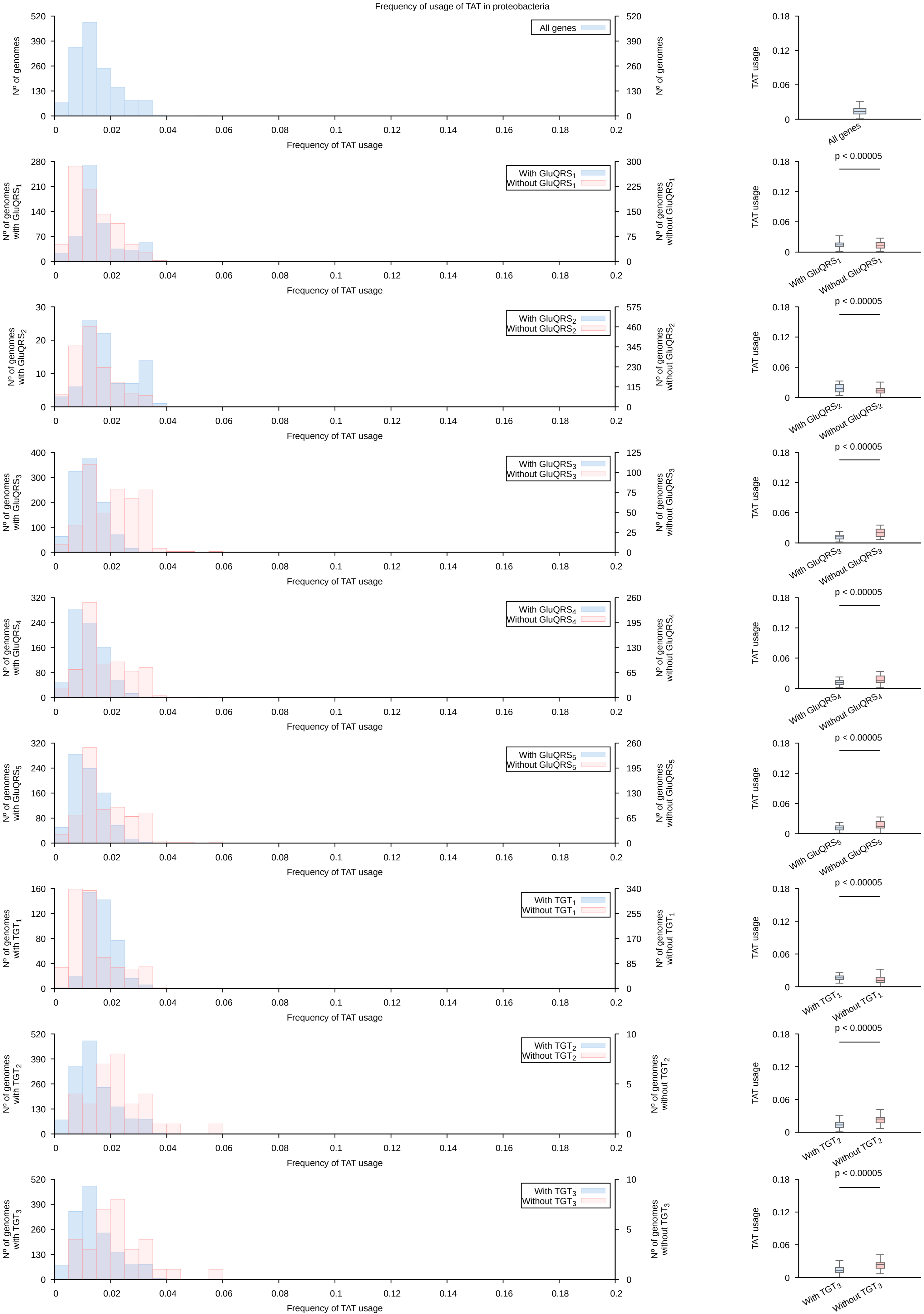
Frequency of usage of TAT in proteobacteria
All genes
With GluQRS$_1$
Without GluQRS$_1$
With GluQRS$_2$
Without GluQRS$_2$
With GluQRS$_3$
Without GluQRS$_3$
With GluQRS$_4$
Without GluQRS$_4$
With GluQRS$_5$
Without GluQRS$_5$
With TGT$_1$
Without TGT$_1$
With TGT$_2$
Without TGT$_2$
With TGT$_3$
Without TGT$_3$
Nº of genomes
Frequency of TAT usage
TAT usage
p < 0.00005

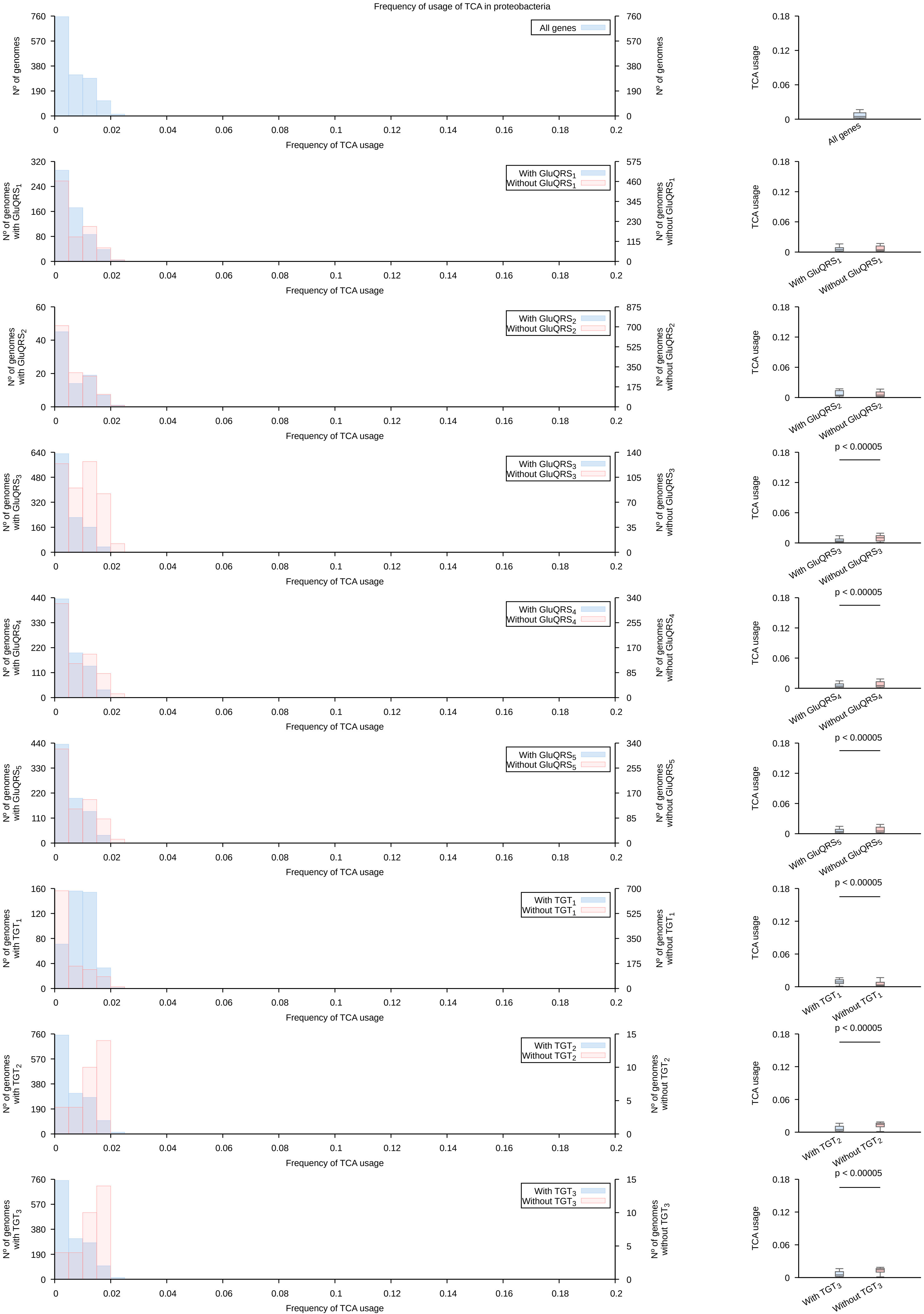

Frequency of usage of TCA in proteobacteria
All genes
With GluQRS$_1$
Without GluQRS$_1$
With GluQRS$_2$
Without GluQRS$_2$
With GluQRS$_3$
Without GluQRS$_3$
With GluQRS$_4$
Without GluQRS$_4$
With GluQRS$_5$
Without GluQRS$_5$
With TGT$_1$
Without TGT$_1$
With TGT$_2$
Without TGT$_2$
With TGT$_3$
Without TGT$_3$
Nº of genomes
Nº of genomes with GluQRS$_1$
Nº of genomes without GluQRS$_1$
Nº of genomes with GluQRS$_2$
Nº of genomes without GluQRS$_2$
Nº of genomes with GluQRS$_3$
Nº of genomes without GluQRS$_3$
Nº of genomes with GluQRS$_4$
Nº of genomes without GluQRS$_4$
Nº of genomes with GluQRS$_5$
Nº of genomes without GluQRS$_5$
Nº of genomes with TGT$_1$
Nº of genomes without TGT$_1$
Nº of genomes with TGT$_2$
Nº of genomes without TGT$_2$
Nº of genomes with TGT$_3$
Nº of genomes without TGT$_3$
Frequency of TCA usage
0
0.02
0.04
0.06
0.08
0.1
0.12
0.14
0.16
0.18
0.2
TCA usage
0.18
0.12
0.06
0
p < 0.00005

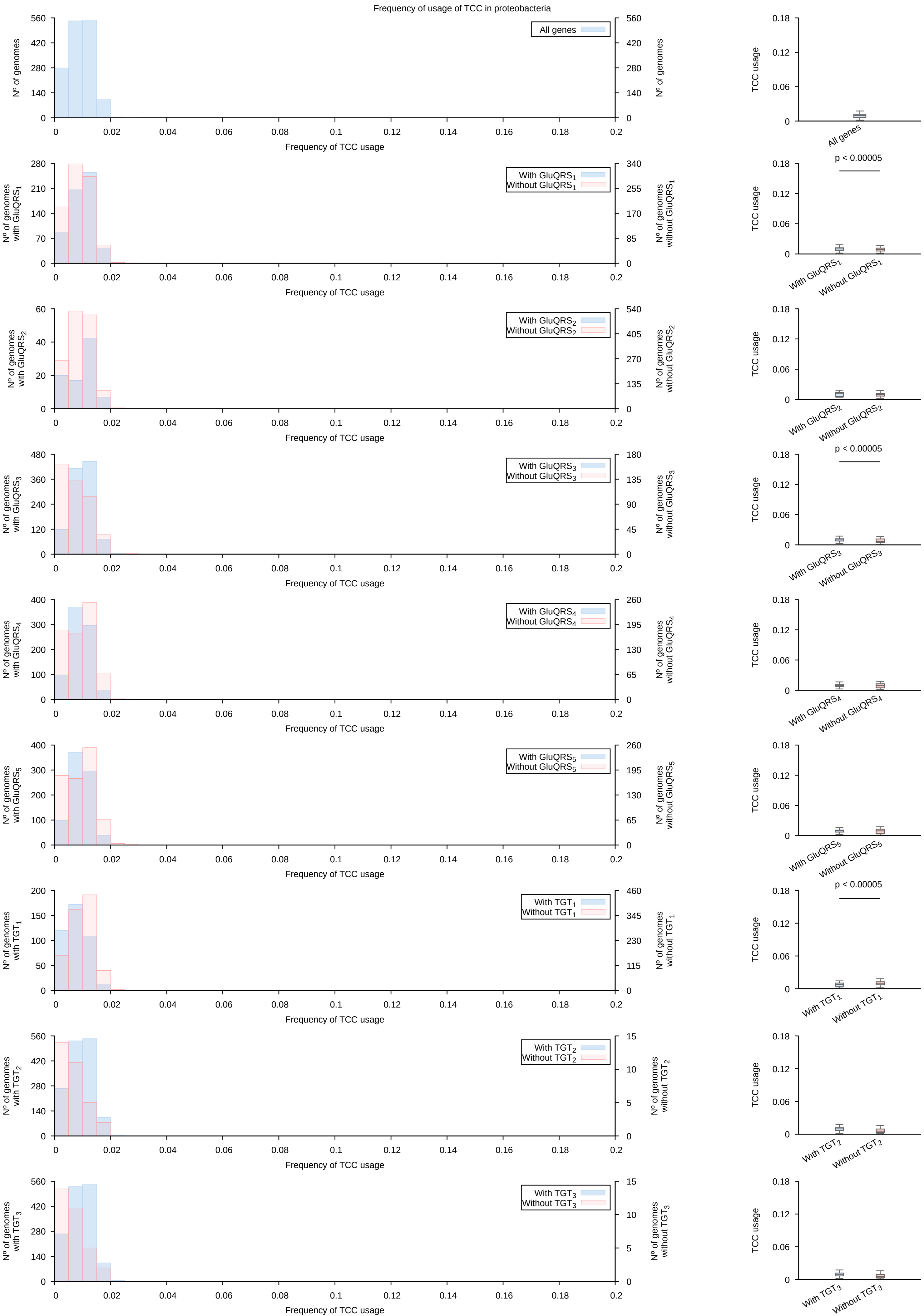
Frequency of usage of TCC in proteobacteria
Nº of genomes
All genes
Frequency of TCC usage
TCC usage
Nº of genomes with GluQRS$_1$
Nº of genomes without GluQRS$_1$
With GluQRS$_1$
Without GluQRS$_1$
p < 0.00005
Nº of genomes with GluQRS$_2$
Nº of genomes without GluQRS$_2$
With GluQRS$_2$
Without GluQRS$_2$
Nº of genomes with GluQRS$_3$
Nº of genomes without GluQRS$_3$
With GluQRS$_3$
Without GluQRS$_3$
p < 0.00005
Nº of genomes with GluQRS$_4$
Nº of genomes without GluQRS$_4$
With GluQRS$_4$
Without GluQRS$_4$
Nº of genomes with GluQRS$_5$
Nº of genomes without GluQRS$_5$
With GluQRS$_5$
Without GluQRS$_5$
Nº of genomes with TGT$_1$
Nº of genomes without TGT$_1$
With TGT$_1$
Without TGT$_1$
p < 0.00005
Nº of genomes with TGT$_2$
Nº of genomes without TGT$_2$
With TGT$_2$
Without TGT$_2$
Nº of genomes with TGT$_3$
Nº of genomes without TGT$_3$
With TGT$_3$
Without TGT$_3$

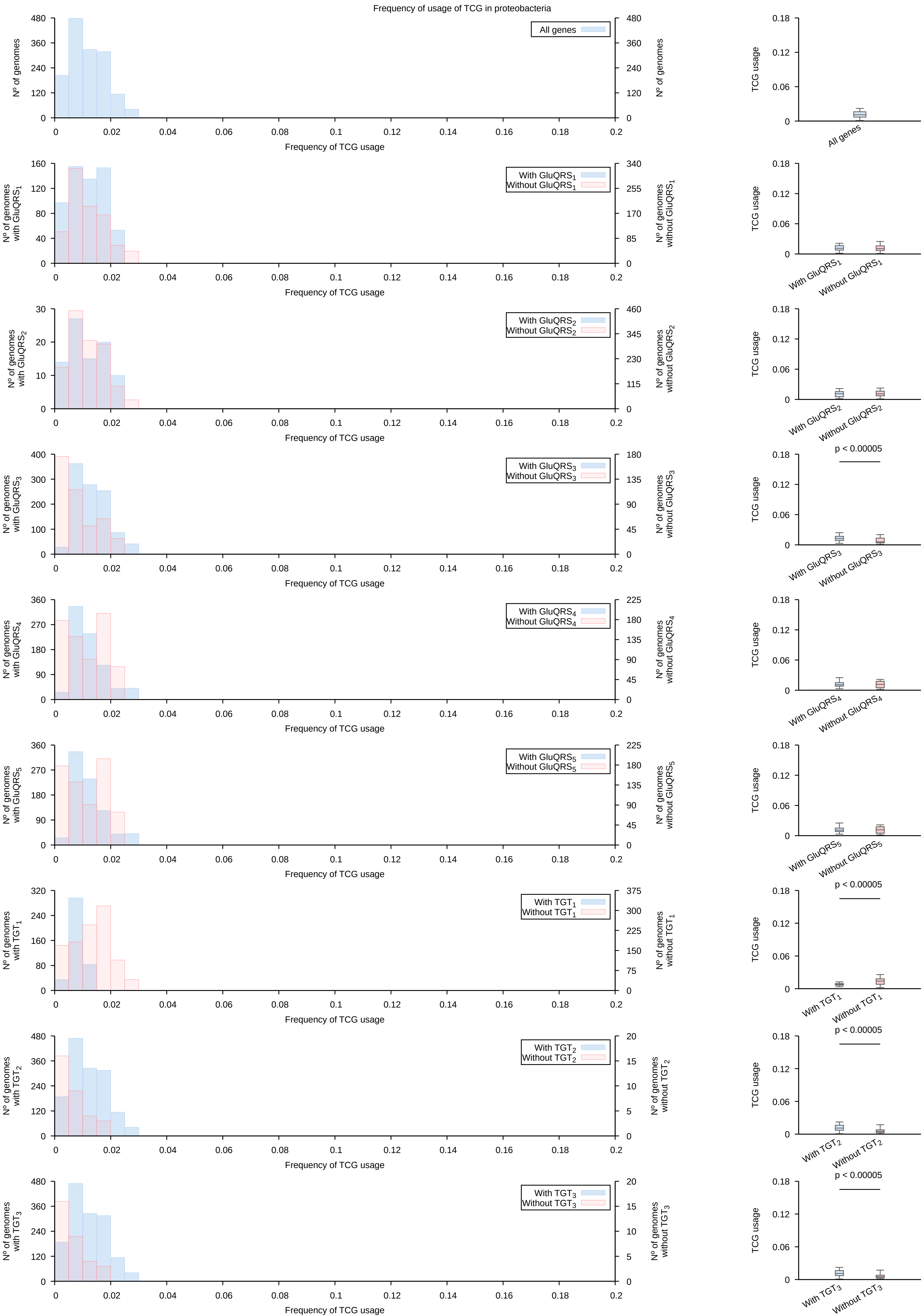
Frequency of usage of TCG in proteobacteria
Nº of genomes
All genes
Frequency of TCG usage
TCG usage
Nº of genomes with GluQRS$_1$
Nº of genomes without GluQRS$_1$
With GluQRS$_1$
Without GluQRS$_1$
Nº of genomes with GluQRS$_2$
Nº of genomes without GluQRS$_2$
With GluQRS$_2$
Without GluQRS$_2$
Nº of genomes with GluQRS$_3$
Nº of genomes without GluQRS$_3$
With GluQRS$_3$
Without GluQRS$_3$
p < 0.00005
Nº of genomes with GluQRS$_4$
Nº of genomes without GluQRS$_4$
With GluQRS$_4$
Without GluQRS$_4$
Nº of genomes with GluQRS$_5$
Nº of genomes without GluQRS$_5$
With GluQRS$_5$
Without GluQRS$_5$
Nº of genomes with TGT$_1$
Nº of genomes without TGT$_1$
With TGT$_1$
Without TGT$_1$
p < 0.00005
Nº of genomes with TGT$_2$
Nº of genomes without TGT$_2$
With TGT$_2$
Without TGT$_2$
p < 0.00005
Nº of genomes with TGT$_3$
Nº of genomes without TGT$_3$
With TGT$_3$
Without TGT$_3$
p < 0.00005

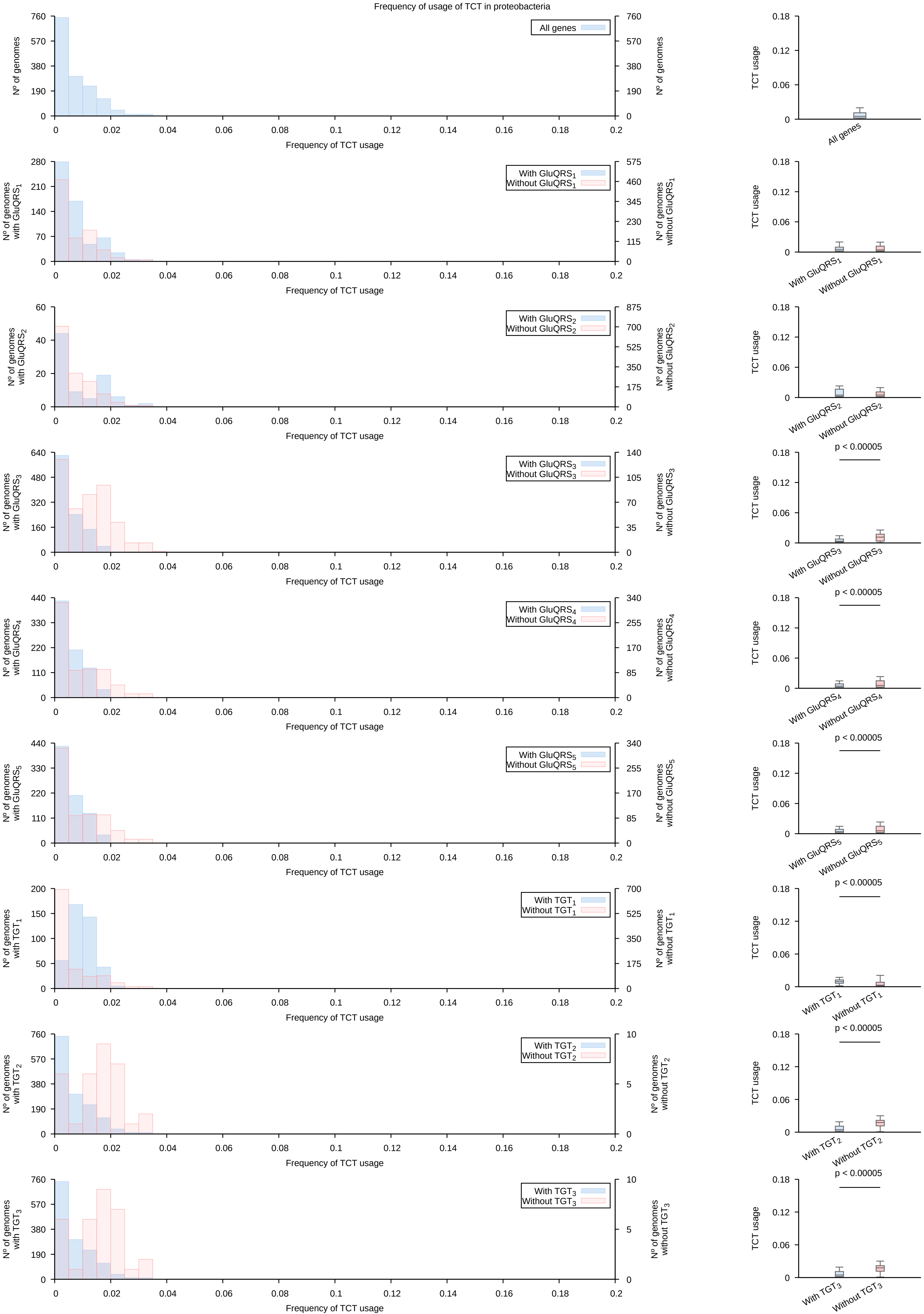

Frequency of usage of TCT in proteobacteria
All genes
With GluQRS1
Without GluQRS1
With GluQRS2
Without GluQRS2
With GluQRS3
Without GluQRS3
With GluQRS4
Without GluQRS4
With GluQRS5
Without GluQRS5
With TGT1
Without TGT1
With TGT2
Without TGT2
With TGT3
Without TGT3
Nº of genomes
Frequency of TCT usage
TCT usage
p < 0.00005

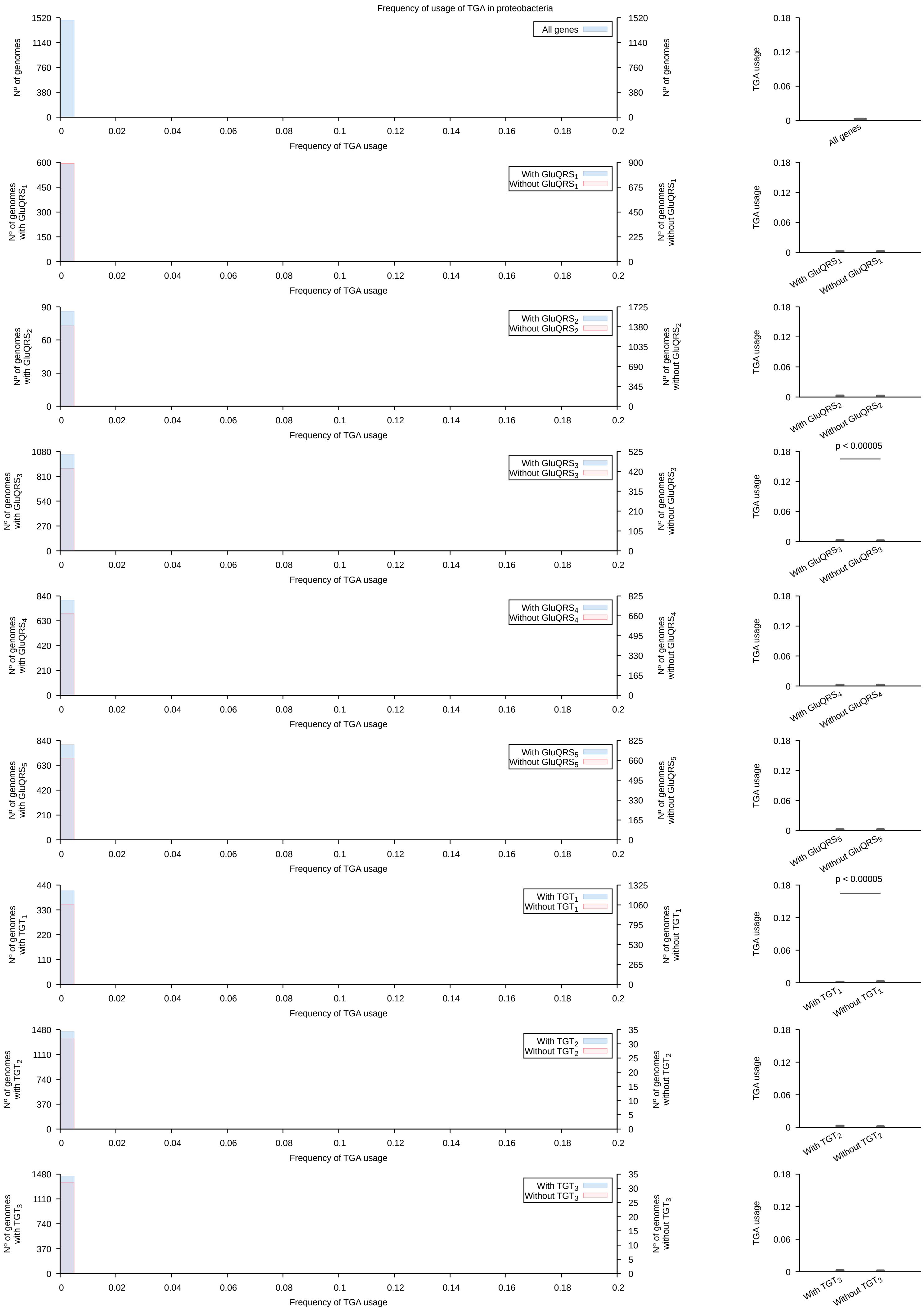
Frequency of usage of TGA in proteobacteria
Nº of genomes
All genes
Frequency of TGA usage
TGA usage
Nº of genomes with GluQRS$_1$
Nº of genomes without GluQRS$_1$
With GluQRS$_1$
Without GluQRS$_1$
Nº of genomes with GluQRS$_2$
Nº of genomes without GluQRS$_2$
With GluQRS$_2$
Without GluQRS$_2$
Nº of genomes with GluQRS$_3$
Nº of genomes without GluQRS$_3$
With GluQRS$_3$
Without GluQRS$_3$
p < 0.00005
Nº of genomes with GluQRS$_4$
Nº of genomes without GluQRS$_4$
With GluQRS$_4$
Without GluQRS$_4$
Nº of genomes with GluQRS$_5$
Nº of genomes without GluQRS$_5$
With GluQRS$_5$
Without GluQRS$_5$
Nº of genomes with TGT$_1$
Nº of genomes without TGT$_1$
With TGT$_1$
Without TGT$_1$
p < 0.00005
Nº of genomes with TGT$_2$
Nº of genomes without TGT$_2$
With TGT$_2$
Without TGT$_2$
Nº of genomes with TGT$_3$
Nº of genomes without TGT$_3$
With TGT$_3$
Without TGT$_3$

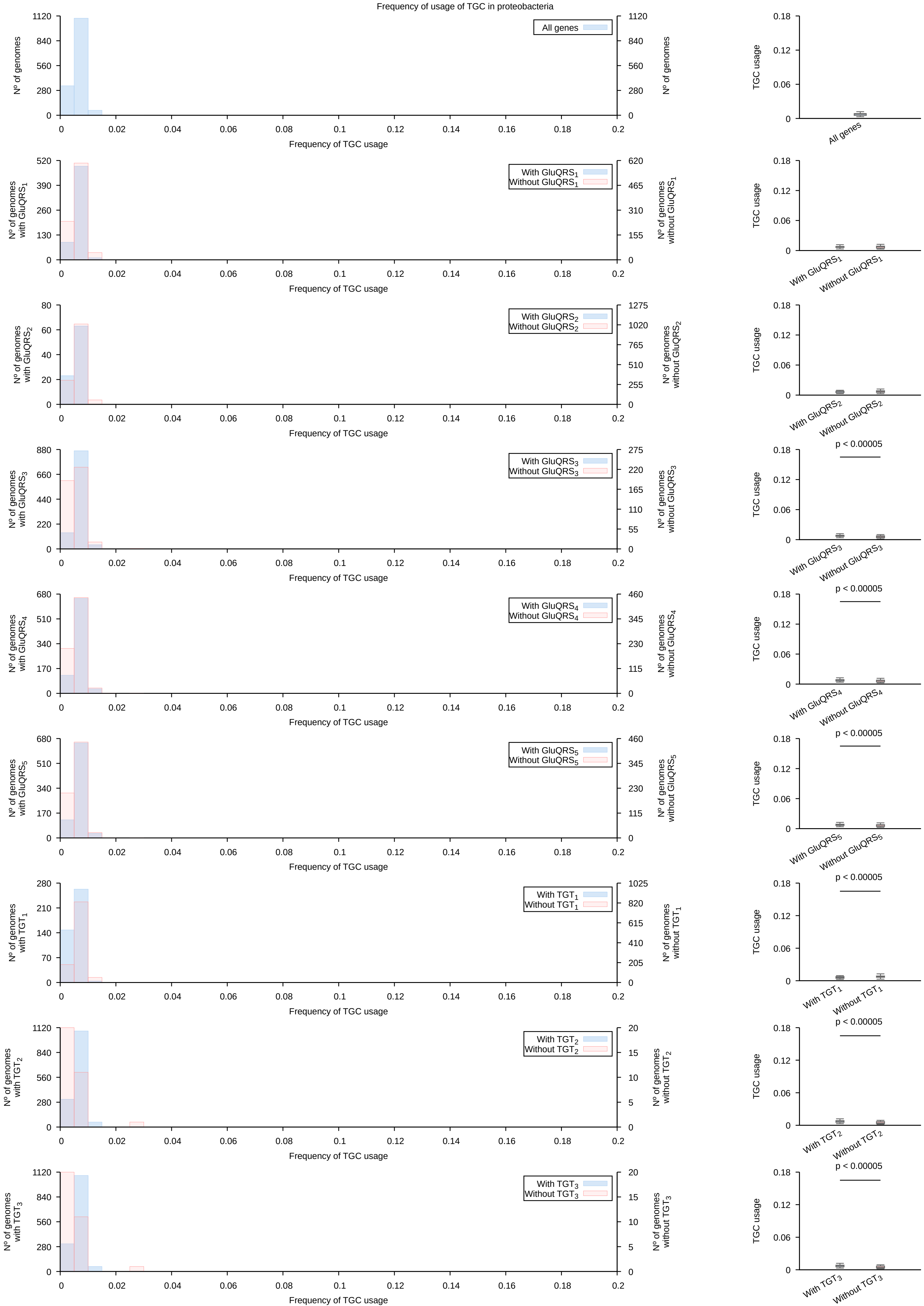
Frequency of usage of TGC in proteobacteria
Nº of genomes
Frequency of TGC usage
TGC usage
All genes
With GluQRS$_1$
Without GluQRS$_1$
Nº of genomes with GluQRS$_1$
Nº of genomes without GluQRS$_1$
With GluQRS$_2$
Without GluQRS$_2$
Nº of genomes with GluQRS$_2$
Nº of genomes without GluQRS$_2$
With GluQRS$_3$
Without GluQRS$_3$
Nº of genomes with GluQRS$_3$
Nº of genomes without GluQRS$_3$
p < 0.00005
With GluQRS$_4$
Without GluQRS$_4$
Nº of genomes with GluQRS$_4$
Nº of genomes without GluQRS$_4$
p < 0.00005
With GluQRS$_5$
Without GluQRS$_5$
Nº of genomes with GluQRS$_5$
Nº of genomes without GluQRS$_5$
p < 0.00005
With TGT$_1$
Without TGT$_1$
Nº of genomes with TGT$_1$
Nº of genomes without TGT$_1$
p < 0.00005
With TGT$_2$
Without TGT$_2$
Nº of genomes with TGT$_2$
Nº of genomes without TGT$_2$
p < 0.00005
With TGT$_3$
Without TGT$_3$
Nº of genomes with TGT$_3$
Nº of genomes without TGT$_3$
p < 0.00005
0
0.02
0.04
0.06
0.08
0.1
0.12
0.14
0.16
0.18
0.2

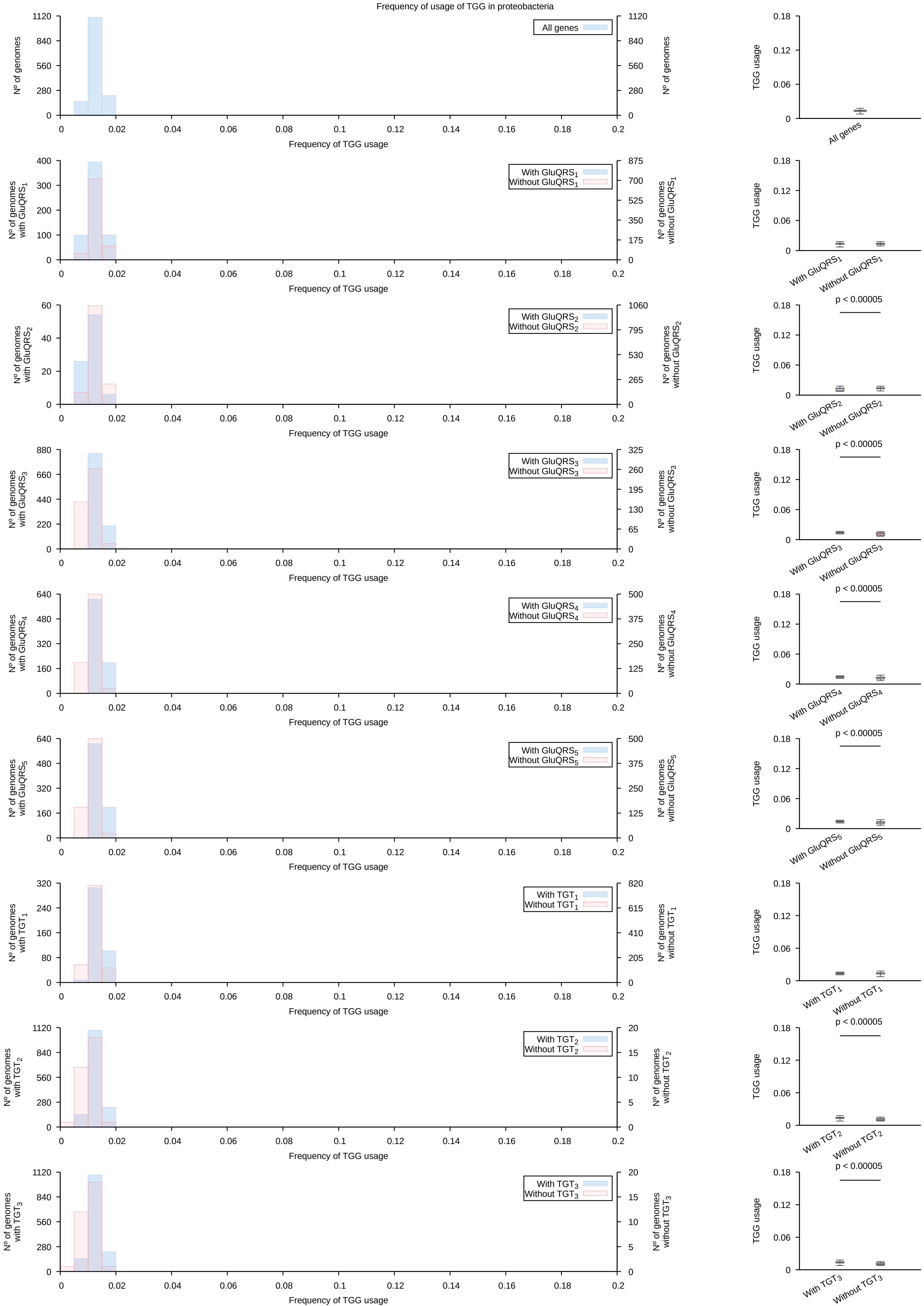
Frequency of usage of TGG in proteobacteria
Nº of genomes
Frequency of TGG usage
TGG usage
All genes
With GluQRS1
Without GluQRS1
Nº of genomes with GluQRS1
Nº of genomes without GluQRS1
With GluQRS2
Without GluQRS2
Nº of genomes with GluQRS2
Nº of genomes without GluQRS2
With GluQRS3
Without GluQRS3
Nº of genomes with GluQRS3
Nº of genomes without GluQRS3
With GluQRS4
Without GluQRS4
Nº of genomes with GluQRS4
Nº of genomes without GluQRS4
With GluQRS5
Without GluQRS5
Nº of genomes with GluQRS5
Nº of genomes without GluQRS5
With TGT1
Without TGT1
Nº of genomes with TGT1
Nº of genomes without TGT1
With TGT2
Without TGT2
Nº of genomes with TGT2
Nº of genomes without TGT2
With TGT3
Without TGT3
Nº of genomes with TGT3
Nº of genomes without TGT3
p < 0.00005

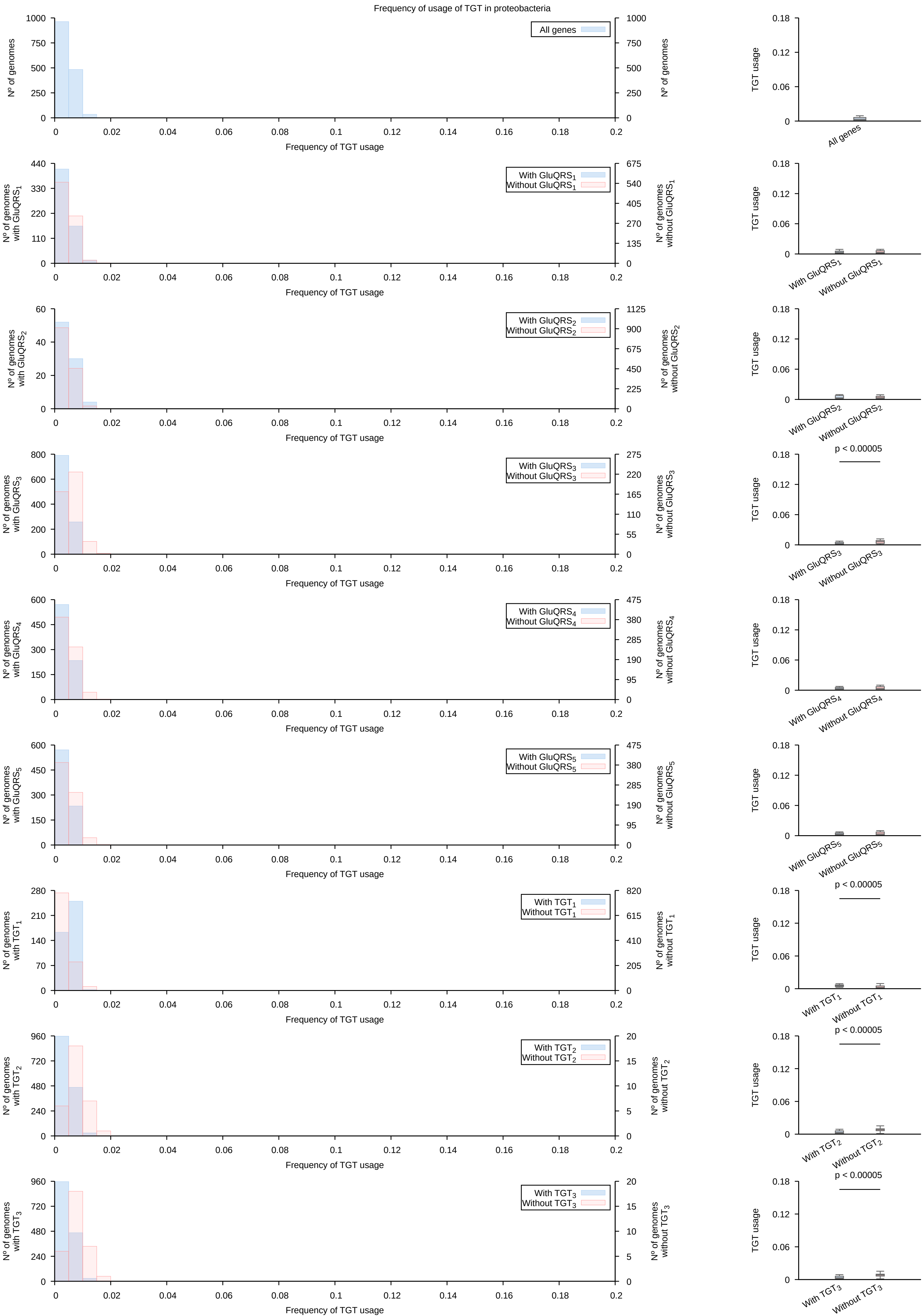
Frequency of usage of TGT in proteobacteria
Nº of genomes
Frequency of TGT usage
TGT usage
All genes
With GluQRS1
Without GluQRS1
Nº of genomes with GluQRS1
Nº of genomes without GluQRS1
With GluQRS2
Without GluQRS2
Nº of genomes with GluQRS2
Nº of genomes without GluQRS2
With GluQRS3
Without GluQRS3
Nº of genomes with GluQRS3
Nº of genomes without GluQRS3
p < 0.00005
With GluQRS4
Without GluQRS4
Nº of genomes with GluQRS4
Nº of genomes without GluQRS4
With GluQRS5
Without GluQRS5
Nº of genomes with GluQRS5
Nº of genomes without GluQRS5
With TGT1
Without TGT1
Nº of genomes with TGT1
Nº of genomes without TGT1
p < 0.00005
With TGT2
Without TGT2
Nº of genomes with TGT2
Nº of genomes without TGT2
p < 0.00005
With TGT3
Without TGT3
Nº of genomes with TGT3
Nº of genomes without TGT3
p < 0.00005

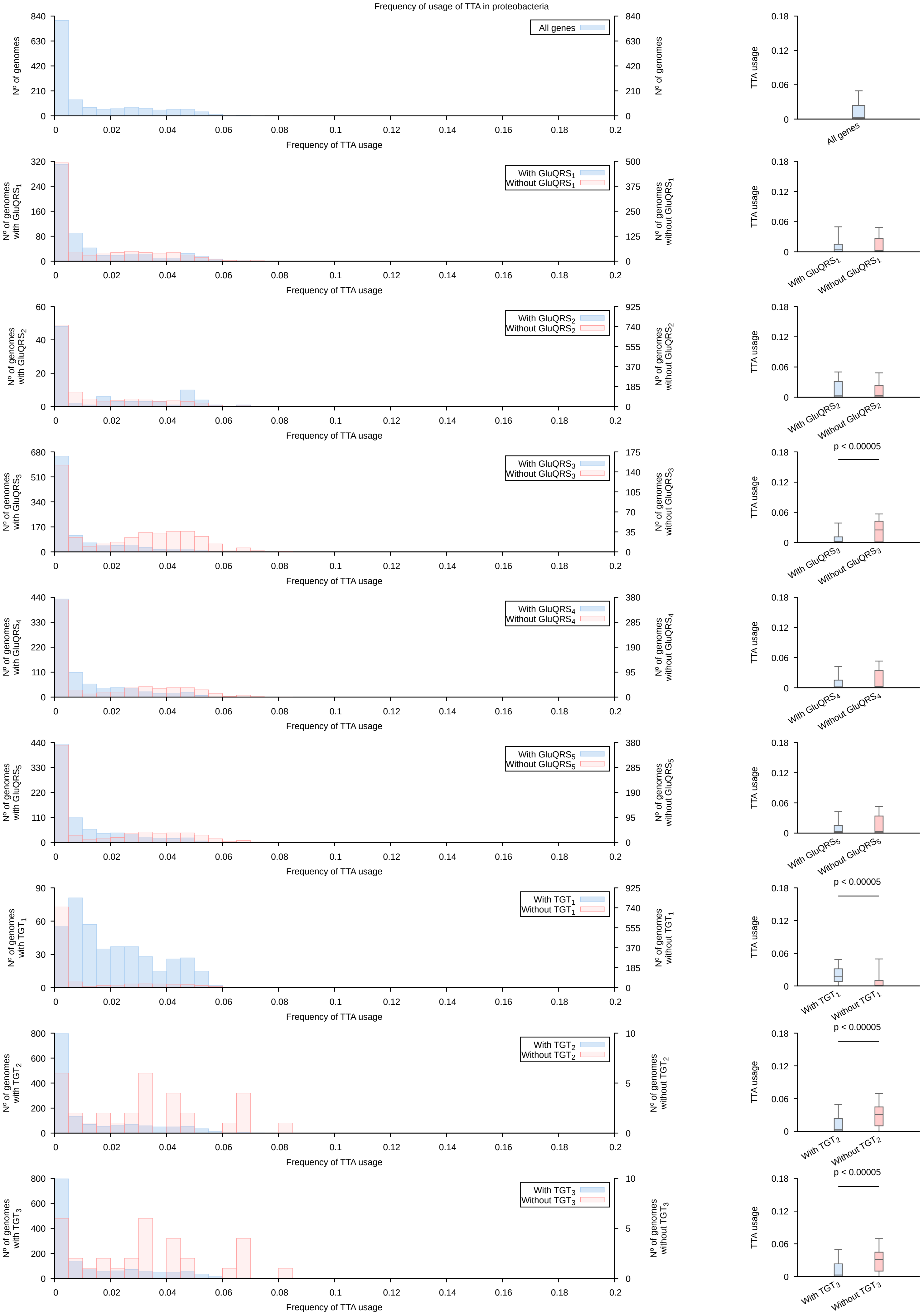

Frequency of usage of TTA in proteobacteria
Nº of genomes
All genes
Frequency of TTA usage
TTA usage
All genes
Nº of genomes with GluQRS1
Nº of genomes without GluQRS1
With GluQRS1
Without GluQRS1
Nº of genomes with GluQRS2
Nº of genomes without GluQRS2
With GluQRS2
Without GluQRS2
Nº of genomes with GluQRS3
Nº of genomes without GluQRS3
With GluQRS3
Without GluQRS3
p < 0.00005
Nº of genomes with GluQRS4
Nº of genomes without GluQRS4
With GluQRS4
Without GluQRS4
Nº of genomes with GluQRS5
Nº of genomes without GluQRS5
With GluQRS5
Without GluQRS5
Nº of genomes with TGT1
Nº of genomes without TGT1
With TGT1
Without TGT1
p < 0.00005
Nº of genomes with TGT2
Nº of genomes without TGT2
With TGT2
Without TGT2
p < 0.00005
Nº of genomes with TGT3
Nº of genomes without TGT3
With TGT3
Without TGT3
p < 0.00005
0
0.02
0.04
0.06
0.08
0.1
0.12
0.14
0.16
0.18
0.2

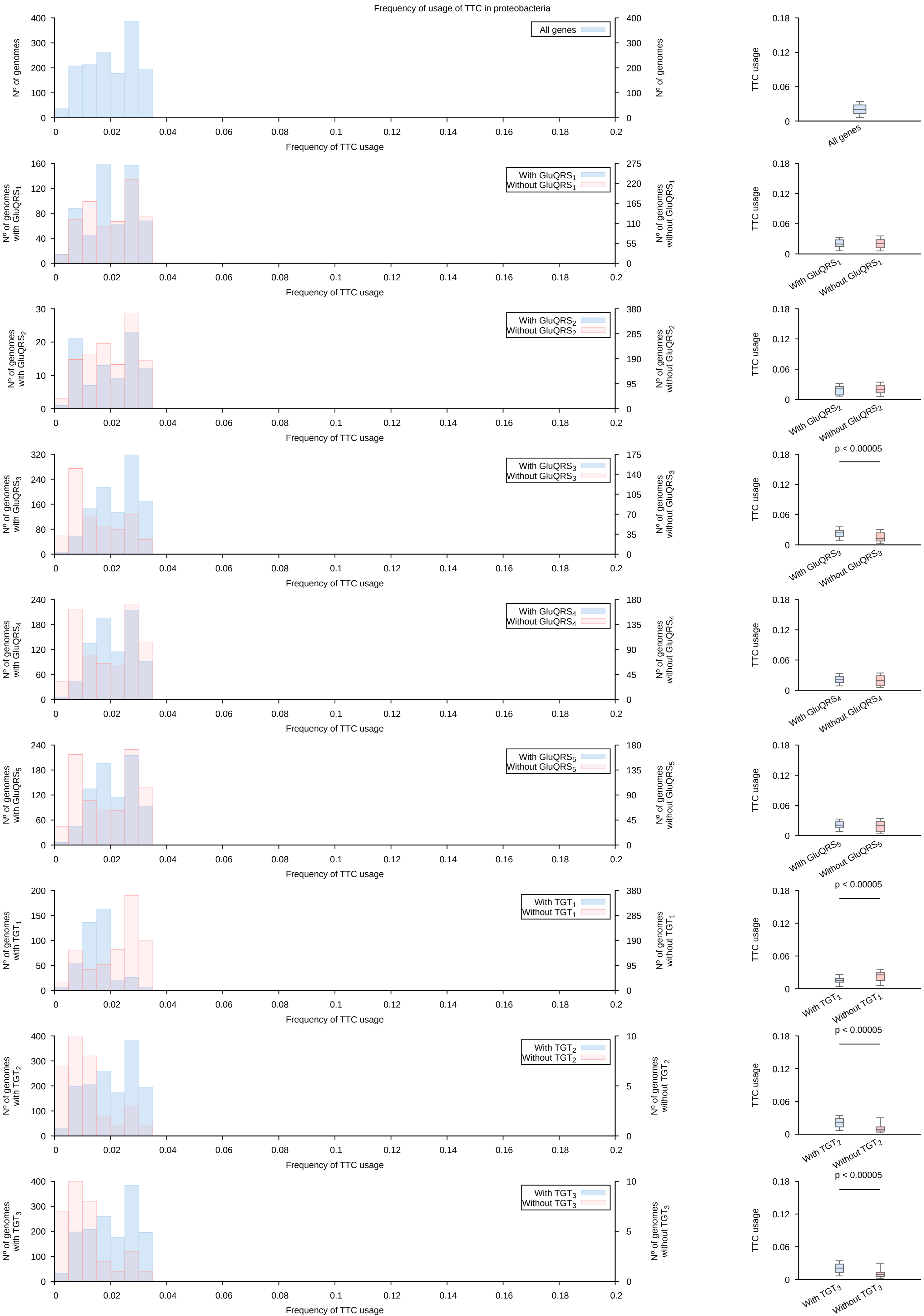
Frequency of usage of TTC in proteobacteria

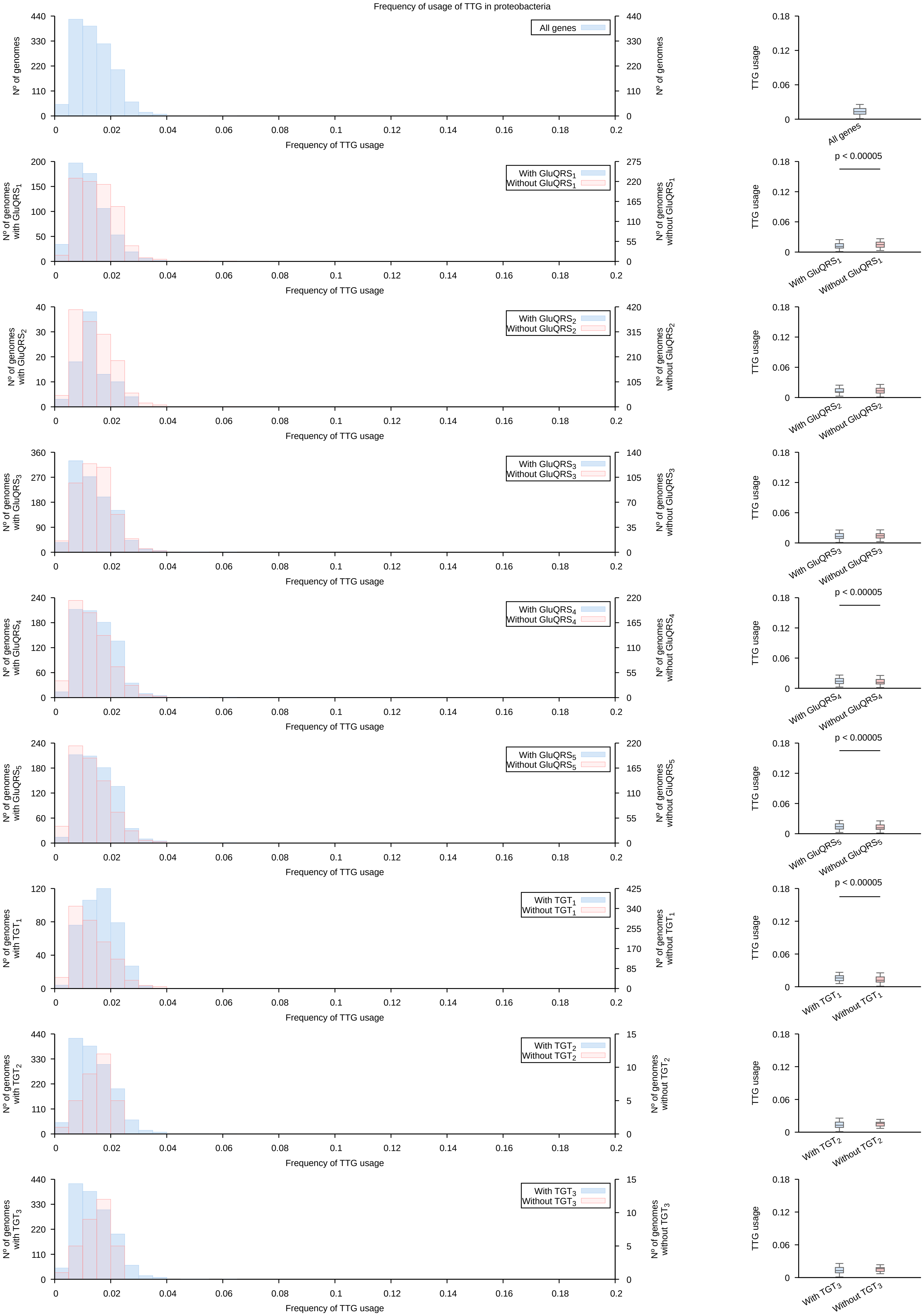
Frequency of usage of TTG in proteobacteria
Nº of genomes
Frequency of TTG usage
All genes
TTG usage
Nº of genomes with GluQRS$_1$
Nº of genomes without GluQRS$_1$
With GluQRS$_1$
Without GluQRS$_1$
p < 0.00005
Nº of genomes with GluQRS$_2$
Nº of genomes without GluQRS$_2$
With GluQRS$_2$
Without GluQRS$_2$
Nº of genomes with GluQRS$_3$
Nº of genomes without GluQRS$_3$
With GluQRS$_3$
Without GluQRS$_3$
Nº of genomes with GluQRS$_4$
Nº of genomes without GluQRS$_4$
With GluQRS$_4$
Without GluQRS$_4$
p < 0.00005
Nº of genomes with GluQRS$_5$
Nº of genomes without GluQRS$_5$
With GluQRS$_5$
Without GluQRS$_5$
p < 0.00005
Nº of genomes with TGT$_1$
Nº of genomes without TGT$_1$
With TGT$_1$
Without TGT$_1$
p < 0.00005
Nº of genomes with TGT$_2$
Nº of genomes without TGT$_2$
With TGT$_2$
Without TGT$_2$
Nº of genomes with TGT$_3$
Nº of genomes without TGT$_3$
With TGT$_3$
Without TGT$_3$

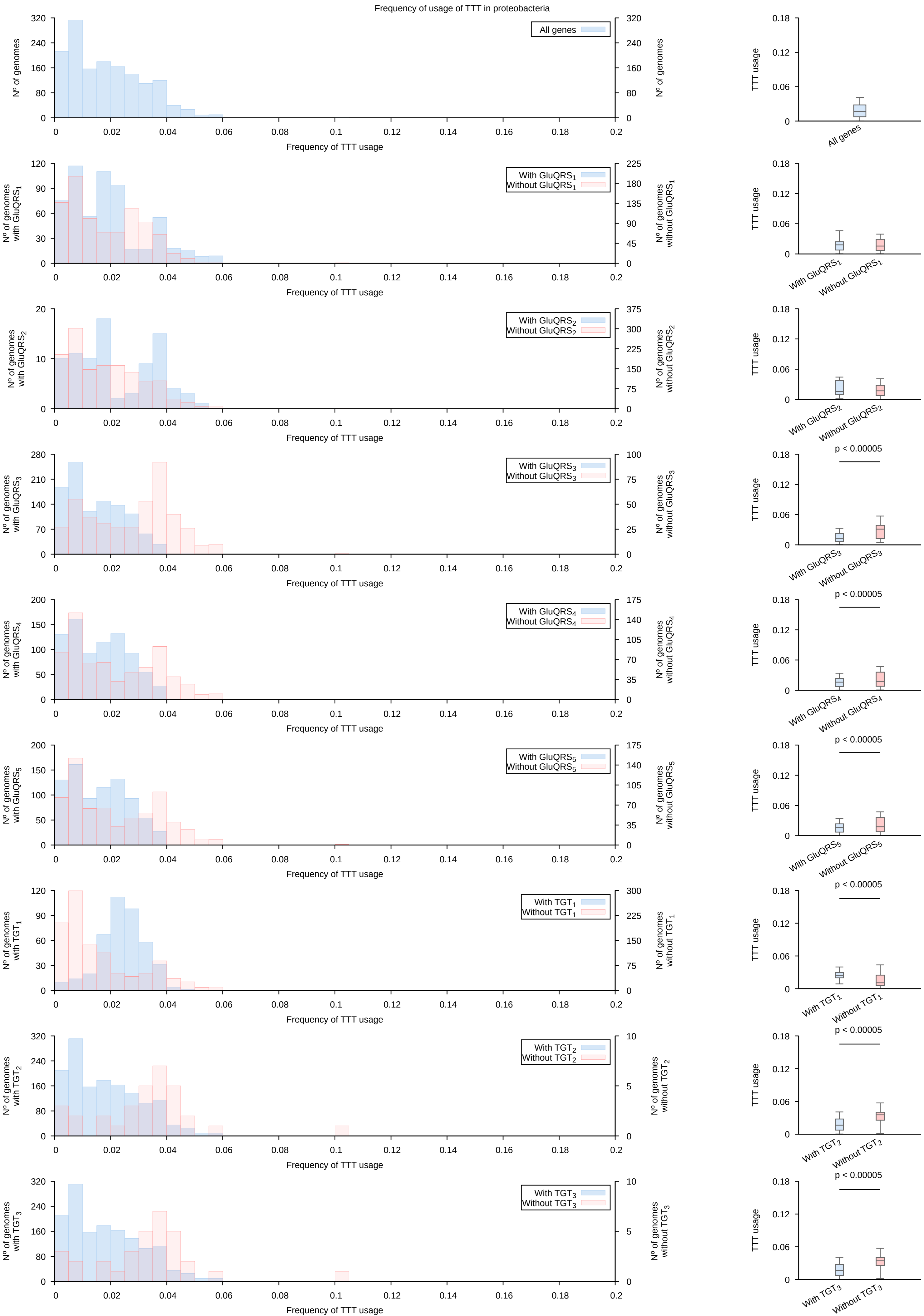
Frequency of usage of TTT in proteobacteria
Nº of genomes
Frequency of TTT usage
TTT usage
All genes
With GluQRS$_1$
Without GluQRS$_1$
Nº of genomes with GluQRS$_1$
Nº of genomes without GluQRS$_1$
With GluQRS$_2$
Without GluQRS$_2$
Nº of genomes with GluQRS$_2$
Nº of genomes without GluQRS$_2$
With GluQRS$_3$
Without GluQRS$_3$
Nº of genomes with GluQRS$_3$
Nº of genomes without GluQRS$_3$
With GluQRS$_4$
Without GluQRS$_4$
Nº of genomes with GluQRS$_4$
Nº of genomes without GluQRS$_4$
With GluQRS$_5$
Without GluQRS$_5$
Nº of genomes with GluQRS$_5$
Nº of genomes without GluQRS$_5$
With TGT$_1$
Without TGT$_1$
Nº of genomes with TGT$_1$
Nº of genomes without TGT$_1$
With TGT$_2$
Without TGT$_2$
Nº of genomes with TGT$_2$
Nº of genomes without TGT$_2$
With TGT$_3$
Without TGT$_3$
Nº of genomes with TGT$_3$
Nº of genomes without TGT$_3$
p < 0.00005

Figure S13. Histogram of codon usage in all genes of genomes that present or lack genes involved in k^2C formation. Top left graph: histogram of the usage of the indicated codon in highly expressed genes of proteobacterial genomes. **Top right graph:** box plot indicating the distribution of codon usage in the same population. **Lower graphs:** The collection of 1484 proteobacterial genomes were separated in two groups, based on the presence (light blue) or absence (orange) of the indicated pattern in any gene of the analyzed genomes. Patterns are indicated using subscript numbers. See table S4 for a detailed description of the used patterns. **Left:** Data is represented in superposed histograms. **Right:** Data is represented in a boxplot. An horizontal bar was added when the two populations are different based on Wilcoxon-Mann-Whitney test and $p < 0.00005$. Whiskers include 95% of data.

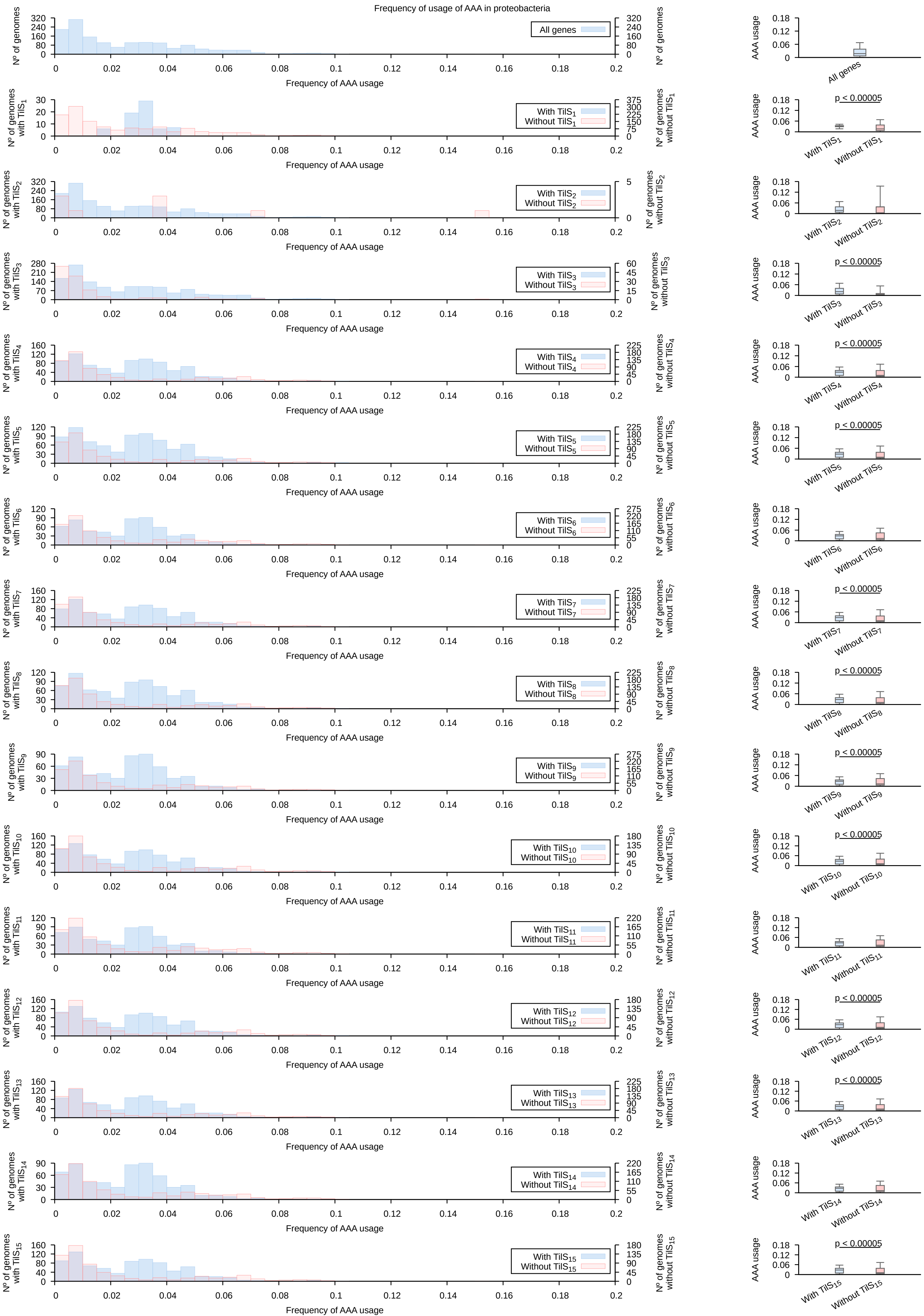
Frequency of usage of AAA in proteobacteria
Nº of genomes
All genes
AAA usage
Frequency of AAA usage
Nº of genomes with TilS1
Nº of genomes without TilS1
With TilS1
Without TilS1
p < 0.00005
Nº of genomes with TilS2
Nº of genomes without TilS2
With TilS2
Without TilS2
Nº of genomes with TilS3
Nº of genomes without TilS3
With TilS3
Without TilS3
p < 0.00005
Nº of genomes with TilS4
Nº of genomes without TilS4
With TilS4
Without TilS4
p < 0.00005
Nº of genomes with TilS5
Nº of genomes without TilS5
With TilS5
Without TilS5
p < 0.00005
Nº of genomes with TilS6
Nº of genomes without TilS6
With TilS6
Without TilS6
Nº of genomes with TilS7
Nº of genomes without TilS7
With TilS7
Without TilS7
p < 0.00005
Nº of genomes with TilS8
Nº of genomes without TilS8
With TilS8
Without TilS8
p < 0.00005
Nº of genomes with TilS9
Nº of genomes without TilS9
With TilS9
Without TilS9
p < 0.00005
Nº of genomes with TilS10
Nº of genomes without TilS10
With TilS10
Without TilS10
p < 0.00005
Nº of genomes with TilS11
Nº of genomes without TilS11
With TilS11
Without TilS11
Nº of genomes with TilS12
Nº of genomes without TilS12
With TilS12
Without TilS12
p < 0.00005
Nº of genomes with TilS13
Nº of genomes without TilS13
With TilS13
Without TilS13
p < 0.00005
Nº of genomes with TilS14
Nº of genomes without TilS14
With TilS14
Without TilS14
Nº of genomes with TilS15
Nº of genomes without TilS15
With TilS15
Without TilS15
p < 0.00005

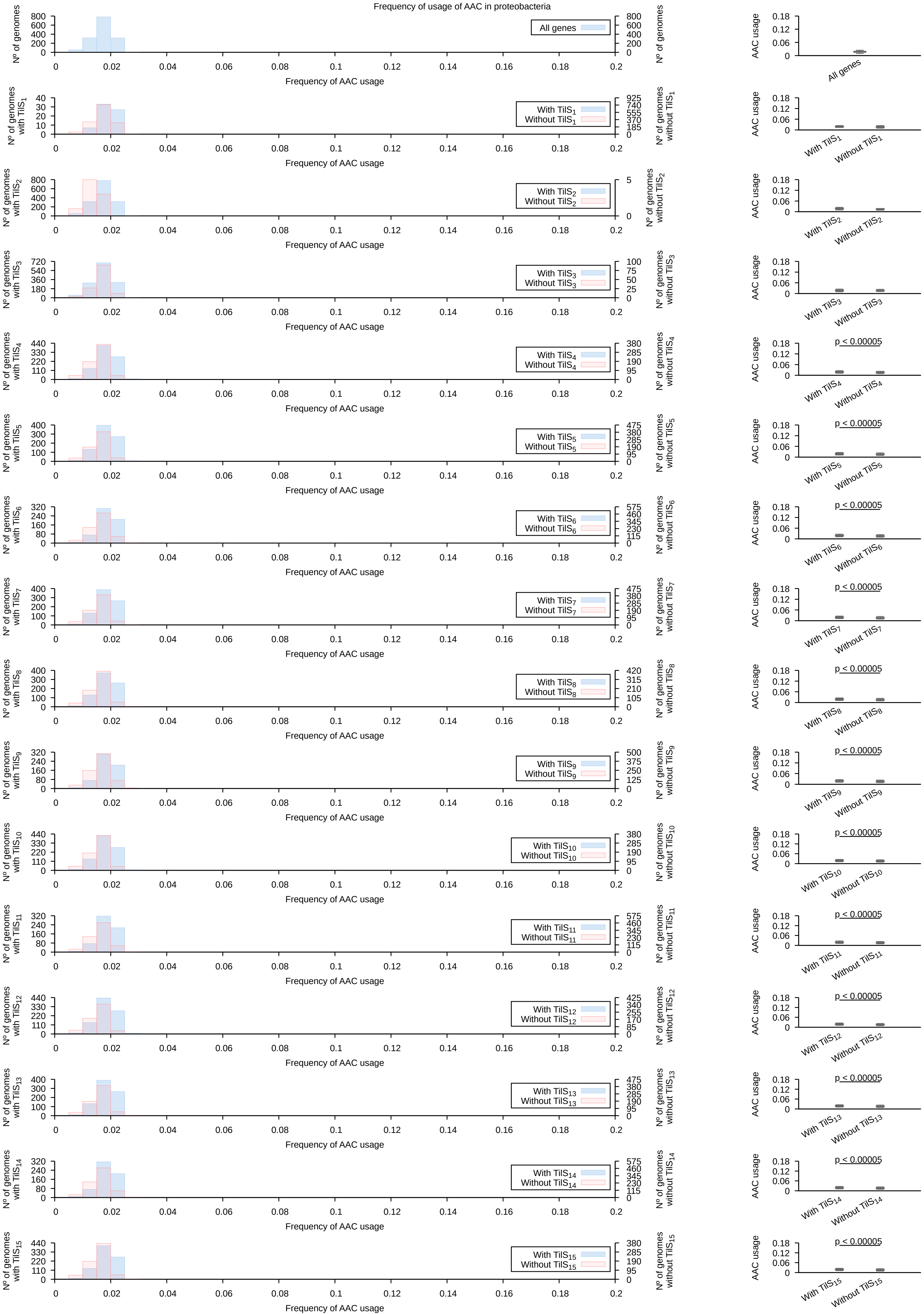

Frequency of usage of AAC in proteobacteria
Nº of genomes
All genes
Frequency of AAC usage
AAC usage
With TilS$_1$
Without TilS$_1$
Nº of genomes with TilS$_1$
Nº of genomes without TilS$_1$
With TilS$_2$
Without TilS$_2$
With TilS$_3$
Without TilS$_3$
With TilS$_4$
Without TilS$_4$
With TilS$_5$
Without TilS$_5$
With TilS$_6$
Without TilS$_6$
With TilS$_7$
Without TilS$_7$
With TilS$_8$
Without TilS$_8$
With TilS$_9$
Without TilS$_9$
With TilS$_{10}$
Without TilS$_{10}$
With TilS$_{11}$
Without TilS$_{11}$
With TilS$_{12}$
Without TilS$_{12}$
With TilS$_{13}$
Without TilS$_{13}$
With TilS$_{14}$
Without TilS$_{14}$
With TilS$_{15}$
Without TilS$_{15}$
p < 0.00005

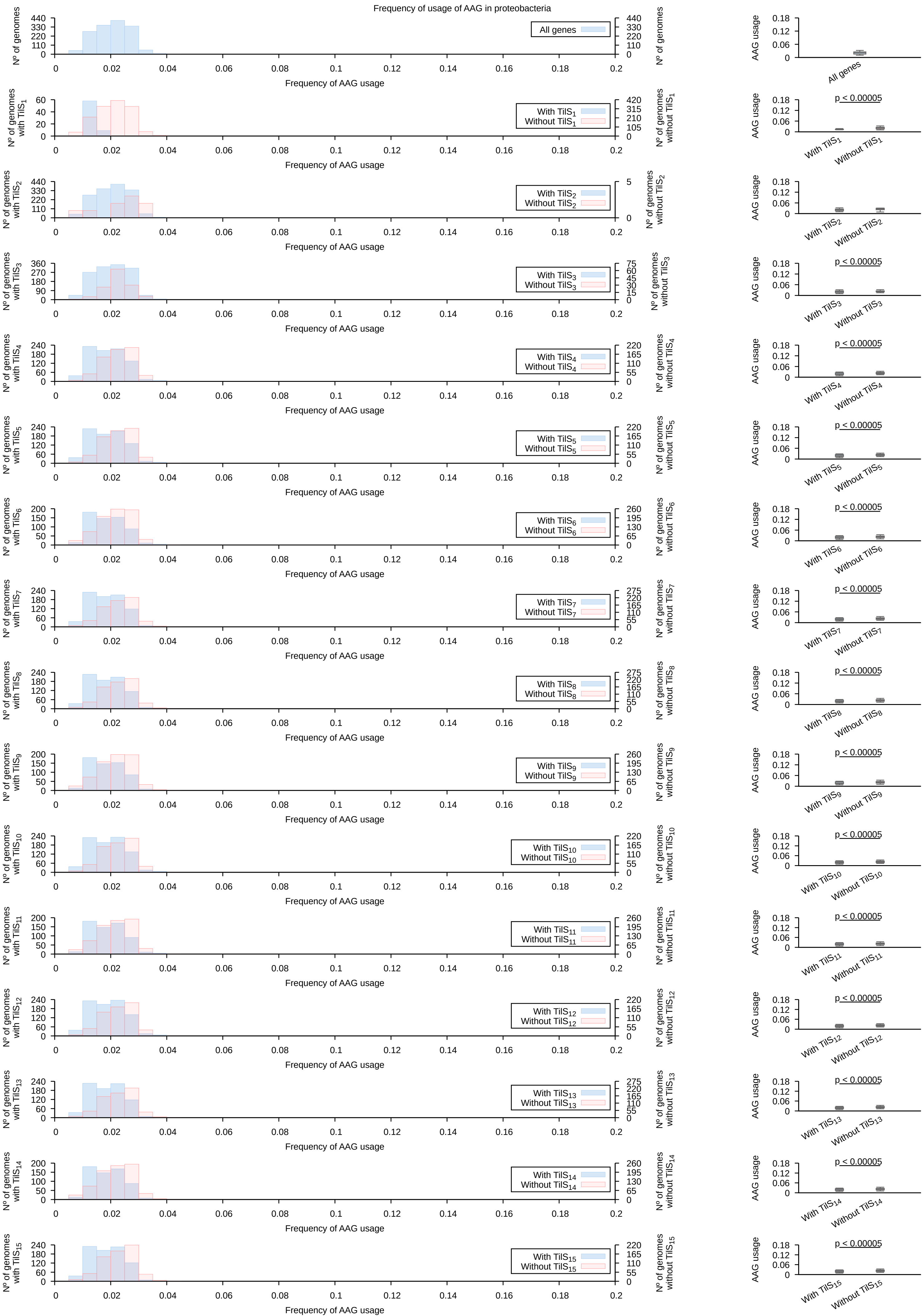
Frequency of usage of AAG in proteobacteria
Nº of genomes
All genes
Frequency of AAG usage
AAG usage
Nº of genomes with TilS$_1$ … Nº of genomes with TilS$_{15}$
Nº of genomes without TilS$_1$ … Nº of genomes without TilS$_{15}$
With TilS$_1$ … With TilS$_{15}$
Without TilS$_1$ … Without TilS$_{15}$
p < 0.00005
0 0.02 0.04 0.06 0.08 0.1 0.12 0.14 0.16 0.18 0.2
0 0.06 0.12 0.18

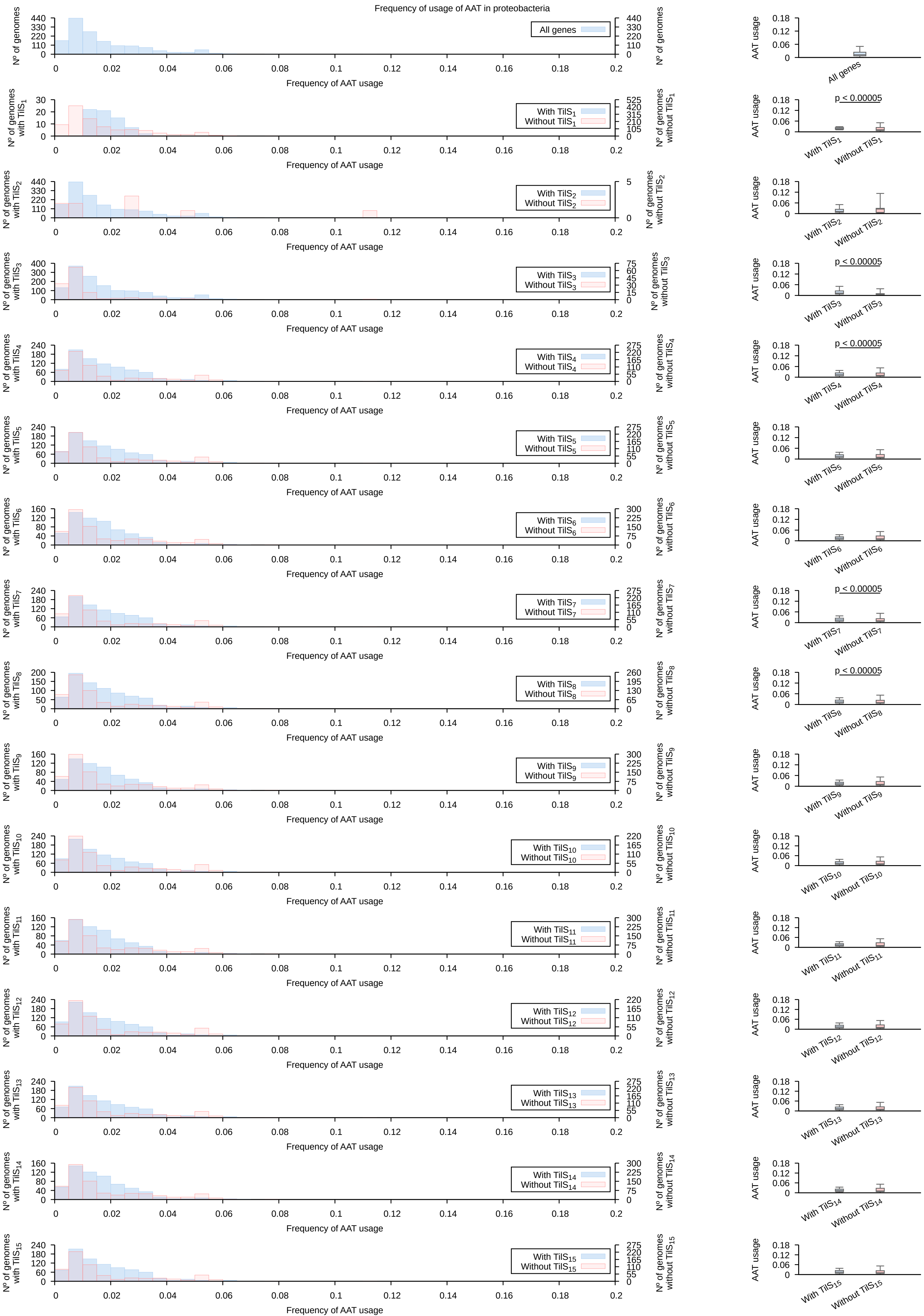

Frequency of usage of AAT in proteobacteria
Nº of genomes
All genes
AAT usage
Frequency of AAT usage
Nº of genomes with TilS$_1$
Nº of genomes without TilS$_1$
With TilS$_1$
Without TilS$_1$
p < 0.00005
Nº of genomes with TilS$_2$
Nº of genomes without TilS$_2$
With TilS$_2$
Without TilS$_2$
Nº of genomes with TilS$_3$
Nº of genomes without TilS$_3$
With TilS$_3$
Without TilS$_3$
p < 0.00005
Nº of genomes with TilS$_4$
Nº of genomes without TilS$_4$
With TilS$_4$
Without TilS$_4$
p < 0.00005
Nº of genomes with TilS$_5$
Nº of genomes without TilS$_5$
With TilS$_5$
Without TilS$_5$
Nº of genomes with TilS$_6$
Nº of genomes without TilS$_6$
With TilS$_6$
Without TilS$_6$
Nº of genomes with TilS$_7$
Nº of genomes without TilS$_7$
With TilS$_7$
Without TilS$_7$
p < 0.00005
Nº of genomes with TilS$_8$
Nº of genomes without TilS$_8$
With TilS$_8$
Without TilS$_8$
p < 0.00005
Nº of genomes with TilS$_9$
Nº of genomes without TilS$_9$
With TilS$_9$
Without TilS$_9$
Nº of genomes with TilS$_{10}$
Nº of genomes without TilS$_{10}$
With TilS$_{10}$
Without TilS$_{10}$
Nº of genomes with TilS$_{11}$
Nº of genomes without TilS$_{11}$
With TilS$_{11}$
Without TilS$_{11}$
Nº of genomes with TilS$_{12}$
Nº of genomes without TilS$_{12}$
With TilS$_{12}$
Without TilS$_{12}$
Nº of genomes with TilS$_{13}$
Nº of genomes without TilS$_{13}$
With TilS$_{13}$
Without TilS$_{13}$
Nº of genomes with TilS$_{14}$
Nº of genomes without TilS$_{14}$
With TilS$_{14}$
Without TilS$_{14}$
Nº of genomes with TilS$_{15}$
Nº of genomes without TilS$_{15}$
With TilS$_{15}$
Without TilS$_{15}$

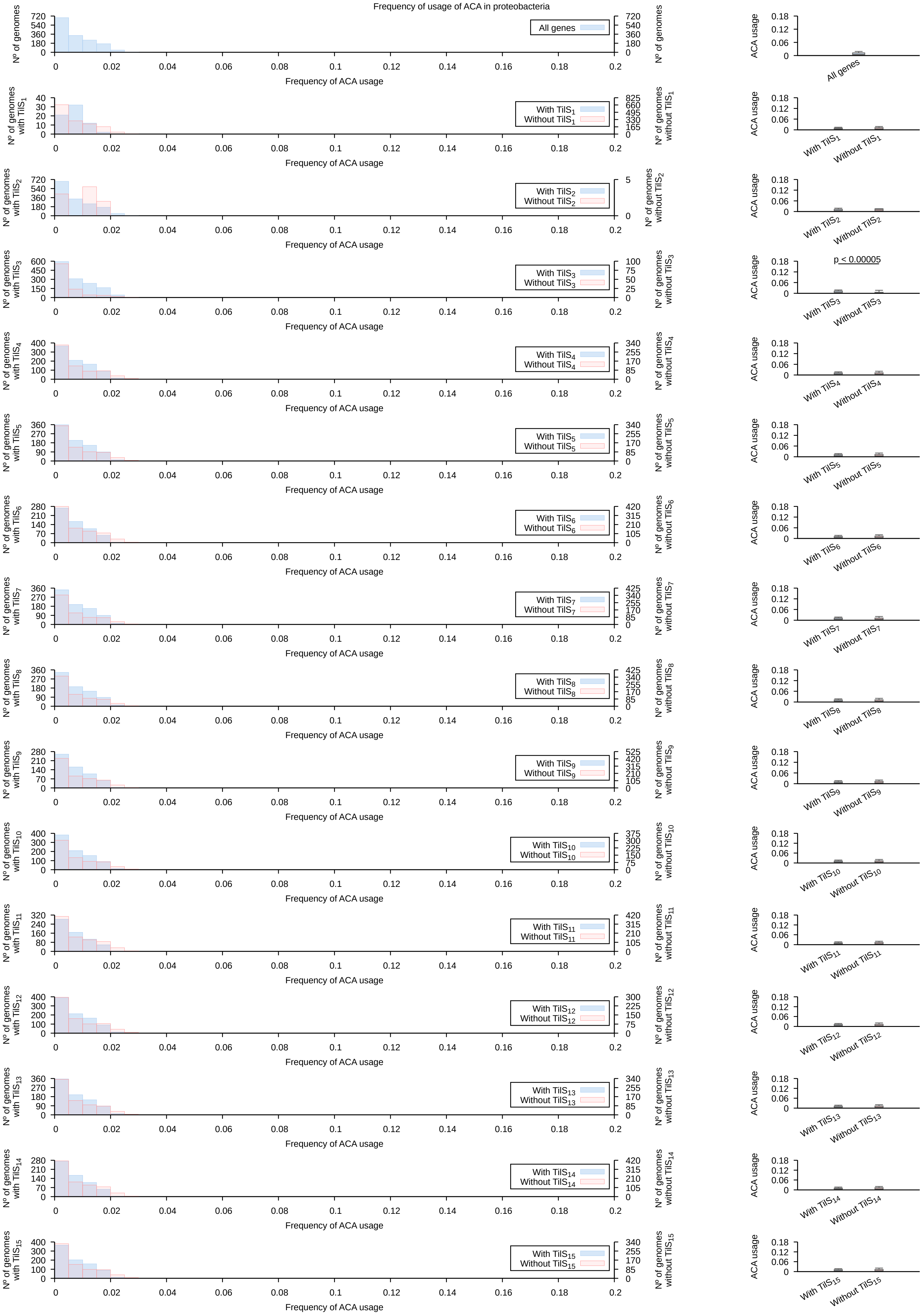
Frequency of usage of ACA in proteobacteria
Nº of genomes
Frequency of ACA usage
ACA usage
All genes
With $TilS_1$
Without $TilS_1$
With $TilS_2$
Without $TilS_2$
With $TilS_3$
Without $TilS_3$
p < 0.00005
With $TilS_4$
Without $TilS_4$
With $TilS_5$
Without $TilS_5$
With $TilS_6$
Without $TilS_6$
With $TilS_7$
Without $TilS_7$
With $TilS_8$
Without $TilS_8$
With $TilS_9$
Without $TilS_9$
With $TilS_{10}$
Without $TilS_{10}$
With $TilS_{11}$
Without $TilS_{11}$
With $TilS_{12}$
Without $TilS_{12}$
With $TilS_{13}$
Without $TilS_{13}$
With $TilS_{14}$
Without $TilS_{14}$
With $TilS_{15}$
Without $TilS_{15}$

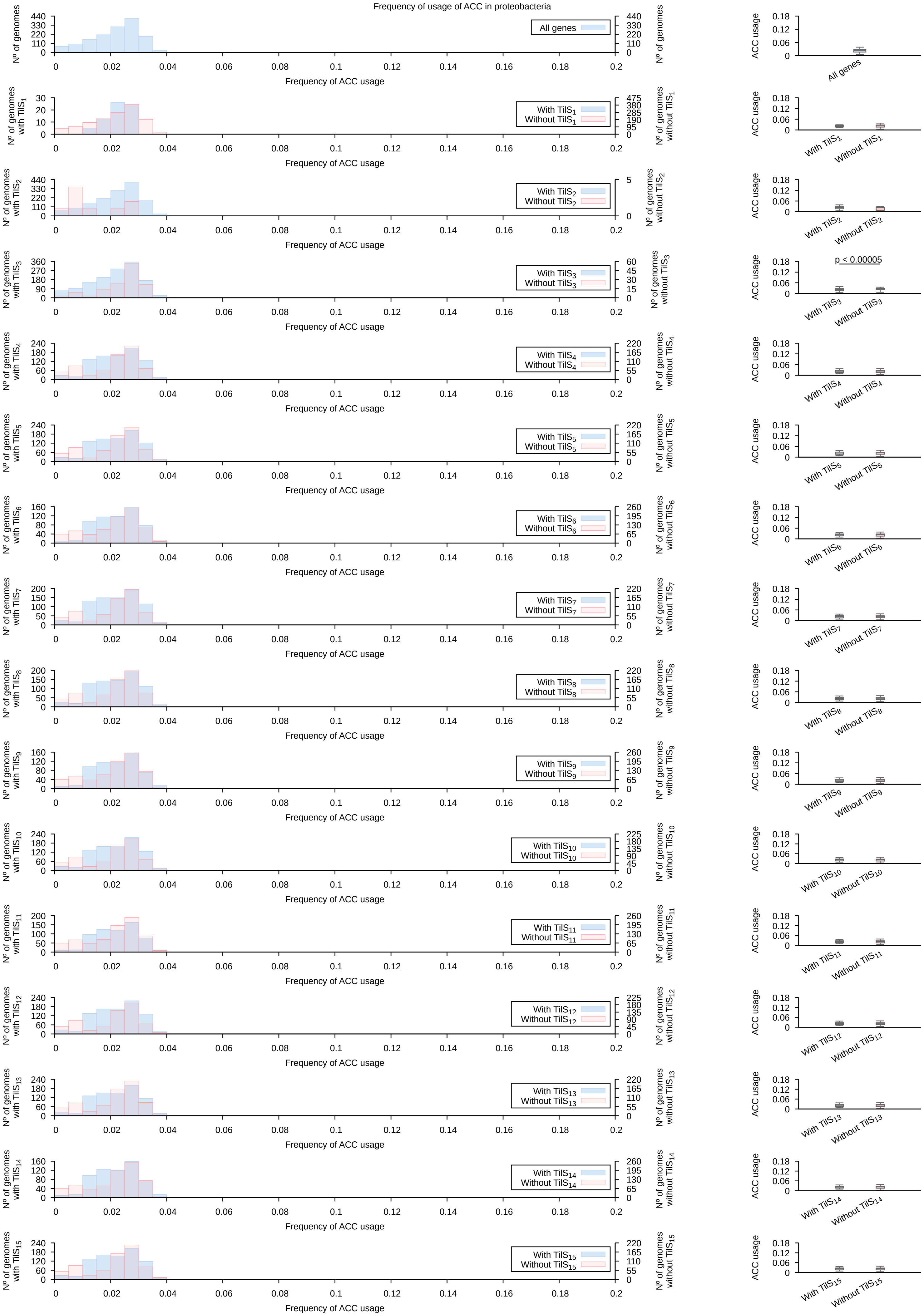

Frequency of usage of ACC in proteobacteria
All genes
Nº of genomes
Frequency of ACC usage
ACC usage
With TilS1
Without TilS1
Nº of genomes with TilS1
Nº of genomes without TilS1
With TilS2
Without TilS2
With TilS3
Without TilS3
p < 0.00005
With TilS4
Without TilS4
With TilS5
Without TilS5
With TilS6
Without TilS6
With TilS7
Without TilS7
With TilS8
Without TilS8
With TilS9
Without TilS9
With TilS10
Without TilS10
With TilS11
Without TilS11
With TilS12
Without TilS12
With TilS13
Without TilS13
With TilS14
Without TilS14
With TilS15
Without TilS15

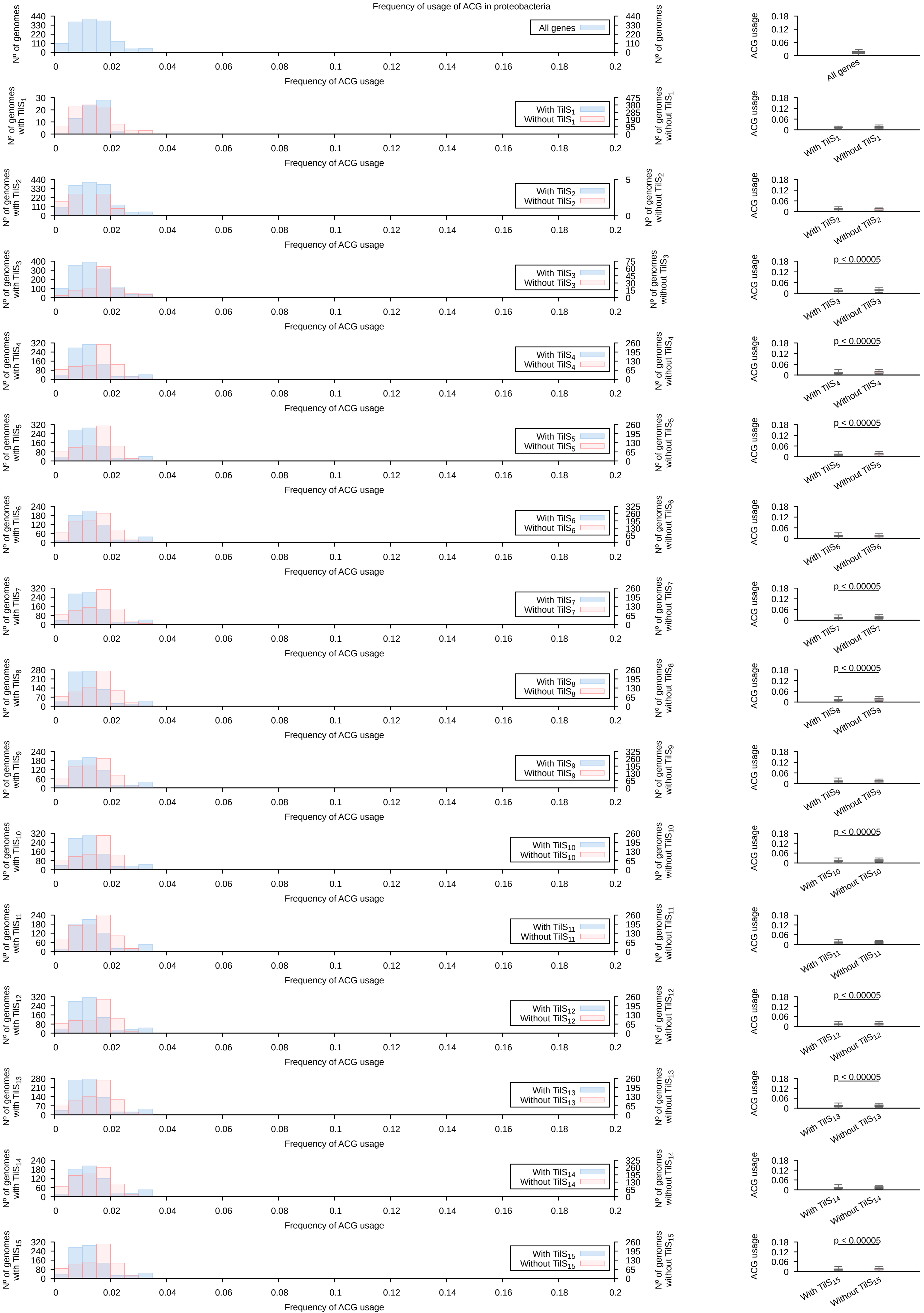

Frequency of usage of ACG in proteobacteria
Nº of genomes
All genes
Frequency of ACG usage
ACG usage
With TilS1
Without TilS1
With TilS2
Without TilS2
With TilS3
Without TilS3
p < 0.00005
With TilS4
Without TilS4
With TilS5
Without TilS5
With TilS6
Without TilS6
With TilS7
Without TilS7
With TilS8
Without TilS8
With TilS9
Without TilS9
With TilS10
Without TilS10
With TilS11
Without TilS11
With TilS12
Without TilS12
With TilS13
Without TilS13
With TilS14
Without TilS14
With TilS15
Without TilS15

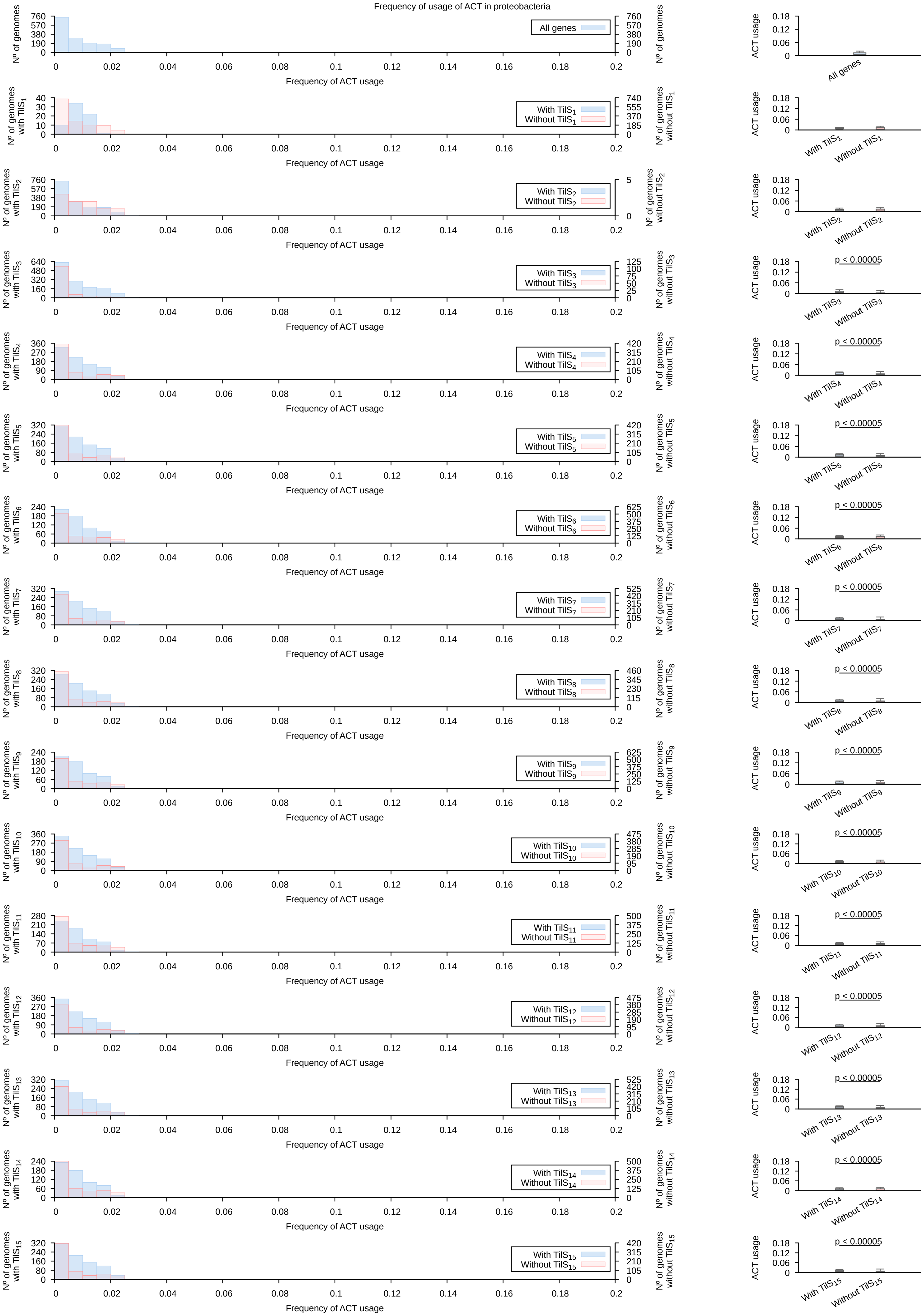

Frequency of usage of ACT in proteobacteria
Nº of genomes
All genes
Frequency of ACT usage
ACT usage
All genes
Nº of genomes with TilS1
Nº of genomes without TilS1
With TilS1
Without TilS1
Nº of genomes with TilS2
Nº of genomes without TilS2
With TilS2
Without TilS2
Nº of genomes with TilS3
Nº of genomes without TilS3
With TilS3
Without TilS3
p < 0.00005
Nº of genomes with TilS4
Nº of genomes without TilS4
With TilS4
Without TilS4
p < 0.00005
Nº of genomes with TilS5
Nº of genomes without TilS5
With TilS5
Without TilS5
p < 0.00005
Nº of genomes with TilS6
Nº of genomes without TilS6
With TilS6
Without TilS6
p < 0.00005
Nº of genomes with TilS7
Nº of genomes without TilS7
With TilS7
Without TilS7
p < 0.00005
Nº of genomes with TilS8
Nº of genomes without TilS8
With TilS8
Without TilS8
p < 0.00005
Nº of genomes with TilS9
Nº of genomes without TilS9
With TilS9
Without TilS9
p < 0.00005
Nº of genomes with TilS10
Nº of genomes without TilS10
With TilS10
Without TilS10
p < 0.00005
Nº of genomes with TilS11
Nº of genomes without TilS11
With TilS11
Without TilS11
p < 0.00005
Nº of genomes with TilS12
Nº of genomes without TilS12
With TilS12
Without TilS12
p < 0.00005
Nº of genomes with TilS13
Nº of genomes without TilS13
With TilS13
Without TilS13
p < 0.00005
Nº of genomes with TilS14
Nº of genomes without TilS14
With TilS14
Without TilS14
p < 0.00005
Nº of genomes with TilS15
Nº of genomes without TilS15
With TilS15
Without TilS15
p < 0.00005

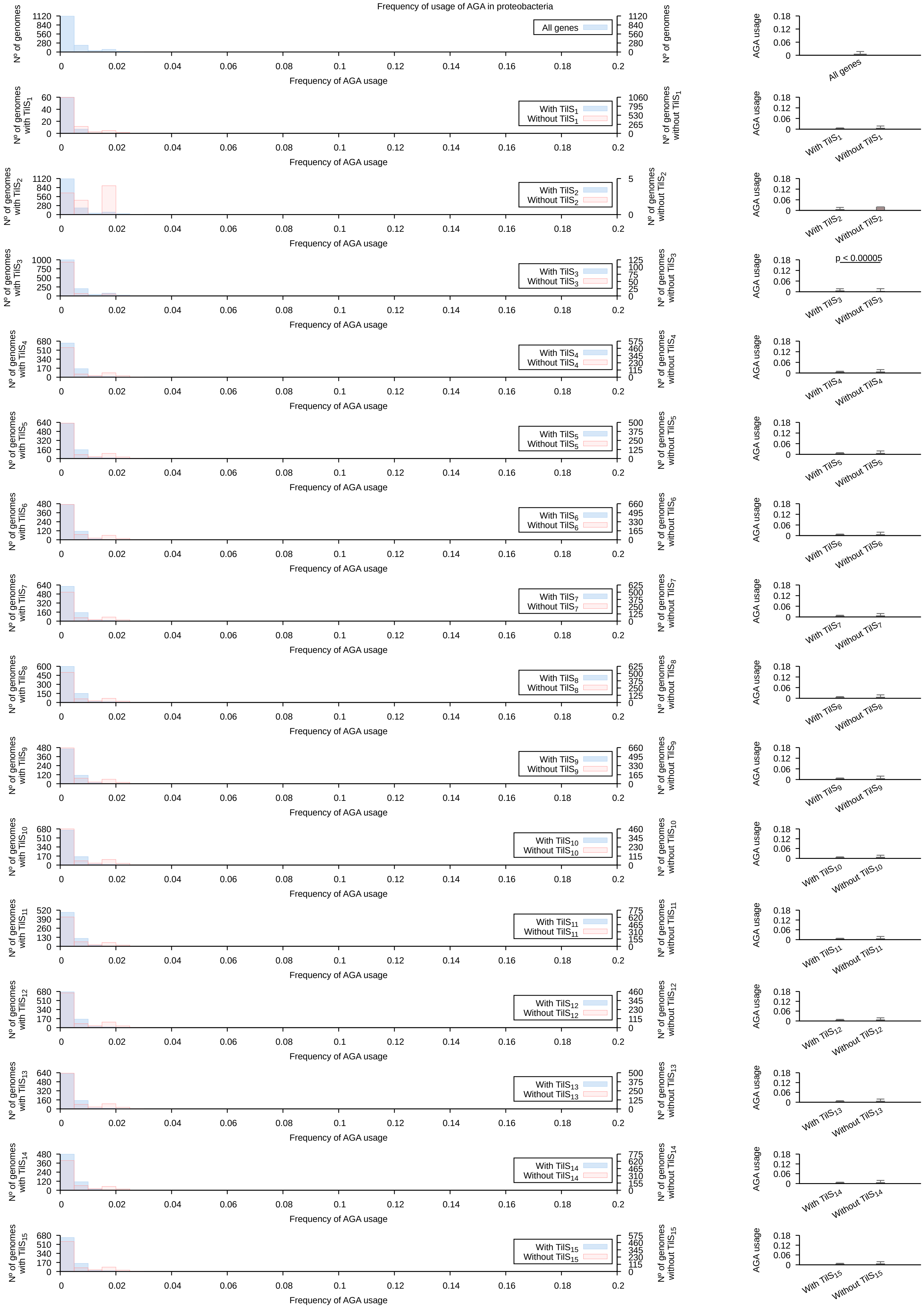
Frequency of usage of AGA in proteobacteria
All genes
Nº of genomes
Frequency of AGA usage
AGA usage
With TilS1
Without TilS1
Nº of genomes with TilS1
Nº of genomes without TilS1
With TilS2
Without TilS2
With TilS3
Without TilS3
p < 0.00005
With TilS4
Without TilS4
With TilS5
Without TilS5
With TilS6
Without TilS6
With TilS7
Without TilS7
With TilS8
Without TilS8
With TilS9
Without TilS9
With TilS10
Without TilS10
With TilS11
Without TilS11
With TilS12
Without TilS12
With TilS13
Without TilS13
With TilS14
Without TilS14
With TilS15
Without TilS15

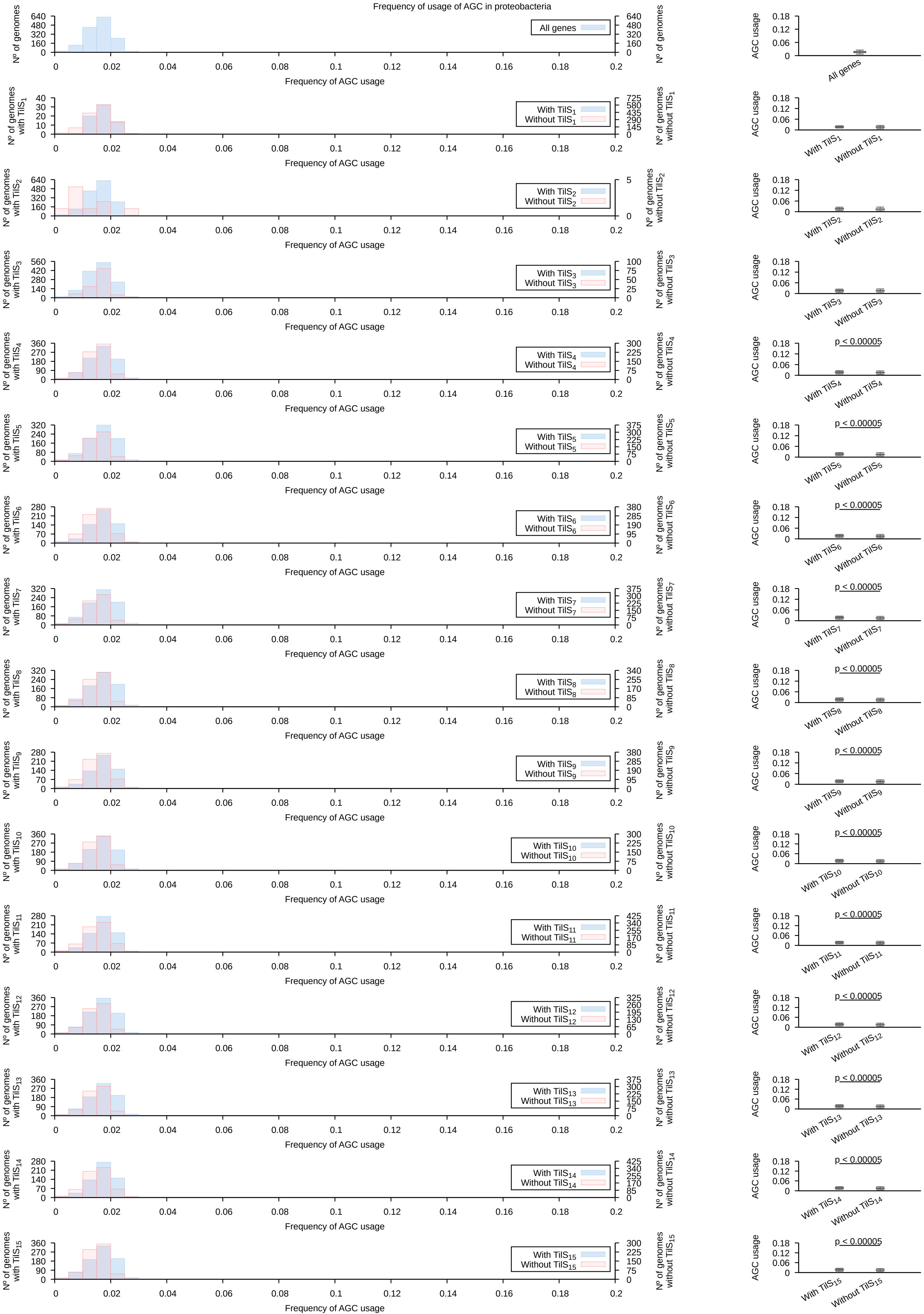

Frequency of usage of AGC in proteobacteria
Nº of genomes
All genes
Frequency of AGC usage
AGC usage
All genes
Nº of genomes with TilS$_1$
With TilS$_1$
Without TilS$_1$
Nº of genomes without TilS$_1$
Nº of genomes with TilS$_2$
With TilS$_2$
Without TilS$_2$
Nº of genomes without TilS$_2$
Nº of genomes with TilS$_3$
With TilS$_3$
Without TilS$_3$
Nº of genomes without TilS$_3$
Nº of genomes with TilS$_4$
With TilS$_4$
Without TilS$_4$
Nº of genomes without TilS$_4$
p < 0.00005
Nº of genomes with TilS$_5$
With TilS$_5$
Without TilS$_5$
Nº of genomes without TilS$_5$
p < 0.00005
Nº of genomes with TilS$_6$
With TilS$_6$
Without TilS$_6$
Nº of genomes without TilS$_6$
p < 0.00005
Nº of genomes with TilS$_7$
With TilS$_7$
Without TilS$_7$
Nº of genomes without TilS$_7$
p < 0.00005
Nº of genomes with TilS$_8$
With TilS$_8$
Without TilS$_8$
Nº of genomes without TilS$_8$
p < 0.00005
Nº of genomes with TilS$_9$
With TilS$_9$
Without TilS$_9$
Nº of genomes without TilS$_9$
p < 0.00005
Nº of genomes with TilS$_{10}$
With TilS$_{10}$
Without TilS$_{10}$
Nº of genomes without TilS$_{10}$
p < 0.00005
Nº of genomes with TilS$_{11}$
With TilS$_{11}$
Without TilS$_{11}$
Nº of genomes without TilS$_{11}$
p < 0.00005
Nº of genomes with TilS$_{12}$
With TilS$_{12}$
Without TilS$_{12}$
Nº of genomes without TilS$_{12}$
p < 0.00005
Nº of genomes with TilS$_{13}$
With TilS$_{13}$
Without TilS$_{13}$
Nº of genomes without TilS$_{13}$
p < 0.00005
Nº of genomes with TilS$_{14}$
With TilS$_{14}$
Without TilS$_{14}$
Nº of genomes without TilS$_{14}$
p < 0.00005
Nº of genomes with TilS$_{15}$
With TilS$_{15}$
Without TilS$_{15}$
Nº of genomes without TilS$_{15}$
p < 0.00005

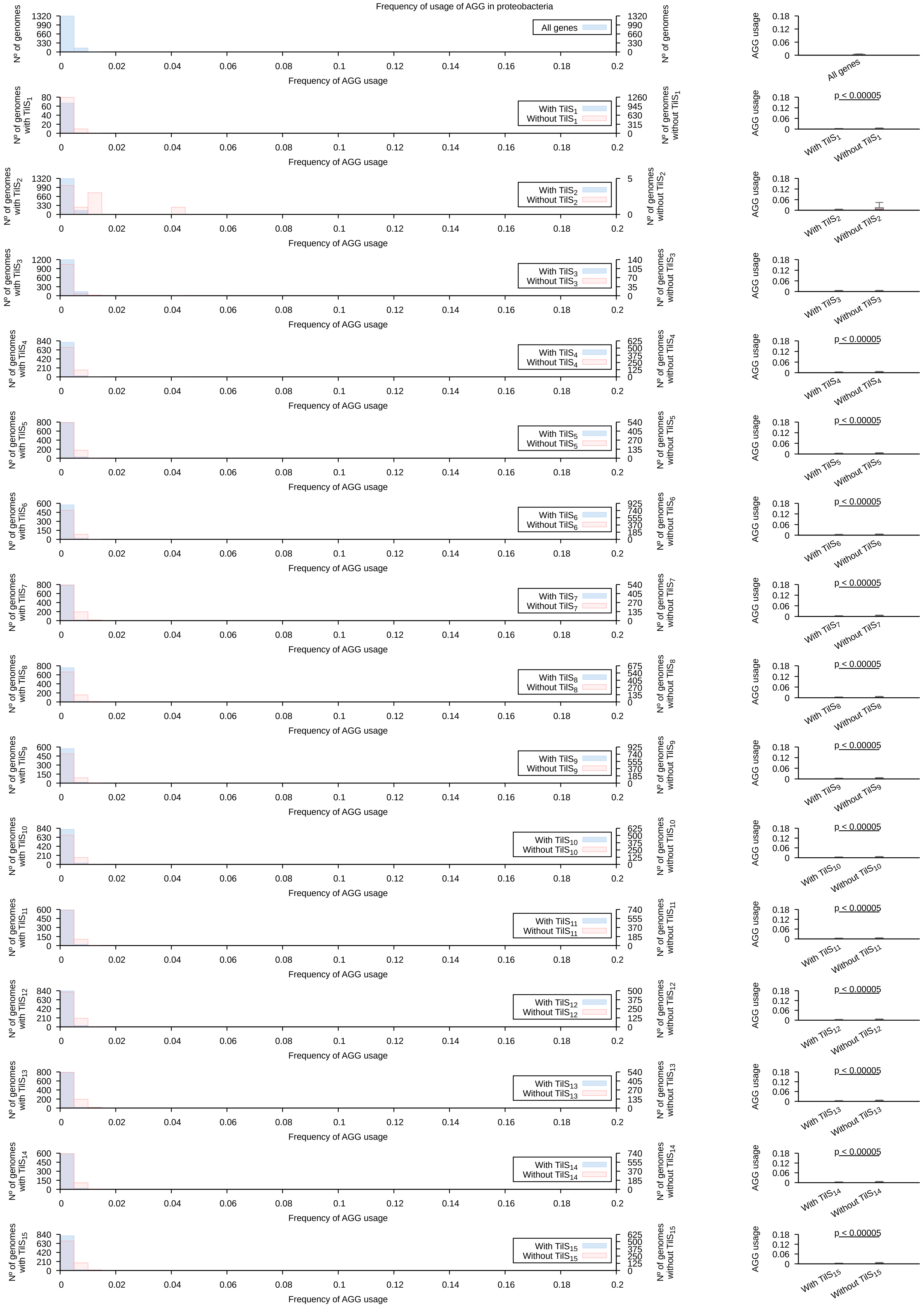

Frequency of usage of AGG in proteobacteria
Nº of genomes
All genes
Frequency of AGG usage
AGG usage
Nº of genomes with TilS1
Nº of genomes without TilS1
With TilS1
Without TilS1
p < 0.00005
With TilS2
Without TilS2
With TilS3
Without TilS3
With TilS4
Without TilS4
With TilS5
Without TilS5
With TilS6
Without TilS6
With TilS7
Without TilS7
With TilS8
Without TilS8
With TilS9
Without TilS9
With TilS10
Without TilS10
With TilS11
Without TilS11
With TilS12
Without TilS12
With TilS13
Without TilS13
With TilS14
Without TilS14
With TilS15
Without TilS15

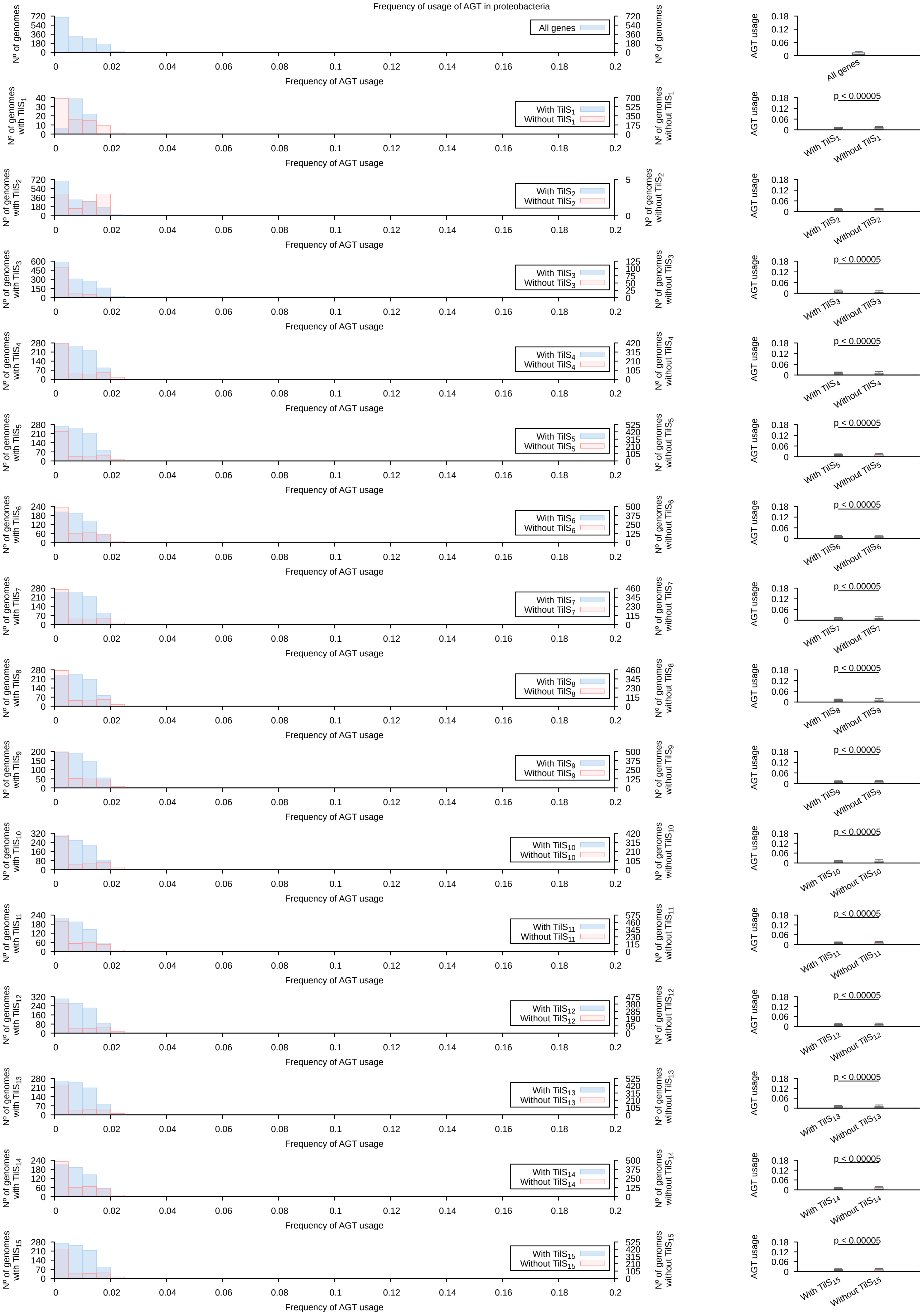
Frequency of usage of AGT in proteobacteria
Nº of genomes
All genes
Frequency of AGT usage
AGT usage
Nº of genomes with $TilS_1$
Nº of genomes without $TilS_1$
With $TilS_1$
Without $TilS_1$
p < 0.00005
Nº of genomes with $TilS_2$
Nº of genomes without $TilS_2$
With $TilS_2$
Without $TilS_2$
Nº of genomes with $TilS_3$
Nº of genomes without $TilS_3$
With $TilS_3$
Without $TilS_3$
Nº of genomes with $TilS_4$
Nº of genomes without $TilS_4$
With $TilS_4$
Without $TilS_4$
Nº of genomes with $TilS_5$
Nº of genomes without $TilS_5$
With $TilS_5$
Without $TilS_5$
Nº of genomes with $TilS_6$
Nº of genomes without $TilS_6$
With $TilS_6$
Without $TilS_6$
Nº of genomes with $TilS_7$
Nº of genomes without $TilS_7$
With $TilS_7$
Without $TilS_7$
Nº of genomes with $TilS_8$
Nº of genomes without $TilS_8$
With $TilS_8$
Without $TilS_8$
Nº of genomes with $TilS_9$
Nº of genomes without $TilS_9$
With $TilS_9$
Without $TilS_9$
Nº of genomes with $TilS_{10}$
Nº of genomes without $TilS_{10}$
With $TilS_{10}$
Without $TilS_{10}$
Nº of genomes with $TilS_{11}$
Nº of genomes without $TilS_{11}$
With $TilS_{11}$
Without $TilS_{11}$
Nº of genomes with $TilS_{12}$
Nº of genomes without $TilS_{12}$
With $TilS_{12}$
Without $TilS_{12}$
Nº of genomes with $TilS_{13}$
Nº of genomes without $TilS_{13}$
With $TilS_{13}$
Without $TilS_{13}$
Nº of genomes with $TilS_{14}$
Nº of genomes without $TilS_{14}$
With $TilS_{14}$
Without $TilS_{14}$
Nº of genomes with $TilS_{15}$
Nº of genomes without $TilS_{15}$
With $TilS_{15}$
Without $TilS_{15}$

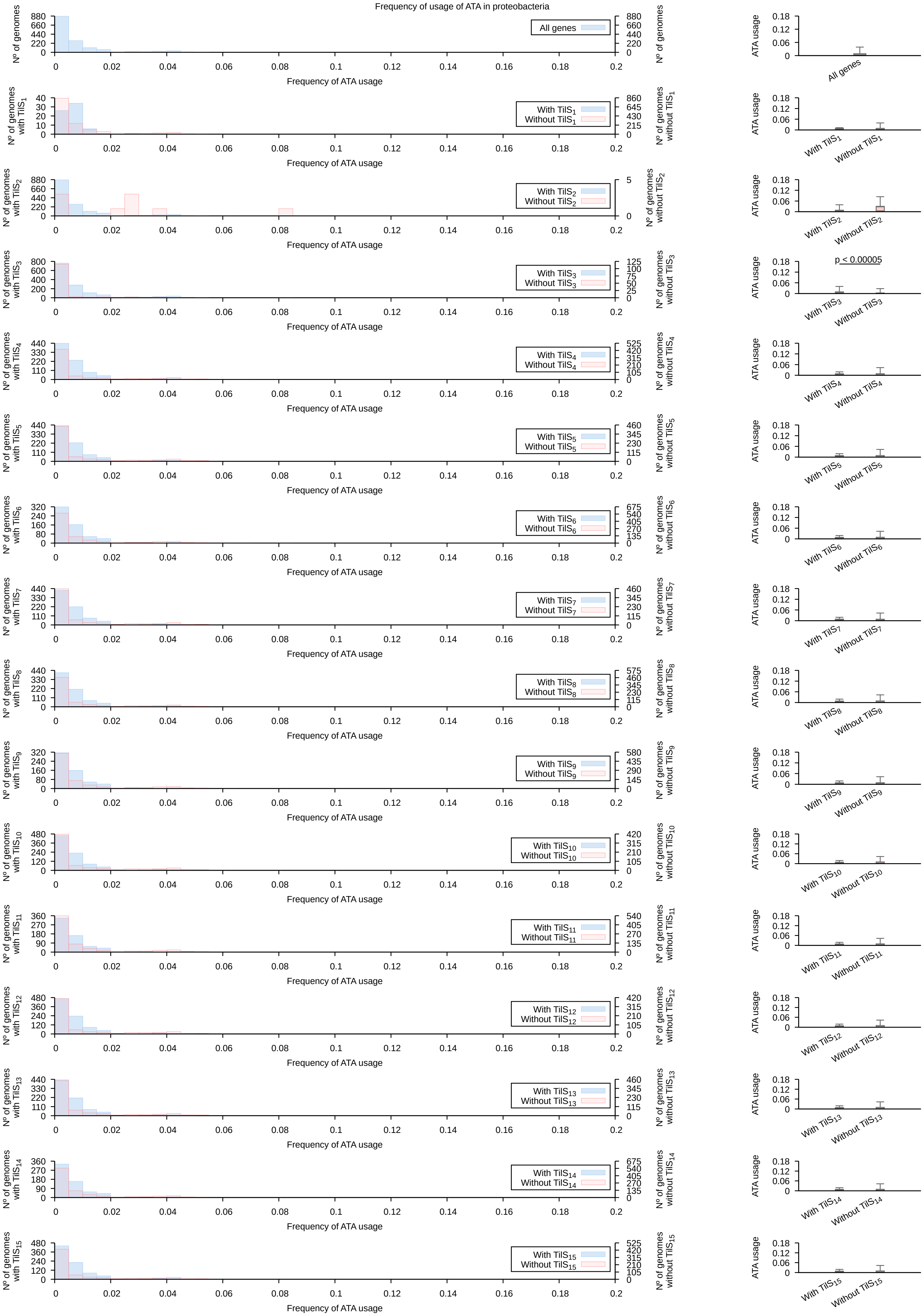
Frequency of usage of ATA in proteobacteria
Nº of genomes
All genes
Frequency of ATA usage
ATA usage
Nº of genomes with TilS1
Nº of genomes without TilS1
With TilS1
Without TilS1
Nº of genomes with TilS2
Nº of genomes without TilS2
With TilS2
Without TilS2
Nº of genomes with TilS3
Nº of genomes without TilS3
With TilS3
Without TilS3
p < 0.00005
Nº of genomes with TilS4
Nº of genomes without TilS4
With TilS4
Without TilS4
Nº of genomes with TilS5
Nº of genomes without TilS5
With TilS5
Without TilS5
Nº of genomes with TilS6
Nº of genomes without TilS6
With TilS6
Without TilS6
Nº of genomes with TilS7
Nº of genomes without TilS7
With TilS7
Without TilS7
Nº of genomes with TilS8
Nº of genomes without TilS8
With TilS8
Without TilS8
Nº of genomes with TilS9
Nº of genomes without TilS9
With TilS9
Without TilS9
Nº of genomes with TilS10
Nº of genomes without TilS10
With TilS10
Without TilS10
Nº of genomes with TilS11
Nº of genomes without TilS11
With TilS11
Without TilS11
Nº of genomes with TilS12
Nº of genomes without TilS12
With TilS12
Without TilS12
Nº of genomes with TilS13
Nº of genomes without TilS13
With TilS13
Without TilS13
Nº of genomes with TilS14
Nº of genomes without TilS14
With TilS14
Without TilS14
Nº of genomes with TilS15
Nº of genomes without TilS15
With TilS15
Without TilS15

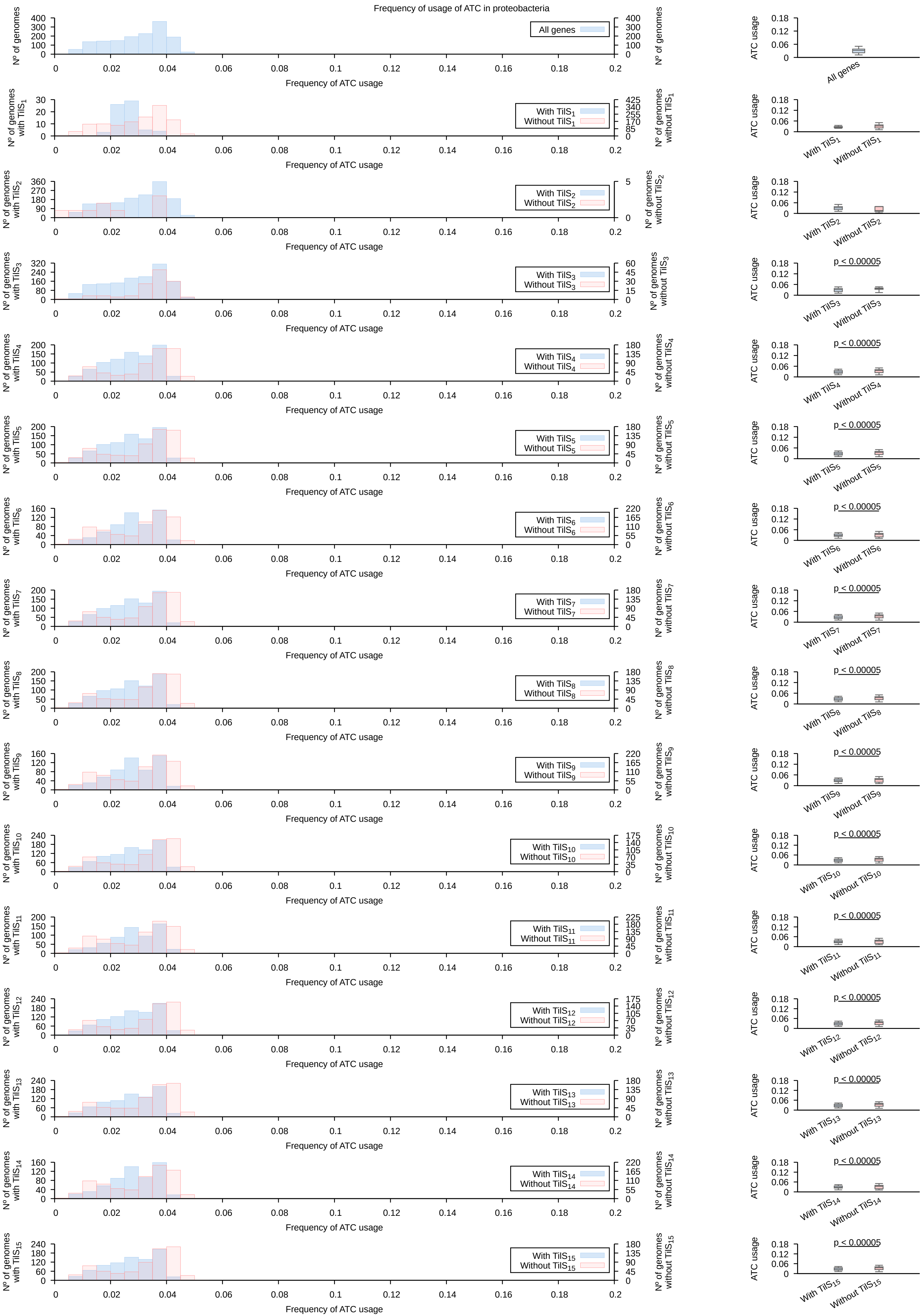

Frequency of usage of ATC in proteobacteria
Nº of genomes
All genes
Frequency of ATC usage
ATC usage
All genes
Nº of genomes with TilS$_1$
Nº of genomes without TilS$_1$
With TilS$_1$
Without TilS$_1$
Nº of genomes with TilS$_2$
Nº of genomes without TilS$_2$
With TilS$_2$
Without TilS$_2$
Nº of genomes with TilS$_3$
Nº of genomes without TilS$_3$
With TilS$_3$
Without TilS$_3$
p < 0.00005
Nº of genomes with TilS$_4$
Nº of genomes without TilS$_4$
With TilS$_4$
Without TilS$_4$
p < 0.00005
Nº of genomes with TilS$_5$
Nº of genomes without TilS$_5$
With TilS$_5$
Without TilS$_5$
p < 0.00005
Nº of genomes with TilS$_6$
Nº of genomes without TilS$_6$
With TilS$_6$
Without TilS$_6$
p < 0.00005
Nº of genomes with TilS$_7$
Nº of genomes without TilS$_7$
With TilS$_7$
Without TilS$_7$
p < 0.00005
Nº of genomes with TilS$_8$
Nº of genomes without TilS$_8$
With TilS$_8$
Without TilS$_8$
p < 0.00005
Nº of genomes with TilS$_9$
Nº of genomes without TilS$_9$
With TilS$_9$
Without TilS$_9$
p < 0.00005
Nº of genomes with TilS$_{10}$
Nº of genomes without TilS$_{10}$
With TilS$_{10}$
Without TilS$_{10}$
p < 0.00005
Nº of genomes with TilS$_{11}$
Nº of genomes without TilS$_{11}$
With TilS$_{11}$
Without TilS$_{11}$
p < 0.00005
Nº of genomes with TilS$_{12}$
Nº of genomes without TilS$_{12}$
With TilS$_{12}$
Without TilS$_{12}$
p < 0.00005
Nº of genomes with TilS$_{13}$
Nº of genomes without TilS$_{13}$
With TilS$_{13}$
Without TilS$_{13}$
p < 0.00005
Nº of genomes with TilS$_{14}$
Nº of genomes without TilS$_{14}$
With TilS$_{14}$
Without TilS$_{14}$
p < 0.00005
Nº of genomes with TilS$_{15}$
Nº of genomes without TilS$_{15}$
With TilS$_{15}$
Without TilS$_{15}$
p < 0.00005
0 0.02 0.04 0.06 0.08 0.1 0.12 0.14 0.16 0.18 0.2
0 0.06 0.12 0.18

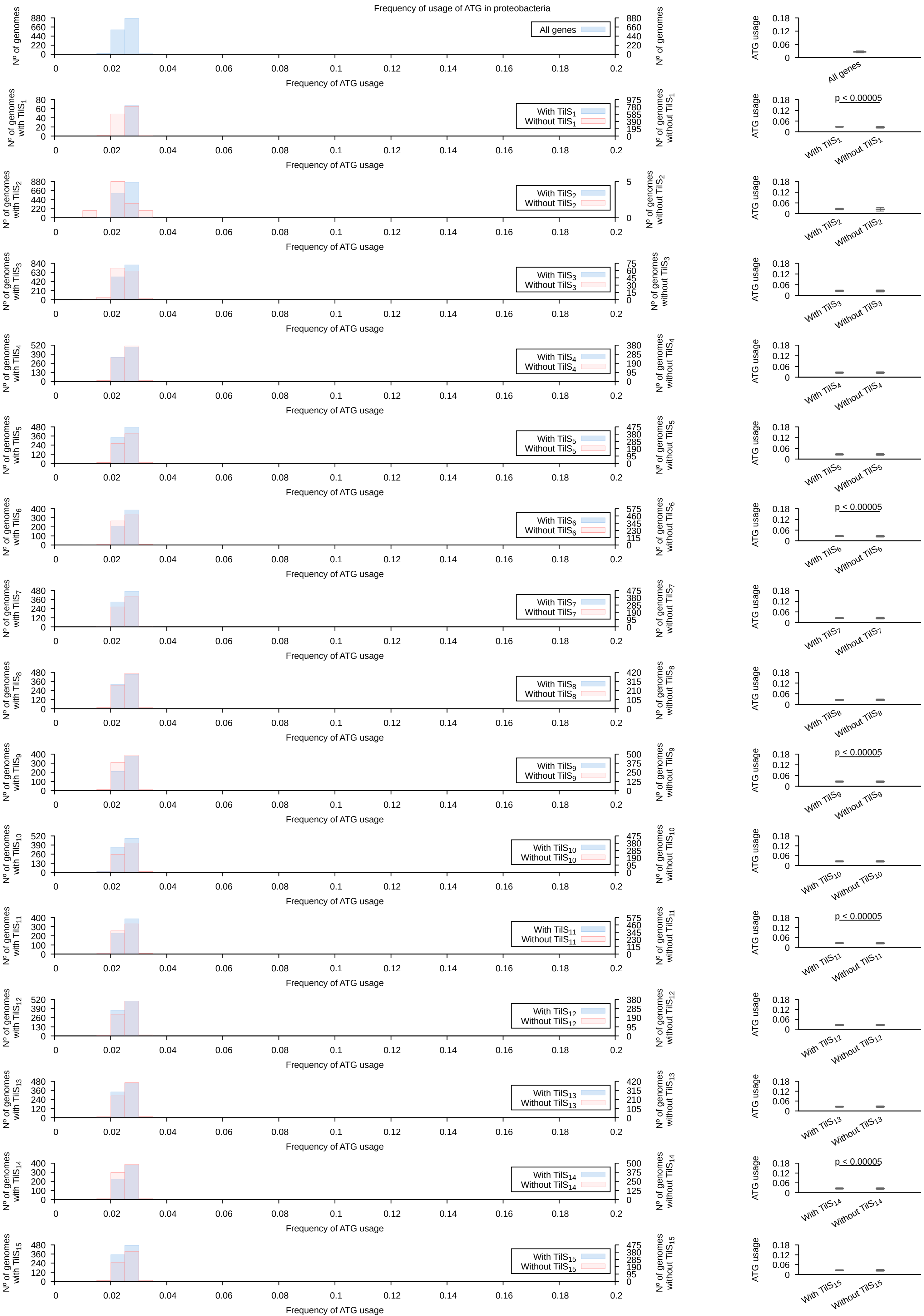
Frequency of usage of ATG in proteobacteria
Nº of genomes
Frequency of ATG usage
ATG usage
All genes
Nº of genomes with TilS$_{1}$
Nº of genomes without TilS$_{1}$
With TilS$_{1}$
Without TilS$_{1}$
p < 0.00005
With TilS$_{2}$
Without TilS$_{2}$
With TilS$_{3}$
Without TilS$_{3}$
With TilS$_{4}$
Without TilS$_{4}$
With TilS$_{5}$
Without TilS$_{5}$
With TilS$_{6}$
Without TilS$_{6}$
p < 0.00005
With TilS$_{7}$
Without TilS$_{7}$
With TilS$_{8}$
Without TilS$_{8}$
With TilS$_{9}$
Without TilS$_{9}$
p < 0.00005
With TilS$_{10}$
Without TilS$_{10}$
With TilS$_{11}$
Without TilS$_{11}$
p < 0.00005
With TilS$_{12}$
Without TilS$_{12}$
With TilS$_{13}$
Without TilS$_{13}$
With TilS$_{14}$
Without TilS$_{14}$
p < 0.00005
With TilS$_{15}$
Without TilS$_{15}$

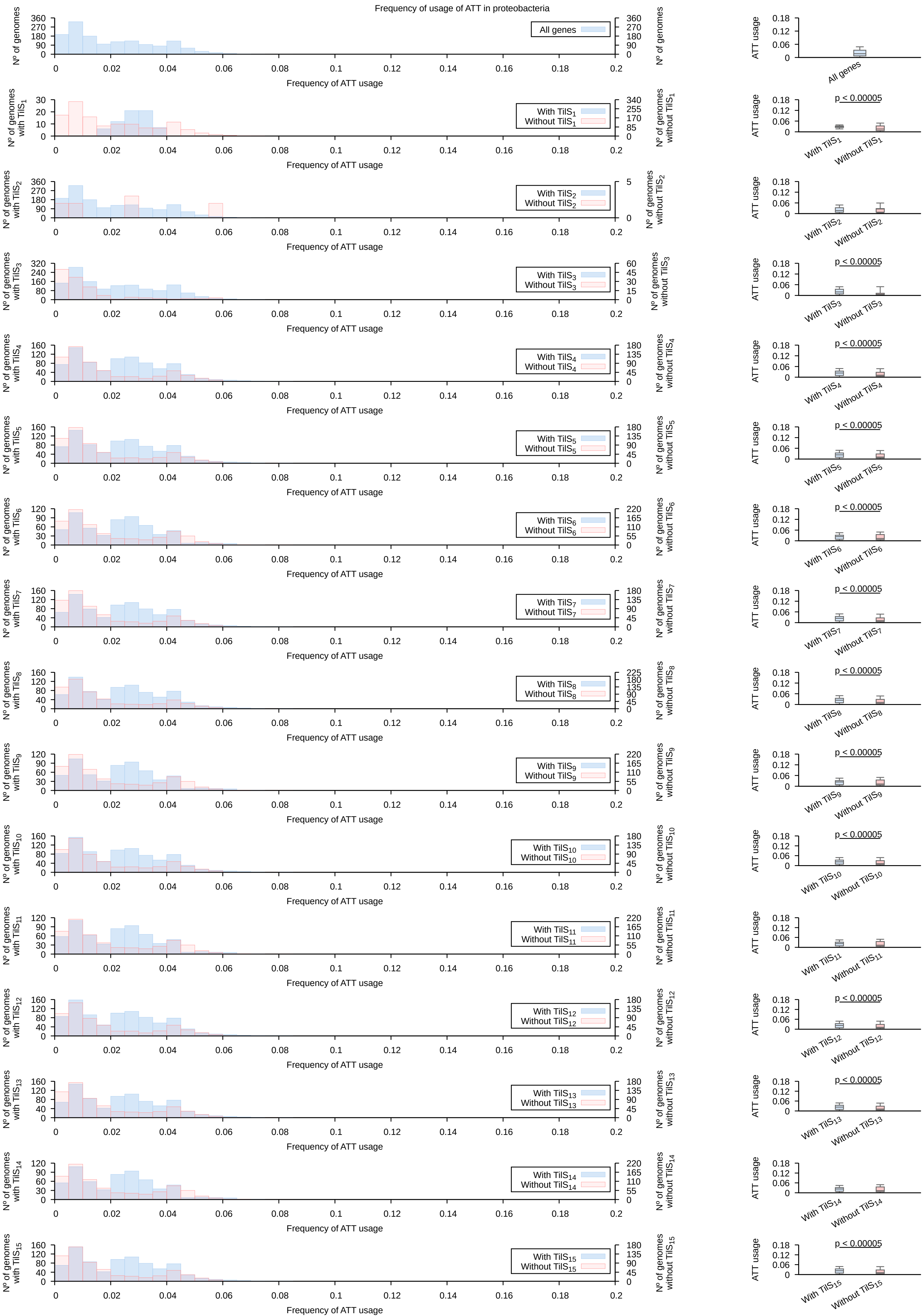

Frequency of usage of ATT in proteobacteria
Nº of genomes
All genes
Frequency of ATT usage
ATT usage
All genes
Nº of genomes with TilS$_1$
Nº of genomes without TilS$_1$
With TilS$_1$
Without TilS$_1$
p < 0.00005
Nº of genomes with TilS$_2$
Nº of genomes without TilS$_2$
With TilS$_2$
Without TilS$_2$
Nº of genomes with TilS$_3$
Nº of genomes without TilS$_3$
With TilS$_3$
Without TilS$_3$
p < 0.00005
Nº of genomes with TilS$_4$
Nº of genomes without TilS$_4$
With TilS$_4$
Without TilS$_4$
p < 0.00005
Nº of genomes with TilS$_5$
Nº of genomes without TilS$_5$
With TilS$_5$
Without TilS$_5$
p < 0.00005
Nº of genomes with TilS$_6$
Nº of genomes without TilS$_6$
With TilS$_6$
Without TilS$_6$
p < 0.00005
Nº of genomes with TilS$_7$
Nº of genomes without TilS$_7$
With TilS$_7$
Without TilS$_7$
p < 0.00005
Nº of genomes with TilS$_8$
Nº of genomes without TilS$_8$
With TilS$_8$
Without TilS$_8$
p < 0.00005
Nº of genomes with TilS$_9$
Nº of genomes without TilS$_9$
With TilS$_9$
Without TilS$_9$
p < 0.00005
Nº of genomes with TilS$_{10}$
Nº of genomes without TilS$_{10}$
With TilS$_{10}$
Without TilS$_{10}$
p < 0.00005
Nº of genomes with TilS$_{11}$
Nº of genomes without TilS$_{11}$
With TilS$_{11}$
Without TilS$_{11}$
Nº of genomes with TilS$_{12}$
Nº of genomes without TilS$_{12}$
With TilS$_{12}$
Without TilS$_{12}$
p < 0.00005
Nº of genomes with TilS$_{13}$
Nº of genomes without TilS$_{13}$
With TilS$_{13}$
Without TilS$_{13}$
p < 0.00005
Nº of genomes with TilS$_{14}$
Nº of genomes without TilS$_{14}$
With TilS$_{14}$
Without TilS$_{14}$
Nº of genomes with TilS$_{15}$
Nº of genomes without TilS$_{15}$
With TilS$_{15}$
Without TilS$_{15}$
p < 0.00005

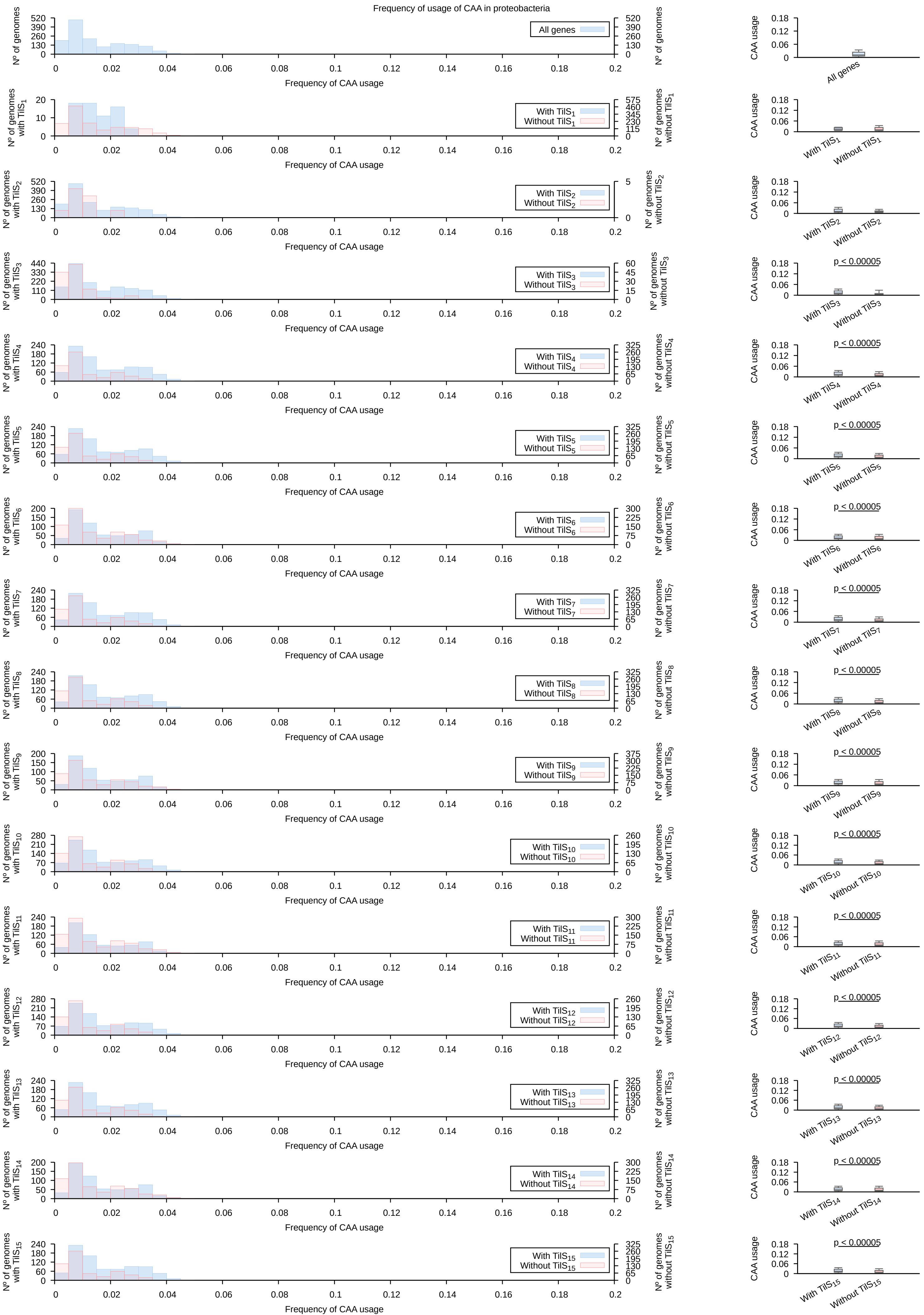
Frequency of usage of CAA in proteobacteria
Nº of genomes
All genes
Frequency of CAA usage
CAA usage
Nº of genomes with $TilS_1$
Nº of genomes without $TilS_1$
With $TilS_1$
Without $TilS_1$
Nº of genomes with $TilS_2$
Nº of genomes without $TilS_2$
With $TilS_2$
Without $TilS_2$
Nº of genomes with $TilS_3$
Nº of genomes without $TilS_3$
With $TilS_3$
Without $TilS_3$
p < 0.00005
Nº of genomes with $TilS_4$
Nº of genomes without $TilS_4$
With $TilS_4$
Without $TilS_4$
p < 0.00005
Nº of genomes with $TilS_5$
Nº of genomes without $TilS_5$
With $TilS_5$
Without $TilS_5$
p < 0.00005
Nº of genomes with $TilS_6$
Nº of genomes without $TilS_6$
With $TilS_6$
Without $TilS_6$
p < 0.00005
Nº of genomes with $TilS_7$
Nº of genomes without $TilS_7$
With $TilS_7$
Without $TilS_7$
p < 0.00005
Nº of genomes with $TilS_8$
Nº of genomes without $TilS_8$
With $TilS_8$
Without $TilS_8$
p < 0.00005
Nº of genomes with $TilS_9$
Nº of genomes without $TilS_9$
With $TilS_9$
Without $TilS_9$
p < 0.00005
Nº of genomes with $TilS_{10}$
Nº of genomes without $TilS_{10}$
With $TilS_{10}$
Without $TilS_{10}$
p < 0.00005
Nº of genomes with $TilS_{11}$
Nº of genomes without $TilS_{11}$
With $TilS_{11}$
Without $TilS_{11}$
p < 0.00005
Nº of genomes with $TilS_{12}$
Nº of genomes without $TilS_{12}$
With $TilS_{12}$
Without $TilS_{12}$
p < 0.00005
Nº of genomes with $TilS_{13}$
Nº of genomes without $TilS_{13}$
With $TilS_{13}$
Without $TilS_{13}$
p < 0.00005
Nº of genomes with $TilS_{14}$
Nº of genomes without $TilS_{14}$
With $TilS_{14}$
Without $TilS_{14}$
p < 0.00005
Nº of genomes with $TilS_{15}$
Nº of genomes without $TilS_{15}$
With $TilS_{15}$
Without $TilS_{15}$
p < 0.00005

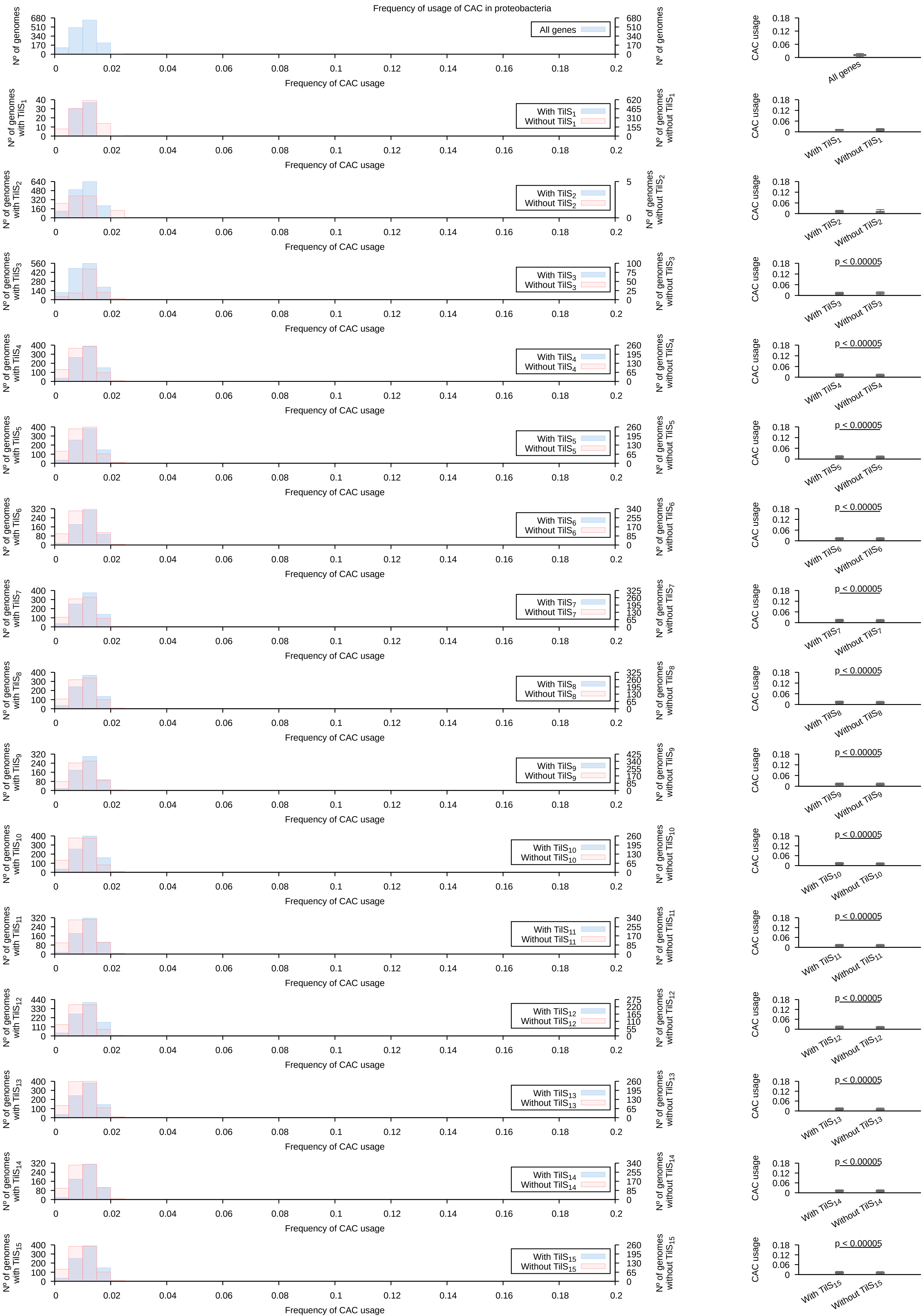
Frequency of usage of CAC in proteobacteria
Nº of genomes
All genes
Frequency of CAC usage
CAC usage
All genes
Nº of genomes with TilS$_1$
Nº of genomes without TilS$_1$
With TilS$_1$
Without TilS$_1$
Nº of genomes with TilS$_2$
Nº of genomes without TilS$_2$
With TilS$_2$
Without TilS$_2$
Nº of genomes with TilS$_3$
Nº of genomes without TilS$_3$
With TilS$_3$
Without TilS$_3$
p < 0.00005
Nº of genomes with TilS$_4$
Nº of genomes without TilS$_4$
With TilS$_4$
Without TilS$_4$
Nº of genomes with TilS$_5$
Nº of genomes without TilS$_5$
With TilS$_5$
Without TilS$_5$
Nº of genomes with TilS$_6$
Nº of genomes without TilS$_6$
With TilS$_6$
Without TilS$_6$
Nº of genomes with TilS$_7$
Nº of genomes without TilS$_7$
With TilS$_7$
Without TilS$_7$
Nº of genomes with TilS$_8$
Nº of genomes without TilS$_8$
With TilS$_8$
Without TilS$_8$
Nº of genomes with TilS$_9$
Nº of genomes without TilS$_9$
With TilS$_9$
Without TilS$_9$
Nº of genomes with TilS$_{10}$
Nº of genomes without TilS$_{10}$
With TilS$_{10}$
Without TilS$_{10}$
Nº of genomes with TilS$_{11}$
Nº of genomes without TilS$_{11}$
With TilS$_{11}$
Without TilS$_{11}$
Nº of genomes with TilS$_{12}$
Nº of genomes without TilS$_{12}$
With TilS$_{12}$
Without TilS$_{12}$
Nº of genomes with TilS$_{13}$
Nº of genomes without TilS$_{13}$
With TilS$_{13}$
Without TilS$_{13}$
Nº of genomes with TilS$_{14}$
Nº of genomes without TilS$_{14}$
With TilS$_{14}$
Without TilS$_{14}$
Nº of genomes with TilS$_{15}$
Nº of genomes without TilS$_{15}$
With TilS$_{15}$
Without TilS$_{15}$

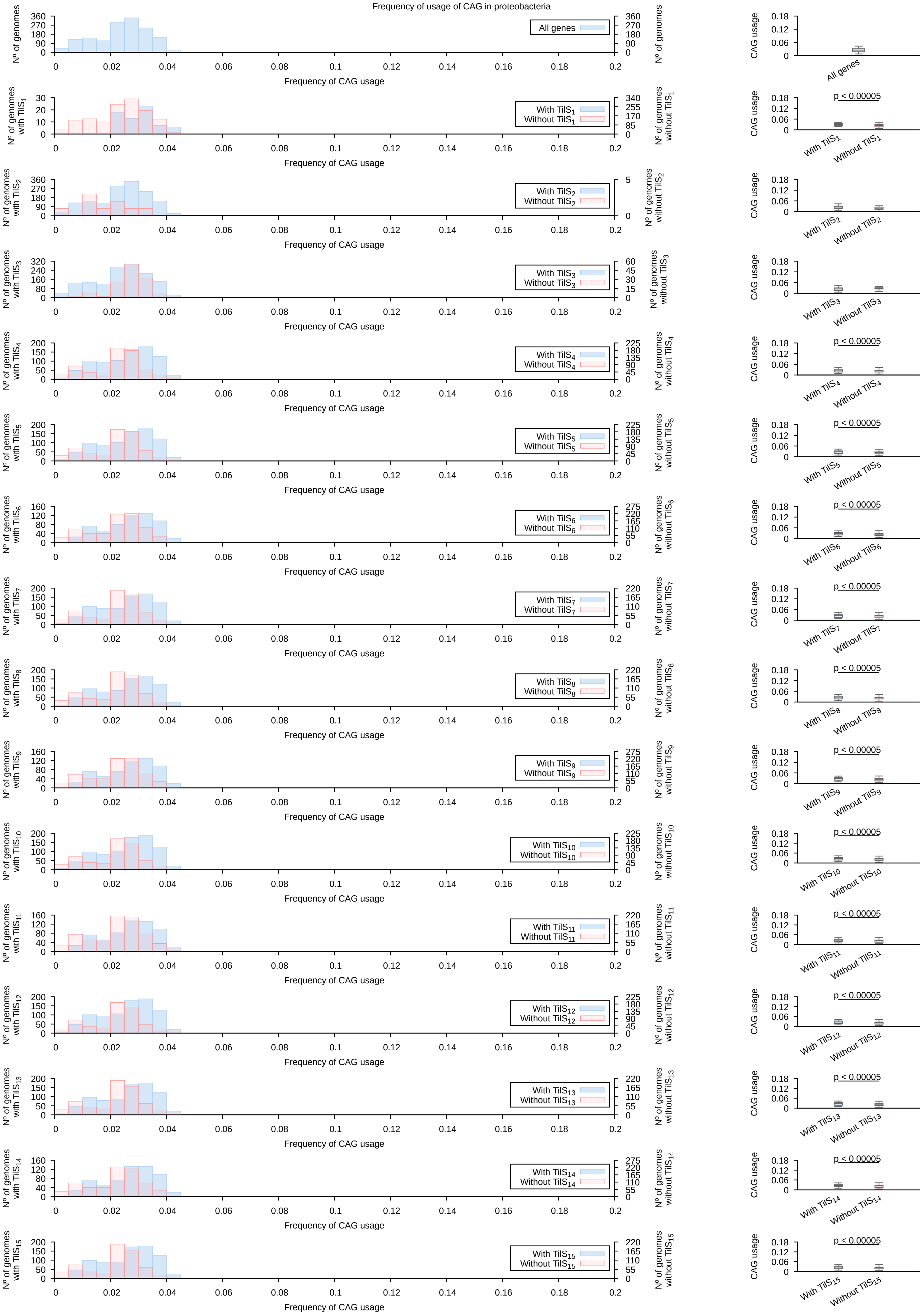
Frequency of usage of CAG in proteobacteria
Nº of genomes
All genes
Frequency of CAG usage
CAG usage
0
0.02
0.04
0.06
0.08
0.1
0.12
0.14
0.16
0.18
0.2
0.06
0.12
0.18
p < 0.00005
Nº of genomes with TilS$_1$
Nº of genomes without TilS$_1$
With TilS$_1$
Without TilS$_1$
Nº of genomes with TilS$_2$
Nº of genomes without TilS$_2$
With TilS$_2$
Without TilS$_2$
Nº of genomes with TilS$_3$
Nº of genomes without TilS$_3$
With TilS$_3$
Without TilS$_3$
Nº of genomes with TilS$_4$
Nº of genomes without TilS$_4$
With TilS$_4$
Without TilS$_4$
Nº of genomes with TilS$_5$
Nº of genomes without TilS$_5$
With TilS$_5$
Without TilS$_5$
Nº of genomes with TilS$_6$
Nº of genomes without TilS$_6$
With TilS$_6$
Without TilS$_6$
Nº of genomes with TilS$_7$
Nº of genomes without TilS$_7$
With TilS$_7$
Without TilS$_7$
Nº of genomes with TilS$_8$
Nº of genomes without TilS$_8$
With TilS$_8$
Without TilS$_8$
Nº of genomes with TilS$_9$
Nº of genomes without TilS$_9$
With TilS$_9$
Without TilS$_9$
Nº of genomes with TilS$_{10}$
Nº of genomes without TilS$_{10}$
With TilS$_{10}$
Without TilS$_{10}$
Nº of genomes with TilS$_{11}$
Nº of genomes without TilS$_{11}$
With TilS$_{11}$
Without TilS$_{11}$
Nº of genomes with TilS$_{12}$
Nº of genomes without TilS$_{12}$
With TilS$_{12}$
Without TilS$_{12}$
Nº of genomes with TilS$_{13}$
Nº of genomes without TilS$_{13}$
With TilS$_{13}$
Without TilS$_{13}$
Nº of genomes with TilS$_{14}$
Nº of genomes without TilS$_{14}$
With TilS$_{14}$
Without TilS$_{14}$
Nº of genomes with TilS$_{15}$
Nº of genomes without TilS$_{15}$
With TilS$_{15}$
Without TilS$_{15}$

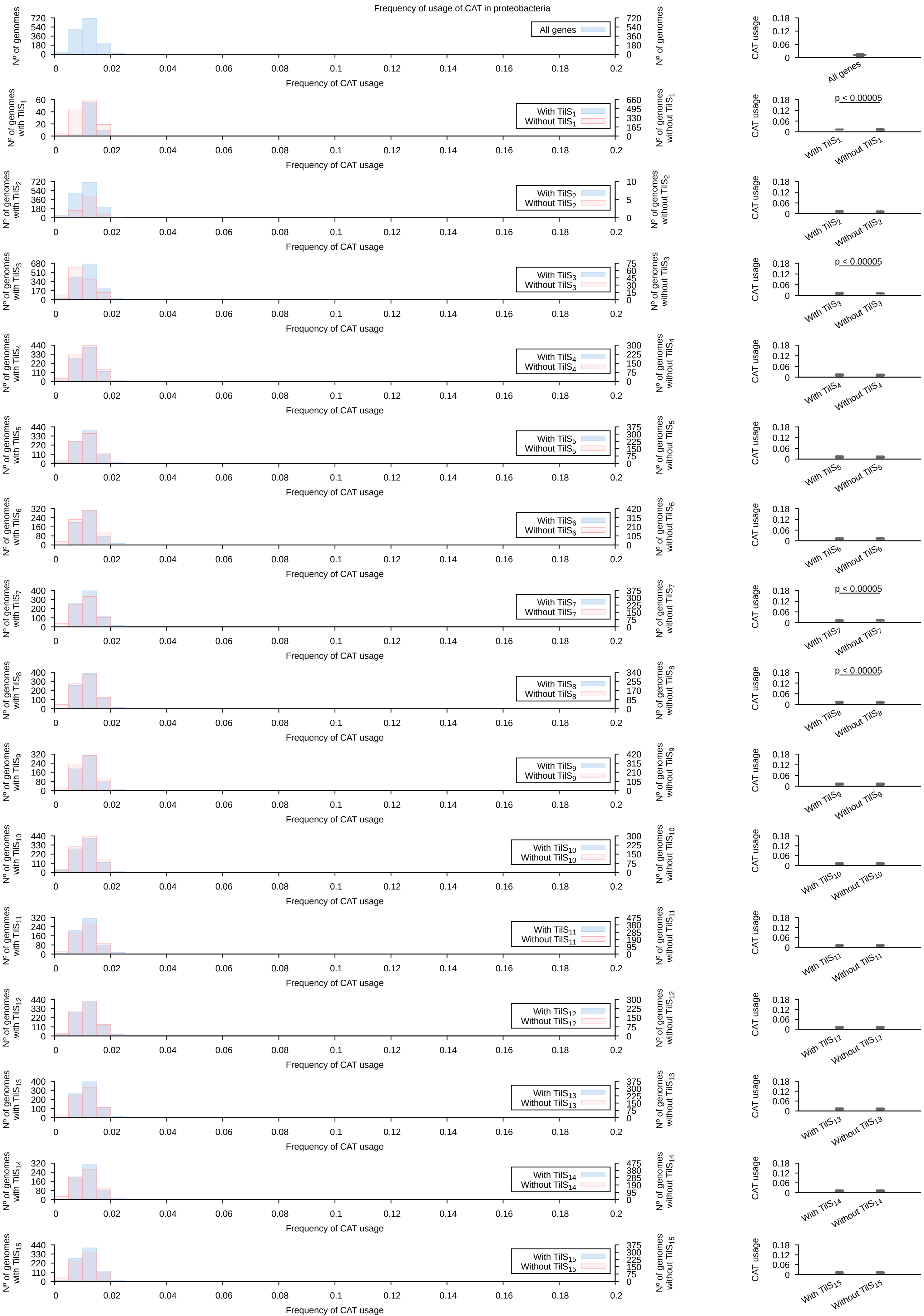

Frequency of usage of CAT in proteobacteria
Nº of genomes
All genes
Frequency of CAT usage
CAT usage
p < 0.00005
Nº of genomes with TilS$_1$
Nº of genomes without TilS$_1$
With TilS$_1$
Without TilS$_1$
With TilS$_2$
Without TilS$_2$
With TilS$_3$
Without TilS$_3$
With TilS$_4$
Without TilS$_4$
With TilS$_5$
Without TilS$_5$
With TilS$_6$
Without TilS$_6$
With TilS$_7$
Without TilS$_7$
With TilS$_8$
Without TilS$_8$
With TilS$_9$
Without TilS$_9$
With TilS$_{10}$
Without TilS$_{10}$
With TilS$_{11}$
Without TilS$_{11}$
With TilS$_{12}$
Without TilS$_{12}$
With TilS$_{13}$
Without TilS$_{13}$
With TilS$_{14}$
Without TilS$_{14}$
With TilS$_{15}$
Without TilS$_{15}$

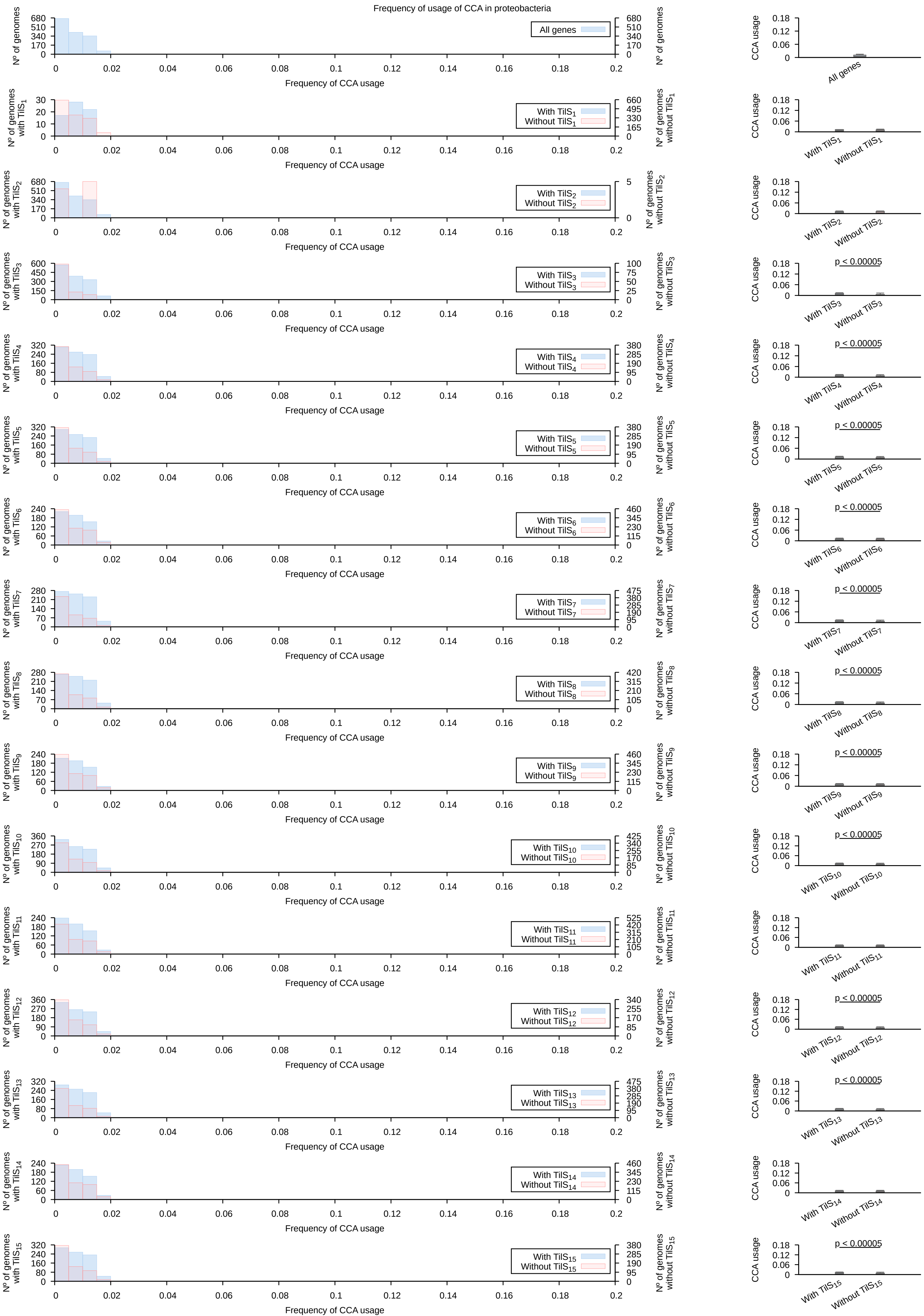

Frequency of usage of CCA in proteobacteria
Nº of genomes
All genes
Frequency of CCA usage
CCA usage
Nº of genomes with TilS$_1$
Nº of genomes without TilS$_1$
With TilS$_1$
Without TilS$_1$
Nº of genomes with TilS$_2$
Nº of genomes without TilS$_2$
With TilS$_2$
Without TilS$_2$
Nº of genomes with TilS$_3$
Nº of genomes without TilS$_3$
With TilS$_3$
Without TilS$_3$
p < 0.00005
Nº of genomes with TilS$_4$
Nº of genomes without TilS$_4$
With TilS$_4$
Without TilS$_4$
p < 0.00005
Nº of genomes with TilS$_5$
Nº of genomes without TilS$_5$
With TilS$_5$
Without TilS$_5$
p < 0.00005
Nº of genomes with TilS$_6$
Nº of genomes without TilS$_6$
With TilS$_6$
Without TilS$_6$
p < 0.00005
Nº of genomes with TilS$_7$
Nº of genomes without TilS$_7$
With TilS$_7$
Without TilS$_7$
p < 0.00005
Nº of genomes with TilS$_8$
Nº of genomes without TilS$_8$
With TilS$_8$
Without TilS$_8$
p < 0.00005
Nº of genomes with TilS$_9$
Nº of genomes without TilS$_9$
With TilS$_9$
Without TilS$_9$
p < 0.00005
Nº of genomes with TilS$_{10}$
Nº of genomes without TilS$_{10}$
With TilS$_{10}$
Without TilS$_{10}$
p < 0.00005
Nº of genomes with TilS$_{11}$
Nº of genomes without TilS$_{11}$
With TilS$_{11}$
Without TilS$_{11}$
Nº of genomes with TilS$_{12}$
Nº of genomes without TilS$_{12}$
With TilS$_{12}$
Without TilS$_{12}$
p < 0.00005
Nº of genomes with TilS$_{13}$
Nº of genomes without TilS$_{13}$
With TilS$_{13}$
Without TilS$_{13}$
p < 0.00005
Nº of genomes with TilS$_{14}$
Nº of genomes without TilS$_{14}$
With TilS$_{14}$
Without TilS$_{14}$
Nº of genomes with TilS$_{15}$
Nº of genomes without TilS$_{15}$
With TilS$_{15}$
Without TilS$_{15}$
p < 0.00005

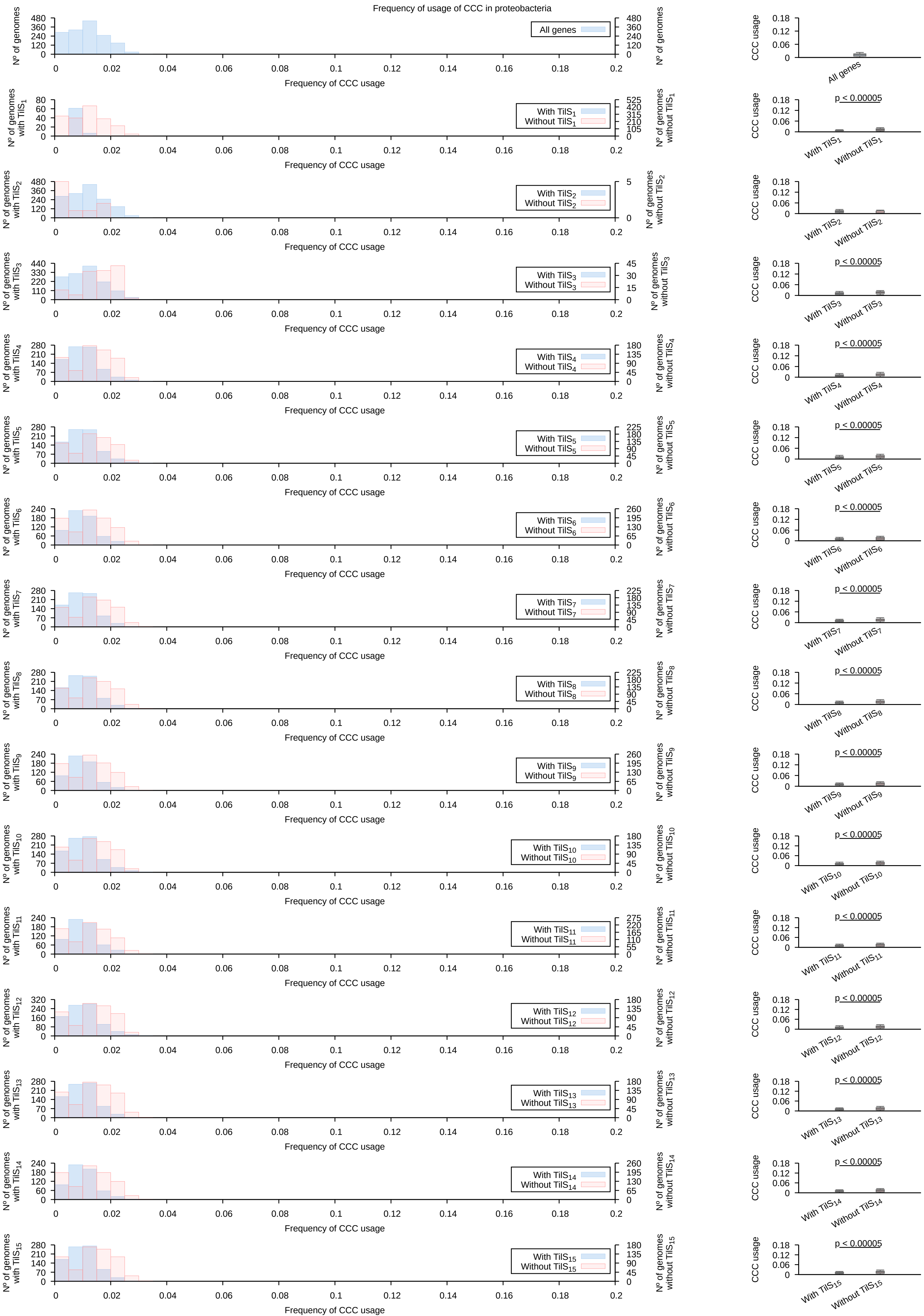
Frequency of usage of CCC in proteobacteria
Nº of genomes
All genes
Frequency of CCC usage
CCC usage
p < 0.00005
Nº of genomes with TilS$_1$
Nº of genomes without TilS$_1$
With TilS$_1$
Without TilS$_1$
Nº of genomes with TilS$_2$
Nº of genomes without TilS$_2$
With TilS$_2$
Without TilS$_2$
Nº of genomes with TilS$_3$
Nº of genomes without TilS$_3$
With TilS$_3$
Without TilS$_3$
Nº of genomes with TilS$_4$
Nº of genomes without TilS$_4$
With TilS$_4$
Without TilS$_4$
Nº of genomes with TilS$_5$
Nº of genomes without TilS$_5$
With TilS$_5$
Without TilS$_5$
Nº of genomes with TilS$_6$
Nº of genomes without TilS$_6$
With TilS$_6$
Without TilS$_6$
Nº of genomes with TilS$_7$
Nº of genomes without TilS$_7$
With TilS$_7$
Without TilS$_7$
Nº of genomes with TilS$_8$
Nº of genomes without TilS$_8$
With TilS$_8$
Without TilS$_8$
Nº of genomes with TilS$_9$
Nº of genomes without TilS$_9$
With TilS$_9$
Without TilS$_9$
Nº of genomes with TilS$_{10}$
Nº of genomes without TilS$_{10}$
With TilS$_{10}$
Without TilS$_{10}$
Nº of genomes with TilS$_{11}$
Nº of genomes without TilS$_{11}$
With TilS$_{11}$
Without TilS$_{11}$
Nº of genomes with TilS$_{12}$
Nº of genomes without TilS$_{12}$
With TilS$_{12}$
Without TilS$_{12}$
Nº of genomes with TilS$_{13}$
Nº of genomes without TilS$_{13}$
With TilS$_{13}$
Without TilS$_{13}$
Nº of genomes with TilS$_{14}$
Nº of genomes without TilS$_{14}$
With TilS$_{14}$
Without TilS$_{14}$
Nº of genomes with TilS$_{15}$
Nº of genomes without TilS$_{15}$
With TilS$_{15}$
Without TilS$_{15}$

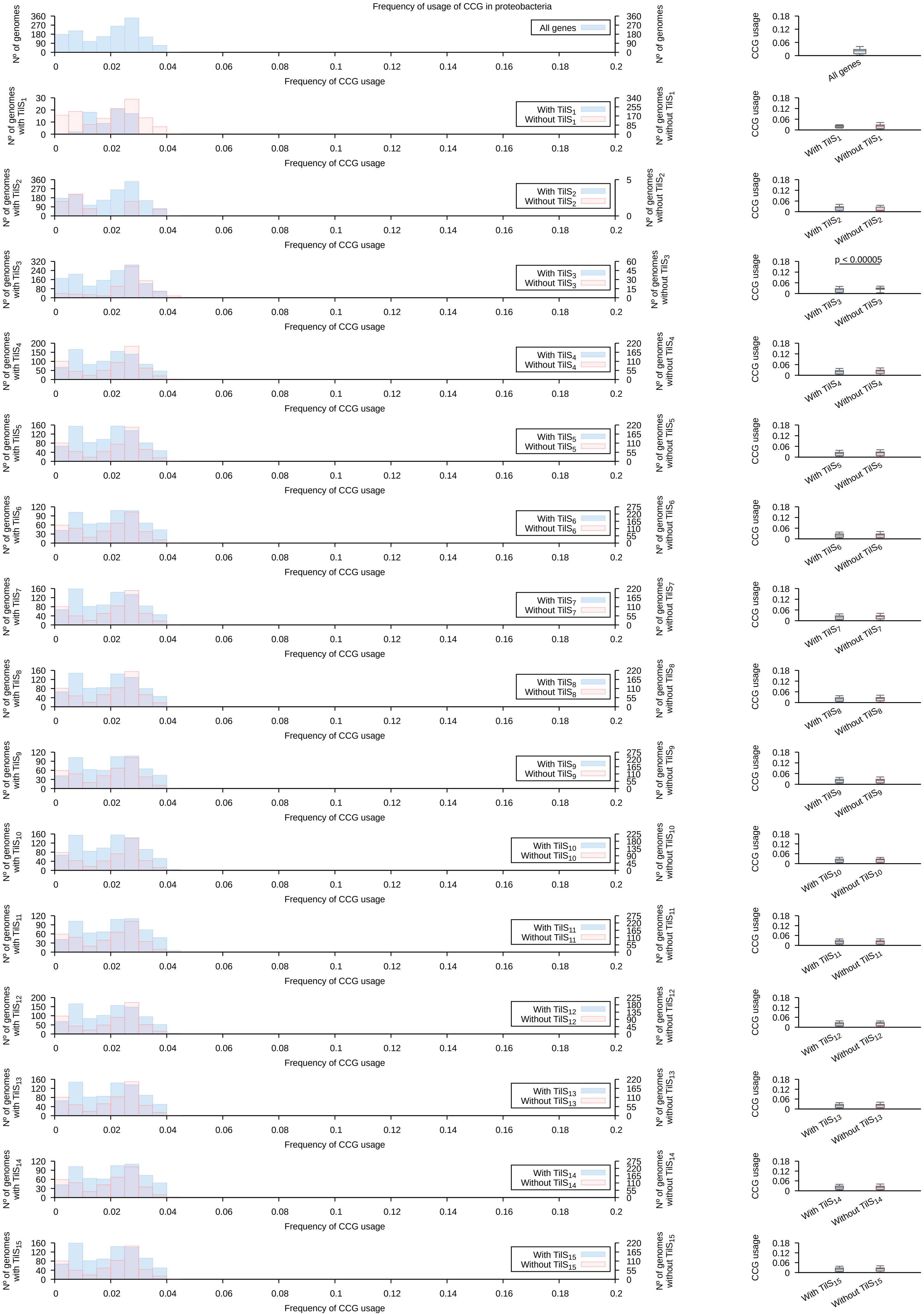

Frequency of usage of CCG in proteobacteria
Nº of genomes
All genes
Frequency of CCG usage
CCG usage
With TilS$_1$
Without TilS$_1$
Nº of genomes with TilS$_1$
Nº of genomes without TilS$_1$
With TilS$_2$
Without TilS$_2$
With TilS$_3$
Without TilS$_3$
p < 0.00005
With TilS$_4$
Without TilS$_4$
With TilS$_5$
Without TilS$_5$
With TilS$_6$
Without TilS$_6$
With TilS$_7$
Without TilS$_7$
With TilS$_8$
Without TilS$_8$
With TilS$_9$
Without TilS$_9$
With TilS$_{10}$
Without TilS$_{10}$
With TilS$_{11}$
Without TilS$_{11}$
With TilS$_{12}$
Without TilS$_{12}$
With TilS$_{13}$
Without TilS$_{13}$
With TilS$_{14}$
Without TilS$_{14}$
With TilS$_{15}$
Without TilS$_{15}$
0 0.02 0.04 0.06 0.08 0.1 0.12 0.14 0.16 0.18 0.2
0 0.06 0.12 0.18

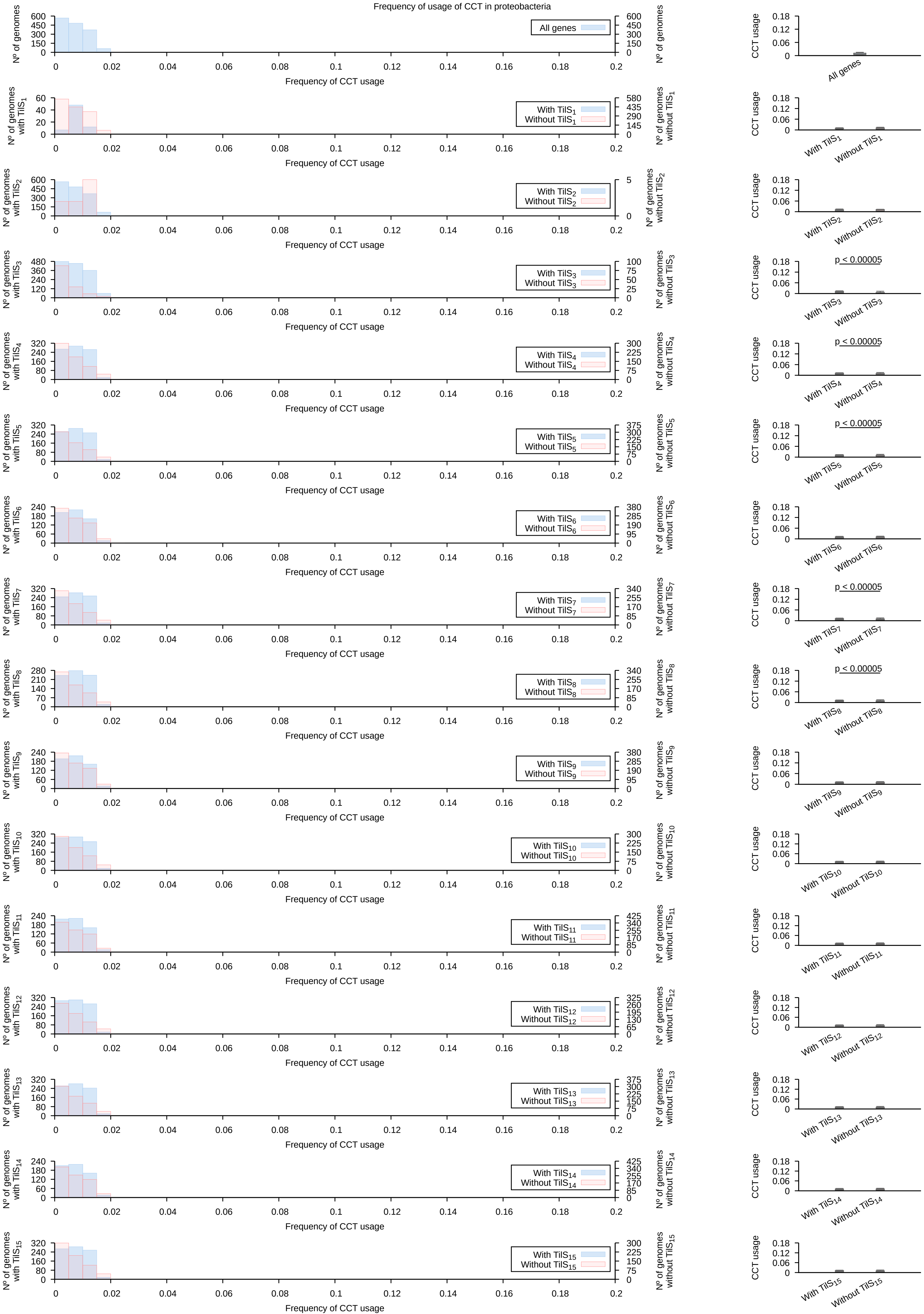
Frequency of usage of CCT in proteobacteria
Nº of genomes
All genes
Frequency of CCT usage
CCT usage
0 0.02 0.04 0.06 0.08 0.1 0.12 0.14 0.16 0.18 0.2
0 0.06 0.12 0.18
Nº of genomes with TilS1
Nº of genomes without TilS1
With TilS1
Without TilS1
Nº of genomes with TilS2
Nº of genomes without TilS2
With TilS2
Without TilS2
Nº of genomes with TilS3
Nº of genomes without TilS3
With TilS3
Without TilS3
p < 0.00005
Nº of genomes with TilS4
Nº of genomes without TilS4
With TilS4
Without TilS4
p < 0.00005
Nº of genomes with TilS5
Nº of genomes without TilS5
With TilS5
Without TilS5
p < 0.00005
Nº of genomes with TilS6
Nº of genomes without TilS6
With TilS6
Without TilS6
Nº of genomes with TilS7
Nº of genomes without TilS7
With TilS7
Without TilS7
p < 0.00005
Nº of genomes with TilS8
Nº of genomes without TilS8
With TilS8
Without TilS8
p < 0.00005
Nº of genomes with TilS9
Nº of genomes without TilS9
With TilS9
Without TilS9
Nº of genomes with TilS10
Nº of genomes without TilS10
With TilS10
Without TilS10
Nº of genomes with TilS11
Nº of genomes without TilS11
With TilS11
Without TilS11
Nº of genomes with TilS12
Nº of genomes without TilS12
With TilS12
Without TilS12
Nº of genomes with TilS13
Nº of genomes without TilS13
With TilS13
Without TilS13
Nº of genomes with TilS14
Nº of genomes without TilS14
With TilS14
Without TilS14
Nº of genomes with TilS15
Nº of genomes without TilS15
With TilS15
Without TilS15

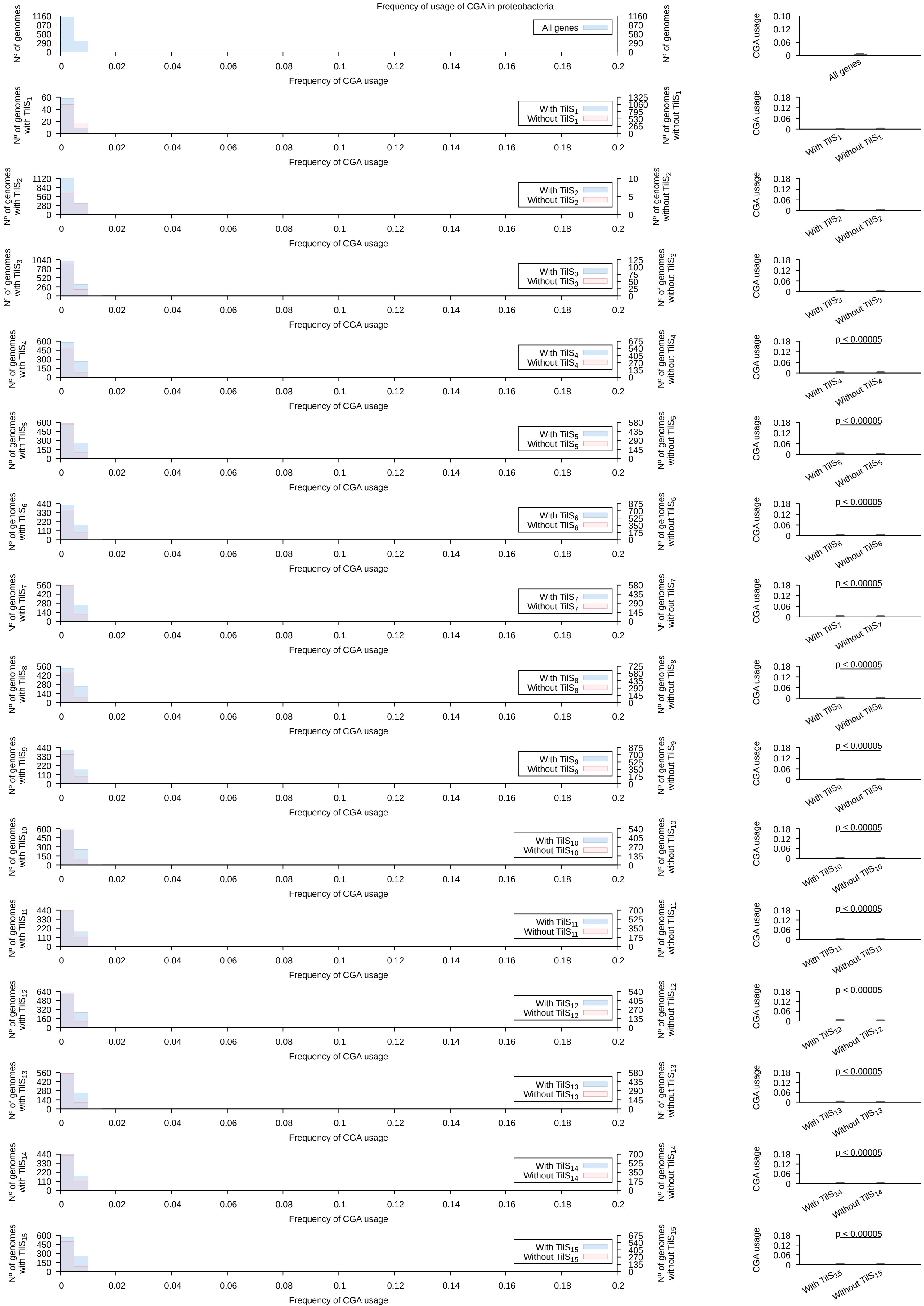

Frequency of usage of CGA in proteobacteria
Nº of genomes
All genes
Frequency of CGA usage
CGA usage
Nº of genomes with TilS1
Nº of genomes without TilS1
With TilS1
Without TilS1
Nº of genomes with TilS2
Nº of genomes without TilS2
With TilS2
Without TilS2
Nº of genomes with TilS3
Nº of genomes without TilS3
With TilS3
Without TilS3
Nº of genomes with TilS4
Nº of genomes without TilS4
With TilS4
Without TilS4
p < 0.00005
Nº of genomes with TilS5
Nº of genomes without TilS5
With TilS5
Without TilS5
Nº of genomes with TilS6
Nº of genomes without TilS6
With TilS6
Without TilS6
Nº of genomes with TilS7
Nº of genomes without TilS7
With TilS7
Without TilS7
Nº of genomes with TilS8
Nº of genomes without TilS8
With TilS8
Without TilS8
Nº of genomes with TilS9
Nº of genomes without TilS9
With TilS9
Without TilS9
Nº of genomes with TilS10
Nº of genomes without TilS10
With TilS10
Without TilS10
Nº of genomes with TilS11
Nº of genomes without TilS11
With TilS11
Without TilS11
Nº of genomes with TilS12
Nº of genomes without TilS12
With TilS12
Without TilS12
Nº of genomes with TilS13
Nº of genomes without TilS13
With TilS13
Without TilS13
Nº of genomes with TilS14
Nº of genomes without TilS14
With TilS14
Without TilS14
Nº of genomes with TilS15
Nº of genomes without TilS15
With TilS15
Without TilS15

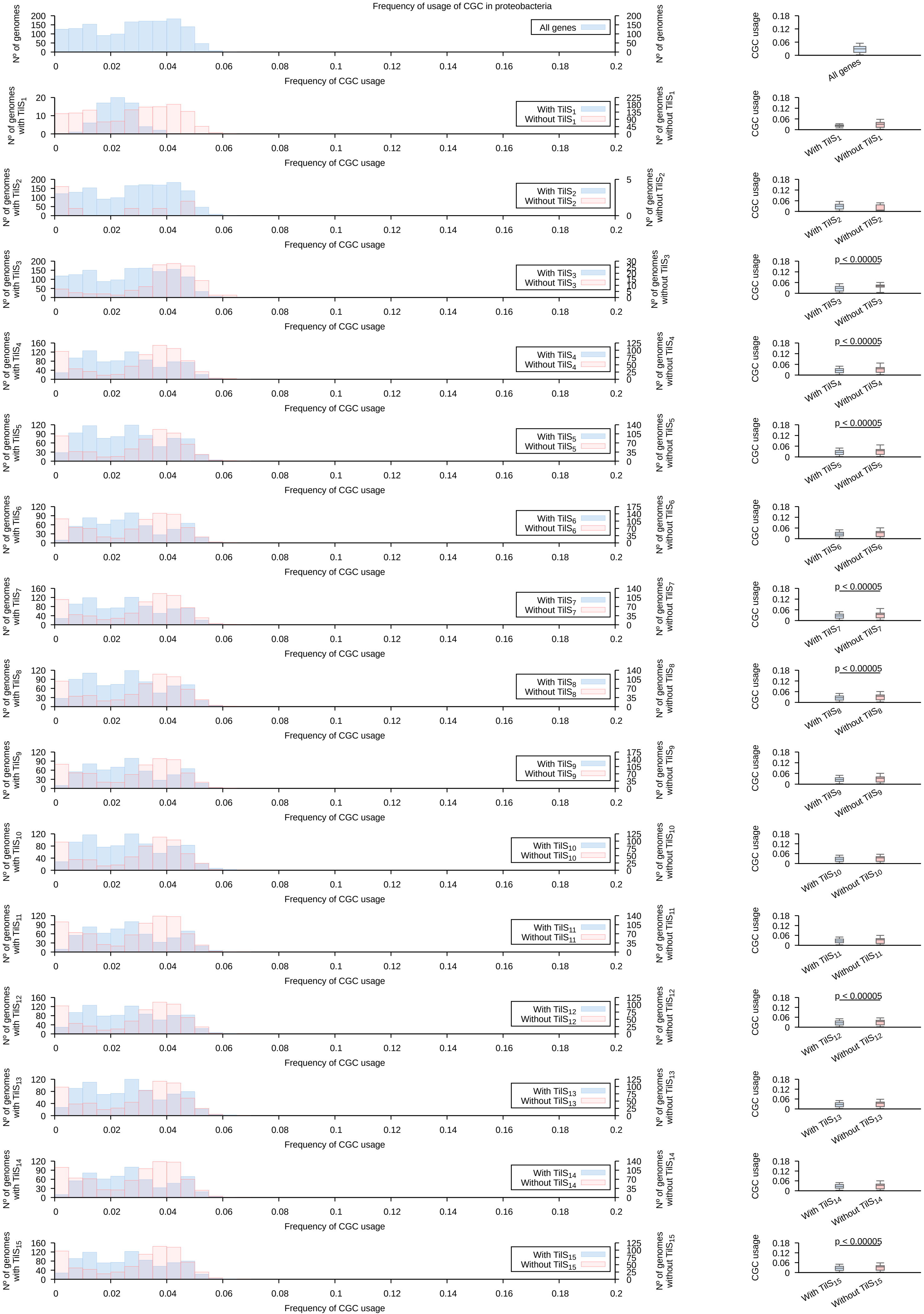
Frequency of usage of CGC in proteobacteria
Nº of genomes
All genes
Frequency of CGC usage
CGC usage
With TilS$_1$
Without TilS$_1$
Nº of genomes with TilS$_1$
Nº of genomes without TilS$_1$
With TilS$_2$
Without TilS$_2$
Nº of genomes with TilS$_2$
Nº of genomes without TilS$_2$
With TilS$_3$
Without TilS$_3$
p < 0.00005
With TilS$_4$
Without TilS$_4$
p < 0.00005
With TilS$_5$
Without TilS$_5$
p < 0.00005
With TilS$_6$
Without TilS$_6$
With TilS$_7$
Without TilS$_7$
p < 0.00005
With TilS$_8$
Without TilS$_8$
p < 0.00005
With TilS$_9$
Without TilS$_9$
With TilS$_{10}$
Without TilS$_{10}$
With TilS$_{11}$
Without TilS$_{11}$
With TilS$_{12}$
Without TilS$_{12}$
p < 0.00005
With TilS$_{13}$
Without TilS$_{13}$
With TilS$_{14}$
Without TilS$_{14}$
With TilS$_{15}$
Without TilS$_{15}$
p < 0.00005

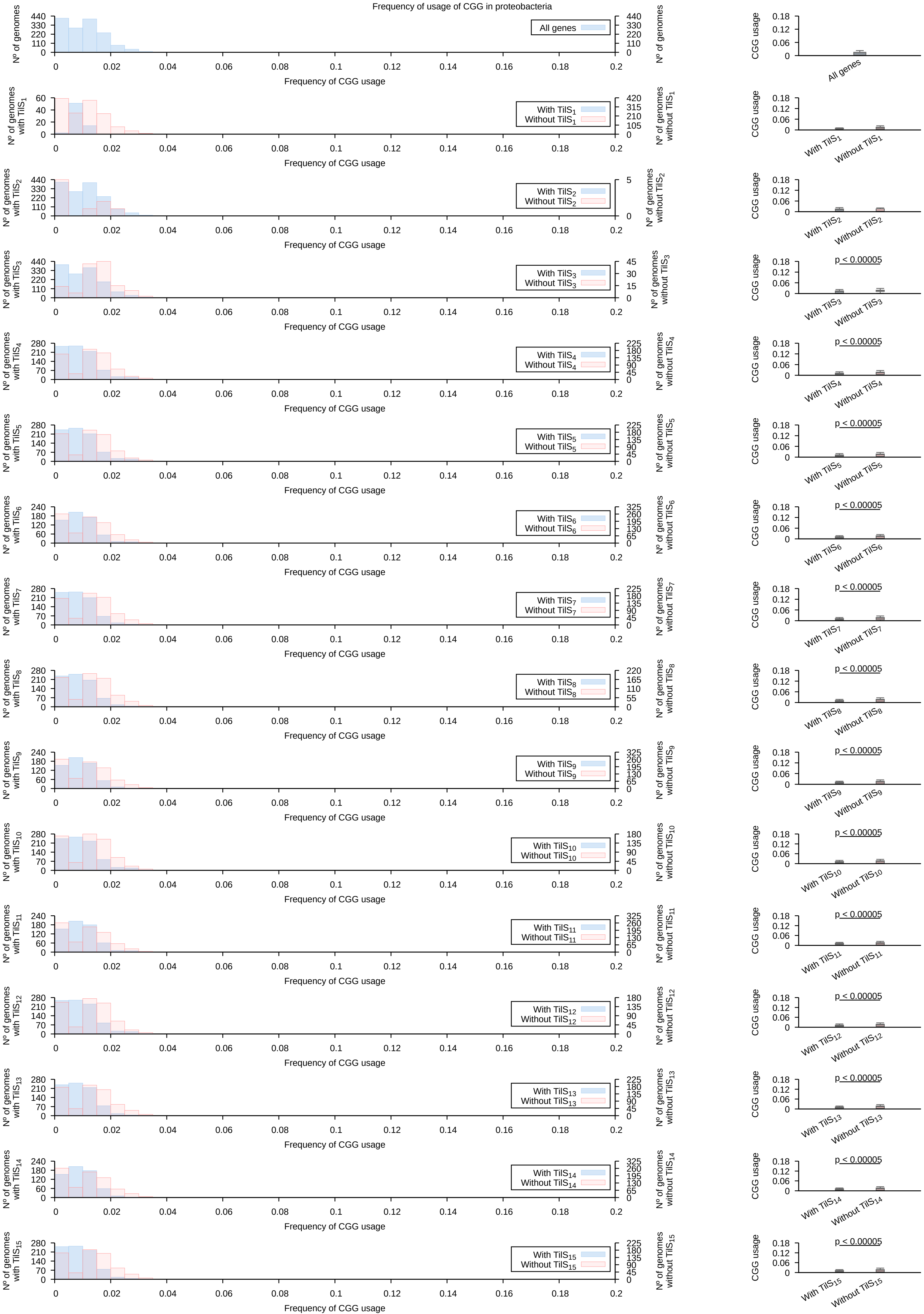

Frequency of usage of CGG in proteobacteria
Nº of genomes
All genes
Frequency of CGG usage
CGG usage
Nº of genomes with TilS1
Nº of genomes without TilS1
With TilS1
Without TilS1
Nº of genomes with TilS2
Nº of genomes without TilS2
With TilS2
Without TilS2
Nº of genomes with TilS3
Nº of genomes without TilS3
With TilS3
Without TilS3
Nº of genomes with TilS4
Nº of genomes without TilS4
With TilS4
Without TilS4
Nº of genomes with TilS5
Nº of genomes without TilS5
With TilS5
Without TilS5
Nº of genomes with TilS6
Nº of genomes without TilS6
With TilS6
Without TilS6
Nº of genomes with TilS7
Nº of genomes without TilS7
With TilS7
Without TilS7
Nº of genomes with TilS8
Nº of genomes without TilS8
With TilS8
Without TilS8
Nº of genomes with TilS9
Nº of genomes without TilS9
With TilS9
Without TilS9
Nº of genomes with TilS10
Nº of genomes without TilS10
With TilS10
Without TilS10
Nº of genomes with TilS11
Nº of genomes without TilS11
With TilS11
Without TilS11
Nº of genomes with TilS12
Nº of genomes without TilS12
With TilS12
Without TilS12
Nº of genomes with TilS13
Nº of genomes without TilS13
With TilS13
Without TilS13
Nº of genomes with TilS14
Nº of genomes without TilS14
With TilS14
Without TilS14
Nº of genomes with TilS15
Nº of genomes without TilS15
With TilS15
Without TilS15
p < 0.00005

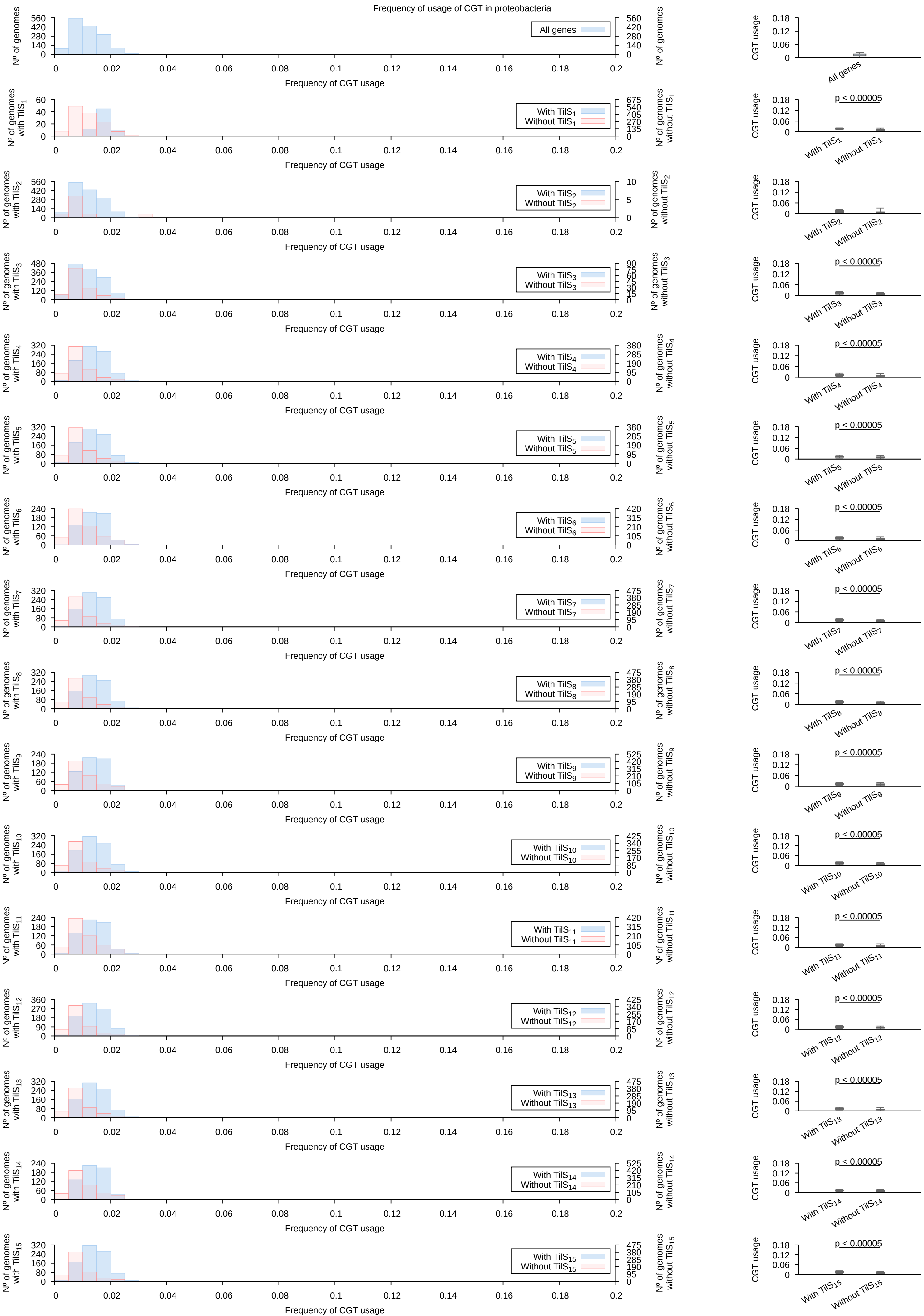
Frequency of usage of CGT in proteobacteria
Nº of genomes
All genes
Frequency of CGT usage
CGT usage
Nº of genomes with TilS1
Nº of genomes without TilS1
With TilS1
Without TilS1
p < 0.00005
Nº of genomes with TilS2
Nº of genomes without TilS2
With TilS2
Without TilS2
Nº of genomes with TilS3
Nº of genomes without TilS3
With TilS3
Without TilS3
Nº of genomes with TilS4
Nº of genomes without TilS4
With TilS4
Without TilS4
Nº of genomes with TilS5
Nº of genomes without TilS5
With TilS5
Without TilS5
Nº of genomes with TilS6
Nº of genomes without TilS6
With TilS6
Without TilS6
Nº of genomes with TilS7
Nº of genomes without TilS7
With TilS7
Without TilS7
Nº of genomes with TilS8
Nº of genomes without TilS8
With TilS8
Without TilS8
Nº of genomes with TilS9
Nº of genomes without TilS9
With TilS9
Without TilS9
Nº of genomes with TilS10
Nº of genomes without TilS10
With TilS10
Without TilS10
Nº of genomes with TilS11
Nº of genomes without TilS11
With TilS11
Without TilS11
Nº of genomes with TilS12
Nº of genomes without TilS12
With TilS12
Without TilS12
Nº of genomes with TilS13
Nº of genomes without TilS13
With TilS13
Without TilS13
Nº of genomes with TilS14
Nº of genomes without TilS14
With TilS14
Without TilS14
Nº of genomes with TilS15
Nº of genomes without TilS15
With TilS15
Without TilS15

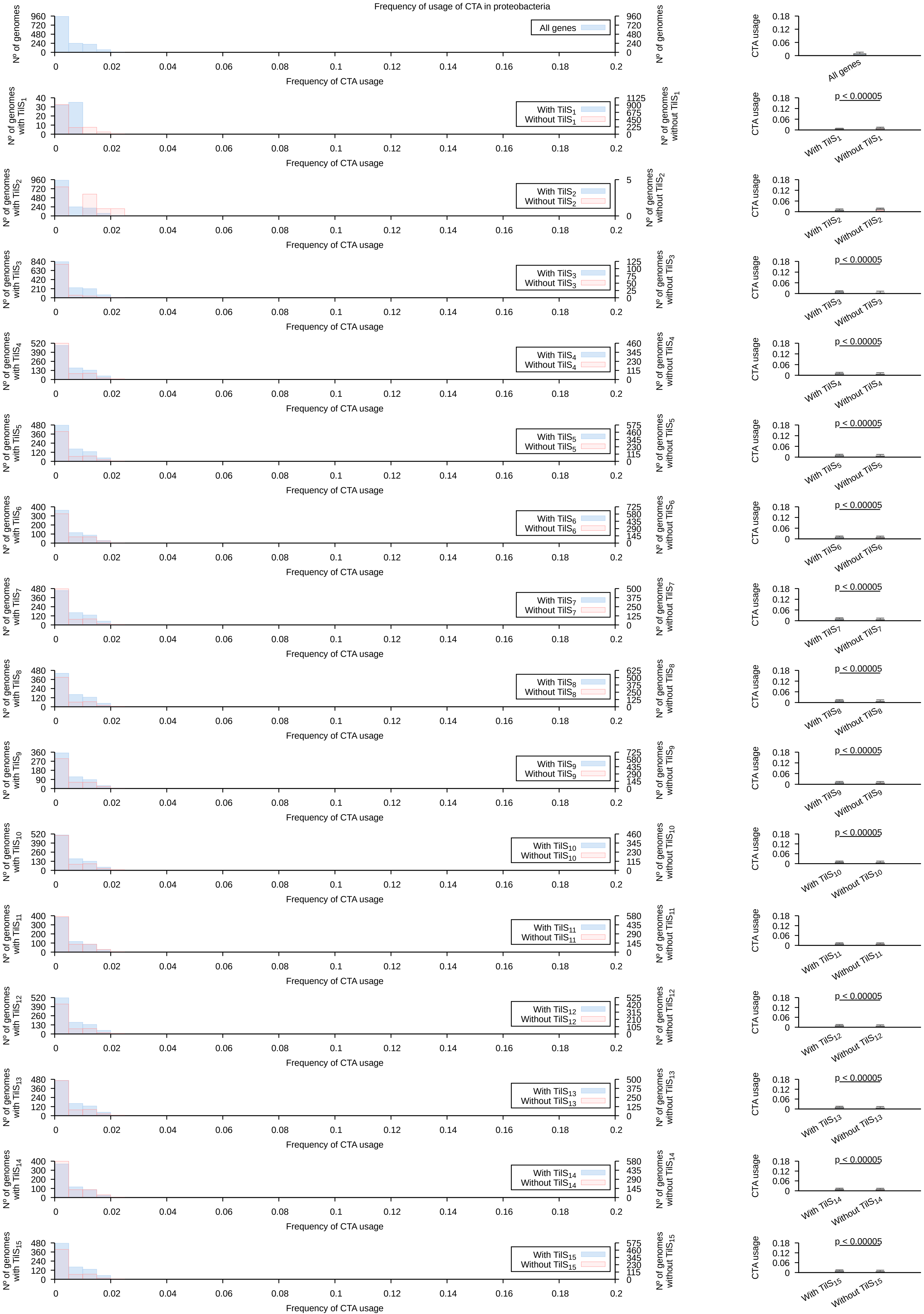
Frequency of usage of CTA in proteobacteria
Nº of genomes
Frequency of CTA usage
CTA usage
All genes
With TilS1
Without TilS1
Nº of genomes with TilS1
Nº of genomes without TilS1
p < 0.00005
With TilS2
Without TilS2
With TilS3
Without TilS3
With TilS4
Without TilS4
With TilS5
Without TilS5
With TilS6
Without TilS6
With TilS7
Without TilS7
With TilS8
Without TilS8
With TilS9
Without TilS9
With TilS10
Without TilS10
With TilS11
Without TilS11
With TilS12
Without TilS12
With TilS13
Without TilS13
With TilS14
Without TilS14
With TilS15
Without TilS15

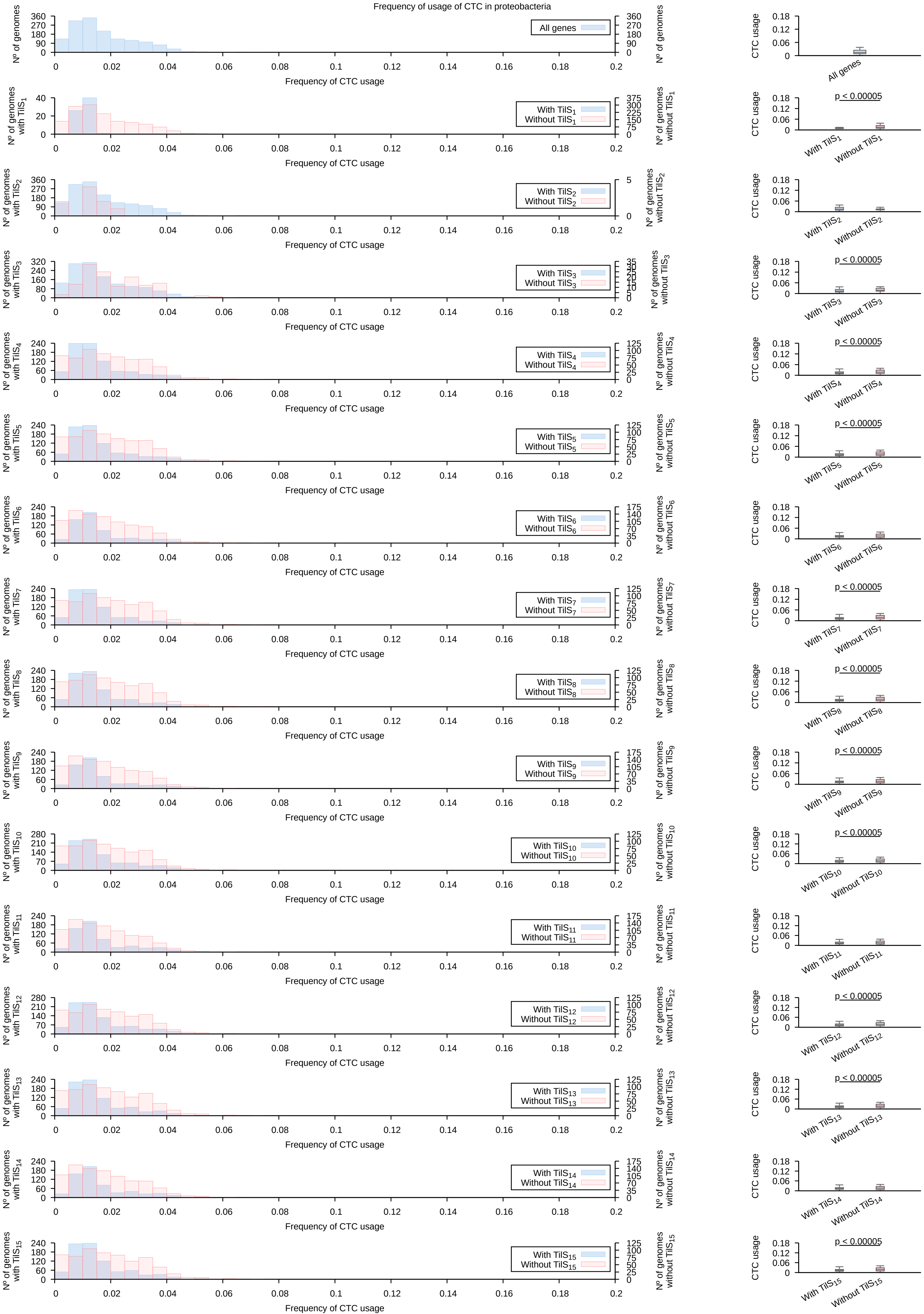

Frequency of usage of CTC in proteobacteria
Nº of genomes
All genes
Frequency of CTC usage
CTC usage
With TilS1
Without TilS1
p < 0.00005
With TilS2
Without TilS2
With TilS3
Without TilS3
With TilS4
Without TilS4
With TilS5
Without TilS5
With TilS6
Without TilS6
With TilS7
Without TilS7
With TilS8
Without TilS8
With TilS9
Without TilS9
With TilS10
Without TilS10
With TilS11
Without TilS11
With TilS12
Without TilS12
With TilS13
Without TilS13
With TilS14
Without TilS14
With TilS15
Without TilS15

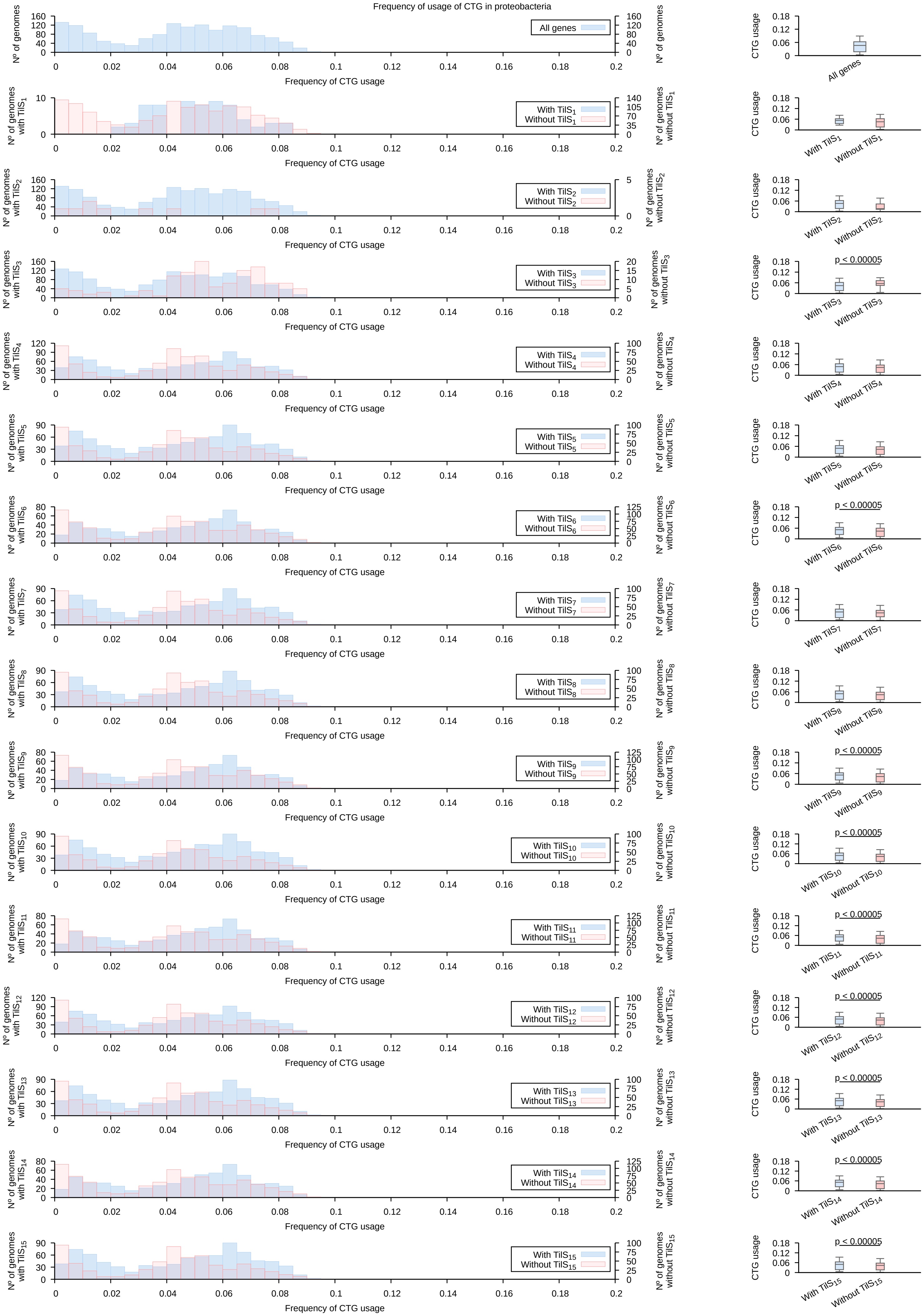
Frequency of usage of CTG in proteobacteria
Nº of genomes
All genes
Frequency of CTG usage
CTG usage
Nº of genomes with TilS$_1$
Nº of genomes without TilS$_1$
With TilS$_1$
Without TilS$_1$
Nº of genomes with TilS$_2$
Nº of genomes without TilS$_2$
With TilS$_2$
Without TilS$_2$
Nº of genomes with TilS$_3$
Nº of genomes without TilS$_3$
With TilS$_3$
Without TilS$_3$
p < 0.00005
Nº of genomes with TilS$_4$
Nº of genomes without TilS$_4$
With TilS$_4$
Without TilS$_4$
Nº of genomes with TilS$_5$
Nº of genomes without TilS$_5$
With TilS$_5$
Without TilS$_5$
Nº of genomes with TilS$_6$
Nº of genomes without TilS$_6$
With TilS$_6$
Without TilS$_6$
p < 0.00005
Nº of genomes with TilS$_7$
Nº of genomes without TilS$_7$
With TilS$_7$
Without TilS$_7$
Nº of genomes with TilS$_8$
Nº of genomes without TilS$_8$
With TilS$_8$
Without TilS$_8$
Nº of genomes with TilS$_9$
Nº of genomes without TilS$_9$
With TilS$_9$
Without TilS$_9$
p < 0.00005
Nº of genomes with TilS$_{10}$
Nº of genomes without TilS$_{10}$
With TilS$_{10}$
Without TilS$_{10}$
p < 0.00005
Nº of genomes with TilS$_{11}$
Nº of genomes without TilS$_{11}$
With TilS$_{11}$
Without TilS$_{11}$
p < 0.00005
Nº of genomes with TilS$_{12}$
Nº of genomes without TilS$_{12}$
With TilS$_{12}$
Without TilS$_{12}$
p < 0.00005
Nº of genomes with TilS$_{13}$
Nº of genomes without TilS$_{13}$
With TilS$_{13}$
Without TilS$_{13}$
p < 0.00005
Nº of genomes with TilS$_{14}$
Nº of genomes without TilS$_{14}$
With TilS$_{14}$
Without TilS$_{14}$
p < 0.00005
Nº of genomes with TilS$_{15}$
Nº of genomes without TilS$_{15}$
With TilS$_{15}$
Without TilS$_{15}$
p < 0.00005

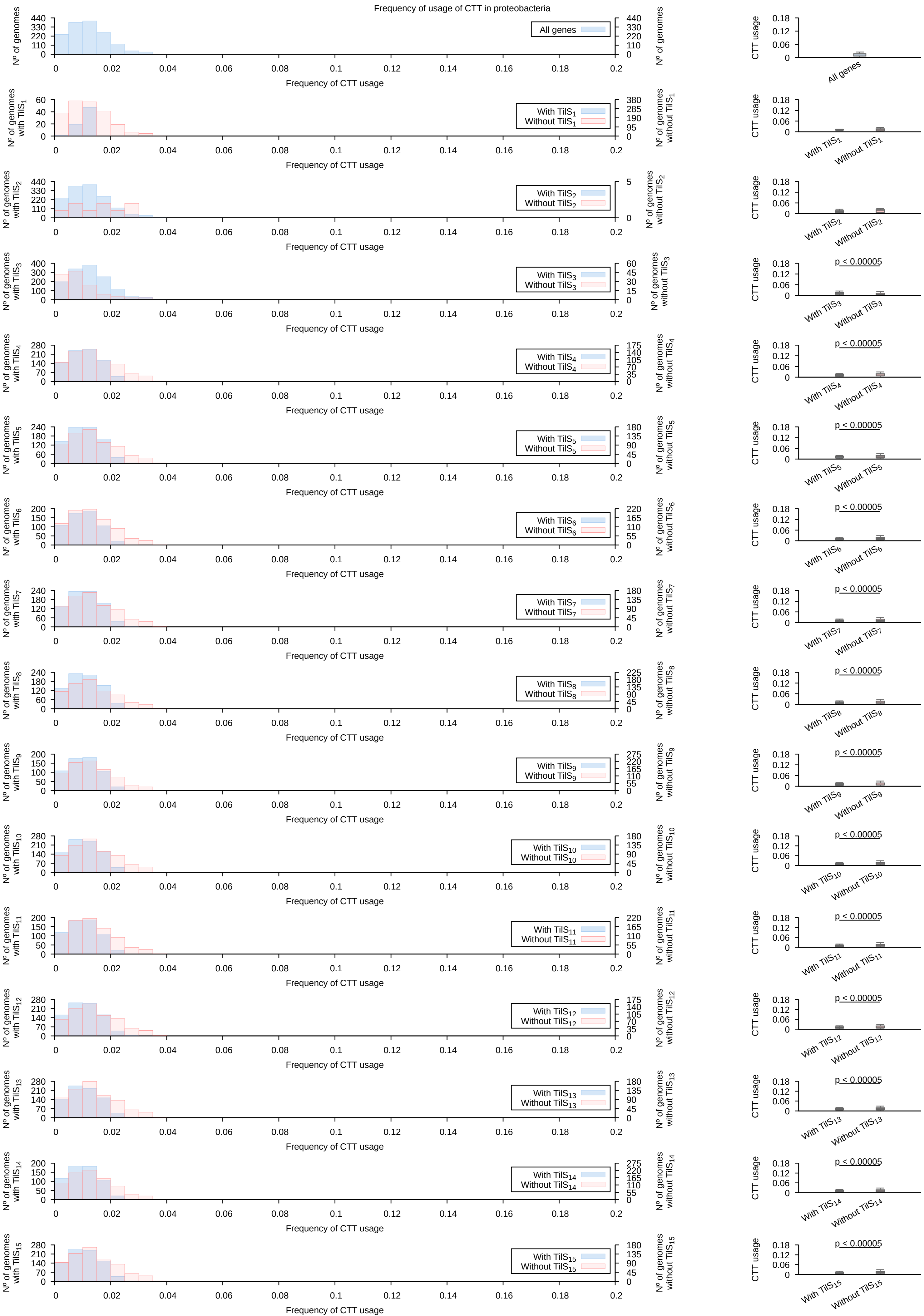

Frequency of usage of CTT in proteobacteria
Nº of genomes
Frequency of CTT usage
CTT usage
All genes
With TilS$_1$
Without TilS$_1$
With TilS$_2$
Without TilS$_2$
With TilS$_3$
Without TilS$_3$
With TilS$_4$
Without TilS$_4$
With TilS$_5$
Without TilS$_5$
With TilS$_6$
Without TilS$_6$
With TilS$_7$
Without TilS$_7$
With TilS$_8$
Without TilS$_8$
With TilS$_9$
Without TilS$_9$
With TilS$_{10}$
Without TilS$_{10}$
With TilS$_{11}$
Without TilS$_{11}$
With TilS$_{12}$
Without TilS$_{12}$
With TilS$_{13}$
Without TilS$_{13}$
With TilS$_{14}$
Without TilS$_{14}$
With TilS$_{15}$
Without TilS$_{15}$
$p < 0.00005$

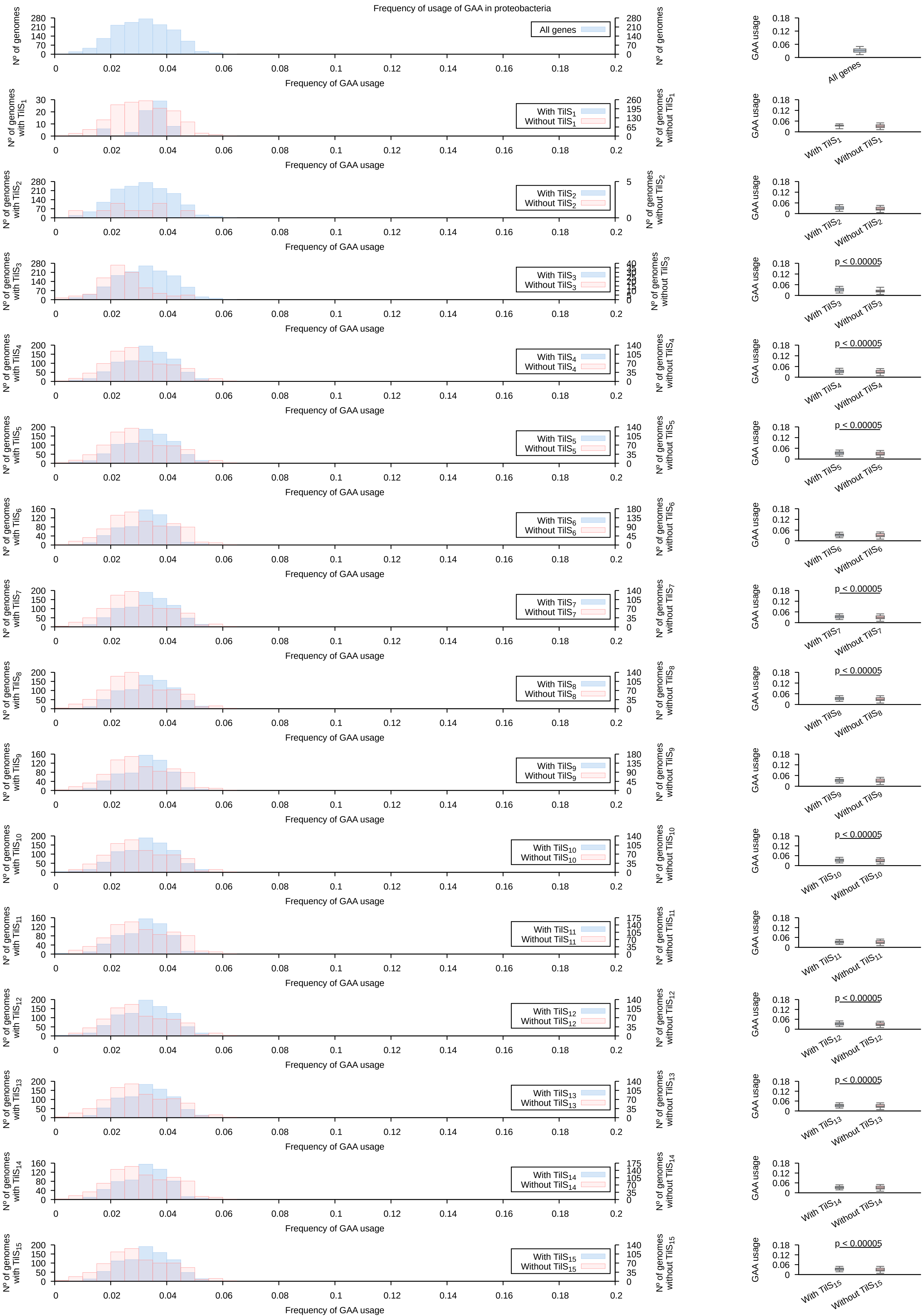

Frequency of usage of GAA in proteobacteria
Nº of genomes
Frequency of GAA usage
All genes
GAA usage
Nº of genomes with TilS1
Nº of genomes without TilS1
With TilS1
Without TilS1
Nº of genomes with TilS2
Nº of genomes without TilS2
With TilS2
Without TilS2
Nº of genomes with TilS3
Nº of genomes without TilS3
With TilS3
Without TilS3
p < 0.00005
Nº of genomes with TilS4
Nº of genomes without TilS4
With TilS4
Without TilS4
p < 0.00005
Nº of genomes with TilS5
Nº of genomes without TilS5
With TilS5
Without TilS5
p < 0.00005
Nº of genomes with TilS6
Nº of genomes without TilS6
With TilS6
Without TilS6
Nº of genomes with TilS7
Nº of genomes without TilS7
With TilS7
Without TilS7
p < 0.00005
Nº of genomes with TilS8
Nº of genomes without TilS8
With TilS8
Without TilS8
p < 0.00005
Nº of genomes with TilS9
Nº of genomes without TilS9
With TilS9
Without TilS9
Nº of genomes with TilS10
Nº of genomes without TilS10
With TilS10
Without TilS10
p < 0.00005
Nº of genomes with TilS11
Nº of genomes without TilS11
With TilS11
Without TilS11
Nº of genomes with TilS12
Nº of genomes without TilS12
With TilS12
Without TilS12
p < 0.00005
Nº of genomes with TilS13
Nº of genomes without TilS13
With TilS13
Without TilS13
p < 0.00005
Nº of genomes with TilS14
Nº of genomes without TilS14
With TilS14
Without TilS14
Nº of genomes with TilS15
Nº of genomes without TilS15
With TilS15
Without TilS15
p < 0.00005

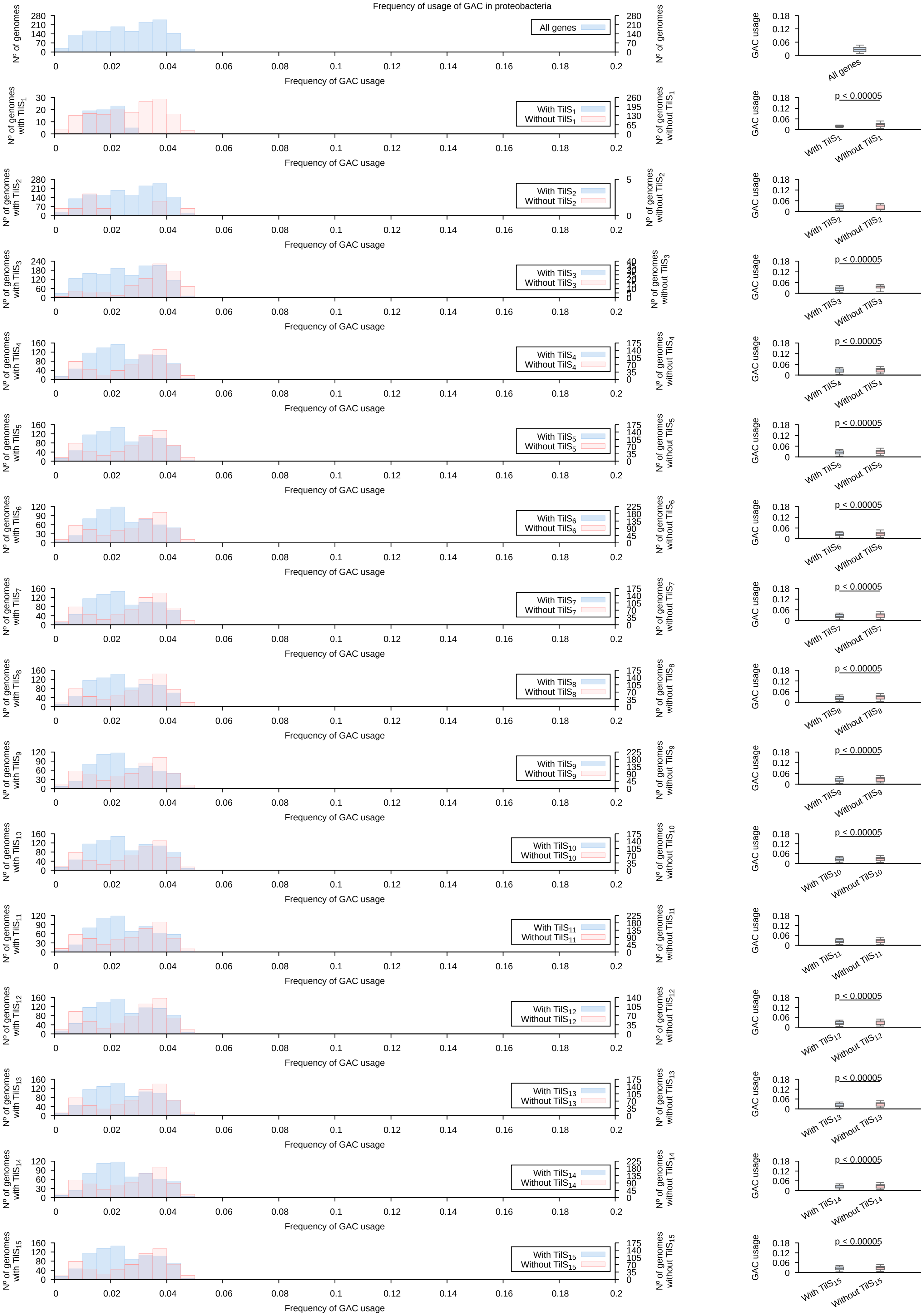

Frequency of usage of GAC in proteobacteria
Nº of genomes
All genes
Frequency of GAC usage
GAC usage
Nº of genomes with TilS1
Nº of genomes without TilS1
With TilS1
Without TilS1
p < 0.00005
With TilS2
Without TilS2
With TilS3
Without TilS3
With TilS4
Without TilS4
With TilS5
Without TilS5
With TilS6
Without TilS6
With TilS7
Without TilS7
With TilS8
Without TilS8
With TilS9
Without TilS9
With TilS10
Without TilS10
With TilS11
Without TilS11
With TilS12
Without TilS12
With TilS13
Without TilS13
With TilS14
Without TilS14
With TilS15
Without TilS15

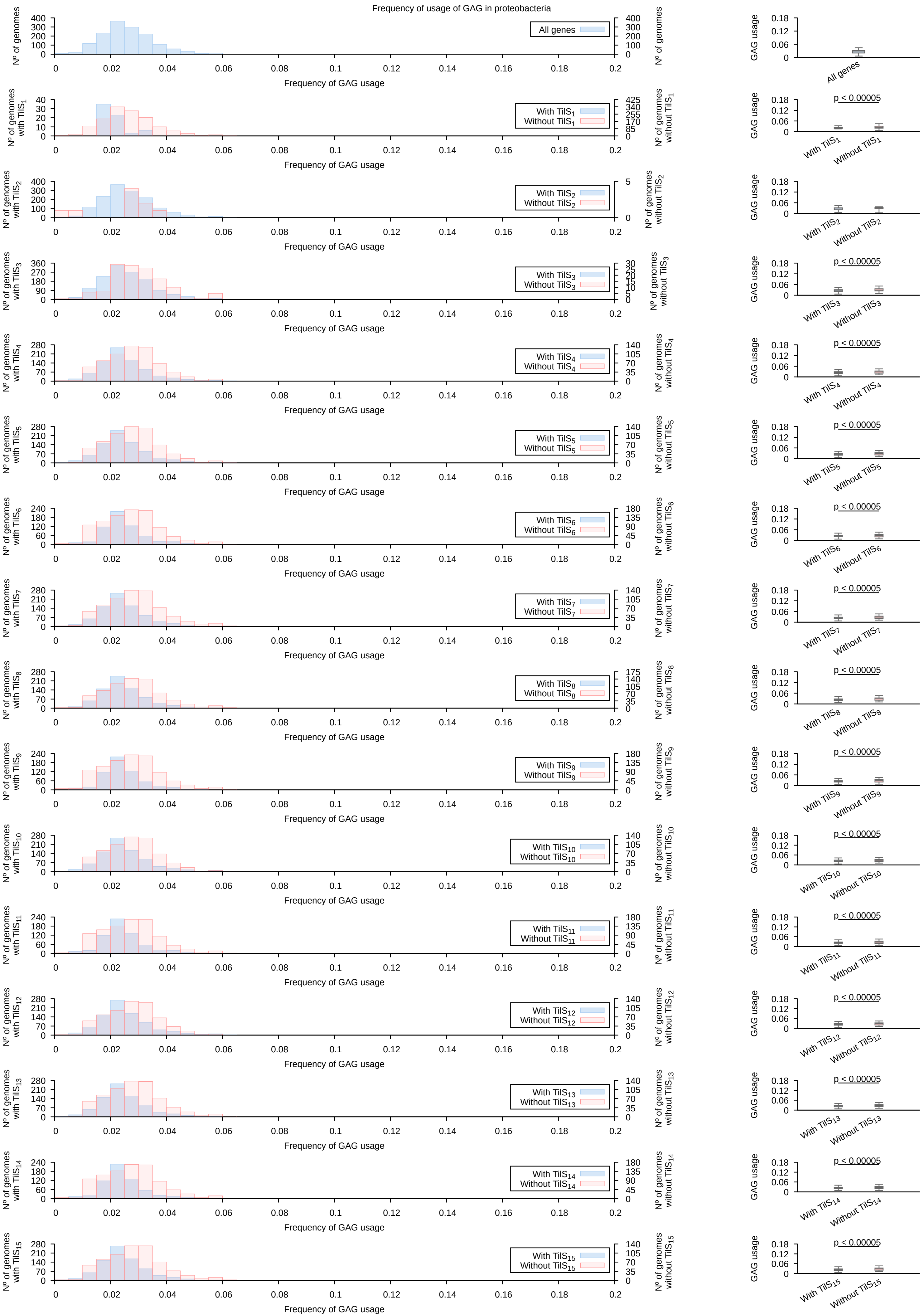

Frequency of usage of GAG in proteobacteria
Nº of genomes
All genes
Frequency of GAG usage
GAG usage
0 0.02 0.04 0.06 0.08 0.1 0.12 0.14 0.16 0.18 0.2
0 0.06 0.12 0.18
Nº of genomes with TilS$_1$
Nº of genomes without TilS$_1$
With TilS$_1$
Without TilS$_1$
p < 0.00005
Nº of genomes with TilS$_2$
Nº of genomes without TilS$_2$
With TilS$_2$
Without TilS$_2$
Nº of genomes with TilS$_3$
Nº of genomes without TilS$_3$
With TilS$_3$
Without TilS$_3$
p < 0.00005
Nº of genomes with TilS$_4$
Nº of genomes without TilS$_4$
With TilS$_4$
Without TilS$_4$
p < 0.00005
Nº of genomes with TilS$_5$
Nº of genomes without TilS$_5$
With TilS$_5$
Without TilS$_5$
p < 0.00005
Nº of genomes with TilS$_6$
Nº of genomes without TilS$_6$
With TilS$_6$
Without TilS$_6$
p < 0.00005
Nº of genomes with TilS$_7$
Nº of genomes without TilS$_7$
With TilS$_7$
Without TilS$_7$
p < 0.00005
Nº of genomes with TilS$_8$
Nº of genomes without TilS$_8$
With TilS$_8$
Without TilS$_8$
p < 0.00005
Nº of genomes with TilS$_9$
Nº of genomes without TilS$_9$
With TilS$_9$
Without TilS$_9$
p < 0.00005
Nº of genomes with TilS$_{10}$
Nº of genomes without TilS$_{10}$
With TilS$_{10}$
Without TilS$_{10}$
p < 0.00005
Nº of genomes with TilS$_{11}$
Nº of genomes without TilS$_{11}$
With TilS$_{11}$
Without TilS$_{11}$
p < 0.00005
Nº of genomes with TilS$_{12}$
Nº of genomes without TilS$_{12}$
With TilS$_{12}$
Without TilS$_{12}$
p < 0.00005
Nº of genomes with TilS$_{13}$
Nº of genomes without TilS$_{13}$
With TilS$_{13}$
Without TilS$_{13}$
p < 0.00005
Nº of genomes with TilS$_{14}$
Nº of genomes without TilS$_{14}$
With TilS$_{14}$
Without TilS$_{14}$
p < 0.00005
Nº of genomes with TilS$_{15}$
Nº of genomes without TilS$_{15}$
With TilS$_{15}$
Without TilS$_{15}$
p < 0.00005

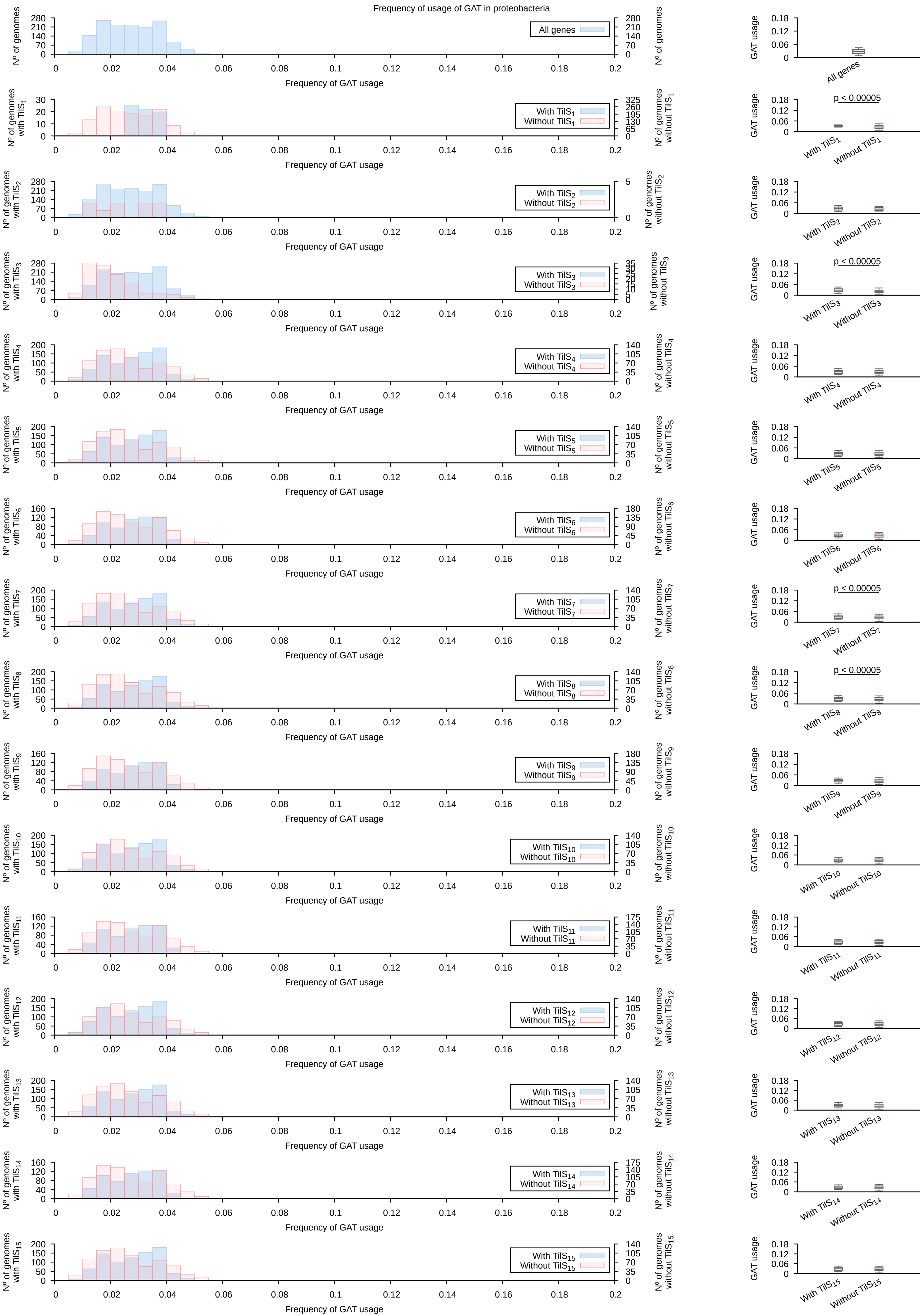

Frequency of usage of GAT in proteobacteria
Nº of genomes
All genes
Frequency of GAT usage
GAT usage
p < 0.00005
Nº of genomes with $TilS_1$
Nº of genomes without $TilS_1$
With $TilS_1$
Without $TilS_1$
With $TilS_2$
Without $TilS_2$
With $TilS_3$
Without $TilS_3$
With $TilS_4$
Without $TilS_4$
With $TilS_5$
Without $TilS_5$
With $TilS_6$
Without $TilS_6$
With $TilS_7$
Without $TilS_7$
With $TilS_8$
Without $TilS_8$
With $TilS_9$
Without $TilS_9$
With $TilS_{10}$
Without $TilS_{10}$
With $TilS_{11}$
Without $TilS_{11}$
With $TilS_{12}$
Without $TilS_{12}$
With $TilS_{13}$
Without $TilS_{13}$
With $TilS_{14}$
Without $TilS_{14}$
With $TilS_{15}$
Without $TilS_{15}$

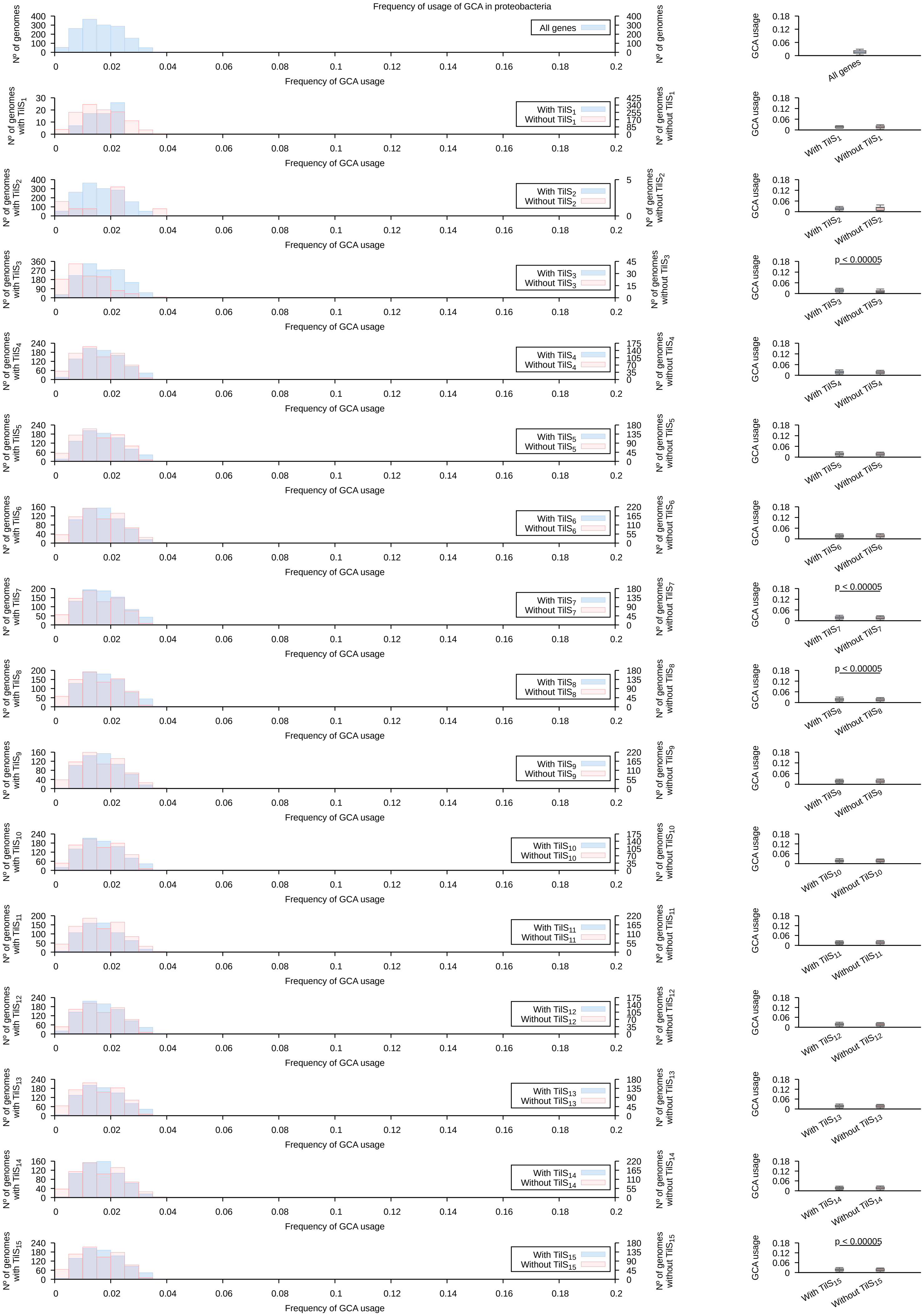

Frequency of usage of GCA in proteobacteria
Nº of genomes
All genes
Frequency of GCA usage
GCA usage
Nº of genomes with TilS$_1$
Nº of genomes without TilS$_1$
With TilS$_1$
Without TilS$_1$
Nº of genomes with TilS$_2$
Nº of genomes without TilS$_2$
With TilS$_2$
Without TilS$_2$
Nº of genomes with TilS$_3$
Nº of genomes without TilS$_3$
With TilS$_3$
Without TilS$_3$
p < 0.00005
Nº of genomes with TilS$_4$
Nº of genomes without TilS$_4$
With TilS$_4$
Without TilS$_4$
Nº of genomes with TilS$_5$
Nº of genomes without TilS$_5$
With TilS$_5$
Without TilS$_5$
Nº of genomes with TilS$_6$
Nº of genomes without TilS$_6$
With TilS$_6$
Without TilS$_6$
Nº of genomes with TilS$_7$
Nº of genomes without TilS$_7$
With TilS$_7$
Without TilS$_7$
p < 0.00005
Nº of genomes with TilS$_8$
Nº of genomes without TilS$_8$
With TilS$_8$
Without TilS$_8$
p < 0.00005
Nº of genomes with TilS$_9$
Nº of genomes without TilS$_9$
With TilS$_9$
Without TilS$_9$
Nº of genomes with TilS$_{10}$
Nº of genomes without TilS$_{10}$
With TilS$_{10}$
Without TilS$_{10}$
Nº of genomes with TilS$_{11}$
Nº of genomes without TilS$_{11}$
With TilS$_{11}$
Without TilS$_{11}$
Nº of genomes with TilS$_{12}$
Nº of genomes without TilS$_{12}$
With TilS$_{12}$
Without TilS$_{12}$
Nº of genomes with TilS$_{13}$
Nº of genomes without TilS$_{13}$
With TilS$_{13}$
Without TilS$_{13}$
Nº of genomes with TilS$_{14}$
Nº of genomes without TilS$_{14}$
With TilS$_{14}$
Without TilS$_{14}$
Nº of genomes with TilS$_{15}$
Nº of genomes without TilS$_{15}$
With TilS$_{15}$
Without TilS$_{15}$
p < 0.00005

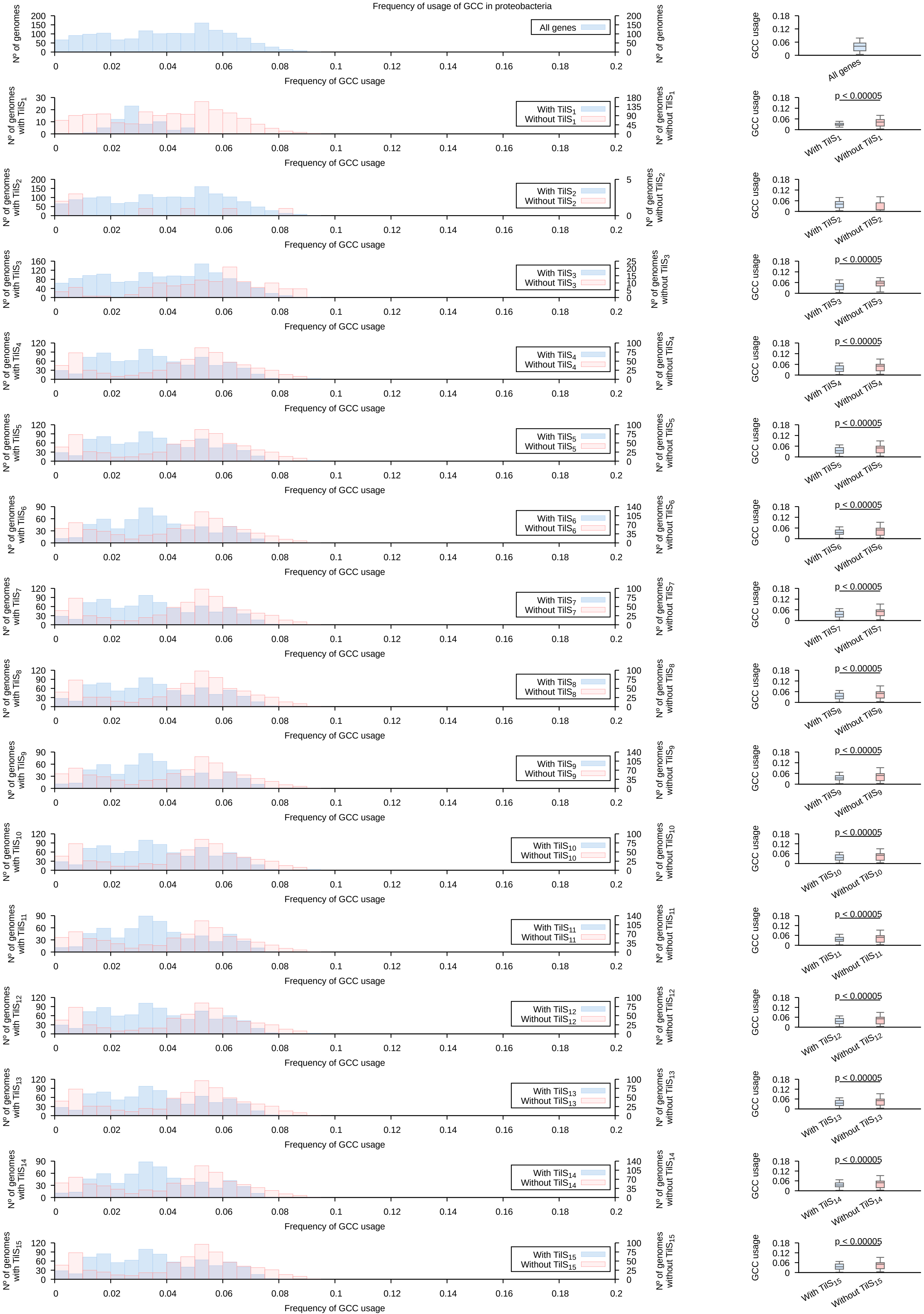
Frequency of usage of GCC in proteobacteria
All genes
Nº of genomes
Frequency of GCC usage
GCC usage
Nº of genomes with TilS$_1$
Nº of genomes without TilS$_1$
With TilS$_1$
Without TilS$_1$
p < 0.00005
With TilS$_2$
Without TilS$_2$
With TilS$_3$
Without TilS$_3$
With TilS$_4$
Without TilS$_4$
With TilS$_5$
Without TilS$_5$
With TilS$_6$
Without TilS$_6$
With TilS$_7$
Without TilS$_7$
With TilS$_8$
Without TilS$_8$
With TilS$_9$
Without TilS$_9$
With TilS$_{10}$
Without TilS$_{10}$
With TilS$_{11}$
Without TilS$_{11}$
With TilS$_{12}$
Without TilS$_{12}$
With TilS$_{13}$
Without TilS$_{13}$
With TilS$_{14}$
Without TilS$_{14}$
With TilS$_{15}$
Without TilS$_{15}$
0
0.02
0.04
0.06
0.08
0.1
0.12
0.14
0.16
0.18
0.2
0.06
0.12
0.18

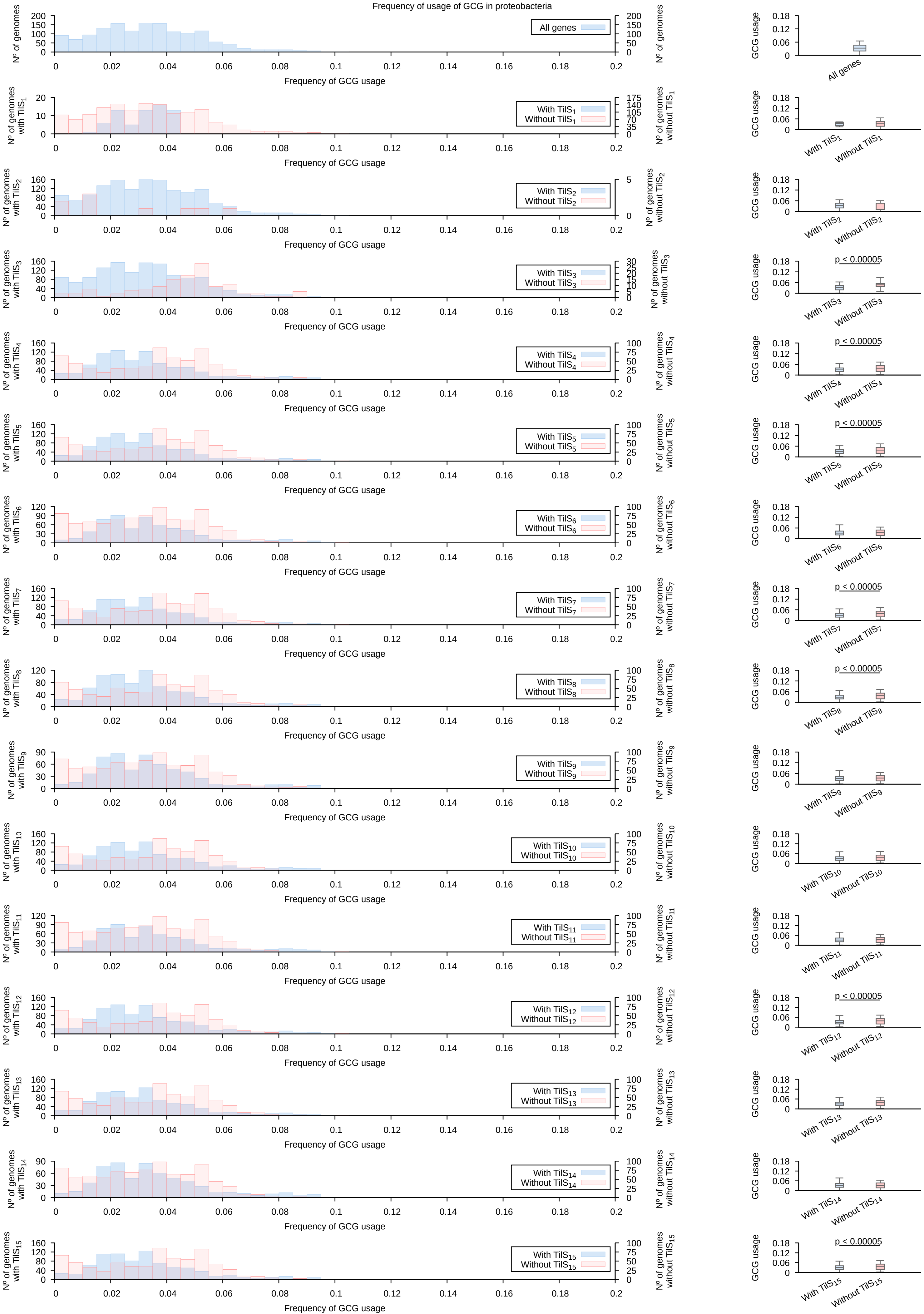
Frequency of usage of GCG in proteobacteria
Nº of genomes
All genes
Frequency of GCG usage
GCG usage
Nº of genomes with TilS1
Nº of genomes without TilS1
With TilS1
Without TilS1
Nº of genomes with TilS2
Nº of genomes without TilS2
With TilS2
Without TilS2
Nº of genomes with TilS3
Nº of genomes without TilS3
With TilS3
Without TilS3
p < 0.00005
Nº of genomes with TilS4
Nº of genomes without TilS4
With TilS4
Without TilS4
p < 0.00005
Nº of genomes with TilS5
Nº of genomes without TilS5
With TilS5
Without TilS5
p < 0.00005
Nº of genomes with TilS6
Nº of genomes without TilS6
With TilS6
Without TilS6
Nº of genomes with TilS7
Nº of genomes without TilS7
With TilS7
Without TilS7
p < 0.00005
Nº of genomes with TilS8
Nº of genomes without TilS8
With TilS8
Without TilS8
p < 0.00005
Nº of genomes with TilS9
Nº of genomes without TilS9
With TilS9
Without TilS9
Nº of genomes with TilS10
Nº of genomes without TilS10
With TilS10
Without TilS10
Nº of genomes with TilS11
Nº of genomes without TilS11
With TilS11
Without TilS11
Nº of genomes with TilS12
Nº of genomes without TilS12
With TilS12
Without TilS12
p < 0.00005
Nº of genomes with TilS13
Nº of genomes without TilS13
With TilS13
Without TilS13
Nº of genomes with TilS14
Nº of genomes without TilS14
With TilS14
Without TilS14
Nº of genomes with TilS15
Nº of genomes without TilS15
With TilS15
Without TilS15
p < 0.00005

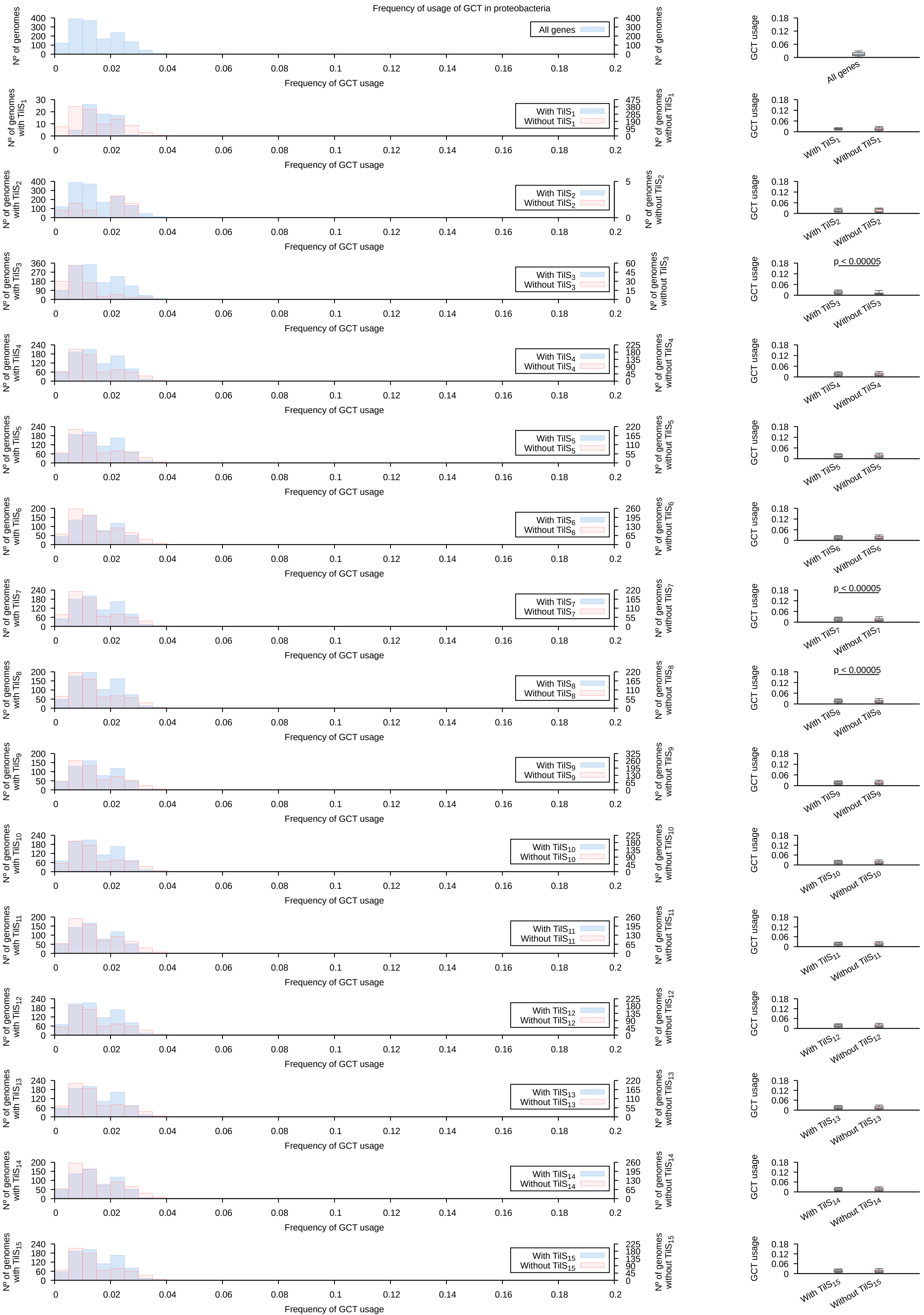
Frequency of usage of GCT in proteobacteria
Nº of genomes
All genes
Frequency of GCT usage
GCT usage
Nº of genomes with TilS$_1$
Nº of genomes without TilS$_1$
With TilS$_1$
Without TilS$_1$
Nº of genomes with TilS$_2$
Nº of genomes without TilS$_2$
With TilS$_2$
Without TilS$_2$
Nº of genomes with TilS$_3$
Nº of genomes without TilS$_3$
With TilS$_3$
Without TilS$_3$
p < 0.00005
Nº of genomes with TilS$_4$
Nº of genomes without TilS$_4$
With TilS$_4$
Without TilS$_4$
Nº of genomes with TilS$_5$
Nº of genomes without TilS$_5$
With TilS$_5$
Without TilS$_5$
Nº of genomes with TilS$_6$
Nº of genomes without TilS$_6$
With TilS$_6$
Without TilS$_6$
Nº of genomes with TilS$_7$
Nº of genomes without TilS$_7$
With TilS$_7$
Without TilS$_7$
p < 0.00005
Nº of genomes with TilS$_8$
Nº of genomes without TilS$_8$
With TilS$_8$
Without TilS$_8$
p < 0.00005
Nº of genomes with TilS$_9$
Nº of genomes without TilS$_9$
With TilS$_9$
Without TilS$_9$
Nº of genomes with TilS$_{10}$
Nº of genomes without TilS$_{10}$
With TilS$_{10}$
Without TilS$_{10}$
Nº of genomes with TilS$_{11}$
Nº of genomes without TilS$_{11}$
With TilS$_{11}$
Without TilS$_{11}$
Nº of genomes with TilS$_{12}$
Nº of genomes without TilS$_{12}$
With TilS$_{12}$
Without TilS$_{12}$
Nº of genomes with TilS$_{13}$
Nº of genomes without TilS$_{13}$
With TilS$_{13}$
Without TilS$_{13}$
Nº of genomes with TilS$_{14}$
Nº of genomes without TilS$_{14}$
With TilS$_{14}$
Without TilS$_{14}$
Nº of genomes with TilS$_{15}$
Nº of genomes without TilS$_{15}$
With TilS$_{15}$
Without TilS$_{15}$

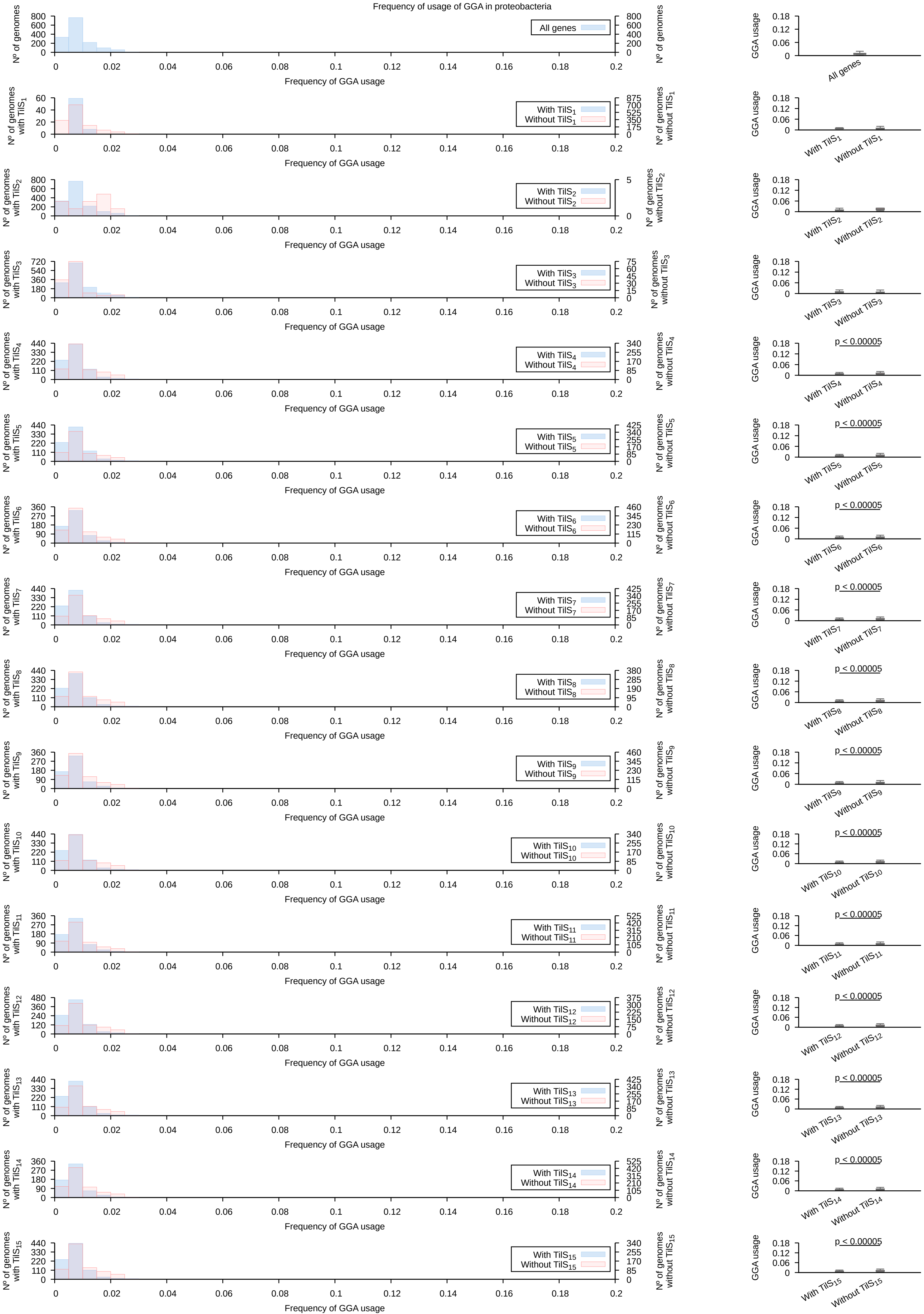
Frequency of usage of GGA in proteobacteria
All genes
Nº of genomes
Frequency of GGA usage
GGA usage
With TilS1
Without TilS1
Nº of genomes with TilS1
Nº of genomes without TilS1
With TilS2
Without TilS2
Nº of genomes with TilS2
Nº of genomes without TilS2
With TilS3
Without TilS3
Nº of genomes with TilS3
Nº of genomes without TilS3
With TilS4
Without TilS4
Nº of genomes with TilS4
Nº of genomes without TilS4
p < 0.00005
With TilS5
Without TilS5
Nº of genomes with TilS5
Nº of genomes without TilS5
p < 0.00005
With TilS6
Without TilS6
Nº of genomes with TilS6
Nº of genomes without TilS6
p < 0.00005
With TilS7
Without TilS7
Nº of genomes with TilS7
Nº of genomes without TilS7
p < 0.00005
With TilS8
Without TilS8
Nº of genomes with TilS8
Nº of genomes without TilS8
p < 0.00005
With TilS9
Without TilS9
Nº of genomes with TilS9
Nº of genomes without TilS9
p < 0.00005
With TilS10
Without TilS10
Nº of genomes with TilS10
Nº of genomes without TilS10
p < 0.00005
With TilS11
Without TilS11
Nº of genomes with TilS11
Nº of genomes without TilS11
p < 0.00005
With TilS12
Without TilS12
Nº of genomes with TilS12
Nº of genomes without TilS12
p < 0.00005
With TilS13
Without TilS13
Nº of genomes with TilS13
Nº of genomes without TilS13
p < 0.00005
With TilS14
Without TilS14
Nº of genomes with TilS14
Nº of genomes without TilS14
p < 0.00005
With TilS15
Without TilS15
Nº of genomes with TilS15
Nº of genomes without TilS15
p < 0.00005

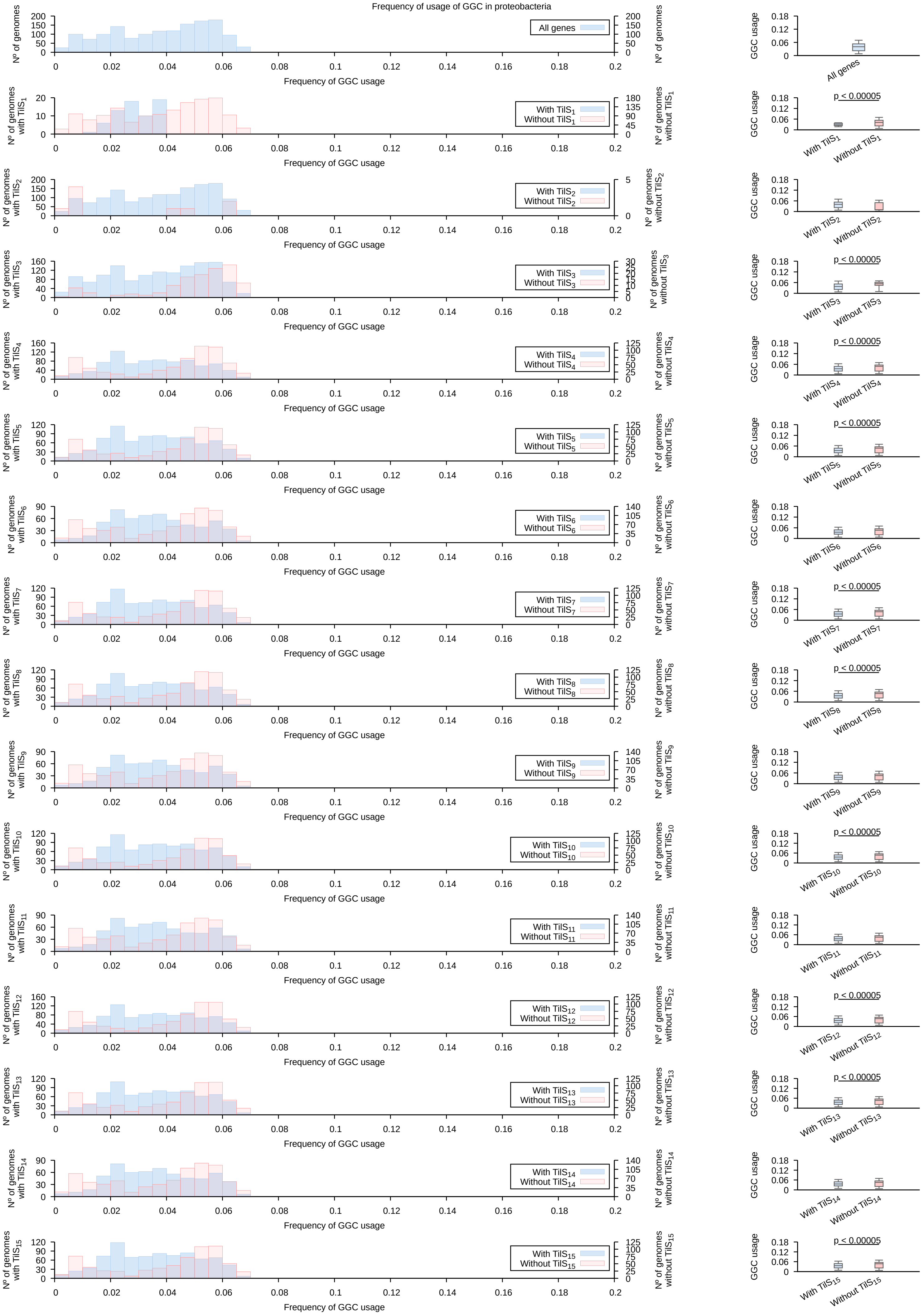
Frequency of usage of GGC in proteobacteria
Nº of genomes
All genes
Frequency of GGC usage
GGC usage
Nº of genomes with $TilS_1$
Nº of genomes without $TilS_1$
With $TilS_1$
Without $TilS_1$
p < 0.00005
Nº of genomes with $TilS_2$
Nº of genomes without $TilS_2$
With $TilS_2$
Without $TilS_2$
Nº of genomes with $TilS_3$
Nº of genomes without $TilS_3$
With $TilS_3$
Without $TilS_3$
p < 0.00005
Nº of genomes with $TilS_4$
Nº of genomes without $TilS_4$
With $TilS_4$
Without $TilS_4$
p < 0.00005
Nº of genomes with $TilS_5$
Nº of genomes without $TilS_5$
With $TilS_5$
Without $TilS_5$
p < 0.00005
Nº of genomes with $TilS_6$
Nº of genomes without $TilS_6$
With $TilS_6$
Without $TilS_6$
Nº of genomes with $TilS_7$
Nº of genomes without $TilS_7$
With $TilS_7$
Without $TilS_7$
p < 0.00005
Nº of genomes with $TilS_8$
Nº of genomes without $TilS_8$
With $TilS_8$
Without $TilS_8$
p < 0.00005
Nº of genomes with $TilS_9$
Nº of genomes without $TilS_9$
With $TilS_9$
Without $TilS_9$
Nº of genomes with $TilS_{10}$
Nº of genomes without $TilS_{10}$
With $TilS_{10}$
Without $TilS_{10}$
p < 0.00005
Nº of genomes with $TilS_{11}$
Nº of genomes without $TilS_{11}$
With $TilS_{11}$
Without $TilS_{11}$
Nº of genomes with $TilS_{12}$
Nº of genomes without $TilS_{12}$
With $TilS_{12}$
Without $TilS_{12}$
p < 0.00005
Nº of genomes with $TilS_{13}$
Nº of genomes without $TilS_{13}$
With $TilS_{13}$
Without $TilS_{13}$
p < 0.00005
Nº of genomes with $TilS_{14}$
Nº of genomes without $TilS_{14}$
With $TilS_{14}$
Without $TilS_{14}$
Nº of genomes with $TilS_{15}$
Nº of genomes without $TilS_{15}$
With $TilS_{15}$
Without $TilS_{15}$
p < 0.00005

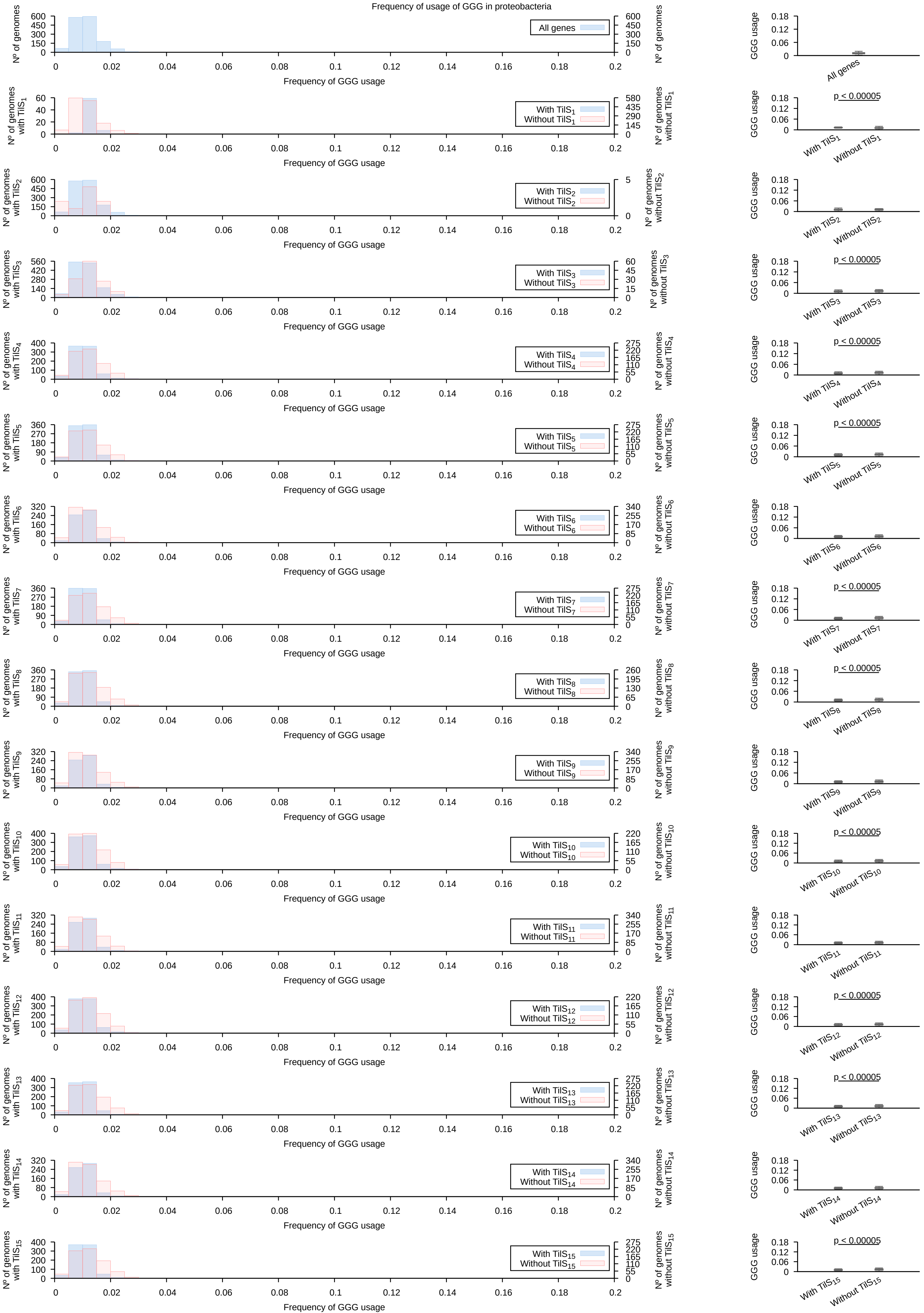
Frequency of usage of GGG in proteobacteria
Nº of genomes
All genes
Frequency of GGG usage
GGG usage
Nº of genomes with TilS1
Nº of genomes without TilS1
With TilS1
Without TilS1
p < 0.00005
Nº of genomes with TilS2
Nº of genomes without TilS2
With TilS2
Without TilS2
Nº of genomes with TilS3
Nº of genomes without TilS3
With TilS3
Without TilS3
p < 0.00005
Nº of genomes with TilS4
Nº of genomes without TilS4
With TilS4
Without TilS4
p < 0.00005
Nº of genomes with TilS5
Nº of genomes without TilS5
With TilS5
Without TilS5
p < 0.00005
Nº of genomes with TilS6
Nº of genomes without TilS6
With TilS6
Without TilS6
Nº of genomes with TilS7
Nº of genomes without TilS7
With TilS7
Without TilS7
p < 0.00005
Nº of genomes with TilS8
Nº of genomes without TilS8
With TilS8
Without TilS8
p < 0.00005
Nº of genomes with TilS9
Nº of genomes without TilS9
With TilS9
Without TilS9
Nº of genomes with TilS10
Nº of genomes without TilS10
With TilS10
Without TilS10
p < 0.00005
Nº of genomes with TilS11
Nº of genomes without TilS11
With TilS11
Without TilS11
Nº of genomes with TilS12
Nº of genomes without TilS12
With TilS12
Without TilS12
p < 0.00005
Nº of genomes with TilS13
Nº of genomes without TilS13
With TilS13
Without TilS13
p < 0.00005
Nº of genomes with TilS14
Nº of genomes without TilS14
With TilS14
Without TilS14
Nº of genomes with TilS15
Nº of genomes without TilS15
With TilS15
Without TilS15
p < 0.00005

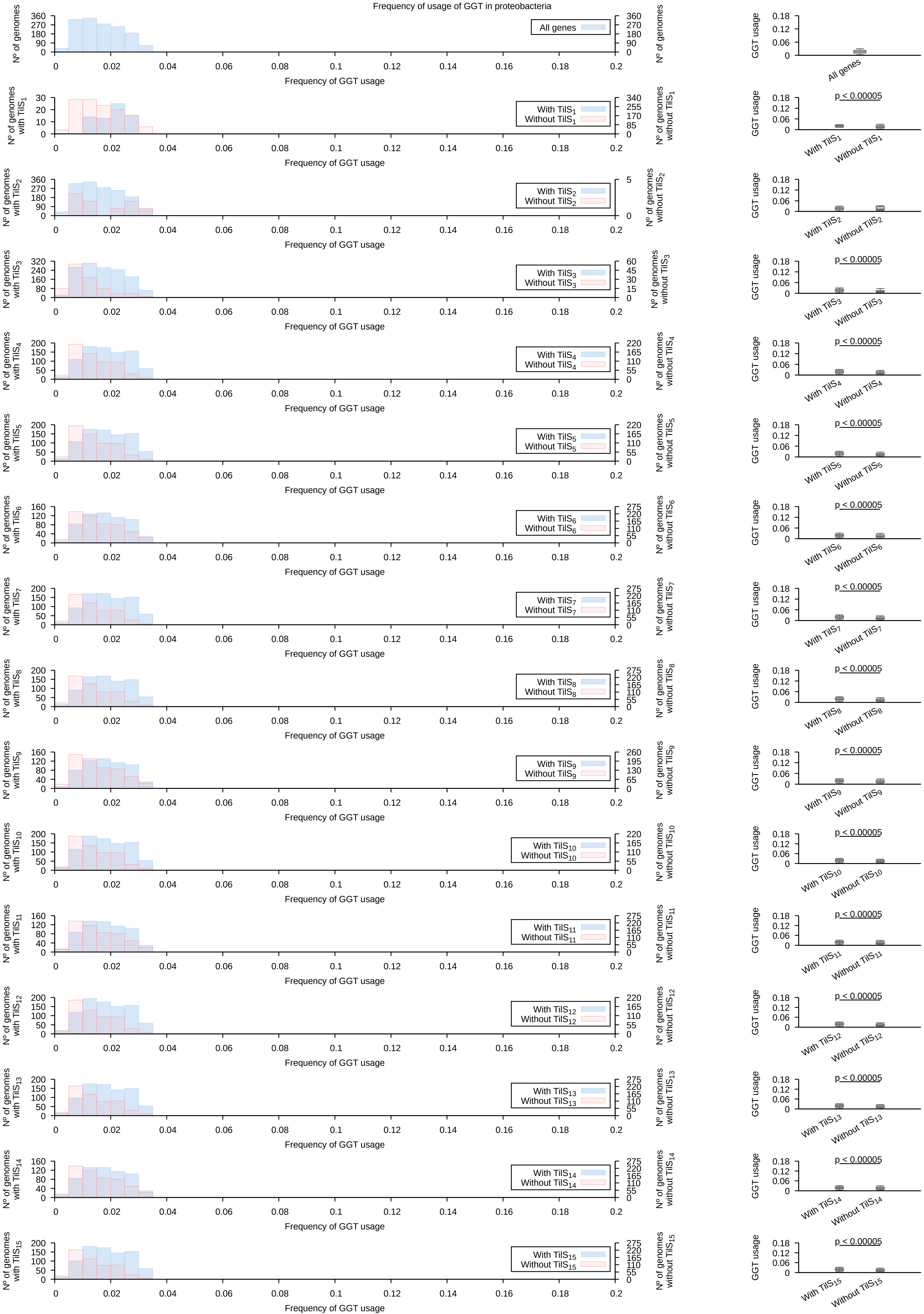
Frequency of usage of GGT in proteobacteria
Nº of genomes
All genes
Frequency of GGT usage
GGT usage
Nº of genomes with TilS1
Nº of genomes without TilS1
With TilS1
Without TilS1
p < 0.00005
Nº of genomes with TilS2
Nº of genomes without TilS2
With TilS2
Without TilS2
Nº of genomes with TilS3
Nº of genomes without TilS3
With TilS3
Without TilS3
p < 0.00005
Nº of genomes with TilS4
Nº of genomes without TilS4
With TilS4
Without TilS4
p < 0.00005
Nº of genomes with TilS5
Nº of genomes without TilS5
With TilS5
Without TilS5
p < 0.00005
Nº of genomes with TilS6
Nº of genomes without TilS6
With TilS6
Without TilS6
p < 0.00005
Nº of genomes with TilS7
Nº of genomes without TilS7
With TilS7
Without TilS7
p < 0.00005
Nº of genomes with TilS8
Nº of genomes without TilS8
With TilS8
Without TilS8
p < 0.00005
Nº of genomes with TilS9
Nº of genomes without TilS9
With TilS9
Without TilS9
p < 0.00005
Nº of genomes with TilS10
Nº of genomes without TilS10
With TilS10
Without TilS10
p < 0.00005
Nº of genomes with TilS11
Nº of genomes without TilS11
With TilS11
Without TilS11
p < 0.00005
Nº of genomes with TilS12
Nº of genomes without TilS12
With TilS12
Without TilS12
p < 0.00005
Nº of genomes with TilS13
Nº of genomes without TilS13
With TilS13
Without TilS13
p < 0.00005
Nº of genomes with TilS14
Nº of genomes without TilS14
With TilS14
Without TilS14
p < 0.00005
Nº of genomes with TilS15
Nº of genomes without TilS15
With TilS15
Without TilS15
p < 0.00005

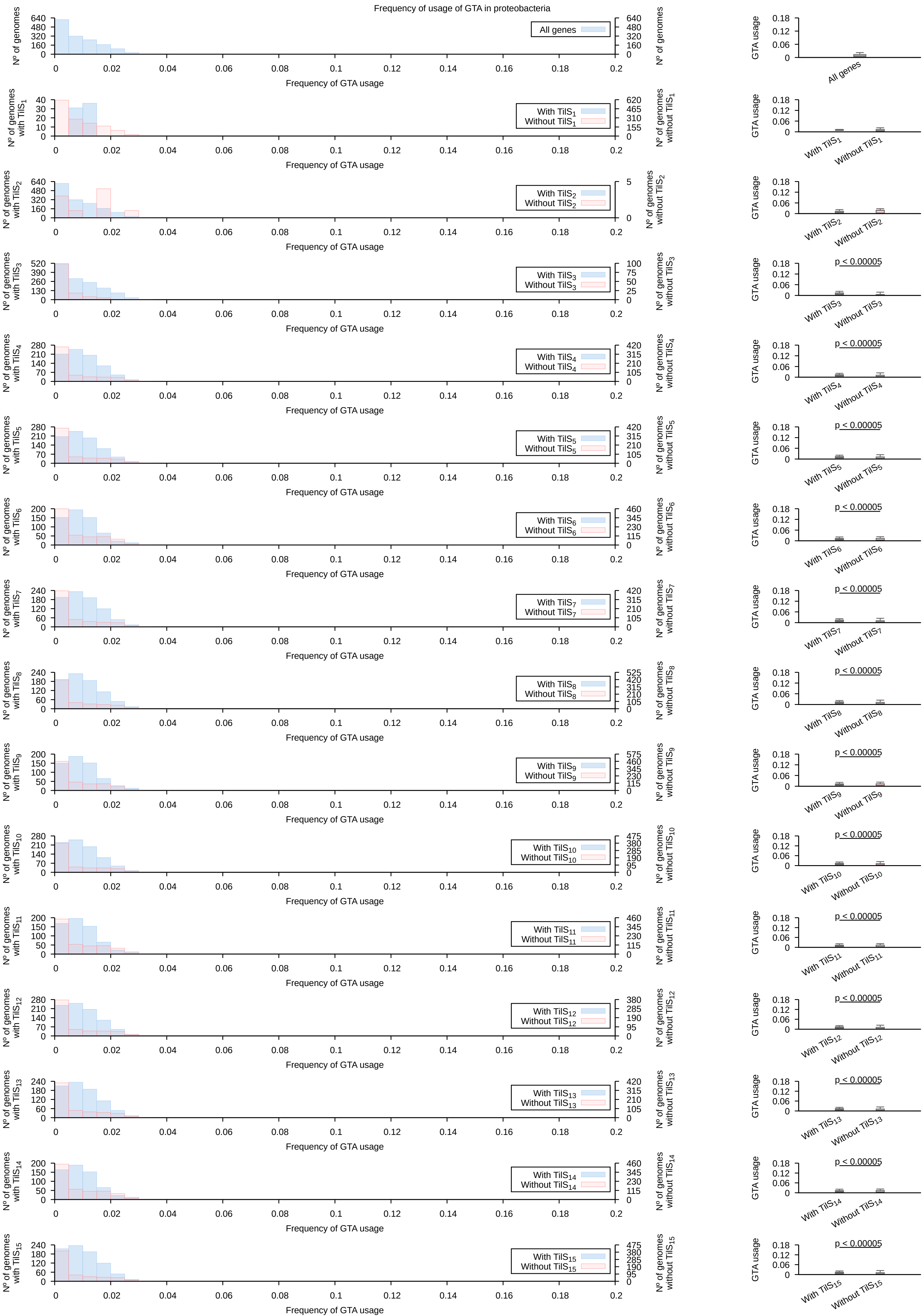
Frequency of usage of GTA in proteobacteria
Nº of genomes
Frequency of GTA usage
All genes
GTA usage
With TilS1
Without TilS1
With TilS2
Without TilS2
With TilS3
Without TilS3
With TilS4
Without TilS4
With TilS5
Without TilS5
With TilS6
Without TilS6
With TilS7
Without TilS7
With TilS8
Without TilS8
With TilS9
Without TilS9
With TilS10
Without TilS10
With TilS11
Without TilS11
With TilS12
Without TilS12
With TilS13
Without TilS13
With TilS14
Without TilS14
With TilS15
Without TilS15
p < 0.00005

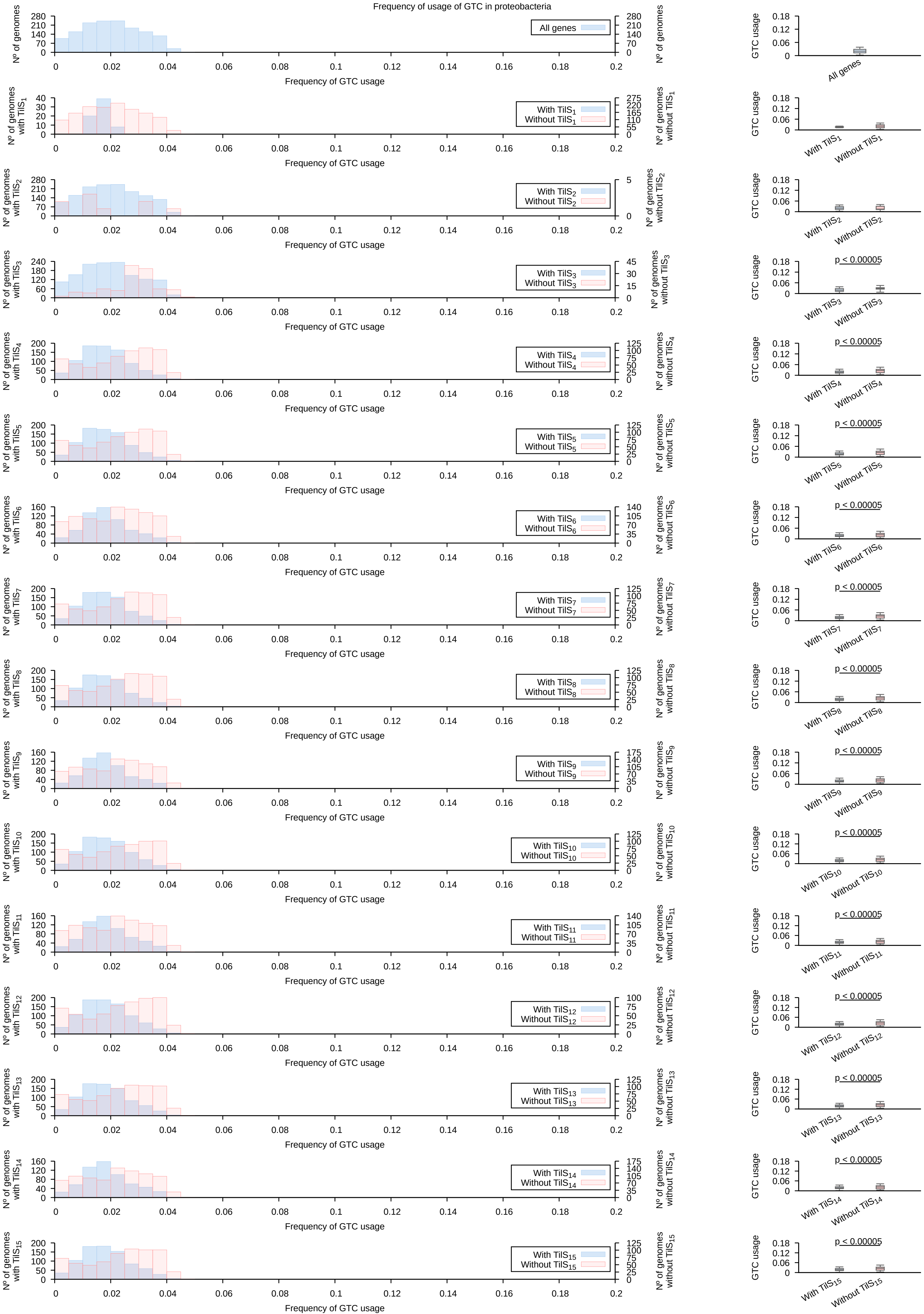
Frequency of usage of GTC in proteobacteria
Nº of genomes
All genes
GTC usage
Frequency of GTC usage
Nº of genomes with TilS1
Nº of genomes without TilS1
With TilS1
Without TilS1
Nº of genomes with TilS2
Nº of genomes without TilS2
With TilS2
Without TilS2
Nº of genomes with TilS3
Nº of genomes without TilS3
With TilS3
Without TilS3
p < 0.00005
Nº of genomes with TilS4
Nº of genomes without TilS4
With TilS4
Without TilS4
Nº of genomes with TilS5
Nº of genomes without TilS5
With TilS5
Without TilS5
Nº of genomes with TilS6
Nº of genomes without TilS6
With TilS6
Without TilS6
Nº of genomes with TilS7
Nº of genomes without TilS7
With TilS7
Without TilS7
Nº of genomes with TilS8
Nº of genomes without TilS8
With TilS8
Without TilS8
Nº of genomes with TilS9
Nº of genomes without TilS9
With TilS9
Without TilS9
Nº of genomes with TilS10
Nº of genomes without TilS10
With TilS10
Without TilS10
Nº of genomes with TilS11
Nº of genomes without TilS11
With TilS11
Without TilS11
Nº of genomes with TilS12
Nº of genomes without TilS12
With TilS12
Without TilS12
Nº of genomes with TilS13
Nº of genomes without TilS13
With TilS13
Without TilS13
Nº of genomes with TilS14
Nº of genomes without TilS14
With TilS14
Without TilS14
Nº of genomes with TilS15
Nº of genomes without TilS15
With TilS15
Without TilS15
0 0.02 0.04 0.06 0.08 0.1 0.12 0.14 0.16 0.18 0.2
0 0.06 0.12 0.18

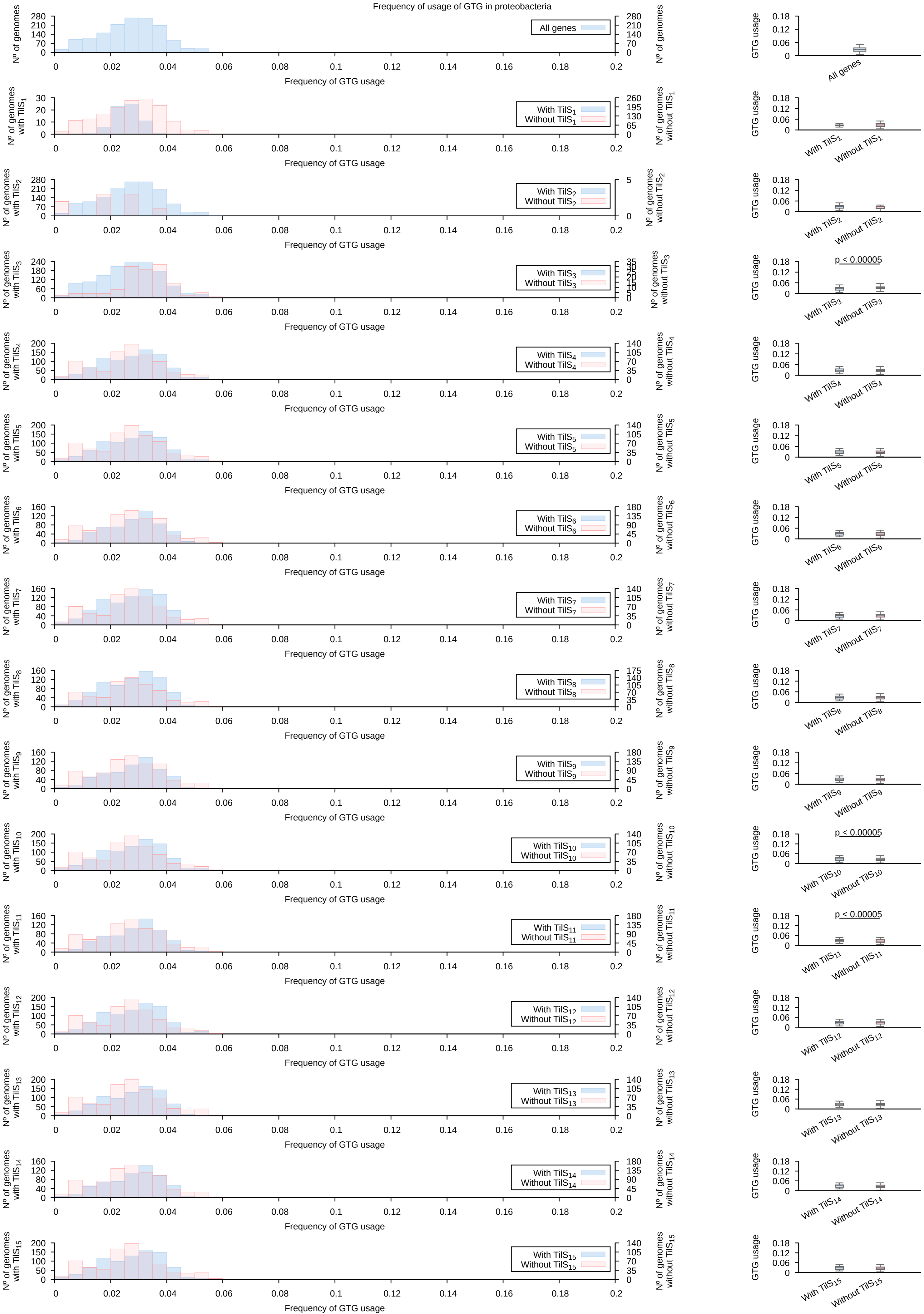

Frequency of usage of GTG in proteobacteria
Nº of genomes
All genes
Frequency of GTG usage
GTG usage
Nº of genomes with TilS$_1$
Nº of genomes without TilS$_1$
With TilS$_1$
Without TilS$_1$
Nº of genomes with TilS$_2$
Nº of genomes without TilS$_2$
With TilS$_2$
Without TilS$_2$
Nº of genomes with TilS$_3$
Nº of genomes without TilS$_3$
With TilS$_3$
Without TilS$_3$
p < 0.00005
Nº of genomes with TilS$_4$
Nº of genomes without TilS$_4$
With TilS$_4$
Without TilS$_4$
Nº of genomes with TilS$_5$
Nº of genomes without TilS$_5$
With TilS$_5$
Without TilS$_5$
Nº of genomes with TilS$_6$
Nº of genomes without TilS$_6$
With TilS$_6$
Without TilS$_6$
Nº of genomes with TilS$_7$
Nº of genomes without TilS$_7$
With TilS$_7$
Without TilS$_7$
Nº of genomes with TilS$_8$
Nº of genomes without TilS$_8$
With TilS$_8$
Without TilS$_8$
Nº of genomes with TilS$_9$
Nº of genomes without TilS$_9$
With TilS$_9$
Without TilS$_9$
Nº of genomes with TilS$_{10}$
Nº of genomes without TilS$_{10}$
With TilS$_{10}$
Without TilS$_{10}$
p < 0.00005
Nº of genomes with TilS$_{11}$
Nº of genomes without TilS$_{11}$
With TilS$_{11}$
Without TilS$_{11}$
p < 0.00005
Nº of genomes with TilS$_{12}$
Nº of genomes without TilS$_{12}$
With TilS$_{12}$
Without TilS$_{12}$
Nº of genomes with TilS$_{13}$
Nº of genomes without TilS$_{13}$
With TilS$_{13}$
Without TilS$_{13}$
Nº of genomes with TilS$_{14}$
Nº of genomes without TilS$_{14}$
With TilS$_{14}$
Without TilS$_{14}$
Nº of genomes with TilS$_{15}$
Nº of genomes without TilS$_{15}$
With TilS$_{15}$
Without TilS$_{15}$

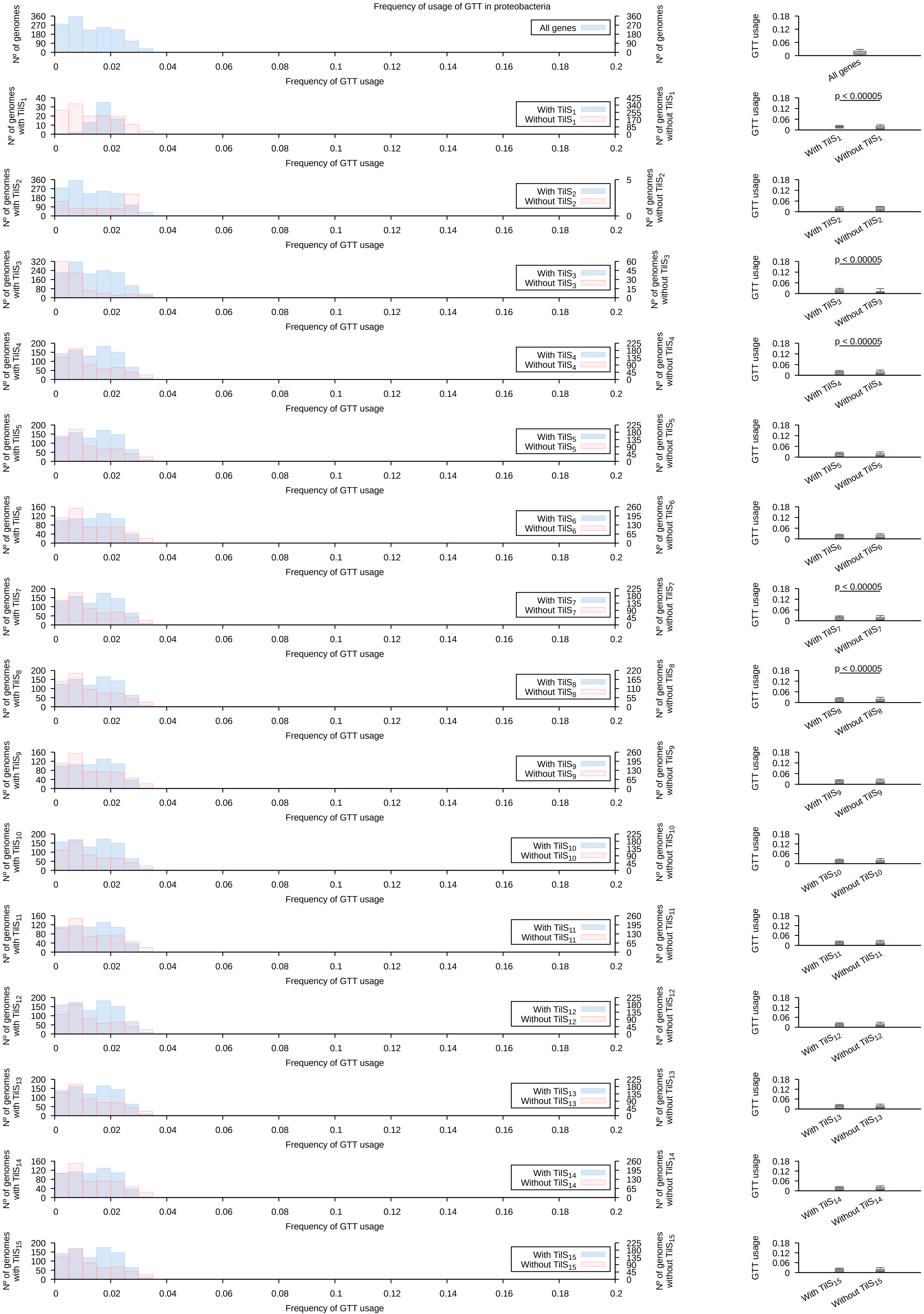

Frequency of usage of GTT in proteobacteria
Nº of genomes
All genes
Frequency of GTT usage
GTT usage
Nº of genomes with TilS1
Nº of genomes without TilS1
With TilS1
Without TilS1
p < 0.00005
Nº of genomes with TilS2
Nº of genomes without TilS2
With TilS2
Without TilS2
Nº of genomes with TilS3
Nº of genomes without TilS3
With TilS3
Without TilS3
p < 0.00005
Nº of genomes with TilS4
Nº of genomes without TilS4
With TilS4
Without TilS4
p < 0.00005
Nº of genomes with TilS5
Nº of genomes without TilS5
With TilS5
Without TilS5
Nº of genomes with TilS6
Nº of genomes without TilS6
With TilS6
Without TilS6
Nº of genomes with TilS7
Nº of genomes without TilS7
With TilS7
Without TilS7
p < 0.00005
Nº of genomes with TilS8
Nº of genomes without TilS8
With TilS8
Without TilS8
p < 0.00005
Nº of genomes with TilS9
Nº of genomes without TilS9
With TilS9
Without TilS9
Nº of genomes with TilS10
Nº of genomes without TilS10
With TilS10
Without TilS10
Nº of genomes with TilS11
Nº of genomes without TilS11
With TilS11
Without TilS11
Nº of genomes with TilS12
Nº of genomes without TilS12
With TilS12
Without TilS12
Nº of genomes with TilS13
Nº of genomes without TilS13
With TilS13
Without TilS13
Nº of genomes with TilS14
Nº of genomes without TilS14
With TilS14
Without TilS14
Nº of genomes with TilS15
Nº of genomes without TilS15
With TilS15
Without TilS15

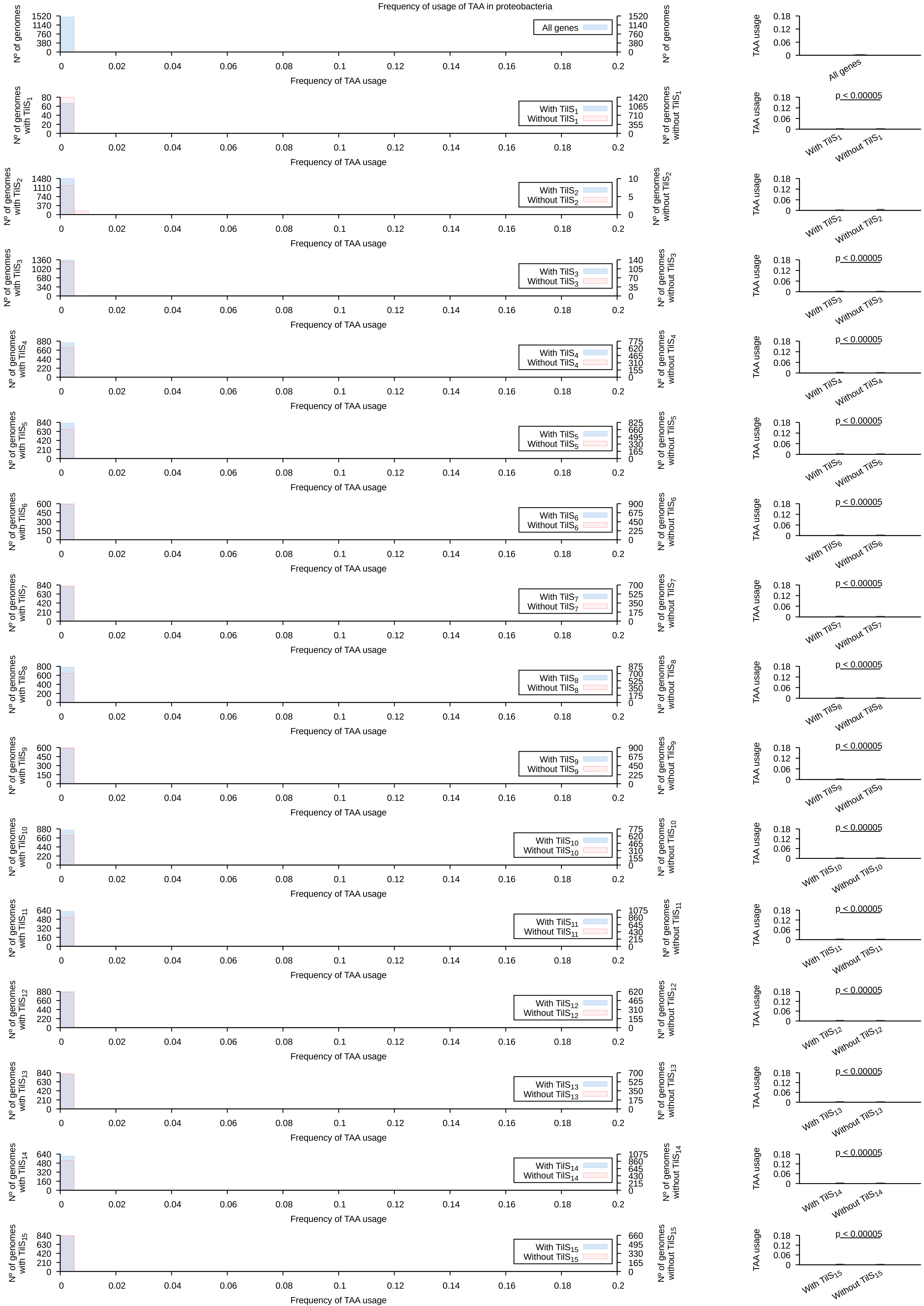

Frequency of usage of TAA in proteobacteria
Nº of genomes
All genes
Frequency of TAA usage
TAA usage
Nº of genomes with TilS$_1$
Nº of genomes without TilS$_1$
With TilS$_1$
Without TilS$_1$
p < 0.00005
Nº of genomes with TilS$_2$
Nº of genomes without TilS$_2$
With TilS$_2$
Without TilS$_2$
Nº of genomes with TilS$_3$
Nº of genomes without TilS$_3$
With TilS$_3$
Without TilS$_3$
p < 0.00005
Nº of genomes with TilS$_4$
Nº of genomes without TilS$_4$
With TilS$_4$
Without TilS$_4$
p < 0.00005
Nº of genomes with TilS$_5$
Nº of genomes without TilS$_5$
With TilS$_5$
Without TilS$_5$
p < 0.00005
Nº of genomes with TilS$_6$
Nº of genomes without TilS$_6$
With TilS$_6$
Without TilS$_6$
p < 0.00005
Nº of genomes with TilS$_7$
Nº of genomes without TilS$_7$
With TilS$_7$
Without TilS$_7$
p < 0.00005
Nº of genomes with TilS$_8$
Nº of genomes without TilS$_8$
With TilS$_8$
Without TilS$_8$
p < 0.00005
Nº of genomes with TilS$_9$
Nº of genomes without TilS$_9$
With TilS$_9$
Without TilS$_9$
p < 0.00005
Nº of genomes with TilS$_{10}$
Nº of genomes without TilS$_{10}$
With TilS$_{10}$
Without TilS$_{10}$
p < 0.00005
Nº of genomes with TilS$_{11}$
Nº of genomes without TilS$_{11}$
With TilS$_{11}$
Without TilS$_{11}$
p < 0.00005
Nº of genomes with TilS$_{12}$
Nº of genomes without TilS$_{12}$
With TilS$_{12}$
Without TilS$_{12}$
p < 0.00005
Nº of genomes with TilS$_{13}$
Nº of genomes without TilS$_{13}$
With TilS$_{13}$
Without TilS$_{13}$
p < 0.00005
Nº of genomes with TilS$_{14}$
Nº of genomes without TilS$_{14}$
With TilS$_{14}$
Without TilS$_{14}$
p < 0.00005
Nº of genomes with TilS$_{15}$
Nº of genomes without TilS$_{15}$
With TilS$_{15}$
Without TilS$_{15}$
p < 0.00005

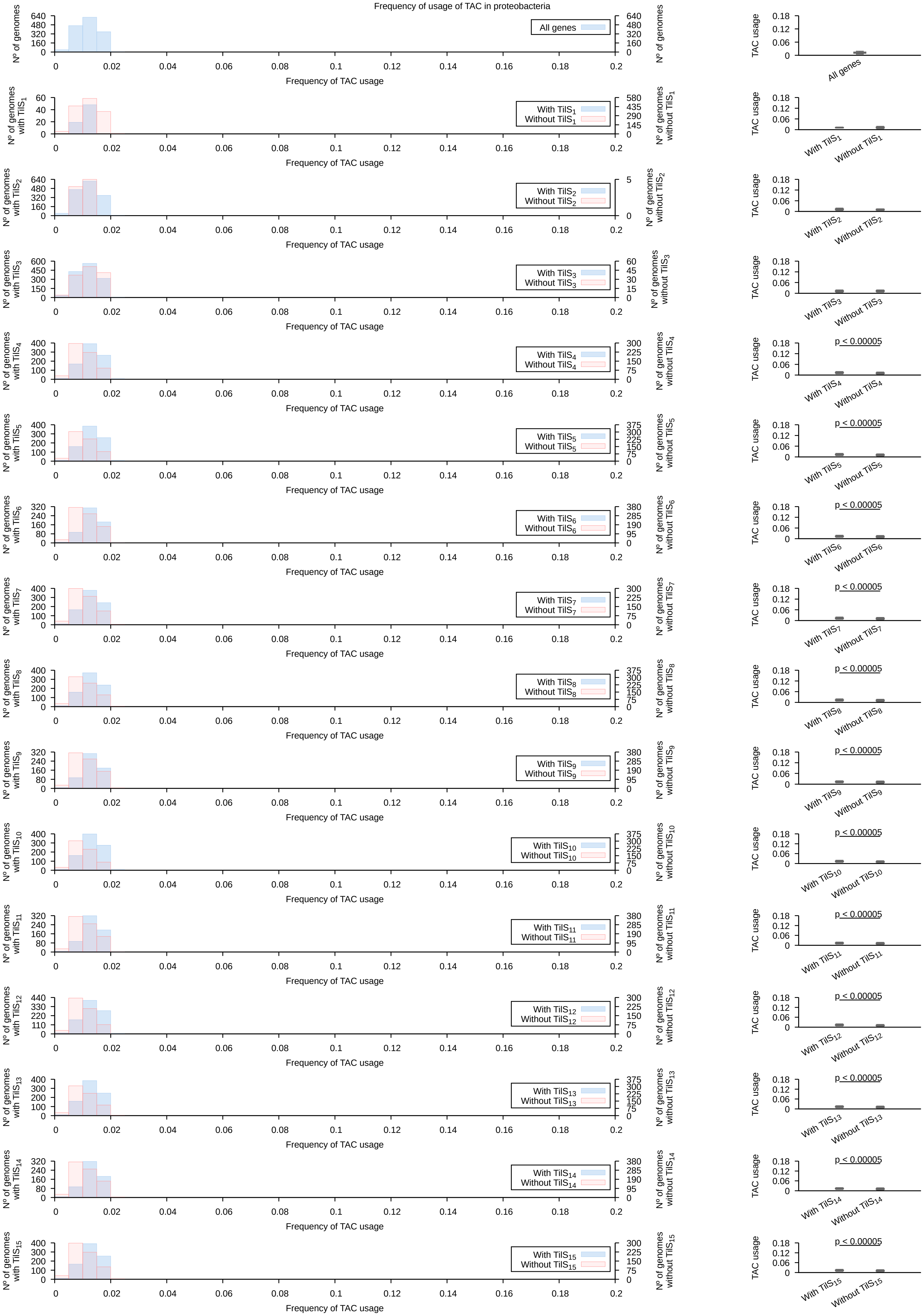

Frequency of usage of TAC in proteobacteria
Nº of genomes
All genes
Frequency of TAC usage
TAC usage
0 0.02 0.04 0.06 0.08 0.1 0.12 0.14 0.16 0.18 0.2
0 0.06 0.12 0.18
Nº of genomes with TilS$_1$
Nº of genomes without TilS$_1$
With TilS$_1$
Without TilS$_1$
Nº of genomes with TilS$_2$
Nº of genomes without TilS$_2$
With TilS$_2$
Without TilS$_2$
Nº of genomes with TilS$_3$
Nº of genomes without TilS$_3$
With TilS$_3$
Without TilS$_3$
Nº of genomes with TilS$_4$
Nº of genomes without TilS$_4$
With TilS$_4$
Without TilS$_4$
p < 0.00005
Nº of genomes with TilS$_5$
Nº of genomes without TilS$_5$
With TilS$_5$
Without TilS$_5$
p < 0.00005
Nº of genomes with TilS$_6$
Nº of genomes without TilS$_6$
With TilS$_6$
Without TilS$_6$
p < 0.00005
Nº of genomes with TilS$_7$
Nº of genomes without TilS$_7$
With TilS$_7$
Without TilS$_7$
p < 0.00005
Nº of genomes with TilS$_8$
Nº of genomes without TilS$_8$
With TilS$_8$
Without TilS$_8$
p < 0.00005
Nº of genomes with TilS$_9$
Nº of genomes without TilS$_9$
With TilS$_9$
Without TilS$_9$
p < 0.00005
Nº of genomes with TilS$_{10}$
Nº of genomes without TilS$_{10}$
With TilS$_{10}$
Without TilS$_{10}$
p < 0.00005
Nº of genomes with TilS$_{11}$
Nº of genomes without TilS$_{11}$
With TilS$_{11}$
Without TilS$_{11}$
p < 0.00005
Nº of genomes with TilS$_{12}$
Nº of genomes without TilS$_{12}$
With TilS$_{12}$
Without TilS$_{12}$
p < 0.00005
Nº of genomes with TilS$_{13}$
Nº of genomes without TilS$_{13}$
With TilS$_{13}$
Without TilS$_{13}$
p < 0.00005
Nº of genomes with TilS$_{14}$
Nº of genomes without TilS$_{14}$
With TilS$_{14}$
Without TilS$_{14}$
p < 0.00005
Nº of genomes with TilS$_{15}$
Nº of genomes without TilS$_{15}$
With TilS$_{15}$
Without TilS$_{15}$
p < 0.00005

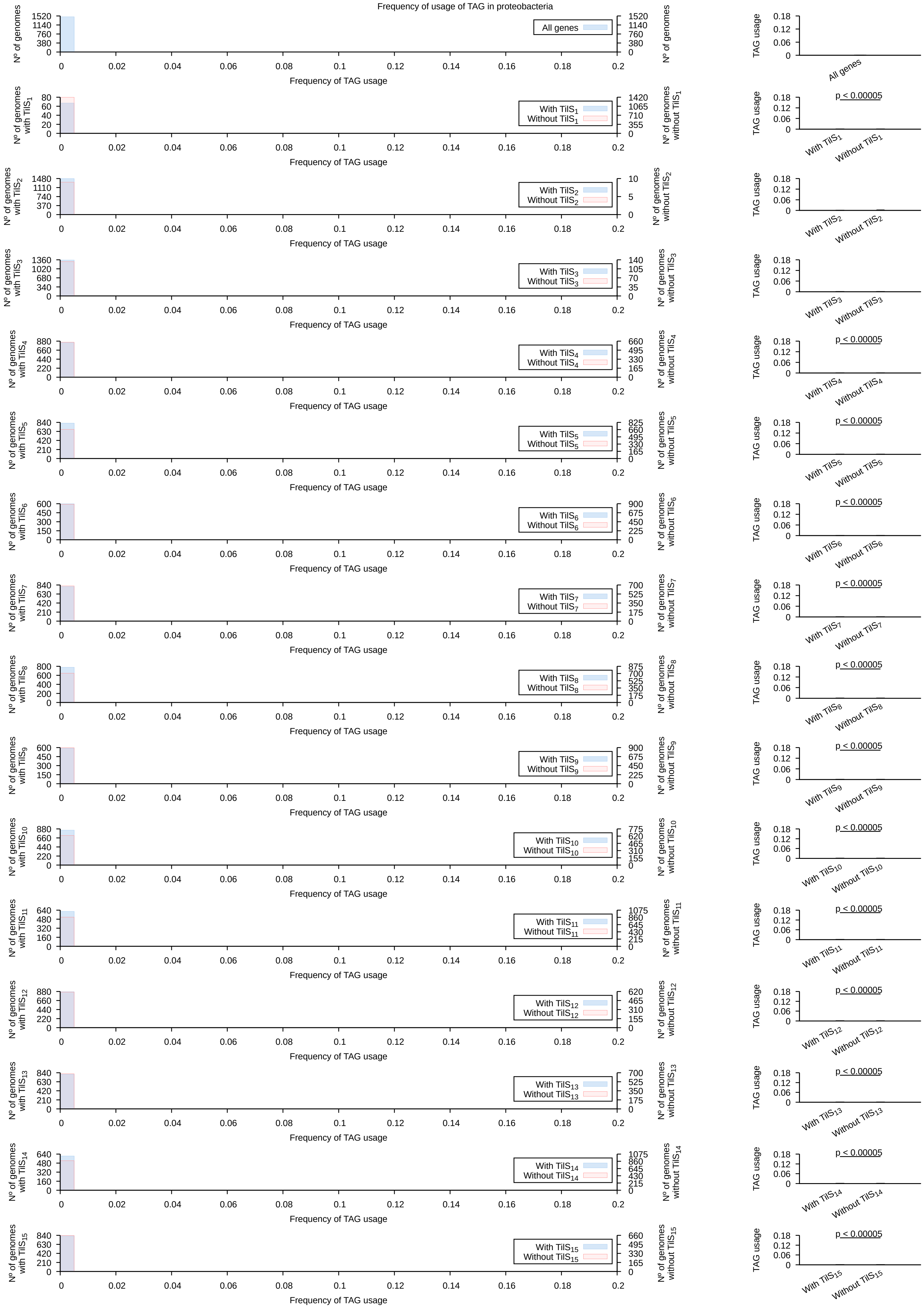
Frequency of usage of TAG in proteobacteria
Nº of genomes
Frequency of TAG usage
All genes
TAG usage
Nº of genomes with TilS1
Nº of genomes without TilS1
With TilS1
Without TilS1
p < 0.00005
Nº of genomes with TilS2
Nº of genomes without TilS2
With TilS2
Without TilS2
Nº of genomes with TilS3
Nº of genomes without TilS3
With TilS3
Without TilS3
Nº of genomes with TilS4
Nº of genomes without TilS4
With TilS4
Without TilS4
p < 0.00005
Nº of genomes with TilS5
Nº of genomes without TilS5
With TilS5
Without TilS5
p < 0.00005
Nº of genomes with TilS6
Nº of genomes without TilS6
With TilS6
Without TilS6
p < 0.00005
Nº of genomes with TilS7
Nº of genomes without TilS7
With TilS7
Without TilS7
p < 0.00005
Nº of genomes with TilS8
Nº of genomes without TilS8
With TilS8
Without TilS8
p < 0.00005
Nº of genomes with TilS9
Nº of genomes without TilS9
With TilS9
Without TilS9
p < 0.00005
Nº of genomes with TilS10
Nº of genomes without TilS10
With TilS10
Without TilS10
p < 0.00005
Nº of genomes with TilS11
Nº of genomes without TilS11
With TilS11
Without TilS11
p < 0.00005
Nº of genomes with TilS12
Nº of genomes without TilS12
With TilS12
Without TilS12
p < 0.00005
Nº of genomes with TilS13
Nº of genomes without TilS13
With TilS13
Without TilS13
p < 0.00005
Nº of genomes with TilS14
Nº of genomes without TilS14
With TilS14
Without TilS14
p < 0.00005
Nº of genomes with TilS15
Nº of genomes without TilS15
With TilS15
Without TilS15
p < 0.00005

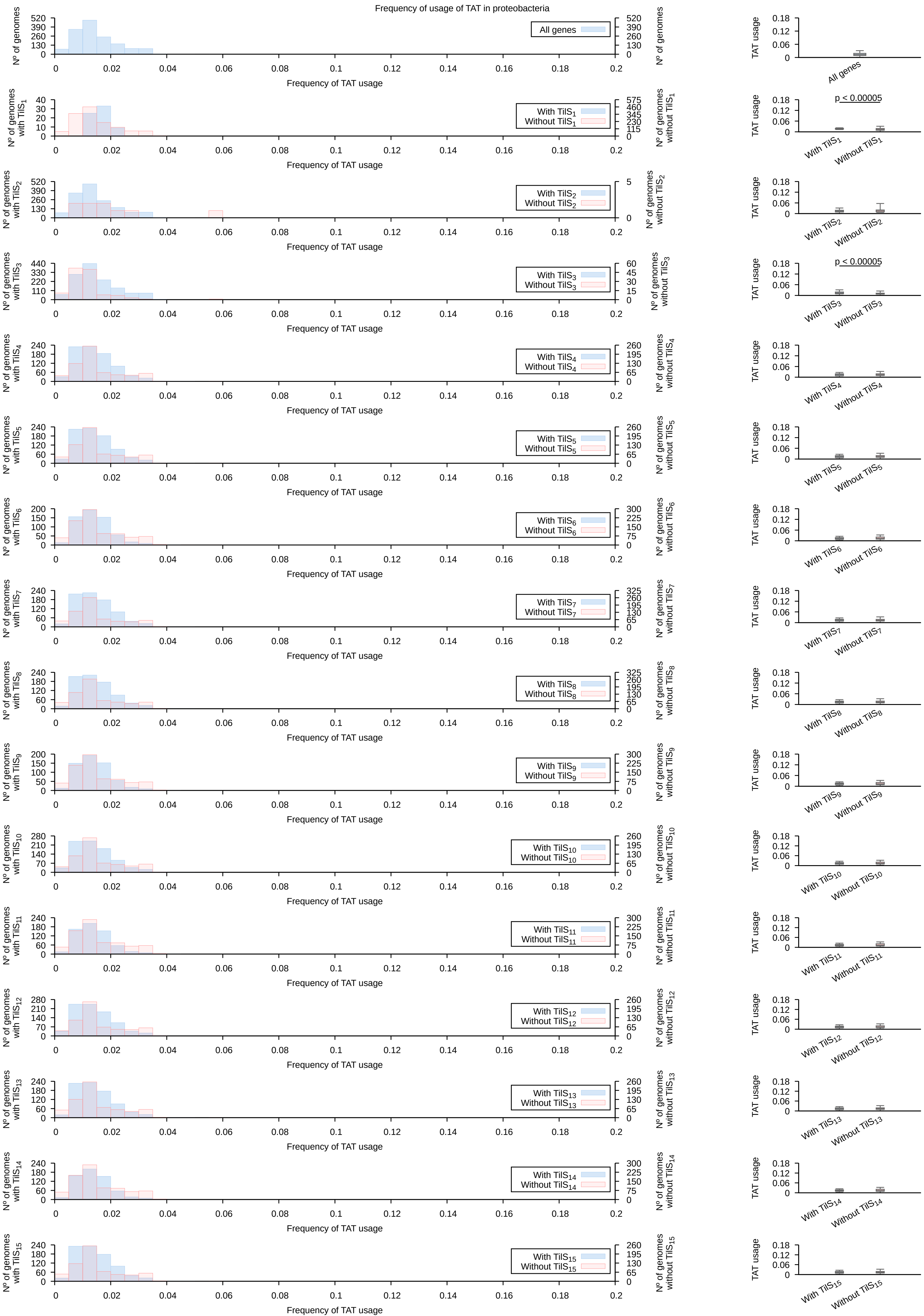
Frequency of usage of TAT in proteobacteria
Nº of genomes
Frequency of TAT usage
TAT usage
All genes
With TilS$_1$
Without TilS$_1$
Nº of genomes with TilS$_1$
Nº of genomes without TilS$_1$
p < 0.00005
With TilS$_2$
Without TilS$_2$
With TilS$_3$
Without TilS$_3$
p < 0.00005
With TilS$_4$
Without TilS$_4$
With TilS$_5$
Without TilS$_5$
With TilS$_6$
Without TilS$_6$
With TilS$_7$
Without TilS$_7$
With TilS$_8$
Without TilS$_8$
With TilS$_9$
Without TilS$_9$
With TilS$_{10}$
Without TilS$_{10}$
With TilS$_{11}$
Without TilS$_{11}$
With TilS$_{12}$
Without TilS$_{12}$
With TilS$_{13}$
Without TilS$_{13}$
With TilS$_{14}$
Without TilS$_{14}$
With TilS$_{15}$
Without TilS$_{15}$

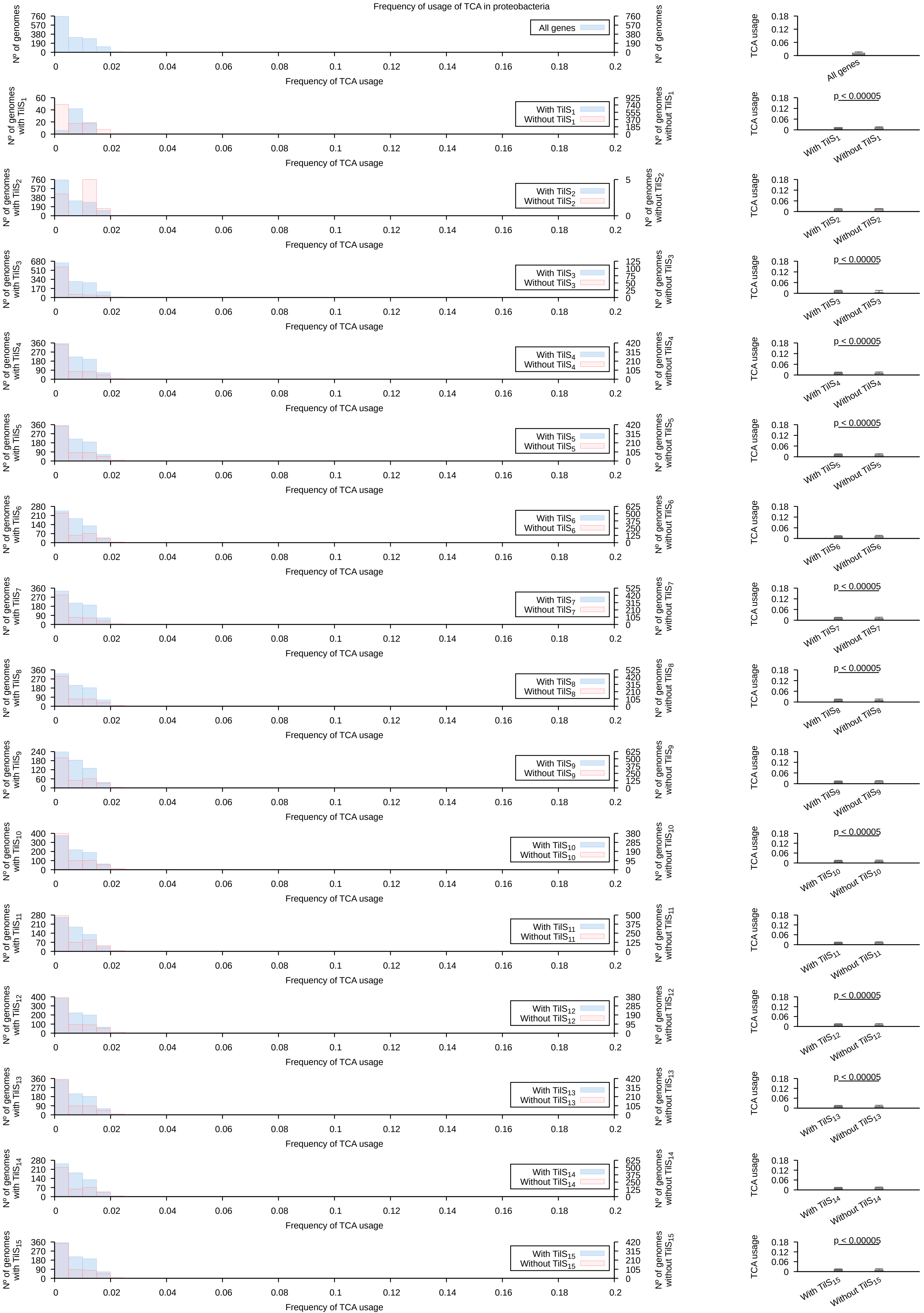

Frequency of usage of TCA in proteobacteria
Nº of genomes
All genes
Frequency of TCA usage
TCA usage
0
0.02
0.04
0.06
0.08
0.1
0.12
0.14
0.16
0.18
0.2
Nº of genomes with TilS$_1$
Nº of genomes without TilS$_1$
With TilS$_1$
Without TilS$_1$
p < 0.00005
Nº of genomes with TilS$_2$
Nº of genomes without TilS$_2$
With TilS$_2$
Without TilS$_2$
Nº of genomes with TilS$_3$
Nº of genomes without TilS$_3$
With TilS$_3$
Without TilS$_3$
p < 0.00005
Nº of genomes with TilS$_4$
Nº of genomes without TilS$_4$
With TilS$_4$
Without TilS$_4$
p < 0.00005
Nº of genomes with TilS$_5$
Nº of genomes without TilS$_5$
With TilS$_5$
Without TilS$_5$
p < 0.00005
Nº of genomes with TilS$_6$
Nº of genomes without TilS$_6$
With TilS$_6$
Without TilS$_6$
Nº of genomes with TilS$_7$
Nº of genomes without TilS$_7$
With TilS$_7$
Without TilS$_7$
p < 0.00005
Nº of genomes with TilS$_8$
Nº of genomes without TilS$_8$
With TilS$_8$
Without TilS$_8$
p < 0.00005
Nº of genomes with TilS$_9$
Nº of genomes without TilS$_9$
With TilS$_9$
Without TilS$_9$
Nº of genomes with TilS$_{10}$
Nº of genomes without TilS$_{10}$
With TilS$_{10}$
Without TilS$_{10}$
p < 0.00005
Nº of genomes with TilS$_{11}$
Nº of genomes without TilS$_{11}$
With TilS$_{11}$
Without TilS$_{11}$
Nº of genomes with TilS$_{12}$
Nº of genomes without TilS$_{12}$
With TilS$_{12}$
Without TilS$_{12}$
p < 0.00005
Nº of genomes with TilS$_{13}$
Nº of genomes without TilS$_{13}$
With TilS$_{13}$
Without TilS$_{13}$
p < 0.00005
Nº of genomes with TilS$_{14}$
Nº of genomes without TilS$_{14}$
With TilS$_{14}$
Without TilS$_{14}$
Nº of genomes with TilS$_{15}$
Nº of genomes without TilS$_{15}$
With TilS$_{15}$
Without TilS$_{15}$
p < 0.00005

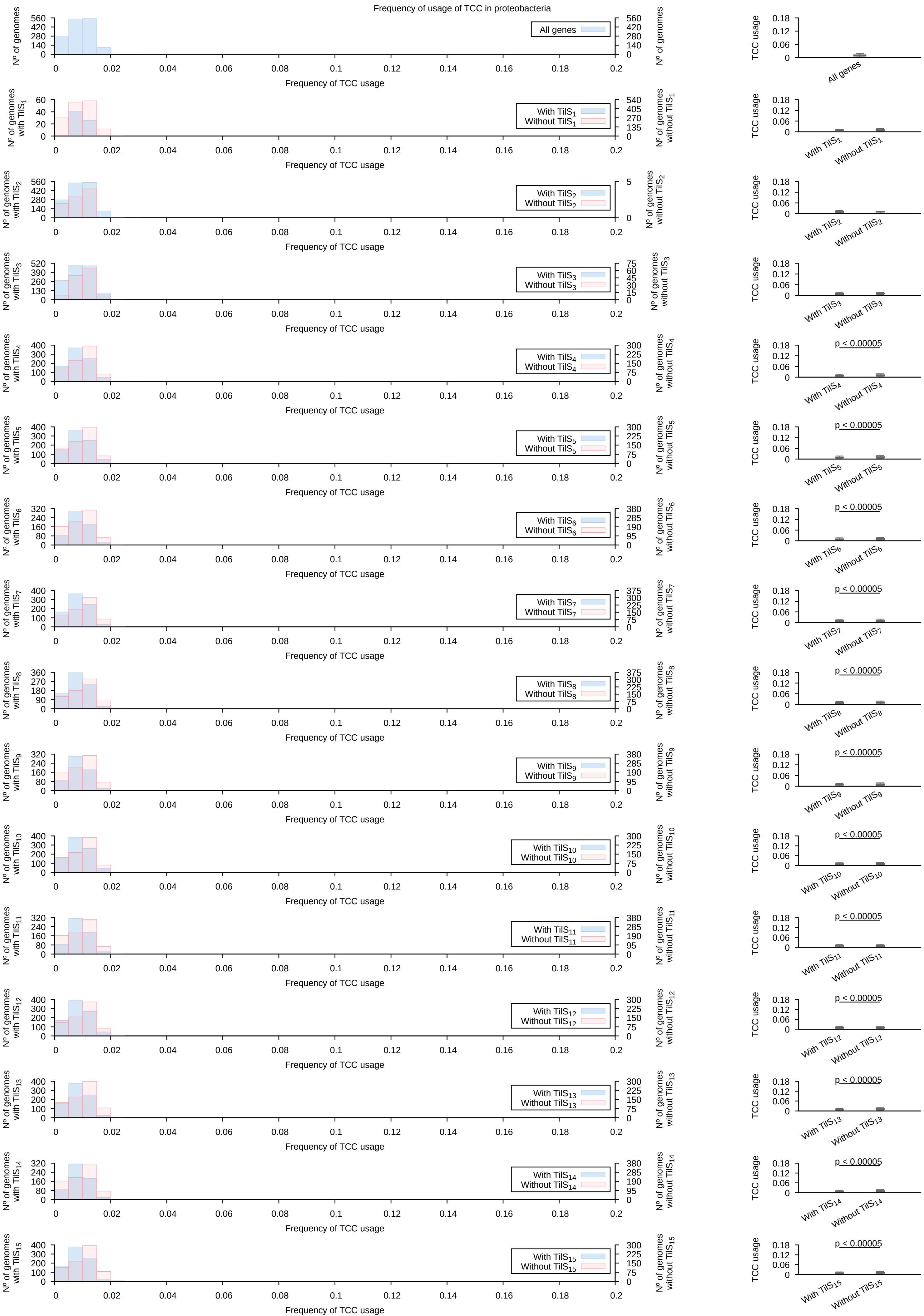

Frequency of usage of TCC in proteobacteria
Nº of genomes
All genes
Frequency of TCC usage
TCC usage
Nº of genomes with TilS$_1$
Nº of genomes without TilS$_1$
With TilS$_1$
Without TilS$_1$
Nº of genomes with TilS$_2$
Nº of genomes without TilS$_2$
With TilS$_2$
Without TilS$_2$
Nº of genomes with TilS$_3$
Nº of genomes without TilS$_3$
With TilS$_3$
Without TilS$_3$
Nº of genomes with TilS$_4$
Nº of genomes without TilS$_4$
With TilS$_4$
Without TilS$_4$
p < 0.00005
Nº of genomes with TilS$_5$
Nº of genomes without TilS$_5$
With TilS$_5$
Without TilS$_5$
Nº of genomes with TilS$_6$
Nº of genomes without TilS$_6$
With TilS$_6$
Without TilS$_6$
Nº of genomes with TilS$_7$
Nº of genomes without TilS$_7$
With TilS$_7$
Without TilS$_7$
Nº of genomes with TilS$_8$
Nº of genomes without TilS$_8$
With TilS$_8$
Without TilS$_8$
Nº of genomes with TilS$_9$
Nº of genomes without TilS$_9$
With TilS$_9$
Without TilS$_9$
Nº of genomes with TilS$_{10}$
Nº of genomes without TilS$_{10}$
With TilS$_{10}$
Without TilS$_{10}$
Nº of genomes with TilS$_{11}$
Nº of genomes without TilS$_{11}$
With TilS$_{11}$
Without TilS$_{11}$
Nº of genomes with TilS$_{12}$
Nº of genomes without TilS$_{12}$
With TilS$_{12}$
Without TilS$_{12}$
Nº of genomes with TilS$_{13}$
Nº of genomes without TilS$_{13}$
With TilS$_{13}$
Without TilS$_{13}$
Nº of genomes with TilS$_{14}$
Nº of genomes without TilS$_{14}$
With TilS$_{14}$
Without TilS$_{14}$
Nº of genomes with TilS$_{15}$
Nº of genomes without TilS$_{15}$
With TilS$_{15}$
Without TilS$_{15}$

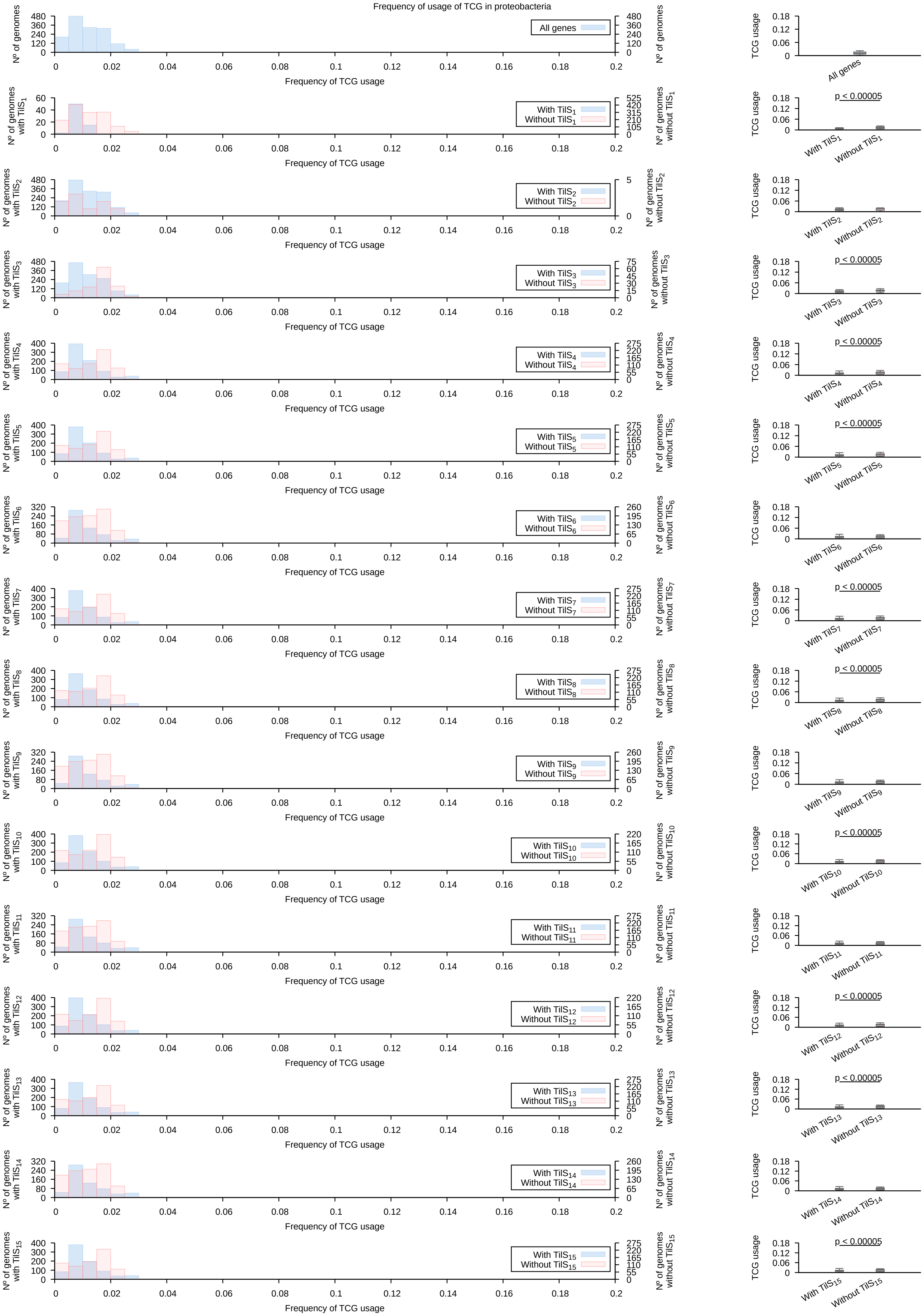

Frequency of usage of TCG in proteobacteria
Nº of genomes
All genes
Frequency of TCG usage
TCG usage
All genes
Nº of genomes with TilS$_1$
Nº of genomes without TilS$_1$
With TilS$_1$
Without TilS$_1$
p < 0.00005
Nº of genomes with TilS$_2$
Nº of genomes without TilS$_2$
With TilS$_2$
Without TilS$_2$
Nº of genomes with TilS$_3$
Nº of genomes without TilS$_3$
With TilS$_3$
Without TilS$_3$
p < 0.00005
Nº of genomes with TilS$_4$
Nº of genomes without TilS$_4$
With TilS$_4$
Without TilS$_4$
p < 0.00005
Nº of genomes with TilS$_5$
Nº of genomes without TilS$_5$
With TilS$_5$
Without TilS$_5$
p < 0.00005
Nº of genomes with TilS$_6$
Nº of genomes without TilS$_6$
With TilS$_6$
Without TilS$_6$
Nº of genomes with TilS$_7$
Nº of genomes without TilS$_7$
With TilS$_7$
Without TilS$_7$
p < 0.00005
Nº of genomes with TilS$_8$
Nº of genomes without TilS$_8$
With TilS$_8$
Without TilS$_8$
p < 0.00005
Nº of genomes with TilS$_9$
Nº of genomes without TilS$_9$
With TilS$_9$
Without TilS$_9$
Nº of genomes with TilS$_{10}$
Nº of genomes without TilS$_{10}$
With TilS$_{10}$
Without TilS$_{10}$
p < 0.00005
Nº of genomes with TilS$_{11}$
Nº of genomes without TilS$_{11}$
With TilS$_{11}$
Without TilS$_{11}$
Nº of genomes with TilS$_{12}$
Nº of genomes without TilS$_{12}$
With TilS$_{12}$
Without TilS$_{12}$
p < 0.00005
Nº of genomes with TilS$_{13}$
Nº of genomes without TilS$_{13}$
With TilS$_{13}$
Without TilS$_{13}$
p < 0.00005
Nº of genomes with TilS$_{14}$
Nº of genomes without TilS$_{14}$
With TilS$_{14}$
Without TilS$_{14}$
Nº of genomes with TilS$_{15}$
Nº of genomes without TilS$_{15}$
With TilS$_{15}$
Without TilS$_{15}$
p < 0.00005

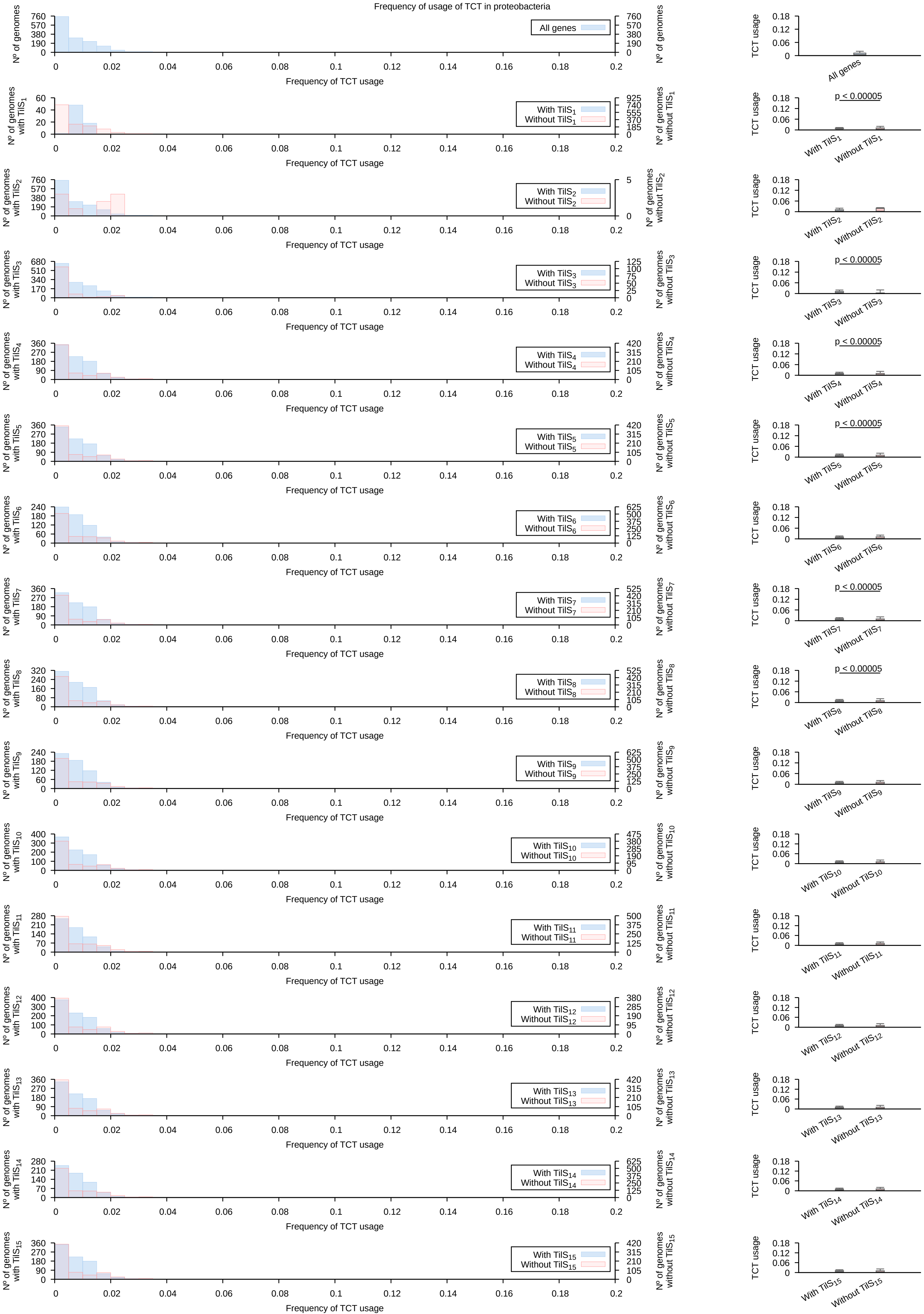
Frequency of usage of TCT in proteobacteria
Nº of genomes
Frequency of TCT usage
All genes
TCT usage
With TilS$_1$
Without TilS$_1$
Nº of genomes with TilS$_1$
Nº of genomes without TilS$_1$
p < 0.00005
With TilS$_2$
Without TilS$_2$
With TilS$_3$
Without TilS$_3$
p < 0.00005
With TilS$_4$
Without TilS$_4$
p < 0.00005
With TilS$_5$
Without TilS$_5$
p < 0.00005
With TilS$_6$
Without TilS$_6$
With TilS$_7$
Without TilS$_7$
p < 0.00005
With TilS$_8$
Without TilS$_8$
p < 0.00005
With TilS$_9$
Without TilS$_9$
With TilS$_{10}$
Without TilS$_{10}$
With TilS$_{11}$
Without TilS$_{11}$
With TilS$_{12}$
Without TilS$_{12}$
With TilS$_{13}$
Without TilS$_{13}$
With TilS$_{14}$
Without TilS$_{14}$
With TilS$_{15}$
Without TilS$_{15}$

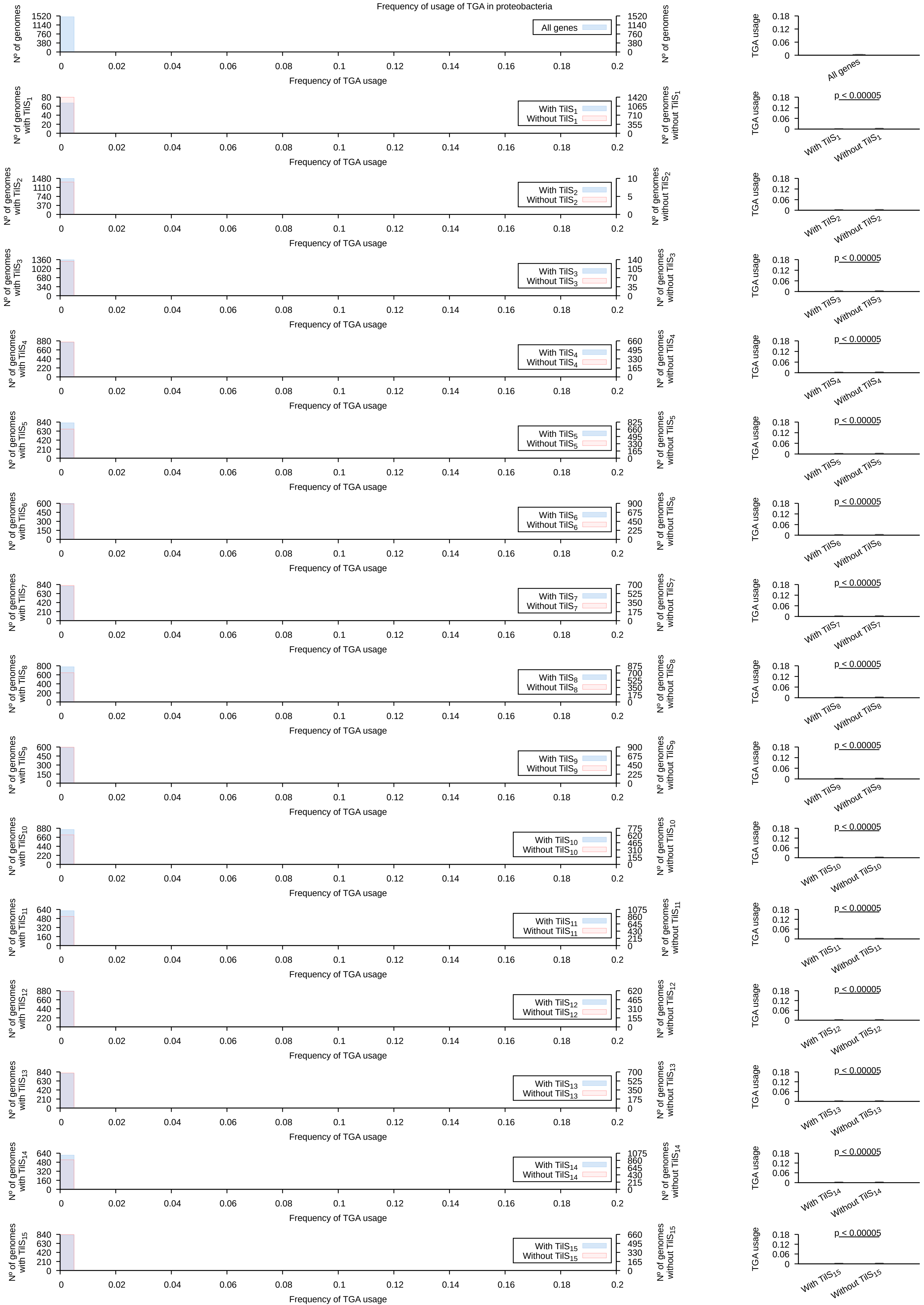
Frequency of usage of TGA in proteobacteria
Nº of genomes
All genes
Frequency of TGA usage
TGA usage
Nº of genomes with TilS$_1$
With TilS$_1$
Without TilS$_1$
Nº of genomes without TilS$_1$
p < 0.00005
Nº of genomes with TilS$_2$
With TilS$_2$
Without TilS$_2$
Nº of genomes without TilS$_2$
Nº of genomes with TilS$_3$
With TilS$_3$
Without TilS$_3$
Nº of genomes without TilS$_3$
Nº of genomes with TilS$_4$
With TilS$_4$
Without TilS$_4$
Nº of genomes without TilS$_4$
Nº of genomes with TilS$_5$
With TilS$_5$
Without TilS$_5$
Nº of genomes without TilS$_5$
Nº of genomes with TilS$_6$
With TilS$_6$
Without TilS$_6$
Nº of genomes without TilS$_6$
Nº of genomes with TilS$_7$
With TilS$_7$
Without TilS$_7$
Nº of genomes without TilS$_7$
Nº of genomes with TilS$_8$
With TilS$_8$
Without TilS$_8$
Nº of genomes without TilS$_8$
Nº of genomes with TilS$_9$
With TilS$_9$
Without TilS$_9$
Nº of genomes without TilS$_9$
Nº of genomes with TilS$_{10}$
With TilS$_{10}$
Without TilS$_{10}$
Nº of genomes without TilS$_{10}$
Nº of genomes with TilS$_{11}$
With TilS$_{11}$
Without TilS$_{11}$
Nº of genomes without TilS$_{11}$
Nº of genomes with TilS$_{12}$
With TilS$_{12}$
Without TilS$_{12}$
Nº of genomes without TilS$_{12}$
Nº of genomes with TilS$_{13}$
With TilS$_{13}$
Without TilS$_{13}$
Nº of genomes without TilS$_{13}$
Nº of genomes with TilS$_{14}$
With TilS$_{14}$
Without TilS$_{14}$
Nº of genomes without TilS$_{14}$
Nº of genomes with TilS$_{15}$
With TilS$_{15}$
Without TilS$_{15}$
Nº of genomes without TilS$_{15}$

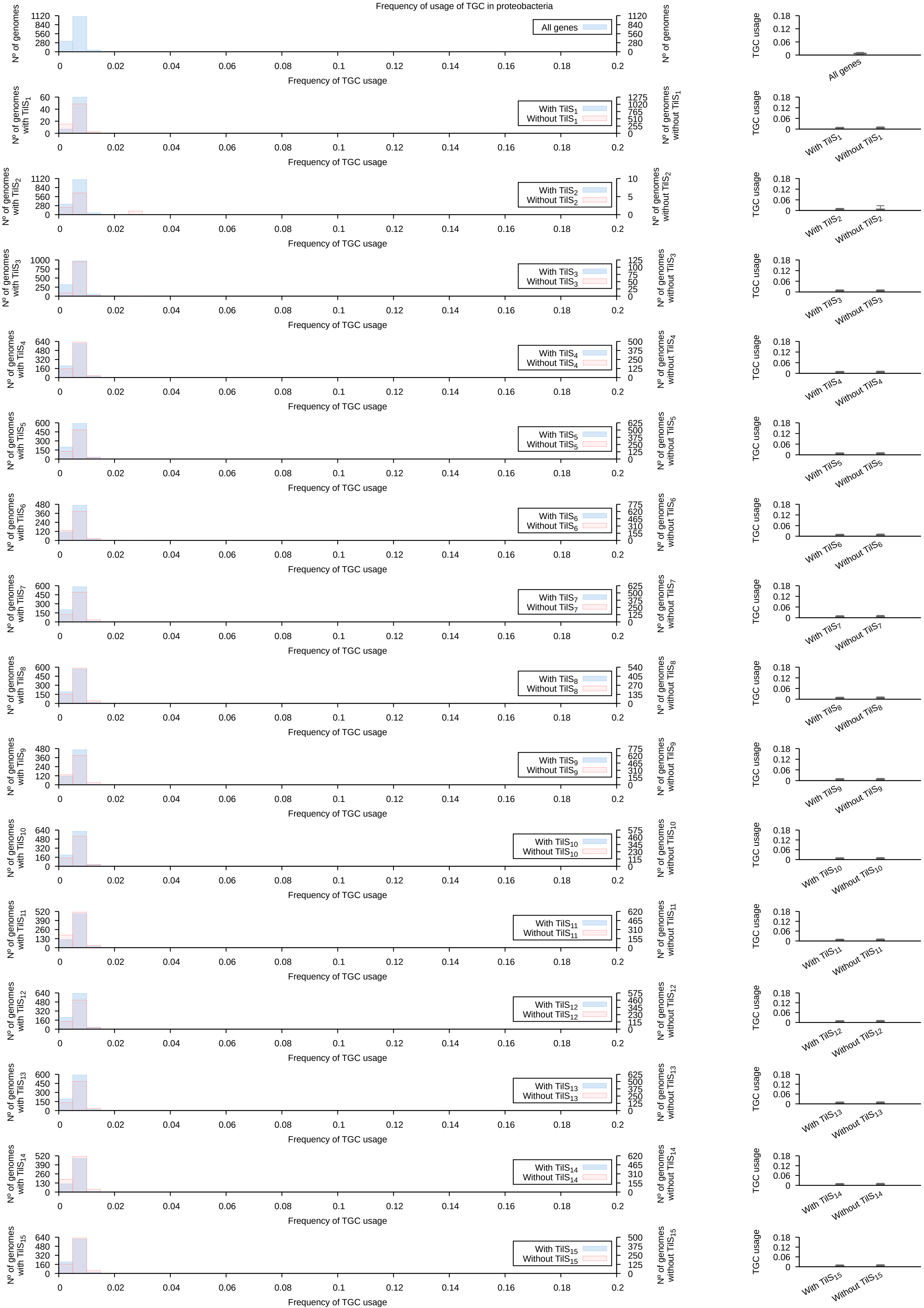
Frequency of usage of TGC in proteobacteria
Nº of genomes
All genes
Frequency of TGC usage
TGC usage
Nº of genomes with TilS$_1$
Nº of genomes without TilS$_1$
With TilS$_1$
Without TilS$_1$
Nº of genomes with TilS$_2$
Nº of genomes without TilS$_2$
With TilS$_2$
Without TilS$_2$
Nº of genomes with TilS$_3$
Nº of genomes without TilS$_3$
With TilS$_3$
Without TilS$_3$
Nº of genomes with TilS$_4$
Nº of genomes without TilS$_4$
With TilS$_4$
Without TilS$_4$
Nº of genomes with TilS$_5$
Nº of genomes without TilS$_5$
With TilS$_5$
Without TilS$_5$
Nº of genomes with TilS$_6$
Nº of genomes without TilS$_6$
With TilS$_6$
Without TilS$_6$
Nº of genomes with TilS$_7$
Nº of genomes without TilS$_7$
With TilS$_7$
Without TilS$_7$
Nº of genomes with TilS$_8$
Nº of genomes without TilS$_8$
With TilS$_8$
Without TilS$_8$
Nº of genomes with TilS$_9$
Nº of genomes without TilS$_9$
With TilS$_9$
Without TilS$_9$
Nº of genomes with TilS$_{10}$
Nº of genomes without TilS$_{10}$
With TilS$_{10}$
Without TilS$_{10}$
Nº of genomes with TilS$_{11}$
Nº of genomes without TilS$_{11}$
With TilS$_{11}$
Without TilS$_{11}$
Nº of genomes with TilS$_{12}$
Nº of genomes without TilS$_{12}$
With TilS$_{12}$
Without TilS$_{12}$
Nº of genomes with TilS$_{13}$
Nº of genomes without TilS$_{13}$
With TilS$_{13}$
Without TilS$_{13}$
Nº of genomes with TilS$_{14}$
Nº of genomes without TilS$_{14}$
With TilS$_{14}$
Without TilS$_{14}$
Nº of genomes with TilS$_{15}$
Nº of genomes without TilS$_{15}$
With TilS$_{15}$
Without TilS$_{15}$

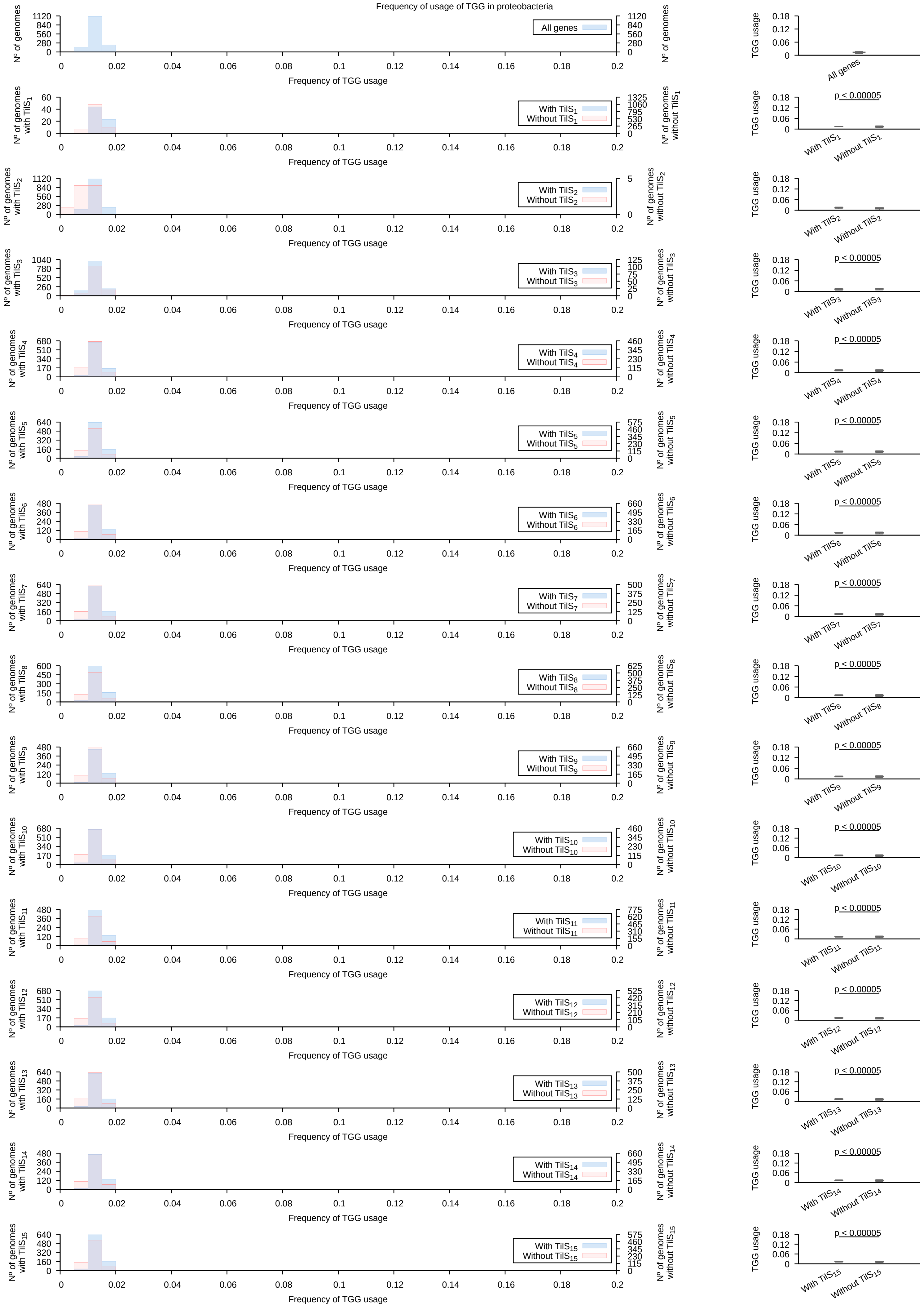
Frequency of usage of TGG in proteobacteria
Nº of genomes
All genes
Frequency of TGG usage
TGG usage
All genes
Nº of genomes with TilS1
Nº of genomes without TilS1
With TilS1
Without TilS1
p < 0.00005
Nº of genomes with TilS2
Nº of genomes without TilS2
With TilS2
Without TilS2
Nº of genomes with TilS3
Nº of genomes without TilS3
With TilS3
Without TilS3
Nº of genomes with TilS4
Nº of genomes without TilS4
With TilS4
Without TilS4
Nº of genomes with TilS5
Nº of genomes without TilS5
With TilS5
Without TilS5
Nº of genomes with TilS6
Nº of genomes without TilS6
With TilS6
Without TilS6
Nº of genomes with TilS7
Nº of genomes without TilS7
With TilS7
Without TilS7
Nº of genomes with TilS8
Nº of genomes without TilS8
With TilS8
Without TilS8
Nº of genomes with TilS9
Nº of genomes without TilS9
With TilS9
Without TilS9
Nº of genomes with TilS10
Nº of genomes without TilS10
With TilS10
Without TilS10
Nº of genomes with TilS11
Nº of genomes without TilS11
With TilS11
Without TilS11
Nº of genomes with TilS12
Nº of genomes without TilS12
With TilS12
Without TilS12
Nº of genomes with TilS13
Nº of genomes without TilS13
With TilS13
Without TilS13
Nº of genomes with TilS14
Nº of genomes without TilS14
With TilS14
Without TilS14
Nº of genomes with TilS15
Nº of genomes without TilS15
With TilS15
Without TilS15

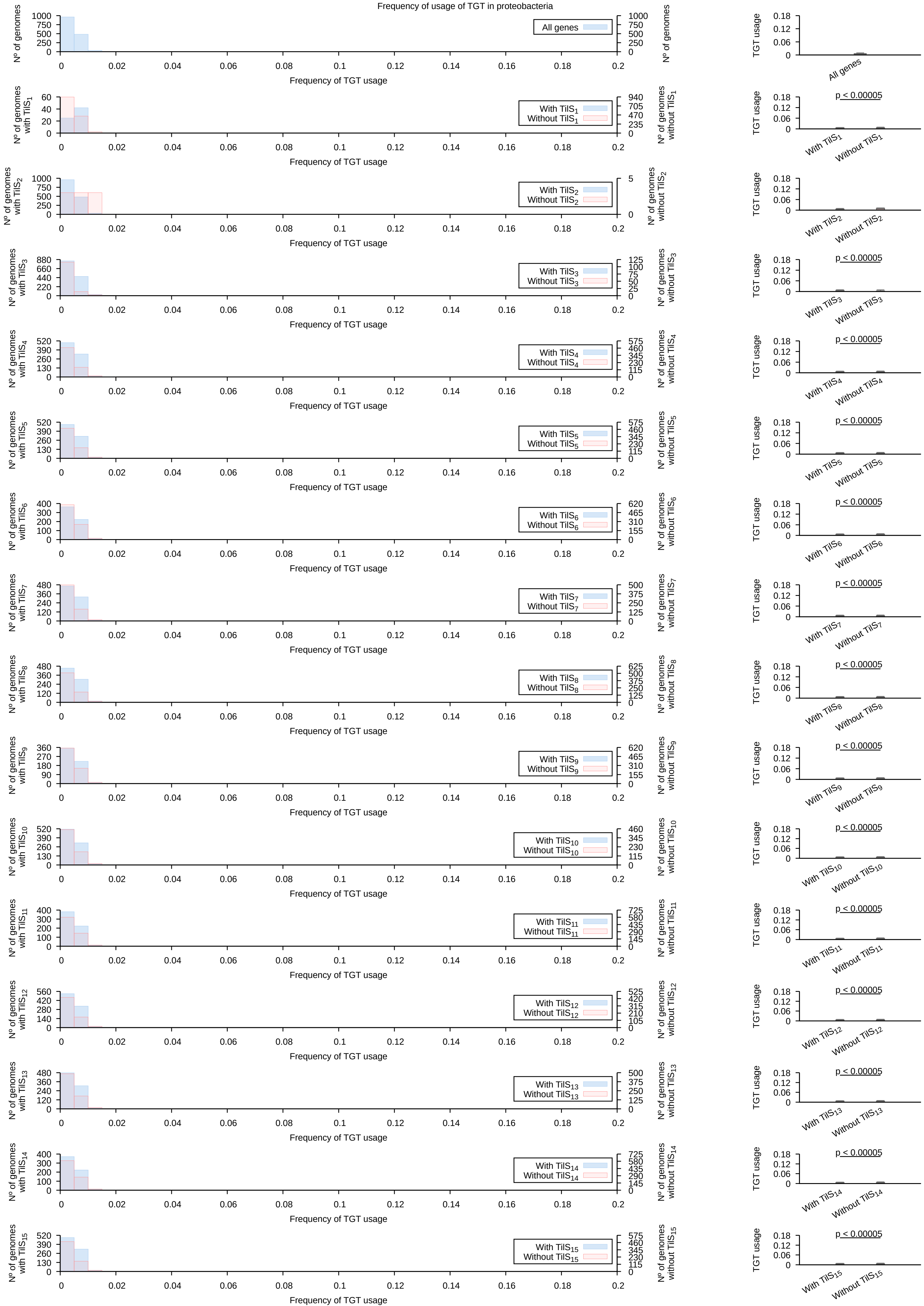
Frequency of usage of TGT in proteobacteria
Nº of genomes
1000 750 500 250 0
All genes
Nº of genomes
0 0.02 0.04 0.06 0.08 0.1 0.12 0.14 0.16 0.18 0.2
Frequency of TGT usage
TGT usage
0.18 0.12 0.06 0
All genes
Nº of genomes with TilS$_{1}$
60 40 20 0
With TilS$_{1}$
Without TilS$_{1}$
940 705 470 235 0
Nº of genomes without TilS$_{1}$
p < 0.00005
With TilS$_{1}$
Without TilS$_{1}$
Nº of genomes with TilS$_{2}$
1000 750 500 250 0
With TilS$_{2}$
Without TilS$_{2}$
5 0
Nº of genomes without TilS$_{2}$
With TilS$_{2}$
Without TilS$_{2}$
Nº of genomes with TilS$_{3}$
880 660 440 220 0
With TilS$_{3}$
Without TilS$_{3}$
125 100 75 50 25 0
Nº of genomes without TilS$_{3}$
p < 0.00005
With TilS$_{3}$
Without TilS$_{3}$
Nº of genomes with TilS$_{4}$
520 390 260 130 0
With TilS$_{4}$
Without TilS$_{4}$
575 460 345 230 115 0
Nº of genomes without TilS$_{4}$
p < 0.00005
With TilS$_{4}$
Without TilS$_{4}$
Nº of genomes with TilS$_{5}$
520 390 260 130 0
With TilS$_{5}$
Without TilS$_{5}$
575 460 345 230 115 0
Nº of genomes without TilS$_{5}$
p < 0.00005
With TilS$_{5}$
Without TilS$_{5}$
Nº of genomes with TilS$_{6}$
400 300 200 100 0
With TilS$_{6}$
Without TilS$_{6}$
620 465 310 155 0
Nº of genomes without TilS$_{6}$
p < 0.00005
With TilS$_{6}$
Without TilS$_{6}$
Nº of genomes with TilS$_{7}$
480 360 240 120 0
With TilS$_{7}$
Without TilS$_{7}$
500 375 250 125 0
Nº of genomes without TilS$_{7}$
p < 0.00005
With TilS$_{7}$
Without TilS$_{7}$
Nº of genomes with TilS$_{8}$
480 360 240 120 0
With TilS$_{8}$
Without TilS$_{8}$
625 500 375 250 125 0
Nº of genomes without TilS$_{8}$
p < 0.00005
With TilS$_{8}$
Without TilS$_{8}$
Nº of genomes with TilS$_{9}$
360 270 180 90 0
With TilS$_{9}$
Without TilS$_{9}$
620 465 310 155 0
Nº of genomes without TilS$_{9}$
p < 0.00005
With TilS$_{9}$
Without TilS$_{9}$
Nº of genomes with TilS$_{10}$
520 390 260 130 0
With TilS$_{10}$
Without TilS$_{10}$
460 345 230 115 0
Nº of genomes without TilS$_{10}$
p < 0.00005
With TilS$_{10}$
Without TilS$_{10}$
Nº of genomes with TilS$_{11}$
400 300 200 100 0
With TilS$_{11}$
Without TilS$_{11}$
725 580 435 290 145 0
Nº of genomes without TilS$_{11}$
p < 0.00005
With TilS$_{11}$
Without TilS$_{11}$
Nº of genomes with TilS$_{12}$
560 420 280 140 0
With TilS$_{12}$
Without TilS$_{12}$
525 420 315 210 105 0
Nº of genomes without TilS$_{12}$
p < 0.00005
With TilS$_{12}$
Without TilS$_{12}$
Nº of genomes with TilS$_{13}$
480 360 240 120 0
With TilS$_{13}$
Without TilS$_{13}$
500 375 250 125 0
Nº of genomes without TilS$_{13}$
p < 0.00005
With TilS$_{13}$
Without TilS$_{13}$
Nº of genomes with TilS$_{14}$
400 300 200 100 0
With TilS$_{14}$
Without TilS$_{14}$
725 580 435 290 145 0
Nº of genomes without TilS$_{14}$
p < 0.00005
With TilS$_{14}$
Without TilS$_{14}$
Nº of genomes with TilS$_{15}$
520 390 260 130 0
With TilS$_{15}$
Without TilS$_{15}$
575 460 345 230 115 0
Nº of genomes without TilS$_{15}$
p < 0.00005
With TilS$_{15}$
Without TilS$_{15}$

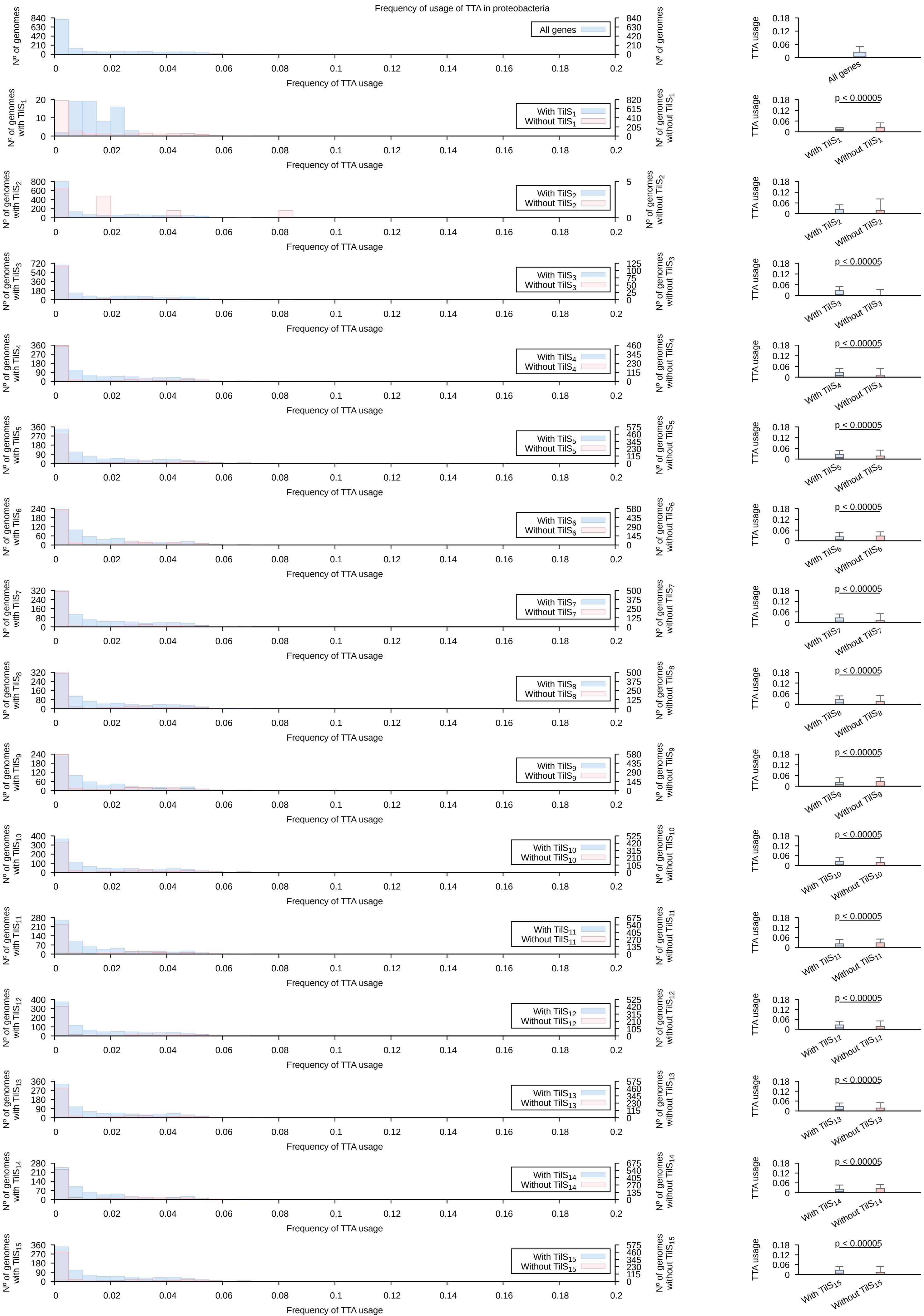

Frequency of usage of TTA in proteobacteria
Nº of genomes
Frequency of TTA usage
TTA usage
All genes
Nº of genomes with TilS$_1$
Nº of genomes without TilS$_1$
With TilS$_1$
Without TilS$_1$
p < 0.00005
Nº of genomes with TilS$_2$
Nº of genomes without TilS$_2$
With TilS$_2$
Without TilS$_2$
Nº of genomes with TilS$_3$
Nº of genomes without TilS$_3$
With TilS$_3$
Without TilS$_3$
p < 0.00005
Nº of genomes with TilS$_4$
Nº of genomes without TilS$_4$
With TilS$_4$
Without TilS$_4$
p < 0.00005
Nº of genomes with TilS$_5$
Nº of genomes without TilS$_5$
With TilS$_5$
Without TilS$_5$
p < 0.00005
Nº of genomes with TilS$_6$
Nº of genomes without TilS$_6$
With TilS$_6$
Without TilS$_6$
p < 0.00005
Nº of genomes with TilS$_7$
Nº of genomes without TilS$_7$
With TilS$_7$
Without TilS$_7$
p < 0.00005
Nº of genomes with TilS$_8$
Nº of genomes without TilS$_8$
With TilS$_8$
Without TilS$_8$
p < 0.00005
Nº of genomes with TilS$_9$
Nº of genomes without TilS$_9$
With TilS$_9$
Without TilS$_9$
p < 0.00005
Nº of genomes with TilS$_{10}$
Nº of genomes without TilS$_{10}$
With TilS$_{10}$
Without TilS$_{10}$
p < 0.00005
Nº of genomes with TilS$_{11}$
Nº of genomes without TilS$_{11}$
With TilS$_{11}$
Without TilS$_{11}$
p < 0.00005
Nº of genomes with TilS$_{12}$
Nº of genomes without TilS$_{12}$
With TilS$_{12}$
Without TilS$_{12}$
p < 0.00005
Nº of genomes with TilS$_{13}$
Nº of genomes without TilS$_{13}$
With TilS$_{13}$
Without TilS$_{13}$
p < 0.00005
Nº of genomes with TilS$_{14}$
Nº of genomes without TilS$_{14}$
With TilS$_{14}$
Without TilS$_{14}$
p < 0.00005
Nº of genomes with TilS$_{15}$
Nº of genomes without TilS$_{15}$
With TilS$_{15}$
Without TilS$_{15}$
p < 0.00005

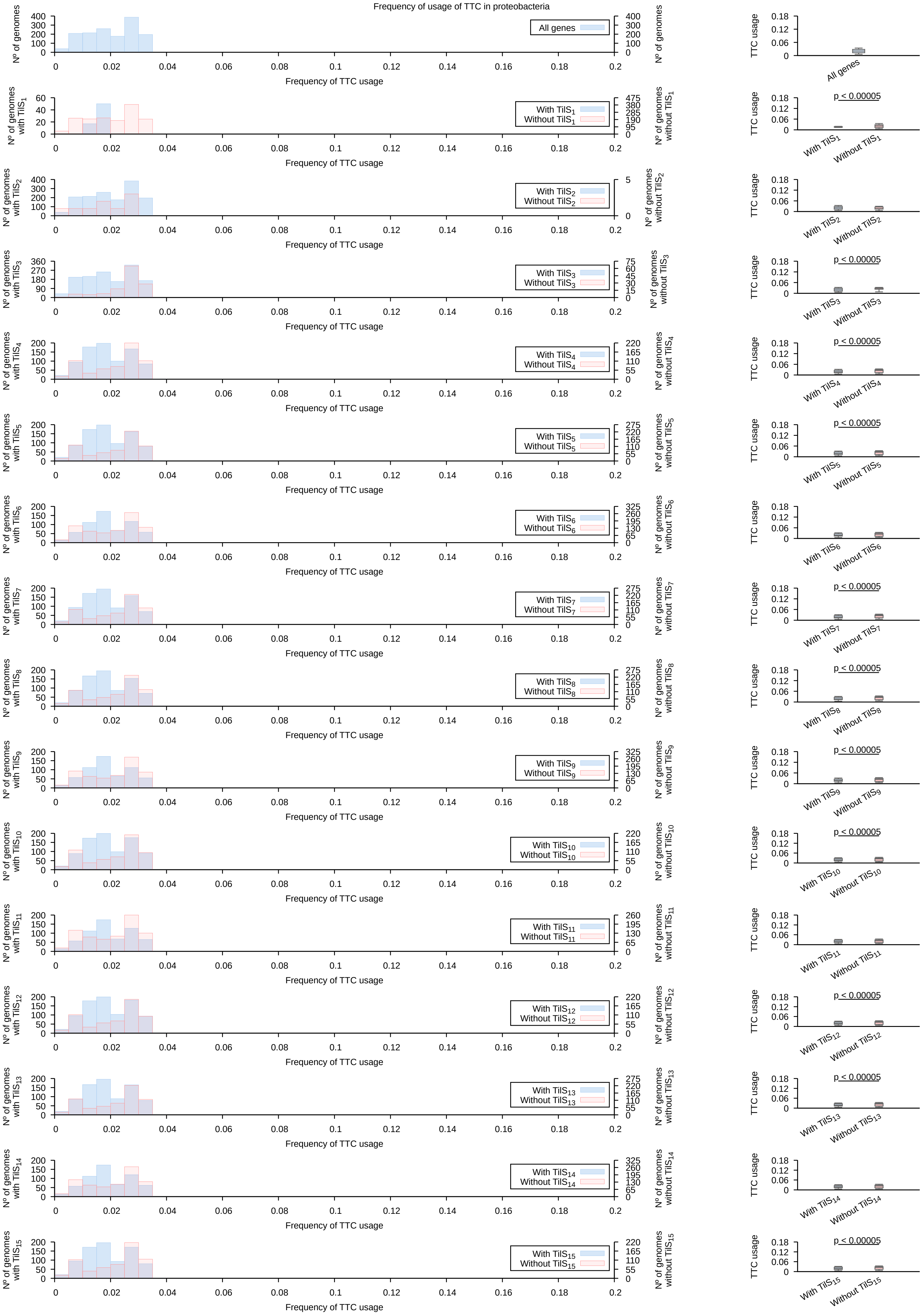

Frequency of usage of TTC in proteobacteria
Nº of genomes
All genes
Frequency of TTC usage
TTC usage
All genes
Nº of genomes with TilS1
Nº of genomes without TilS1
With TilS1
Without TilS1
p < 0.00005
Nº of genomes with TilS2
Nº of genomes without TilS2
With TilS2
Without TilS2
Nº of genomes with TilS3
Nº of genomes without TilS3
With TilS3
Without TilS3
p < 0.00005
Nº of genomes with TilS4
Nº of genomes without TilS4
With TilS4
Without TilS4
p < 0.00005
Nº of genomes with TilS5
Nº of genomes without TilS5
With TilS5
Without TilS5
p < 0.00005
Nº of genomes with TilS6
Nº of genomes without TilS6
With TilS6
Without TilS6
Nº of genomes with TilS7
Nº of genomes without TilS7
With TilS7
Without TilS7
p < 0.00005
Nº of genomes with TilS8
Nº of genomes without TilS8
With TilS8
Without TilS8
p < 0.00005
Nº of genomes with TilS9
Nº of genomes without TilS9
With TilS9
Without TilS9
p < 0.00005
Nº of genomes with TilS10
Nº of genomes without TilS10
With TilS10
Without TilS10
p < 0.00005
Nº of genomes with TilS11
Nº of genomes without TilS11
With TilS11
Without TilS11
Nº of genomes with TilS12
Nº of genomes without TilS12
With TilS12
Without TilS12
p < 0.00005
Nº of genomes with TilS13
Nº of genomes without TilS13
With TilS13
Without TilS13
p < 0.00005
Nº of genomes with TilS14
Nº of genomes without TilS14
With TilS14
Without TilS14
Nº of genomes with TilS15
Nº of genomes without TilS15
With TilS15
Without TilS15
p < 0.00005

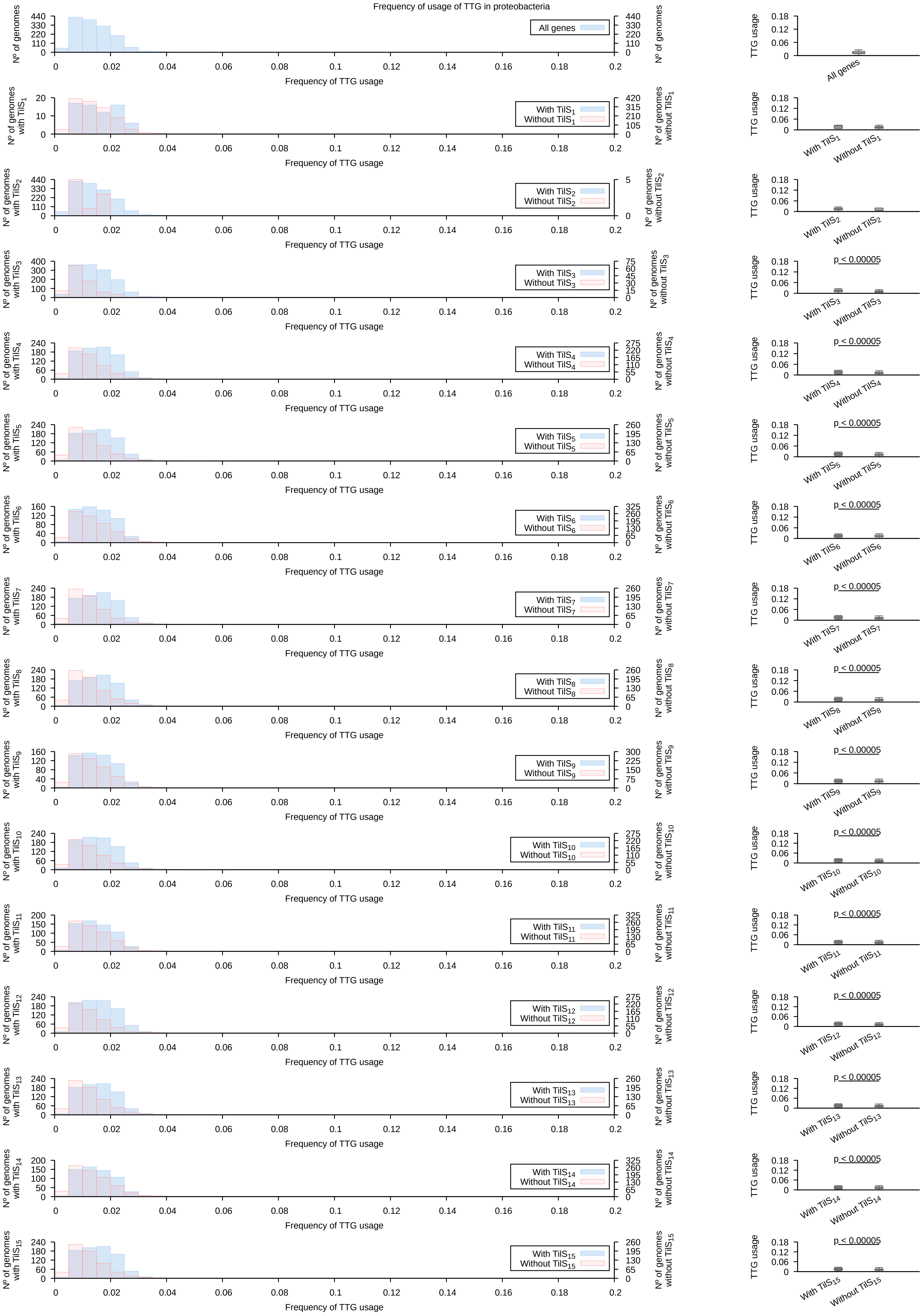

Frequency of usage of TTG in proteobacteria
Nº of genomes
Frequency of TTG usage
TTG usage
All genes
With TilS1
Without TilS1
With TilS2
Without TilS2
With TilS3
Without TilS3
With TilS4
Without TilS4
With TilS5
Without TilS5
With TilS6
Without TilS6
With TilS7
Without TilS7
With TilS8
Without TilS8
With TilS9
Without TilS9
With TilS10
Without TilS10
With TilS11
Without TilS11
With TilS12
Without TilS12
With TilS13
Without TilS13
With TilS14
Without TilS14
With TilS15
Without TilS15
p < 0.00005

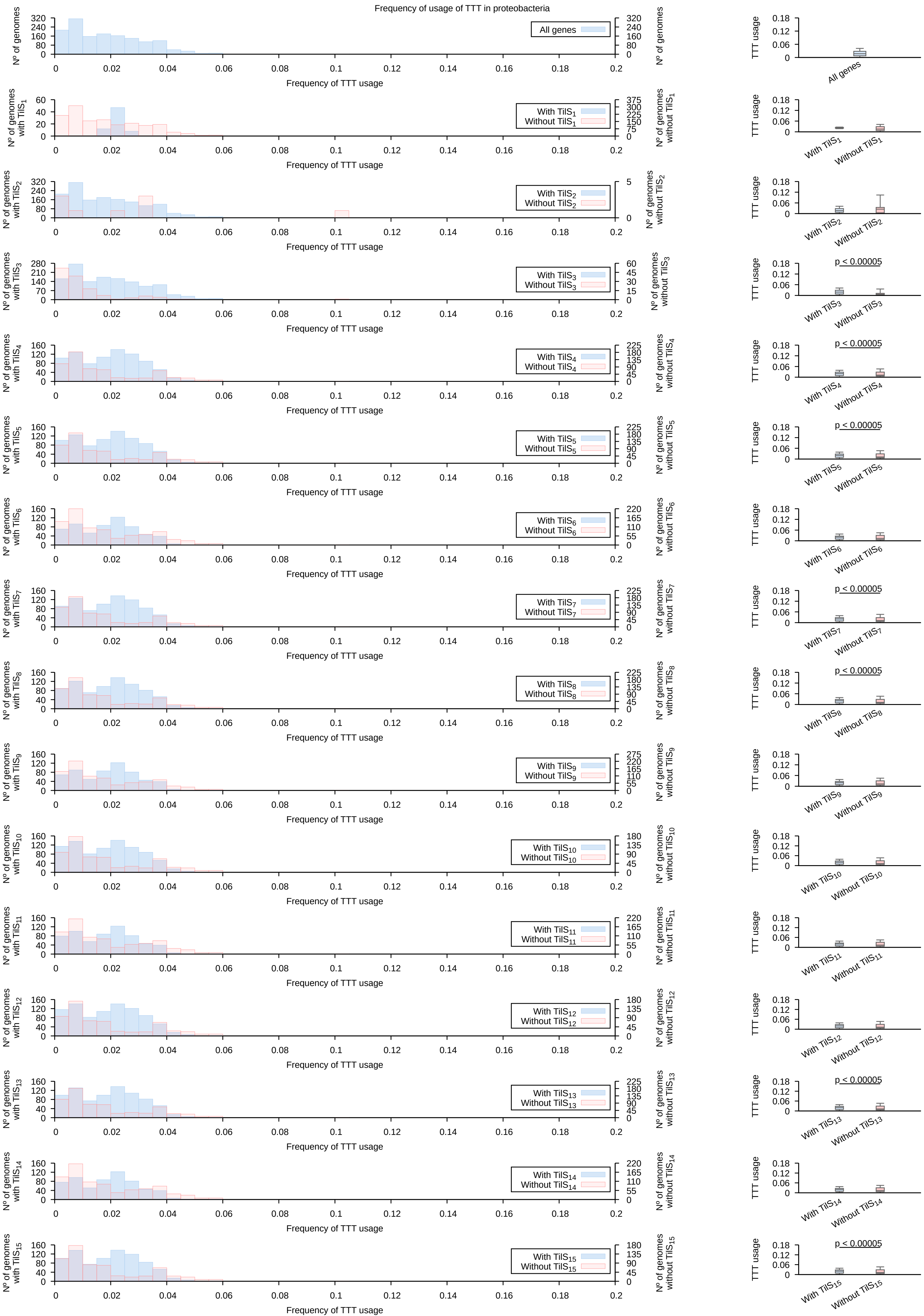

Frequency of usage of TTT in proteobacteria
Nº of genomes
All genes
Frequency of TTT usage
TTT usage
All genes
Nº of genomes with TilS$_1$
Nº of genomes without TilS$_1$
With TilS$_1$
Without TilS$_1$
Nº of genomes with TilS$_2$
Nº of genomes without TilS$_2$
With TilS$_2$
Without TilS$_2$
Nº of genomes with TilS$_3$
Nº of genomes without TilS$_3$
With TilS$_3$
Without TilS$_3$
p < 0.00005
Nº of genomes with TilS$_4$
Nº of genomes without TilS$_4$
With TilS$_4$
Without TilS$_4$
p < 0.00005
Nº of genomes with TilS$_5$
Nº of genomes without TilS$_5$
With TilS$_5$
Without TilS$_5$
p < 0.00005
Nº of genomes with TilS$_6$
Nº of genomes without TilS$_6$
With TilS$_6$
Without TilS$_6$
Nº of genomes with TilS$_7$
Nº of genomes without TilS$_7$
With TilS$_7$
Without TilS$_7$
p < 0.00005
Nº of genomes with TilS$_8$
Nº of genomes without TilS$_8$
With TilS$_8$
Without TilS$_8$
p < 0.00005
Nº of genomes with TilS$_9$
Nº of genomes without TilS$_9$
With TilS$_9$
Without TilS$_9$
Nº of genomes with TilS$_{10}$
Nº of genomes without TilS$_{10}$
With TilS$_{10}$
Without TilS$_{10}$
Nº of genomes with TilS$_{11}$
Nº of genomes without TilS$_{11}$
With TilS$_{11}$
Without TilS$_{11}$
Nº of genomes with TilS$_{12}$
Nº of genomes without TilS$_{12}$
With TilS$_{12}$
Without TilS$_{12}$
Nº of genomes with TilS$_{13}$
Nº of genomes without TilS$_{13}$
With TilS$_{13}$
Without TilS$_{13}$
p < 0.00005
Nº of genomes with TilS$_{14}$
Nº of genomes without TilS$_{14}$
With TilS$_{14}$
Without TilS$_{14}$
Nº of genomes with TilS$_{15}$
Nº of genomes without TilS$_{15}$
With TilS$_{15}$
Without TilS$_{15}$
p < 0.00005

Figure S14. Histogram of codon usage in all genes of genomes that present or lack genes involved in s^2U formation. Top left graph: histogram of the usage of the indicated codon in highly expressed genes of proteobacterial genomes. **Top right graph:** box plot indicating the distribution of codon usage in the same population. **Lower graphs:** The collection of 1484 proteobacterial genomes were separated in two groups, based on the presence (light blue) or absence (orange) of the indicated pattern in any gene of the analyzed genomes. Patterns are indicated using subscript numbers. See table S4 for a detailed description of the used patterns. **Left:** Data is represented in superposed histograms. **Right:** Data is represented in a boxplot. An horizontal bar was added when the two populations are different based on Wilcoxon-Mann-Whitney test and $p < 0.00005$. Whiskers include 95% of data.

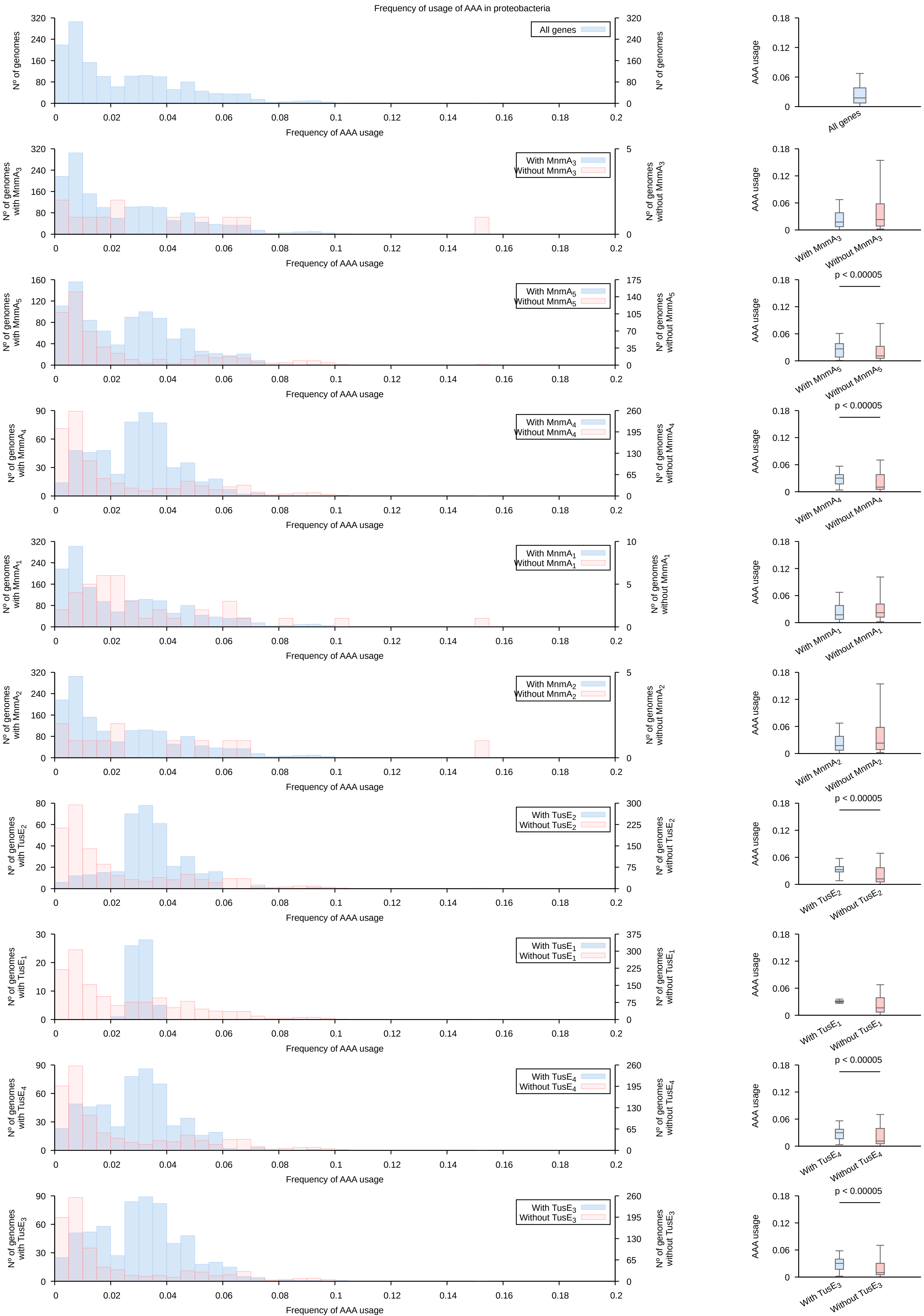

Frequency of usage of AAA in proteobacteria
All genes
Nº of genomes
Frequency of AAA usage
AAA usage
With MnmA$_3$
Without MnmA$_3$
Nº of genomes with MnmA$_3$
Nº of genomes without MnmA$_3$
With MnmA$_5$
Without MnmA$_5$
Nº of genomes with MnmA$_5$
Nº of genomes without MnmA$_5$
p < 0.00005
With MnmA$_4$
Without MnmA$_4$
Nº of genomes with MnmA$_4$
Nº of genomes without MnmA$_4$
p < 0.00005
With MnmA$_1$
Without MnmA$_1$
Nº of genomes with MnmA$_1$
Nº of genomes without MnmA$_1$
With MnmA$_2$
Without MnmA$_2$
Nº of genomes with MnmA$_2$
Nº of genomes without MnmA$_2$
With TusE$_2$
Without TusE$_2$
Nº of genomes with TusE$_2$
Nº of genomes without TusE$_2$
p < 0.00005
With TusE$_1$
Without TusE$_1$
Nº of genomes with TusE$_1$
Nº of genomes without TusE$_1$
With TusE$_4$
Without TusE$_4$
Nº of genomes with TusE$_4$
Nº of genomes without TusE$_4$
p < 0.00005
With TusE$_3$
Without TusE$_3$
Nº of genomes with TusE$_3$
Nº of genomes without TusE$_3$
p < 0.00005

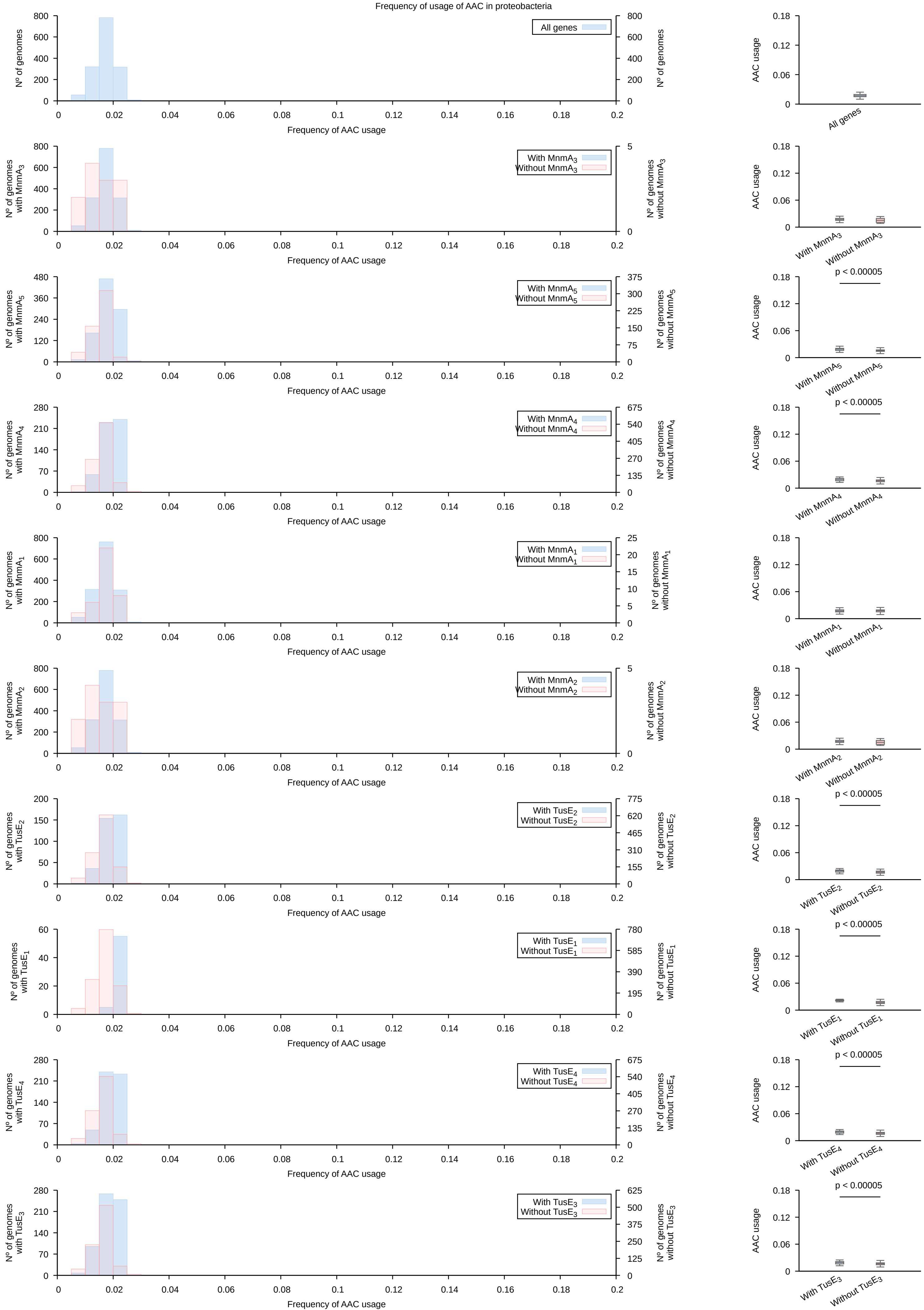
Frequency of usage of AAC in proteobacteria
Nº of genomes
All genes
Frequency of AAC usage
AAC usage
Nº of genomes with MnmA$_3$
With MnmA$_3$
Without MnmA$_3$
Nº of genomes without MnmA$_3$
Nº of genomes with MnmA$_5$
With MnmA$_5$
Without MnmA$_5$
Nº of genomes without MnmA$_5$
p < 0.00005
Nº of genomes with MnmA$_4$
With MnmA$_4$
Without MnmA$_4$
Nº of genomes without MnmA$_4$
p < 0.00005
Nº of genomes with MnmA$_1$
With MnmA$_1$
Without MnmA$_1$
Nº of genomes without MnmA$_1$
Nº of genomes with MnmA$_2$
With MnmA$_2$
Without MnmA$_2$
Nº of genomes without MnmA$_2$
Nº of genomes with TusE$_2$
With TusE$_2$
Without TusE$_2$
Nº of genomes without TusE$_2$
p < 0.00005
Nº of genomes with TusE$_1$
With TusE$_1$
Without TusE$_1$
Nº of genomes without TusE$_1$
p < 0.00005
Nº of genomes with TusE$_4$
With TusE$_4$
Without TusE$_4$
Nº of genomes without TusE$_4$
p < 0.00005
Nº of genomes with TusE$_3$
With TusE$_3$
Without TusE$_3$
Nº of genomes without TusE$_3$
p < 0.00005

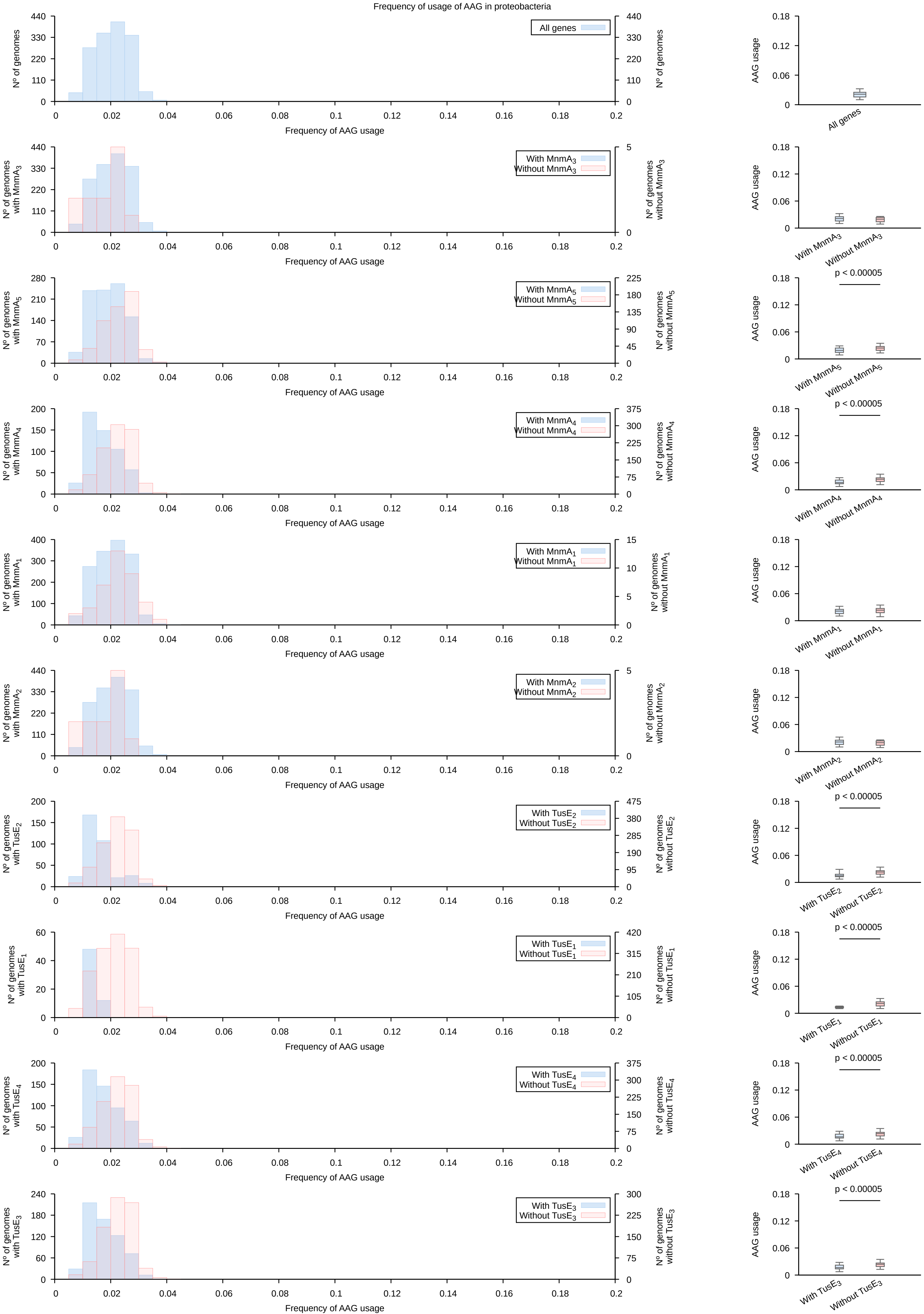
Frequency of usage of AAG in proteobacteria
All genes
Nº of genomes
Frequency of AAG usage
AAG usage
With MnmA$_3$
Without MnmA$_3$
Nº of genomes with MnmA$_3$
Nº of genomes without MnmA$_3$
With MnmA$_5$
Without MnmA$_5$
Nº of genomes with MnmA$_5$
Nº of genomes without MnmA$_5$
p < 0.00005
With MnmA$_4$
Without MnmA$_4$
Nº of genomes with MnmA$_4$
Nº of genomes without MnmA$_4$
p < 0.00005
With MnmA$_1$
Without MnmA$_1$
Nº of genomes with MnmA$_1$
Nº of genomes without MnmA$_1$
With MnmA$_2$
Without MnmA$_2$
Nº of genomes with MnmA$_2$
Nº of genomes without MnmA$_2$
With TusE$_2$
Without TusE$_2$
Nº of genomes with TusE$_2$
Nº of genomes without TusE$_2$
p < 0.00005
With TusE$_1$
Without TusE$_1$
Nº of genomes with TusE$_1$
Nº of genomes without TusE$_1$
p < 0.00005
With TusE$_4$
Without TusE$_4$
Nº of genomes with TusE$_4$
Nº of genomes without TusE$_4$
p < 0.00005
With TusE$_3$
Without TusE$_3$
Nº of genomes with TusE$_3$
Nº of genomes without TusE$_3$
p < 0.00005

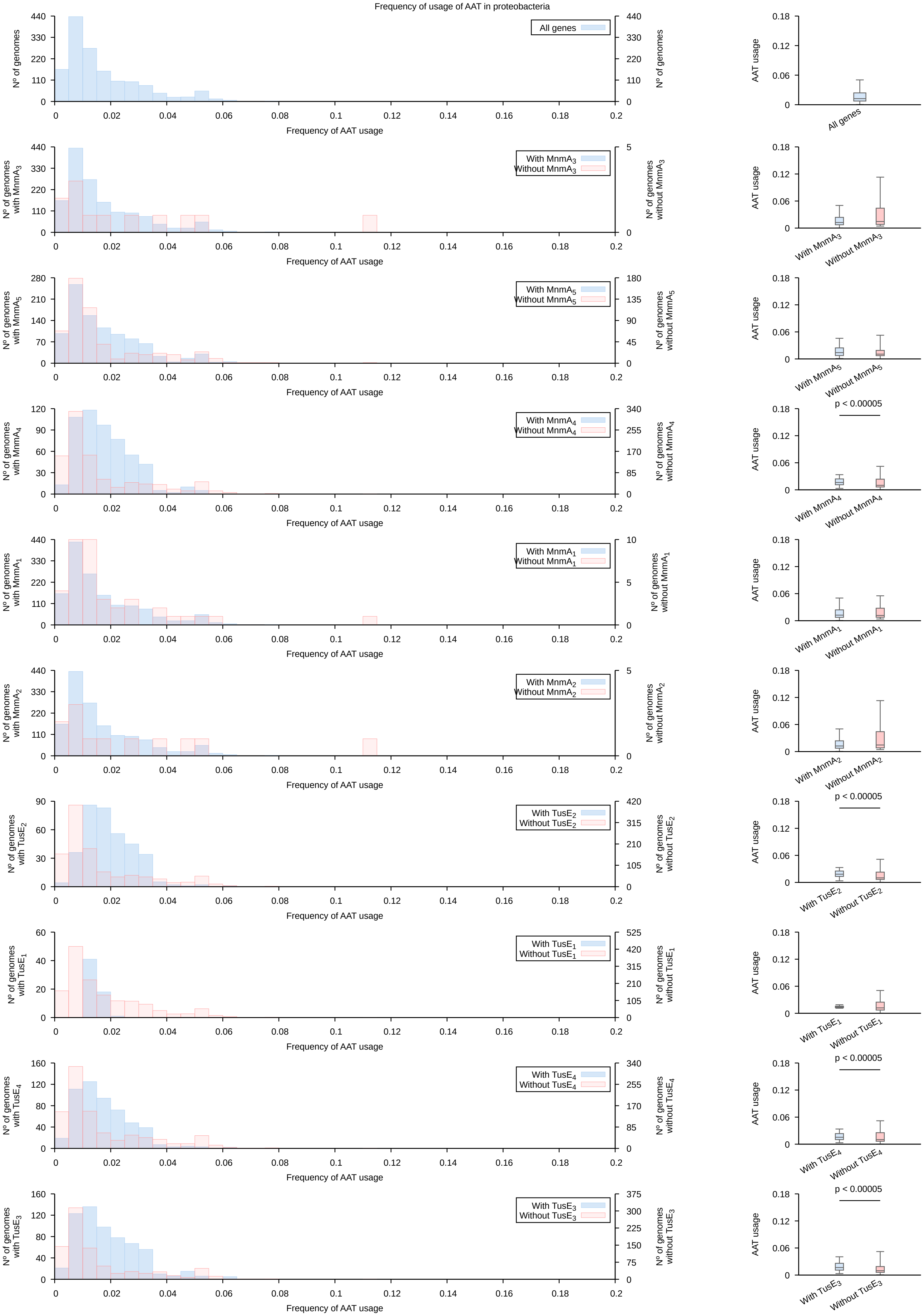

Frequency of usage of AAT in proteobacteria
Nº of genomes
Frequency of AAT usage
AAT usage
All genes
With MnmA3
Without MnmA3
With MnmA5
Without MnmA5
With MnmA4
Without MnmA4
With MnmA1
Without MnmA1
With MnmA2
Without MnmA2
With TusE2
Without TusE2
With TusE1
Without TusE1
With TusE4
Without TusE4
With TusE3
Without TusE3
p < 0.00005

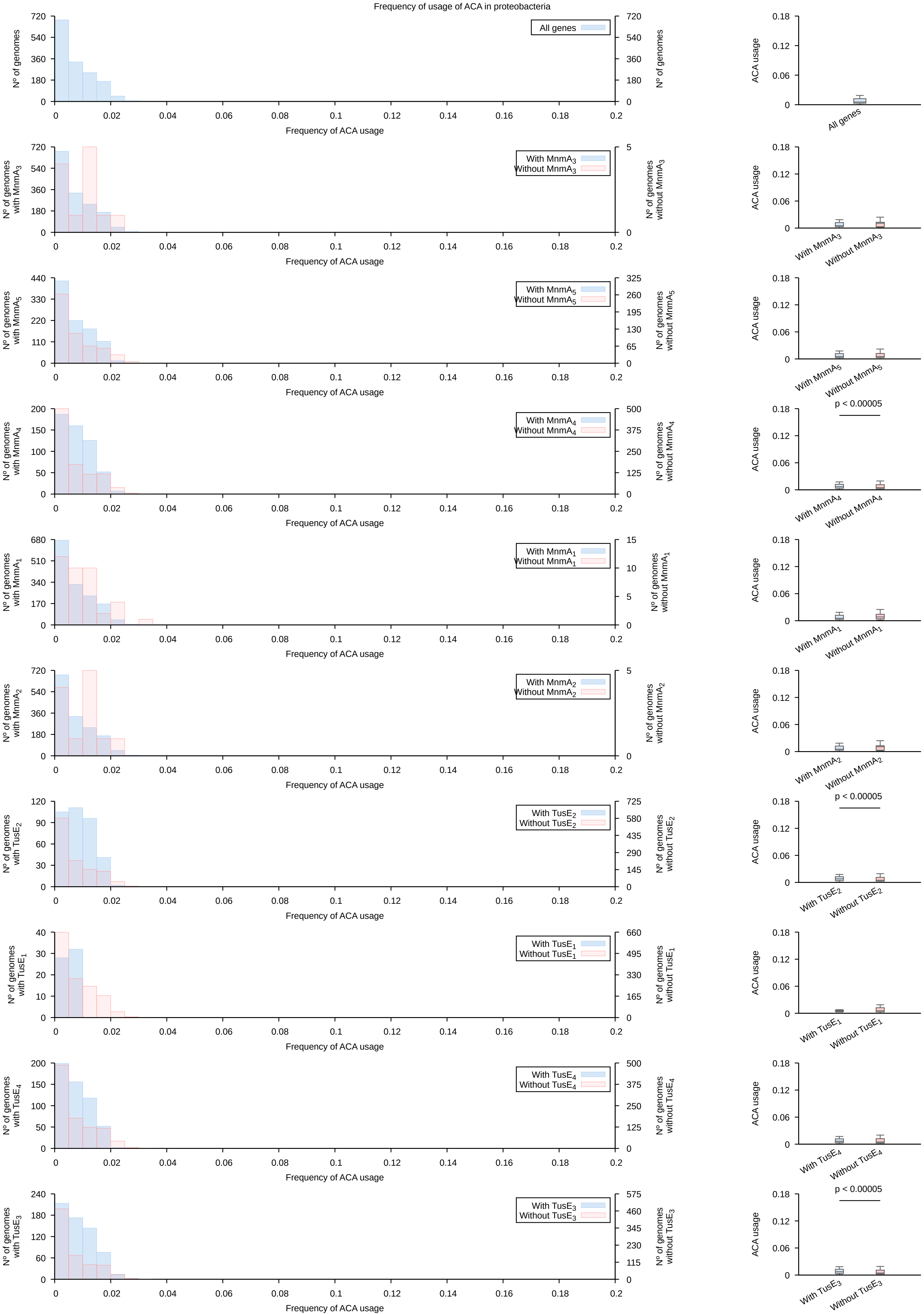
Frequency of usage of ACA in proteobacteria
All genes
Nº of genomes
Frequency of ACA usage
ACA usage
With MnmA$_3$
Without MnmA$_3$
Nº of genomes with MnmA$_3$
Nº of genomes without MnmA$_3$
With MnmA$_5$
Without MnmA$_5$
Nº of genomes with MnmA$_5$
Nº of genomes without MnmA$_5$
p < 0.00005
With MnmA$_4$
Without MnmA$_4$
Nº of genomes with MnmA$_4$
Nº of genomes without MnmA$_4$
With MnmA$_1$
Without MnmA$_1$
Nº of genomes with MnmA$_1$
Nº of genomes without MnmA$_1$
With MnmA$_2$
Without MnmA$_2$
Nº of genomes with MnmA$_2$
Nº of genomes without MnmA$_2$
With TusE$_2$
Without TusE$_2$
Nº of genomes with TusE$_2$
Nº of genomes without TusE$_2$
With TusE$_1$
Without TusE$_1$
Nº of genomes with TusE$_1$
Nº of genomes without TusE$_1$
With TusE$_4$
Without TusE$_4$
Nº of genomes with TusE$_4$
Nº of genomes without TusE$_4$
With TusE$_3$
Without TusE$_3$
Nº of genomes with TusE$_3$
Nº of genomes without TusE$_3$

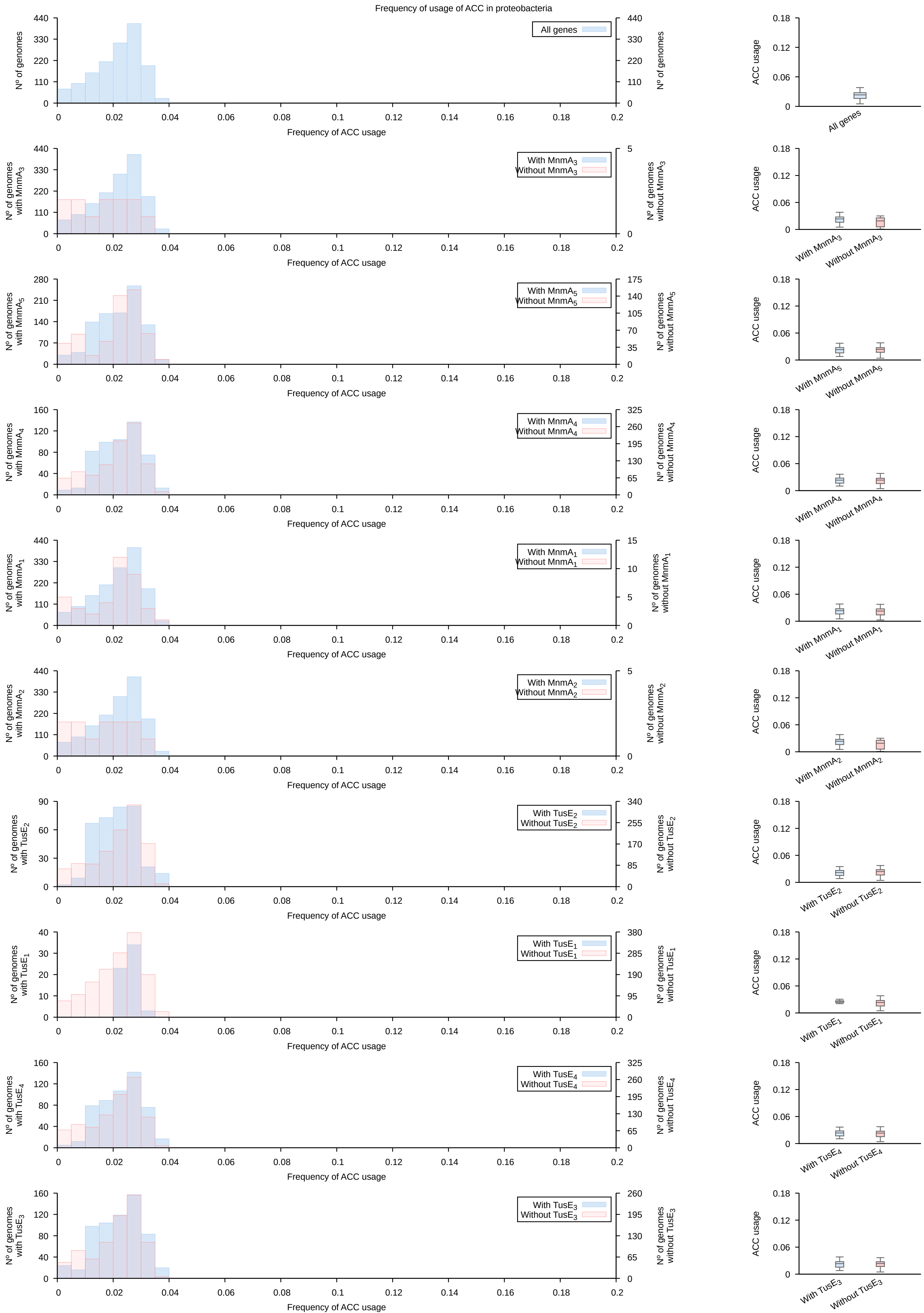
Frequency of usage of ACC in proteobacteria
Nº of genomes
Frequency of ACC usage
ACC usage
All genes
Nº of genomes with MnmA3
Nº of genomes without MnmA3
With MnmA3
Without MnmA3
Nº of genomes with MnmA5
Nº of genomes without MnmA5
With MnmA5
Without MnmA5
Nº of genomes with MnmA4
Nº of genomes without MnmA4
With MnmA4
Without MnmA4
Nº of genomes with MnmA1
Nº of genomes without MnmA1
With MnmA1
Without MnmA1
Nº of genomes with MnmA2
Nº of genomes without MnmA2
With MnmA2
Without MnmA2
Nº of genomes with TusE2
Nº of genomes without TusE2
With TusE2
Without TusE2
Nº of genomes with TusE1
Nº of genomes without TusE1
With TusE1
Without TusE1
Nº of genomes with TusE4
Nº of genomes without TusE4
With TusE4
Without TusE4
Nº of genomes with TusE3
Nº of genomes without TusE3
With TusE3
Without TusE3

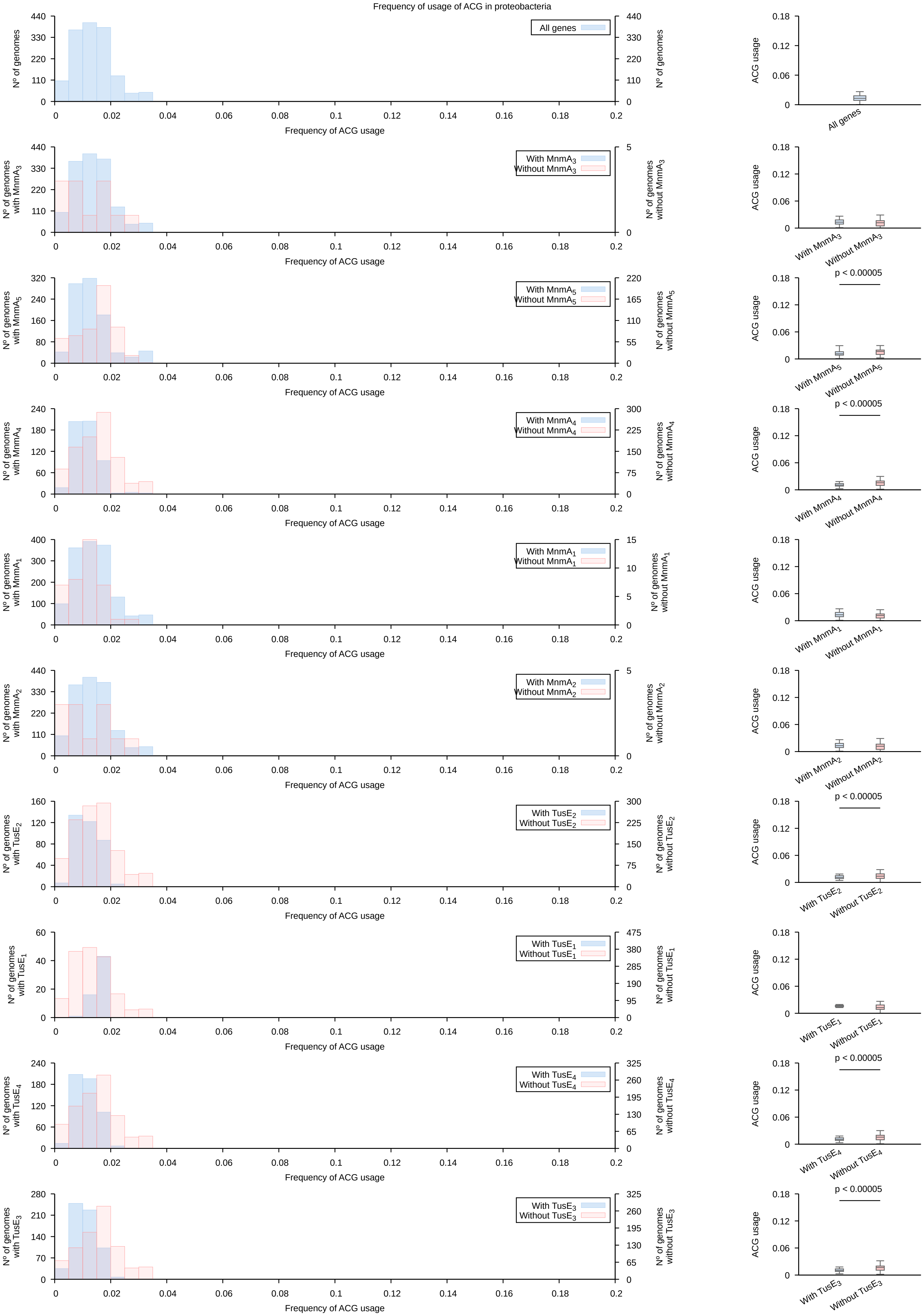
Frequency of usage of ACG in proteobacteria
All genes
Nº of genomes
Frequency of ACG usage
ACG usage
With MnmA3
Without MnmA3
Nº of genomes with MnmA3
Nº of genomes without MnmA3
With MnmA5
Without MnmA5
Nº of genomes with MnmA5
Nº of genomes without MnmA5
p < 0.00005
With MnmA4
Without MnmA4
Nº of genomes with MnmA4
Nº of genomes without MnmA4
p < 0.00005
With MnmA1
Without MnmA1
Nº of genomes with MnmA1
Nº of genomes without MnmA1
With MnmA2
Without MnmA2
Nº of genomes with MnmA2
Nº of genomes without MnmA2
With TusE2
Without TusE2
Nº of genomes with TusE2
Nº of genomes without TusE2
p < 0.00005
With TusE1
Without TusE1
Nº of genomes with TusE1
Nº of genomes without TusE1
With TusE4
Without TusE4
Nº of genomes with TusE4
Nº of genomes without TusE4
p < 0.00005
With TusE3
Without TusE3
Nº of genomes with TusE3
Nº of genomes without TusE3
p < 0.00005
0 0.02 0.04 0.06 0.08 0.1 0.12 0.14 0.16 0.18 0.2
0 0.06 0.12 0.18

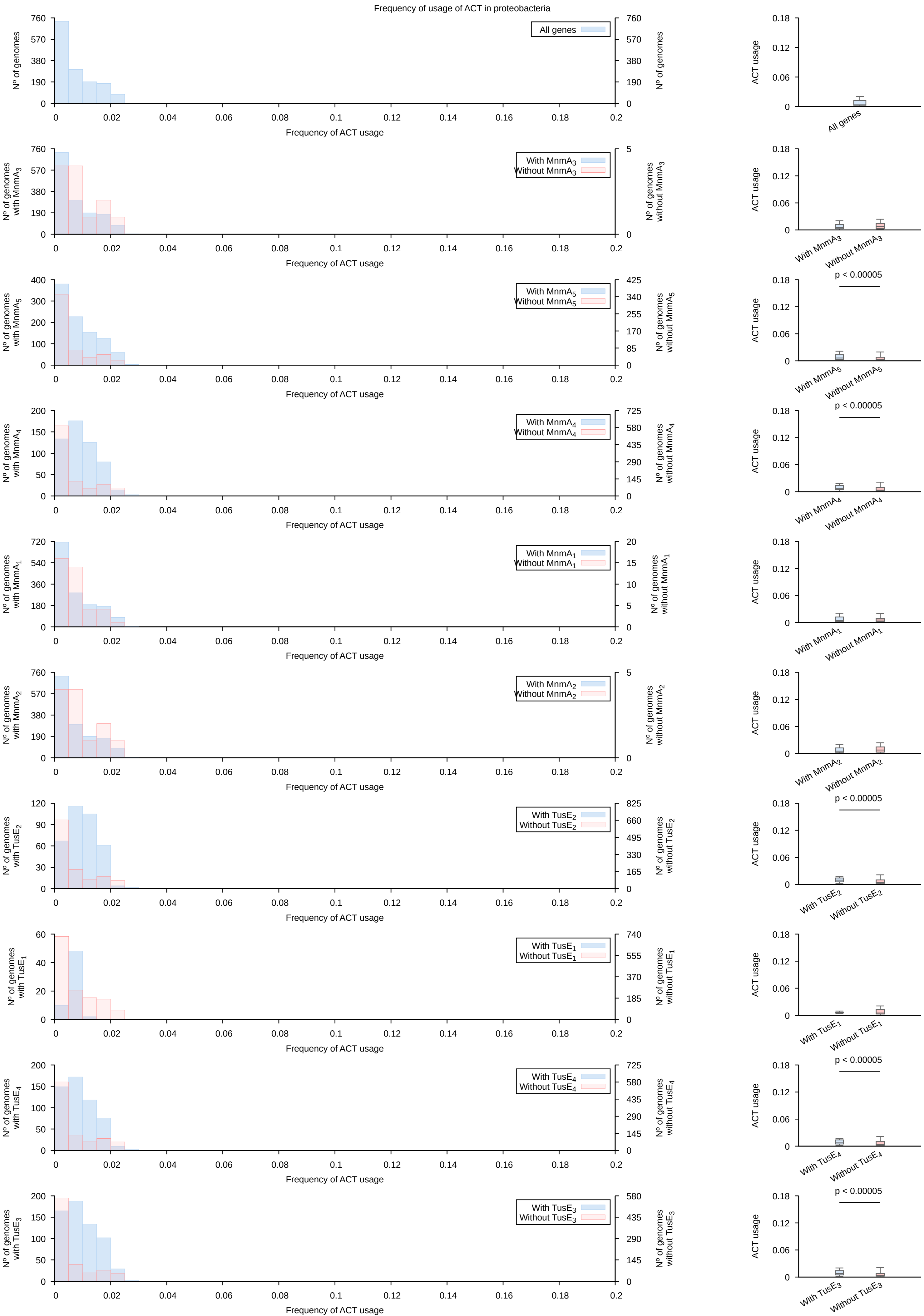

Frequency of usage of ACT in proteobacteria
All genes
Nº of genomes
Frequency of ACT usage
ACT usage
With MnmA3
Without MnmA3
Nº of genomes with MnmA3
Nº of genomes without MnmA3
With MnmA5
Without MnmA5
Nº of genomes with MnmA5
Nº of genomes without MnmA5
p < 0.00005
With MnmA4
Without MnmA4
Nº of genomes with MnmA4
Nº of genomes without MnmA4
p < 0.00005
With MnmA1
Without MnmA1
Nº of genomes with MnmA1
Nº of genomes without MnmA1
With MnmA2
Without MnmA2
Nº of genomes with MnmA2
Nº of genomes without MnmA2
With TusE2
Without TusE2
Nº of genomes with TusE2
Nº of genomes without TusE2
p < 0.00005
With TusE1
Without TusE1
Nº of genomes with TusE1
Nº of genomes without TusE1
With TusE4
Without TusE4
Nº of genomes with TusE4
Nº of genomes without TusE4
p < 0.00005
With TusE3
Without TusE3
Nº of genomes with TusE3
Nº of genomes without TusE3
p < 0.00005

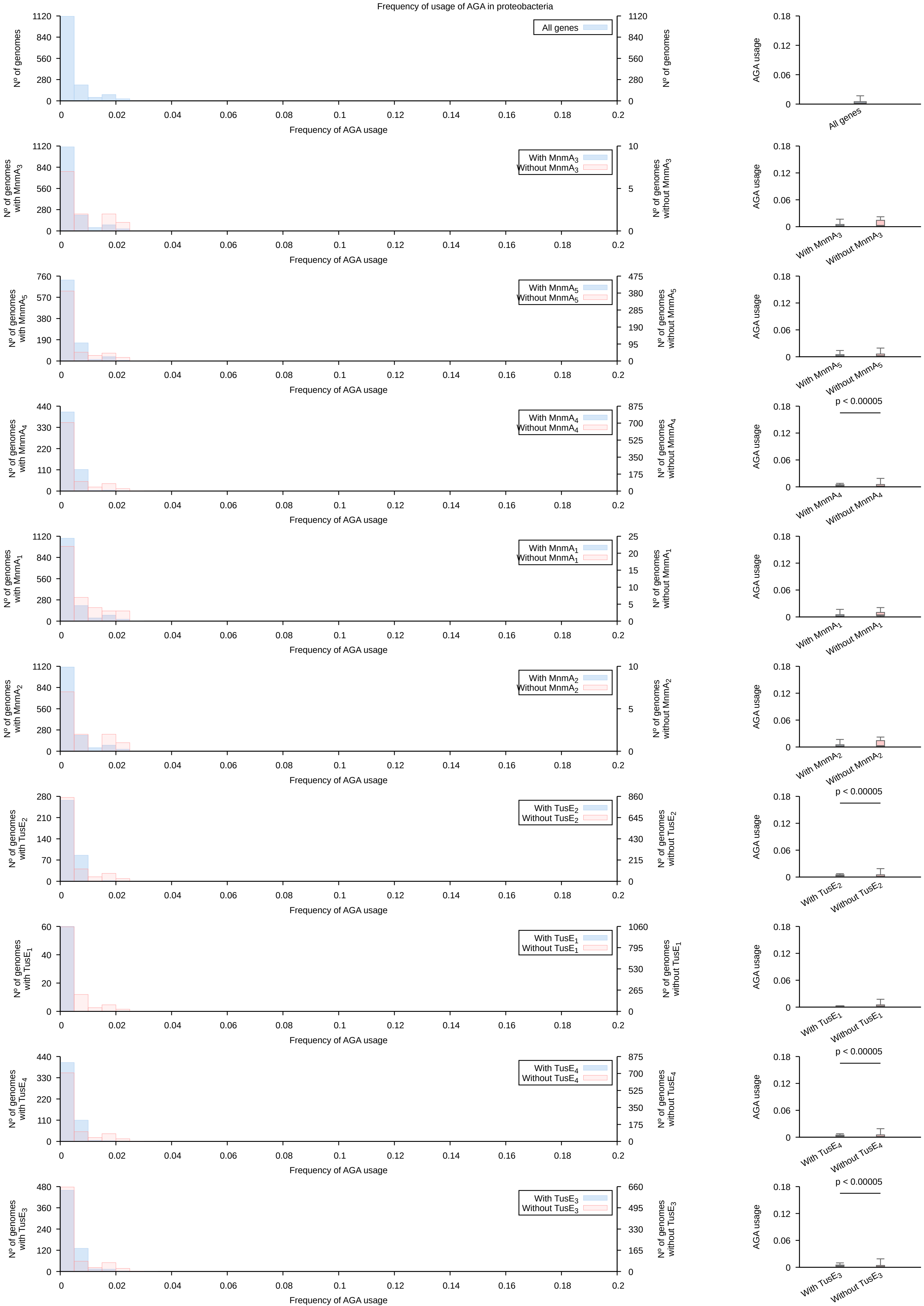

Frequency of usage of AGA in proteobacteria
Nº of genomes
All genes
AGA usage
Frequency of AGA usage
Nº of genomes with MnmA$_3$
Nº of genomes without MnmA$_3$
With MnmA$_3$
Without MnmA$_3$
Nº of genomes with MnmA$_5$
Nº of genomes without MnmA$_5$
With MnmA$_5$
Without MnmA$_5$
Nº of genomes with MnmA$_4$
Nº of genomes without MnmA$_4$
With MnmA$_4$
Without MnmA$_4$
p < 0.00005
Nº of genomes with MnmA$_1$
Nº of genomes without MnmA$_1$
With MnmA$_1$
Without MnmA$_1$
Nº of genomes with MnmA$_2$
Nº of genomes without MnmA$_2$
With MnmA$_2$
Without MnmA$_2$
Nº of genomes with TusE$_2$
Nº of genomes without TusE$_2$
With TusE$_2$
Without TusE$_2$
p < 0.00005
Nº of genomes with TusE$_1$
Nº of genomes without TusE$_1$
With TusE$_1$
Without TusE$_1$
Nº of genomes with TusE$_4$
Nº of genomes without TusE$_4$
With TusE$_4$
Without TusE$_4$
p < 0.00005
Nº of genomes with TusE$_3$
Nº of genomes without TusE$_3$
With TusE$_3$
Without TusE$_3$
p < 0.00005

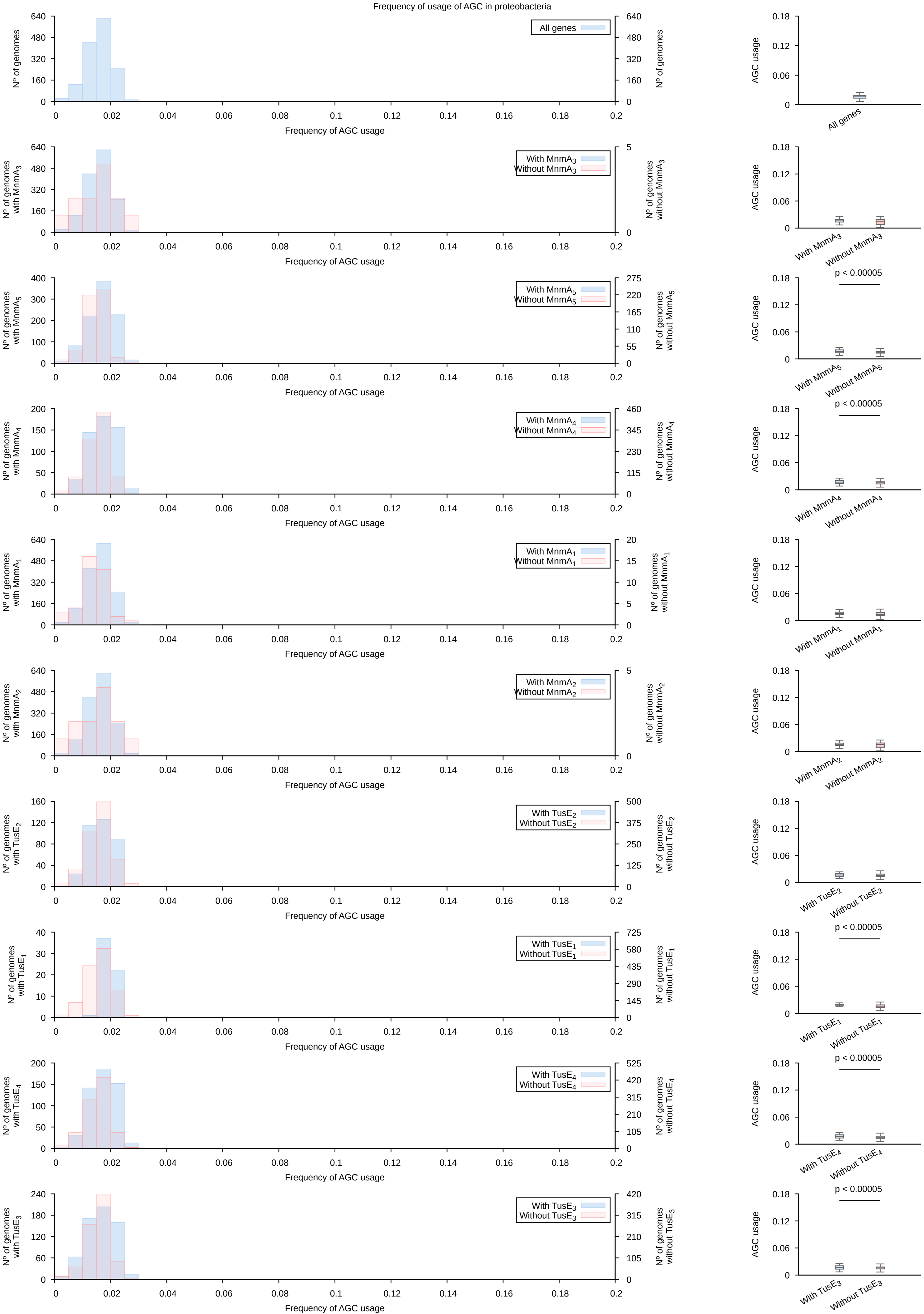

Frequency of usage of AGC in proteobacteria
Nº of genomes
Frequency of AGC usage
AGC usage
All genes
With MnmA3
Without MnmA3
With MnmA5
Without MnmA5
With MnmA4
Without MnmA4
With MnmA1
Without MnmA1
With MnmA2
Without MnmA2
With TusE2
Without TusE2
With TusE1
Without TusE1
With TusE4
Without TusE4
With TusE3
Without TusE3
p < 0.00005

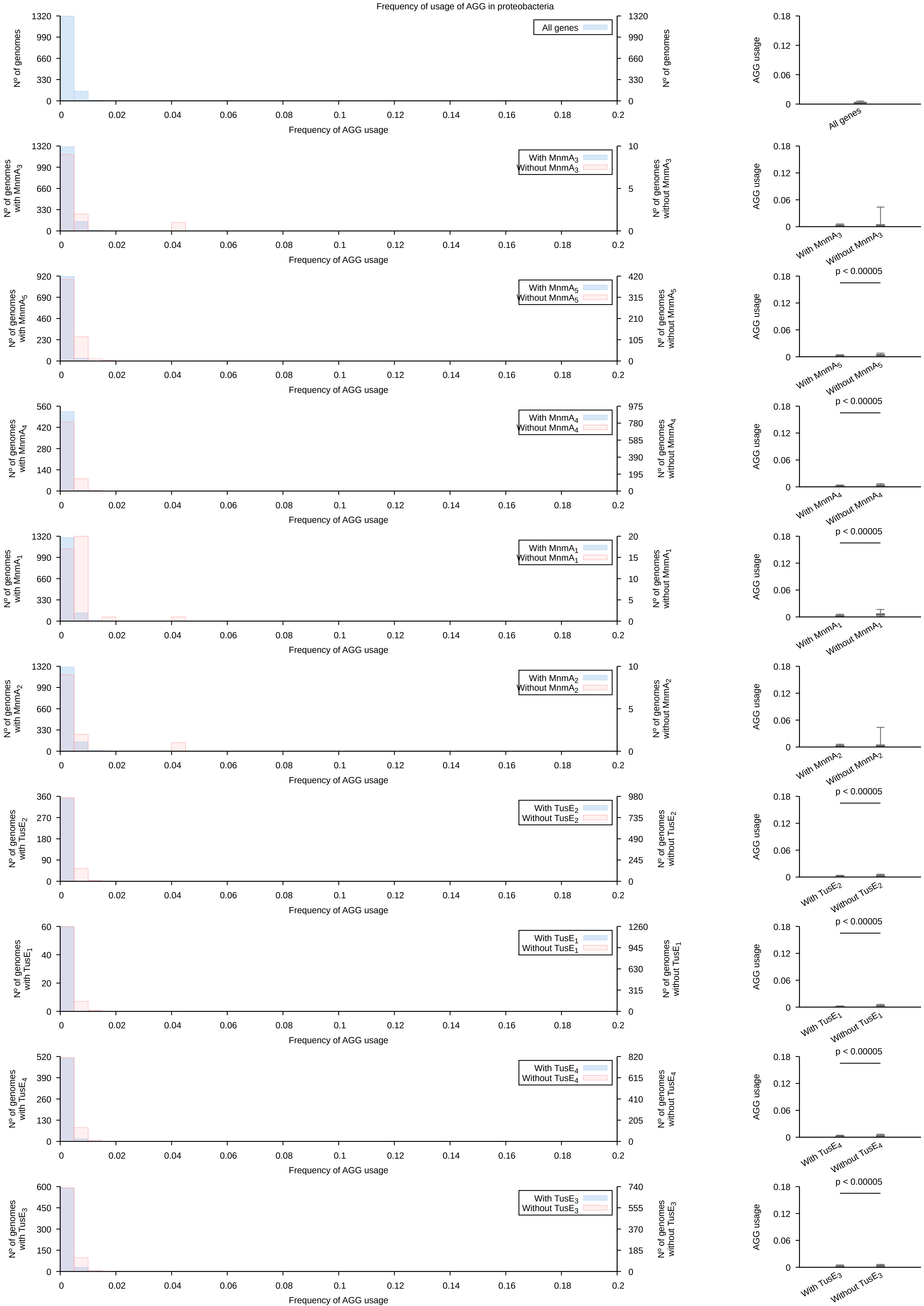

Frequency of usage of AGG in proteobacteria
All genes
Nº of genomes
Frequency of AGG usage
AGG usage
With MnmA$_3$
Without MnmA$_3$
Nº of genomes with MnmA$_3$
Nº of genomes without MnmA$_3$
With MnmA$_5$
Without MnmA$_5$
Nº of genomes with MnmA$_5$
Nº of genomes without MnmA$_5$
p < 0.00005
With MnmA$_4$
Without MnmA$_4$
Nº of genomes with MnmA$_4$
Nº of genomes without MnmA$_4$
p < 0.00005
With MnmA$_1$
Without MnmA$_1$
Nº of genomes with MnmA$_1$
Nº of genomes without MnmA$_1$
p < 0.00005
With MnmA$_2$
Without MnmA$_2$
Nº of genomes with MnmA$_2$
Nº of genomes without MnmA$_2$
With TusE$_2$
Without TusE$_2$
Nº of genomes with TusE$_2$
Nº of genomes without TusE$_2$
p < 0.00005
With TusE$_1$
Without TusE$_1$
Nº of genomes with TusE$_1$
Nº of genomes without TusE$_1$
p < 0.00005
With TusE$_4$
Without TusE$_4$
Nº of genomes with TusE$_4$
Nº of genomes without TusE$_4$
p < 0.00005
With TusE$_3$
Without TusE$_3$
Nº of genomes with TusE$_3$
Nº of genomes without TusE$_3$
p < 0.00005

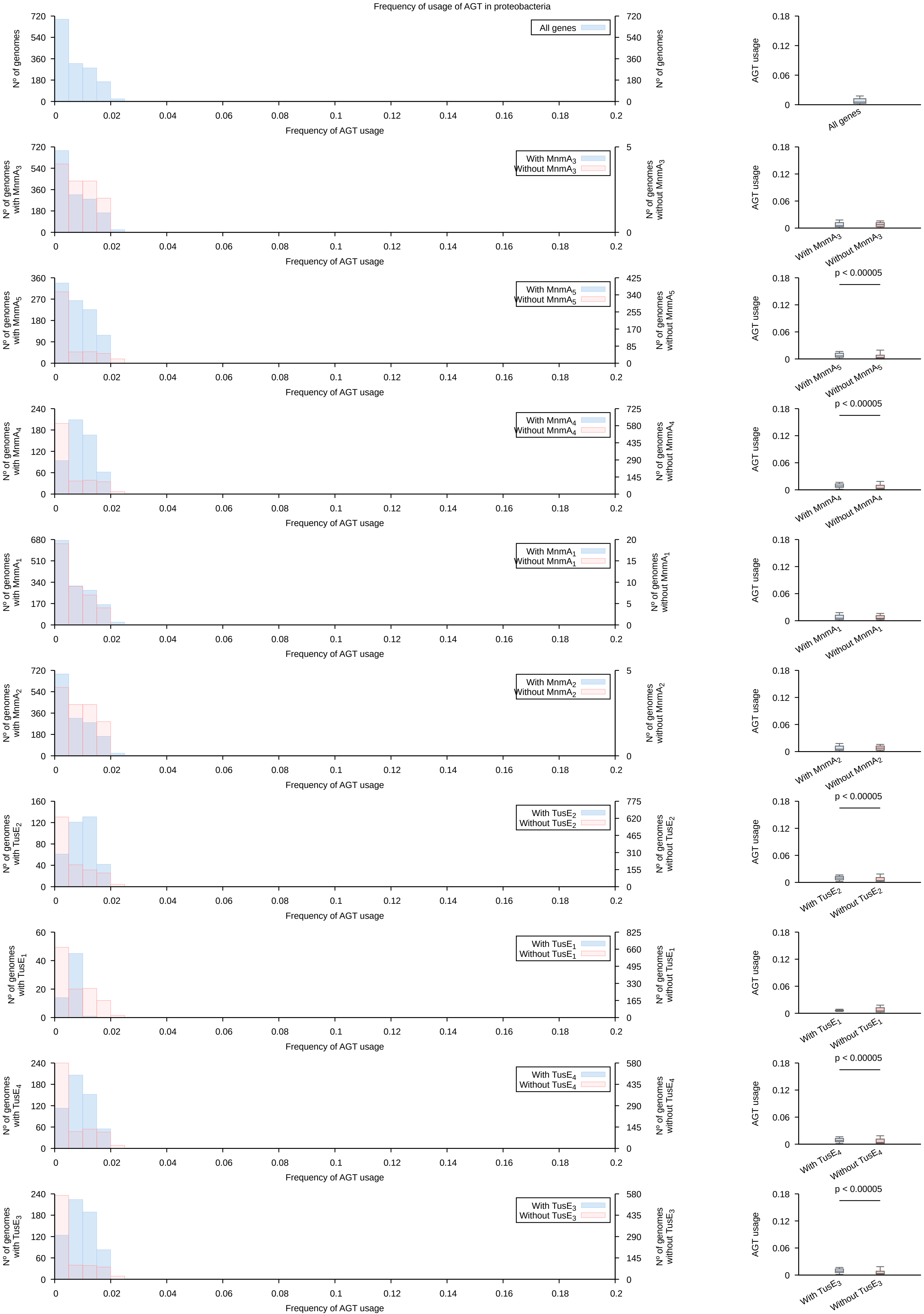

Frequency of usage of AGT in proteobacteria
All genes
Nº of genomes
Frequency of AGT usage
AGT usage
0 0.02 0.04 0.06 0.08 0.1 0.12 0.14 0.16 0.18 0.2
0 180 360 540 720
0 0.06 0.12 0.18
With $MnmA_3$
Without $MnmA_3$
Nº of genomes with $MnmA_3$
Nº of genomes without $MnmA_3$
With $MnmA_5$
Without $MnmA_5$
Nº of genomes with $MnmA_5$
Nº of genomes without $MnmA_5$
0 85 170 255 340 425
0 90 180 270 360
p < 0.00005
With $MnmA_4$
Without $MnmA_4$
Nº of genomes with $MnmA_4$
Nº of genomes without $MnmA_4$
0 60 120 180 240
0 145 290 435 580 725
p < 0.00005
With $MnmA_1$
Without $MnmA_1$
Nº of genomes with $MnmA_1$
Nº of genomes without $MnmA_1$
0 170 340 510 680
0 5 10 15 20
With $MnmA_2$
Without $MnmA_2$
Nº of genomes with $MnmA_2$
Nº of genomes without $MnmA_2$
With $TusE_2$
Without $TusE_2$
Nº of genomes with $TusE_2$
Nº of genomes without $TusE_2$
0 40 80 120 160
0 155 310 465 620 775
p < 0.00005
With $TusE_1$
Without $TusE_1$
Nº of genomes with $TusE_1$
Nº of genomes without $TusE_1$
0 20 40 60
0 165 330 495 660 825
With $TusE_4$
Without $TusE_4$
Nº of genomes with $TusE_4$
Nº of genomes without $TusE_4$
0 60 120 180 240
0 145 290 435 580
p < 0.00005
With $TusE_3$
Without $TusE_3$
Nº of genomes with $TusE_3$
Nº of genomes without $TusE_3$
p < 0.00005

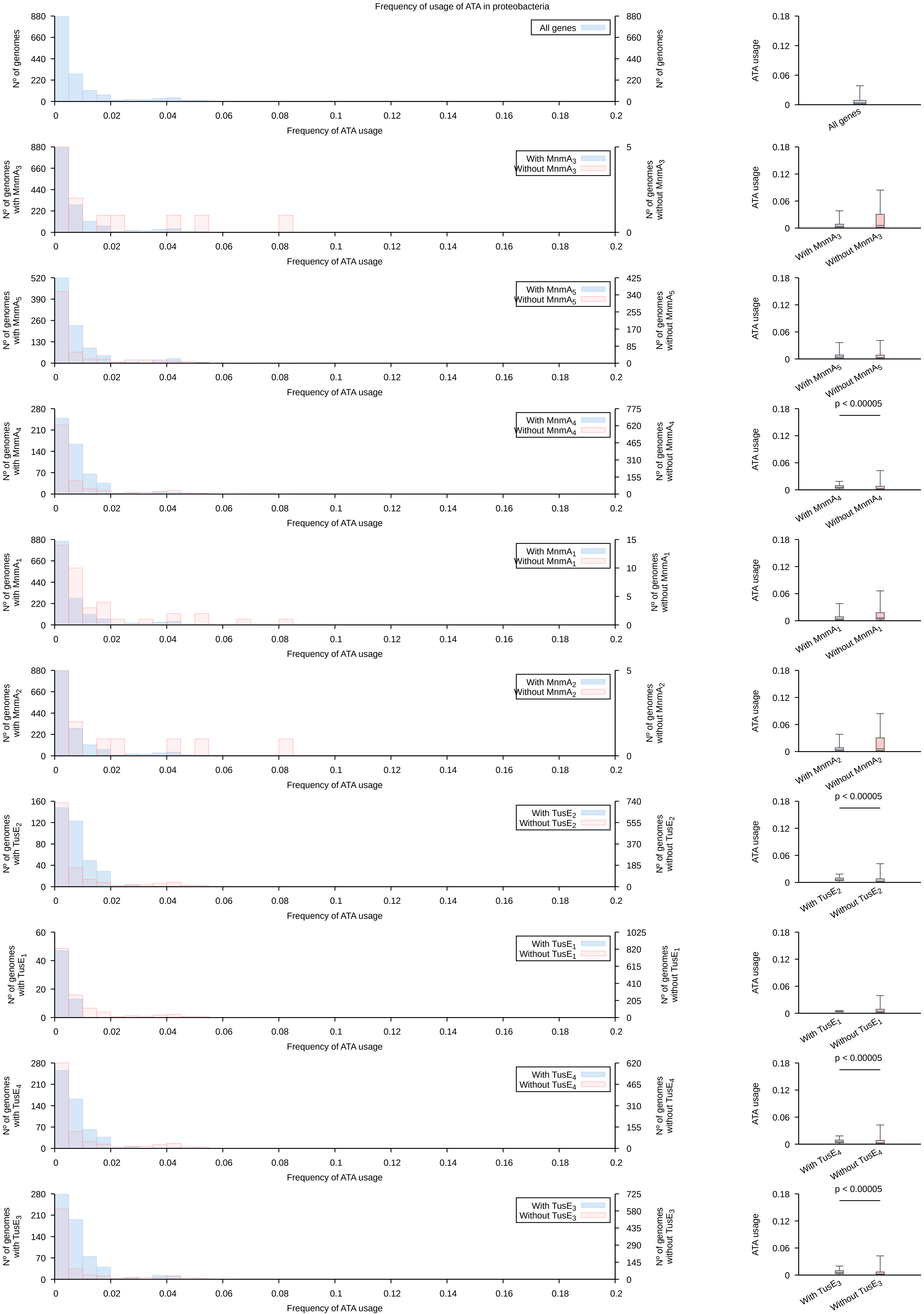

Frequency of usage of ATA in proteobacteria
All genes
Nº of genomes
Frequency of ATA usage
ATA usage
With MnmA3
Without MnmA3
Nº of genomes with MnmA3
Nº of genomes without MnmA3
With MnmA5
Without MnmA5
Nº of genomes with MnmA5
Nº of genomes without MnmA5
With MnmA4
Without MnmA4
Nº of genomes with MnmA4
Nº of genomes without MnmA4
p < 0.00005
With MnmA1
Without MnmA1
Nº of genomes with MnmA1
Nº of genomes without MnmA1
With MnmA2
Without MnmA2
Nº of genomes with MnmA2
Nº of genomes without MnmA2
With TusE2
Without TusE2
Nº of genomes with TusE2
Nº of genomes without TusE2
p < 0.00005
With TusE1
Without TusE1
Nº of genomes with TusE1
Nº of genomes without TusE1
With TusE4
Without TusE4
Nº of genomes with TusE4
Nº of genomes without TusE4
p < 0.00005
With TusE3
Without TusE3
Nº of genomes with TusE3
Nº of genomes without TusE3
p < 0.00005

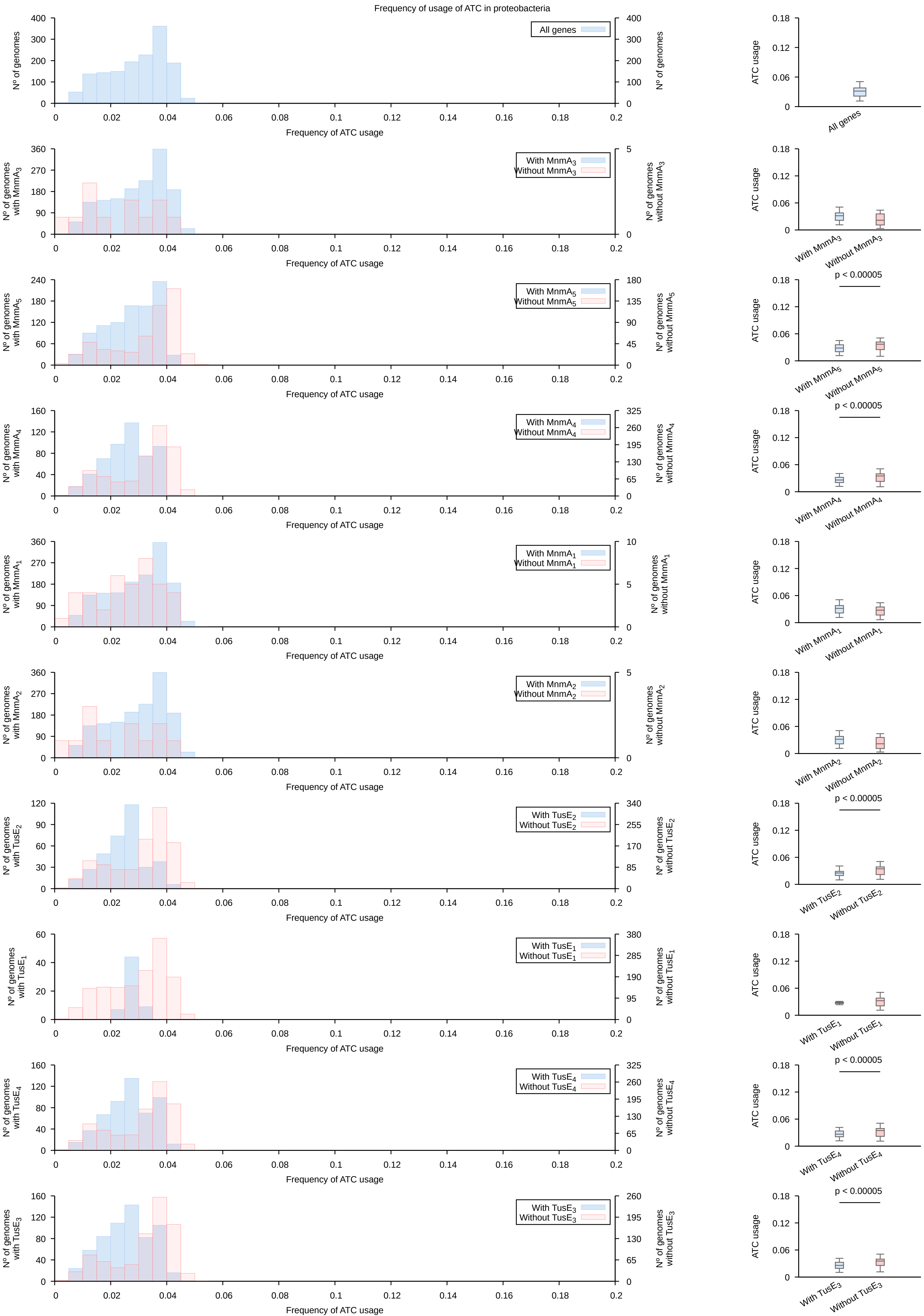
Frequency of usage of ATC in proteobacteria
Nº of genomes
All genes
Frequency of ATC usage
ATC usage
Nº of genomes with MnmA3
Nº of genomes without MnmA3
With MnmA3
Without MnmA3
Nº of genomes with MnmA5
Nº of genomes without MnmA5
With MnmA5
Without MnmA5
p < 0.00005
Nº of genomes with MnmA4
Nº of genomes without MnmA4
With MnmA4
Without MnmA4
p < 0.00005
Nº of genomes with MnmA1
Nº of genomes without MnmA1
With MnmA1
Without MnmA1
Nº of genomes with MnmA2
Nº of genomes without MnmA2
With MnmA2
Without MnmA2
Nº of genomes with TusE2
Nº of genomes without TusE2
With TusE2
Without TusE2
p < 0.00005
Nº of genomes with TusE1
Nº of genomes without TusE1
With TusE1
Without TusE1
Nº of genomes with TusE4
Nº of genomes without TusE4
With TusE4
Without TusE4
p < 0.00005
Nº of genomes with TusE3
Nº of genomes without TusE3
With TusE3
Without TusE3
p < 0.00005

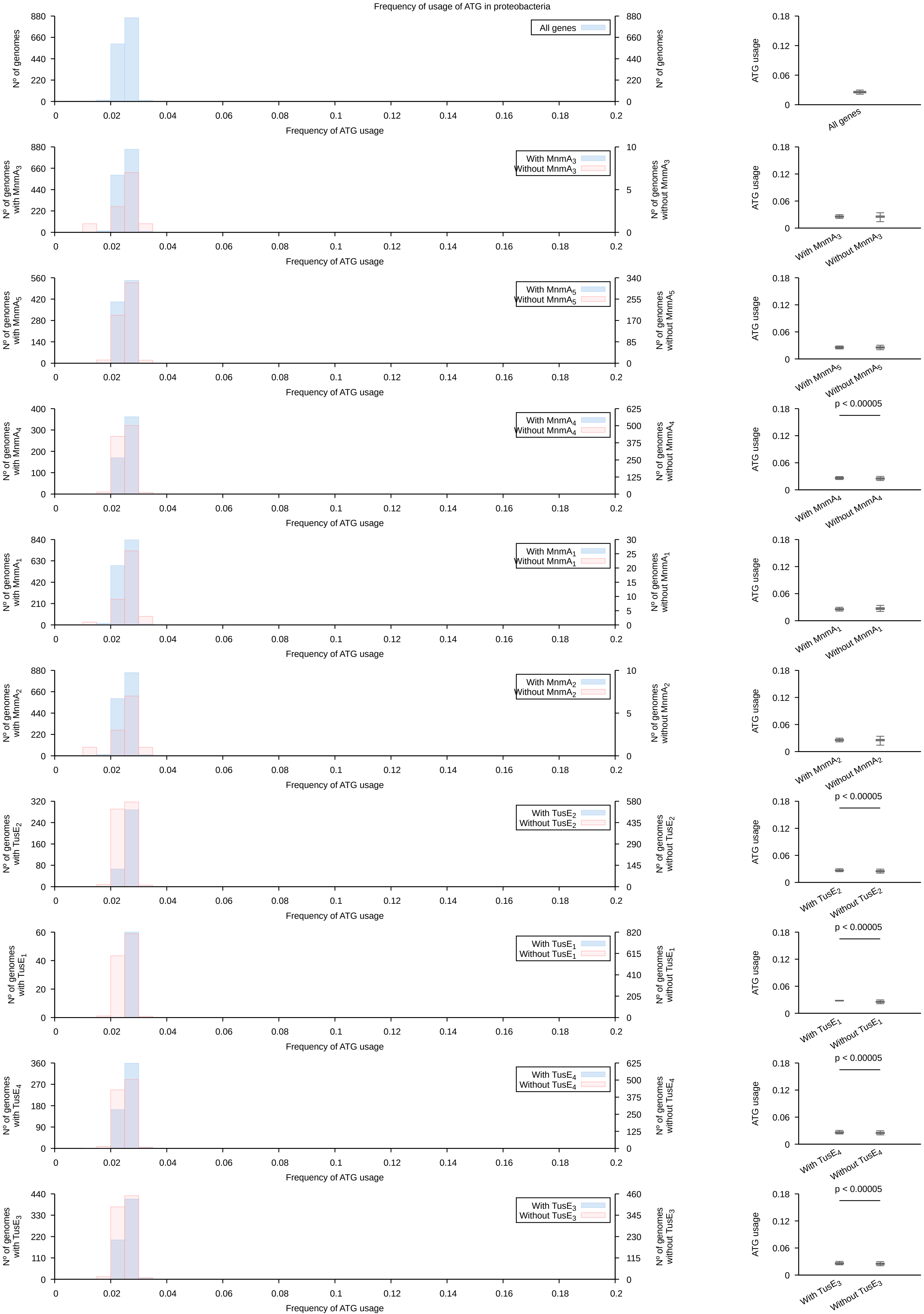
Frequency of usage of ATG in proteobacteria
All genes
With MnmA3
Without MnmA3
With MnmA5
Without MnmA5
With MnmA4
Without MnmA4
With MnmA1
Without MnmA1
With MnmA2
Without MnmA2
With TusE2
Without TusE2
With TusE1
Without TusE1
With TusE4
Without TusE4
With TusE3
Without TusE3
Nº of genomes
Frequency of ATG usage
ATG usage
p < 0.00005

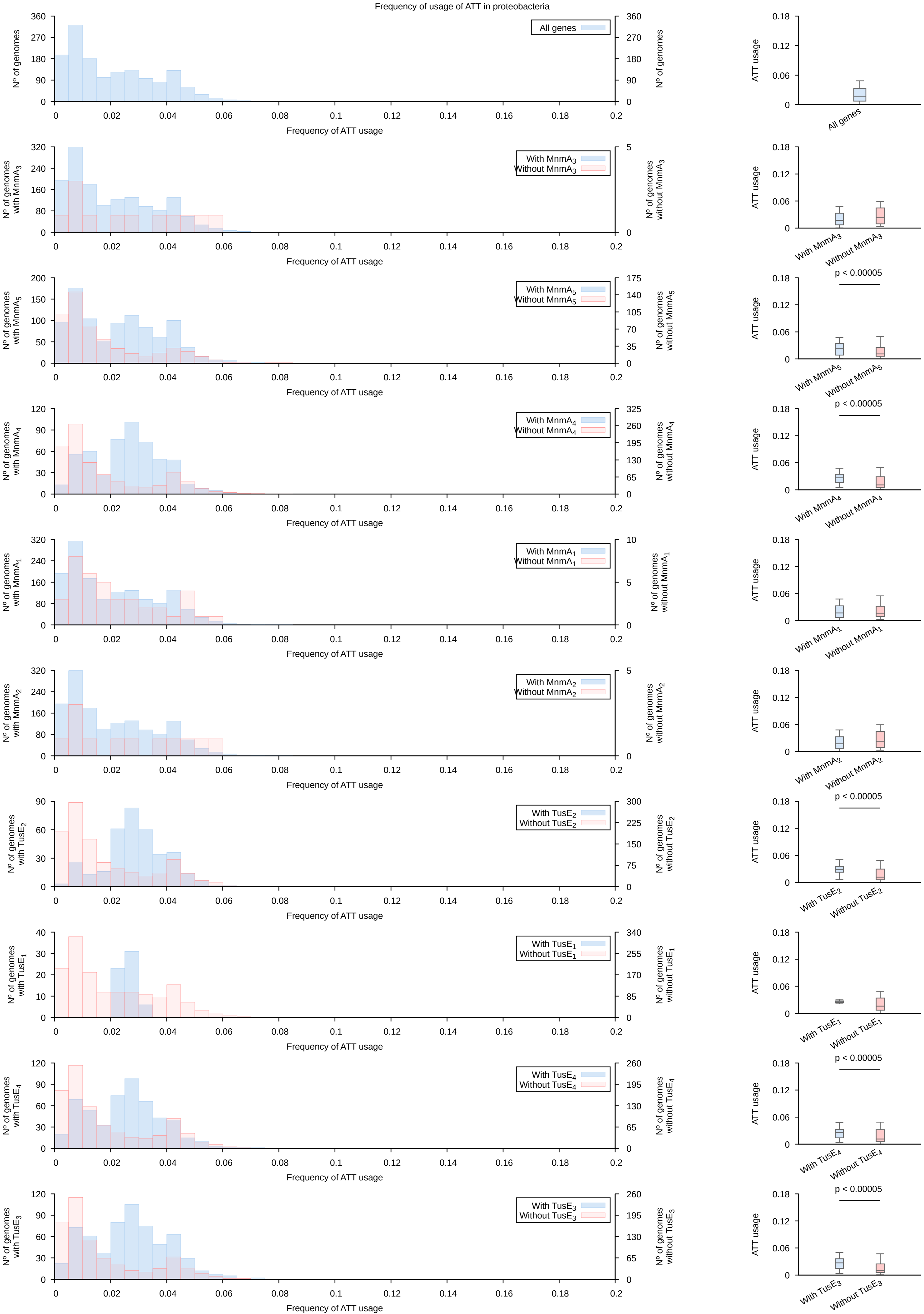

Frequency of usage of ATT in proteobacteria
Nº of genomes
All genes
Frequency of ATT usage
ATT usage
All genes
Nº of genomes with MnmA$_3$
Nº of genomes without MnmA$_3$
With MnmA$_3$
Without MnmA$_3$
Nº of genomes with MnmA$_5$
Nº of genomes without MnmA$_5$
With MnmA$_5$
Without MnmA$_5$
p < 0.00005
Nº of genomes with MnmA$_4$
Nº of genomes without MnmA$_4$
With MnmA$_4$
Without MnmA$_4$
p < 0.00005
Nº of genomes with MnmA$_1$
Nº of genomes without MnmA$_1$
With MnmA$_1$
Without MnmA$_1$
Nº of genomes with MnmA$_2$
Nº of genomes without MnmA$_2$
With MnmA$_2$
Without MnmA$_2$
Nº of genomes with TusE$_2$
Nº of genomes without TusE$_2$
With TusE$_2$
Without TusE$_2$
p < 0.00005
Nº of genomes with TusE$_1$
Nº of genomes without TusE$_1$
With TusE$_1$
Without TusE$_1$
Nº of genomes with TusE$_4$
Nº of genomes without TusE$_4$
With TusE$_4$
Without TusE$_4$
p < 0.00005
Nº of genomes with TusE$_3$
Nº of genomes without TusE$_3$
With TusE$_3$
Without TusE$_3$
p < 0.00005

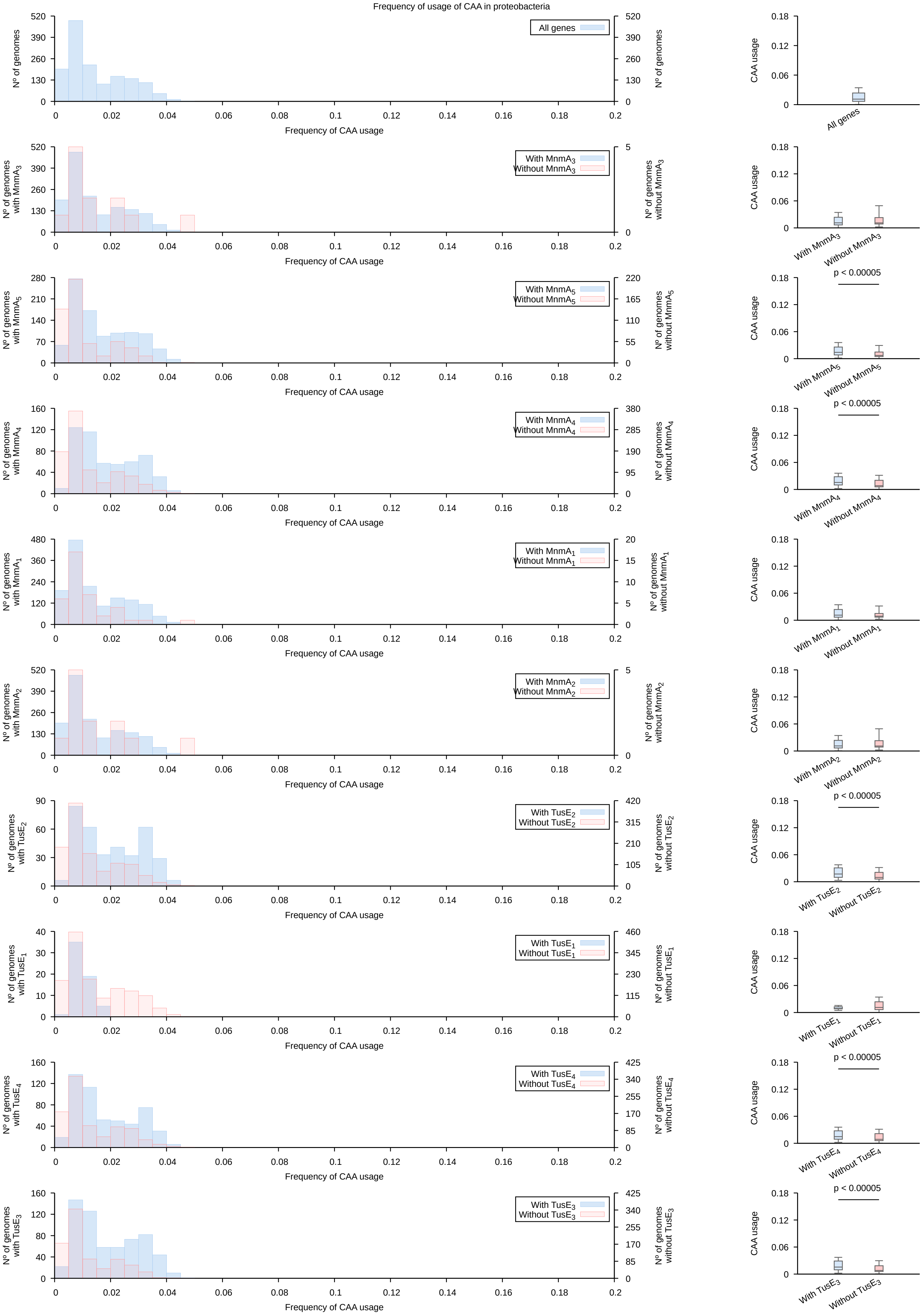
Frequency of usage of CAA in proteobacteria
All genes
Nº of genomes
CAA usage
Frequency of CAA usage
With MnmA$_3$
Without MnmA$_3$
Nº of genomes with MnmA$_3$
Nº of genomes without MnmA$_3$
With MnmA$_5$
Without MnmA$_5$
Nº of genomes with MnmA$_5$
Nº of genomes without MnmA$_5$
p < 0.00005
With MnmA$_4$
Without MnmA$_4$
Nº of genomes with MnmA$_4$
Nº of genomes without MnmA$_4$
p < 0.00005
With MnmA$_1$
Without MnmA$_1$
Nº of genomes with MnmA$_1$
Nº of genomes without MnmA$_1$
With MnmA$_2$
Without MnmA$_2$
Nº of genomes with MnmA$_2$
Nº of genomes without MnmA$_2$
With TusE$_2$
Without TusE$_2$
Nº of genomes with TusE$_2$
Nº of genomes without TusE$_2$
p < 0.00005
With TusE$_1$
Without TusE$_1$
Nº of genomes with TusE$_1$
Nº of genomes without TusE$_1$
With TusE$_4$
Without TusE$_4$
Nº of genomes with TusE$_4$
Nº of genomes without TusE$_4$
p < 0.00005
With TusE$_3$
Without TusE$_3$
Nº of genomes with TusE$_3$
Nº of genomes without TusE$_3$
p < 0.00005

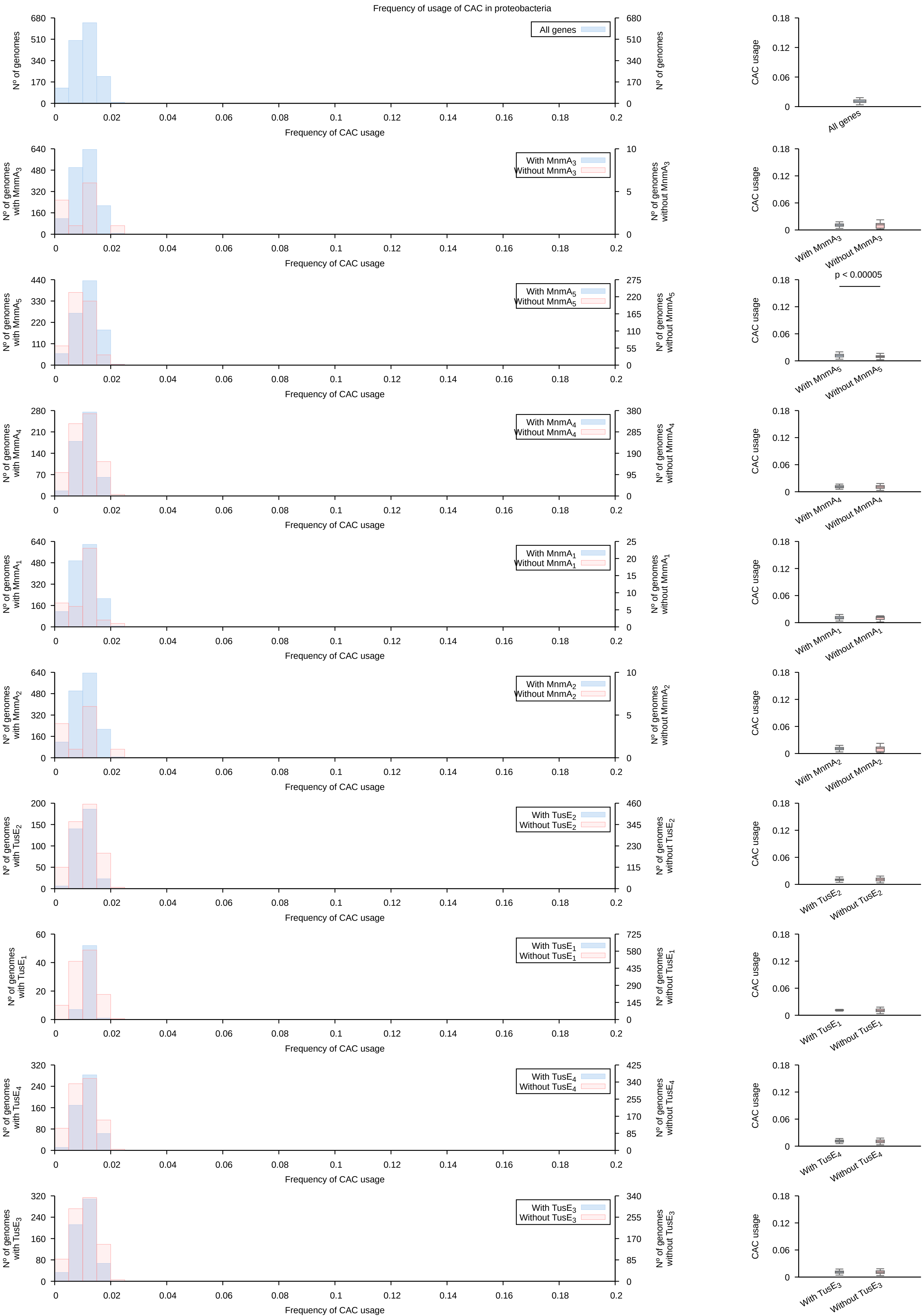
Frequency of usage of CAC in proteobacteria
All genes
Nº of genomes
Frequency of CAC usage
CAC usage
With MnmA$_3$
Without MnmA$_3$
Nº of genomes with MnmA$_3$
Nº of genomes without MnmA$_3$
With MnmA$_5$
Without MnmA$_5$
Nº of genomes with MnmA$_5$
Nº of genomes without MnmA$_5$
p < 0.00005
With MnmA$_4$
Without MnmA$_4$
Nº of genomes with MnmA$_4$
Nº of genomes without MnmA$_4$
With MnmA$_1$
Without MnmA$_1$
Nº of genomes with MnmA$_1$
Nº of genomes without MnmA$_1$
With MnmA$_2$
Without MnmA$_2$
Nº of genomes with MnmA$_2$
Nº of genomes without MnmA$_2$
With TusE$_2$
Without TusE$_2$
Nº of genomes with TusE$_2$
Nº of genomes without TusE$_2$
With TusE$_1$
Without TusE$_1$
Nº of genomes with TusE$_1$
Nº of genomes without TusE$_1$
With TusE$_4$
Without TusE$_4$
Nº of genomes with TusE$_4$
Nº of genomes without TusE$_4$
With TusE$_3$
Without TusE$_3$
Nº of genomes with TusE$_3$
Nº of genomes without TusE$_3$

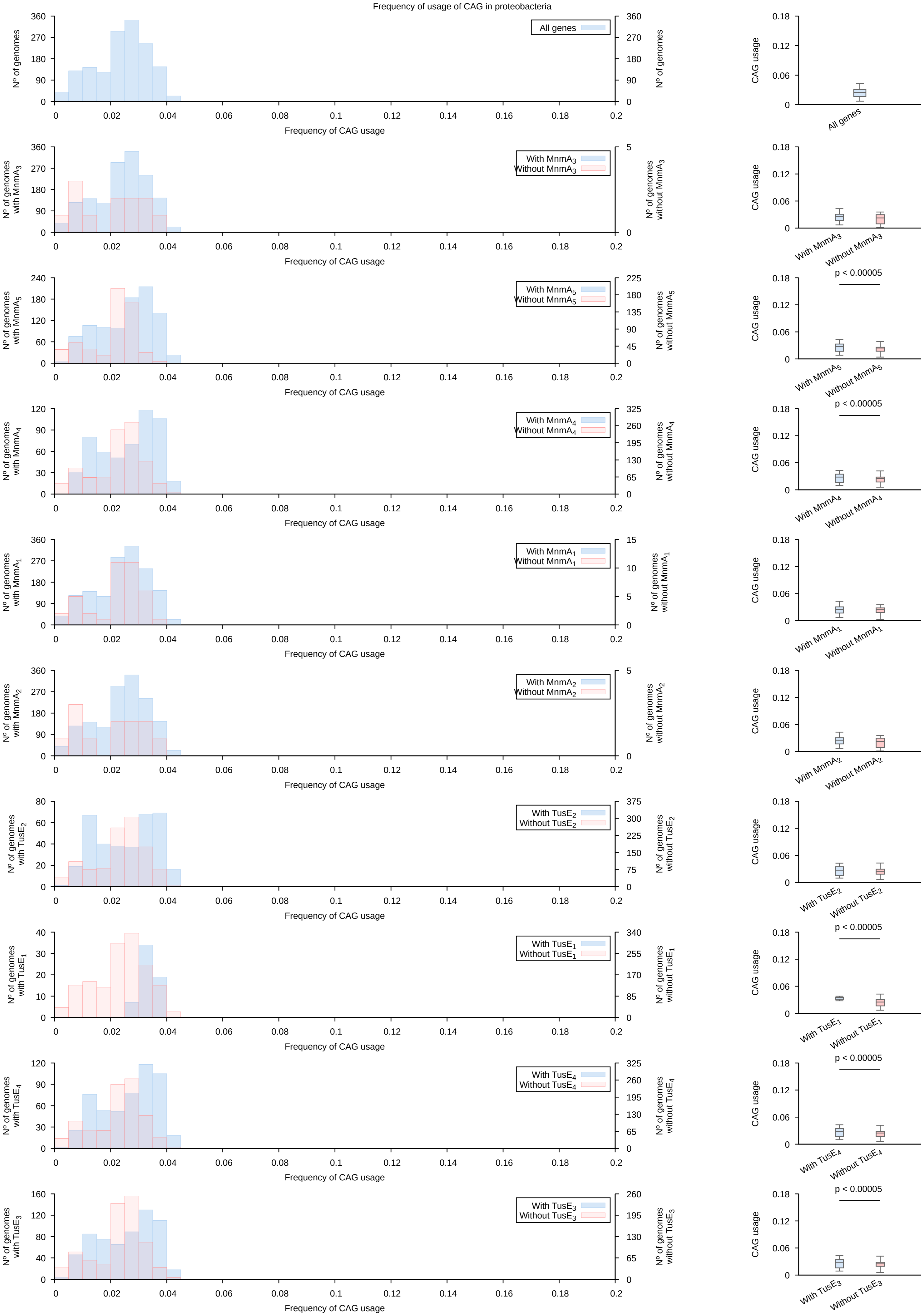
Frequency of usage of CAG in proteobacteria
Nº of genomes
All genes
Frequency of CAG usage
CAG usage
Nº of genomes with MnmA$_3$
Nº of genomes without MnmA$_3$
With MnmA$_3$
Without MnmA$_3$
Nº of genomes with MnmA$_5$
Nº of genomes without MnmA$_5$
With MnmA$_5$
Without MnmA$_5$
p < 0.00005
Nº of genomes with MnmA$_4$
Nº of genomes without MnmA$_4$
With MnmA$_4$
Without MnmA$_4$
p < 0.00005
Nº of genomes with MnmA$_1$
Nº of genomes without MnmA$_1$
With MnmA$_1$
Without MnmA$_1$
Nº of genomes with MnmA$_2$
Nº of genomes without MnmA$_2$
With MnmA$_2$
Without MnmA$_2$
Nº of genomes with TusE$_2$
Nº of genomes without TusE$_2$
With TusE$_2$
Without TusE$_2$
Nº of genomes with TusE$_1$
Nº of genomes without TusE$_1$
With TusE$_1$
Without TusE$_1$
p < 0.00005
Nº of genomes with TusE$_4$
Nº of genomes without TusE$_4$
With TusE$_4$
Without TusE$_4$
p < 0.00005
Nº of genomes with TusE$_3$
Nº of genomes without TusE$_3$
With TusE$_3$
Without TusE$_3$
p < 0.00005

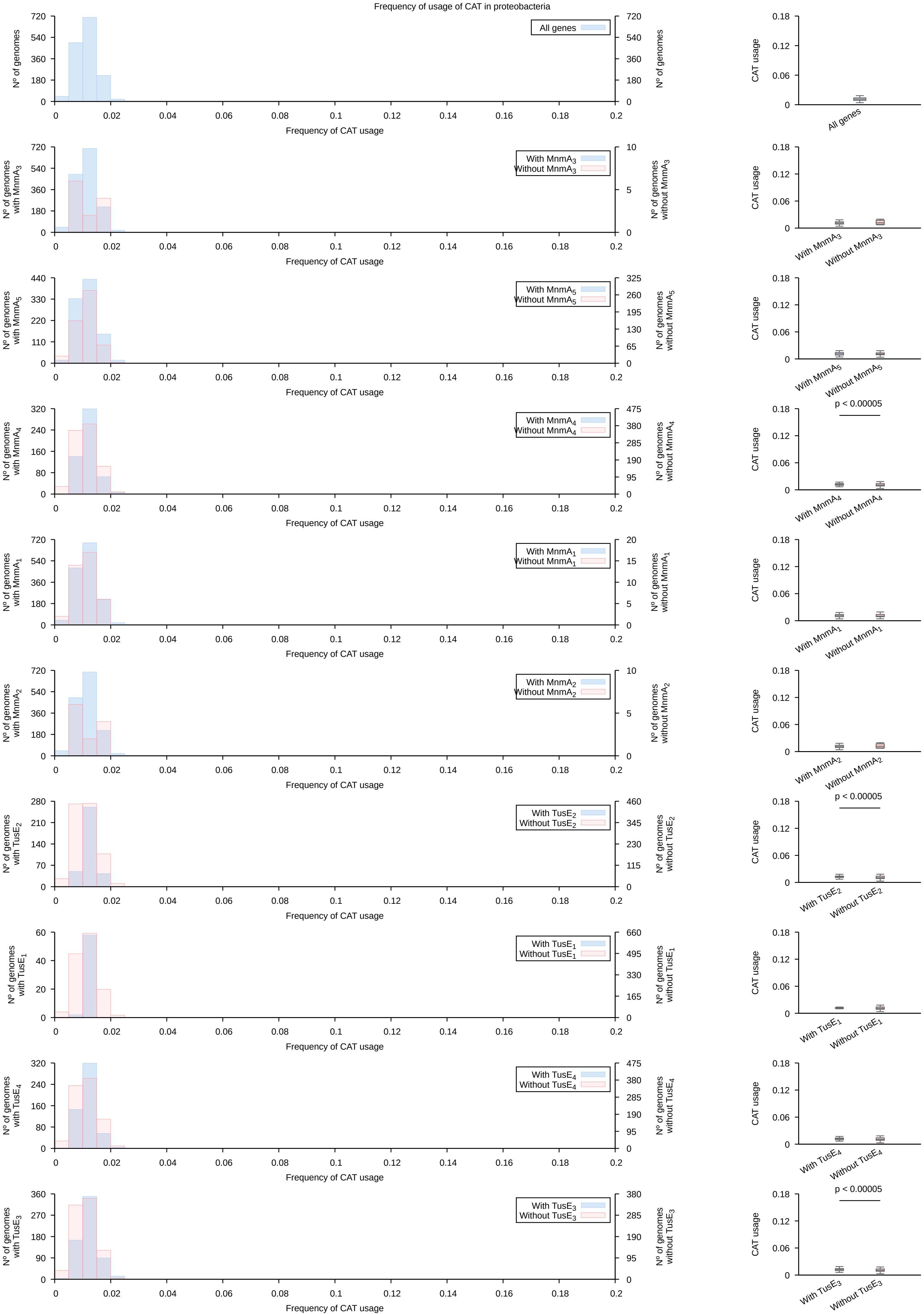
Frequency of usage of CAT in proteobacteria
All genes
Nº of genomes
Frequency of CAT usage
CAT usage
With MnmA$_3$
Without MnmA$_3$
Nº of genomes with MnmA$_3$
Nº of genomes without MnmA$_3$
With MnmA$_5$
Without MnmA$_5$
Nº of genomes with MnmA$_5$
Nº of genomes without MnmA$_5$
With MnmA$_4$
Without MnmA$_4$
Nº of genomes with MnmA$_4$
Nº of genomes without MnmA$_4$
p < 0.00005
With MnmA$_1$
Without MnmA$_1$
Nº of genomes with MnmA$_1$
Nº of genomes without MnmA$_1$
With MnmA$_2$
Without MnmA$_2$
Nº of genomes with MnmA$_2$
Nº of genomes without MnmA$_2$
With TusE$_2$
Without TusE$_2$
Nº of genomes with TusE$_2$
Nº of genomes without TusE$_2$
p < 0.00005
With TusE$_1$
Without TusE$_1$
Nº of genomes with TusE$_1$
Nº of genomes without TusE$_1$
With TusE$_4$
Without TusE$_4$
Nº of genomes with TusE$_4$
Nº of genomes without TusE$_4$
With TusE$_3$
Without TusE$_3$
Nº of genomes with TusE$_3$
Nº of genomes without TusE$_3$
p < 0.00005

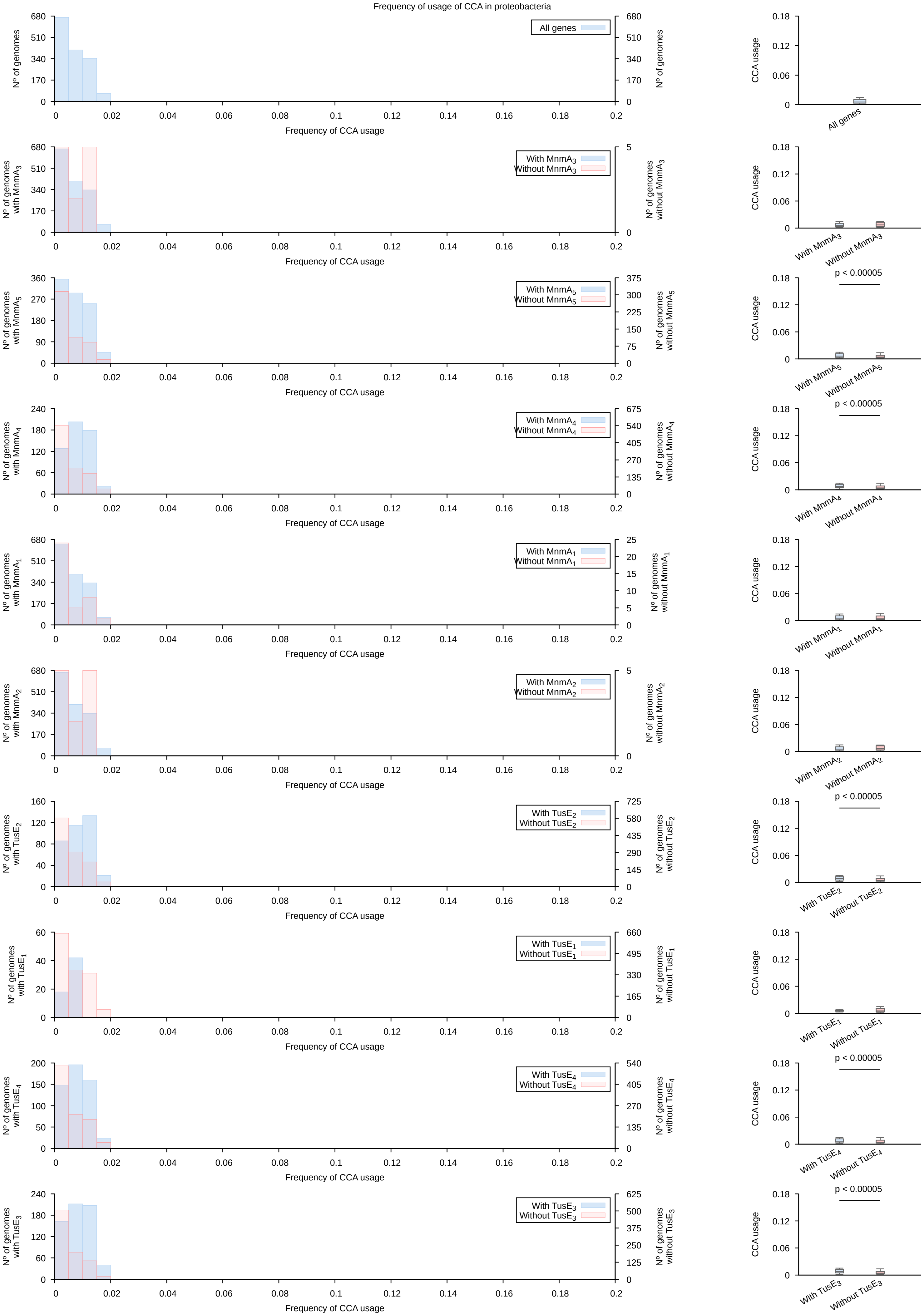

Frequency of usage of CCA in proteobacteria
All genes
Nº of genomes
Frequency of CCA usage
CCA usage
With MnmA$_3$
Without MnmA$_3$
Nº of genomes with MnmA$_3$
Nº of genomes without MnmA$_3$
With MnmA$_5$
Without MnmA$_5$
Nº of genomes with MnmA$_5$
Nº of genomes without MnmA$_5$
p < 0.00005
With MnmA$_4$
Without MnmA$_4$
Nº of genomes with MnmA$_4$
Nº of genomes without MnmA$_4$
p < 0.00005
With MnmA$_1$
Without MnmA$_1$
Nº of genomes with MnmA$_1$
Nº of genomes without MnmA$_1$
With MnmA$_2$
Without MnmA$_2$
Nº of genomes with MnmA$_2$
Nº of genomes without MnmA$_2$
With TusE$_2$
Without TusE$_2$
Nº of genomes with TusE$_2$
Nº of genomes without TusE$_2$
p < 0.00005
With TusE$_1$
Without TusE$_1$
Nº of genomes with TusE$_1$
Nº of genomes without TusE$_1$
With TusE$_4$
Without TusE$_4$
Nº of genomes with TusE$_4$
Nº of genomes without TusE$_4$
p < 0.00005
With TusE$_3$
Without TusE$_3$
Nº of genomes with TusE$_3$
Nº of genomes without TusE$_3$
p < 0.00005
0 0.02 0.04 0.06 0.08 0.1 0.12 0.14 0.16 0.18 0.2
0 0.06 0.12 0.18

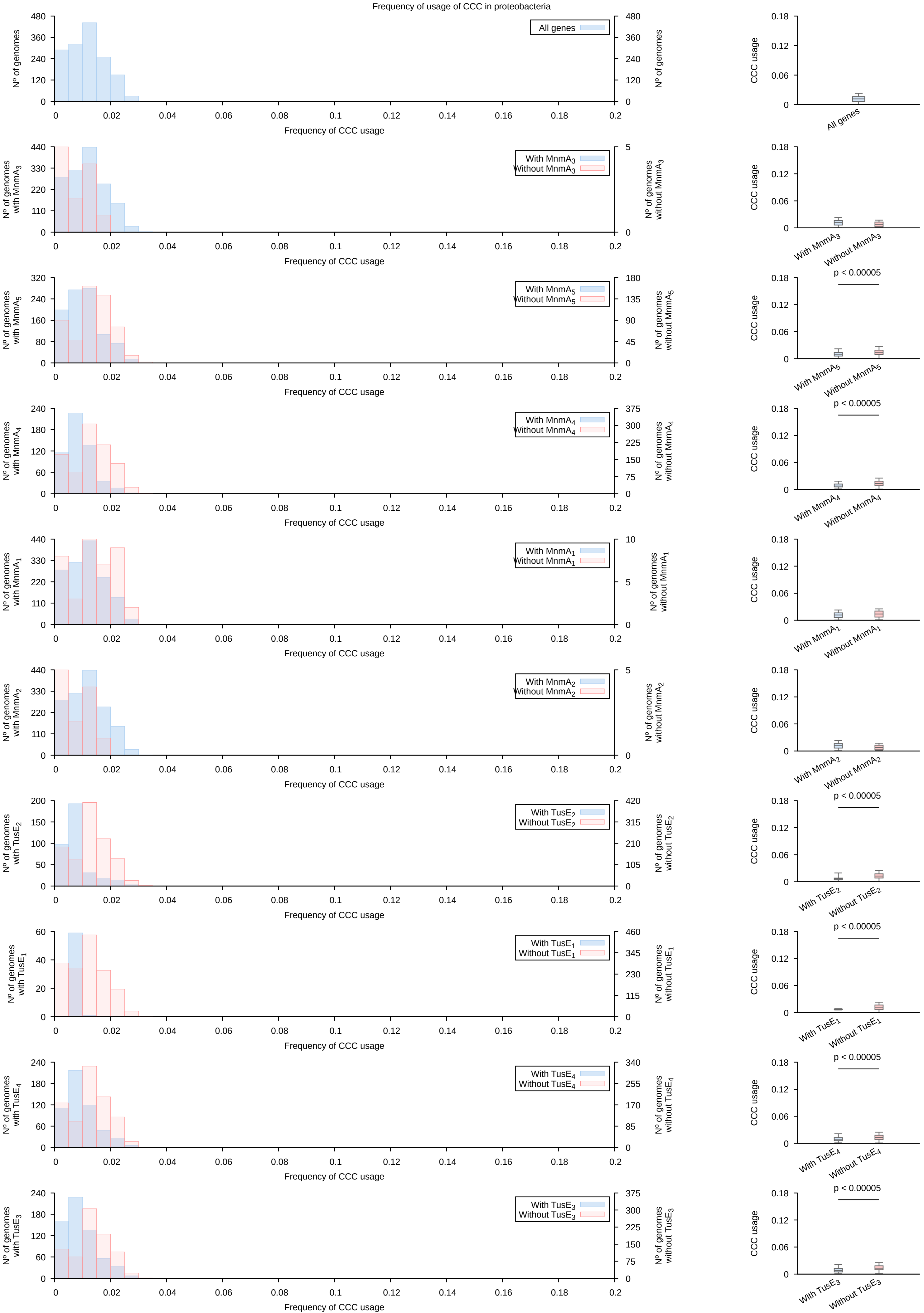
Frequency of usage of CCC in proteobacteria
All genes
Nº of genomes
Frequency of CCC usage
CCC usage
With MnmA$_3$
Without MnmA$_3$
Nº of genomes with MnmA$_3$
Nº of genomes without MnmA$_3$
With MnmA$_5$
Without MnmA$_5$
Nº of genomes with MnmA$_5$
Nº of genomes without MnmA$_5$
p < 0.00005
With MnmA$_4$
Without MnmA$_4$
Nº of genomes with MnmA$_4$
Nº of genomes without MnmA$_4$
p < 0.00005
With MnmA$_1$
Without MnmA$_1$
Nº of genomes with MnmA$_1$
Nº of genomes without MnmA$_1$
With MnmA$_2$
Without MnmA$_2$
Nº of genomes with MnmA$_2$
Nº of genomes without MnmA$_2$
With TusE$_2$
Without TusE$_2$
Nº of genomes with TusE$_2$
Nº of genomes without TusE$_2$
p < 0.00005
With TusE$_1$
Without TusE$_1$
Nº of genomes with TusE$_1$
Nº of genomes without TusE$_1$
p < 0.00005
With TusE$_4$
Without TusE$_4$
Nº of genomes with TusE$_4$
Nº of genomes without TusE$_4$
p < 0.00005
With TusE$_3$
Without TusE$_3$
Nº of genomes with TusE$_3$
Nº of genomes without TusE$_3$
p < 0.00005

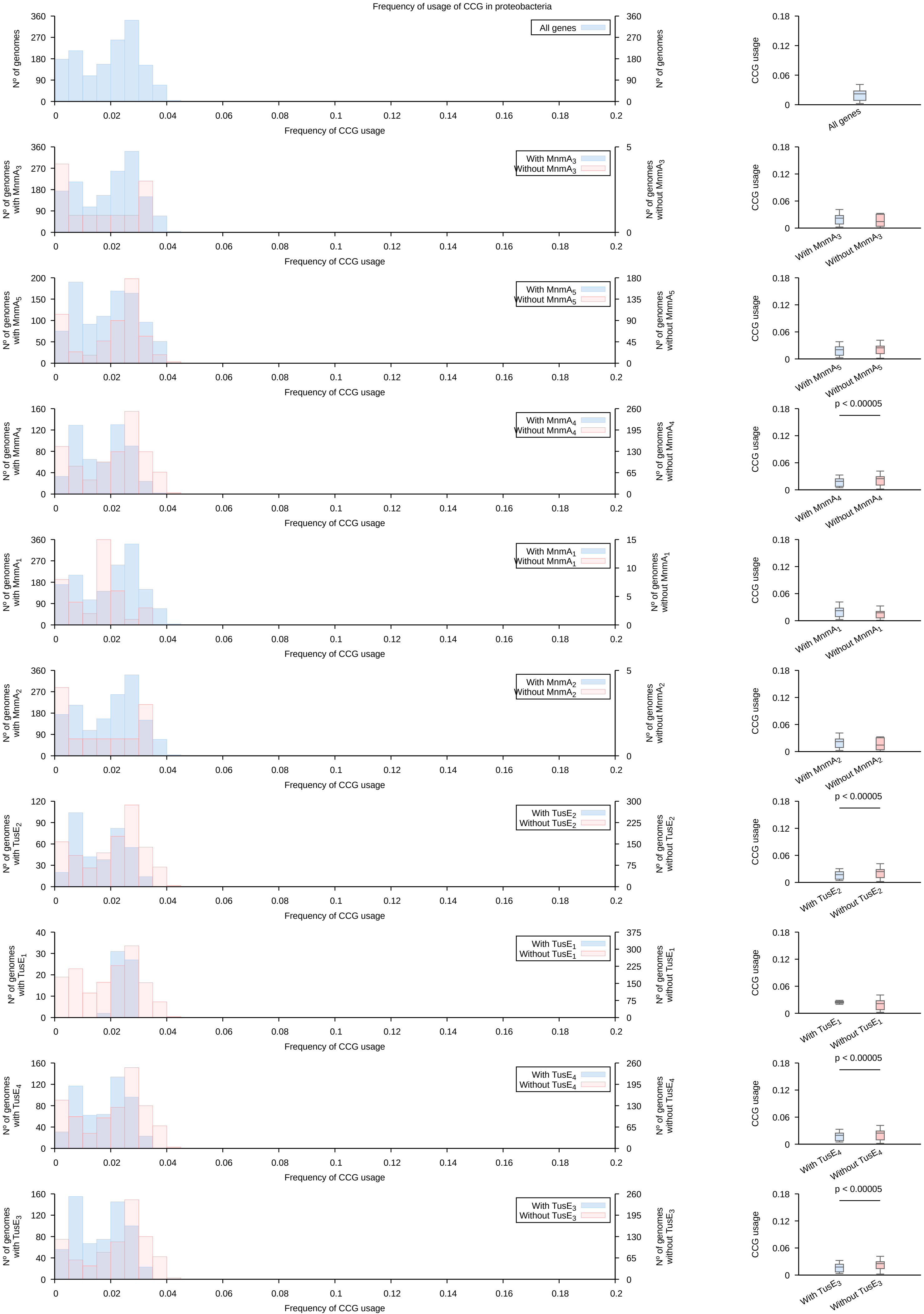

Frequency of usage of CCG in proteobacteria
Nº of genomes
All genes
Frequency of CCG usage
CCG usage
With MnmA3
Without MnmA3
With MnmA5
Without MnmA5
With MnmA4
Without MnmA4
p < 0.00005
With MnmA1
Without MnmA1
With MnmA2
Without MnmA2
With TusE2
Without TusE2
With TusE1
Without TusE1
With TusE4
Without TusE4
With TusE3
Without TusE3

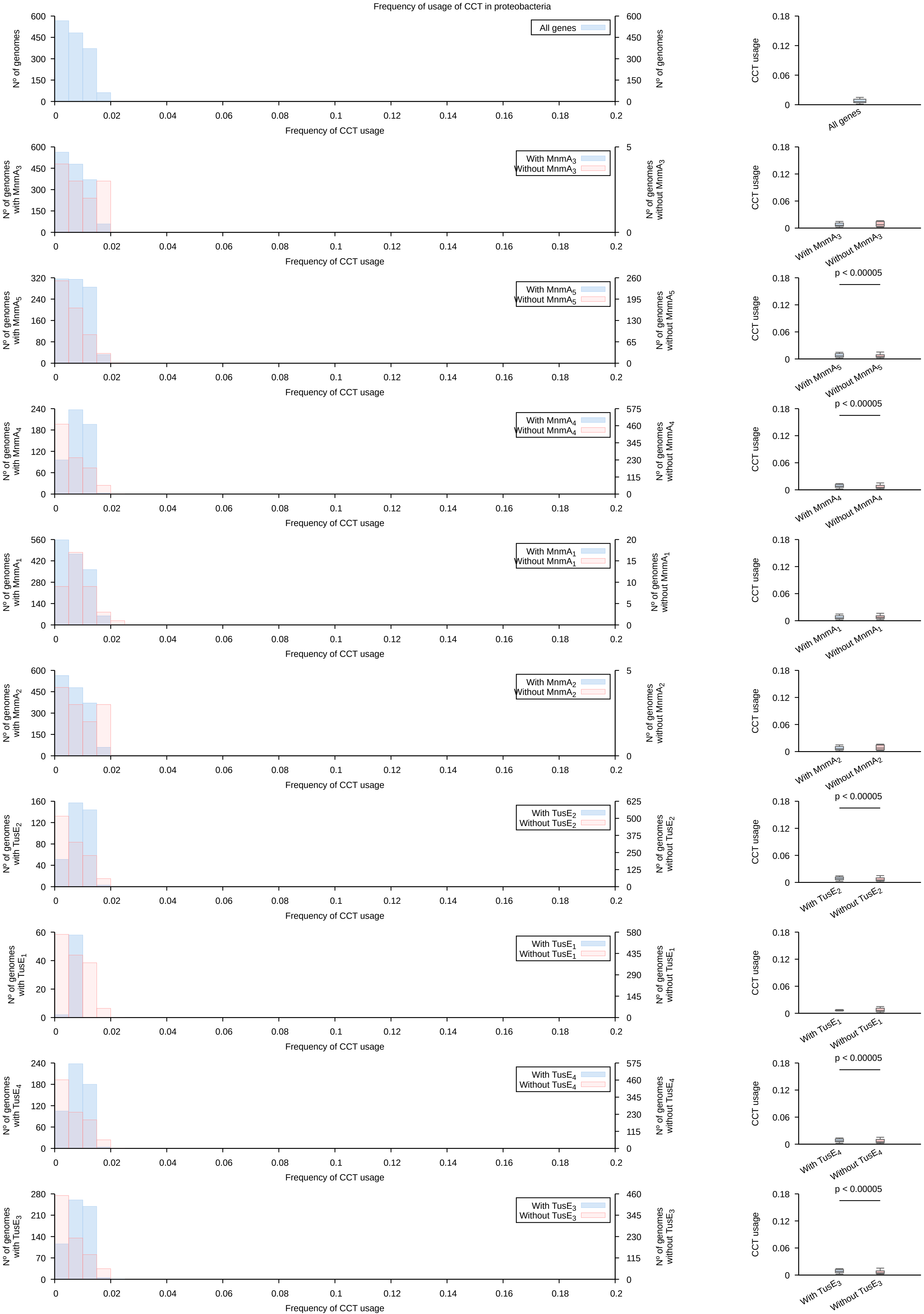
Frequency of usage of CCT in proteobacteria
All genes
Nº of genomes
Frequency of CCT usage
CCT usage
With MnmA$_3$
Without MnmA$_3$
Nº of genomes with MnmA$_3$
Nº of genomes without MnmA$_3$
With MnmA$_5$
Without MnmA$_5$
Nº of genomes with MnmA$_5$
Nº of genomes without MnmA$_5$
p < 0.00005
With MnmA$_4$
Without MnmA$_4$
Nº of genomes with MnmA$_4$
Nº of genomes without MnmA$_4$
p < 0.00005
With MnmA$_1$
Without MnmA$_1$
Nº of genomes with MnmA$_1$
Nº of genomes without MnmA$_1$
With MnmA$_2$
Without MnmA$_2$
Nº of genomes with MnmA$_2$
Nº of genomes without MnmA$_2$
With TusE$_2$
Without TusE$_2$
Nº of genomes with TusE$_2$
Nº of genomes without TusE$_2$
p < 0.00005
With TusE$_1$
Without TusE$_1$
Nº of genomes with TusE$_1$
Nº of genomes without TusE$_1$
With TusE$_4$
Without TusE$_4$
Nº of genomes with TusE$_4$
Nº of genomes without TusE$_4$
p < 0.00005
With TusE$_3$
Without TusE$_3$
Nº of genomes with TusE$_3$
Nº of genomes without TusE$_3$
p < 0.00005

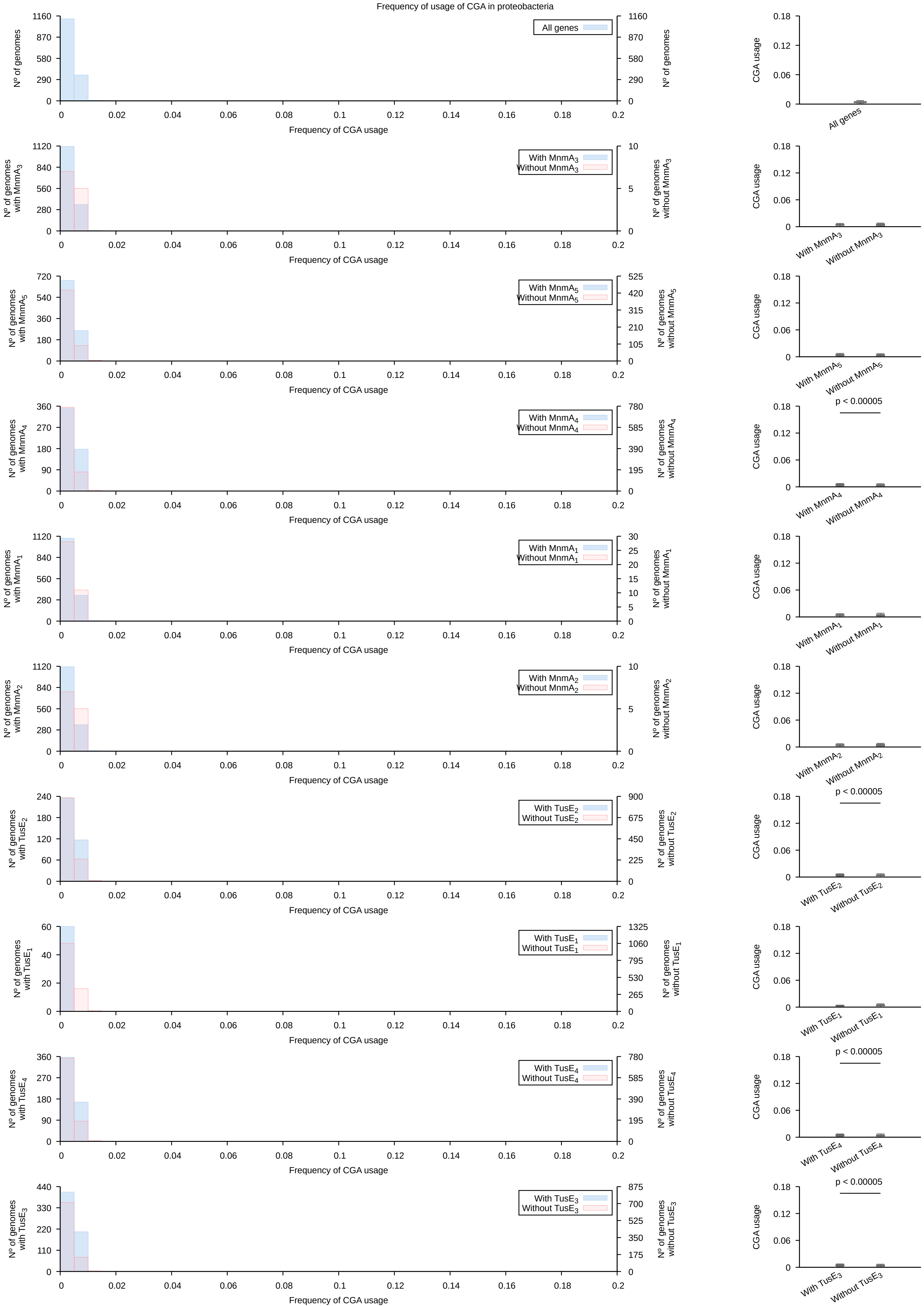
Frequency of usage of CGA in proteobacteria
All genes
Nº of genomes
Frequency of CGA usage
CGA usage
With MnmA3
Without MnmA3
Nº of genomes with MnmA3
Nº of genomes without MnmA3
With MnmA5
Without MnmA5
Nº of genomes with MnmA5
Nº of genomes without MnmA5
With MnmA4
Without MnmA4
Nº of genomes with MnmA4
Nº of genomes without MnmA4
p < 0.00005
With MnmA1
Without MnmA1
Nº of genomes with MnmA1
Nº of genomes without MnmA1
With MnmA2
Without MnmA2
Nº of genomes with MnmA2
Nº of genomes without MnmA2
With TusE2
Without TusE2
Nº of genomes with TusE2
Nº of genomes without TusE2
p < 0.00005
With TusE1
Without TusE1
Nº of genomes with TusE1
Nº of genomes without TusE1
With TusE4
Without TusE4
Nº of genomes with TusE4
Nº of genomes without TusE4
p < 0.00005
With TusE3
Without TusE3
Nº of genomes with TusE3
Nº of genomes without TusE3
p < 0.00005

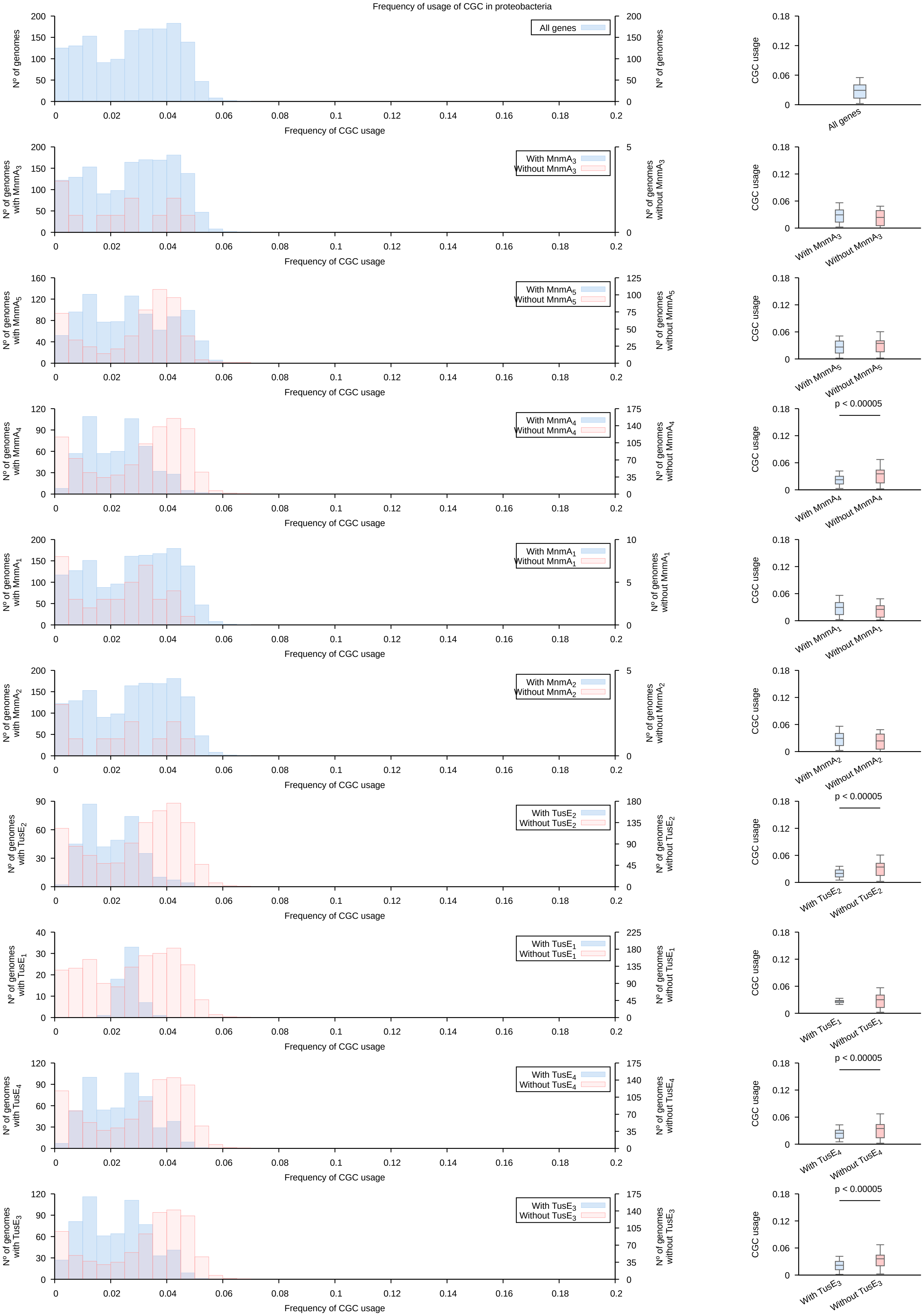

Frequency of usage of CGC in proteobacteria
Nº of genomes
Frequency of CGC usage
All genes
CGC usage
Nº of genomes with $MnmA_3$
Nº of genomes without $MnmA_3$
With $MnmA_3$
Without $MnmA_3$
Nº of genomes with $MnmA_5$
Nº of genomes without $MnmA_5$
With $MnmA_5$
Without $MnmA_5$
Nº of genomes with $MnmA_4$
Nº of genomes without $MnmA_4$
With $MnmA_4$
Without $MnmA_4$
$p < 0.00005$
Nº of genomes with $MnmA_1$
Nº of genomes without $MnmA_1$
With $MnmA_1$
Without $MnmA_1$
Nº of genomes with $MnmA_2$
Nº of genomes without $MnmA_2$
With $MnmA_2$
Without $MnmA_2$
Nº of genomes with $TusE_2$
Nº of genomes without $TusE_2$
With $TusE_2$
Without $TusE_2$
$p < 0.00005$
Nº of genomes with $TusE_1$
Nº of genomes without $TusE_1$
With $TusE_1$
Without $TusE_1$
Nº of genomes with $TusE_4$
Nº of genomes without $TusE_4$
With $TusE_4$
Without $TusE_4$
$p < 0.00005$
Nº of genomes with $TusE_3$
Nº of genomes without $TusE_3$
With $TusE_3$
Without $TusE_3$
$p < 0.00005$

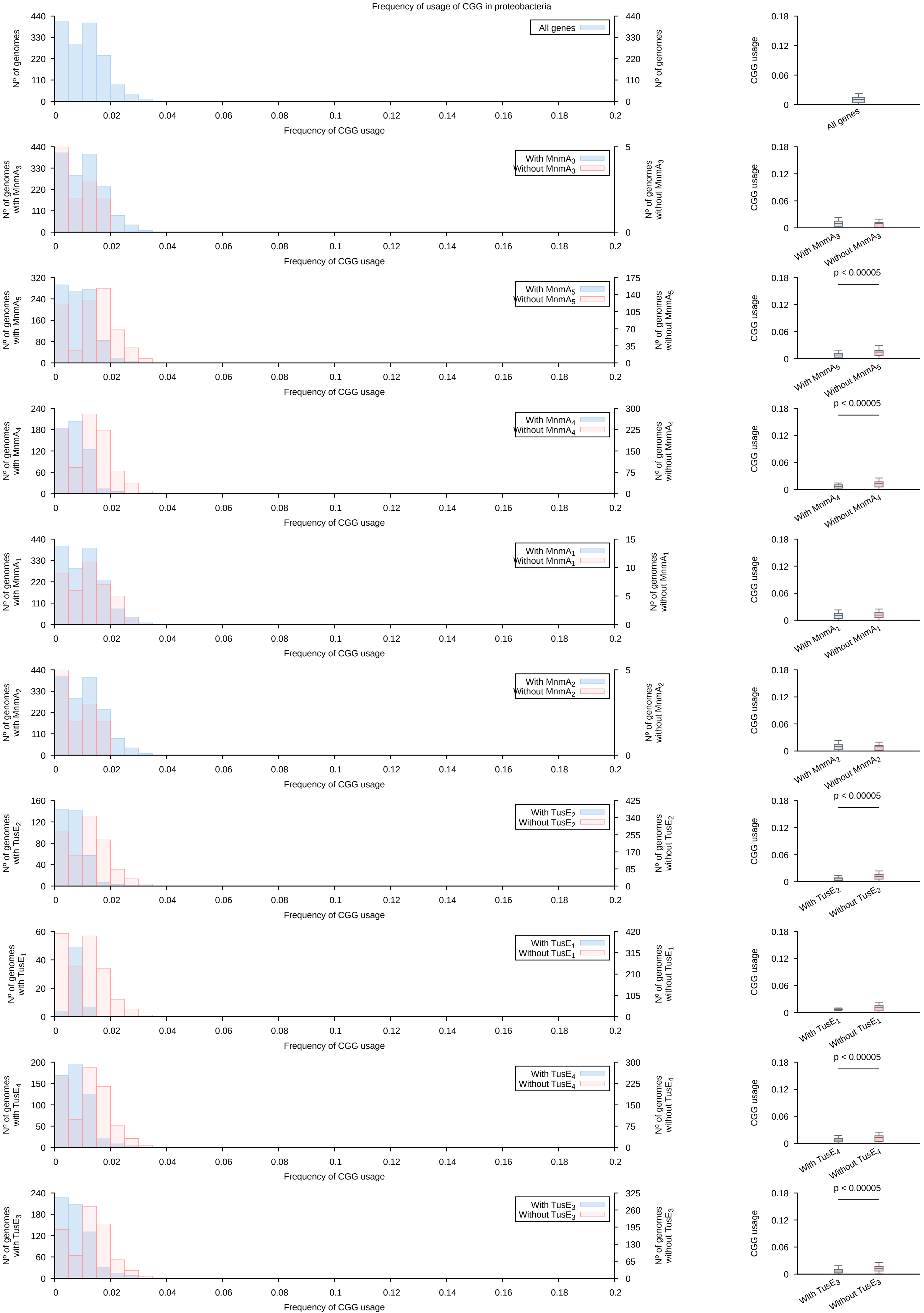

Frequency of usage of CGG in proteobacteria
All genes
Nº of genomes
Frequency of CGG usage
CGG usage
With MnmA$_3$
Without MnmA$_3$
Nº of genomes with MnmA$_3$
Nº of genomes without MnmA$_3$
With MnmA$_5$
Without MnmA$_5$
Nº of genomes with MnmA$_5$
Nº of genomes without MnmA$_5$
p < 0.00005
With MnmA$_4$
Without MnmA$_4$
Nº of genomes with MnmA$_4$
Nº of genomes without MnmA$_4$
p < 0.00005
With MnmA$_1$
Without MnmA$_1$
Nº of genomes with MnmA$_1$
Nº of genomes without MnmA$_1$
With MnmA$_2$
Without MnmA$_2$
Nº of genomes with MnmA$_2$
Nº of genomes without MnmA$_2$
With TusE$_2$
Without TusE$_2$
Nº of genomes with TusE$_2$
Nº of genomes without TusE$_2$
p < 0.00005
With TusE$_1$
Without TusE$_1$
Nº of genomes with TusE$_1$
Nº of genomes without TusE$_1$
With TusE$_4$
Without TusE$_4$
Nº of genomes with TusE$_4$
Nº of genomes without TusE$_4$
p < 0.00005
With TusE$_3$
Without TusE$_3$
Nº of genomes with TusE$_3$
Nº of genomes without TusE$_3$
p < 0.00005

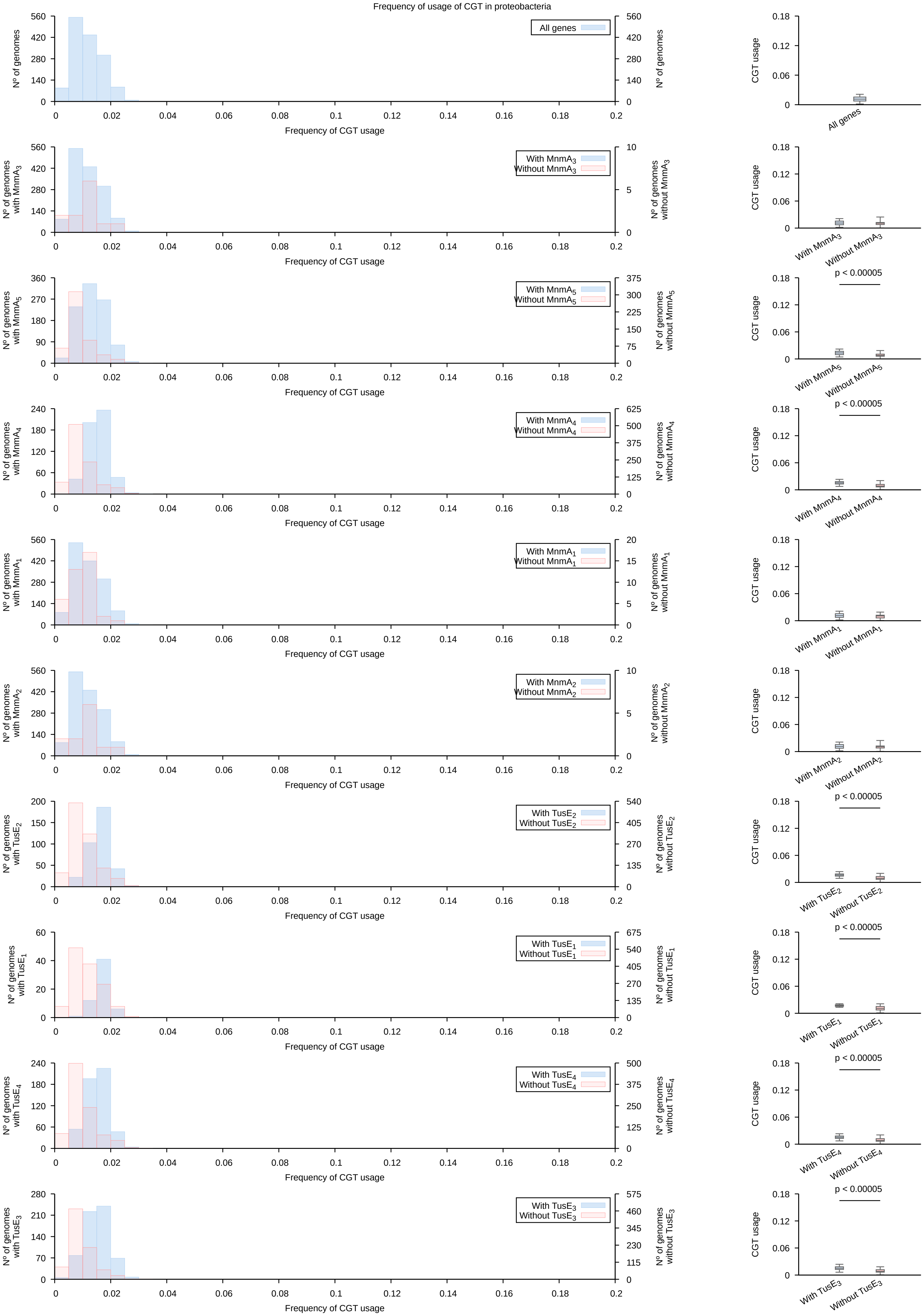

Frequency of usage of CGT in proteobacteria
Nº of genomes
All genes
Frequency of CGT usage
CGT usage
Nº of genomes with MnmA3
Nº of genomes without MnmA3
With MnmA3
Without MnmA3
Nº of genomes with MnmA5
Nº of genomes without MnmA5
With MnmA5
Without MnmA5
p < 0.00005
Nº of genomes with MnmA4
Nº of genomes without MnmA4
With MnmA4
Without MnmA4
p < 0.00005
Nº of genomes with MnmA1
Nº of genomes without MnmA1
With MnmA1
Without MnmA1
Nº of genomes with MnmA2
Nº of genomes without MnmA2
With MnmA2
Without MnmA2
Nº of genomes with TusE2
Nº of genomes without TusE2
With TusE2
Without TusE2
p < 0.00005
Nº of genomes with TusE1
Nº of genomes without TusE1
With TusE1
Without TusE1
p < 0.00005
Nº of genomes with TusE4
Nº of genomes without TusE4
With TusE4
Without TusE4
p < 0.00005
Nº of genomes with TusE3
Nº of genomes without TusE3
With TusE3
Without TusE3
p < 0.00005

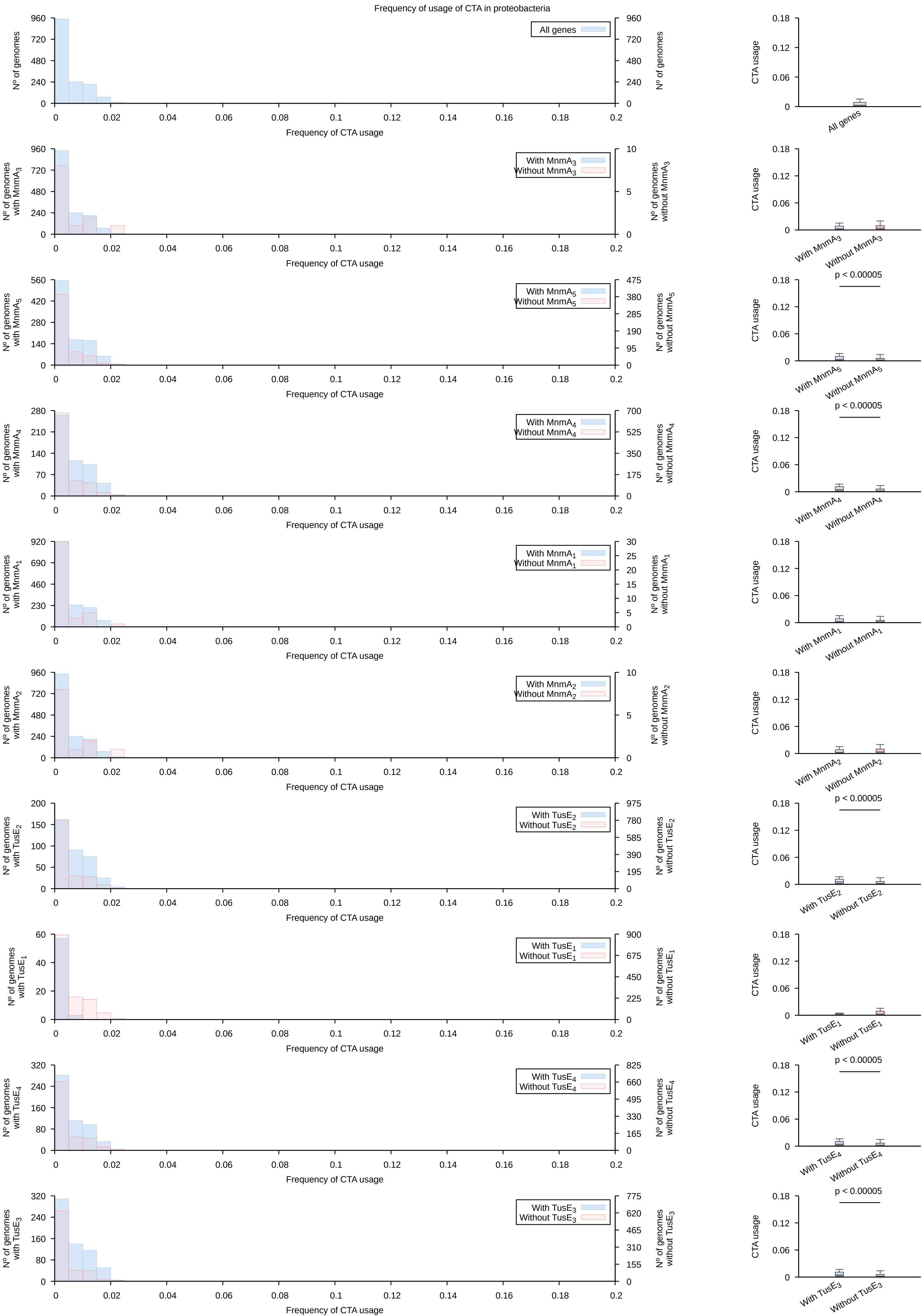
Frequency of usage of CTA in proteobacteria
All genes
With MnmA3
Without MnmA3
With MnmA5
Without MnmA5
With MnmA4
Without MnmA4
With MnmA1
Without MnmA1
With MnmA2
Without MnmA2
With TusE2
Without TusE2
With TusE1
Without TusE1
With TusE4
Without TusE4
With TusE3
Without TusE3
Nº of genomes
Nº of genomes with MnmA3
Nº of genomes without MnmA3
Nº of genomes with MnmA5
Nº of genomes without MnmA5
Nº of genomes with MnmA4
Nº of genomes without MnmA4
Nº of genomes with MnmA1
Nº of genomes without MnmA1
Nº of genomes with MnmA2
Nº of genomes without MnmA2
Nº of genomes with TusE2
Nº of genomes without TusE2
Nº of genomes with TusE1
Nº of genomes without TusE1
Nº of genomes with TusE4
Nº of genomes without TusE4
Nº of genomes with TusE3
Nº of genomes without TusE3
Frequency of CTA usage
CTA usage
p < 0.00005
0
0.02
0.04
0.06
0.08
0.1
0.12
0.14
0.16
0.18
0.2

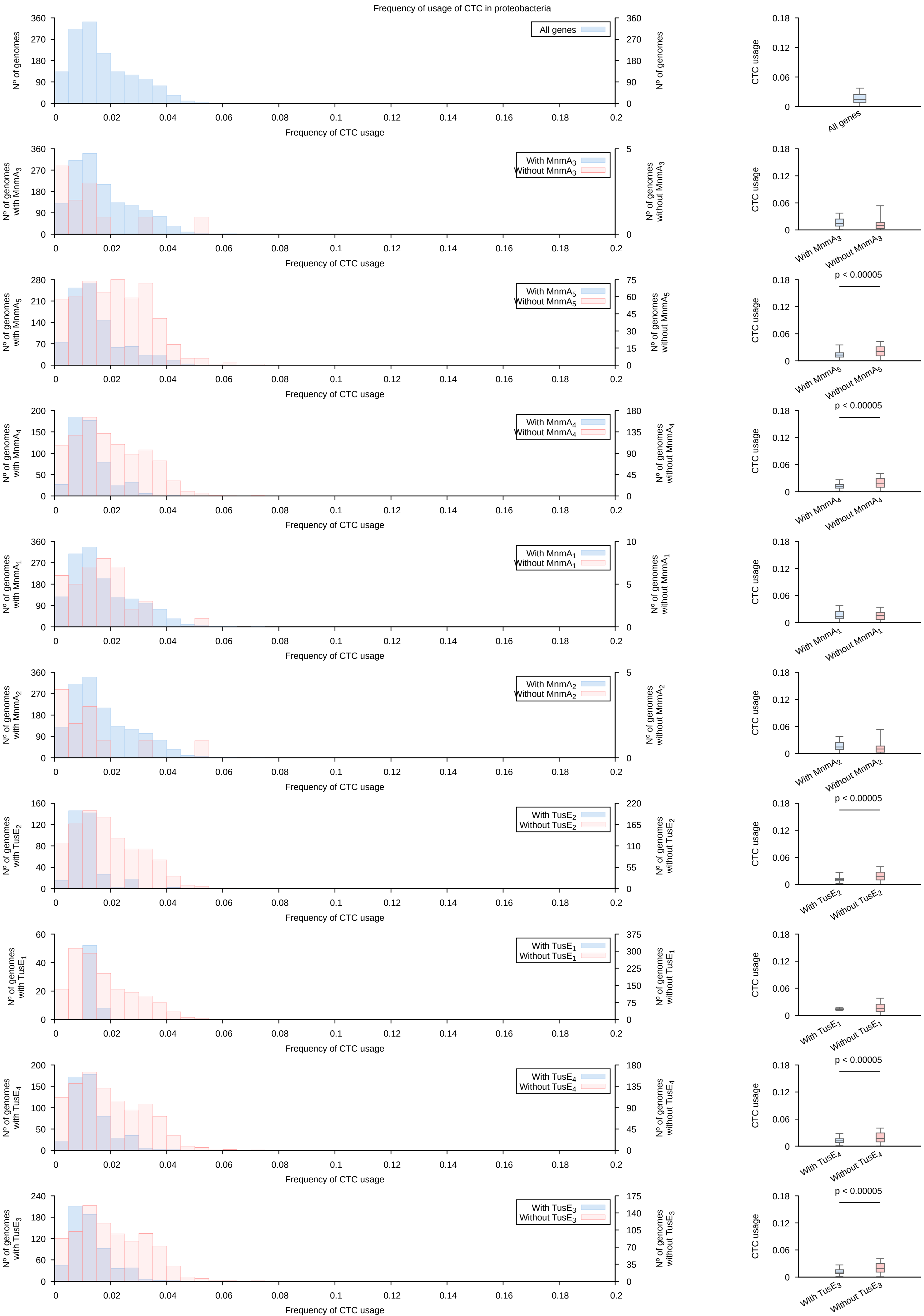
Frequency of usage of CTC in proteobacteria
Nº of genomes
All genes
Frequency of CTC usage
CTC usage
0 0.02 0.04 0.06 0.08 0.1 0.12 0.14 0.16 0.18 0.2
0 0.06 0.12 0.18
Nº of genomes with MnmA$_3$
Nº of genomes without MnmA$_3$
With MnmA$_3$
Without MnmA$_3$
Nº of genomes with MnmA$_5$
Nº of genomes without MnmA$_5$
With MnmA$_5$
Without MnmA$_5$
p < 0.00005
Nº of genomes with MnmA$_4$
Nº of genomes without MnmA$_4$
With MnmA$_4$
Without MnmA$_4$
p < 0.00005
Nº of genomes with MnmA$_1$
Nº of genomes without MnmA$_1$
With MnmA$_1$
Without MnmA$_1$
Nº of genomes with MnmA$_2$
Nº of genomes without MnmA$_2$
With MnmA$_2$
Without MnmA$_2$
Nº of genomes with TusE$_2$
Nº of genomes without TusE$_2$
With TusE$_2$
Without TusE$_2$
p < 0.00005
Nº of genomes with TusE$_1$
Nº of genomes without TusE$_1$
With TusE$_1$
Without TusE$_1$
Nº of genomes with TusE$_4$
Nº of genomes without TusE$_4$
With TusE$_4$
Without TusE$_4$
p < 0.00005
Nº of genomes with TusE$_3$
Nº of genomes without TusE$_3$
With TusE$_3$
Without TusE$_3$
p < 0.00005

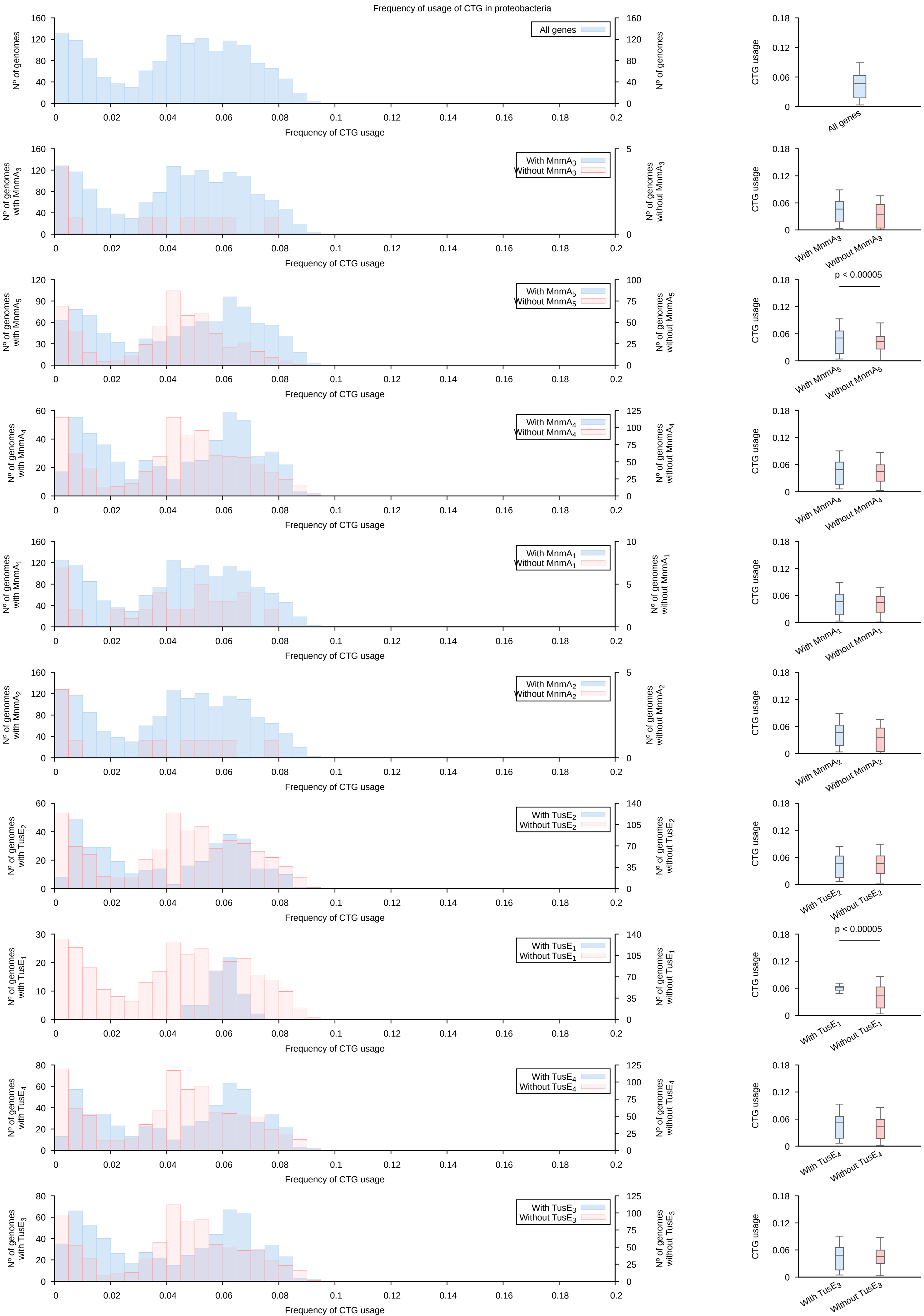
Frequency of usage of CTG in proteobacteria
Nº of genomes
Frequency of CTG usage
All genes
CTG usage
With MnmA3
Without MnmA3
Nº of genomes with MnmA3
Nº of genomes without MnmA3
With MnmA5
Without MnmA5
Nº of genomes with MnmA5
Nº of genomes without MnmA5
p < 0.00005
With MnmA4
Without MnmA4
Nº of genomes with MnmA4
Nº of genomes without MnmA4
With MnmA1
Without MnmA1
Nº of genomes with MnmA1
Nº of genomes without MnmA1
With MnmA2
Without MnmA2
Nº of genomes with MnmA2
Nº of genomes without MnmA2
With TusE2
Without TusE2
Nº of genomes with TusE2
Nº of genomes without TusE2
With TusE1
Without TusE1
Nº of genomes with TusE1
Nº of genomes without TusE1
p < 0.00005
With TusE4
Without TusE4
Nº of genomes with TusE4
Nº of genomes without TusE4
With TusE3
Without TusE3
Nº of genomes with TusE3
Nº of genomes without TusE3
0
0.02
0.04
0.06
0.08
0.1
0.12
0.14
0.16
0.18
0.2

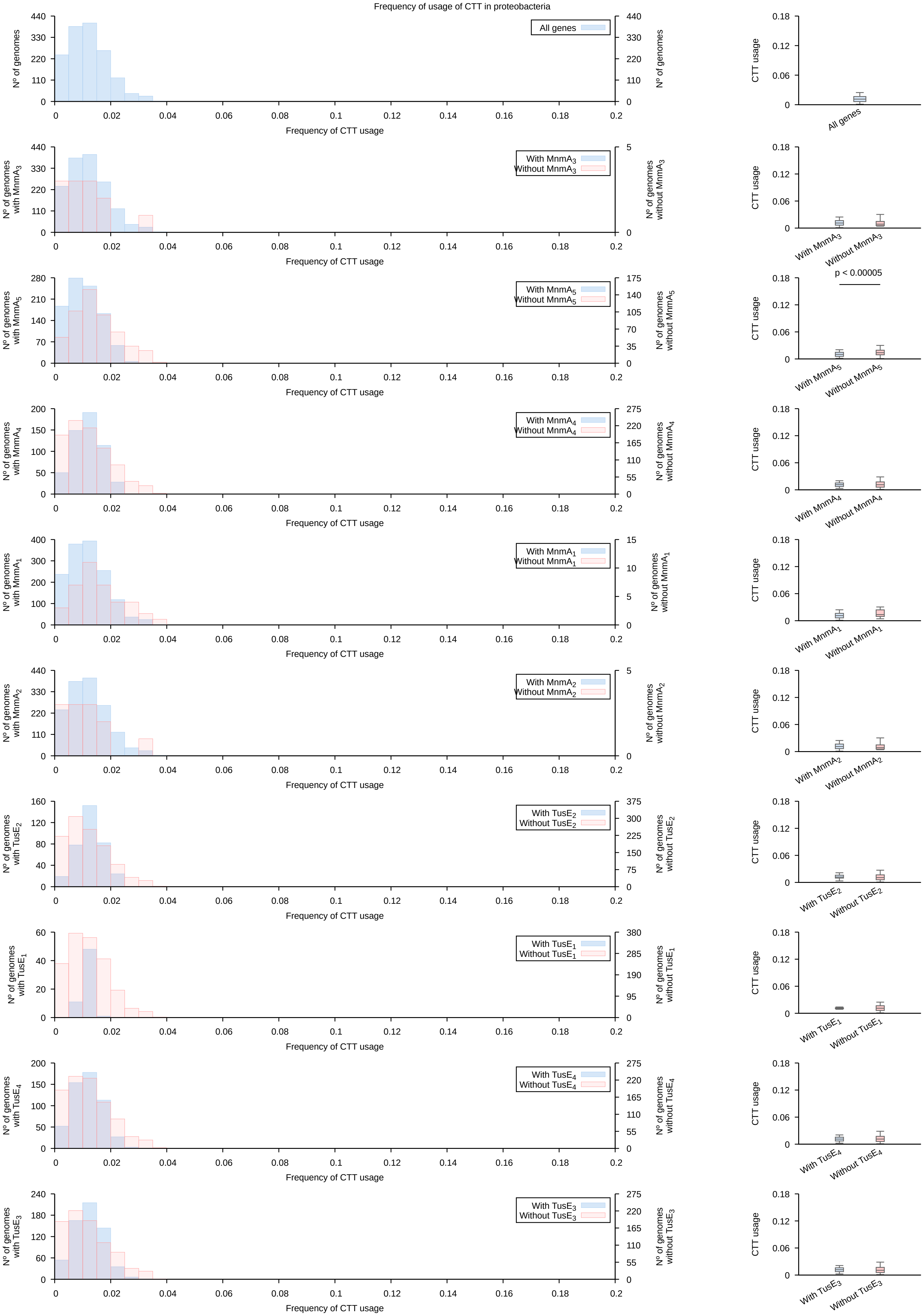

Frequency of usage of CTT in proteobacteria
Nº of genomes
Frequency of CTT usage
CTT usage
All genes
With MnmA$_3$
Without MnmA$_3$
With MnmA$_5$
Without MnmA$_5$
p < 0.00005
With MnmA$_4$
Without MnmA$_4$
With MnmA$_1$
Without MnmA$_1$
With MnmA$_2$
Without MnmA$_2$
With TusE$_2$
Without TusE$_2$
With TusE$_1$
Without TusE$_1$
With TusE$_4$
Without TusE$_4$
With TusE$_3$
Without TusE$_3$

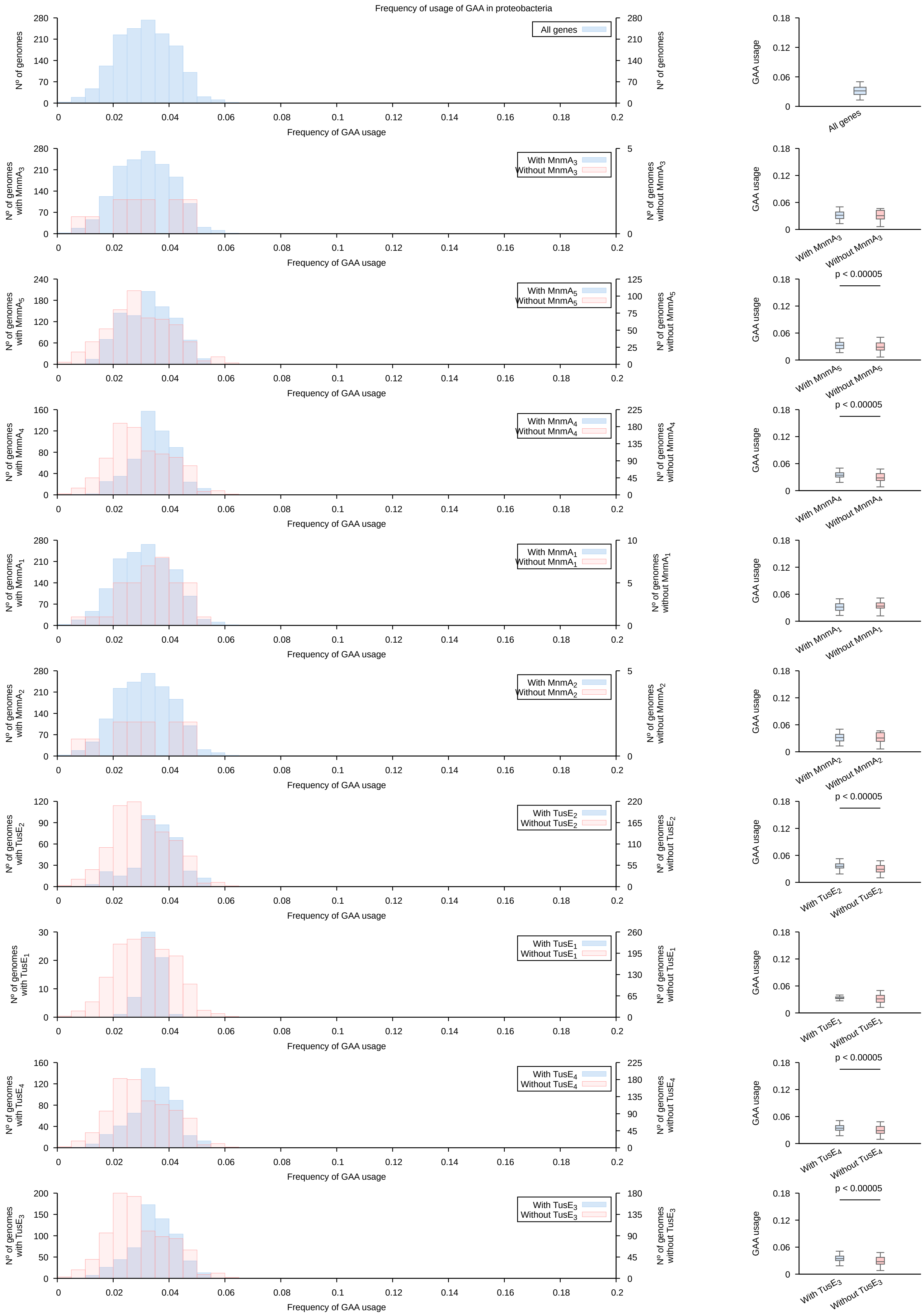
Frequency of usage of GAA in proteobacteria
Nº of genomes
All genes
Frequency of GAA usage
GAA usage
Nº of genomes with MnmA$_3$
Nº of genomes without MnmA$_3$
With MnmA$_3$
Without MnmA$_3$
Nº of genomes with MnmA$_5$
Nº of genomes without MnmA$_5$
With MnmA$_5$
Without MnmA$_5$
p < 0.00005
Nº of genomes with MnmA$_4$
Nº of genomes without MnmA$_4$
With MnmA$_4$
Without MnmA$_4$
p < 0.00005
Nº of genomes with MnmA$_1$
Nº of genomes without MnmA$_1$
With MnmA$_1$
Without MnmA$_1$
Nº of genomes with MnmA$_2$
Nº of genomes without MnmA$_2$
With MnmA$_2$
Without MnmA$_2$
Nº of genomes with TusE$_2$
Nº of genomes without TusE$_2$
With TusE$_2$
Without TusE$_2$
p < 0.00005
Nº of genomes with TusE$_1$
Nº of genomes without TusE$_1$
With TusE$_1$
Without TusE$_1$
Nº of genomes with TusE$_4$
Nº of genomes without TusE$_4$
With TusE$_4$
Without TusE$_4$
p < 0.00005
Nº of genomes with TusE$_3$
Nº of genomes without TusE$_3$
With TusE$_3$
Without TusE$_3$
p < 0.00005

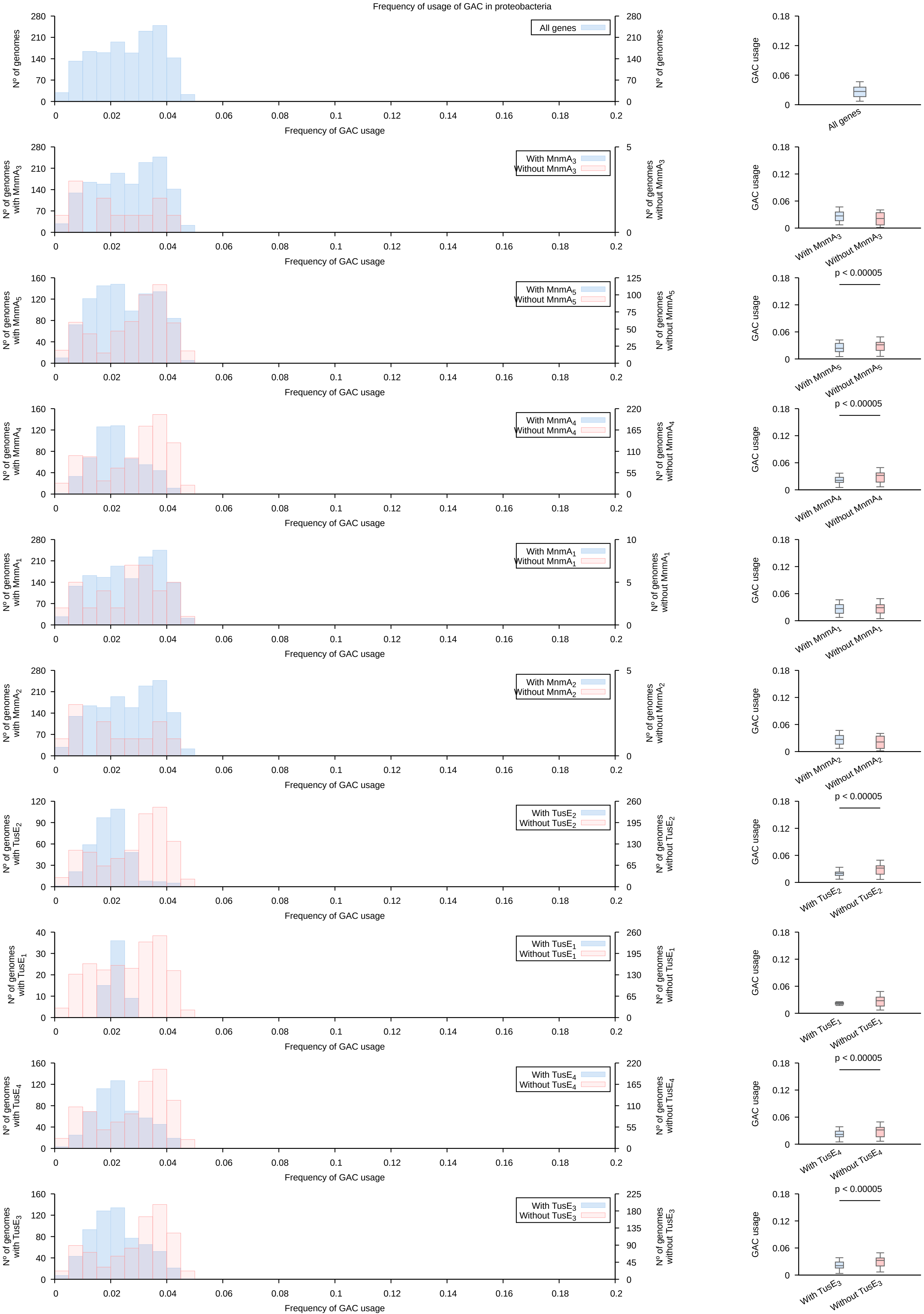
Frequency of usage of GAC in proteobacteria
Nº of genomes
All genes
GAC usage
Frequency of GAC usage
Nº of genomes with $MnmA_3$
Nº of genomes without $MnmA_3$
With $MnmA_3$
Without $MnmA_3$
Nº of genomes with $MnmA_5$
Nº of genomes without $MnmA_5$
With $MnmA_5$
Without $MnmA_5$
p < 0.00005
Nº of genomes with $MnmA_4$
Nº of genomes without $MnmA_4$
With $MnmA_4$
Without $MnmA_4$
p < 0.00005
Nº of genomes with $MnmA_1$
Nº of genomes without $MnmA_1$
With $MnmA_1$
Without $MnmA_1$
Nº of genomes with $MnmA_2$
Nº of genomes without $MnmA_2$
With $MnmA_2$
Without $MnmA_2$
Nº of genomes with $TusE_2$
Nº of genomes without $TusE_2$
With $TusE_2$
Without $TusE_2$
p < 0.00005
Nº of genomes with $TusE_1$
Nº of genomes without $TusE_1$
With $TusE_1$
Without $TusE_1$
Nº of genomes with $TusE_4$
Nº of genomes without $TusE_4$
With $TusE_4$
Without $TusE_4$
p < 0.00005
Nº of genomes with $TusE_3$
Nº of genomes without $TusE_3$
With $TusE_3$
Without $TusE_3$
p < 0.00005

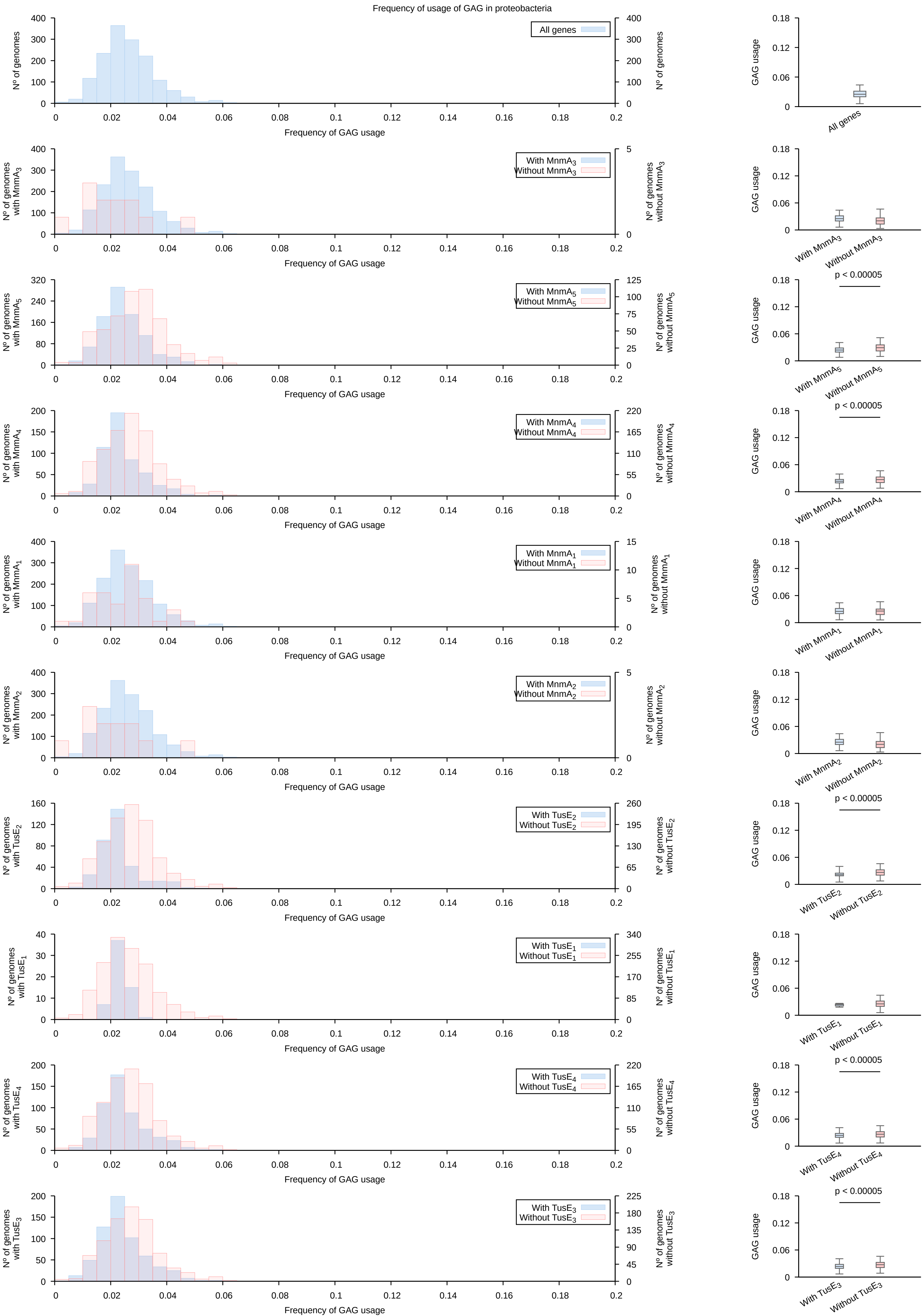
Frequency of usage of GAG in proteobacteria
Nº of genomes
Frequency of GAG usage
GAG usage
All genes
With MnmA3
Without MnmA3
Nº of genomes with MnmA3
Nº of genomes without MnmA3
With MnmA5
Without MnmA5
Nº of genomes with MnmA5
Nº of genomes without MnmA5
With MnmA4
Without MnmA4
Nº of genomes with MnmA4
Nº of genomes without MnmA4
With MnmA1
Without MnmA1
Nº of genomes with MnmA1
Nº of genomes without MnmA1
With MnmA2
Without MnmA2
Nº of genomes with MnmA2
Nº of genomes without MnmA2
With TusE2
Without TusE2
Nº of genomes with TusE2
Nº of genomes without TusE2
With TusE1
Without TusE1
Nº of genomes with TusE1
Nº of genomes without TusE1
With TusE4
Without TusE4
Nº of genomes with TusE4
Nº of genomes without TusE4
With TusE3
Without TusE3
Nº of genomes with TusE3
Nº of genomes without TusE3
p < 0.00005

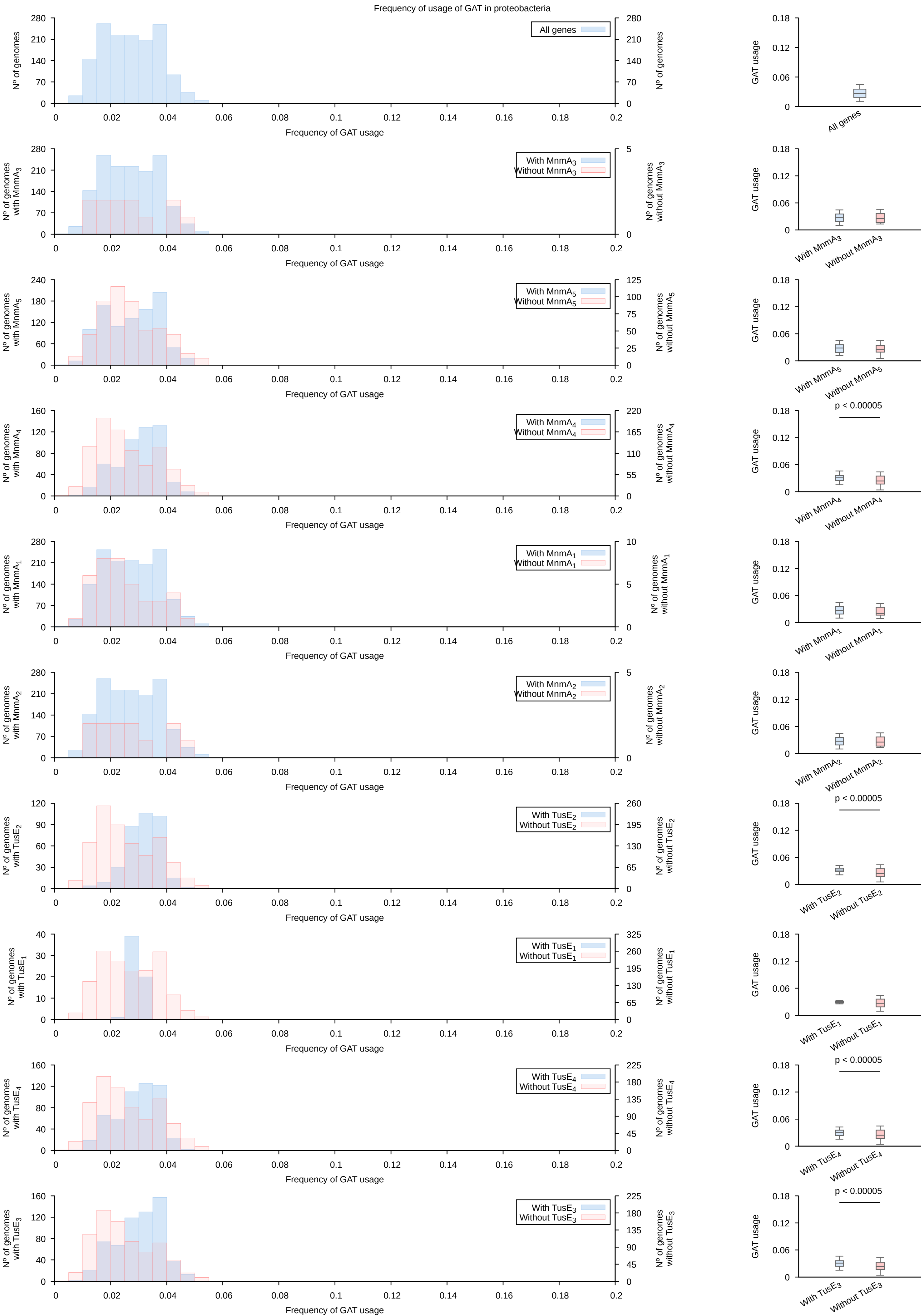

Frequency of usage of GAT in proteobacteria
Nº of genomes
Frequency of GAT usage
GAT usage
All genes
Nº of genomes with $MnmA_3$
Nº of genomes without $MnmA_3$
With $MnmA_3$
Without $MnmA_3$
Nº of genomes with $MnmA_5$
Nº of genomes without $MnmA_5$
With $MnmA_5$
Without $MnmA_5$
Nº of genomes with $MnmA_4$
Nº of genomes without $MnmA_4$
With $MnmA_4$
Without $MnmA_4$
p < 0.00005
Nº of genomes with $MnmA_1$
Nº of genomes without $MnmA_1$
With $MnmA_1$
Without $MnmA_1$
Nº of genomes with $MnmA_2$
Nº of genomes without $MnmA_2$
With $MnmA_2$
Without $MnmA_2$
Nº of genomes with $TusE_2$
Nº of genomes without $TusE_2$
With $TusE_2$
Without $TusE_2$
p < 0.00005
Nº of genomes with $TusE_1$
Nº of genomes without $TusE_1$
With $TusE_1$
Without $TusE_1$
Nº of genomes with $TusE_4$
Nº of genomes without $TusE_4$
With $TusE_4$
Without $TusE_4$
p < 0.00005
Nº of genomes with $TusE_3$
Nº of genomes without $TusE_3$
With $TusE_3$
Without $TusE_3$
p < 0.00005

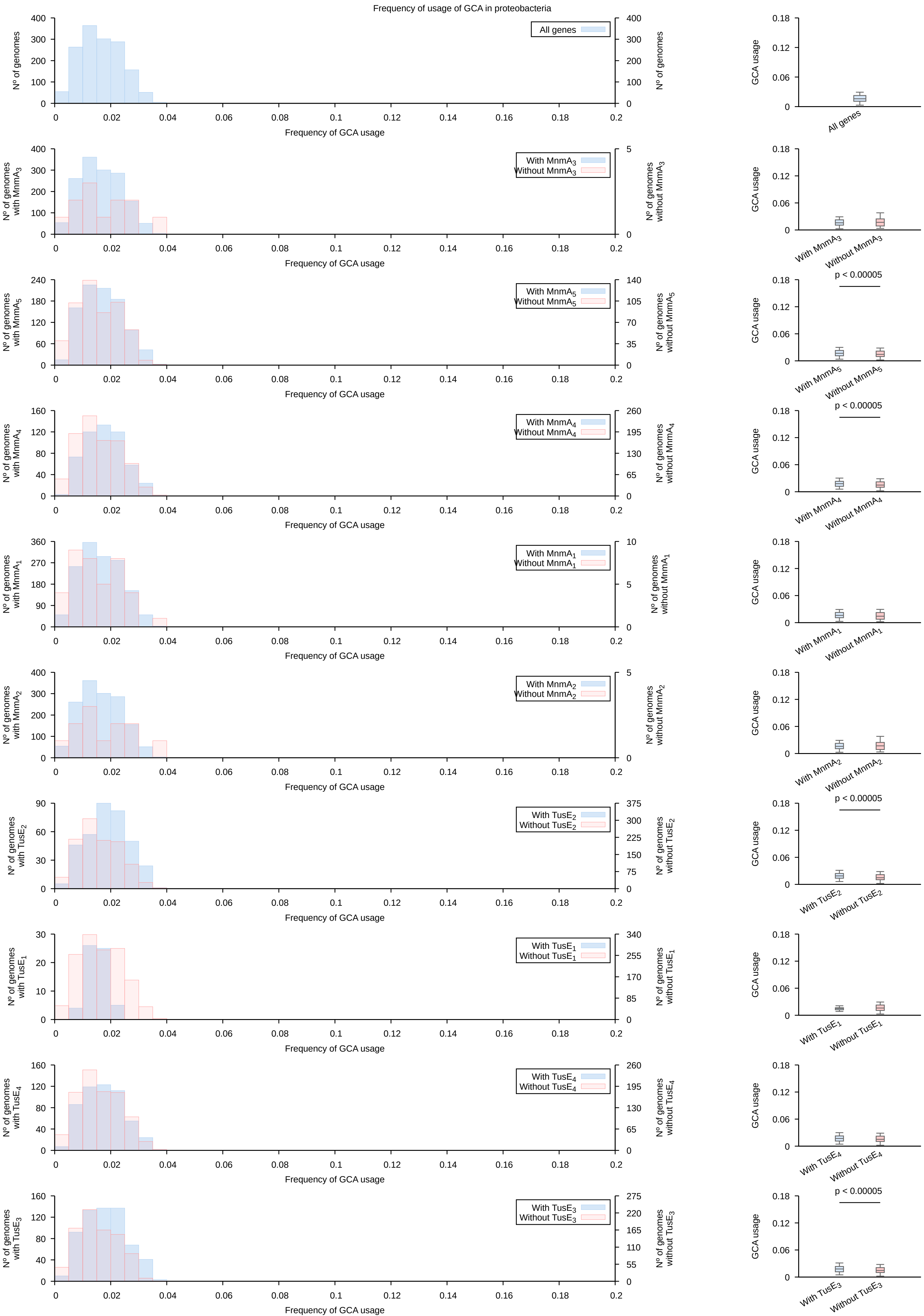
Frequency of usage of GCA in proteobacteria
All genes
Nº of genomes
Frequency of GCA usage
GCA usage
With MnmA$_3$
Without MnmA$_3$
Nº of genomes with MnmA$_3$
Nº of genomes without MnmA$_3$
With MnmA$_5$
Without MnmA$_5$
Nº of genomes with MnmA$_5$
Nº of genomes without MnmA$_5$
p < 0.00005
With MnmA$_4$
Without MnmA$_4$
Nº of genomes with MnmA$_4$
Nº of genomes without MnmA$_4$
p < 0.00005
With MnmA$_1$
Without MnmA$_1$
Nº of genomes with MnmA$_1$
Nº of genomes without MnmA$_1$
With MnmA$_2$
Without MnmA$_2$
Nº of genomes with MnmA$_2$
Nº of genomes without MnmA$_2$
With TusE$_2$
Without TusE$_2$
Nº of genomes with TusE$_2$
Nº of genomes without TusE$_2$
p < 0.00005
With TusE$_1$
Without TusE$_1$
Nº of genomes with TusE$_1$
Nº of genomes without TusE$_1$
With TusE$_4$
Without TusE$_4$
Nº of genomes with TusE$_4$
Nº of genomes without TusE$_4$
With TusE$_3$
Without TusE$_3$
Nº of genomes with TusE$_3$
Nº of genomes without TusE$_3$
p < 0.00005

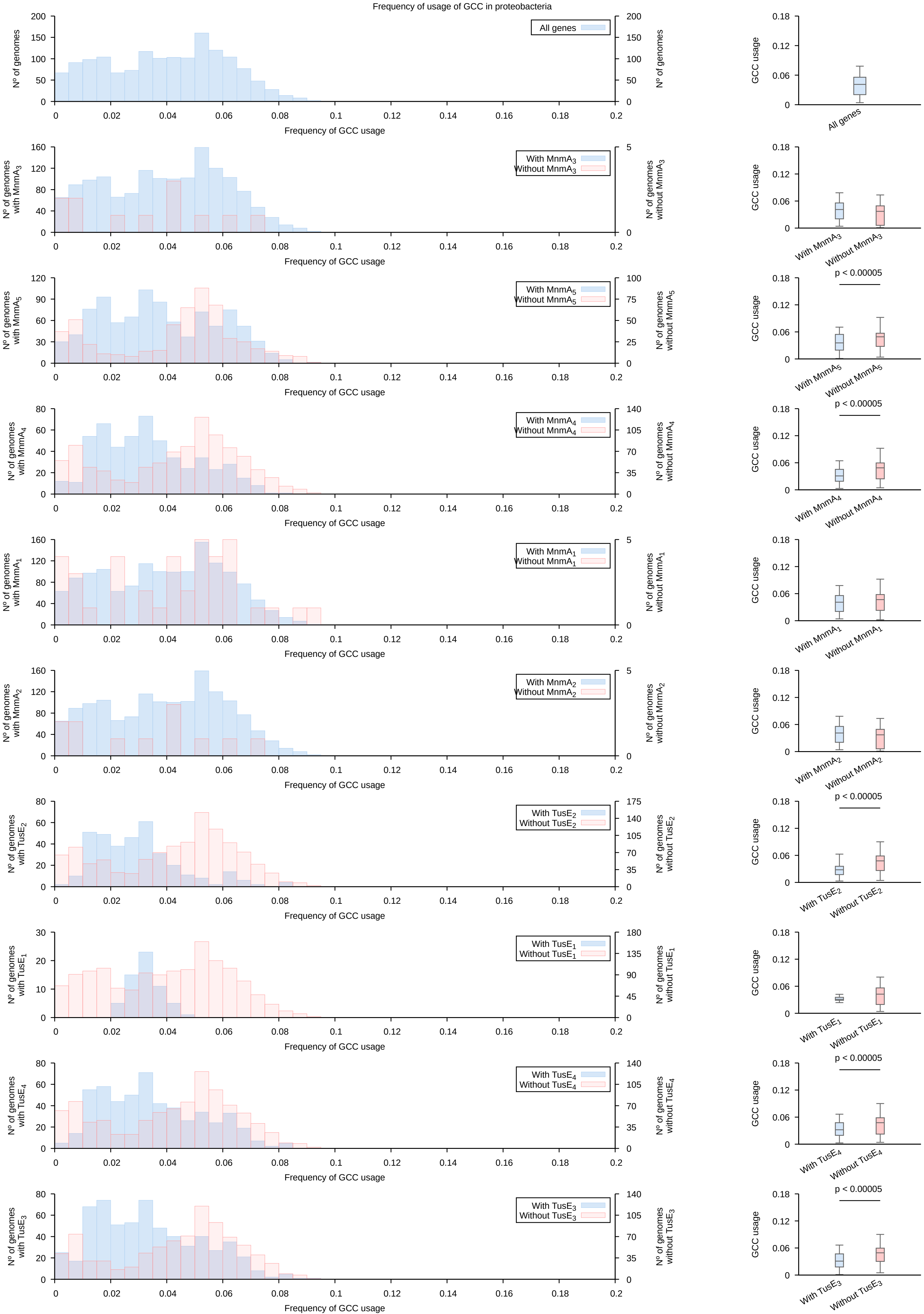
Frequency of usage of GCC in proteobacteria
All genes
Nº of genomes
Frequency of GCC usage
GCC usage
With MnmA$_3$
Without MnmA$_3$
Nº of genomes with MnmA$_3$
Nº of genomes without MnmA$_3$
With MnmA$_5$
Without MnmA$_5$
Nº of genomes with MnmA$_5$
Nº of genomes without MnmA$_5$
p < 0.00005
With MnmA$_4$
Without MnmA$_4$
Nº of genomes with MnmA$_4$
Nº of genomes without MnmA$_4$
p < 0.00005
With MnmA$_1$
Without MnmA$_1$
Nº of genomes with MnmA$_1$
Nº of genomes without MnmA$_1$
With MnmA$_2$
Without MnmA$_2$
Nº of genomes with MnmA$_2$
Nº of genomes without MnmA$_2$
With TusE$_2$
Without TusE$_2$
Nº of genomes with TusE$_2$
Nº of genomes without TusE$_2$
p < 0.00005
With TusE$_1$
Without TusE$_1$
Nº of genomes with TusE$_1$
Nº of genomes without TusE$_1$
With TusE$_4$
Without TusE$_4$
Nº of genomes with TusE$_4$
Nº of genomes without TusE$_4$
p < 0.00005
With TusE$_3$
Without TusE$_3$
Nº of genomes with TusE$_3$
Nº of genomes without TusE$_3$
p < 0.00005

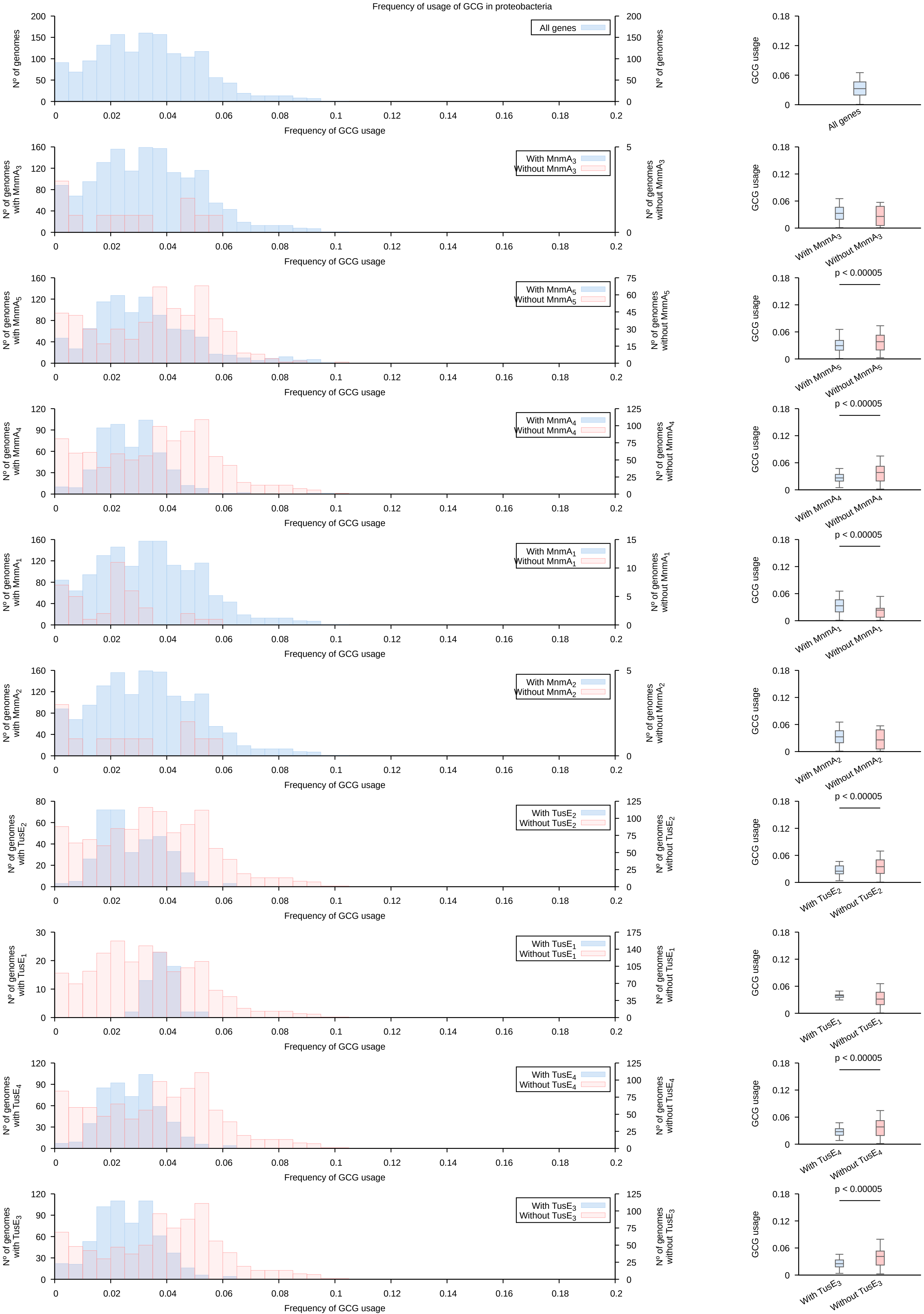
Frequency of usage of GCG in proteobacteria
Nº of genomes
All genes
Frequency of GCG usage
GCG usage
All genes
Nº of genomes with MnmA$_3$
Nº of genomes without MnmA$_3$
With MnmA$_3$
Without MnmA$_3$
Nº of genomes with MnmA$_5$
Nº of genomes without MnmA$_5$
With MnmA$_5$
Without MnmA$_5$
p < 0.00005
Nº of genomes with MnmA$_4$
Nº of genomes without MnmA$_4$
With MnmA$_4$
Without MnmA$_4$
p < 0.00005
Nº of genomes with MnmA$_1$
Nº of genomes without MnmA$_1$
With MnmA$_1$
Without MnmA$_1$
p < 0.00005
Nº of genomes with MnmA$_2$
Nº of genomes without MnmA$_2$
With MnmA$_2$
Without MnmA$_2$
Nº of genomes with TusE$_2$
Nº of genomes without TusE$_2$
With TusE$_2$
Without TusE$_2$
p < 0.00005
Nº of genomes with TusE$_1$
Nº of genomes without TusE$_1$
With TusE$_1$
Without TusE$_1$
Nº of genomes with TusE$_4$
Nº of genomes without TusE$_4$
With TusE$_4$
Without TusE$_4$
p < 0.00005
Nº of genomes with TusE$_3$
Nº of genomes without TusE$_3$
With TusE$_3$
Without TusE$_3$
p < 0.00005

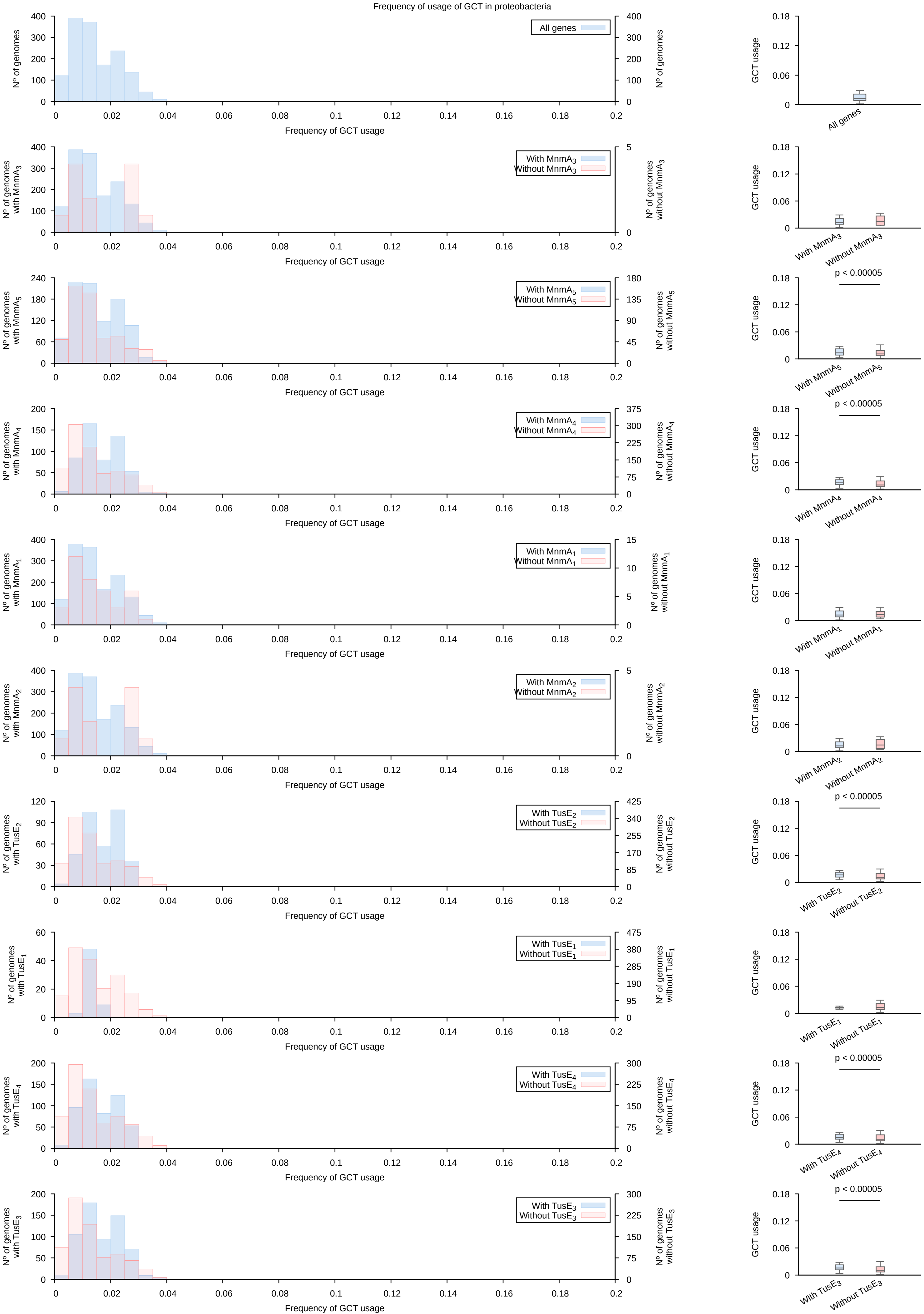
Frequency of usage of GCT in proteobacteria
All genes
Nº of genomes
Frequency of GCT usage
GCT usage
With $MnmA_3$
Without $MnmA_3$
Nº of genomes with $MnmA_3$
Nº of genomes without $MnmA_3$
With $MnmA_5$
Without $MnmA_5$
Nº of genomes with $MnmA_5$
Nº of genomes without $MnmA_5$
p < 0.00005
With $MnmA_4$
Without $MnmA_4$
Nº of genomes with $MnmA_4$
Nº of genomes without $MnmA_4$
p < 0.00005
With $MnmA_1$
Without $MnmA_1$
Nº of genomes with $MnmA_1$
Nº of genomes without $MnmA_1$
With $MnmA_2$
Without $MnmA_2$
Nº of genomes with $MnmA_2$
Nº of genomes without $MnmA_2$
With $TusE_2$
Without $TusE_2$
Nº of genomes with $TusE_2$
Nº of genomes without $TusE_2$
p < 0.00005
With $TusE_1$
Without $TusE_1$
Nº of genomes with $TusE_1$
Nº of genomes without $TusE_1$
With $TusE_4$
Without $TusE_4$
Nº of genomes with $TusE_4$
Nº of genomes without $TusE_4$
p < 0.00005
With $TusE_3$
Without $TusE_3$
Nº of genomes with $TusE_3$
Nº of genomes without $TusE_3$
p < 0.00005

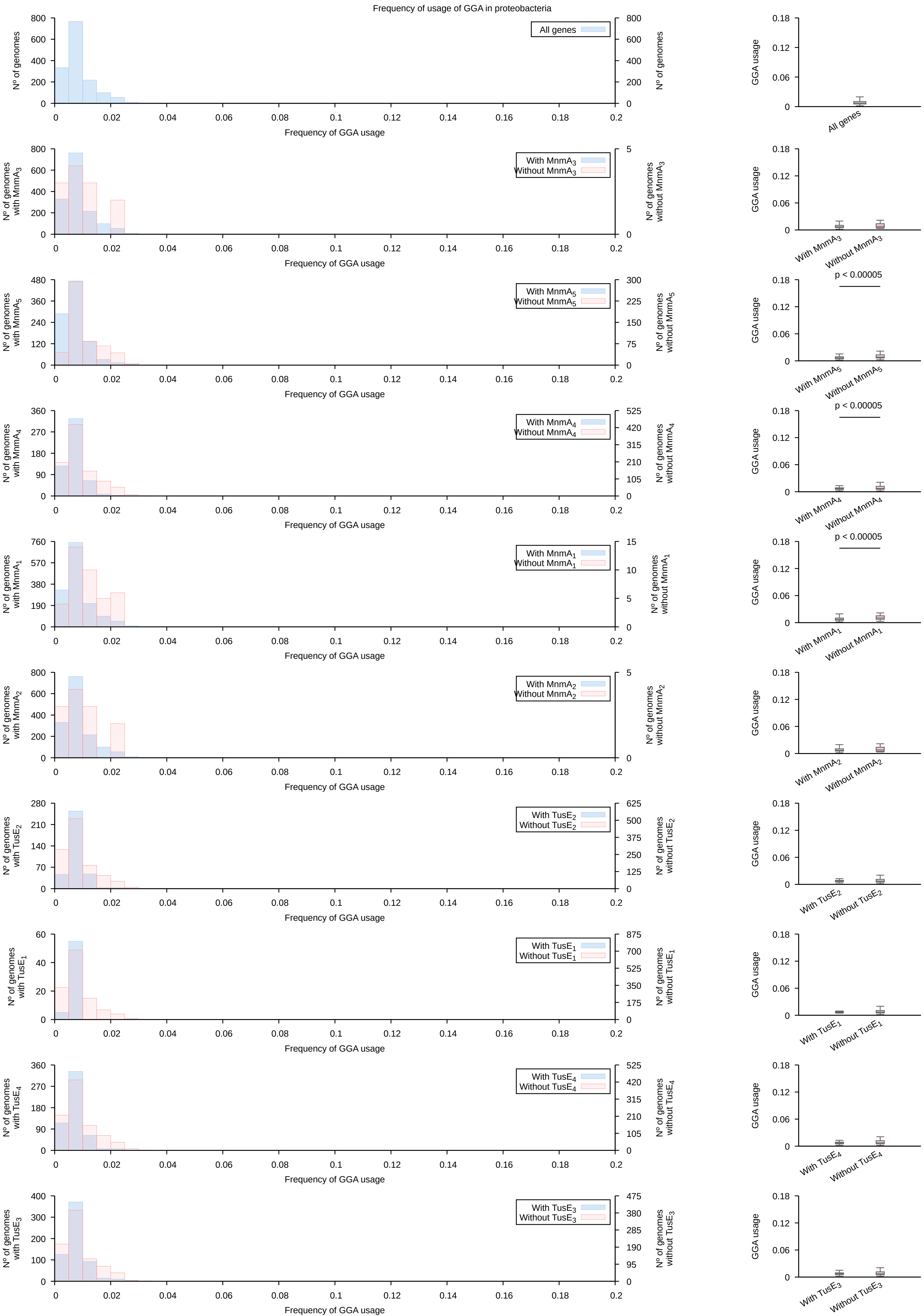
Frequency of usage of GGA in proteobacteria
All genes
Nº of genomes
Frequency of GGA usage
GGA usage
With MnmA$_3$
Without MnmA$_3$
Nº of genomes with MnmA$_3$
Nº of genomes without MnmA$_3$
With MnmA$_5$
Without MnmA$_5$
Nº of genomes with MnmA$_5$
Nº of genomes without MnmA$_5$
p < 0.00005
With MnmA$_4$
Without MnmA$_4$
Nº of genomes with MnmA$_4$
Nº of genomes without MnmA$_4$
p < 0.00005
With MnmA$_1$
Without MnmA$_1$
Nº of genomes with MnmA$_1$
Nº of genomes without MnmA$_1$
p < 0.00005
With MnmA$_2$
Without MnmA$_2$
Nº of genomes with MnmA$_2$
Nº of genomes without MnmA$_2$
With TusE$_2$
Without TusE$_2$
Nº of genomes with TusE$_2$
Nº of genomes without TusE$_2$
With TusE$_1$
Without TusE$_1$
Nº of genomes with TusE$_1$
Nº of genomes without TusE$_1$
With TusE$_4$
Without TusE$_4$
Nº of genomes with TusE$_4$
Nº of genomes without TusE$_4$
With TusE$_3$
Without TusE$_3$
Nº of genomes with TusE$_3$
Nº of genomes without TusE$_3$

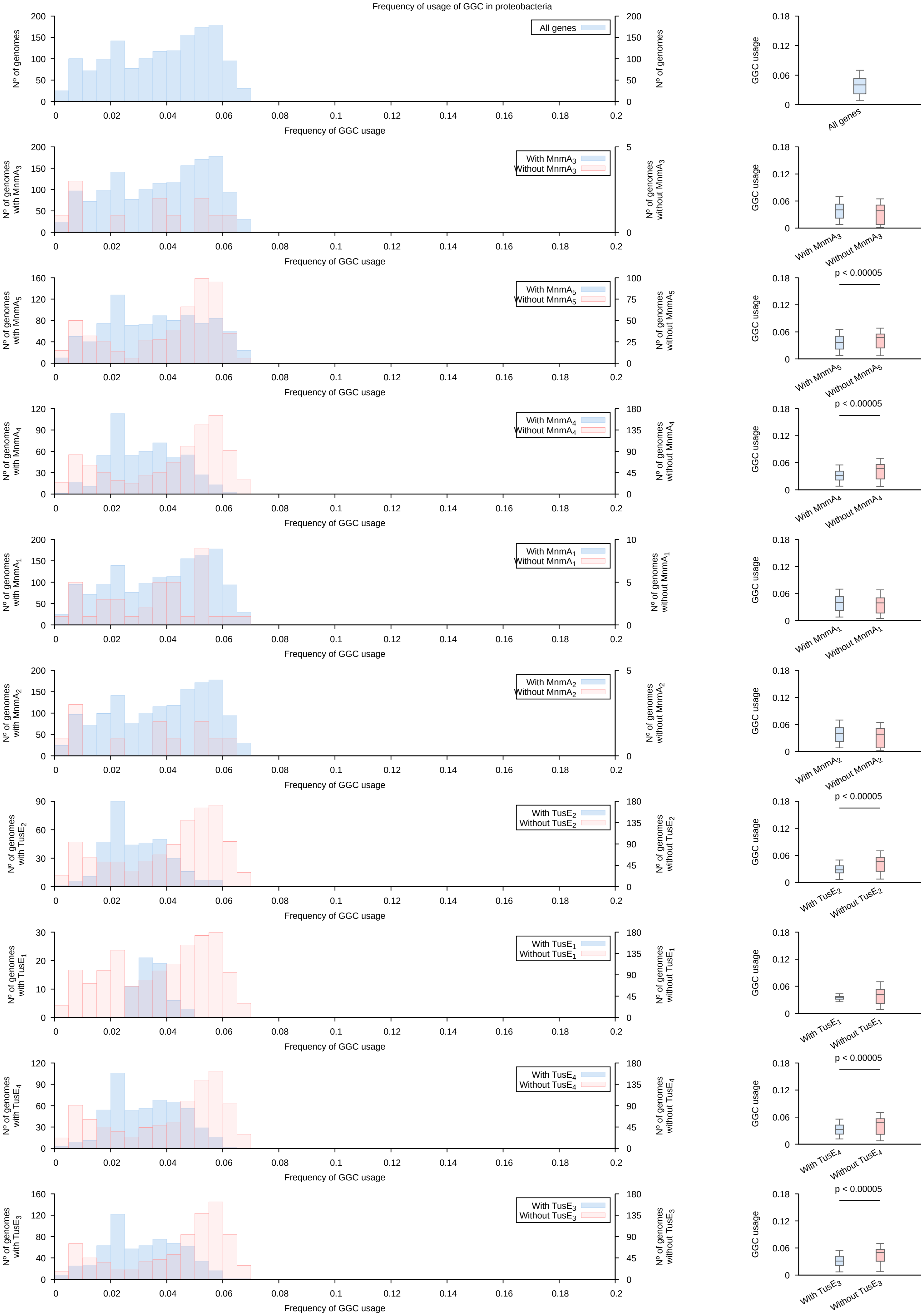
Frequency of usage of GGC in proteobacteria
All genes
Nº of genomes
Frequency of GGC usage
GGC usage
With MnmA3
Without MnmA3
Nº of genomes with MnmA3
Nº of genomes without MnmA3
With MnmA5
Without MnmA5
Nº of genomes with MnmA5
Nº of genomes without MnmA5
p < 0.00005
With MnmA4
Without MnmA4
Nº of genomes with MnmA4
Nº of genomes without MnmA4
p < 0.00005
With MnmA1
Without MnmA1
Nº of genomes with MnmA1
Nº of genomes without MnmA1
With MnmA2
Without MnmA2
Nº of genomes with MnmA2
Nº of genomes without MnmA2
With TusE2
Without TusE2
Nº of genomes with TusE2
Nº of genomes without TusE2
p < 0.00005
With TusE1
Without TusE1
Nº of genomes with TusE1
Nº of genomes without TusE1
With TusE4
Without TusE4
Nº of genomes with TusE4
Nº of genomes without TusE4
p < 0.00005
With TusE3
Without TusE3
Nº of genomes with TusE3
Nº of genomes without TusE3
p < 0.00005

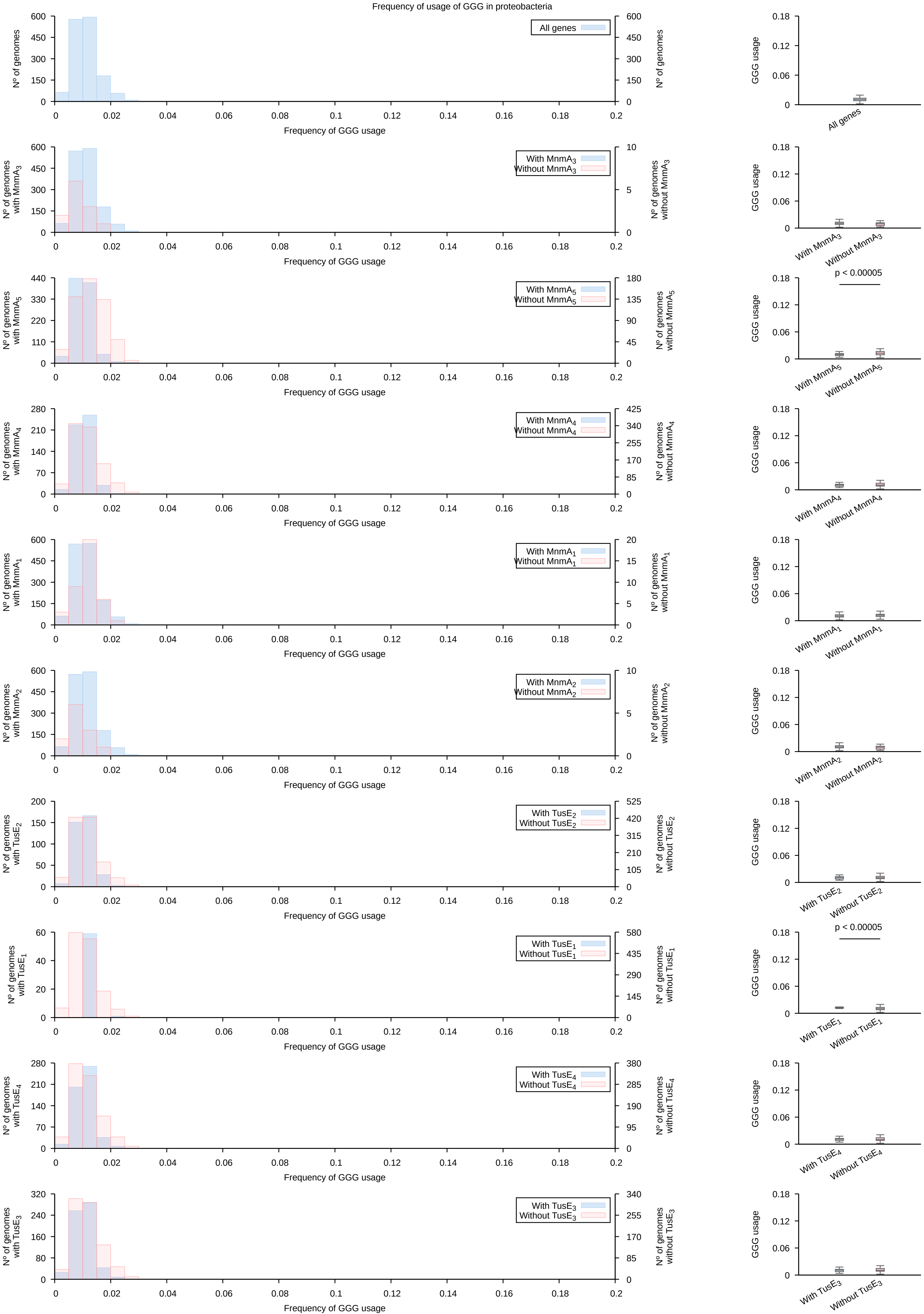

Frequency of usage of GGG in proteobacteria
All genes
Nº of genomes
Frequency of GGG usage
GGG usage
With MnmA3
Without MnmA3
Nº of genomes with MnmA3
Nº of genomes without MnmA3
With MnmA5
Without MnmA5
Nº of genomes with MnmA5
Nº of genomes without MnmA5
p < 0.00005
With MnmA4
Without MnmA4
Nº of genomes with MnmA4
Nº of genomes without MnmA4
With MnmA1
Without MnmA1
Nº of genomes with MnmA1
Nº of genomes without MnmA1
With MnmA2
Without MnmA2
Nº of genomes with MnmA2
Nº of genomes without MnmA2
With TusE2
Without TusE2
Nº of genomes with TusE2
Nº of genomes without TusE2
With TusE1
Without TusE1
Nº of genomes with TusE1
Nº of genomes without TusE1
p < 0.00005
With TusE4
Without TusE4
Nº of genomes with TusE4
Nº of genomes without TusE4
With TusE3
Without TusE3
Nº of genomes with TusE3
Nº of genomes without TusE3

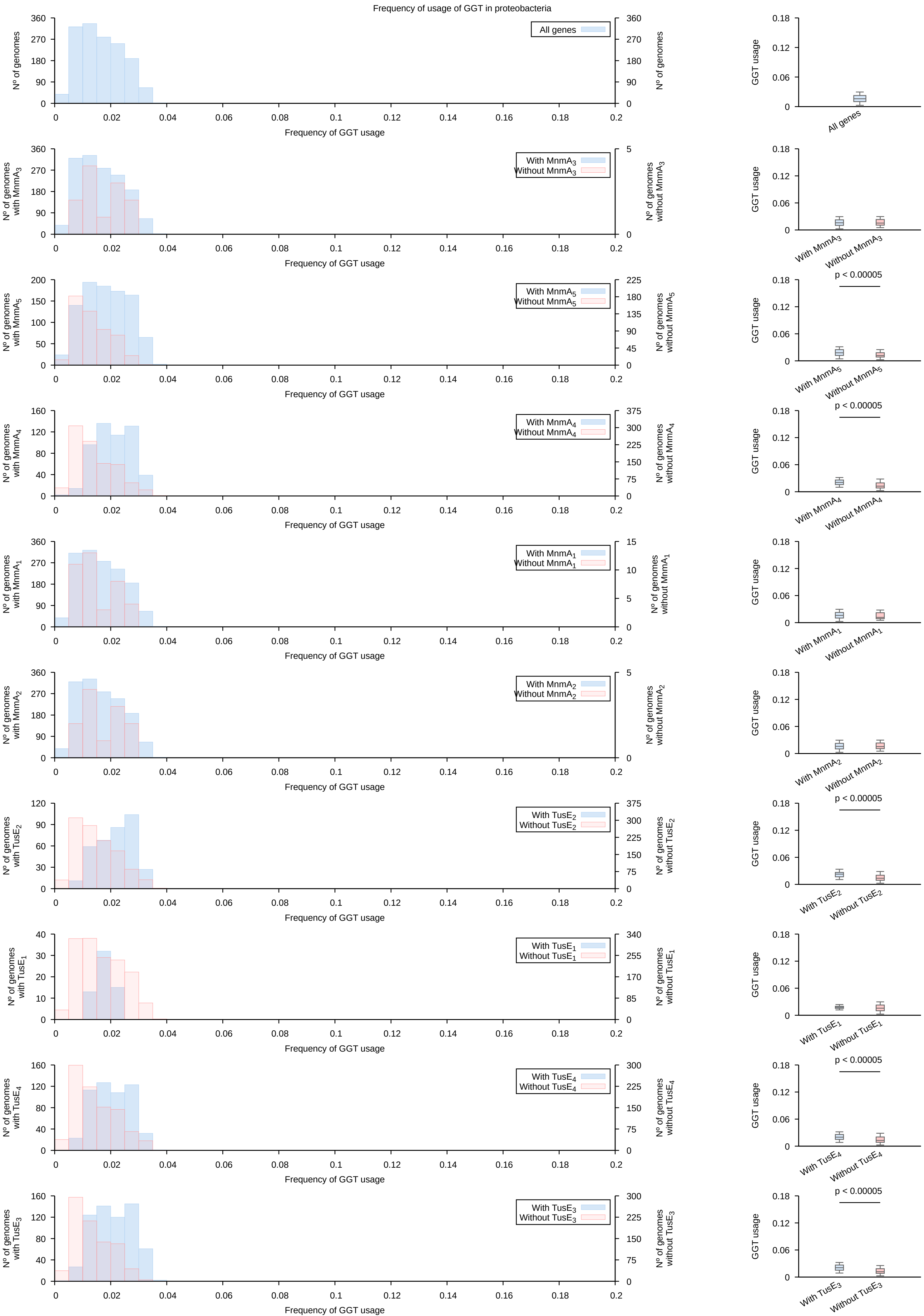

Frequency of usage of GGT in proteobacteria
Nº of genomes
All genes
Frequency of GGT usage
GGT usage
Nº of genomes with MnmA$_3$
Nº of genomes without MnmA$_3$
With MnmA$_3$
Without MnmA$_3$
Nº of genomes with MnmA$_5$
Nº of genomes without MnmA$_5$
With MnmA$_5$
Without MnmA$_5$
p < 0.00005
Nº of genomes with MnmA$_4$
Nº of genomes without MnmA$_4$
With MnmA$_4$
Without MnmA$_4$
p < 0.00005
Nº of genomes with MnmA$_1$
Nº of genomes without MnmA$_1$
With MnmA$_1$
Without MnmA$_1$
Nº of genomes with MnmA$_2$
Nº of genomes without MnmA$_2$
With MnmA$_2$
Without MnmA$_2$
Nº of genomes with TusE$_2$
Nº of genomes without TusE$_2$
With TusE$_2$
Without TusE$_2$
p < 0.00005
Nº of genomes with TusE$_1$
Nº of genomes without TusE$_1$
With TusE$_1$
Without TusE$_1$
Nº of genomes with TusE$_4$
Nº of genomes without TusE$_4$
With TusE$_4$
Without TusE$_4$
p < 0.00005
Nº of genomes with TusE$_3$
Nº of genomes without TusE$_3$
With TusE$_3$
Without TusE$_3$
p < 0.00005

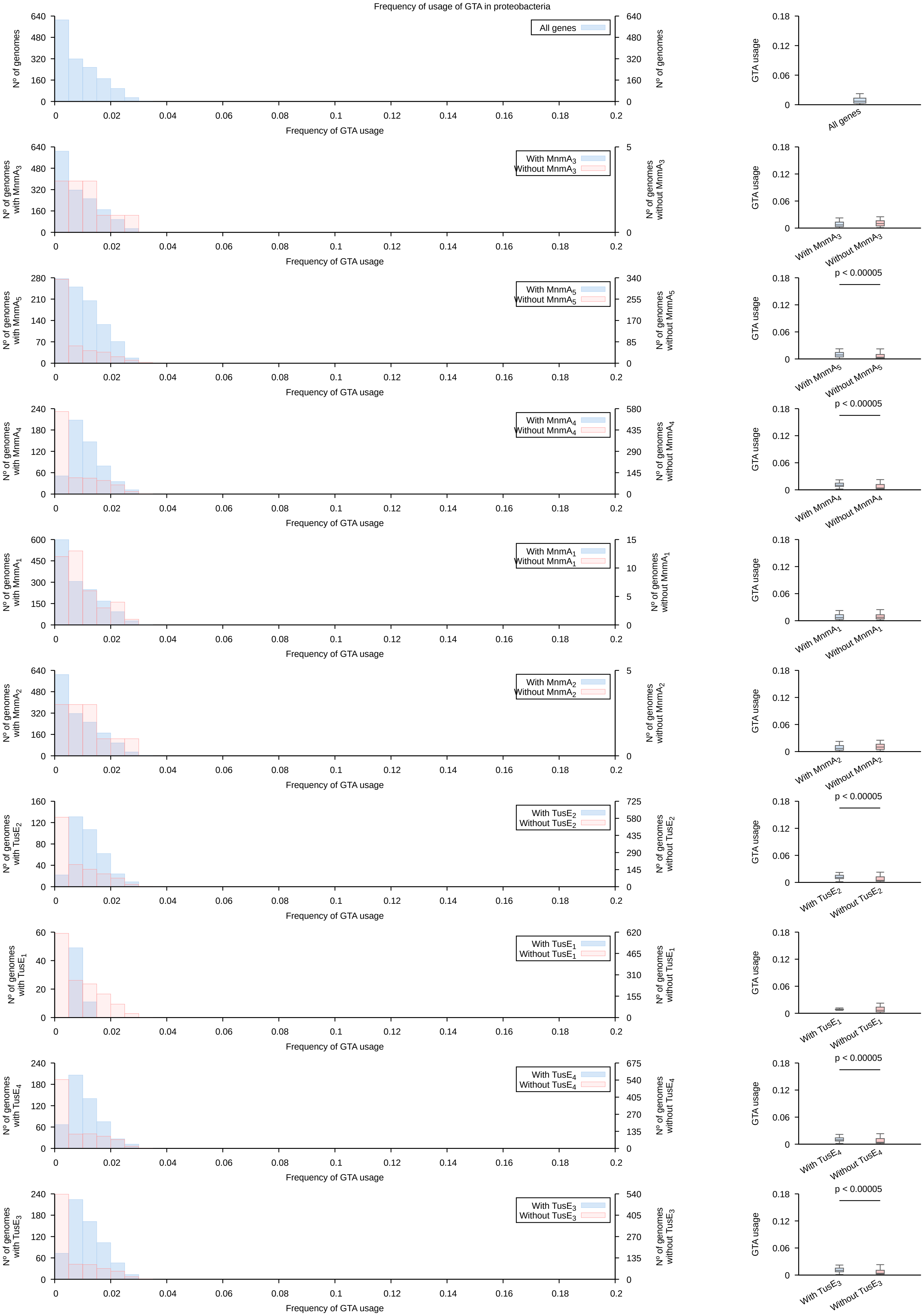
Frequency of usage of GTA in proteobacteria
All genes
Nº of genomes
Frequency of GTA usage
GTA usage
With MnmA$_3$
Without MnmA$_3$
Nº of genomes with MnmA$_3$
Nº of genomes without MnmA$_3$
With MnmA$_5$
Without MnmA$_5$
Nº of genomes with MnmA$_5$
Nº of genomes without MnmA$_5$
p < 0.00005
With MnmA$_4$
Without MnmA$_4$
Nº of genomes with MnmA$_4$
Nº of genomes without MnmA$_4$
p < 0.00005
With MnmA$_1$
Without MnmA$_1$
Nº of genomes with MnmA$_1$
Nº of genomes without MnmA$_1$
With MnmA$_2$
Without MnmA$_2$
Nº of genomes with MnmA$_2$
Nº of genomes without MnmA$_2$
With TusE$_2$
Without TusE$_2$
Nº of genomes with TusE$_2$
Nº of genomes without TusE$_2$
p < 0.00005
With TusE$_1$
Without TusE$_1$
Nº of genomes with TusE$_1$
Nº of genomes without TusE$_1$
With TusE$_4$
Without TusE$_4$
Nº of genomes with TusE$_4$
Nº of genomes without TusE$_4$
p < 0.00005
With TusE$_3$
Without TusE$_3$
Nº of genomes with TusE$_3$
Nº of genomes without TusE$_3$
p < 0.00005

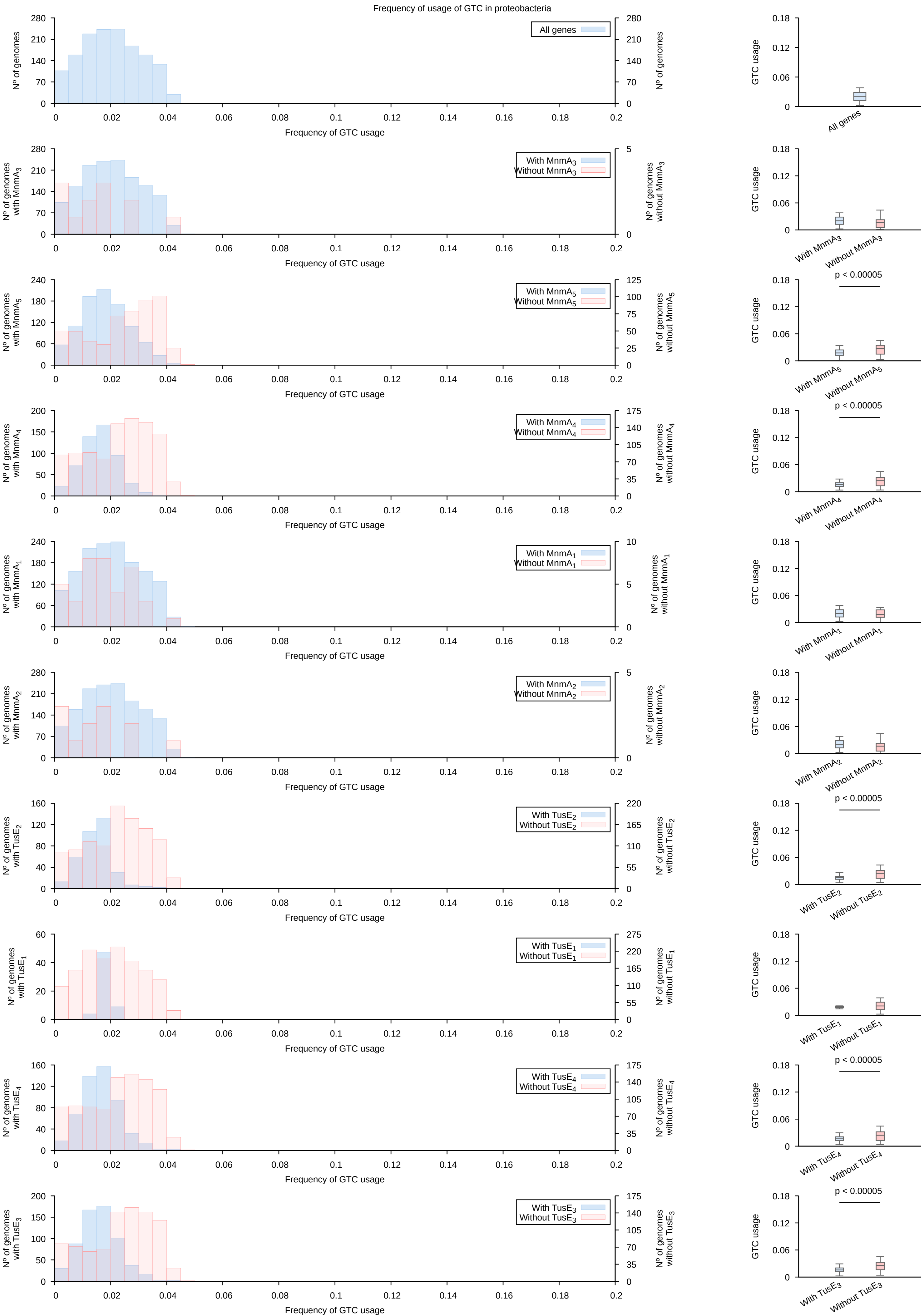

Frequency of usage of GTC in proteobacteria
All genes
With MnmA$_3$
Without MnmA$_3$
With MnmA$_5$
Without MnmA$_5$
With MnmA$_4$
Without MnmA$_4$
With MnmA$_1$
Without MnmA$_1$
With MnmA$_2$
Without MnmA$_2$
With TusE$_2$
Without TusE$_2$
With TusE$_1$
Without TusE$_1$
With TusE$_4$
Without TusE$_4$
With TusE$_3$
Without TusE$_3$
Nº of genomes
Nº of genomes with MnmA$_3$
Nº of genomes without MnmA$_3$
Nº of genomes with MnmA$_5$
Nº of genomes without MnmA$_5$
Nº of genomes with MnmA$_4$
Nº of genomes without MnmA$_4$
Nº of genomes with MnmA$_1$
Nº of genomes without MnmA$_1$
Nº of genomes with MnmA$_2$
Nº of genomes without MnmA$_2$
Nº of genomes with TusE$_2$
Nº of genomes without TusE$_2$
Nº of genomes with TusE$_1$
Nº of genomes without TusE$_1$
Nº of genomes with TusE$_4$
Nº of genomes without TusE$_4$
Nº of genomes with TusE$_3$
Nº of genomes without TusE$_3$
Frequency of GTC usage
GTC usage
p < 0.00005

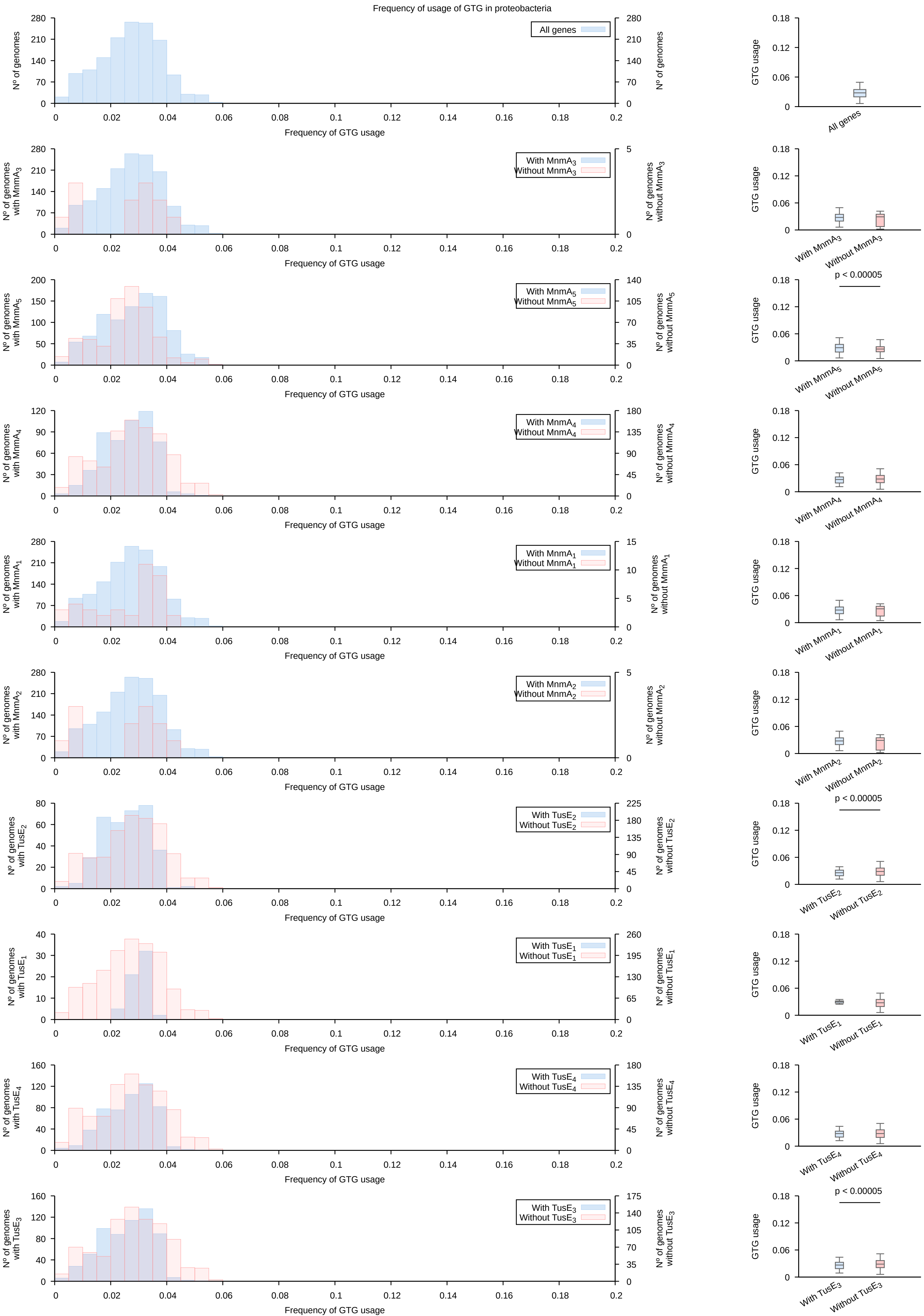
Frequency of usage of GTG in proteobacteria
Nº of genomes
All genes
Frequency of GTG usage
GTG usage
Nº of genomes with $MnmA_3$
Nº of genomes without $MnmA_3$
With $MnmA_3$
Without $MnmA_3$
Nº of genomes with $MnmA_5$
Nº of genomes without $MnmA_5$
With $MnmA_5$
Without $MnmA_5$
p < 0.00005
Nº of genomes with $MnmA_4$
Nº of genomes without $MnmA_4$
With $MnmA_4$
Without $MnmA_4$
Nº of genomes with $MnmA_1$
Nº of genomes without $MnmA_1$
With $MnmA_1$
Without $MnmA_1$
Nº of genomes with $MnmA_2$
Nº of genomes without $MnmA_2$
With $MnmA_2$
Without $MnmA_2$
Nº of genomes with $TusE_2$
Nº of genomes without $TusE_2$
With $TusE_2$
Without $TusE_2$
p < 0.00005
Nº of genomes with $TusE_1$
Nº of genomes without $TusE_1$
With $TusE_1$
Without $TusE_1$
Nº of genomes with $TusE_4$
Nº of genomes without $TusE_4$
With $TusE_4$
Without $TusE_4$
Nº of genomes with $TusE_3$
Nº of genomes without $TusE_3$
With $TusE_3$
Without $TusE_3$
p < 0.00005

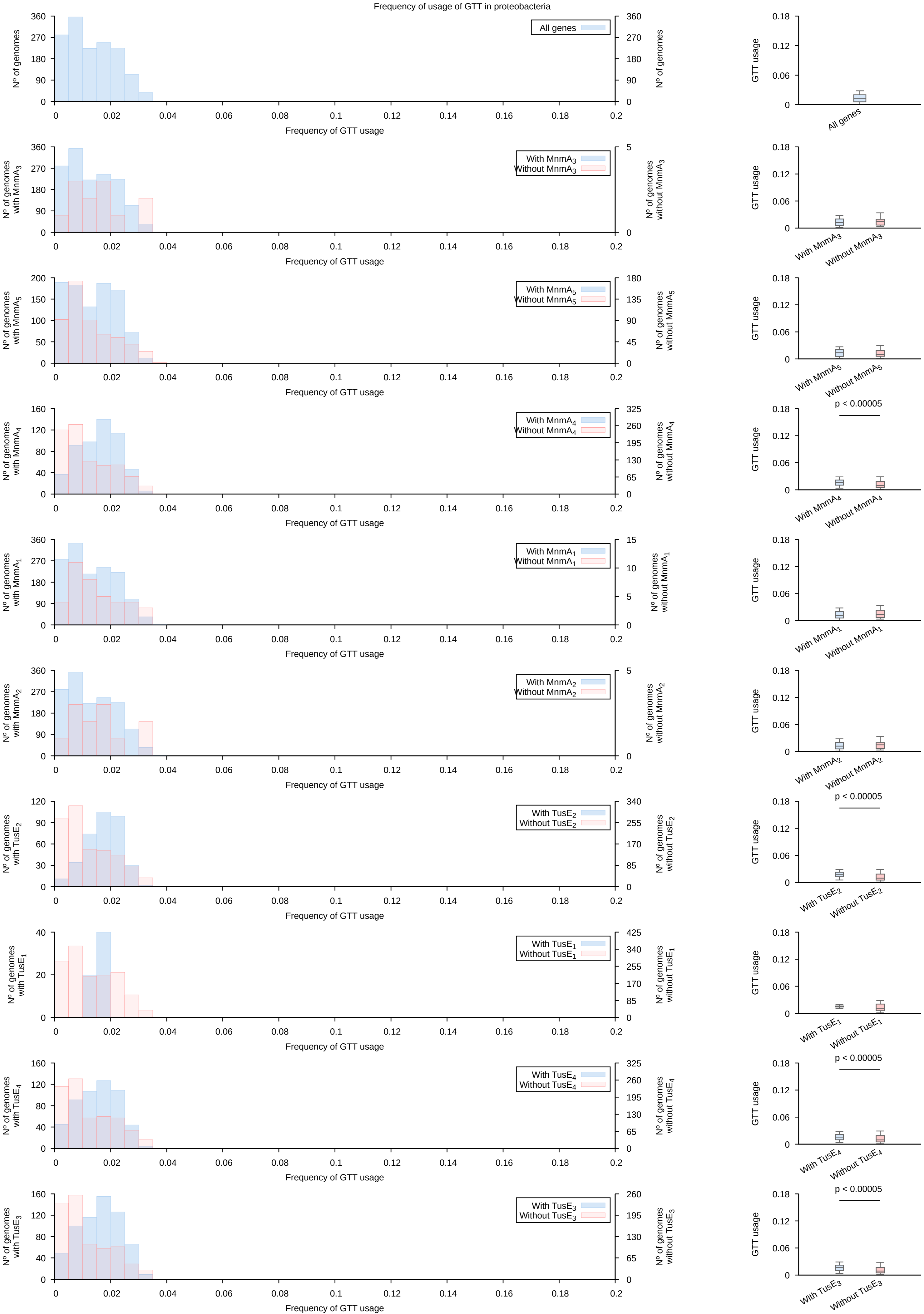
Frequency of usage of GTT in proteobacteria
All genes
Nº of genomes
GTT usage
Frequency of GTT usage
With $MnmA_3$
Without $MnmA_3$
Nº of genomes with $MnmA_3$
Nº of genomes without $MnmA_3$
With $MnmA_5$
Without $MnmA_5$
Nº of genomes with $MnmA_5$
Nº of genomes without $MnmA_5$
With $MnmA_4$
Without $MnmA_4$
Nº of genomes with $MnmA_4$
Nº of genomes without $MnmA_4$
p < 0.00005
With $MnmA_1$
Without $MnmA_1$
Nº of genomes with $MnmA_1$
Nº of genomes without $MnmA_1$
With $MnmA_2$
Without $MnmA_2$
Nº of genomes with $MnmA_2$
Nº of genomes without $MnmA_2$
With $TusE_2$
Without $TusE_2$
Nº of genomes with $TusE_2$
Nº of genomes without $TusE_2$
p < 0.00005
With $TusE_1$
Without $TusE_1$
Nº of genomes with $TusE_1$
Nº of genomes without $TusE_1$
With $TusE_4$
Without $TusE_4$
Nº of genomes with $TusE_4$
Nº of genomes without $TusE_4$
p < 0.00005
With $TusE_3$
Without $TusE_3$
Nº of genomes with $TusE_3$
Nº of genomes without $TusE_3$
p < 0.00005

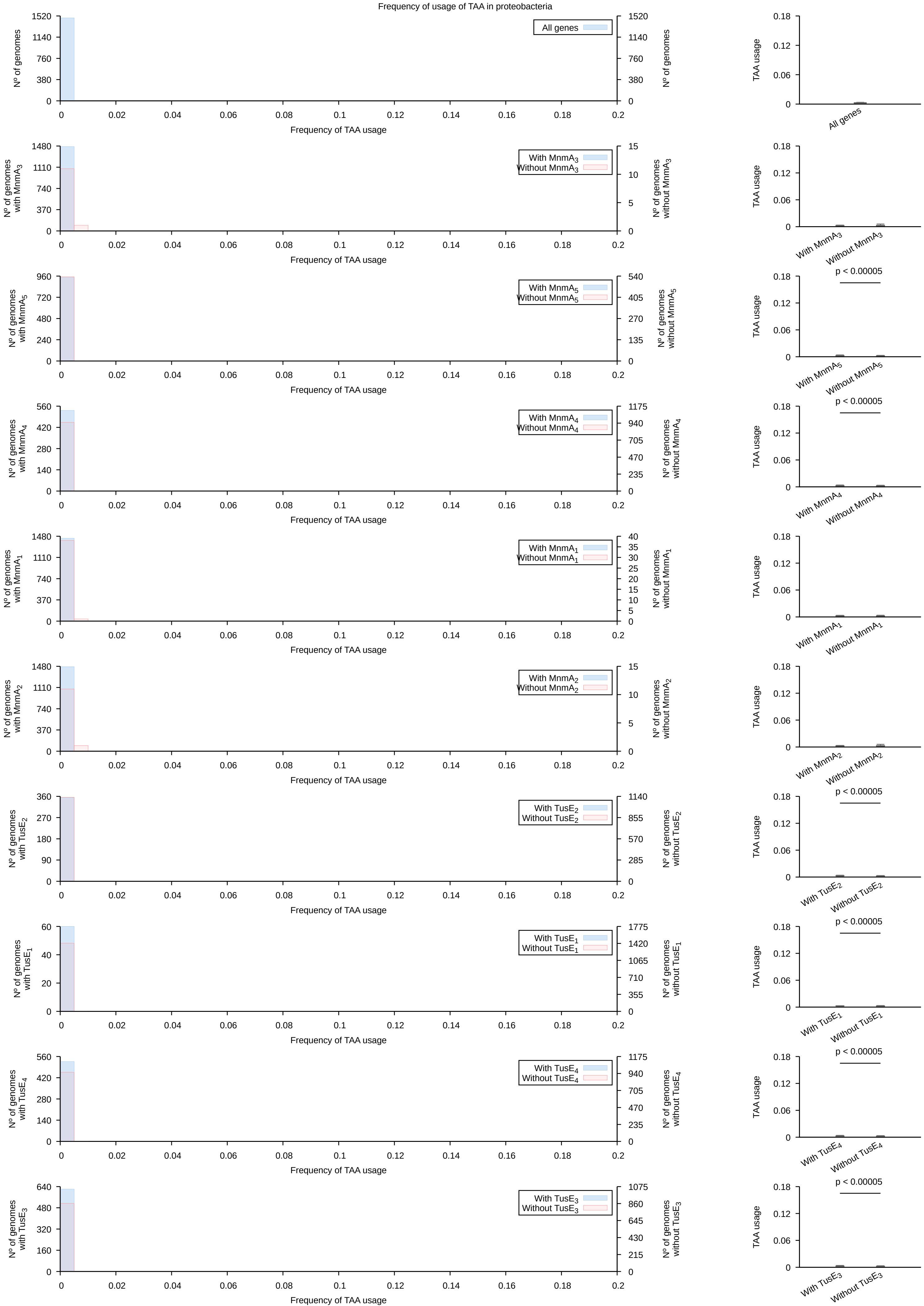
Frequency of usage of TAA in proteobacteria
All genes
Nº of genomes
Frequency of TAA usage
TAA usage
With MnmA3
Without MnmA3
Nº of genomes with MnmA3
Nº of genomes without MnmA3
With MnmA5
Without MnmA5
Nº of genomes with MnmA5
Nº of genomes without MnmA5
p < 0.00005
With MnmA4
Without MnmA4
Nº of genomes with MnmA4
Nº of genomes without MnmA4
p < 0.00005
With MnmA1
Without MnmA1
Nº of genomes with MnmA1
Nº of genomes without MnmA1
With MnmA2
Without MnmA2
Nº of genomes with MnmA2
Nº of genomes without MnmA2
With TusE2
Without TusE2
Nº of genomes with TusE2
Nº of genomes without TusE2
p < 0.00005
With TusE1
Without TusE1
Nº of genomes with TusE1
Nº of genomes without TusE1
p < 0.00005
With TusE4
Without TusE4
Nº of genomes with TusE4
Nº of genomes without TusE4
p < 0.00005
With TusE3
Without TusE3
Nº of genomes with TusE3
Nº of genomes without TusE3
p < 0.00005

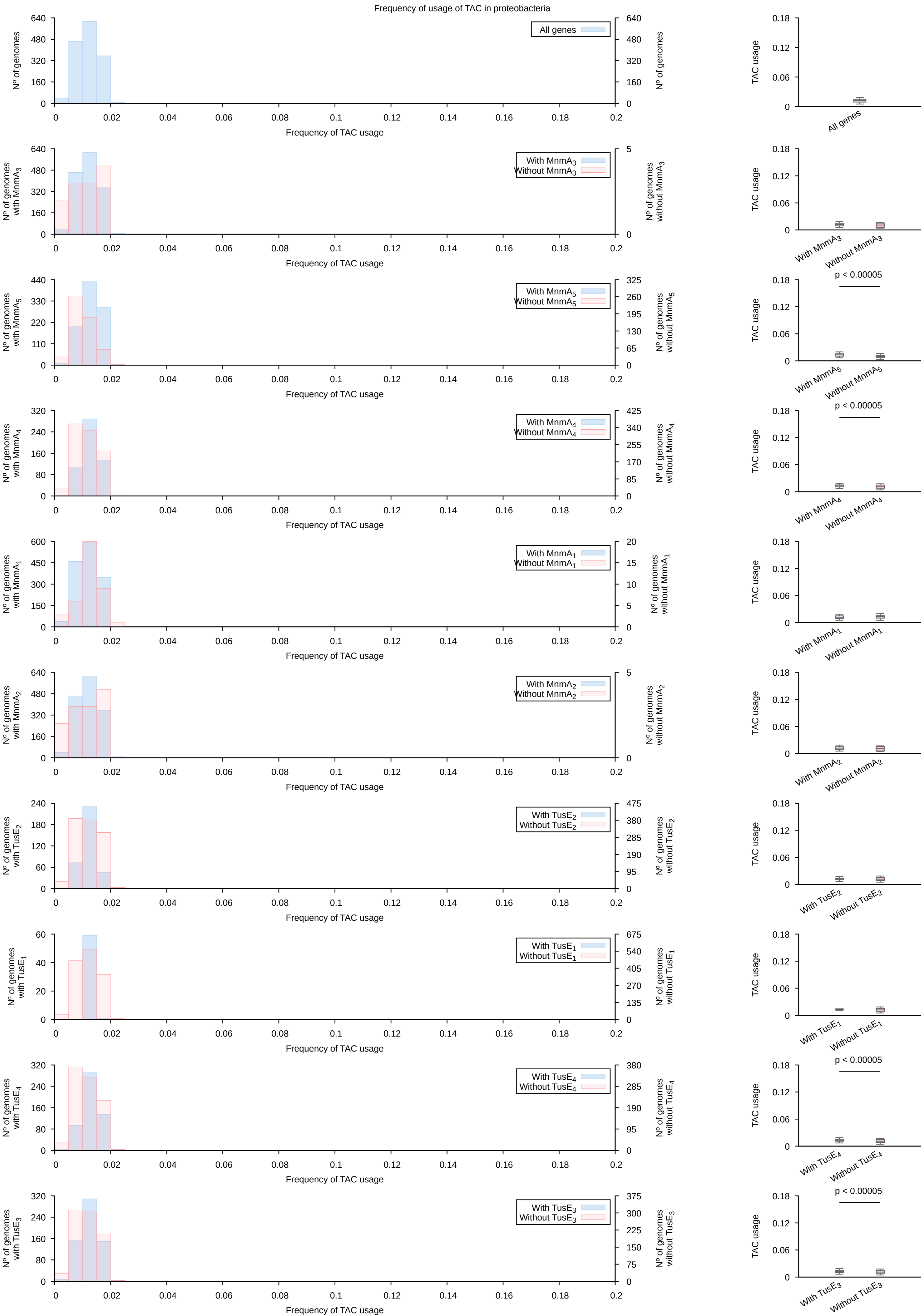

Frequency of usage of TAC in proteobacteria
All genes
Nº of genomes
Frequency of TAC usage
TAC usage
With MnmA3
Without MnmA3
Nº of genomes with MnmA3
Nº of genomes without MnmA3
With MnmA5
Without MnmA5
Nº of genomes with MnmA5
Nº of genomes without MnmA5
p < 0.00005
With MnmA4
Without MnmA4
Nº of genomes with MnmA4
Nº of genomes without MnmA4
p < 0.00005
With MnmA1
Without MnmA1
Nº of genomes with MnmA1
Nº of genomes without MnmA1
With MnmA2
Without MnmA2
Nº of genomes with MnmA2
Nº of genomes without MnmA2
With TusE2
Without TusE2
Nº of genomes with TusE2
Nº of genomes without TusE2
With TusE1
Without TusE1
Nº of genomes with TusE1
Nº of genomes without TusE1
With TusE4
Without TusE4
Nº of genomes with TusE4
Nº of genomes without TusE4
p < 0.00005
With TusE3
Without TusE3
Nº of genomes with TusE3
Nº of genomes without TusE3
p < 0.00005

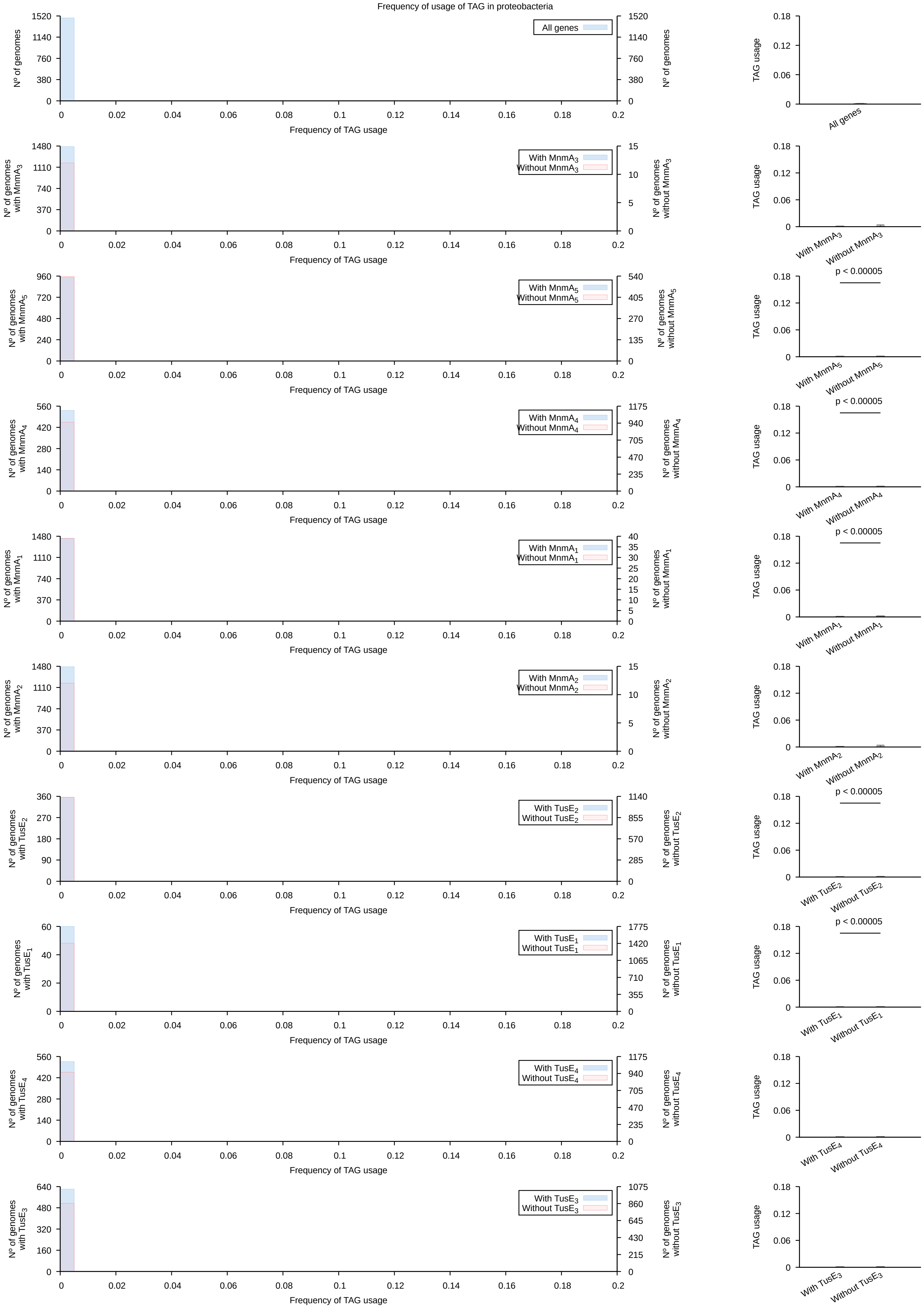

Frequency of usage of TAG in proteobacteria
All genes
Nº of genomes
Frequency of TAG usage
TAG usage
With MnmA$_3$
Without MnmA$_3$
Nº of genomes with MnmA$_3$
Nº of genomes without MnmA$_3$
With MnmA$_5$
Without MnmA$_5$
Nº of genomes with MnmA$_5$
Nº of genomes without MnmA$_5$
p < 0.00005
With MnmA$_4$
Without MnmA$_4$
Nº of genomes with MnmA$_4$
Nº of genomes without MnmA$_4$
p < 0.00005
With MnmA$_1$
Without MnmA$_1$
Nº of genomes with MnmA$_1$
Nº of genomes without MnmA$_1$
p < 0.00005
With MnmA$_2$
Without MnmA$_2$
Nº of genomes with MnmA$_2$
Nº of genomes without MnmA$_2$
With TusE$_2$
Without TusE$_2$
Nº of genomes with TusE$_2$
Nº of genomes without TusE$_2$
p < 0.00005
With TusE$_1$
Without TusE$_1$
Nº of genomes with TusE$_1$
Nº of genomes without TusE$_1$
p < 0.00005
With TusE$_4$
Without TusE$_4$
Nº of genomes with TusE$_4$
Nº of genomes without TusE$_4$
With TusE$_3$
Without TusE$_3$
Nº of genomes with TusE$_3$
Nº of genomes without TusE$_3$

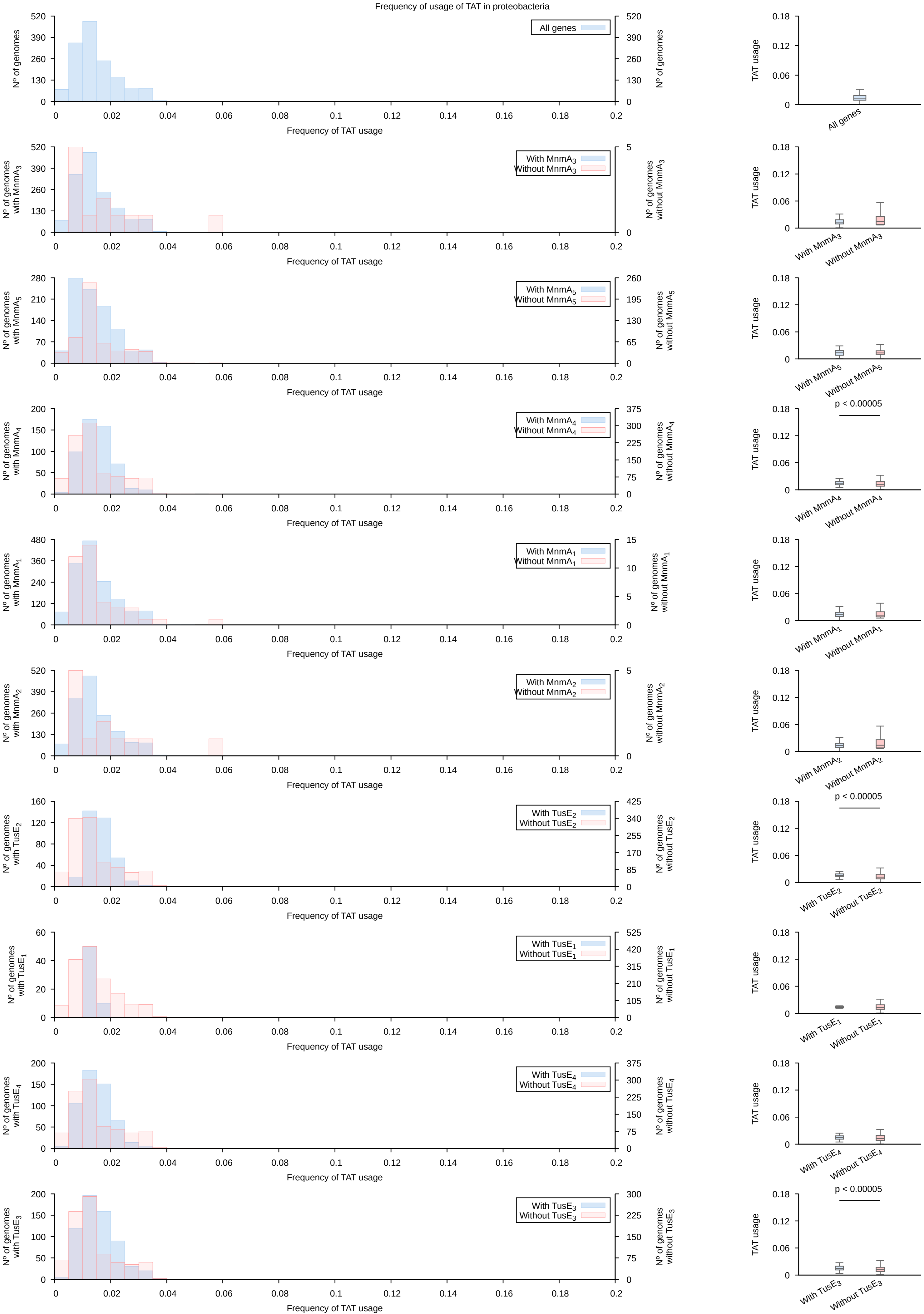
Frequency of usage of TAT in proteobacteria
Nº of genomes
Frequency of TAT usage
TAT usage
All genes
Nº of genomes with $MnmA_3$
Nº of genomes without $MnmA_3$
With $MnmA_3$
Without $MnmA_3$
Nº of genomes with $MnmA_5$
Nº of genomes without $MnmA_5$
With $MnmA_5$
Without $MnmA_5$
Nº of genomes with $MnmA_4$
Nº of genomes without $MnmA_4$
With $MnmA_4$
Without $MnmA_4$
$p < 0.00005$
Nº of genomes with $MnmA_1$
Nº of genomes without $MnmA_1$
With $MnmA_1$
Without $MnmA_1$
Nº of genomes with $MnmA_2$
Nº of genomes without $MnmA_2$
With $MnmA_2$
Without $MnmA_2$
Nº of genomes with $TusE_2$
Nº of genomes without $TusE_2$
With $TusE_2$
Without $TusE_2$
$p < 0.00005$
Nº of genomes with $TusE_1$
Nº of genomes without $TusE_1$
With $TusE_1$
Without $TusE_1$
Nº of genomes with $TusE_4$
Nº of genomes without $TusE_4$
With $TusE_4$
Without $TusE_4$
Nº of genomes with $TusE_3$
Nº of genomes without $TusE_3$
With $TusE_3$
Without $TusE_3$
$p < 0.00005$

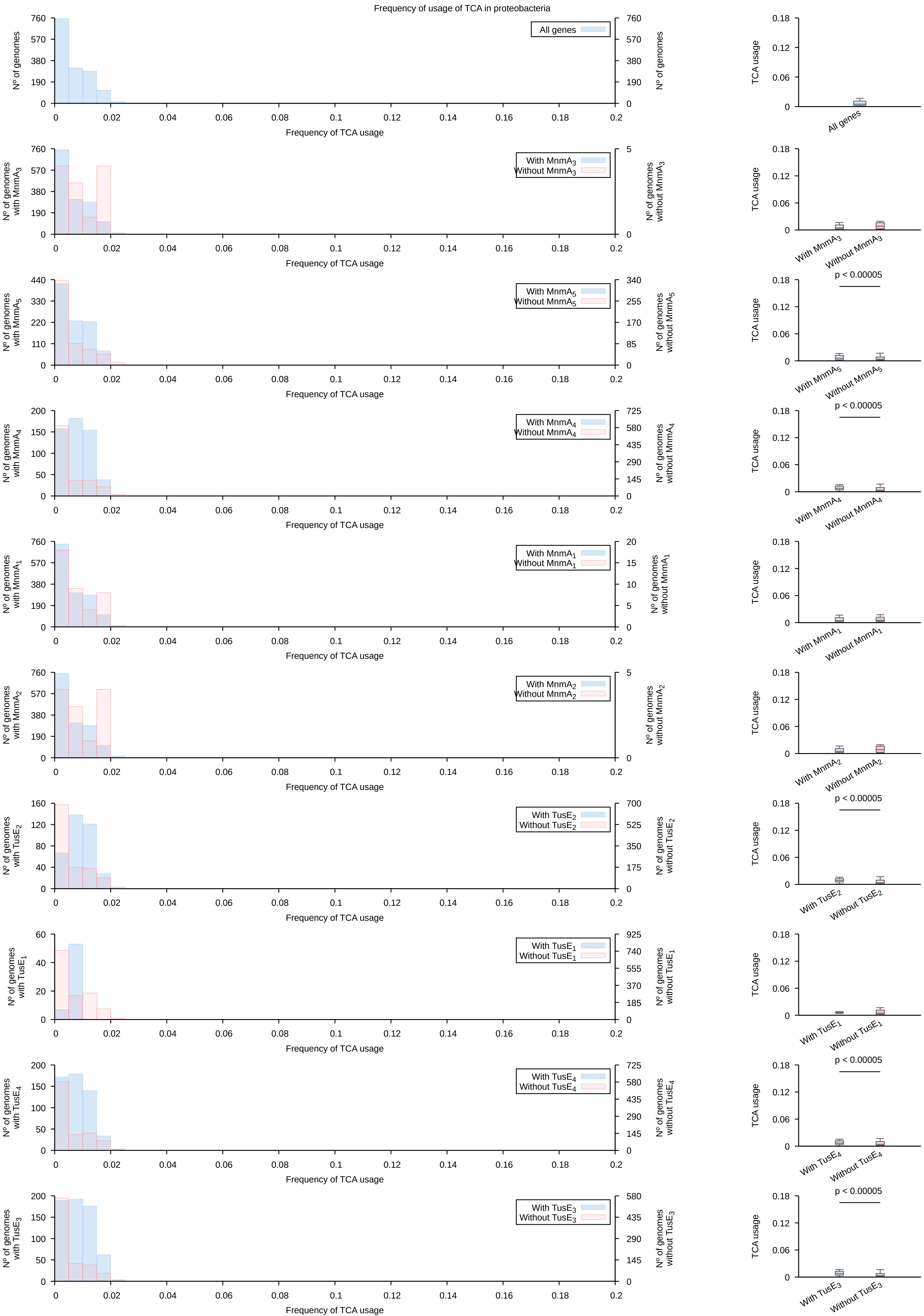
Frequency of usage of TCA in proteobacteria
All genes
Nº of genomes
Frequency of TCA usage
TCA usage
With MnmA$_3$
Without MnmA$_3$
Nº of genomes with MnmA$_3$
Nº of genomes without MnmA$_3$
With MnmA$_5$
Without MnmA$_5$
Nº of genomes with MnmA$_5$
Nº of genomes without MnmA$_5$
p < 0.00005
With MnmA$_4$
Without MnmA$_4$
Nº of genomes with MnmA$_4$
Nº of genomes without MnmA$_4$
p < 0.00005
With MnmA$_1$
Without MnmA$_1$
Nº of genomes with MnmA$_1$
Nº of genomes without MnmA$_1$
With MnmA$_2$
Without MnmA$_2$
Nº of genomes with MnmA$_2$
Nº of genomes without MnmA$_2$
With TusE$_2$
Without TusE$_2$
Nº of genomes with TusE$_2$
Nº of genomes without TusE$_2$
p < 0.00005
With TusE$_1$
Without TusE$_1$
Nº of genomes with TusE$_1$
Nº of genomes without TusE$_1$
With TusE$_4$
Without TusE$_4$
Nº of genomes with TusE$_4$
Nº of genomes without TusE$_4$
p < 0.00005
With TusE$_3$
Without TusE$_3$
Nº of genomes with TusE$_3$
Nº of genomes without TusE$_3$
p < 0.00005

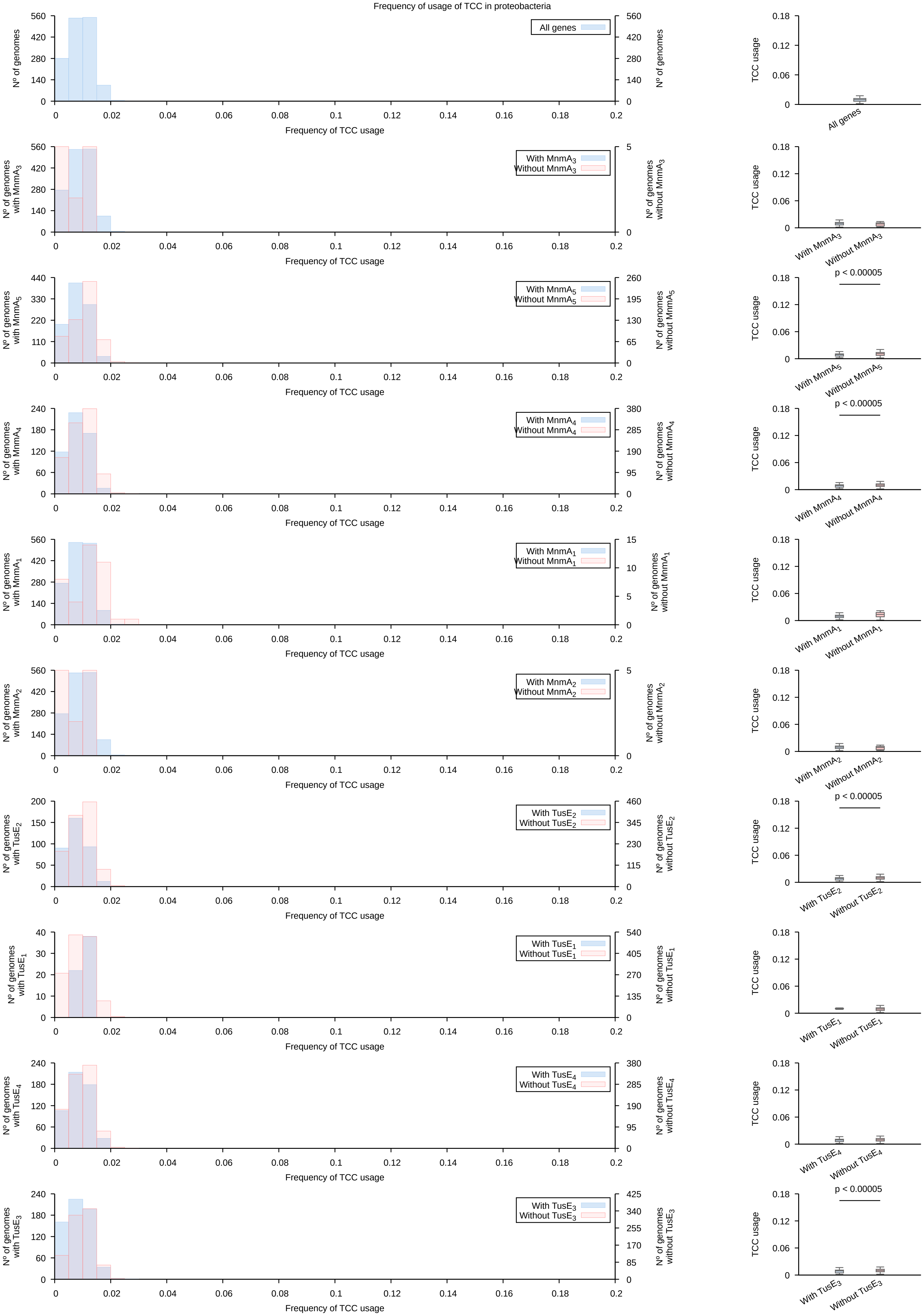
Frequency of usage of TCC in proteobacteria
All genes
Nº of genomes
Frequency of TCC usage
TCC usage
With MnmA$_3$
Without MnmA$_3$
Nº of genomes with MnmA$_3$
Nº of genomes without MnmA$_3$
With MnmA$_5$
Without MnmA$_5$
Nº of genomes with MnmA$_5$
Nº of genomes without MnmA$_5$
p < 0.00005
With MnmA$_4$
Without MnmA$_4$
Nº of genomes with MnmA$_4$
Nº of genomes without MnmA$_4$
p < 0.00005
With MnmA$_1$
Without MnmA$_1$
Nº of genomes with MnmA$_1$
Nº of genomes without MnmA$_1$
With MnmA$_2$
Without MnmA$_2$
Nº of genomes with MnmA$_2$
Nº of genomes without MnmA$_2$
With TusE$_2$
Without TusE$_2$
Nº of genomes with TusE$_2$
Nº of genomes without TusE$_2$
p < 0.00005
With TusE$_1$
Without TusE$_1$
Nº of genomes with TusE$_1$
Nº of genomes without TusE$_1$
With TusE$_4$
Without TusE$_4$
Nº of genomes with TusE$_4$
Nº of genomes without TusE$_4$
With TusE$_3$
Without TusE$_3$
Nº of genomes with TusE$_3$
Nº of genomes without TusE$_3$
p < 0.00005

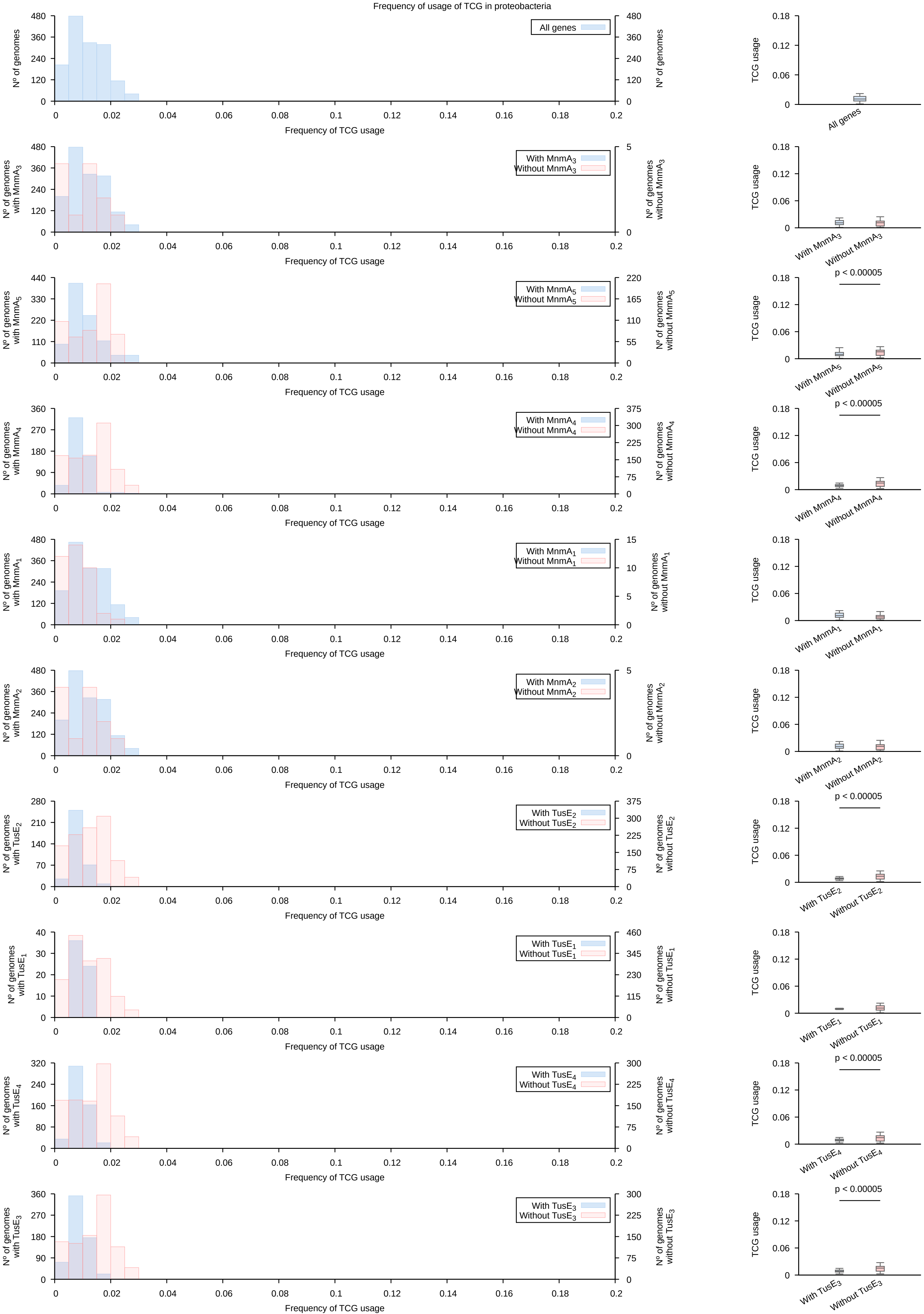
Frequency of usage of TCG in proteobacteria
All genes
Nº of genomes
Frequency of TCG usage
TCG usage
All genes
With $MnmA_3$
Without $MnmA_3$
Nº of genomes with $MnmA_3$
Nº of genomes without $MnmA_3$
With $MnmA_5$
Without $MnmA_5$
Nº of genomes with $MnmA_5$
Nº of genomes without $MnmA_5$
$p < 0.00005$
With $MnmA_4$
Without $MnmA_4$
Nº of genomes with $MnmA_4$
Nº of genomes without $MnmA_4$
$p < 0.00005$
With $MnmA_1$
Without $MnmA_1$
Nº of genomes with $MnmA_1$
Nº of genomes without $MnmA_1$
With $MnmA_2$
Without $MnmA_2$
Nº of genomes with $MnmA_2$
Nº of genomes without $MnmA_2$
With $TusE_2$
Without $TusE_2$
Nº of genomes with $TusE_2$
Nº of genomes without $TusE_2$
$p < 0.00005$
With $TusE_1$
Without $TusE_1$
Nº of genomes with $TusE_1$
Nº of genomes without $TusE_1$
With $TusE_4$
Without $TusE_4$
Nº of genomes with $TusE_4$
Nº of genomes without $TusE_4$
$p < 0.00005$
With $TusE_3$
Without $TusE_3$
Nº of genomes with $TusE_3$
Nº of genomes without $TusE_3$
$p < 0.00005$

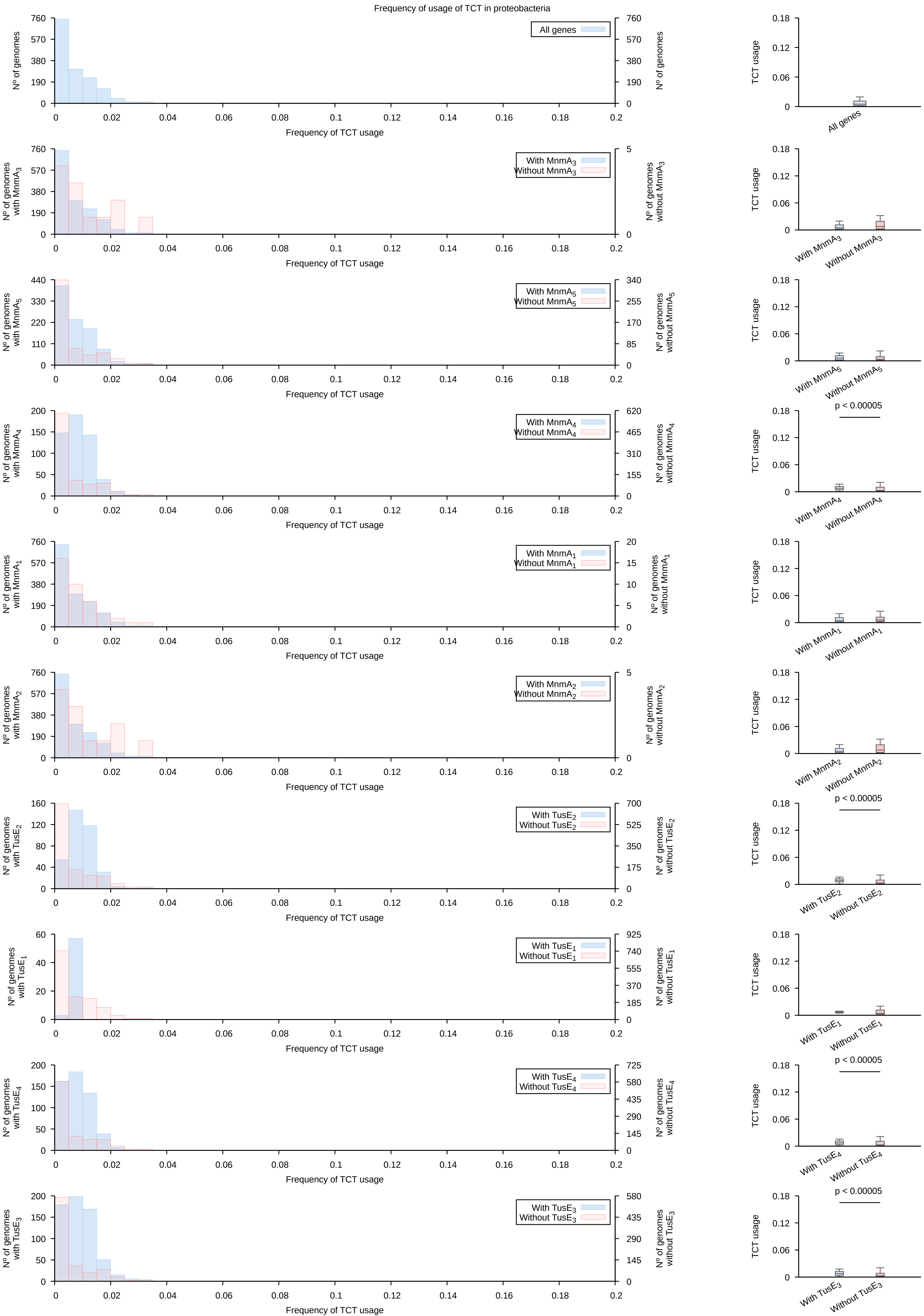
Frequency of usage of TCT in proteobacteria
Nº of genomes
Frequency of TCT usage
TCT usage
All genes
0
190
380
570
760
0.02
0.04
0.06
0.08
0.1
0.12
0.14
0.16
0.18
0.2
Nº of genomes with MnmA$_3$
Nº of genomes without MnmA$_3$
With MnmA$_3$
Without MnmA$_3$
Nº of genomes with MnmA$_5$
Nº of genomes without MnmA$_5$
With MnmA$_5$
Without MnmA$_5$
Nº of genomes with MnmA$_4$
Nº of genomes without MnmA$_4$
With MnmA$_4$
Without MnmA$_4$
Nº of genomes with MnmA$_1$
Nº of genomes without MnmA$_1$
With MnmA$_1$
Without MnmA$_1$
Nº of genomes with MnmA$_2$
Nº of genomes without MnmA$_2$
With MnmA$_2$
Without MnmA$_2$
Nº of genomes with TusE$_2$
Nº of genomes without TusE$_2$
With TusE$_2$
Without TusE$_2$
Nº of genomes with TusE$_1$
Nº of genomes without TusE$_1$
With TusE$_1$
Without TusE$_1$
Nº of genomes with TusE$_4$
Nº of genomes without TusE$_4$
With TusE$_4$
Without TusE$_4$
Nº of genomes with TusE$_3$
Nº of genomes without TusE$_3$
With TusE$_3$
Without TusE$_3$
p < 0.00005

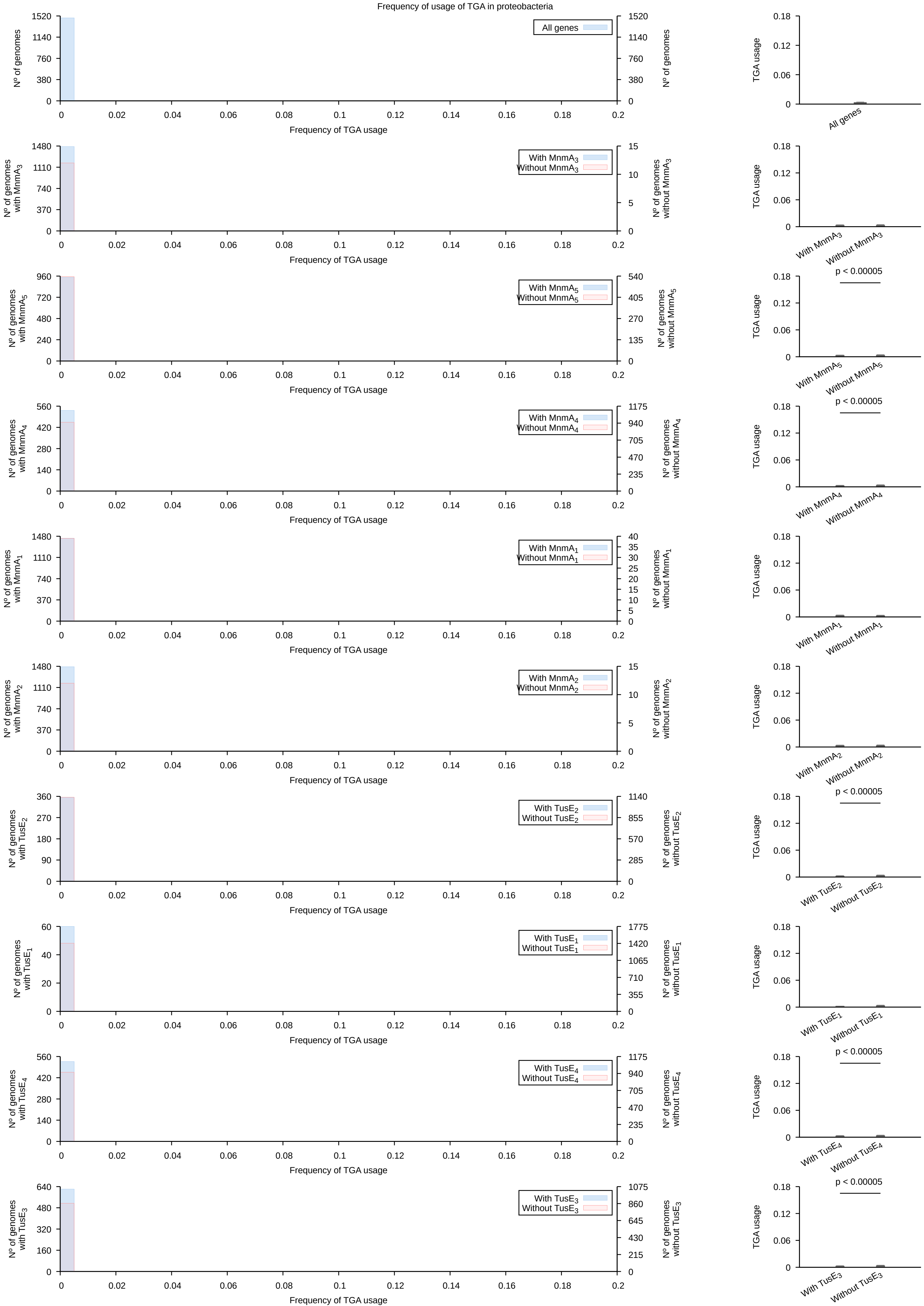
Frequency of usage of TGA in proteobacteria
All genes
With MnmA$_3$
Without MnmA$_3$
With MnmA$_5$
Without MnmA$_5$
With MnmA$_4$
Without MnmA$_4$
With MnmA$_1$
Without MnmA$_1$
With MnmA$_2$
Without MnmA$_2$
With TusE$_2$
Without TusE$_2$
With TusE$_1$
Without TusE$_1$
With TusE$_4$
Without TusE$_4$
With TusE$_3$
Without TusE$_3$
Frequency of TGA usage
Nº of genomes
TGA usage
p < 0.00005

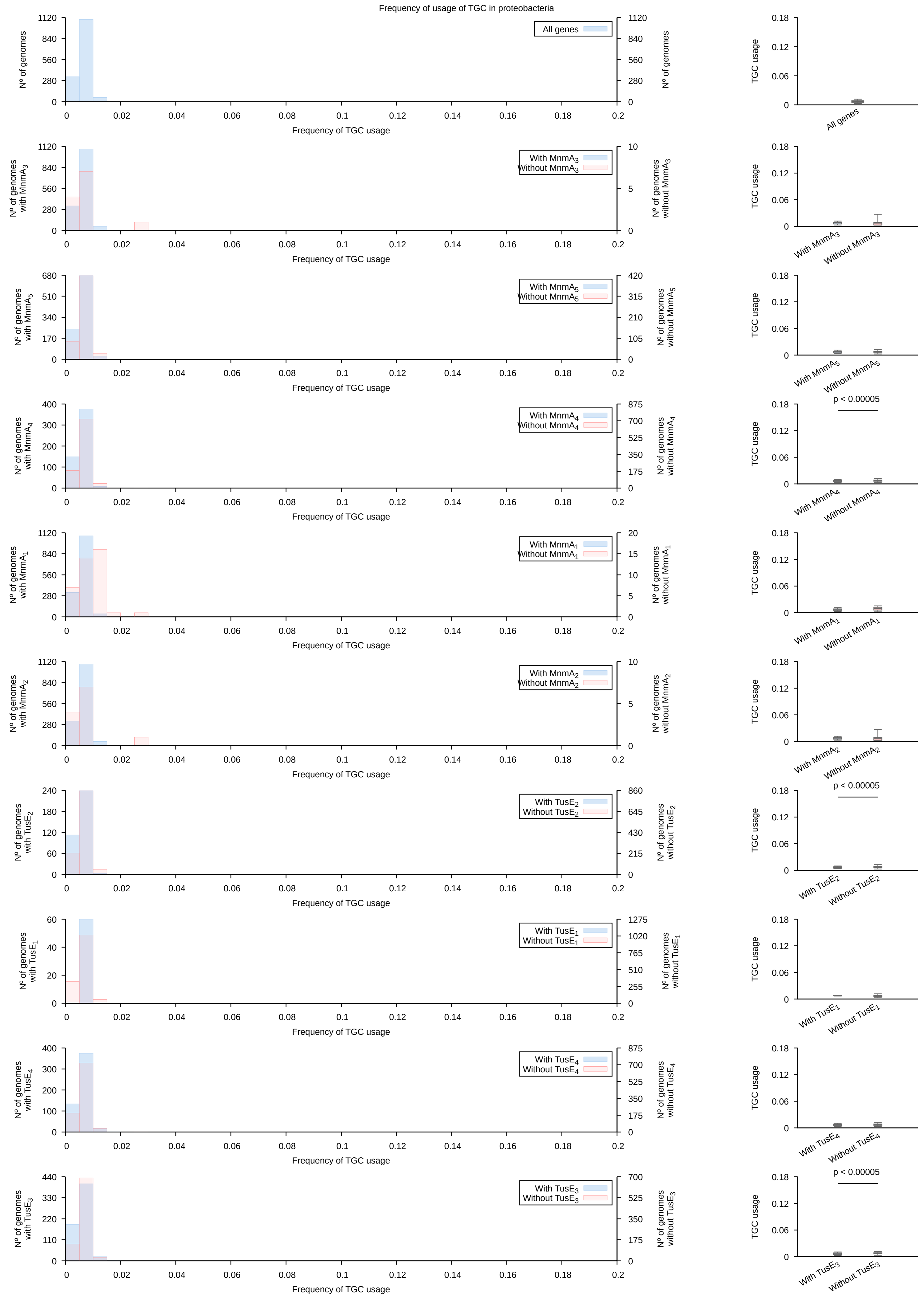
Frequency of usage of TGC in proteobacteria
All genes
Nº of genomes
Frequency of TGC usage
TGC usage
With MnmA3
Without MnmA3
Nº of genomes with MnmA3
Nº of genomes without MnmA3
With MnmA5
Without MnmA5
Nº of genomes with MnmA5
Nº of genomes without MnmA5
With MnmA4
Without MnmA4
Nº of genomes with MnmA4
Nº of genomes without MnmA4
p < 0.00005
With MnmA1
Without MnmA1
Nº of genomes with MnmA1
Nº of genomes without MnmA1
With MnmA2
Without MnmA2
Nº of genomes with MnmA2
Nº of genomes without MnmA2
With TusE2
Without TusE2
Nº of genomes with TusE2
Nº of genomes without TusE2
p < 0.00005
With TusE1
Without TusE1
Nº of genomes with TusE1
Nº of genomes without TusE1
With TusE4
Without TusE4
Nº of genomes with TusE4
Nº of genomes without TusE4
With TusE3
Without TusE3
Nº of genomes with TusE3
Nº of genomes without TusE3
p < 0.00005

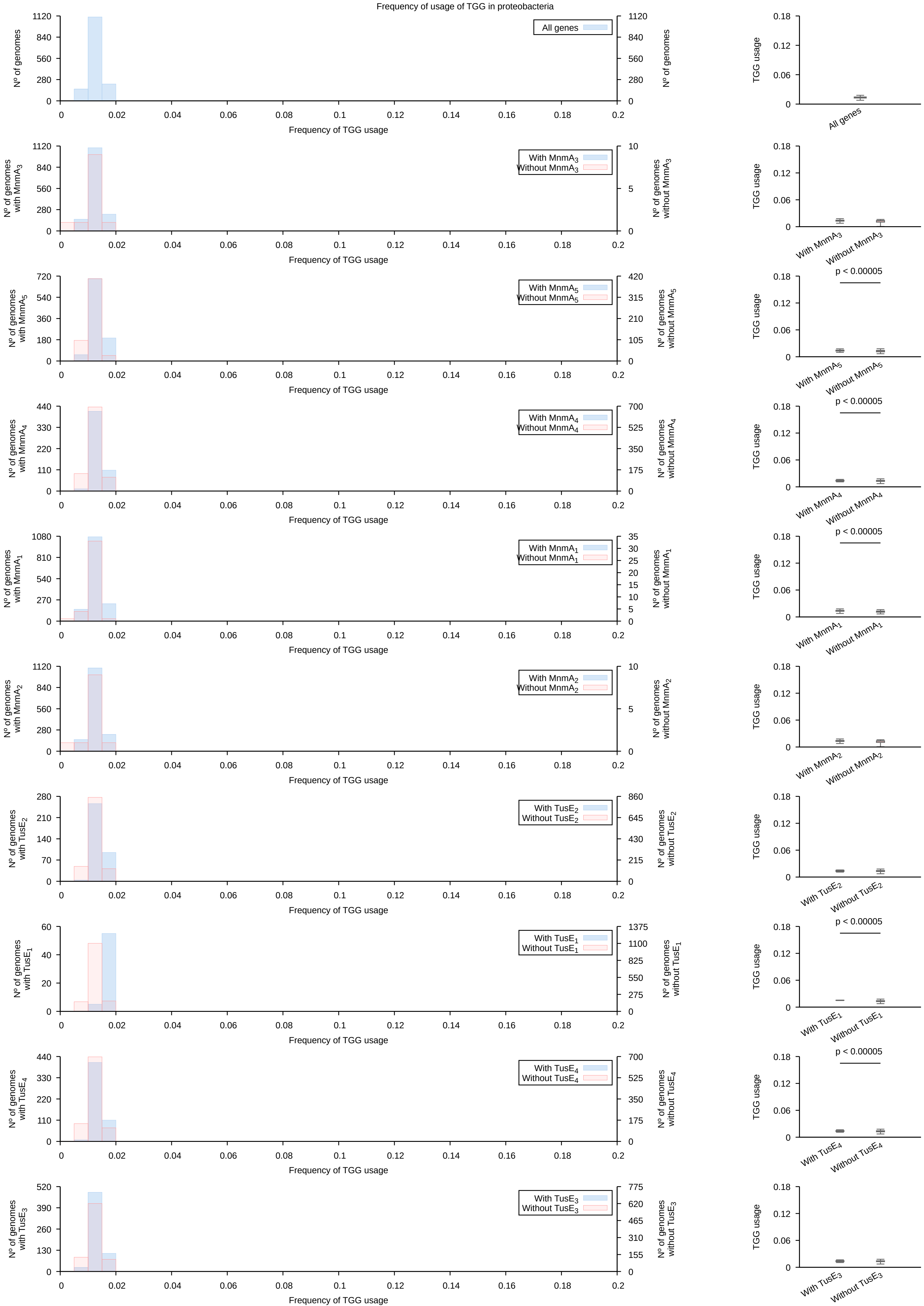
Frequency of usage of TGG in proteobacteria
All genes
Nº of genomes
Frequency of TGG usage
TGG usage
With $MnmA_3$
Without $MnmA_3$
Nº of genomes with $MnmA_3$
Nº of genomes without $MnmA_3$
With $MnmA_5$
Without $MnmA_5$
Nº of genomes with $MnmA_5$
Nº of genomes without $MnmA_5$
p < 0.00005
With $MnmA_4$
Without $MnmA_4$
Nº of genomes with $MnmA_4$
Nº of genomes without $MnmA_4$
p < 0.00005
With $MnmA_1$
Without $MnmA_1$
Nº of genomes with $MnmA_1$
Nº of genomes without $MnmA_1$
p < 0.00005
With $MnmA_2$
Without $MnmA_2$
Nº of genomes with $MnmA_2$
Nº of genomes without $MnmA_2$
With $TusE_2$
Without $TusE_2$
Nº of genomes with $TusE_2$
Nº of genomes without $TusE_2$
With $TusE_1$
Without $TusE_1$
Nº of genomes with $TusE_1$
Nº of genomes without $TusE_1$
p < 0.00005
With $TusE_4$
Without $TusE_4$
Nº of genomes with $TusE_4$
Nº of genomes without $TusE_4$
p < 0.00005
With $TusE_3$
Without $TusE_3$
Nº of genomes with $TusE_3$
Nº of genomes without $TusE_3$

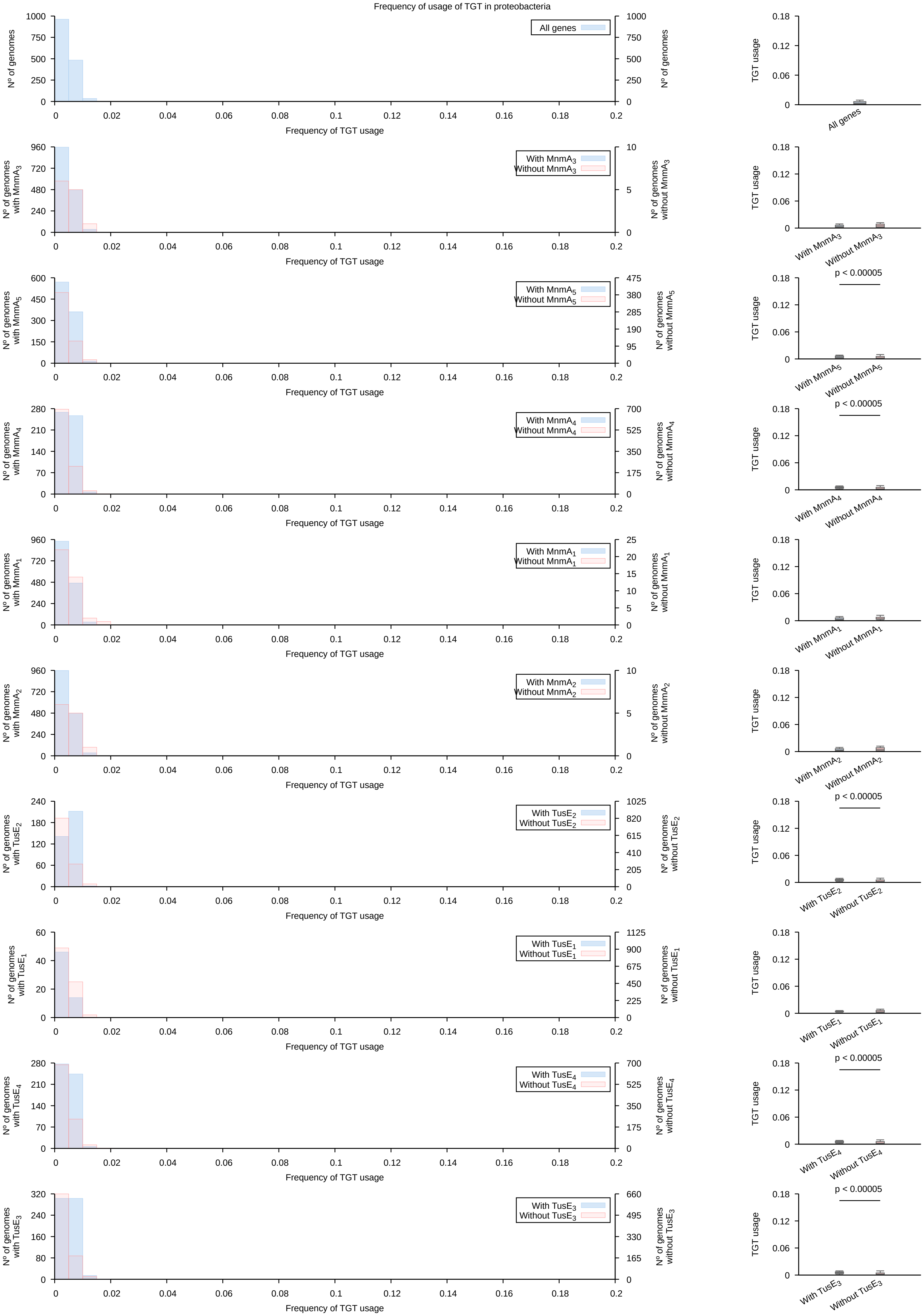

Frequency of usage of TGT in proteobacteria
All genes
With $MnmA_3$
Without $MnmA_3$
With $MnmA_5$
Without $MnmA_5$
With $MnmA_4$
Without $MnmA_4$
With $MnmA_1$
Without $MnmA_1$
With $MnmA_2$
Without $MnmA_2$
With $TusE_2$
Without $TusE_2$
With $TusE_1$
Without $TusE_1$
With $TusE_4$
Without $TusE_4$
With $TusE_3$
Without $TusE_3$
Nº of genomes
Frequency of TGT usage
TGT usage
p < 0.00005

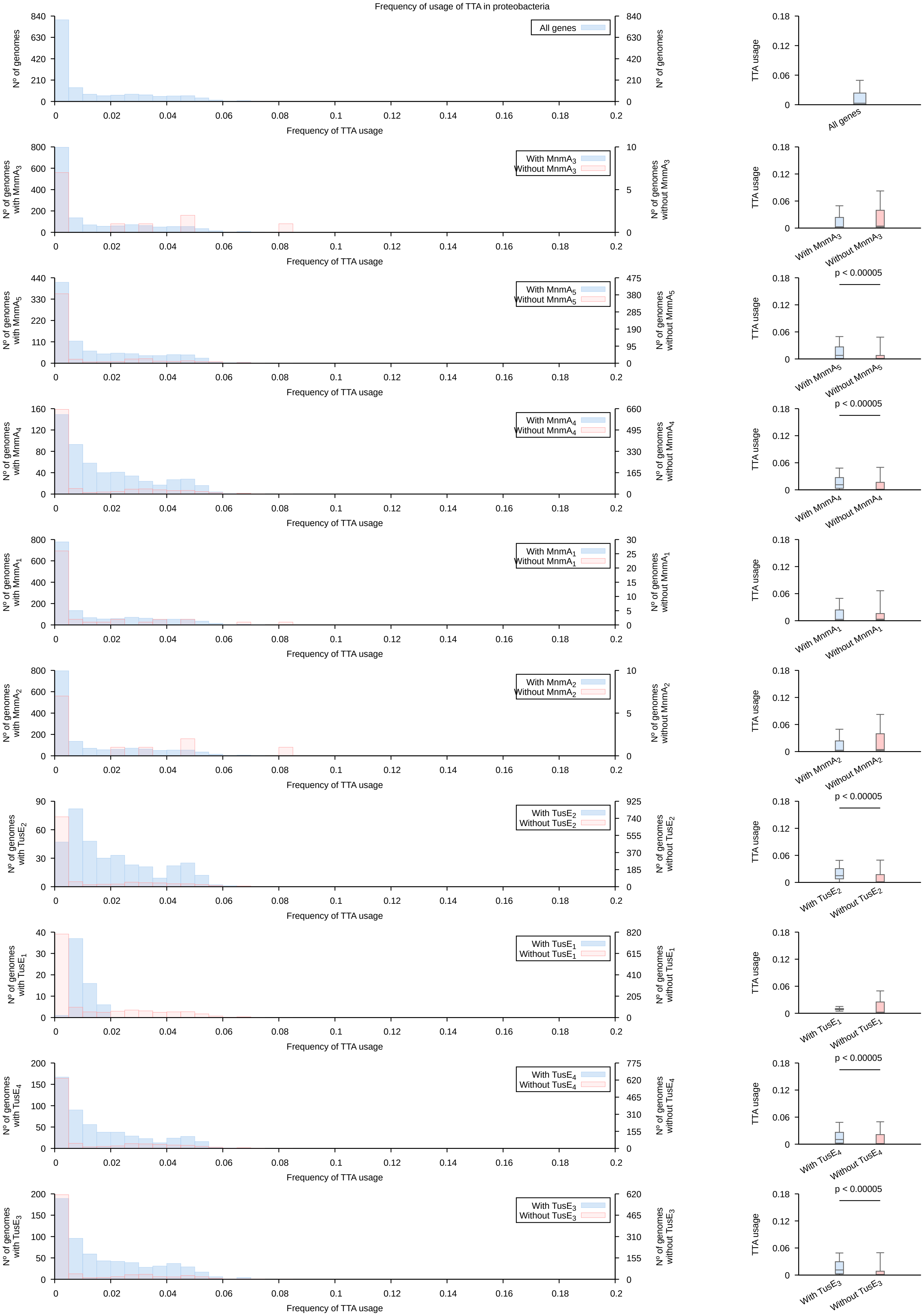

Frequency of usage of TTA in proteobacteria
Nº of genomes
Frequency of TTA usage
All genes
TTA usage
Nº of genomes with $MnmA_3$
Nº of genomes without $MnmA_3$
With $MnmA_3$
Without $MnmA_3$
Nº of genomes with $MnmA_5$
Nº of genomes without $MnmA_5$
With $MnmA_5$
Without $MnmA_5$
p < 0.00005
Nº of genomes with $MnmA_4$
Nº of genomes without $MnmA_4$
With $MnmA_4$
Without $MnmA_4$
p < 0.00005
Nº of genomes with $MnmA_1$
Nº of genomes without $MnmA_1$
With $MnmA_1$
Without $MnmA_1$
Nº of genomes with $MnmA_2$
Nº of genomes without $MnmA_2$
With $MnmA_2$
Without $MnmA_2$
Nº of genomes with $TusE_2$
Nº of genomes without $TusE_2$
With $TusE_2$
Without $TusE_2$
p < 0.00005
Nº of genomes with $TusE_1$
Nº of genomes without $TusE_1$
With $TusE_1$
Without $TusE_1$
Nº of genomes with $TusE_4$
Nº of genomes without $TusE_4$
With $TusE_4$
Without $TusE_4$
p < 0.00005
Nº of genomes with $TusE_3$
Nº of genomes without $TusE_3$
With $TusE_3$
Without $TusE_3$
p < 0.00005

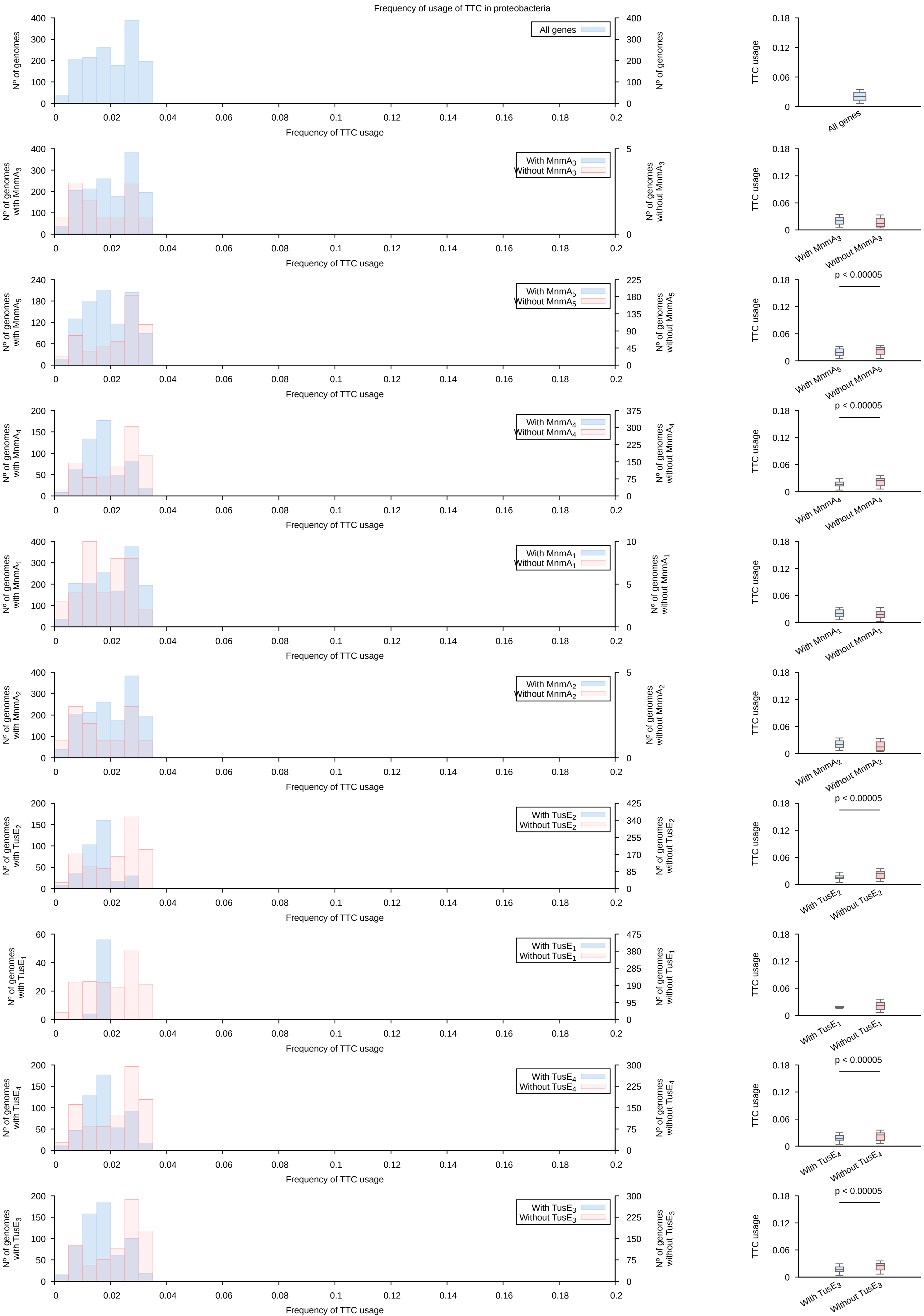

Frequency of usage of TTC in proteobacteria
All genes
Nº of genomes
Frequency of TTC usage
TTC usage
With MnmA$_3$
Without MnmA$_3$
Nº of genomes with MnmA$_3$
Nº of genomes without MnmA$_3$
With MnmA$_5$
Without MnmA$_5$
Nº of genomes with MnmA$_5$
Nº of genomes without MnmA$_5$
p < 0.00005
With MnmA$_4$
Without MnmA$_4$
Nº of genomes with MnmA$_4$
Nº of genomes without MnmA$_4$
p < 0.00005
With MnmA$_1$
Without MnmA$_1$
Nº of genomes with MnmA$_1$
Nº of genomes without MnmA$_1$
With MnmA$_2$
Without MnmA$_2$
Nº of genomes with MnmA$_2$
Nº of genomes without MnmA$_2$
With TusE$_2$
Without TusE$_2$
Nº of genomes with TusE$_2$
Nº of genomes without TusE$_2$
p < 0.00005
With TusE$_1$
Without TusE$_1$
Nº of genomes with TusE$_1$
Nº of genomes without TusE$_1$
With TusE$_4$
Without TusE$_4$
Nº of genomes with TusE$_4$
Nº of genomes without TusE$_4$
p < 0.00005
With TusE$_3$
Without TusE$_3$
Nº of genomes with TusE$_3$
Nº of genomes without TusE$_3$
p < 0.00005

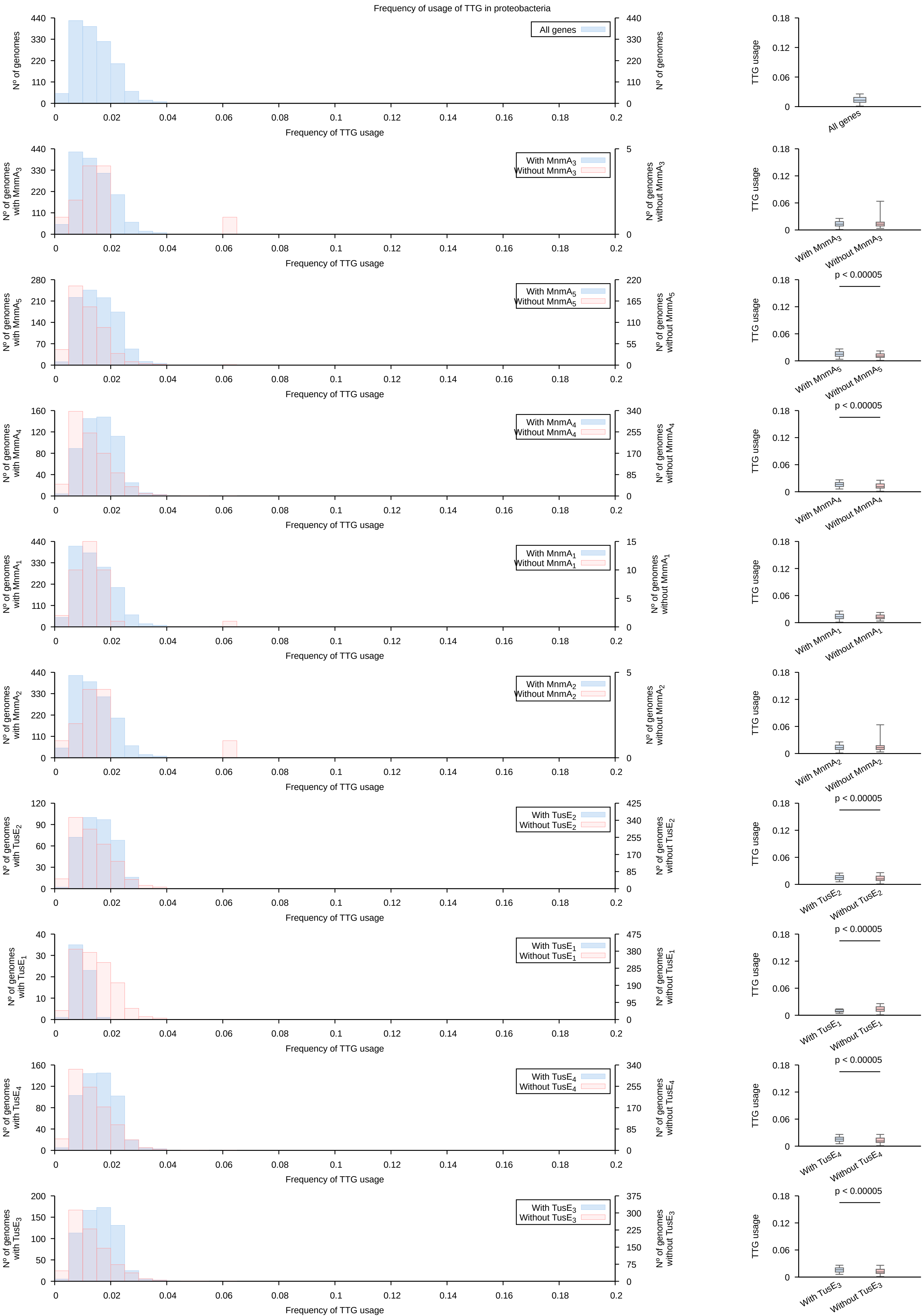

Frequency of usage of TTG in proteobacteria
All genes
Nº of genomes
Frequency of TTG usage
TTG usage
With MnmA$_3$
Without MnmA$_3$
Nº of genomes with MnmA$_3$
Nº of genomes without MnmA$_3$
With MnmA$_5$
Without MnmA$_5$
Nº of genomes with MnmA$_5$
Nº of genomes without MnmA$_5$
p < 0.00005
With MnmA$_4$
Without MnmA$_4$
Nº of genomes with MnmA$_4$
Nº of genomes without MnmA$_4$
p < 0.00005
With MnmA$_1$
Without MnmA$_1$
Nº of genomes with MnmA$_1$
Nº of genomes without MnmA$_1$
With MnmA$_2$
Without MnmA$_2$
Nº of genomes with MnmA$_2$
Nº of genomes without MnmA$_2$
With TusE$_2$
Without TusE$_2$
Nº of genomes with TusE$_2$
Nº of genomes without TusE$_2$
p < 0.00005
With TusE$_1$
Without TusE$_1$
Nº of genomes with TusE$_1$
Nº of genomes without TusE$_1$
p < 0.00005
With TusE$_4$
Without TusE$_4$
Nº of genomes with TusE$_4$
Nº of genomes without TusE$_4$
p < 0.00005
With TusE$_3$
Without TusE$_3$
Nº of genomes with TusE$_3$
Nº of genomes without TusE$_3$
p < 0.00005

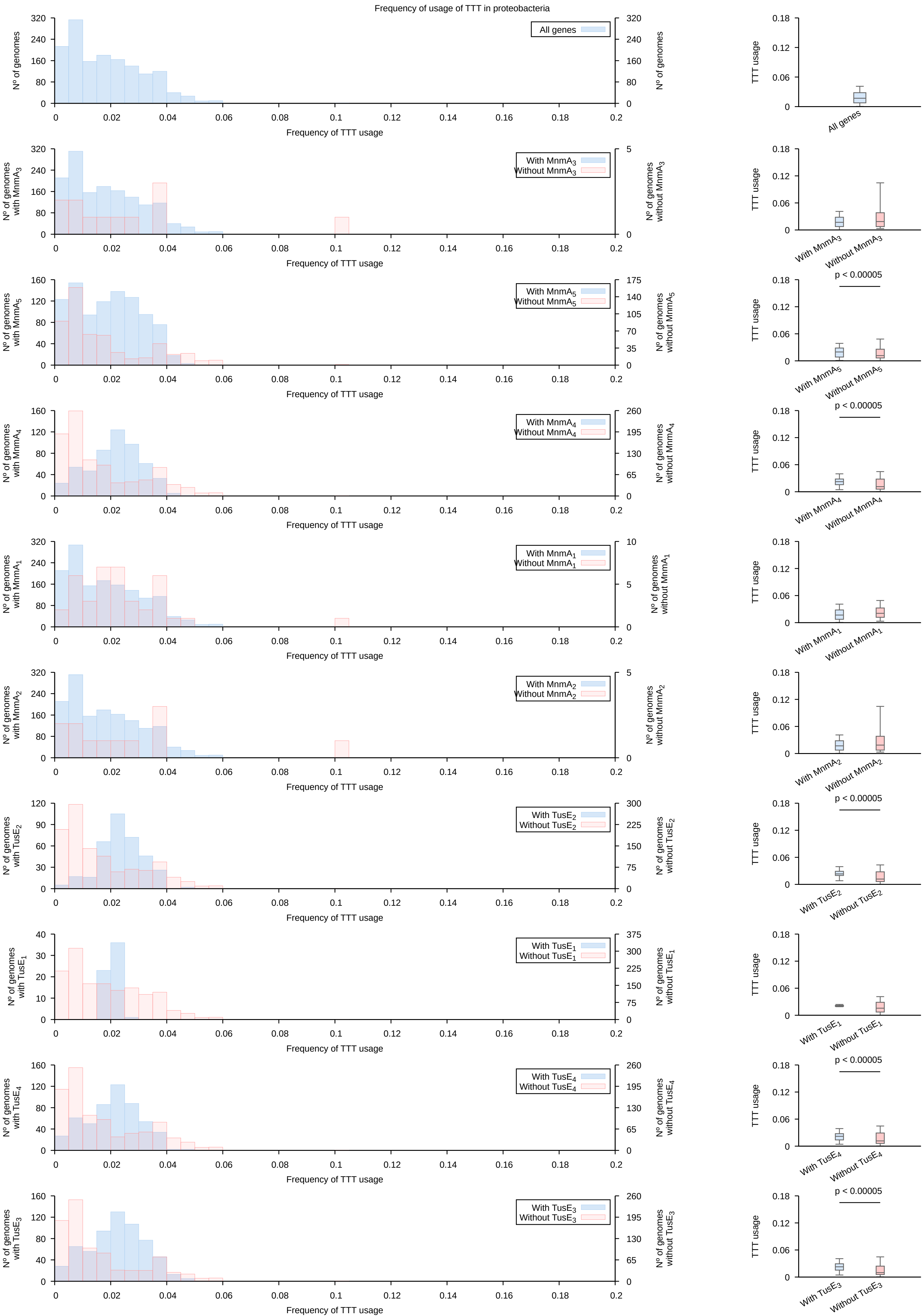
Frequency of usage of TTT in proteobacteria
Nº of genomes
All genes
Frequency of TTT usage
TTT usage
Nº of genomes with MnmA3
Nº of genomes without MnmA3
With MnmA3
Without MnmA3
Nº of genomes with MnmA5
Nº of genomes without MnmA5
With MnmA5
Without MnmA5
p < 0.00005
Nº of genomes with MnmA4
Nº of genomes without MnmA4
With MnmA4
Without MnmA4
Nº of genomes with MnmA1
Nº of genomes without MnmA1
With MnmA1
Without MnmA1
Nº of genomes with MnmA2
Nº of genomes without MnmA2
With MnmA2
Without MnmA2
Nº of genomes with TusE2
Nº of genomes without TusE2
With TusE2
Without TusE2
Nº of genomes with TusE1
Nº of genomes without TusE1
With TusE1
Without TusE1
Nº of genomes with TusE4
Nº of genomes without TusE4
With TusE4
Without TusE4
Nº of genomes with TusE3
Nº of genomes without TusE3
With TusE3
Without TusE3

Figure S15. Histogram of GC content in proteobacterias genomes that present or lack genes involved in t^6A, GluQ, k^2C and s^2U modifications. The 1484 proteobacterial genomes were separated in groups depending on the presence or absence of the indicated genes and the GC content of genome sequences was analyzed. **Left:** Data is represented in superposed histograms. **Right:** Data is represented in a boxplot. An horizontal bar was added when the two populations are different based on Wilcoxon-Mann-Whitney test and $p < 0.00005$. Whiskers include 95% of data. Patterns are indicated using subscript numbers.

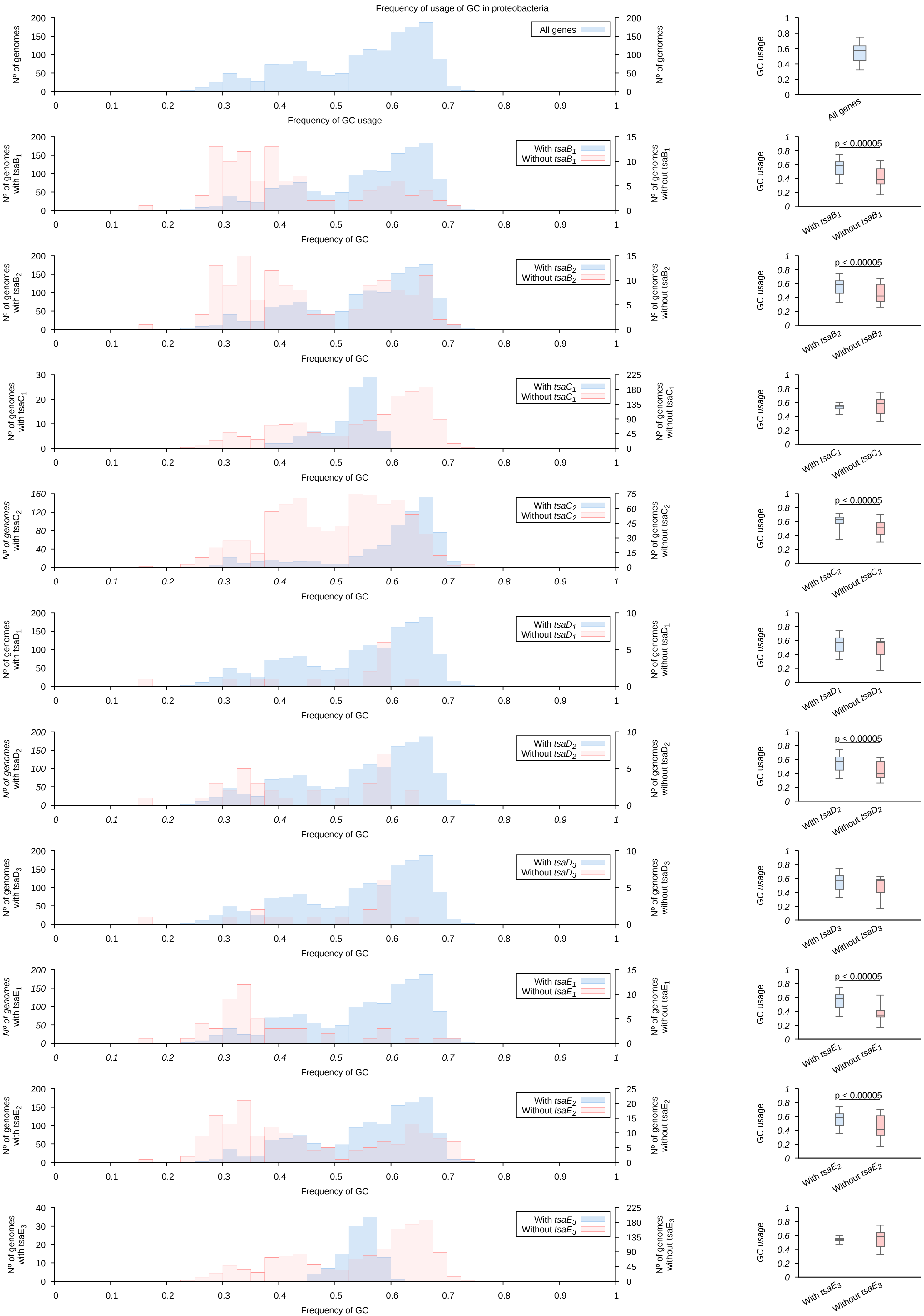
Frequency of usage of GC in proteobacteria
Nº of genomes
All genes
Frequency of GC usage
GC usage
Nº of genomes with tsaB1
Nº of genomes without tsaB1
With tsaB1
Without tsaB1
Frequency of GC
p < 0.00005
Nº of genomes with tsaB2
Nº of genomes without tsaB2
With tsaB2
Without tsaB2
Nº of genomes with tsaC1
Nº of genomes without tsaC1
With tsaC1
Without tsaC1
Nº of genomes with tsaC2
Nº of genomes without tsaC2
With tsaC2
Without tsaC2
Nº of genomes with tsaD1
Nº of genomes without tsaD1
With tsaD1
Without tsaD1
Nº of genomes with tsaD2
Nº of genomes without tsaD2
With tsaD2
Without tsaD2
Nº of genomes with tsaD3
Nº of genomes without tsaD3
With tsaD3
Without tsaD3
Nº of genomes with tsaE1
Nº of genomes without tsaE1
With tsaE1
Without tsaE1
Nº of genomes with tsaE2
Nº of genomes without tsaE2
With tsaE2
Without tsaE2
Nº of genomes with tsaE3
Nº of genomes without tsaE3
With tsaE3
Without tsaE3

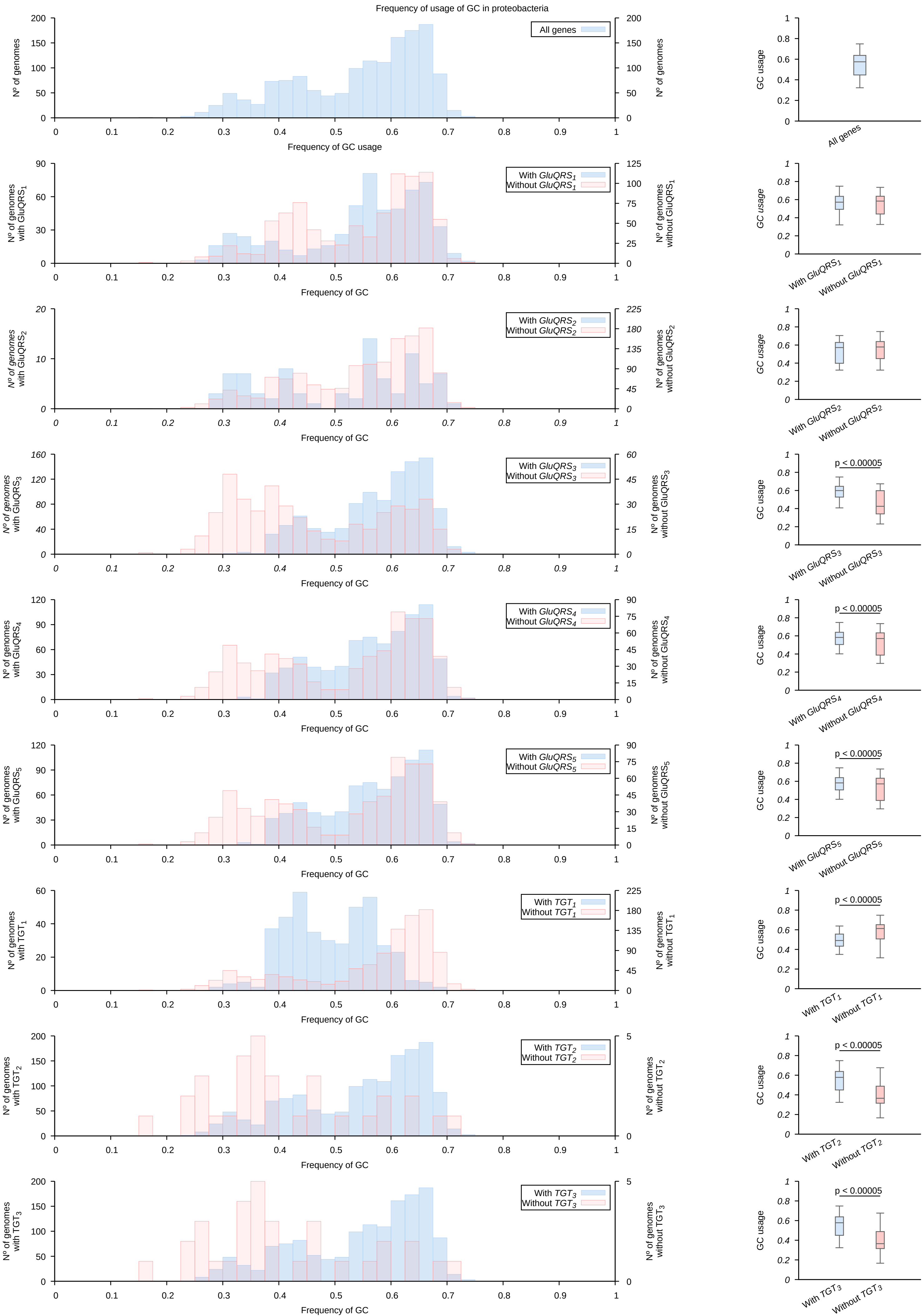
Frequency of usage of GC in proteobacteria
All genes
Nº of genomes
Frequency of GC usage
GC usage
With GluQRS1
Without GluQRS1
Nº of genomes with GluQRS1
Nº of genomes without GluQRS1
Frequency of GC
With GluQRS2
Without GluQRS2
Nº of genomes with GluQRS2
Nº of genomes without GluQRS2
With GluQRS3
Without GluQRS3
Nº of genomes with GluQRS3
Nº of genomes without GluQRS3
With GluQRS4
Without GluQRS4
Nº of genomes with GluQRS4
Nº of genomes without GluQRS4
With GluQRS5
Without GluQRS5
Nº of genomes with GluQRS5
Nº of genomes without GluQRS5
With TGT1
Without TGT1
Nº of genomes with TGT1
Nº of genomes without TGT1
With TGT2
Without TGT2
Nº of genomes with TGT2
Nº of genomes without TGT2
With TGT3
Without TGT3
Nº of genomes with TGT3
Nº of genomes without TGT3
p < 0.00005

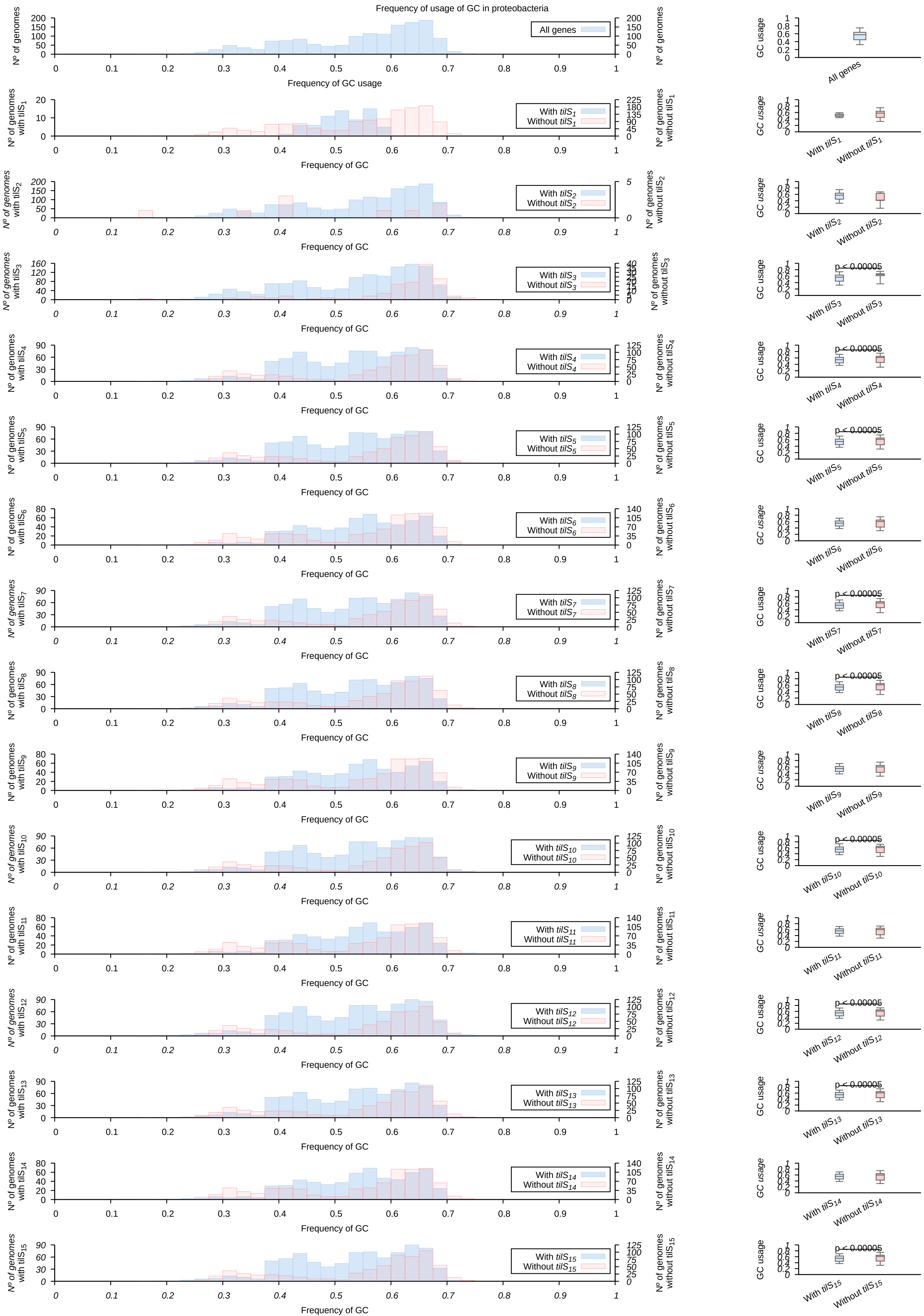

Frequency of usage of GC in proteobacteria
Nº of genomes
All genes
Frequency of GC usage
GC usage
Nº of genomes with tilS$_1$
Nº of genomes without tilS$_1$
With tilS$_1$
Without tilS$_1$
Frequency of GC
With tilS$_2$
Without tilS$_2$
With tilS$_3$
Without tilS$_3$
p < 0.00005
With tilS$_4$
Without tilS$_4$
With tilS$_5$
Without tilS$_5$
With tilS$_6$
Without tilS$_6$
With tilS$_7$
Without tilS$_7$
With tilS$_8$
Without tilS$_8$
With tilS$_9$
Without tilS$_9$
With tilS$_{10}$
Without tilS$_{10}$
With tilS$_{11}$
Without tilS$_{11}$
With tilS$_{12}$
Without tilS$_{12}$
With tilS$_{13}$
Without tilS$_{13}$
With tilS$_{14}$
Without tilS$_{14}$
With tilS$_{15}$
Without tilS$_{15}$

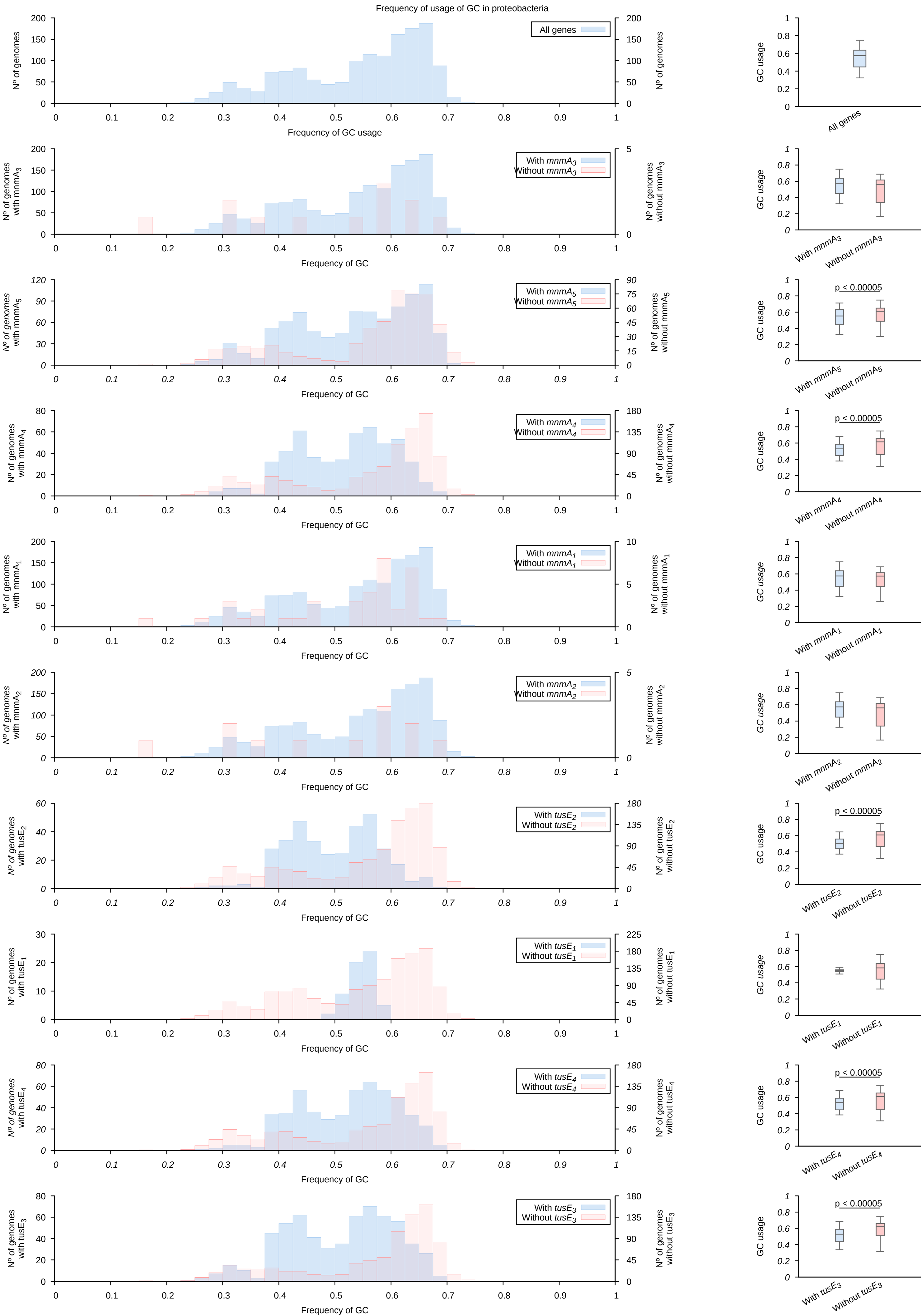
Frequency of usage of GC in proteobacteria
Nº of genomes
All genes
Frequency of GC usage
GC usage
Nº of genomes with mnmA$_3$
Nº of genomes without mnmA$_3$
With *mnmA$_3$*
Without *mnmA$_3$*
Frequency of GC
Nº of genomes with mnmA$_5$
Nº of genomes without mnmA$_5$
With *mnmA$_5$*
Without *mnmA$_5$*
p < 0.00005
Nº of genomes with mnmA$_4$
Nº of genomes without mnmA$_4$
With *mnmA$_4$*
Without *mnmA$_4$*
Nº of genomes with mnmA$_1$
Nº of genomes without mnmA$_1$
With *mnmA$_1$*
Without *mnmA$_1$*
Nº of genomes with mnmA$_2$
Nº of genomes without mnmA$_2$
With *mnmA$_2$*
Without *mnmA$_2$*
Nº of genomes with tusE$_2$
Nº of genomes without tusE$_2$
With *tusE$_2$*
Without *tusE$_2$*
Nº of genomes with tusE$_1$
Nº of genomes without tusE$_1$
With *tusE$_1$*
Without *tusE$_1$*
Nº of genomes with tusE$_4$
Nº of genomes without tusE$_4$
With *tusE$_4$*
Without *tusE$_4$*
Nº of genomes with tusE$_3$
Nº of genomes without tusE$_3$
With *tusE$_3$*
Without *tusE$_3$*

Figure S16. Histogram of GC content in proteobacterias genomes that present or lack genes *tsaB* and *gluQ* involved in t^6A and GluQ modifications. The 1484 proteobacterial genomes were separated in groups depending on the presence or absence of *tsaB* and *gluQ* (indicated as GluQRS to avoid confusions with the modification) and the GC content of genome sequences was analyzed. Data is represented in histograms superposed to the histogram of the complete database of proteobacterial genomes. **A)** Gray: all genomes. Blue: genomes that present *tsaB*, but lack *gluQ*. **B)** Gray: all genomes. Orange: genomes that lack *tsaB* and present *gluQ*. **C)** Gray: all genomes. Purple: genomes that present *tsaB* and *gluQ*. **D)** Gray: all genomes. Pink: genomes that lack *tsaB* and *gluQ*. Motifs are indicated using subscript numbers.

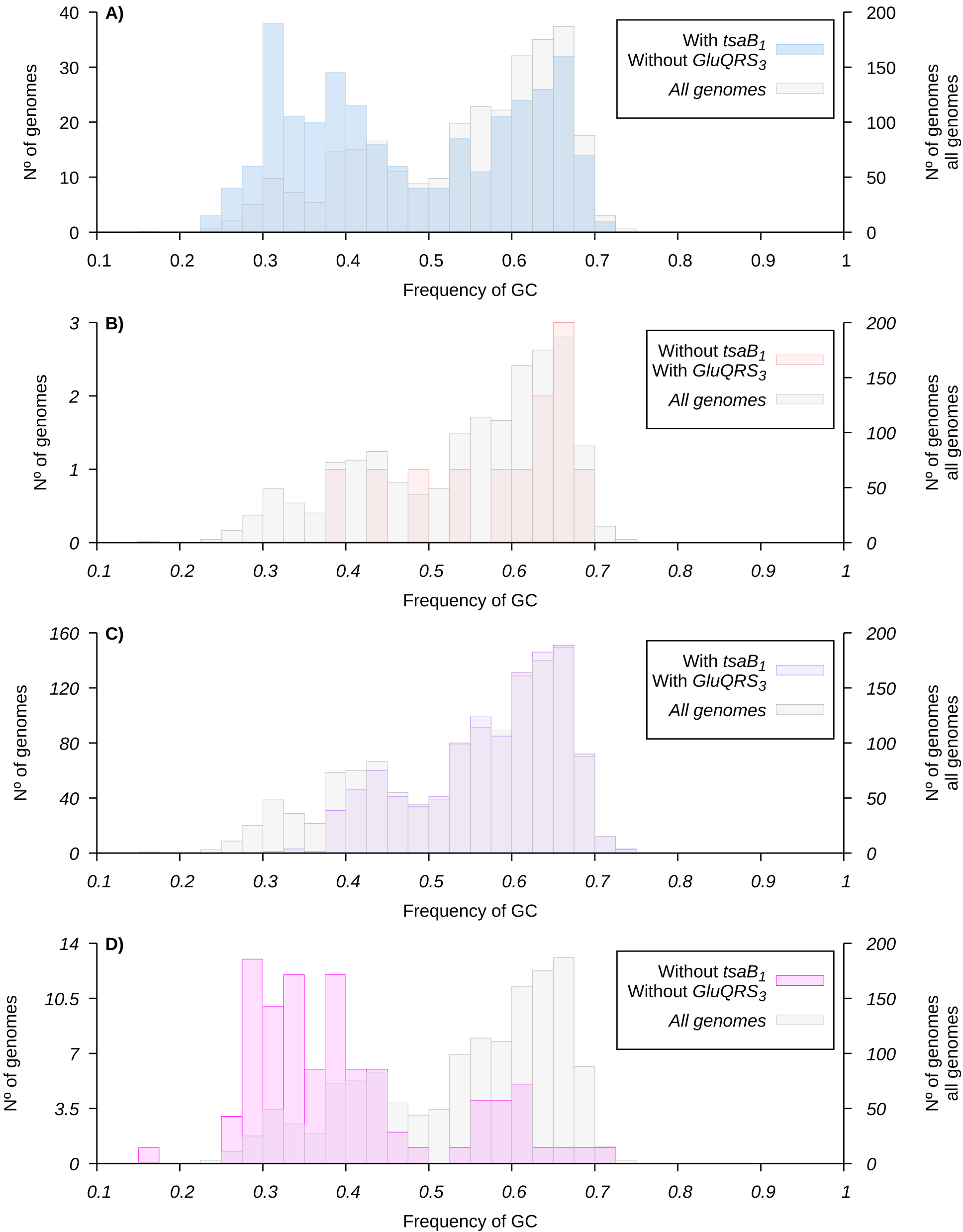

A)
With tsaB1
Without GluQRS3
All genomes
Nº of genomes
Nº of genomes all genomes
Frequency of GC
B)
Without tsaB1
With GluQRS3
All genomes
Nº of genomes
Nº of genomes all genomes
Frequency of GC
C)
With tsaB1
With GluQRS3
All genomes
Nº of genomes
Nº of genomes all genomes
Frequency of GC
D)
Without tsaB1
Without GluQRS3
All genomes
Nº of genomes
Nº of genomes all genomes
Frequency of GC

Figure S17. Histogram of genome sizes in species that present or lack genes involved in tRNA modification. Top left graph: histogram of the sizes of proteobacterial genomes. **Top right graph:** box plot indicating the distribution of genomes sizes in the same population. **Lower graphs:** The collection of 1484 proteobacterial genomes were separated in two groups, based on the presence (light blue) or absence (orange) of the indicated pattern in any gene of the analyzed genomes. Patterns are indicated using subscript numbers. See table S4 for a detailed description of the used patterns. **Left:** Data is represented in superposed histograms. **Right:** Data is represented in a boxplot. An horizontal bar was added when the two populations are different based on Wilcoxon-Mann-Whitney test and $p < 0.00005$. Whiskers include 95% of data.

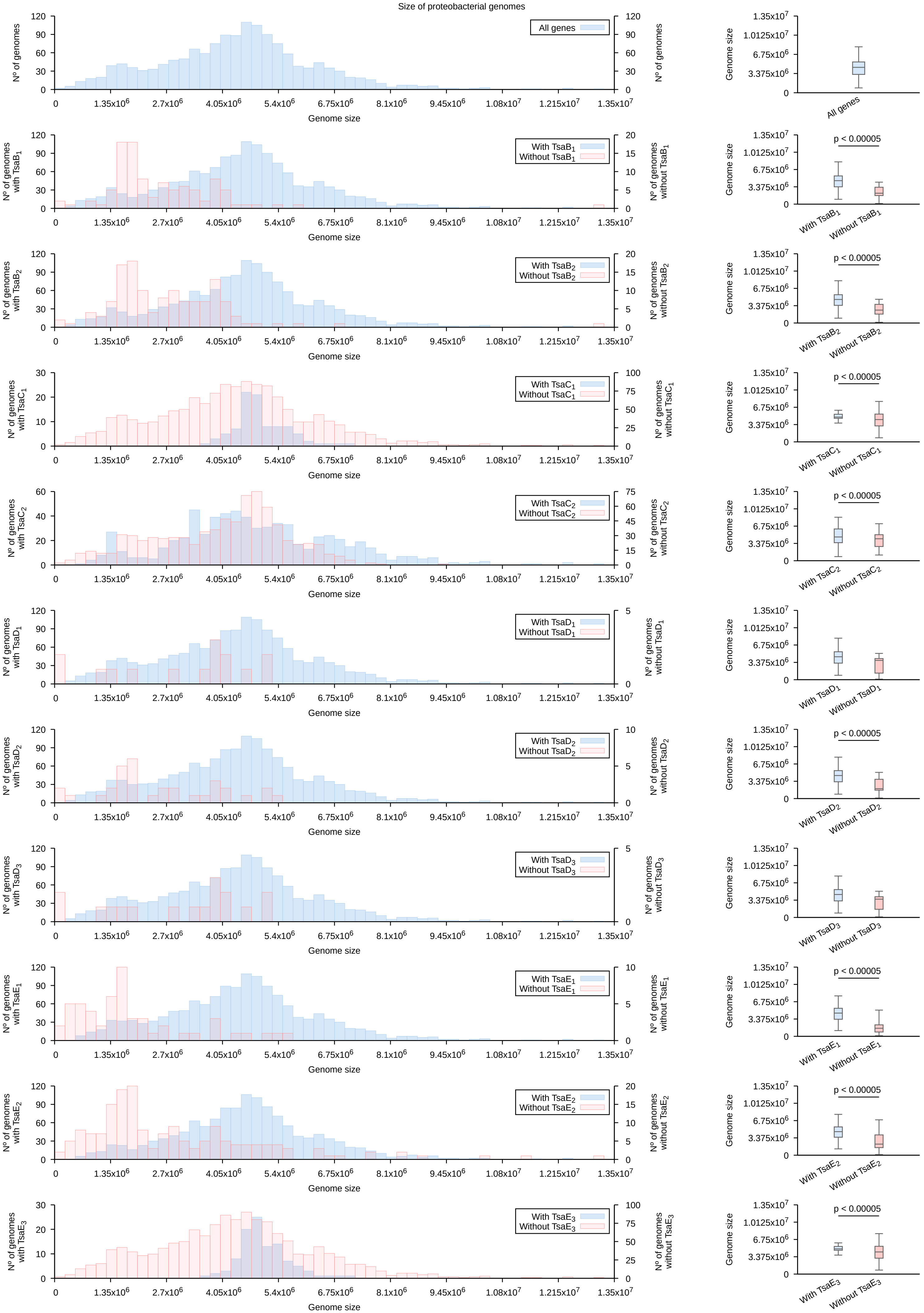
Size of proteobacterial genomes
All genes
Nº of genomes
Genome size
With TsaB1
Without TsaB1
Nº of genomes with TsaB1
Nº of genomes without TsaB1
p < 0.00005
With TsaB2
Without TsaB2
Nº of genomes with TsaB2
Nº of genomes without TsaB2
With TsaC1
Without TsaC1
Nº of genomes with TsaC1
Nº of genomes without TsaC1
With TsaC2
Without TsaC2
Nº of genomes with TsaC2
Nº of genomes without TsaC2
With TsaD1
Without TsaD1
Nº of genomes with TsaD1
Nº of genomes without TsaD1
With TsaD2
Without TsaD2
Nº of genomes with TsaD2
Nº of genomes without TsaD2
With TsaD3
Without TsaD3
Nº of genomes with TsaD3
Nº of genomes without TsaD3
With TsaE1
Without TsaE1
Nº of genomes with TsaE1
Nº of genomes without TsaE1
With TsaE2
Without TsaE2
Nº of genomes with TsaE2
Nº of genomes without TsaE2
With TsaE3
Without TsaE3
Nº of genomes with TsaE3
Nº of genomes without TsaE3
0 1.35x10^6 2.7x10^6 4.05x10^6 5.4x10^6 6.75x10^6 8.1x10^6 9.45x10^6 1.08x10^7 1.215x10^7 1.35x10^7
0 3.375x10^6 6.75x10^6 1.0125x10^7 1.35x10^7

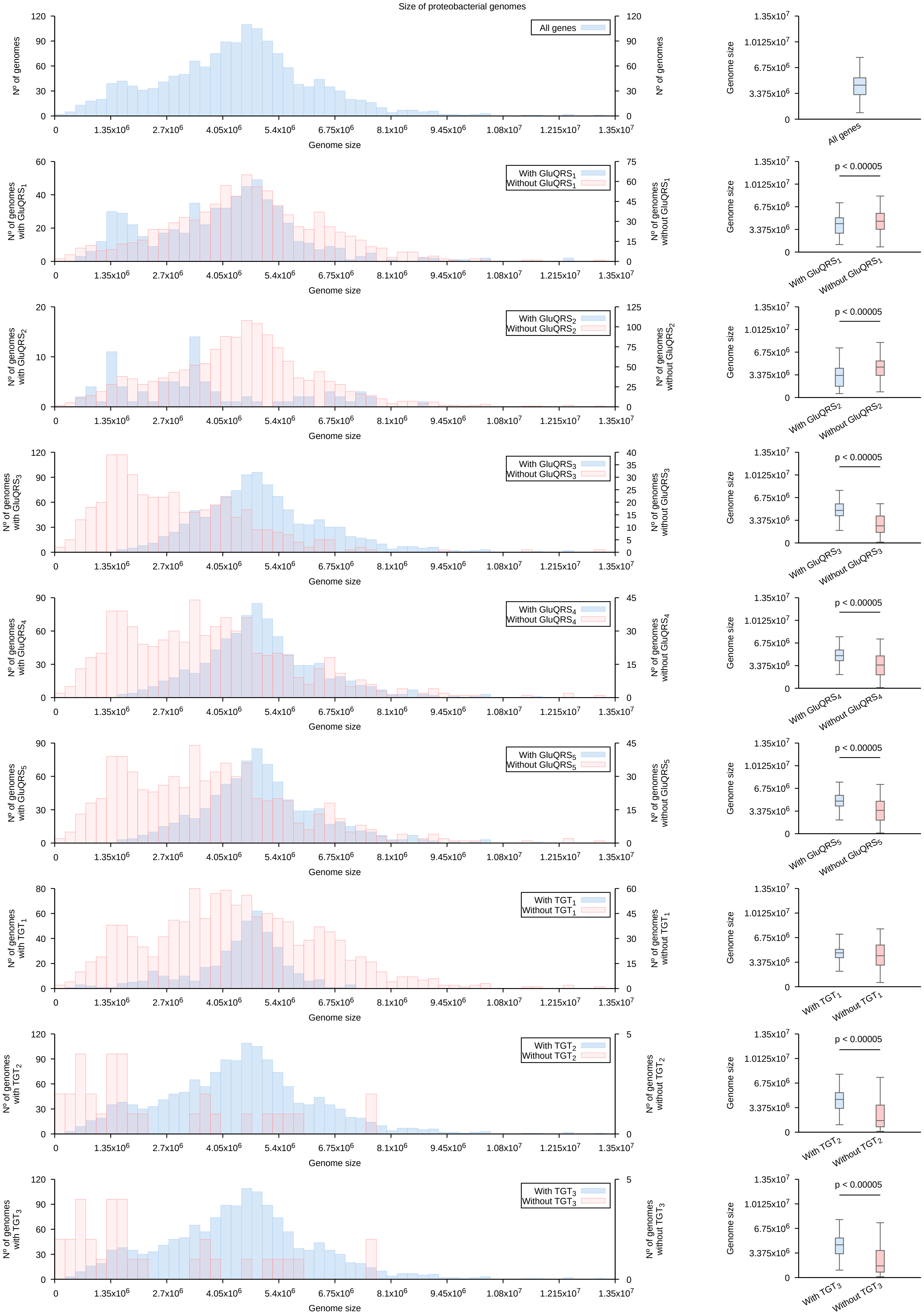

Size of proteobacterial genomes
All genes
Nº of genomes
Genome size
With GluQRS$_1$
Without GluQRS$_1$
Nº of genomes with GluQRS$_1$
Nº of genomes without GluQRS$_1$
With GluQRS$_2$
Without GluQRS$_2$
Nº of genomes with GluQRS$_2$
Nº of genomes without GluQRS$_2$
With GluQRS$_3$
Without GluQRS$_3$
Nº of genomes with GluQRS$_3$
Nº of genomes without GluQRS$_3$
With GluQRS$_4$
Without GluQRS$_4$
Nº of genomes with GluQRS$_4$
Nº of genomes without GluQRS$_4$
With GluQRS$_5$
Without GluQRS$_5$
Nº of genomes with GluQRS$_5$
Nº of genomes without GluQRS$_5$
With TGT$_1$
Without TGT$_1$
Nº of genomes with TGT$_1$
Nº of genomes without TGT$_1$
With TGT$_2$
Without TGT$_2$
Nº of genomes with TGT$_2$
Nº of genomes without TGT$_2$
With TGT$_3$
Without TGT$_3$
Nº of genomes with TGT$_3$
Nº of genomes without TGT$_3$
p < 0.00005
0
1.35x10^6
2.7x10^6
4.05x10^6
5.4x10^6
6.75x10^6
8.1x10^6
9.45x10^6
1.08x10^7
1.215x10^7
1.35x10^7
3.375x10^6
1.0125x10^7

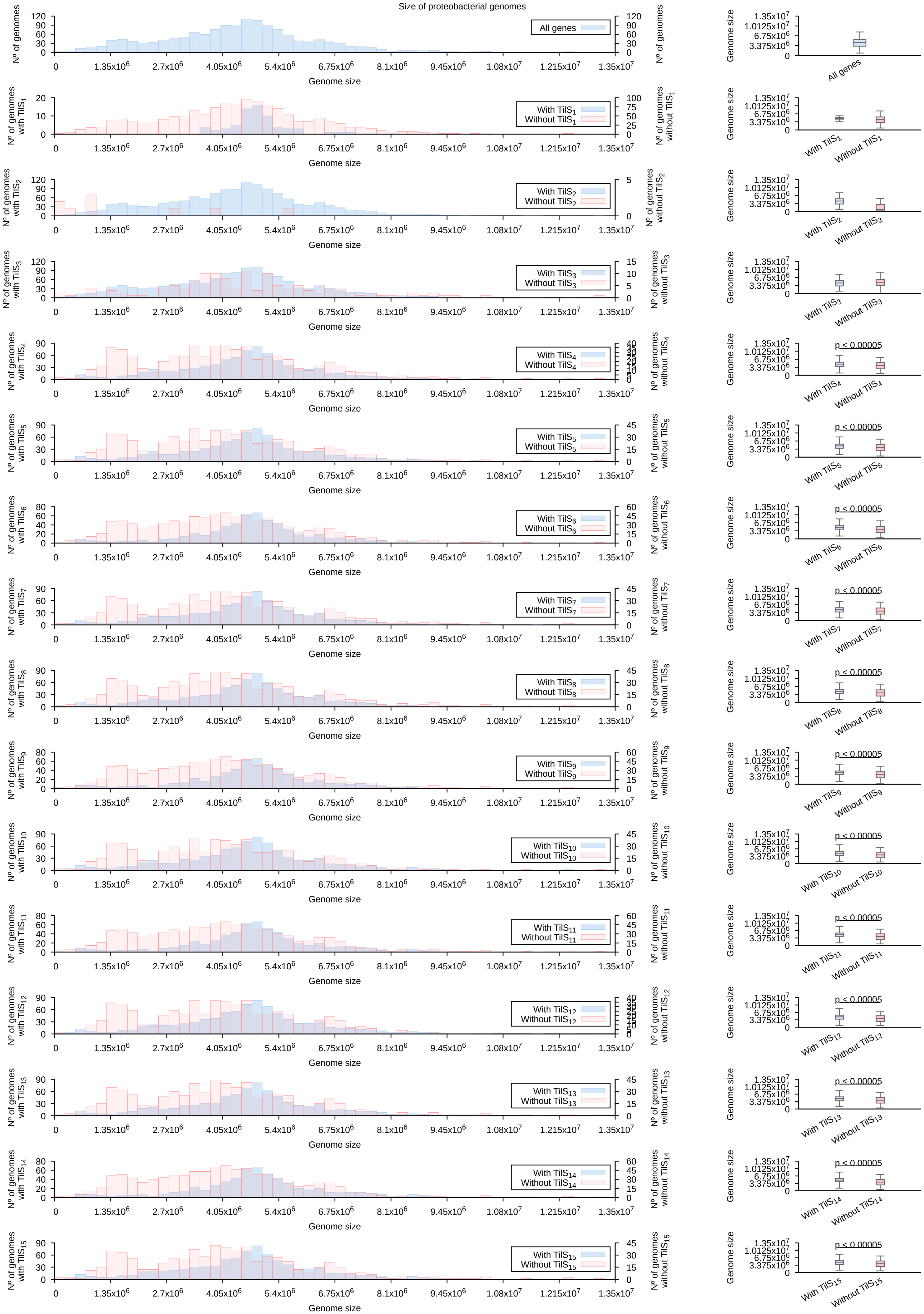

Size of proteobacterial genomes
Nº of genomes
Genome size
All genes
0
1.35x10^6
2.7x10^6
4.05x10^6
5.4x10^6
6.75x10^6
8.1x10^6
9.45x10^6
1.08x10^7
1.215x10^7
1.35x10^7
3.375x10^6
1.0125x10^7
Nº of genomes with TilS1
Nº of genomes without TilS1
With TilS1
Without TilS1
Nº of genomes with TilS2
Nº of genomes without TilS2
With TilS2
Without TilS2
Nº of genomes with TilS3
Nº of genomes without TilS3
With TilS3
Without TilS3
Nº of genomes with TilS4
Nº of genomes without TilS4
With TilS4
Without TilS4
p < 0.00005
Nº of genomes with TilS5
Nº of genomes without TilS5
With TilS5
Without TilS5
Nº of genomes with TilS6
Nº of genomes without TilS6
With TilS6
Without TilS6
Nº of genomes with TilS7
Nº of genomes without TilS7
With TilS7
Without TilS7
Nº of genomes with TilS8
Nº of genomes without TilS8
With TilS8
Without TilS8
Nº of genomes with TilS9
Nº of genomes without TilS9
With TilS9
Without TilS9
Nº of genomes with TilS10
Nº of genomes without TilS10
With TilS10
Without TilS10
Nº of genomes with TilS11
Nº of genomes without TilS11
With TilS11
Without TilS11
Nº of genomes with TilS12
Nº of genomes without TilS12
With TilS12
Without TilS12
Nº of genomes with TilS13
Nº of genomes without TilS13
With TilS13
Without TilS13
Nº of genomes with TilS14
Nº of genomes without TilS14
With TilS14
Without TilS14
Nº of genomes with TilS15
Nº of genomes without TilS15
With TilS15
Without TilS15

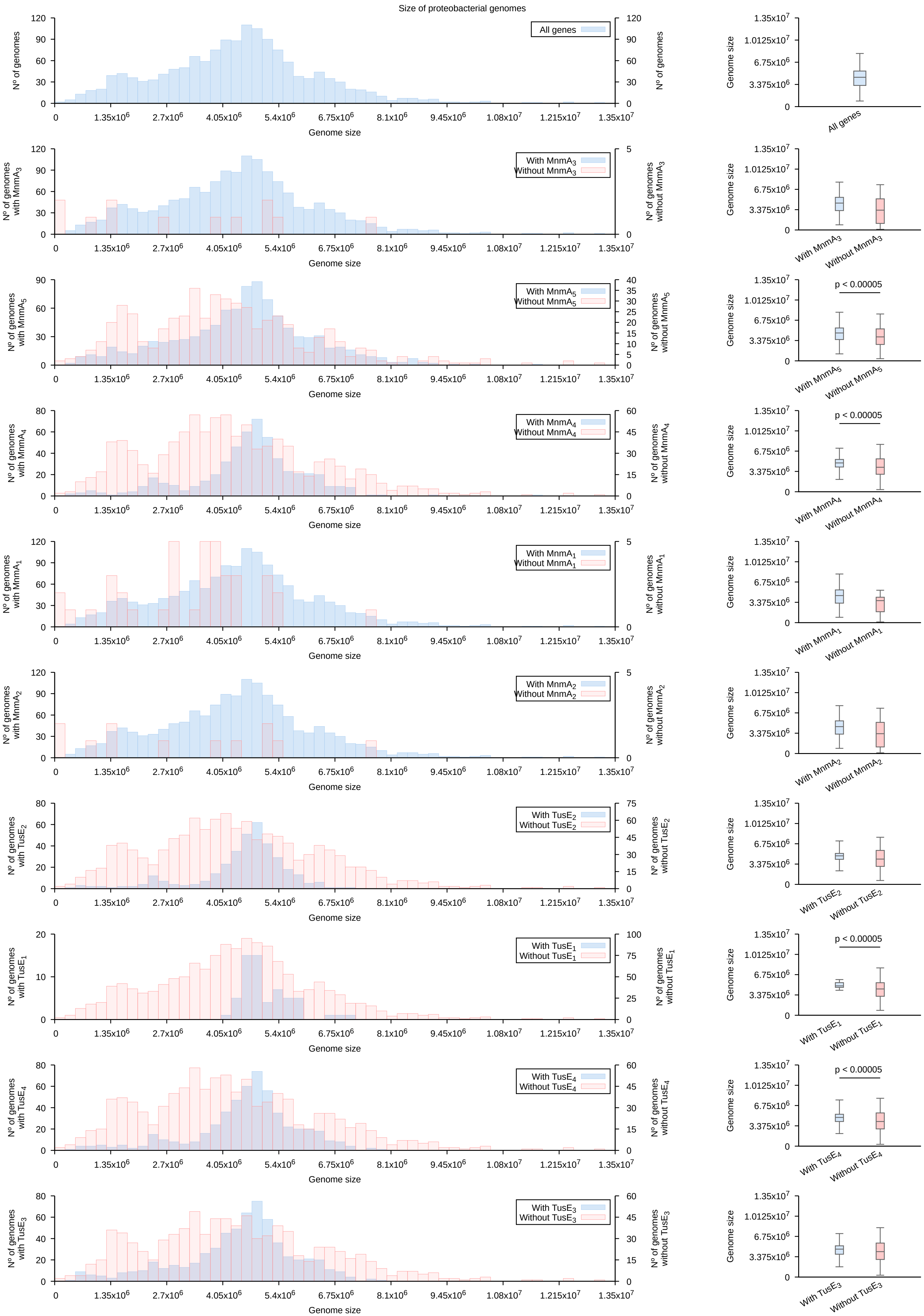
Size of proteobacterial genomes
Nº of genomes
Genome size
All genes
Nº of genomes with MnmA3
Nº of genomes without MnmA3
With MnmA3
Without MnmA3
Nº of genomes with MnmA5
Nº of genomes without MnmA5
With MnmA5
Without MnmA5
p < 0.00005
Nº of genomes with MnmA4
Nº of genomes without MnmA4
With MnmA4
Without MnmA4
p < 0.00005
Nº of genomes with MnmA1
Nº of genomes without MnmA1
With MnmA1
Without MnmA1
Nº of genomes with MnmA2
Nº of genomes without MnmA2
With MnmA2
Without MnmA2
Nº of genomes with TusE2
Nº of genomes without TusE2
With TusE2
Without TusE2
Nº of genomes with TusE1
Nº of genomes without TusE1
With TusE1
Without TusE1
p < 0.00005
Nº of genomes with TusE4
Nº of genomes without TusE4
With TusE4
Without TusE4
p < 0.00005
Nº of genomes with TusE3
Nº of genomes without TusE3
With TusE3
Without TusE3

Figure S18. GC content and genome size in proteobacterias genomes that present or lack genes *tsaB* and *gluQ* involved in t^6A and GluQ modifications. A) The 1484 proteobacterial genomes were separated in groups depending on the presence or absence of *tsaB* and *gluQ* (indicated as GluQRS to avoid confusions with the modification) as indicated in the inset legends of each graph. Each genome is represented as a point representing its GC content and genome size. Genome sizes consider all chromosomes and plasmids if present. Additionally, points are colored based of the presence of genes coding for TsaB or GluQRS. Light blue: Genomes that present a gene coding for TsaB, but not a gene coding for GluQRS. Black: Genomes that present a gene coding for GluQRS, but not a gene coding for TsaB. Green: Genomes that present a gene coding for TsaB and a gene coding for GluQRS. Magenta: Genomes that do not present a gene coding for TsaB or a gene coding for GluQRS. In all plots, GluQRS was identified based on the presence of motif 3 and TsaB based on motif 1 as indicated in table S4. An horizontal gray line was added to all plots at a GC fraction of 0.4 (40% GC%) and a similar vertical line was added at 2Mbp. **B)** Similar to **(A)**, but eliminating 4 potential false negative determinations of the *tsaB* presence in genomes where a GluQRS coding gene was found.

(A)

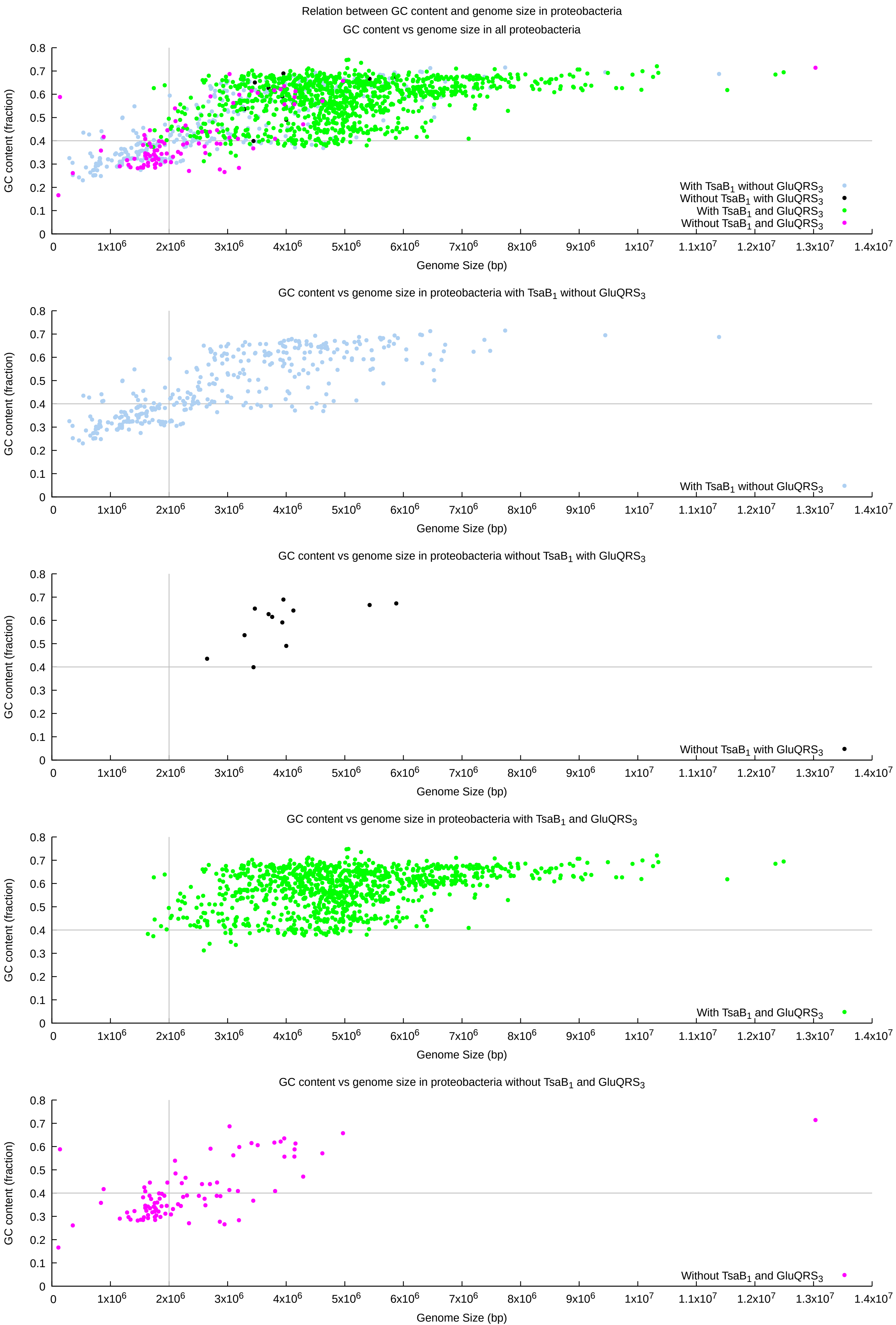
Relation between GC content and genome size in proteobacteria
GC content vs genome size in all proteobacteria
GC content (fraction)
Genome Size (bp)
0 0.1 0.2 0.3 0.4 0.5 0.6 0.7 0.8
0 1x10^6 2x10^6 3x10^6 4x10^6 5x10^6 6x10^6 7x10^6 8x10^6 9x10^6 1x10^7 1.1x10^7 1.2x10^7 1.3x10^7 1.4x10^7
With TsaB1 without GluQRS3
Without TsaB1 with GluQRS3
With TsaB1 and GluQRS3
Without TsaB1 and GluQRS3
GC content vs genome size in proteobacteria with TsaB1 without GluQRS3
With TsaB1 without GluQRS3
GC content vs genome size in proteobacteria without TsaB1 with GluQRS3
Without TsaB1 with GluQRS3
GC content vs genome size in proteobacteria with TsaB1 and GluQRS3
With TsaB1 and GluQRS3
GC content vs genome size in proteobacteria without TsaB1 and GluQRS3
Without TsaB1 and GluQRS3

(B)

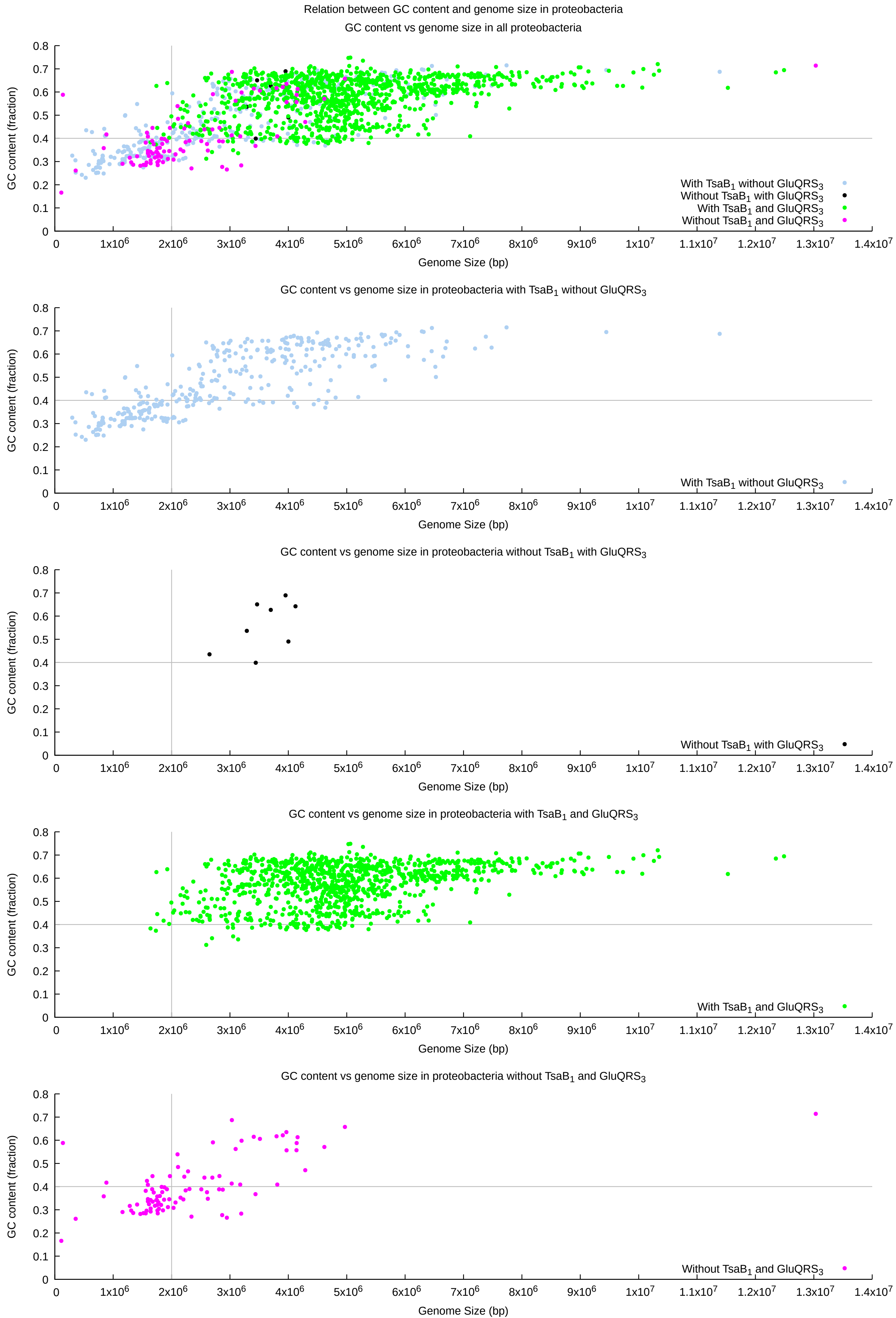
Relation between GC content and genome size in proteobacteria
GC content vs genome size in all proteobacteria
GC content (fraction)
Genome Size (bp)
With $TsaB_1$ without $GluQRS_3$
Without $TsaB_1$ with $GluQRS_3$
With $TsaB_1$ and $GluQRS_3$
Without $TsaB_1$ and $GluQRS_3$
GC content vs genome size in proteobacteria with $TsaB_1$ without $GluQRS_3$
GC content (fraction)
Genome Size (bp)
With $TsaB_1$ without $GluQRS_3$
GC content vs genome size in proteobacteria without $TsaB_1$ with $GluQRS_3$
GC content (fraction)
Genome Size (bp)
Without $TsaB_1$ with $GluQRS_3$
GC content vs genome size in proteobacteria with $TsaB_1$ and $GluQRS_3$
GC content (fraction)
Genome Size (bp)
With $TsaB_1$ and $GluQRS_3$
GC content vs genome size in proteobacteria without $TsaB_1$ and $GluQRS_3$
GC content (fraction)
Genome Size (bp)
Without $TsaB_1$ and $GluQRS_3$

Figure S19. Histogram of codon usage in high expression genes of genomes that present or lack genes involved in t^6A formation excluding small genomes. Top left graph: histogram of the usage of the indicated codon in highly expressed genes of proteobacterial genomes with more than 400 CDS. **Top right graph:** box plot indicating the distribution of codon usage in the same population. **Lower graphs:** The collection of 1477 proteobacterial genomes, excluding those with less than 400 CDS, were separated in two groups, based on the presence (light blue) or absence (orange) of the indicated pattern in any gene of the analyzed genomes. Patterns are indicated using subscript numbers. See table S4 for a detailed description of the used patterns. **Left:** Data is represented in superposed histograms. **Right:** Data is represented in a boxplot. An horizontal bar was added when the two populations are different based on Wilcoxon-Mann-Whitney test and $p < 0.00005$. Whiskers include 95% of data.

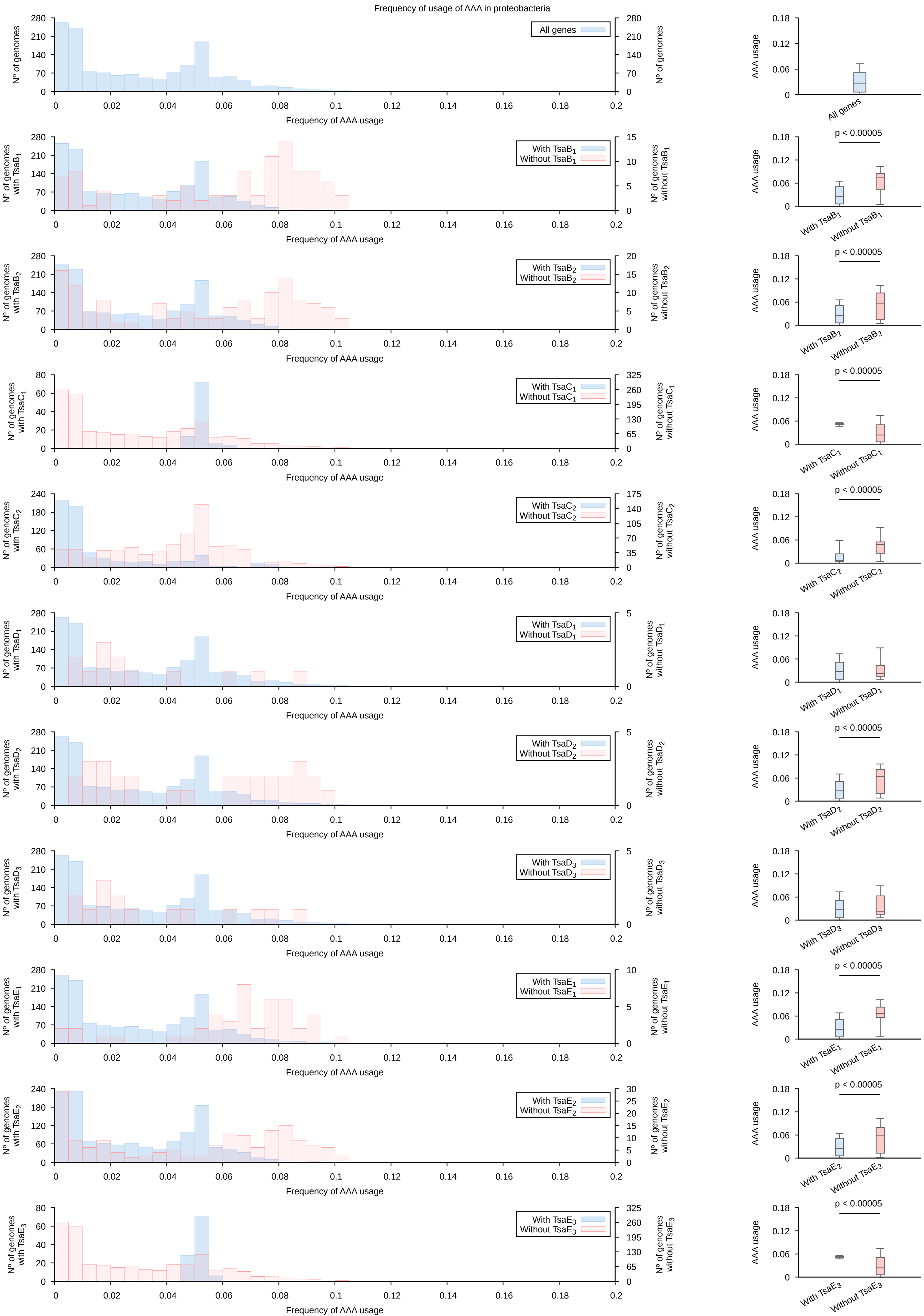
Frequency of usage of AAA in proteobacteria
All genes
Nº of genomes
Frequency of AAA usage
AAA usage
With TsaB$_1$
Without TsaB$_1$
Nº of genomes with TsaB$_1$
Nº of genomes without TsaB$_1$
With TsaB$_2$
Without TsaB$_2$
Nº of genomes with TsaB$_2$
Nº of genomes without TsaB$_2$
With TsaC$_1$
Without TsaC$_1$
Nº of genomes with TsaC$_1$
Nº of genomes without TsaC$_1$
With TsaC$_2$
Without TsaC$_2$
Nº of genomes with TsaC$_2$
Nº of genomes without TsaC$_2$
With TsaD$_1$
Without TsaD$_1$
Nº of genomes with TsaD$_1$
Nº of genomes without TsaD$_1$
With TsaD$_2$
Without TsaD$_2$
Nº of genomes with TsaD$_2$
Nº of genomes without TsaD$_2$
With TsaD$_3$
Without TsaD$_3$
Nº of genomes with TsaD$_3$
Nº of genomes without TsaD$_3$
With TsaE$_1$
Without TsaE$_1$
Nº of genomes with TsaE$_1$
Nº of genomes without TsaE$_1$
With TsaE$_2$
Without TsaE$_2$
Nº of genomes with TsaE$_2$
Nº of genomes without TsaE$_2$
With TsaE$_3$
Without TsaE$_3$
Nº of genomes with TsaE$_3$
Nº of genomes without TsaE$_3$
p < 0.00005

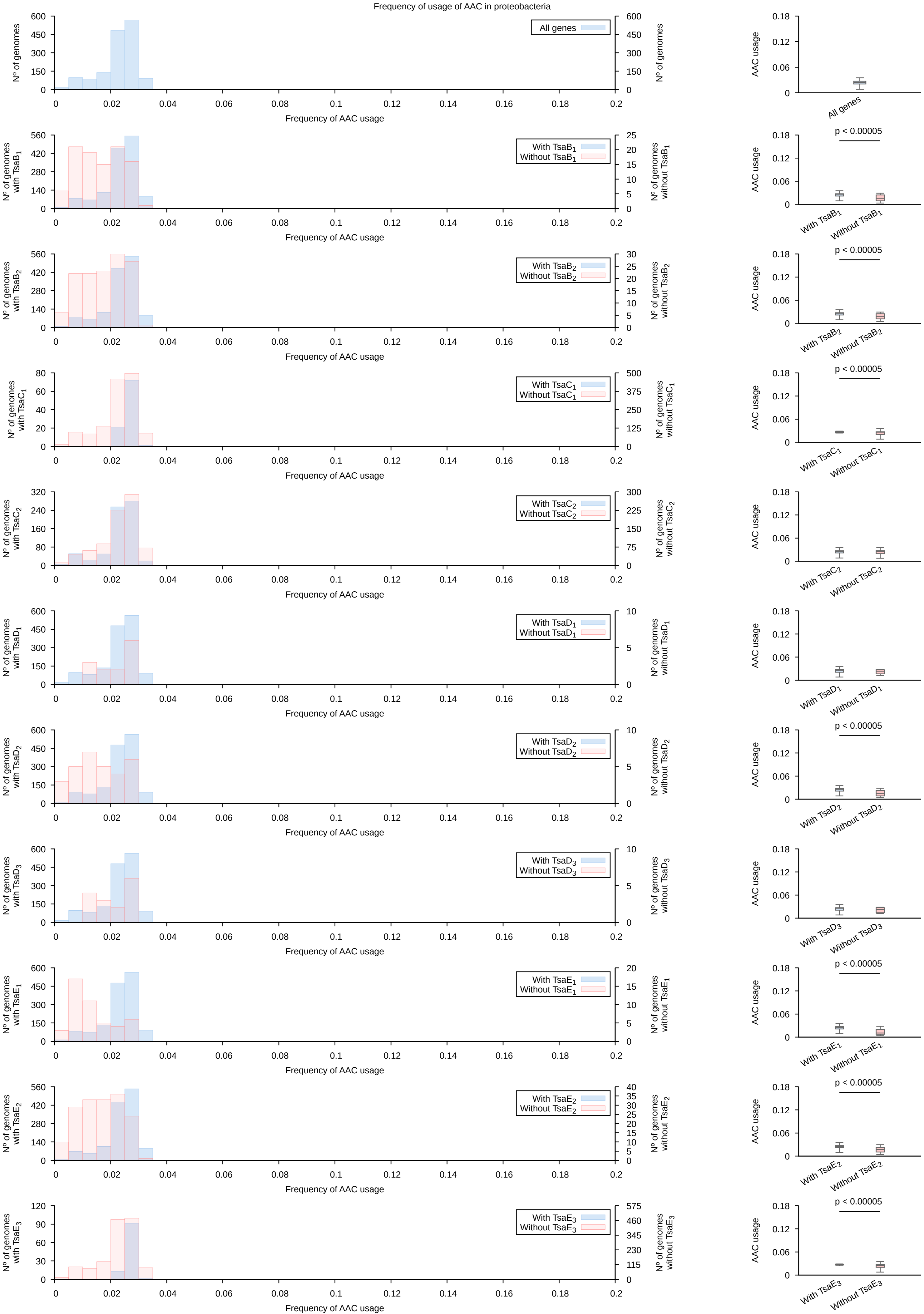
Frequency of usage of AAC in proteobacteria
All genes
Nº of genomes
Frequency of AAC usage
AAC usage
With TsaB$_1$
Without TsaB$_1$
Nº of genomes with TsaB$_1$
Nº of genomes without TsaB$_1$
With TsaB$_2$
Without TsaB$_2$
Nº of genomes with TsaB$_2$
Nº of genomes without TsaB$_2$
With TsaC$_1$
Without TsaC$_1$
Nº of genomes with TsaC$_1$
Nº of genomes without TsaC$_1$
With TsaC$_2$
Without TsaC$_2$
Nº of genomes with TsaC$_2$
Nº of genomes without TsaC$_2$
With TsaD$_1$
Without TsaD$_1$
Nº of genomes with TsaD$_1$
Nº of genomes without TsaD$_1$
With TsaD$_2$
Without TsaD$_2$
Nº of genomes with TsaD$_2$
Nº of genomes without TsaD$_2$
With TsaD$_3$
Without TsaD$_3$
Nº of genomes with TsaD$_3$
Nº of genomes without TsaD$_3$
With TsaE$_1$
Without TsaE$_1$
Nº of genomes with TsaE$_1$
Nº of genomes without TsaE$_1$
With TsaE$_2$
Without TsaE$_2$
Nº of genomes with TsaE$_2$
Nº of genomes without TsaE$_2$
With TsaE$_3$
Without TsaE$_3$
Nº of genomes with TsaE$_3$
Nº of genomes without TsaE$_3$
p < 0.00005
0 0.02 0.04 0.06 0.08 0.1 0.12 0.14 0.16 0.18 0.2
0 0.06 0.12 0.18

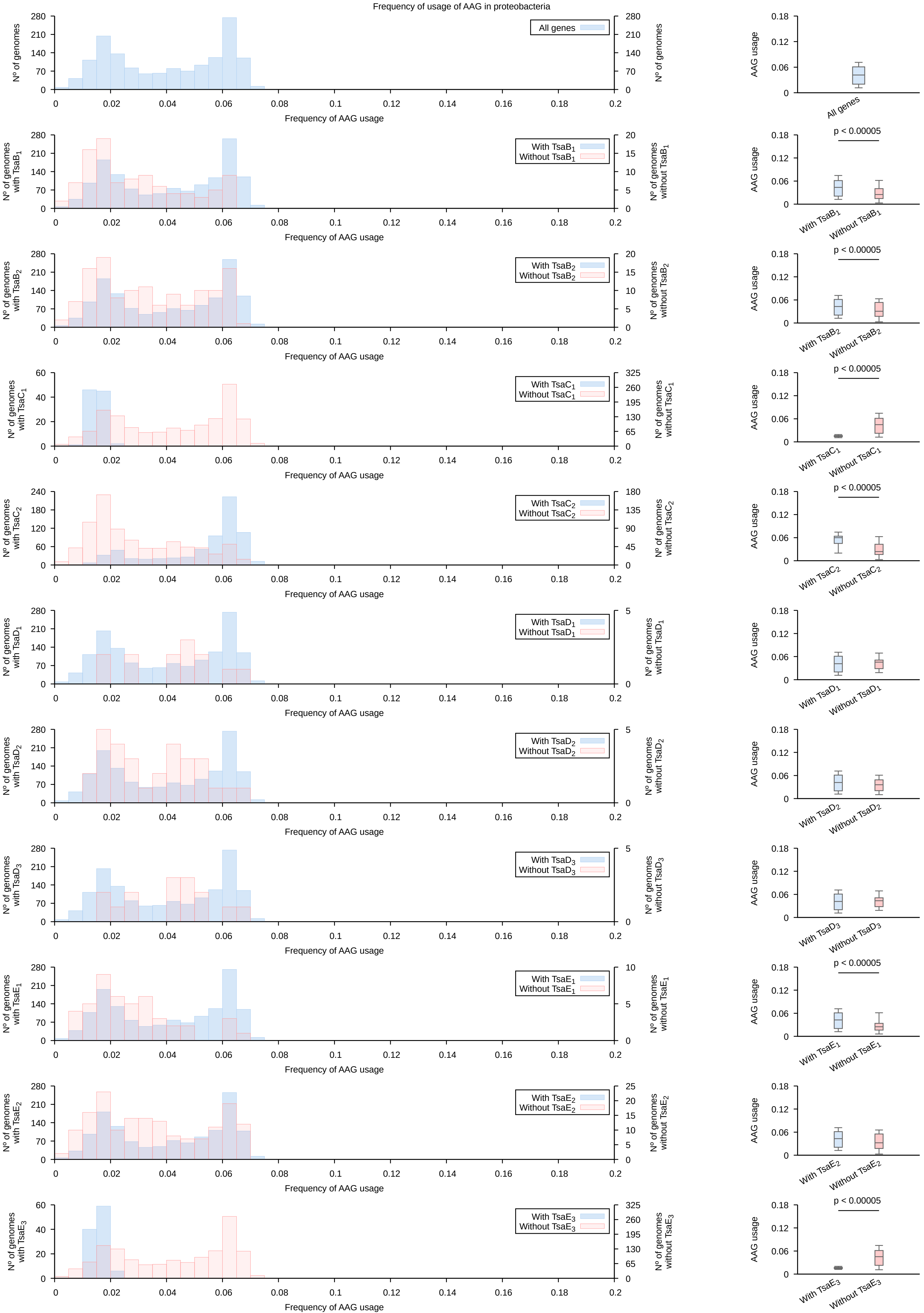

Frequency of usage of AAG in proteobacteria
All genes
Nº of genomes
Frequency of AAG usage
AAG usage
With $TsaB_1$
Without $TsaB_1$
Nº of genomes with $TsaB_1$
Nº of genomes without $TsaB_1$
With $TsaB_2$
Without $TsaB_2$
Nº of genomes with $TsaB_2$
Nº of genomes without $TsaB_2$
With $TsaC_1$
Without $TsaC_1$
Nº of genomes with $TsaC_1$
Nº of genomes without $TsaC_1$
With $TsaC_2$
Without $TsaC_2$
Nº of genomes with $TsaC_2$
Nº of genomes without $TsaC_2$
With $TsaD_1$
Without $TsaD_1$
Nº of genomes with $TsaD_1$
Nº of genomes without $TsaD_1$
With $TsaD_2$
Without $TsaD_2$
Nº of genomes with $TsaD_2$
Nº of genomes without $TsaD_2$
With $TsaD_3$
Without $TsaD_3$
Nº of genomes with $TsaD_3$
Nº of genomes without $TsaD_3$
With $TsaE_1$
Without $TsaE_1$
Nº of genomes with $TsaE_1$
Nº of genomes without $TsaE_1$
With $TsaE_2$
Without $TsaE_2$
Nº of genomes with $TsaE_2$
Nº of genomes without $TsaE_2$
With $TsaE_3$
Without $TsaE_3$
Nº of genomes with $TsaE_3$
Nº of genomes without $TsaE_3$
$p < 0.00005$

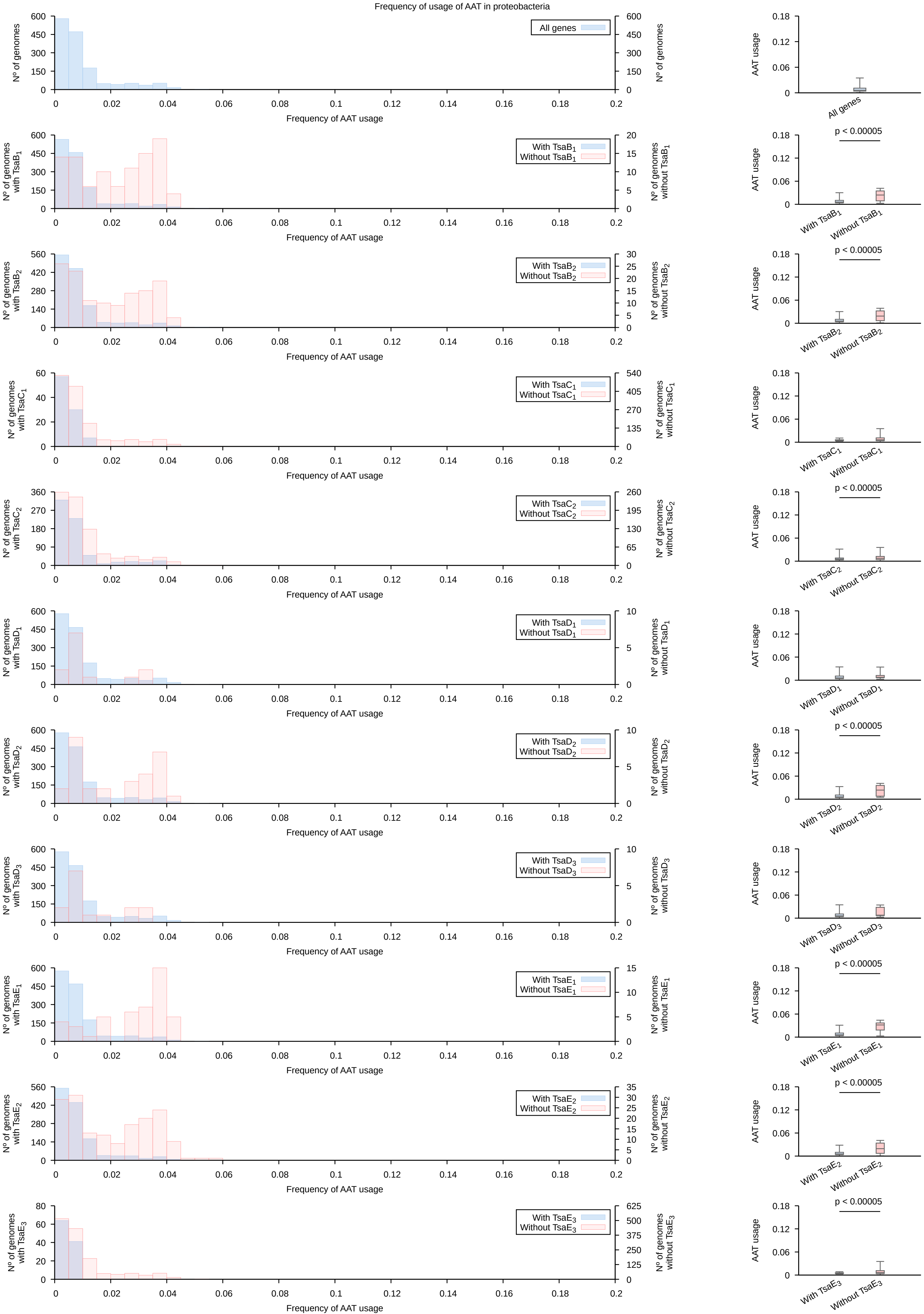
Frequency of usage of AAT in proteobacteria
Nº of genomes
Frequency of AAT usage
AAT usage
All genes
Nº of genomes with TsaB$_1$
Nº of genomes without TsaB$_1$
With TsaB$_1$
Without TsaB$_1$
p < 0.00005
Nº of genomes with TsaB$_2$
Nº of genomes without TsaB$_2$
With TsaB$_2$
Without TsaB$_2$
p < 0.00005
Nº of genomes with TsaC$_1$
Nº of genomes without TsaC$_1$
With TsaC$_1$
Without TsaC$_1$
Nº of genomes with TsaC$_2$
Nº of genomes without TsaC$_2$
With TsaC$_2$
Without TsaC$_2$
p < 0.00005
Nº of genomes with TsaD$_1$
Nº of genomes without TsaD$_1$
With TsaD$_1$
Without TsaD$_1$
Nº of genomes with TsaD$_2$
Nº of genomes without TsaD$_2$
With TsaD$_2$
Without TsaD$_2$
p < 0.00005
Nº of genomes with TsaD$_3$
Nº of genomes without TsaD$_3$
With TsaD$_3$
Without TsaD$_3$
Nº of genomes with TsaE$_1$
Nº of genomes without TsaE$_1$
With TsaE$_1$
Without TsaE$_1$
p < 0.00005
Nº of genomes with TsaE$_2$
Nº of genomes without TsaE$_2$
With TsaE$_2$
Without TsaE$_2$
p < 0.00005
Nº of genomes with TsaE$_3$
Nº of genomes without TsaE$_3$
With TsaE$_3$
Without TsaE$_3$
p < 0.00005

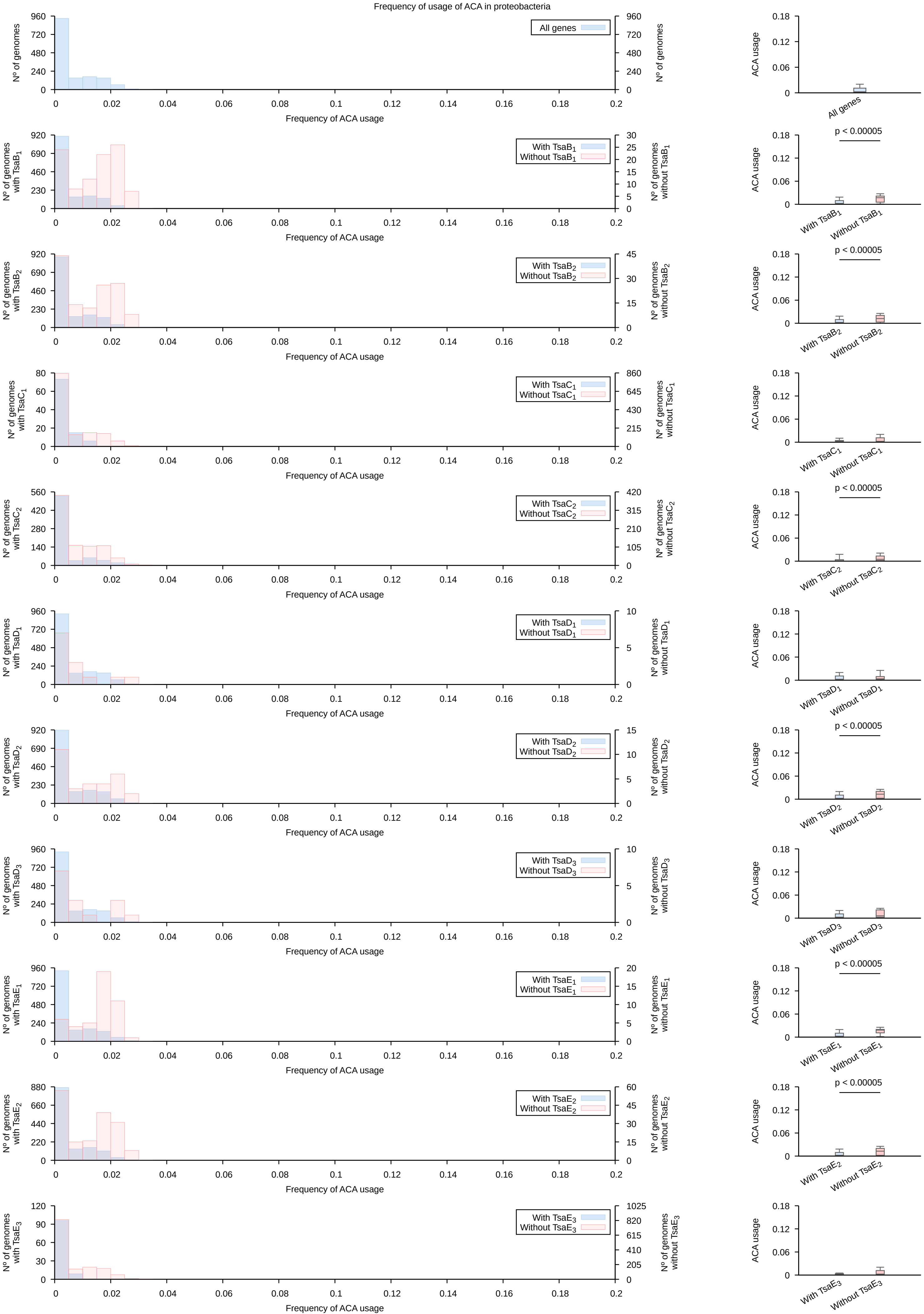
Frequency of usage of ACA in proteobacteria
All genes
Nº of genomes
Frequency of ACA usage
ACA usage
With TsaB$_1$
Without TsaB$_1$
Nº of genomes with TsaB$_1$
Nº of genomes without TsaB$_1$
p < 0.00005
With TsaB$_2$
Without TsaB$_2$
Nº of genomes with TsaB$_2$
Nº of genomes without TsaB$_2$
p < 0.00005
With TsaC$_1$
Without TsaC$_1$
Nº of genomes with TsaC$_1$
Nº of genomes without TsaC$_1$
With TsaC$_2$
Without TsaC$_2$
Nº of genomes with TsaC$_2$
Nº of genomes without TsaC$_2$
p < 0.00005
With TsaD$_1$
Without TsaD$_1$
Nº of genomes with TsaD$_1$
Nº of genomes without TsaD$_1$
With TsaD$_2$
Without TsaD$_2$
Nº of genomes with TsaD$_2$
Nº of genomes without TsaD$_2$
p < 0.00005
With TsaD$_3$
Without TsaD$_3$
Nº of genomes with TsaD$_3$
Nº of genomes without TsaD$_3$
With TsaE$_1$
Without TsaE$_1$
Nº of genomes with TsaE$_1$
Nº of genomes without TsaE$_1$
p < 0.00005
With TsaE$_2$
Without TsaE$_2$
Nº of genomes with TsaE$_2$
Nº of genomes without TsaE$_2$
p < 0.00005
With TsaE$_3$
Without TsaE$_3$
Nº of genomes with TsaE$_3$
Nº of genomes without TsaE$_3$

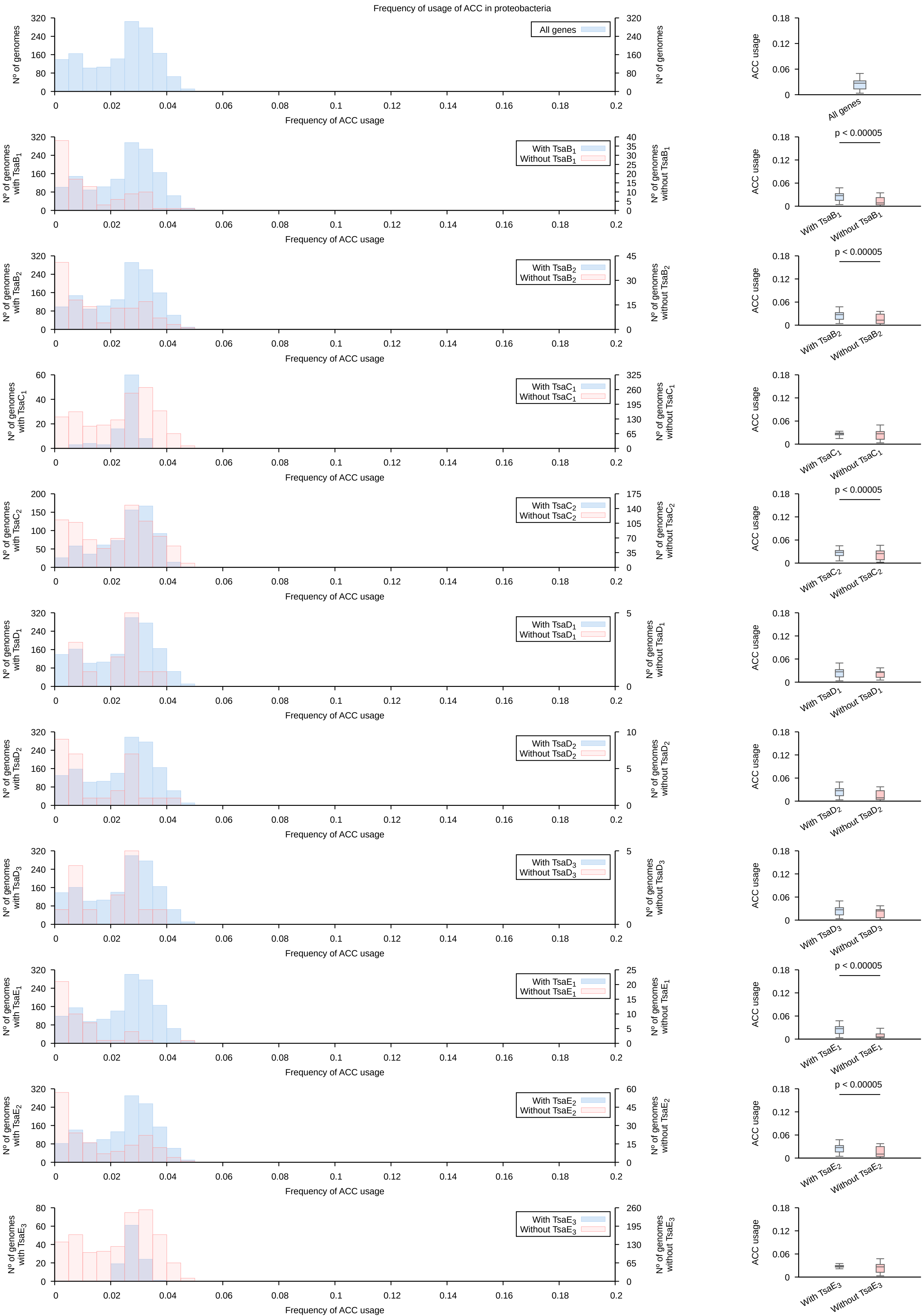

Frequency of usage of ACC in proteobacteria
All genes
Nº of genomes
Frequency of ACC usage
ACC usage
With TsaB1
Without TsaB1
Nº of genomes with TsaB1
Nº of genomes without TsaB1
p < 0.00005
With TsaB2
Without TsaB2
Nº of genomes with TsaB2
Nº of genomes without TsaB2
p < 0.00005
With TsaC1
Without TsaC1
Nº of genomes with TsaC1
Nº of genomes without TsaC1
With TsaC2
Without TsaC2
Nº of genomes with TsaC2
Nº of genomes without TsaC2
p < 0.00005
With TsaD1
Without TsaD1
Nº of genomes with TsaD1
Nº of genomes without TsaD1
With TsaD2
Without TsaD2
Nº of genomes with TsaD2
Nº of genomes without TsaD2
With TsaD3
Without TsaD3
Nº of genomes with TsaD3
Nº of genomes without TsaD3
With TsaE1
Without TsaE1
Nº of genomes with TsaE1
Nº of genomes without TsaE1
p < 0.00005
With TsaE2
Without TsaE2
Nº of genomes with TsaE2
Nº of genomes without TsaE2
p < 0.00005
With TsaE3
Without TsaE3
Nº of genomes with TsaE3
Nº of genomes without TsaE3

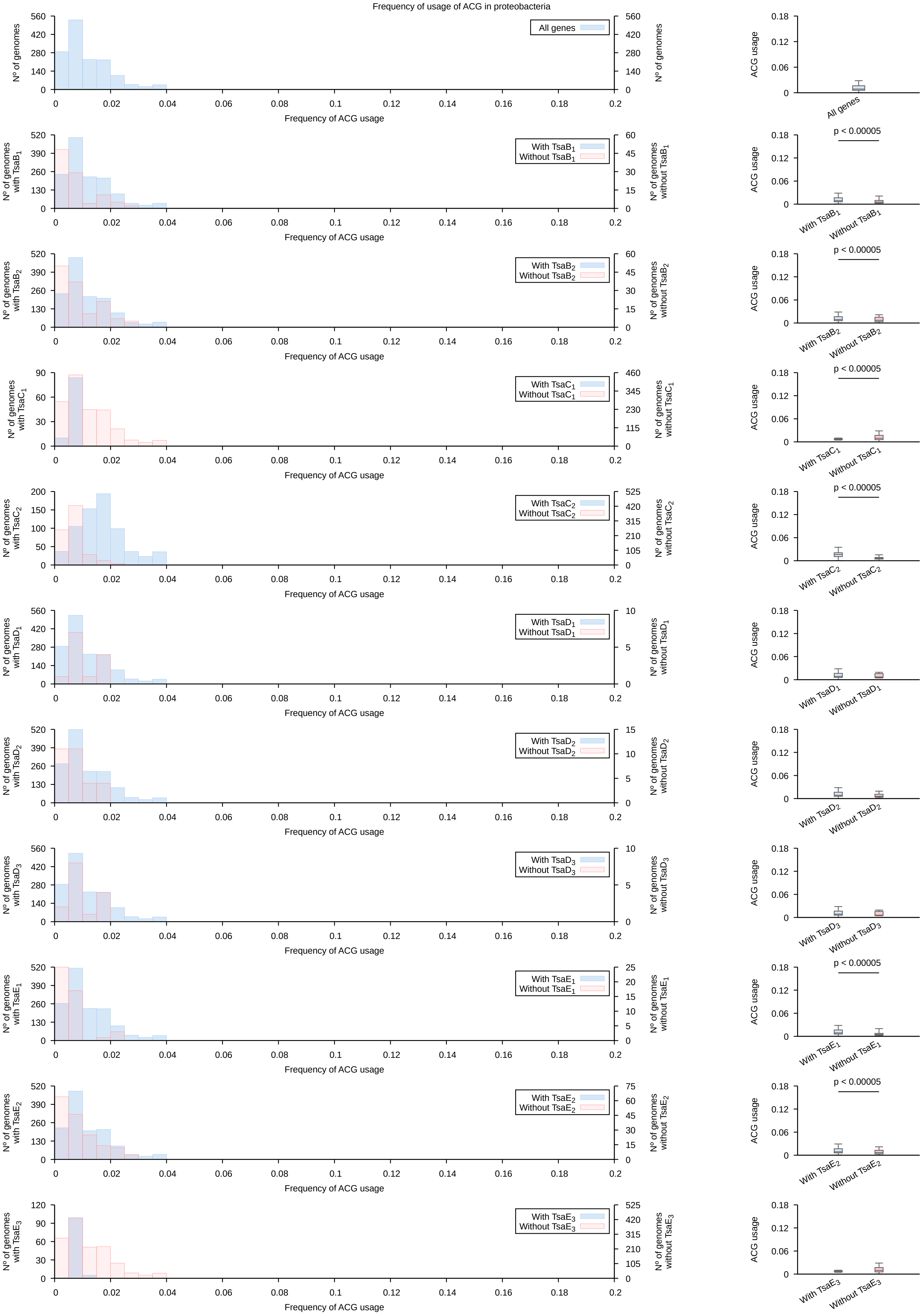
Frequency of usage of ACG in proteobacteria
Nº of genomes
Frequency of ACG usage
All genes
ACG usage
Nº of genomes with TsaB1
Nº of genomes without TsaB1
With TsaB1
Without TsaB1
p < 0.00005
Nº of genomes with TsaB2
Nº of genomes without TsaB2
With TsaB2
Without TsaB2
p < 0.00005
Nº of genomes with TsaC1
Nº of genomes without TsaC1
With TsaC1
Without TsaC1
p < 0.00005
Nº of genomes with TsaC2
Nº of genomes without TsaC2
With TsaC2
Without TsaC2
p < 0.00005
Nº of genomes with TsaD1
Nº of genomes without TsaD1
With TsaD1
Without TsaD1
Nº of genomes with TsaD2
Nº of genomes without TsaD2
With TsaD2
Without TsaD2
Nº of genomes with TsaD3
Nº of genomes without TsaD3
With TsaD3
Without TsaD3
Nº of genomes with TsaE1
Nº of genomes without TsaE1
With TsaE1
Without TsaE1
p < 0.00005
Nº of genomes with TsaE2
Nº of genomes without TsaE2
With TsaE2
Without TsaE2
p < 0.00005
Nº of genomes with TsaE3
Nº of genomes without TsaE3
With TsaE3
Without TsaE3
0
0.02
0.04
0.06
0.08
0.1
0.12
0.14
0.16
0.18
0.2

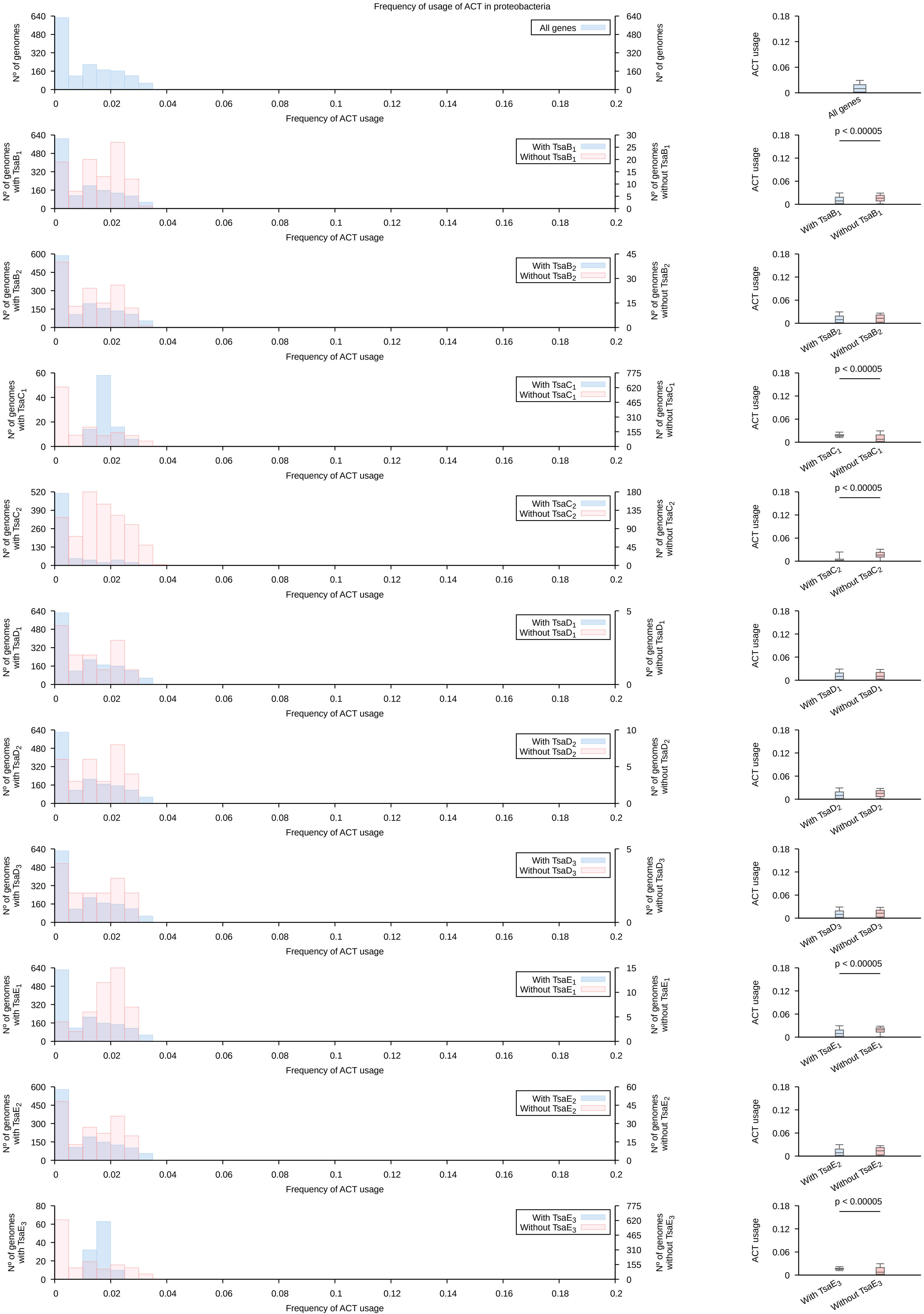

Frequency of usage of ACT in proteobacteria
Nº of genomes
All genes
Frequency of ACT usage
ACT usage
0
0.02
0.04
0.06
0.08
0.1
0.12
0.14
0.16
0.18
0.2
Nº of genomes with $TsaB_1$
Nº of genomes without $TsaB_1$
With $TsaB_1$
Without $TsaB_1$
p < 0.00005
Nº of genomes with $TsaB_2$
Nº of genomes without $TsaB_2$
With $TsaB_2$
Without $TsaB_2$
Nº of genomes with $TsaC_1$
Nº of genomes without $TsaC_1$
With $TsaC_1$
Without $TsaC_1$
p < 0.00005
Nº of genomes with $TsaC_2$
Nº of genomes without $TsaC_2$
With $TsaC_2$
Without $TsaC_2$
p < 0.00005
Nº of genomes with $TsaD_1$
Nº of genomes without $TsaD_1$
With $TsaD_1$
Without $TsaD_1$
Nº of genomes with $TsaD_2$
Nº of genomes without $TsaD_2$
With $TsaD_2$
Without $TsaD_2$
Nº of genomes with $TsaD_3$
Nº of genomes without $TsaD_3$
With $TsaD_3$
Without $TsaD_3$
Nº of genomes with $TsaE_1$
Nº of genomes without $TsaE_1$
With $TsaE_1$
Without $TsaE_1$
p < 0.00005
Nº of genomes with $TsaE_2$
Nº of genomes without $TsaE_2$
With $TsaE_2$
Without $TsaE_2$
Nº of genomes with $TsaE_3$
Nº of genomes without $TsaE_3$
With $TsaE_3$
Without $TsaE_3$
p < 0.00005

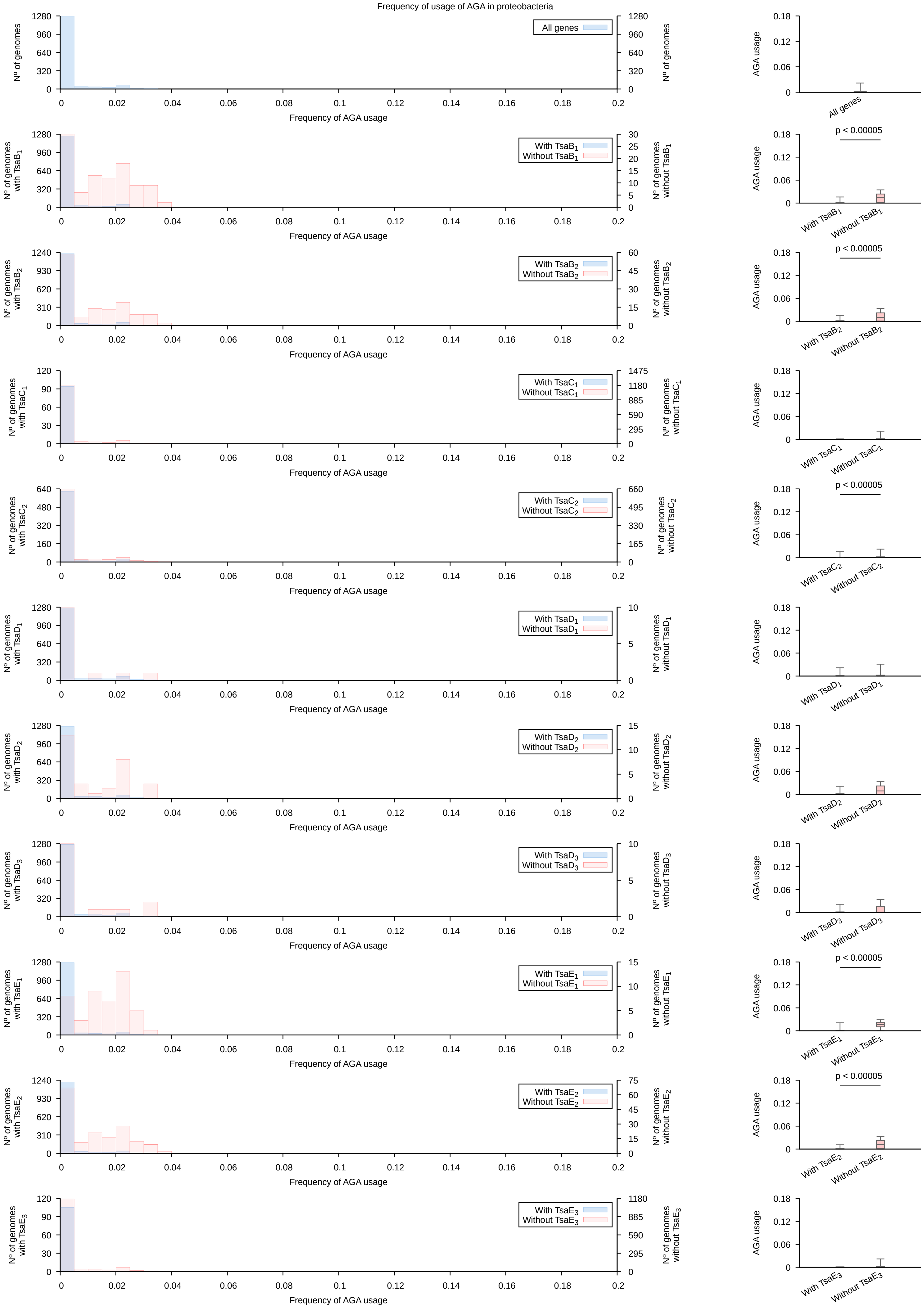

Frequency of usage of AGA in proteobacteria
All genes
Nº of genomes
Frequency of AGA usage
AGA usage
All genes
With TsaB1
Without TsaB1
Nº of genomes with TsaB1
Nº of genomes without TsaB1
p < 0.00005
With TsaB2
Without TsaB2
Nº of genomes with TsaB2
Nº of genomes without TsaB2
p < 0.00005
With TsaC1
Without TsaC1
Nº of genomes with TsaC1
Nº of genomes without TsaC1
With TsaC2
Without TsaC2
Nº of genomes with TsaC2
Nº of genomes without TsaC2
p < 0.00005
With TsaD1
Without TsaD1
Nº of genomes with TsaD1
Nº of genomes without TsaD1
With TsaD2
Without TsaD2
Nº of genomes with TsaD2
Nº of genomes without TsaD2
With TsaD3
Without TsaD3
Nº of genomes with TsaD3
Nº of genomes without TsaD3
With TsaE1
Without TsaE1
Nº of genomes with TsaE1
Nº of genomes without TsaE1
p < 0.00005
With TsaE2
Without TsaE2
Nº of genomes with TsaE2
Nº of genomes without TsaE2
p < 0.00005
With TsaE3
Without TsaE3
Nº of genomes with TsaE3
Nº of genomes without TsaE3

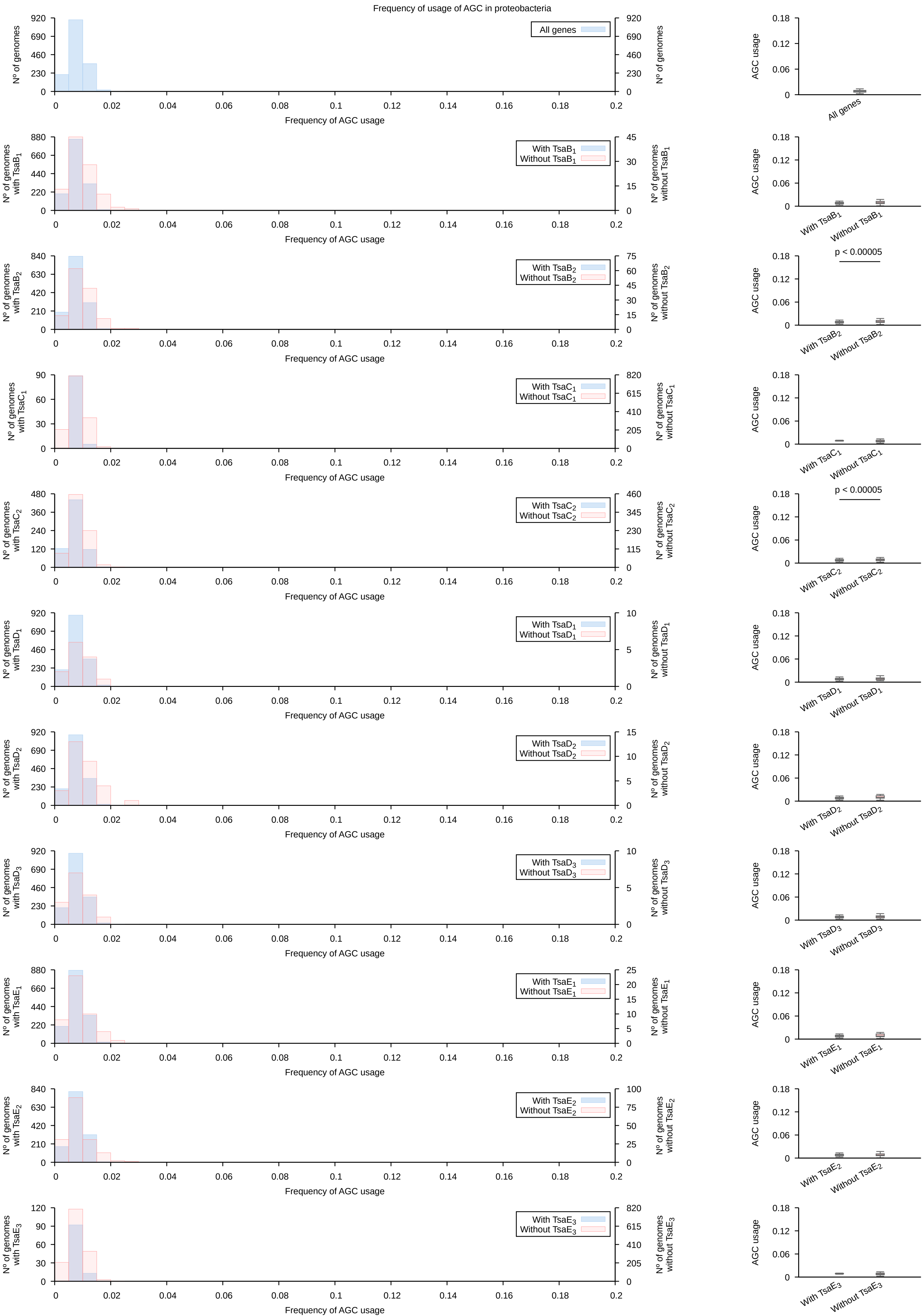
Frequency of usage of AGC in proteobacteria
All genes
With TsaB1
Without TsaB1
With TsaB2
Without TsaB2
With TsaC1
Without TsaC1
With TsaC2
Without TsaC2
With TsaD1
Without TsaD1
With TsaD2
Without TsaD2
With TsaD3
Without TsaD3
With TsaE1
Without TsaE1
With TsaE2
Without TsaE2
With TsaE3
Without TsaE3
Nº of genomes
Frequency of AGC usage
AGC usage
p < 0.00005
p < 0.00005

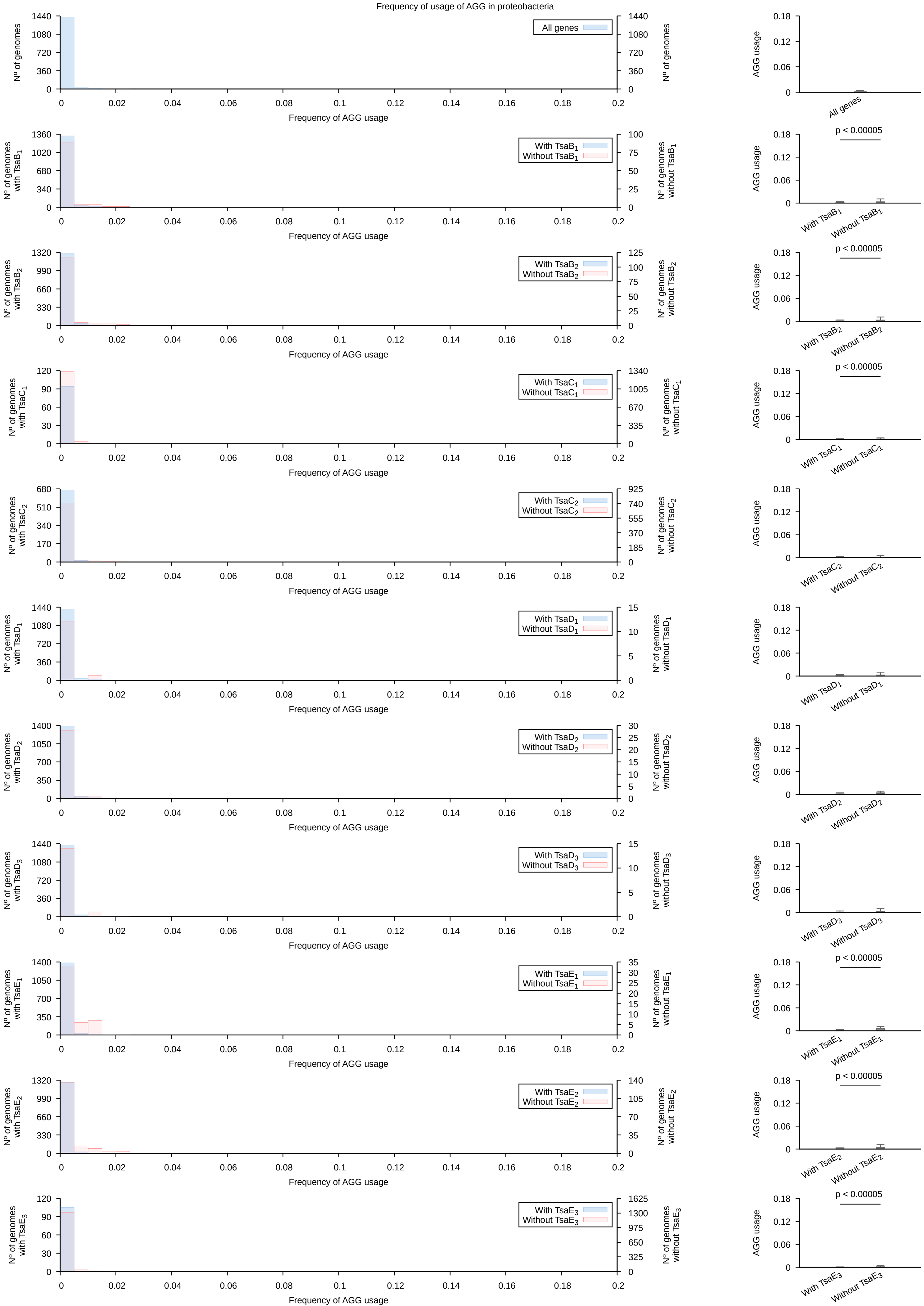
Frequency of usage of AGG in proteobacteria
Nº of genomes
All genes
Frequency of AGG usage
AGG usage
Nº of genomes with $TsaB_1$
Nº of genomes without $TsaB_1$
With $TsaB_1$
Without $TsaB_1$
p < 0.00005
Nº of genomes with $TsaB_2$
Nº of genomes without $TsaB_2$
With $TsaB_2$
Without $TsaB_2$
p < 0.00005
Nº of genomes with $TsaC_1$
Nº of genomes without $TsaC_1$
With $TsaC_1$
Without $TsaC_1$
p < 0.00005
Nº of genomes with $TsaC_2$
Nº of genomes without $TsaC_2$
With $TsaC_2$
Without $TsaC_2$
Nº of genomes with $TsaD_1$
Nº of genomes without $TsaD_1$
With $TsaD_1$
Without $TsaD_1$
Nº of genomes with $TsaD_2$
Nº of genomes without $TsaD_2$
With $TsaD_2$
Without $TsaD_2$
Nº of genomes with $TsaD_3$
Nº of genomes without $TsaD_3$
With $TsaD_3$
Without $TsaD_3$
Nº of genomes with $TsaE_1$
Nº of genomes without $TsaE_1$
With $TsaE_1$
Without $TsaE_1$
p < 0.00005
Nº of genomes with $TsaE_2$
Nº of genomes without $TsaE_2$
With $TsaE_2$
Without $TsaE_2$
p < 0.00005
Nº of genomes with $TsaE_3$
Nº of genomes without $TsaE_3$
With $TsaE_3$
Without $TsaE_3$
p < 0.00005

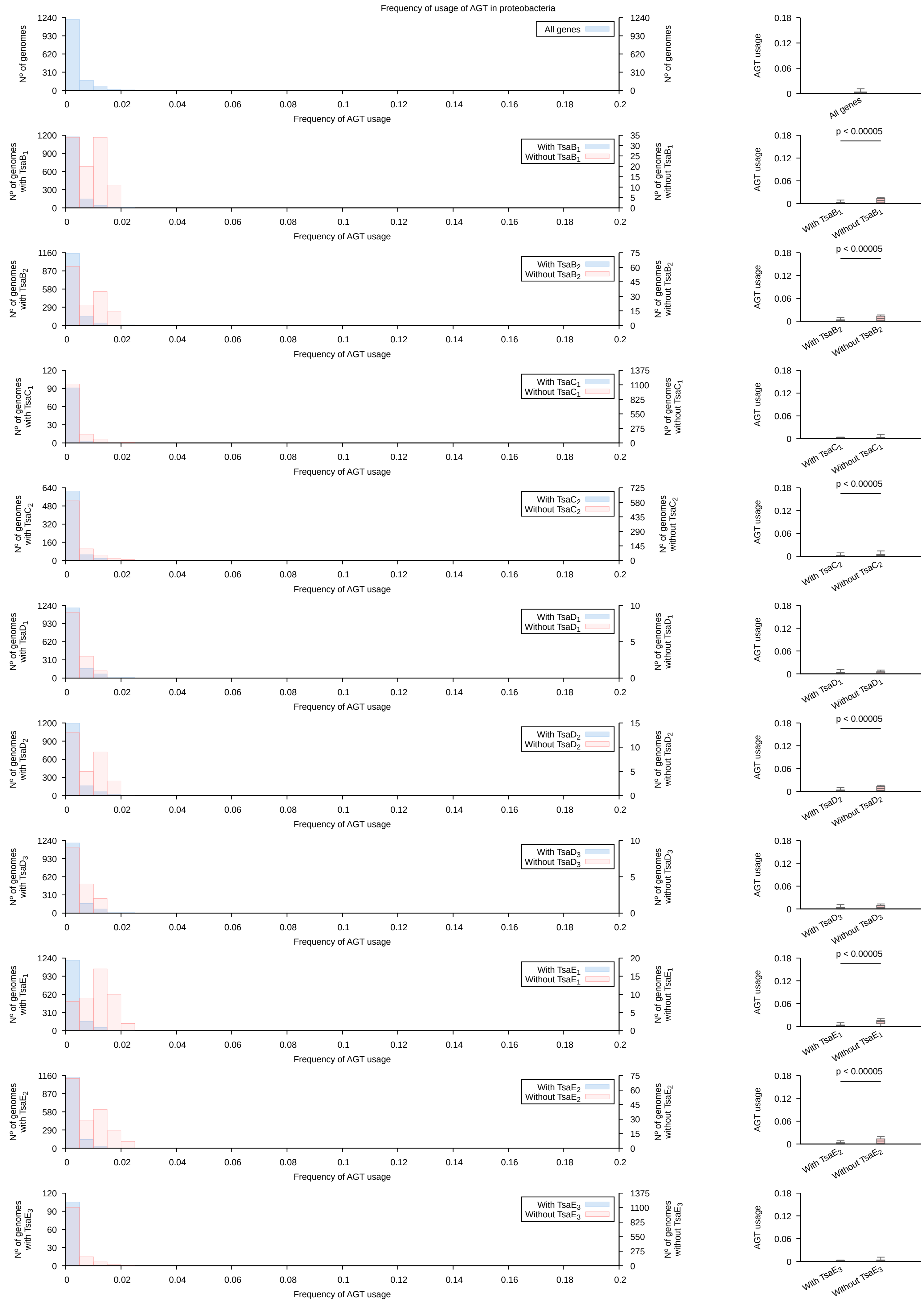
Frequency of usage of AGT in proteobacteria
Nº of genomes
All genes
Frequency of AGT usage
AGT usage
With TsaB1
Without TsaB1
Nº of genomes with TsaB1
Nº of genomes without TsaB1
p < 0.00005
With TsaB2
Without TsaB2
Nº of genomes with TsaB2
Nº of genomes without TsaB2
With TsaC1
Without TsaC1
Nº of genomes with TsaC1
Nº of genomes without TsaC1
With TsaC2
Without TsaC2
Nº of genomes with TsaC2
Nº of genomes without TsaC2
With TsaD1
Without TsaD1
Nº of genomes with TsaD1
Nº of genomes without TsaD1
With TsaD2
Without TsaD2
Nº of genomes with TsaD2
Nº of genomes without TsaD2
With TsaD3
Without TsaD3
Nº of genomes with TsaD3
Nº of genomes without TsaD3
With TsaE1
Without TsaE1
Nº of genomes with TsaE1
Nº of genomes without TsaE1
With TsaE2
Without TsaE2
Nº of genomes with TsaE2
Nº of genomes without TsaE2
With TsaE3
Without TsaE3
Nº of genomes with TsaE3
Nº of genomes without TsaE3

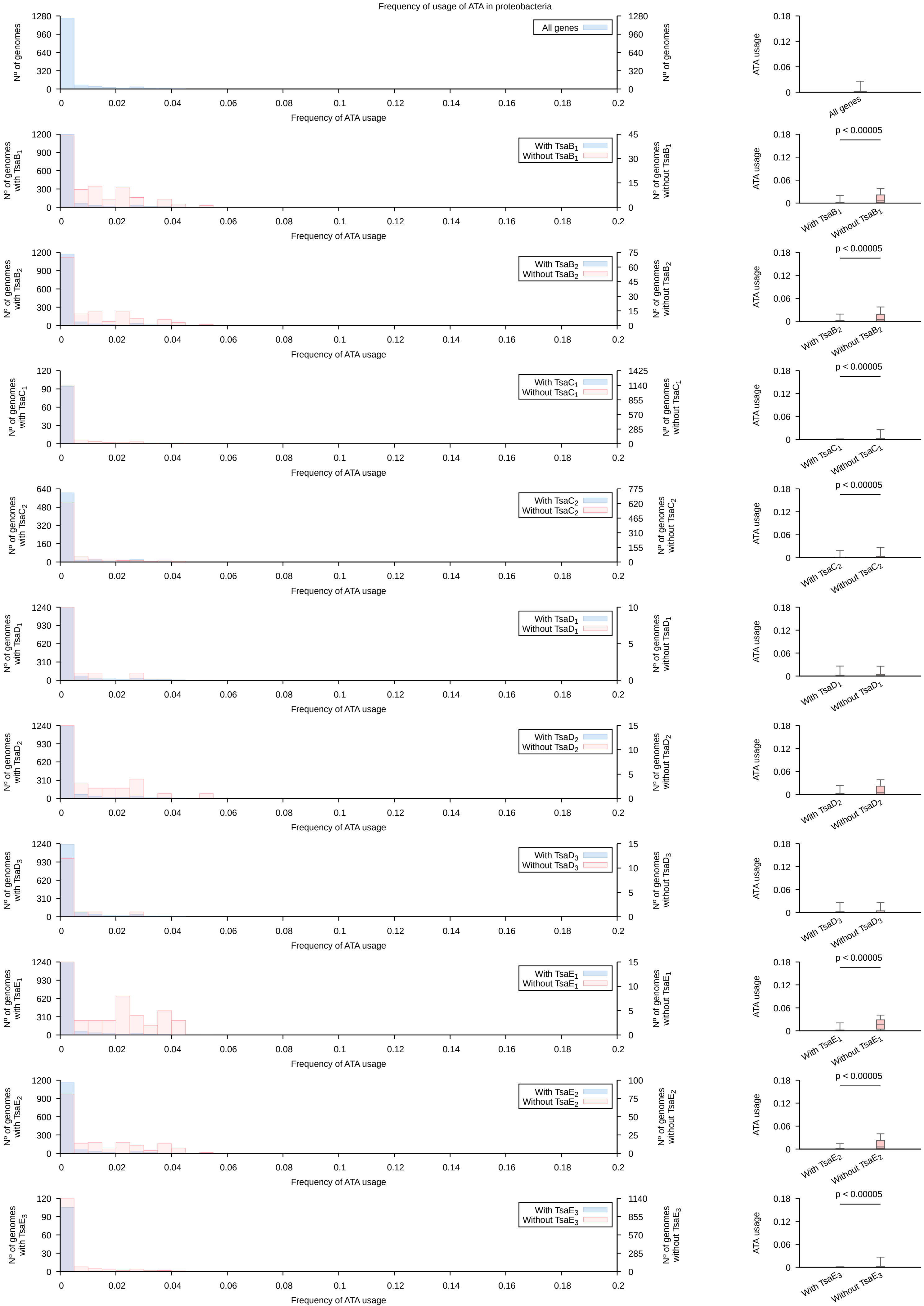

Frequency of usage of ATA in proteobacteria
Nº of genomes
All genes
Frequency of ATA usage
ATA usage
All genes
Nº of genomes with TsaB$_1$
Nº of genomes without TsaB$_1$
With TsaB$_1$
Without TsaB$_1$
p < 0.00005
Nº of genomes with TsaB$_2$
Nº of genomes without TsaB$_2$
With TsaB$_2$
Without TsaB$_2$
p < 0.00005
Nº of genomes with TsaC$_1$
Nº of genomes without TsaC$_1$
With TsaC$_1$
Without TsaC$_1$
p < 0.00005
Nº of genomes with TsaC$_2$
Nº of genomes without TsaC$_2$
With TsaC$_2$
Without TsaC$_2$
p < 0.00005
Nº of genomes with TsaD$_1$
Nº of genomes without TsaD$_1$
With TsaD$_1$
Without TsaD$_1$
Nº of genomes with TsaD$_2$
Nº of genomes without TsaD$_2$
With TsaD$_2$
Without TsaD$_2$
Nº of genomes with TsaD$_3$
Nº of genomes without TsaD$_3$
With TsaD$_3$
Without TsaD$_3$
Nº of genomes with TsaE$_1$
Nº of genomes without TsaE$_1$
With TsaE$_1$
Without TsaE$_1$
p < 0.00005
Nº of genomes with TsaE$_2$
Nº of genomes without TsaE$_2$
With TsaE$_2$
Without TsaE$_2$
p < 0.00005
Nº of genomes with TsaE$_3$
Nº of genomes without TsaE$_3$
With TsaE$_3$
Without TsaE$_3$
p < 0.00005

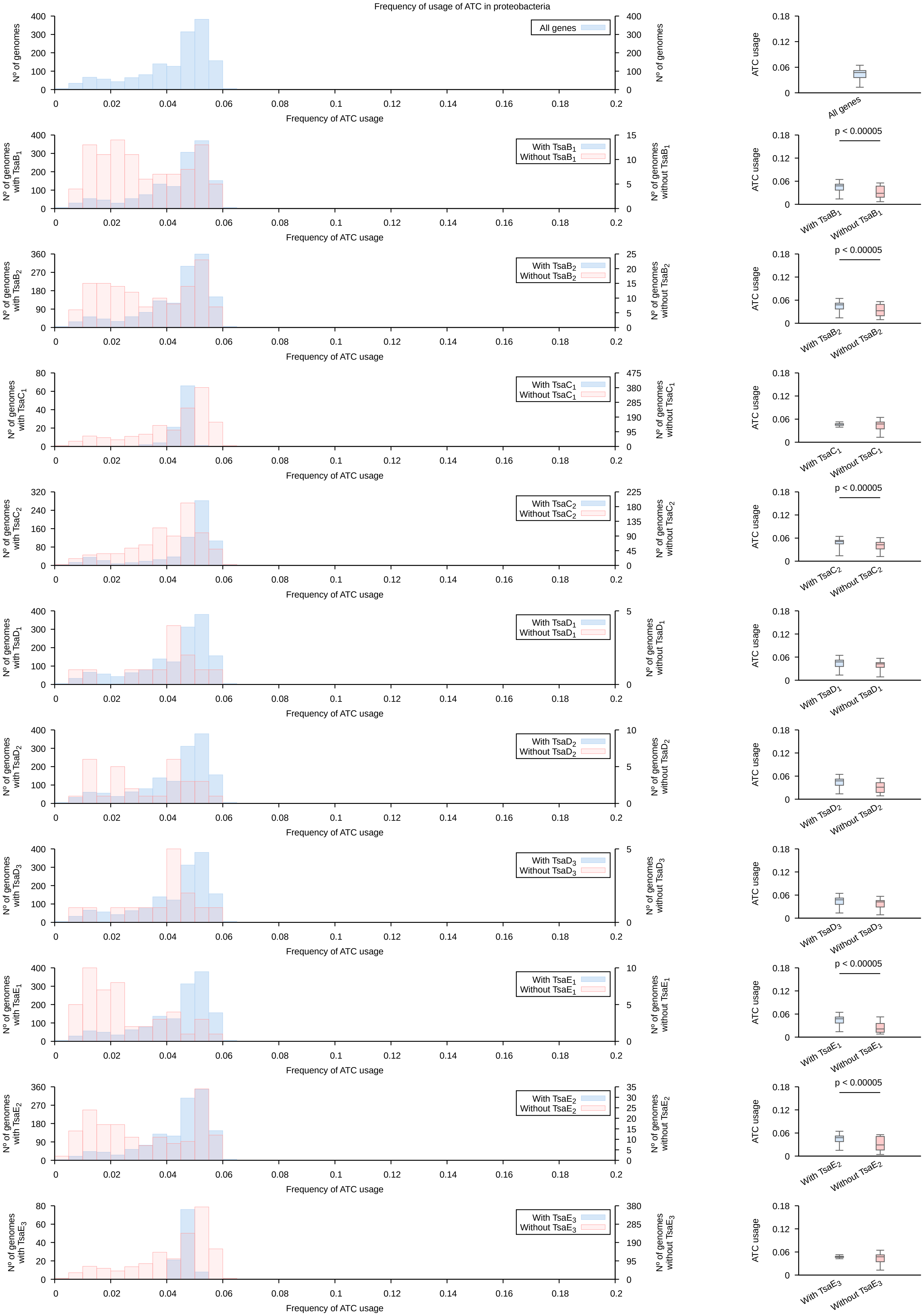

Frequency of usage of ATC in proteobacteria
All genes
Nº of genomes
Frequency of ATC usage
ATC usage
With TsaB$_1$
Without TsaB$_1$
Nº of genomes with TsaB$_1$
Nº of genomes without TsaB$_1$
p < 0.00005
With TsaB$_2$
Without TsaB$_2$
Nº of genomes with TsaB$_2$
Nº of genomes without TsaB$_2$
p < 0.00005
With TsaC$_1$
Without TsaC$_1$
Nº of genomes with TsaC$_1$
Nº of genomes without TsaC$_1$
With TsaC$_2$
Without TsaC$_2$
Nº of genomes with TsaC$_2$
Nº of genomes without TsaC$_2$
p < 0.00005
With TsaD$_1$
Without TsaD$_1$
Nº of genomes with TsaD$_1$
Nº of genomes without TsaD$_1$
With TsaD$_2$
Without TsaD$_2$
Nº of genomes with TsaD$_2$
Nº of genomes without TsaD$_2$
With TsaD$_3$
Without TsaD$_3$
Nº of genomes with TsaD$_3$
Nº of genomes without TsaD$_3$
With TsaE$_1$
Without TsaE$_1$
Nº of genomes with TsaE$_1$
Nº of genomes without TsaE$_1$
p < 0.00005
With TsaE$_2$
Without TsaE$_2$
Nº of genomes with TsaE$_2$
Nº of genomes without TsaE$_2$
p < 0.00005
With TsaE$_3$
Without TsaE$_3$
Nº of genomes with TsaE$_3$
Nº of genomes without TsaE$_3$

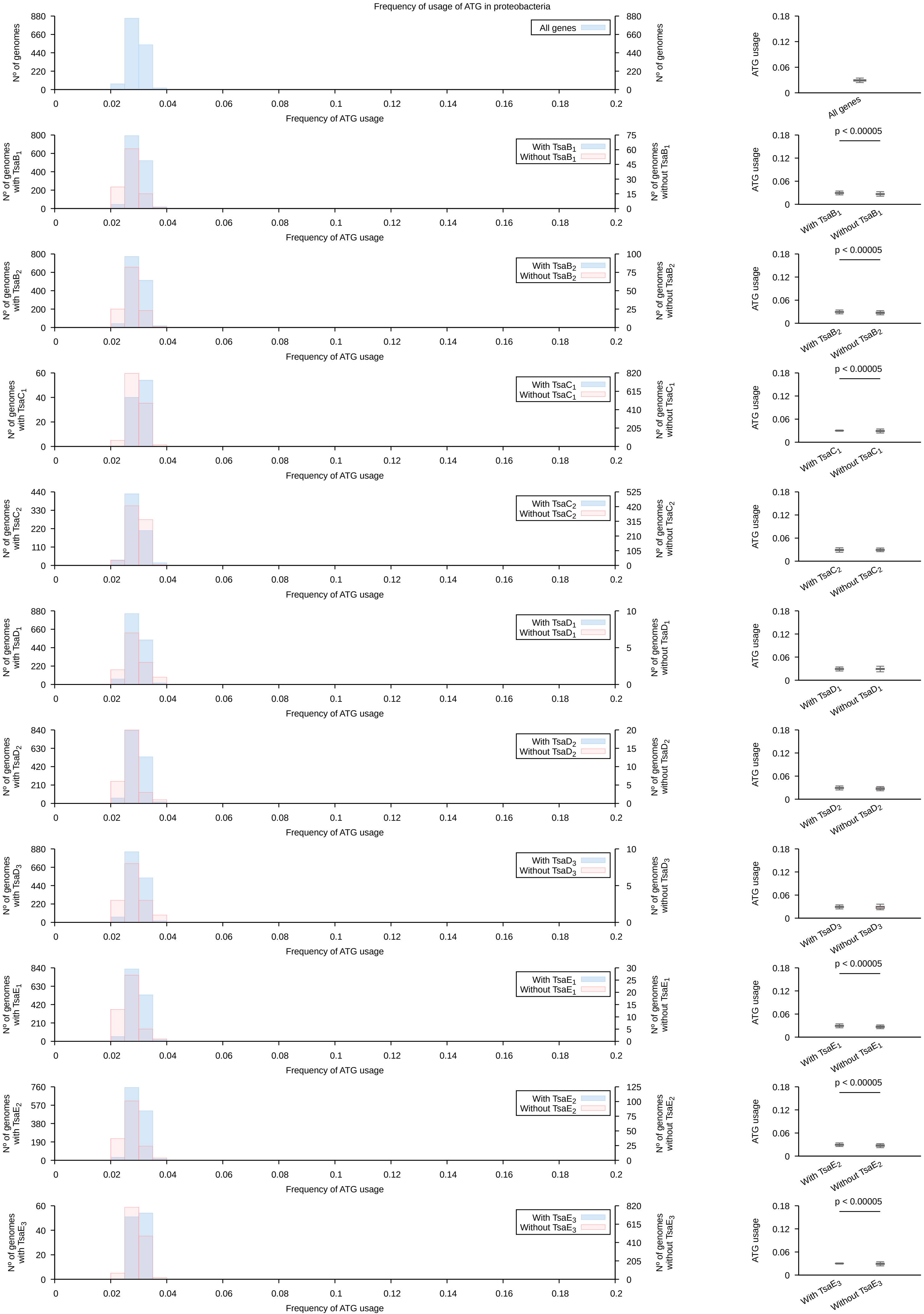

Frequency of usage of ATG in proteobacteria
All genes
Nº of genomes
Frequency of ATG usage
ATG usage
All genes
With TsaB$_1$
Without TsaB$_1$
Nº of genomes with TsaB$_1$
Nº of genomes without TsaB$_1$
p < 0.00005
With TsaB$_2$
Without TsaB$_2$
Nº of genomes with TsaB$_2$
Nº of genomes without TsaB$_2$
p < 0.00005
With TsaC$_1$
Without TsaC$_1$
Nº of genomes with TsaC$_1$
Nº of genomes without TsaC$_1$
p < 0.00005
With TsaC$_2$
Without TsaC$_2$
Nº of genomes with TsaC$_2$
Nº of genomes without TsaC$_2$
With TsaD$_1$
Without TsaD$_1$
Nº of genomes with TsaD$_1$
Nº of genomes without TsaD$_1$
With TsaD$_2$
Without TsaD$_2$
Nº of genomes with TsaD$_2$
Nº of genomes without TsaD$_2$
With TsaD$_3$
Without TsaD$_3$
Nº of genomes with TsaD$_3$
Nº of genomes without TsaD$_3$
With TsaE$_1$
Without TsaE$_1$
Nº of genomes with TsaE$_1$
Nº of genomes without TsaE$_1$
p < 0.00005
With TsaE$_2$
Without TsaE$_2$
Nº of genomes with TsaE$_2$
Nº of genomes without TsaE$_2$
p < 0.00005
With TsaE$_3$
Without TsaE$_3$
Nº of genomes with TsaE$_3$
Nº of genomes without TsaE$_3$
p < 0.00005

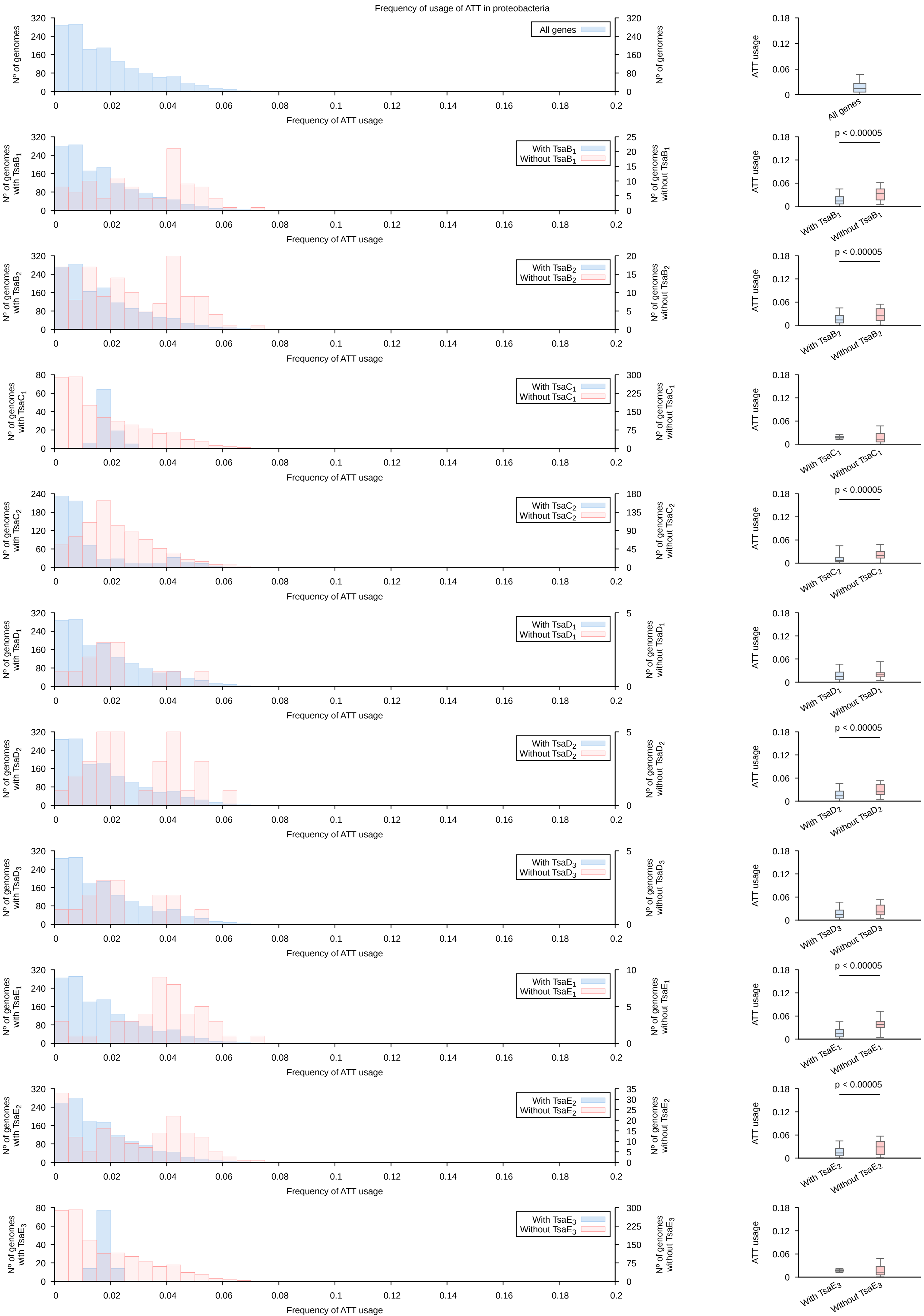
Frequency of usage of ATT in proteobacteria
Nº of genomes
All genes
Frequency of ATT usage
ATT usage
Nº of genomes with $TsaB_1$
Nº of genomes without $TsaB_1$
With $TsaB_1$
Without $TsaB_1$
p < 0.00005
Nº of genomes with $TsaB_2$
Nº of genomes without $TsaB_2$
With $TsaB_2$
Without $TsaB_2$
p < 0.00005
Nº of genomes with $TsaC_1$
Nº of genomes without $TsaC_1$
With $TsaC_1$
Without $TsaC_1$
Nº of genomes with $TsaC_2$
Nº of genomes without $TsaC_2$
With $TsaC_2$
Without $TsaC_2$
p < 0.00005
Nº of genomes with $TsaD_1$
Nº of genomes without $TsaD_1$
With $TsaD_1$
Without $TsaD_1$
Nº of genomes with $TsaD_2$
Nº of genomes without $TsaD_2$
With $TsaD_2$
Without $TsaD_2$
p < 0.00005
Nº of genomes with $TsaD_3$
Nº of genomes without $TsaD_3$
With $TsaD_3$
Without $TsaD_3$
Nº of genomes with $TsaE_1$
Nº of genomes without $TsaE_1$
With $TsaE_1$
Without $TsaE_1$
p < 0.00005
Nº of genomes with $TsaE_2$
Nº of genomes without $TsaE_2$
With $TsaE_2$
Without $TsaE_2$
p < 0.00005
Nº of genomes with $TsaE_3$
Nº of genomes without $TsaE_3$
With $TsaE_3$
Without $TsaE_3$

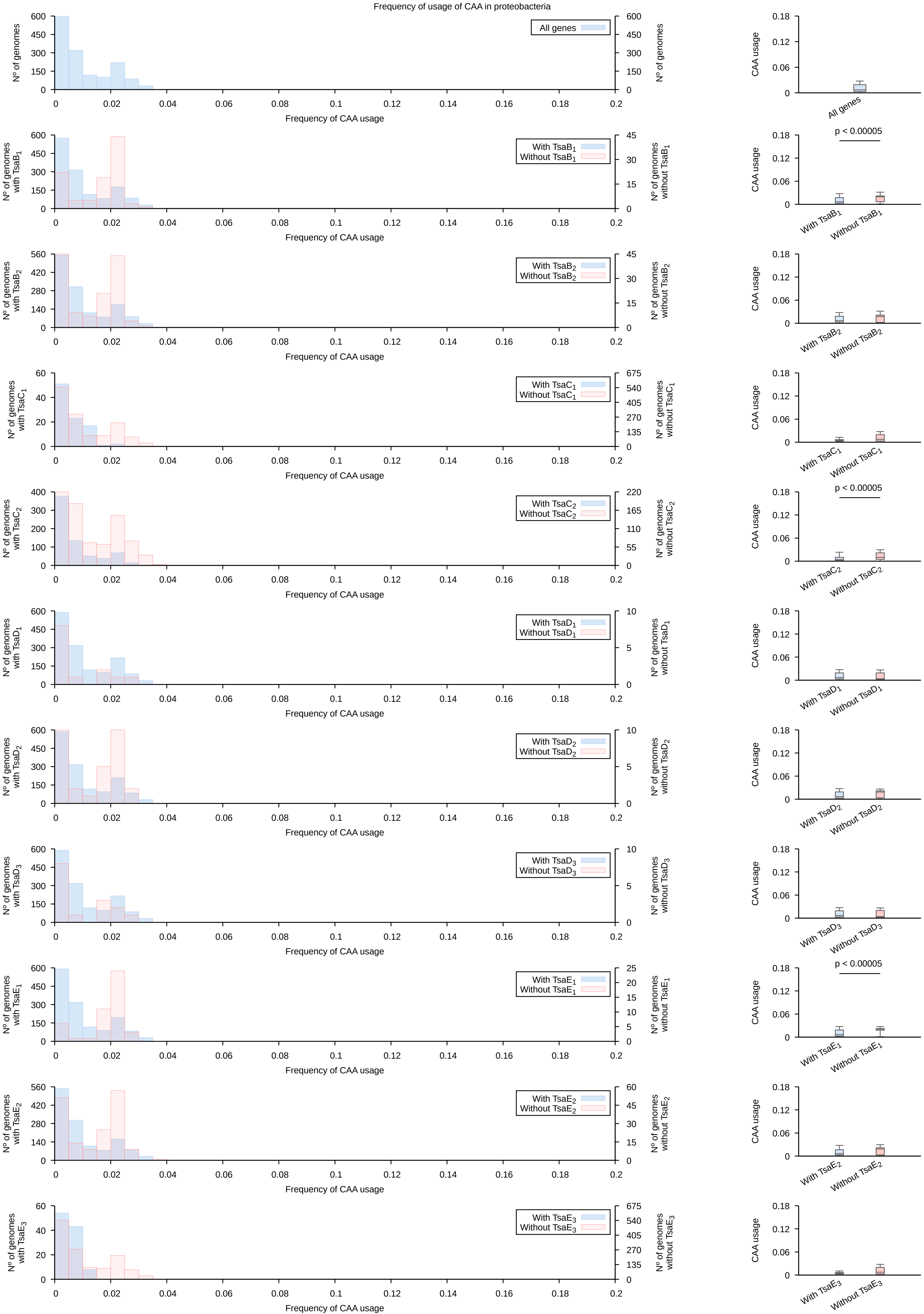

Frequency of usage of CAA in proteobacteria
Nº of genomes
All genes
Frequency of CAA usage
CAA usage
Nº of genomes with TsaB1
Nº of genomes without TsaB1
With TsaB1
Without TsaB1
p < 0.00005
Nº of genomes with TsaB2
Nº of genomes without TsaB2
With TsaB2
Without TsaB2
Nº of genomes with TsaC1
Nº of genomes without TsaC1
With TsaC1
Without TsaC1
Nº of genomes with TsaC2
Nº of genomes without TsaC2
With TsaC2
Without TsaC2
p < 0.00005
Nº of genomes with TsaD1
Nº of genomes without TsaD1
With TsaD1
Without TsaD1
Nº of genomes with TsaD2
Nº of genomes without TsaD2
With TsaD2
Without TsaD2
Nº of genomes with TsaD3
Nº of genomes without TsaD3
With TsaD3
Without TsaD3
Nº of genomes with TsaE1
Nº of genomes without TsaE1
With TsaE1
Without TsaE1
p < 0.00005
Nº of genomes with TsaE2
Nº of genomes without TsaE2
With TsaE2
Without TsaE2
Nº of genomes with TsaE3
Nº of genomes without TsaE3
With TsaE3
Without TsaE3

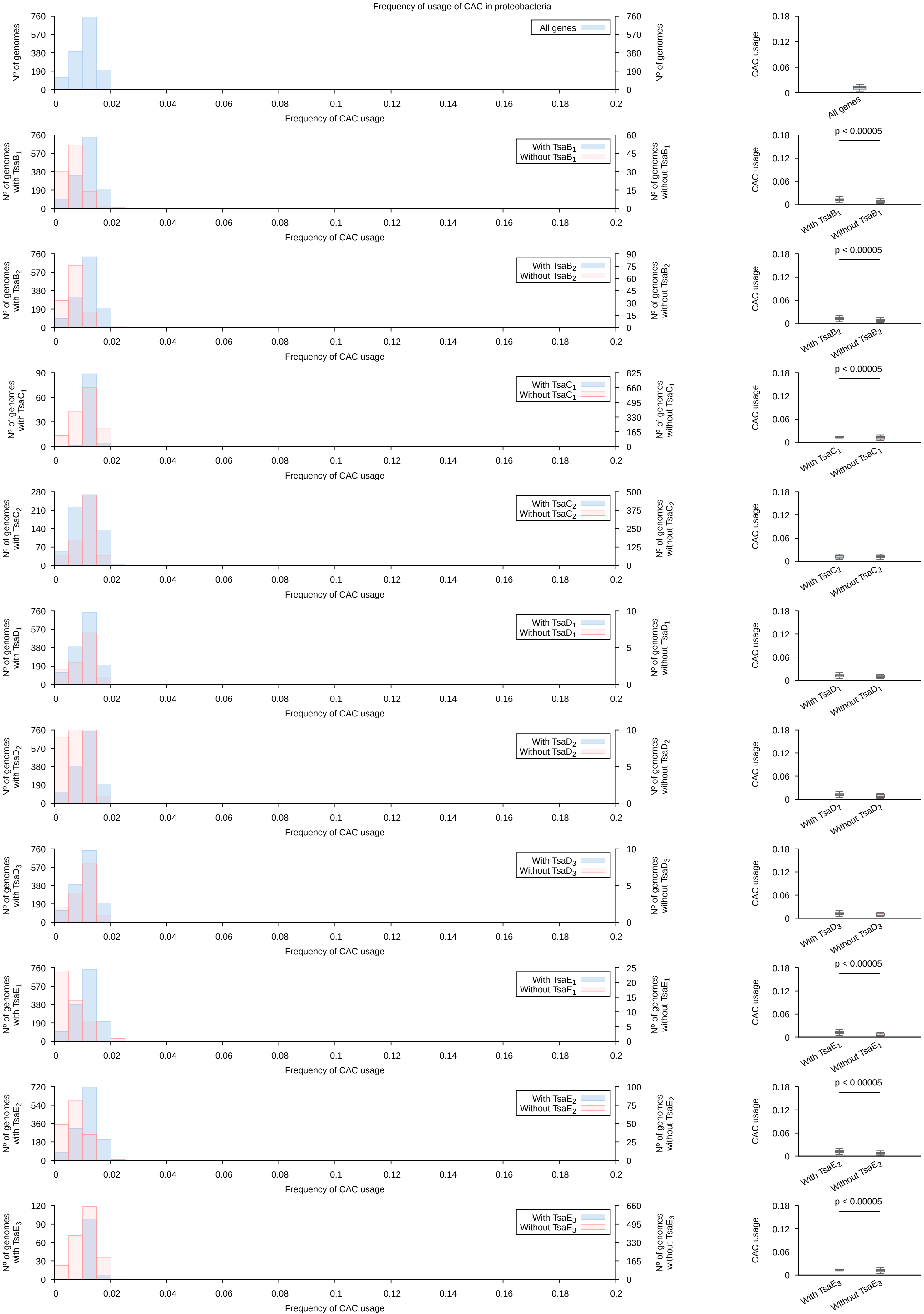
Frequency of usage of CAC in proteobacteria
Nº of genomes
Frequency of CAC usage
CAC usage
All genes
With TsaB$_1$
Without TsaB$_1$
With TsaB$_2$
Without TsaB$_2$
With TsaC$_1$
Without TsaC$_1$
With TsaC$_2$
Without TsaC$_2$
With TsaD$_1$
Without TsaD$_1$
With TsaD$_2$
Without TsaD$_2$
With TsaD$_3$
Without TsaD$_3$
With TsaE$_1$
Without TsaE$_1$
With TsaE$_2$
Without TsaE$_2$
With TsaE$_3$
Without TsaE$_3$
p < 0.00005

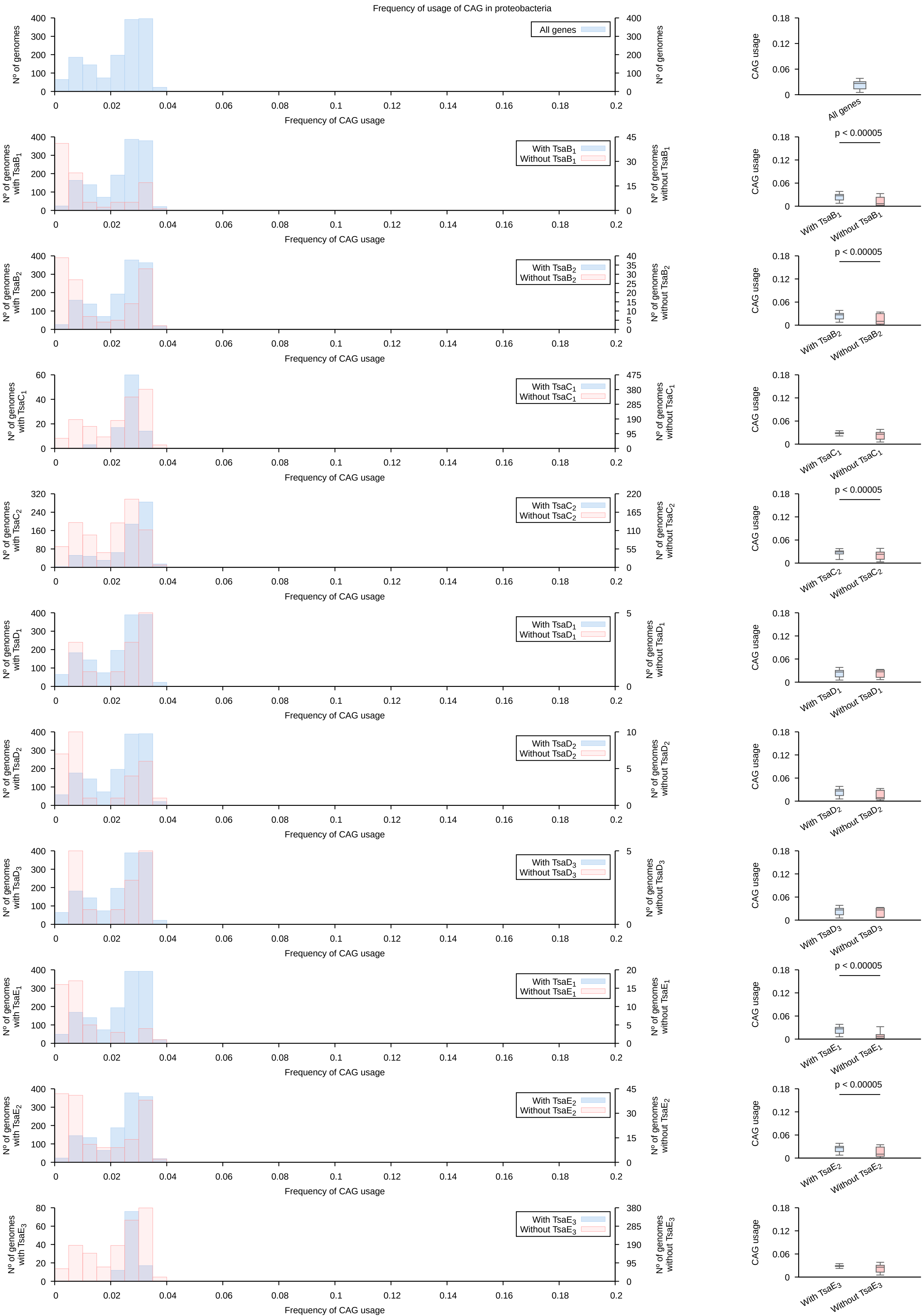
Frequency of usage of CAG in proteobacteria
Nº of genomes
All genes
Frequency of CAG usage
CAG usage
Nº of genomes with TsaB$_1$
Nº of genomes without TsaB$_1$
With TsaB$_1$
Without TsaB$_1$
p < 0.00005
Nº of genomes with TsaB$_2$
Nº of genomes without TsaB$_2$
With TsaB$_2$
Without TsaB$_2$
p < 0.00005
Nº of genomes with TsaC$_1$
Nº of genomes without TsaC$_1$
With TsaC$_1$
Without TsaC$_1$
Nº of genomes with TsaC$_2$
Nº of genomes without TsaC$_2$
With TsaC$_2$
Without TsaC$_2$
p < 0.00005
Nº of genomes with TsaD$_1$
Nº of genomes without TsaD$_1$
With TsaD$_1$
Without TsaD$_1$
Nº of genomes with TsaD$_2$
Nº of genomes without TsaD$_2$
With TsaD$_2$
Without TsaD$_2$
Nº of genomes with TsaD$_3$
Nº of genomes without TsaD$_3$
With TsaD$_3$
Without TsaD$_3$
Nº of genomes with TsaE$_1$
Nº of genomes without TsaE$_1$
With TsaE$_1$
Without TsaE$_1$
p < 0.00005
Nº of genomes with TsaE$_2$
Nº of genomes without TsaE$_2$
With TsaE$_2$
Without TsaE$_2$
p < 0.00005
Nº of genomes with TsaE$_3$
Nº of genomes without TsaE$_3$
With TsaE$_3$
Without TsaE$_3$

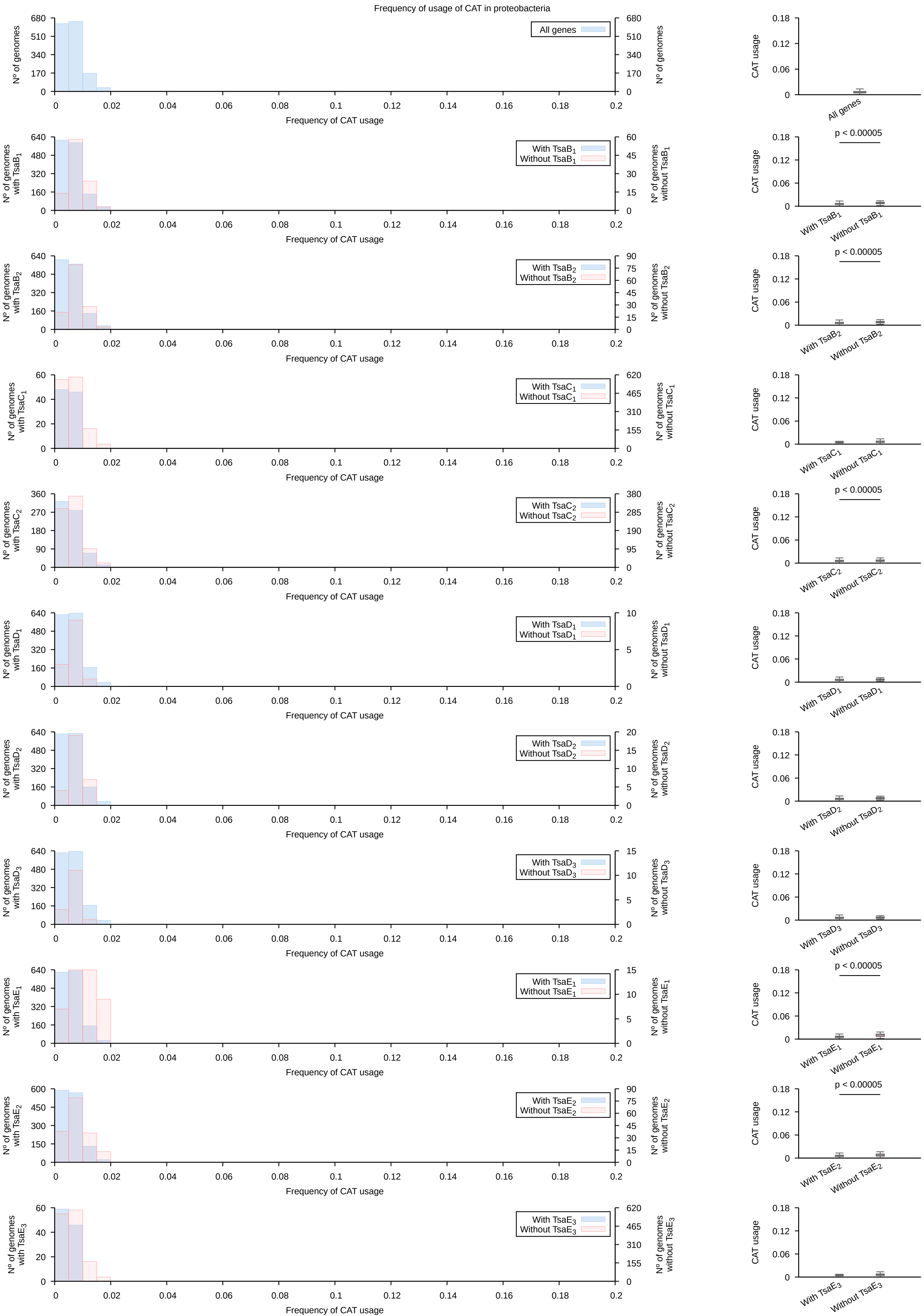
Frequency of usage of CAT in proteobacteria
All genes
Nº of genomes
Frequency of CAT usage
CAT usage
With TsaB$_1$
Without TsaB$_1$
Nº of genomes with TsaB$_1$
Nº of genomes without TsaB$_1$
p < 0.00005
With TsaB$_2$
Without TsaB$_2$
Nº of genomes with TsaB$_2$
Nº of genomes without TsaB$_2$
p < 0.00005
With TsaC$_1$
Without TsaC$_1$
Nº of genomes with TsaC$_1$
Nº of genomes without TsaC$_1$
With TsaC$_2$
Without TsaC$_2$
Nº of genomes with TsaC$_2$
Nº of genomes without TsaC$_2$
p < 0.00005
With TsaD$_1$
Without TsaD$_1$
Nº of genomes with TsaD$_1$
Nº of genomes without TsaD$_1$
With TsaD$_2$
Without TsaD$_2$
Nº of genomes with TsaD$_2$
Nº of genomes without TsaD$_2$
With TsaD$_3$
Without TsaD$_3$
Nº of genomes with TsaD$_3$
Nº of genomes without TsaD$_3$
With TsaE$_1$
Without TsaE$_1$
Nº of genomes with TsaE$_1$
Nº of genomes without TsaE$_1$
p < 0.00005
With TsaE$_2$
Without TsaE$_2$
Nº of genomes with TsaE$_2$
Nº of genomes without TsaE$_2$
p < 0.00005
With TsaE$_3$
Without TsaE$_3$
Nº of genomes with TsaE$_3$
Nº of genomes without TsaE$_3$

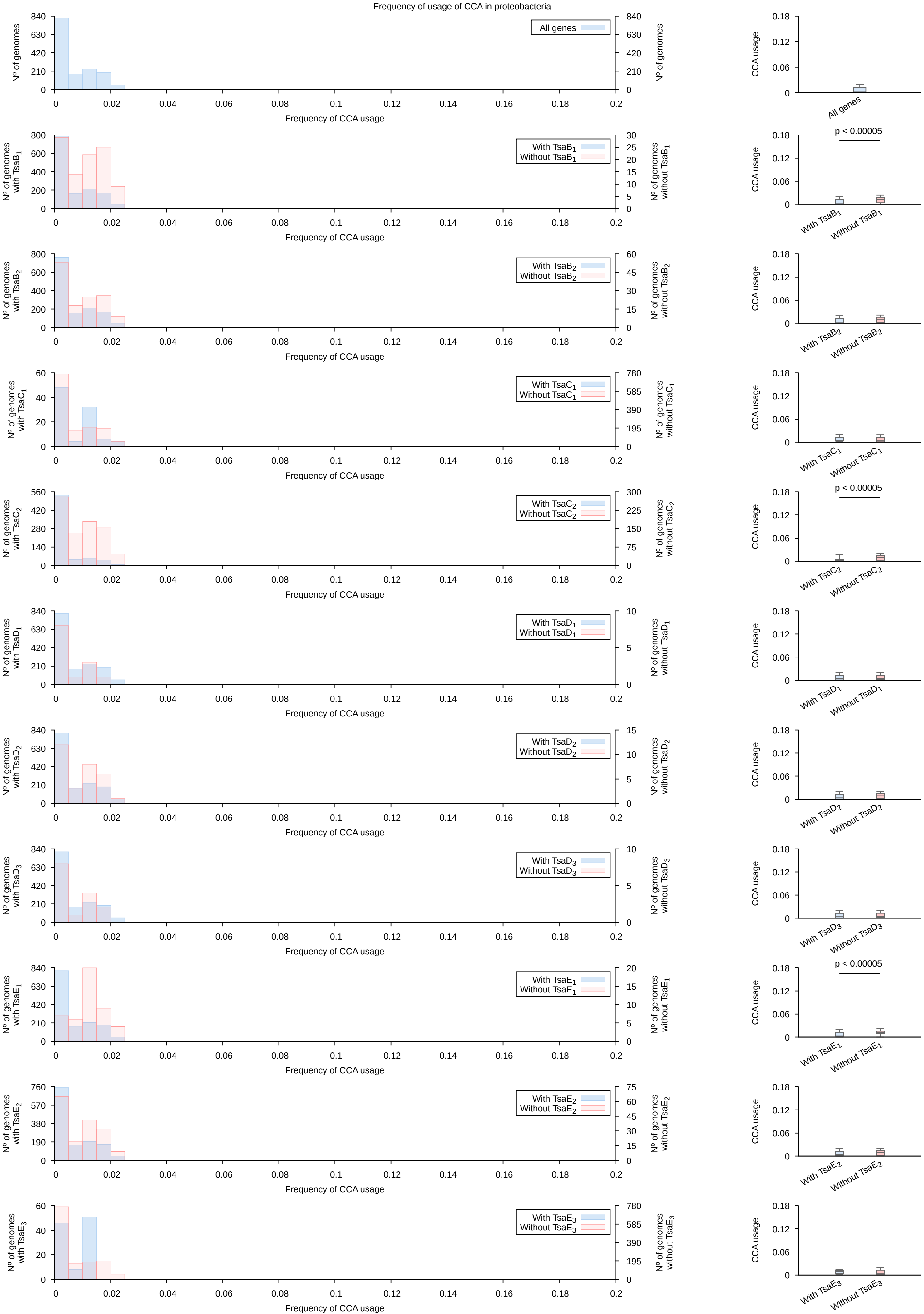

Frequency of usage of CCA in proteobacteria
All genes
Nº of genomes
Frequency of CCA usage
CCA usage
With TsaB1
Without TsaB1
Nº of genomes with TsaB1
Nº of genomes without TsaB1
p < 0.00005
With TsaB2
Without TsaB2
Nº of genomes with TsaB2
Nº of genomes without TsaB2
With TsaC1
Without TsaC1
Nº of genomes with TsaC1
Nº of genomes without TsaC1
With TsaC2
Without TsaC2
Nº of genomes with TsaC2
Nº of genomes without TsaC2
p < 0.00005
With TsaD1
Without TsaD1
Nº of genomes with TsaD1
Nº of genomes without TsaD1
With TsaD2
Without TsaD2
Nº of genomes with TsaD2
Nº of genomes without TsaD2
With TsaD3
Without TsaD3
Nº of genomes with TsaD3
Nº of genomes without TsaD3
With TsaE1
Without TsaE1
Nº of genomes with TsaE1
Nº of genomes without TsaE1
p < 0.00005
With TsaE2
Without TsaE2
Nº of genomes with TsaE2
Nº of genomes without TsaE2
With TsaE3
Without TsaE3
Nº of genomes with TsaE3
Nº of genomes without TsaE3

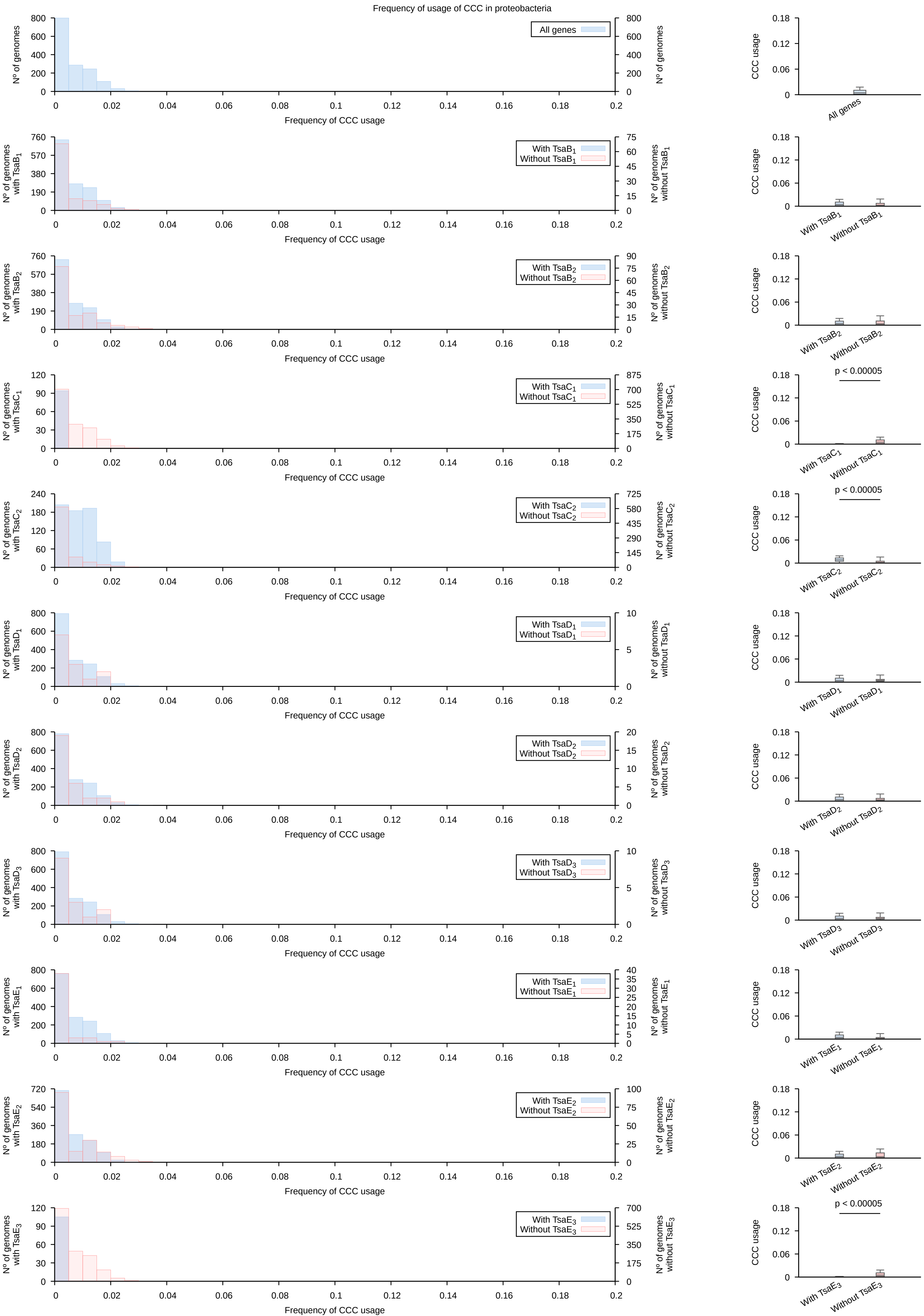
Frequency of usage of CCC in proteobacteria
All genes
Nº of genomes
Frequency of CCC usage
CCC usage
With TsaB$_1$
Without TsaB$_1$
Nº of genomes with TsaB$_1$
Nº of genomes without TsaB$_1$
With TsaB$_2$
Without TsaB$_2$
Nº of genomes with TsaB$_2$
Nº of genomes without TsaB$_2$
With TsaC$_1$
Without TsaC$_1$
Nº of genomes with TsaC$_1$
Nº of genomes without TsaC$_1$
p < 0.00005
With TsaC$_2$
Without TsaC$_2$
Nº of genomes with TsaC$_2$
Nº of genomes without TsaC$_2$
p < 0.00005
With TsaD$_1$
Without TsaD$_1$
Nº of genomes with TsaD$_1$
Nº of genomes without TsaD$_1$
With TsaD$_2$
Without TsaD$_2$
Nº of genomes with TsaD$_2$
Nº of genomes without TsaD$_2$
With TsaD$_3$
Without TsaD$_3$
Nº of genomes with TsaD$_3$
Nº of genomes without TsaD$_3$
With TsaE$_1$
Without TsaE$_1$
Nº of genomes with TsaE$_1$
Nº of genomes without TsaE$_1$
With TsaE$_2$
Without TsaE$_2$
Nº of genomes with TsaE$_2$
Nº of genomes without TsaE$_2$
With TsaE$_3$
Without TsaE$_3$
Nº of genomes with TsaE$_3$
Nº of genomes without TsaE$_3$
p < 0.00005

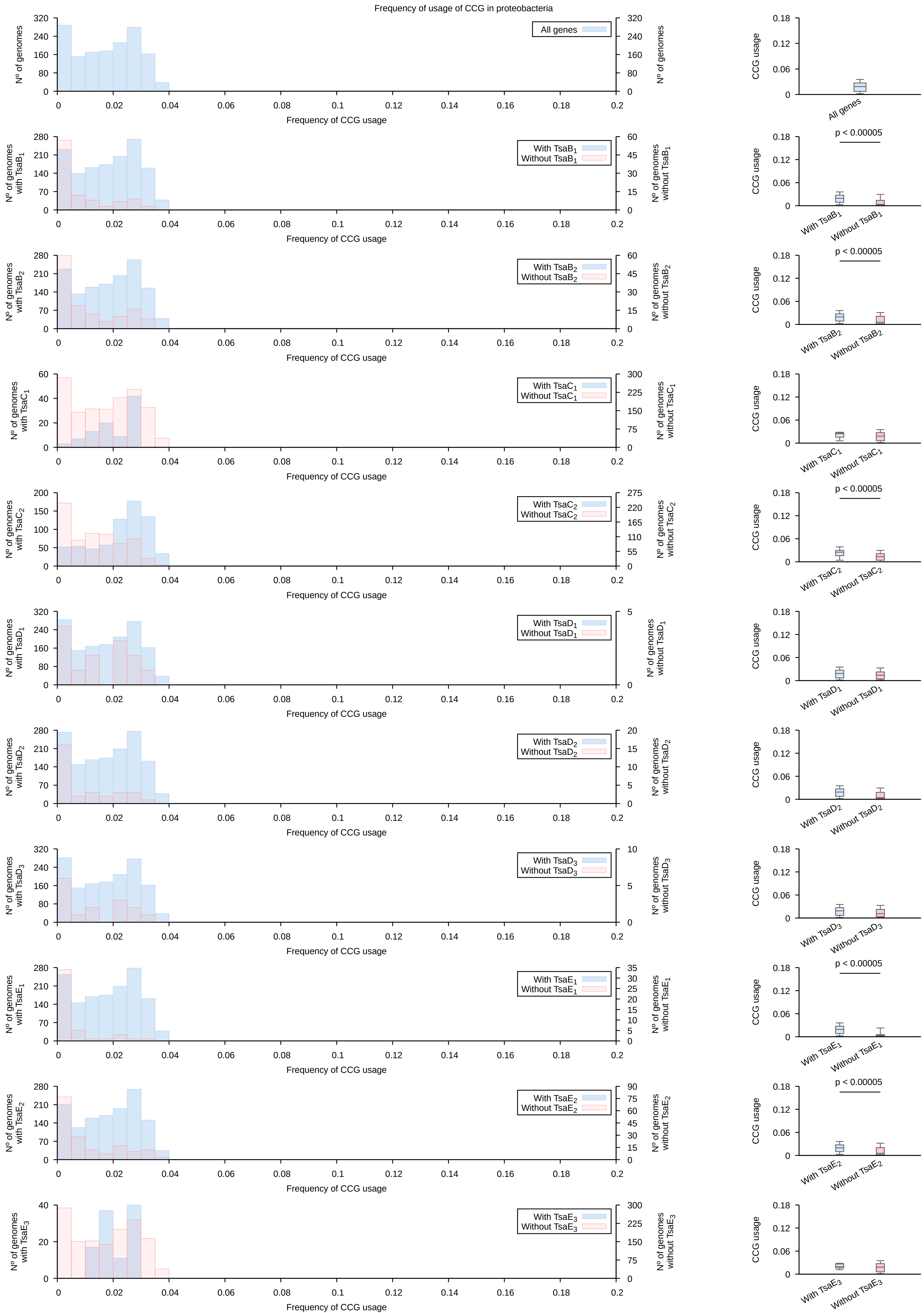

Frequency of usage of CCG in proteobacteria
Nº of genomes
All genes
Frequency of CCG usage
CCG usage
Nº of genomes with TsaB1
Nº of genomes without TsaB1
With TsaB1
Without TsaB1
p < 0.00005
Nº of genomes with TsaB2
Nº of genomes without TsaB2
With TsaB2
Without TsaB2
p < 0.00005
Nº of genomes with TsaC1
Nº of genomes without TsaC1
With TsaC1
Without TsaC1
Nº of genomes with TsaC2
Nº of genomes without TsaC2
With TsaC2
Without TsaC2
p < 0.00005
Nº of genomes with TsaD1
Nº of genomes without TsaD1
With TsaD1
Without TsaD1
Nº of genomes with TsaD2
Nº of genomes without TsaD2
With TsaD2
Without TsaD2
Nº of genomes with TsaD3
Nº of genomes without TsaD3
With TsaD3
Without TsaD3
Nº of genomes with TsaE1
Nº of genomes without TsaE1
With TsaE1
Without TsaE1
p < 0.00005
Nº of genomes with TsaE2
Nº of genomes without TsaE2
With TsaE2
Without TsaE2
p < 0.00005
Nº of genomes with TsaE3
Nº of genomes without TsaE3
With TsaE3
Without TsaE3

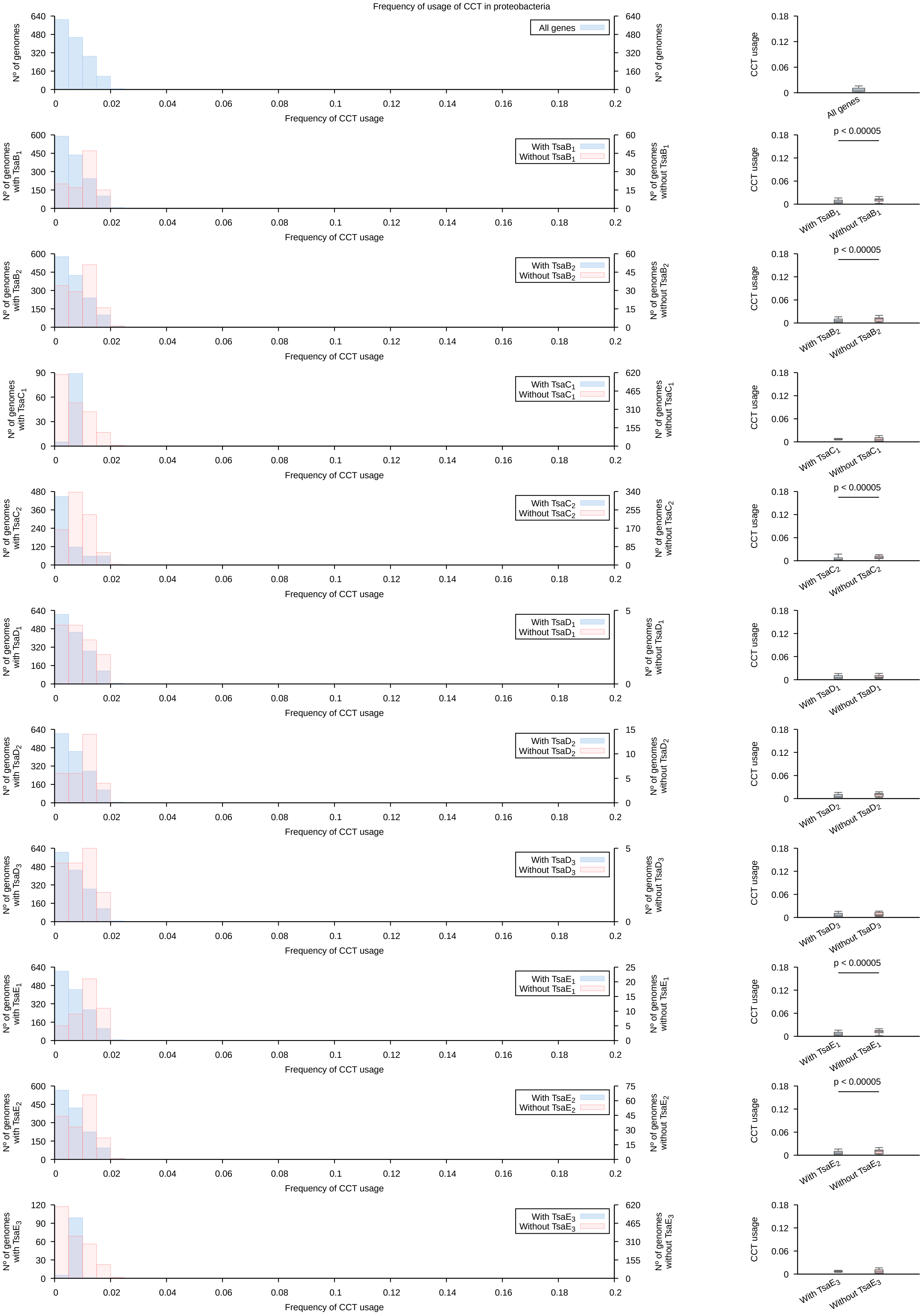
Frequency of usage of CCT in proteobacteria
Nº of genomes
Frequency of CCT usage
All genes
CCT usage
Nº of genomes with TsaB$_1$
Nº of genomes without TsaB$_1$
With TsaB$_1$
Without TsaB$_1$
p < 0.00005
Nº of genomes with TsaB$_2$
Nº of genomes without TsaB$_2$
With TsaB$_2$
Without TsaB$_2$
p < 0.00005
Nº of genomes with TsaC$_1$
Nº of genomes without TsaC$_1$
With TsaC$_1$
Without TsaC$_1$
Nº of genomes with TsaC$_2$
Nº of genomes without TsaC$_2$
With TsaC$_2$
Without TsaC$_2$
p < 0.00005
Nº of genomes with TsaD$_1$
Nº of genomes without TsaD$_1$
With TsaD$_1$
Without TsaD$_1$
Nº of genomes with TsaD$_2$
Nº of genomes without TsaD$_2$
With TsaD$_2$
Without TsaD$_2$
Nº of genomes with TsaD$_3$
Nº of genomes without TsaD$_3$
With TsaD$_3$
Without TsaD$_3$
Nº of genomes with TsaE$_1$
Nº of genomes without TsaE$_1$
With TsaE$_1$
Without TsaE$_1$
p < 0.00005
Nº of genomes with TsaE$_2$
Nº of genomes without TsaE$_2$
With TsaE$_2$
Without TsaE$_2$
p < 0.00005
Nº of genomes with TsaE$_3$
Nº of genomes without TsaE$_3$
With TsaE$_3$
Without TsaE$_3$

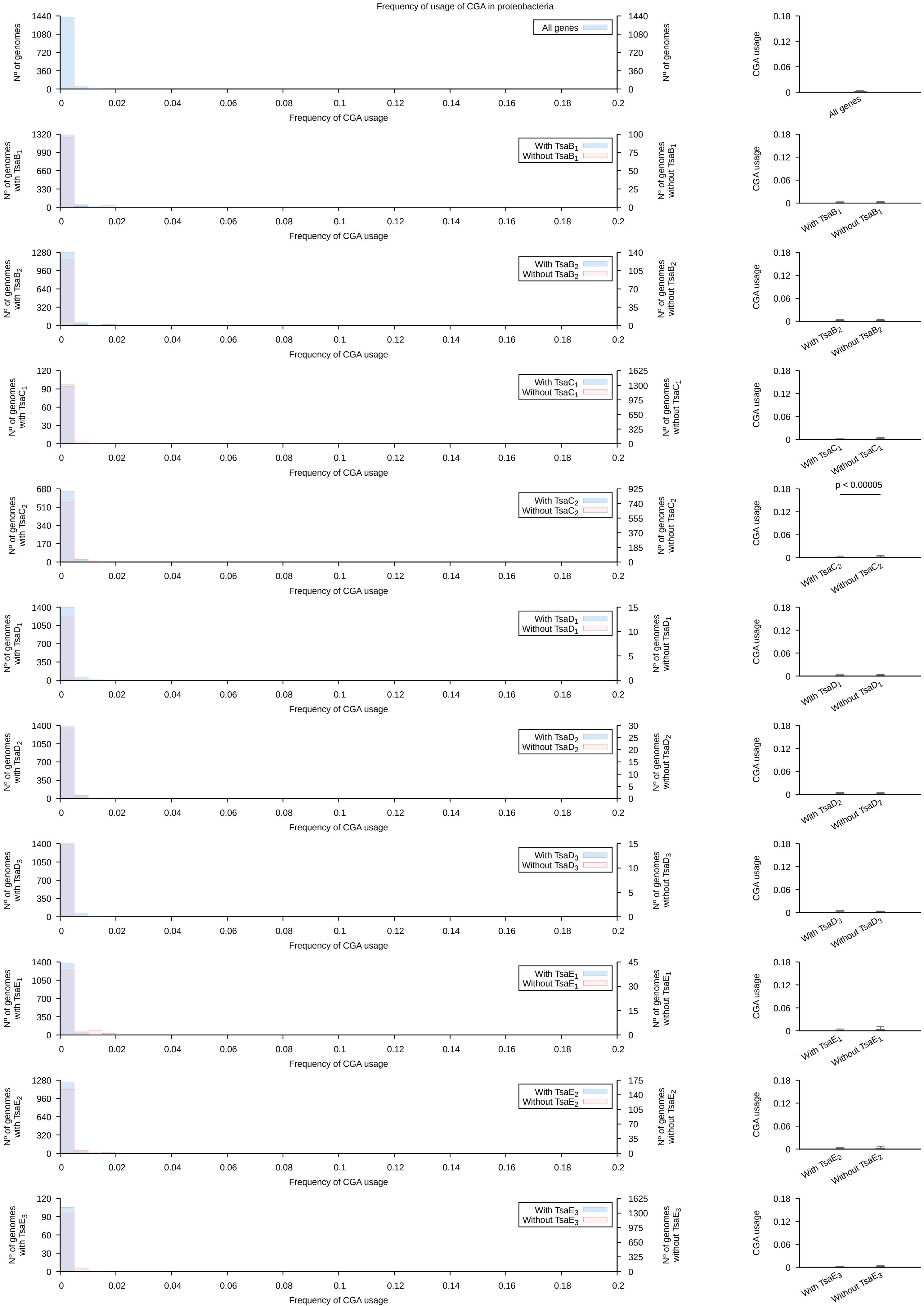
Frequency of usage of CGA in proteobacteria
All genes
Nº of genomes
Frequency of CGA usage
CGA usage
With TsaB$_1$
Without TsaB$_1$
Nº of genomes with TsaB$_1$
Nº of genomes without TsaB$_1$
With TsaB$_2$
Without TsaB$_2$
Nº of genomes with TsaB$_2$
Nº of genomes without TsaB$_2$
With TsaC$_1$
Without TsaC$_1$
Nº of genomes with TsaC$_1$
Nº of genomes without TsaC$_1$
With TsaC$_2$
Without TsaC$_2$
Nº of genomes with TsaC$_2$
Nº of genomes without TsaC$_2$
p < 0.00005
With TsaD$_1$
Without TsaD$_1$
Nº of genomes with TsaD$_1$
Nº of genomes without TsaD$_1$
With TsaD$_2$
Without TsaD$_2$
Nº of genomes with TsaD$_2$
Nº of genomes without TsaD$_2$
With TsaD$_3$
Without TsaD$_3$
Nº of genomes with TsaD$_3$
Nº of genomes without TsaD$_3$
With TsaE$_1$
Without TsaE$_1$
Nº of genomes with TsaE$_1$
Nº of genomes without TsaE$_1$
With TsaE$_2$
Without TsaE$_2$
Nº of genomes with TsaE$_2$
Nº of genomes without TsaE$_2$
With TsaE$_3$
Without TsaE$_3$
Nº of genomes with TsaE$_3$
Nº of genomes without TsaE$_3$

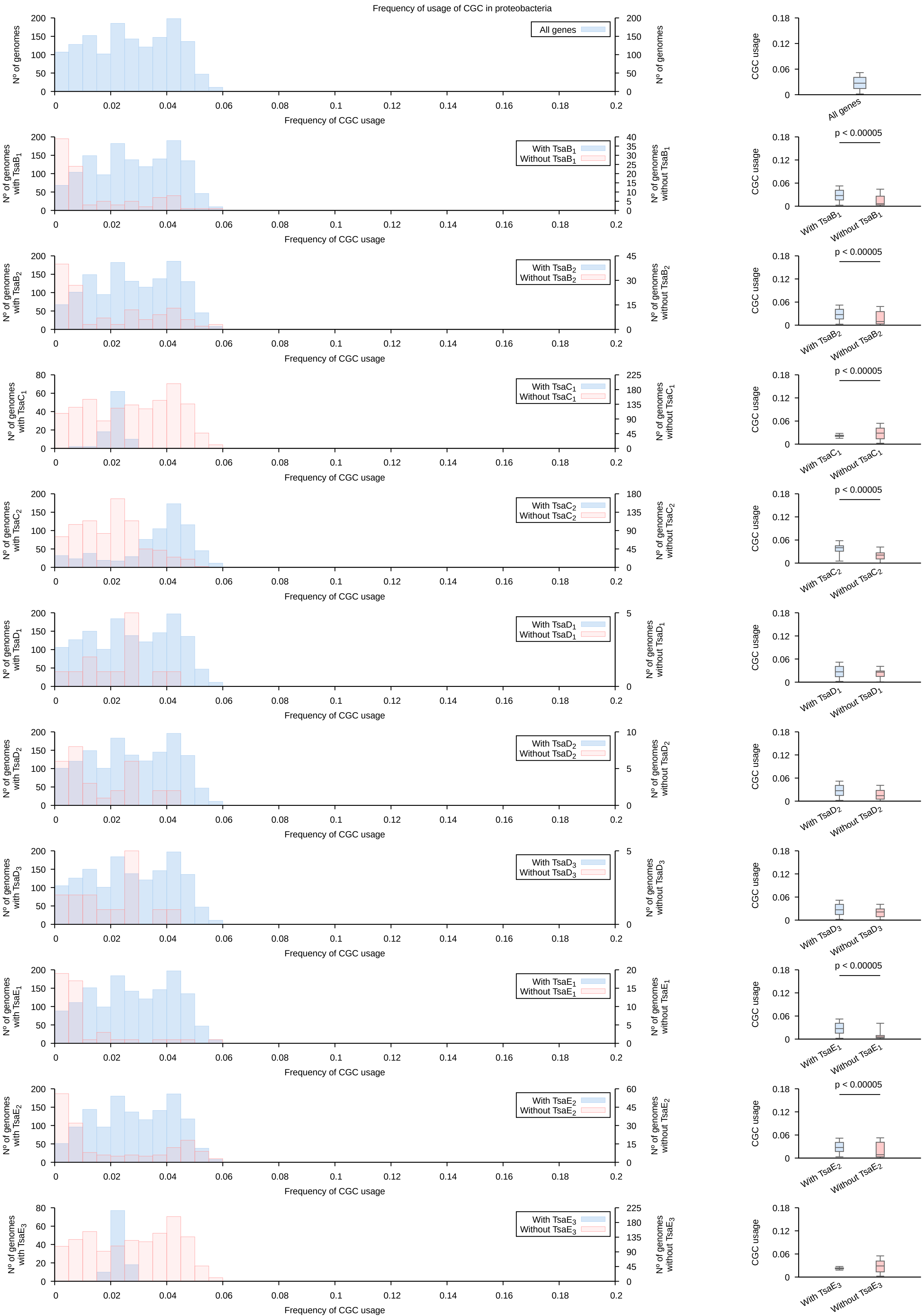
Frequency of usage of CGC in proteobacteria
All genes
Nº of genomes
Frequency of CGC usage
CGC usage
With TsaB1
Without TsaB1
Nº of genomes with TsaB1
Nº of genomes without TsaB1
p < 0.00005
With TsaB2
Without TsaB2
Nº of genomes with TsaB2
Nº of genomes without TsaB2
p < 0.00005
With TsaC1
Without TsaC1
Nº of genomes with TsaC1
Nº of genomes without TsaC1
p < 0.00005
With TsaC2
Without TsaC2
Nº of genomes with TsaC2
Nº of genomes without TsaC2
p < 0.00005
With TsaD1
Without TsaD1
Nº of genomes with TsaD1
Nº of genomes without TsaD1
With TsaD2
Without TsaD2
Nº of genomes with TsaD2
Nº of genomes without TsaD2
With TsaD3
Without TsaD3
Nº of genomes with TsaD3
Nº of genomes without TsaD3
With TsaE1
Without TsaE1
Nº of genomes with TsaE1
Nº of genomes without TsaE1
p < 0.00005
With TsaE2
Without TsaE2
Nº of genomes with TsaE2
Nº of genomes without TsaE2
p < 0.00005
With TsaE3
Without TsaE3
Nº of genomes with TsaE3
Nº of genomes without TsaE3

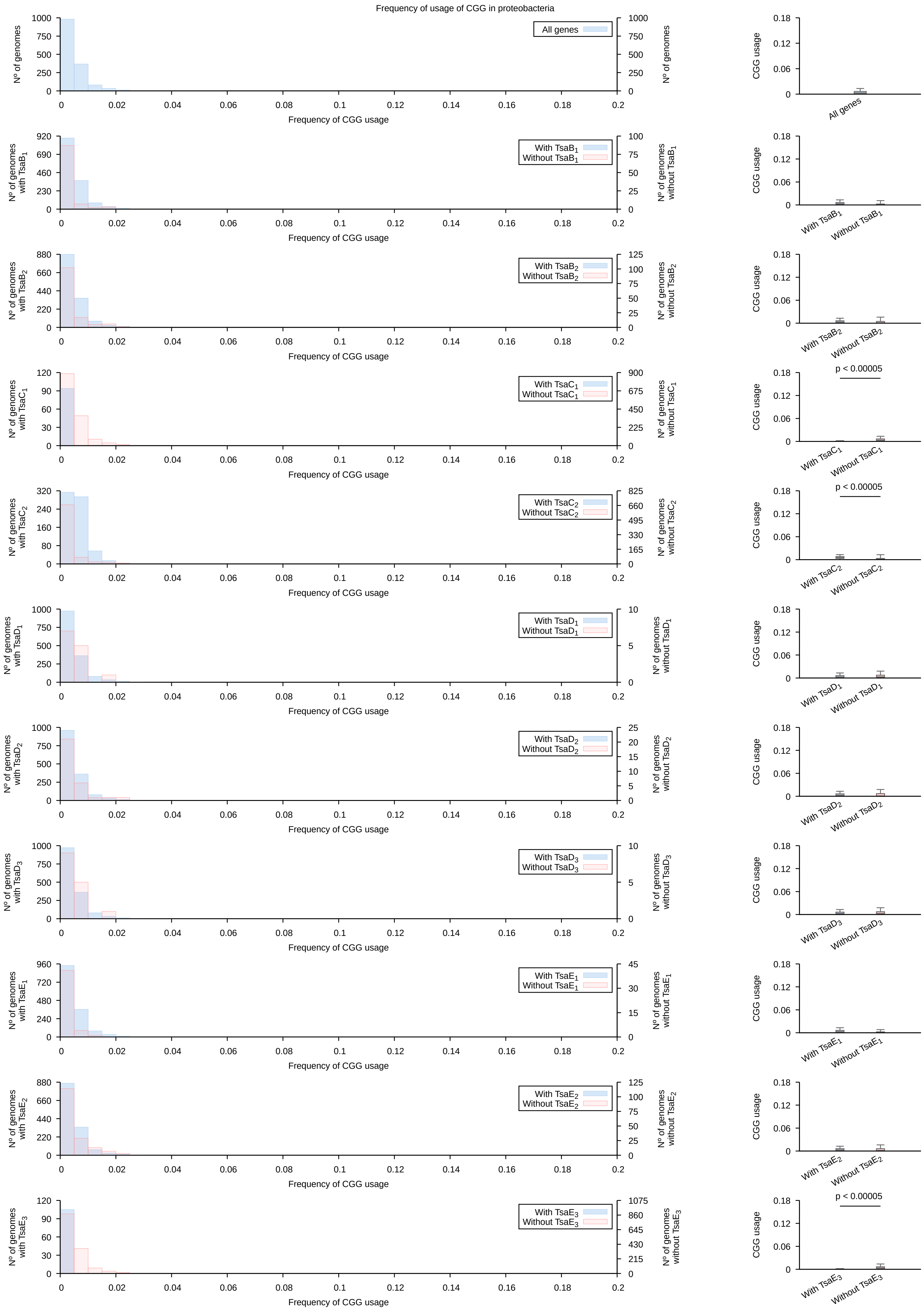

Frequency of usage of CGG in proteobacteria
All genes
Nº of genomes
Frequency of CGG usage
CGG usage
With TsaB$_1$
Without TsaB$_1$
Nº of genomes with TsaB$_1$
Nº of genomes without TsaB$_1$
With TsaB$_2$
Without TsaB$_2$
Nº of genomes with TsaB$_2$
Nº of genomes without TsaB$_2$
With TsaC$_1$
Without TsaC$_1$
Nº of genomes with TsaC$_1$
Nº of genomes without TsaC$_1$
p < 0.00005
With TsaC$_2$
Without TsaC$_2$
Nº of genomes with TsaC$_2$
Nº of genomes without TsaC$_2$
p < 0.00005
With TsaD$_1$
Without TsaD$_1$
Nº of genomes with TsaD$_1$
Nº of genomes without TsaD$_1$
With TsaD$_2$
Without TsaD$_2$
Nº of genomes with TsaD$_2$
Nº of genomes without TsaD$_2$
With TsaD$_3$
Without TsaD$_3$
Nº of genomes with TsaD$_3$
Nº of genomes without TsaD$_3$
With TsaE$_1$
Without TsaE$_1$
Nº of genomes with TsaE$_1$
Nº of genomes without TsaE$_1$
With TsaE$_2$
Without TsaE$_2$
Nº of genomes with TsaE$_2$
Nº of genomes without TsaE$_2$
With TsaE$_3$
Without TsaE$_3$
Nº of genomes with TsaE$_3$
Nº of genomes without TsaE$_3$
p < 0.00005

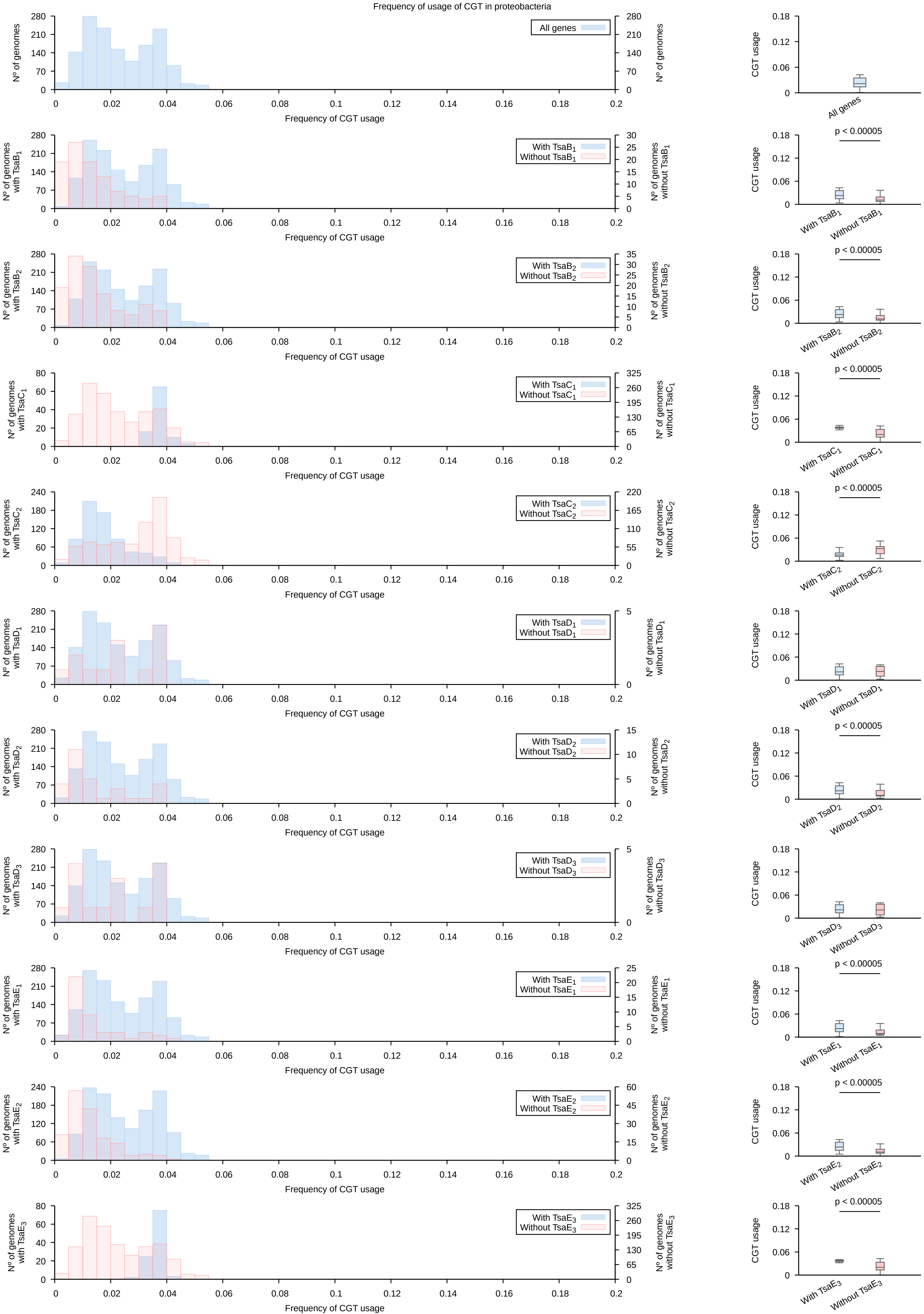
Frequency of usage of CGT in proteobacteria
All genes
With TsaB1
Without TsaB1
With TsaB2
Without TsaB2
With TsaC1
Without TsaC1
With TsaC2
Without TsaC2
With TsaD1
Without TsaD1
With TsaD2
Without TsaD2
With TsaD3
Without TsaD3
With TsaE1
Without TsaE1
With TsaE2
Without TsaE2
With TsaE3
Without TsaE3
Nº of genomes
Frequency of CGT usage
CGT usage
p < 0.00005

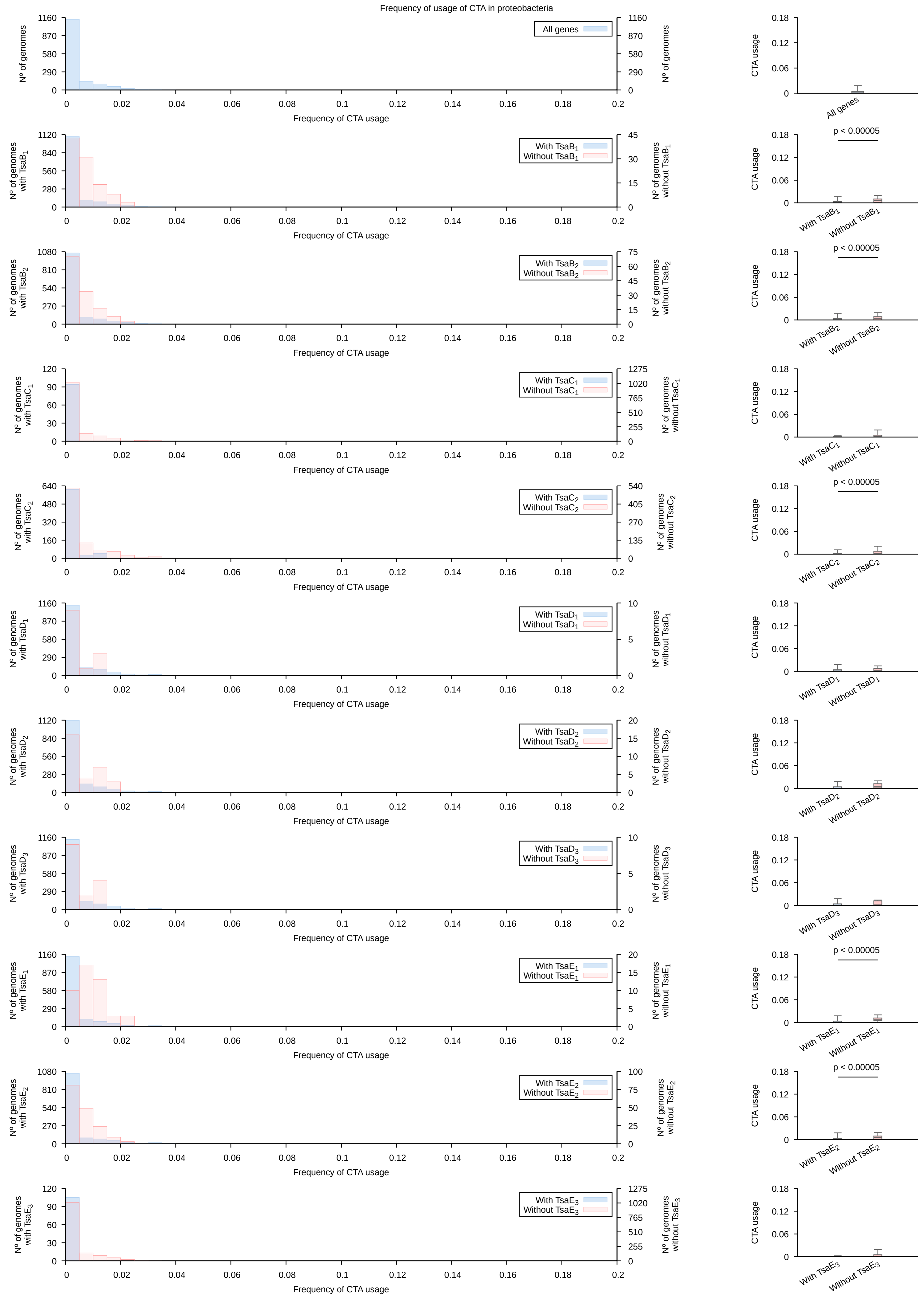
Frequency of usage of CTA in proteobacteria
All genes
Nº of genomes
Frequency of CTA usage
CTA usage
With TsaB1
Without TsaB1
With TsaB2
Without TsaB2
With TsaC1
Without TsaC1
With TsaC2
Without TsaC2
With TsaD1
Without TsaD1
With TsaD2
Without TsaD2
With TsaD3
Without TsaD3
With TsaE1
Without TsaE1
With TsaE2
Without TsaE2
With TsaE3
Without TsaE3
p < 0.00005

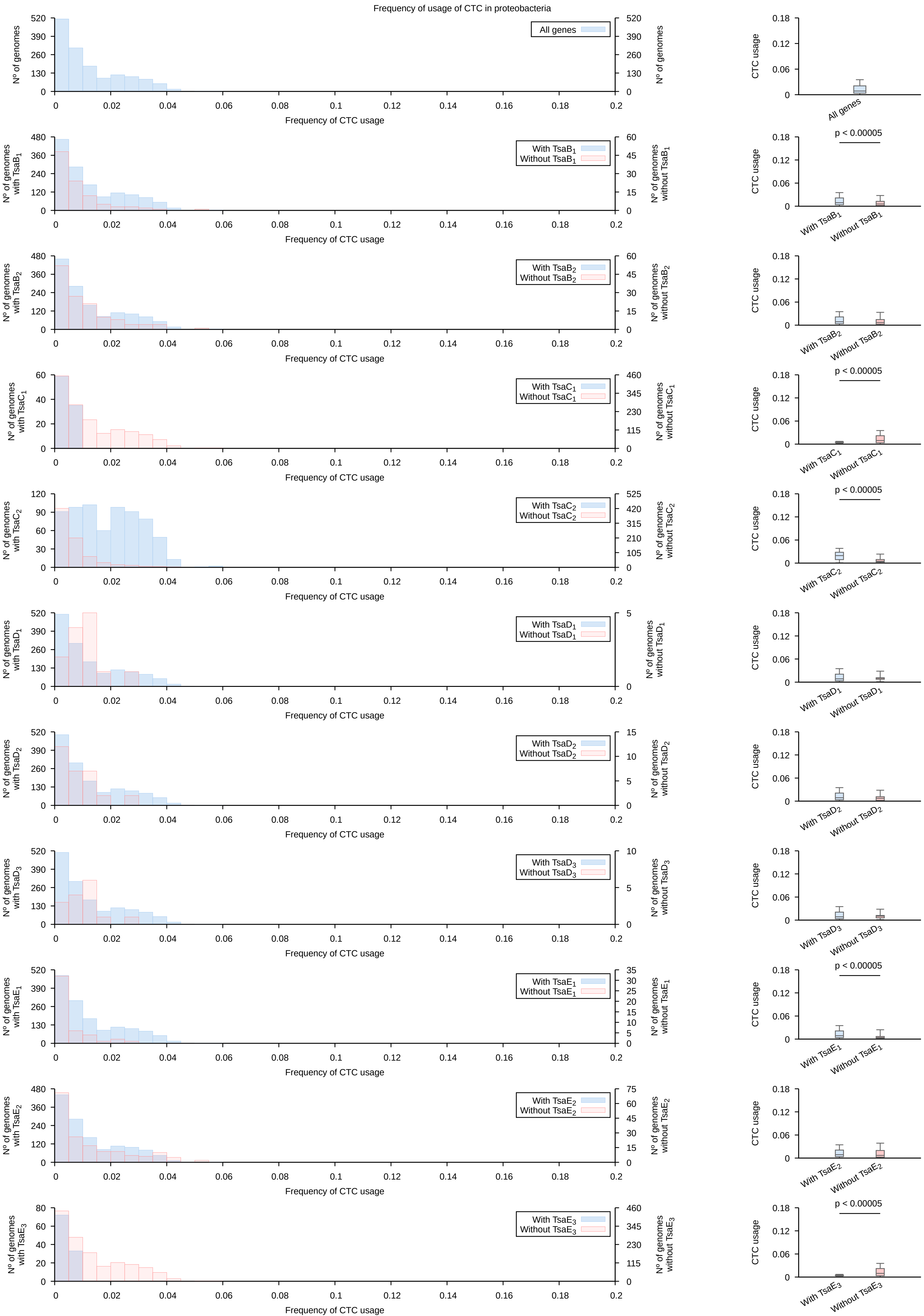

Frequency of usage of CTC in proteobacteria
All genes
Nº of genomes
CTC usage
Frequency of CTC usage
With TsaB$_1$
Without TsaB$_1$
Nº of genomes with TsaB$_1$
Nº of genomes without TsaB$_1$
p < 0.00005
With TsaB$_2$
Without TsaB$_2$
Nº of genomes with TsaB$_2$
Nº of genomes without TsaB$_2$
With TsaC$_1$
Without TsaC$_1$
Nº of genomes with TsaC$_1$
Nº of genomes without TsaC$_1$
p < 0.00005
With TsaC$_2$
Without TsaC$_2$
Nº of genomes with TsaC$_2$
Nº of genomes without TsaC$_2$
p < 0.00005
With TsaD$_1$
Without TsaD$_1$
Nº of genomes with TsaD$_1$
Nº of genomes without TsaD$_1$
With TsaD$_2$
Without TsaD$_2$
Nº of genomes with TsaD$_2$
Nº of genomes without TsaD$_2$
With TsaD$_3$
Without TsaD$_3$
Nº of genomes with TsaD$_3$
Nº of genomes without TsaD$_3$
With TsaE$_1$
Without TsaE$_1$
Nº of genomes with TsaE$_1$
Nº of genomes without TsaE$_1$
p < 0.00005
With TsaE$_2$
Without TsaE$_2$
Nº of genomes with TsaE$_2$
Nº of genomes without TsaE$_2$
With TsaE$_3$
Without TsaE$_3$
Nº of genomes with TsaE$_3$
Nº of genomes without TsaE$_3$
p < 0.00005

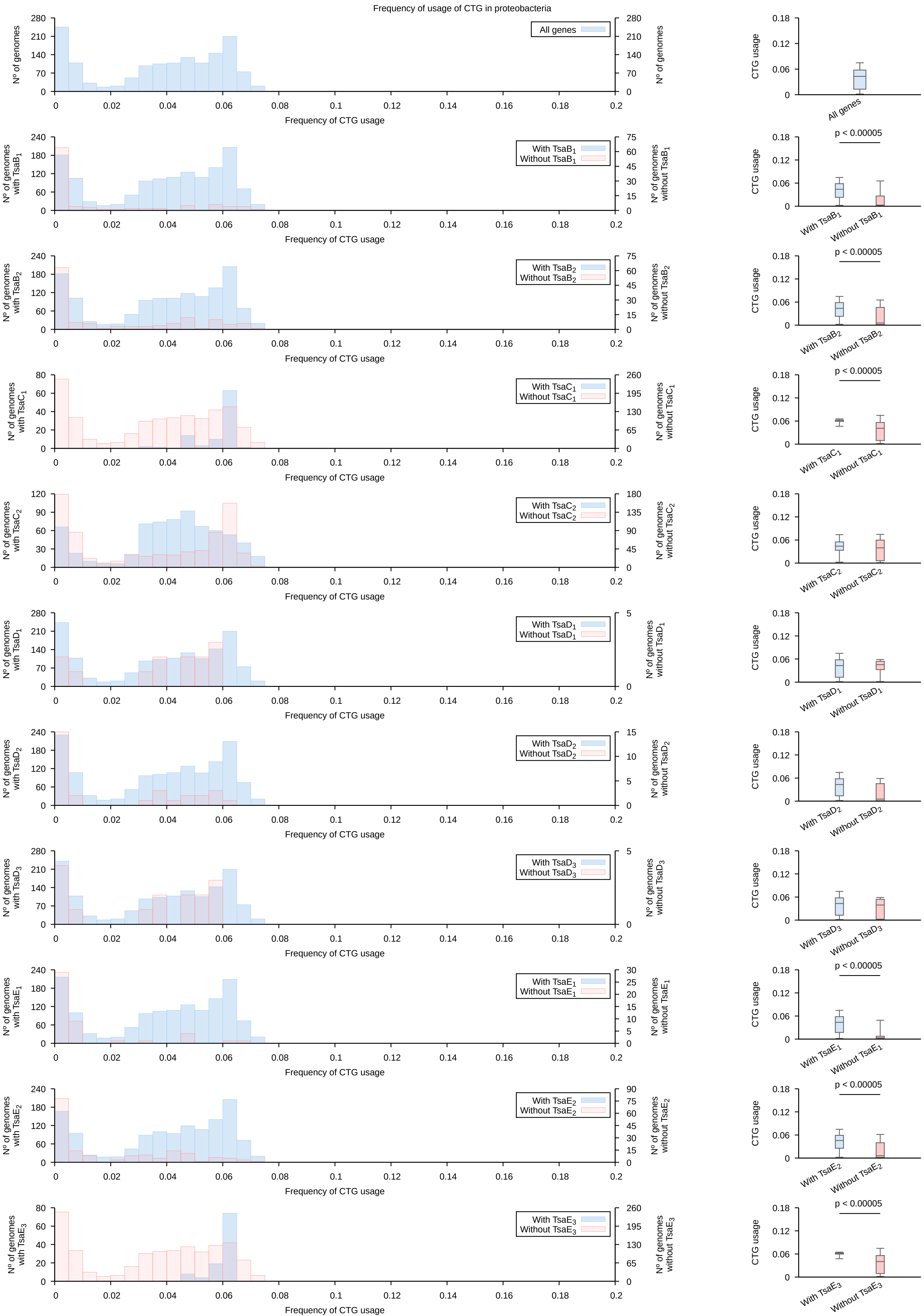
Frequency of usage of CTG in proteobacteria
Nº of genomes
All genes
Frequency of CTG usage
CTG usage
Nº of genomes with TsaB$_1$
Nº of genomes without TsaB$_1$
With TsaB$_1$
Without TsaB$_1$
p < 0.00005
Nº of genomes with TsaB$_2$
Nº of genomes without TsaB$_2$
With TsaB$_2$
Without TsaB$_2$
p < 0.00005
Nº of genomes with TsaC$_1$
Nº of genomes without TsaC$_1$
With TsaC$_1$
Without TsaC$_1$
p < 0.00005
Nº of genomes with TsaC$_2$
Nº of genomes without TsaC$_2$
With TsaC$_2$
Without TsaC$_2$
Nº of genomes with TsaD$_1$
Nº of genomes without TsaD$_1$
With TsaD$_1$
Without TsaD$_1$
Nº of genomes with TsaD$_2$
Nº of genomes without TsaD$_2$
With TsaD$_2$
Without TsaD$_2$
Nº of genomes with TsaD$_3$
Nº of genomes without TsaD$_3$
With TsaD$_3$
Without TsaD$_3$
Nº of genomes with TsaE$_1$
Nº of genomes without TsaE$_1$
With TsaE$_1$
Without TsaE$_1$
p < 0.00005
Nº of genomes with TsaE$_2$
Nº of genomes without TsaE$_2$
With TsaE$_2$
Without TsaE$_2$
p < 0.00005
Nº of genomes with TsaE$_3$
Nº of genomes without TsaE$_3$
With TsaE$_3$
Without TsaE$_3$
p < 0.00005

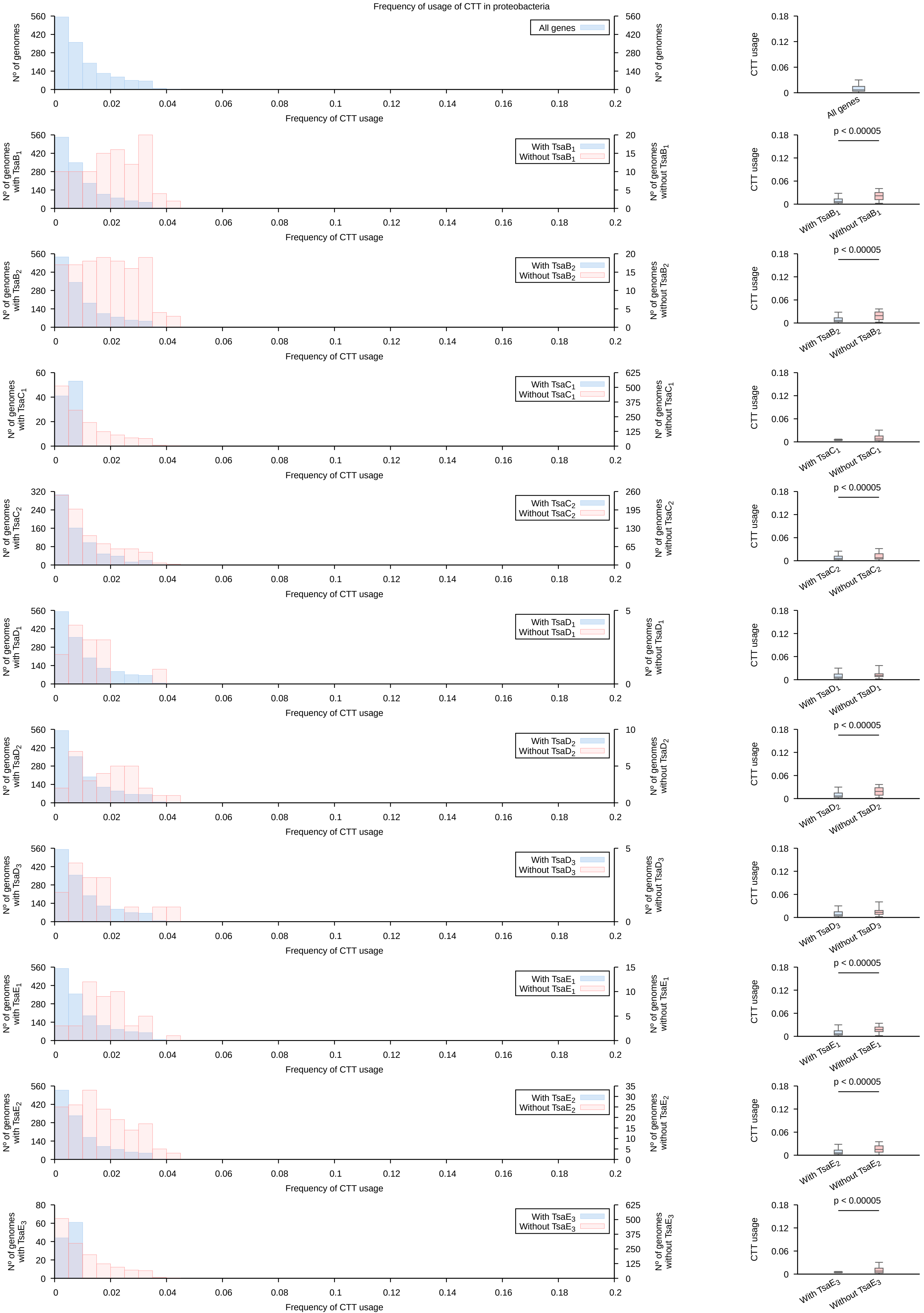
Frequency of usage of CTT in proteobacteria
Nº of genomes
All genes
Frequency of CTT usage
CTT usage
p < 0.00005
Nº of genomes with TsaB1
Nº of genomes without TsaB1
With TsaB1
Without TsaB1
Nº of genomes with TsaB2
Nº of genomes without TsaB2
With TsaB2
Without TsaB2
Nº of genomes with TsaC1
Nº of genomes without TsaC1
With TsaC1
Without TsaC1
Nº of genomes with TsaC2
Nº of genomes without TsaC2
With TsaC2
Without TsaC2
Nº of genomes with TsaD1
Nº of genomes without TsaD1
With TsaD1
Without TsaD1
Nº of genomes with TsaD2
Nº of genomes without TsaD2
With TsaD2
Without TsaD2
Nº of genomes with TsaD3
Nº of genomes without TsaD3
With TsaD3
Without TsaD3
Nº of genomes with TsaE1
Nº of genomes without TsaE1
With TsaE1
Without TsaE1
Nº of genomes with TsaE2
Nº of genomes without TsaE2
With TsaE2
Without TsaE2
Nº of genomes with TsaE3
Nº of genomes without TsaE3
With TsaE3
Without TsaE3

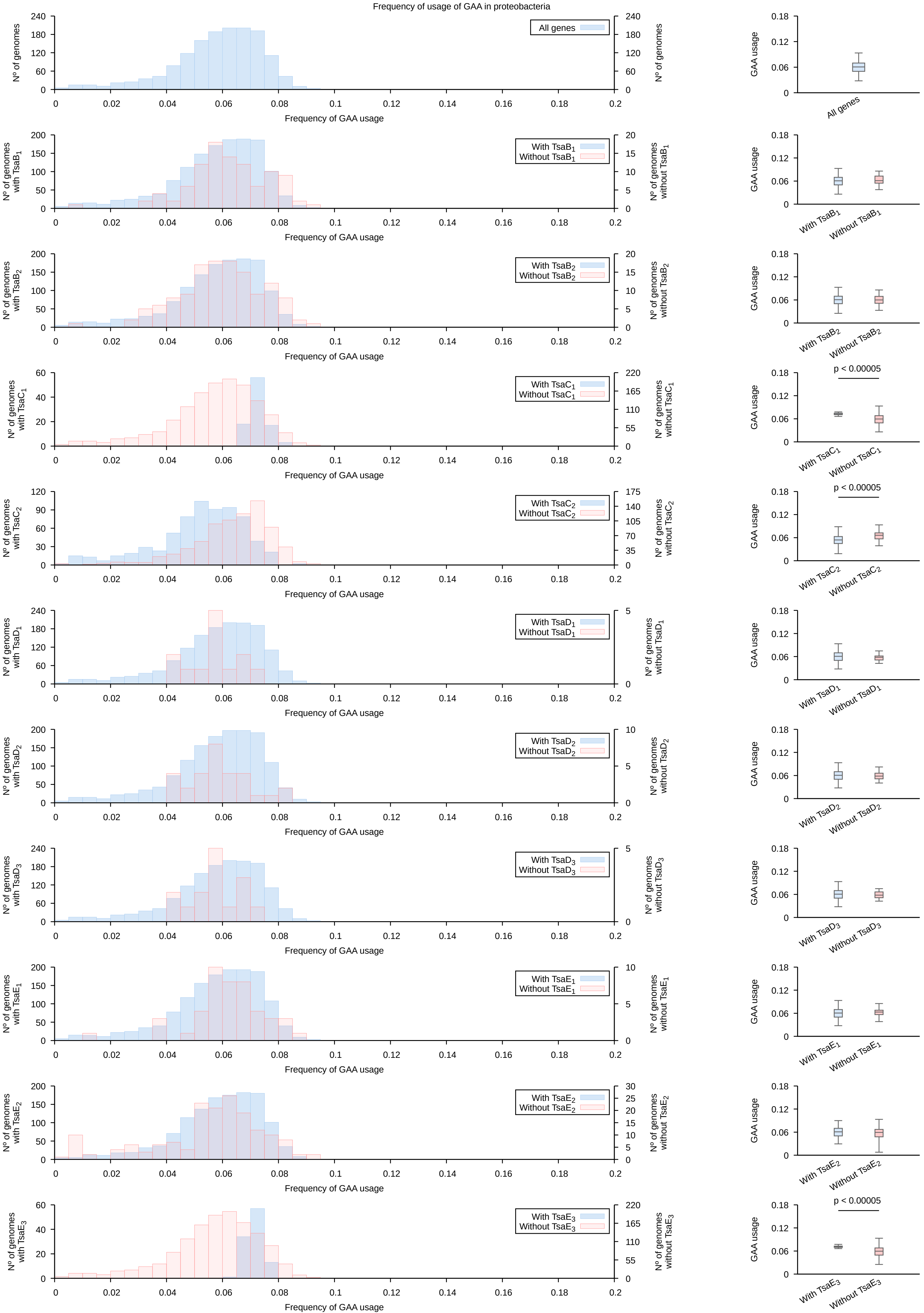

Frequency of usage of GAA in proteobacteria
Nº of genomes
Frequency of GAA usage
All genes
GAA usage
Nº of genomes with TsaB$_1$
Nº of genomes without TsaB$_1$
With TsaB$_1$
Without TsaB$_1$
Nº of genomes with TsaB$_2$
Nº of genomes without TsaB$_2$
With TsaB$_2$
Without TsaB$_2$
Nº of genomes with TsaC$_1$
Nº of genomes without TsaC$_1$
With TsaC$_1$
Without TsaC$_1$
p < 0.00005
Nº of genomes with TsaC$_2$
Nº of genomes without TsaC$_2$
With TsaC$_2$
Without TsaC$_2$
p < 0.00005
Nº of genomes with TsaD$_1$
Nº of genomes without TsaD$_1$
With TsaD$_1$
Without TsaD$_1$
Nº of genomes with TsaD$_2$
Nº of genomes without TsaD$_2$
With TsaD$_2$
Without TsaD$_2$
Nº of genomes with TsaD$_3$
Nº of genomes without TsaD$_3$
With TsaD$_3$
Without TsaD$_3$
Nº of genomes with TsaE$_1$
Nº of genomes without TsaE$_1$
With TsaE$_1$
Without TsaE$_1$
Nº of genomes with TsaE$_2$
Nº of genomes without TsaE$_2$
With TsaE$_2$
Without TsaE$_2$
Nº of genomes with TsaE$_3$
Nº of genomes without TsaE$_3$
With TsaE$_3$
Without TsaE$_3$
p < 0.00005

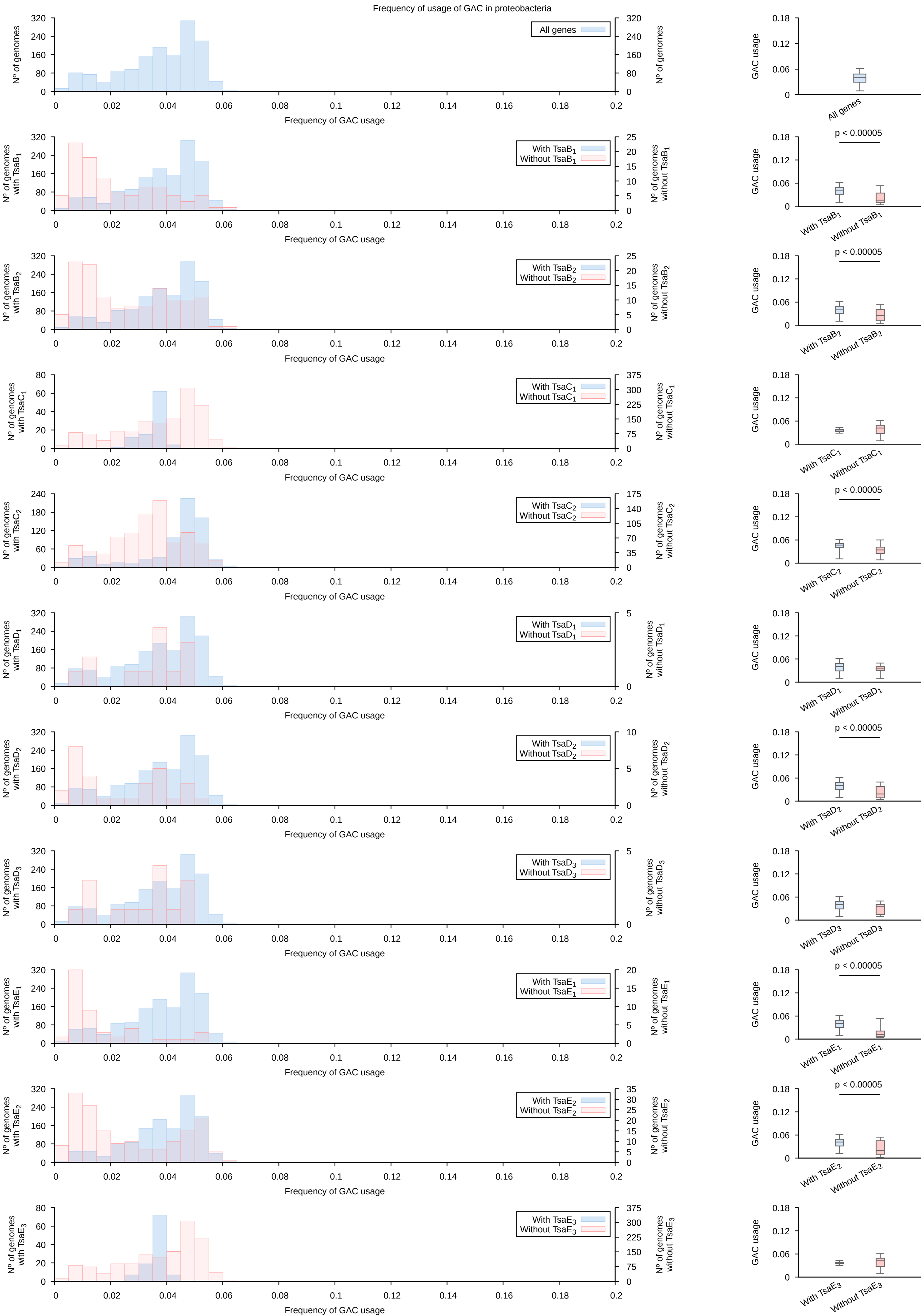
Frequency of usage of GAC in proteobacteria
All genes
Nº of genomes
Frequency of GAC usage
GAC usage
With TsaB1
Without TsaB1
Nº of genomes with TsaB1
Nº of genomes without TsaB1
p < 0.00005
With TsaB2
Without TsaB2
Nº of genomes with TsaB2
Nº of genomes without TsaB2
p < 0.00005
With TsaC1
Without TsaC1
Nº of genomes with TsaC1
Nº of genomes without TsaC1
With TsaC2
Without TsaC2
Nº of genomes with TsaC2
Nº of genomes without TsaC2
p < 0.00005
With TsaD1
Without TsaD1
Nº of genomes with TsaD1
Nº of genomes without TsaD1
With TsaD2
Without TsaD2
Nº of genomes with TsaD2
Nº of genomes without TsaD2
p < 0.00005
With TsaD3
Without TsaD3
Nº of genomes with TsaD3
Nº of genomes without TsaD3
With TsaE1
Without TsaE1
Nº of genomes with TsaE1
Nº of genomes without TsaE1
p < 0.00005
With TsaE2
Without TsaE2
Nº of genomes with TsaE2
Nº of genomes without TsaE2
p < 0.00005
With TsaE3
Without TsaE3
Nº of genomes with TsaE3
Nº of genomes without TsaE3

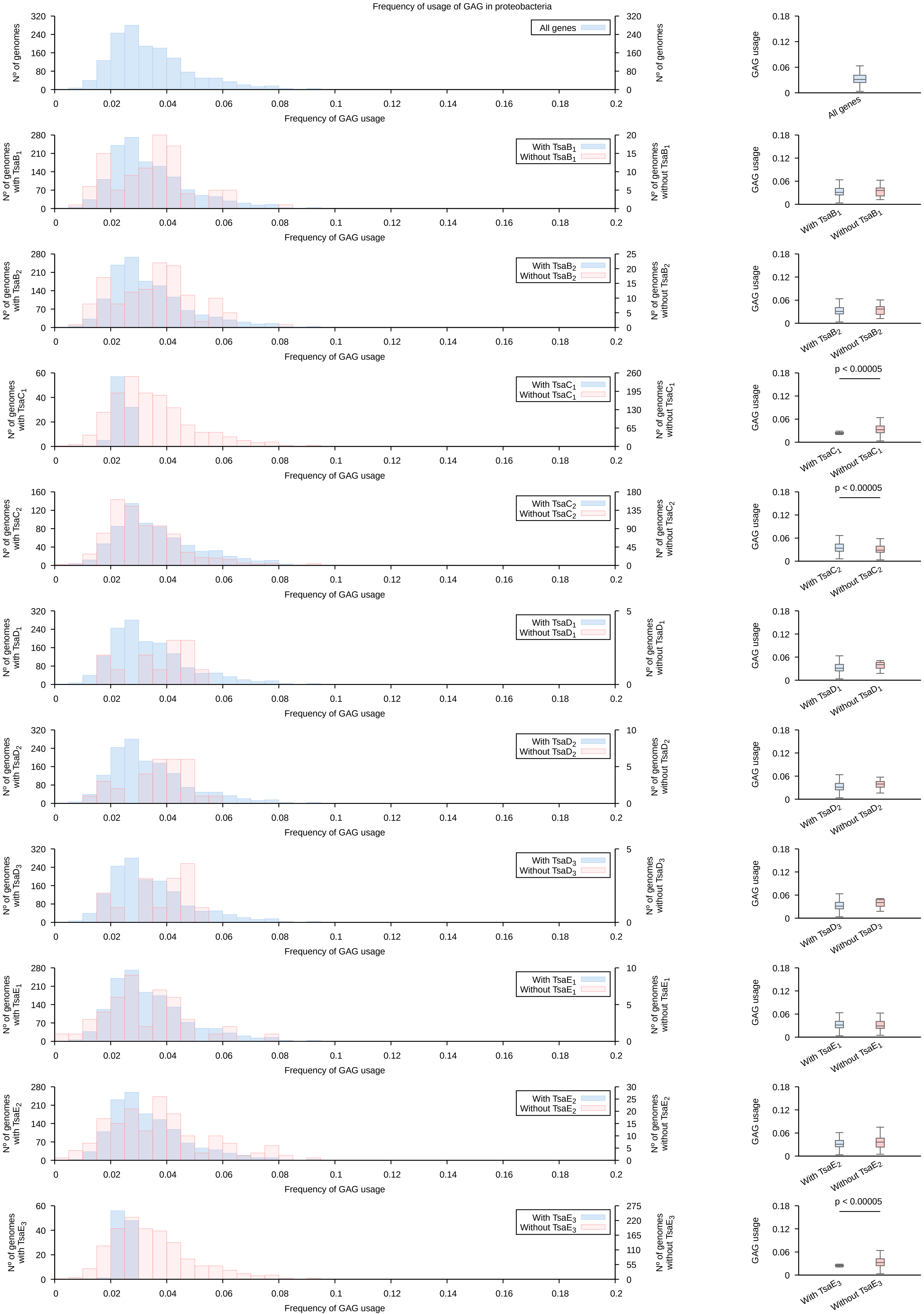
Frequency of usage of GAG in proteobacteria
Nº of genomes
All genes
Frequency of GAG usage
GAG usage
Nº of genomes with TsaB$_1$
Nº of genomes without TsaB$_1$
With TsaB$_1$
Without TsaB$_1$
Nº of genomes with TsaB$_2$
Nº of genomes without TsaB$_2$
With TsaB$_2$
Without TsaB$_2$
Nº of genomes with TsaC$_1$
Nº of genomes without TsaC$_1$
With TsaC$_1$
Without TsaC$_1$
p < 0.00005
Nº of genomes with TsaC$_2$
Nº of genomes without TsaC$_2$
With TsaC$_2$
Without TsaC$_2$
p < 0.00005
Nº of genomes with TsaD$_1$
Nº of genomes without TsaD$_1$
With TsaD$_1$
Without TsaD$_1$
Nº of genomes with TsaD$_2$
Nº of genomes without TsaD$_2$
With TsaD$_2$
Without TsaD$_2$
Nº of genomes with TsaD$_3$
Nº of genomes without TsaD$_3$
With TsaD$_3$
Without TsaD$_3$
Nº of genomes with TsaE$_1$
Nº of genomes without TsaE$_1$
With TsaE$_1$
Without TsaE$_1$
Nº of genomes with TsaE$_2$
Nº of genomes without TsaE$_2$
With TsaE$_2$
Without TsaE$_2$
Nº of genomes with TsaE$_3$
Nº of genomes without TsaE$_3$
With TsaE$_3$
Without TsaE$_3$
p < 0.00005

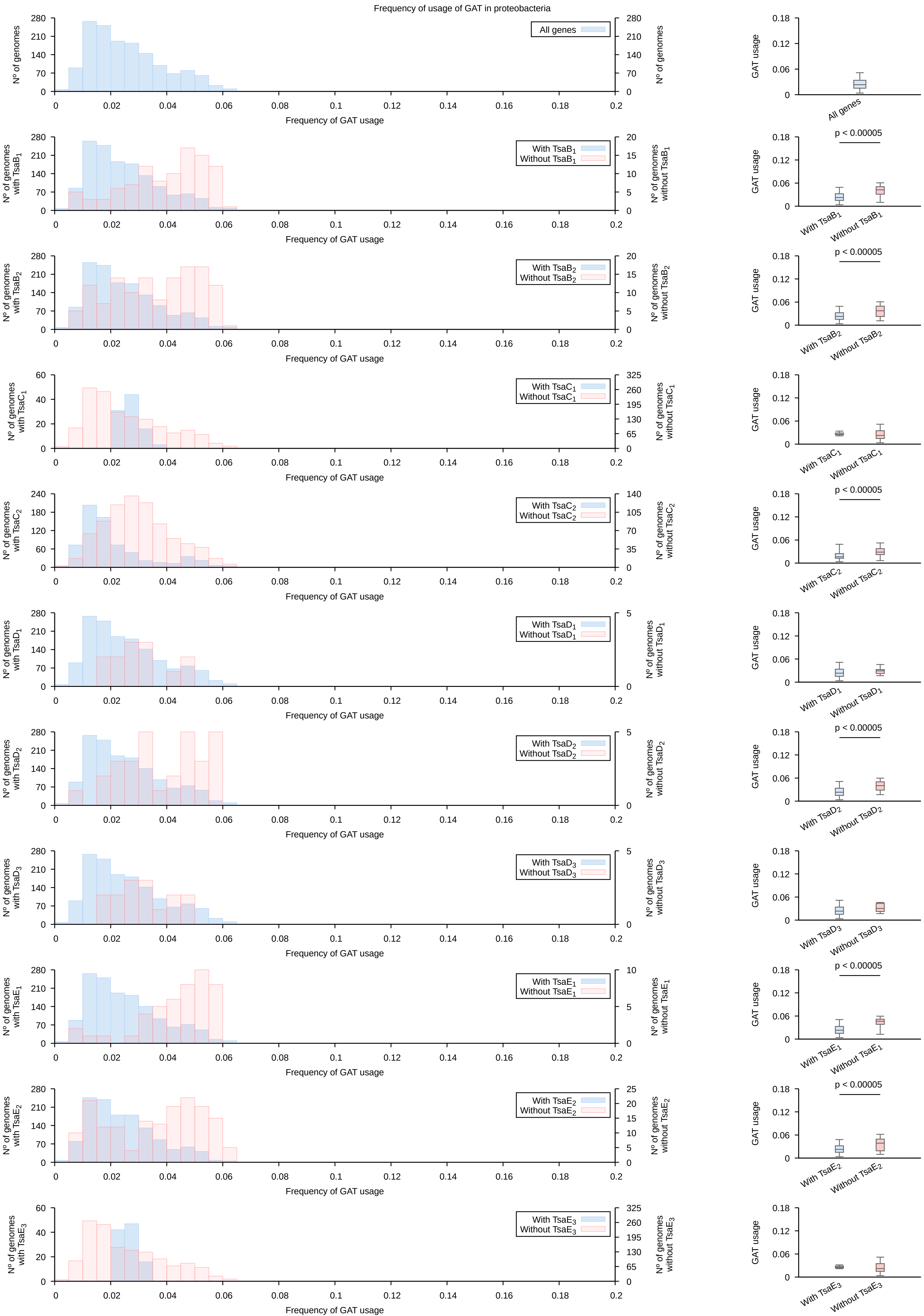
Frequency of usage of GAT in proteobacteria
Nº of genomes
All genes
Frequency of GAT usage
GAT usage
All genes
Nº of genomes with $TsaB_1$
Nº of genomes without $TsaB_1$
With $TsaB_1$
Without $TsaB_1$
p < 0.00005
Nº of genomes with $TsaB_2$
Nº of genomes without $TsaB_2$
With $TsaB_2$
Without $TsaB_2$
p < 0.00005
Nº of genomes with $TsaC_1$
Nº of genomes without $TsaC_1$
With $TsaC_1$
Without $TsaC_1$
Nº of genomes with $TsaC_2$
Nº of genomes without $TsaC_2$
With $TsaC_2$
Without $TsaC_2$
p < 0.00005
Nº of genomes with $TsaD_1$
Nº of genomes without $TsaD_1$
With $TsaD_1$
Without $TsaD_1$
Nº of genomes with $TsaD_2$
Nº of genomes without $TsaD_2$
With $TsaD_2$
Without $TsaD_2$
p < 0.00005
Nº of genomes with $TsaD_3$
Nº of genomes without $TsaD_3$
With $TsaD_3$
Without $TsaD_3$
Nº of genomes with $TsaE_1$
Nº of genomes without $TsaE_1$
With $TsaE_1$
Without $TsaE_1$
p < 0.00005
Nº of genomes with $TsaE_2$
Nº of genomes without $TsaE_2$
With $TsaE_2$
Without $TsaE_2$
p < 0.00005
Nº of genomes with $TsaE_3$
Nº of genomes without $TsaE_3$
With $TsaE_3$
Without $TsaE_3$

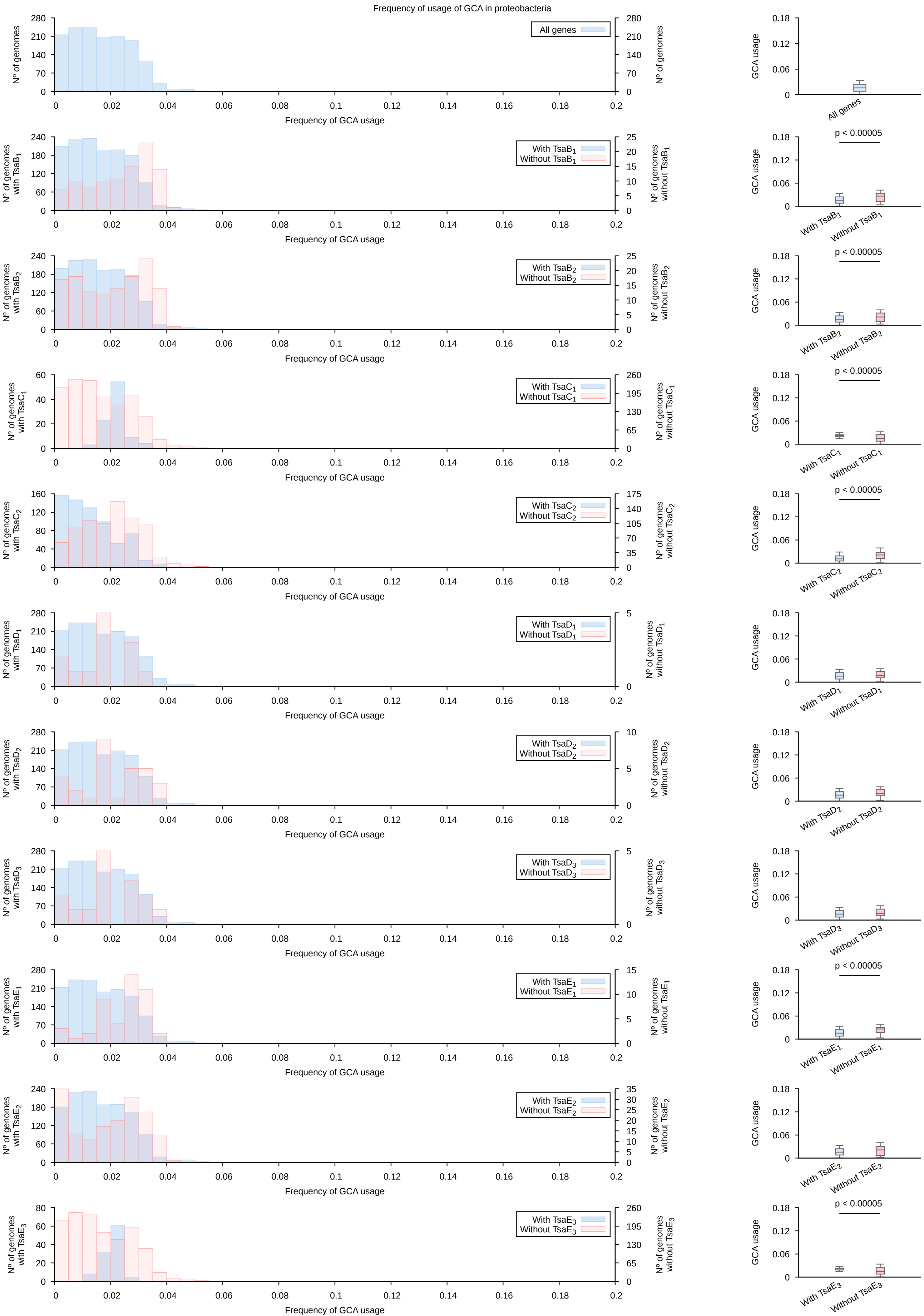
Frequency of usage of GCA in proteobacteria
Nº of genomes
All genes
Frequency of GCA usage
GCA usage
Nº of genomes with TsaB$_1$
Nº of genomes without TsaB$_1$
With TsaB$_1$
Without TsaB$_1$
p < 0.00005
Nº of genomes with TsaB$_2$
Nº of genomes without TsaB$_2$
With TsaB$_2$
Without TsaB$_2$
p < 0.00005
Nº of genomes with TsaC$_1$
Nº of genomes without TsaC$_1$
With TsaC$_1$
Without TsaC$_1$
p < 0.00005
Nº of genomes with TsaC$_2$
Nº of genomes without TsaC$_2$
With TsaC$_2$
Without TsaC$_2$
p < 0.00005
Nº of genomes with TsaD$_1$
Nº of genomes without TsaD$_1$
With TsaD$_1$
Without TsaD$_1$
Nº of genomes with TsaD$_2$
Nº of genomes without TsaD$_2$
With TsaD$_2$
Without TsaD$_2$
Nº of genomes with TsaD$_3$
Nº of genomes without TsaD$_3$
With TsaD$_3$
Without TsaD$_3$
Nº of genomes with TsaE$_1$
Nº of genomes without TsaE$_1$
With TsaE$_1$
Without TsaE$_1$
p < 0.00005
Nº of genomes with TsaE$_2$
Nº of genomes without TsaE$_2$
With TsaE$_2$
Without TsaE$_2$
Nº of genomes with TsaE$_3$
Nº of genomes without TsaE$_3$
With TsaE$_3$
Without TsaE$_3$
p < 0.00005

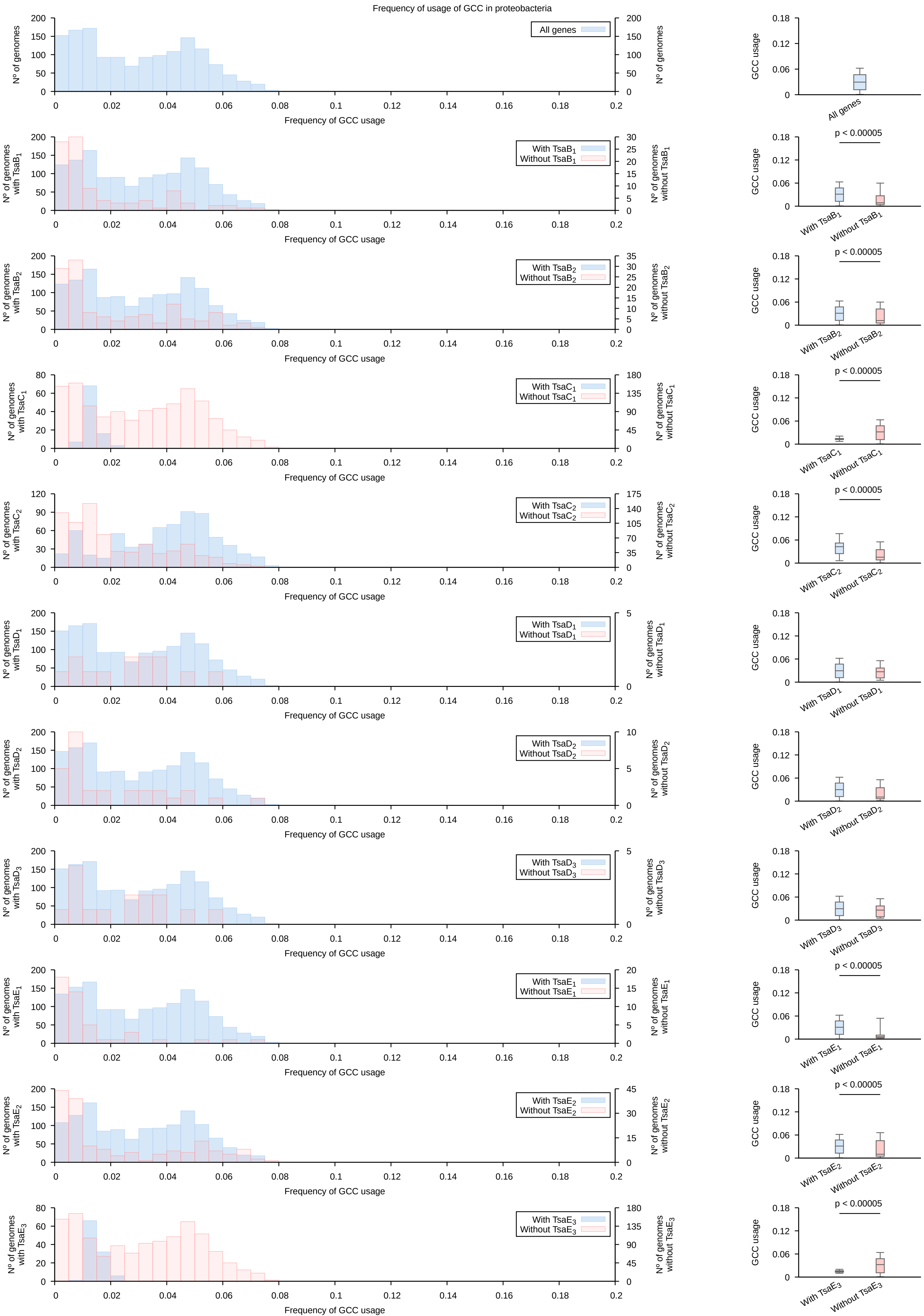

Frequency of usage of GCC in proteobacteria
Nº of genomes
All genes
Frequency of GCC usage
0 0.02 0.04 0.06 0.08 0.1 0.12 0.14 0.16 0.18 0.2
GCC usage
0.18 0.12 0.06 0
All genes
Nº of genomes with TsaB1
Nº of genomes without TsaB1
With TsaB1
Without TsaB1
p < 0.00005
Nº of genomes with TsaB2
Nº of genomes without TsaB2
With TsaB2
Without TsaB2
p < 0.00005
Nº of genomes with TsaC1
Nº of genomes without TsaC1
With TsaC1
Without TsaC1
p < 0.00005
Nº of genomes with TsaC2
Nº of genomes without TsaC2
With TsaC2
Without TsaC2
p < 0.00005
Nº of genomes with TsaD1
Nº of genomes without TsaD1
With TsaD1
Without TsaD1
Nº of genomes with TsaD2
Nº of genomes without TsaD2
With TsaD2
Without TsaD2
Nº of genomes with TsaD3
Nº of genomes without TsaD3
With TsaD3
Without TsaD3
Nº of genomes with TsaE1
Nº of genomes without TsaE1
With TsaE1
Without TsaE1
p < 0.00005
Nº of genomes with TsaE2
Nº of genomes without TsaE2
With TsaE2
Without TsaE2
p < 0.00005
Nº of genomes with TsaE3
Nº of genomes without TsaE3
With TsaE3
Without TsaE3
p < 0.00005

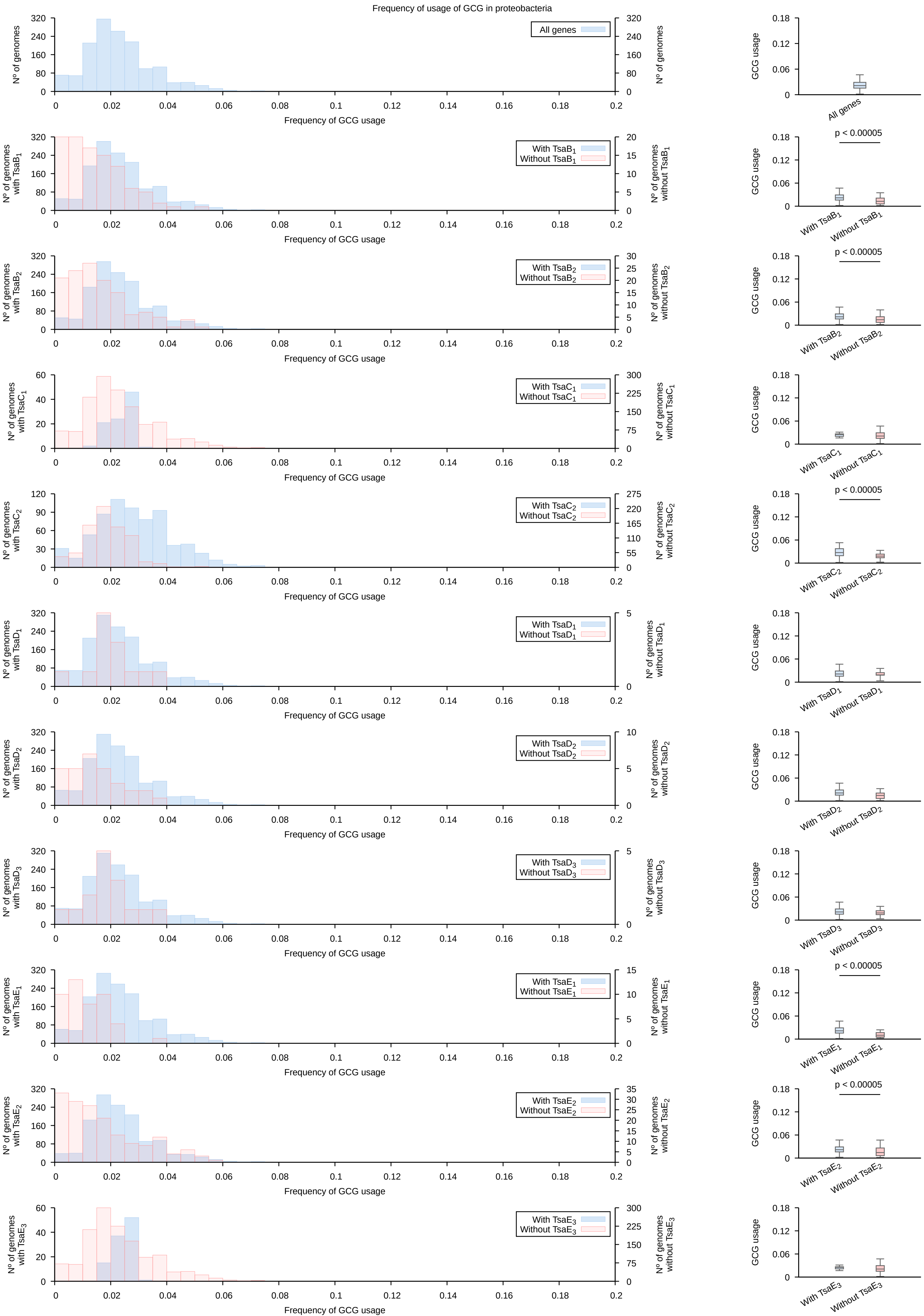
Frequency of usage of GCG in proteobacteria
Nº of genomes
All genes
Frequency of GCG usage
GCG usage
All genes
Nº of genomes with $TsaB_1$
Nº of genomes without $TsaB_1$
With $TsaB_1$
Without $TsaB_1$
p < 0.00005
Nº of genomes with $TsaB_2$
Nº of genomes without $TsaB_2$
With $TsaB_2$
Without $TsaB_2$
p < 0.00005
Nº of genomes with $TsaC_1$
Nº of genomes without $TsaC_1$
With $TsaC_1$
Without $TsaC_1$
Nº of genomes with $TsaC_2$
Nº of genomes without $TsaC_2$
With $TsaC_2$
Without $TsaC_2$
p < 0.00005
Nº of genomes with $TsaD_1$
Nº of genomes without $TsaD_1$
With $TsaD_1$
Without $TsaD_1$
Nº of genomes with $TsaD_2$
Nº of genomes without $TsaD_2$
With $TsaD_2$
Without $TsaD_2$
Nº of genomes with $TsaD_3$
Nº of genomes without $TsaD_3$
With $TsaD_3$
Without $TsaD_3$
Nº of genomes with $TsaE_1$
Nº of genomes without $TsaE_1$
With $TsaE_1$
Without $TsaE_1$
p < 0.00005
Nº of genomes with $TsaE_2$
Nº of genomes without $TsaE_2$
With $TsaE_2$
Without $TsaE_2$
p < 0.00005
Nº of genomes with $TsaE_3$
Nº of genomes without $TsaE_3$
With $TsaE_3$
Without $TsaE_3$

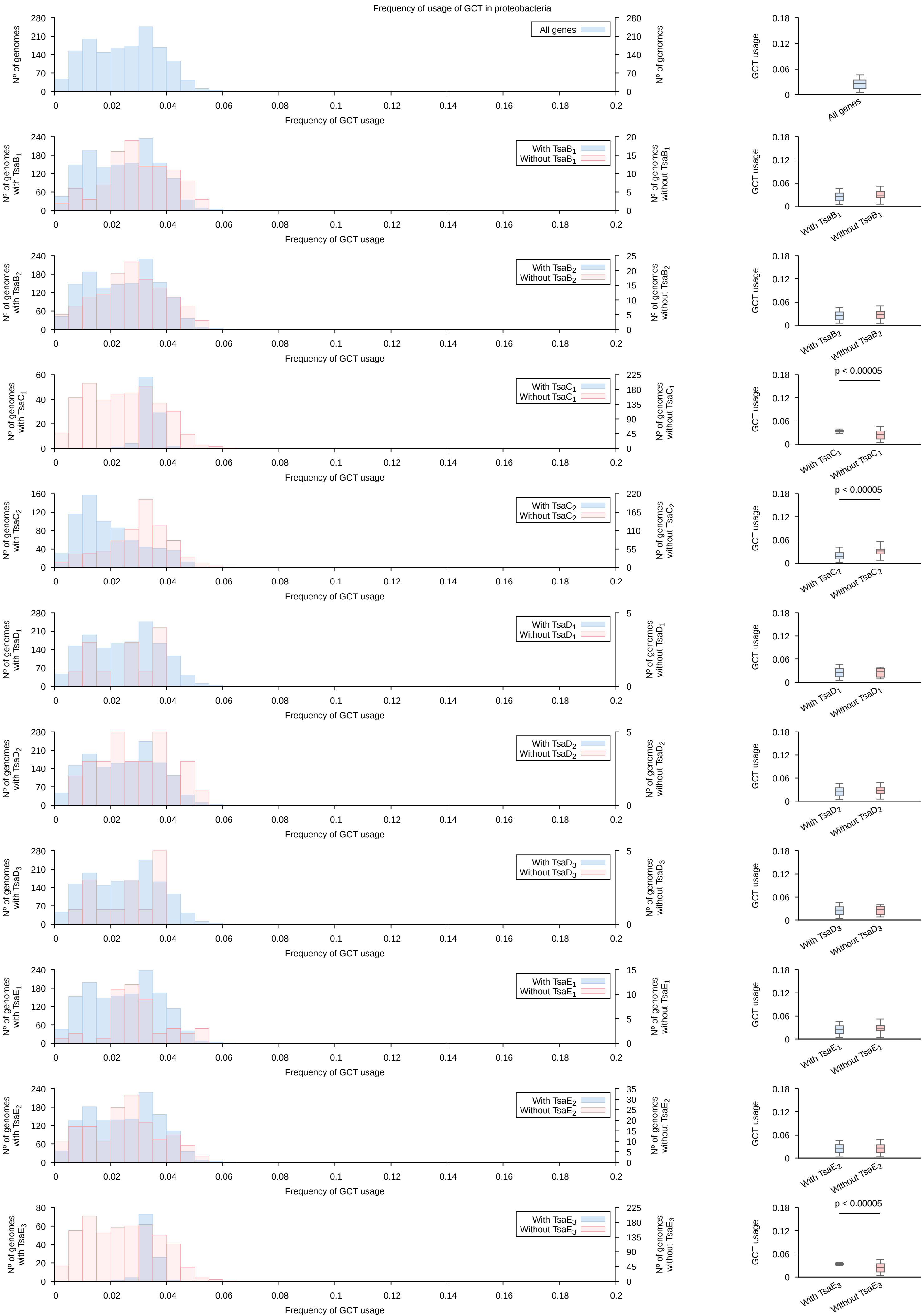
Frequency of usage of GCT in proteobacteria
All genes
Nº of genomes
Frequency of GCT usage
GCT usage
With TsaB1
Without TsaB1
Nº of genomes with TsaB1
Nº of genomes without TsaB1
With TsaB2
Without TsaB2
Nº of genomes with TsaB2
Nº of genomes without TsaB2
With TsaC1
Without TsaC1
Nº of genomes with TsaC1
Nº of genomes without TsaC1
p < 0.00005
With TsaC2
Without TsaC2
Nº of genomes with TsaC2
Nº of genomes without TsaC2
p < 0.00005
With TsaD1
Without TsaD1
Nº of genomes with TsaD1
Nº of genomes without TsaD1
With TsaD2
Without TsaD2
Nº of genomes with TsaD2
Nº of genomes without TsaD2
With TsaD3
Without TsaD3
Nº of genomes with TsaD3
Nº of genomes without TsaD3
With TsaE1
Without TsaE1
Nº of genomes with TsaE1
Nº of genomes without TsaE1
With TsaE2
Without TsaE2
Nº of genomes with TsaE2
Nº of genomes without TsaE2
With TsaE3
Without TsaE3
Nº of genomes with TsaE3
Nº of genomes without TsaE3
p < 0.00005

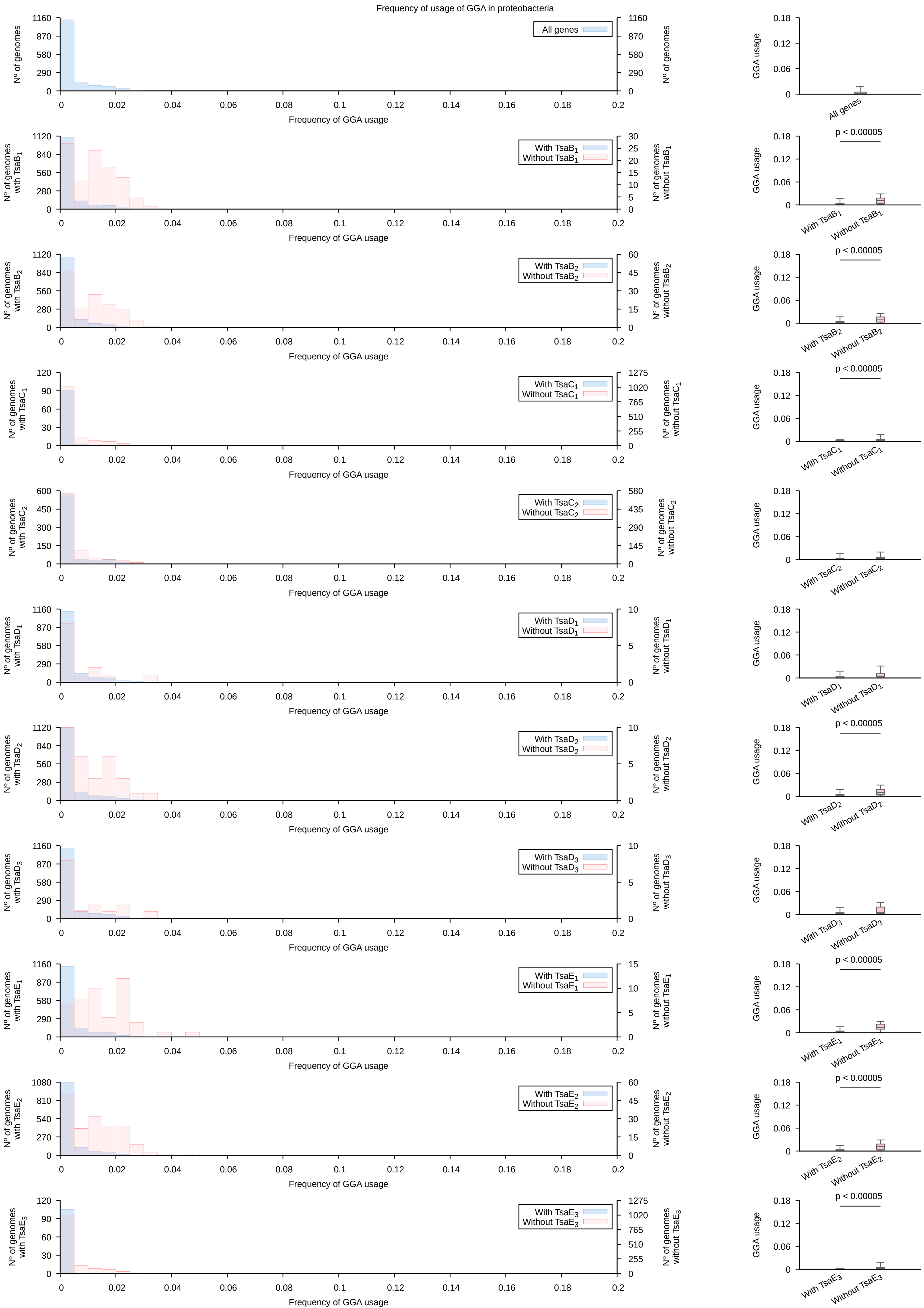
Frequency of usage of GGA in proteobacteria
All genes
Nº of genomes
Frequency of GGA usage
GGA usage
With TsaB1
Without TsaB1
Nº of genomes with TsaB1
Nº of genomes without TsaB1
p < 0.00005
With TsaB2
Without TsaB2
Nº of genomes with TsaB2
Nº of genomes without TsaB2
p < 0.00005
With TsaC1
Without TsaC1
Nº of genomes with TsaC1
Nº of genomes without TsaC1
p < 0.00005
With TsaC2
Without TsaC2
Nº of genomes with TsaC2
Nº of genomes without TsaC2
With TsaD1
Without TsaD1
Nº of genomes with TsaD1
Nº of genomes without TsaD1
With TsaD2
Without TsaD2
Nº of genomes with TsaD2
Nº of genomes without TsaD2
p < 0.00005
With TsaD3
Without TsaD3
Nº of genomes with TsaD3
Nº of genomes without TsaD3
With TsaE1
Without TsaE1
Nº of genomes with TsaE1
Nº of genomes without TsaE1
p < 0.00005
With TsaE2
Without TsaE2
Nº of genomes with TsaE2
Nº of genomes without TsaE2
p < 0.00005
With TsaE3
Without TsaE3
Nº of genomes with TsaE3
Nº of genomes without TsaE3
p < 0.00005

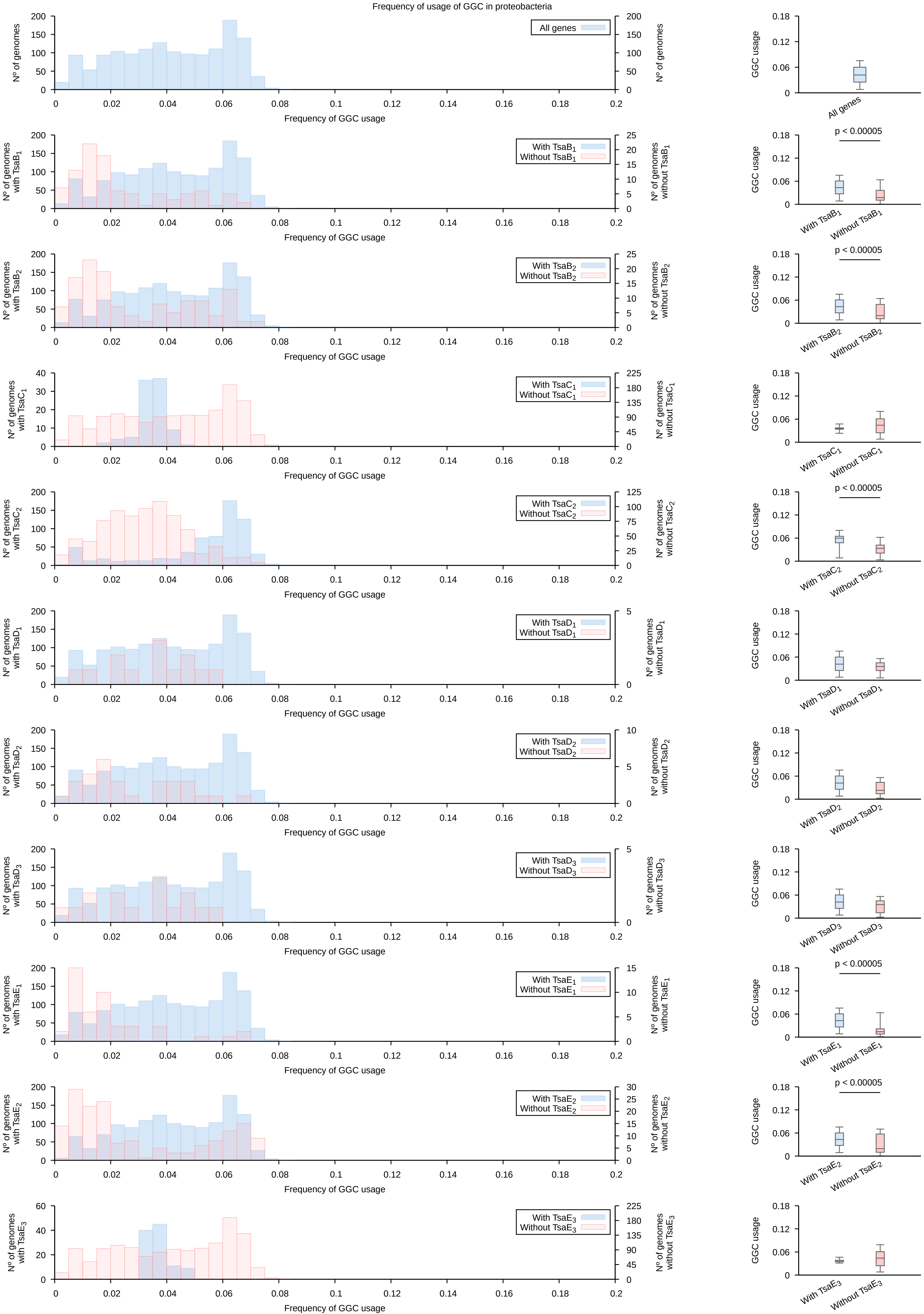
Frequency of usage of GGC in proteobacteria
All genes
Nº of genomes
Frequency of GGC usage
GGC usage
With TsaB$_1$
Without TsaB$_1$
Nº of genomes with TsaB$_1$
Nº of genomes without TsaB$_1$
p < 0.00005
With TsaB$_2$
Without TsaB$_2$
Nº of genomes with TsaB$_2$
Nº of genomes without TsaB$_2$
p < 0.00005
With TsaC$_1$
Without TsaC$_1$
Nº of genomes with TsaC$_1$
Nº of genomes without TsaC$_1$
With TsaC$_2$
Without TsaC$_2$
Nº of genomes with TsaC$_2$
Nº of genomes without TsaC$_2$
p < 0.00005
With TsaD$_1$
Without TsaD$_1$
Nº of genomes with TsaD$_1$
Nº of genomes without TsaD$_1$
With TsaD$_2$
Without TsaD$_2$
Nº of genomes with TsaD$_2$
Nº of genomes without TsaD$_2$
With TsaD$_3$
Without TsaD$_3$
Nº of genomes with TsaD$_3$
Nº of genomes without TsaD$_3$
With TsaE$_1$
Without TsaE$_1$
Nº of genomes with TsaE$_1$
Nº of genomes without TsaE$_1$
p < 0.00005
With TsaE$_2$
Without TsaE$_2$
Nº of genomes with TsaE$_2$
Nº of genomes without TsaE$_2$
p < 0.00005
With TsaE$_3$
Without TsaE$_3$
Nº of genomes with TsaE$_3$
Nº of genomes without TsaE$_3$

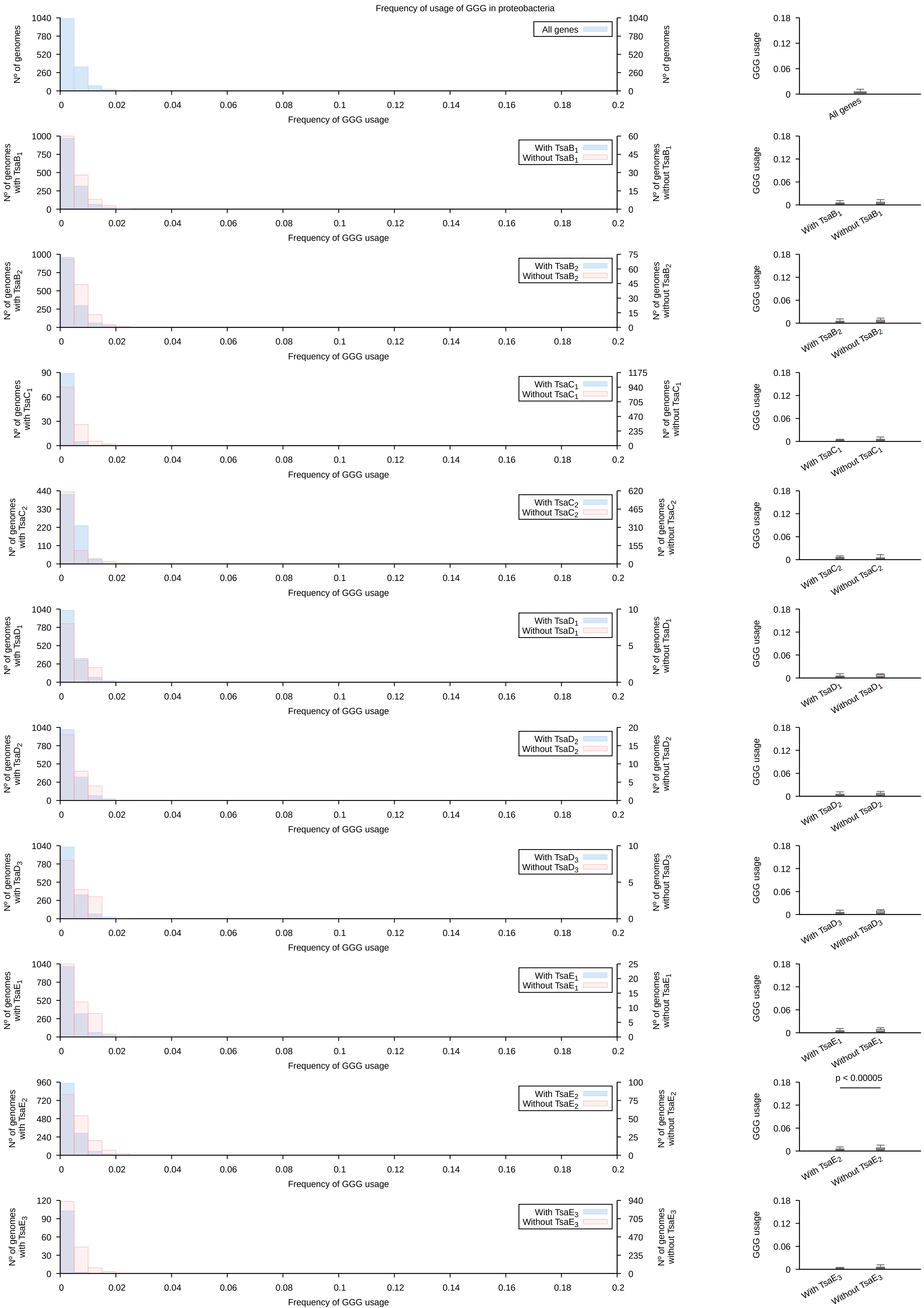
Frequency of usage of GGG in proteobacteria
Frequency of GGG usage
Nº of genomes
GGG usage
All genes
With TsaB1
Without TsaB1
With TsaB2
Without TsaB2
With TsaC1
Without TsaC1
With TsaC2
Without TsaC2
With TsaD1
Without TsaD1
With TsaD2
Without TsaD2
With TsaD3
Without TsaD3
With TsaE1
Without TsaE1
With TsaE2
Without TsaE2
p < 0.00005
With TsaE3
Without TsaE3

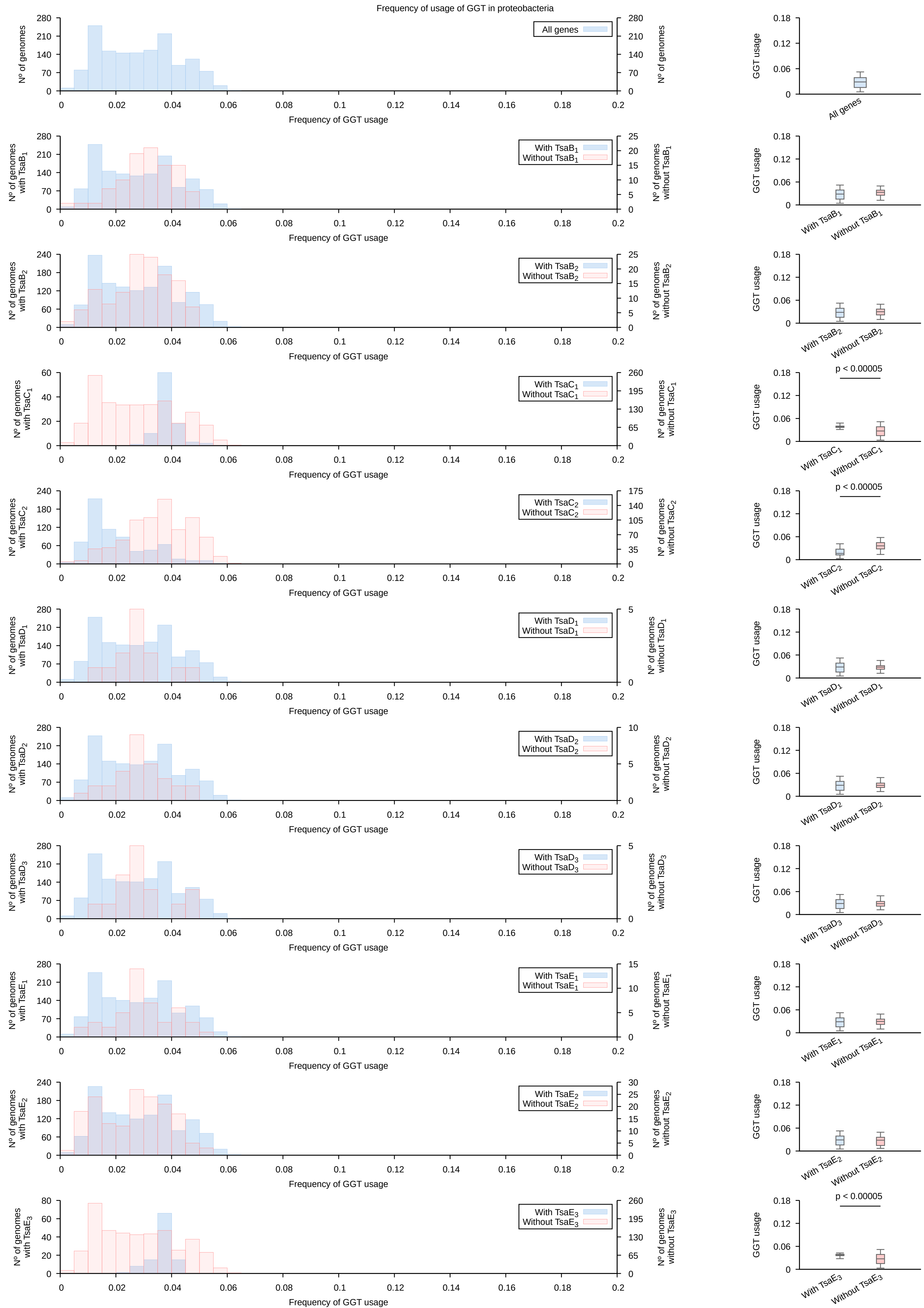
Frequency of usage of GGT in proteobacteria
All genes
Nº of genomes
Frequency of GGT usage
GGT usage
With TsaB$_1$
Without TsaB$_1$
Nº of genomes with TsaB$_1$
Nº of genomes without TsaB$_1$
With TsaB$_2$
Without TsaB$_2$
Nº of genomes with TsaB$_2$
Nº of genomes without TsaB$_2$
With TsaC$_1$
Without TsaC$_1$
Nº of genomes with TsaC$_1$
Nº of genomes without TsaC$_1$
p < 0.00005
With TsaC$_2$
Without TsaC$_2$
Nº of genomes with TsaC$_2$
Nº of genomes without TsaC$_2$
p < 0.00005
With TsaD$_1$
Without TsaD$_1$
Nº of genomes with TsaD$_1$
Nº of genomes without TsaD$_1$
With TsaD$_2$
Without TsaD$_2$
Nº of genomes with TsaD$_2$
Nº of genomes without TsaD$_2$
With TsaD$_3$
Without TsaD$_3$
Nº of genomes with TsaD$_3$
Nº of genomes without TsaD$_3$
With TsaE$_1$
Without TsaE$_1$
Nº of genomes with TsaE$_1$
Nº of genomes without TsaE$_1$
With TsaE$_2$
Without TsaE$_2$
Nº of genomes with TsaE$_2$
Nº of genomes without TsaE$_2$
With TsaE$_3$
Without TsaE$_3$
Nº of genomes with TsaE$_3$
Nº of genomes without TsaE$_3$
p < 0.00005

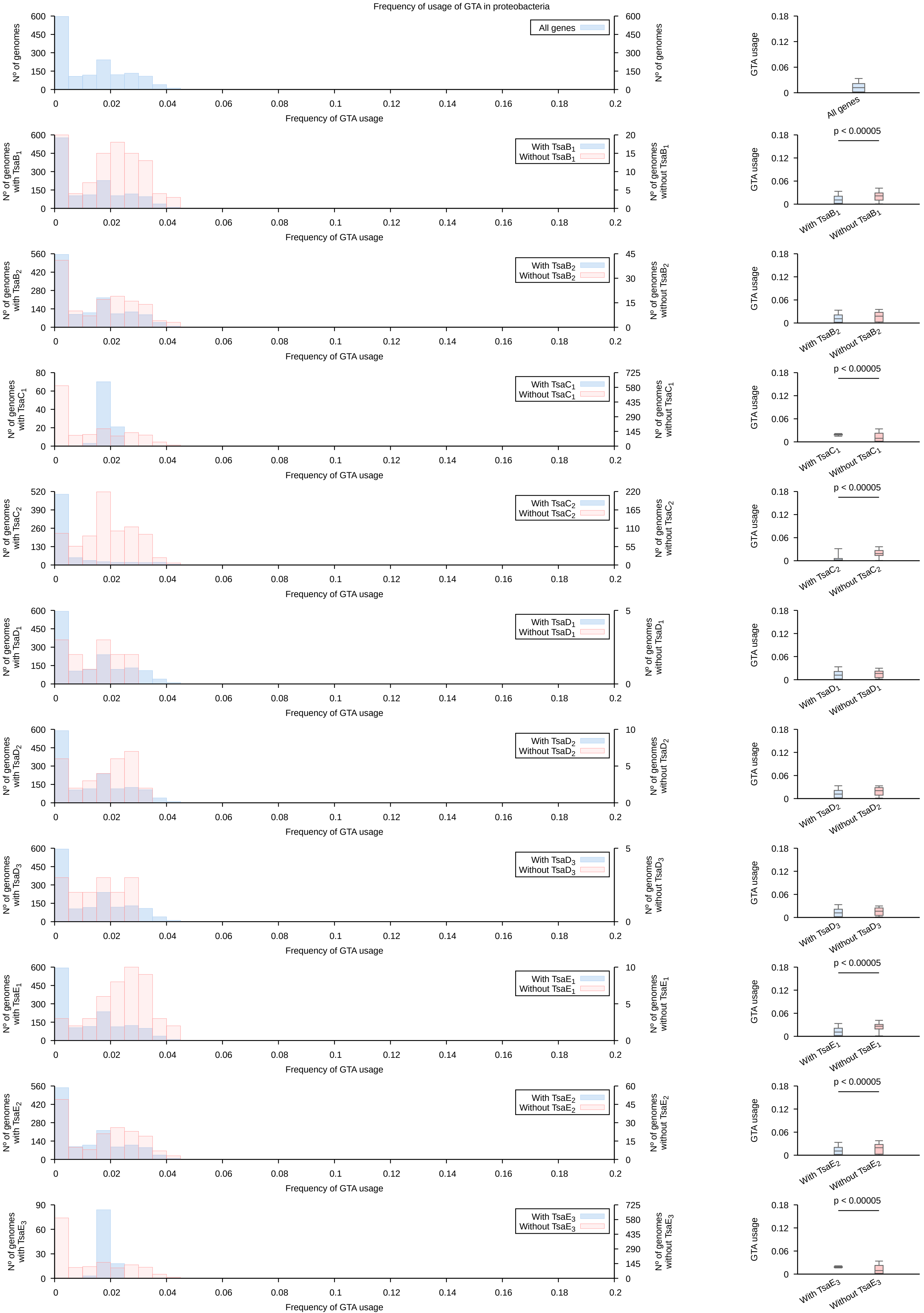

Frequency of usage of GTA in proteobacteria
All genes
With TsaB$_1$
Without TsaB$_1$
With TsaB$_2$
Without TsaB$_2$
With TsaC$_1$
Without TsaC$_1$
With TsaC$_2$
Without TsaC$_2$
With TsaD$_1$
Without TsaD$_1$
With TsaD$_2$
Without TsaD$_2$
With TsaD$_3$
Without TsaD$_3$
With TsaE$_1$
Without TsaE$_1$
With TsaE$_2$
Without TsaE$_2$
With TsaE$_3$
Without TsaE$_3$
Nº of genomes
Nº of genomes with TsaB$_1$
Nº of genomes without TsaB$_1$
Nº of genomes with TsaB$_2$
Nº of genomes without TsaB$_2$
Nº of genomes with TsaC$_1$
Nº of genomes without TsaC$_1$
Nº of genomes with TsaC$_2$
Nº of genomes without TsaC$_2$
Nº of genomes with TsaD$_1$
Nº of genomes without TsaD$_1$
Nº of genomes with TsaD$_2$
Nº of genomes without TsaD$_2$
Nº of genomes with TsaD$_3$
Nº of genomes without TsaD$_3$
Nº of genomes with TsaE$_1$
Nº of genomes without TsaE$_1$
Nº of genomes with TsaE$_2$
Nº of genomes without TsaE$_2$
Nº of genomes with TsaE$_3$
Nº of genomes without TsaE$_3$
Frequency of GTA usage
GTA usage
p < 0.00005
0 0.02 0.04 0.06 0.08 0.1 0.12 0.14 0.16 0.18 0.2
0 0.06 0.12 0.18

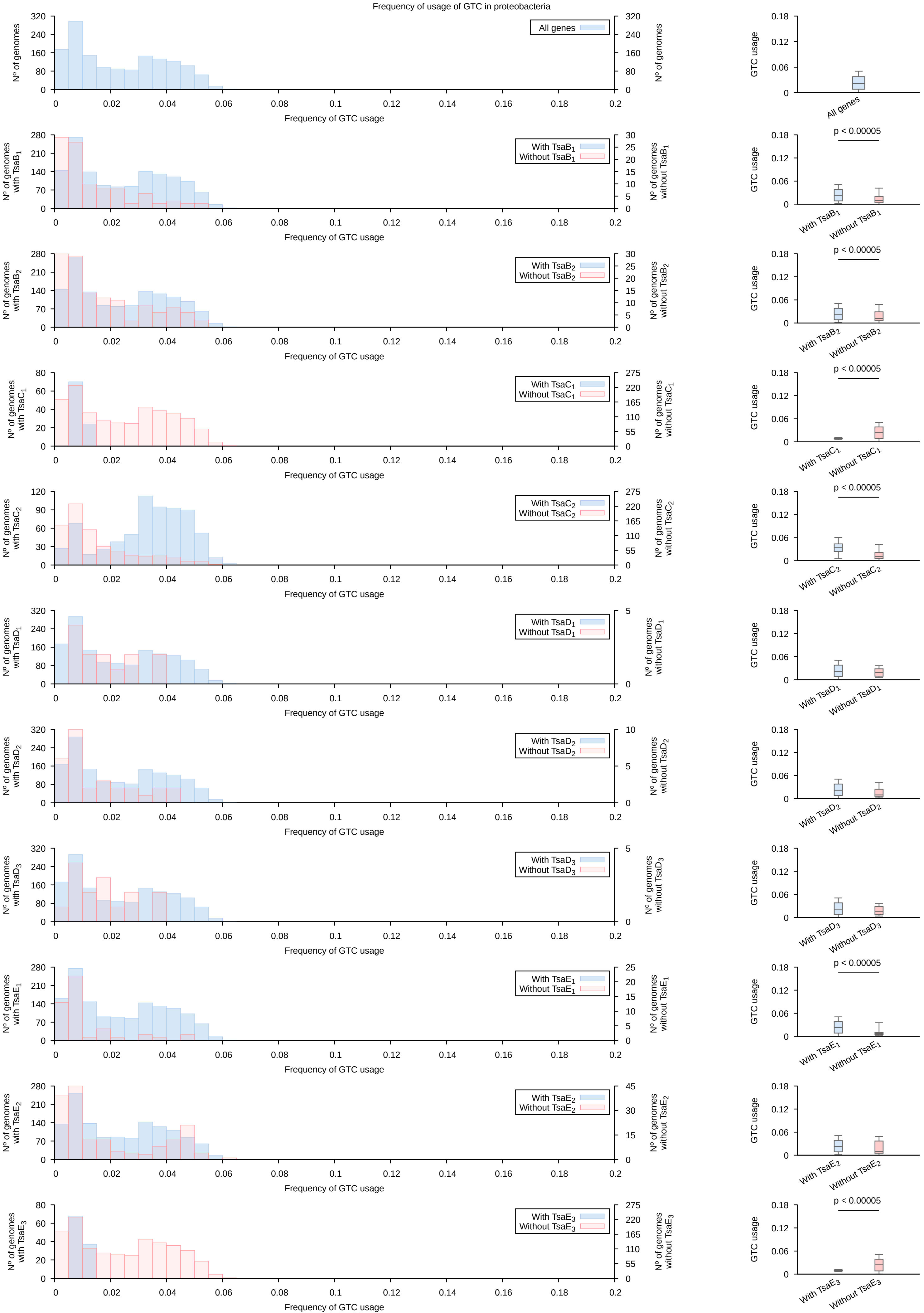
Frequency of usage of GTC in proteobacteria
Nº of genomes
All genes
Frequency of GTC usage
GTC usage
All genes
Nº of genomes with TsaB1
Nº of genomes without TsaB1
With TsaB1
Without TsaB1
p < 0.00005
Nº of genomes with TsaB2
Nº of genomes without TsaB2
With TsaB2
Without TsaB2
p < 0.00005
Nº of genomes with TsaC1
Nº of genomes without TsaC1
With TsaC1
Without TsaC1
p < 0.00005
Nº of genomes with TsaC2
Nº of genomes without TsaC2
With TsaC2
Without TsaC2
p < 0.00005
Nº of genomes with TsaD1
Nº of genomes without TsaD1
With TsaD1
Without TsaD1
Nº of genomes with TsaD2
Nº of genomes without TsaD2
With TsaD2
Without TsaD2
Nº of genomes with TsaD3
Nº of genomes without TsaD3
With TsaD3
Without TsaD3
Nº of genomes with TsaE1
Nº of genomes without TsaE1
With TsaE1
Without TsaE1
p < 0.00005
Nº of genomes with TsaE2
Nº of genomes without TsaE2
With TsaE2
Without TsaE2
Nº of genomes with TsaE3
Nº of genomes without TsaE3
With TsaE3
Without TsaE3
p < 0.00005

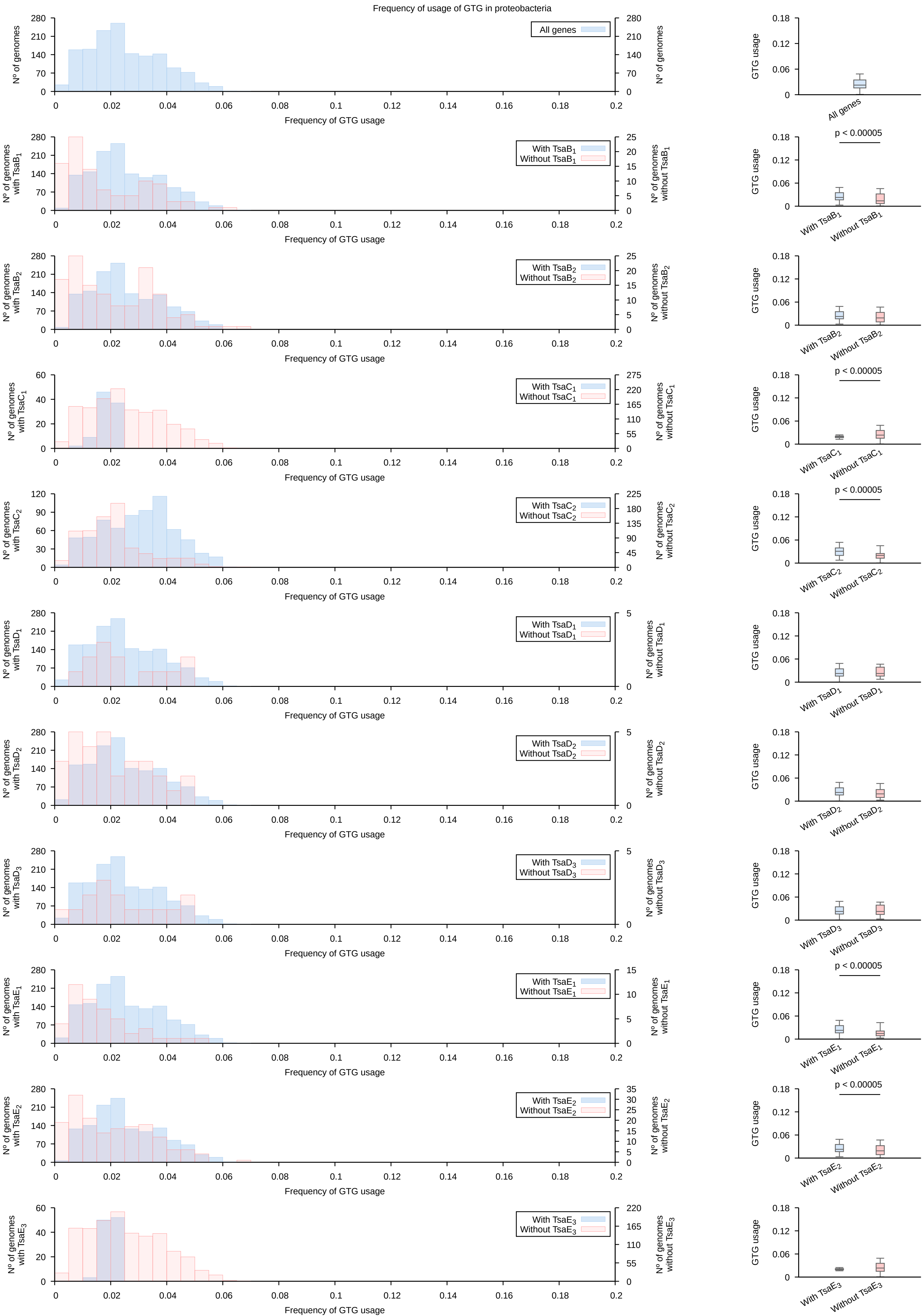
Frequency of usage of GTG in proteobacteria
Nº of genomes
All genes
Frequency of GTG usage
GTG usage
Nº of genomes with TsaB1
Nº of genomes without TsaB1
With TsaB1
Without TsaB1
p < 0.00005
Nº of genomes with TsaB2
Nº of genomes without TsaB2
With TsaB2
Without TsaB2
Nº of genomes with TsaC1
Nº of genomes without TsaC1
With TsaC1
Without TsaC1
p < 0.00005
Nº of genomes with TsaC2
Nº of genomes without TsaC2
With TsaC2
Without TsaC2
p < 0.00005
Nº of genomes with TsaD1
Nº of genomes without TsaD1
With TsaD1
Without TsaD1
Nº of genomes with TsaD2
Nº of genomes without TsaD2
With TsaD2
Without TsaD2
Nº of genomes with TsaD3
Nº of genomes without TsaD3
With TsaD3
Without TsaD3
Nº of genomes with TsaE1
Nº of genomes without TsaE1
With TsaE1
Without TsaE1
p < 0.00005
Nº of genomes with TsaE2
Nº of genomes without TsaE2
With TsaE2
Without TsaE2
p < 0.00005
Nº of genomes with TsaE3
Nº of genomes without TsaE3
With TsaE3
Without TsaE3

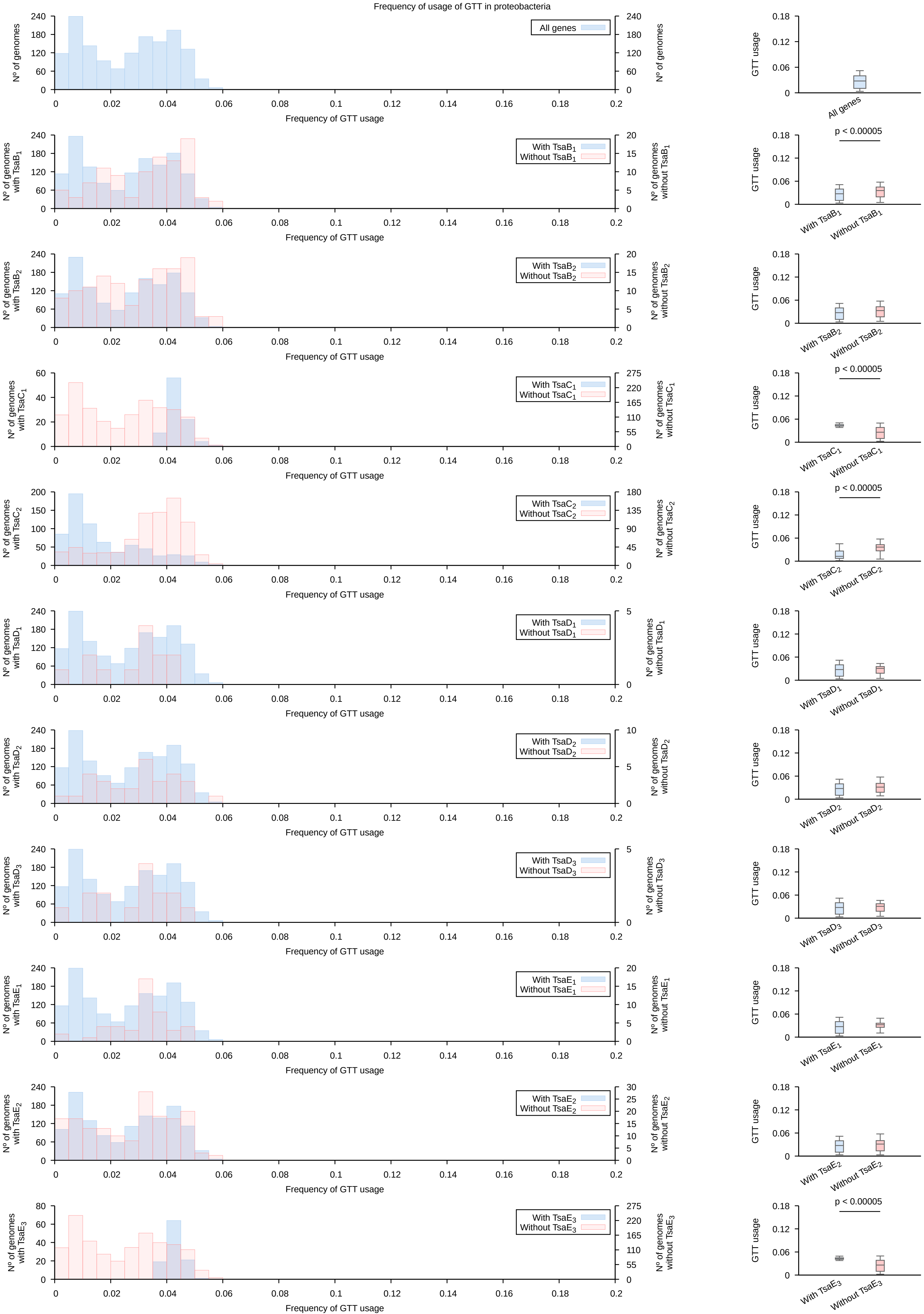

Frequency of usage of GTT in proteobacteria
Nº of genomes
All genes
Frequency of GTT usage
GTT usage
Nº of genomes with TsaB$_1$
Nº of genomes without TsaB$_1$
With TsaB$_1$
Without TsaB$_1$
p < 0.00005
Nº of genomes with TsaB$_2$
Nº of genomes without TsaB$_2$
With TsaB$_2$
Without TsaB$_2$
Nº of genomes with TsaC$_1$
Nº of genomes without TsaC$_1$
With TsaC$_1$
Without TsaC$_1$
p < 0.00005
Nº of genomes with TsaC$_2$
Nº of genomes without TsaC$_2$
With TsaC$_2$
Without TsaC$_2$
p < 0.00005
Nº of genomes with TsaD$_1$
Nº of genomes without TsaD$_1$
With TsaD$_1$
Without TsaD$_1$
Nº of genomes with TsaD$_2$
Nº of genomes without TsaD$_2$
With TsaD$_2$
Without TsaD$_2$
Nº of genomes with TsaD$_3$
Nº of genomes without TsaD$_3$
With TsaD$_3$
Without TsaD$_3$
Nº of genomes with TsaE$_1$
Nº of genomes without TsaE$_1$
With TsaE$_1$
Without TsaE$_1$
Nº of genomes with TsaE$_2$
Nº of genomes without TsaE$_2$
With TsaE$_2$
Without TsaE$_2$
Nº of genomes with TsaE$_3$
Nº of genomes without TsaE$_3$
With TsaE$_3$
Without TsaE$_3$
p < 0.00005

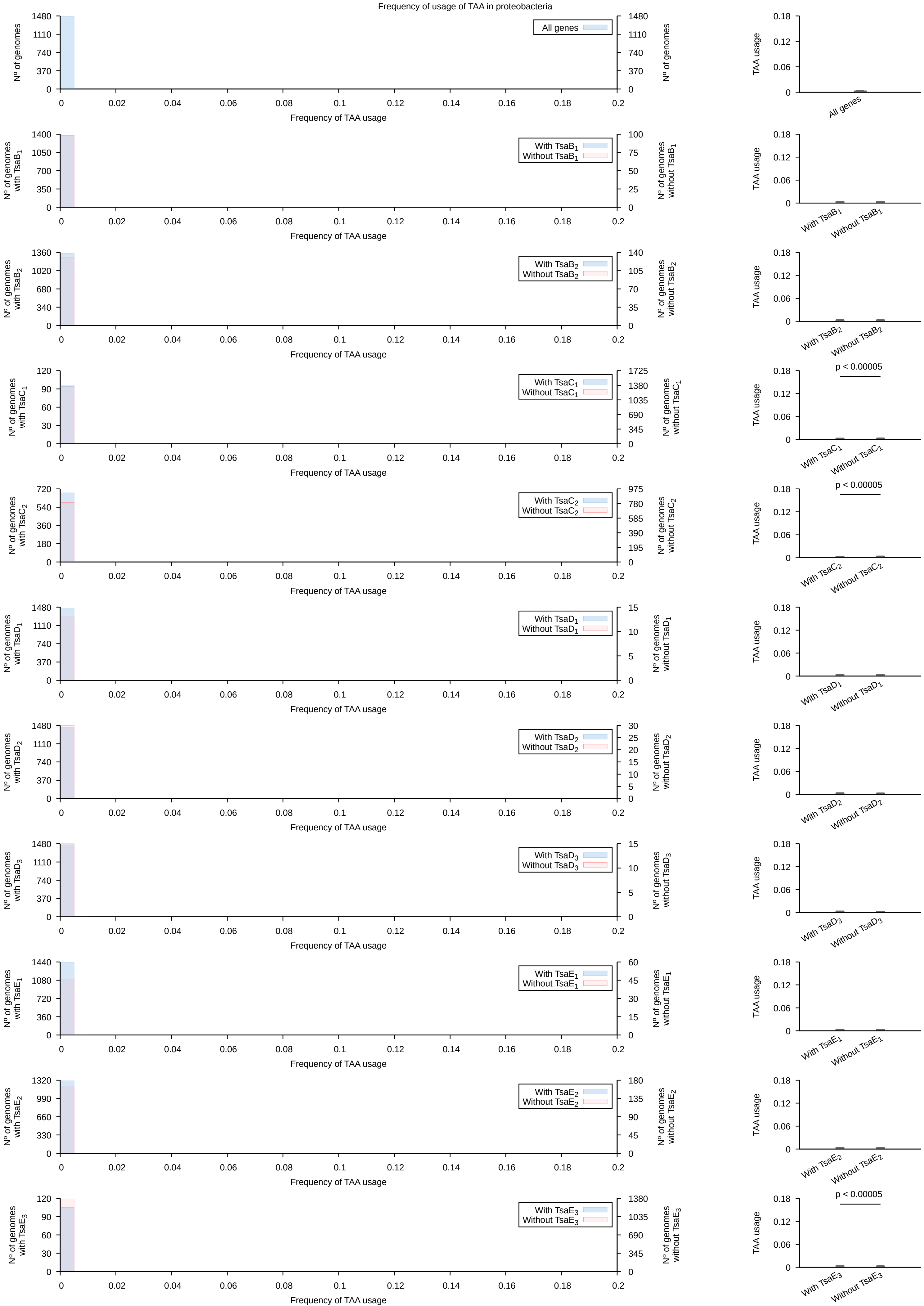
Frequency of usage of TAA in proteobacteria
Nº of genomes
Frequency of TAA usage
TAA usage
All genes
With TsaB1
Without TsaB1
With TsaB2
Without TsaB2
With TsaC1
Without TsaC1
p < 0.00005
With TsaC2
Without TsaC2
p < 0.00005
With TsaD1
Without TsaD1
With TsaD2
Without TsaD2
With TsaD3
Without TsaD3
With TsaE1
Without TsaE1
With TsaE2
Without TsaE2
With TsaE3
Without TsaE3
p < 0.00005

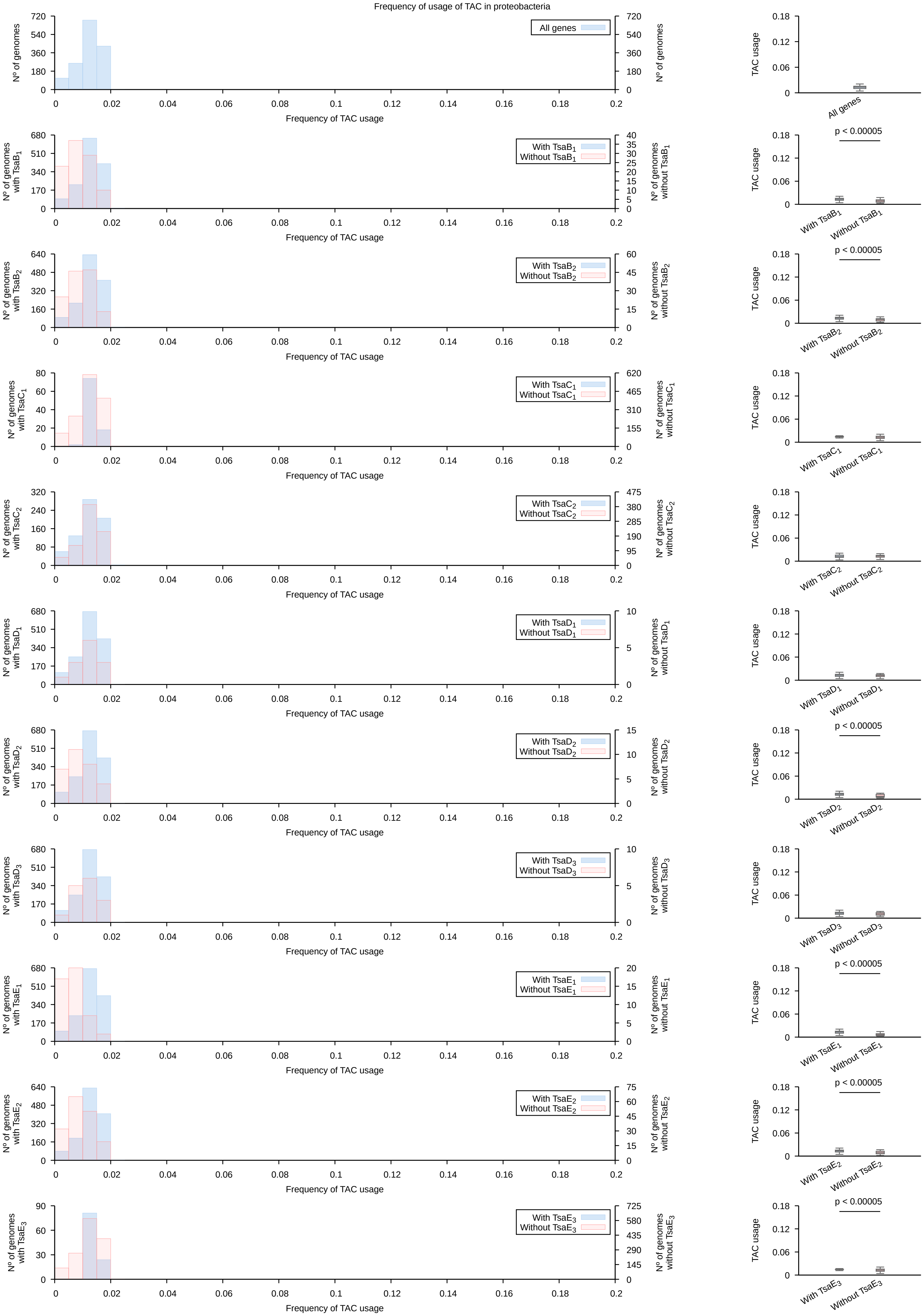

Frequency of usage of TAC in proteobacteria
Frequency of TAC usage
Nº of genomes
TAC usage
All genes
With TsaB1
Without TsaB1
Nº of genomes with TsaB1
Nº of genomes without TsaB1
p < 0.00005
With TsaB2
Without TsaB2
Nº of genomes with TsaB2
Nº of genomes without TsaB2
p < 0.00005
With TsaC1
Without TsaC1
Nº of genomes with TsaC1
Nº of genomes without TsaC1
With TsaC2
Without TsaC2
Nº of genomes with TsaC2
Nº of genomes without TsaC2
With TsaD1
Without TsaD1
Nº of genomes with TsaD1
Nº of genomes without TsaD1
With TsaD2
Without TsaD2
Nº of genomes with TsaD2
Nº of genomes without TsaD2
p < 0.00005
With TsaD3
Without TsaD3
Nº of genomes with TsaD3
Nº of genomes without TsaD3
With TsaE1
Without TsaE1
Nº of genomes with TsaE1
Nº of genomes without TsaE1
p < 0.00005
With TsaE2
Without TsaE2
Nº of genomes with TsaE2
Nº of genomes without TsaE2
p < 0.00005
With TsaE3
Without TsaE3
Nº of genomes with TsaE3
Nº of genomes without TsaE3
p < 0.00005

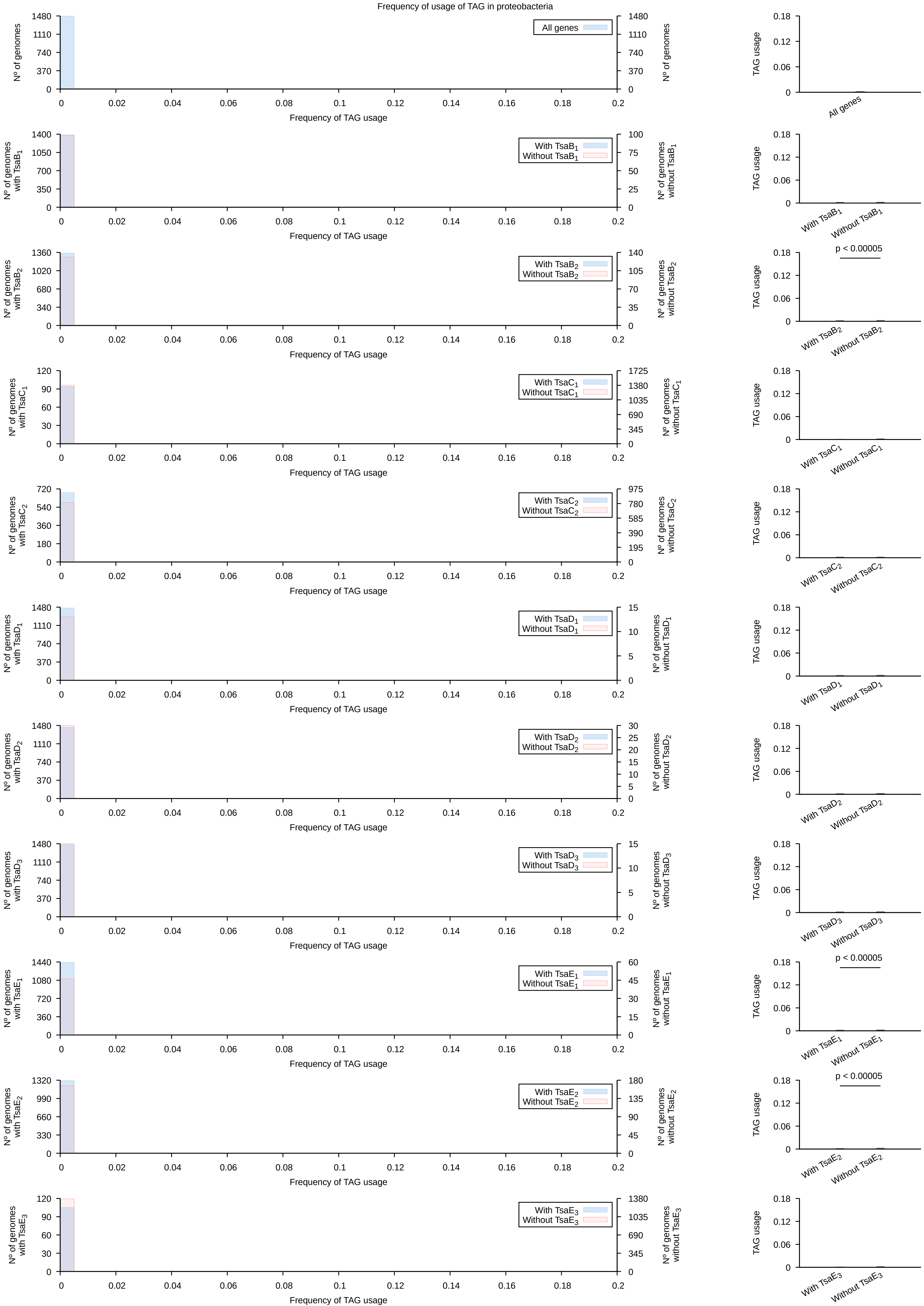

Frequency of usage of TAG in proteobacteria
All genes
Nº of genomes
Frequency of TAG usage
TAG usage
0 0.02 0.04 0.06 0.08 0.1 0.12 0.14 0.16 0.18 0.2
0 0.06 0.12 0.18
With $TsaB_1$
Without $TsaB_1$
Nº of genomes with $TsaB_1$
Nº of genomes without $TsaB_1$
With $TsaB_2$
Without $TsaB_2$
Nº of genomes with $TsaB_2$
Nº of genomes without $TsaB_2$
p < 0.00005
With $TsaC_1$
Without $TsaC_1$
Nº of genomes with $TsaC_1$
Nº of genomes without $TsaC_1$
With $TsaC_2$
Without $TsaC_2$
Nº of genomes with $TsaC_2$
Nº of genomes without $TsaC_2$
With $TsaD_1$
Without $TsaD_1$
Nº of genomes with $TsaD_1$
Nº of genomes without $TsaD_1$
With $TsaD_2$
Without $TsaD_2$
Nº of genomes with $TsaD_2$
Nº of genomes without $TsaD_2$
With $TsaD_3$
Without $TsaD_3$
Nº of genomes with $TsaD_3$
Nº of genomes without $TsaD_3$
With $TsaE_1$
Without $TsaE_1$
Nº of genomes with $TsaE_1$
Nº of genomes without $TsaE_1$
p < 0.00005
With $TsaE_2$
Without $TsaE_2$
Nº of genomes with $TsaE_2$
Nº of genomes without $TsaE_2$
p < 0.00005
With $TsaE_3$
Without $TsaE_3$
Nº of genomes with $TsaE_3$
Nº of genomes without $TsaE_3$

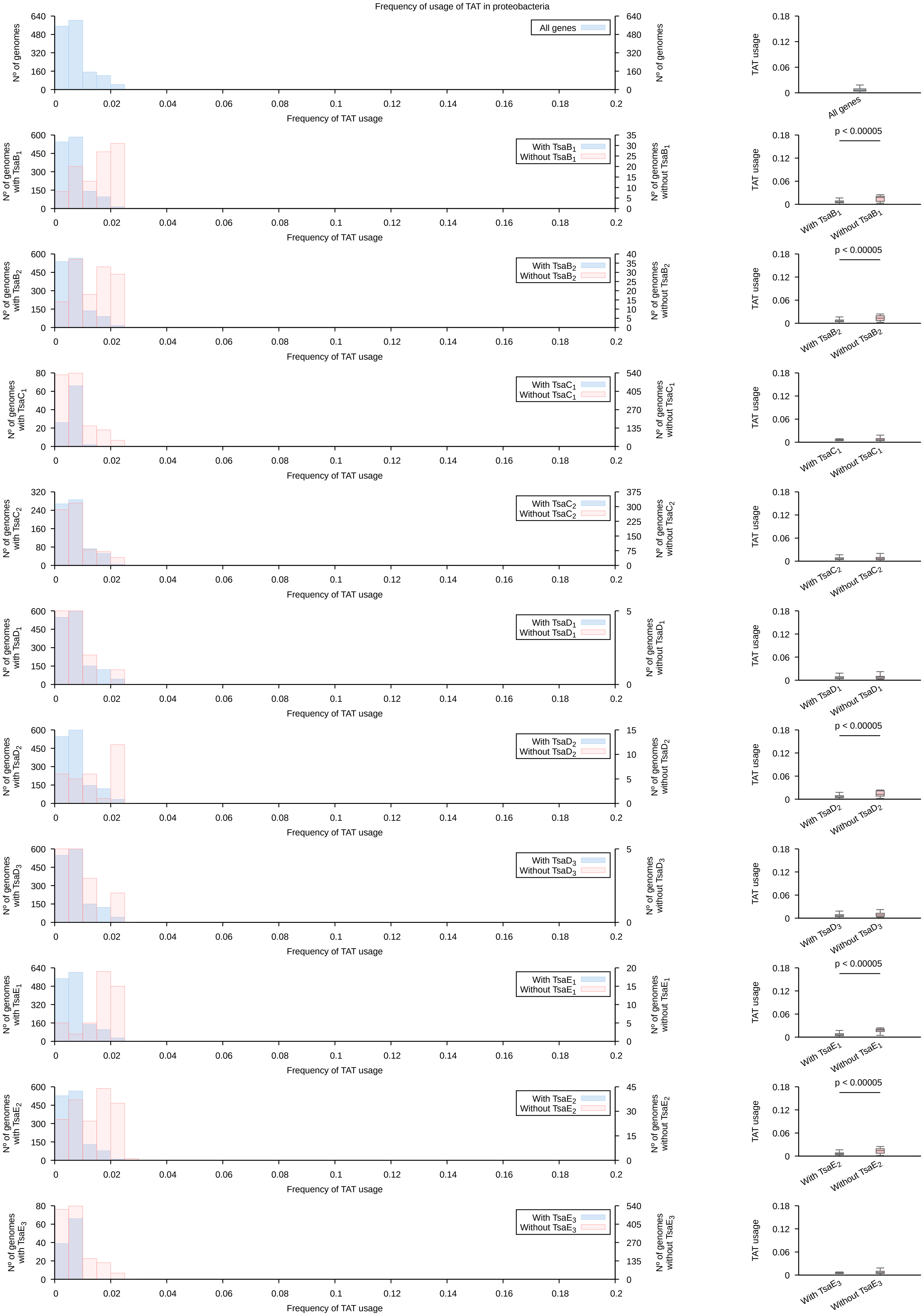
Frequency of usage of TAT in proteobacteria
All genes
Nº of genomes
Frequency of TAT usage
TAT usage
With TsaB$_1$
Without TsaB$_1$
Nº of genomes with TsaB$_1$
Nº of genomes without TsaB$_1$
p < 0.00005
With TsaB$_2$
Without TsaB$_2$
Nº of genomes with TsaB$_2$
Nº of genomes without TsaB$_2$
p < 0.00005
With TsaC$_1$
Without TsaC$_1$
Nº of genomes with TsaC$_1$
Nº of genomes without TsaC$_1$
With TsaC$_2$
Without TsaC$_2$
Nº of genomes with TsaC$_2$
Nº of genomes without TsaC$_2$
With TsaD$_1$
Without TsaD$_1$
Nº of genomes with TsaD$_1$
Nº of genomes without TsaD$_1$
With TsaD$_2$
Without TsaD$_2$
Nº of genomes with TsaD$_2$
Nº of genomes without TsaD$_2$
p < 0.00005
With TsaD$_3$
Without TsaD$_3$
Nº of genomes with TsaD$_3$
Nº of genomes without TsaD$_3$
With TsaE$_1$
Without TsaE$_1$
Nº of genomes with TsaE$_1$
Nº of genomes without TsaE$_1$
p < 0.00005
With TsaE$_2$
Without TsaE$_2$
Nº of genomes with TsaE$_2$
Nº of genomes without TsaE$_2$
p < 0.00005
With TsaE$_3$
Without TsaE$_3$
Nº of genomes with TsaE$_3$
Nº of genomes without TsaE$_3$

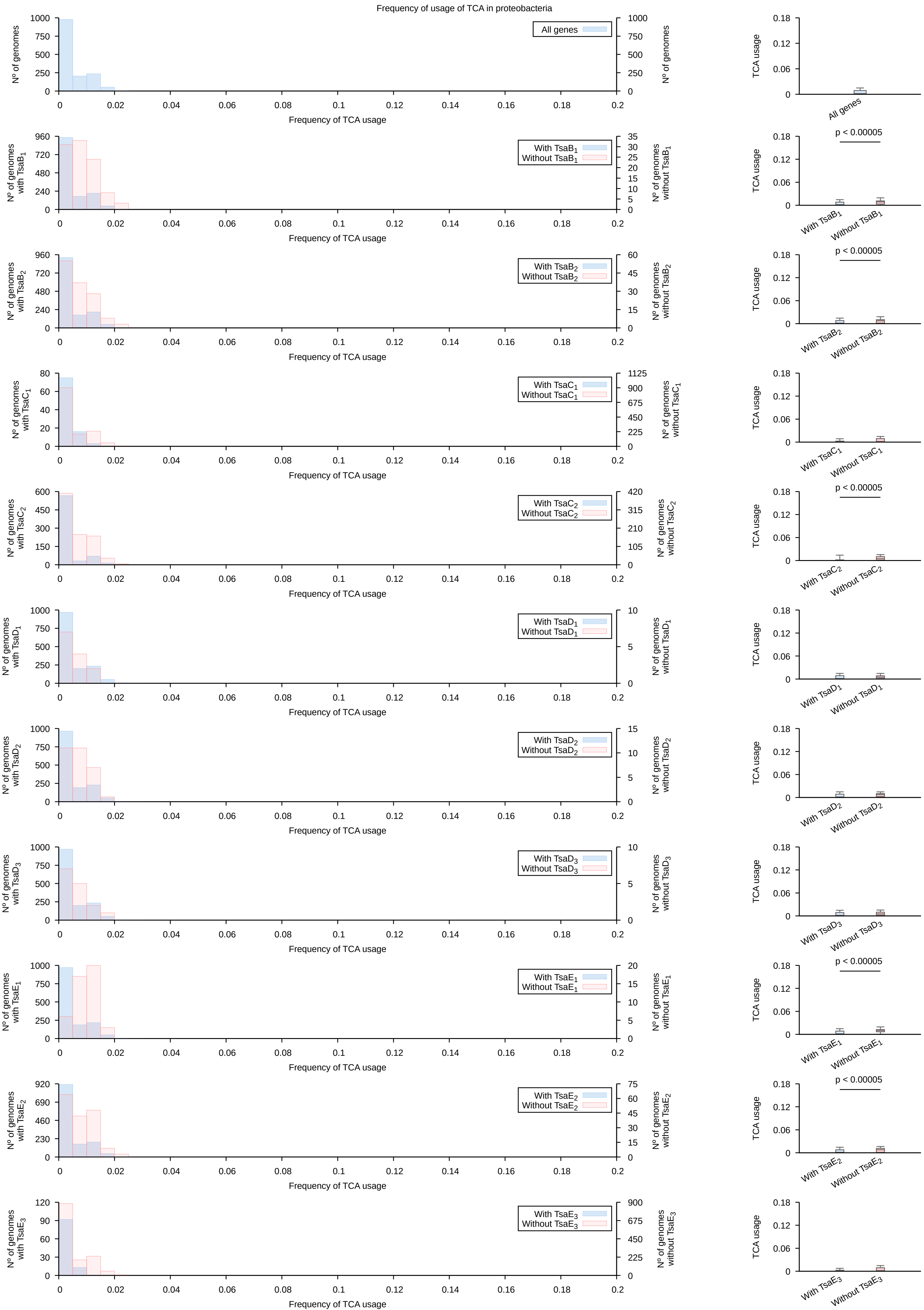
Frequency of usage of TCA in proteobacteria
All genes
Nº of genomes
Frequency of TCA usage
TCA usage
With TsaB1
Without TsaB1
Nº of genomes with TsaB1
Nº of genomes without TsaB1
p < 0.00005
With TsaB2
Without TsaB2
Nº of genomes with TsaB2
Nº of genomes without TsaB2
p < 0.00005
With TsaC1
Without TsaC1
Nº of genomes with TsaC1
Nº of genomes without TsaC1
With TsaC2
Without TsaC2
Nº of genomes with TsaC2
Nº of genomes without TsaC2
p < 0.00005
With TsaD1
Without TsaD1
Nº of genomes with TsaD1
Nº of genomes without TsaD1
With TsaD2
Without TsaD2
Nº of genomes with TsaD2
Nº of genomes without TsaD2
With TsaD3
Without TsaD3
Nº of genomes with TsaD3
Nº of genomes without TsaD3
With TsaE1
Without TsaE1
Nº of genomes with TsaE1
Nº of genomes without TsaE1
p < 0.00005
With TsaE2
Without TsaE2
Nº of genomes with TsaE2
Nº of genomes without TsaE2
p < 0.00005
With TsaE3
Without TsaE3
Nº of genomes with TsaE3
Nº of genomes without TsaE3

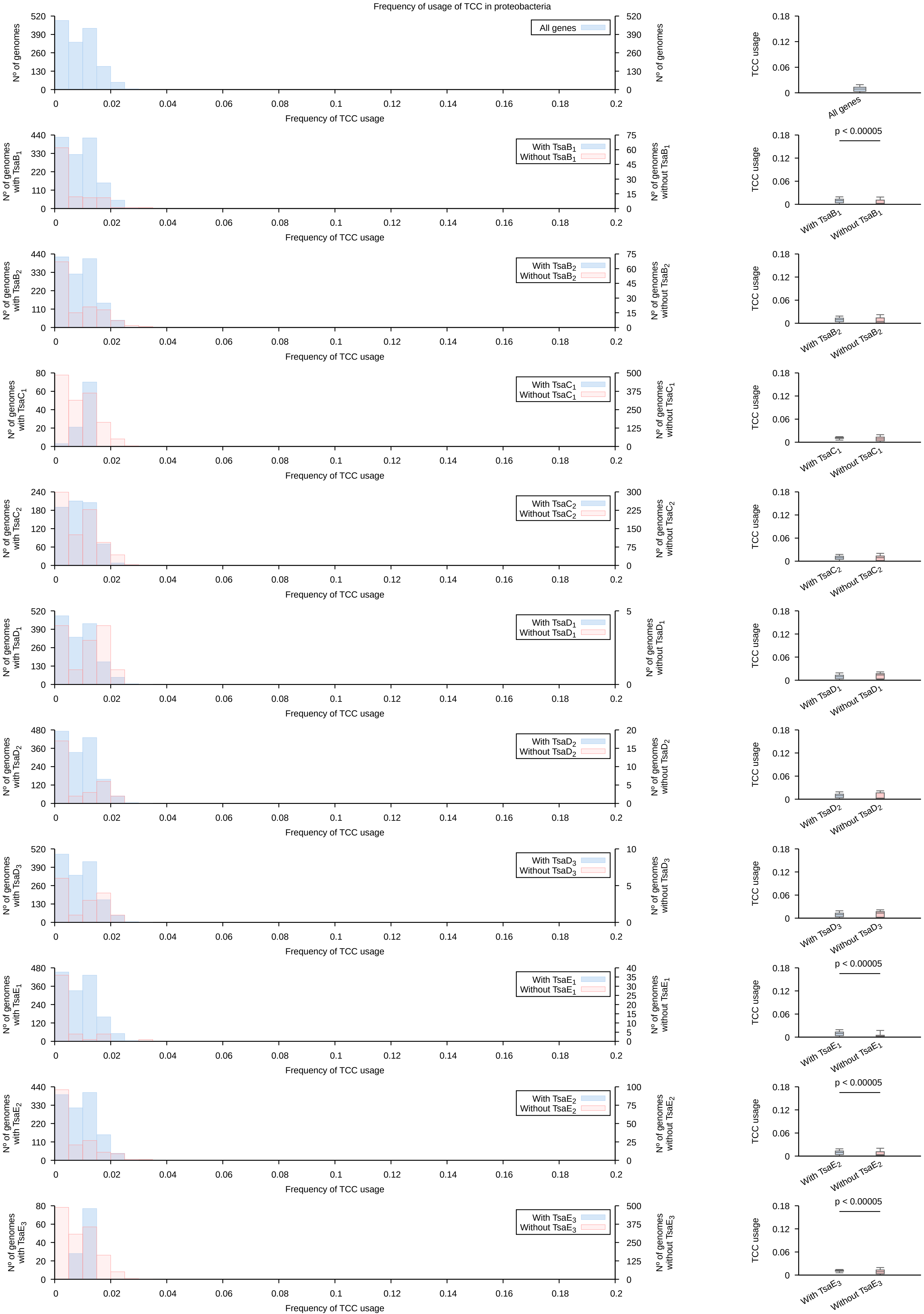

Frequency of usage of TCC in proteobacteria
Nº of genomes
Frequency of TCC usage
TCC usage
All genes
Nº of genomes with TsaB1
Nº of genomes without TsaB1
With TsaB1
Without TsaB1
p < 0.00005
Nº of genomes with TsaB2
Nº of genomes without TsaB2
With TsaB2
Without TsaB2
Nº of genomes with TsaC1
Nº of genomes without TsaC1
With TsaC1
Without TsaC1
Nº of genomes with TsaC2
Nº of genomes without TsaC2
With TsaC2
Without TsaC2
Nº of genomes with TsaD1
Nº of genomes without TsaD1
With TsaD1
Without TsaD1
Nº of genomes with TsaD2
Nº of genomes without TsaD2
With TsaD2
Without TsaD2
Nº of genomes with TsaD3
Nº of genomes without TsaD3
With TsaD3
Without TsaD3
Nº of genomes with TsaE1
Nº of genomes without TsaE1
With TsaE1
Without TsaE1
p < 0.00005
Nº of genomes with TsaE2
Nº of genomes without TsaE2
With TsaE2
Without TsaE2
p < 0.00005
Nº of genomes with TsaE3
Nº of genomes without TsaE3
With TsaE3
Without TsaE3
p < 0.00005

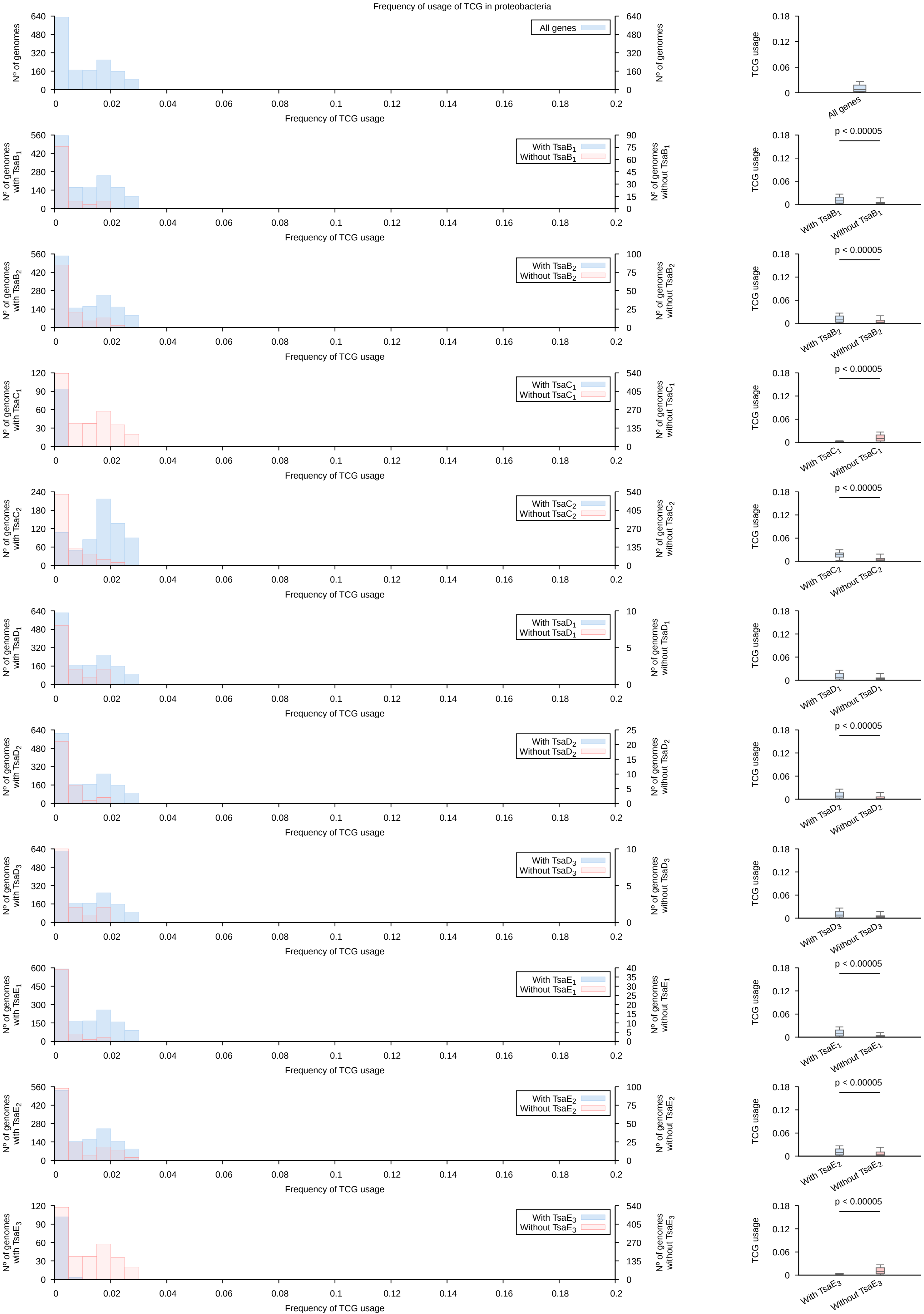

Frequency of usage of TCG in proteobacteria
All genes
Nº of genomes
Frequency of TCG usage
TCG usage
With TsaB$_1$
Without TsaB$_1$
Nº of genomes with TsaB$_1$
Nº of genomes without TsaB$_1$
With TsaB$_2$
Without TsaB$_2$
Nº of genomes with TsaB$_2$
Nº of genomes without TsaB$_2$
With TsaC$_1$
Without TsaC$_1$
Nº of genomes with TsaC$_1$
Nº of genomes without TsaC$_1$
With TsaC$_2$
Without TsaC$_2$
Nº of genomes with TsaC$_2$
Nº of genomes without TsaC$_2$
With TsaD$_1$
Without TsaD$_1$
Nº of genomes with TsaD$_1$
Nº of genomes without TsaD$_1$
With TsaD$_2$
Without TsaD$_2$
Nº of genomes with TsaD$_2$
Nº of genomes without TsaD$_2$
With TsaD$_3$
Without TsaD$_3$
Nº of genomes with TsaD$_3$
Nº of genomes without TsaD$_3$
With TsaE$_1$
Without TsaE$_1$
Nº of genomes with TsaE$_1$
Nº of genomes without TsaE$_1$
With TsaE$_2$
Without TsaE$_2$
Nº of genomes with TsaE$_2$
Nº of genomes without TsaE$_2$
With TsaE$_3$
Without TsaE$_3$
Nº of genomes with TsaE$_3$
Nº of genomes without TsaE$_3$
p < 0.00005

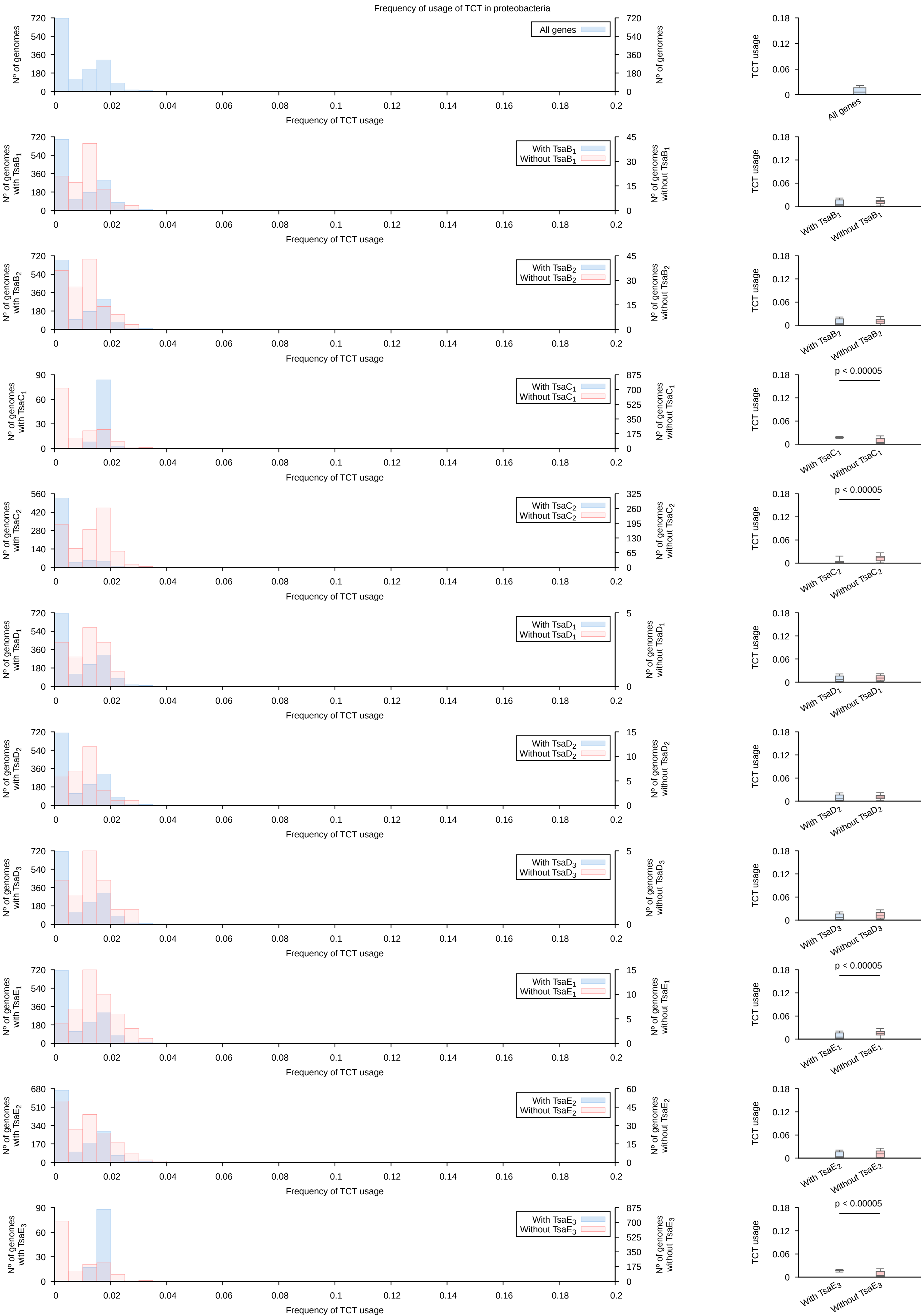
Frequency of usage of TCT in proteobacteria
Nº of genomes
All genes
Frequency of TCT usage
0
0.02
0.04
0.06
0.08
0.1
0.12
0.14
0.16
0.18
0.2
720
540
360
180
TCT usage
0.18
0.12
0.06
Nº of genomes with TsaB$_1$
Nº of genomes without TsaB$_1$
With TsaB$_1$
Without TsaB$_1$
Nº of genomes with TsaB$_2$
Nº of genomes without TsaB$_2$
With TsaB$_2$
Without TsaB$_2$
Nº of genomes with TsaC$_1$
Nº of genomes without TsaC$_1$
With TsaC$_1$
Without TsaC$_1$
p < 0.00005
Nº of genomes with TsaC$_2$
Nº of genomes without TsaC$_2$
With TsaC$_2$
Without TsaC$_2$
p < 0.00005
Nº of genomes with TsaD$_1$
Nº of genomes without TsaD$_1$
With TsaD$_1$
Without TsaD$_1$
Nº of genomes with TsaD$_2$
Nº of genomes without TsaD$_2$
With TsaD$_2$
Without TsaD$_2$
Nº of genomes with TsaD$_3$
Nº of genomes without TsaD$_3$
With TsaD$_3$
Without TsaD$_3$
Nº of genomes with TsaE$_1$
Nº of genomes without TsaE$_1$
With TsaE$_1$
Without TsaE$_1$
p < 0.00005
Nº of genomes with TsaE$_2$
Nº of genomes without TsaE$_2$
With TsaE$_2$
Without TsaE$_2$
Nº of genomes with TsaE$_3$
Nº of genomes without TsaE$_3$
With TsaE$_3$
Without TsaE$_3$
p < 0.00005

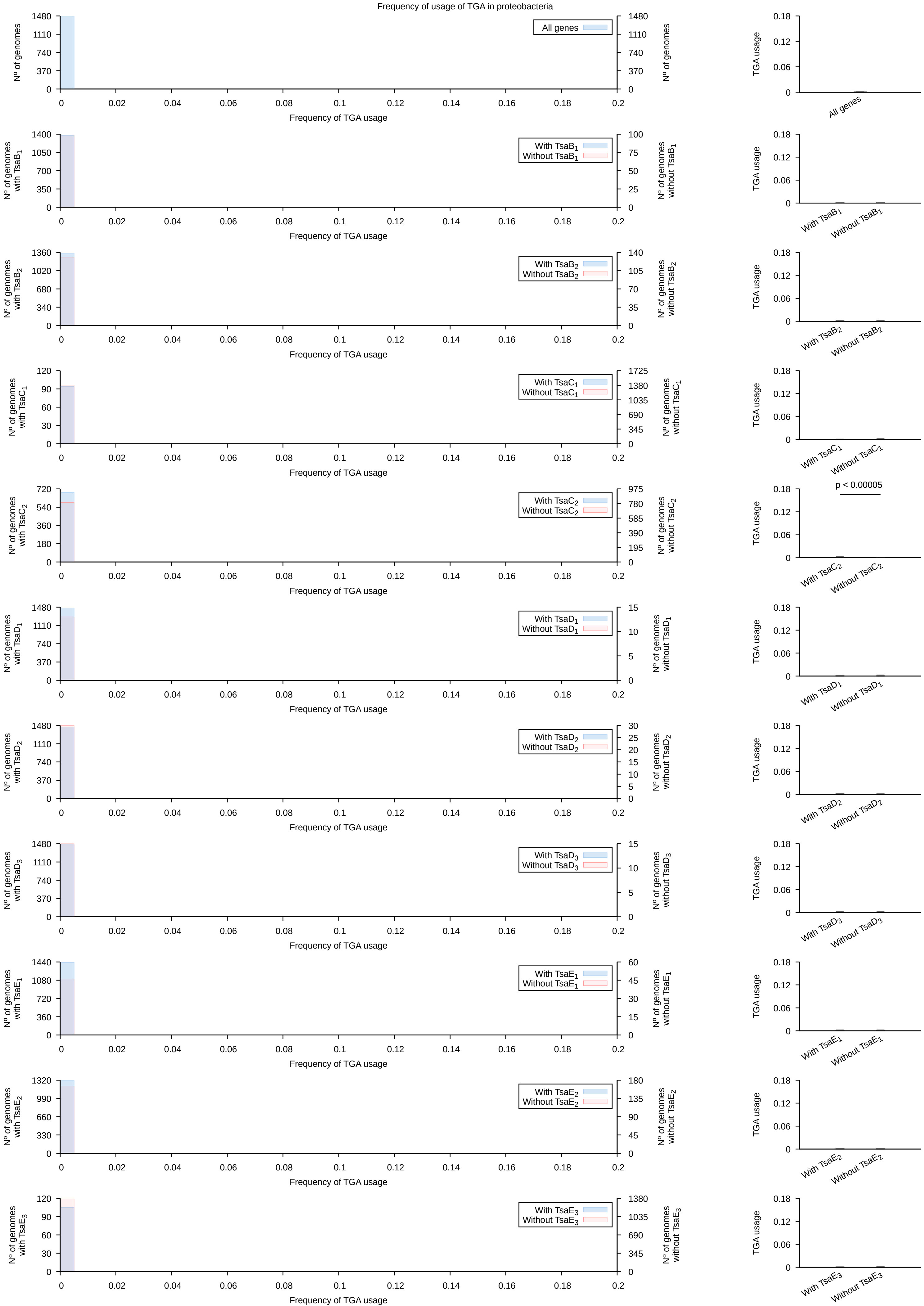
Frequency of usage of TGA in proteobacteria
Nº of genomes
Frequency of TGA usage
TGA usage
All genes
With TsaB$_1$
Without TsaB$_1$
Nº of genomes with TsaB$_1$
Nº of genomes without TsaB$_1$
With TsaB$_2$
Without TsaB$_2$
Nº of genomes with TsaB$_2$
Nº of genomes without TsaB$_2$
With TsaC$_1$
Without TsaC$_1$
Nº of genomes with TsaC$_1$
Nº of genomes without TsaC$_1$
With TsaC$_2$
Without TsaC$_2$
Nº of genomes with TsaC$_2$
Nº of genomes without TsaC$_2$
p < 0.00005
With TsaD$_1$
Without TsaD$_1$
Nº of genomes with TsaD$_1$
Nº of genomes without TsaD$_1$
With TsaD$_2$
Without TsaD$_2$
Nº of genomes with TsaD$_2$
Nº of genomes without TsaD$_2$
With TsaD$_3$
Without TsaD$_3$
Nº of genomes with TsaD$_3$
Nº of genomes without TsaD$_3$
With TsaE$_1$
Without TsaE$_1$
Nº of genomes with TsaE$_1$
Nº of genomes without TsaE$_1$
With TsaE$_2$
Without TsaE$_2$
Nº of genomes with TsaE$_2$
Nº of genomes without TsaE$_2$
With TsaE$_3$
Without TsaE$_3$
Nº of genomes with TsaE$_3$
Nº of genomes without TsaE$_3$
0 0.02 0.04 0.06 0.08 0.1 0.12 0.14 0.16 0.18 0.2
0 0.06 0.12 0.18

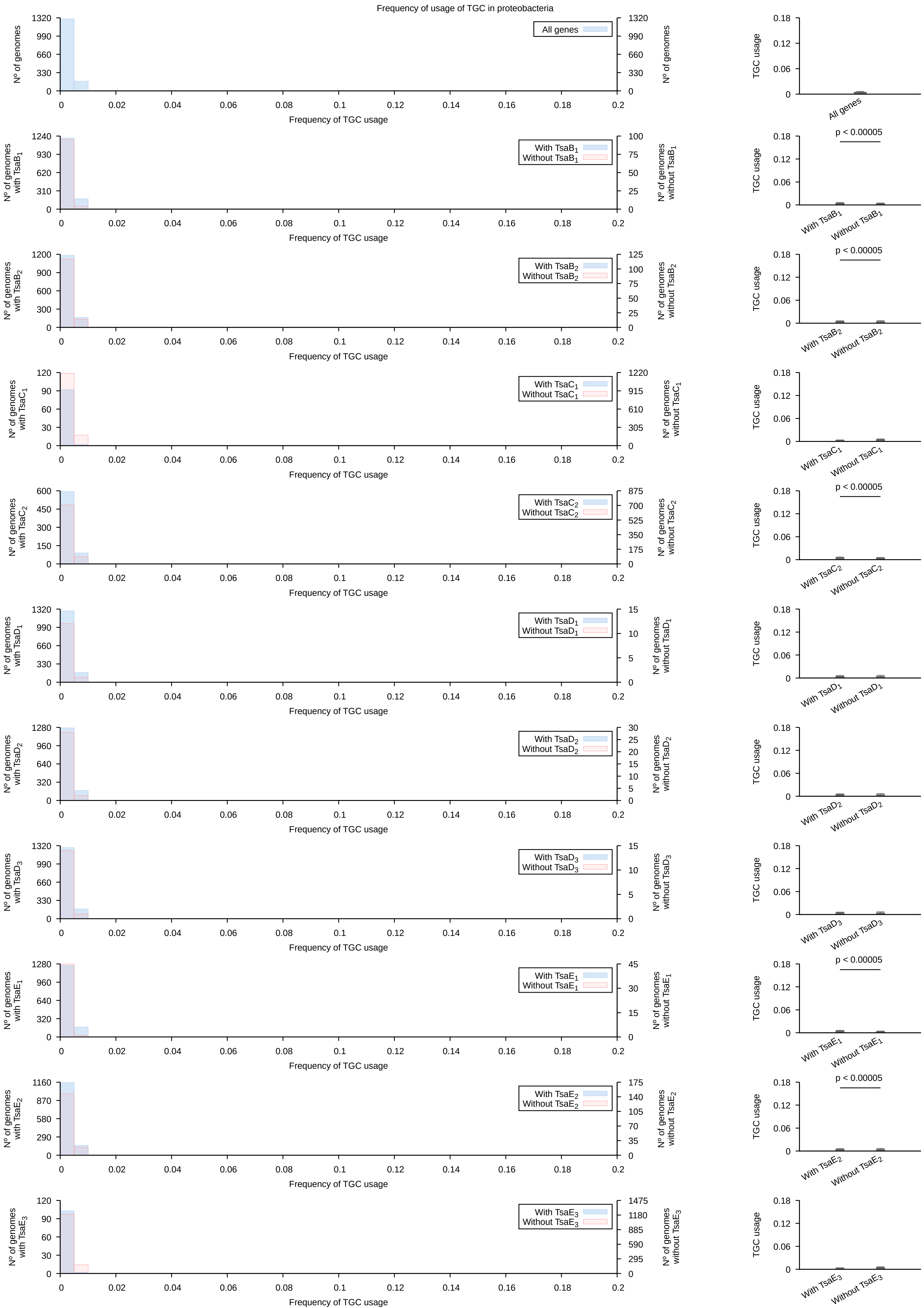

Frequency of usage of TGC in proteobacteria
All genes
Nº of genomes
Frequency of TGC usage
TGC usage
With TsaB$_1$
Without TsaB$_1$
With TsaB$_2$
Without TsaB$_2$
With TsaC$_1$
Without TsaC$_1$
With TsaC$_2$
Without TsaC$_2$
With TsaD$_1$
Without TsaD$_1$
With TsaD$_2$
Without TsaD$_2$
With TsaD$_3$
Without TsaD$_3$
With TsaE$_1$
Without TsaE$_1$
With TsaE$_2$
Without TsaE$_2$
With TsaE$_3$
Without TsaE$_3$
p < 0.00005

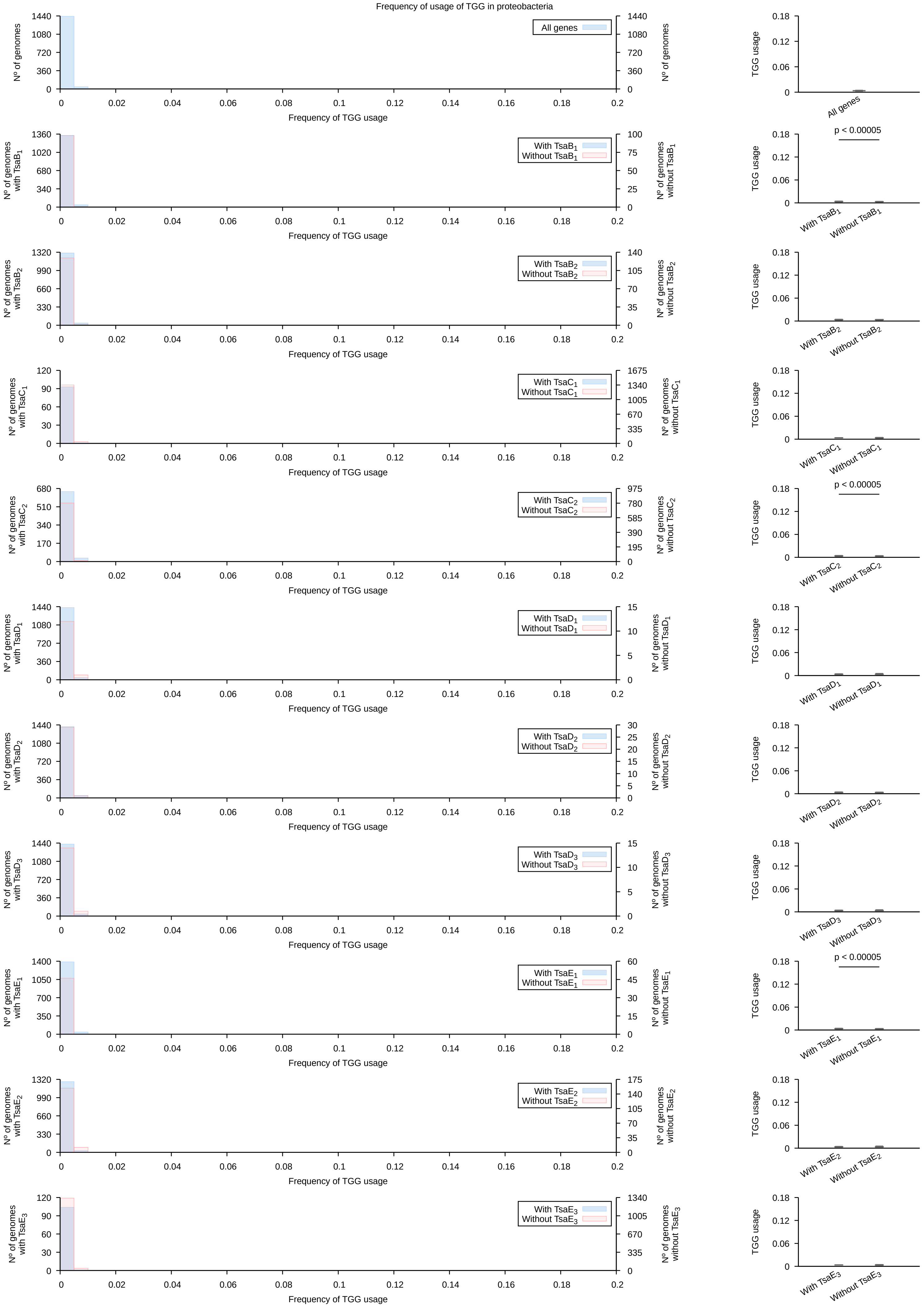

Frequency of usage of TGG in proteobacteria
All genes
Nº of genomes
Frequency of TGG usage
TGG usage
With TsaB$_1$
Without TsaB$_1$
Nº of genomes with TsaB$_1$
Nº of genomes without TsaB$_1$
p < 0.00005
With TsaB$_2$
Without TsaB$_2$
Nº of genomes with TsaB$_2$
Nº of genomes without TsaB$_2$
With TsaC$_1$
Without TsaC$_1$
Nº of genomes with TsaC$_1$
Nº of genomes without TsaC$_1$
With TsaC$_2$
Without TsaC$_2$
Nº of genomes with TsaC$_2$
Nº of genomes without TsaC$_2$
p < 0.00005
With TsaD$_1$
Without TsaD$_1$
Nº of genomes with TsaD$_1$
Nº of genomes without TsaD$_1$
With TsaD$_2$
Without TsaD$_2$
Nº of genomes with TsaD$_2$
Nº of genomes without TsaD$_2$
With TsaD$_3$
Without TsaD$_3$
Nº of genomes with TsaD$_3$
Nº of genomes without TsaD$_3$
With TsaE$_1$
Without TsaE$_1$
Nº of genomes with TsaE$_1$
Nº of genomes without TsaE$_1$
p < 0.00005
With TsaE$_2$
Without TsaE$_2$
Nº of genomes with TsaE$_2$
Nº of genomes without TsaE$_2$
With TsaE$_3$
Without TsaE$_3$
Nº of genomes with TsaE$_3$
Nº of genomes without TsaE$_3$

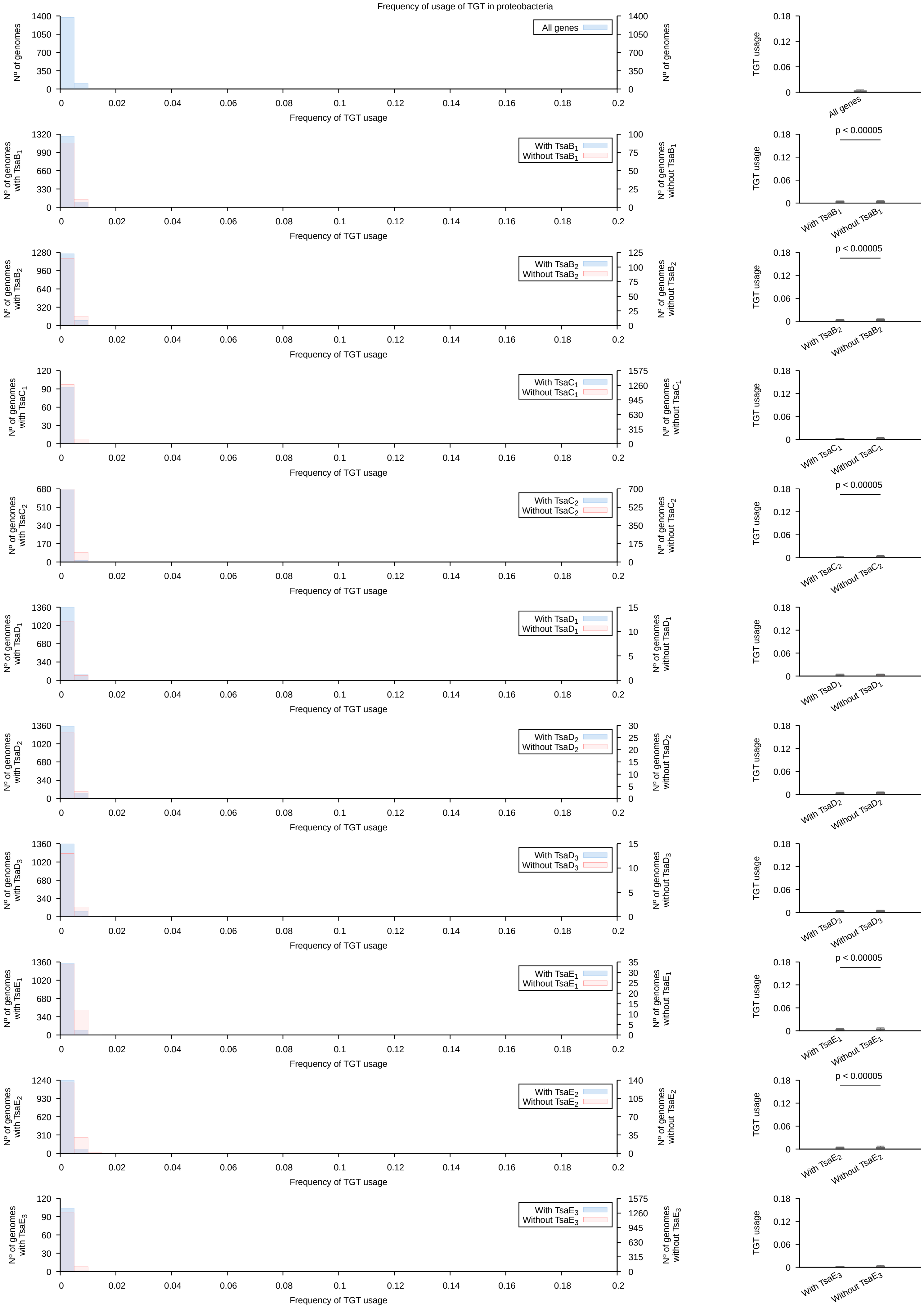
Frequency of usage of TGT in proteobacteria
All genes
Nº of genomes
Frequency of TGT usage
TGT usage
p < 0.00005
With $TsaB_1$
Without $TsaB_1$
With $TsaB_2$
Without $TsaB_2$
With $TsaC_1$
Without $TsaC_1$
With $TsaC_2$
Without $TsaC_2$
With $TsaD_1$
Without $TsaD_1$
With $TsaD_2$
Without $TsaD_2$
With $TsaD_3$
Without $TsaD_3$
With $TsaE_1$
Without $TsaE_1$
With $TsaE_2$
Without $TsaE_2$
With $TsaE_3$
Without $TsaE_3$

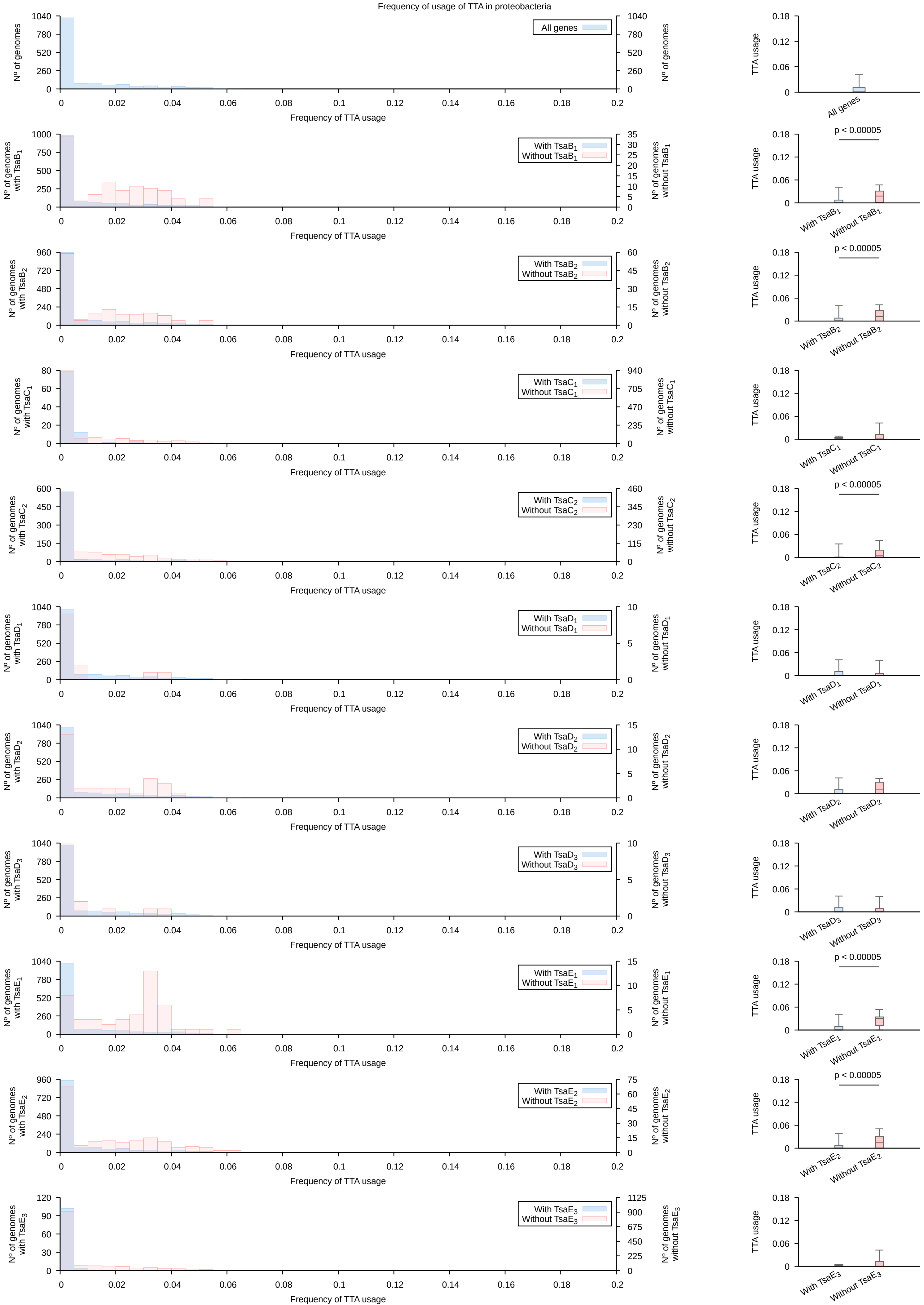

Frequency of usage of TTA in proteobacteria
All genes
Nº of genomes
Frequency of TTA usage
TTA usage
With TsaB$_1$
Without TsaB$_1$
Nº of genomes with TsaB$_1$
Nº of genomes without TsaB$_1$
p < 0.00005
With TsaB$_2$
Without TsaB$_2$
Nº of genomes with TsaB$_2$
Nº of genomes without TsaB$_2$
p < 0.00005
With TsaC$_1$
Without TsaC$_1$
Nº of genomes with TsaC$_1$
Nº of genomes without TsaC$_1$
With TsaC$_2$
Without TsaC$_2$
Nº of genomes with TsaC$_2$
Nº of genomes without TsaC$_2$
p < 0.00005
With TsaD$_1$
Without TsaD$_1$
Nº of genomes with TsaD$_1$
Nº of genomes without TsaD$_1$
With TsaD$_2$
Without TsaD$_2$
Nº of genomes with TsaD$_2$
Nº of genomes without TsaD$_2$
With TsaD$_3$
Without TsaD$_3$
Nº of genomes with TsaD$_3$
Nº of genomes without TsaD$_3$
With TsaE$_1$
Without TsaE$_1$
Nº of genomes with TsaE$_1$
Nº of genomes without TsaE$_1$
p < 0.00005
With TsaE$_2$
Without TsaE$_2$
Nº of genomes with TsaE$_2$
Nº of genomes without TsaE$_2$
p < 0.00005
With TsaE$_3$
Without TsaE$_3$
Nº of genomes with TsaE$_3$
Nº of genomes without TsaE$_3$

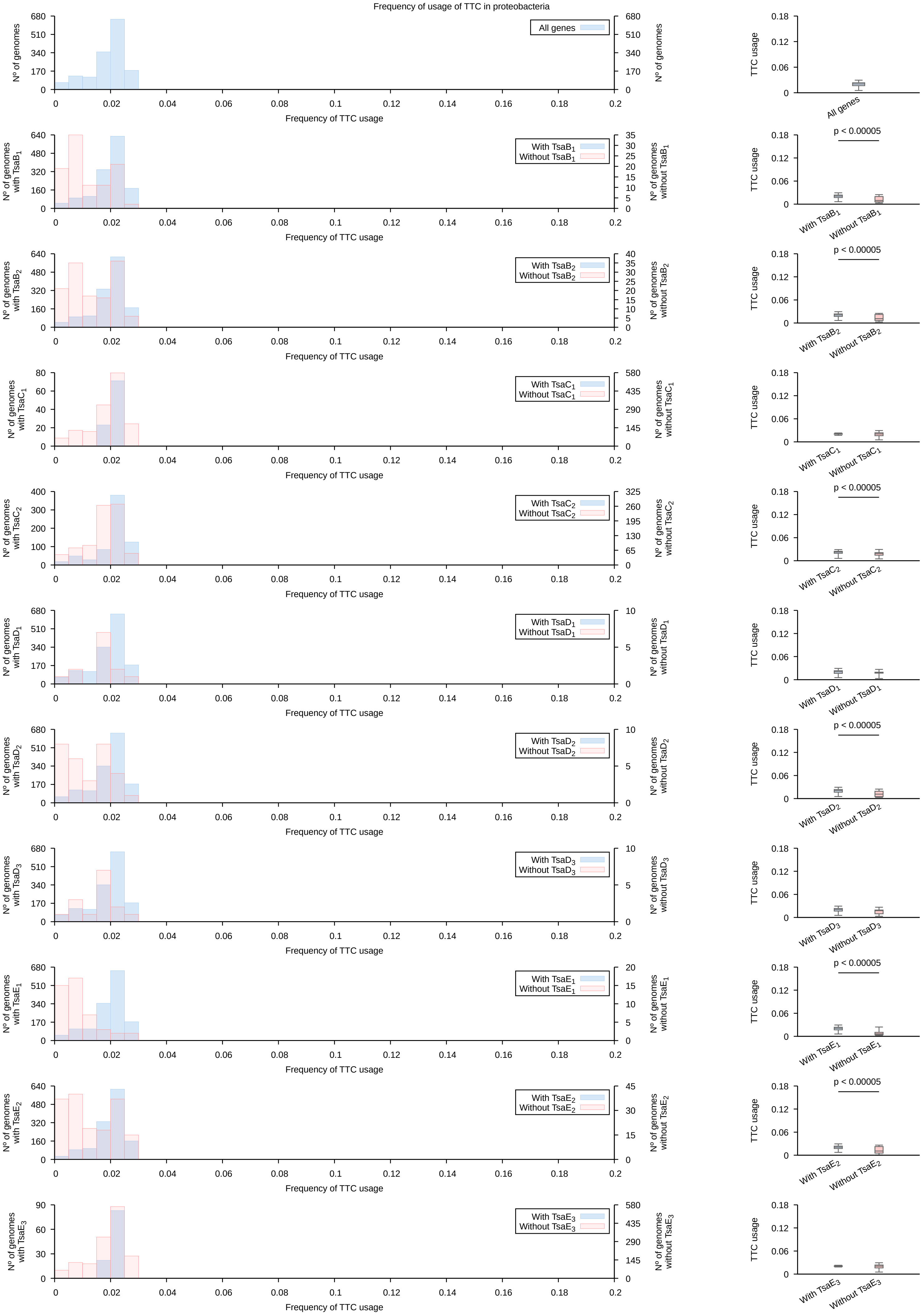

Frequency of usage of TTC in proteobacteria
All genes
Nº of genomes
Frequency of TTC usage
TTC usage
With TsaB$_1$
Without TsaB$_1$
Nº of genomes with TsaB$_1$
Nº of genomes without TsaB$_1$
p < 0.00005
With TsaB$_2$
Without TsaB$_2$
Nº of genomes with TsaB$_2$
Nº of genomes without TsaB$_2$
p < 0.00005
With TsaC$_1$
Without TsaC$_1$
Nº of genomes with TsaC$_1$
Nº of genomes without TsaC$_1$
With TsaC$_2$
Without TsaC$_2$
Nº of genomes with TsaC$_2$
Nº of genomes without TsaC$_2$
p < 0.00005
With TsaD$_1$
Without TsaD$_1$
Nº of genomes with TsaD$_1$
Nº of genomes without TsaD$_1$
With TsaD$_2$
Without TsaD$_2$
Nº of genomes with TsaD$_2$
Nº of genomes without TsaD$_2$
p < 0.00005
With TsaD$_3$
Without TsaD$_3$
Nº of genomes with TsaD$_3$
Nº of genomes without TsaD$_3$
With TsaE$_1$
Without TsaE$_1$
Nº of genomes with TsaE$_1$
Nº of genomes without TsaE$_1$
p < 0.00005
With TsaE$_2$
Without TsaE$_2$
Nº of genomes with TsaE$_2$
Nº of genomes without TsaE$_2$
p < 0.00005
With TsaE$_3$
Without TsaE$_3$
Nº of genomes with TsaE$_3$
Nº of genomes without TsaE$_3$

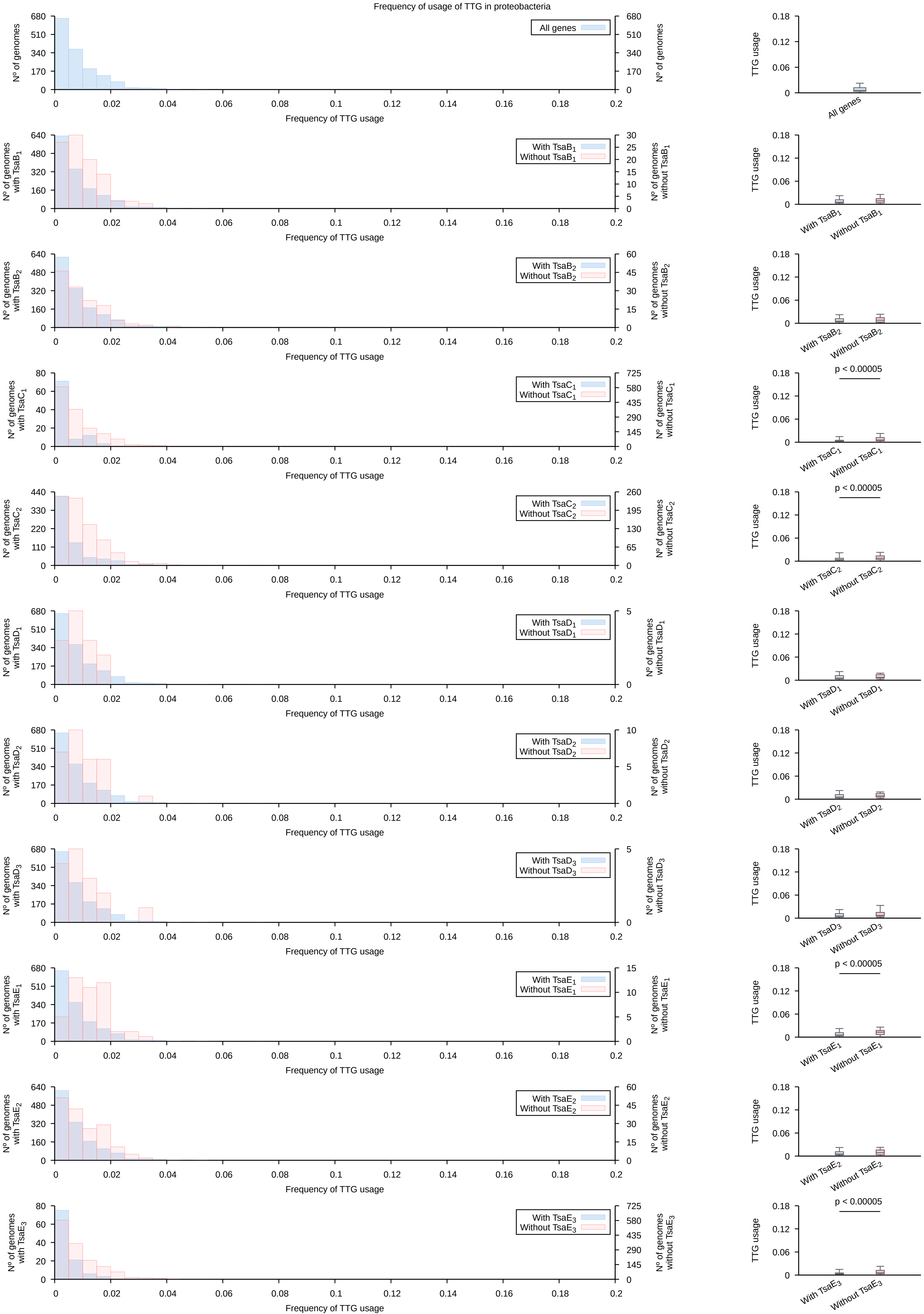
Frequency of usage of TTG in proteobacteria
All genes
Nº of genomes
Frequency of TTG usage
TTG usage
With TsaB$_1$
Without TsaB$_1$
Nº of genomes with TsaB$_1$
Nº of genomes without TsaB$_1$
With TsaB$_2$
Without TsaB$_2$
Nº of genomes with TsaB$_2$
Nº of genomes without TsaB$_2$
With TsaC$_1$
Without TsaC$_1$
Nº of genomes with TsaC$_1$
Nº of genomes without TsaC$_1$
p < 0.00005
With TsaC$_2$
Without TsaC$_2$
Nº of genomes with TsaC$_2$
Nº of genomes without TsaC$_2$
p < 0.00005
With TsaD$_1$
Without TsaD$_1$
Nº of genomes with TsaD$_1$
Nº of genomes without TsaD$_1$
With TsaD$_2$
Without TsaD$_2$
Nº of genomes with TsaD$_2$
Nº of genomes without TsaD$_2$
With TsaD$_3$
Without TsaD$_3$
Nº of genomes with TsaD$_3$
Nº of genomes without TsaD$_3$
With TsaE$_1$
Without TsaE$_1$
Nº of genomes with TsaE$_1$
Nº of genomes without TsaE$_1$
p < 0.00005
With TsaE$_2$
Without TsaE$_2$
Nº of genomes with TsaE$_2$
Nº of genomes without TsaE$_2$
With TsaE$_3$
Without TsaE$_3$
Nº of genomes with TsaE$_3$
Nº of genomes without TsaE$_3$
p < 0.00005

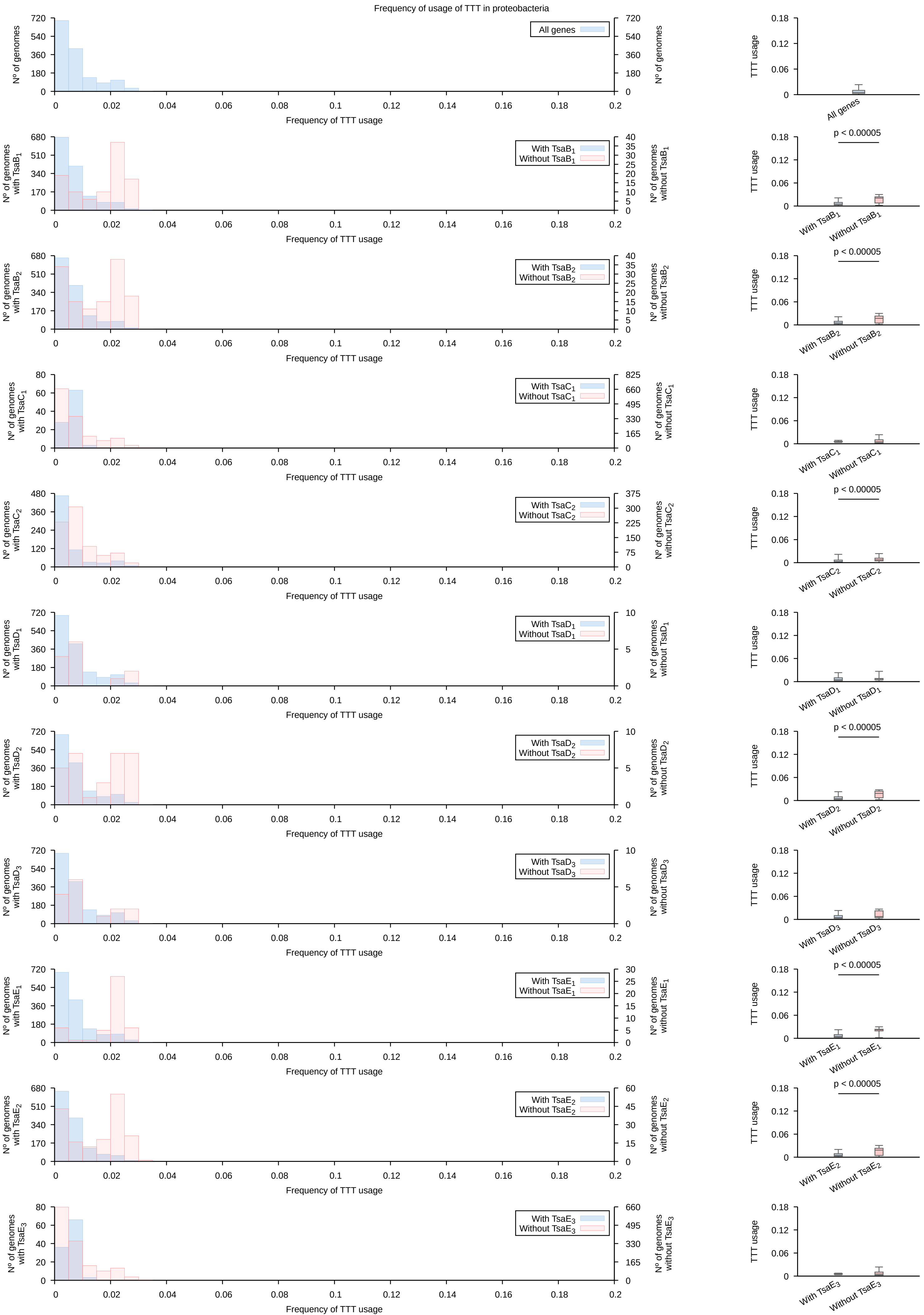
Frequency of usage of TTT in proteobacteria
Nº of genomes
Frequency of TTT usage
TTT usage
All genes
Nº of genomes with TsaB$_1$
Nº of genomes without TsaB$_1$
With TsaB$_1$
Without TsaB$_1$
p < 0.00005
Nº of genomes with TsaB$_2$
Nº of genomes without TsaB$_2$
With TsaB$_2$
Without TsaB$_2$
p < 0.00005
Nº of genomes with TsaC$_1$
Nº of genomes without TsaC$_1$
With TsaC$_1$
Without TsaC$_1$
Nº of genomes with TsaC$_2$
Nº of genomes without TsaC$_2$
With TsaC$_2$
Without TsaC$_2$
p < 0.00005
Nº of genomes with TsaD$_1$
Nº of genomes without TsaD$_1$
With TsaD$_1$
Without TsaD$_1$
Nº of genomes with TsaD$_2$
Nº of genomes without TsaD$_2$
With TsaD$_2$
Without TsaD$_2$
p < 0.00005
Nº of genomes with TsaD$_3$
Nº of genomes without TsaD$_3$
With TsaD$_3$
Without TsaD$_3$
Nº of genomes with TsaE$_1$
Nº of genomes without TsaE$_1$
With TsaE$_1$
Without TsaE$_1$
p < 0.00005
Nº of genomes with TsaE$_2$
Nº of genomes without TsaE$_2$
With TsaE$_2$
Without TsaE$_2$
p < 0.00005
Nº of genomes with TsaE$_3$
Nº of genomes without TsaE$_3$
With TsaE$_3$
Without TsaE$_3$

Figure S20. Histogram of codon usage in high expression genes of genomes that present or lack genes involved in GluQ formation excluding small genomes. Top left graph: histogram of the usage of the indicated codon in highly expressed genes of proteobacterial genomes with more than 400 CDS. **Top right graph:** box plot indicating the distribution of codon usage in the same population. **Lower graphs:** The collection of 1477 proteobacterial genomes, excluding those with less than 400 CDS, were separated in two groups, based on the presence (light blue) or absence (orange) of the indicated pattern in any gene of the analyzed genomes. Patterns are indicated using subscript numbers. See table S4 for a detailed description of the used patterns. **Left:** Data is represented in superposed histograms. **Right:** Data is represented in a boxplot. An horizontal bar was added when the two populations are different based on Wilcoxon-Mann-Whitney test and $p < 0.00005$. Whiskers include 95% of data.

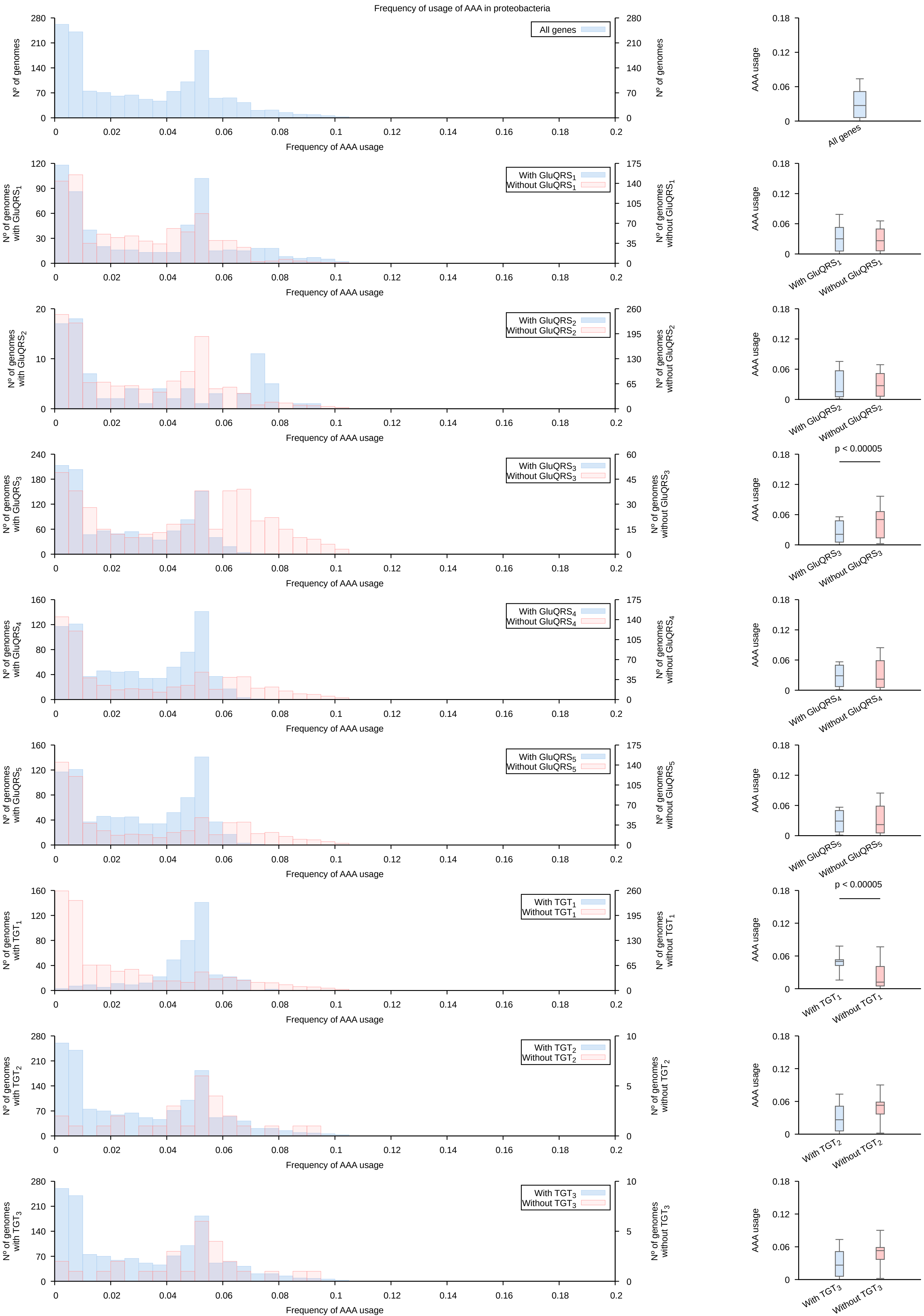
Frequency of usage of AAA in proteobacteria
All genes
Nº of genomes
Frequency of AAA usage
AAA usage
All genes
With GluQRS1
Without GluQRS1
Nº of genomes with GluQRS1
Nº of genomes without GluQRS1
With GluQRS2
Without GluQRS2
Nº of genomes with GluQRS2
Nº of genomes without GluQRS2
With GluQRS3
Without GluQRS3
Nº of genomes with GluQRS3
Nº of genomes without GluQRS3
p < 0.00005
With GluQRS4
Without GluQRS4
Nº of genomes with GluQRS4
Nº of genomes without GluQRS4
With GluQRS5
Without GluQRS5
Nº of genomes with GluQRS5
Nº of genomes without GluQRS5
With TGT1
Without TGT1
Nº of genomes with TGT1
Nº of genomes without TGT1
p < 0.00005
With TGT2
Without TGT2
Nº of genomes with TGT2
Nº of genomes without TGT2
With TGT3
Without TGT3
Nº of genomes with TGT3
Nº of genomes without TGT3

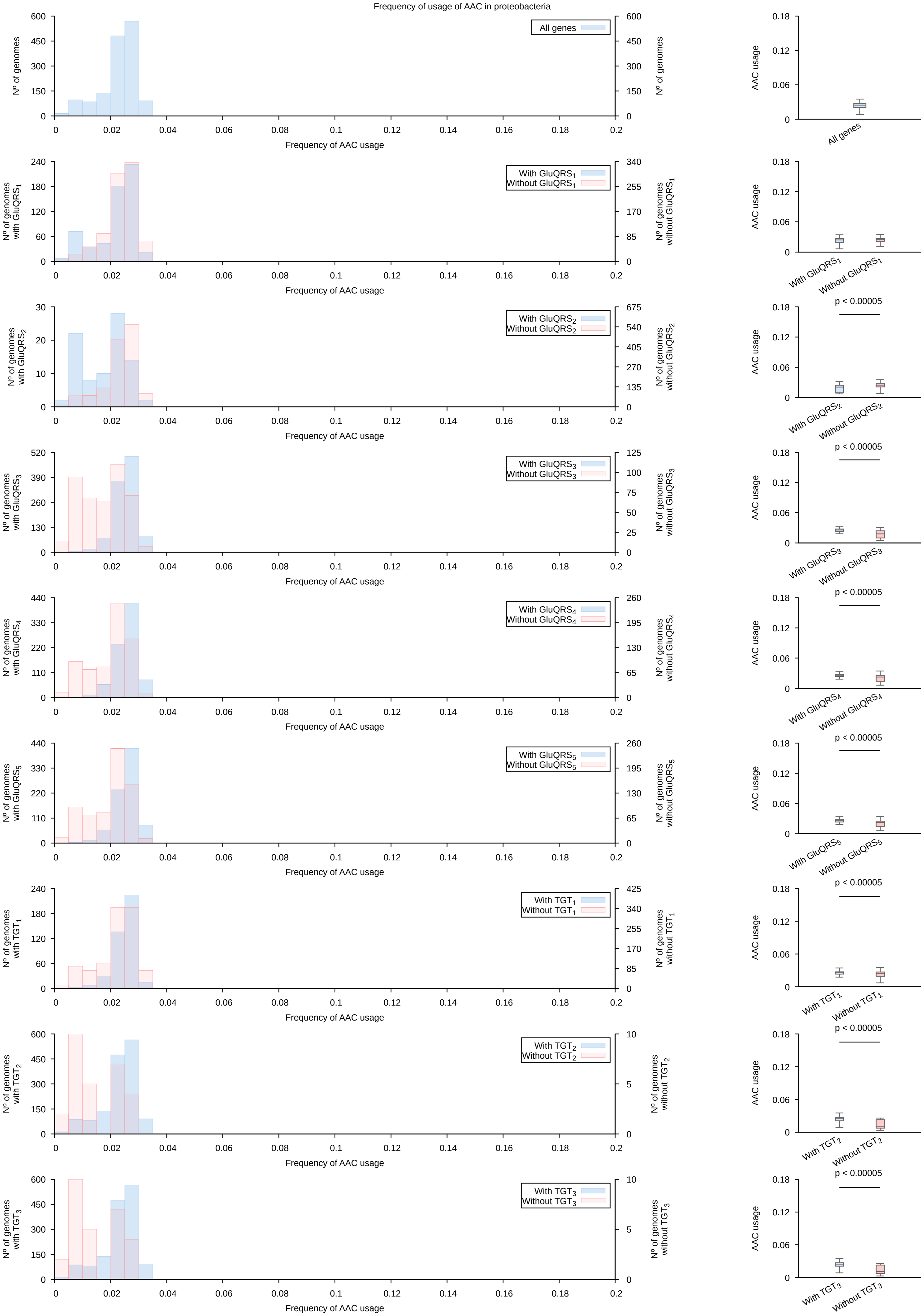

Frequency of usage of AAC in proteobacteria
Nº of genomes
Frequency of AAC usage
AAC usage
All genes
With GluQRS1
Without GluQRS1
With GluQRS2
Without GluQRS2
With GluQRS3
Without GluQRS3
With GluQRS4
Without GluQRS4
With GluQRS5
Without GluQRS5
With TGT1
Without TGT1
With TGT2
Without TGT2
With TGT3
Without TGT3
p < 0.00005

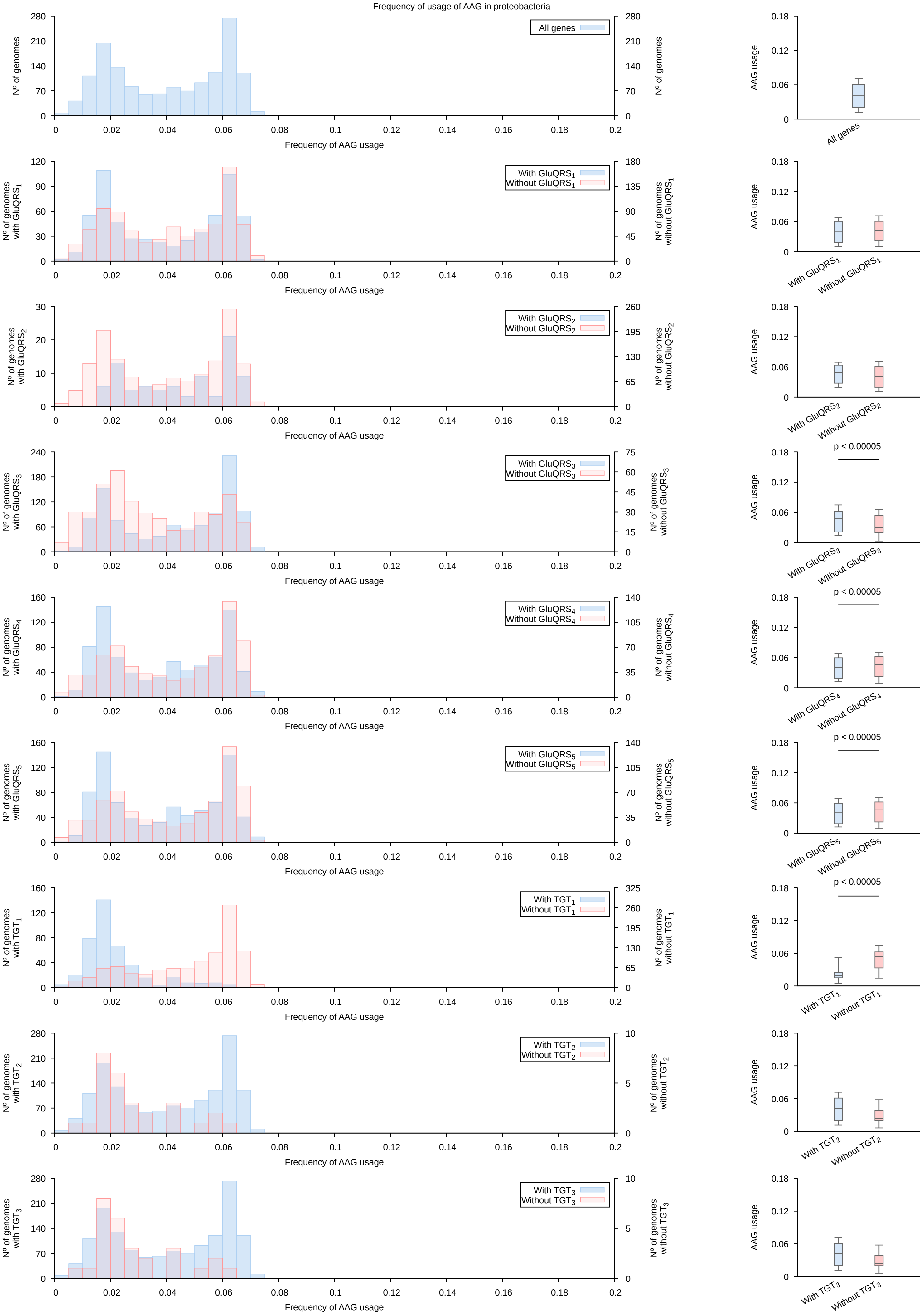

Frequency of usage of AAG in proteobacteria
All genes
Nº of genomes
AAG usage
Frequency of AAG usage
With GluQRS1
Without GluQRS1
Nº of genomes with GluQRS1
Nº of genomes without GluQRS1
With GluQRS2
Without GluQRS2
Nº of genomes with GluQRS2
Nº of genomes without GluQRS2
With GluQRS3
Without GluQRS3
Nº of genomes with GluQRS3
Nº of genomes without GluQRS3
p < 0.00005
With GluQRS4
Without GluQRS4
Nº of genomes with GluQRS4
Nº of genomes without GluQRS4
p < 0.00005
With GluQRS5
Without GluQRS5
Nº of genomes with GluQRS5
Nº of genomes without GluQRS5
p < 0.00005
With TGT1
Without TGT1
Nº of genomes with TGT1
Nº of genomes without TGT1
p < 0.00005
With TGT2
Without TGT2
Nº of genomes with TGT2
Nº of genomes without TGT2
With TGT3
Without TGT3
Nº of genomes with TGT3
Nº of genomes without TGT3

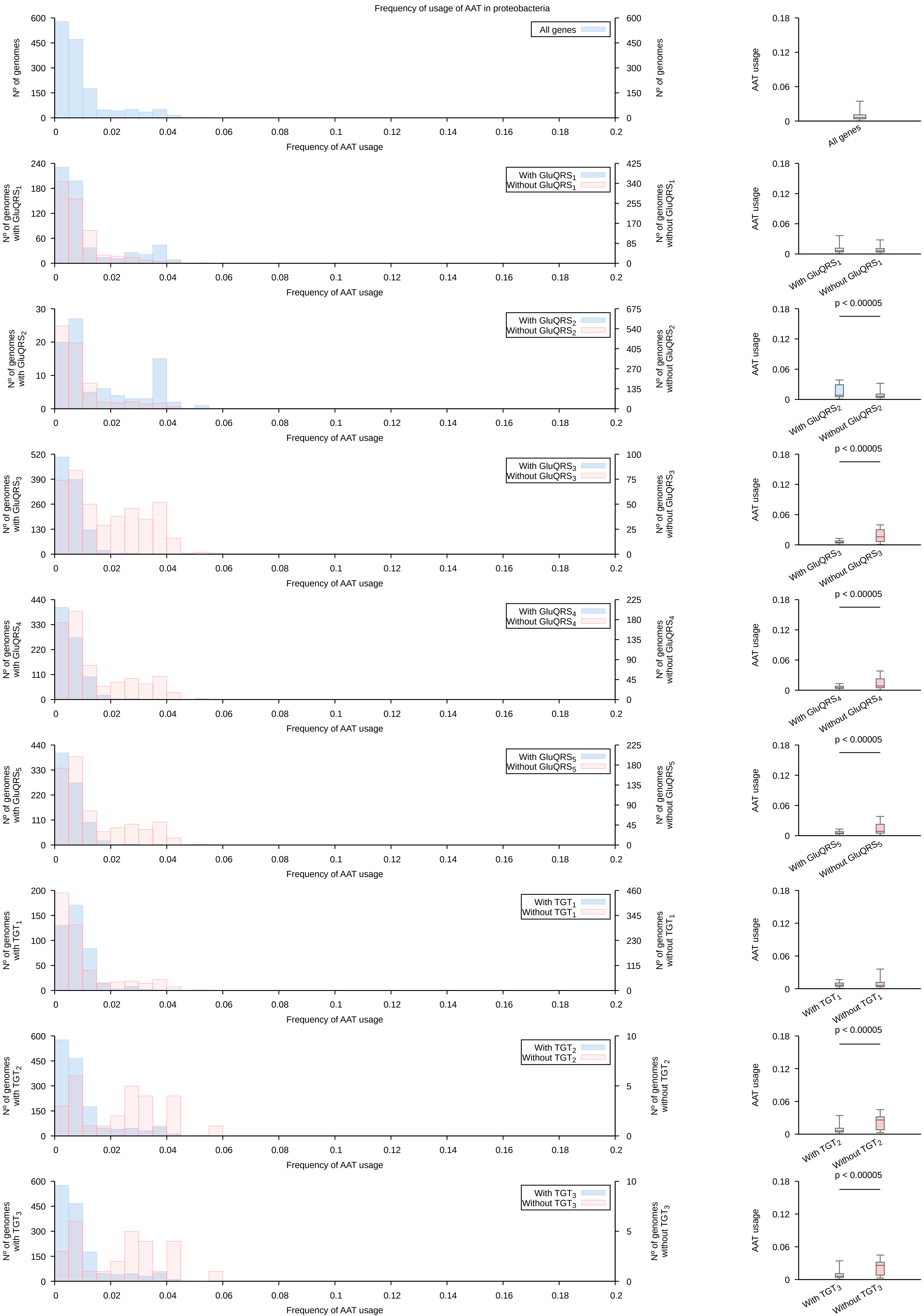

Frequency of usage of AAT in proteobacteria
All genes
Nº of genomes
Frequency of AAT usage
AAT usage
With $GluQRS_1$
Without $GluQRS_1$
Nº of genomes with $GluQRS_1$
Nº of genomes without $GluQRS_1$
With $GluQRS_2$
Without $GluQRS_2$
Nº of genomes with $GluQRS_2$
Nº of genomes without $GluQRS_2$
With $GluQRS_3$
Without $GluQRS_3$
Nº of genomes with $GluQRS_3$
Nº of genomes without $GluQRS_3$
With $GluQRS_4$
Without $GluQRS_4$
Nº of genomes with $GluQRS_4$
Nº of genomes without $GluQRS_4$
With $GluQRS_5$
Without $GluQRS_5$
Nº of genomes with $GluQRS_5$
Nº of genomes without $GluQRS_5$
With TGT_1
Without TGT_1
Nº of genomes with TGT_1
Nº of genomes without TGT_1
With TGT_2
Without TGT_2
Nº of genomes with TGT_2
Nº of genomes without TGT_2
With TGT_3
Without TGT_3
Nº of genomes with TGT_3
Nº of genomes without TGT_3
p < 0.00005

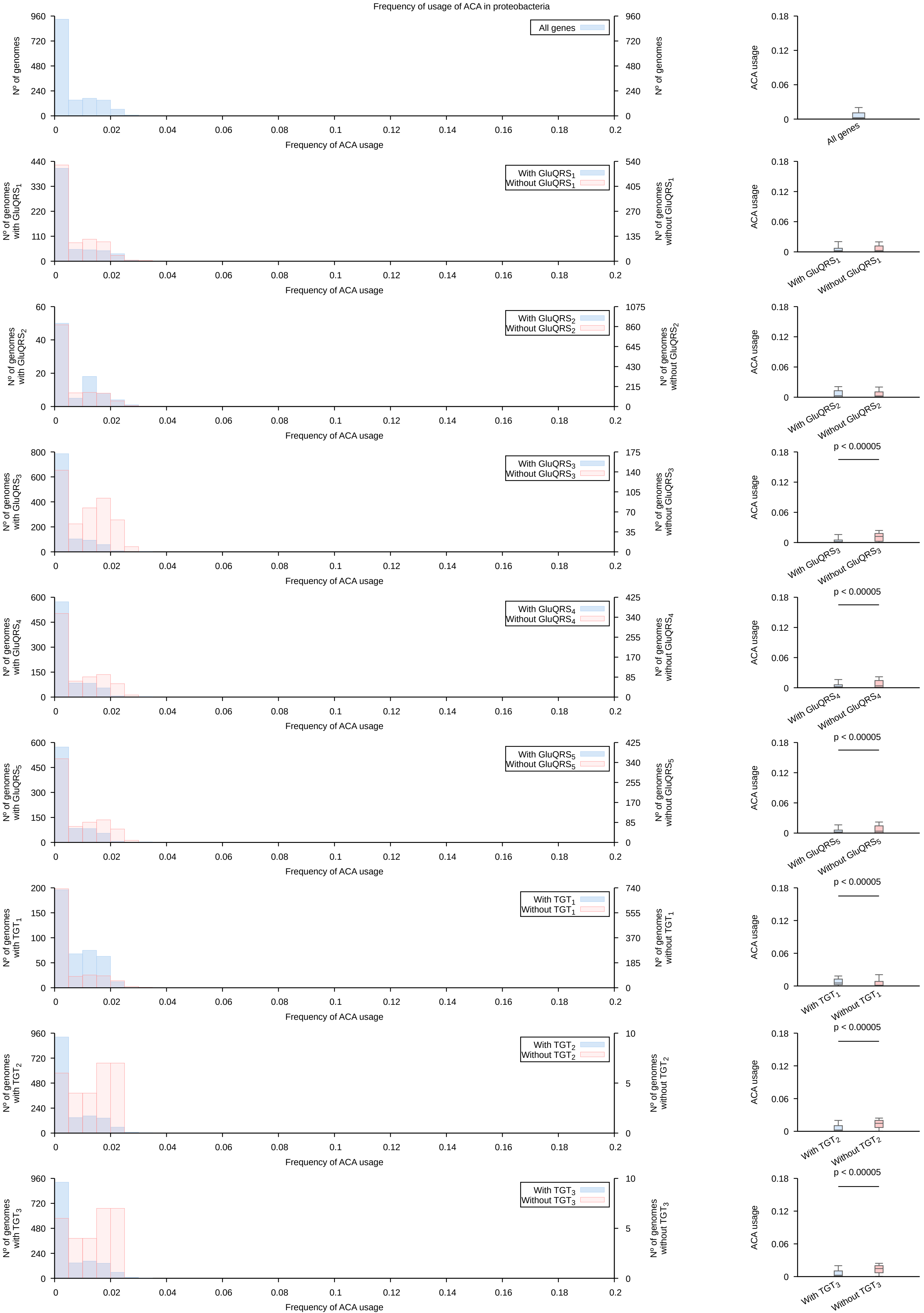
Frequency of usage of ACA in proteobacteria
All genes
Nº of genomes
Frequency of ACA usage
ACA usage
With GluQRS1
Without GluQRS1
Nº of genomes with GluQRS1
Nº of genomes without GluQRS1
With GluQRS2
Without GluQRS2
Nº of genomes with GluQRS2
Nº of genomes without GluQRS2
With GluQRS3
Without GluQRS3
Nº of genomes with GluQRS3
Nº of genomes without GluQRS3
p < 0.00005
With GluQRS4
Without GluQRS4
Nº of genomes with GluQRS4
Nº of genomes without GluQRS4
p < 0.00005
With GluQRS5
Without GluQRS5
Nº of genomes with GluQRS5
Nº of genomes without GluQRS5
p < 0.00005
With TGT1
Without TGT1
Nº of genomes with TGT1
Nº of genomes without TGT1
p < 0.00005
With TGT2
Without TGT2
Nº of genomes with TGT2
Nº of genomes without TGT2
p < 0.00005
With TGT3
Without TGT3
Nº of genomes with TGT3
Nº of genomes without TGT3
p < 0.00005

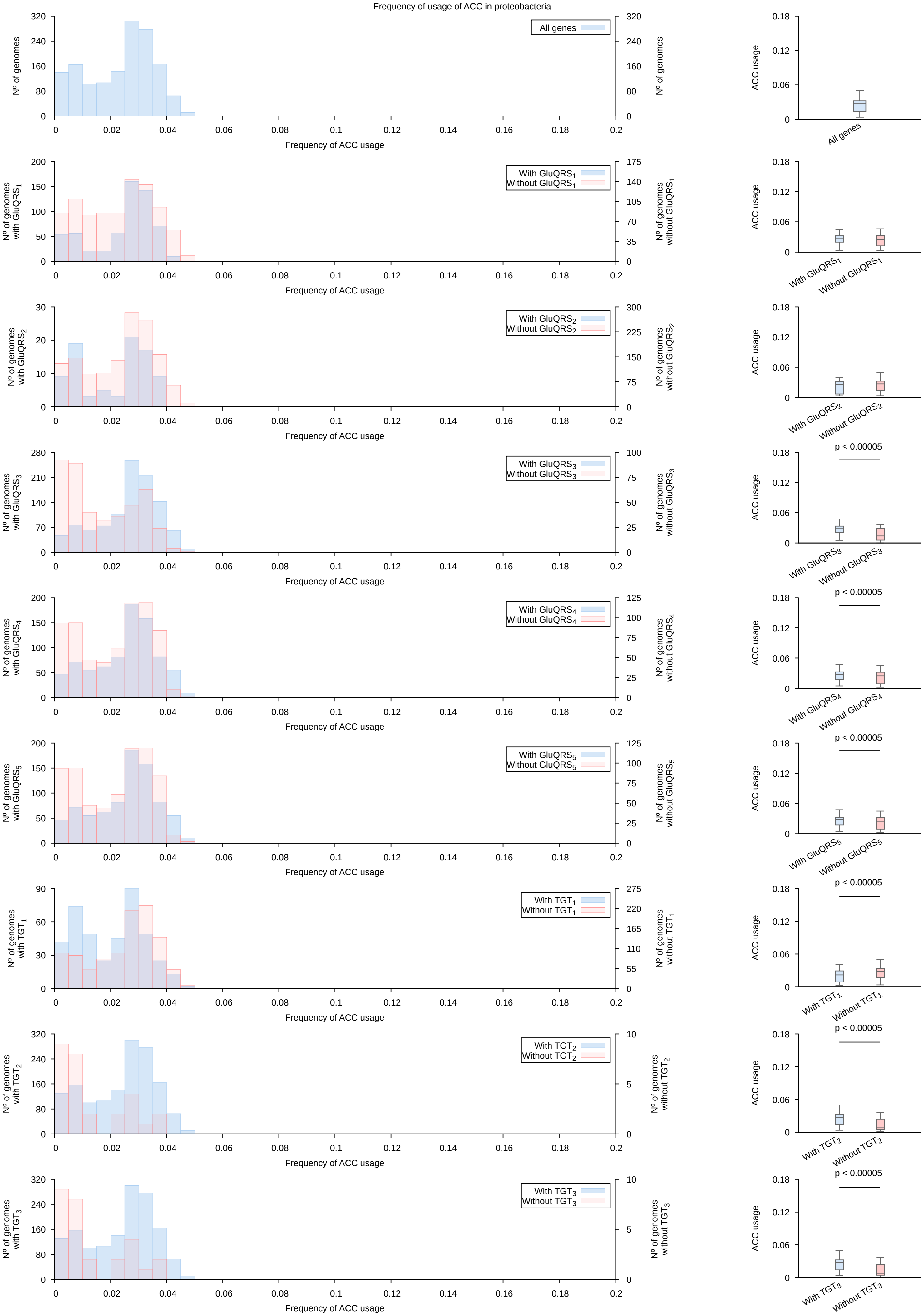
Frequency of usage of ACC in proteobacteria
All genes
With GluQRS1
Without GluQRS1
With GluQRS2
Without GluQRS2
With GluQRS3
Without GluQRS3
With GluQRS4
Without GluQRS4
With GluQRS5
Without GluQRS5
With TGT1
Without TGT1
With TGT2
Without TGT2
With TGT3
Without TGT3
Nº of genomes
Frequency of ACC usage
ACC usage
p < 0.00005

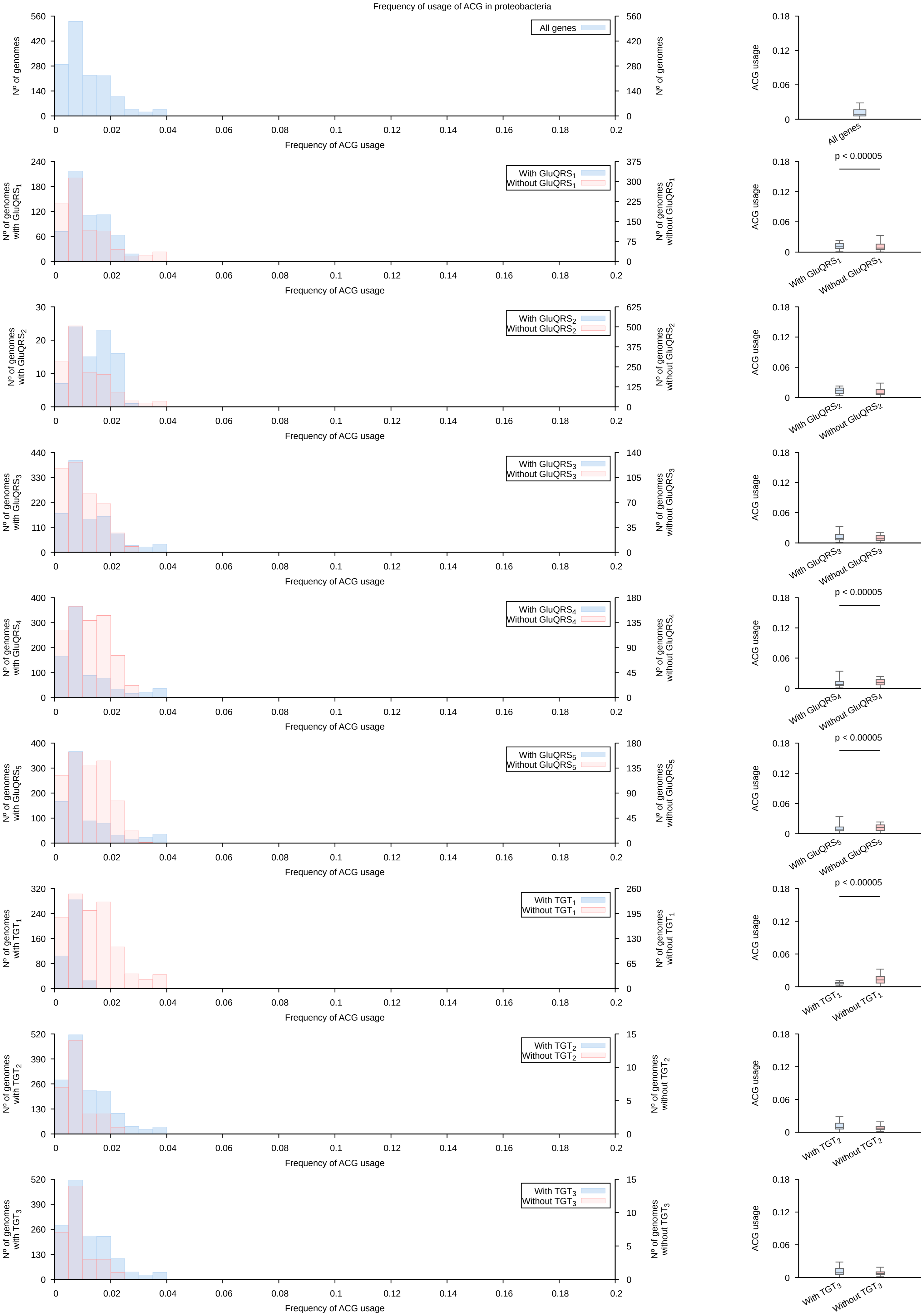

Frequency of usage of ACG in proteobacteria
Nº of genomes
All genes
Frequency of ACG usage
ACG usage
All genes
Nº of genomes with GluQRS1
Nº of genomes without GluQRS1
With GluQRS1
Without GluQRS1
p < 0.00005
Nº of genomes with GluQRS2
Nº of genomes without GluQRS2
With GluQRS2
Without GluQRS2
Nº of genomes with GluQRS3
Nº of genomes without GluQRS3
With GluQRS3
Without GluQRS3
Nº of genomes with GluQRS4
Nº of genomes without GluQRS4
With GluQRS4
Without GluQRS4
p < 0.00005
Nº of genomes with GluQRS5
Nº of genomes without GluQRS5
With GluQRS5
Without GluQRS5
p < 0.00005
Nº of genomes with TGT1
Nº of genomes without TGT1
With TGT1
Without TGT1
p < 0.00005
Nº of genomes with TGT2
Nº of genomes without TGT2
With TGT2
Without TGT2
Nº of genomes with TGT3
Nº of genomes without TGT3
With TGT3
Without TGT3

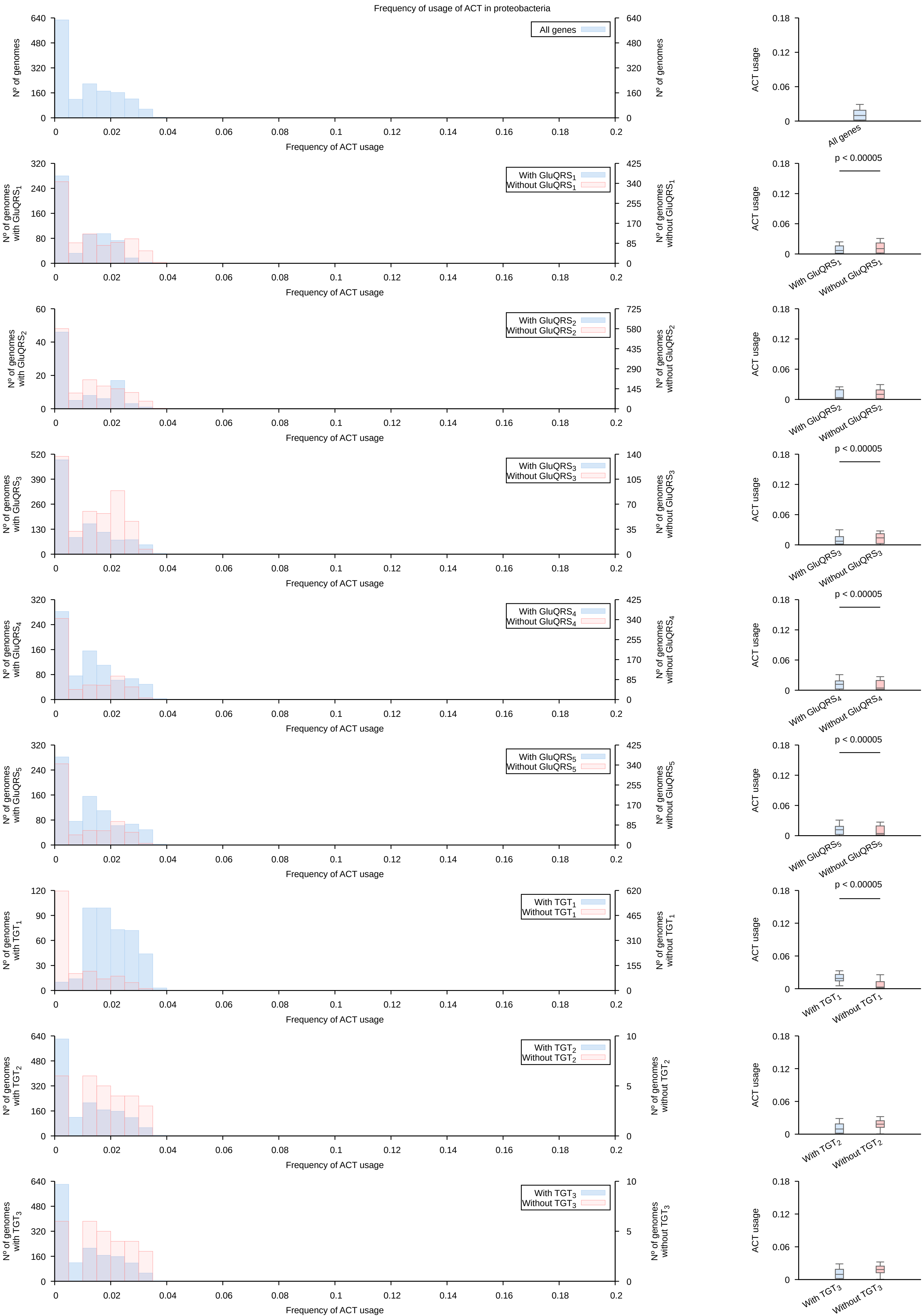

Frequency of usage of ACT in proteobacteria
All genes
Nº of genomes
ACT usage
Frequency of ACT usage
p < 0.00005
With GluQRS1
Without GluQRS1
Nº of genomes with GluQRS1
Nº of genomes without GluQRS1
With GluQRS2
Without GluQRS2
Nº of genomes with GluQRS2
Nº of genomes without GluQRS2
With GluQRS3
Without GluQRS3
Nº of genomes with GluQRS3
Nº of genomes without GluQRS3
With GluQRS4
Without GluQRS4
Nº of genomes with GluQRS4
Nº of genomes without GluQRS4
With GluQRS5
Without GluQRS5
Nº of genomes with GluQRS5
Nº of genomes without GluQRS5
With TGT1
Without TGT1
Nº of genomes with TGT1
Nº of genomes without TGT1
With TGT2
Without TGT2
Nº of genomes with TGT2
Nº of genomes without TGT2
With TGT3
Without TGT3
Nº of genomes with TGT3
Nº of genomes without TGT3

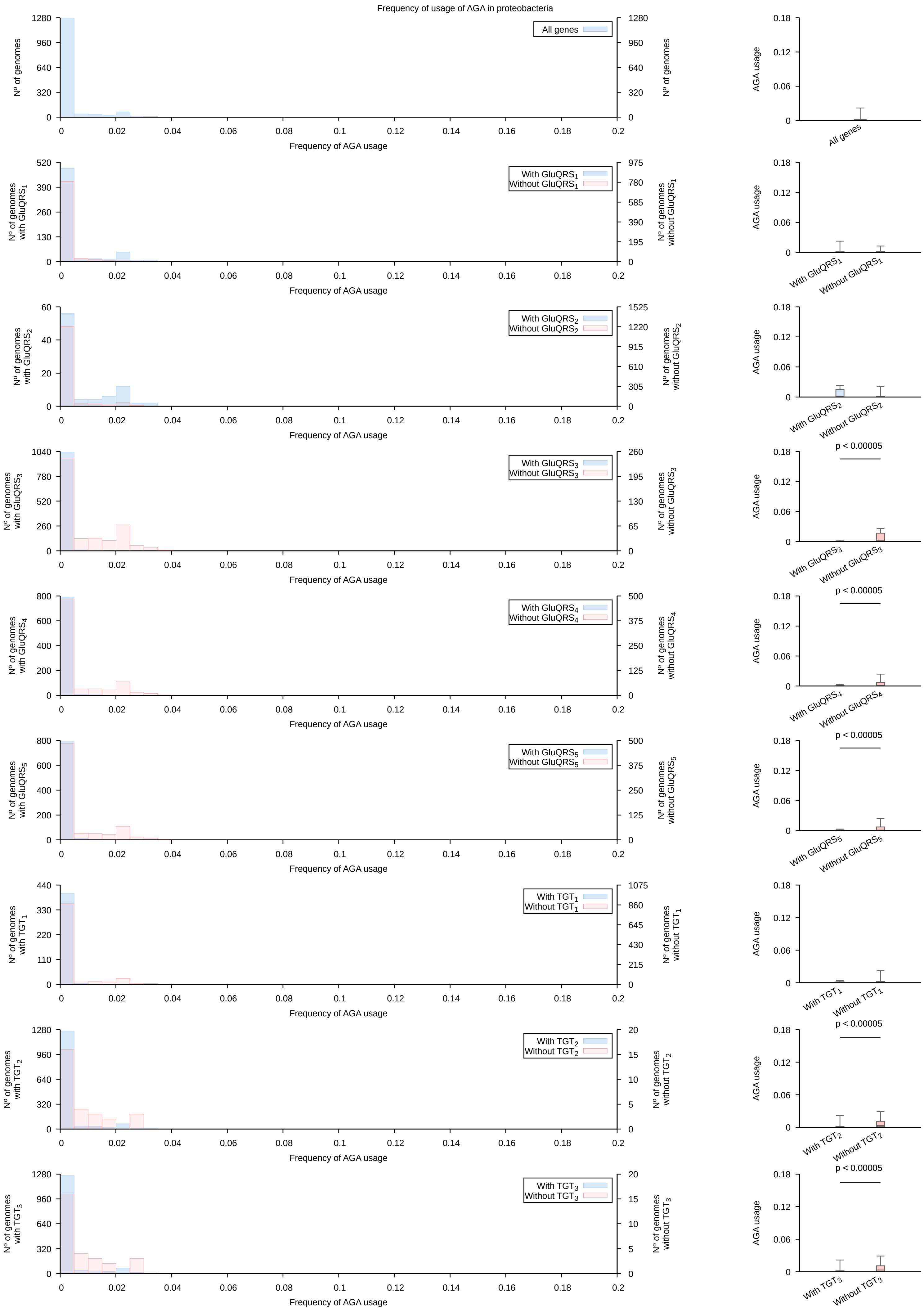
Frequency of usage of AGA in proteobacteria
All genes
Nº of genomes
Frequency of AGA usage
AGA usage
With GluQRS$_1$
Without GluQRS$_1$
Nº of genomes with GluQRS$_1$
Nº of genomes without GluQRS$_1$
With GluQRS$_2$
Without GluQRS$_2$
Nº of genomes with GluQRS$_2$
Nº of genomes without GluQRS$_2$
With GluQRS$_3$
Without GluQRS$_3$
Nº of genomes with GluQRS$_3$
Nº of genomes without GluQRS$_3$
p < 0.00005
With GluQRS$_4$
Without GluQRS$_4$
Nº of genomes with GluQRS$_4$
Nº of genomes without GluQRS$_4$
p < 0.00005
With GluQRS$_5$
Without GluQRS$_5$
Nº of genomes with GluQRS$_5$
Nº of genomes without GluQRS$_5$
p < 0.00005
With TGT$_1$
Without TGT$_1$
Nº of genomes with TGT$_1$
Nº of genomes without TGT$_1$
With TGT$_2$
Without TGT$_2$
Nº of genomes with TGT$_2$
Nº of genomes without TGT$_2$
p < 0.00005
With TGT$_3$
Without TGT$_3$
Nº of genomes with TGT$_3$
Nº of genomes without TGT$_3$
p < 0.00005

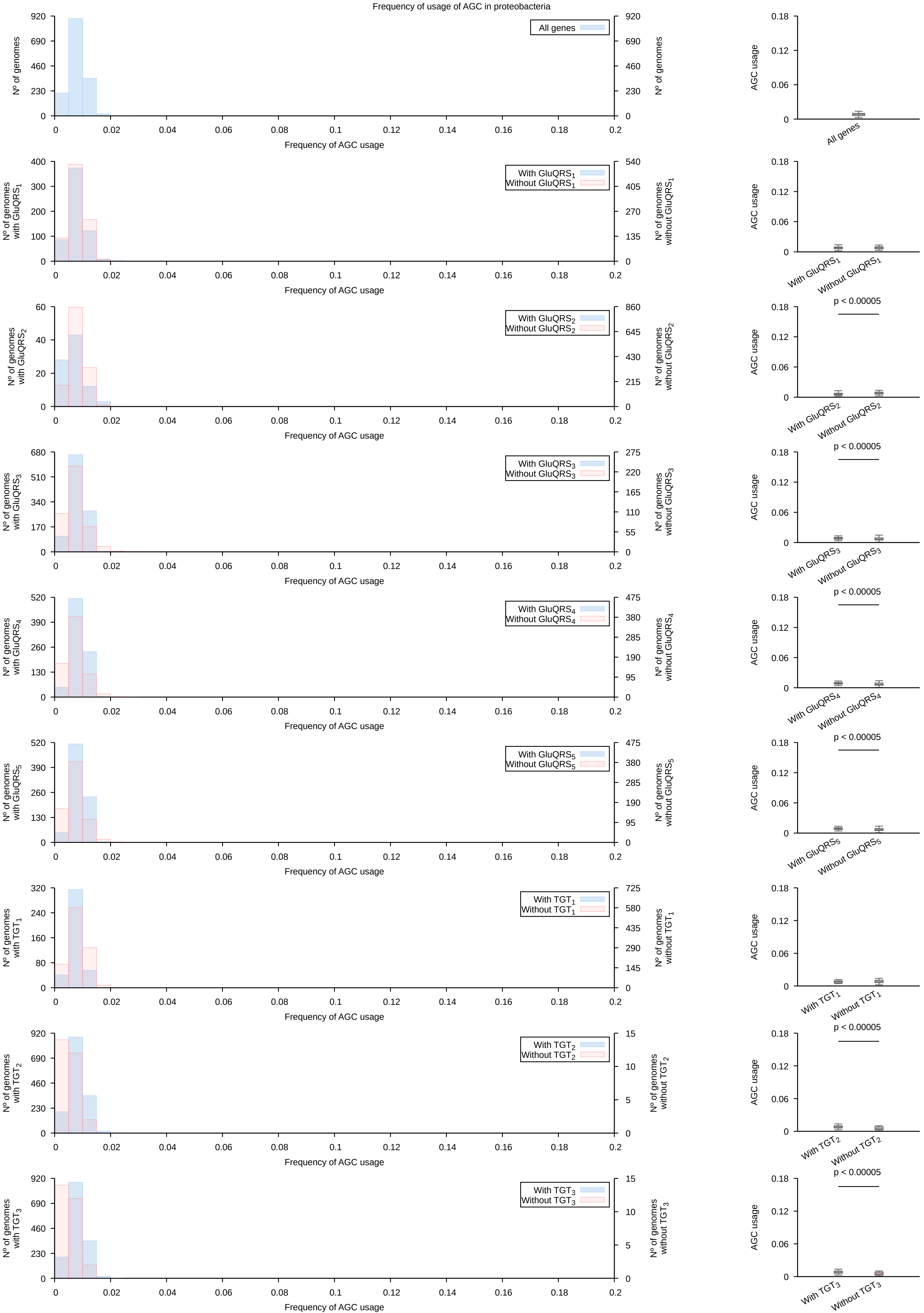
Frequency of usage of AGC in proteobacteria
Nº of genomes
All genes
Frequency of AGC usage
AGC usage
Nº of genomes with GluQRS$_1$
Nº of genomes without GluQRS$_1$
With GluQRS$_1$
Without GluQRS$_1$
Nº of genomes with GluQRS$_2$
Nº of genomes without GluQRS$_2$
With GluQRS$_2$
Without GluQRS$_2$
p < 0.00005
Nº of genomes with GluQRS$_3$
Nº of genomes without GluQRS$_3$
With GluQRS$_3$
Without GluQRS$_3$
p < 0.00005
Nº of genomes with GluQRS$_4$
Nº of genomes without GluQRS$_4$
With GluQRS$_4$
Without GluQRS$_4$
p < 0.00005
Nº of genomes with GluQRS$_5$
Nº of genomes without GluQRS$_5$
With GluQRS$_5$
Without GluQRS$_5$
p < 0.00005
Nº of genomes with TGT$_1$
Nº of genomes without TGT$_1$
With TGT$_1$
Without TGT$_1$
Nº of genomes with TGT$_2$
Nº of genomes without TGT$_2$
With TGT$_2$
Without TGT$_2$
p < 0.00005
Nº of genomes with TGT$_3$
Nº of genomes without TGT$_3$
With TGT$_3$
Without TGT$_3$
p < 0.00005

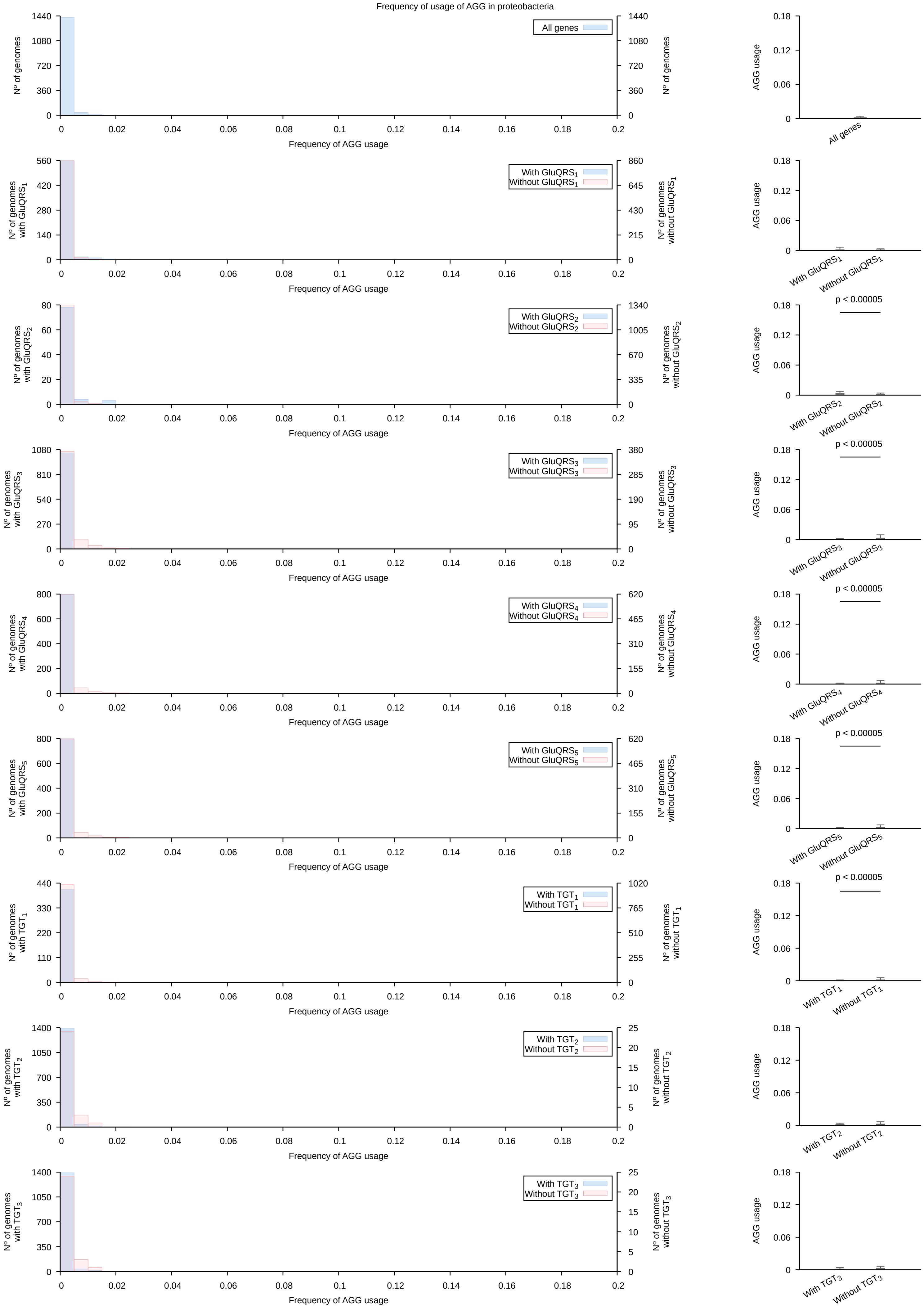
Frequency of usage of AGG in proteobacteria
Nº of genomes
Frequency of AGG usage
AGG usage
All genes
With GluQRS1
Without GluQRS1
Nº of genomes with GluQRS1
Nº of genomes without GluQRS1
With GluQRS2
Without GluQRS2
Nº of genomes with GluQRS2
Nº of genomes without GluQRS2
p < 0.00005
With GluQRS3
Without GluQRS3
Nº of genomes with GluQRS3
Nº of genomes without GluQRS3
p < 0.00005
With GluQRS4
Without GluQRS4
Nº of genomes with GluQRS4
Nº of genomes without GluQRS4
p < 0.00005
With GluQRS5
Without GluQRS5
Nº of genomes with GluQRS5
Nº of genomes without GluQRS5
p < 0.00005
With TGT1
Without TGT1
Nº of genomes with TGT1
Nº of genomes without TGT1
p < 0.00005
With TGT2
Without TGT2
Nº of genomes with TGT2
Nº of genomes without TGT2
With TGT3
Without TGT3
Nº of genomes with TGT3
Nº of genomes without TGT3

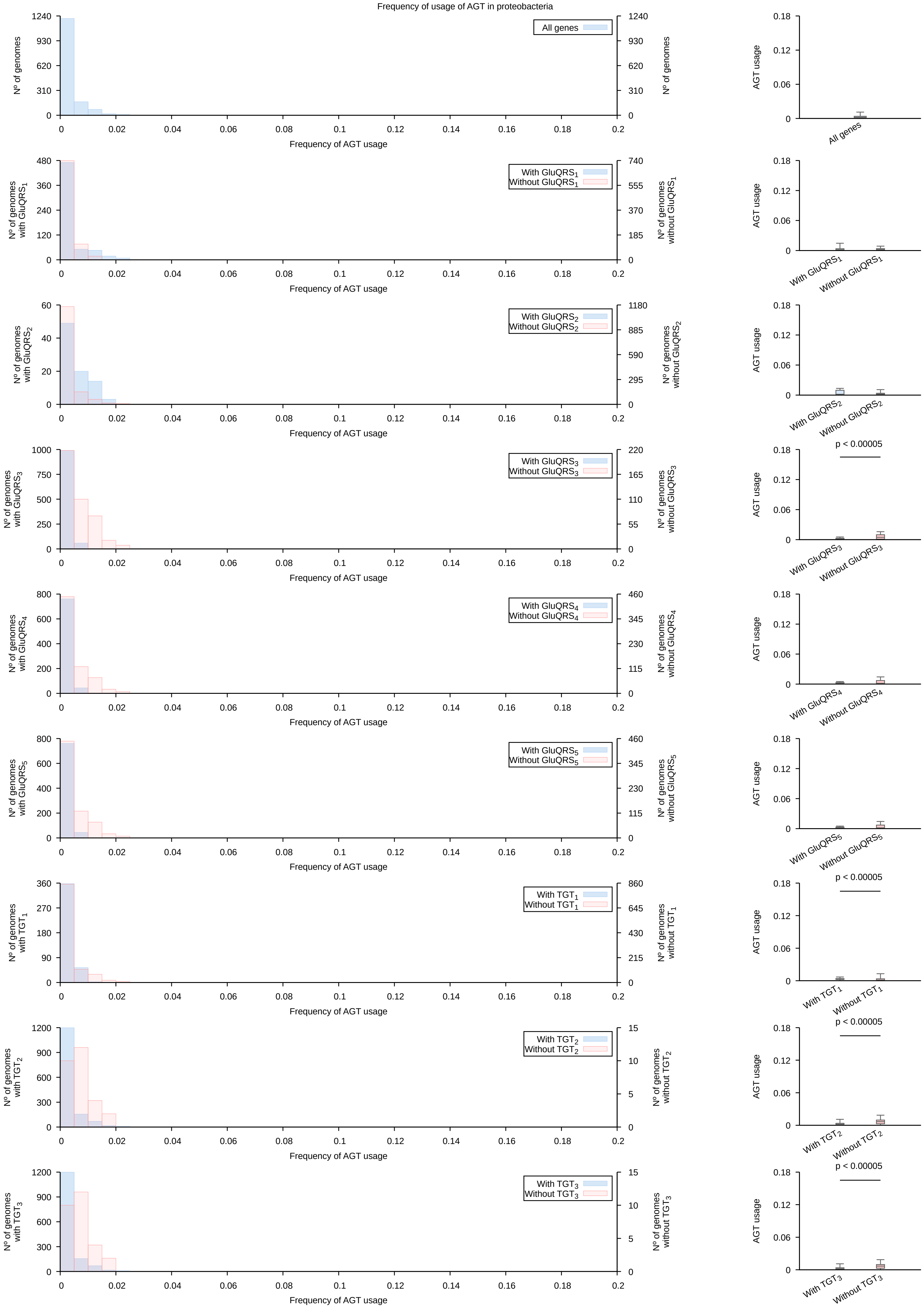
Frequency of usage of AGT in proteobacteria
All genes
Nº of genomes
Frequency of AGT usage
AGT usage
With GluQRS$_1$
Without GluQRS$_1$
Nº of genomes with GluQRS$_1$
Nº of genomes without GluQRS$_1$
With GluQRS$_2$
Without GluQRS$_2$
Nº of genomes with GluQRS$_2$
Nº of genomes without GluQRS$_2$
With GluQRS$_3$
Without GluQRS$_3$
Nº of genomes with GluQRS$_3$
Nº of genomes without GluQRS$_3$
p < 0.00005
With GluQRS$_4$
Without GluQRS$_4$
Nº of genomes with GluQRS$_4$
Nº of genomes without GluQRS$_4$
With GluQRS$_5$
Without GluQRS$_5$
Nº of genomes with GluQRS$_5$
Nº of genomes without GluQRS$_5$
With TGT$_1$
Without TGT$_1$
Nº of genomes with TGT$_1$
Nº of genomes without TGT$_1$
p < 0.00005
With TGT$_2$
Without TGT$_2$
Nº of genomes with TGT$_2$
Nº of genomes without TGT$_2$
p < 0.00005
With TGT$_3$
Without TGT$_3$
Nº of genomes with TGT$_3$
Nº of genomes without TGT$_3$
p < 0.00005

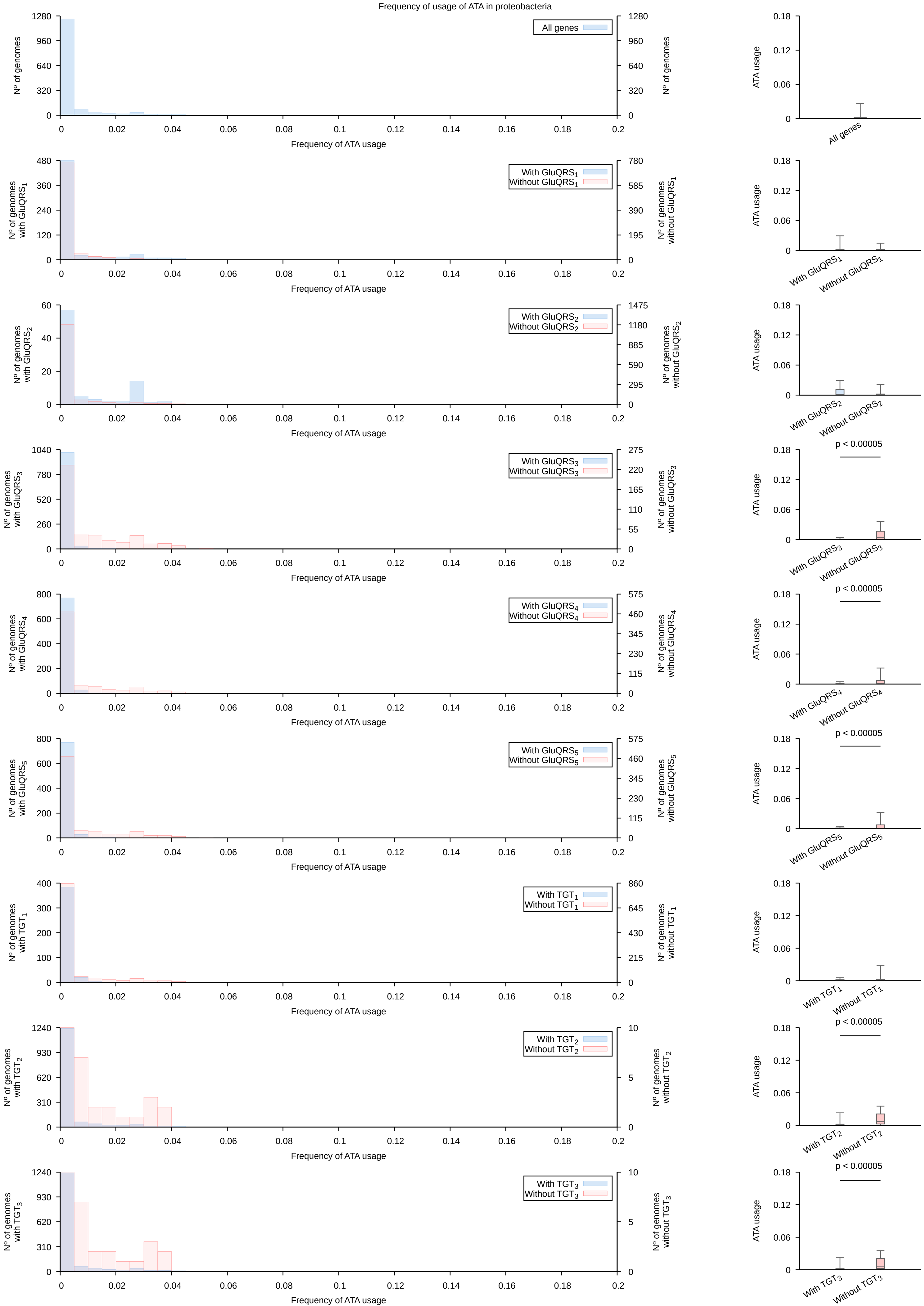
Frequency of usage of ATA in proteobacteria
All genes
With GluQRS1
Without GluQRS1
With GluQRS2
Without GluQRS2
With GluQRS3
Without GluQRS3
With GluQRS4
Without GluQRS4
With GluQRS5
Without GluQRS5
With TGT1
Without TGT1
With TGT2
Without TGT2
With TGT3
Without TGT3
Nº of genomes
Frequency of ATA usage
ATA usage
p < 0.00005

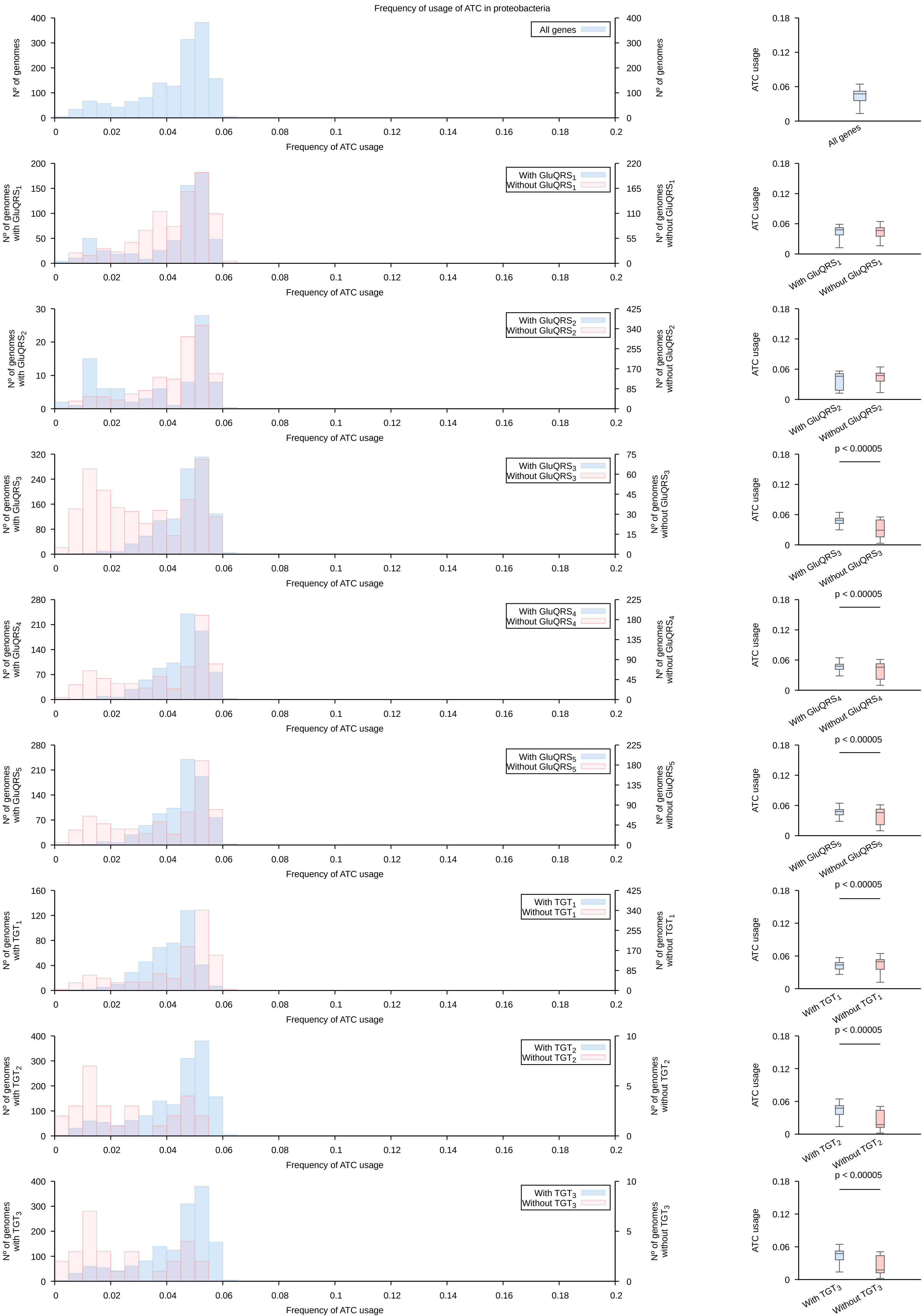
Frequency of usage of ATC in proteobacteria
All genes
Nº of genomes
Frequency of ATC usage
ATC usage
With GluQRS$_1$
Without GluQRS$_1$
Nº of genomes with GluQRS$_1$
Nº of genomes without GluQRS$_1$
With GluQRS$_2$
Without GluQRS$_2$
Nº of genomes with GluQRS$_2$
Nº of genomes without GluQRS$_2$
With GluQRS$_3$
Without GluQRS$_3$
Nº of genomes with GluQRS$_3$
Nº of genomes without GluQRS$_3$
p < 0.00005
With GluQRS$_4$
Without GluQRS$_4$
Nº of genomes with GluQRS$_4$
Nº of genomes without GluQRS$_4$
p < 0.00005
With GluQRS$_5$
Without GluQRS$_5$
Nº of genomes with GluQRS$_5$
Nº of genomes without GluQRS$_5$
p < 0.00005
With TGT$_1$
Without TGT$_1$
Nº of genomes with TGT$_1$
Nº of genomes without TGT$_1$
p < 0.00005
With TGT$_2$
Without TGT$_2$
Nº of genomes with TGT$_2$
Nº of genomes without TGT$_2$
p < 0.00005
With TGT$_3$
Without TGT$_3$
Nº of genomes with TGT$_3$
Nº of genomes without TGT$_3$
p < 0.00005

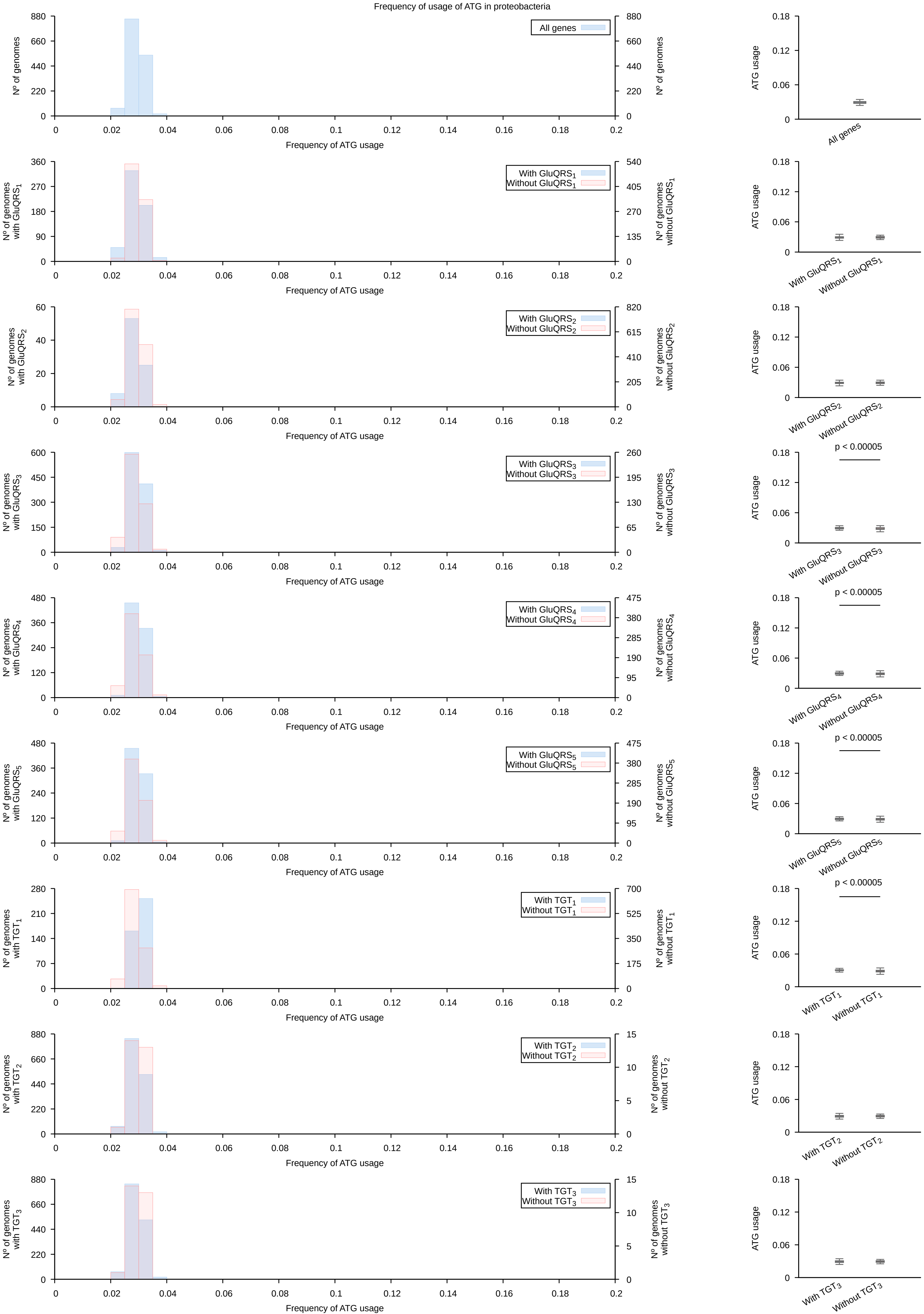
Frequency of usage of ATG in proteobacteria
Nº of genomes
Frequency of ATG usage
All genes
ATG usage
Nº of genomes with $GluQRS_1$
Nº of genomes without $GluQRS_1$
With $GluQRS_1$
Without $GluQRS_1$
Nº of genomes with $GluQRS_2$
Nº of genomes without $GluQRS_2$
With $GluQRS_2$
Without $GluQRS_2$
Nº of genomes with $GluQRS_3$
Nº of genomes without $GluQRS_3$
With $GluQRS_3$
Without $GluQRS_3$
$p < 0.00005$
Nº of genomes with $GluQRS_4$
Nº of genomes without $GluQRS_4$
With $GluQRS_4$
Without $GluQRS_4$
$p < 0.00005$
Nº of genomes with $GluQRS_5$
Nº of genomes without $GluQRS_5$
With $GluQRS_5$
Without $GluQRS_5$
$p < 0.00005$
Nº of genomes with TGT_1
Nº of genomes without TGT_1
With TGT_1
Without TGT_1
$p < 0.00005$
Nº of genomes with TGT_2
Nº of genomes without TGT_2
With TGT_2
Without TGT_2
Nº of genomes with TGT_3
Nº of genomes without TGT_3
With TGT_3
Without TGT_3

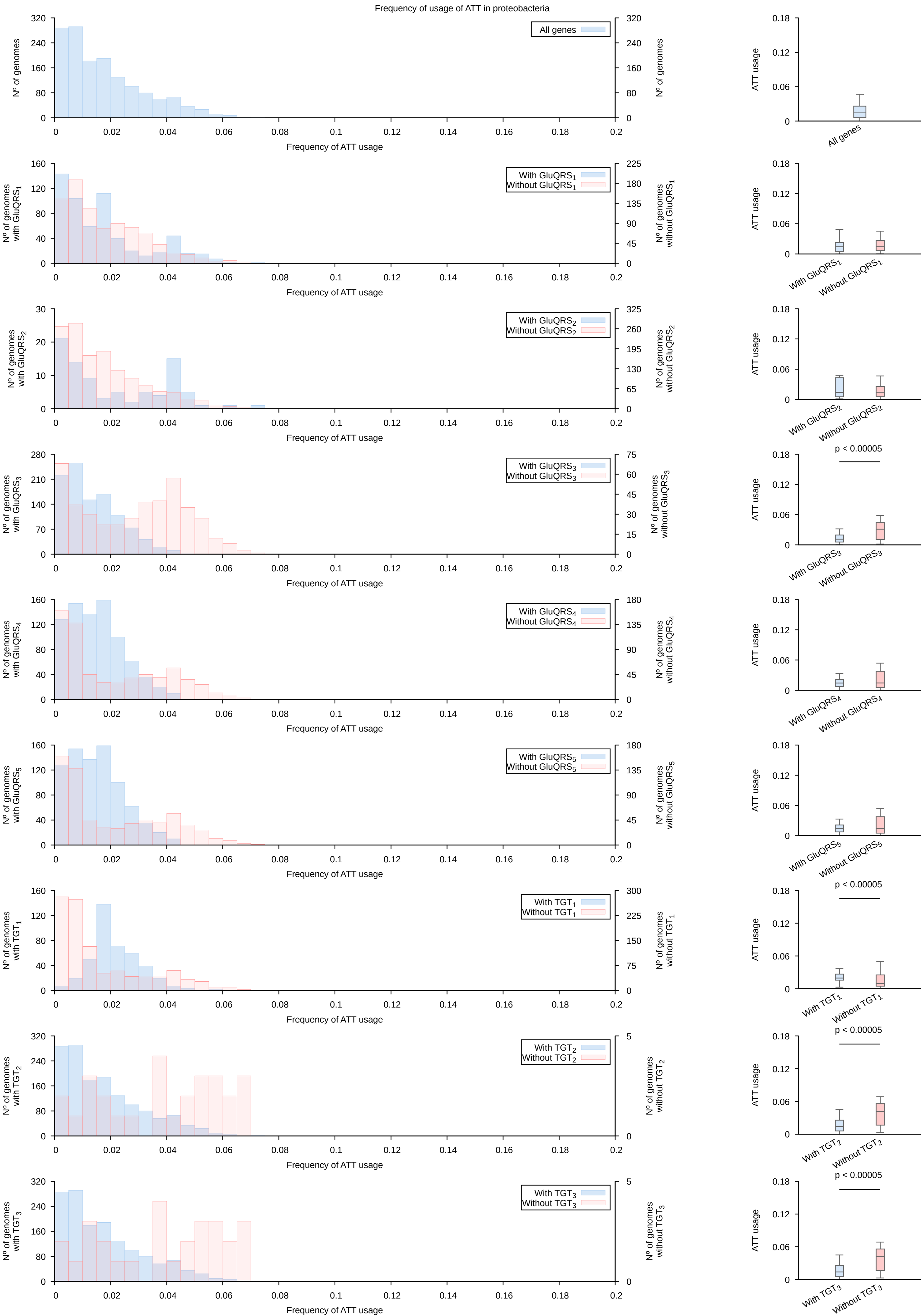
Frequency of usage of ATT in proteobacteria
Nº of genomes
All genes
Frequency of ATT usage
ATT usage
All genes
Nº of genomes with GluQRS$_1$
Nº of genomes without GluQRS$_1$
With GluQRS$_1$
Without GluQRS$_1$
Nº of genomes with GluQRS$_2$
Nº of genomes without GluQRS$_2$
With GluQRS$_2$
Without GluQRS$_2$
Nº of genomes with GluQRS$_3$
Nº of genomes without GluQRS$_3$
With GluQRS$_3$
Without GluQRS$_3$
p < 0.00005
Nº of genomes with GluQRS$_4$
Nº of genomes without GluQRS$_4$
With GluQRS$_4$
Without GluQRS$_4$
Nº of genomes with GluQRS$_5$
Nº of genomes without GluQRS$_5$
With GluQRS$_5$
Without GluQRS$_5$
Nº of genomes with TGT$_1$
Nº of genomes without TGT$_1$
With TGT$_1$
Without TGT$_1$
p < 0.00005
Nº of genomes with TGT$_2$
Nº of genomes without TGT$_2$
With TGT$_2$
Without TGT$_2$
p < 0.00005
Nº of genomes with TGT$_3$
Nº of genomes without TGT$_3$
With TGT$_3$
Without TGT$_3$
p < 0.00005

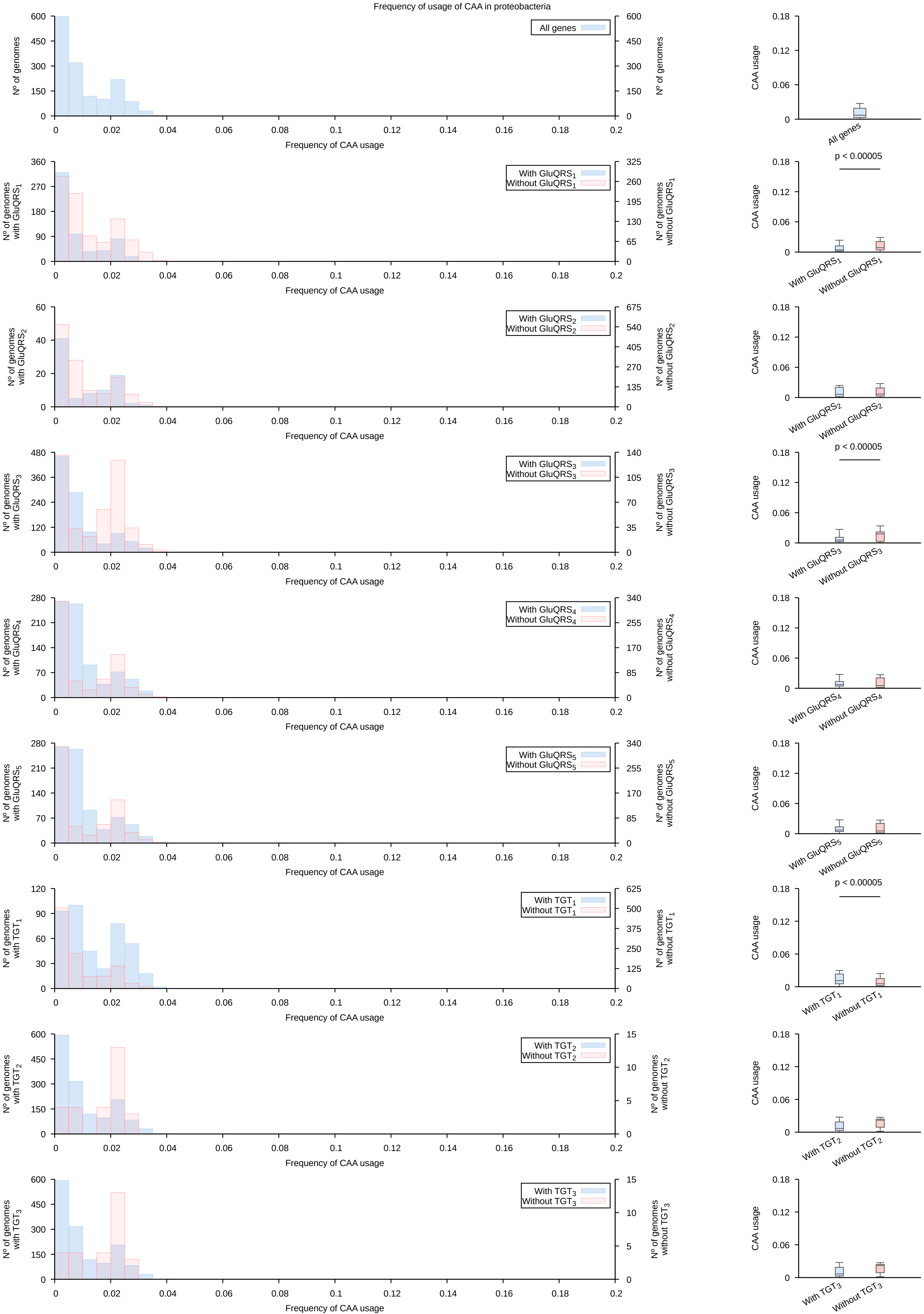

Frequency of usage of CAA in proteobacteria
All genes
Nº of genomes
Frequency of CAA usage
CAA usage
With GluQRS1
Without GluQRS1
Nº of genomes with GluQRS1
Nº of genomes without GluQRS1
p < 0.00005
With GluQRS2
Without GluQRS2
Nº of genomes with GluQRS2
Nº of genomes without GluQRS2
With GluQRS3
Without GluQRS3
Nº of genomes with GluQRS3
Nº of genomes without GluQRS3
p < 0.00005
With GluQRS4
Without GluQRS4
Nº of genomes with GluQRS4
Nº of genomes without GluQRS4
With GluQRS5
Without GluQRS5
Nº of genomes with GluQRS5
Nº of genomes without GluQRS5
With TGT1
Without TGT1
Nº of genomes with TGT1
Nº of genomes without TGT1
p < 0.00005
With TGT2
Without TGT2
Nº of genomes with TGT2
Nº of genomes without TGT2
With TGT3
Without TGT3
Nº of genomes with TGT3
Nº of genomes without TGT3

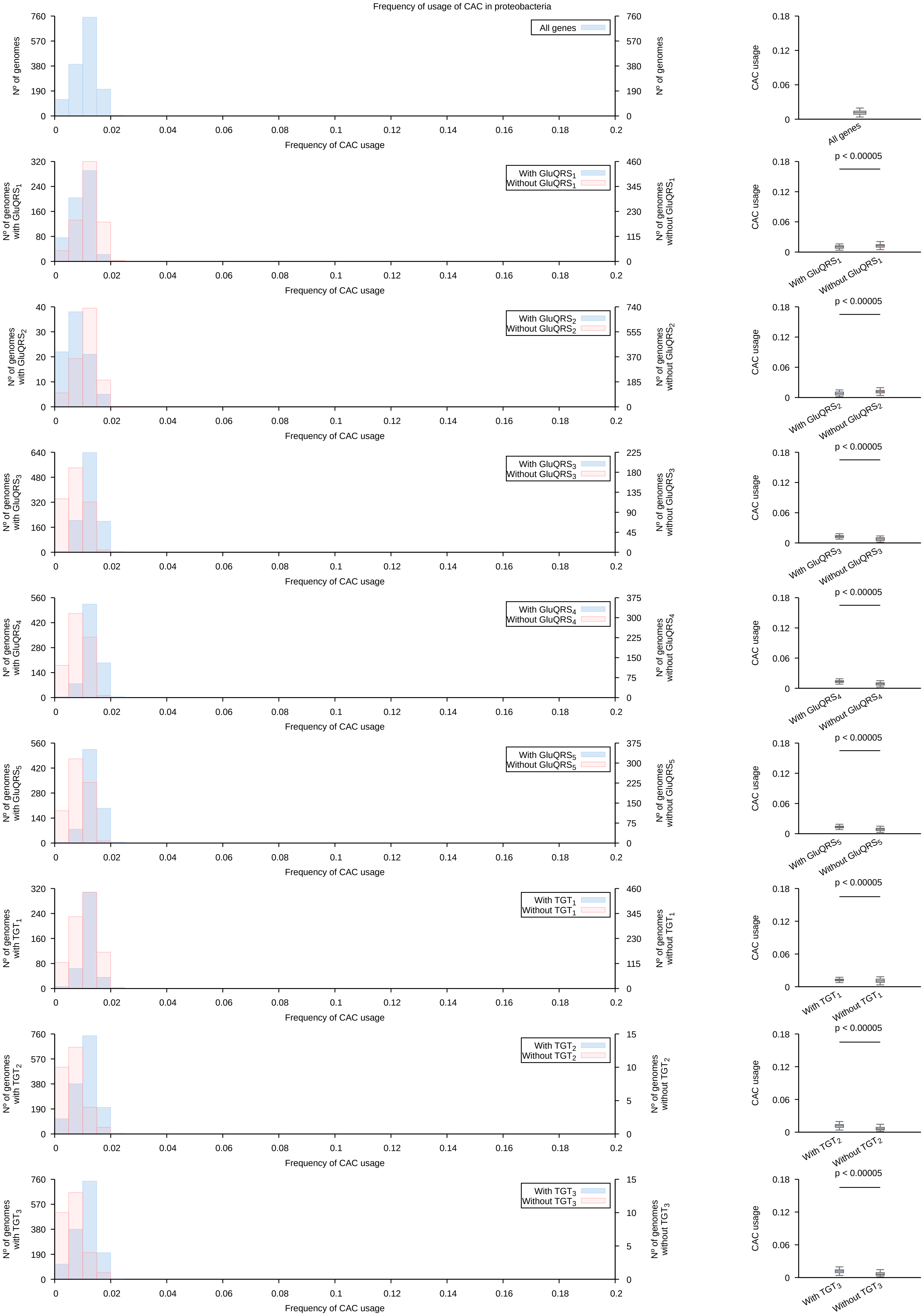

Frequency of usage of CAC in proteobacteria
Nº of genomes
All genes
Frequency of CAC usage
CAC usage
All genes
Nº of genomes with GluQRS$_1$
Nº of genomes without GluQRS$_1$
With GluQRS$_1$
Without GluQRS$_1$
p < 0.00005
Nº of genomes with GluQRS$_2$
Nº of genomes without GluQRS$_2$
With GluQRS$_2$
Without GluQRS$_2$
Nº of genomes with GluQRS$_3$
Nº of genomes without GluQRS$_3$
With GluQRS$_3$
Without GluQRS$_3$
Nº of genomes with GluQRS$_4$
Nº of genomes without GluQRS$_4$
With GluQRS$_4$
Without GluQRS$_4$
Nº of genomes with GluQRS$_5$
Nº of genomes without GluQRS$_5$
With GluQRS$_5$
Without GluQRS$_5$
Nº of genomes with TGT$_1$
Nº of genomes without TGT$_1$
With TGT$_1$
Without TGT$_1$
Nº of genomes with TGT$_2$
Nº of genomes without TGT$_2$
With TGT$_2$
Without TGT$_2$
Nº of genomes with TGT$_3$
Nº of genomes without TGT$_3$
With TGT$_3$
Without TGT$_3$

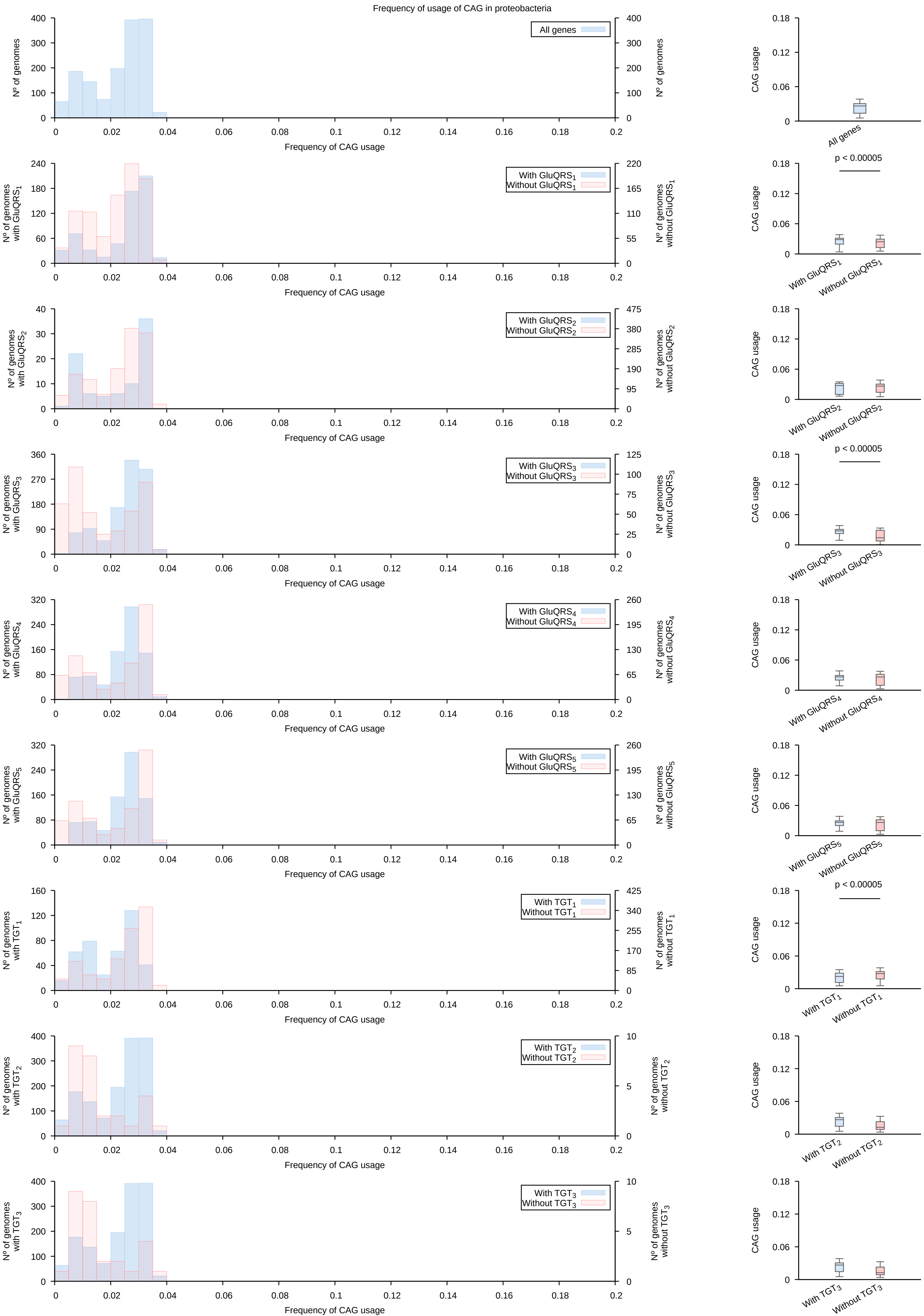
Frequency of usage of CAG in proteobacteria
Nº of genomes
Frequency of CAG usage
All genes
CAG usage
With GluQRS$_1$
Without GluQRS$_1$
Nº of genomes with GluQRS$_1$
Nº of genomes without GluQRS$_1$
p < 0.00005
With GluQRS$_2$
Without GluQRS$_2$
Nº of genomes with GluQRS$_2$
Nº of genomes without GluQRS$_2$
With GluQRS$_3$
Without GluQRS$_3$
Nº of genomes with GluQRS$_3$
Nº of genomes without GluQRS$_3$
p < 0.00005
With GluQRS$_4$
Without GluQRS$_4$
Nº of genomes with GluQRS$_4$
Nº of genomes without GluQRS$_4$
With GluQRS$_5$
Without GluQRS$_5$
Nº of genomes with GluQRS$_5$
Nº of genomes without GluQRS$_5$
With TGT$_1$
Without TGT$_1$
Nº of genomes with TGT$_1$
Nº of genomes without TGT$_1$
p < 0.00005
With TGT$_2$
Without TGT$_2$
Nº of genomes with TGT$_2$
Nº of genomes without TGT$_2$
With TGT$_3$
Without TGT$_3$
Nº of genomes with TGT$_3$
Nº of genomes without TGT$_3$

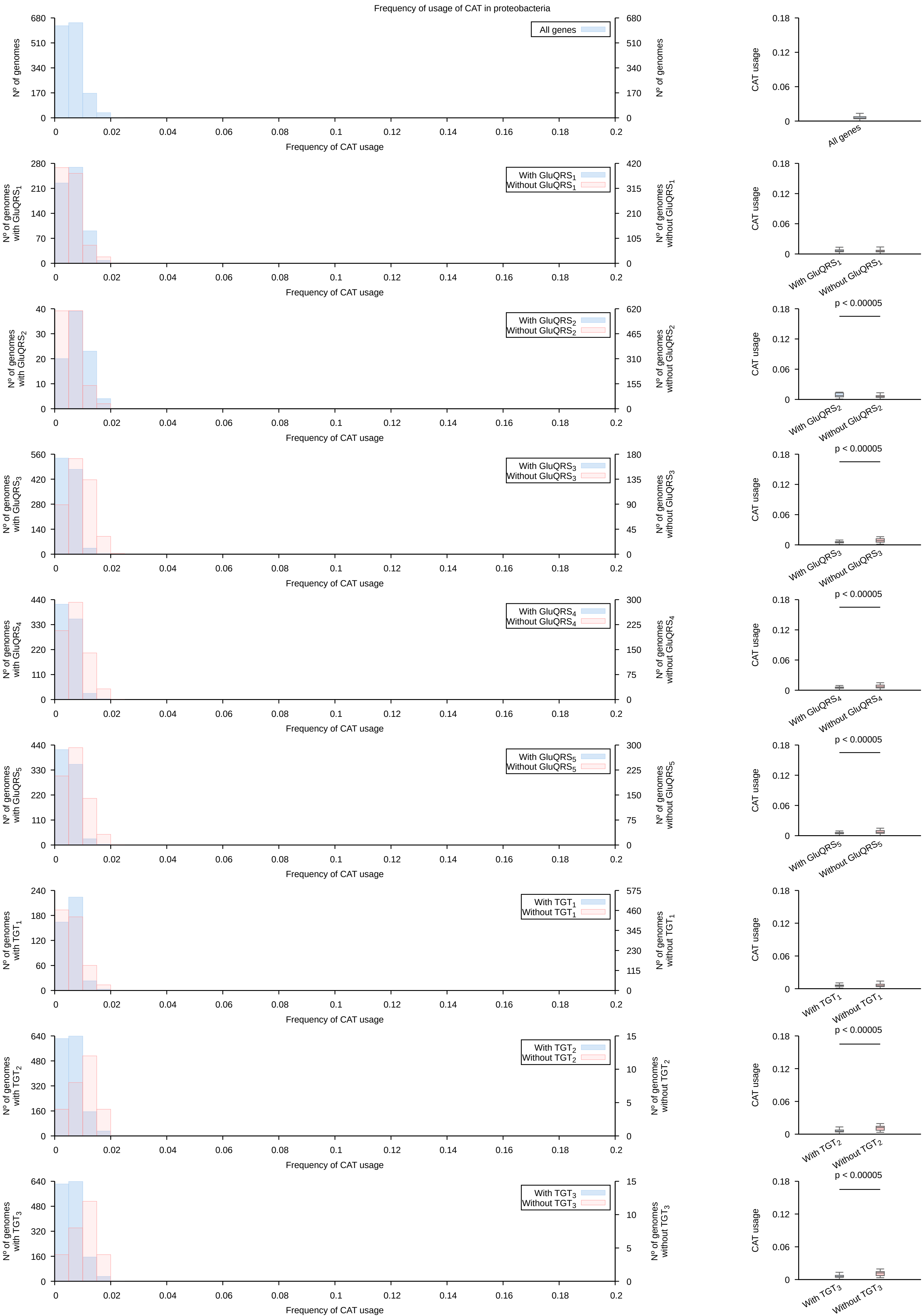

Frequency of usage of CAT in proteobacteria
Nº of genomes
All genes
Frequency of CAT usage
CAT usage
Nº of genomes with GluQRS$_1$
Nº of genomes without GluQRS$_1$
With GluQRS$_1$
Without GluQRS$_1$
Nº of genomes with GluQRS$_2$
Nº of genomes without GluQRS$_2$
With GluQRS$_2$
Without GluQRS$_2$
p < 0.00005
Nº of genomes with GluQRS$_3$
Nº of genomes without GluQRS$_3$
With GluQRS$_3$
Without GluQRS$_3$
Nº of genomes with GluQRS$_4$
Nº of genomes without GluQRS$_4$
With GluQRS$_4$
Without GluQRS$_4$
Nº of genomes with GluQRS$_5$
Nº of genomes without GluQRS$_5$
With GluQRS$_5$
Without GluQRS$_5$
Nº of genomes with TGT$_1$
Nº of genomes without TGT$_1$
With TGT$_1$
Without TGT$_1$
Nº of genomes with TGT$_2$
Nº of genomes without TGT$_2$
With TGT$_2$
Without TGT$_2$
Nº of genomes with TGT$_3$
Nº of genomes without TGT$_3$
With TGT$_3$
Without TGT$_3$

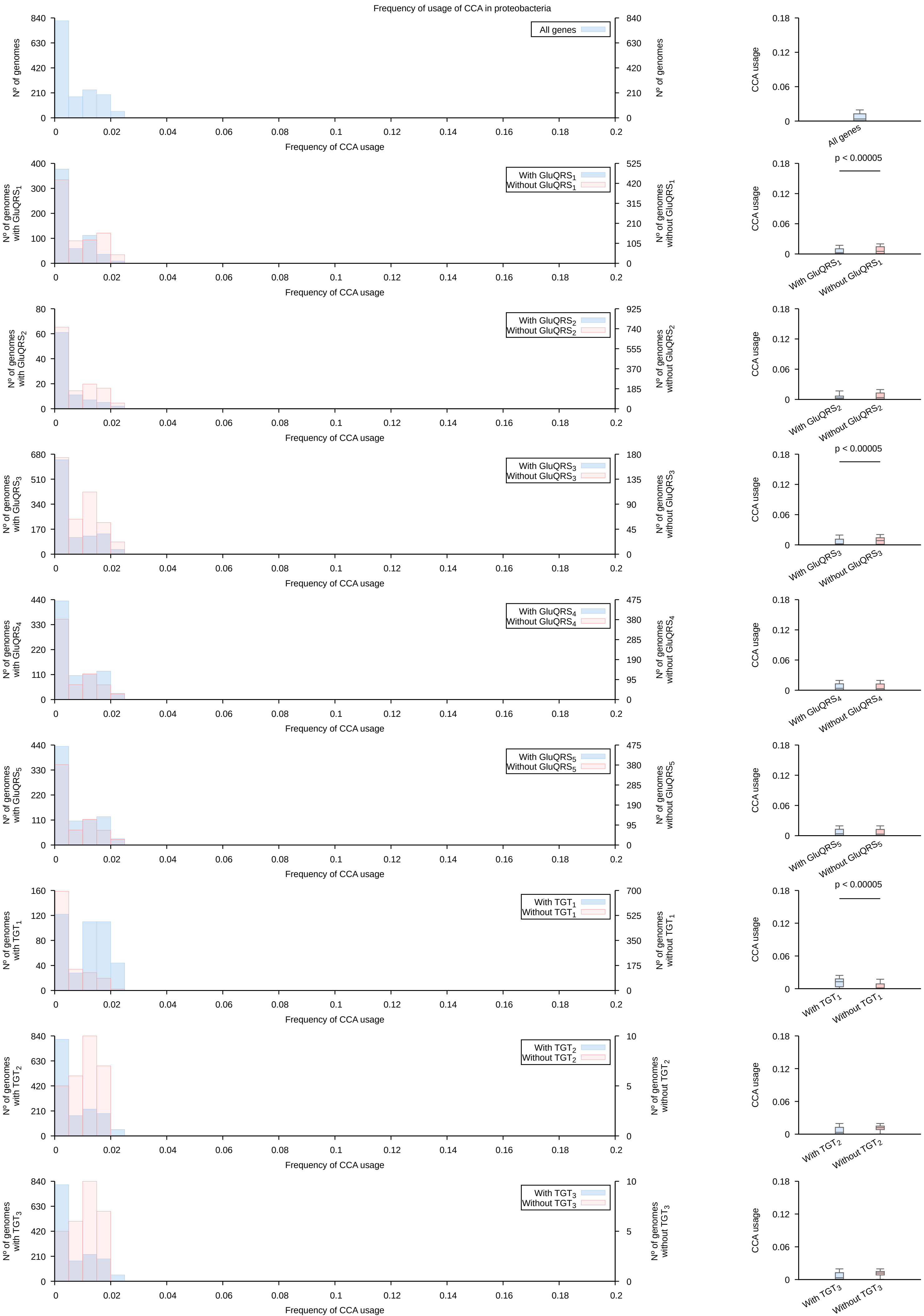
Frequency of usage of CCA in proteobacteria
All genes
Nº of genomes
Frequency of CCA usage
CCA usage
With GluQRS$_1$
Without GluQRS$_1$
Nº of genomes with GluQRS$_1$
Nº of genomes without GluQRS$_1$
p < 0.00005
With GluQRS$_2$
Without GluQRS$_2$
Nº of genomes with GluQRS$_2$
Nº of genomes without GluQRS$_2$
With GluQRS$_3$
Without GluQRS$_3$
Nº of genomes with GluQRS$_3$
Nº of genomes without GluQRS$_3$
p < 0.00005
With GluQRS$_4$
Without GluQRS$_4$
Nº of genomes with GluQRS$_4$
Nº of genomes without GluQRS$_4$
With GluQRS$_5$
Without GluQRS$_5$
Nº of genomes with GluQRS$_5$
Nº of genomes without GluQRS$_5$
With TGT$_1$
Without TGT$_1$
Nº of genomes with TGT$_1$
Nº of genomes without TGT$_1$
p < 0.00005
With TGT$_2$
Without TGT$_2$
Nº of genomes with TGT$_2$
Nº of genomes without TGT$_2$
With TGT$_3$
Without TGT$_3$
Nº of genomes with TGT$_3$
Nº of genomes without TGT$_3$

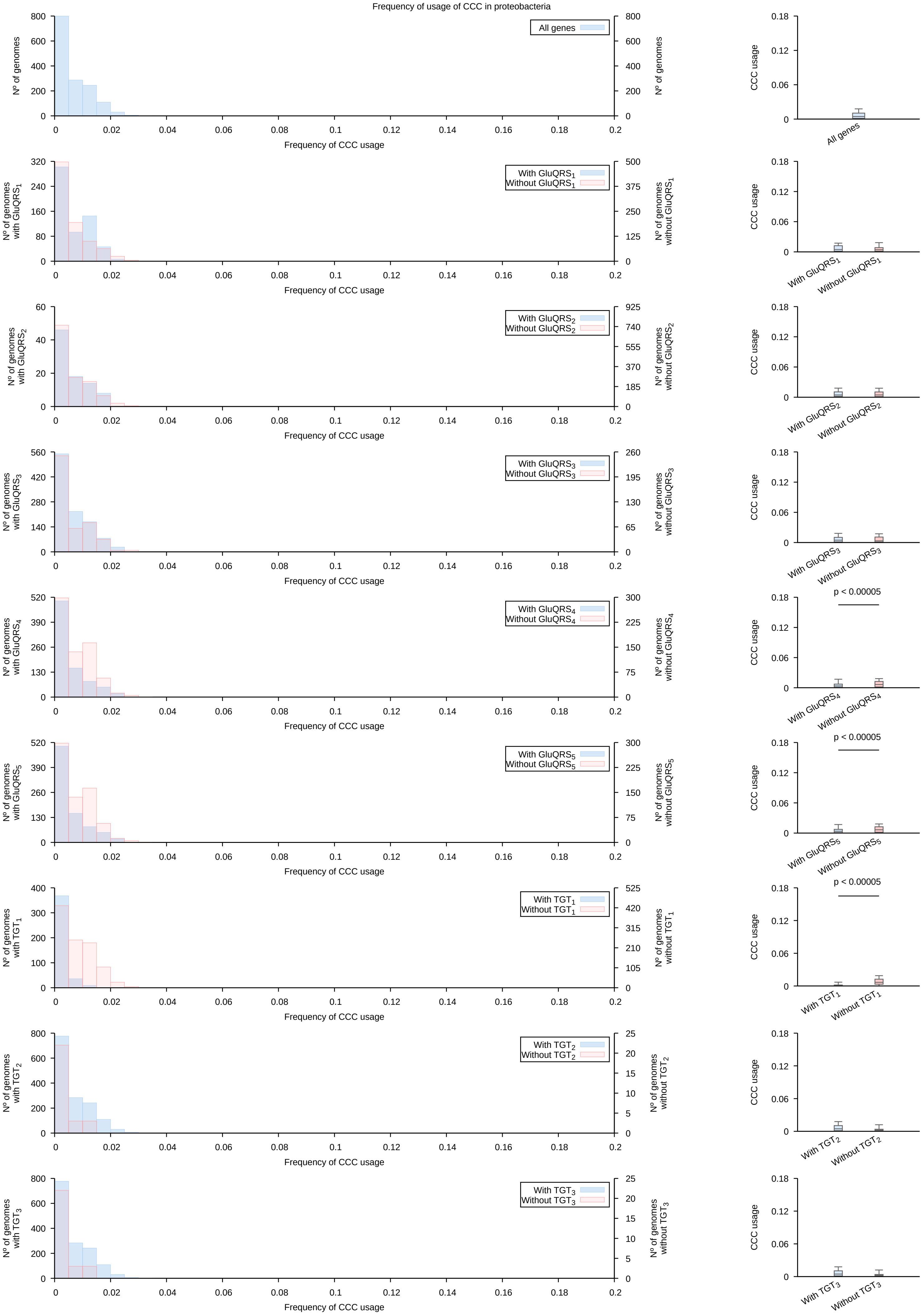
Frequency of usage of CCC in proteobacteria
All genes
Nº of genomes
Frequency of CCC usage
CCC usage
Nº of genomes with $GluQRS_1$
Nº of genomes without $GluQRS_1$
With $GluQRS_1$
Without $GluQRS_1$
Nº of genomes with $GluQRS_2$
Nº of genomes without $GluQRS_2$
With $GluQRS_2$
Without $GluQRS_2$
Nº of genomes with $GluQRS_3$
Nº of genomes without $GluQRS_3$
With $GluQRS_3$
Without $GluQRS_3$
Nº of genomes with $GluQRS_4$
Nº of genomes without $GluQRS_4$
With $GluQRS_4$
Without $GluQRS_4$
p < 0.00005
Nº of genomes with $GluQRS_5$
Nº of genomes without $GluQRS_5$
With $GluQRS_5$
Without $GluQRS_5$
p < 0.00005
Nº of genomes with TGT_1
Nº of genomes without TGT_1
With TGT_1
Without TGT_1
p < 0.00005
Nº of genomes with TGT_2
Nº of genomes without TGT_2
With TGT_2
Without TGT_2
Nº of genomes with TGT_3
Nº of genomes without TGT_3
With TGT_3
Without TGT_3

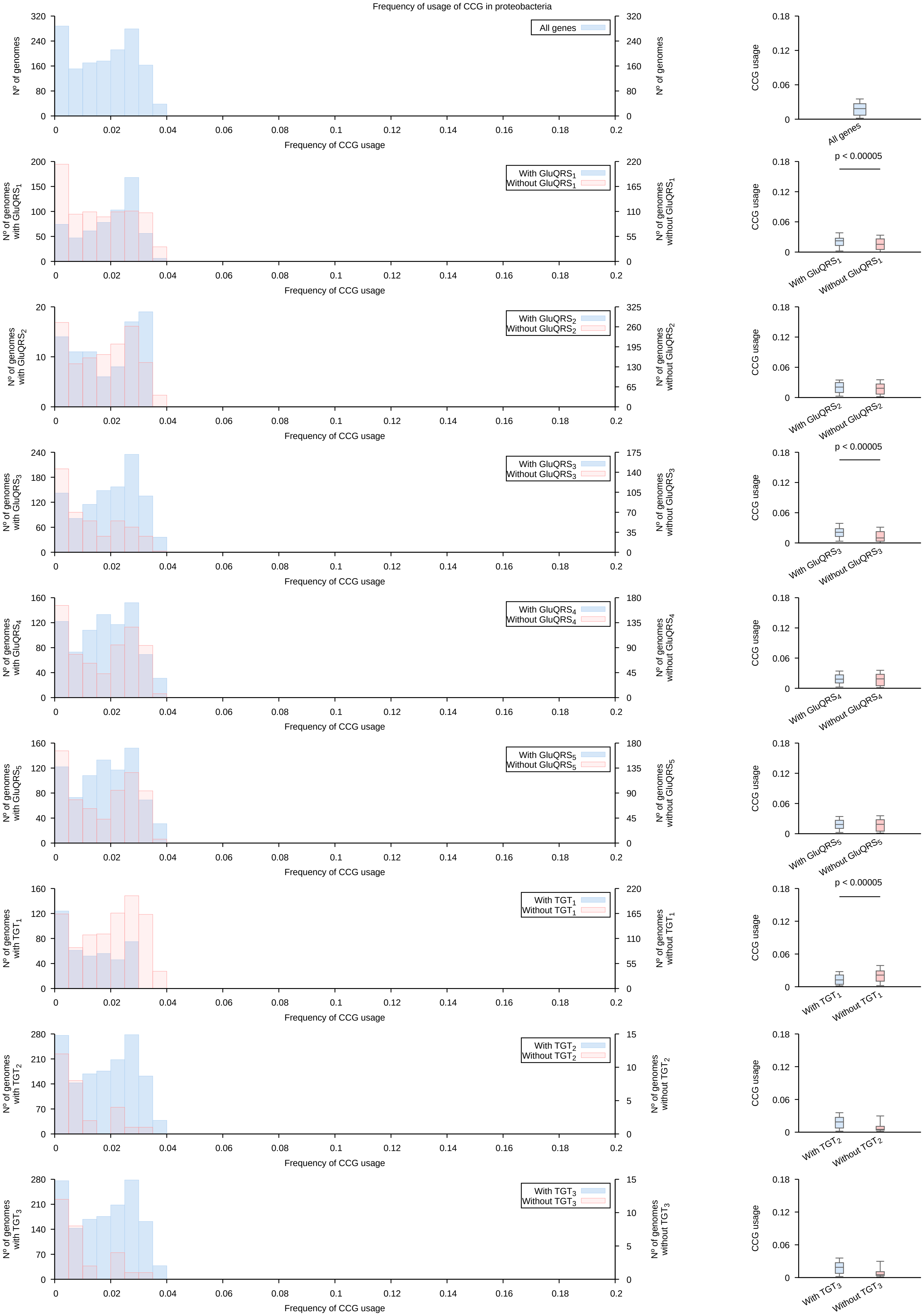
Frequency of usage of CCG in proteobacteria
All genes
Nº of genomes
Frequency of CCG usage
CCG usage
p < 0.00005
With GluQRS$_1$
Without GluQRS$_1$
Nº of genomes with GluQRS$_1$
Nº of genomes without GluQRS$_1$
With GluQRS$_2$
Without GluQRS$_2$
Nº of genomes with GluQRS$_2$
Nº of genomes without GluQRS$_2$
With GluQRS$_3$
Without GluQRS$_3$
Nº of genomes with GluQRS$_3$
Nº of genomes without GluQRS$_3$
With GluQRS$_4$
Without GluQRS$_4$
Nº of genomes with GluQRS$_4$
Nº of genomes without GluQRS$_4$
With GluQRS$_5$
Without GluQRS$_5$
Nº of genomes with GluQRS$_5$
Nº of genomes without GluQRS$_5$
With TGT$_1$
Without TGT$_1$
Nº of genomes with TGT$_1$
Nº of genomes without TGT$_1$
With TGT$_2$
Without TGT$_2$
Nº of genomes with TGT$_2$
Nº of genomes without TGT$_2$
With TGT$_3$
Without TGT$_3$
Nº of genomes with TGT$_3$
Nº of genomes without TGT$_3$

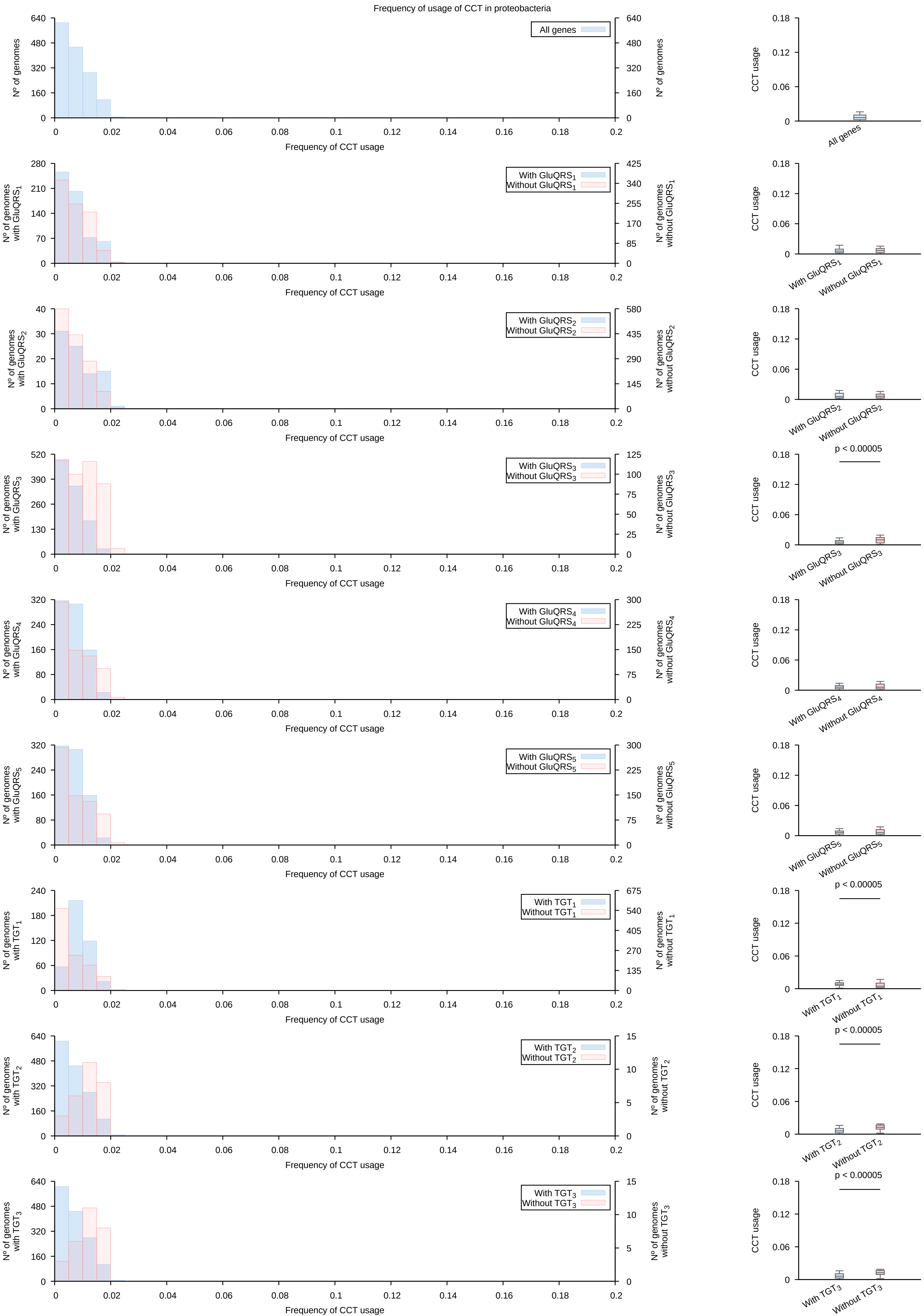
Frequency of usage of CCT in proteobacteria
All genes
Nº of genomes
Frequency of CCT usage
CCT usage
With GluQRS$_1$
Without GluQRS$_1$
Nº of genomes with GluQRS$_1$
Nº of genomes without GluQRS$_1$
With GluQRS$_2$
Without GluQRS$_2$
Nº of genomes with GluQRS$_2$
Nº of genomes without GluQRS$_2$
With GluQRS$_3$
Without GluQRS$_3$
Nº of genomes with GluQRS$_3$
Nº of genomes without GluQRS$_3$
p < 0.00005
With GluQRS$_4$
Without GluQRS$_4$
Nº of genomes with GluQRS$_4$
Nº of genomes without GluQRS$_4$
With GluQRS$_5$
Without GluQRS$_5$
Nº of genomes with GluQRS$_5$
Nº of genomes without GluQRS$_5$
With TGT$_1$
Without TGT$_1$
Nº of genomes with TGT$_1$
Nº of genomes without TGT$_1$
p < 0.00005
With TGT$_2$
Without TGT$_2$
Nº of genomes with TGT$_2$
Nº of genomes without TGT$_2$
p < 0.00005
With TGT$_3$
Without TGT$_3$
Nº of genomes with TGT$_3$
Nº of genomes without TGT$_3$
p < 0.00005

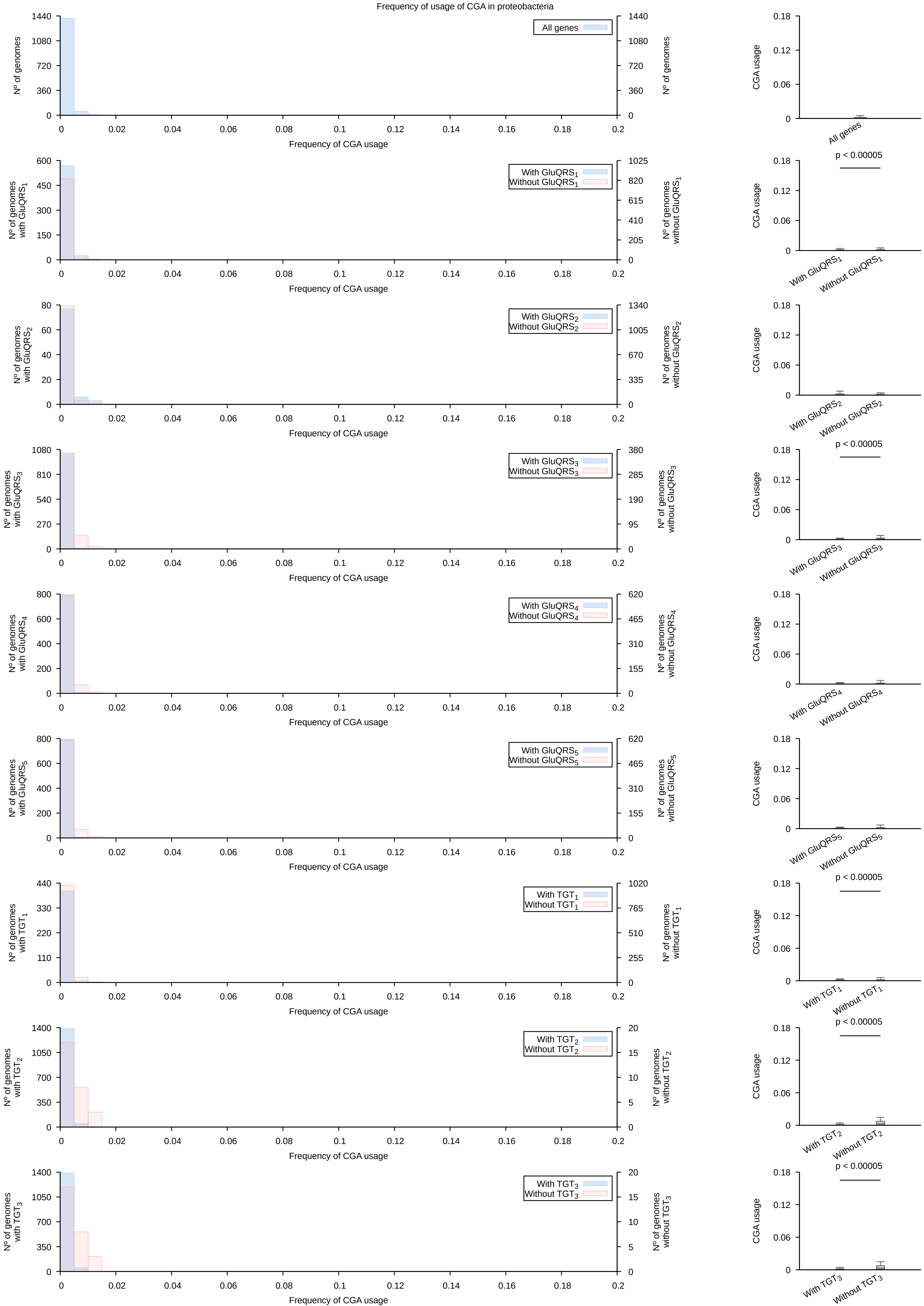
Frequency of usage of CGA in proteobacteria
All genes
Nº of genomes
Frequency of CGA usage
CGA usage
With GluQRS$_1$
Without GluQRS$_1$
Nº of genomes with GluQRS$_1$
Nº of genomes without GluQRS$_1$
p < 0.00005
With GluQRS$_2$
Without GluQRS$_2$
Nº of genomes with GluQRS$_2$
Nº of genomes without GluQRS$_2$
With GluQRS$_3$
Without GluQRS$_3$
Nº of genomes with GluQRS$_3$
Nº of genomes without GluQRS$_3$
p < 0.00005
With GluQRS$_4$
Without GluQRS$_4$
Nº of genomes with GluQRS$_4$
Nº of genomes without GluQRS$_4$
With GluQRS$_5$
Without GluQRS$_5$
Nº of genomes with GluQRS$_5$
Nº of genomes without GluQRS$_5$
With TGT$_1$
Without TGT$_1$
Nº of genomes with TGT$_1$
Nº of genomes without TGT$_1$
p < 0.00005
With TGT$_2$
Without TGT$_2$
Nº of genomes with TGT$_2$
Nº of genomes without TGT$_2$
p < 0.00005
With TGT$_3$
Without TGT$_3$
Nº of genomes with TGT$_3$
Nº of genomes without TGT$_3$
p < 0.00005

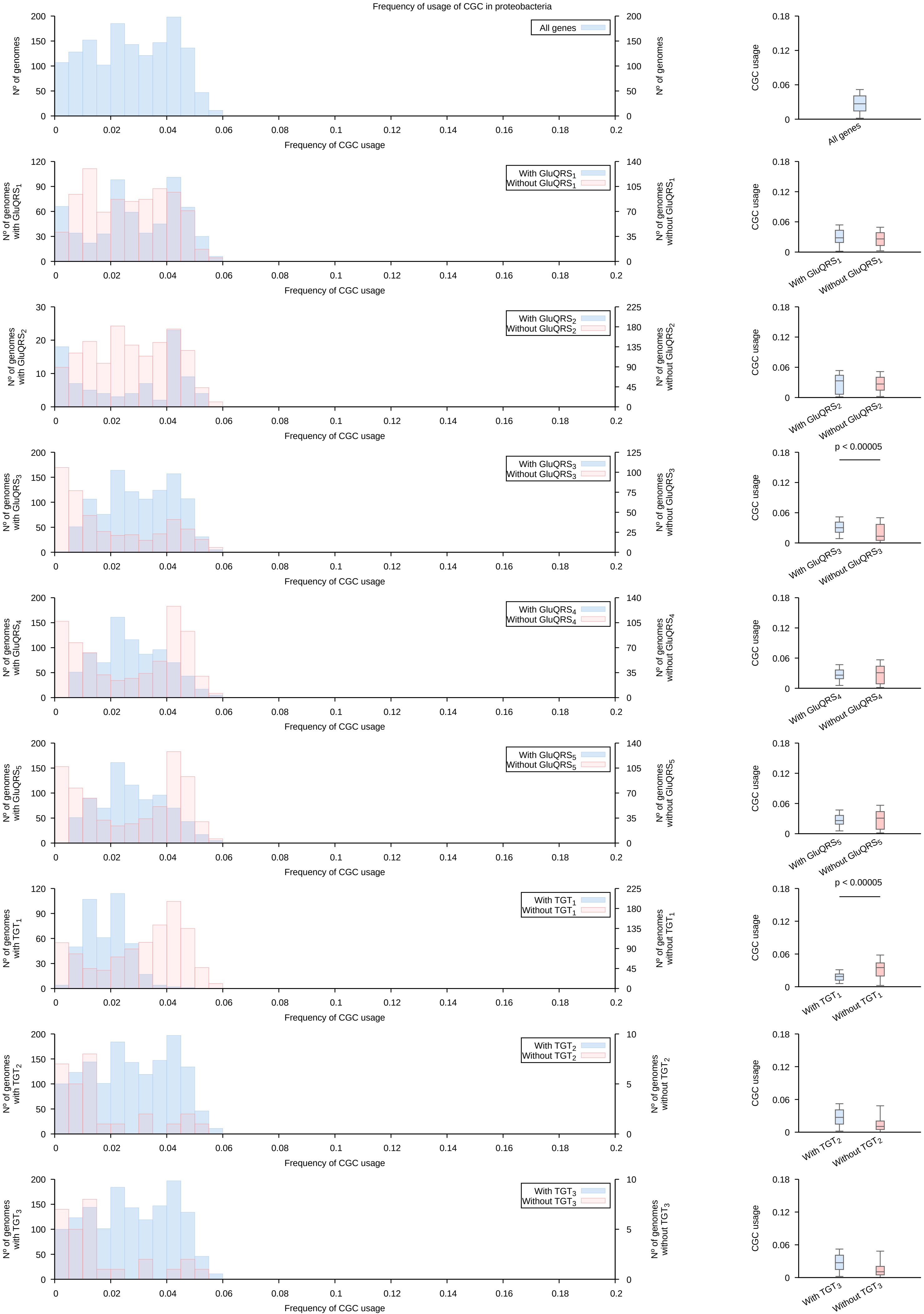
Frequency of usage of CGC in proteobacteria
All genes
With GluQRS$_1$
Without GluQRS$_1$
With GluQRS$_2$
Without GluQRS$_2$
With GluQRS$_3$
Without GluQRS$_3$
With GluQRS$_4$
Without GluQRS$_4$
With GluQRS$_5$
Without GluQRS$_5$
With TGT$_1$
Without TGT$_1$
With TGT$_2$
Without TGT$_2$
With TGT$_3$
Without TGT$_3$
Nº of genomes
Nº of genomes with GluQRS$_1$
Nº of genomes without GluQRS$_1$
Nº of genomes with GluQRS$_2$
Nº of genomes without GluQRS$_2$
Nº of genomes with GluQRS$_3$
Nº of genomes without GluQRS$_3$
Nº of genomes with GluQRS$_4$
Nº of genomes without GluQRS$_4$
Nº of genomes with GluQRS$_5$
Nº of genomes without GluQRS$_5$
Nº of genomes with TGT$_1$
Nº of genomes without TGT$_1$
Nº of genomes with TGT$_2$
Nº of genomes without TGT$_2$
Nº of genomes with TGT$_3$
Nº of genomes without TGT$_3$
Frequency of CGC usage
CGC usage
$p < 0.00005$
$p < 0.00005$

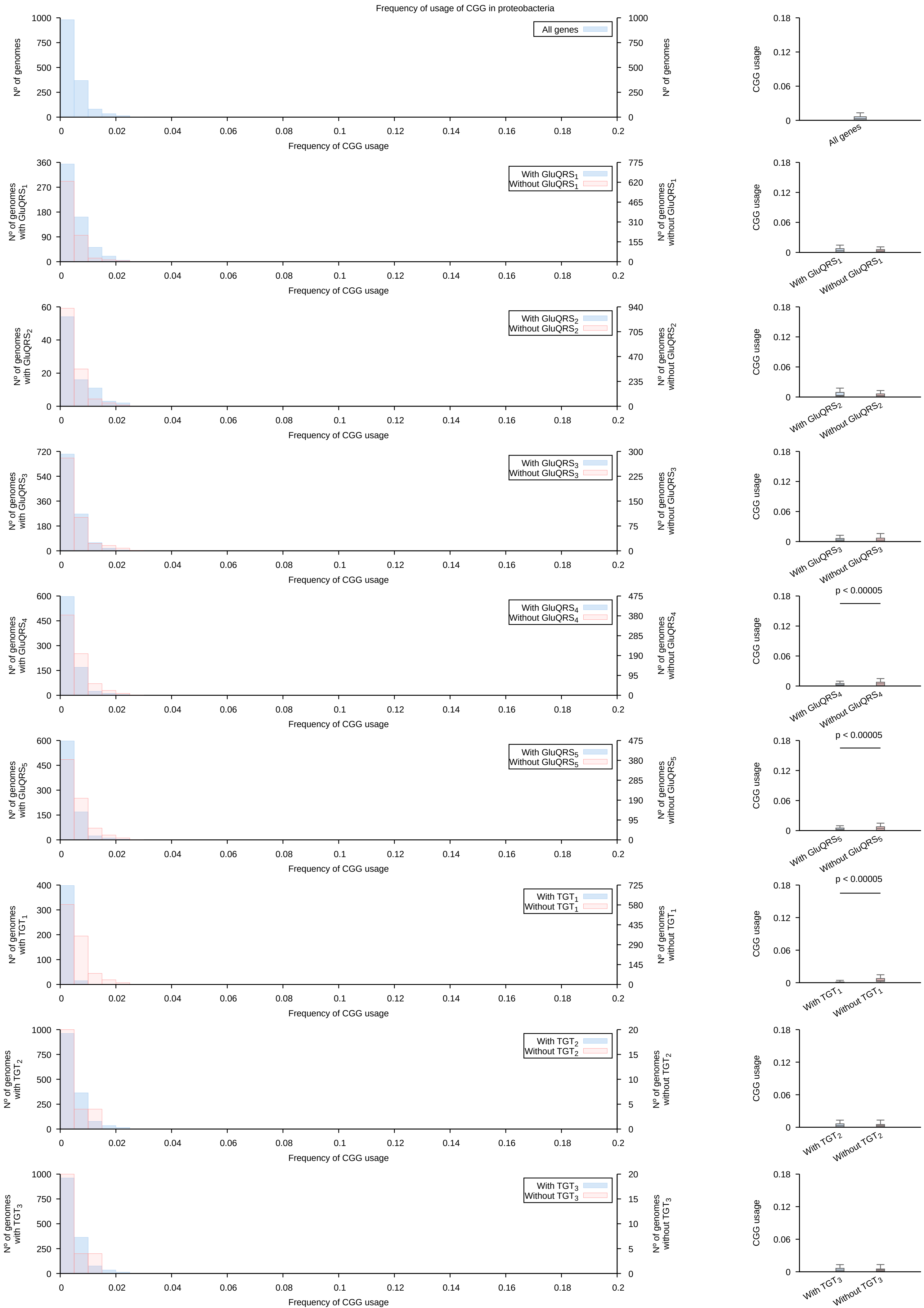

Frequency of usage of CGG in proteobacteria
Nº of genomes
Frequency of CGG usage
CGG usage
All genes
With GluQRS$_1$
Without GluQRS$_1$
Nº of genomes with GluQRS$_1$
Nº of genomes without GluQRS$_1$
With GluQRS$_2$
Without GluQRS$_2$
Nº of genomes with GluQRS$_2$
Nº of genomes without GluQRS$_2$
With GluQRS$_3$
Without GluQRS$_3$
Nº of genomes with GluQRS$_3$
Nº of genomes without GluQRS$_3$
With GluQRS$_4$
Without GluQRS$_4$
Nº of genomes with GluQRS$_4$
Nº of genomes without GluQRS$_4$
p < 0.00005
With GluQRS$_5$
Without GluQRS$_5$
Nº of genomes with GluQRS$_5$
Nº of genomes without GluQRS$_5$
p < 0.00005
With TGT$_1$
Without TGT$_1$
Nº of genomes with TGT$_1$
Nº of genomes without TGT$_1$
p < 0.00005
With TGT$_2$
Without TGT$_2$
Nº of genomes with TGT$_2$
Nº of genomes without TGT$_2$
With TGT$_3$
Without TGT$_3$
Nº of genomes with TGT$_3$
Nº of genomes without TGT$_3$

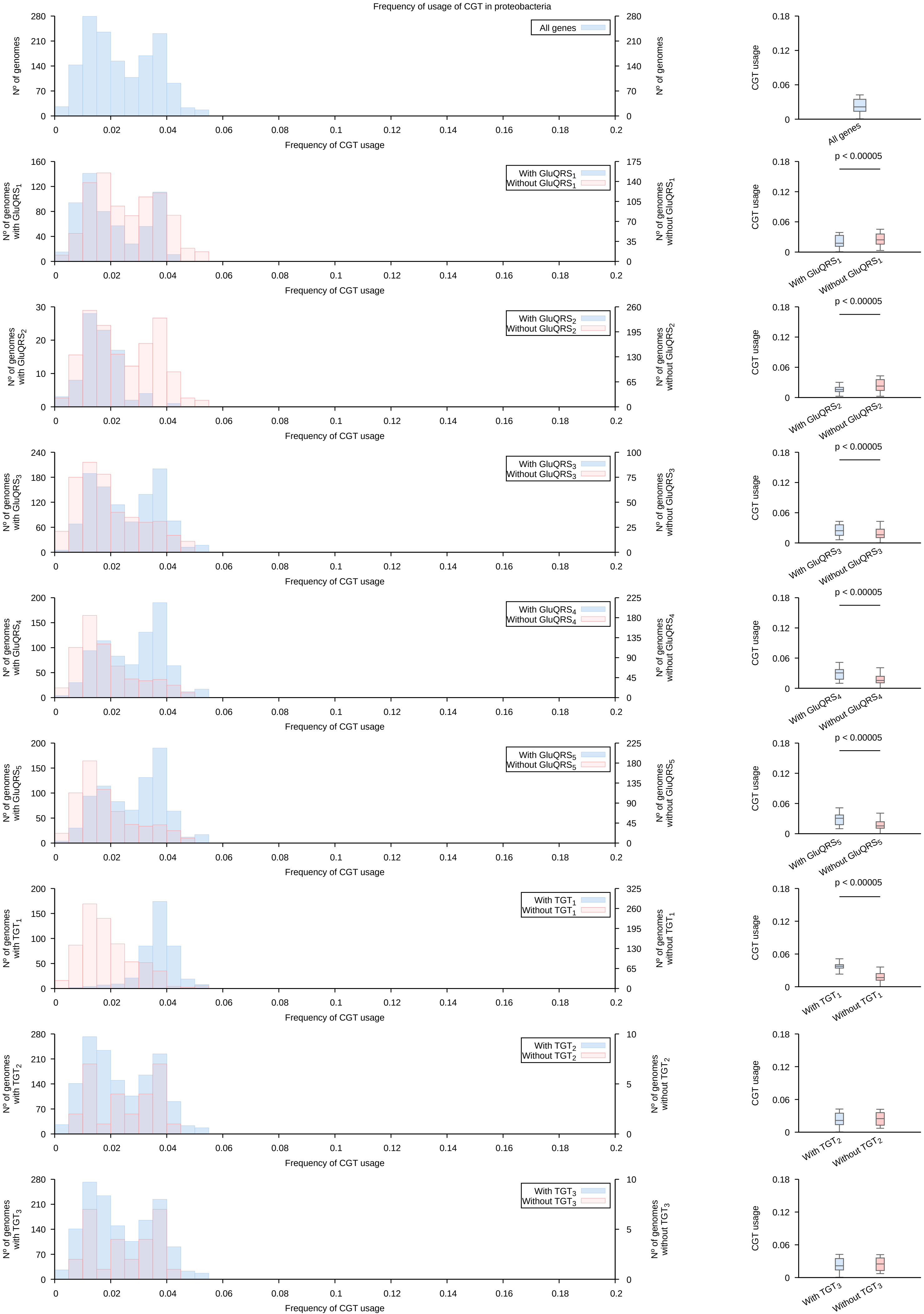
Frequency of usage of CGT in proteobacteria
All genes
Frequency of CGT usage
Nº of genomes
CGT usage
p < 0.00005
With GluQRS1
Without GluQRS1
With GluQRS2
Without GluQRS2
With GluQRS3
Without GluQRS3
With GluQRS4
Without GluQRS4
With GluQRS5
Without GluQRS5
With TGT1
Without TGT1
With TGT2
Without TGT2
With TGT3
Without TGT3

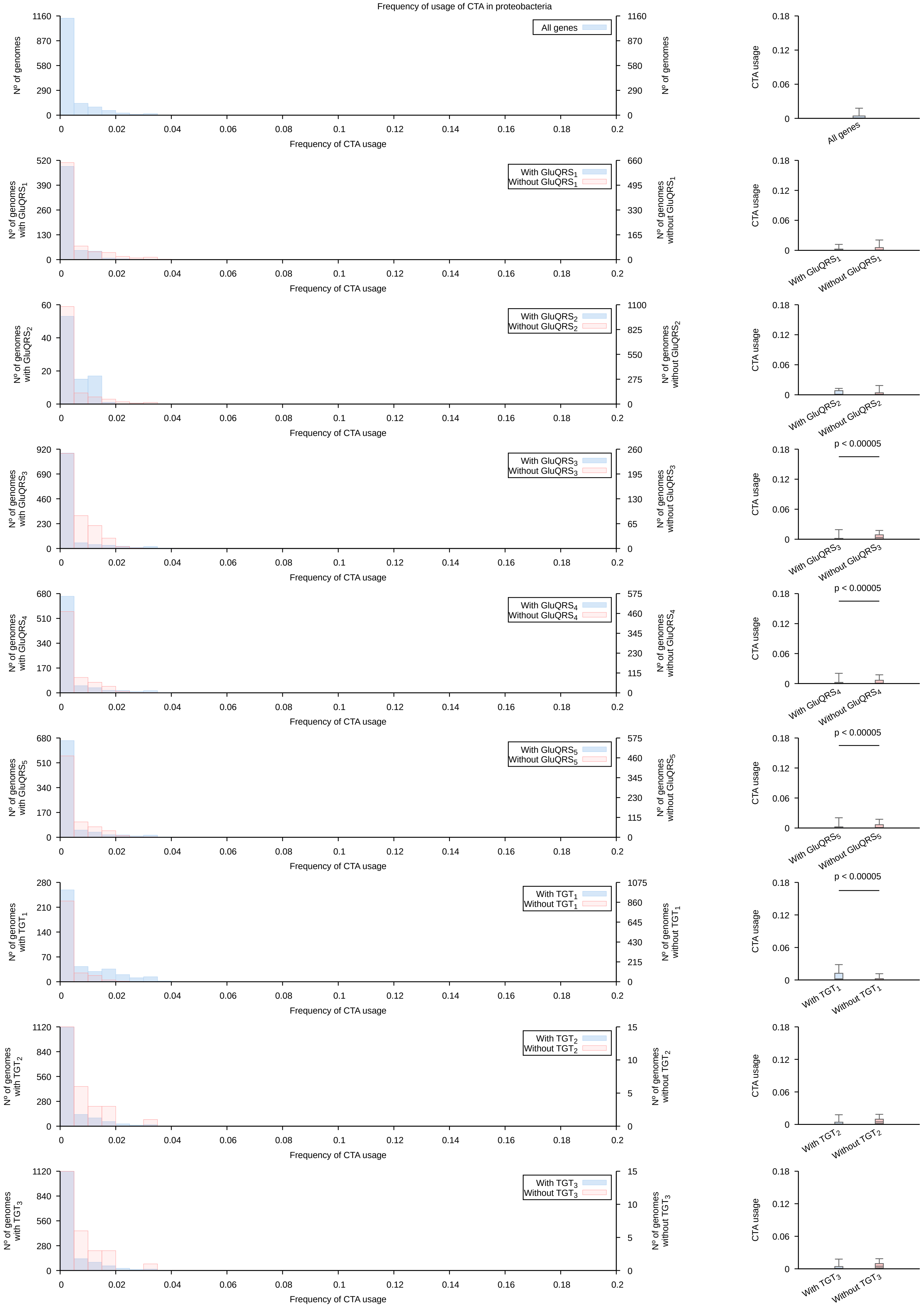
Frequency of usage of CTA in proteobacteria
Nº of genomes
Frequency of CTA usage
CTA usage
All genes
With GluQRS$_1$
Without GluQRS$_1$
Nº of genomes with GluQRS$_1$
Nº of genomes without GluQRS$_1$
With GluQRS$_2$
Without GluQRS$_2$
Nº of genomes with GluQRS$_2$
Nº of genomes without GluQRS$_2$
With GluQRS$_3$
Without GluQRS$_3$
Nº of genomes with GluQRS$_3$
Nº of genomes without GluQRS$_3$
p < 0.00005
With GluQRS$_4$
Without GluQRS$_4$
Nº of genomes with GluQRS$_4$
Nº of genomes without GluQRS$_4$
p < 0.00005
With GluQRS$_5$
Without GluQRS$_5$
Nº of genomes with GluQRS$_5$
Nº of genomes without GluQRS$_5$
p < 0.00005
With TGT$_1$
Without TGT$_1$
Nº of genomes with TGT$_1$
Nº of genomes without TGT$_1$
p < 0.00005
With TGT$_2$
Without TGT$_2$
Nº of genomes with TGT$_2$
Nº of genomes without TGT$_2$
With TGT$_3$
Without TGT$_3$
Nº of genomes with TGT$_3$
Nº of genomes without TGT$_3$

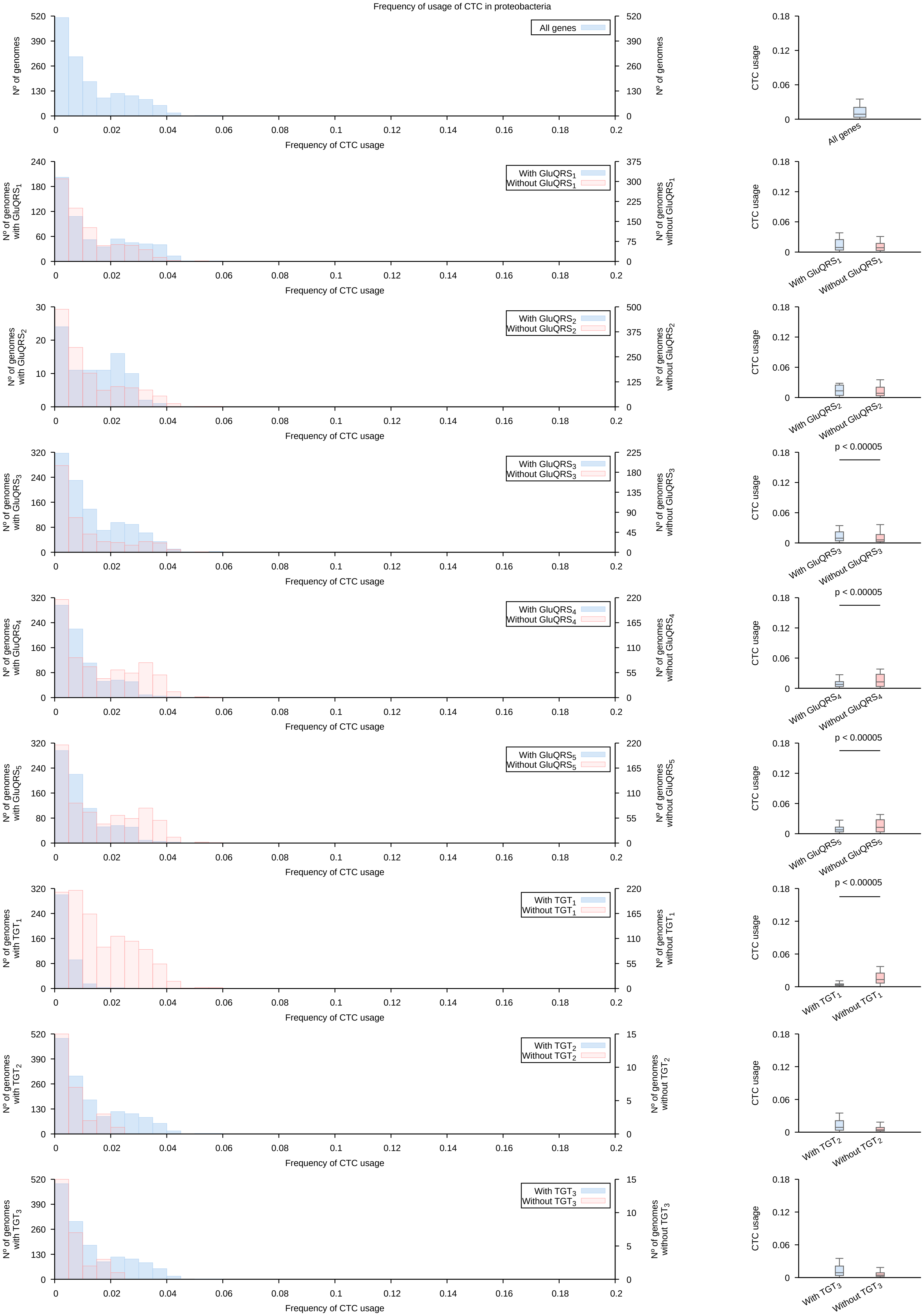

Frequency of usage of CTC in proteobacteria
All genes
Nº of genomes
Frequency of CTC usage
CTC usage
With GluQRS1
Without GluQRS1
Nº of genomes with GluQRS1
Nº of genomes without GluQRS1
With GluQRS2
Without GluQRS2
Nº of genomes with GluQRS2
Nº of genomes without GluQRS2
With GluQRS3
Without GluQRS3
Nº of genomes with GluQRS3
Nº of genomes without GluQRS3
p < 0.00005
With GluQRS4
Without GluQRS4
Nº of genomes with GluQRS4
Nº of genomes without GluQRS4
p < 0.00005
With GluQRS5
Without GluQRS5
Nº of genomes with GluQRS5
Nº of genomes without GluQRS5
p < 0.00005
With TGT1
Without TGT1
Nº of genomes with TGT1
Nº of genomes without TGT1
p < 0.00005
With TGT2
Without TGT2
Nº of genomes with TGT2
Nº of genomes without TGT2
With TGT3
Without TGT3
Nº of genomes with TGT3
Nº of genomes without TGT3
0 0.02 0.04 0.06 0.08 0.1 0.12 0.14 0.16 0.18 0.2
0 0.06 0.12 0.18

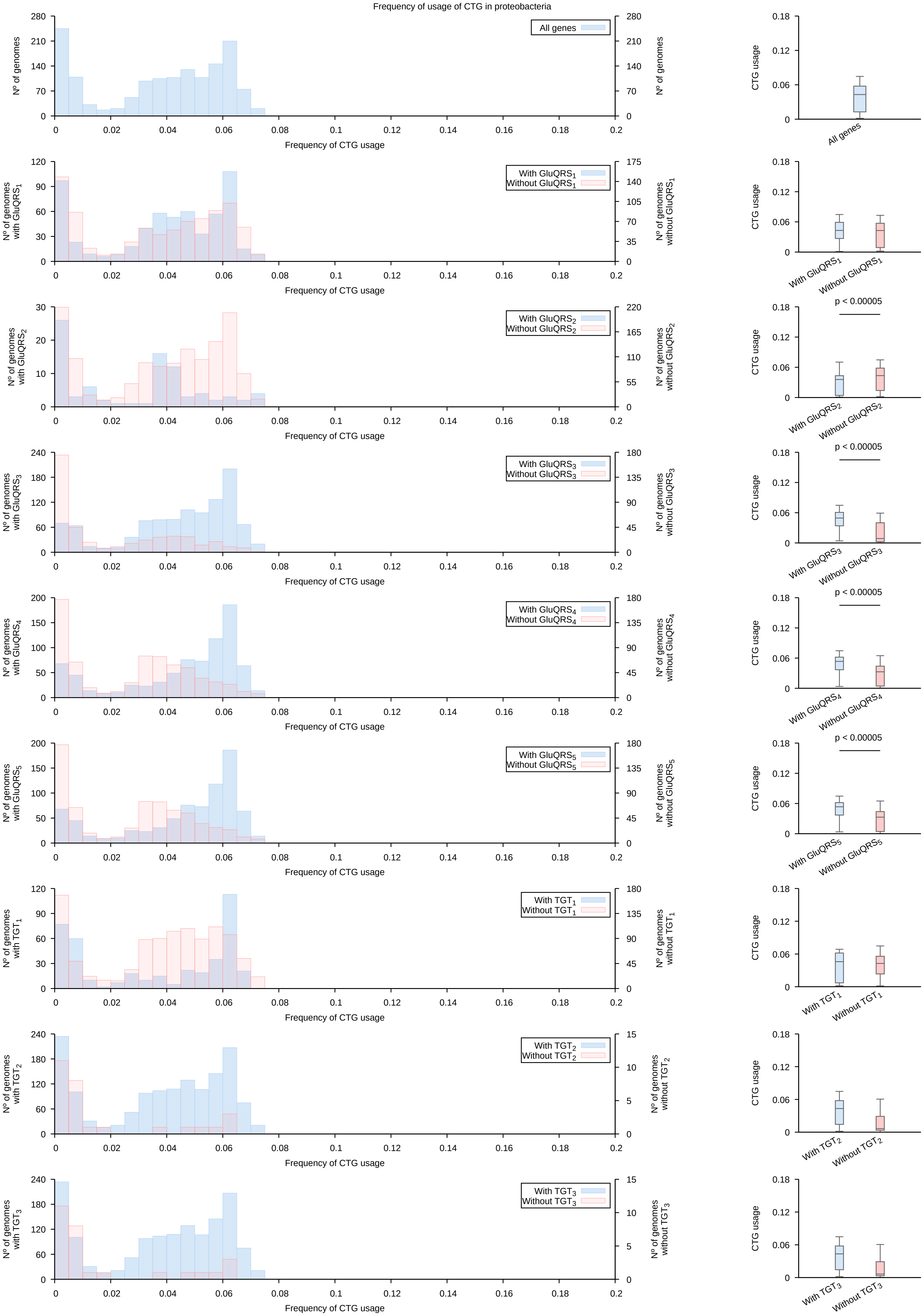
Frequency of usage of CTG in proteobacteria
All genes
Nº of genomes
Frequency of CTG usage
CTG usage
With GluQRS$_1$
Without GluQRS$_1$
Nº of genomes with GluQRS$_1$
Nº of genomes without GluQRS$_1$
With GluQRS$_2$
Without GluQRS$_2$
Nº of genomes with GluQRS$_2$
Nº of genomes without GluQRS$_2$
p < 0.00005
With GluQRS$_3$
Without GluQRS$_3$
Nº of genomes with GluQRS$_3$
Nº of genomes without GluQRS$_3$
p < 0.00005
With GluQRS$_4$
Without GluQRS$_4$
Nº of genomes with GluQRS$_4$
Nº of genomes without GluQRS$_4$
p < 0.00005
With GluQRS$_5$
Without GluQRS$_5$
Nº of genomes with GluQRS$_5$
Nº of genomes without GluQRS$_5$
p < 0.00005
With TGT$_1$
Without TGT$_1$
Nº of genomes with TGT$_1$
Nº of genomes without TGT$_1$
With TGT$_2$
Without TGT$_2$
Nº of genomes with TGT$_2$
Nº of genomes without TGT$_2$
With TGT$_3$
Without TGT$_3$
Nº of genomes with TGT$_3$
Nº of genomes without TGT$_3$

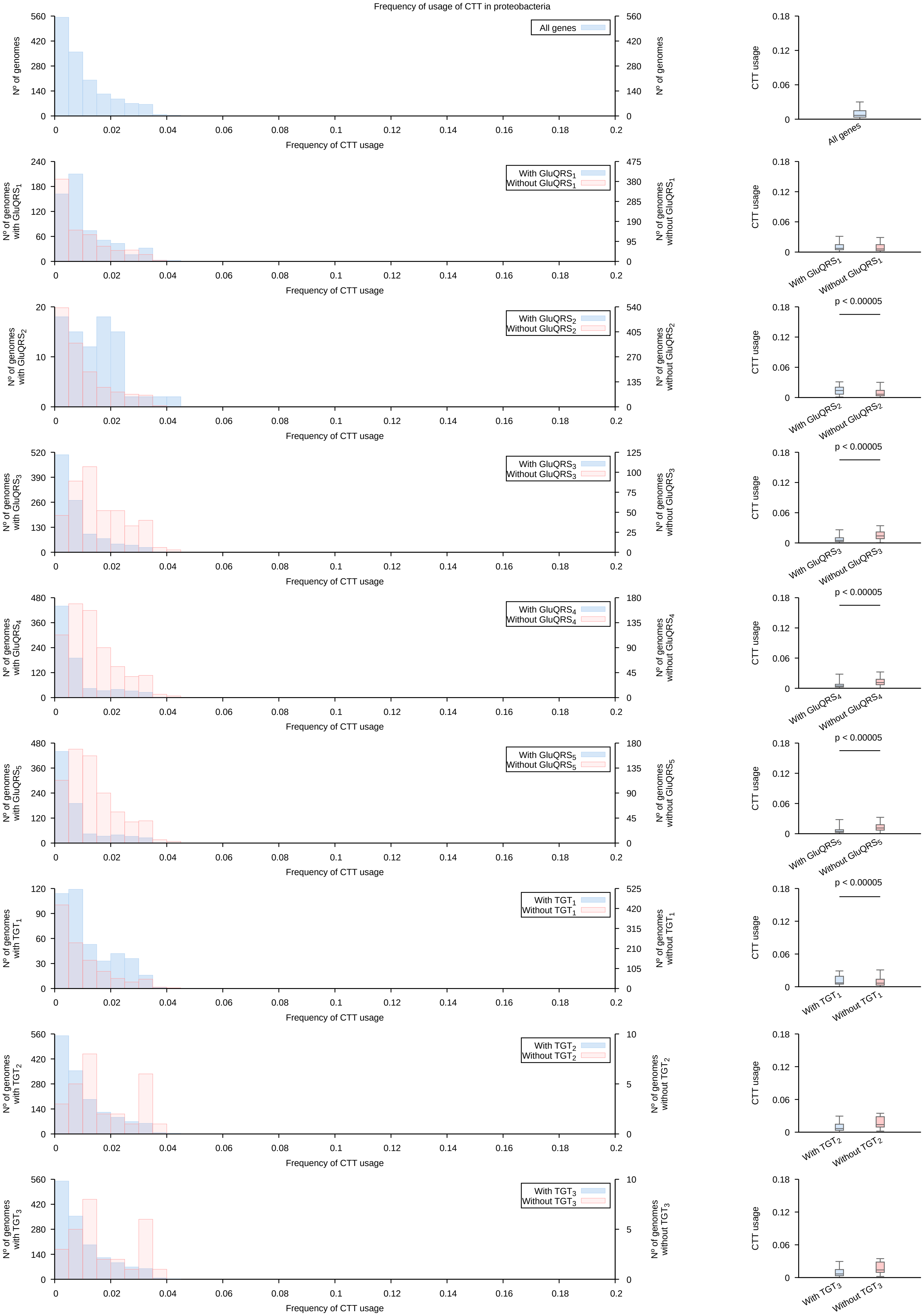
Frequency of usage of CTT in proteobacteria
All genes
Nº of genomes
Frequency of CTT usage
CTT usage
With GluQRS$_1$
Without GluQRS$_1$
Nº of genomes with GluQRS$_1$
Nº of genomes without GluQRS$_1$
With GluQRS$_2$
Without GluQRS$_2$
Nº of genomes with GluQRS$_2$
Nº of genomes without GluQRS$_2$
p < 0.00005
With GluQRS$_3$
Without GluQRS$_3$
Nº of genomes with GluQRS$_3$
Nº of genomes without GluQRS$_3$
p < 0.00005
With GluQRS$_4$
Without GluQRS$_4$
Nº of genomes with GluQRS$_4$
Nº of genomes without GluQRS$_4$
p < 0.00005
With GluQRS$_5$
Without GluQRS$_5$
Nº of genomes with GluQRS$_5$
Nº of genomes without GluQRS$_5$
p < 0.00005
With TGT$_1$
Without TGT$_1$
Nº of genomes with TGT$_1$
Nº of genomes without TGT$_1$
p < 0.00005
With TGT$_2$
Without TGT$_2$
Nº of genomes with TGT$_2$
Nº of genomes without TGT$_2$
With TGT$_3$
Without TGT$_3$
Nº of genomes with TGT$_3$
Nº of genomes without TGT$_3$

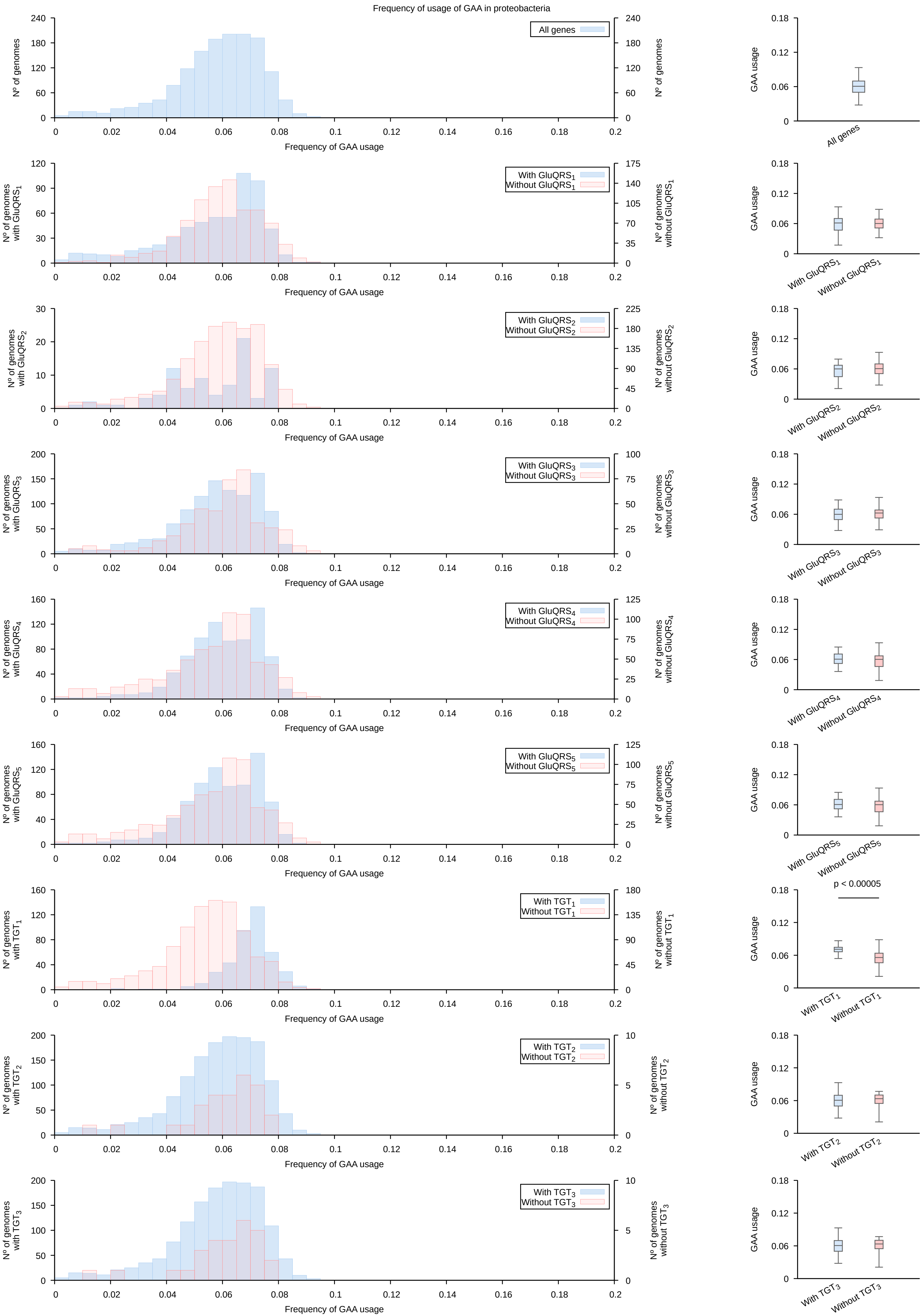
Frequency of usage of GAA in proteobacteria
All genes
Nº of genomes
Frequency of GAA usage
GAA usage
With $GluQRS_1$
Without $GluQRS_1$
Nº of genomes with $GluQRS_1$
Nº of genomes without $GluQRS_1$
With $GluQRS_2$
Without $GluQRS_2$
Nº of genomes with $GluQRS_2$
Nº of genomes without $GluQRS_2$
With $GluQRS_3$
Without $GluQRS_3$
Nº of genomes with $GluQRS_3$
Nº of genomes without $GluQRS_3$
With $GluQRS_4$
Without $GluQRS_4$
Nº of genomes with $GluQRS_4$
Nº of genomes without $GluQRS_4$
With $GluQRS_5$
Without $GluQRS_5$
Nº of genomes with $GluQRS_5$
Nº of genomes without $GluQRS_5$
With TGT_1
Without TGT_1
Nº of genomes with TGT_1
Nº of genomes without TGT_1
$p < 0.00005$
With TGT_2
Without TGT_2
Nº of genomes with TGT_2
Nº of genomes without TGT_2
With TGT_3
Without TGT_3
Nº of genomes with TGT_3
Nº of genomes without TGT_3

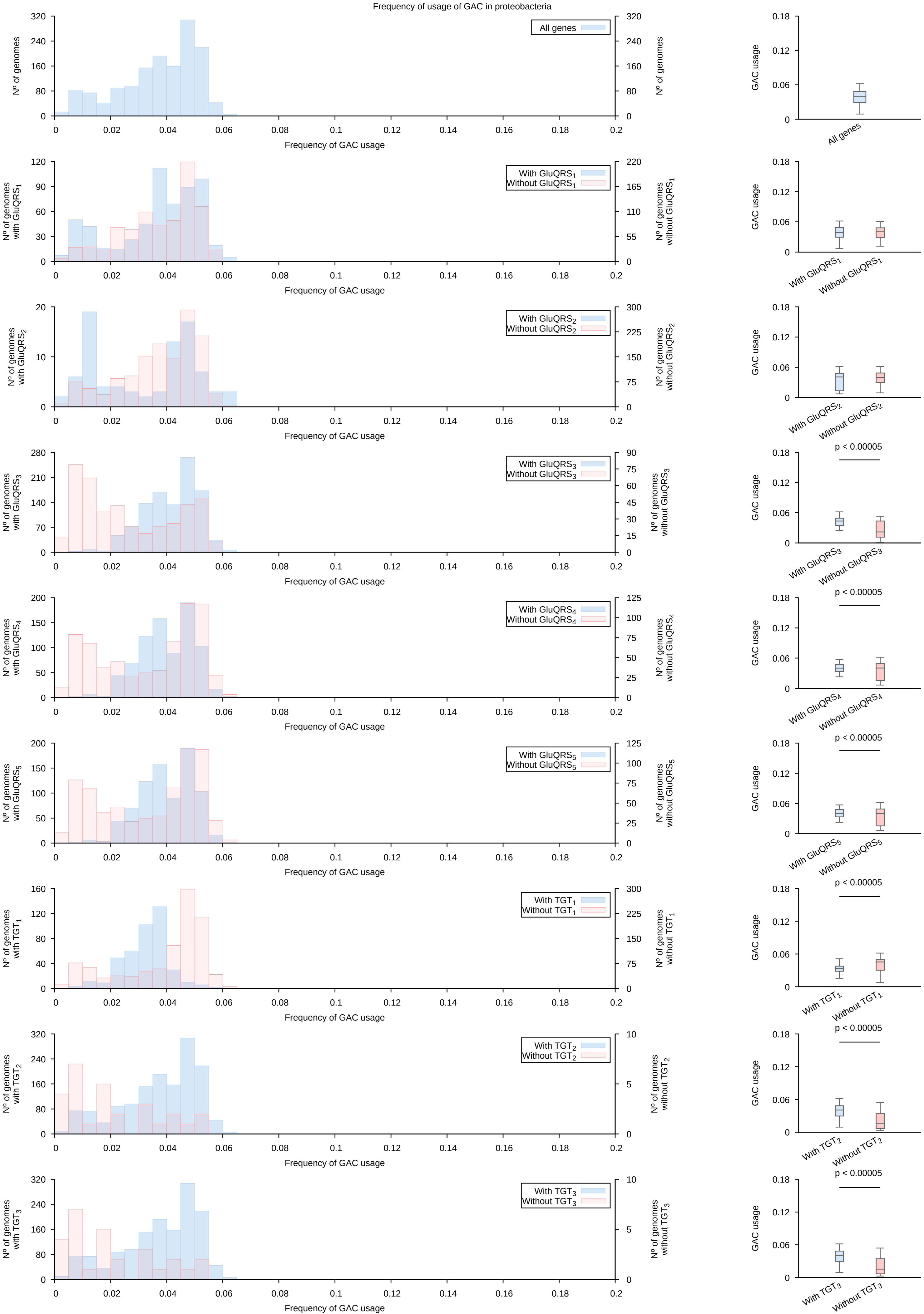

Frequency of usage of GAC in proteobacteria
Nº of genomes
Frequency of GAC usage
GAC usage
All genes
Nº of genomes with GluQRS$_1$
Nº of genomes without GluQRS$_1$
With GluQRS$_1$
Without GluQRS$_1$
Nº of genomes with GluQRS$_2$
Nº of genomes without GluQRS$_2$
With GluQRS$_2$
Without GluQRS$_2$
Nº of genomes with GluQRS$_3$
Nº of genomes without GluQRS$_3$
With GluQRS$_3$
Without GluQRS$_3$
p < 0.00005
Nº of genomes with GluQRS$_4$
Nº of genomes without GluQRS$_4$
With GluQRS$_4$
Without GluQRS$_4$
p < 0.00005
Nº of genomes with GluQRS$_5$
Nº of genomes without GluQRS$_5$
With GluQRS$_5$
Without GluQRS$_5$
p < 0.00005
Nº of genomes with TGT$_1$
Nº of genomes without TGT$_1$
With TGT$_1$
Without TGT$_1$
p < 0.00005
Nº of genomes with TGT$_2$
Nº of genomes without TGT$_2$
With TGT$_2$
Without TGT$_2$
p < 0.00005
Nº of genomes with TGT$_3$
Nº of genomes without TGT$_3$
With TGT$_3$
Without TGT$_3$
p < 0.00005

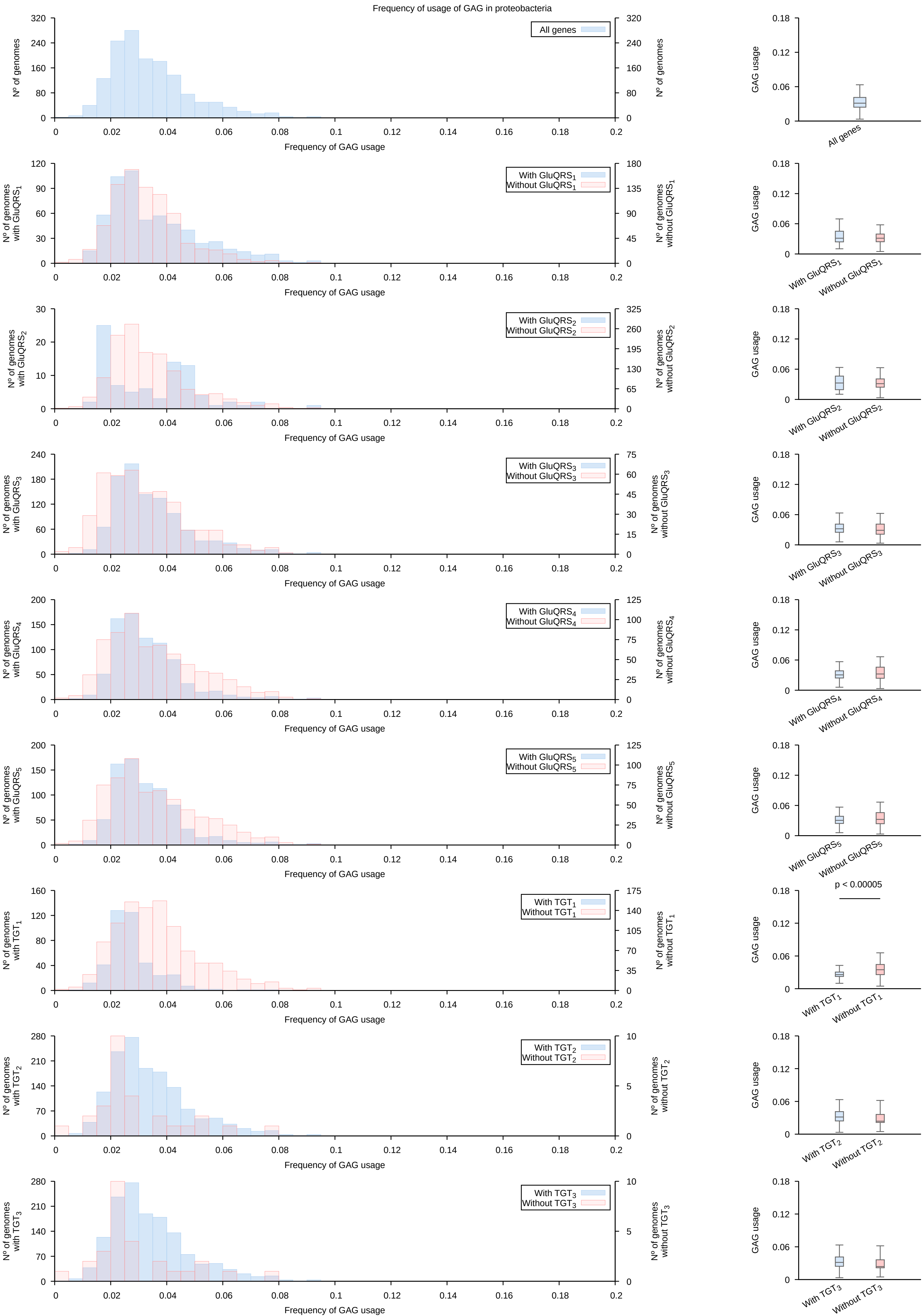

Frequency of usage of GAG in proteobacteria
All genes
Nº of genomes
Frequency of GAG usage
GAG usage
With GluQRS1
Without GluQRS1
Nº of genomes with GluQRS1
Nº of genomes without GluQRS1
With GluQRS2
Without GluQRS2
Nº of genomes with GluQRS2
Nº of genomes without GluQRS2
With GluQRS3
Without GluQRS3
Nº of genomes with GluQRS3
Nº of genomes without GluQRS3
With GluQRS4
Without GluQRS4
Nº of genomes with GluQRS4
Nº of genomes without GluQRS4
With GluQRS5
Without GluQRS5
Nº of genomes with GluQRS5
Nº of genomes without GluQRS5
With TGT1
Without TGT1
Nº of genomes with TGT1
Nº of genomes without TGT1
p < 0.00005
With TGT2
Without TGT2
Nº of genomes with TGT2
Nº of genomes without TGT2
With TGT3
Without TGT3
Nº of genomes with TGT3
Nº of genomes without TGT3

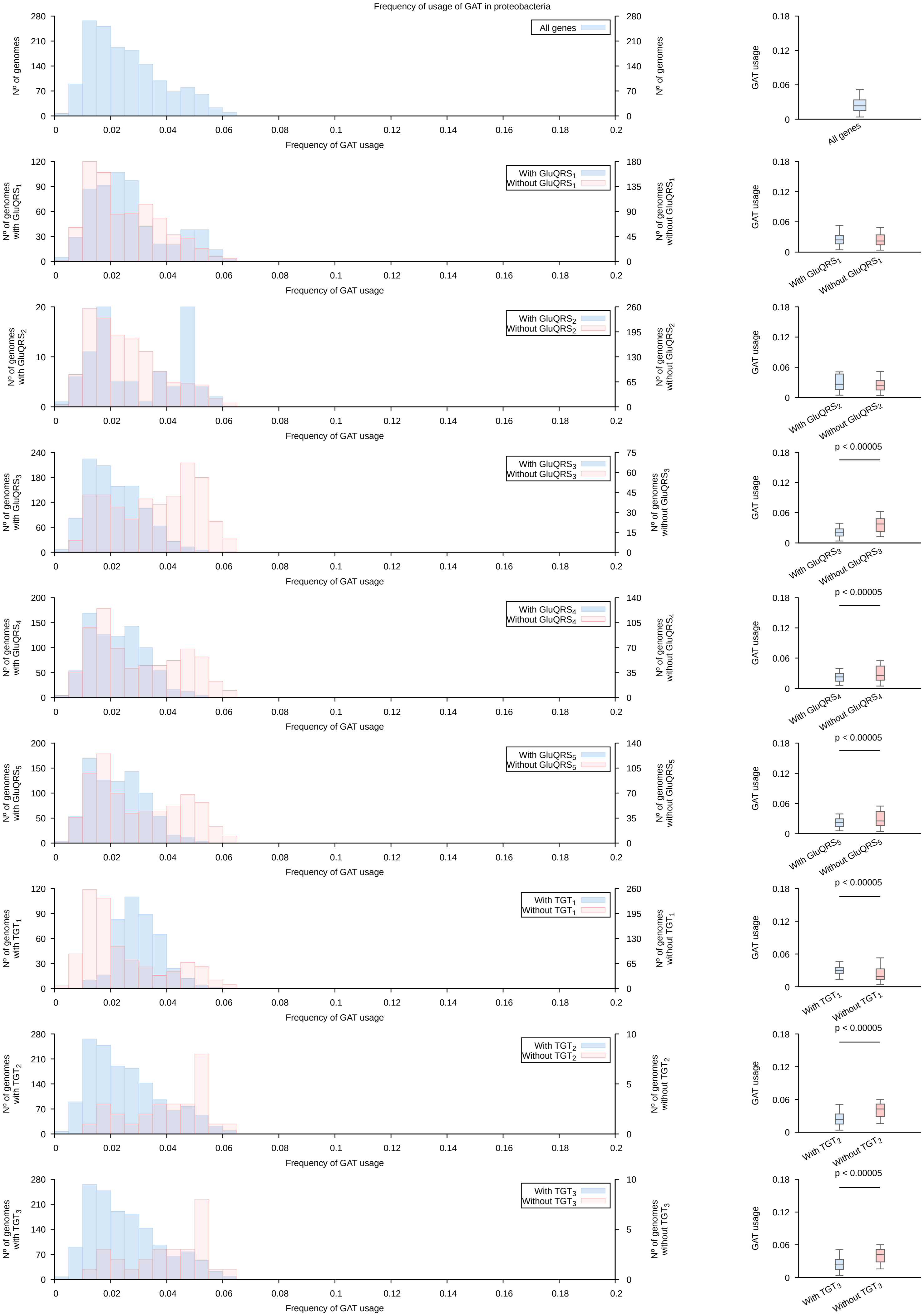
Frequency of usage of GAT in proteobacteria
All genes
With GluQRS1
Without GluQRS1
With GluQRS2
Without GluQRS2
With GluQRS3
Without GluQRS3
With GluQRS4
Without GluQRS4
With GluQRS5
Without GluQRS5
With TGT1
Without TGT1
With TGT2
Without TGT2
With TGT3
Without TGT3
Nº of genomes
Frequency of GAT usage
GAT usage
p < 0.00005

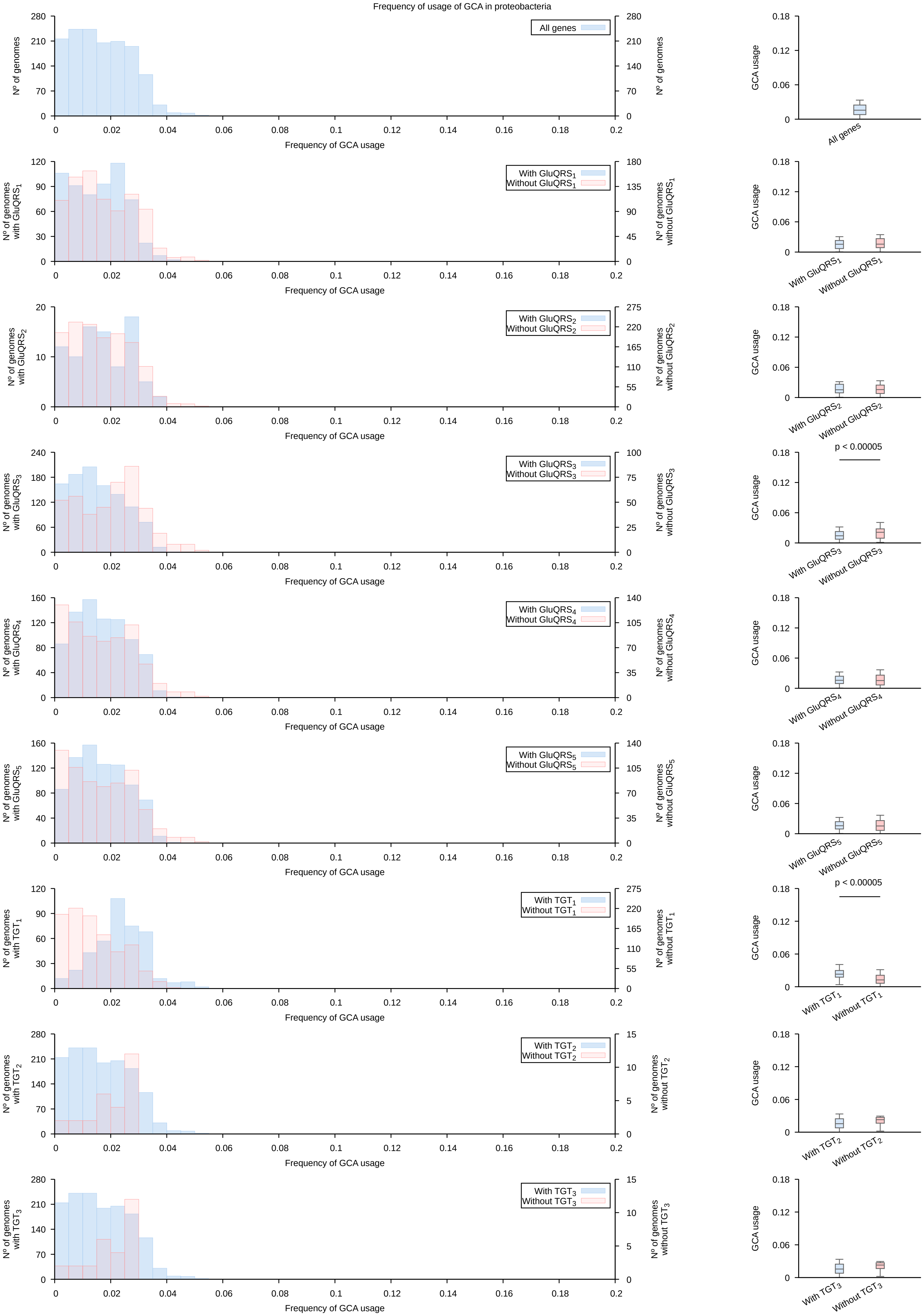

Frequency of usage of GCA in proteobacteria
Nº of genomes
All genes
GCA usage
Frequency of GCA usage
Nº of genomes with GluQRS$_1$
Nº of genomes without GluQRS$_1$
With GluQRS$_1$
Without GluQRS$_1$
Nº of genomes with GluQRS$_2$
Nº of genomes without GluQRS$_2$
With GluQRS$_2$
Without GluQRS$_2$
Nº of genomes with GluQRS$_3$
Nº of genomes without GluQRS$_3$
With GluQRS$_3$
Without GluQRS$_3$
p < 0.00005
Nº of genomes with GluQRS$_4$
Nº of genomes without GluQRS$_4$
With GluQRS$_4$
Without GluQRS$_4$
Nº of genomes with GluQRS$_5$
Nº of genomes without GluQRS$_5$
With GluQRS$_5$
Without GluQRS$_5$
Nº of genomes with TGT$_1$
Nº of genomes without TGT$_1$
With TGT$_1$
Without TGT$_1$
p < 0.00005
Nº of genomes with TGT$_2$
Nº of genomes without TGT$_2$
With TGT$_2$
Without TGT$_2$
Nº of genomes with TGT$_3$
Nº of genomes without TGT$_3$
With TGT$_3$
Without TGT$_3$

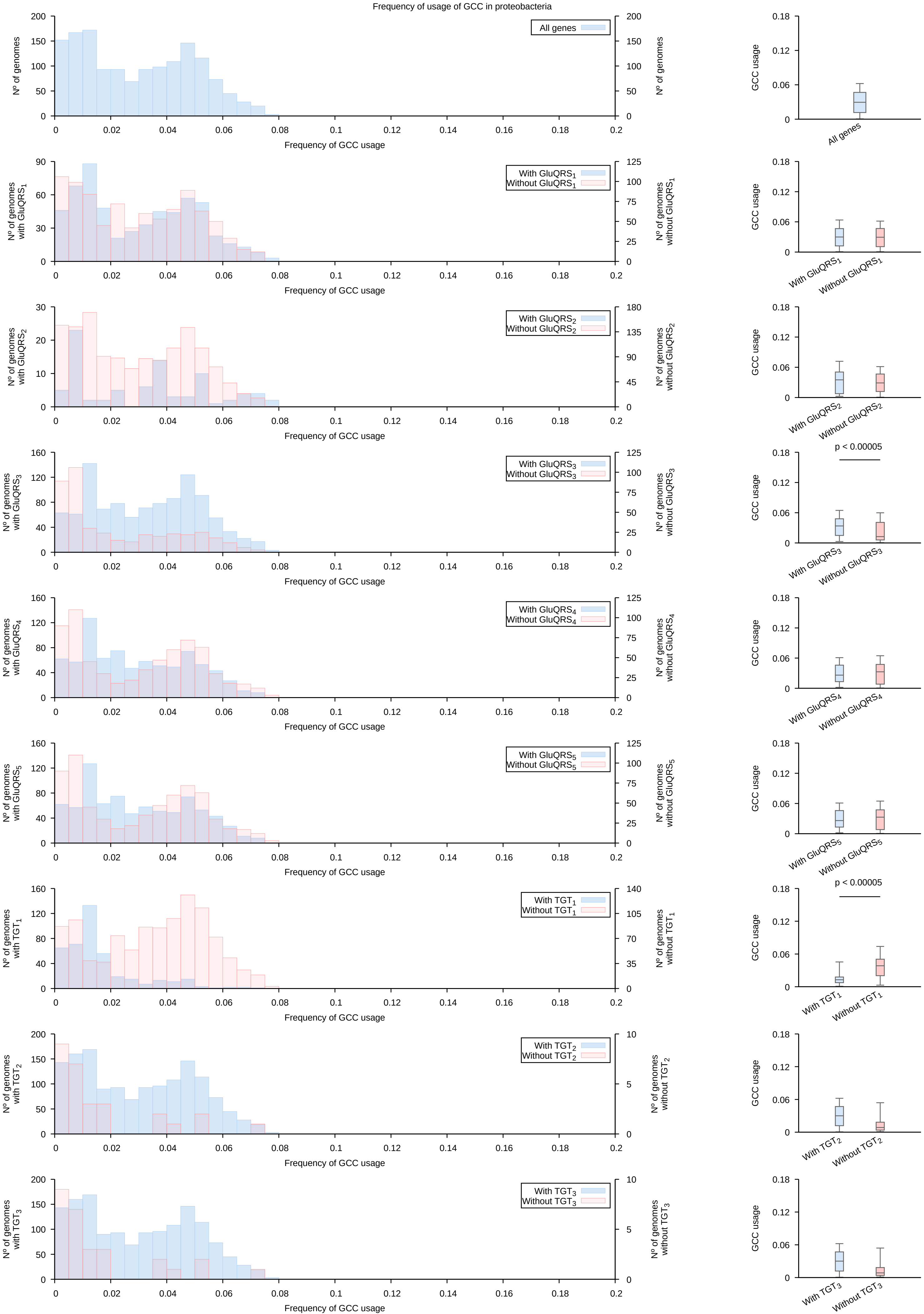
Frequency of usage of GCC in proteobacteria
All genes
With GluQRS1
Without GluQRS1
With GluQRS2
Without GluQRS2
With GluQRS3
Without GluQRS3
With GluQRS4
Without GluQRS4
With GluQRS5
Without GluQRS5
With TGT1
Without TGT1
With TGT2
Without TGT2
With TGT3
Without TGT3
Nº of genomes
Frequency of GCC usage
GCC usage
p < 0.00005
p < 0.00005

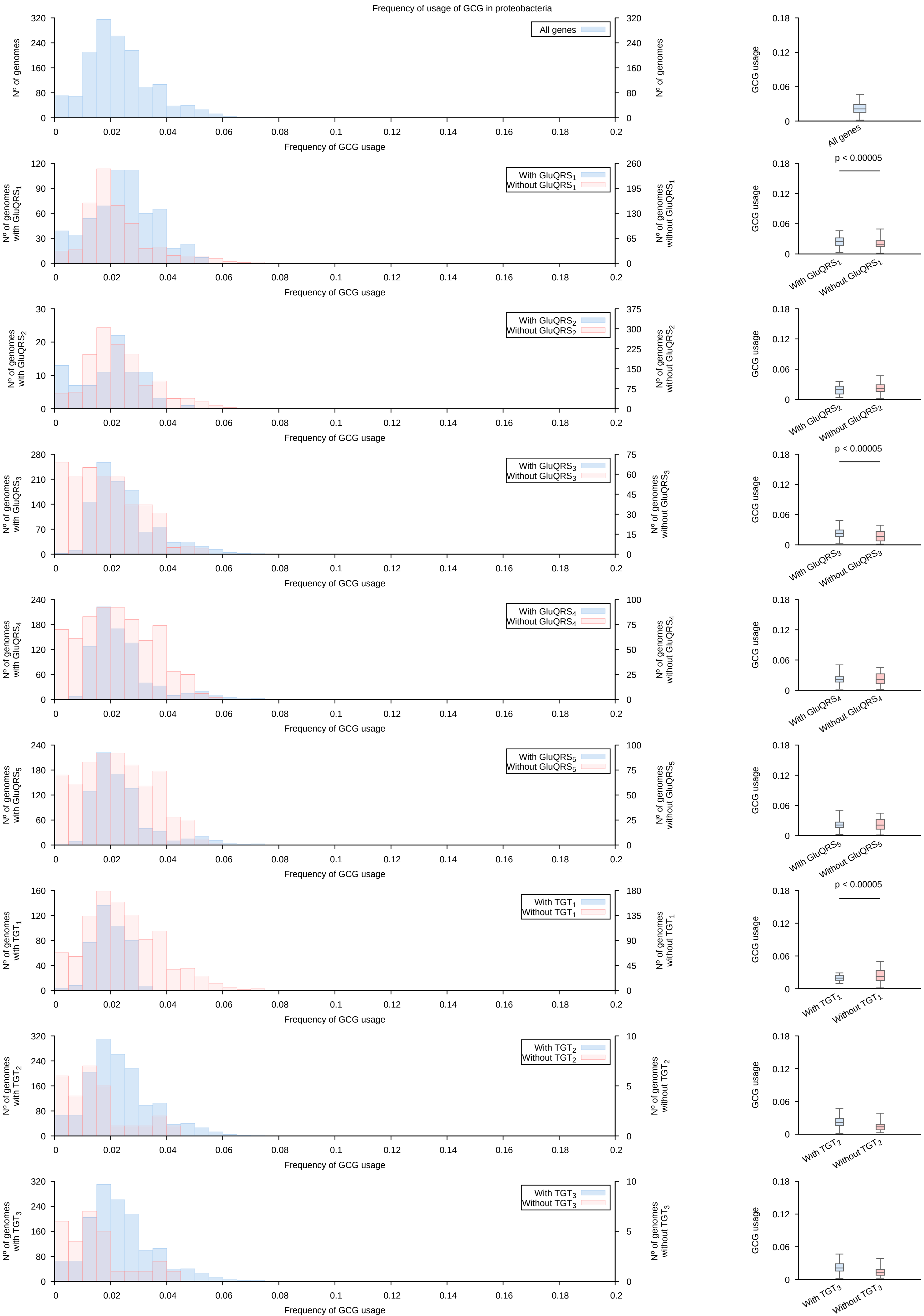

Frequency of usage of GCG in proteobacteria
All genes
Nº of genomes
Frequency of GCG usage
GCG usage
With GluQRS$_1$
Without GluQRS$_1$
Nº of genomes with GluQRS$_1$
Nº of genomes without GluQRS$_1$
p < 0.00005
With GluQRS$_2$
Without GluQRS$_2$
Nº of genomes with GluQRS$_2$
Nº of genomes without GluQRS$_2$
With GluQRS$_3$
Without GluQRS$_3$
Nº of genomes with GluQRS$_3$
Nº of genomes without GluQRS$_3$
p < 0.00005
With GluQRS$_4$
Without GluQRS$_4$
Nº of genomes with GluQRS$_4$
Nº of genomes without GluQRS$_4$
With GluQRS$_5$
Without GluQRS$_5$
Nº of genomes with GluQRS$_5$
Nº of genomes without GluQRS$_5$
With TGT$_1$
Without TGT$_1$
Nº of genomes with TGT$_1$
Nº of genomes without TGT$_1$
p < 0.00005
With TGT$_2$
Without TGT$_2$
Nº of genomes with TGT$_2$
Nº of genomes without TGT$_2$
With TGT$_3$
Without TGT$_3$
Nº of genomes with TGT$_3$
Nº of genomes without TGT$_3$

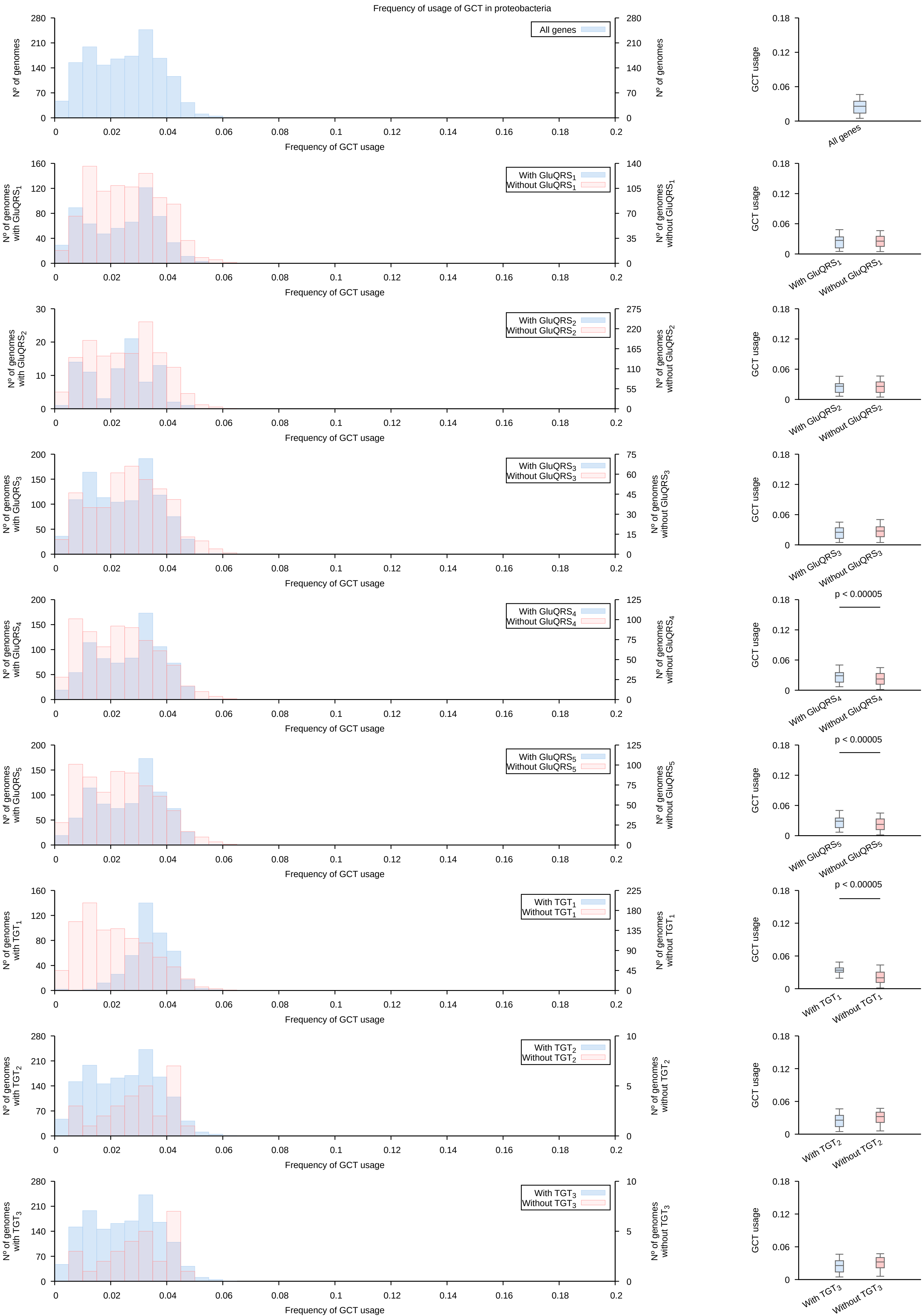
Frequency of usage of GCT in proteobacteria
All genes
Nº of genomes
Frequency of GCT usage
GCT usage
With GluQRS$_1$
Without GluQRS$_1$
Nº of genomes with GluQRS$_1$
Nº of genomes without GluQRS$_1$
With GluQRS$_2$
Without GluQRS$_2$
Nº of genomes with GluQRS$_2$
Nº of genomes without GluQRS$_2$
With GluQRS$_3$
Without GluQRS$_3$
Nº of genomes with GluQRS$_3$
Nº of genomes without GluQRS$_3$
With GluQRS$_4$
Without GluQRS$_4$
Nº of genomes with GluQRS$_4$
Nº of genomes without GluQRS$_4$
p < 0.00005
With GluQRS$_5$
Without GluQRS$_5$
Nº of genomes with GluQRS$_5$
Nº of genomes without GluQRS$_5$
p < 0.00005
With TGT$_1$
Without TGT$_1$
Nº of genomes with TGT$_1$
Nº of genomes without TGT$_1$
p < 0.00005
With TGT$_2$
Without TGT$_2$
Nº of genomes with TGT$_2$
Nº of genomes without TGT$_2$
With TGT$_3$
Without TGT$_3$
Nº of genomes with TGT$_3$
Nº of genomes without TGT$_3$

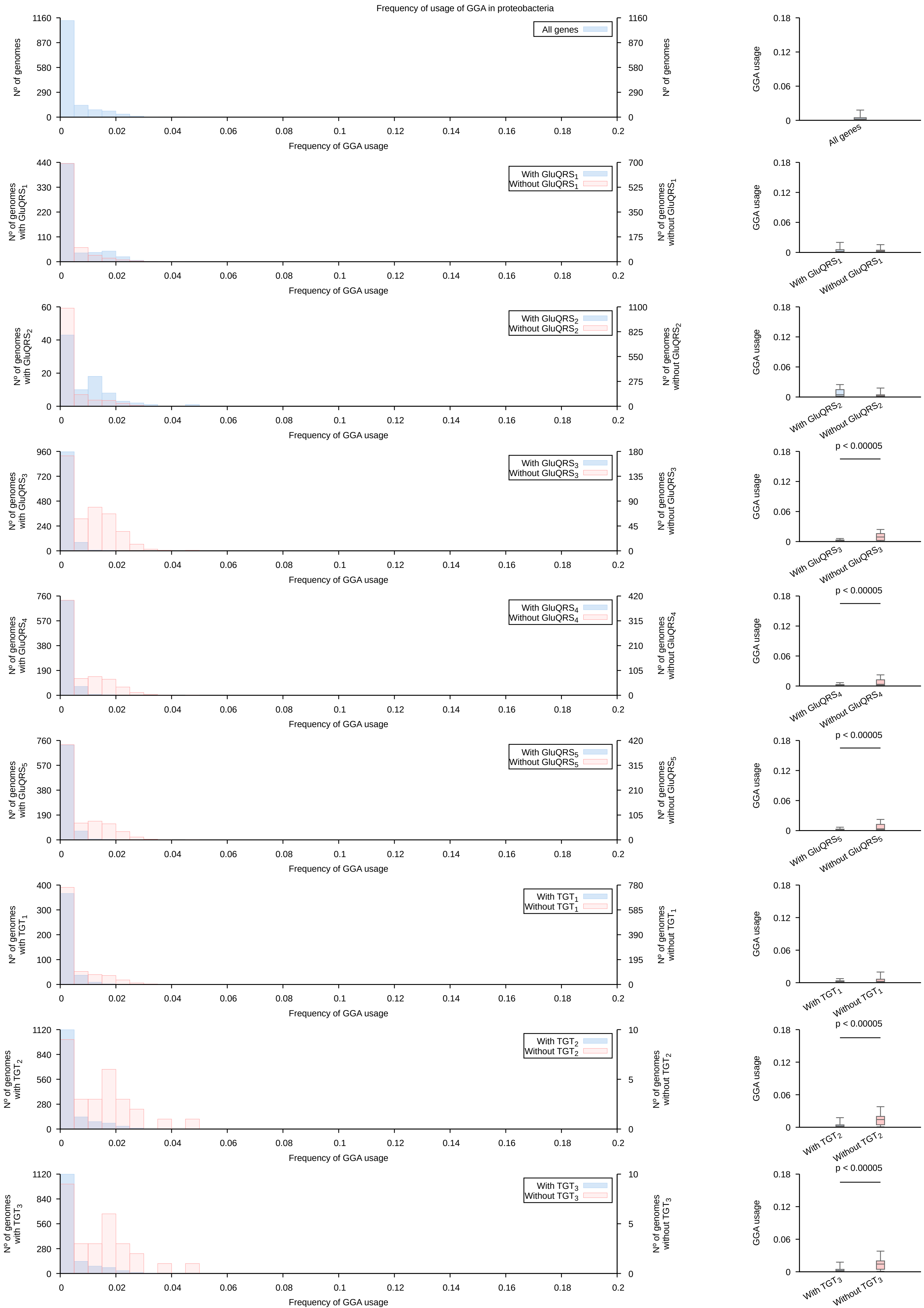

Frequency of usage of GGA in proteobacteria
All genes
With $GluQRS_1$
Without $GluQRS_1$
With $GluQRS_2$
Without $GluQRS_2$
With $GluQRS_3$
Without $GluQRS_3$
With $GluQRS_4$
Without $GluQRS_4$
With $GluQRS_5$
Without $GluQRS_5$
With TGT_1
Without TGT_1
With TGT_2
Without TGT_2
With TGT_3
Without TGT_3
Nº of genomes
Frequency of GGA usage
GGA usage
$p < 0.00005$

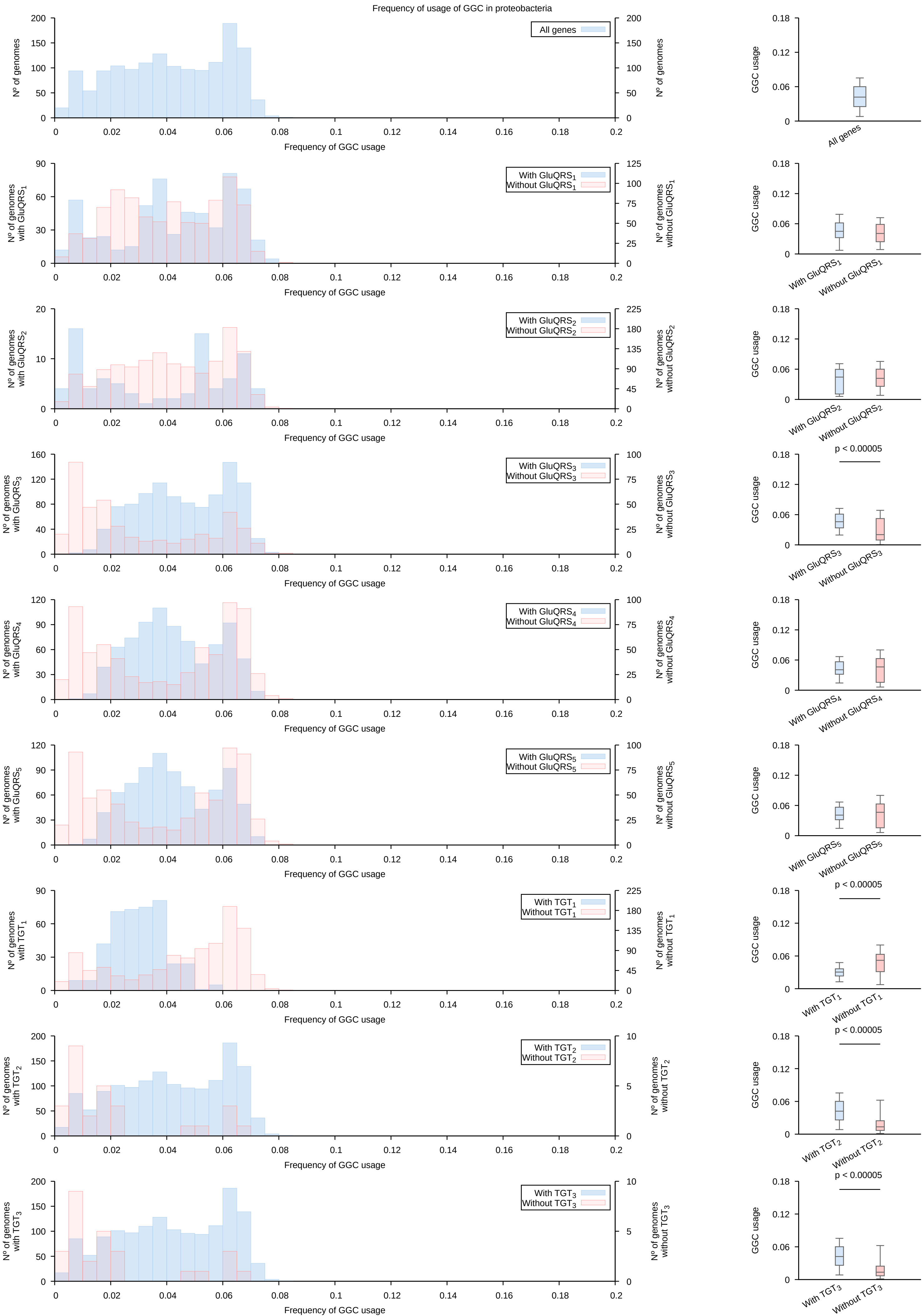

Frequency of usage of GGC in proteobacteria
Nº of genomes
All genes
Frequency of GGC usage
GGC usage
All genes
Nº of genomes with GluQRS$_1$
Nº of genomes without GluQRS$_1$
With GluQRS$_1$
Without GluQRS$_1$
Nº of genomes with GluQRS$_2$
Nº of genomes without GluQRS$_2$
With GluQRS$_2$
Without GluQRS$_2$
Nº of genomes with GluQRS$_3$
Nº of genomes without GluQRS$_3$
With GluQRS$_3$
Without GluQRS$_3$
p < 0.00005
Nº of genomes with GluQRS$_4$
Nº of genomes without GluQRS$_4$
With GluQRS$_4$
Without GluQRS$_4$
Nº of genomes with GluQRS$_5$
Nº of genomes without GluQRS$_5$
With GluQRS$_5$
Without GluQRS$_5$
Nº of genomes with TGT$_1$
Nº of genomes without TGT$_1$
With TGT$_1$
Without TGT$_1$
p < 0.00005
Nº of genomes with TGT$_2$
Nº of genomes without TGT$_2$
With TGT$_2$
Without TGT$_2$
p < 0.00005
Nº of genomes with TGT$_3$
Nº of genomes without TGT$_3$
With TGT$_3$
Without TGT$_3$
p < 0.00005

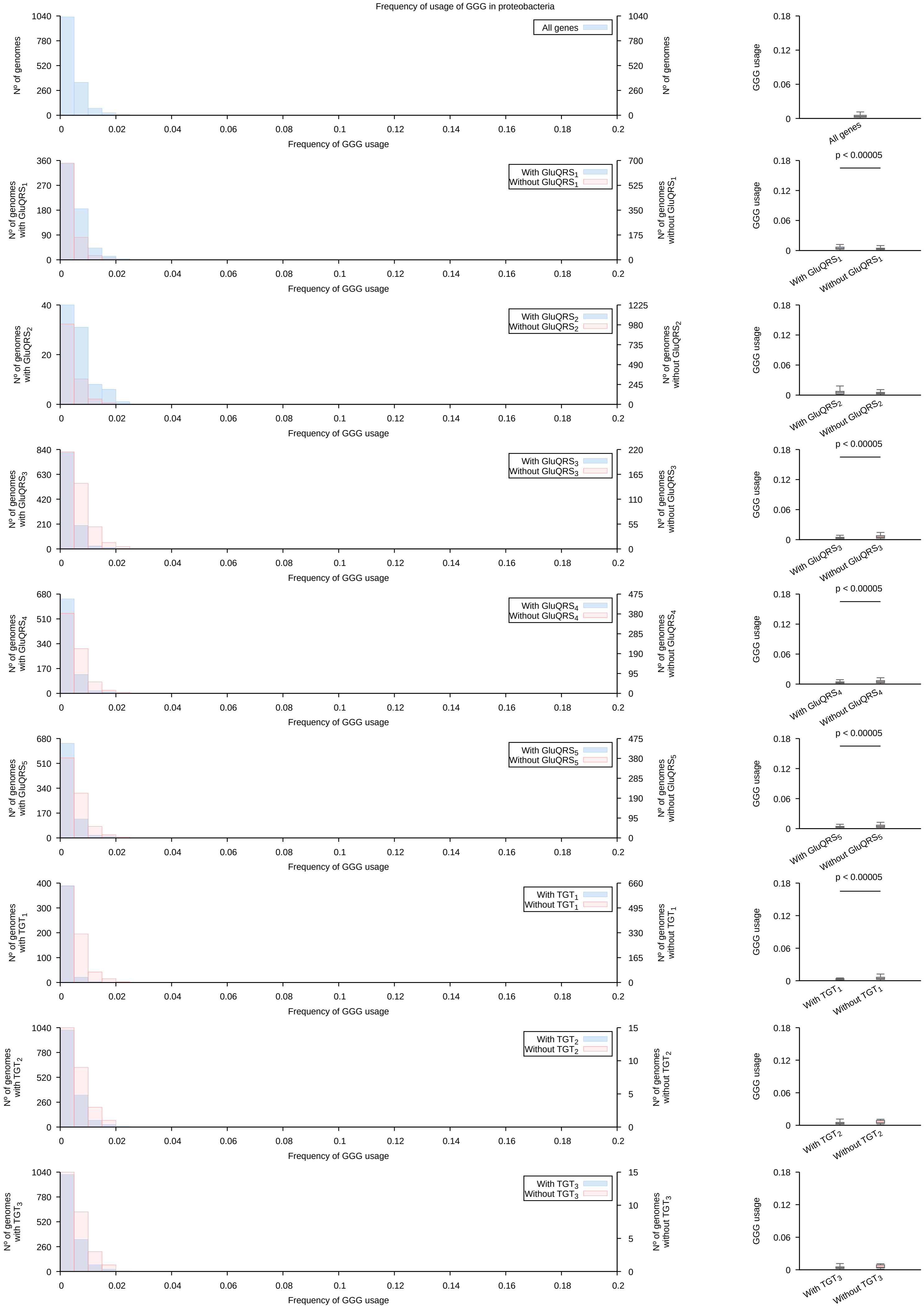
Frequency of usage of GGG in proteobacteria
All genes
Nº of genomes
Frequency of GGG usage
GGG usage
With GluQRS$_1$
Without GluQRS$_1$
Nº of genomes with GluQRS$_1$
Nº of genomes without GluQRS$_1$
p < 0.00005
With GluQRS$_2$
Without GluQRS$_2$
Nº of genomes with GluQRS$_2$
Nº of genomes without GluQRS$_2$
With GluQRS$_3$
Without GluQRS$_3$
Nº of genomes with GluQRS$_3$
Nº of genomes without GluQRS$_3$
p < 0.00005
With GluQRS$_4$
Without GluQRS$_4$
Nº of genomes with GluQRS$_4$
Nº of genomes without GluQRS$_4$
p < 0.00005
With GluQRS$_5$
Without GluQRS$_5$
Nº of genomes with GluQRS$_5$
Nº of genomes without GluQRS$_5$
p < 0.00005
With TGT$_1$
Without TGT$_1$
Nº of genomes with TGT$_1$
Nº of genomes without TGT$_1$
p < 0.00005
With TGT$_2$
Without TGT$_2$
Nº of genomes with TGT$_2$
Nº of genomes without TGT$_2$
With TGT$_3$
Without TGT$_3$
Nº of genomes with TGT$_3$
Nº of genomes without TGT$_3$

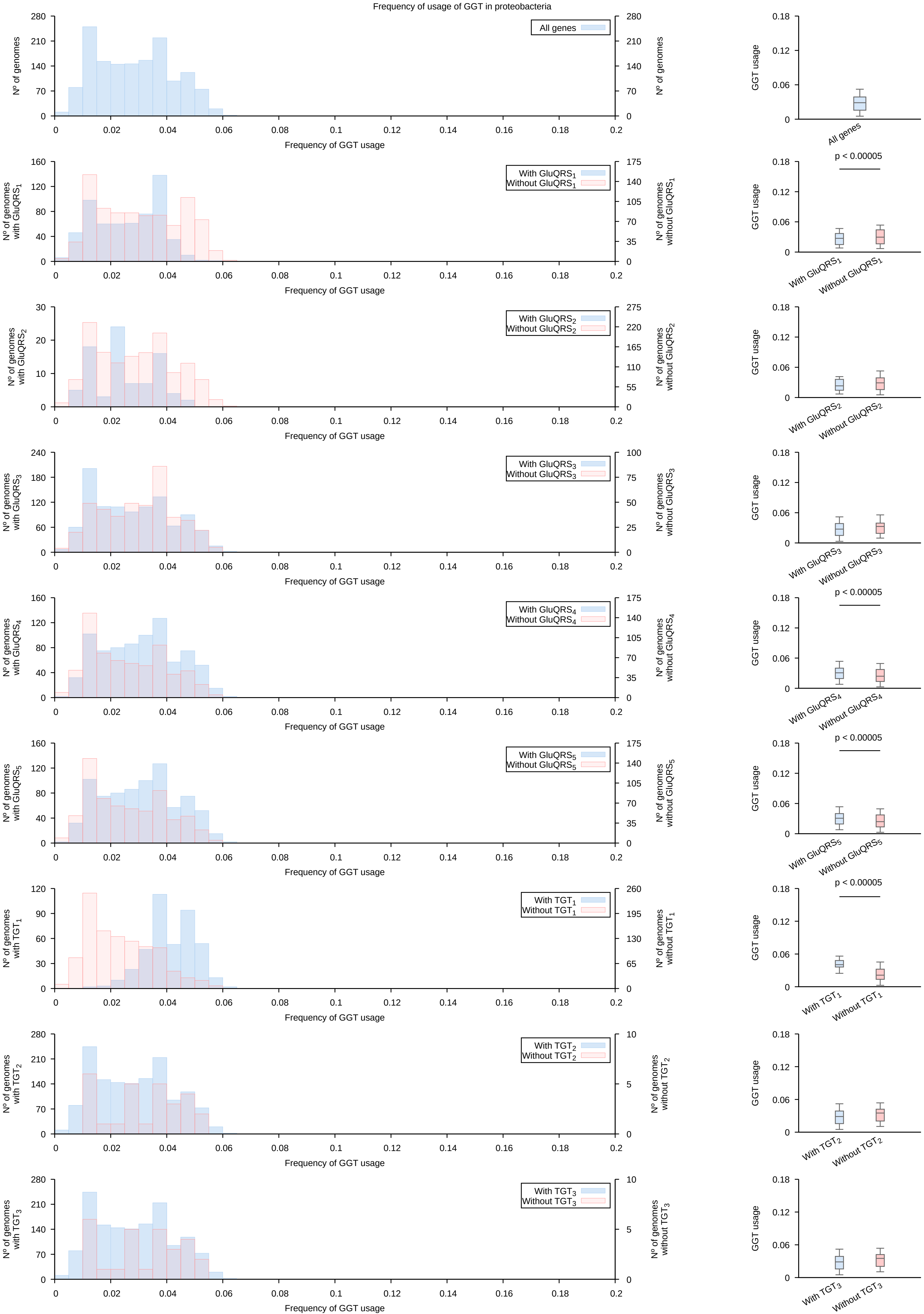
Frequency of usage of GGT in proteobacteria
All genes
Nº of genomes
Frequency of GGT usage
GGT usage
With GluQRS$_1$
Without GluQRS$_1$
Nº of genomes with GluQRS$_1$
Nº of genomes without GluQRS$_1$
p < 0.00005
With GluQRS$_2$
Without GluQRS$_2$
Nº of genomes with GluQRS$_2$
Nº of genomes without GluQRS$_2$
With GluQRS$_3$
Without GluQRS$_3$
Nº of genomes with GluQRS$_3$
Nº of genomes without GluQRS$_3$
With GluQRS$_4$
Without GluQRS$_4$
Nº of genomes with GluQRS$_4$
Nº of genomes without GluQRS$_4$
p < 0.00005
With GluQRS$_5$
Without GluQRS$_5$
Nº of genomes with GluQRS$_5$
Nº of genomes without GluQRS$_5$
p < 0.00005
With TGT$_1$
Without TGT$_1$
Nº of genomes with TGT$_1$
Nº of genomes without TGT$_1$
p < 0.00005
With TGT$_2$
Without TGT$_2$
Nº of genomes with TGT$_2$
Nº of genomes without TGT$_2$
With TGT$_3$
Without TGT$_3$
Nº of genomes with TGT$_3$
Nº of genomes without TGT$_3$

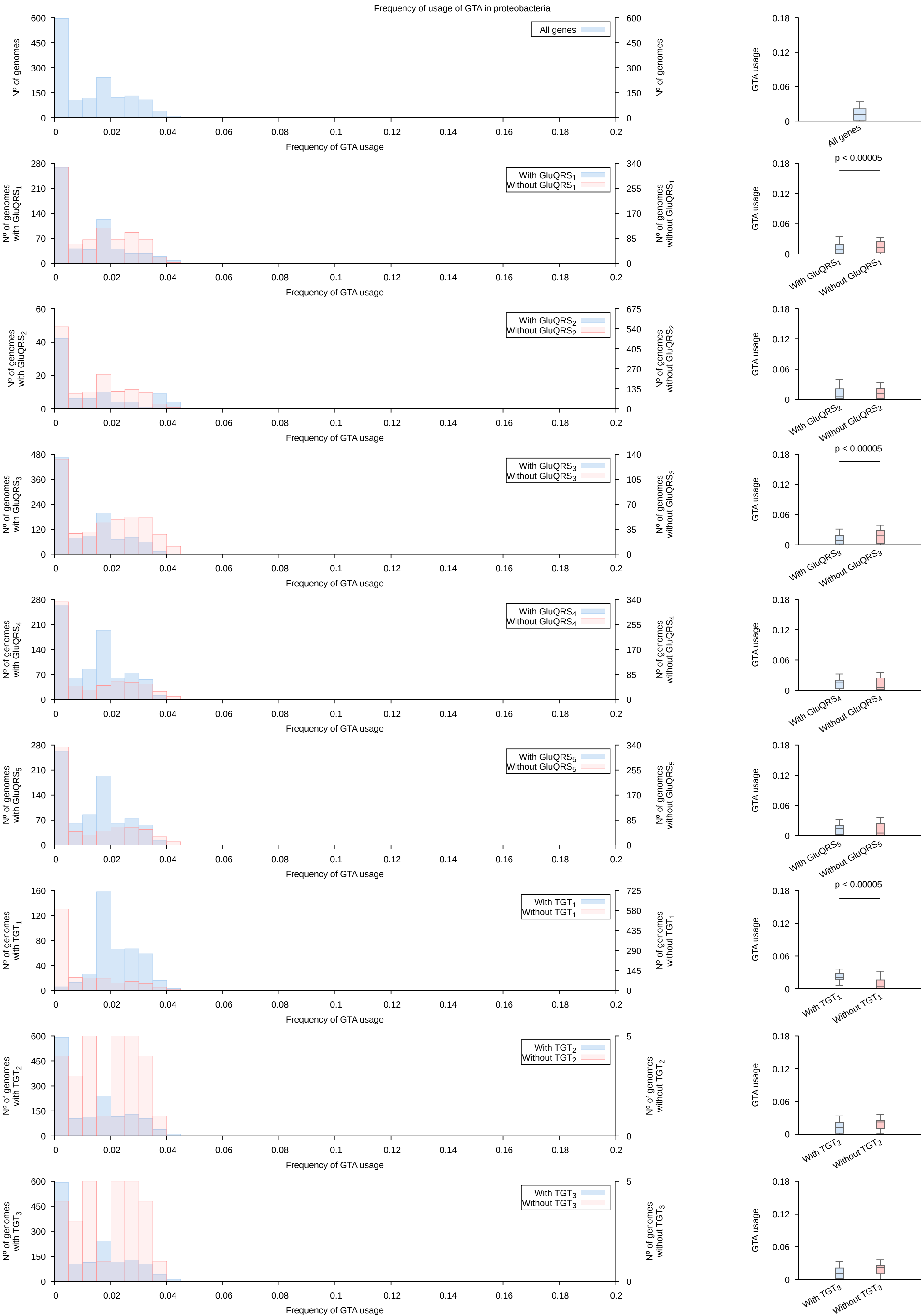
Frequency of usage of GTA in proteobacteria
All genes
With GluQRS$_1$
Without GluQRS$_1$
With GluQRS$_2$
Without GluQRS$_2$
With GluQRS$_3$
Without GluQRS$_3$
With GluQRS$_4$
Without GluQRS$_4$
With GluQRS$_5$
Without GluQRS$_5$
With TGT$_1$
Without TGT$_1$
With TGT$_2$
Without TGT$_2$
With TGT$_3$
Without TGT$_3$
Nº of genomes
Nº of genomes with GluQRS$_1$
Nº of genomes without GluQRS$_1$
Nº of genomes with GluQRS$_2$
Nº of genomes without GluQRS$_2$
Nº of genomes with GluQRS$_3$
Nº of genomes without GluQRS$_3$
Nº of genomes with GluQRS$_4$
Nº of genomes without GluQRS$_4$
Nº of genomes with GluQRS$_5$
Nº of genomes without GluQRS$_5$
Nº of genomes with TGT$_1$
Nº of genomes without TGT$_1$
Nº of genomes with TGT$_2$
Nº of genomes without TGT$_2$
Nº of genomes with TGT$_3$
Nº of genomes without TGT$_3$
Frequency of GTA usage
GTA usage
p < 0.00005
p < 0.00005
p < 0.00005
0 0.02 0.04 0.06 0.08 0.1 0.12 0.14 0.16 0.18 0.2
0 0.06 0.12 0.18

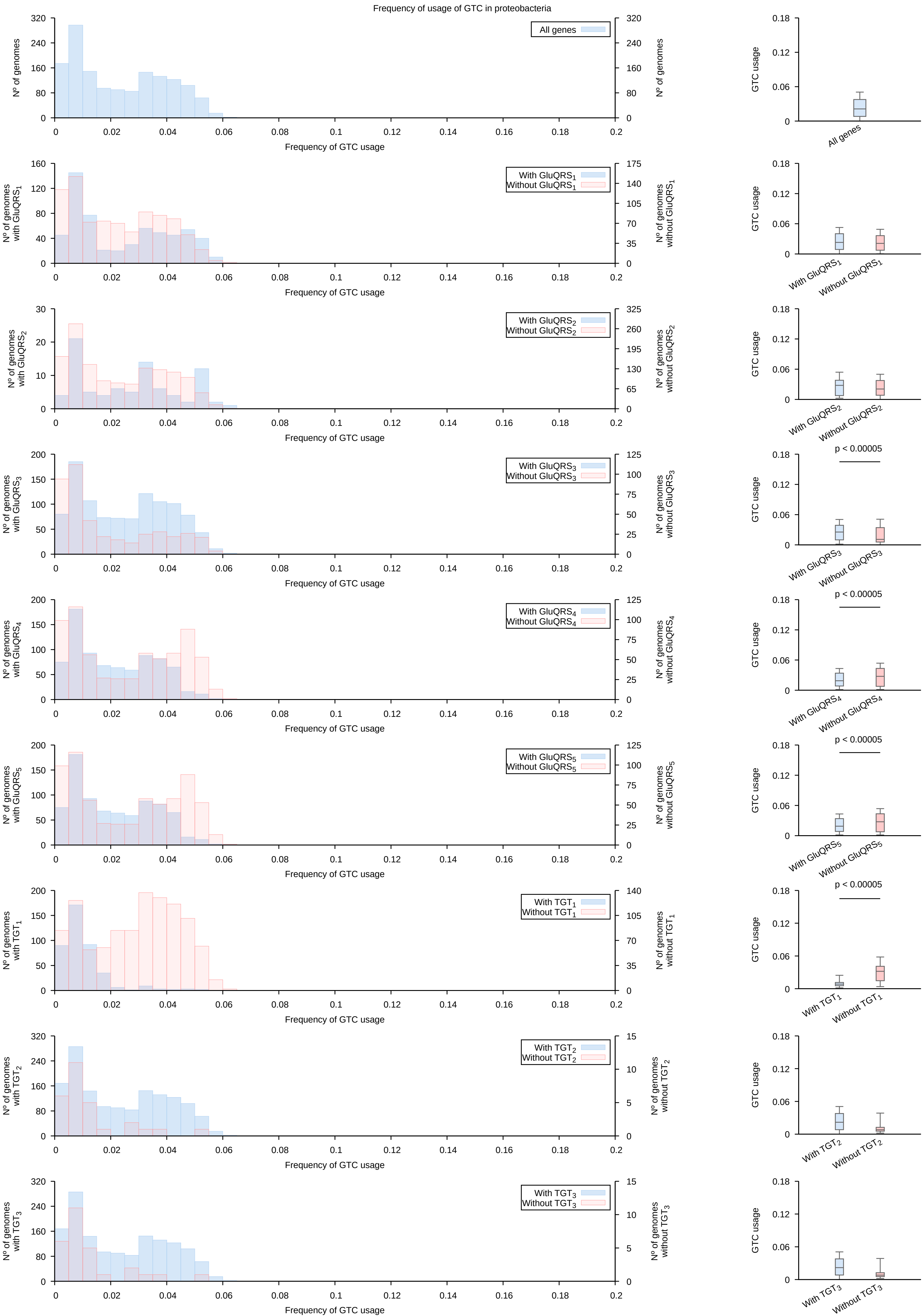
Frequency of usage of GTC in proteobacteria
All genes
Nº of genomes
Frequency of GTC usage
GTC usage
With GluQRS$_1$
Without GluQRS$_1$
Nº of genomes with GluQRS$_1$
Nº of genomes without GluQRS$_1$
With GluQRS$_2$
Without GluQRS$_2$
Nº of genomes with GluQRS$_2$
Nº of genomes without GluQRS$_2$
With GluQRS$_3$
Without GluQRS$_3$
Nº of genomes with GluQRS$_3$
Nº of genomes without GluQRS$_3$
p < 0.00005
With GluQRS$_4$
Without GluQRS$_4$
Nº of genomes with GluQRS$_4$
Nº of genomes without GluQRS$_4$
p < 0.00005
With GluQRS$_5$
Without GluQRS$_5$
Nº of genomes with GluQRS$_5$
Nº of genomes without GluQRS$_5$
p < 0.00005
With TGT$_1$
Without TGT$_1$
Nº of genomes with TGT$_1$
Nº of genomes without TGT$_1$
p < 0.00005
With TGT$_2$
Without TGT$_2$
Nº of genomes with TGT$_2$
Nº of genomes without TGT$_2$
With TGT$_3$
Without TGT$_3$
Nº of genomes with TGT$_3$
Nº of genomes without TGT$_3$

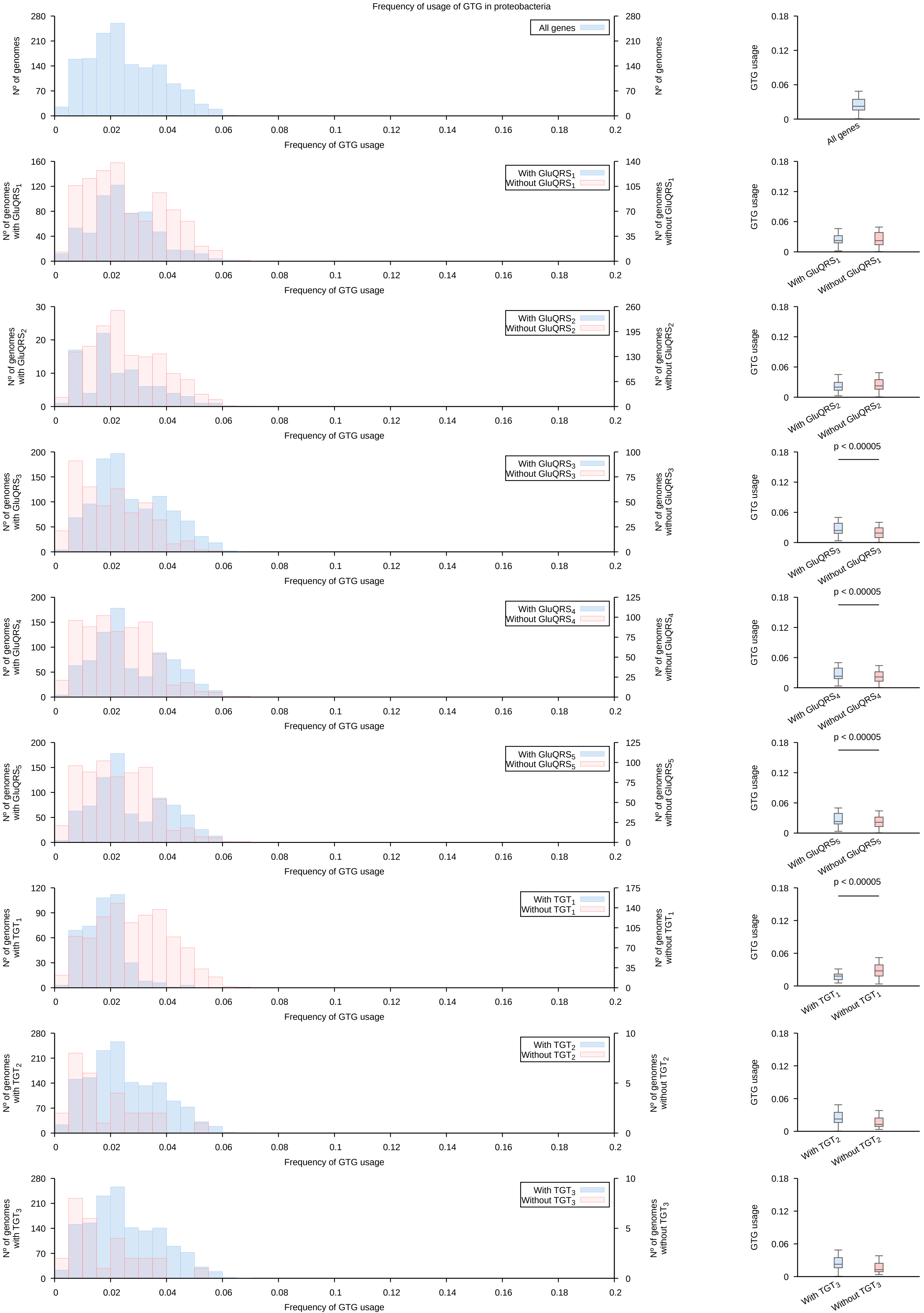
Frequency of usage of GTG in proteobacteria
All genes
Nº of genomes
Frequency of GTG usage
GTG usage
With GluQRS$_1$
Without GluQRS$_1$
Nº of genomes with GluQRS$_1$
Nº of genomes without GluQRS$_1$
With GluQRS$_2$
Without GluQRS$_2$
Nº of genomes with GluQRS$_2$
Nº of genomes without GluQRS$_2$
With GluQRS$_3$
Without GluQRS$_3$
Nº of genomes with GluQRS$_3$
Nº of genomes without GluQRS$_3$
p < 0.00005
With GluQRS$_4$
Without GluQRS$_4$
Nº of genomes with GluQRS$_4$
Nº of genomes without GluQRS$_4$
p < 0.00005
With GluQRS$_5$
Without GluQRS$_5$
Nº of genomes with GluQRS$_5$
Nº of genomes without GluQRS$_5$
p < 0.00005
With TGT$_1$
Without TGT$_1$
Nº of genomes with TGT$_1$
Nº of genomes without TGT$_1$
p < 0.00005
With TGT$_2$
Without TGT$_2$
Nº of genomes with TGT$_2$
Nº of genomes without TGT$_2$
With TGT$_3$
Without TGT$_3$
Nº of genomes with TGT$_3$
Nº of genomes without TGT$_3$

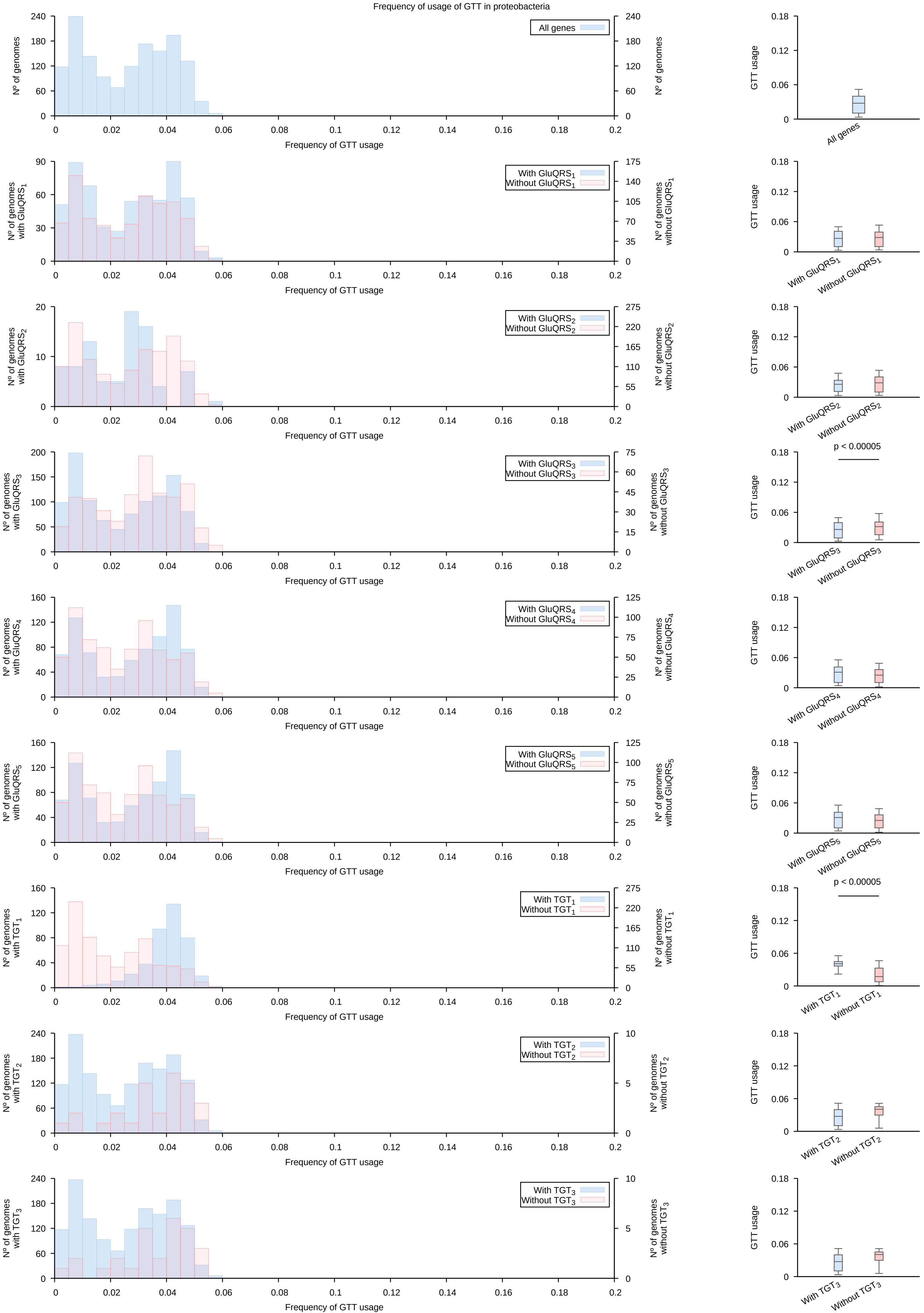

Frequency of usage of GTT in proteobacteria
Nº of genomes
Frequency of GTT usage
GTT usage
All genes
With GluQRS1
Without GluQRS1
Nº of genomes with GluQRS1
Nº of genomes without GluQRS1
With GluQRS2
Without GluQRS2
Nº of genomes with GluQRS2
Nº of genomes without GluQRS2
With GluQRS3
Without GluQRS3
Nº of genomes with GluQRS3
Nº of genomes without GluQRS3
p < 0.00005
With GluQRS4
Without GluQRS4
Nº of genomes with GluQRS4
Nº of genomes without GluQRS4
With GluQRS5
Without GluQRS5
Nº of genomes with GluQRS5
Nº of genomes without GluQRS5
With TGT1
Without TGT1
Nº of genomes with TGT1
Nº of genomes without TGT1
p < 0.00005
With TGT2
Without TGT2
Nº of genomes with TGT2
Nº of genomes without TGT2
With TGT3
Without TGT3
Nº of genomes with TGT3
Nº of genomes without TGT3

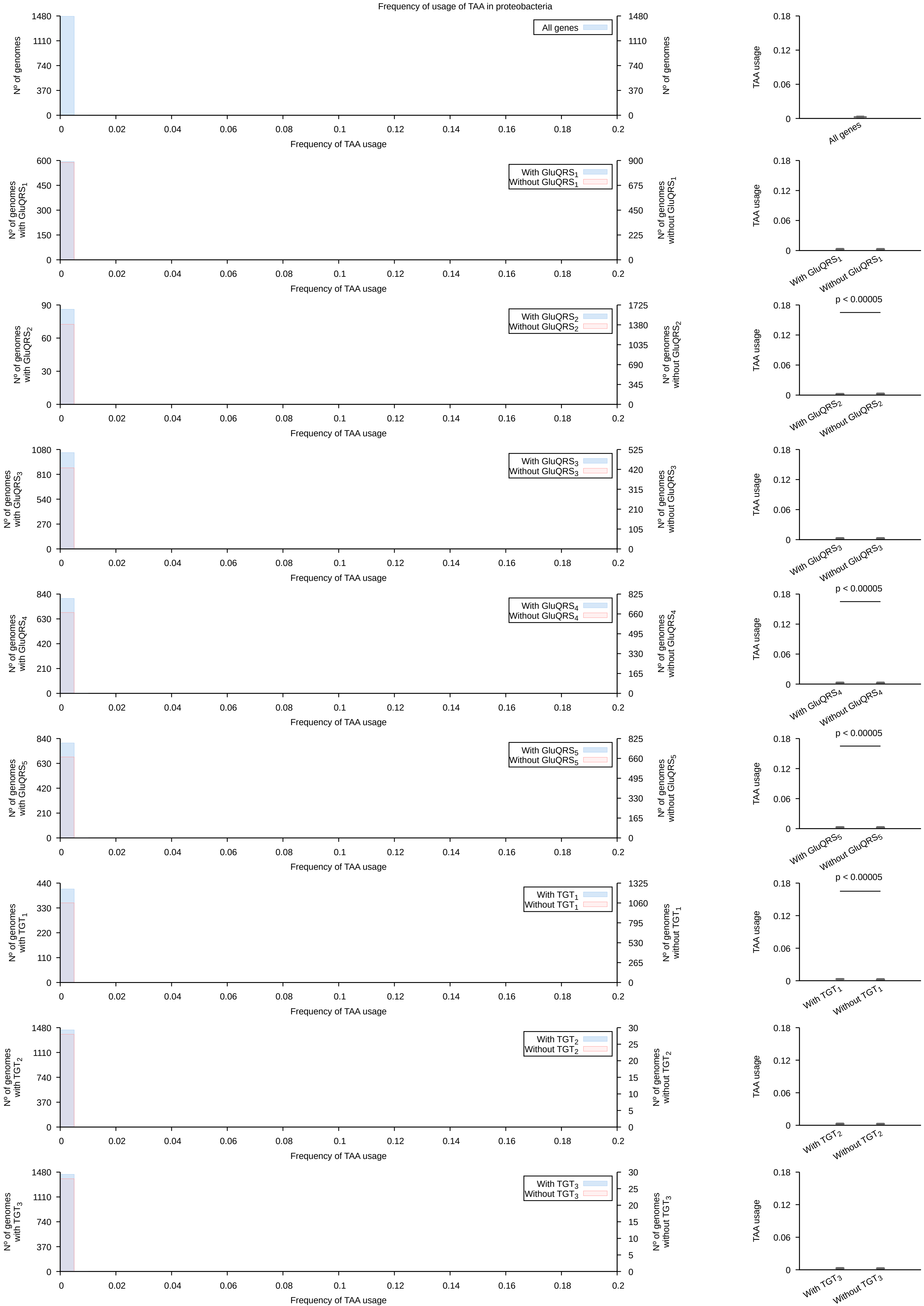
Frequency of usage of TAA in proteobacteria
All genes
Nº of genomes
Frequency of TAA usage
TAA usage
With GluQRS$_1$
Without GluQRS$_1$
Nº of genomes with GluQRS$_1$
Nº of genomes without GluQRS$_1$
With GluQRS$_2$
Without GluQRS$_2$
Nº of genomes with GluQRS$_2$
Nº of genomes without GluQRS$_2$
p < 0.00005
With GluQRS$_3$
Without GluQRS$_3$
Nº of genomes with GluQRS$_3$
Nº of genomes without GluQRS$_3$
With GluQRS$_4$
Without GluQRS$_4$
Nº of genomes with GluQRS$_4$
Nº of genomes without GluQRS$_4$
p < 0.00005
With GluQRS$_5$
Without GluQRS$_5$
Nº of genomes with GluQRS$_5$
Nº of genomes without GluQRS$_5$
p < 0.00005
With TGT$_1$
Without TGT$_1$
Nº of genomes with TGT$_1$
Nº of genomes without TGT$_1$
p < 0.00005
With TGT$_2$
Without TGT$_2$
Nº of genomes with TGT$_2$
Nº of genomes without TGT$_2$
With TGT$_3$
Without TGT$_3$
Nº of genomes with TGT$_3$
Nº of genomes without TGT$_3$

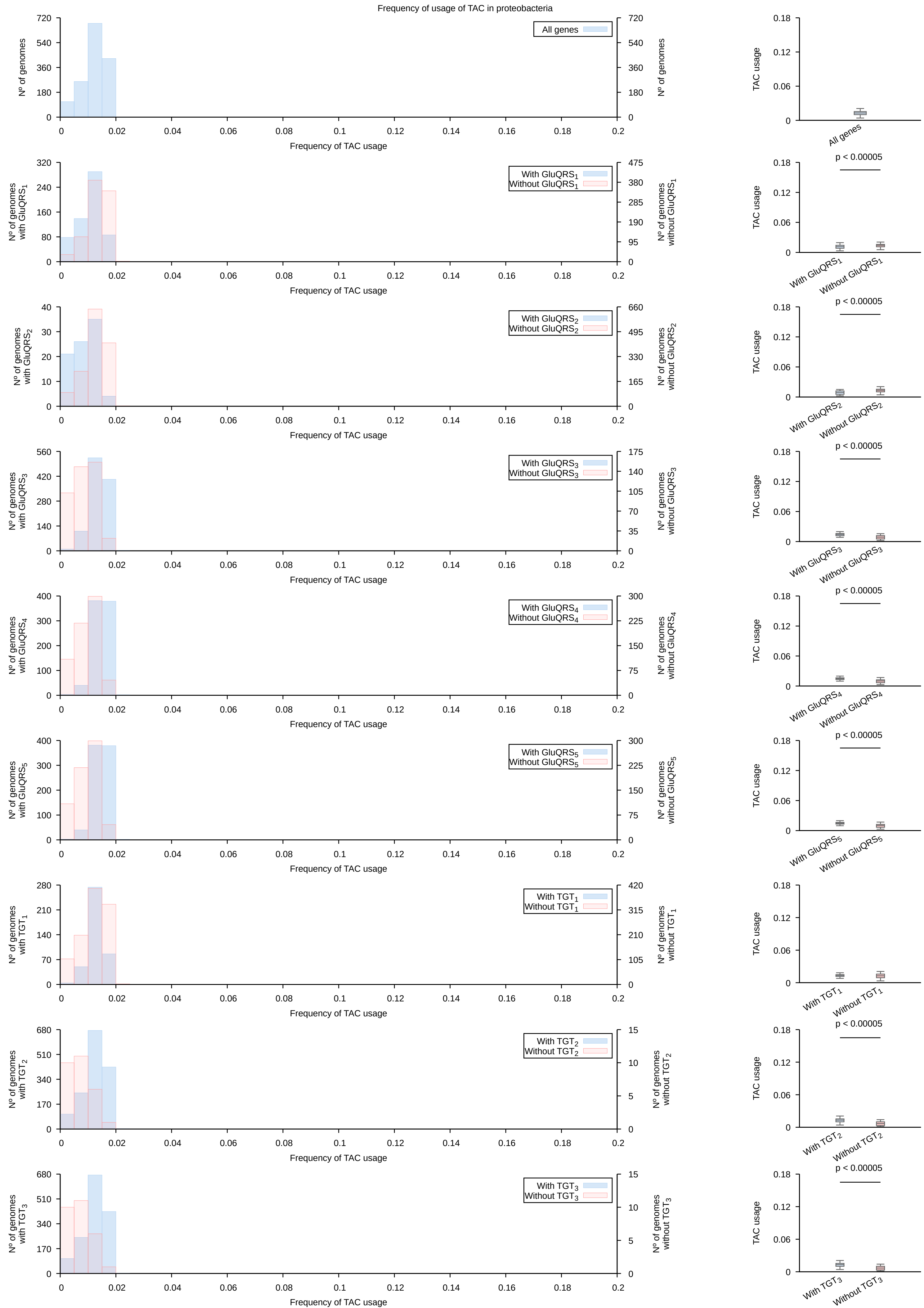
Frequency of usage of TAC in proteobacteria
All genes
Nº of genomes
Frequency of TAC usage
TAC usage
With GluQRS1
Without GluQRS1
Nº of genomes with GluQRS1
Nº of genomes without GluQRS1
p < 0.00005
With GluQRS2
Without GluQRS2
Nº of genomes with GluQRS2
Nº of genomes without GluQRS2
With GluQRS3
Without GluQRS3
Nº of genomes with GluQRS3
Nº of genomes without GluQRS3
With GluQRS4
Without GluQRS4
Nº of genomes with GluQRS4
Nº of genomes without GluQRS4
With GluQRS5
Without GluQRS5
Nº of genomes with GluQRS5
Nº of genomes without GluQRS5
With TGT1
Without TGT1
Nº of genomes with TGT1
Nº of genomes without TGT1
With TGT2
Without TGT2
Nº of genomes with TGT2
Nº of genomes without TGT2
With TGT3
Without TGT3
Nº of genomes with TGT3
Nº of genomes without TGT3

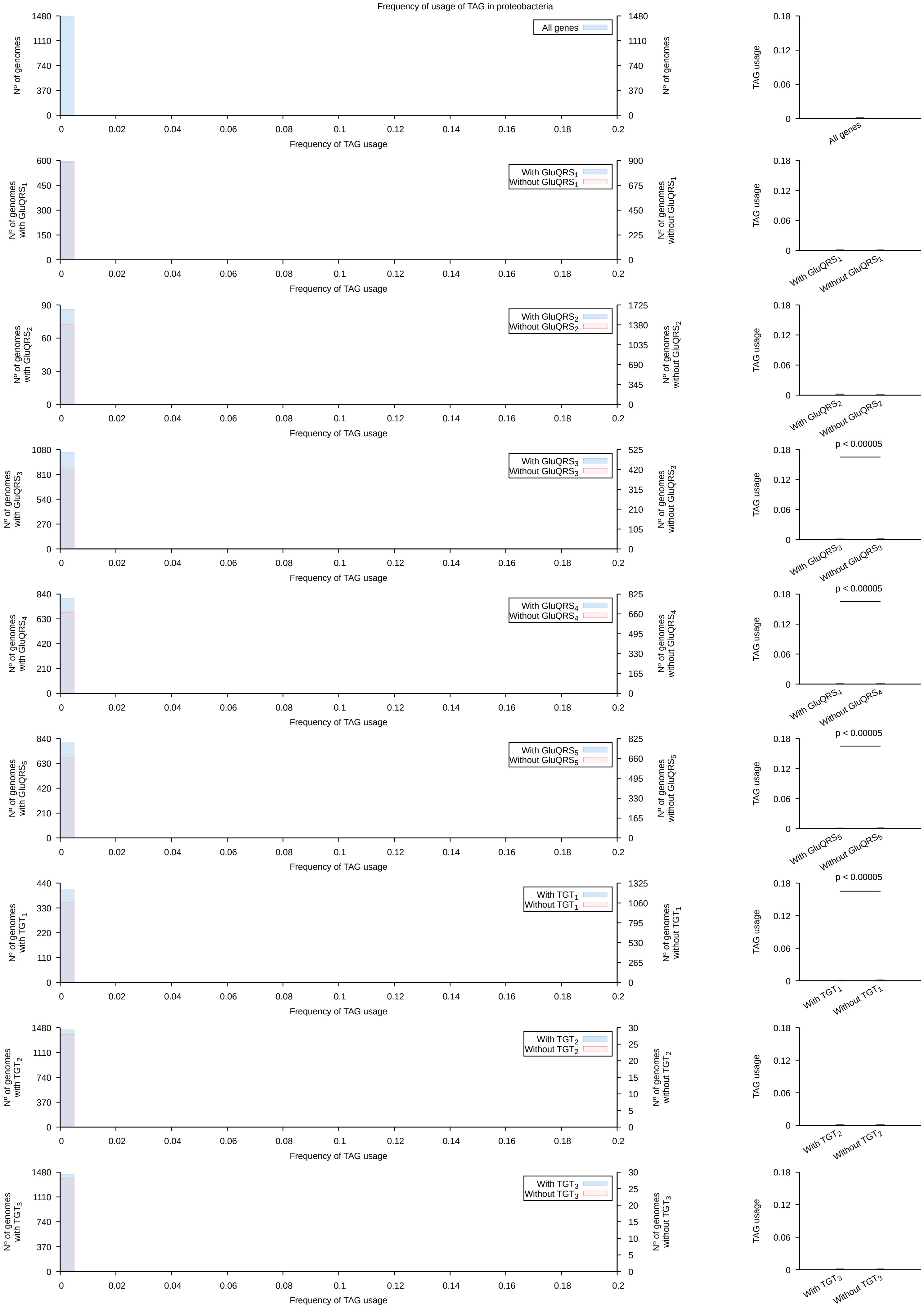
Frequency of usage of TAG in proteobacteria
Nº of genomes
Frequency of TAG usage
TAG usage
All genes
With GluQRS$_1$
Without GluQRS$_1$
Nº of genomes with GluQRS$_1$
Nº of genomes without GluQRS$_1$
With GluQRS$_2$
Without GluQRS$_2$
Nº of genomes with GluQRS$_2$
Nº of genomes without GluQRS$_2$
With GluQRS$_3$
Without GluQRS$_3$
Nº of genomes with GluQRS$_3$
Nº of genomes without GluQRS$_3$
p < 0.00005
With GluQRS$_4$
Without GluQRS$_4$
Nº of genomes with GluQRS$_4$
Nº of genomes without GluQRS$_4$
p < 0.00005
With GluQRS$_5$
Without GluQRS$_5$
Nº of genomes with GluQRS$_5$
Nº of genomes without GluQRS$_5$
p < 0.00005
With TGT$_1$
Without TGT$_1$
Nº of genomes with TGT$_1$
Nº of genomes without TGT$_1$
p < 0.00005
With TGT$_2$
Without TGT$_2$
Nº of genomes with TGT$_2$
Nº of genomes without TGT$_2$
With TGT$_3$
Without TGT$_3$
Nº of genomes with TGT$_3$
Nº of genomes without TGT$_3$

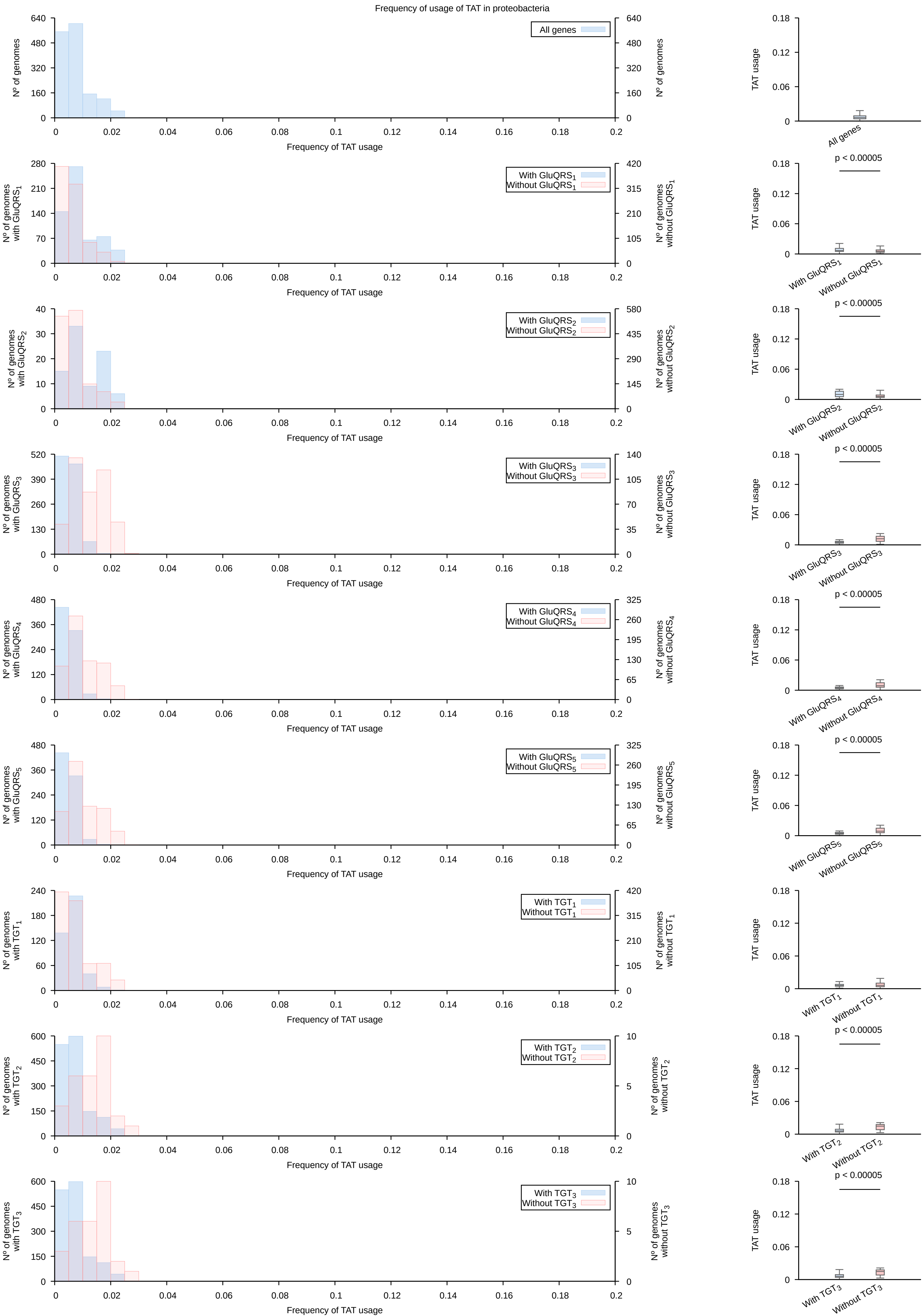
Frequency of usage of TAT in proteobacteria
Nº of genomes
Frequency of TAT usage
All genes
TAT usage
Nº of genomes with GluQRS$_1$
Nº of genomes without GluQRS$_1$
With GluQRS$_1$
Without GluQRS$_1$
p < 0.00005
Nº of genomes with GluQRS$_2$
Nº of genomes without GluQRS$_2$
With GluQRS$_2$
Without GluQRS$_2$
p < 0.00005
Nº of genomes with GluQRS$_3$
Nº of genomes without GluQRS$_3$
With GluQRS$_3$
Without GluQRS$_3$
p < 0.00005
Nº of genomes with GluQRS$_4$
Nº of genomes without GluQRS$_4$
With GluQRS$_4$
Without GluQRS$_4$
p < 0.00005
Nº of genomes with GluQRS$_5$
Nº of genomes without GluQRS$_5$
With GluQRS$_5$
Without GluQRS$_5$
p < 0.00005
Nº of genomes with TGT$_1$
Nº of genomes without TGT$_1$
With TGT$_1$
Without TGT$_1$
Nº of genomes with TGT$_2$
Nº of genomes without TGT$_2$
With TGT$_2$
Without TGT$_2$
p < 0.00005
Nº of genomes with TGT$_3$
Nº of genomes without TGT$_3$
With TGT$_3$
Without TGT$_3$
p < 0.00005

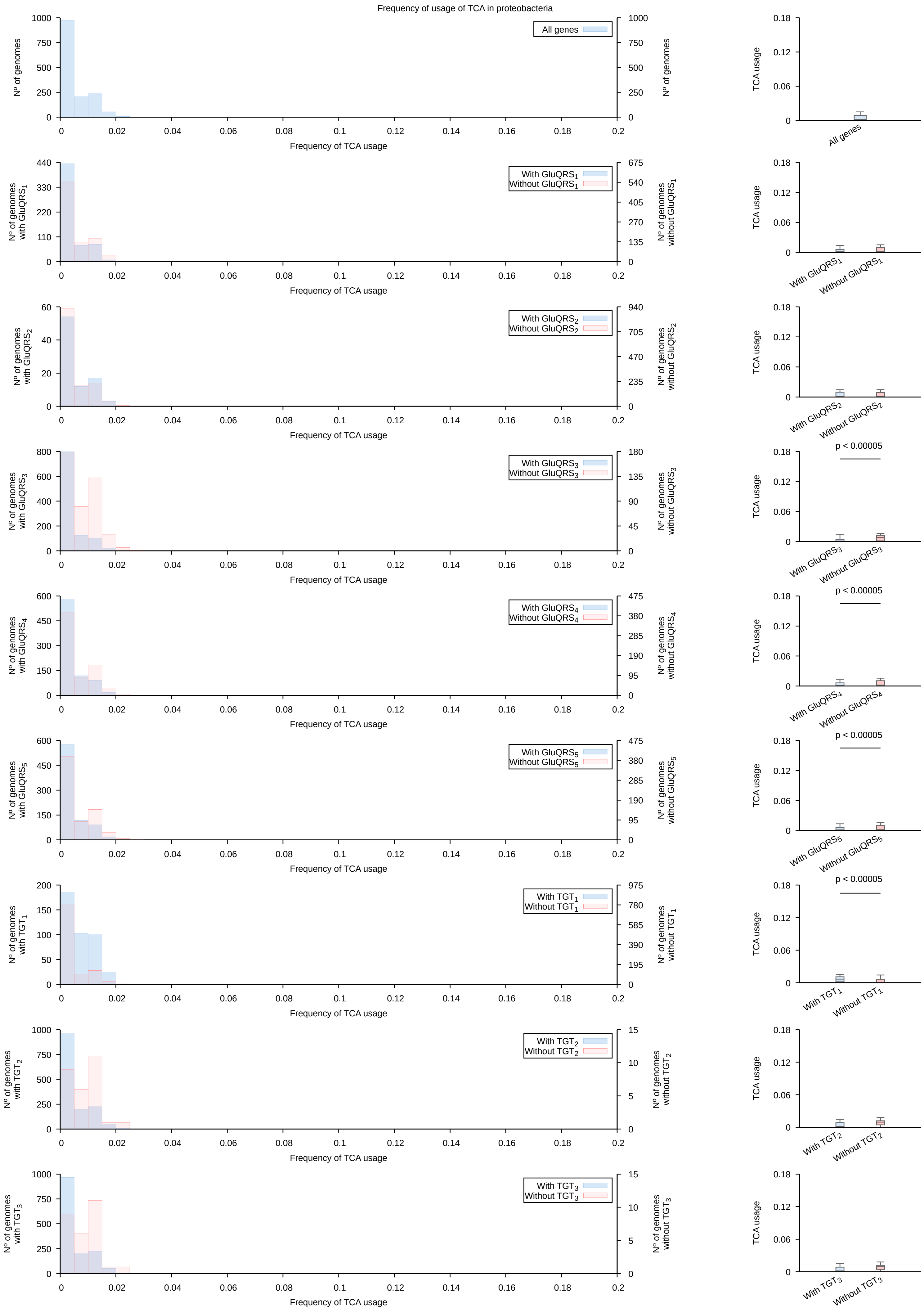

Frequency of usage of TCA in proteobacteria
All genes
Nº of genomes
Frequency of TCA usage
TCA usage
With GluQRS1
Without GluQRS1
Nº of genomes with GluQRS1
Nº of genomes without GluQRS1
With GluQRS2
Without GluQRS2
Nº of genomes with GluQRS2
Nº of genomes without GluQRS2
With GluQRS3
Without GluQRS3
Nº of genomes with GluQRS3
Nº of genomes without GluQRS3
p < 0.00005
With GluQRS4
Without GluQRS4
Nº of genomes with GluQRS4
Nº of genomes without GluQRS4
p < 0.00005
With GluQRS5
Without GluQRS5
Nº of genomes with GluQRS5
Nº of genomes without GluQRS5
p < 0.00005
With TGT1
Without TGT1
Nº of genomes with TGT1
Nº of genomes without TGT1
p < 0.00005
With TGT2
Without TGT2
Nº of genomes with TGT2
Nº of genomes without TGT2
With TGT3
Without TGT3
Nº of genomes with TGT3
Nº of genomes without TGT3

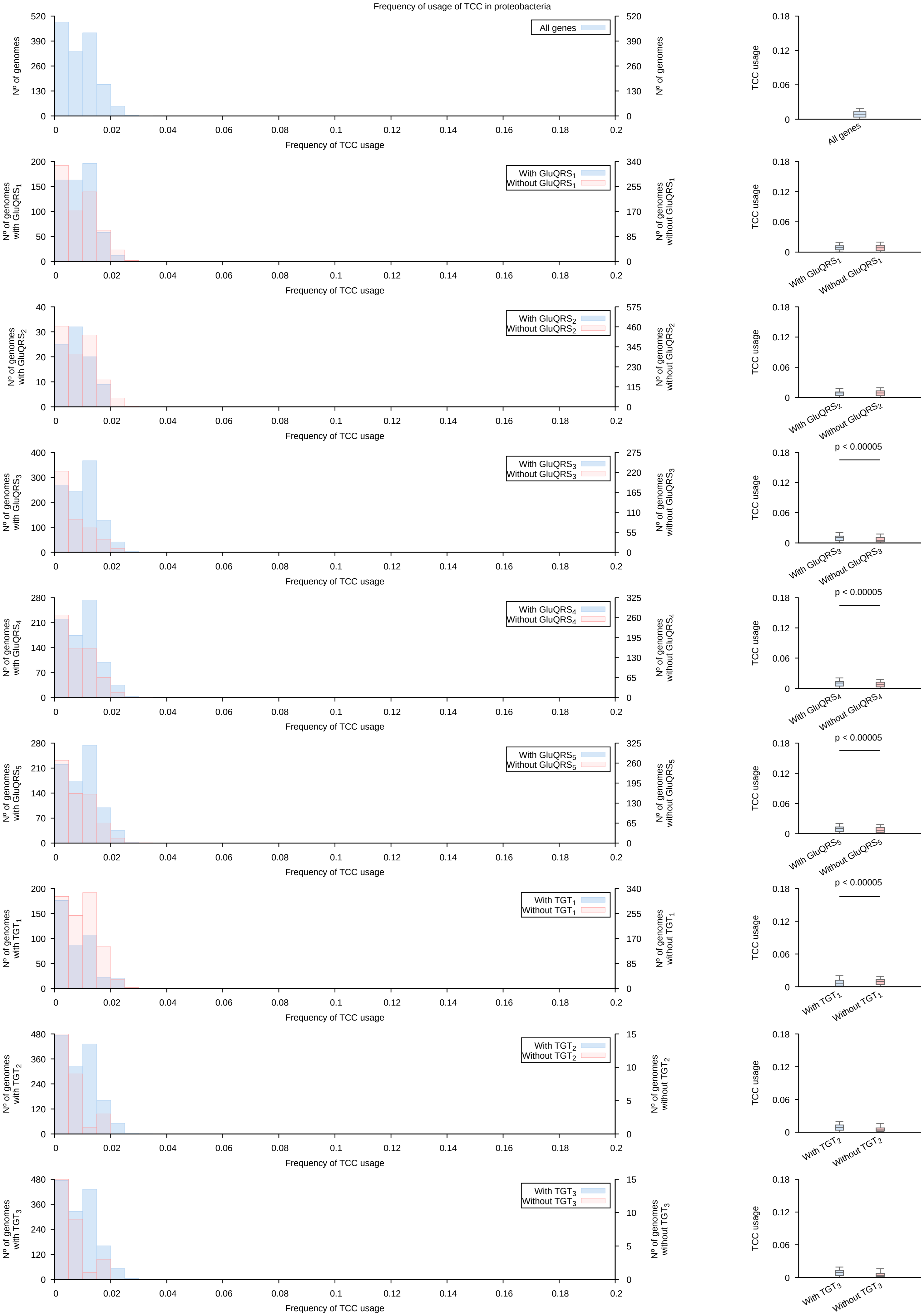
Frequency of usage of TCC in proteobacteria
All genes
With GluQRS1
Without GluQRS1
With GluQRS2
Without GluQRS2
With GluQRS3
Without GluQRS3
With GluQRS4
Without GluQRS4
With GluQRS5
Without GluQRS5
With TGT1
Without TGT1
With TGT2
Without TGT2
With TGT3
Without TGT3
Nº of genomes
Nº of genomes with GluQRS1
Nº of genomes without GluQRS1
Nº of genomes with GluQRS2
Nº of genomes without GluQRS2
Nº of genomes with GluQRS3
Nº of genomes without GluQRS3
Nº of genomes with GluQRS4
Nº of genomes without GluQRS4
Nº of genomes with GluQRS5
Nº of genomes without GluQRS5
Nº of genomes with TGT1
Nº of genomes without TGT1
Nº of genomes with TGT2
Nº of genomes without TGT2
Nº of genomes with TGT3
Nº of genomes without TGT3
Frequency of TCC usage
TCC usage
p < 0.00005

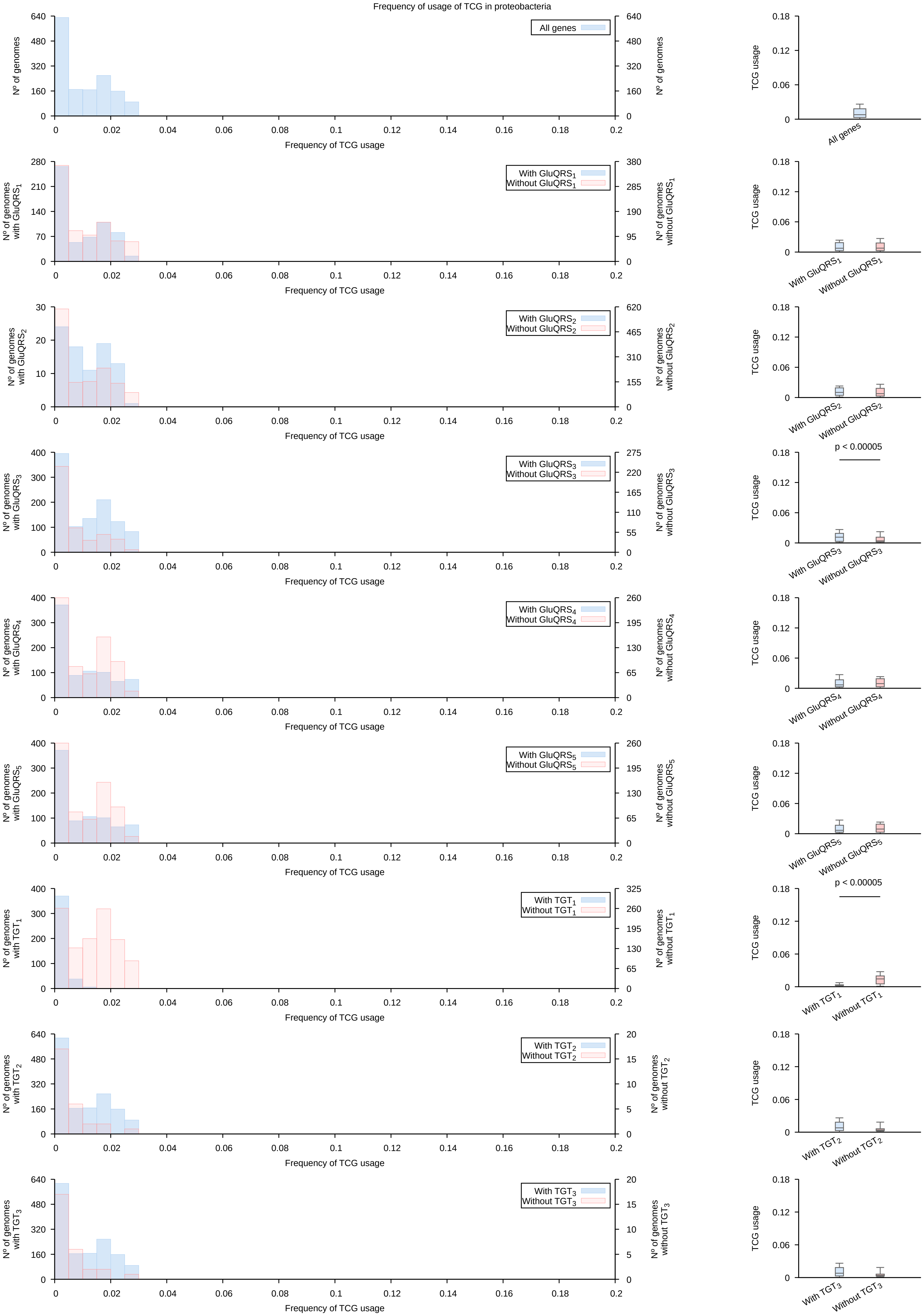

Frequency of usage of TCG in proteobacteria
All genes
Nº of genomes
Frequency of TCG usage
TCG usage
With GluQRS$_1$
Without GluQRS$_1$
Nº of genomes with GluQRS$_1$
Nº of genomes without GluQRS$_1$
With GluQRS$_2$
Without GluQRS$_2$
Nº of genomes with GluQRS$_2$
Nº of genomes without GluQRS$_2$
With GluQRS$_3$
Without GluQRS$_3$
Nº of genomes with GluQRS$_3$
Nº of genomes without GluQRS$_3$
p < 0.00005
With GluQRS$_4$
Without GluQRS$_4$
Nº of genomes with GluQRS$_4$
Nº of genomes without GluQRS$_4$
With GluQRS$_5$
Without GluQRS$_5$
Nº of genomes with GluQRS$_5$
Nº of genomes without GluQRS$_5$
With TGT$_1$
Without TGT$_1$
Nº of genomes with TGT$_1$
Nº of genomes without TGT$_1$
p < 0.00005
With TGT$_2$
Without TGT$_2$
Nº of genomes with TGT$_2$
Nº of genomes without TGT$_2$
With TGT$_3$
Without TGT$_3$
Nº of genomes with TGT$_3$
Nº of genomes without TGT$_3$

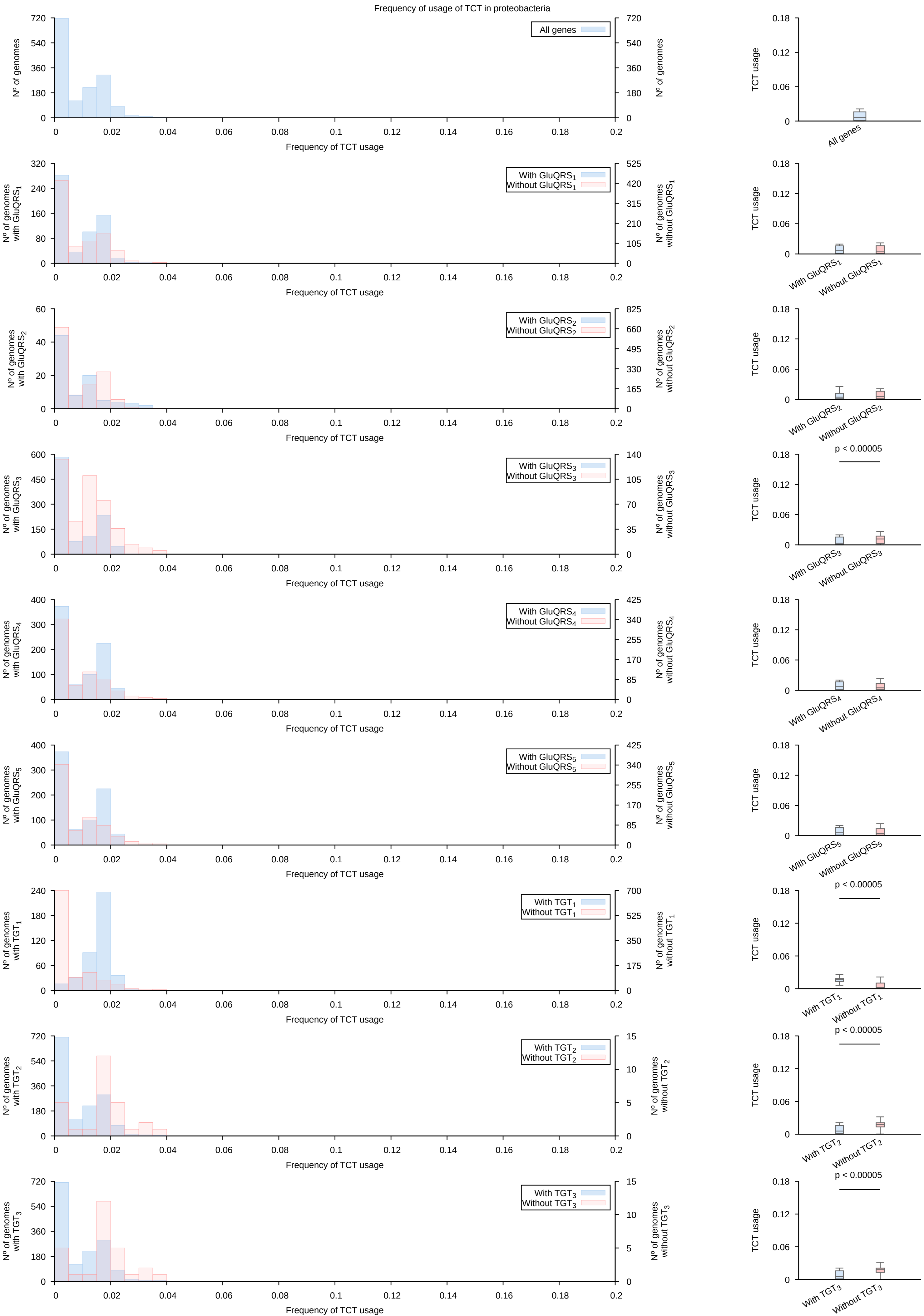
Frequency of usage of TCT in proteobacteria
All genes
Nº of genomes
Frequency of TCT usage
TCT usage
With GluQRS$_1$
Without GluQRS$_1$
Nº of genomes with GluQRS$_1$
Nº of genomes without GluQRS$_1$
With GluQRS$_2$
Without GluQRS$_2$
Nº of genomes with GluQRS$_2$
Nº of genomes without GluQRS$_2$
With GluQRS$_3$
Without GluQRS$_3$
Nº of genomes with GluQRS$_3$
Nº of genomes without GluQRS$_3$
p < 0.00005
With GluQRS$_4$
Without GluQRS$_4$
Nº of genomes with GluQRS$_4$
Nº of genomes without GluQRS$_4$
With GluQRS$_5$
Without GluQRS$_5$
Nº of genomes with GluQRS$_5$
Nº of genomes without GluQRS$_5$
With TGT$_1$
Without TGT$_1$
Nº of genomes with TGT$_1$
Nº of genomes without TGT$_1$
p < 0.00005
With TGT$_2$
Without TGT$_2$
Nº of genomes with TGT$_2$
Nº of genomes without TGT$_2$
p < 0.00005
With TGT$_3$
Without TGT$_3$
Nº of genomes with TGT$_3$
Nº of genomes without TGT$_3$
p < 0.00005

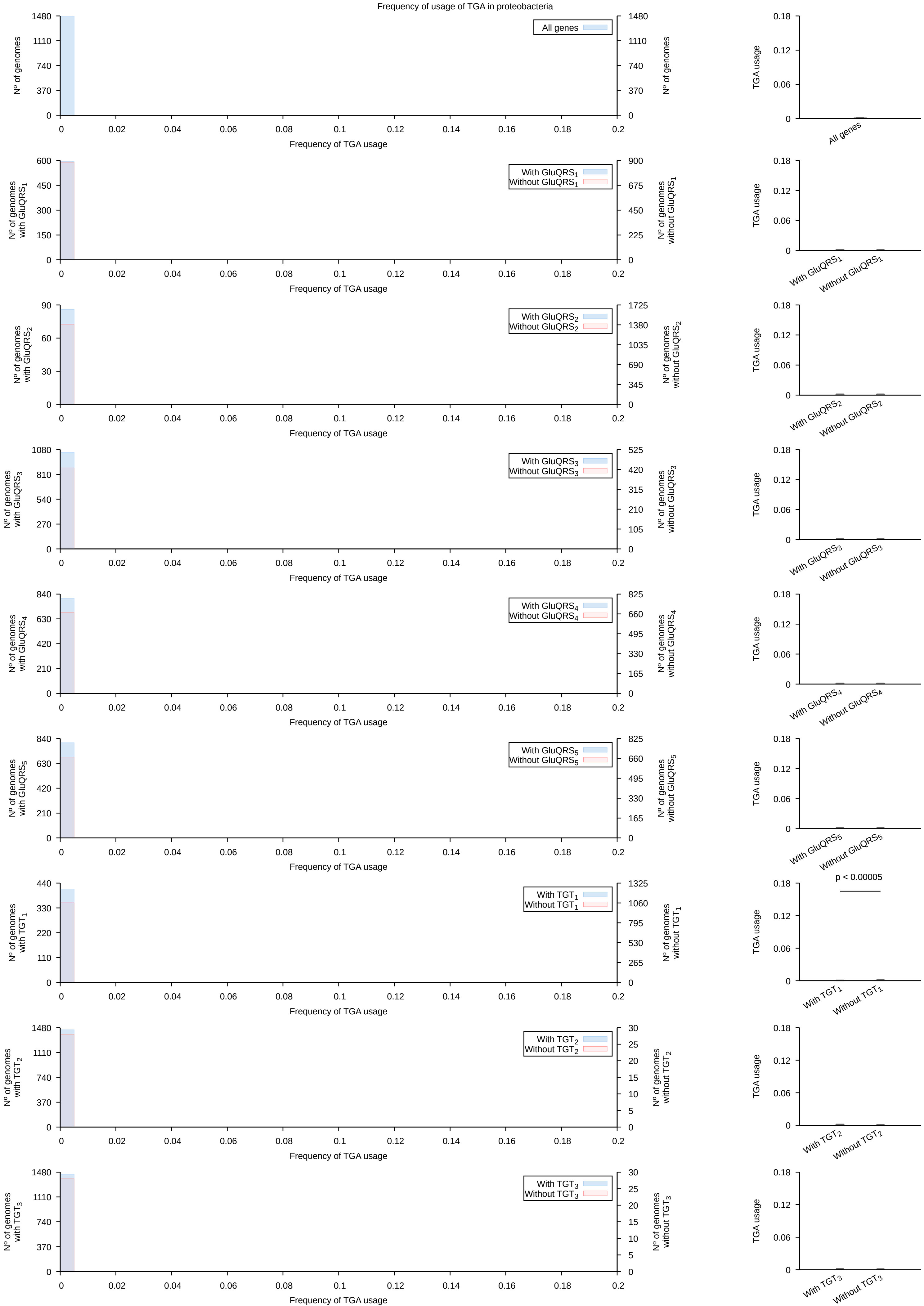
Frequency of usage of TGA in proteobacteria
Nº of genomes
0
370
740
1110
1480
All genes
Frequency of TGA usage
0
0.02
0.04
0.06
0.08
0.1
0.12
0.14
0.16
0.18
0.2
TGA usage
0.06
0.12
0.18
All genes
Nº of genomes with $GluQRS_1$
Nº of genomes without $GluQRS_1$
With $GluQRS_1$
Without $GluQRS_1$
0
150
300
450
600
225
675
900
Nº of genomes with $GluQRS_2$
Nº of genomes without $GluQRS_2$
With $GluQRS_2$
Without $GluQRS_2$
30
60
90
345
690
1035
1380
1725
Nº of genomes with $GluQRS_3$
Nº of genomes without $GluQRS_3$
With $GluQRS_3$
Without $GluQRS_3$
270
540
810
1080
105
210
315
420
525
Nº of genomes with $GluQRS_4$
Nº of genomes without $GluQRS_4$
With $GluQRS_4$
Without $GluQRS_4$
210
420
630
840
165
330
495
660
825
Nº of genomes with $GluQRS_5$
Nº of genomes without $GluQRS_5$
With $GluQRS_5$
Without $GluQRS_5$
Nº of genomes with TGT_1
Nº of genomes without TGT_1
With TGT_1
Without TGT_1
110
220
330
440
265
530
795
1060
1325
p < 0.00005
Nº of genomes with TGT_2
Nº of genomes without TGT_2
With TGT_2
Without TGT_2
5
10
15
20
25
30
Nº of genomes with TGT_3
Nº of genomes without TGT_3
With TGT_3
Without TGT_3

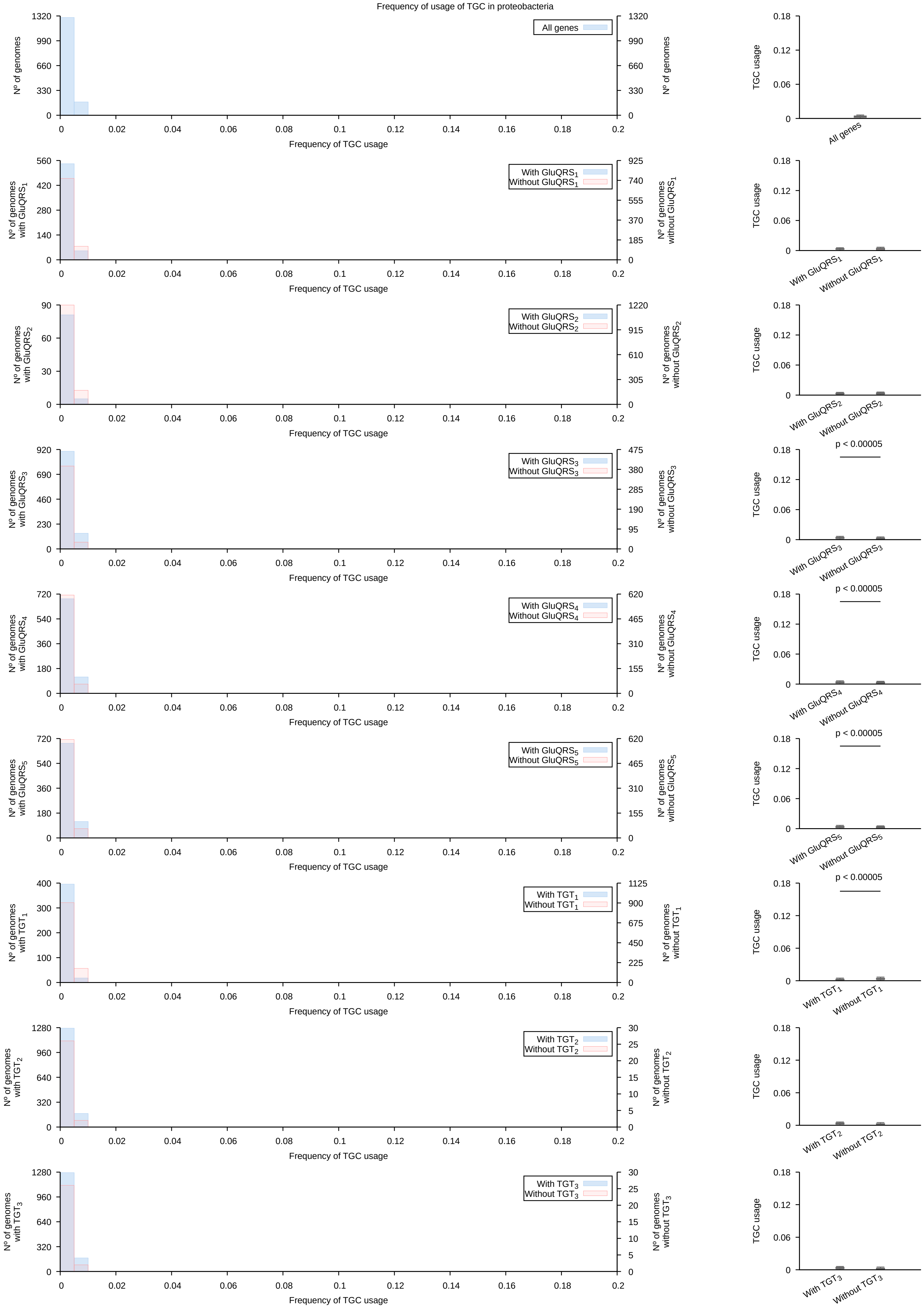
Frequency of usage of TGC in proteobacteria
All genes
With GluQRS$_1$
Without GluQRS$_1$
With GluQRS$_2$
Without GluQRS$_2$
With GluQRS$_3$
Without GluQRS$_3$
With GluQRS$_4$
Without GluQRS$_4$
With GluQRS$_5$
Without GluQRS$_5$
With TGT$_1$
Without TGT$_1$
With TGT$_2$
Without TGT$_2$
With TGT$_3$
Without TGT$_3$
Nº of genomes
Nº of genomes with GluQRS$_1$
Nº of genomes without GluQRS$_1$
Nº of genomes with GluQRS$_2$
Nº of genomes without GluQRS$_2$
Nº of genomes with GluQRS$_3$
Nº of genomes without GluQRS$_3$
Nº of genomes with GluQRS$_4$
Nº of genomes without GluQRS$_4$
Nº of genomes with GluQRS$_5$
Nº of genomes without GluQRS$_5$
Nº of genomes with TGT$_1$
Nº of genomes without TGT$_1$
Nº of genomes with TGT$_2$
Nº of genomes without TGT$_2$
Nº of genomes with TGT$_3$
Nº of genomes without TGT$_3$
Frequency of TGC usage
TGC usage
p < 0.00005

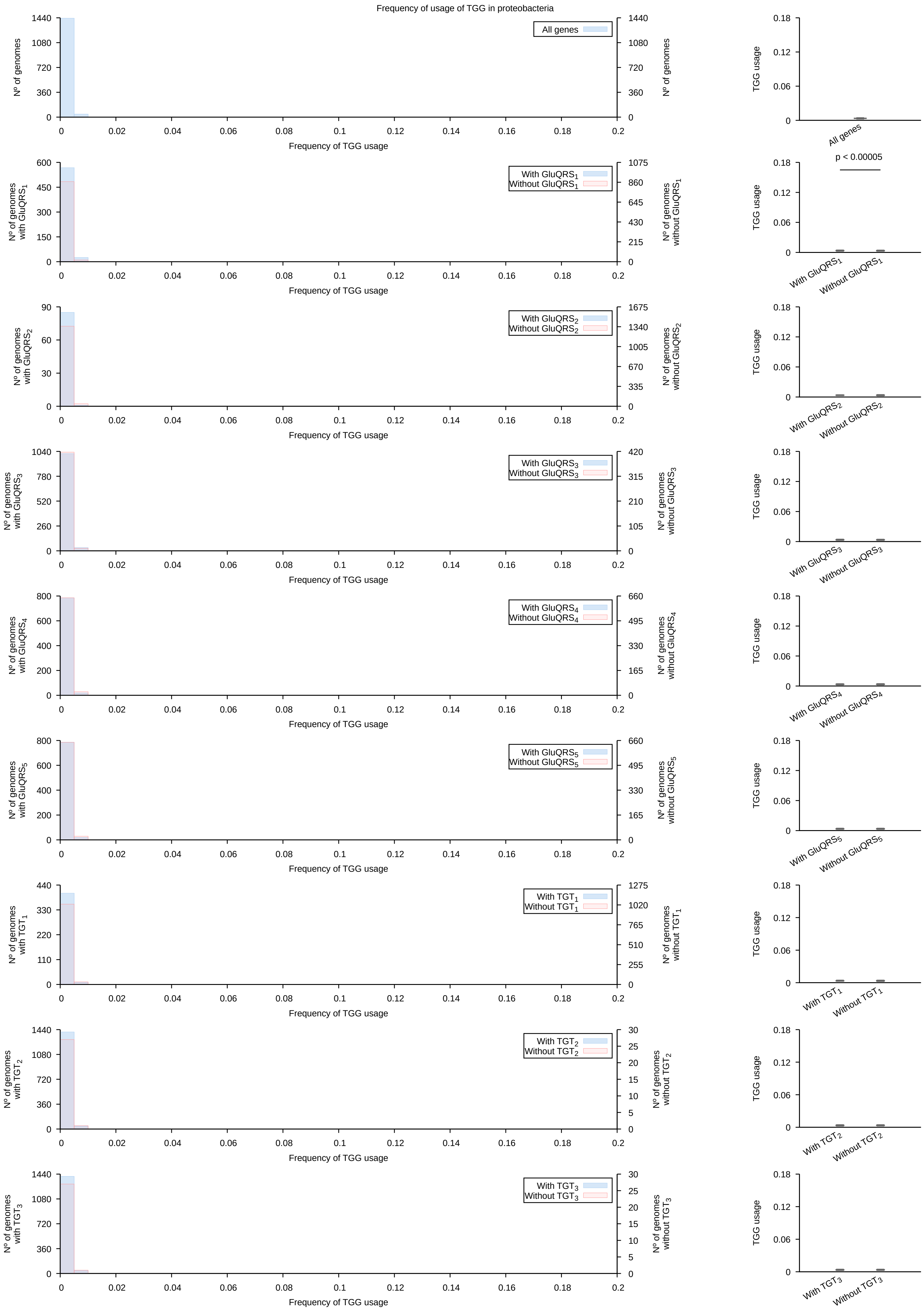
Frequency of usage of TGG in proteobacteria
All genes
Nº of genomes
Frequency of TGG usage
TGG usage
p < 0.00005
With GluQRS1
Without GluQRS1
Nº of genomes with GluQRS1
Nº of genomes without GluQRS1
With GluQRS2
Without GluQRS2
Nº of genomes with GluQRS2
Nº of genomes without GluQRS2
With GluQRS3
Without GluQRS3
Nº of genomes with GluQRS3
Nº of genomes without GluQRS3
With GluQRS4
Without GluQRS4
Nº of genomes with GluQRS4
Nº of genomes without GluQRS4
With GluQRS5
Without GluQRS5
Nº of genomes with GluQRS5
Nº of genomes without GluQRS5
With TGT1
Without TGT1
Nº of genomes with TGT1
Nº of genomes without TGT1
With TGT2
Without TGT2
Nº of genomes with TGT2
Nº of genomes without TGT2
With TGT3
Without TGT3
Nº of genomes with TGT3
Nº of genomes without TGT3

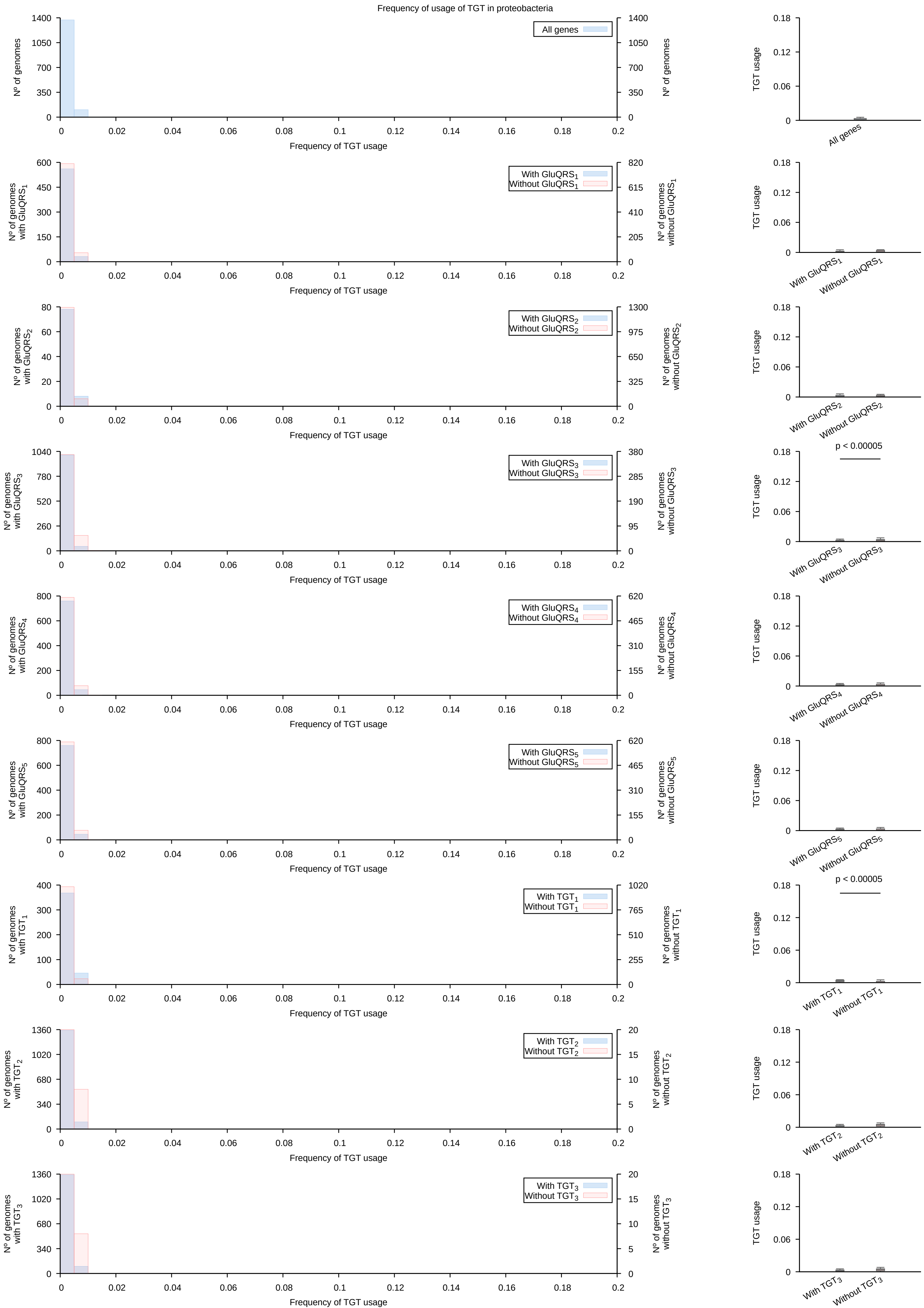

Frequency of usage of TGT in proteobacteria
All genes
Nº of genomes
Frequency of TGT usage
TGT usage
With GluQRS$_1$
Without GluQRS$_1$
Nº of genomes with GluQRS$_1$
Nº of genomes without GluQRS$_1$
With GluQRS$_2$
Without GluQRS$_2$
Nº of genomes with GluQRS$_2$
Nº of genomes without GluQRS$_2$
With GluQRS$_3$
Without GluQRS$_3$
Nº of genomes with GluQRS$_3$
Nº of genomes without GluQRS$_3$
p < 0.00005
With GluQRS$_4$
Without GluQRS$_4$
Nº of genomes with GluQRS$_4$
Nº of genomes without GluQRS$_4$
With GluQRS$_5$
Without GluQRS$_5$
Nº of genomes with GluQRS$_5$
Nº of genomes without GluQRS$_5$
With TGT$_1$
Without TGT$_1$
Nº of genomes with TGT$_1$
Nº of genomes without TGT$_1$
p < 0.00005
With TGT$_2$
Without TGT$_2$
Nº of genomes with TGT$_2$
Nº of genomes without TGT$_2$
With TGT$_3$
Without TGT$_3$
Nº of genomes with TGT$_3$
Nº of genomes without TGT$_3$

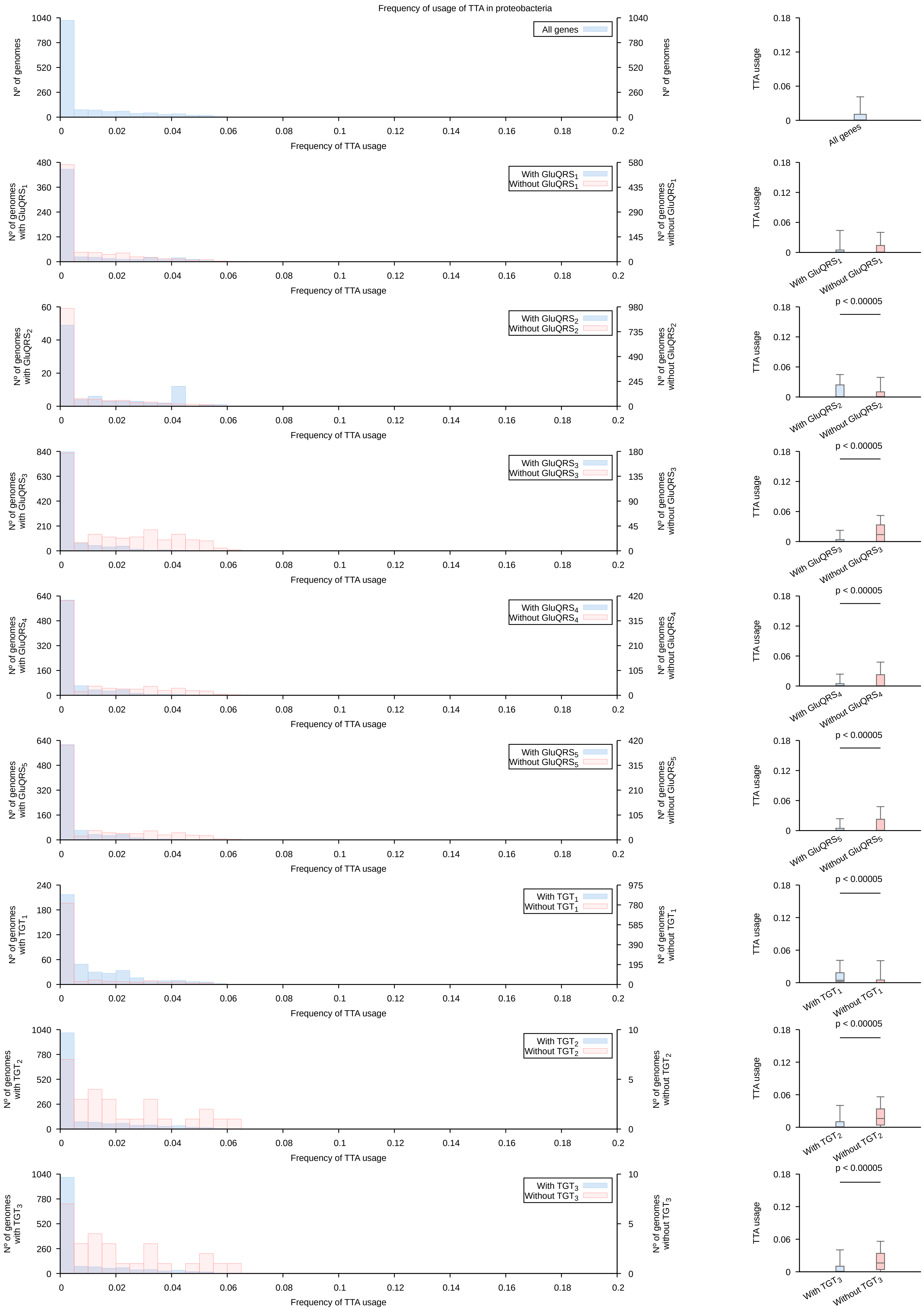

Frequency of usage of TTA in proteobacteria
All genes
Nº of genomes
Frequency of TTA usage
TTA usage
With GluQRS$_1$
Without GluQRS$_1$
Nº of genomes with GluQRS$_1$
Nº of genomes without GluQRS$_1$
With GluQRS$_2$
Without GluQRS$_2$
Nº of genomes with GluQRS$_2$
Nº of genomes without GluQRS$_2$
p < 0.00005
With GluQRS$_3$
Without GluQRS$_3$
Nº of genomes with GluQRS$_3$
Nº of genomes without GluQRS$_3$
p < 0.00005
With GluQRS$_4$
Without GluQRS$_4$
Nº of genomes with GluQRS$_4$
Nº of genomes without GluQRS$_4$
p < 0.00005
With GluQRS$_5$
Without GluQRS$_5$
Nº of genomes with GluQRS$_5$
Nº of genomes without GluQRS$_5$
p < 0.00005
With TGT$_1$
Without TGT$_1$
Nº of genomes with TGT$_1$
Nº of genomes without TGT$_1$
p < 0.00005
With TGT$_2$
Without TGT$_2$
Nº of genomes with TGT$_2$
Nº of genomes without TGT$_2$
p < 0.00005
With TGT$_3$
Without TGT$_3$
Nº of genomes with TGT$_3$
Nº of genomes without TGT$_3$

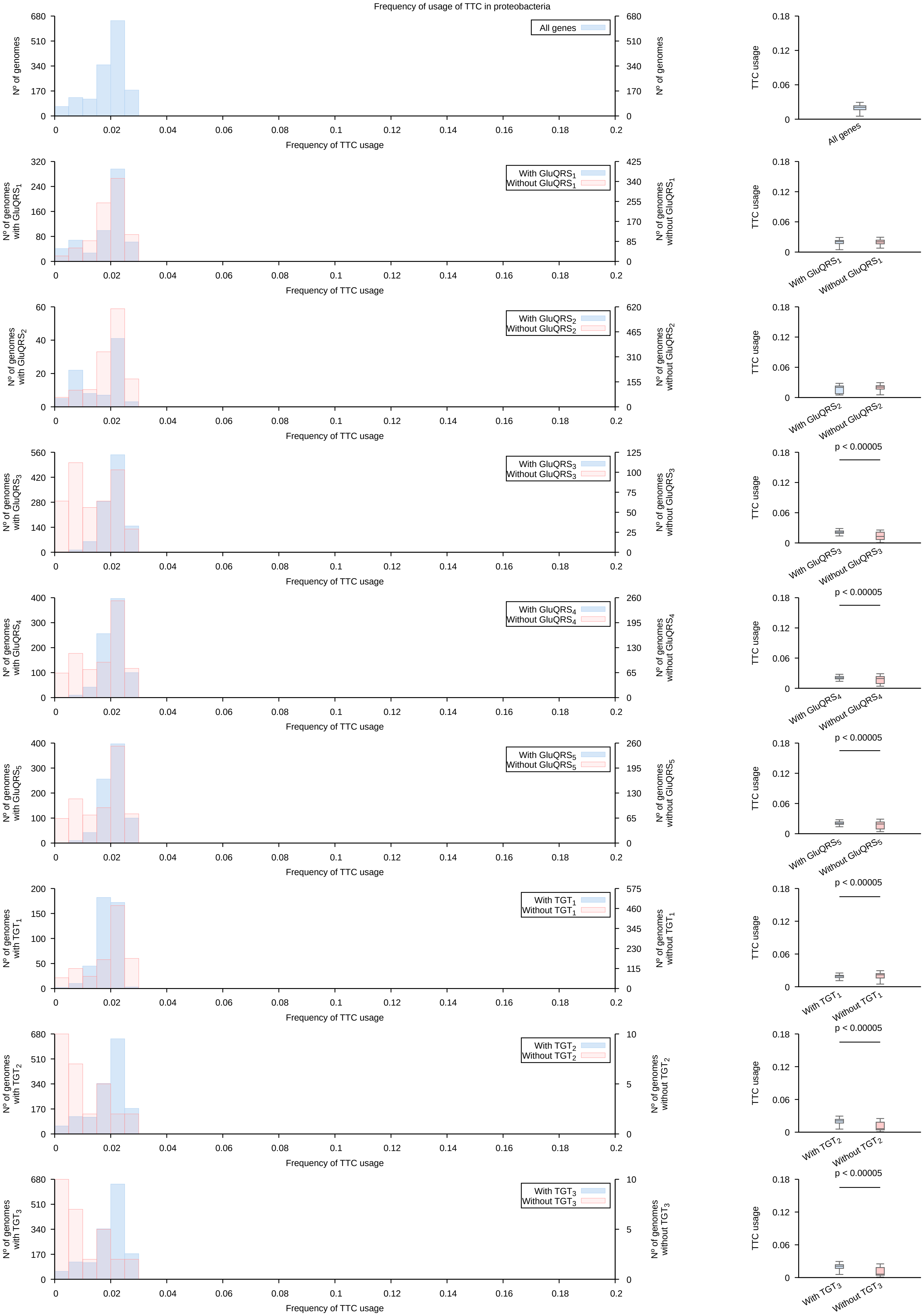

Frequency of usage of TTC in proteobacteria
All genes
With GluQRS1
Without GluQRS1
With GluQRS2
Without GluQRS2
With GluQRS3
Without GluQRS3
With GluQRS4
Without GluQRS4
With GluQRS5
Without GluQRS5
With TGT1
Without TGT1
With TGT2
Without TGT2
With TGT3
Without TGT3
Nº of genomes
Nº of genomes with GluQRS1
Nº of genomes without GluQRS1
Nº of genomes with GluQRS2
Nº of genomes without GluQRS2
Nº of genomes with GluQRS3
Nº of genomes without GluQRS3
Nº of genomes with GluQRS4
Nº of genomes without GluQRS4
Nº of genomes with GluQRS5
Nº of genomes without GluQRS5
Nº of genomes with TGT1
Nº of genomes without TGT1
Nº of genomes with TGT2
Nº of genomes without TGT2
Nº of genomes with TGT3
Nº of genomes without TGT3
Frequency of TTC usage
TTC usage
p < 0.00005

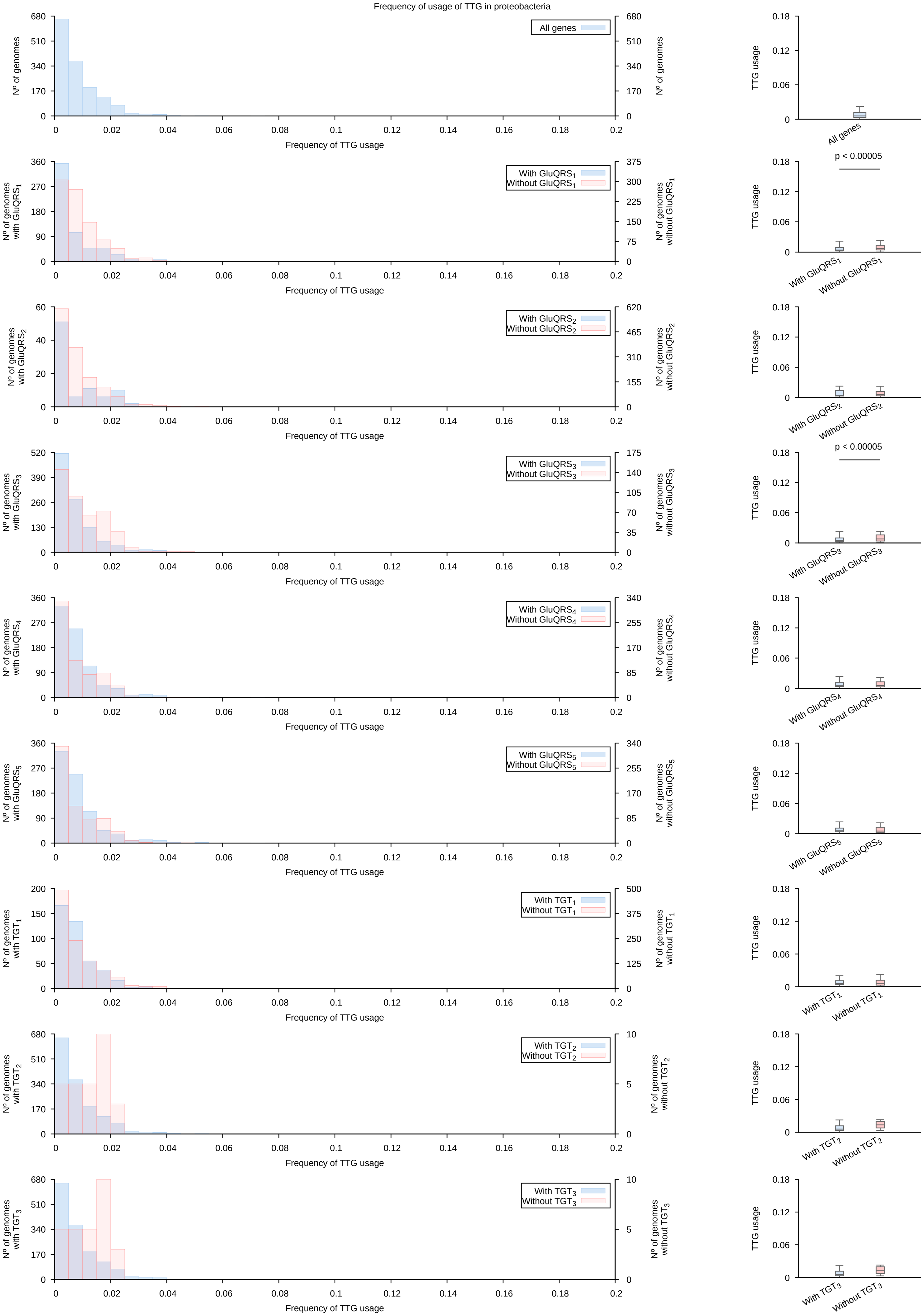
Frequency of usage of TTG in proteobacteria
All genes
With GluQRS1
Without GluQRS1
With GluQRS2
Without GluQRS2
With GluQRS3
Without GluQRS3
With GluQRS4
Without GluQRS4
With GluQRS5
Without GluQRS5
With TGT1
Without TGT1
With TGT2
Without TGT2
With TGT3
Without TGT3
Nº of genomes
Nº of genomes with GluQRS1
Nº of genomes without GluQRS1
Nº of genomes with GluQRS2
Nº of genomes without GluQRS2
Nº of genomes with GluQRS3
Nº of genomes without GluQRS3
Nº of genomes with GluQRS4
Nº of genomes without GluQRS4
Nº of genomes with GluQRS5
Nº of genomes without GluQRS5
Nº of genomes with TGT1
Nº of genomes without TGT1
Nº of genomes with TGT2
Nº of genomes without TGT2
Nº of genomes with TGT3
Nº of genomes without TGT3
Frequency of TTG usage
TTG usage
p < 0.00005
p < 0.00005

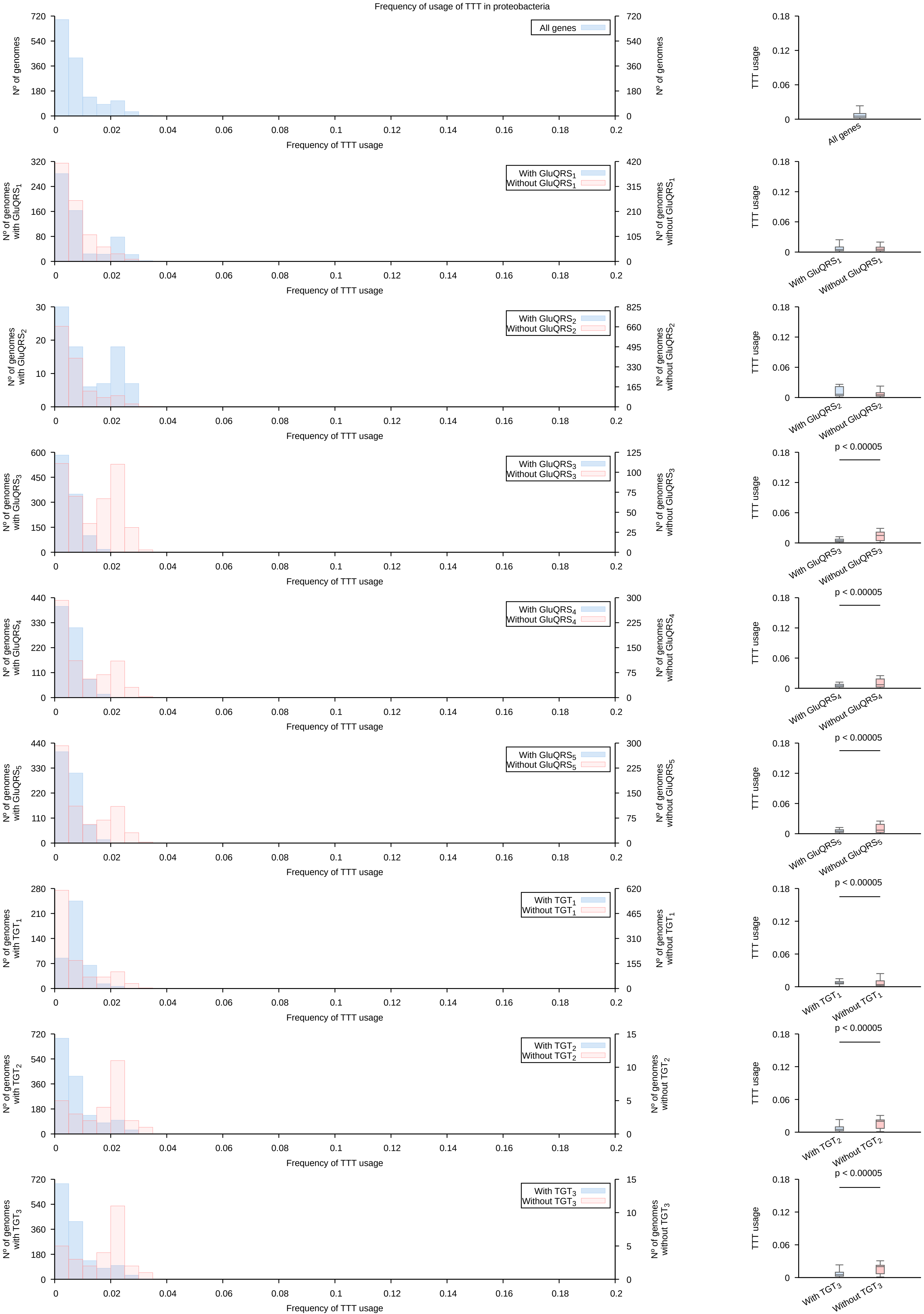
Frequency of usage of TTT in proteobacteria
All genes
Nº of genomes
Frequency of TTT usage
TTT usage
With GluQRS$_1$
Without GluQRS$_1$
Nº of genomes with GluQRS$_1$
Nº of genomes without GluQRS$_1$
With GluQRS$_2$
Without GluQRS$_2$
Nº of genomes with GluQRS$_2$
Nº of genomes without GluQRS$_2$
With GluQRS$_3$
Without GluQRS$_3$
Nº of genomes with GluQRS$_3$
Nº of genomes without GluQRS$_3$
p < 0.00005
With GluQRS$_4$
Without GluQRS$_4$
Nº of genomes with GluQRS$_4$
Nº of genomes without GluQRS$_4$
p < 0.00005
With GluQRS$_5$
Without GluQRS$_5$
Nº of genomes with GluQRS$_5$
Nº of genomes without GluQRS$_5$
p < 0.00005
With TGT$_1$
Without TGT$_1$
Nº of genomes with TGT$_1$
Nº of genomes without TGT$_1$
p < 0.00005
With TGT$_2$
Without TGT$_2$
Nº of genomes with TGT$_2$
Nº of genomes without TGT$_2$
p < 0.00005
With TGT$_3$
Without TGT$_3$
Nº of genomes with TGT$_3$
Nº of genomes without TGT$_3$

Figure S21. Histogram of codon usage in high expression genes of genomes that present or lack genes involved in k^2C formation excluding small genomes. Top left graph: histogram of the usage of the indicated codon in highly expressed genes of proteobacterial genomes with more than 400 CDS. **Top right graph:** box plot indicating the distribution of codon usage in the same population. **Lower graphs:** The collection of 1477 proteobacterial genomes, excluding those with less than 400 CDS, were separated in two groups, based on the presence (light blue) or absence (orange) of the indicated pattern in any gene of the analyzed genomes. Patterns are indicated using subscript numbers. See table S4 for a detailed description of the used patterns. **Left:** Data is represented in superposed histograms. **Right:** Data is represented in a boxplot. An horizontal bar was added when the two populations are different based on Wilcoxon-Mann-Whitney test and $p < 0.00005$. Whiskers include 95% of data.

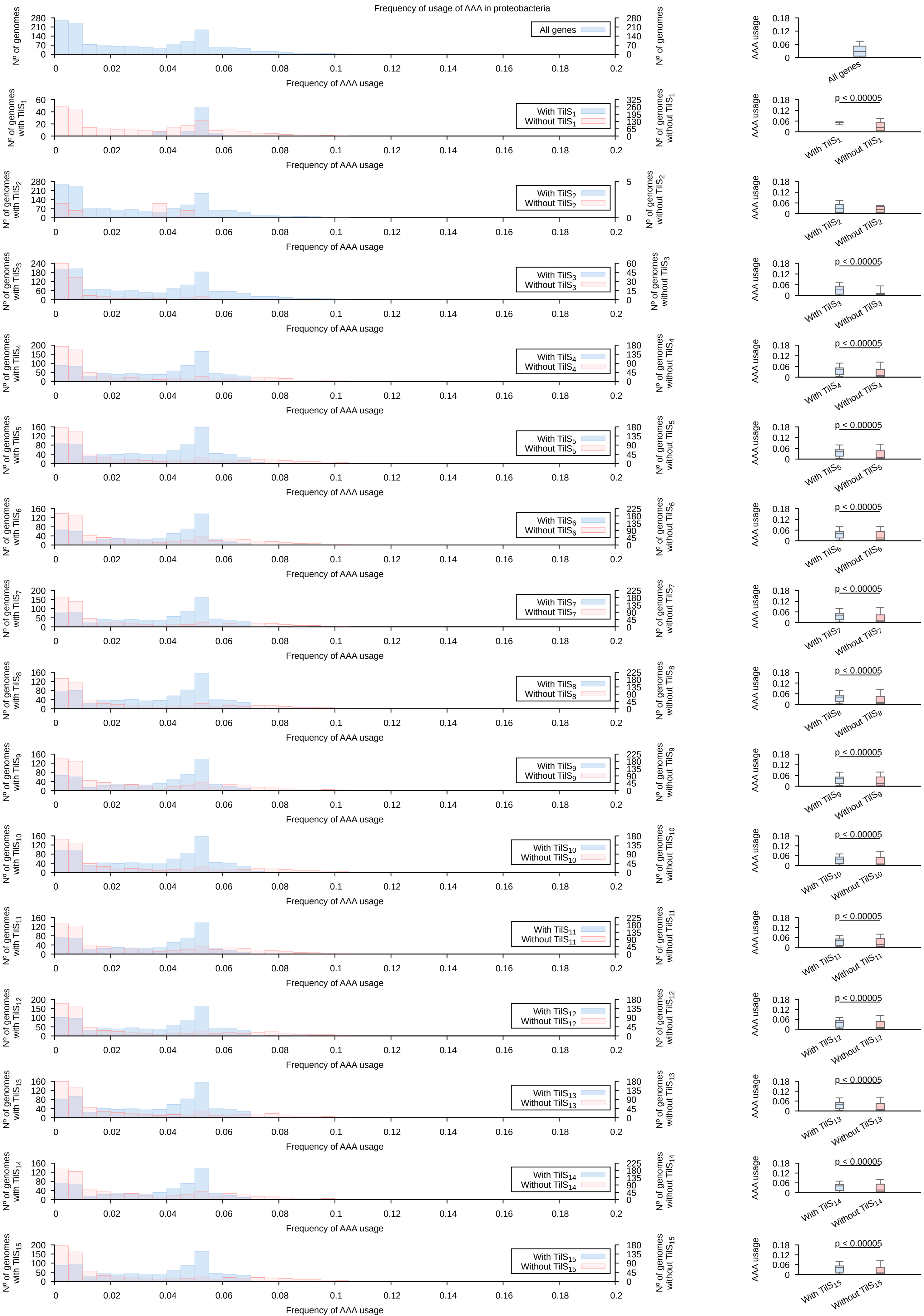

Frequency of usage of AAA in proteobacteria
Nº of genomes
All genes
Frequency of AAA usage
AAA usage
All genes
Nº of genomes with $TilS_1$
Nº of genomes without $TilS_1$
With $TilS_1$
Without $TilS_1$
p < 0.00005
Nº of genomes with $TilS_2$
Nº of genomes without $TilS_2$
With $TilS_2$
Without $TilS_2$
Nº of genomes with $TilS_3$
Nº of genomes without $TilS_3$
With $TilS_3$
Without $TilS_3$
p < 0.00005
Nº of genomes with $TilS_4$
Nº of genomes without $TilS_4$
With $TilS_4$
Without $TilS_4$
p < 0.00005
Nº of genomes with $TilS_5$
Nº of genomes without $TilS_5$
With $TilS_5$
Without $TilS_5$
p < 0.00005
Nº of genomes with $TilS_6$
Nº of genomes without $TilS_6$
With $TilS_6$
Without $TilS_6$
p < 0.00005
Nº of genomes with $TilS_7$
Nº of genomes without $TilS_7$
With $TilS_7$
Without $TilS_7$
p < 0.00005
Nº of genomes with $TilS_8$
Nº of genomes without $TilS_8$
With $TilS_8$
Without $TilS_8$
p < 0.00005
Nº of genomes with $TilS_9$
Nº of genomes without $TilS_9$
With $TilS_9$
Without $TilS_9$
p < 0.00005
Nº of genomes with $TilS_{10}$
Nº of genomes without $TilS_{10}$
With $TilS_{10}$
Without $TilS_{10}$
p < 0.00005
Nº of genomes with $TilS_{11}$
Nº of genomes without $TilS_{11}$
With $TilS_{11}$
Without $TilS_{11}$
p < 0.00005
Nº of genomes with $TilS_{12}$
Nº of genomes without $TilS_{12}$
With $TilS_{12}$
Without $TilS_{12}$
p < 0.00005
Nº of genomes with $TilS_{13}$
Nº of genomes without $TilS_{13}$
With $TilS_{13}$
Without $TilS_{13}$
p < 0.00005
Nº of genomes with $TilS_{14}$
Nº of genomes without $TilS_{14}$
With $TilS_{14}$
Without $TilS_{14}$
p < 0.00005
Nº of genomes with $TilS_{15}$
Nº of genomes without $TilS_{15}$
With $TilS_{15}$
Without $TilS_{15}$
p < 0.00005

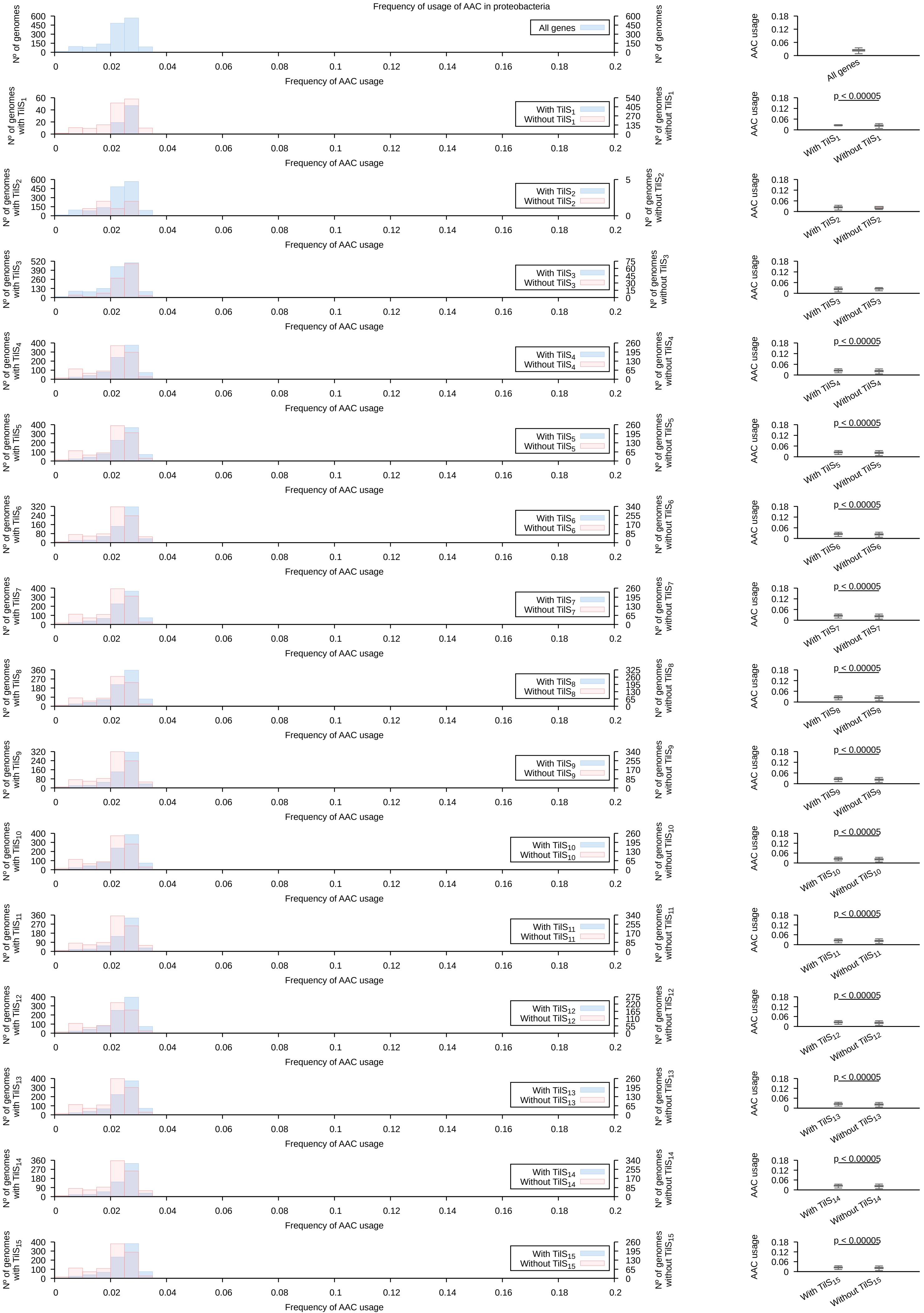
Frequency of usage of AAC in proteobacteria
Nº of genomes
Frequency of AAC usage
All genes
AAC usage
Nº of genomes with TilS$_1$
Nº of genomes without TilS$_1$
With TilS$_1$
Without TilS$_1$
p < 0.00005
Nº of genomes with TilS$_2$
Nº of genomes without TilS$_2$
With TilS$_2$
Without TilS$_2$
Nº of genomes with TilS$_3$
Nº of genomes without TilS$_3$
With TilS$_3$
Without TilS$_3$
Nº of genomes with TilS$_4$
Nº of genomes without TilS$_4$
With TilS$_4$
Without TilS$_4$
p < 0.00005
Nº of genomes with TilS$_5$
Nº of genomes without TilS$_5$
With TilS$_5$
Without TilS$_5$
p < 0.00005
Nº of genomes with TilS$_6$
Nº of genomes without TilS$_6$
With TilS$_6$
Without TilS$_6$
p < 0.00005
Nº of genomes with TilS$_7$
Nº of genomes without TilS$_7$
With TilS$_7$
Without TilS$_7$
p < 0.00005
Nº of genomes with TilS$_8$
Nº of genomes without TilS$_8$
With TilS$_8$
Without TilS$_8$
p < 0.00005
Nº of genomes with TilS$_9$
Nº of genomes without TilS$_9$
With TilS$_9$
Without TilS$_9$
p < 0.00005
Nº of genomes with TilS$_{10}$
Nº of genomes without TilS$_{10}$
With TilS$_{10}$
Without TilS$_{10}$
p < 0.00005
Nº of genomes with TilS$_{11}$
Nº of genomes without TilS$_{11}$
With TilS$_{11}$
Without TilS$_{11}$
p < 0.00005
Nº of genomes with TilS$_{12}$
Nº of genomes without TilS$_{12}$
With TilS$_{12}$
Without TilS$_{12}$
p < 0.00005
Nº of genomes with TilS$_{13}$
Nº of genomes without TilS$_{13}$
With TilS$_{13}$
Without TilS$_{13}$
p < 0.00005
Nº of genomes with TilS$_{14}$
Nº of genomes without TilS$_{14}$
With TilS$_{14}$
Without TilS$_{14}$
p < 0.00005
Nº of genomes with TilS$_{15}$
Nº of genomes without TilS$_{15}$
With TilS$_{15}$
Without TilS$_{15}$
p < 0.00005

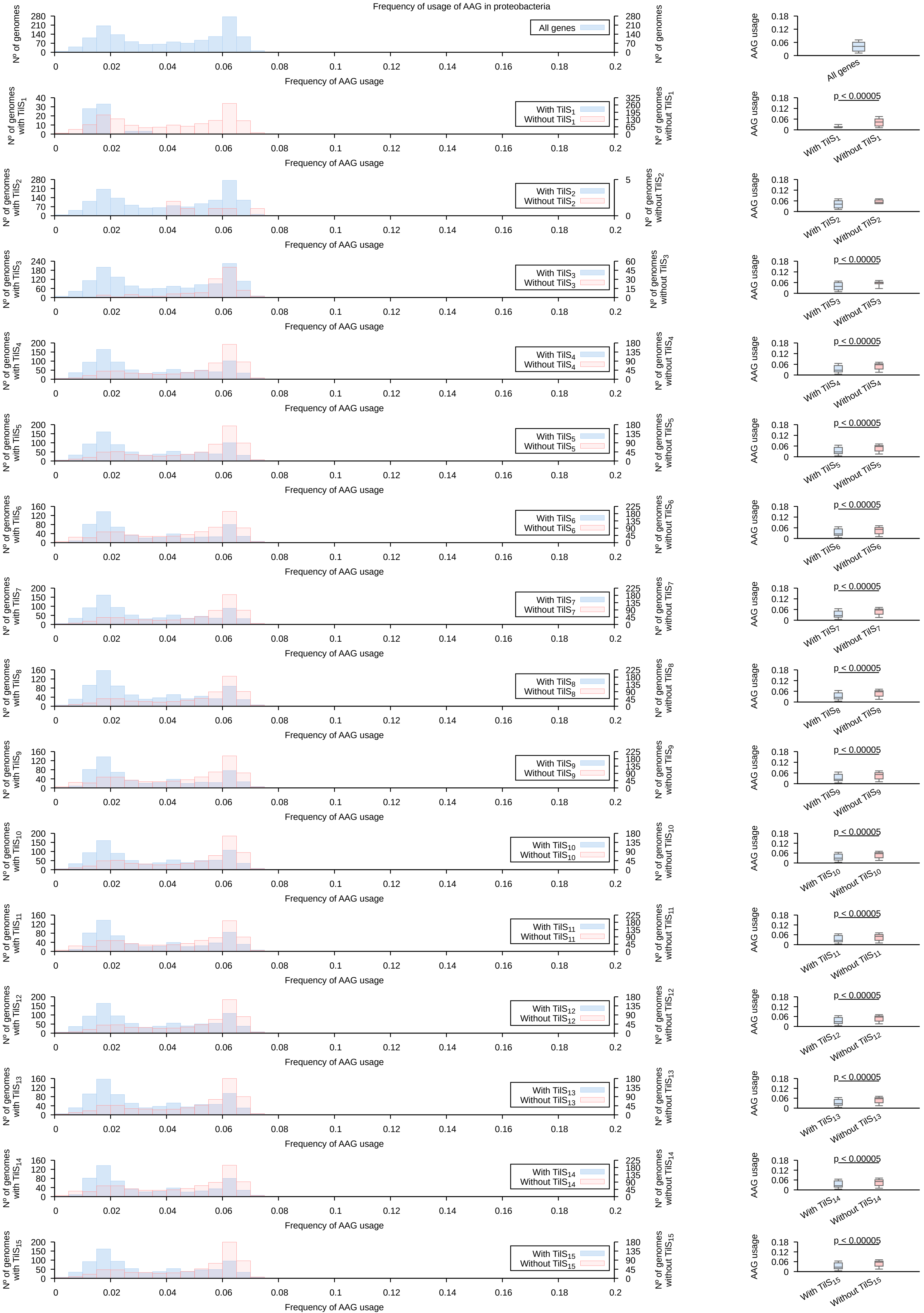
Frequency of usage of AAG in proteobacteria
Nº of genomes
All genes
Frequency of AAG usage
AAG usage
p < 0.00005
Nº of genomes with TilS$_1$
Nº of genomes without TilS$_1$
With TilS$_1$
Without TilS$_1$
Nº of genomes with TilS$_2$
Nº of genomes without TilS$_2$
With TilS$_2$
Without TilS$_2$
Nº of genomes with TilS$_3$
Nº of genomes without TilS$_3$
With TilS$_3$
Without TilS$_3$
Nº of genomes with TilS$_4$
Nº of genomes without TilS$_4$
With TilS$_4$
Without TilS$_4$
Nº of genomes with TilS$_5$
Nº of genomes without TilS$_5$
With TilS$_5$
Without TilS$_5$
Nº of genomes with TilS$_6$
Nº of genomes without TilS$_6$
With TilS$_6$
Without TilS$_6$
Nº of genomes with TilS$_7$
Nº of genomes without TilS$_7$
With TilS$_7$
Without TilS$_7$
Nº of genomes with TilS$_8$
Nº of genomes without TilS$_8$
With TilS$_8$
Without TilS$_8$
Nº of genomes with TilS$_9$
Nº of genomes without TilS$_9$
With TilS$_9$
Without TilS$_9$
Nº of genomes with TilS$_{10}$
Nº of genomes without TilS$_{10}$
With TilS$_{10}$
Without TilS$_{10}$
Nº of genomes with TilS$_{11}$
Nº of genomes without TilS$_{11}$
With TilS$_{11}$
Without TilS$_{11}$
Nº of genomes with TilS$_{12}$
Nº of genomes without TilS$_{12}$
With TilS$_{12}$
Without TilS$_{12}$
Nº of genomes with TilS$_{13}$
Nº of genomes without TilS$_{13}$
With TilS$_{13}$
Without TilS$_{13}$
Nº of genomes with TilS$_{14}$
Nº of genomes without TilS$_{14}$
With TilS$_{14}$
Without TilS$_{14}$
Nº of genomes with TilS$_{15}$
Nº of genomes without TilS$_{15}$
With TilS$_{15}$
Without TilS$_{15}$

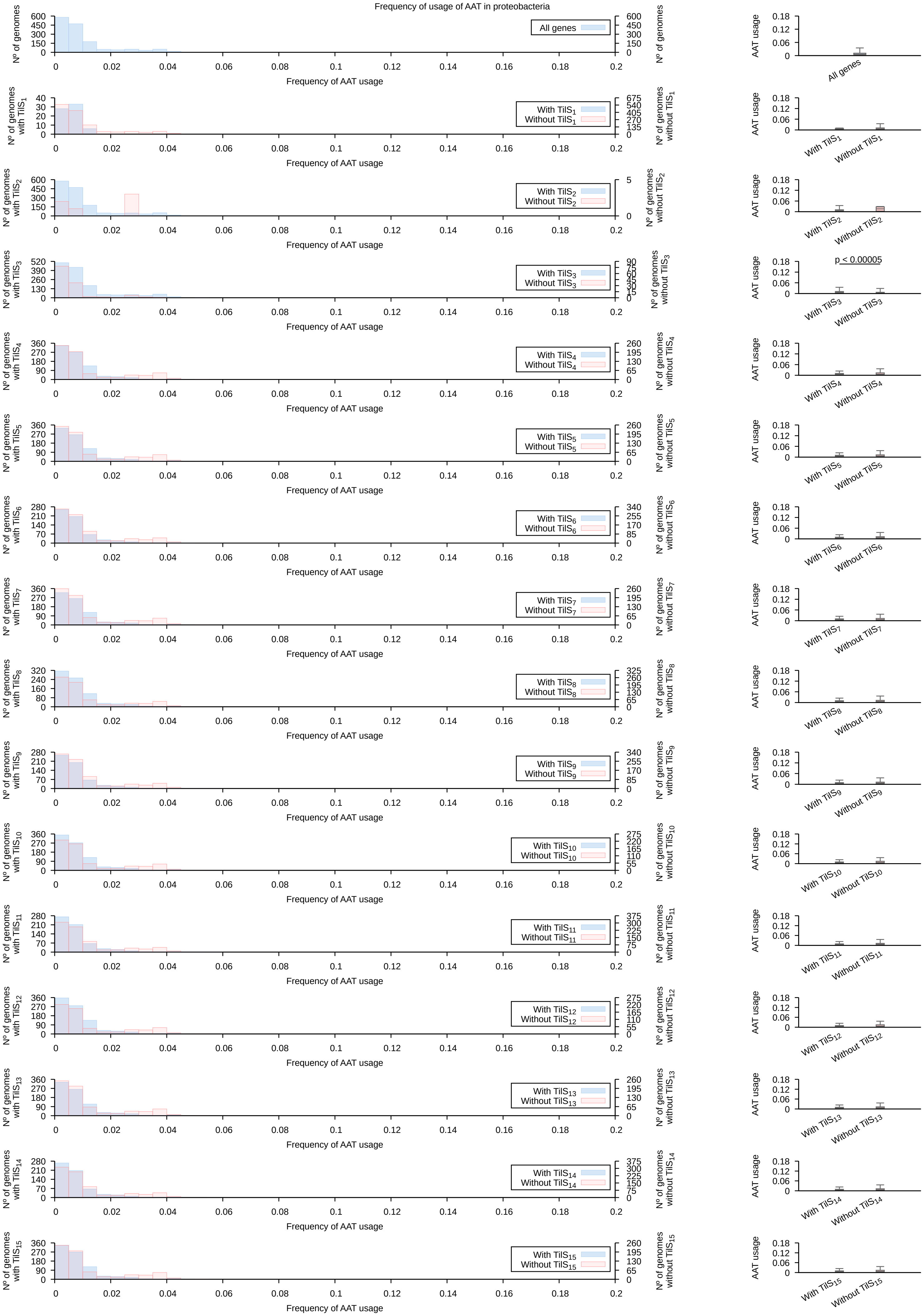

Frequency of usage of AAT in proteobacteria
Nº of genomes
All genes
Frequency of AAT usage
AAT usage
Nº of genomes with TilS$_1$
Nº of genomes without TilS$_1$
With TilS$_1$
Without TilS$_1$
Nº of genomes with TilS$_2$
Nº of genomes without TilS$_2$
With TilS$_2$
Without TilS$_2$
Nº of genomes with TilS$_3$
Nº of genomes without TilS$_3$
With TilS$_3$
Without TilS$_3$
p < 0.00005
Nº of genomes with TilS$_4$
Nº of genomes without TilS$_4$
With TilS$_4$
Without TilS$_4$
Nº of genomes with TilS$_5$
Nº of genomes without TilS$_5$
With TilS$_5$
Without TilS$_5$
Nº of genomes with TilS$_6$
Nº of genomes without TilS$_6$
With TilS$_6$
Without TilS$_6$
Nº of genomes with TilS$_7$
Nº of genomes without TilS$_7$
With TilS$_7$
Without TilS$_7$
Nº of genomes with TilS$_8$
Nº of genomes without TilS$_8$
With TilS$_8$
Without TilS$_8$
Nº of genomes with TilS$_9$
Nº of genomes without TilS$_9$
With TilS$_9$
Without TilS$_9$
Nº of genomes with TilS$_{10}$
Nº of genomes without TilS$_{10}$
With TilS$_{10}$
Without TilS$_{10}$
Nº of genomes with TilS$_{11}$
Nº of genomes without TilS$_{11}$
With TilS$_{11}$
Without TilS$_{11}$
Nº of genomes with TilS$_{12}$
Nº of genomes without TilS$_{12}$
With TilS$_{12}$
Without TilS$_{12}$
Nº of genomes with TilS$_{13}$
Nº of genomes without TilS$_{13}$
With TilS$_{13}$
Without TilS$_{13}$
Nº of genomes with TilS$_{14}$
Nº of genomes without TilS$_{14}$
With TilS$_{14}$
Without TilS$_{14}$
Nº of genomes with TilS$_{15}$
Nº of genomes without TilS$_{15}$
With TilS$_{15}$
Without TilS$_{15}$

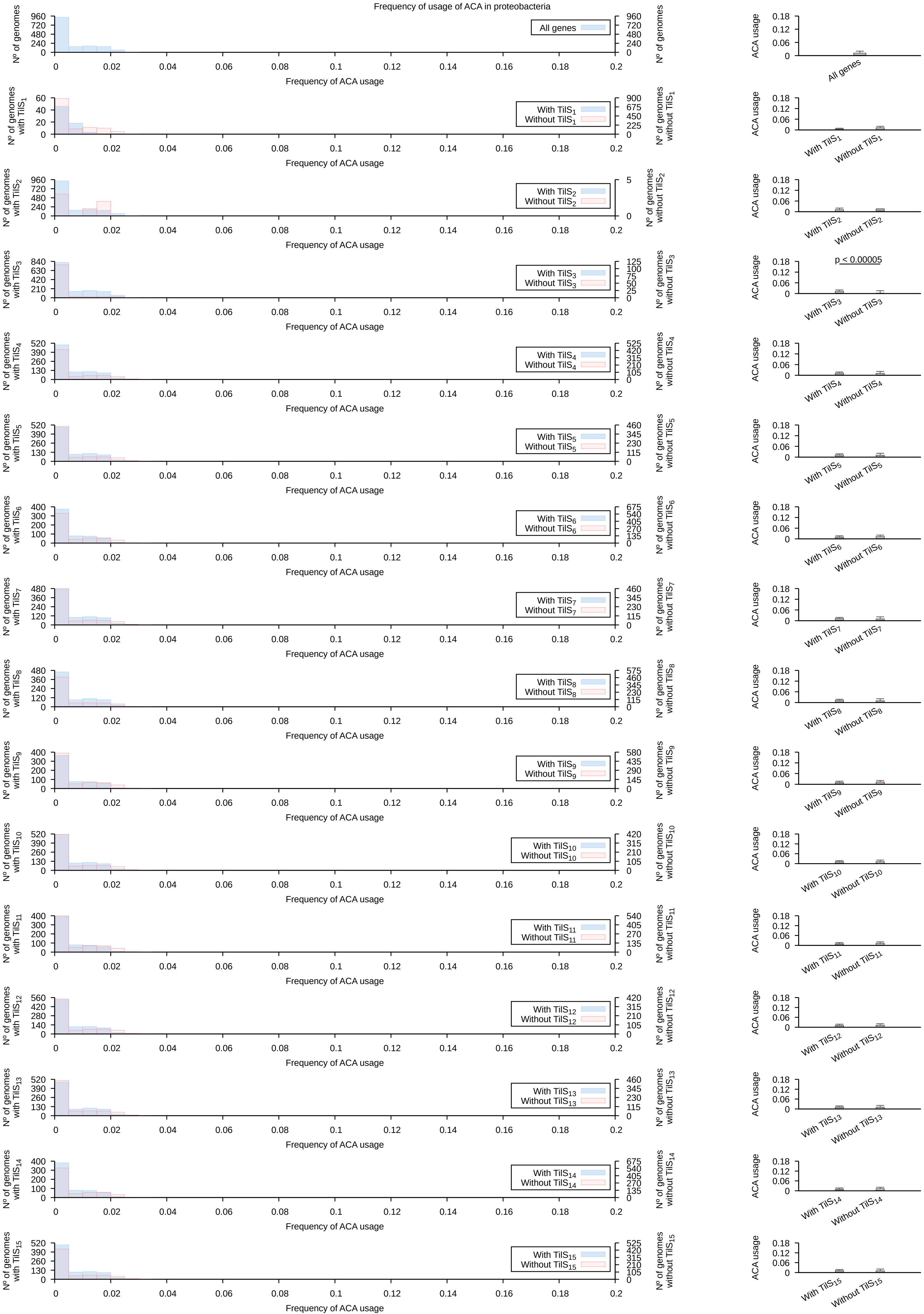
Frequency of usage of ACA in proteobacteria
Nº of genomes
All genes
Frequency of ACA usage
ACA usage
Nº of genomes with TilS$_1$
With TilS$_1$
Without TilS$_1$
Nº of genomes without TilS$_1$
Nº of genomes with TilS$_2$
With TilS$_2$
Without TilS$_2$
Nº of genomes without TilS$_2$
Nº of genomes with TilS$_3$
With TilS$_3$
Without TilS$_3$
Nº of genomes without TilS$_3$
p < 0.00005
Nº of genomes with TilS$_4$
With TilS$_4$
Without TilS$_4$
Nº of genomes without TilS$_4$
Nº of genomes with TilS$_5$
With TilS$_5$
Without TilS$_5$
Nº of genomes without TilS$_5$
Nº of genomes with TilS$_6$
With TilS$_6$
Without TilS$_6$
Nº of genomes without TilS$_6$
Nº of genomes with TilS$_7$
With TilS$_7$
Without TilS$_7$
Nº of genomes without TilS$_7$
Nº of genomes with TilS$_8$
With TilS$_8$
Without TilS$_8$
Nº of genomes without TilS$_8$
Nº of genomes with TilS$_9$
With TilS$_9$
Without TilS$_9$
Nº of genomes without TilS$_9$
Nº of genomes with TilS$_{10}$
With TilS$_{10}$
Without TilS$_{10}$
Nº of genomes without TilS$_{10}$
Nº of genomes with TilS$_{11}$
With TilS$_{11}$
Without TilS$_{11}$
Nº of genomes without TilS$_{11}$
Nº of genomes with TilS$_{12}$
With TilS$_{12}$
Without TilS$_{12}$
Nº of genomes without TilS$_{12}$
Nº of genomes with TilS$_{13}$
With TilS$_{13}$
Without TilS$_{13}$
Nº of genomes without TilS$_{13}$
Nº of genomes with TilS$_{14}$
With TilS$_{14}$
Without TilS$_{14}$
Nº of genomes without TilS$_{14}$
Nº of genomes with TilS$_{15}$
With TilS$_{15}$
Without TilS$_{15}$
Nº of genomes without TilS$_{15}$

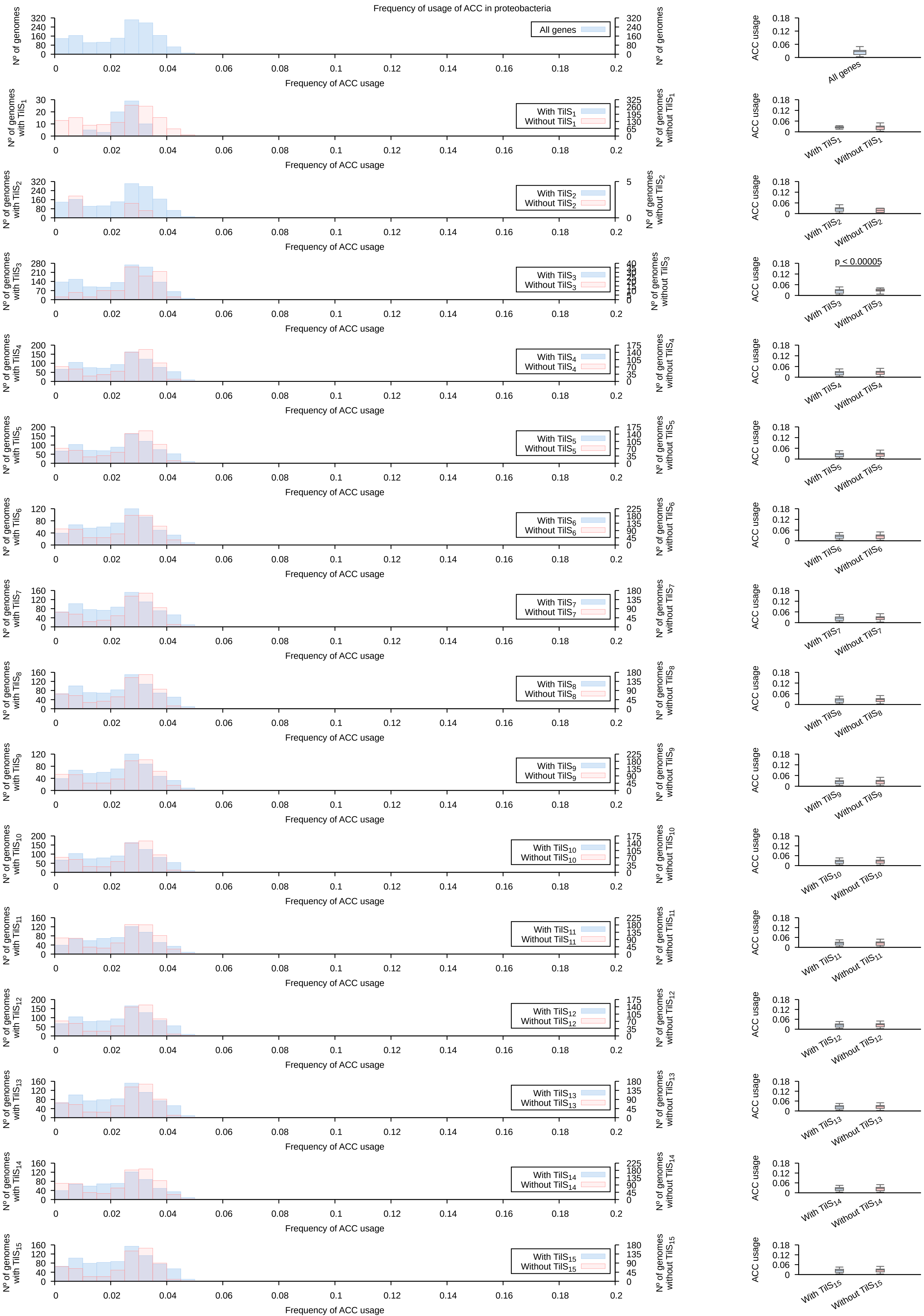

Frequency of usage of ACC in proteobacteria
Nº of genomes
All genes
Frequency of ACC usage
ACC usage
Nº of genomes with TilS$_1$
With TilS$_1$
Without TilS$_1$
Nº of genomes without TilS$_1$
Nº of genomes with TilS$_2$
With TilS$_2$
Without TilS$_2$
Nº of genomes without TilS$_2$
Nº of genomes with TilS$_3$
With TilS$_3$
Without TilS$_3$
Nº of genomes without TilS$_3$
p < 0.00005
Nº of genomes with TilS$_4$
With TilS$_4$
Without TilS$_4$
Nº of genomes without TilS$_4$
Nº of genomes with TilS$_5$
With TilS$_5$
Without TilS$_5$
Nº of genomes without TilS$_5$
Nº of genomes with TilS$_6$
With TilS$_6$
Without TilS$_6$
Nº of genomes without TilS$_6$
Nº of genomes with TilS$_7$
With TilS$_7$
Without TilS$_7$
Nº of genomes without TilS$_7$
Nº of genomes with TilS$_8$
With TilS$_8$
Without TilS$_8$
Nº of genomes without TilS$_8$
Nº of genomes with TilS$_9$
With TilS$_9$
Without TilS$_9$
Nº of genomes without TilS$_9$
Nº of genomes with TilS$_{10}$
With TilS$_{10}$
Without TilS$_{10}$
Nº of genomes without TilS$_{10}$
Nº of genomes with TilS$_{11}$
With TilS$_{11}$
Without TilS$_{11}$
Nº of genomes without TilS$_{11}$
Nº of genomes with TilS$_{12}$
With TilS$_{12}$
Without TilS$_{12}$
Nº of genomes without TilS$_{12}$
Nº of genomes with TilS$_{13}$
With TilS$_{13}$
Without TilS$_{13}$
Nº of genomes without TilS$_{13}$
Nº of genomes with TilS$_{14}$
With TilS$_{14}$
Without TilS$_{14}$
Nº of genomes without TilS$_{14}$
Nº of genomes with TilS$_{15}$
With TilS$_{15}$
Without TilS$_{15}$
Nº of genomes without TilS$_{15}$

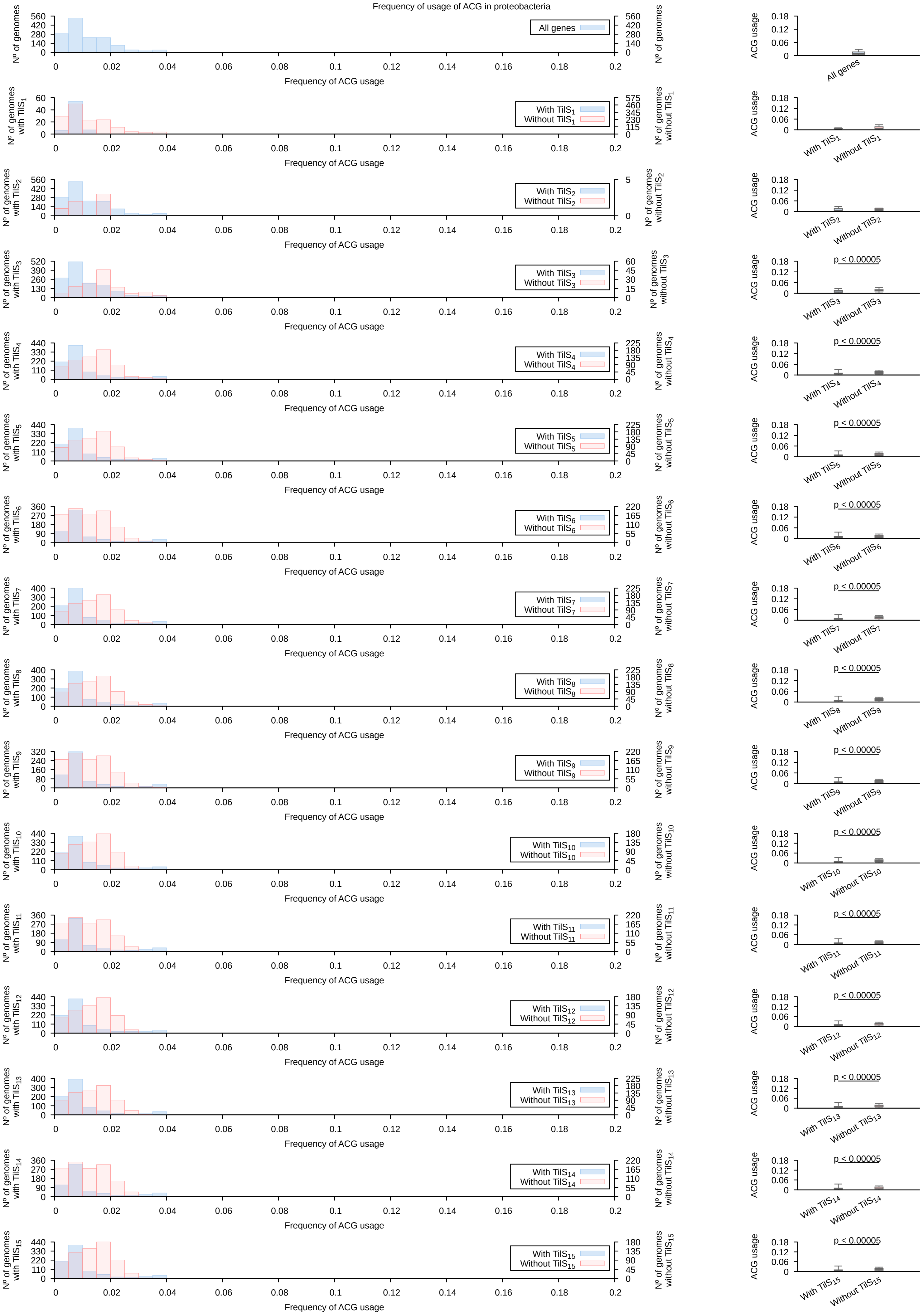

Frequency of usage of ACG in proteobacteria
Nº of genomes
Frequency of ACG usage
All genes
ACG usage
0 0.02 0.04 0.06 0.08 0.1 0.12 0.14 0.16 0.18 0.2
0 0.06 0.12 0.18
Nº of genomes with TilS1
Nº of genomes without TilS1
With TilS1
Without TilS1
Nº of genomes with TilS2
Nº of genomes without TilS2
With TilS2
Without TilS2
Nº of genomes with TilS3
Nº of genomes without TilS3
With TilS3
Without TilS3
p < 0.00005
Nº of genomes with TilS4
Nº of genomes without TilS4
With TilS4
Without TilS4
p < 0.00005
Nº of genomes with TilS5
Nº of genomes without TilS5
With TilS5
Without TilS5
p < 0.00005
Nº of genomes with TilS6
Nº of genomes without TilS6
With TilS6
Without TilS6
p < 0.00005
Nº of genomes with TilS7
Nº of genomes without TilS7
With TilS7
Without TilS7
p < 0.00005
Nº of genomes with TilS8
Nº of genomes without TilS8
With TilS8
Without TilS8
p < 0.00005
Nº of genomes with TilS9
Nº of genomes without TilS9
With TilS9
Without TilS9
p < 0.00005
Nº of genomes with TilS10
Nº of genomes without TilS10
With TilS10
Without TilS10
p < 0.00005
Nº of genomes with TilS11
Nº of genomes without TilS11
With TilS11
Without TilS11
p < 0.00005
Nº of genomes with TilS12
Nº of genomes without TilS12
With TilS12
Without TilS12
p < 0.00005
Nº of genomes with TilS13
Nº of genomes without TilS13
With TilS13
Without TilS13
p < 0.00005
Nº of genomes with TilS14
Nº of genomes without TilS14
With TilS14
Without TilS14
p < 0.00005
Nº of genomes with TilS15
Nº of genomes without TilS15
With TilS15
Without TilS15
p < 0.00005

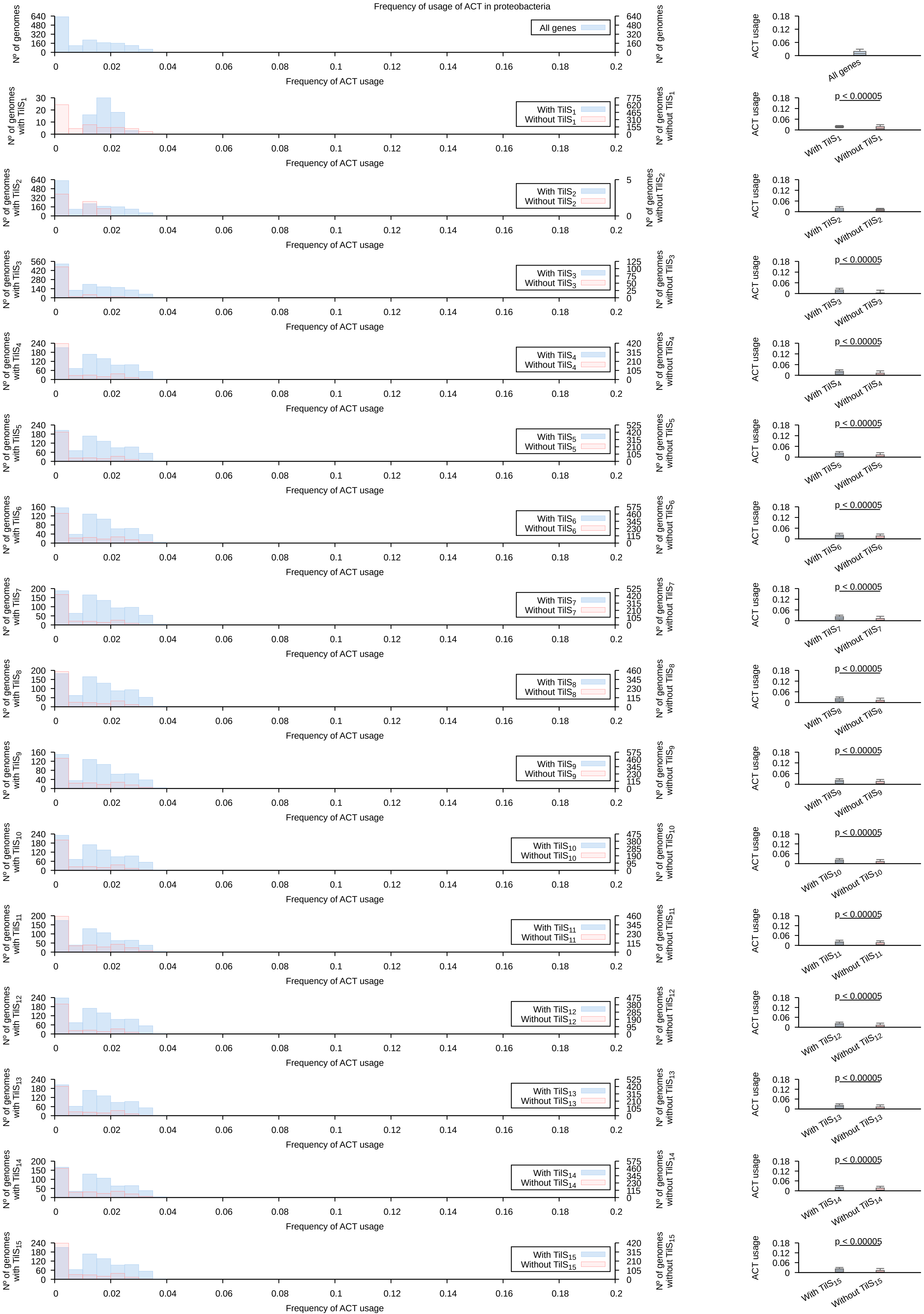
Frequency of usage of ACT in proteobacteria
Nº of genomes
All genes
Frequency of ACT usage
ACT usage
Nº of genomes with TilS$_1$
Nº of genomes without TilS$_1$
With TilS$_1$
Without TilS$_1$
p < 0.00005
Nº of genomes with TilS$_2$
Nº of genomes without TilS$_2$
With TilS$_2$
Without TilS$_2$
Nº of genomes with TilS$_3$
Nº of genomes without TilS$_3$
With TilS$_3$
Without TilS$_3$
p < 0.00005
Nº of genomes with TilS$_4$
Nº of genomes without TilS$_4$
With TilS$_4$
Without TilS$_4$
p < 0.00005
Nº of genomes with TilS$_5$
Nº of genomes without TilS$_5$
With TilS$_5$
Without TilS$_5$
p < 0.00005
Nº of genomes with TilS$_6$
Nº of genomes without TilS$_6$
With TilS$_6$
Without TilS$_6$
p < 0.00005
Nº of genomes with TilS$_7$
Nº of genomes without TilS$_7$
With TilS$_7$
Without TilS$_7$
p < 0.00005
Nº of genomes with TilS$_8$
Nº of genomes without TilS$_8$
With TilS$_8$
Without TilS$_8$
p < 0.00005
Nº of genomes with TilS$_9$
Nº of genomes without TilS$_9$
With TilS$_9$
Without TilS$_9$
p < 0.00005
Nº of genomes with TilS$_{10}$
Nº of genomes without TilS$_{10}$
With TilS$_{10}$
Without TilS$_{10}$
p < 0.00005
Nº of genomes with TilS$_{11}$
Nº of genomes without TilS$_{11}$
With TilS$_{11}$
Without TilS$_{11}$
p < 0.00005
Nº of genomes with TilS$_{12}$
Nº of genomes without TilS$_{12}$
With TilS$_{12}$
Without TilS$_{12}$
p < 0.00005
Nº of genomes with TilS$_{13}$
Nº of genomes without TilS$_{13}$
With TilS$_{13}$
Without TilS$_{13}$
p < 0.00005
Nº of genomes with TilS$_{14}$
Nº of genomes without TilS$_{14}$
With TilS$_{14}$
Without TilS$_{14}$
p < 0.00005
Nº of genomes with TilS$_{15}$
Nº of genomes without TilS$_{15}$
With TilS$_{15}$
Without TilS$_{15}$
p < 0.00005

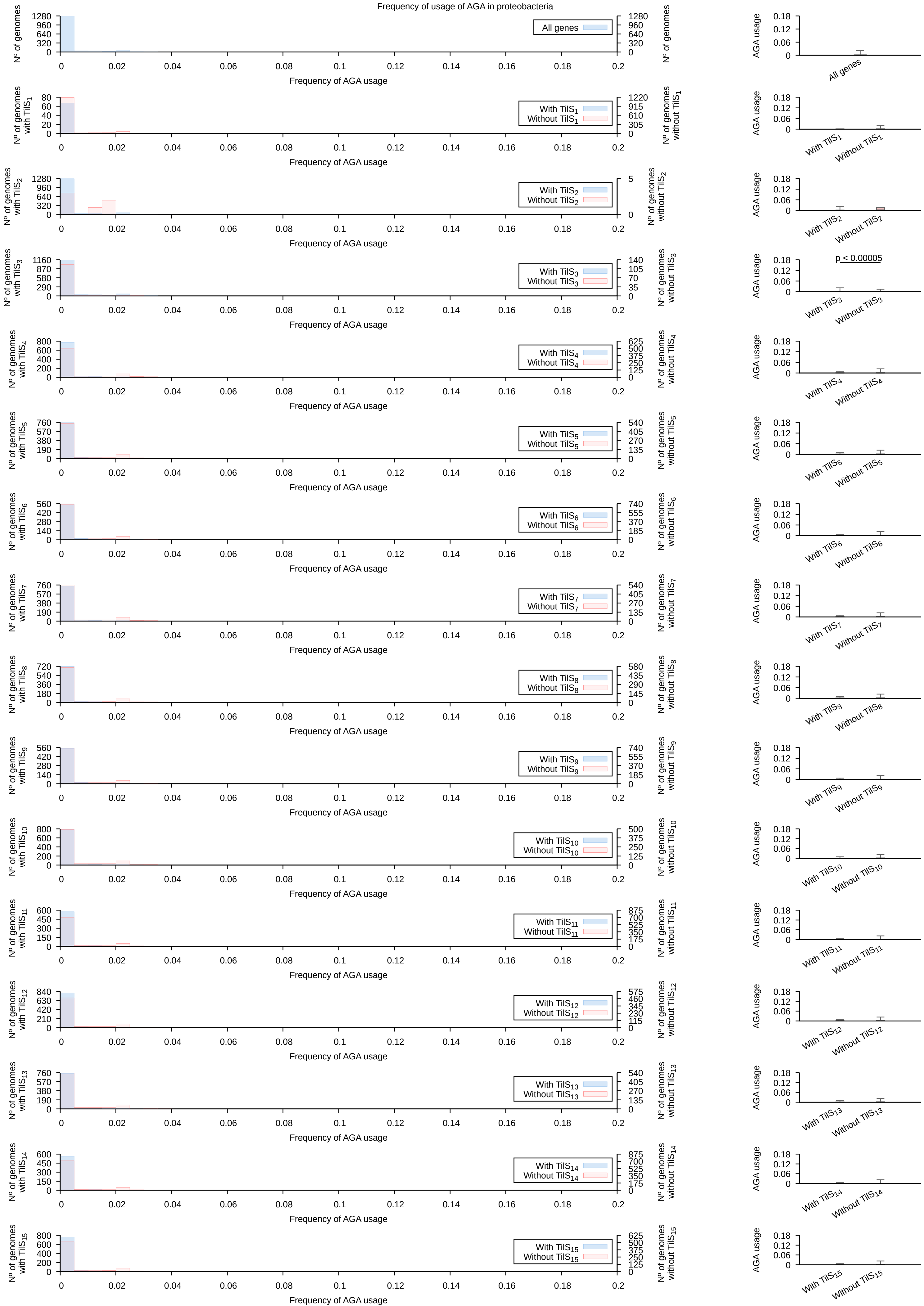
Frequency of usage of AGA in proteobacteria
Nº of genomes
All genes
Frequency of AGA usage
AGA usage
Nº of genomes with TilS1
Nº of genomes without TilS1
With TilS1
Without TilS1
Nº of genomes with TilS2
Nº of genomes without TilS2
With TilS2
Without TilS2
Nº of genomes with TilS3
Nº of genomes without TilS3
With TilS3
Without TilS3
p < 0.00005
Nº of genomes with TilS4
Nº of genomes without TilS4
With TilS4
Without TilS4
Nº of genomes with TilS5
Nº of genomes without TilS5
With TilS5
Without TilS5
Nº of genomes with TilS6
Nº of genomes without TilS6
With TilS6
Without TilS6
Nº of genomes with TilS7
Nº of genomes without TilS7
With TilS7
Without TilS7
Nº of genomes with TilS8
Nº of genomes without TilS8
With TilS8
Without TilS8
Nº of genomes with TilS9
Nº of genomes without TilS9
With TilS9
Without TilS9
Nº of genomes with TilS10
Nº of genomes without TilS10
With TilS10
Without TilS10
Nº of genomes with TilS11
Nº of genomes without TilS11
With TilS11
Without TilS11
Nº of genomes with TilS12
Nº of genomes without TilS12
With TilS12
Without TilS12
Nº of genomes with TilS13
Nº of genomes without TilS13
With TilS13
Without TilS13
Nº of genomes with TilS14
Nº of genomes without TilS14
With TilS14
Without TilS14
Nº of genomes with TilS15
Nº of genomes without TilS15
With TilS15
Without TilS15

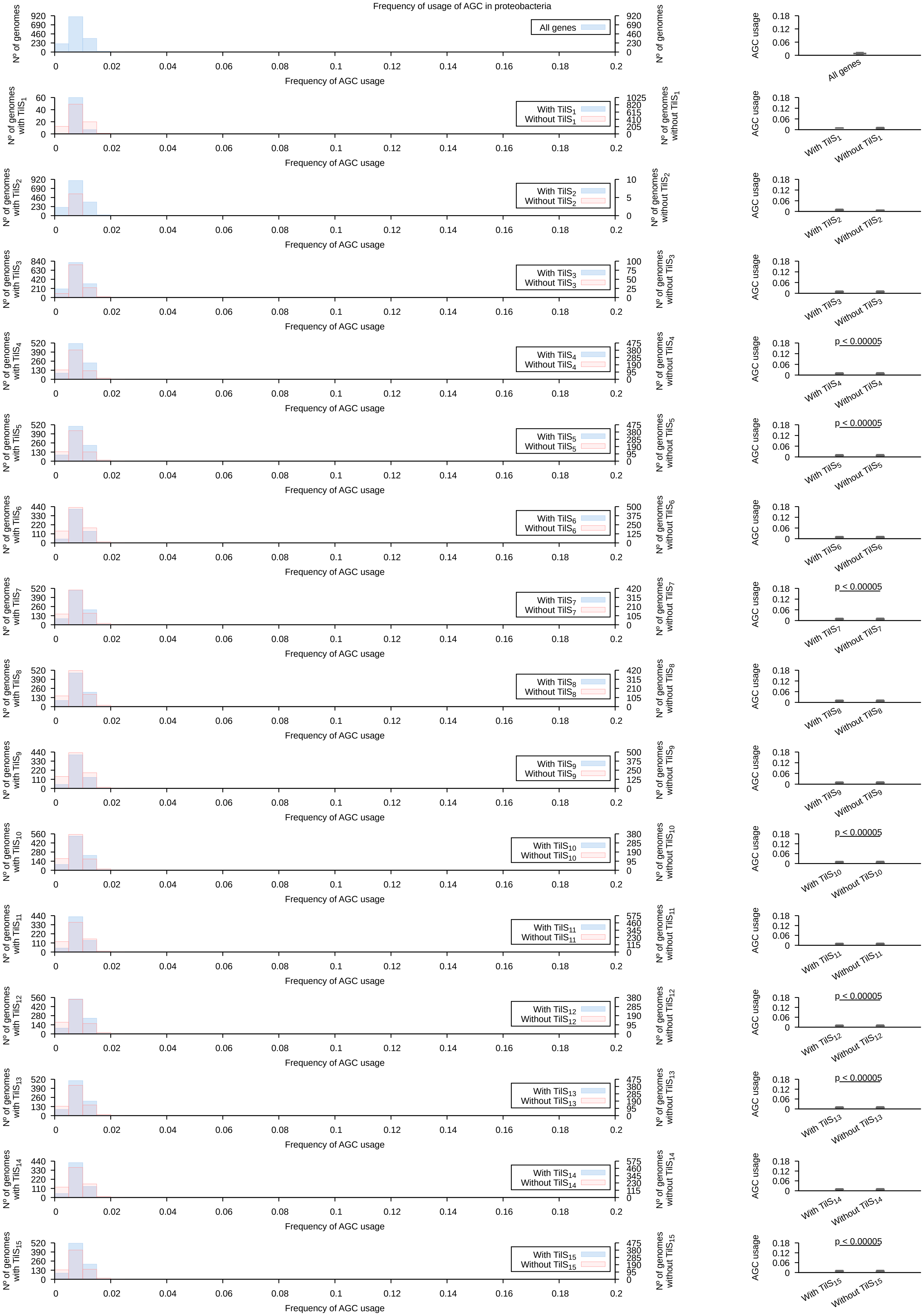
Frequency of usage of AGC in proteobacteria
Nº of genomes
All genes
Frequency of AGC usage
AGC usage
All genes
Nº of genomes with TilS$_1$
Nº of genomes without TilS$_1$
With TilS$_1$
Without TilS$_1$
Nº of genomes with TilS$_2$
Nº of genomes without TilS$_2$
With TilS$_2$
Without TilS$_2$
Nº of genomes with TilS$_3$
Nº of genomes without TilS$_3$
With TilS$_3$
Without TilS$_3$
Nº of genomes with TilS$_4$
Nº of genomes without TilS$_4$
With TilS$_4$
Without TilS$_4$
p < 0.00005
Nº of genomes with TilS$_5$
Nº of genomes without TilS$_5$
With TilS$_5$
Without TilS$_5$
p < 0.00005
Nº of genomes with TilS$_6$
Nº of genomes without TilS$_6$
With TilS$_6$
Without TilS$_6$
Nº of genomes with TilS$_7$
Nº of genomes without TilS$_7$
With TilS$_7$
Without TilS$_7$
p < 0.00005
Nº of genomes with TilS$_8$
Nº of genomes without TilS$_8$
With TilS$_8$
Without TilS$_8$
Nº of genomes with TilS$_9$
Nº of genomes without TilS$_9$
With TilS$_9$
Without TilS$_9$
Nº of genomes with TilS$_{10}$
Nº of genomes without TilS$_{10}$
With TilS$_{10}$
Without TilS$_{10}$
p < 0.00005
Nº of genomes with TilS$_{11}$
Nº of genomes without TilS$_{11}$
With TilS$_{11}$
Without TilS$_{11}$
Nº of genomes with TilS$_{12}$
Nº of genomes without TilS$_{12}$
With TilS$_{12}$
Without TilS$_{12}$
p < 0.00005
Nº of genomes with TilS$_{13}$
Nº of genomes without TilS$_{13}$
With TilS$_{13}$
Without TilS$_{13}$
p < 0.00005
Nº of genomes with TilS$_{14}$
Nº of genomes without TilS$_{14}$
With TilS$_{14}$
Without TilS$_{14}$
Nº of genomes with TilS$_{15}$
Nº of genomes without TilS$_{15}$
With TilS$_{15}$
Without TilS$_{15}$
p < 0.00005

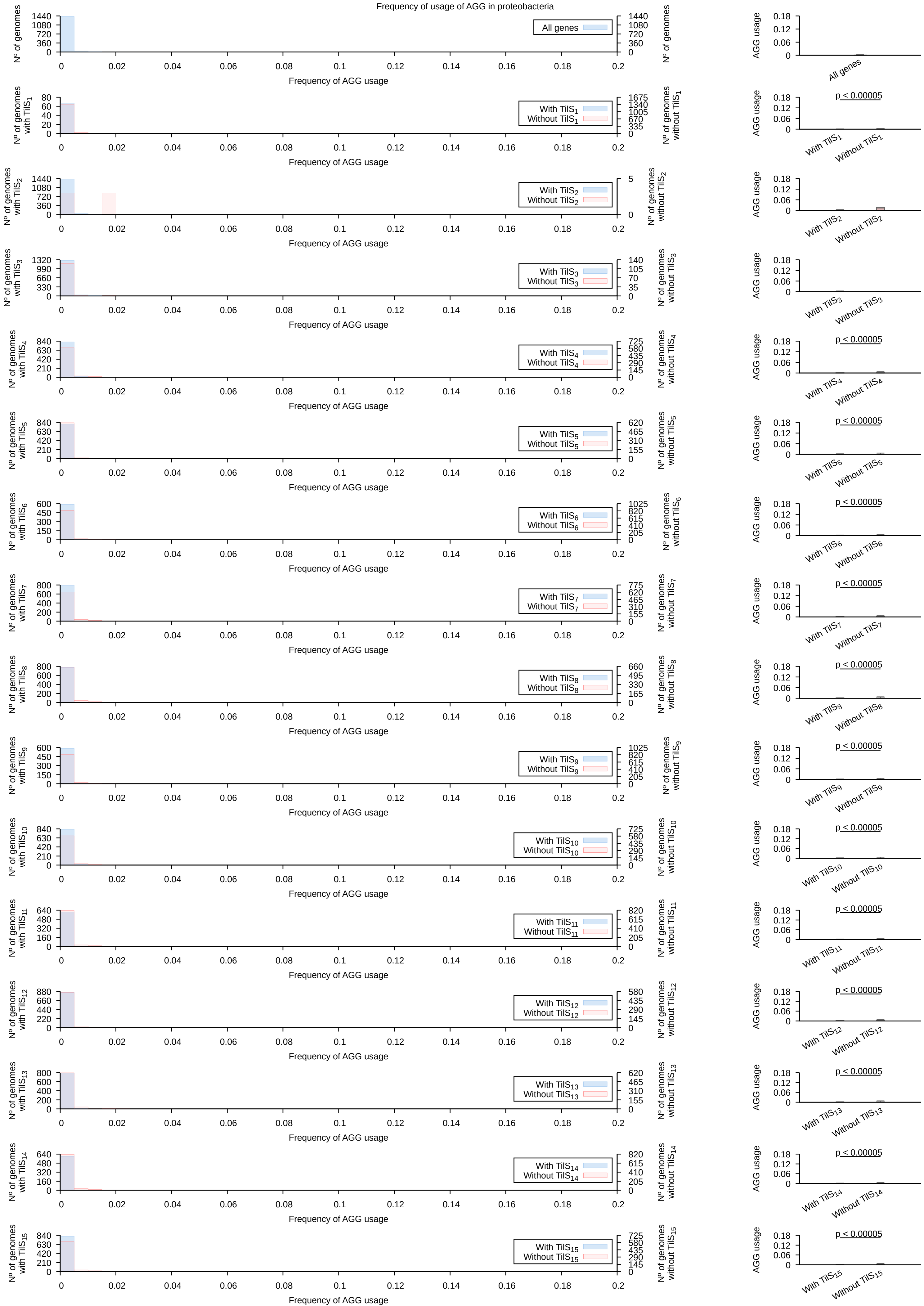

Frequency of usage of AGG in proteobacteria
Nº of genomes
All genes
Frequency of AGG usage
AGG usage
All genes
Nº of genomes with $TilS_1$
Nº of genomes without $TilS_1$
With $TilS_1$
Without $TilS_1$
$p < 0.00005$
Nº of genomes with $TilS_2$
Nº of genomes without $TilS_2$
With $TilS_2$
Without $TilS_2$
Nº of genomes with $TilS_3$
Nº of genomes without $TilS_3$
With $TilS_3$
Without $TilS_3$
Nº of genomes with $TilS_4$
Nº of genomes without $TilS_4$
With $TilS_4$
Without $TilS_4$
$p < 0.00005$
Nº of genomes with $TilS_5$
Nº of genomes without $TilS_5$
With $TilS_5$
Without $TilS_5$
$p < 0.00005$
Nº of genomes with $TilS_6$
Nº of genomes without $TilS_6$
With $TilS_6$
Without $TilS_6$
$p < 0.00005$
Nº of genomes with $TilS_7$
Nº of genomes without $TilS_7$
With $TilS_7$
Without $TilS_7$
$p < 0.00005$
Nº of genomes with $TilS_8$
Nº of genomes without $TilS_8$
With $TilS_8$
Without $TilS_8$
$p < 0.00005$
Nº of genomes with $TilS_9$
Nº of genomes without $TilS_9$
With $TilS_9$
Without $TilS_9$
$p < 0.00005$
Nº of genomes with $TilS_{10}$
Nº of genomes without $TilS_{10}$
With $TilS_{10}$
Without $TilS_{10}$
$p < 0.00005$
Nº of genomes with $TilS_{11}$
Nº of genomes without $TilS_{11}$
With $TilS_{11}$
Without $TilS_{11}$
$p < 0.00005$
Nº of genomes with $TilS_{12}$
Nº of genomes without $TilS_{12}$
With $TilS_{12}$
Without $TilS_{12}$
$p < 0.00005$
Nº of genomes with $TilS_{13}$
Nº of genomes without $TilS_{13}$
With $TilS_{13}$
Without $TilS_{13}$
$p < 0.00005$
Nº of genomes with $TilS_{14}$
Nº of genomes without $TilS_{14}$
With $TilS_{14}$
Without $TilS_{14}$
$p < 0.00005$
Nº of genomes with $TilS_{15}$
Nº of genomes without $TilS_{15}$
With $TilS_{15}$
Without $TilS_{15}$
$p < 0.00005$

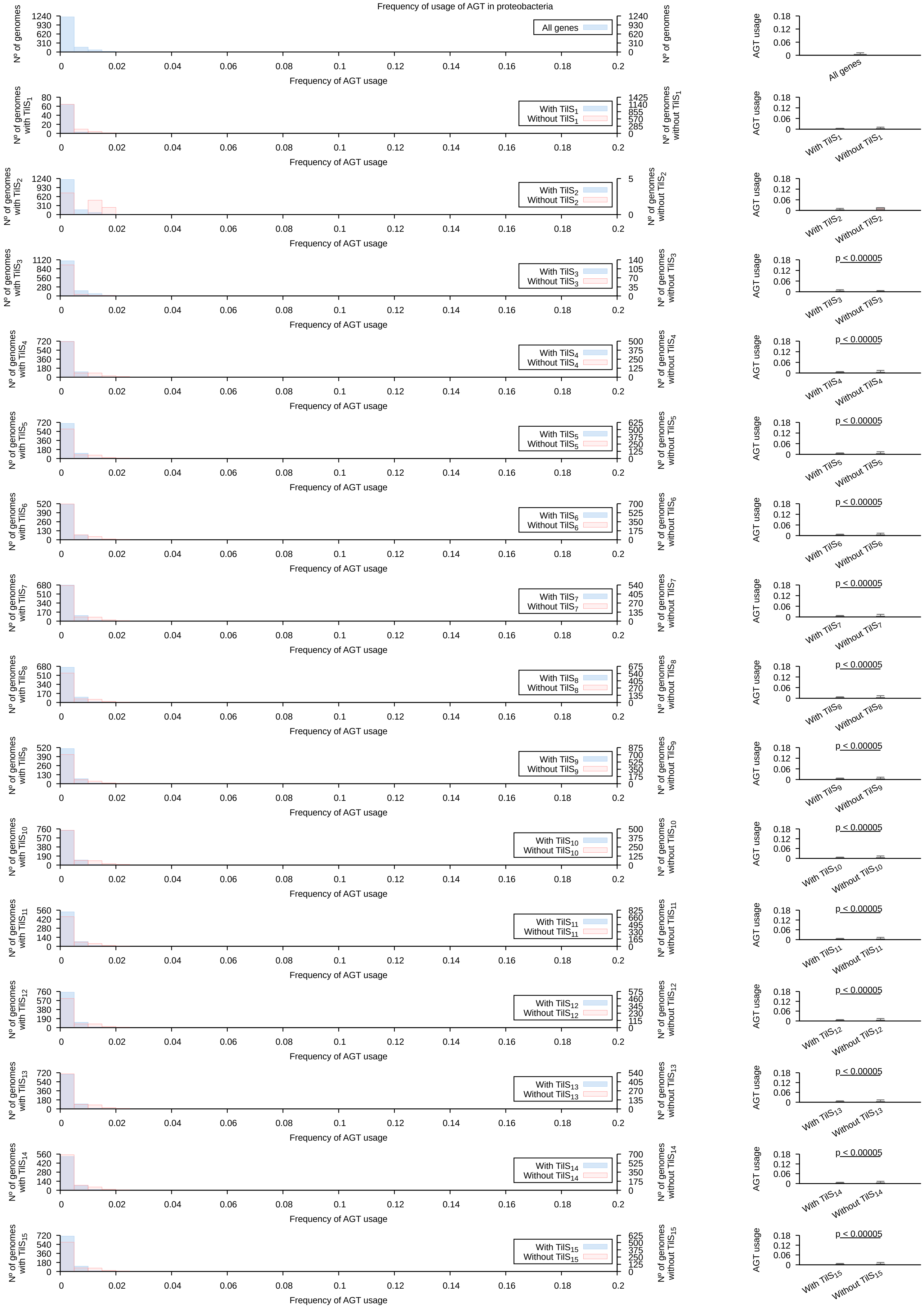

Frequency of usage of AGT in proteobacteria
Nº of genomes
All genes
Frequency of AGT usage
AGT usage
Nº of genomes with TilS1
Nº of genomes without TilS1
With TilS1
Without TilS1
Nº of genomes with TilS2
Nº of genomes without TilS2
With TilS2
Without TilS2
Nº of genomes with TilS3
Nº of genomes without TilS3
With TilS3
Without TilS3
Nº of genomes with TilS4
Nº of genomes without TilS4
With TilS4
Without TilS4
Nº of genomes with TilS5
Nº of genomes without TilS5
With TilS5
Without TilS5
Nº of genomes with TilS6
Nº of genomes without TilS6
With TilS6
Without TilS6
Nº of genomes with TilS7
Nº of genomes without TilS7
With TilS7
Without TilS7
Nº of genomes with TilS8
Nº of genomes without TilS8
With TilS8
Without TilS8
Nº of genomes with TilS9
Nº of genomes without TilS9
With TilS9
Without TilS9
Nº of genomes with TilS10
Nº of genomes without TilS10
With TilS10
Without TilS10
Nº of genomes with TilS11
Nº of genomes without TilS11
With TilS11
Without TilS11
Nº of genomes with TilS12
Nº of genomes without TilS12
With TilS12
Without TilS12
Nº of genomes with TilS13
Nº of genomes without TilS13
With TilS13
Without TilS13
Nº of genomes with TilS14
Nº of genomes without TilS14
With TilS14
Without TilS14
Nº of genomes with TilS15
Nº of genomes without TilS15
With TilS15
Without TilS15
p < 0.00005
0 0.02 0.04 0.06 0.08 0.1 0.12 0.14 0.16 0.18 0.2
0 0.06 0.12 0.18

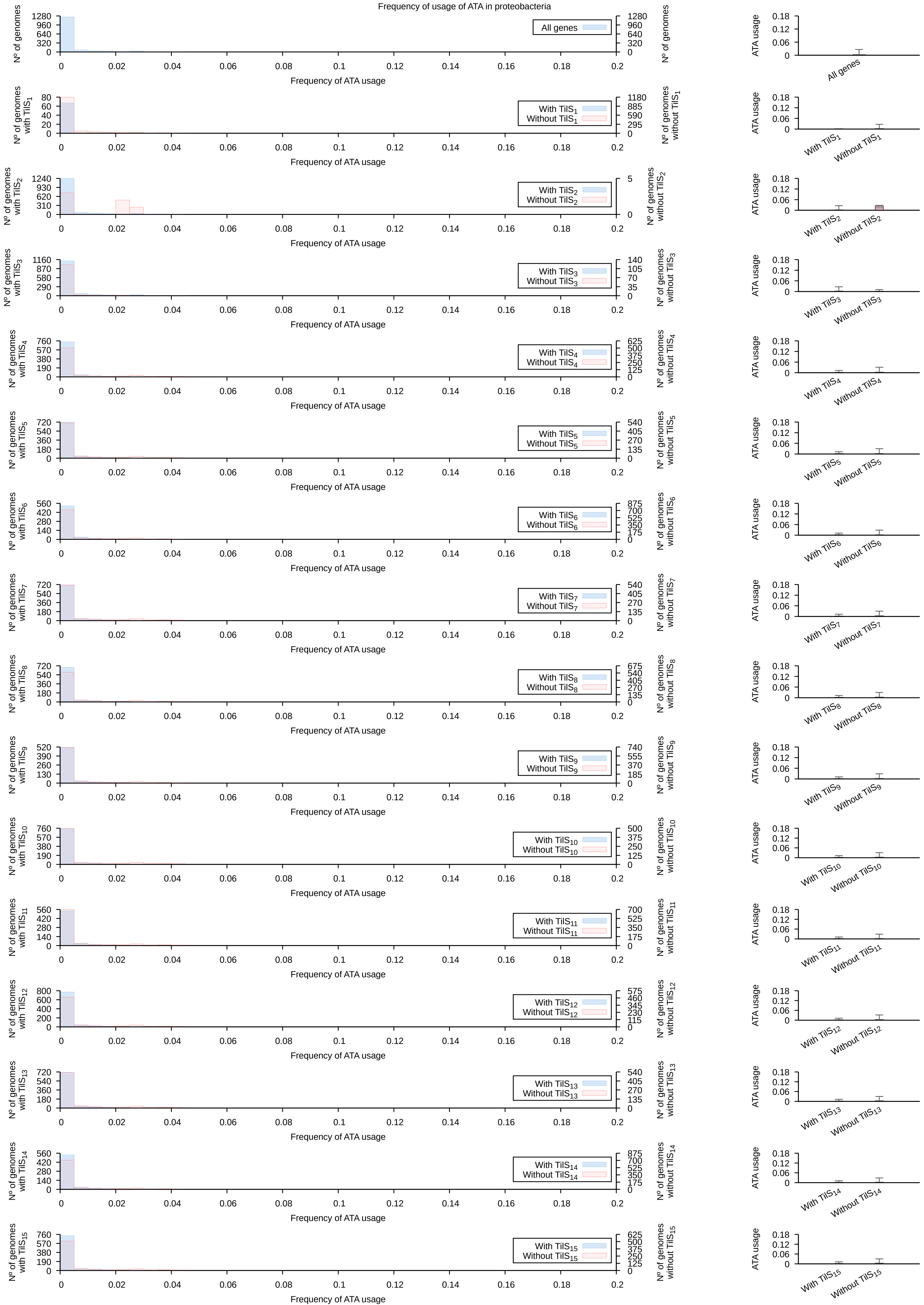

Frequency of usage of ATA in proteobacteria
Nº of genomes
All genes
Frequency of ATA usage
ATA usage
Nº of genomes with TilS1
Nº of genomes without TilS1
With TilS1
Without TilS1
With TilS2
Without TilS2
With TilS3
Without TilS3
With TilS4
Without TilS4
With TilS5
Without TilS5
With TilS6
Without TilS6
With TilS7
Without TilS7
With TilS8
Without TilS8
With TilS9
Without TilS9
With TilS10
Without TilS10
With TilS11
Without TilS11
With TilS12
Without TilS12
With TilS13
Without TilS13
With TilS14
Without TilS14
With TilS15
Without TilS15

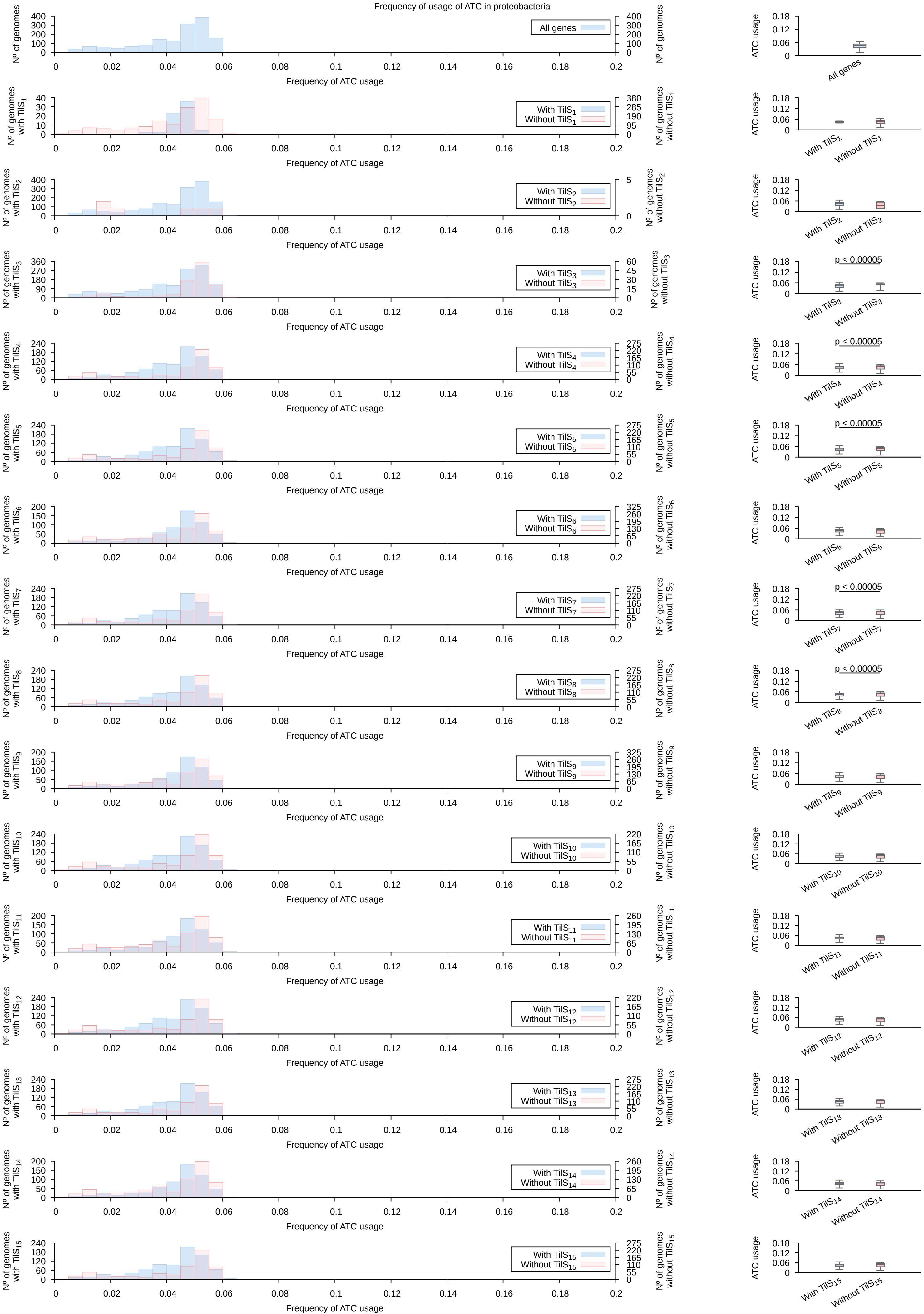

Frequency of usage of ATC in proteobacteria
Nº of genomes
All genes
Frequency of ATC usage
ATC usage
Nº of genomes with TilS1
Nº of genomes without TilS1
With TilS1
Without TilS1
Nº of genomes with TilS2
Nº of genomes without TilS2
With TilS2
Without TilS2
Nº of genomes with TilS3
Nº of genomes without TilS3
With TilS3
Without TilS3
p < 0.00005
Nº of genomes with TilS4
Nº of genomes without TilS4
With TilS4
Without TilS4
p < 0.00005
Nº of genomes with TilS5
Nº of genomes without TilS5
With TilS5
Without TilS5
p < 0.00005
Nº of genomes with TilS6
Nº of genomes without TilS6
With TilS6
Without TilS6
Nº of genomes with TilS7
Nº of genomes without TilS7
With TilS7
Without TilS7
p < 0.00005
Nº of genomes with TilS8
Nº of genomes without TilS8
With TilS8
Without TilS8
p < 0.00005
Nº of genomes with TilS9
Nº of genomes without TilS9
With TilS9
Without TilS9
Nº of genomes with TilS10
Nº of genomes without TilS10
With TilS10
Without TilS10
Nº of genomes with TilS11
Nº of genomes without TilS11
With TilS11
Without TilS11
Nº of genomes with TilS12
Nº of genomes without TilS12
With TilS12
Without TilS12
Nº of genomes with TilS13
Nº of genomes without TilS13
With TilS13
Without TilS13
Nº of genomes with TilS14
Nº of genomes without TilS14
With TilS14
Without TilS14
Nº of genomes with TilS15
Nº of genomes without TilS15
With TilS15
Without TilS15

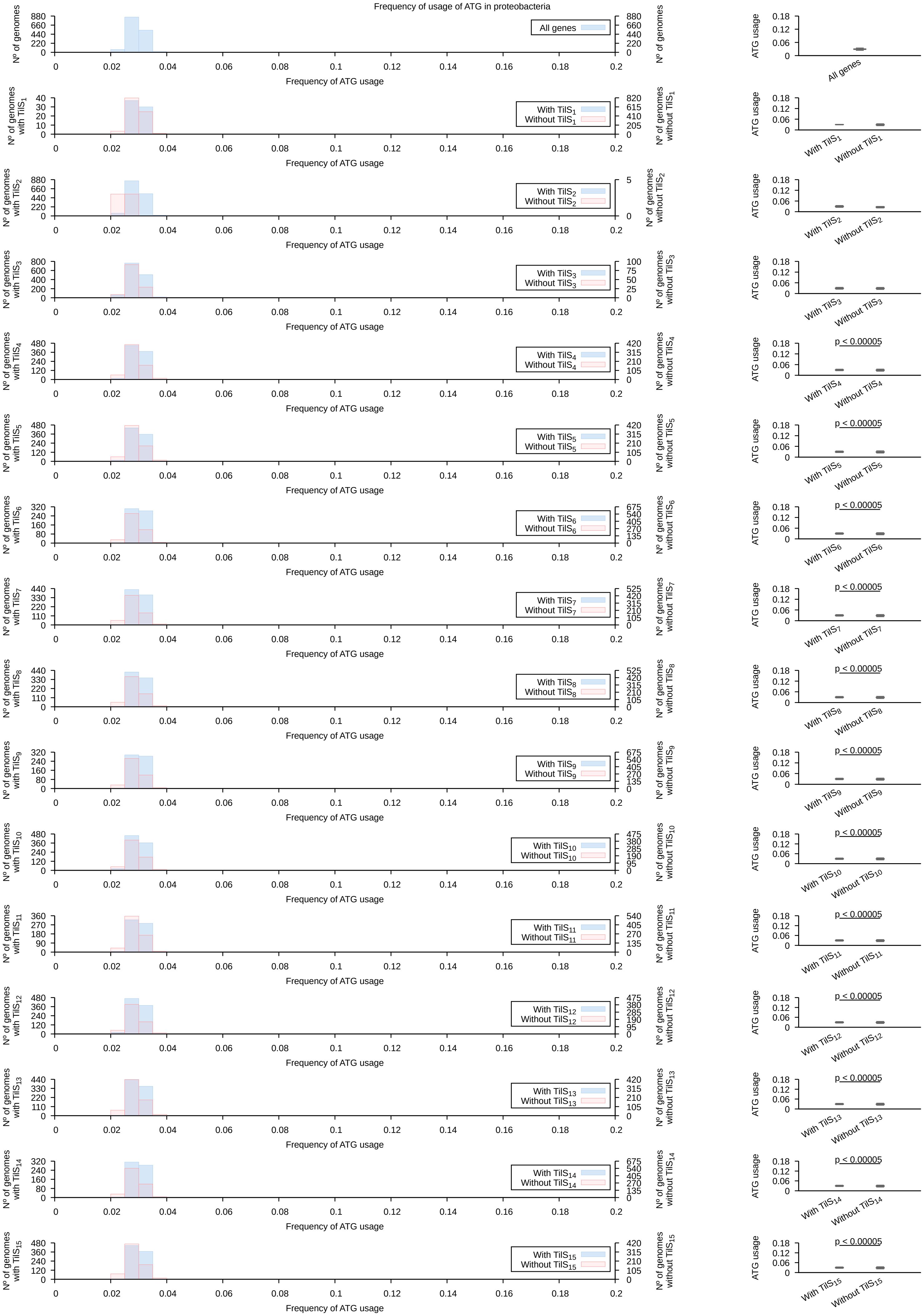

Frequency of usage of ATG in proteobacteria
Nº of genomes
Frequency of ATG usage
All genes
ATG usage
With TilS$_1$
Without TilS$_1$
Nº of genomes with TilS$_1$
Nº of genomes without TilS$_1$
With TilS$_2$
Without TilS$_2$
With TilS$_3$
Without TilS$_3$
With TilS$_4$
Without TilS$_4$
With TilS$_5$
Without TilS$_5$
With TilS$_6$
Without TilS$_6$
With TilS$_7$
Without TilS$_7$
With TilS$_8$
Without TilS$_8$
With TilS$_9$
Without TilS$_9$
With TilS$_{10}$
Without TilS$_{10}$
With TilS$_{11}$
Without TilS$_{11}$
With TilS$_{12}$
Without TilS$_{12}$
With TilS$_{13}$
Without TilS$_{13}$
With TilS$_{14}$
Without TilS$_{14}$
With TilS$_{15}$
Without TilS$_{15}$
$p < 0.00005$

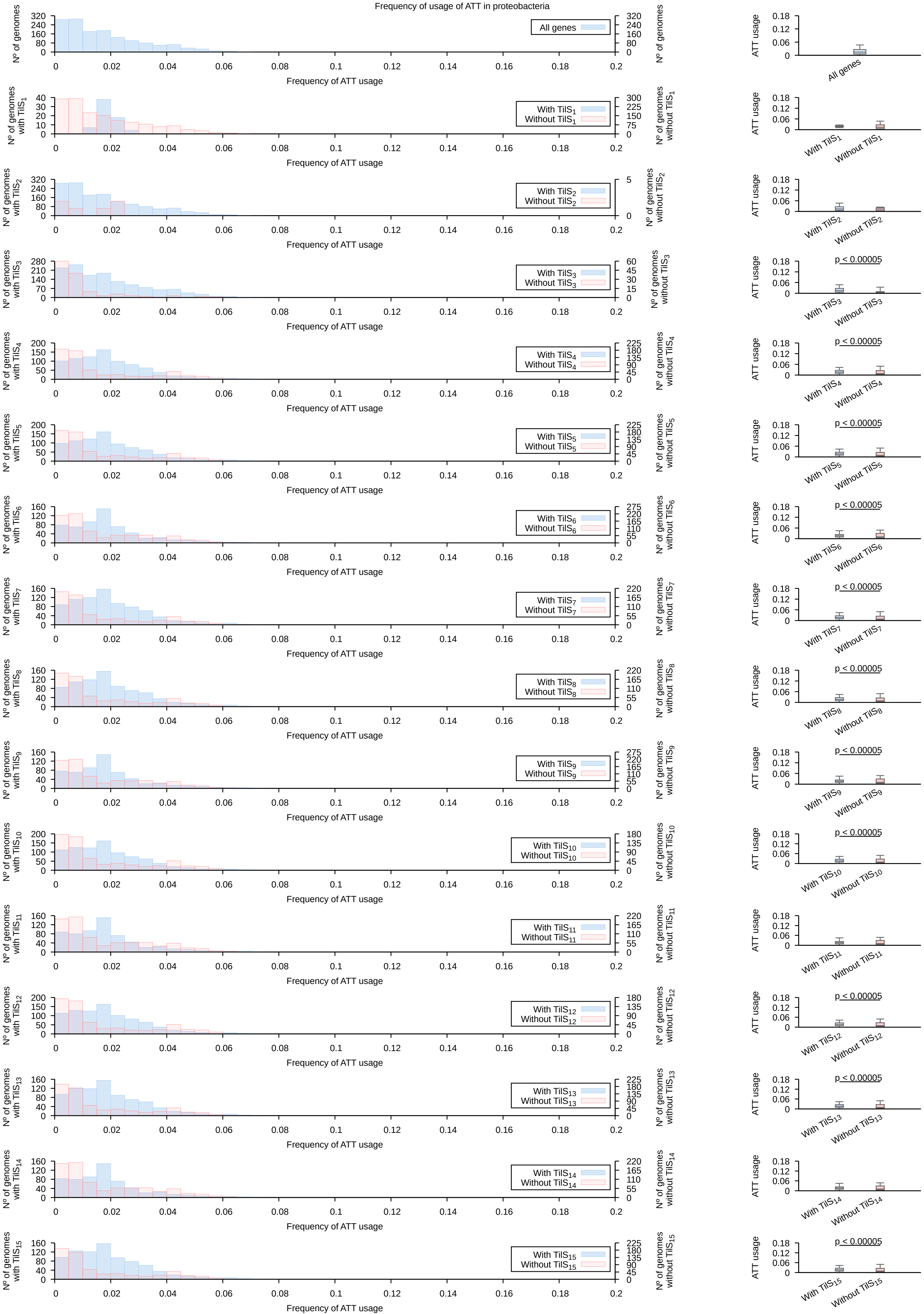
Frequency of usage of ATT in proteobacteria
Nº of genomes
All genes
Frequency of ATT usage
ATT usage
All genes
Nº of genomes with TilS$_1$
With TilS$_1$
Without TilS$_1$
Nº of genomes without TilS$_1$
Nº of genomes with TilS$_2$
With TilS$_2$
Without TilS$_2$
Nº of genomes without TilS$_2$
Nº of genomes with TilS$_3$
With TilS$_3$
Without TilS$_3$
Nº of genomes without TilS$_3$
p < 0.00005
Nº of genomes with TilS$_4$
With TilS$_4$
Without TilS$_4$
Nº of genomes without TilS$_4$
p < 0.00005
Nº of genomes with TilS$_5$
With TilS$_5$
Without TilS$_5$
Nº of genomes without TilS$_5$
p < 0.00005
Nº of genomes with TilS$_6$
With TilS$_6$
Without TilS$_6$
Nº of genomes without TilS$_6$
p < 0.00005
Nº of genomes with TilS$_7$
With TilS$_7$
Without TilS$_7$
Nº of genomes without TilS$_7$
p < 0.00005
Nº of genomes with TilS$_8$
With TilS$_8$
Without TilS$_8$
Nº of genomes without TilS$_8$
p < 0.00005
Nº of genomes with TilS$_9$
With TilS$_9$
Without TilS$_9$
Nº of genomes without TilS$_9$
p < 0.00005
Nº of genomes with TilS$_{10}$
With TilS$_{10}$
Without TilS$_{10}$
Nº of genomes without TilS$_{10}$
p < 0.00005
Nº of genomes with TilS$_{11}$
With TilS$_{11}$
Without TilS$_{11}$
Nº of genomes without TilS$_{11}$
Nº of genomes with TilS$_{12}$
With TilS$_{12}$
Without TilS$_{12}$
Nº of genomes without TilS$_{12}$
p < 0.00005
Nº of genomes with TilS$_{13}$
With TilS$_{13}$
Without TilS$_{13}$
Nº of genomes without TilS$_{13}$
p < 0.00005
Nº of genomes with TilS$_{14}$
With TilS$_{14}$
Without TilS$_{14}$
Nº of genomes without TilS$_{14}$
Nº of genomes with TilS$_{15}$
With TilS$_{15}$
Without TilS$_{15}$
Nº of genomes without TilS$_{15}$
p < 0.00005

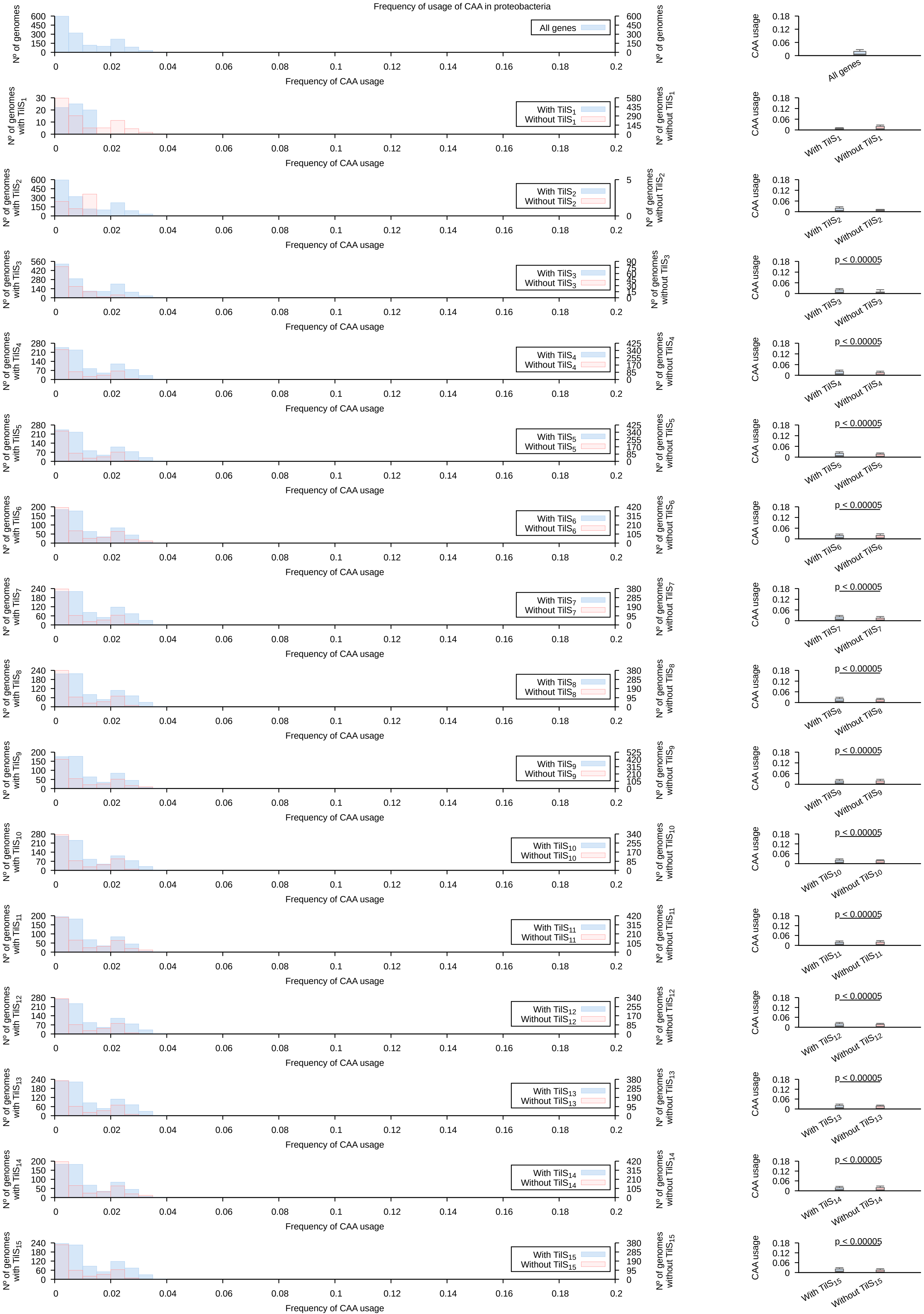
Frequency of usage of CAA in proteobacteria
Nº of genomes
Frequency of CAA usage
CAA usage
All genes
With TilS$_1$
Without TilS$_1$
With TilS$_2$
Without TilS$_2$
With TilS$_3$
Without TilS$_3$
With TilS$_4$
Without TilS$_4$
With TilS$_5$
Without TilS$_5$
With TilS$_6$
Without TilS$_6$
With TilS$_7$
Without TilS$_7$
With TilS$_8$
Without TilS$_8$
With TilS$_9$
Without TilS$_9$
With TilS$_{10}$
Without TilS$_{10}$
With TilS$_{11}$
Without TilS$_{11}$
With TilS$_{12}$
Without TilS$_{12}$
With TilS$_{13}$
Without TilS$_{13}$
With TilS$_{14}$
Without TilS$_{14}$
With TilS$_{15}$
Without TilS$_{15}$
$p < 0.00005$

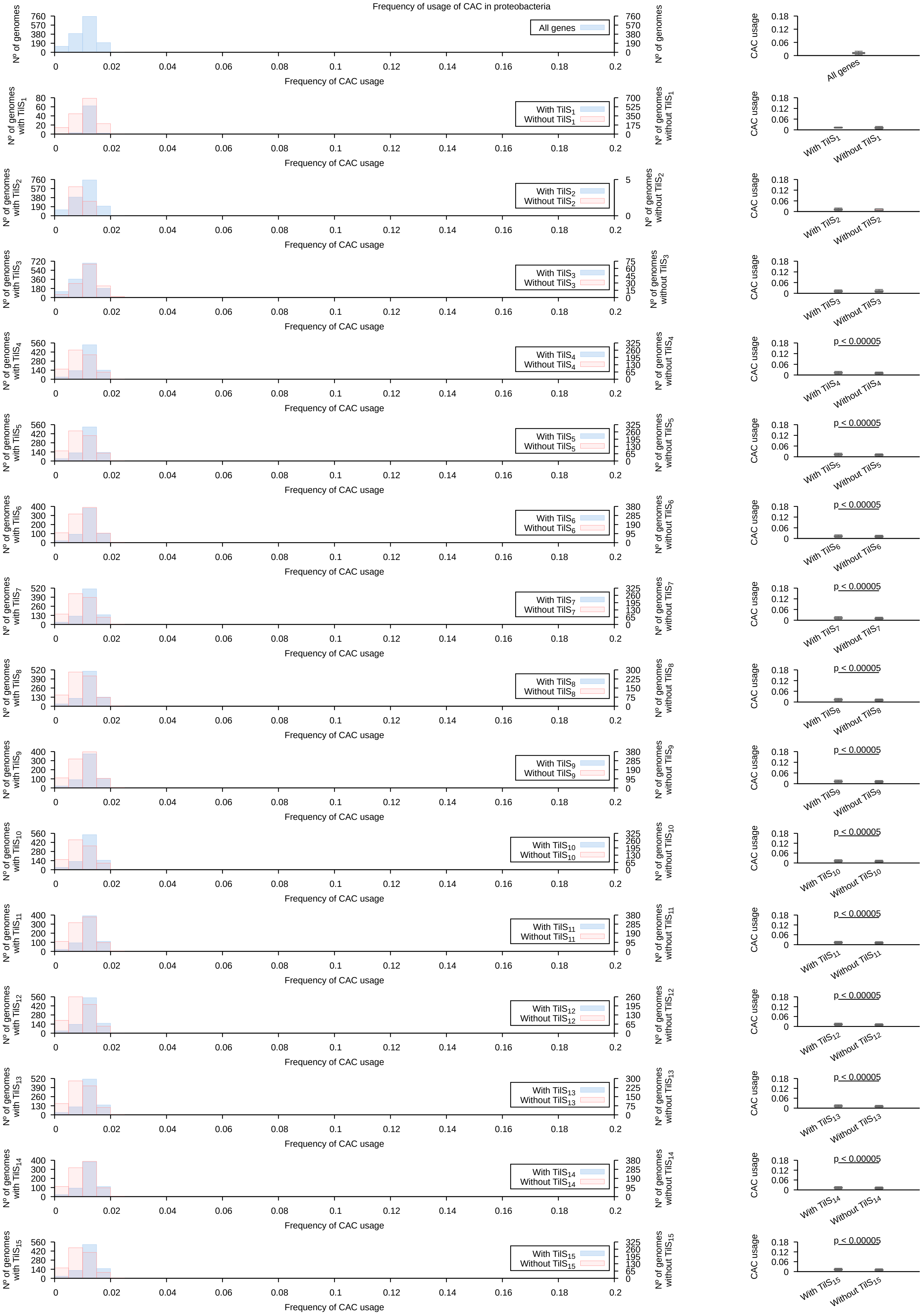

Frequency of usage of CAC in proteobacteria
Nº of genomes
All genes
Frequency of CAC usage
CAC usage
p < 0.00005
Nº of genomes with TilS$_1$
Nº of genomes without TilS$_1$
With TilS$_1$
Without TilS$_1$
With TilS$_2$
Without TilS$_2$
With TilS$_3$
Without TilS$_3$
With TilS$_4$
Without TilS$_4$
With TilS$_5$
Without TilS$_5$
With TilS$_6$
Without TilS$_6$
With TilS$_7$
Without TilS$_7$
With TilS$_8$
Without TilS$_8$
With TilS$_9$
Without TilS$_9$
With TilS$_{10}$
Without TilS$_{10}$
With TilS$_{11}$
Without TilS$_{11}$
With TilS$_{12}$
Without TilS$_{12}$
With TilS$_{13}$
Without TilS$_{13}$
With TilS$_{14}$
Without TilS$_{14}$
With TilS$_{15}$
Without TilS$_{15}$

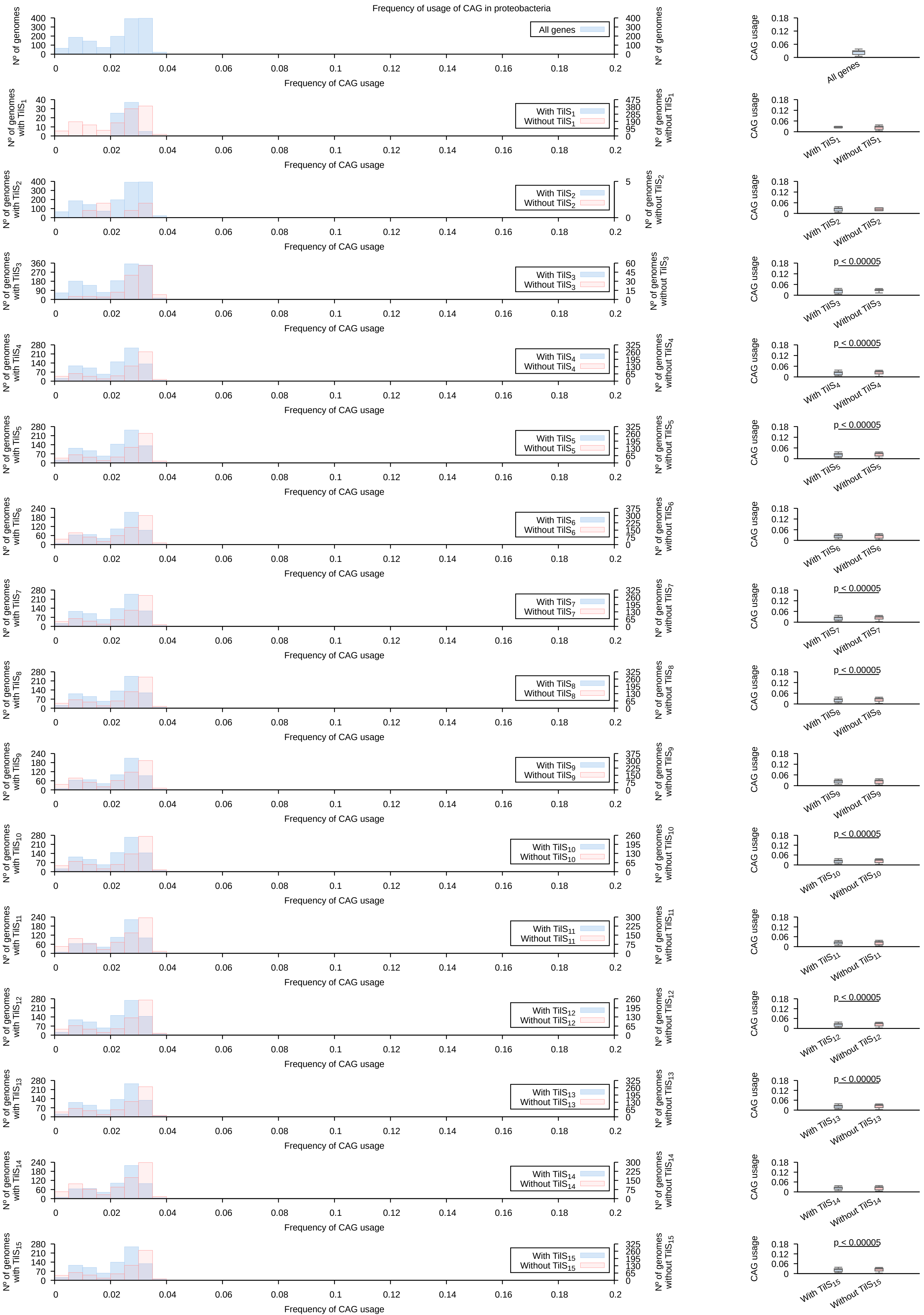
Frequency of usage of CAG in proteobacteria
Nº of genomes
All genes
Frequency of CAG usage
CAG usage
With TilS1
Without TilS1
Nº of genomes with TilS1
Nº of genomes without TilS1
With TilS2
Without TilS2
With TilS3
Without TilS3
p < 0.00005
With TilS4
Without TilS4
With TilS5
Without TilS5
With TilS6
Without TilS6
With TilS7
Without TilS7
With TilS8
Without TilS8
With TilS9
Without TilS9
With TilS10
Without TilS10
With TilS11
Without TilS11
With TilS12
Without TilS12
With TilS13
Without TilS13
With TilS14
Without TilS14
With TilS15
Without TilS15

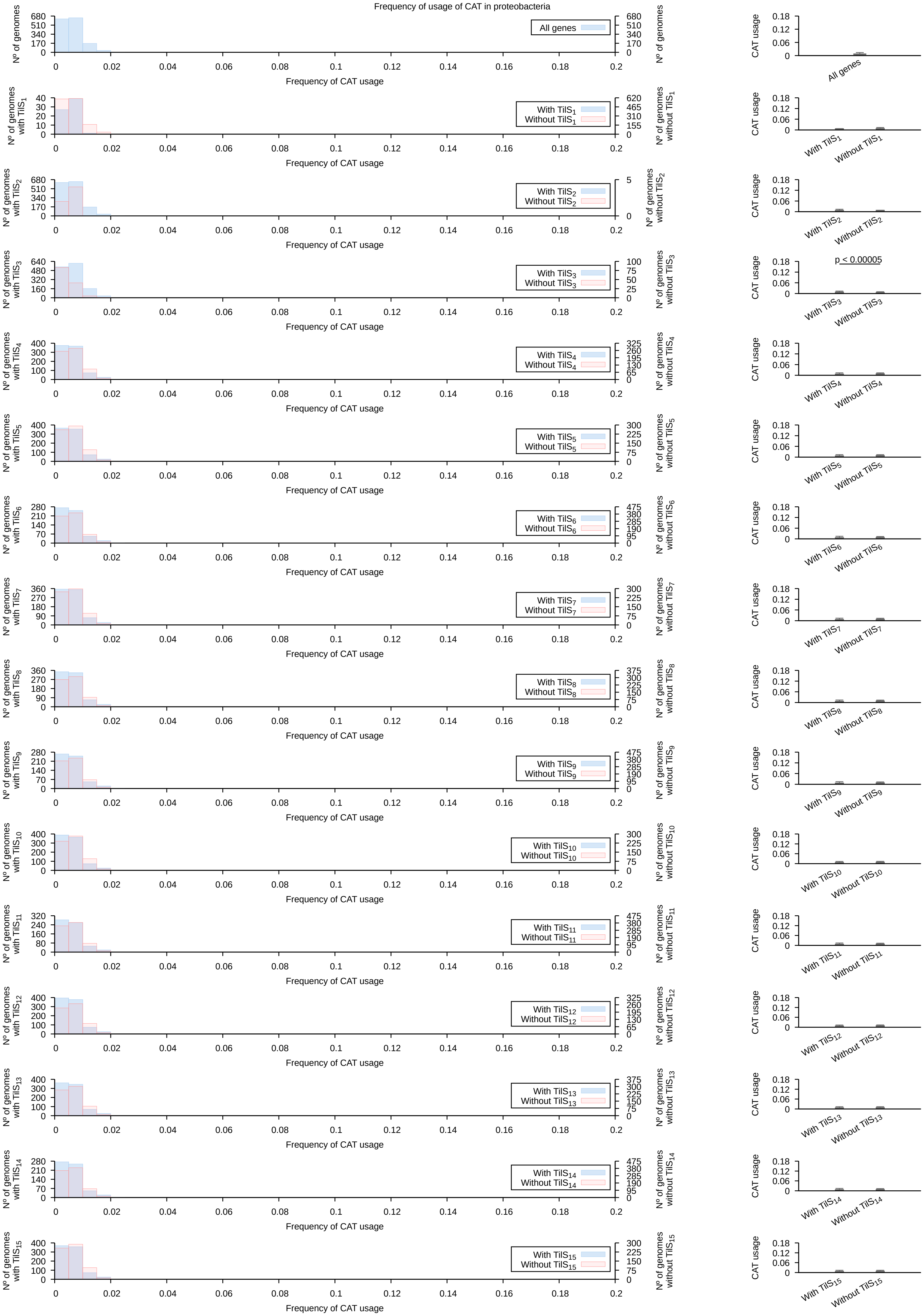
Frequency of usage of CAT in proteobacteria
Nº of genomes
All genes
Frequency of CAT usage
CAT usage
Nº of genomes with TilS1
Nº of genomes without TilS1
With TilS1
Without TilS1
Nº of genomes with TilS2
Nº of genomes without TilS2
With TilS2
Without TilS2
Nº of genomes with TilS3
Nº of genomes without TilS3
With TilS3
Without TilS3
p < 0.00005
Nº of genomes with TilS4
Nº of genomes without TilS4
With TilS4
Without TilS4
Nº of genomes with TilS5
Nº of genomes without TilS5
With TilS5
Without TilS5
Nº of genomes with TilS6
Nº of genomes without TilS6
With TilS6
Without TilS6
Nº of genomes with TilS7
Nº of genomes without TilS7
With TilS7
Without TilS7
Nº of genomes with TilS8
Nº of genomes without TilS8
With TilS8
Without TilS8
Nº of genomes with TilS9
Nº of genomes without TilS9
With TilS9
Without TilS9
Nº of genomes with TilS10
Nº of genomes without TilS10
With TilS10
Without TilS10
Nº of genomes with TilS11
Nº of genomes without TilS11
With TilS11
Without TilS11
Nº of genomes with TilS12
Nº of genomes without TilS12
With TilS12
Without TilS12
Nº of genomes with TilS13
Nº of genomes without TilS13
With TilS13
Without TilS13
Nº of genomes with TilS14
Nº of genomes without TilS14
With TilS14
Without TilS14
Nº of genomes with TilS15
Nº of genomes without TilS15
With TilS15
Without TilS15

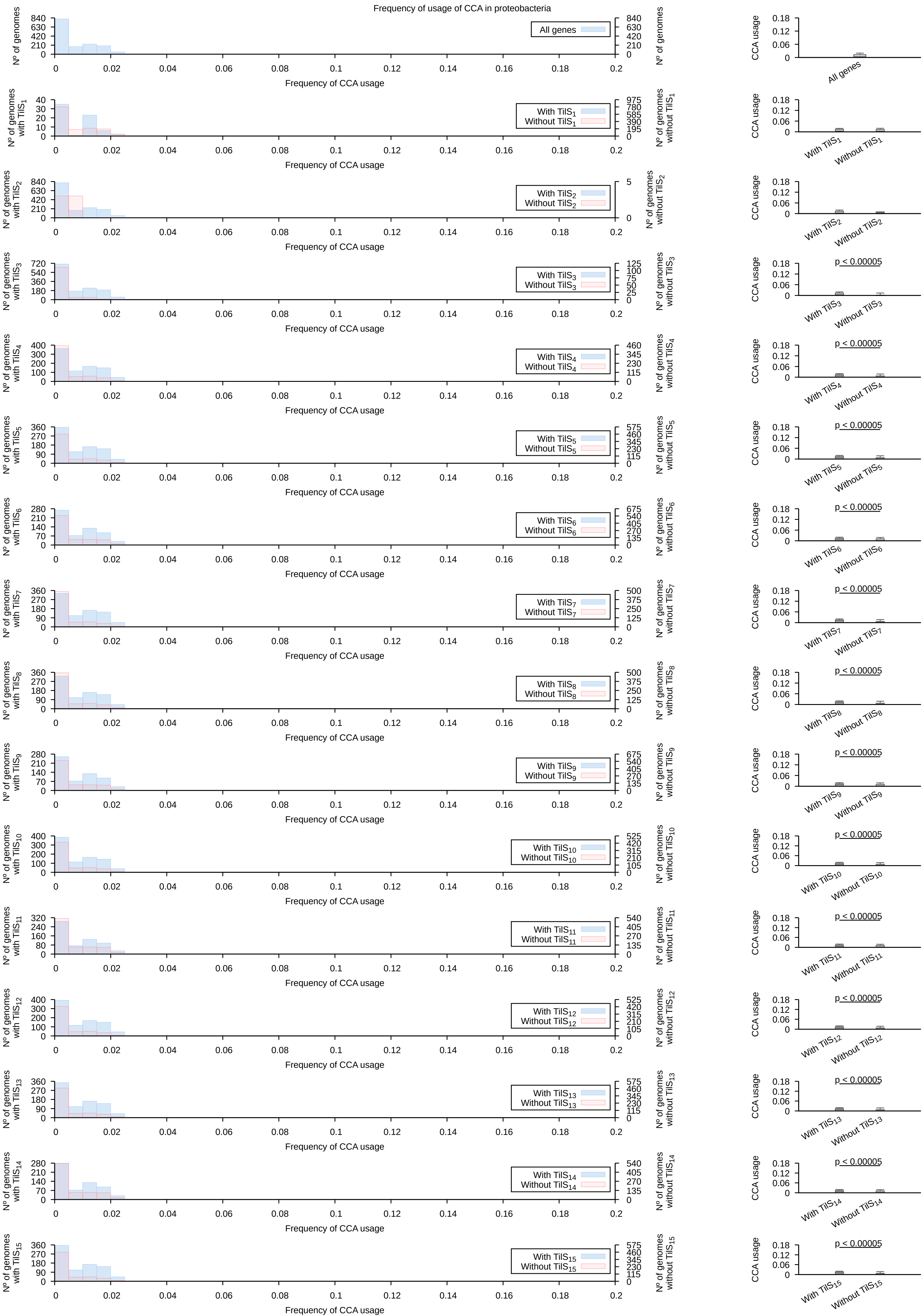

Frequency of usage of CCA in proteobacteria
All genes
With TilS1
Without TilS1
With TilS2
Without TilS2
With TilS3
Without TilS3
With TilS4
Without TilS4
With TilS5
Without TilS5
With TilS6
Without TilS6
With TilS7
Without TilS7
With TilS8
Without TilS8
With TilS9
Without TilS9
With TilS10
Without TilS10
With TilS11
Without TilS11
With TilS12
Without TilS12
With TilS13
Without TilS13
With TilS14
Without TilS14
With TilS15
Without TilS15
Frequency of CCA usage
Nº of genomes
CCA usage
p < 0.00005

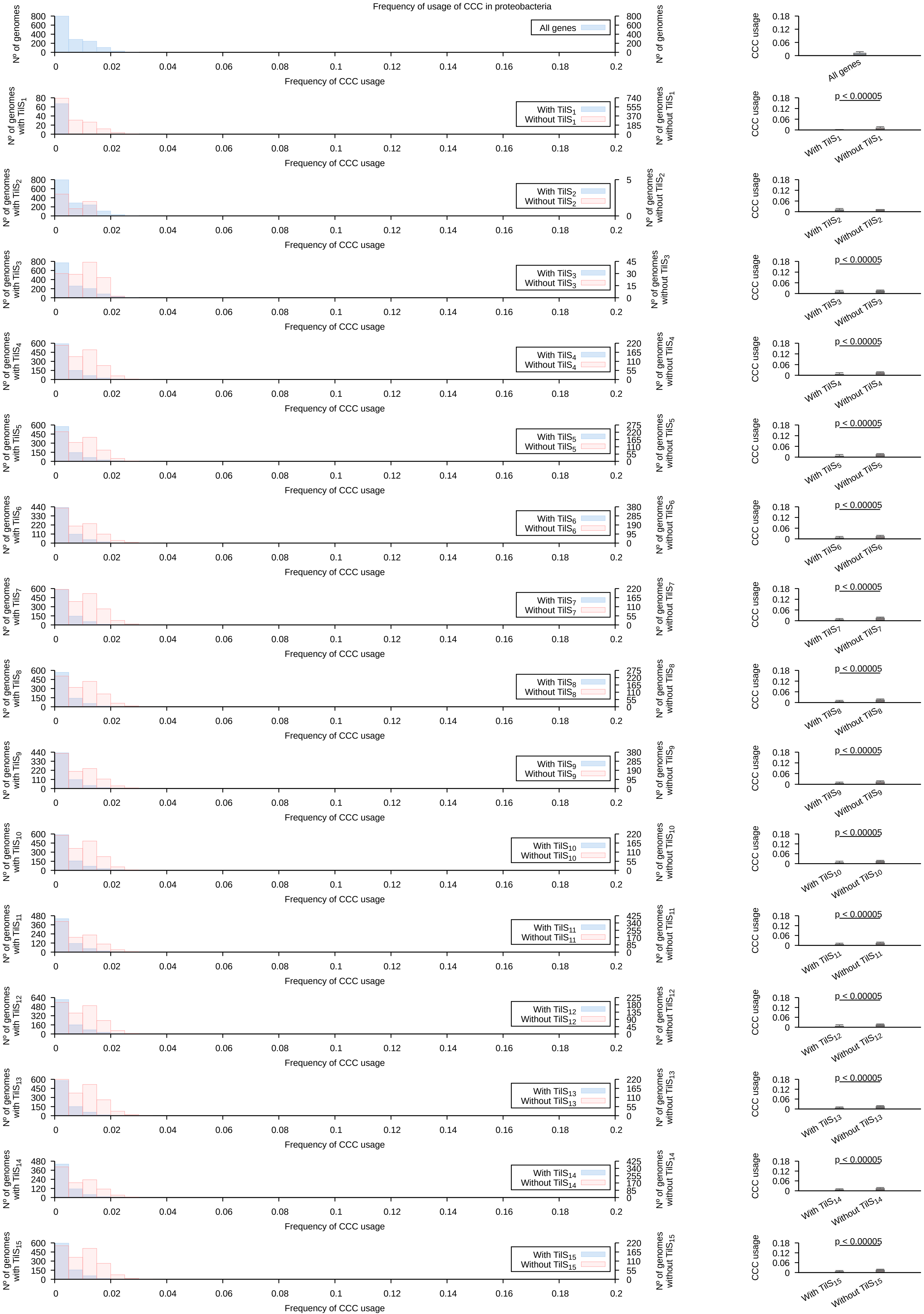

Frequency of usage of CCC in proteobacteria
Nº of genomes
All genes
Frequency of CCC usage
CCC usage
With TilS1
Without TilS1
With TilS2
Without TilS2
With TilS3
Without TilS3
With TilS4
Without TilS4
With TilS5
Without TilS5
With TilS6
Without TilS6
With TilS7
Without TilS7
With TilS8
Without TilS8
With TilS9
Without TilS9
With TilS10
Without TilS10
With TilS11
Without TilS11
With TilS12
Without TilS12
With TilS13
Without TilS13
With TilS14
Without TilS14
With TilS15
Without TilS15
Nº of genomes with TilS
Nº of genomes without TilS
p < 0.00005

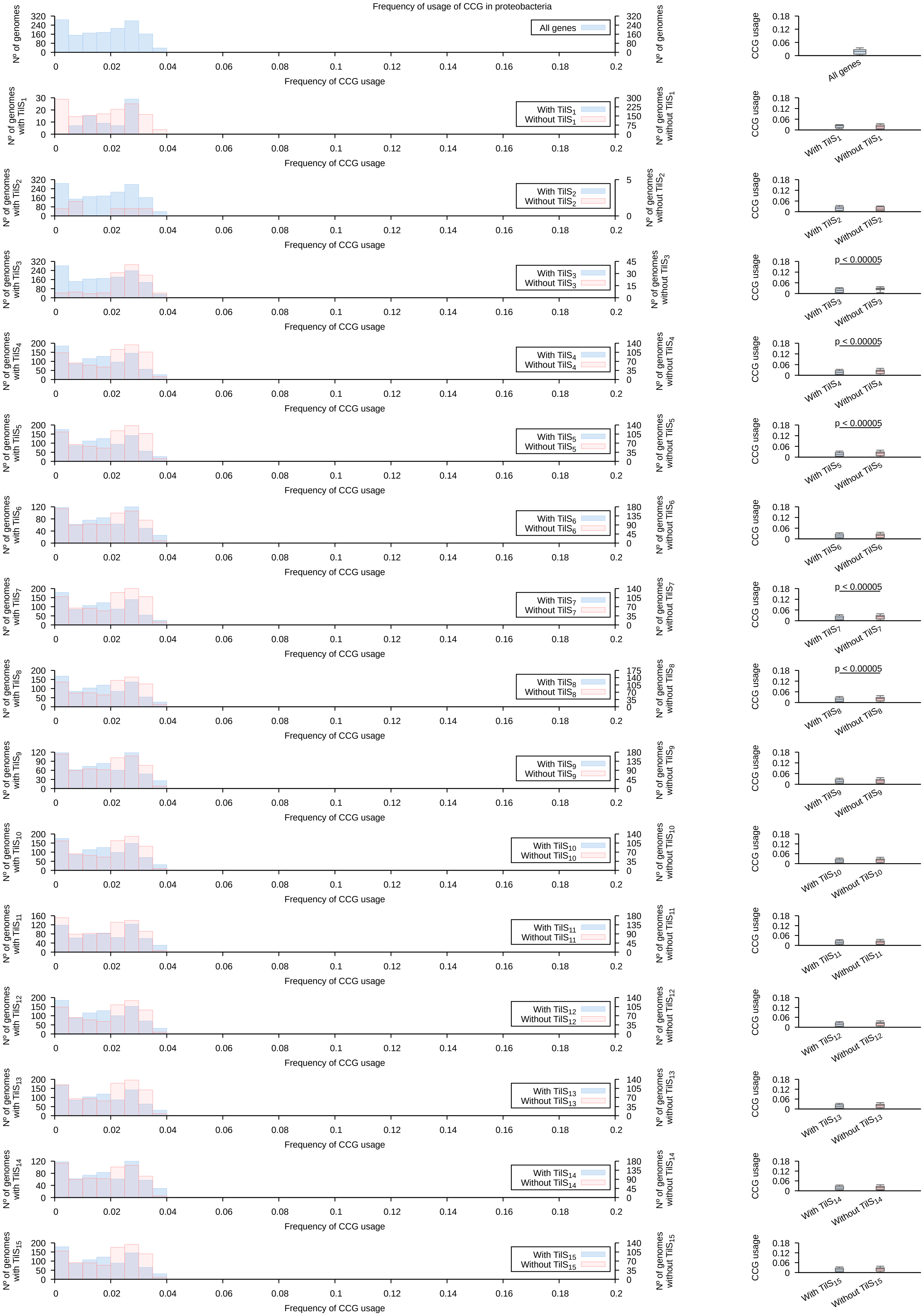

Frequency of usage of CCG in proteobacteria
Nº of genomes
All genes
Frequency of CCG usage
CCG usage
Nº of genomes with TilS$_1$
With TilS$_1$
Without TilS$_1$
Nº of genomes without TilS$_1$
Nº of genomes with TilS$_2$
With TilS$_2$
Without TilS$_2$
Nº of genomes without TilS$_2$
Nº of genomes with TilS$_3$
With TilS$_3$
Without TilS$_3$
Nº of genomes without TilS$_3$
p < 0.00005
Nº of genomes with TilS$_4$
With TilS$_4$
Without TilS$_4$
Nº of genomes without TilS$_4$
p < 0.00005
Nº of genomes with TilS$_5$
With TilS$_5$
Without TilS$_5$
Nº of genomes without TilS$_5$
p < 0.00005
Nº of genomes with TilS$_6$
With TilS$_6$
Without TilS$_6$
Nº of genomes without TilS$_6$
Nº of genomes with TilS$_7$
With TilS$_7$
Without TilS$_7$
Nº of genomes without TilS$_7$
p < 0.00005
Nº of genomes with TilS$_8$
With TilS$_8$
Without TilS$_8$
Nº of genomes without TilS$_8$
p < 0.00005
Nº of genomes with TilS$_9$
With TilS$_9$
Without TilS$_9$
Nº of genomes without TilS$_9$
Nº of genomes with TilS$_{10}$
With TilS$_{10}$
Without TilS$_{10}$
Nº of genomes without TilS$_{10}$
Nº of genomes with TilS$_{11}$
With TilS$_{11}$
Without TilS$_{11}$
Nº of genomes without TilS$_{11}$
Nº of genomes with TilS$_{12}$
With TilS$_{12}$
Without TilS$_{12}$
Nº of genomes without TilS$_{12}$
Nº of genomes with TilS$_{13}$
With TilS$_{13}$
Without TilS$_{13}$
Nº of genomes without TilS$_{13}$
Nº of genomes with TilS$_{14}$
With TilS$_{14}$
Without TilS$_{14}$
Nº of genomes without TilS$_{14}$
Nº of genomes with TilS$_{15}$
With TilS$_{15}$
Without TilS$_{15}$
Nº of genomes without TilS$_{15}$

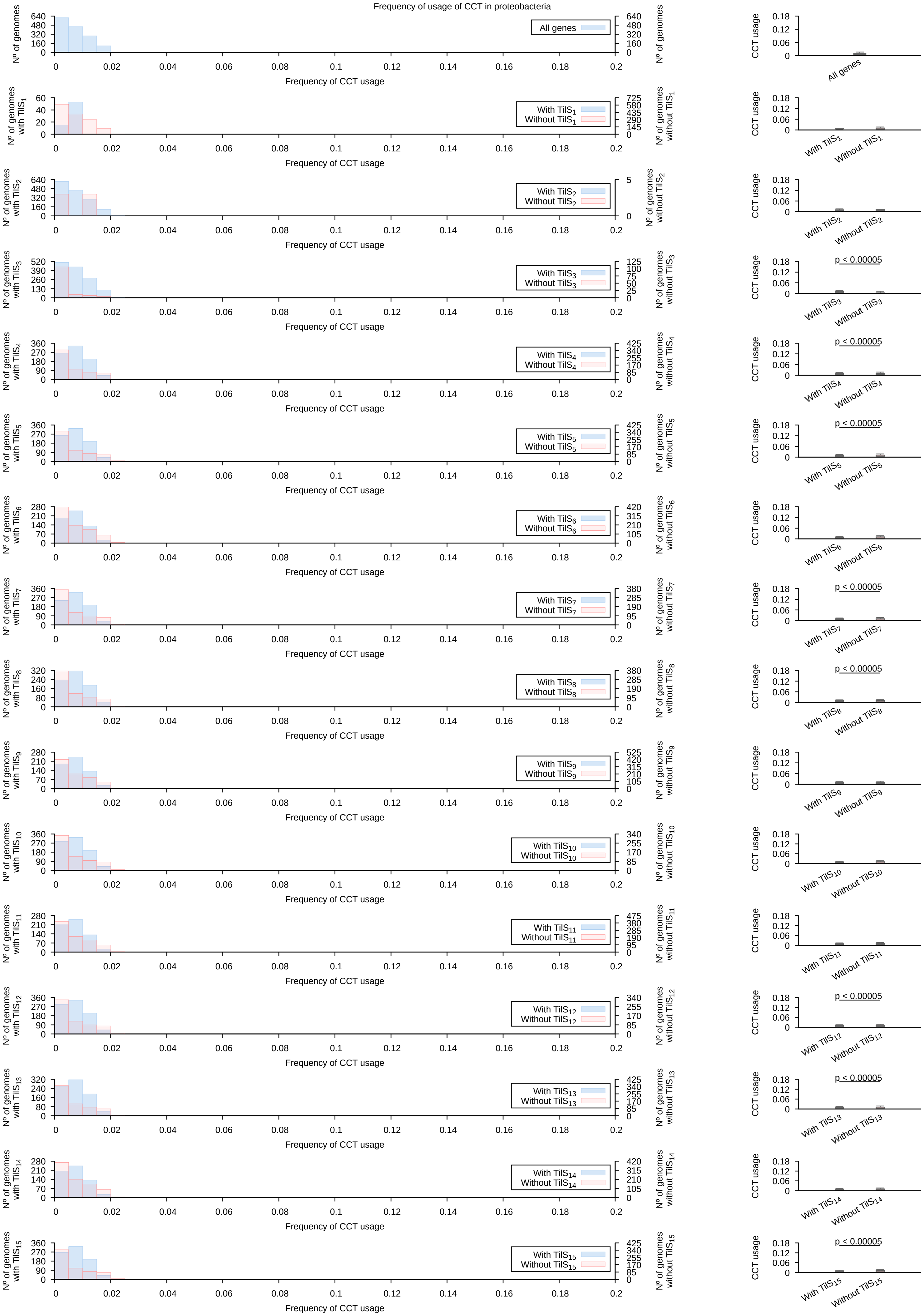

Frequency of usage of CCT in proteobacteria
Nº of genomes
All genes
Frequency of CCT usage
CCT usage
Nº of genomes with TilS1
Nº of genomes without TilS1
With TilS1
Without TilS1
Nº of genomes with TilS2
Nº of genomes without TilS2
With TilS2
Without TilS2
Nº of genomes with TilS3
Nº of genomes without TilS3
With TilS3
Without TilS3
p < 0.00005
Nº of genomes with TilS4
Nº of genomes without TilS4
With TilS4
Without TilS4
p < 0.00005
Nº of genomes with TilS5
Nº of genomes without TilS5
With TilS5
Without TilS5
p < 0.00005
Nº of genomes with TilS6
Nº of genomes without TilS6
With TilS6
Without TilS6
Nº of genomes with TilS7
Nº of genomes without TilS7
With TilS7
Without TilS7
p < 0.00005
Nº of genomes with TilS8
Nº of genomes without TilS8
With TilS8
Without TilS8
p < 0.00005
Nº of genomes with TilS9
Nº of genomes without TilS9
With TilS9
Without TilS9
Nº of genomes with TilS10
Nº of genomes without TilS10
With TilS10
Without TilS10
Nº of genomes with TilS11
Nº of genomes without TilS11
With TilS11
Without TilS11
Nº of genomes with TilS12
Nº of genomes without TilS12
With TilS12
Without TilS12
p < 0.00005
Nº of genomes with TilS13
Nº of genomes without TilS13
With TilS13
Without TilS13
p < 0.00005
Nº of genomes with TilS14
Nº of genomes without TilS14
With TilS14
Without TilS14
Nº of genomes with TilS15
Nº of genomes without TilS15
With TilS15
Without TilS15
p < 0.00005

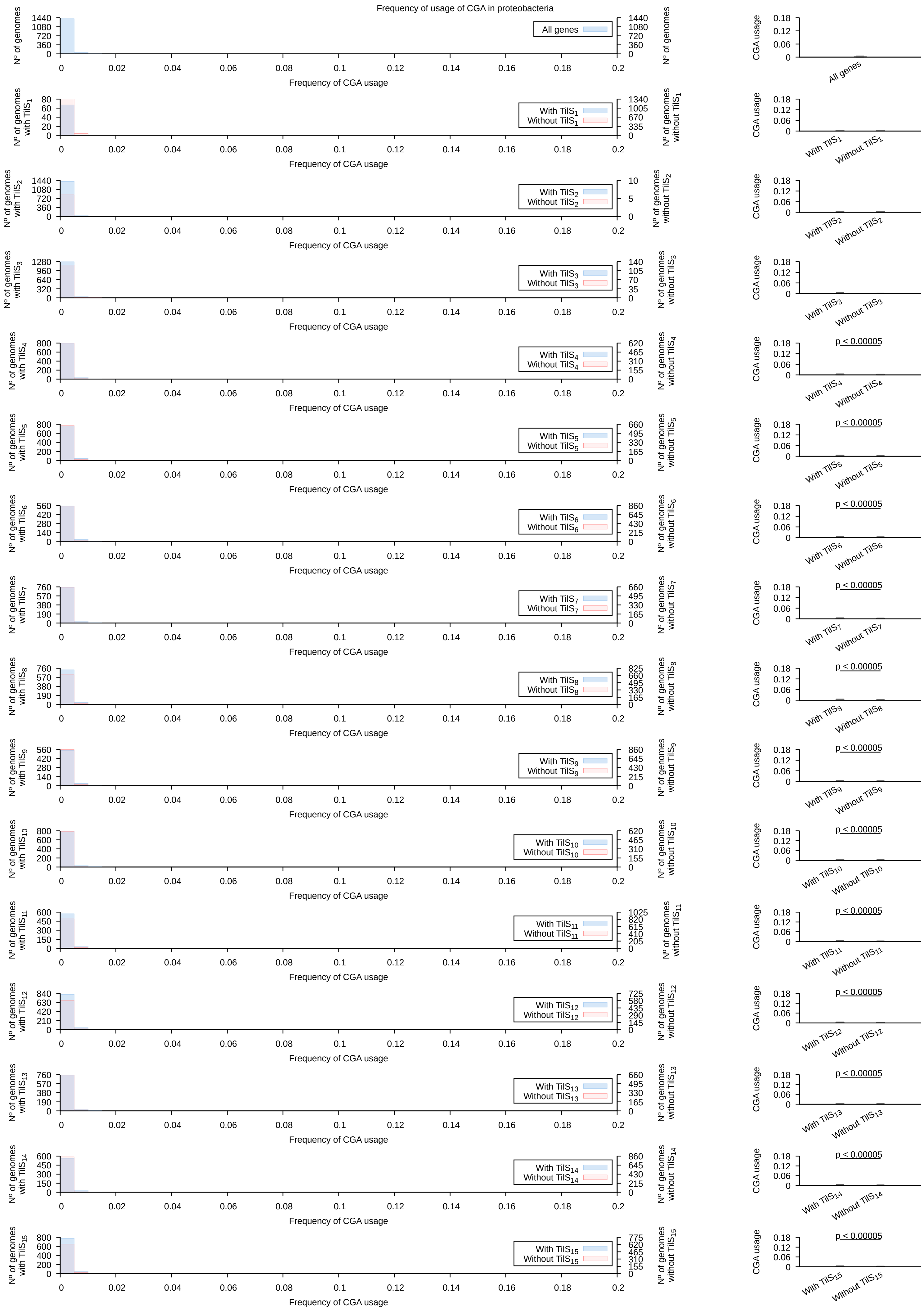

Frequency of usage of CGA in proteobacteria
Nº of genomes
1440
1080
720
360
0
All genes
Frequency of CGA usage
0
0.02
0.04
0.06
0.08
0.1
0.12
0.14
0.16
0.18
0.2
CGA usage
0.18
0.12
0.06
0
All genes
Nº of genomes with TilS1
80
60
40
20
0
With TilS1
Without TilS1
1340
1005
670
335
0
Nº of genomes without TilS1
Nº of genomes with TilS2
With TilS2
Without TilS2
10
5
0
Nº of genomes without TilS2
Nº of genomes with TilS3
1280
960
640
320
0
With TilS3
Without TilS3
140
105
70
35
0
Nº of genomes without TilS3
Nº of genomes with TilS4
800
600
400
200
0
With TilS4
Without TilS4
620
465
310
155
0
Nº of genomes without TilS4
p < 0.00005
Nº of genomes with TilS5
With TilS5
Without TilS5
660
495
330
165
Nº of genomes without TilS5
Nº of genomes with TilS6
560
420
280
140
With TilS6
Without TilS6
860
645
430
215
Nº of genomes without TilS6
Nº of genomes with TilS7
760
570
380
190
With TilS7
Without TilS7
Nº of genomes without TilS7
Nº of genomes with TilS8
With TilS8
Without TilS8
825
660
495
330
165
Nº of genomes without TilS8
Nº of genomes with TilS9
With TilS9
Without TilS9
Nº of genomes without TilS9
Nº of genomes with TilS10
With TilS10
Without TilS10
Nº of genomes without TilS10
Nº of genomes with TilS11
600
450
300
150
With TilS11
Without TilS11
1025
820
615
410
205
Nº of genomes without TilS11
Nº of genomes with TilS12
840
630
420
210
With TilS12
Without TilS12
725
580
435
290
145
Nº of genomes without TilS12
Nº of genomes with TilS13
With TilS13
Without TilS13
Nº of genomes without TilS13
Nº of genomes with TilS14
With TilS14
Without TilS14
Nº of genomes without TilS14
Nº of genomes with TilS15
With TilS15
Without TilS15
775
620
465
310
155
Nº of genomes without TilS15

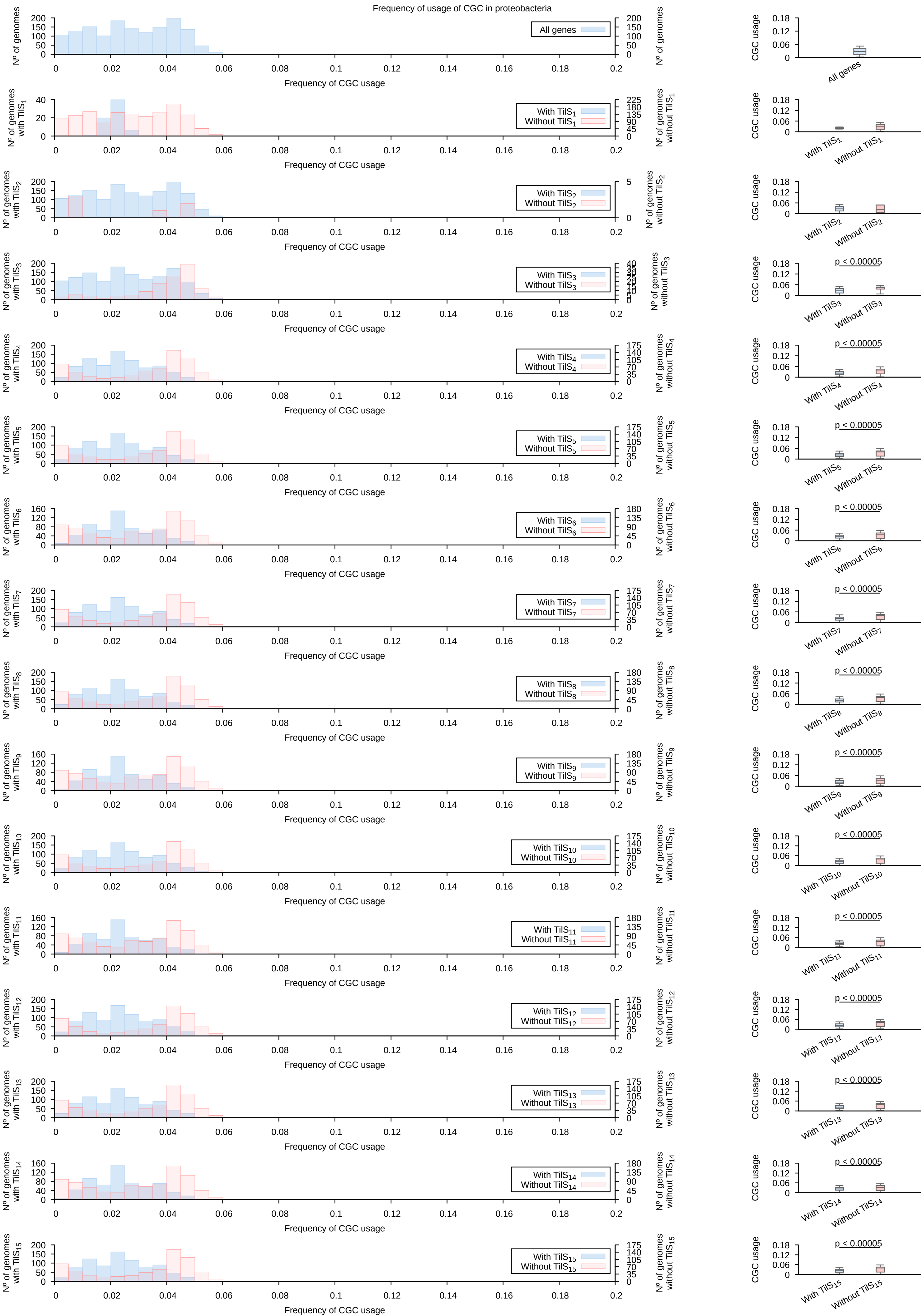
Frequency of usage of CGC in proteobacteria
Nº of genomes
All genes
Frequency of CGC usage
CGC usage
All genes
Nº of genomes with TilS$_1$
Nº of genomes without TilS$_1$
With TilS$_1$
Without TilS$_1$
Nº of genomes with TilS$_2$
Nº of genomes without TilS$_2$
With TilS$_2$
Without TilS$_2$
Nº of genomes with TilS$_3$
Nº of genomes without TilS$_3$
With TilS$_3$
Without TilS$_3$
p < 0.00005
Nº of genomes with TilS$_4$
Nº of genomes without TilS$_4$
With TilS$_4$
Without TilS$_4$
p < 0.00005
Nº of genomes with TilS$_5$
Nº of genomes without TilS$_5$
With TilS$_5$
Without TilS$_5$
p < 0.00005
Nº of genomes with TilS$_6$
Nº of genomes without TilS$_6$
With TilS$_6$
Without TilS$_6$
p < 0.00005
Nº of genomes with TilS$_7$
Nº of genomes without TilS$_7$
With TilS$_7$
Without TilS$_7$
p < 0.00005
Nº of genomes with TilS$_8$
Nº of genomes without TilS$_8$
With TilS$_8$
Without TilS$_8$
p < 0.00005
Nº of genomes with TilS$_9$
Nº of genomes without TilS$_9$
With TilS$_9$
Without TilS$_9$
p < 0.00005
Nº of genomes with TilS$_{10}$
Nº of genomes without TilS$_{10}$
With TilS$_{10}$
Without TilS$_{10}$
p < 0.00005
Nº of genomes with TilS$_{11}$
Nº of genomes without TilS$_{11}$
With TilS$_{11}$
Without TilS$_{11}$
p < 0.00005
Nº of genomes with TilS$_{12}$
Nº of genomes without TilS$_{12}$
With TilS$_{12}$
Without TilS$_{12}$
p < 0.00005
Nº of genomes with TilS$_{13}$
Nº of genomes without TilS$_{13}$
With TilS$_{13}$
Without TilS$_{13}$
p < 0.00005
Nº of genomes with TilS$_{14}$
Nº of genomes without TilS$_{14}$
With TilS$_{14}$
Without TilS$_{14}$
p < 0.00005
Nº of genomes with TilS$_{15}$
Nº of genomes without TilS$_{15}$
With TilS$_{15}$
Without TilS$_{15}$
p < 0.00005

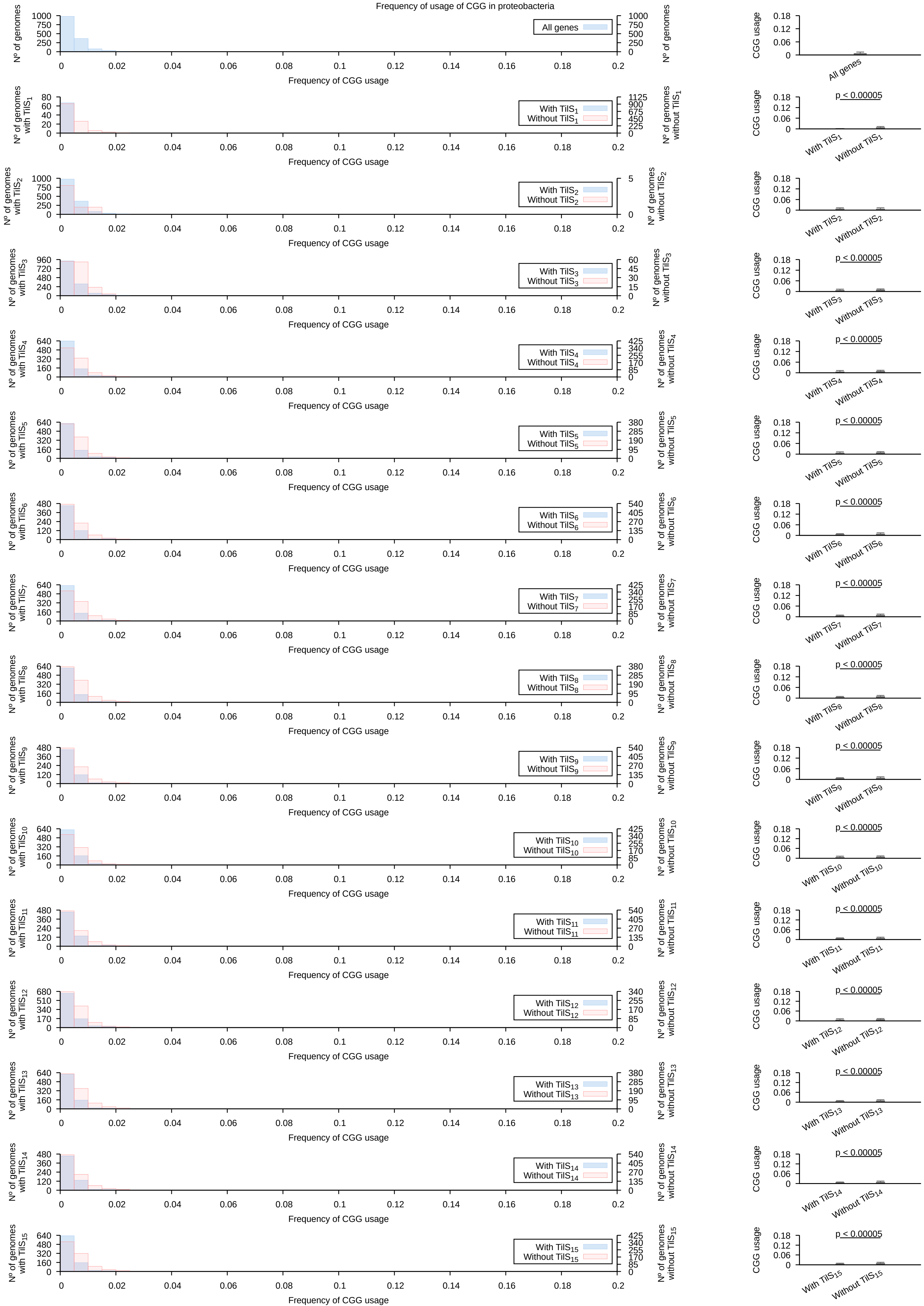
Frequency of usage of CGG in proteobacteria
Nº of genomes
All genes
Frequency of CGG usage
CGG usage
All genes
Nº of genomes with $TilS_1$
Nº of genomes without $TilS_1$
With $TilS_1$
Without $TilS_1$
p < 0.00005
Nº of genomes with $TilS_2$
Nº of genomes without $TilS_2$
With $TilS_2$
Without $TilS_2$
Nº of genomes with $TilS_3$
Nº of genomes without $TilS_3$
With $TilS_3$
Without $TilS_3$
p < 0.00005
Nº of genomes with $TilS_4$
Nº of genomes without $TilS_4$
With $TilS_4$
Without $TilS_4$
p < 0.00005
Nº of genomes with $TilS_5$
Nº of genomes without $TilS_5$
With $TilS_5$
Without $TilS_5$
p < 0.00005
Nº of genomes with $TilS_6$
Nº of genomes without $TilS_6$
With $TilS_6$
Without $TilS_6$
p < 0.00005
Nº of genomes with $TilS_7$
Nº of genomes without $TilS_7$
With $TilS_7$
Without $TilS_7$
p < 0.00005
Nº of genomes with $TilS_8$
Nº of genomes without $TilS_8$
With $TilS_8$
Without $TilS_8$
p < 0.00005
Nº of genomes with $TilS_9$
Nº of genomes without $TilS_9$
With $TilS_9$
Without $TilS_9$
p < 0.00005
Nº of genomes with $TilS_{10}$
Nº of genomes without $TilS_{10}$
With $TilS_{10}$
Without $TilS_{10}$
p < 0.00005
Nº of genomes with $TilS_{11}$
Nº of genomes without $TilS_{11}$
With $TilS_{11}$
Without $TilS_{11}$
p < 0.00005
Nº of genomes with $TilS_{12}$
Nº of genomes without $TilS_{12}$
With $TilS_{12}$
Without $TilS_{12}$
p < 0.00005
Nº of genomes with $TilS_{13}$
Nº of genomes without $TilS_{13}$
With $TilS_{13}$
Without $TilS_{13}$
p < 0.00005
Nº of genomes with $TilS_{14}$
Nº of genomes without $TilS_{14}$
With $TilS_{14}$
Without $TilS_{14}$
p < 0.00005
Nº of genomes with $TilS_{15}$
Nº of genomes without $TilS_{15}$
With $TilS_{15}$
Without $TilS_{15}$
p < 0.00005

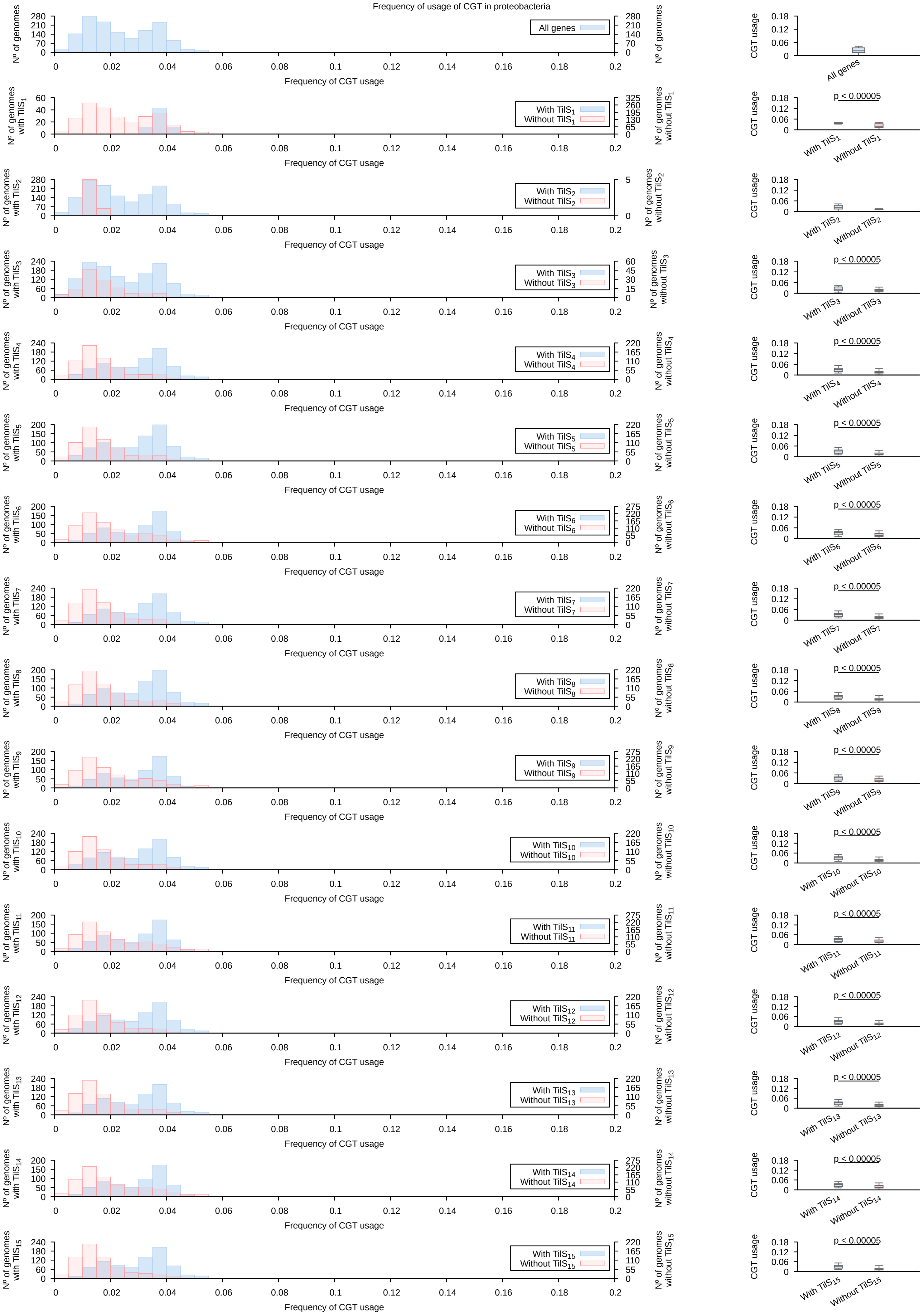
Frequency of usage of CGT in proteobacteria
Nº of genomes
All genes
Frequency of CGT usage
CGT usage
Nº of genomes with TilS$_1$
Nº of genomes without TilS$_1$
With TilS$_1$
Without TilS$_1$
p < 0.00005
Nº of genomes with TilS$_2$
Nº of genomes without TilS$_2$
With TilS$_2$
Without TilS$_2$
Nº of genomes with TilS$_3$
Nº of genomes without TilS$_3$
With TilS$_3$
Without TilS$_3$
p < 0.00005
Nº of genomes with TilS$_4$
Nº of genomes without TilS$_4$
With TilS$_4$
Without TilS$_4$
p < 0.00005
Nº of genomes with TilS$_5$
Nº of genomes without TilS$_5$
With TilS$_5$
Without TilS$_5$
p < 0.00005
Nº of genomes with TilS$_6$
Nº of genomes without TilS$_6$
With TilS$_6$
Without TilS$_6$
p < 0.00005
Nº of genomes with TilS$_7$
Nº of genomes without TilS$_7$
With TilS$_7$
Without TilS$_7$
p < 0.00005
Nº of genomes with TilS$_8$
Nº of genomes without TilS$_8$
With TilS$_8$
Without TilS$_8$
p < 0.00005
Nº of genomes with TilS$_9$
Nº of genomes without TilS$_9$
With TilS$_9$
Without TilS$_9$
p < 0.00005
Nº of genomes with TilS$_{10}$
Nº of genomes without TilS$_{10}$
With TilS$_{10}$
Without TilS$_{10}$
p < 0.00005
Nº of genomes with TilS$_{11}$
Nº of genomes without TilS$_{11}$
With TilS$_{11}$
Without TilS$_{11}$
p < 0.00005
Nº of genomes with TilS$_{12}$
Nº of genomes without TilS$_{12}$
With TilS$_{12}$
Without TilS$_{12}$
p < 0.00005
Nº of genomes with TilS$_{13}$
Nº of genomes without TilS$_{13}$
With TilS$_{13}$
Without TilS$_{13}$
p < 0.00005
Nº of genomes with TilS$_{14}$
Nº of genomes without TilS$_{14}$
With TilS$_{14}$
Without TilS$_{14}$
p < 0.00005
Nº of genomes with TilS$_{15}$
Nº of genomes without TilS$_{15}$
With TilS$_{15}$
Without TilS$_{15}$
p < 0.00005

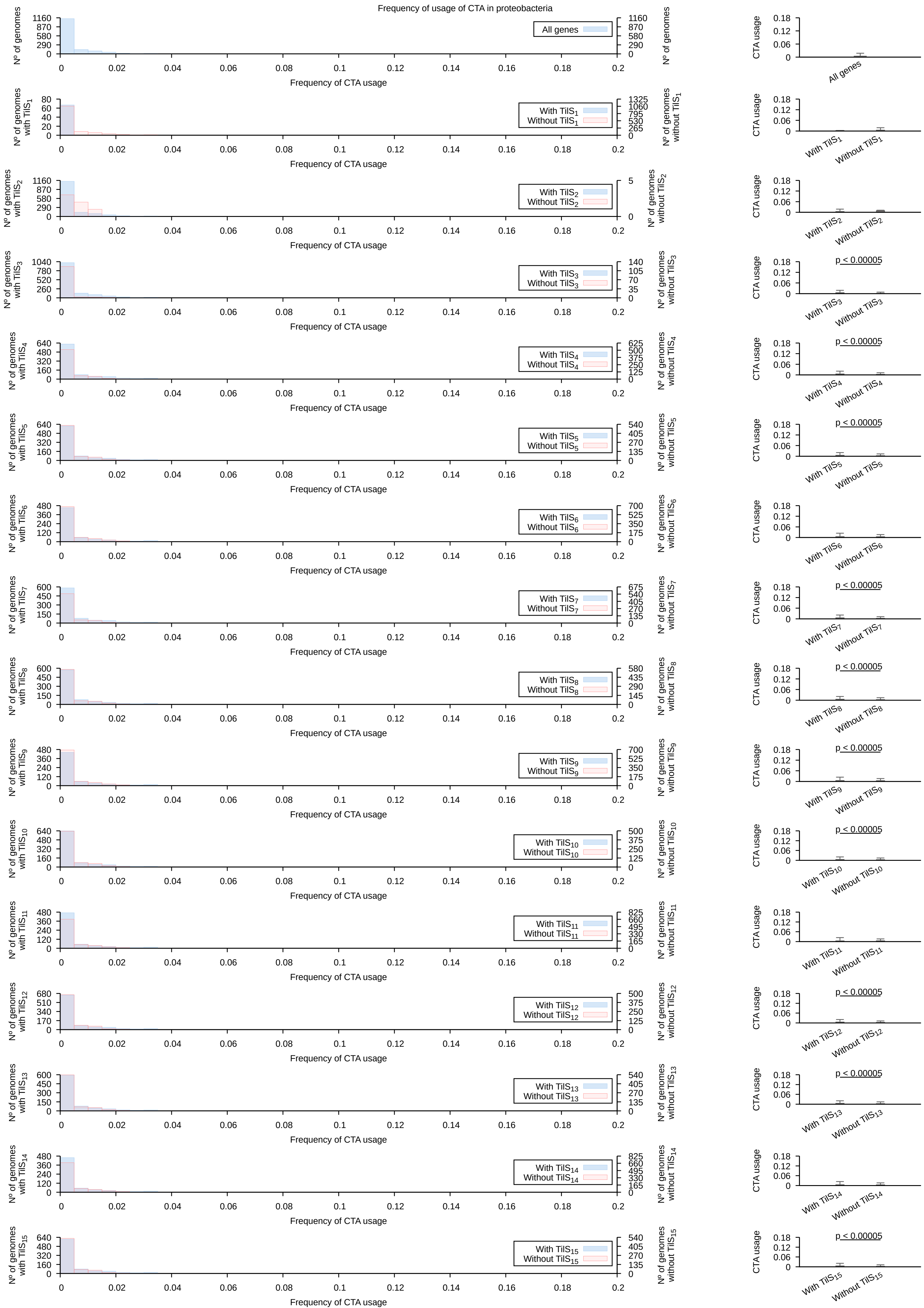

Frequency of usage of CTA in proteobacteria
Nº of genomes
All genes
Frequency of CTA usage
CTA usage
0
0.02
0.04
0.06
0.08
0.1
0.12
0.14
0.16
0.18
0.2
0.06
0.12
0.18
Nº of genomes with TilS1
Nº of genomes without TilS1
With TilS1
Without TilS1
Nº of genomes with TilS2
Nº of genomes without TilS2
With TilS2
Without TilS2
Nº of genomes with TilS3
Nº of genomes without TilS3
With TilS3
Without TilS3
p < 0.00005
Nº of genomes with TilS4
Nº of genomes without TilS4
With TilS4
Without TilS4
p < 0.00005
Nº of genomes with TilS5
Nº of genomes without TilS5
With TilS5
Without TilS5
p < 0.00005
Nº of genomes with TilS6
Nº of genomes without TilS6
With TilS6
Without TilS6
Nº of genomes with TilS7
Nº of genomes without TilS7
With TilS7
Without TilS7
p < 0.00005
Nº of genomes with TilS8
Nº of genomes without TilS8
With TilS8
Without TilS8
p < 0.00005
Nº of genomes with TilS9
Nº of genomes without TilS9
With TilS9
Without TilS9
p < 0.00005
Nº of genomes with TilS10
Nº of genomes without TilS10
With TilS10
Without TilS10
p < 0.00005
Nº of genomes with TilS11
Nº of genomes without TilS11
With TilS11
Without TilS11
Nº of genomes with TilS12
Nº of genomes without TilS12
With TilS12
Without TilS12
p < 0.00005
Nº of genomes with TilS13
Nº of genomes without TilS13
With TilS13
Without TilS13
p < 0.00005
Nº of genomes with TilS14
Nº of genomes without TilS14
With TilS14
Without TilS14
Nº of genomes with TilS15
Nº of genomes without TilS15
With TilS15
Without TilS15
p < 0.00005

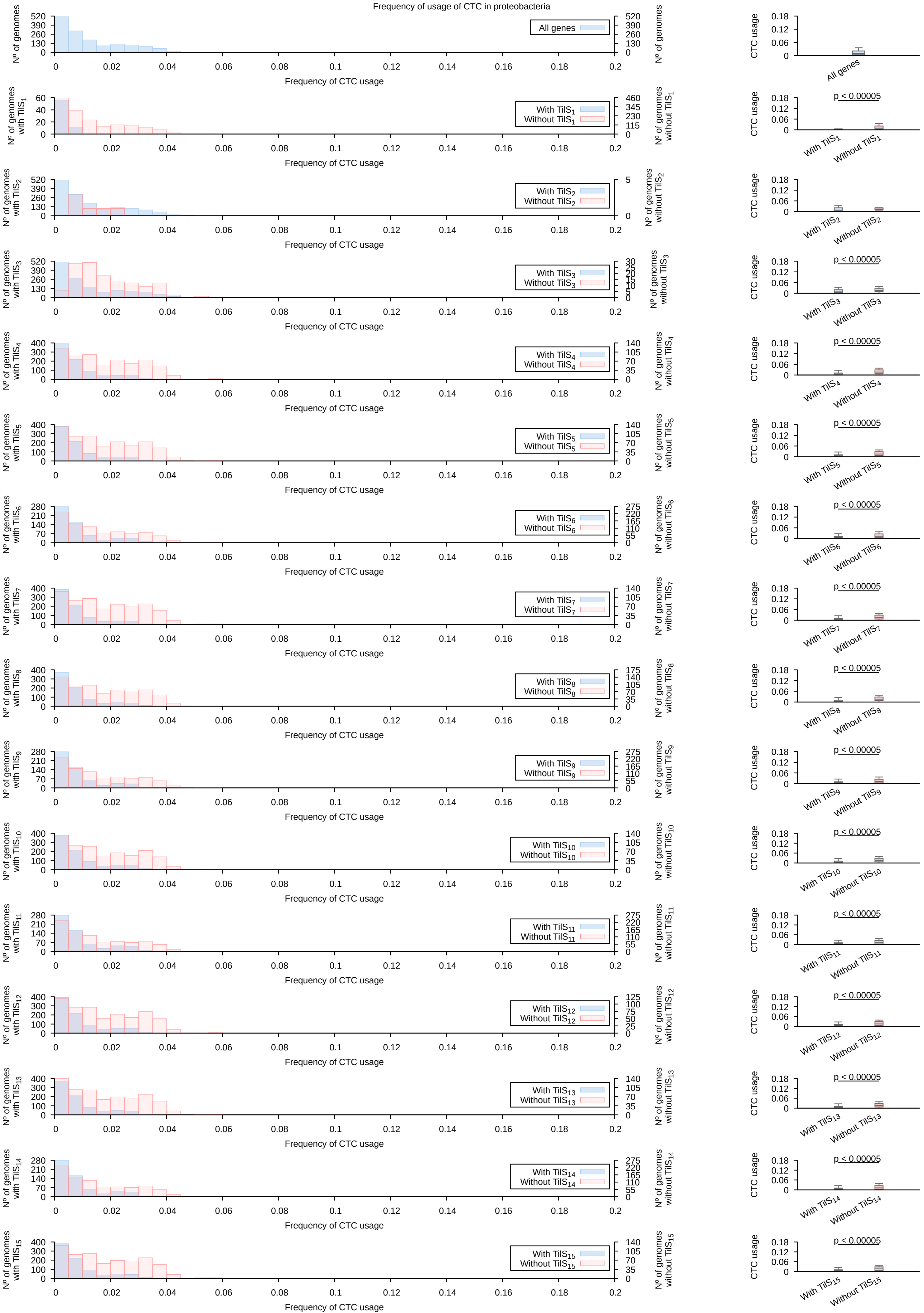

Frequency of usage of CTC in proteobacteria
Nº of genomes
All genes
Frequency of CTC usage
CTC usage
Nº of genomes with TilS1
Nº of genomes without TilS1
With TilS1
Without TilS1
p < 0.00005
Nº of genomes with TilS2
Nº of genomes without TilS2
With TilS2
Without TilS2
Nº of genomes with TilS3
Nº of genomes without TilS3
With TilS3
Without TilS3
Nº of genomes with TilS4
Nº of genomes without TilS4
With TilS4
Without TilS4
Nº of genomes with TilS5
Nº of genomes without TilS5
With TilS5
Without TilS5
Nº of genomes with TilS6
Nº of genomes without TilS6
With TilS6
Without TilS6
Nº of genomes with TilS7
Nº of genomes without TilS7
With TilS7
Without TilS7
Nº of genomes with TilS8
Nº of genomes without TilS8
With TilS8
Without TilS8
Nº of genomes with TilS9
Nº of genomes without TilS9
With TilS9
Without TilS9
Nº of genomes with TilS10
Nº of genomes without TilS10
With TilS10
Without TilS10
Nº of genomes with TilS11
Nº of genomes without TilS11
With TilS11
Without TilS11
Nº of genomes with TilS12
Nº of genomes without TilS12
With TilS12
Without TilS12
Nº of genomes with TilS13
Nº of genomes without TilS13
With TilS13
Without TilS13
Nº of genomes with TilS14
Nº of genomes without TilS14
With TilS14
Without TilS14
Nº of genomes with TilS15
Nº of genomes without TilS15
With TilS15
Without TilS15

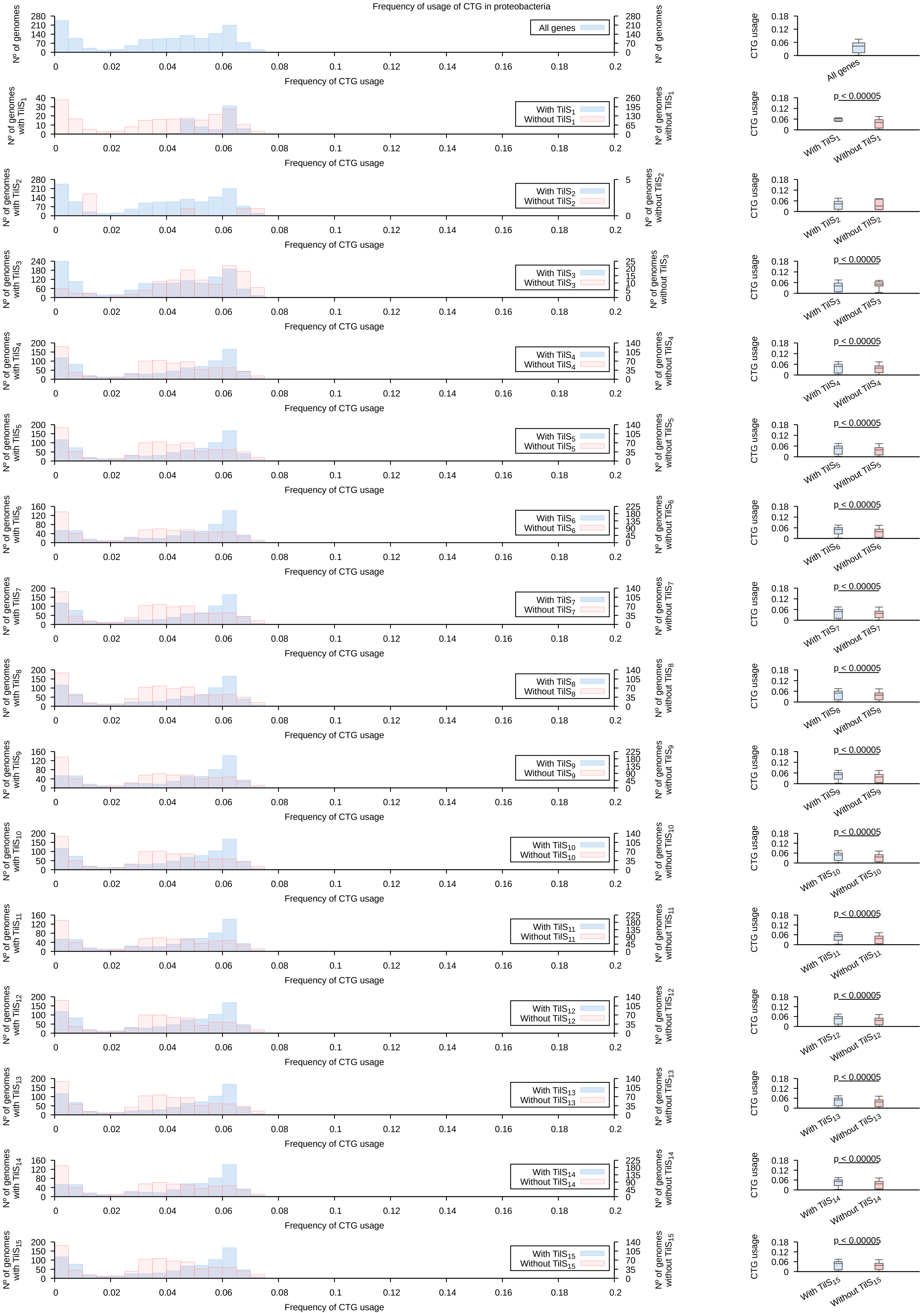

Frequency of usage of CTG in proteobacteria
Nº of genomes
All genes
Frequency of CTG usage
CTG usage
0 0.02 0.04 0.06 0.08 0.1 0.12 0.14 0.16 0.18 0.2
0 0.06 0.12 0.18
Nº of genomes with TilS1
Nº of genomes without TilS1
With TilS1
Without TilS1
p < 0.00005
Nº of genomes with TilS2
Nº of genomes without TilS2
With TilS2
Without TilS2
Nº of genomes with TilS3
Nº of genomes without TilS3
With TilS3
Without TilS3
Nº of genomes with TilS4
Nº of genomes without TilS4
With TilS4
Without TilS4
Nº of genomes with TilS5
Nº of genomes without TilS5
With TilS5
Without TilS5
Nº of genomes with TilS6
Nº of genomes without TilS6
With TilS6
Without TilS6
Nº of genomes with TilS7
Nº of genomes without TilS7
With TilS7
Without TilS7
Nº of genomes with TilS8
Nº of genomes without TilS8
With TilS8
Without TilS8
Nº of genomes with TilS9
Nº of genomes without TilS9
With TilS9
Without TilS9
Nº of genomes with TilS10
Nº of genomes without TilS10
With TilS10
Without TilS10
Nº of genomes with TilS11
Nº of genomes without TilS11
With TilS11
Without TilS11
Nº of genomes with TilS12
Nº of genomes without TilS12
With TilS12
Without TilS12
Nº of genomes with TilS13
Nº of genomes without TilS13
With TilS13
Without TilS13
Nº of genomes with TilS14
Nº of genomes without TilS14
With TilS14
Without TilS14
Nº of genomes with TilS15
Nº of genomes without TilS15
With TilS15
Without TilS15

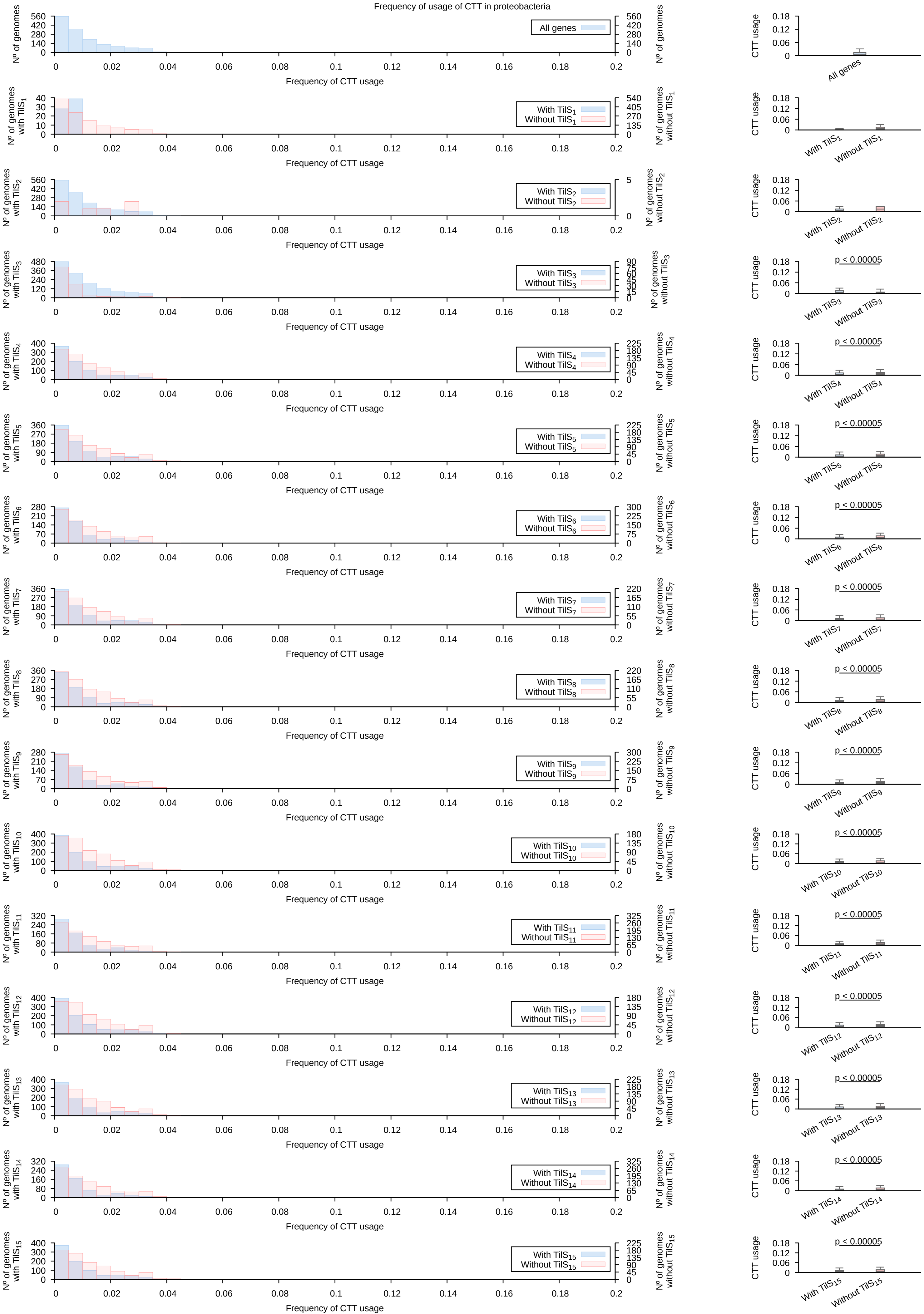
Frequency of usage of CTT in proteobacteria
Nº of genomes
Frequency of CTT usage
CTT usage
All genes
With $TilS_1$
Without $TilS_1$
With $TilS_2$
Without $TilS_2$
With $TilS_3$
Without $TilS_3$
With $TilS_4$
Without $TilS_4$
With $TilS_5$
Without $TilS_5$
With $TilS_6$
Without $TilS_6$
With $TilS_7$
Without $TilS_7$
With $TilS_8$
Without $TilS_8$
With $TilS_9$
Without $TilS_9$
With $TilS_{10}$
Without $TilS_{10}$
With $TilS_{11}$
Without $TilS_{11}$
With $TilS_{12}$
Without $TilS_{12}$
With $TilS_{13}$
Without $TilS_{13}$
With $TilS_{14}$
Without $TilS_{14}$
With $TilS_{15}$
Without $TilS_{15}$
$p < 0.00005$

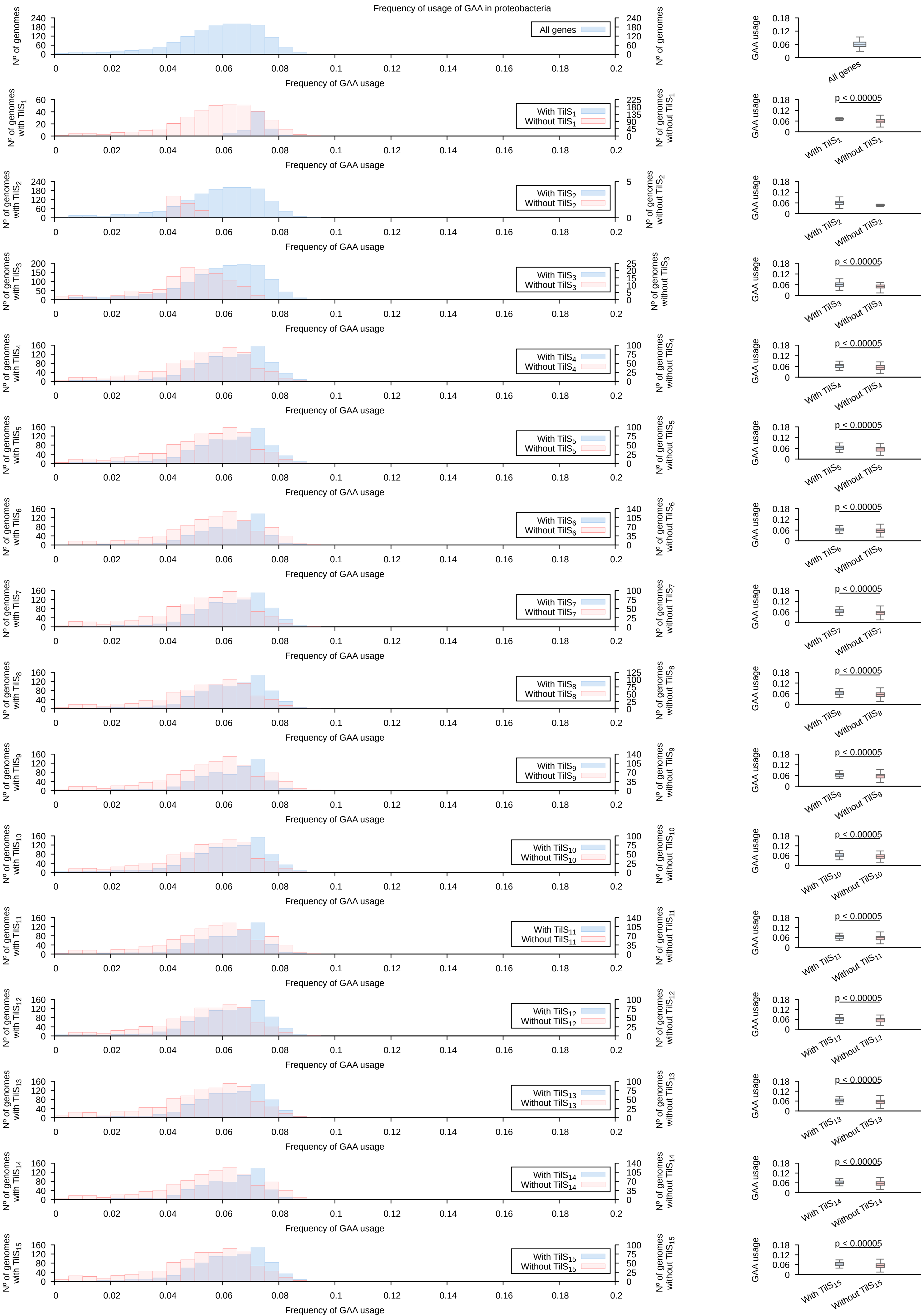

Frequency of usage of GAA in proteobacteria
Nº of genomes
All genes
Frequency of GAA usage
GAA usage
All genes
Nº of genomes with TilS$_1$
Nº of genomes without TilS$_1$
With TilS$_1$
Without TilS$_1$
p < 0.00005
Nº of genomes with TilS$_2$
Nº of genomes without TilS$_2$
With TilS$_2$
Without TilS$_2$
Nº of genomes with TilS$_3$
Nº of genomes without TilS$_3$
With TilS$_3$
Without TilS$_3$
Nº of genomes with TilS$_4$
Nº of genomes without TilS$_4$
With TilS$_4$
Without TilS$_4$
Nº of genomes with TilS$_5$
Nº of genomes without TilS$_5$
With TilS$_5$
Without TilS$_5$
Nº of genomes with TilS$_6$
Nº of genomes without TilS$_6$
With TilS$_6$
Without TilS$_6$
Nº of genomes with TilS$_7$
Nº of genomes without TilS$_7$
With TilS$_7$
Without TilS$_7$
Nº of genomes with TilS$_8$
Nº of genomes without TilS$_8$
With TilS$_8$
Without TilS$_8$
Nº of genomes with TilS$_9$
Nº of genomes without TilS$_9$
With TilS$_9$
Without TilS$_9$
Nº of genomes with TilS$_{10}$
Nº of genomes without TilS$_{10}$
With TilS$_{10}$
Without TilS$_{10}$
Nº of genomes with TilS$_{11}$
Nº of genomes without TilS$_{11}$
With TilS$_{11}$
Without TilS$_{11}$
Nº of genomes with TilS$_{12}$
Nº of genomes without TilS$_{12}$
With TilS$_{12}$
Without TilS$_{12}$
Nº of genomes with TilS$_{13}$
Nº of genomes without TilS$_{13}$
With TilS$_{13}$
Without TilS$_{13}$
Nº of genomes with TilS$_{14}$
Nº of genomes without TilS$_{14}$
With TilS$_{14}$
Without TilS$_{14}$
Nº of genomes with TilS$_{15}$
Nº of genomes without TilS$_{15}$
With TilS$_{15}$
Without TilS$_{15}$

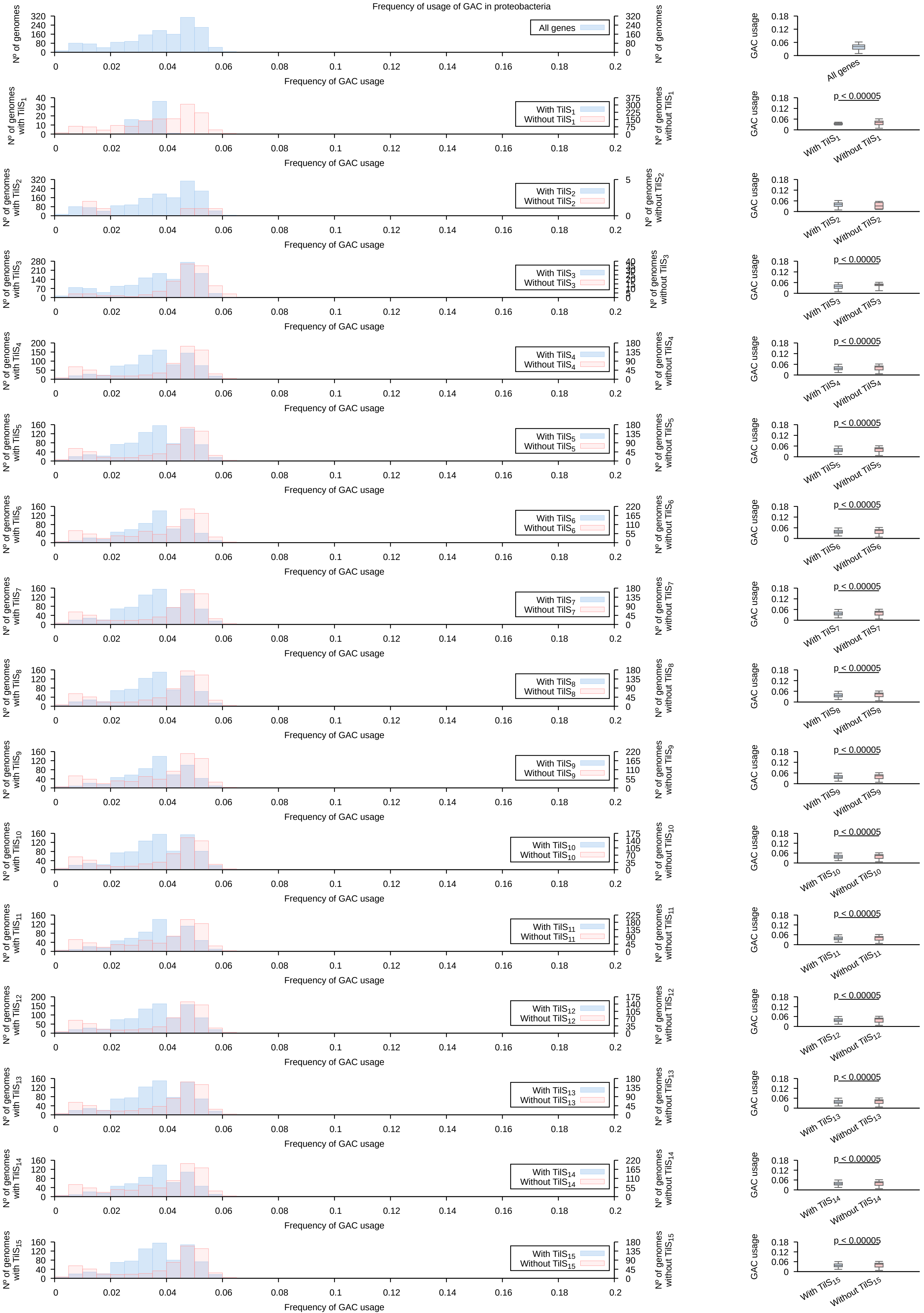

Frequency of usage of GAC in proteobacteria
Nº of genomes
All genes
Frequency of GAC usage
GAC usage
0 0.02 0.04 0.06 0.08 0.1 0.12 0.14 0.16 0.18 0.2
0 0.06 0.12 0.18
Nº of genomes with TilS1
Nº of genomes without TilS1
With TilS1
Without TilS1
p < 0.00005
Nº of genomes with TilS2
Nº of genomes without TilS2
With TilS2
Without TilS2
Nº of genomes with TilS3
Nº of genomes without TilS3
With TilS3
Without TilS3
p < 0.00005
Nº of genomes with TilS4
Nº of genomes without TilS4
With TilS4
Without TilS4
p < 0.00005
Nº of genomes with TilS5
Nº of genomes without TilS5
With TilS5
Without TilS5
p < 0.00005
Nº of genomes with TilS6
Nº of genomes without TilS6
With TilS6
Without TilS6
p < 0.00005
Nº of genomes with TilS7
Nº of genomes without TilS7
With TilS7
Without TilS7
p < 0.00005
Nº of genomes with TilS8
Nº of genomes without TilS8
With TilS8
Without TilS8
p < 0.00005
Nº of genomes with TilS9
Nº of genomes without TilS9
With TilS9
Without TilS9
p < 0.00005
Nº of genomes with TilS10
Nº of genomes without TilS10
With TilS10
Without TilS10
p < 0.00005
Nº of genomes with TilS11
Nº of genomes without TilS11
With TilS11
Without TilS11
p < 0.00005
Nº of genomes with TilS12
Nº of genomes without TilS12
With TilS12
Without TilS12
p < 0.00005
Nº of genomes with TilS13
Nº of genomes without TilS13
With TilS13
Without TilS13
p < 0.00005
Nº of genomes with TilS14
Nº of genomes without TilS14
With TilS14
Without TilS14
p < 0.00005
Nº of genomes with TilS15
Nº of genomes without TilS15
With TilS15
Without TilS15
p < 0.00005

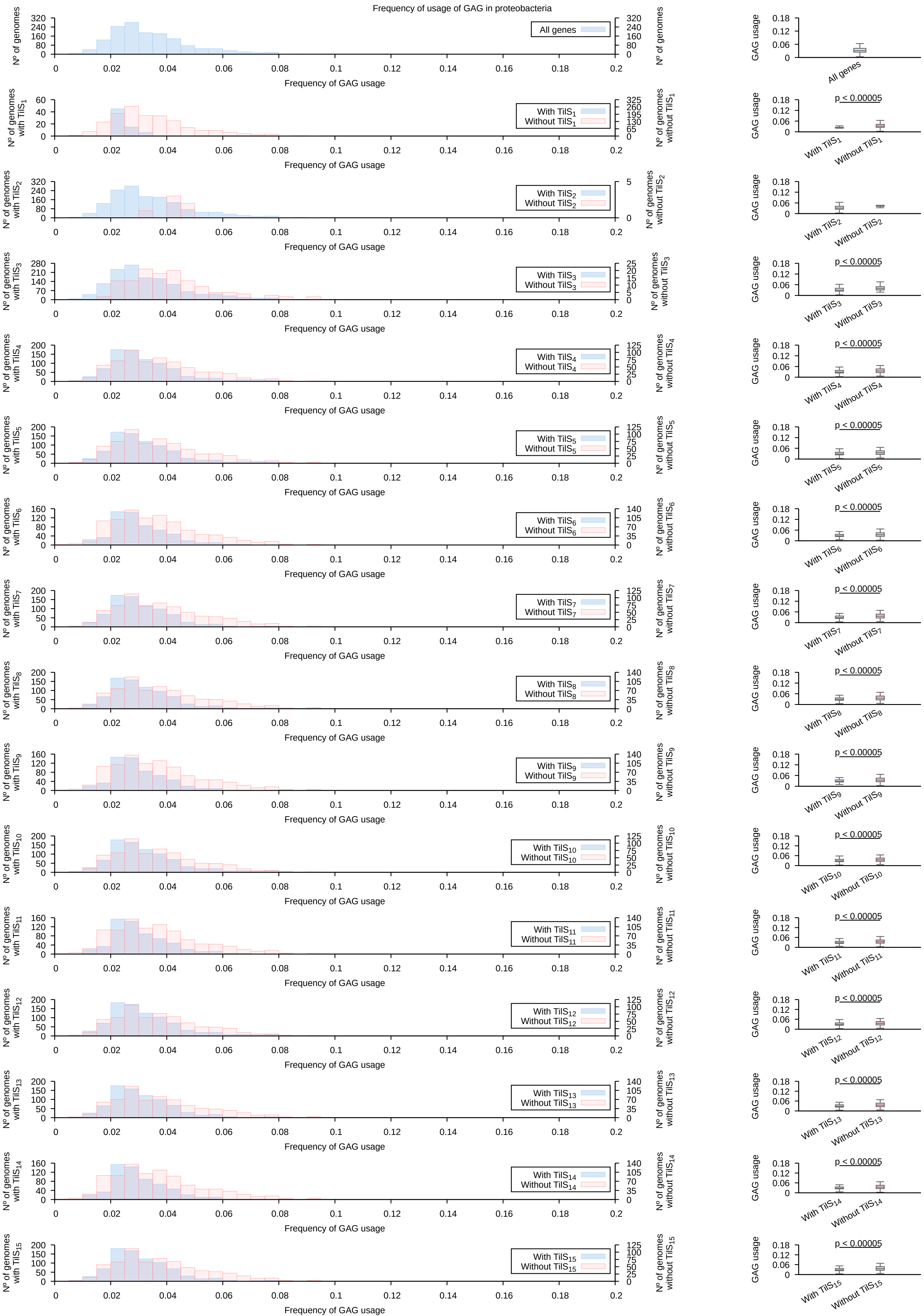
Frequency of usage of GAG in proteobacteria
Nº of genomes
All genes
Frequency of GAG usage
GAG usage
Nº of genomes with TilS1
Nº of genomes without TilS1
With TilS1
Without TilS1
p < 0.00005
Nº of genomes with TilS2
Nº of genomes without TilS2
With TilS2
Without TilS2
Nº of genomes with TilS3
Nº of genomes without TilS3
With TilS3
Without TilS3
Nº of genomes with TilS4
Nº of genomes without TilS4
With TilS4
Without TilS4
Nº of genomes with TilS5
Nº of genomes without TilS5
With TilS5
Without TilS5
Nº of genomes with TilS6
Nº of genomes without TilS6
With TilS6
Without TilS6
Nº of genomes with TilS7
Nº of genomes without TilS7
With TilS7
Without TilS7
Nº of genomes with TilS8
Nº of genomes without TilS8
With TilS8
Without TilS8
Nº of genomes with TilS9
Nº of genomes without TilS9
With TilS9
Without TilS9
Nº of genomes with TilS10
Nº of genomes without TilS10
With TilS10
Without TilS10
Nº of genomes with TilS11
Nº of genomes without TilS11
With TilS11
Without TilS11
Nº of genomes with TilS12
Nº of genomes without TilS12
With TilS12
Without TilS12
Nº of genomes with TilS13
Nº of genomes without TilS13
With TilS13
Without TilS13
Nº of genomes with TilS14
Nº of genomes without TilS14
With TilS14
Without TilS14
Nº of genomes with TilS15
Nº of genomes without TilS15
With TilS15
Without TilS15

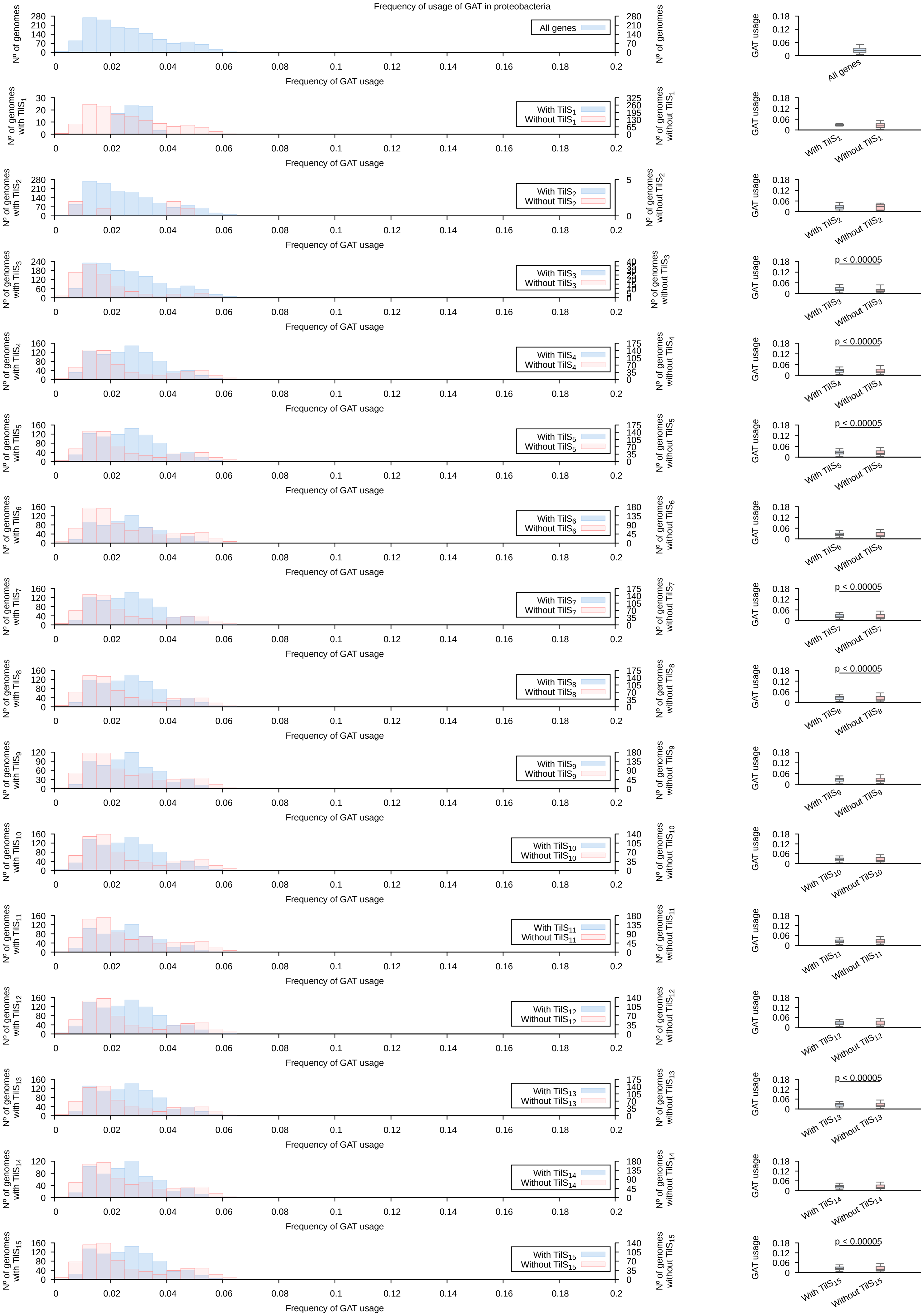

Frequency of usage of GAT in proteobacteria
Nº of genomes
All genes
Frequency of GAT usage
GAT usage
Nº of genomes with TilS1
Nº of genomes without TilS1
With TilS1
Without TilS1
Nº of genomes with TilS2
Nº of genomes without TilS2
With TilS2
Without TilS2
Nº of genomes with TilS3
Nº of genomes without TilS3
With TilS3
Without TilS3
p < 0.00005
Nº of genomes with TilS4
Nº of genomes without TilS4
With TilS4
Without TilS4
p < 0.00005
Nº of genomes with TilS5
Nº of genomes without TilS5
With TilS5
Without TilS5
p < 0.00005
Nº of genomes with TilS6
Nº of genomes without TilS6
With TilS6
Without TilS6
Nº of genomes with TilS7
Nº of genomes without TilS7
With TilS7
Without TilS7
p < 0.00005
Nº of genomes with TilS8
Nº of genomes without TilS8
With TilS8
Without TilS8
p < 0.00005
Nº of genomes with TilS9
Nº of genomes without TilS9
With TilS9
Without TilS9
Nº of genomes with TilS10
Nº of genomes without TilS10
With TilS10
Without TilS10
Nº of genomes with TilS11
Nº of genomes without TilS11
With TilS11
Without TilS11
Nº of genomes with TilS12
Nº of genomes without TilS12
With TilS12
Without TilS12
Nº of genomes with TilS13
Nº of genomes without TilS13
With TilS13
Without TilS13
p < 0.00005
Nº of genomes with TilS14
Nº of genomes without TilS14
With TilS14
Without TilS14
Nº of genomes with TilS15
Nº of genomes without TilS15
With TilS15
Without TilS15
p < 0.00005

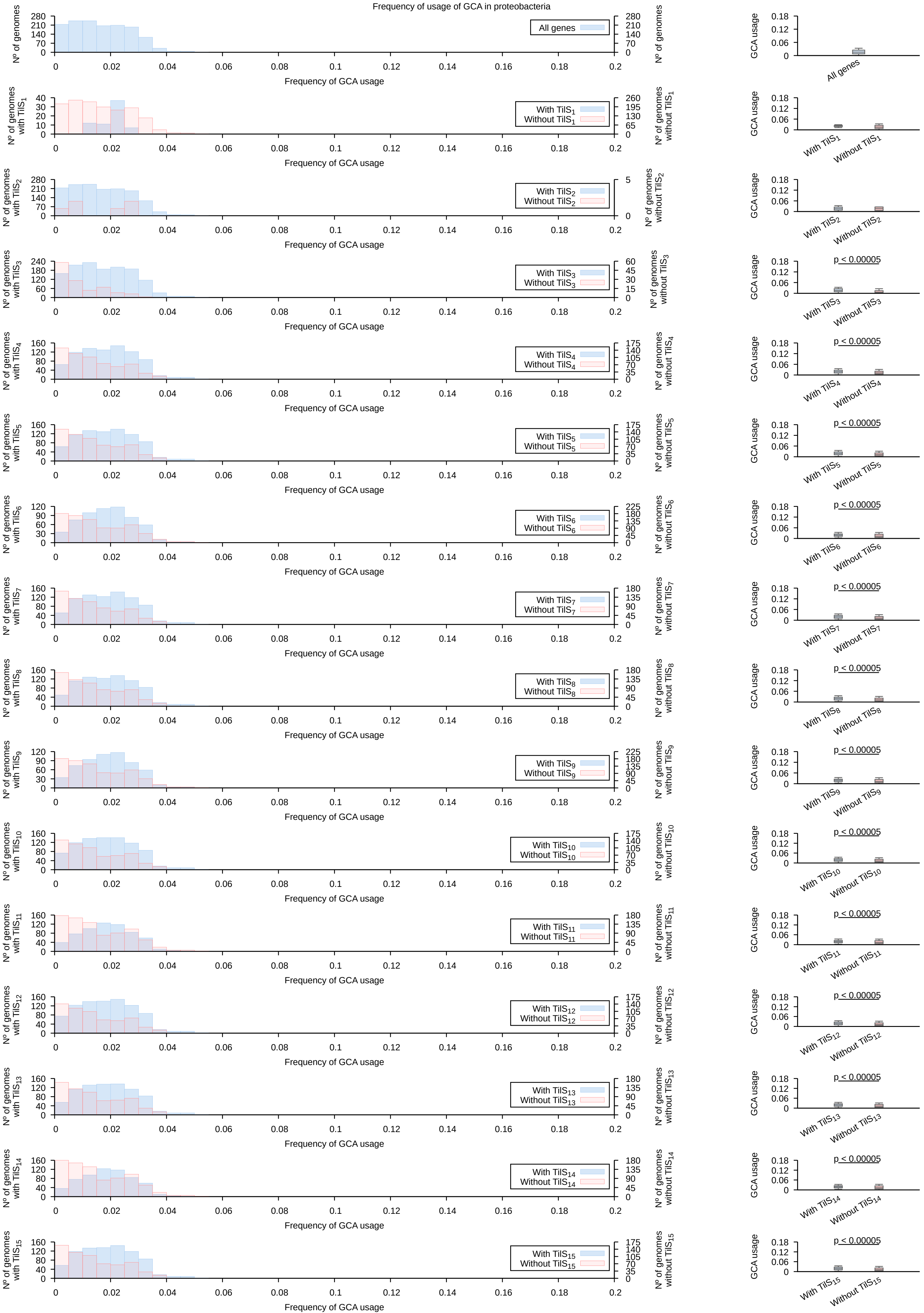

Frequency of usage of GCA in proteobacteria
Nº of genomes
All genes
Frequency of GCA usage
GCA usage
All genes
Nº of genomes with TilS$_1$
Nº of genomes without TilS$_1$
With TilS$_1$
Without TilS$_1$
Nº of genomes with TilS$_2$
Nº of genomes without TilS$_2$
With TilS$_2$
Without TilS$_2$
Nº of genomes with TilS$_3$
Nº of genomes without TilS$_3$
With TilS$_3$
Without TilS$_3$
p < 0.00005
Nº of genomes with TilS$_4$
Nº of genomes without TilS$_4$
With TilS$_4$
Without TilS$_4$
p < 0.00005
Nº of genomes with TilS$_5$
Nº of genomes without TilS$_5$
With TilS$_5$
Without TilS$_5$
p < 0.00005
Nº of genomes with TilS$_6$
Nº of genomes without TilS$_6$
With TilS$_6$
Without TilS$_6$
p < 0.00005
Nº of genomes with TilS$_7$
Nº of genomes without TilS$_7$
With TilS$_7$
Without TilS$_7$
p < 0.00005
Nº of genomes with TilS$_8$
Nº of genomes without TilS$_8$
With TilS$_8$
Without TilS$_8$
p < 0.00005
Nº of genomes with TilS$_9$
Nº of genomes without TilS$_9$
With TilS$_9$
Without TilS$_9$
p < 0.00005
Nº of genomes with TilS$_{10}$
Nº of genomes without TilS$_{10}$
With TilS$_{10}$
Without TilS$_{10}$
p < 0.00005
Nº of genomes with TilS$_{11}$
Nº of genomes without TilS$_{11}$
With TilS$_{11}$
Without TilS$_{11}$
p < 0.00005
Nº of genomes with TilS$_{12}$
Nº of genomes without TilS$_{12}$
With TilS$_{12}$
Without TilS$_{12}$
p < 0.00005
Nº of genomes with TilS$_{13}$
Nº of genomes without TilS$_{13}$
With TilS$_{13}$
Without TilS$_{13}$
p < 0.00005
Nº of genomes with TilS$_{14}$
Nº of genomes without TilS$_{14}$
With TilS$_{14}$
Without TilS$_{14}$
p < 0.00005
Nº of genomes with TilS$_{15}$
Nº of genomes without TilS$_{15}$
With TilS$_{15}$
Without TilS$_{15}$
p < 0.00005

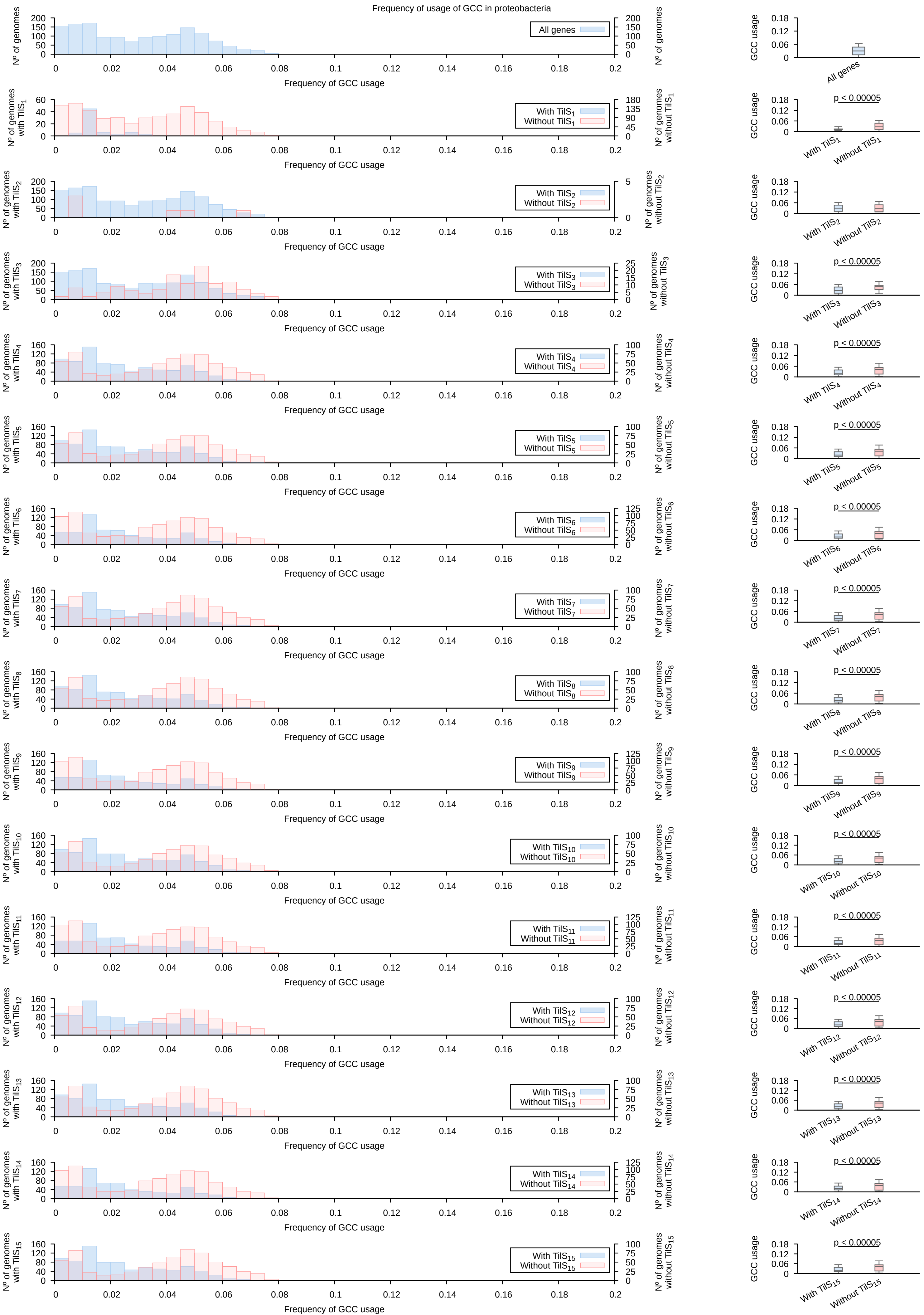

Frequency of usage of GCC in proteobacteria
Nº of genomes
All genes
Frequency of GCC usage
GCC usage
Nº of genomes with TilS$_1$
Nº of genomes without TilS$_1$
With TilS$_1$
Without TilS$_1$
p < 0.00005
Nº of genomes with TilS$_2$
Nº of genomes without TilS$_2$
With TilS$_2$
Without TilS$_2$
Nº of genomes with TilS$_3$
Nº of genomes without TilS$_3$
With TilS$_3$
Without TilS$_3$
Nº of genomes with TilS$_4$
Nº of genomes without TilS$_4$
With TilS$_4$
Without TilS$_4$
Nº of genomes with TilS$_5$
Nº of genomes without TilS$_5$
With TilS$_5$
Without TilS$_5$
Nº of genomes with TilS$_6$
Nº of genomes without TilS$_6$
With TilS$_6$
Without TilS$_6$
Nº of genomes with TilS$_7$
Nº of genomes without TilS$_7$
With TilS$_7$
Without TilS$_7$
Nº of genomes with TilS$_8$
Nº of genomes without TilS$_8$
With TilS$_8$
Without TilS$_8$
Nº of genomes with TilS$_9$
Nº of genomes without TilS$_9$
With TilS$_9$
Without TilS$_9$
Nº of genomes with TilS$_{10}$
Nº of genomes without TilS$_{10}$
With TilS$_{10}$
Without TilS$_{10}$
Nº of genomes with TilS$_{11}$
Nº of genomes without TilS$_{11}$
With TilS$_{11}$
Without TilS$_{11}$
Nº of genomes with TilS$_{12}$
Nº of genomes without TilS$_{12}$
With TilS$_{12}$
Without TilS$_{12}$
Nº of genomes with TilS$_{13}$
Nº of genomes without TilS$_{13}$
With TilS$_{13}$
Without TilS$_{13}$
Nº of genomes with TilS$_{14}$
Nº of genomes without TilS$_{14}$
With TilS$_{14}$
Without TilS$_{14}$
Nº of genomes with TilS$_{15}$
Nº of genomes without TilS$_{15}$
With TilS$_{15}$
Without TilS$_{15}$

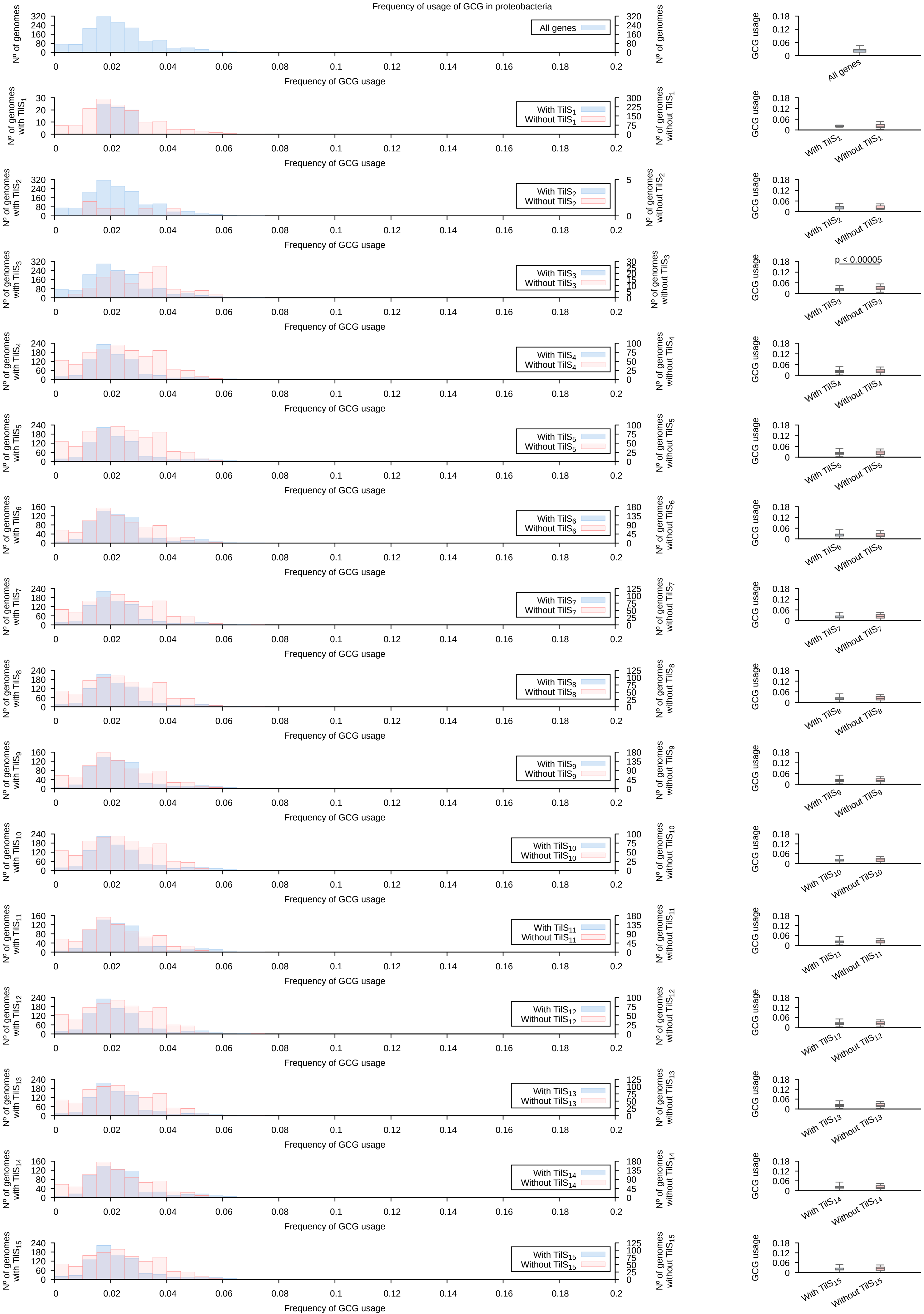
Frequency of usage of GCG in proteobacteria
Nº of genomes
All genes
Frequency of GCG usage
GCG usage
All genes
Nº of genomes with $TilS_1$
Nº of genomes without $TilS_1$
With $TilS_1$
Without $TilS_1$
Nº of genomes with $TilS_2$
Nº of genomes without $TilS_2$
With $TilS_2$
Without $TilS_2$
Nº of genomes with $TilS_3$
Nº of genomes without $TilS_3$
With $TilS_3$
Without $TilS_3$
p < 0.00005
Nº of genomes with $TilS_4$
Nº of genomes without $TilS_4$
With $TilS_4$
Without $TilS_4$
Nº of genomes with $TilS_5$
Nº of genomes without $TilS_5$
With $TilS_5$
Without $TilS_5$
Nº of genomes with $TilS_6$
Nº of genomes without $TilS_6$
With $TilS_6$
Without $TilS_6$
Nº of genomes with $TilS_7$
Nº of genomes without $TilS_7$
With $TilS_7$
Without $TilS_7$
Nº of genomes with $TilS_8$
Nº of genomes without $TilS_8$
With $TilS_8$
Without $TilS_8$
Nº of genomes with $TilS_9$
Nº of genomes without $TilS_9$
With $TilS_9$
Without $TilS_9$
Nº of genomes with $TilS_{10}$
Nº of genomes without $TilS_{10}$
With $TilS_{10}$
Without $TilS_{10}$
Nº of genomes with $TilS_{11}$
Nº of genomes without $TilS_{11}$
With $TilS_{11}$
Without $TilS_{11}$
Nº of genomes with $TilS_{12}$
Nº of genomes without $TilS_{12}$
With $TilS_{12}$
Without $TilS_{12}$
Nº of genomes with $TilS_{13}$
Nº of genomes without $TilS_{13}$
With $TilS_{13}$
Without $TilS_{13}$
Nº of genomes with $TilS_{14}$
Nº of genomes without $TilS_{14}$
With $TilS_{14}$
Without $TilS_{14}$
Nº of genomes with $TilS_{15}$
Nº of genomes without $TilS_{15}$
With $TilS_{15}$
Without $TilS_{15}$

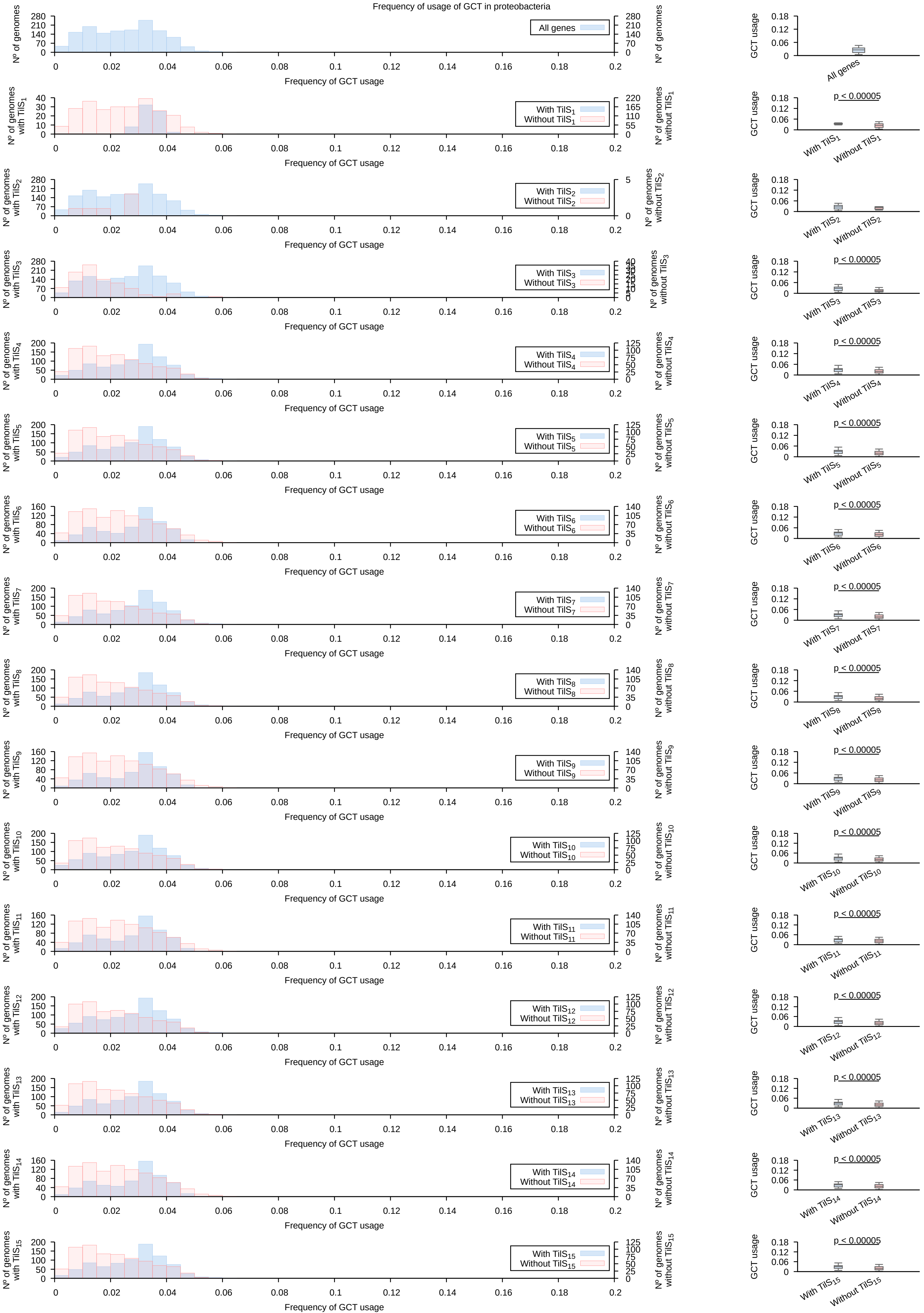

Frequency of usage of GCT in proteobacteria
Nº of genomes
All genes
Frequency of GCT usage
GCT usage
Nº of genomes with TilS$_1$
Nº of genomes without TilS$_1$
With TilS$_1$
Without TilS$_1$
p < 0.00005
With TilS$_2$
Without TilS$_2$
With TilS$_3$
Without TilS$_3$
With TilS$_4$
Without TilS$_4$
With TilS$_5$
Without TilS$_5$
With TilS$_6$
Without TilS$_6$
With TilS$_7$
Without TilS$_7$
With TilS$_8$
Without TilS$_8$
With TilS$_9$
Without TilS$_9$
With TilS$_{10}$
Without TilS$_{10}$
With TilS$_{11}$
Without TilS$_{11}$
With TilS$_{12}$
Without TilS$_{12}$
With TilS$_{13}$
Without TilS$_{13}$
With TilS$_{14}$
Without TilS$_{14}$
With TilS$_{15}$
Without TilS$_{15}$

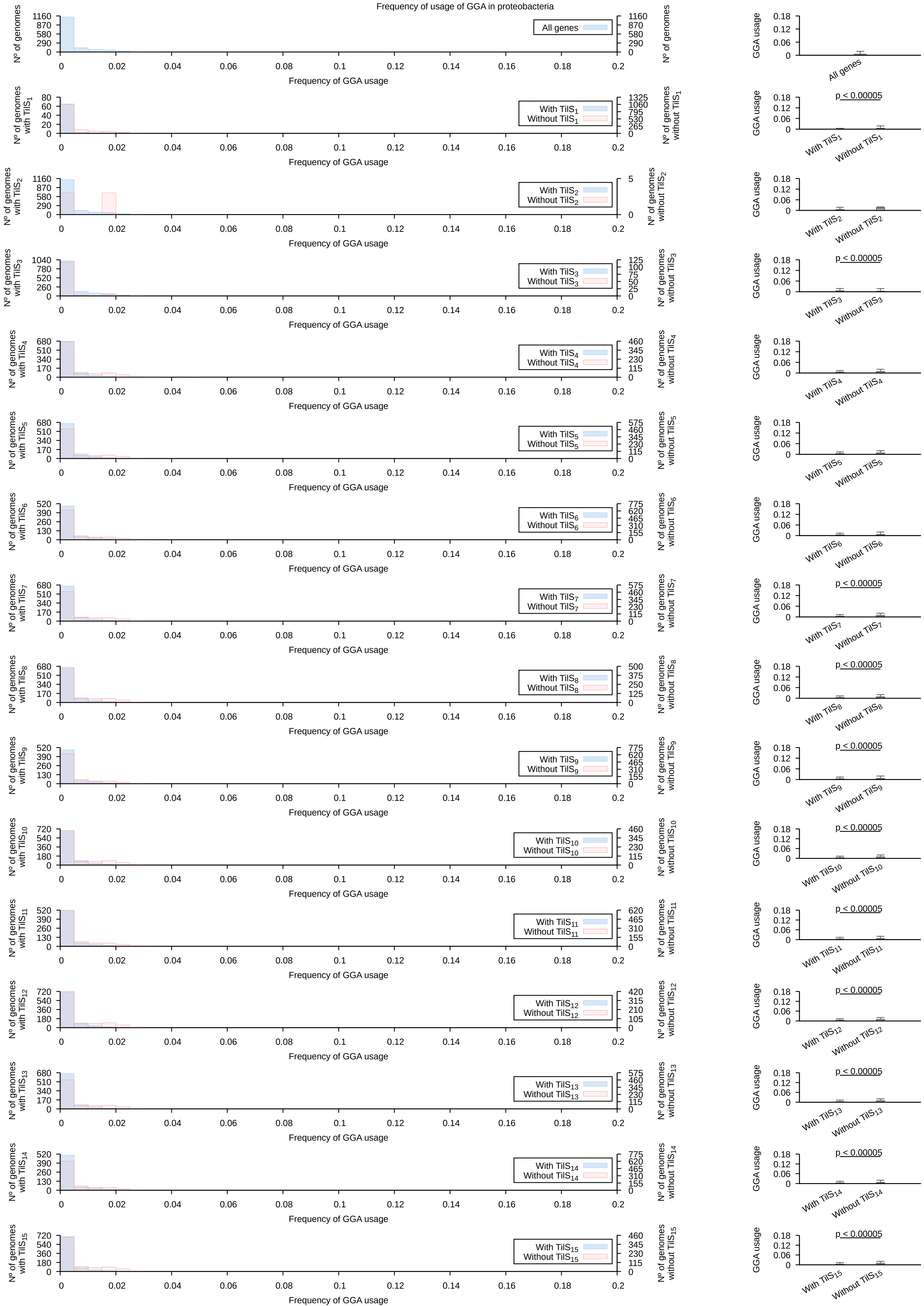

Frequency of usage of GGA in proteobacteria
Nº of genomes
All genes
Frequency of GGA usage
GGA usage
All genes
Nº of genomes with TilS1
Nº of genomes without TilS1
With TilS1
Without TilS1
p < 0.00005
Nº of genomes with TilS2
Nº of genomes without TilS2
With TilS2
Without TilS2
Nº of genomes with TilS3
Nº of genomes without TilS3
With TilS3
Without TilS3
p < 0.00005
Nº of genomes with TilS4
Nº of genomes without TilS4
With TilS4
Without TilS4
Nº of genomes with TilS5
Nº of genomes without TilS5
With TilS5
Without TilS5
Nº of genomes with TilS6
Nº of genomes without TilS6
With TilS6
Without TilS6
Nº of genomes with TilS7
Nº of genomes without TilS7
With TilS7
Without TilS7
p < 0.00005
Nº of genomes with TilS8
Nº of genomes without TilS8
With TilS8
Without TilS8
p < 0.00005
Nº of genomes with TilS9
Nº of genomes without TilS9
With TilS9
Without TilS9
p < 0.00005
Nº of genomes with TilS10
Nº of genomes without TilS10
With TilS10
Without TilS10
p < 0.00005
Nº of genomes with TilS11
Nº of genomes without TilS11
With TilS11
Without TilS11
p < 0.00005
Nº of genomes with TilS12
Nº of genomes without TilS12
With TilS12
Without TilS12
p < 0.00005
Nº of genomes with TilS13
Nº of genomes without TilS13
With TilS13
Without TilS13
p < 0.00005
Nº of genomes with TilS14
Nº of genomes without TilS14
With TilS14
Without TilS14
p < 0.00005
Nº of genomes with TilS15
Nº of genomes without TilS15
With TilS15
Without TilS15
p < 0.00005

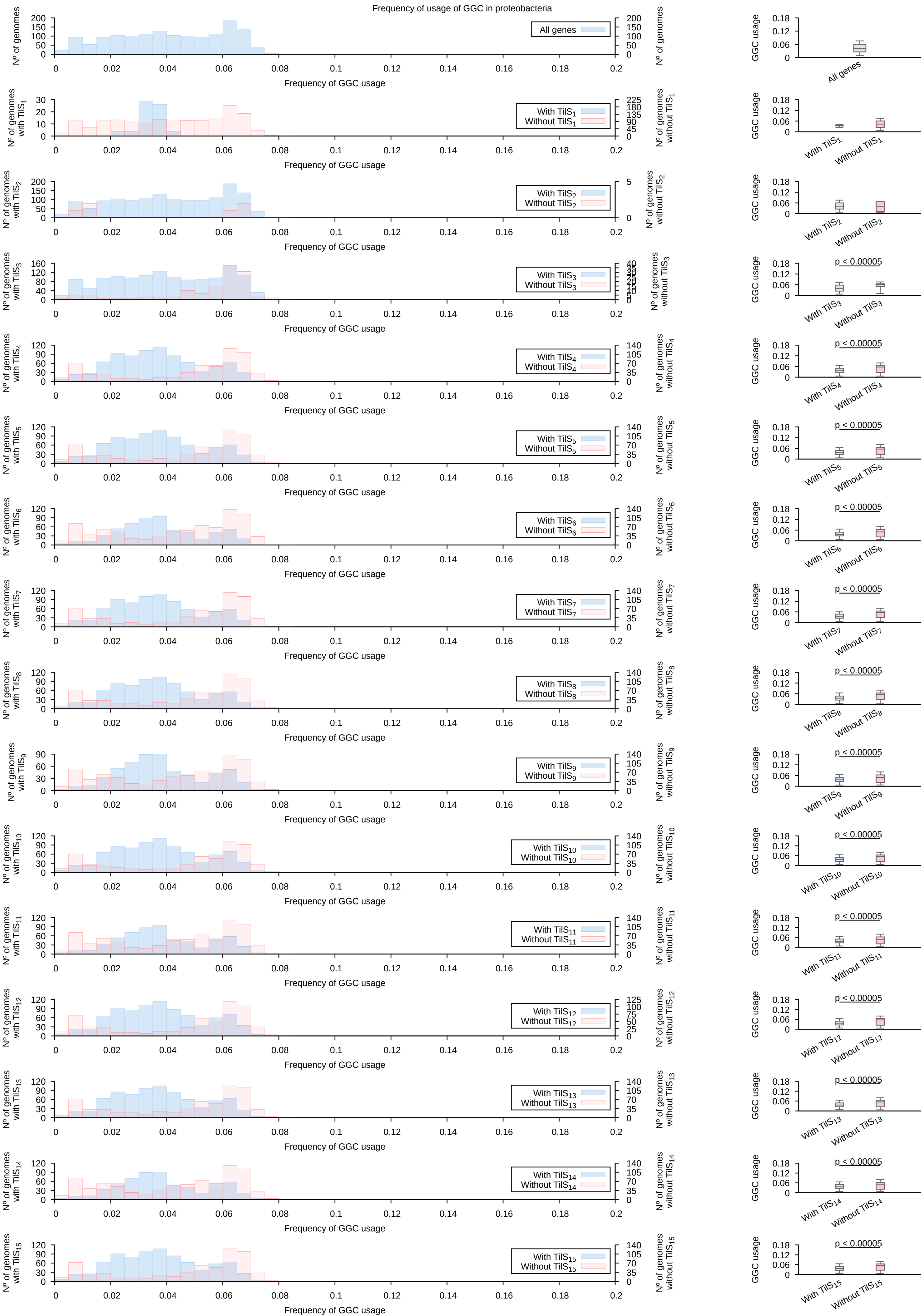
Frequency of usage of GGC in proteobacteria
Nº of genomes
All genes
Frequency of GGC usage
GGC usage
Nº of genomes with TilS$_1$
Nº of genomes without TilS$_1$
With TilS$_1$
Without TilS$_1$
Nº of genomes with TilS$_2$
Nº of genomes without TilS$_2$
With TilS$_2$
Without TilS$_2$
Nº of genomes with TilS$_3$
Nº of genomes without TilS$_3$
With TilS$_3$
Without TilS$_3$
p < 0.00005
Nº of genomes with TilS$_4$
Nº of genomes without TilS$_4$
With TilS$_4$
Without TilS$_4$
Nº of genomes with TilS$_5$
Nº of genomes without TilS$_5$
With TilS$_5$
Without TilS$_5$
Nº of genomes with TilS$_6$
Nº of genomes without TilS$_6$
With TilS$_6$
Without TilS$_6$
Nº of genomes with TilS$_7$
Nº of genomes without TilS$_7$
With TilS$_7$
Without TilS$_7$
Nº of genomes with TilS$_8$
Nº of genomes without TilS$_8$
With TilS$_8$
Without TilS$_8$
Nº of genomes with TilS$_9$
Nº of genomes without TilS$_9$
With TilS$_9$
Without TilS$_9$
Nº of genomes with TilS$_{10}$
Nº of genomes without TilS$_{10}$
With TilS$_{10}$
Without TilS$_{10}$
Nº of genomes with TilS$_{11}$
Nº of genomes without TilS$_{11}$
With TilS$_{11}$
Without TilS$_{11}$
Nº of genomes with TilS$_{12}$
Nº of genomes without TilS$_{12}$
With TilS$_{12}$
Without TilS$_{12}$
Nº of genomes with TilS$_{13}$
Nº of genomes without TilS$_{13}$
With TilS$_{13}$
Without TilS$_{13}$
Nº of genomes with TilS$_{14}$
Nº of genomes without TilS$_{14}$
With TilS$_{14}$
Without TilS$_{14}$
Nº of genomes with TilS$_{15}$
Nº of genomes without TilS$_{15}$
With TilS$_{15}$
Without TilS$_{15}$

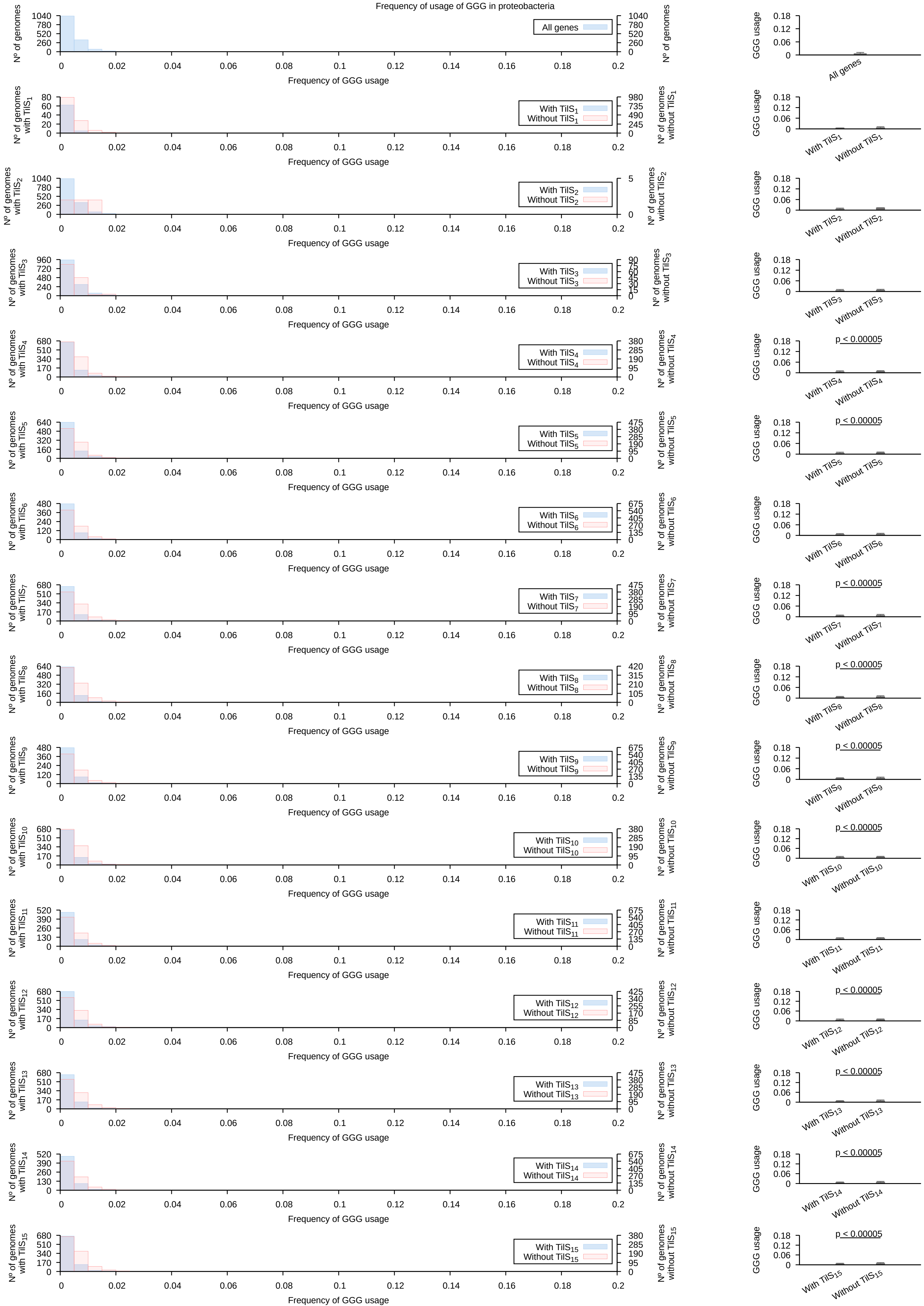
Frequency of usage of GGG in proteobacteria
Nº of genomes
All genes
Frequency of GGG usage
GGG usage
With TilS1
Without TilS1
With TilS2
Without TilS2
With TilS3
Without TilS3
With TilS4
Without TilS4
With TilS5
Without TilS5
With TilS6
Without TilS6
With TilS7
Without TilS7
With TilS8
Without TilS8
With TilS9
Without TilS9
With TilS10
Without TilS10
With TilS11
Without TilS11
With TilS12
Without TilS12
With TilS13
Without TilS13
With TilS14
Without TilS14
With TilS15
Without TilS15
p < 0.00005

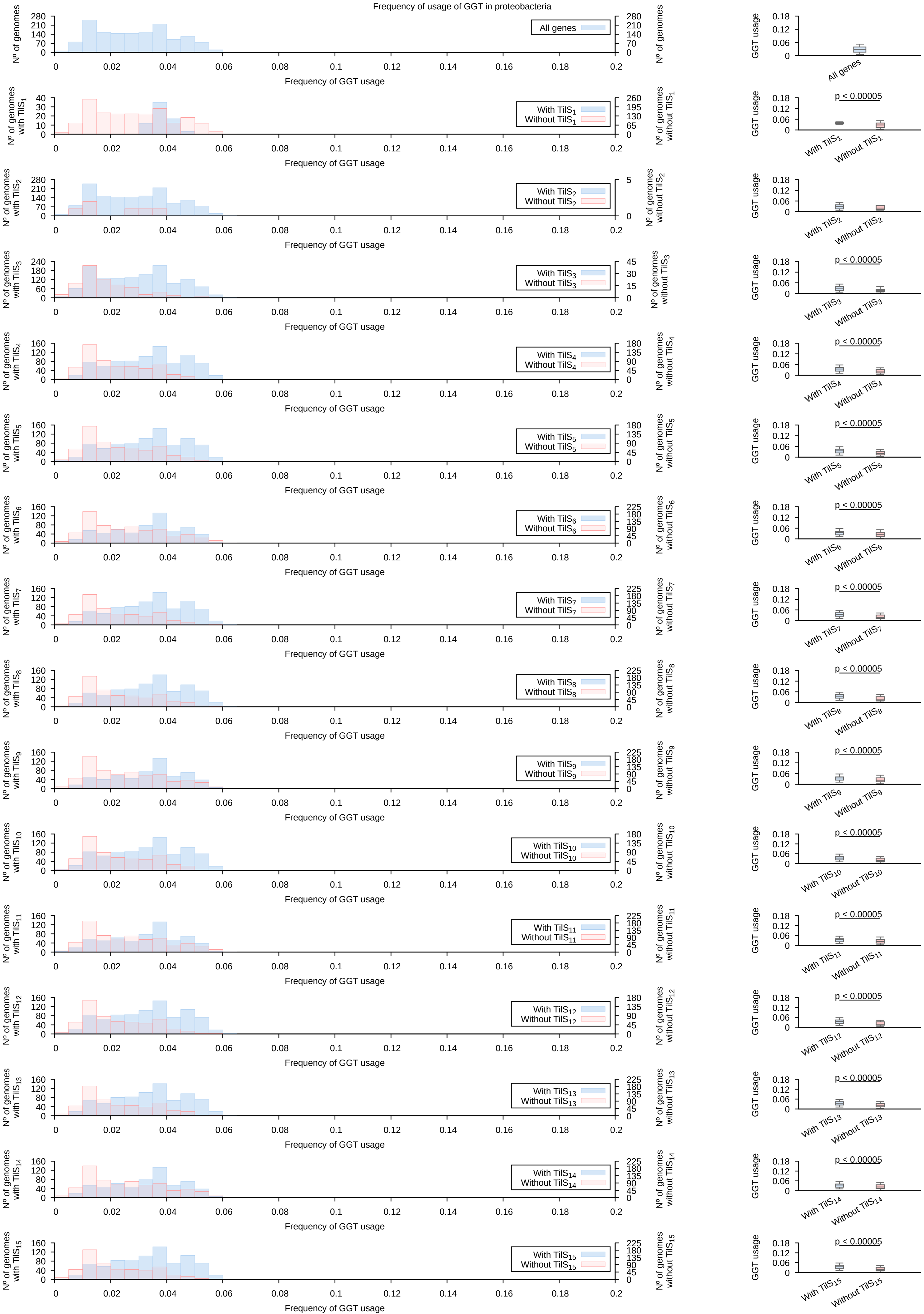
Frequency of usage of GGT in proteobacteria
Nº of genomes
All genes
Frequency of GGT usage
GGT usage
All genes
Nº of genomes with TilS$_1$
Nº of genomes without TilS$_1$
With TilS$_1$
Without TilS$_1$
p < 0.00005
Nº of genomes with TilS$_2$
Nº of genomes without TilS$_2$
With TilS$_2$
Without TilS$_2$
Nº of genomes with TilS$_3$
Nº of genomes without TilS$_3$
With TilS$_3$
Without TilS$_3$
p < 0.00005
Nº of genomes with TilS$_4$
Nº of genomes without TilS$_4$
With TilS$_4$
Without TilS$_4$
p < 0.00005
Nº of genomes with TilS$_5$
Nº of genomes without TilS$_5$
With TilS$_5$
Without TilS$_5$
p < 0.00005
Nº of genomes with TilS$_6$
Nº of genomes without TilS$_6$
With TilS$_6$
Without TilS$_6$
p < 0.00005
Nº of genomes with TilS$_7$
Nº of genomes without TilS$_7$
With TilS$_7$
Without TilS$_7$
p < 0.00005
Nº of genomes with TilS$_8$
Nº of genomes without TilS$_8$
With TilS$_8$
Without TilS$_8$
p < 0.00005
Nº of genomes with TilS$_9$
Nº of genomes without TilS$_9$
With TilS$_9$
Without TilS$_9$
p < 0.00005
Nº of genomes with TilS$_{10}$
Nº of genomes without TilS$_{10}$
With TilS$_{10}$
Without TilS$_{10}$
p < 0.00005
Nº of genomes with TilS$_{11}$
Nº of genomes without TilS$_{11}$
With TilS$_{11}$
Without TilS$_{11}$
p < 0.00005
Nº of genomes with TilS$_{12}$
Nº of genomes without TilS$_{12}$
With TilS$_{12}$
Without TilS$_{12}$
p < 0.00005
Nº of genomes with TilS$_{13}$
Nº of genomes without TilS$_{13}$
With TilS$_{13}$
Without TilS$_{13}$
p < 0.00005
Nº of genomes with TilS$_{14}$
Nº of genomes without TilS$_{14}$
With TilS$_{14}$
Without TilS$_{14}$
p < 0.00005
Nº of genomes with TilS$_{15}$
Nº of genomes without TilS$_{15}$
With TilS$_{15}$
Without TilS$_{15}$
p < 0.00005

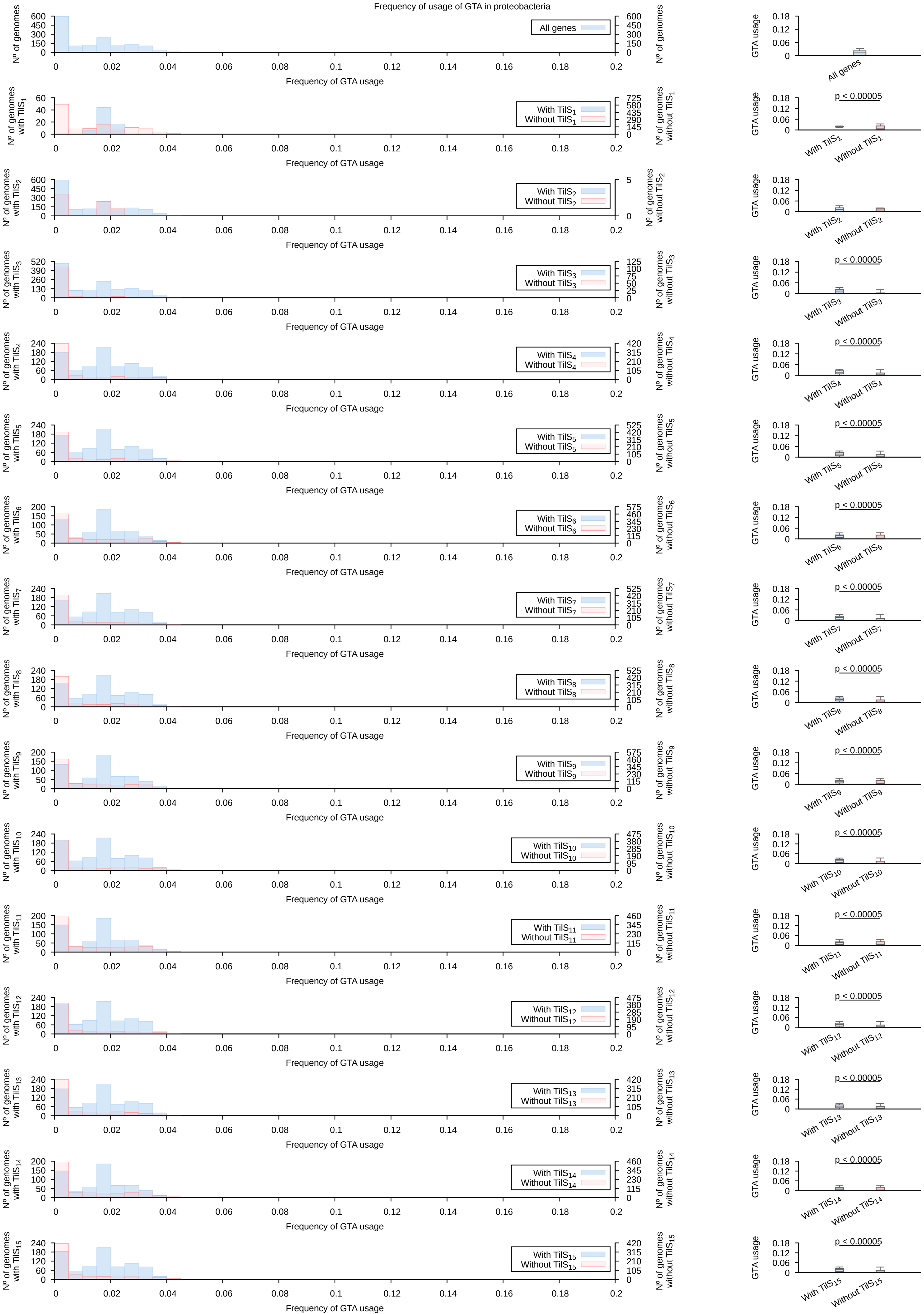

Frequency of usage of GTA in proteobacteria
Nº of genomes
All genes
Frequency of GTA usage
GTA usage
All genes
Nº of genomes with $TilS_1$
With $TilS_1$
Without $TilS_1$
Nº of genomes without $TilS_1$
$p < 0.00005$
Nº of genomes with $TilS_2$
With $TilS_2$
Without $TilS_2$
Nº of genomes without $TilS_2$
Nº of genomes with $TilS_3$
With $TilS_3$
Without $TilS_3$
Nº of genomes without $TilS_3$
Nº of genomes with $TilS_4$
With $TilS_4$
Without $TilS_4$
Nº of genomes without $TilS_4$
Nº of genomes with $TilS_5$
With $TilS_5$
Without $TilS_5$
Nº of genomes without $TilS_5$
Nº of genomes with $TilS_6$
With $TilS_6$
Without $TilS_6$
Nº of genomes without $TilS_6$
Nº of genomes with $TilS_7$
With $TilS_7$
Without $TilS_7$
Nº of genomes without $TilS_7$
Nº of genomes with $TilS_8$
With $TilS_8$
Without $TilS_8$
Nº of genomes without $TilS_8$
Nº of genomes with $TilS_9$
With $TilS_9$
Without $TilS_9$
Nº of genomes without $TilS_9$
Nº of genomes with $TilS_{10}$
With $TilS_{10}$
Without $TilS_{10}$
Nº of genomes without $TilS_{10}$
Nº of genomes with $TilS_{11}$
With $TilS_{11}$
Without $TilS_{11}$
Nº of genomes without $TilS_{11}$
Nº of genomes with $TilS_{12}$
With $TilS_{12}$
Without $TilS_{12}$
Nº of genomes without $TilS_{12}$
Nº of genomes with $TilS_{13}$
With $TilS_{13}$
Without $TilS_{13}$
Nº of genomes without $TilS_{13}$
Nº of genomes with $TilS_{14}$
With $TilS_{14}$
Without $TilS_{14}$
Nº of genomes without $TilS_{14}$
Nº of genomes with $TilS_{15}$
With $TilS_{15}$
Without $TilS_{15}$
Nº of genomes without $TilS_{15}$

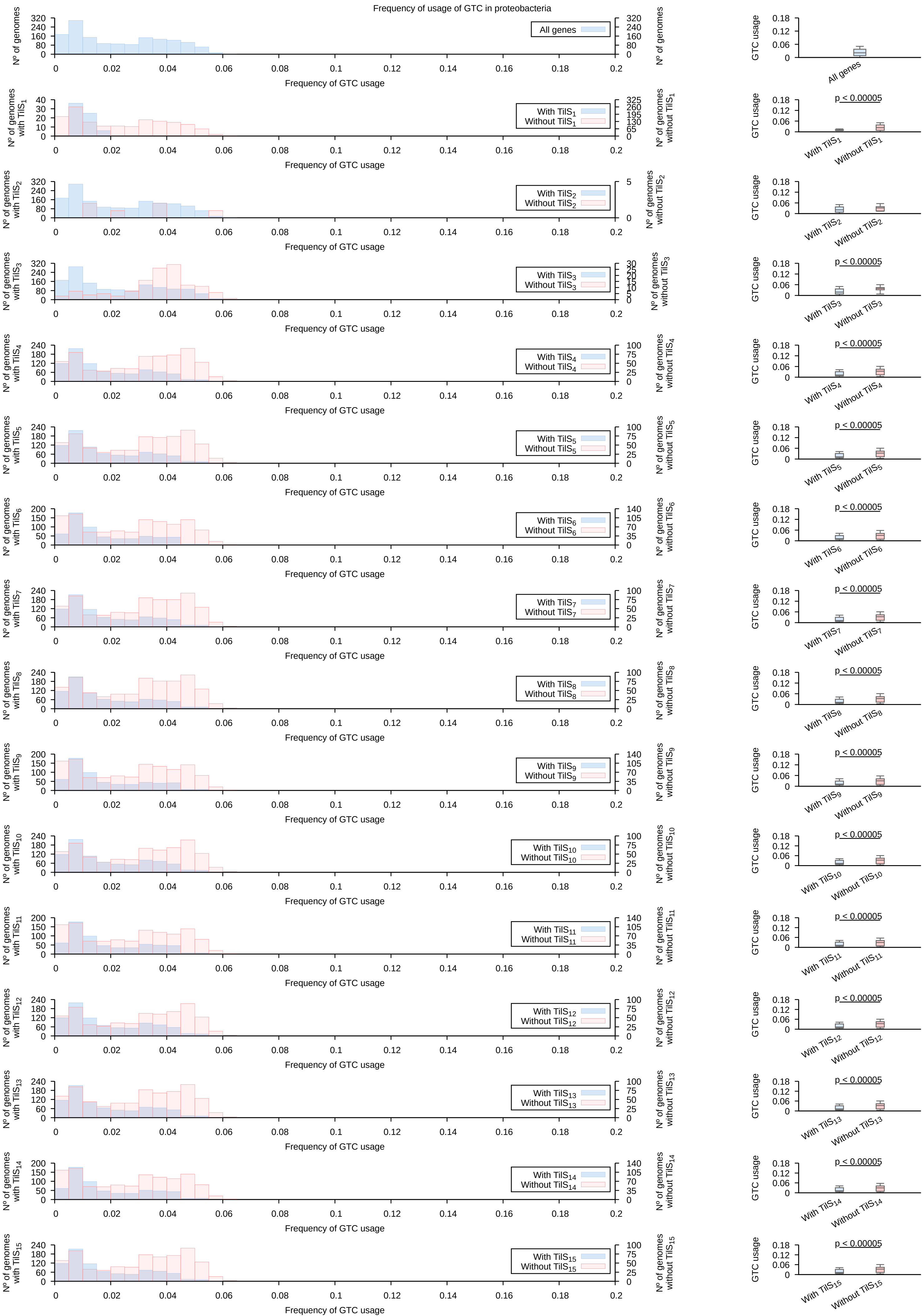
Frequency of usage of GTC in proteobacteria
Nº of genomes
All genes
Frequency of GTC usage
GTC usage
Nº of genomes with TilS$_1$
Nº of genomes without TilS$_1$
With TilS$_1$
Without TilS$_1$
p < 0.00005
Nº of genomes with TilS$_2$
Nº of genomes without TilS$_2$
With TilS$_2$
Without TilS$_2$
Nº of genomes with TilS$_3$
Nº of genomes without TilS$_3$
With TilS$_3$
Without TilS$_3$
Nº of genomes with TilS$_4$
Nº of genomes without TilS$_4$
With TilS$_4$
Without TilS$_4$
Nº of genomes with TilS$_5$
Nº of genomes without TilS$_5$
With TilS$_5$
Without TilS$_5$
Nº of genomes with TilS$_6$
Nº of genomes without TilS$_6$
With TilS$_6$
Without TilS$_6$
Nº of genomes with TilS$_7$
Nº of genomes without TilS$_7$
With TilS$_7$
Without TilS$_7$
Nº of genomes with TilS$_8$
Nº of genomes without TilS$_8$
With TilS$_8$
Without TilS$_8$
Nº of genomes with TilS$_9$
Nº of genomes without TilS$_9$
With TilS$_9$
Without TilS$_9$
Nº of genomes with TilS$_{10}$
Nº of genomes without TilS$_{10}$
With TilS$_{10}$
Without TilS$_{10}$
Nº of genomes with TilS$_{11}$
Nº of genomes without TilS$_{11}$
With TilS$_{11}$
Without TilS$_{11}$
Nº of genomes with TilS$_{12}$
Nº of genomes without TilS$_{12}$
With TilS$_{12}$
Without TilS$_{12}$
Nº of genomes with TilS$_{13}$
Nº of genomes without TilS$_{13}$
With TilS$_{13}$
Without TilS$_{13}$
Nº of genomes with TilS$_{14}$
Nº of genomes without TilS$_{14}$
With TilS$_{14}$
Without TilS$_{14}$
Nº of genomes with TilS$_{15}$
Nº of genomes without TilS$_{15}$
With TilS$_{15}$
Without TilS$_{15}$

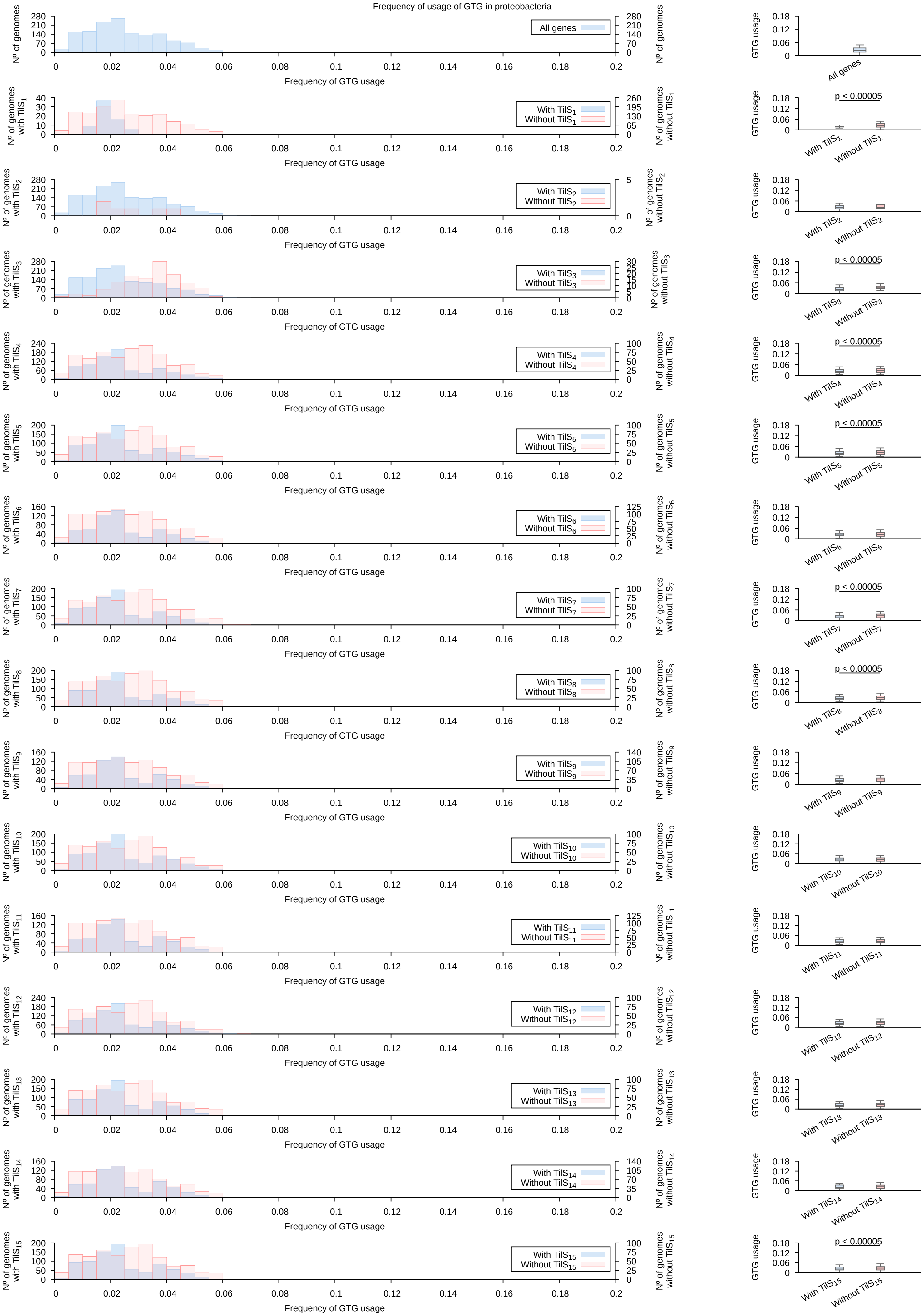
Frequency of usage of GTG in proteobacteria
Nº of genomes
All genes
Frequency of GTG usage
GTG usage
Nº of genomes with TilS$_1$
Nº of genomes without TilS$_1$
With TilS$_1$
Without TilS$_1$
p < 0.00005
With TilS$_2$
Without TilS$_2$
With TilS$_3$
Without TilS$_3$
p < 0.00005
With TilS$_4$
Without TilS$_4$
p < 0.00005
With TilS$_5$
Without TilS$_5$
p < 0.00005
With TilS$_6$
Without TilS$_6$
With TilS$_7$
Without TilS$_7$
p < 0.00005
With TilS$_8$
Without TilS$_8$
p < 0.00005
With TilS$_9$
Without TilS$_9$
With TilS$_{10}$
Without TilS$_{10}$
With TilS$_{11}$
Without TilS$_{11}$
With TilS$_{12}$
Without TilS$_{12}$
With TilS$_{13}$
Without TilS$_{13}$
With TilS$_{14}$
Without TilS$_{14}$
With TilS$_{15}$
Without TilS$_{15}$
p < 0.00005

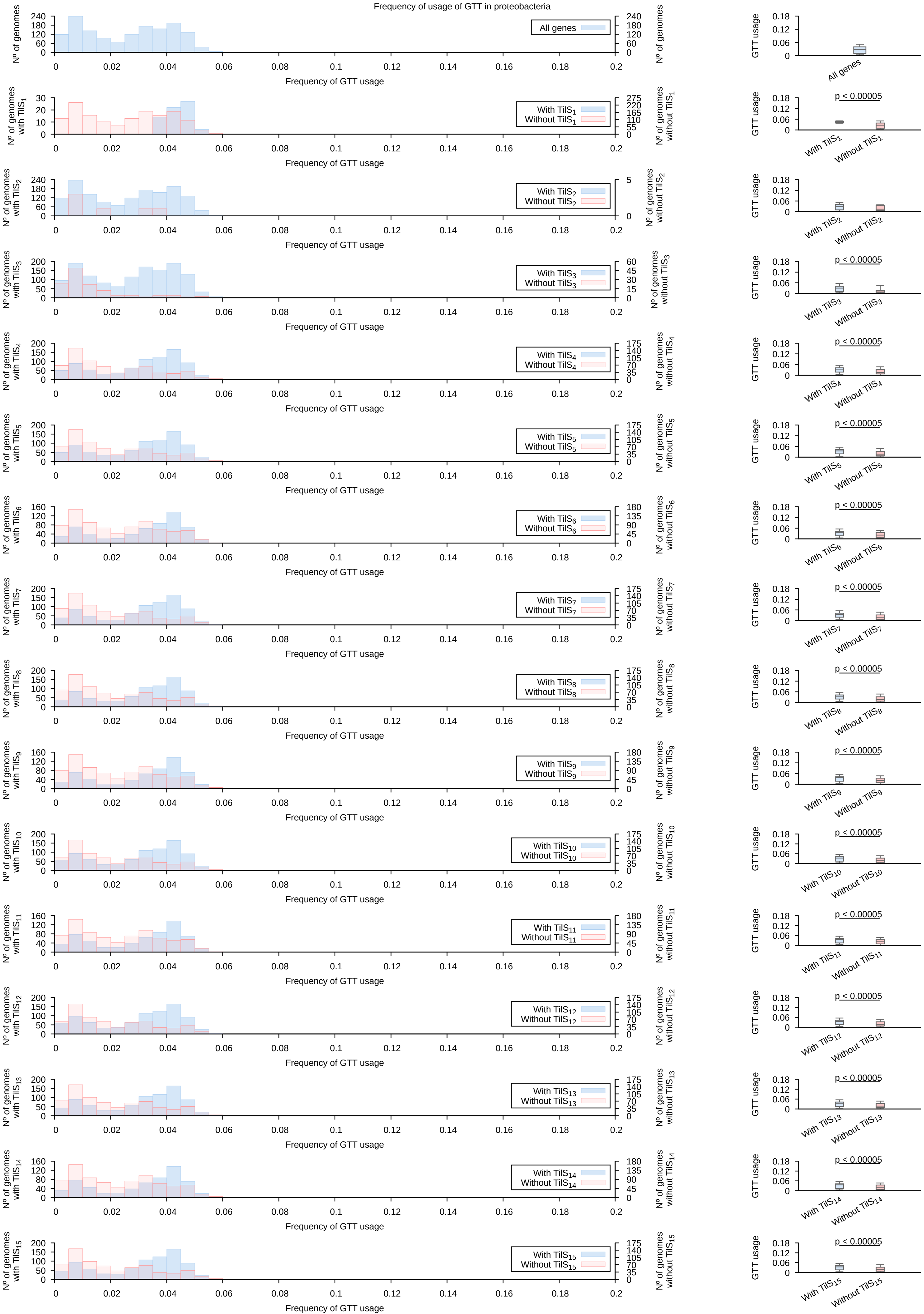
Frequency of usage of GTT in proteobacteria
Nº of genomes
All genes
Frequency of GTT usage
GTT usage
p < 0.00005
Nº of genomes with TilS$_1$
Nº of genomes without TilS$_1$
With TilS$_1$
Without TilS$_1$
Nº of genomes with TilS$_2$
Nº of genomes without TilS$_2$
With TilS$_2$
Without TilS$_2$
Nº of genomes with TilS$_3$
Nº of genomes without TilS$_3$
With TilS$_3$
Without TilS$_3$
Nº of genomes with TilS$_4$
Nº of genomes without TilS$_4$
With TilS$_4$
Without TilS$_4$
Nº of genomes with TilS$_5$
Nº of genomes without TilS$_5$
With TilS$_5$
Without TilS$_5$
Nº of genomes with TilS$_6$
Nº of genomes without TilS$_6$
With TilS$_6$
Without TilS$_6$
Nº of genomes with TilS$_7$
Nº of genomes without TilS$_7$
With TilS$_7$
Without TilS$_7$
Nº of genomes with TilS$_8$
Nº of genomes without TilS$_8$
With TilS$_8$
Without TilS$_8$
Nº of genomes with TilS$_9$
Nº of genomes without TilS$_9$
With TilS$_9$
Without TilS$_9$
Nº of genomes with TilS$_{10}$
Nº of genomes without TilS$_{10}$
With TilS$_{10}$
Without TilS$_{10}$
Nº of genomes with TilS$_{11}$
Nº of genomes without TilS$_{11}$
With TilS$_{11}$
Without TilS$_{11}$
Nº of genomes with TilS$_{12}$
Nº of genomes without TilS$_{12}$
With TilS$_{12}$
Without TilS$_{12}$
Nº of genomes with TilS$_{13}$
Nº of genomes without TilS$_{13}$
With TilS$_{13}$
Without TilS$_{13}$
Nº of genomes with TilS$_{14}$
Nº of genomes without TilS$_{14}$
With TilS$_{14}$
Without TilS$_{14}$
Nº of genomes with TilS$_{15}$
Nº of genomes without TilS$_{15}$
With TilS$_{15}$
Without TilS$_{15}$
0 0.02 0.04 0.06 0.08 0.1 0.12 0.14 0.16 0.18 0.2
0 0.06 0.12 0.18

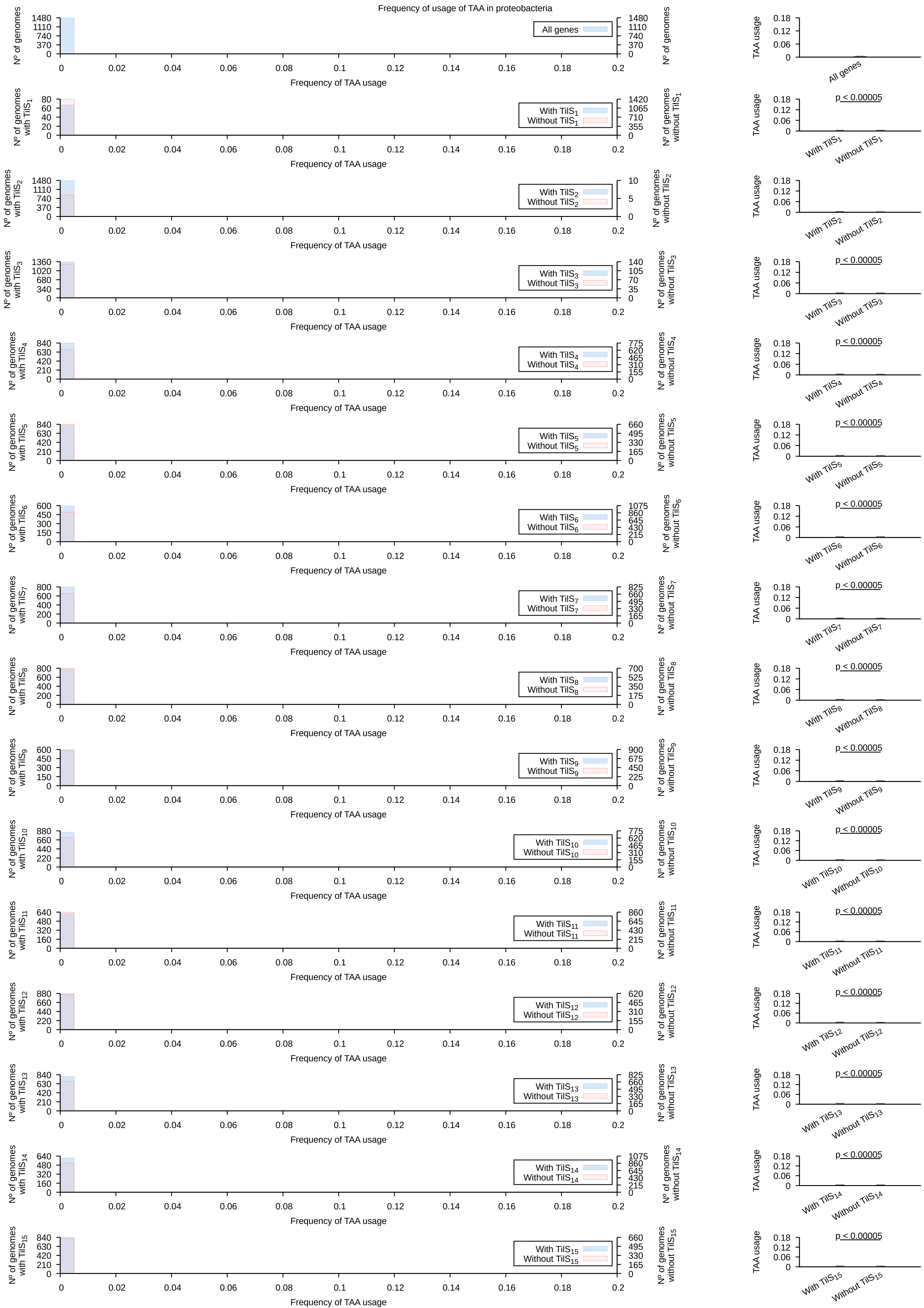

Frequency of usage of TAA in proteobacteria
Nº of genomes
All genes
TAA usage
Frequency of TAA usage
Nº of genomes with TilS1
Nº of genomes without TilS1
With TilS1
Without TilS1
p < 0.00005
Nº of genomes with TilS2
Nº of genomes without TilS2
With TilS2
Without TilS2
Nº of genomes with TilS3
Nº of genomes without TilS3
With TilS3
Without TilS3
Nº of genomes with TilS4
Nº of genomes without TilS4
With TilS4
Without TilS4
Nº of genomes with TilS5
Nº of genomes without TilS5
With TilS5
Without TilS5
Nº of genomes with TilS6
Nº of genomes without TilS6
With TilS6
Without TilS6
Nº of genomes with TilS7
Nº of genomes without TilS7
With TilS7
Without TilS7
Nº of genomes with TilS8
Nº of genomes without TilS8
With TilS8
Without TilS8
Nº of genomes with TilS9
Nº of genomes without TilS9
With TilS9
Without TilS9
Nº of genomes with TilS10
Nº of genomes without TilS10
With TilS10
Without TilS10
Nº of genomes with TilS11
Nº of genomes without TilS11
With TilS11
Without TilS11
Nº of genomes with TilS12
Nº of genomes without TilS12
With TilS12
Without TilS12
Nº of genomes with TilS13
Nº of genomes without TilS13
With TilS13
Without TilS13
Nº of genomes with TilS14
Nº of genomes without TilS14
With TilS14
Without TilS14
Nº of genomes with TilS15
Nº of genomes without TilS15
With TilS15
Without TilS15

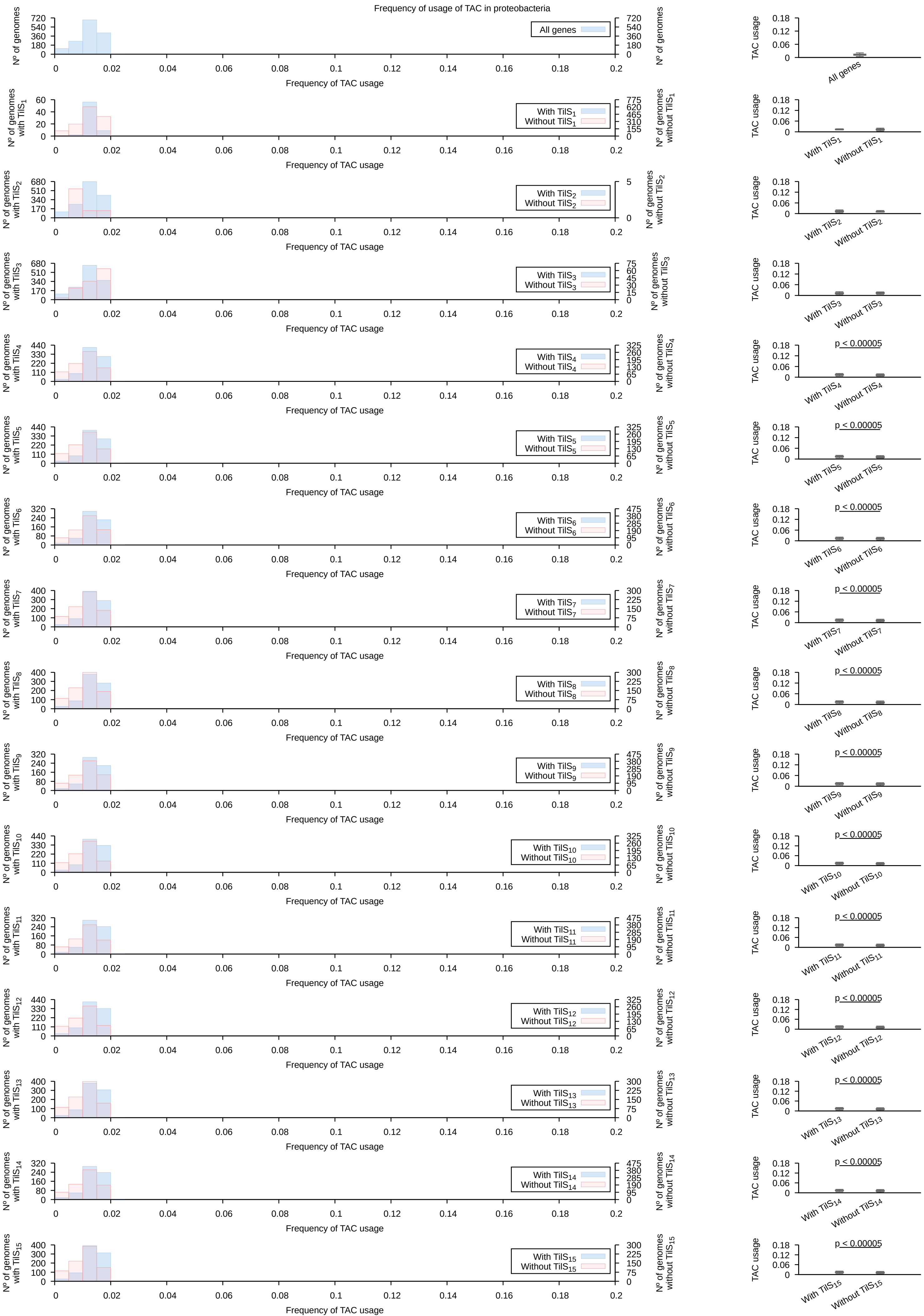

Frequency of usage of TAC in proteobacteria
Nº of genomes
All genes
Frequency of TAC usage
TAC usage
Nº of genomes with TilS$_1$
Nº of genomes without TilS$_1$
With TilS$_1$
Without TilS$_1$
Nº of genomes with TilS$_2$
Nº of genomes without TilS$_2$
With TilS$_2$
Without TilS$_2$
Nº of genomes with TilS$_3$
Nº of genomes without TilS$_3$
With TilS$_3$
Without TilS$_3$
Nº of genomes with TilS$_4$
Nº of genomes without TilS$_4$
With TilS$_4$
Without TilS$_4$
p < 0.00005
Nº of genomes with TilS$_5$
Nº of genomes without TilS$_5$
With TilS$_5$
Without TilS$_5$
Nº of genomes with TilS$_6$
Nº of genomes without TilS$_6$
With TilS$_6$
Without TilS$_6$
Nº of genomes with TilS$_7$
Nº of genomes without TilS$_7$
With TilS$_7$
Without TilS$_7$
Nº of genomes with TilS$_8$
Nº of genomes without TilS$_8$
With TilS$_8$
Without TilS$_8$
Nº of genomes with TilS$_9$
Nº of genomes without TilS$_9$
With TilS$_9$
Without TilS$_9$
Nº of genomes with TilS$_{10}$
Nº of genomes without TilS$_{10}$
With TilS$_{10}$
Without TilS$_{10}$
Nº of genomes with TilS$_{11}$
Nº of genomes without TilS$_{11}$
With TilS$_{11}$
Without TilS$_{11}$
Nº of genomes with TilS$_{12}$
Nº of genomes without TilS$_{12}$
With TilS$_{12}$
Without TilS$_{12}$
Nº of genomes with TilS$_{13}$
Nº of genomes without TilS$_{13}$
With TilS$_{13}$
Without TilS$_{13}$
Nº of genomes with TilS$_{14}$
Nº of genomes without TilS$_{14}$
With TilS$_{14}$
Without TilS$_{14}$
Nº of genomes with TilS$_{15}$
Nº of genomes without TilS$_{15}$
With TilS$_{15}$
Without TilS$_{15}$

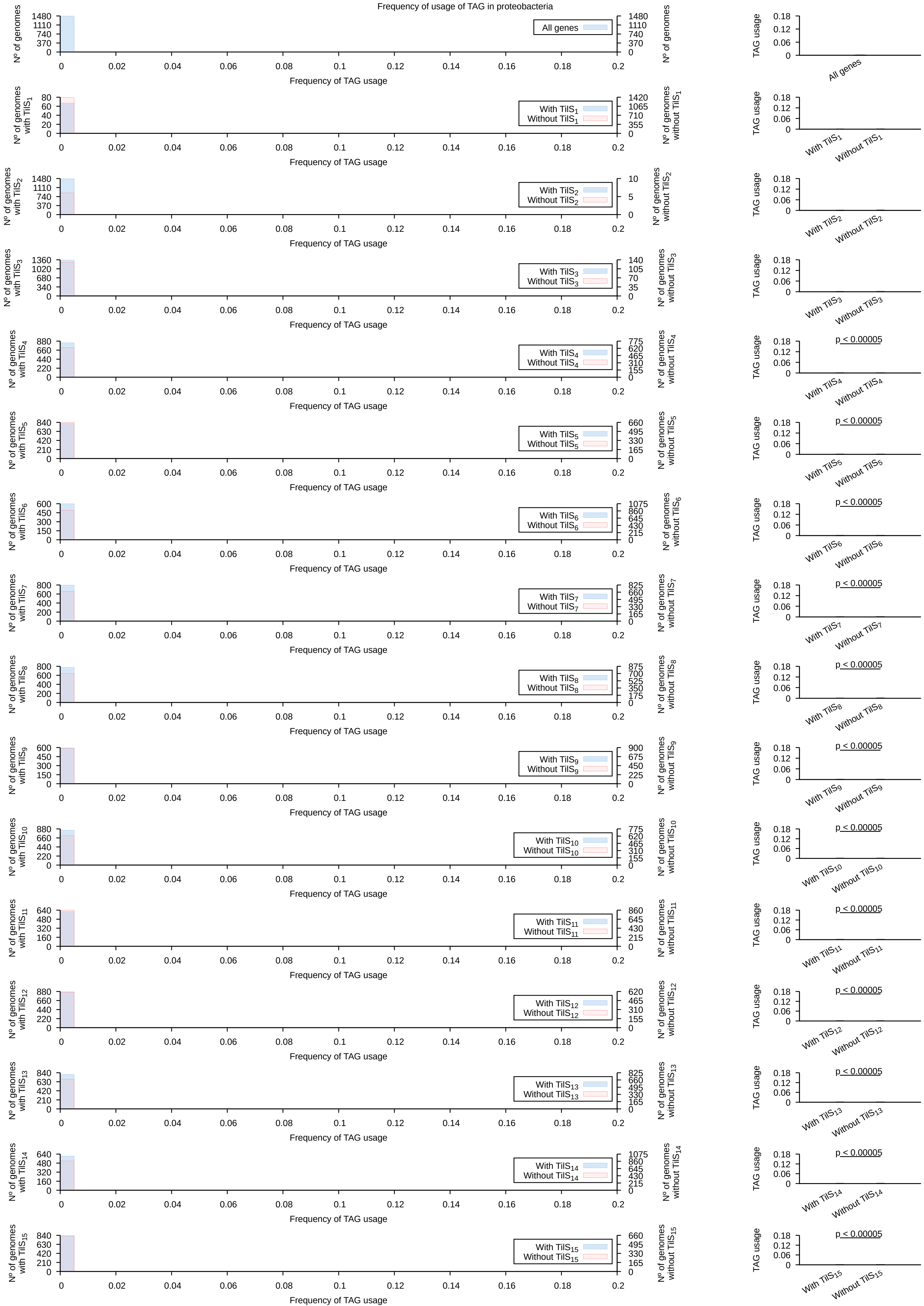
Frequency of usage of TAG in proteobacteria
Nº of genomes
All genes
Frequency of TAG usage
TAG usage
With TilS$_1$
Without TilS$_1$
Nº of genomes with TilS$_1$
Nº of genomes without TilS$_1$
With TilS$_2$
Without TilS$_2$
With TilS$_3$
Without TilS$_3$
With TilS$_4$
Without TilS$_4$
With TilS$_5$
Without TilS$_5$
With TilS$_6$
Without TilS$_6$
With TilS$_7$
Without TilS$_7$
With TilS$_8$
Without TilS$_8$
With TilS$_9$
Without TilS$_9$
With TilS$_{10}$
Without TilS$_{10}$
With TilS$_{11}$
Without TilS$_{11}$
With TilS$_{12}$
Without TilS$_{12}$
With TilS$_{13}$
Without TilS$_{13}$
With TilS$_{14}$
Without TilS$_{14}$
With TilS$_{15}$
Without TilS$_{15}$
p < 0.00005

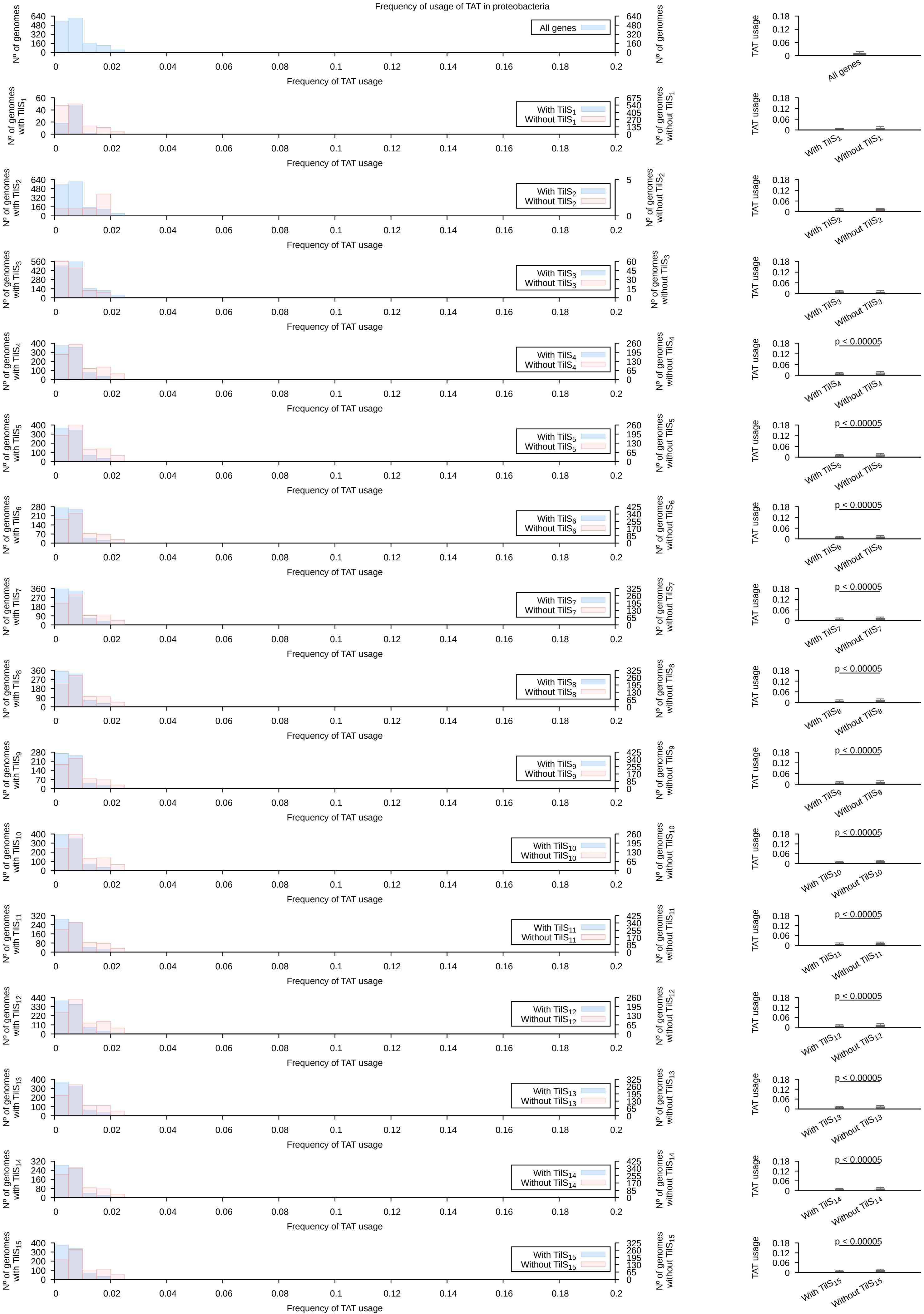

Frequency of usage of TAT in proteobacteria
Nº of genomes
Frequency of TAT usage
TAT usage
All genes
With TilS$_1$
Without TilS$_1$
Nº of genomes with TilS$_1$
Nº of genomes without TilS$_1$
With TilS$_2$
Without TilS$_2$
With TilS$_3$
Without TilS$_3$
With TilS$_4$
Without TilS$_4$
With TilS$_5$
Without TilS$_5$
With TilS$_6$
Without TilS$_6$
With TilS$_7$
Without TilS$_7$
With TilS$_8$
Without TilS$_8$
With TilS$_9$
Without TilS$_9$
With TilS$_{10}$
Without TilS$_{10}$
With TilS$_{11}$
Without TilS$_{11}$
With TilS$_{12}$
Without TilS$_{12}$
With TilS$_{13}$
Without TilS$_{13}$
With TilS$_{14}$
Without TilS$_{14}$
With TilS$_{15}$
Without TilS$_{15}$
p < 0.00005
0 0.02 0.04 0.06 0.08 0.1 0.12 0.14 0.16 0.18 0.2
0 0.06 0.12 0.18

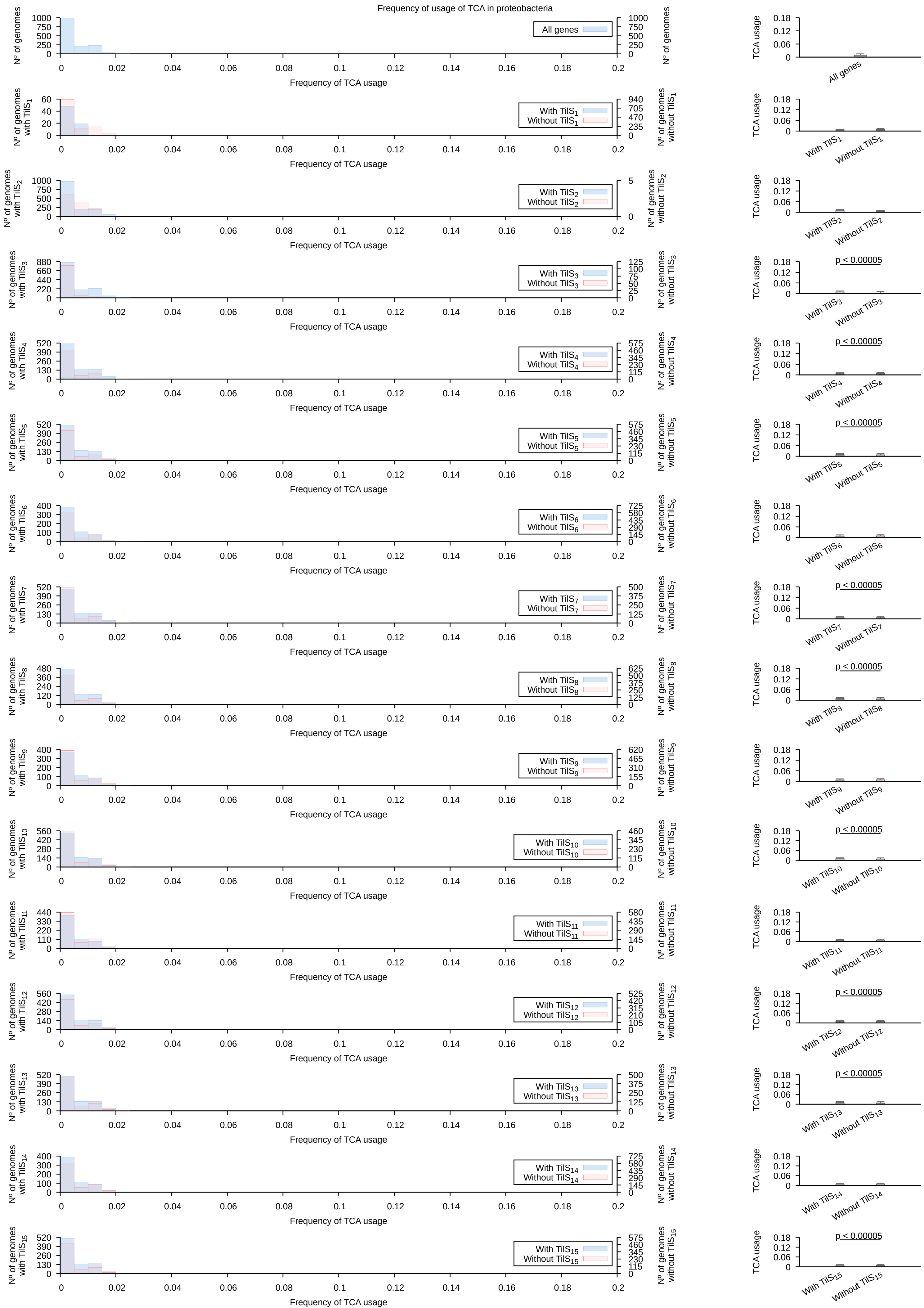

Frequency of usage of TCA in proteobacteria
Nº of genomes
All genes
Frequency of TCA usage
TCA usage
All genes
Nº of genomes with TilS$_1$
With TilS$_1$
Without TilS$_1$
Nº of genomes without TilS$_1$
Nº of genomes with TilS$_2$
With TilS$_2$
Without TilS$_2$
Nº of genomes without TilS$_2$
Nº of genomes with TilS$_3$
With TilS$_3$
Without TilS$_3$
Nº of genomes without TilS$_3$
p < 0.00005
Nº of genomes with TilS$_4$
With TilS$_4$
Without TilS$_4$
Nº of genomes without TilS$_4$
p < 0.00005
Nº of genomes with TilS$_5$
With TilS$_5$
Without TilS$_5$
Nº of genomes without TilS$_5$
p < 0.00005
Nº of genomes with TilS$_6$
With TilS$_6$
Without TilS$_6$
Nº of genomes without TilS$_6$
Nº of genomes with TilS$_7$
With TilS$_7$
Without TilS$_7$
Nº of genomes without TilS$_7$
p < 0.00005
Nº of genomes with TilS$_8$
With TilS$_8$
Without TilS$_8$
Nº of genomes without TilS$_8$
p < 0.00005
Nº of genomes with TilS$_9$
With TilS$_9$
Without TilS$_9$
Nº of genomes without TilS$_9$
Nº of genomes with TilS$_{10}$
With TilS$_{10}$
Without TilS$_{10}$
Nº of genomes without TilS$_{10}$
p < 0.00005
Nº of genomes with TilS$_{11}$
With TilS$_{11}$
Without TilS$_{11}$
Nº of genomes without TilS$_{11}$
Nº of genomes with TilS$_{12}$
With TilS$_{12}$
Without TilS$_{12}$
Nº of genomes without TilS$_{12}$
p < 0.00005
Nº of genomes with TilS$_{13}$
With TilS$_{13}$
Without TilS$_{13}$
Nº of genomes without TilS$_{13}$
p < 0.00005
Nº of genomes with TilS$_{14}$
With TilS$_{14}$
Without TilS$_{14}$
Nº of genomes without TilS$_{14}$
Nº of genomes with TilS$_{15}$
With TilS$_{15}$
Without TilS$_{15}$
Nº of genomes without TilS$_{15}$
p < 0.00005

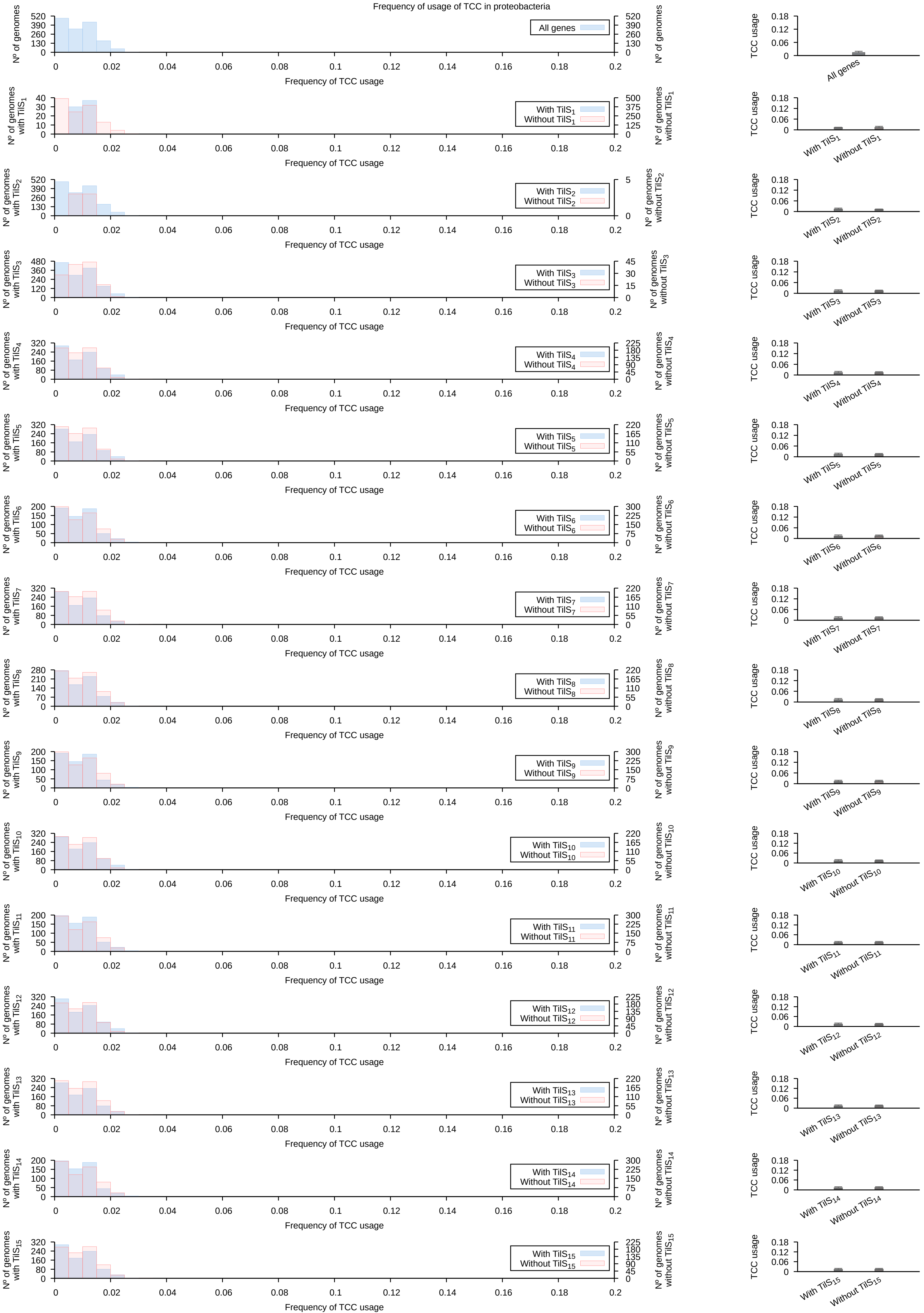

Frequency of usage of TCC in proteobacteria
Nº of genomes
All genes
Frequency of TCC usage
TCC usage
Nº of genomes with TilS1
Nº of genomes without TilS1
With TilS1
Without TilS1
Nº of genomes with TilS2
Nº of genomes without TilS2
With TilS2
Without TilS2
Nº of genomes with TilS3
Nº of genomes without TilS3
With TilS3
Without TilS3
Nº of genomes with TilS4
Nº of genomes without TilS4
With TilS4
Without TilS4
Nº of genomes with TilS5
Nº of genomes without TilS5
With TilS5
Without TilS5
Nº of genomes with TilS6
Nº of genomes without TilS6
With TilS6
Without TilS6
Nº of genomes with TilS7
Nº of genomes without TilS7
With TilS7
Without TilS7
Nº of genomes with TilS8
Nº of genomes without TilS8
With TilS8
Without TilS8
Nº of genomes with TilS9
Nº of genomes without TilS9
With TilS9
Without TilS9
Nº of genomes with TilS10
Nº of genomes without TilS10
With TilS10
Without TilS10
Nº of genomes with TilS11
Nº of genomes without TilS11
With TilS11
Without TilS11
Nº of genomes with TilS12
Nº of genomes without TilS12
With TilS12
Without TilS12
Nº of genomes with TilS13
Nº of genomes without TilS13
With TilS13
Without TilS13
Nº of genomes with TilS14
Nº of genomes without TilS14
With TilS14
Without TilS14
Nº of genomes with TilS15
Nº of genomes without TilS15
With TilS15
Without TilS15

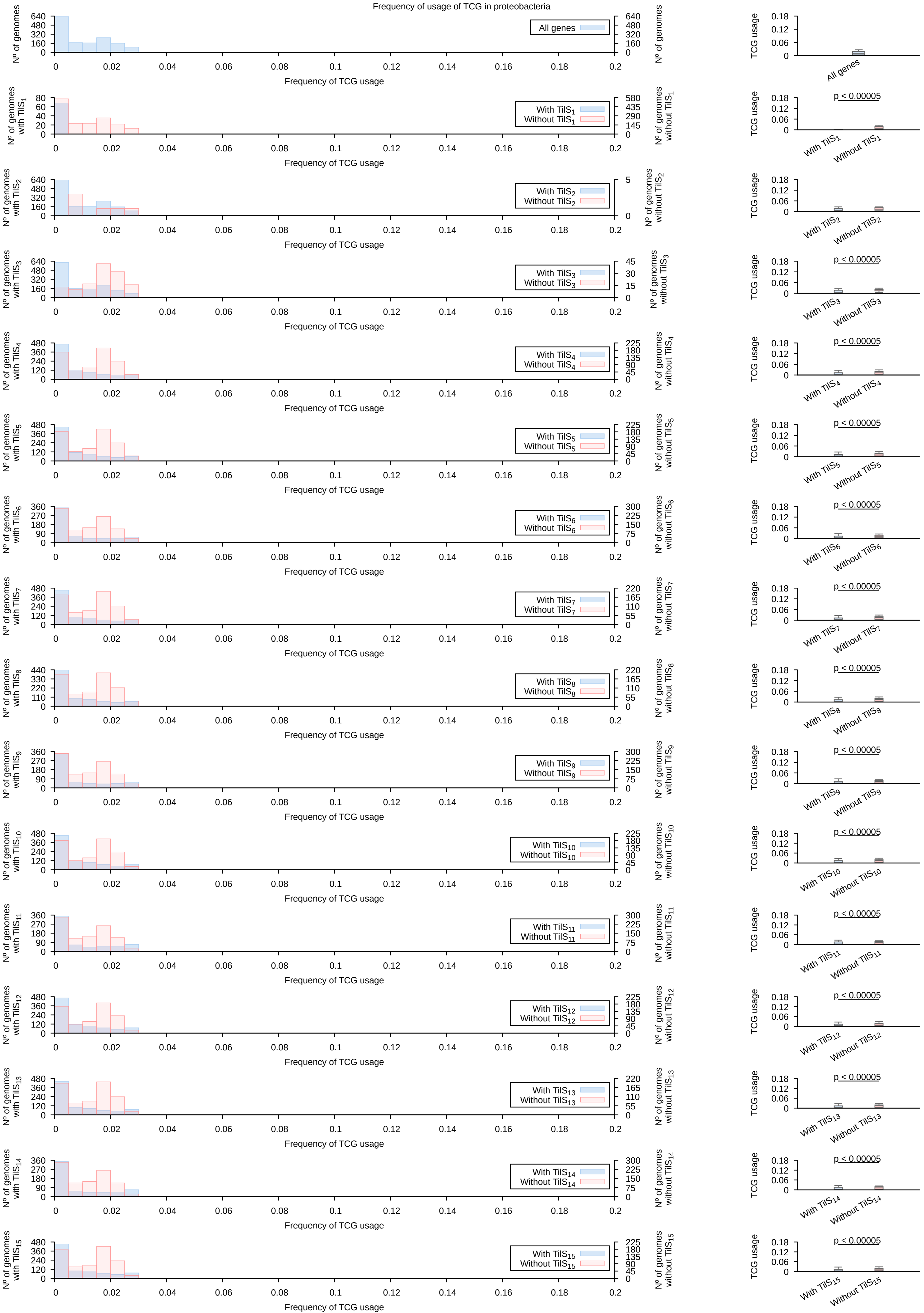

Frequency of usage of TCG in proteobacteria
Nº of genomes
All genes
Frequency of TCG usage
TCG usage
0
0.02
0.04
0.06
0.08
0.1
0.12
0.14
0.16
0.18
0.2
0.06
0.12
0.18
Nº of genomes with TilS$_1$
Nº of genomes without TilS$_1$
With TilS$_1$
Without TilS$_1$
p < 0.00005
Nº of genomes with TilS$_2$
Nº of genomes without TilS$_2$
With TilS$_2$
Without TilS$_2$
Nº of genomes with TilS$_3$
Nº of genomes without TilS$_3$
With TilS$_3$
Without TilS$_3$
p < 0.00005
Nº of genomes with TilS$_4$
Nº of genomes without TilS$_4$
With TilS$_4$
Without TilS$_4$
p < 0.00005
Nº of genomes with TilS$_5$
Nº of genomes without TilS$_5$
With TilS$_5$
Without TilS$_5$
p < 0.00005
Nº of genomes with TilS$_6$
Nº of genomes without TilS$_6$
With TilS$_6$
Without TilS$_6$
p < 0.00005
Nº of genomes with TilS$_7$
Nº of genomes without TilS$_7$
With TilS$_7$
Without TilS$_7$
p < 0.00005
Nº of genomes with TilS$_8$
Nº of genomes without TilS$_8$
With TilS$_8$
Without TilS$_8$
p < 0.00005
Nº of genomes with TilS$_9$
Nº of genomes without TilS$_9$
With TilS$_9$
Without TilS$_9$
p < 0.00005
Nº of genomes with TilS$_{10}$
Nº of genomes without TilS$_{10}$
With TilS$_{10}$
Without TilS$_{10}$
p < 0.00005
Nº of genomes with TilS$_{11}$
Nº of genomes without TilS$_{11}$
With TilS$_{11}$
Without TilS$_{11}$
p < 0.00005
Nº of genomes with TilS$_{12}$
Nº of genomes without TilS$_{12}$
With TilS$_{12}$
Without TilS$_{12}$
p < 0.00005
Nº of genomes with TilS$_{13}$
Nº of genomes without TilS$_{13}$
With TilS$_{13}$
Without TilS$_{13}$
p < 0.00005
Nº of genomes with TilS$_{14}$
Nº of genomes without TilS$_{14}$
With TilS$_{14}$
Without TilS$_{14}$
p < 0.00005
Nº of genomes with TilS$_{15}$
Nº of genomes without TilS$_{15}$
With TilS$_{15}$
Without TilS$_{15}$
p < 0.00005

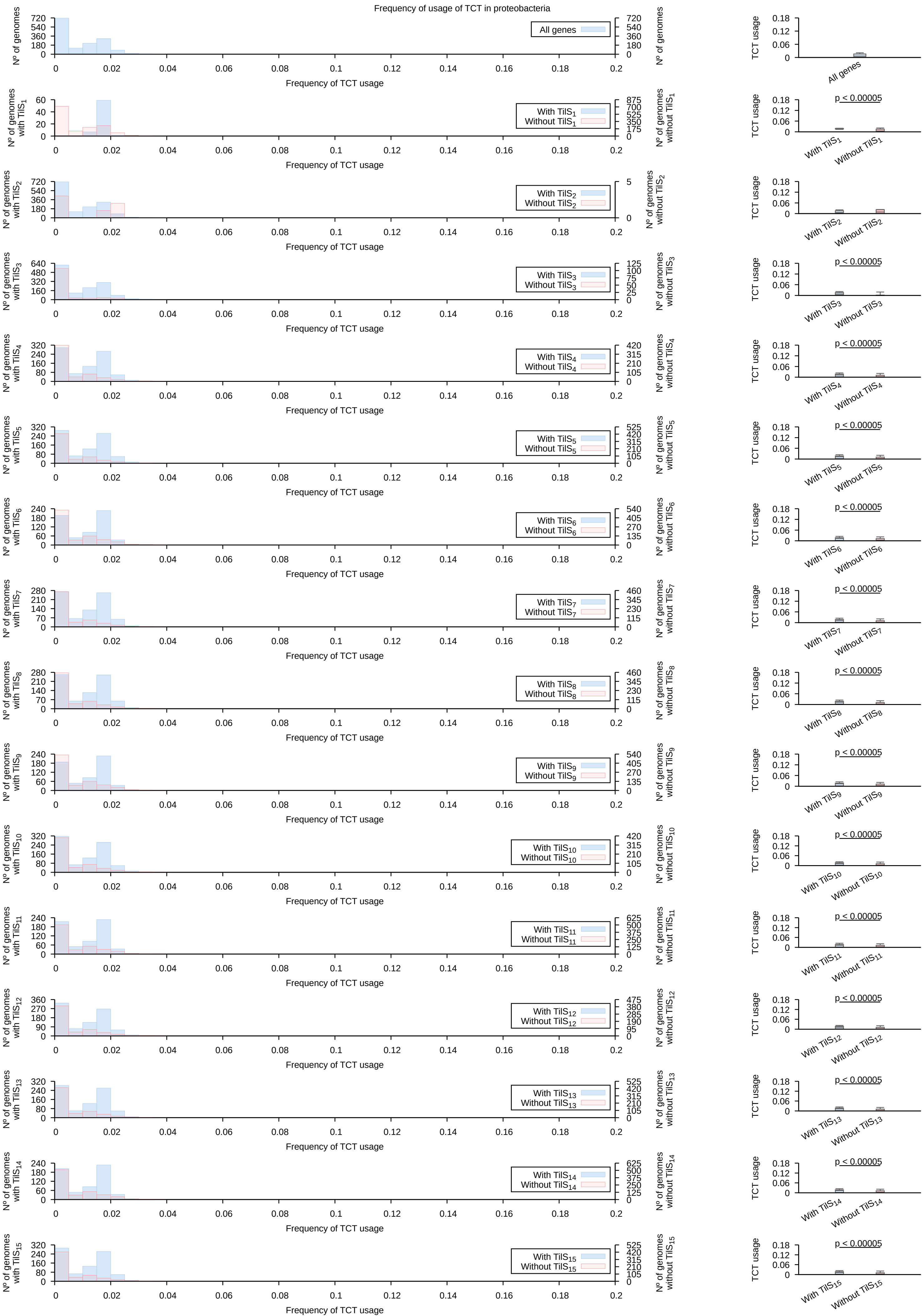
Frequency of usage of TCT in proteobacteria
Nº of genomes
All genes
Frequency of TCT usage
TCT usage
Nº of genomes with TilS$_1$
Nº of genomes without TilS$_1$
With TilS$_1$
Without TilS$_1$
p < 0.00005
Nº of genomes with TilS$_2$
Nº of genomes without TilS$_2$
With TilS$_2$
Without TilS$_2$
Nº of genomes with TilS$_3$
Nº of genomes without TilS$_3$
With TilS$_3$
Without TilS$_3$
p < 0.00005
Nº of genomes with TilS$_4$
Nº of genomes without TilS$_4$
With TilS$_4$
Without TilS$_4$
p < 0.00005
Nº of genomes with TilS$_5$
Nº of genomes without TilS$_5$
With TilS$_5$
Without TilS$_5$
p < 0.00005
Nº of genomes with TilS$_6$
Nº of genomes without TilS$_6$
With TilS$_6$
Without TilS$_6$
p < 0.00005
Nº of genomes with TilS$_7$
Nº of genomes without TilS$_7$
With TilS$_7$
Without TilS$_7$
p < 0.00005
Nº of genomes with TilS$_8$
Nº of genomes without TilS$_8$
With TilS$_8$
Without TilS$_8$
p < 0.00005
Nº of genomes with TilS$_9$
Nº of genomes without TilS$_9$
With TilS$_9$
Without TilS$_9$
p < 0.00005
Nº of genomes with TilS$_{10}$
Nº of genomes without TilS$_{10}$
With TilS$_{10}$
Without TilS$_{10}$
p < 0.00005
Nº of genomes with TilS$_{11}$
Nº of genomes without TilS$_{11}$
With TilS$_{11}$
Without TilS$_{11}$
p < 0.00005
Nº of genomes with TilS$_{12}$
Nº of genomes without TilS$_{12}$
With TilS$_{12}$
Without TilS$_{12}$
p < 0.00005
Nº of genomes with TilS$_{13}$
Nº of genomes without TilS$_{13}$
With TilS$_{13}$
Without TilS$_{13}$
p < 0.00005
Nº of genomes with TilS$_{14}$
Nº of genomes without TilS$_{14}$
With TilS$_{14}$
Without TilS$_{14}$
p < 0.00005
Nº of genomes with TilS$_{15}$
Nº of genomes without TilS$_{15}$
With TilS$_{15}$
Without TilS$_{15}$
p < 0.00005

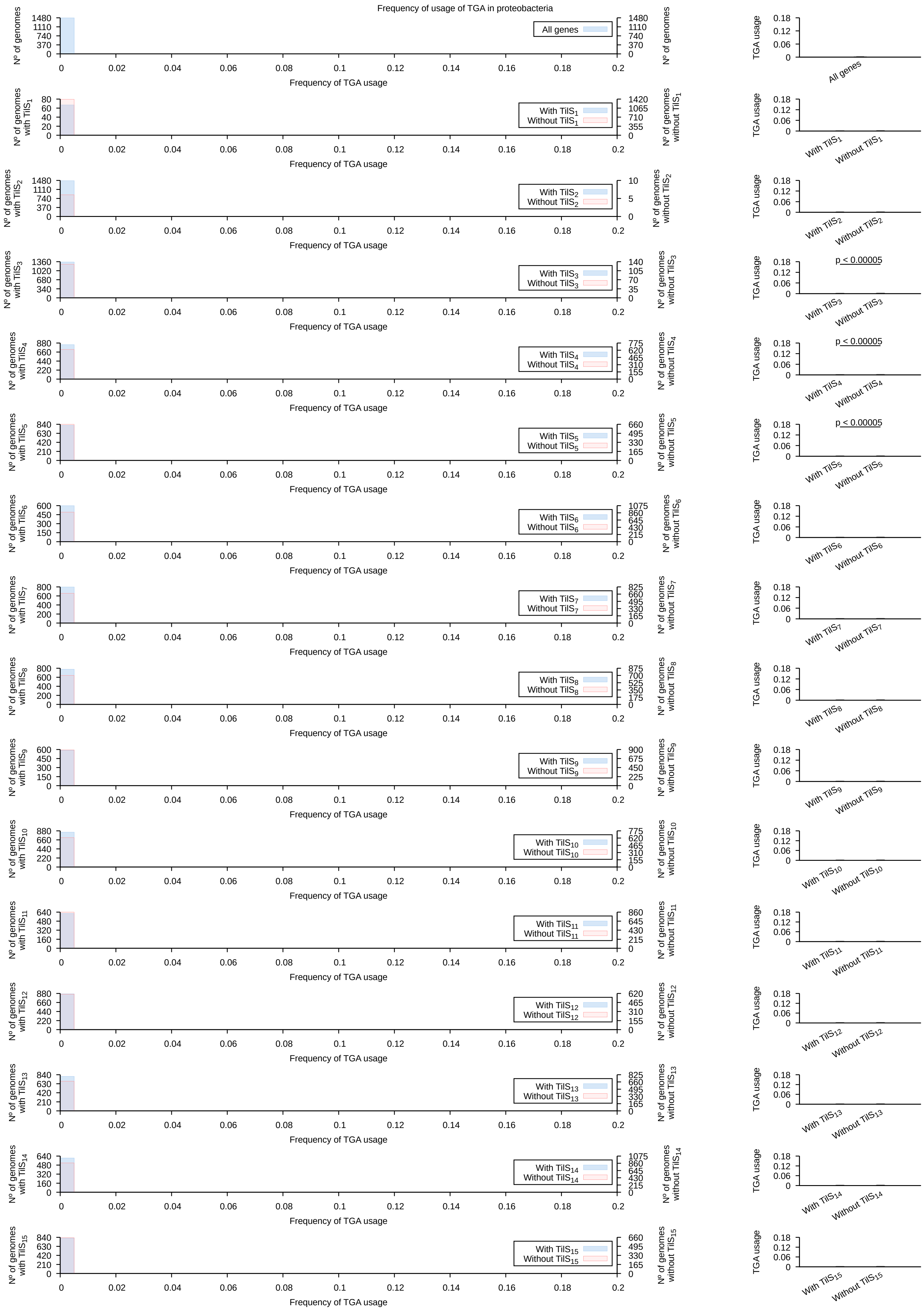
Frequency of usage of TGA in proteobacteria
Nº of genomes
All genes
Frequency of TGA usage
TGA usage
Nº of genomes with TilS$_1$
With TilS$_1$
Without TilS$_1$
Nº of genomes without TilS$_1$
Nº of genomes with TilS$_2$
With TilS$_2$
Without TilS$_2$
Nº of genomes without TilS$_2$
Nº of genomes with TilS$_3$
With TilS$_3$
Without TilS$_3$
Nº of genomes without TilS$_3$
p < 0.00005
Nº of genomes with TilS$_4$
With TilS$_4$
Without TilS$_4$
Nº of genomes without TilS$_4$
p < 0.00005
Nº of genomes with TilS$_5$
With TilS$_5$
Without TilS$_5$
Nº of genomes without TilS$_5$
p < 0.00005
Nº of genomes with TilS$_6$
With TilS$_6$
Without TilS$_6$
Nº of genomes without TilS$_6$
Nº of genomes with TilS$_7$
With TilS$_7$
Without TilS$_7$
Nº of genomes without TilS$_7$
Nº of genomes with TilS$_8$
With TilS$_8$
Without TilS$_8$
Nº of genomes without TilS$_8$
Nº of genomes with TilS$_9$
With TilS$_9$
Without TilS$_9$
Nº of genomes without TilS$_9$
Nº of genomes with TilS$_{10}$
With TilS$_{10}$
Without TilS$_{10}$
Nº of genomes without TilS$_{10}$
Nº of genomes with TilS$_{11}$
With TilS$_{11}$
Without TilS$_{11}$
Nº of genomes without TilS$_{11}$
Nº of genomes with TilS$_{12}$
With TilS$_{12}$
Without TilS$_{12}$
Nº of genomes without TilS$_{12}$
Nº of genomes with TilS$_{13}$
With TilS$_{13}$
Without TilS$_{13}$
Nº of genomes without TilS$_{13}$
Nº of genomes with TilS$_{14}$
With TilS$_{14}$
Without TilS$_{14}$
Nº of genomes without TilS$_{14}$
Nº of genomes with TilS$_{15}$
With TilS$_{15}$
Without TilS$_{15}$
Nº of genomes without TilS$_{15}$

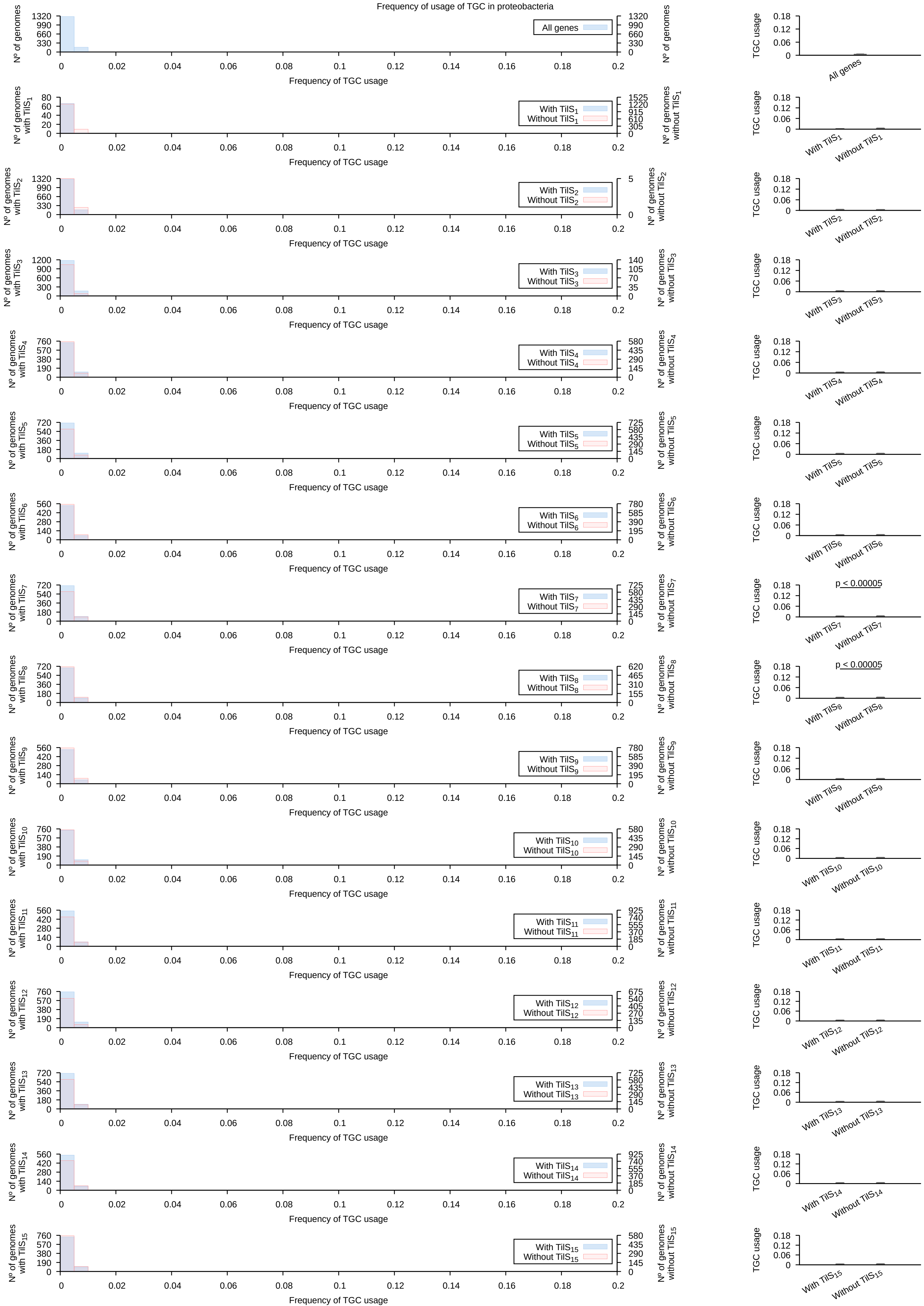
Frequency of usage of TGC in proteobacteria
Nº of genomes
All genes
Frequency of TGC usage
TGC usage
Nº of genomes with TilS$_1$
With TilS$_1$
Without TilS$_1$
Nº of genomes without TilS$_1$
Nº of genomes with TilS$_2$
With TilS$_2$
Without TilS$_2$
Nº of genomes without TilS$_2$
Nº of genomes with TilS$_3$
With TilS$_3$
Without TilS$_3$
Nº of genomes without TilS$_3$
Nº of genomes with TilS$_4$
With TilS$_4$
Without TilS$_4$
Nº of genomes without TilS$_4$
Nº of genomes with TilS$_5$
With TilS$_5$
Without TilS$_5$
Nº of genomes without TilS$_5$
Nº of genomes with TilS$_6$
With TilS$_6$
Without TilS$_6$
Nº of genomes without TilS$_6$
Nº of genomes with TilS$_7$
With TilS$_7$
Without TilS$_7$
Nº of genomes without TilS$_7$
p < 0.00005
Nº of genomes with TilS$_8$
With TilS$_8$
Without TilS$_8$
Nº of genomes without TilS$_8$
p < 0.00005
Nº of genomes with TilS$_9$
With TilS$_9$
Without TilS$_9$
Nº of genomes without TilS$_9$
Nº of genomes with TilS$_{10}$
With TilS$_{10}$
Without TilS$_{10}$
Nº of genomes without TilS$_{10}$
Nº of genomes with TilS$_{11}$
With TilS$_{11}$
Without TilS$_{11}$
Nº of genomes without TilS$_{11}$
Nº of genomes with TilS$_{12}$
With TilS$_{12}$
Without TilS$_{12}$
Nº of genomes without TilS$_{12}$
Nº of genomes with TilS$_{13}$
With TilS$_{13}$
Without TilS$_{13}$
Nº of genomes without TilS$_{13}$
Nº of genomes with TilS$_{14}$
With TilS$_{14}$
Without TilS$_{14}$
Nº of genomes without TilS$_{14}$
Nº of genomes with TilS$_{15}$
With TilS$_{15}$
Without TilS$_{15}$
Nº of genomes without TilS$_{15}$

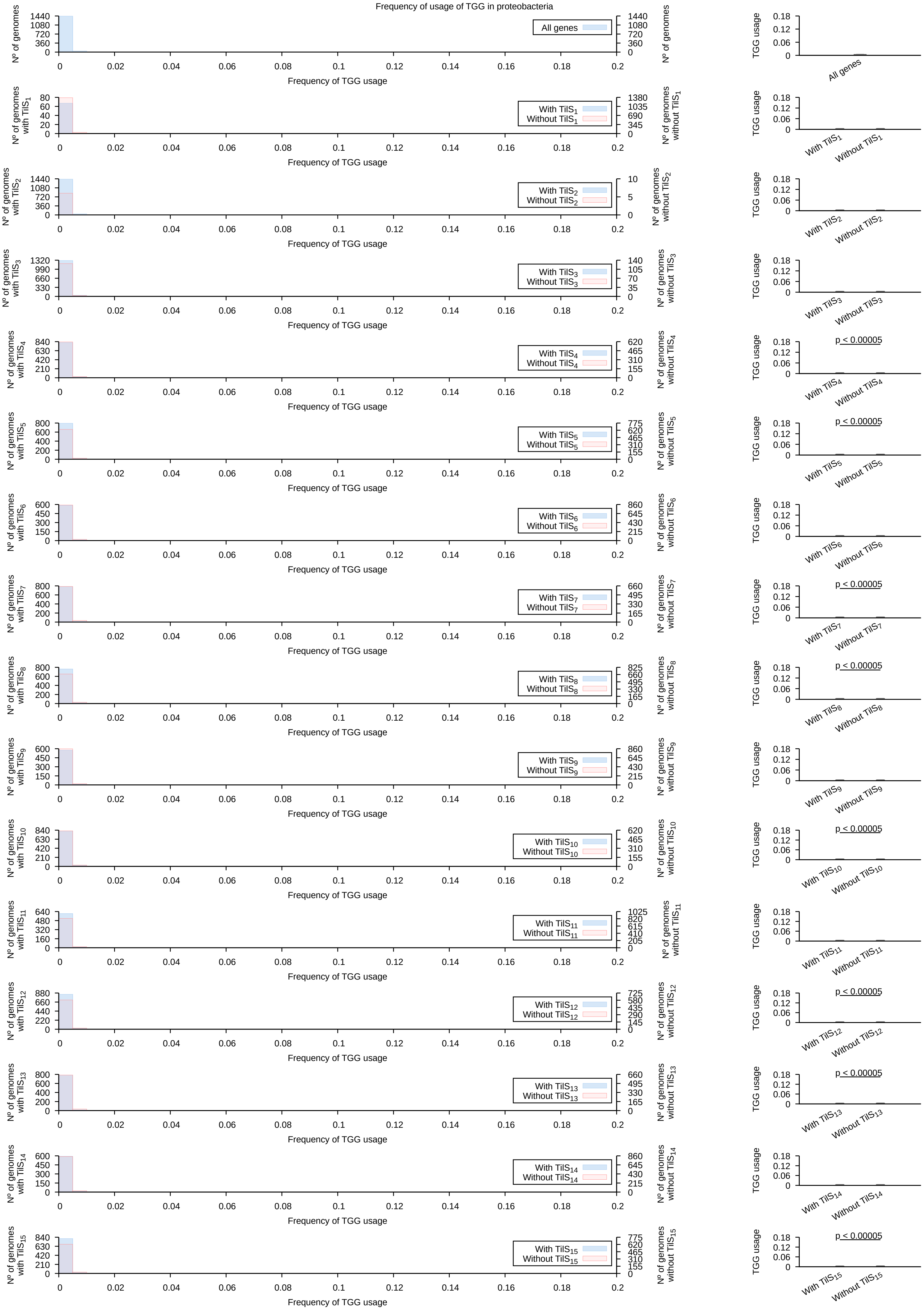

Frequency of usage of TGG in proteobacteria
Nº of genomes
Frequency of TGG usage
All genes
TGG usage
Nº of genomes with TilS$_1$
Nº of genomes without TilS$_1$
With TilS$_1$
Without TilS$_1$
With TilS$_2$
Without TilS$_2$
With TilS$_3$
Without TilS$_3$
With TilS$_4$
Without TilS$_4$
p < 0.00005
With TilS$_5$
Without TilS$_5$
p < 0.00005
With TilS$_6$
Without TilS$_6$
With TilS$_7$
Without TilS$_7$
p < 0.00005
With TilS$_8$
Without TilS$_8$
p < 0.00005
With TilS$_9$
Without TilS$_9$
With TilS$_{10}$
Without TilS$_{10}$
p < 0.00005
With TilS$_{11}$
Without TilS$_{11}$
With TilS$_{12}$
Without TilS$_{12}$
p < 0.00005
With TilS$_{13}$
Without TilS$_{13}$
p < 0.00005
With TilS$_{14}$
Without TilS$_{14}$
With TilS$_{15}$
Without TilS$_{15}$
p < 0.00005

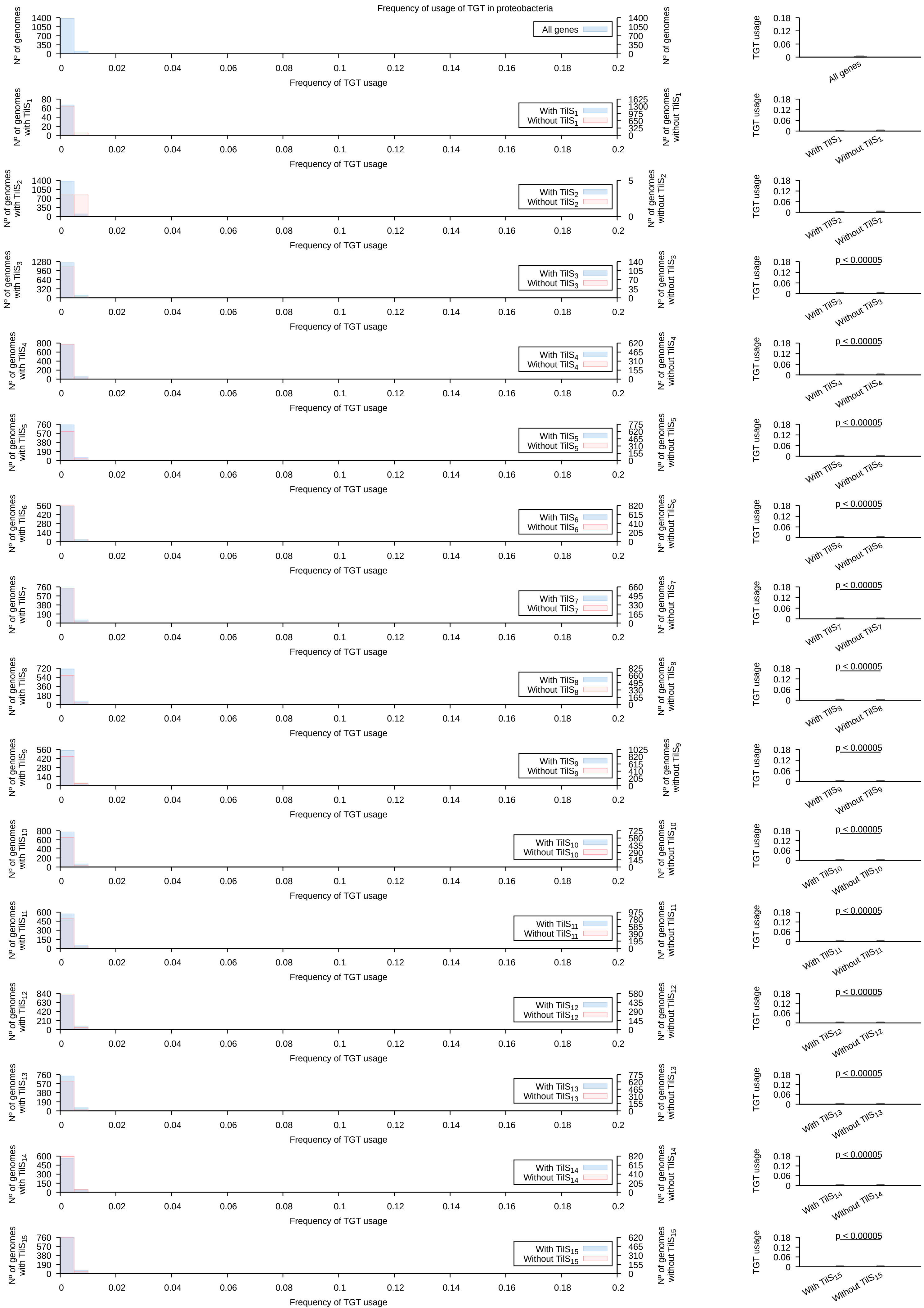
Frequency of usage of TGT in proteobacteria
Nº of genomes
All genes
Frequency of TGT usage
TGT usage
Nº of genomes with TilS1
With TilS1
Without TilS1
Nº of genomes without TilS1
Nº of genomes with TilS2
With TilS2
Without TilS2
Nº of genomes without TilS2
Nº of genomes with TilS3
With TilS3
Without TilS3
Nº of genomes without TilS3
p < 0.00005
Nº of genomes with TilS4
With TilS4
Without TilS4
Nº of genomes without TilS4
p < 0.00005
Nº of genomes with TilS5
With TilS5
Without TilS5
Nº of genomes without TilS5
p < 0.00005
Nº of genomes with TilS6
With TilS6
Without TilS6
Nº of genomes without TilS6
p < 0.00005
Nº of genomes with TilS7
With TilS7
Without TilS7
Nº of genomes without TilS7
p < 0.00005
Nº of genomes with TilS8
With TilS8
Without TilS8
Nº of genomes without TilS8
p < 0.00005
Nº of genomes with TilS9
With TilS9
Without TilS9
Nº of genomes without TilS9
p < 0.00005
Nº of genomes with TilS10
With TilS10
Without TilS10
Nº of genomes without TilS10
p < 0.00005
Nº of genomes with TilS11
With TilS11
Without TilS11
Nº of genomes without TilS11
p < 0.00005
Nº of genomes with TilS12
With TilS12
Without TilS12
Nº of genomes without TilS12
p < 0.00005
Nº of genomes with TilS13
With TilS13
Without TilS13
Nº of genomes without TilS13
p < 0.00005
Nº of genomes with TilS14
With TilS14
Without TilS14
Nº of genomes without TilS14
p < 0.00005
Nº of genomes with TilS15
With TilS15
Without TilS15
Nº of genomes without TilS15
p < 0.00005

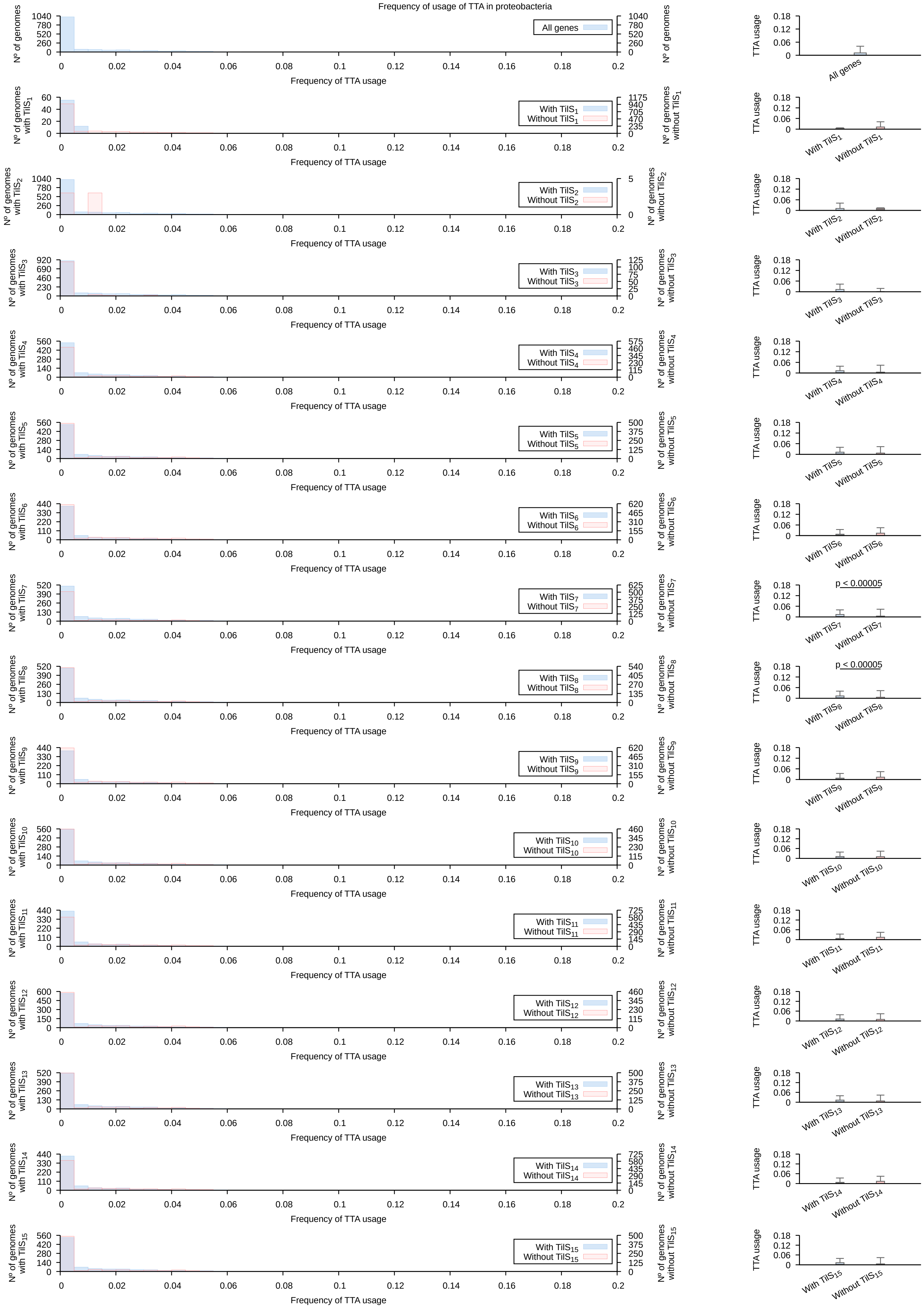

Frequency of usage of TTA in proteobacteria
Nº of genomes
Frequency of TTA usage
TTA usage
All genes
With TilS$_1$
Without TilS$_1$
With TilS$_2$
Without TilS$_2$
With TilS$_3$
Without TilS$_3$
With TilS$_4$
Without TilS$_4$
With TilS$_5$
Without TilS$_5$
With TilS$_6$
Without TilS$_6$
With TilS$_7$
Without TilS$_7$
p < 0.00005
With TilS$_8$
Without TilS$_8$
p < 0.00005
With TilS$_9$
Without TilS$_9$
With TilS$_{10}$
Without TilS$_{10}$
With TilS$_{11}$
Without TilS$_{11}$
With TilS$_{12}$
Without TilS$_{12}$
With TilS$_{13}$
Without TilS$_{13}$
With TilS$_{14}$
Without TilS$_{14}$
With TilS$_{15}$
Without TilS$_{15}$

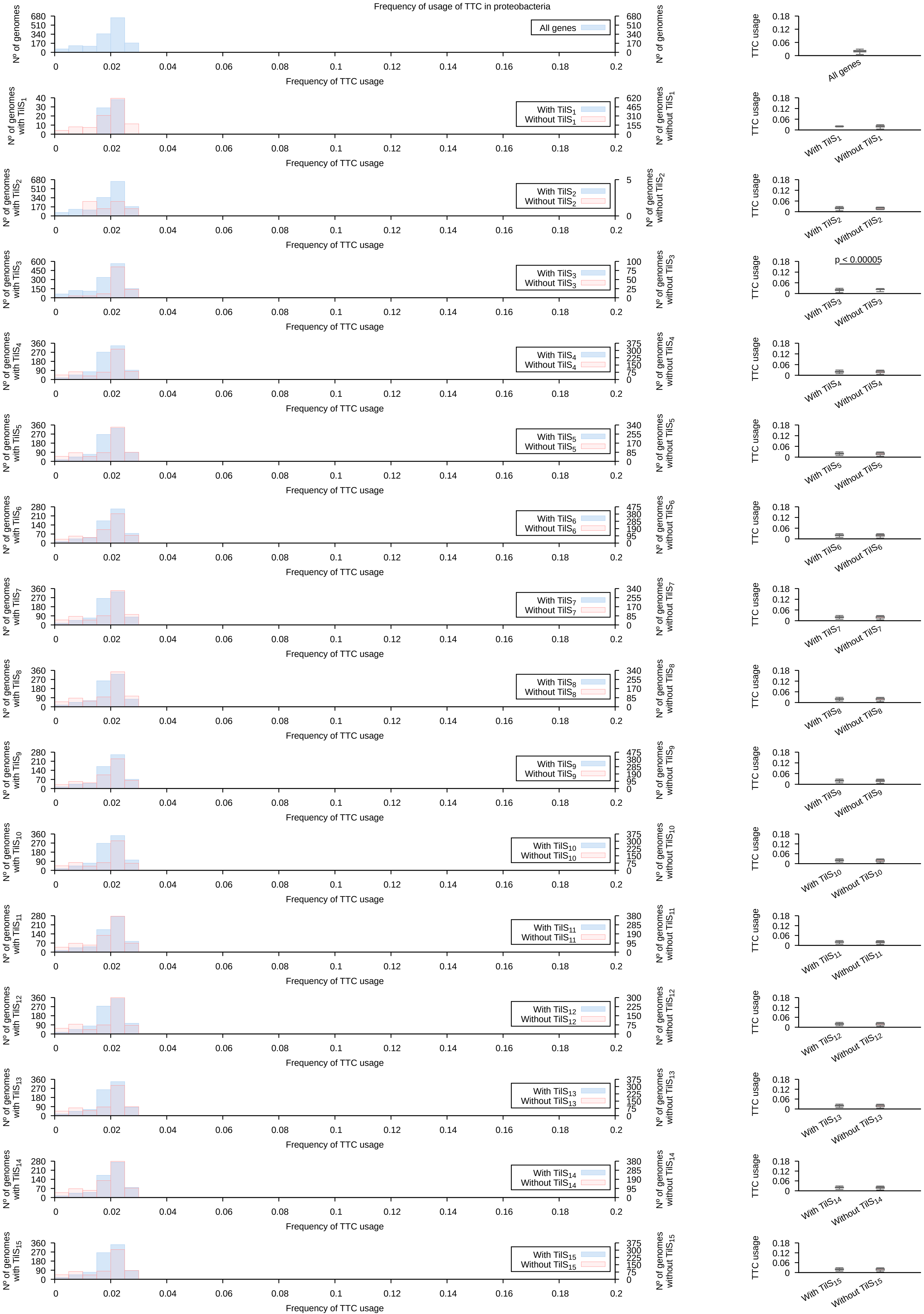
Frequency of usage of TTC in proteobacteria
Nº of genomes
All genes
Frequency of TTC usage
TTC usage
Nº of genomes with TilS$_1$
With TilS$_1$
Without TilS$_1$
Nº of genomes without TilS$_1$
Nº of genomes with TilS$_2$
With TilS$_2$
Without TilS$_2$
Nº of genomes without TilS$_2$
Nº of genomes with TilS$_3$
With TilS$_3$
Without TilS$_3$
Nº of genomes without TilS$_3$
p < 0.00005
Nº of genomes with TilS$_4$
With TilS$_4$
Without TilS$_4$
Nº of genomes without TilS$_4$
Nº of genomes with TilS$_5$
With TilS$_5$
Without TilS$_5$
Nº of genomes without TilS$_5$
Nº of genomes with TilS$_6$
With TilS$_6$
Without TilS$_6$
Nº of genomes without TilS$_6$
Nº of genomes with TilS$_7$
With TilS$_7$
Without TilS$_7$
Nº of genomes without TilS$_7$
Nº of genomes with TilS$_8$
With TilS$_8$
Without TilS$_8$
Nº of genomes without TilS$_8$
Nº of genomes with TilS$_9$
With TilS$_9$
Without TilS$_9$
Nº of genomes without TilS$_9$
Nº of genomes with TilS$_{10}$
With TilS$_{10}$
Without TilS$_{10}$
Nº of genomes without TilS$_{10}$
Nº of genomes with TilS$_{11}$
With TilS$_{11}$
Without TilS$_{11}$
Nº of genomes without TilS$_{11}$
Nº of genomes with TilS$_{12}$
With TilS$_{12}$
Without TilS$_{12}$
Nº of genomes without TilS$_{12}$
Nº of genomes with TilS$_{13}$
With TilS$_{13}$
Without TilS$_{13}$
Nº of genomes without TilS$_{13}$
Nº of genomes with TilS$_{14}$
With TilS$_{14}$
Without TilS$_{14}$
Nº of genomes without TilS$_{14}$
Nº of genomes with TilS$_{15}$
With TilS$_{15}$
Without TilS$_{15}$
Nº of genomes without TilS$_{15}$

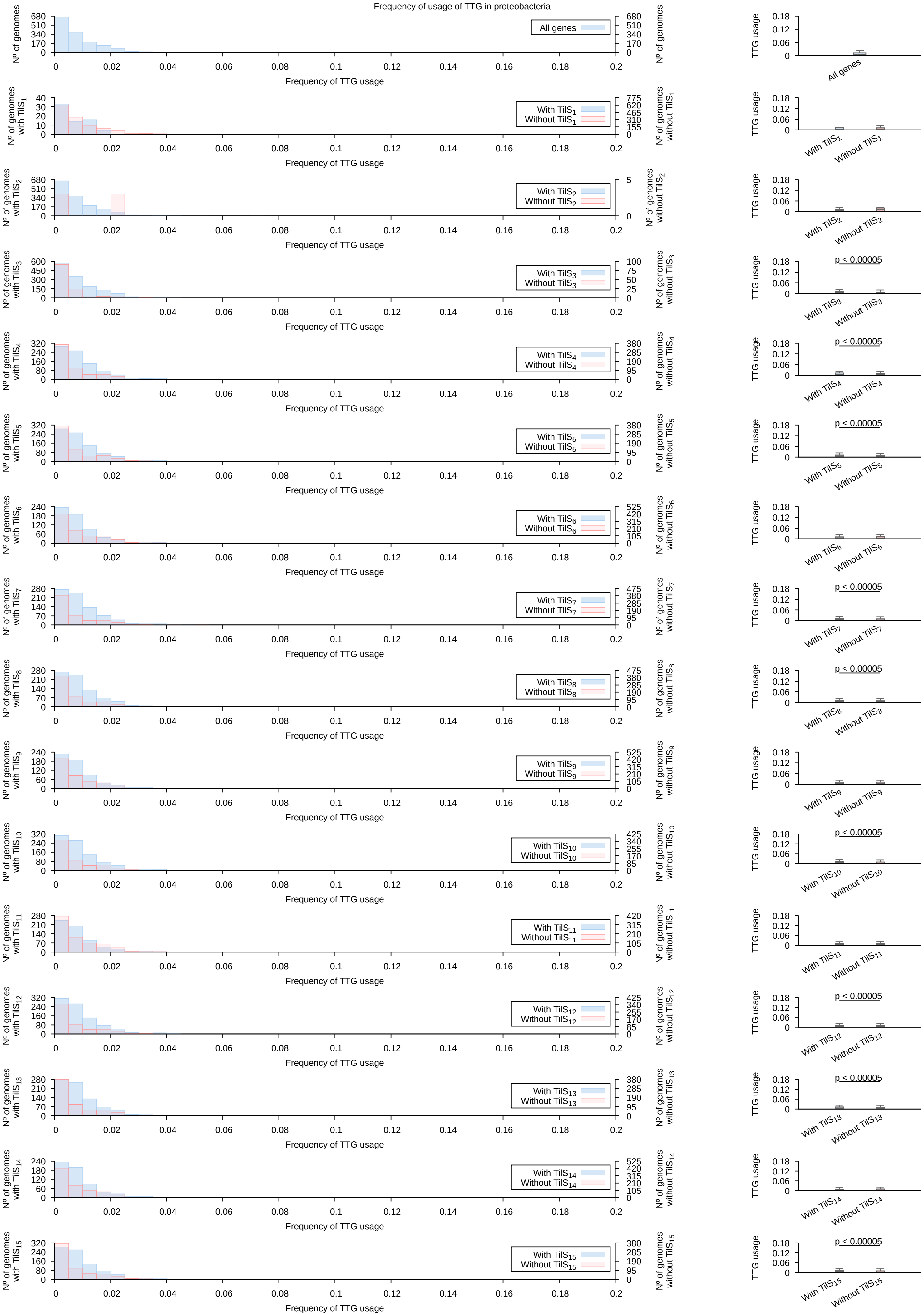
Frequency of usage of TTG in proteobacteria
Nº of genomes
All genes
Frequency of TTG usage
TTG usage
Nº of genomes with TilS$_1$
Nº of genomes without TilS$_1$
With TilS$_1$
Without TilS$_1$
With TilS$_2$
Without TilS$_2$
p < 0.00005
With TilS$_3$
Without TilS$_3$
With TilS$_4$
Without TilS$_4$
With TilS$_5$
Without TilS$_5$
With TilS$_6$
Without TilS$_6$
With TilS$_7$
Without TilS$_7$
With TilS$_8$
Without TilS$_8$
With TilS$_9$
Without TilS$_9$
With TilS$_{10}$
Without TilS$_{10}$
With TilS$_{11}$
Without TilS$_{11}$
With TilS$_{12}$
Without TilS$_{12}$
With TilS$_{13}$
Without TilS$_{13}$
With TilS$_{14}$
Without TilS$_{14}$
With TilS$_{15}$
Without TilS$_{15}$

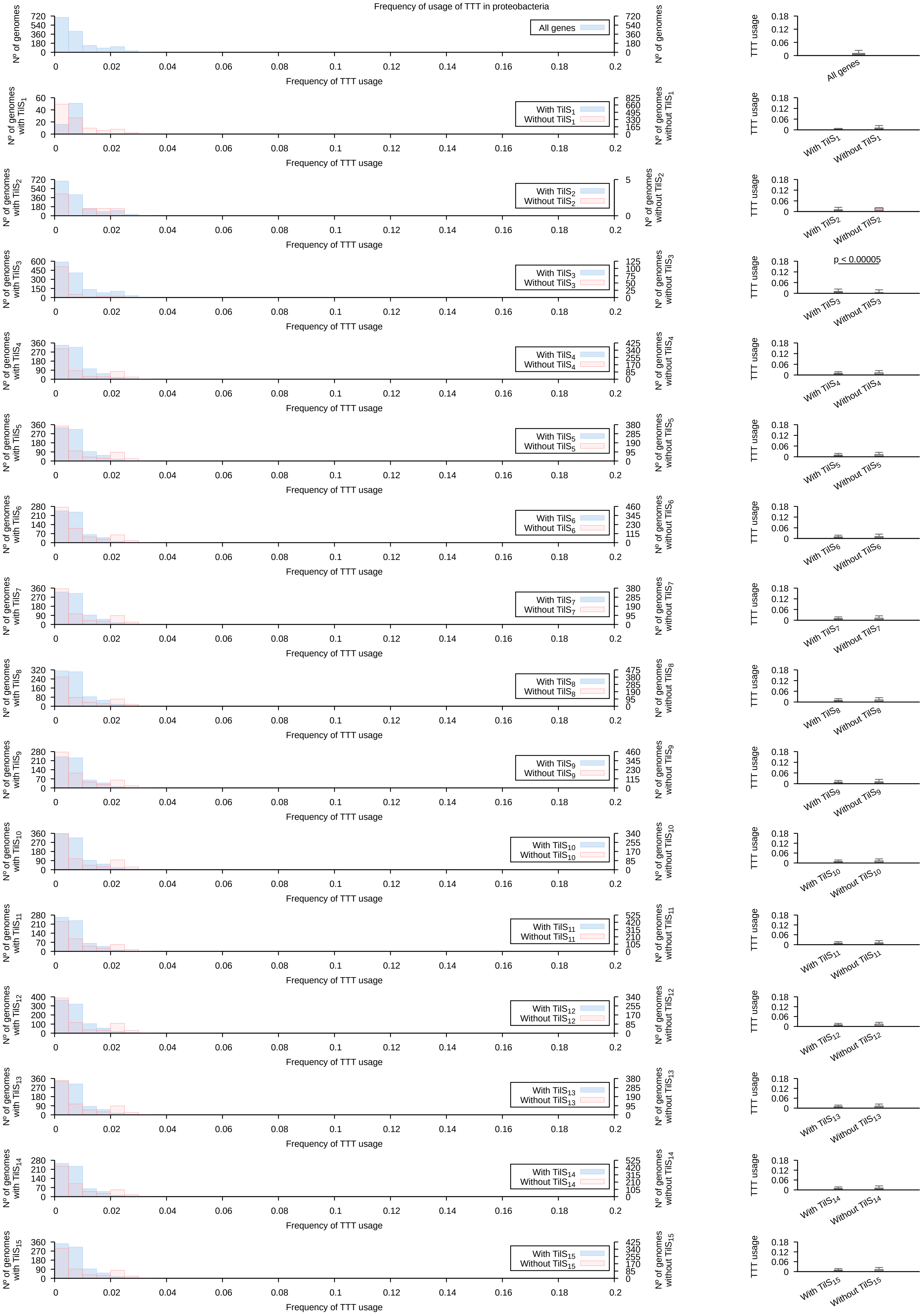

Frequency of usage of TTT in proteobacteria
Nº of genomes
All genes
Frequency of TTT usage
TTT usage
Nº of genomes with TilS1
Nº of genomes without TilS1
With TilS1
Without TilS1
Nº of genomes with TilS2
Nº of genomes without TilS2
With TilS2
Without TilS2
Nº of genomes with TilS3
Nº of genomes without TilS3
With TilS3
Without TilS3
p < 0.00005
Nº of genomes with TilS4
Nº of genomes without TilS4
With TilS4
Without TilS4
Nº of genomes with TilS5
Nº of genomes without TilS5
With TilS5
Without TilS5
Nº of genomes with TilS6
Nº of genomes without TilS6
With TilS6
Without TilS6
Nº of genomes with TilS7
Nº of genomes without TilS7
With TilS7
Without TilS7
Nº of genomes with TilS8
Nº of genomes without TilS8
With TilS8
Without TilS8
Nº of genomes with TilS9
Nº of genomes without TilS9
With TilS9
Without TilS9
Nº of genomes with TilS10
Nº of genomes without TilS10
With TilS10
Without TilS10
Nº of genomes with TilS11
Nº of genomes without TilS11
With TilS11
Without TilS11
Nº of genomes with TilS12
Nº of genomes without TilS12
With TilS12
Without TilS12
Nº of genomes with TilS13
Nº of genomes without TilS13
With TilS13
Without TilS13
Nº of genomes with TilS14
Nº of genomes without TilS14
With TilS14
Without TilS14
Nº of genomes with TilS15
Nº of genomes without TilS15
With TilS15
Without TilS15

Figure S22. Histogram of codon usage in high expression genes of genomes that present or lack genes involved in s^2U formation excluding small genomes. Top left graph: histogram of the usage of the indicated codon in highly expressed genes of proteobacterial genomes with more than 400 CDS. **Top right graph:** box plot indicating the distribution of codon usage in the same population. **Lower graphs:** The collection of 1477 proteobacterial genomes, excluding those with less than 400 CDS, were separated in two groups, based on the presence (light blue) or absence (orange) of the indicated pattern in any gene of the analyzed genomes. Patterns are indicated using subscript numbers. See table S4 for a detailed description of the used patterns. **Left:** Data is represented in superposed histograms. **Right:** Data is represented in a boxplot. An horizontal bar was added when the two populations are different based on Wilcoxon-Mann-Whitney test and $p < 0.00005$. Whiskers include 95% of data.

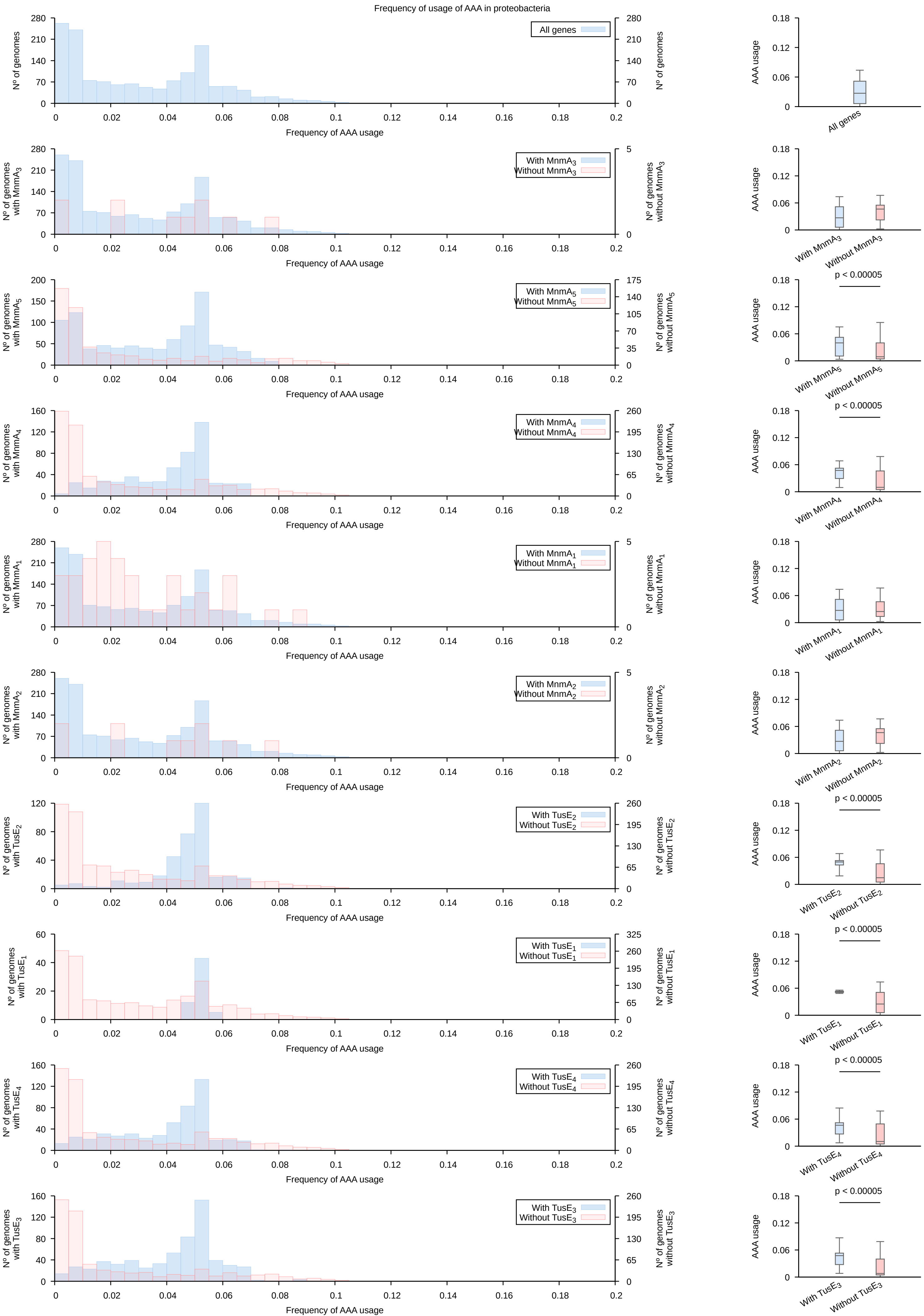
Frequency of usage of AAA in proteobacteria
Nº of genomes
Frequency of AAA usage
All genes
AAA usage
Nº of genomes with MnmA$_3$
Nº of genomes without MnmA$_3$
With MnmA$_3$
Without MnmA$_3$
Nº of genomes with MnmA$_5$
Nº of genomes without MnmA$_5$
With MnmA$_5$
Without MnmA$_5$
p < 0.00005
Nº of genomes with MnmA$_4$
Nº of genomes without MnmA$_4$
With MnmA$_4$
Without MnmA$_4$
p < 0.00005
Nº of genomes with MnmA$_1$
Nº of genomes without MnmA$_1$
With MnmA$_1$
Without MnmA$_1$
Nº of genomes with MnmA$_2$
Nº of genomes without MnmA$_2$
With MnmA$_2$
Without MnmA$_2$
Nº of genomes with TusE$_2$
Nº of genomes without TusE$_2$
With TusE$_2$
Without TusE$_2$
p < 0.00005
Nº of genomes with TusE$_1$
Nº of genomes without TusE$_1$
With TusE$_1$
Without TusE$_1$
p < 0.00005
Nº of genomes with TusE$_4$
Nº of genomes without TusE$_4$
With TusE$_4$
Without TusE$_4$
p < 0.00005
Nº of genomes with TusE$_3$
Nº of genomes without TusE$_3$
With TusE$_3$
Without TusE$_3$
p < 0.00005

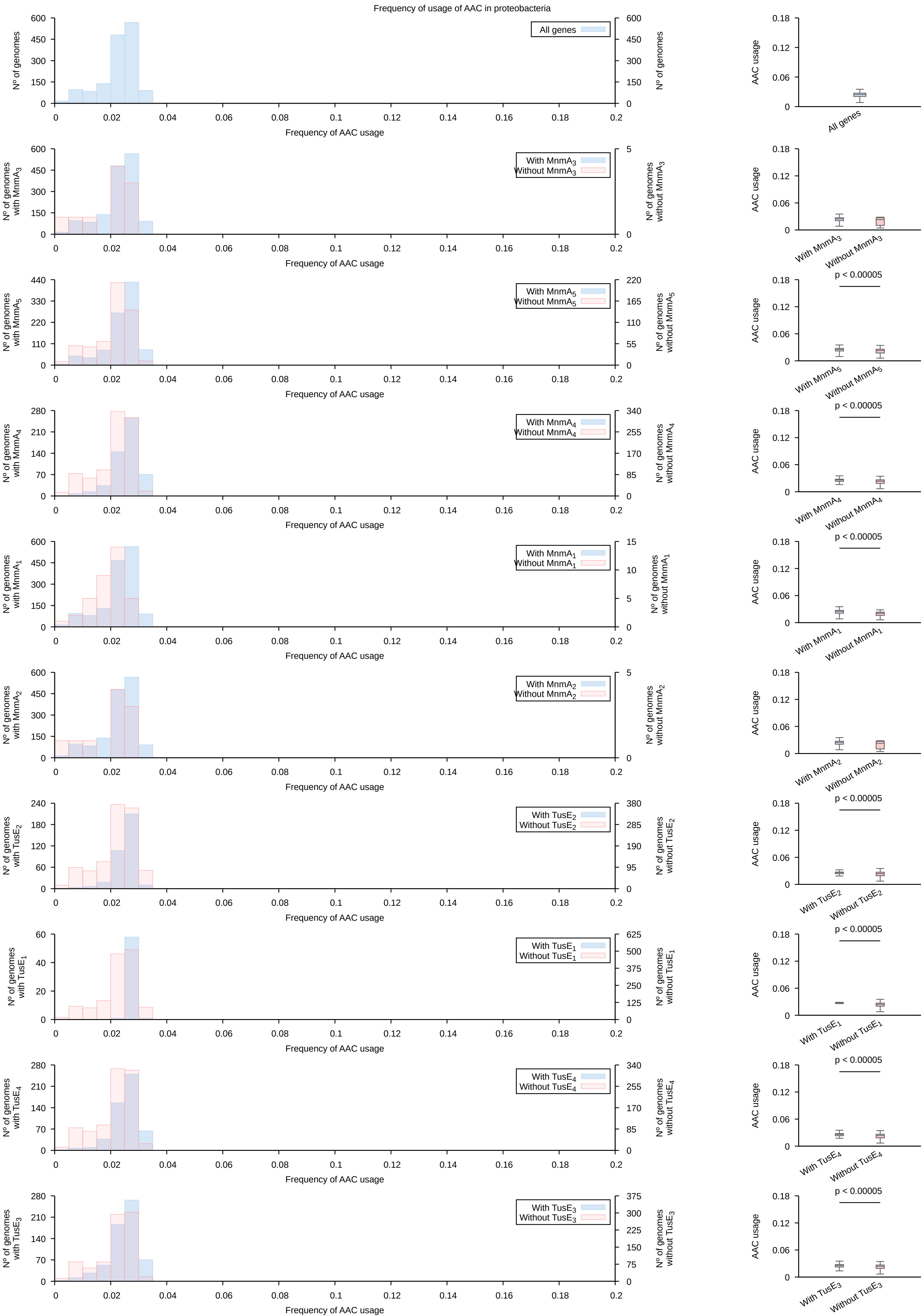
Frequency of usage of AAC in proteobacteria
Nº of genomes
Frequency of AAC usage
AAC usage
All genes
With $MnmA_3$
Without $MnmA_3$
Nº of genomes with $MnmA_3$
Nº of genomes without $MnmA_3$
With $MnmA_5$
Without $MnmA_5$
Nº of genomes with $MnmA_5$
Nº of genomes without $MnmA_5$
With $MnmA_4$
Without $MnmA_4$
Nº of genomes with $MnmA_4$
Nº of genomes without $MnmA_4$
With $MnmA_1$
Without $MnmA_1$
Nº of genomes with $MnmA_1$
Nº of genomes without $MnmA_1$
With $MnmA_2$
Without $MnmA_2$
Nº of genomes with $MnmA_2$
Nº of genomes without $MnmA_2$
With $TusE_2$
Without $TusE_2$
Nº of genomes with $TusE_2$
Nº of genomes without $TusE_2$
With $TusE_1$
Without $TusE_1$
Nº of genomes with $TusE_1$
Nº of genomes without $TusE_1$
With $TusE_4$
Without $TusE_4$
Nº of genomes with $TusE_4$
Nº of genomes without $TusE_4$
With $TusE_3$
Without $TusE_3$
Nº of genomes with $TusE_3$
Nº of genomes without $TusE_3$
$p < 0.00005$

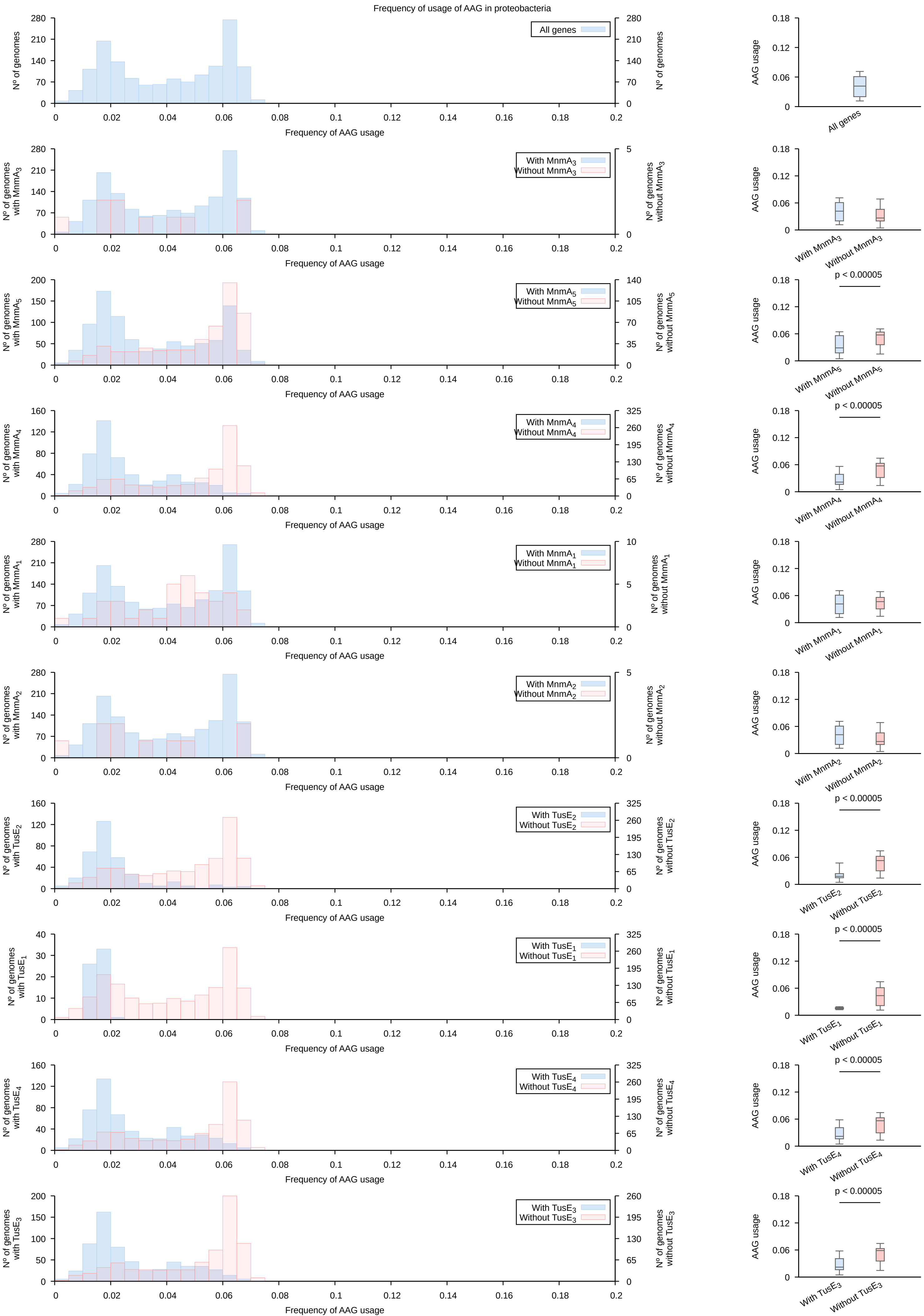
Frequency of usage of AAG in proteobacteria
Nº of genomes
Frequency of AAG usage
AAG usage
All genes
Nº of genomes with MnmA$_3$
Nº of genomes without MnmA$_3$
With MnmA$_3$
Without MnmA$_3$
Nº of genomes with MnmA$_5$
Nº of genomes without MnmA$_5$
With MnmA$_5$
Without MnmA$_5$
p < 0.00005
Nº of genomes with MnmA$_4$
Nº of genomes without MnmA$_4$
With MnmA$_4$
Without MnmA$_4$
p < 0.00005
Nº of genomes with MnmA$_1$
Nº of genomes without MnmA$_1$
With MnmA$_1$
Without MnmA$_1$
Nº of genomes with MnmA$_2$
Nº of genomes without MnmA$_2$
With MnmA$_2$
Without MnmA$_2$
Nº of genomes with TusE$_2$
Nº of genomes without TusE$_2$
With TusE$_2$
Without TusE$_2$
p < 0.00005
Nº of genomes with TusE$_1$
Nº of genomes without TusE$_1$
With TusE$_1$
Without TusE$_1$
p < 0.00005
Nº of genomes with TusE$_4$
Nº of genomes without TusE$_4$
With TusE$_4$
Without TusE$_4$
p < 0.00005
Nº of genomes with TusE$_3$
Nº of genomes without TusE$_3$
With TusE$_3$
Without TusE$_3$
p < 0.00005

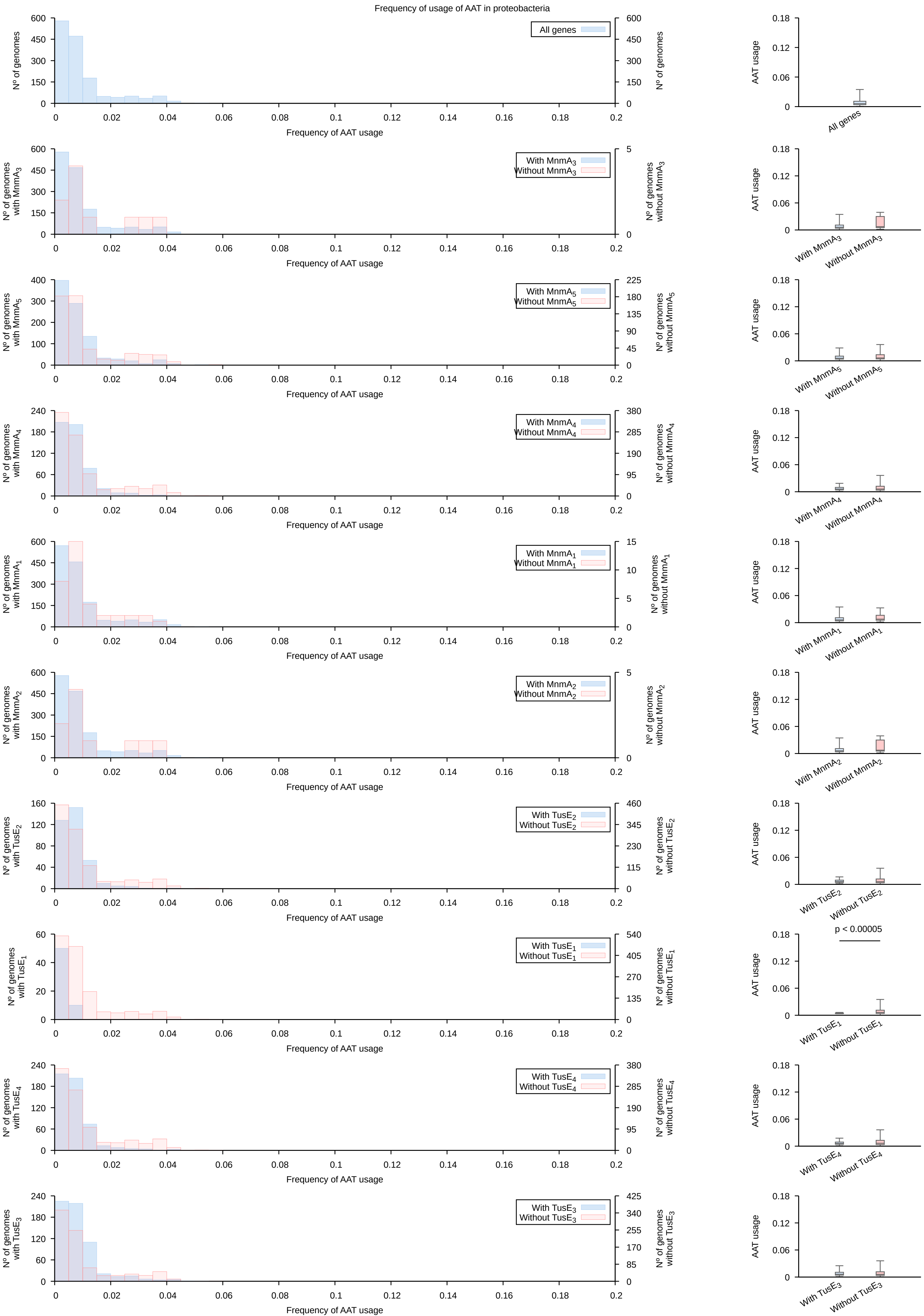
Frequency of usage of AAT in proteobacteria
All genes
With $MnmA_3$
Without $MnmA_3$
With $MnmA_5$
Without $MnmA_5$
With $MnmA_4$
Without $MnmA_4$
With $MnmA_1$
Without $MnmA_1$
With $MnmA_2$
Without $MnmA_2$
With $TusE_2$
Without $TusE_2$
With $TusE_1$
Without $TusE_1$
With $TusE_4$
Without $TusE_4$
With $TusE_3$
Without $TusE_3$
Nº of genomes
Frequency of AAT usage
AAT usage
$p < 0.00005$

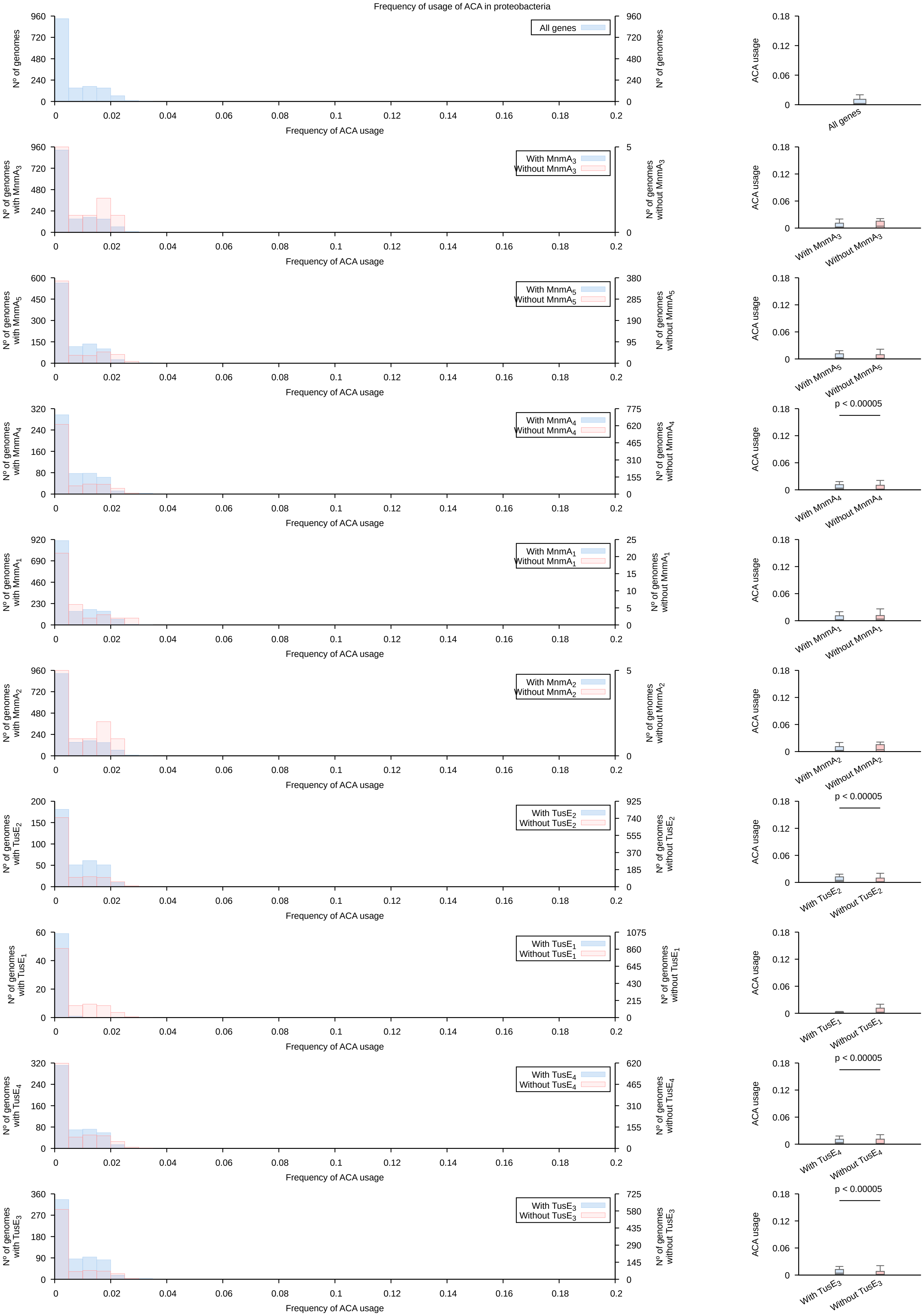
Frequency of usage of ACA in proteobacteria
Nº of genomes
Frequency of ACA usage
All genes
ACA usage
p < 0.00005
Nº of genomes with MnmA$_3$
Nº of genomes without MnmA$_3$
With MnmA$_3$
Without MnmA$_3$
Nº of genomes with MnmA$_5$
Nº of genomes without MnmA$_5$
With MnmA$_5$
Without MnmA$_5$
Nº of genomes with MnmA$_4$
Nº of genomes without MnmA$_4$
With MnmA$_4$
Without MnmA$_4$
Nº of genomes with MnmA$_1$
Nº of genomes without MnmA$_1$
With MnmA$_1$
Without MnmA$_1$
Nº of genomes with MnmA$_2$
Nº of genomes without MnmA$_2$
With MnmA$_2$
Without MnmA$_2$
Nº of genomes with TusE$_2$
Nº of genomes without TusE$_2$
With TusE$_2$
Without TusE$_2$
Nº of genomes with TusE$_1$
Nº of genomes without TusE$_1$
With TusE$_1$
Without TusE$_1$
Nº of genomes with TusE$_4$
Nº of genomes without TusE$_4$
With TusE$_4$
Without TusE$_4$
Nº of genomes with TusE$_3$
Nº of genomes without TusE$_3$
With TusE$_3$
Without TusE$_3$

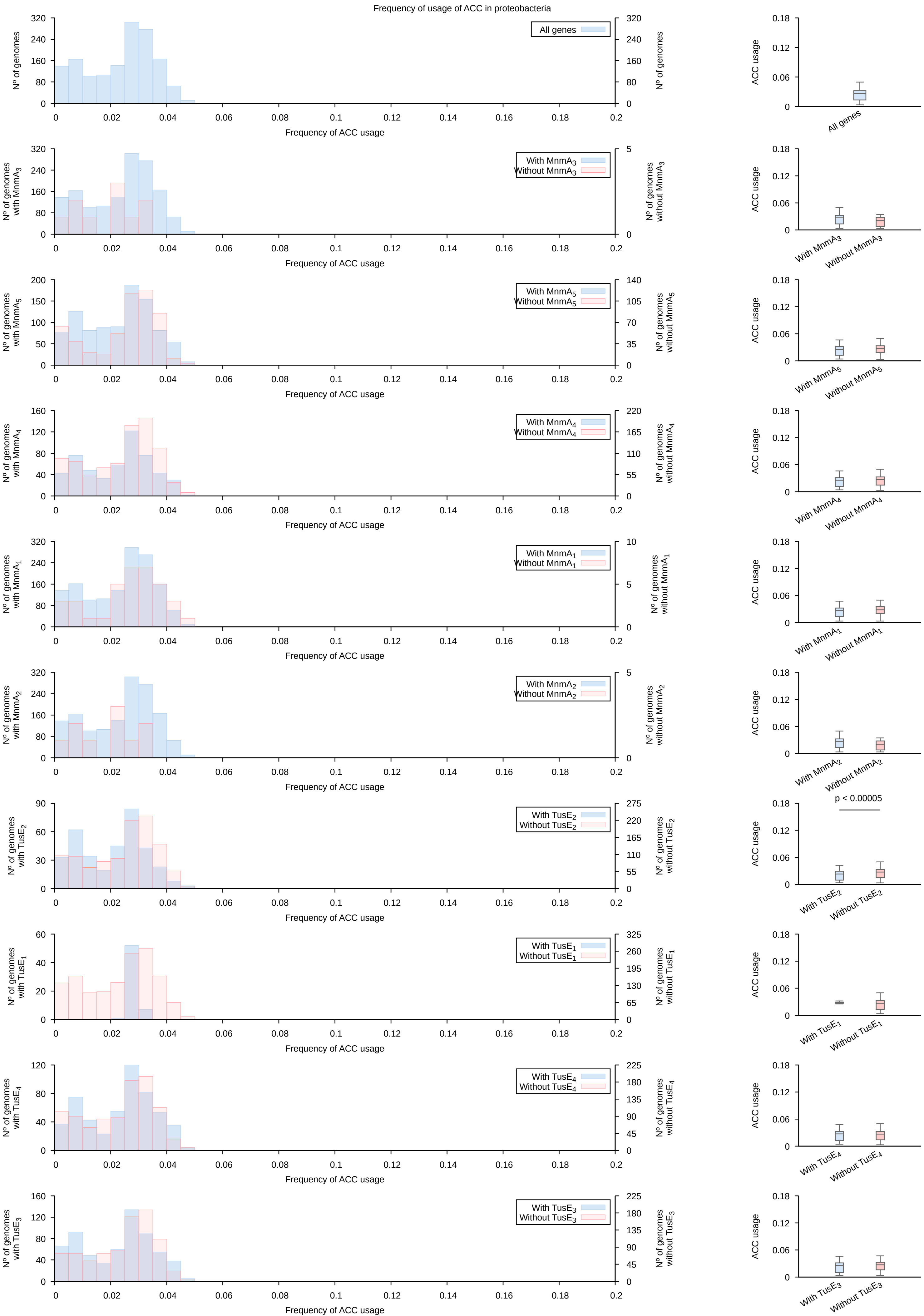

Frequency of usage of ACC in proteobacteria
Nº of genomes
All genes
Frequency of ACC usage
ACC usage
All genes
Nº of genomes with $MnmA_3$
Nº of genomes without $MnmA_3$
With $MnmA_3$
Without $MnmA_3$
Nº of genomes with $MnmA_5$
Nº of genomes without $MnmA_5$
With $MnmA_5$
Without $MnmA_5$
Nº of genomes with $MnmA_4$
Nº of genomes without $MnmA_4$
With $MnmA_4$
Without $MnmA_4$
Nº of genomes with $MnmA_1$
Nº of genomes without $MnmA_1$
With $MnmA_1$
Without $MnmA_1$
Nº of genomes with $MnmA_2$
Nº of genomes without $MnmA_2$
With $MnmA_2$
Without $MnmA_2$
Nº of genomes with $TusE_2$
Nº of genomes without $TusE_2$
With $TusE_2$
Without $TusE_2$
p < 0.00005
Nº of genomes with $TusE_1$
Nº of genomes without $TusE_1$
With $TusE_1$
Without $TusE_1$
Nº of genomes with $TusE_4$
Nº of genomes without $TusE_4$
With $TusE_4$
Without $TusE_4$
Nº of genomes with $TusE_3$
Nº of genomes without $TusE_3$
With $TusE_3$
Without $TusE_3$

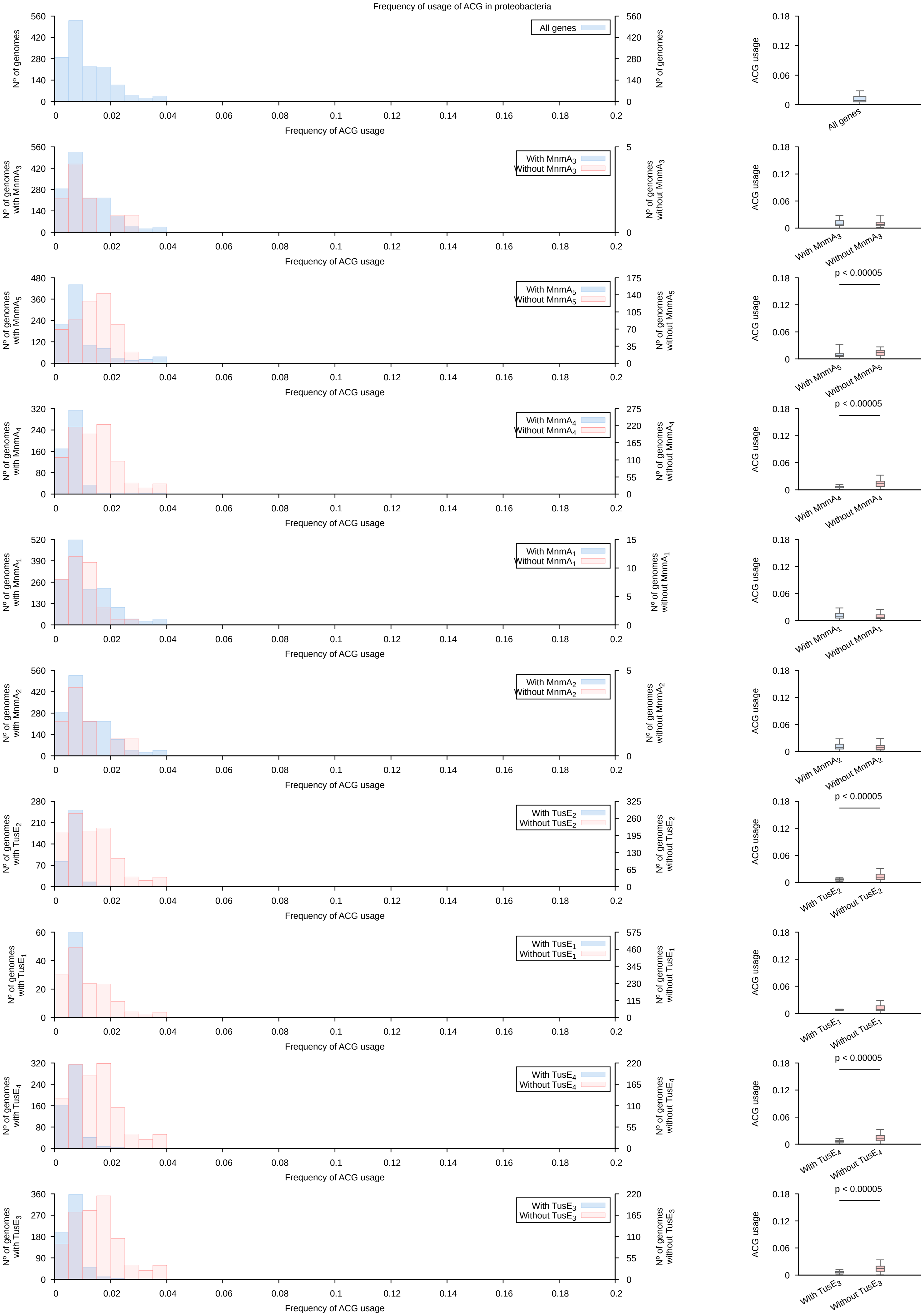
Frequency of usage of ACG in proteobacteria
All genes
With MnmA3
Without MnmA3
With MnmA5
Without MnmA5
With MnmA4
Without MnmA4
With MnmA1
Without MnmA1
With MnmA2
Without MnmA2
With TusE2
Without TusE2
With TusE1
Without TusE1
With TusE4
Without TusE4
With TusE3
Without TusE3
Frequency of ACG usage
Nº of genomes
ACG usage
p < 0.00005

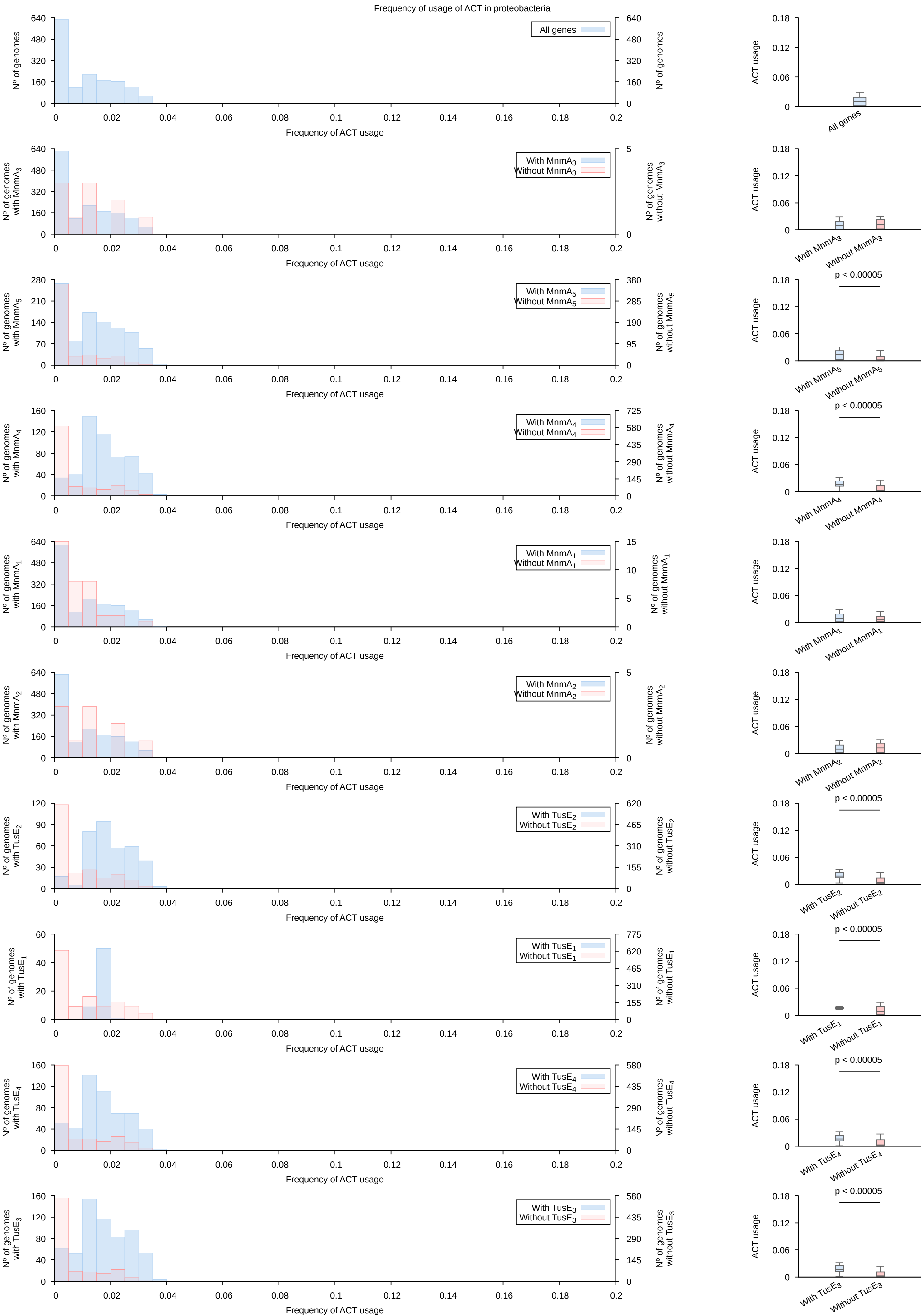
Frequency of usage of ACT in proteobacteria
Nº of genomes
Frequency of ACT usage
All genes
ACT usage
Nº of genomes with MnmA3
Nº of genomes without MnmA3
With MnmA3
Without MnmA3
Nº of genomes with MnmA5
Nº of genomes without MnmA5
With MnmA5
Without MnmA5
p < 0.00005
Nº of genomes with MnmA4
Nº of genomes without MnmA4
With MnmA4
Without MnmA4
p < 0.00005
Nº of genomes with MnmA1
Nº of genomes without MnmA1
With MnmA1
Without MnmA1
Nº of genomes with MnmA2
Nº of genomes without MnmA2
With MnmA2
Without MnmA2
Nº of genomes with TusE2
Nº of genomes without TusE2
With TusE2
Without TusE2
p < 0.00005
Nº of genomes with TusE1
Nº of genomes without TusE1
With TusE1
Without TusE1
p < 0.00005
Nº of genomes with TusE4
Nº of genomes without TusE4
With TusE4
Without TusE4
p < 0.00005
Nº of genomes with TusE3
Nº of genomes without TusE3
With TusE3
Without TusE3
p < 0.00005

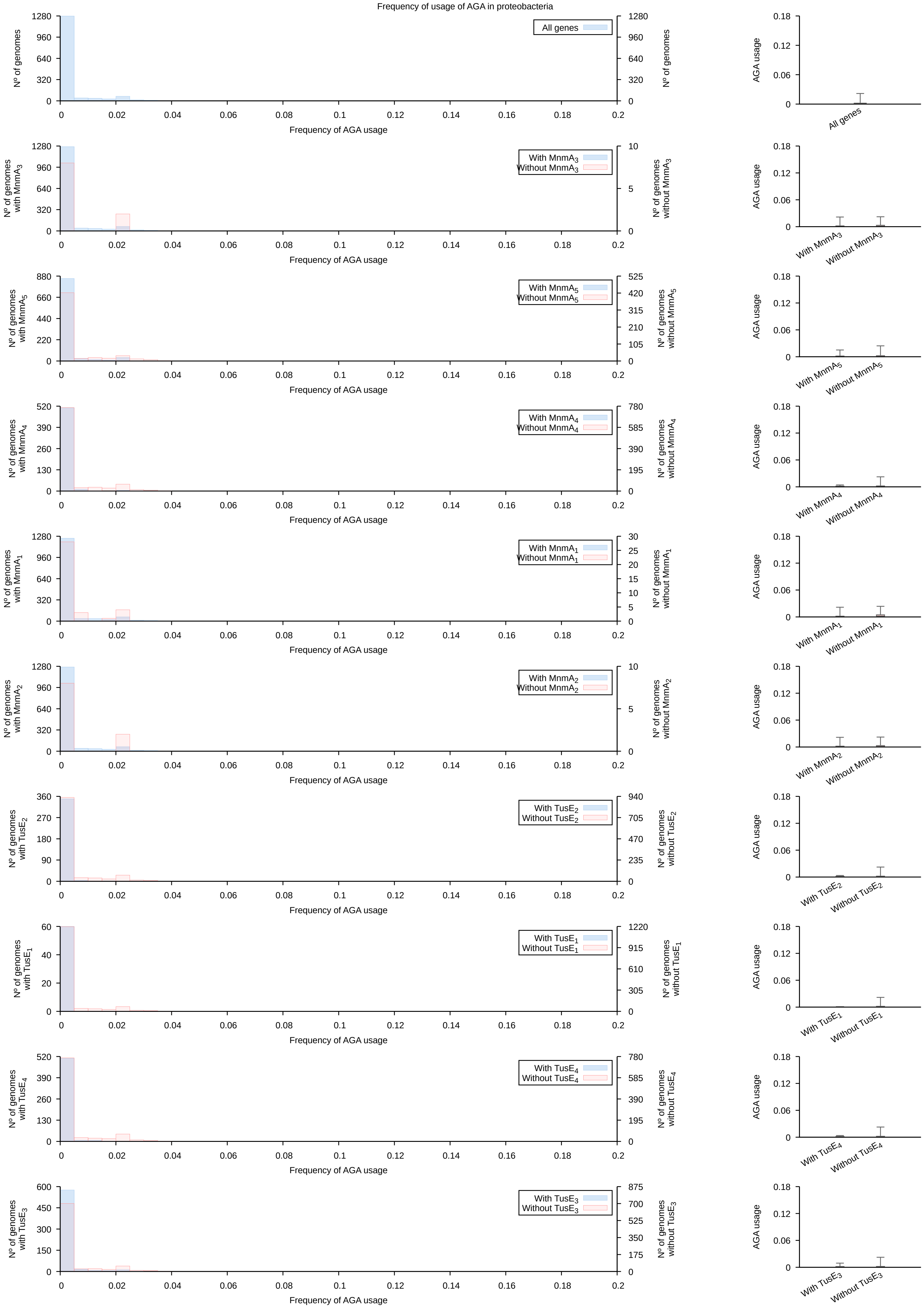

Frequency of usage of AGA in proteobacteria
All genes
Nº of genomes
Frequency of AGA usage
AGA usage
With MnmA3
Without MnmA3
Nº of genomes with MnmA3
Nº of genomes without MnmA3
With MnmA5
Without MnmA5
Nº of genomes with MnmA5
Nº of genomes without MnmA5
With MnmA4
Without MnmA4
Nº of genomes with MnmA4
Nº of genomes without MnmA4
With MnmA1
Without MnmA1
Nº of genomes with MnmA1
Nº of genomes without MnmA1
With MnmA2
Without MnmA2
Nº of genomes with MnmA2
Nº of genomes without MnmA2
With TusE2
Without TusE2
Nº of genomes with TusE2
Nº of genomes without TusE2
With TusE1
Without TusE1
Nº of genomes with TusE1
Nº of genomes without TusE1
With TusE4
Without TusE4
Nº of genomes with TusE4
Nº of genomes without TusE4
With TusE3
Without TusE3
Nº of genomes with TusE3
Nº of genomes without TusE3

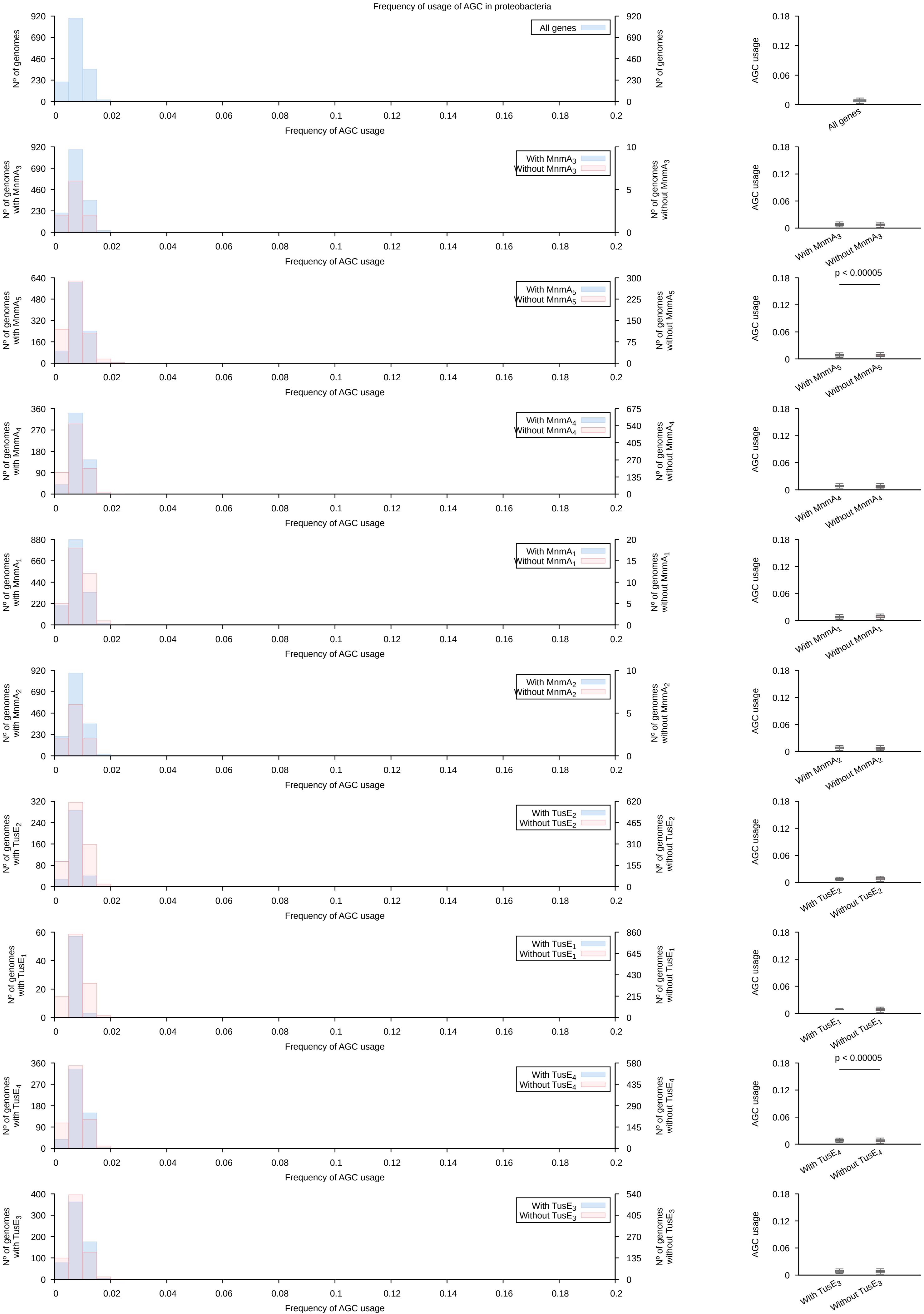
Frequency of usage of AGC in proteobacteria
Nº of genomes
Frequency of AGC usage
AGC usage
All genes
With MnmA3
Without MnmA3
With MnmA5
Without MnmA5
p < 0.00005
With MnmA4
Without MnmA4
With MnmA1
Without MnmA1
With MnmA2
Without MnmA2
With TusE2
Without TusE2
With TusE1
Without TusE1
With TusE4
Without TusE4
p < 0.00005
With TusE3
Without TusE3

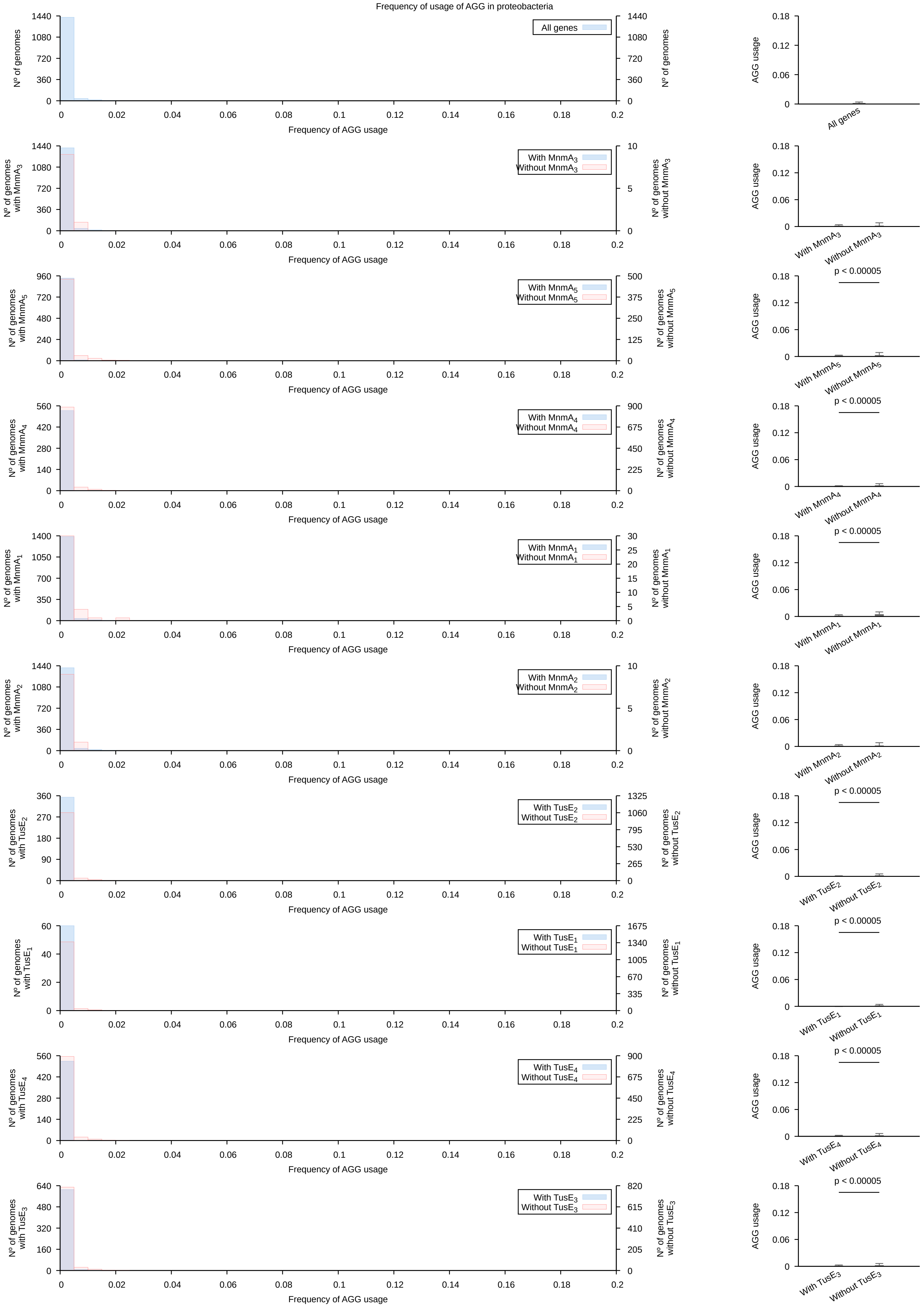

Frequency of usage of AGG in proteobacteria
All genes
Nº of genomes
Frequency of AGG usage
AGG usage
With MnmA3
Without MnmA3
Nº of genomes with MnmA3
Nº of genomes without MnmA3
With MnmA5
Without MnmA5
Nº of genomes with MnmA5
Nº of genomes without MnmA5
p < 0.00005
With MnmA4
Without MnmA4
Nº of genomes with MnmA4
Nº of genomes without MnmA4
p < 0.00005
With MnmA1
Without MnmA1
Nº of genomes with MnmA1
Nº of genomes without MnmA1
p < 0.00005
With MnmA2
Without MnmA2
Nº of genomes with MnmA2
Nº of genomes without MnmA2
With TusE2
Without TusE2
Nº of genomes with TusE2
Nº of genomes without TusE2
p < 0.00005
With TusE1
Without TusE1
Nº of genomes with TusE1
Nº of genomes without TusE1
p < 0.00005
With TusE4
Without TusE4
Nº of genomes with TusE4
Nº of genomes without TusE4
p < 0.00005
With TusE3
Without TusE3
Nº of genomes with TusE3
Nº of genomes without TusE3
p < 0.00005

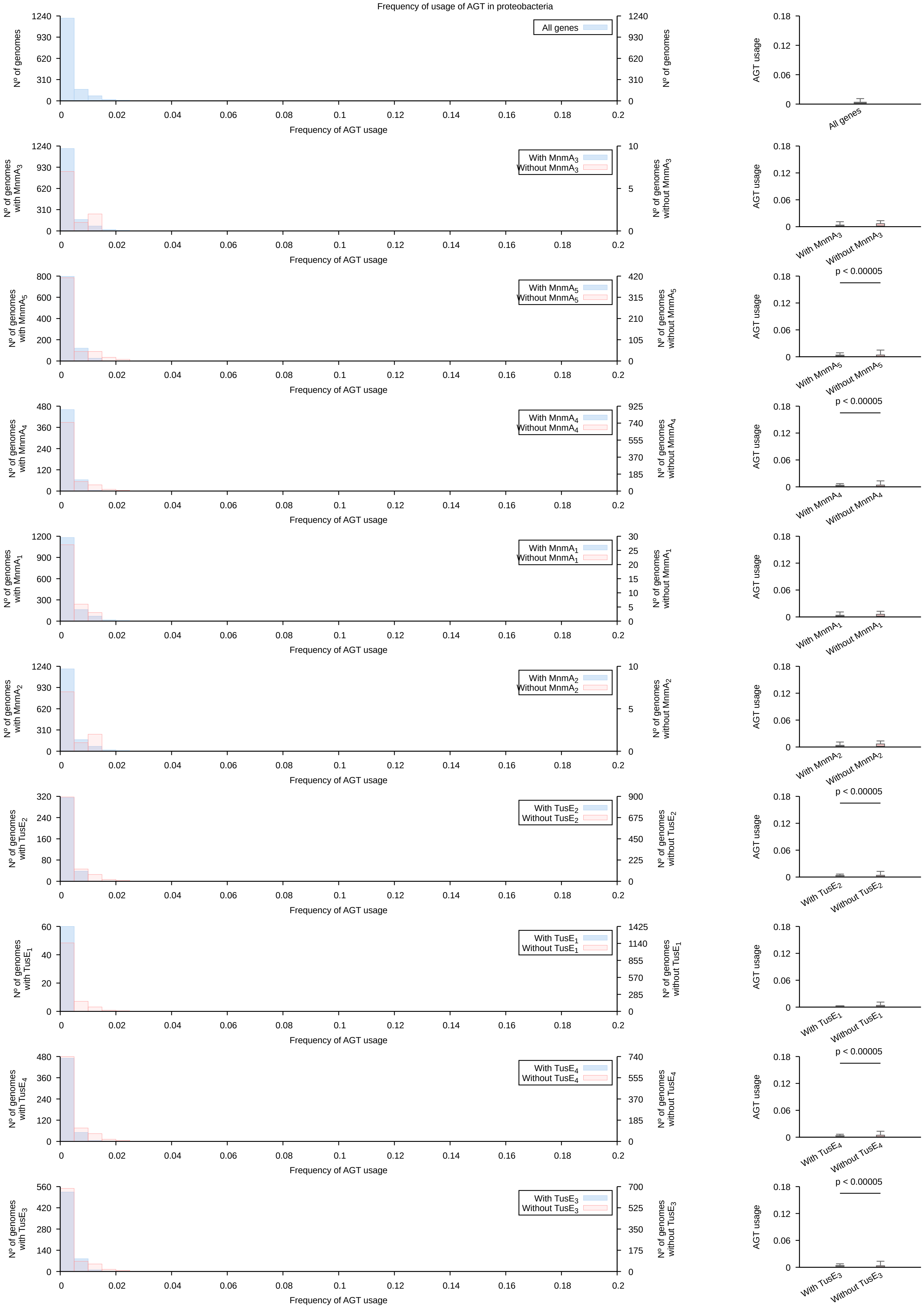
Frequency of usage of AGT in proteobacteria
All genes
With MnmA$_3$
Without MnmA$_3$
With MnmA$_5$
Without MnmA$_5$
With MnmA$_4$
Without MnmA$_4$
With MnmA$_1$
Without MnmA$_1$
With MnmA$_2$
Without MnmA$_2$
With TusE$_2$
Without TusE$_2$
With TusE$_1$
Without TusE$_1$
With TusE$_4$
Without TusE$_4$
With TusE$_3$
Without TusE$_3$
Frequency of AGT usage
Nº of genomes
AGT usage
p < 0.00005

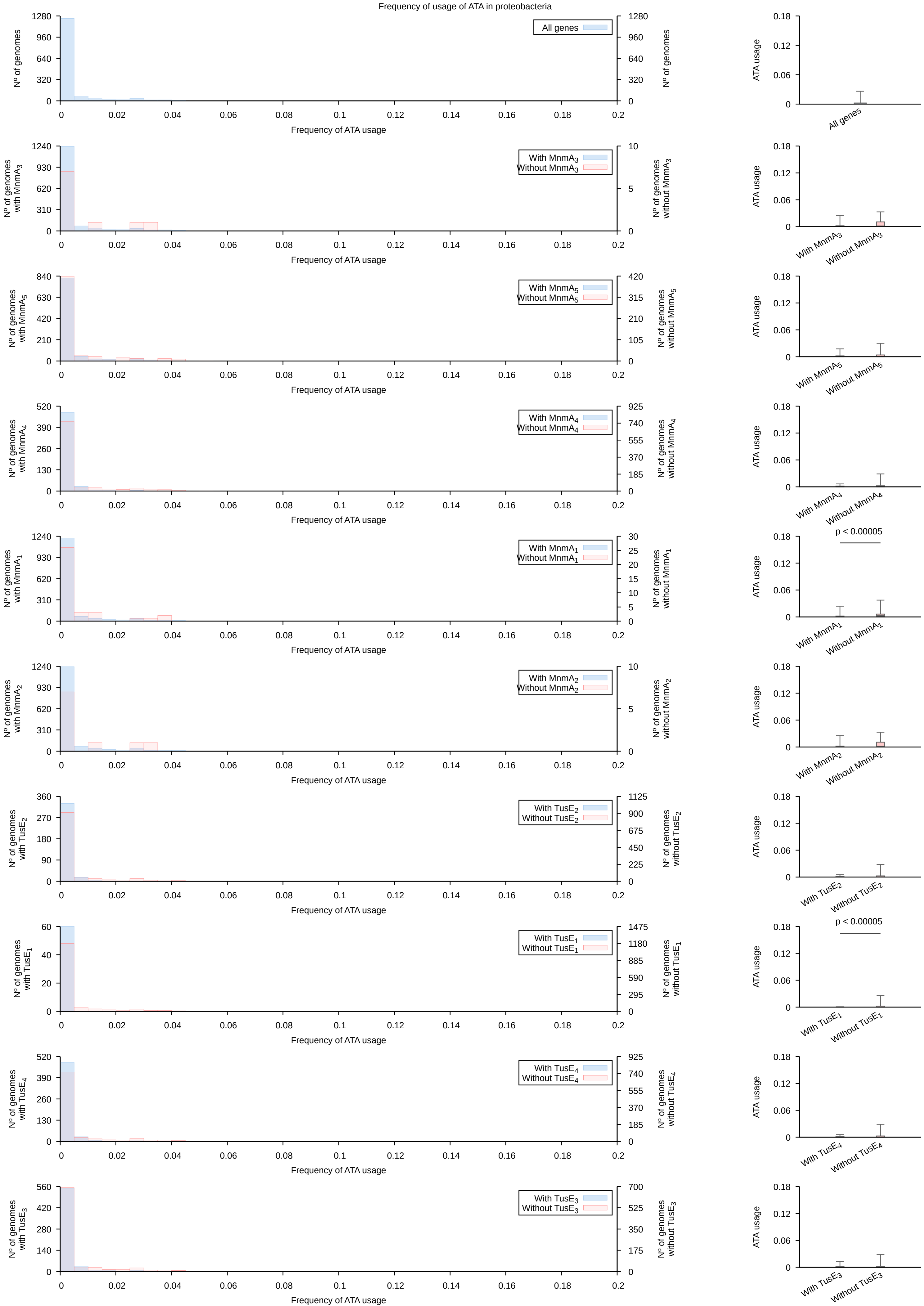
Frequency of usage of ATA in proteobacteria
All genes
With MnmA3
Without MnmA3
With MnmA5
Without MnmA5
With MnmA4
Without MnmA4
With MnmA1
Without MnmA1
With MnmA2
Without MnmA2
With TusE2
Without TusE2
With TusE1
Without TusE1
With TusE4
Without TusE4
With TusE3
Without TusE3
p < 0.00005
p < 0.00005
Frequency of ATA usage
Nº of genomes
ATA usage

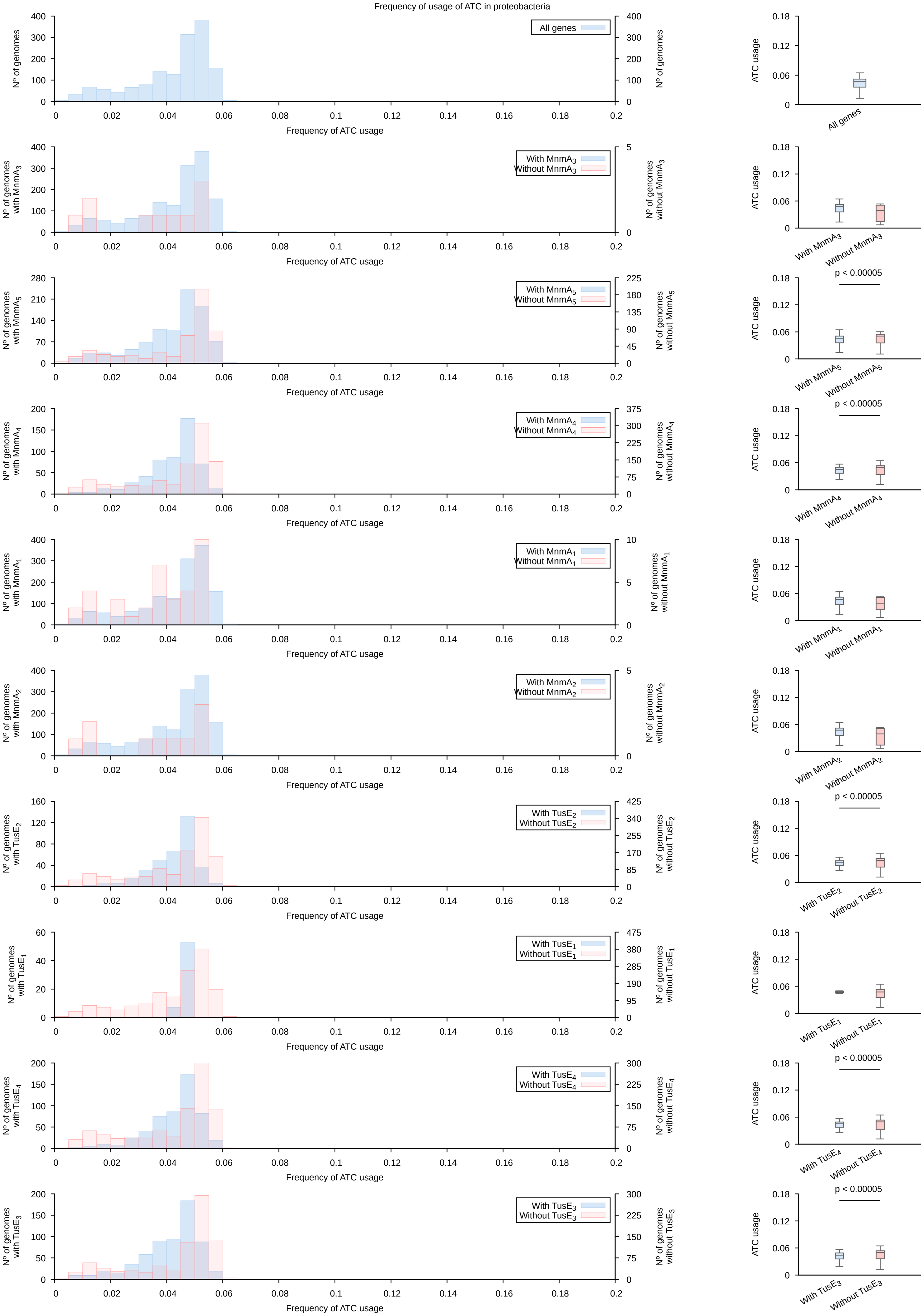

Frequency of usage of ATC in proteobacteria
All genes
Nº of genomes
Frequency of ATC usage
ATC usage
With MnmA3
Without MnmA3
Nº of genomes with MnmA3
Nº of genomes without MnmA3
With MnmA5
Without MnmA5
Nº of genomes with MnmA5
Nº of genomes without MnmA5
p < 0.00005
With MnmA4
Without MnmA4
Nº of genomes with MnmA4
Nº of genomes without MnmA4
p < 0.00005
With MnmA1
Without MnmA1
Nº of genomes with MnmA1
Nº of genomes without MnmA1
With MnmA2
Without MnmA2
Nº of genomes with MnmA2
Nº of genomes without MnmA2
With TusE2
Without TusE2
Nº of genomes with TusE2
Nº of genomes without TusE2
p < 0.00005
With TusE1
Without TusE1
Nº of genomes with TusE1
Nº of genomes without TusE1
With TusE4
Without TusE4
Nº of genomes with TusE4
Nº of genomes without TusE4
p < 0.00005
With TusE3
Without TusE3
Nº of genomes with TusE3
Nº of genomes without TusE3
p < 0.00005

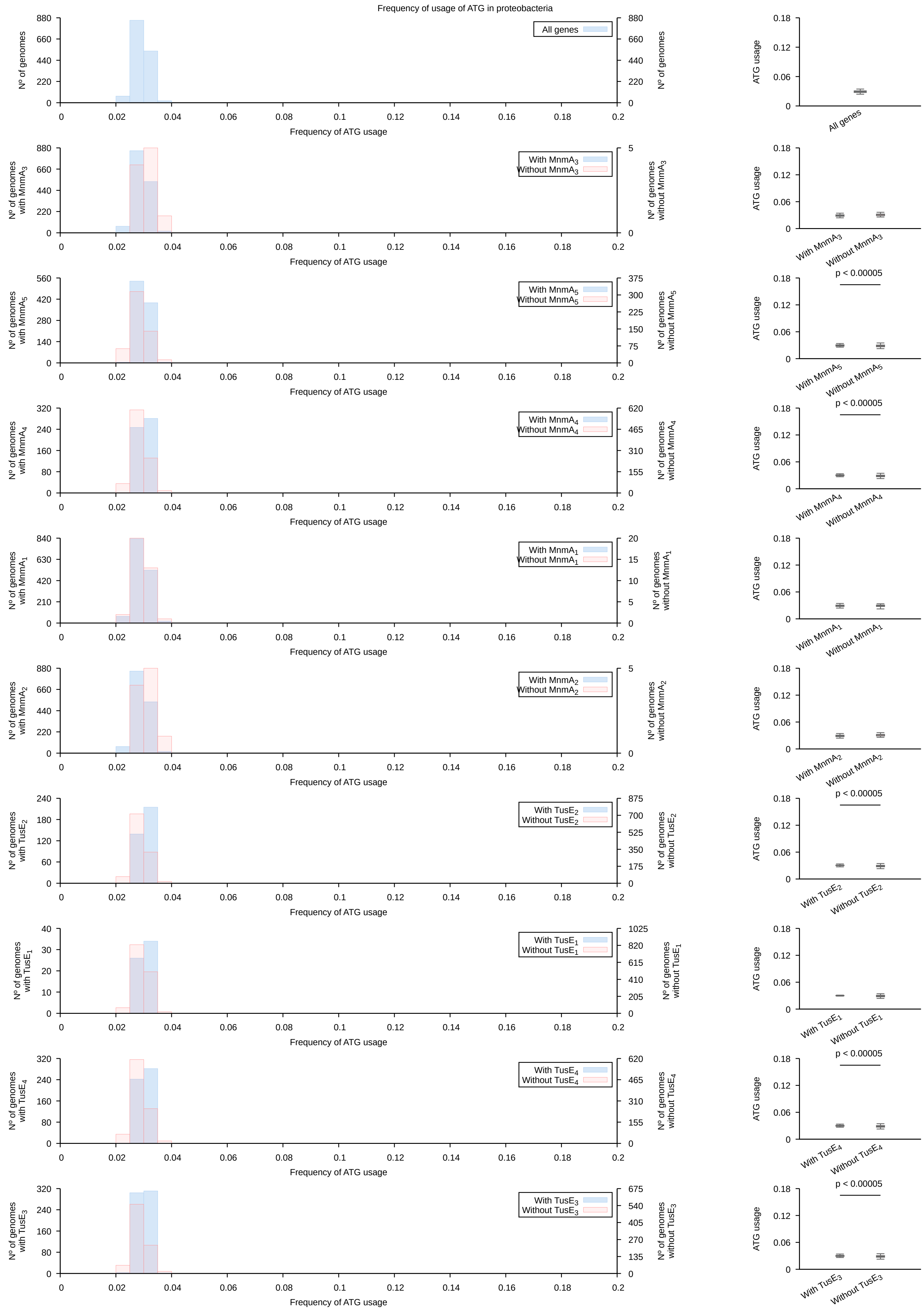

Frequency of usage of ATG in proteobacteria
All genes
Nº of genomes
Frequency of ATG usage
ATG usage
With MnmA3
Without MnmA3
Nº of genomes with MnmA3
Nº of genomes without MnmA3
With MnmA5
Without MnmA5
Nº of genomes with MnmA5
Nº of genomes without MnmA5
p < 0.00005
With MnmA4
Without MnmA4
Nº of genomes with MnmA4
Nº of genomes without MnmA4
p < 0.00005
With MnmA1
Without MnmA1
Nº of genomes with MnmA1
Nº of genomes without MnmA1
With MnmA2
Without MnmA2
Nº of genomes with MnmA2
Nº of genomes without MnmA2
With TusE2
Without TusE2
Nº of genomes with TusE2
Nº of genomes without TusE2
p < 0.00005
With TusE1
Without TusE1
Nº of genomes with TusE1
Nº of genomes without TusE1
With TusE4
Without TusE4
Nº of genomes with TusE4
Nº of genomes without TusE4
p < 0.00005
With TusE3
Without TusE3
Nº of genomes with TusE3
Nº of genomes without TusE3
p < 0.00005

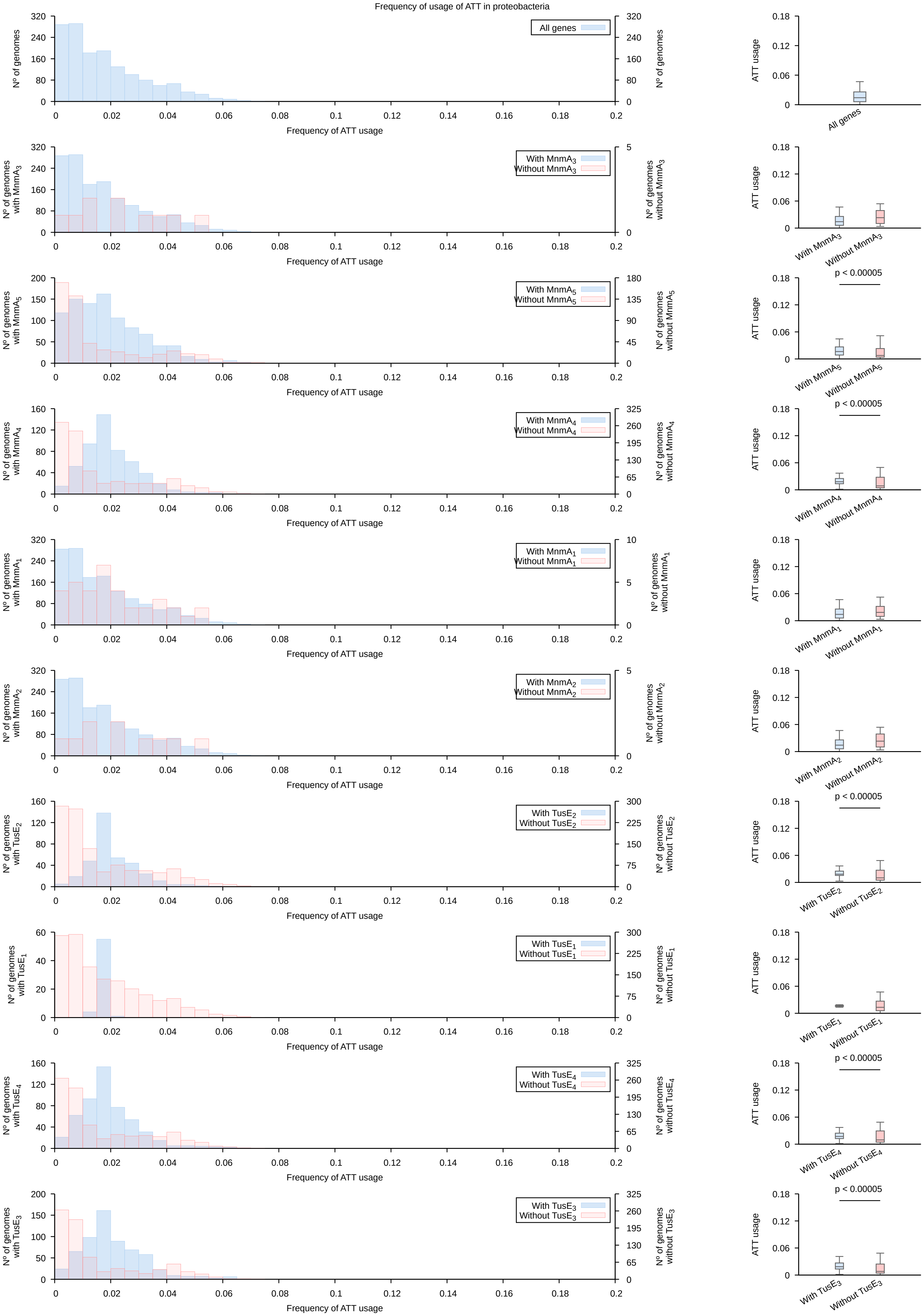

Frequency of usage of ATT in proteobacteria
All genes
Nº of genomes
Frequency of ATT usage
ATT usage
With MnmA$_3$
Without MnmA$_3$
Nº of genomes with MnmA$_3$
Nº of genomes without MnmA$_3$
With MnmA$_5$
Without MnmA$_5$
Nº of genomes with MnmA$_5$
Nº of genomes without MnmA$_5$
p < 0.00005
With MnmA$_4$
Without MnmA$_4$
Nº of genomes with MnmA$_4$
Nº of genomes without MnmA$_4$
p < 0.00005
With MnmA$_1$
Without MnmA$_1$
Nº of genomes with MnmA$_1$
Nº of genomes without MnmA$_1$
With MnmA$_2$
Without MnmA$_2$
Nº of genomes with MnmA$_2$
Nº of genomes without MnmA$_2$
With TusE$_2$
Without TusE$_2$
Nº of genomes with TusE$_2$
Nº of genomes without TusE$_2$
p < 0.00005
With TusE$_1$
Without TusE$_1$
Nº of genomes with TusE$_1$
Nº of genomes without TusE$_1$
With TusE$_4$
Without TusE$_4$
Nº of genomes with TusE$_4$
Nº of genomes without TusE$_4$
p < 0.00005
With TusE$_3$
Without TusE$_3$
Nº of genomes with TusE$_3$
Nº of genomes without TusE$_3$
p < 0.00005

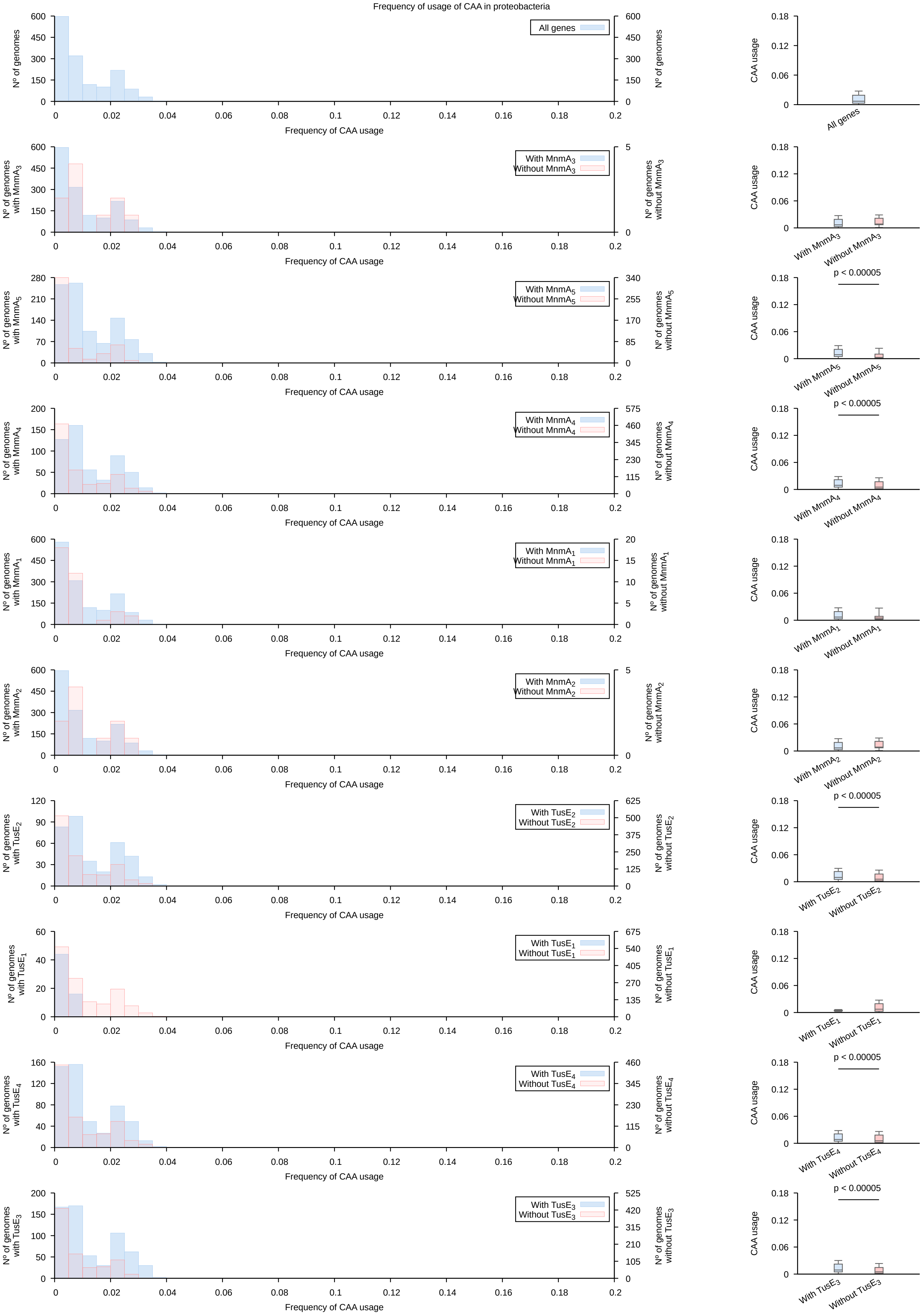

Frequency of usage of CAA in proteobacteria
Nº of genomes
All genes
Frequency of CAA usage
CAA usage
Nº of genomes with $MnmA_3$
Nº of genomes without $MnmA_3$
With $MnmA_3$
Without $MnmA_3$
Nº of genomes with $MnmA_5$
Nº of genomes without $MnmA_5$
With $MnmA_5$
Without $MnmA_5$
p < 0.00005
Nº of genomes with $MnmA_4$
Nº of genomes without $MnmA_4$
With $MnmA_4$
Without $MnmA_4$
p < 0.00005
Nº of genomes with $MnmA_1$
Nº of genomes without $MnmA_1$
With $MnmA_1$
Without $MnmA_1$
Nº of genomes with $MnmA_2$
Nº of genomes without $MnmA_2$
With $MnmA_2$
Without $MnmA_2$
Nº of genomes with $TusE_2$
Nº of genomes without $TusE_2$
With $TusE_2$
Without $TusE_2$
p < 0.00005
Nº of genomes with $TusE_1$
Nº of genomes without $TusE_1$
With $TusE_1$
Without $TusE_1$
Nº of genomes with $TusE_4$
Nº of genomes without $TusE_4$
With $TusE_4$
Without $TusE_4$
p < 0.00005
Nº of genomes with $TusE_3$
Nº of genomes without $TusE_3$
With $TusE_3$
Without $TusE_3$
p < 0.00005

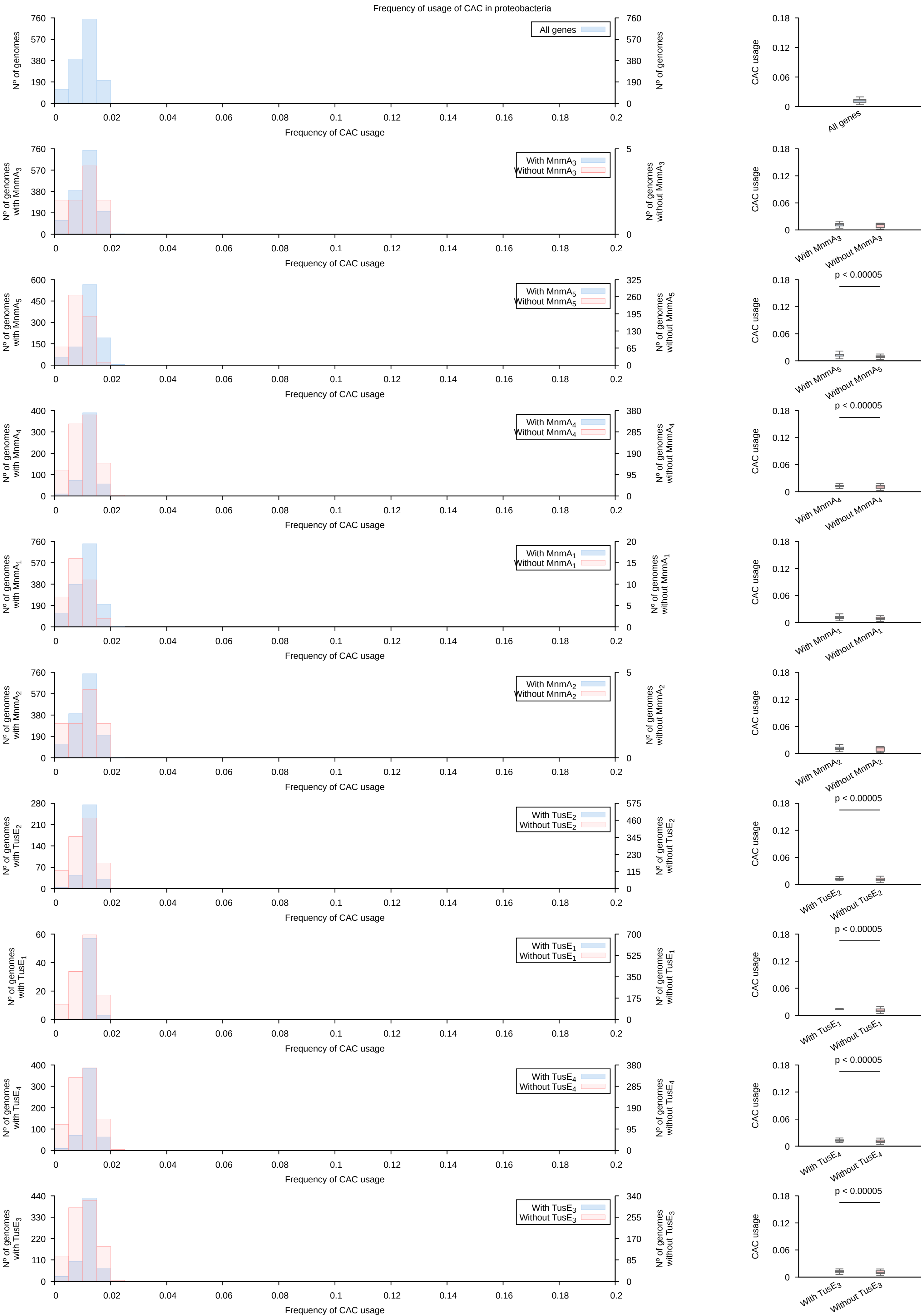
Frequency of usage of CAC in proteobacteria
Nº of genomes
Frequency of CAC usage
CAC usage
All genes
Nº of genomes with MnmA$_3$
Nº of genomes without MnmA$_3$
With MnmA$_3$
Without MnmA$_3$
Nº of genomes with MnmA$_5$
Nº of genomes without MnmA$_5$
With MnmA$_5$
Without MnmA$_5$
p < 0.00005
Nº of genomes with MnmA$_4$
Nº of genomes without MnmA$_4$
With MnmA$_4$
Without MnmA$_4$
p < 0.00005
Nº of genomes with MnmA$_1$
Nº of genomes without MnmA$_1$
With MnmA$_1$
Without MnmA$_1$
Nº of genomes with MnmA$_2$
Nº of genomes without MnmA$_2$
With MnmA$_2$
Without MnmA$_2$
Nº of genomes with TusE$_2$
Nº of genomes without TusE$_2$
With TusE$_2$
Without TusE$_2$
p < 0.00005
Nº of genomes with TusE$_1$
Nº of genomes without TusE$_1$
With TusE$_1$
Without TusE$_1$
p < 0.00005
Nº of genomes with TusE$_4$
Nº of genomes without TusE$_4$
With TusE$_4$
Without TusE$_4$
p < 0.00005
Nº of genomes with TusE$_3$
Nº of genomes without TusE$_3$
With TusE$_3$
Without TusE$_3$
p < 0.00005

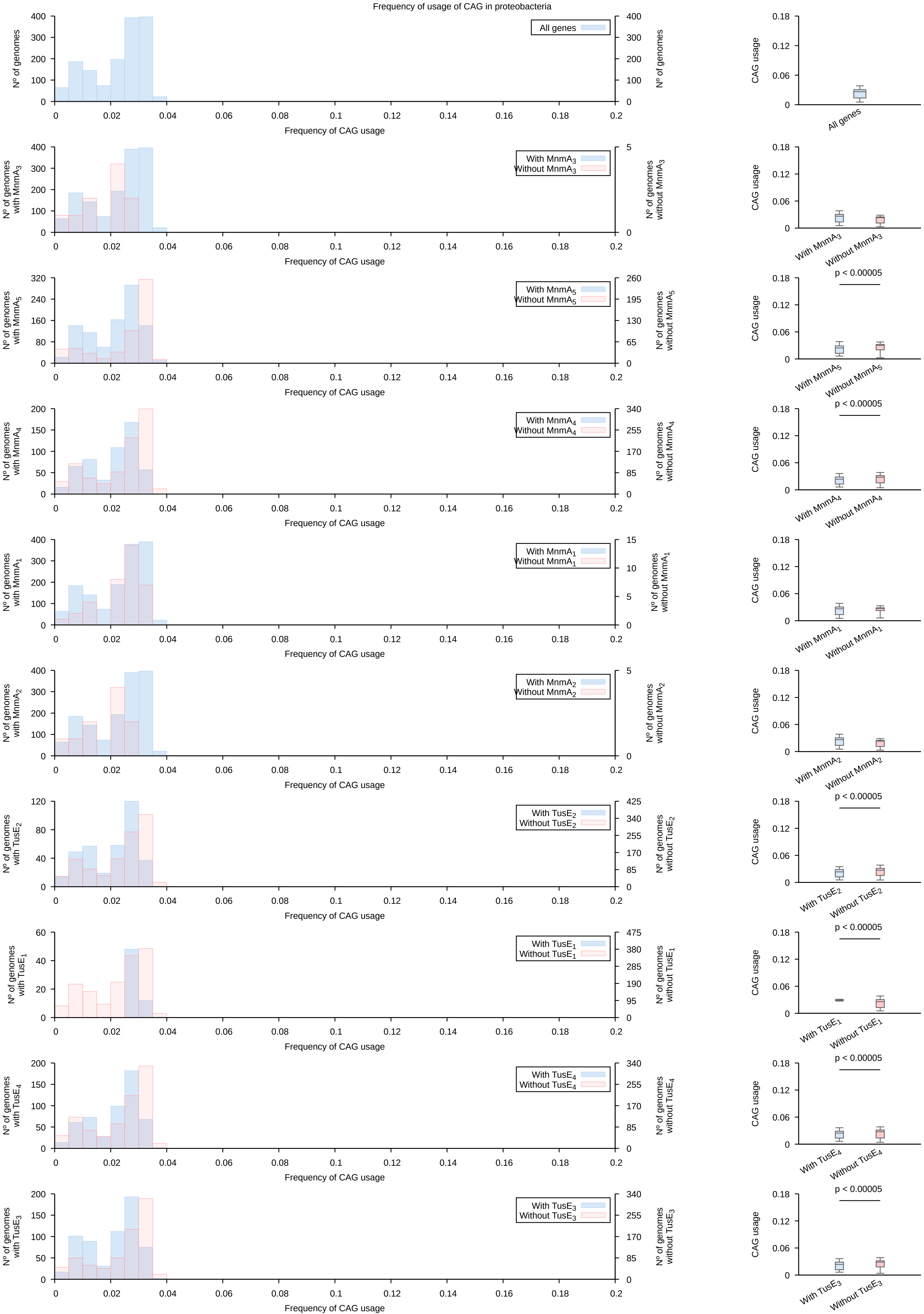

Frequency of usage of CAG in proteobacteria
Nº of genomes
All genes
Frequency of CAG usage
CAG usage
Nº of genomes with MnmA$_3$
Nº of genomes without MnmA$_3$
With MnmA$_3$
Without MnmA$_3$
Nº of genomes with MnmA$_5$
Nº of genomes without MnmA$_5$
With MnmA$_5$
Without MnmA$_5$
p < 0.00005
Nº of genomes with MnmA$_4$
Nº of genomes without MnmA$_4$
With MnmA$_4$
Without MnmA$_4$
p < 0.00005
Nº of genomes with MnmA$_1$
Nº of genomes without MnmA$_1$
With MnmA$_1$
Without MnmA$_1$
Nº of genomes with MnmA$_2$
Nº of genomes without MnmA$_2$
With MnmA$_2$
Without MnmA$_2$
Nº of genomes with TusE$_2$
Nº of genomes without TusE$_2$
With TusE$_2$
Without TusE$_2$
p < 0.00005
Nº of genomes with TusE$_1$
Nº of genomes without TusE$_1$
With TusE$_1$
Without TusE$_1$
p < 0.00005
Nº of genomes with TusE$_4$
Nº of genomes without TusE$_4$
With TusE$_4$
Without TusE$_4$
p < 0.00005
Nº of genomes with TusE$_3$
Nº of genomes without TusE$_3$
With TusE$_3$
Without TusE$_3$
p < 0.00005

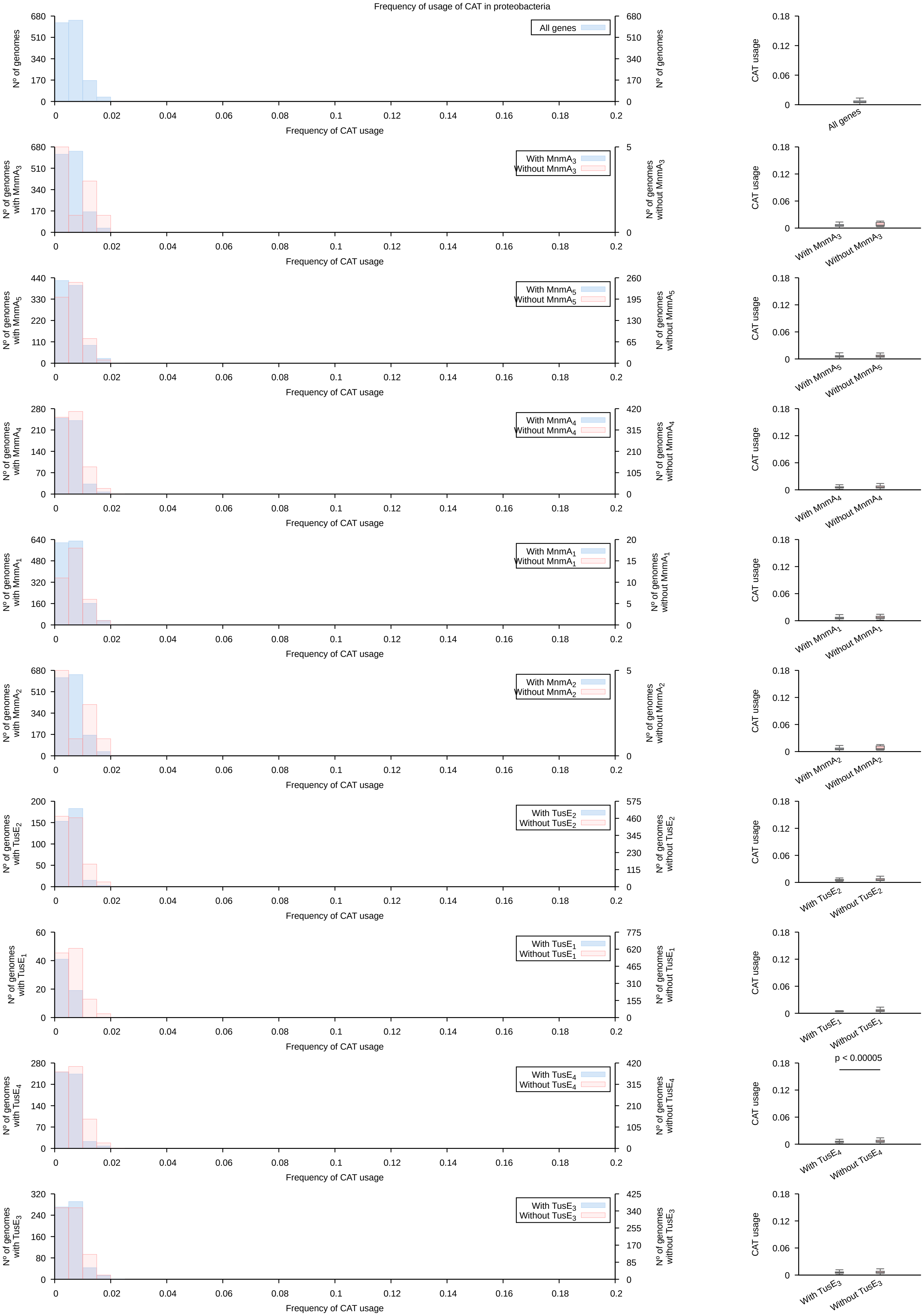
Frequency of usage of CAT in proteobacteria
Nº of genomes
All genes
Frequency of CAT usage
CAT usage
All genes
Nº of genomes with MnmA3
Nº of genomes without MnmA3
With MnmA3
Without MnmA3
Nº of genomes with MnmA5
Nº of genomes without MnmA5
With MnmA5
Without MnmA5
Nº of genomes with MnmA4
Nº of genomes without MnmA4
With MnmA4
Without MnmA4
Nº of genomes with MnmA1
Nº of genomes without MnmA1
With MnmA1
Without MnmA1
Nº of genomes with MnmA2
Nº of genomes without MnmA2
With MnmA2
Without MnmA2
Nº of genomes with TusE2
Nº of genomes without TusE2
With TusE2
Without TusE2
Nº of genomes with TusE1
Nº of genomes without TusE1
With TusE1
Without TusE1
Nº of genomes with TusE4
Nº of genomes without TusE4
With TusE4
Without TusE4
p < 0.00005
Nº of genomes with TusE3
Nº of genomes without TusE3
With TusE3
Without TusE3

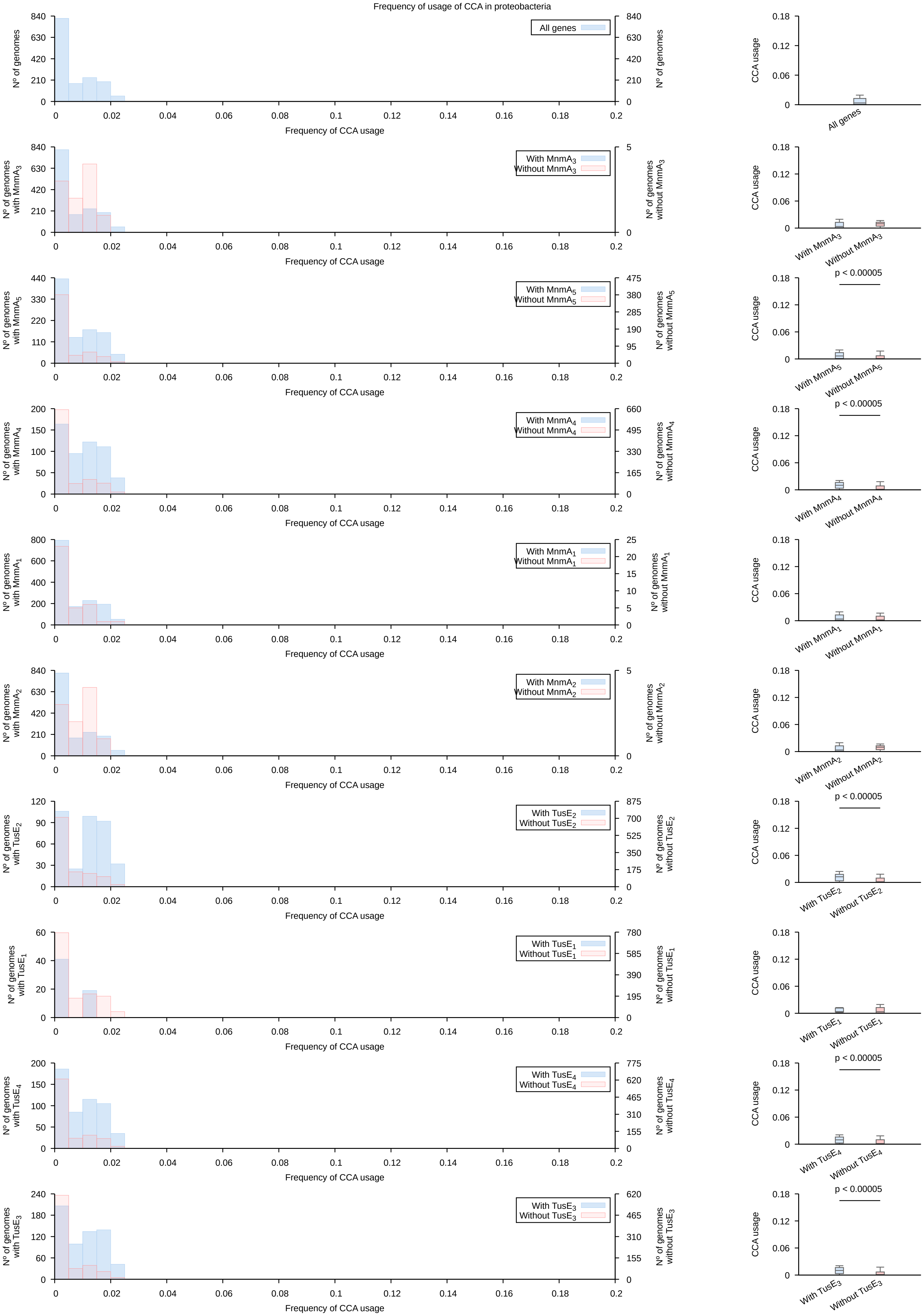
Frequency of usage of CCA in proteobacteria
Nº of genomes
All genes
Frequency of CCA usage
CCA usage
All genes
Nº of genomes with MnmA$_3$
With MnmA$_3$
Without MnmA$_3$
Nº of genomes without MnmA$_3$
Frequency of CCA usage
CCA usage
With MnmA$_3$
Without MnmA$_3$
Nº of genomes with MnmA$_5$
With MnmA$_5$
Without MnmA$_5$
Nº of genomes without MnmA$_5$
Frequency of CCA usage
p < 0.00005
CCA usage
With MnmA$_5$
Without MnmA$_5$
Nº of genomes with MnmA$_4$
With MnmA$_4$
Without MnmA$_4$
Nº of genomes without MnmA$_4$
Frequency of CCA usage
p < 0.00005
CCA usage
With MnmA$_4$
Without MnmA$_4$
Nº of genomes with MnmA$_1$
With MnmA$_1$
Without MnmA$_1$
Nº of genomes without MnmA$_1$
Frequency of CCA usage
CCA usage
With MnmA$_1$
Without MnmA$_1$
Nº of genomes with MnmA$_2$
With MnmA$_2$
Without MnmA$_2$
Nº of genomes without MnmA$_2$
Frequency of CCA usage
CCA usage
With MnmA$_2$
Without MnmA$_2$
Nº of genomes with TusE$_2$
With TusE$_2$
Without TusE$_2$
Nº of genomes without TusE$_2$
Frequency of CCA usage
p < 0.00005
CCA usage
With TusE$_2$
Without TusE$_2$
Nº of genomes with TusE$_1$
With TusE$_1$
Without TusE$_1$
Nº of genomes without TusE$_1$
Frequency of CCA usage
CCA usage
With TusE$_1$
Without TusE$_1$
Nº of genomes with TusE$_4$
With TusE$_4$
Without TusE$_4$
Nº of genomes without TusE$_4$
Frequency of CCA usage
p < 0.00005
CCA usage
With TusE$_4$
Without TusE$_4$
Nº of genomes with TusE$_3$
With TusE$_3$
Without TusE$_3$
Nº of genomes without TusE$_3$
Frequency of CCA usage
p < 0.00005
CCA usage
With TusE$_3$
Without TusE$_3$

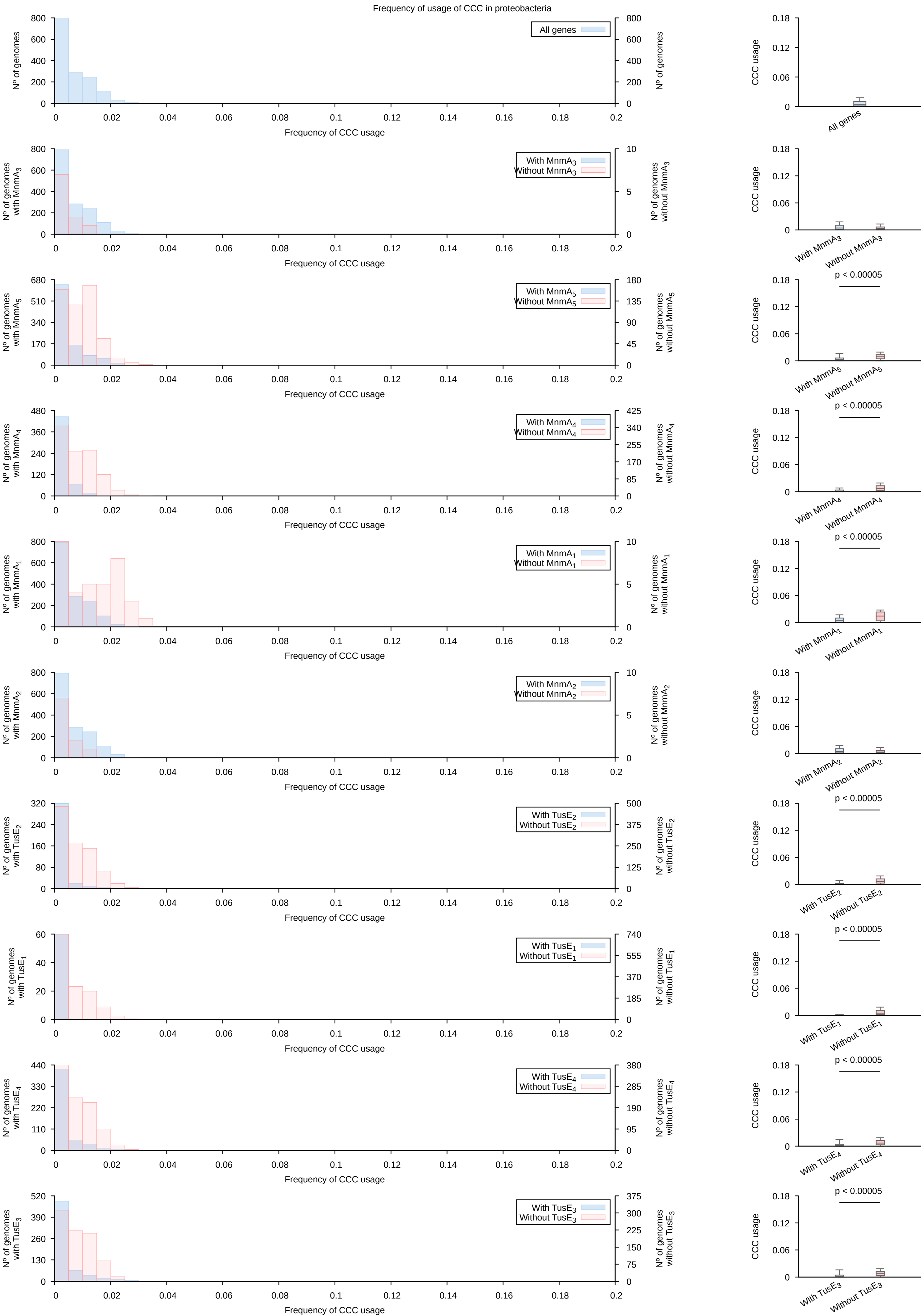

Frequency of usage of CCC in proteobacteria
All genes
Nº of genomes
Frequency of CCC usage
CCC usage
With MnmA$_3$
Without MnmA$_3$
Nº of genomes with MnmA$_3$
Nº of genomes without MnmA$_3$
With MnmA$_5$
Without MnmA$_5$
Nº of genomes with MnmA$_5$
Nº of genomes without MnmA$_5$
p < 0.00005
With MnmA$_4$
Without MnmA$_4$
Nº of genomes with MnmA$_4$
Nº of genomes without MnmA$_4$
p < 0.00005
With MnmA$_1$
Without MnmA$_1$
Nº of genomes with MnmA$_1$
Nº of genomes without MnmA$_1$
p < 0.00005
With MnmA$_2$
Without MnmA$_2$
Nº of genomes with MnmA$_2$
Nº of genomes without MnmA$_2$
With TusE$_2$
Without TusE$_2$
Nº of genomes with TusE$_2$
Nº of genomes without TusE$_2$
p < 0.00005
With TusE$_1$
Without TusE$_1$
Nº of genomes with TusE$_1$
Nº of genomes without TusE$_1$
p < 0.00005
With TusE$_4$
Without TusE$_4$
Nº of genomes with TusE$_4$
Nº of genomes without TusE$_4$
p < 0.00005
With TusE$_3$
Without TusE$_3$
Nº of genomes with TusE$_3$
Nº of genomes without TusE$_3$
p < 0.00005

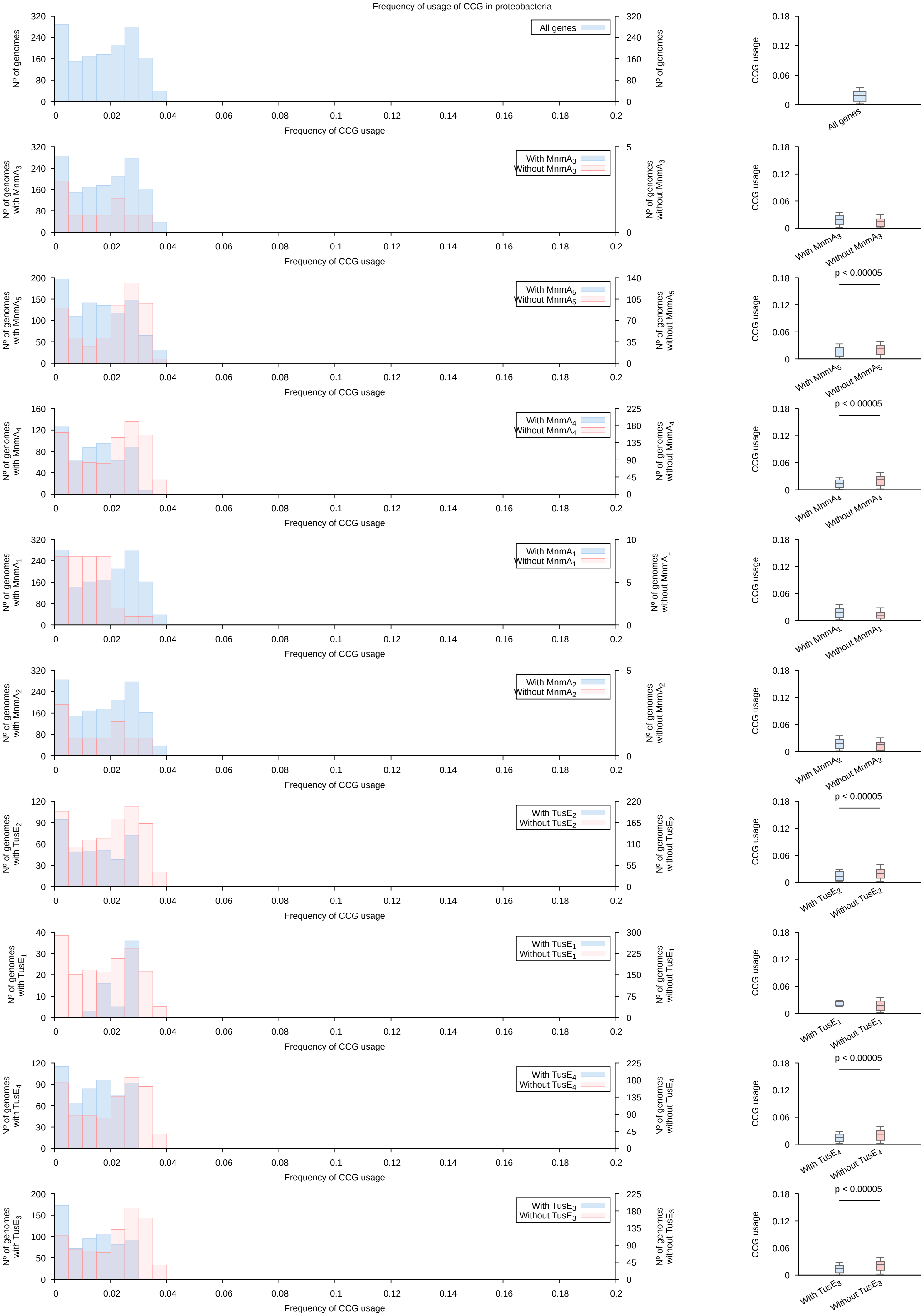
Frequency of usage of CCG in proteobacteria
Nº of genomes
Frequency of CCG usage
CCG usage
All genes
With MnmA$_3$
Without MnmA$_3$
Nº of genomes with MnmA$_3$
Nº of genomes without MnmA$_3$
With MnmA$_5$
Without MnmA$_5$
Nº of genomes with MnmA$_5$
Nº of genomes without MnmA$_5$
p < 0.00005
With MnmA$_4$
Without MnmA$_4$
Nº of genomes with MnmA$_4$
Nº of genomes without MnmA$_4$
p < 0.00005
With MnmA$_1$
Without MnmA$_1$
Nº of genomes with MnmA$_1$
Nº of genomes without MnmA$_1$
With MnmA$_2$
Without MnmA$_2$
Nº of genomes with MnmA$_2$
Nº of genomes without MnmA$_2$
With TusE$_2$
Without TusE$_2$
Nº of genomes with TusE$_2$
Nº of genomes without TusE$_2$
p < 0.00005
With TusE$_1$
Without TusE$_1$
Nº of genomes with TusE$_1$
Nº of genomes without TusE$_1$
With TusE$_4$
Without TusE$_4$
Nº of genomes with TusE$_4$
Nº of genomes without TusE$_4$
p < 0.00005
With TusE$_3$
Without TusE$_3$
Nº of genomes with TusE$_3$
Nº of genomes without TusE$_3$
p < 0.00005

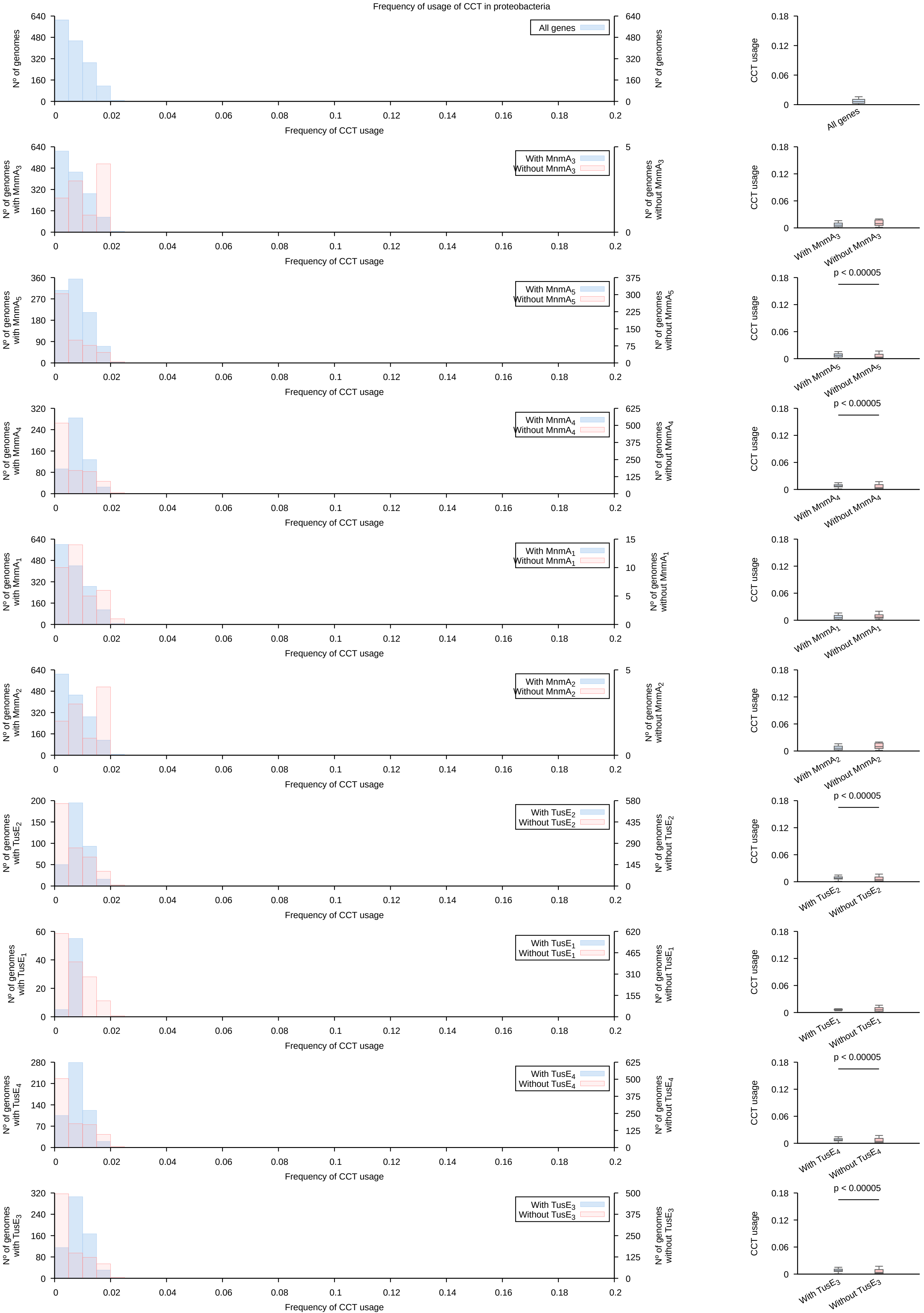
Frequency of usage of CCT in proteobacteria
Nº of genomes
All genes
Frequency of CCT usage
CCT usage
0
0.02
0.04
0.06
0.08
0.1
0.12
0.14
0.16
0.18
0.2
Nº of genomes with MnmA$_3$
Nº of genomes without MnmA$_3$
With MnmA$_3$
Without MnmA$_3$
Nº of genomes with MnmA$_5$
Nº of genomes without MnmA$_5$
With MnmA$_5$
Without MnmA$_5$
p < 0.00005
Nº of genomes with MnmA$_4$
Nº of genomes without MnmA$_4$
With MnmA$_4$
Without MnmA$_4$
p < 0.00005
Nº of genomes with MnmA$_1$
Nº of genomes without MnmA$_1$
With MnmA$_1$
Without MnmA$_1$
Nº of genomes with MnmA$_2$
Nº of genomes without MnmA$_2$
With MnmA$_2$
Without MnmA$_2$
Nº of genomes with TusE$_2$
Nº of genomes without TusE$_2$
With TusE$_2$
Without TusE$_2$
p < 0.00005
Nº of genomes with TusE$_1$
Nº of genomes without TusE$_1$
With TusE$_1$
Without TusE$_1$
Nº of genomes with TusE$_4$
Nº of genomes without TusE$_4$
With TusE$_4$
Without TusE$_4$
p < 0.00005
Nº of genomes with TusE$_3$
Nº of genomes without TusE$_3$
With TusE$_3$
Without TusE$_3$
p < 0.00005

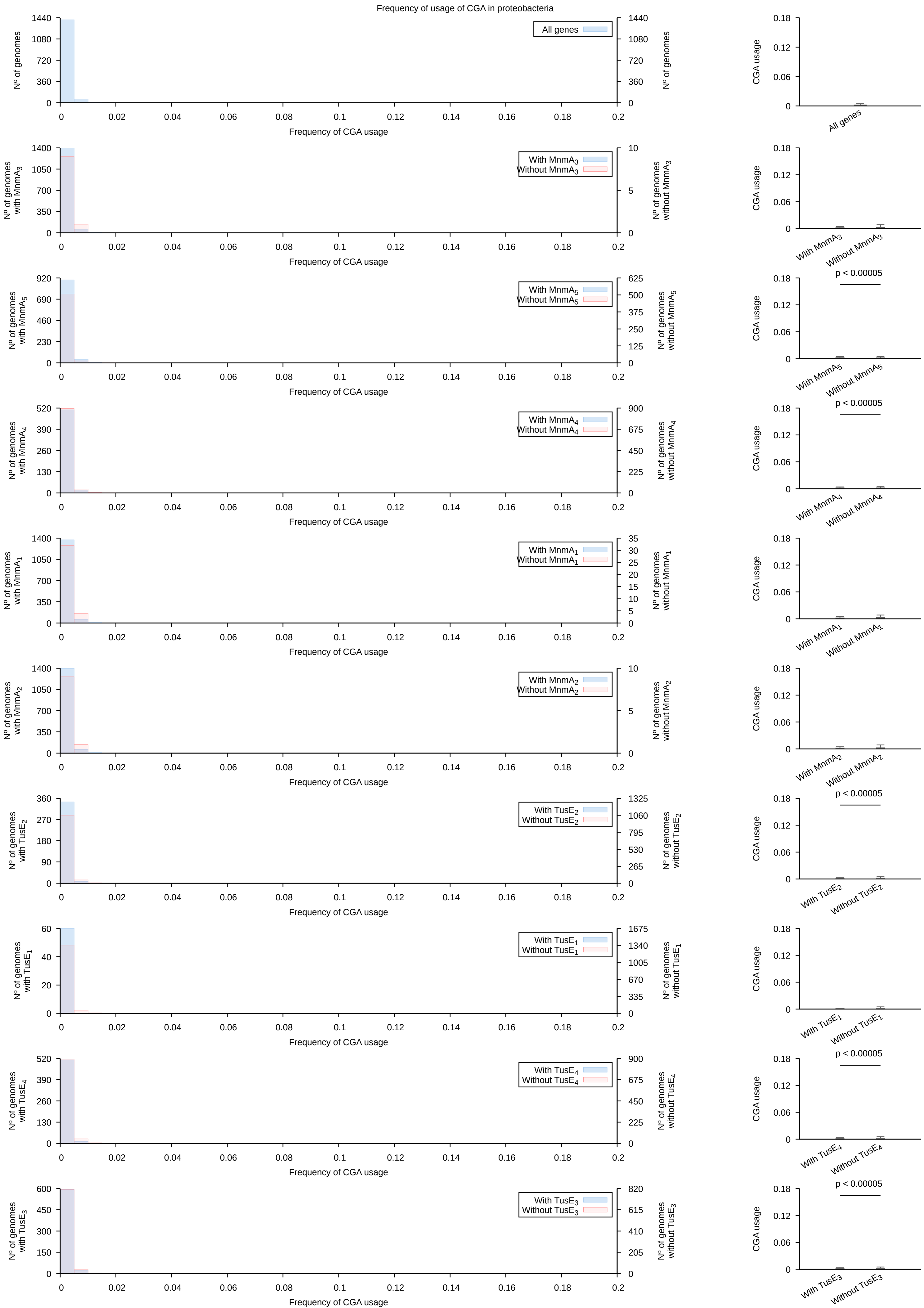
Frequency of usage of CGA in proteobacteria
All genes
Nº of genomes
Frequency of CGA usage
CGA usage
With MnmA3
Without MnmA3
Nº of genomes with MnmA3
Nº of genomes without MnmA3
With MnmA5
Without MnmA5
Nº of genomes with MnmA5
Nº of genomes without MnmA5
p < 0.00005
With MnmA4
Without MnmA4
Nº of genomes with MnmA4
Nº of genomes without MnmA4
With MnmA1
Without MnmA1
Nº of genomes with MnmA1
Nº of genomes without MnmA1
With MnmA2
Without MnmA2
Nº of genomes with MnmA2
Nº of genomes without MnmA2
With TusE2
Without TusE2
Nº of genomes with TusE2
Nº of genomes without TusE2
With TusE1
Without TusE1
Nº of genomes with TusE1
Nº of genomes without TusE1
With TusE4
Without TusE4
Nº of genomes with TusE4
Nº of genomes without TusE4
With TusE3
Without TusE3
Nº of genomes with TusE3
Nº of genomes without TusE3

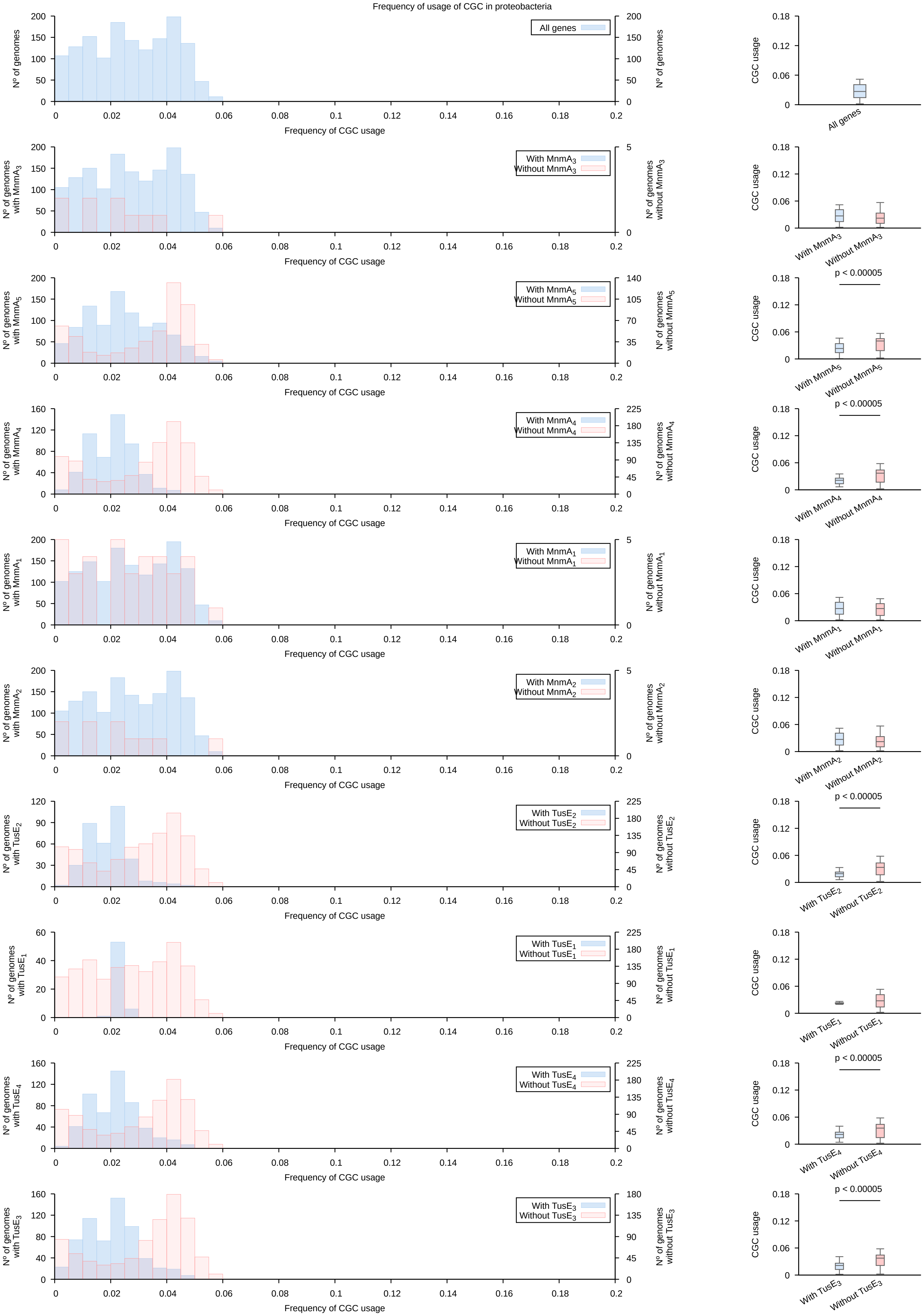
Frequency of usage of CGC in proteobacteria
Nº of genomes
All genes
Frequency of CGC usage
CGC usage
All genes
Nº of genomes with $MnmA_3$
Nº of genomes without $MnmA_3$
With $MnmA_3$
Without $MnmA_3$
Nº of genomes with $MnmA_5$
Nº of genomes without $MnmA_5$
With $MnmA_5$
Without $MnmA_5$
p < 0.00005
Nº of genomes with $MnmA_4$
Nº of genomes without $MnmA_4$
With $MnmA_4$
Without $MnmA_4$
p < 0.00005
Nº of genomes with $MnmA_1$
Nº of genomes without $MnmA_1$
With $MnmA_1$
Without $MnmA_1$
Nº of genomes with $MnmA_2$
Nº of genomes without $MnmA_2$
With $MnmA_2$
Without $MnmA_2$
Nº of genomes with $TusE_2$
Nº of genomes without $TusE_2$
With $TusE_2$
Without $TusE_2$
p < 0.00005
Nº of genomes with $TusE_1$
Nº of genomes without $TusE_1$
With $TusE_1$
Without $TusE_1$
Nº of genomes with $TusE_4$
Nº of genomes without $TusE_4$
With $TusE_4$
Without $TusE_4$
p < 0.00005
Nº of genomes with $TusE_3$
Nº of genomes without $TusE_3$
With $TusE_3$
Without $TusE_3$
p < 0.00005

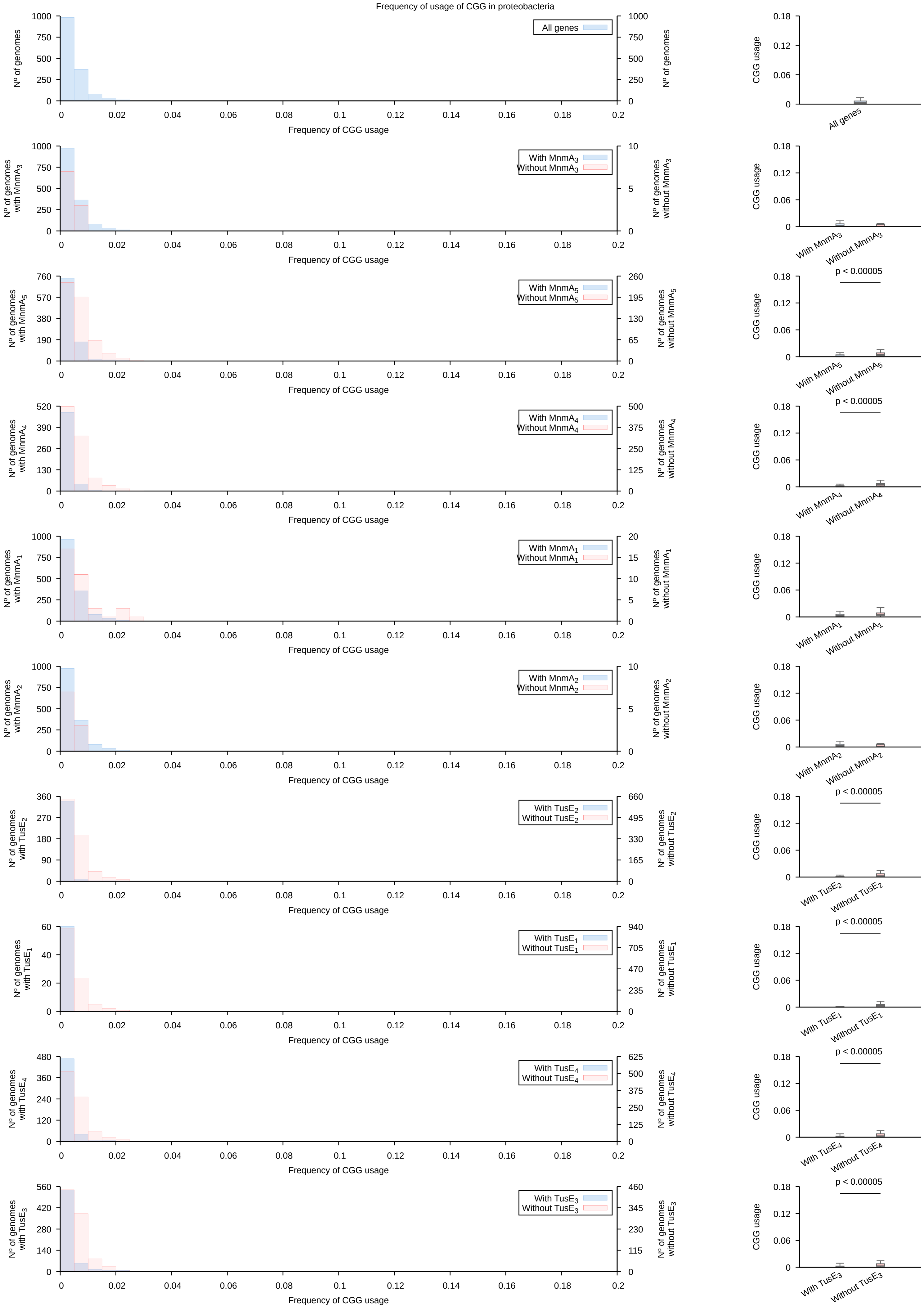
Frequency of usage of CGG in proteobacteria
Nº of genomes
Frequency of CGG usage
All genes
CGG usage
Nº of genomes with MnmA3
Nº of genomes without MnmA3
With MnmA3
Without MnmA3
Nº of genomes with MnmA5
Nº of genomes without MnmA5
With MnmA5
Without MnmA5
p < 0.00005
Nº of genomes with MnmA4
Nº of genomes without MnmA4
With MnmA4
Without MnmA4
p < 0.00005
Nº of genomes with MnmA1
Nº of genomes without MnmA1
With MnmA1
Without MnmA1
Nº of genomes with MnmA2
Nº of genomes without MnmA2
With MnmA2
Without MnmA2
Nº of genomes with TusE2
Nº of genomes without TusE2
With TusE2
Without TusE2
p < 0.00005
Nº of genomes with TusE1
Nº of genomes without TusE1
With TusE1
Without TusE1
p < 0.00005
Nº of genomes with TusE4
Nº of genomes without TusE4
With TusE4
Without TusE4
p < 0.00005
Nº of genomes with TusE3
Nº of genomes without TusE3
With TusE3
Without TusE3
p < 0.00005

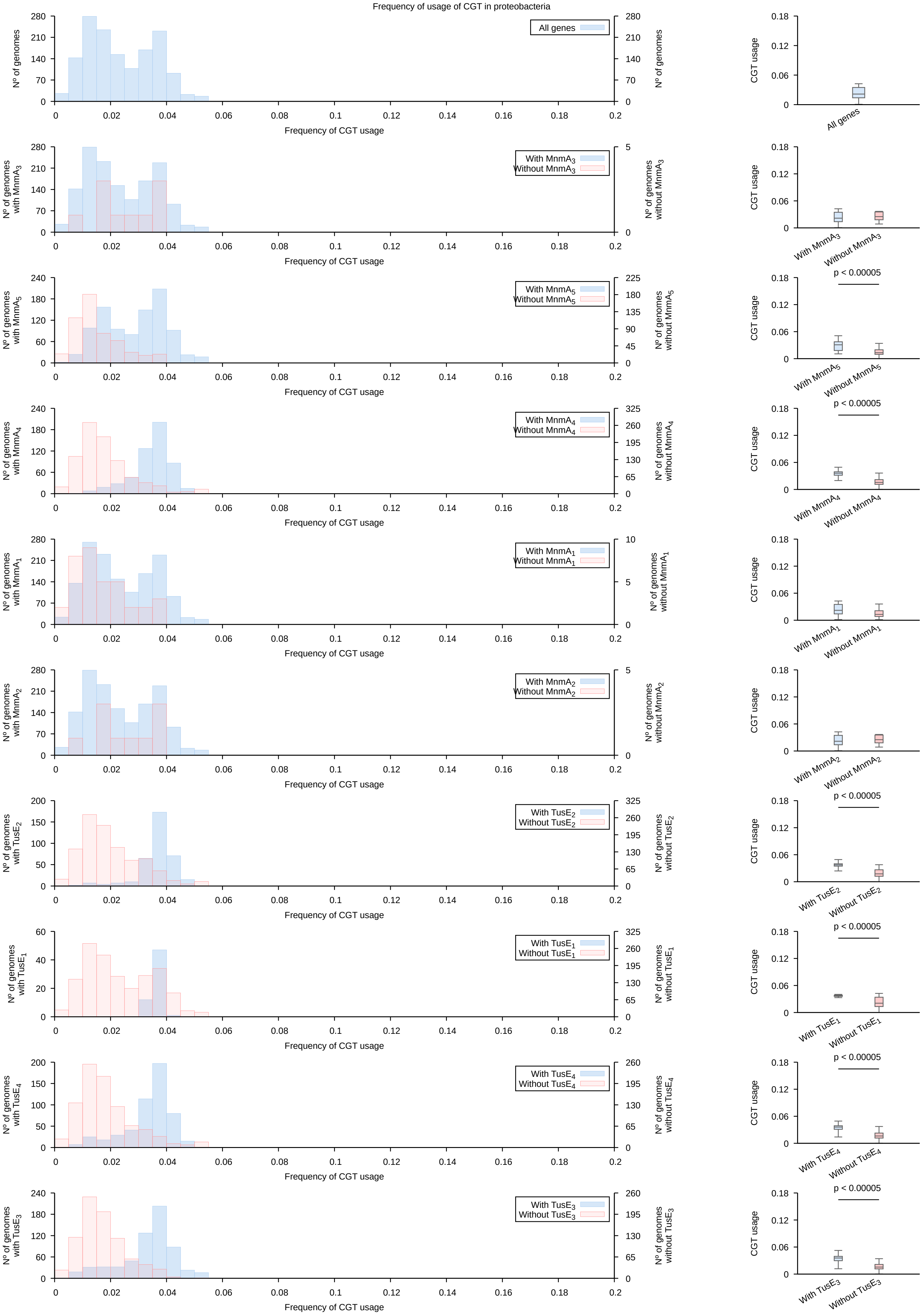
Frequency of usage of CGT in proteobacteria
All genes
Nº of genomes
Frequency of CGT usage
CGT usage
With $MnmA_3$
Without $MnmA_3$
Nº of genomes with $MnmA_3$
Nº of genomes without $MnmA_3$
With $MnmA_5$
Without $MnmA_5$
Nº of genomes with $MnmA_5$
Nº of genomes without $MnmA_5$
$p < 0.00005$
With $MnmA_4$
Without $MnmA_4$
Nº of genomes with $MnmA_4$
Nº of genomes without $MnmA_4$
With $MnmA_1$
Without $MnmA_1$
Nº of genomes with $MnmA_1$
Nº of genomes without $MnmA_1$
With $MnmA_2$
Without $MnmA_2$
Nº of genomes with $MnmA_2$
Nº of genomes without $MnmA_2$
With $TusE_2$
Without $TusE_2$
Nº of genomes with $TusE_2$
Nº of genomes without $TusE_2$
With $TusE_1$
Without $TusE_1$
Nº of genomes with $TusE_1$
Nº of genomes without $TusE_1$
With $TusE_4$
Without $TusE_4$
Nº of genomes with $TusE_4$
Nº of genomes without $TusE_4$
With $TusE_3$
Without $TusE_3$
Nº of genomes with $TusE_3$
Nº of genomes without $TusE_3$

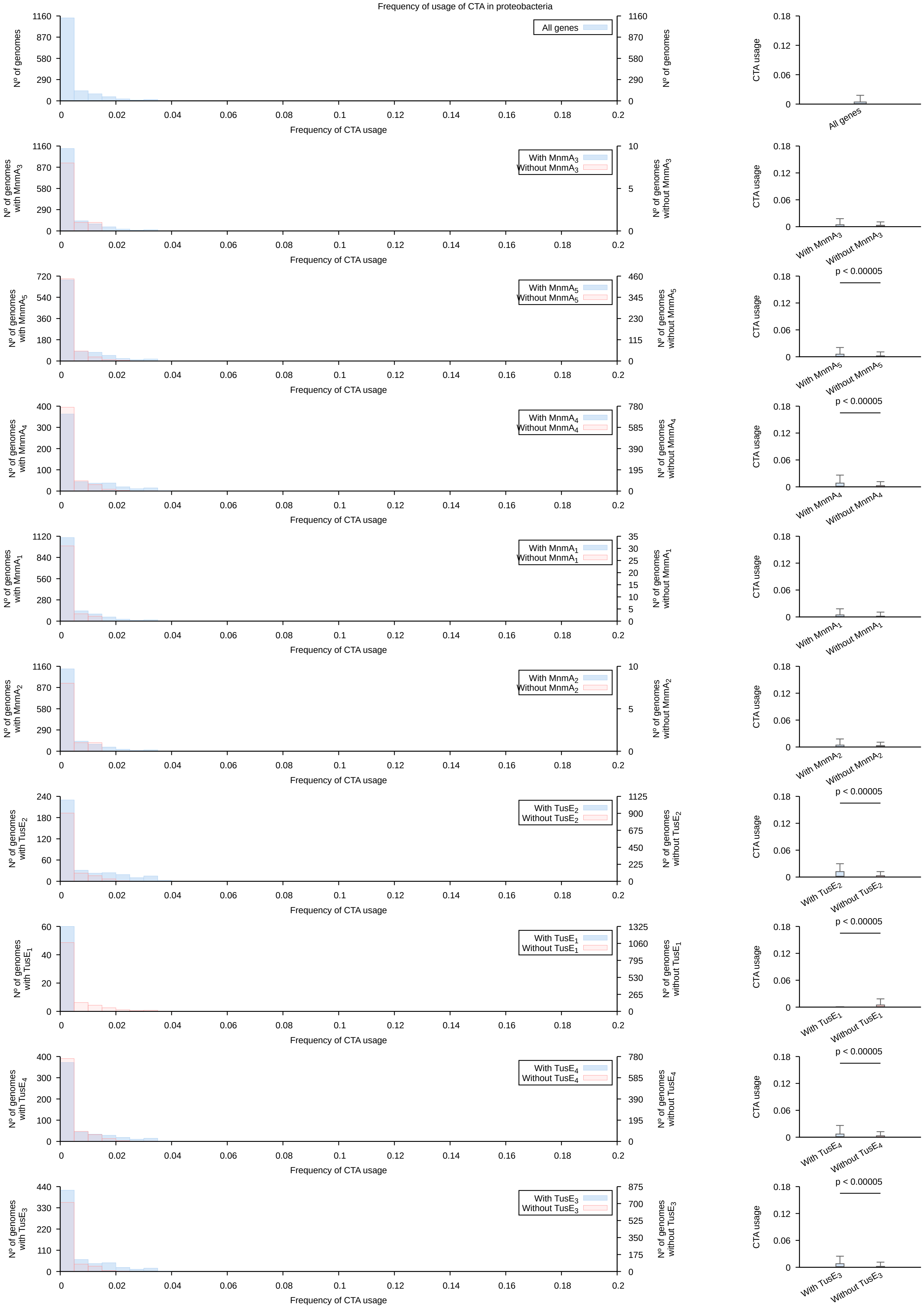
Frequency of usage of CTA in proteobacteria
All genes
Nº of genomes
Frequency of CTA usage
CTA usage
With MnmA3
Without MnmA3
Nº of genomes with MnmA3
Nº of genomes without MnmA3
With MnmA5
Without MnmA5
Nº of genomes with MnmA5
Nº of genomes without MnmA5
p < 0.00005
With MnmA4
Without MnmA4
Nº of genomes with MnmA4
Nº of genomes without MnmA4
p < 0.00005
With MnmA1
Without MnmA1
Nº of genomes with MnmA1
Nº of genomes without MnmA1
With MnmA2
Without MnmA2
Nº of genomes with MnmA2
Nº of genomes without MnmA2
With TusE2
Without TusE2
Nº of genomes with TusE2
Nº of genomes without TusE2
p < 0.00005
With TusE1
Without TusE1
Nº of genomes with TusE1
Nº of genomes without TusE1
p < 0.00005
With TusE4
Without TusE4
Nº of genomes with TusE4
Nº of genomes without TusE4
p < 0.00005
With TusE3
Without TusE3
Nº of genomes with TusE3
Nº of genomes without TusE3
p < 0.00005

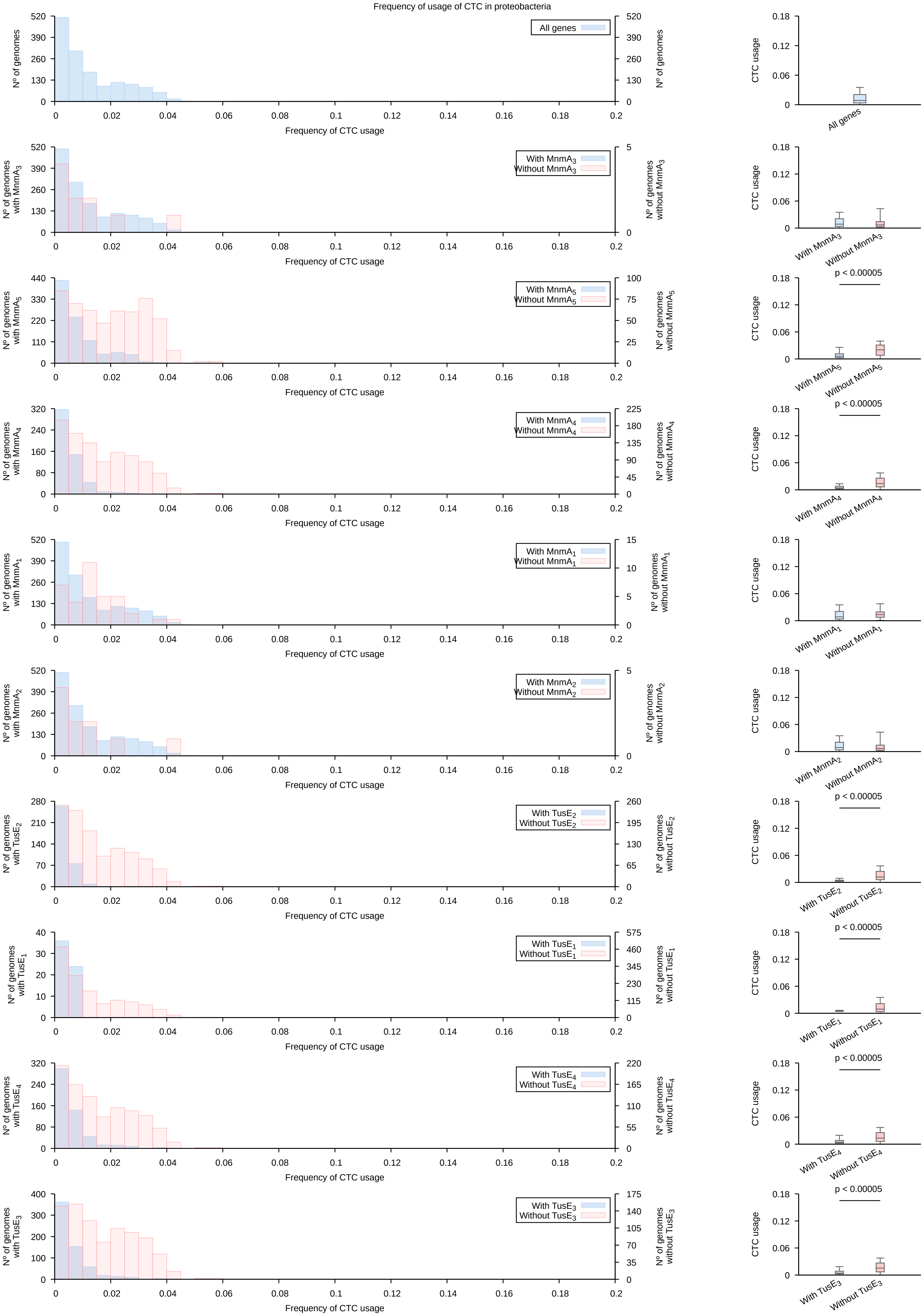

Frequency of usage of CTC in proteobacteria
All genes
With $MnmA_3$
Without $MnmA_3$
With $MnmA_5$
Without $MnmA_5$
With $MnmA_4$
Without $MnmA_4$
With $MnmA_1$
Without $MnmA_1$
With $MnmA_2$
Without $MnmA_2$
With $TusE_2$
Without $TusE_2$
With $TusE_1$
Without $TusE_1$
With $TusE_4$
Without $TusE_4$
With $TusE_3$
Without $TusE_3$
Nº of genomes
Frequency of CTC usage
CTC usage
p < 0.00005

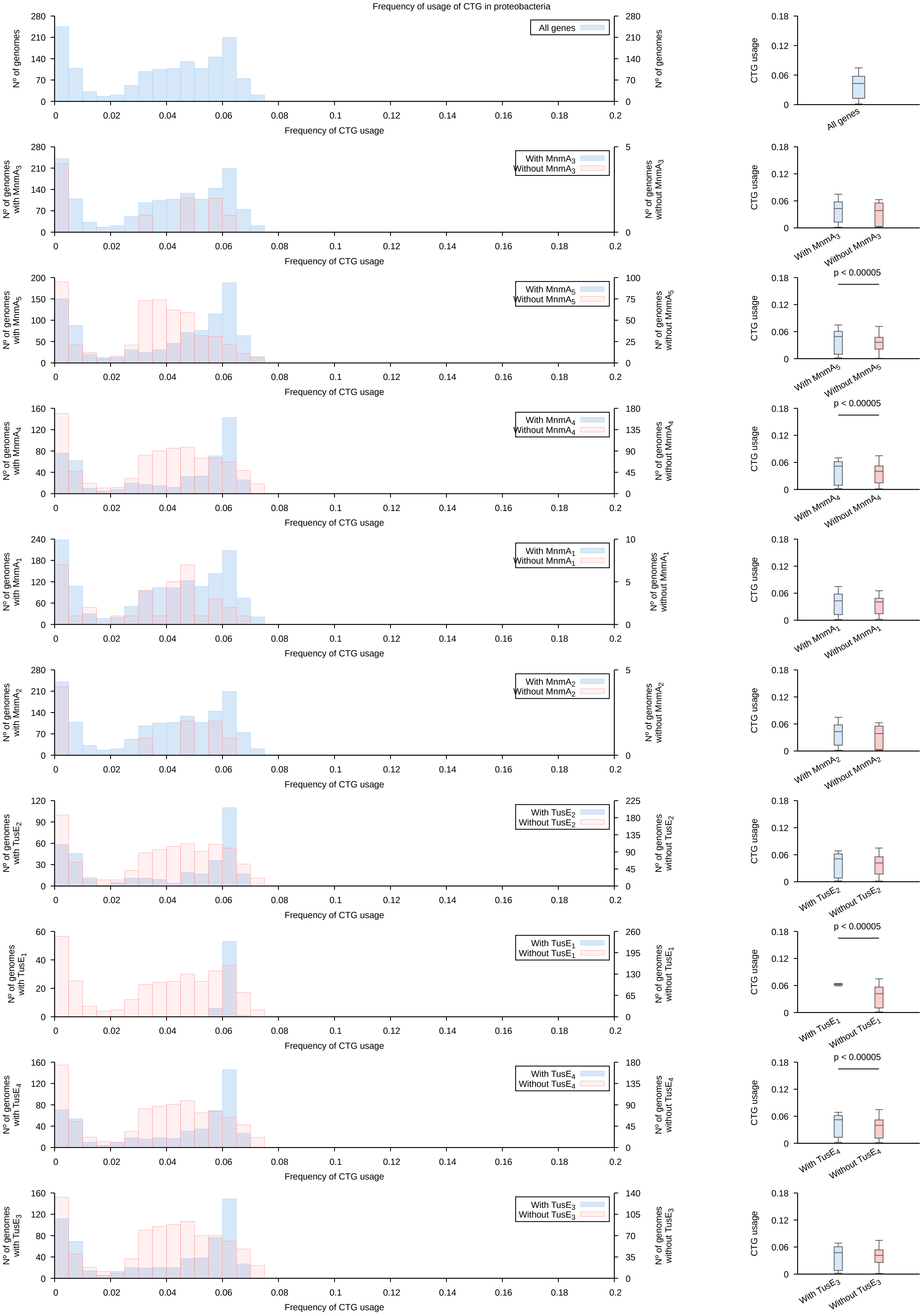
Frequency of usage of CTG in proteobacteria
All genes
Nº of genomes
Frequency of CTG usage
CTG usage
With MnmA3
Without MnmA3
Nº of genomes with MnmA3
Nº of genomes without MnmA3
With MnmA5
Without MnmA5
Nº of genomes with MnmA5
Nº of genomes without MnmA5
p < 0.00005
With MnmA4
Without MnmA4
Nº of genomes with MnmA4
Nº of genomes without MnmA4
p < 0.00005
With MnmA1
Without MnmA1
Nº of genomes with MnmA1
Nº of genomes without MnmA1
With MnmA2
Without MnmA2
Nº of genomes with MnmA2
Nº of genomes without MnmA2
With TusE2
Without TusE2
Nº of genomes with TusE2
Nº of genomes without TusE2
With TusE1
Without TusE1
Nº of genomes with TusE1
Nº of genomes without TusE1
p < 0.00005
With TusE4
Without TusE4
Nº of genomes with TusE4
Nº of genomes without TusE4
p < 0.00005
With TusE3
Without TusE3
Nº of genomes with TusE3
Nº of genomes without TusE3

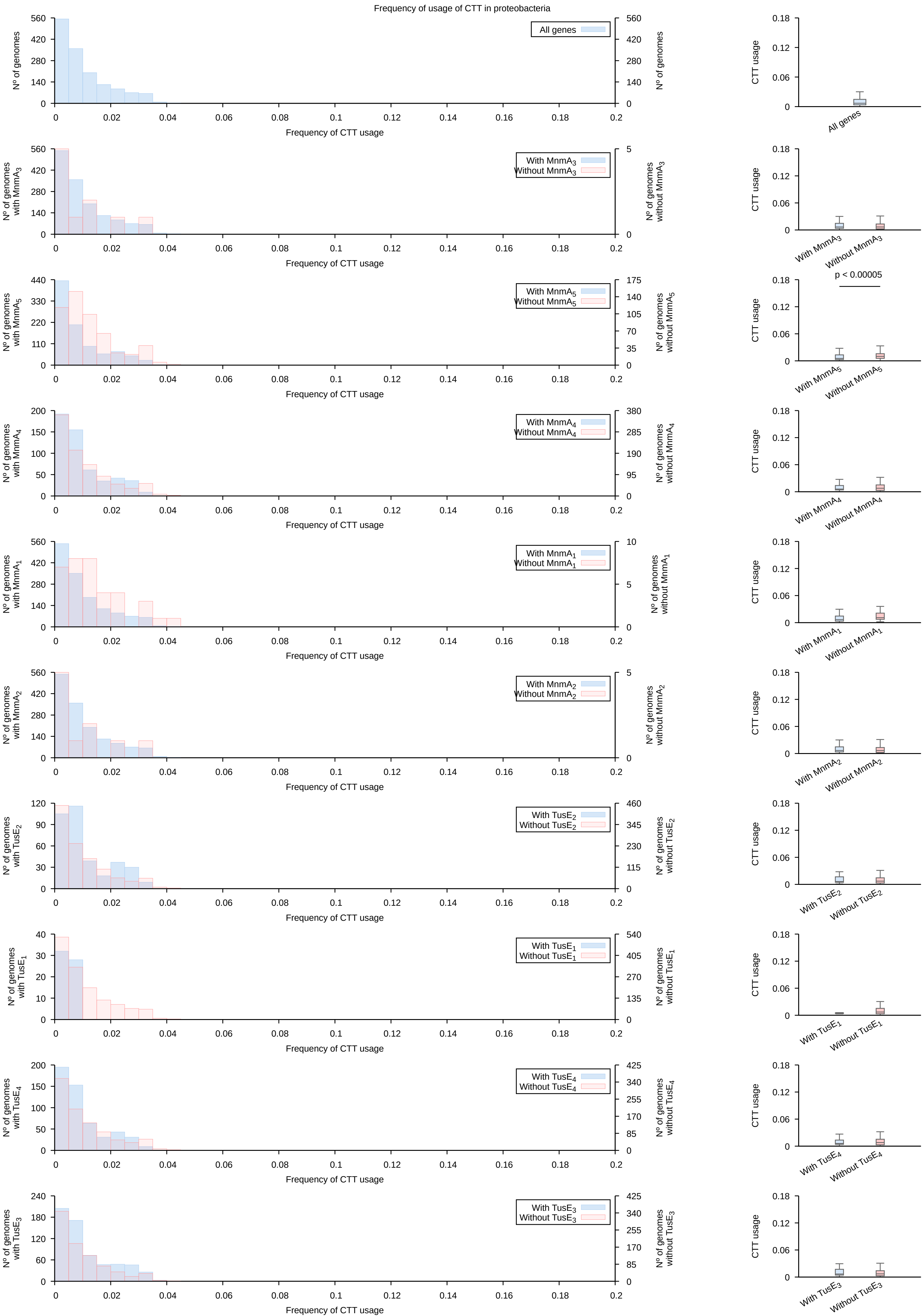

Frequency of usage of CTT in proteobacteria
All genes
Nº of genomes
Frequency of CTT usage
CTT usage
With MnmA3
Without MnmA3
Nº of genomes with MnmA3
Nº of genomes without MnmA3
With MnmA5
Without MnmA5
Nº of genomes with MnmA5
Nº of genomes without MnmA5
p < 0.00005
With MnmA4
Without MnmA4
Nº of genomes with MnmA4
Nº of genomes without MnmA4
With MnmA1
Without MnmA1
Nº of genomes with MnmA1
Nº of genomes without MnmA1
With MnmA2
Without MnmA2
Nº of genomes with MnmA2
Nº of genomes without MnmA2
With TusE2
Without TusE2
Nº of genomes with TusE2
Nº of genomes without TusE2
With TusE1
Without TusE1
Nº of genomes with TusE1
Nº of genomes without TusE1
With TusE4
Without TusE4
Nº of genomes with TusE4
Nº of genomes without TusE4
With TusE3
Without TusE3
Nº of genomes with TusE3
Nº of genomes without TusE3

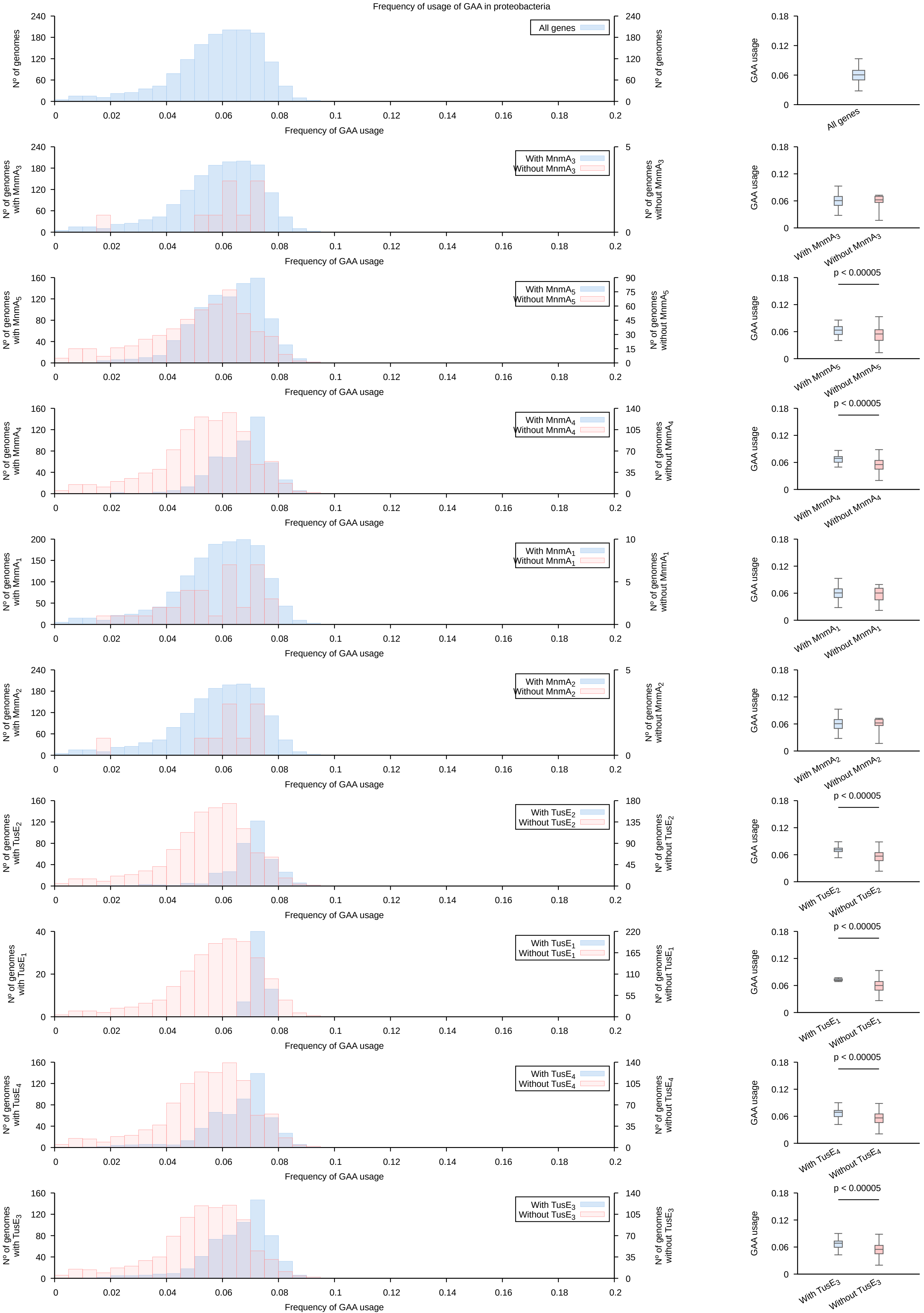
Frequency of usage of GAA in proteobacteria
Nº of genomes
All genes
Frequency of GAA usage
GAA usage
Nº of genomes with $MnmA_3$
Nº of genomes without $MnmA_3$
With $MnmA_3$
Without $MnmA_3$
Nº of genomes with $MnmA_5$
Nº of genomes without $MnmA_5$
With $MnmA_5$
Without $MnmA_5$
p < 0.00005
Nº of genomes with $MnmA_4$
Nº of genomes without $MnmA_4$
With $MnmA_4$
Without $MnmA_4$
p < 0.00005
Nº of genomes with $MnmA_1$
Nº of genomes without $MnmA_1$
With $MnmA_1$
Without $MnmA_1$
Nº of genomes with $MnmA_2$
Nº of genomes without $MnmA_2$
With $MnmA_2$
Without $MnmA_2$
Nº of genomes with $TusE_2$
Nº of genomes without $TusE_2$
With $TusE_2$
Without $TusE_2$
p < 0.00005
Nº of genomes with $TusE_1$
Nº of genomes without $TusE_1$
With $TusE_1$
Without $TusE_1$
p < 0.00005
Nº of genomes with $TusE_4$
Nº of genomes without $TusE_4$
With $TusE_4$
Without $TusE_4$
p < 0.00005
Nº of genomes with $TusE_3$
Nº of genomes without $TusE_3$
With $TusE_3$
Without $TusE_3$
p < 0.00005

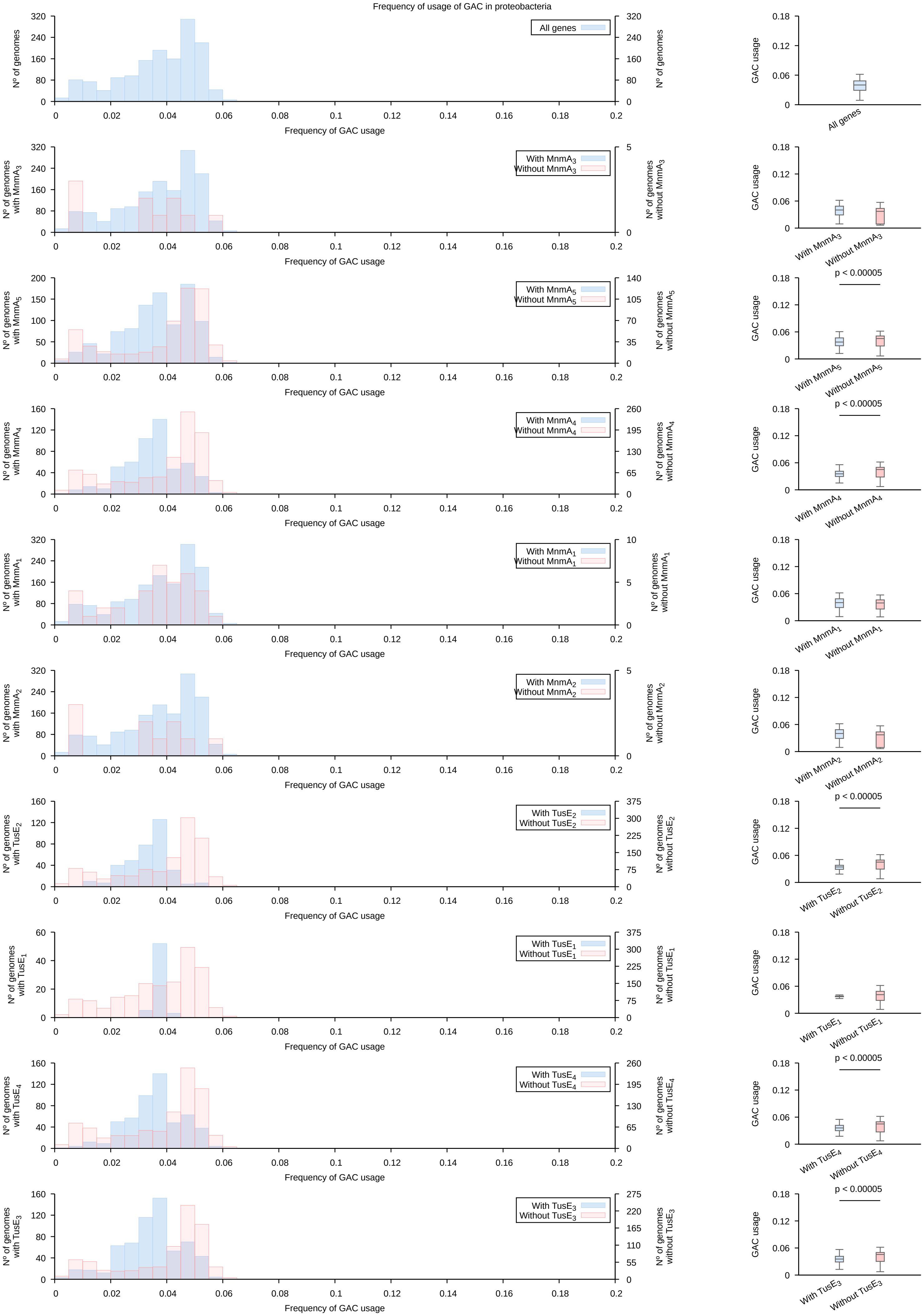

Frequency of usage of GAC in proteobacteria
Nº of genomes
All genes
Frequency of GAC usage
GAC usage
All genes
Nº of genomes with MnmA$_3$
Nº of genomes without MnmA$_3$
With MnmA$_3$
Without MnmA$_3$
Nº of genomes with MnmA$_5$
Nº of genomes without MnmA$_5$
With MnmA$_5$
Without MnmA$_5$
p < 0.00005
Nº of genomes with MnmA$_4$
Nº of genomes without MnmA$_4$
With MnmA$_4$
Without MnmA$_4$
p < 0.00005
Nº of genomes with MnmA$_1$
Nº of genomes without MnmA$_1$
With MnmA$_1$
Without MnmA$_1$
Nº of genomes with MnmA$_2$
Nº of genomes without MnmA$_2$
With MnmA$_2$
Without MnmA$_2$
Nº of genomes with TusE$_2$
Nº of genomes without TusE$_2$
With TusE$_2$
Without TusE$_2$
p < 0.00005
Nº of genomes with TusE$_1$
Nº of genomes without TusE$_1$
With TusE$_1$
Without TusE$_1$
Nº of genomes with TusE$_4$
Nº of genomes without TusE$_4$
With TusE$_4$
Without TusE$_4$
p < 0.00005
Nº of genomes with TusE$_3$
Nº of genomes without TusE$_3$
With TusE$_3$
Without TusE$_3$
p < 0.00005

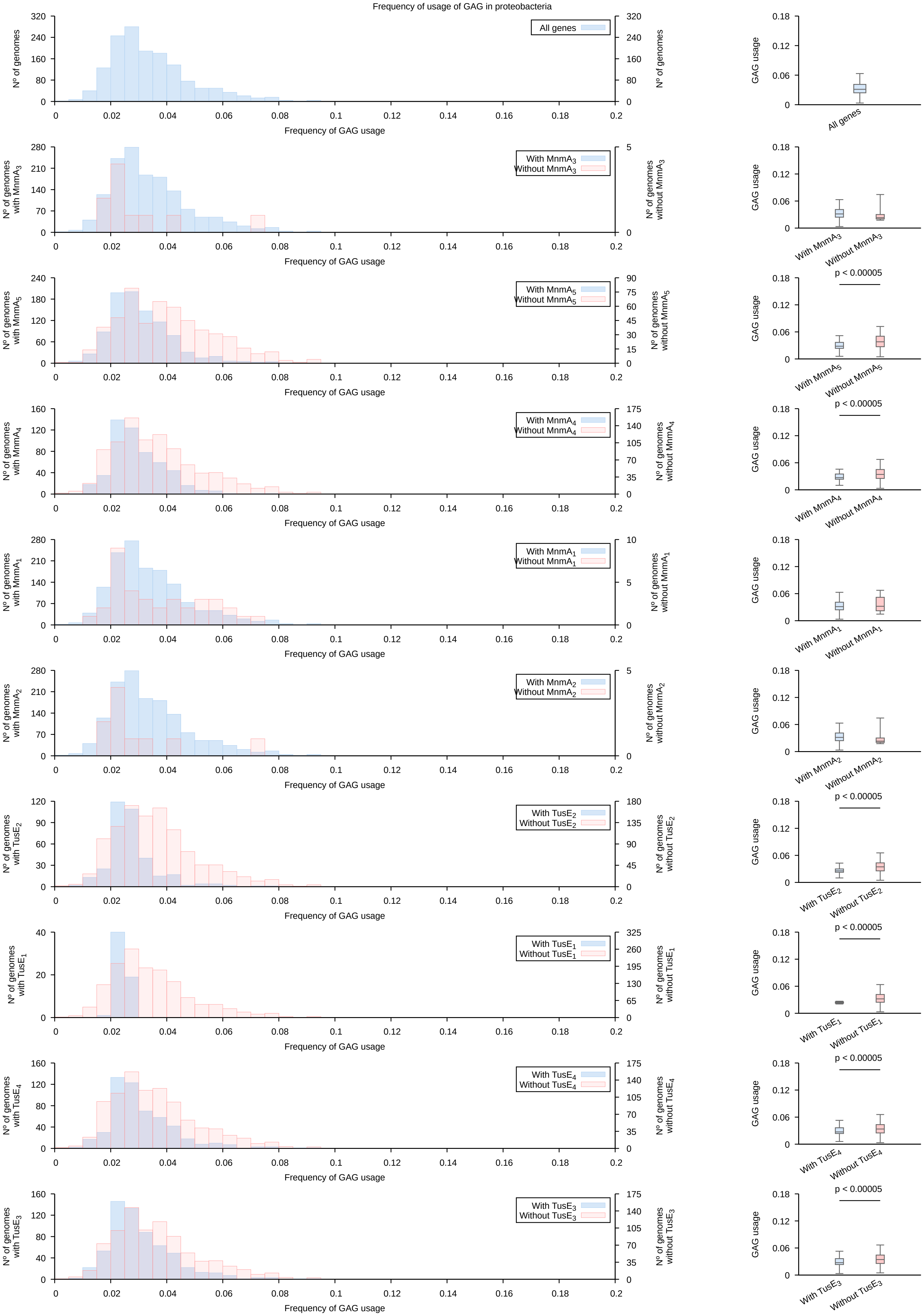
Frequency of usage of GAG in proteobacteria
Nº of genomes
Frequency of GAG usage
GAG usage
All genes
With MnmA$_3$
Without MnmA$_3$
Nº of genomes with MnmA$_3$
Nº of genomes without MnmA$_3$
With MnmA$_5$
Without MnmA$_5$
Nº of genomes with MnmA$_5$
Nº of genomes without MnmA$_5$
With MnmA$_4$
Without MnmA$_4$
Nº of genomes with MnmA$_4$
Nº of genomes without MnmA$_4$
With MnmA$_1$
Without MnmA$_1$
Nº of genomes with MnmA$_1$
Nº of genomes without MnmA$_1$
With MnmA$_2$
Without MnmA$_2$
Nº of genomes with MnmA$_2$
Nº of genomes without MnmA$_2$
With TusE$_2$
Without TusE$_2$
Nº of genomes with TusE$_2$
Nº of genomes without TusE$_2$
With TusE$_1$
Without TusE$_1$
Nº of genomes with TusE$_1$
Nº of genomes without TusE$_1$
With TusE$_4$
Without TusE$_4$
Nº of genomes with TusE$_4$
Nº of genomes without TusE$_4$
With TusE$_3$
Without TusE$_3$
Nº of genomes with TusE$_3$
Nº of genomes without TusE$_3$
p < 0.00005

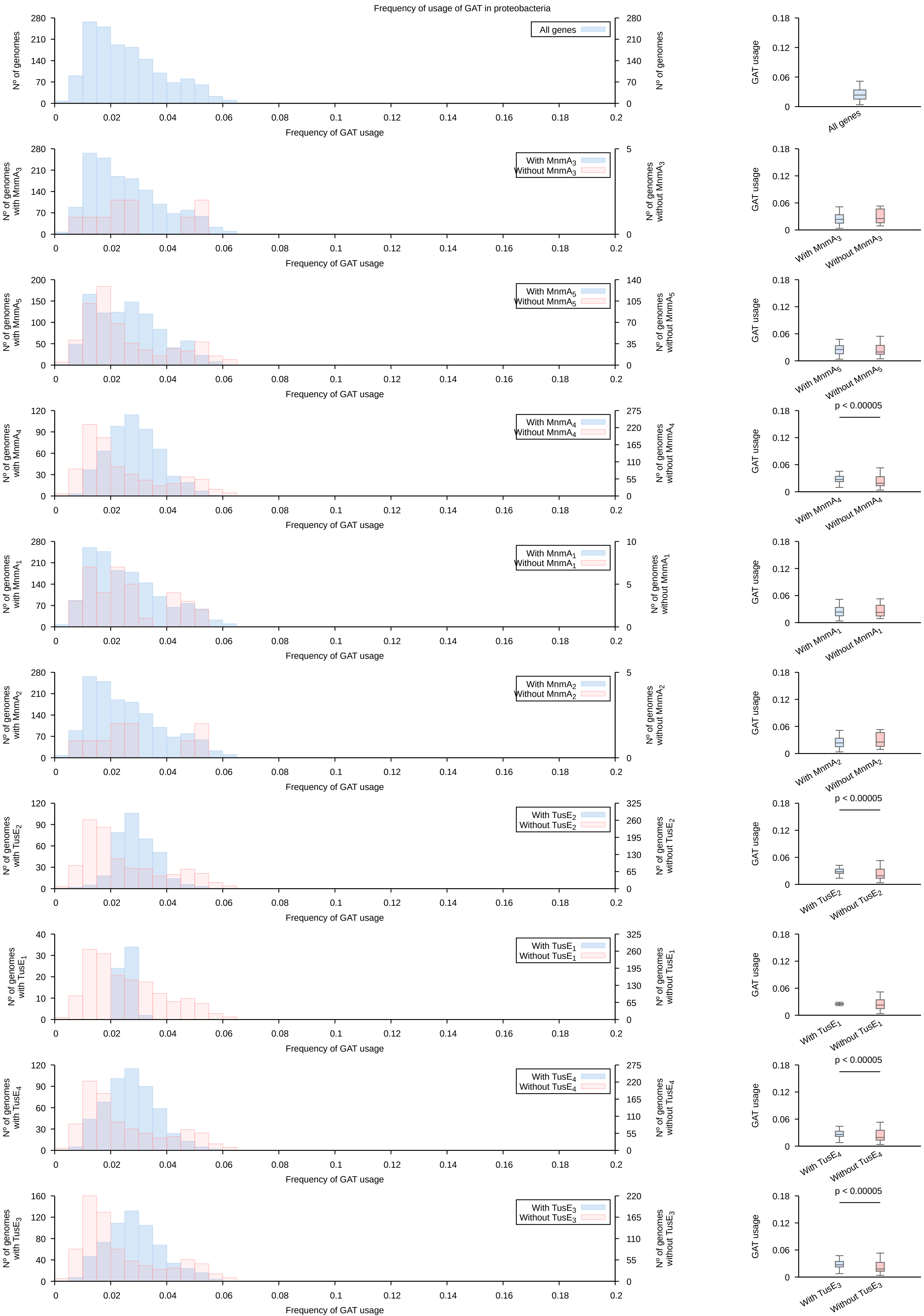

Frequency of usage of GAT in proteobacteria
Nº of genomes
All genes
Frequency of GAT usage
GAT usage
All genes
Nº of genomes with $MnmA_3$
Nº of genomes without $MnmA_3$
With $MnmA_3$
Without $MnmA_3$
Nº of genomes with $MnmA_5$
Nº of genomes without $MnmA_5$
With $MnmA_5$
Without $MnmA_5$
Nº of genomes with $MnmA_4$
Nº of genomes without $MnmA_4$
With $MnmA_4$
Without $MnmA_4$
p < 0.00005
Nº of genomes with $MnmA_1$
Nº of genomes without $MnmA_1$
With $MnmA_1$
Without $MnmA_1$
Nº of genomes with $MnmA_2$
Nº of genomes without $MnmA_2$
With $MnmA_2$
Without $MnmA_2$
Nº of genomes with $TusE_2$
Nº of genomes without $TusE_2$
With $TusE_2$
Without $TusE_2$
p < 0.00005
Nº of genomes with $TusE_1$
Nº of genomes without $TusE_1$
With $TusE_1$
Without $TusE_1$
Nº of genomes with $TusE_4$
Nº of genomes without $TusE_4$
With $TusE_4$
Without $TusE_4$
p < 0.00005
Nº of genomes with $TusE_3$
Nº of genomes without $TusE_3$
With $TusE_3$
Without $TusE_3$
p < 0.00005

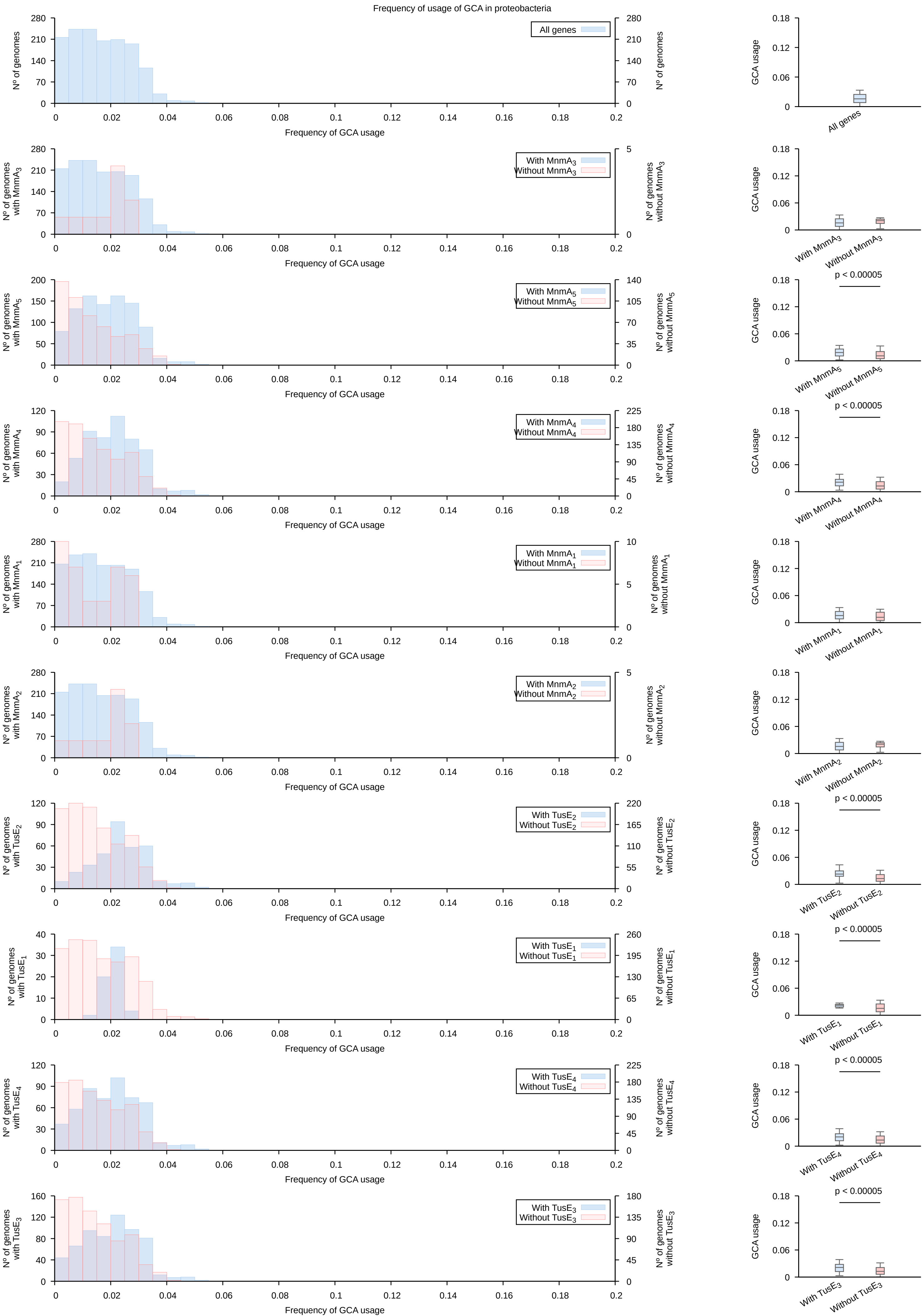

Frequency of usage of GCA in proteobacteria
All genes
With MnmA3
Without MnmA3
With MnmA5
Without MnmA5
With MnmA4
Without MnmA4
With MnmA1
Without MnmA1
With MnmA2
Without MnmA2
With TusE2
Without TusE2
With TusE1
Without TusE1
With TusE4
Without TusE4
With TusE3
Without TusE3
Nº of genomes
Frequency of GCA usage
GCA usage
p < 0.00005

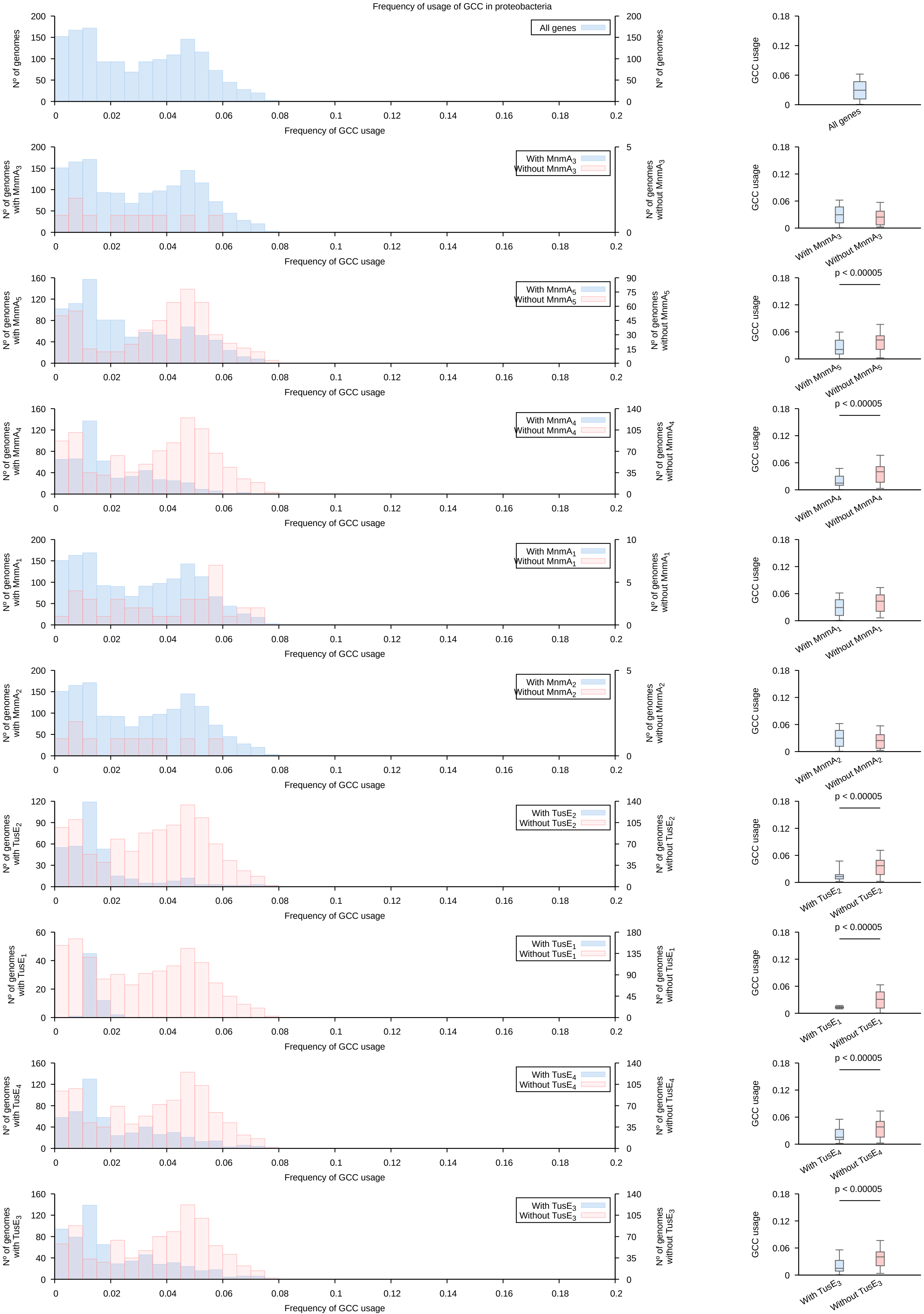
Frequency of usage of GCC in proteobacteria
Nº of genomes
All genes
Frequency of GCC usage
GCC usage
All genes
Nº of genomes with MnmA$_3$
Nº of genomes without MnmA$_3$
With MnmA$_3$
Without MnmA$_3$
Nº of genomes with MnmA$_5$
Nº of genomes without MnmA$_5$
With MnmA$_5$
Without MnmA$_5$
p < 0.00005
Nº of genomes with MnmA$_4$
Nº of genomes without MnmA$_4$
With MnmA$_4$
Without MnmA$_4$
p < 0.00005
Nº of genomes with MnmA$_1$
Nº of genomes without MnmA$_1$
With MnmA$_1$
Without MnmA$_1$
Nº of genomes with MnmA$_2$
Nº of genomes without MnmA$_2$
With MnmA$_2$
Without MnmA$_2$
Nº of genomes with TusE$_2$
Nº of genomes without TusE$_2$
With TusE$_2$
Without TusE$_2$
p < 0.00005
Nº of genomes with TusE$_1$
Nº of genomes without TusE$_1$
With TusE$_1$
Without TusE$_1$
p < 0.00005
Nº of genomes with TusE$_4$
Nº of genomes without TusE$_4$
With TusE$_4$
Without TusE$_4$
p < 0.00005
Nº of genomes with TusE$_3$
Nº of genomes without TusE$_3$
With TusE$_3$
Without TusE$_3$
p < 0.00005
Frequency of GCC usage

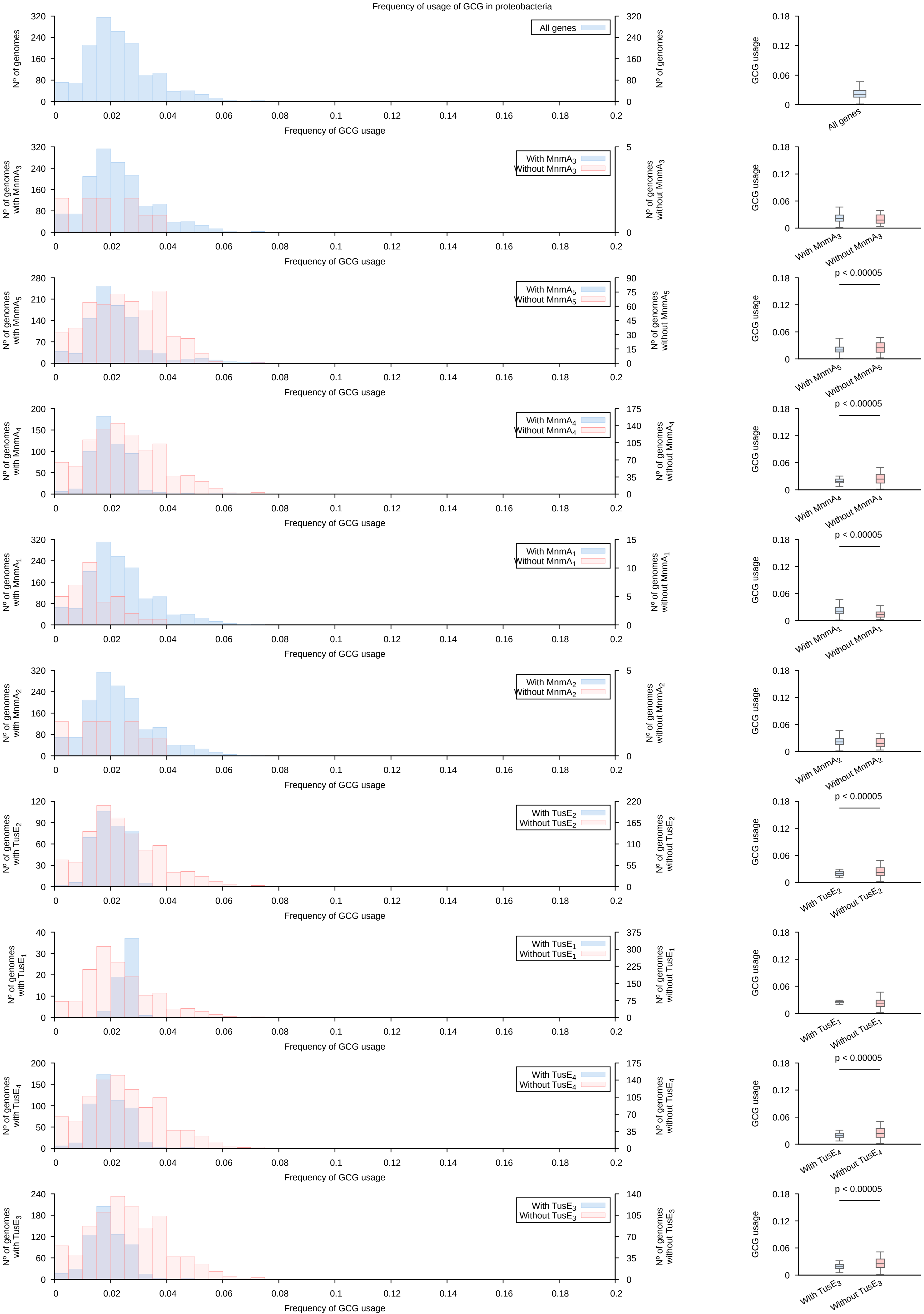

Frequency of usage of GCG in proteobacteria
Nº of genomes
All genes
Frequency of GCG usage
GCG usage
All genes
Nº of genomes with MnmA$_3$
With MnmA$_3$
Without MnmA$_3$
Nº of genomes without MnmA$_3$
Nº of genomes with MnmA$_5$
With MnmA$_5$
Without MnmA$_5$
Nº of genomes without MnmA$_5$
p < 0.00005
Nº of genomes with MnmA$_4$
With MnmA$_4$
Without MnmA$_4$
Nº of genomes without MnmA$_4$
p < 0.00005
Nº of genomes with MnmA$_1$
With MnmA$_1$
Without MnmA$_1$
Nº of genomes without MnmA$_1$
p < 0.00005
Nº of genomes with MnmA$_2$
With MnmA$_2$
Without MnmA$_2$
Nº of genomes without MnmA$_2$
Nº of genomes with TusE$_2$
With TusE$_2$
Without TusE$_2$
Nº of genomes without TusE$_2$
p < 0.00005
Nº of genomes with TusE$_1$
With TusE$_1$
Without TusE$_1$
Nº of genomes without TusE$_1$
Nº of genomes with TusE$_4$
With TusE$_4$
Without TusE$_4$
Nº of genomes without TusE$_4$
p < 0.00005
Nº of genomes with TusE$_3$
With TusE$_3$
Without TusE$_3$
Nº of genomes without TusE$_3$
p < 0.00005

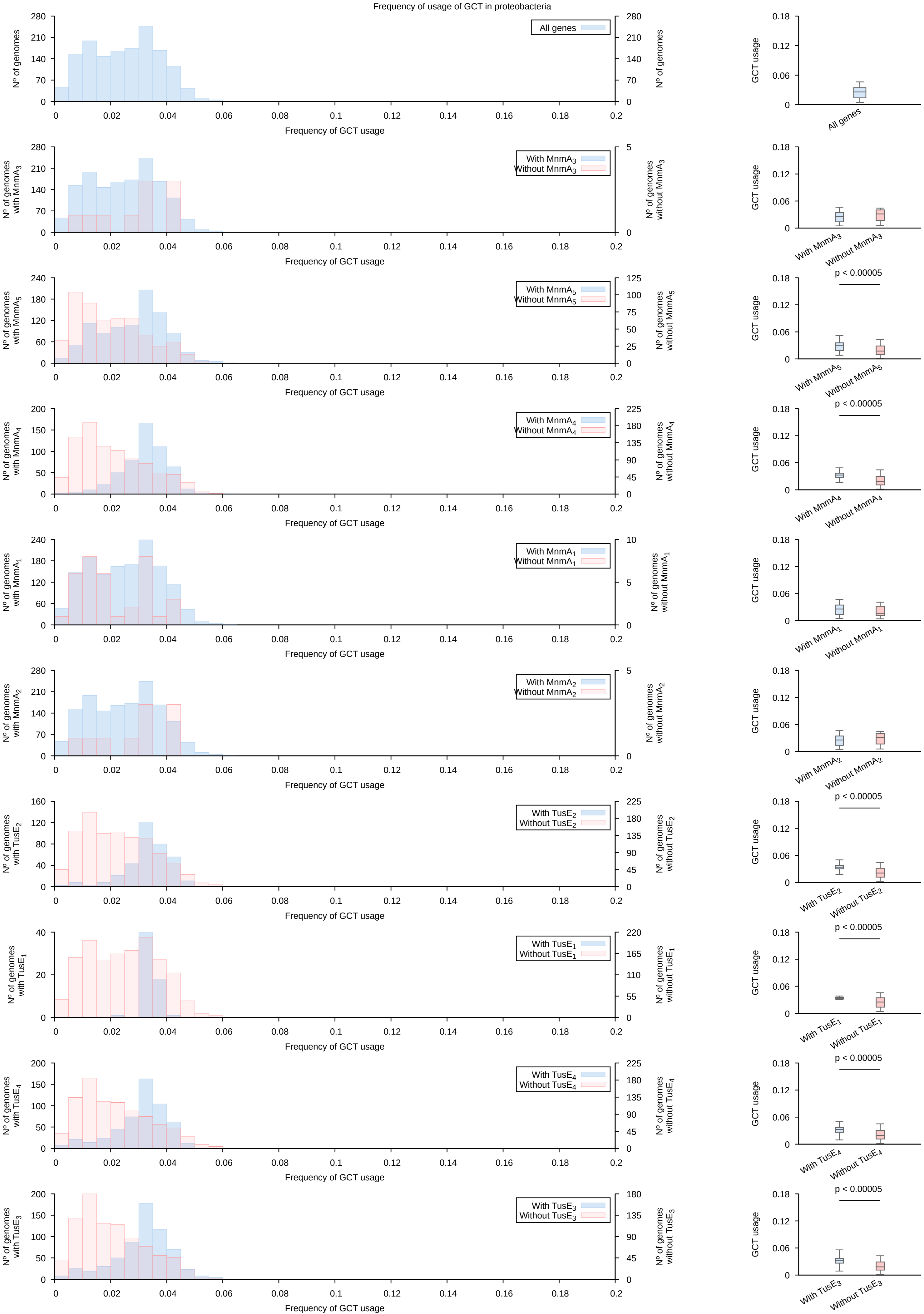

Frequency of usage of GCT in proteobacteria
Nº of genomes
All genes
Frequency of GCT usage
GCT usage
Nº of genomes with MnmA3
Nº of genomes without MnmA3
With MnmA3
Without MnmA3
Nº of genomes with MnmA5
Nº of genomes without MnmA5
With MnmA5
Without MnmA5
p < 0.00005
Nº of genomes with MnmA4
Nº of genomes without MnmA4
With MnmA4
Without MnmA4
Nº of genomes with MnmA1
Nº of genomes without MnmA1
With MnmA1
Without MnmA1
Nº of genomes with MnmA2
Nº of genomes without MnmA2
With MnmA2
Without MnmA2
Nº of genomes with TusE2
Nº of genomes without TusE2
With TusE2
Without TusE2
Nº of genomes with TusE1
Nº of genomes without TusE1
With TusE1
Without TusE1
Nº of genomes with TusE4
Nº of genomes without TusE4
With TusE4
Without TusE4
Nº of genomes with TusE3
Nº of genomes without TusE3
With TusE3
Without TusE3

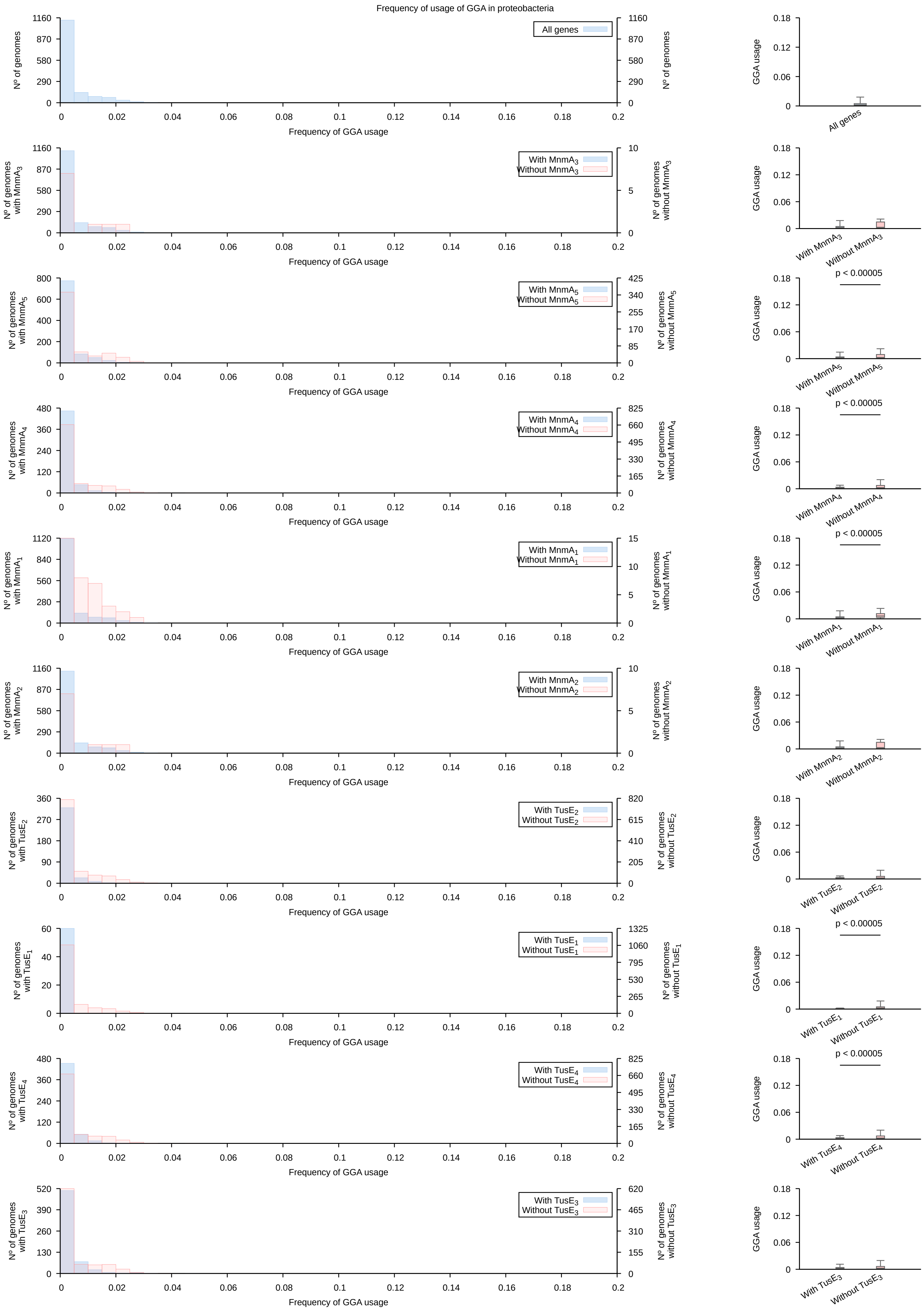

Frequency of usage of GGA in proteobacteria
All genes
Nº of genomes
Frequency of GGA usage
GGA usage
With MnmA$_3$
Without MnmA$_3$
Nº of genomes with MnmA$_3$
Nº of genomes without MnmA$_3$
With MnmA$_5$
Without MnmA$_5$
Nº of genomes with MnmA$_5$
Nº of genomes without MnmA$_5$
p < 0.00005
With MnmA$_4$
Without MnmA$_4$
Nº of genomes with MnmA$_4$
Nº of genomes without MnmA$_4$
p < 0.00005
With MnmA$_1$
Without MnmA$_1$
Nº of genomes with MnmA$_1$
Nº of genomes without MnmA$_1$
p < 0.00005
With MnmA$_2$
Without MnmA$_2$
Nº of genomes with MnmA$_2$
Nº of genomes without MnmA$_2$
With TusE$_2$
Without TusE$_2$
Nº of genomes with TusE$_2$
Nº of genomes without TusE$_2$
With TusE$_1$
Without TusE$_1$
Nº of genomes with TusE$_1$
Nº of genomes without TusE$_1$
p < 0.00005
With TusE$_4$
Without TusE$_4$
Nº of genomes with TusE$_4$
Nº of genomes without TusE$_4$
p < 0.00005
With TusE$_3$
Without TusE$_3$
Nº of genomes with TusE$_3$
Nº of genomes without TusE$_3$

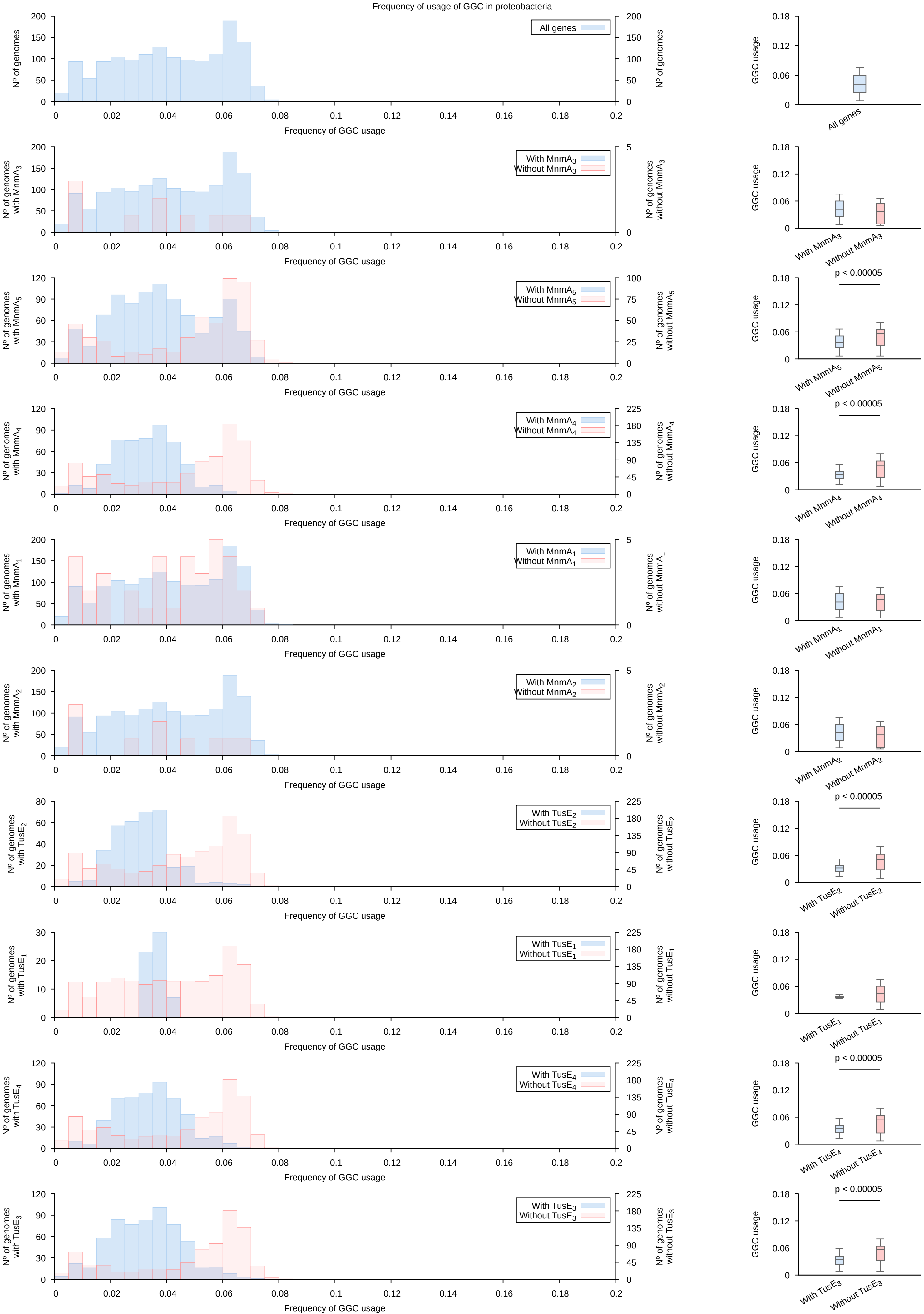
Frequency of usage of GGC in proteobacteria
All genes
Nº of genomes
Frequency of GGC usage
GGC usage
With MnmA$_3$
Without MnmA$_3$
Nº of genomes with MnmA$_3$
Nº of genomes without MnmA$_3$
With MnmA$_5$
Without MnmA$_5$
Nº of genomes with MnmA$_5$
Nº of genomes without MnmA$_5$
p < 0.00005
With MnmA$_4$
Without MnmA$_4$
Nº of genomes with MnmA$_4$
Nº of genomes without MnmA$_4$
p < 0.00005
With MnmA$_1$
Without MnmA$_1$
Nº of genomes with MnmA$_1$
Nº of genomes without MnmA$_1$
With MnmA$_2$
Without MnmA$_2$
Nº of genomes with MnmA$_2$
Nº of genomes without MnmA$_2$
With TusE$_2$
Without TusE$_2$
Nº of genomes with TusE$_2$
Nº of genomes without TusE$_2$
p < 0.00005
With TusE$_1$
Without TusE$_1$
Nº of genomes with TusE$_1$
Nº of genomes without TusE$_1$
With TusE$_4$
Without TusE$_4$
Nº of genomes with TusE$_4$
Nº of genomes without TusE$_4$
p < 0.00005
With TusE$_3$
Without TusE$_3$
Nº of genomes with TusE$_3$
Nº of genomes without TusE$_3$
p < 0.00005
0 0.02 0.04 0.06 0.08 0.1 0.12 0.14 0.16 0.18 0.2
0 0.06 0.12 0.18

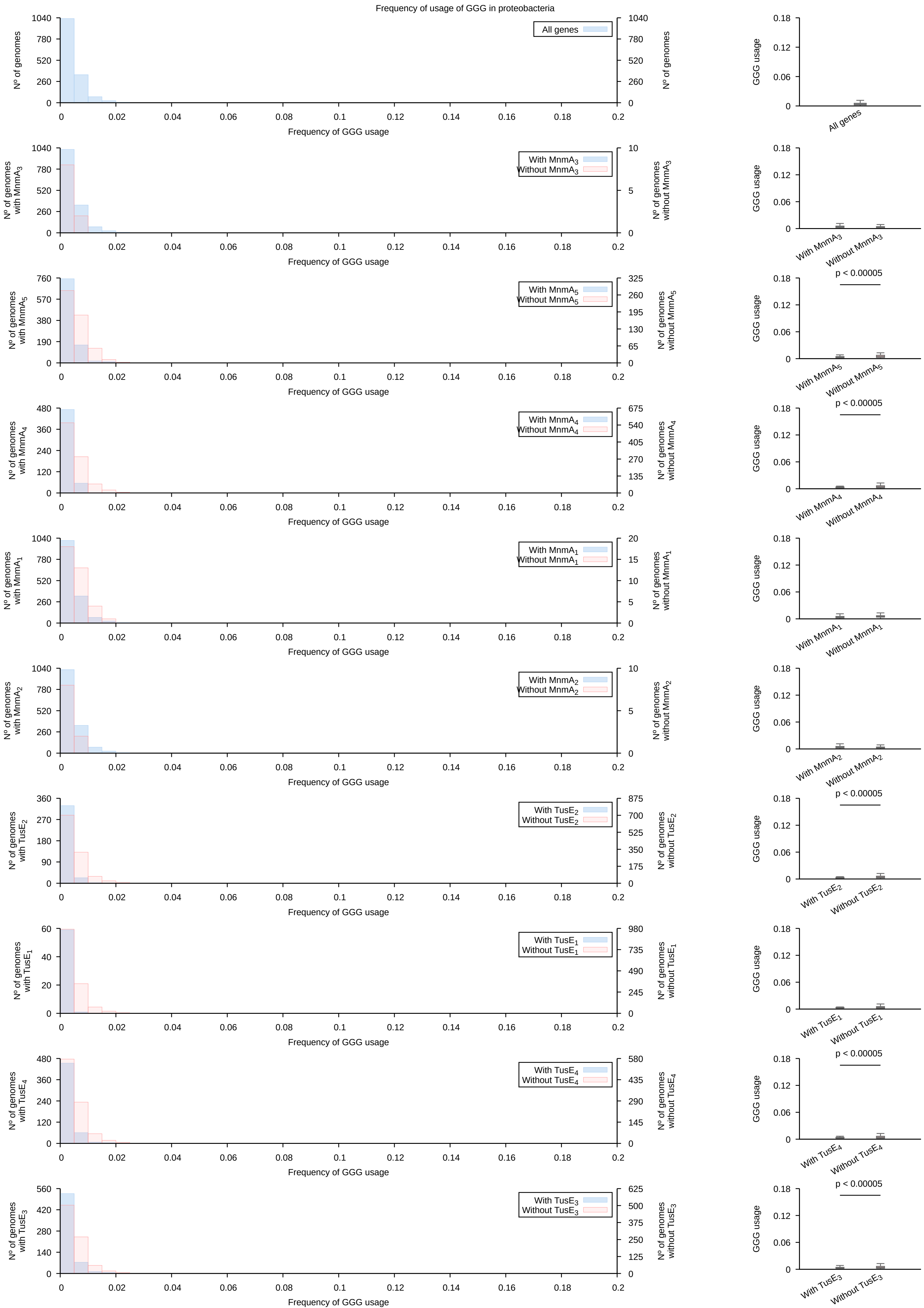
Frequency of usage of GGG in proteobacteria
All genes
Nº of genomes
Frequency of GGG usage
GGG usage
With MnmA3
Without MnmA3
Nº of genomes with MnmA3
Nº of genomes without MnmA3
With MnmA5
Without MnmA5
Nº of genomes with MnmA5
Nº of genomes without MnmA5
p < 0.00005
With MnmA4
Without MnmA4
Nº of genomes with MnmA4
Nº of genomes without MnmA4
p < 0.00005
With MnmA1
Without MnmA1
Nº of genomes with MnmA1
Nº of genomes without MnmA1
With MnmA2
Without MnmA2
Nº of genomes with MnmA2
Nº of genomes without MnmA2
With TusE2
Without TusE2
Nº of genomes with TusE2
Nº of genomes without TusE2
p < 0.00005
With TusE1
Without TusE1
Nº of genomes with TusE1
Nº of genomes without TusE1
With TusE4
Without TusE4
Nº of genomes with TusE4
Nº of genomes without TusE4
p < 0.00005
With TusE3
Without TusE3
Nº of genomes with TusE3
Nº of genomes without TusE3
p < 0.00005

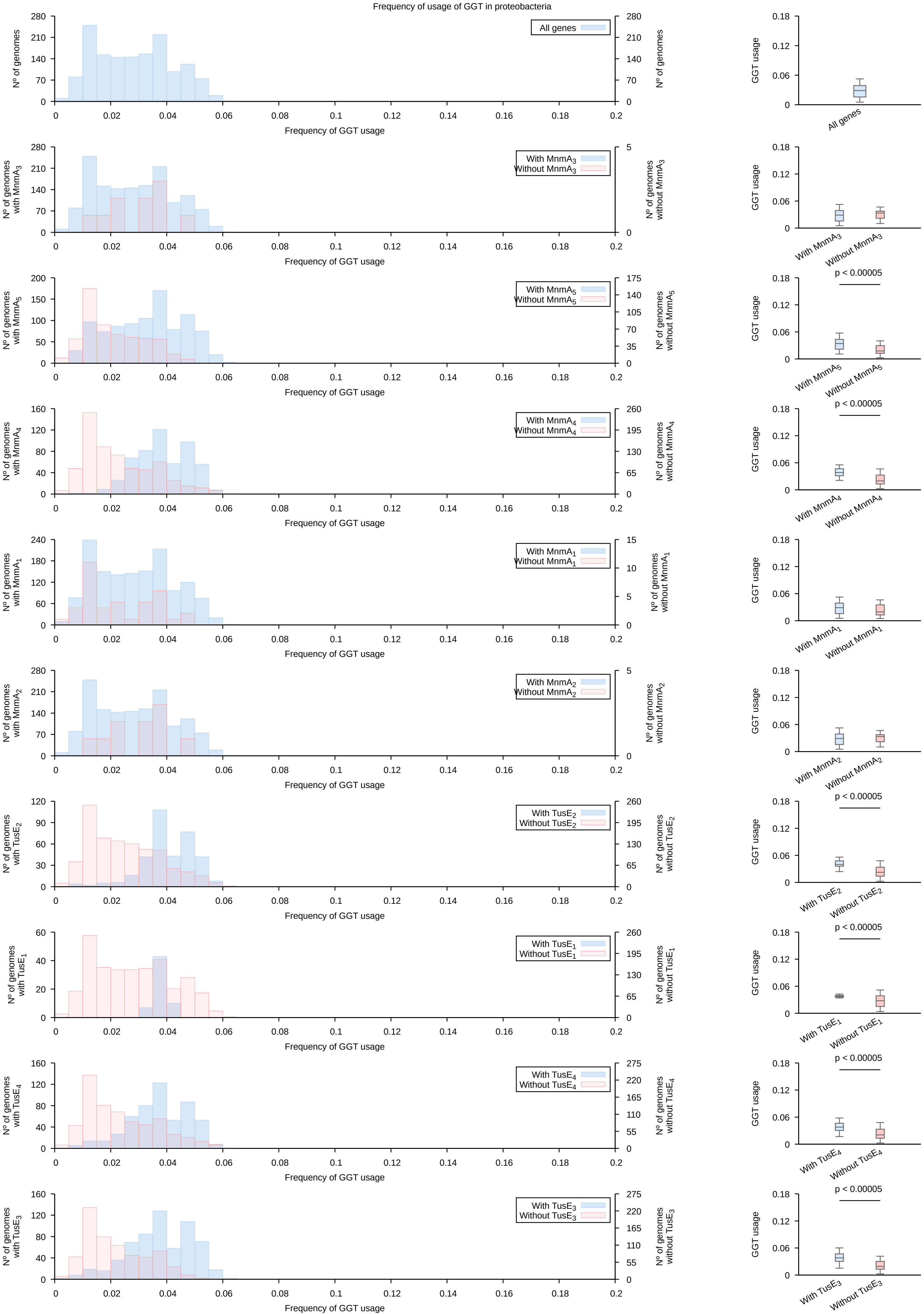
Frequency of usage of GGT in proteobacteria
All genes
With MnmA3
Without MnmA3
With MnmA5
Without MnmA5
With MnmA4
Without MnmA4
With MnmA1
Without MnmA1
With MnmA2
Without MnmA2
With TusE2
Without TusE2
With TusE1
Without TusE1
With TusE4
Without TusE4
With TusE3
Without TusE3
Nº of genomes
Frequency of GGT usage
GGT usage
p < 0.00005

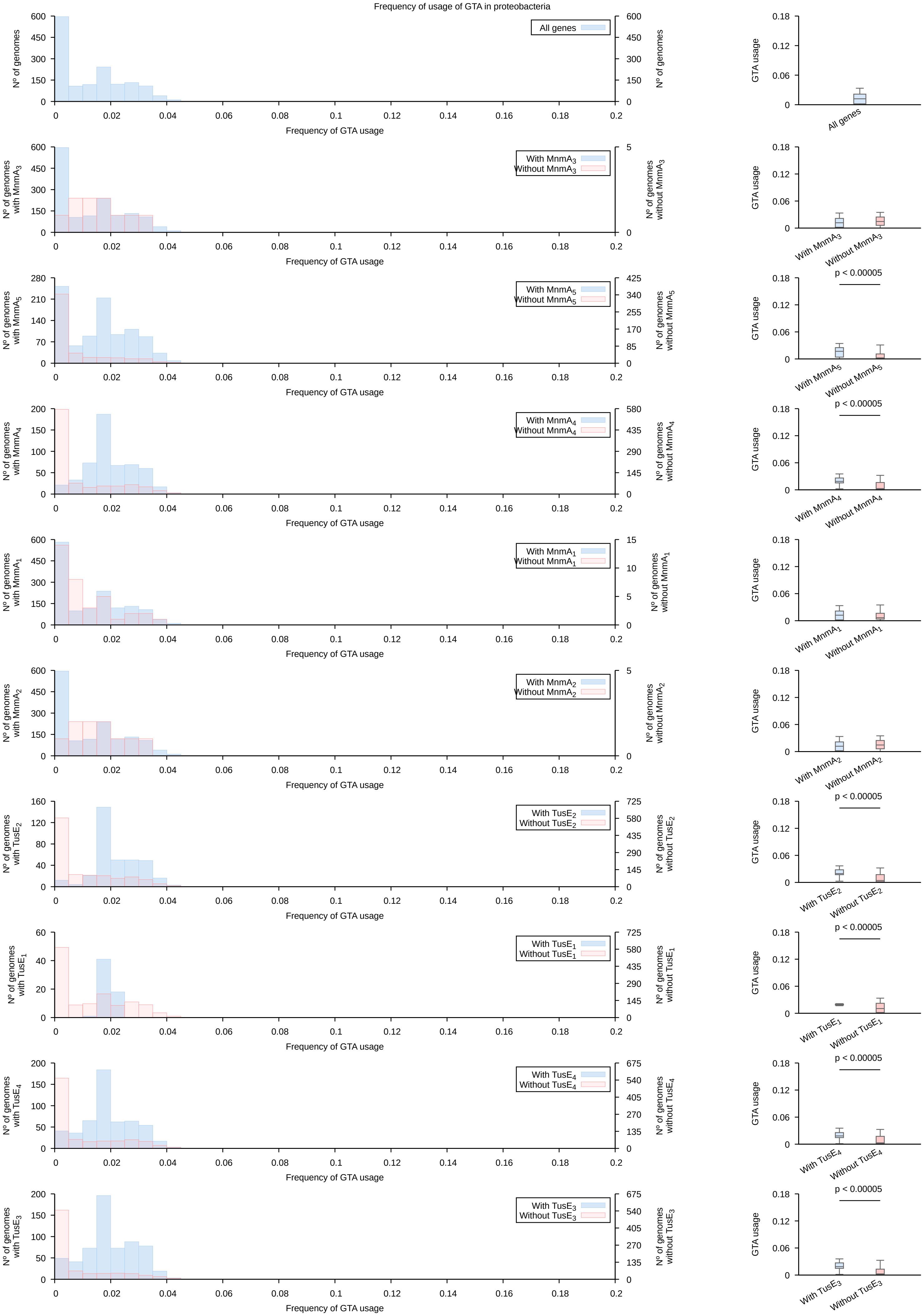

Frequency of usage of GTA in proteobacteria
Nº of genomes
All genes
Frequency of GTA usage
GTA usage
Nº of genomes with MnmA3
Nº of genomes without MnmA3
With MnmA3
Without MnmA3
Nº of genomes with MnmA5
Nº of genomes without MnmA5
With MnmA5
Without MnmA5
p < 0.00005
Nº of genomes with MnmA4
Nº of genomes without MnmA4
With MnmA4
Without MnmA4
p < 0.00005
Nº of genomes with MnmA1
Nº of genomes without MnmA1
With MnmA1
Without MnmA1
Nº of genomes with MnmA2
Nº of genomes without MnmA2
With MnmA2
Without MnmA2
Nº of genomes with TusE2
Nº of genomes without TusE2
With TusE2
Without TusE2
p < 0.00005
Nº of genomes with TusE1
Nº of genomes without TusE1
With TusE1
Without TusE1
p < 0.00005
Nº of genomes with TusE4
Nº of genomes without TusE4
With TusE4
Without TusE4
p < 0.00005
Nº of genomes with TusE3
Nº of genomes without TusE3
With TusE3
Without TusE3
p < 0.00005

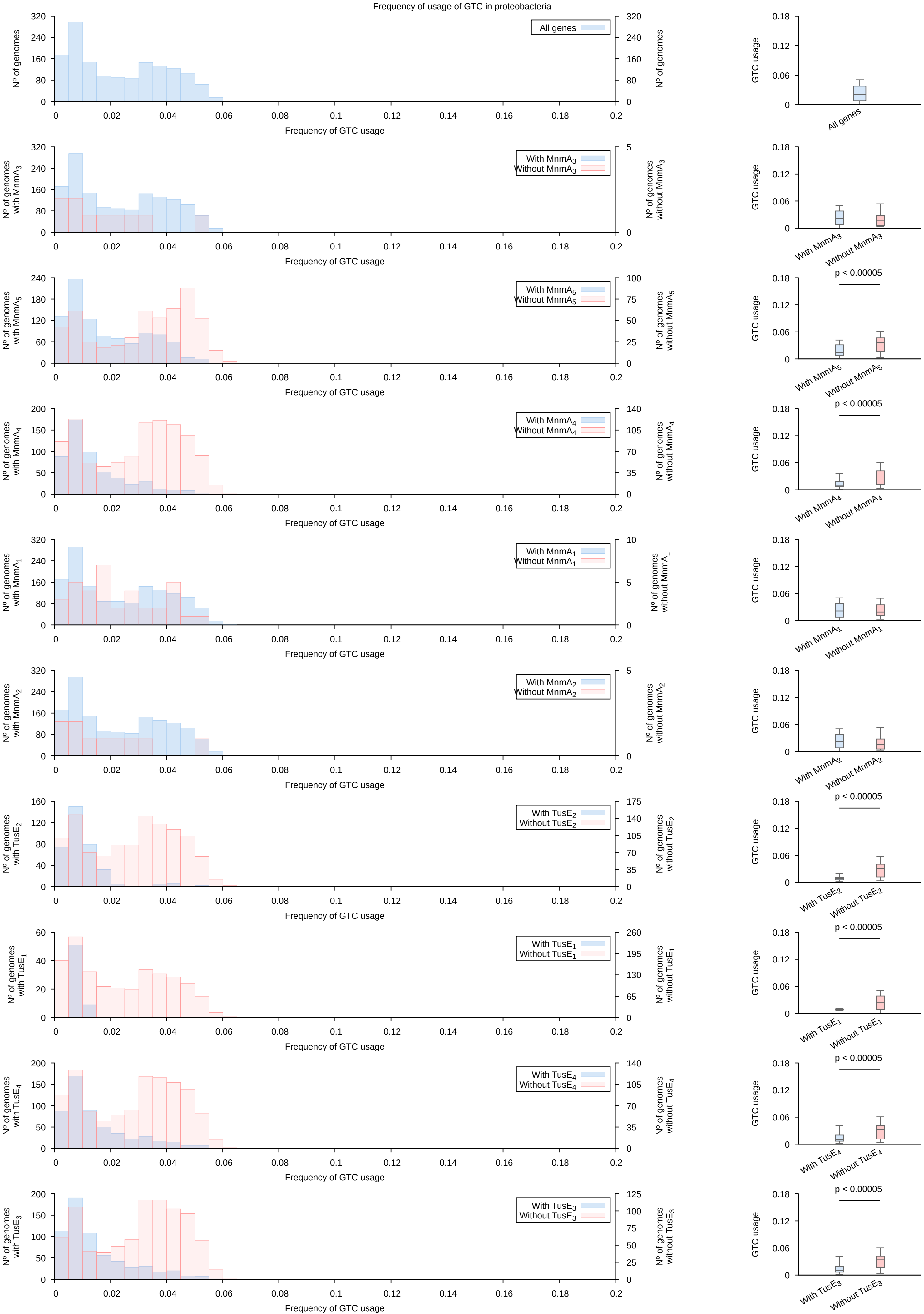
Frequency of usage of GTC in proteobacteria
All genes
Nº of genomes
Frequency of GTC usage
GTC usage
With MnmA$_3$
Without MnmA$_3$
Nº of genomes with MnmA$_3$
Nº of genomes without MnmA$_3$
With MnmA$_5$
Without MnmA$_5$
Nº of genomes with MnmA$_5$
Nº of genomes without MnmA$_5$
p < 0.00005
With MnmA$_4$
Without MnmA$_4$
Nº of genomes with MnmA$_4$
Nº of genomes without MnmA$_4$
p < 0.00005
With MnmA$_1$
Without MnmA$_1$
Nº of genomes with MnmA$_1$
Nº of genomes without MnmA$_1$
With MnmA$_2$
Without MnmA$_2$
Nº of genomes with MnmA$_2$
Nº of genomes without MnmA$_2$
With TusE$_2$
Without TusE$_2$
Nº of genomes with TusE$_2$
Nº of genomes without TusE$_2$
p < 0.00005
With TusE$_1$
Without TusE$_1$
Nº of genomes with TusE$_1$
Nº of genomes without TusE$_1$
p < 0.00005
With TusE$_4$
Without TusE$_4$
Nº of genomes with TusE$_4$
Nº of genomes without TusE$_4$
p < 0.00005
With TusE$_3$
Without TusE$_3$
Nº of genomes with TusE$_3$
Nº of genomes without TusE$_3$
p < 0.00005

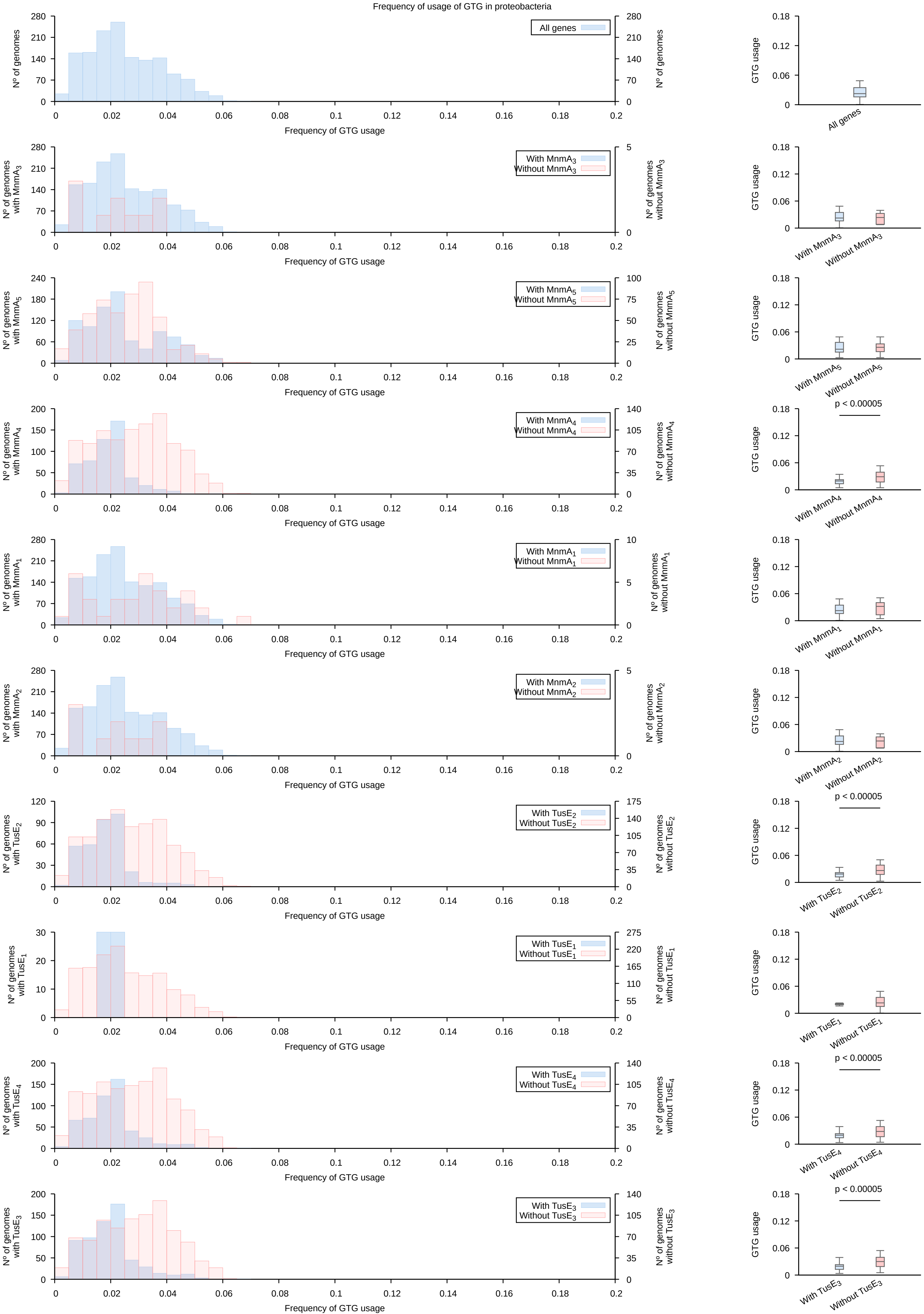
Frequency of usage of GTG in proteobacteria
Nº of genomes
All genes
Frequency of GTG usage
GTG usage
Nº of genomes with MnmA3
Nº of genomes without MnmA3
With MnmA3
Without MnmA3
Nº of genomes with MnmA5
Nº of genomes without MnmA5
With MnmA5
Without MnmA5
Nº of genomes with MnmA4
Nº of genomes without MnmA4
With MnmA4
Without MnmA4
p < 0.00005
Nº of genomes with MnmA1
Nº of genomes without MnmA1
With MnmA1
Without MnmA1
Nº of genomes with MnmA2
Nº of genomes without MnmA2
With MnmA2
Without MnmA2
Nº of genomes with TusE2
Nº of genomes without TusE2
With TusE2
Without TusE2
p < 0.00005
Nº of genomes with TusE1
Nº of genomes without TusE1
With TusE1
Without TusE1
Nº of genomes with TusE4
Nº of genomes without TusE4
With TusE4
Without TusE4
p < 0.00005
Nº of genomes with TusE3
Nº of genomes without TusE3
With TusE3
Without TusE3
p < 0.00005

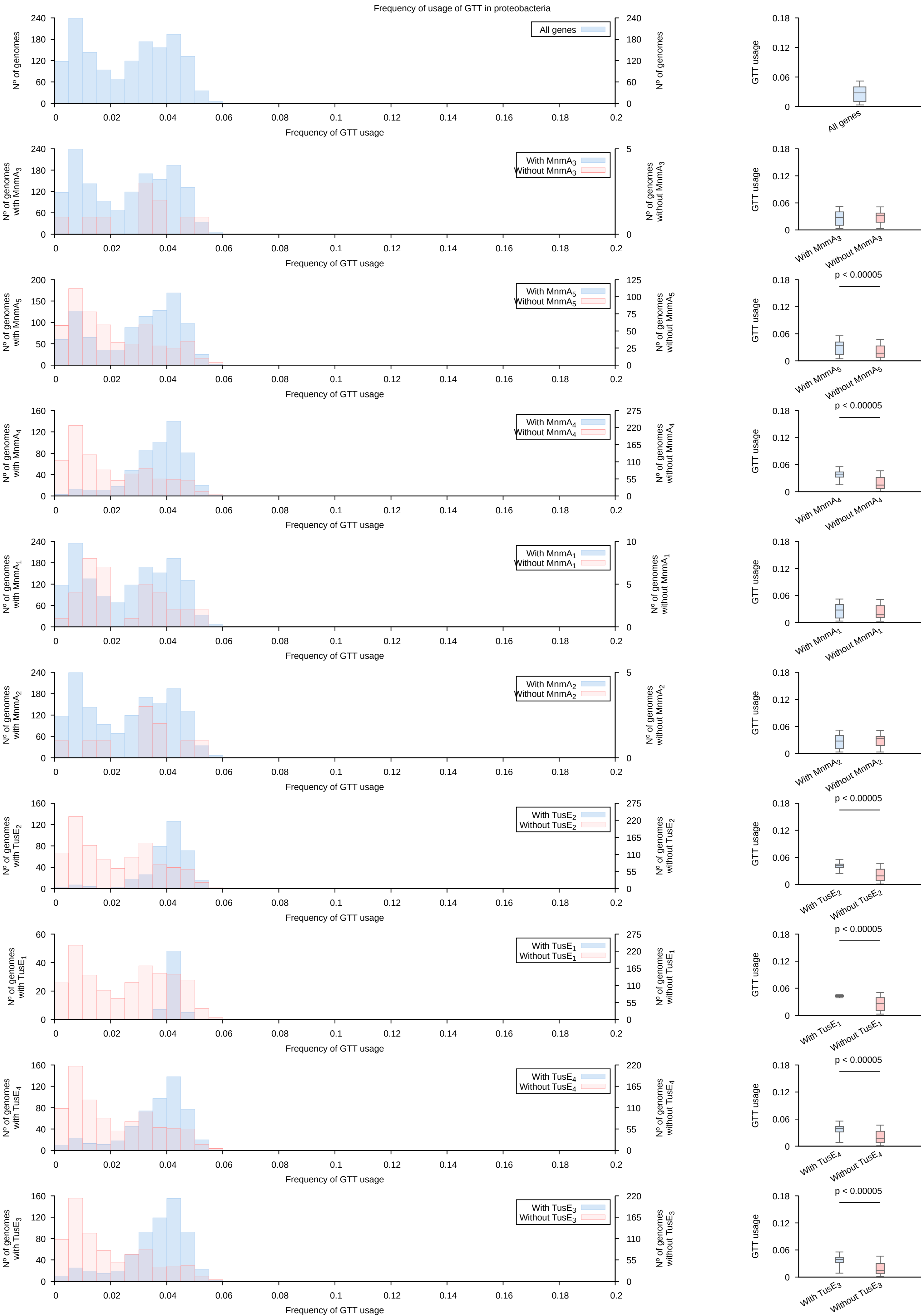

Frequency of usage of GTT in proteobacteria
All genes
Nº of genomes
Frequency of GTT usage
GTT usage
With MnmA$_3$
Without MnmA$_3$
Nº of genomes with MnmA$_3$
Nº of genomes without MnmA$_3$
With MnmA$_5$
Without MnmA$_5$
Nº of genomes with MnmA$_5$
Nº of genomes without MnmA$_5$
p < 0.00005
With MnmA$_4$
Without MnmA$_4$
Nº of genomes with MnmA$_4$
Nº of genomes without MnmA$_4$
p < 0.00005
With MnmA$_1$
Without MnmA$_1$
Nº of genomes with MnmA$_1$
Nº of genomes without MnmA$_1$
With MnmA$_2$
Without MnmA$_2$
Nº of genomes with MnmA$_2$
Nº of genomes without MnmA$_2$
With TusE$_2$
Without TusE$_2$
Nº of genomes with TusE$_2$
Nº of genomes without TusE$_2$
p < 0.00005
With TusE$_1$
Without TusE$_1$
Nº of genomes with TusE$_1$
Nº of genomes without TusE$_1$
p < 0.00005
With TusE$_4$
Without TusE$_4$
Nº of genomes with TusE$_4$
Nº of genomes without TusE$_4$
p < 0.00005
With TusE$_3$
Without TusE$_3$
Nº of genomes with TusE$_3$
Nº of genomes without TusE$_3$
p < 0.00005

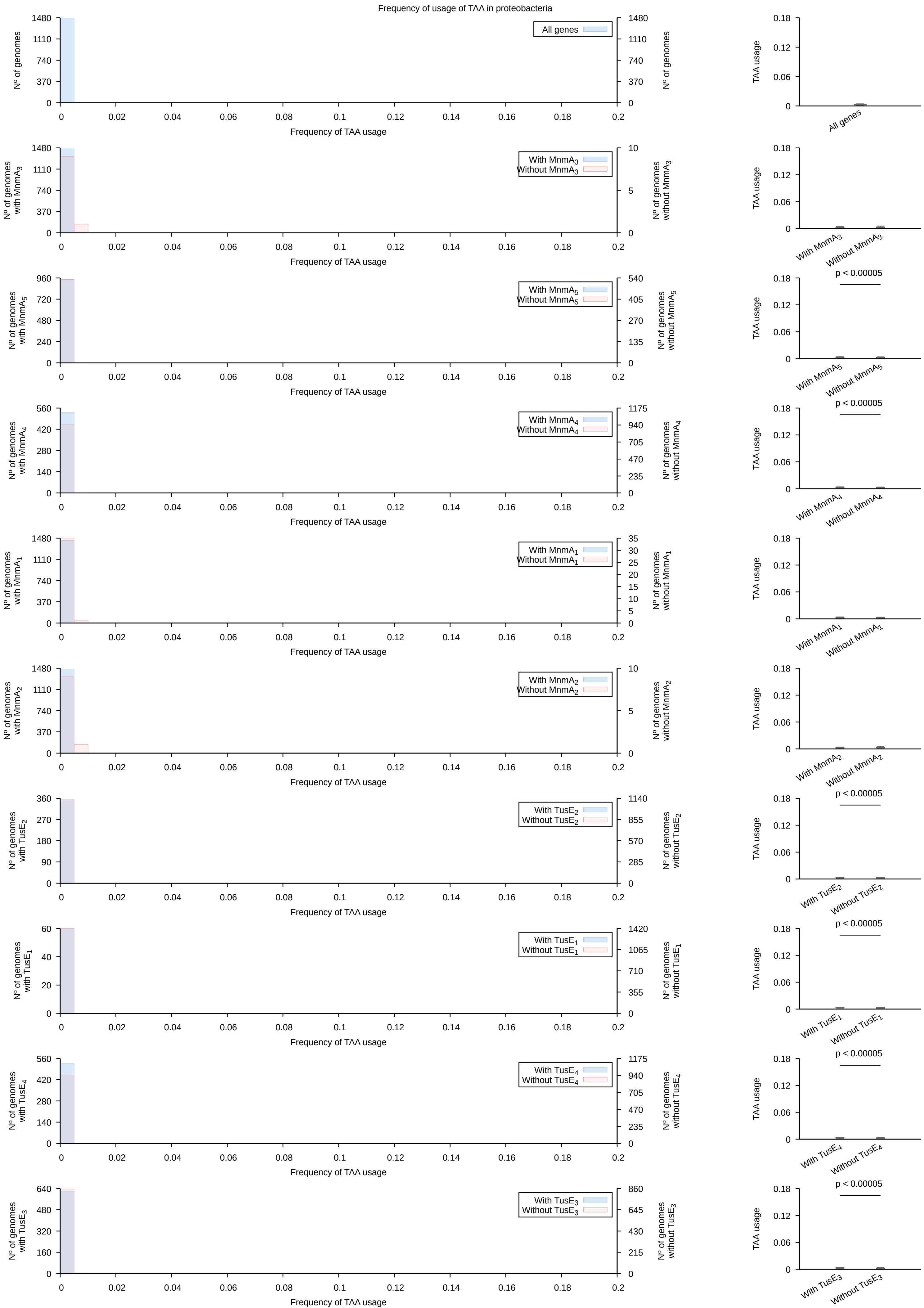

Frequency of usage of TAA in proteobacteria
Nº of genomes
All genes
Frequency of TAA usage
TAA usage
With MnmA3
Without MnmA3
Nº of genomes with MnmA3
Nº of genomes without MnmA3
With MnmA5
Without MnmA5
Nº of genomes with MnmA5
Nº of genomes without MnmA5
p < 0.00005
With MnmA4
Without MnmA4
Nº of genomes with MnmA4
Nº of genomes without MnmA4
With MnmA1
Without MnmA1
Nº of genomes with MnmA1
Nº of genomes without MnmA1
With MnmA2
Without MnmA2
Nº of genomes with MnmA2
Nº of genomes without MnmA2
With TusE2
Without TusE2
Nº of genomes with TusE2
Nº of genomes without TusE2
With TusE1
Without TusE1
Nº of genomes with TusE1
Nº of genomes without TusE1
With TusE4
Without TusE4
Nº of genomes with TusE4
Nº of genomes without TusE4
With TusE3
Without TusE3
Nº of genomes with TusE3
Nº of genomes without TusE3

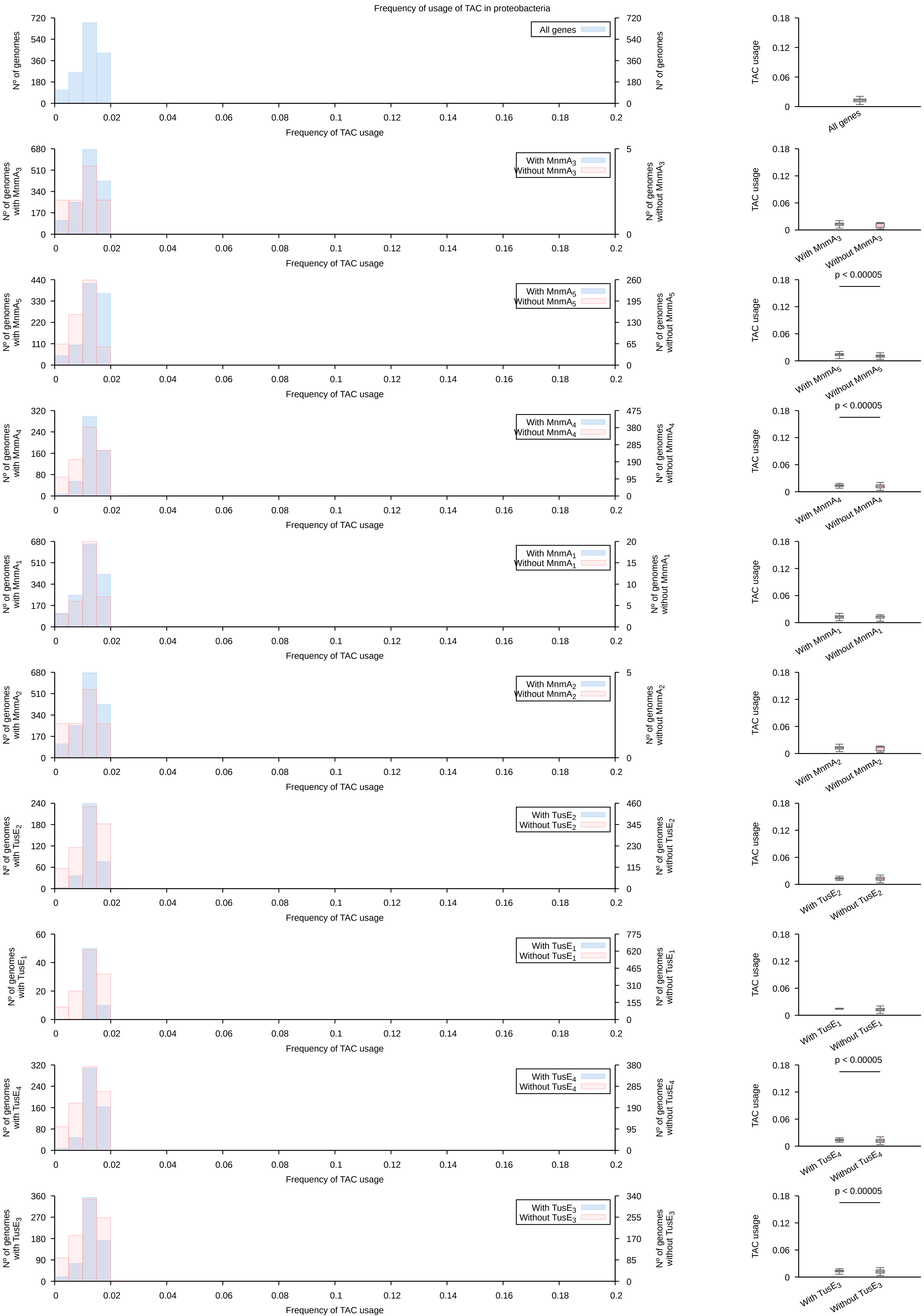

Frequency of usage of TAC in proteobacteria
All genes
Nº of genomes
Frequency of TAC usage
TAC usage
With MnmA$_3$
Without MnmA$_3$
Nº of genomes with MnmA$_3$
Nº of genomes without MnmA$_3$
With MnmA$_5$
Without MnmA$_5$
Nº of genomes with MnmA$_5$
Nº of genomes without MnmA$_5$
p < 0.00005
With MnmA$_4$
Without MnmA$_4$
Nº of genomes with MnmA$_4$
Nº of genomes without MnmA$_4$
p < 0.00005
With MnmA$_1$
Without MnmA$_1$
Nº of genomes with MnmA$_1$
Nº of genomes without MnmA$_1$
With MnmA$_2$
Without MnmA$_2$
Nº of genomes with MnmA$_2$
Nº of genomes without MnmA$_2$
With TusE$_2$
Without TusE$_2$
Nº of genomes with TusE$_2$
Nº of genomes without TusE$_2$
With TusE$_1$
Without TusE$_1$
Nº of genomes with TusE$_1$
Nº of genomes without TusE$_1$
With TusE$_4$
Without TusE$_4$
Nº of genomes with TusE$_4$
Nº of genomes without TusE$_4$
p < 0.00005
With TusE$_3$
Without TusE$_3$
Nº of genomes with TusE$_3$
Nº of genomes without TusE$_3$
p < 0.00005

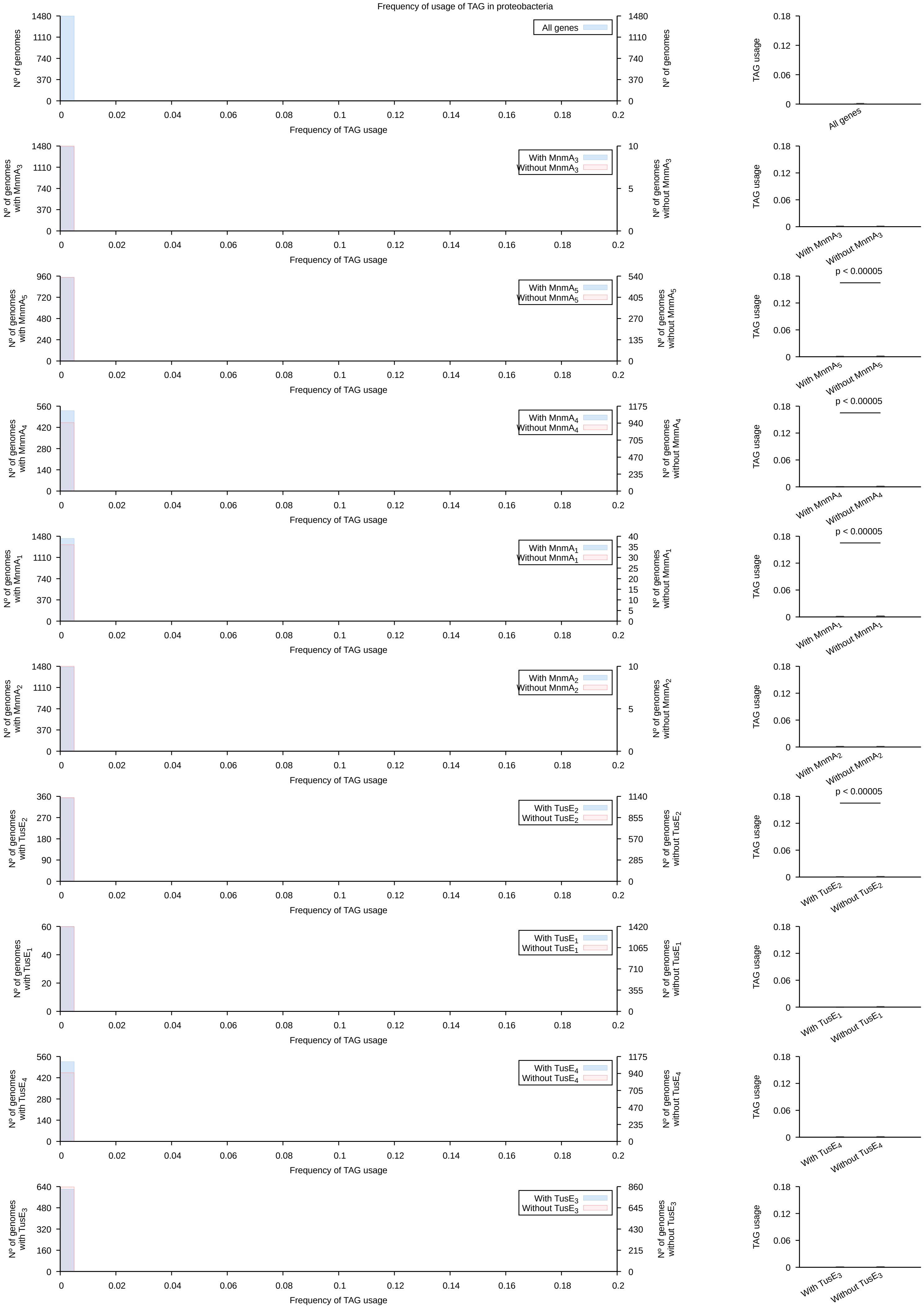
Frequency of usage of TAG in proteobacteria
All genes
Nº of genomes
Frequency of TAG usage
TAG usage
With MnmA$_3$
Without MnmA$_3$
Nº of genomes with MnmA$_3$
Nº of genomes without MnmA$_3$
With MnmA$_5$
Without MnmA$_5$
Nº of genomes with MnmA$_5$
Nº of genomes without MnmA$_5$
p < 0.00005
With MnmA$_4$
Without MnmA$_4$
Nº of genomes with MnmA$_4$
Nº of genomes without MnmA$_4$
p < 0.00005
With MnmA$_1$
Without MnmA$_1$
Nº of genomes with MnmA$_1$
Nº of genomes without MnmA$_1$
p < 0.00005
With MnmA$_2$
Without MnmA$_2$
Nº of genomes with MnmA$_2$
Nº of genomes without MnmA$_2$
With TusE$_2$
Without TusE$_2$
Nº of genomes with TusE$_2$
Nº of genomes without TusE$_2$
p < 0.00005
With TusE$_1$
Without TusE$_1$
Nº of genomes with TusE$_1$
Nº of genomes without TusE$_1$
With TusE$_4$
Without TusE$_4$
Nº of genomes with TusE$_4$
Nº of genomes without TusE$_4$
With TusE$_3$
Without TusE$_3$
Nº of genomes with TusE$_3$
Nº of genomes without TusE$_3$

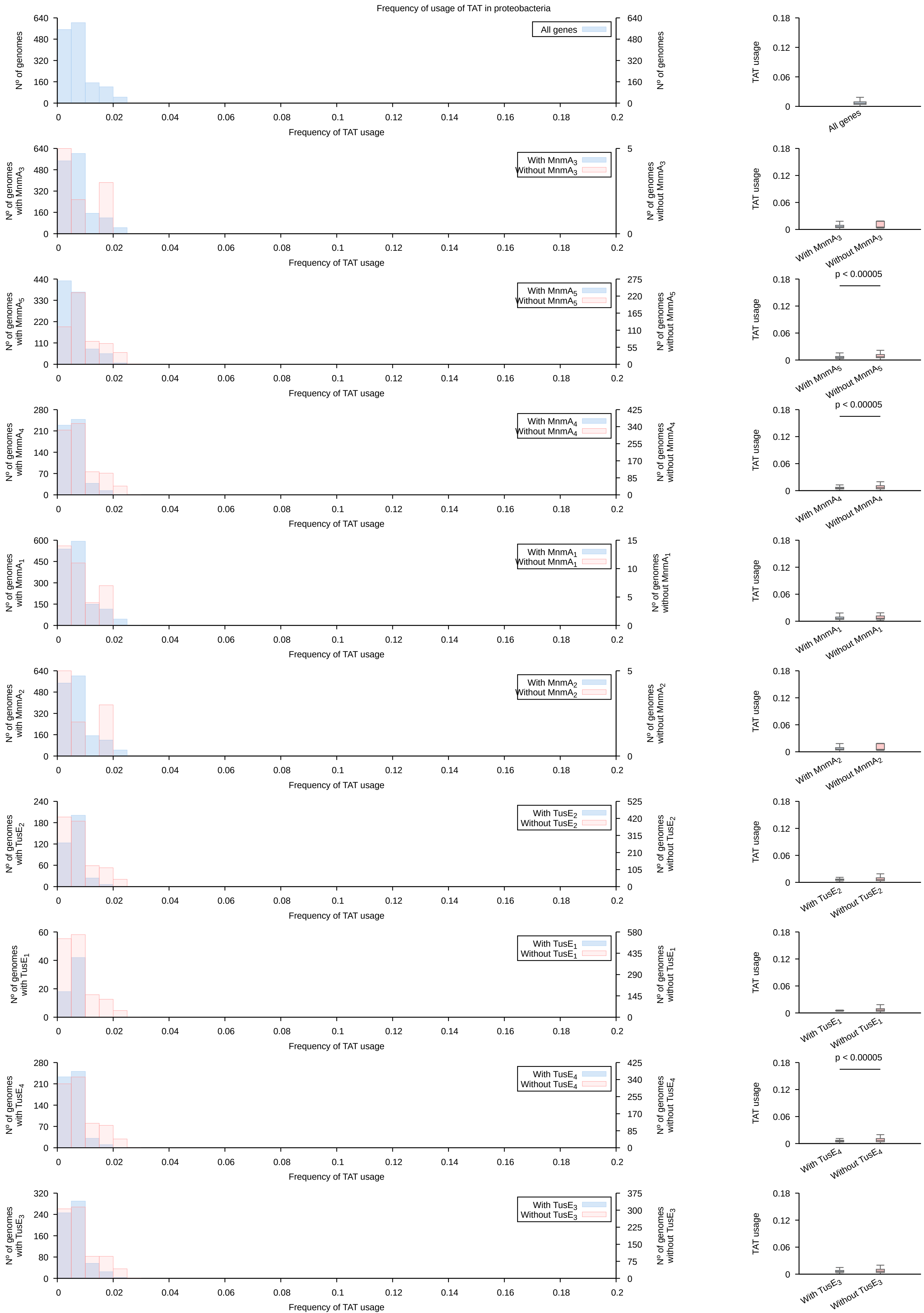
Frequency of usage of TAT in proteobacteria
All genes
Nº of genomes
Frequency of TAT usage
TAT usage
0
0.02
0.04
0.06
0.08
0.1
0.12
0.14
0.16
0.18
0.2
With MnmA$_3$
Without MnmA$_3$
Nº of genomes with MnmA$_3$
Nº of genomes without MnmA$_3$
With MnmA$_5$
Without MnmA$_5$
Nº of genomes with MnmA$_5$
Nº of genomes without MnmA$_5$
p < 0.00005
With MnmA$_4$
Without MnmA$_4$
Nº of genomes with MnmA$_4$
Nº of genomes without MnmA$_4$
p < 0.00005
With MnmA$_1$
Without MnmA$_1$
Nº of genomes with MnmA$_1$
Nº of genomes without MnmA$_1$
With MnmA$_2$
Without MnmA$_2$
Nº of genomes with MnmA$_2$
Nº of genomes without MnmA$_2$
With TusE$_2$
Without TusE$_2$
Nº of genomes with TusE$_2$
Nº of genomes without TusE$_2$
With TusE$_1$
Without TusE$_1$
Nº of genomes with TusE$_1$
Nº of genomes without TusE$_1$
With TusE$_4$
Without TusE$_4$
Nº of genomes with TusE$_4$
Nº of genomes without TusE$_4$
p < 0.00005
With TusE$_3$
Without TusE$_3$
Nº of genomes with TusE$_3$
Nº of genomes without TusE$_3$

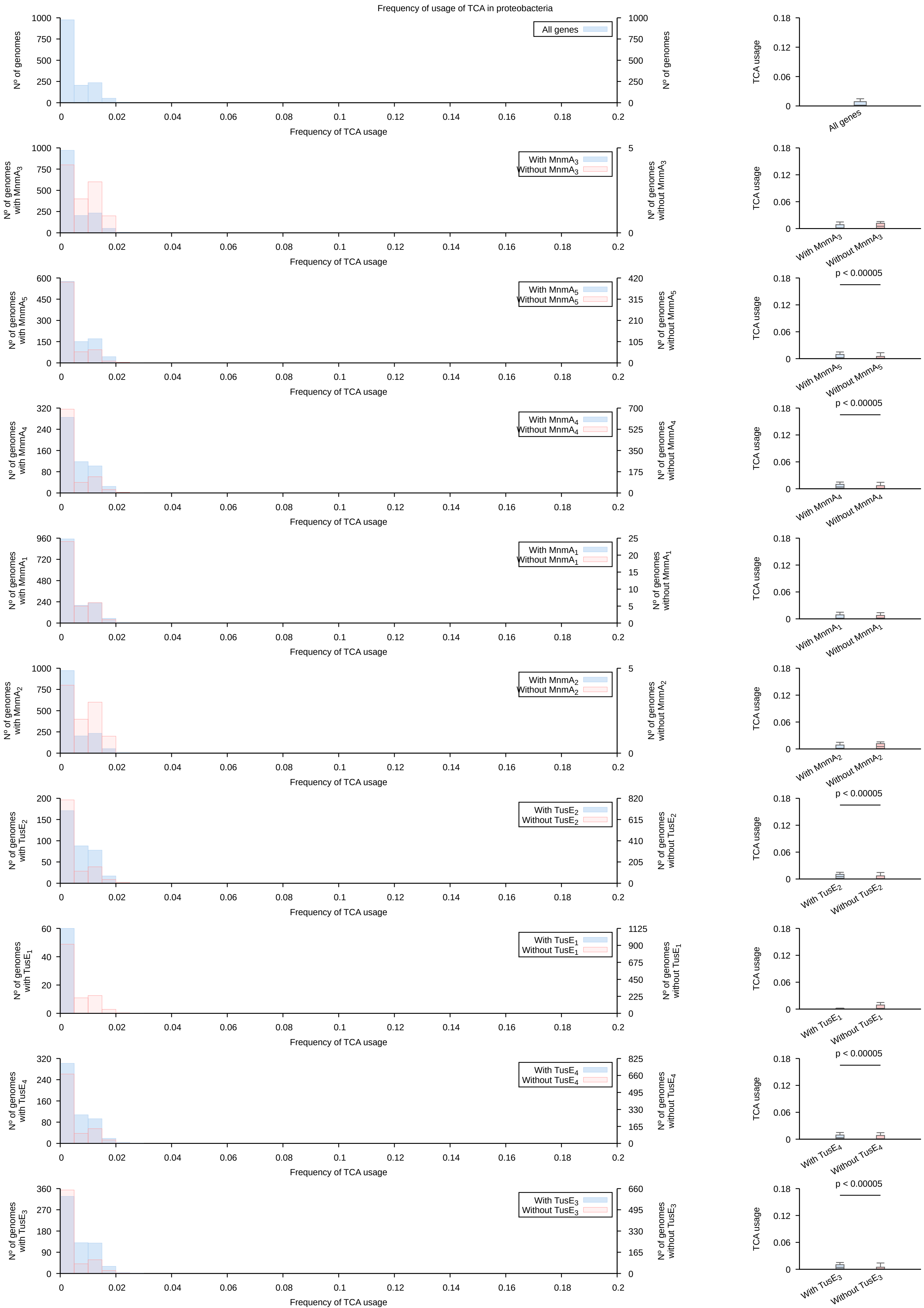
Frequency of usage of TCA in proteobacteria
Nº of genomes
Frequency of TCA usage
All genes
TCA usage
Nº of genomes with MnmA$_3$
Nº of genomes without MnmA$_3$
With MnmA$_3$
Without MnmA$_3$
Nº of genomes with MnmA$_5$
Nº of genomes without MnmA$_5$
With MnmA$_5$
Without MnmA$_5$
p < 0.00005
Nº of genomes with MnmA$_4$
Nº of genomes without MnmA$_4$
With MnmA$_4$
Without MnmA$_4$
p < 0.00005
Nº of genomes with MnmA$_1$
Nº of genomes without MnmA$_1$
With MnmA$_1$
Without MnmA$_1$
Nº of genomes with MnmA$_2$
Nº of genomes without MnmA$_2$
With MnmA$_2$
Without MnmA$_2$
Nº of genomes with TusE$_2$
Nº of genomes without TusE$_2$
With TusE$_2$
Without TusE$_2$
p < 0.00005
Nº of genomes with TusE$_1$
Nº of genomes without TusE$_1$
With TusE$_1$
Without TusE$_1$
Nº of genomes with TusE$_4$
Nº of genomes without TusE$_4$
With TusE$_4$
Without TusE$_4$
p < 0.00005
Nº of genomes with TusE$_3$
Nº of genomes without TusE$_3$
With TusE$_3$
Without TusE$_3$
p < 0.00005

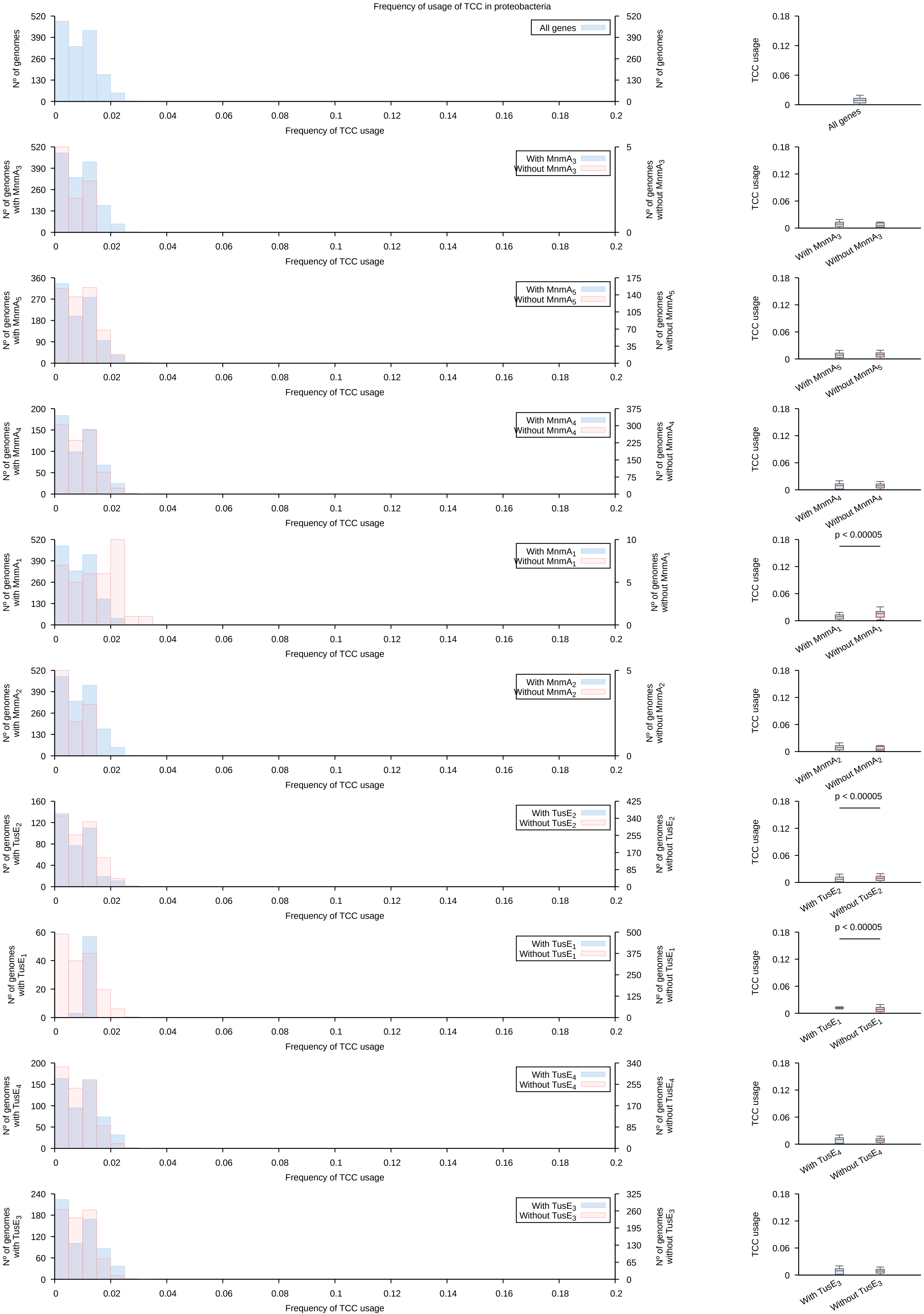

Frequency of usage of TCC in proteobacteria
All genes
Nº of genomes
Frequency of TCC usage
TCC usage
With MnmA3
Without MnmA3
Nº of genomes with MnmA3
Nº of genomes without MnmA3
With MnmA5
Without MnmA5
Nº of genomes with MnmA5
Nº of genomes without MnmA5
With MnmA4
Without MnmA4
Nº of genomes with MnmA4
Nº of genomes without MnmA4
With MnmA1
Without MnmA1
Nº of genomes with MnmA1
Nº of genomes without MnmA1
p < 0.00005
With MnmA2
Without MnmA2
Nº of genomes with MnmA2
Nº of genomes without MnmA2
With TusE2
Without TusE2
Nº of genomes with TusE2
Nº of genomes without TusE2
p < 0.00005
With TusE1
Without TusE1
Nº of genomes with TusE1
Nº of genomes without TusE1
p < 0.00005
With TusE4
Without TusE4
Nº of genomes with TusE4
Nº of genomes without TusE4
With TusE3
Without TusE3
Nº of genomes with TusE3
Nº of genomes without TusE3

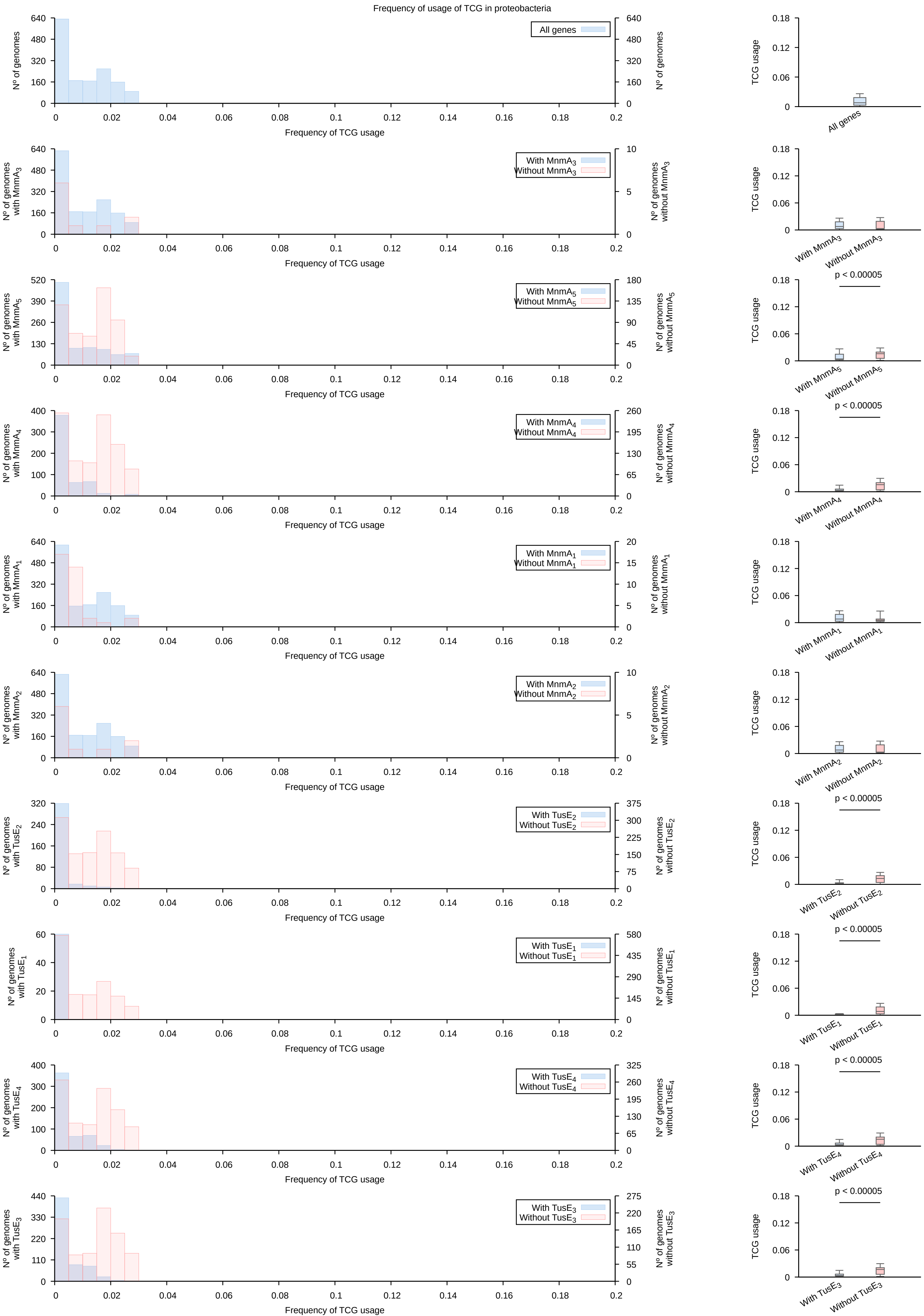

Frequency of usage of TCG in proteobacteria
Nº of genomes
All genes
Frequency of TCG usage
TCG usage
All genes
Nº of genomes with $MnmA_3$
Nº of genomes without $MnmA_3$
With $MnmA_3$
Without $MnmA_3$
Nº of genomes with $MnmA_5$
Nº of genomes without $MnmA_5$
With $MnmA_5$
Without $MnmA_5$
p < 0.00005
Nº of genomes with $MnmA_4$
Nº of genomes without $MnmA_4$
With $MnmA_4$
Without $MnmA_4$
p < 0.00005
Nº of genomes with $MnmA_1$
Nº of genomes without $MnmA_1$
With $MnmA_1$
Without $MnmA_1$
Nº of genomes with $MnmA_2$
Nº of genomes without $MnmA_2$
With $MnmA_2$
Without $MnmA_2$
Nº of genomes with $TusE_2$
Nº of genomes without $TusE_2$
With $TusE_2$
Without $TusE_2$
p < 0.00005
Nº of genomes with $TusE_1$
Nº of genomes without $TusE_1$
With $TusE_1$
Without $TusE_1$
p < 0.00005
Nº of genomes with $TusE_4$
Nº of genomes without $TusE_4$
With $TusE_4$
Without $TusE_4$
p < 0.00005
Nº of genomes with $TusE_3$
Nº of genomes without $TusE_3$
With $TusE_3$
Without $TusE_3$
p < 0.00005

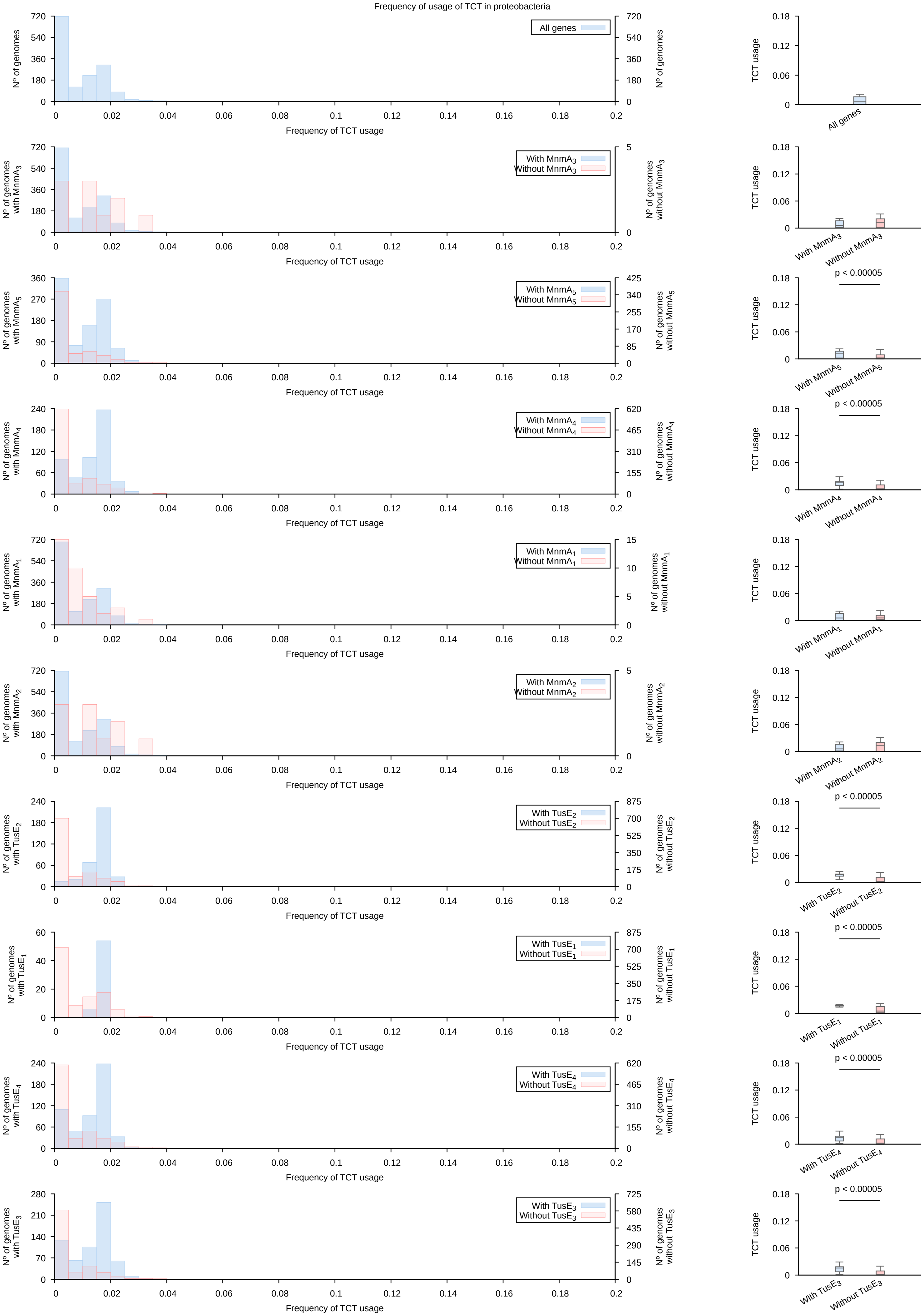
Frequency of usage of TCT in proteobacteria
All genes
With MnmA$_3$
Without MnmA$_3$
With MnmA$_5$
Without MnmA$_5$
With MnmA$_4$
Without MnmA$_4$
With MnmA$_1$
Without MnmA$_1$
With MnmA$_2$
Without MnmA$_2$
With TusE$_2$
Without TusE$_2$
With TusE$_1$
Without TusE$_1$
With TusE$_4$
Without TusE$_4$
With TusE$_3$
Without TusE$_3$
Nº of genomes
Frequency of TCT usage
TCT usage
p < 0.00005

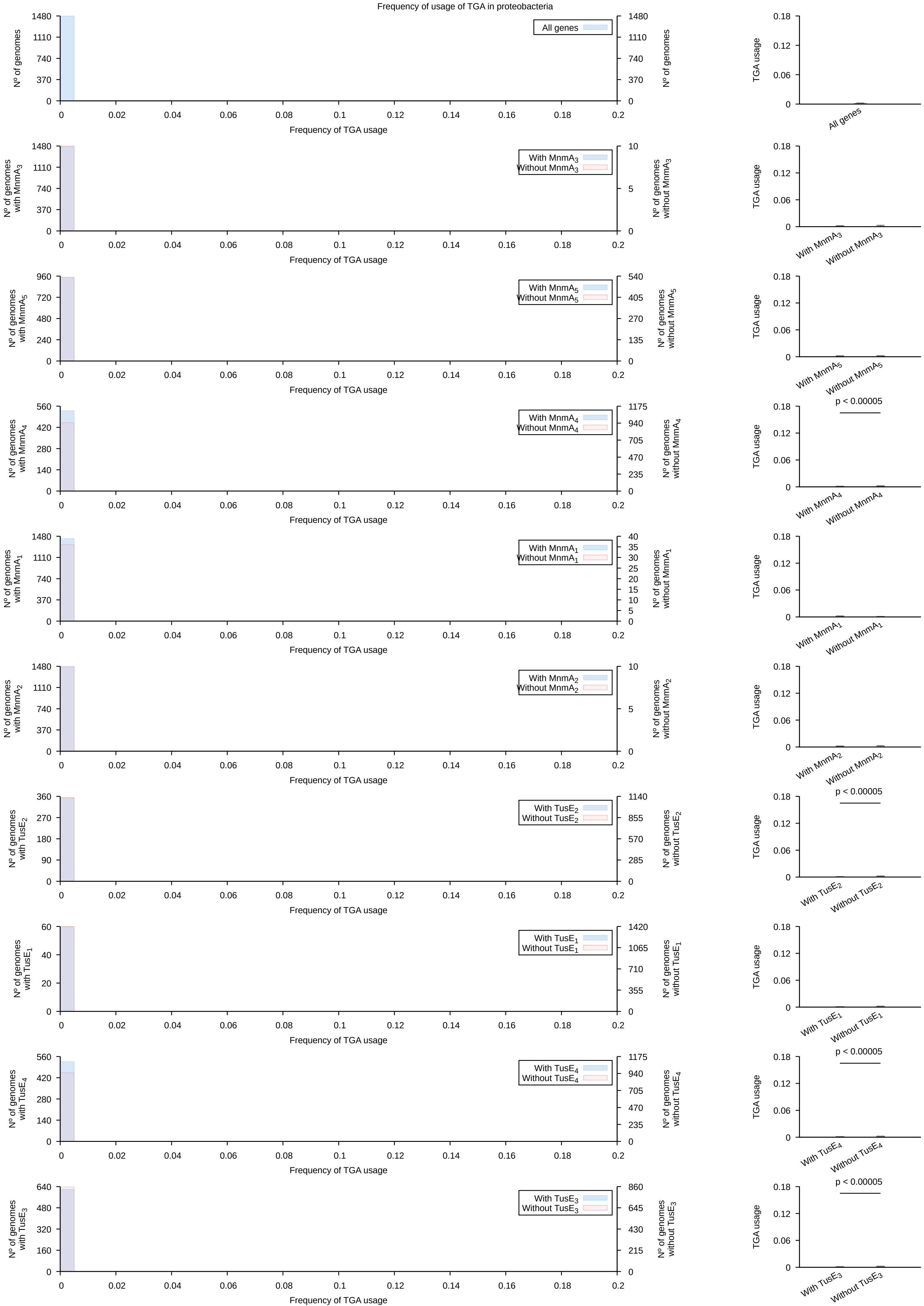

Frequency of usage of TGA in proteobacteria
All genes
With MnmA3
Without MnmA3
With MnmA5
Without MnmA5
With MnmA4
Without MnmA4
With MnmA1
Without MnmA1
With MnmA2
Without MnmA2
With TusE2
Without TusE2
With TusE1
Without TusE1
With TusE4
Without TusE4
With TusE3
Without TusE3
Nº of genomes
Frequency of TGA usage
TGA usage
p < 0.00005

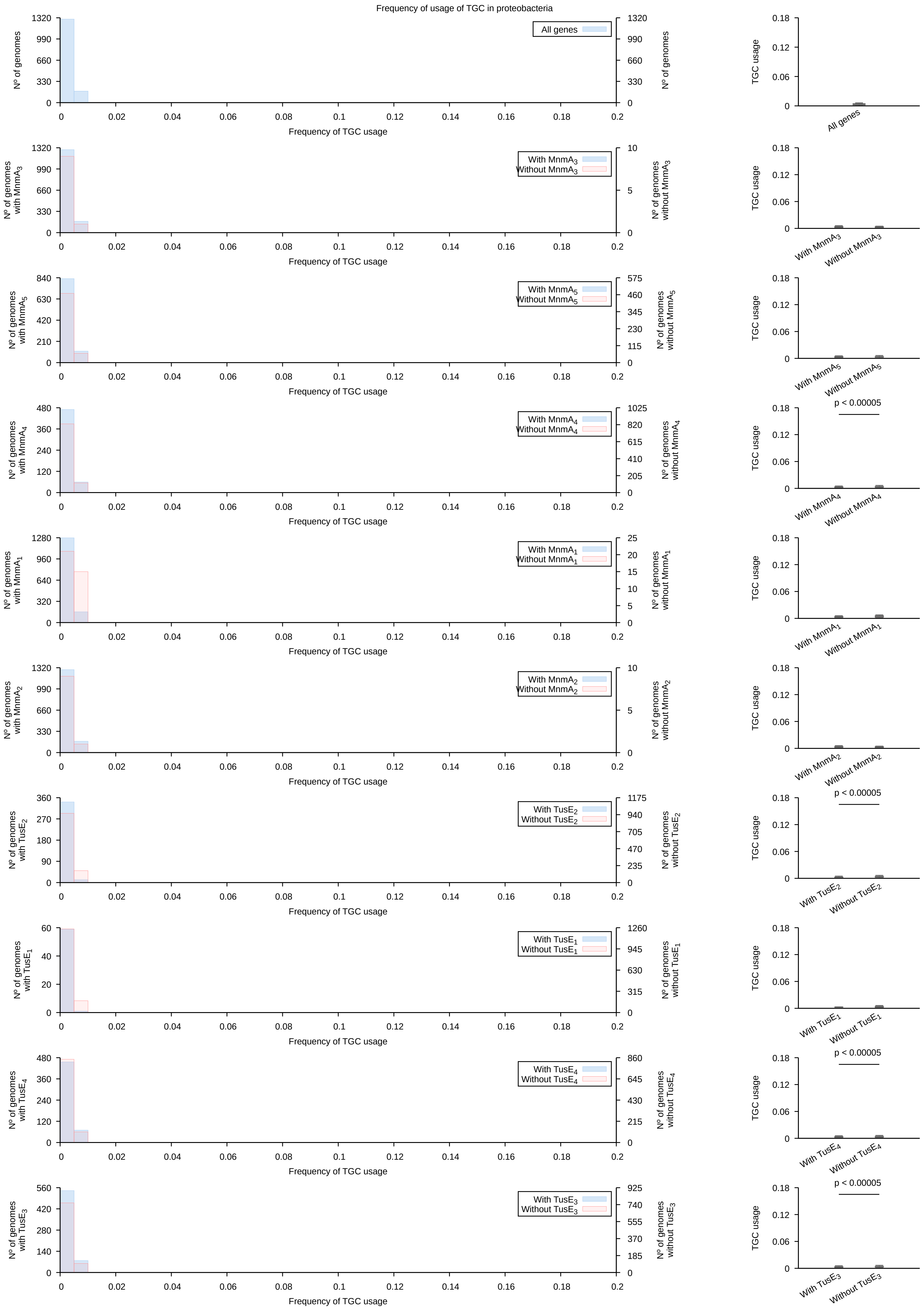
Frequency of usage of TGC in proteobacteria
Nº of genomes
All genes
Frequency of TGC usage
TGC usage
Nº of genomes with MnmA3
Nº of genomes without MnmA3
With MnmA3
Without MnmA3
Nº of genomes with MnmA5
Nº of genomes without MnmA5
With MnmA5
Without MnmA5
Nº of genomes with MnmA4
Nº of genomes without MnmA4
With MnmA4
Without MnmA4
p < 0.00005
Nº of genomes with MnmA1
Nº of genomes without MnmA1
With MnmA1
Without MnmA1
Nº of genomes with MnmA2
Nº of genomes without MnmA2
With MnmA2
Without MnmA2
Nº of genomes with TusE2
Nº of genomes without TusE2
With TusE2
Without TusE2
p < 0.00005
Nº of genomes with TusE1
Nº of genomes without TusE1
With TusE1
Without TusE1
Nº of genomes with TusE4
Nº of genomes without TusE4
With TusE4
Without TusE4
p < 0.00005
Nº of genomes with TusE3
Nº of genomes without TusE3
With TusE3
Without TusE3
p < 0.00005

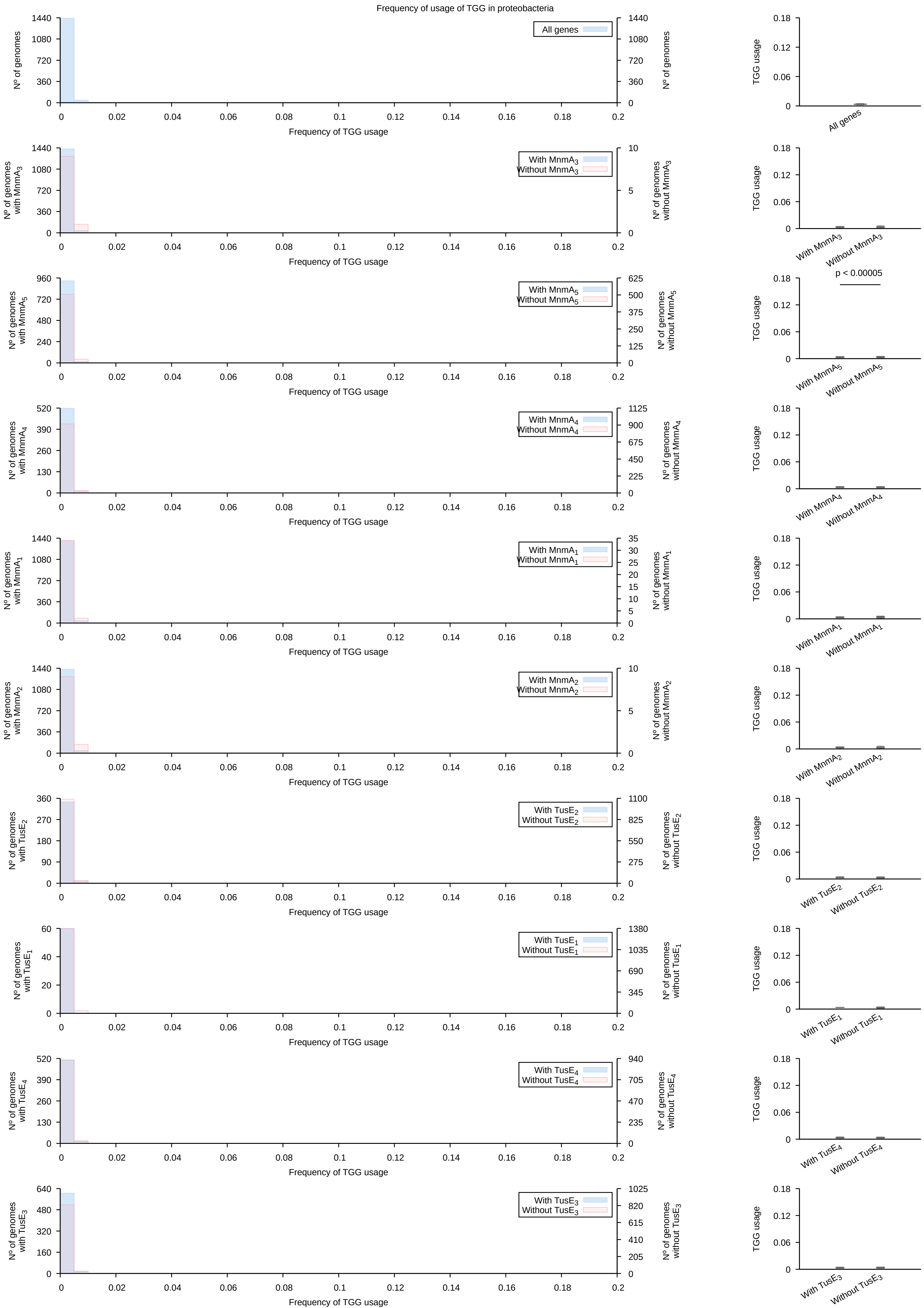

Frequency of usage of TGG in proteobacteria
All genes
Nº of genomes
Frequency of TGG usage
TGG usage
With $MnmA_3$
Without $MnmA_3$
Nº of genomes with $MnmA_3$
Nº of genomes without $MnmA_3$
With $MnmA_5$
Without $MnmA_5$
Nº of genomes with $MnmA_5$
Nº of genomes without $MnmA_5$
p < 0.00005
With $MnmA_4$
Without $MnmA_4$
Nº of genomes with $MnmA_4$
Nº of genomes without $MnmA_4$
With $MnmA_1$
Without $MnmA_1$
Nº of genomes with $MnmA_1$
Nº of genomes without $MnmA_1$
With $MnmA_2$
Without $MnmA_2$
Nº of genomes with $MnmA_2$
Nº of genomes without $MnmA_2$
With $TusE_2$
Without $TusE_2$
Nº of genomes with $TusE_2$
Nº of genomes without $TusE_2$
With $TusE_1$
Without $TusE_1$
Nº of genomes with $TusE_1$
Nº of genomes without $TusE_1$
With $TusE_4$
Without $TusE_4$
Nº of genomes with $TusE_4$
Nº of genomes without $TusE_4$
With $TusE_3$
Without $TusE_3$
Nº of genomes with $TusE_3$
Nº of genomes without $TusE_3$

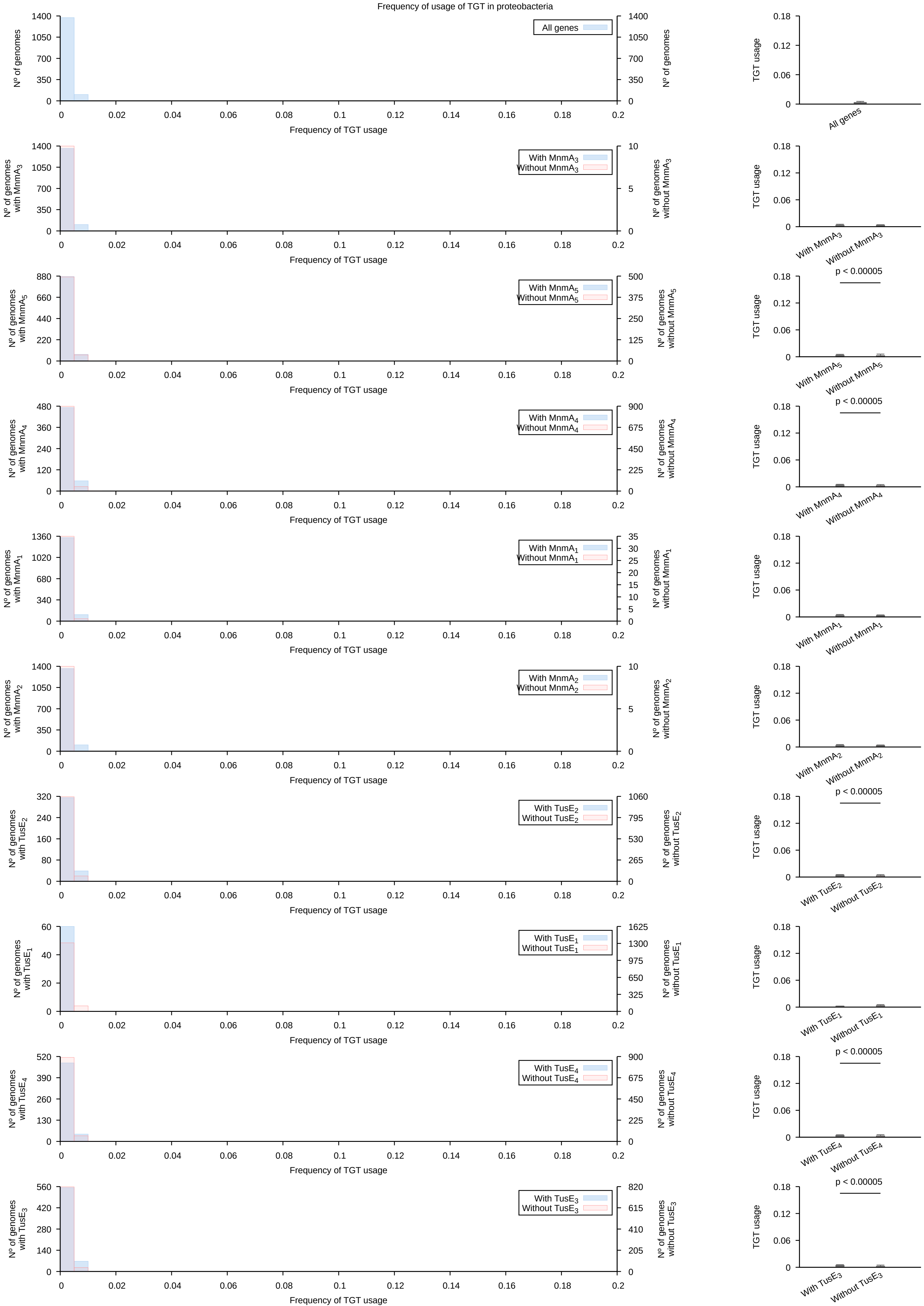
Frequency of usage of TGT in proteobacteria
All genes
Nº of genomes
Frequency of TGT usage
TGT usage
All genes
With MnmA$_3$
Without MnmA$_3$
Nº of genomes with MnmA$_3$
Nº of genomes without MnmA$_3$
With MnmA$_5$
Without MnmA$_5$
Nº of genomes with MnmA$_5$
Nº of genomes without MnmA$_5$
p < 0.00005
With MnmA$_4$
Without MnmA$_4$
Nº of genomes with MnmA$_4$
Nº of genomes without MnmA$_4$
p < 0.00005
With MnmA$_1$
Without MnmA$_1$
Nº of genomes with MnmA$_1$
Nº of genomes without MnmA$_1$
With MnmA$_2$
Without MnmA$_2$
Nº of genomes with MnmA$_2$
Nº of genomes without MnmA$_2$
With TusE$_2$
Without TusE$_2$
Nº of genomes with TusE$_2$
Nº of genomes without TusE$_2$
p < 0.00005
With TusE$_1$
Without TusE$_1$
Nº of genomes with TusE$_1$
Nº of genomes without TusE$_1$
With TusE$_4$
Without TusE$_4$
Nº of genomes with TusE$_4$
Nº of genomes without TusE$_4$
p < 0.00005
With TusE$_3$
Without TusE$_3$
Nº of genomes with TusE$_3$
Nº of genomes without TusE$_3$
p < 0.00005

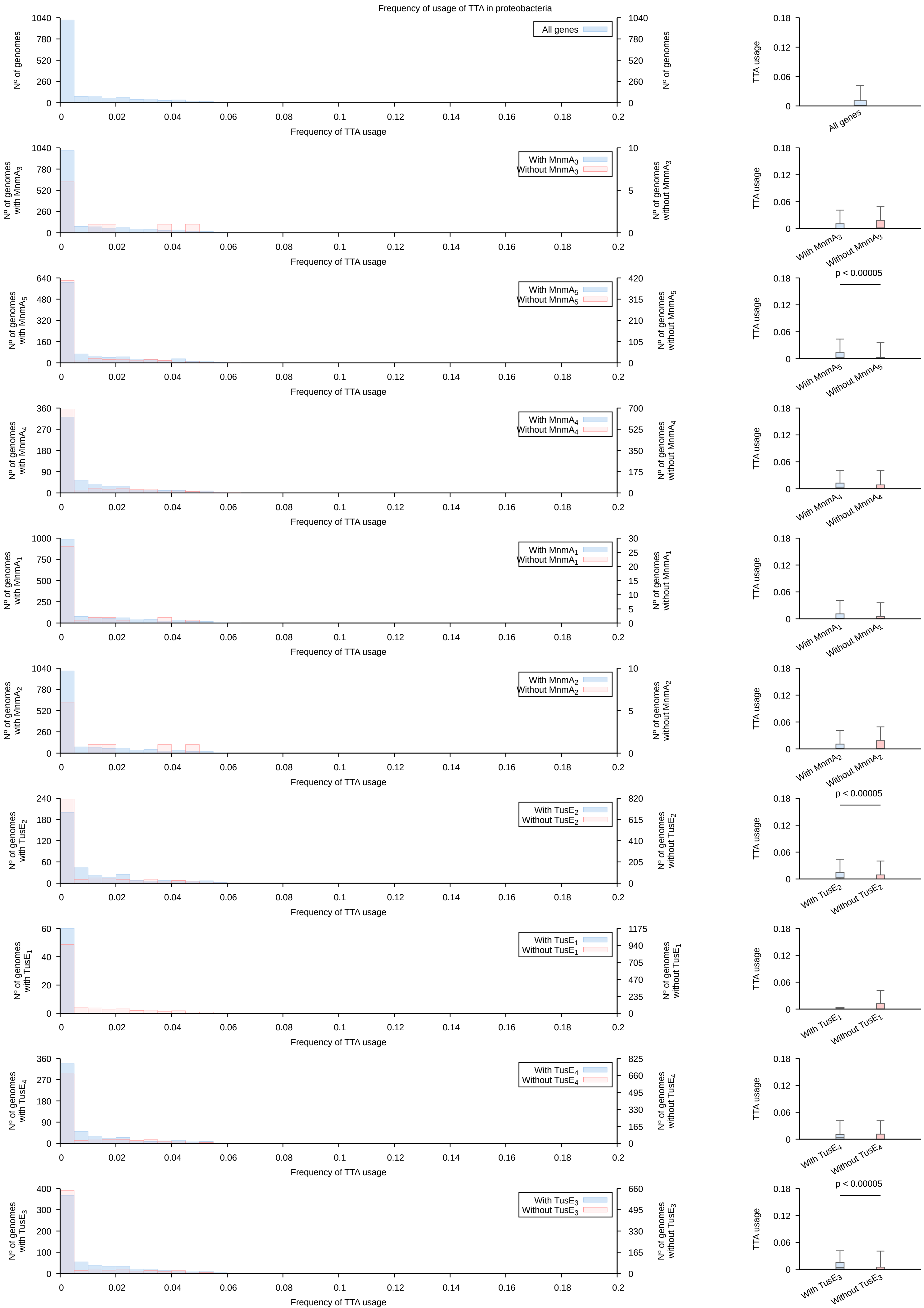

Frequency of usage of TTA in proteobacteria
Nº of genomes
Frequency of TTA usage
All genes
TTA usage
With MnmA3
Without MnmA3
Nº of genomes with MnmA3
Nº of genomes without MnmA3
With MnmA5
Without MnmA5
Nº of genomes with MnmA5
Nº of genomes without MnmA5
p < 0.00005
With MnmA4
Without MnmA4
Nº of genomes with MnmA4
Nº of genomes without MnmA4
With MnmA1
Without MnmA1
Nº of genomes with MnmA1
Nº of genomes without MnmA1
With MnmA2
Without MnmA2
Nº of genomes with MnmA2
Nº of genomes without MnmA2
With TusE2
Without TusE2
Nº of genomes with TusE2
Nº of genomes without TusE2
p < 0.00005
With TusE1
Without TusE1
Nº of genomes with TusE1
Nº of genomes without TusE1
With TusE4
Without TusE4
Nº of genomes with TusE4
Nº of genomes without TusE4
With TusE3
Without TusE3
Nº of genomes with TusE3
Nº of genomes without TusE3
p < 0.00005

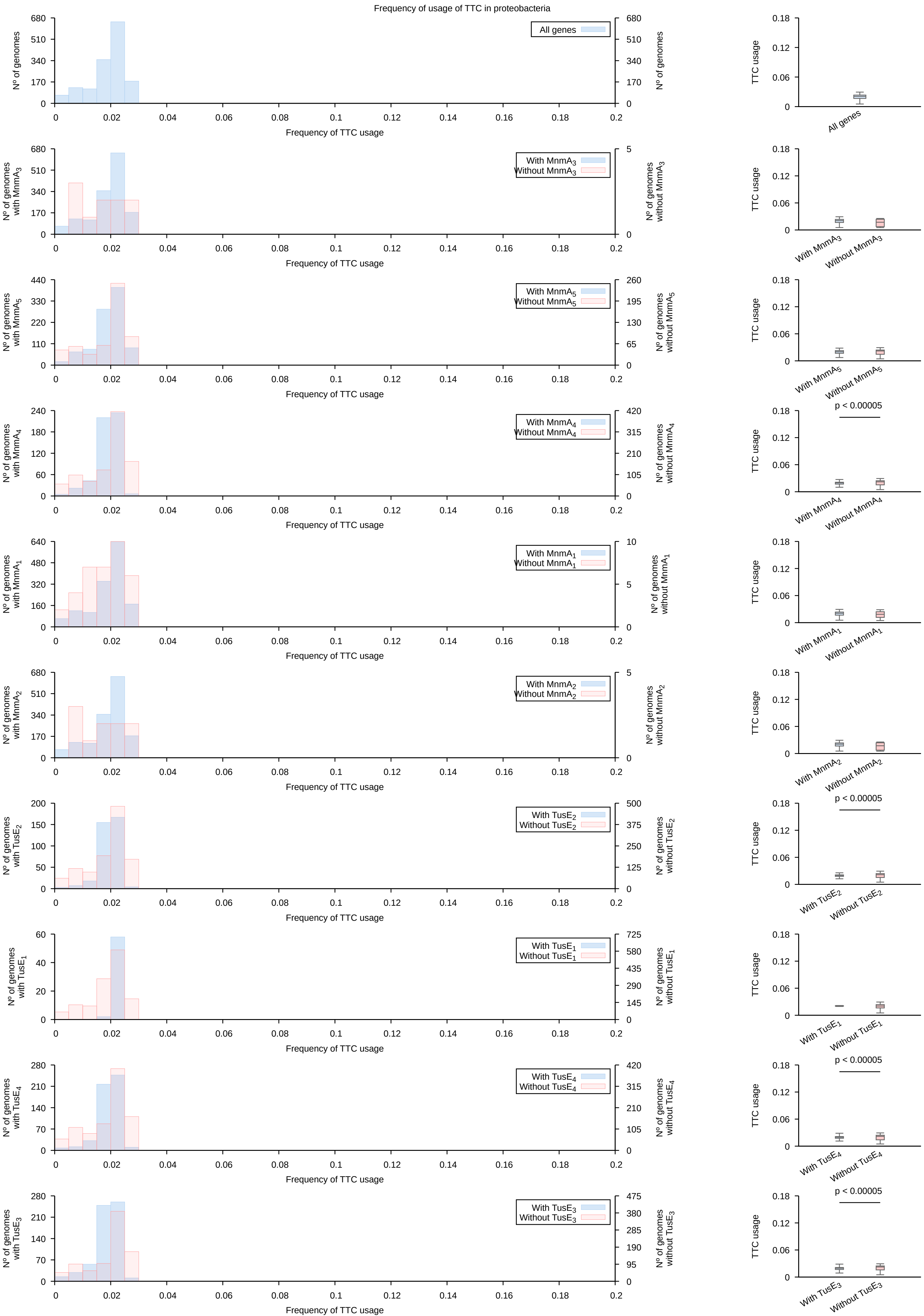

Frequency of usage of TTC in proteobacteria
Nº of genomes
All genes
Frequency of TTC usage
TTC usage
All genes
Nº of genomes with MnmA3
Nº of genomes without MnmA3
With MnmA3
Without MnmA3
Nº of genomes with MnmA5
Nº of genomes without MnmA5
With MnmA5
Without MnmA5
Nº of genomes with MnmA4
Nº of genomes without MnmA4
With MnmA4
Without MnmA4
p < 0.00005
Nº of genomes with MnmA1
Nº of genomes without MnmA1
With MnmA1
Without MnmA1
Nº of genomes with MnmA2
Nº of genomes without MnmA2
With MnmA2
Without MnmA2
Nº of genomes with TusE2
Nº of genomes without TusE2
With TusE2
Without TusE2
p < 0.00005
Nº of genomes with TusE1
Nº of genomes without TusE1
With TusE1
Without TusE1
Nº of genomes with TusE4
Nº of genomes without TusE4
With TusE4
Without TusE4
p < 0.00005
Nº of genomes with TusE3
Nº of genomes without TusE3
With TusE3
Without TusE3
p < 0.00005

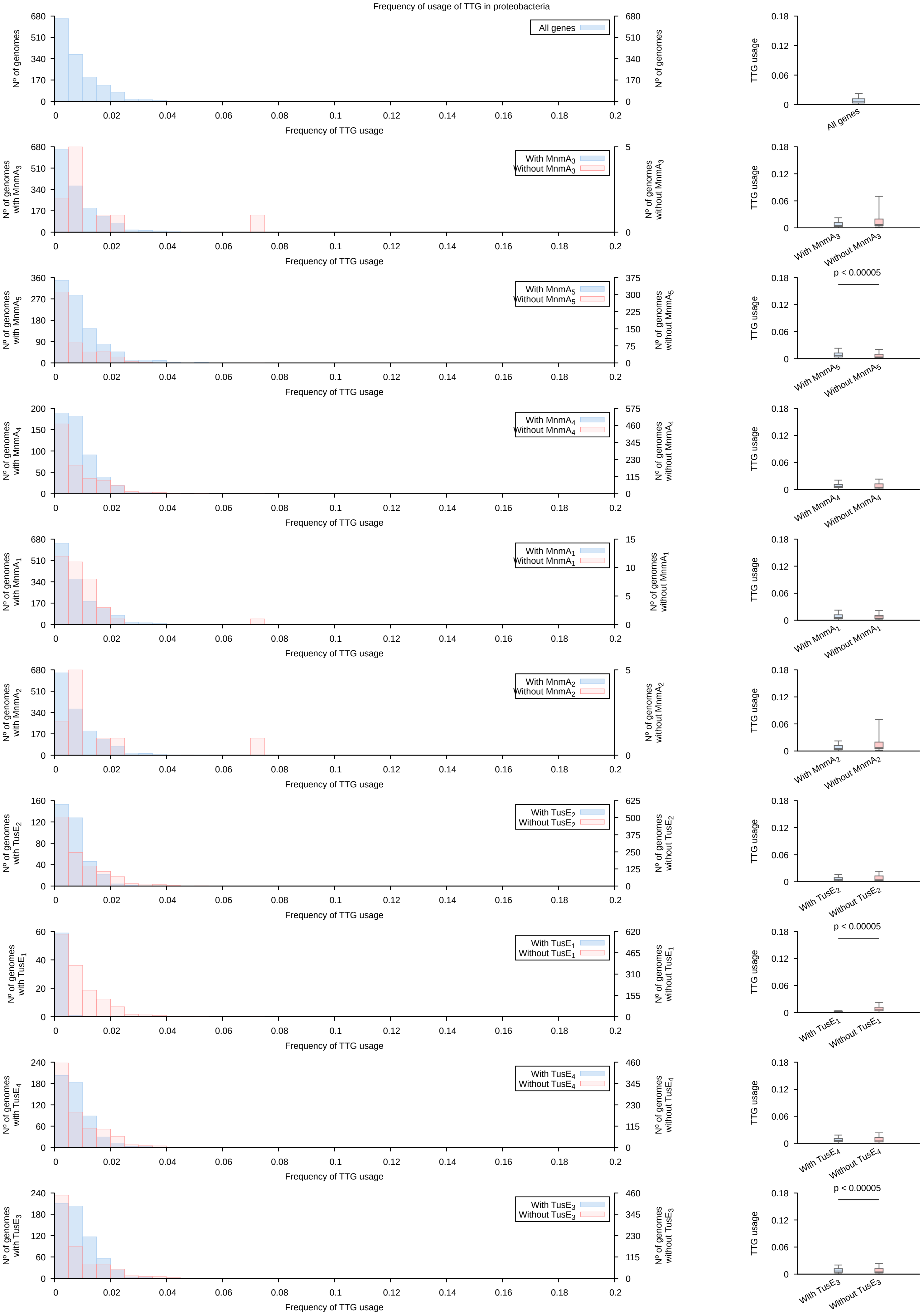
Frequency of usage of TTG in proteobacteria
Nº of genomes
All genes
Frequency of TTG usage
TTG usage
0
0.02
0.04
0.06
0.08
0.1
0.12
0.14
0.16
0.18
0.2
Nº of genomes with MnmA$_3$
Nº of genomes without MnmA$_3$
With MnmA$_3$
Without MnmA$_3$
Nº of genomes with MnmA$_5$
Nº of genomes without MnmA$_5$
With MnmA$_5$
Without MnmA$_5$
p < 0.00005
Nº of genomes with MnmA$_4$
Nº of genomes without MnmA$_4$
With MnmA$_4$
Without MnmA$_4$
Nº of genomes with MnmA$_1$
Nº of genomes without MnmA$_1$
With MnmA$_1$
Without MnmA$_1$
Nº of genomes with MnmA$_2$
Nº of genomes without MnmA$_2$
With MnmA$_2$
Without MnmA$_2$
Nº of genomes with TusE$_2$
Nº of genomes without TusE$_2$
With TusE$_2$
Without TusE$_2$
Nº of genomes with TusE$_1$
Nº of genomes without TusE$_1$
With TusE$_1$
Without TusE$_1$
p < 0.00005
Nº of genomes with TusE$_4$
Nº of genomes without TusE$_4$
With TusE$_4$
Without TusE$_4$
Nº of genomes with TusE$_3$
Nº of genomes without TusE$_3$
With TusE$_3$
Without TusE$_3$
p < 0.00005

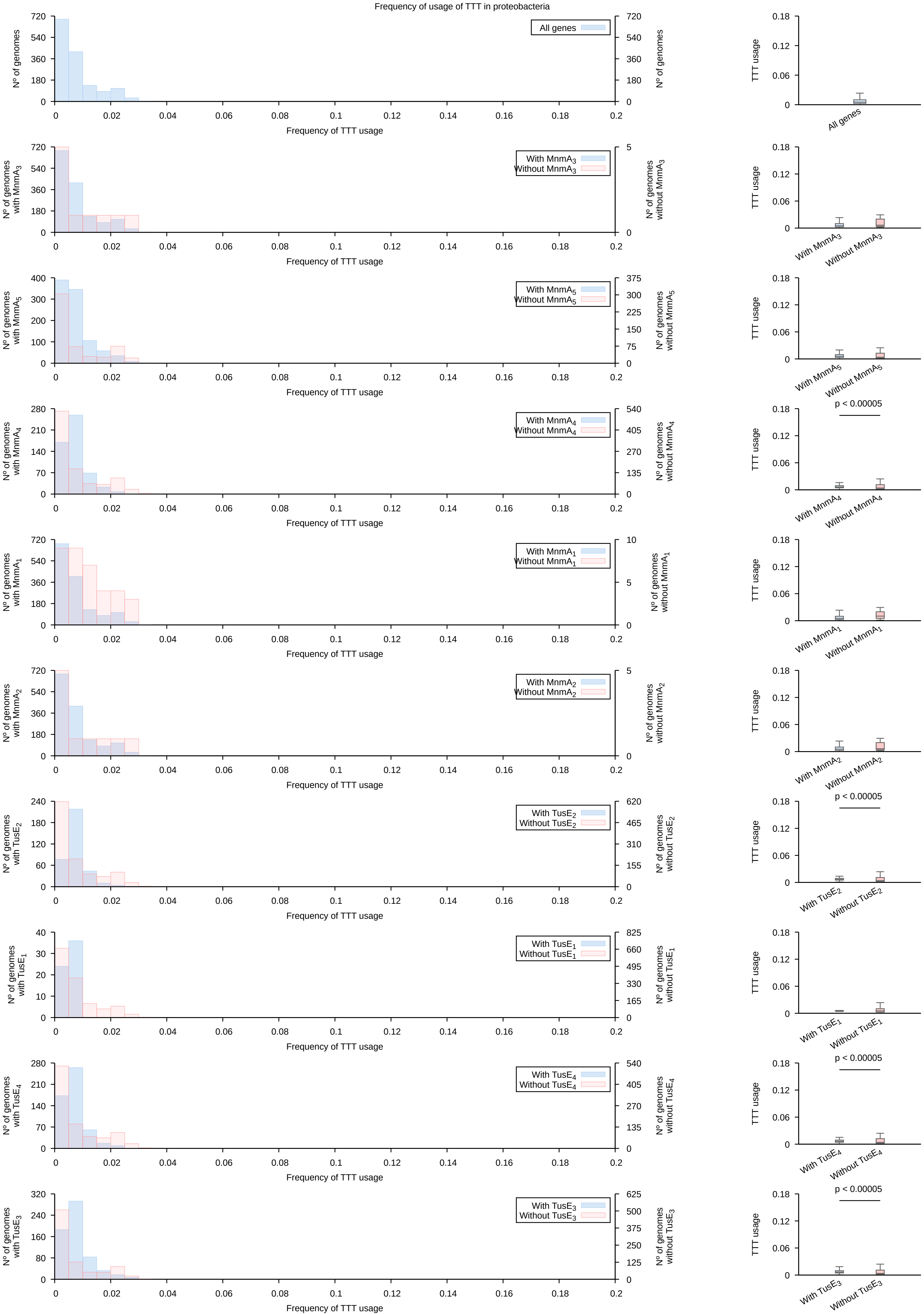

Frequency of usage of TTT in proteobacteria
All genes
Nº of genomes
Frequency of TTT usage
TTT usage
With MnmA3
Without MnmA3
With MnmA5
Without MnmA5
With MnmA4
Without MnmA4
With MnmA1
Without MnmA1
With MnmA2
Without MnmA2
With TusE2
Without TusE2
With TusE1
Without TusE1
With TusE4
Without TusE4
With TusE3
Without TusE3
p < 0.00005

Figure S23. The frequency of usage of some codons is independent of genomic GC%. The frequency of usage of each codon in 1484 genomes was compared to the GC% of the corresponding genome considering codon usage in all genes of genomes (purple dots) or in a set of genes expected to be highly expressed (green dots). Although some distributions are not perfectly linear, linear regressions (light blue and orange lines) were used to allow approximate comparisons of the impact of GC% on codon usage based on the observed slopes.

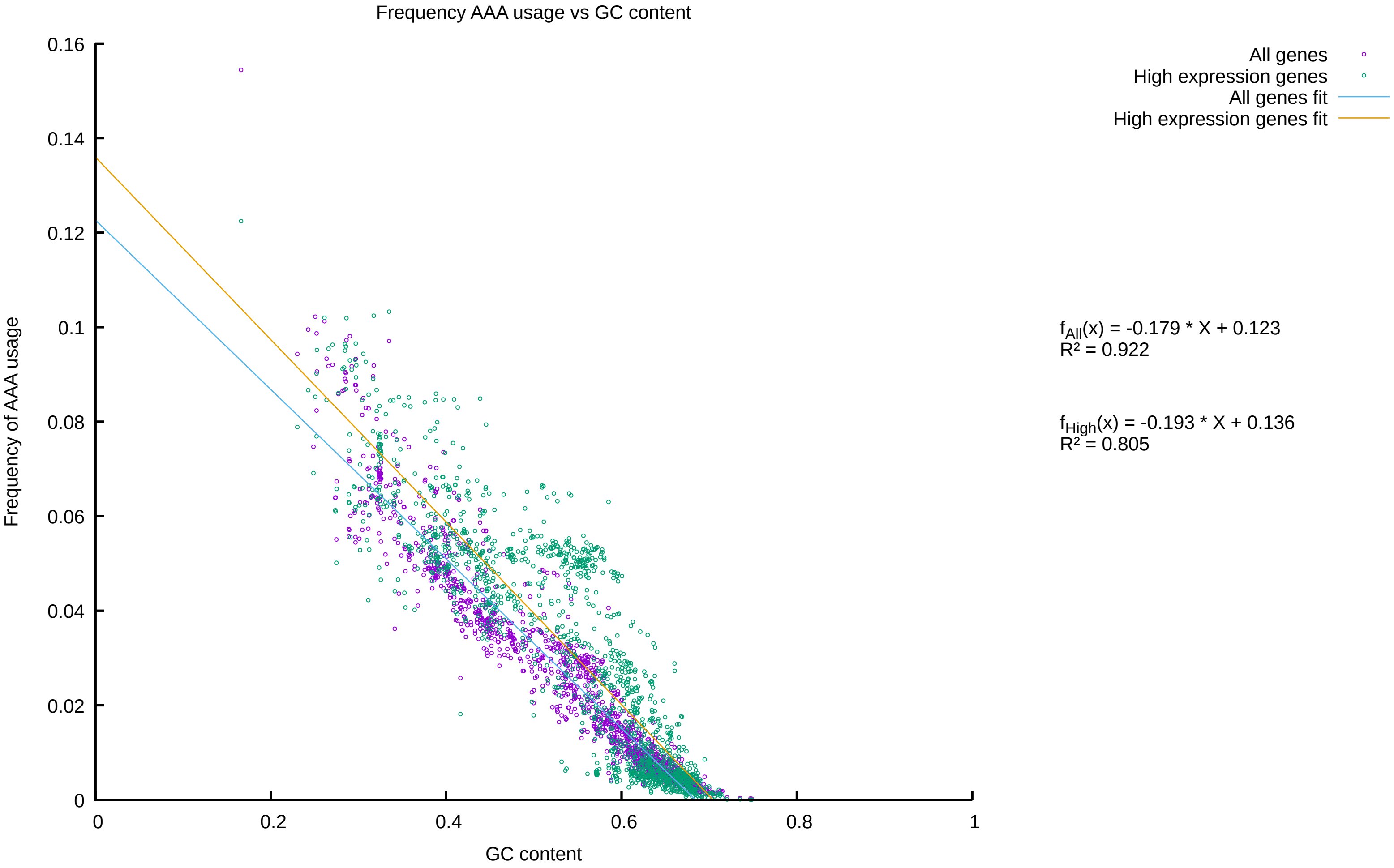
Frequency AAA usage vs GC content
0.16
0.14
0.12
0.1
0.08
0.06
0.04
0.02
0
Frequency of AAA usage
0
0.2
0.4
0.6
0.8
1
GC content
All genes
High expression genes
All genes fit
High expression genes fit
$f_{All}(x) = -0.179 * X + 0.123$
$R^2 = 0.922$
$f_{High}(x) = -0.193 * X + 0.136$
$R^2 = 0.805$

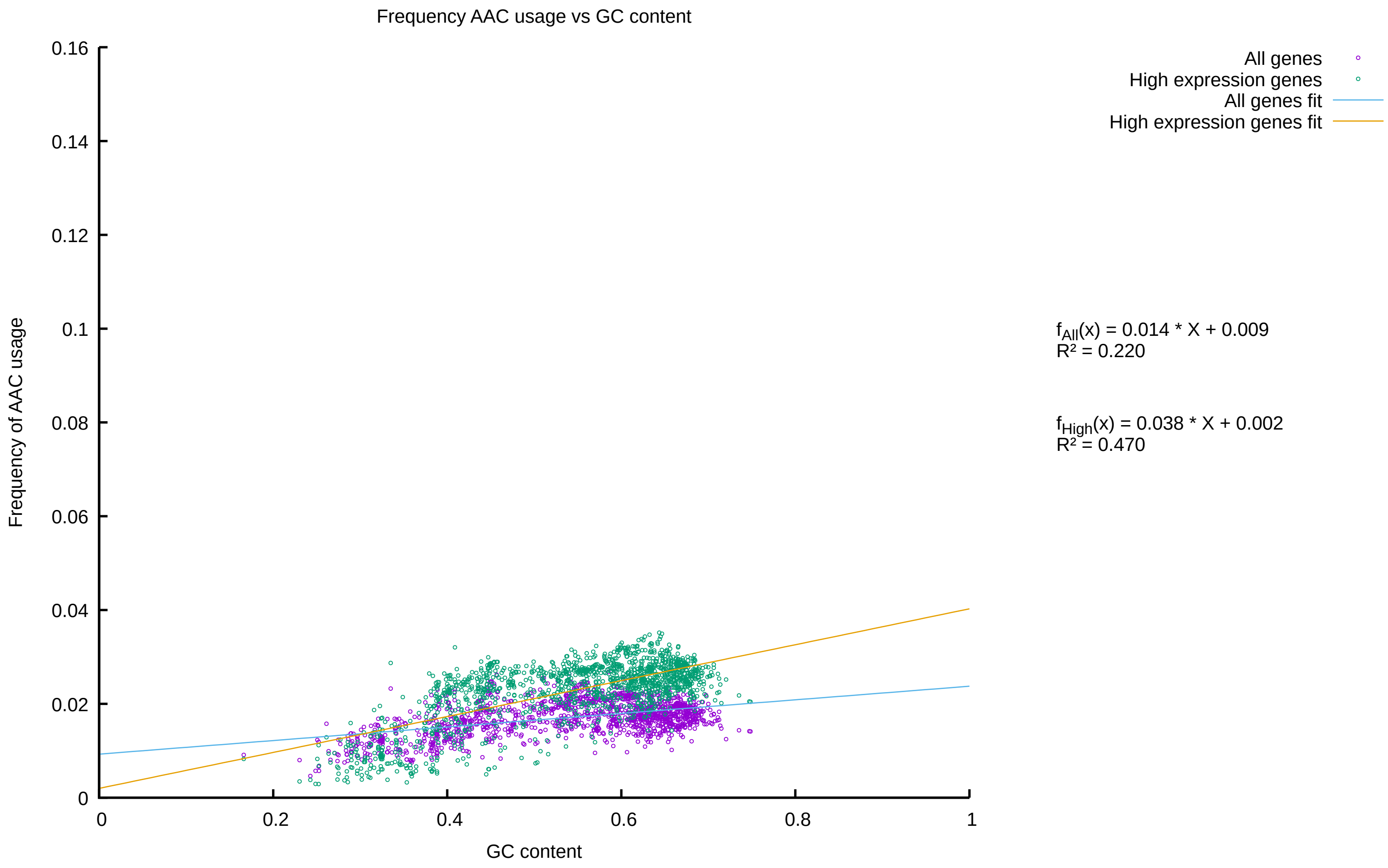
Frequency AAC usage vs GC content
All genes
High expression genes
All genes fit
High expression genes fit
$f_{All}(x) = 0.014 * X + 0.009$
$R^2 = 0.220$
$f_{High}(x) = 0.038 * X + 0.002$
$R^2 = 0.470$
Frequency of AAC usage
GC content
0.16
0.14
0.12
0.1
0.08
0.06
0.04
0.02
0
0
0.2
0.4
0.6
0.8
1

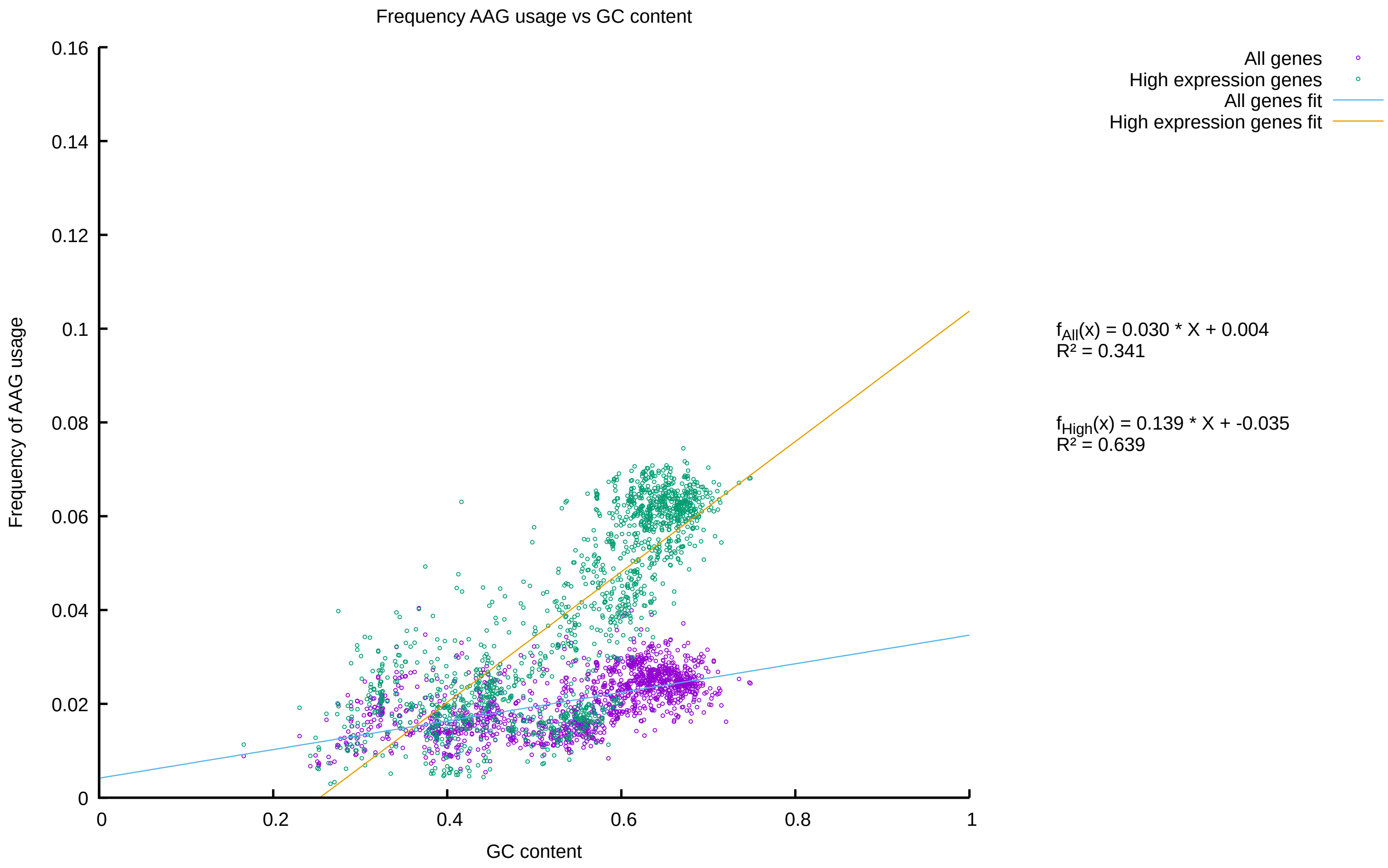
Frequency AAG usage vs GC content
All genes
High expression genes
All genes fit
High expression genes fit
$f_{All}(x) = 0.030 * X + 0.004$
$R^2 = 0.341$
$f_{High}(x) = 0.139 * X + -0.035$
$R^2 = 0.639$
Frequency of AAG usage
GC content
0
0.02
0.04
0.06
0.08
0.1
0.12
0.14
0.16
0.2
0.4
0.6
0.8
1

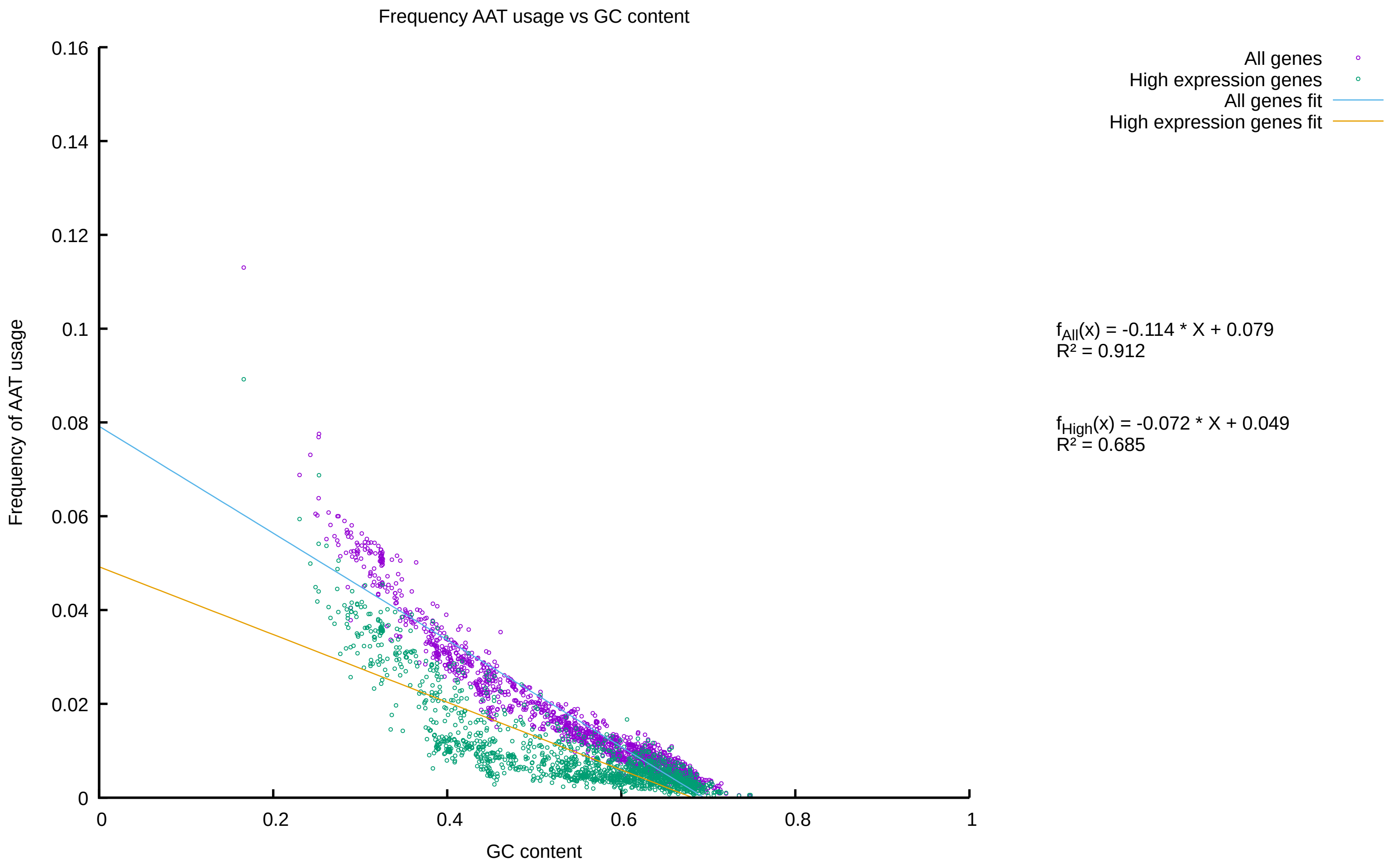

Frequency AAT usage vs GC content
0.16
0.14
0.12
0.1
0.08
0.06
0.04
0.02
0
Frequency of AAT usage
0
0.2
0.4
0.6
0.8
1
GC content
All genes
High expression genes
All genes fit
High expression genes fit
f_All(x) = -0.114 * X + 0.079
R² = 0.912
f_High(x) = -0.072 * X + 0.049
R² = 0.685

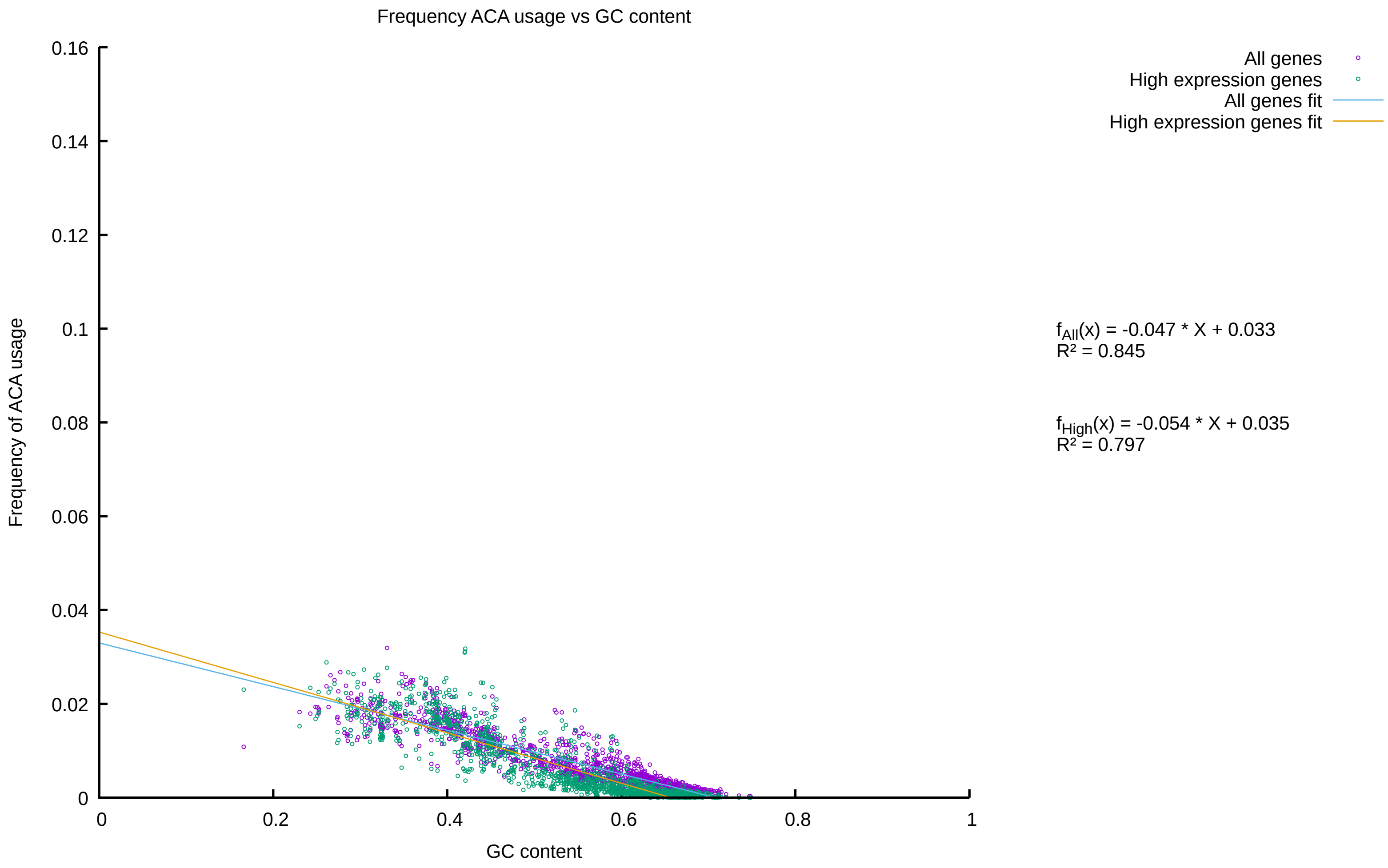
Frequency ACA usage vs GC content
Frequency of ACA usage
GC content
0.16
0.14
0.12
0.1
0.08
0.06
0.04
0.02
0
0
0.2
0.4
0.6
0.8
1
All genes
High expression genes
All genes fit
High expression genes fit
f_All(x) = -0.047 * X + 0.033
R² = 0.845
f_High(x) = -0.054 * X + 0.035
R² = 0.797

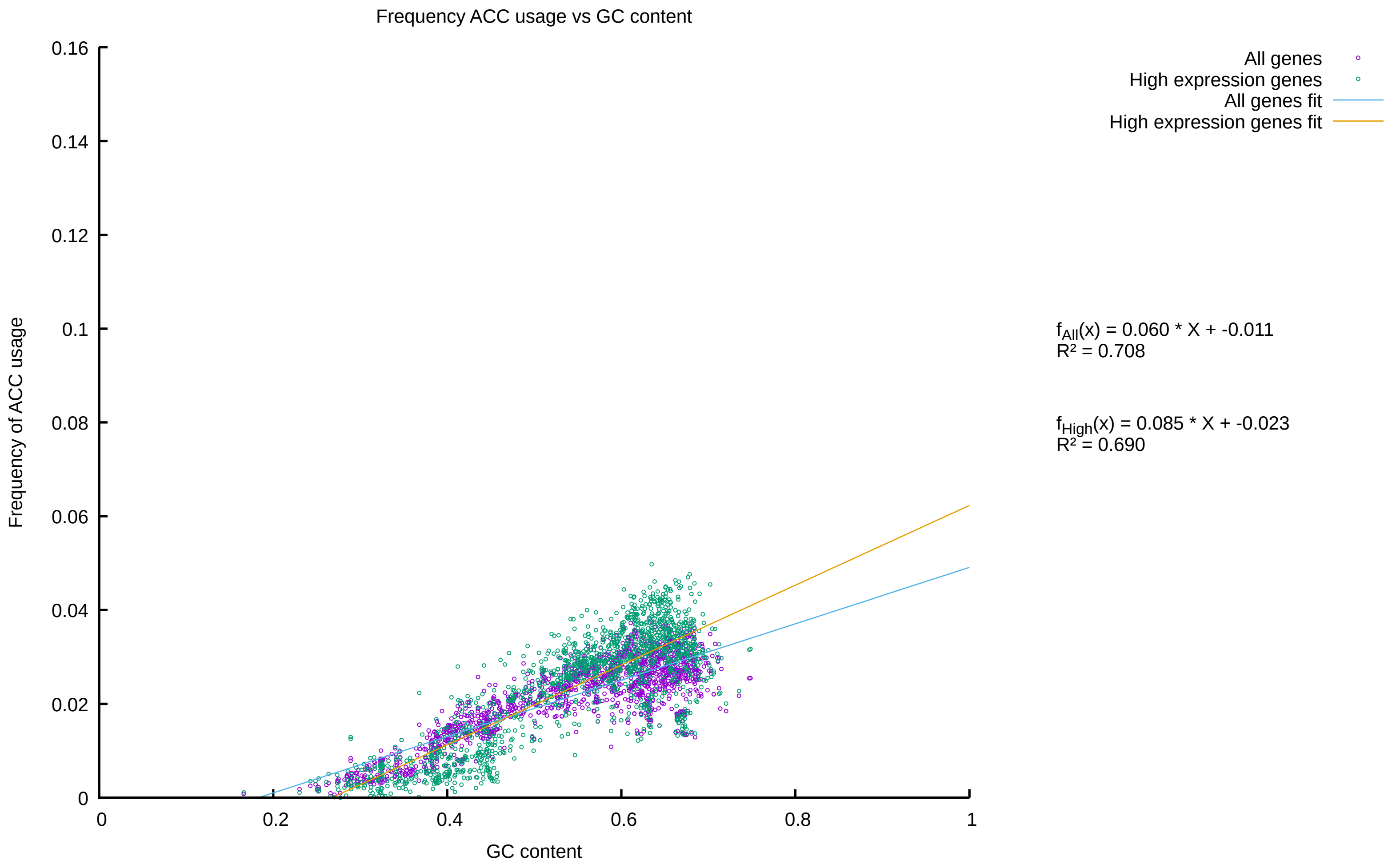

Frequency ACC usage vs GC content
0.16
0.14
0.12
0.1
0.08
0.06
0.04
0.02
0
0
0.2
0.4
0.6
0.8
1
GC content
Frequency of ACC usage
All genes
High expression genes
All genes fit
High expression genes fit
$f_{All}(x) = 0.060 * X + -0.011$
$R^2 = 0.708$
$f_{High}(x) = 0.085 * X + -0.023$
$R^2 = 0.690$

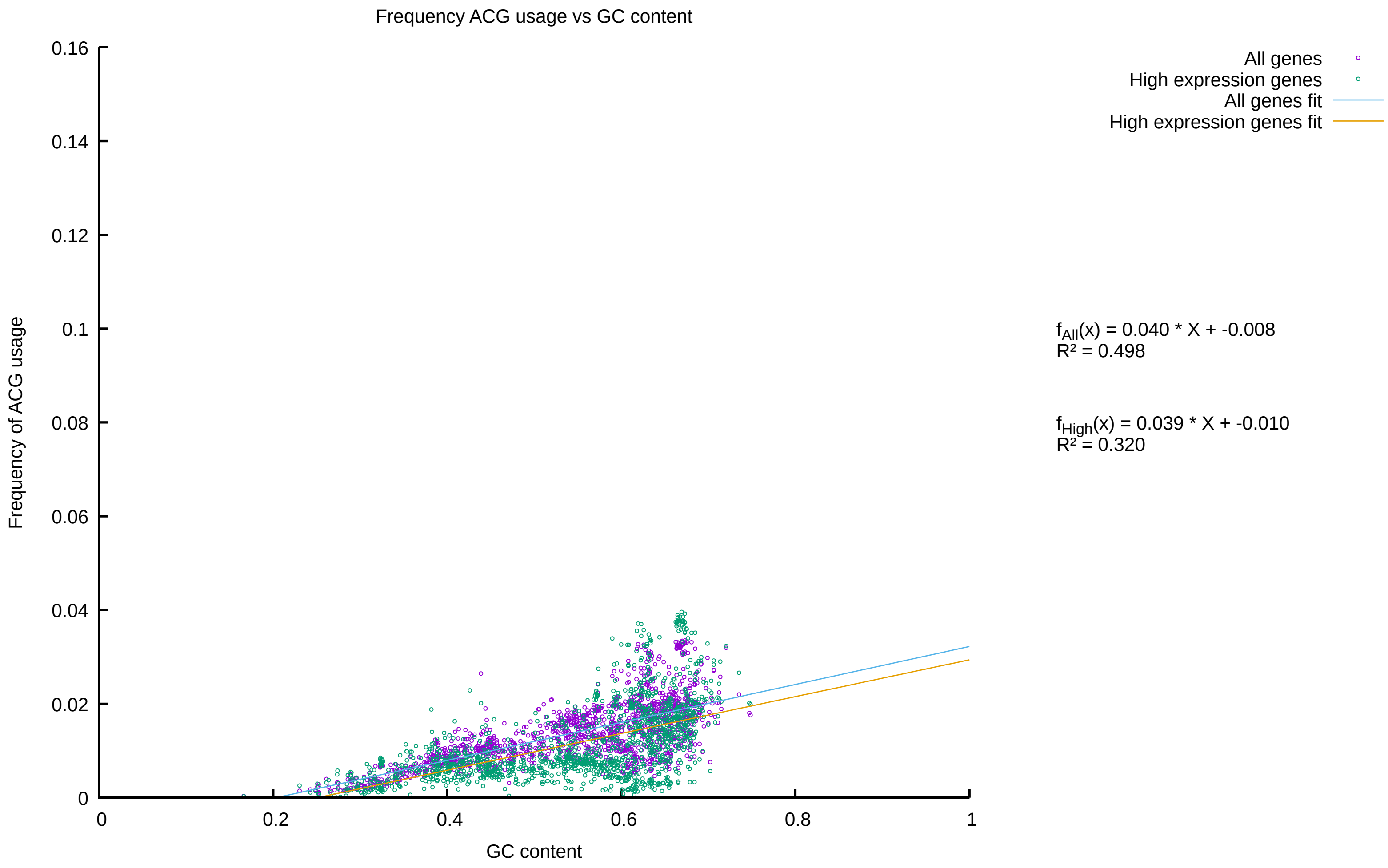
Frequency ACG usage vs GC content
Frequency of ACG usage
GC content
0
0.02
0.04
0.06
0.08
0.1
0.12
0.14
0.16
0
0.2
0.4
0.6
0.8
1
All genes
High expression genes
All genes fit
High expression genes fit
$f_{All}(x) = 0.040 * X + -0.008$
$R^2 = 0.498$
$f_{High}(x) = 0.039 * X + -0.010$
$R^2 = 0.320$

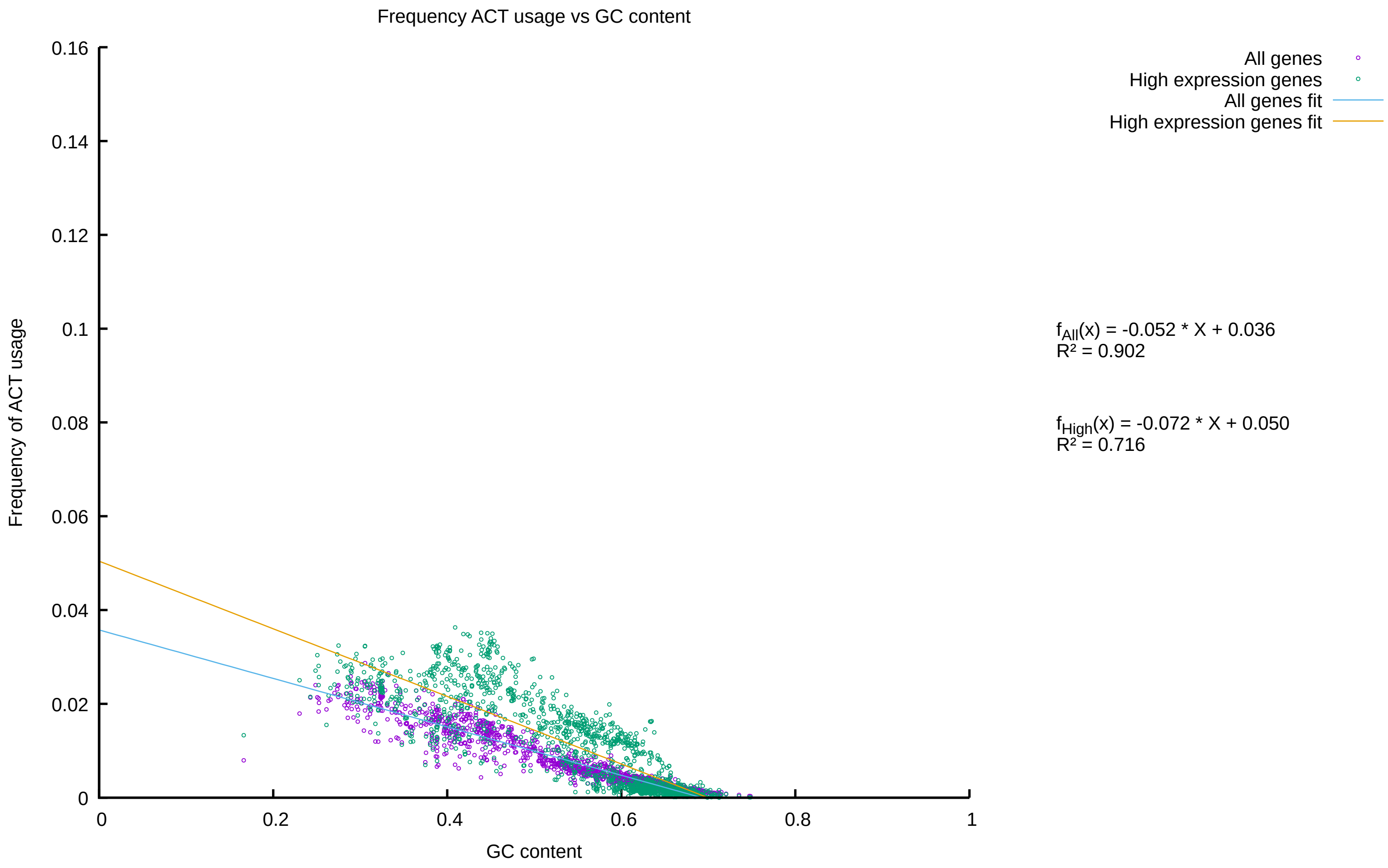

Frequency ACT usage vs GC content
0.16
0.14
0.12
0.1
0.08
0.06
0.04
0.02
0
Frequency of ACT usage
0
0.2
0.4
0.6
0.8
1
GC content
All genes
High expression genes
All genes fit
High expression genes fit
$f_{All}(x) = -0.052 * X + 0.036$
$R^2 = 0.902$
$f_{High}(x) = -0.072 * X + 0.050$
$R^2 = 0.716$

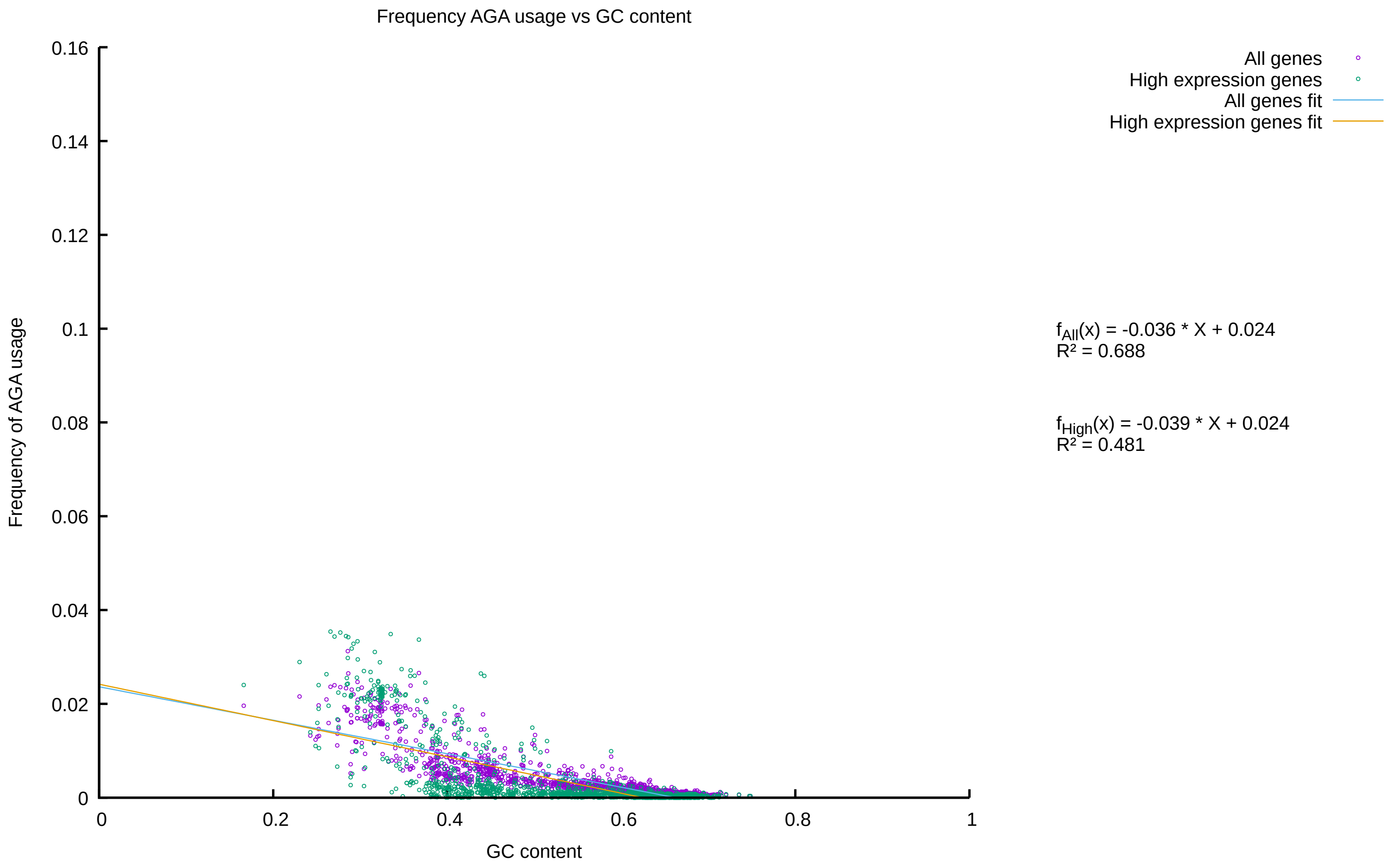

Frequency AGA usage vs GC content
Frequency of AGA usage
GC content
0.16
0.14
0.12
0.1
0.08
0.06
0.04
0.02
0
0
0.2
0.4
0.6
0.8
1
All genes
High expression genes
All genes fit
High expression genes fit
$f_{All}(x) = -0.036 * X + 0.024$
$R^2 = 0.688$
$f_{High}(x) = -0.039 * X + 0.024$
$R^2 = 0.481$

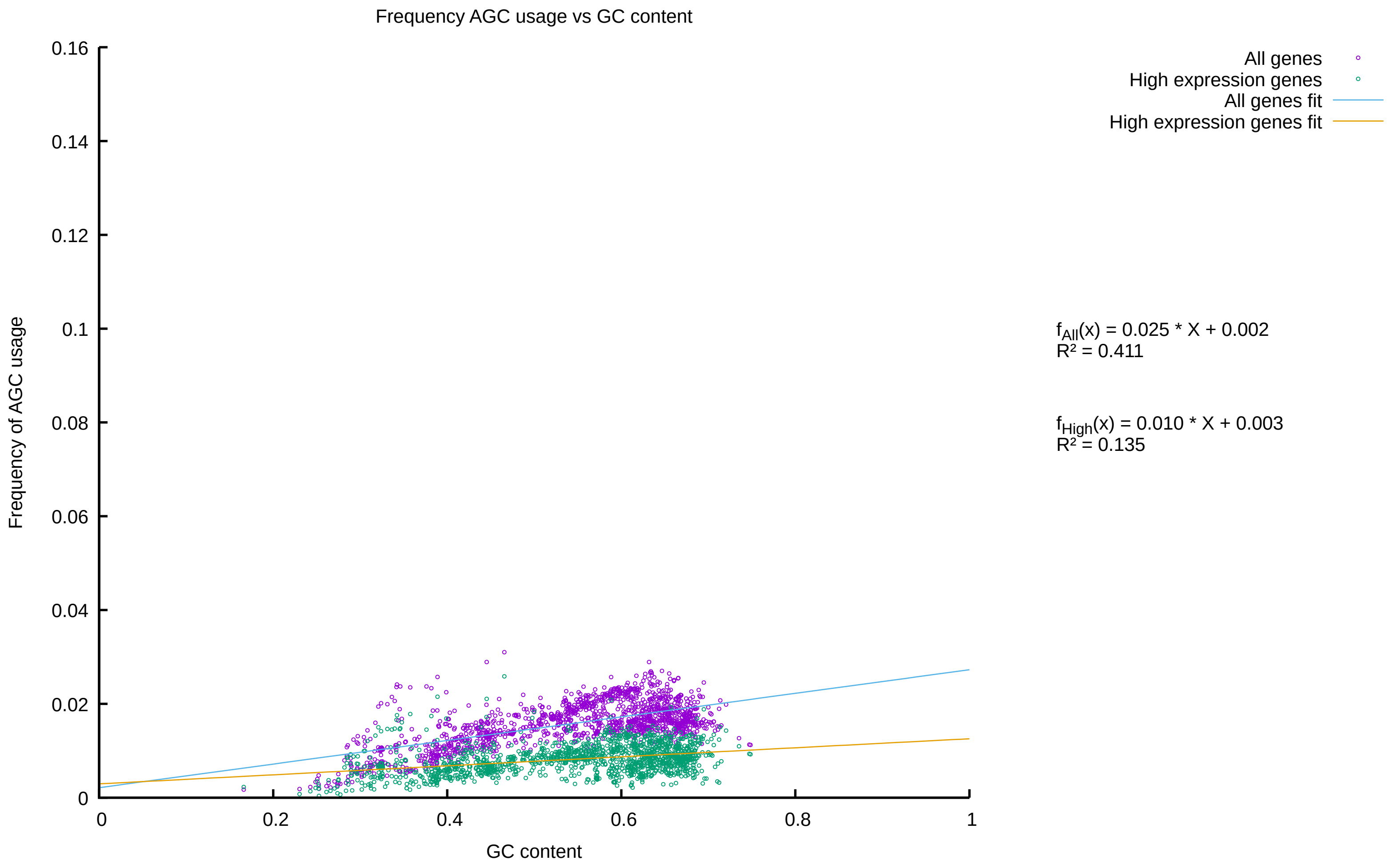
Frequency AGC usage vs GC content
0.16
0.14
0.12
0.1
0.08
0.06
0.04
0.02
0
Frequency of AGC usage
0
0.2
0.4
0.6
0.8
1
GC content
All genes
High expression genes
All genes fit
High expression genes fit
$f_{All}(x) = 0.025 * X + 0.002$
$R^2 = 0.411$
$f_{High}(x) = 0.010 * X + 0.003$
$R^2 = 0.135$

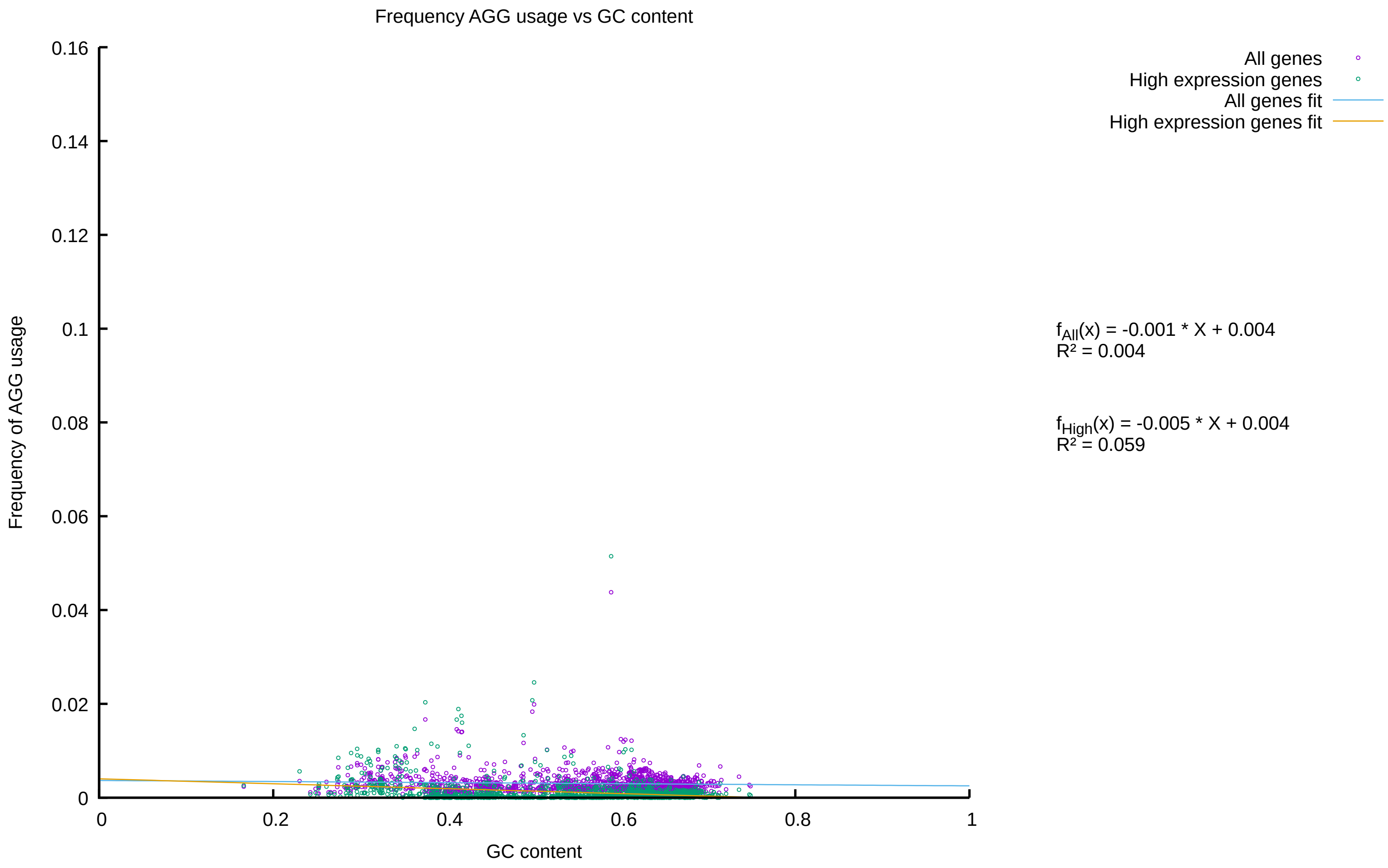

$f_{All}(x) = -0.001 * X + 0.004$
$R^2 = 0.004$

$f_{High}(x) = -0.005 * X + 0.004$
$R^2 = 0.059$

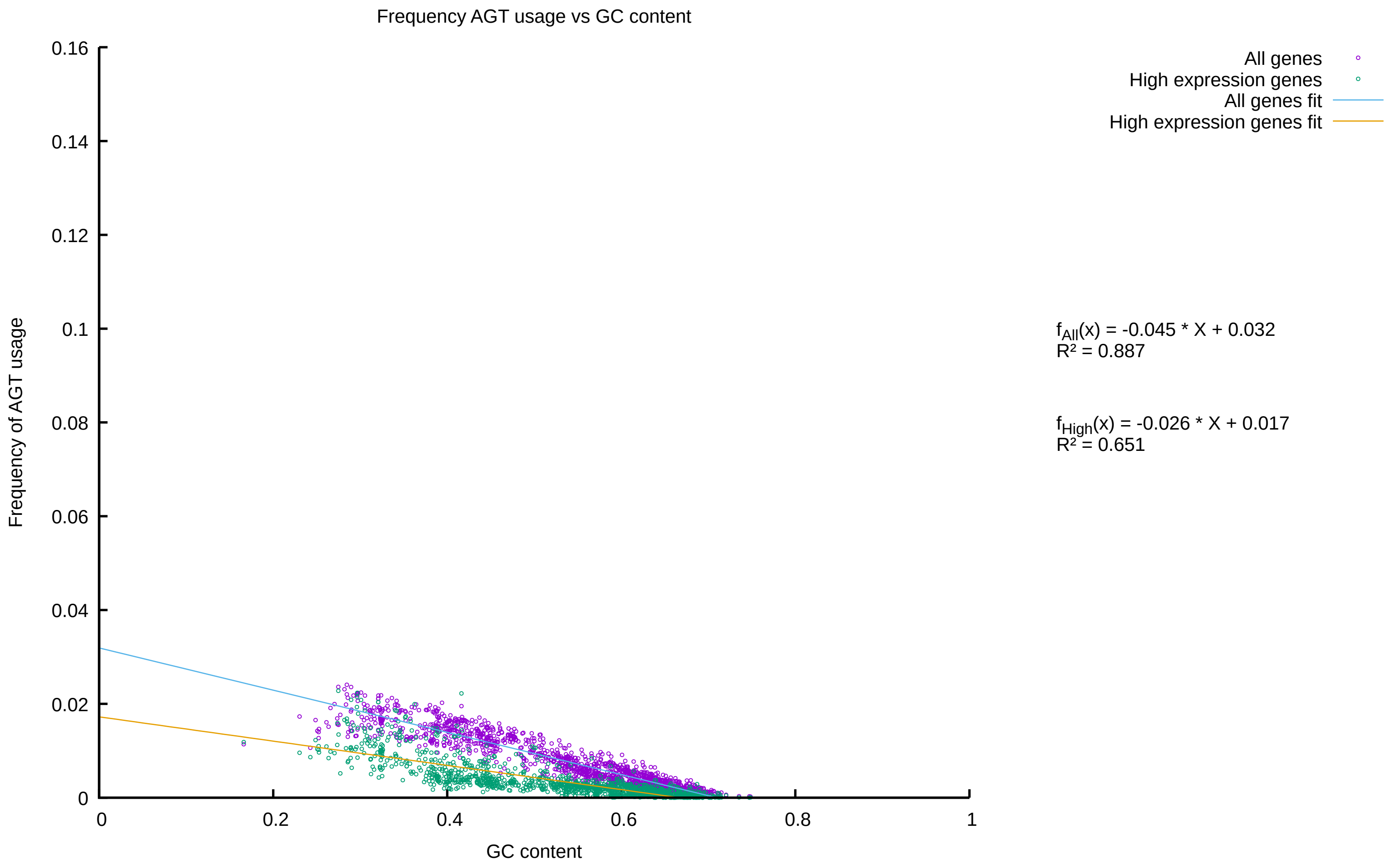
Frequency AGT usage vs GC content
0.16
0.14
0.12
0.1
0.08
0.06
0.04
0.02
0
0
0.2
0.4
0.6
0.8
1
GC content
Frequency of AGT usage
All genes
High expression genes
All genes fit
High expression genes fit
$f_{All}(x) = -0.045 * X + 0.032$
$R^2 = 0.887$
$f_{High}(x) = -0.026 * X + 0.017$
$R^2 = 0.651$

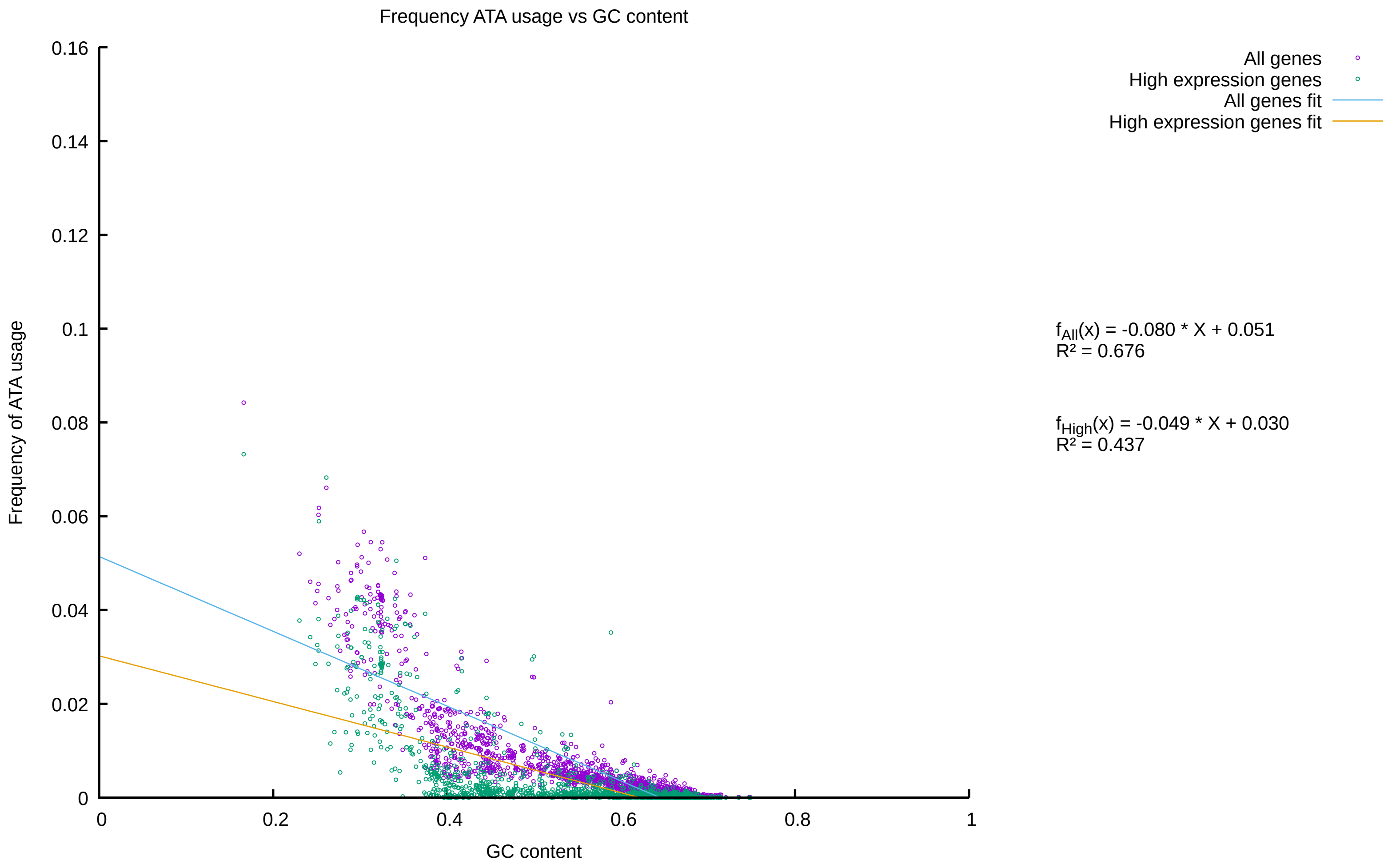

Frequency ATA usage vs GC content
Frequency of ATA usage
GC content
0.16
0.14
0.12
0.1
0.08
0.06
0.04
0.02
0
0.2
0.4
0.6
0.8
1
All genes
High expression genes
All genes fit
High expression genes fit
f_All(x) = -0.080 * X + 0.051
R² = 0.676
f_High(x) = -0.049 * X + 0.030
R² = 0.437

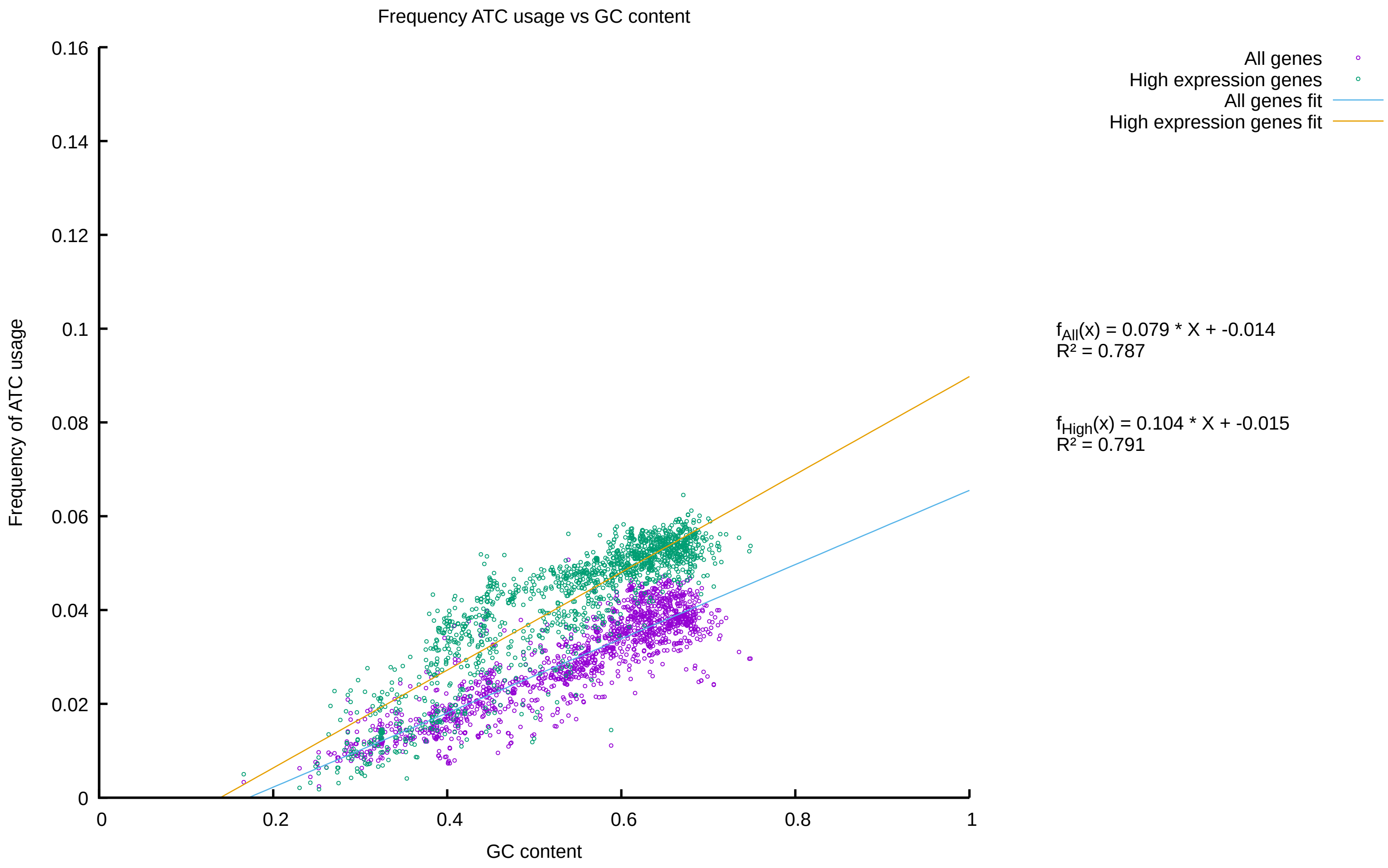

Frequency ATC usage vs GC content
All genes
High expression genes
All genes fit
High expression genes fit
fAll(x) = 0.079 * X + -0.014
R² = 0.787
fHigh(x) = 0.104 * X + -0.015
R² = 0.791
Frequency of ATC usage
GC content
0
0.02
0.04
0.06
0.08
0.1
0.12
0.14
0.16
0
0.2
0.4
0.6
0.8
1

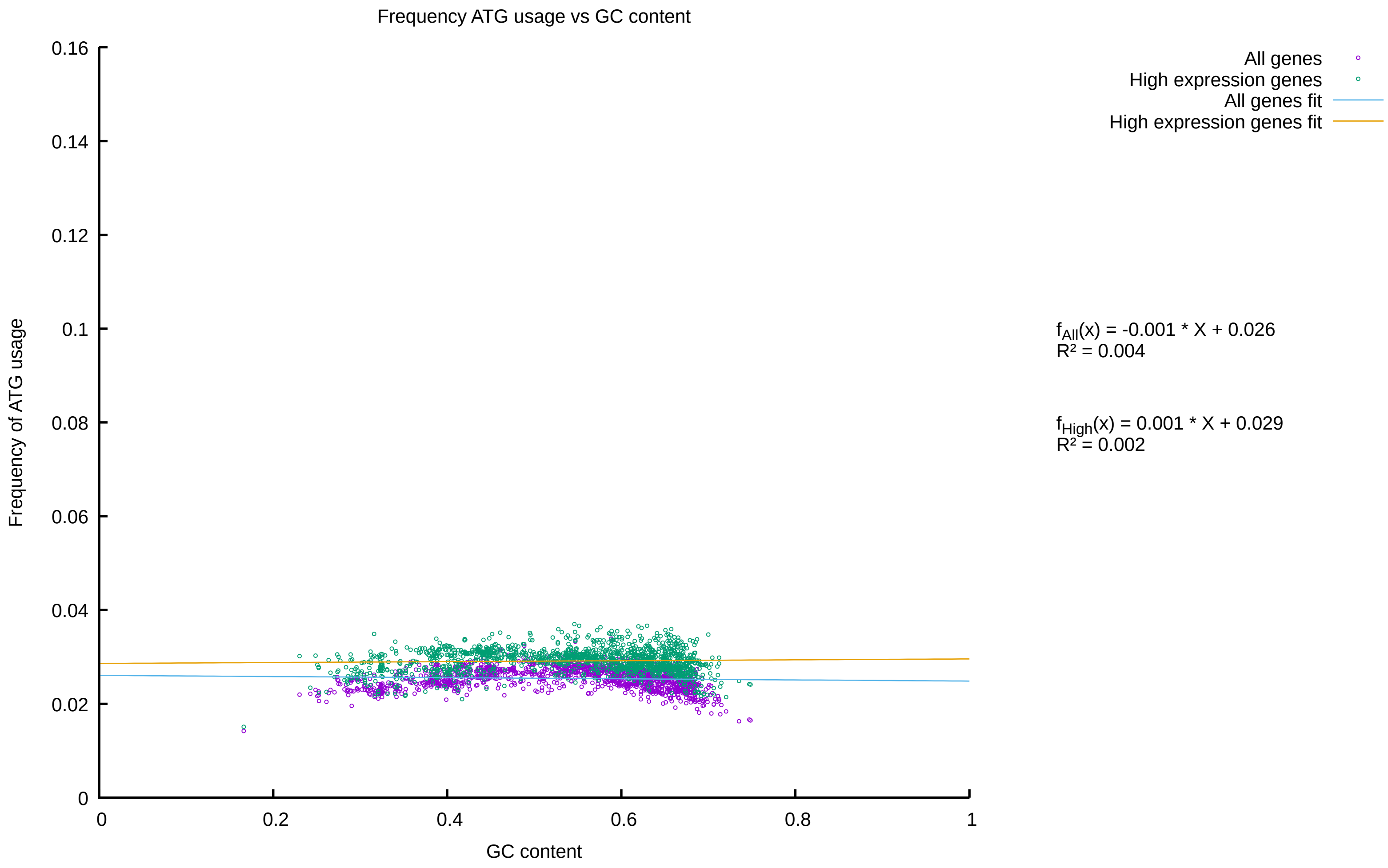
Frequency ATG usage vs GC content
Frequency of ATG usage
GC content
0.16
0.14
0.12
0.1
0.08
0.06
0.04
0.02
0
0
0.2
0.4
0.6
0.8
1
All genes
High expression genes
All genes fit
High expression genes fit
$f_{All}(x) = -0.001 * X + 0.026$
$R^2 = 0.004$
$f_{High}(x) = 0.001 * X + 0.029$
$R^2 = 0.002$

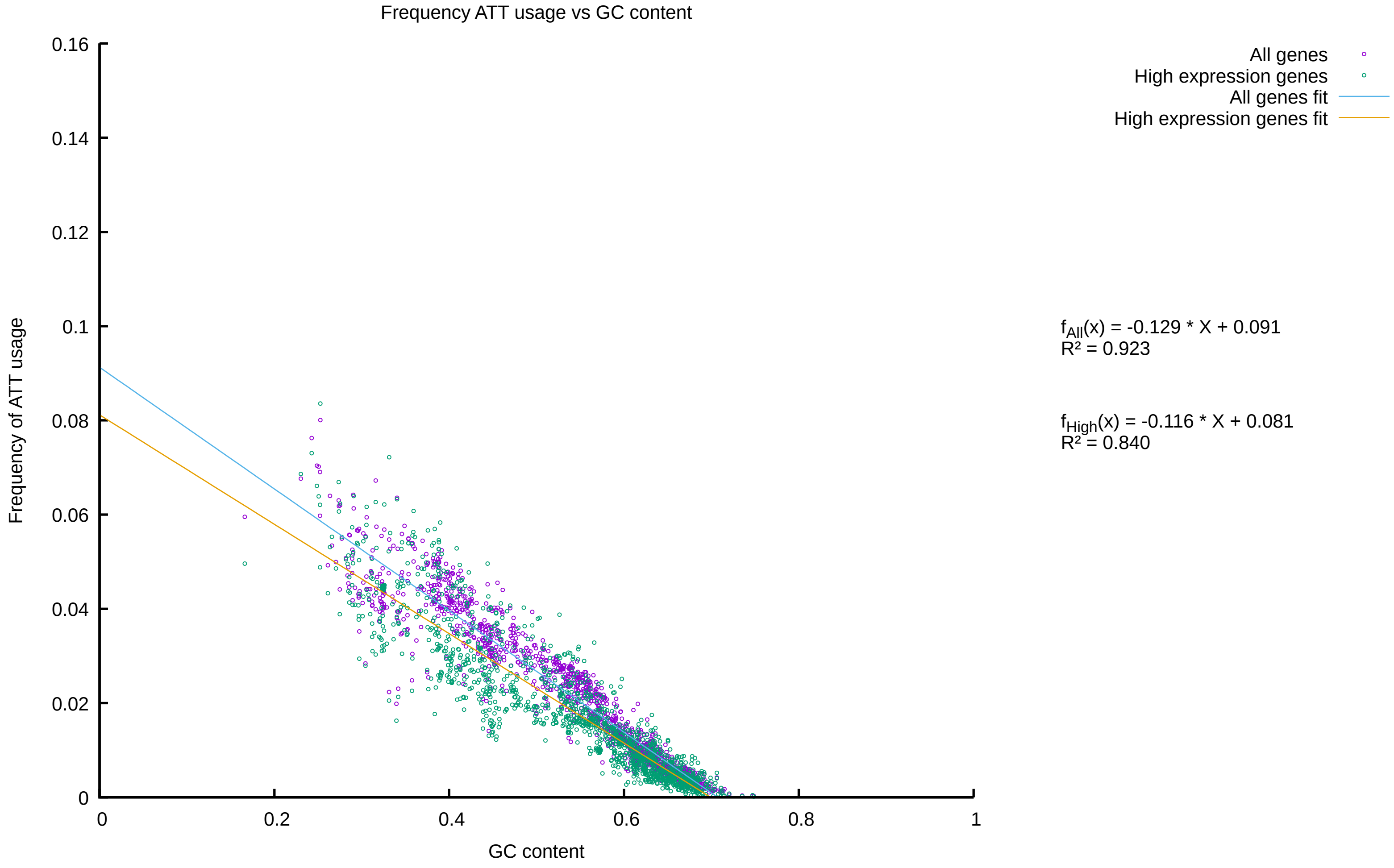

$f_{All}(x) = -0.129 * X + 0.091$
$R^2 = 0.923$

$f_{High}(x) = -0.116 * X + 0.081$
$R^2 = 0.840$

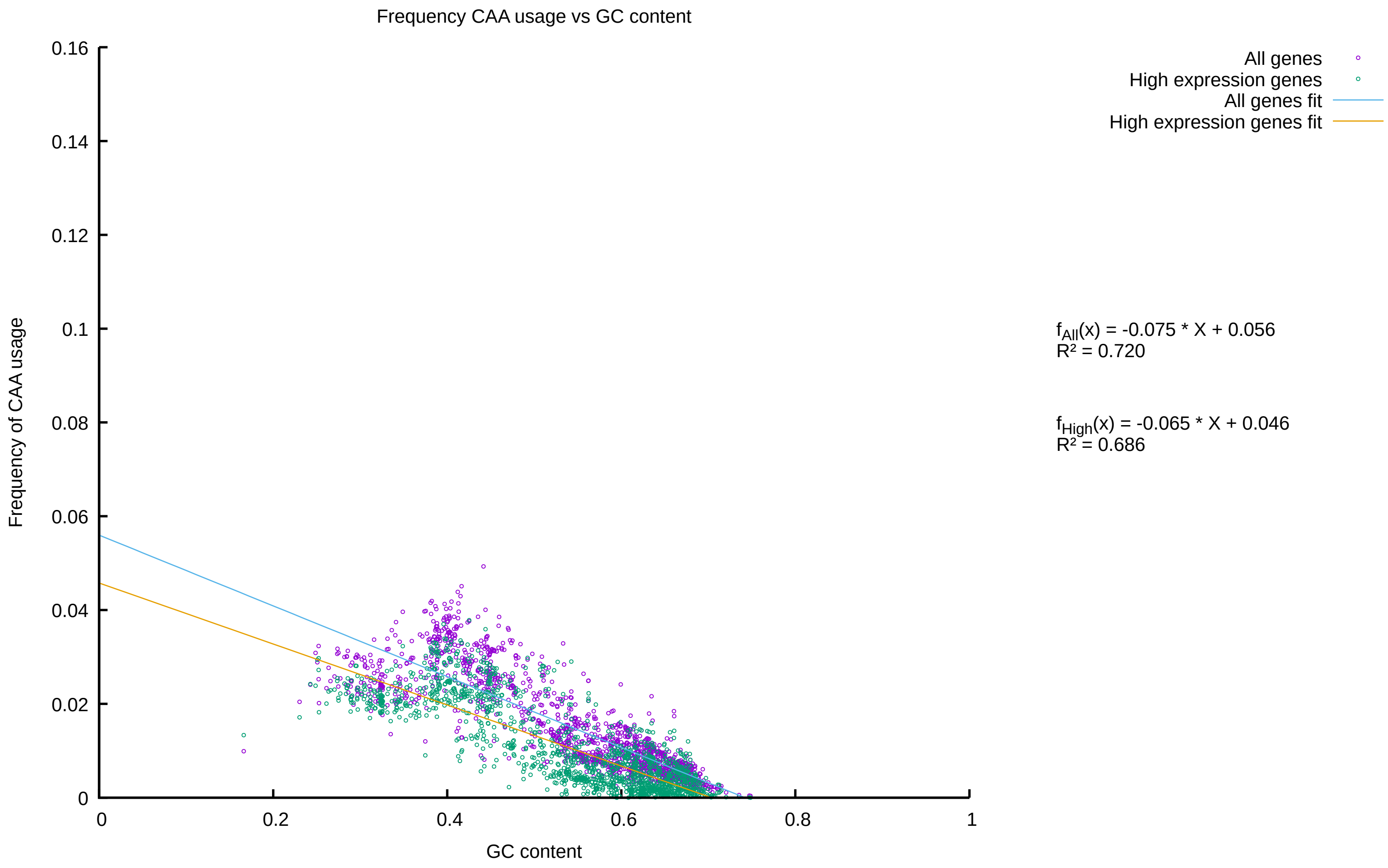
Frequency CAA usage vs GC content
Frequency of CAA usage
0.16
0.14
0.12
0.1
0.08
0.06
0.04
0.02
0
0.2
0.4
0.6
0.8
1
GC content
All genes
High expression genes
All genes fit
High expression genes fit
$f_{All}(x) = -0.075 * X + 0.056$
$R^2 = 0.720$
$f_{High}(x) = -0.065 * X + 0.046$
$R^2 = 0.686$

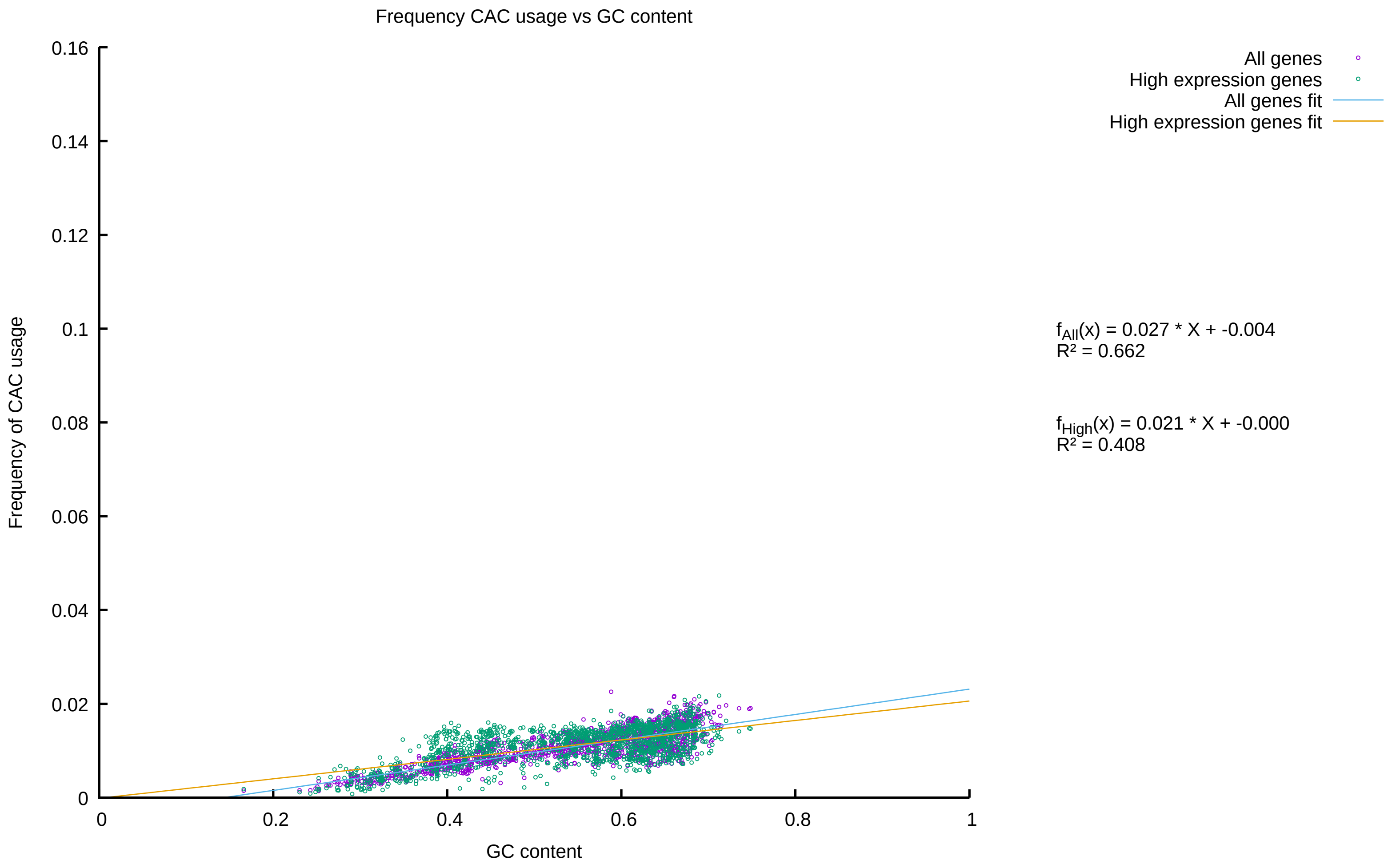

Frequency CAC usage vs GC content
Frequency of CAC usage
GC content
0.16
0.14
0.12
0.1
0.08
0.06
0.04
0.02
0
0
0.2
0.4
0.6
0.8
1
All genes
High expression genes
All genes fit
High expression genes fit
$f_{All}(x) = 0.027 * X + -0.004$
$R^2 = 0.662$
$f_{High}(x) = 0.021 * X + -0.000$
$R^2 = 0.408$

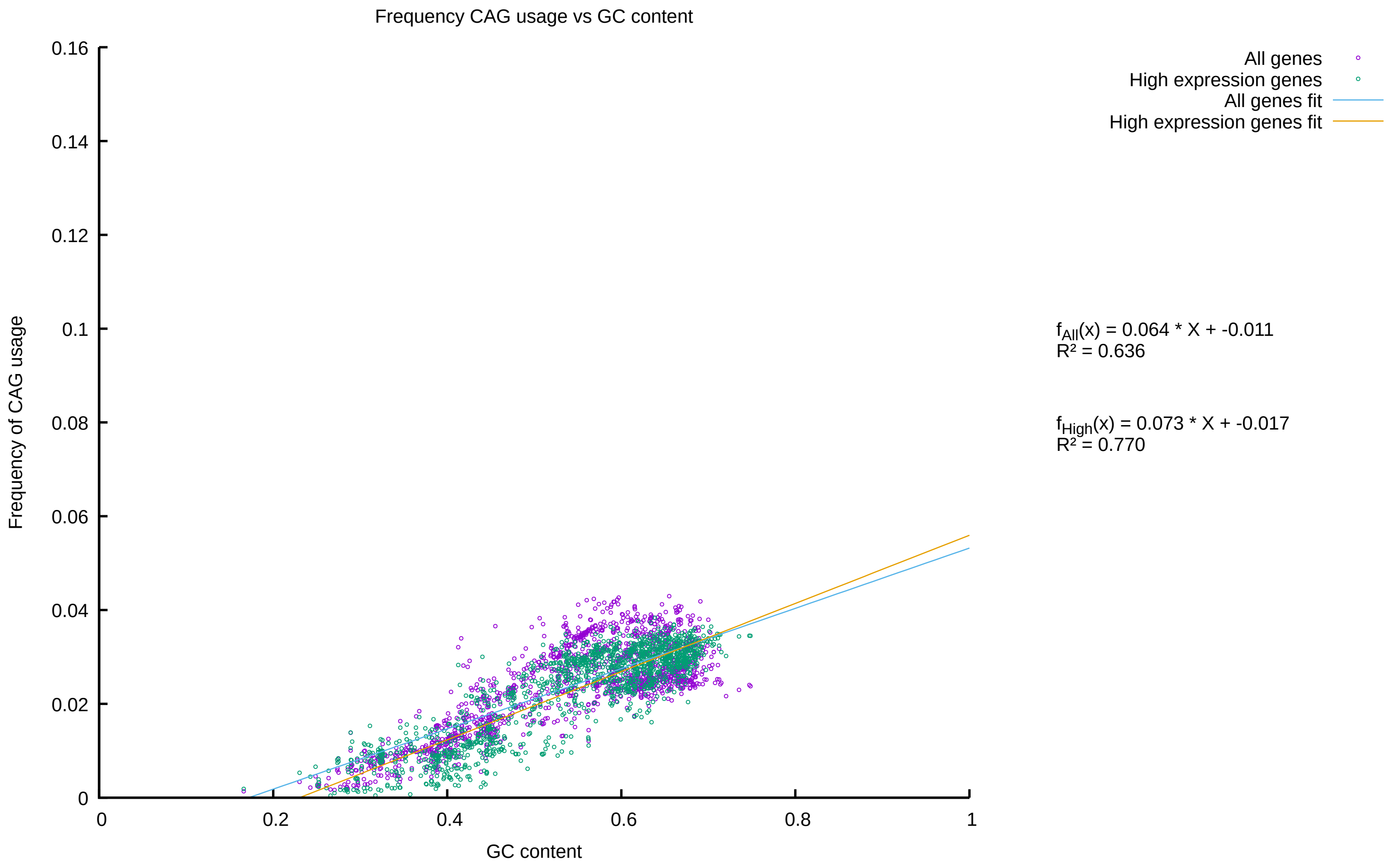

Frequency CAG usage vs GC content
All genes
High expression genes
All genes fit
High expression genes fit
f_All(x) = 0.064 * X + -0.011
R² = 0.636
f_High(x) = 0.073 * X + -0.017
R² = 0.770
Frequency of CAG usage
GC content
0.16
0.14
0.12
0.1
0.08
0.06
0.04
0.02
0
0.2
0.4
0.6
0.8
1

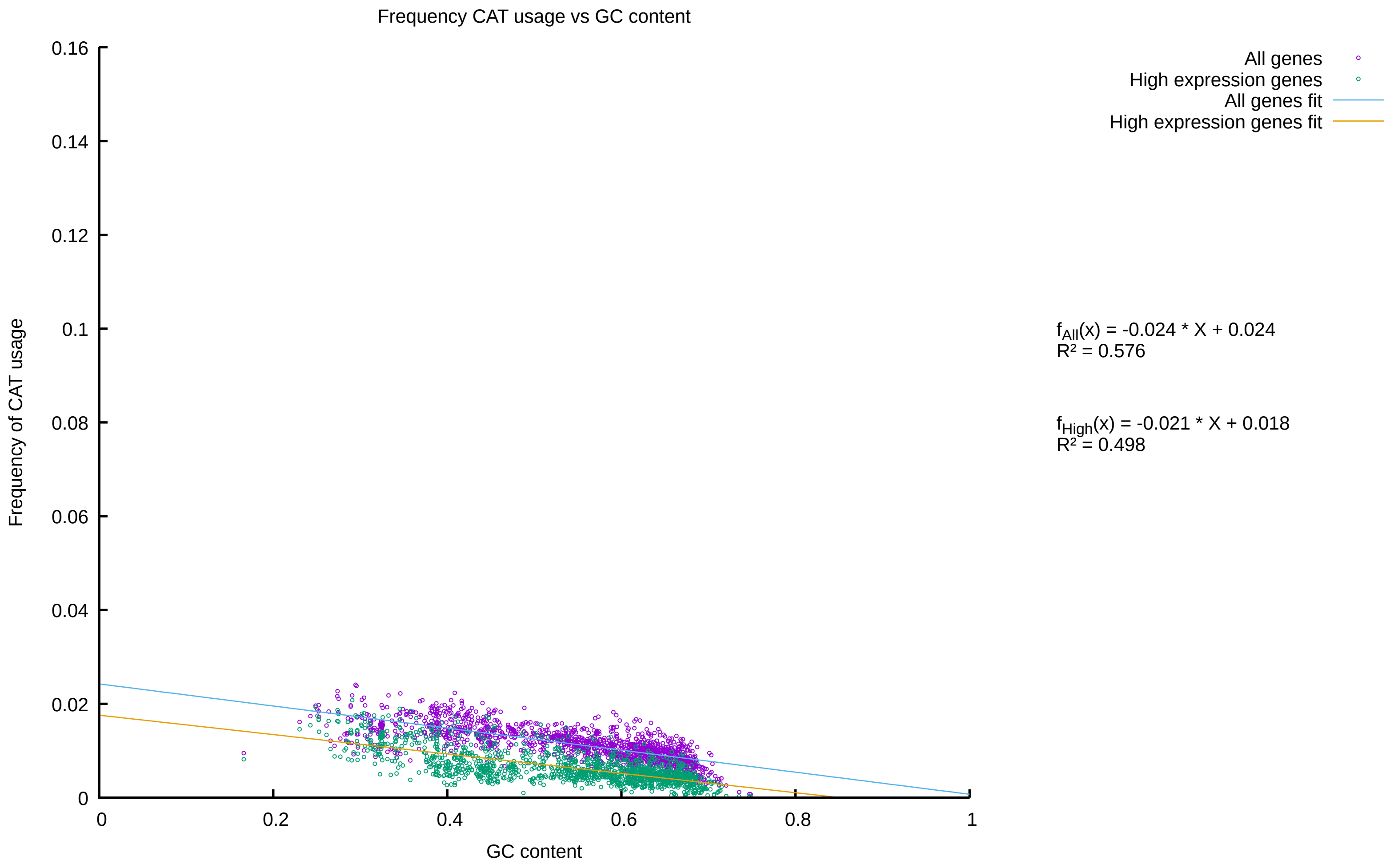

Frequency CAT usage vs GC content
All genes
High expression genes
All genes fit
High expression genes fit
Frequency of CAT usage
GC content
0
0.02
0.04
0.06
0.08
0.1
0.12
0.14
0.16
0
0.2
0.4
0.6
0.8
1
$f_{All}(x) = -0.024 * X + 0.024$
$R^2 = 0.576$
$f_{High}(x) = -0.021 * X + 0.018$
$R^2 = 0.498$

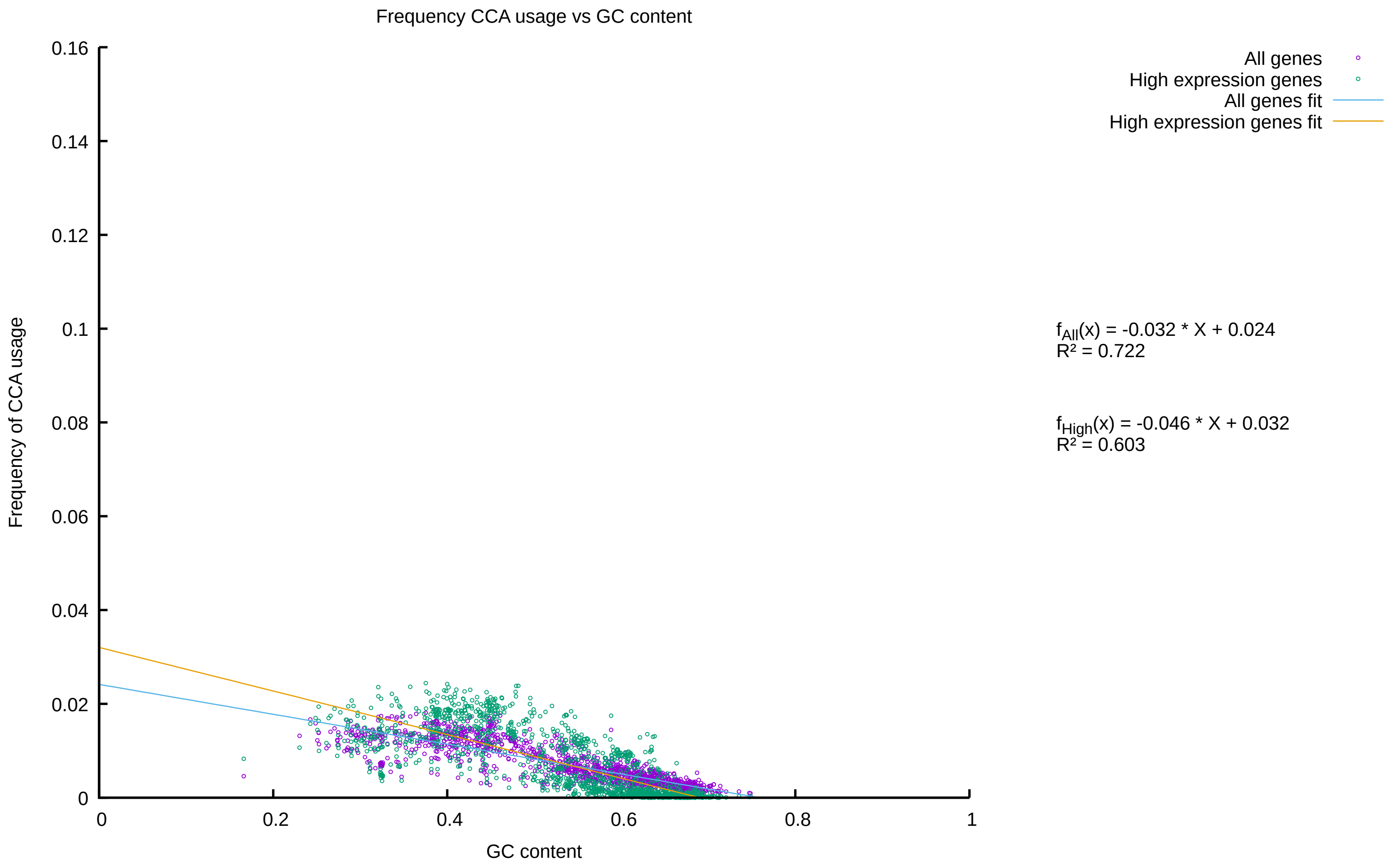

Frequency CCA usage vs GC content
0.16
0.14
0.12
0.1
0.08
0.06
0.04
0.02
0
Frequency of CCA usage
0
0.2
0.4
0.6
0.8
1
GC content
All genes
High expression genes
All genes fit
High expression genes fit
$f_{All}(x) = -0.032 * X + 0.024$
$R^2 = 0.722$
$f_{High}(x) = -0.046 * X + 0.032$
$R^2 = 0.603$

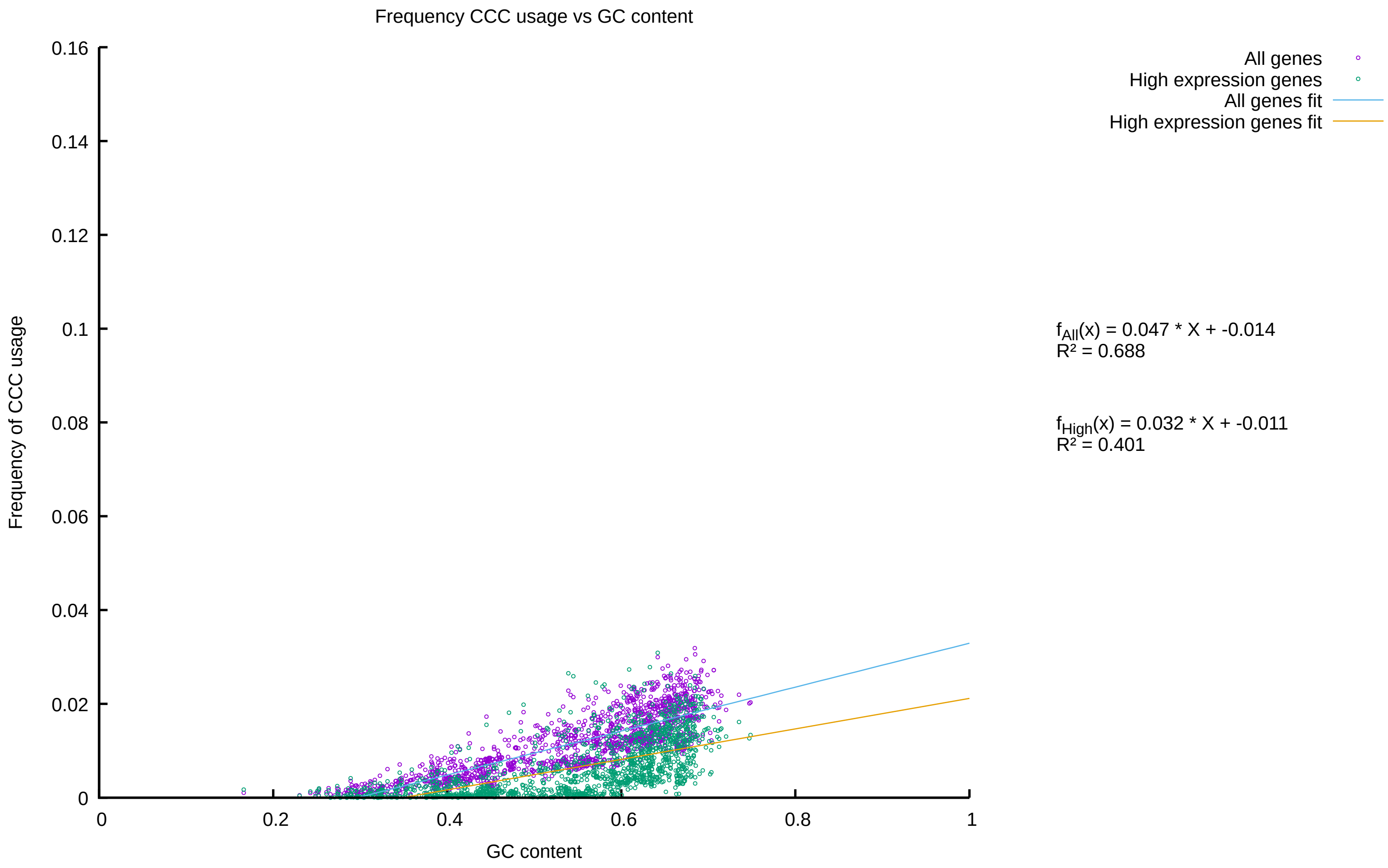

Frequency CCC usage vs GC content
All genes
High expression genes
All genes fit
High expression genes fit
$f_{All}(x) = 0.047 * X + -0.014$
$R^2 = 0.688$
$f_{High}(x) = 0.032 * X + -0.011$
$R^2 = 0.401$
Frequency of CCC usage
GC content
0
0.02
0.04
0.06
0.08
0.1
0.12
0.14
0.16
0.2
0.4
0.6
0.8
1

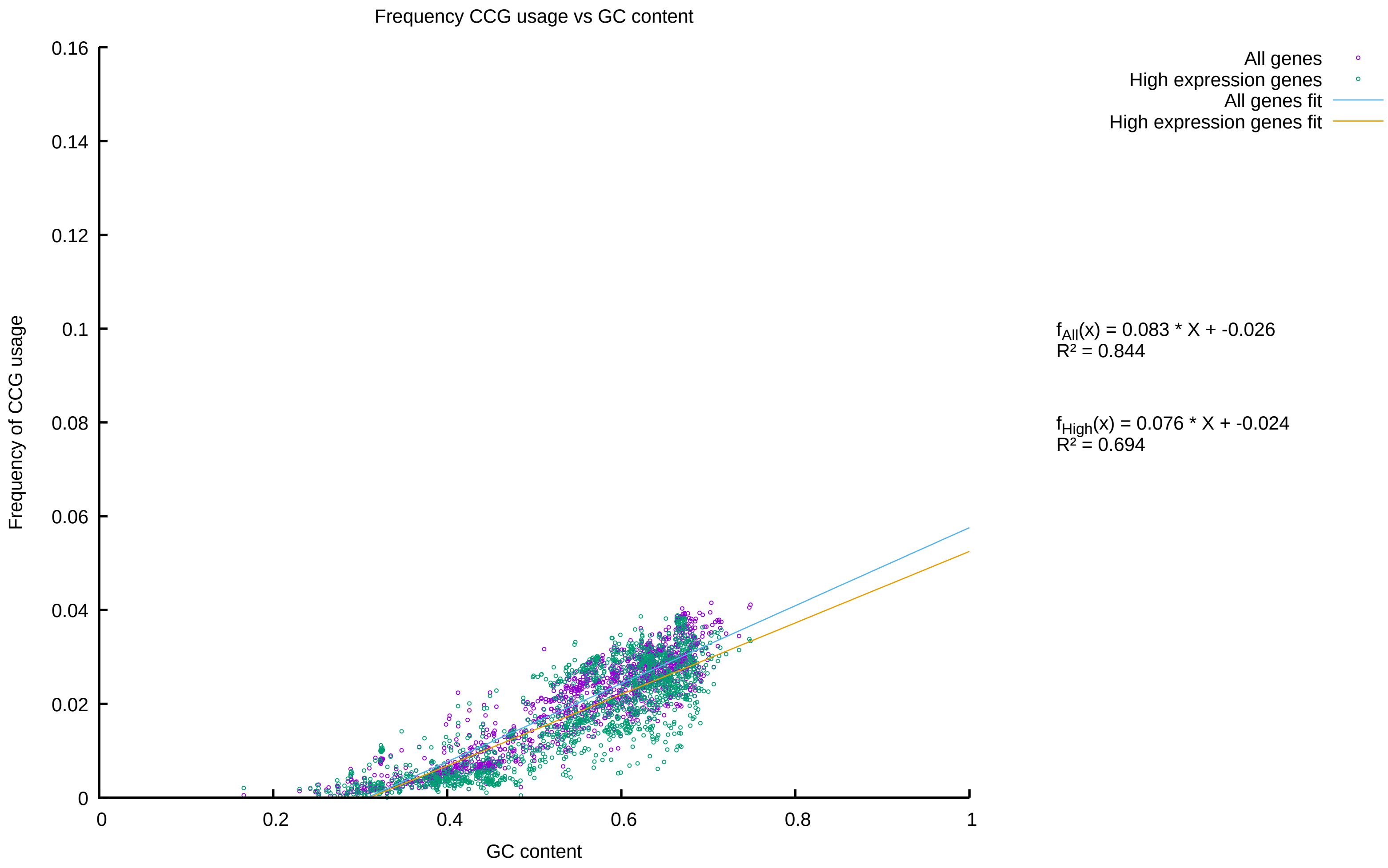

Frequency CCG usage vs GC content
Frequency of CCG usage
GC content
0.16
0.14
0.12
0.1
0.08
0.06
0.04
0.02
0
0.2
0.4
0.6
0.8
1
All genes
High expression genes
All genes fit
High expression genes fit
$f_{All}(x) = 0.083 * X + -0.026$
$R^2 = 0.844$
$f_{High}(x) = 0.076 * X + -0.024$
$R^2 = 0.694$

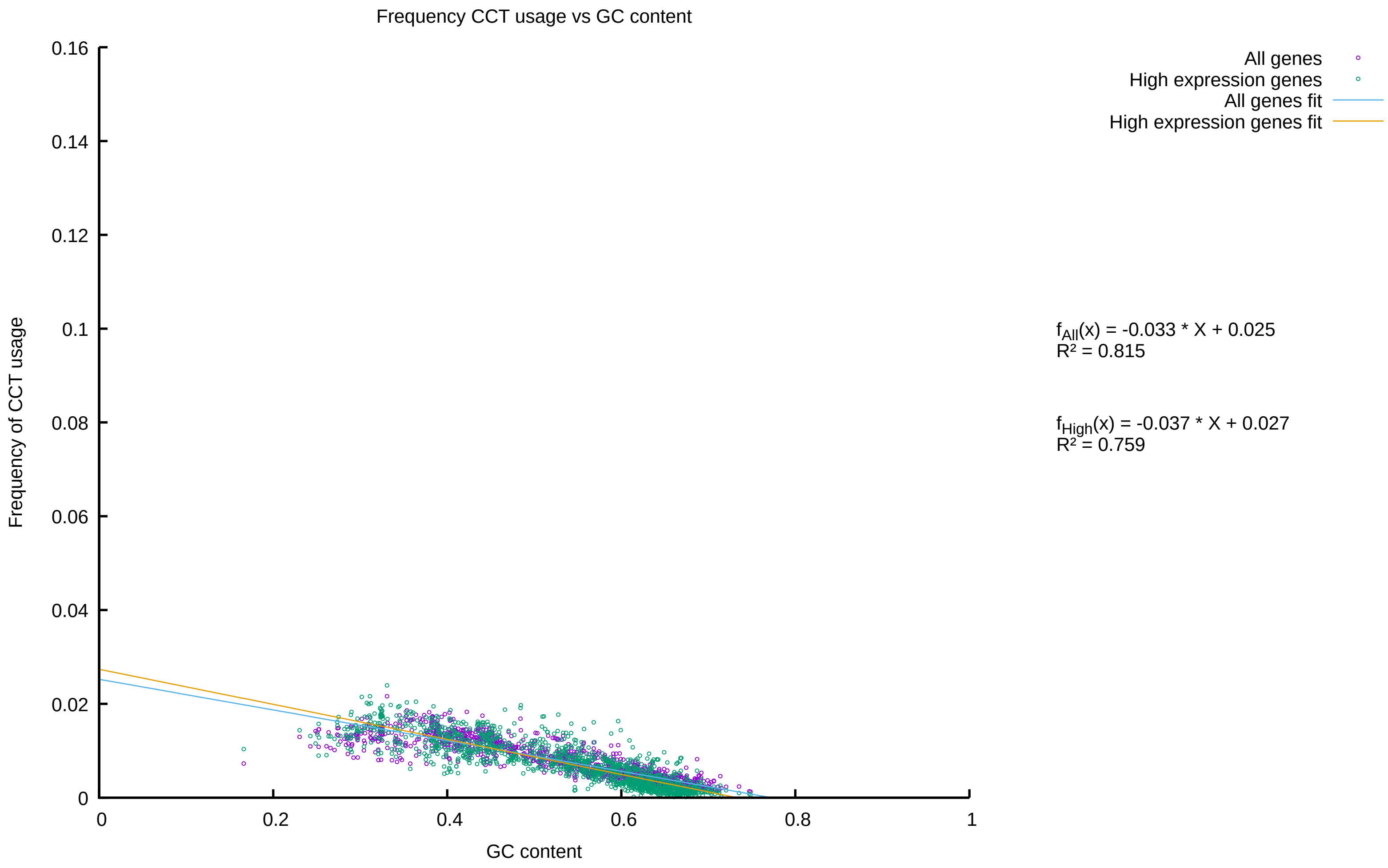
Frequency CCT usage vs GC content
All genes
High expression genes
All genes fit
High expression genes fit
f_All(x) = -0.033 * X + 0.025
R² = 0.815
f_High(x) = -0.037 * X + 0.027
R² = 0.759
Frequency of CCT usage
GC content
0.16
0.14
0.12
0.1
0.08
0.06
0.04
0.02
0
0
0.2
0.4
0.6
0.8
1

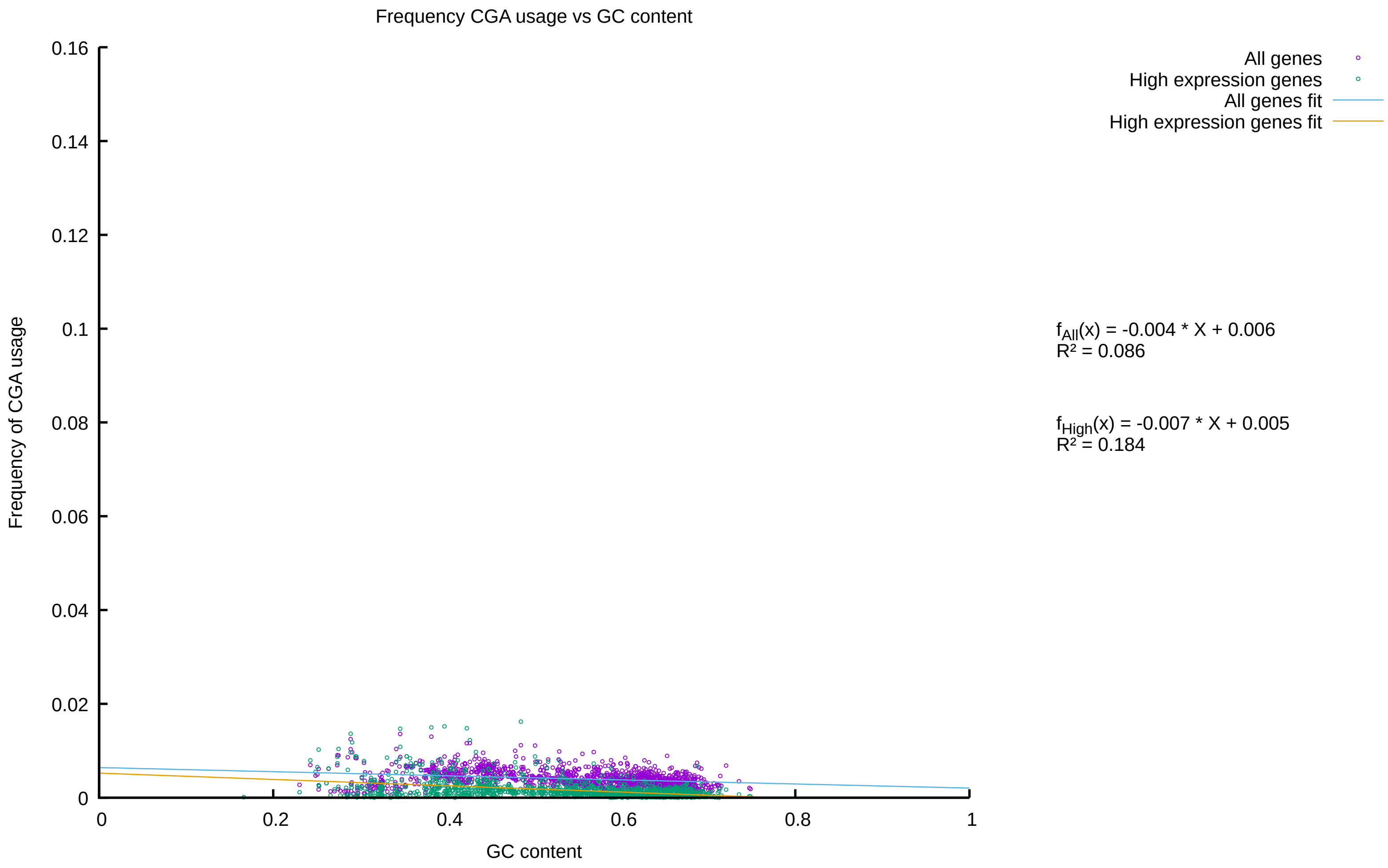

Frequency CGA usage vs GC content
0.16
0.14
0.12
0.1
0.08
0.06
0.04
0.02
0
Frequency of CGA usage
0
0.2
0.4
0.6
0.8
1
GC content
All genes
High expression genes
All genes fit
High expression genes fit
$f_{All}(x) = -0.004 * X + 0.006$
$R^2 = 0.086$
$f_{High}(x) = -0.007 * X + 0.005$
$R^2 = 0.184$

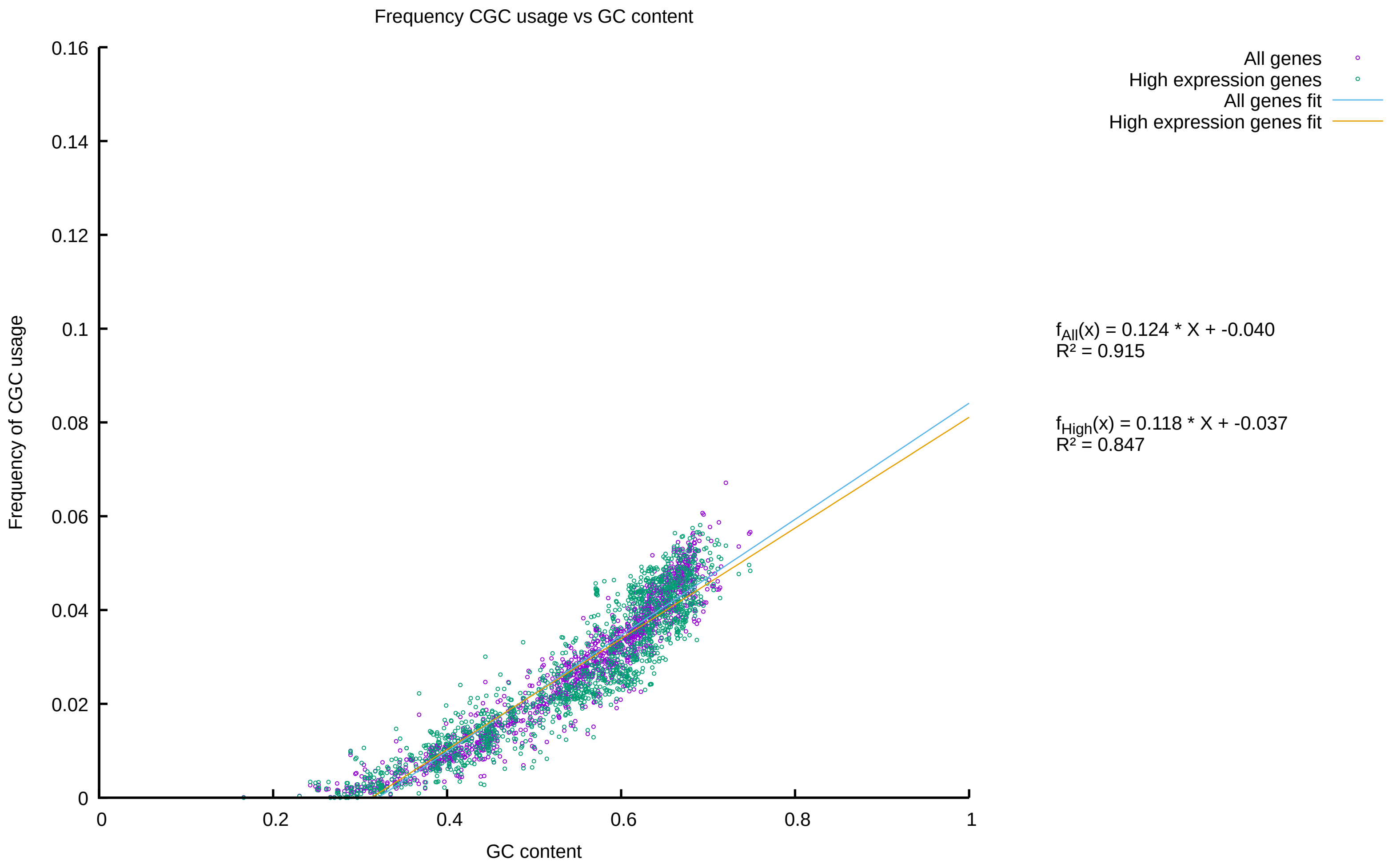
Frequency CGC usage vs GC content
Frequency of CGC usage
GC content
All genes
High expression genes
All genes fit
High expression genes fit
$f_{All}(x) = 0.124 * X + -0.040$
$R^2 = 0.915$
$f_{High}(x) = 0.118 * X + -0.037$
$R^2 = 0.847$
0
0.02
0.04
0.06
0.08
0.1
0.12
0.14
0.16
0
0.2
0.4
0.6
0.8
1

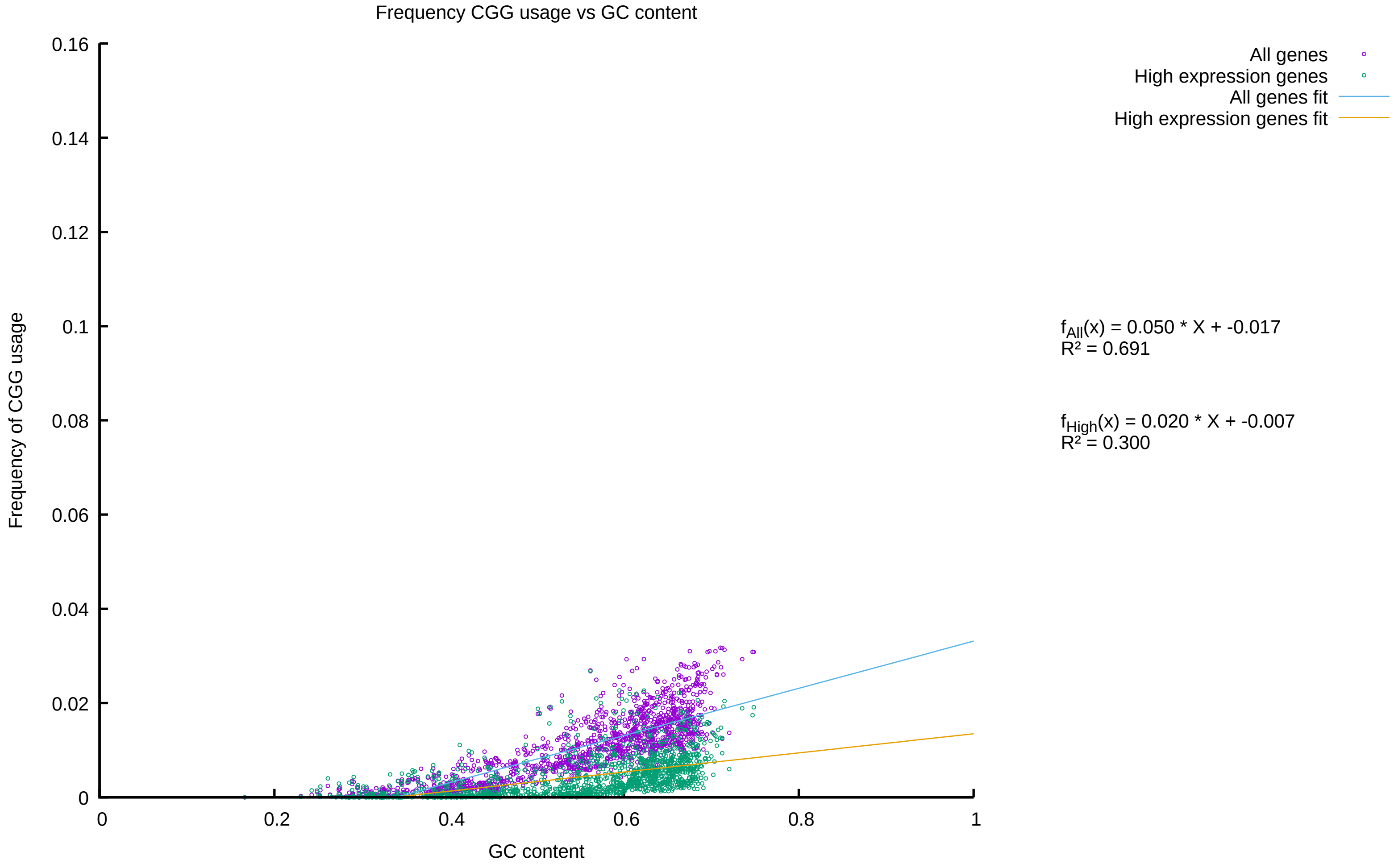

$f_{All}(x) = 0.050 * X + -0.017$
$R^2 = 0.691$

$f_{High}(x) = 0.020 * X + -0.007$
$R^2 = 0.300$

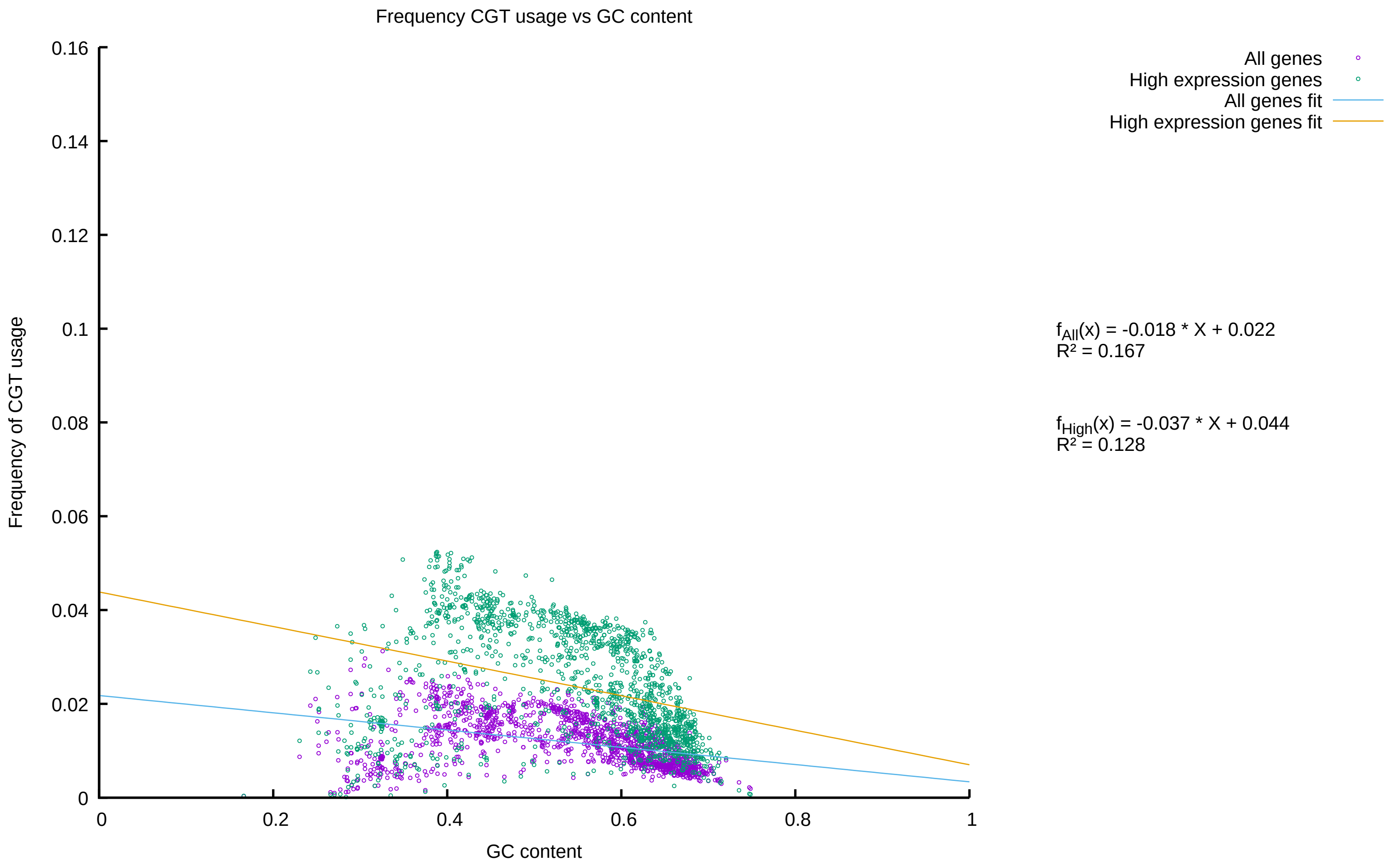

Frequency CGT usage vs GC content
Frequency of CGT usage
GC content
0
0.02
0.04
0.06
0.08
0.1
0.12
0.14
0.16
0
0.2
0.4
0.6
0.8
1
All genes
High expression genes
All genes fit
High expression genes fit
$f_{All}(x) = -0.018 * X + 0.022$
$R^2 = 0.167$
$f_{High}(x) = -0.037 * X + 0.044$
$R^2 = 0.128$

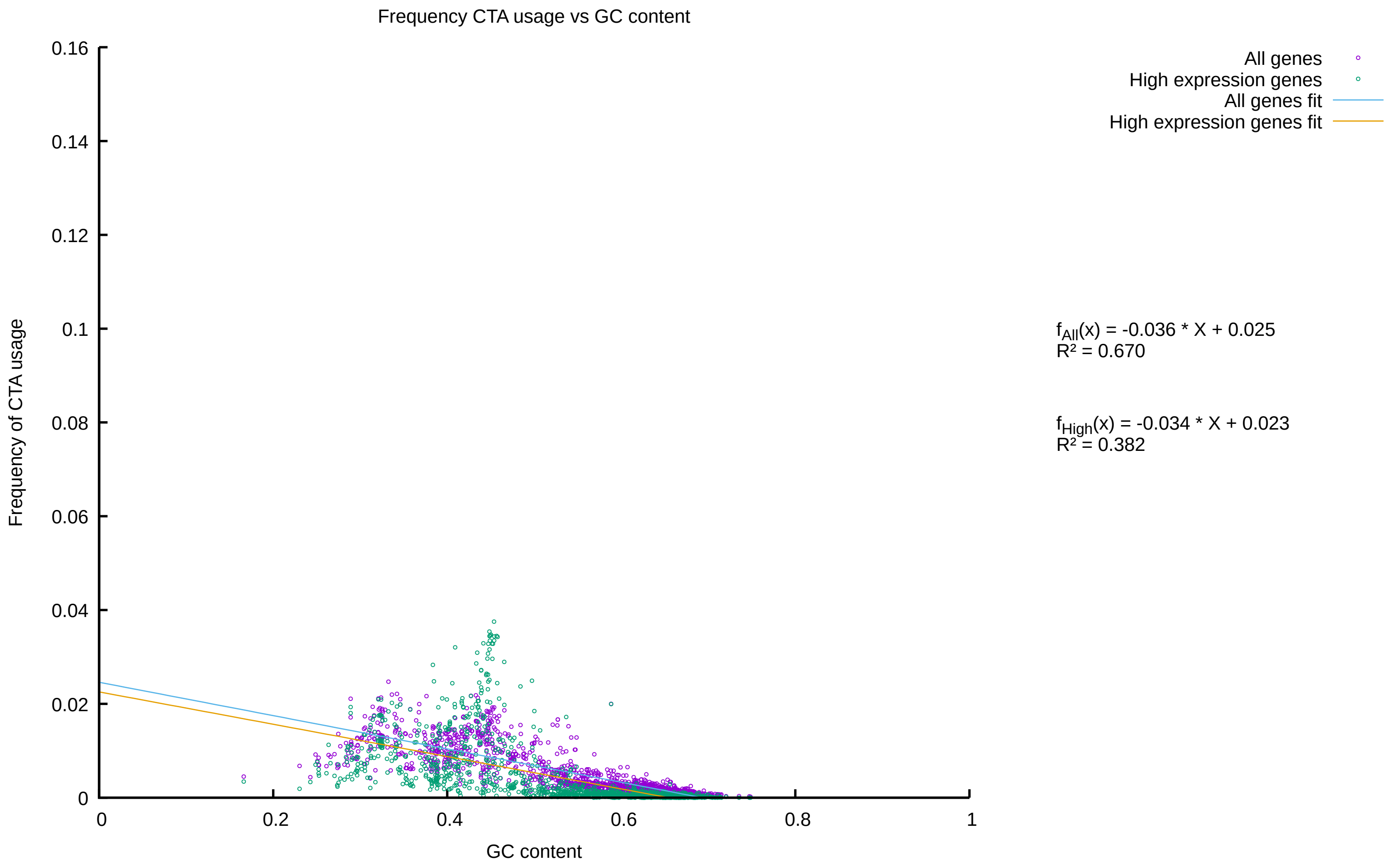

Frequency CTA usage vs GC content
0.16
0.14
0.12
0.1
0.08
0.06
0.04
0.02
0
0
0.2
0.4
0.6
0.8
1
GC content
Frequency of CTA usage
All genes
High expression genes
All genes fit
High expression genes fit
$f_{All}(x) = -0.036 * X + 0.025$
$R^2 = 0.670$
$f_{High}(x) = -0.034 * X + 0.023$
$R^2 = 0.382$

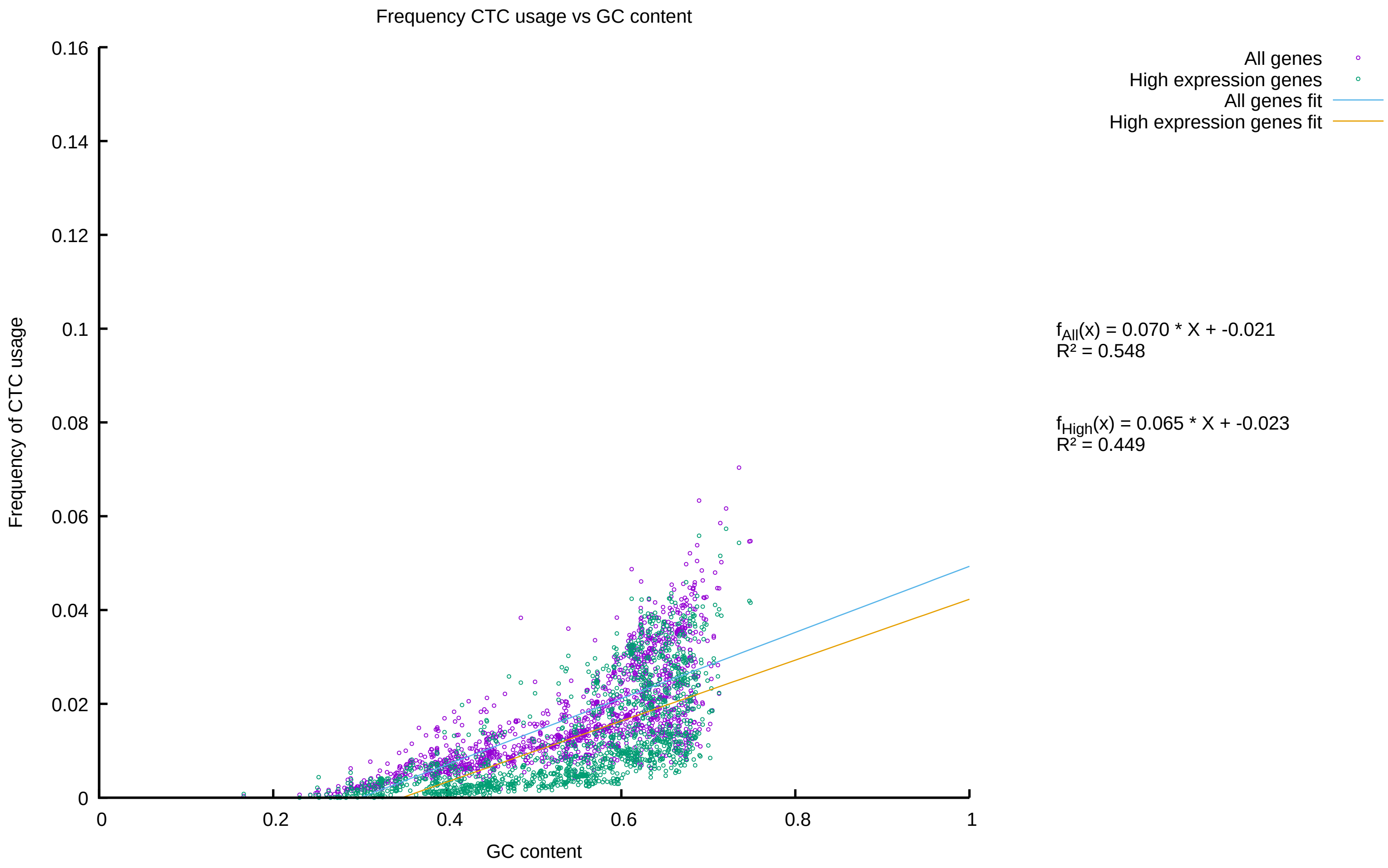

Frequency CTC usage vs GC content
Frequency of CTC usage
GC content
0.16
0.14
0.12
0.1
0.08
0.06
0.04
0.02
0
0.2
0.4
0.6
0.8
1
All genes
High expression genes
All genes fit
High expression genes fit
$f_{All}(x) = 0.070 * X + -0.021$
$R^2 = 0.548$
$f_{High}(x) = 0.065 * X + -0.023$
$R^2 = 0.449$

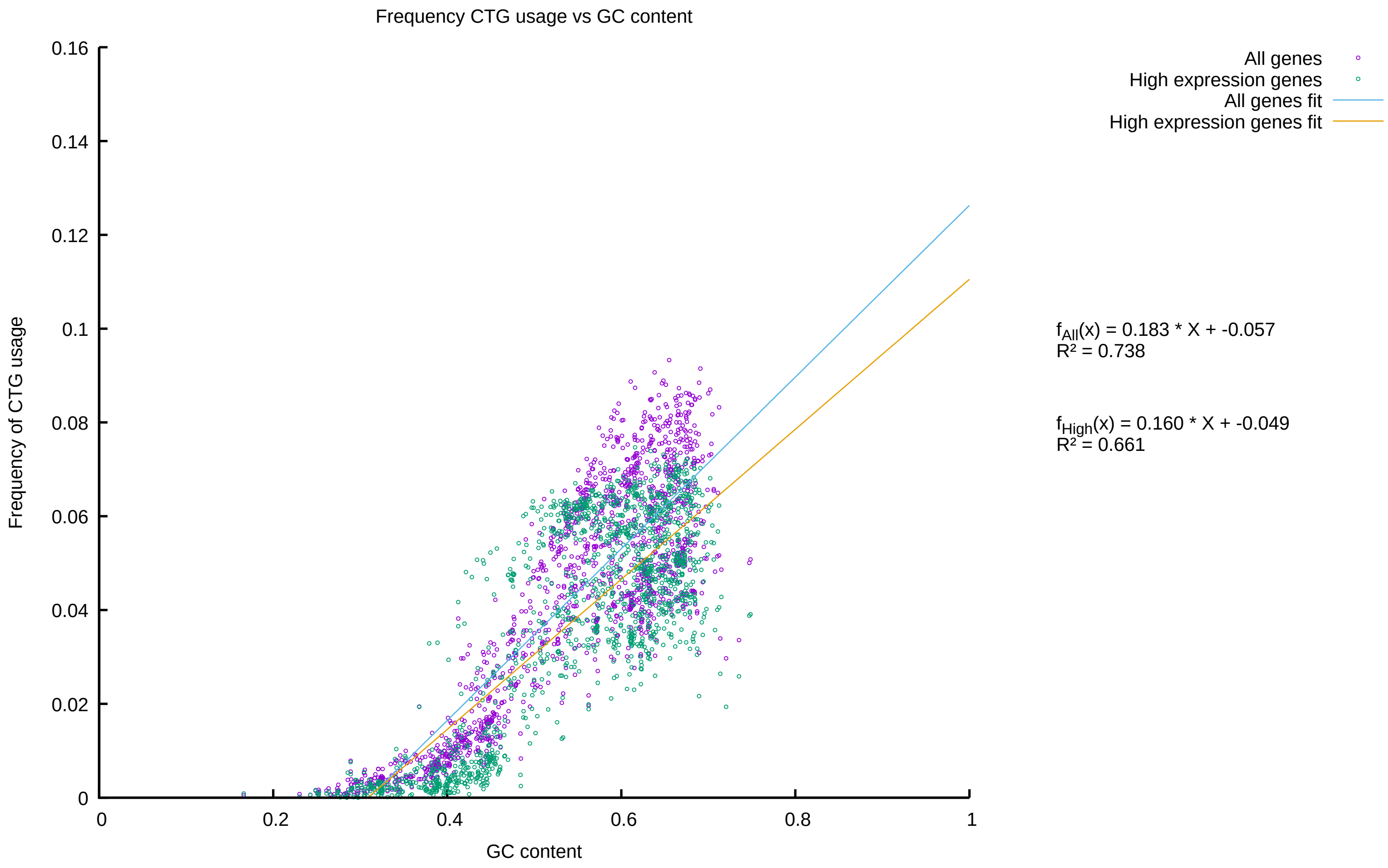

Frequency CTG usage vs GC content
Frequency of CTG usage
GC content
0.16
0.14
0.12
0.1
0.08
0.06
0.04
0.02
0
0
0.2
0.4
0.6
0.8
1
All genes
High expression genes
All genes fit
High expression genes fit
$f_{All}(x) = 0.183 * X + -0.057$
$R^2 = 0.738$
$f_{High}(x) = 0.160 * X + -0.049$
$R^2 = 0.661$

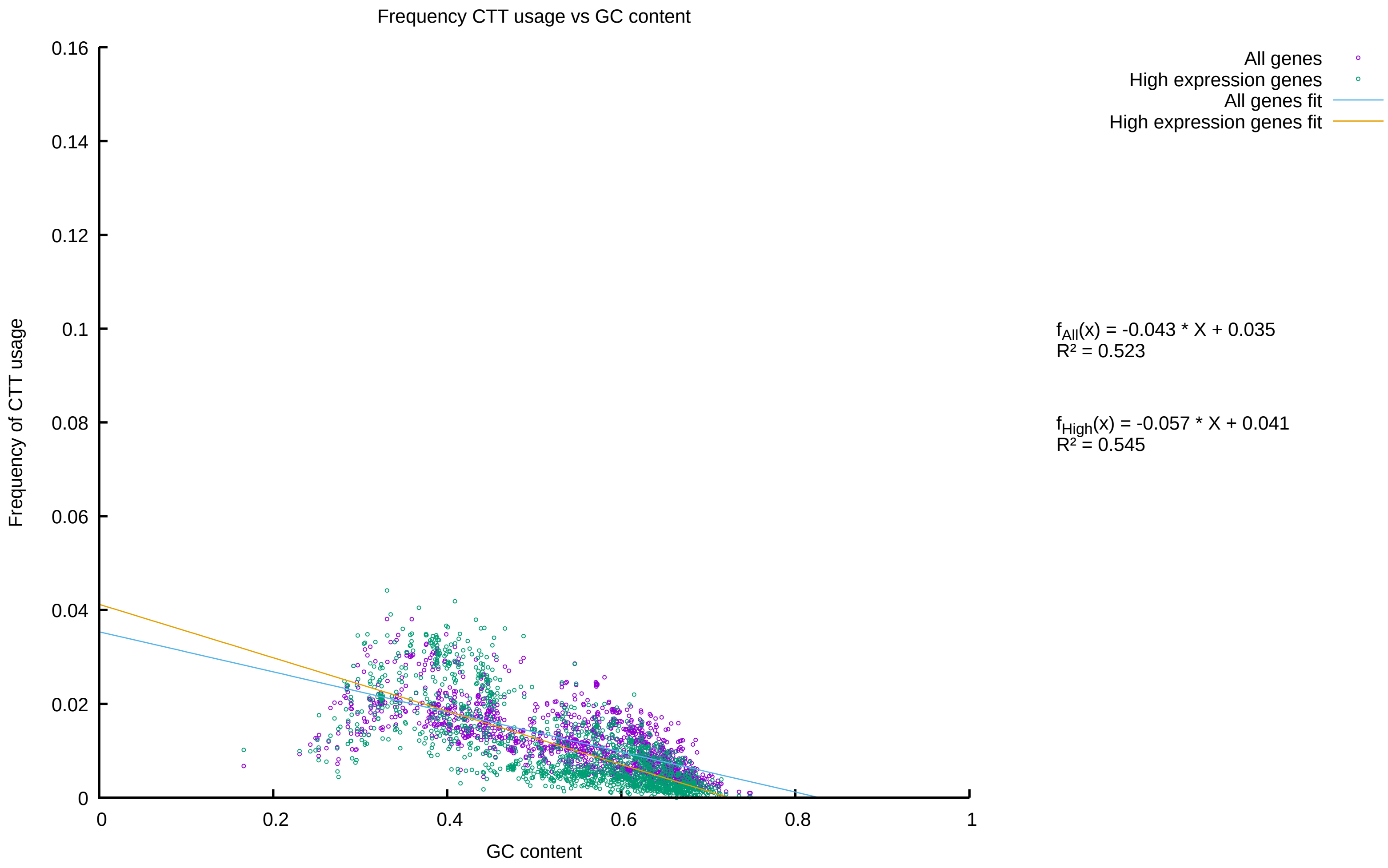

Frequency CTT usage vs GC content
0.16
0.14
0.12
0.1
0.08
0.06
0.04
0.02
0
0
0.2
0.4
0.6
0.8
1
Frequency of CTT usage
GC content
All genes
High expression genes
All genes fit
High expression genes fit
$f_{All}(x) = -0.043 * X + 0.035$
$R^2 = 0.523$
$f_{High}(x) = -0.057 * X + 0.041$
$R^2 = 0.545$

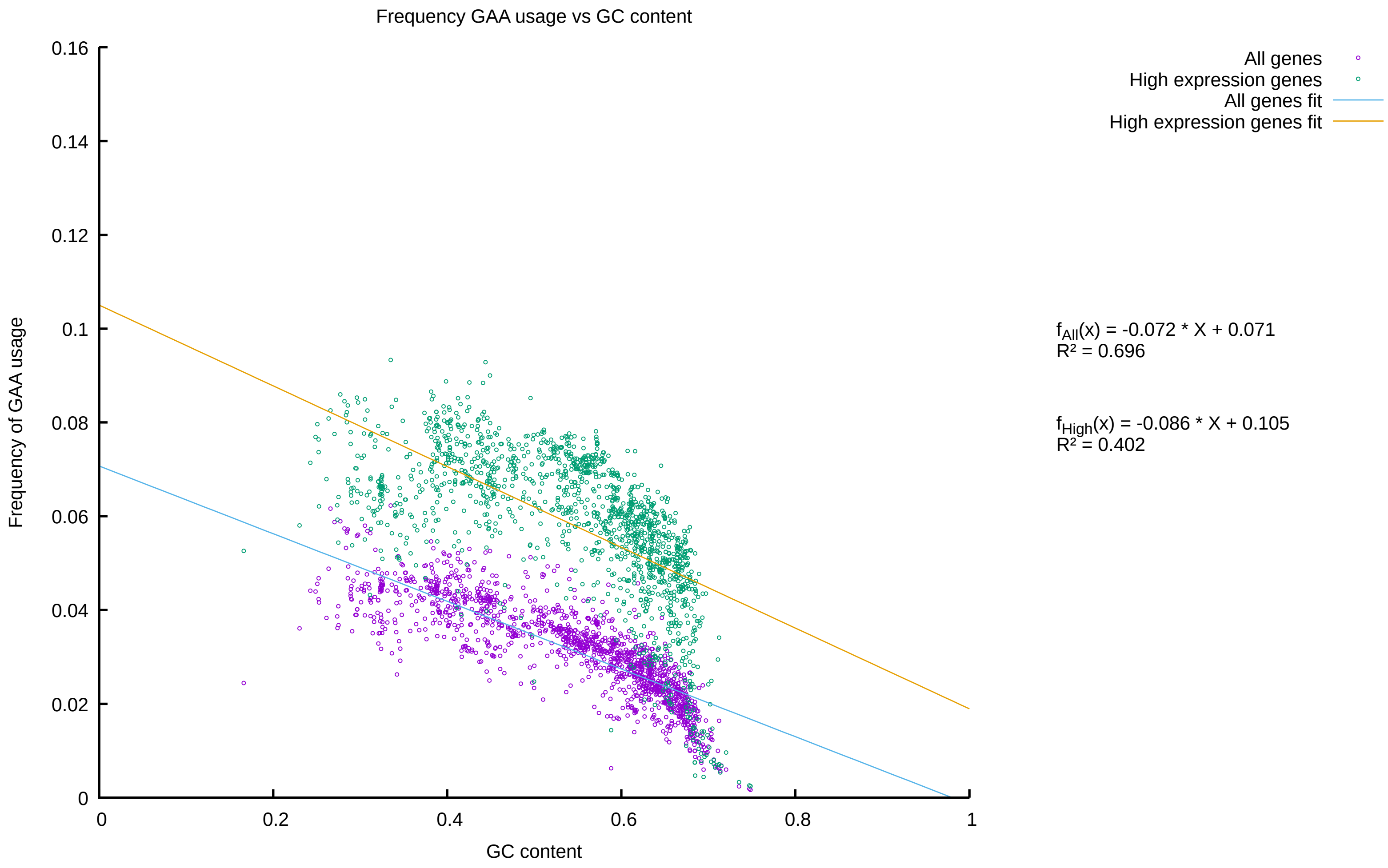

Frequency GAA usage vs GC content
0.16
0.14
0.12
0.1
0.08
0.06
0.04
0.02
0
Frequency of GAA usage
0
0.2
0.4
0.6
0.8
1
GC content
All genes
High expression genes
All genes fit
High expression genes fit
$f_{All}(x) = -0.072 * X + 0.071$
$R^2 = 0.696$
$f_{High}(x) = -0.086 * X + 0.105$
$R^2 = 0.402$

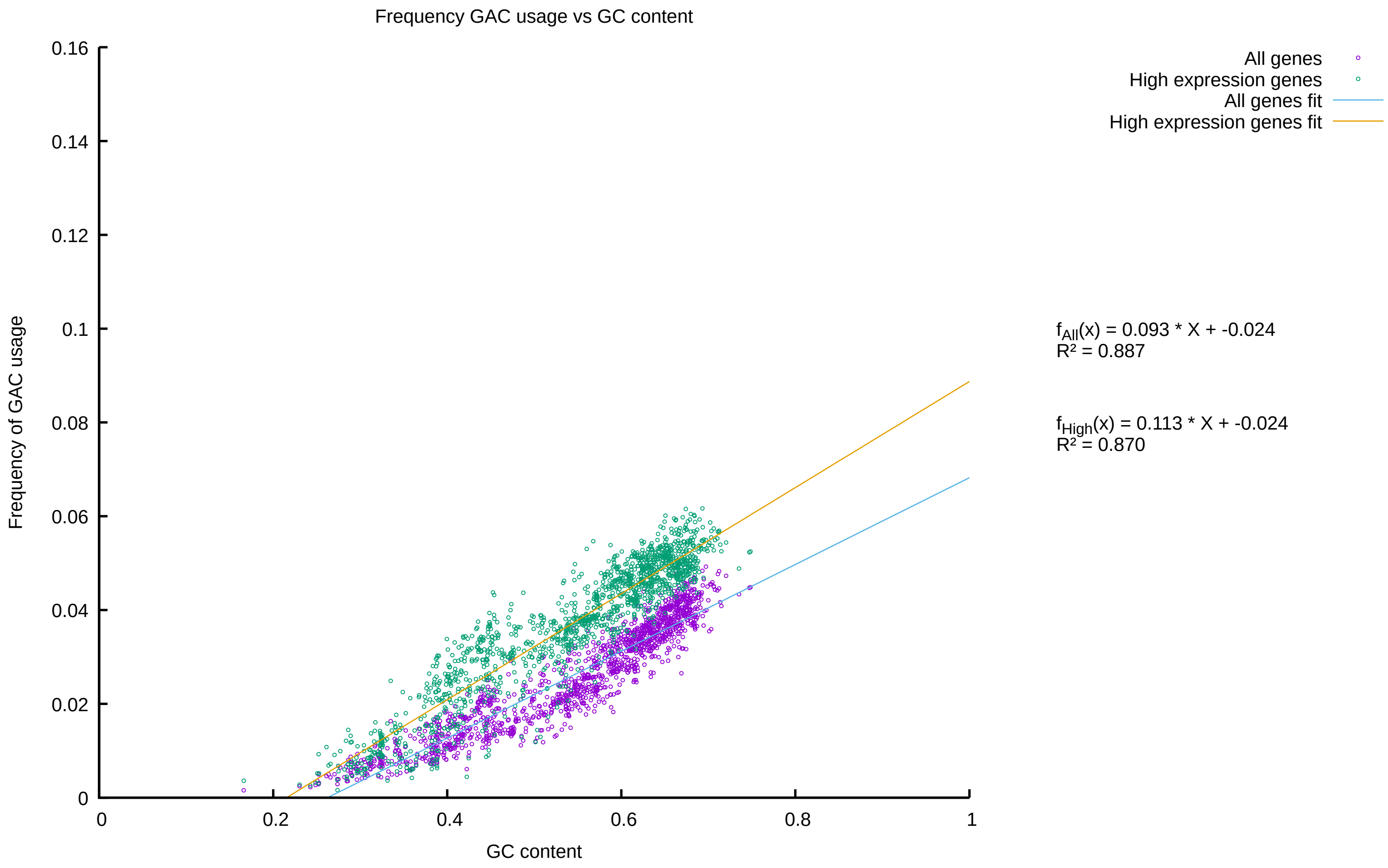
Frequency GAC usage vs GC content
Frequency of GAC usage
GC content
0.16
0.14
0.12
0.1
0.08
0.06
0.04
0.02
0
0
0.2
0.4
0.6
0.8
1
All genes
High expression genes
All genes fit
High expression genes fit
$f_{All}(x) = 0.093 * X + -0.024$
$R^2 = 0.887$
$f_{High}(x) = 0.113 * X + -0.024$
$R^2 = 0.870$

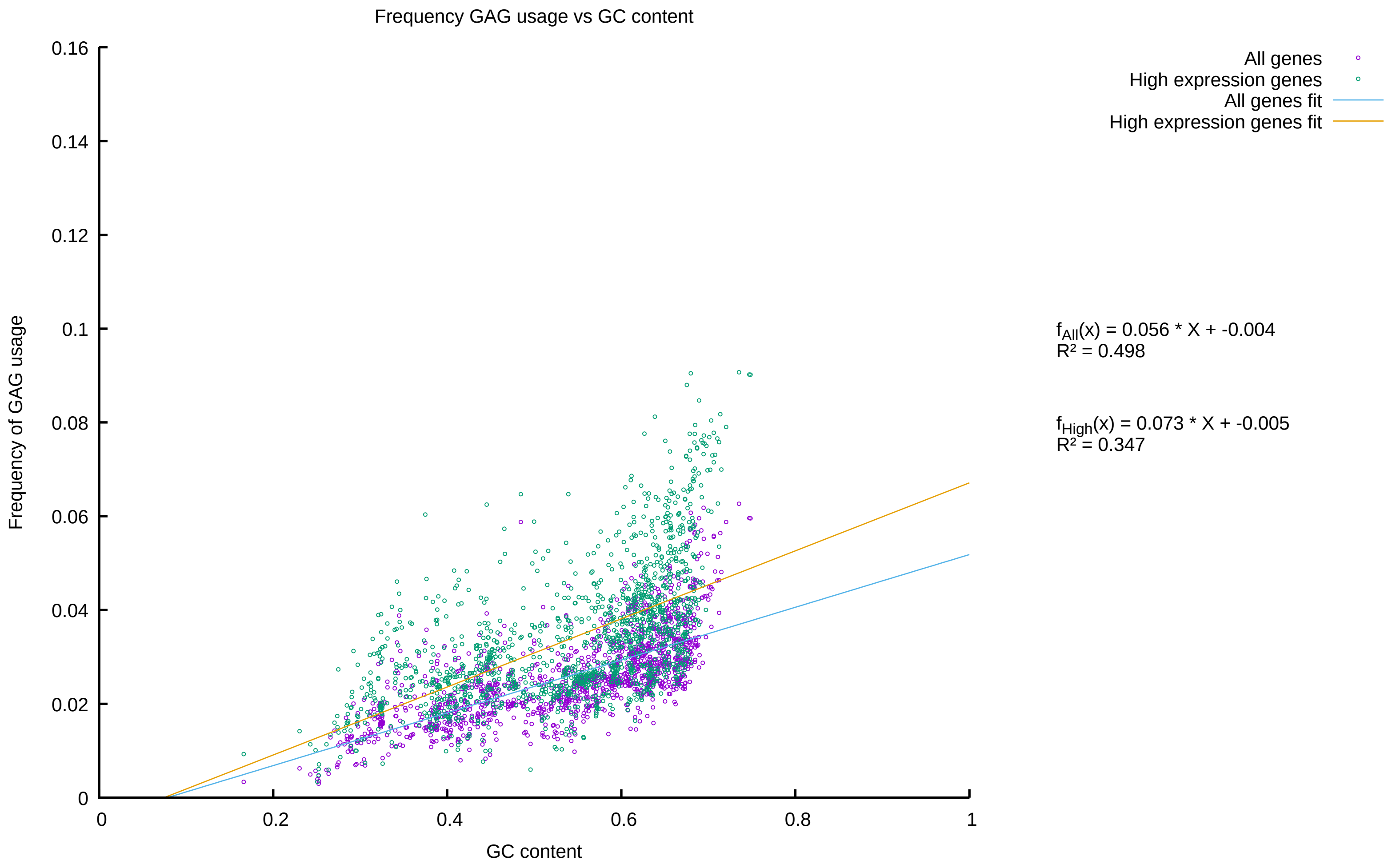

Frequency GAG usage vs GC content
All genes
High expression genes
All genes fit
High expression genes fit
Frequency of GAG usage
GC content
0.16
0.14
0.12
0.1
0.08
0.06
0.04
0.02
0
0
0.2
0.4
0.6
0.8
1
$f_{All}(x) = 0.056 * X + -0.004$
$R^2 = 0.498$
$f_{High}(x) = 0.073 * X + -0.005$
$R^2 = 0.347$

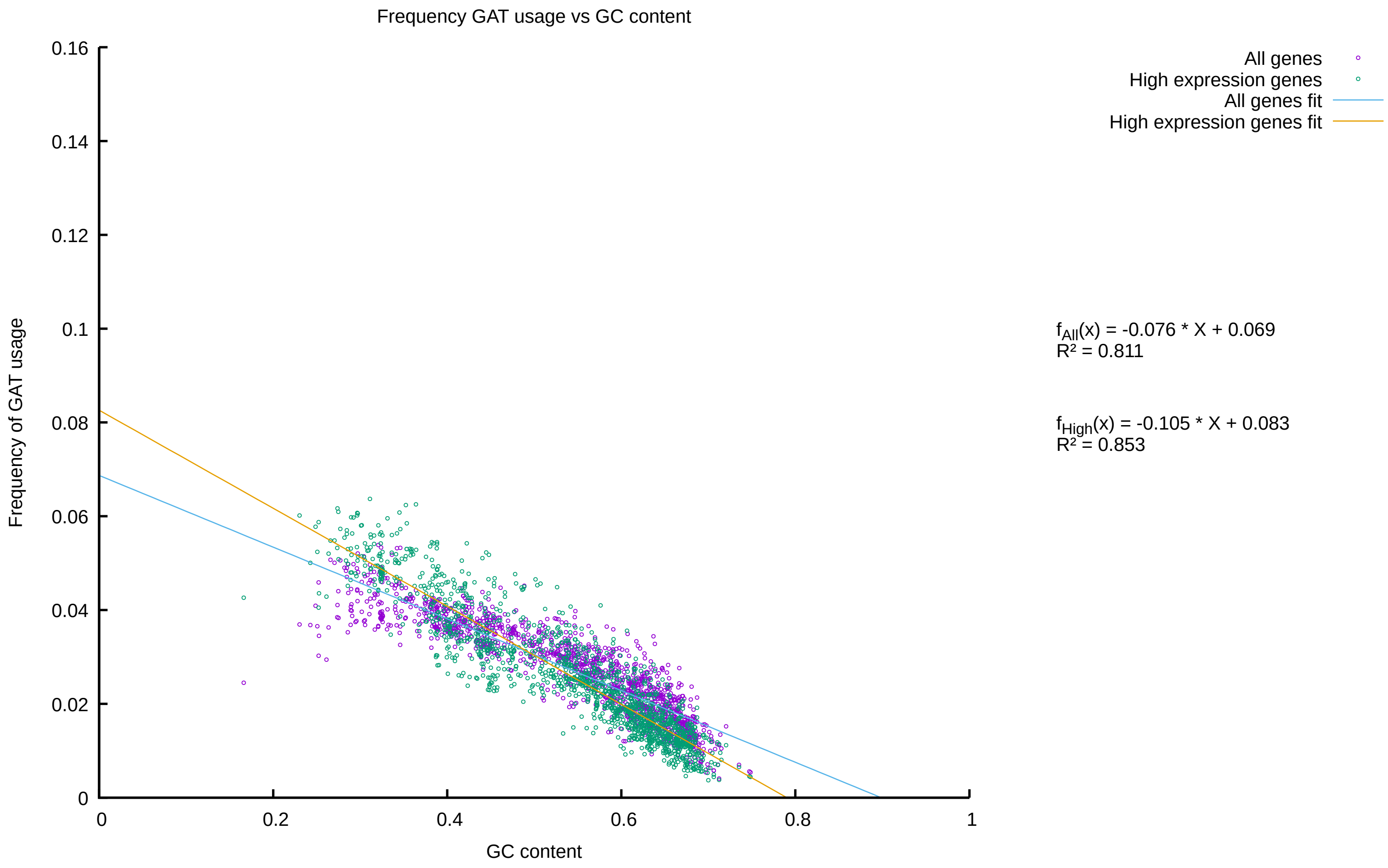

Frequency GAT usage vs GC content
0.16
0.14
0.12
0.1
0.08
0.06
0.04
0.02
0
0
0.2
0.4
0.6
0.8
1
Frequency of GAT usage
GC content
All genes
High expression genes
All genes fit
High expression genes fit
$f_{All}(x) = -0.076 * X + 0.069$
$R^2 = 0.811$
$f_{High}(x) = -0.105 * X + 0.083$
$R^2 = 0.853$

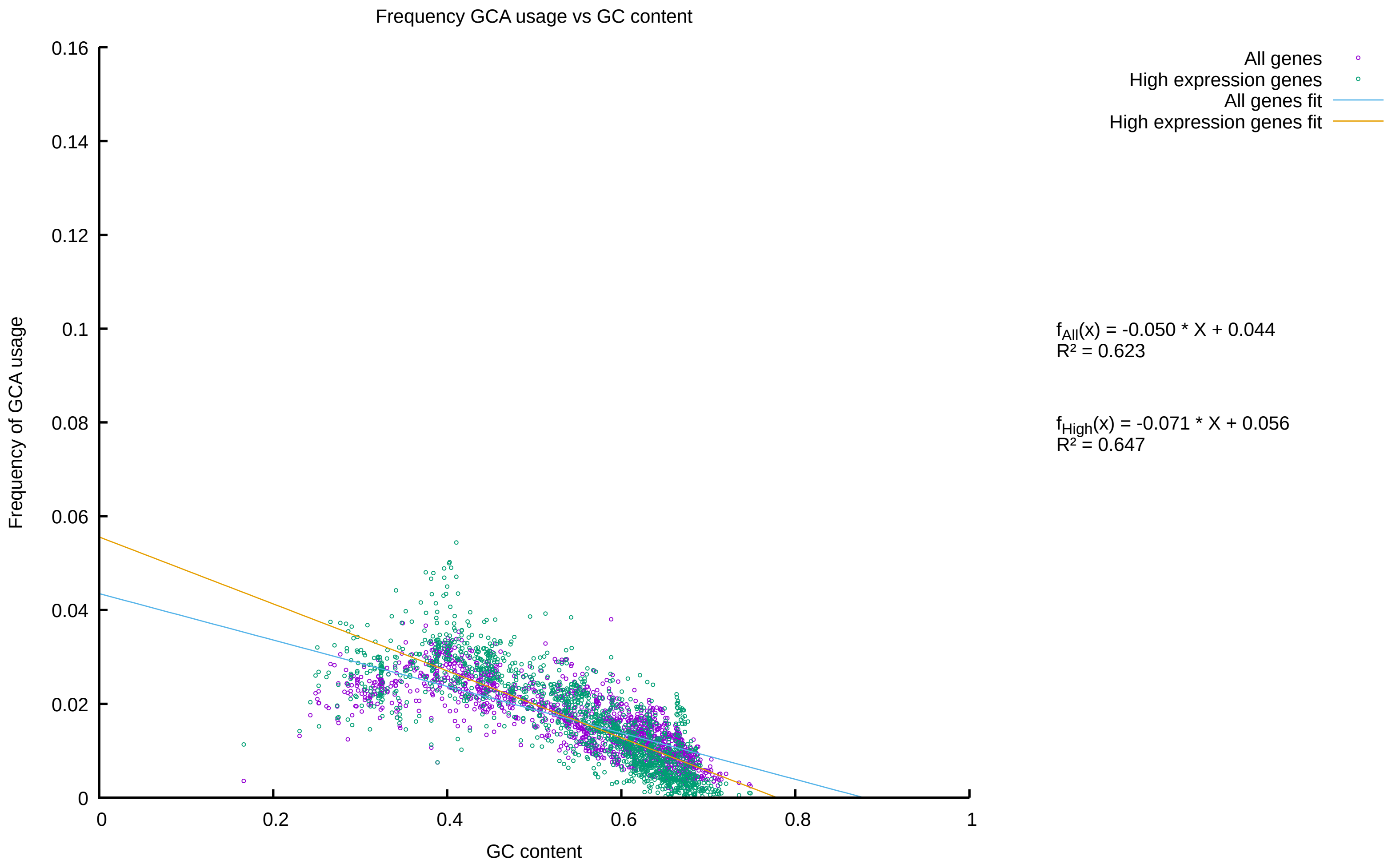

Frequency GCA usage vs GC content
All genes
High expression genes
All genes fit
High expression genes fit
$f_{All}(x) = -0.050 * X + 0.044$
$R^2 = 0.623$
$f_{High}(x) = -0.071 * X + 0.056$
$R^2 = 0.647$
Frequency of GCA usage
GC content
0.16
0.14
0.12
0.1
0.08
0.06
0.04
0.02
0
0
0.2
0.4
0.6
0.8
1

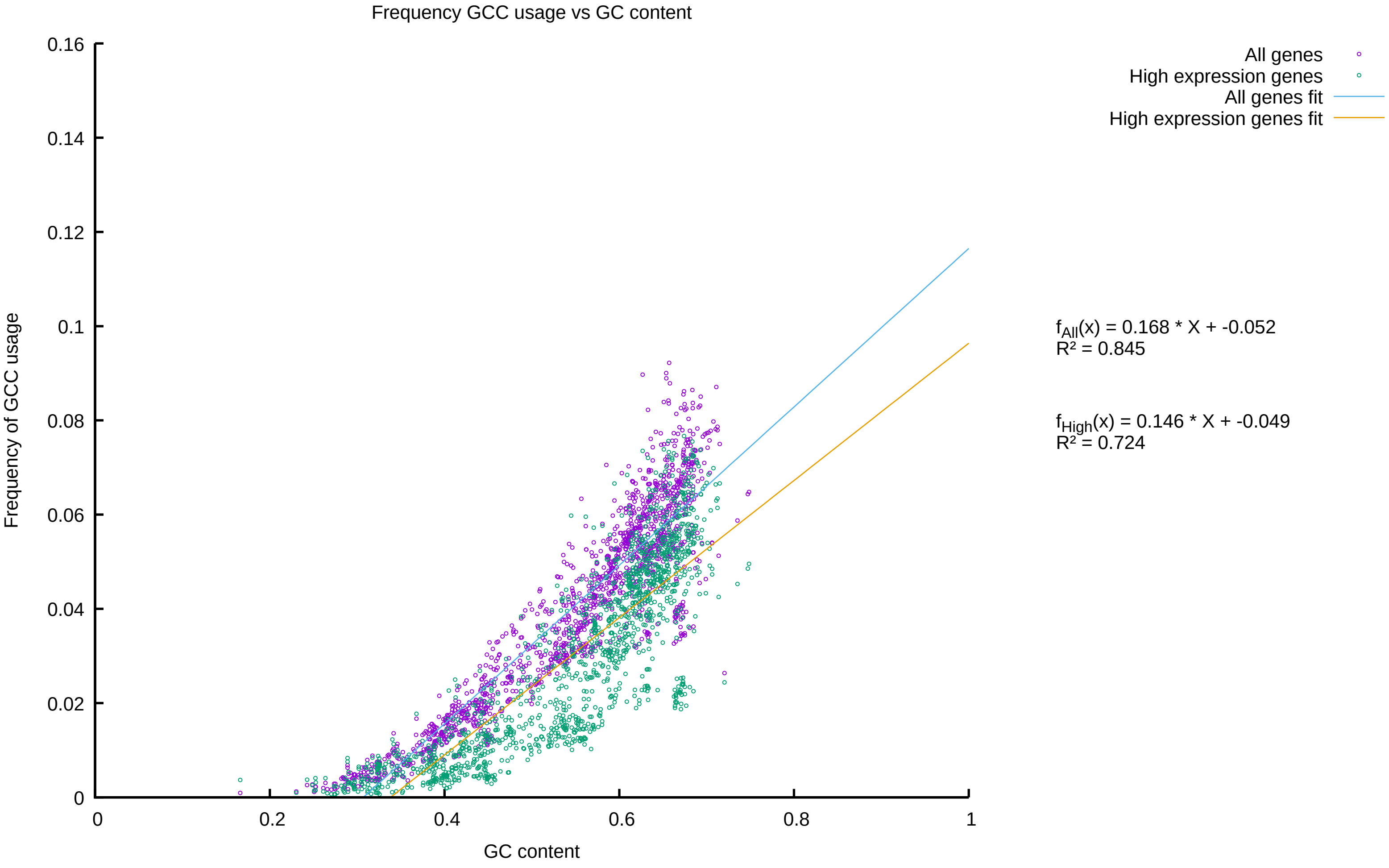

$f_{All}(x) = 0.168 * X + -0.052$
$R^2 = 0.845$

$f_{High}(x) = 0.146 * X + -0.049$
$R^2 = 0.724$

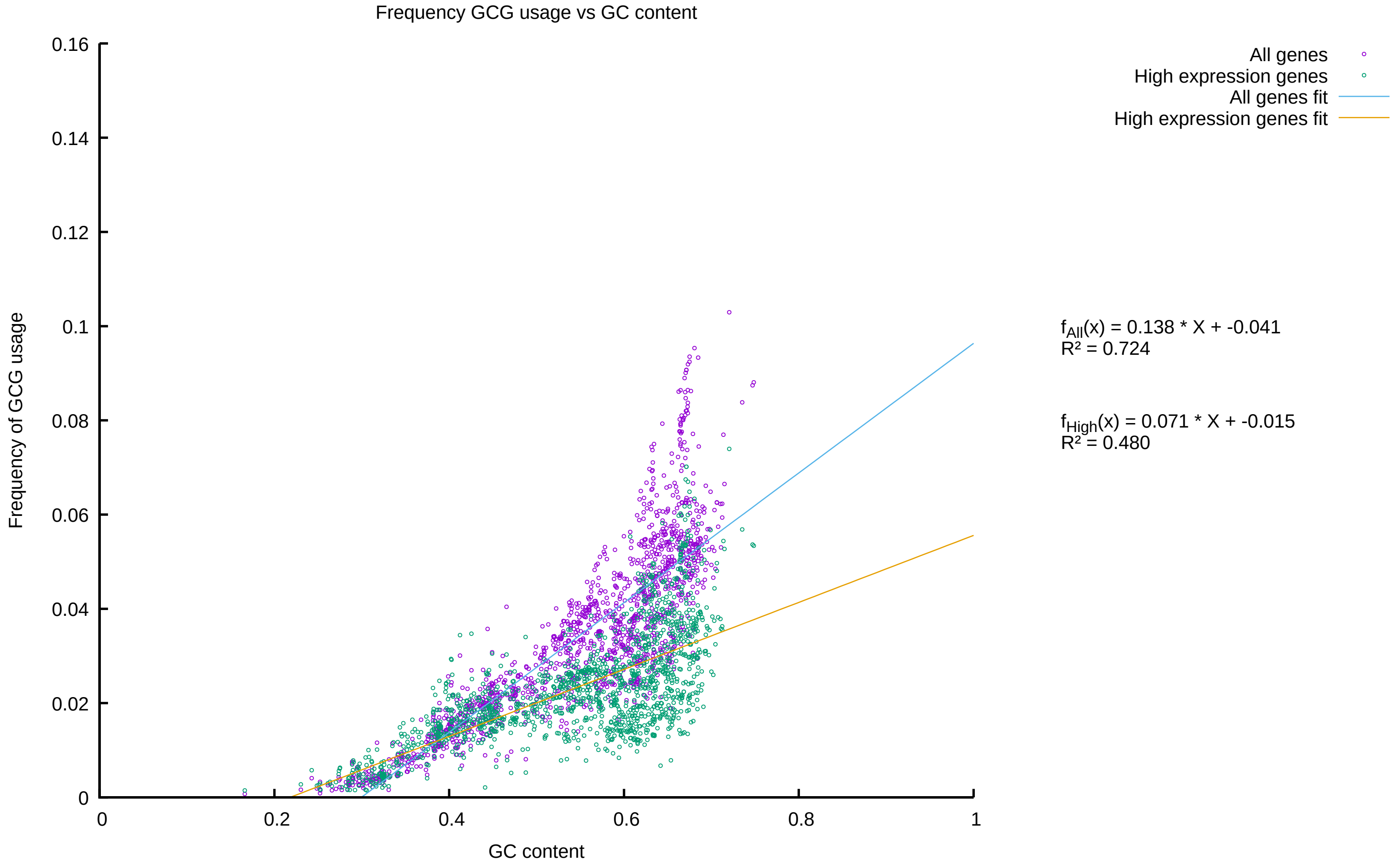
Frequency GCG usage vs GC content
All genes
High expression genes
All genes fit
High expression genes fit
$f_{All}(x) = 0.138 * X + -0.041$
$R^2 = 0.724$
$f_{High}(x) = 0.071 * X + -0.015$
$R^2 = 0.480$
Frequency of GCG usage
GC content
0.16
0.14
0.12
0.1
0.08
0.06
0.04
0.02
0
0
0.2
0.4
0.6
0.8
1

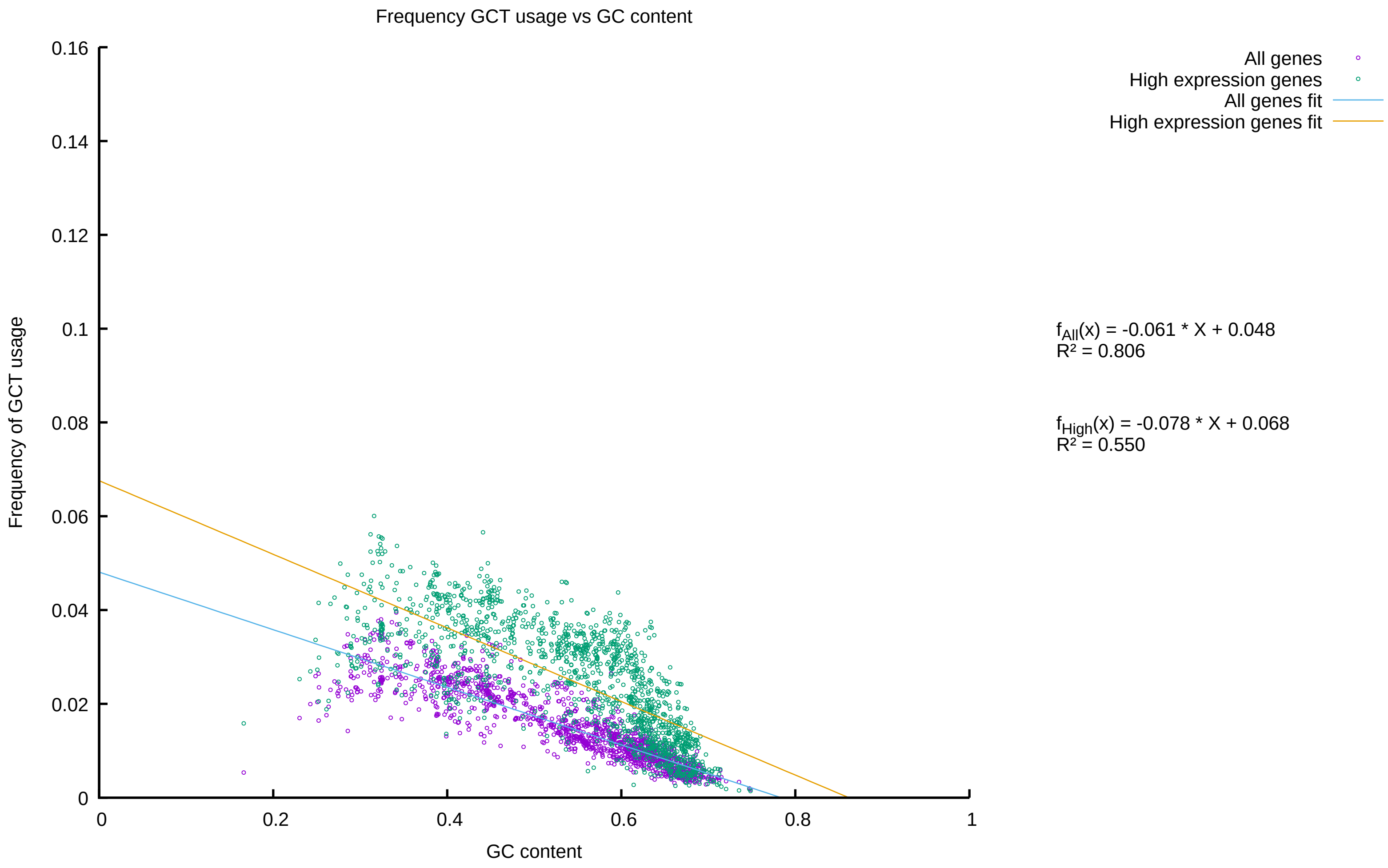

Frequency GCT usage vs GC content
Frequency of GCT usage
GC content
0
0.02
0.04
0.06
0.08
0.1
0.12
0.14
0.16
0
0.2
0.4
0.6
0.8
1
All genes
High expression genes
All genes fit
High expression genes fit
$f_{All}(x) = -0.061 * X + 0.048$
$R^2 = 0.806$
$f_{High}(x) = -0.078 * X + 0.068$
$R^2 = 0.550$

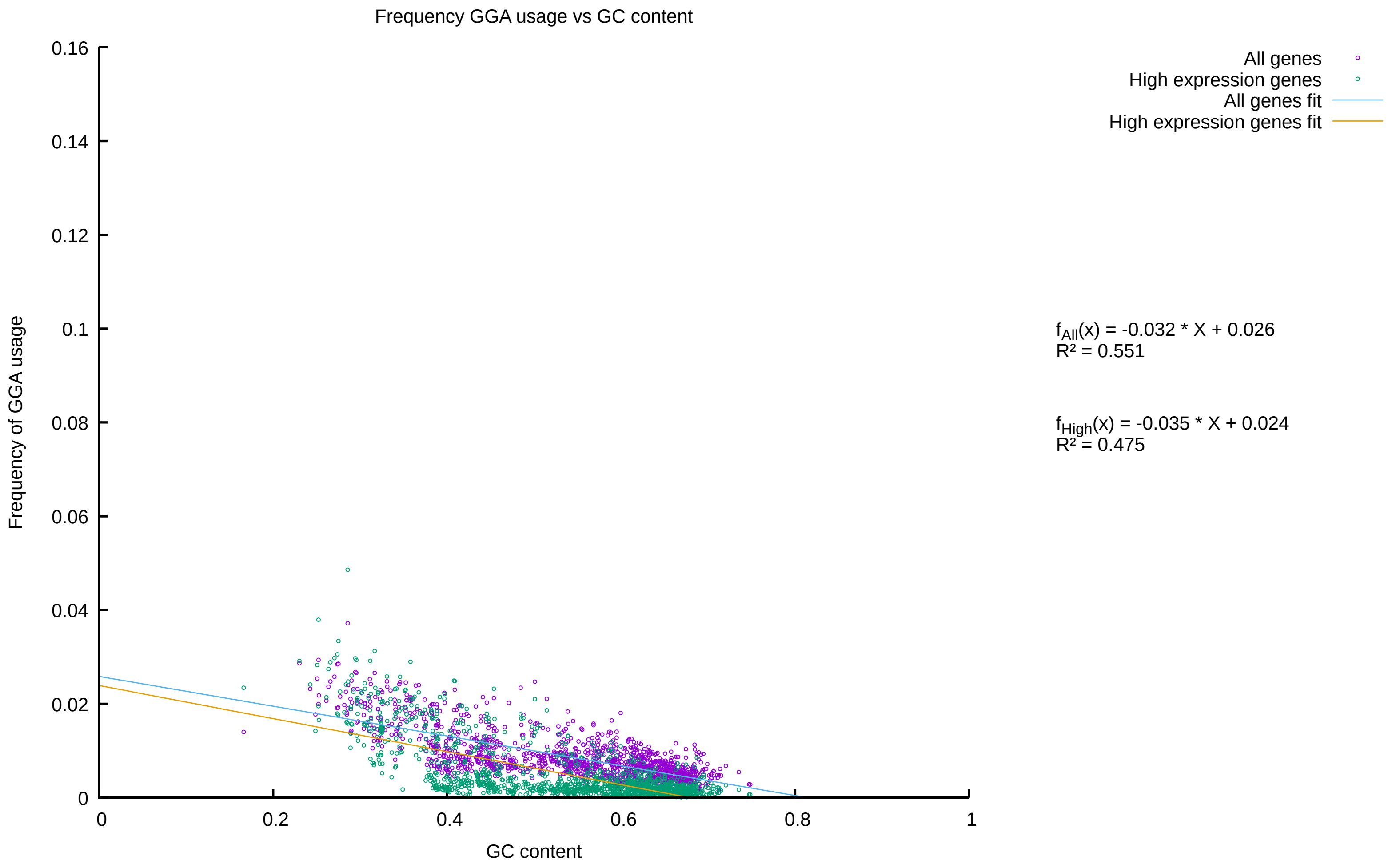
Frequency GGA usage vs GC content
Frequency of GGA usage
GC content
0.16
0.14
0.12
0.1
0.08
0.06
0.04
0.02
0
0.2
0.4
0.6
0.8
1
All genes
High expression genes
All genes fit
High expression genes fit
$f_{All}(x) = -0.032 * X + 0.026$
$R^2 = 0.551$
$f_{High}(x) = -0.035 * X + 0.024$
$R^2 = 0.475$

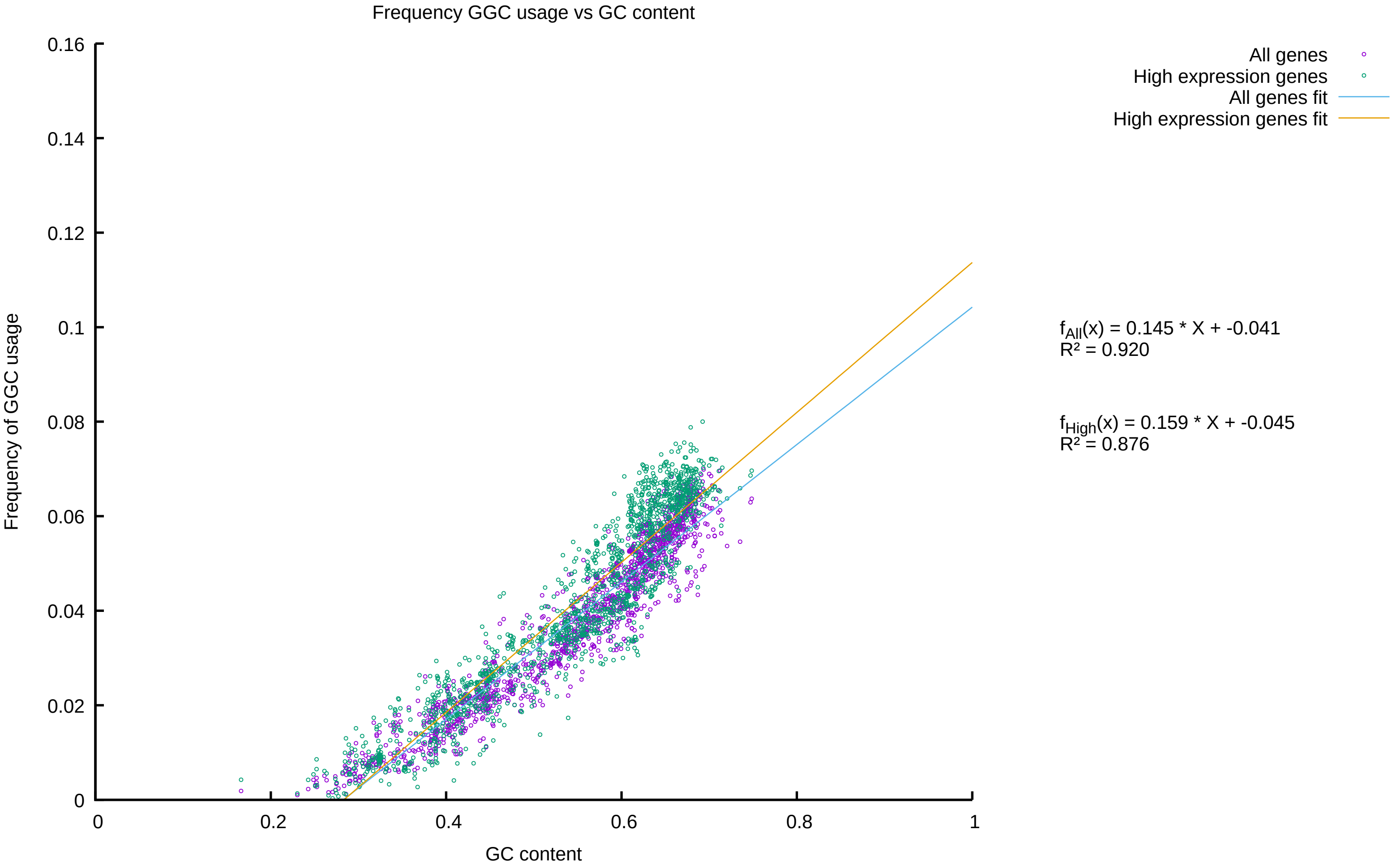
Frequency GGC usage vs GC content
0.16
0.14
0.12
0.1
0.08
0.06
0.04
0.02
0
0
0.2
0.4
0.6
0.8
1
GC content
Frequency of GGC usage
All genes
High expression genes
All genes fit
High expression genes fit
$f_{All}(x) = 0.145 * X + -0.041$
$R^2 = 0.920$
$f_{High}(x) = 0.159 * X + -0.045$
$R^2 = 0.876$

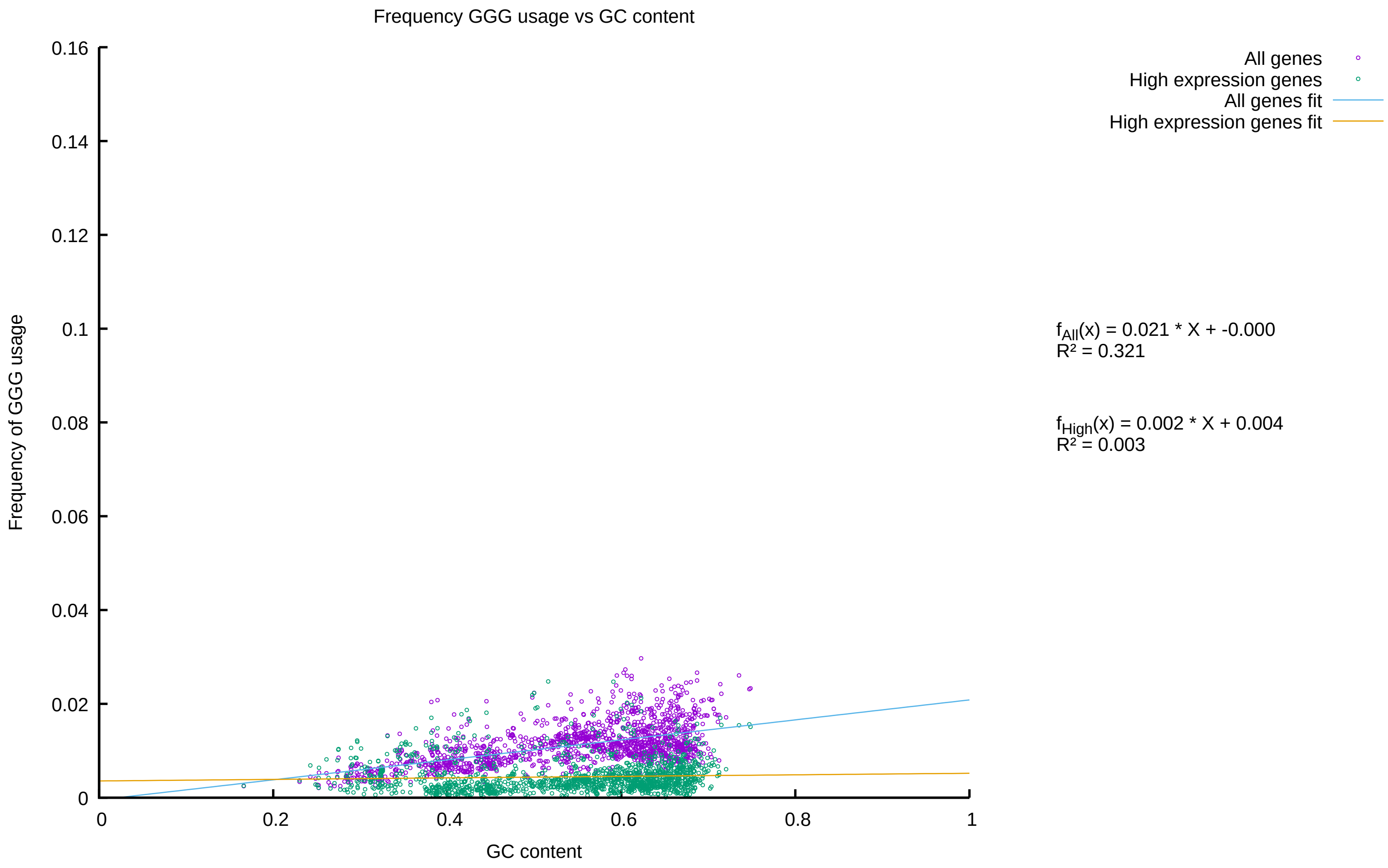

Frequency GGG usage vs GC content
Frequency of GGG usage
GC content
0.16
0.14
0.12
0.1
0.08
0.06
0.04
0.02
0
0
0.2
0.4
0.6
0.8
1
All genes
High expression genes
All genes fit
High expression genes fit
$f_{All}(x) = 0.021 * X + -0.000$
$R^2 = 0.321$
$f_{High}(x) = 0.002 * X + 0.004$
$R^2 = 0.003$

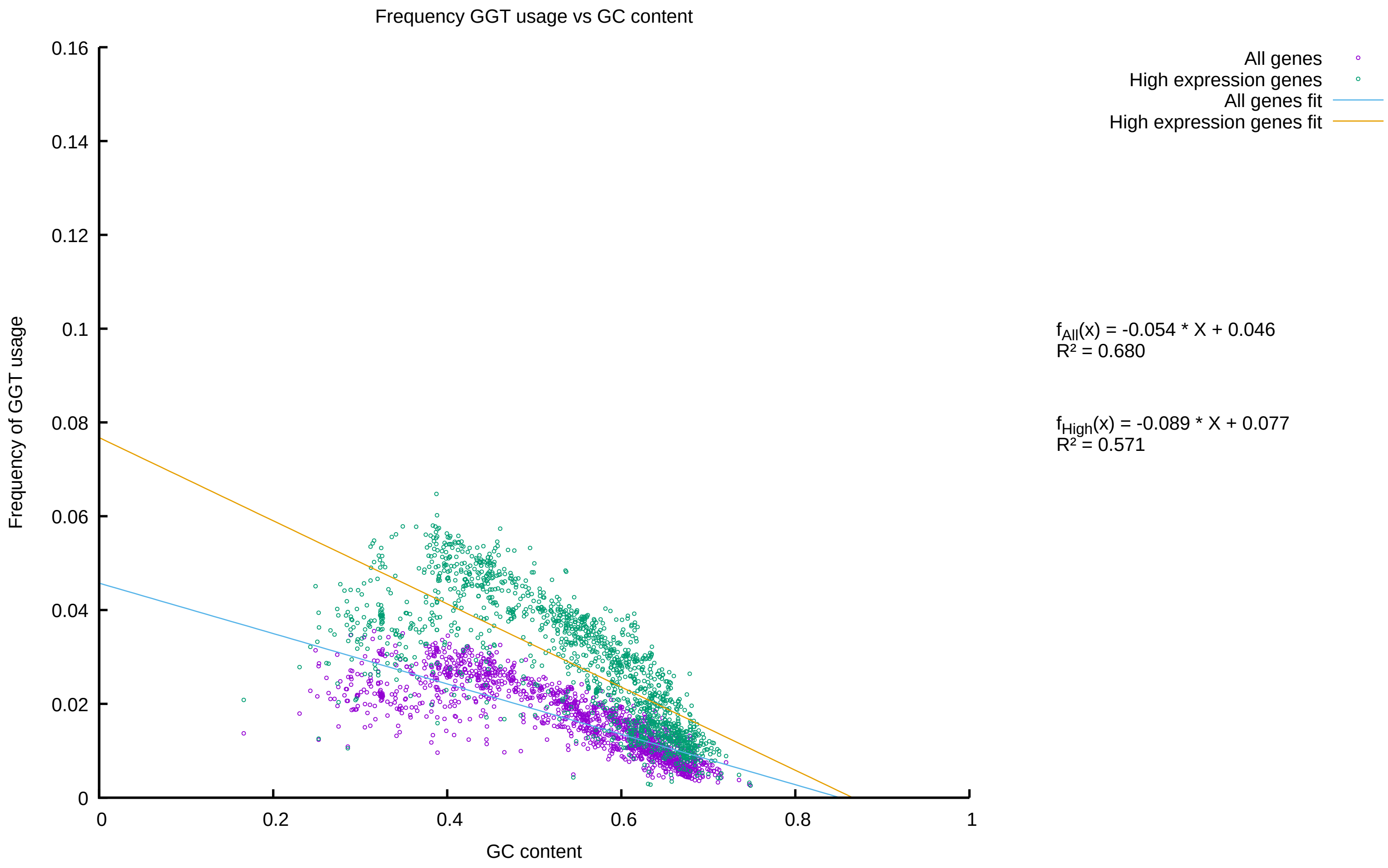

$f_{All}(x) = -0.054 * X + 0.046$
$R^2 = 0.680$

$f_{High}(x) = -0.089 * X + 0.077$
$R^2 = 0.571$

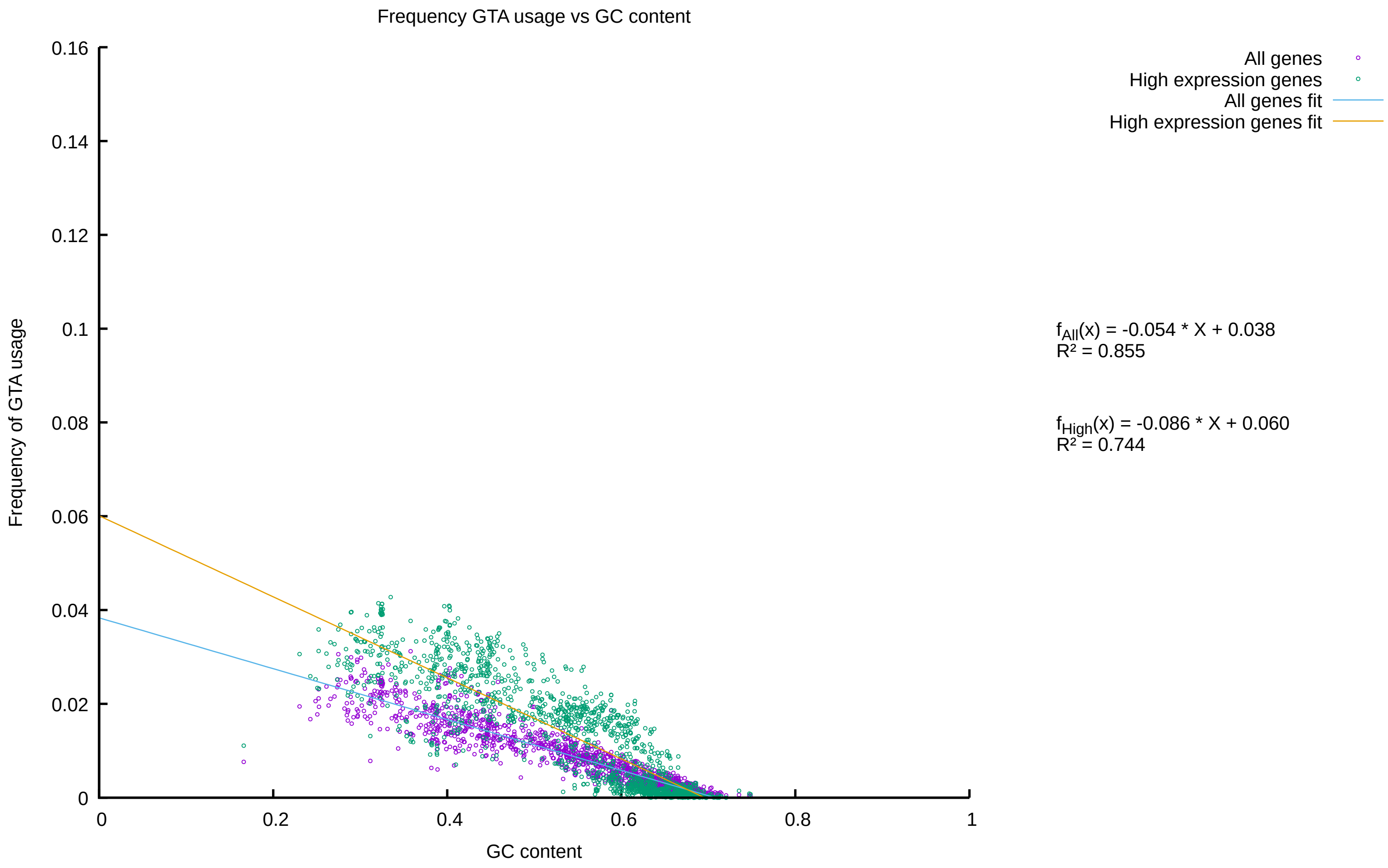

Frequency GTA usage vs GC content
Frequency of GTA usage
GC content
0.16
0.14
0.12
0.1
0.08
0.06
0.04
0.02
0
0
0.2
0.4
0.6
0.8
1
All genes
High expression genes
All genes fit
High expression genes fit
$f_{All}(x) = -0.054 * X + 0.038$
$R^2 = 0.855$
$f_{High}(x) = -0.086 * X + 0.060$
$R^2 = 0.744$

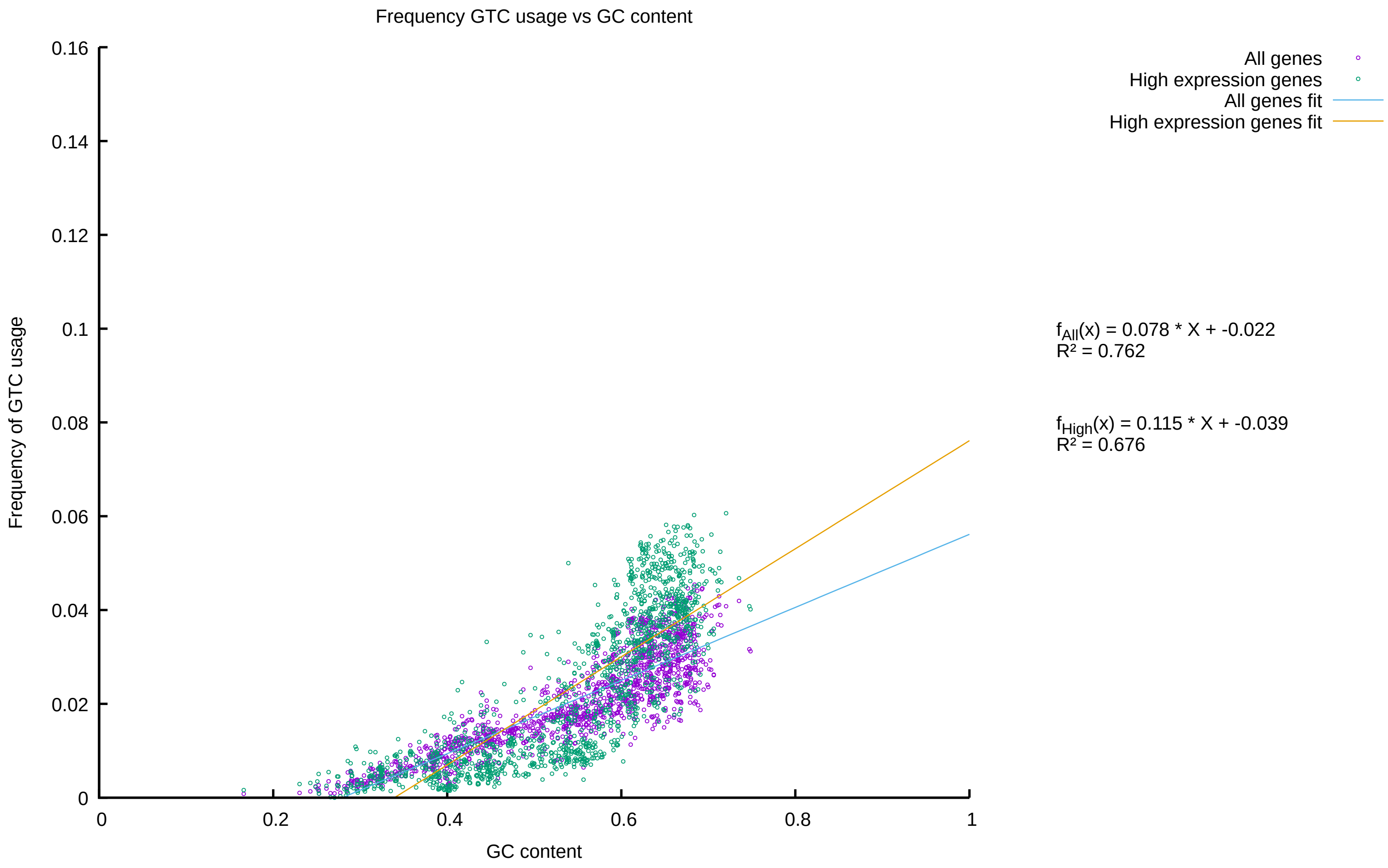

Frequency GTC usage vs GC content
0.16
0.14
0.12
0.1
0.08
0.06
0.04
0.02
0
0
0.2
0.4
0.6
0.8
1
GC content
Frequency of GTC usage
All genes
High expression genes
All genes fit
High expression genes fit
$f_{All}(x) = 0.078 * X + -0.022$
$R^2 = 0.762$
$f_{High}(x) = 0.115 * X + -0.039$
$R^2 = 0.676$

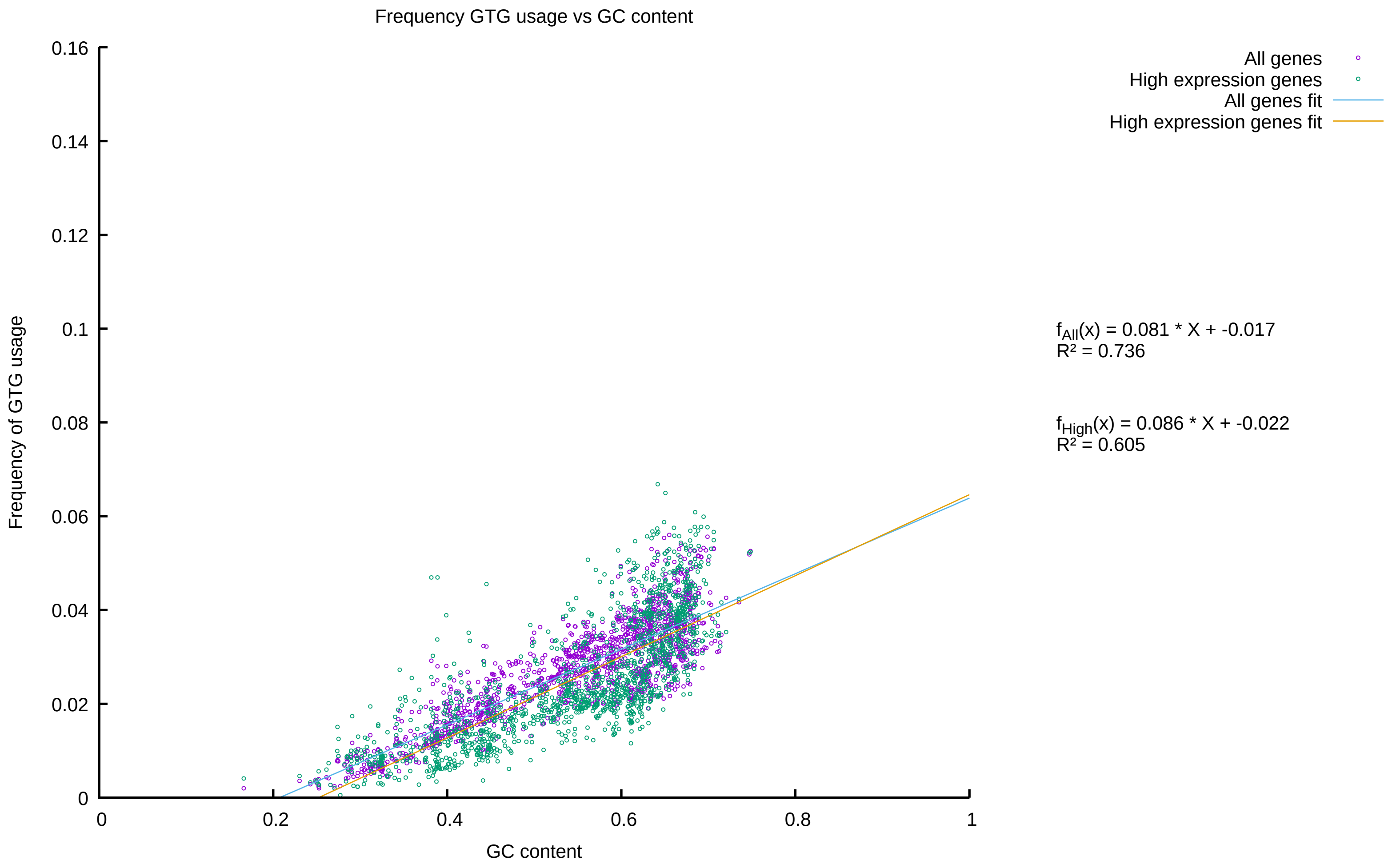

Frequency GTG usage vs GC content
All genes
High expression genes
All genes fit
High expression genes fit
$f_{All}(x) = 0.081 * X + -0.017$
$R^2 = 0.736$
$f_{High}(x) = 0.086 * X + -0.022$
$R^2 = 0.605$
Frequency of GTG usage
GC content
0.16
0.14
0.12
0.1
0.08
0.06
0.04
0.02
0
0
0.2
0.4
0.6
0.8
1

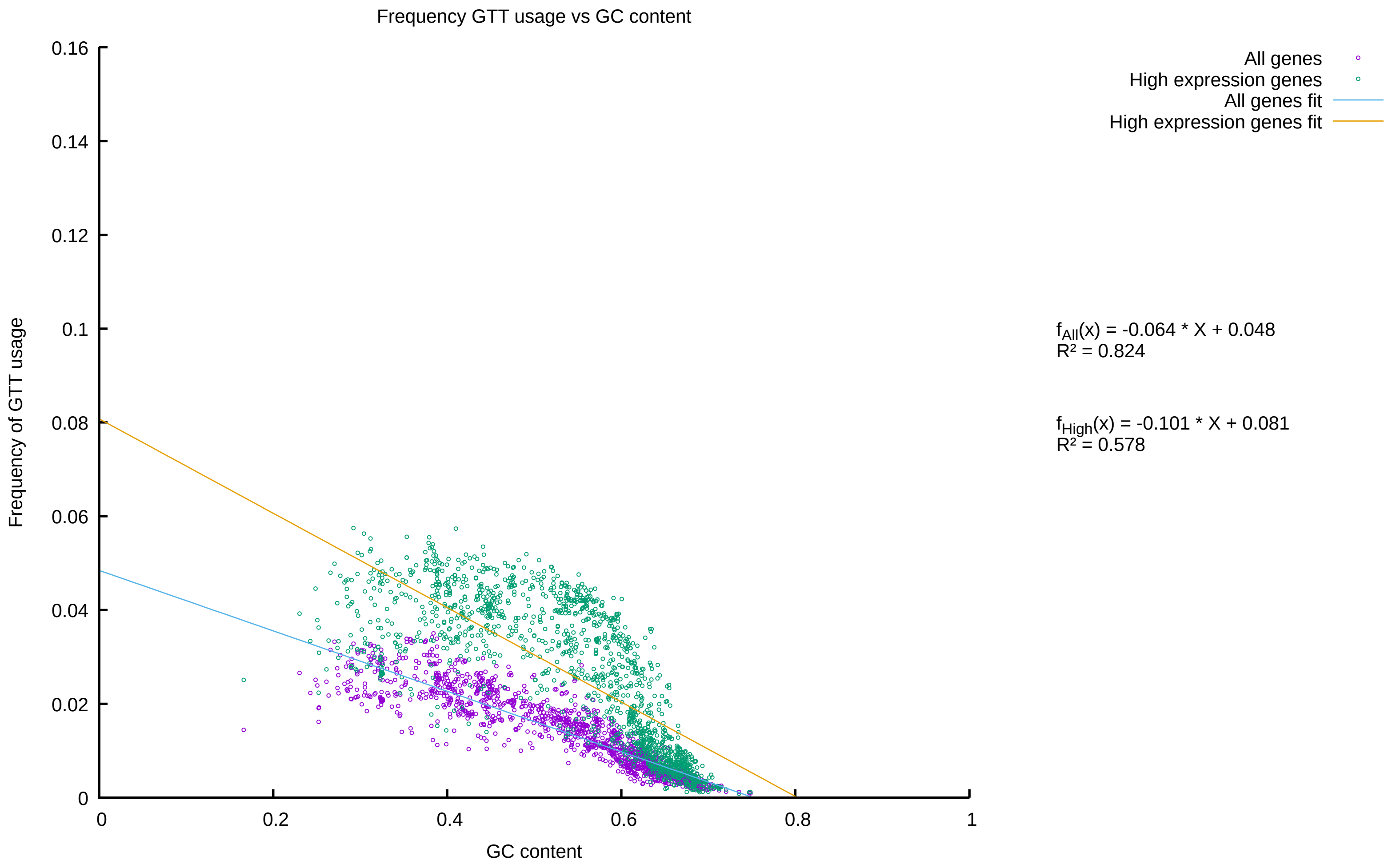
Frequency GTT usage vs GC content
Frequency of GTT usage
GC content
0.16
0.14
0.12
0.1
0.08
0.06
0.04
0.02
0
0
0.2
0.4
0.6
0.8
1
All genes
High expression genes
All genes fit
High expression genes fit
$f_{All}(x) = -0.064 * X + 0.048$
$R^2 = 0.824$
$f_{High}(x) = -0.101 * X + 0.081$
$R^2 = 0.578$

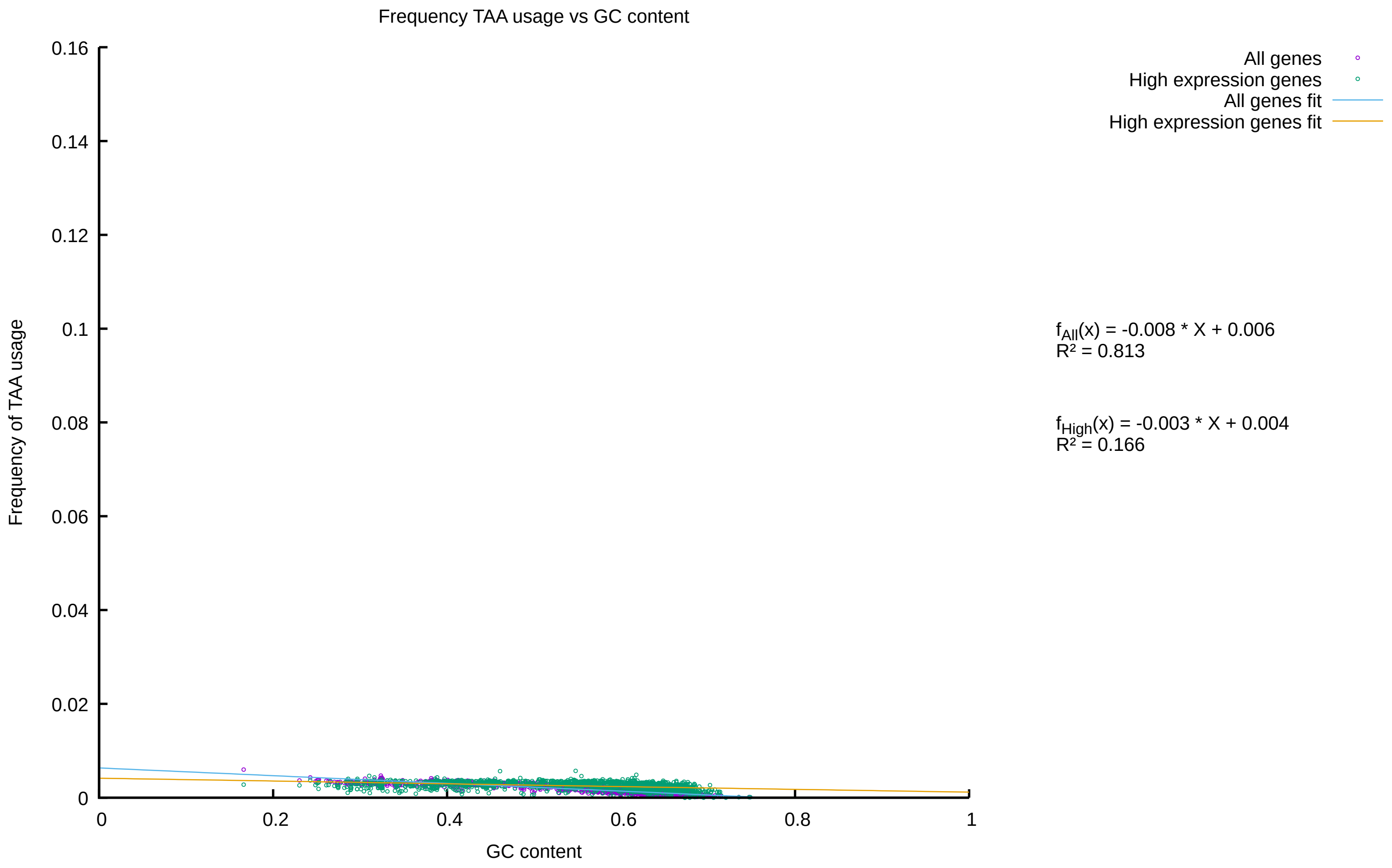

Frequency TAA usage vs GC content
0.16
0.14
0.12
0.1
0.08
0.06
0.04
0.02
0
0
0.2
0.4
0.6
0.8
1
GC content
Frequency of TAA usage
All genes
High expression genes
All genes fit
High expression genes fit
$f_{All}(x) = -0.008 * X + 0.006$
$R^2 = 0.813$
$f_{High}(x) = -0.003 * X + 0.004$
$R^2 = 0.166$

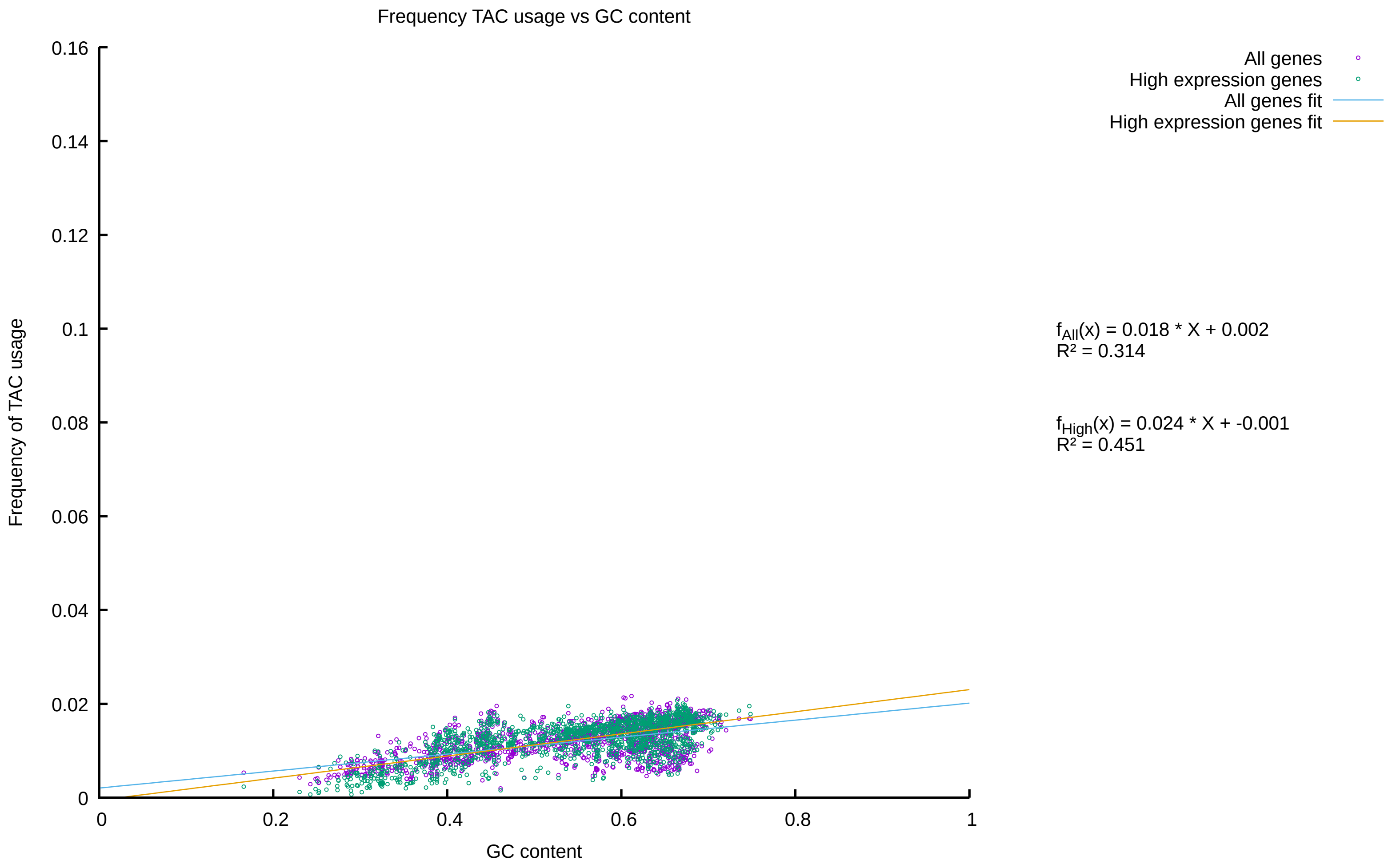

Frequency TAC usage vs GC content
Frequency of TAC usage
GC content
0.16
0.14
0.12
0.1
0.08
0.06
0.04
0.02
0
0.2
0.4
0.6
0.8
1
All genes
High expression genes
All genes fit
High expression genes fit
f_All(x) = 0.018 * X + 0.002
R² = 0.314
f_High(x) = 0.024 * X + -0.001
R² = 0.451

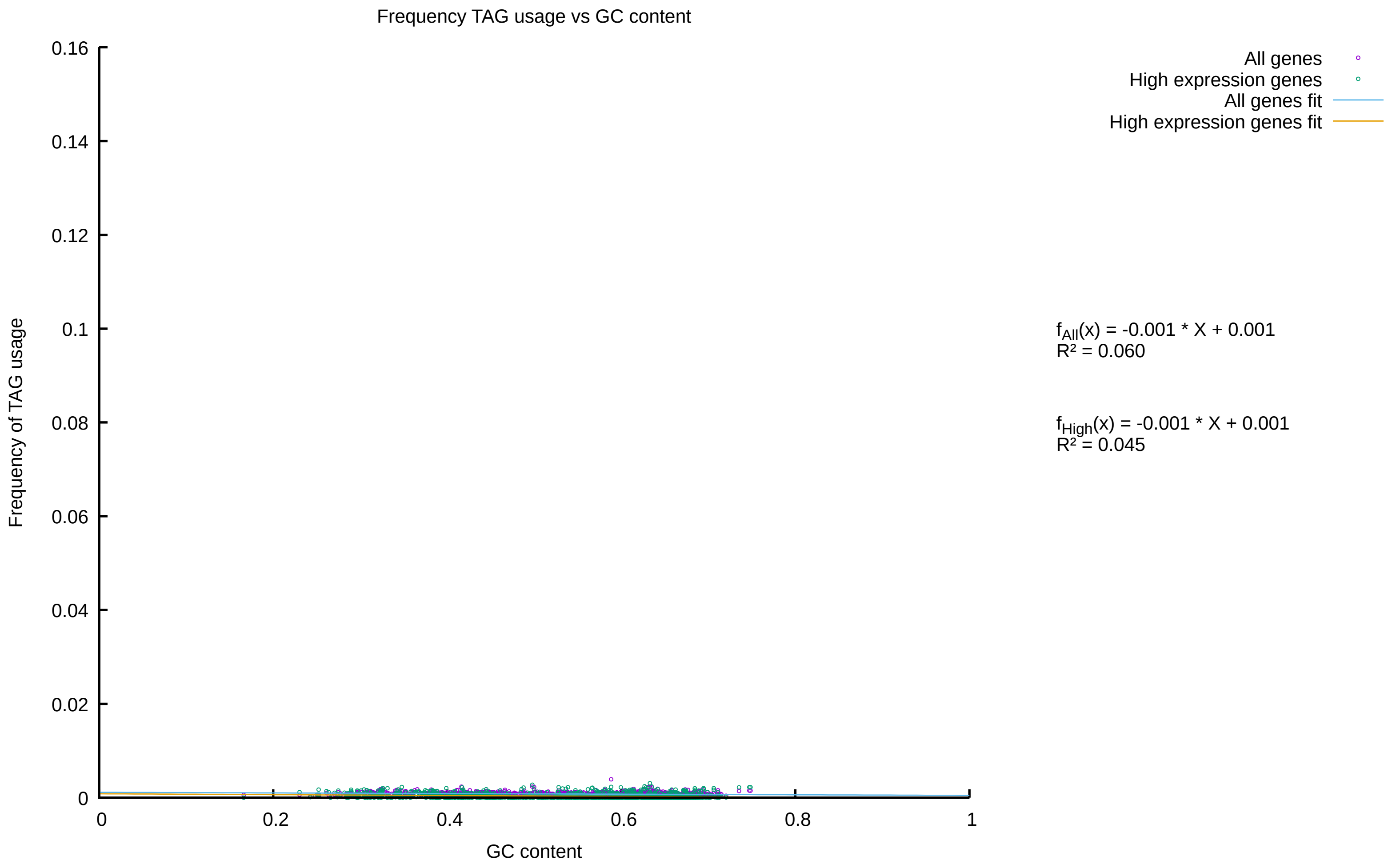

Frequency TAG usage vs GC content
Frequency of TAG usage
GC content
0.16
0.14
0.12
0.1
0.08
0.06
0.04
0.02
0
0
0.2
0.4
0.6
0.8
1
All genes
High expression genes
All genes fit
High expression genes fit
$f_{All}(x) = -0.001 * X + 0.001$
$R^2 = 0.060$
$f_{High}(x) = -0.001 * X + 0.001$
$R^2 = 0.045$

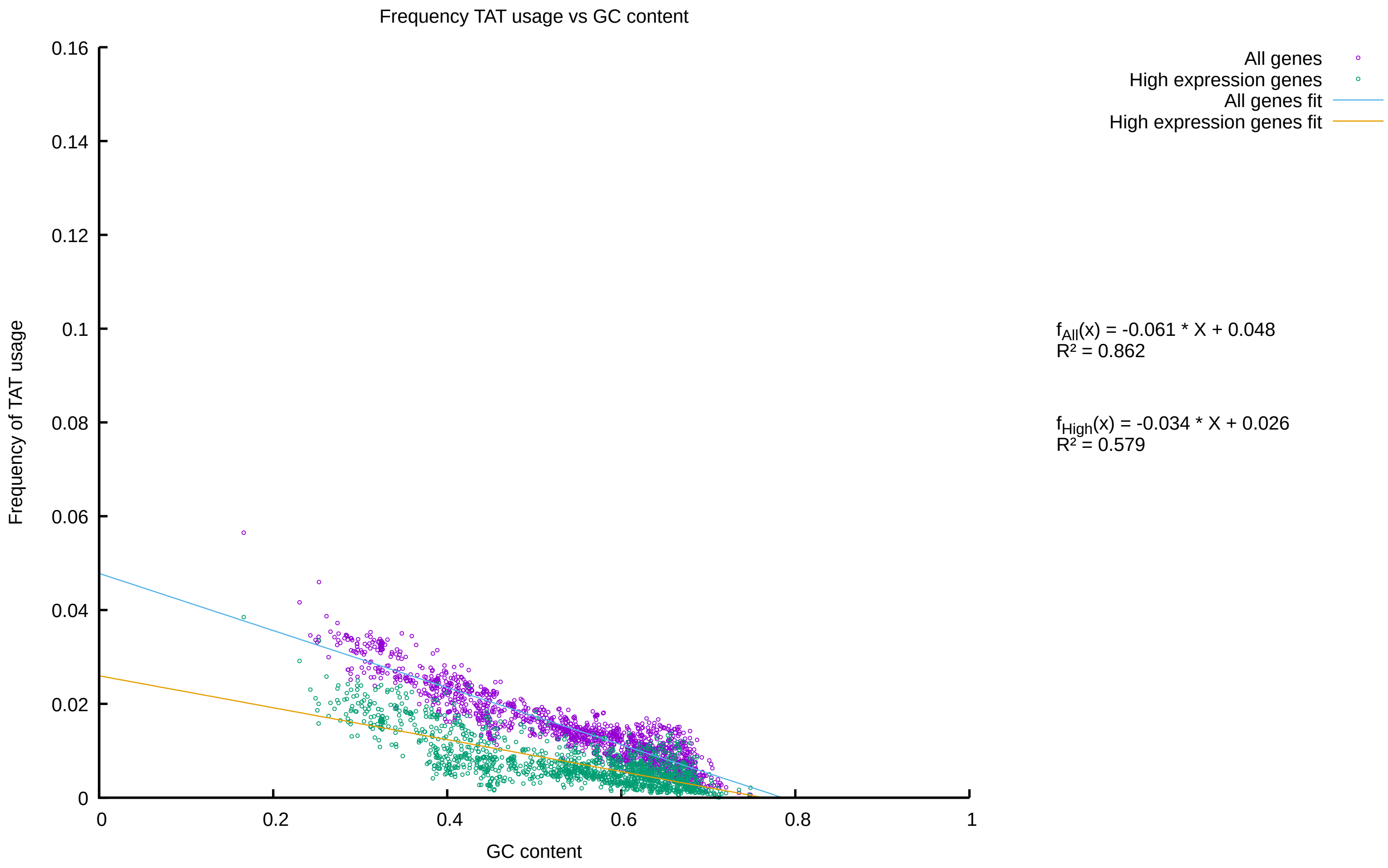

Frequency TAT usage vs GC content
All genes
High expression genes
All genes fit
High expression genes fit
$f_{All}(x) = -0.061 * X + 0.048$
$R^2 = 0.862$
$f_{High}(x) = -0.034 * X + 0.026$
$R^2 = 0.579$
Frequency of TAT usage
GC content
0.16
0.14
0.12
0.1
0.08
0.06
0.04
0.02
0
0.2
0.4
0.6
0.8
1

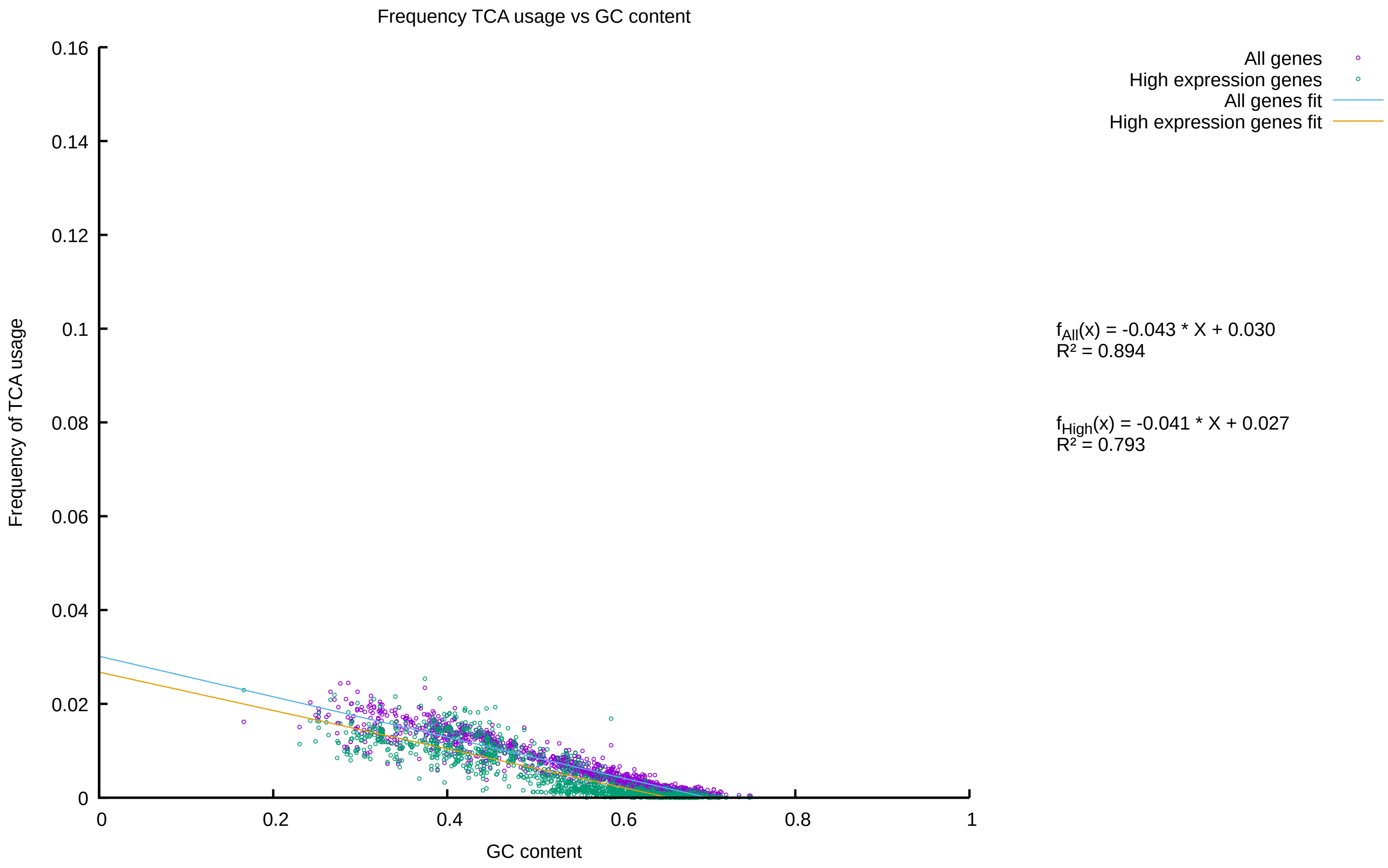
Frequency TCA usage vs GC content
0.16
0.14
0.12
0.1
0.08
0.06
0.04
0.02
0
0
0.2
0.4
0.6
0.8
1
GC content
Frequency of TCA usage
All genes
High expression genes
All genes fit
High expression genes fit
f_All(x) = -0.043 * X + 0.030
R² = 0.894
f_High(x) = -0.041 * X + 0.027
R² = 0.793

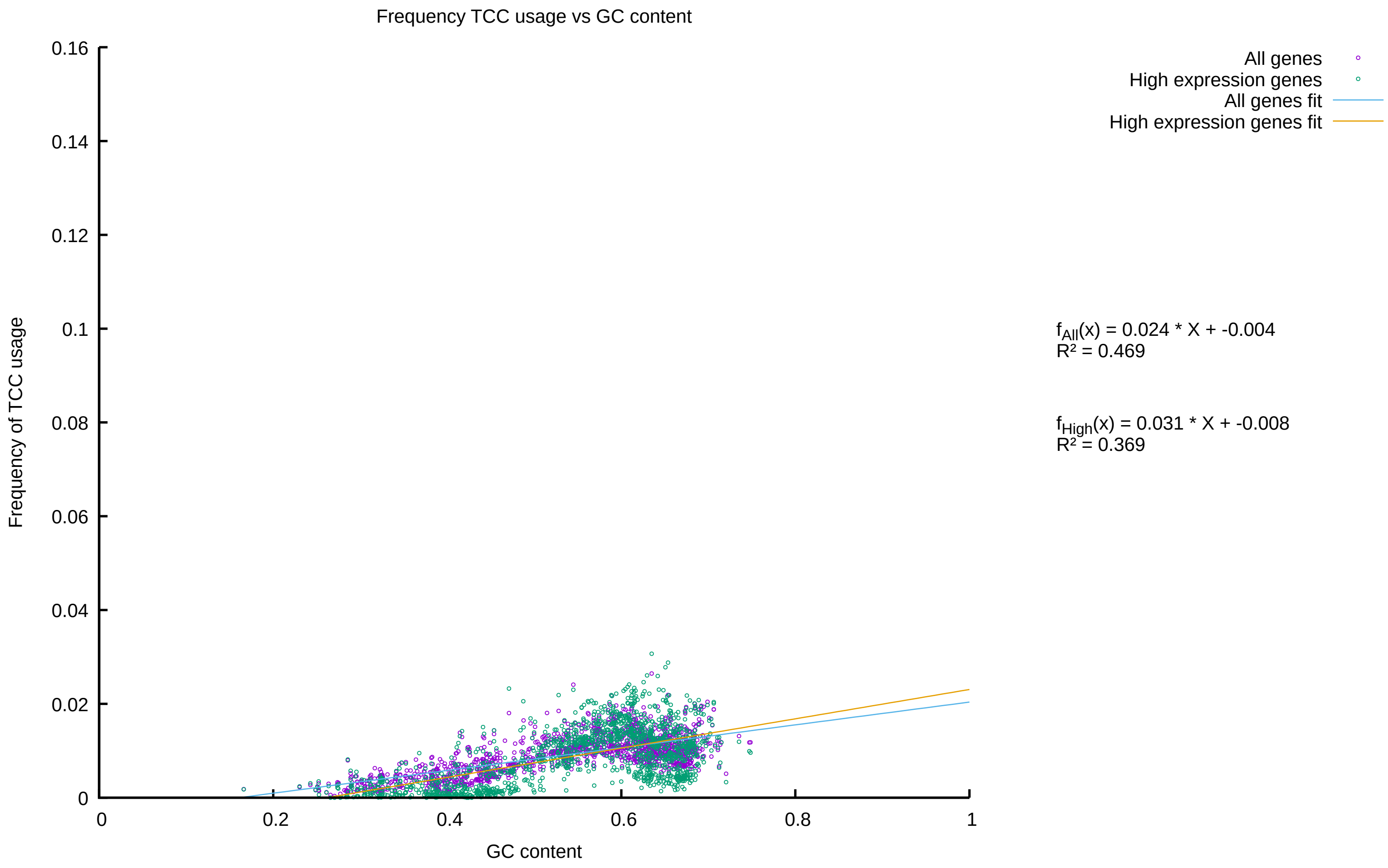

Frequency TCC usage vs GC content
Frequency of TCC usage
GC content
0.16
0.14
0.12
0.1
0.08
0.06
0.04
0.02
0
0
0.2
0.4
0.6
0.8
1
All genes
High expression genes
All genes fit
High expression genes fit
f_All(x) = 0.024 * X + -0.004
R² = 0.469
f_High(x) = 0.031 * X + -0.008
R² = 0.369

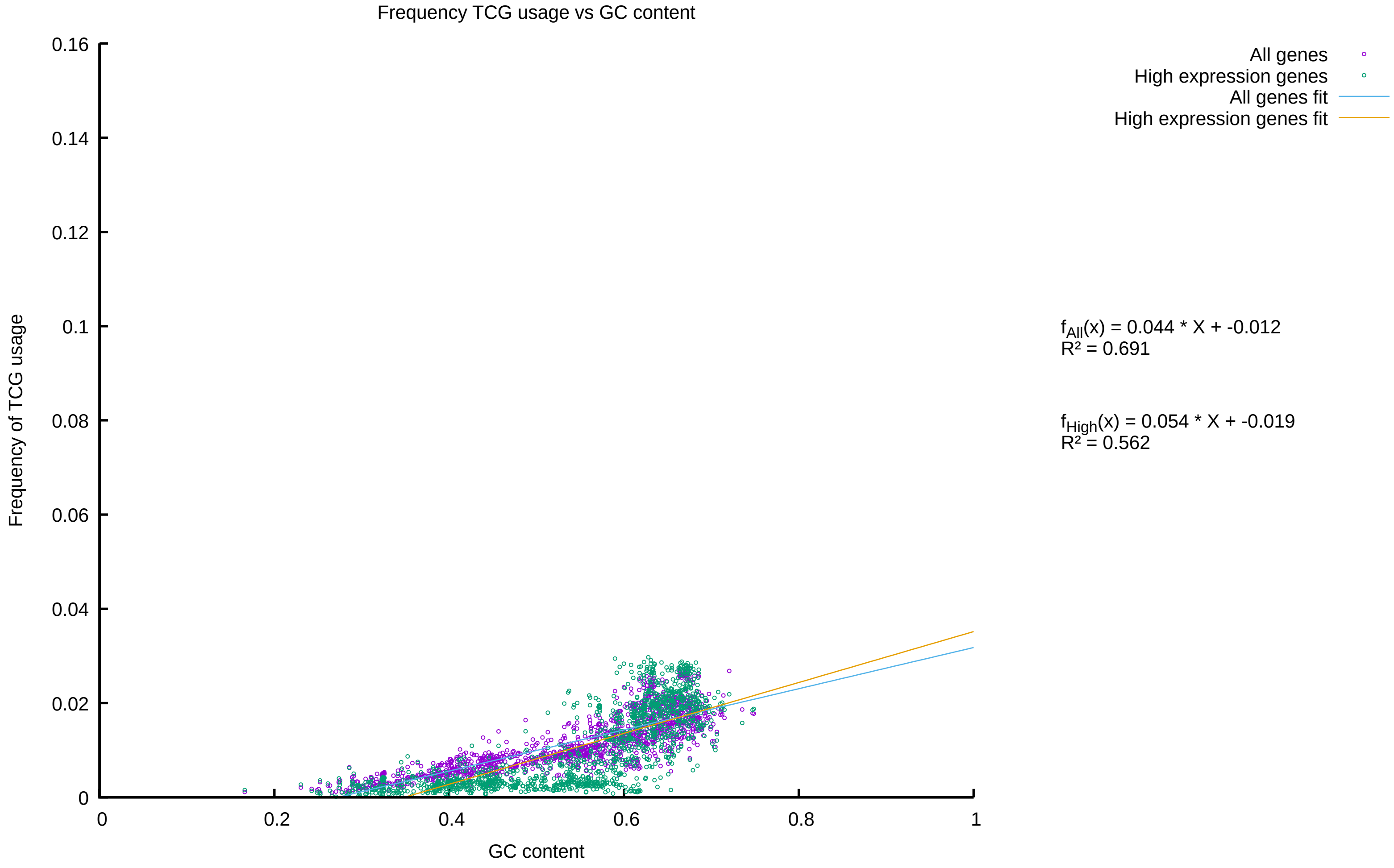

$f_{All}(x) = 0.044 * X + -0.012$
$R^2 = 0.691$

$f_{High}(x) = 0.054 * X + -0.019$
$R^2 = 0.562$

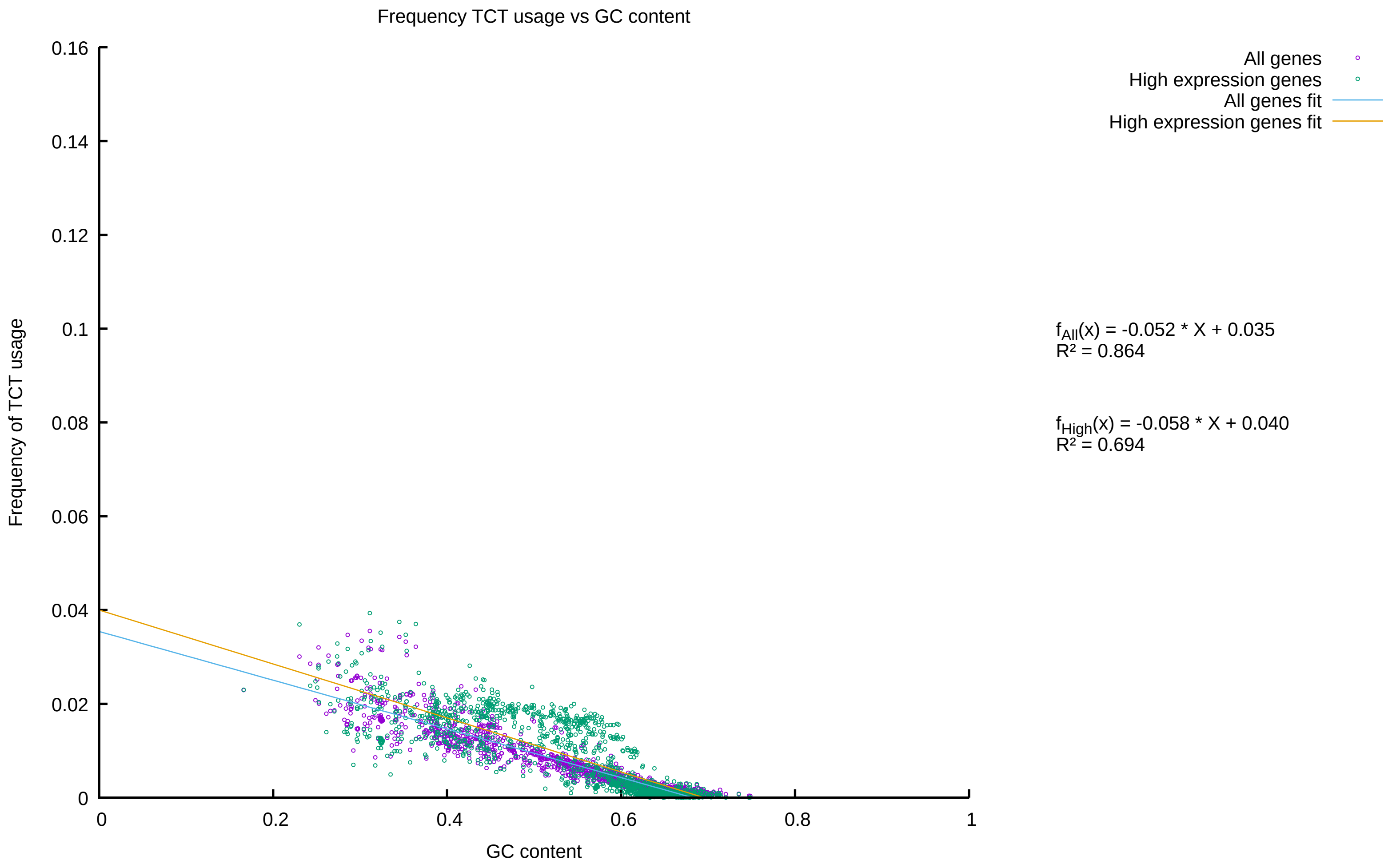

Frequency TCT usage vs GC content
0.16
0.14
0.12
0.1
0.08
0.06
0.04
0.02
0
Frequency of TCT usage
0
0.2
0.4
0.6
0.8
1
GC content
All genes
High expression genes
All genes fit
High expression genes fit
$f_{All}(x) = -0.052 * X + 0.035$
$R^2 = 0.864$
$f_{High}(x) = -0.058 * X + 0.040$
$R^2 = 0.694$

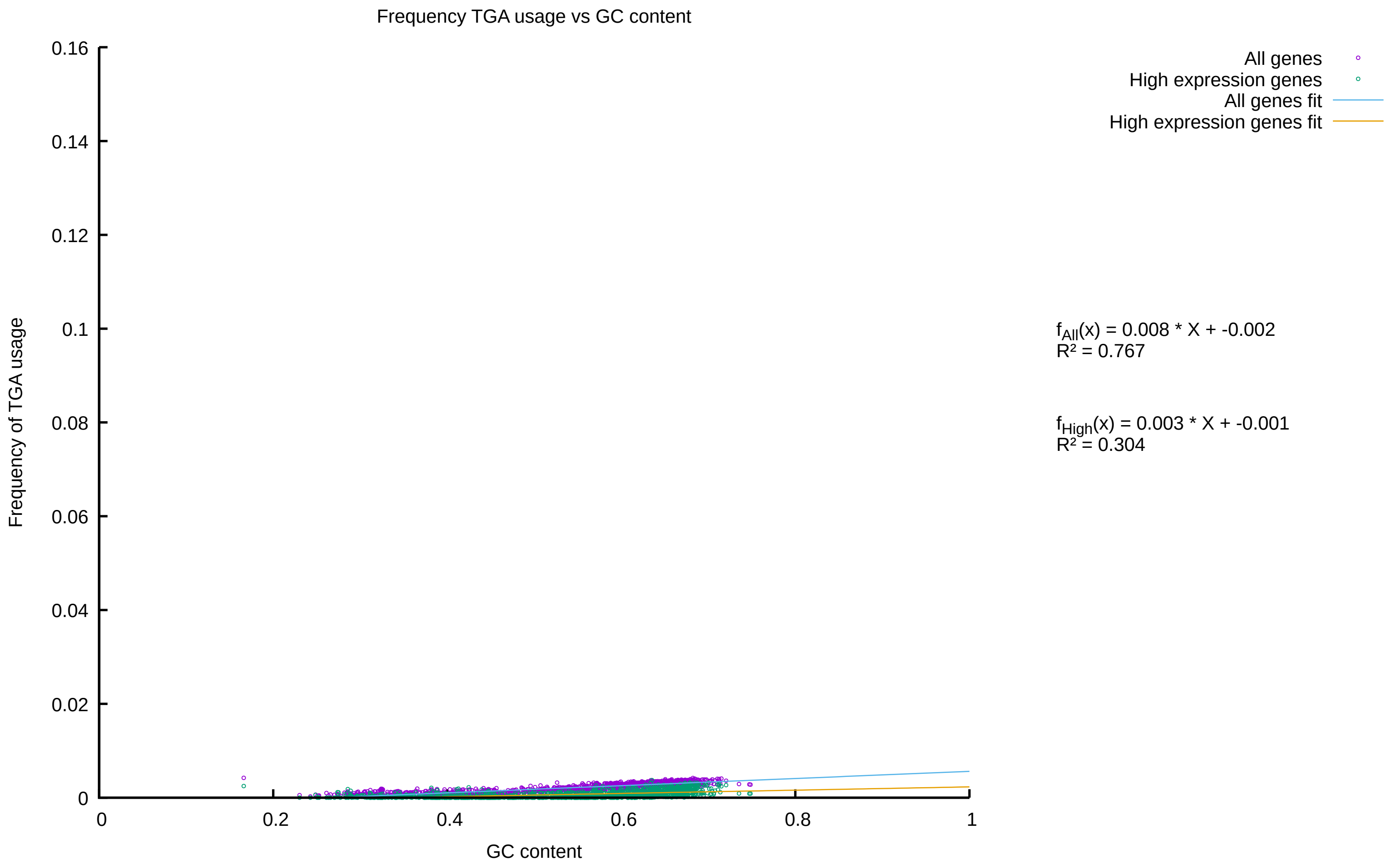

Frequency TGA usage vs GC content
Frequency of TGA usage
GC content
0.16
0.14
0.12
0.1
0.08
0.06
0.04
0.02
0
0.2
0.4
0.6
0.8
1
All genes
High expression genes
All genes fit
High expression genes fit
$f_{All}(x) = 0.008 * X + -0.002$
$R^2 = 0.767$
$f_{High}(x) = 0.003 * X + -0.001$
$R^2 = 0.304$

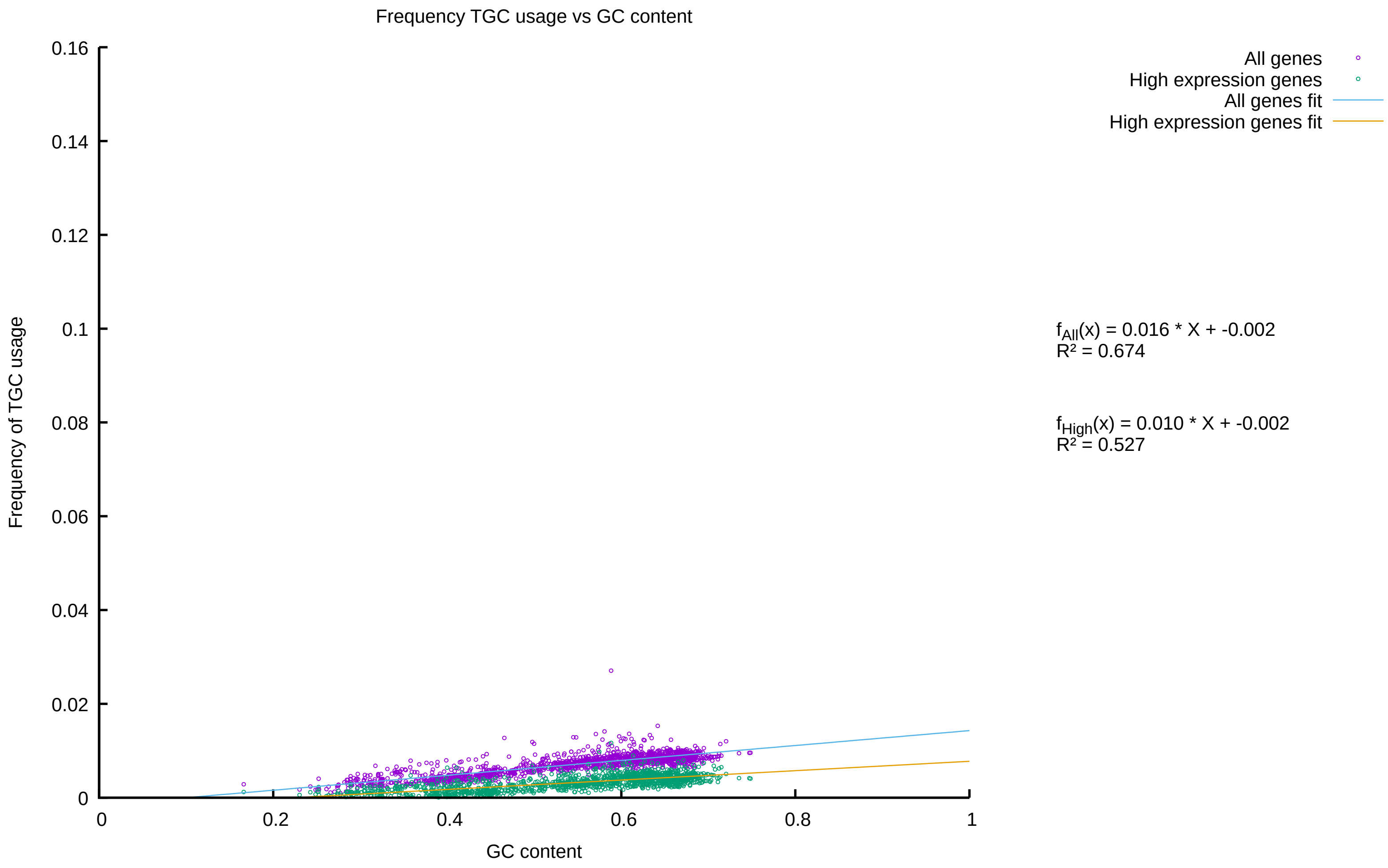

Frequency TGC usage vs GC content
0.16
0.14
0.12
0.1
0.08
0.06
0.04
0.02
0
Frequency of TGC usage
0
0.2
0.4
0.6
0.8
1
GC content
All genes
High expression genes
All genes fit
High expression genes fit
$f_{All}(x) = 0.016 * X + -0.002$
$R^2 = 0.674$
$f_{High}(x) = 0.010 * X + -0.002$
$R^2 = 0.527$

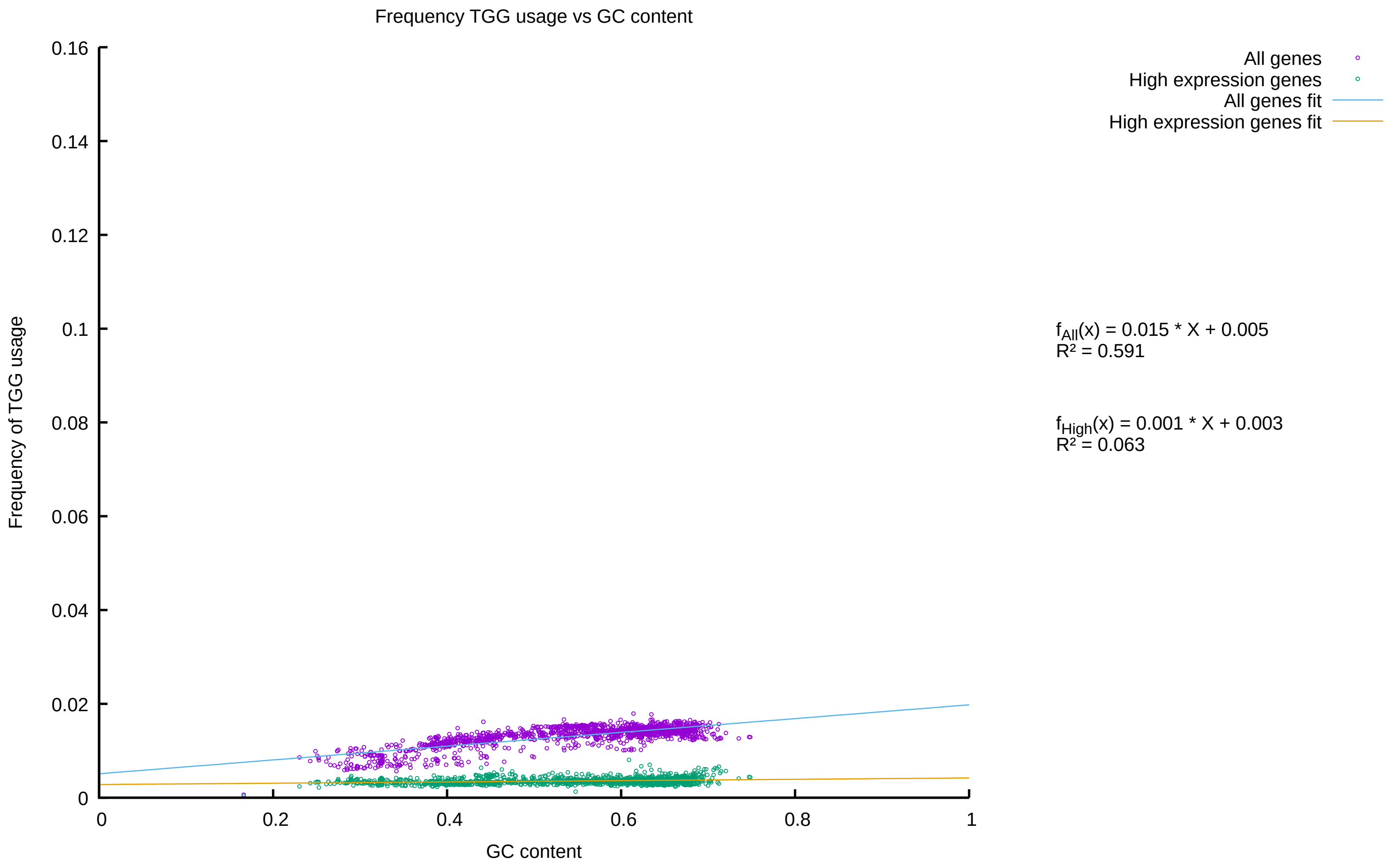

Frequency TGG usage vs GC content
0.16
0.14
0.12
0.1
0.08
0.06
0.04
0.02
0
Frequency of TGG usage
0
0.2
0.4
0.6
0.8
1
GC content
All genes
High expression genes
All genes fit
High expression genes fit
$f_{All}(x) = 0.015 * X + 0.005$
$R^2 = 0.591$
$f_{High}(x) = 0.001 * X + 0.003$
$R^2 = 0.063$

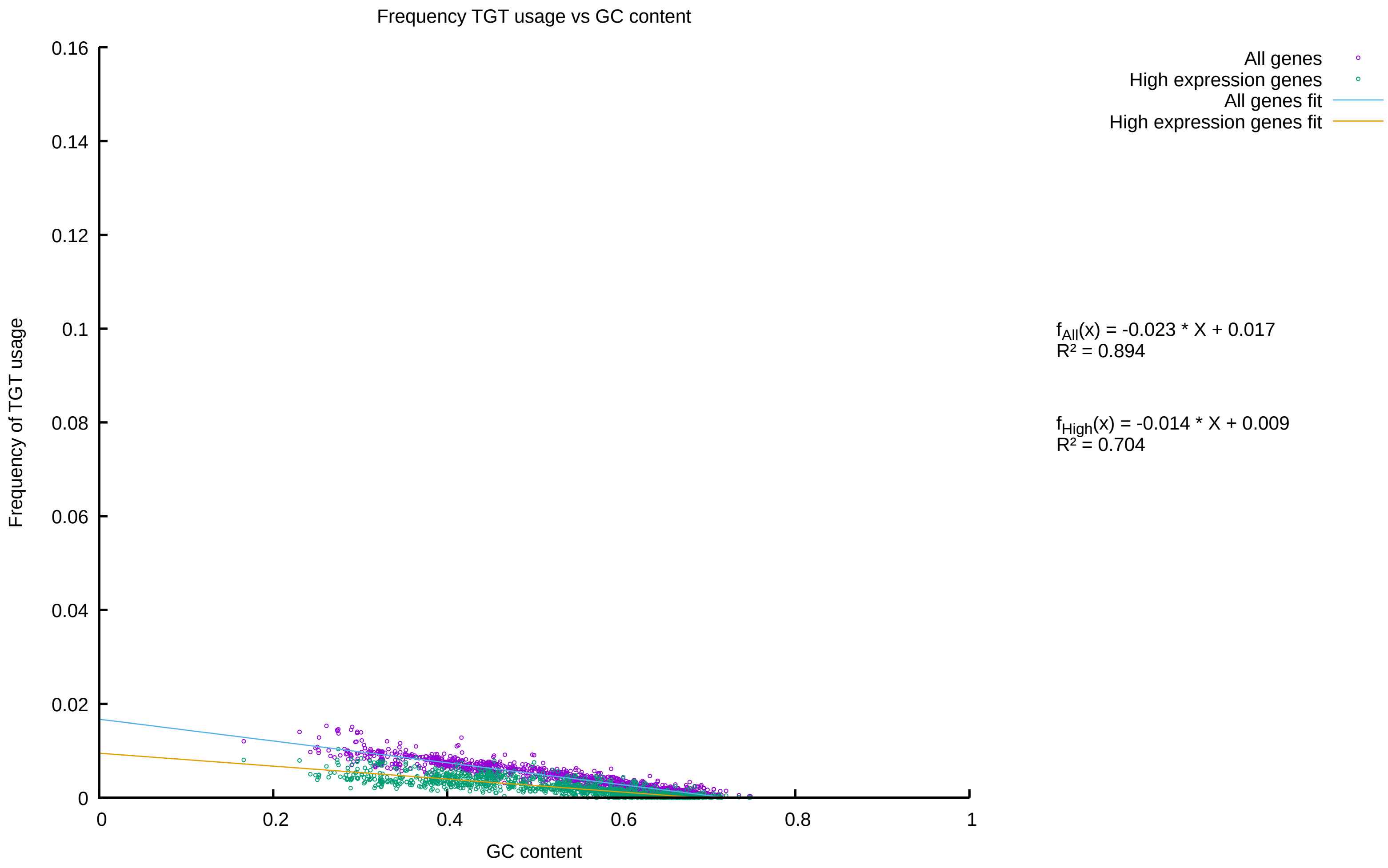

Frequency TGT usage vs GC content
Frequency of TGT usage
GC content
0.16
0.14
0.12
0.1
0.08
0.06
0.04
0.02
0
0
0.2
0.4
0.6
0.8
1
All genes
High expression genes
All genes fit
High expression genes fit
$f_{All}(x) = -0.023 * X + 0.017$
$R^2 = 0.894$
$f_{High}(x) = -0.014 * X + 0.009$
$R^2 = 0.704$

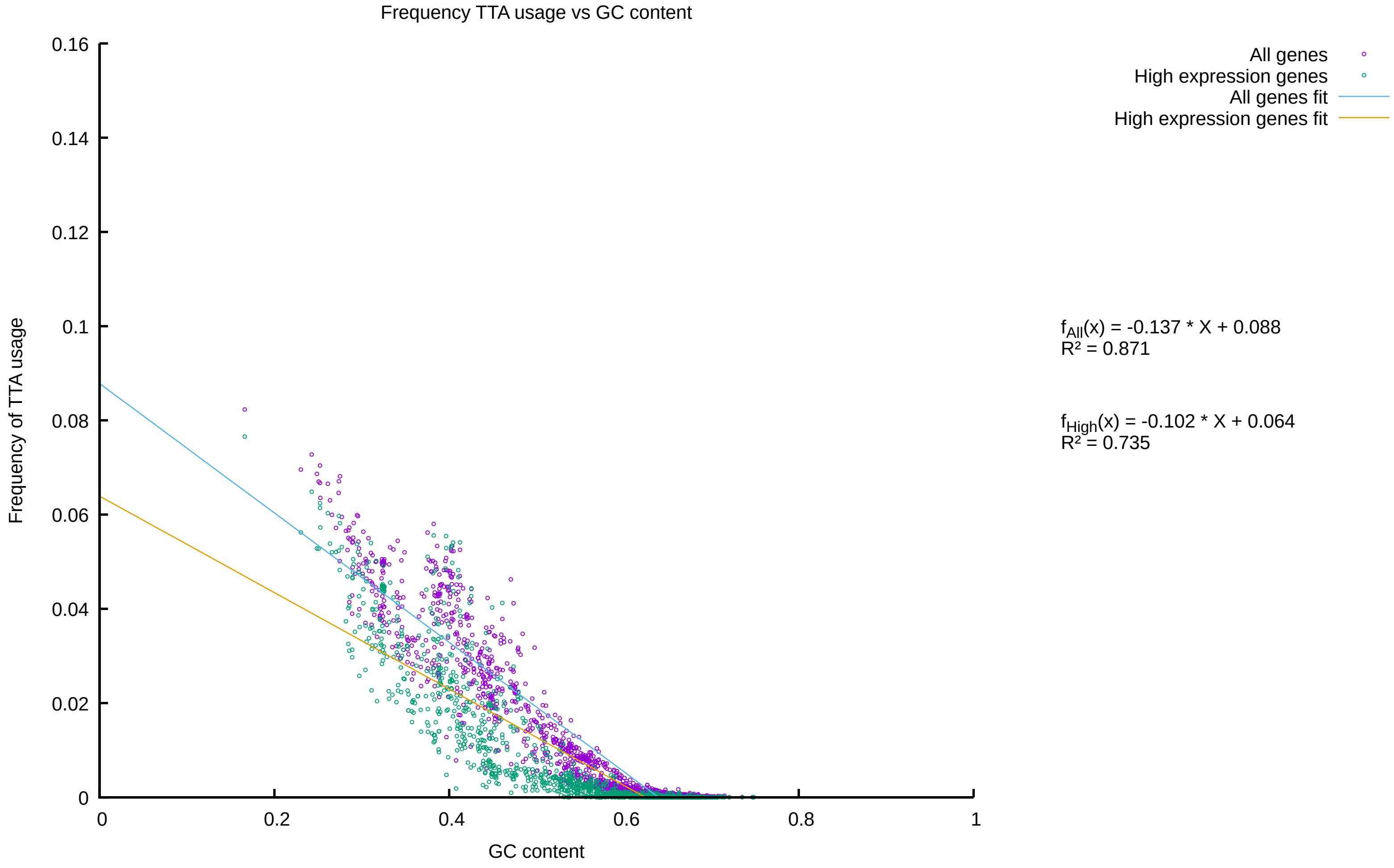
Frequency TTA usage vs GC content
Frequency of TTA usage
GC content
0
0.02
0.04
0.06
0.08
0.1
0.12
0.14
0.16
0
0.2
0.4
0.6
0.8
1
All genes
High expression genes
All genes fit
High expression genes fit
$f_{All}(x) = -0.137 * X + 0.088$
$R^2 = 0.871$
$f_{High}(x) = -0.102 * X + 0.064$
$R^2 = 0.735$

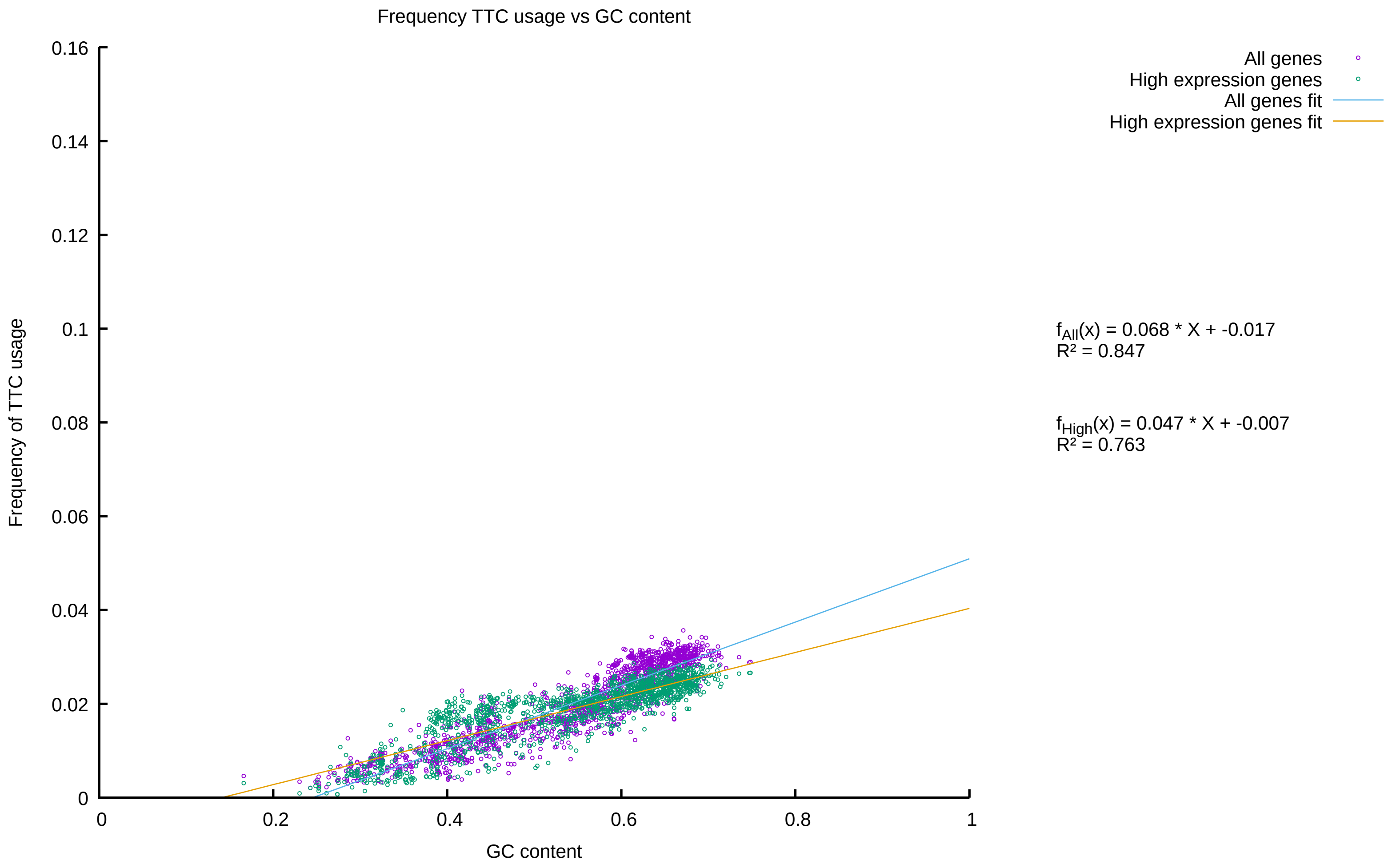

Frequency TTC usage vs GC content
Frequency of TTC usage
GC content
0.16
0.14
0.12
0.1
0.08
0.06
0.04
0.02
0
0
0.2
0.4
0.6
0.8
1
All genes
High expression genes
All genes fit
High expression genes fit
f_All(x) = 0.068 * X + -0.017
R² = 0.847
f_High(x) = 0.047 * X + -0.007
R² = 0.763

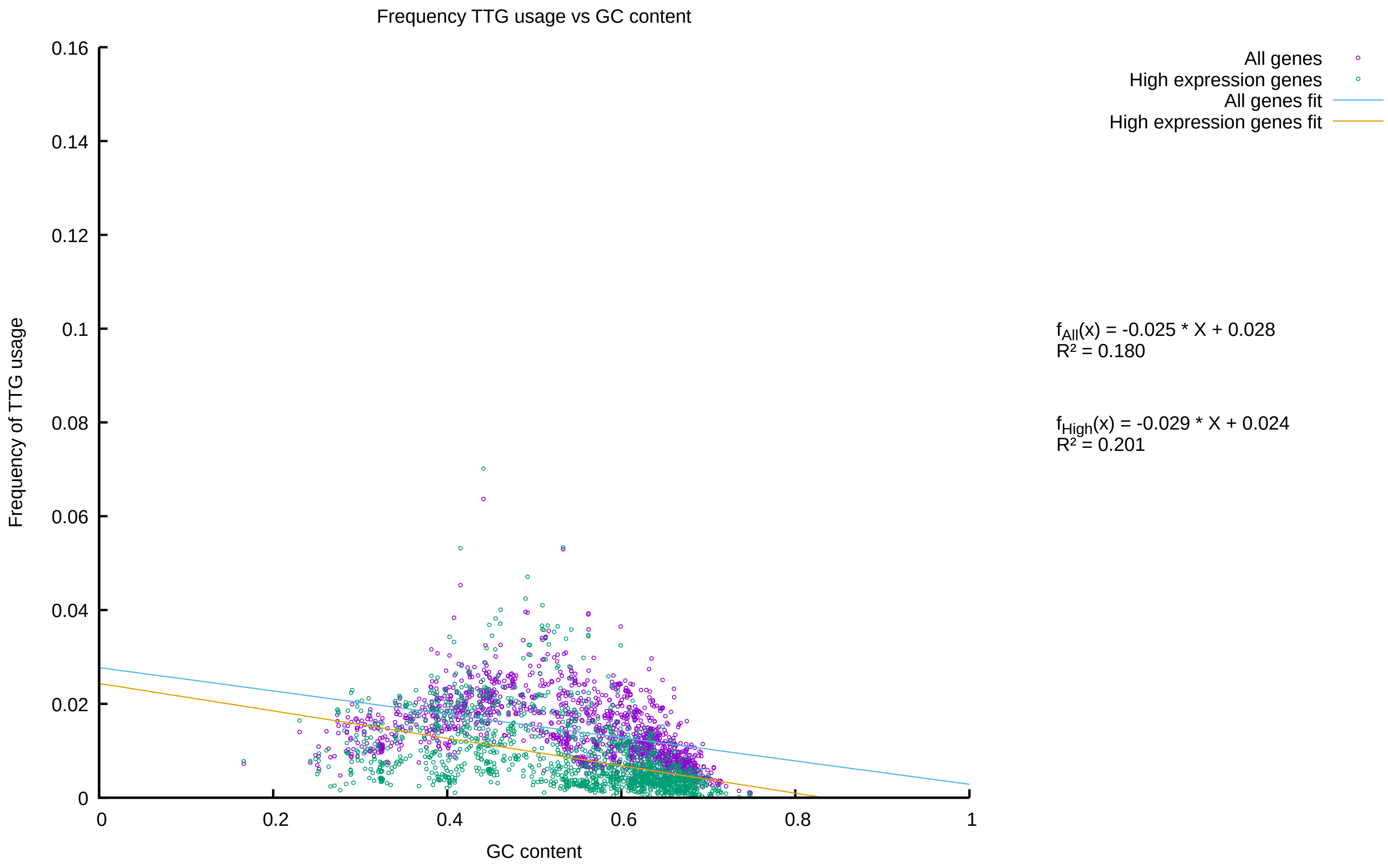

Frequency TTG usage vs GC content
Frequency of TTG usage
GC content
0.16
0.14
0.12
0.1
0.08
0.06
0.04
0.02
0
0
0.2
0.4
0.6
0.8
1
All genes
High expression genes
All genes fit
High expression genes fit
$f_{All}(x) = -0.025 * X + 0.028$
$R^2 = 0.180$
$f_{High}(x) = -0.029 * X + 0.024$
$R^2 = 0.201$

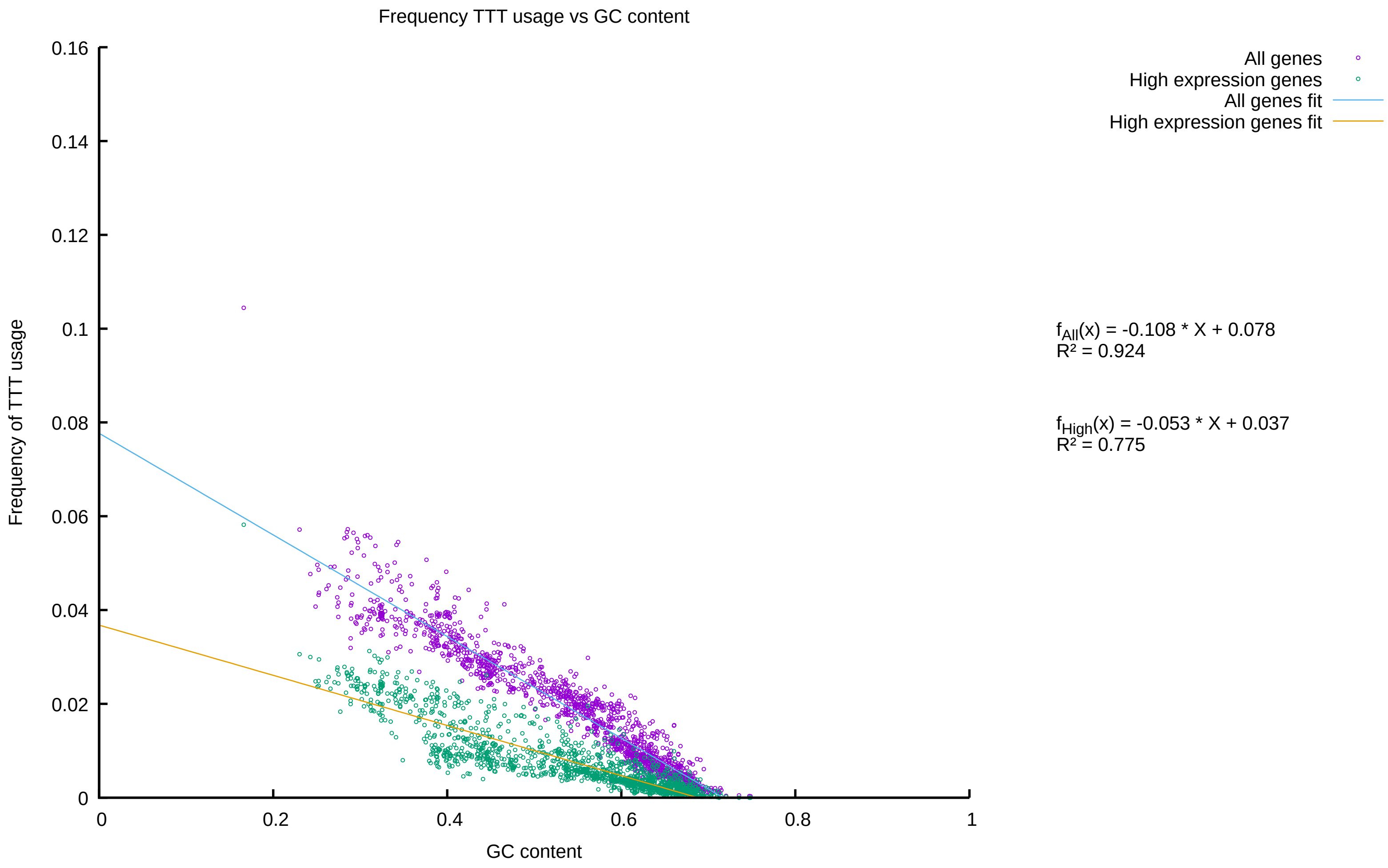
Frequency TTT usage vs GC content
0.16
0.14
0.12
0.1
0.08
0.06
0.04
0.02
0
0
0.2
0.4
0.6
0.8
1
Frequency of TTT usage
GC content
All genes
High expression genes
All genes fit
High expression genes fit
f_All(x) = -0.108 * X + 0.078
R² = 0.924
f_High(x) = -0.053 * X + 0.037
R² = 0.775

Figure S24. Codon usage is not correlated to the number of genes coding for complementary tRNAs. The frequency of codon usage in all genes of each of the analyzed genomes **(A)** or a subset of genes expected to be highly expressed **(B)** is compared to the number of genes coding for tRNAs with perfectly complementary anticodons. Linear regressions are included in plots, but care should be taken when analyzing these trend lines as most correlation coefficients are low.

(A)

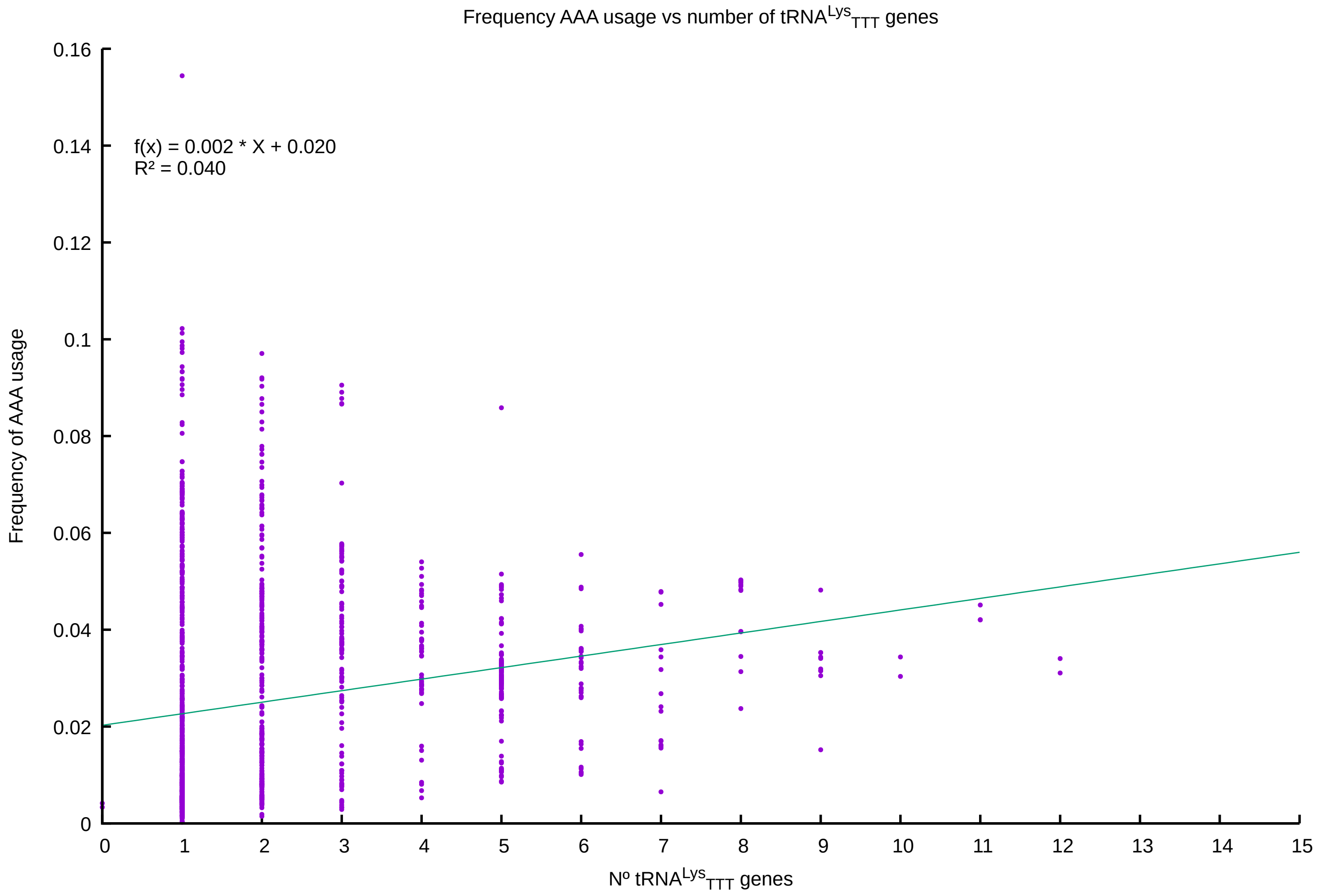

Frequency AAA usage vs number of tRNA$^{Lys}_{TTT}$ genes
f(x) = 0.002 * X + 0.020
R^2 = 0.040
Frequency of AAA usage
0.16
0.14
0.12
0.1
0.08
0.06
0.04
0.02
0
0 1 2 3 4 5 6 7 8 9 10 11 12 13 14 15
Nº tRNA$^{Lys}_{TTT}$ genes

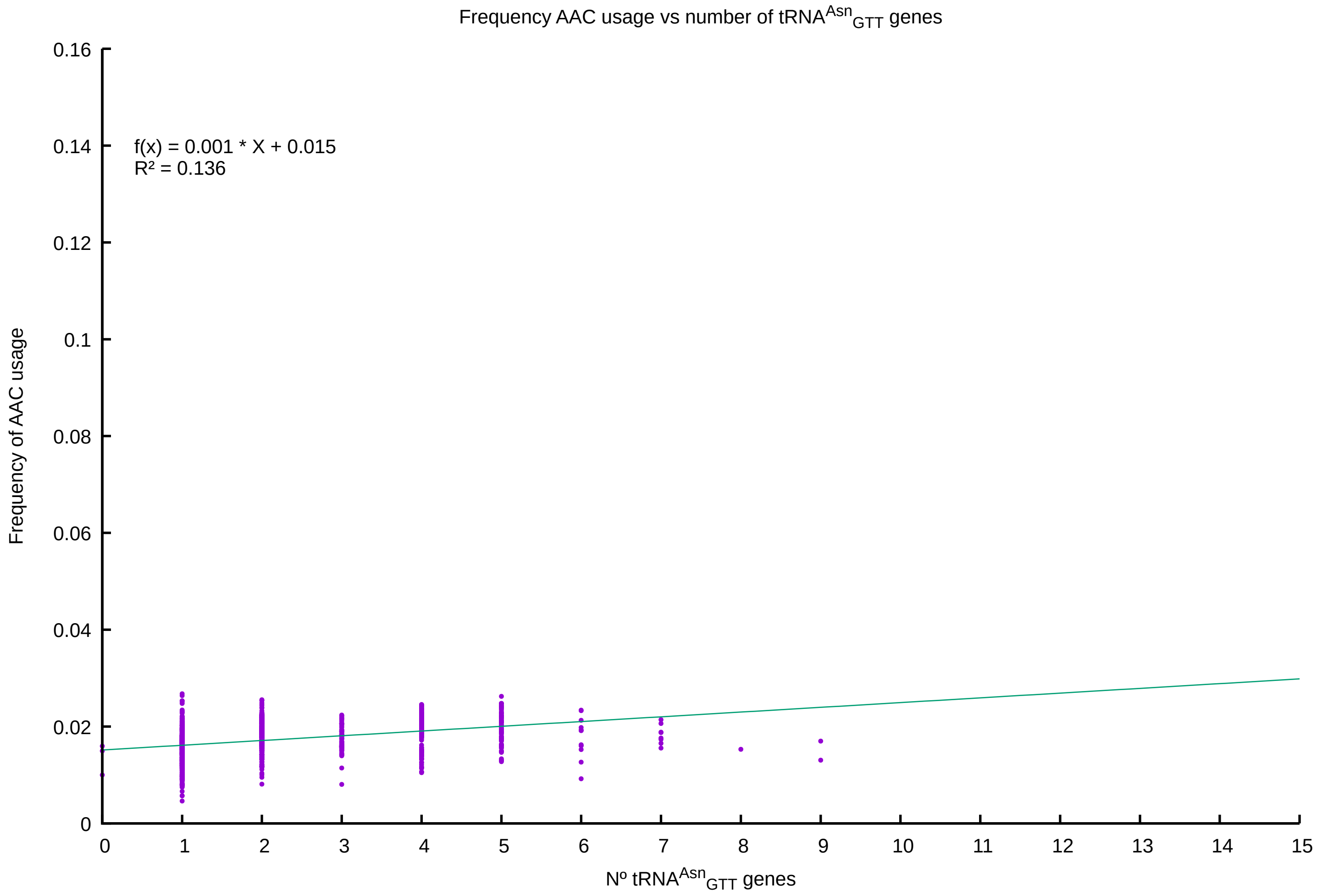

Frequency AAC usage vs number of tRNA$^{Asn}_{GTT}$ genes
f(x) = 0.001 * X + 0.015
R² = 0.136
Frequency of AAC usage
0.16
0.14
0.12
0.1
0.08
0.06
0.04
0.02
0
0 1 2 3 4 5 6 7 8 9 10 11 12 13 14 15
Nº tRNA$^{Asn}_{GTT}$ genes

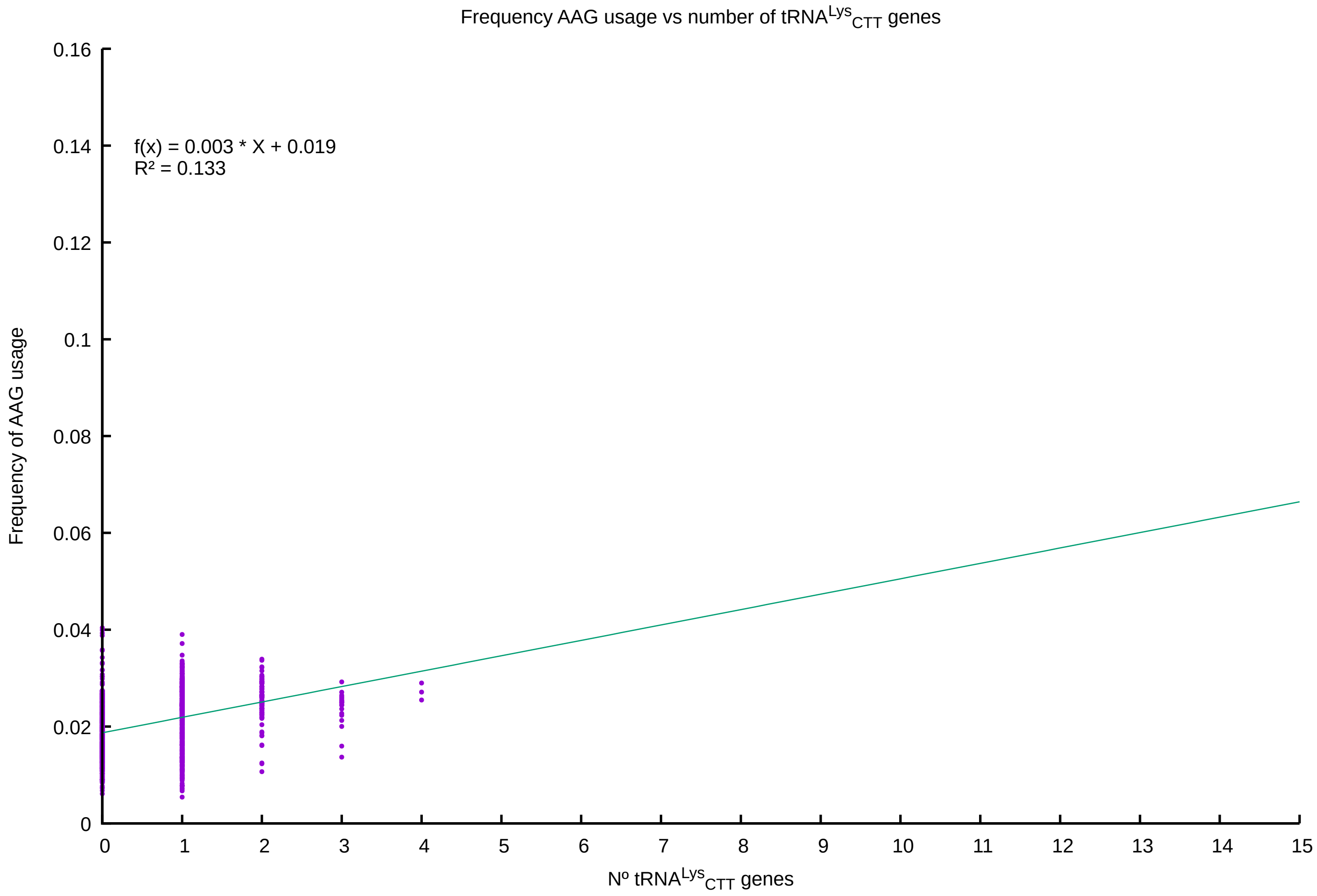

Frequency AAG usage vs number of tRNALysCTT genes
f(x) = 0.003 * X + 0.019
R² = 0.133
Frequency of AAG usage
0.16
0.14
0.12
0.1
0.08
0.06
0.04
0.02
0
0 1 2 3 4 5 6 7 8 9 10 11 12 13 14 15
Nº tRNALysCTT genes

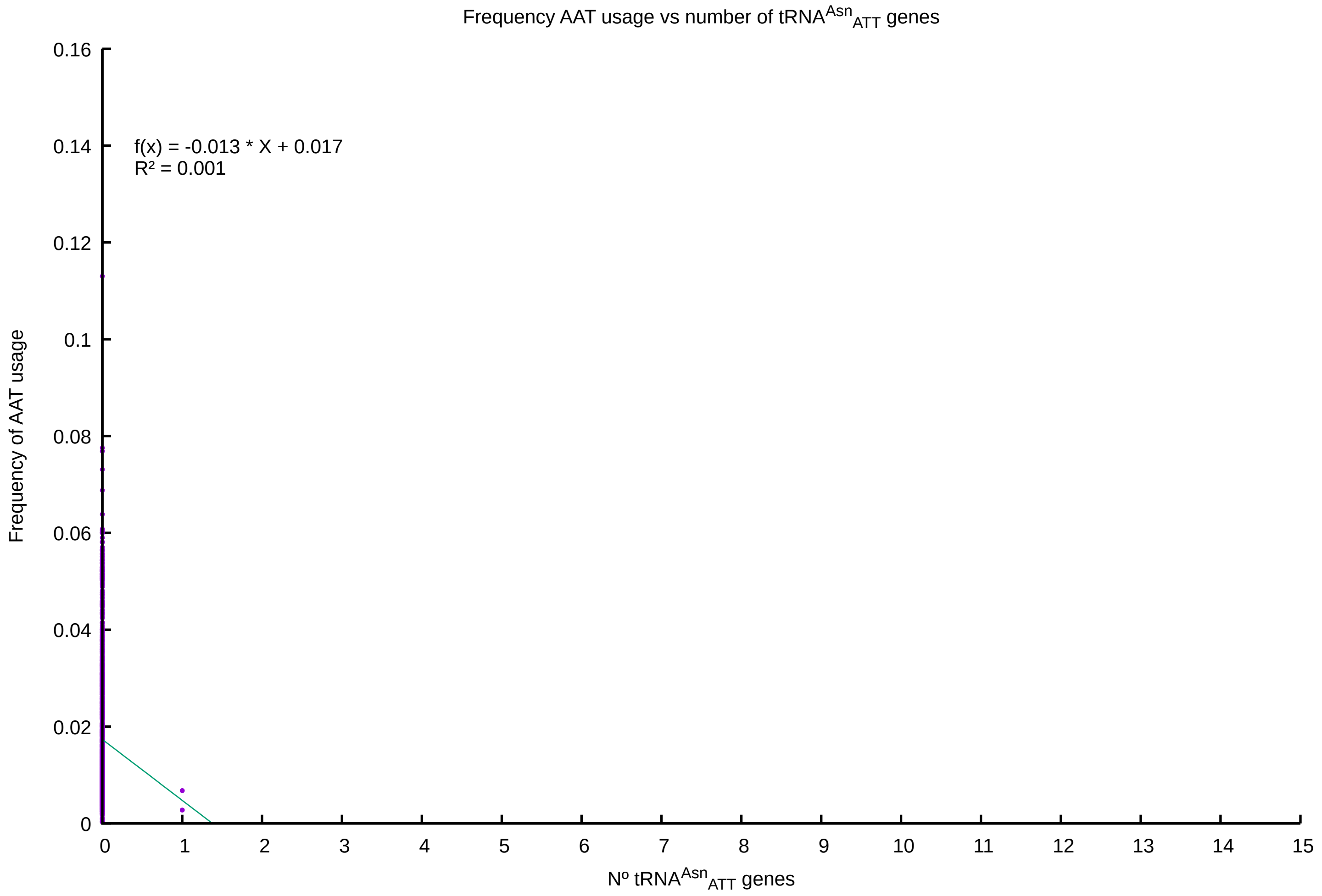
Frequency AAT usage vs number of tRNA$^{Asn}_{ATT}$ genes
f(x) = -0.013 * X + 0.017
R² = 0.001
Frequency of AAT usage
0.16
0.14
0.12
0.1
0.08
0.06
0.04
0.02
0
0 1 2 3 4 5 6 7 8 9 10 11 12 13 14 15
Nº tRNA$^{Asn}_{ATT}$ genes

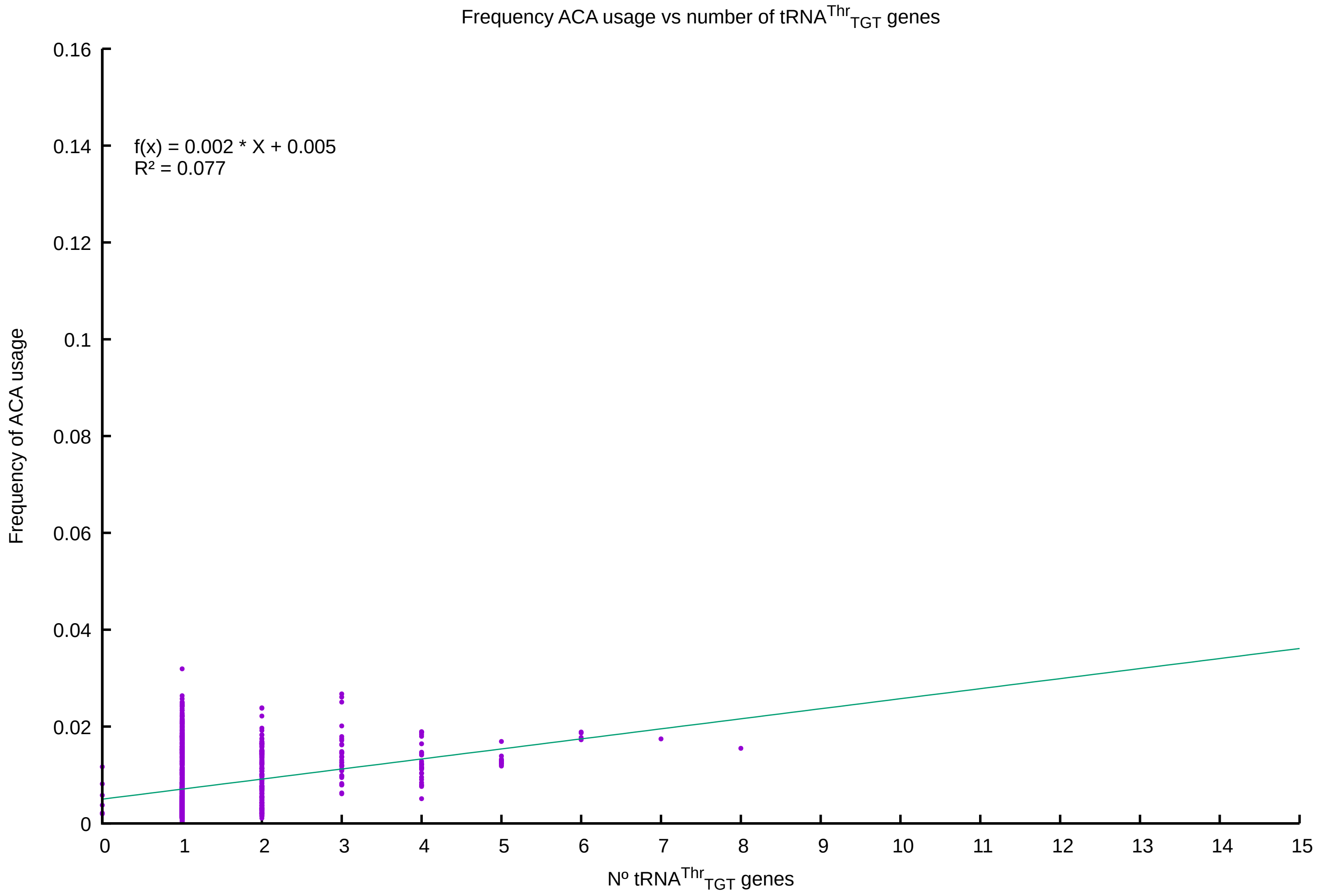
Frequency ACA usage vs number of tRNAThrTGT genes
f(x) = 0.002 * X + 0.005
R² = 0.077
Frequency of ACA usage
0.16
0.14
0.12
0.1
0.08
0.06
0.04
0.02
0
0 1 2 3 4 5 6 7 8 9 10 11 12 13 14 15
Nº tRNAThrTGT genes

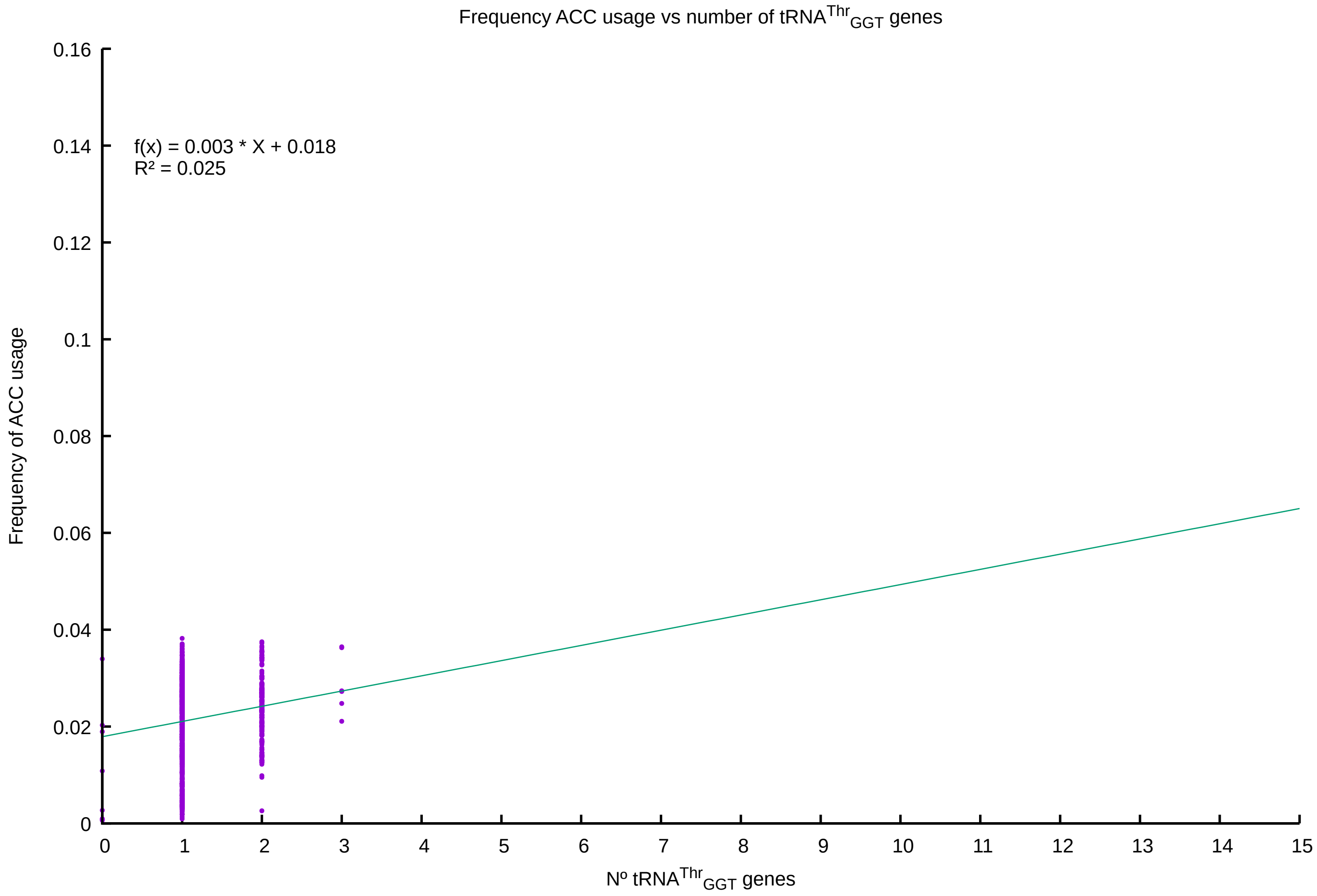
Frequency ACC usage vs number of tRNAThrGGT genes
f(x) = 0.003 * X + 0.018
R² = 0.025
Frequency of ACC usage
0.16
0.14
0.12
0.1
0.08
0.06
0.04
0.02
0
0 1 2 3 4 5 6 7 8 9 10 11 12 13 14 15
Nº tRNAThrGGT genes

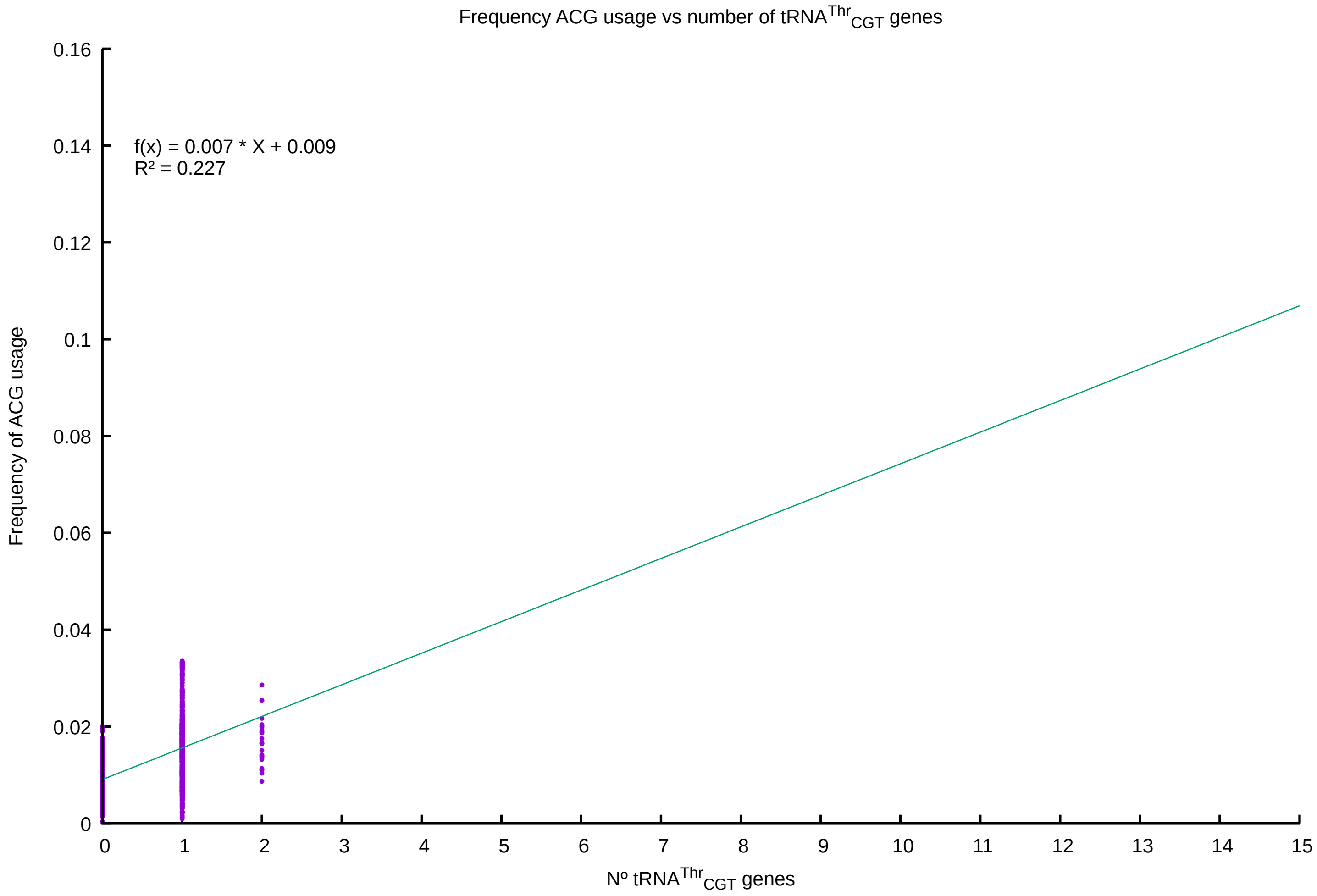

Frequency ACG usage vs number of tRNAThrCGT genes
f(x) = 0.007 * X + 0.009
R² = 0.227
Frequency of ACG usage
0.16
0.14
0.12
0.1
0.08
0.06
0.04
0.02
0
0 1 2 3 4 5 6 7 8 9 10 11 12 13 14 15
Nº tRNAThrCGT genes

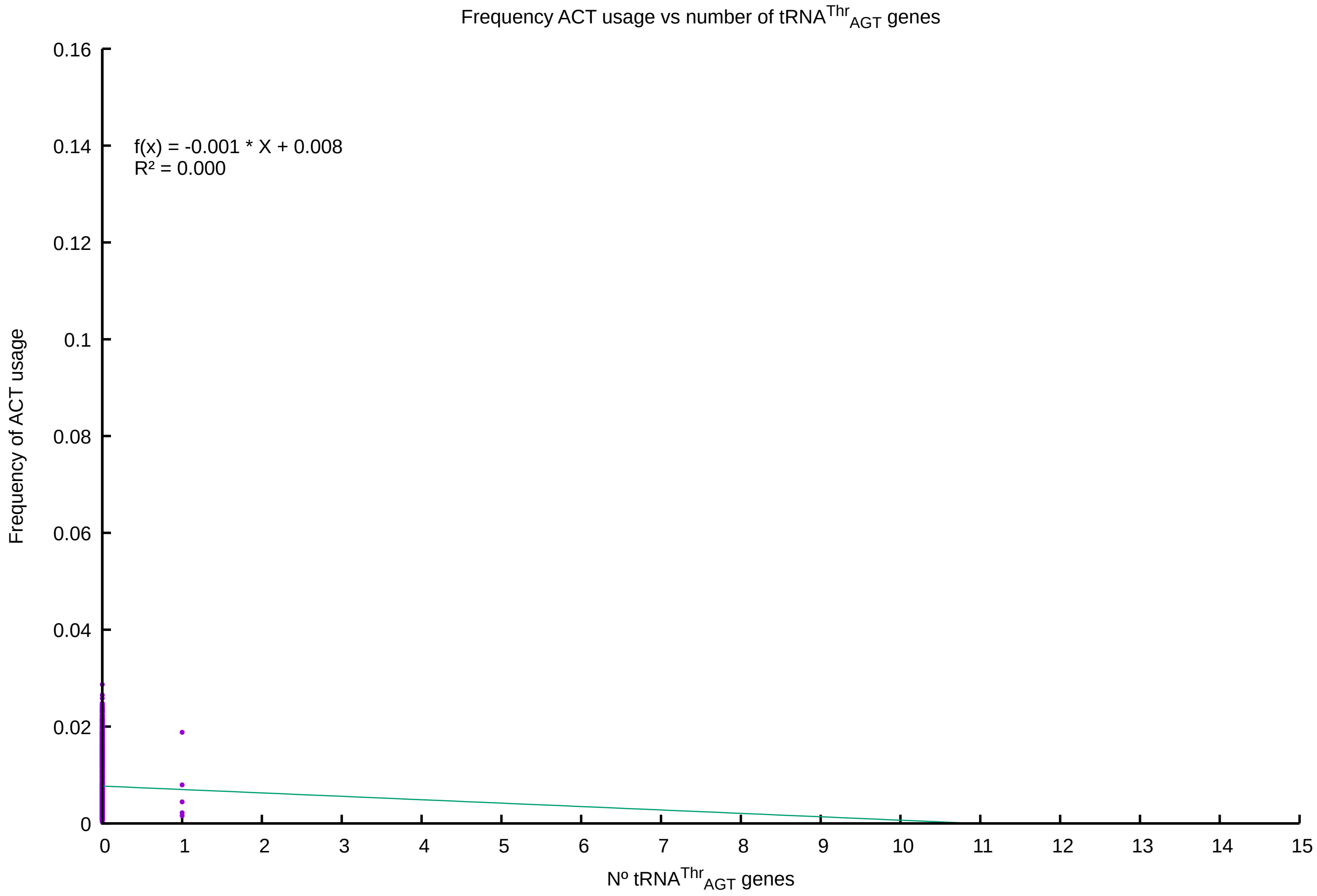

Frequency ACT usage vs number of tRNAThrAGT genes
f(x) = -0.001 * X + 0.008
R² = 0.000
Frequency of ACT usage
Nº tRNAThrAGT genes
0.16
0.14
0.12
0.1
0.08
0.06
0.04
0.02
0
0 1 2 3 4 5 6 7 8 9 10 11 12 13 14 15

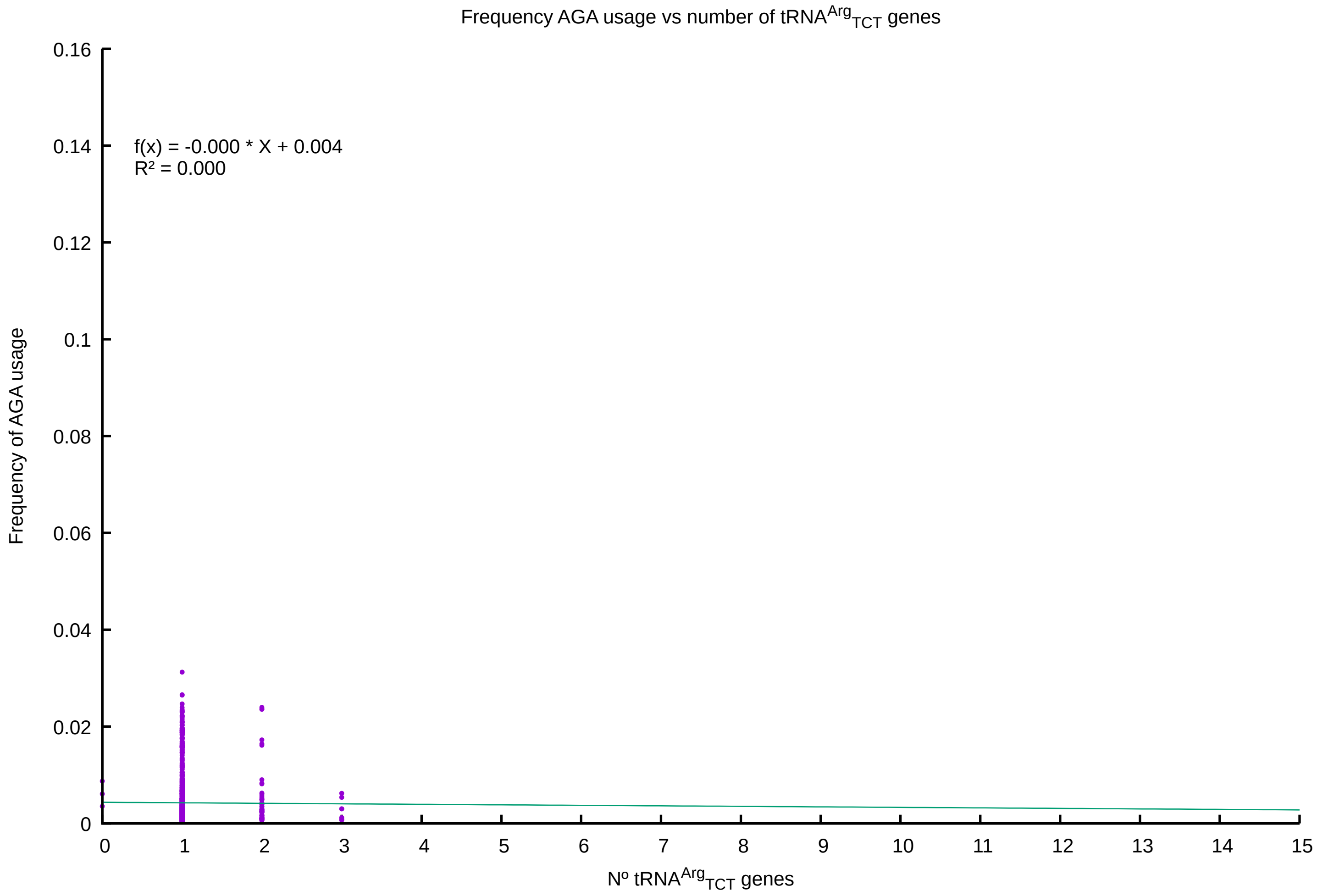

Frequency AGA usage vs number of tRNA^Arg_TCT genes
f(x) = -0.000 * X + 0.004
R² = 0.000
Frequency of AGA usage
0.16
0.14
0.12
0.1
0.08
0.06
0.04
0.02
0
0 1 2 3 4 5 6 7 8 9 10 11 12 13 14 15
Nº tRNA^Arg_TCT genes

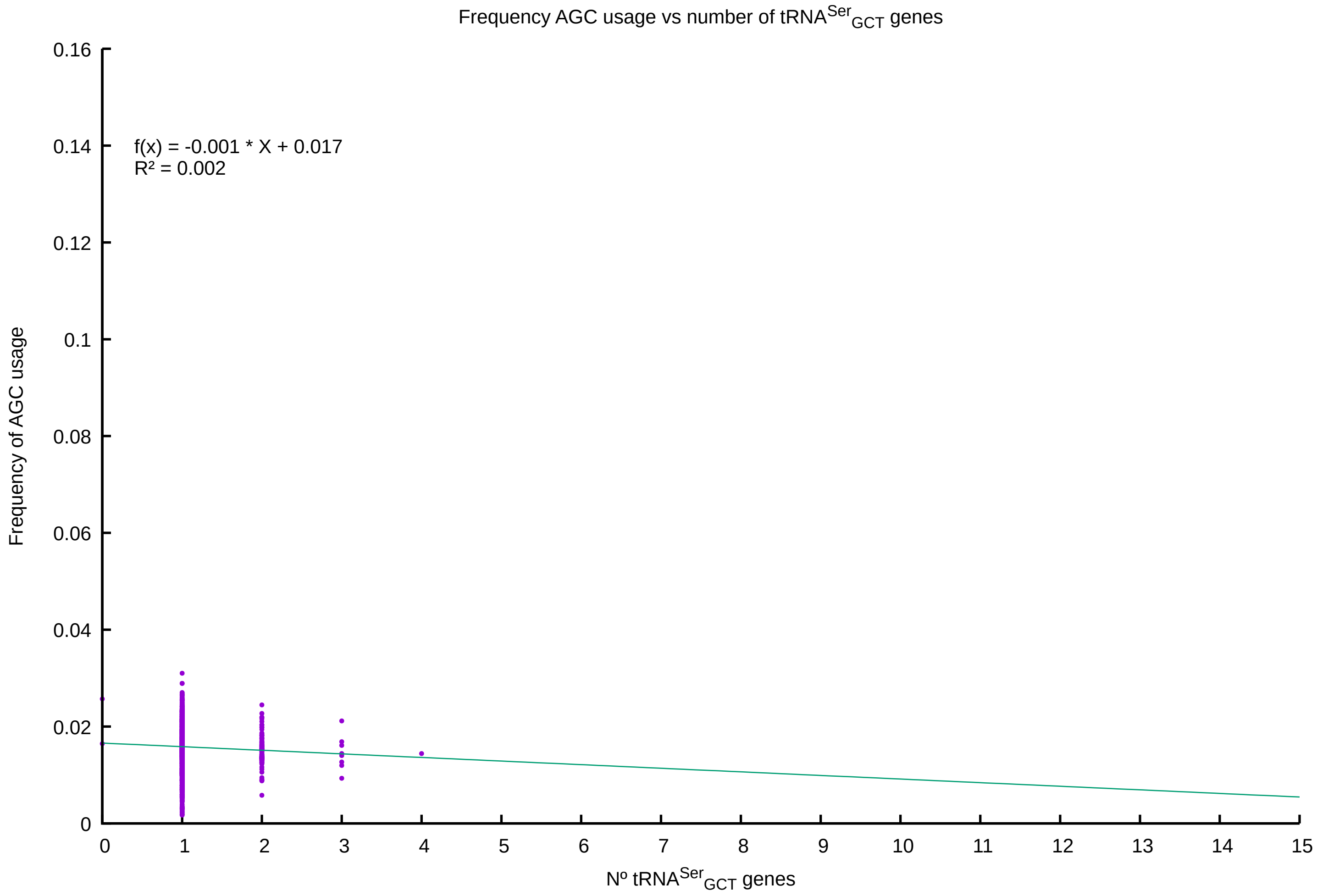
Frequency AGC usage vs number of tRNA$^{Ser}_{GCT}$ genes
f(x) = -0.001 * X + 0.017
R^2 = 0.002
Frequency of AGC usage
0.16
0.14
0.12
0.1
0.08
0.06
0.04
0.02
0
0 1 2 3 4 5 6 7 8 9 10 11 12 13 14 15
Nº tRNA$^{Ser}_{GCT}$ genes

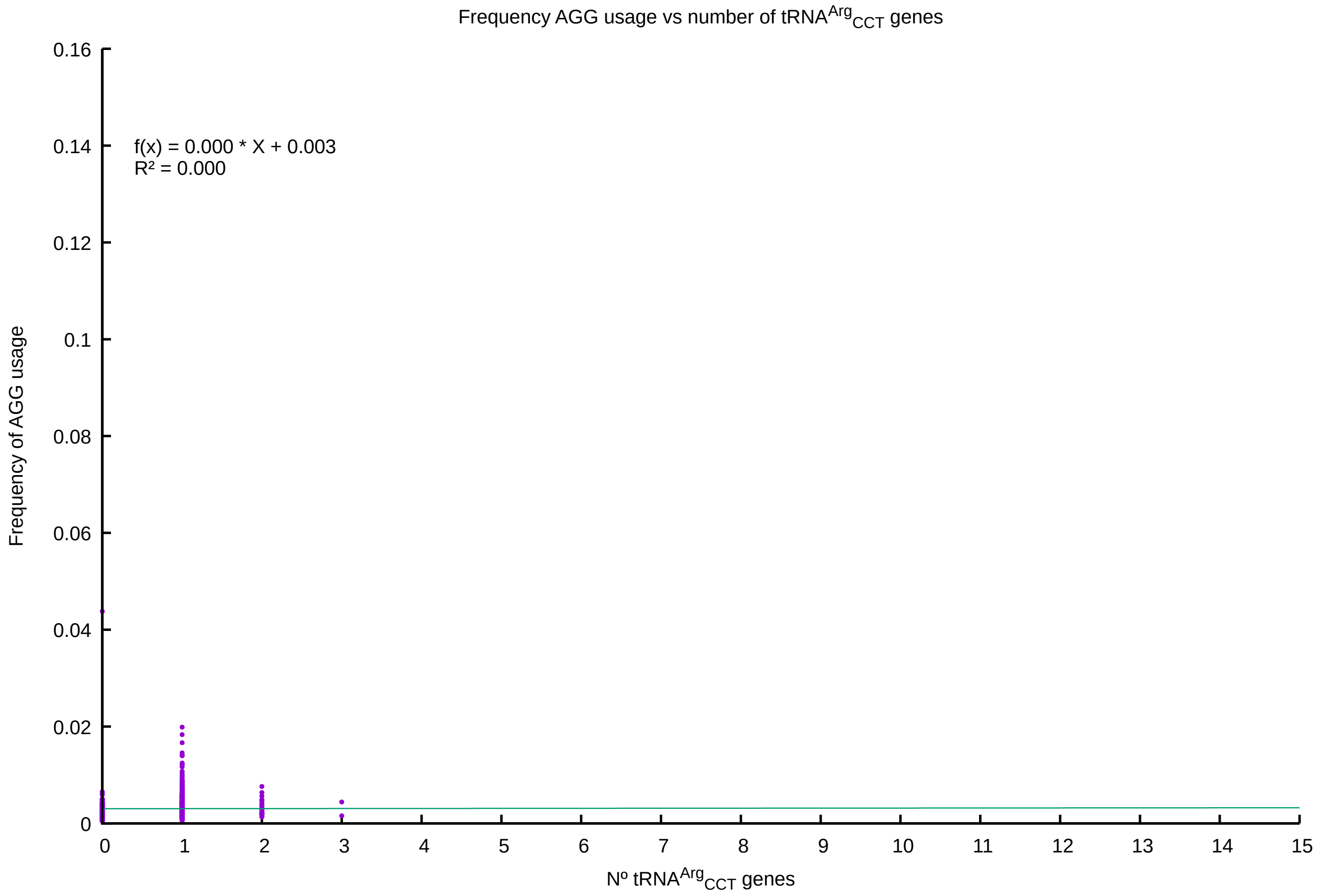

Frequency AGG usage vs number of tRNAArgCCT genes
f(x) = 0.000 * X + 0.003
R² = 0.000
Frequency of AGG usage
0.16
0.14
0.12
0.1
0.08
0.06
0.04
0.02
0
0 1 2 3 4 5 6 7 8 9 10 11 12 13 14 15
Nº tRNAArgCCT genes

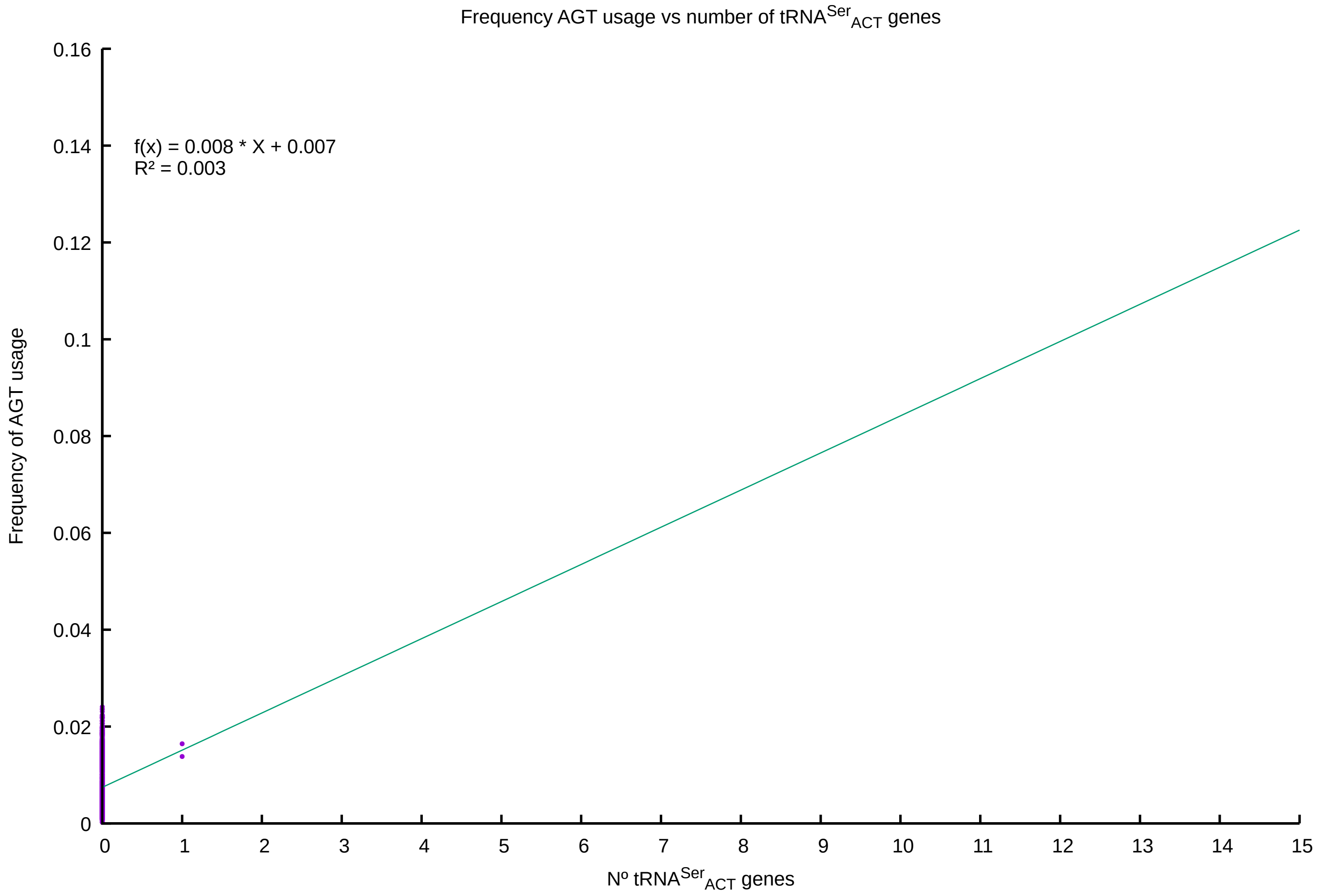

Frequency AGT usage vs number of tRNASerACT genes
f(x) = 0.008 * X + 0.007
R² = 0.003
Frequency of AGT usage
Nº tRNASerACT genes
0.16
0.14
0.12
0.1
0.08
0.06
0.04
0.02
0
0 1 2 3 4 5 6 7 8 9 10 11 12 13 14 15

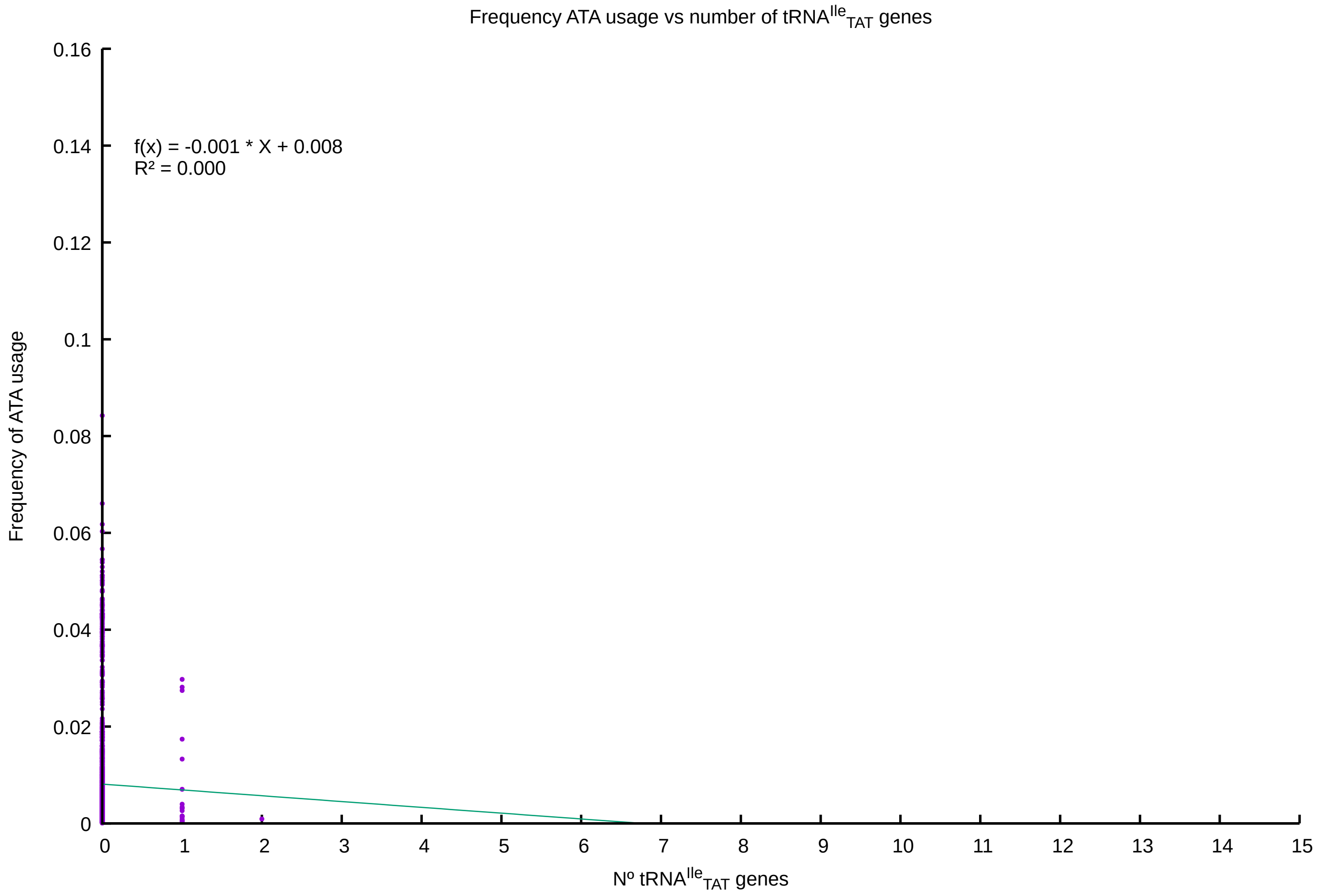

Frequency ATA usage vs number of tRNA^Ile_TAT genes
f(x) = -0.001 * X + 0.008
R² = 0.000
Frequency of ATA usage
0.16
0.14
0.12
0.1
0.08
0.06
0.04
0.02
0
0 1 2 3 4 5 6 7 8 9 10 11 12 13 14 15
Nº tRNA^Ile_TAT genes

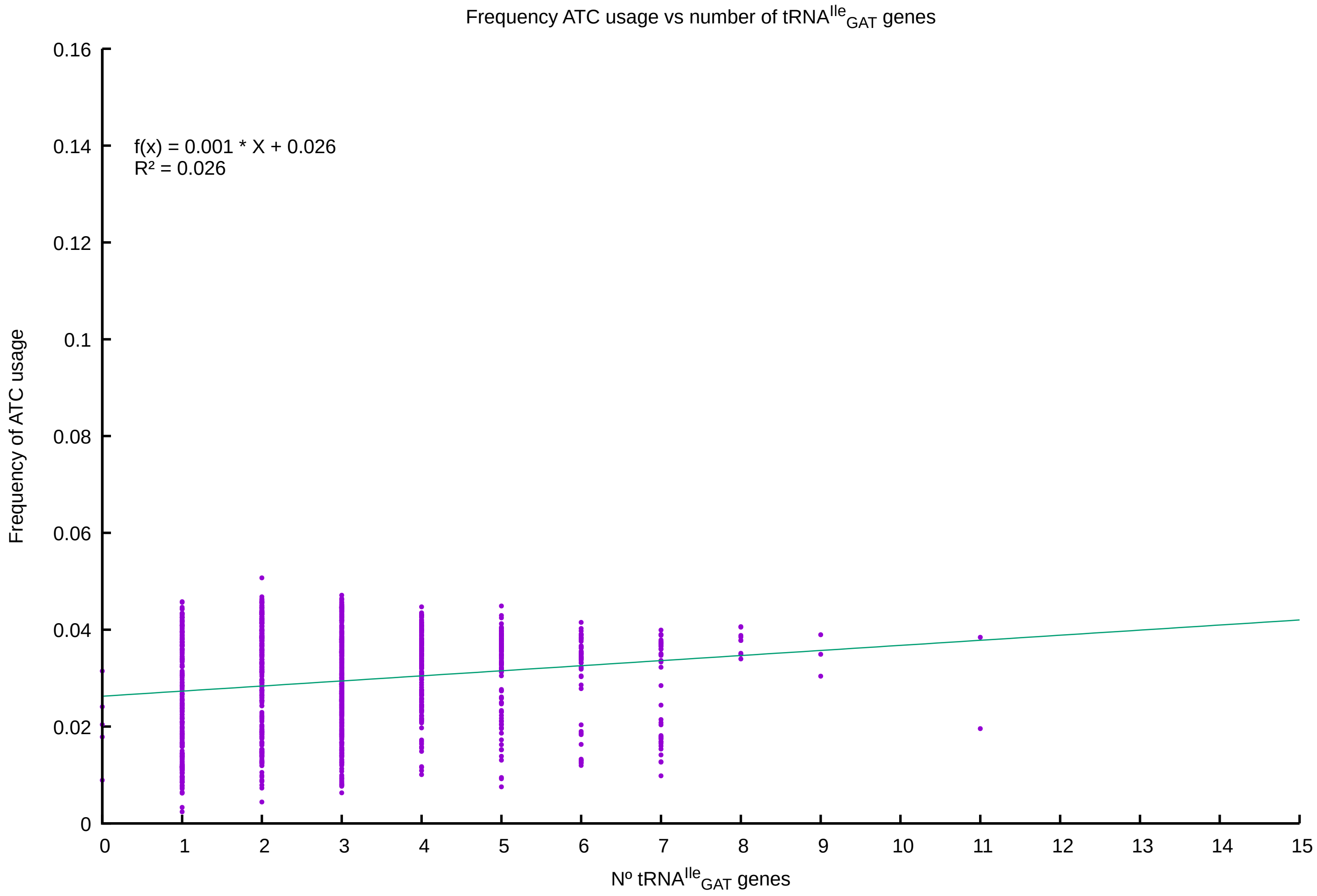

Frequency ATC usage vs number of tRNA^Ile_GAT genes
f(x) = 0.001 * X + 0.026
R² = 0.026
Frequency of ATC usage
0.16
0.14
0.12
0.1
0.08
0.06
0.04
0.02
0
0 1 2 3 4 5 6 7 8 9 10 11 12 13 14 15
Nº tRNA^Ile_GAT genes

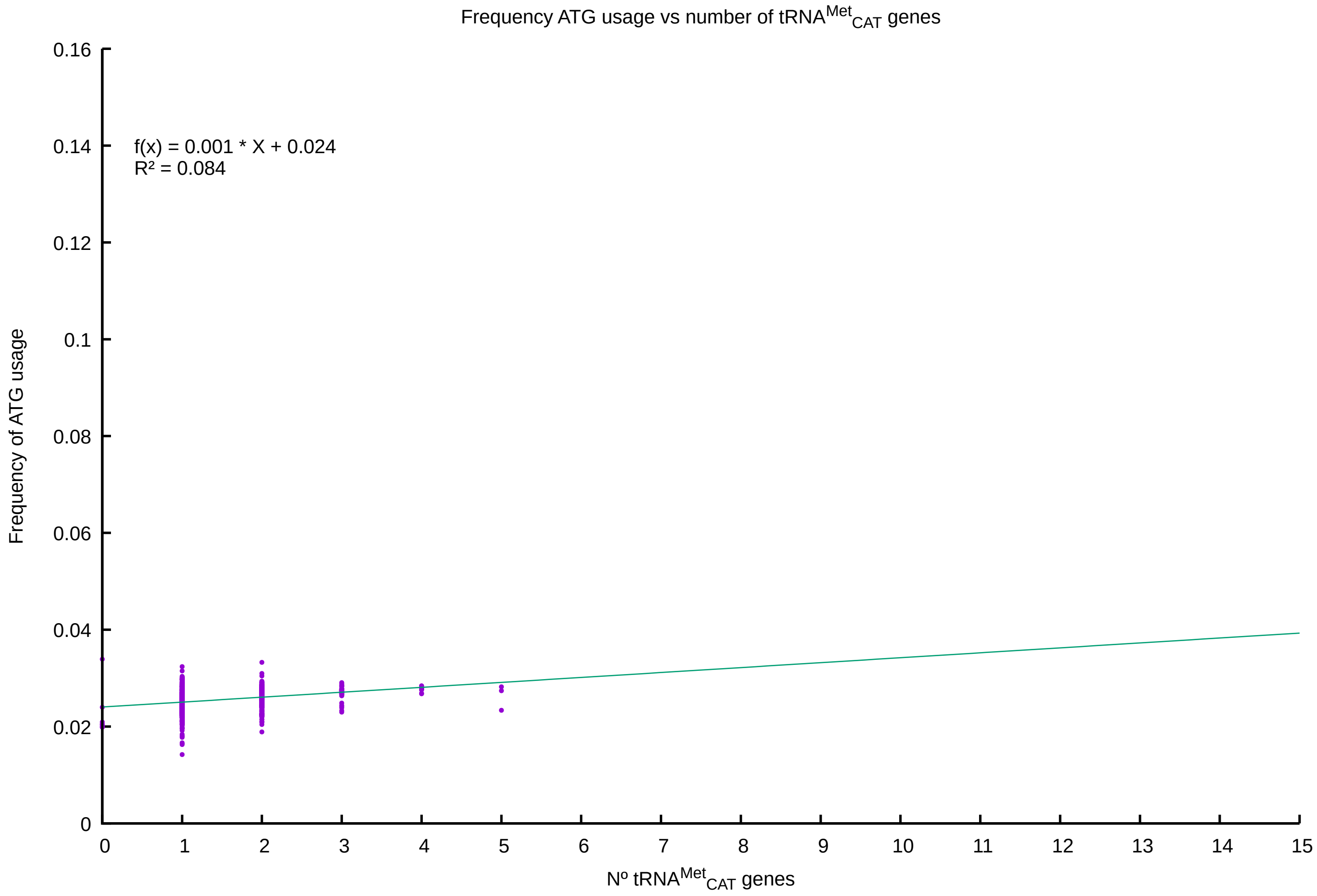

Frequency ATG usage vs number of tRNA Met CAT genes
f(x) = 0.001 * X + 0.024
R² = 0.084
Frequency of ATG usage
0.16
0.14
0.12
0.1
0.08
0.06
0.04
0.02
0
0 1 2 3 4 5 6 7 8 9 10 11 12 13 14 15
Nº tRNA Met CAT genes

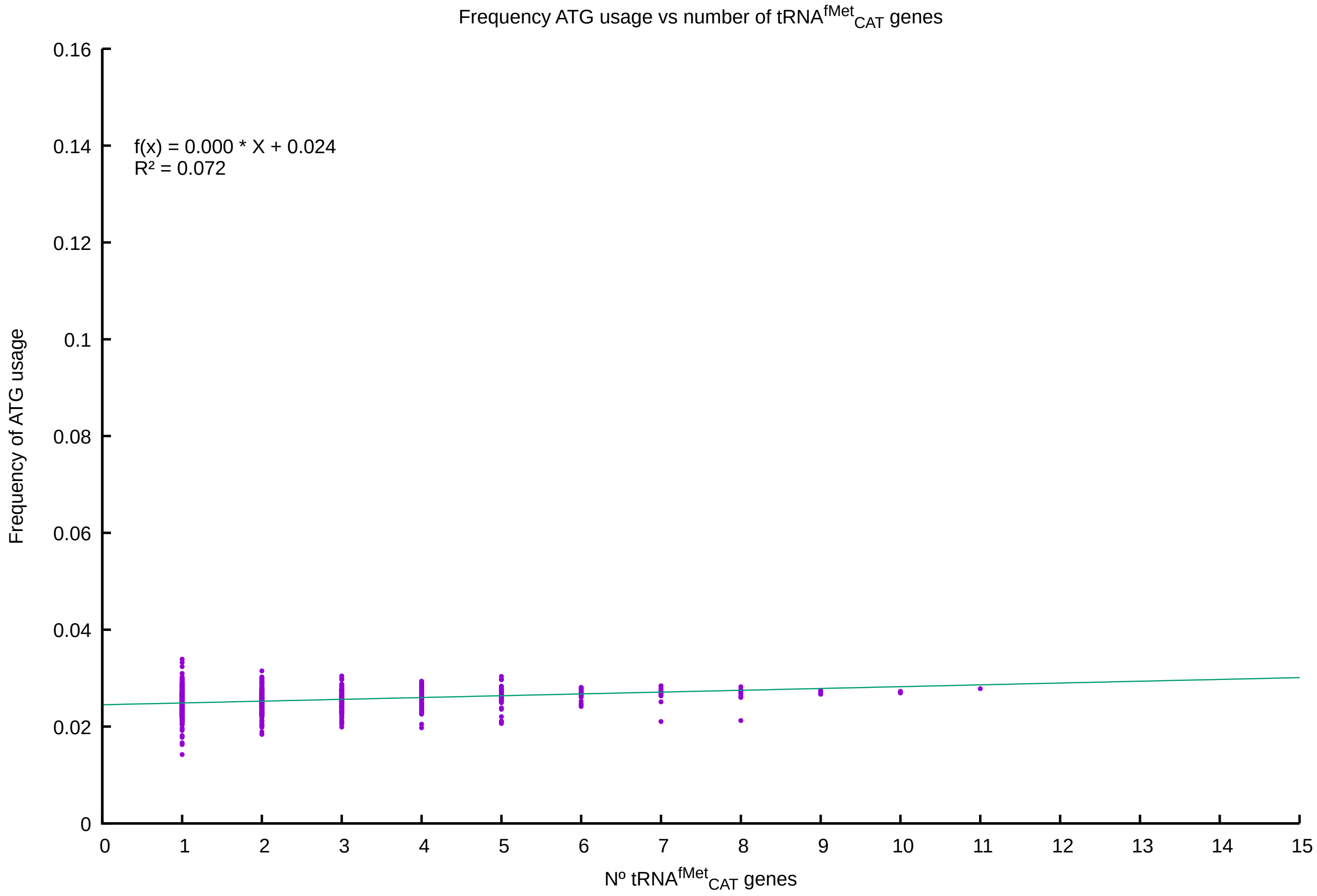

Frequency ATG usage vs number of $tRNA^{fMet}_{CAT}$ genes
f(x) = 0.000 * X + 0.024
R^2 = 0.072
Frequency of ATG usage
0.16
0.14
0.12
0.1
0.08
0.06
0.04
0.02
0
0 1 2 3 4 5 6 7 8 9 10 11 12 13 14 15
Nº $tRNA^{fMet}_{CAT}$ genes

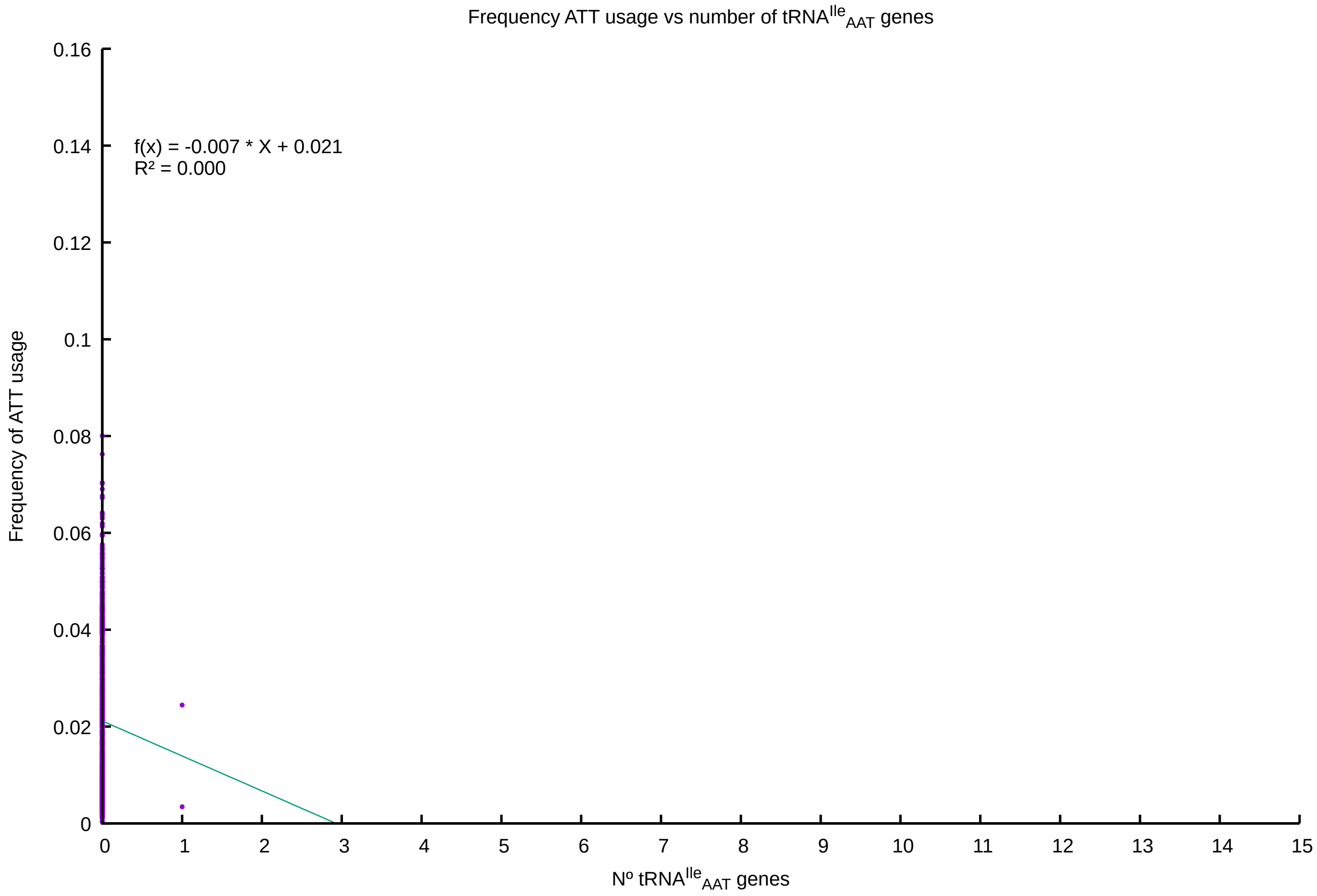

Frequency ATT usage vs number of tRNA$^{Ile}_{AAT}$ genes
f(x) = -0.007 * X + 0.021
R² = 0.000
Frequency of ATT usage
0.16
0.14
0.12
0.1
0.08
0.06
0.04
0.02
0
0 1 2 3 4 5 6 7 8 9 10 11 12 13 14 15
Nº tRNA$^{Ile}_{AAT}$ genes

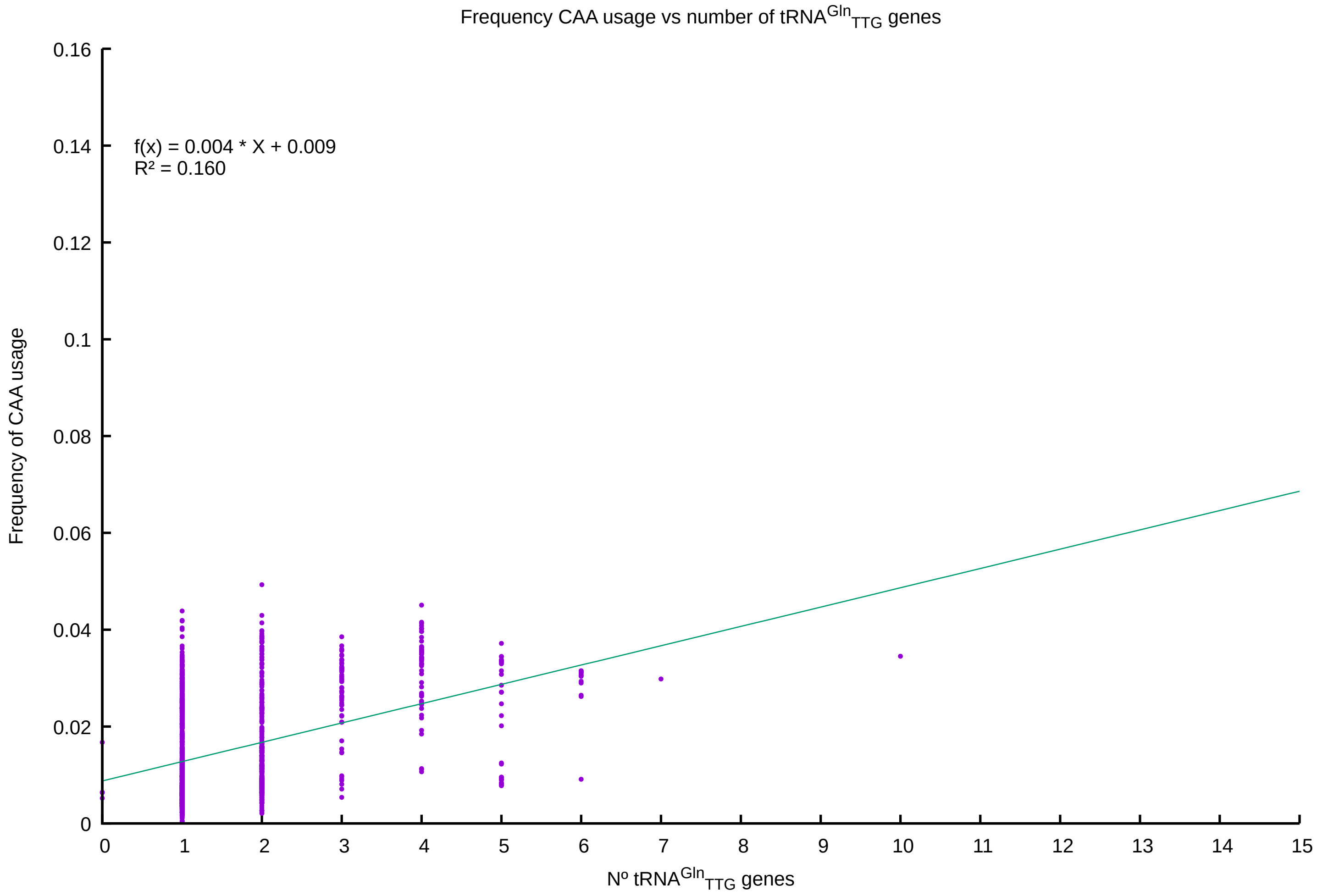
Frequency CAA usage vs number of tRNA^Gln_TTG genes
f(x) = 0.004 * X + 0.009
R² = 0.160
Frequency of CAA usage
0
0.02
0.04
0.06
0.08
0.1
0.12
0.14
0.16
0 1 2 3 4 5 6 7 8 9 10 11 12 13 14 15
Nº tRNA^Gln_TTG genes

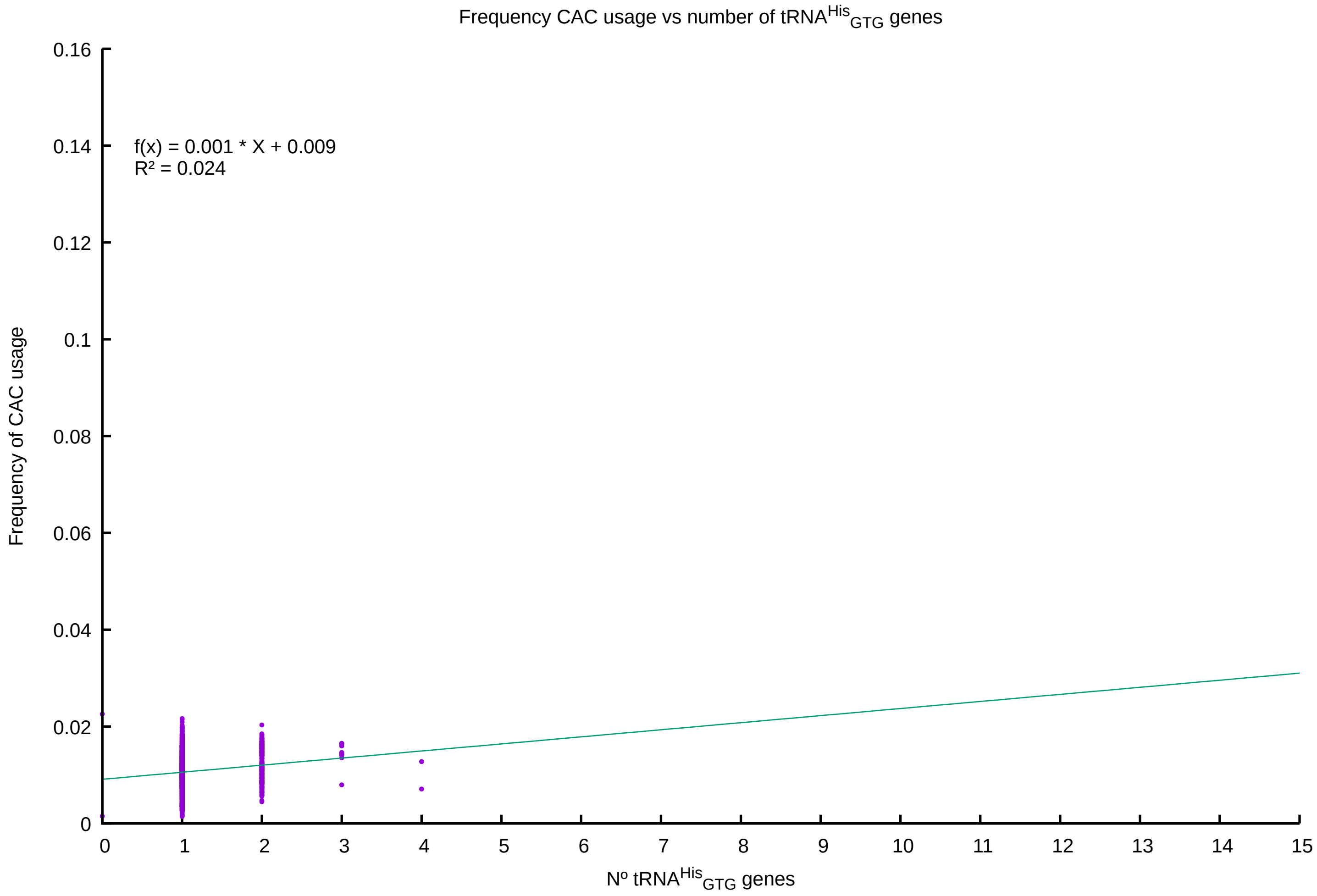

Frequency CAC usage vs number of tRNA His GTG genes
f(x) = 0.001 * X + 0.009
R² = 0.024
Frequency of CAC usage
0.16
0.14
0.12
0.1
0.08
0.06
0.04
0.02
0
0 1 2 3 4 5 6 7 8 9 10 11 12 13 14 15
Nº tRNA His GTG genes

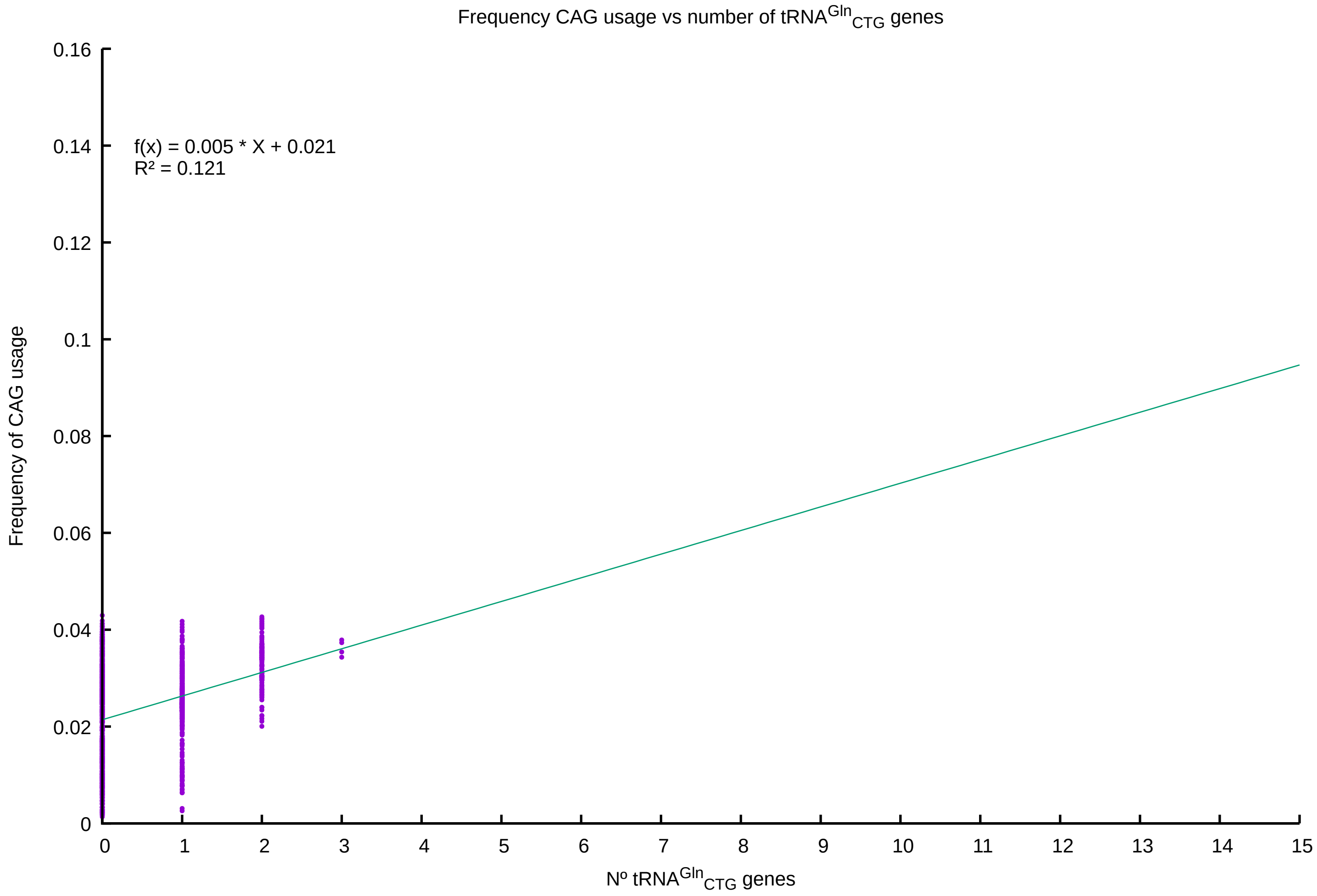

Frequency CAG usage vs number of tRNAGlnCTG genes
f(x) = 0.005 * X + 0.021
R² = 0.121
Frequency of CAG usage
0.16
0.14
0.12
0.1
0.08
0.06
0.04
0.02
0
0 1 2 3 4 5 6 7 8 9 10 11 12 13 14 15
Nº tRNAGlnCTG genes

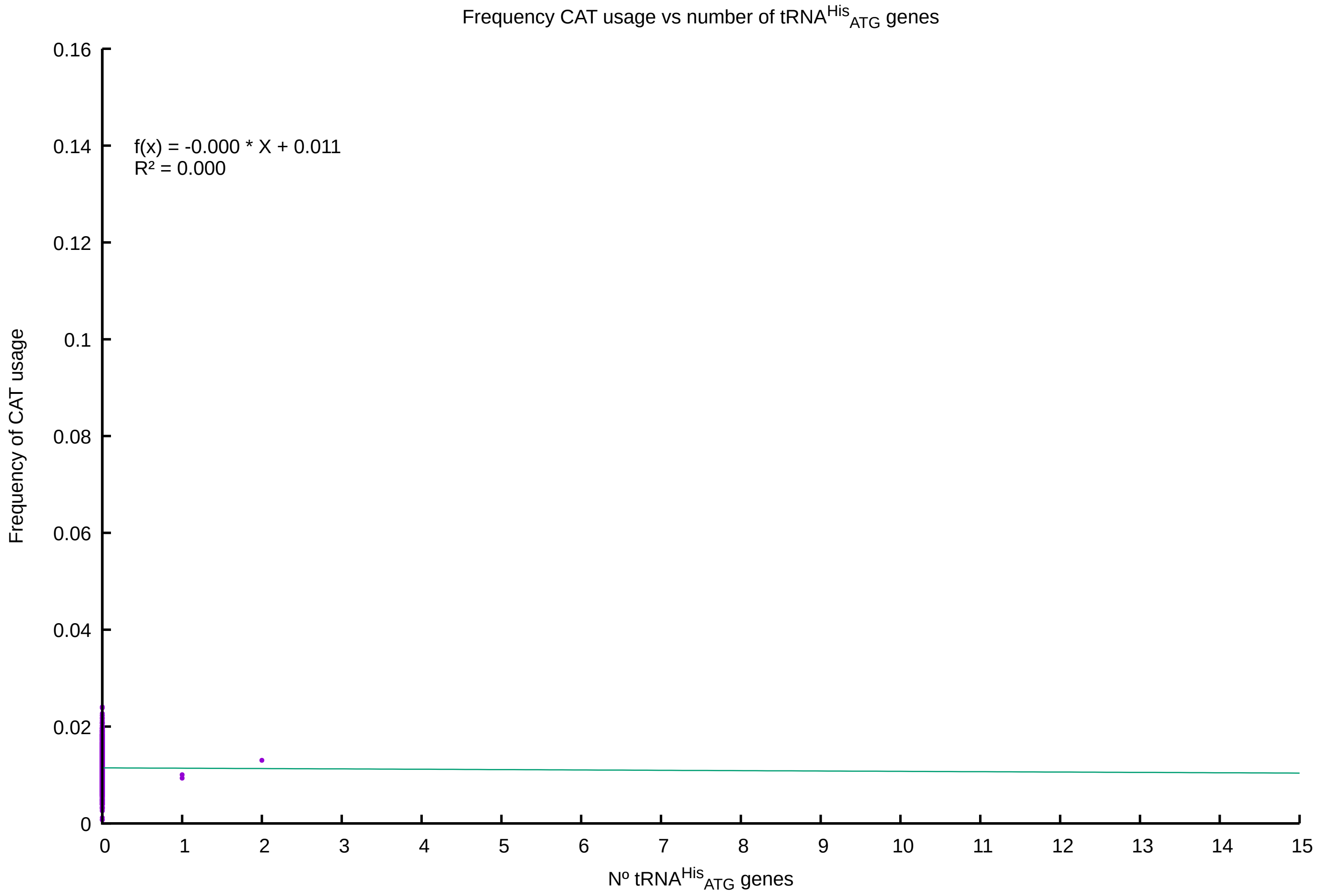

Frequency CAT usage vs number of tRNAHisATG genes
f(x) = -0.000 * X + 0.011
R² = 0.000
Frequency of CAT usage
0.16
0.14
0.12
0.1
0.08
0.06
0.04
0.02
0
0 1 2 3 4 5 6 7 8 9 10 11 12 13 14 15
Nº tRNAHisATG genes

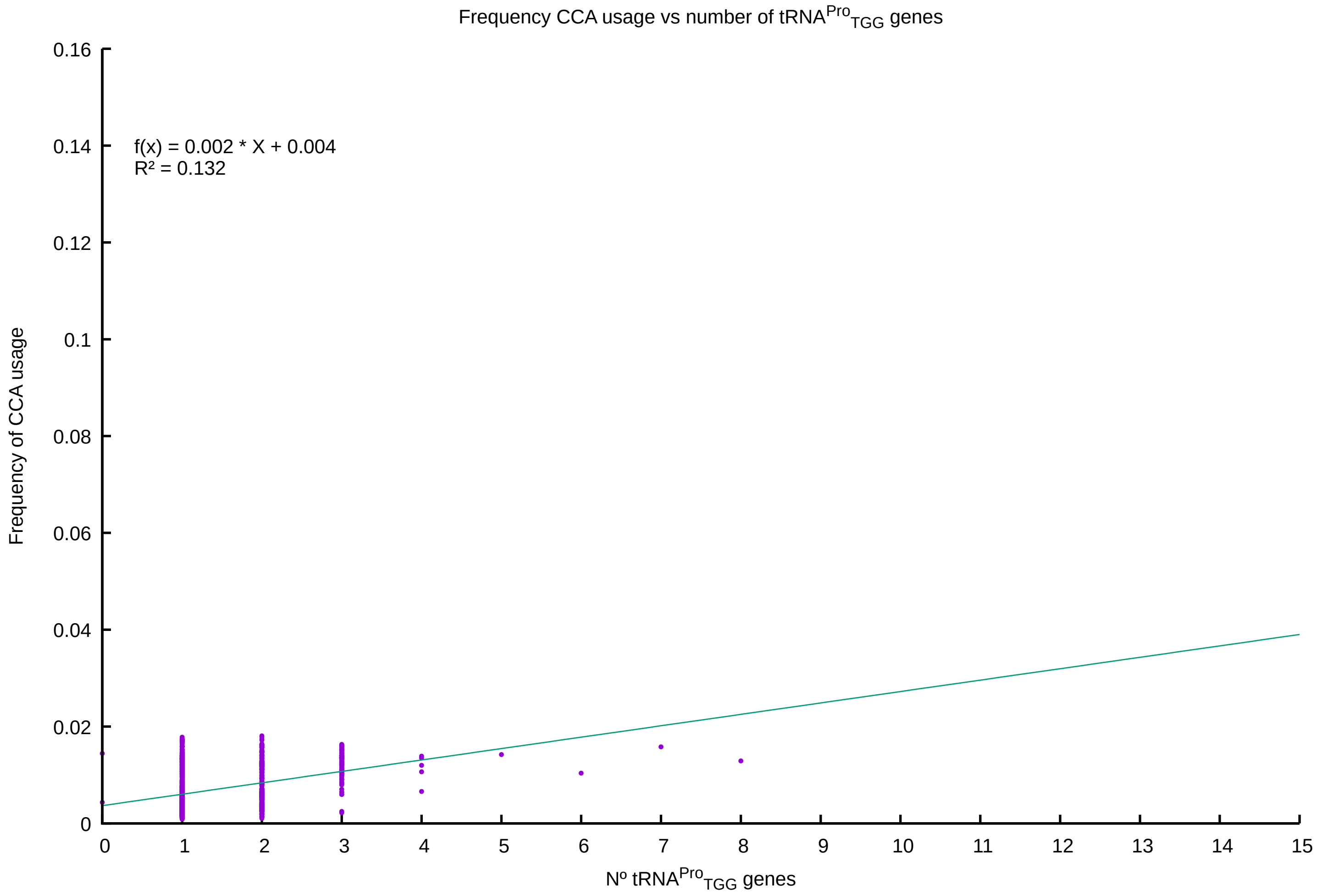

Frequency CCA usage vs number of $tRNA^{Pro}_{TGG}$ genes
f(x) = 0.002 * X + 0.004
R^2 = 0.132
Frequency of CCA usage
0.16
0.14
0.12
0.1
0.08
0.06
0.04
0.02
0
0 1 2 3 4 5 6 7 8 9 10 11 12 13 14 15
Nº $tRNA^{Pro}_{TGG}$ genes

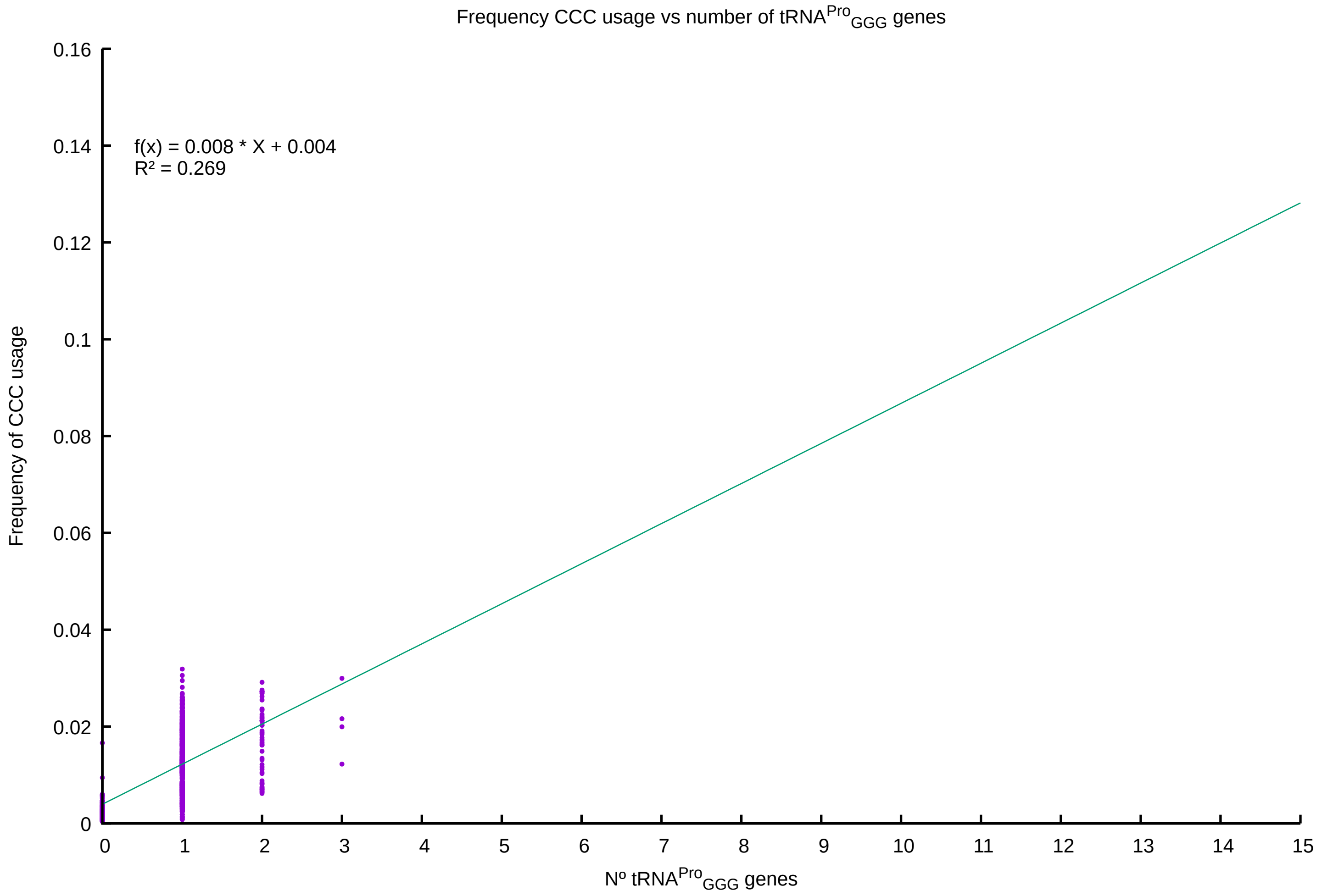

Frequency CCC usage vs number of tRNAProGGG genes
f(x) = 0.008 * X + 0.004
R² = 0.269
Frequency of CCC usage
0.16
0.14
0.12
0.1
0.08
0.06
0.04
0.02
0
0 1 2 3 4 5 6 7 8 9 10 11 12 13 14 15
Nº tRNAProGGG genes

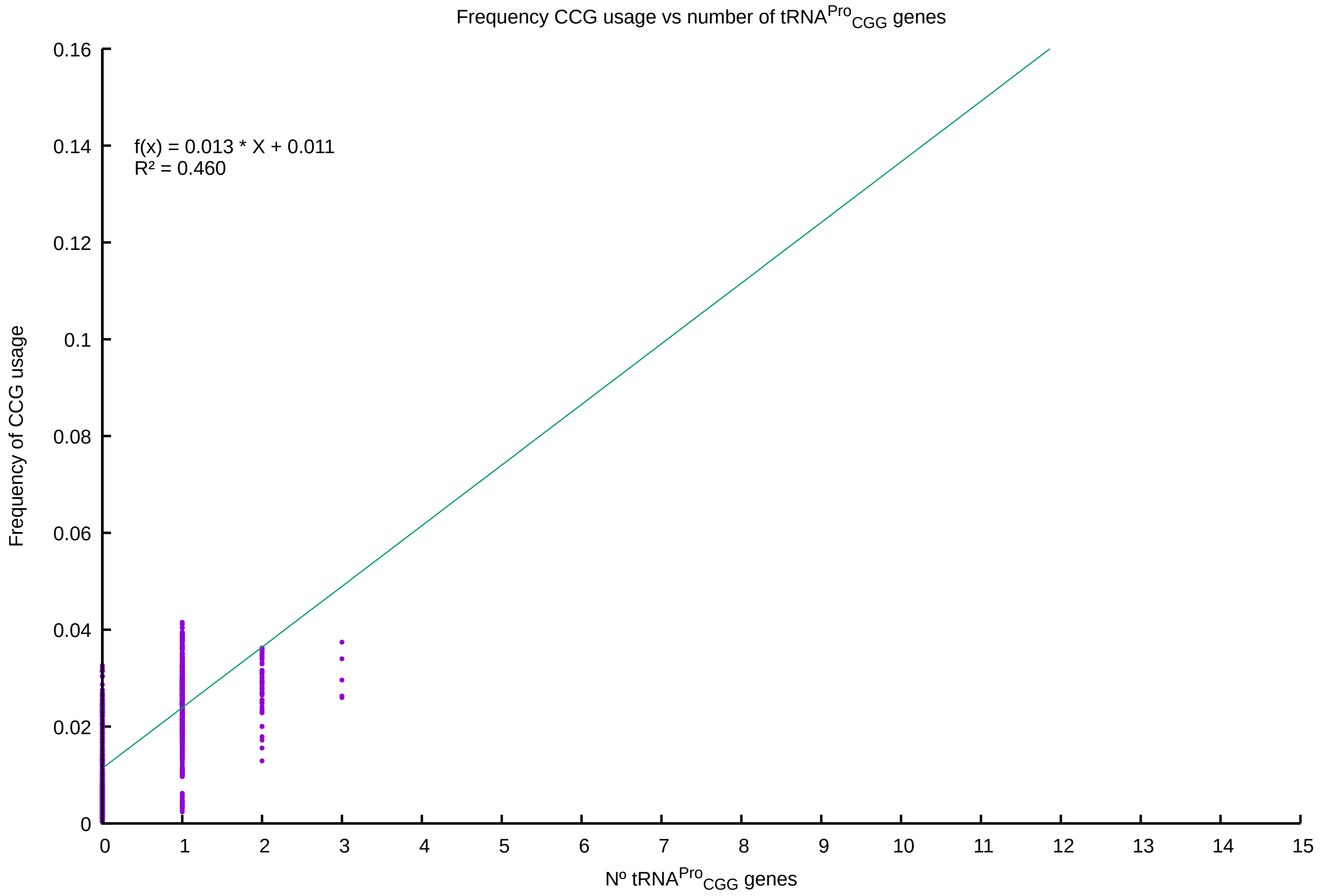

Frequency CCG usage vs number of tRNAProCGG genes
f(x) = 0.013 * X + 0.011
R² = 0.460
Frequency of CCG usage
0.16
0.14
0.12
0.1
0.08
0.06
0.04
0.02
0
0 1 2 3 4 5 6 7 8 9 10 11 12 13 14 15
Nº tRNAProCGG genes

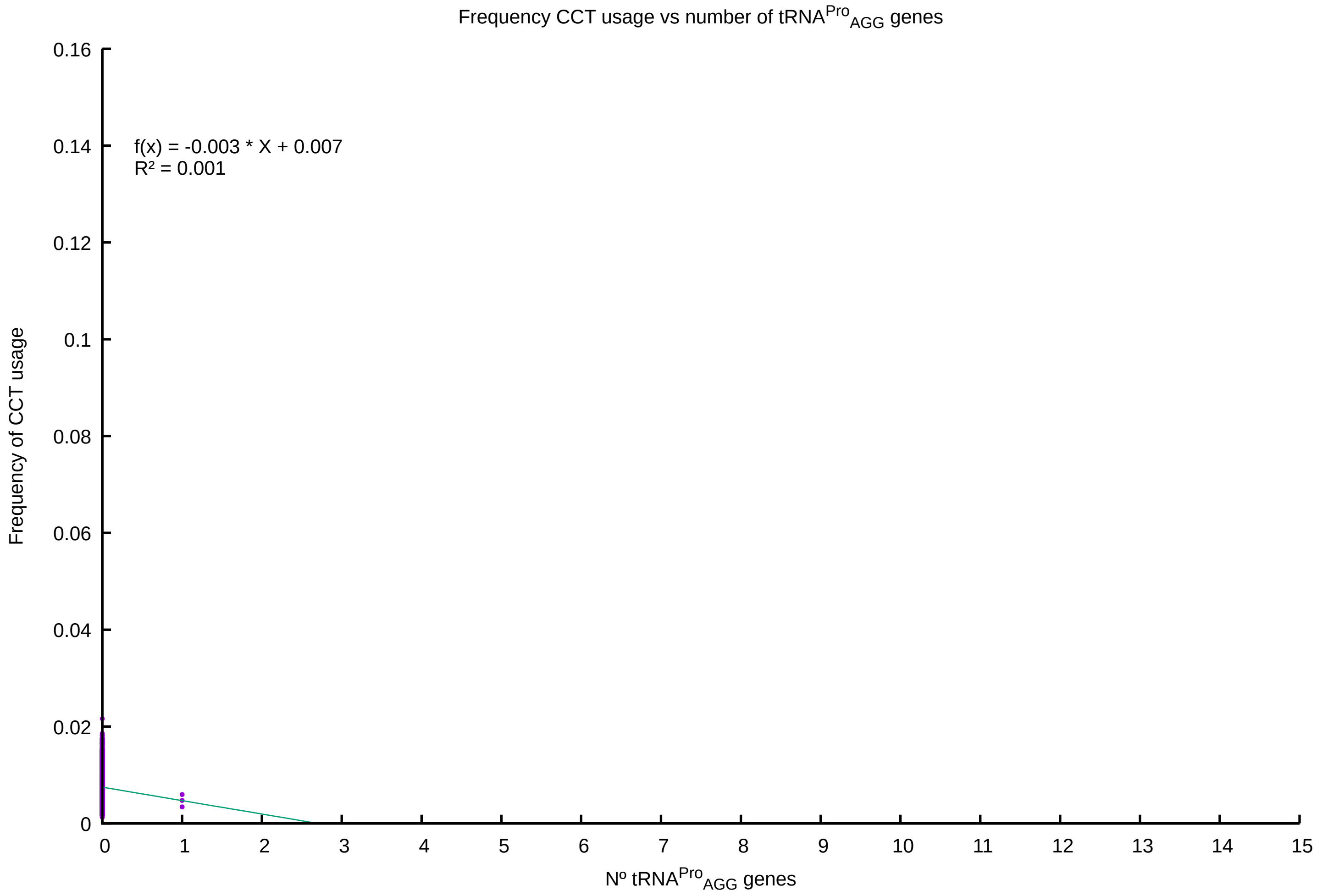

Frequency CCT usage vs number of tRNA$^{Pro}_{AGG}$ genes
f(x) = -0.003 * X + 0.007
R^2 = 0.001
Frequency of CCT usage
0.16
0.14
0.12
0.1
0.08
0.06
0.04
0.02
0
0
1
2
3
4
5
6
7
8
9
10
11
12
13
14
15
Nº tRNA$^{Pro}_{AGG}$ genes

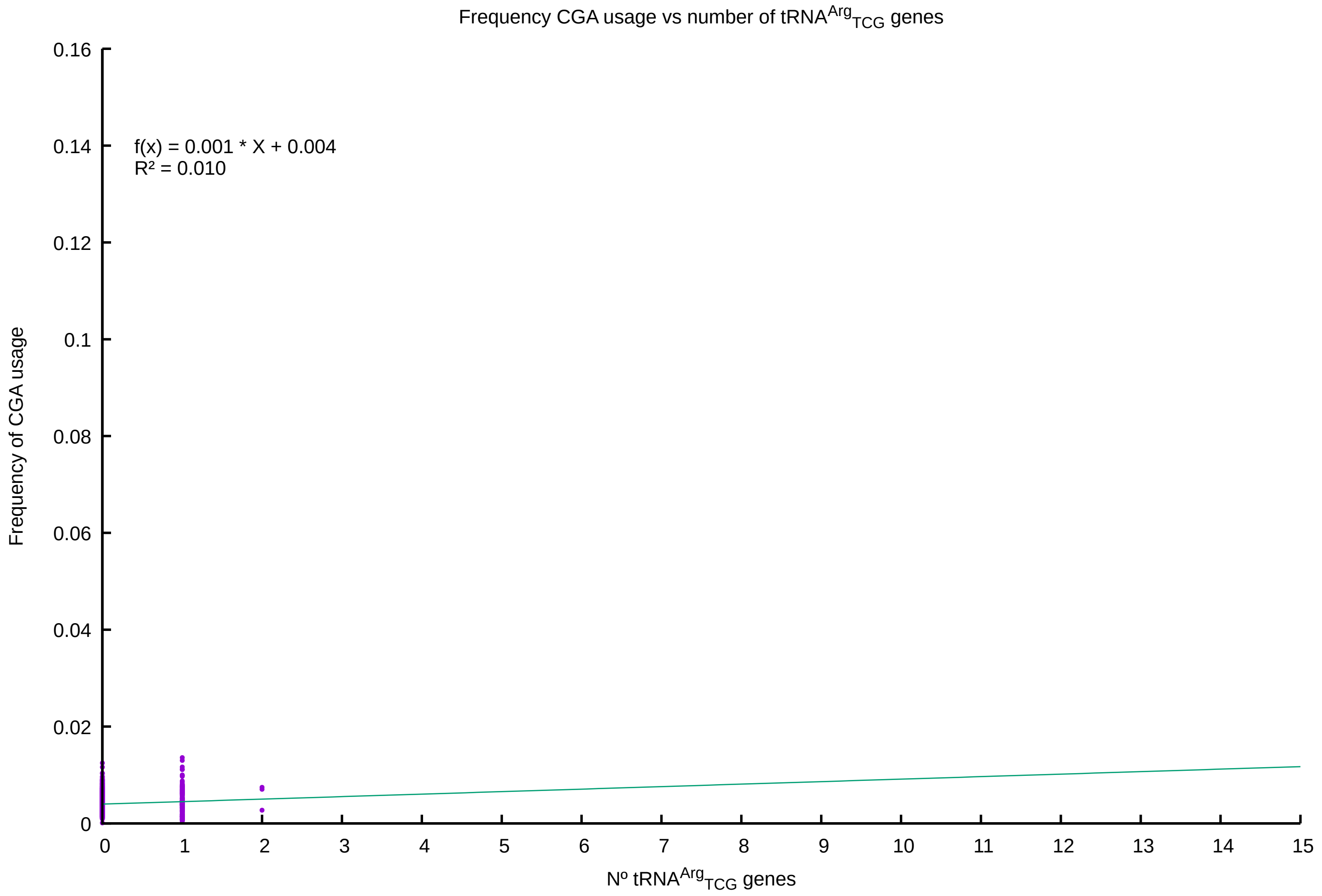

Frequency CGA usage vs number of tRNAArgTCG genes
f(x) = 0.001 * X + 0.004
R² = 0.010
Frequency of CGA usage
0.16
0.14
0.12
0.1
0.08
0.06
0.04
0.02
0
0 1 2 3 4 5 6 7 8 9 10 11 12 13 14 15
Nº tRNAArgTCG genes

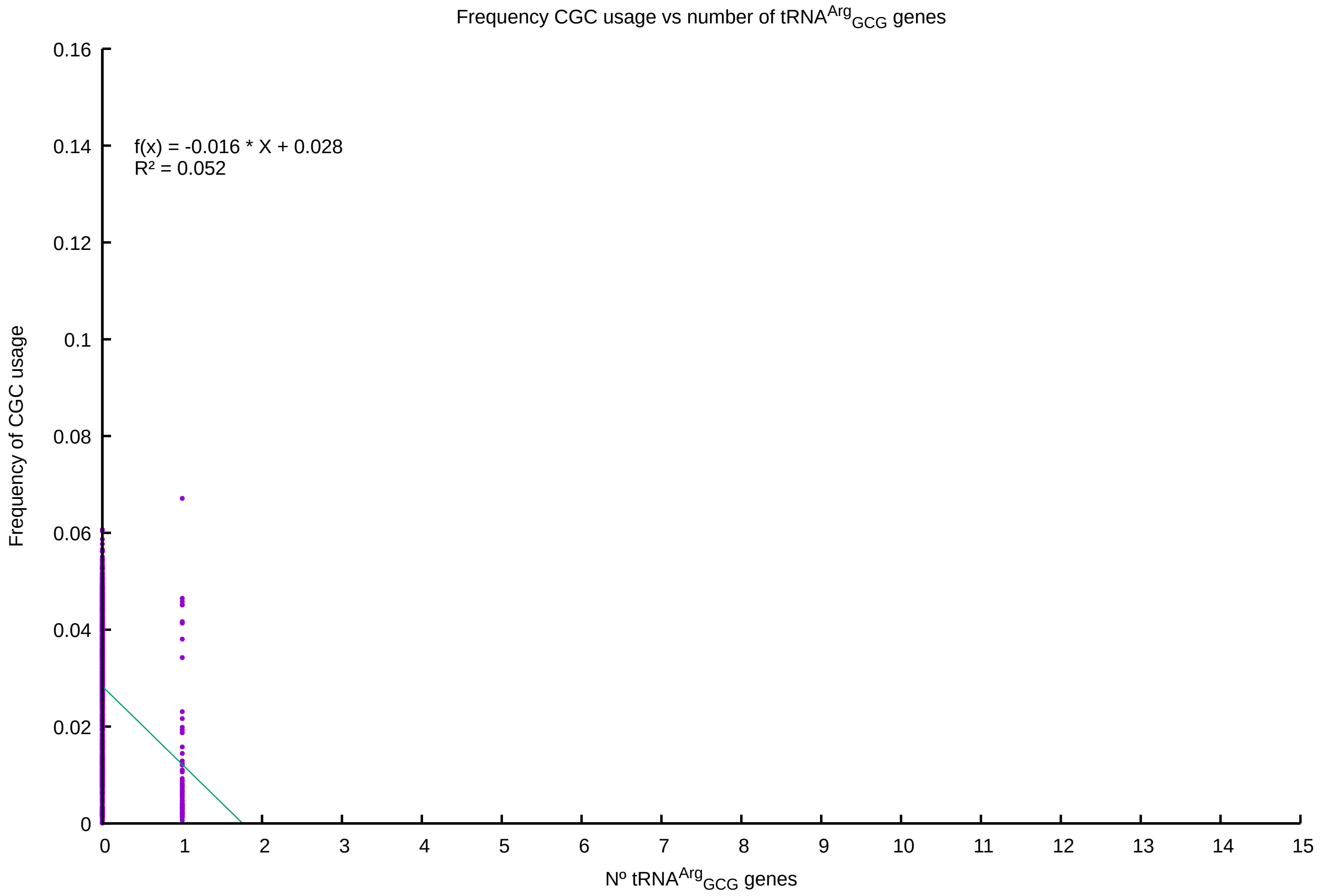

Frequency CGC usage vs number of tRNAArgGCG genes
f(x) = -0.016 * X + 0.028
R² = 0.052
Frequency of CGC usage
0.16
0.14
0.12
0.1
0.08
0.06
0.04
0.02
0
0 1 2 3 4 5 6 7 8 9 10 11 12 13 14 15
Nº tRNAArgGCG genes

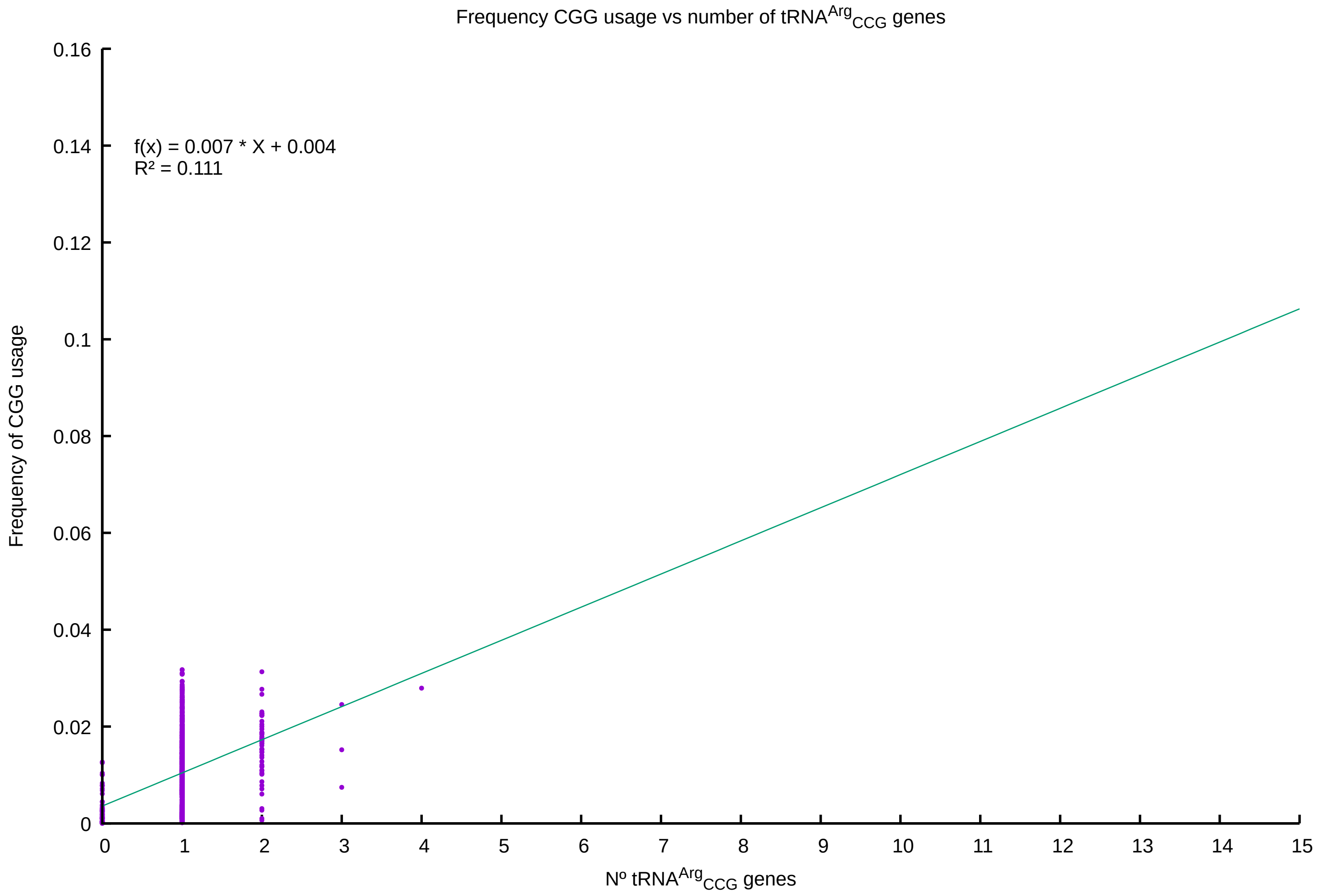

Frequency CGG usage vs number of tRNA^Arg_CCG genes
f(x) = 0.007 * X + 0.004
R² = 0.111
Frequency of CGG usage
0
0.02
0.04
0.06
0.08
0.1
0.12
0.14
0.16
0
1
2
3
4
5
6
7
8
9
10
11
12
13
14
15
Nº tRNA^Arg_CCG genes

Frequency CGT usage vs number of $tRNA^{Arg}_{ACG}$ genes

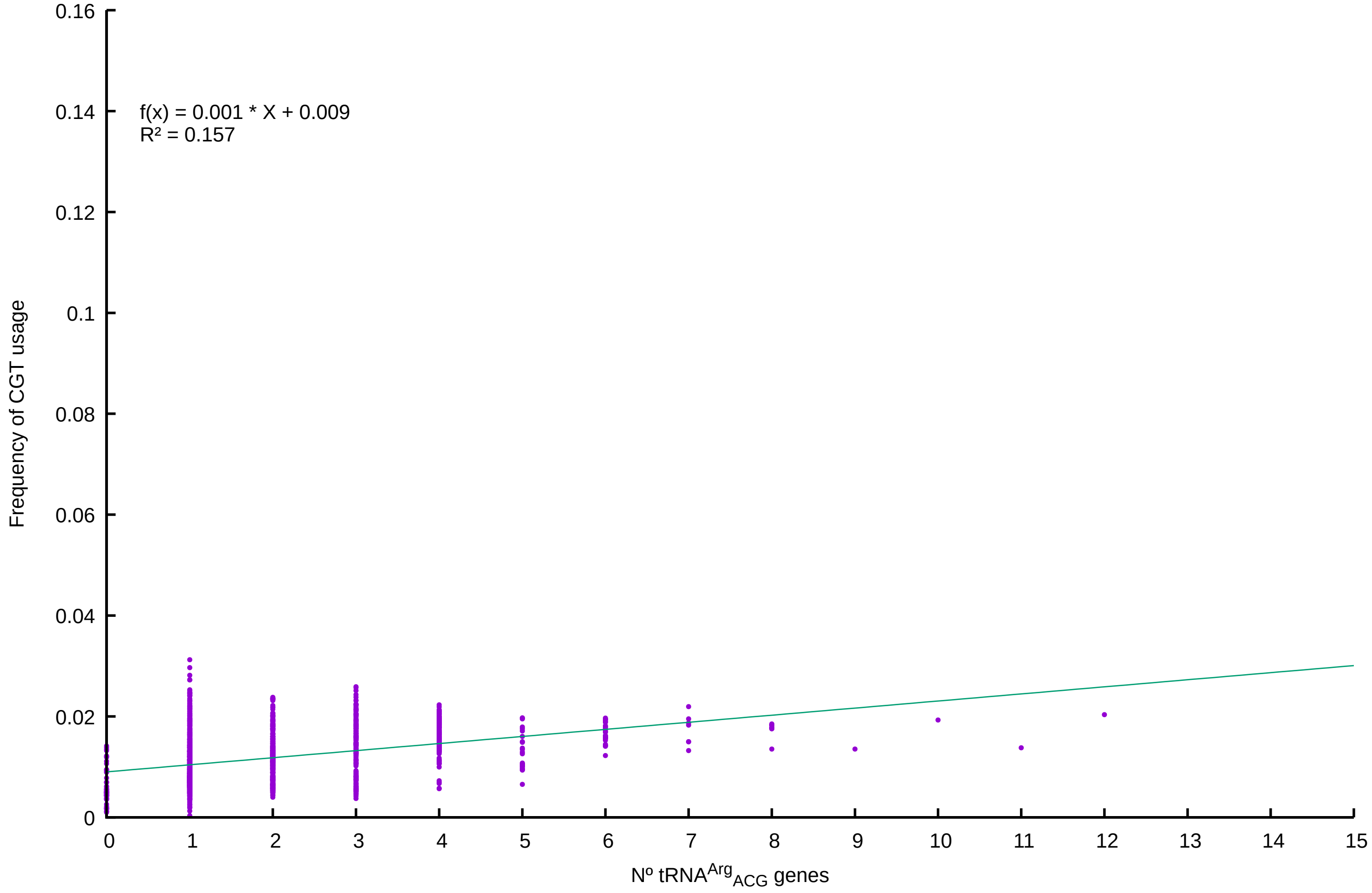

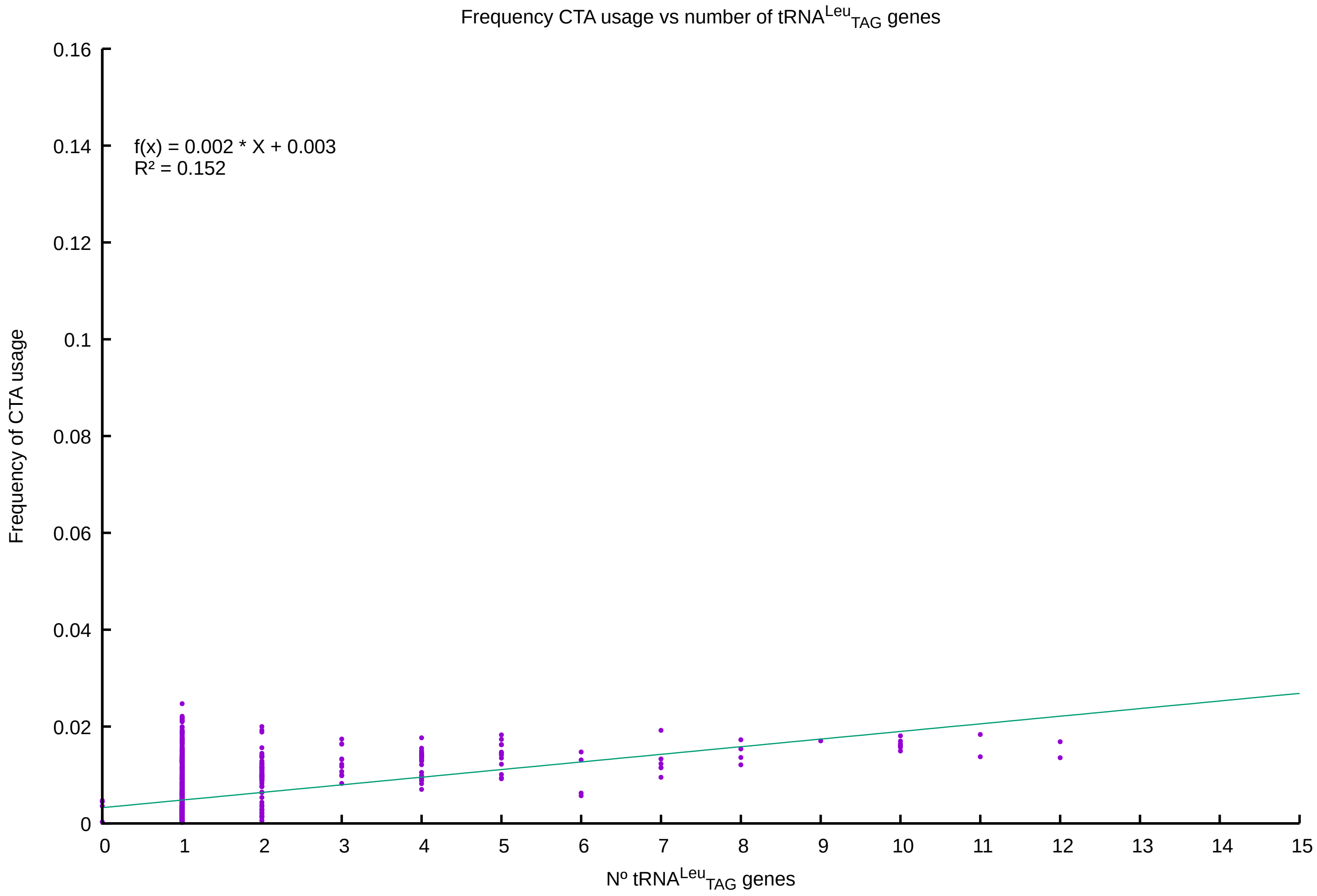

Frequency CTA usage vs number of tRNALeuTAG genes
f(x) = 0.002 * X + 0.003
R² = 0.152
Frequency of CTA usage
0.16
0.14
0.12
0.1
0.08
0.06
0.04
0.02
0
0 1 2 3 4 5 6 7 8 9 10 11 12 13 14 15
Nº tRNALeuTAG genes

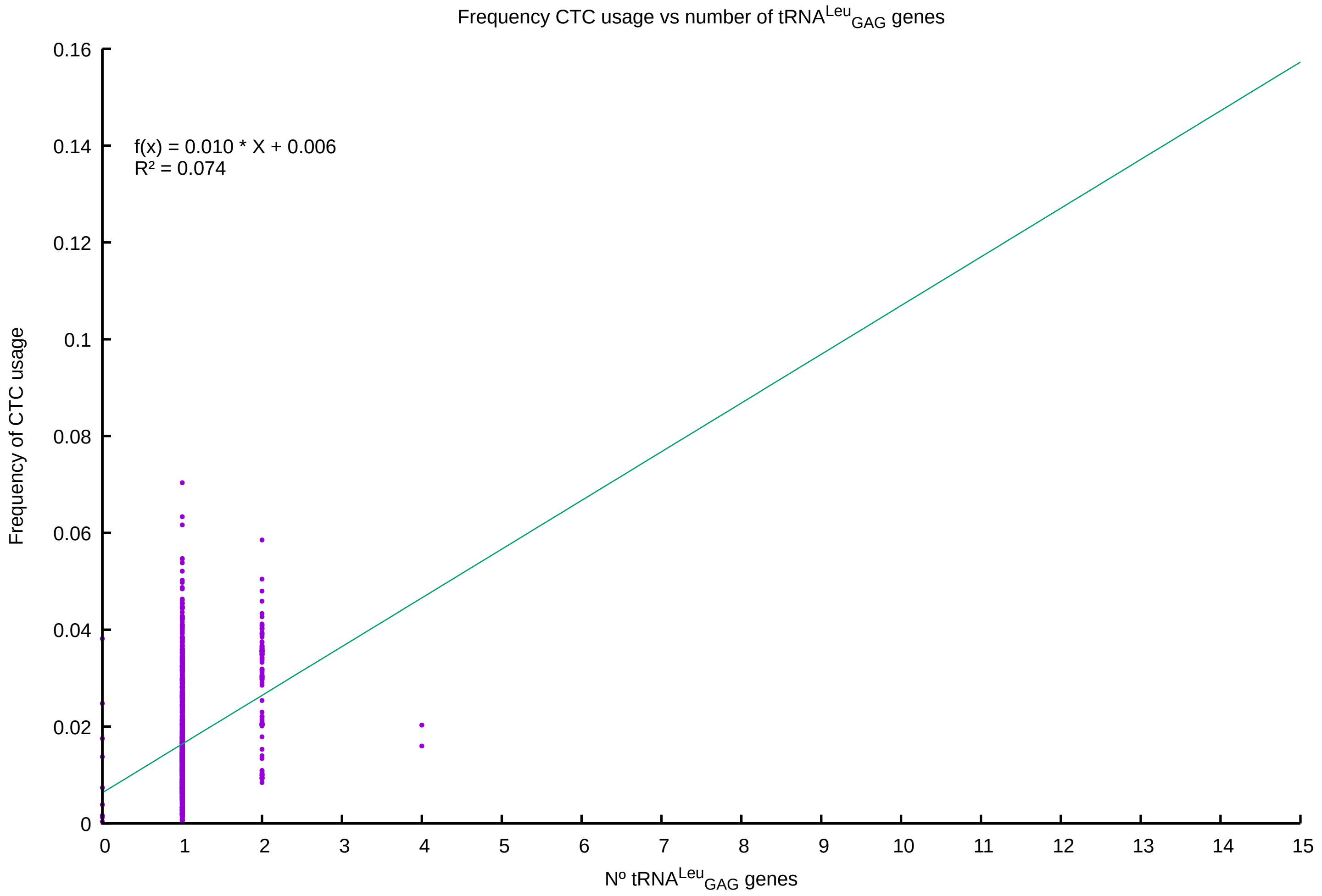
Frequency CTC usage vs number of tRNALeuGAG genes
f(x) = 0.010 * X + 0.006
R² = 0.074
Frequency of CTC usage
0.16
0.14
0.12
0.1
0.08
0.06
0.04
0.02
0
0 1 2 3 4 5 6 7 8 9 10 11 12 13 14 15
Nº tRNALeuGAG genes

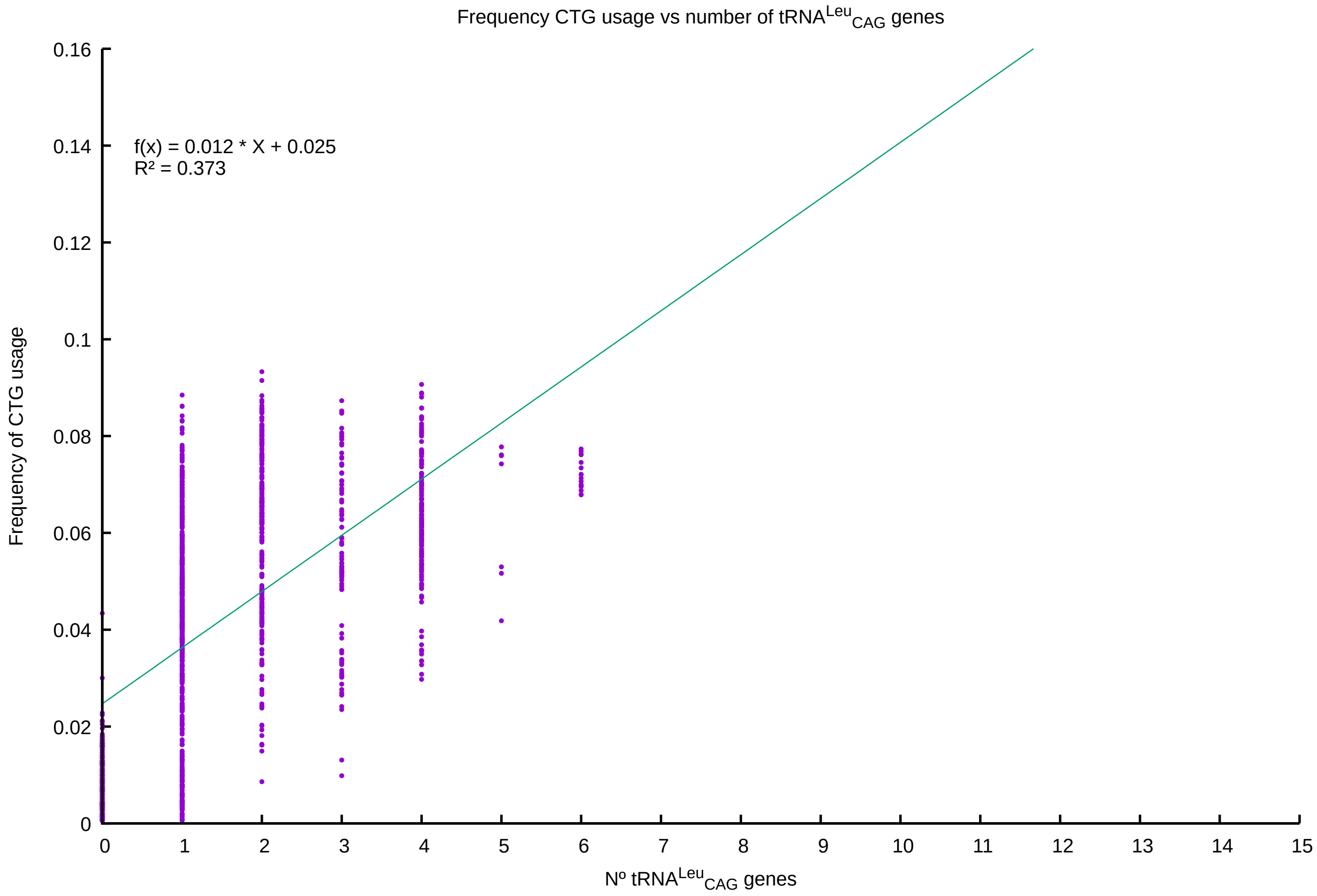

Frequency CTG usage vs number of tRNALeuCAG genes
f(x) = 0.012 * X + 0.025
R² = 0.373
Frequency of CTG usage
0.16
0.14
0.12
0.1
0.08
0.06
0.04
0.02
0
0 1 2 3 4 5 6 7 8 9 10 11 12 13 14 15
Nº tRNALeuCAG genes

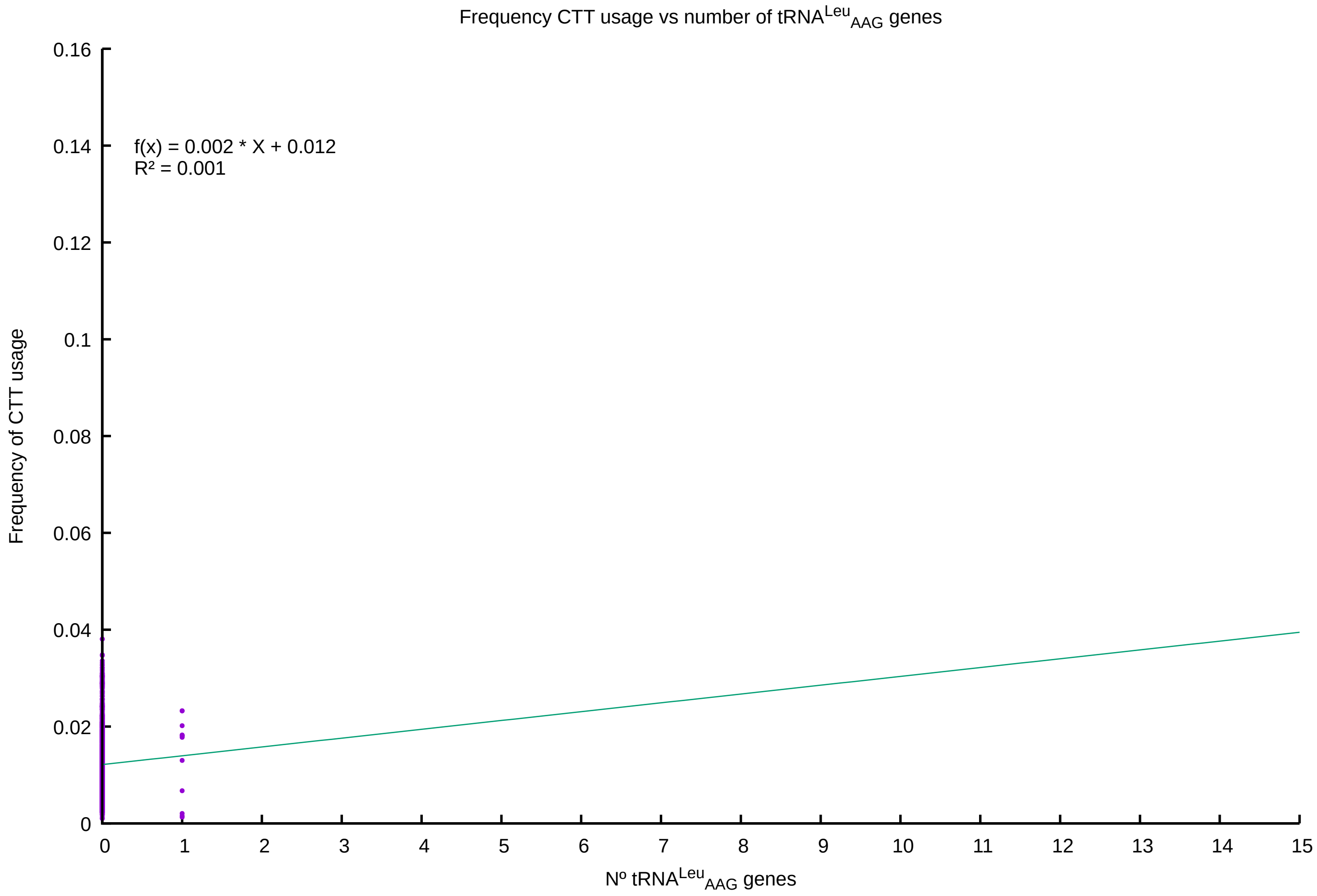
Frequency CTT usage vs number of tRNA^Leu_AAG genes
f(x) = 0.002 * X + 0.012
R² = 0.001
Frequency of CTT usage
0.16
0.14
0.12
0.1
0.08
0.06
0.04
0.02
0
0 1 2 3 4 5 6 7 8 9 10 11 12 13 14 15
Nº tRNA^Leu_AAG genes

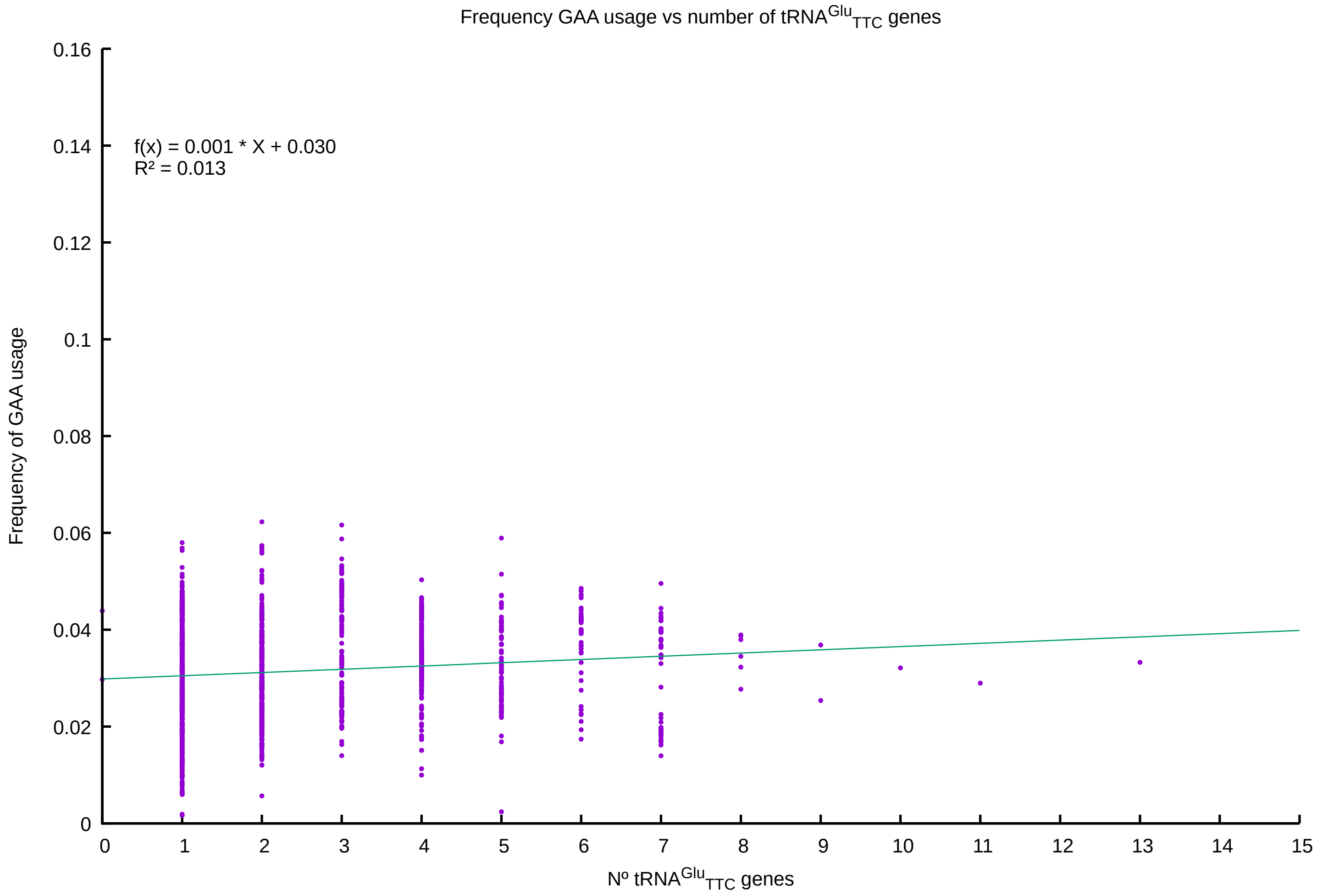

Frequency GAA usage vs number of tRNA^Glu_TTC genes
f(x) = 0.001 * X + 0.030
R² = 0.013
Frequency of GAA usage
0.16
0.14
0.12
0.1
0.08
0.06
0.04
0.02
0
0
1
2
3
4
5
6
7
8
9
10
11
12
13
14
15
Nº tRNA^Glu_TTC genes

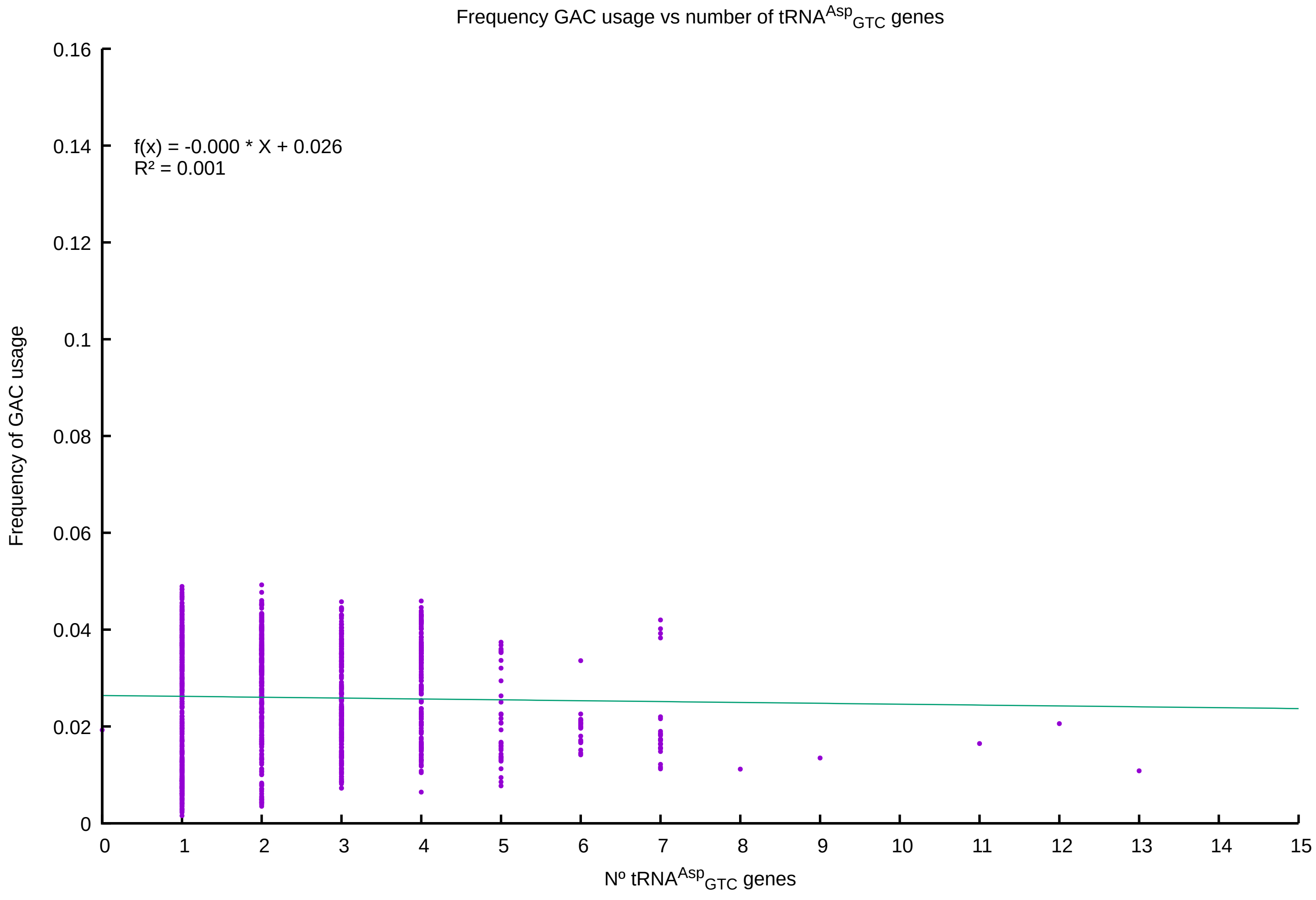
Frequency GAC usage vs number of tRNA^Asp_GTC genes
f(x) = -0.000 * X + 0.026
R² = 0.001
Frequency of GAC usage
0.16
0.14
0.12
0.1
0.08
0.06
0.04
0.02
0
0 1 2 3 4 5 6 7 8 9 10 11 12 13 14 15
Nº tRNA^Asp_GTC genes

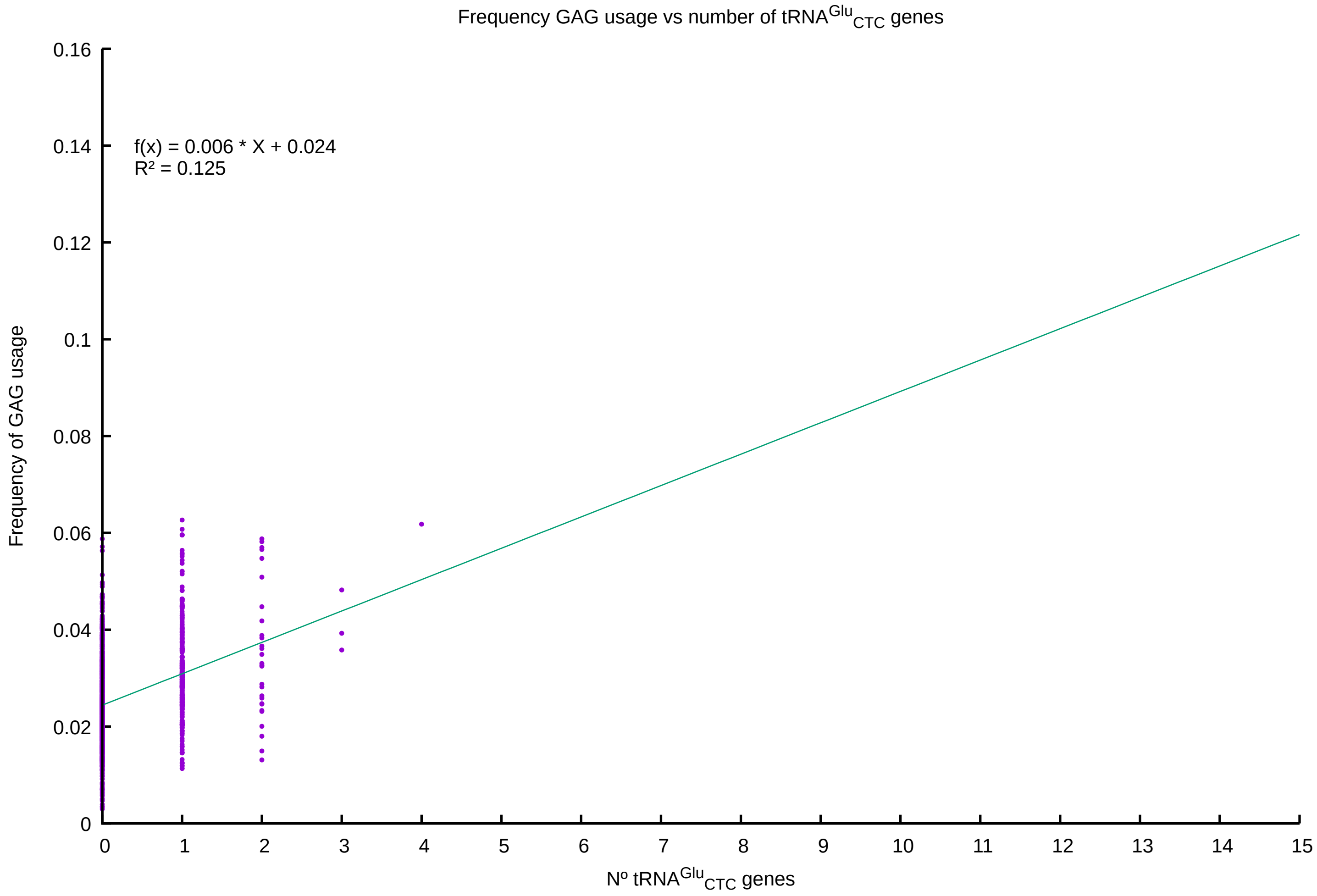
Frequency GAG usage vs number of tRNAGluCTC genes
f(x) = 0.006 * X + 0.024
R² = 0.125
Frequency of GAG usage
Nº tRNAGluCTC genes
0
0.02
0.04
0.06
0.08
0.1
0.12
0.14
0.16
0
1
2
3
4
5
6
7
8
9
10
11
12
13
14
15

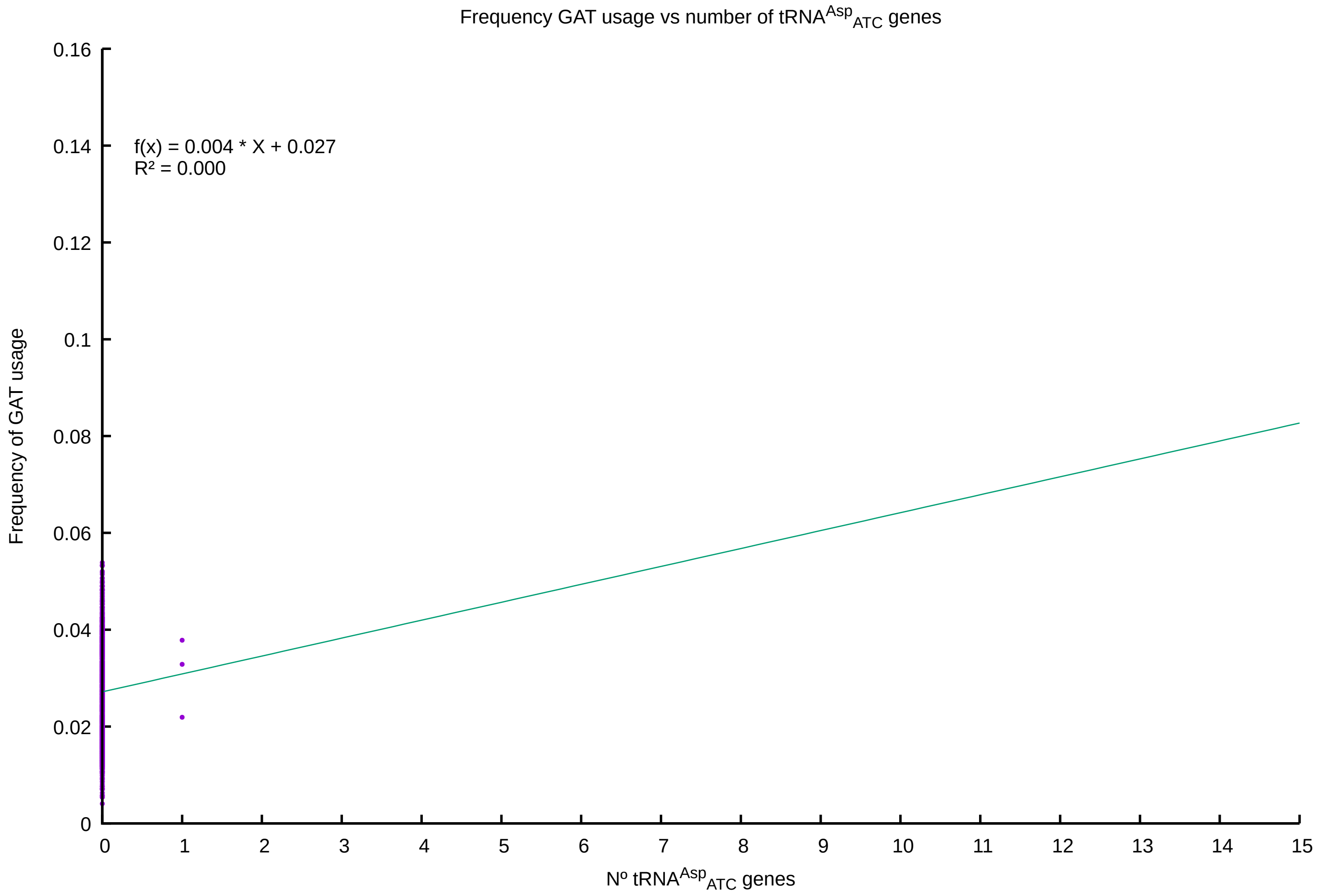
Frequency GAT usage vs number of tRNA$^{Asp}_{ATC}$ genes
f(x) = 0.004 * X + 0.027
R² = 0.000
Frequency of GAT usage
0
0.02
0.04
0.06
0.08
0.1
0.12
0.14
0.16
0
1
2
3
4
5
6
7
8
9
10
11
12
13
14
15
Nº tRNA$^{Asp}_{ATC}$ genes

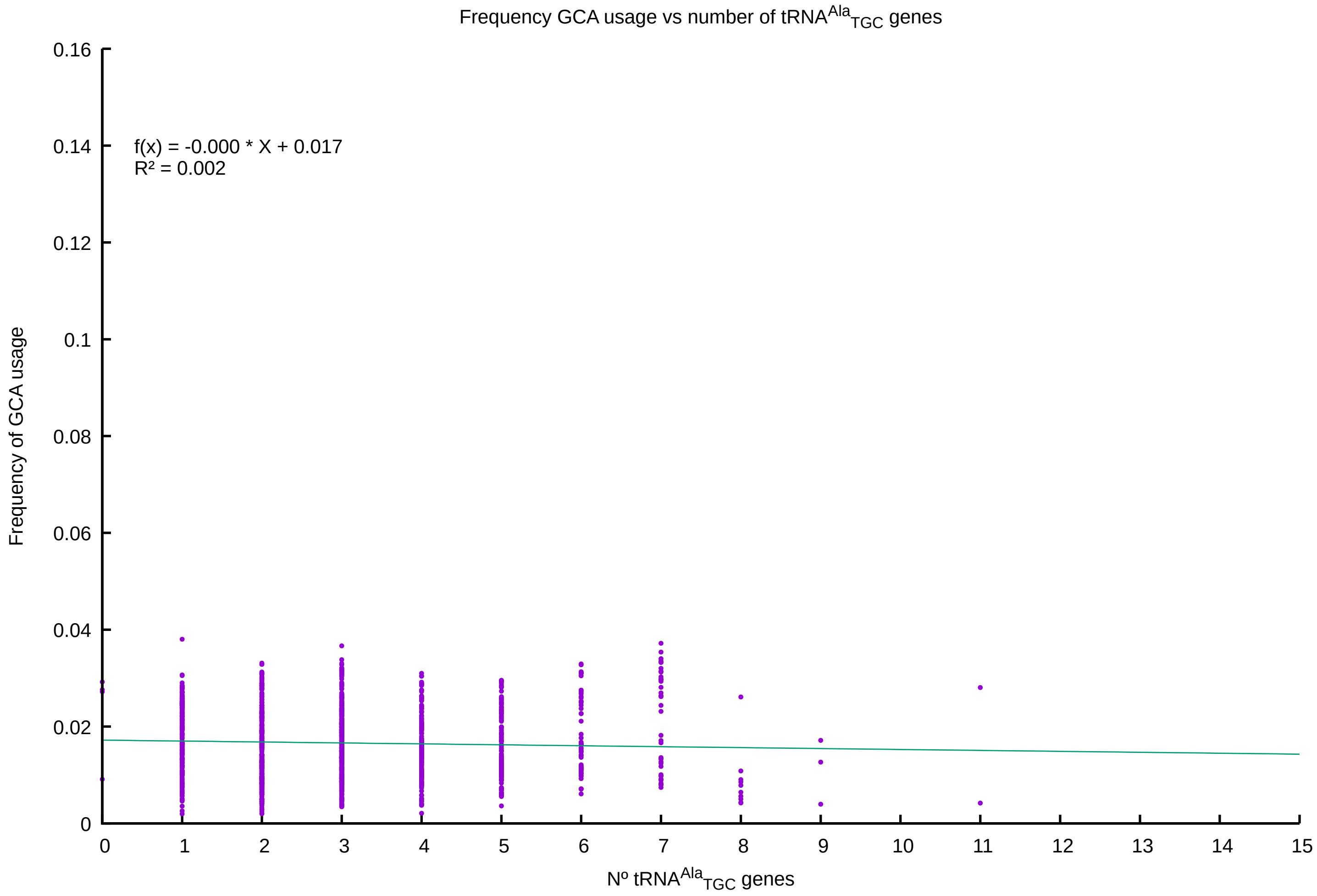
Frequency GCA usage vs number of tRNA^Ala_TGC genes
f(x) = -0.000 * X + 0.017
R² = 0.002
Frequency of GCA usage
0.16
0.14
0.12
0.1
0.08
0.06
0.04
0.02
0
0 1 2 3 4 5 6 7 8 9 10 11 12 13 14 15
Nº tRNA^Ala_TGC genes

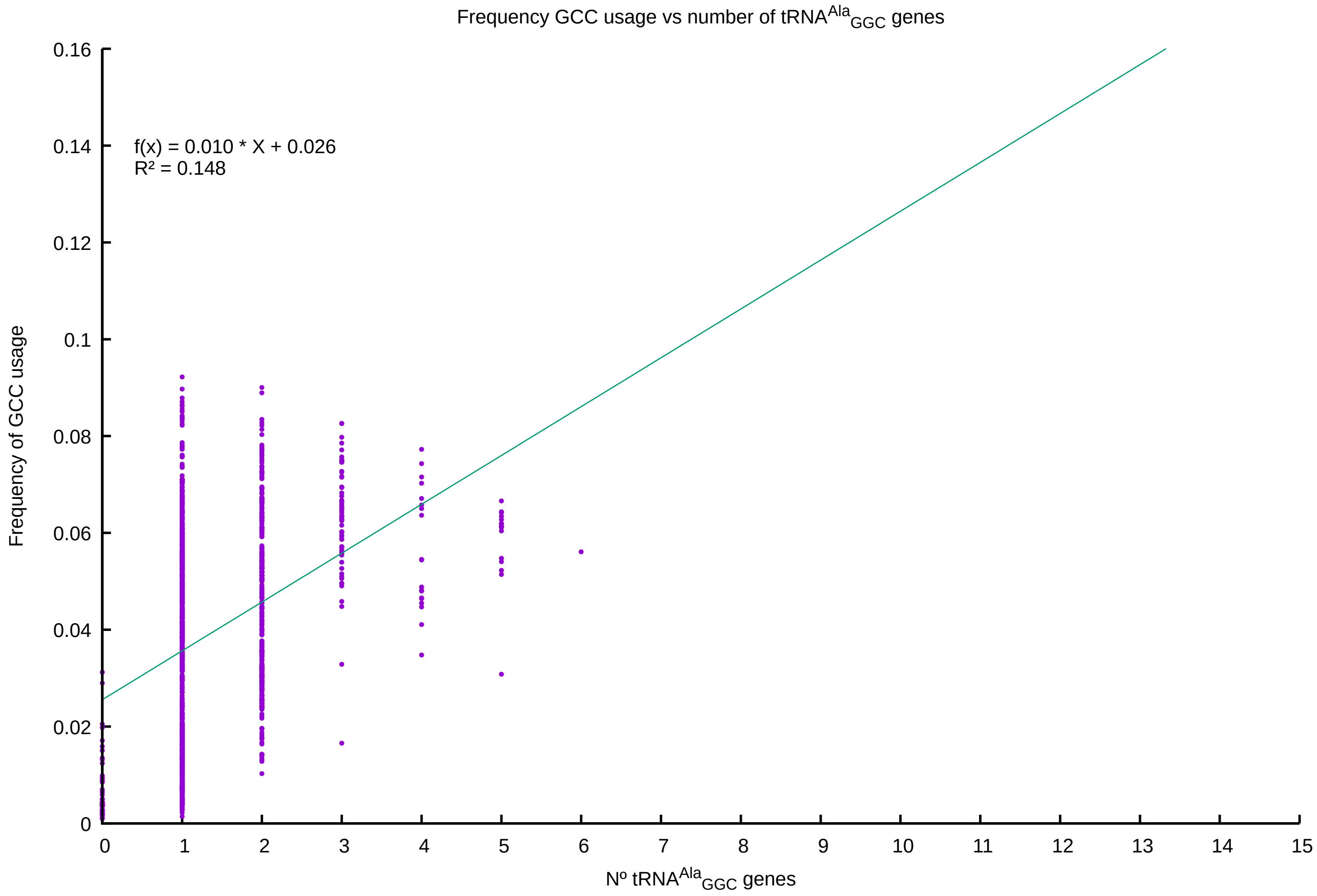

Frequency GCC usage vs number of tRNA^Ala_GGC genes
f(x) = 0.010 * X + 0.026
R² = 0.148
Frequency of GCC usage
0.16
0.14
0.12
0.1
0.08
0.06
0.04
0.02
0
0 1 2 3 4 5 6 7 8 9 10 11 12 13 14 15
Nº tRNA^Ala_GGC genes

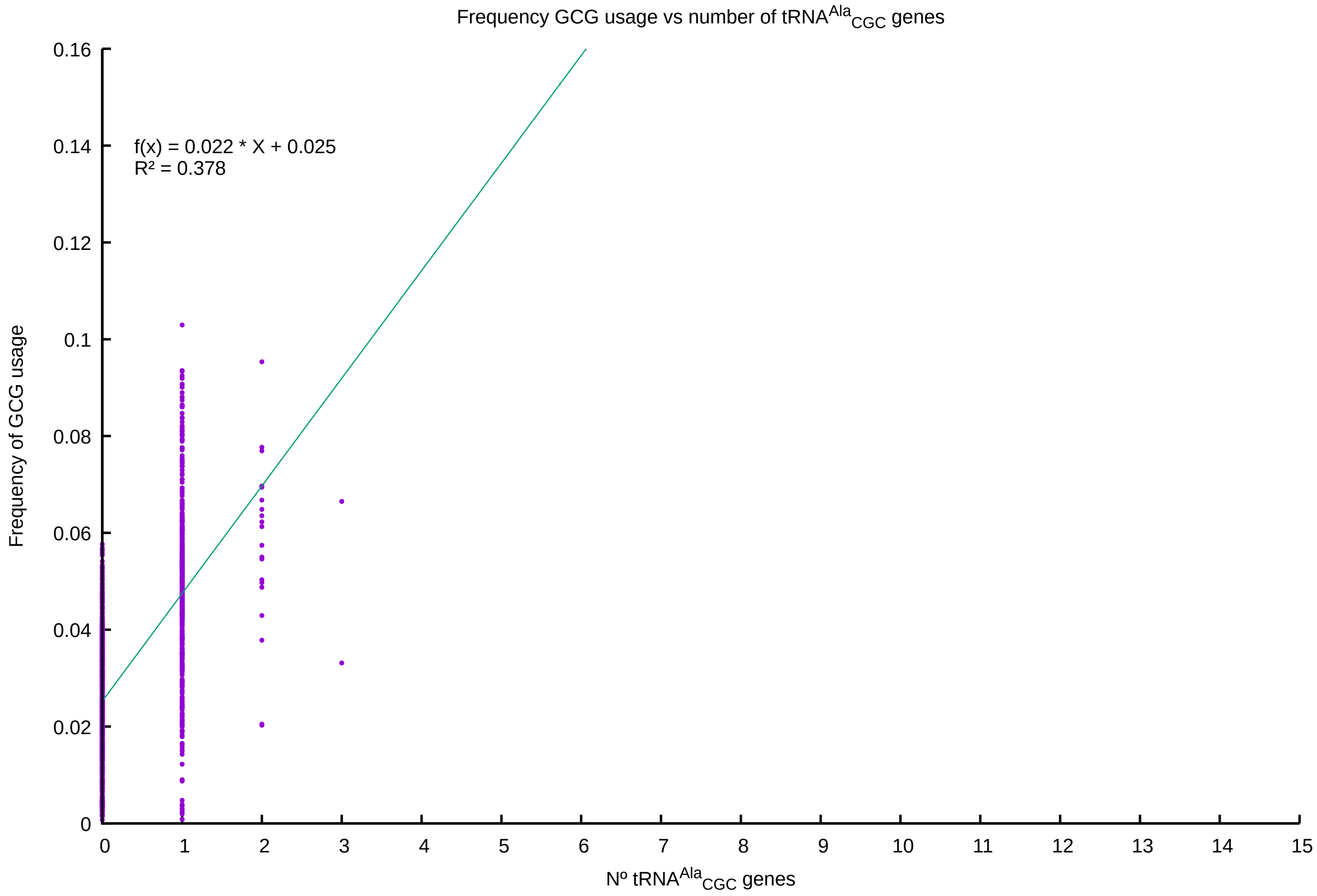

Frequency GCG usage vs number of tRNA^Ala_CGC genes
f(x) = 0.022 * X + 0.025
R² = 0.378
Frequency of GCG usage
0.16
0.14
0.12
0.1
0.08
0.06
0.04
0.02
0
0 1 2 3 4 5 6 7 8 9 10 11 12 13 14 15
Nº tRNA^Ala_CGC genes

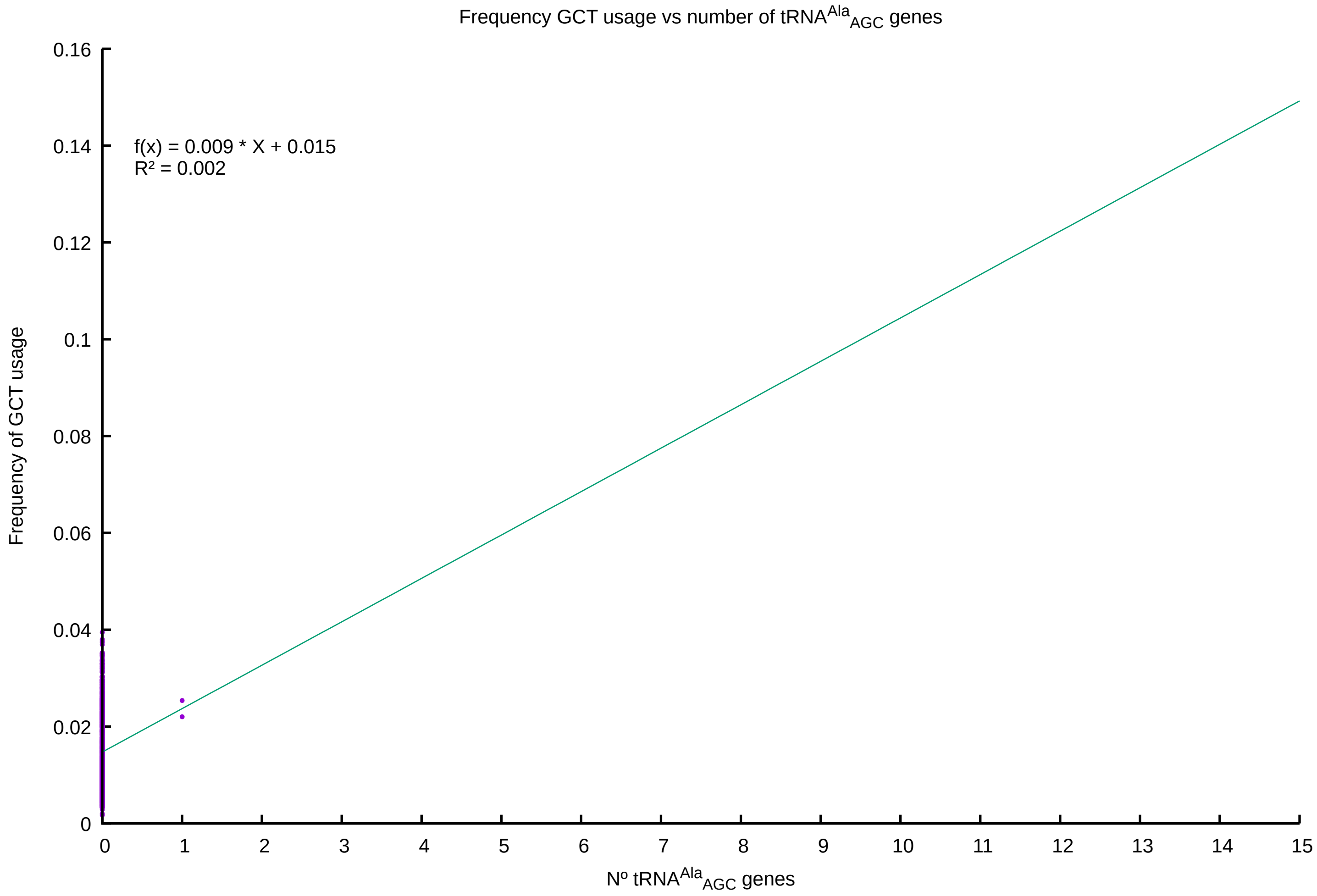

Frequency GCT usage vs number of tRNA Ala AGC genes
f(x) = 0.009 * X + 0.015
R² = 0.002
Frequency of GCT usage
0.16
0.14
0.12
0.1
0.08
0.06
0.04
0.02
0
0 1 2 3 4 5 6 7 8 9 10 11 12 13 14 15
Nº tRNA Ala AGC genes

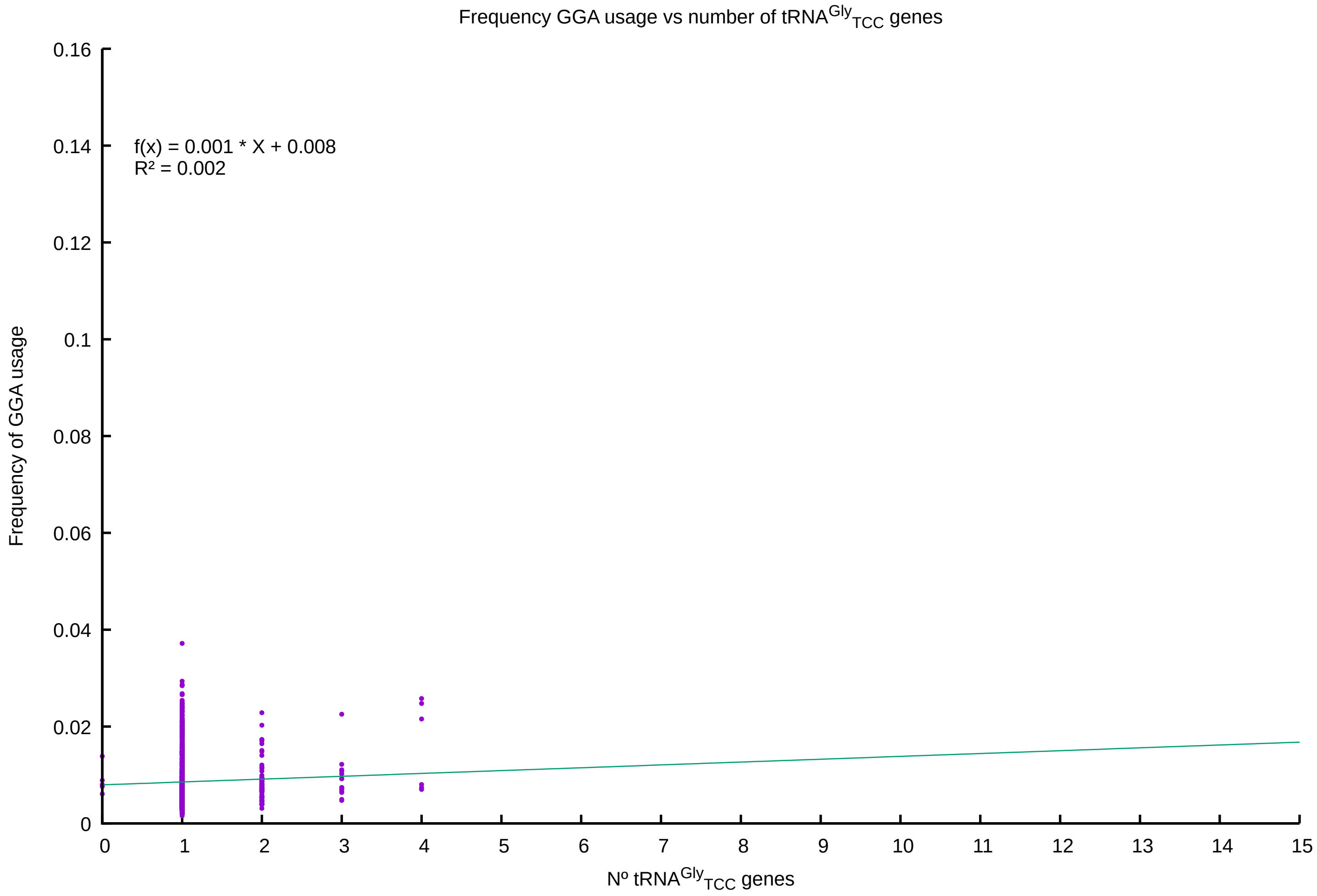
Frequency GGA usage vs number of tRNA^Gly_TCC genes
f(x) = 0.001 * X + 0.008
R² = 0.002
Frequency of GGA usage
0.16
0.14
0.12
0.1
0.08
0.06
0.04
0.02
0
0 1 2 3 4 5 6 7 8 9 10 11 12 13 14 15
Nº tRNA^Gly_TCC genes

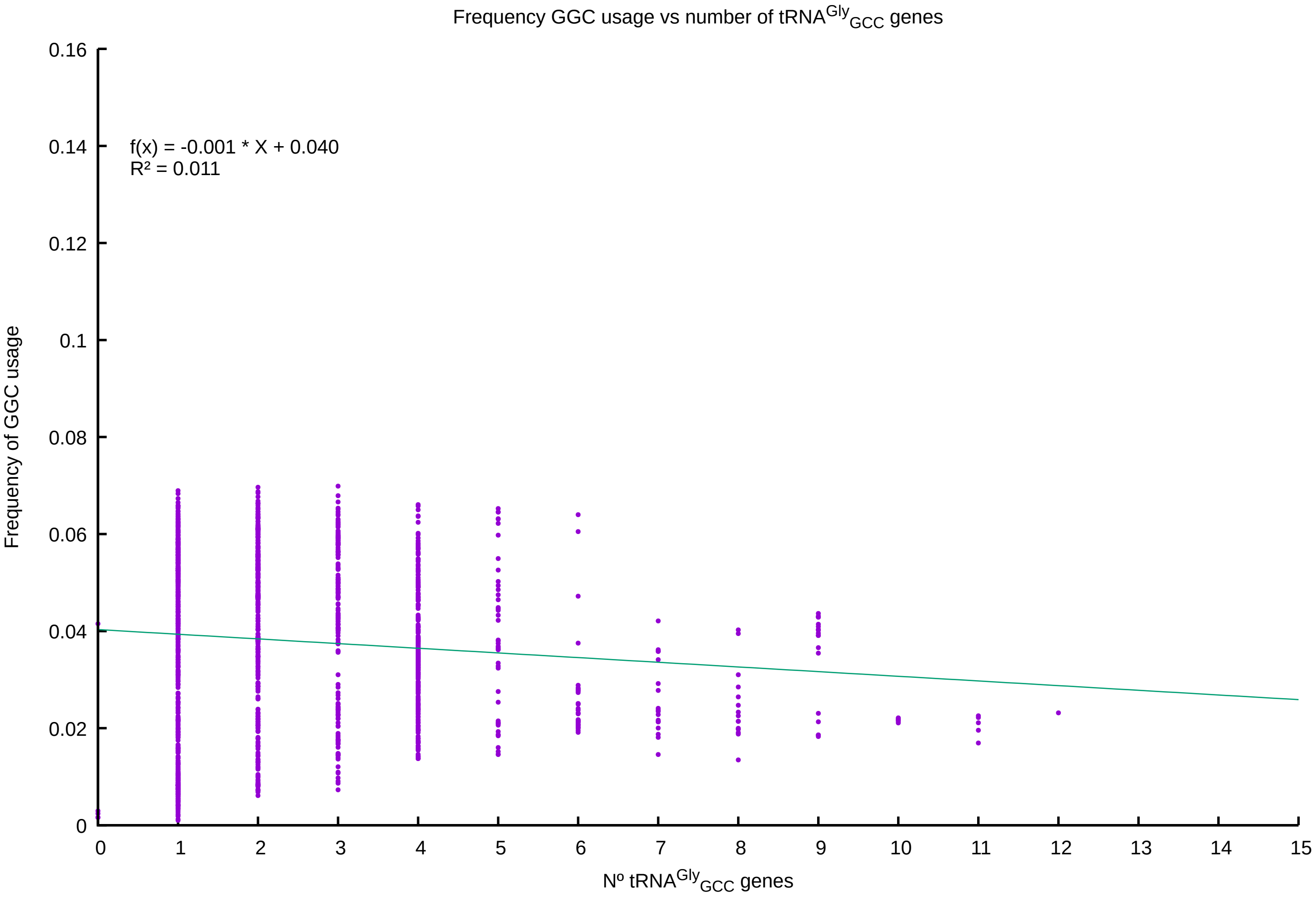

Frequency GGC usage vs number of tRNA$^{Gly}_{GCC}$ genes
f(x) = -0.001 * X + 0.040
R^2 = 0.011
Frequency of GGC usage
0
0.02
0.04
0.06
0.08
0.1
0.12
0.14
0.16
0
1
2
3
4
5
6
7
8
9
10
11
12
13
14
15
Nº tRNA$^{Gly}_{GCC}$ genes

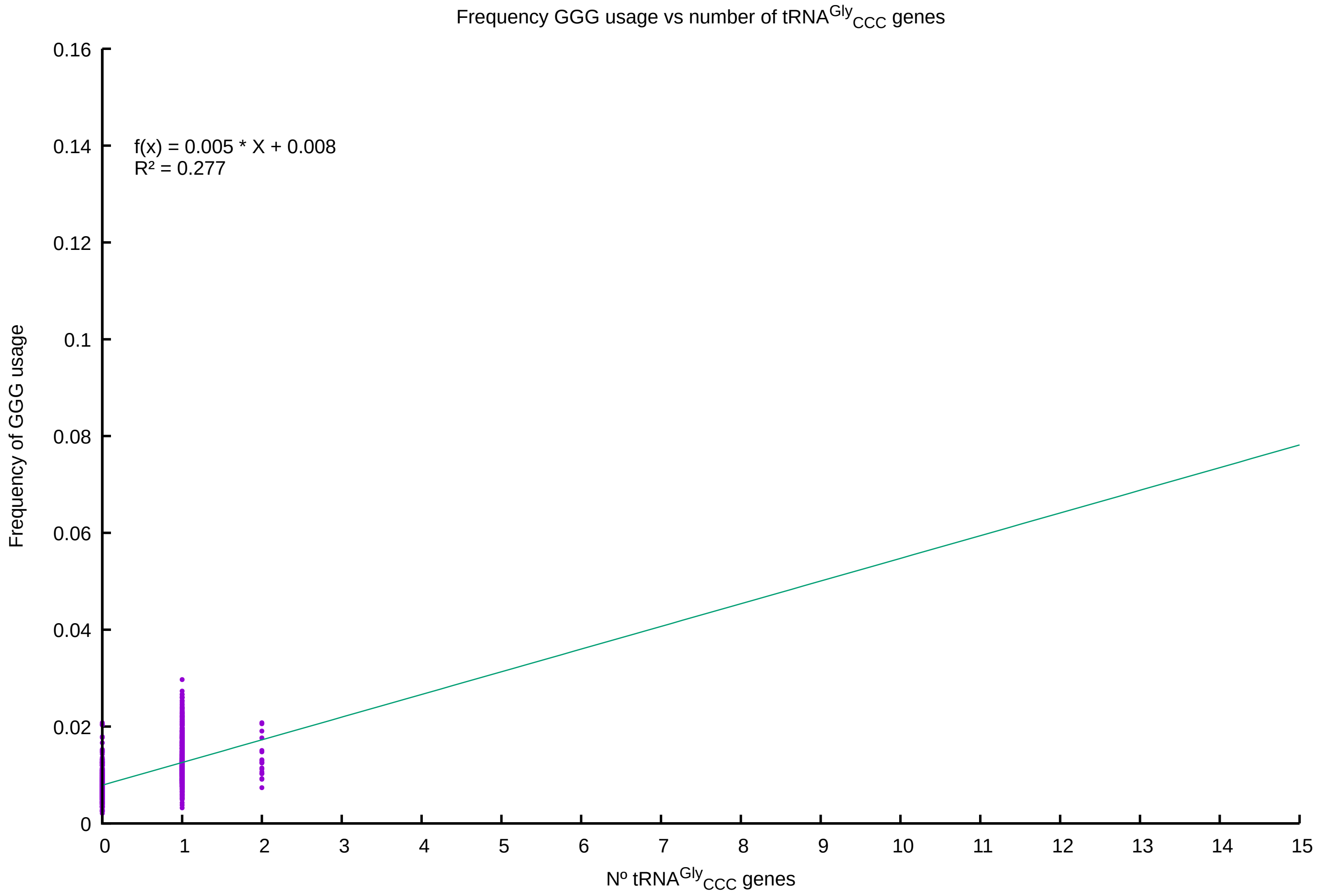
Frequency GGG usage vs number of tRNA$^{Gly}_{CCC}$ genes
f(x) = 0.005 * X + 0.008
R^2 = 0.277
Frequency of GGG usage
0.16
0.14
0.12
0.1
0.08
0.06
0.04
0.02
0
0 1 2 3 4 5 6 7 8 9 10 11 12 13 14 15
Nº tRNA$^{Gly}_{CCC}$ genes

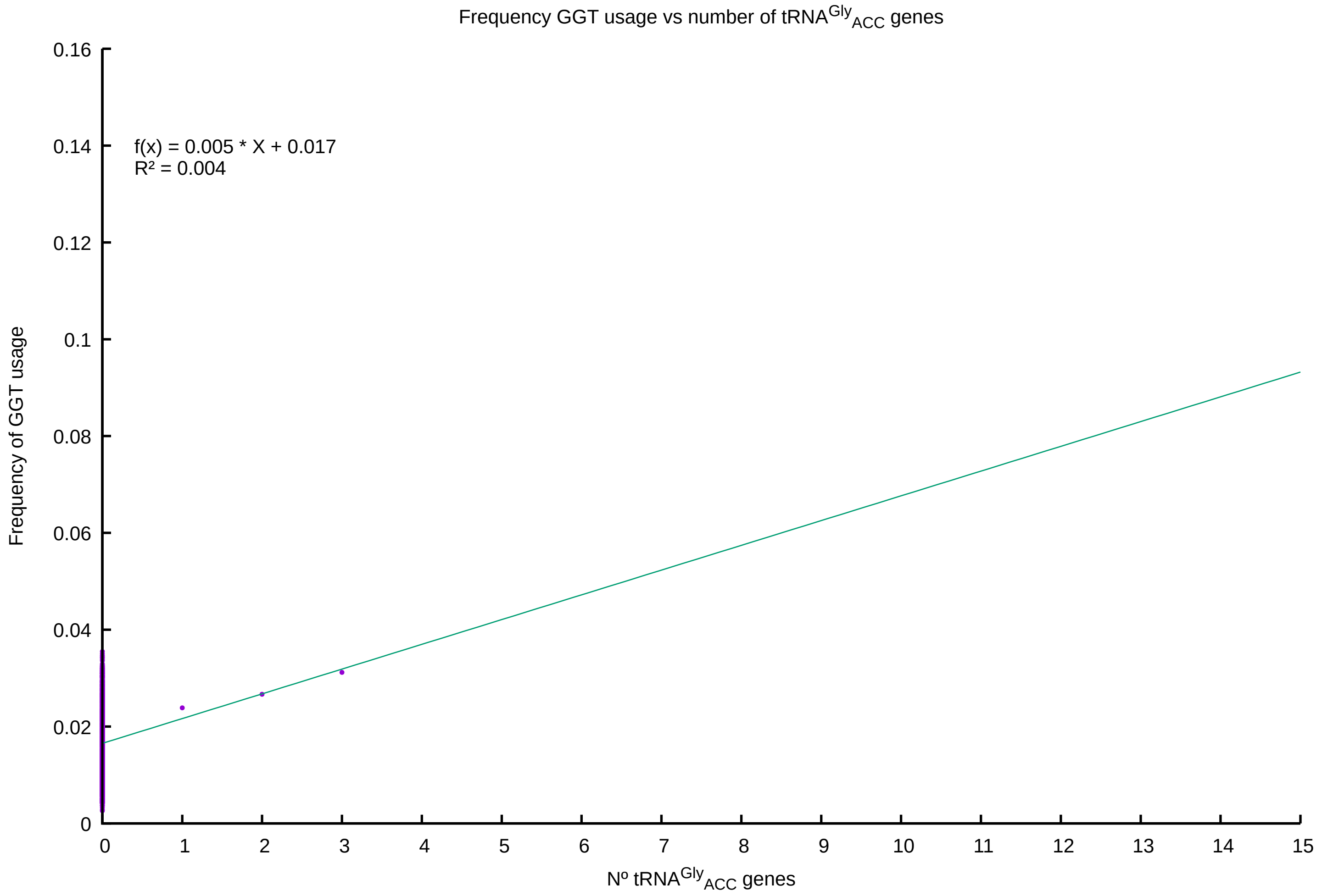

Frequency GGT usage vs number of tRNA^Gly_ACC genes
f(x) = 0.005 * X + 0.017
R² = 0.004
Frequency of GGT usage
0.16
0.14
0.12
0.1
0.08
0.06
0.04
0.02
0
0 1 2 3 4 5 6 7 8 9 10 11 12 13 14 15
Nº tRNA^Gly_ACC genes

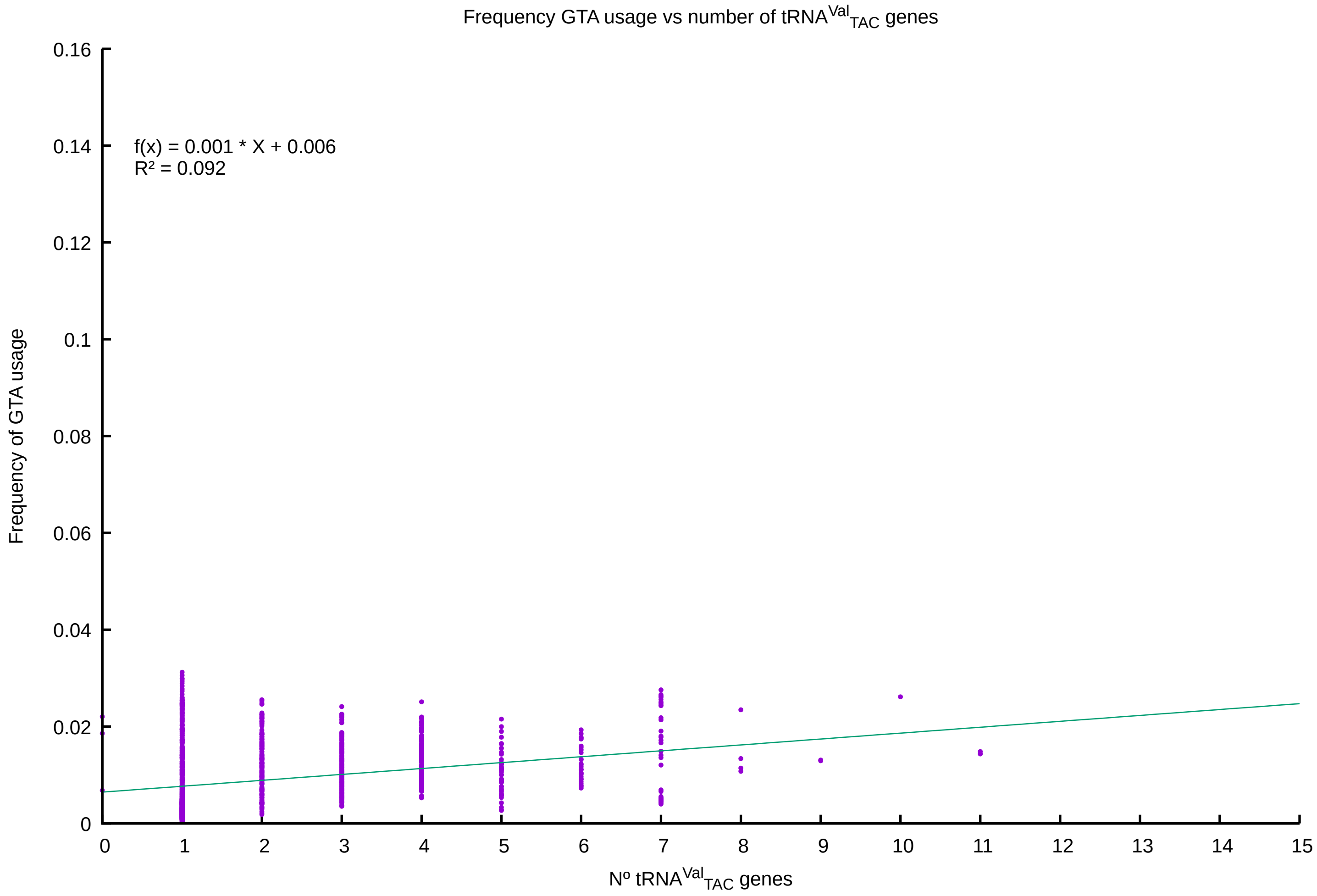

Frequency GTA usage vs number of tRNA$^{Val}_{TAC}$ genes
f(x) = 0.001 * X + 0.006
R^2 = 0.092
Frequency of GTA usage
0
0.02
0.04
0.06
0.08
0.1
0.12
0.14
0.16
0
1
2
3
4
5
6
7
8
9
10
11
12
13
14
15
Nº tRNA$^{Val}_{TAC}$ genes

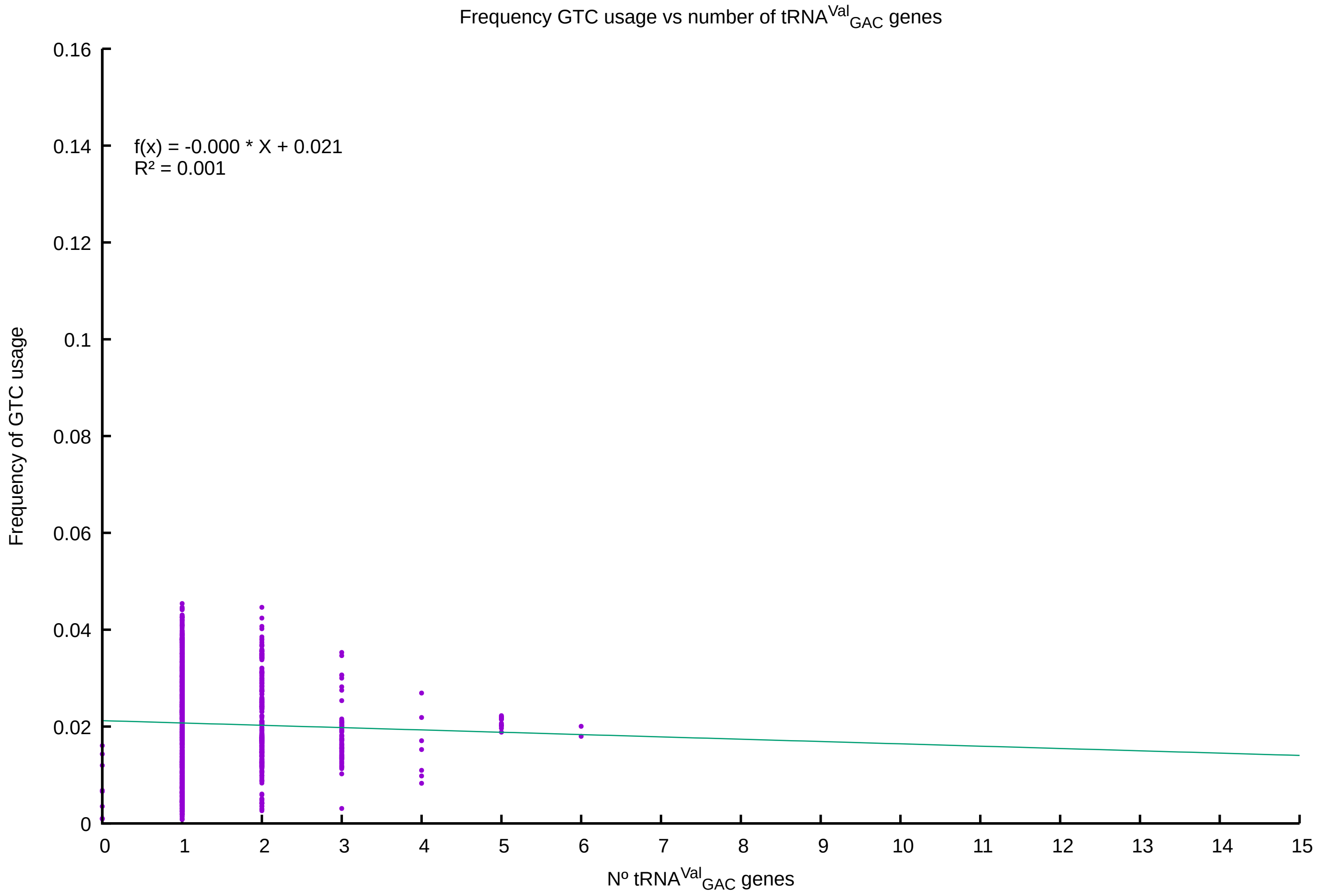

Frequency GTC usage vs number of tRNA^Val_GAC genes
f(x) = -0.000 * X + 0.021
R² = 0.001
Frequency of GTC usage
0.16
0.14
0.12
0.1
0.08
0.06
0.04
0.02
0
0 1 2 3 4 5 6 7 8 9 10 11 12 13 14 15
Nº tRNA^Val_GAC genes

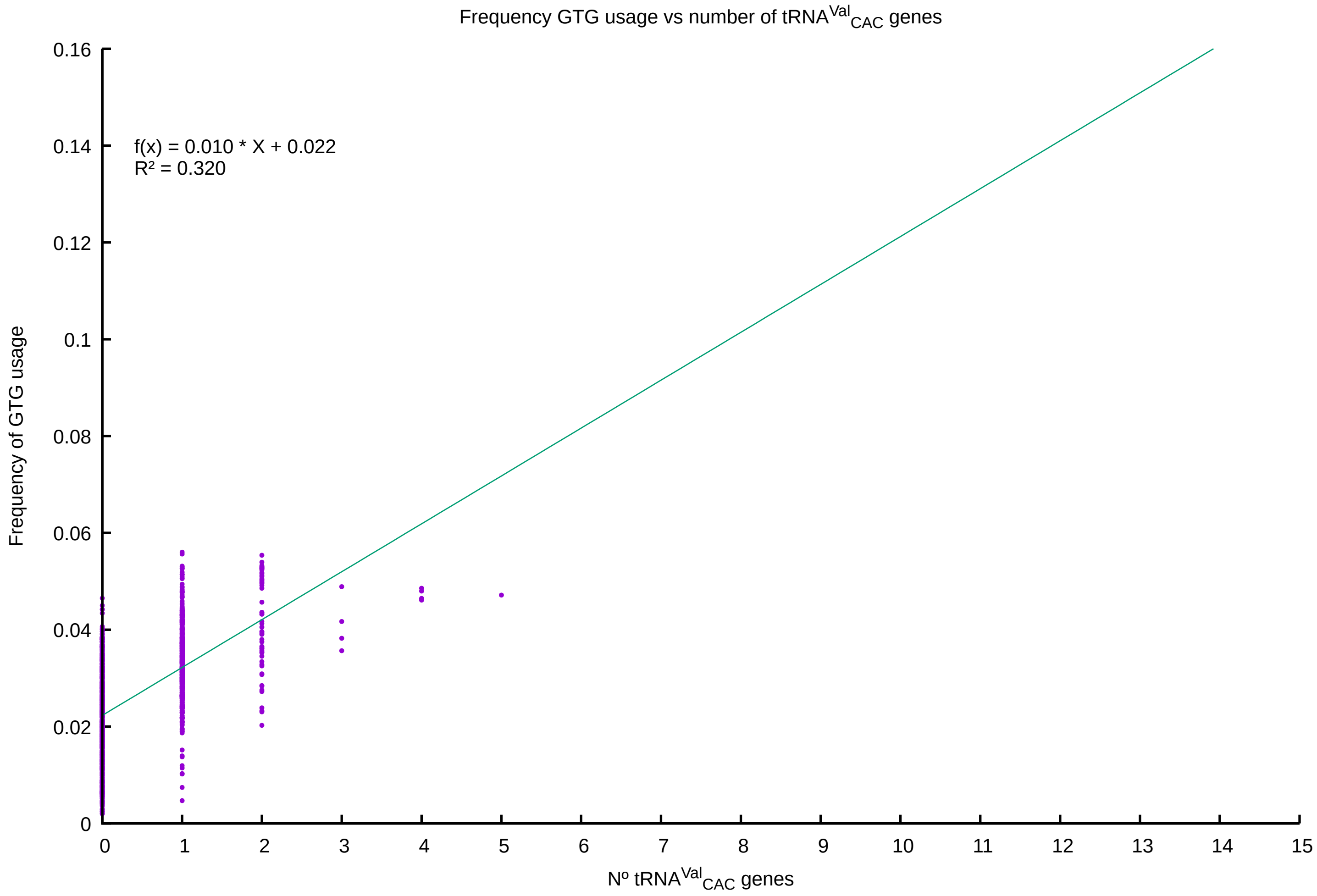

Frequency GTG usage vs number of tRNA Val CAC genes
f(x) = 0.010 * X + 0.022
R² = 0.320
Frequency of GTG usage
Nº tRNA Val CAC genes
0
0.02
0.04
0.06
0.08
0.1
0.12
0.14
0.16
0
1
2
3
4
5
6
7
8
9
10
11
12
13
14
15

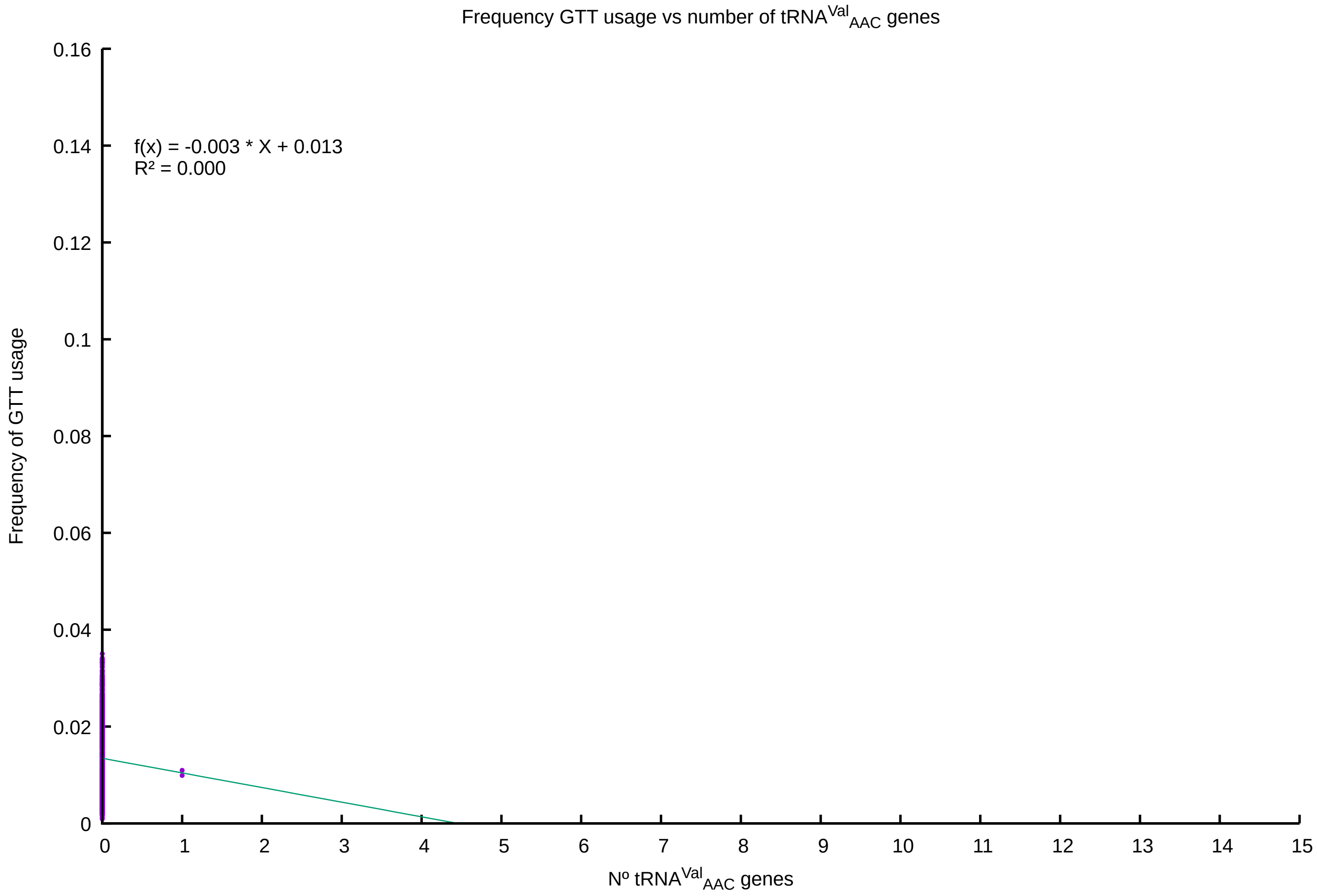
Frequency GTT usage vs number of tRNA^Val_AAC genes
f(x) = -0.003 * X + 0.013
R² = 0.000
Frequency of GTT usage
0.16
0.14
0.12
0.1
0.08
0.06
0.04
0.02
0
0 1 2 3 4 5 6 7 8 9 10 11 12 13 14 15
Nº tRNA^Val_AAC genes

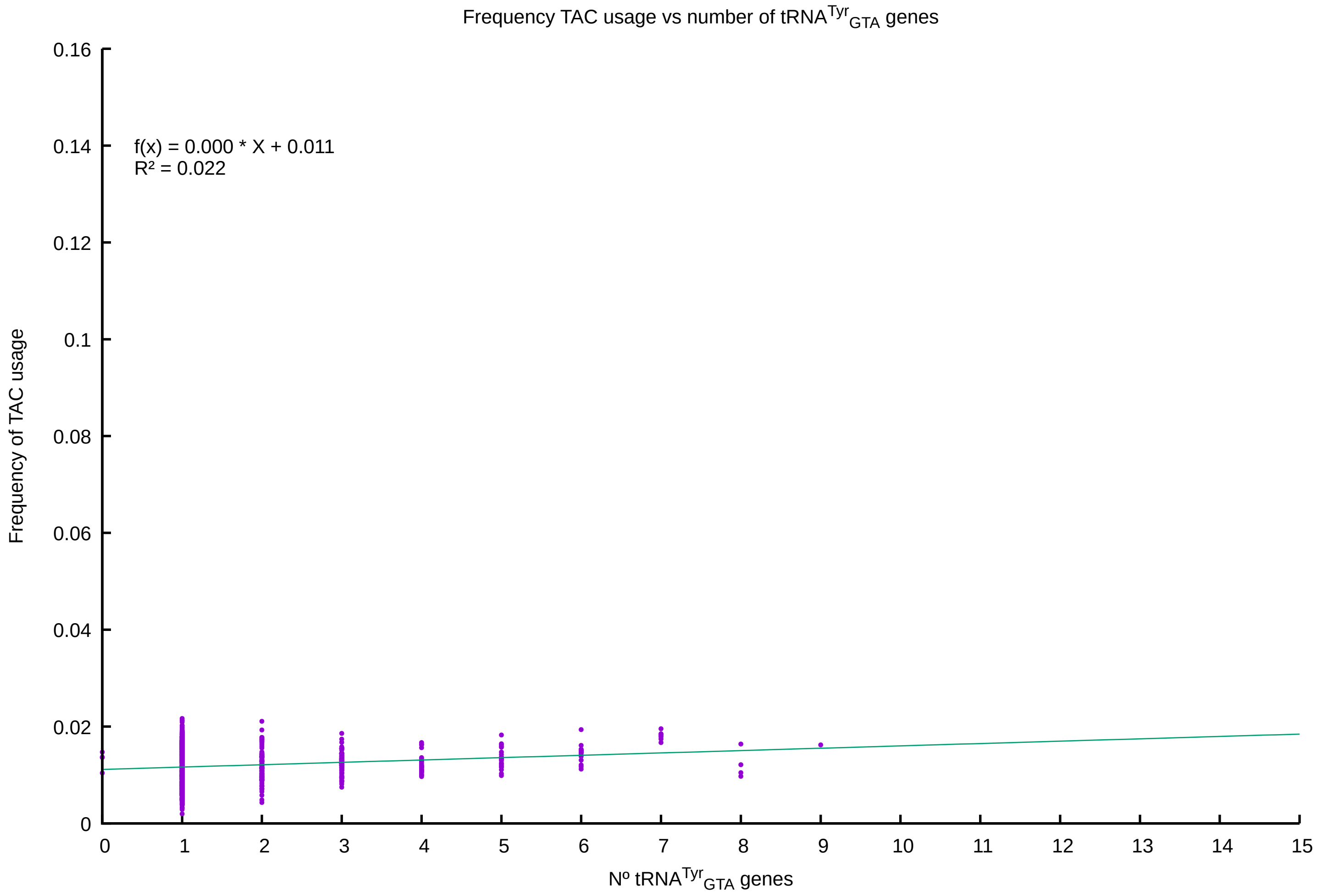
Frequency TAC usage vs number of tRNA$^{Tyr}_{GTA}$ genes
f(x) = 0.000 * X + 0.011
R^2 = 0.022
Frequency of TAC usage
0.16
0.14
0.12
0.1
0.08
0.06
0.04
0.02
0
0 1 2 3 4 5 6 7 8 9 10 11 12 13 14 15
Nº tRNA$^{Tyr}_{GTA}$ genes

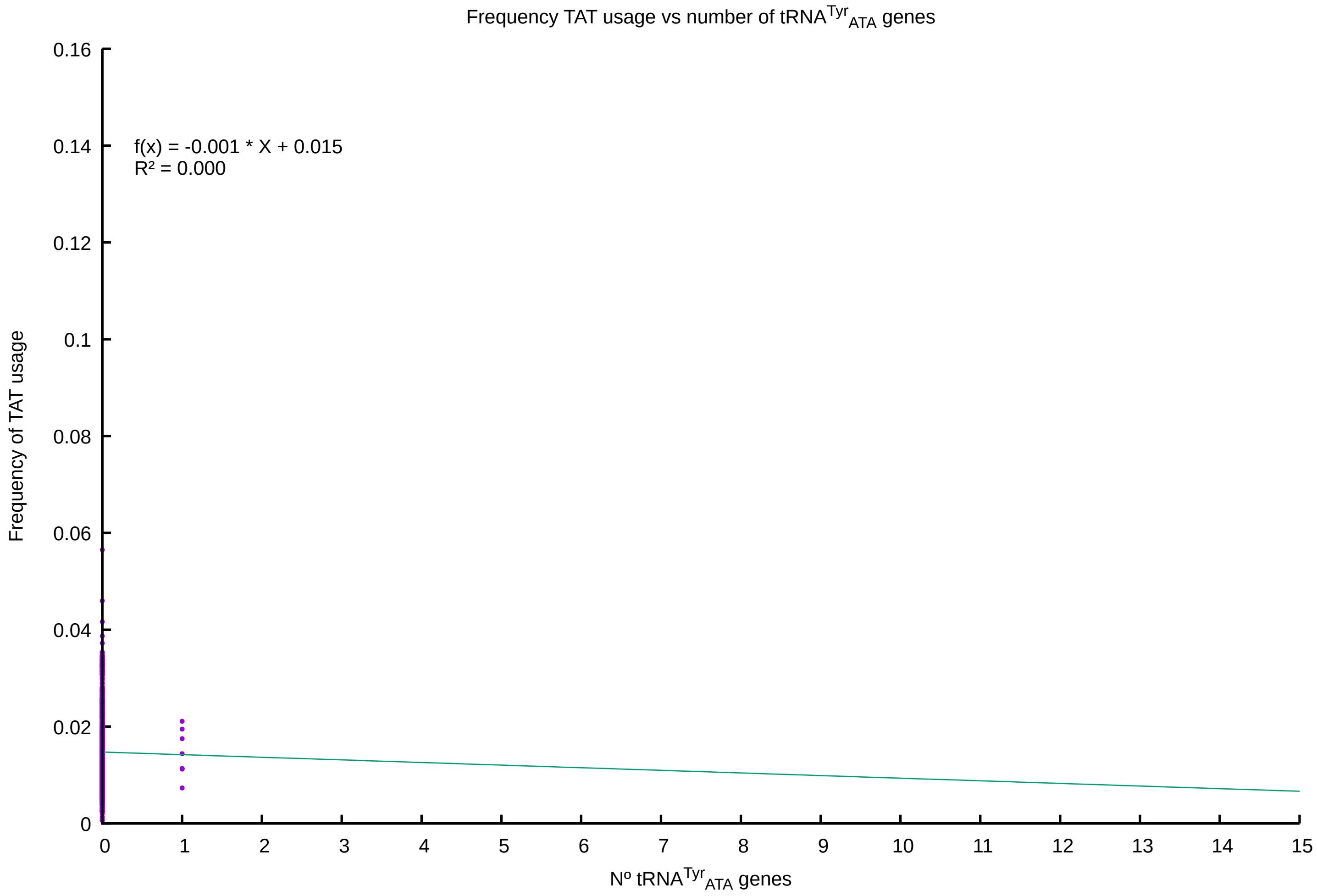
Frequency TAT usage vs number of tRNA$^{Tyr}_{ATA}$ genes
f(x) = -0.001 * X + 0.015
R² = 0.000
Frequency of TAT usage
Nº tRNA$^{Tyr}_{ATA}$ genes
0
0.02
0.04
0.06
0.08
0.1
0.12
0.14
0.16
0
1
2
3
4
5
6
7
8
9
10
11
12
13
14
15

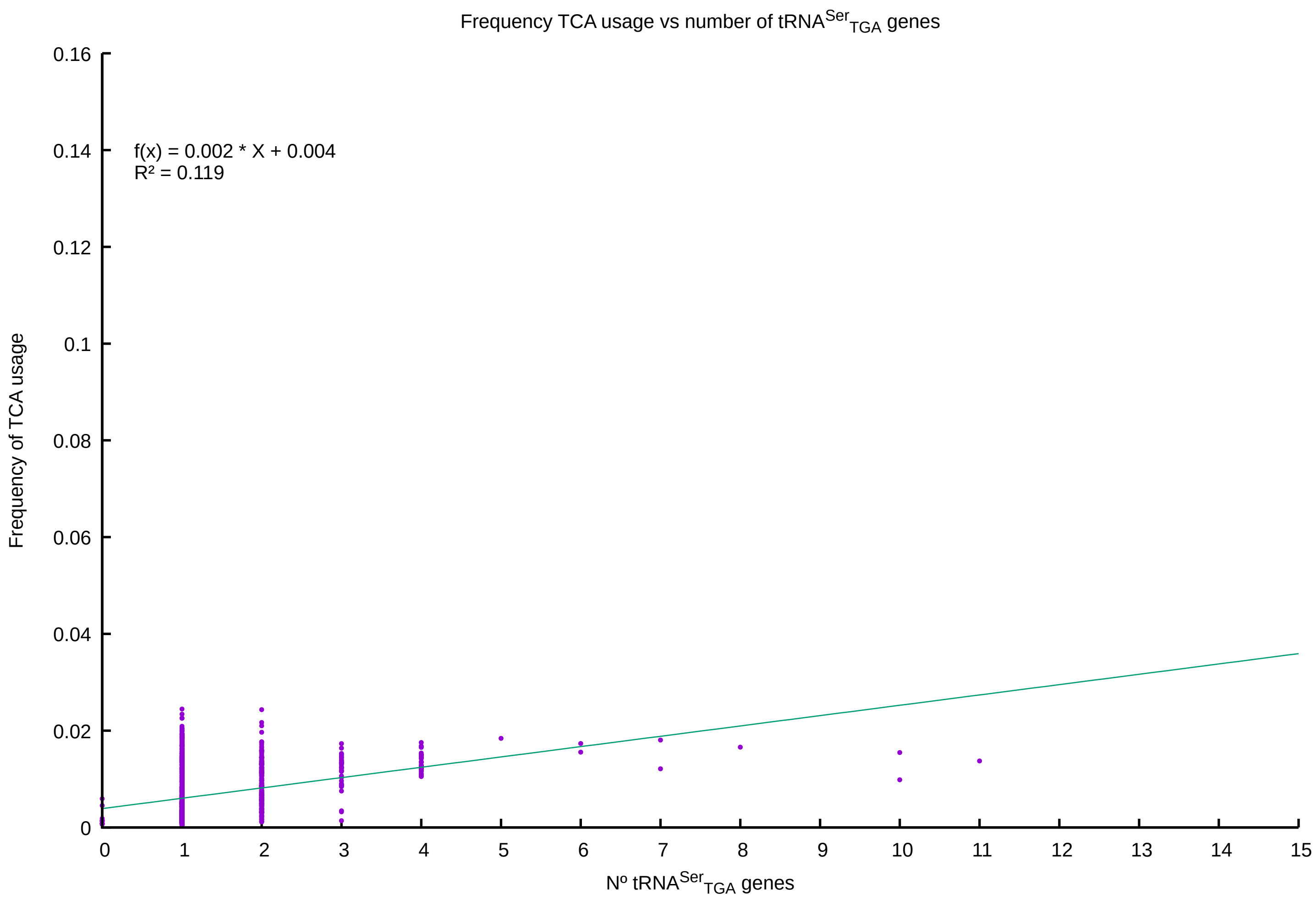
Frequency TCA usage vs number of $tRNA^{Ser}_{TGA}$ genes
f(x) = 0.002 * X + 0.004
R^2 = 0.119
Frequency of TCA usage
0.16
0.14
0.12
0.1
0.08
0.06
0.04
0.02
0
0 1 2 3 4 5 6 7 8 9 10 11 12 13 14 15
Nº $tRNA^{Ser}_{TGA}$ genes

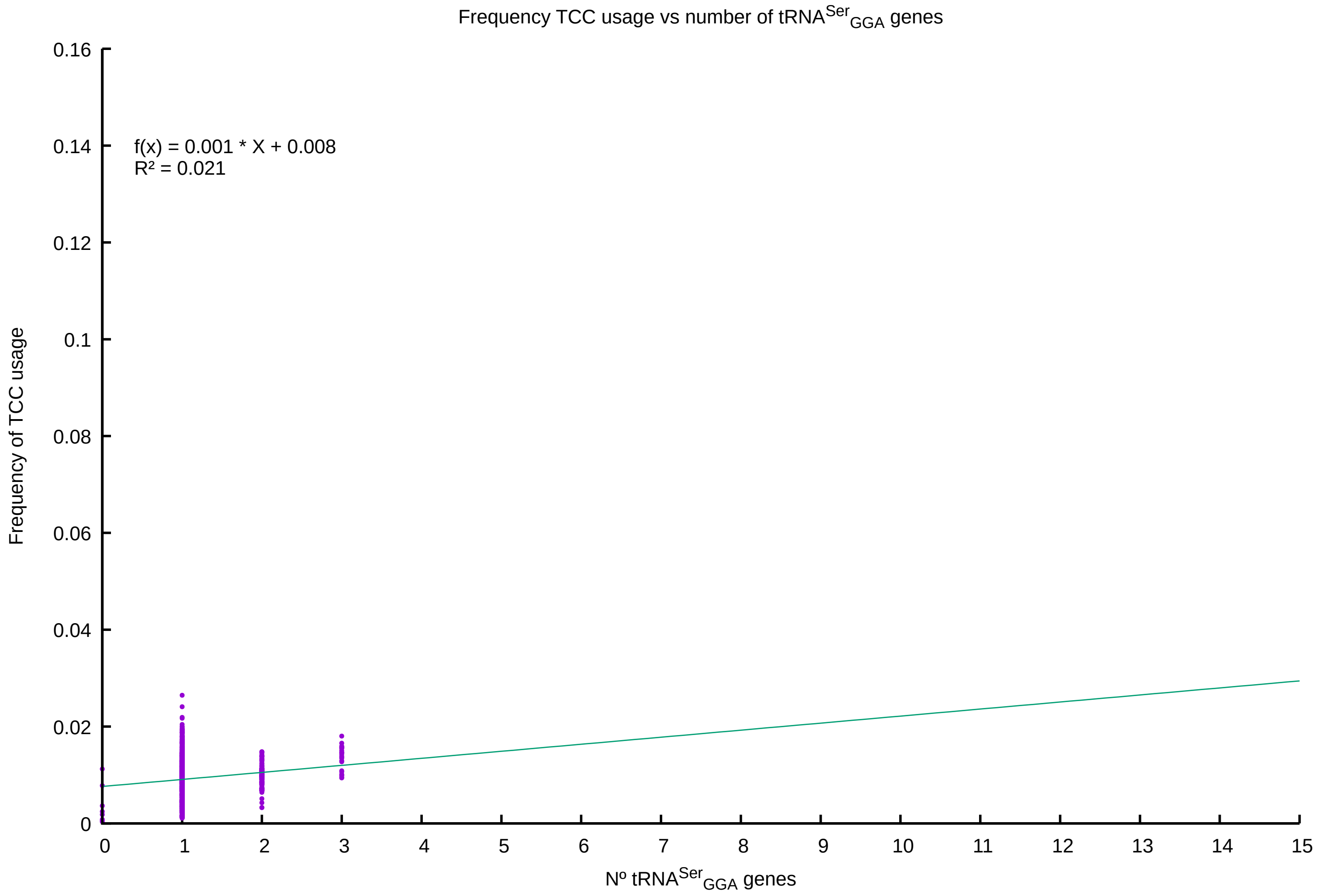

Frequency TCC usage vs number of tRNASerGGA genes
f(x) = 0.001 * X + 0.008
R² = 0.021
Frequency of TCC usage
Nº tRNASerGGA genes
0
0.02
0.04
0.06
0.08
0.1
0.12
0.14
0.16
0
1
2
3
4
5
6
7
8
9
10
11
12
13
14
15

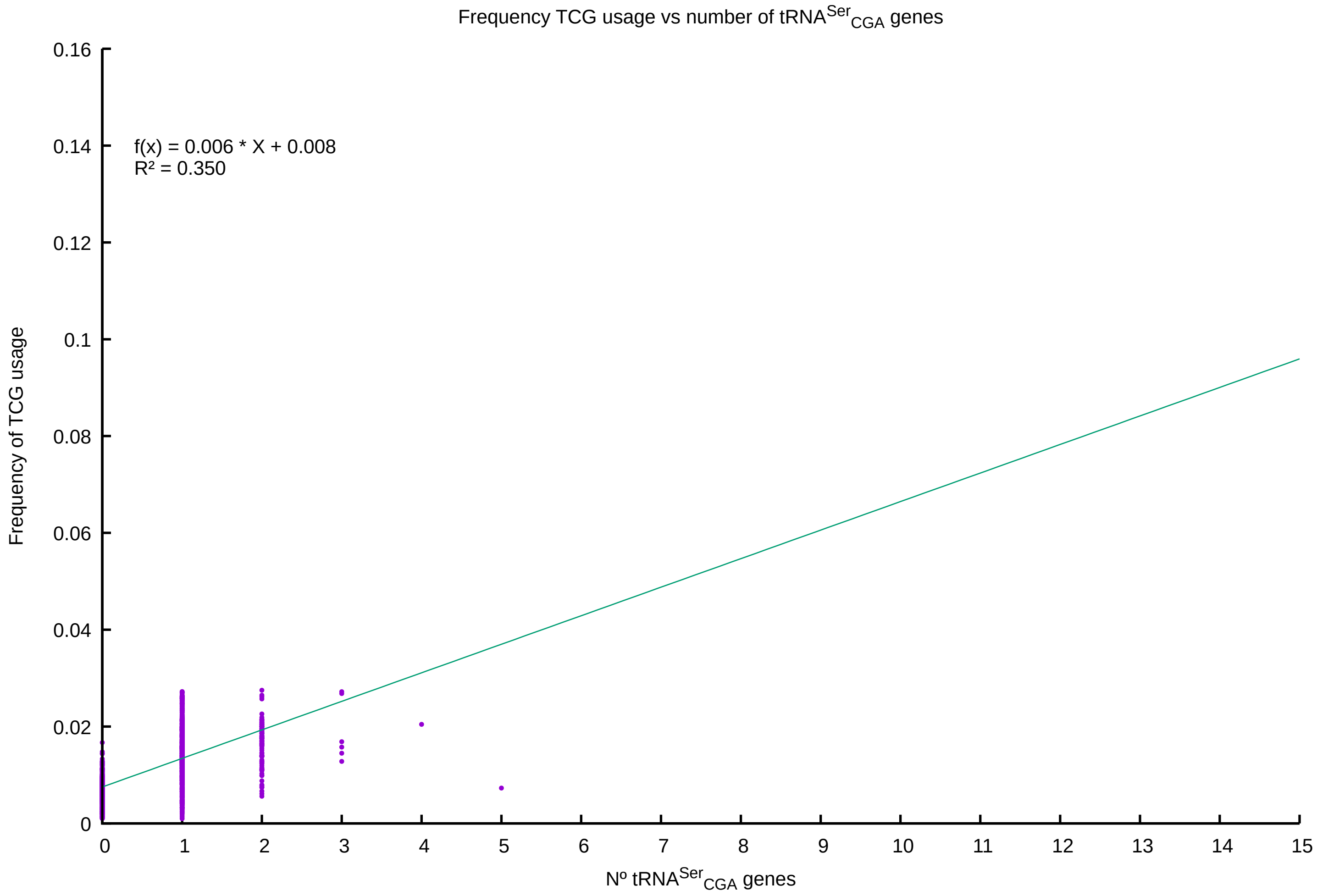

Frequency TCG usage vs number of tRNA^Ser_CGA genes
f(x) = 0.006 * X + 0.008
R² = 0.350
Frequency of TCG usage
0.16
0.14
0.12
0.1
0.08
0.06
0.04
0.02
0
0 1 2 3 4 5 6 7 8 9 10 11 12 13 14 15
Nº tRNA^Ser_CGA genes

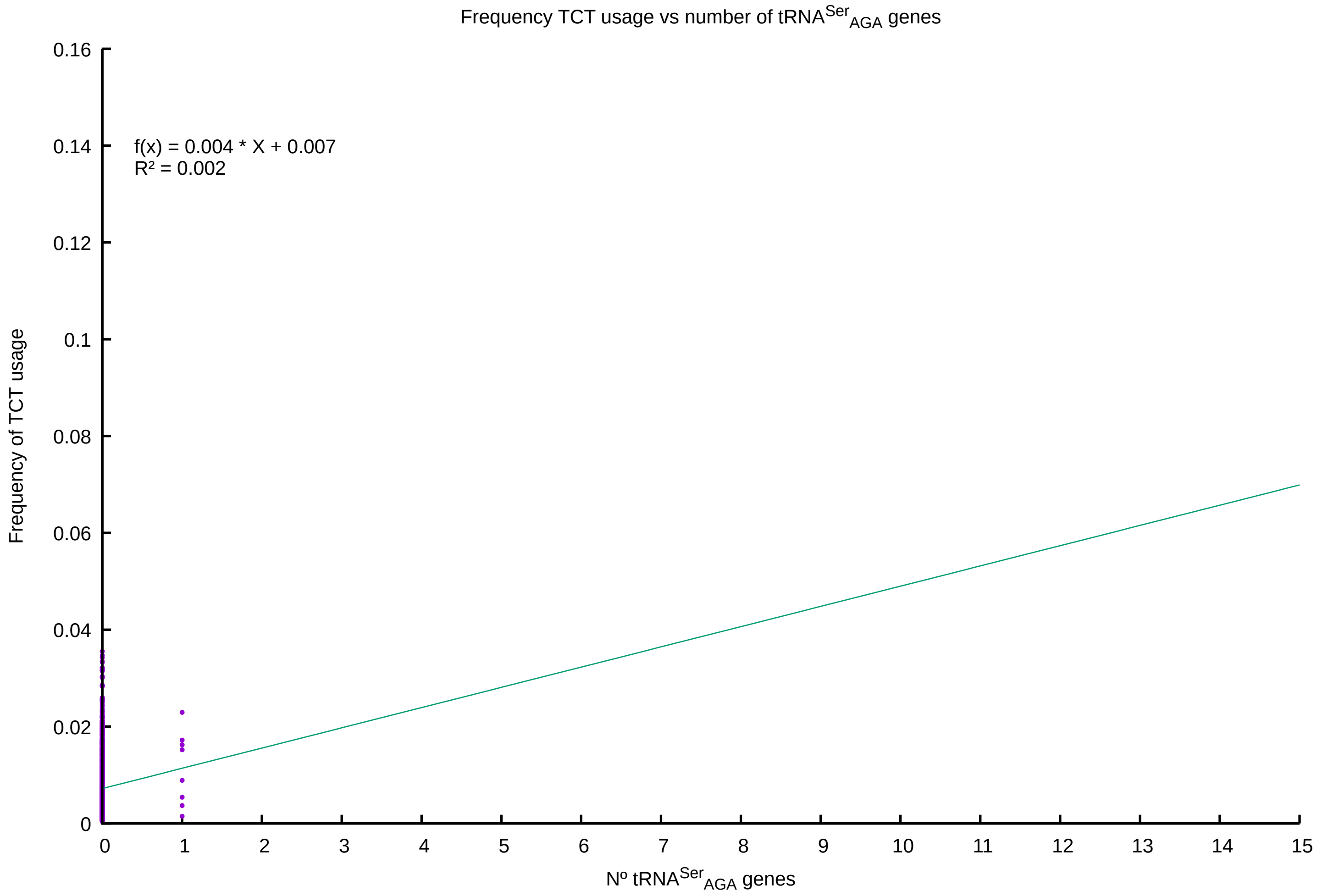

Frequency TCT usage vs number of tRNASerAGA genes
f(x) = 0.004 * X + 0.007
R² = 0.002
Frequency of TCT usage
0.16
0.14
0.12
0.1
0.08
0.06
0.04
0.02
0
0 1 2 3 4 5 6 7 8 9 10 11 12 13 14 15
Nº tRNASerAGA genes

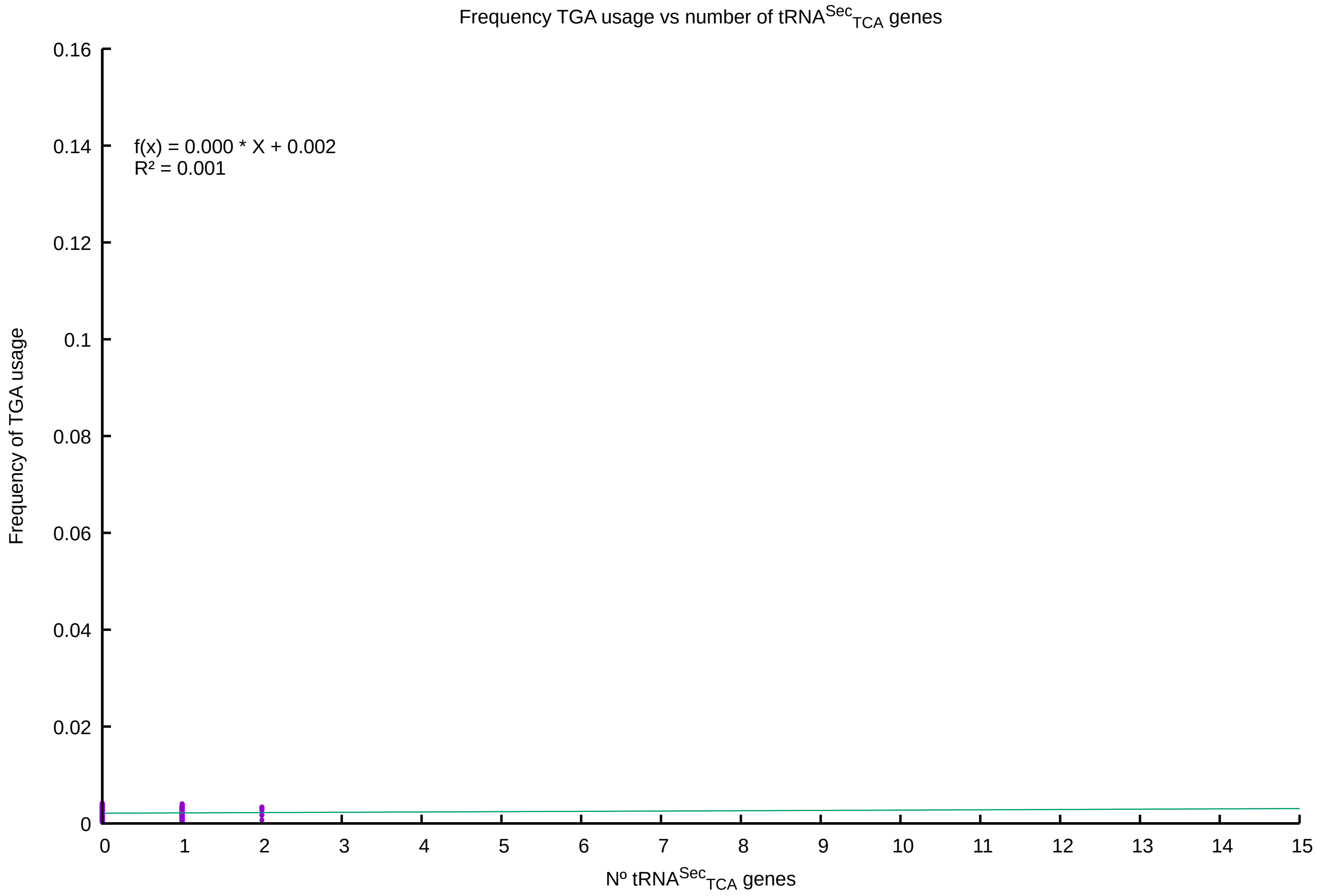
Frequency TGA usage vs number of tRNASecTCA genes
f(x) = 0.000 * X + 0.002
R² = 0.001
Frequency of TGA usage
0.16
0.14
0.12
0.1
0.08
0.06
0.04
0.02
0
0 1 2 3 4 5 6 7 8 9 10 11 12 13 14 15
Nº tRNASecTCA genes

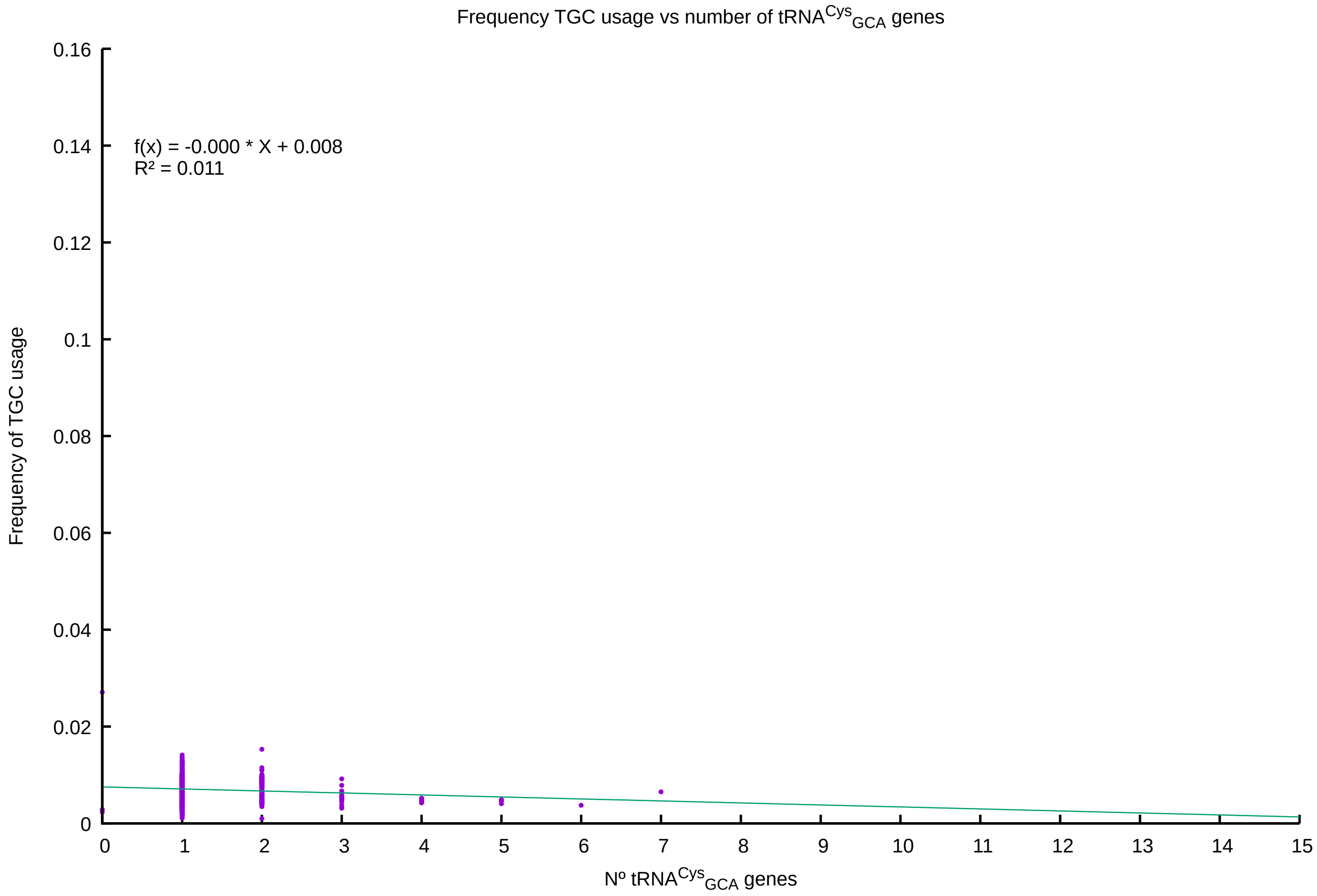
Frequency TGC usage vs number of tRNA Cys GCA genes
f(x) = -0.000 * X + 0.008
R² = 0.011
Frequency of TGC usage
0.16
0.14
0.12
0.1
0.08
0.06
0.04
0.02
0
0 1 2 3 4 5 6 7 8 9 10 11 12 13 14 15
Nº tRNA Cys GCA genes

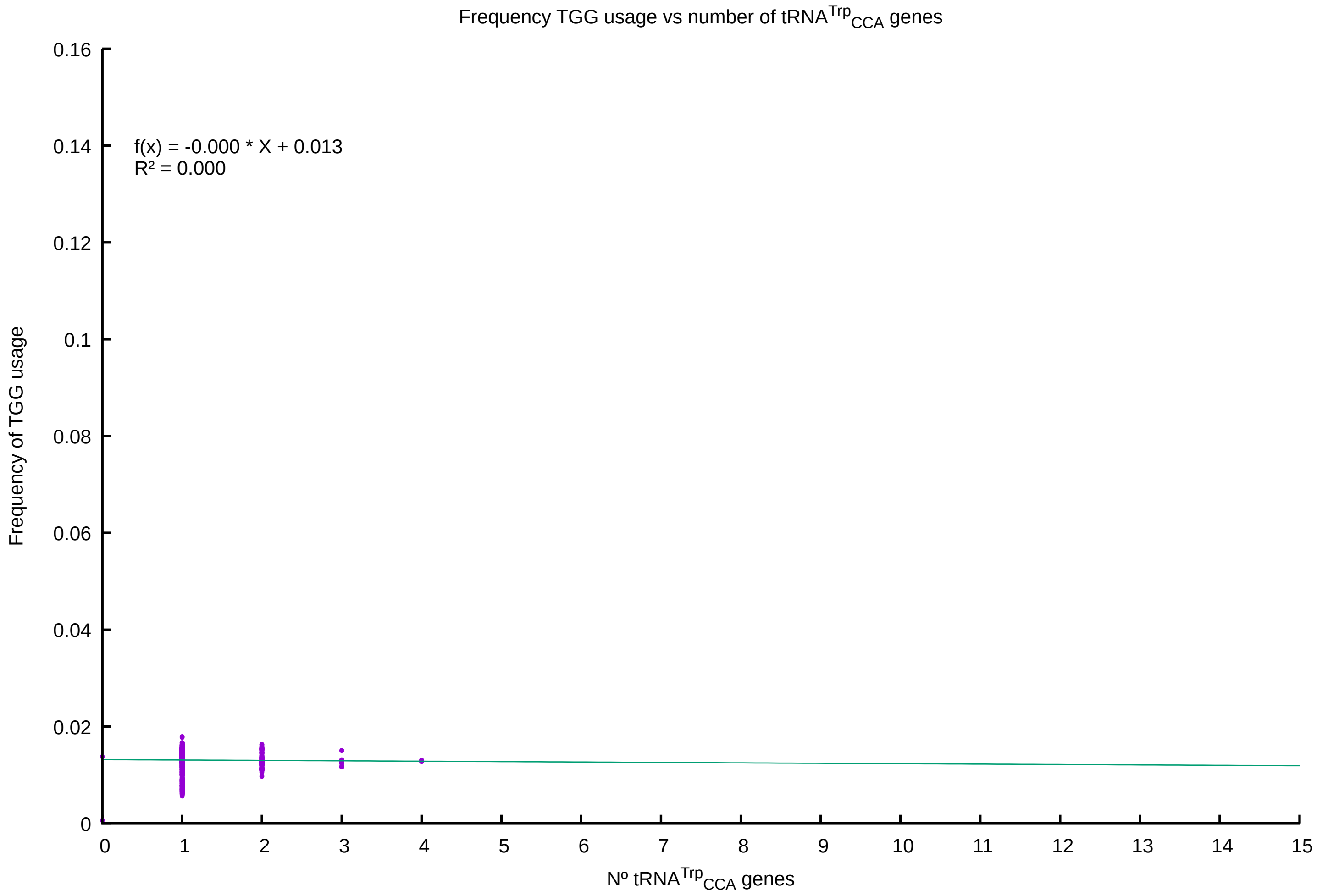
Frequency TGG usage vs number of tRNA$^{Trp}_{CCA}$ genes
f(x) = -0.000 * X + 0.013
R^2 = 0.000
Frequency of TGG usage
0.16
0.14
0.12
0.1
0.08
0.06
0.04
0.02
0
0 1 2 3 4 5 6 7 8 9 10 11 12 13 14 15
Nº tRNA$^{Trp}_{CCA}$ genes

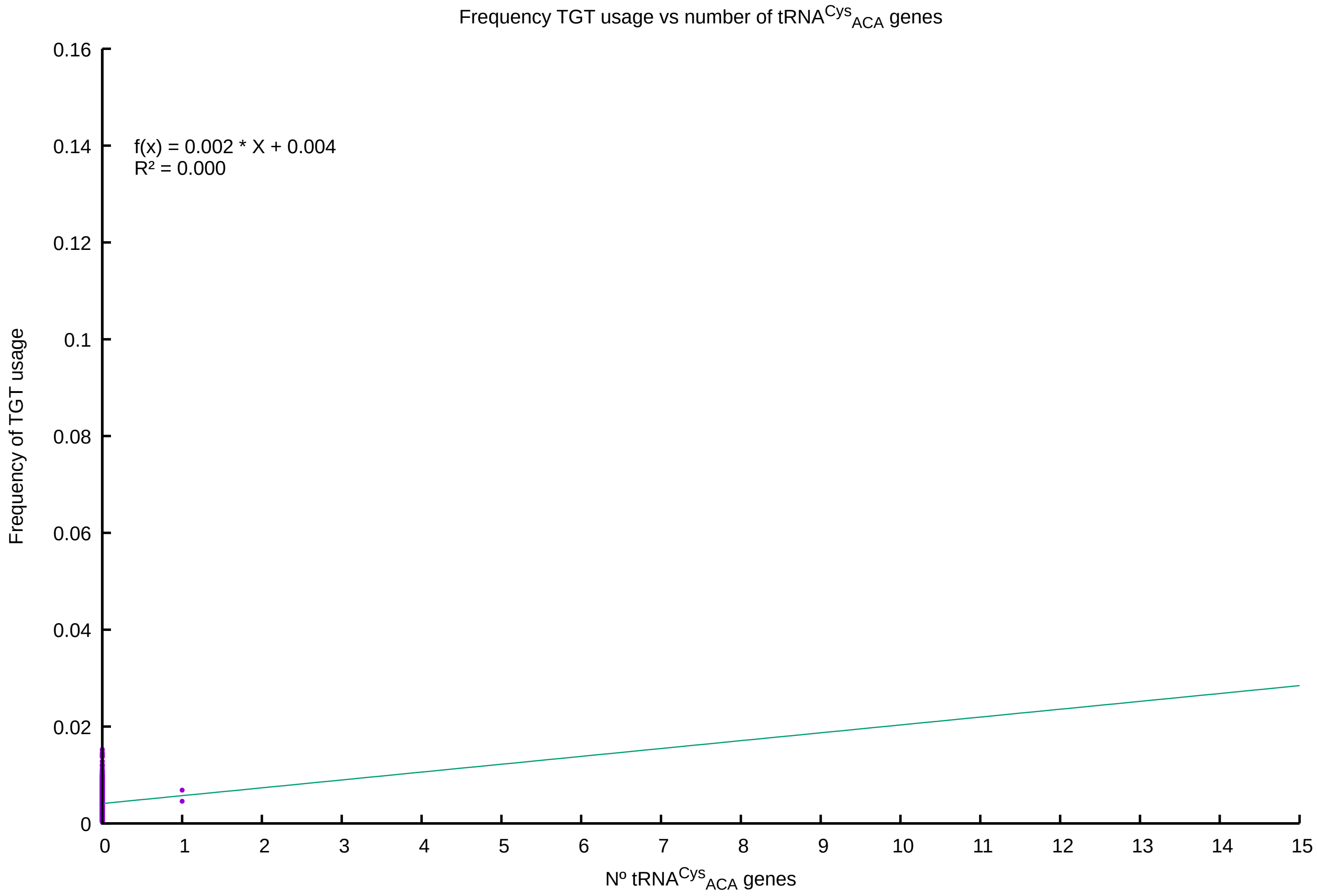

Frequency TGT usage vs number of tRNA$^{Cys}_{ACA}$ genes
f(x) = 0.002 * X + 0.004
R^2 = 0.000
Frequency of TGT usage
0.16
0.14
0.12
0.1
0.08
0.06
0.04
0.02
0
0 1 2 3 4 5 6 7 8 9 10 11 12 13 14 15
Nº tRNA$^{Cys}_{ACA}$ genes

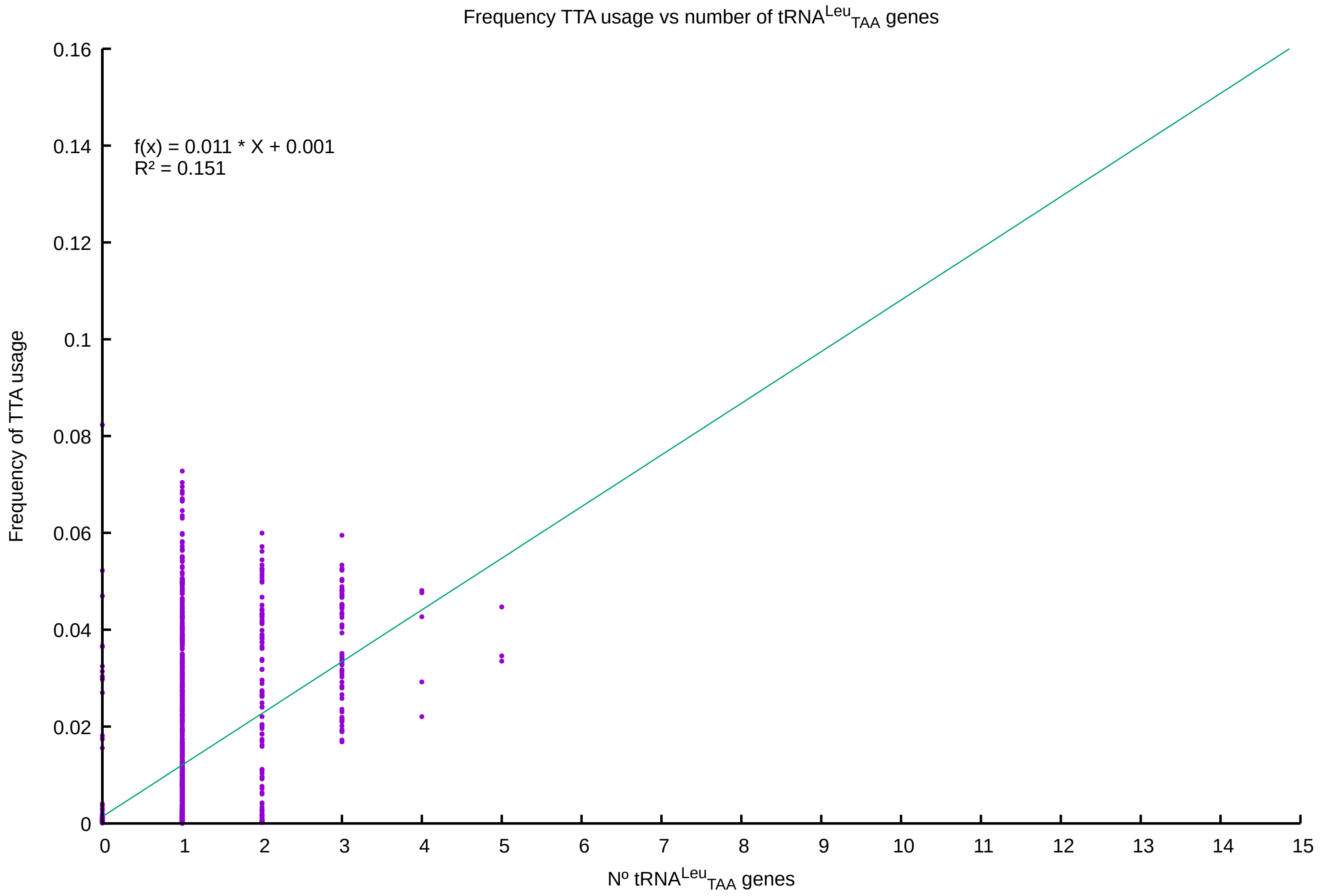

Frequency TTA usage vs number of tRNA^Leu_TAA genes
f(x) = 0.011 * X + 0.001
R² = 0.151
0.16
0.14
0.12
0.1
0.08
0.06
0.04
0.02
0
Frequency of TTA usage
0 1 2 3 4 5 6 7 8 9 10 11 12 13 14 15
Nº tRNA^Leu_TAA genes

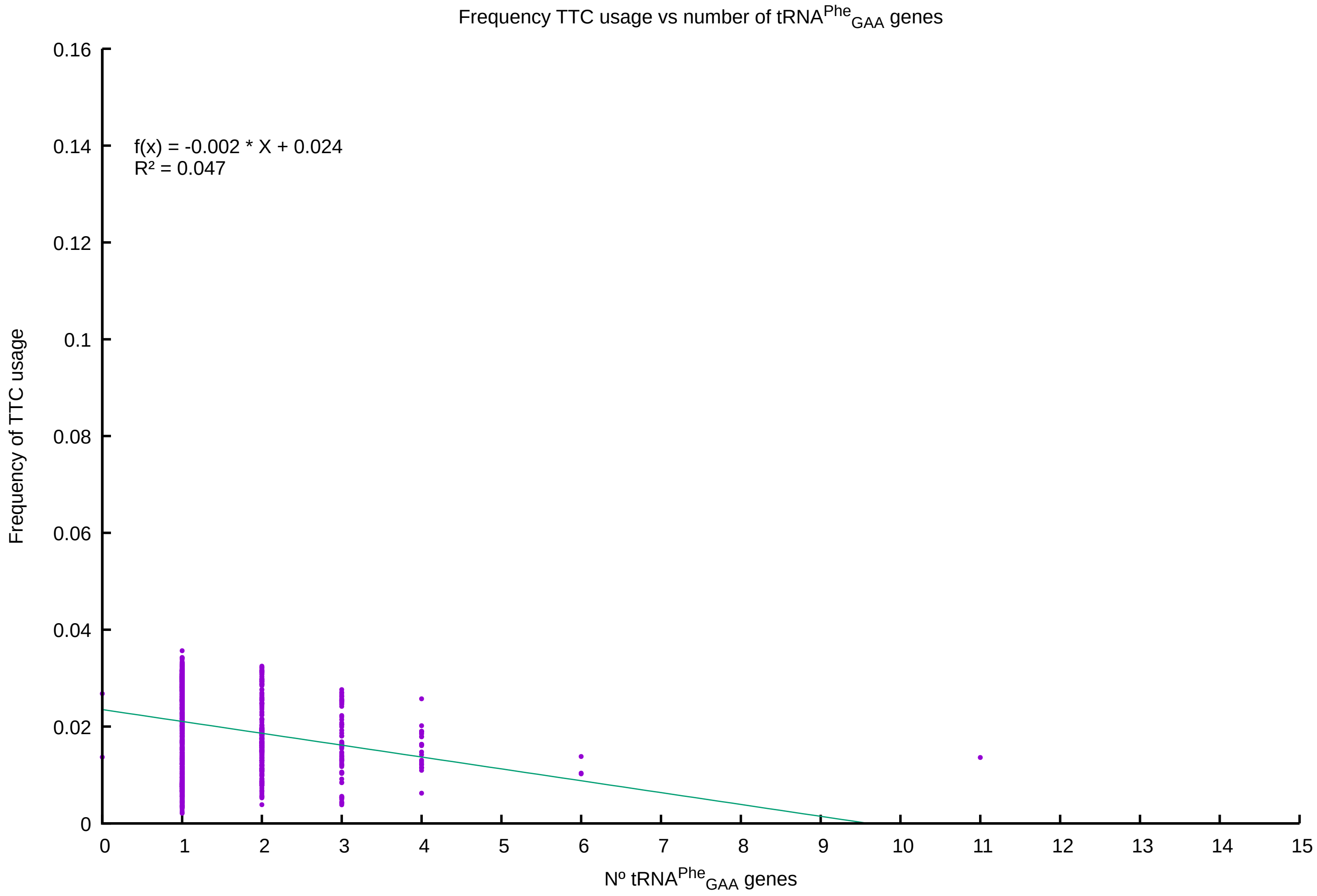

Frequency TTC usage vs number of tRNA$^{Phe}_{GAA}$ genes
f(x) = -0.002 * X + 0.024
R^2 = 0.047
Frequency of TTC usage
0.16
0.14
0.12
0.1
0.08
0.06
0.04
0.02
0
0 1 2 3 4 5 6 7 8 9 10 11 12 13 14 15
Nº tRNA$^{Phe}_{GAA}$ genes

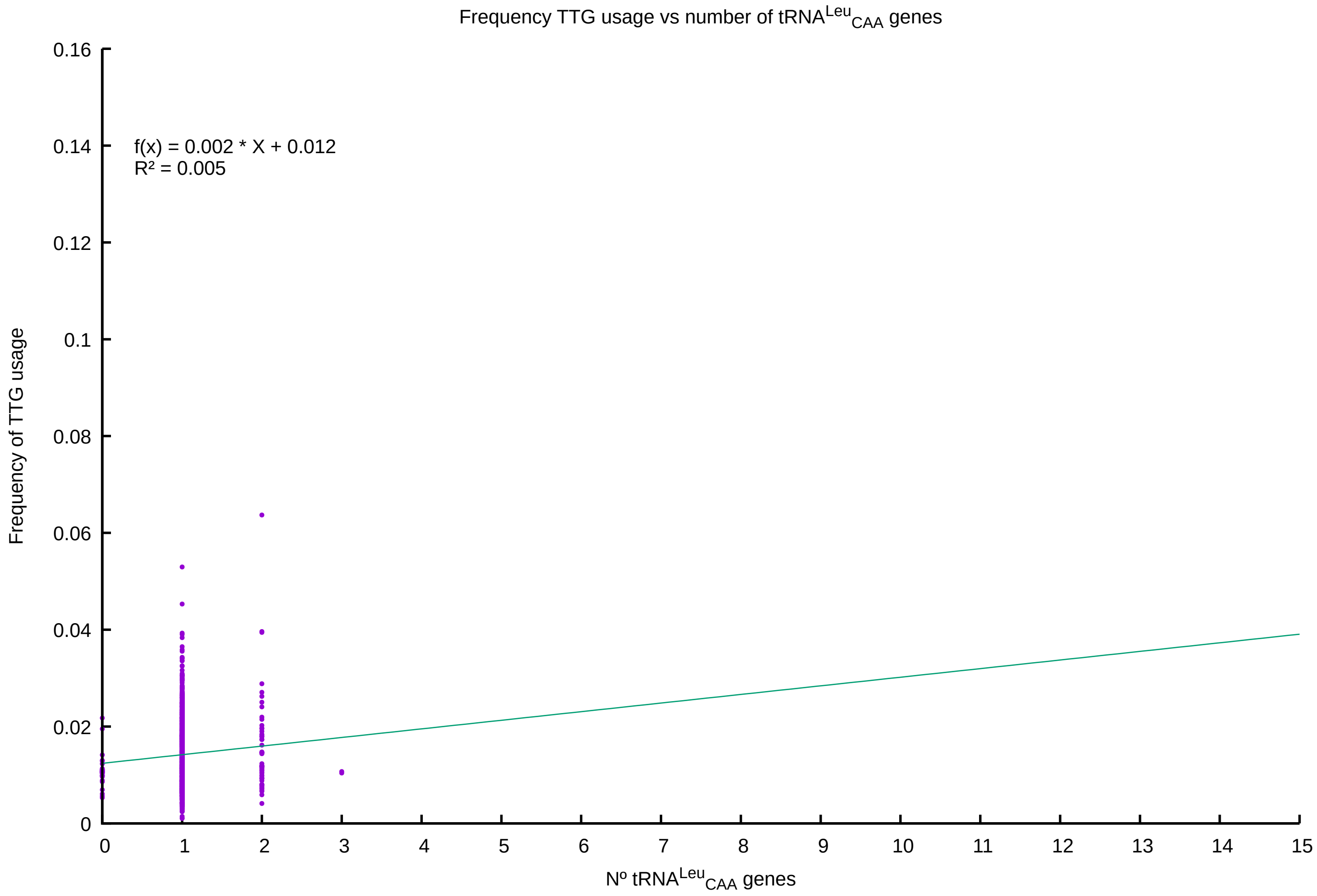

Frequency TTG usage vs number of tRNA^Leu_CAA genes
f(x) = 0.002 * X + 0.012
R² = 0.005
Frequency of TTG usage
0.16
0.14
0.12
0.1
0.08
0.06
0.04
0.02
0
0 1 2 3 4 5 6 7 8 9 10 11 12 13 14 15
Nº tRNA^Leu_CAA genes

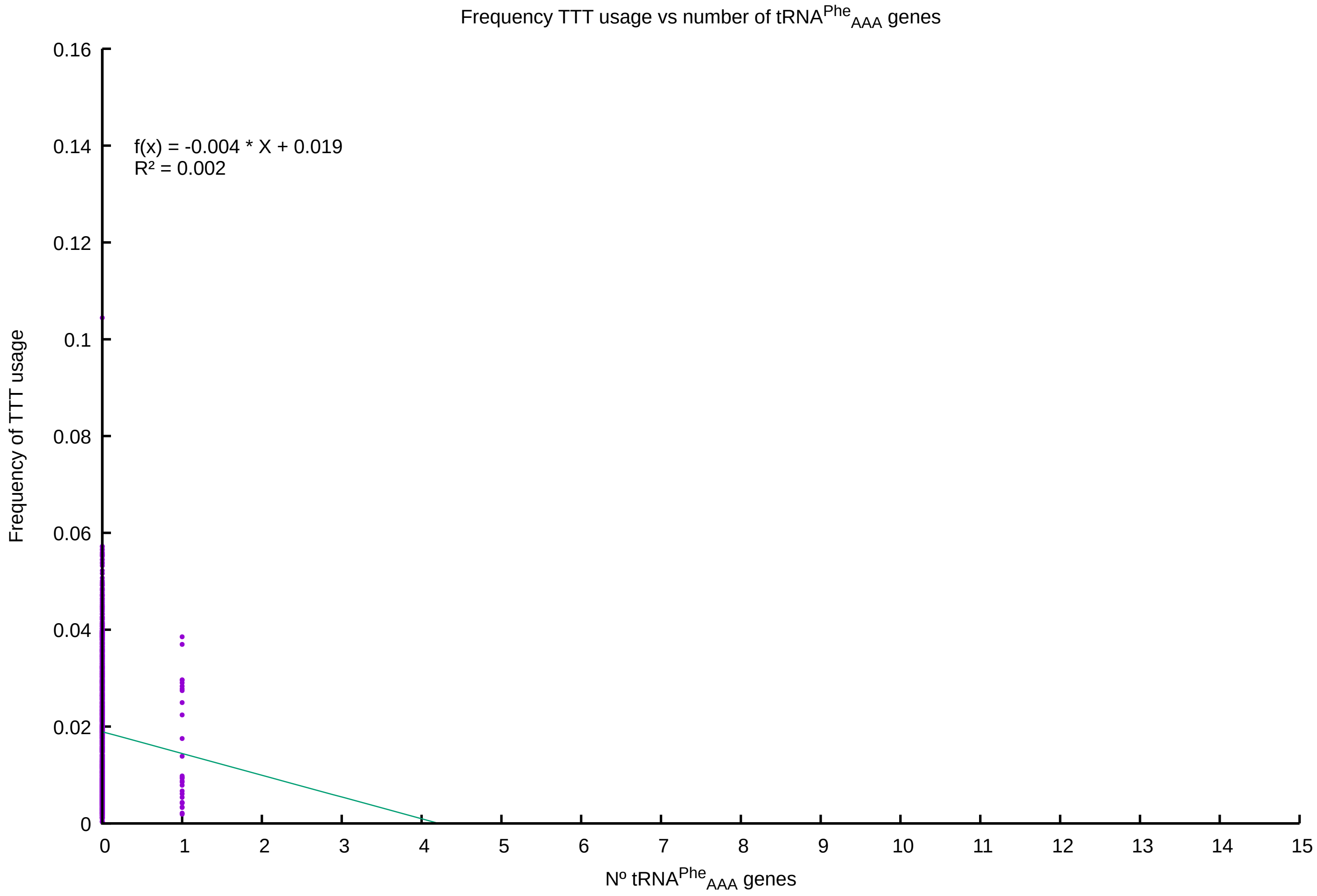

Frequency TTT usage vs number of tRNAPheAAA genes
f(x) = -0.004 * X + 0.019
R² = 0.002
Frequency of TTT usage
0.16
0.14
0.12
0.1
0.08
0.06
0.04
0.02
0
0 1 2 3 4 5 6 7 8 9 10 11 12 13 14 15
Nº tRNAPheAAA genes

(B)

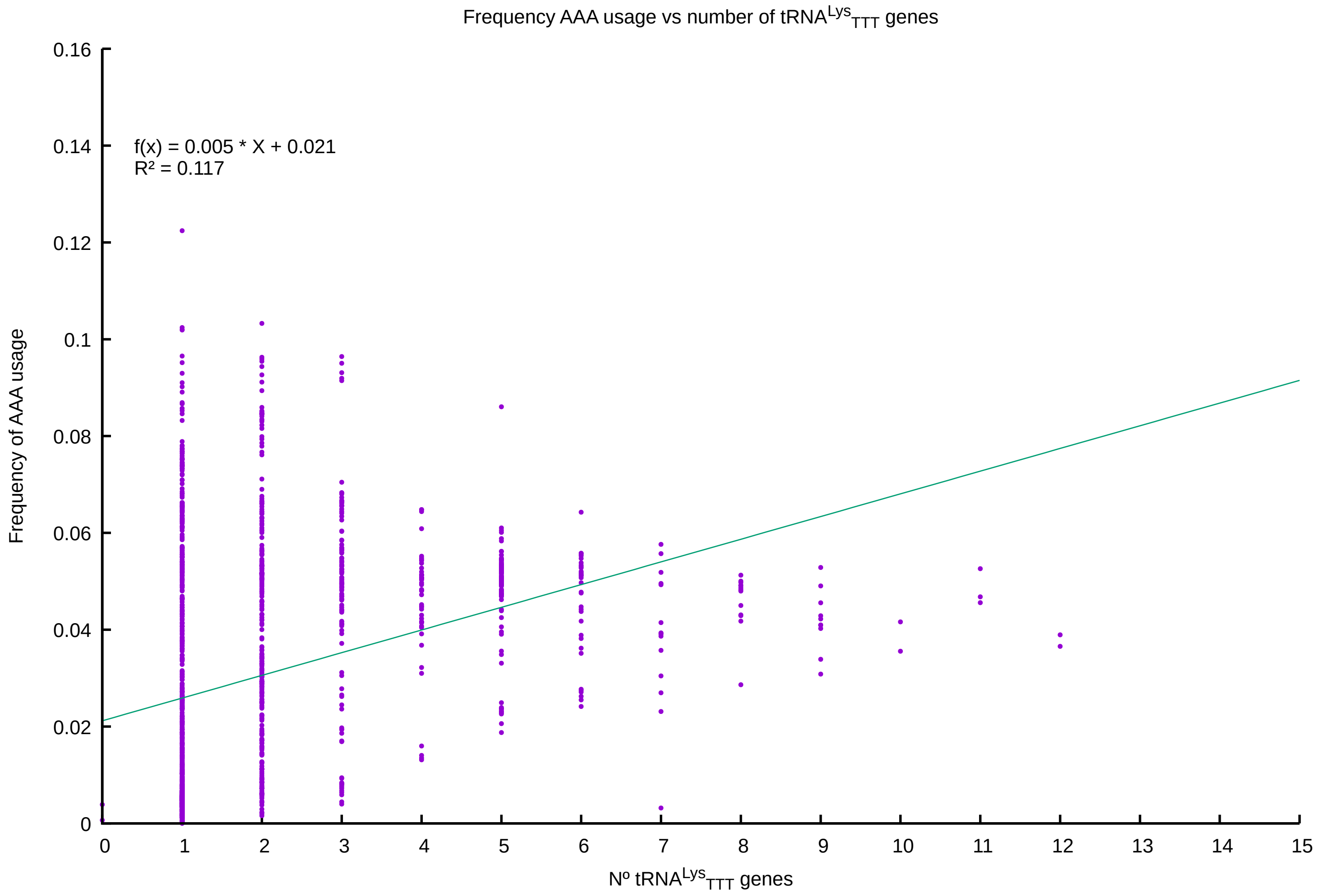

Frequency AAA usage vs number of tRNA^Lys_TTT genes
f(x) = 0.005 * X + 0.021
R² = 0.117
Frequency of AAA usage
0.16
0.14
0.12
0.1
0.08
0.06
0.04
0.02
0
0 1 2 3 4 5 6 7 8 9 10 11 12 13 14 15
Nº tRNA^Lys_TTT genes

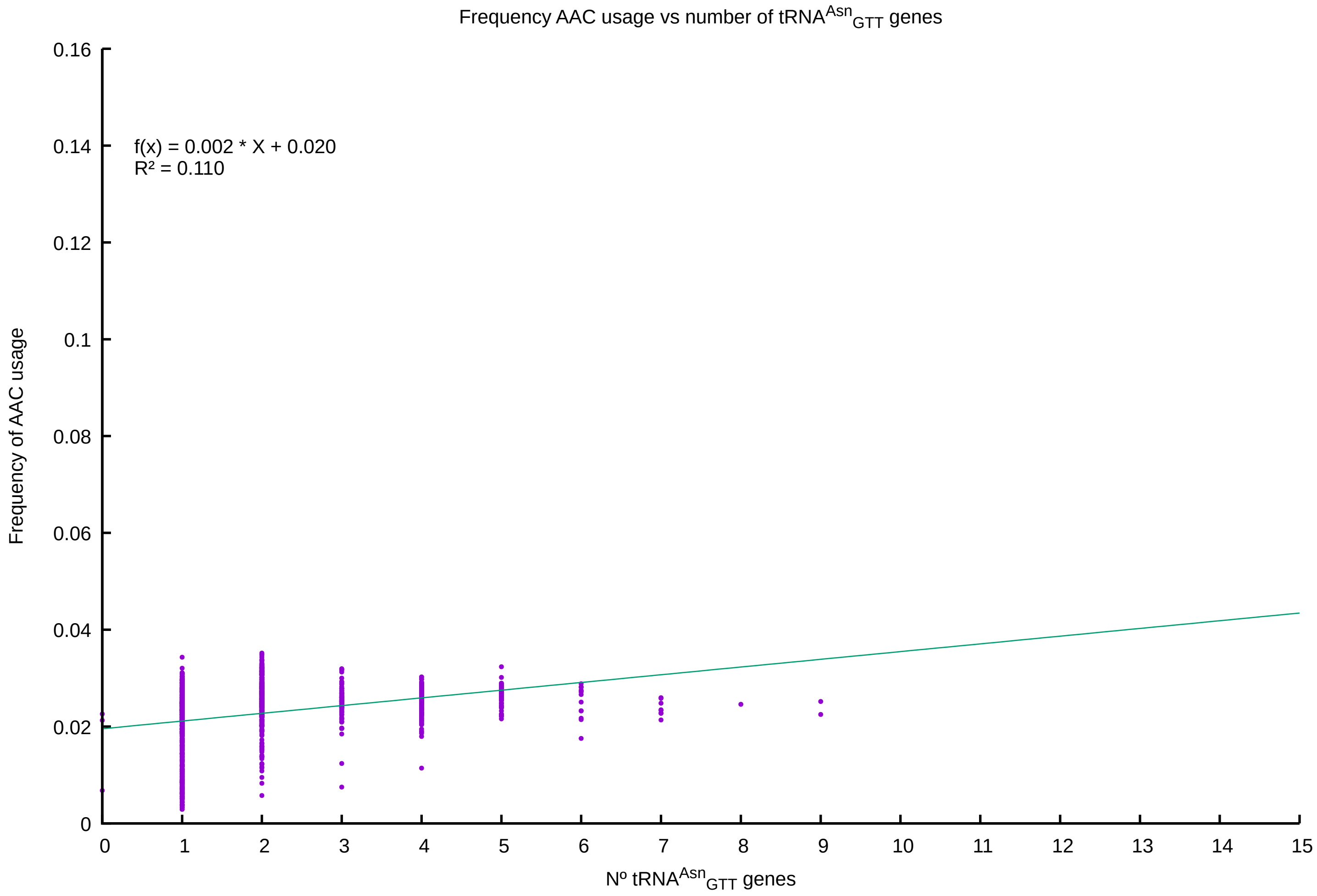

Frequency AAC usage vs number of tRNA^Asn_GTT genes
f(x) = 0.002 * X + 0.020
R² = 0.110
Frequency of AAC usage
0.16
0.14
0.12
0.1
0.08
0.06
0.04
0.02
0
0 1 2 3 4 5 6 7 8 9 10 11 12 13 14 15
Nº tRNA^Asn_GTT genes

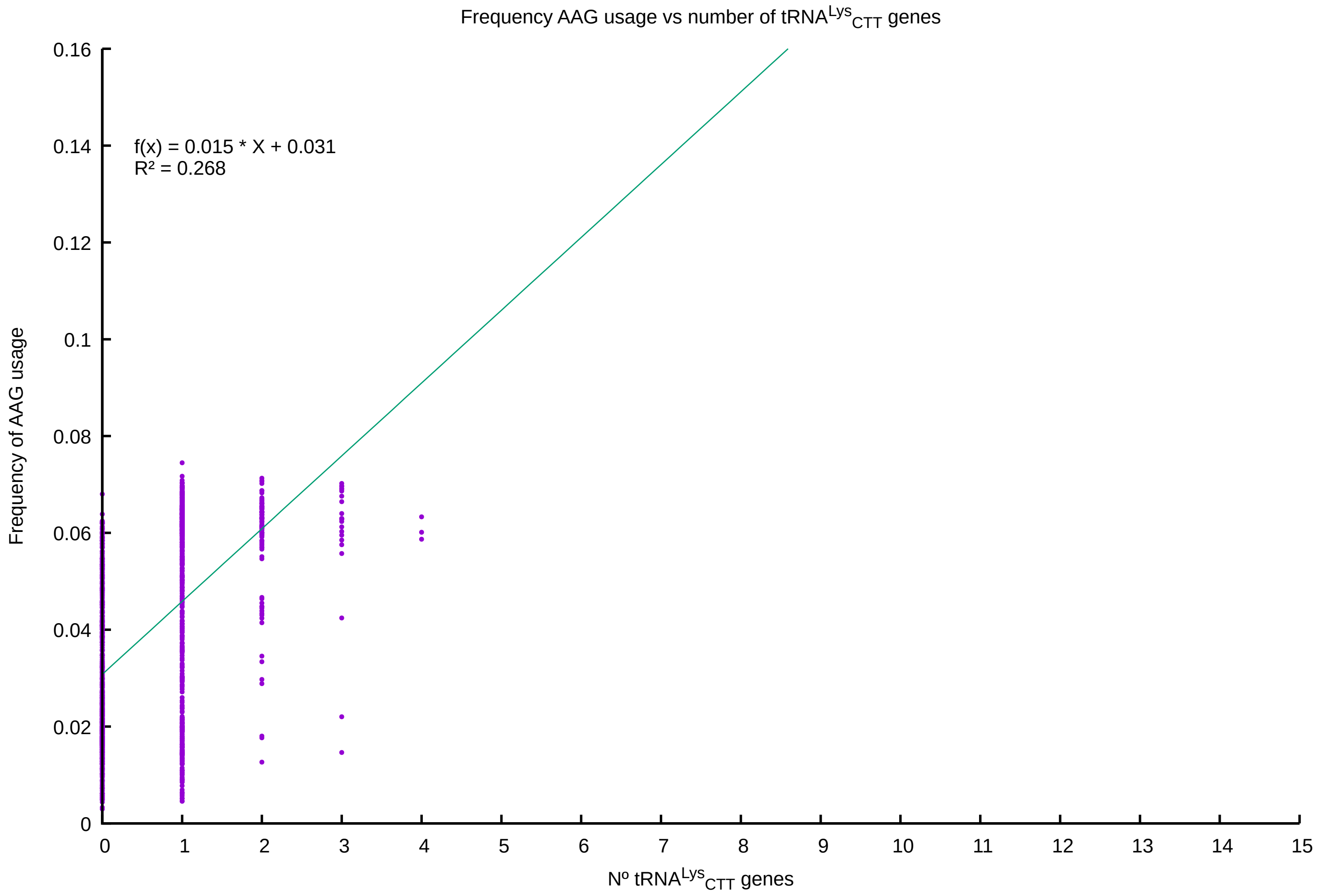

Frequency AAG usage vs number of tRNA$^{Lys}_{CTT}$ genes
f(x) = 0.015 * X + 0.031
R^2 = 0.268
Frequency of AAG usage
Nº tRNA$^{Lys}_{CTT}$ genes
0.16
0.14
0.12
0.1
0.08
0.06
0.04
0.02
0
0 1 2 3 4 5 6 7 8 9 10 11 12 13 14 15

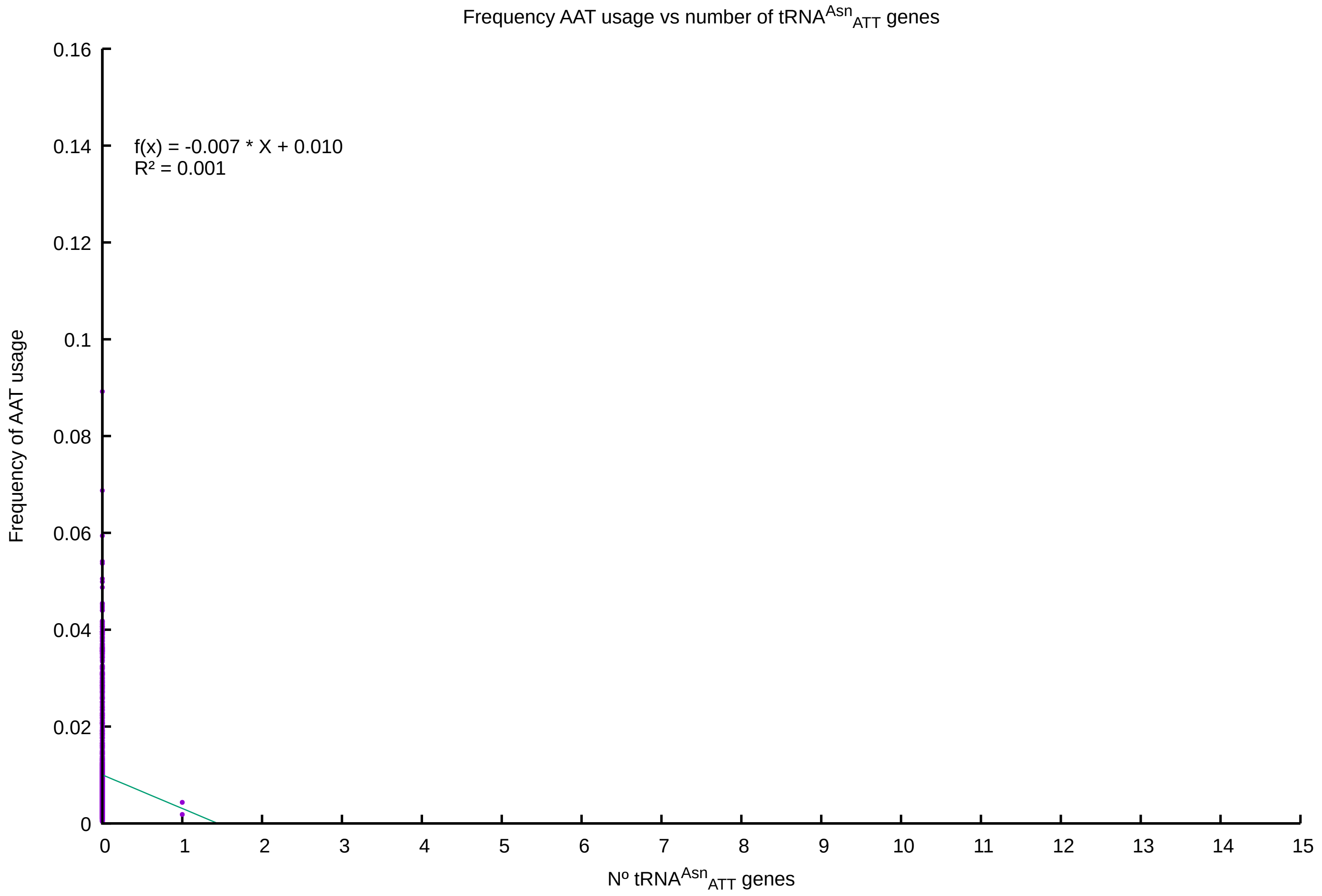

Frequency AAT usage vs number of tRNA^Asn_ATT genes
f(x) = -0.007 * X + 0.010
R² = 0.001
Frequency of AAT usage
0.16
0.14
0.12
0.1
0.08
0.06
0.04
0.02
0
0 1 2 3 4 5 6 7 8 9 10 11 12 13 14 15
Nº tRNA^Asn_ATT genes

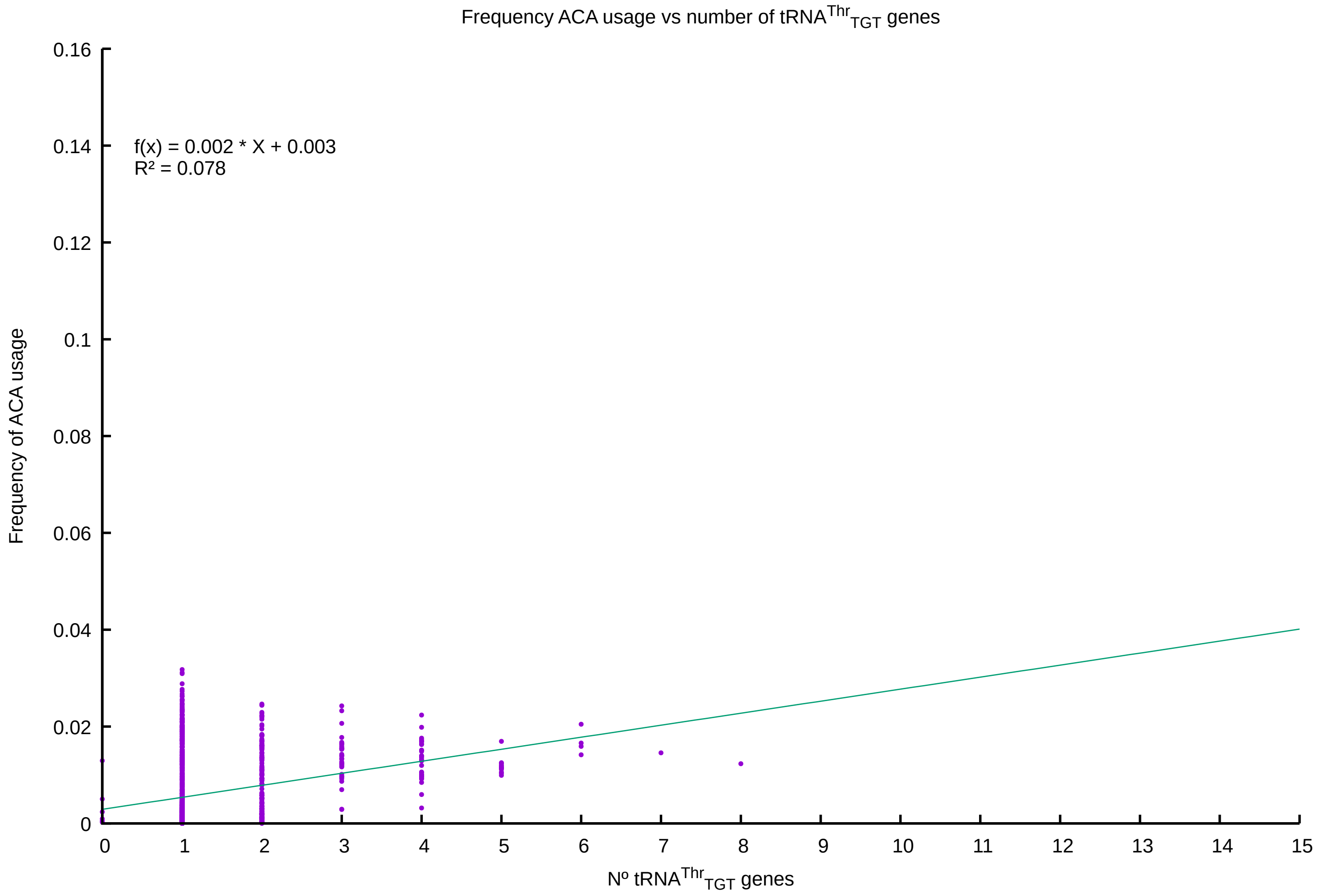
Frequency ACA usage vs number of tRNA$^{Thr}_{TGT}$ genes
f(x) = 0.002 * X + 0.003
R^2 = 0.078
Frequency of ACA usage
0.16
0.14
0.12
0.1
0.08
0.06
0.04
0.02
0
0 1 2 3 4 5 6 7 8 9 10 11 12 13 14 15
Nº tRNA$^{Thr}_{TGT}$ genes

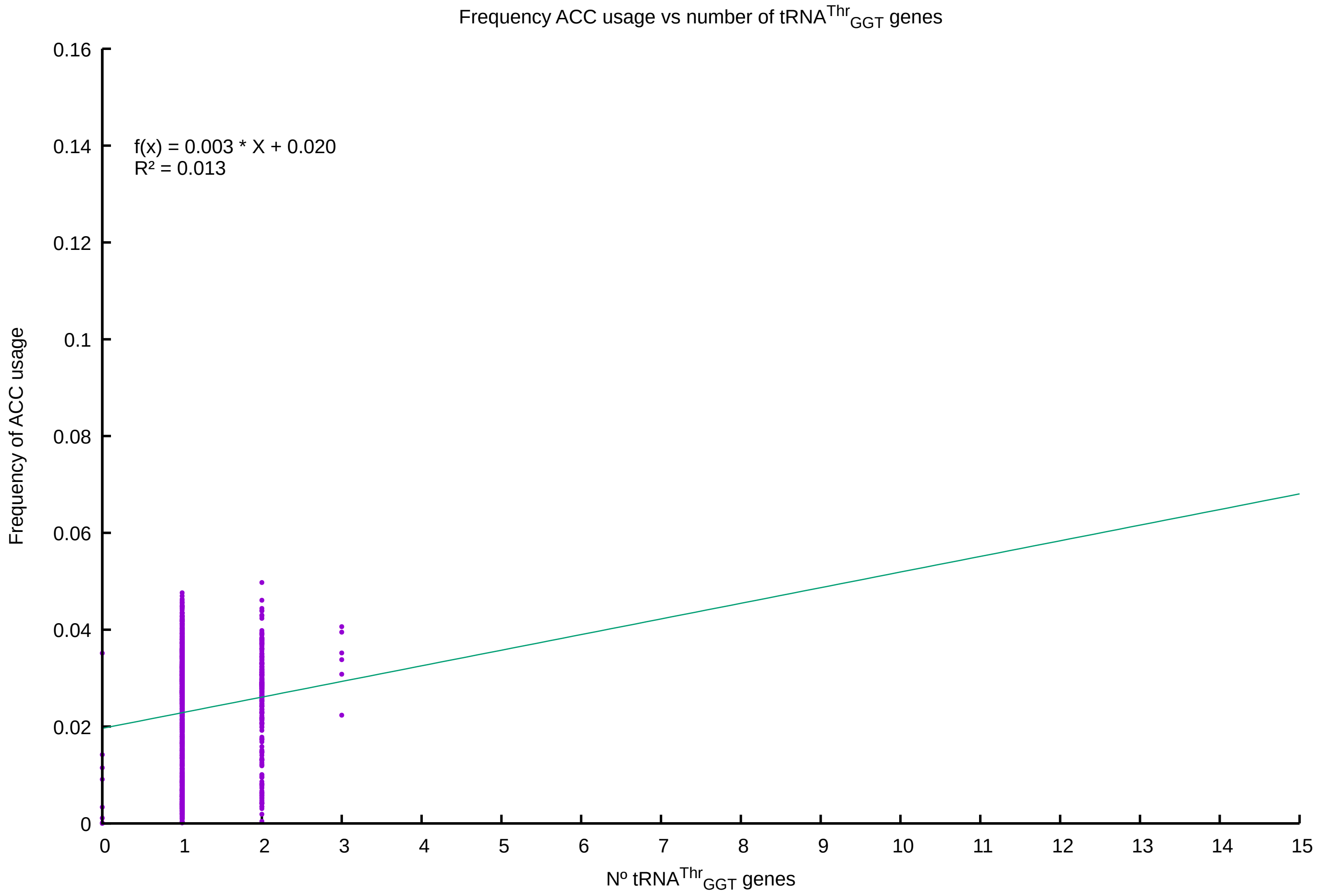

Frequency ACC usage vs number of tRNAThrGGT genes
f(x) = 0.003 * X + 0.020
R² = 0.013
Frequency of ACC usage
0.16
0.14
0.12
0.1
0.08
0.06
0.04
0.02
0
0 1 2 3 4 5 6 7 8 9 10 11 12 13 14 15
Nº tRNAThrGGT genes

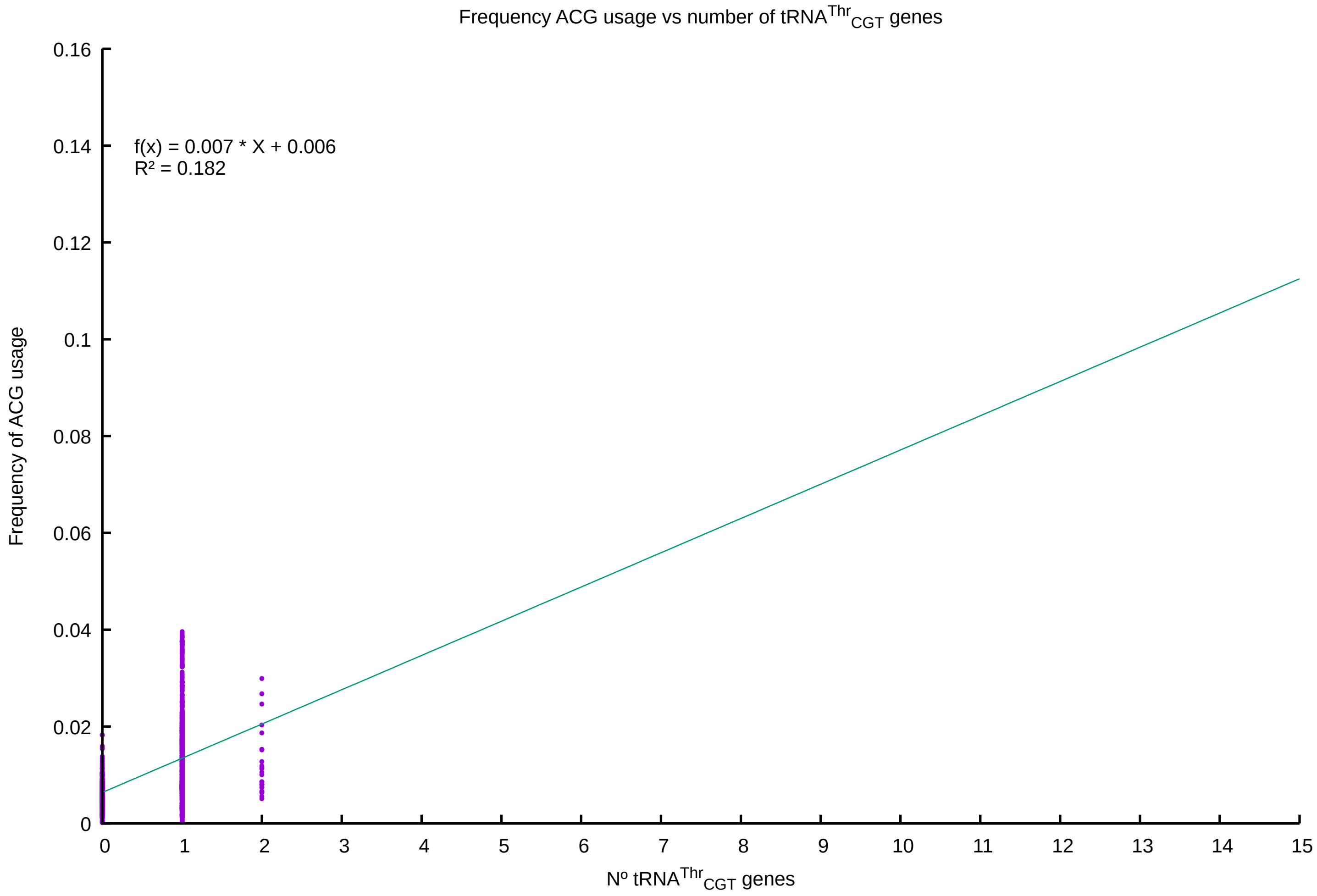

Frequency ACG usage vs number of tRNAThrCGT genes
f(x) = 0.007 * X + 0.006
R² = 0.182
Frequency of ACG usage
0.16
0.14
0.12
0.1
0.08
0.06
0.04
0.02
0
0 1 2 3 4 5 6 7 8 9 10 11 12 13 14 15
Nº tRNAThrCGT genes

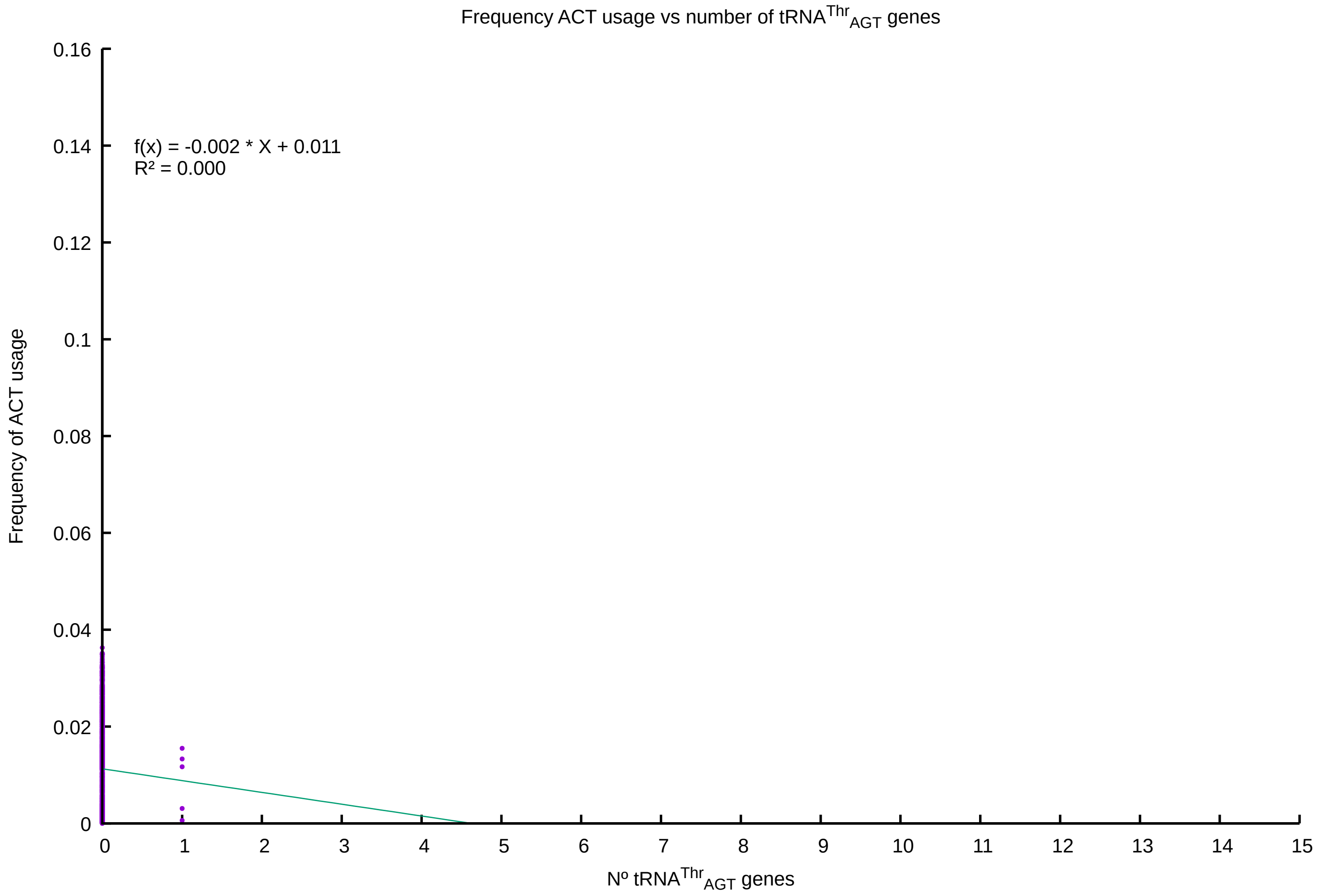

Frequency ACT usage vs number of tRNAThrAGT genes
f(x) = -0.002 * X + 0.011
R² = 0.000
0.16
0.14
0.12
0.1
0.08
0.06
0.04
0.02
0
Frequency of ACT usage
0 1 2 3 4 5 6 7 8 9 10 11 12 13 14 15
Nº tRNAThrAGT genes

Frequency AGA usage vs number of $tRNA^{Arg}_{TCT}$ genes

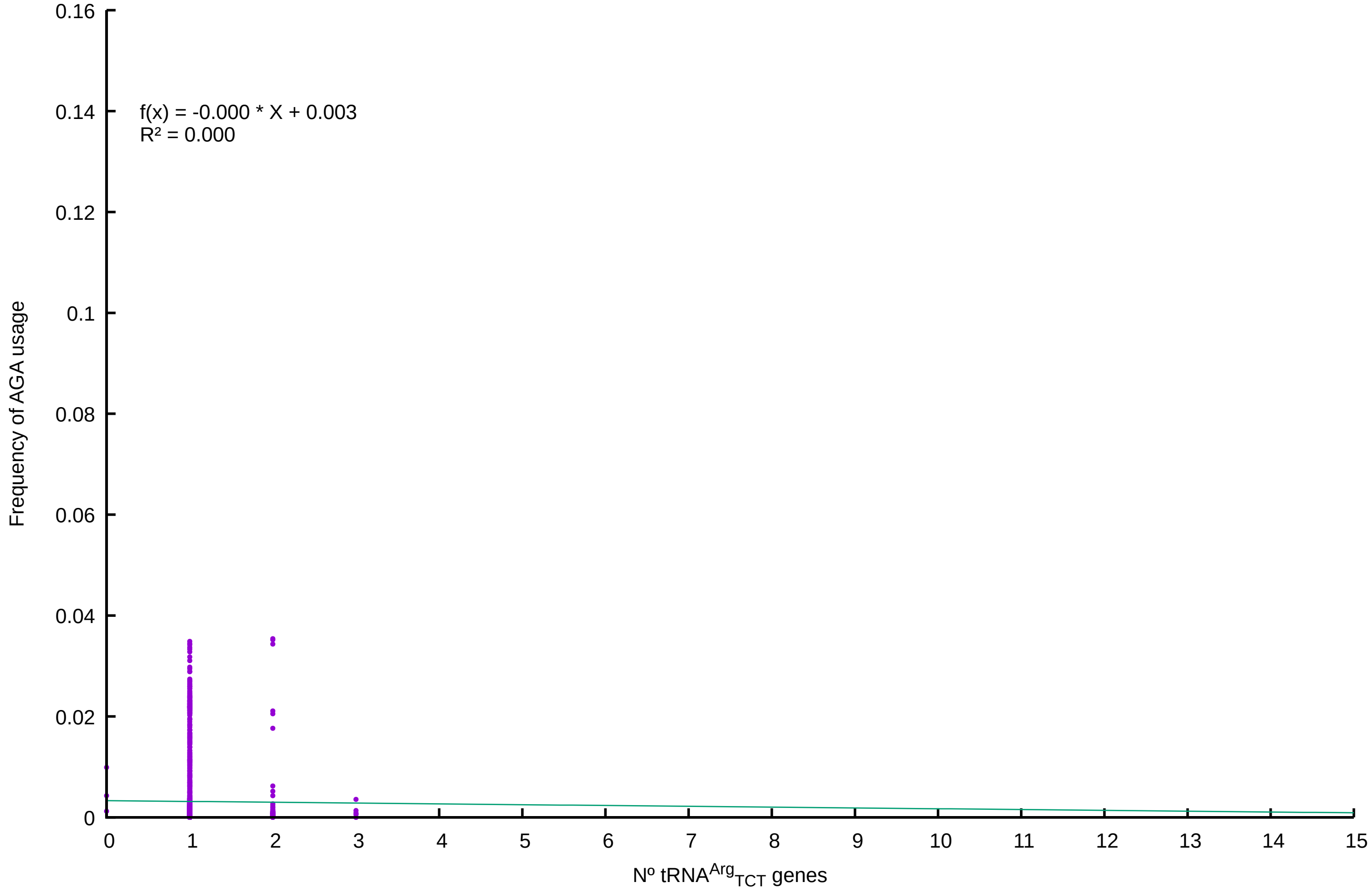

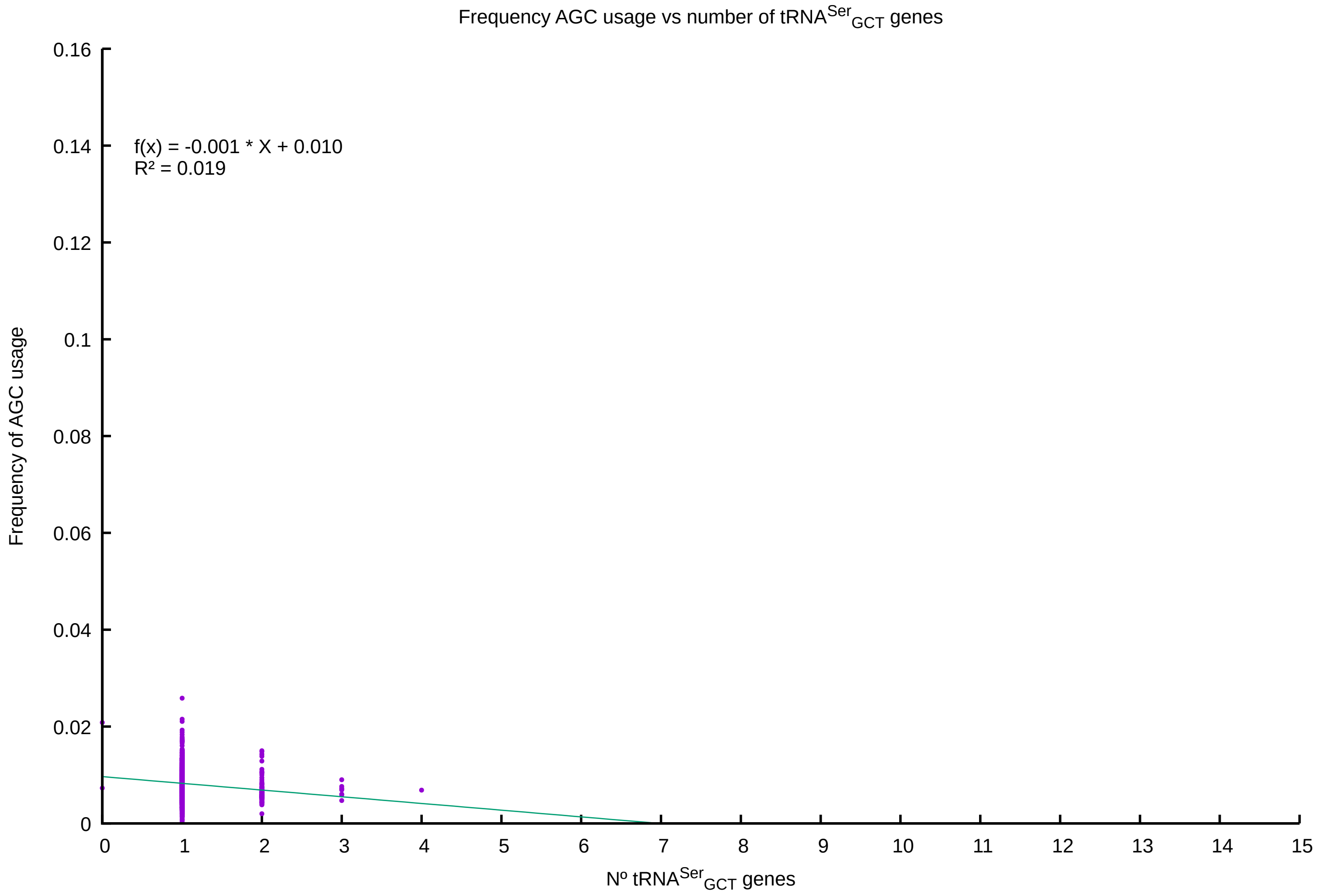

Frequency AGC usage vs number of tRNA^Ser_GCT genes
f(x) = -0.001 * X + 0.010
R² = 0.019
Frequency of AGC usage
0.16
0.14
0.12
0.1
0.08
0.06
0.04
0.02
0
0 1 2 3 4 5 6 7 8 9 10 11 12 13 14 15
Nº tRNA^Ser_GCT genes

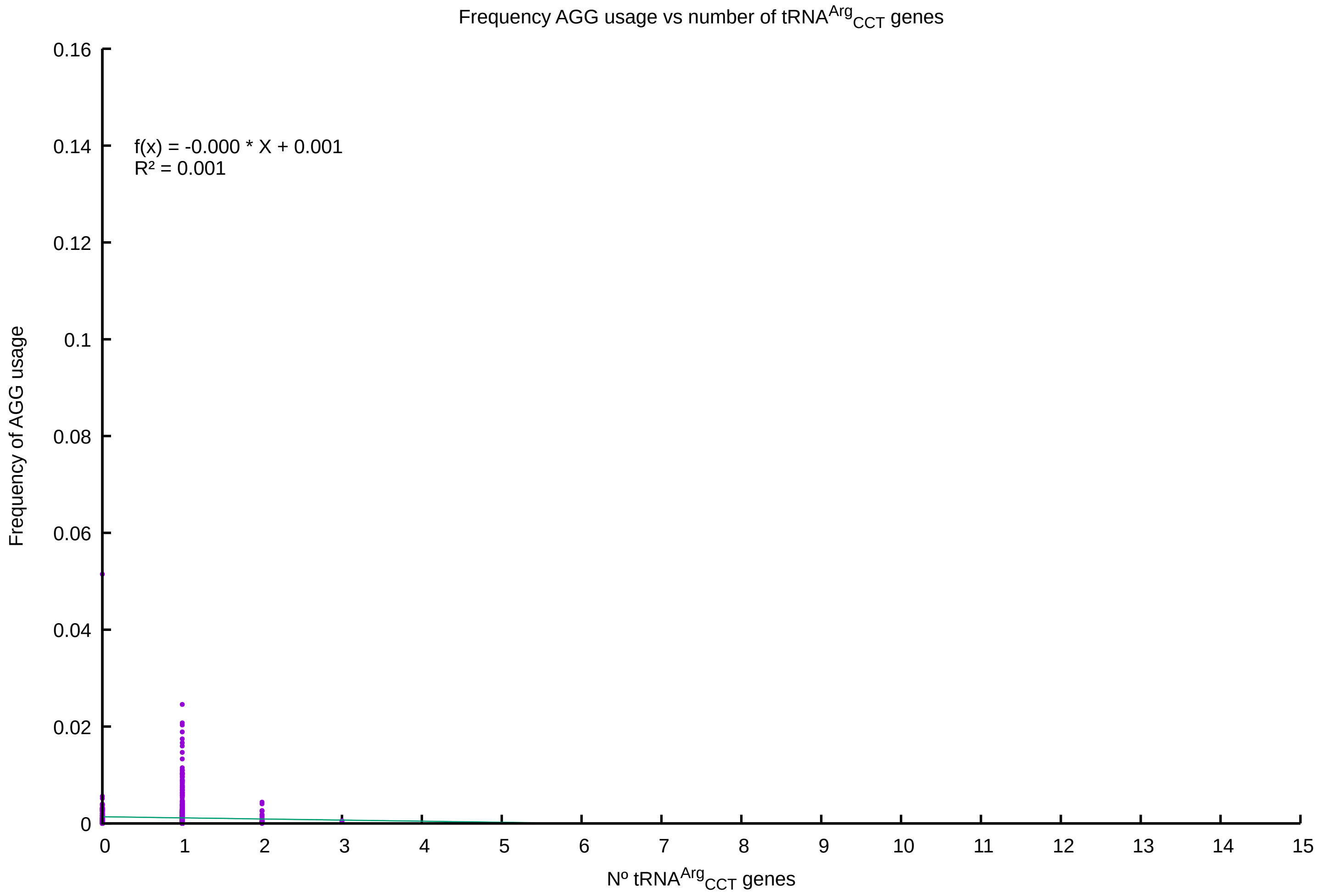

Frequency AGG usage vs number of tRNA$^{Arg}_{CCT}$ genes
f(x) = -0.000 * X + 0.001
R² = 0.001
Frequency of AGG usage
0.16
0.14
0.12
0.1
0.08
0.06
0.04
0.02
0
0 1 2 3 4 5 6 7 8 9 10 11 12 13 14 15
Nº tRNA$^{Arg}_{CCT}$ genes

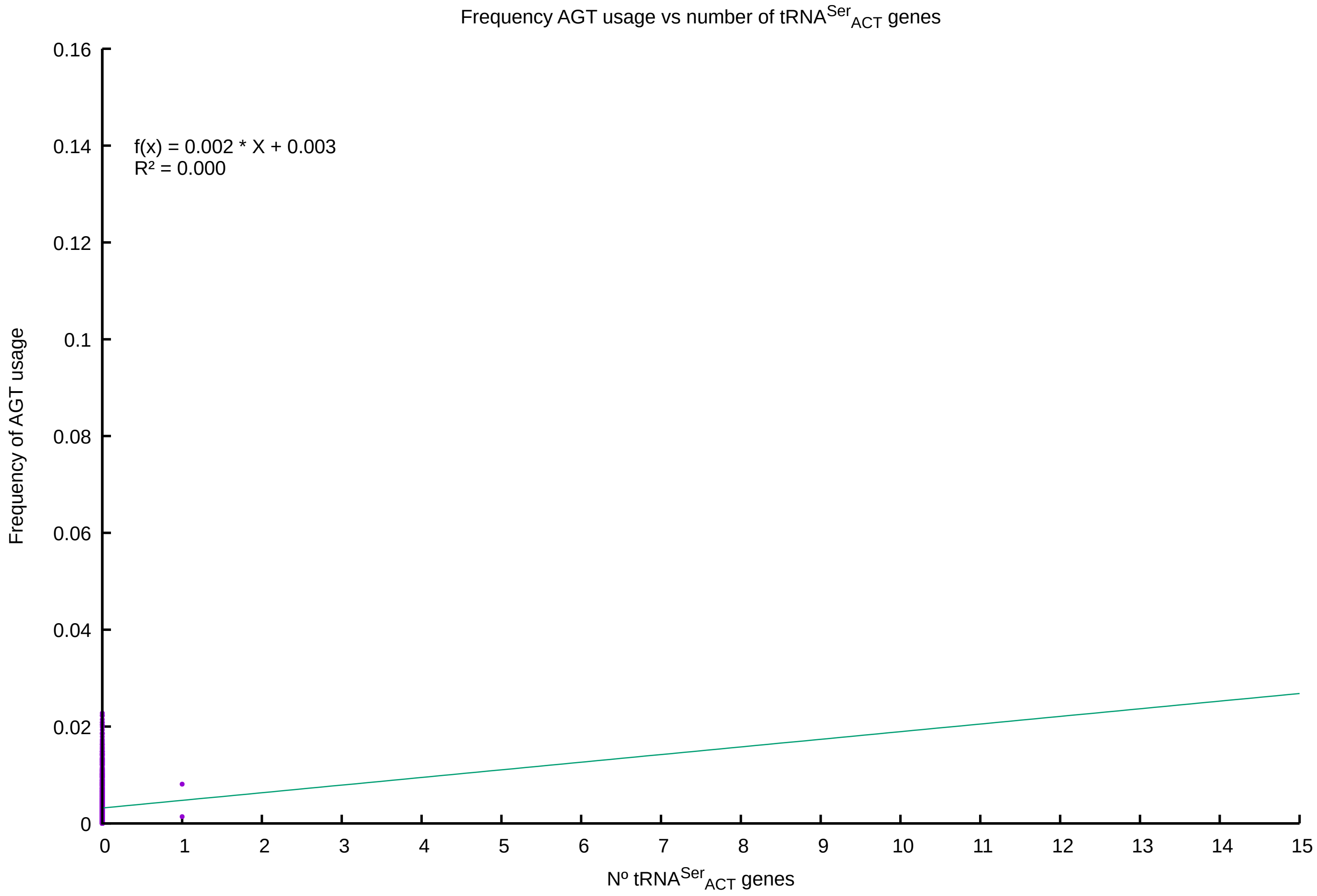

Frequency AGT usage vs number of tRNA^Ser_ACT genes
f(x) = 0.002 * X + 0.003
R² = 0.000
Frequency of AGT usage
0.16
0.14
0.12
0.1
0.08
0.06
0.04
0.02
0
0 1 2 3 4 5 6 7 8 9 10 11 12 13 14 15
Nº tRNA^Ser_ACT genes

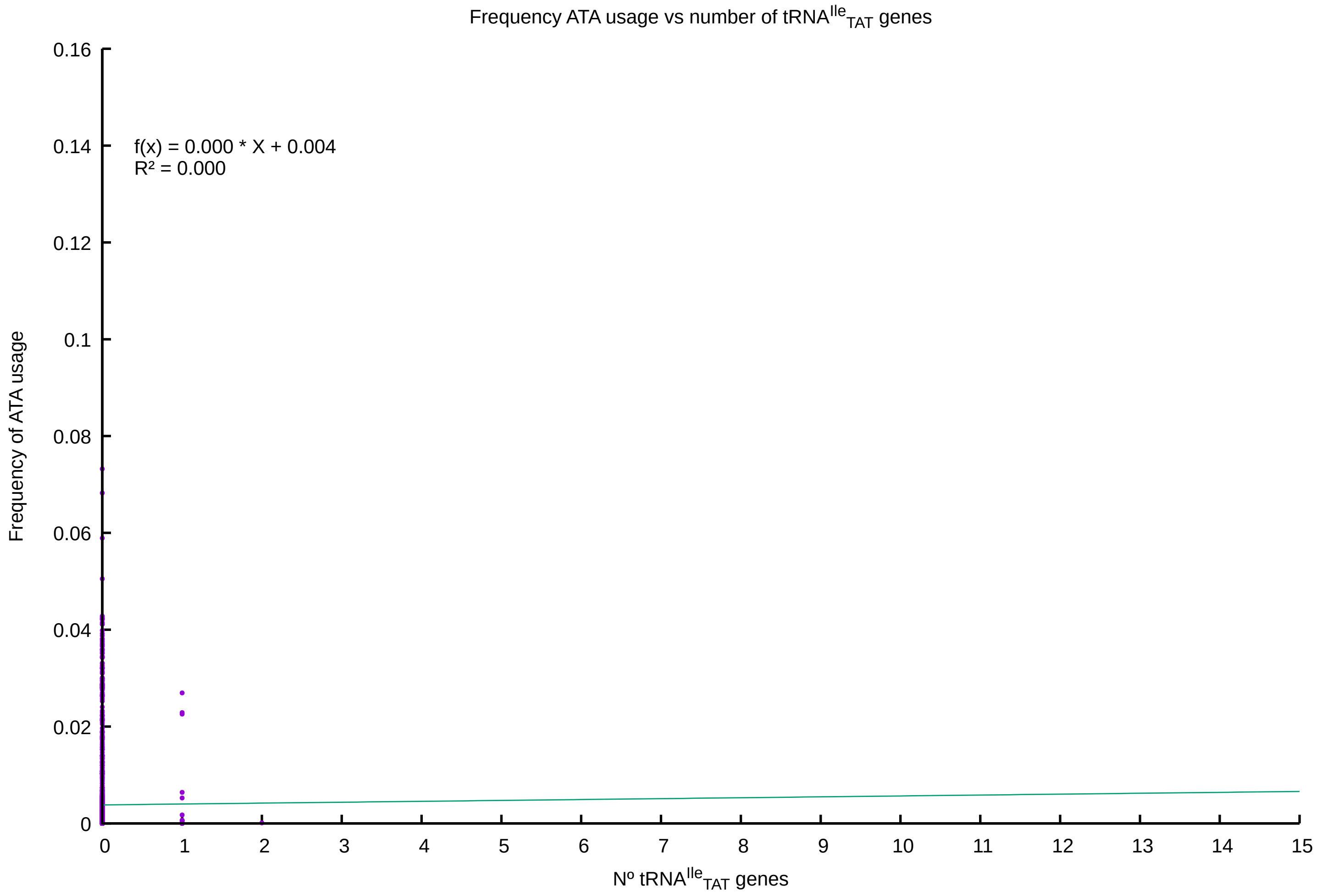
Frequency ATA usage vs number of tRNA$^{Ile}_{TAT}$ genes
f(x) = 0.000 * X + 0.004
R² = 0.000
Frequency of ATA usage
Nº tRNA$^{Ile}_{TAT}$ genes
0
0.02
0.04
0.06
0.08
0.1
0.12
0.14
0.16
0
1
2
3
4
5
6
7
8
9
10
11
12
13
14
15

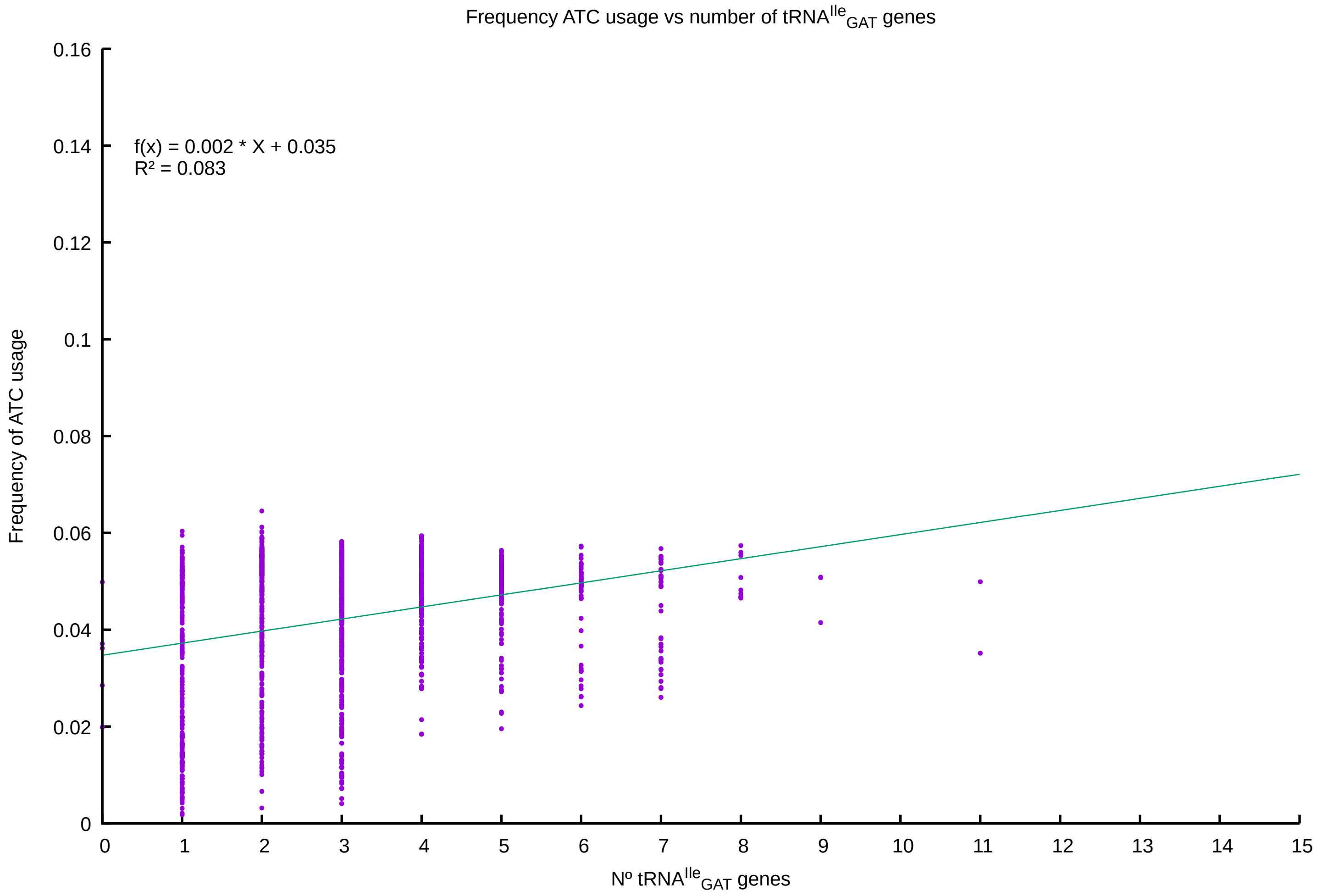
Frequency ATC usage vs number of tRNA^Ile_GAT genes
f(x) = 0.002 * X + 0.035
R² = 0.083
Frequency of ATC usage
0.16
0.14
0.12
0.1
0.08
0.06
0.04
0.02
0
0 1 2 3 4 5 6 7 8 9 10 11 12 13 14 15
Nº tRNA^Ile_GAT genes

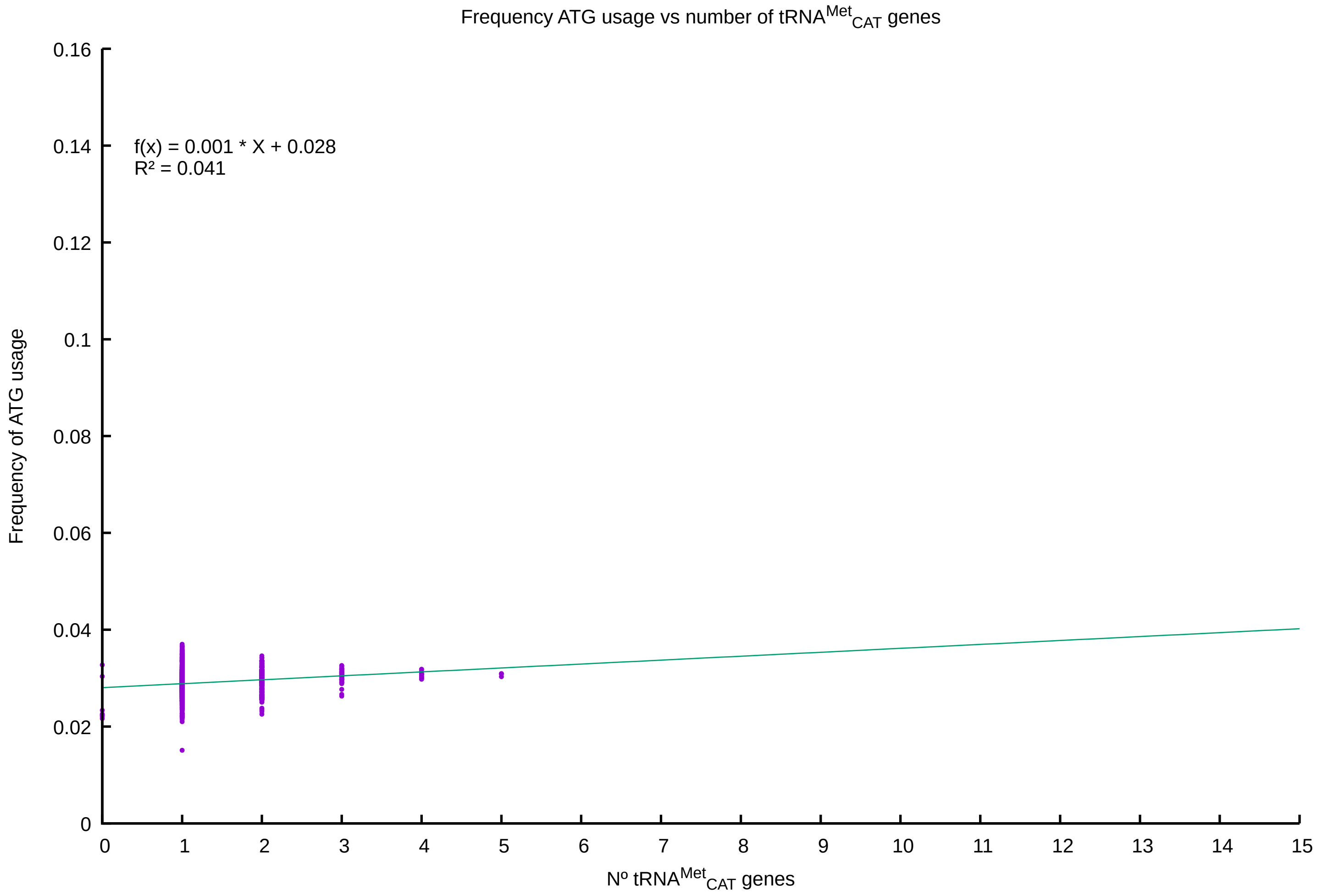

Frequency ATG usage vs number of tRNA^Met_CAT genes
f(x) = 0.001 * X + 0.028
R² = 0.041
Frequency of ATG usage
0.16
0.14
0.12
0.1
0.08
0.06
0.04
0.02
0
0 1 2 3 4 5 6 7 8 9 10 11 12 13 14 15
Nº tRNA^Met_CAT genes

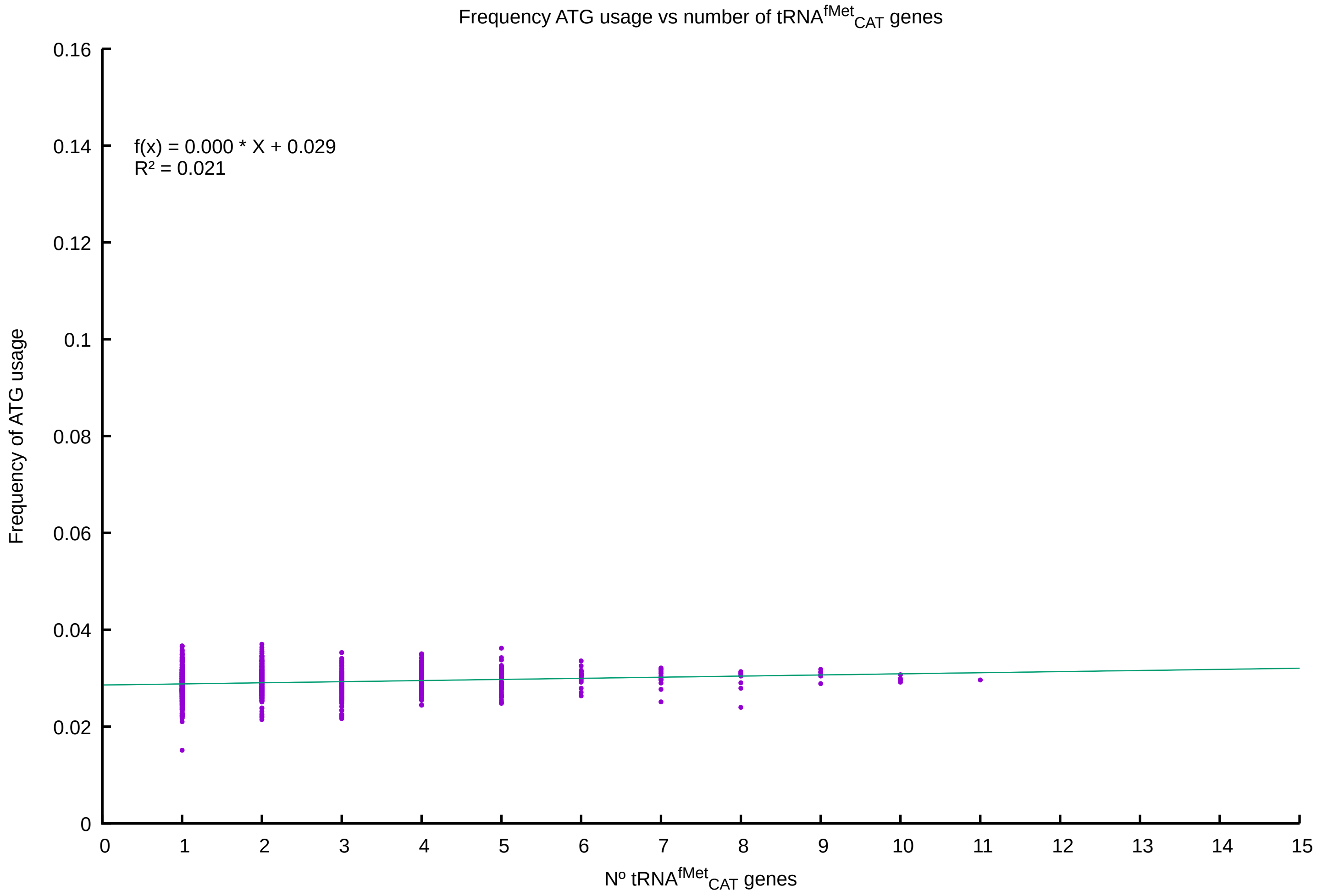
Frequency ATG usage vs number of tRNA$^{fMet}_{CAT}$ genes
f(x) = 0.000 * X + 0.029
R^2 = 0.021
Frequency of ATG usage
0.16
0.14
0.12
0.1
0.08
0.06
0.04
0.02
0
0
1
2
3
4
5
6
7
8
9
10
11
12
13
14
15
Nº tRNA$^{fMet}_{CAT}$ genes

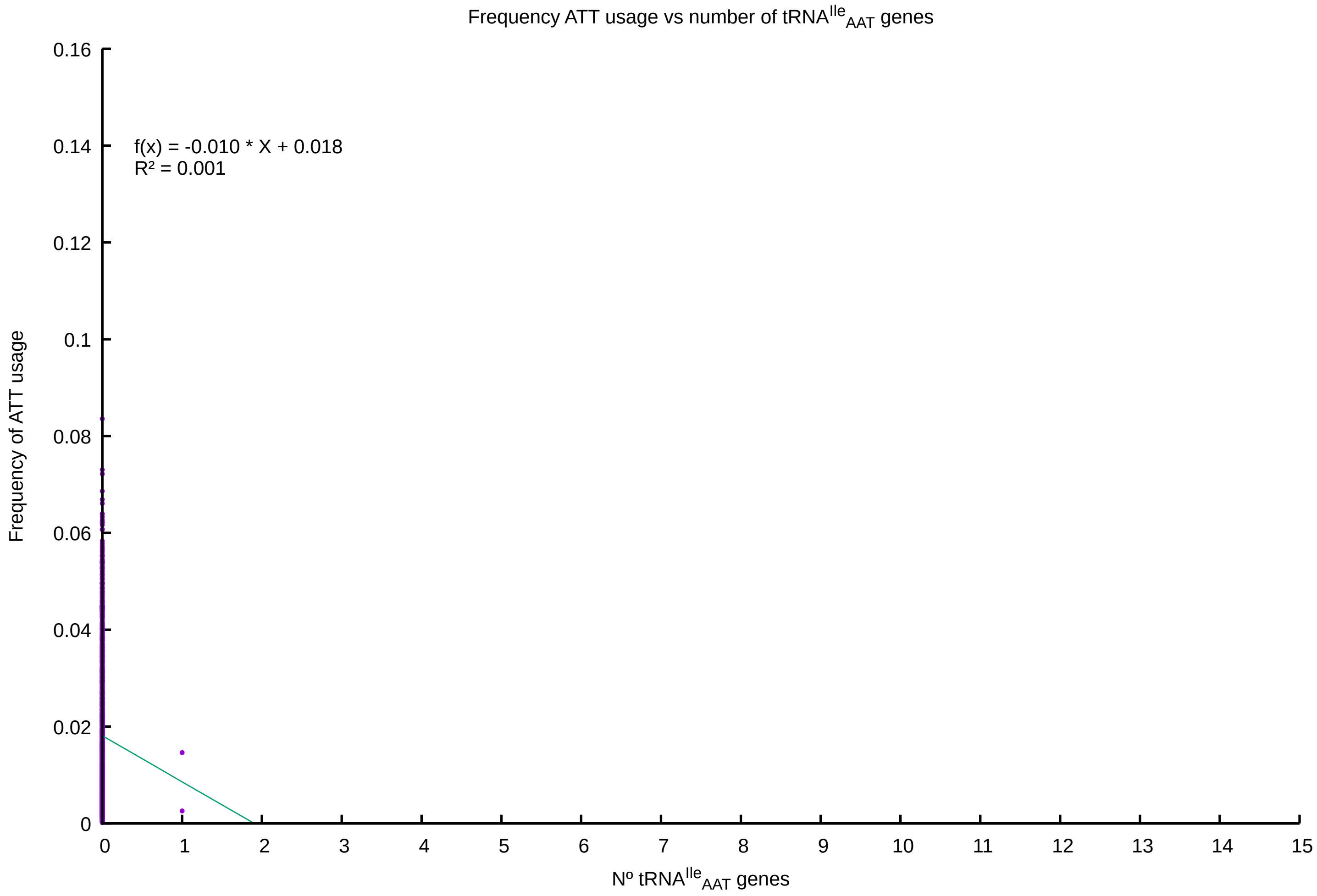
Frequency ATT usage vs number of tRNA^Ile_AAT genes
f(x) = -0.010 * X + 0.018
R² = 0.001
Frequency of ATT usage
0.16
0.14
0.12
0.1
0.08
0.06
0.04
0.02
0
0 1 2 3 4 5 6 7 8 9 10 11 12 13 14 15
Nº tRNA^Ile_AAT genes

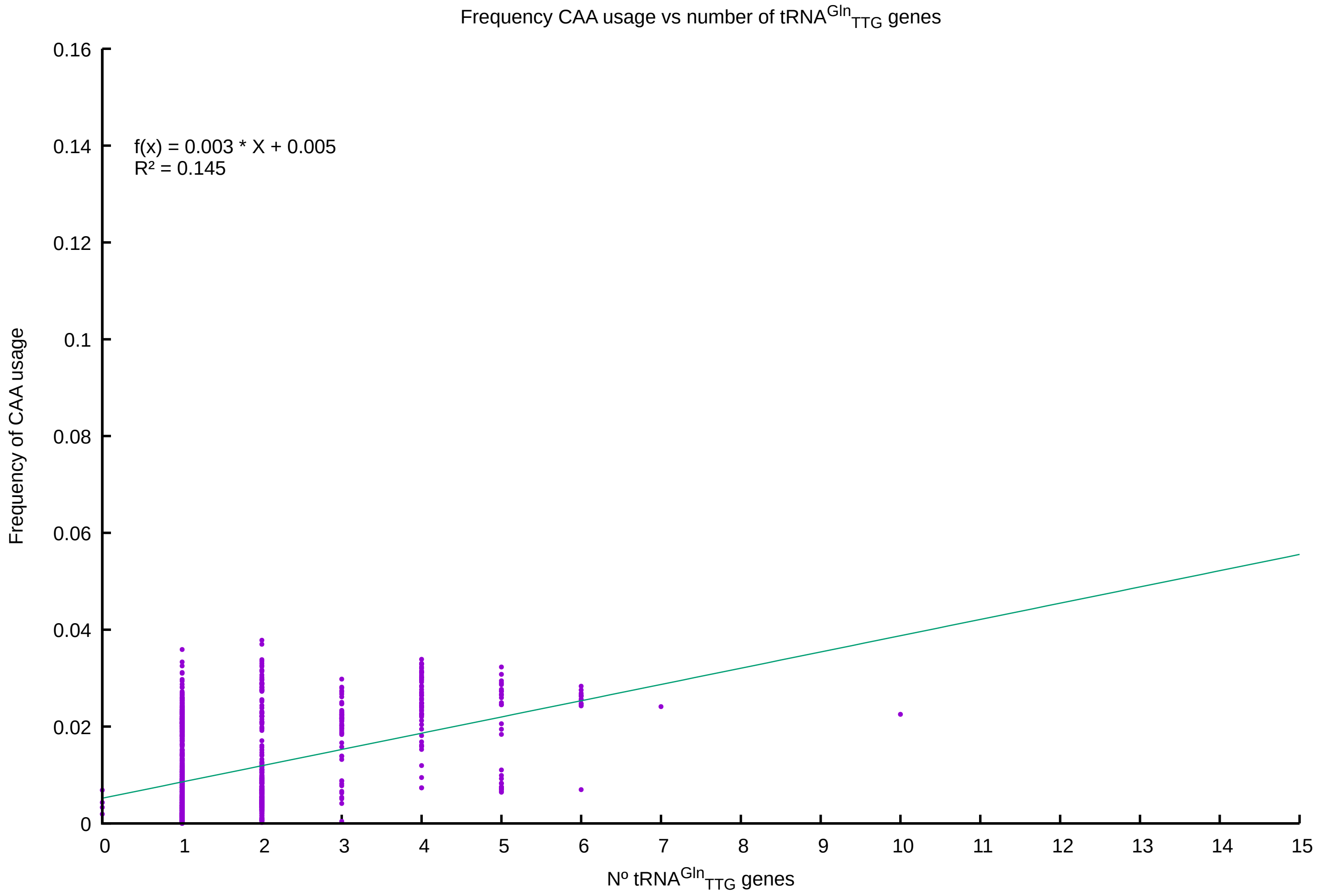

Frequency CAA usage vs number of tRNAGlnTTG genes
f(x) = 0.003 * X + 0.005
R² = 0.145
Frequency of CAA usage
0.16
0.14
0.12
0.1
0.08
0.06
0.04
0.02
0
0 1 2 3 4 5 6 7 8 9 10 11 12 13 14 15
Nº tRNAGlnTTG genes

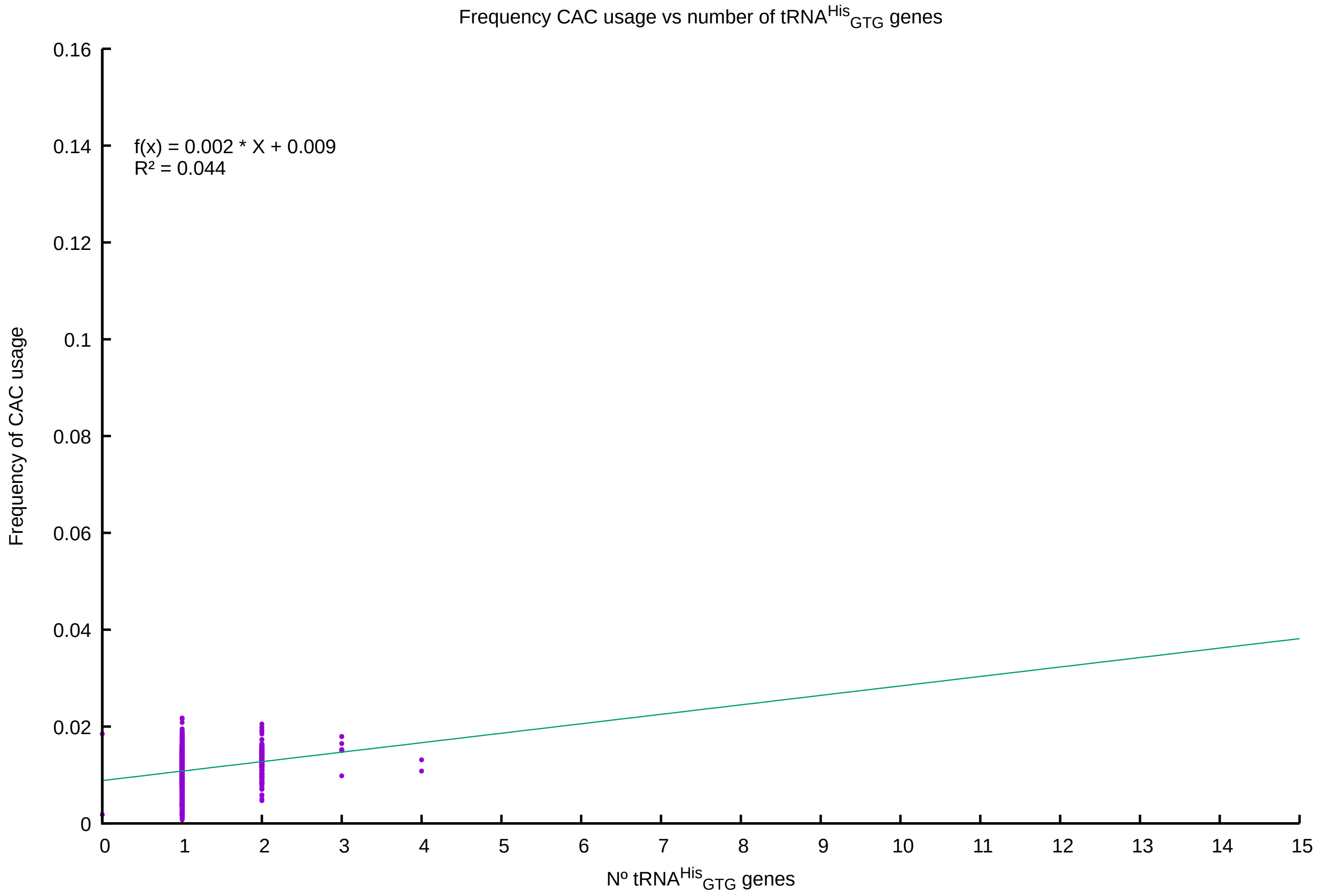

Frequency CAC usage vs number of tRNA^His_GTG genes
f(x) = 0.002 * X + 0.009
R² = 0.044
Frequency of CAC usage
0.16
0.14
0.12
0.1
0.08
0.06
0.04
0.02
0
0 1 2 3 4 5 6 7 8 9 10 11 12 13 14 15
Nº tRNA^His_GTG genes

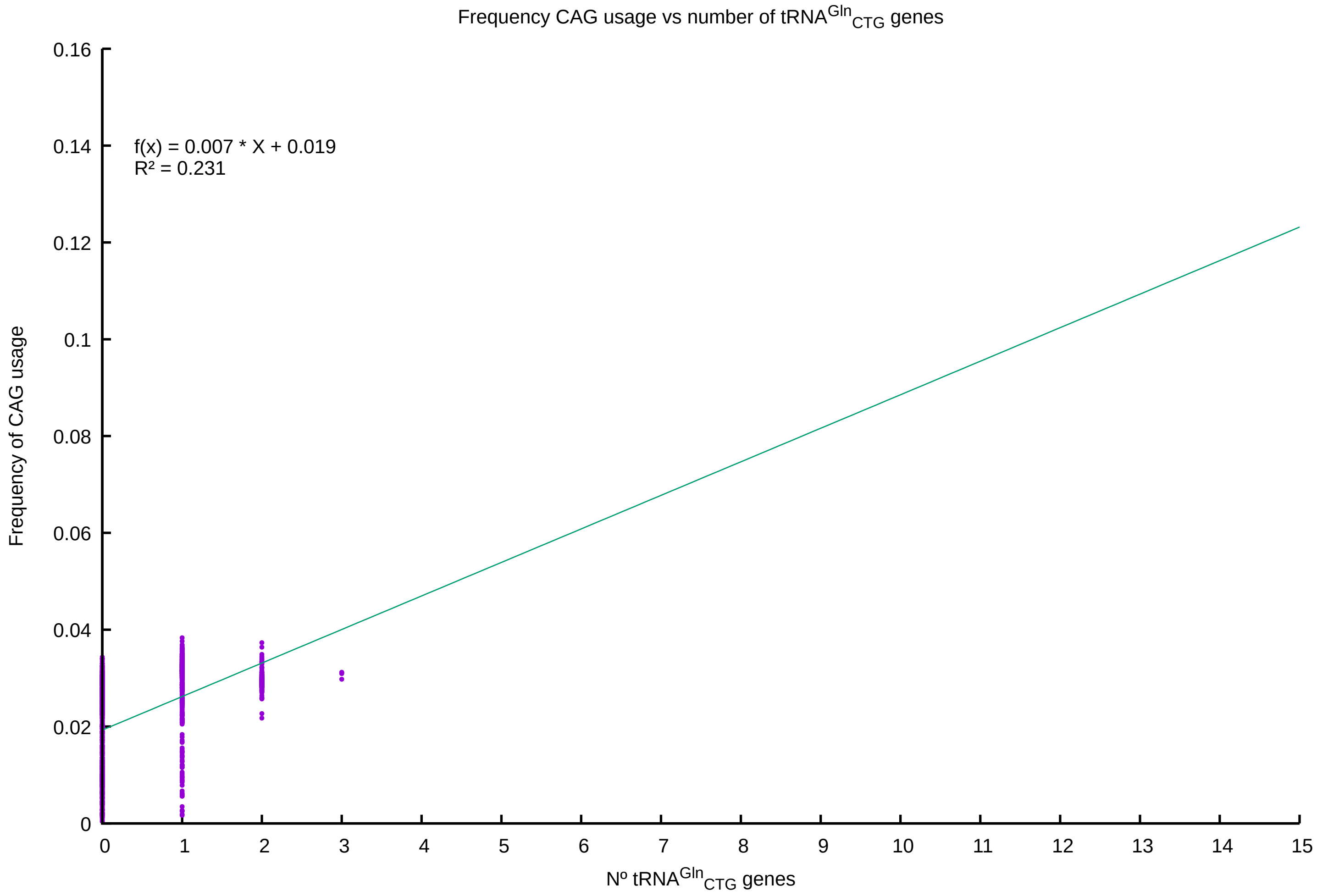

Frequency CAG usage vs number of tRNAGlnCTG genes
f(x) = 0.007 * X + 0.019
R² = 0.231
Frequency of CAG usage
0.16
0.14
0.12
0.1
0.08
0.06
0.04
0.02
0
0 1 2 3 4 5 6 7 8 9 10 11 12 13 14 15
Nº tRNAGlnCTG genes

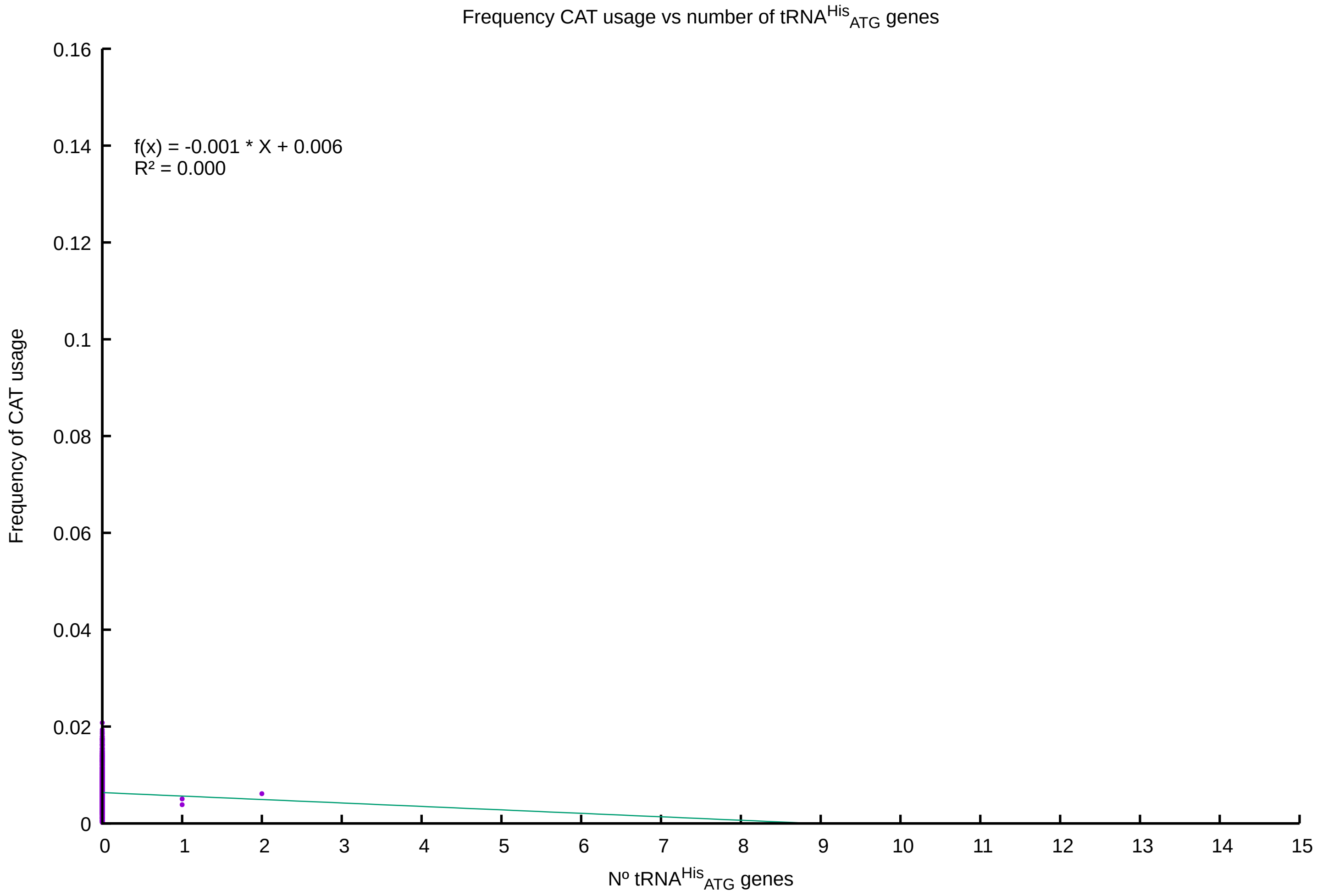

Frequency CAT usage vs number of tRNA His ATG genes
f(x) = -0.001 * X + 0.006
R² = 0.000
Frequency of CAT usage
0.16
0.14
0.12
0.1
0.08
0.06
0.04
0.02
0
0
1
2
3
4
5
6
7
8
9
10
11
12
13
14
15
Nº tRNA His ATG genes

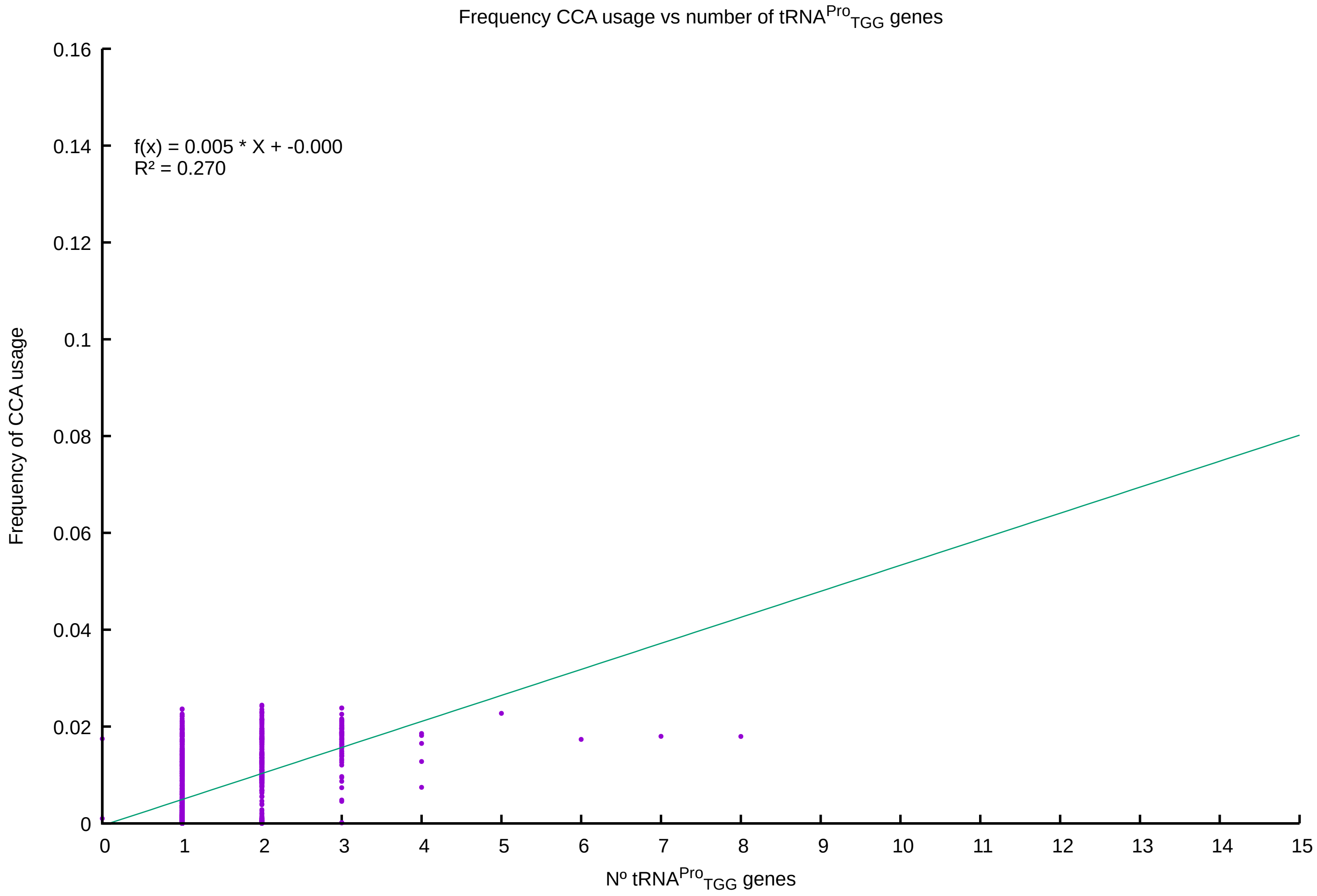

Frequency CCA usage vs number of tRNA^Pro_TGG genes
f(x) = 0.005 * X + -0.000
R² = 0.270
Frequency of CCA usage
0.16
0.14
0.12
0.1
0.08
0.06
0.04
0.02
0
0 1 2 3 4 5 6 7 8 9 10 11 12 13 14 15
Nº tRNA^Pro_TGG genes

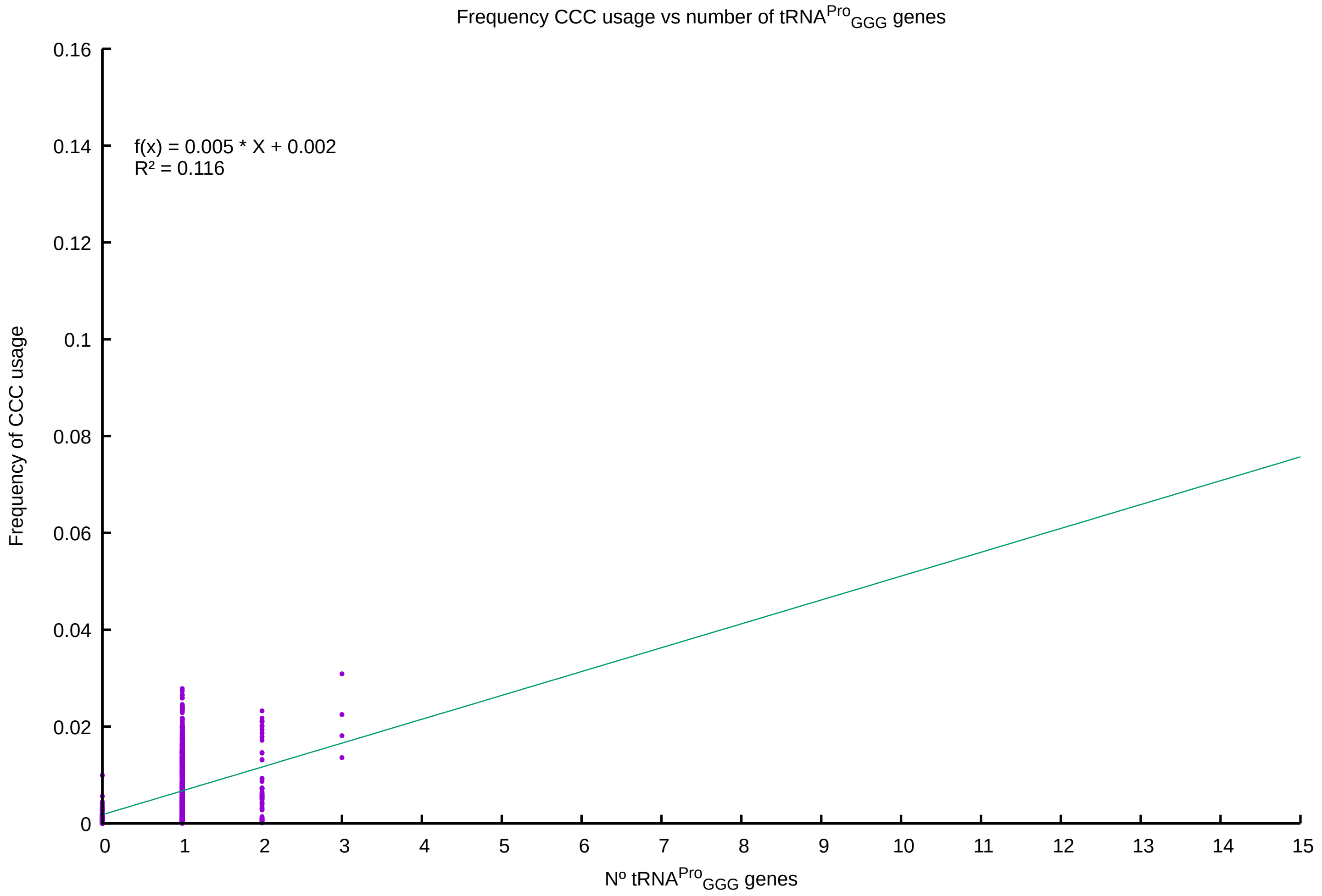

Frequency CCC usage vs number of tRNAProGGG genes
f(x) = 0.005 * X + 0.002
R² = 0.116
Frequency of CCC usage
0
0.02
0.04
0.06
0.08
0.1
0.12
0.14
0.16
0
1
2
3
4
5
6
7
8
9
10
11
12
13
14
15
Nº tRNAProGGG genes

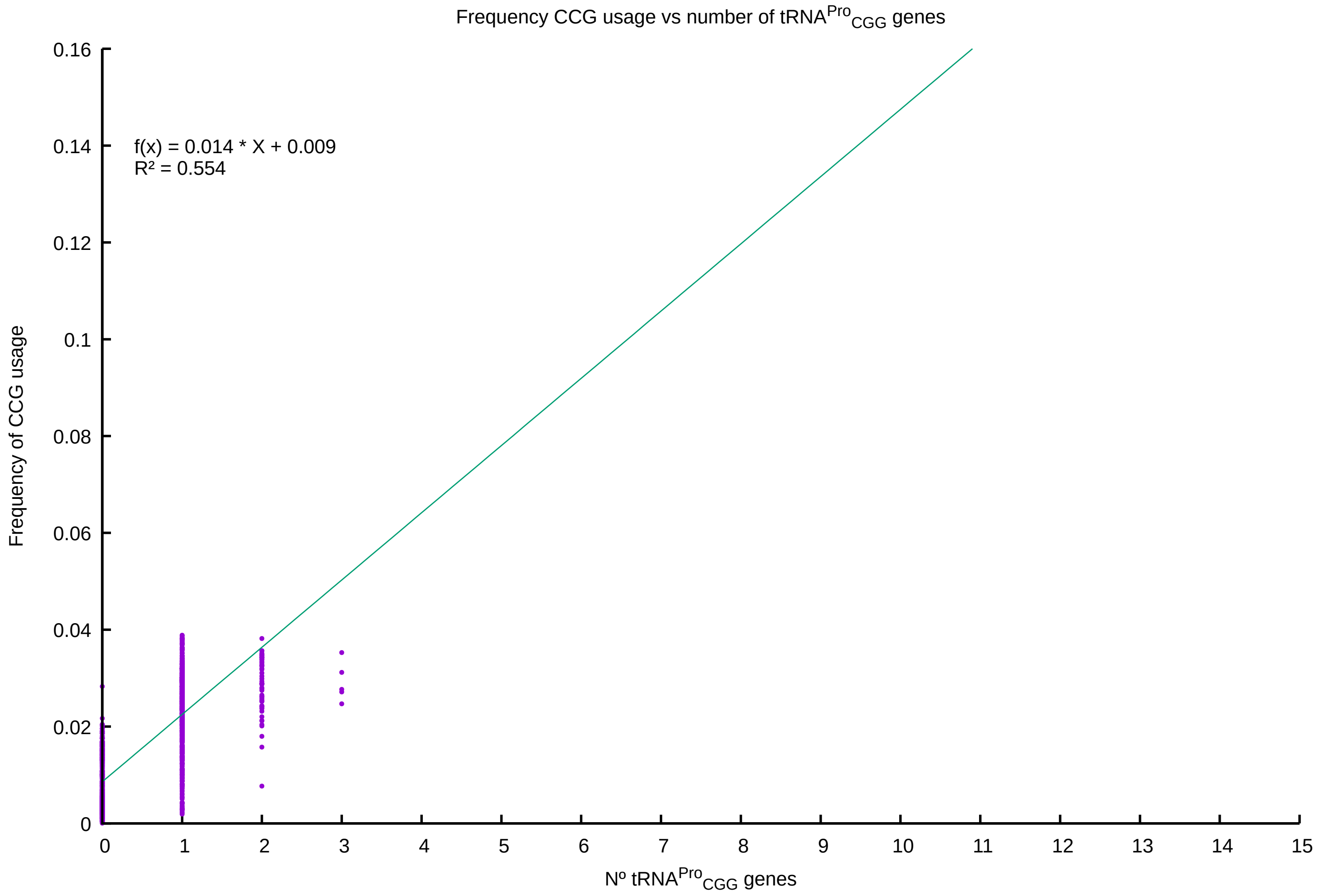
Frequency CCG usage vs number of tRNAProCGG genes
f(x) = 0.014 * X + 0.009
R² = 0.554
Frequency of CCG usage
0.16
0.14
0.12
0.1
0.08
0.06
0.04
0.02
0
0 1 2 3 4 5 6 7 8 9 10 11 12 13 14 15
Nº tRNAProCGG genes

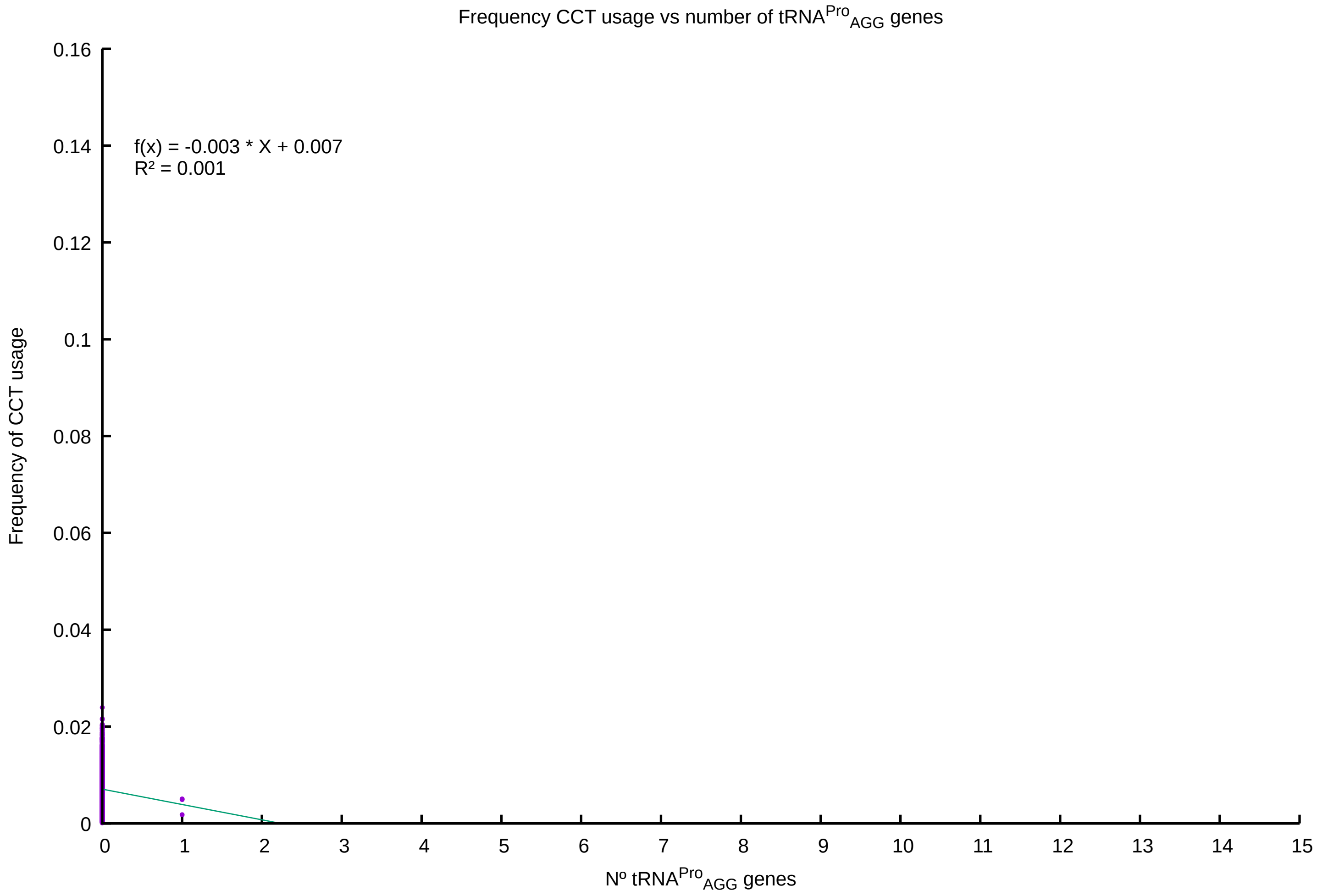

Frequency CCT usage vs number of tRNAPro AGG genes
f(x) = -0.003 * X + 0.007
R² = 0.001
Frequency of CCT usage
0.16
0.14
0.12
0.1
0.08
0.06
0.04
0.02
0
0 1 2 3 4 5 6 7 8 9 10 11 12 13 14 15
Nº tRNAPro AGG genes

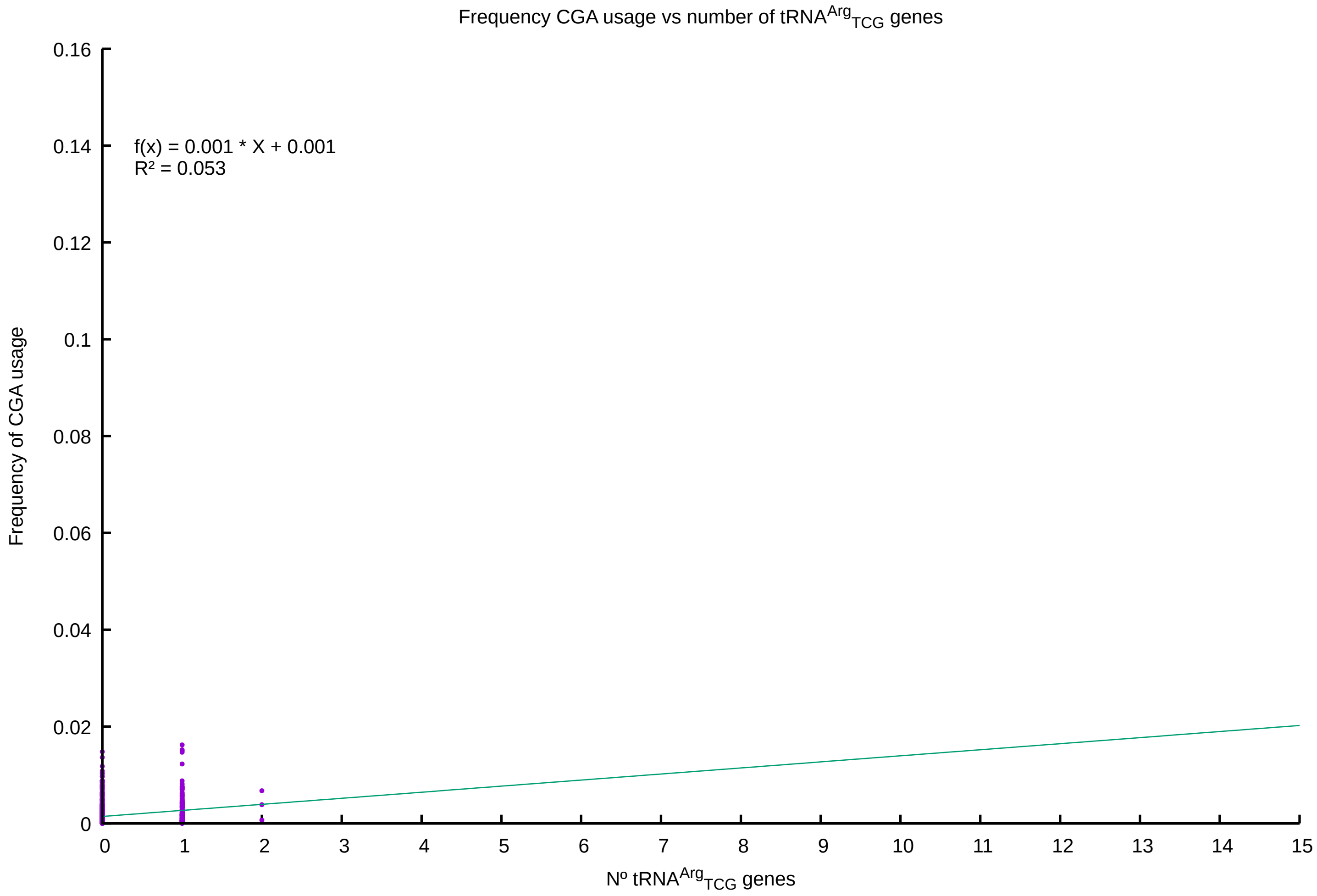

Frequency CGA usage vs number of tRNA^Arg_TCG genes
f(x) = 0.001 * X + 0.001
R² = 0.053
Frequency of CGA usage
0.16
0.14
0.12
0.1
0.08
0.06
0.04
0.02
0
0 1 2 3 4 5 6 7 8 9 10 11 12 13 14 15
Nº tRNA^Arg_TCG genes

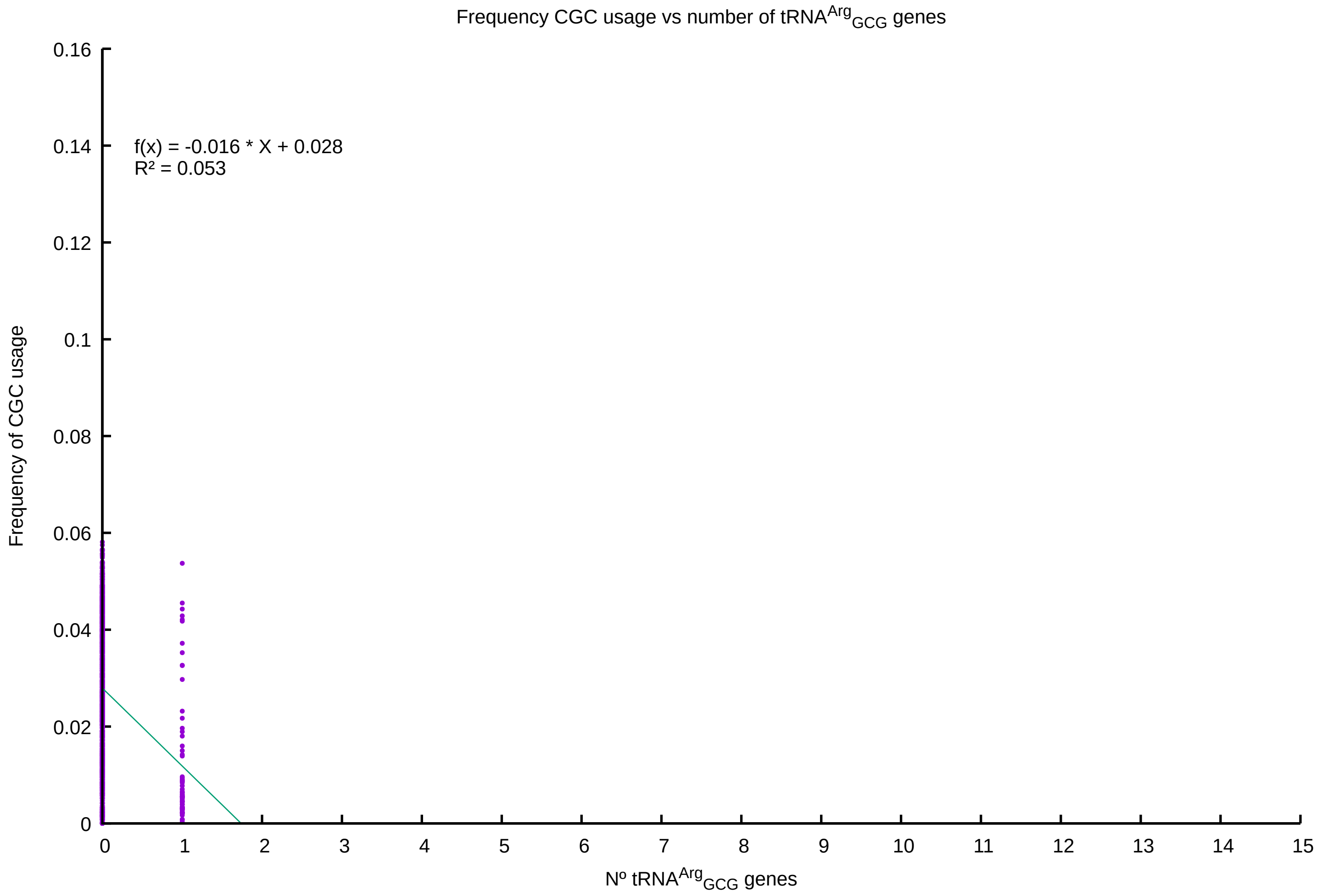

Frequency CGC usage vs number of tRNA^Arg_GCG genes
f(x) = -0.016 * X + 0.028
R² = 0.053
Frequency of CGC usage
0.16
0.14
0.12
0.1
0.08
0.06
0.04
0.02
0
0 1 2 3 4 5 6 7 8 9 10 11 12 13 14 15
Nº tRNA^Arg_GCG genes

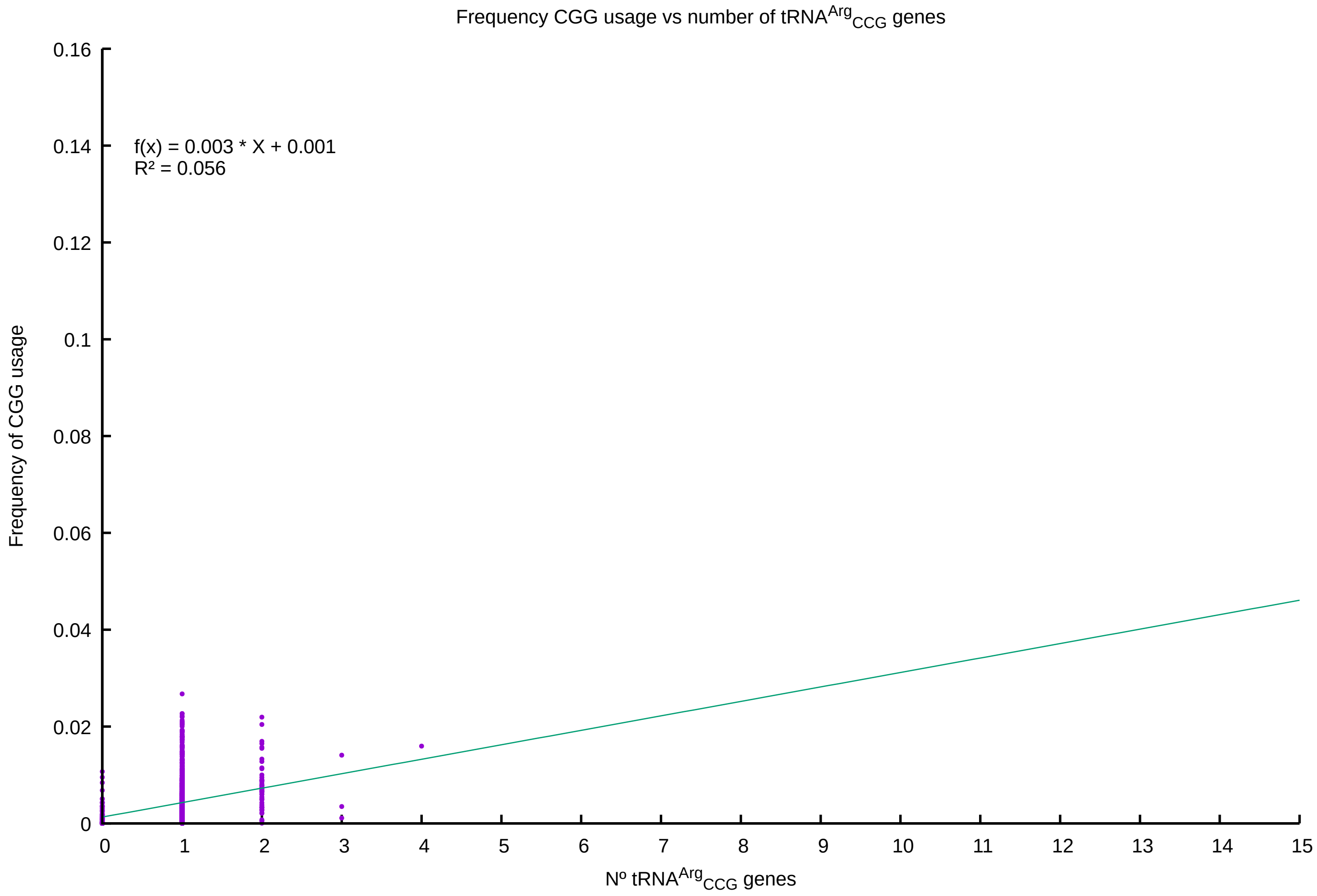
Frequency CGG usage vs number of tRNA$^{Arg}_{CCG}$ genes
f(x) = 0.003 * X + 0.001
R^2 = 0.056
Frequency of CGG usage
0.16
0.14
0.12
0.1
0.08
0.06
0.04
0.02
0
0 1 2 3 4 5 6 7 8 9 10 11 12 13 14 15
Nº tRNA$^{Arg}_{CCG}$ genes

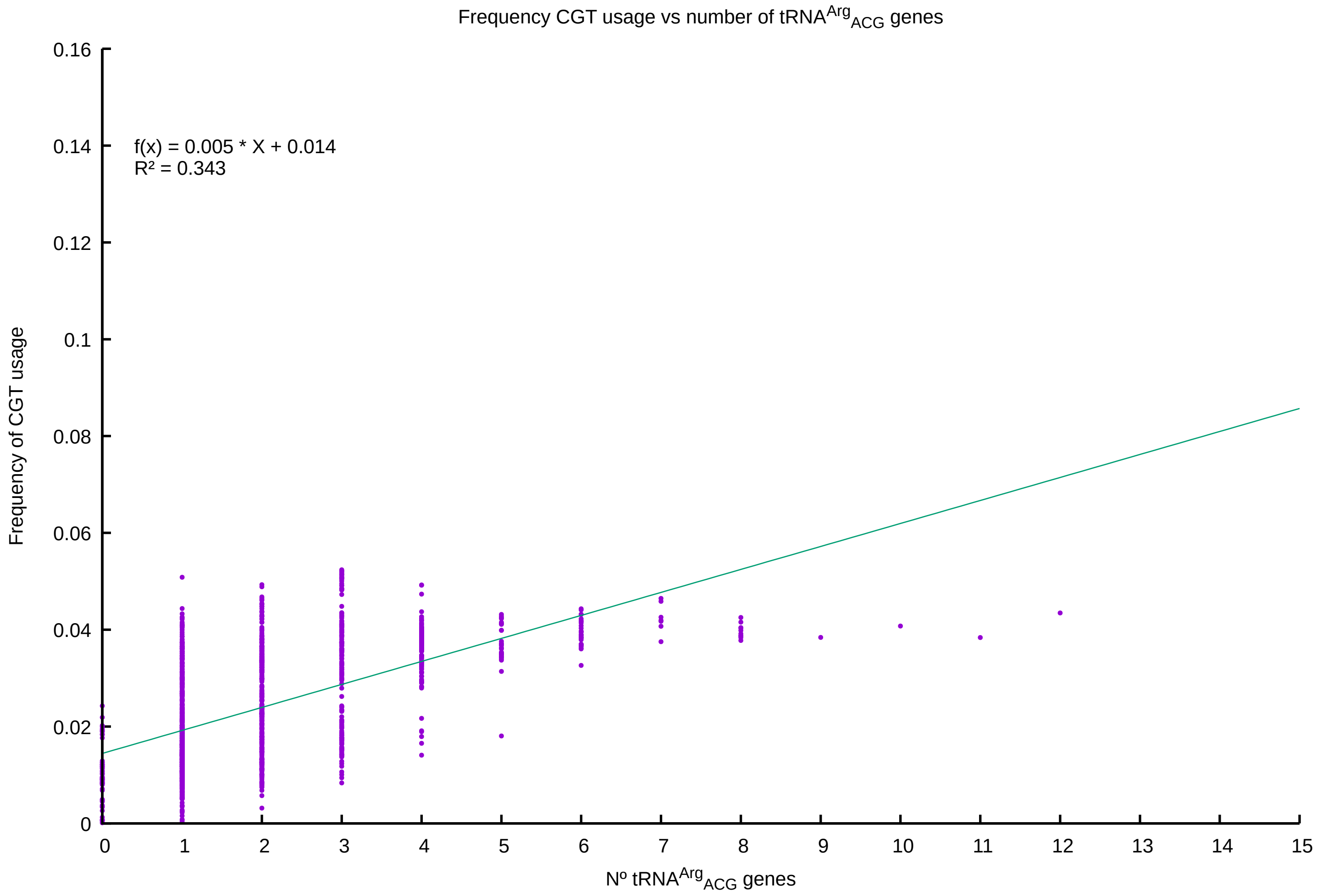

Frequency CGT usage vs number of tRNA^Arg_ACG genes
f(x) = 0.005 * X + 0.014
R² = 0.343
Frequency of CGT usage
0.16
0.14
0.12
0.1
0.08
0.06
0.04
0.02
0
0 1 2 3 4 5 6 7 8 9 10 11 12 13 14 15
Nº tRNA^Arg_ACG genes

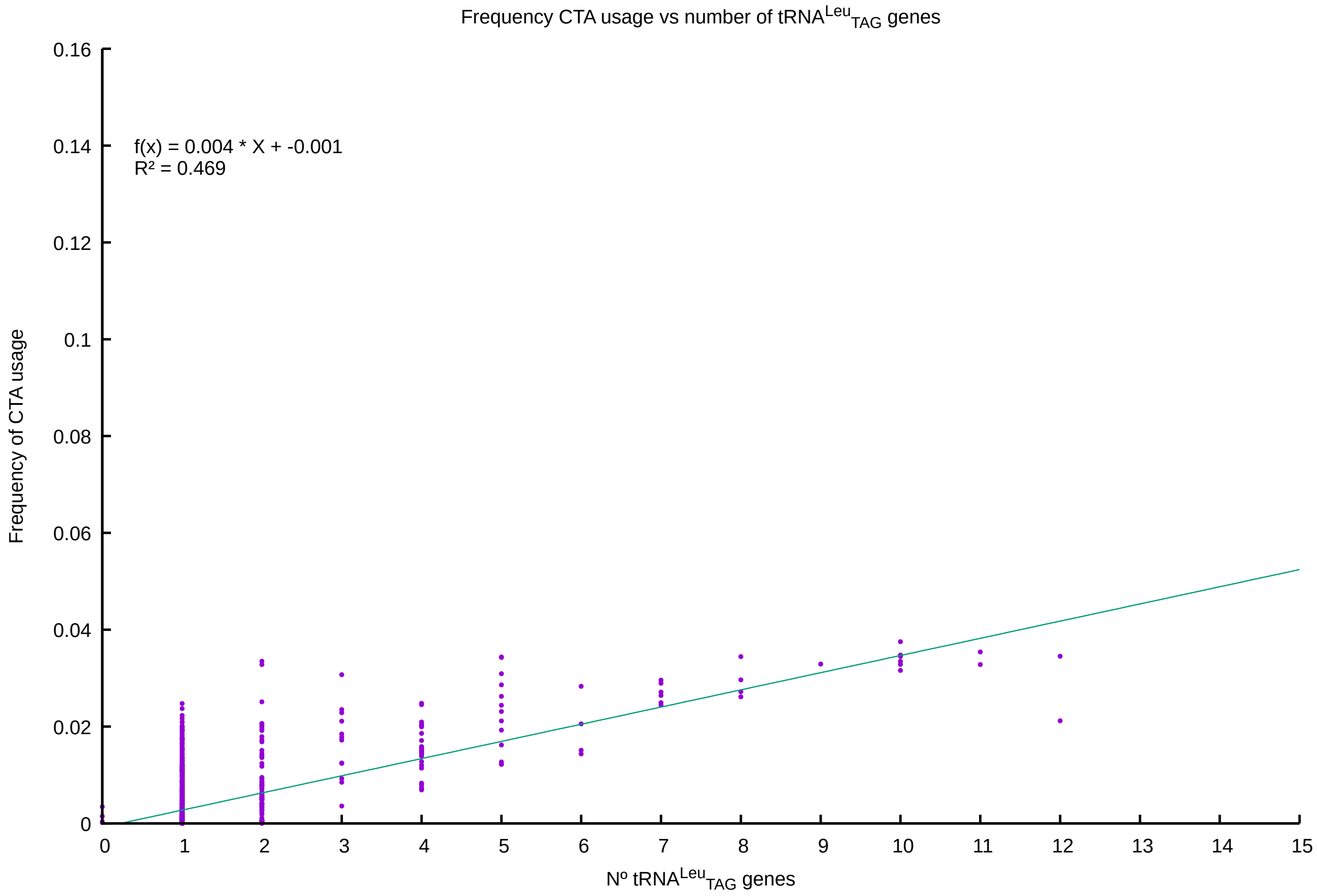

Frequency CTA usage vs number of tRNA^Leu_TAG genes
f(x) = 0.004 * X + -0.001
R² = 0.469
Frequency of CTA usage
0.16
0.14
0.12
0.1
0.08
0.06
0.04
0.02
0
0 1 2 3 4 5 6 7 8 9 10 11 12 13 14 15
Nº tRNA^Leu_TAG genes

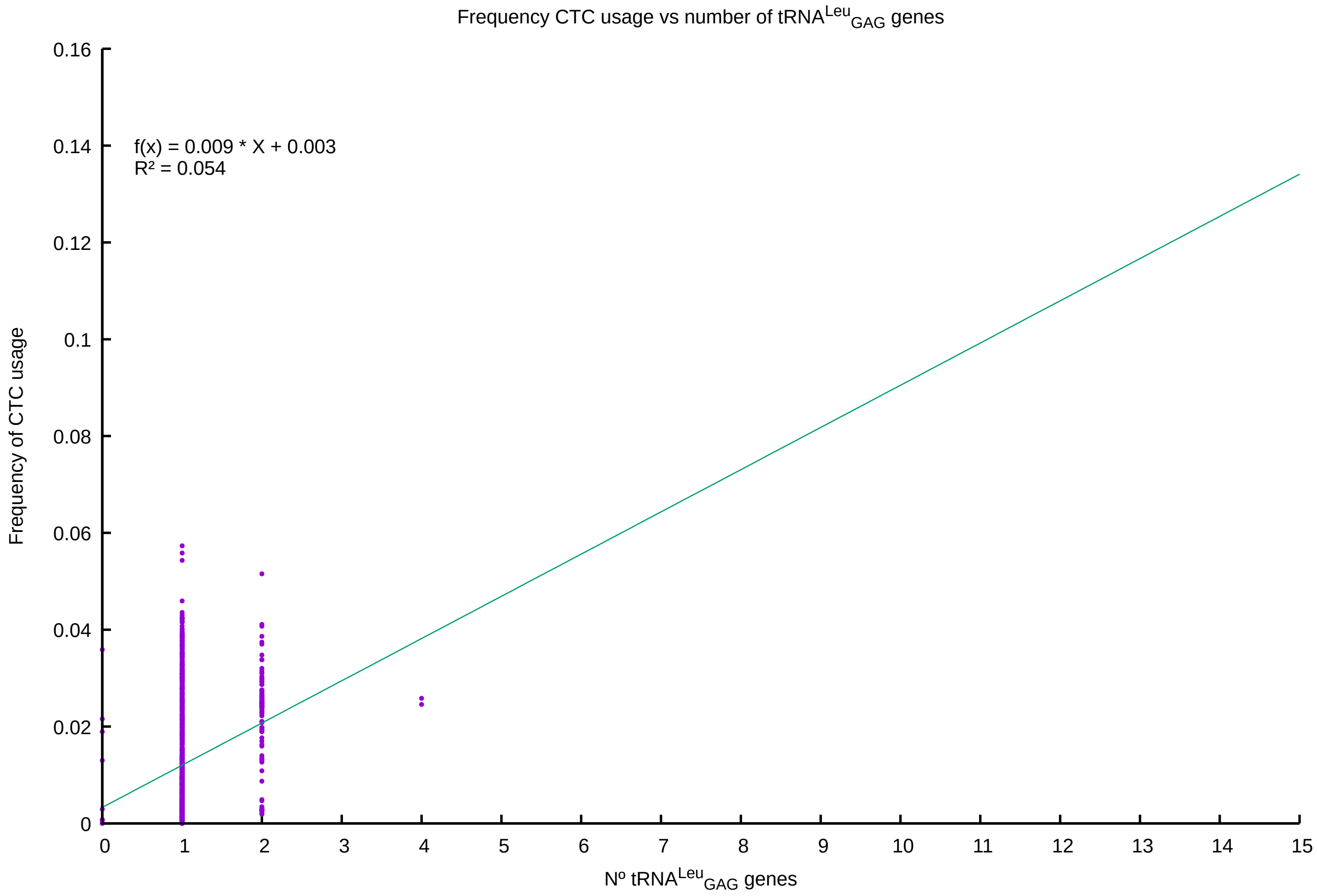

Frequency CTC usage vs number of tRNA^Leu_GAG genes
f(x) = 0.009 * X + 0.003
R² = 0.054
Frequency of CTC usage
0.16
0.14
0.12
0.1
0.08
0.06
0.04
0.02
0
0 1 2 3 4 5 6 7 8 9 10 11 12 13 14 15
Nº tRNA^Leu_GAG genes

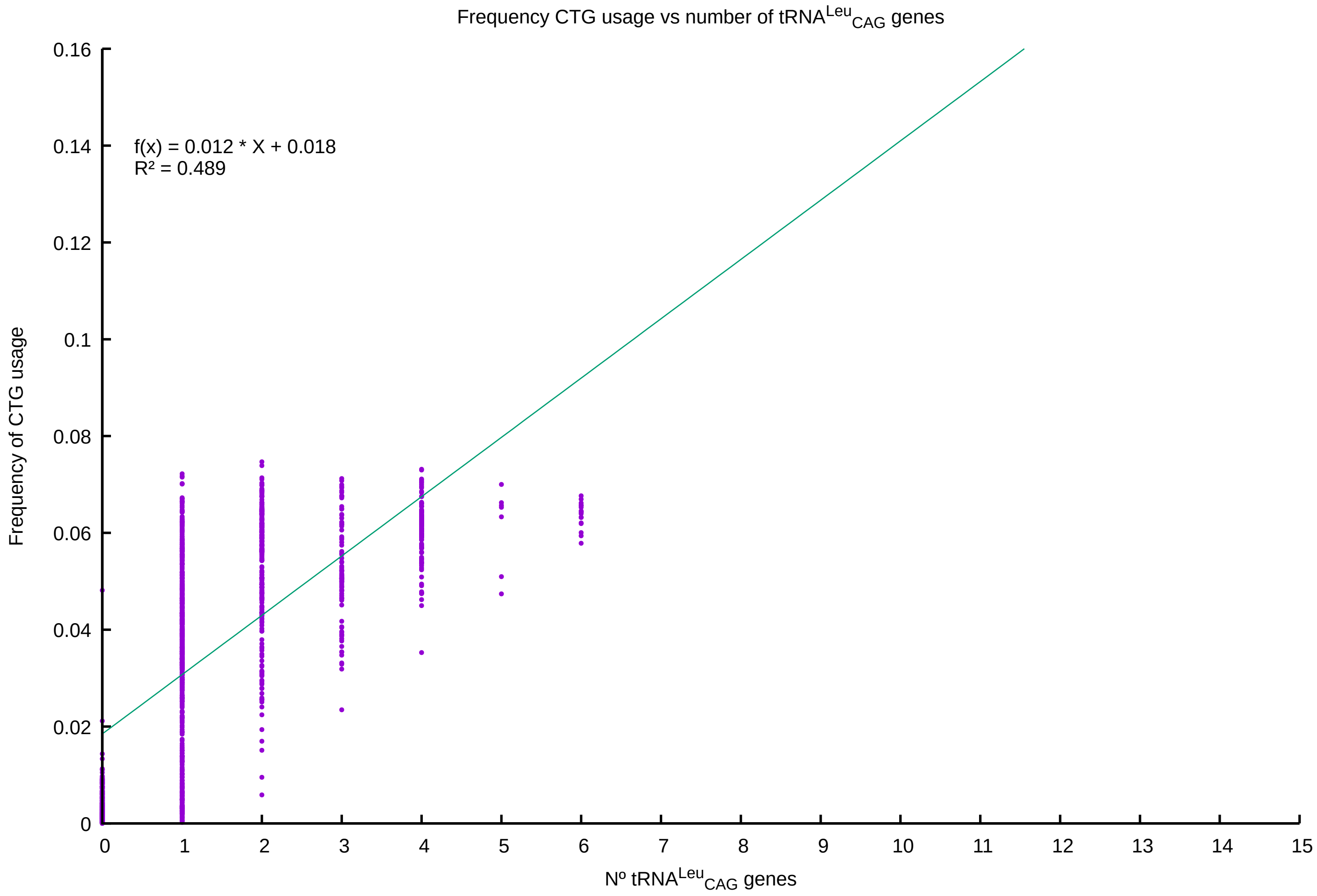

Frequency CTG usage vs number of tRNA$^{Leu}_{CAG}$ genes
f(x) = 0.012 * X + 0.018
R^2 = 0.489
Frequency of CTG usage
0.16
0.14
0.12
0.1
0.08
0.06
0.04
0.02
0
0 1 2 3 4 5 6 7 8 9 10 11 12 13 14 15
Nº tRNA$^{Leu}_{CAG}$ genes

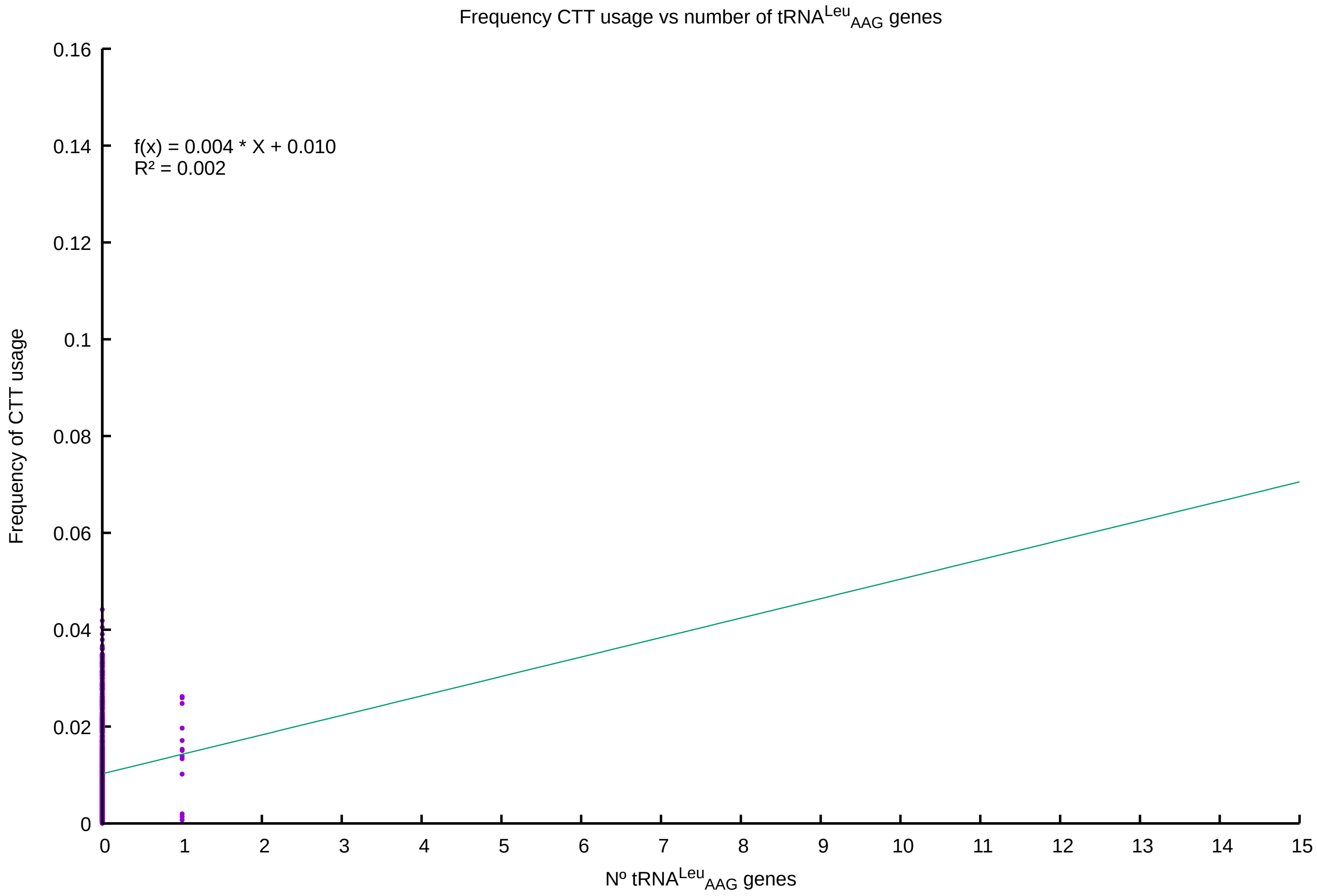

Frequency CTT usage vs number of tRNALeuAAG genes
f(x) = 0.004 * X + 0.010
R² = 0.002
Frequency of CTT usage
0.16
0.14
0.12
0.1
0.08
0.06
0.04
0.02
0
0 1 2 3 4 5 6 7 8 9 10 11 12 13 14 15
Nº tRNALeuAAG genes

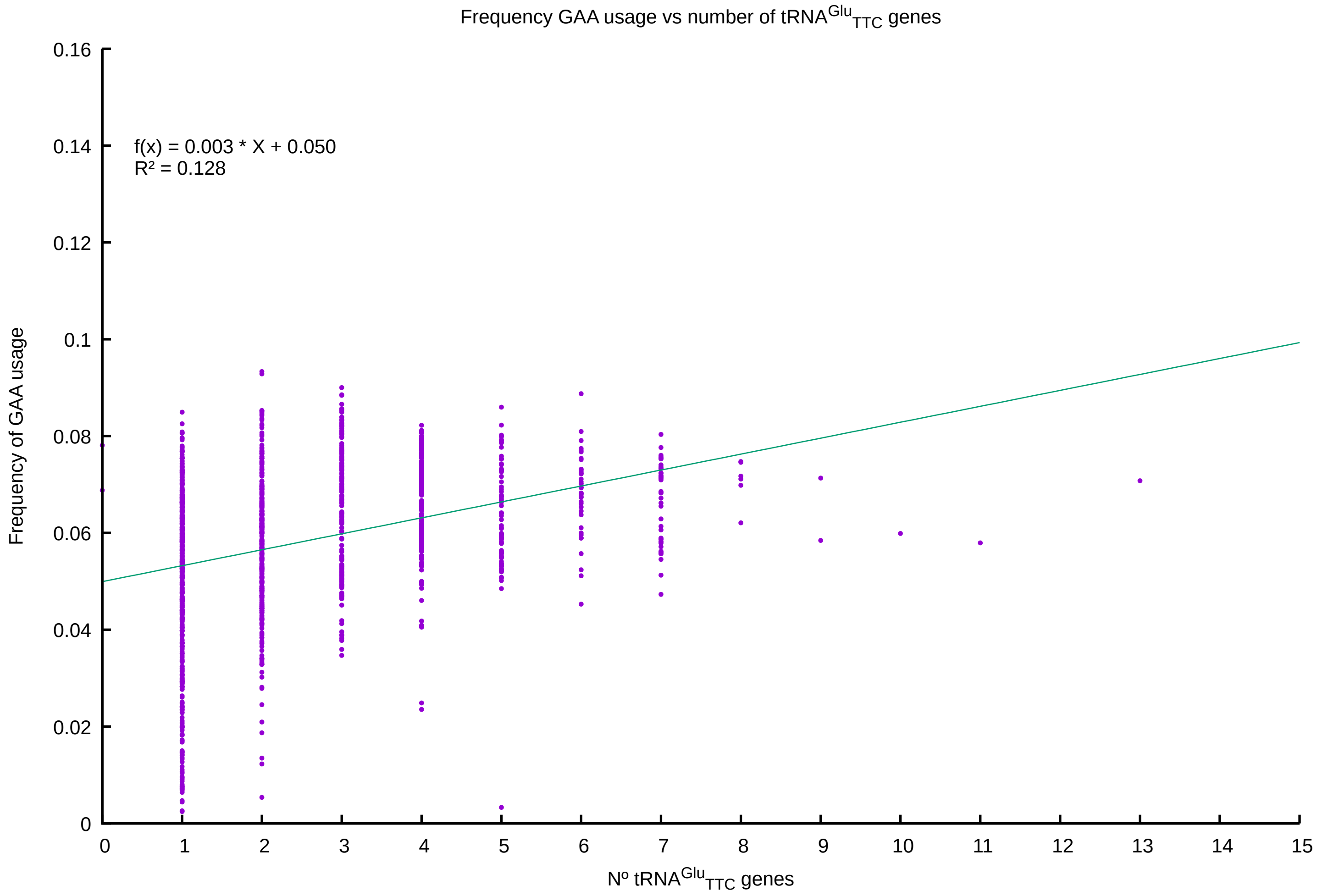

Frequency GAA usage vs number of tRNA Glu TTC genes
f(x) = 0.003 * X + 0.050
R² = 0.128
Frequency of GAA usage
0.16
0.14
0.12
0.1
0.08
0.06
0.04
0.02
0
0 1 2 3 4 5 6 7 8 9 10 11 12 13 14 15
Nº tRNA Glu TTC genes

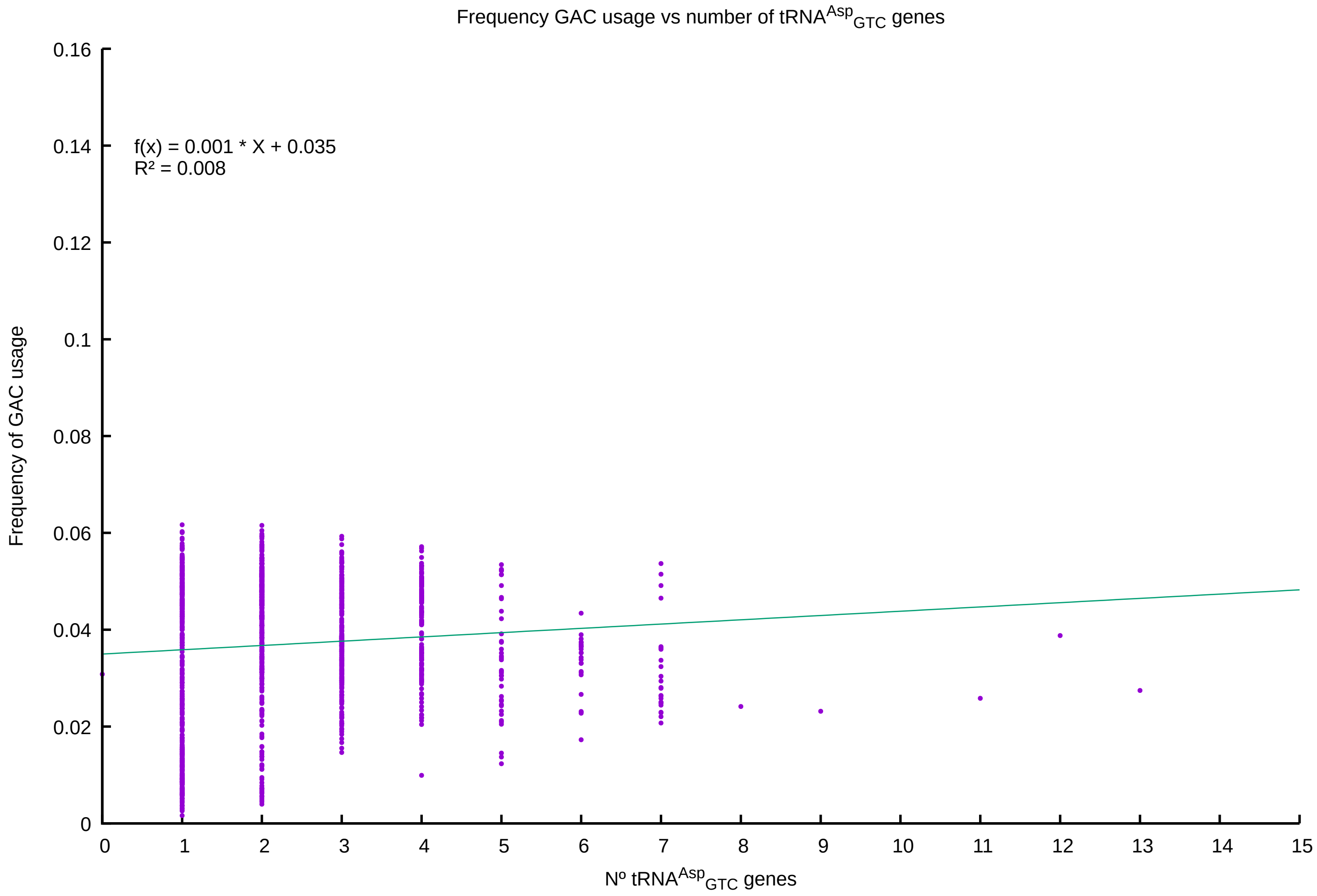
Frequency GAC usage vs number of tRNA^Asp_GTC genes
f(x) = 0.001 * X + 0.035
R² = 0.008
Frequency of GAC usage
Nº tRNA^Asp_GTC genes
0.16
0.14
0.12
0.1
0.08
0.06
0.04
0.02
0
0 1 2 3 4 5 6 7 8 9 10 11 12 13 14 15

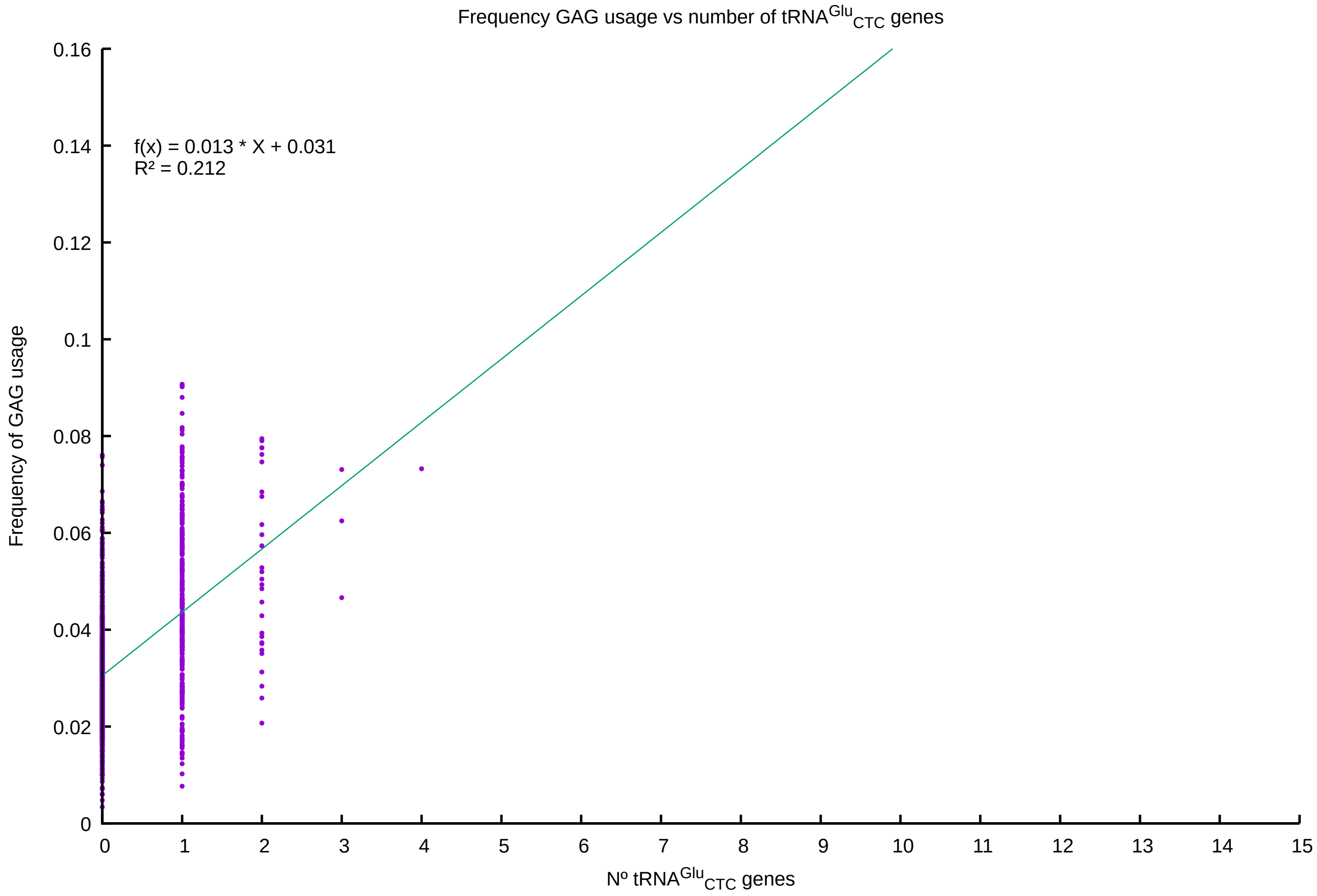

Frequency GAG usage vs number of tRNA^Glu_CTC genes
f(x) = 0.013 * X + 0.031
R² = 0.212
Frequency of GAG usage
0.16
0.14
0.12
0.1
0.08
0.06
0.04
0.02
0
0 1 2 3 4 5 6 7 8 9 10 11 12 13 14 15
Nº tRNA^Glu_CTC genes

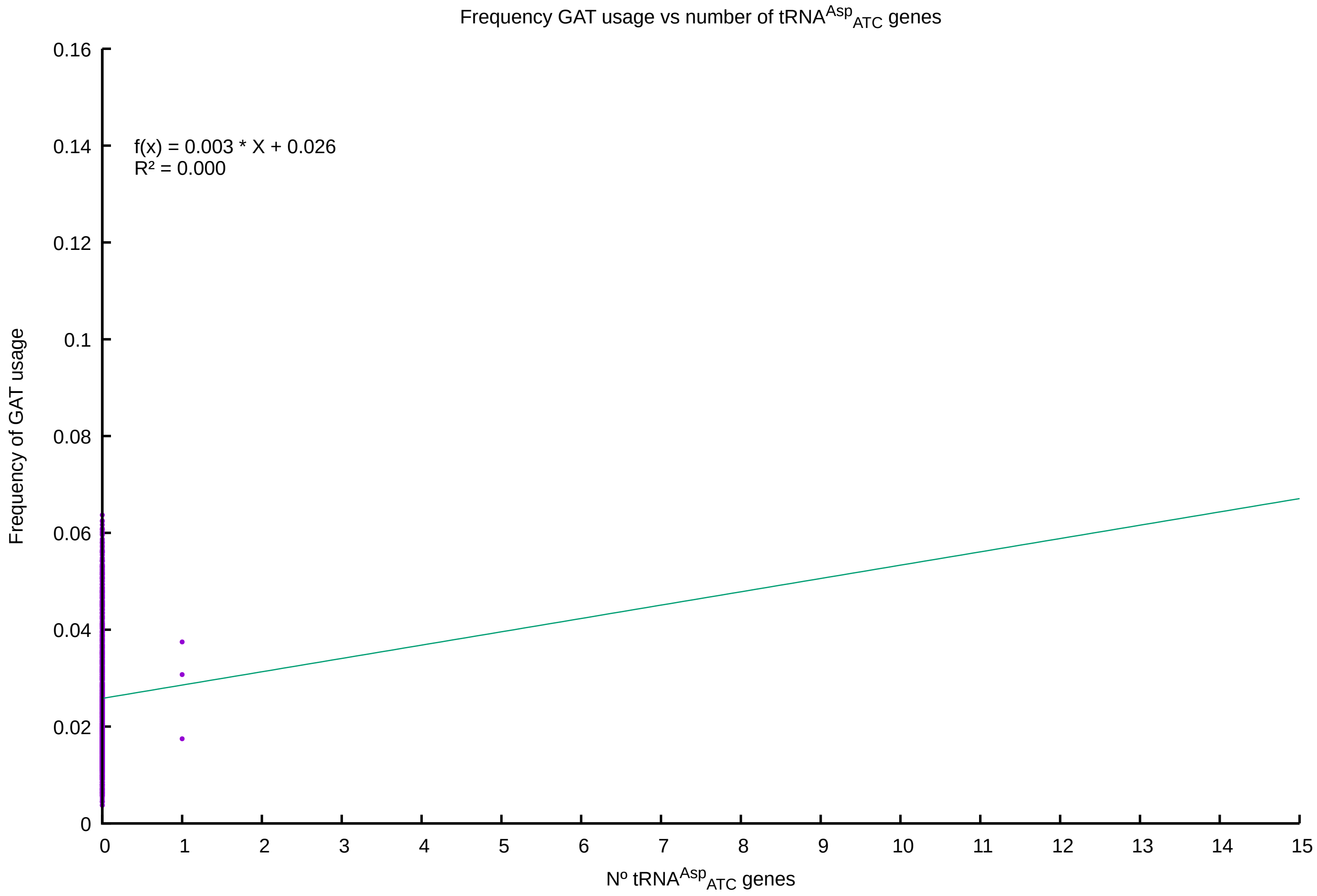
Frequency GAT usage vs number of tRNA^Asp_ATC genes
f(x) = 0.003 * X + 0.026
R² = 0.000
Frequency of GAT usage
Nº tRNA^Asp_ATC genes
0.16
0.14
0.12
0.1
0.08
0.06
0.04
0.02
0
0 1 2 3 4 5 6 7 8 9 10 11 12 13 14 15

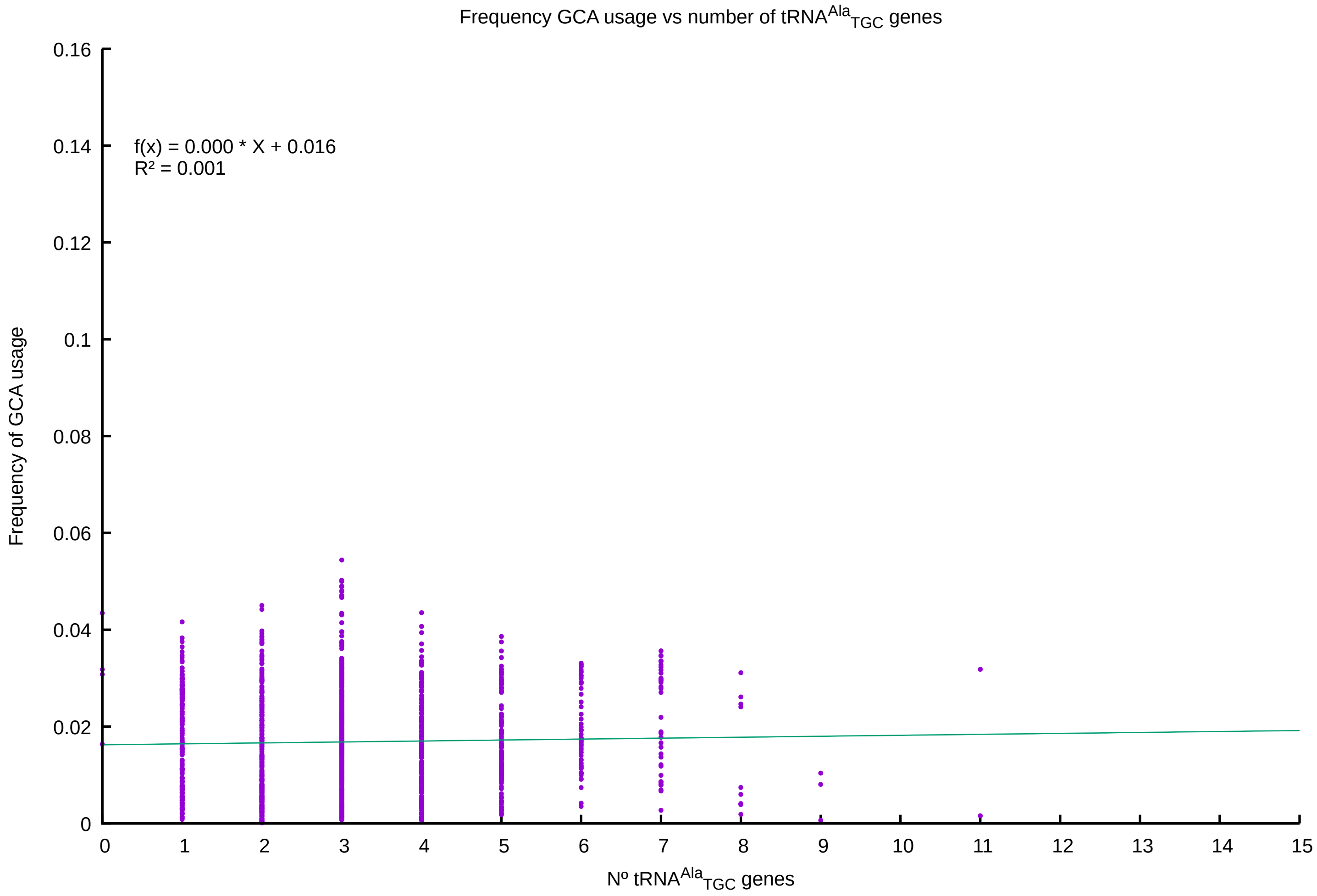

Frequency GCA usage vs number of tRNA^Ala_TGC genes
f(x) = 0.000 * X + 0.016
R² = 0.001
Frequency of GCA usage
0.16
0.14
0.12
0.1
0.08
0.06
0.04
0.02
0
0 1 2 3 4 5 6 7 8 9 10 11 12 13 14 15
Nº tRNA^Ala_TGC genes

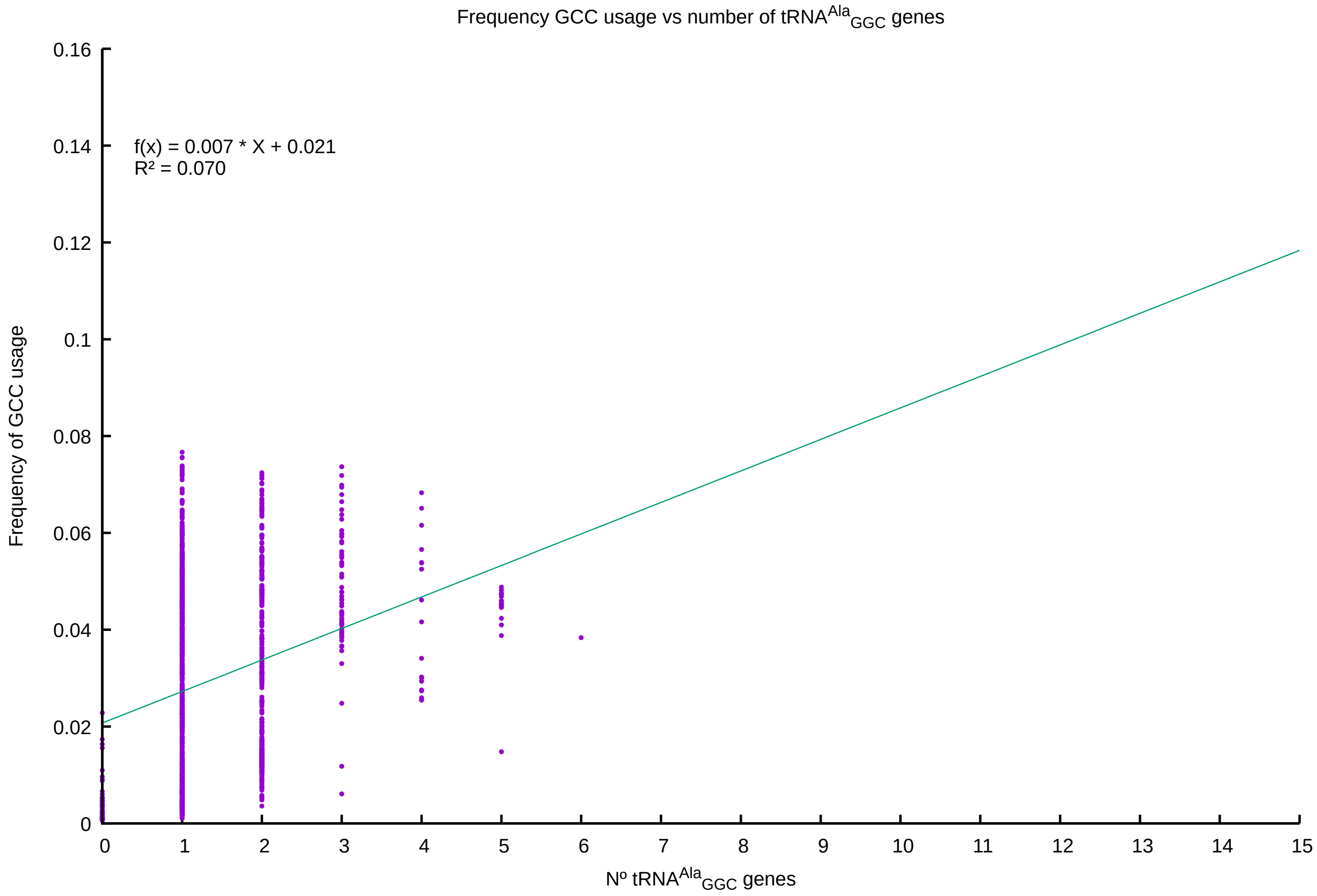
Frequency GCC usage vs number of tRNA$^{Ala}_{GGC}$ genes
f(x) = 0.007 * X + 0.021
R^2 = 0.070
Frequency of GCC usage
0.16
0.14
0.12
0.1
0.08
0.06
0.04
0.02
0
0 1 2 3 4 5 6 7 8 9 10 11 12 13 14 15
Nº tRNA$^{Ala}_{GGC}$ genes

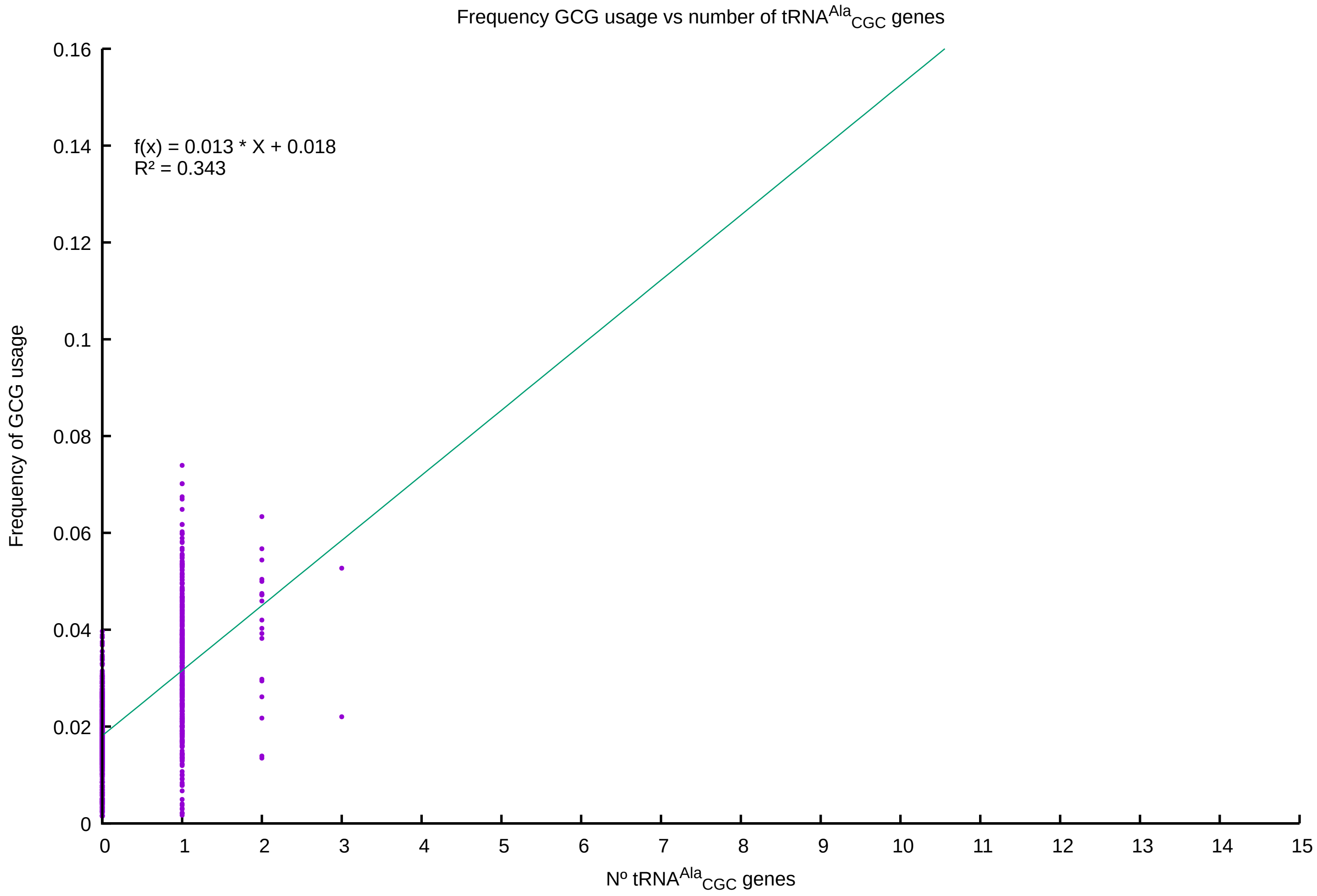

Frequency GCG usage vs number of tRNA$^{Ala}_{CGC}$ genes
f(x) = 0.013 * X + 0.018
R^2 = 0.343
Frequency of GCG usage
0.16
0.14
0.12
0.1
0.08
0.06
0.04
0.02
0
0 1 2 3 4 5 6 7 8 9 10 11 12 13 14 15
Nº tRNA$^{Ala}_{CGC}$ genes

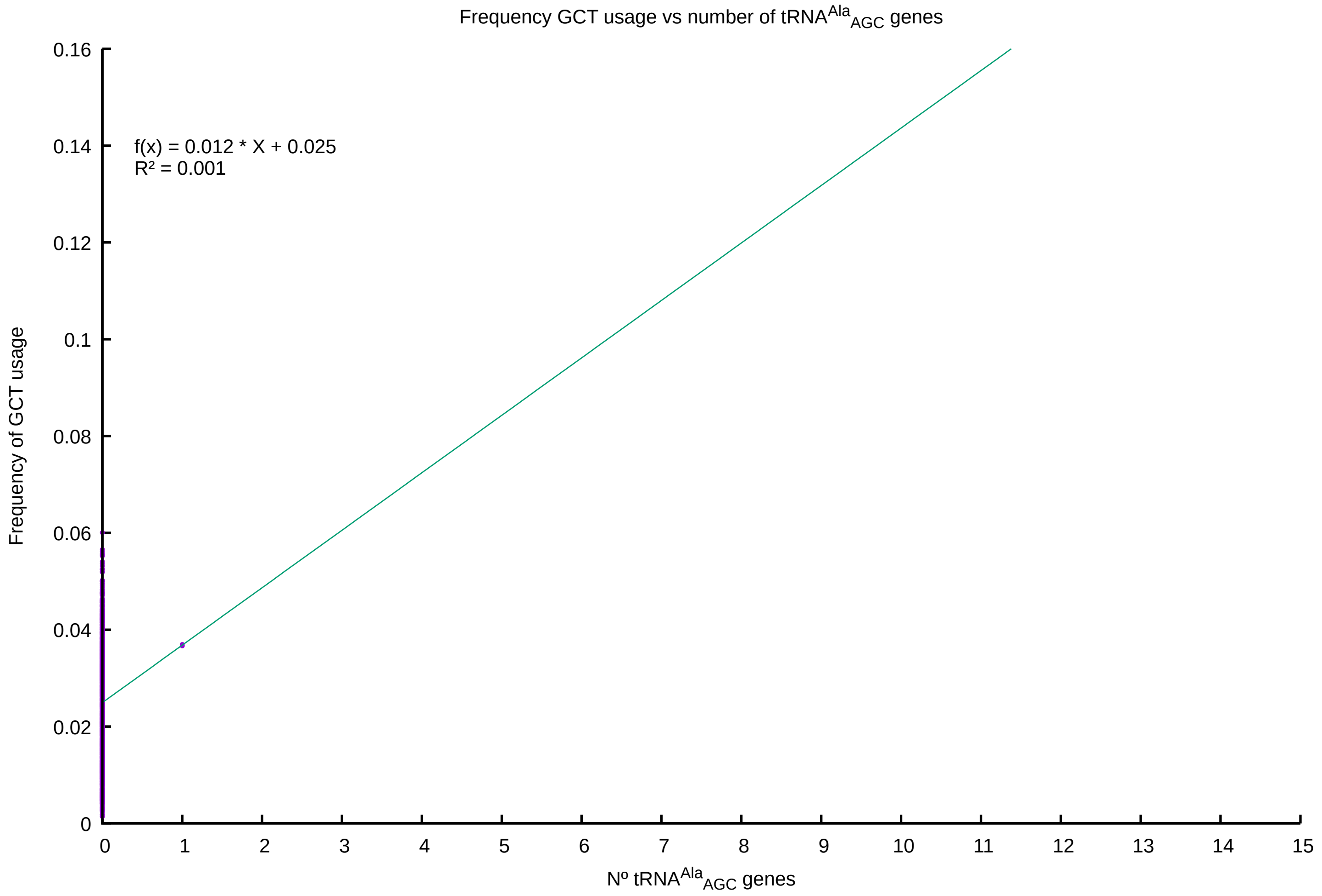
Frequency GCT usage vs number of tRNA^Ala_AGC genes
f(x) = 0.012 * X + 0.025
R² = 0.001
Frequency of GCT usage
Nº tRNA^Ala_AGC genes
0
0.02
0.04
0.06
0.08
0.1
0.12
0.14
0.16
0
1
2
3
4
5
6
7
8
9
10
11
12
13
14
15

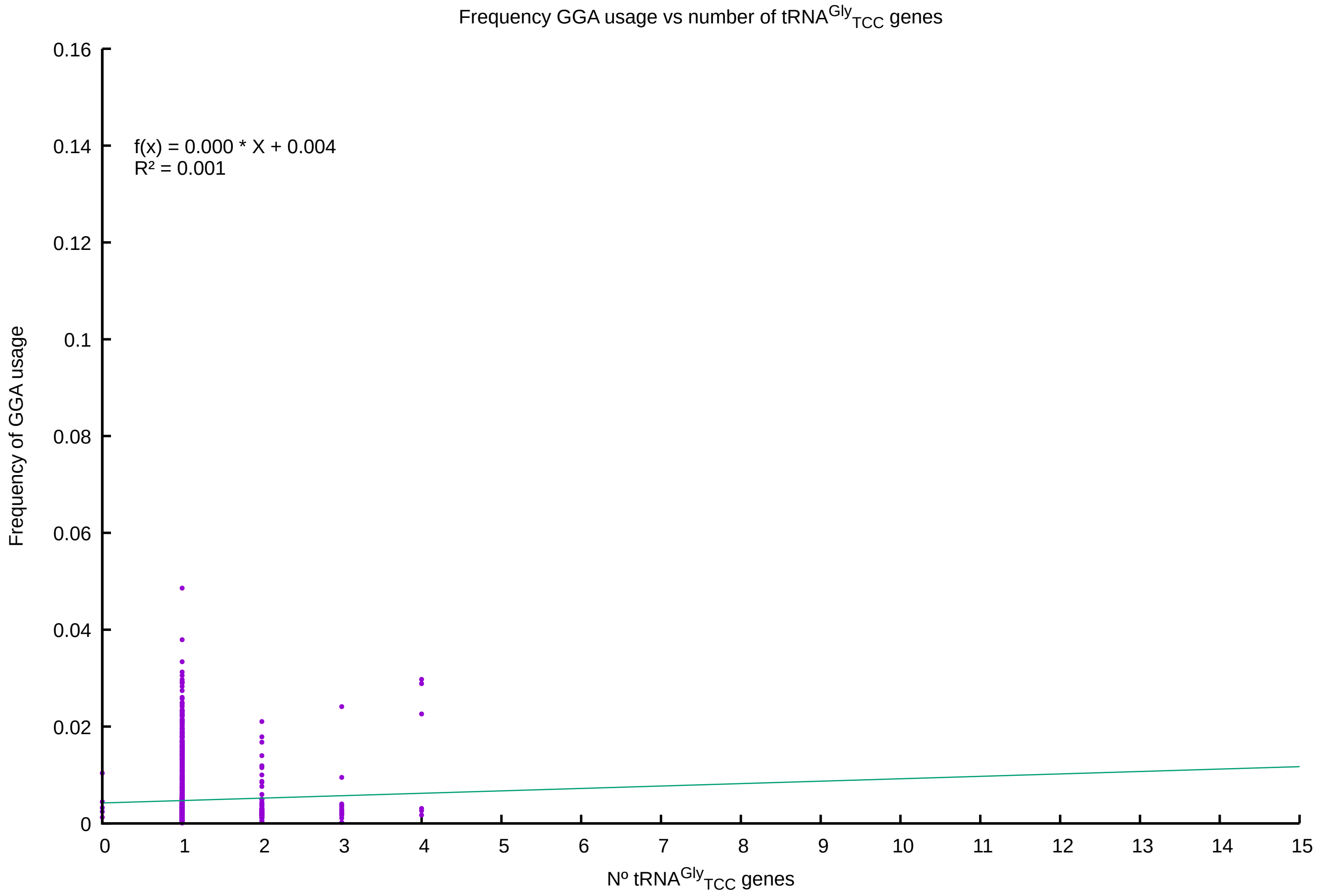

Frequency GGA usage vs number of tRNA^Gly_TCC genes
f(x) = 0.000 * X + 0.004
R² = 0.001
Frequency of GGA usage
Nº tRNA^Gly_TCC genes
0.16
0.14
0.12
0.1
0.08
0.06
0.04
0.02
0
0 1 2 3 4 5 6 7 8 9 10 11 12 13 14 15

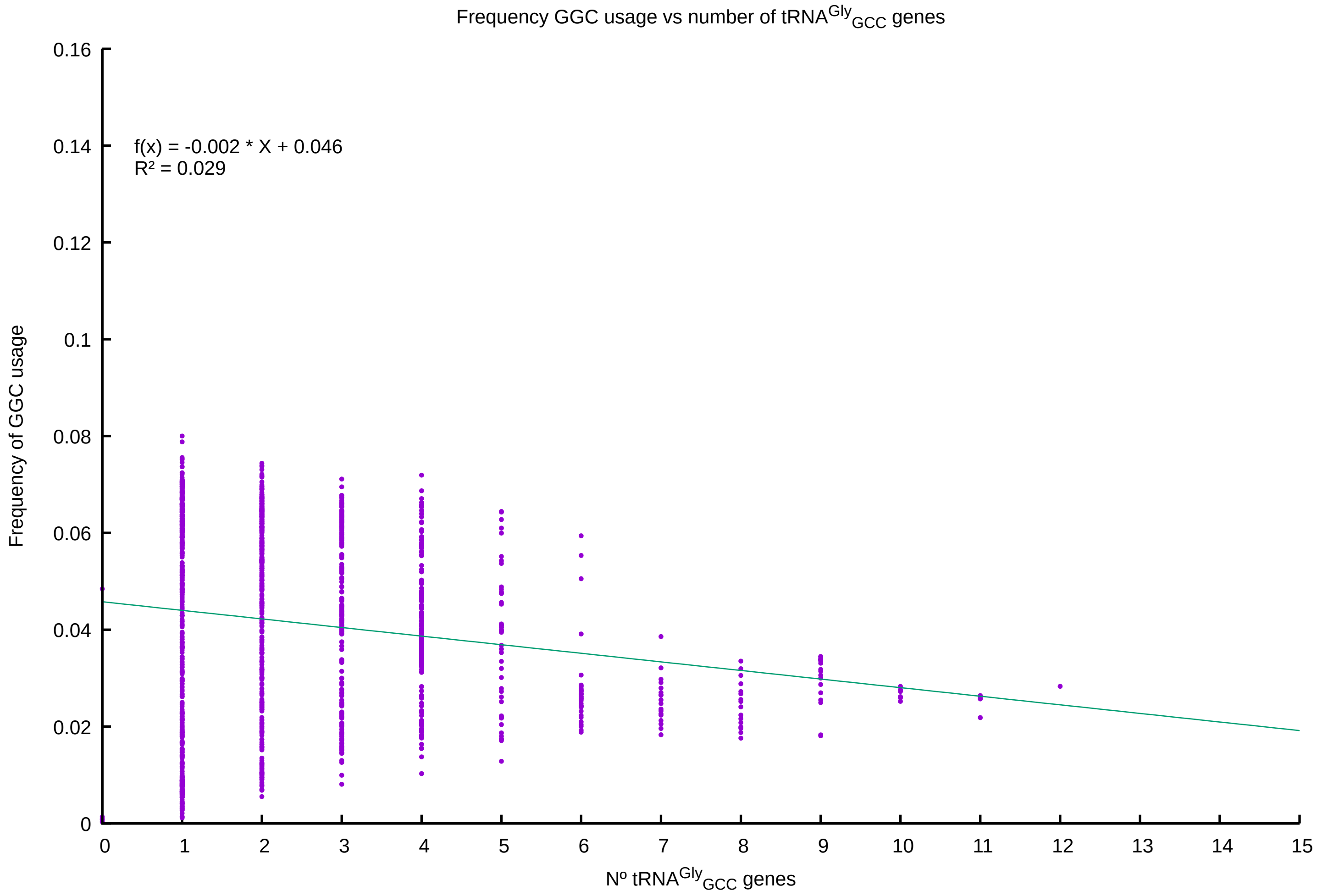

Frequency GGC usage vs number of tRNA$^{Gly}_{GCC}$ genes
f(x) = -0.002 * X + 0.046
R^2 = 0.029
Frequency of GGC usage
0.16
0.14
0.12
0.1
0.08
0.06
0.04
0.02
0
0 1 2 3 4 5 6 7 8 9 10 11 12 13 14 15
Nº tRNA$^{Gly}_{GCC}$ genes

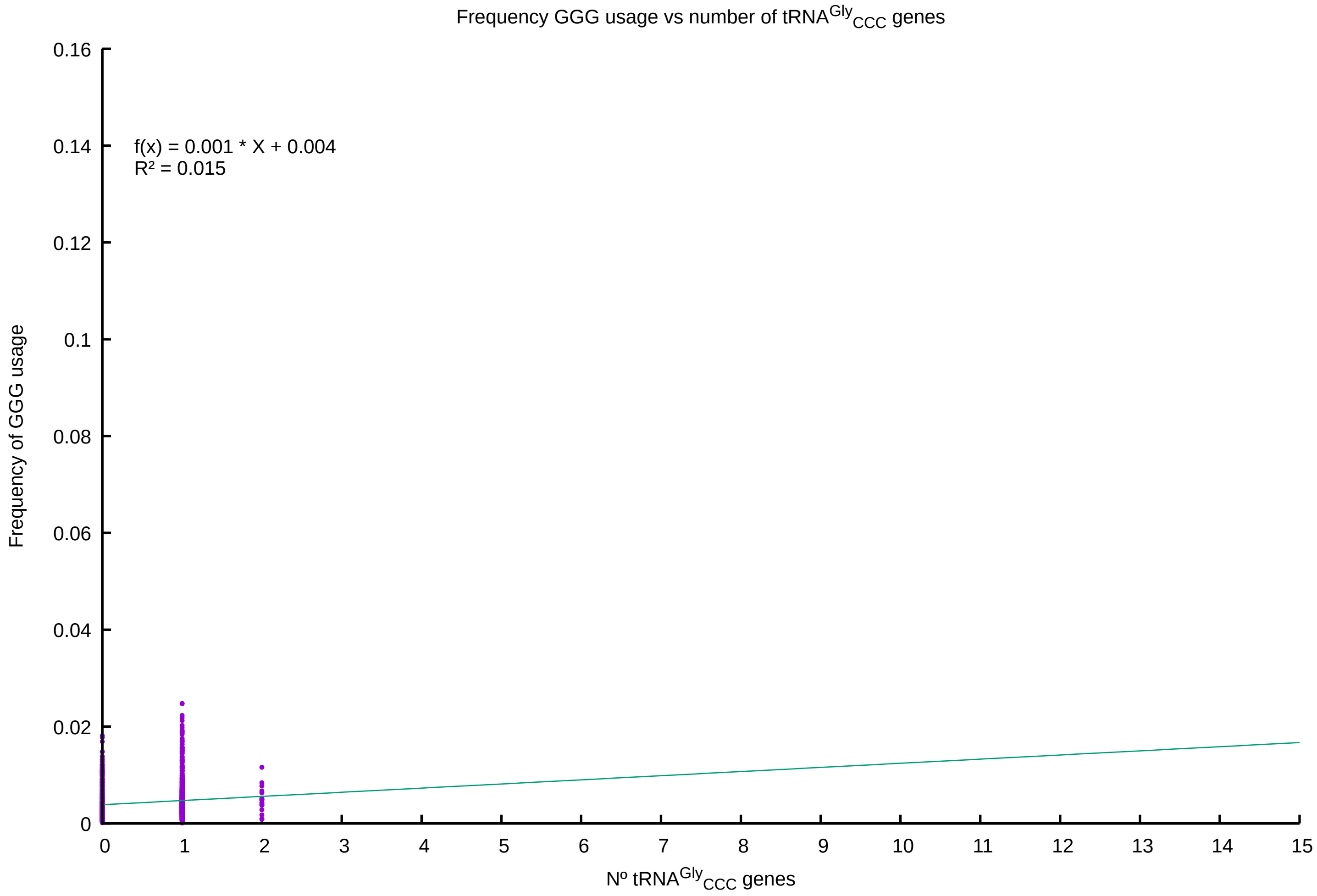

Frequency GGG usage vs number of tRNA$^{Gly}_{CCC}$ genes
f(x) = 0.001 * X + 0.004
R^2 = 0.015
Frequency of GGG usage
0.16
0.14
0.12
0.1
0.08
0.06
0.04
0.02
0
0 1 2 3 4 5 6 7 8 9 10 11 12 13 14 15
Nº tRNA$^{Gly}_{CCC}$ genes

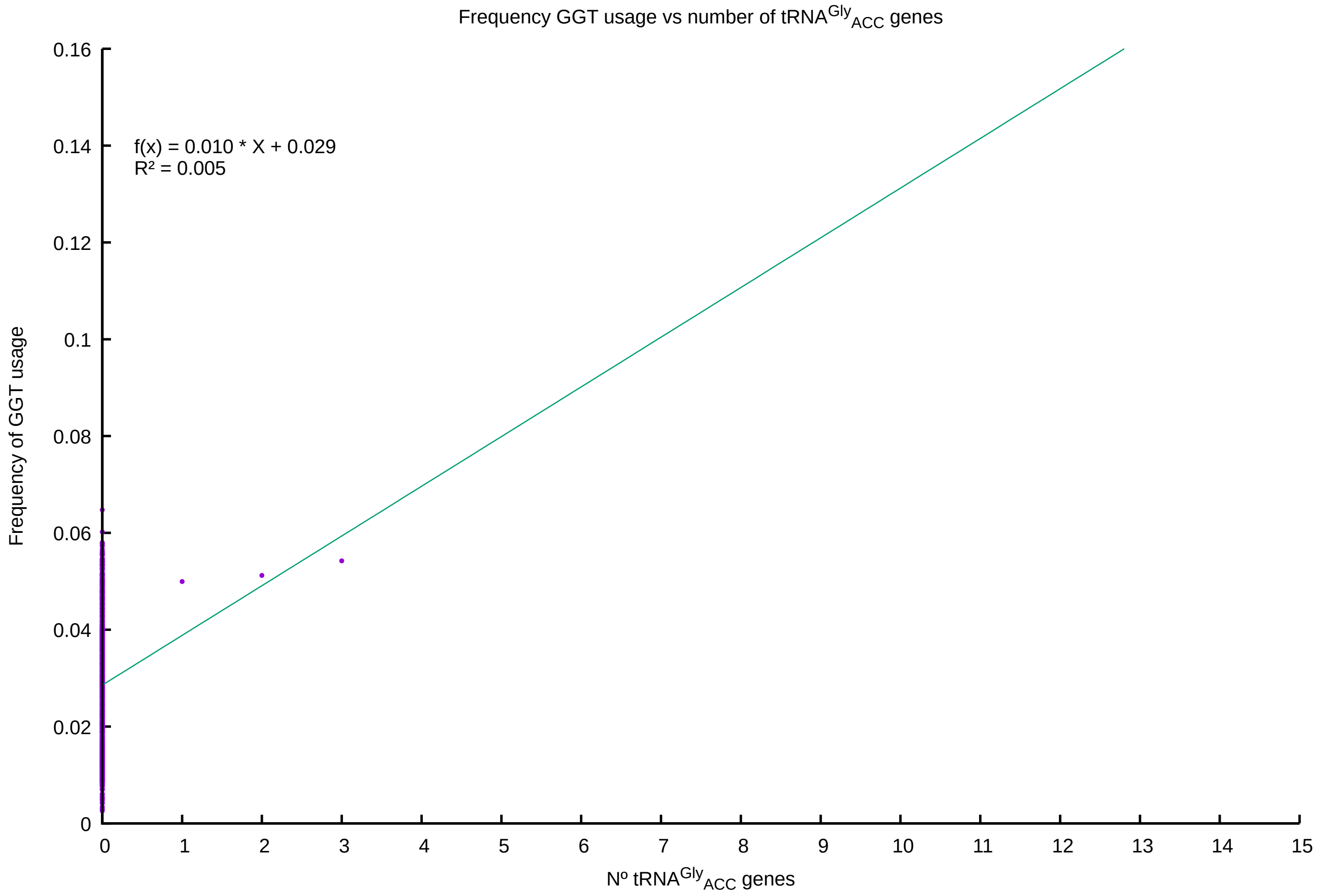

Frequency GGT usage vs number of tRNA^Gly_ACC genes
f(x) = 0.010 * X + 0.029
R² = 0.005
Frequency of GGT usage
Nº tRNA^Gly_ACC genes
0.16
0.14
0.12
0.1
0.08
0.06
0.04
0.02
0
0 1 2 3 4 5 6 7 8 9 10 11 12 13 14 15

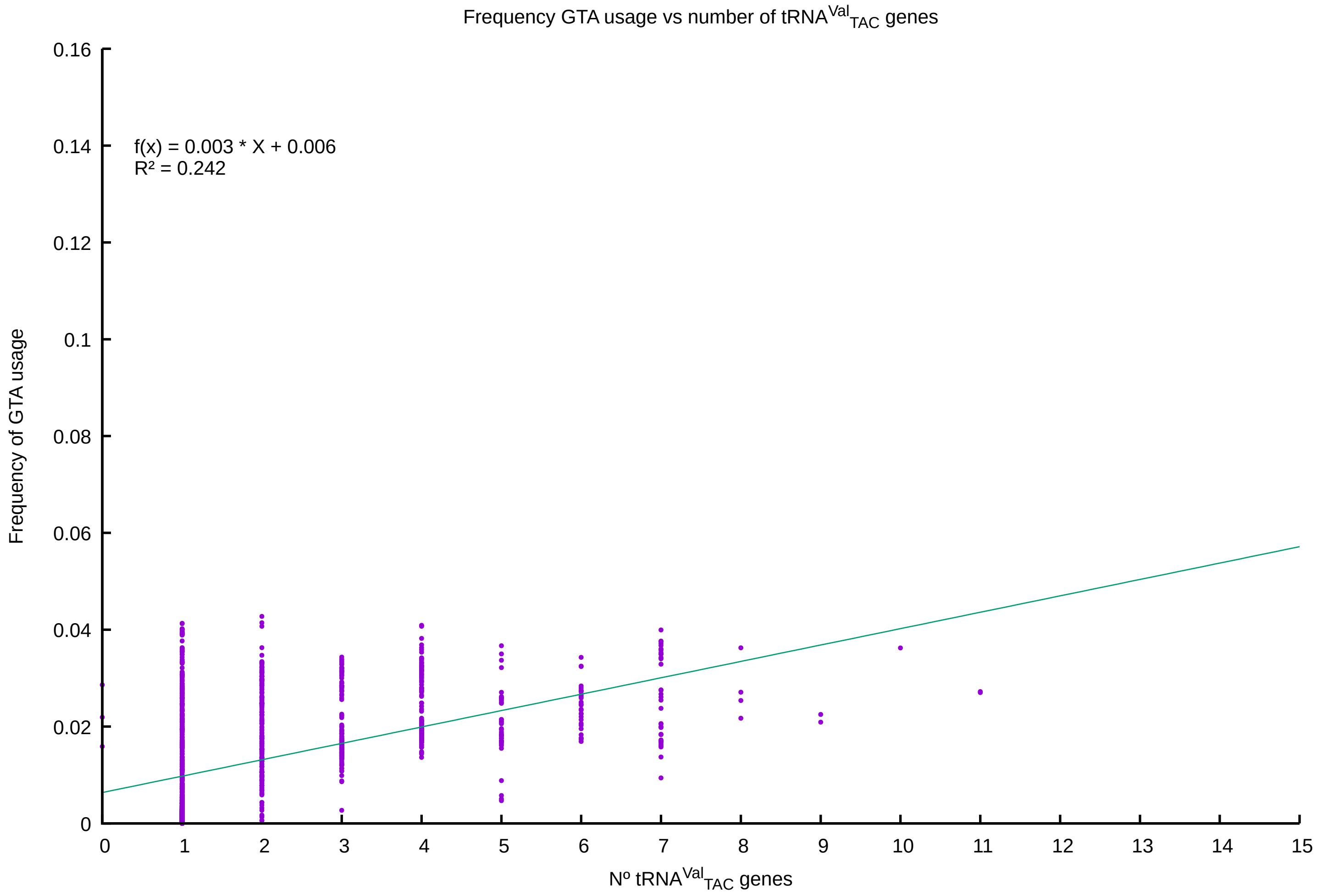
Frequency GTA usage vs number of tRNAValTAC genes
f(x) = 0.003 * X + 0.006
R² = 0.242
Frequency of GTA usage
0.16
0.14
0.12
0.1
0.08
0.06
0.04
0.02
0
0 1 2 3 4 5 6 7 8 9 10 11 12 13 14 15
Nº tRNAValTAC genes

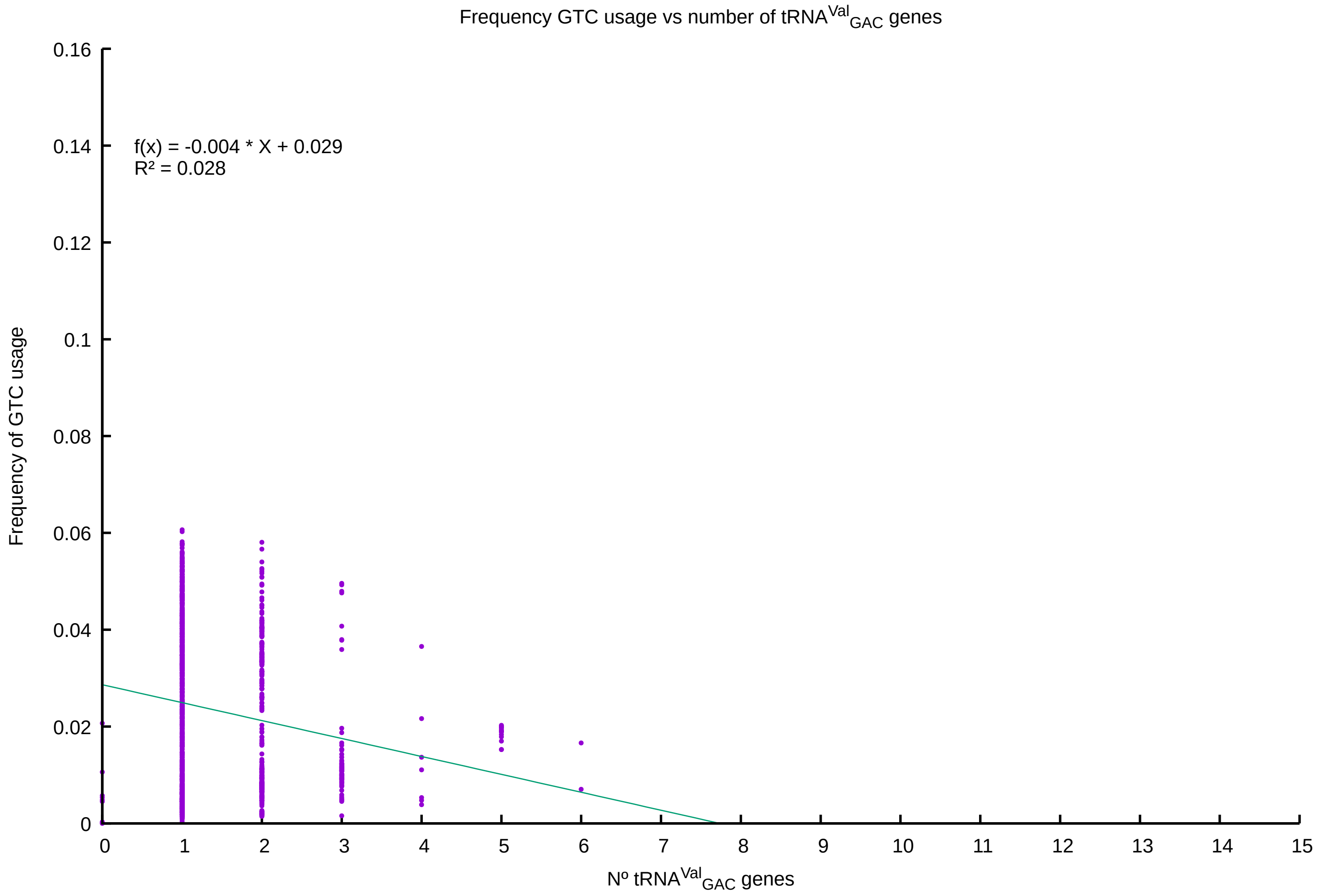
Frequency GTC usage vs number of tRNA Val GAC genes
f(x) = -0.004 * X + 0.029
R² = 0.028
Frequency of GTC usage
0.16
0.14
0.12
0.1
0.08
0.06
0.04
0.02
0
0 1 2 3 4 5 6 7 8 9 10 11 12 13 14 15
Nº tRNA Val GAC genes

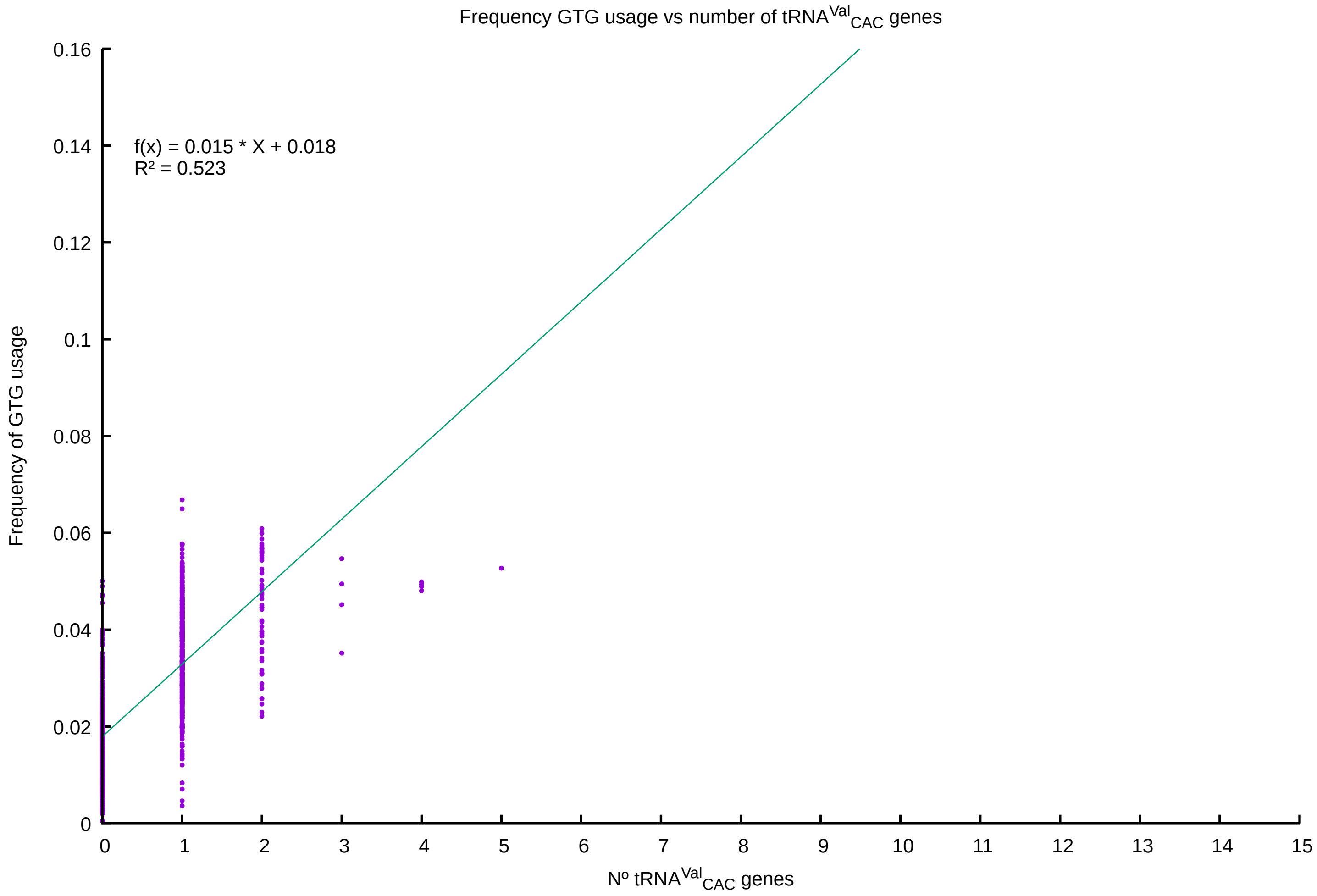
Frequency GTG usage vs number of tRNA Val CAC genes
f(x) = 0.015 * X + 0.018
R² = 0.523
Frequency of GTG usage
0.16
0.14
0.12
0.1
0.08
0.06
0.04
0.02
0
0 1 2 3 4 5 6 7 8 9 10 11 12 13 14 15
Nº tRNA Val CAC genes

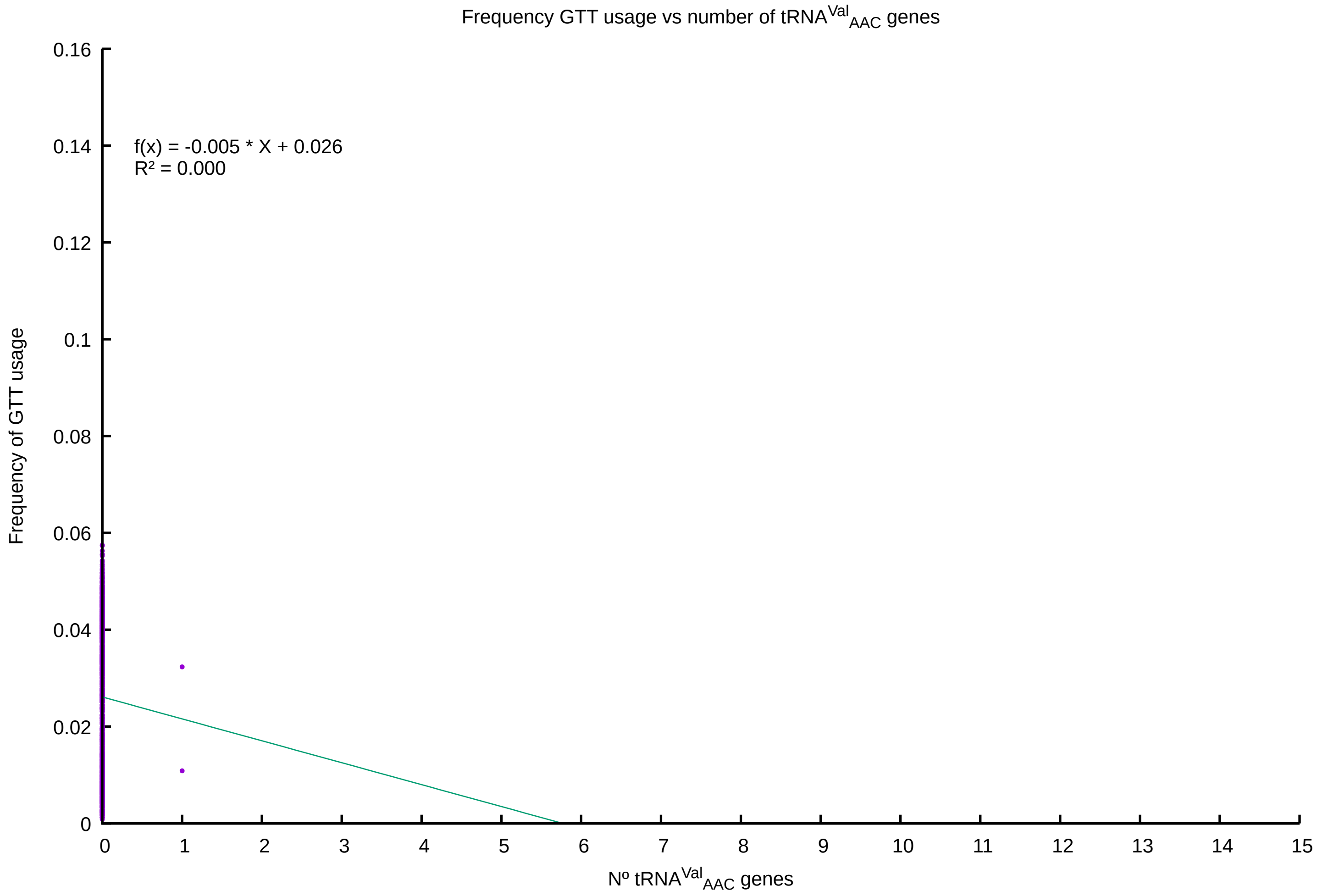
Frequency GTT usage vs number of tRNAValAAC genes
f(x) = -0.005 * X + 0.026
R² = 0.000
Frequency of GTT usage
0.16
0.14
0.12
0.1
0.08
0.06
0.04
0.02
0
0 1 2 3 4 5 6 7 8 9 10 11 12 13 14 15
Nº tRNAValAAC genes

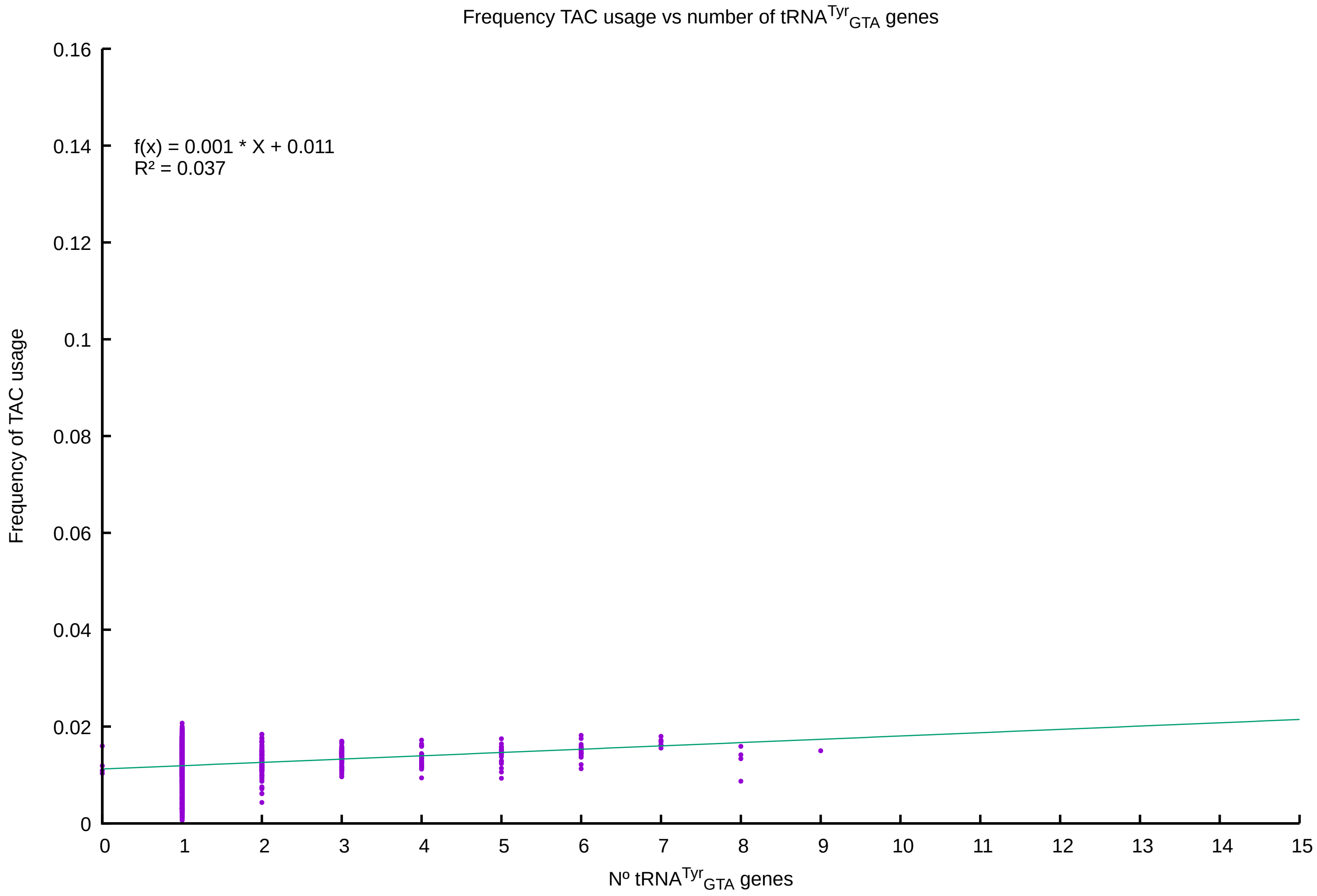

Frequency TAC usage vs number of tRNATyrGTA genes
f(x) = 0.001 * X + 0.011
R² = 0.037
Frequency of TAC usage
0.16
0.14
0.12
0.1
0.08
0.06
0.04
0.02
0
0 1 2 3 4 5 6 7 8 9 10 11 12 13 14 15
Nº tRNATyrGTA genes

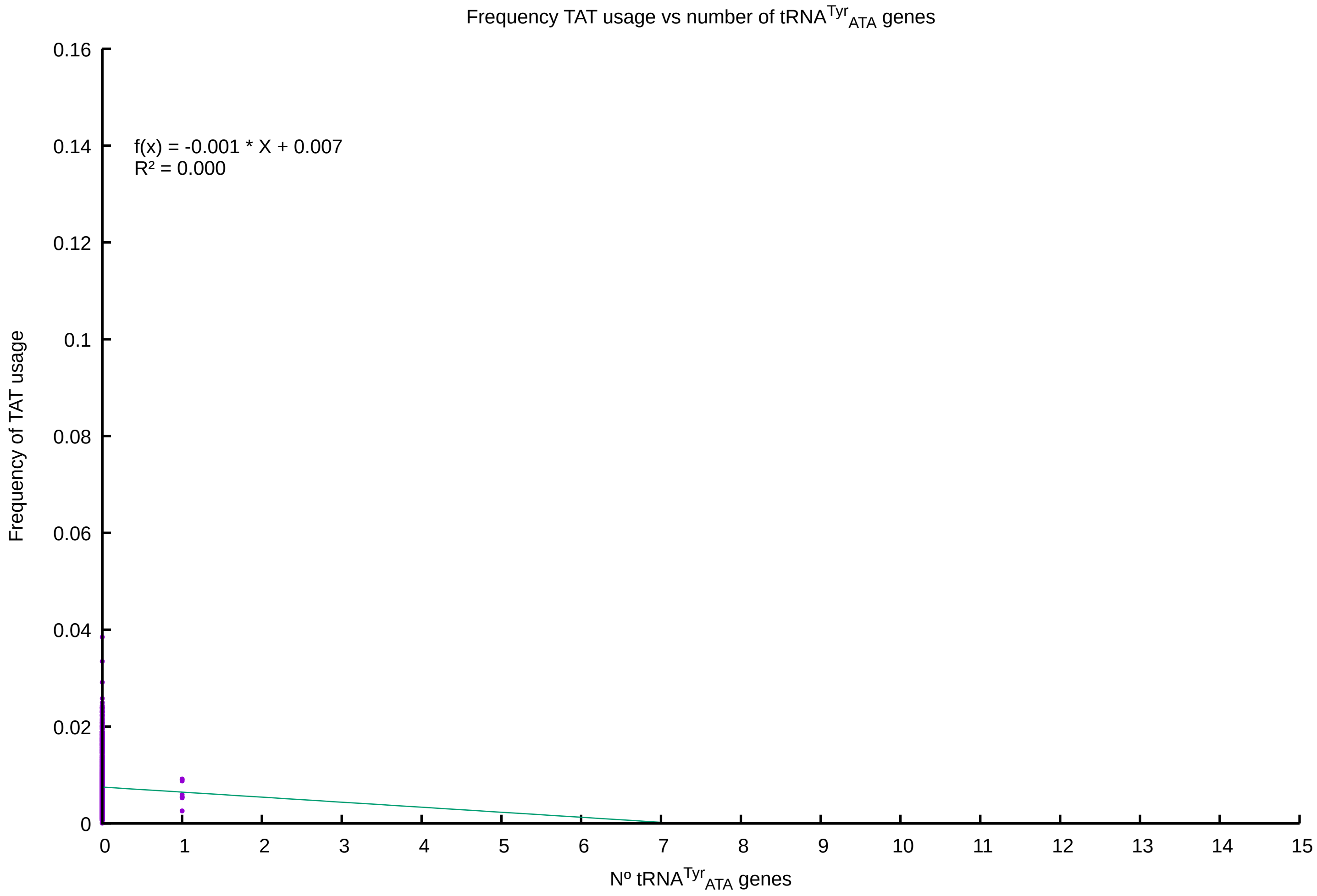

Frequency TAT usage vs number of tRNA^Tyr_ATA genes
f(x) = -0.001 * X + 0.007
R² = 0.000
Frequency of TAT usage
0.16
0.14
0.12
0.1
0.08
0.06
0.04
0.02
0
0 1 2 3 4 5 6 7 8 9 10 11 12 13 14 15
Nº tRNA^Tyr_ATA genes

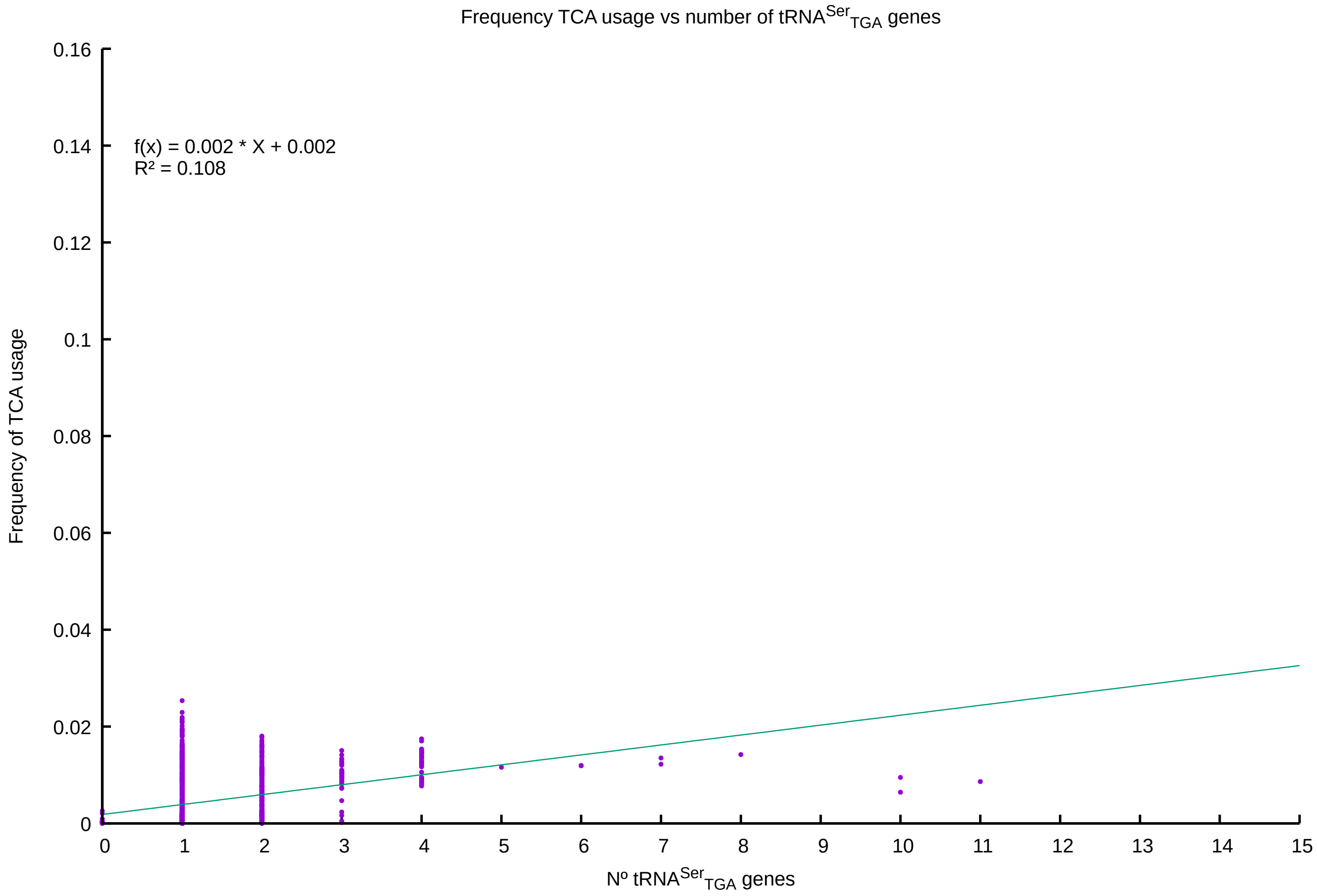

Frequency TCA usage vs number of tRNA Ser TGA genes
f(x) = 0.002 * X + 0.002
R² = 0.108
Frequency of TCA usage
0.16
0.14
0.12
0.1
0.08
0.06
0.04
0.02
0
0 1 2 3 4 5 6 7 8 9 10 11 12 13 14 15
Nº tRNA Ser TGA genes

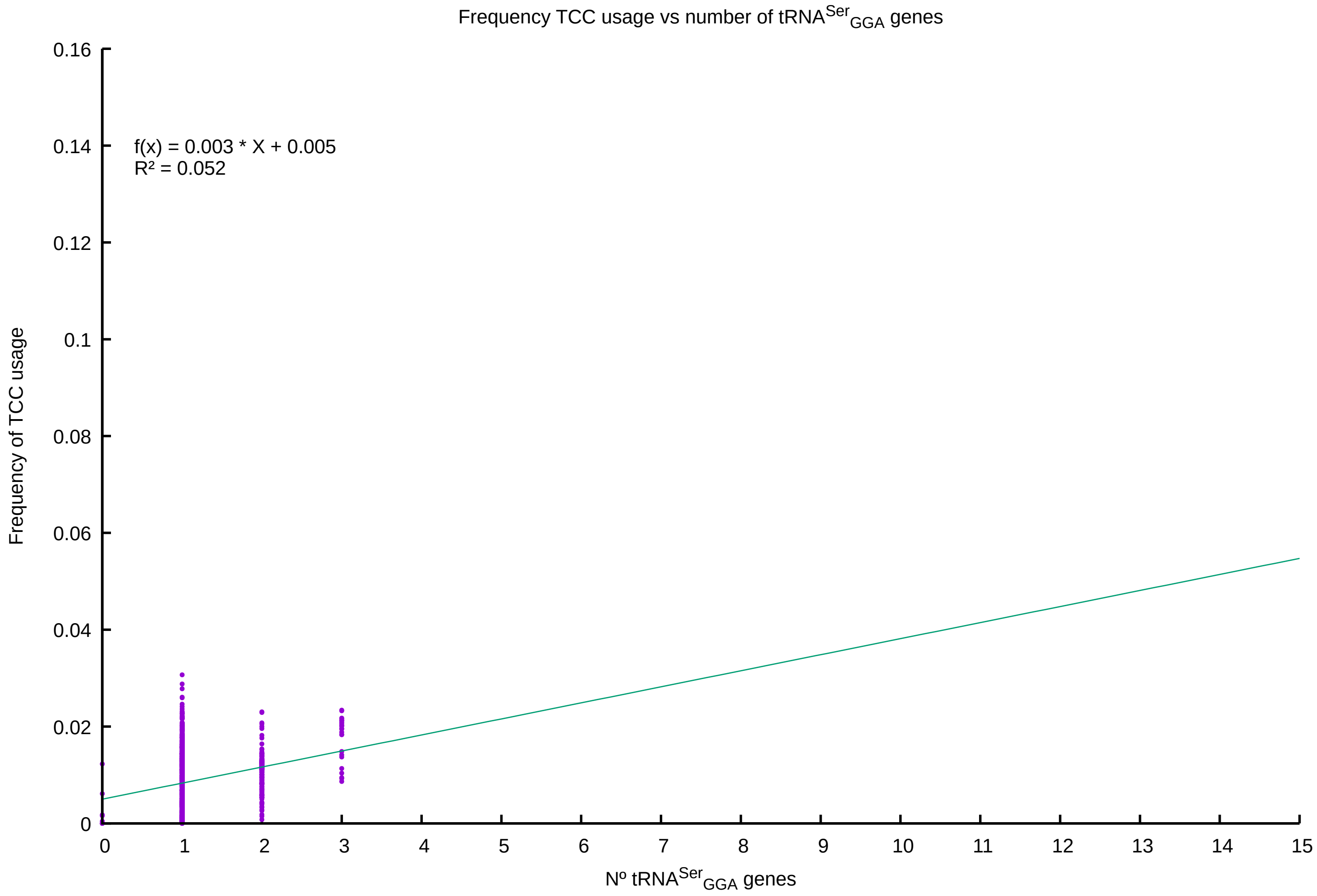

Frequency TCC usage vs number of tRNA$^{Ser}_{GGA}$ genes
f(x) = 0.003 * X + 0.005
R^2 = 0.052
Frequency of TCC usage
0
0.02
0.04
0.06
0.08
0.1
0.12
0.14
0.16
0
1
2
3
4
5
6
7
8
9
10
11
12
13
14
15
Nº tRNA$^{Ser}_{GGA}$ genes

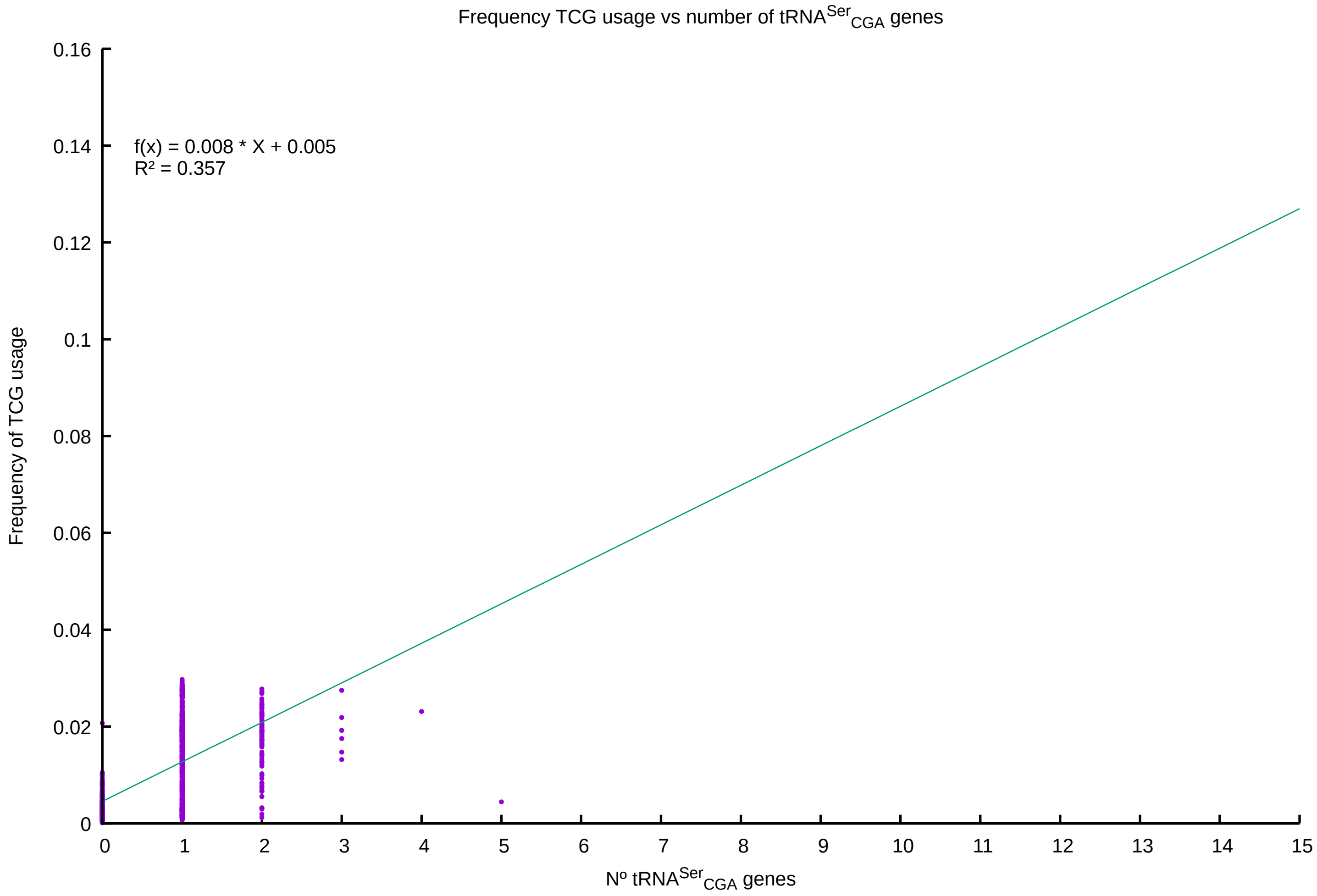
Frequency TCG usage vs number of tRNA^Ser_CGA genes
f(x) = 0.008 * X + 0.005
R² = 0.357
Frequency of TCG usage
0.16
0.14
0.12
0.1
0.08
0.06
0.04
0.02
0
0 1 2 3 4 5 6 7 8 9 10 11 12 13 14 15
Nº tRNA^Ser_CGA genes

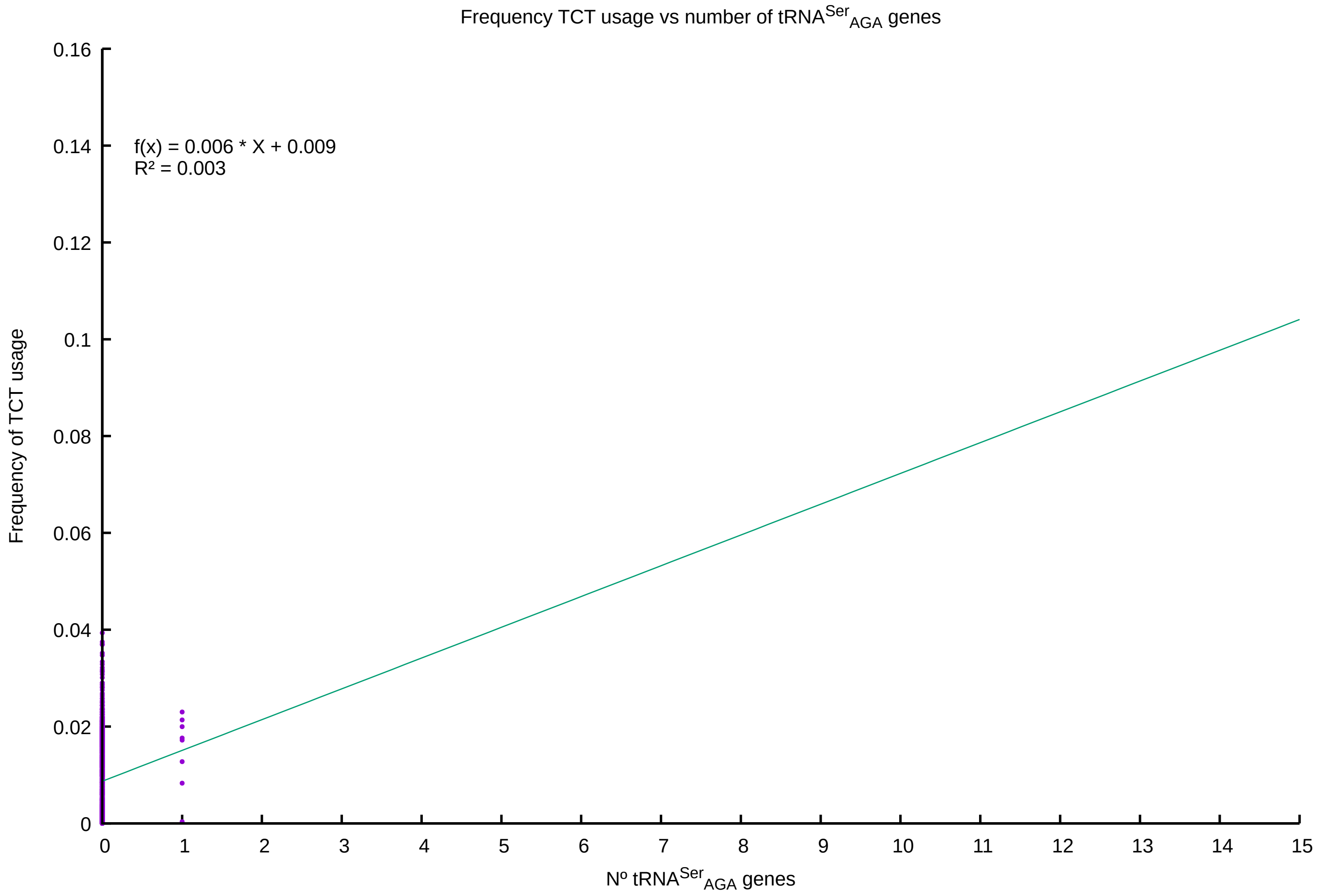
Frequency TCT usage vs number of tRNA^Ser_AGA genes
f(x) = 0.006 * X + 0.009
R² = 0.003
Frequency of TCT usage
0.16
0.14
0.12
0.1
0.08
0.06
0.04
0.02
0
0 1 2 3 4 5 6 7 8 9 10 11 12 13 14 15
Nº tRNA^Ser_AGA genes

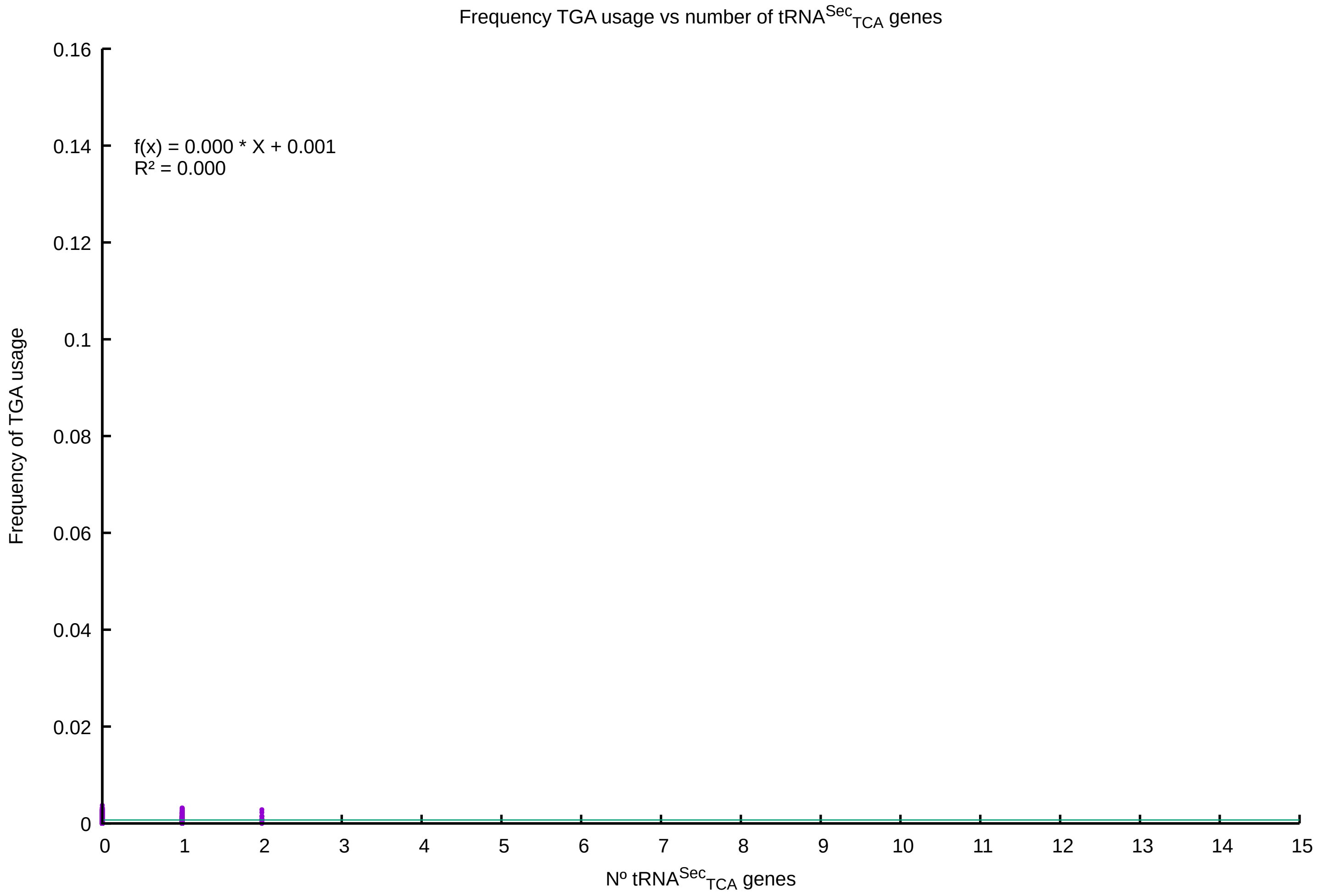
Frequency TGA usage vs number of tRNA^Sec_TCA genes
f(x) = 0.000 * X + 0.001
R² = 0.000
Frequency of TGA usage
0.16
0.14
0.12
0.1
0.08
0.06
0.04
0.02
0
0 1 2 3 4 5 6 7 8 9 10 11 12 13 14 15
Nº tRNA^Sec_TCA genes

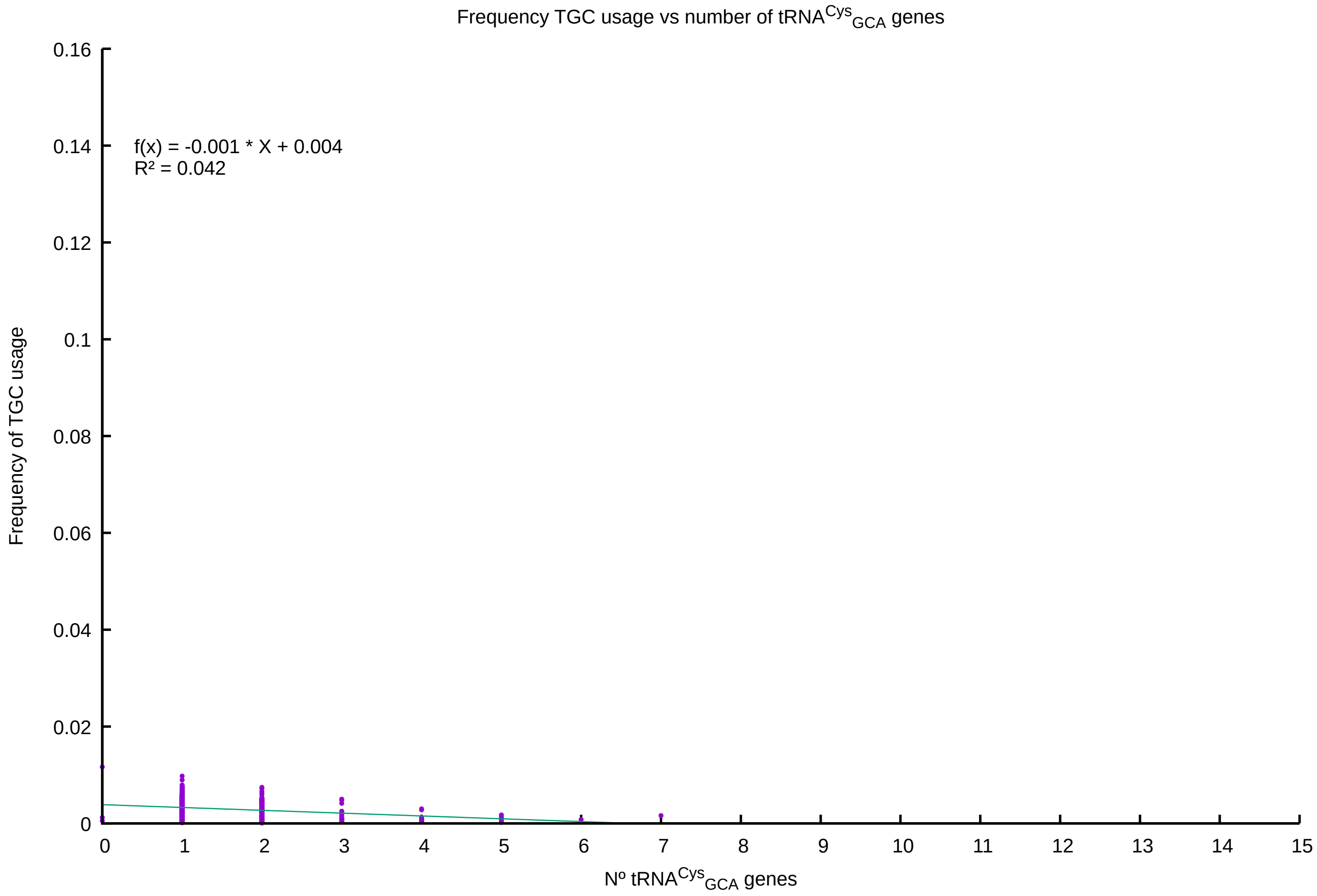
Frequency TGC usage vs number of tRNA$^{Cys}_{GCA}$ genes
f(x) = -0.001 * X + 0.004
R^2 = 0.042
Frequency of TGC usage
0.16
0.14
0.12
0.1
0.08
0.06
0.04
0.02
0
0 1 2 3 4 5 6 7 8 9 10 11 12 13 14 15
Nº tRNA$^{Cys}_{GCA}$ genes

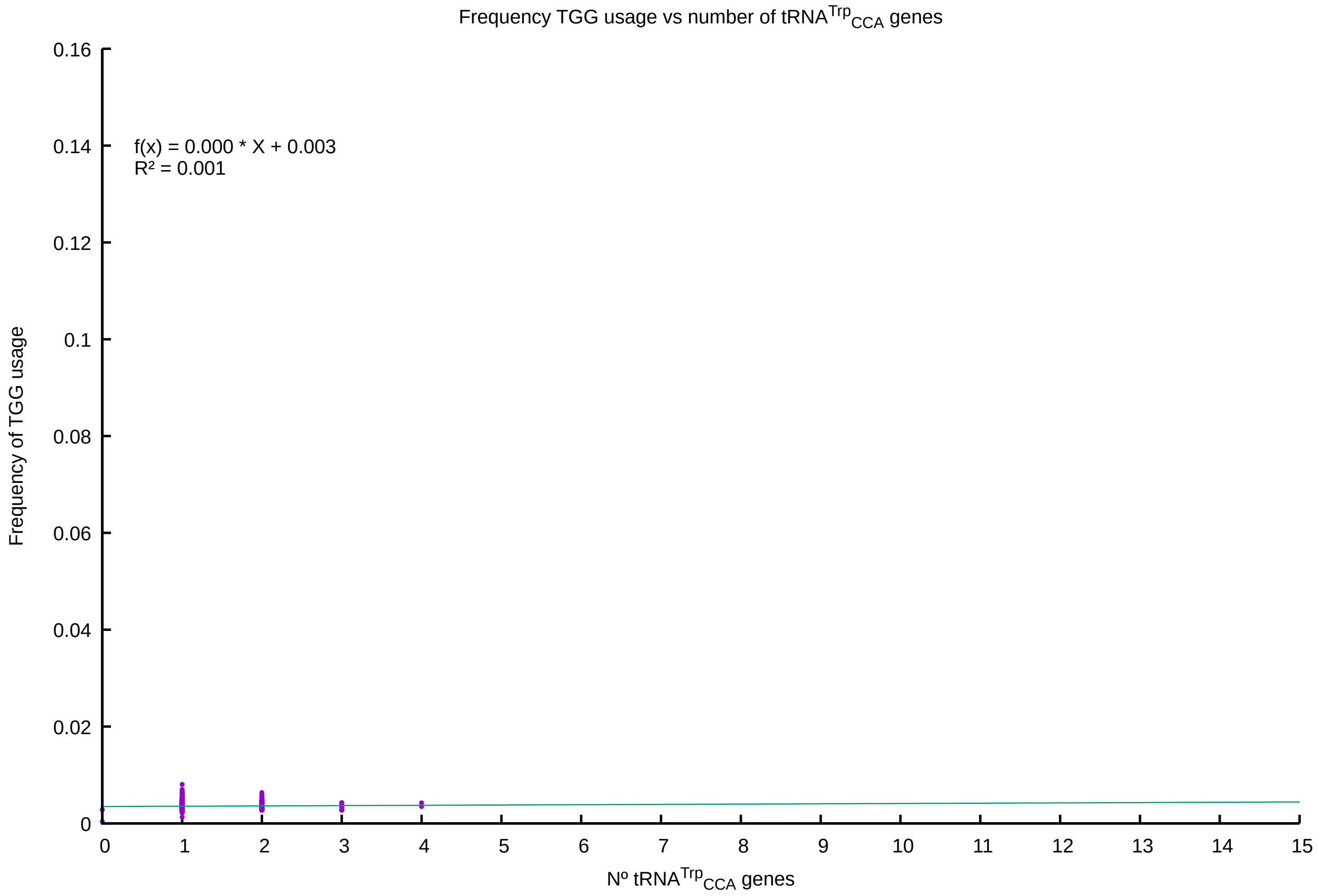

Frequency TGG usage vs number of tRNA$^{Trp}_{CCA}$ genes
f(x) = 0.000 * X + 0.003
R^2 = 0.001
Frequency of TGG usage
0.16
0.14
0.12
0.1
0.08
0.06
0.04
0.02
0
0 1 2 3 4 5 6 7 8 9 10 11 12 13 14 15
Nº tRNA$^{Trp}_{CCA}$ genes

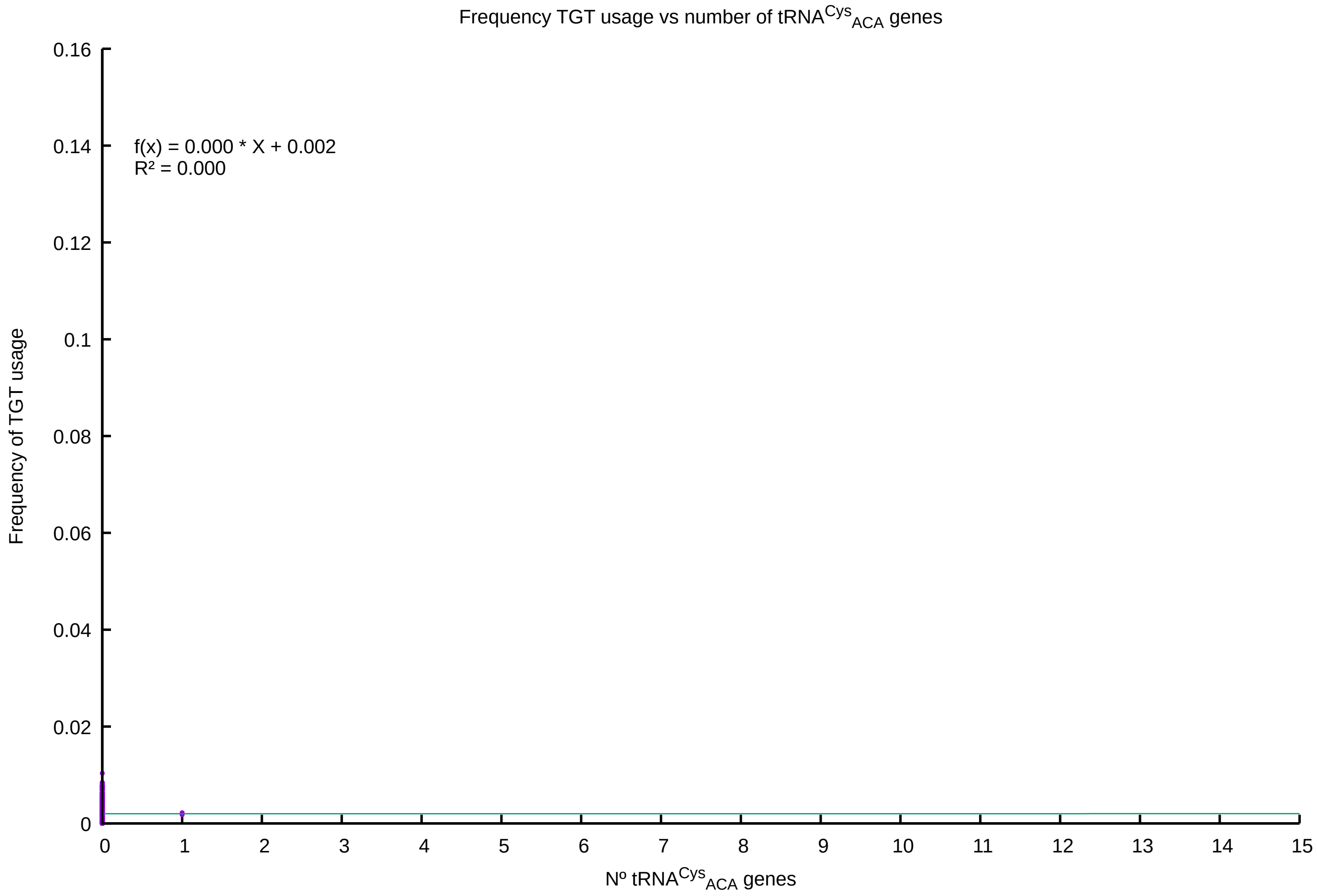

Frequency TGT usage vs number of tRNA^Cys_ACA genes
f(x) = 0.000 * X + 0.002
R² = 0.000
Frequency of TGT usage
0.16
0.14
0.12
0.1
0.08
0.06
0.04
0.02
0
0 1 2 3 4 5 6 7 8 9 10 11 12 13 14 15
Nº tRNA^Cys_ACA genes

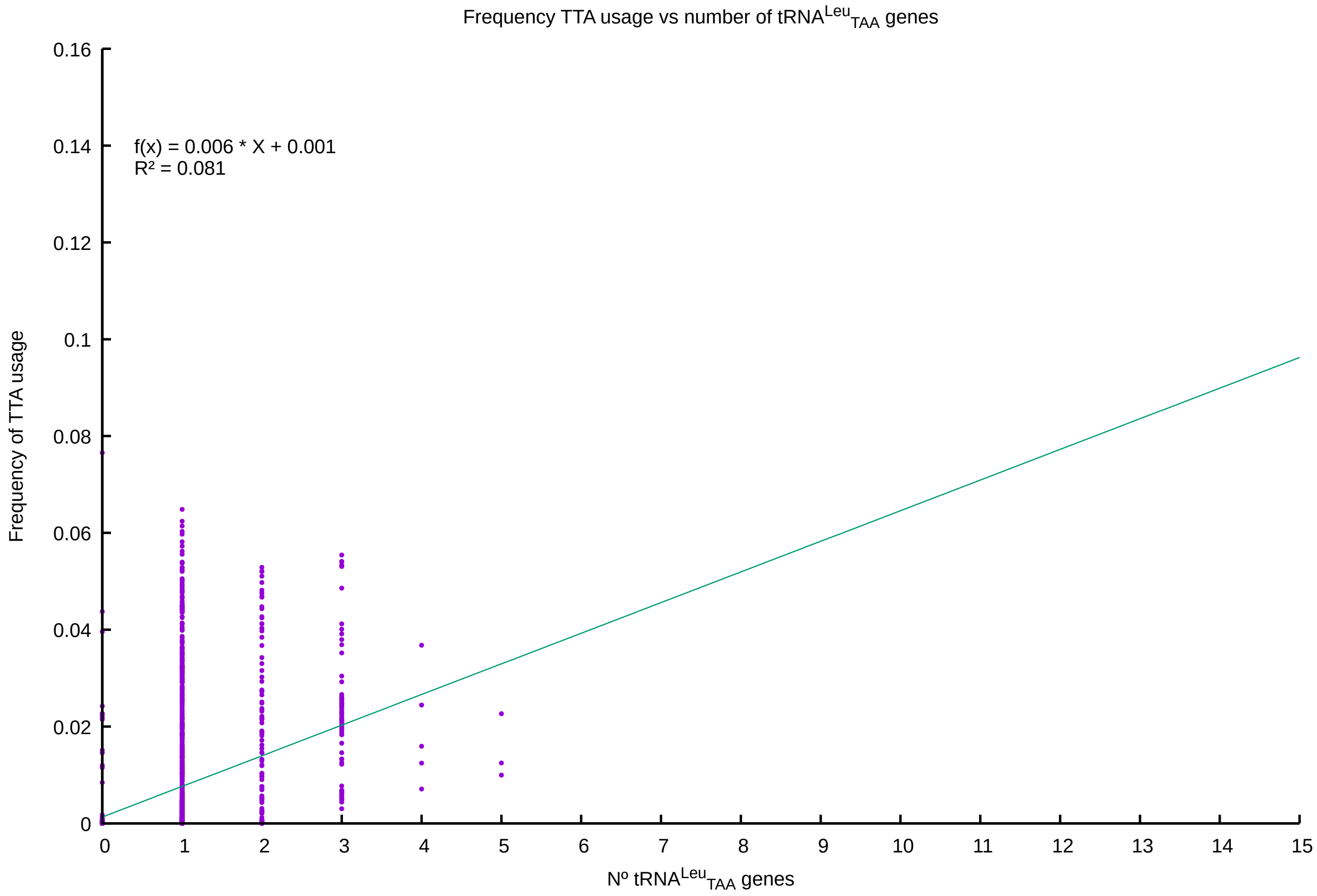

Frequency TTA usage vs number of tRNA Leu TAA genes
f(x) = 0.006 * X + 0.001
R² = 0.081
Frequency of TTA usage
0.16
0.14
0.12
0.1
0.08
0.06
0.04
0.02
0
0 1 2 3 4 5 6 7 8 9 10 11 12 13 14 15
Nº tRNA Leu TAA genes

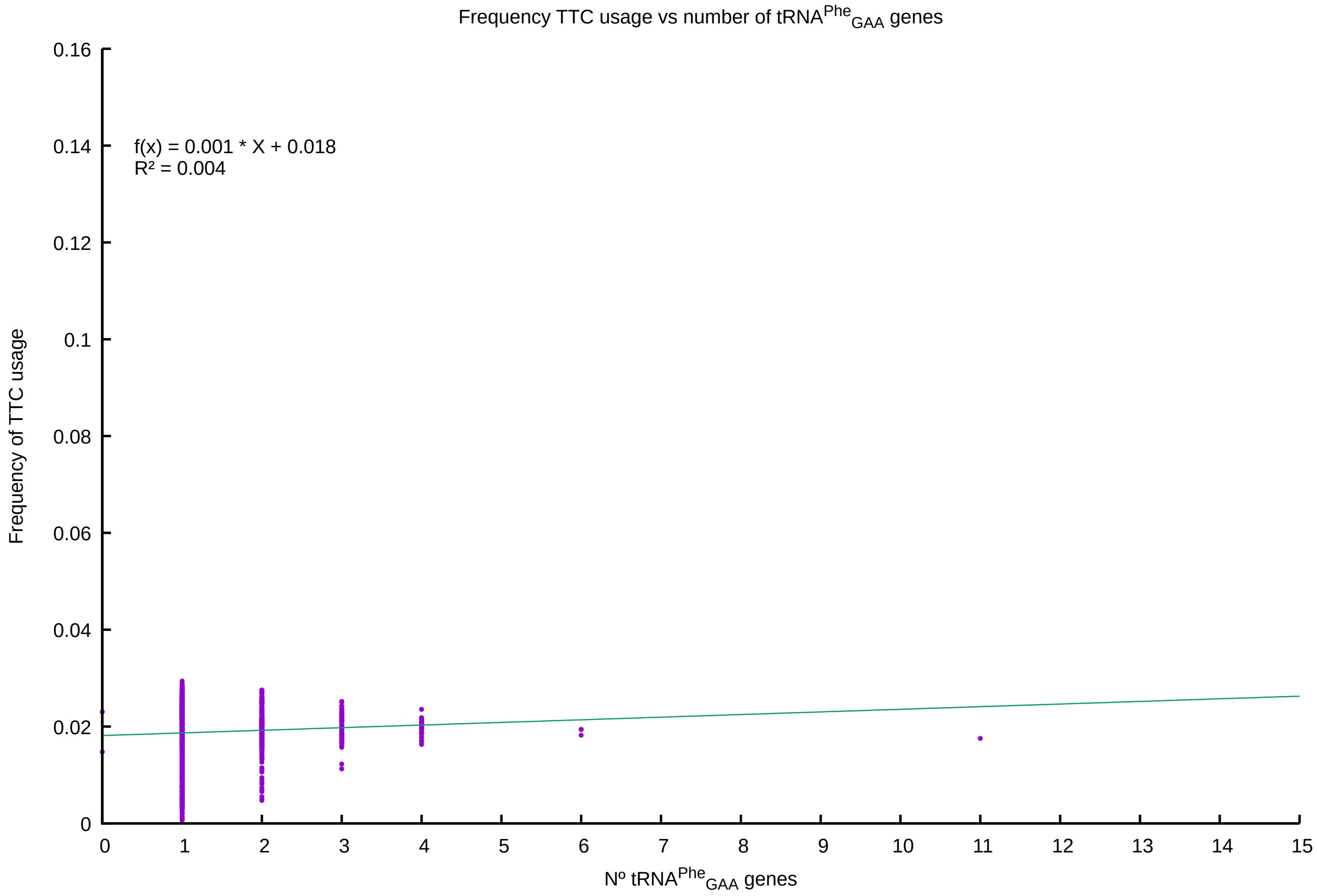

Frequency TTC usage vs number of tRNA Phe GAA genes
f(x) = 0.001 * X + 0.018
R² = 0.004
Frequency of TTC usage
Nº tRNA Phe GAA genes
0.16
0.14
0.12
0.1
0.08
0.06
0.04
0.02
0
0 1 2 3 4 5 6 7 8 9 10 11 12 13 14 15

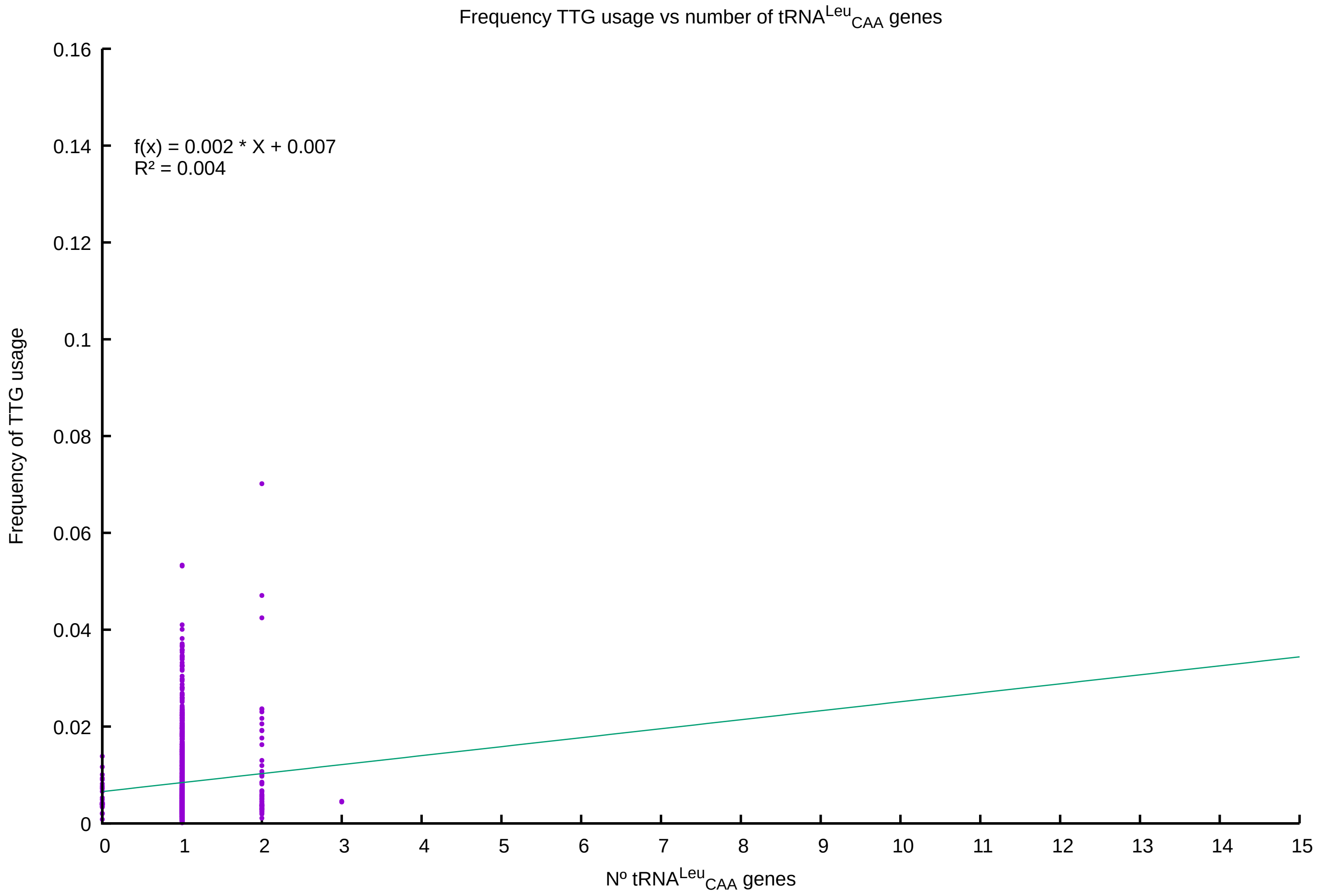
Frequency TTG usage vs number of tRNA$^{Leu}_{CAA}$ genes
f(x) = 0.002 * X + 0.007
R^2 = 0.004
Frequency of TTG usage
0.16
0.14
0.12
0.1
0.08
0.06
0.04
0.02
0
0 1 2 3 4 5 6 7 8 9 10 11 12 13 14 15
Nº tRNA$^{Leu}_{CAA}$ genes

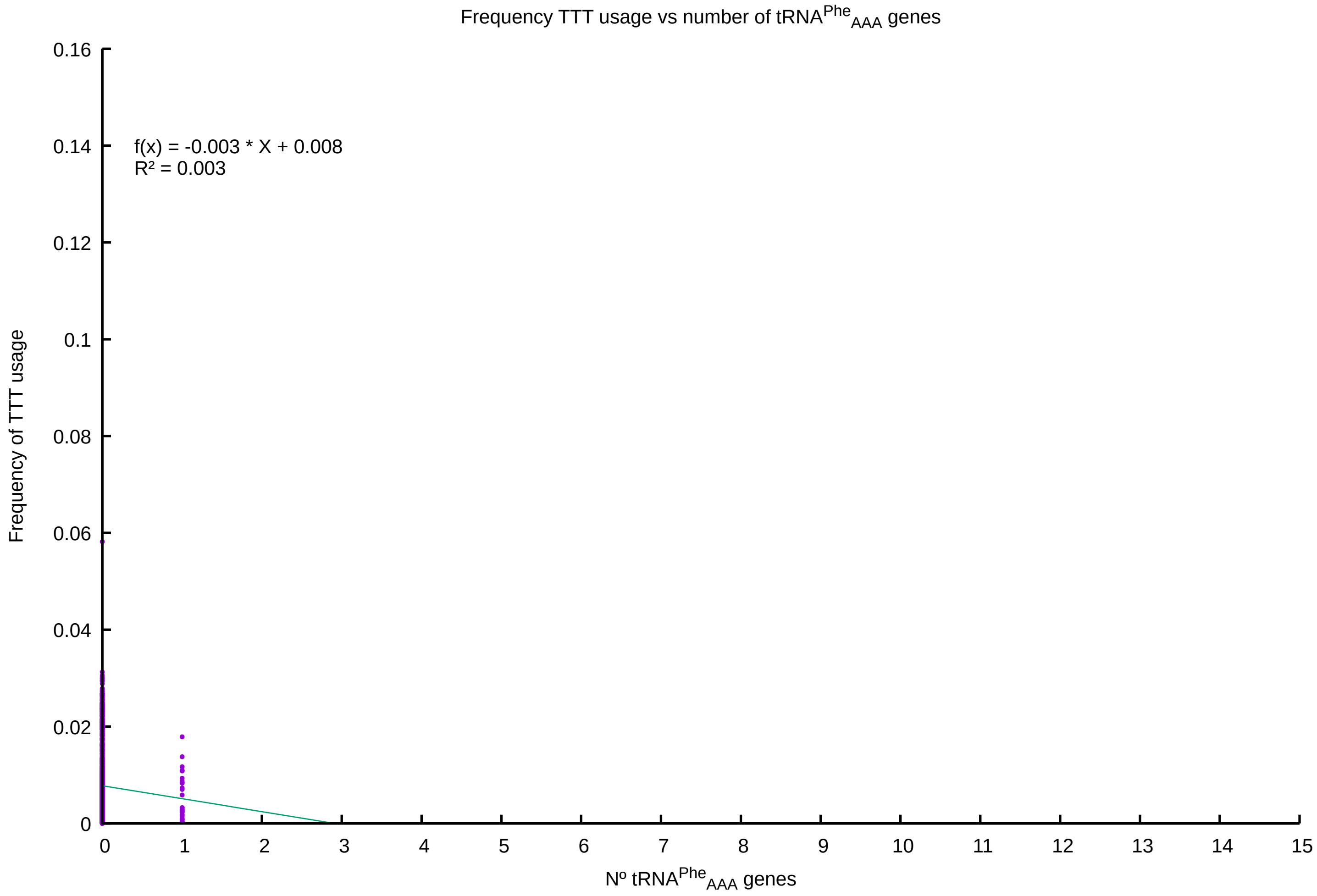

Frequency TTT usage vs number of tRNA^Phe_AAA genes
f(x) = -0.003 * X + 0.008
R² = 0.003
Frequency of TTT usage
0.16
0.14
0.12
0.1
0.08
0.06
0.04
0.02
0
0 1 2 3 4 5 6 7 8 9 10 11 12 13 14 15
Nº tRNA^Phe_AAA genes

Figure S25. Histogram of codon usage in high expression genes of genomes that present or lack genes involved in t^6A formation excluding potential false negatives. Top left graph: histogram of the usage of the indicated codon in highly expressed genes of proteobacterial genomes excluding 6 genomes where a potential false negative *tsaB* gene was found by tblastn analyses. **Top right graph:** box plot indicating the distribution of codon usage in the same population. **Lower graphs:** The collection of 1478 proteobacterial genomes, excluding those with potential a false negative *tsaB* gene, were separated in two groups, based on the presence (light blue) or absence (orange) of the indicated pattern in any gene of the analyzed genomes. Patterns are indicated using subscript numbers. See table S4 for a detailed description of the used patterns. **Left:** Data is represented in superposed histograms. **Right:** Data is represented in a boxplot. An horizontal bar was added when the two populations are different based on Wilcoxon-Mann-Whitney test and $p < 0.00005$. Whiskers include 95% of data.

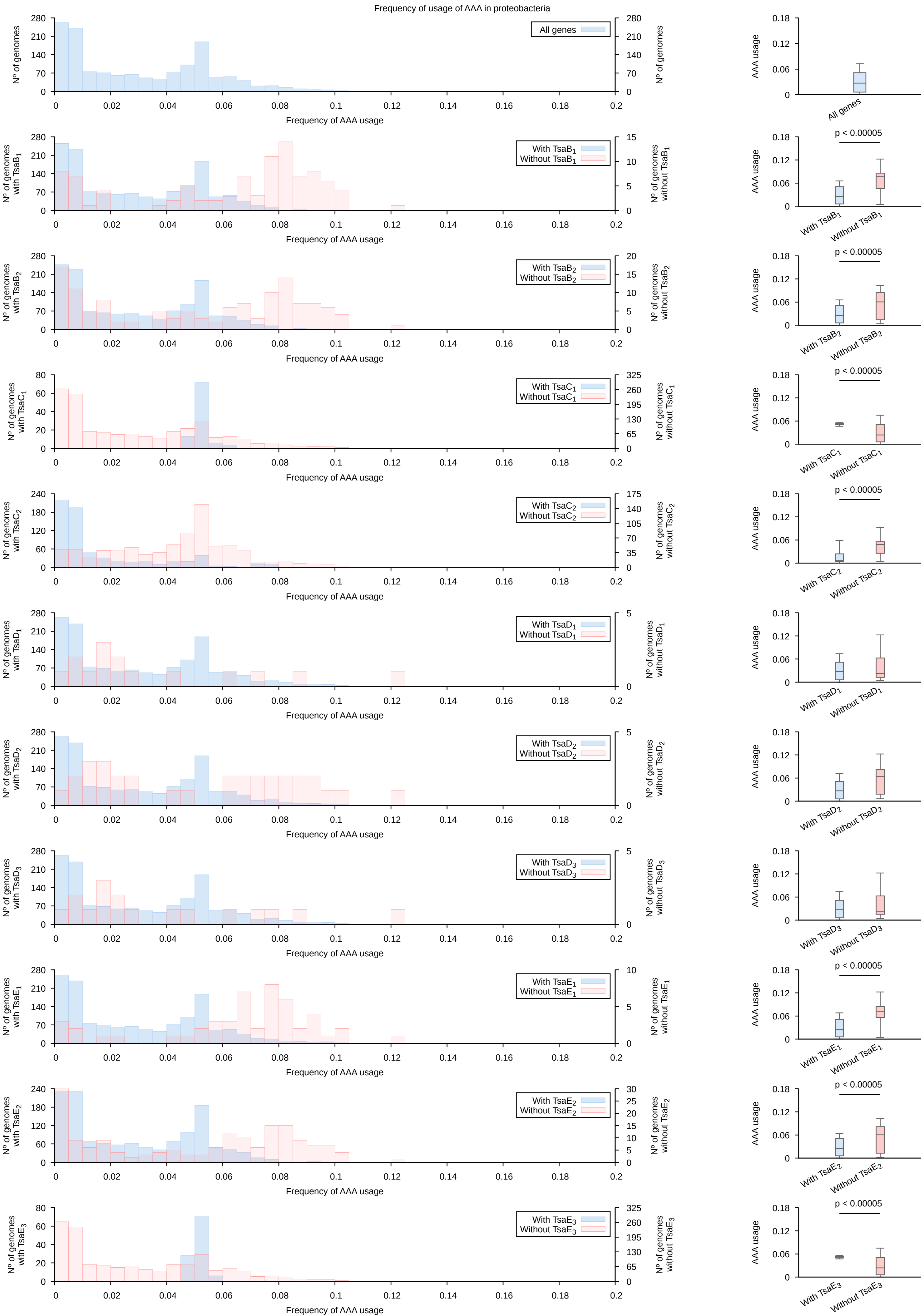
Frequency of usage of AAA in proteobacteria
All genes
Nº of genomes
Frequency of AAA usage
AAA usage
With TsaB1
Without TsaB1
Nº of genomes with TsaB1
Nº of genomes without TsaB1
p < 0.00005
With TsaB2
Without TsaB2
Nº of genomes with TsaB2
Nº of genomes without TsaB2
With TsaC1
Without TsaC1
Nº of genomes with TsaC1
Nº of genomes without TsaC1
With TsaC2
Without TsaC2
Nº of genomes with TsaC2
Nº of genomes without TsaC2
With TsaD1
Without TsaD1
Nº of genomes with TsaD1
Nº of genomes without TsaD1
With TsaD2
Without TsaD2
Nº of genomes with TsaD2
Nº of genomes without TsaD2
With TsaD3
Without TsaD3
Nº of genomes with TsaD3
Nº of genomes without TsaD3
With TsaE1
Without TsaE1
Nº of genomes with TsaE1
Nº of genomes without TsaE1
With TsaE2
Without TsaE2
Nº of genomes with TsaE2
Nº of genomes without TsaE2
With TsaE3
Without TsaE3
Nº of genomes with TsaE3
Nº of genomes without TsaE3

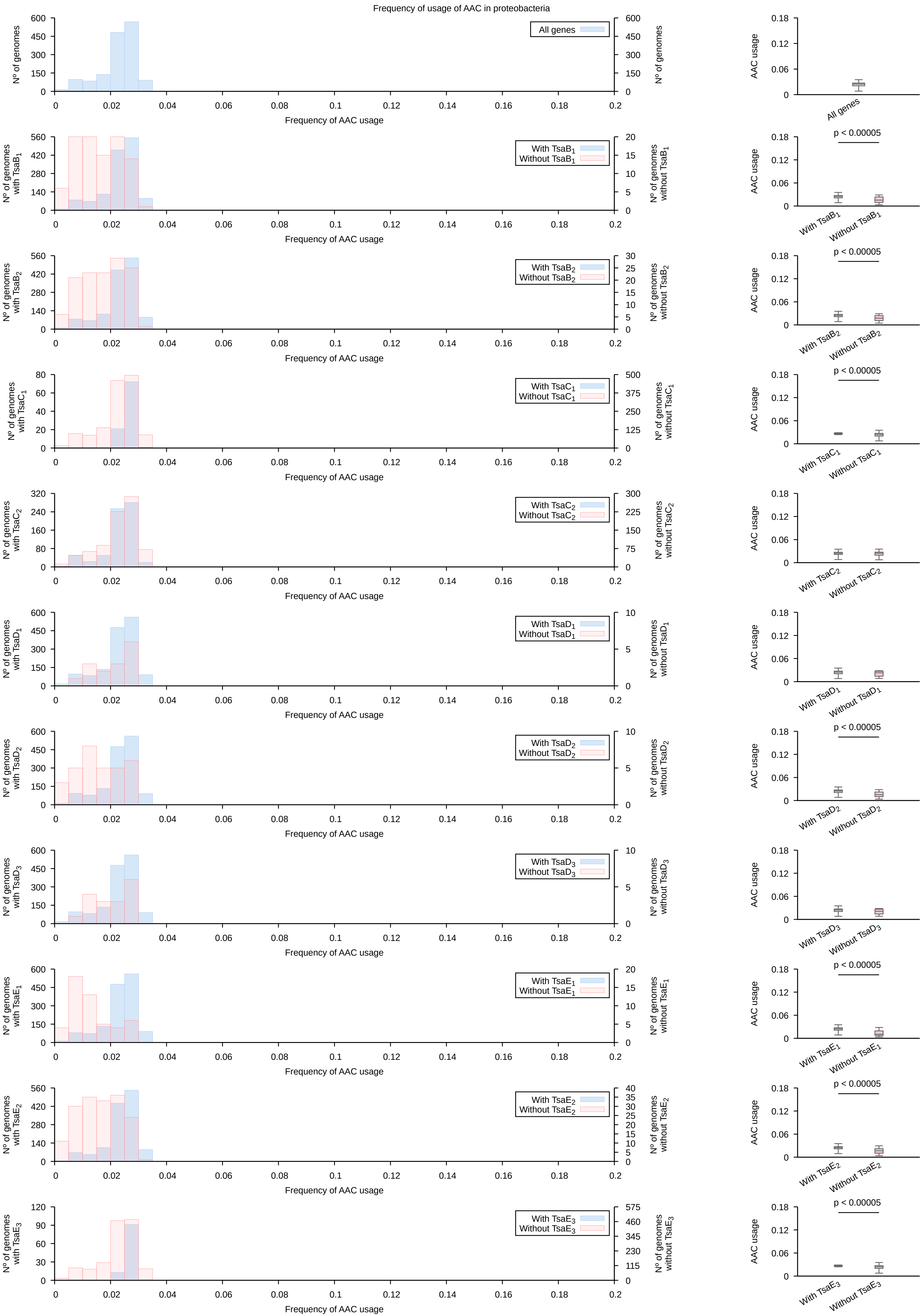
Frequency of usage of AAC in proteobacteria
Nº of genomes
All genes
Frequency of AAC usage
AAC usage
All genes
Nº of genomes with $TsaB_1$
Nº of genomes without $TsaB_1$
With $TsaB_1$
Without $TsaB_1$
p < 0.00005
Nº of genomes with $TsaB_2$
Nº of genomes without $TsaB_2$
With $TsaB_2$
Without $TsaB_2$
p < 0.00005
Nº of genomes with $TsaC_1$
Nº of genomes without $TsaC_1$
With $TsaC_1$
Without $TsaC_1$
p < 0.00005
Nº of genomes with $TsaC_2$
Nº of genomes without $TsaC_2$
With $TsaC_2$
Without $TsaC_2$
Nº of genomes with $TsaD_1$
Nº of genomes without $TsaD_1$
With $TsaD_1$
Without $TsaD_1$
Nº of genomes with $TsaD_2$
Nº of genomes without $TsaD_2$
With $TsaD_2$
Without $TsaD_2$
p < 0.00005
Nº of genomes with $TsaD_3$
Nº of genomes without $TsaD_3$
With $TsaD_3$
Without $TsaD_3$
Nº of genomes with $TsaE_1$
Nº of genomes without $TsaE_1$
With $TsaE_1$
Without $TsaE_1$
p < 0.00005
Nº of genomes with $TsaE_2$
Nº of genomes without $TsaE_2$
With $TsaE_2$
Without $TsaE_2$
p < 0.00005
Nº of genomes with $TsaE_3$
Nº of genomes without $TsaE_3$
With $TsaE_3$
Without $TsaE_3$
p < 0.00005

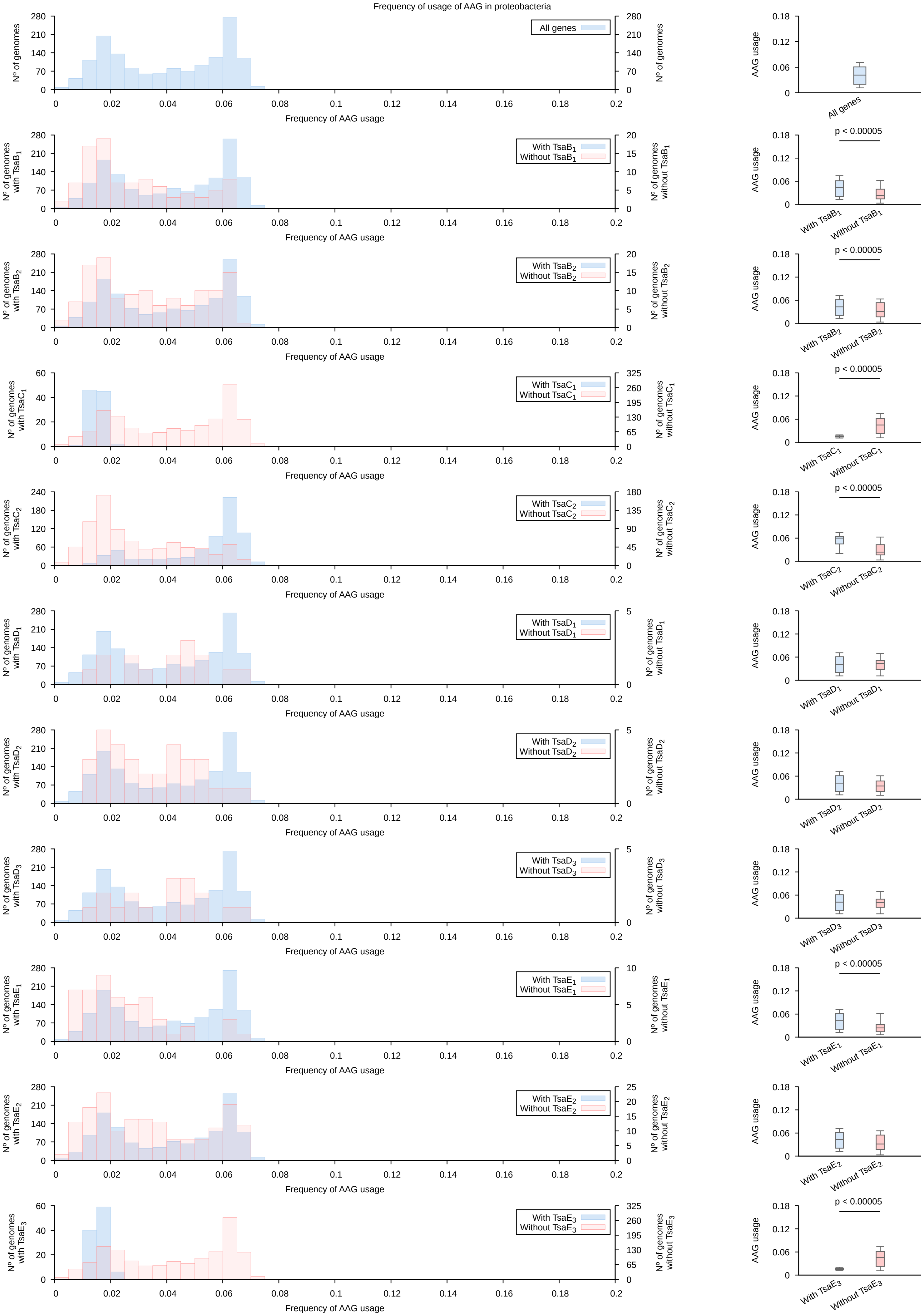

Frequency of usage of AAG in proteobacteria
All genes
Nº of genomes
Frequency of AAG usage
AAG usage
With TsaB1
Without TsaB1
Nº of genomes with TsaB1
Nº of genomes without TsaB1
With TsaB2
Without TsaB2
Nº of genomes with TsaB2
Nº of genomes without TsaB2
With TsaC1
Without TsaC1
Nº of genomes with TsaC1
Nº of genomes without TsaC1
With TsaC2
Without TsaC2
Nº of genomes with TsaC2
Nº of genomes without TsaC2
With TsaD1
Without TsaD1
Nº of genomes with TsaD1
Nº of genomes without TsaD1
With TsaD2
Without TsaD2
Nº of genomes with TsaD2
Nº of genomes without TsaD2
With TsaD3
Without TsaD3
Nº of genomes with TsaD3
Nº of genomes without TsaD3
With TsaE1
Without TsaE1
Nº of genomes with TsaE1
Nº of genomes without TsaE1
With TsaE2
Without TsaE2
Nº of genomes with TsaE2
Nº of genomes without TsaE2
With TsaE3
Without TsaE3
Nº of genomes with TsaE3
Nº of genomes without TsaE3
p < 0.00005

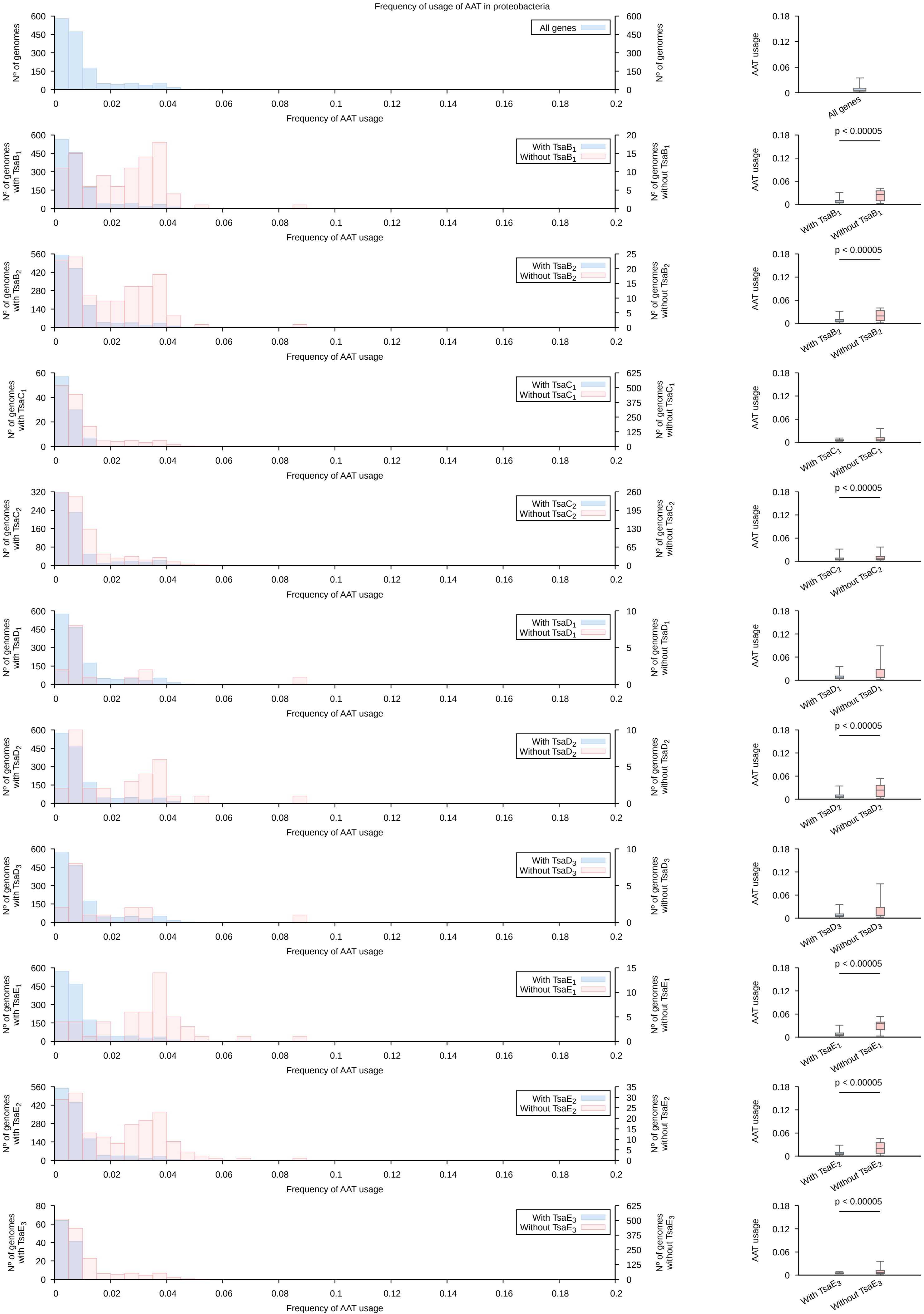

Frequency of usage of AAT in proteobacteria
Nº of genomes
All genes
Frequency of AAT usage
AAT usage
All genes
Nº of genomes with TsaB1
With TsaB1
Without TsaB1
Nº of genomes without TsaB1
p < 0.00005
Nº of genomes with TsaB2
With TsaB2
Without TsaB2
Nº of genomes without TsaB2
Nº of genomes with TsaC1
With TsaC1
Without TsaC1
Nº of genomes without TsaC1
Nº of genomes with TsaC2
With TsaC2
Without TsaC2
Nº of genomes without TsaC2
Nº of genomes with TsaD1
With TsaD1
Without TsaD1
Nº of genomes without TsaD1
Nº of genomes with TsaD2
With TsaD2
Without TsaD2
Nº of genomes without TsaD2
Nº of genomes with TsaD3
With TsaD3
Without TsaD3
Nº of genomes without TsaD3
Nº of genomes with TsaE1
With TsaE1
Without TsaE1
Nº of genomes without TsaE1
Nº of genomes with TsaE2
With TsaE2
Without TsaE2
Nº of genomes without TsaE2
Nº of genomes with TsaE3
With TsaE3
Without TsaE3
Nº of genomes without TsaE3
0
0.02
0.04
0.06
0.08
0.1
0.12
0.14
0.16
0.18
0.2

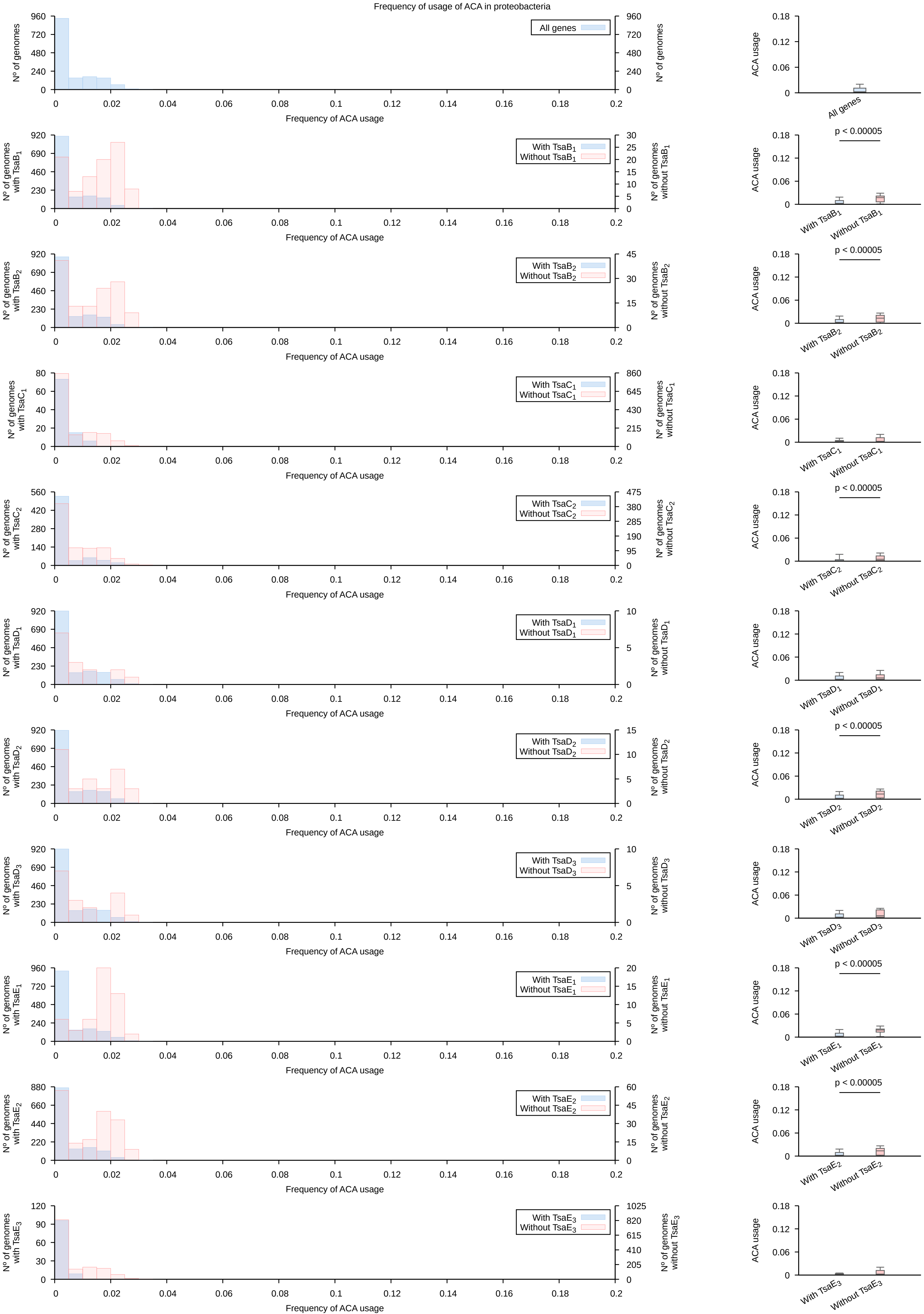

Frequency of usage of ACA in proteobacteria
Nº of genomes
Frequency of ACA usage
ACA usage
All genes
Nº of genomes with TsaB1
Nº of genomes without TsaB1
With TsaB1
Without TsaB1
p < 0.00005
With TsaB2
Without TsaB2
With TsaC1
Without TsaC1
With TsaC2
Without TsaC2
With TsaD1
Without TsaD1
With TsaD2
Without TsaD2
With TsaD3
Without TsaD3
With TsaE1
Without TsaE1
With TsaE2
Without TsaE2
With TsaE3
Without TsaE3

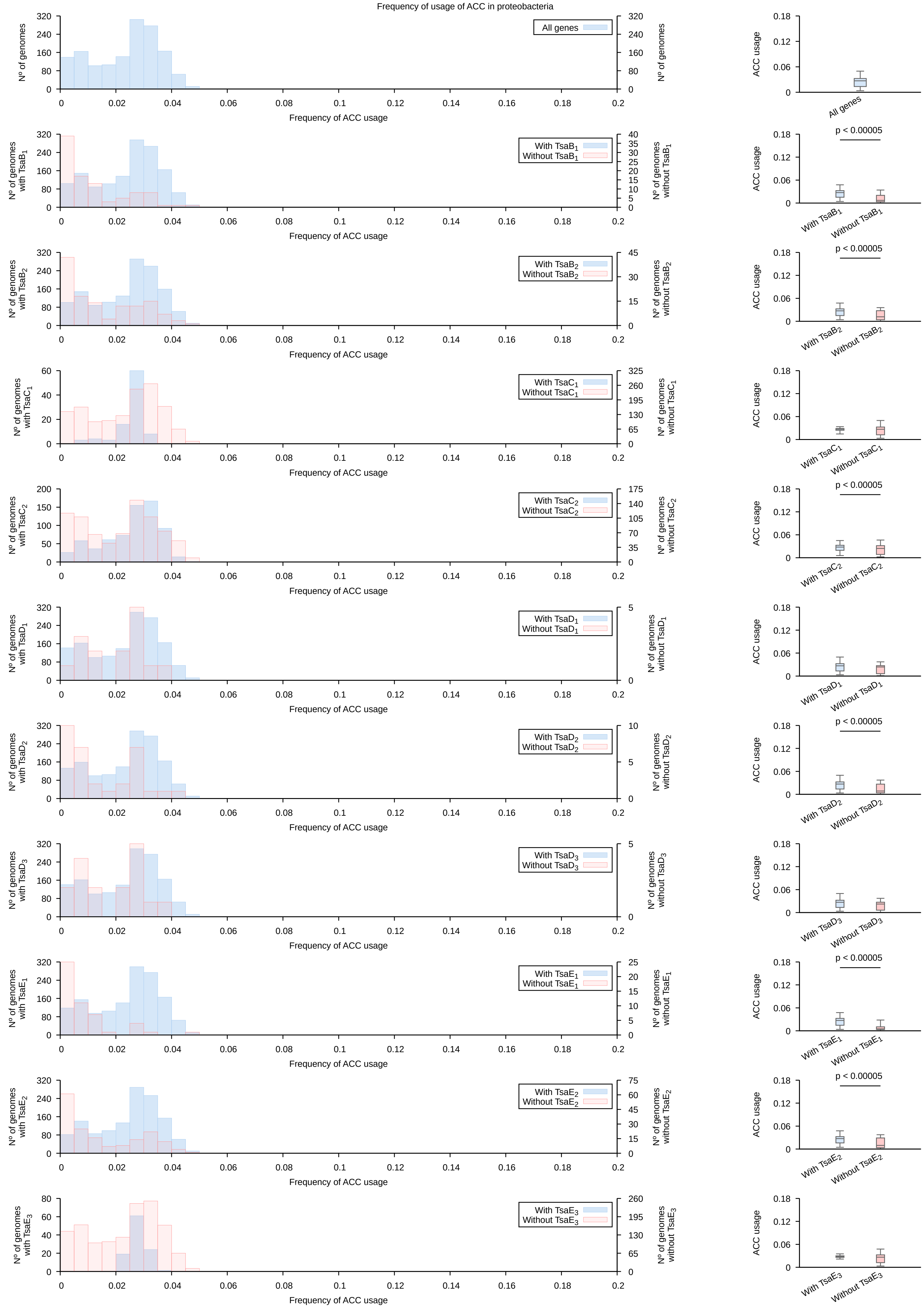

Frequency of usage of ACC in proteobacteria
All genes
Nº of genomes
Frequency of ACC usage
ACC usage
With $TsaB_1$
Without $TsaB_1$
Nº of genomes with $TsaB_1$
Nº of genomes without $TsaB_1$
p < 0.00005
With $TsaB_2$
Without $TsaB_2$
Nº of genomes with $TsaB_2$
Nº of genomes without $TsaB_2$
p < 0.00005
With $TsaC_1$
Without $TsaC_1$
Nº of genomes with $TsaC_1$
Nº of genomes without $TsaC_1$
With $TsaC_2$
Without $TsaC_2$
Nº of genomes with $TsaC_2$
Nº of genomes without $TsaC_2$
p < 0.00005
With $TsaD_1$
Without $TsaD_1$
Nº of genomes with $TsaD_1$
Nº of genomes without $TsaD_1$
With $TsaD_2$
Without $TsaD_2$
Nº of genomes with $TsaD_2$
Nº of genomes without $TsaD_2$
p < 0.00005
With $TsaD_3$
Without $TsaD_3$
Nº of genomes with $TsaD_3$
Nº of genomes without $TsaD_3$
With $TsaE_1$
Without $TsaE_1$
Nº of genomes with $TsaE_1$
Nº of genomes without $TsaE_1$
p < 0.00005
With $TsaE_2$
Without $TsaE_2$
Nº of genomes with $TsaE_2$
Nº of genomes without $TsaE_2$
p < 0.00005
With $TsaE_3$
Without $TsaE_3$
Nº of genomes with $TsaE_3$
Nº of genomes without $TsaE_3$

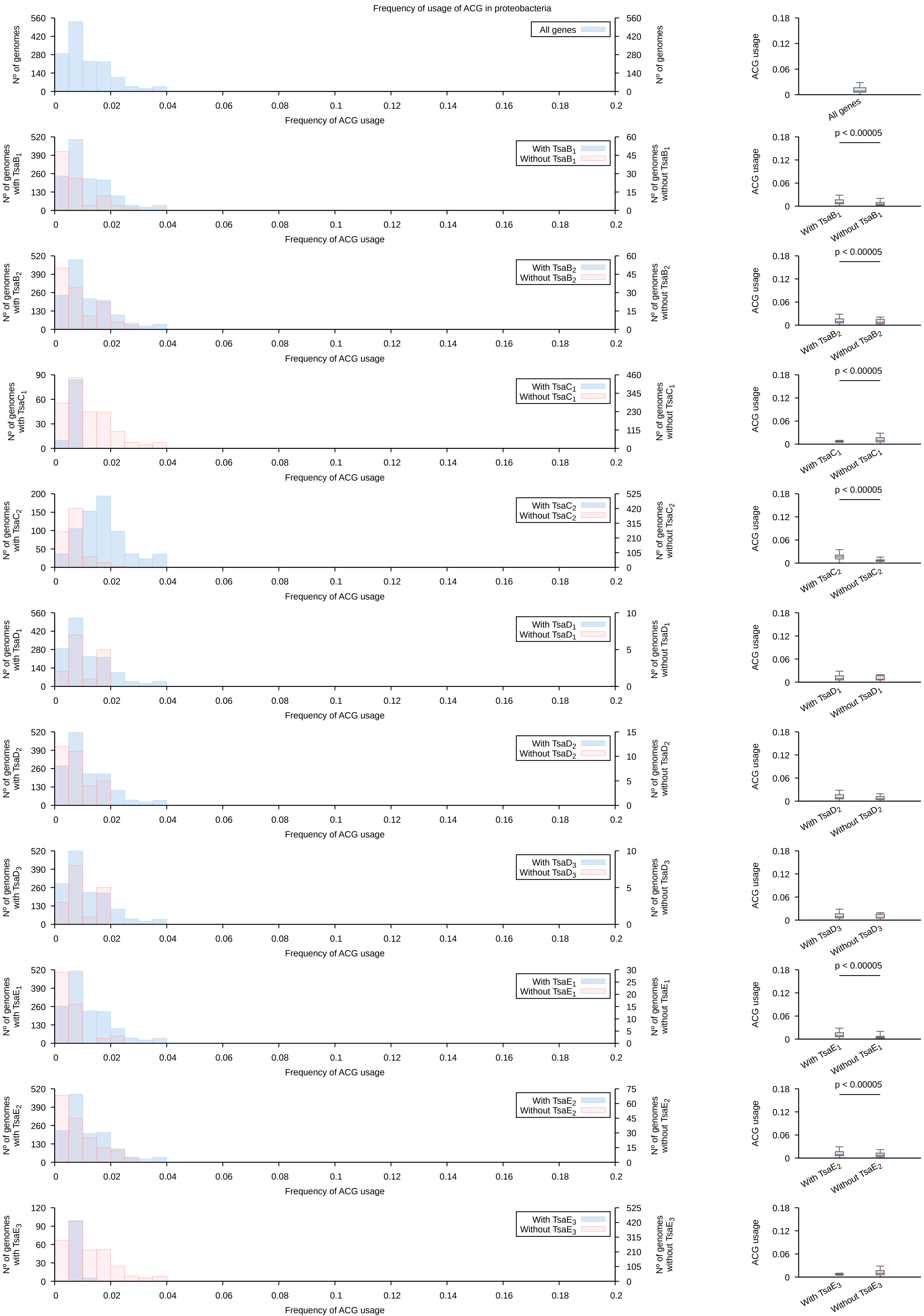
Frequency of usage of ACG in proteobacteria
Nº of genomes
Frequency of ACG usage
ACG usage
All genes
p < 0.00005
Nº of genomes with TsaB1
Nº of genomes without TsaB1
With TsaB1
Without TsaB1
Nº of genomes with TsaB2
Nº of genomes without TsaB2
With TsaB2
Without TsaB2
Nº of genomes with TsaC1
Nº of genomes without TsaC1
With TsaC1
Without TsaC1
Nº of genomes with TsaC2
Nº of genomes without TsaC2
With TsaC2
Without TsaC2
Nº of genomes with TsaD1
Nº of genomes without TsaD1
With TsaD1
Without TsaD1
Nº of genomes with TsaD2
Nº of genomes without TsaD2
With TsaD2
Without TsaD2
Nº of genomes with TsaD3
Nº of genomes without TsaD3
With TsaD3
Without TsaD3
Nº of genomes with TsaE1
Nº of genomes without TsaE1
With TsaE1
Without TsaE1
Nº of genomes with TsaE2
Nº of genomes without TsaE2
With TsaE2
Without TsaE2
Nº of genomes with TsaE3
Nº of genomes without TsaE3
With TsaE3
Without TsaE3

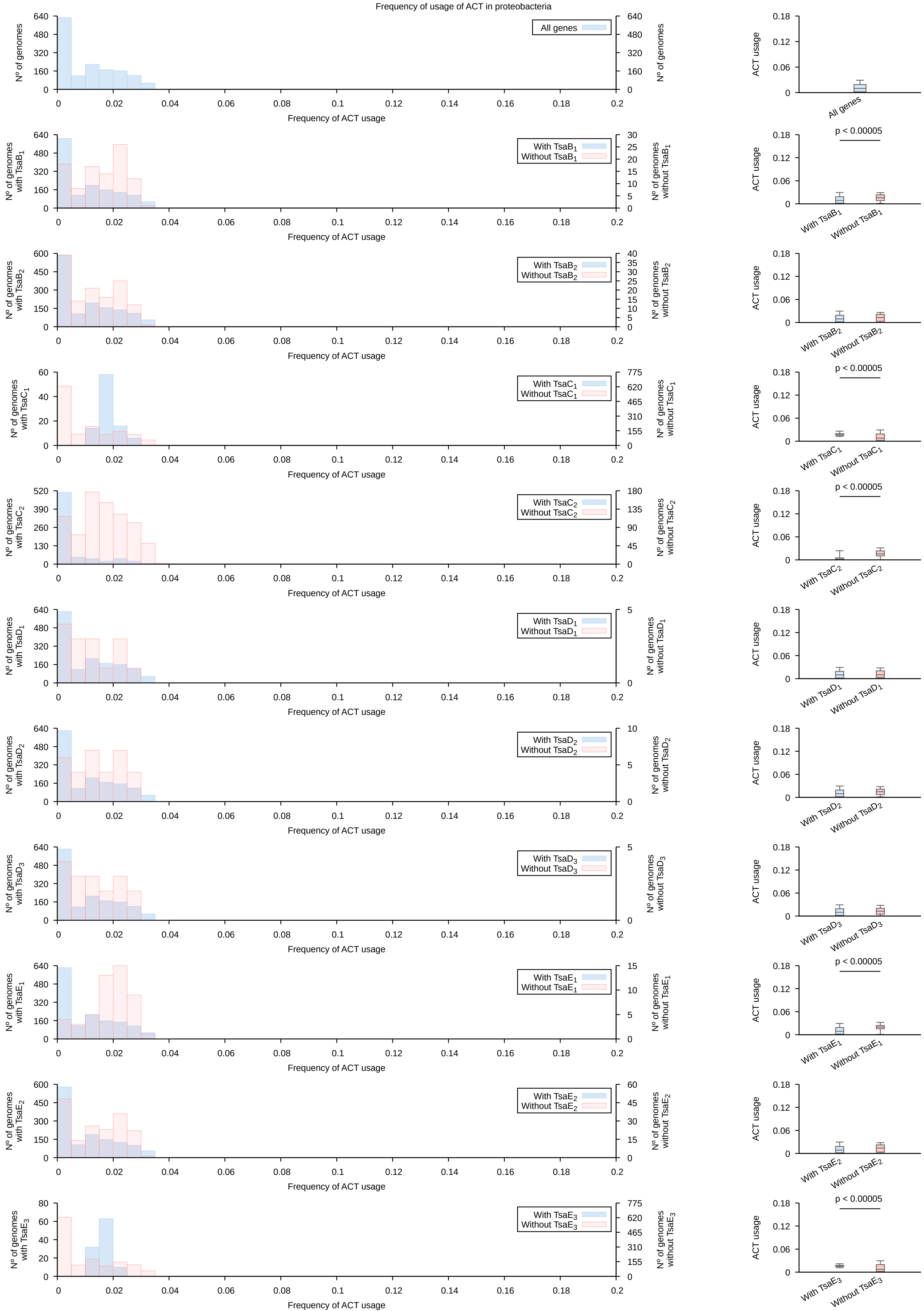
Frequency of usage of ACT in proteobacteria
All genes
Nº of genomes
Frequency of ACT usage
ACT usage
All genes
With $TsaB_1$
Without $TsaB_1$
Nº of genomes with $TsaB_1$
Nº of genomes without $TsaB_1$
p < 0.00005
With $TsaB_2$
Without $TsaB_2$
Nº of genomes with $TsaB_2$
Nº of genomes without $TsaB_2$
With $TsaC_1$
Without $TsaC_1$
Nº of genomes with $TsaC_1$
Nº of genomes without $TsaC_1$
p < 0.00005
With $TsaC_2$
Without $TsaC_2$
Nº of genomes with $TsaC_2$
Nº of genomes without $TsaC_2$
p < 0.00005
With $TsaD_1$
Without $TsaD_1$
Nº of genomes with $TsaD_1$
Nº of genomes without $TsaD_1$
With $TsaD_2$
Without $TsaD_2$
Nº of genomes with $TsaD_2$
Nº of genomes without $TsaD_2$
With $TsaD_3$
Without $TsaD_3$
Nº of genomes with $TsaD_3$
Nº of genomes without $TsaD_3$
With $TsaE_1$
Without $TsaE_1$
Nº of genomes with $TsaE_1$
Nº of genomes without $TsaE_1$
p < 0.00005
With $TsaE_2$
Without $TsaE_2$
Nº of genomes with $TsaE_2$
Nº of genomes without $TsaE_2$
With $TsaE_3$
Without $TsaE_3$
Nº of genomes with $TsaE_3$
Nº of genomes without $TsaE_3$
p < 0.00005

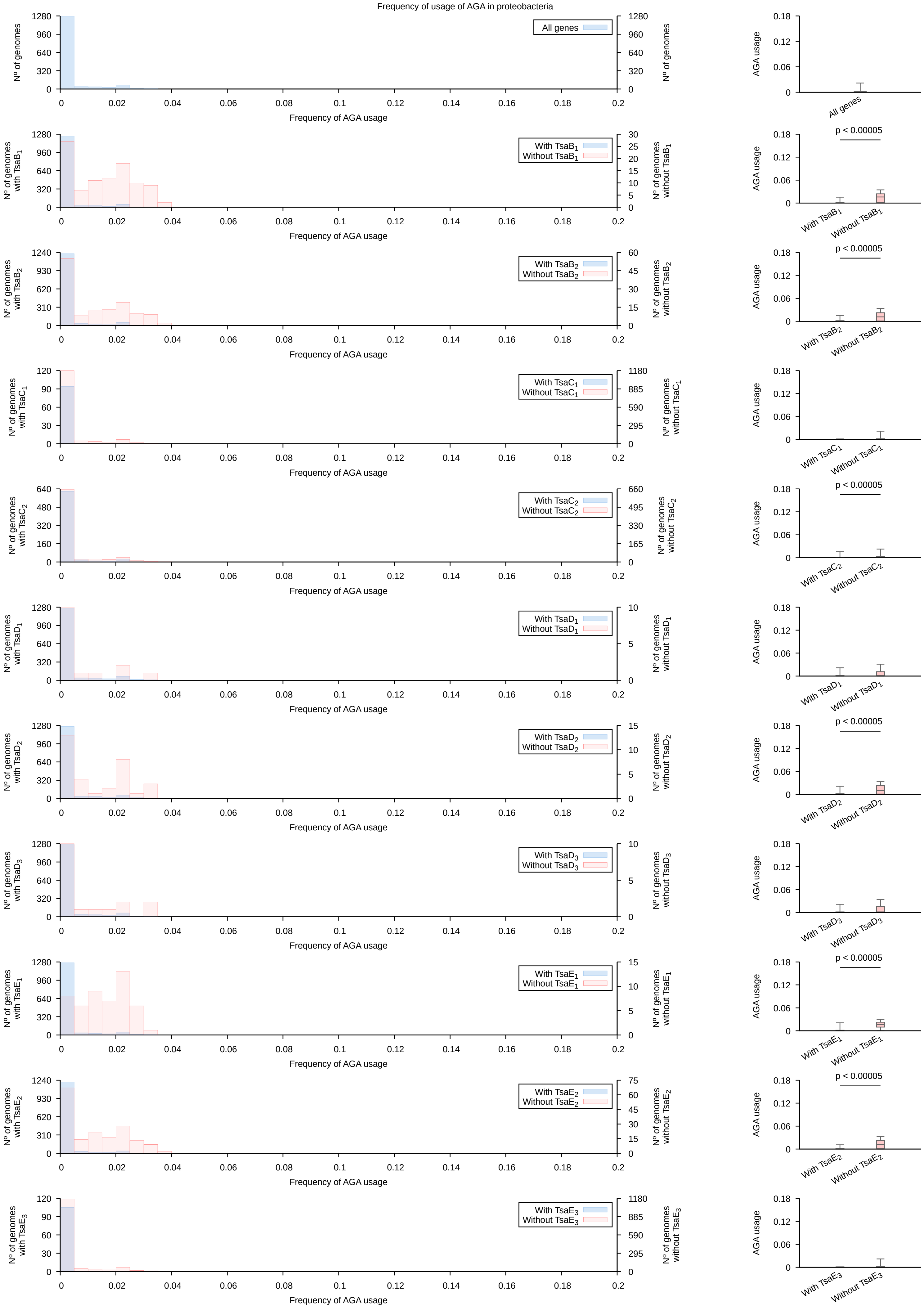

Frequency of usage of AGA in proteobacteria
All genes
Nº of genomes
Frequency of AGA usage
AGA usage
With TsaB$_1$
Without TsaB$_1$
Nº of genomes with TsaB$_1$
Nº of genomes without TsaB$_1$
p < 0.00005
With TsaB$_2$
Without TsaB$_2$
Nº of genomes with TsaB$_2$
Nº of genomes without TsaB$_2$
p < 0.00005
With TsaC$_1$
Without TsaC$_1$
Nº of genomes with TsaC$_1$
Nº of genomes without TsaC$_1$
With TsaC$_2$
Without TsaC$_2$
Nº of genomes with TsaC$_2$
Nº of genomes without TsaC$_2$
p < 0.00005
With TsaD$_1$
Without TsaD$_1$
Nº of genomes with TsaD$_1$
Nº of genomes without TsaD$_1$
With TsaD$_2$
Without TsaD$_2$
Nº of genomes with TsaD$_2$
Nº of genomes without TsaD$_2$
p < 0.00005
With TsaD$_3$
Without TsaD$_3$
Nº of genomes with TsaD$_3$
Nº of genomes without TsaD$_3$
With TsaE$_1$
Without TsaE$_1$
Nº of genomes with TsaE$_1$
Nº of genomes without TsaE$_1$
p < 0.00005
With TsaE$_2$
Without TsaE$_2$
Nº of genomes with TsaE$_2$
Nº of genomes without TsaE$_2$
p < 0.00005
With TsaE$_3$
Without TsaE$_3$
Nº of genomes with TsaE$_3$
Nº of genomes without TsaE$_3$

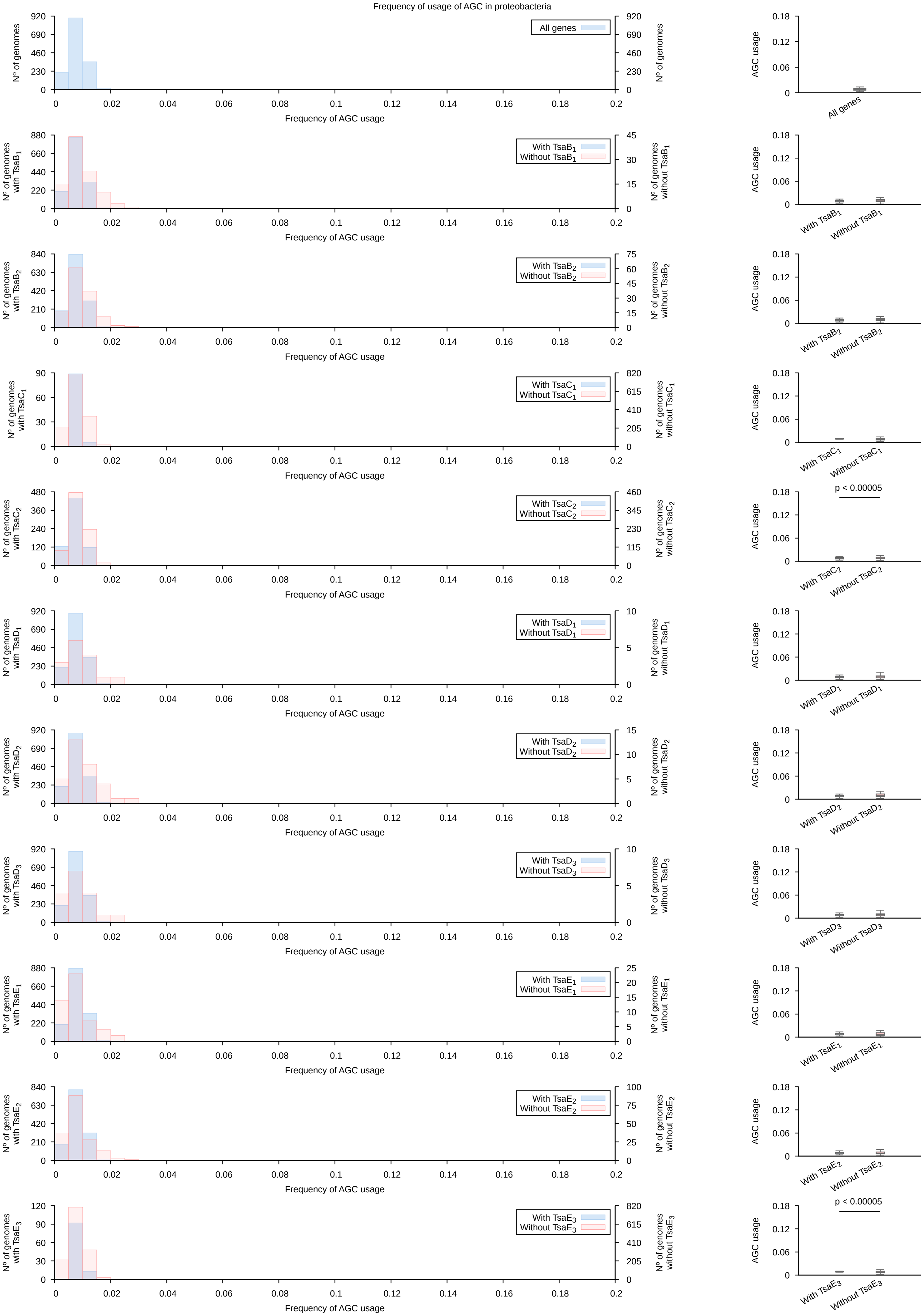

Frequency of usage of AGC in proteobacteria
Nº of genomes
All genes
Frequency of AGC usage
AGC usage
Nº of genomes with $TsaB_1$
Nº of genomes without $TsaB_1$
With $TsaB_1$
Without $TsaB_1$
Nº of genomes with $TsaB_2$
Nº of genomes without $TsaB_2$
With $TsaB_2$
Without $TsaB_2$
Nº of genomes with $TsaC_1$
Nº of genomes without $TsaC_1$
With $TsaC_1$
Without $TsaC_1$
Nº of genomes with $TsaC_2$
Nº of genomes without $TsaC_2$
With $TsaC_2$
Without $TsaC_2$
$p < 0.00005$
Nº of genomes with $TsaD_1$
Nº of genomes without $TsaD_1$
With $TsaD_1$
Without $TsaD_1$
Nº of genomes with $TsaD_2$
Nº of genomes without $TsaD_2$
With $TsaD_2$
Without $TsaD_2$
Nº of genomes with $TsaD_3$
Nº of genomes without $TsaD_3$
With $TsaD_3$
Without $TsaD_3$
Nº of genomes with $TsaE_1$
Nº of genomes without $TsaE_1$
With $TsaE_1$
Without $TsaE_1$
Nº of genomes with $TsaE_2$
Nº of genomes without $TsaE_2$
With $TsaE_2$
Without $TsaE_2$
Nº of genomes with $TsaE_3$
Nº of genomes without $TsaE_3$
With $TsaE_3$
Without $TsaE_3$
$p < 0.00005$

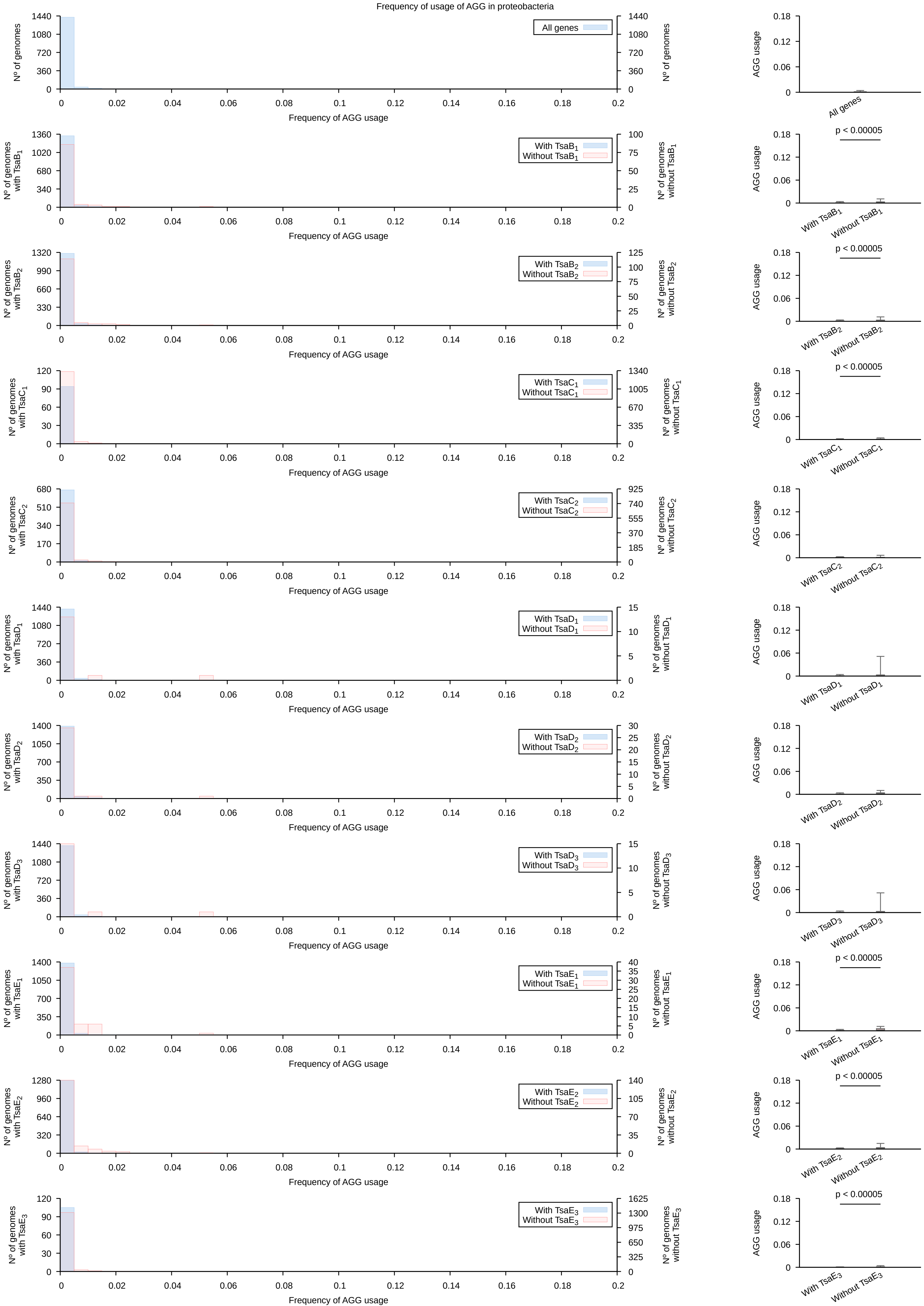
Frequency of usage of AGG in proteobacteria
All genes
Nº of genomes
Frequency of AGG usage
AGG usage
With TsaB1
Without TsaB1
Nº of genomes with TsaB1
Nº of genomes without TsaB1
p < 0.00005
With TsaB2
Without TsaB2
Nº of genomes with TsaB2
Nº of genomes without TsaB2
p < 0.00005
With TsaC1
Without TsaC1
Nº of genomes with TsaC1
Nº of genomes without TsaC1
p < 0.00005
With TsaC2
Without TsaC2
Nº of genomes with TsaC2
Nº of genomes without TsaC2
With TsaD1
Without TsaD1
Nº of genomes with TsaD1
Nº of genomes without TsaD1
With TsaD2
Without TsaD2
Nº of genomes with TsaD2
Nº of genomes without TsaD2
With TsaD3
Without TsaD3
Nº of genomes with TsaD3
Nº of genomes without TsaD3
With TsaE1
Without TsaE1
Nº of genomes with TsaE1
Nº of genomes without TsaE1
p < 0.00005
With TsaE2
Without TsaE2
Nº of genomes with TsaE2
Nº of genomes without TsaE2
p < 0.00005
With TsaE3
Without TsaE3
Nº of genomes with TsaE3
Nº of genomes without TsaE3
p < 0.00005

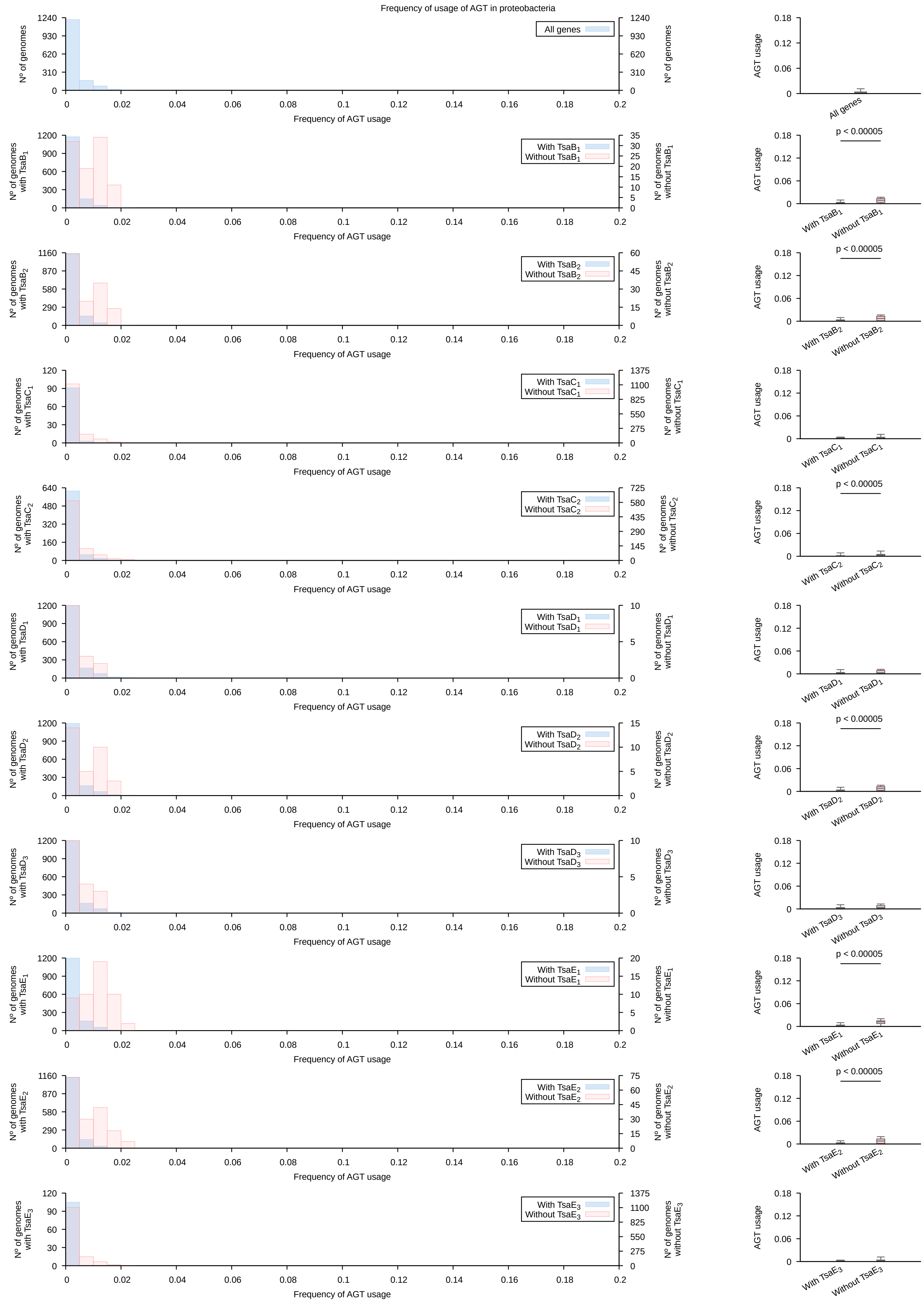
Frequency of usage of AGT in proteobacteria
Nº of genomes
All genes
Frequency of AGT usage
AGT usage
Nº of genomes with TsaB$_1$
Nº of genomes without TsaB$_1$
With TsaB$_1$
Without TsaB$_1$
p < 0.00005
Nº of genomes with TsaB$_2$
Nº of genomes without TsaB$_2$
With TsaB$_2$
Without TsaB$_2$
p < 0.00005
Nº of genomes with TsaC$_1$
Nº of genomes without TsaC$_1$
With TsaC$_1$
Without TsaC$_1$
Nº of genomes with TsaC$_2$
Nº of genomes without TsaC$_2$
With TsaC$_2$
Without TsaC$_2$
p < 0.00005
Nº of genomes with TsaD$_1$
Nº of genomes without TsaD$_1$
With TsaD$_1$
Without TsaD$_1$
Nº of genomes with TsaD$_2$
Nº of genomes without TsaD$_2$
With TsaD$_2$
Without TsaD$_2$
p < 0.00005
Nº of genomes with TsaD$_3$
Nº of genomes without TsaD$_3$
With TsaD$_3$
Without TsaD$_3$
Nº of genomes with TsaE$_1$
Nº of genomes without TsaE$_1$
With TsaE$_1$
Without TsaE$_1$
p < 0.00005
Nº of genomes with TsaE$_2$
Nº of genomes without TsaE$_2$
With TsaE$_2$
Without TsaE$_2$
p < 0.00005
Nº of genomes with TsaE$_3$
Nº of genomes without TsaE$_3$
With TsaE$_3$
Without TsaE$_3$

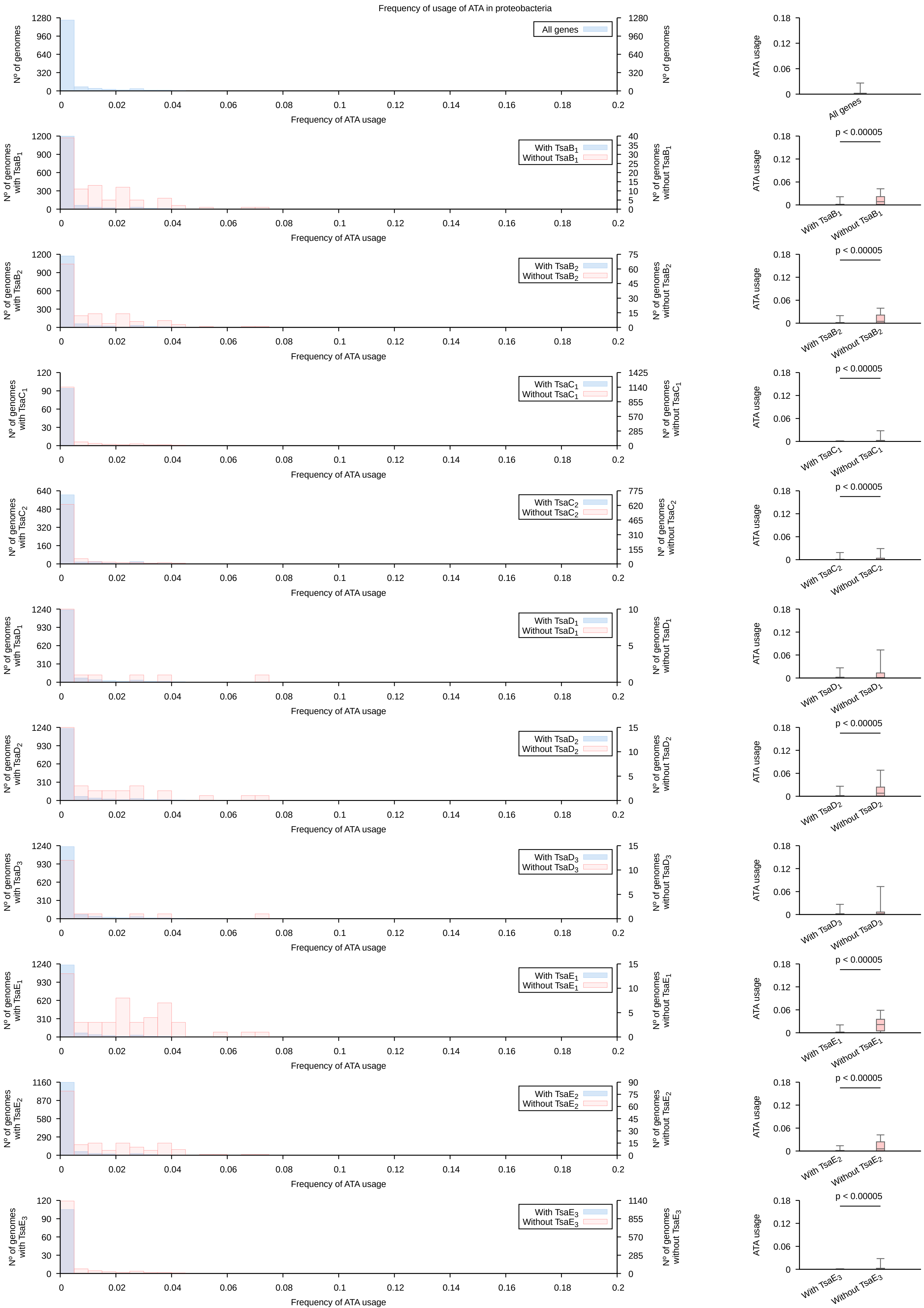

Frequency of usage of ATA in proteobacteria
All genes
Nº of genomes
Frequency of ATA usage
ATA usage
With $TsaB_1$
Without $TsaB_1$
Nº of genomes with $TsaB_1$
Nº of genomes without $TsaB_1$
p < 0.00005
With $TsaB_2$
Without $TsaB_2$
Nº of genomes with $TsaB_2$
Nº of genomes without $TsaB_2$
p < 0.00005
With $TsaC_1$
Without $TsaC_1$
Nº of genomes with $TsaC_1$
Nº of genomes without $TsaC_1$
p < 0.00005
With $TsaC_2$
Without $TsaC_2$
Nº of genomes with $TsaC_2$
Nº of genomes without $TsaC_2$
p < 0.00005
With $TsaD_1$
Without $TsaD_1$
Nº of genomes with $TsaD_1$
Nº of genomes without $TsaD_1$
With $TsaD_2$
Without $TsaD_2$
Nº of genomes with $TsaD_2$
Nº of genomes without $TsaD_2$
p < 0.00005
With $TsaD_3$
Without $TsaD_3$
Nº of genomes with $TsaD_3$
Nº of genomes without $TsaD_3$
With $TsaE_1$
Without $TsaE_1$
Nº of genomes with $TsaE_1$
Nº of genomes without $TsaE_1$
p < 0.00005
With $TsaE_2$
Without $TsaE_2$
Nº of genomes with $TsaE_2$
Nº of genomes without $TsaE_2$
p < 0.00005
With $TsaE_3$
Without $TsaE_3$
Nº of genomes with $TsaE_3$
Nº of genomes without $TsaE_3$
p < 0.00005

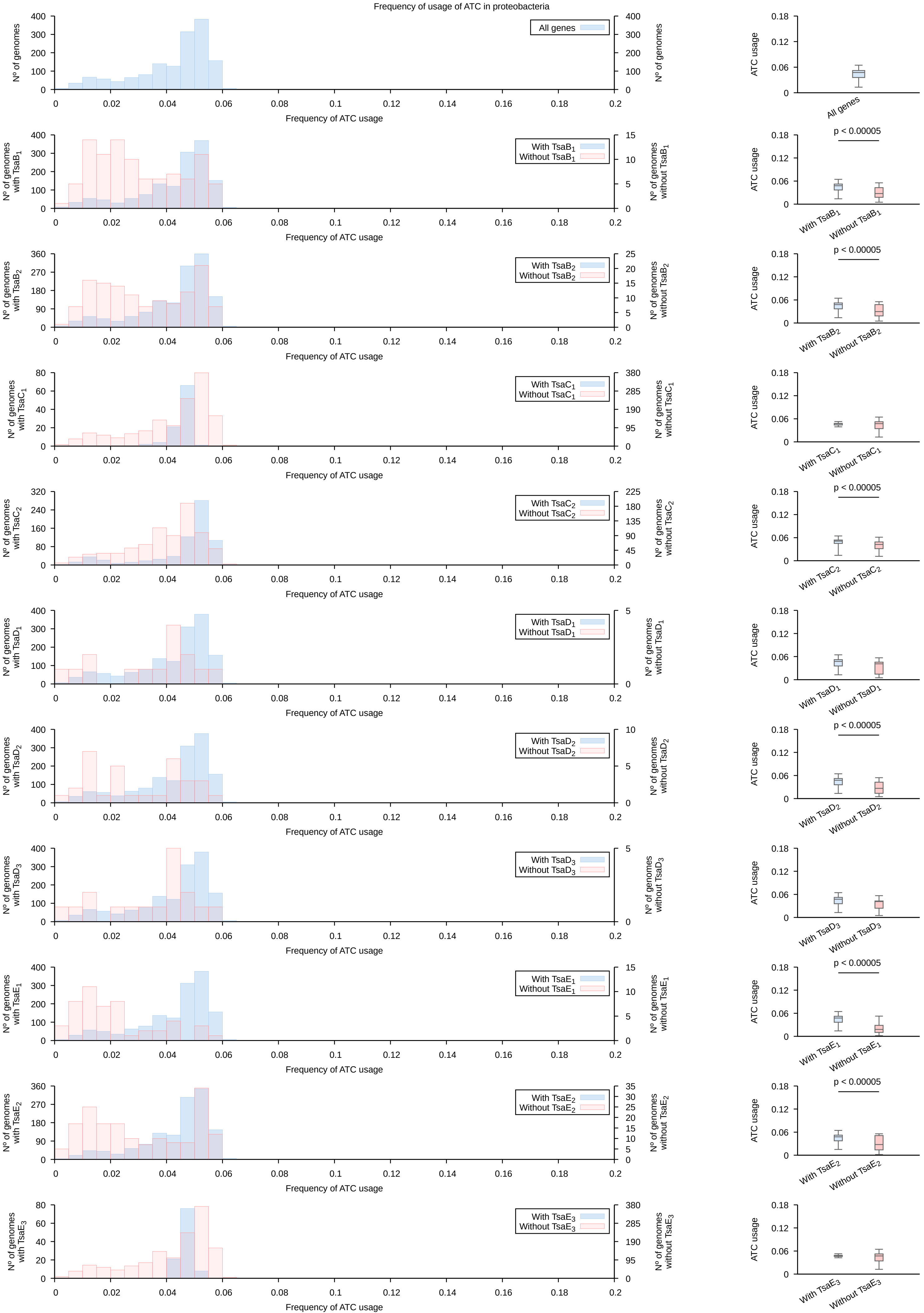
Frequency of usage of ATC in proteobacteria
All genes
Nº of genomes
Frequency of ATC usage
ATC usage
With TsaB$_1$
Without TsaB$_1$
Nº of genomes with TsaB$_1$
Nº of genomes without TsaB$_1$
p < 0.00005
With TsaB$_2$
Without TsaB$_2$
Nº of genomes with TsaB$_2$
Nº of genomes without TsaB$_2$
p < 0.00005
With TsaC$_1$
Without TsaC$_1$
Nº of genomes with TsaC$_1$
Nº of genomes without TsaC$_1$
With TsaC$_2$
Without TsaC$_2$
Nº of genomes with TsaC$_2$
Nº of genomes without TsaC$_2$
p < 0.00005
With TsaD$_1$
Without TsaD$_1$
Nº of genomes with TsaD$_1$
Nº of genomes without TsaD$_1$
With TsaD$_2$
Without TsaD$_2$
Nº of genomes with TsaD$_2$
Nº of genomes without TsaD$_2$
p < 0.00005
With TsaD$_3$
Without TsaD$_3$
Nº of genomes with TsaD$_3$
Nº of genomes without TsaD$_3$
With TsaE$_1$
Without TsaE$_1$
Nº of genomes with TsaE$_1$
Nº of genomes without TsaE$_1$
p < 0.00005
With TsaE$_2$
Without TsaE$_2$
Nº of genomes with TsaE$_2$
Nº of genomes without TsaE$_2$
p < 0.00005
With TsaE$_3$
Without TsaE$_3$
Nº of genomes with TsaE$_3$
Nº of genomes without TsaE$_3$

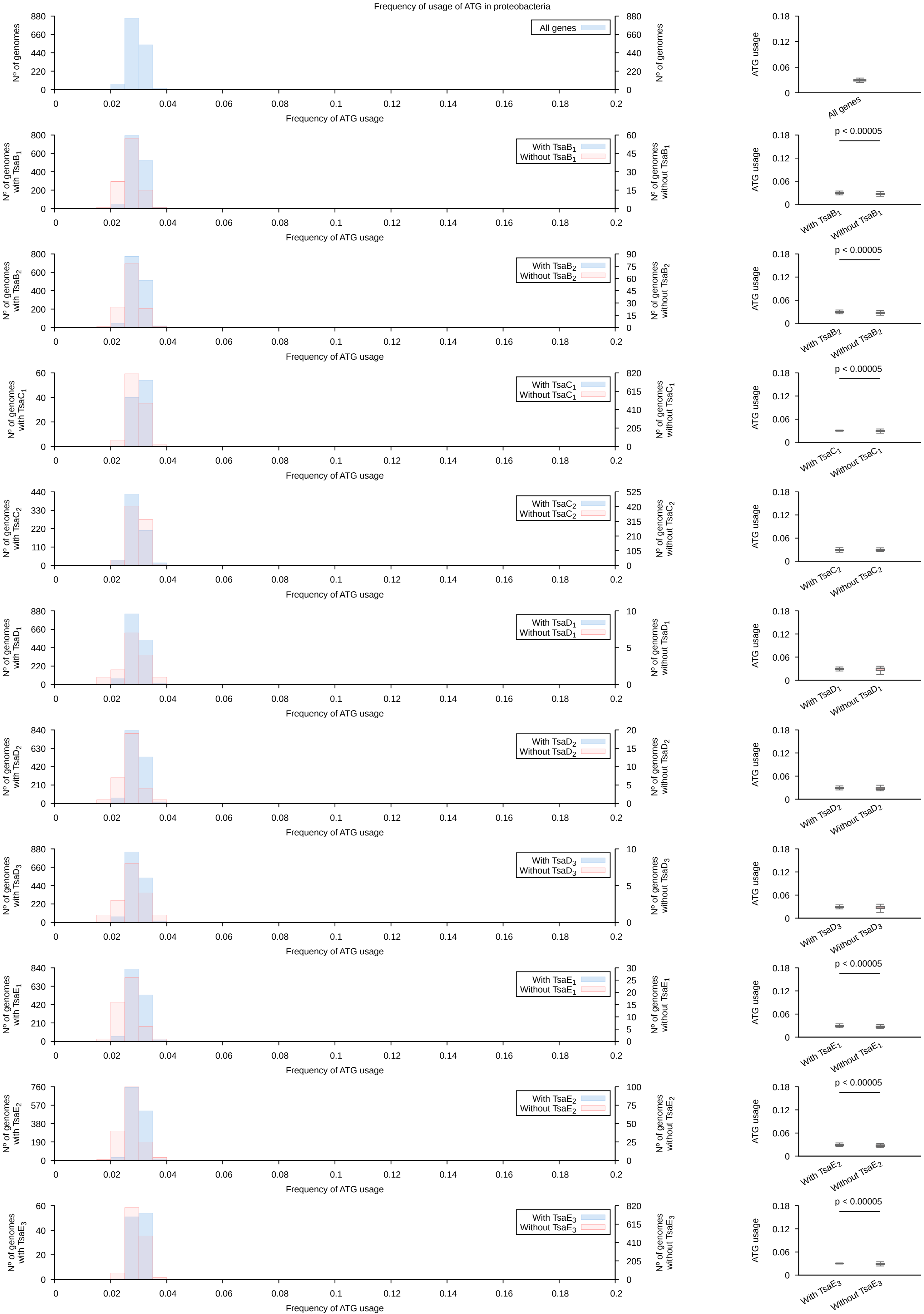
Frequency of usage of ATG in proteobacteria
All genes
Nº of genomes
Frequency of ATG usage
ATG usage
With TsaB$_1$
Without TsaB$_1$
Nº of genomes with TsaB$_1$
Nº of genomes without TsaB$_1$
p < 0.00005
With TsaB$_2$
Without TsaB$_2$
Nº of genomes with TsaB$_2$
Nº of genomes without TsaB$_2$
p < 0.00005
With TsaC$_1$
Without TsaC$_1$
Nº of genomes with TsaC$_1$
Nº of genomes without TsaC$_1$
p < 0.00005
With TsaC$_2$
Without TsaC$_2$
Nº of genomes with TsaC$_2$
Nº of genomes without TsaC$_2$
With TsaD$_1$
Without TsaD$_1$
Nº of genomes with TsaD$_1$
Nº of genomes without TsaD$_1$
With TsaD$_2$
Without TsaD$_2$
Nº of genomes with TsaD$_2$
Nº of genomes without TsaD$_2$
With TsaD$_3$
Without TsaD$_3$
Nº of genomes with TsaD$_3$
Nº of genomes without TsaD$_3$
With TsaE$_1$
Without TsaE$_1$
Nº of genomes with TsaE$_1$
Nº of genomes without TsaE$_1$
p < 0.00005
With TsaE$_2$
Without TsaE$_2$
Nº of genomes with TsaE$_2$
Nº of genomes without TsaE$_2$
p < 0.00005
With TsaE$_3$
Without TsaE$_3$
Nº of genomes with TsaE$_3$
Nº of genomes without TsaE$_3$
p < 0.00005

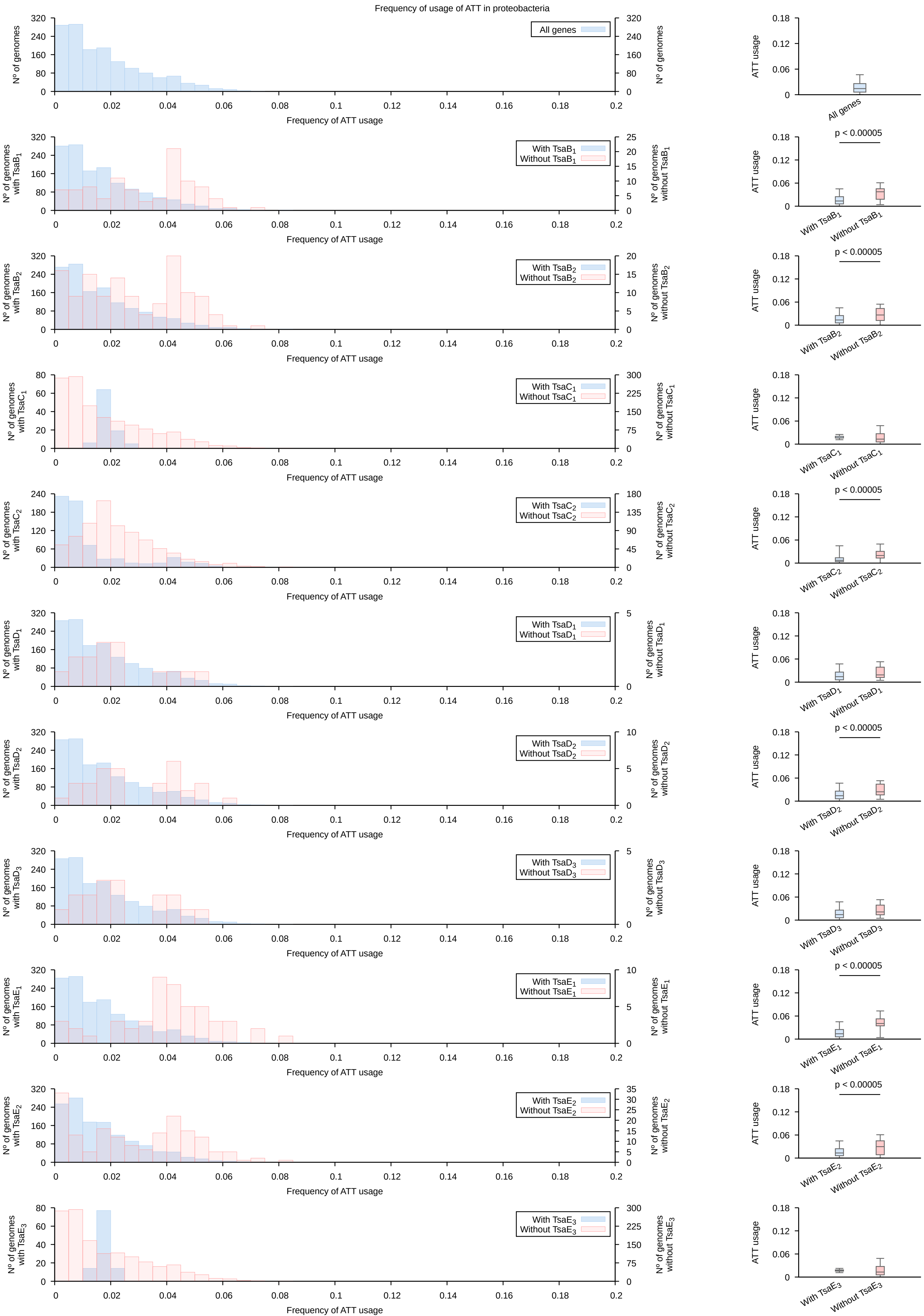
Frequency of usage of ATT in proteobacteria
All genes
Nº of genomes
Frequency of ATT usage
ATT usage
With TsaB$_1$
Without TsaB$_1$
Nº of genomes with TsaB$_1$
Nº of genomes without TsaB$_1$
p < 0.00005
With TsaB$_2$
Without TsaB$_2$
Nº of genomes with TsaB$_2$
Nº of genomes without TsaB$_2$
p < 0.00005
With TsaC$_1$
Without TsaC$_1$
Nº of genomes with TsaC$_1$
Nº of genomes without TsaC$_1$
With TsaC$_2$
Without TsaC$_2$
Nº of genomes with TsaC$_2$
Nº of genomes without TsaC$_2$
p < 0.00005
With TsaD$_1$
Without TsaD$_1$
Nº of genomes with TsaD$_1$
Nº of genomes without TsaD$_1$
With TsaD$_2$
Without TsaD$_2$
Nº of genomes with TsaD$_2$
Nº of genomes without TsaD$_2$
p < 0.00005
With TsaD$_3$
Without TsaD$_3$
Nº of genomes with TsaD$_3$
Nº of genomes without TsaD$_3$
With TsaE$_1$
Without TsaE$_1$
Nº of genomes with TsaE$_1$
Nº of genomes without TsaE$_1$
p < 0.00005
With TsaE$_2$
Without TsaE$_2$
Nº of genomes with TsaE$_2$
Nº of genomes without TsaE$_2$
p < 0.00005
With TsaE$_3$
Without TsaE$_3$
Nº of genomes with TsaE$_3$
Nº of genomes without TsaE$_3$

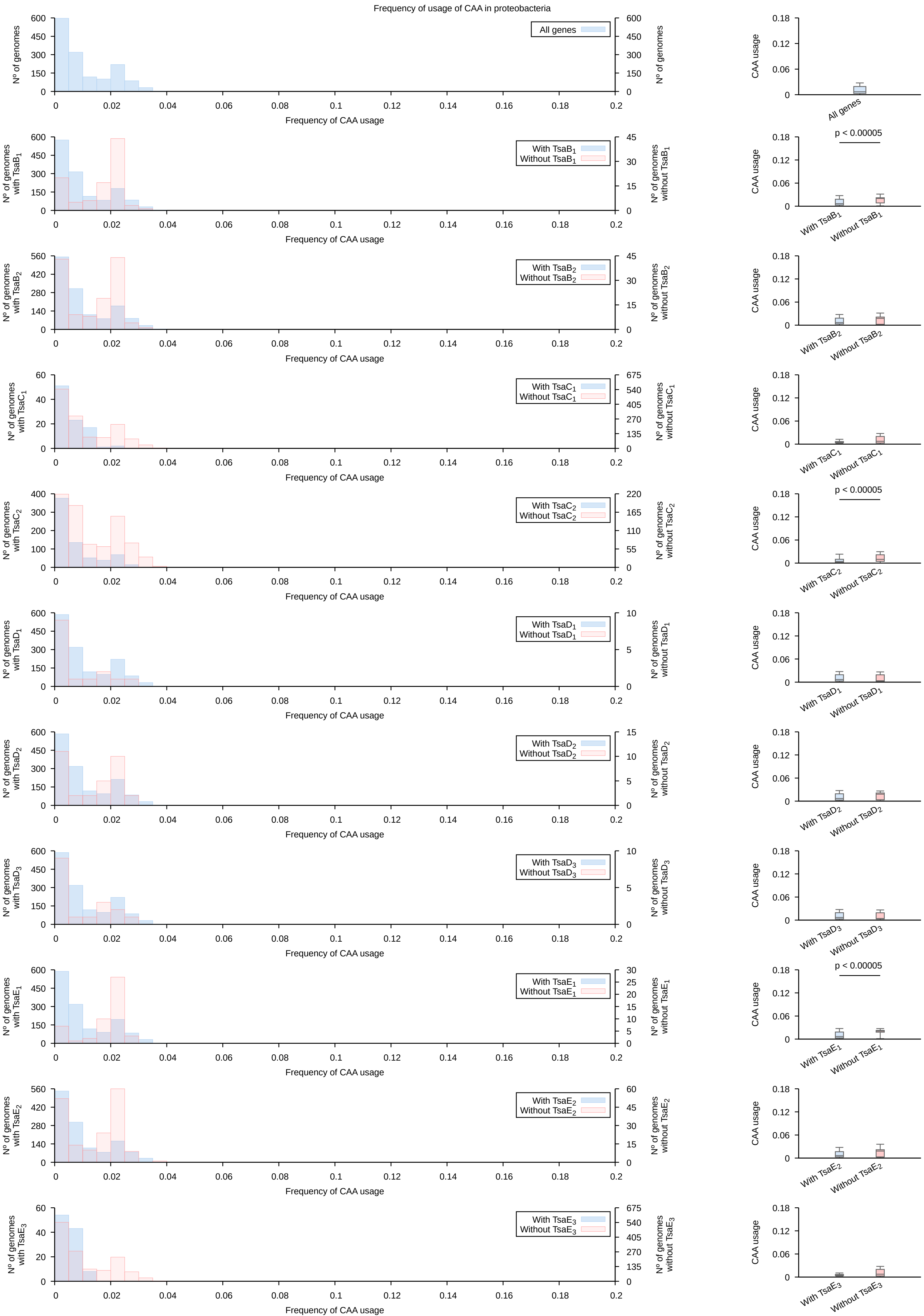
Frequency of usage of CAA in proteobacteria
All genes
Nº of genomes
Frequency of CAA usage
CAA usage
With TsaB$_1$
Without TsaB$_1$
p < 0.00005
With TsaB$_2$
Without TsaB$_2$
With TsaC$_1$
Without TsaC$_1$
With TsaC$_2$
Without TsaC$_2$
p < 0.00005
With TsaD$_1$
Without TsaD$_1$
With TsaD$_2$
Without TsaD$_2$
With TsaD$_3$
Without TsaD$_3$
With TsaE$_1$
Without TsaE$_1$
p < 0.00005
With TsaE$_2$
Without TsaE$_2$
With TsaE$_3$
Without TsaE$_3$

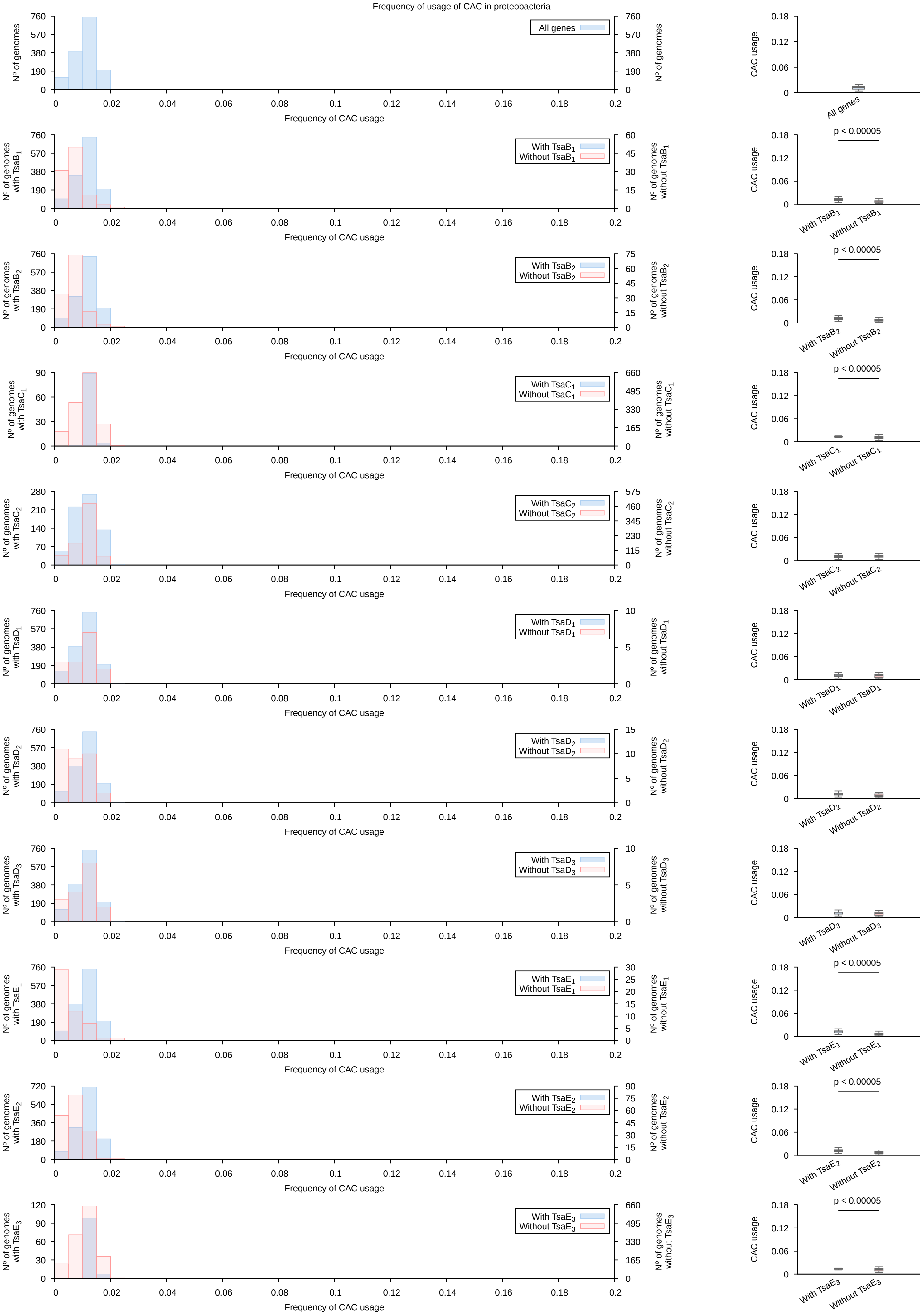
Frequency of usage of CAC in proteobacteria
All genes
Nº of genomes
CAC usage
Frequency of CAC usage
With TsaB$_1$
Without TsaB$_1$
Nº of genomes with TsaB$_1$
Nº of genomes without TsaB$_1$
With TsaB$_2$
Without TsaB$_2$
Nº of genomes with TsaB$_2$
Nº of genomes without TsaB$_2$
With TsaC$_1$
Without TsaC$_1$
Nº of genomes with TsaC$_1$
Nº of genomes without TsaC$_1$
With TsaC$_2$
Without TsaC$_2$
Nº of genomes with TsaC$_2$
Nº of genomes without TsaC$_2$
With TsaD$_1$
Without TsaD$_1$
Nº of genomes with TsaD$_1$
Nº of genomes without TsaD$_1$
With TsaD$_2$
Without TsaD$_2$
Nº of genomes with TsaD$_2$
Nº of genomes without TsaD$_2$
With TsaD$_3$
Without TsaD$_3$
Nº of genomes with TsaD$_3$
Nº of genomes without TsaD$_3$
With TsaE$_1$
Without TsaE$_1$
Nº of genomes with TsaE$_1$
Nº of genomes without TsaE$_1$
With TsaE$_2$
Without TsaE$_2$
Nº of genomes with TsaE$_2$
Nº of genomes without TsaE$_2$
With TsaE$_3$
Without TsaE$_3$
Nº of genomes with TsaE$_3$
Nº of genomes without TsaE$_3$
p < 0.00005

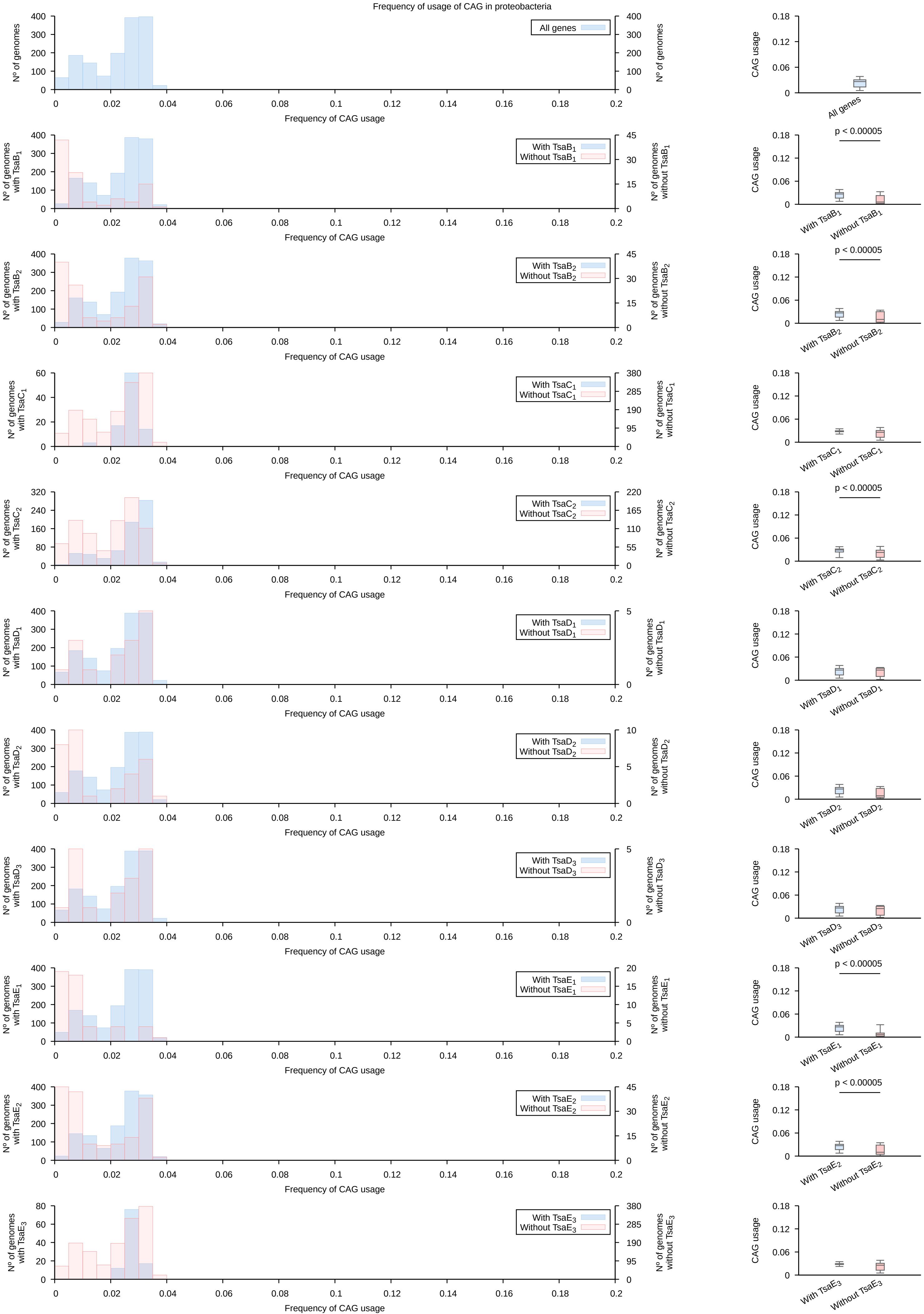

Frequency of usage of CAG in proteobacteria
All genes
Nº of genomes
Frequency of CAG usage
CAG usage
With TsaB$_1$
Without TsaB$_1$
Nº of genomes with TsaB$_1$
Nº of genomes without TsaB$_1$
p < 0.00005
With TsaB$_2$
Without TsaB$_2$
Nº of genomes with TsaB$_2$
Nº of genomes without TsaB$_2$
p < 0.00005
With TsaC$_1$
Without TsaC$_1$
Nº of genomes with TsaC$_1$
Nº of genomes without TsaC$_1$
With TsaC$_2$
Without TsaC$_2$
Nº of genomes with TsaC$_2$
Nº of genomes without TsaC$_2$
p < 0.00005
With TsaD$_1$
Without TsaD$_1$
Nº of genomes with TsaD$_1$
Nº of genomes without TsaD$_1$
With TsaD$_2$
Without TsaD$_2$
Nº of genomes with TsaD$_2$
Nº of genomes without TsaD$_2$
With TsaD$_3$
Without TsaD$_3$
Nº of genomes with TsaD$_3$
Nº of genomes without TsaD$_3$
With TsaE$_1$
Without TsaE$_1$
Nº of genomes with TsaE$_1$
Nº of genomes without TsaE$_1$
p < 0.00005
With TsaE$_2$
Without TsaE$_2$
Nº of genomes with TsaE$_2$
Nº of genomes without TsaE$_2$
p < 0.00005
With TsaE$_3$
Without TsaE$_3$
Nº of genomes with TsaE$_3$
Nº of genomes without TsaE$_3$

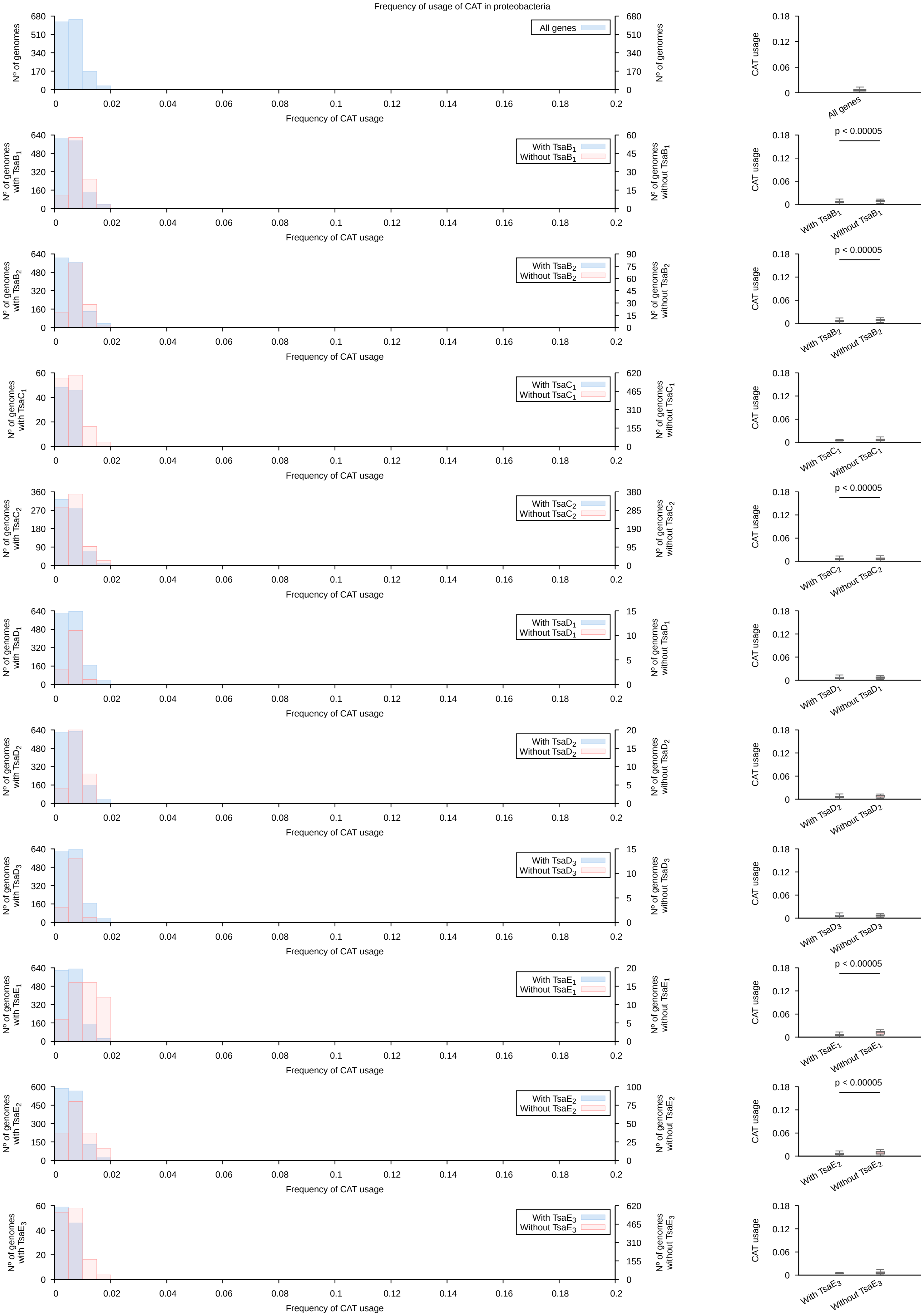
Frequency of usage of CAT in proteobacteria
All genes
Nº of genomes
Frequency of CAT usage
CAT usage
With TsaB1
Without TsaB1
Nº of genomes with TsaB1
Nº of genomes without TsaB1
p < 0.00005
With TsaB2
Without TsaB2
Nº of genomes with TsaB2
Nº of genomes without TsaB2
p < 0.00005
With TsaC1
Without TsaC1
Nº of genomes with TsaC1
Nº of genomes without TsaC1
With TsaC2
Without TsaC2
Nº of genomes with TsaC2
Nº of genomes without TsaC2
p < 0.00005
With TsaD1
Without TsaD1
Nº of genomes with TsaD1
Nº of genomes without TsaD1
With TsaD2
Without TsaD2
Nº of genomes with TsaD2
Nº of genomes without TsaD2
With TsaD3
Without TsaD3
Nº of genomes with TsaD3
Nº of genomes without TsaD3
With TsaE1
Without TsaE1
Nº of genomes with TsaE1
Nº of genomes without TsaE1
p < 0.00005
With TsaE2
Without TsaE2
Nº of genomes with TsaE2
Nº of genomes without TsaE2
p < 0.00005
With TsaE3
Without TsaE3
Nº of genomes with TsaE3
Nº of genomes without TsaE3

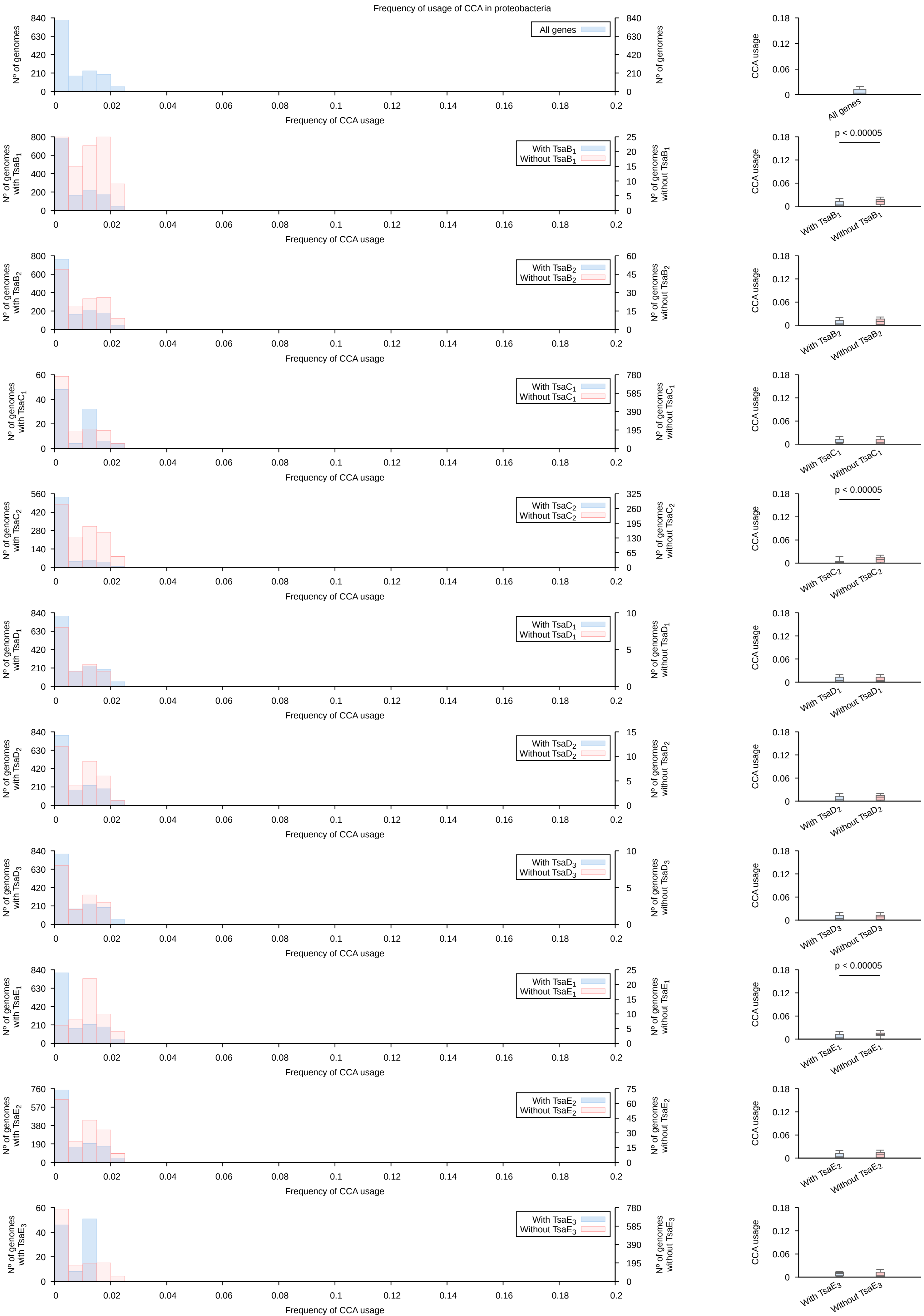
Frequency of usage of CCA in proteobacteria
All genes
Nº of genomes
Frequency of CCA usage
CCA usage
With TsaB$_1$
Without TsaB$_1$
Nº of genomes with TsaB$_1$
Nº of genomes without TsaB$_1$
p < 0.00005
With TsaB$_2$
Without TsaB$_2$
Nº of genomes with TsaB$_2$
Nº of genomes without TsaB$_2$
With TsaC$_1$
Without TsaC$_1$
Nº of genomes with TsaC$_1$
Nº of genomes without TsaC$_1$
With TsaC$_2$
Without TsaC$_2$
Nº of genomes with TsaC$_2$
Nº of genomes without TsaC$_2$
p < 0.00005
With TsaD$_1$
Without TsaD$_1$
Nº of genomes with TsaD$_1$
Nº of genomes without TsaD$_1$
With TsaD$_2$
Without TsaD$_2$
Nº of genomes with TsaD$_2$
Nº of genomes without TsaD$_2$
With TsaD$_3$
Without TsaD$_3$
Nº of genomes with TsaD$_3$
Nº of genomes without TsaD$_3$
With TsaE$_1$
Without TsaE$_1$
Nº of genomes with TsaE$_1$
Nº of genomes without TsaE$_1$
p < 0.00005
With TsaE$_2$
Without TsaE$_2$
Nº of genomes with TsaE$_2$
Nº of genomes without TsaE$_2$
With TsaE$_3$
Without TsaE$_3$
Nº of genomes with TsaE$_3$
Nº of genomes without TsaE$_3$

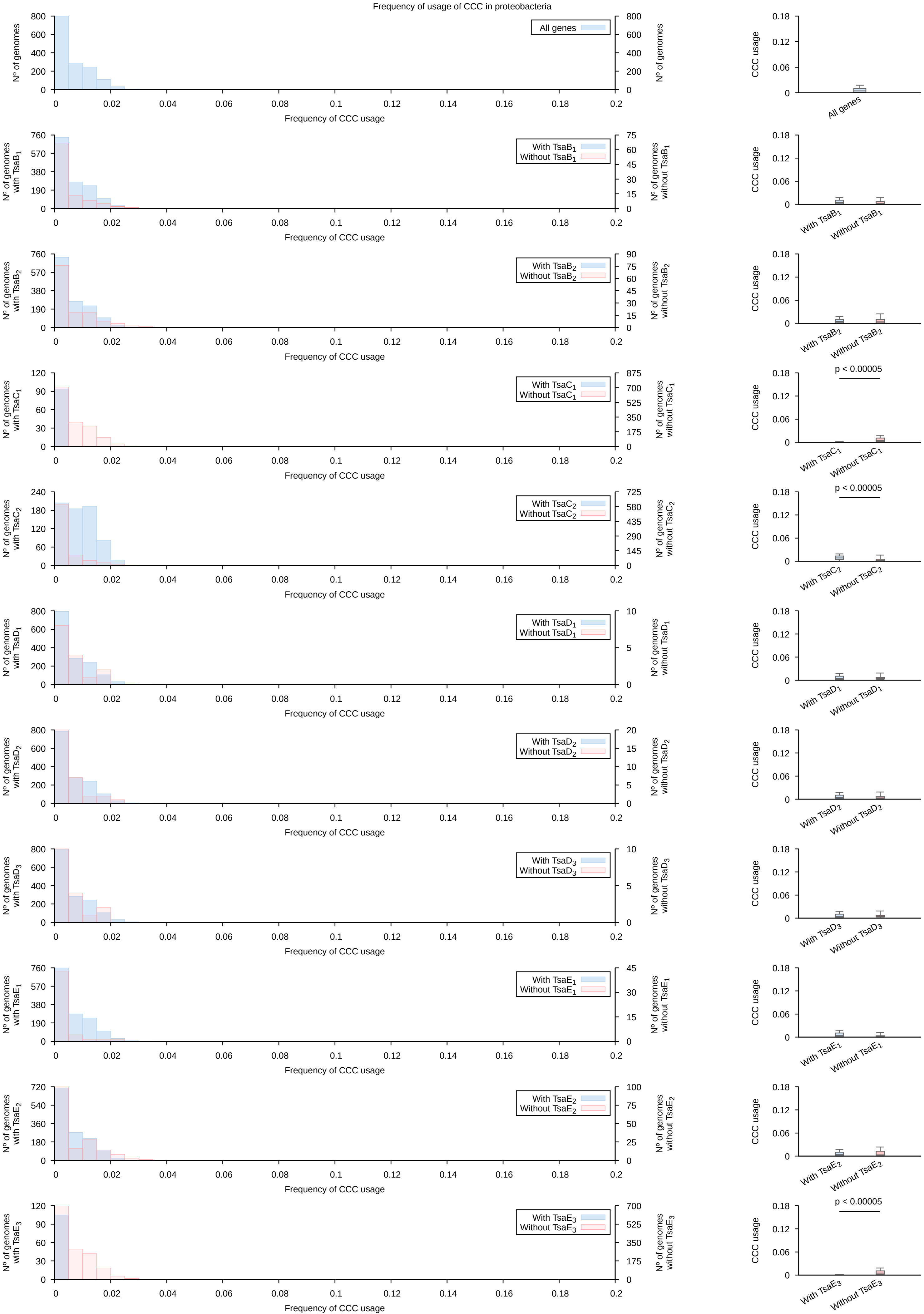

Frequency of usage of CCC in proteobacteria
All genes
Nº of genomes
Frequency of CCC usage
CCC usage
With TsaB$_1$
Without TsaB$_1$
Nº of genomes with TsaB$_1$
Nº of genomes without TsaB$_1$
With TsaB$_2$
Without TsaB$_2$
Nº of genomes with TsaB$_2$
Nº of genomes without TsaB$_2$
With TsaC$_1$
Without TsaC$_1$
Nº of genomes with TsaC$_1$
Nº of genomes without TsaC$_1$
p < 0.00005
With TsaC$_2$
Without TsaC$_2$
Nº of genomes with TsaC$_2$
Nº of genomes without TsaC$_2$
p < 0.00005
With TsaD$_1$
Without TsaD$_1$
Nº of genomes with TsaD$_1$
Nº of genomes without TsaD$_1$
With TsaD$_2$
Without TsaD$_2$
Nº of genomes with TsaD$_2$
Nº of genomes without TsaD$_2$
With TsaD$_3$
Without TsaD$_3$
Nº of genomes with TsaD$_3$
Nº of genomes without TsaD$_3$
With TsaE$_1$
Without TsaE$_1$
Nº of genomes with TsaE$_1$
Nº of genomes without TsaE$_1$
With TsaE$_2$
Without TsaE$_2$
Nº of genomes with TsaE$_2$
Nº of genomes without TsaE$_2$
With TsaE$_3$
Without TsaE$_3$
Nº of genomes with TsaE$_3$
Nº of genomes without TsaE$_3$
p < 0.00005

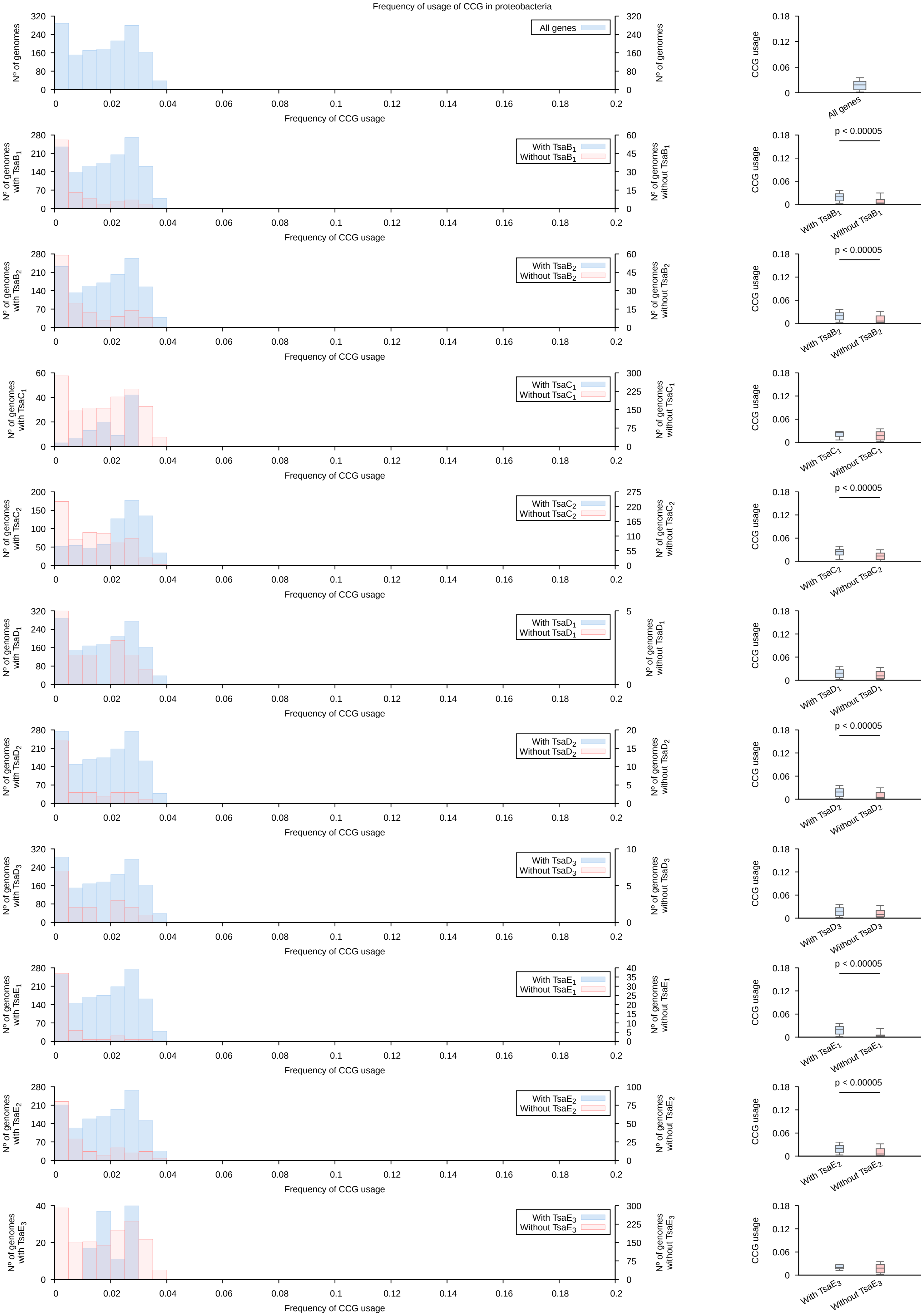

Frequency of usage of CCG in proteobacteria
All genes
Nº of genomes
CCG usage
Frequency of CCG usage
With TsaB1
Without TsaB1
Nº of genomes with TsaB1
Nº of genomes without TsaB1
p < 0.00005
With TsaB2
Without TsaB2
Nº of genomes with TsaB2
Nº of genomes without TsaB2
p < 0.00005
With TsaC1
Without TsaC1
Nº of genomes with TsaC1
Nº of genomes without TsaC1
With TsaC2
Without TsaC2
Nº of genomes with TsaC2
Nº of genomes without TsaC2
p < 0.00005
With TsaD1
Without TsaD1
Nº of genomes with TsaD1
Nº of genomes without TsaD1
With TsaD2
Without TsaD2
Nº of genomes with TsaD2
Nº of genomes without TsaD2
p < 0.00005
With TsaD3
Without TsaD3
Nº of genomes with TsaD3
Nº of genomes without TsaD3
With TsaE1
Without TsaE1
Nº of genomes with TsaE1
Nº of genomes without TsaE1
p < 0.00005
With TsaE2
Without TsaE2
Nº of genomes with TsaE2
Nº of genomes without TsaE2
p < 0.00005
With TsaE3
Without TsaE3
Nº of genomes with TsaE3
Nº of genomes without TsaE3

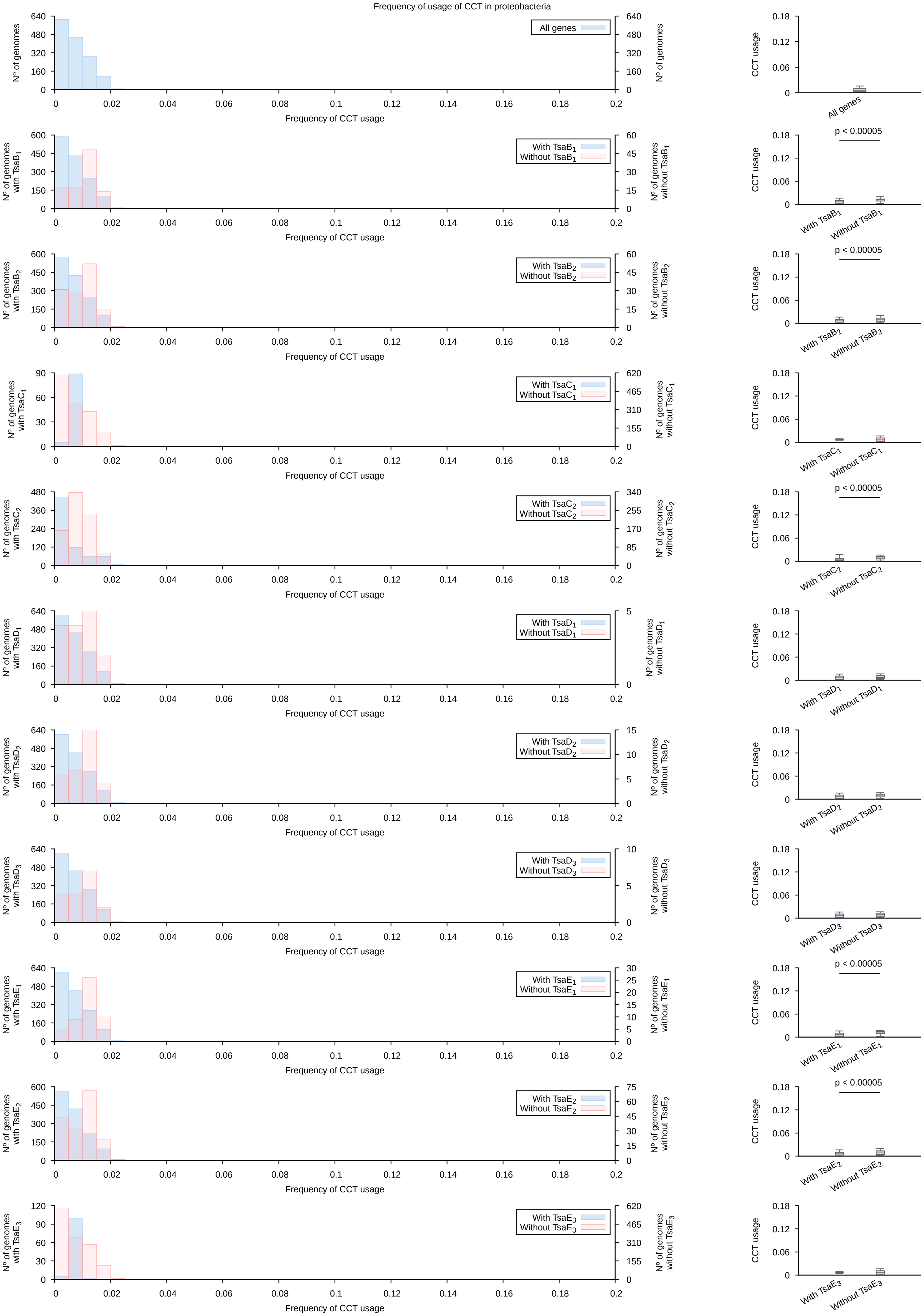

Frequency of usage of CCT in proteobacteria
All genes
Nº of genomes
Frequency of CCT usage
CCT usage
With TsaB1
Without TsaB1
With TsaB2
Without TsaB2
With TsaC1
Without TsaC1
With TsaC2
Without TsaC2
With TsaD1
Without TsaD1
With TsaD2
Without TsaD2
With TsaD3
Without TsaD3
With TsaE1
Without TsaE1
With TsaE2
Without TsaE2
With TsaE3
Without TsaE3
p < 0.00005

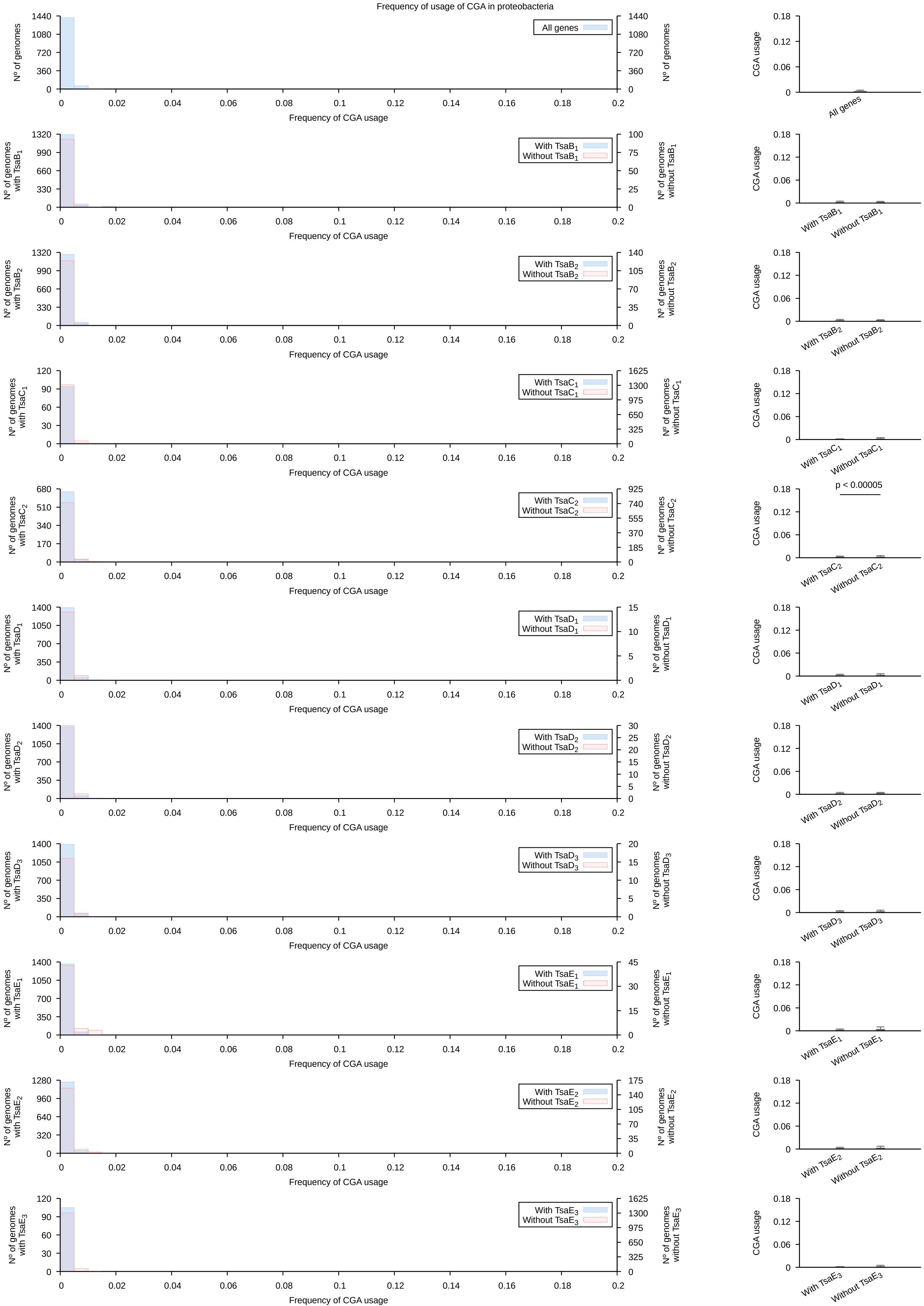

Frequency of usage of CGA in proteobacteria
Nº of genomes
All genes
Frequency of CGA usage
CGA usage
All genes
Nº of genomes with TsaB1
Nº of genomes without TsaB1
With TsaB1
Without TsaB1
Nº of genomes with TsaB2
Nº of genomes without TsaB2
With TsaB2
Without TsaB2
Nº of genomes with TsaC1
Nº of genomes without TsaC1
With TsaC1
Without TsaC1
Nº of genomes with TsaC2
Nº of genomes without TsaC2
With TsaC2
Without TsaC2
p < 0.00005
Nº of genomes with TsaD1
Nº of genomes without TsaD1
With TsaD1
Without TsaD1
Nº of genomes with TsaD2
Nº of genomes without TsaD2
With TsaD2
Without TsaD2
Nº of genomes with TsaD3
Nº of genomes without TsaD3
With TsaD3
Without TsaD3
Nº of genomes with TsaE1
Nº of genomes without TsaE1
With TsaE1
Without TsaE1
Nº of genomes with TsaE2
Nº of genomes without TsaE2
With TsaE2
Without TsaE2
Nº of genomes with TsaE3
Nº of genomes without TsaE3
With TsaE3
Without TsaE3

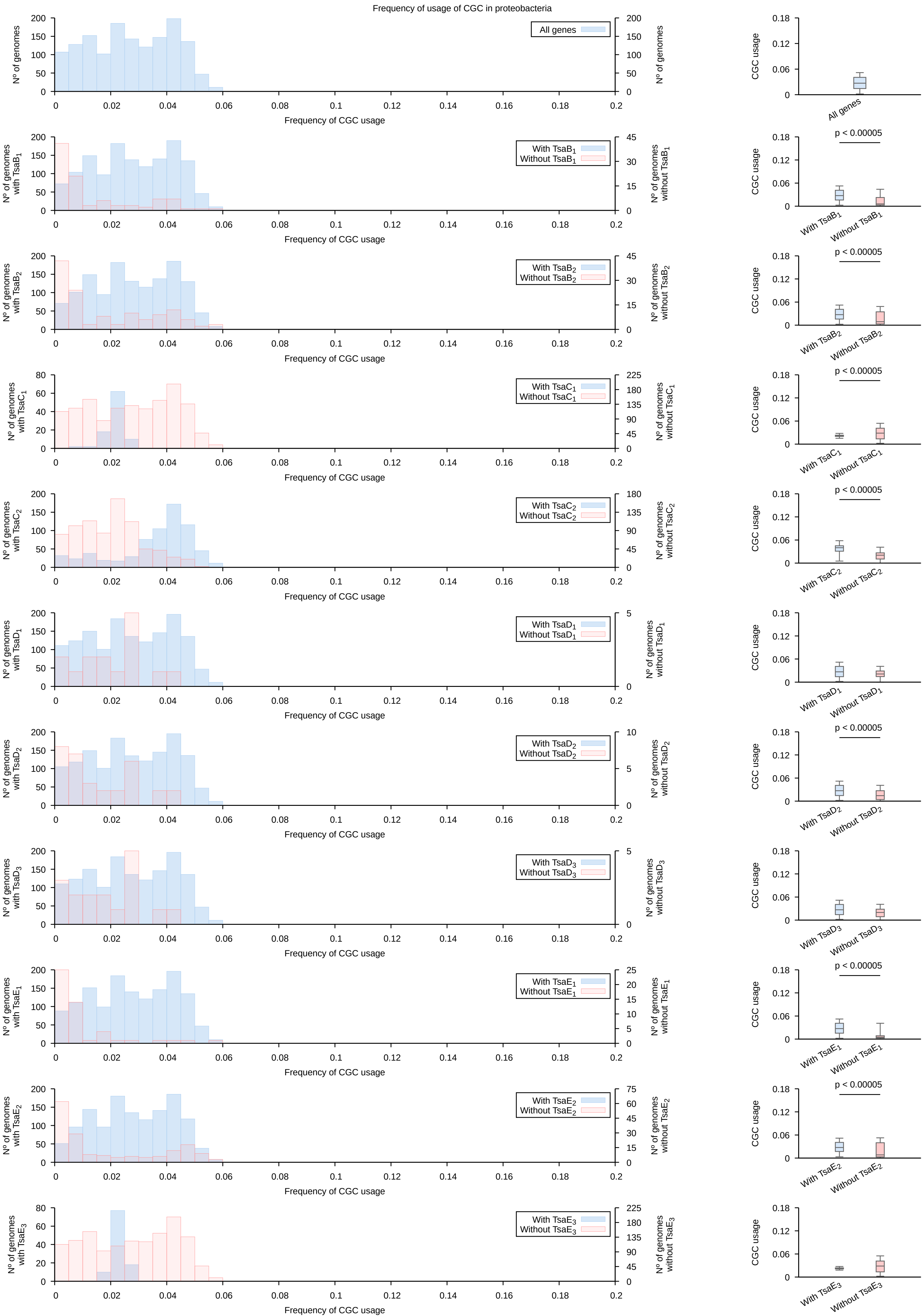

Frequency of usage of CGC in proteobacteria
Nº of genomes
All genes
Frequency of CGC usage
CGC usage
All genes
Nº of genomes with $TsaB_1$
Nº of genomes without $TsaB_1$
With $TsaB_1$
Without $TsaB_1$
p < 0.00005
Nº of genomes with $TsaB_2$
Nº of genomes without $TsaB_2$
With $TsaB_2$
Without $TsaB_2$
p < 0.00005
Nº of genomes with $TsaC_1$
Nº of genomes without $TsaC_1$
With $TsaC_1$
Without $TsaC_1$
p < 0.00005
Nº of genomes with $TsaC_2$
Nº of genomes without $TsaC_2$
With $TsaC_2$
Without $TsaC_2$
p < 0.00005
Nº of genomes with $TsaD_1$
Nº of genomes without $TsaD_1$
With $TsaD_1$
Without $TsaD_1$
Nº of genomes with $TsaD_2$
Nº of genomes without $TsaD_2$
With $TsaD_2$
Without $TsaD_2$
p < 0.00005
Nº of genomes with $TsaD_3$
Nº of genomes without $TsaD_3$
With $TsaD_3$
Without $TsaD_3$
Nº of genomes with $TsaE_1$
Nº of genomes without $TsaE_1$
With $TsaE_1$
Without $TsaE_1$
p < 0.00005
Nº of genomes with $TsaE_2$
Nº of genomes without $TsaE_2$
With $TsaE_2$
Without $TsaE_2$
p < 0.00005
Nº of genomes with $TsaE_3$
Nº of genomes without $TsaE_3$
With $TsaE_3$
Without $TsaE_3$

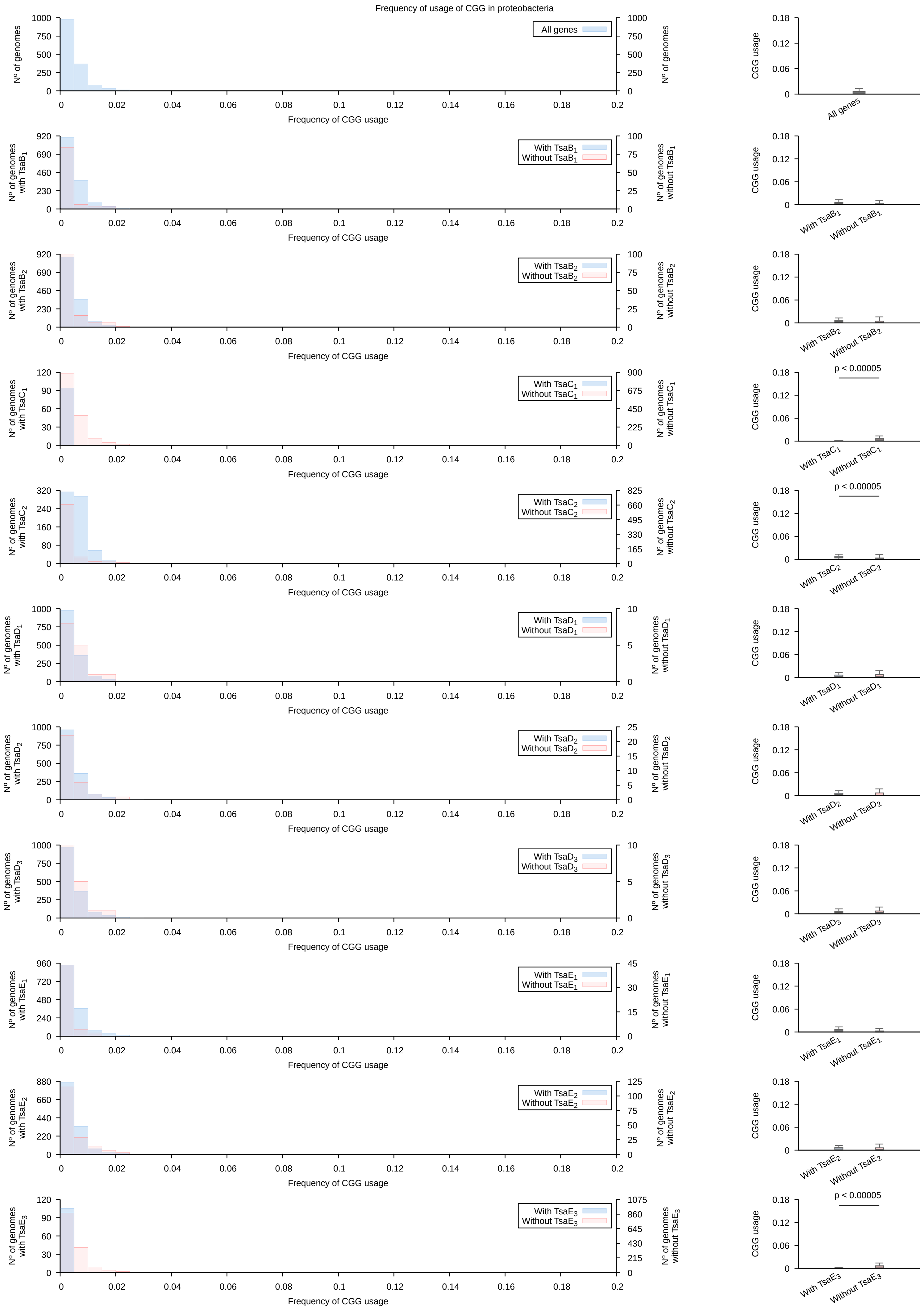
Frequency of usage of CGG in proteobacteria
Nº of genomes
All genes
Frequency of CGG usage
CGG usage
Nº of genomes with TsaB$_1$
Nº of genomes without TsaB$_1$
With TsaB$_1$
Without TsaB$_1$
Nº of genomes with TsaB$_2$
Nº of genomes without TsaB$_2$
With TsaB$_2$
Without TsaB$_2$
Nº of genomes with TsaC$_1$
Nº of genomes without TsaC$_1$
With TsaC$_1$
Without TsaC$_1$
p < 0.00005
Nº of genomes with TsaC$_2$
Nº of genomes without TsaC$_2$
With TsaC$_2$
Without TsaC$_2$
p < 0.00005
Nº of genomes with TsaD$_1$
Nº of genomes without TsaD$_1$
With TsaD$_1$
Without TsaD$_1$
Nº of genomes with TsaD$_2$
Nº of genomes without TsaD$_2$
With TsaD$_2$
Without TsaD$_2$
Nº of genomes with TsaD$_3$
Nº of genomes without TsaD$_3$
With TsaD$_3$
Without TsaD$_3$
Nº of genomes with TsaE$_1$
Nº of genomes without TsaE$_1$
With TsaE$_1$
Without TsaE$_1$
Nº of genomes with TsaE$_2$
Nº of genomes without TsaE$_2$
With TsaE$_2$
Without TsaE$_2$
Nº of genomes with TsaE$_3$
Nº of genomes without TsaE$_3$
With TsaE$_3$
Without TsaE$_3$
p < 0.00005

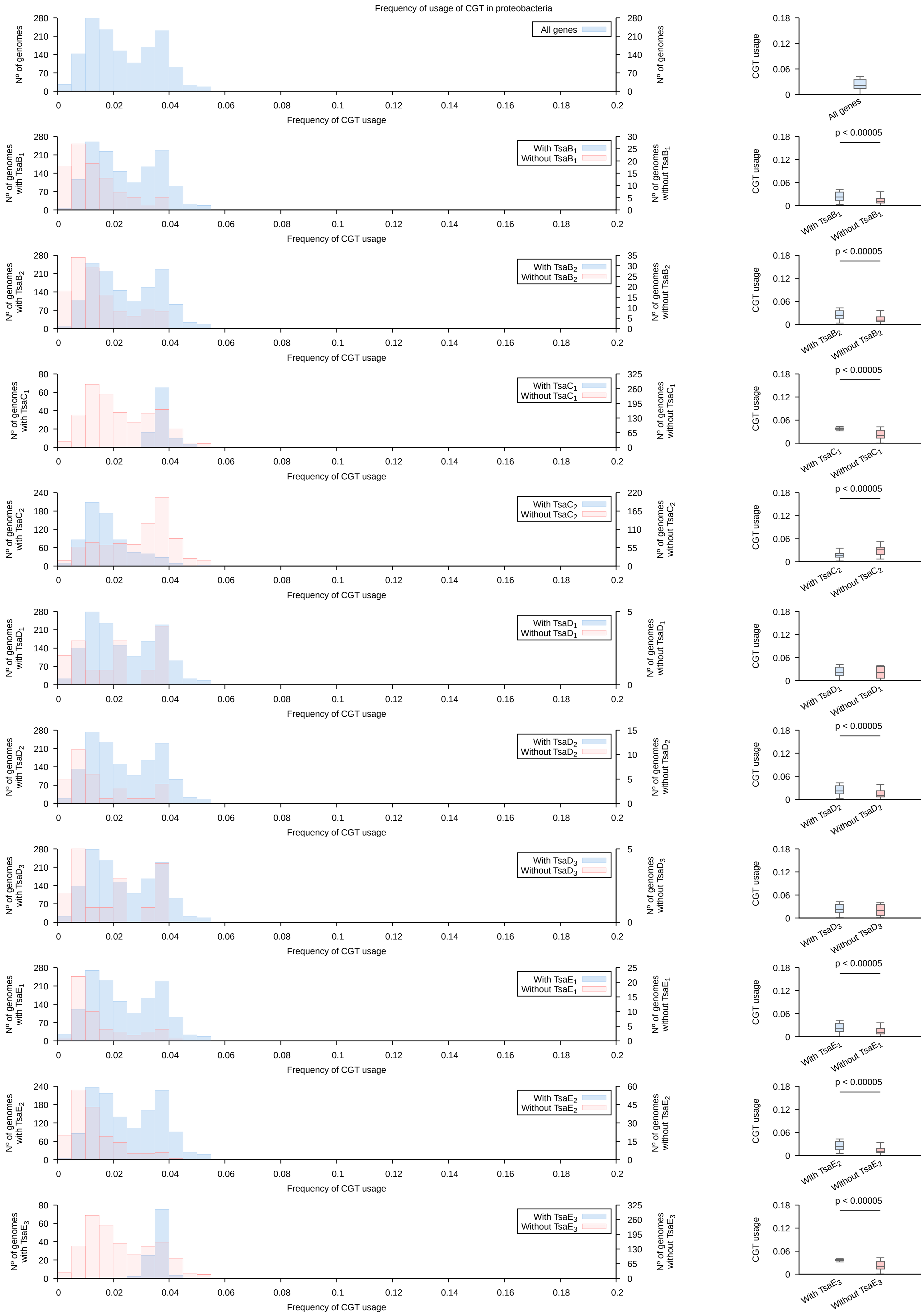

Frequency of usage of CGT in proteobacteria
All genes
With TsaB1
Without TsaB1
With TsaB2
Without TsaB2
With TsaC1
Without TsaC1
With TsaC2
Without TsaC2
With TsaD1
Without TsaD1
With TsaD2
Without TsaD2
With TsaD3
Without TsaD3
With TsaE1
Without TsaE1
With TsaE2
Without TsaE2
With TsaE3
Without TsaE3
Nº of genomes
Frequency of CGT usage
CGT usage
p < 0.00005

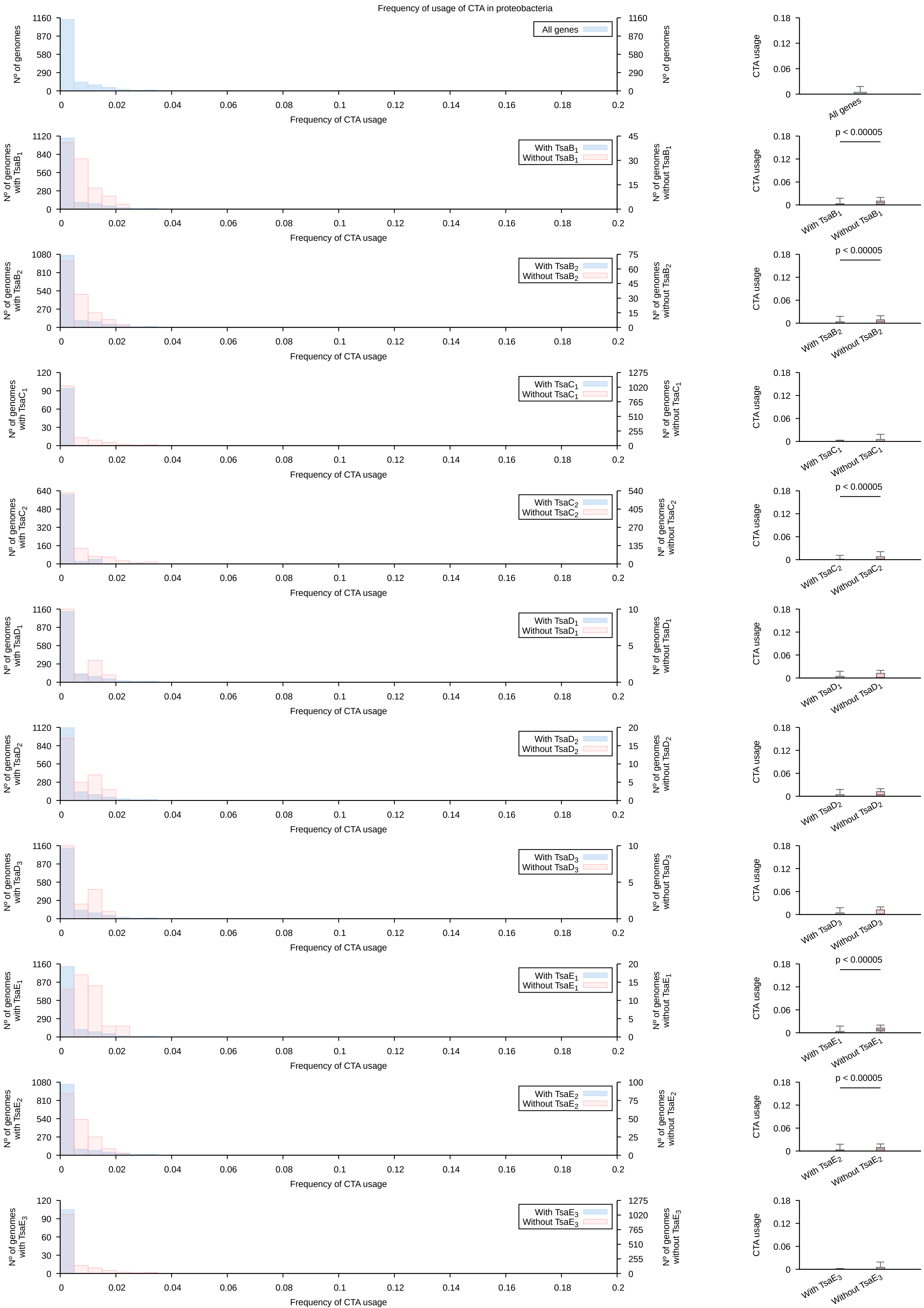

Frequency of usage of CTA in proteobacteria
Nº of genomes
Frequency of CTA usage
All genes
CTA usage
With TsaB1
Without TsaB1
With TsaB2
Without TsaB2
With TsaC1
Without TsaC1
With TsaC2
Without TsaC2
With TsaD1
Without TsaD1
With TsaD2
Without TsaD2
With TsaD3
Without TsaD3
With TsaE1
Without TsaE1
With TsaE2
Without TsaE2
With TsaE3
Without TsaE3
p < 0.00005

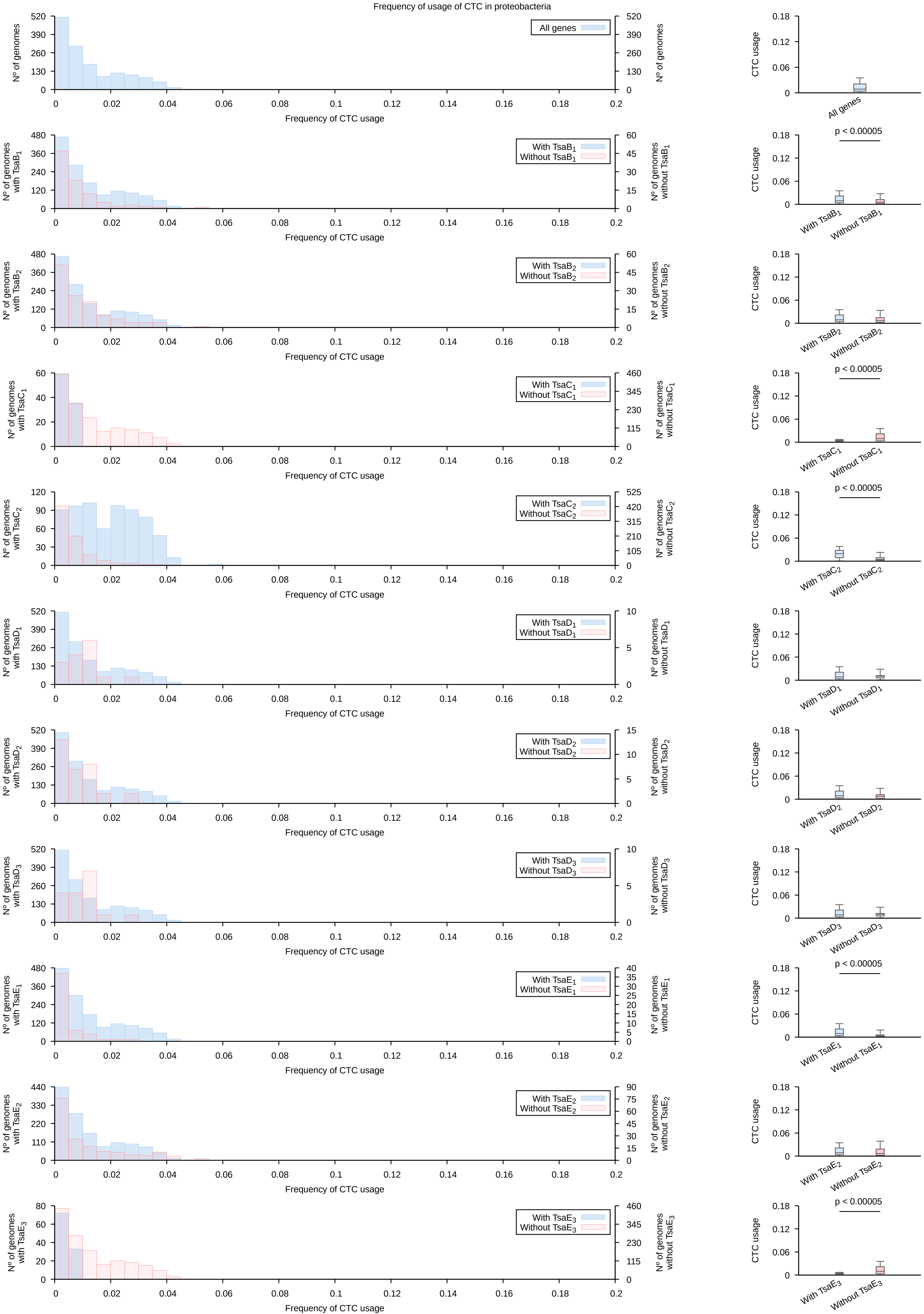

Frequency of usage of CTC in proteobacteria
All genes
Nº of genomes
Frequency of CTC usage
CTC usage
With TsaB$_1$
Without TsaB$_1$
Nº of genomes with TsaB$_1$
Nº of genomes without TsaB$_1$
p < 0.00005
With TsaB$_2$
Without TsaB$_2$
Nº of genomes with TsaB$_2$
Nº of genomes without TsaB$_2$
With TsaC$_1$
Without TsaC$_1$
Nº of genomes with TsaC$_1$
Nº of genomes without TsaC$_1$
p < 0.00005
With TsaC$_2$
Without TsaC$_2$
Nº of genomes with TsaC$_2$
Nº of genomes without TsaC$_2$
p < 0.00005
With TsaD$_1$
Without TsaD$_1$
Nº of genomes with TsaD$_1$
Nº of genomes without TsaD$_1$
With TsaD$_2$
Without TsaD$_2$
Nº of genomes with TsaD$_2$
Nº of genomes without TsaD$_2$
With TsaD$_3$
Without TsaD$_3$
Nº of genomes with TsaD$_3$
Nº of genomes without TsaD$_3$
With TsaE$_1$
Without TsaE$_1$
Nº of genomes with TsaE$_1$
Nº of genomes without TsaE$_1$
p < 0.00005
With TsaE$_2$
Without TsaE$_2$
Nº of genomes with TsaE$_2$
Nº of genomes without TsaE$_2$
With TsaE$_3$
Without TsaE$_3$
Nº of genomes with TsaE$_3$
Nº of genomes without TsaE$_3$
p < 0.00005

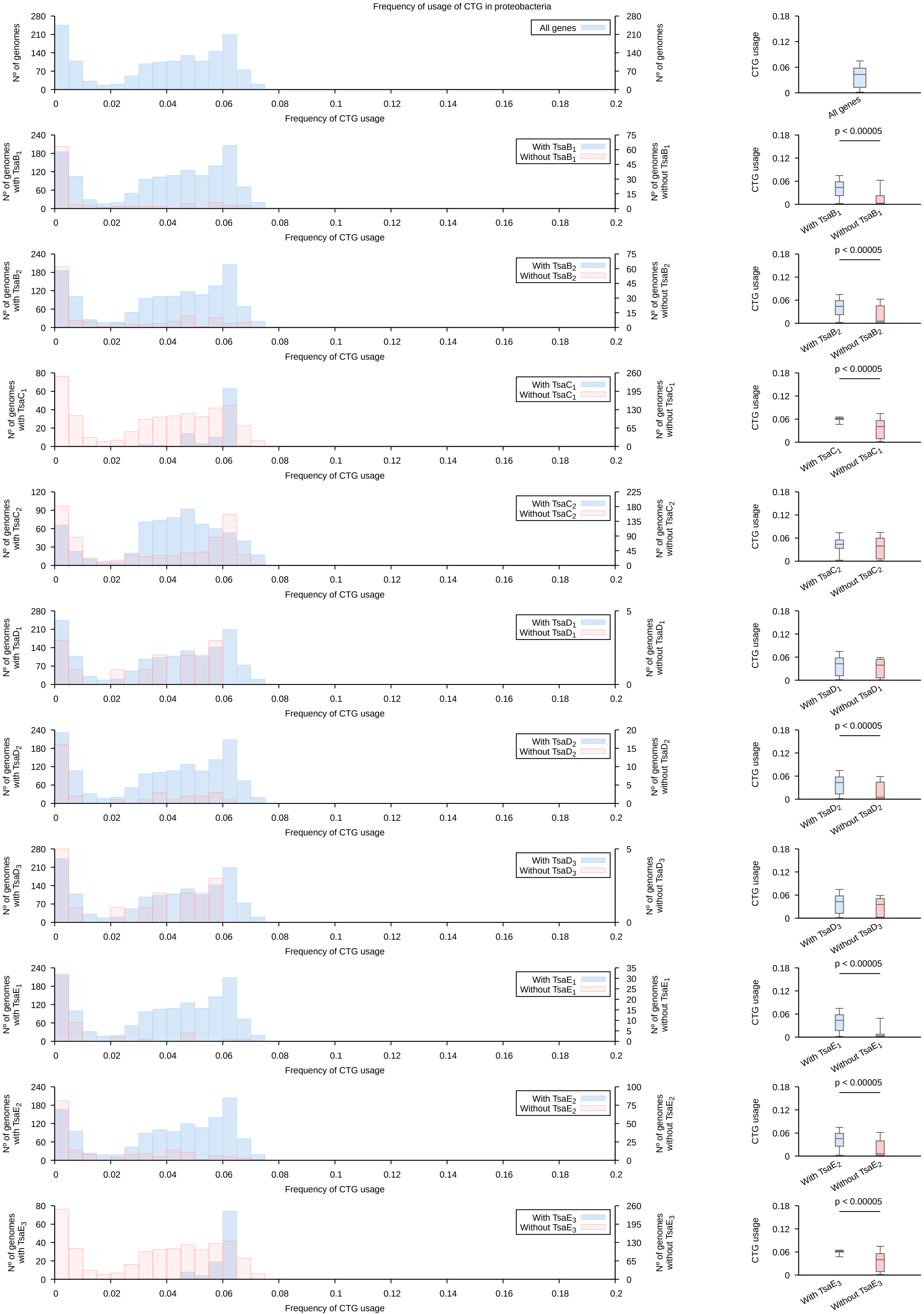

Frequency of usage of CTG in proteobacteria
Nº of genomes
All genes
CTG usage
Frequency of CTG usage
Nº of genomes with TsaB$_1$
Nº of genomes without TsaB$_1$
With TsaB$_1$
Without TsaB$_1$
Nº of genomes with TsaB$_2$
Nº of genomes without TsaB$_2$
With TsaB$_2$
Without TsaB$_2$
Nº of genomes with TsaC$_1$
Nº of genomes without TsaC$_1$
With TsaC$_1$
Without TsaC$_1$
Nº of genomes with TsaC$_2$
Nº of genomes without TsaC$_2$
With TsaC$_2$
Without TsaC$_2$
Nº of genomes with TsaD$_1$
Nº of genomes without TsaD$_1$
With TsaD$_1$
Without TsaD$_1$
Nº of genomes with TsaD$_2$
Nº of genomes without TsaD$_2$
With TsaD$_2$
Without TsaD$_2$
Nº of genomes with TsaD$_3$
Nº of genomes without TsaD$_3$
With TsaD$_3$
Without TsaD$_3$
Nº of genomes with TsaE$_1$
Nº of genomes without TsaE$_1$
With TsaE$_1$
Without TsaE$_1$
Nº of genomes with TsaE$_2$
Nº of genomes without TsaE$_2$
With TsaE$_2$
Without TsaE$_2$
Nº of genomes with TsaE$_3$
Nº of genomes without TsaE$_3$
With TsaE$_3$
Without TsaE$_3$
$p < 0.00005$

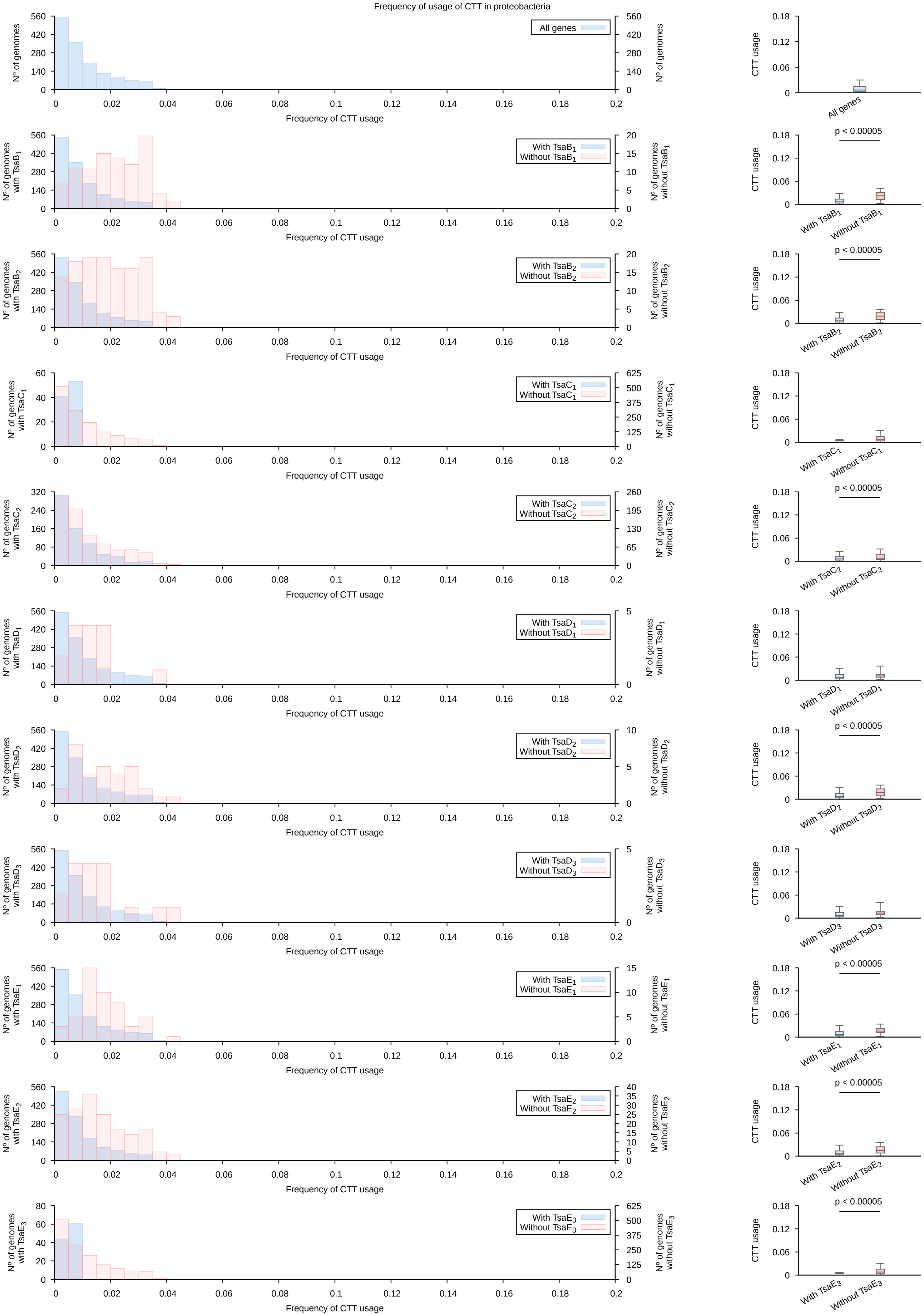

Frequency of usage of CTT in proteobacteria
Nº of genomes
All genes
Frequency of CTT usage
CTT usage
All genes
Nº of genomes with TsaB$_1$
With TsaB$_1$
Without TsaB$_1$
Nº of genomes without TsaB$_1$
p < 0.00005
Nº of genomes with TsaB$_2$
With TsaB$_2$
Without TsaB$_2$
Nº of genomes without TsaB$_2$
p < 0.00005
Nº of genomes with TsaC$_1$
With TsaC$_1$
Without TsaC$_1$
Nº of genomes without TsaC$_1$
Nº of genomes with TsaC$_2$
With TsaC$_2$
Without TsaC$_2$
Nº of genomes without TsaC$_2$
p < 0.00005
Nº of genomes with TsaD$_1$
With TsaD$_1$
Without TsaD$_1$
Nº of genomes without TsaD$_1$
Nº of genomes with TsaD$_2$
With TsaD$_2$
Without TsaD$_2$
Nº of genomes without TsaD$_2$
p < 0.00005
Nº of genomes with TsaD$_3$
With TsaD$_3$
Without TsaD$_3$
Nº of genomes without TsaD$_3$
Nº of genomes with TsaE$_1$
With TsaE$_1$
Without TsaE$_1$
Nº of genomes without TsaE$_1$
p < 0.00005
Nº of genomes with TsaE$_2$
With TsaE$_2$
Without TsaE$_2$
Nº of genomes without TsaE$_2$
p < 0.00005
Nº of genomes with TsaE$_3$
With TsaE$_3$
Without TsaE$_3$
Nº of genomes without TsaE$_3$
p < 0.00005

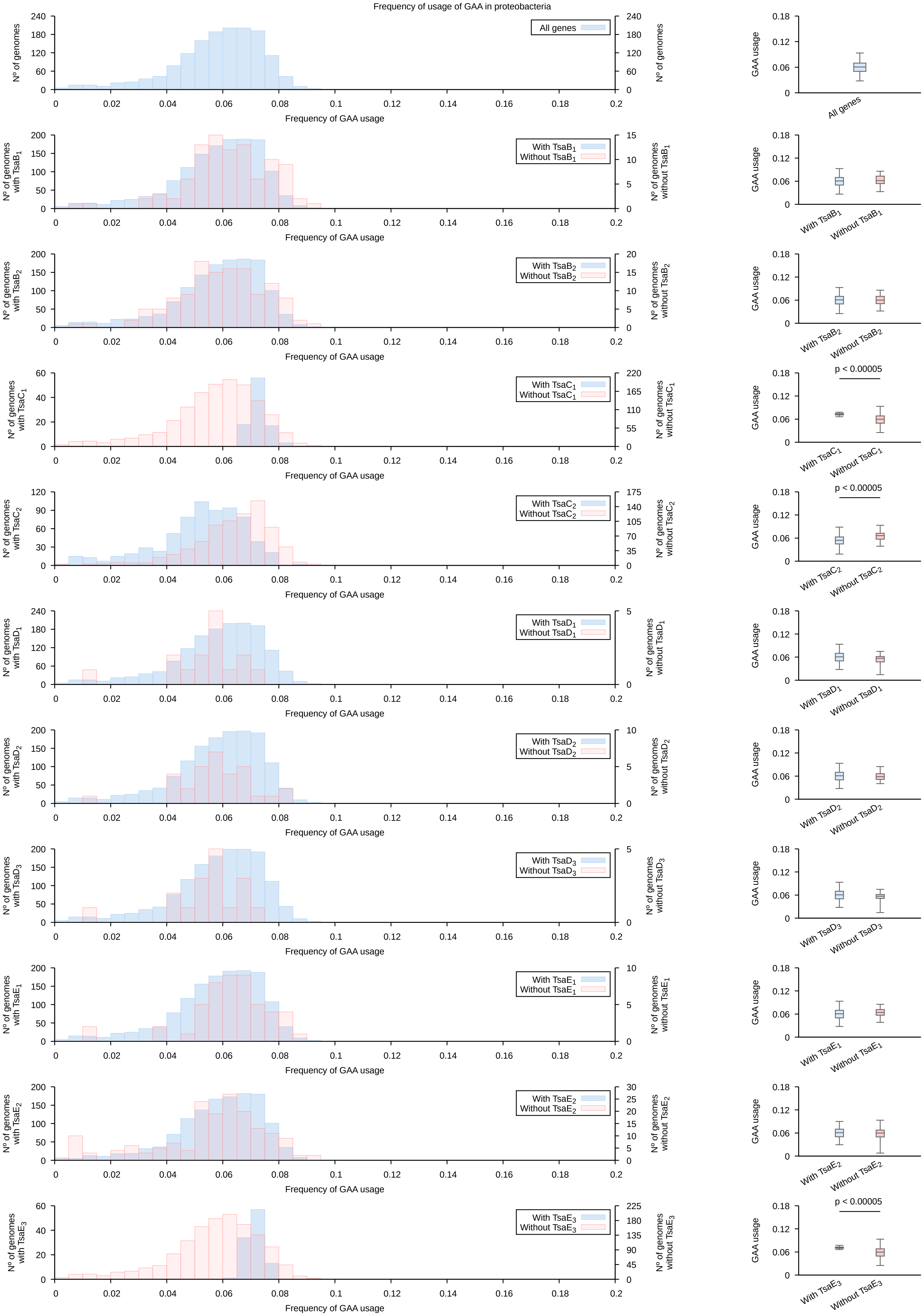

Frequency of usage of GAA in proteobacteria
Nº of genomes
All genes
Frequency of GAA usage
GAA usage
With TsaB$_1$
Without TsaB$_1$
Nº of genomes with TsaB$_1$
Nº of genomes without TsaB$_1$
With TsaB$_2$
Without TsaB$_2$
Nº of genomes with TsaB$_2$
Nº of genomes without TsaB$_2$
With TsaC$_1$
Without TsaC$_1$
Nº of genomes with TsaC$_1$
Nº of genomes without TsaC$_1$
p < 0.00005
With TsaC$_2$
Without TsaC$_2$
Nº of genomes with TsaC$_2$
Nº of genomes without TsaC$_2$
p < 0.00005
With TsaD$_1$
Without TsaD$_1$
Nº of genomes with TsaD$_1$
Nº of genomes without TsaD$_1$
With TsaD$_2$
Without TsaD$_2$
Nº of genomes with TsaD$_2$
Nº of genomes without TsaD$_2$
With TsaD$_3$
Without TsaD$_3$
Nº of genomes with TsaD$_3$
Nº of genomes without TsaD$_3$
With TsaE$_1$
Without TsaE$_1$
Nº of genomes with TsaE$_1$
Nº of genomes without TsaE$_1$
With TsaE$_2$
Without TsaE$_2$
Nº of genomes with TsaE$_2$
Nº of genomes without TsaE$_2$
With TsaE$_3$
Without TsaE$_3$
Nº of genomes with TsaE$_3$
Nº of genomes without TsaE$_3$
p < 0.00005

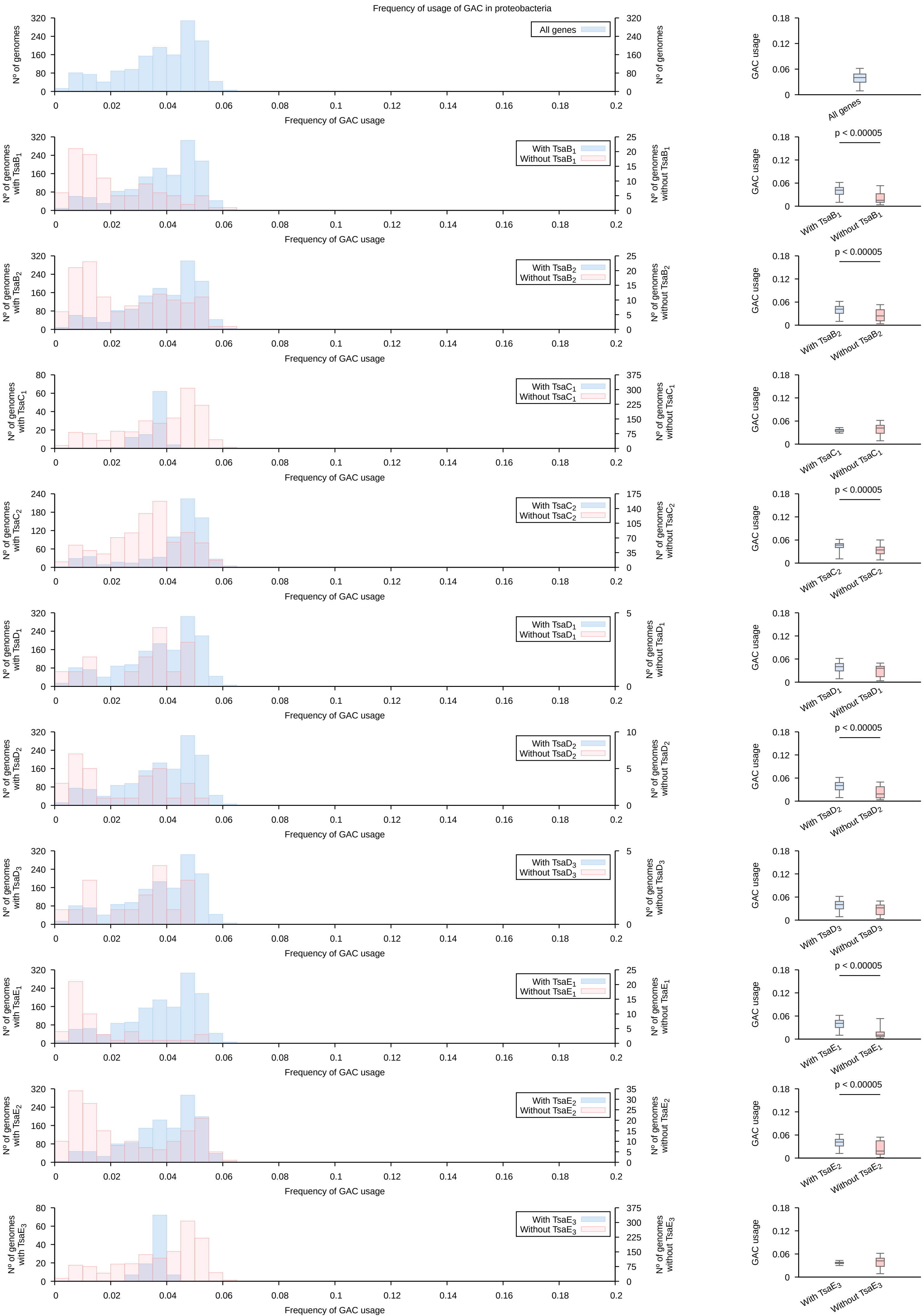
Frequency of usage of GAC in proteobacteria
All genes
Nº of genomes
Frequency of GAC usage
GAC usage
With TsaB$_1$
Without TsaB$_1$
Nº of genomes with TsaB$_1$
Nº of genomes without TsaB$_1$
p < 0.00005
With TsaB$_2$
Without TsaB$_2$
Nº of genomes with TsaB$_2$
Nº of genomes without TsaB$_2$
p < 0.00005
With TsaC$_1$
Without TsaC$_1$
Nº of genomes with TsaC$_1$
Nº of genomes without TsaC$_1$
With TsaC$_2$
Without TsaC$_2$
Nº of genomes with TsaC$_2$
Nº of genomes without TsaC$_2$
p < 0.00005
With TsaD$_1$
Without TsaD$_1$
Nº of genomes with TsaD$_1$
Nº of genomes without TsaD$_1$
With TsaD$_2$
Without TsaD$_2$
Nº of genomes with TsaD$_2$
Nº of genomes without TsaD$_2$
p < 0.00005
With TsaD$_3$
Without TsaD$_3$
Nº of genomes with TsaD$_3$
Nº of genomes without TsaD$_3$
With TsaE$_1$
Without TsaE$_1$
Nº of genomes with TsaE$_1$
Nº of genomes without TsaE$_1$
p < 0.00005
With TsaE$_2$
Without TsaE$_2$
Nº of genomes with TsaE$_2$
Nº of genomes without TsaE$_2$
p < 0.00005
With TsaE$_3$
Without TsaE$_3$
Nº of genomes with TsaE$_3$
Nº of genomes without TsaE$_3$

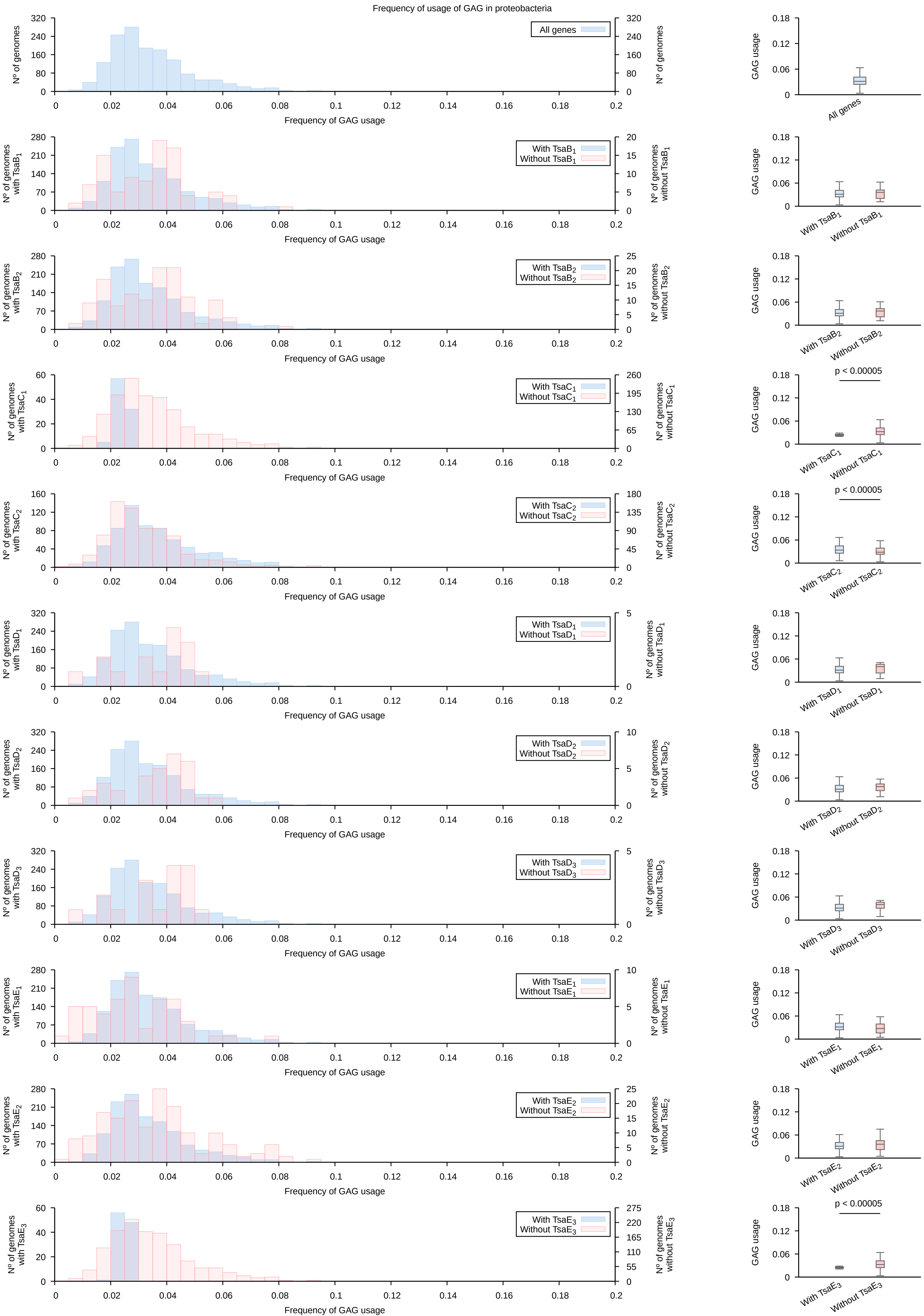
Frequency of usage of GAG in proteobacteria
All genes
With TsaB1
Without TsaB1
With TsaB2
Without TsaB2
With TsaC1
Without TsaC1
With TsaC2
Without TsaC2
With TsaD1
Without TsaD1
With TsaD2
Without TsaD2
With TsaD3
Without TsaD3
With TsaE1
Without TsaE1
With TsaE2
Without TsaE2
With TsaE3
Without TsaE3
Nº of genomes
Frequency of GAG usage
GAG usage
p < 0.00005

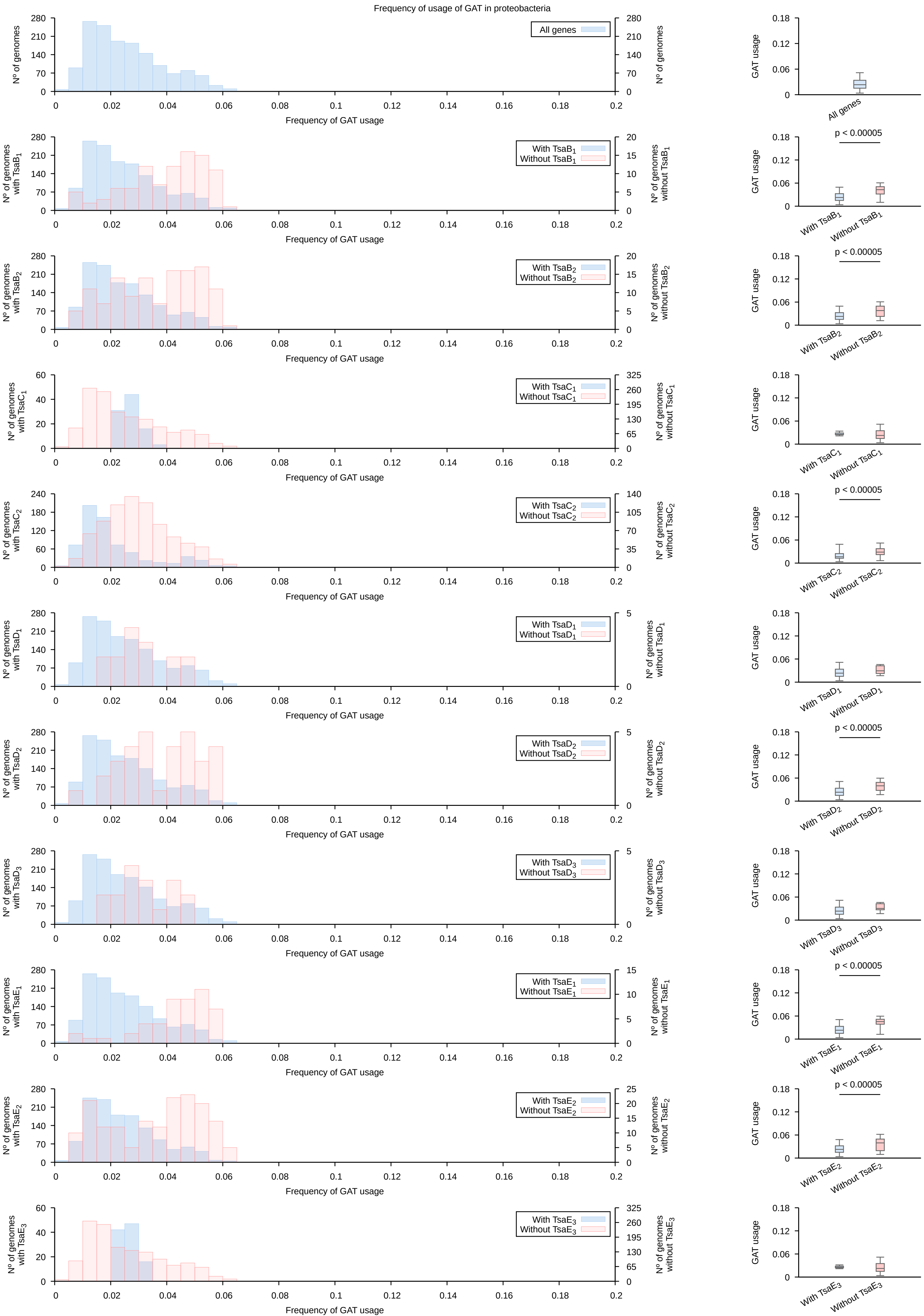
Frequency of usage of GAT in proteobacteria
Nº of genomes
All genes
Frequency of GAT usage
GAT usage
Nº of genomes with $TsaB_1$
Nº of genomes without $TsaB_1$
With $TsaB_1$
Without $TsaB_1$
p < 0.00005
Nº of genomes with $TsaB_2$
Nº of genomes without $TsaB_2$
With $TsaB_2$
Without $TsaB_2$
p < 0.00005
Nº of genomes with $TsaC_1$
Nº of genomes without $TsaC_1$
With $TsaC_1$
Without $TsaC_1$
Nº of genomes with $TsaC_2$
Nº of genomes without $TsaC_2$
With $TsaC_2$
Without $TsaC_2$
p < 0.00005
Nº of genomes with $TsaD_1$
Nº of genomes without $TsaD_1$
With $TsaD_1$
Without $TsaD_1$
Nº of genomes with $TsaD_2$
Nº of genomes without $TsaD_2$
With $TsaD_2$
Without $TsaD_2$
p < 0.00005
Nº of genomes with $TsaD_3$
Nº of genomes without $TsaD_3$
With $TsaD_3$
Without $TsaD_3$
Nº of genomes with $TsaE_1$
Nº of genomes without $TsaE_1$
With $TsaE_1$
Without $TsaE_1$
p < 0.00005
Nº of genomes with $TsaE_2$
Nº of genomes without $TsaE_2$
With $TsaE_2$
Without $TsaE_2$
p < 0.00005
Nº of genomes with $TsaE_3$
Nº of genomes without $TsaE_3$
With $TsaE_3$
Without $TsaE_3$

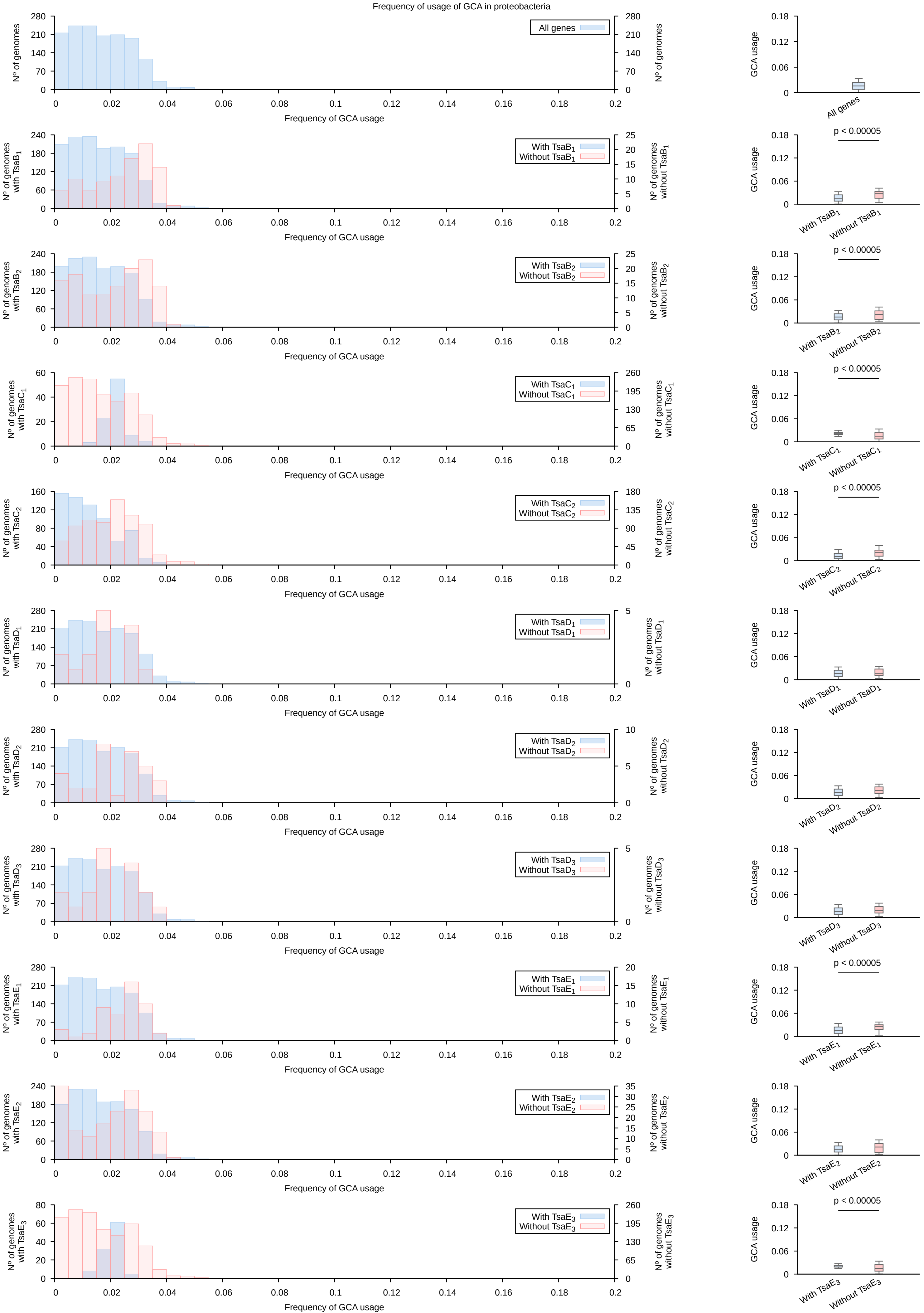

Frequency of usage of GCA in proteobacteria
Nº of genomes
All genes
Frequency of GCA usage
GCA usage
All genes
Nº of genomes with TsaB1
Nº of genomes without TsaB1
With TsaB1
Without TsaB1
p < 0.00005
Nº of genomes with TsaB2
Nº of genomes without TsaB2
With TsaB2
Without TsaB2
p < 0.00005
Nº of genomes with TsaC1
Nº of genomes without TsaC1
With TsaC1
Without TsaC1
p < 0.00005
Nº of genomes with TsaC2
Nº of genomes without TsaC2
With TsaC2
Without TsaC2
p < 0.00005
Nº of genomes with TsaD1
Nº of genomes without TsaD1
With TsaD1
Without TsaD1
Nº of genomes with TsaD2
Nº of genomes without TsaD2
With TsaD2
Without TsaD2
Nº of genomes with TsaD3
Nº of genomes without TsaD3
With TsaD3
Without TsaD3
Nº of genomes with TsaE1
Nº of genomes without TsaE1
With TsaE1
Without TsaE1
p < 0.00005
Nº of genomes with TsaE2
Nº of genomes without TsaE2
With TsaE2
Without TsaE2
Nº of genomes with TsaE3
Nº of genomes without TsaE3
With TsaE3
Without TsaE3
p < 0.00005

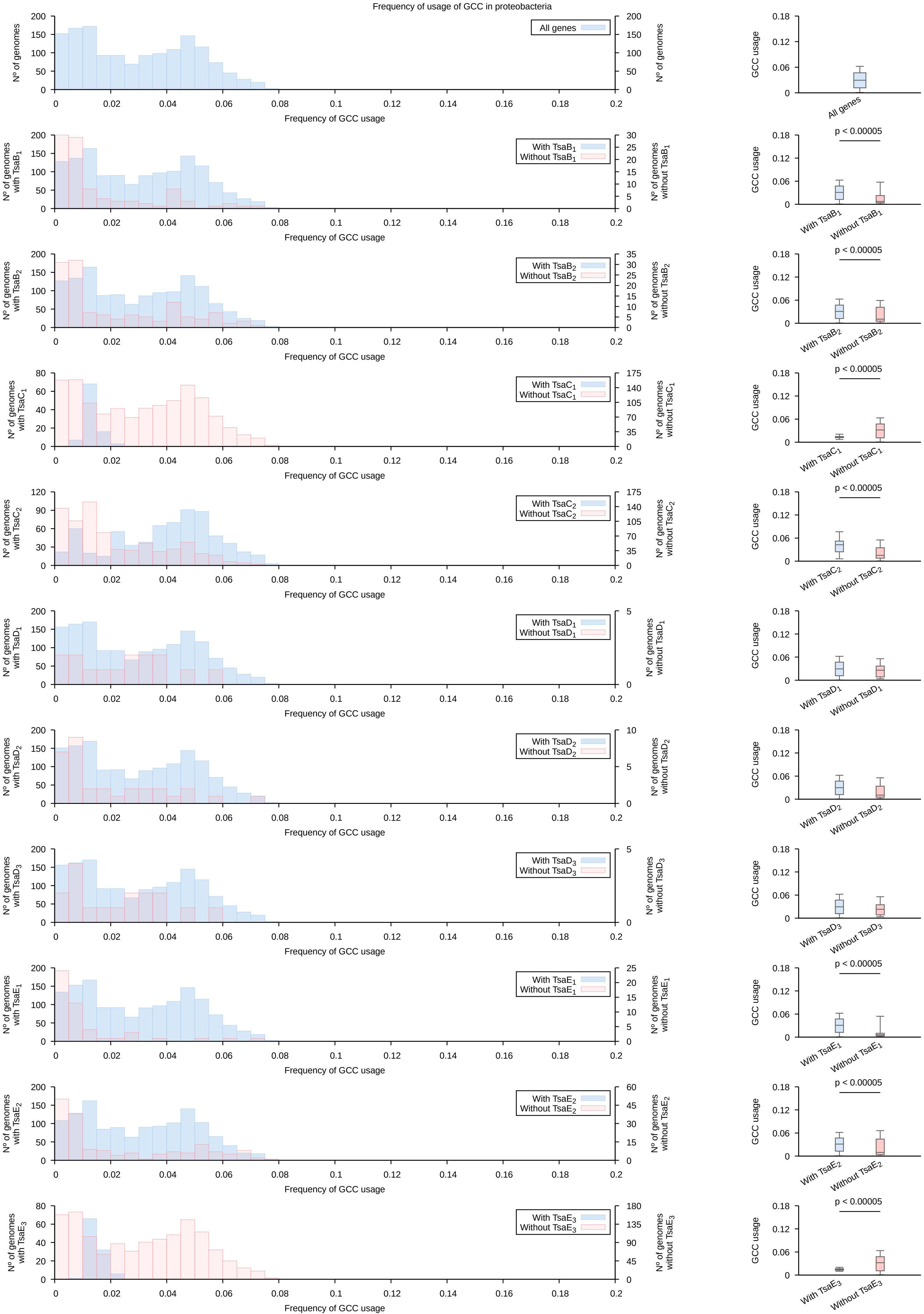

Frequency of usage of GCC in proteobacteria
Nº of genomes
All genes
Frequency of GCC usage
GCC usage
Nº of genomes with TsaB1
Nº of genomes without TsaB1
With TsaB1
Without TsaB1
p < 0.00005
Nº of genomes with TsaB2
Nº of genomes without TsaB2
With TsaB2
Without TsaB2
p < 0.00005
Nº of genomes with TsaC1
Nº of genomes without TsaC1
With TsaC1
Without TsaC1
p < 0.00005
Nº of genomes with TsaC2
Nº of genomes without TsaC2
With TsaC2
Without TsaC2
p < 0.00005
Nº of genomes with TsaD1
Nº of genomes without TsaD1
With TsaD1
Without TsaD1
Nº of genomes with TsaD2
Nº of genomes without TsaD2
With TsaD2
Without TsaD2
Nº of genomes with TsaD3
Nº of genomes without TsaD3
With TsaD3
Without TsaD3
Nº of genomes with TsaE1
Nº of genomes without TsaE1
With TsaE1
Without TsaE1
p < 0.00005
Nº of genomes with TsaE2
Nº of genomes without TsaE2
With TsaE2
Without TsaE2
p < 0.00005
Nº of genomes with TsaE3
Nº of genomes without TsaE3
With TsaE3
Without TsaE3
p < 0.00005

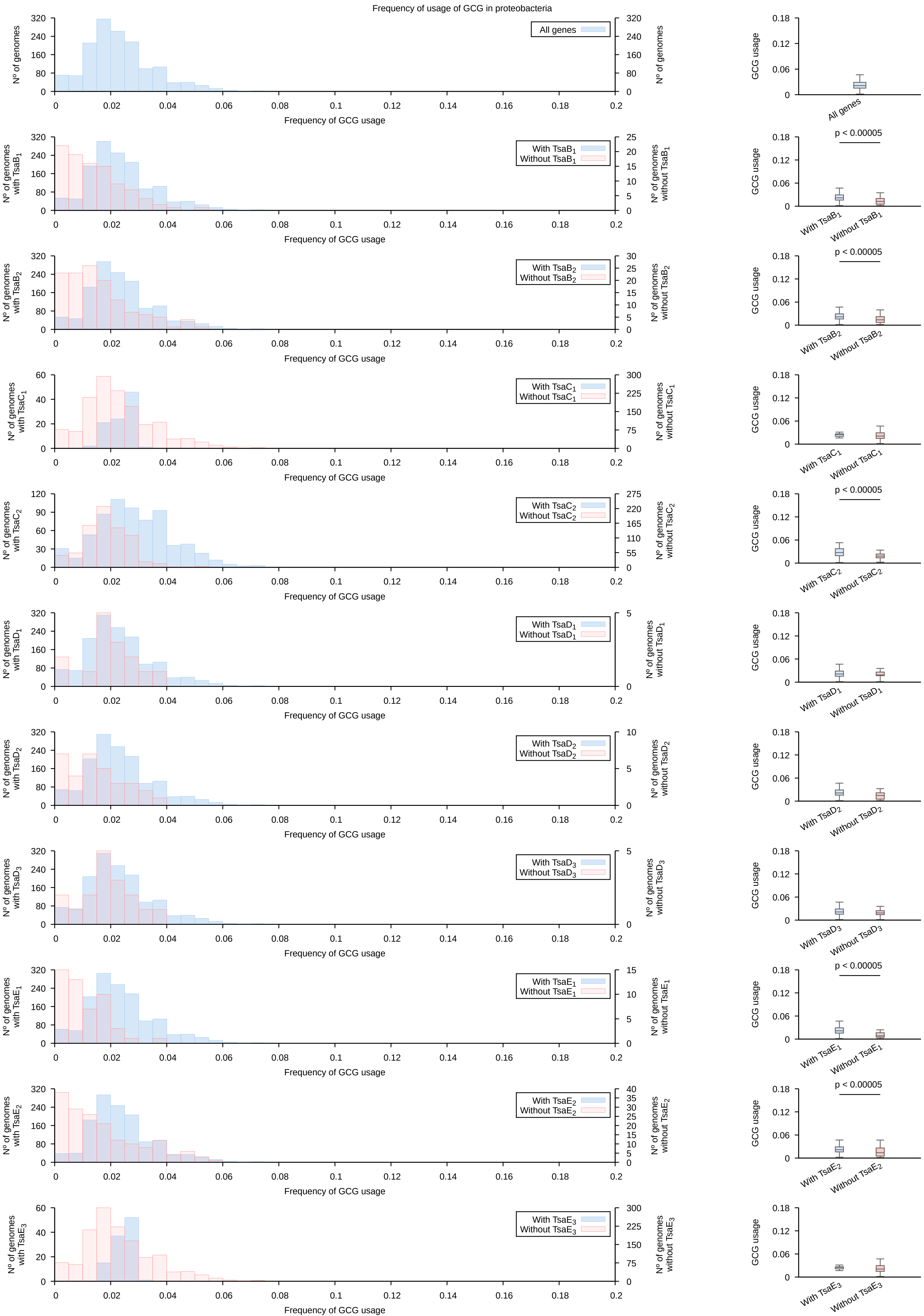

Frequency of usage of GCG in proteobacteria
Nº of genomes
All genes
Frequency of GCG usage
GCG usage
Nº of genomes with $TsaB_1$
Nº of genomes without $TsaB_1$
With $TsaB_1$
Without $TsaB_1$
p < 0.00005
Nº of genomes with $TsaB_2$
Nº of genomes without $TsaB_2$
With $TsaB_2$
Without $TsaB_2$
p < 0.00005
Nº of genomes with $TsaC_1$
Nº of genomes without $TsaC_1$
With $TsaC_1$
Without $TsaC_1$
Nº of genomes with $TsaC_2$
Nº of genomes without $TsaC_2$
With $TsaC_2$
Without $TsaC_2$
p < 0.00005
Nº of genomes with $TsaD_1$
Nº of genomes without $TsaD_1$
With $TsaD_1$
Without $TsaD_1$
Nº of genomes with $TsaD_2$
Nº of genomes without $TsaD_2$
With $TsaD_2$
Without $TsaD_2$
Nº of genomes with $TsaD_3$
Nº of genomes without $TsaD_3$
With $TsaD_3$
Without $TsaD_3$
Nº of genomes with $TsaE_1$
Nº of genomes without $TsaE_1$
With $TsaE_1$
Without $TsaE_1$
p < 0.00005
Nº of genomes with $TsaE_2$
Nº of genomes without $TsaE_2$
With $TsaE_2$
Without $TsaE_2$
p < 0.00005
Nº of genomes with $TsaE_3$
Nº of genomes without $TsaE_3$
With $TsaE_3$
Without $TsaE_3$

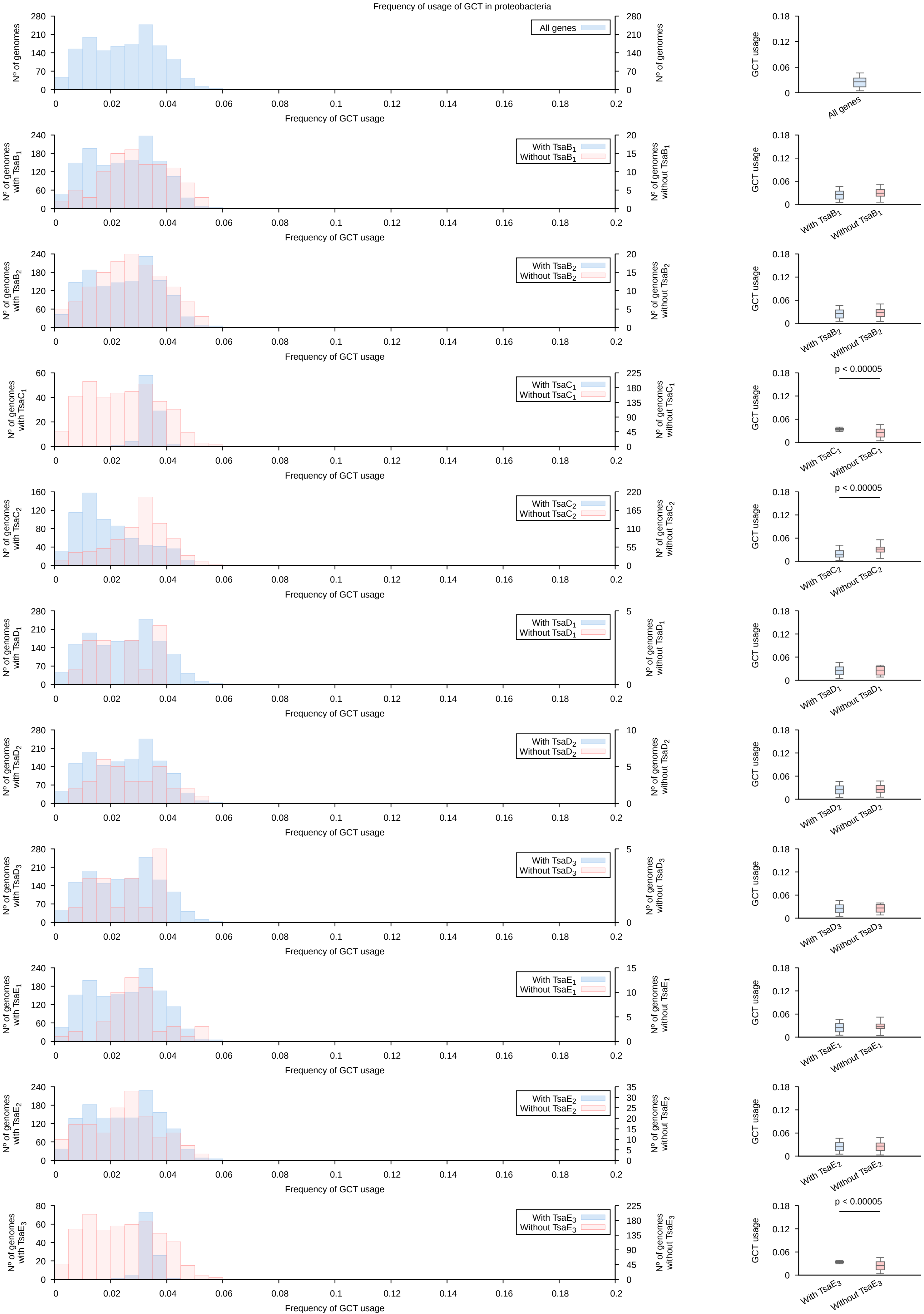

Frequency of usage of GCT in proteobacteria
All genes
Nº of genomes
Frequency of GCT usage
GCT usage
With TsaB1
Without TsaB1
Nº of genomes with TsaB1
Nº of genomes without TsaB1
With TsaB2
Without TsaB2
Nº of genomes with TsaB2
Nº of genomes without TsaB2
With TsaC1
Without TsaC1
Nº of genomes with TsaC1
Nº of genomes without TsaC1
p < 0.00005
With TsaC2
Without TsaC2
Nº of genomes with TsaC2
Nº of genomes without TsaC2
p < 0.00005
With TsaD1
Without TsaD1
Nº of genomes with TsaD1
Nº of genomes without TsaD1
With TsaD2
Without TsaD2
Nº of genomes with TsaD2
Nº of genomes without TsaD2
With TsaD3
Without TsaD3
Nº of genomes with TsaD3
Nº of genomes without TsaD3
With TsaE1
Without TsaE1
Nº of genomes with TsaE1
Nº of genomes without TsaE1
With TsaE2
Without TsaE2
Nº of genomes with TsaE2
Nº of genomes without TsaE2
With TsaE3
Without TsaE3
Nº of genomes with TsaE3
Nº of genomes without TsaE3
p < 0.00005

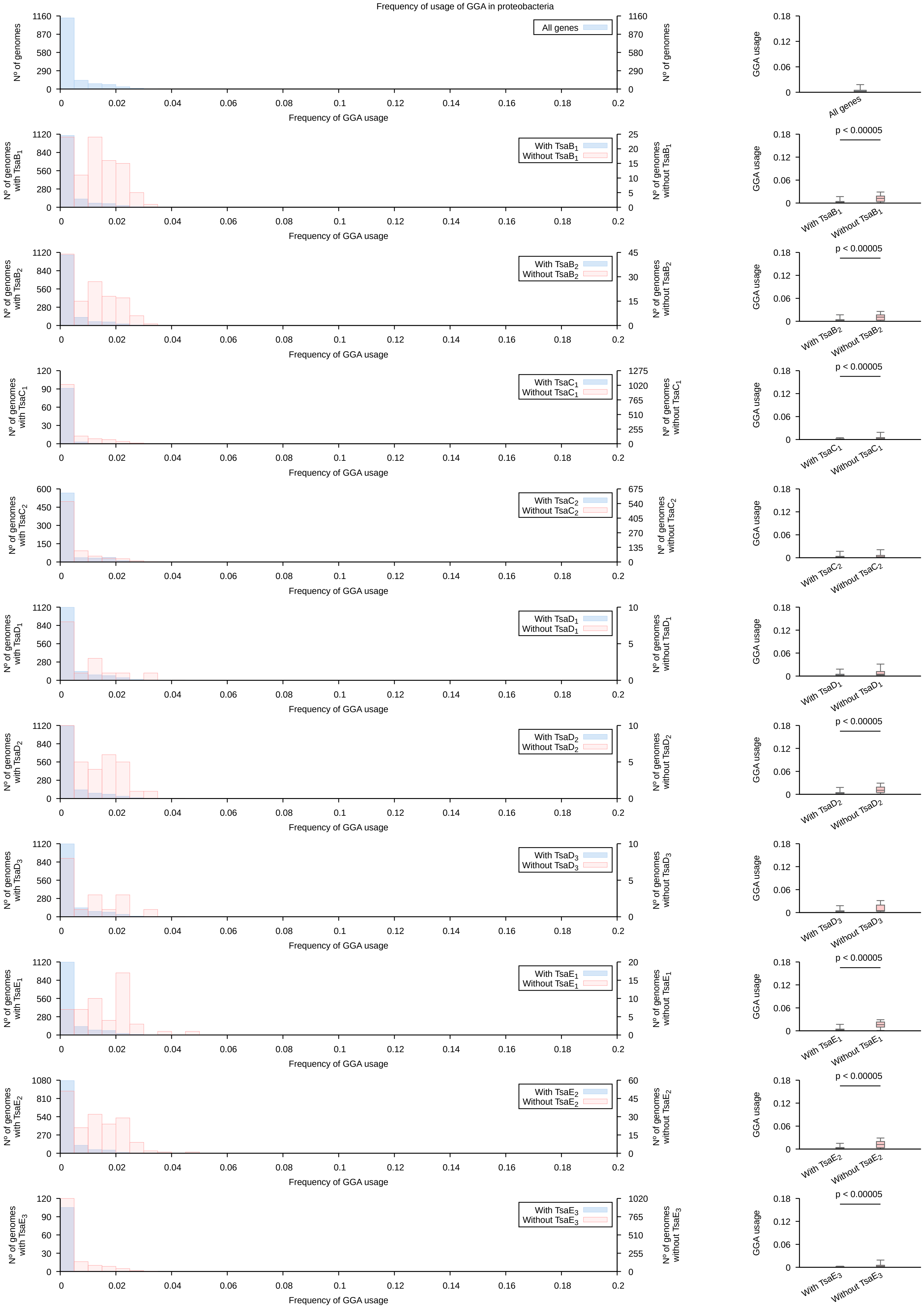

Frequency of usage of GGA in proteobacteria
All genes
Nº of genomes
Frequency of GGA usage
GGA usage
With TsaB1
Without TsaB1
Nº of genomes with TsaB1
Nº of genomes without TsaB1
p < 0.00005
With TsaB2
Without TsaB2
Nº of genomes with TsaB2
Nº of genomes without TsaB2
p < 0.00005
With TsaC1
Without TsaC1
Nº of genomes with TsaC1
Nº of genomes without TsaC1
p < 0.00005
With TsaC2
Without TsaC2
Nº of genomes with TsaC2
Nº of genomes without TsaC2
With TsaD1
Without TsaD1
Nº of genomes with TsaD1
Nº of genomes without TsaD1
With TsaD2
Without TsaD2
Nº of genomes with TsaD2
Nº of genomes without TsaD2
p < 0.00005
With TsaD3
Without TsaD3
Nº of genomes with TsaD3
Nº of genomes without TsaD3
With TsaE1
Without TsaE1
Nº of genomes with TsaE1
Nº of genomes without TsaE1
p < 0.00005
With TsaE2
Without TsaE2
Nº of genomes with TsaE2
Nº of genomes without TsaE2
p < 0.00005
With TsaE3
Without TsaE3
Nº of genomes with TsaE3
Nº of genomes without TsaE3
p < 0.00005

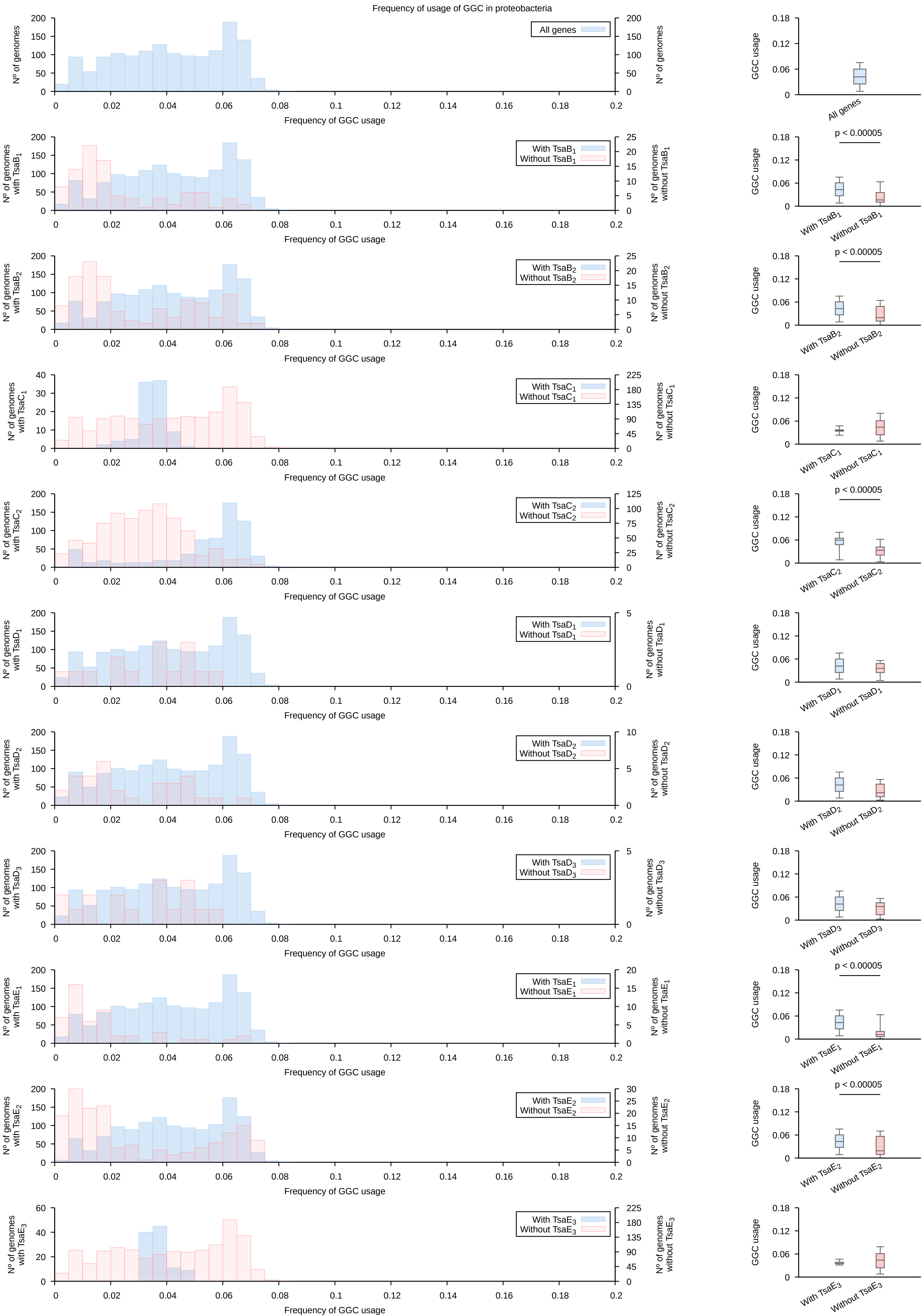
Frequency of usage of GGC in proteobacteria
Nº of genomes
Frequency of GGC usage
All genes
GGC usage
With TsaB$_1$
Without TsaB$_1$
Nº of genomes with TsaB$_1$
Nº of genomes without TsaB$_1$
p < 0.00005
With TsaB$_2$
Without TsaB$_2$
Nº of genomes with TsaB$_2$
Nº of genomes without TsaB$_2$
p < 0.00005
With TsaC$_1$
Without TsaC$_1$
Nº of genomes with TsaC$_1$
Nº of genomes without TsaC$_1$
With TsaC$_2$
Without TsaC$_2$
Nº of genomes with TsaC$_2$
Nº of genomes without TsaC$_2$
p < 0.00005
With TsaD$_1$
Without TsaD$_1$
Nº of genomes with TsaD$_1$
Nº of genomes without TsaD$_1$
With TsaD$_2$
Without TsaD$_2$
Nº of genomes with TsaD$_2$
Nº of genomes without TsaD$_2$
With TsaD$_3$
Without TsaD$_3$
Nº of genomes with TsaD$_3$
Nº of genomes without TsaD$_3$
With TsaE$_1$
Without TsaE$_1$
Nº of genomes with TsaE$_1$
Nº of genomes without TsaE$_1$
p < 0.00005
With TsaE$_2$
Without TsaE$_2$
Nº of genomes with TsaE$_2$
Nº of genomes without TsaE$_2$
p < 0.00005
With TsaE$_3$
Without TsaE$_3$
Nº of genomes with TsaE$_3$
Nº of genomes without TsaE$_3$

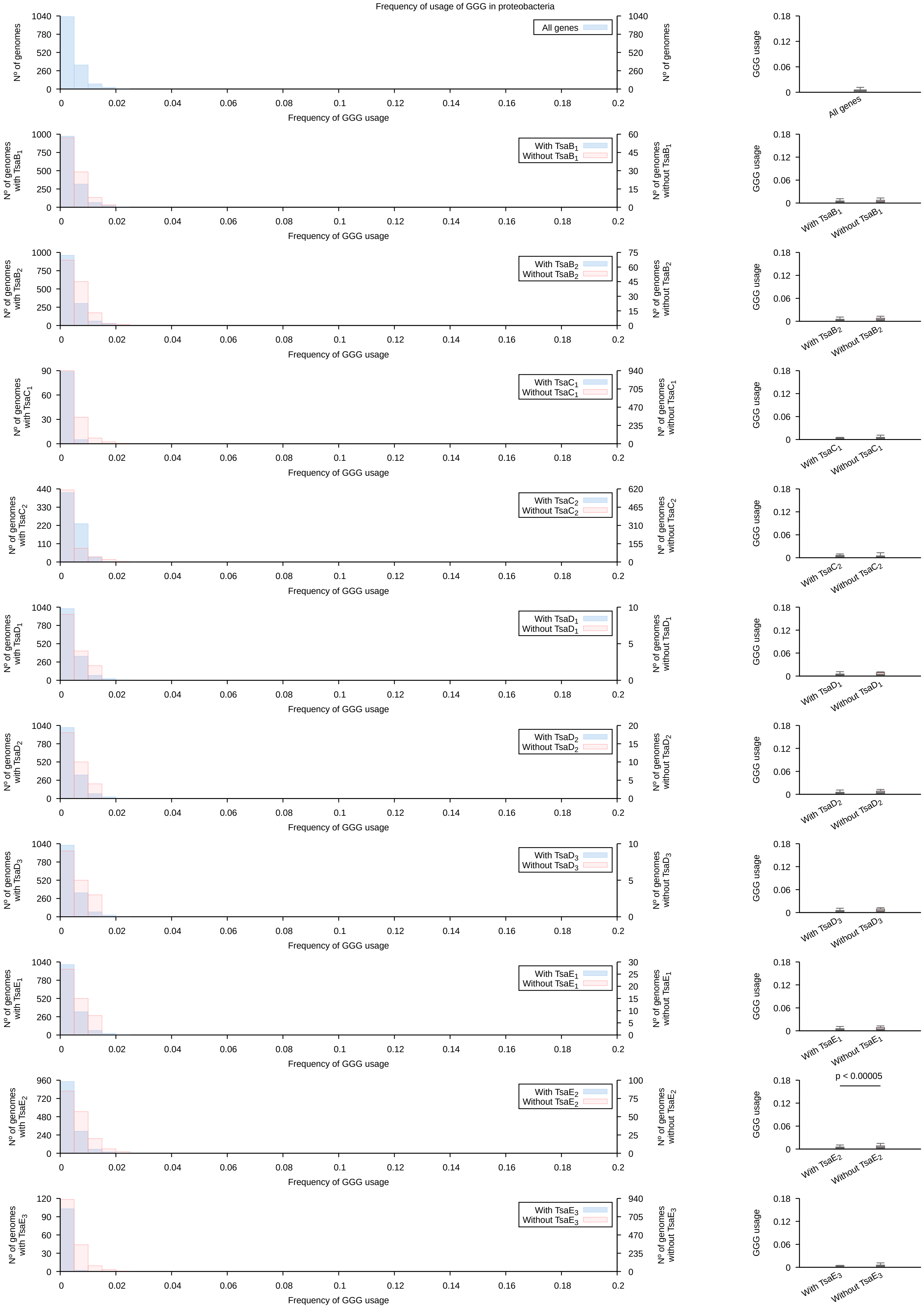
Frequency of usage of GGG in proteobacteria
Nº of genomes
Frequency of GGG usage
GGG usage
All genes
With TsaB$_1$
Without TsaB$_1$
With TsaB$_2$
Without TsaB$_2$
With TsaC$_1$
Without TsaC$_1$
With TsaC$_2$
Without TsaC$_2$
With TsaD$_1$
Without TsaD$_1$
With TsaD$_2$
Without TsaD$_2$
With TsaD$_3$
Without TsaD$_3$
With TsaE$_1$
Without TsaE$_1$
With TsaE$_2$
Without TsaE$_2$
p < 0.00005
With TsaE$_3$
Without TsaE$_3$

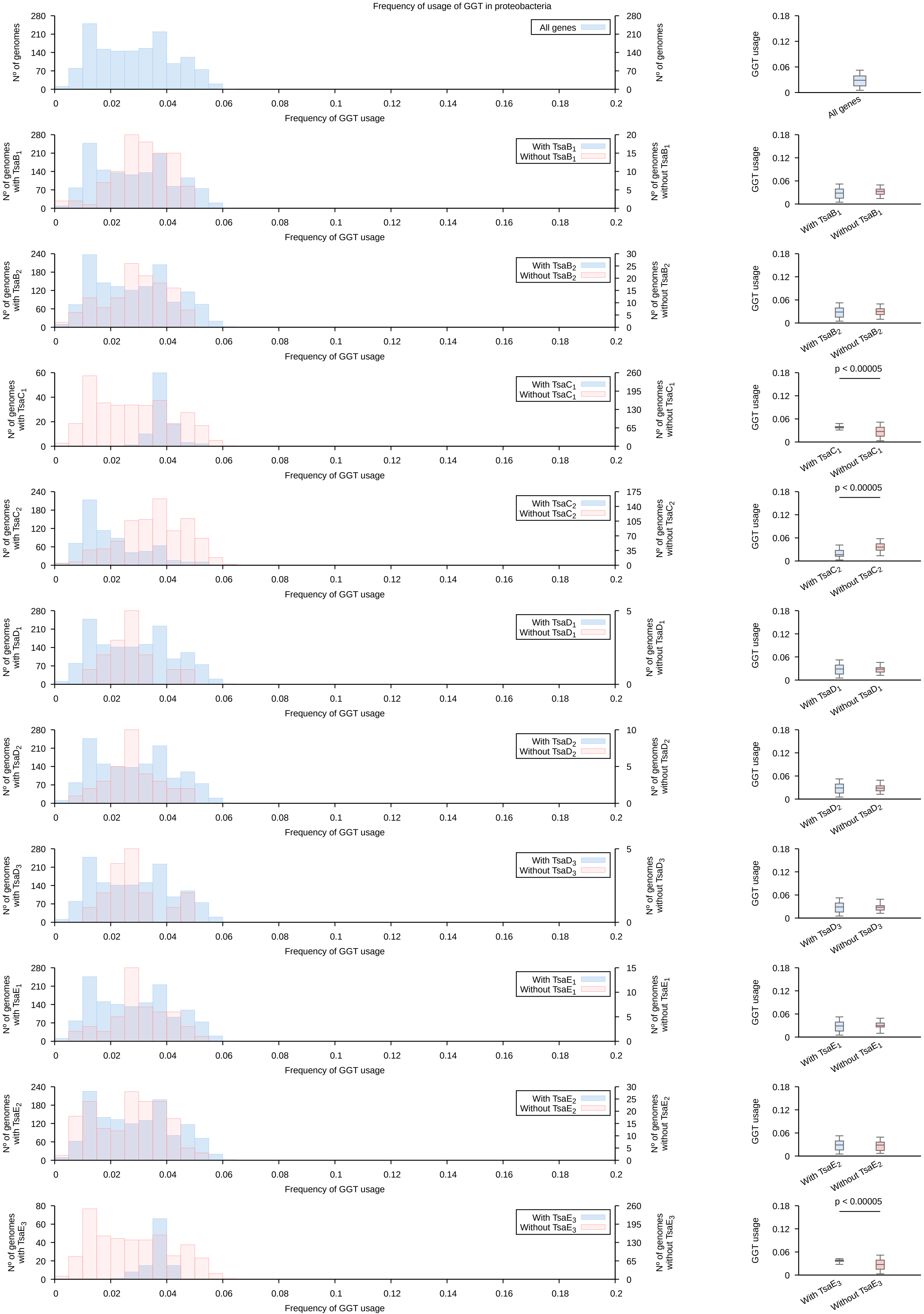

Frequency of usage of GGT in proteobacteria
Nº of genomes
All genes
Frequency of GGT usage
GGT usage
All genes
Nº of genomes with TsaB$_1$
Nº of genomes without TsaB$_1$
With TsaB$_1$
Without TsaB$_1$
Nº of genomes with TsaB$_2$
Nº of genomes without TsaB$_2$
With TsaB$_2$
Without TsaB$_2$
Nº of genomes with TsaC$_1$
Nº of genomes without TsaC$_1$
With TsaC$_1$
Without TsaC$_1$
p < 0.00005
Nº of genomes with TsaC$_2$
Nº of genomes without TsaC$_2$
With TsaC$_2$
Without TsaC$_2$
p < 0.00005
Nº of genomes with TsaD$_1$
Nº of genomes without TsaD$_1$
With TsaD$_1$
Without TsaD$_1$
Nº of genomes with TsaD$_2$
Nº of genomes without TsaD$_2$
With TsaD$_2$
Without TsaD$_2$
Nº of genomes with TsaD$_3$
Nº of genomes without TsaD$_3$
With TsaD$_3$
Without TsaD$_3$
Nº of genomes with TsaE$_1$
Nº of genomes without TsaE$_1$
With TsaE$_1$
Without TsaE$_1$
Nº of genomes with TsaE$_2$
Nº of genomes without TsaE$_2$
With TsaE$_2$
Without TsaE$_2$
Nº of genomes with TsaE$_3$
Nº of genomes without TsaE$_3$
With TsaE$_3$
Without TsaE$_3$
p < 0.00005

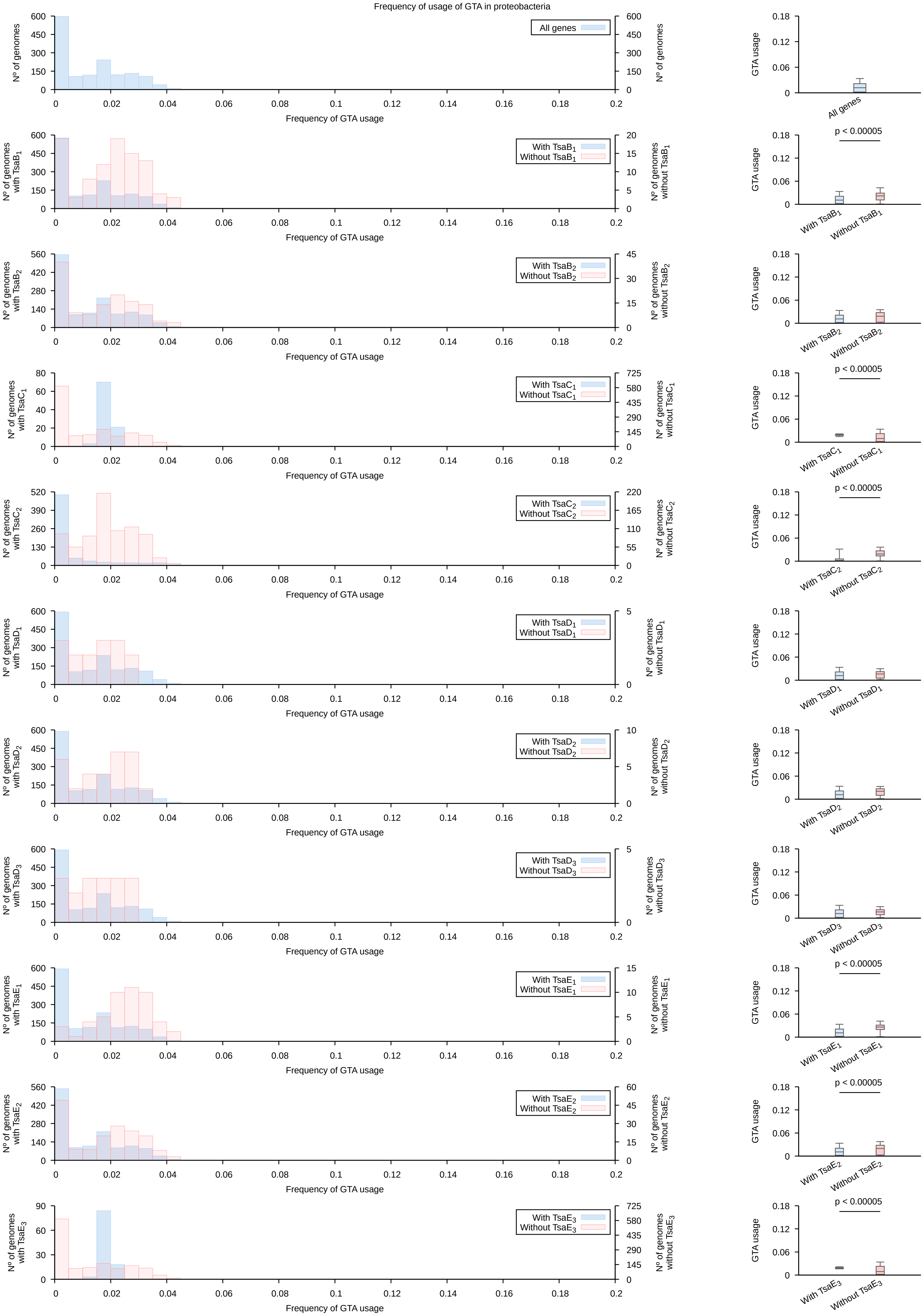

Frequency of usage of GTA in proteobacteria
All genes
Nº of genomes
Frequency of GTA usage
GTA usage
All genes
With TsaB$_1$
Without TsaB$_1$
Nº of genomes with TsaB$_1$
Nº of genomes without TsaB$_1$
p < 0.00005
With TsaB$_2$
Without TsaB$_2$
Nº of genomes with TsaB$_2$
Nº of genomes without TsaB$_2$
With TsaC$_1$
Without TsaC$_1$
Nº of genomes with TsaC$_1$
Nº of genomes without TsaC$_1$
p < 0.00005
With TsaC$_2$
Without TsaC$_2$
Nº of genomes with TsaC$_2$
Nº of genomes without TsaC$_2$
p < 0.00005
With TsaD$_1$
Without TsaD$_1$
Nº of genomes with TsaD$_1$
Nº of genomes without TsaD$_1$
With TsaD$_2$
Without TsaD$_2$
Nº of genomes with TsaD$_2$
Nº of genomes without TsaD$_2$
With TsaD$_3$
Without TsaD$_3$
Nº of genomes with TsaD$_3$
Nº of genomes without TsaD$_3$
With TsaE$_1$
Without TsaE$_1$
Nº of genomes with TsaE$_1$
Nº of genomes without TsaE$_1$
p < 0.00005
With TsaE$_2$
Without TsaE$_2$
Nº of genomes with TsaE$_2$
Nº of genomes without TsaE$_2$
p < 0.00005
With TsaE$_3$
Without TsaE$_3$
Nº of genomes with TsaE$_3$
Nº of genomes without TsaE$_3$
p < 0.00005

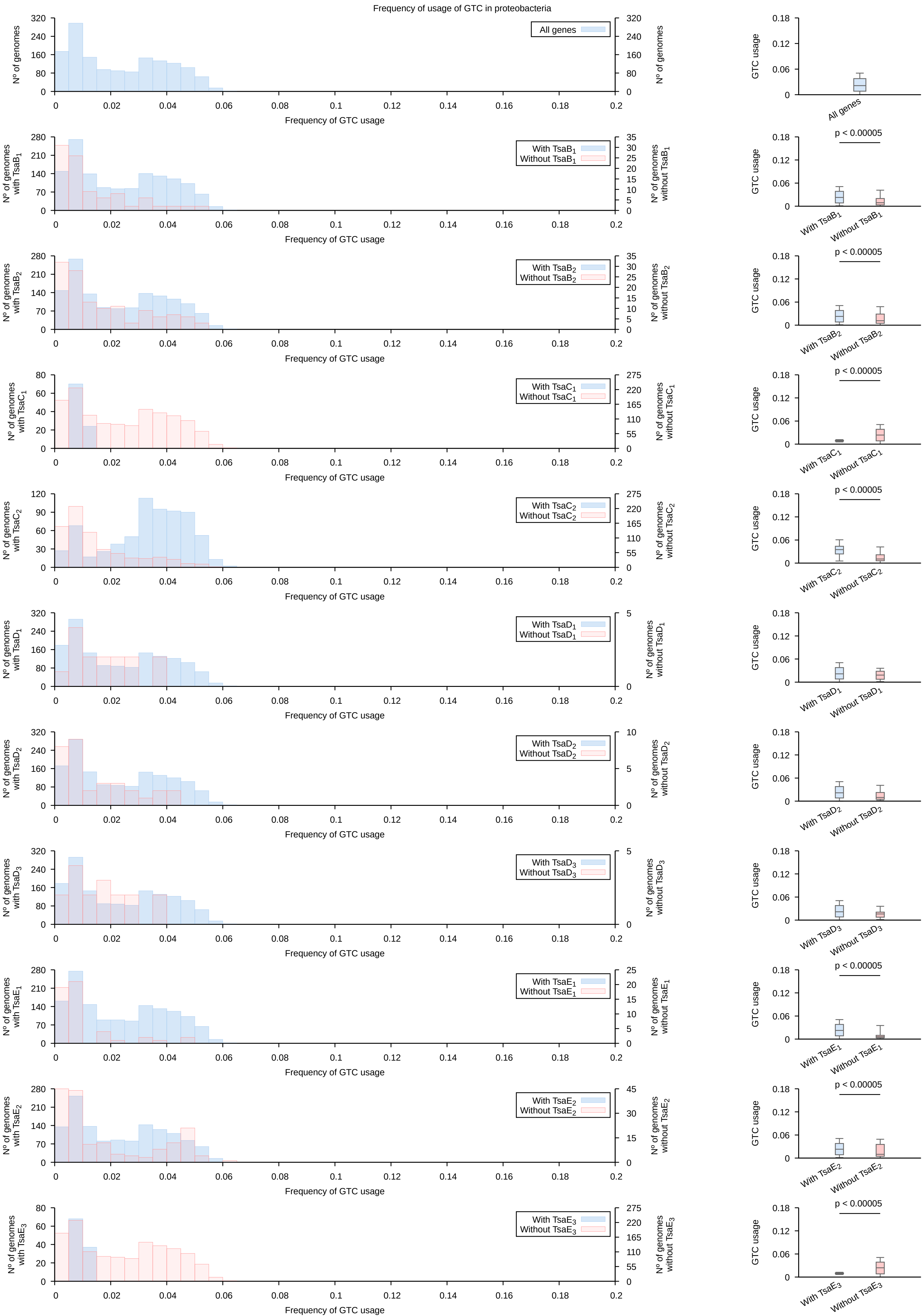
Frequency of usage of GTC in proteobacteria
Nº of genomes
All genes
Frequency of GTC usage
GTC usage
Nº of genomes with TsaB1
Nº of genomes without TsaB1
With TsaB1
Without TsaB1
p < 0.00005
Nº of genomes with TsaB2
Nº of genomes without TsaB2
With TsaB2
Without TsaB2
p < 0.00005
Nº of genomes with TsaC1
Nº of genomes without TsaC1
With TsaC1
Without TsaC1
p < 0.00005
Nº of genomes with TsaC2
Nº of genomes without TsaC2
With TsaC2
Without TsaC2
p < 0.00005
Nº of genomes with TsaD1
Nº of genomes without TsaD1
With TsaD1
Without TsaD1
Nº of genomes with TsaD2
Nº of genomes without TsaD2
With TsaD2
Without TsaD2
Nº of genomes with TsaD3
Nº of genomes without TsaD3
With TsaD3
Without TsaD3
Nº of genomes with TsaE1
Nº of genomes without TsaE1
With TsaE1
Without TsaE1
p < 0.00005
Nº of genomes with TsaE2
Nº of genomes without TsaE2
With TsaE2
Without TsaE2
p < 0.00005
Nº of genomes with TsaE3
Nº of genomes without TsaE3
With TsaE3
Without TsaE3
p < 0.00005

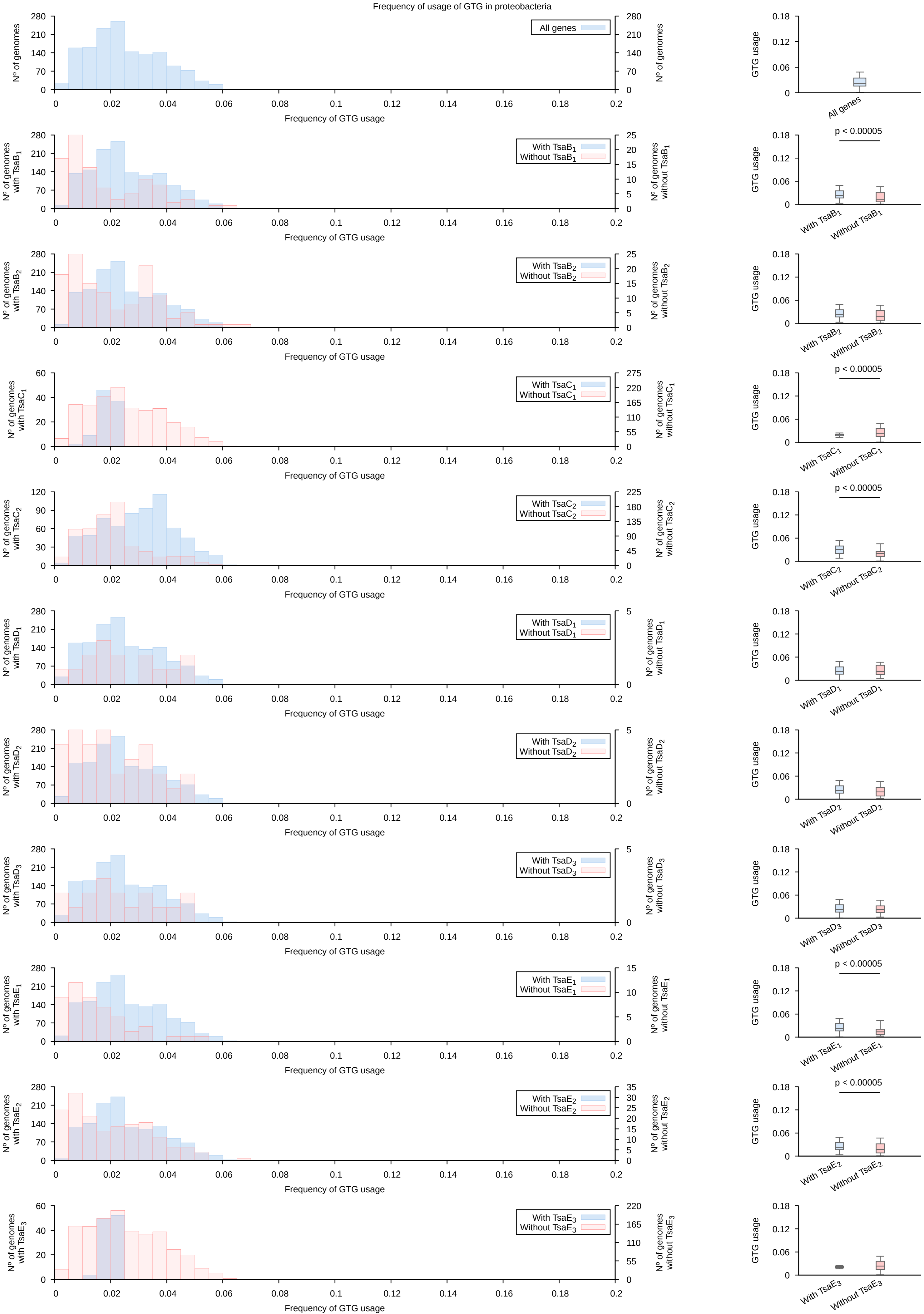
Frequency of usage of GTG in proteobacteria
Nº of genomes
All genes
Frequency of GTG usage
GTG usage
Nº of genomes with TsaB1
Nº of genomes without TsaB1
With TsaB1
Without TsaB1
p < 0.00005
Nº of genomes with TsaB2
Nº of genomes without TsaB2
With TsaB2
Without TsaB2
Nº of genomes with TsaC1
Nº of genomes without TsaC1
With TsaC1
Without TsaC1
Nº of genomes with TsaC2
Nº of genomes without TsaC2
With TsaC2
Without TsaC2
Nº of genomes with TsaD1
Nº of genomes without TsaD1
With TsaD1
Without TsaD1
Nº of genomes with TsaD2
Nº of genomes without TsaD2
With TsaD2
Without TsaD2
Nº of genomes with TsaD3
Nº of genomes without TsaD3
With TsaD3
Without TsaD3
Nº of genomes with TsaE1
Nº of genomes without TsaE1
With TsaE1
Without TsaE1
Nº of genomes with TsaE2
Nº of genomes without TsaE2
With TsaE2
Without TsaE2
Nº of genomes with TsaE3
Nº of genomes without TsaE3
With TsaE3
Without TsaE3

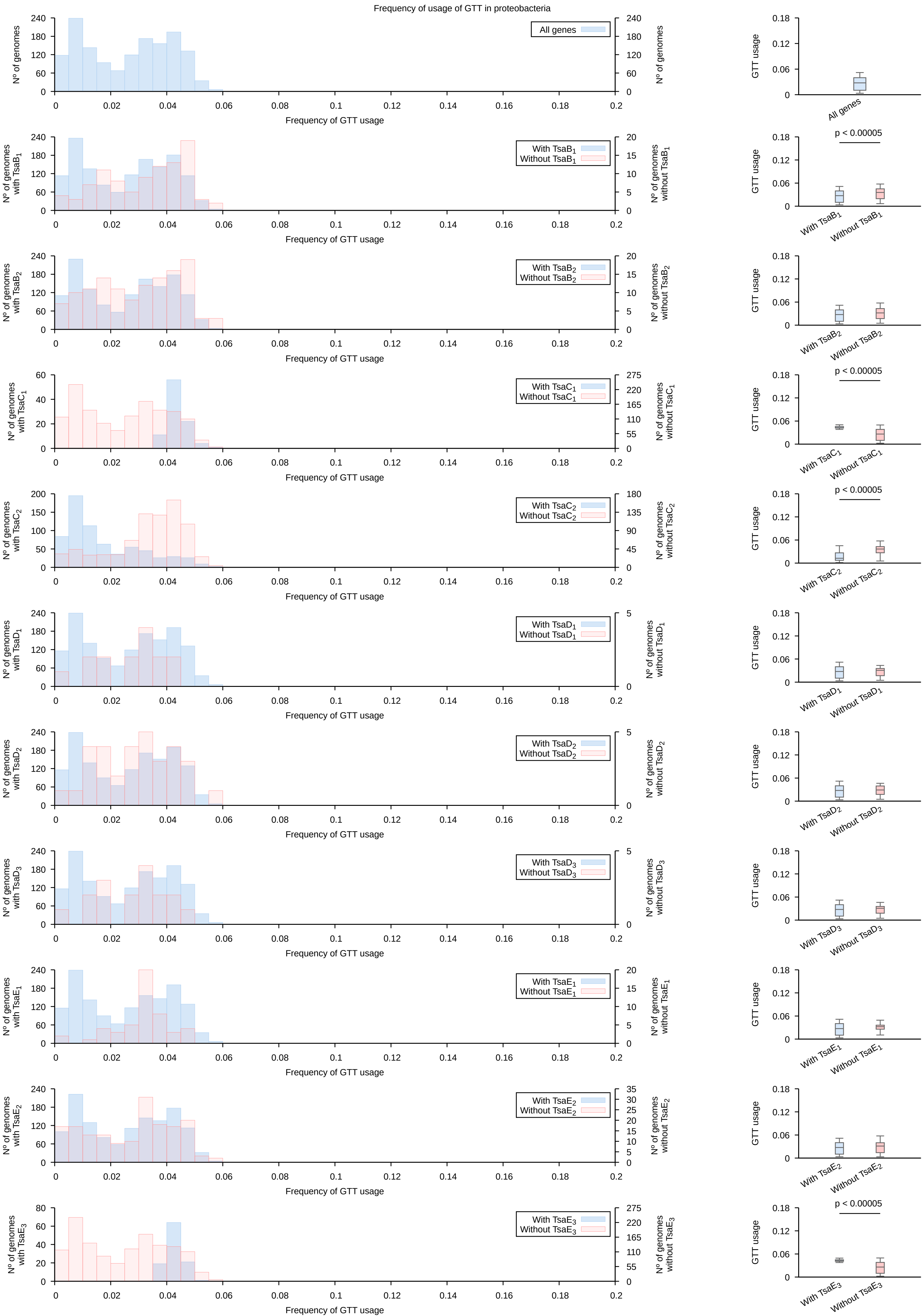
Frequency of usage of GTT in proteobacteria
All genes
Nº of genomes
Frequency of GTT usage
GTT usage
With TsaB1
Without TsaB1
Nº of genomes with TsaB1
Nº of genomes without TsaB1
p < 0.00005
With TsaB2
Without TsaB2
Nº of genomes with TsaB2
Nº of genomes without TsaB2
With TsaC1
Without TsaC1
Nº of genomes with TsaC1
Nº of genomes without TsaC1
p < 0.00005
With TsaC2
Without TsaC2
Nº of genomes with TsaC2
Nº of genomes without TsaC2
p < 0.00005
With TsaD1
Without TsaD1
Nº of genomes with TsaD1
Nº of genomes without TsaD1
With TsaD2
Without TsaD2
Nº of genomes with TsaD2
Nº of genomes without TsaD2
With TsaD3
Without TsaD3
Nº of genomes with TsaD3
Nº of genomes without TsaD3
With TsaE1
Without TsaE1
Nº of genomes with TsaE1
Nº of genomes without TsaE1
With TsaE2
Without TsaE2
Nº of genomes with TsaE2
Nº of genomes without TsaE2
With TsaE3
Without TsaE3
Nº of genomes with TsaE3
Nº of genomes without TsaE3
p < 0.00005

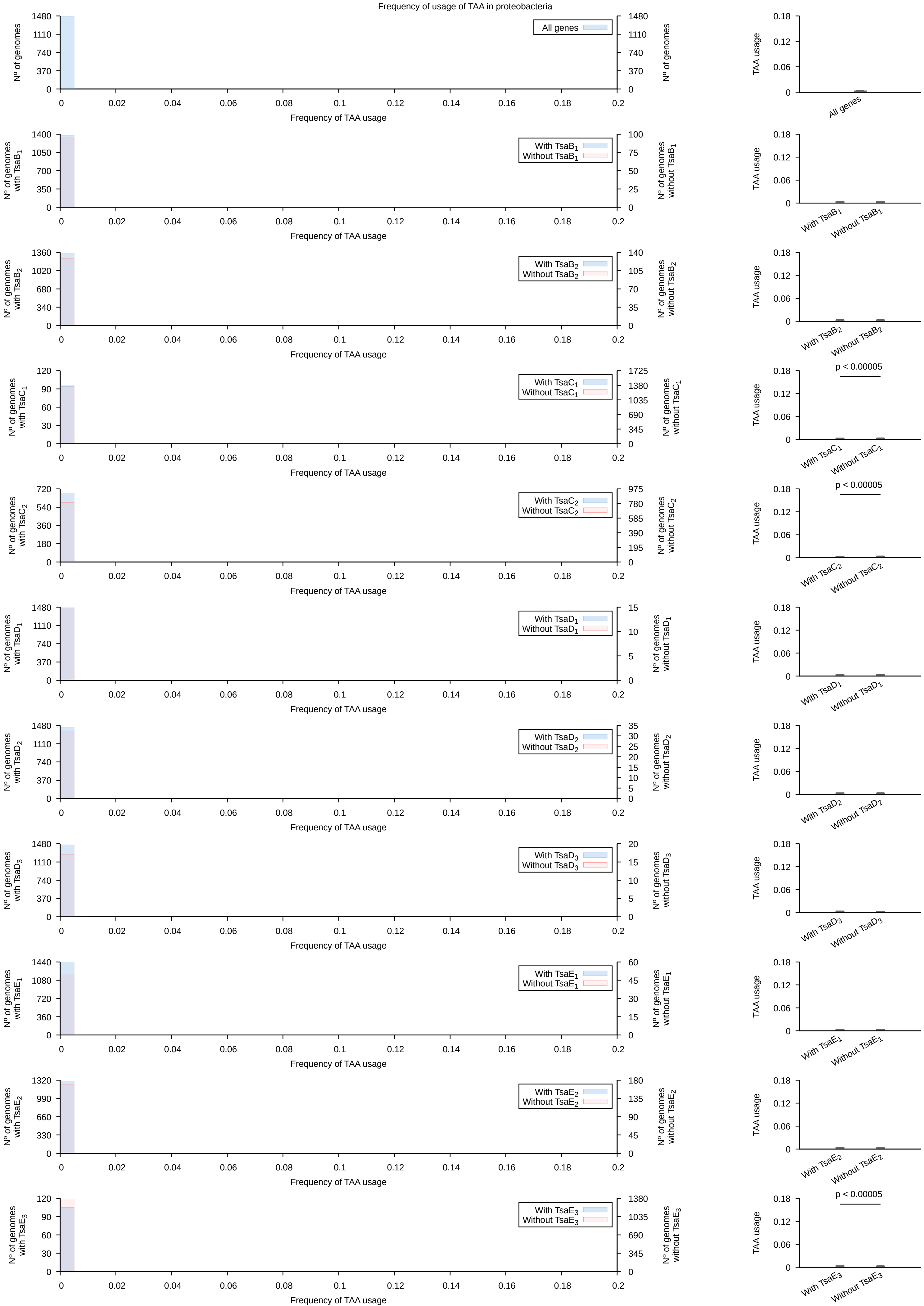
Frequency of usage of TAA in proteobacteria
All genes
Nº of genomes
Frequency of TAA usage
TAA usage
With TsaB1
Without TsaB1
Nº of genomes with TsaB1
Nº of genomes without TsaB1
With TsaB2
Without TsaB2
Nº of genomes with TsaB2
Nº of genomes without TsaB2
With TsaC1
Without TsaC1
Nº of genomes with TsaC1
Nº of genomes without TsaC1
p < 0.00005
With TsaC2
Without TsaC2
Nº of genomes with TsaC2
Nº of genomes without TsaC2
p < 0.00005
With TsaD1
Without TsaD1
Nº of genomes with TsaD1
Nº of genomes without TsaD1
With TsaD2
Without TsaD2
Nº of genomes with TsaD2
Nº of genomes without TsaD2
With TsaD3
Without TsaD3
Nº of genomes with TsaD3
Nº of genomes without TsaD3
With TsaE1
Without TsaE1
Nº of genomes with TsaE1
Nº of genomes without TsaE1
With TsaE2
Without TsaE2
Nº of genomes with TsaE2
Nº of genomes without TsaE2
With TsaE3
Without TsaE3
Nº of genomes with TsaE3
Nº of genomes without TsaE3
p < 0.00005

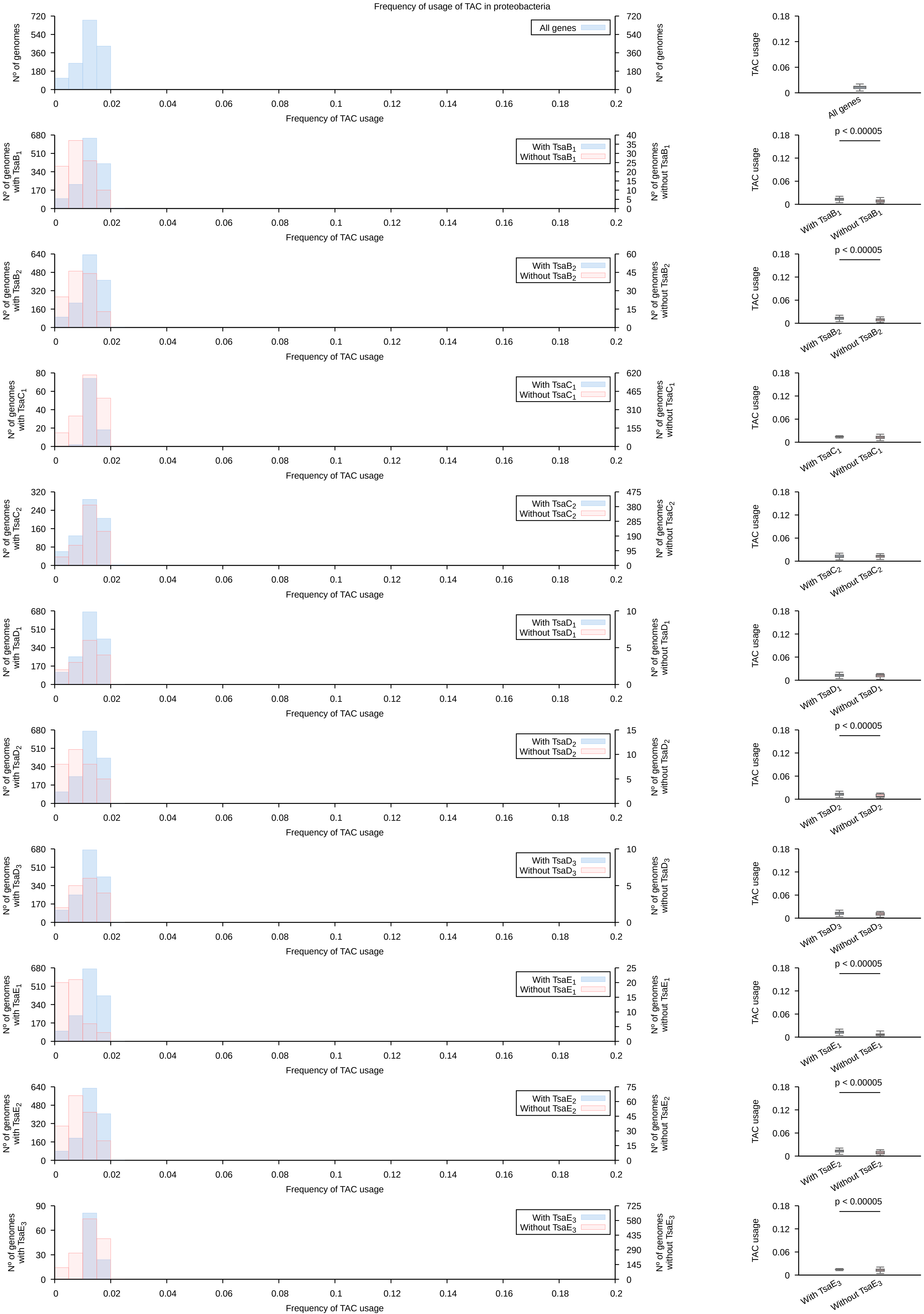
Frequency of usage of TAC in proteobacteria
Nº of genomes
Frequency of TAC usage
TAC usage
All genes
With TsaB1
Without TsaB1
With TsaB2
Without TsaB2
With TsaC1
Without TsaC1
With TsaC2
Without TsaC2
With TsaD1
Without TsaD1
With TsaD2
Without TsaD2
With TsaD3
Without TsaD3
With TsaE1
Without TsaE1
With TsaE2
Without TsaE2
With TsaE3
Without TsaE3
p < 0.00005

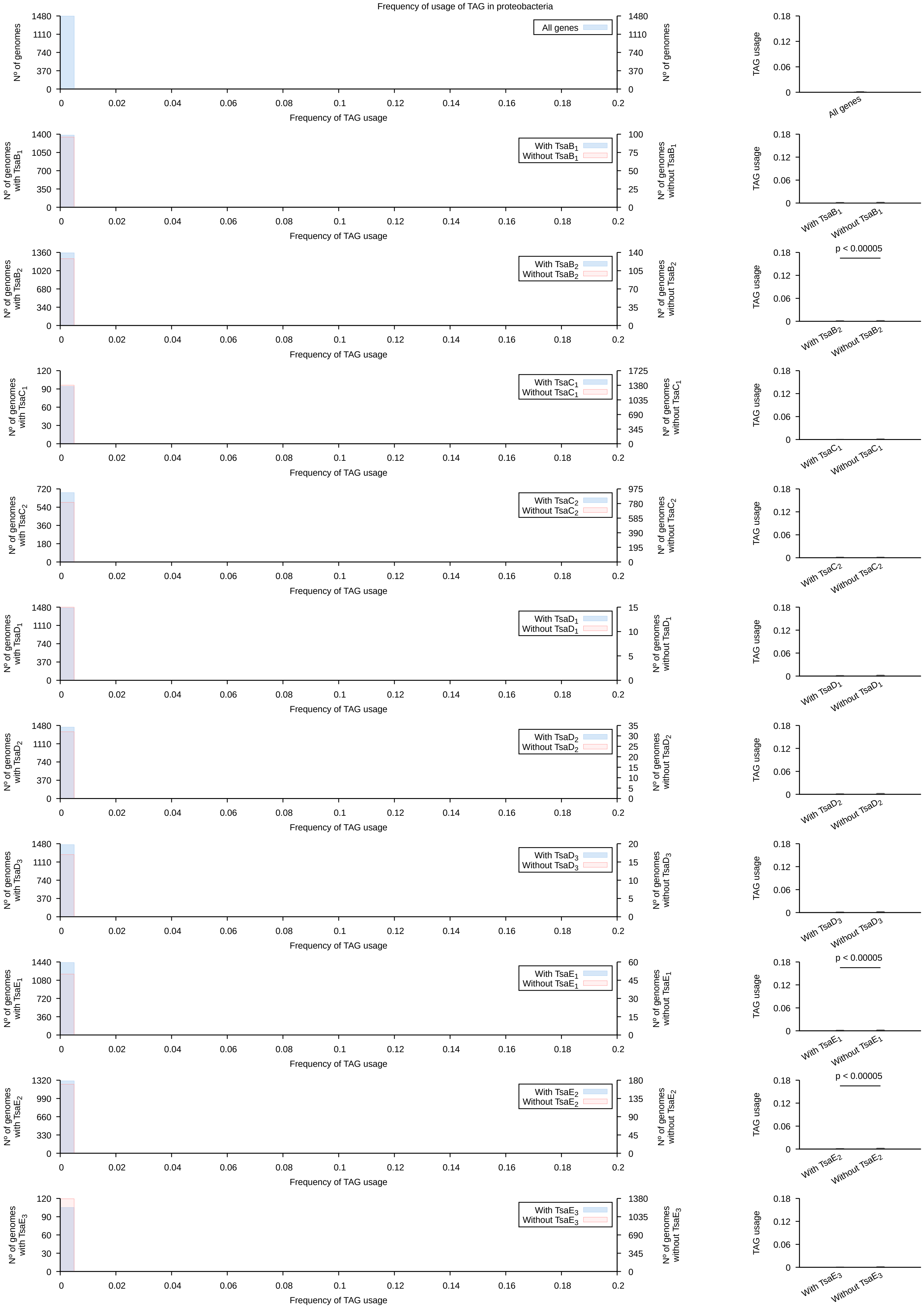

Frequency of usage of TAG in proteobacteria
All genes
Nº of genomes
Frequency of TAG usage
TAG usage
With $TsaB_1$
Without $TsaB_1$
Nº of genomes with $TsaB_1$
Nº of genomes without $TsaB_1$
With $TsaB_2$
Without $TsaB_2$
Nº of genomes with $TsaB_2$
Nº of genomes without $TsaB_2$
$p < 0.00005$
With $TsaC_1$
Without $TsaC_1$
Nº of genomes with $TsaC_1$
Nº of genomes without $TsaC_1$
With $TsaC_2$
Without $TsaC_2$
Nº of genomes with $TsaC_2$
Nº of genomes without $TsaC_2$
With $TsaD_1$
Without $TsaD_1$
Nº of genomes with $TsaD_1$
Nº of genomes without $TsaD_1$
With $TsaD_2$
Without $TsaD_2$
Nº of genomes with $TsaD_2$
Nº of genomes without $TsaD_2$
With $TsaD_3$
Without $TsaD_3$
Nº of genomes with $TsaD_3$
Nº of genomes without $TsaD_3$
With $TsaE_1$
Without $TsaE_1$
Nº of genomes with $TsaE_1$
Nº of genomes without $TsaE_1$
$p < 0.00005$
With $TsaE_2$
Without $TsaE_2$
Nº of genomes with $TsaE_2$
Nº of genomes without $TsaE_2$
$p < 0.00005$
With $TsaE_3$
Without $TsaE_3$
Nº of genomes with $TsaE_3$
Nº of genomes without $TsaE_3$

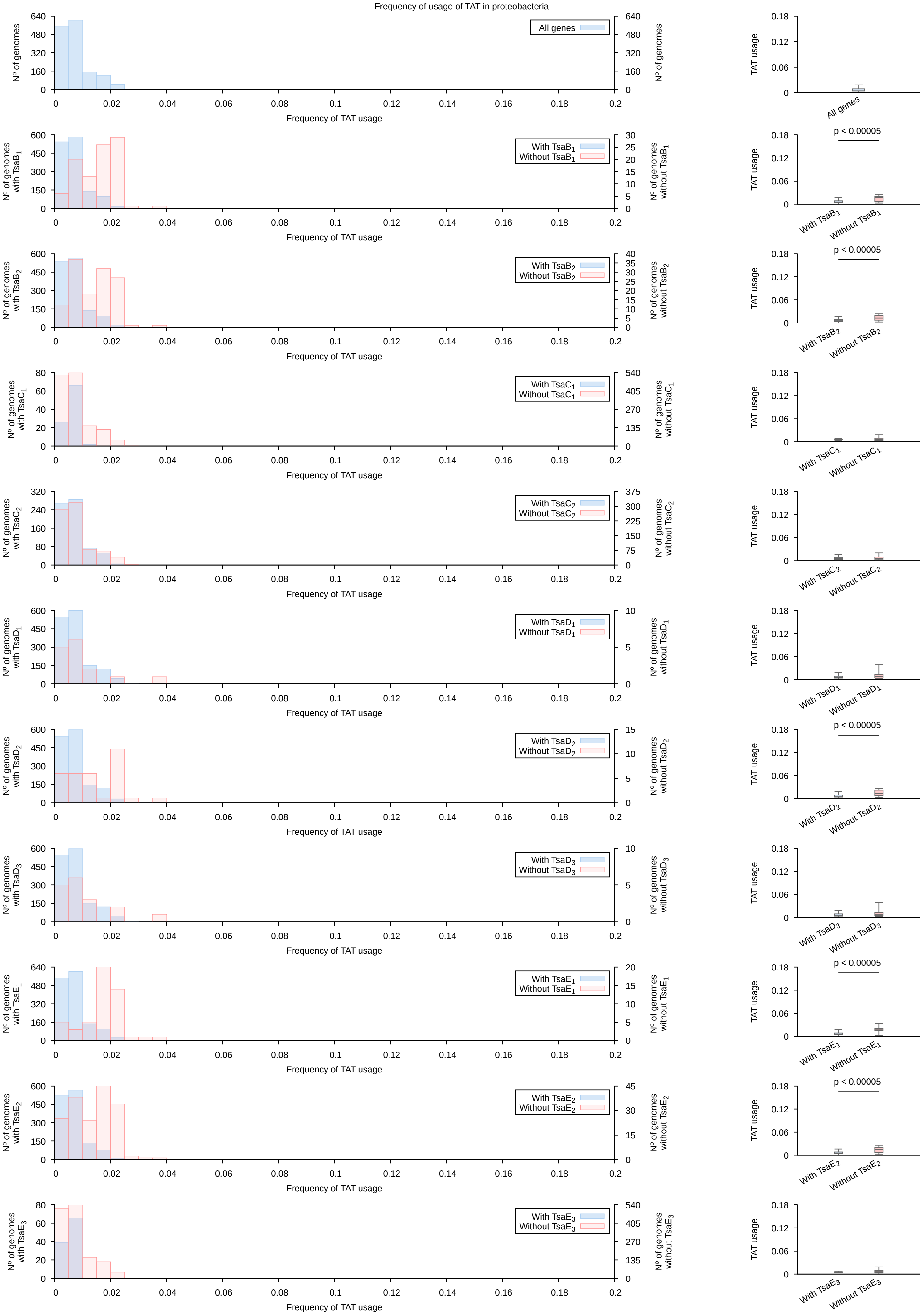

Frequency of usage of TAT in proteobacteria
Nº of genomes
Frequency of TAT usage
TAT usage
All genes
Nº of genomes with TsaB1
Nº of genomes without TsaB1
With TsaB1
Without TsaB1
p < 0.00005
Nº of genomes with TsaB2
Nº of genomes without TsaB2
With TsaB2
Without TsaB2
p < 0.00005
Nº of genomes with TsaC1
Nº of genomes without TsaC1
With TsaC1
Without TsaC1
Nº of genomes with TsaC2
Nº of genomes without TsaC2
With TsaC2
Without TsaC2
Nº of genomes with TsaD1
Nº of genomes without TsaD1
With TsaD1
Without TsaD1
Nº of genomes with TsaD2
Nº of genomes without TsaD2
With TsaD2
Without TsaD2
p < 0.00005
Nº of genomes with TsaD3
Nº of genomes without TsaD3
With TsaD3
Without TsaD3
Nº of genomes with TsaE1
Nº of genomes without TsaE1
With TsaE1
Without TsaE1
p < 0.00005
Nº of genomes with TsaE2
Nº of genomes without TsaE2
With TsaE2
Without TsaE2
p < 0.00005
Nº of genomes with TsaE3
Nº of genomes without TsaE3
With TsaE3
Without TsaE3

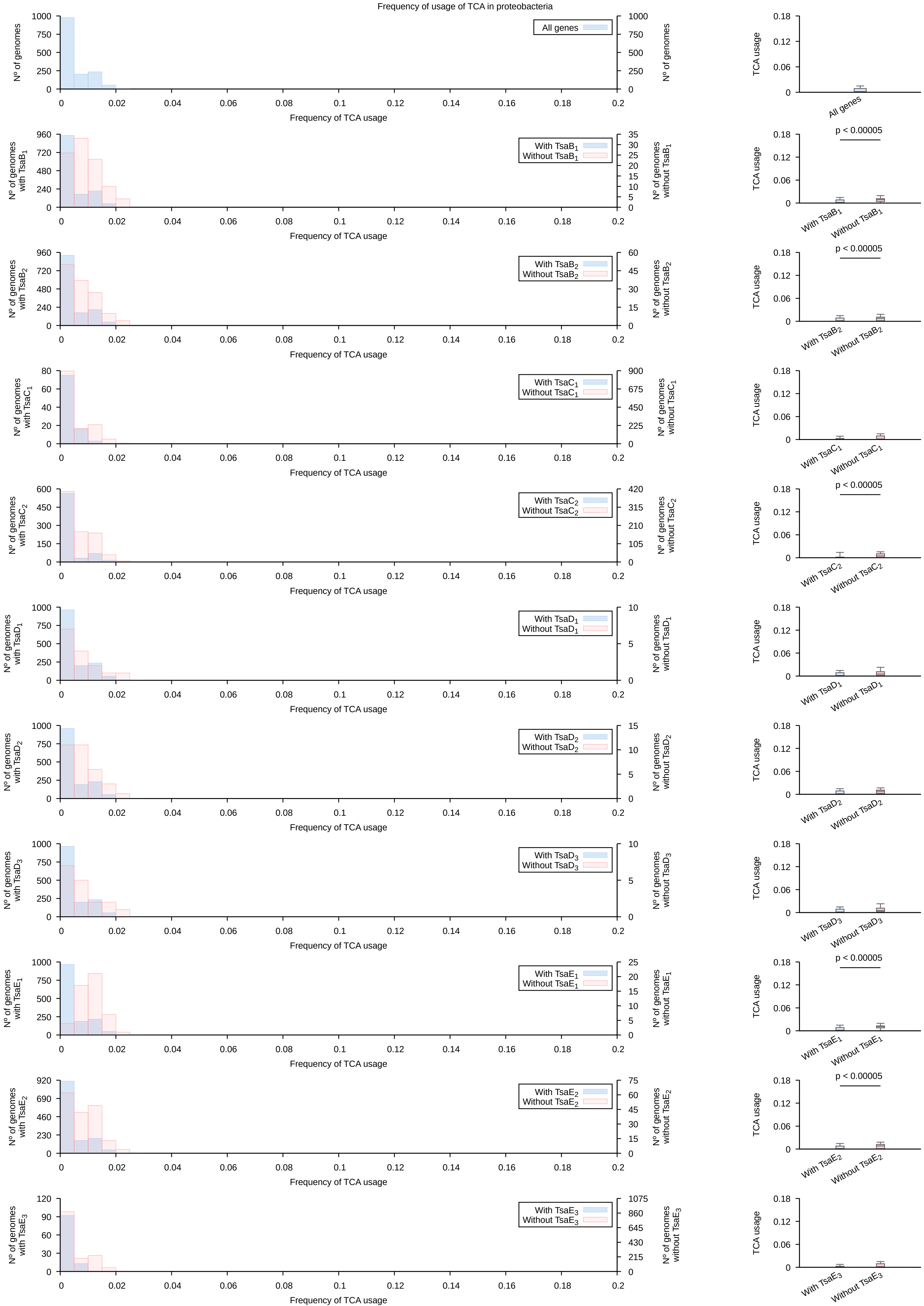

Frequency of usage of TCA in proteobacteria
All genes
Nº of genomes
Frequency of TCA usage
TCA usage
With TsaB$_1$
Without TsaB$_1$
With TsaB$_2$
Without TsaB$_2$
With TsaC$_1$
Without TsaC$_1$
With TsaC$_2$
Without TsaC$_2$
With TsaD$_1$
Without TsaD$_1$
With TsaD$_2$
Without TsaD$_2$
With TsaD$_3$
Without TsaD$_3$
With TsaE$_1$
Without TsaE$_1$
With TsaE$_2$
Without TsaE$_2$
With TsaE$_3$
Without TsaE$_3$
p < 0.00005

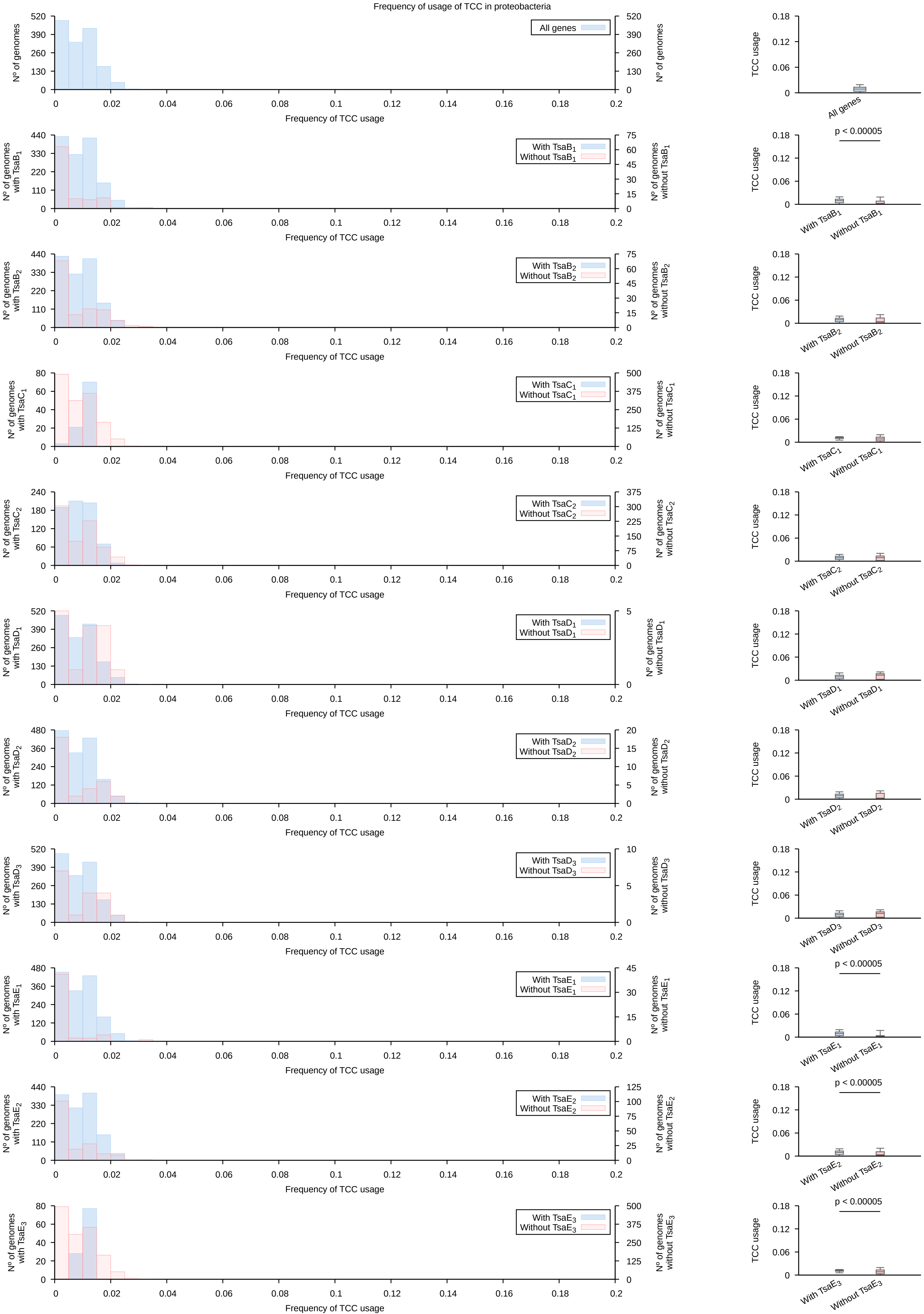
Frequency of usage of TCC in proteobacteria
Nº of genomes
Frequency of TCC usage
All genes
TCC usage
Nº of genomes with TsaB1
Nº of genomes without TsaB1
With TsaB1
Without TsaB1
p < 0.00005
Nº of genomes with TsaB2
Nº of genomes without TsaB2
With TsaB2
Without TsaB2
Nº of genomes with TsaC1
Nº of genomes without TsaC1
With TsaC1
Without TsaC1
Nº of genomes with TsaC2
Nº of genomes without TsaC2
With TsaC2
Without TsaC2
Nº of genomes with TsaD1
Nº of genomes without TsaD1
With TsaD1
Without TsaD1
Nº of genomes with TsaD2
Nº of genomes without TsaD2
With TsaD2
Without TsaD2
Nº of genomes with TsaD3
Nº of genomes without TsaD3
With TsaD3
Without TsaD3
Nº of genomes with TsaE1
Nº of genomes without TsaE1
With TsaE1
Without TsaE1
p < 0.00005
Nº of genomes with TsaE2
Nº of genomes without TsaE2
With TsaE2
Without TsaE2
p < 0.00005
Nº of genomes with TsaE3
Nº of genomes without TsaE3
With TsaE3
Without TsaE3
p < 0.00005

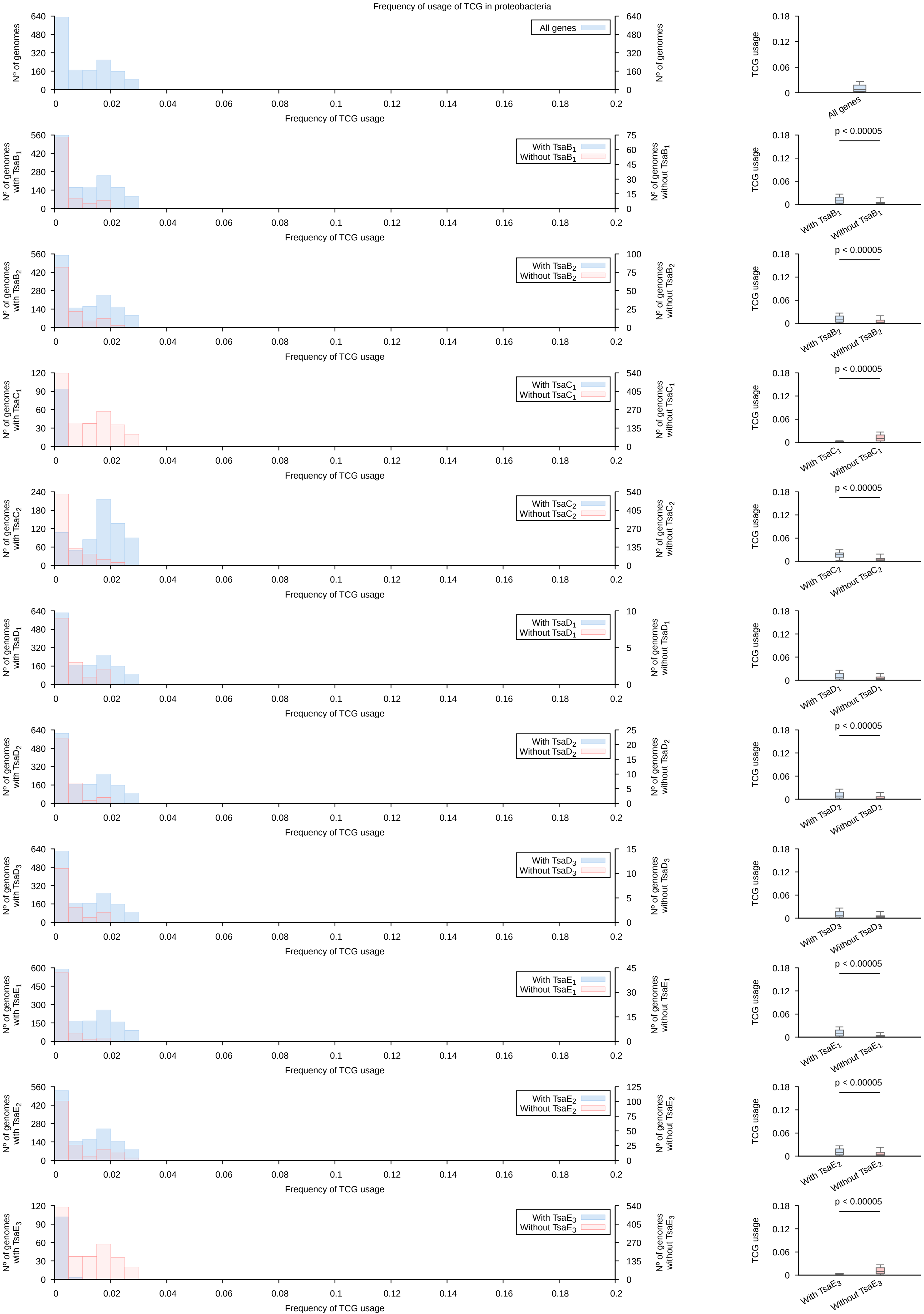

Frequency of usage of TCG in proteobacteria
Nº of genomes
All genes
Frequency of TCG usage
TCG usage
All genes
Nº of genomes with TsaB$_1$
Nº of genomes without TsaB$_1$
With TsaB$_1$
Without TsaB$_1$
p < 0.00005
Nº of genomes with TsaB$_2$
Nº of genomes without TsaB$_2$
With TsaB$_2$
Without TsaB$_2$
p < 0.00005
Nº of genomes with TsaC$_1$
Nº of genomes without TsaC$_1$
With TsaC$_1$
Without TsaC$_1$
p < 0.00005
Nº of genomes with TsaC$_2$
Nº of genomes without TsaC$_2$
With TsaC$_2$
Without TsaC$_2$
p < 0.00005
Nº of genomes with TsaD$_1$
Nº of genomes without TsaD$_1$
With TsaD$_1$
Without TsaD$_1$
Nº of genomes with TsaD$_2$
Nº of genomes without TsaD$_2$
With TsaD$_2$
Without TsaD$_2$
p < 0.00005
Nº of genomes with TsaD$_3$
Nº of genomes without TsaD$_3$
With TsaD$_3$
Without TsaD$_3$
Nº of genomes with TsaE$_1$
Nº of genomes without TsaE$_1$
With TsaE$_1$
Without TsaE$_1$
p < 0.00005
Nº of genomes with TsaE$_2$
Nº of genomes without TsaE$_2$
With TsaE$_2$
Without TsaE$_2$
p < 0.00005
Nº of genomes with TsaE$_3$
Nº of genomes without TsaE$_3$
With TsaE$_3$
Without TsaE$_3$
p < 0.00005

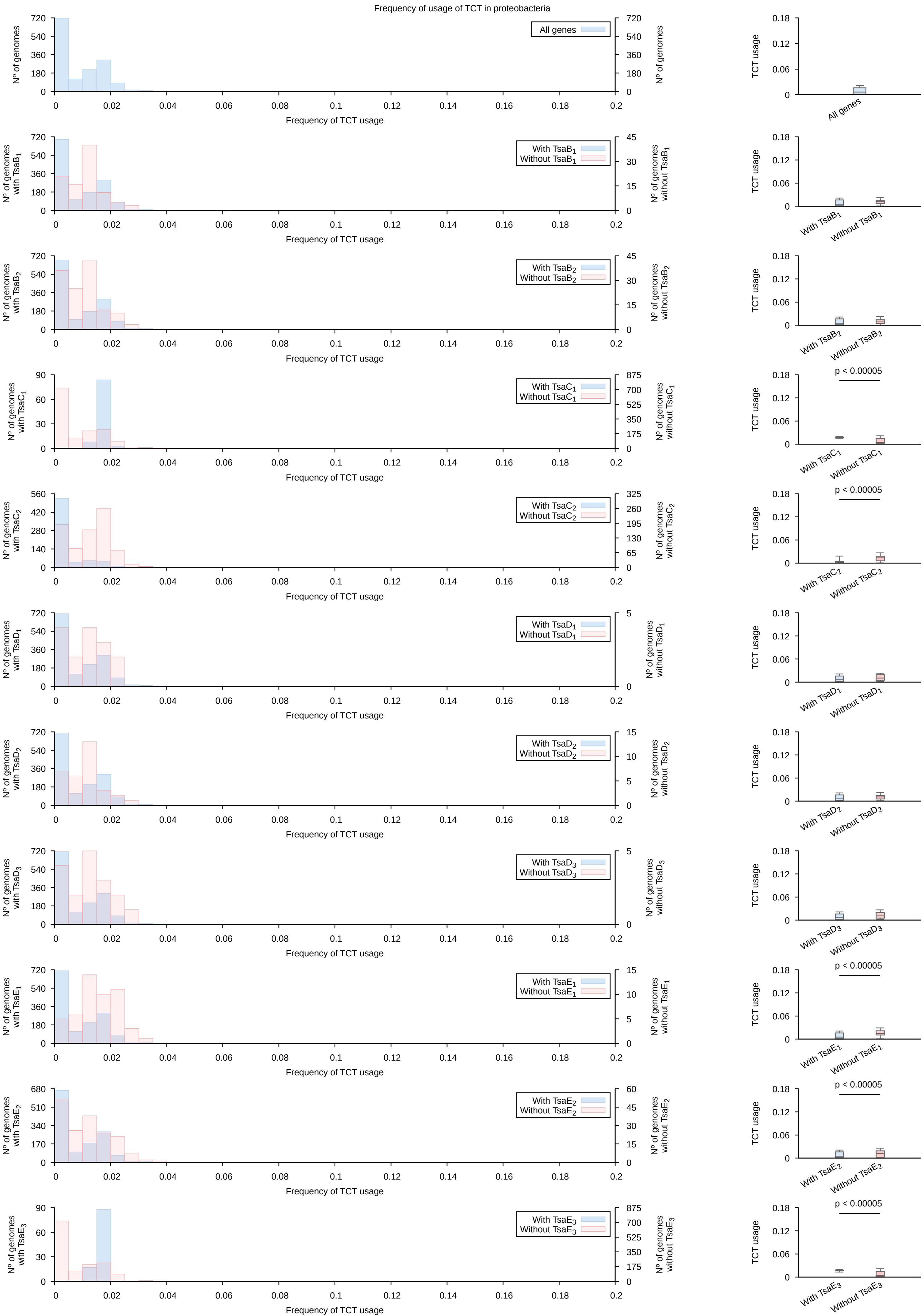

Frequency of usage of TCT in proteobacteria
All genes
With $TsaB_1$
Without $TsaB_1$
With $TsaB_2$
Without $TsaB_2$
With $TsaC_1$
Without $TsaC_1$
With $TsaC_2$
Without $TsaC_2$
With $TsaD_1$
Without $TsaD_1$
With $TsaD_2$
Without $TsaD_2$
With $TsaD_3$
Without $TsaD_3$
With $TsaE_1$
Without $TsaE_1$
With $TsaE_2$
Without $TsaE_2$
With $TsaE_3$
Without $TsaE_3$
Nº of genomes
Frequency of TCT usage
TCT usage
$p < 0.00005$

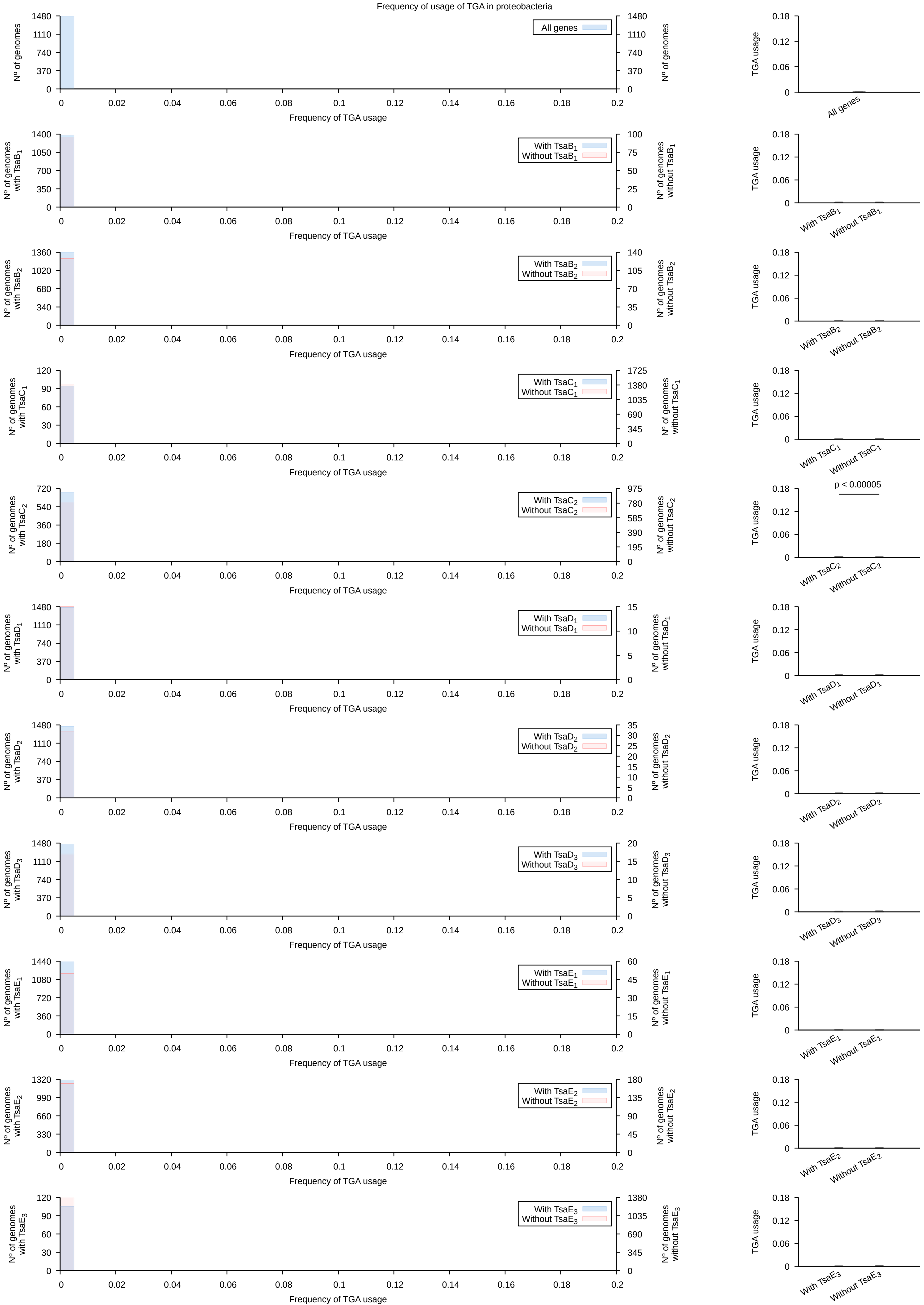
Frequency of usage of TGA in proteobacteria
All genes
Nº of genomes
Frequency of TGA usage
TGA usage
With TsaB$_1$
Without TsaB$_1$
Nº of genomes with TsaB$_1$
Nº of genomes without TsaB$_1$
With TsaB$_2$
Without TsaB$_2$
Nº of genomes with TsaB$_2$
Nº of genomes without TsaB$_2$
With TsaC$_1$
Without TsaC$_1$
Nº of genomes with TsaC$_1$
Nº of genomes without TsaC$_1$
With TsaC$_2$
Without TsaC$_2$
Nº of genomes with TsaC$_2$
Nº of genomes without TsaC$_2$
p < 0.00005
With TsaD$_1$
Without TsaD$_1$
Nº of genomes with TsaD$_1$
Nº of genomes without TsaD$_1$
With TsaD$_2$
Without TsaD$_2$
Nº of genomes with TsaD$_2$
Nº of genomes without TsaD$_2$
With TsaD$_3$
Without TsaD$_3$
Nº of genomes with TsaD$_3$
Nº of genomes without TsaD$_3$
With TsaE$_1$
Without TsaE$_1$
Nº of genomes with TsaE$_1$
Nº of genomes without TsaE$_1$
With TsaE$_2$
Without TsaE$_2$
Nº of genomes with TsaE$_2$
Nº of genomes without TsaE$_2$
With TsaE$_3$
Without TsaE$_3$
Nº of genomes with TsaE$_3$
Nº of genomes without TsaE$_3$

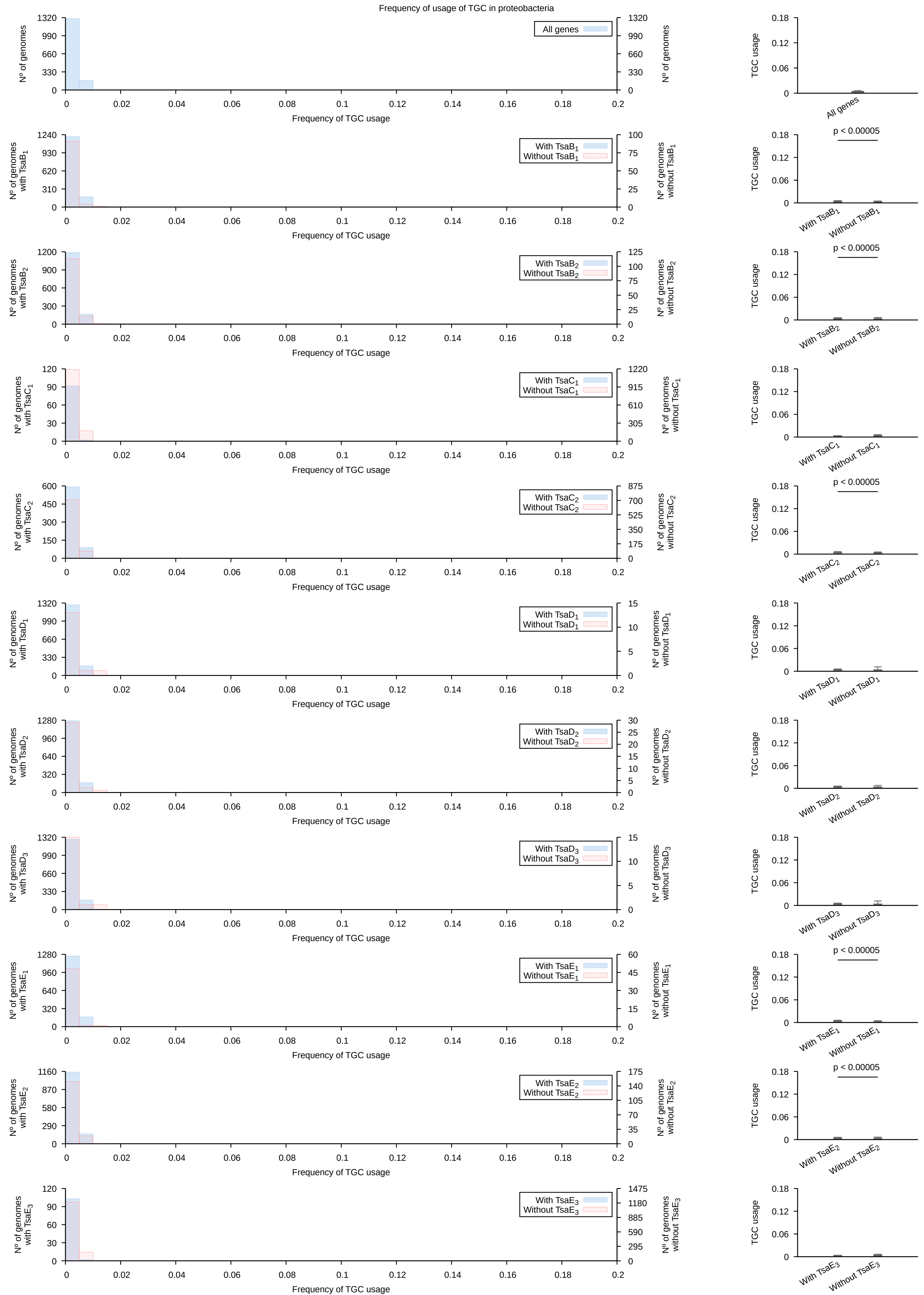
Frequency of usage of TGC in proteobacteria
Nº of genomes
All genes
Frequency of TGC usage
TGC usage
All genes
Nº of genomes with $TsaB_1$
Nº of genomes without $TsaB_1$
With $TsaB_1$
Without $TsaB_1$
$p < 0.00005$
Nº of genomes with $TsaB_2$
Nº of genomes without $TsaB_2$
With $TsaB_2$
Without $TsaB_2$
$p < 0.00005$
Nº of genomes with $TsaC_1$
Nº of genomes without $TsaC_1$
With $TsaC_1$
Without $TsaC_1$
Nº of genomes with $TsaC_2$
Nº of genomes without $TsaC_2$
With $TsaC_2$
Without $TsaC_2$
$p < 0.00005$
Nº of genomes with $TsaD_1$
Nº of genomes without $TsaD_1$
With $TsaD_1$
Without $TsaD_1$
Nº of genomes with $TsaD_2$
Nº of genomes without $TsaD_2$
With $TsaD_2$
Without $TsaD_2$
Nº of genomes with $TsaD_3$
Nº of genomes without $TsaD_3$
With $TsaD_3$
Without $TsaD_3$
Nº of genomes with $TsaE_1$
Nº of genomes without $TsaE_1$
With $TsaE_1$
Without $TsaE_1$
$p < 0.00005$
Nº of genomes with $TsaE_2$
Nº of genomes without $TsaE_2$
With $TsaE_2$
Without $TsaE_2$
$p < 0.00005$
Nº of genomes with $TsaE_3$
Nº of genomes without $TsaE_3$
With $TsaE_3$
Without $TsaE_3$

Frequency of usage of TGG in proteobacteria

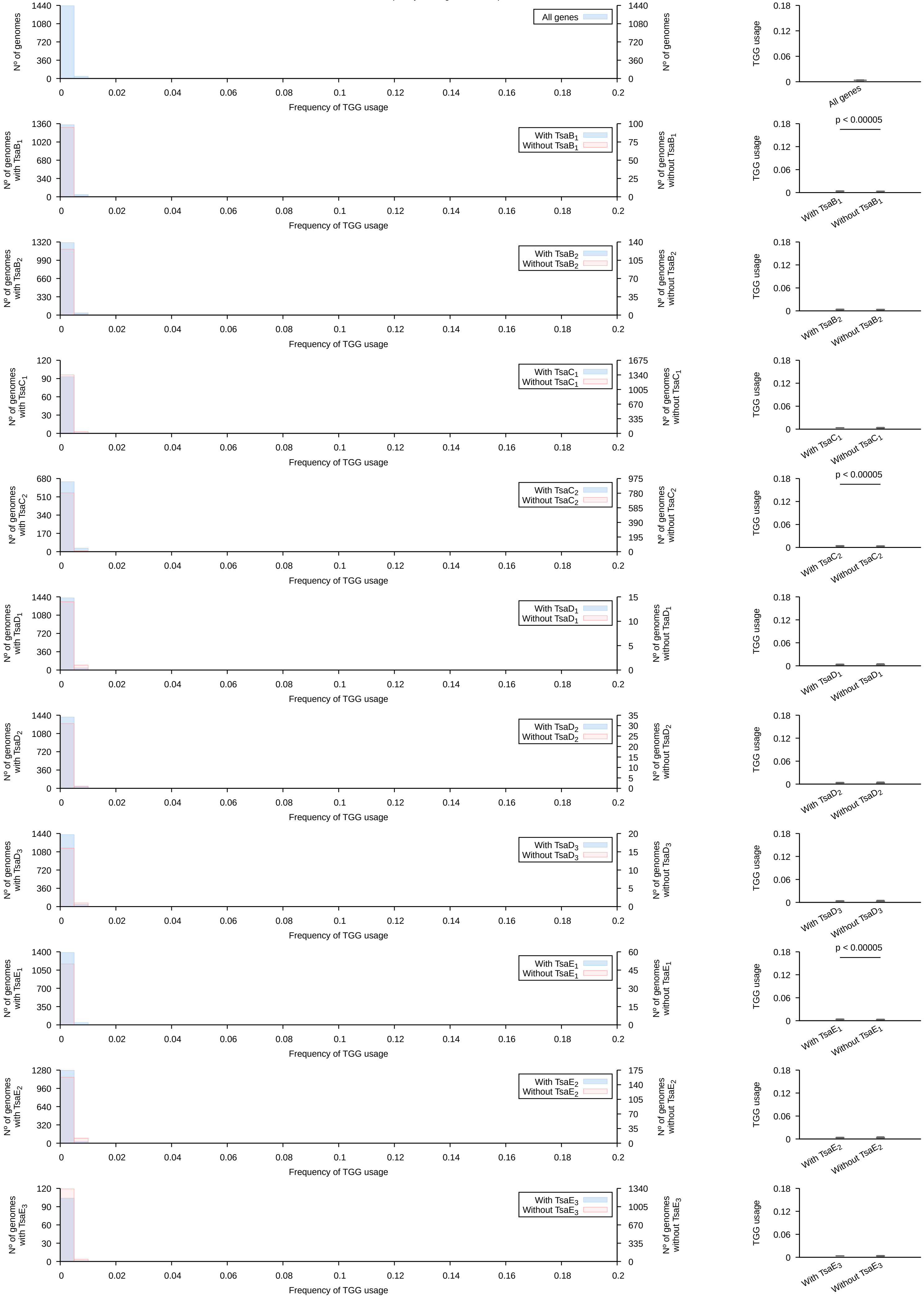

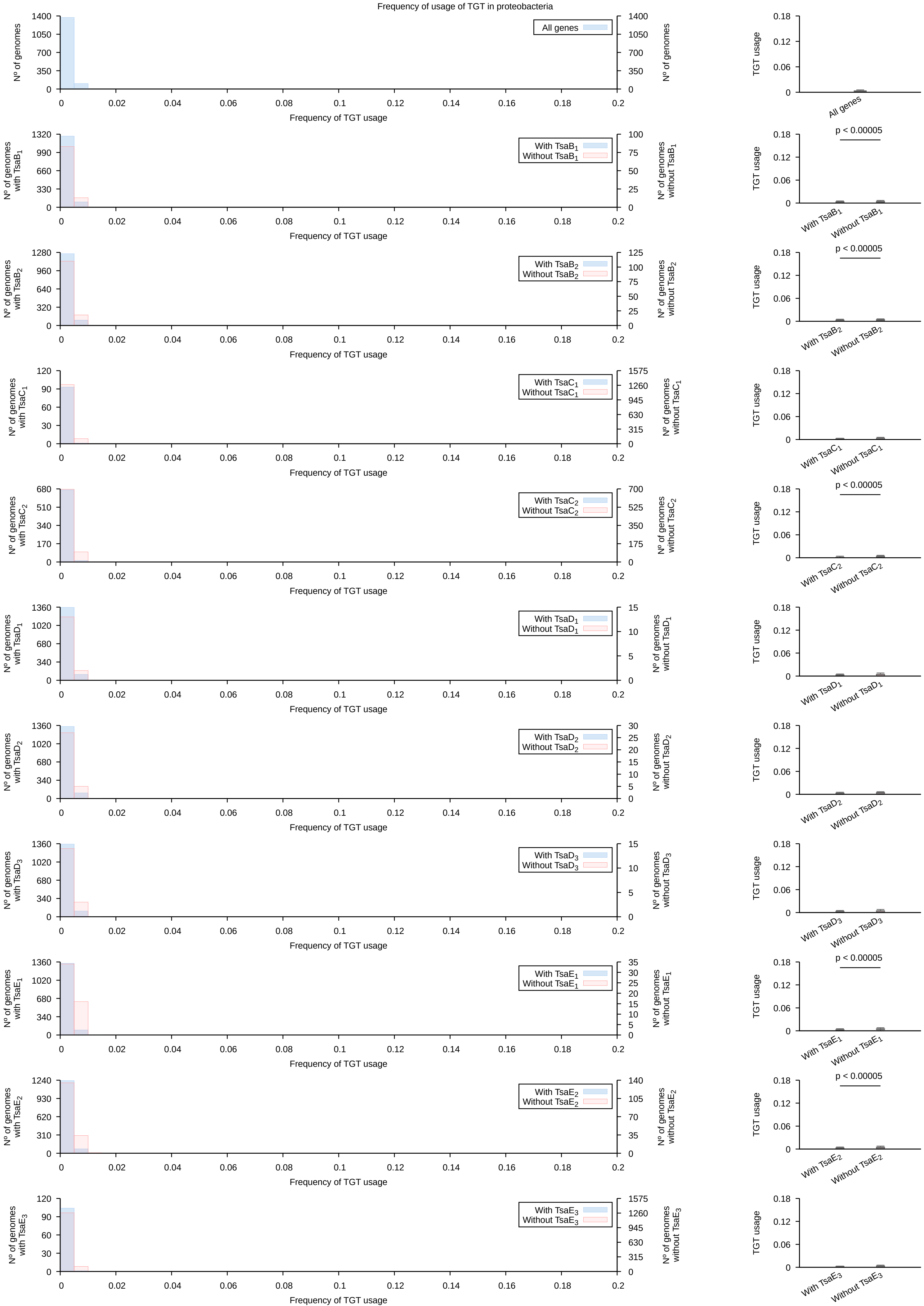

Frequency of usage of TGT in proteobacteria
All genes
Nº of genomes
Frequency of TGT usage
TGT usage
With TsaB1
Without TsaB1
Nº of genomes with TsaB1
Nº of genomes without TsaB1
p < 0.00005
With TsaB2
Without TsaB2
Nº of genomes with TsaB2
Nº of genomes without TsaB2
p < 0.00005
With TsaC1
Without TsaC1
Nº of genomes with TsaC1
Nº of genomes without TsaC1
With TsaC2
Without TsaC2
Nº of genomes with TsaC2
Nº of genomes without TsaC2
p < 0.00005
With TsaD1
Without TsaD1
Nº of genomes with TsaD1
Nº of genomes without TsaD1
With TsaD2
Without TsaD2
Nº of genomes with TsaD2
Nº of genomes without TsaD2
With TsaD3
Without TsaD3
Nº of genomes with TsaD3
Nº of genomes without TsaD3
With TsaE1
Without TsaE1
Nº of genomes with TsaE1
Nº of genomes without TsaE1
p < 0.00005
With TsaE2
Without TsaE2
Nº of genomes with TsaE2
Nº of genomes without TsaE2
p < 0.00005
With TsaE3
Without TsaE3
Nº of genomes with TsaE3
Nº of genomes without TsaE3

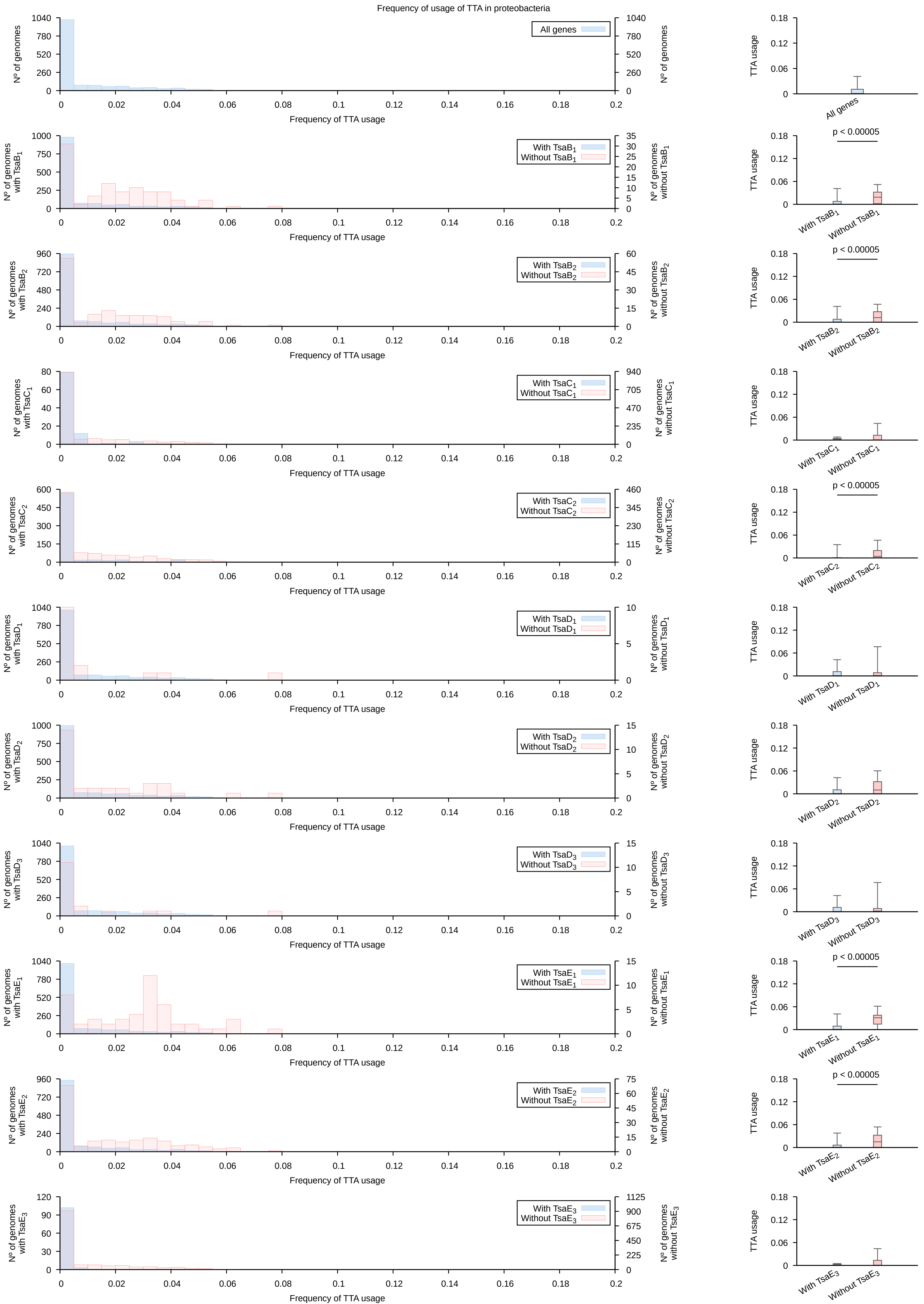

Frequency of usage of TTA in proteobacteria
All genes
Nº of genomes
Frequency of TTA usage
TTA usage
With TsaB1
Without TsaB1
Nº of genomes with TsaB1
Nº of genomes without TsaB1
p < 0.00005
With TsaB2
Without TsaB2
Nº of genomes with TsaB2
Nº of genomes without TsaB2
p < 0.00005
With TsaC1
Without TsaC1
Nº of genomes with TsaC1
Nº of genomes without TsaC1
With TsaC2
Without TsaC2
Nº of genomes with TsaC2
Nº of genomes without TsaC2
p < 0.00005
With TsaD1
Without TsaD1
Nº of genomes with TsaD1
Nº of genomes without TsaD1
With TsaD2
Without TsaD2
Nº of genomes with TsaD2
Nº of genomes without TsaD2
With TsaD3
Without TsaD3
Nº of genomes with TsaD3
Nº of genomes without TsaD3
With TsaE1
Without TsaE1
Nº of genomes with TsaE1
Nº of genomes without TsaE1
p < 0.00005
With TsaE2
Without TsaE2
Nº of genomes with TsaE2
Nº of genomes without TsaE2
p < 0.00005
With TsaE3
Without TsaE3
Nº of genomes with TsaE3
Nº of genomes without TsaE3

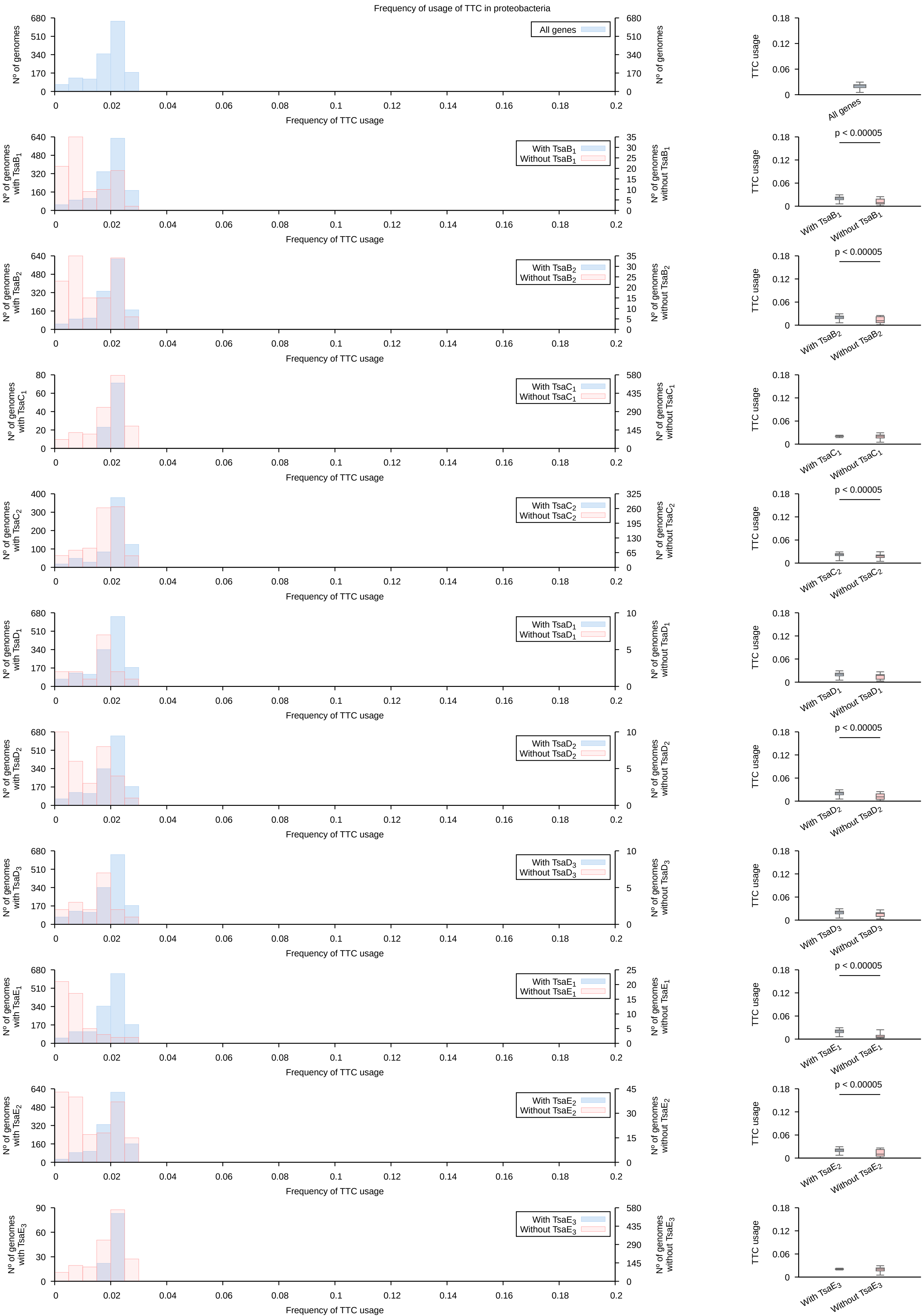

Frequency of usage of TTC in proteobacteria
All genes
Nº of genomes
TTC usage
Frequency of TTC usage
With TsaB1
Without TsaB1
Nº of genomes with TsaB1
Nº of genomes without TsaB1
p < 0.00005
With TsaB2
Without TsaB2
Nº of genomes with TsaB2
Nº of genomes without TsaB2
p < 0.00005
With TsaC1
Without TsaC1
Nº of genomes with TsaC1
Nº of genomes without TsaC1
With TsaC2
Without TsaC2
Nº of genomes with TsaC2
Nº of genomes without TsaC2
p < 0.00005
With TsaD1
Without TsaD1
Nº of genomes with TsaD1
Nº of genomes without TsaD1
With TsaD2
Without TsaD2
Nº of genomes with TsaD2
Nº of genomes without TsaD2
p < 0.00005
With TsaD3
Without TsaD3
Nº of genomes with TsaD3
Nº of genomes without TsaD3
With TsaE1
Without TsaE1
Nº of genomes with TsaE1
Nº of genomes without TsaE1
p < 0.00005
With TsaE2
Without TsaE2
Nº of genomes with TsaE2
Nº of genomes without TsaE2
p < 0.00005
With TsaE3
Without TsaE3
Nº of genomes with TsaE3
Nº of genomes without TsaE3

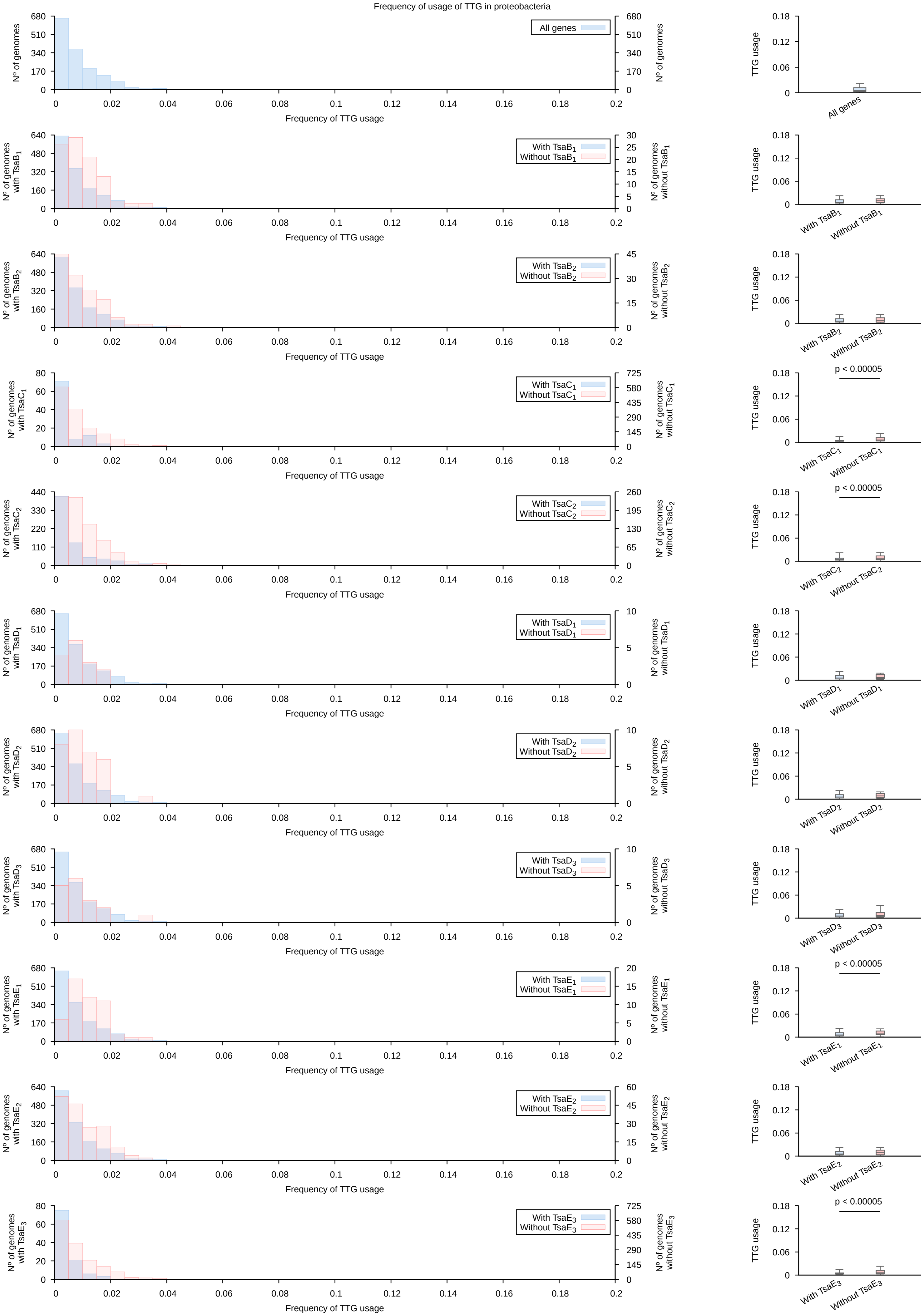
Frequency of usage of TTG in proteobacteria
All genes
Nº of genomes
Frequency of TTG usage
TTG usage
With TsaB$_1$
Without TsaB$_1$
Nº of genomes with TsaB$_1$
Nº of genomes without TsaB$_1$
With TsaB$_2$
Without TsaB$_2$
Nº of genomes with TsaB$_2$
Nº of genomes without TsaB$_2$
With TsaC$_1$
Without TsaC$_1$
Nº of genomes with TsaC$_1$
Nº of genomes without TsaC$_1$
p < 0.00005
With TsaC$_2$
Without TsaC$_2$
Nº of genomes with TsaC$_2$
Nº of genomes without TsaC$_2$
p < 0.00005
With TsaD$_1$
Without TsaD$_1$
Nº of genomes with TsaD$_1$
Nº of genomes without TsaD$_1$
With TsaD$_2$
Without TsaD$_2$
Nº of genomes with TsaD$_2$
Nº of genomes without TsaD$_2$
With TsaD$_3$
Without TsaD$_3$
Nº of genomes with TsaD$_3$
Nº of genomes without TsaD$_3$
With TsaE$_1$
Without TsaE$_1$
Nº of genomes with TsaE$_1$
Nº of genomes without TsaE$_1$
p < 0.00005
With TsaE$_2$
Without TsaE$_2$
Nº of genomes with TsaE$_2$
Nº of genomes without TsaE$_2$
With TsaE$_3$
Without TsaE$_3$
Nº of genomes with TsaE$_3$
Nº of genomes without TsaE$_3$
p < 0.00005

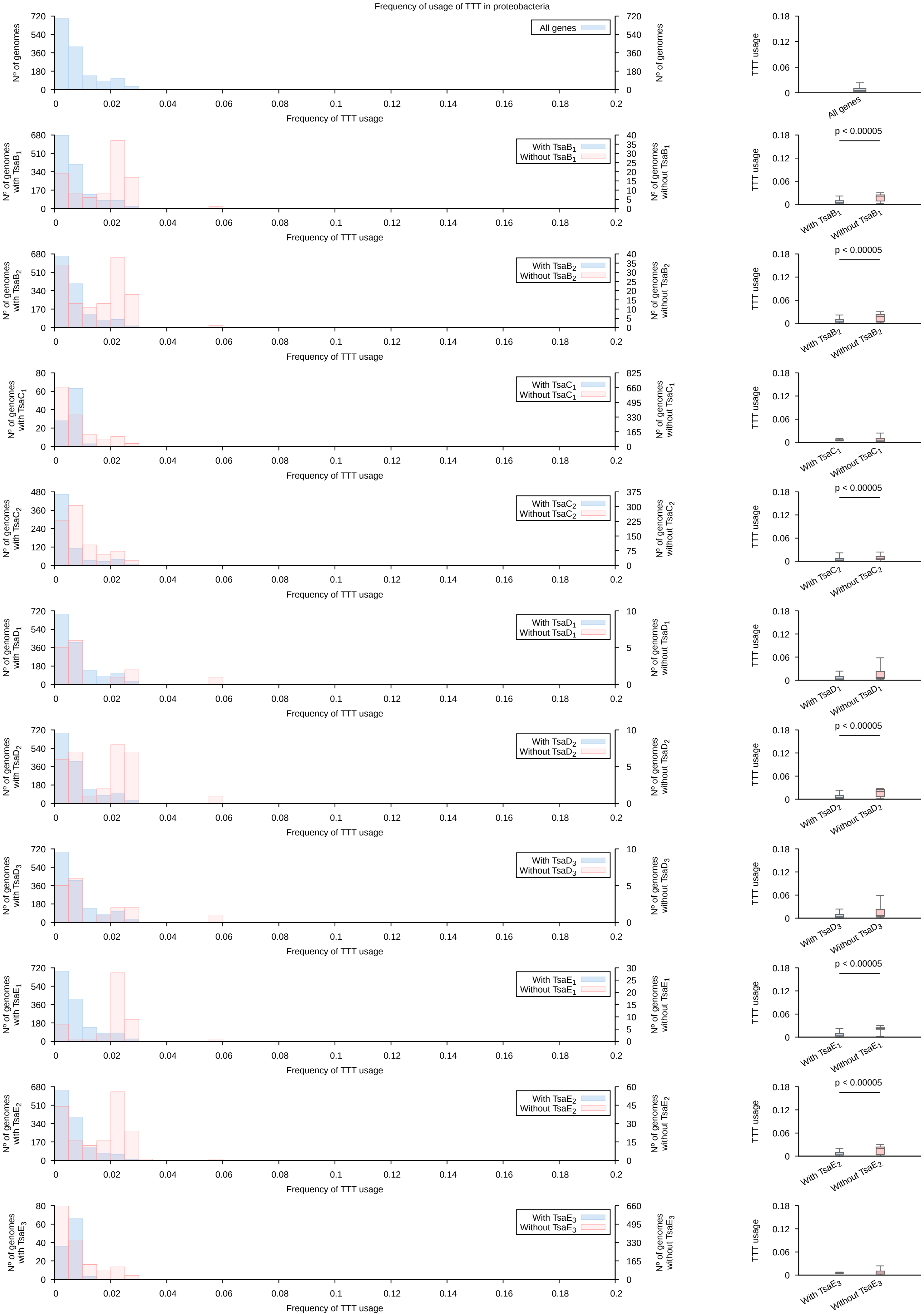

Frequency of usage of TTT in proteobacteria
All genes
With TsaB1
Without TsaB1
With TsaB2
Without TsaB2
With TsaC1
Without TsaC1
With TsaC2
Without TsaC2
With TsaD1
Without TsaD1
With TsaD2
Without TsaD2
With TsaD3
Without TsaD3
With TsaE1
Without TsaE1
With TsaE2
Without TsaE2
With TsaE3
Without TsaE3
Nº of genomes
Frequency of TTT usage
TTT usage
p < 0.00005
